# In Silico Exploration of Staphylococcal Cassette Chromosome *mec* (SCC*mec*) Evolution Based on Phylogenetic Relationship of *ccrAB/C*

**DOI:** 10.3390/microorganisms13010153

**Published:** 2025-01-13

**Authors:** Huawei Wang, Jinxing He

**Affiliations:** College of Food Science and Engineering, Qilu University of Technology (Shandong Academy of Sciences), Jinan 250353, China

**Keywords:** *ccrAB/C*, evolution, staphylococci, in silico

## Abstract

As the mobile cassette carrier of the methicillin resistance gene *mecA* that is transported across staphylococci species, the evolution and origin of Staphylococcal Cassette Chromosome *mec* (SCC*mec*)—and in particular, the composition of *mecA* and SCC*mec*—have been extensively discussed in the scientific literature; however, information regarding its dissemination across geographical limits and evolution over decades remains limited. In addition, whole-genome sequencing-based macro-analysis was unable to provide sufficiently detailed evolutionary information on SCC*mec*. Herein, the cassette chromosome recombinase genes *ccrAB/C*, as essential components of SCC*mec*, were employed to explore the evolution of SCC*mec*. This work established the basic taxonomy of 33 staphylococci species. The CUB of *mecA*, *ccrAB/C* of 12 SCC*mec* types and core genome of 33 staphylococci species were subsequently compared; the phylogenetic relationship of *ccrAB/C* was observed via SCC*mec* typing on a temporal and geographical scale; and the duplicate appearance of *ccrAB/C* was illustrated by comparing SCC*mec* compositions. The results highlighted a deviation in the CUB of *mecA* and *ccrAB/C*, which evidenced their exogenous characteristics to staphylococci, and provided theological support for the phylogenetic analysis of *ccrAB/C* as representative of SCC*mec*. Importantly, the phylogenetic relationship of *ccrAB/C* did not exhibit centralization over time; instead, similarly to *mecA*, *ccrAB/C* with similar identities had close clades across decades and geographical limits and different SCC*mec* types, which enabled us to discriminate SCC*mec* based on the sequence identity of *ccrAB/C*. In addition, the duplicate appearance of *ccrAB/C* and fixed composition of the *ccrAB/C* complex among different strains were indicative of more complicated transmission mechanisms than targeting direct repeats of SCC*mec*.

## 1. Introduction

Methicillin-resistant *Staphylococcus aureus* (MRSA) has long been recognized as a serious nosocomial pathogen due to its broad-spectrum resistance to β-lactam antimicrobial agents [1]. MRSA has been the subject of constant study over the last few decades; as such, Staphylococcal Cassette Chromosome *mec* (SCC*mec*) has been identified as a well-known carrier of the methicillin resistance gene *mec*, which has been transferred within the staphylococcal genus [2], causing ubiquitous dissemination in staphylococci. The increasing appearance of SCC*mec* in *S. aureus* can be attributed to the methicillin-resistant non-aureus staphylococci involved in the exploration of the evolution and origin of SCC*mec* [1,2,3,4]. It has been established that, due to the more common dissemination of coagulase-negative staphylococci (CoNS) in natural environments [5], methicillin-resistant coagulase-negative staphylococci (MR-CoNS) serve as reservoirs of SCCmec for *S. aureus* [6], facilitating the increasing emergence of new types of MRSA.

The evolution and origin of methicillin resistance genetic determinants should be considered based on the following factors:

Firstly, as methicillin resistance dominates gene-encoding penicillin-binding protein 2a (PBP2a), *mecA* was primarily analyzed to explore its dissemination and evolution within staphylococci. Incipient investigations demonstrated a ubiquitous presence and high identity of *mecA* in *Mammaliicoccus sciuri* (reassigned from *Staphylococcus* species to *Mammaliicoccus* species [7]) compared with MRSA [8,9]. Subsequently, modern Bayesian informatics and selective pressure analysis were utilized to evaluate the evolution and adaption of *mecA* in staphylococci, and it was proposed that *Mammaliicoccus vitulinus*, *Mammaliicoccus fleurettii* and *M. sciuri* constitute the phased evolution of *mecA*; more importantly, *M. sciuri* was inferred to be a precursor of SCC*mec* [10].

Secondly, since the initial designation of SCC*mec* composed of *mecA* and cassette chromosome recombinase *ccrAB* [11], the evolutionary analysis of methicillin resistance genetic determinants has been largely dependent on SCC*mec* structural comparison. For instance, based on the common presence of *M. vitulinus*, *M. fleurettii* and *M. sciuri* originating from mammals, through a sequence identity comparison of multiple serial Open Reading Frames (ORFs) within SCC*mec*, *M. fleurettii* was proposed as the evolutionary origin of SCC*mec* [3]. In addition, the evolutionary steps of SCC*mec* were hypothesized among *M. vitulinus*, *M. fleurettii* and *M. sciuri*; it was found that *M. vitulinus* and *M. fleurettii* constitute the *mec* complex, and *M. sciuri* is the precursor of SCC*mec* [4].

Thirdly, *ccrAB/C* (*ccrC* was later discovered [12]), as another determinant of SCC*mec* function and in the excision and reintegration of SCC*mec* [2], were employed to analyze the evolution and phylogenic relationship of SCC*mec* variants. A previous report identified sequence variations among different isolates of the same *ccrAB* type, and high sequence conservation of *ccrAB* was observed within same geographical region, which led the authors to hypothesize that SCC*mec* may be transmitted horizontally [13]. However, in light of the increasing emergence of methicillin-resistant staphylococci and novel types of SCC*mec*, the underlying homology of novel types of *ccrAB/C* compared with previously established types is in need of updating. Notably, the close relationship of *ccrAB* of the SCC*mec*-like element in *Macrococcus caseolyticus* with *ccrAB* of *S. aureus* was emphasized via phylogenetic analysis, and the potential transmission of SCC*mec* across *M. caseolyticus* and *S. aureus* was elucidated [14].

Currently, high-resolution sequence polymorphisms (e.g., multi-locus sequence typing and single-nucleotide polymorphisms) and evolutionary deduction (e.g., Bayesian analysis) on methicillin-resistant staphylococci provide more detailed information based on whole-genome sequencing (WGS) [10,15]. For instance, through the application of WGS and Bayesian analysis, the evolutionary route of MRSA (sequence type (ST) 225) was observed within central Europe on a temporal and geographical scale [16]; additionally, the evolution, expansion and transmission routes of pandemic EMRSA-16 were observed within the United Kingdom [17]. Inspiringly, the origin of USA300 was observed in terms of the evolutionary route of ST8 and acquisition history of pathogenic elements (e.g., SCC*mec* and Panton–Valentine Leukocidin (PVL)) on a global scale [18]. Subsequently, the genome evolution of MRSA (involving multiple features, e.g., SCC*mec*, ST, antibiotic resistance genes, PVL and arginine catabolic mobile element) was observed on a larger scale (i.e., transcontinental dissemination, diverged time point and acquisition of SCC*mec*) [19]. The above evidence and exploration shed light on the evolutionary route of MRSA across time and geographical scale. Nevertheless, SCC*mec* acts as an independent transmission cassette transferred within staphylococci [2], and is usually of ancestral lineage and is unaccompanied by other pathogenic elements [20]. Regarding the potential emergence of new methicillin-resistant staphylococci in native surroundings and transmission of SCC*mec* across geographical limits, in our opinion, these macro-analyses on the evolution of SCC*mec* remain incomplete.

Regarding the potential difference in Codon Usage Bias (CUB) between exogenous and endogenous genes, SCC*mec*, as a mobile chromosome cassette, likely exhibits distinctive CUB compared to the core genome of staphylococci, since it has been suggested that CUB is capable of revealing a horizontal gene transfer within closely related organisms [21]. A related study performed evolutionary analysis of horizontally transferred genes by examining their CUB and tRNA profiles, and suggested that horizontally transferred genes exhibited varied CUB adaption frequency and extended residence times, and several horizontally transferred genes maintained atypical CUB compared with the core genome [22]. These findings support our hypothesis that CUB could be employed to observe the homologous relationship between SCC*mec* and the core genome of staphylococci, and genes within SCC*mec* are likely to maintain stable CUB/high sequence conservation over long-term evolution (as demonstrated by the high sequence conservation of *mecA*). Generally speaking, with the exception of two essential complexes (including *mecA* and *ccrAB/C* complexes), SCC*me*c commonly harbors multiple regulatory factors (e.g., *mecR1*, *mecI*), accessory genes (e.g., metabolic genes, insertion sequences, transposons), and other antibiotic resistance genes (e.g., *fusC*, *ant4′*, *tetK*, *spc*, *ermA*) [23,24], especially for the varied composition of genes within J1, J2, and J3 regions (separated by *mecA* and *ccrAB/C* complexes) [25], which constituted a complex exogenous mobile cassette accompanied by the loss or addition of regulatory, accessory, and other antibiotic resistance genes. In contrast with the occasional absence of these additional genes, *mecA* and *ccrAB/C* were commonly identified in methicillin-resistant staphylococci [26], and thus were established as reliable references for evolutionary exploration. However, to the best of our knowledge, recent works have predominantly focused on the evolution of *mecA* [10,27]. The high sequence conservation of *mecA* (observed in this study) among different SCC*mec* types and methicillin-resistant staphylococci limited its capacity for discriminating SCC*mec* variants and evolutionary routes within staphylococcal genus. Comparatively, *ccrAB/C* was rarely identified; thus, when it comes to the increasing emergence of novel types of SCC*mec* accompanied by existing or new types of *ccrAB/C* [26], the evolution of *ccrAB/C* is likely better able to elucidate the emergence of novel types of SCC*mec* [28] and transmission of SCC*mec*.

To date, of the 15 SCC*mec* types identified according to the organization diversity of *mecA* and *ccrAB/C* complexes [26,29], the *ccrAB/C* complex was generally categorized by *ccrA1B1* (in SCC*mec* types I and IX), *ccrA2B2* (II and IV), *ccrA3B3* (III), *ccrA4B4* (VI and VIII), *ccrA1B6* (X and XV), *ccrA1B3* (XI), *ccrC1* (V, VII and XIV) and *ccrC2* (XII and XIII), while *mecA* complexes were generally characterized by *mecA*, *mecR1*, and *mecI*. Compared with the common presence of *ccrAB/C*, the *mecA* complex usually exhibited an absence of or variation in mecR1 and *mecI* [30]. As such, our work exploited *ccrAB/C* as a reference for SCC*mec*. To establish a solid CUB for the core genome, a tandem sequence of 31 housekeeping genes of staphylococci was deemed to be representative of the core genome, instead of a single housekeeping gene. Subsequently, the CUB of *mecA* and *ccrAB/C* was compared with the CUB of the core genome, and the phylogenetic relationship of *ccrAB/C* was constructed in terms of SCC*mec* typing, time, and geographical scale.

## 2. Materials and Methods

### 2.1. Collection of mecA-Positive Staphylococci and SCCmec Screening

As shown in Table 1, 176 *mecA*-positive staphylococci were obtained via conserved domain sequence alignment of *mecA*, which was achieved using the Basic Local Alignment Search Tool (BLAST) provided by the National Center for Biological Information (NCBI). The presence of coagulase gene coa was confirmed only in *S. aureus*, with the exception of coagulase-variable *Staphylococcus pseudintermedius* [5]; therefore, 8 *S. pseudintermedius* strains were classified as CoNS. According to previous demonstrations [28,31], the SCC*mec* region was generally designated within upstream *attR* at the end of *orfX* (namely *rlmH*, encoding 23S rRNA methyltransferase [32]) to downstream *attL* at the end of SCC*mec*; therefore, the conserved sequence of *attR/L*, i.e., direct repeats (DRs) (5′-GARGCDTATCATAAVT-3′) was utilized to extract SCC*mec*. According to our extraction method, 61 strains exhibited incomplete SCC*mec* structures (e.g., missing *orfX* or DRs). The SCC*mec*-like elements of these strains were found, at the very least, to cover the *ccrAB/C* and *mecA* complex. Subsequently, the SCC*mec* sequence was submitted to SCC*mec*Finder (https://cge.food.dtu.dk (accessed on 12 April 2024)) and assigned a 90% threshold identity and 60% gene length [33]. ORFs of SCC*mec* were annotated by RAST (https://rast.nmpdr.org/ (accessed on 15 April 2024)) [34,35,36]. The SCC*mec* types and sequences of 176 strains are described in detail in the Supplementary Materials.

### 2.2. Basic Taxonomy of Staphylococci Species

The basic taxonomy of 33 staphylococci species was constructed based on 16S rRNA phylogeny (the tandem sequence of 31 housekeeping genes failed this phylogenetic analysis due to excessive sequence variation) to provide a phylogenetic background for further analysis. Briefly, 16S rRNA sequences of 33 staphylococci species were aligned by Multiple Sequence Comparison by Log-Expression (MUSCLE), and a Neighbor-Joining (NJ) tree (1000 bootstrap) was constructed using Molecular Evolutionary Genetic Analysis (MEGA) 11 software. The 16S rRNA sequences of 33 staphylococci species are listed in the Supplementary Materials.

### 2.3. CUB Assay

As mentioned above, CUB comparison of core genome and SCC*mec* was performed to determine the differences between endogenous and exogenous genes. According to our SCC*mec* typing result, 12 SCC*mec* types were observed and subsequently subjected to this CUB assay. Briefly, Relative Synonymous Codon Usage (RSCU) values of the tandem sequences of 31 housekeeping genes (*rsmE*, *glpE*, *gmk*, *tpi*, *gyrA*, *gyrB*, *rpoB*, *dnaG*, *frr*, *infA*, *infB*, *infC*, *nusA*, *pgk*, *pyrG*, *rplA*, *rplB*, *rplC*, *rplD*, *rplE*, *rplF*, *rpmA*, *rpsA*, *rpsB*, *rpsC*, *rpsE*, *smpB*, *tsf*, *recA*, *ftsZ* and *pyk*), *mecA*, and *ccrAB/C* of 12 SCC*mec* types were calculated using CodonW 1.3 software with default parameters applied. A cluster dendrogram of RSCU profiles of core genome, *mecA* and *ccrAB/C* was plotted using an online tool (www.omicshare.com (accessed on 25 April 2024)). Details of the sequences and RSCU values of 31 housekeeping genes, mecA and ccrAB/C of 12 SCCmec types, are provided in the Supplementary Materials.

### 2.4. Phylogenetic Tree Construction of ccrAB/C

According to the preliminary screening of the SCC*mec* structures of these 176 staphylococcal strains, occasional absences of accessory and regulatory genes were observed within SCC*mec* (e.g., *glpQ*, *merA*, *walK*, *IS431*, *Tn554*, *maoC*, *mecI* and *mecR1*). In contrast, the *ccrAB/C* complex functioned as essential recombinases, which were commonly present within SCC*mec*. Meanwhile, *mecA* (with less than a 3-point mutation) sustained high sequence conservation in these 176 strains; comparatively, *ccrAB/C* exhibited sequence variation even within the same SCC*mec* type. Therefore, the phylogenetic relationship of *ccrAB/C* was constructed to reflect the phylogenetic correlation of SCC*mec* among these strains. A complete *ccrAB/C* sequence was subjected to a CD search of NCBI (https://www.ncbi.nlm.nih.gov/Structure/cdd/wrpsb.cgi (accessed on 7 May 2024)), and conserved domain regions and sequences of *ccrAB/C* were extracted separately and combined in tandem. Subsequently, a phylogenetic tree of the tandem conserved sequence of *ccrAB/C* was constructed via MUSCLE alignment and an NJ (1000 bootstrap) algorithm. The collection year and locus were obtained from the source information of the corresponding strain in the NCBI. iTol (https://itol.embl.de/, accessed on 9 May 2024) and Adobe Illustrator (AI) 26.0 software were employed to visualize and optimize the phylogenetic tree. The collection years, locus, and conserved domain sequences of ccrAB/C of 176 staphylococcal strains are provided in the Supplementary Materials.

### 2.5. Comparison of SCCmec Structures

During our screening of *ccrAB/C* within SCC*mec* or SCC*mec*-like elements, 35 strains tested positive for multiple *ccrAB/C* genes. Specifically, 2 strains contained *ccrAB*, *ccrC1* and *ccrC2*, 12 strains contained *ccrAB* and *ccrC1*, and 21 strains contained *ccrC1* and *ccrC2*, simultaneously. To highlight the characteristics of multiple *ccrAB/C* within these staphylococcal strains, the *mecA* and *ccrAB/C* complexes of SCC*mec* with multiple *ccrAB/C* genes were graphed by SnapGene 8, and compared with the regular SCC*mec* structure by AI 26.0 software. The characteristics of multiple ccrAB/C genes identified within 35 staphylococcal strains are described in detail in the Supplementary Materials.

## 3. Results and Discussion

### 3.1. Basic Taxonomy of 33 Staphylococci Species

**Figure 1:**
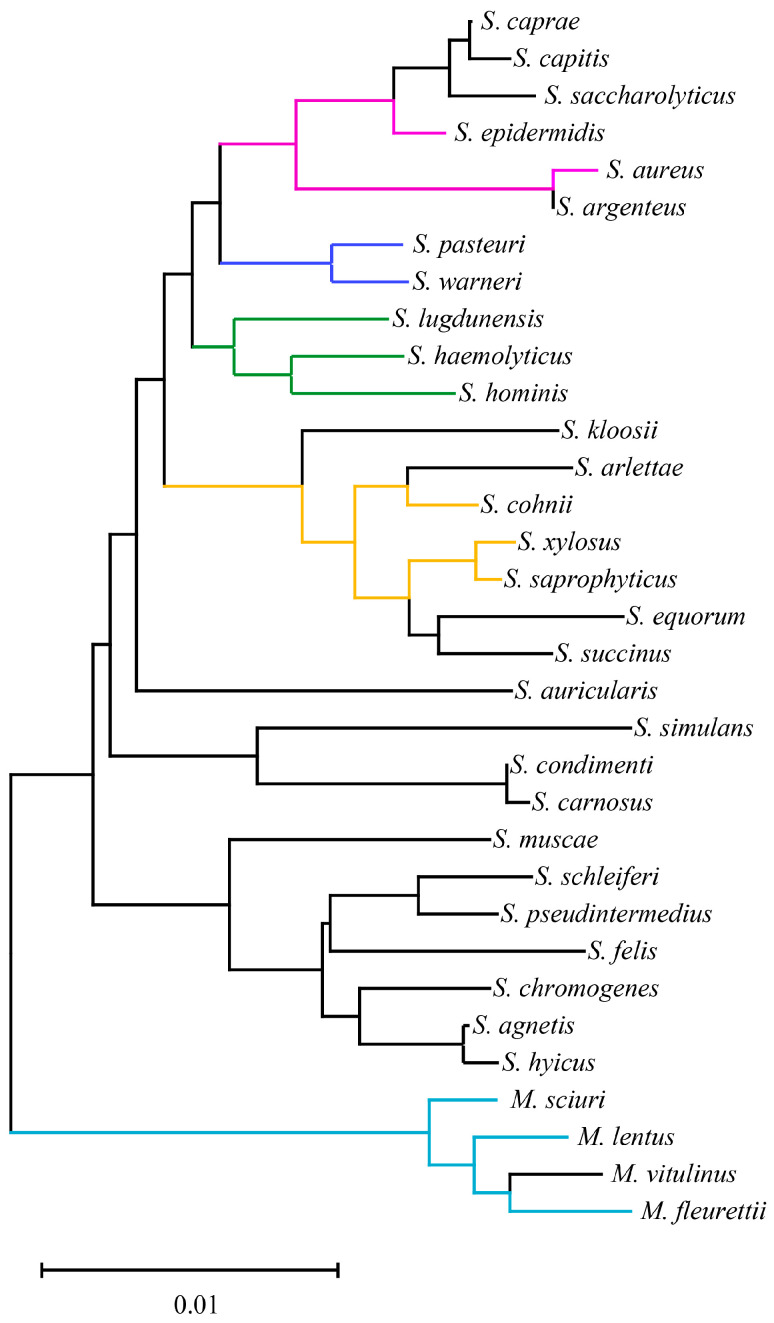
Basic taxonomy of 33 staphylococci species based on 16S rRNA. Colored clades represent the dominant emergence of methicillin-resistant staphylococci in animals.

As shown in Figure 1, the clade length remained within the range of 0–0.02 for 33 staphylococci species. Notably, according to previous research [7], the reassigned *M. sciuri*, *Mammaliicoccus lentus*, *M. vitulinus* and *M. fleurettii* exhibited greater taxonomic distances from other staphylococci species. Interestingly, the current emerging methicillin-resistant staphylococci species generally showed close taxonomic relationships as opposed to random distribution within the staphylococci genus. For instance, according to previous reports involving MR-CoNS investigations in animals, the emergence of MR-CoNS disseminated within *Staphylococcus cohnii*, *Staphylococcus epidermidis*, *Staphylococcus haemolyticus*, *M. sciuri*, *Staphylococcus warneri*, *Staphylococcus xylosus*, *M. lentus*, *Staphylococcus lugdunensis*, *Staphylococcus hominis*, *Staphylococcus saprophyticus*, *M. fleurettii*, and *Staphylococcus pasteuri* [5,37,38,39,40,41,42]. This could explain the greater prevalence of these CoNS in animal-origin environments and increase the likelihood of MR-CoNS emergence.

### 3.2. RSCU Profiles of mecA, ccrAB/C, and Core Genome

**Figure 2:**
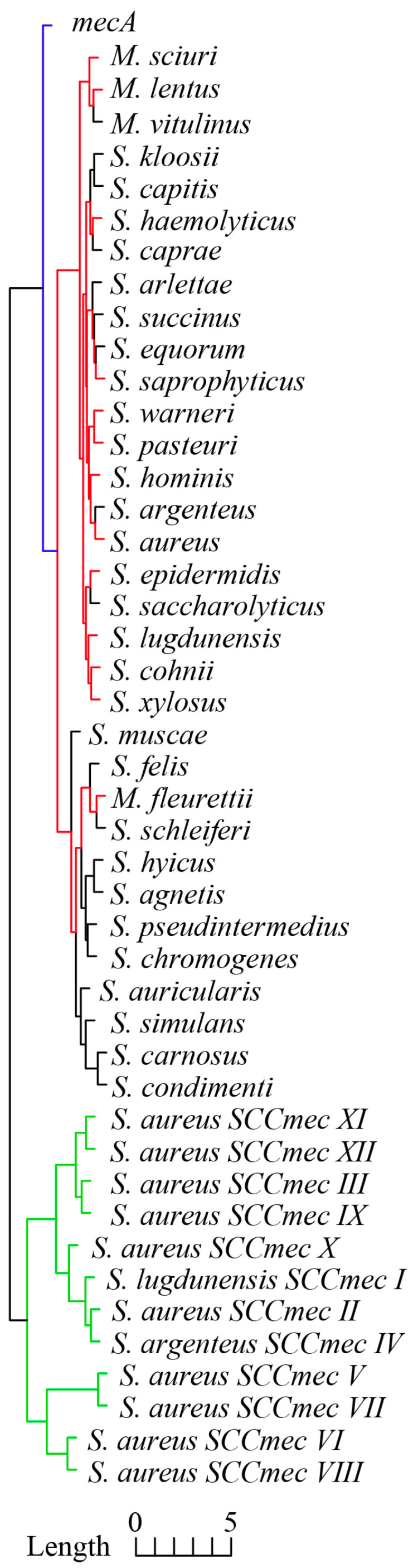
Cluster dendrogram of RSCU profiles of *mecA*, 31 tandem housekeeping genes of 33 staphylococci species, and *ccrAB/C* of 12 SCC*mec* types. Colored clades represent the similarity clustering of RSCU profiles.

As shown in Figure 2, *mecA* and *ccrAB/C* genes within 12 SCC*mec* types and the core genome of 33 staphylococci species exhibited distinctive RSCU clustering. Generally speaking, the core genome of 33 staphylococci species had the most similar RSCU profiles compared with *mecA* and *ccrAB/C*, and the dominant species of the emerged MR-CoNS mentioned above exhibited more similar RSCU profiles (red branches) compared with other CoNS. This clustering result concurred with the basic taxonomy analysis; unfortunately, to the best of our knowledge, there is currently no theoretical statement or experimental evidence to explain this phenomenon. Importantly, *mecA* exhibited a more similar RSCU profile to the staphylococcal core genome than *ccrAB/C*; in other words, *mecA* exhibited more similar CUB to the core genome than *ccrAB/C*. The other two key CUB indexes, including the Codon Adaption Index (CAI) and GC3s [21], exhibited similar tendencies. In detail, the average CAI and GC3s values of *ccrAB/C* of 12 SCC*mec* types were 0.211 (95% CI: 0.205–0.217) and 0.265 (95% CI: 0.252–0.280), respectively, and the CAI and GC3s values of highly conserved *mecA* were 0.261 and 0.191, respectively. In contrast, the average CAI and GC3s values of the core genome were 0.300 (95% CI: 0.295–0.305) and 0.192 (95% CI: 0.181–0.202), respectively (the CAI and GC3s values are described in detail in the Supplementary Materials).

The results presented above indicate that *ccrAB/C* exhibited more explicit exogenous characteristics than *mecA* and support the previous assertion that exogenous *ccrAB/C* was later introduced into *mecA*-positive staphylococci, leading to the formation of SCC*mec* [4]. More importantly, exogenous *mecA* and *ccrAB/C* exhibited deviated CUB compared to the core genome, and *ccrAB/C* provided clearer discrimination than *mecA* among 12 SCC*mec* types [26], which can be employed to explore the horizontal transmission route of SCC*mec* based on sequence identity.

To our knowledge, there are few reports on CUB of SCC*mec* in the staphylococcal genome; however, a previous analysis of horizontal transfer-acquired genes of Pseudomonas aeruginosa proposed that the CUB deviation of acquired genes exhibited relatively stable long-term retention, and the synonymous mutations led to insignificant changes in the CUB of acquired genes [22]. This explained the high sequence conservation of *mecA* in these 176 strains across different countries and decades of evolution and provided solid theoretical support for further phylogenetic analysis in which *ccrAB/C* was representative of SCC*mec*, without implementing the sequence mutation of *ccrAB/C* to adapt the CUB of different receiving species.

### 3.3. Phylogenetic Relationship of ccrAB/C

**Figure 3:**
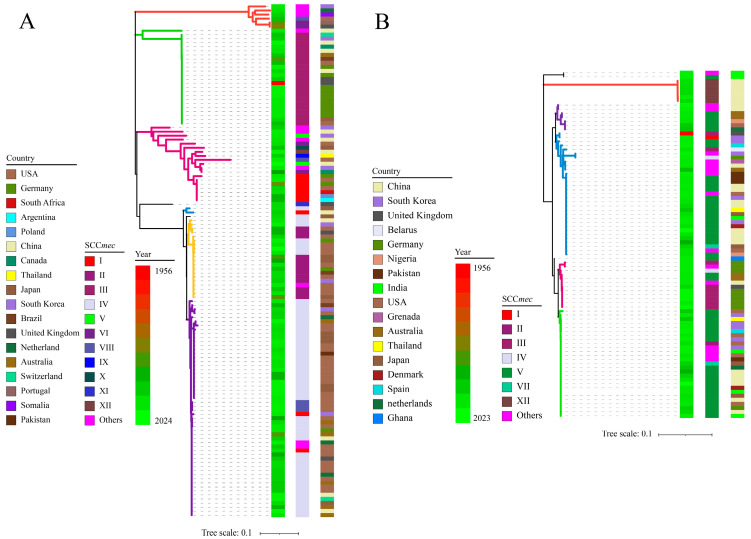
Phylogenic relationship of 213 *ccrAB/C* corresponding to yearly, geographical, and SCC*mec*-type characteristics. (**A**,**B**) represent *ccrAB* and *ccrC*, respectively. The color strips in turn represent collection years, SCC*mec* types, and geographical locations from left to right.

A total of 127 *ccrAB* and 86 *ccrC* of 176 *mecA*-positive staphylococcal strains (including 127 *ccrAB* strains and 63 *ccrC* strains (23 strains harboring 2 *ccrC*)) were sought to construct a phylogenetic tree, and were used in combination with information about SCC*mec* types, collection year, and locus. As shown in Figure 3, in general, there were no differentiable clades between *S. aureus* and CoNS; instead, the only correlation was observed between the sequence identities of *ccrAB/C*. *S. aureus*. NCTC9944 was the oldest strain (collected in 1956) that harbored *ccrAB* and *ccrC* simultaneously; however, other strains have been collected since the 1980s, and the majority of strains were obtained and sequenced in the 2000s. Clearly, *ccrAB/C* did not show a change in clade concentration as the number of collection years increased, which supported our CUB hypothesis for *ccrAB/C*, which states that exogenous genes can maintain stable sequence conservation in the long-term (this also explained our initial failed attempts to perform Bayesian analysis to explore the evolution of *ccrAB/C*).

Based on the classification of SCC*mec* types, generally, close phylogenetic relationships were observed within the same SCC*mec* type. As shown in Figure 3, regular phylogenetic distribution should be achieved for 12 SCC*mec* types, according to the classification rule for *ccrAB/C* of SCC*mec* mentioned above; nevertheless, more distant clades appeared in several SCC*mec* types, and nearby clades appeared among other SCC*mec* types. For instance, SCC*mec* IV (*ccrA2B2*) displayed two major clades, i.e., yellow and purple, and had nearby clades with II (*ccrA2B2*) and VIII (ccrA4B4), respectively. Meanwhile, SCC*mec* V (*ccrC1*) and VII (*ccrC1*) exhibited divergent distribution due to significant sequence variation in ccrC1, with the exception of XII (*ccrC2*). In addition, SCC*mec* V and XII appeared in the *ccrAB* phylogenetic tree, and SCC*mec* I, II, III, and IV appeared in the *ccrC* phylogenetic tree. The presence of a certain number of untyped SCC*mec* genes (others) limited further typing analysis for SCC*mec* in this study. These unexpected divergences could contribute to the low-resolution discrimination or ambiguous outputs for SCC*mec* typing achieved using SCC*mec*Finder, resulting from sequence variation in ccrAB*/C* within the same SCC*mec* type and the presence of multiple forms of *ccrAB/C* within SCC*mec*. According to the preliminary sequence screening for *ccrAB/C*, dramatic sequence variation occurred within the same SCC*mec* type; conversely, high sequence identity was observed among different SCC*mec* types (the conserved sequences of ccrAB/C are described in detail in the Supplementary Materials). This also explained the more distant clades within same SCC*mec* types. Based on the high sequence conservation of *mecA* [27], the phylogenetic relationship of *ccrAB/C* provided more phylogenetic information to balance the SCC*mec* types and sequence identity of *ccrAB/C*.

Regarding the collection locus of these 176 strains, the distribution of these strains on a geographical scale was mainly dependent on the number of isolates obtained from native environments. As shown in Figure 3A, the majority of the *ccrAB* strains (110/127) were obtained from the USA (46), Germany (15), China (13), Japan (13), South Korea (10), Australia (7), and the United Kingdom (6), with the USA exhibiting centralized distribution of SCC*mec* II and IV. A previous study on the genomic evolution of 386 MRSA in America showed that the two dominant linkages were cluster 7 (all composed of SCC*mec* IV) and cluster 8 (containing 146/209 SCC*mec* II and 37/209 SCC*mec* IV) [19]. Meanwhile, a review emphasized the dominant presence of hospital-associated MRSA (HA-MRSA) SCC*mec* II in the USA/Canada [25]. Additionally, 10 *S. epidermidis* strains from Germany were grouped into type III, which was mainly attributed to our centralized selection of *S. epidermidis* strains from the same environments [43,44]. Notably, 5 SCC*mec* IV strains from Australia exhibited nearby clades, which could be attributed to the dominant community-associated MRSA (CA-MRSA) SCC*mec* IV disseminated in Australia/New Zealand [25]. In contrast, China, Japan, South Korea, and the United Kingdom exhibited dispersed distribution of either SCC*mec* types or phylogenetic clades, as summarized in terms of the varied presence of SCC*mec* types in Asia and Europe [25].

As for *ccrC* distribution (Figure 3B), due to the sequence variation of ccrC1 and high sequence identity of *ccrC2*, *ccrC1* exhibited diverged clades, with the exception of *ccrC2*, which was distributed into a single clade (red). Interestingly, the 20 additional *ccrC* of 23 strains were mainly distributed in another major clade (green). Regarding the collection countries, most of the ccrC strains (44/63) were obtained from China (17), Germany (9), South Korea (6), India (6), and the USA (6). However, due to the presence of *ccrAB* and *ccrC*, eight strains from Germany exhibited unexpected corresponding relationships with SCC*mec* types. Disregarding the ambiguous SCC*mec* types observed via SCC*mec*Finder, 13 strains from China had centralized distribution and close clades in SCC*mec* V (7) and XII (6), which were mainly attributed to the high sequence identity of *ccrC* [45,46]. Comparatively, *ccrC* strains from other countries commonly exhibited sequence variation of ccrC and the presence of multiple forms of *ccr*, leading to the more distant phylogenetic clades and dispersed distribution of SCC*mec* types. The original phylogenetic trees of ccrAB and ccrC are listed in the Supplementary Materials.

**Figure 4:**
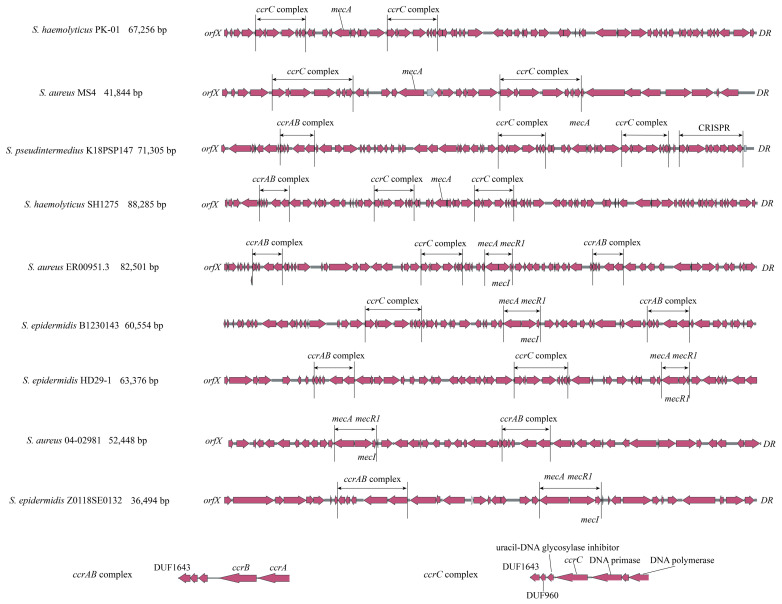
Duplicate appearance of *ccrAB/C* within complete SCC*mec* and incomplete SCC*mec* structures in staphylococci. *S. haemolyticus* PK-01, *S. aureus* MS4, *S. pseudintermedius* K18PSP147, *S. haemolyticus* SH1275, and *S. aureus* ER00951.3 have duplicate *ccrAB/C* complexes, while *S. epidermidis* B1230143 and *S. epidermidis* HD29-1 have incomplete SCCmec structures compared with regular SCC*mec* structures of *S. aureus* 04-02981 and *S. epidermidis* Z0118SE0132.

As mentioned above, there are multiple forms of *ccrAB/C* (e.g., *ccrAB*-*ccrC*, *ccrC*-*ccrC*, *ccrAB*-*ccrAB*-*ccrC*, *ccrAB*-*ccrC*-*ccrC*) close to *mecA* in 35 strains with complete or incomplete SCC*mec* structures. This could explain the low identity or ambiguous outputs observed using SCC*mec*Finder, resulting in the presence of unexpected SCC*mec* types in phylogenetic trees. As shown in Figure 4, nine representative strains were selected to demonstrate these phenomena. *S. haemolyticus* PK-01 and *S. aureus* MS4 exhibit *ccrC*-*ccrC* and a complete SCC*mec* structure; *S. pseudintermedius* K18PSP147 and *S. haemolyticus* SH1275 exhibit *ccrAB*-*ccrC*-*ccrC* and a complete SCC*mec* structure, whereas all four strains exhibit a loss of *mecR1* and *mecI* in the *mecA* complex. *S. aureus* ER00951.3 exhibits *ccrAB*-*ccrC*-*ccrAB* and complete SCC*mec* structure with a complete *mecA* complex. *S. epidermidis* B1230143 and *S. epidermidis* HD29-1 exhibit *ccrAB*-*ccrC* and an incomplete SCC*mec* structure with a complete *mecA* complex. *S. aureus* 04-02981 and *S. epidermidis* Z0118SE0132 were introduced as regular SCC*mec* types that exhibit *ccrAB* and *ccrC* with a complete *mecA* complex, respectively. The *ccrAB* and *ccrC* complexes in these nine strains have the same ORF structures. Regarding the distant geographical locus and collection years of these nine strains, it is suggested that there may have been a fixed transmission model for *ccrAB* and *ccrC* complexes compared with the *mecA* complex. The excision and reintegration of *ccrAB/C* might be more complicated than previously believed (i.e., these processes may involve targeting upstream and downstream DRs of SCC*mec*).

## 4. Conclusions

In this work, we made a novel attempt to explore the phylogenetic relationship of SCC*mec* in terms of *ccrAB/C* and demonstrated the sequence conservation and exogenous characteristics of *ccrAB/C* across SCC*mec* types, geographical limits, and decades of evolution. Currently, SCC*mec* typing is commonly based on the composition of a single *mecA* complex and a single *ccrAB/C* complex, and the SCC*mec* region is commonly located from upstream *attR* to downstream *attL*. Due to the presence of multiple forms of *ccrAB/C* near *mecA* loci and incomplete SCC*mec* structures observed in this study, the variation in ccrAB*/C* could be utilized as an additional or alternative classification marker for SCC*mec*, especially for the ambiguous definition of SCC*mec* types and omission of incomplete SCC*mec* structures. The fixed composition of the *ccrAB/C* complex in multi-*ccrAB/C* SCC*mec* suggests there might be an undiscovered transmission mechanism for *ccrAB/C*. More importantly, regarding the large proportion (60/176) of incomplete SCC*mec* structures observed in this study, we have ascertained that there might be other potential recognition sites for *ccrAB/C* except *attR/L*. However, these observations are in need of further experimental validation to identify the variation in ccrAB*/C* within same the SCC*mec* type, the same recombinase function of multiple forms of *ccrAB/C* within SCC*mec*, and the possible new recognition sites of *ccrAB/C*.

## Figures and Tables

**Table 1:** *mecA*-positive staphylococci used in this study.

Staphylococci Species	Number
*S. aureus*	116
*S. epidermidis*	32
*S. pseudintermedius*	8
*S. haemolyticus*	5
*S. lugdunensis*	4
*S. argenteus*	3
*S. hominis*	2
*M. sciuri*	2
*S. caprae*	1
*S. coagulans*	1
*S. arlettae*	1
*S. schleiferi*	1
Total	176

## Data Availability

The data presented in this study are available within the article and in Supplementary Materials.

## Supplementary Materials

The following supporting information can be downloaded at https://www.mdpi.com/article/10.3390/microorganisms13010153/s1: The 16S rRNA and 33 housekeeping genes sequences of 33 staphylococci species, RSCU values of *mecA*, *ccrAB/C* of 12 SCC*mec* types and 31 tandem housekeeping genes of 33 staphylococci species, and SCC*mec* types, conserved domain sequences of 213 *ccrAB/C*, collection year and locus of 176 *mecA*-positive staphylococcal strains are provided in Supplementary Data S1. xlsx. The original phylogenetic trees of *ccrAB* and *ccrC* are provided in Supplementary Data S2. docx. The SCC*mec* sequences of 176 staphylococcal strains are provided in Supplementary Data S3. docx. The annotation information of 35 multiple *ccrAB/C* staphylococcal strains is provided in Supplementary Data S4. docx.
