# Supplementary material for: In Silico Exploration of Staphylococcal Cassette Chromosome mec (SCCmec) Evolution Based on Phylogenetic Relationship of ccrAB/C
Source: Microorganisms. 2025 Jan 13;13(1):153. doi: 10.3390/microorganisms13010153 (PMC11767417; doi:10.3390/microorganisms13010153)
Supplement: Supplementary file 1 [file microorganisms-13-00153-s001.zip › microorganisms-3393230-supplementary/Supplementary Data S2.pdf]

The original phylogenetic tree of *ccrAB*:

(((((((((((((((((((((((((((((((((Staphylococcusepidermidisc034:0.00000000,
StaphylococcusaureusstrainFizz:0.00055234):0.00000002,Staphylococcu
saureusstrain515798:0.00000000):0.00000001,Staphylococcusepidermidi
sstraintMDU-
41:0.00000000):0.00000001,Staphylococcusepidermidisstrain41DSE01:0
.00000000):0.00000000,StaphylococcusaureusstrainFJ0318:0.00000000):
0.00000000,Staphylococcusaureussubsp.aureus3020.c01:0.00000000):0.0
0000000,Staphylococcusaureussubsp.aureus1969.N:0.00000000):0.00000
000,Staphylococcusaureussubsp.aureusJKD6159:0.00000000):0.0000000
0,StaphylococcusaureusUtSWMRSa55:0.00000000):0.00000000,Staphyl
ococcusaureusUSa300-
SUR9:0.00000000):0.00000000,StaphylococcusaureusJE2:0.00000000):0
.00000000,StaphylococcusaureusFDaaRgOS140:0.00000000):0.0000000
0,Staphylococcusaureus25bMRSa:0.00000000):0.00000000,Staphylococ
cusargenteusMSHR1132:0.00000000):0.00000000,Staphylococcusschleif
eri5909-
02:0.00000000):0.00000027,Staphylococcusaureusstrainpt174:0.0000000
0):0.00000027,StaphylococcusaureusUSA3002014.C01:0.00000000):0.0
0000052,StaphylococcusargenteusstrainRIVMM046968:0.00000000):0.0
0000098,Staphylococcusaureussubsp.aureusSa268:0.00000000):0.000001
85,StaphylococcusaureusstrainWBG8287:0.00000000):0.00000351,Staph

ylococcusepidermidisstrainHD29-
1:0.00000000):0.00007734,(StaphylococcusaureusNRS120:0.00000000,(StaphylococcusaureusNRS271:0.00055242,Staphylococcusaureusstrainc308:0.00000055):0.00000053):0.00102275):0.00025311,StaphylococcusepidermidisZ0118SE0272:0.00466628):0.00078024,(Staphylococcusaureusstrain16445:0.00000000,(StaphylococcusaureusstrainBSN14S3:0.00000000,StaphylococcusaureusstrainBSN14R1:0.00000000):0.00000000):0.00226684):0.00075065,(StaphylococcusargenteusstrainRIVMM020832:0.00325209,((Staphylococcusaureusstrainpt185:0.00551477,StaphylococcusaureusstrainWA121-
202115363:0.00059097):0.00436509,(StaphylococcusaureusstrainER01109.3:0.00320778,(StaphylococcusaureusCN05:0.00000000,(StaphylococcusaureusCN09:0.00000000,(Staphylococcusaureus59731:0.00000000,(StaphylococcusaureusstrainAR0226:0.00000000,(StaphylococcusaureusstrainER01560.3:0.00000000,(StaphylococcusaureusstrainR46:0.00000000,(StaphylococcusaureusstrainBSN9R:0.00000000,(Staphylococcusaureusstrainpt265:0.00000000,(Staphylococcusaureusstrainpt248:0.00000000,(StaphylococcusaureusLAC:0.00000000,(StaphylococcusaureusstrainVMRSA-WC121:0.00000000,(StaphylococcusaureusstrainVMRSA-
WC081:0.00000000,(StaphylococcusaureusstrainUNCSA54:0.00000000,(StaphylococcusaureusstrainTUM20929:0.00000000,(StaphylococcusaureusstrainTUM20825:0.00000000,(StaphylococcusaureusstrainBSN07S:0.

00000000,StaphylococcusaureusstrainBSN42:0.00000000):0.00000000):
0.00000000):0.00000000):0.00000000):0.00000000):0.00000000):0.0000
0000):0.00000000):0.00000000):0.00000000):0.00000000):0.00000000):
0.00000000):0.00000000):0.00000000):0.00011911):0.00010865):0.0068
1404):0.00055304):0.00169758,(Staphylococcusaureusstrain07-
059:0.00078081,(Staphylococcusaureussubsp.aureuscN1:0.00032019,Sta
phylococcusaureusstrainHL16278:0.00134482):0.00590838):0.00472685
):0.00197679,StaphylococcuspseudintermediusstrainME4692:0.0064420
5):0.00744342,((((StaphylococcusaureusMRSa252:0.00000000,Staphyloc
occusepidermidisRP62a:0.00000000):0.00368278,Staphylococcusaureuss
trainKNIH6268:0.00300962):0.00231087,Staphylococcuslugdunensisstra
inRMLUg3:0.00439346):0.00338880,(Staphylococcusaureusca-
347:0.00072100,((((StaphylococcusaureusstrainER09654.3:0.00000000,S
taphylococcusaureusstrainCUBIST-
2:0.00000000):0.00000000,StaphylococcusaureusstrainER11501.3:0.000
00000):0.00000000,StaphylococcusaureusstrainUP1525:0.00000000):0.0
0136152,(Staphylococcusaureussubsp.aureusMu3DNa:0.00000000,(Stap
hylococcusaureussubsp.aureusN315DNa:0.00000000,(Staphylococcusaur
eusstraintcH32767:0.00000000,(Staphylococcusaureus04-
02981:0.00000000,(StaphylococcusaureusstrainCHU15-
080:0.00000000,(StaphylococcusaureusstrainER00951.3:0.00000000,(Sta
phylococcusaureusNccP14562:0.00000000,(Staphylococcusaureusstrain

AR0469:0.00000000,(Staphylococcusaureussubsp.aureusUcI62:0.00000000,(StaphylococcusaureusNRS1:0.00000000,Staphylococcusaureusstrain pt279:0.00113021):0.00001297):0.00000658):0.00000334):0.00000169):0.00000086):0.00000044):0.00000022):0.00000011):0.00000006):0.00030350):0.00038799):0.01207109):0.00488516):0.00061888,StaphylococcusepidermidisstraintSM-47:0.00998972):0.00802242,(StaphylococcuslugdunensisstraincgMH-SL138:0.01806513,StaphylococcusepidermidisstraintMDU-323:0.00842236):0.00792144):0.11312001,StaphylococcusaureusZta09/03698-9St:0.08508231):0.00766995,(StaphylococcushaemolyticusS167:0.04727869,(StaphylococcusarlettaestrainSA283:0.07613933,(StaphylococcuspseudintermediusstrainK18PSP147:0.07275032,(StaphylococcusaureusstrainNV1:0.04369061,(StaphylococcusaureusstrainER06690.3:0.03734183,(StaphylococcusaureusJcSc6945:0.05112657,(((((StaphylococcushominisstrainC34847:0.00000000,StaphylococcusepidermidisstrainZ0118SE0260:0.00000000):0.00322014,StaphylococcushaemolyticusstrainSH1275:0.00794370):0.01291359,StaphylococcushominisstrainFDAARGOS661:0.09205408):0.00828981,(StaphylococcusaureusstrainJcSc6690:0.02538966,StaphylococcusaureusstraingD4Sa159-1:0.01785888):0.00895981):0.00991685,(StaphylococcusaureusstrainCC1153:0.02080480,(Staphylococcusaureussubsp.aureusCOL:0.00000000,(

Staphylococcusaureusstrain628:0.00000000,(Staphylococcusaureussubsp.
aureusFDaaRgOS5:0.00000000,(StaphylococcusaureusstrainMIN-
175:0.00000000,(StaphylococcusaureusisolateHU-
14:0.00000000,StaphylococcusaureusstrainWCUH29:0.00000000):0.000
00000):0.00000000):0.00000000):0.00000000):0.00816564):0.01392114)
:0.01032940):0.00934944):0.03795038):0.02048505):0.01259903):0.004
79697):0.03799900):0.00398129,(StaphylococcussciuristraingDK8D6P:0
.10134292,(StaphylococcusaureusM92:0.00000000,(Staphylococcusaure
usV521:0.00000000,(StaphylococcusaureusstrainZed:0.00000000,(Staph
ylococcusaureusstrainCMRSA-
3:0.00000000,(StaphylococcusaureusstrainMRSA107:0.00000000,(Staph
ylococcusaureussubsp.aureusstr.JKD6008:0.00000000,(Staphylococcusau
reussubsp.aureusgv69:0.00000000,(StaphylococcusepidermidisDaR1907:
0.00000000,(StaphylococcusepidermidisV1950266:0.00000000,(Staphyl
ococcussciuristrain82104:0.00000000,(Staphylococcusepidermidisstrain
HD12-
2:0.00000000,(StaphylococcusepidermidisstrainNCTC13924:0.00000000
,(StaphylococcusaureusstrainNCTC9944:0.00000000,(Staphylococcusepi
dermidisstrainB1230143:0.00000000,(StaphylococcusepidermidisstrainB
1265603:0.00000000,(StaphylococcusepidermidisstrainB1276296:0.0000
0000,(StaphylococcusepidermidisstrainHD31-
1:0.00000000,(StaphylococcusepidermidisstrainHD47-

1:0.00000000,(StaphylococcusepidermidisstrainHD21-
2:0.00000000,(StaphylococcusepidermidisstrainHD12-
4:0.00000000,(StaphylococcusepidermidisstrainHD12-
9:0.00000000,(Staphylococcuspseudintermedius081661:0.00008302,Stap
hylococcusepidermidisstraintSM-
51:0.00000000):0.00004191):0.00002116):0.00001068):0.00000539):0.0
0000272):0.00000138):0.00000069):0.00000035):0.00000018):0.000000
09):0.00000005):0.00000002):0.00000001):0.00000001):0.00000000):0.
00000000):0.00055790):0.00000296):0.00000150):0.00000076):0.00000
038):0.10132554):0.01894581,(StaphylococcusepidermidisZ0118SE0132
:0.04301481,((StaphylococcusepidermidisstrainIVB6208:0.03468645,Sta
phylococcushaemolyticus83131a:0.03904203):0.00630619,(Staphylococc
usaureusstrainBK20781:0.03371240,(StaphylococcusaureusstrainNCTC1
3140:0.00000000,StaphylococcusaureusstrainFDAARGOS1:0.00000000)
:0.03547698):0.00863849):0.01043330):0.29441804);

The original phylogenetic tree of *ccrC*:

(((((((((((((((((((((((((((Staphylococcushaemolyticusstrain4:0.00000000,St
aphylococcusaureusstrainVGC2:0.00066450):0.00000000,Staphylococcu
saureusstrain12-
03119:0.00000000):0.00000000,StaphylococcusaureusstrainER11501.3:0
.00000000):0.00000000,StaphylococcusaureusstrainZY05C2:0.00000000

):0.00000000,StaphylococcuscapraestrainJMUB145C2:0.00000000):0.00
000000,Staphylococcuscoagulansstrain1031373C2:0.00000000):0.00000
000,Staphylococcusaureusstrain110900C2:0.00000000):0.00000000,Stap
hylococcushaemolyticusstrainSH1275C2:0.00000000):0.00000000,Staph
ylococcusaureusHZW450C2:0.00000000):0.00000000,Staphylococcusau
reusMS4C2:0.00000000):0.00000000,Staphylococcusaureussubsp.aureus
M013C2:0.00000000):0.00000000,Staphylococcusaureussubsp.aureusSt3
98C2:0.00000000):0.00000000,StaphylococcusaureusPM1C2:0.0000000
0):0.00000000,StaphylococcushaemolyticusstrainPK-
01C2:0.00000000):0.00000001,StaphylococcusepidermidisstrainFDAAR
GOS529C2:0.00000000):0.00000002,Staphylococcusepidermidisstrain7
C2:0.00000000):0.00000003,StaphylococcusaureusstrainHL24830C2:0.0
0000000):0.00000006,StaphylococcusaureusstrainER00951.3:0.0000000
0):0.00000011,StaphylococcusaureusBDH17:0.00000000):0.00000021,St
aphylococcusaureusstrainER09654.3:0.00000000):0.00022529,(Staphylo
coccuspseudintermediusstrainAI14C2:0.00000000,(Staphylococcuspseud
intermediusstrainZ0118SP0108C2:0.00000000,(Staphylococcuspseudinte
rmediusstrainHSP235C2:0.00000000,Staphylococcuspseudintermediusstr
ainK18PSP147C2:0.00000000):0.00000000):0.00000000):0.00043889):0
.00031324,StaphylococcusaureusisolateHL20709C2:0.00014675):0.0015
7657,Staphylococcusaureussubsp.aureusISU926C2:0.00583800):0.00267
503,((StaphylococcusepidermidisstrainHD21N4:0.00000000,Staphylococ

cusepidermidisstrainHD47-
1:0.00066954):0.00934512,(StaphylococcusepidermidisstrainHD29-
1:0.00051289,(StaphylococcusepidermidisstrainC101C2:0.00000000,(Sta
phylococcusepidermidisstrainC99C2:0.00000000,(Staphylococcusaureuss
trainJL28:0.00000000,(StaphylococcusepidermidisstrainNCTC13924:0.0
0000000,(StaphylococcusepidermidisstrainB1200343:0.00000000,(Staph
ylococcusepidermidisstrainB1230143:0.00000000,(Staphylococcusepider
midisstrainB1265603:0.00000000,(StaphylococcusepidermidisstrainB127
6296:0.00000000,StaphylococcusepidermidisstrainHD21-
2:0.00000000):0.00000000):0.00000000):0.00000000):0.00000000):0.00
067079):0.00000322):0.00000167):0.00215238):0.00148113):0.0030394
9):0.00741609,StaphylococcusaureusstrainGHA8:0.00000000):0.003334
38,(((((Staphylococcusaureussubsp.aureusSt398C1:0.00000000,Staphylo
coccusaureussubsp.aureusISU926C1:0.00000000):0.00000000,Staphyloc
occusaureusstrainNga71:0.00000000):0.00788396,(Staphylococcusepider
midisstrainC101C1:0.00000000,StaphylococcusepidermidisstrainC99C1:
0.00000000):0.00217440):0.00643101,(Staphylococcuslugdunensisstrain
RMLUg1:0.00000000,StaphylococcuslugdunensisstrainRMLUg6:0.0000
0000):0.00134042):0.00339560,((StaphylococcusepidermidisZ0118SE02
72:0.00040844,StaphylococcusaureusstrainNCTC9944:0.00025448):0.00
560505,(StaphylococcuspseudintermediusstrainK18PSP147C1:0.001953
67,(StaphylococcuspseudintermediusstrainHSP235C1:0.00291895,(((((St

aphylococcuspseudintermediusstrainMAD627:0.00000000,Staphylococc
usaureusstrain0213-M-
4A:0.00001240):0.00000628,StaphylococcuspseudintermediusstrainMA
D568:0.00000000):0.00348913,(StaphylococcusepidermidisstrainHD04-
1:0.00000000,StaphylococcusaureusstrainHL24830C1:0.00000000):0.02
263079):0.00024902,StaphylococcusaureusstrainBLR-
DV:0.00584112):0.00063956,(StaphylococcushaemolyticusstrainPK-
01C1:0.00062822,(StaphylococcusaureusstrainR50:0.00000000,(Staphyl
ococcusaureusstrainLr3:0.00000000,(StaphylococcusaureusstrainWH39:0
.00000000,(StaphylococcusaureusstrainS36:0.00000000,(Staphylococcus
aureusstrainAR0473:0.00000000,(StaphylococcusaureusisolateHL20709
C1:0.00000000,(StaphylococcusaureusstrainZY05C1:0.00000000,(Staph
ylococcusaureusstrainVGC1:0.00000000,(Staphylococcuspseudintermedi
usstrainAI14C1:0.00000000,(StaphylococcuscapraestrainJMUB145C1:0.
00000000,(Staphylococcuscoagulansstrain1031373C1:0.00000000,(Staph
ylococcuspseudintermediusstrainZ0118SP0108C1:0.00000000,(Staphylo
coccusaureusstrain110900C1:0.00000000,(Staphylococcushaemolyticusst
rainSH1275C1:0.00000000,(StaphylococcusaureusHZW450C1:0.000000
00,(StaphylococcusaureusMS4C1:0.00000000,(Staphylococcusaureussub
sp.aureusM013C1:0.00000000,(StaphylococcusaureusPM1C1:0.0000000
0,(StaphylococcusaureusstrainNga102:0.00069946,Staphylococcusepider
midisstrainFDAARGOS529C1:0.00000000):0.00001853):0.00000940):0.

00000477):0.00000242):0.00000123):0.00000062):0.00000032):0.00000
016):0.00000008):0.00000004):0.00000002):0.00000001):0.00000001):0
.00000000):0.00000000):0.00000000):0.00000000):0.00000000):0.00003
470):0.00611216):0.00406750):0.00482736):0.00312004):0.00172177):0.
00576162):0.01672955,(Staphylococcusepidermidisstrain9:0.00000000,S
taphylococcusepidermidisstrain7C1:0.00000000):0.03775987,(Staphyloc
occusaureusstrainN28cSa05:0.00000000,(StaphylococcusaureusstrainN2
9cSa11:0.00000000,(StaphylococcusaureusstrainQD-
CD9:0.00000000,(StaphylococcusaureusstrainBA01611:0.00000000,(Sta
phylococcusaureusstrainS57:0.00110335,StaphylococcusaureusstrainWH
C09:0.00000000):0.00022496):0.00011385):0.00005763):0.00002918):0.
25167275);
