# Supplementary material for: In Silico Exploration of Staphylococcal Cassette Chromosome mec (SCCmec) Evolution Based on Phylogenetic Relationship of ccrAB/C
Source: Microorganisms. 2025 Jan 13;13(1):153. doi: 10.3390/microorganisms13010153 (PMC11767417; doi:10.3390/microorganisms13010153)
Supplement: Supplementary file 1 [file microorganisms-13-00153-s001.zip › microorganisms-3393230-supplementary/Supplementary Data S3.pdf]

>Mammaliicoccus sciuri strain 82104
GAAGCTTATCATAAATGATGCGGTTTTACCTACGTTTAGCTTCTATATGAGATATTAATAAAACTGTAATTTATTA
AGAAAGAAGGTTACAATAGTTTACAAAGGTGAATTAACATTAGAAAATTCAGAATTAGTGGCATTATGATAA
GAAATTTTTAATGAAAACAGTAAATATGATATCTTAAAATATCGATGTACATGTACATTTTAAAAAATTATTAGA
AATCATGAGGATACAAACCTAGATATGGAGGACTTATAGTTGTATGAATTGGCAAGTAAAGAAATAGTGATTT
ATTTTTGGGTGATAAAAGAGTAAATAGTGAGTCTGATGATAAATTTGAACCAAACGGATATACAATGCCTTAC
ATGACTCATAGTGATATAAACGCACTAGCACCTATATTTGGGATTACAAATATTGAAAATGTTAGTGATTACAAT
AGATTAAATAAAATGGCATGGTTGATAGAACAGTGTTATAAATTAAATAAAGAGAATAAGTTATTAAAGTATTT
TTTAGATGTAACCCGATATATACCAAATAAGCGAGAAGAGACTTCTATATATACAAATGAAGAACTAAAAAAA
GAAACCCTTAAAGAGGTTGAAGCTTATTTTAATCATATAAATAATATATTGAACTTTCAGATAAAAAATTAATAT
ACTTAAATAAGAAATATAAAATTATAGATTTAATAGAGGATTCACAAGTCACACATGATATTGATAAACATATTA
ATTTTGATTATGTAAGGAACTTCCCATTCAGATTAAAGGATAATTTAGAAGATGAAAATTACGACACAGTTATT
ACGCAATCTAGAACTTTATTAGAAGAAGTATATATACATATTTTAGAATACTCAAATAAAGACTATAGTTCGGCA
AAAGGAAATTTACAAAAATTGAATTCTATGGTTAAAACAGAATTAAAAATGAAAAATAATAGAGATTATGATA
AGCGGATAAATGAATTATTATCTGGTCTAAATAAAATTAATGATGCTATAGGGAAAATGAGAAATGAGAATAG
TGATAGTCATGGTGTAGTTCTAAGAGAATAAATATCAATAGACGTGAGGCAAAATTAGTGATGAATTCAGCCA
TAATAATTTGTGATTATATTTTAGAAATATTGAAGACAACAAAATAATAACAAAATATAAAATGAAAATAAGCTA
AGAGTGGATTGGCATAATCAAGGAGAATATTCTTAATAATTATAGCTCTTAGCAATTGAATAGAGGGATACAA
TATGGCAATTAAAGGAATTGGCTTAAAAGATAGTATGAATAAGTTTGTTCAAACTATCATGCCTTTAGATGAA
GAAAAAGAAATATTTAATCAAAGGCTTGTAGATTATTTAACACATCTTCGAGACAATAAAAATGAGAGTGAA
GAATATCAGAAAAATATTTTGAAAAATCTTTTAGAAAAGACTTTGCCTAATAATTTTATCAATACTTCAAATAG
AATTGATCTTGCTATTTATAATGGTAAAAGTGCTAATTCTTCATTAGGAGTTCTTTTTGAATGTAAAAGTTTAAC
AAATAAATCTGAAATGATGAGTAAGGAGAAACTTAATACTAAGGCTTTTCAGGAAATTATCGCTTATTATCTCA
ATGAGCGATTAATTTACAAAAATTTAGAAATTAAAAAGTAATTATTACTAACGGATTATCATGGTTTATTATTGA
AGCTAAAGAAATAGAAAAATATTTTTTCAAAAATAAGAATCTTATTGATTTAGTTACTAAATTTTATAATAATCA
ACTATCAAGTAATAAACAGATTTTTGTATTCAGAAGTTATAGCACCTGAAATTGATAAGTCTATTGAAAAAGGT
ATTACAATTGCTCATTTTGACTTGAGACATGCTCTAAAACAAGGTGACAAAATTGAGATTAAAAAAATAATCT
AACTCAATTATATCGTTTTTTTACTGCAGAAAATCTTTTGAATAAAGAAATTTTTACAGATTCTAACAAATTGA
ATAAAGCATTTTATGATGAATTACTTTATCTTATGGACTAGAAGAAATACAACAAGGAACGTCTAAGATTATCT
CTCGATTAAAGCCTGATAAACGACAATATTTTTCTTTTGTTGAAAATATTATTAACAAACTAGAATTAAAAGAT
GTTTCTAAAGAAAAACAAGAAGAGATTGCTATCCAATTAACTGTTATTTGGATGAATAGAATATTATTTTTAAA
ATTGTTAGAATCACAGCTAGTTCTTTTCAATAAGGATGATTCTTATCGTTTTTTAACTTTTGAAAAACTAAATA
GTTTTGAAGAGATTTATAATCTCTTTTTGCAGTTTTAGCTAAGAAAATTCCTGATAGAAATAATCGAATTCAAG
AAAAATATAATTATATTCCTTATTTAAATTCATCTCTTTTTGAAGAAACAGAATTGGAAATGTCAAGAGATGGA
TTGGTATTGATCGTCTGCCAGAAGGAGATATTTATATTTTTGACAAGACAATATTAAAGGGTATGGATAAGAA
ACGAAAAAAGGGAAAAATTAATTTTTTAGAATATTTATTTGAATTTCTCGACTCCTATGACTTTTCTACCACTAT
TTCTCATCAGCAAAAGTCAAAAATGCTTTGATTAATGCTTCAGTTCTTGGATTGATTTTTGAAAAAATAAATG
GTTATAAAGATGGTTCATTTTACACTCCTGGTCATATTACAATGTATATGTCGAAGAAAGCAATAAGAACAACT
ATAGTAGAAAAAATAAACGAGCACCTTGGATGGTCTTGTAATTCAATTGAGGATATAAAATTTCAAATTAGAA
ATATTGAGGTAGCAAAAAAAGTTAGCAAGGCAATAGATAATTTGAAAATTTGTGACCCAGCTGTTGGTTCGG
GCCATTTTTTAGTTTCTATTCTTAATGAGATTATTGCTCTAAAAAGCGAACTGAATGTTCTTTTTGATAATGATG
GAAATTATATTGGAAATTTGATTCAATGTTATGTTATAAATGATGAATTGATTATTCAAGACATGCTTGGTAATA
ATTTTATTTATCAGGCAGGTAACAAATTATCCGAACAGATTCAAAAAGCTATTTTTGATATGAAACGTCATATAT

TAGAAAATAGTCTATTTGGAGTTGACATTAACCCAAGTTCTGTTAATATCTGTCGTTTAAGATTGTGGATTGAA
CTTCTGAAGTCATCATATTATTATGAGGATAAAGTAATTCAAAAACAAGTGTTGACAACATTACCTAATATAGAT
ATTAATATCAAAGTGGGTGATAGTCTACTTTATAAATTTGATATAAACTATGAGTTTGATATGCGACGTAAGGAT
CTTAAAGAATATTTAGCTTTGGTAAAAAAATATAAGTCTACAAATAATAAGAGGATAAAAACTGATATATGGGA
AAAAATTGAAAAAATTAAAAGTTCTTTTGATGATATTGTTTCAAGTCCTGAACTCAAACAATTAAAATTACTTG
AAAAAGAATTGAAAAAAGCTAGCCAGATTAGCCTTTTTGAGGATGACACTATTGAAAGAATAAGATATGAAC
AAGTCCAAAAACAGGTGAAAGAGGCGAAAAAAAAACTTGGAGTTCGCTTGAAGAATCCAATGTATAGTGG
GGGATGGAATGGCGAATGGAATTTCCAGAAATACTTGGAGATAATGGAGAATTCATAGGATTTGATCTTTTA
ATTGGGAATCCACCATATATTTTCTCAAAAAATCAATCGTTTACCGAAGAAATGAAAACTTATTATATGAAAAC
TTATCCACTGAATCATTATCAAGCAAATACATTCGGTTTGTTTTTGGAATTAGCATTTTCATTAGTCAAAAAAG
GTGGCGGAATTTCATTTATTATTCCAAATACATTTTTAATGATTAATCAATATAAAACATTAAGACATTATATATT
AAATAATTTTAATGAATTGACATTTATTAATTCTAAAGAACGGATCTTTGATGAAGCTTCAGTAGATGTTTGTAT
AATTGATATGTATACTCGTGGAACAAAGAAAATAGGTTTAGGTGAAATTGAATCAGGGGAAGTTACTATATTA
ACAGAAACTGATGCAGATACTCTCCTCAAAAATGATGTTATAGTAGTTTCAGATAACAAAAACATTAATATTTT
ACCTCAAATAGAAAAAAAATCAAAAGTATTGGAAGGAAATTATGCTAATGTTAAAGACGGATTAAAAGTATT
TGAAAGAGGAAAAGGCACACCAAAACAGCCTGAAGATAAGAATGAATTTGATTTATTTAAAGAAAATAAAC
CGTTCTTTGATATAGAAAAAAAAGATGACTCTTATCGCATGTTTCTTTCTGGTAGAGATTTACAAAGATACAA
GATAAATTGGAGTGGCCAATATTTAAAATATGGAAAACATTTAGCTGCGCCAAGGAATCCTGAAATTTTTGAA
GGAGAAAGAATATTAATAGCCAGAATACCAGTGAAATCTAGCTACTCATTCAGAGCGACACTTGTTTCAAGTA
ATTATGTTCATGAACAAAGTATTGAGAGTATTTGTAACATAAAATCTAATCCACTATTTTTAATTGGGGTTCTTA
ATTCTAAAATATTATCTTACTATACTATCAAAAATTTAATTTCTTACAGAGAAATACATTCCCTCAAATGAGATTG
ACTCAAATAAAAGAATTTCCAATTCCAGACGCTACTGATGAACAAGAATTAGAACTTGCTAAAAAAGTAGAT
ACACTTATGGAAGAGATTAGGAAAGAGAATGGTAATCAAGAGTTAATCGACACTCTTAATTCACAAGTTGAT
GAATTTGTTATGGATTTGTTTTGTCTAGCAGAAAAAGAAAAAAAGATCATTAGAGAATTTGAGATTTAATTTA
ACTGATATAATTTGTATAGATCAAAATATTTTAAAAATTTCAATATCATAAAGTTAGTCAGTCATACTTTTAAATT
AAAGTAGCTCAATCTACATAGATTAGATTGAGCTGTTTTTTATATAATCTCATTATAAAATACATATTTAACCTTA
ATTACACGCTGAGGCTGTTCCAATATTTTTTGAAATTTAAACACATCGTAATAGGCACCTAATTCATTATTAATC
ATTTTGAATACACGACGATCCATATCTTTCATATAAGGATATTTCTCTTTTAAATCCAAACGTTCTCTAAATGCTT
CAATACTAAATGTGTATGTTTCTTCATCTATATATTGTAAAAACAAACGATATAAATATTTGGAATAAATCGACTT
TAAGTGCGTCAATCTTGCTATTGGGAAAGCAACAGAATGTTTATCAAAATGATTGAGAATATACGAGAAACG
CGGTGTTAGACCTATCGATAACTTTTTACCATCAAAATCTATCTTTGTCTTTTTTACATCAAATAAATCCATTTTT
TGACCCGCAAACTGACTATACTCACTAAAAGGCAATGCCATTACTTTTTCAATCACATTCATTAAATCAGGCTC
GAATCGTTTTTTACTACGACTAGTATAATGTGCAATTTTCTTGATTTCATCAATGTCTAAAATGATACGTTCAGA
ACCACGGTCTTTTAATAAACTACATATCGCAAAAAATAAATTTAACTCTACTGCTTTAAAACCTTTAAATACGAT
GTCATTTAAATCTGTATGATATTGTACAATTGTTTGTTCATGATGTTTTGTCATCTTAATCACCTTCCTTACATCTC
ATTATAAAATGTGAGTACGTCAATTTTTGACGCCCTCAAACATTTGATAAATCATGAAGAAAATGATGAGGTA
ACCCCCAAAAAAATAATATATAAATCCTCCCAACAAAACTAATTGTTGGGGATATTTTCATGTTGGTAAGCCTT
AGGCTTGTCTTATGTTATACTTCGGGCTGTCTATTTGATTGTCCATGCTTTCCTCGCCATAGGATTTAATGGATG
CATGATTTCATTGGTTTGTGGATTTGTTAATGTACTAATCTTTTTTTGATGCGGTTTCAACATCTCTAACACCTC
ATTGACACGTACTTCTACCATTGCTTTCTTACCATAAGCACCCCAAGCTAATAAAACAGTATCAGCTTCTTTGA
CAGCTTTCATAATATGGATATCCGTATGTTTATCATGACTATTTTCGATATGTTTTAAATTGATAGGTGTATCGAT
ATTCGAATAGAGATTTACAAAATTTATACTTCCATATGCTTCTTTCTCAATAACCTTATTCATAATAAGTTGTGTT
GTTAGGTCTATATTAAATACACCATCATAATGTGGATACATGGTAATAATCGTCAGTGTTTGTTTATCGCGATCC
CAGATTTTCTTTAGTAAGTAACGATGTTGTTGATCATCACTAAATATCGCTTCTGTTTCTAATGTGCTTTGTATAT

TTTTCATTATCTTTTACCTCTCTTCTATTAATATTCTTCTGGCAACAACATGACGTAATAAGAACAGTCTATATCA
TCTTGTCGGATAATATAGACCTTATTAATATTGATAGGTTGTTTATAATGTGCTTGATGAATTTTTACGTACTCTG
GACGTTCTTGCTTATGTTTGATATATAATTGACTATTATGCATATTGAACTGGAATATATGAAAGTAATCAATCTC
TTCTAGTTCCTTGAATTGTTCGTTTTCGCGTTGCGCTACAAGTTTCCATAACTGTTGCTGTAACATTGCTGGCA
AGTAATTGGCAATACTACGTGTAATATAACGTTCCATGCTCATTTCTCCTTTTCTATATCTTTCATTATTGTAATAA
CTTCTGTAATGACTGTGACGCTTATCTGAGCTATGTGTATCCATTTATTCATTATTATAATTCTCCTTTTCAAGTA
AGTGTCTTTGATCTAGTAGTGGCATAACTGTGTTATGAAGATATAAGAAGTCTATATCGTTTGTTTCATCAATAT
AACCAATCGCAATAAGTTGTTTTTGATAGTAGAGGTAAAGGGGTATATATGCCTTTCATGAAGTGTATCAGAG
TAGTAAGTATACGTATGAAGGATTTTGTTGGTAAGTGGACCAAGTCTGACATATTGATCAGGAAGGATATTAT
CTGCCACTTCCTCGATAGATTCACATTCCCACTTTTCATCTTTTGGCAGTTCGAATAAGTTGGTTATATAGTGCT
TGAGTTGTTGTTCTAGTGTCATCATTATGACCTCCTAATGTGTTTGATGGATGTATGCATGTGATACATCATTTT
GATAATATAAGTTTGAAATACCATTTGTTTCGCCTAGAAATCCCACTGAAATATAGAAAATATCCCAATCGAAA
GTCATGACAATTGTTGGTATGACAAGGAAATCCCGTTATCCCGCTCATTTATCGGGAAATATAGCTATATATTAT
TTGGATTTGTAGATGTCAGTACGACTTACTTTTTCTTTTTATATTATTTATTTATAAATAATAATGGGATTTCGGG
ATTACGCTTGCGTAATCCTTTTCTTATATAGATTTATCAAATCCCATTATCATCCCGTTCATTTTTTATTTTGGGAT
GTTCTTTCGTCATACAATATAAGCATGCGTGTTTGTTAGGCTAGCTTCATCTATGGATAGAATATATGAGATGTG
TGTTATTGGATGACGAAAGGACAGTATCCATTATGAGATAGGCAAATAGAGTGTTTGAGATAATAATAAAGAC
GAAGTGCTGAGTTGCACTTCGTCGAGAGGGGTATTATGGAAGATTTGTTTAATGATTTCATGATTGAGTTTTA
ATGTGACGTAGAACTGTTTTTTATGATGTTCGTCTTTACGAATATCAATGTGGTCTATGACCGTAAGGTATAGT
GCTTTGAGTTGTGCTTTATCCATGGTTTCGATATTTTTGAATATATGACGTAATATAGTCGCAATCTCATTAGCG
TCATATATTGTTTTGGGAACACTTTGTTGTTGCTTAAGTTGATTGATTTGATTTGTGATGTCATTGAGTTGTTTT
TTATACTGATGAATCGTATTTTTAAGCACAGATGTTAAGTCTGGATTATCTTCAAGTGTTTTGATCAAGTTATCT
AATTTGATTTGGACCTCATCATATTGTTGTTGCTTATATGCGATATCATGATTGAGTGCAGACGTATCAATTTGA
TGTTCTTGATTGACACGCTCAACGACTTGTTTGATAACTTTGTCACTTTTAACGATTTCGAGTATTTGATCCAT
AACATATTTTTCAATTACATCGGCTCTGACACTATTCGCAGAACATACTTTTGACCCTTTATTACGAAAGTTACT
ACACGAATAGTAGCGAATGCGTTTTTTAGTACCATCTTTAAGTGTATTCGTTGTATTCGATGCTGCGTATGCCG
CTCCACATCTTTCACACGAAATTAATCCAGTCAAAAGGTTGGTTCCTTTACCATGTACTTGTGGTTTTTCACTT
ACTTGTTTTTTACGTGCTTGTACTTTATCCCATAATGTTTGGCTAATAATAGGGGTGTGTTTGCCTTCGGCAATT
ACGGGTTTATCATTTAACCCTTTACGTCGTTTATCATTCCAATCTTTGTATTTCGCGAATTGTATTTTACCGACAT
AGAAGGGATTAGAGAGAATATAAGTAACAGCTGAAATACTAAATGGATTCCCTTTCTTTGTGACATAACCTTT
ATGGTTGAGCGCATTGGCAATTTTACGATAGCCATGCCCTTTGGCATATGATTCGAAGATATACTTTACAACAT
TGGCTTCATGTTGATTAATCATAAGTTCGTGTTTACTGTCAGGTACTTTATCATACGCATAGGGAATATTACCCT
GATAATACCCTTCTTTCGCTCTAATTGTCTGGCCCATAAATATATTGTCTACAATATTATTGCGTTCAAATTCTGC
GAAGCTCGCAAGCACCTGGAGCATGAGCTTACCACTACTCGTGCTAACTTCCATACGTTCAGACAGACTGAA
AAATTCGACATTTTGTTTATGCAAATCTTCAACGATTTTGAGTAGGTCAGATGTATTACGTGCTAATCGATTTG
TTTTGTAGACCATAACACAATCGATATGACCATTTTTAGCGTCTTGTAACATACGTTGTAGTGCAGGGCGGTTC
ATAGATTTTCCGGAAATACCACGATCTGCGTATACATCTACCACTTTGAAGTTATTAAATTGACAGTATTCTTCA
ATCTGATTGATCTGGCCTGTTATCGAGTAGCCTTCTGAGCTCTGCATCTCAGTCGATACGCGTACATAAATTCC
CACACGCTTTTGTTTAAGTTTTTCCATGATGTTTCATCCTTTCTTATGATACAATCGATGATTGTTTAGTTTGATT
AACGATATTCAGTGGTTCATTTTTGAAATAAATACCTGTAAGTGTCTTGTTTTCAGTAATATGAATGACATCAAT
ATAAGGATACAAGATGTTTAACGTGAAACATTTTTGTATTACATTATGAAATGCGCTTTGGATGCGCTTAGAAT
TGATAGATGTCAGTGTATTTTGTTGGTGTAAGTTTTGTATTTCTGTTTTGAATATTTCTGCATCTATCGTTCCTTT
GGCTAACTTATTGATGAGTTGTGCTTGGGTGTACGTGCGCTTAGTTTCCAGTGTGTTTTGTTTTTGGATGTGT
TGTTGAATGGCATCATTTATTTTGGAATAGACCTGTTGGTCTTGAAAGAAGTGTTGACATGTTTTTAAAACAC

TTGTTTCTATATCTTTAGCGTTAATCCCTTTAAAGTCACAGACAAAACGAGATTCATTCATATTTCGAGGACAG
ACATAATAACGTAATGTATTATATTGCTTTCTTCTAATTGTCATATTTGTAAGTGTGGCGCCACAGCATGGACAT
TTGATTTTTTGTTTCAGTTGATTAGCTGAAGGTGTTCGTTTGCGATGTTTTTCAGAACGTAAAATCTGCGCTT
GTTCATAGCTACTGATAGAAATAATAGGTGGAAACATGTTGTCATATTGTCCATATTGATTGATAACGCGACCA
CAGTAATTAGGATTCATGATAATAGCGCGTACTTGGTAGGGTTTACGATTGATGTTTTGATTATCTGTTTCTAG
GTATTGCGCAATTTTTTTATAACCATAGCCTTGTAGGTAGTAATGGAATATTGCCTTCACCGTTGGAGCTTCAT
CTTGATTAATTGTGAATGTACCATTGTGATAGGTATAGCCAAATGGTGCATGTGTCGTGATGAGTTTACCTTGT
CTAGCTTTTTCTTTAAGGCCATTCTTGACTTGTTCACCAATATTATCAGACTCTAATTCTGCGAGGCTTATGAAT
ATATTGAGCTTTAGGCGATCAAACGCTTTATCCATATCAAAATAGCCATCATGAACACTTAGAATATGAACATG
ATATTTTTGACAGAGTTTCATAAGTTTTAATGCATTTTTCAAATTACGATGTAGTCGATTCAAGCGATAACAAC
ATAATACATCACATTGTCTTTGTTGAATGCGTTCGATAACTTGTTGGTAACCCGTTCGATTATCCGTTCTGCCTG
ATTGCTTATCACTATAAAAGGTGATGTGTTGGATATTGTATTTTTTAGCGAGCGTTTCGATAGTTTGTTTCTGTG
CAGTGAGTGATTGTTGTTTGAGTGTACTTTGACGTAAGTAACCGATAGCTTGTTTCATGTTTTTCCTCCTTTCA
TTAGGATAATATATGTTTATGAACGAATTTATAGATGAGCCCAACACCAGTCAGGTGTTGGGCGATGTGATTA
GTCATCAGTGCGATTGATTTCTTCAATCACTAAATCAGCGAGTAATGTGATGAATTCGTCCATATGAATAAACC
TCCAAATATTTAATTTATAATTCTAAATCATCAGTAGCTTCTATTTCTCTGGCTTTTTTAACAAATTCATTAAGCA
AATCTGGCTTTGTATTACCGTCAAATTGTGGTGGTGTGAAAGGTTCAACATGAGCGTTTAAGCCTAAAATAG
GTGCAAGTTCTTTATCCAATTTAAGATGGTAGAATACAATCGTTTTGGACTTACCATTGGAATCTTTGATACTG
CGTTTAGTTGTCTTTCTGTTAGGGTCAGATTGAATATGTCCTTTATCGCGTAGTGCATCTATAACGTTATTCACA
TCTTGATATTGGTTTTCTTCCAACATACGTTTAAAGACATCTTTAAGGATTTTGACTTGAATACAATTATCCTTT
AATTCAATAAGTCCATAATTTTCTACCATGGTTGATAATCTATTATCATCAGAAAATTTCCCTCTATTCTGTGCAA
CAAATTGTGTAATCGTTTCGAGTGCTTTATCAGCAAGAGACCGCTCACTTACTGAATCTGAATGGTAAGTGAG
TAAGTAATTTCTAATCGCATCCAAATCTATGGGCGTCGCAATAACACGTTCGAATATACGAGCAGATGTCGTAA
TCGCTGCATAGCGTTTGAACATACGAATACCTGTATGACTCGTTTCATTTTCAAGTTGTGTTTTAAACCATTGA
TGTTCAGTATGGAACGATTTTATGACTTCTTGTTCACGATTGAGTAGATATTGTGCGACTAATGGCATCACATG
ACCGTAATTAGCTGATGTTGCCTTTTTAATGGCATCTGCATTATCAGCACTCGTCGTAAAAGCTTCTGATATTTC
AATGGTACGAACATTTAATCCATCATTACGTGCACTATTATTTAAAACACTGTGTTCTGCCGTACTTATCACTGA
GGTCCCCCAATTTTTGAGTGCTTTGACATTACCATCTATATTAGATCGTTGTCGGCCTTGTCCTTCTGCCAGAC
TGTACAACAACCCAGTAGTGTCTTTGAAGGTTGCAGCCGATAATTCGTCCAATACGATAGGCAAACCATAATT
GTTACTTAAATATCCTTCAAGCGCGTTTCTCGTACCATTCCAAGACCTAAAAAGTGTATTACTACCTTTAGTAG
GATTGCCGGCTATTGAAACAGCTAAAGCGGCTGCAGTCGATTTACCTGTGCTTGAATTTCCCATAAATGAGA
AGATGGTTCCCGCAAACTCGACCTCATGTTTTGATTTTAGGAATGCGGTTACTAGTGATGAGACGCCAAAAG
TAACTGCAAGCTCTAACAACAAGTTGCCTTTTACTTCGTCAAGATACATTTGCCACCAGCCTTCAAAAGTACC
TTTTGGTCGTAAATCATATTGTGTATCGCATAGAATATCTTCTTTTAATGAAGATAGAACCTTTTTAGATACATAA
GGTTCATCTAAAGATATGATAGGATAGGTCTCAGTGTTAATGACACCTACACCTTGATACATAGGTGATAGGG
GTGTTTGAGCACGCATTTGTTGAAGAATGAATCCTAAGTCGCGTATATATTTTTCATTAATATTAAAGCCGAAT
TTGATAATGGTTGGAAGCTTTTGAGATGACAAAATATCAGCTTCTTCAATTAATTCTGCCTGATTGAAGTCTG
ATAGAATAATCTTTTCGACTTTAGAGTTAGGCTCAATGAATCTGGCTGTTATGACAATTGGACTTGCGATAAAT
ACATCTTGATTATCTGCATCTTTCTTTTTTGAAGGGATGGTTAAAAACCATCCCGCCATTGTCAAAGTTGCATT
TCCATACTTTAAAGGTAAGTGTGAAGTGTTCATTAGAACCTCCCCCTTTATGATAGTTACTATTGTGATGAGGA
TTAGGACCTACTTTACGGTAAATTAAATTACCGCTAGAATCCTTGCCAATAATAATGAATGGAACACGTGGTG
CGTGCTTCACAAAATACGCAAACCAGCGACCAACATCCTGTTGAACCTTAATTGAACAGATAACATGAGCCT
TCATATTTAAATCGTGAAATGTAAACTGAGTACCCACAGGTAAATTAAATGCGACTCCCATAGCGCGTTTTTTT
AATAGATAAAATTCTTTCGTTTTCATCATAAAAACCCTCCCAATAATTATTAAGCGTCAATCAATATTACATGTGC

TTAACACGTCAATGATTGATATAATAAGCATAAATGAAGCATTTTAATTAGACGCGGAACAAAAAATAGGGAG
GGGGGGTATATATGATACCTACTTTAGAAGAACTTTTTGACAAGTATGGCAGCCTGACTGATATTTTAATATTT
GATGTGATAGATAAGGTTTATGAAGATTTAGTGTTATTAAGAAGTTTAAGCGATTTAAATGAACATCAAAAGG
TGGATAGAGTTTTATTTATTCAAAAATACTTTAGCCCTCATTTTGATTATTTGTTAGATGACTCTGAATTTAGAA
GTATGAATAAAATTTACAGTAATCTAAATATCGTGACGTATTTCAATAGTCAAAACAATGAATTTAAACTTAAAC
CATTTATAAATGAAGAACAATTAAAATCATTACTTGCGATAAAGAAAGTAGATAAACATTTAAGCTATGATCCA
ACTATTACTTCAAAAAATTTAGCAGATATAAACAAAACGCGAAAAAGATTAGTTACAAAATTAGACAAACTTT
ATAATCGGTCAAAAAAAGAAAATGAATATGTACGGTTAGGAGAAAAGATATCATATACAAAATTGGAGAAGC
TTTGGAAGTATTTATATAATGAGATTCAGTATTATAATCTTACTAATCAATTAGTTAGTTATGTTGCTTTAGAAAA
GGAATATGCATGGGCTTTTTTAAATGAATTATTTTATTTGATAGAATTATATTTGAAGGCATTTAAAAGACAAA
AGAAAACGGAGCATGAATTTGAAGAAAGATTGAATGCATTTATTCAAACATACGTCATTAGTTTACTTATCGA
TATAAGATTACCGACTGTTAGGTTATATATTATTAGACATATTGTGGATGCATGCGAAAAAGAACCAGATAATC
AAAAAAGAATTAAACTGATAGAGGAATCCATTAAAAAGTACAGATATGTAAAAGAACAGATTGAAAGATTTG
GAAATGGGTTGAAAGAAGAATTATCGATCGCGACTTTGTCTATTGAACAGAATAAGTTAGAATCTAAAATTG
ATCATTATAAGGATTACTTCTATCCTAGAGAAAAAACAAAGTATAACAATATTGAATATAATGTATCATTGTTTTT
TAAAGCTGTAGATGCTTTAAAAAAATAGTTTTTAGCTGACAATATTTATAGATATAATTAAATTTAGGCAATTTT
AAGTTAGTCTAGTCTTCGAGGATTTGAATCGCGTGAAATAAGTTGAGAAGTTGCTTGAAATCATAGTAATAAT
CGCGCGAAAATCGTGTGAAATAAGTTGAGAAGTGGTTGGAATCATAGTAATAATCGCGTGAAAATCGCGTGA
AATAAGTTATGAAATTGCTTAGGATCATAGTAATAATCGTGCGAAAATCGCGTGAAATACTATAGCAGATGGCT
TAAGTGAAATCAGAAGAGTATTTTTATAGTTTTGATTCGGAGGGATTGCATTGTACAAAGAAAGTAAATATAT
AGAGATTAAGAAATCACAAAAAGGGCTATCTAATGATATTTGGTCAACATACAGTGCTTTTGCGAATACTGAA
GGTGGCACAATATATTTAGGAATTGAAGAAAAAAAGATTGAGGGTAACAAAGTCTTTGTTCCAGTTGGTGTT
GAAAATCCAGAGAAAATGATTGAAGATTTTTGGAATGCATTATATGGCAGAAATAAAGTTAGTCAAAATATTT
TATCAGACAAAGATGTCAAAATTGTAAGTATTGAAAATAAAGCGTGTATTGAAATTCATGTACCTGAAGCGCC
TTATACTAAGAAGCCGATATATGTAGATAATAAAAAAGATTTAGTATATAAAAGAGTAGATGATGCTGATAGAA
TCGCGACTGAAGAAGAGTACAAATTTATGATTGTAAATTCTCAAGATGATATAGATACAGAATTATTAGATAAC
TACGATATGTCTGATTTAAACCATGAATCTATAGAAGATTATAGGAAACTTTTATTAAAGAATACTAATGATGAG
AGATATGCGAATATGAGCCAACTGGATTTAATGATAGATTTAGGAGCATATCGCAAAGATAGAAGTTCGAAA
GATAAGCAATATAAAATGACAGTAGCATGTTTGTTGTTTTTTGGTAATTATAATGCGATTAGTGATAGATTTCC
AGGGTTTCAATTAGATTATTTTAAGAAAACAAATTATTTAGATACTGATTGGAAAGATAGAGTATCAAGTGGG
GATTTAGGCAATGAAAATTTAAATGTGTATAGTTTTTTTGAAAAAGTGTTGATAAAATTGACAGATAATATTGA
GGAATCATTTACTTTAAACGCTGATTTGACTAGACAGAATTACGCAAATGATTTAAAAGTAGCAATTCGTGAA
GCATTGATTAATACATTAATGCATGCGTATTATGATACTAAGCAAAGTATTAAAATAGTTAATTGTGAAGATTTT
ATAGAGTTTTATAACCCGGGTAATATGAGAATAAATAAAGAAGATTTTATTCATGGAGGACATTCAAAAGTTA
GAAATAGTATATTGTCGACACTTTTCAGAAGAGTAGGGTATTCAGAGAAAGCTGGTTCTGGAGGACCGAGG
ATATTTGATGTAGTAAATAAGCATAGGCTTAAAGCGCCAGAAATAAAATTAACAGACATGGATACTAATGTAAT
ACTTTGGAAACAGAATTTGATGGTAGAGTTTGAAAAATACCCGGAATTAGATAAAAAAGTAATCAAGTATATT
ATTGATTTTGGATCAATAAGTAAATCTGAAGCCTTAAGAATGGAAAACATGACAGAATATAAATTTAGAAATAT
TTTAAAAGAACTCCAATCAGATAAATTGATAGAAAAACAAGGCGATGGTCGATCAACTAAATATGTATTGATA
GAATCACAAGAAGCAGATATATTGAGGACTAAAAAAGTGATCAAAGGTTTAGAGTCATTCTTTAGAAATAAA
TGAATCAAGGTGCGAAGTATTATGATTATATAAATGCTTCGCACCTTATTTTTTACTAATAAATATTATAAAAAAC
CGCATCATCAACTGATAAGCAGAGCCCTATAATAAATAAAACAAGAAATTAAGTTGTACTTAACTTAGAAGTG
GATGTGAAGACTATTAAACTTTTTTAGGTAAGCTTTAACCTGTAAATATCTTTTTAGAAAAAGGTGAAGATTT
ATTAGATTATCAACATATTAAATCACAAATAAAAAGTAGAGACTTACAGATAAATAATAAATTTACGAAAGATC

CACAAGTTGCTTTTATTCATAATGCACGTAAGTTTTTAGGAGATAAATTTATTAGAACTGCAAAACCACATAAA
TTACTAAGTAATAATAAATTAATTGCTGTTAAACTTCATTTAATTAGTCGAAAAACATTAGGGGGTATTCAAAC
CAACCTAGATGGACAAGCATTTAACCAAGATAATCAATTAATTGAAGGGCTATACGCTGCTGGAGAAGCATC
AGGTTTTGGTGGTGGTGGTGTACATGGATATCGATCTTTAGAAGGTACTTTTTTAGGTGGTTGTATATTTAGT
GGTATTCGTGTTGCACATGGTATAAGTAAAAAATGAAATAATGGAGGAACATATATGTCTAATAACTCTGAGTT
AAAAGAAATTAAAAAAAGCGTAATTTTAAATGAAAGTAAAGATAAAGTCTGGGATGCTATTGCAACATCTGA
AGGTATTGCTGGTTGGTGGATGCCAAATGACTTTGTAGCAGAAGAAGGGAAAGATTTCACATTACAAGCTG
GTAATTTTGGTGATTCTCCATGTAAAGTAACTAAAATTGAACCAAAAAGTATAGTTAGCTTTGATTGGGGGAA
AGATTGGAATCTTACCTTCAAATTGAAAGAAATTGATCAGCACACAACTGAATTTACATTGATTCATTCTGGAT
GGAATGAAAATAAAAAAACAGAATTTGGGCAACCTCATTCTGTAGTACAAAGTATTATGGACGATGGTTGGG
AAGAAATAGTTCAAAAGCACTTAGTAAATTATGTGTCGGCATATTAGTATTACTATTATATACAGTACTTAAGAG
TATTTAAATTGGATGGTGTAAAAATTAAATCGATCATAAATATAGAGGCGAGGATATCATCTAATATCCTTGCCT
CTATATTTATTATTTAAATGCTAGTTAATTCAACATCTAATTTTATGAGTAATAATAATGTCCCTTTAGTTATCTTTT
GAACGTTATTAGATTCTTAACTTGTAATGAATTTTTTTGAAAATAGCCCATTAAATGTATACACCCTATGTGTAA
TAAGGAATGACACTAATATCAAAAATTCAGAAATCAGTAATATTATTTTGAAAATATTACAACTTTTTATTTAGT
ATACACAAATCGATTACAAGTATTTATTTTCATTTAAGCACGTGATAGATTGAGTTCTATAGTTTAAGAAAAGG
AAGGTTTATTAAAATGAAAAAAATACTAACAACTTCAATAGCGACTATTGGAATTCTAACTATGAGTATTGCTC
AACAGGATGCACAAGCTGAAGAACAAAGTAGTGTATCTACTCAAAATACTCAAACCTCTACGGGAATTGAAA
ATGGTGTTTATTATACAATAGATAGTAAAGGGAACTATCACCACACATTAGATGGCAATTGGAATCAATCTATG
TTTGGTCATAAAGAATATAAATCGTATGAAGTAGATCAAAATGGTGTTACGCATTATTACTATTTTGAAAAAAA
TGATAATGACTATTCACCATCTCAATCAAATCAATCAATTAAGGATAAAGGCTATCAAGTAAATAATGGAGATG
AAAATCAAACATCTGAAACAAATAAAGAAACGATAGCAAATAATAATCAAACAGCTAAGCATAATGAAACAG
GAAGTAATGGACATGTAGCGACTTCTAATGAAACAAATGATAATCAAGAAACAGCACAATATAACAATACTGC
TTCTAAAGAACAATCAACTCCTAACAACGATGCAGCAAGCACTAAGGAAGCTAAAGGTAATGCAGAAGCTT
CATCAAATTGGCTTACTAAAAATAAAAAGTTACAAGAATATGGTCAATATCACGGCGGTGGTGCACATTACGG
TGTAGATTATGCGATGGAAGAAAATACACCTGTATATTCTTTAGCAGATGGAACTGTGATTCAAAGTGGTTGG
AGTAACTACGGTGGTGGTAACCAAGTAACAATTAAAGAGAAAAATAGTGACTATTATCAATGGTACATGCATA
TGAATAAATTAAATGTAAATAAAGGTAACGAAGTAAAAGCAGGTCAACAAATTGGACAATCAGGTAATACTG
GTAATTCAACGGCACCACACTTACATTTCCAACGTATGCAAGGTGGCGTAGGTAATGAATATTCAGTTAATCC
AGATTCTTATGTCAACAACAATCAATAATTTTATCAGAAAAAAATCACGTCTTATTATAGGTGTGATTTTTTTAT
TTGAACTTAAGAAGTAGACATGTTTAAATGTAATTATAATAGTTACATTTAGAAAAAGGAGATGATTCATTGAA
TTTAGAATTTAATATCGCAGTTCATGCATTAACTTTCTTAAGTAAACATTCATCTGAAAGGTTTAGTAGTGTCG
AATTGTCATCTAAAGTTTGTGTTAATCCCGTACAATTAAGACGAGTTATGAGCAAACTATTAGAAGAAGGTTA
CATTACTACACAAAAGGGTAAATATGGAGGGTATCGTATAAATGGTAATGTGTTAGATACGCCATTATCCAATT
TATTTAAATTATTCGCTTCTAGTCAATCATTTGGCAGAATTTACACAGGTACAAAAGAAAGTGACTGTAAAATT
TCAAAGGAAATGAGTCGAGTAATGTCAAACTACCATCGAAAAGAATTTGAAATAATAGAAAAATATTATCAAA
ATATTTCTATTGGAGATATATTAAACGATATATTAATGGAGGGTTACAATGAAAAAGTATGATTTATTAATTTTA
GGGTTTGGTAAAGGTGGAAAAACACTAGCAAAAACAGCTTCGGCTCAAGGAAAACAAATAGCAGTTGTTG
AACAATCCCAAAAAATGTATGGTGGCACATGTATCAATATCGGCTGTATTCCATCTAAAACATTAGTGCACGAA
GGATTAGAAAGTGGTTCGTTCAATCAAGCATTTGAGCTTAAAGAAGAAGTCGTAACGGCCCTTAATAAAAA
GAATTATCATAACTTAGCAGACGATGAAAATATAACAGTATTAGATTACAAGGCTAGCTTTAAATCCAATACGG
AAGTAGACTTATTAGATGAGCAAGGCAAAGTAGTAGATACTTTAACAGCAGATGACATTGTGATAAACACTG
GTGCAATACCAGTTATTCCTGATATTAAAGGTATTAAAGAATCTAAGCATTTATACGATTCTACAGGAATAATGA
ATTTACGTGAACAACCTAAACGATTAGTGATTGTTGGCGGAGGTTATATTTCTTTAGAATTTGCATCTATGTTT

TCTAACTTTGGTACAGAAGTGACTGTATTAGAAGCGAATGACAAACTAATGGCAAGAGAAGATAAAGAAATC
GTTTCACATGCGATTAAAGATTTAGAAGATAAAGGTGTAAAATTCGAGTTAGGTGCTCAAACTTCAGAATTT
GAAGATAAAGATGGATATACAATCGCTAAAACGAATAAAGGTGATTTTGAAGCAGAAGCGGTATTACTCGCA
ATTGGTAGAAAGCCAAATACTGACTTAAATTTAGAAAACACTGATATTAAACTAGGTGAACGTGGCGAAATA
GAAGTCAATAATCAATTGGAAACAGCTGTCAAACATATCTATGCAATAGGTGATGTAAAAGGTGGCATGCAAT
TCACTTATATTTCTTTAGATGACTTTAGAATTGTTAAAGACAAATTATTCGGTTCAGGTGCTCGCACTACAGAA
AATAGAGGTGCGGTACCTTATACGGTATTTATAGATCCTCCATTATCTCGTGTAGGATTGACTGCTAAAGAAGC
AAAAGAACAAGGGTATGAAATTAAAGAAGGTAAACTACCAGTAAGTAATATACCTCGTCACAAAATTAATAAT
GATCCTAGAGGTCTGTTTAAAGTTGTGATAGACGCAACAACAAATAAAGTACTTGGCGCTACTTTATATGGTT
TACAATCCGAGGAAATCATTAATATAGTAAAACTGGCAATTGACCAAAATATTGACTATACAGTTTTACGTGAC
AATATATATACACATCCTACTATGATAGAATCATTTAATGATTTATTTAATATTTAATTATATATATAAATGTAATAAAG
GCACATCACATATTGACGAATTGTTCAATAATTTAGATGTGCCTTTTTTTATTGAAACGACATTAACTACTCTTC
ATCCCGATTTTAATACTTGTAATTACGATACAGTCAAACATATATATAGACATATATCTATATAGATATTAATCTATT
TAATATCTAGGAGGTACATATATGTCTTATAAAGAACTCTCAACAATATTAAAAGTGTTATCAGATCCAAGCAG
ATTGGAAATATTAGACTTACTTTCTTGTGGTGAATTATGTGCTTGTGATTTGTTGGAGCACTTTCAATTCTCAC
AACCAACACTCAGTCATCATATGAAGTTATTAGTAGATAACTAATTAGTTTATACACGTAAAGATGGTAACAAA
CATATGTATCAACTTAATCATGTTATTTTAGATGATATCAATCAAAATATAAACATTATTAACACATATAACCAAC
GTTGTGTATGTAAAAATATAAAATCAGGTGAATGTTGATGATGACGACTTTAGCAATTGTAATTTTTCTTTTAA
CTTTAATCTTTGTGATATGGCAACCTAAAGGTTTAGATATTGGTATTACAGCTTTAATTGGGGCGGTCATCTCT
ATTATTACAGGTGTTGTTAGCTTTTCTGATGTACTAGAGGTAACAAGTATCGTTTGGAATGCCACTTTAACATT
TGTAGCTGTTATTCTTATTTCATTAATATTAGACGAGATTGGATTTTTTGAATGGTCTGCAATACATATGGTCAA
AGCTTCAAACGGTAACGGCTTAAAAATGTTTGTATATATTATGCTATTAGGATCAATAGTAGCTGCATTTTTCG
CAAATGATGGTGCAGCTTTAATCTTAACGCCAATTGTATTAGCGATGGTAAGAAATTTGGGTTTCAGTAAAAA
AACGATATTCCCTTTTATTATTGCAAGTGGATTTATTGCTGATACTACATCACTTCCATTAATTGTGAGTAATTTA
GTAAATATCGTTTCTGCTGATTACTTCAATATTGGATTTATTGAGTACTTTAGTCGAATGATTATTCCAAATATAT
TCTCTCTGATCGCTAGCATATTTGTATTATGGCTTTATTTCAGAACATCTATACCAAAAACTTTCGAAAAAAAGA
ATCTTATGAACCCAAATGATGCAATTAAAGACTTAAAATTATTTAAGATTTCTTGGCTAGTATTAGCAATATTAC
TTGTAGGCTATCTTGTTAGTGAGTTTATTCAAATCCCTGTATCGATCATTGCTTGTACGATTGCACTTATCTTTG
TGATCTTAGCTCGTAATTCTCCTGCAGTTCATACTAAACAAGTGATTAAAGGTGCACTATGGAATATTGTTCTAT
TTTCTATAGGTATGTACTTAGTTGTATTTGGATTAAAAAACGTTGGCATCACATCAATTTTAGCTGAAGTATTAT
CTAATATTTCAAGTCATGGATTATTCAGTAGTGTTATGGGCATGGGCTTTATATCAGCATTTTTATCATCCATTAT
GAATAATATGCCTACTGTCTTAATCGATGCTATAGCGATTGGACAATCTAATGTAGTAGGAACCATTAAAGAAG
GTATGGTTTATGCCAATATAATAGGTTCTGATTTAGGACCGAAAATCACACCGATTGGTTCTTTAGCAACATTA
TTGTGGCTACATGTGTTAACACAAAAAGGTGTAAAGATTTCGTGGGGAATATATTTTAAAACTGGAATCATTA
TTACTATTCCGGTACTATTTGTCACACTCTTAGGTTTATATCTCACACTAATTATATTTTAAGAAAAGAGGCTTTA
ACTATGGATAAGAAAACAATTTATTTTTTATGTACTGGCAACTCTTGTCGTAGCCAAATGGCAGAAGGTTGGG
GAAAGAAAATTTTAGGTGACAGTTTTTATGTCTATTCTGCTGGAATTGAAACACATGGTGTTAATCCAAAAGC
AGTAGAAGCTATGAAAGAAGTAGGTATTGATATCTCAAATCATACATCAGACTTGATTGATAATGATATCTTAA
GACAATCAGATTTAGTCATAACATTATGTAGCAATGCAGATGATAATTGTCCCATCATACCACCTAACGTCAAA
AAAGAACACTGGGGCTTTGATGATCCAGCAGGAAAAGAATGGTCAGAATTTCAACGTGTTAGAGATGAAAT
CGGAAGTAGAATACAAGAATATAAAGAAACGCTCGTATAATGCGAGCGTTTCTTTATATAGTTGATACAGTTG
AAATTTTAAAAAATTATAGCATATTGCTGAATTTATAGAGATATATTCCTTAAAAATTGAATAGGGTGCAATTAT
ATGATATATAATACTTGATACATATATAAATAAGTAAAATTTATCTTACTTTATAAACCGCATCATTAACTGATAAG
CAGAAGCGTATCACAAATAAAACTAAAAAATAAGGTGCTTTAGAAGAAGTGTAATTTTAAAATTGTCATAGTA

TTTATGGTTAATTAACTATCAAAATTAAACTAAAAGGGCGTGTTAATATTGGATTTAGAATCAAAGATAAAATT
AAAAAAGGAATTGAGTATACTTTATAATCTTTATAAATTTTCAATGGATGAGTTACTACAATATAAAGCTTTGA
ATTATTCAGAAATAGATTTATCAAAAGAAGAAATTCATGAAGCTATCACAAAAAAGATTGATCCAATTTATGCA
CTTGCTGTTGGTGATAAGTTTGATAGATTATATAGTTTATCAAATAGGTATTTTATAAATTTAGAGTCAAAAAAT
TTAGGTAACGACTTTATAGATAAAGTAAAACGATACCAAATTAGTCATTTAGAAAATATTCATATATATATCGATA
AATACTTTAGAAAAGTGTTTATGGATCCAAACATACATAAATTTTTGAAGAACGAACCCATTATAATAAAACAT
ATGTACATTAAGAAGAAAAATAATAACTATATCCTTGAATTTGAATCAAATGGATTAGATGCGCTTTCTAAACA
AATTACTGGAAATAAAGGAGGAAAAAACAATAATGTAATAGATTCACTCTTATTTAGTGAAATCGAGTTACAG
AATATCGTTAATAAGAATGTGTCTAAAGGTATTACAATTAATAAAGTTATTAAAGACCTAAATTTAAGCTTAAAA
AATGTTAAAGTCGAAGTTCCATATATCTACTTTGGACAAGATATCAGTCAAACAAGTTTGCAATTTAAATTAAG
CCAAATAAGTCAAAAGAAAACGATTTATAAAGGTAAAAACATTGAAGGGAAATATGGTTATAAACGTGATGA
AATGACCCAGCGGATATTAAAACTAAAAAATACATATACATTTGATCCTAATATGTTTAGAAACAGTTATGGTG
AAGAAAAAGCTCAATTTGATTTGACTAGGTATGAAAACGACATCTTAGATTTTCTAGTTACATCAGTTGTAGA
TTATCCGTTTCCTAATACACCTAAAAAGCGAAATGATGAAAAATCAATTAATAAAGTTTATCATGATCTATCTAA
TAGTGAGTTTTTAAAATATACAAACAATCAAGTGAAAATAATTAGTTCATTACCCAATAATGAACTAAAAAAGA
ATCTTACTAATGATGATACATATAAATATATTGAAGAATTTACTAATCGAAATCAATCAAAGCTTCAAACTCAAC
TATCCAAATTTTTTAATAAAGTAGATGAAGTAAACTATTATGAAGCCAATCTACTTTATAGTAGATTGGCTCAAA
AGATAGATGAAGTTTTGGTGGACCATGATATAGAAAAGACTAATTATGATTTTGAAGATAAAGCATTTAATGA
AGCTCCACATACATATTCCATTGAATACCTTGATTTTGTGTCTGATTATTATAAATATATACAAAATGTATTCTTTA
GTTCTAATGATGAAAGTAATGAAGAAATAGCTAATAGACCTTATAAAATGAGTTTTATTGAGGTGTATGAAAG
GGTTAAATTTCTTGATAACATTAAAAAAACAAAAATTTCTACATCAAATGGTAAGCAAGAATATCCGGTTTATA
AATATCACGCTATTGATAAATATGCTAAAGCGGAAGCTACTATAAATGATATCATGGTTAAAAATATATTGAATA
TTCCAAAAAGTATATATAGAAAAGAAAACGATGAAATAGCAAAAATAATAAAAAATAAAAAACACAGTGAAA
CAGAGATTGAAGAAAAGCCAGTAGAAAATGATAAGTCATTAAAATACGATGAGGACACTAAAGTTAAAGGA
AATAGTAACGACTATAATCAACTTAATAGTAGAGAATATTATGAAAATTTATTGTATAGTGAAAGGTTAAAGTAT
GAATTAAAAACTGCTGATGAAATGAAGGATATAAATGAAAAAATAAATCTAATTTACCGAGAAACAATAAAAT
CTATAGAGGATTTAGCTTTTTATATCTTATCAGAATATTTATCAATTTCTGAGTATAATGATTCTAAGTTCTTAACT
AATATTTGTTTAACAGATAATAAAAGTATAAGTGAACTGAAAGAAATTTATTTCAAGGCCAATCCTTTATATCA
GTTTTCTGAAGAACATAAAGTCGAAAGAGATAGTGAATTCATCCCCCTTCTATCACTCTTAGATAAAGTGATA
AAGCAACTTAACTCTTATTTCGATAAGTATAAAGAAAATTATGATATACATCATAAATTAATGGATGAAAATGTT
GCAAATCAACTAATAAATGCGAACTCTTATAAAGGGAATAATTTATTAGTTGAACAAGCTATAGAAGCAATAA
CGAGTATGAAACTTAGAGAAGACCTATACAAGTATCACATAAAAACGCAAAATCAAATCAAAGAATAGACTAT
ATTAACCAATACACAATTACGTGTATTGGTTTTTTTAATCAGAAAAAAAATAAAAATTTCTGATGTCATTATTTA
TTAAGAGAATGATGACATACTTAAAGTGTTGATTGGCCAATCGACTATTATATTAAATAAATAATAGGAGTGAT
GACAGATGTTAGAAAGACTAGCATTTGAAATGGAGTTTAACAAAACTGAGAAGTGGTTGATATCTGGTAATT
CAGATCAGAGTGAAGTACTTGAAATGAGAAACGCGCTGTTACTTAATATCAATAGAAAAATTCAACAGCTTG
ATGATGAAAGTAAAAAAATACTAAATCAGTACAATGATTTACACAACGAGTTGTTGCCACTAGTCGATGATAT
AGAACAAGTTGGGATAGACTTTGATATCGATAAACTAAGAAGAACTGGCGAGTTGCTAATAGATAGATCAAA
AAATGAAAGTAATCGCTATCGAATAGAAAAATCGCTAAAAAGCCTTGCAAATTCATTGGAAGGTAACAAGCT
TAAAACTAAATTGAATCTGATTGGCGGTGTTACACATCGCATAACGACAGCTTCATATAATATTCAAGGGCTTC
CTAATGAAATTAAGGCATGCATTATACCGTCTCAATTTAAGCGTGTATATATGATTGATTTCAGTGCATTTGAAC
CTTCTGTAGCAGCTTACATGTCAGATGATAAGACTTTGATAGCGTACTTGAAAAGTGATGATGGTTTATATGA
GCGTTTATTGAAAGATCTAAACTTATCCTCTGATTATAGGAATGCTGTGAAAGTATGTTTTATCGGATCATTCTT
ATTCGGAGGTAACTATCACAATTTTAAATTGAGTGAGGAAGTACCTGAAGTAGAGTGGCATAACTTAATAAA

GAAATTCAGTAATTATATGCTTCTAAGGGTTCTGTTGCAAAGTAAAAAAATATAGCTAACCACTAATTTATCAT
GTCAGTGTTCGCTTAACTTGCTAGCATGATGCTAATTTCGTGGCATGGCGAAAATCCGTAGATCTGAAGAGA
CCTGCGGTTCTTTTTATATAGAGCGTAAATACATTCAATACCTTTTAAAGTATTCTTTGCTGTATTGATACTTTGA
TACCTTGTCTTTCTTACTTTAATATGACGGTGATCTTGCTCAATGAGGTTATTCAGATATTTCGATGTACAATGA
CAGTCAGGTTTAAGTTTAAAAGCTTTAATTACTTTAGCCATTGCTACCTTCGTTGAAGGTGCCTGATCTGTAAT
TACCTTTTGAGGTTTACCAAATTGTTTAATGAGACGTTTGATAAACGCATATGCTGAATGATTATCTCGTTGCT
TACGCAACCAAATATCTAATGTATGTCCCTCTGCATCAATGGCACGATATAAATAGCTCCATTTTCCTTTTATTTT
GATGTACGTCTCATCAATACGCCATTTGTAATAAGCTTTTTTATGCTTTTTCTTCCAAATTTGATACAAAATTGG
GGCATATTCTTGAACCCAACGGTAGACCGTTGAATGATGAACGTTTACACCACGTTCCCTTAATATTTCAGATA
TATCACGATAACTCAATGTATATCTTAGATAGTAGCCAACGGCTACAGTGATAACATCCTTGTTAAATTGTTTAT
ATCTGAAATAGTTCATACAGAAGACTCCTTTTTGTTAAAATTATACTATAAATTCAACTTTGCAACAGAACCGT
ATTATGGAATAGAGATGTTGGTAACATTTATACAGGATCATTATACTTAAGTTTAATTTCGTTATTACAGAACCA
CACATTCCAACCAGAAGAGAAAGTATGTCTATTTAGTTATGGTTCAGGAGCAGTAGGAGAAATCTTTAGTGG
TTCAATCGTTAAAGGATATGACAAAGCATTAGATAAAGAGAAACACTTAAATATGCTAGAATCTAGAGAGCA
ATTATCAGTCGAAGAATACGAAACATTCTTTAACAGATTTGATAATCAAGAATTTGATTTCGAACGTGAATTG
ACACAAGATCCATATTCAAAAGTATACTTATACAGTATAGAAGACCATATCAGAACATATAAGATAGAGAAATA
AACTAGTGGCCGATTGTGCTTGATGAGCTTGGGACATAAATCCTAACTCGAAATAAATAAGCATATCACTAAA
CTGATTTTTTAAAGTTTACAGTGATATGCTTATTTTTTTATCTTACGATTTTGTACGTGCATGCTTGCCTAGGGG
TATGGCTCGAGCCATTAGTCTCTCGCACATACTATTCCCTCAGGCGTCAGCACTTACAAAATCGGTTGTAATTT
TCATTTTTATACGCATTCTTACTGAGATTATACTAATAAGAGGAATAGTAAAAGCAATTCTAAGTAAAATTGCA
GATAAGAGGTTTGTTAAAAGCAGTTCTAAGTAAAATTGCAGATAAGAGGTTTGTTAAAAGCAGTTCTAAGTA
AAATTACAGATAAGAGGTACGTTAAAAGCAGTTCTAAGTAAAATTGCAGATAAGAGGTTTGTTAAAAGCAGT
TCTAAGTAAAATTGCAGATAAGAGGTACGTTAAAAGCAATTCCATGCAAAATTGCTGATAAGGGGTAAGTTA
AAAGCAGTTCTCAGTAAAATTGCAGATAAGAGGTACGTTAAAAGCAGTTCTAGGCAAAATTGTAGATAAGA
GGTGCGTTAAAAGCAGTTCTCAGTAAAATTGCTGATAAGGGGTAAGTTAAAAGCAATCCTAAGTAAAATTGC
AGATAAGAGGTAAGTTAAAAGCAATCCTAAGTAAAATTGCAGATAAGGGGTACAGAAAAACTAGACTTGAT
TACAAAATGGAGCTTGGGACATAAATGATTTTTTAAAAATGAGATGAGACGTAGATTAACTCCATAATCAATA
CGAATCTATCGACTTCTTTATTTATGATATTCATCTCTTTTTAATGGAAATAAAAGTGCGATTAATGTGATAATAC
AGTTACGTTAATTAAAAAAATAAAAATGCAAGGAGAGGTAATATGCTAACTGTATATGGACATAGAGGATTAC
CTAGTAAAGCTCCGGAAAATACAATTGCATCATTTAAAGCTGCTTCAGAAGTAGAAGGTATAAACTGGTTGG
AGTTAGATGTTGCAATTACAAAAGATGAACAACTGATTATCATTCATGATGATTATTTAGAACGGACTACAAAT
ATGTCCGGGGAAATAACTGAATTGAATTATGATGAAATTAAAGATGCTTCTGCAGGATCTTGGTTTGGTGAA
AAATTCAAAGATGAACATTTGCCAACTTTCGATGATGTAGTAAAAATAGCAAATGAATATAATATGAATTTAAA
TGTAGAATTAAAAGGTATTACTGGACCGAATGGACTAGCACTTTCTAAAAGTATGGTTAAGCAAGTGGAAGA
ACAATTAACAAACTTAAATCAGAATCAAGAAGTGCTCATTTCAAGCTTTAATGTTGTGCTTGTTAAACTTGCA
GAAGAAATCATGCCACAATATAACAGAGCAGTTATATTCCATACAACTTCGTTTCGTGAAGACTGGAGAACAC
TTTTAGATTACTGTAATGCTAAAATAGTAAACACTGAAGATGCCAAACTTACTAAAGCAAAAGTAAAAATGGT
AAAAGAAGCGGGTTATGAATTGAACGTATGGACTGTAAACAAACCAGCACGTGCAAACCAACTTGCTAATT
GGGGAGTTGATGGTATCTTTACAGACAATGCAGATAAAATGGTGCATTTGTCTCAATAGAAAGTTAGAGGTG
AGTCTTACGTTTCAGTGACGGTAGACTTACCTTTAACATGTTACATACTAAAAAATTAATTTGAATAAGAAAGA
GAGACATATATGAAATACGATGATTTTATAGTAGGAGAAACATTCAAAACAAAAAGCCTTCATATTACAGAAG
AAGAAATTATCCAATTTGCAACAACTTTTGATCCTCAATATATGCATATAGATAAAGAAAAAGCAGAACAAAG
TAGATTTAAAGGTATCATTGCATCTGGCATGCATACACTTTCAATATCATTTAAATTATGGGTAGAAGAAGGTA
AATACGGAGAAGAAGTTGTAGCAGGAACACAAATGAATAACGTTAAATTTATTAAACCTGTATACCCAGGTA

ATACATTGTACGTTATCGCTGAAATTACAAATAAGAAATCCATAAAAAAAGAAAATGGACTCGTTACAGTGTC
ACTTTCAACATACAATGAAAATGAAGAAATTGTATTTAAGGGAGAAGTAACAGCACTTATTAATAATTCATAAT
AAAACAGTGAAGCAACCATCGTTACGGATTGCTTCACTGTTTTGTTATTCATCTATATCGTATTTTTTATTACCG
TTCTCATATAGCTCATCATACACTTTACCTGAGATTTTGGCATTGTAGCTAGCCATTCCTTTATCTTGTACATCTT
TAACATTAATAGCCATCATCATGTTTGGATTATCTTTATCATATGATATAAACCACCCAATTTGTCTGCCTGTTTC
TCCTTGTTTCATTTTGAGTTCTGCAGTACCGGATTTGCCAATTAAGTTTGCATAAGATCTATAAATATCTTCTTT
ATGTGTTTTATTTACGACTTGTTGCATACCATCAGTTAATAGATTGATATTTTCTTTGGAAATAATATTTTTCTTC
CAAACTTTGTTTTTCGTGTCTTTTAATAAGTGAGGTGCGTTAATATTGCCATTATTTTCTAATGCGCTATAGATT
GAAAGGATCTGTACTGGGTTAATCAGTATTTCACCTTGTCCGTAACCTGAATCAGCTAATAATATTTCATTATCT
AAATTTTTGTTTGAAATTTGAGCATTATAAAATGGATAATCACTTGGTATATCTTCACCAACACCTAGTTTTTTC
ATGCCTTTTTCAAATTTCTTACTGCCTAATTCGAGTGCTACTCTAGCAAAGAAAATGTTATCTGATGATTCTATT
GCTTGTTTTAAGTCGATATTACCATTTACCACTTCATATCTTGTAACGTTGTAACCACCCCAAGATTTATCTTTTT
GCCAACCTTTACCATCGATTTTATAACTTGTTTTATCGTCTAATGTTTTGTTATTTAACCCAATCATTGCTGTTAA
TATTTTTTGAGTTGAACCTGGTGAAGTTGTAATCTGGAACTTGTTGAGCAGAGGTTCTTTTTTATCTTCGGTT
AATTTATTATATTCTTCGTTACTCATGCCATACATAAATGGATAGACGTCATATGAAGGTGTGCTTACAAGTGCT
AATAATTCACCTGTTTGAGGGTGGATAGCAGTACCTGAGCCATAATCATTTTTCATGTTGTTATAAATACTCTTT
TGAACTTTAGCATCAATAGTTAGTTGAATATCTTTGCCATCTTTTTTCTTTTTCTCTATTAATGTATGTGCGATTG
TATTGCTATTATCGTCAACGATTGTGACACGATAGCCATCTTCATGTTGGAGCTTTTTATCGTAAAGTTTTTCGA
GTCCCTTTTTACCAATAACTGCATCATCTTTATAGCCTTTATATTCTTTTTGTTTTAATTCTTCAGAGTTAATGGG
ACCAACATAACCTAATAGATGTGAAGTCGCTTTTTCTAGAGGATAGTTACGACTTTCTGTTTCATTAGTTGTAA
GATGAAATTTTTTTGCGAAATCACTTAAATATTCATCCATTTTTTTAACGGTTTTAAGTGGAACGAAGGTATCA
TCTTGTACCCAATTTTGATCCATTTGTTGTTTGATATAGTCTTCAGAAATACTTAGTTCTTTAGCGATTGCTTTAT
AATCTTTTTTAGATACATTCTTTGGAACGATGCCTATCTCATATGCTGTTCCTGTATTGGCCAATTCCACATTGT
TTCGGTCTAAAATTTTACCACGTTCTGATTTTAAATTTTCAATATGTATGCTTTGGTCTTTCTGCATTCCTGGAA
TAATGACGCTATGATCCCAATCTAACTTCCACATACCATCTTCTTTAACAAAATTAAATTGAACGTTGCGATCAA
TGTTACCGTAGTTTGTTTTAATTTTATATTGAGCATCTACTCGTTTTTTATTTTTAGATACTTTTTTTATTTTACGA
TCCTGAATGTTTATATCTTTAACGCCTAAACTATTATATATTTTTATCGGACGTTCAGTCATTTCTACTTCACCATT
ATCGCTTTTAGAAATATAACTGCTATCTTTATAAACTTGTTTGAAATTTTTATCTTCAATTGCATCAATAGTATTAT
TAATTTCTTTATCTTTTGAAGCATAAAAATATATACCAAACCCGACAACTACAACTATTAAAATAAGTGGAACA
ATTTTTATCTTTTTCATCAATATCCTCCTTATATAAGACTACATTTGTAGTATATTACAAATGTAGTATTTATGTCA
AAATAATGTTATAATTTTTGTGATATGGAGGTGTAGAAGGTGTTATCATCTTTTTTAATGTTAAGTATAATCAGT
TCATTGCTCACGATATGTGTAATTTTTTTAGTGAGAATGCTCTATATAAAATATACTCAAAATATTATGTCACATA
AGATTTGGTTATTAGTGCTCGTCTCCACGTTAATTCCATTAATACCATTTTACAAAATATCGAATTTTACATTTTC
AAAAGATATGATGAATCGAAATGTATCTGACACGACTTCTTCGGTTAGTCATATGTTAGATGGTCAACAATCAT
CTGTTACGAAAGACTTAGCAATTAATGTTAATCAGTTTGAGACCTCAAATATAACGTATATGATTCTTTTGATAT
GGGTATTTGGTAGTTTGTTGTGCTTATTTTATATGATTAAGGCATTCCGACAAATTGATGTTATTAAAAGTTCGT
CATTGGAATCGTCATATCTTAATGAACGACTTAAAGTATGTCAAAGTAAGATGCAGTTCTACAAAAAGCATATA
ACAATTAGTTATAGTTCAAACATTGATAATCCGATGGTATTTGGTTTAGTGAAATCCCAAATTGTACTACCAAC
TGTCGTAGTCGAAACCATGAATGACAAAGAAATTGAATATATTATTCTACATGAACTATCACATGTGAAAAGTC
ATGACTTAATATTCAACCAGCTTTATGTTGTTTTTAAAATGATATTCTGGTTTAATCCTGCACTATATATAAGTAA
AACAATGATGGACAATGACTGTGAAAAAGTATGTGATAGAAACGTTTTAAAAATTTTGAATCGCCATGAACA
TATACGTTATGGTGAATCGATATTAAAATGCTCTATTTTAAAATCTCAGCACATAAATAATGTGGCAGCACAATA
TTTACTAGGTTTTAATTCAAATATTAAAGAACGTGTTAAGTATATTGCACTTTATGATTCAATGCCTAAACCTAA
TCGAAACAAGCGTATTGTTGCGTATATTGTATGTAGTATATCGCTTTTAATACAAGCACCGTTACTATCTGCACA

TGTTCAACAAGACAAATATGAAACAAATGTATCATATAAAAAATTAAATCAACTAGCTCCGTATTTCAAAGGAT
TTGATGGAAGTTTTGTGCTTTATAATGAACGGGAGCAAGCTTATTCTATTTATAATGAACCAGAAAGTAAACA
ACGATATTCACCTAATTCTACTTACAAAATTTATTTAGCGTTAATGGCATTCGACCAAAATTTACTCTCATTAAAT
CATACTGAACAACAATGGGATAAACATCAATATCCATTTAAAGAATGGAACCAAGATCAAAATTTAAATTCTTC
AATGAAATATTCAGTAAATTGGTATTACGAAAATTTAAACAAACATTTAAGACAAGATGAGGTTAAATCTTATT
TAGATCTAATTGAATATGGTAATGAAGAAATATCAGGGAATGAAAATTATTGGAATGAATCTTCATTAAAAATT
TCTGCAATAGAACAGGTTAATTTGTTGAAAAATATGAAACAACATAACATGCATTTTGATAATAAGGCTATTGA
AAAAGTTGAAAATAGTATGACTTTGAAACAAAAAGATACTTATAAATATGTAGGTAAAACTGGAACAGGAAT
CGTGAATCACAAAGAAGCAAATGGATGGTTCGTAGGTTATGTTGAAACGAAAGATAATACGTATTATTTTGCT
ACACATTTAAAAGGCGAAGACAATGCGAATGGCGAAAAAGCACAACAAATTTCTGAGCGTATTTTAAAAGA
AATGGAGTTAATATAATGGATAATAAAACGTATGAAATATCATCTGCAGAATGGGAAGTTATGAATATCATTTG
GATGAAAAAATATGCAAGTGCGAATAATATAATAGAAGAAATACAAATGCAAAAGGACTGGAGTCCAAAAAC
CATTCGTACACTTATAACGAGATTGTATAAAAAGGGATTTATAGATCGTAAAAAAGACAATAAAATTTTTCAAT
ATTACTCTCTTGTAGAAGAAAGTGATATAAAATATAAAACATCTAAAAACTTTATCAATAAAGTATACAAAGGC
GGTTTCAATTCACTTGTCTTAAACTTTGTAGAAAAAGAAGATCTATCACAAGATGAAATAGAAGAATTGAGA
AATATATTGAATAAAAAATAAAATTGTTGTGTTTACAACAATACATAGAAAACAGAGGAAACAATCAAGTCGT
TGAATATTTCCTCTGTTTTTTAGTTGAAAAAATTAACCGAAAGCCTGAATGCAAGTCTTGATTAAATCAATAAT
GCTTGTAATAACACCAGTGAAATCCATATGCATACCCTCTTTCTATTTAAGATACATTAAGTATAATATCAAACA
AATAAAAAATGTTAAAAATTCCCTAATTGGCTATTTAGATTGCATAAATGTCAAAAATTTGAAAAACATACAAC
GACTTTGCATAAAAAAATCGTCATATTGGAAATACGTAATTTATTGAAATAATAAAAAAAATAAAAGAACGAAG
ATGATAACCTAAGTGAGGTTTTAAGTTGTTCTAAGGTTTAATTTAATTTATGTTAAAATAGTTGGTATAAAAATA
CATGATAAACTATAAACTAAATTCAAAATAACTTATGGGGTAGGCAATTATGGAAAATATTTTAAATATAAATGA
TAATGAAAAAAGAGTGCTAAGGGAAATTTATAACCATCATAATATTTCGCGTACTCAAATATCTAAAAATCTTG
AGATTAATAAGGCAACGATTTCTAGTATTTTGAATAAGTTAAAGTATAAATCTCTTGTTAATGAGGTTGGTGAG
GGTGATAGCACGAAGAGTGGTGGTAGAAAACCTATTCTTCTGAAGGTTAATCATCTTTATGGTTATTTTATTTC
TTTGGATTTAACTTATAGTTCTGTTGAAGTGATGTACAATTATTTTGATGGTAATGTCATTAAGCATGAATCTTA
TGATTTACCTGATGAAAAGGTTAGTAGTATATTAAGCATAATAAAAAAACATATTGATATTCAGGAGAAACTTG
ATACTTATAACGGACTATTAGGTGTGTCTGTTTCTATACATGGAGTTGTGGATAATGAGCAGCATGTGACATAT
TTACCATTCCATGAAACTGAAGGAATTTCAATTGCTAAGAAAATAAAAGAAATTACTAATGTTCCAGTCGTAG
TTGAAAATGAAGCGAATCTTTCAGCGTTATATGAACGTAATTTTAATCATAATTTATCCTACAATAATCTTATTGC
TTTAAGTATACATAAAGGTATTGGTGCTGGGCTTATTATTAATAATCAATTGTATCGTGGTGCAAATGGGGAAG
CGGGTGAAATTGGAAAAACACTTGTCTCAAAAGTTAGCGATAATGTGGAGATCTTTCATAAGATTGAAGATA
TTTTTTCACAAGAAGCTTTACTGCATAATTTAAGTAATCAACTAAATGAGAAGATGACGCTTAGCAAATTAATT
CAATTTTATAATGAAAAAAAATCCAGTCGTAGTTGAAGAAATGGAACAATTTATAAATAAAATTGCTGTTTTAAT
ACATAATTTAAACACCCAGTTTAATCCGAATGCAATTTACATTAACTGTCCATTGTTCAATGAAATGCCTGAAA
TATTAGAAGCAATTAAGAACCAGTTCAAACAATATTCACGTAACGAAATTCAAATAAAGTTAACATCTAATGTC
AAATTTGCAACTTTGCTAGGTGGTACATTAGCAATTATCCAAAAAGTACTACAGATTAATGATATTTACTTAGA
TATAAAAGCATAAAAAACTAATTCAAATGAATAATCAAAGTTCGTAATTGTCTTTATAAAAAAATCCCTCAATCC
GAATTGAATTTTCGGATTGAGGGATTTTTATAGTTCTATTGCAGAAGAAAACTATTTTAAAAATGCTGGTAAA
TGTTGATAGCCACCTCTAACGTTAACAATATTCGTAAATCCTTTATATTCTAATATTCCTACCGCTATTGAACTTC
TAACACCTGATTGACAATGTACATAAATTAGGTCATTTTTATCGAAAGGTATATCTTCATTTAAAAGTTTACCGT
GAGGAATATGAATTGCTTGTTTTAAATGACCTTTACGCCATTCATCATCATTACGAACATCTAATACATTATGTT
CTTCACCAGTCATTTCAGAACTATGAATAGATGATGTGACGATATTTGTTTGTGGCAAACGGTAACTTTTTACA
TTTTCAAAACCAATTAATTGTAAAGCATGAATAGCTTGTTGAACGGTAGATTTATCGCCAATTAATTCAATATCT

TAGTCATAATCTAAATACCAACCAATTTGATTTATAAAAGTTTTATTAAAAGGAATATTGATAGTTCCATGCATAT
GACCACCATGGAATGCTTCTTTACTTCGAAGATCAAAAGCAGTTTGTGTATTGCTTGAACTAGGGTAAACATT
ATATGGTTGGTACATTTGCATACCAAATTGATTTATTTTTTTCATTTGTGAAAAATGGTGTGGTGGAGCTGGCT
GATTGAGTGTTAAAGTTTCGATAAATGAAGTTTCATCTTTAACATTAAAAGCCCAGTTGTTTATTTTCTCATAA
CCCAAAGTAGTTGTAGGTAATGAACCTAGCGCTTTACCACAAGGACTCCCTGCACCATGACCTGGCCAAATT
TGAATATAGTCTGGTAATGTTGCAGCAAATTGTATGGACTGATACATTTGTTTTGCTCCGATTTTTGTAGAACC
TTCAACATTTACAGCTTTTTCTAATAGATCTGGTCTACCTACATCACCAACAAAGATGAAGTCACCGCTAAATA
ATCCCATTGGTATACTGGAACCCCCACCTTCGTCAGTAAGTAAAAAACTAATACTCTCAGGGGTATGGCCTGG
AGTGTGTAAGACTTCTAATTTAATCTTTCCTAAATAGATAATATCTTGATGCTTAACGAAATGTGTTTGTTTAGG
CATATTTTTATAATTAAATTCATCTTTACCTTCATCAGATACGTATATACTTGCATTCAATTTATTTGCCACATCTCT
AATACCTGAAGCAAAATCAGCATGAATATGTGTTTCTGCAGCTTTAGTAATTGTGAATCCTTCTTTATCTGCAA
CTTTTAAATATTTTGTTAAATCTCGTATAGGGTCAATAATCATTGCTTCTCCTGTACGTTGACATCCAATTAAATA
AGATGCTTGTGAAAAATTGTCTTCATAAAATTGTTTGAAAAACAAAAAAAAACTCCTTTTTAAAATAGATTTT
ATTGATTAGATAAATAAGTTATGATTTGCTTGCTCAGTATGTCCAATATAAGTACCTACGCCACCATAATCGACT
TCATCTCTTAATTCTTCTTTTGAAATTCCCATAACATCCATACTCATGGTACAAGCAATTAACTTTATATCTTGAT
CGATTGCTTGATCGATAAGTGAGTATAAAGAATCAACATTTTTCTTGTTCATTACATAACGCATCATAATATTAC
CTAGTCCAAACATATTCATTTTTGATAATGGCATATGTATTGGATCCTTAGGTAACATAAGGTCAAACATTTTGG
AAATACCTTTCTTTTTAACGCGAGTTGATTGCGCTTTTTTTAATGCGTTGAGGCCCCAAAAAGTAAAGAAAAT
AGTTACATCTTTACCTGCTGCTTTAGCGCCATTTGCGATGATCATTGCTGCTACTGCCTTATCTAACTCACCGCT
AAATAAAACAATTGTTGTACCTGTAGCAGTGTCATTGATTTCAAATTCTTTTGGCTTTTCTTTTTGAATAATTGC
ATTAATTACATTTGCTTCTTCAGTAAGATTTACAAGGGTATTCCCTGTTTGTTTCGCCCAACTTTTAATATCACT
ATTGAAACCAGGATCTGTAACTGTTACCTCGATTTGCTCACCCGTTGAAATATTGTTAATTTCTTTACTGATATT
AACAATAGGTCCAGGGCACTGAAGACCTCTAAAATCAAATTGTTTACGATTCTCTTTGATTTCAATATCTTTTT
CTATTAAAGGAGCACTATTGAAGTTCTTTGCTTCATAATCTTTATATCCACCCTTTAAATTCACGACATCATAAC
CTTGTTTGGCTAAATAATCGCAAGCTTTAGTGCTTCGGTTACCGCTTTTACAATGTATATAATACGTTTTGTTGC
TATTCTTATTGAATGATTTAATCTCTTCTACTGGGTGTAAAGTTGAACCGTTAATGTGTCCTAATTCATATTCTTC
TTTTGTTCTAACATCAATCAATTGACCCATTTTTGCCAATTTTTCTAATTCTTCTTTGTTTAATGAATTAATGTGT
ACTTCTTTGTATTGTTCCATACTTACCTCCTATAAATACCTATGAGGGTATAATAAAACGGATAGAATCATTTGC
CAAATACCTATAGGGGTATTTGACAATTTTGTTTTAATTTATTATTATTAACTTAATCAATTTATGTGGAGGAAAT
GAATATGACTTATGATAAAAAAATGATTAATCGTATAAATAGAATACAAGGTCAATTAAATGGTGTCGTAAAAA
TGATGGAAGAAGAAAAAGATTGCAAAGATATAATTACGCAACTTAGTGCATCTAAAGGTTCTATACAACGTTT
AATGGGGATTATAATTAGTGAAAATTTAATAGAATGCGTTAAAACAGCAGAAGAAAATAATGAAAGTTCTCA
AGAATTAATTAATGAAGCAGTTAATTTATTAGTTAAAAGTAAATAATGGATATAGCAAATATGACTATTATGTTG
CTAATTGGCGTACTGGGTGGATTTATATCTGGATTAATAGGTATTGGGGGCGCAATTATTATTTACCCAGCTAT
TCTTATATTGCCACCATTAATAGGTATACCTGCGTATAGTGCATATATTGCTTCGGGACTTACCTCTAGTCAAGT
ATTTTTCAGTACACTTAGTGGATCATTAAATGCAAGAAAACAACCAGCTTTCTCTCCTAAACTTGTTATATATAT
GGGAGGGGGTATGTTGATTGGAAGCATGTTAGGGGCAATTTTAGCTAGTTTGTTTAATGCTACTTTTGTAAA
TATGGTATATGTAATAATCGCCATACTTGCTTTAATATTGATGTTTATTAAAGTTAAACCTACTACACAAGAGAC
GAAATCTAAACCTTTGCTATTTATTATAGTTGGATTTGGAATTGGTGTAATTTCGGGAATTGTGGGTGCAGGT
GGAGCATTTATCATCATTCCTGTATTATTAGCATTATTTAAATTACCAATGAATACGGTAGTGAACAATAGCATA
GCAATTGCTTTTATATCTTCAGTAGGGGCATTTTTTATAAAATTAATGCAAGGATATATACCAGTAGAAAGTGC
AATTTTTTTGATAATTGAATGATGATCATTTTCTGAAAATATTATGTGGTCATATAATATAATGCCCATCATTTCA
CTAATCTCTTTTATTCTCTGAGTTATTTTGATATCTCCTGGAGAAGGTGTCGGATCTTATCAAGAGTAAATTATG
AAAAAAATGGTTTAAATCAATGTTCTTACTTTAAGTACATGTGTTTAACGTAAATGATAATTAGTTAAATATAAA

AAGGTTATCCCAATCAATAGGATAACCTCTTTATACTTTACTTTATGGTTTGGATTCGCCACTTCAAAGAAGAA
ATGAAATCAGAGCATTGAATGTTCCATTTTCAAACAATATATAAATACCTAGCCCAATGAATACAATTGGTACA
ATCCAACGTTCATATTTCTCAATTGTTTCCGATATAAAATCGAAGGAAGCTAGACGGTAACTGACATAGCACA
AAACTCCAACCATAATTAGAAAGACAATAGTGACAATAAAGATTTCAGACATACTTAAGGTCGTGAAGTACG
GTATATAAATGGAAAAGTCATCCGCACTGGAAGCCAATACGATGAAAATCATCGTCAAAAATAACTGATTAAA
TTTTCCAGAGGAGAATAAAGATAAAATGCTACTTTCATCTTCATCCTCTTCTCCTTTAATCCATATTTTCACGCC
TAGGTAAAGTGGTAAAAGTCCAAGTAGTCCGATAACCCATTGCTGAGGAATTAAATTTACAACCCCCTGTGC
AACTAAAAGACTTGCTCCTATCACAATTGCAGTCCCTATATATTGTCCTATCCAAATATGTTTTACCTGACCTTTT
TTTACTTGCGAAAACAAAAGAATTAATATGACGAGATAATCAATTCCTGTTGCTACATATACCGCAGTAGCCGT
CAGTATCGTCGCGATCATTTTATCATCTCCAAATATTTTAGGGATAGGACTTTTCTTCAAAATGAAAAGTCCTT
CCGTAAATTGCACACATATAGTACCTATTTATCCTTCACTCTCATCAGTCGCAAACTATTTAATGCTACCAAAATA
GTGGCTCCCATATCGGAAAGAATCGCAATCCAAAGGGTTAGCCAGCCTGGAATAACCAATAGTAAGGCAATT
ATCTTAATTCCGATGGCAAACGTGATGTTCGCTTTGATGATATTTAGCGTTTTCCTGCTAAGTCTTACTGCAAA
TGGAAGCTTACTTAAATCATCTCCCATTAATGCAATATCAGCTGTCTCGATGGCAGTATCTGTTCCAGCACCGC
CCATTGCAATGCCAACAGTGGATGCAGCAAGTGCAGGAGCATCATTGACGCCATCGCCAATCATAGCTACAT
TACCATGCTCGGCTTTCATTTTTTTAATATAGTCCAACTTATCCTGTGGCAACAATTCGGACTGAATATCAGAA
ACGCCTACATGAGCACCGATTGCTTCTGCGGTACCTTGATTATCACCTGTCAGCATAATTGTTTGCTTGATTCC
TAACTGATGAAGTTTTAGAATCACATTTTTACTTGTTTCGCGGACCTCATCTGCTACAGCAATCACGCCGAGG
ATTGTTTGGTCCGTTCCAATAATCATGGCCGTTTTCCCTTGGTTTTGTAAAACTTTCACTTTATTTTCAAACTCA
AGGCTAAAATCGGAAACATTTAATTCTTTAAAAAGCCTTGGACTGCCAATGTAATAGGTTGTTCCATCTATATT
CCCTTGAATGCCCCGACCTGTAATAGAAGTGAAGTCCTTCACTCTAACATCGGAATAAGTAATATTATCTTGCT
CTGCTTTCTTCATTATTGCTGAAGCAAGTGGATGTTGTGATCGATATTCTAAAGCTGTAATAATGGAAAACAG
CTCTTTTTCTTCCACTTGATCATTTAACACTTTAAAATCTGTTACCACTGGTACACCTTTTGTCAGTGTTCCTGT
TTTATCAAATGCGATTGCCTTAATGGCTCCTAATTCCTCTAGATAGACACCGCCTTTAATCAACACACCTTTTTT
AGCTGCATTTCCAATTGCCGAGACAATCGAGATTGGAGTAGTAATAACTAATGCACACGGACATCCAACTAC
AAGTACCGCTAATCCTTGATAAACCCAAGTATCCCAACTTCCACCAAAGAATAAAGGTGGAACGACTGCAAC
GAGCGCCGCAATAACCATAATGATCGGCGTATAATATTTCGCAAATTTATCTACGAATGCTTGCGCTGGAGCG
CGCTCCCCTTGTGCTTCCTCAACCAGATGAATAATCTTGGAGATAGTTGTATCCTCTACGTATTTGGTGATTTTT
ACTTCAAGTAGTCCCTCTTCGTTAAGCGTGCCTGCAAATACTTCATCATCTACCGTTTTGGCAACAGGGACAG
ATTCTCCTGTTATAGCAGCCTGGTTGACAGCCGACACACCATTTATAATGATCCCATCCATGGCAATTTTCTCC
CCTGGTTTGACGATCATAATATCACCCACGGCAATATCGTCCACATGGATCATTATTTCCTGACCATTCCGCCTA
ACAAGTGCTTCTTTTGGGGCAATATCCATCAATGAACGAATGGACTGTCTTGCTCTATCCATAGAAAAACGTT
CAAGTGCTTCACTGATTGCAAAGAGAATGACAACAATGGATGCCTCTGCCCATTCACCAATGATGGCAGCTC
CAATAACTGCAACGGTCATCAGGGTTTTCATGTCGAAATCAAAGCGTATCAAATTTTGAAAACCAACTTTAAA
TAGTGAATATCCGCCAATTACAATCGAACTTACAAATAACATGGACGTTACAAGGTTATCTTCTCCATTTACAA
AGTGAGAAAGGTAACCAAAAGCAATCAGTAATGTGGCAAACAGCAATGTGCTGTGTTTTTTATAAAACGGTA
TTTTCTCTTCTTTAGGAGCCTTAGTGTCTTCTTTGACCGCTTGTATCGATGGATTCGCCAGTTTTTCAGGAATT
ACCTTAAGATTCTCGAAAGCACCTGCTTTTTCAAGCTCTTCAACCGATGCGTTTCCATATACATCAATTTTAGA
AGCGCCAAAGTTCACTTTTGCATCCTGAACTCCAGCTAGTTGTTTTACATTTTTTTCAAACTTCCCAGCACAG
TTTGCGCAAGAAAATCCCTCCACACGGTAAACATTTTTATCTTCTGTTACCACTGGTTCGACCCTTCTTCTAGC
CTTCTCTGGTGCCACTTTAAGATTCTCGAAAGCACCAGCCTTTTCCAGATCTTCAACGGTTGCACTGCCAAA
GACATCAATTTTGGAAGCTCCGAAATTGACTTTAGCATCATGCACCCCTGATAGTTCTTTTACATTTTTTTCAA
ACTTCCCAGCACAATTCGCACACGAGAAACCCTCCACACGGTAAACCTGTTTATCTTCTGTTAATGTTTTTGT
TGAACTATCCAATACTAGCAACCTCCCTTTGATGCAAGAAAGCTTTTTCTACAAGCTGTTTAACATGCTCATCA

TCTAGTGAATAATAGACTAATTTTCCTTCTTTACGGTATTTTGCTATACCTAAATTTTTCAATAATCTTAAATGAT
GGGATGCCGTAGCCGTTGAAGATTCAATGATATTAGCTACATCACAAACACATAACTCTCCCTCTAAAGACAA
AACATAAGCAATTTTAACTCTTGTATCATCTGATAGAGCCTTAAAAACTTTCGCTACATCCATAGGATTCTGTTT
AGCAAGGTCTTTTTTAGCCCTGTTTACCTTATCTTCATGAATATAGGTAACTTCACACATATCTTTTGTCATAATT
ACCCTCCTTATTCAAATGACTGTTTGTTTGATGATTATAATATATCCCACTATAAACAAATAGTCAAATGATTGTT
TGAATGATATATAATTAATATTAAAAAGGATTGGTTTCTAATGTTAGAAACCAATCCTTTCGGAGATTTTAACCA
AATTTTAAAGTATCTTAAACATAACTGCCCCGTTAGTTTAAGTGCATCCTTTCACAATCTGTCTACAGATTAATA
ATAAAAACTACTCTTTATTATACAGATCTCCATATAATTTTTGAATTTGGTTCTGTAATTTTTTATTTTCTTTTTCT
AATTCCATAACTCTTCTTTTTAAGGTTTTTATAAGGATTTCCTCCGAACGAGAACTTTTCTTGGGTTTTGAGAC
TACATTTGATGTTATTTGACGCTCACGAAGGGATTCGATTCTTTGCCTAATATCGTGTTCCTTATAAAGCCATG
ATTTAGAAACATTAGCTTCCTTTGCTATTGAATTAAAATTAATAGCTTTACCTTCAATCGAAAATTTAGAAATCG
CTTTGTCTACTTTTTCTCTTGTCTTTTGTGATTTCTGCTTCGCCAAACGTACAATTTCTGTTGTATTTCTAACTTG
TTTATTCATTGATAATTACCCCGTTAAACTTCCAATGATTTGTTCTAAACGCTCTTTAATACGGCTATTAGTCTCT
ATTTGTCTTTGCCATTGTTTATCCTTTGCTATGGTTAATAACTCTTCTGTACGCTCTAACTGTTCTTCGTGCTGTG
GTAAGAATTGCTTACTGGTACAGAAGTGAGTGCAATCTAAGCATGCATTCGCATGTGGACAACCACCTGCTAT
TACTGGCAATCTACAATAACCATTTGGAAGCACTTGTGCATTTATATTTTTCTTGAACCATTGAAGCTCTACATC
ATCGACTTCACTATCATCATCTAGATTGAGCACATCTCCATTATTGGTAACCAGTTTTTCTTGAAATTTAGTAAA
TTCATTTTTTAGAGTTTCATCAAAGATATGAGCGTATCTGCTTGTCATTTCTGGGCTTTCATGTCCCAAAAATTT
CTGCACAATATGCTTGGGCACCCCGTTGTTAATCATTCTTGTTCCTACTGAATGGCGAAAGGCATGGGCATGG
AATCTATAAATCTCGCCTAATTTATCCACTATATTTTGCTCATAAGCTAATTTATTTAATTCGCCTCTAAATGTTTC
TTGTTTTAATGGCGATCCATCTTTTCTTGGAAAGAGGTATTCACTATCTGGAAATTCCTCTGAAACTTTATCTTC
CCGAACTTTAATAAGTAAAACTACCTCTTTAGATATTGGAACTATATGCTCCTTTTTCATTTTCCATTGATAATAC
TTTAAAAAGTAATCTCCATCTTTGTCCTCTAATAAACAGCCTTTTTTCAAGGTGCACAATTCACTTATCCTCATT
CCACATTCTTGAACAATCATAGTCATCGTAGCTATATATTCGGGTAATTTATCAAGATGACTGTTCAATTGCTCT
AGAACGAATTCGTCTATAAAGCGAGGTTTTGCTCTTGGTATTTTCGGATAGTCCTCGGGATAAATTAATAATTT
GGAAGGAACATCATCCCATTCTAGCCTATGAAGGGTACTAAATAGTCCTTCCAATATAGAGATCCTCCCAGTTA
TTGTACTAGGTTTTATCCCCATCATGTTTAGTTCACTTAAATATGCTTCAATTTCCACTCTCGTTAATTGGTGTAC
TCTCTGAACGTGTTTAAACTTTATGTCCAGAAAATTAAAGAACTCTTTAAGTTTTTGGGCAATATCACTTACAT
AGGAAAAGCTATCCATGTTCAATCTTAACTTACAATATCTTTTTACAAGCTGTTTAAAATATGTATTCCGAAACC
CTTTAAAGTTAATTGTATATTCATATTGTGTTGGGTTAACCTTATCATCTGGCAAAGGTAAGTTTCGTCTATCCC
AAACGTCTTTATCCCACTCCTCTCCATCAAAATAAAAATCCTCATAAAACTCAATAAATTGTTTAAGATTAGTAA
CATAGTAAGAATTAGCTTTTACAGGTGTTTTTTCTTGATTAGCAGTAATCTTATAATTAGTGGTGGTAATTCTAA
CACCCTGTTTTGTCAAATATGTTCTATACTCCGTCATTGCTTTATCAATAGGAACTTCAGTAATTGAAGTAATGC
TGGGATACTTTAAATCTAAAAAATCCAACATTTTATTAATTACTGTTCCTTTTCTAATCCAGACAGTTTTTGCAT
TCCATATTCCATTGTTTAAATGGTAAAAATAAAAATATTTCAATTCTGTTCTTAACCACAGATTTTTAACACGTT
CAAAACGAACCCAACGATTTCTTAAAGCAGGATTCTTACTTAATTCTATGGCAGAAGGATGTGGACATTTTCT
TATATCCCAACTATTATTAGCCCAAAACCCTTGCATTTCTTCATTCATTACAGCTATTTTTTTGCTGATCTCACTC
TGACTGATAATTTTCCTTTTACTAGAAGCATTCATTTAGCTGGTATTCTTTATCTAGTAATAAATACAATGGATAC
GGCTTATTCTCCACTTCTATCCTTTGAACCCTCACACGCGCCCCCCTCTTTAAACACTTTAAGTACTACAAATTA
TAAACCTTGTTAATTTTTATTTAAAGTAAAATCTCCCTTTAACATCTCCTCCTGAACTACTTTAACTACTATTATT
TATTATACTATGGTTAATATGTCTCCACTTGGGTGGTTATGGCCAATAATCATACCCGTTGCGTTACTTAATATGG
CTGTTTTAAATATTTCTCGAGGATGTATAACCGCAAAATTAATGGTTCCTATAGAGACGGTTTCAATATGTGTA
GGTTCATTTTTGCTATTCAAGCAAATTAAAATGAAGTGTTCTCGGTCAGCATTTCCTATGAAGTCACGCATAAT
ATCAGCAGCGTCTTTTGGTTCTTCAATACGTCTTTTTAAATACCAAAGTGTATCTTCTTTAACCATTTGGACTTT

GACAACATCTATTCTTTTTCTACTCATTATGGATAAGCTCTATACTTTCTATTAAGTCAGGGTGAACAATGGATG
TGATAGAATCATCATTCATATAAATGGAATATAGCTCATCTAATTCTGAATTATTTTTTTTGTTTGATGAAAAGG
CGATAATGCCTTTAATAACATCGATGTGTGGTTGATGCATTAAATATTGGTATACTTCTTTCCATGTTTGATTCAT
TCTATTTAGTCTCCTTTAATGGTTTAATTGTCCATGCCTTTCTAGCGTATGGATTTAATGGATGCATGATTTCATT
TGTTTGAGGATTTGTTAATATACTGATTTTCTTACTGTGAGGTTTTAACATGTCTAATACTTCATTCACTCGATTT
TCCACAAGAGGTTTTTTTCCGTAGGAACCCCAAGCCAATAAAACACTATCGGCTTCTTTGACAGCTTTCATAA
TGTGAATATCCGTGTGCTTGTCATGGCTGTTTTCGATATGTTTTAAATTAATGGGGGTATCAATATTTGAAAAA
AGATTTATAAAATTAACGCCACCATATTCATCTTTTTCAGCTACTTTATTGACAATAAGTTGTGTTGTAAGGTCA
ACGTTGATAACACCATCATAATGAGGATACATCGTTATAATTGTAAGTACTTCTTTATCAATATCCCAAGTTTTCT
TGAGAAGATAACGATGCATTTTATCCTGACTAAATATCGCTTCAGTTTCTAATACACTTTTGATTGATTTCATTT
TATCTAACTCCTTTATTTAATATTCTTCAGGTAACAACATGACGTAATAAGAAAGGTCTACATCATCTTCTCGGA
TAATGTAGACCTTATTAATATTGATAGCTTTTGAATAATTTGCCTTATGAATTTTTACGTACTCTGGACGTTCTTG
CTTATGTTTGATATATAATTGACTATTATGCATATTGAACTGGAATATATGAAAGTAATCAATCTCTTCTAGTTCCT
TGAATTGTTCGTTTTCGCGTTGCGCTACAAGTTTCCATAACTGTTGCTGCAACATTGCTGGCAAGTAATTGGC
AATACTACGTGTAATATAACGTTTCATGCTCATTTCTCCTTTTCTATATCTTTCATTATTGTAATAACTTCTGTAAT
GACTGTGACGCTTATCTGAGCTATGTGTATCCATTTATTCATTATTATAATTCTCCTTTTCAAGTAAGTGTCTTTG
ATCTAGTAGTGGCATAACTGTGTTATGAAGATATAAGAAGTCCATATCGTTTGTTTCATCAATATAACCAATCGC
AATAAGTTGTTTTTGATAGTAGAGGATAAAGGGGTATATATGCCTTTCATGAAGTGTGTCAGAGTAGTAAGTA
TACGTATGAAGGATTTTGTTGGTAAGCGGACCAAGTCTGATATATTGATCAGGAAGGATATTATCTGCCACTT
CCTCGATAGATTCACATTCCCATTTTTCGTCCTTTGGCAGATTGAATAAGTTAGTTATATACTCTTTGAGTTGTT
TTTCTAGTGTCATGGTTATGACCTCCTATAATGTTTTTGATGTATACATGTAATACACCATATAGATAATATATGCT
TCAAAAAAATTTTGTTTCGTTTGTGAATCCCATTGTTATTAAGAAAATATCCCAATAGAAAATTATCACAATTGT
TGATATGACAAGGAAATCCCATTATCCCGCTCATTTCTCAGGAAATATAGCGATATATTTATATATCATTTGGATT
TGGTGGTGTCAGTACGACTTACTTTTTCTTTTTATATTATTTATTTATAAATAATAATGGGATTTCGGGATTACGC
TTGCGTAATCCTTATCCTACATGTATTTATCAAATCCCATTGCTATCCCGTTCATATTTTATTTTGGGATGTTCTTT
CGTCATACAATATAAGCTTCACATTGAGTTGTGACTTGTTAGTATGTTGAAAAGATTCGTTAGATGGGTGTTAT
TGAATGACGAAAGGACAGTACATAGATATTAGATTTGAAGAAAGAGCGTTTGAGGCAAAAATAAAGACGAA
GTGCTGAGGAGCACTTCGTCGAGAGGGGTATTATTGAAAAGTTGTTTAATAATTTCATTATTGAGTTTTAGTG
TAACGTAGAACTGTTTTTTATGATTACCGTCTTTACGAATATCAATACGGTCAATGACTGTAAGATATAATGCTT
TGAGTTGTGCTTTATCCATTGATTCTATATTTTGAAATATTCGTTGTAATAGGGCAGCGATTTGTTTCGTATCAT
AAGATAGTTTCTCTTGATTTTGTTGCTGTTTGAGTTGATTCATTTGATTTGTAATGTCATTGAGTTGTGTTTCAT
ATTGATGAATAGTTGCTTTCAATGCAGATGTTAGGTCCGGATTATCTTCAATGGTTTTAACTAAATTATGGAGT
TTCCCGCTGACTTCATCGTATTGTTGTTGTTTATAAGCGATATCGTGGTTCAATGCACCAATATCGACTTTATTT
TCTTGATTGACACGTTCTAAGACTTGGTTAATGACTTTATCACTTTTGACAATTTCGAGTATTTGATCCATGAC
GTATTTCTCAATCACATCAGCTCTAACGCTATTCGCAGAACATACTTTTGAGCCTTTGTTTCGGAAGTTACTGC
AAGAATAATAACGTATTCGCTTCTTGGTACCATCTTTCAATGTGTTCGTTGTGTTACTAGCTGCCATTGGTGCA
CCACATTGTGGACAATGAACGATACCTGTTAATAGATTAGTTCCTTTACCGTGGACTTGAGGTTTTTGACTGA
CTTGTTTTTTACGTAATTGGACTTTATCCCATAAGTCTTGAATAATAATAGGGGAATGCTTACCTTCAGCTATTA
TTGGTTTATCATTCAGCCCTTTACGACGCTTTTCATTCCAATCTTTGTACTTTGCGAATTGAATTTTACCAACAT
AGAATGGATTAGATAAGATATAGGTCACTGAACCAATACTGAAAGGCTTTCCTTTTTTAGTCACGTATCCTTTG
TGATTGAGTGCATTCGCAATTTTACGATATCCGTGGCCTTTAGCATATGACTCAAATATATATTTGACAATATTC
GCTTCATGTTGGTTTATCATGAGTTCATGCTTGCTATCCGGTATTTTGTCATAGCCCAGCGGCAAATTGCCTTG
ATAATAGCCTTCTTGAGCGCGTCGGGTTTGACCCATGAATACATTTTCGACAATATTATTTCTTTCAAATTCTG
AAAAACTCGCTAGAATTTGTAGCATCAATTTACCACTGCTTGTATTGACTTCCATACGCTCAGATAAGCTGAA

GAATTCGACATTTTGACGATGAAGGTCTTCAACAATTTTGAGTAAGTCAGAAGTGTTACGTGCTAGTCGGTT
TGTTTTGTAGACCATAACAGAATCAATCTGACCTTCGTTCGCATCTTTTAACAAACGTTGTAGTTCTGGTCGGT
TCATAGATTTTCCAGAGATACCTCTATCCGCGTATACATCTACAACAACAAAGTTATTGAAATCACAATATTCTC
GAATTTGATTGATTTGTCCATCGATACTATAGCCTTCAGTACTTTGGATTTCCGTTGATACACGAACATAGATAC
CGACACGTTTTTGTTTGAGTTGTTGCATGATTTTTTCATCCTTTCTGATTAAGCAATCGATGATTGCGAGGTTT
GGTTCACAATGTTCAATGGTTCATTTTTGAAATAGATCCCAACAAGGGCTTTATTTTTTGTAATGCGAATTTCA
TCAATATAGGGATGCAGCATGTTTAACGTGAAACTTTTCTGTATAACCTTTTGTAGTGATGTTTGTAACTGATT
ATCACTTATGGAGGATATGGTTTTGTGCTTTTGATTCATCAAATGAGTCTGTTTTCTGAATGATTCAGCATCAA
TCATACCTTTGGCAAGTTTATCTATCAGTTGTTCTTGAGTTAGCGTACTTTTAGCTTCTATCACTCTTTGTTTTTT
GAGGCGTTGATGAATTGCATTATTAATTTTTGAATAGAGCTGTTGGTTTTGAAAGAAGTTCTGACATGTAGCT
AAGACTTGAACTTCTAATTTTTGTGCATTTATTCCTTTGAATGAACAGACAAAGCGAGATTCATTCATATTTTT
AGGACAAATATAATATCGCAATGTATGTTTTTTTCTTATTGTCATATTTGTCAGTGTTGAGTCACAACAAGGAC
ATTTGATCTTTTGTTTCAGCTGATTCTCTGAAGGTATACAGTGAAGTTGCTTCTTATTACGGATTGCTTGAGCA
TGTTCATATTTCGTTGCCGAAACAATAGGTGGTACCATATTGTTATATTGACCATATTGATTGATGACACGACCA
CAATAATTTGGGTTCATAATTATATTTCGTACCTGATAAGGCTTGCGGGTAATAAGTTTATTATCGTCTTCTAAA
TATTGTGCAATCTTCTTGTAGCCATATCCTTGAAGATAATAATTGAATACAGCTTTGACGGTAGGTGATTCATCA
TTATTAATGATGAAAGTACCATTTTGATAGTGATAACCGAAAGGCGCATGGGTCGTTATGAGTTTACCTTGTTT
TGCCTTTTCTCTAAGTCCATTTTTGACTTGTTCTCCAATATTATCGGATTCAAGTTCAGCCAGACTCATGAATAT
ATTGAGTTTTAGGCGATCAAACGCTTTATCCATATCAAAATAGCCATCATGAACACTTAGAATATGAACATGAT
ATTTTTGACAGAGTTTCATGAGTTTTAATGCATTTTTCAAGTTGCGATGAAGTCGATTCAAGCGATAACAACA
TAATACGTCACATTGTCTTTGTTGGATGCGTTCGGTGACTTGTTGATAGCCTGTTCGATTATCTGTTCTGCCTG
ATTGCTTATCACTAATGTATTGGATATTTTGAATATTGTGCTTTGGAGCTAATAATTCTATTGCTTGCTTTTGAGC
TGGGAGTGATTGTTGTTTCGTTGTACTTTGGCGCAAATAACCAATGGCTCGTTTCATAGTATTTCCTCCTTTCA
AAGAGATAATATATATTTATGAACGAAATTATAGATAAGCCCAACACCAGTCGGGTGTTGGGCGGTATTATTAG
TCATCAGCGTGATTGATTTCTTCAATAACCAGATCGGCTAATAGTATGATTAATTCGTCCATGTATATCACCTCAT
TATTCATAGTATTAACAACAATTCTTTAGTTGCATCTCAAAAGTAGTAACGAATTCGATATTCTTCTTTGAGCAA
ATTTTTTTAAATGAATGTAATCTTCATATTCTCCATATGCAACAATGGACGTAGGTTTGATTACTTTCTCCAAAA
TGTCATTTATTAACTTGTACTCCTCAGTGTAAGTGTTACTTATTTGACGACCTTCTTGTTTACAGCATTCCTTCA
CAGTATCTAGGGGTGTGCTTTCTAAGTATTCAAATCTTTCATCTTCAATTTCGTGAGCTAGTGCTACTGTATTCA
TAGTACAATTCCTCCATTGATTTAATATTTGTGCTACAACACCTTTAAATAGGTGTTGTAGCAAATTTATTTAATA
ACATATTTTAAGCTTTAAAAGTCAATATCACCGTTTTTATCCCATTCTCTTGCTTTTGAAATCATGTCTCGTAGTA
TTGGATGTTGGTGTTCAGAGATAAATGATAAGGGTATATTGAATTTACTTTTTTCAGATAGACCAAAAATAGG
AGCGTATGATTTATCAAGCTTAAGATGATAAAAATACGATGGTTTTTGGTTTGCCTTCTTTGTCTTTGACACTAC
GTTTTGTTGTTTTACGATTGGCGTCTGACTGGATGTAATCTTTATCCCTCAGTGCATCAATGACATTATTTGTAT
CTTGGAAATTATGTTCTTTTAACATATTTTTAAAAACCGAGGCAATCATTTTCACCTGGATATGATCATCTTCAA
GTGAAATCATTCCATAATTTTCAATAAGCGTTGATAACCTAGTGTCGTCAGAAAACTTACCACGATTTTGTGCT
ACAAATTGGACAATGACTTCAATTGCTTTATCAGCTAGGCTTCGCTCACTAACAGAATCTAAATGATAGTTAAT
AAGATAGTCCCTTACAGCATTCAAATCAACAGGTGTGGCAATGACACGTTCCAATATACGTGCTGATGTTGTA
ATGGTTGCATAGCGTTTGAACATCCGAATTCCTGTATTGCTGGTTTCCTTTTCTAGTTGAGTTTTGAACCTTAG
GTGCTCATTTTGGAACCATTTAATGACTTCTTGTTCACGATTGAGTAGATATTCAGCTACTAATGGCATGATAT
GACCATAATTAGCTGATGTTGCTTTTTTAATAGTATCTGCGTTGTCAGCGTTAGTTGTAAAAGCTTCAGATATT
TCAATGGTACGTACATTCAAACCATCATTACGTGCACTATTATTGAAAATACTGTGTTCGGCAGTACTAATTACT
GAGGTTCCCCAGTTTTTTAGTGACTTGACTTCTCCGTGTATATTAGAACGTTGTCTTCCTTGACCTTCGGCAAT
TGAATAGAGCAAACCAGTTGTATCTTTAAAGGTTGCAGATGACAGTTCGTCCAATACAATAGGTACACCATAG

TTGTTACTTAGATATCCTTCAAGCGCGTTTCTAGTGCCATTCCATGATCTAAACAGCGTATTACTACCTTTAGTA
GGGTTGCCGGTTATTGAGACTCCTAAAGCCGCTGCAGTTGATTTACCCGTGCTTGAATTTCCCGTAAATGAG
AAAATCGTACCTGCGAATTCAACCTCATGTTTTGTTTTTAAAAACGCAGTTACTAATGAAGAAATACCAAACA
CTACAGCAAGTTCGAGTAACAAGTTTCCTTTTACTTCATCAAGATACATTTGCCACCAGTCTTCAAAAGTGCC
TTTAGGTTGTAGATCGTACTGTGTTTCACAAATGATTTCATCCGCCTGCGACTGTGTAATTTCTTTTGAAAGAT
ATGGTTCATCTAATGAAATGACGACACCTTTATCAGTATTTAACACACCGACACCCGTATACAACGATGACAG
AGGCAGTGATTGGCGCATTAATTGTAGTGCATTGCTTAATGATTTAATGTACTTCTCATTGATGCTGAAGCCAT
ACATGATTAGTGAAGGTAACTTTTGCGATGTTAAAATATCTGATGTTTCAATACGTTCAATATTTTGTCCGTTG
GAAATAACTAGTTTCTCAACCCCAGATTTGGGGTCAAGAAACTTGTTTTTAATTAAAATTGGACTAGATAAGC
GTATGGACTTTTGCTGATCATTTTTACCTTTCGGTGGTATAACTTCATACCATGAATCTTCAGTAAGCCAGTATG
GATATTCACTAAAGATGATGTTAGTCATTAGTTGTTACCACCTTTCCAGTTATTTTGATGATTCATCGGATTTGG
TGCAGTTTTAAGGTAGAGCAAAGATGAACCTTTTTTTCCAACTACAATGAATGGAAGGTTAGGTGTATATTTT
ACAAAATGTGCAAACCAACGACCAACATCTTGTTGAATTTCAGTCGAGCACAAAACATTAGCCCTCACATTC
AAATCGTGATACATAAACATGGTGCCTACAGGTAAATTAAATGCGATGCTTGTAACAGCACGTTTTAGTGTATA
AAATTCTTTTTCTTTCATCATAAGTTCCTCCAAATAAGTCTTATTAGTCAATCAATACTGTAGATGCTTAACACCT
CAATGGTTGATTGCTATAATAAGCATAAATGAAGCATTTTAATTAGACGCGGAACAAAAAATAGGGAGGGGG
GTATATATGATACCTACGATAGAAGAAATTATTGACAAGTATGGCAGTTTAGTAGATTGTCTAAAGTTTGATATA
TCAGGTGAGGTTTATGAAGACTTATTGTTATTAAGAAGTTTAAGCGATTTAAATGAACACCAAAAGATGCATA
GAGTTTCATTTATACAAAAACACTTTAACCCTAATTTTGATTATTTGTTAGATGACTCAGAATTTAGAAGTATG
AATAAAATTTATAGAAATTTAAATACTATGACGTATATCAATAGTCAAAACAATGAATTTAAACTTAAACCATTT
ATAAATGAAGAACAATTAAAATCACTACTTGCGATAAAGAAAGTAGATGAACATTTGAGTTATGACCCATCTA
TTACCTCAAAAGCATTAGCAGATATAAACAAGACGCAAAAAAGATTGGTTACAAAGCCAAATATTCTCTATAA
TCGATCCAAAAAAGAAAATGAGTATGTACGGTTAGGAGGAAAAATATCATATACAAAGATGGAGAAGCATTG
GGAATATTTACATAATGAGATTCAGTATTATAATCTTACTAATCAATTAATTAGTTATGTTGCTTTAGAAAAGGA
ATATGCATGGGCTTTTTTAAATGAATTATTTTATTTAATAGAATTATATTTGAAGGCATTTAAAGGACAAAAGA
AAACTGGCCATGAATTTGAAGAAGGATTGAACGCATTTATTCAATCATATGTCATTATTTTACTTATAGATATAA
GATTACCGACTGTTAGGTTATATATTATTAGGCGTATTGTGGATGAATGCGAAAAAGAATCAGATAATCAAAA
AAGAATTAAACTGATAGAGGAATCCATTAAAAAGTACAGATATGTAAAAGAACAGATTGAAAGATTTGGAAA
TGCGTTGAAAGAAGAATTATCAATCGCGACTTTGTCTATTGAACAGAATATGTTAGAAGCTAAAATTGATTATT
ATAAGAATTACTACTATCCTAGAAAAAAAACAAAGCATAACAATATTGAGTACAACGTATCAATGTTTTTTAAA
GCTGTAGATTCTTTAAAGAAATAGTATTCGACGGACAATATTTATAGATAATAATTAATTAGAGAGGTTTTAAG
TTGTGTTTAGATTAAAAAAATGGTATTTAAAACAAAAAGGCTATTTTTGCCGTGTCGGTGAAAAGTGGGACA
ATAGTAATTTATCCCATGAAAATATACATAGTTATGTAGATAGAGTACTTGAATATGAGACTGAAACACGTAGA
AACGGCCAACCAACTGAAGAATATATATTAGCTAGTAGTGGGGAAAAAATAAATGAAGATTTAAGAAGTGGT
ACTAATGATCACAAAGAAATTCTAGAGGTATTTGATAAATATAAAGTAAAAGAGGATATAACTACTTATAGAAC
GGTTAGTCCTCGGATATACAAATTAATGGAGAAAAATGCTAAAAACTTAAATGGAGTAGATTTGTTTGAACTT
GGAATTTTACACACTTCATTAATAAAAGGGTACGAATCAAGAGAAGAAGGTTACAAATTACGGGTAAAAGTA
AAAAAAGGAACACCAGCTTTCTATGTAGGTAATTTAACAGGCGAAGAAAGCCATTATTATGAGGTGATTGTA
GTTAATAATTTAAAATTAAAAATCATATCTATTGAAGATTACTATATAAATTGTGAAGTAGTATAATTGGAATCAA
TTCTGTTAGTTGTTCATAATAAGCCAATTATTTTTTTAATGAACTTATTACTTAAAAACCGCATCATCAACTGATA
AGCAGAAGCGTATCATAAAT
>Mammaliicoccus sciuri strain GDK8D6P
GAAGCTTATCATAAATGATGCGGTTTTTTTAGCCCCTTTGTAAAGGGATTTTGACCGTATCAGAACGTATGAG
GTTTTTAAAAAAAGTACTTAAGTTTACTTGAAGCATATCATAAATGATGCGGTTTTTTCAGCCGCTTCATAAAG

GGATTTTGAACGTATCGGAACGGGTTCTGTTGCAAAGTAAAAAAATATAGCTAACCACTAATTTATCATGTCA
GTGTTCGCTTAACTTGCTAGCATGATGCTAATTTCGTGGCATGGCGAAAATCCGTAGATCTGAAGAGACCTGC
GGTTCTTTTTATATAGAGCGTAAATACATTCAATACCTTTTAAAGTATTCTTTGCTGTATTGATACTTTGATACCT
TGTCTTTCTTACTTTAATATGACGGTGATCTTGCTCAATGAGGTTATTCAGATATTTCGATGTACAATGACAGTC
AGGTTTAAGTTTAAAAGCTTTAATTACTTTAGCCATTGCTACCTTCGTTGAAGGTGCCTGATCTGTAATTACCT
TTTGAGGTTTACCAAATTGTTTAATGAGACGTTTGATAAACGCATATGCTGAATGATTATCTCGTTGCTTACGC
AACCAAATATCTAATGTATGTCCCTCTGCATCAATGGCACGATATAAATAGCTCCATTTTCCTTTTATTTTGATGT
ACGTCTCATCAATACGCCATTTGTAATAAGCTTTTTTATGCTTTTTCTTCCAAATTTGATACAAAATTGGGGCAT
ATTCTTGAACCCAACGGTAGACCGTTGAATGATGAACGTTTACACCACGTTCCCTTAATATTTCAGATATATCA
CGATAACTCAATGTATATCTTAGATAGTAGCCAACGGCTACAGTGATAACATCCTTGTTAAATTGTTTATATCTG
AAATAGTTCATACAGAAGACTCCTTTTTGTTAAAATTATACTATAAATTCAACTTTGCAACAGAACCGTATTATG
GAATAGAGATGTTGGTAACATTTATACAGGATCATTATACTTAAGTTTAATTTCGTTATTACAGAACCACACATT
CCAACCAGAAGAGAAAGTATGTCTATTTAGTTATGGTTCAGGAGCAGTAGGAGAAATCTTTAGTGGTTCAAT
CGTTAAAGGATATGACAAAGCATTAGATAAAGAGAAACACTTAAATATGCTAGAATCTAGAGAGCAATTATCA
GTCGAAGAATACGAAACATTCTTTAACAGATTTGATAATCAAGAATTTGATTTCGAACGTGAATTGACACAAG
ATCCATATTCAAAAGTATACTTATACAGTATAGAAGACCATATCAGAACATATAAGATAGAGAAATAAACTAGT
GGCCGATTGTGCTTGATGAGCTTGGGACATAAATCCTAACTCGAAATAAATAAGCATATCACTAAACTGATTT
TTTAAAGTTTACAGTGATATGCTTATTTTTTTATCTTACGATTTTGTACGTGCATGCTTGCCTAGGGGTATGGCT
CGAGCCATTAGTCTCTCGCACATACTATTCCCTCAGGCGTCAGCACTTACAAAATCGGTTGTAATTTTCATTTT
TATACGCATTCTTACTGAGATTATACTAATAAGAGGAATAGTAAAAGCAATTCTAAGTAAAATTGCAGATAAGA
GGTTTGTTAAAAGCAGTTCTAAGTAAAATTGCAGATAAGAGGTTTGTTAAAAGCAGTTCTCAGTAAAATTAC
AGATAAGAGGTACGTTAAAAGCAGTTCTCAGTAAAATTGCAGATAAGAGGTTTGTTAAAAGCAGTTCTAAGT
AAAATTGCAGATAAGAGGTACGTTAAAAGCAATTCCATGCAAAATTGCTGATAAGGGGTAAGTTAAAAGCA
GTTCTCAGTAAAATTGCAGATAAGAGGTACGTTAAAAGCAGTTCTAGGCAAAATTGCAGATAAGAGGTGCG
TTAAAAGCAGTTCTAAGTAAAATTGCTGATAAGGGGTAAGTTAAAAGCAATCCTAAGTAAAATTGCAGATAA
GAGGTAAGTTAAAAGCAATCCTAAGTAAAATTGCAGATAAGGGGTACAGAAAAACTAGACTTGATTACAAA
ATGGAGCTTGGGACATAAATGATTTTTTAAAAATGAGATGAGACGTAGATTAACTCCATAATCAATACGAATC
TATCGACTTCTTTATTTATGATATTCATCTCTTTTTAATGGAAATAAAAGTGCGATTAATGTGATAATACAGTTAC
GTTAATTAAAAAAATAAAAATGCAAGGAGAGGTAATATGCTAACTGTATATGGACATAGAGGATTACCTAGTA
AAGCTCCGGAAAATACAATTGCATCATTTAAAGCTGCTTCAGAAGTAGAAGGTATAAACTGGTTGGAGTTAG
ATGTTGCAATTACAAAAGATGAACAACTGATTATCATTCATGATGATTATTTAGAACGGACTACAAATATGTCC
GGGGAAATAACTGAATTGAATTATGATGAAATTAAAGATGCTTCTGCAGGATCTTGGTTTGGTGAAAAATTC
AAAGATGAACATTTGCCAACTTTCGATGATGTAGTAAAAATAGCAAATGAATATAATATGAATTTAAATGTAGA
ATTAAAAGGTATTACTGGACCGAATGGACTAGCACTTTCTAAAAGTATGGTTAAGCAAGTGGAAGAACAATT
AACAAACTTAAATCAGAATCAAGAAGTGCTCATTTCAAGCTTTAATGTTGTGCTTGTTAAACTTGCAGAAGA
AATCATGCCACAATATAACAGAGCAGTTATATTCCATACAACTTCGTTTCGTGAAGACTGGAGAACACTTTTA
GATTACTGTAATGCTAAAATAGTAAACACTGAAGATGCCAAACTTACTAAAGCAAAAGTAAAAATGGTAAAA
GAAGCGGGTTATGAATTGAACGTATGGACTGTAAACAAACCAGCACGTGCAAACCAACTTGCTAATTGGGG
AGTTGATGGTATCTTTACAGACAATGCAGATAAAATGGTGCATTTGTCTCAATAGAAAGTTAGAGGTGAGTCT
TACGTTTCAGTGACGGTAGACTTACCTTTAACATGTTACATACTAAAAAATTAATTTGAATAAGAAAGAGAGA
CATATATGAAATACGATGATTTTATAGTAGGAGAAACATTCAAAACAAAAAGCCTTCATATTACAGAAGAAGA
AATTATCCAATTTGCAACAACTTTTGATCCTCAATATATGCATATAGATAAAGAAAAAGCAGAACAAAGTAGAT
TTAAAGGTATCATTGCATCTGGCATGCATACACTTTCAATATCATTTAAATTATGGGTAGAAGAAGGTAAATAC
GGAGAAGAAGTTGTAGCAGGAACACAAATGAATAACGTTAAATTTATTAAACCTGTATACCCAGGTAATACAT

TGTACGTTATCGCTGAAATTACAAATAAGAAATCCATAAAAAAAGAAAATGGACTCGTTACAGTGTCACTTTC
AACATACAATGAAAATGAAGAAATTGTATTTAAGGGAGAAGTAACAGCACTTATTAATAATTCATAATAAAAC
AGTGAAGCAACCATCGTTACGGATTGCTTCACTGTTTTGTTATTCATCTATATCGTATTTTTTATTACCGTTCTCA
TATAGCTCATCATACACTTTACCTGAGATTTTGGCATTGTAGCTAGCCATTCCTTTATCTTGTACATCTTTAACAT
TAATAGCCATCATCATGTTTGGATTATCTTTATCATATGATATAAACCACCCAATTTGTCTGCCAGTTTCTCCTTG
TTTCATTTTGAGTTCTGCAGTACCGGATTTGCCAATTAAGTTTGCATAAGATCTATAAATATCTTCTTTATGTGT
TTTATTTACGACTTGTTGCATACCATCAGTTAATAGATTGATATTTTCTTTGGAAATAATATTTTTCTTCCAAACT
TTGTTTTTCGTGTCTTTTAATAAGTGAGGTGCGTTAATATTGCCATTATTTTCTAATGCGCTATAGATTGAAAGG
ATCTGTACTGGGTTAATCAGTATTTCACCTTGTCCGTAACCTGAATCAGCTAATAATATTTCATTATCTAAATTTT
TGTTTGAAATTTGAGCATTATAAAATGGATAATCACTTGGTATATCTTCACCAACACCTAGTTTTTTCATGCCTT
TTTCAAATTTCTTACTGCCTAATTCGAGTGCTACTCTAGCAAAGAAAATGTTATCTGATGATTCTATTGCTTGTT
TTAAGTCGATATTACCATTTACCACTTCATATCTTGTAACATTGTAACCACCCCAAGATTTATCTTTTTGCCAACC
TTTACCATCGATTTTATAACTTGTTTTATCGTCTAATGTTTTGTTATTTAACCCAATCATTGCTGTTAATATTTTTT
GAGTTGAACCTGGTGAAGTTGTAATCTGGAACTTGTTGAGCAGAGGTTCTTTTTTATCTTCGGTTAATTTATT
ATATTCTTCGTTACTCATGCCATACATAAATGGATAGACGTCATATGAAGGTGTGCTTACAAGTGCTAATAATTC
ACCTGTTTGAGGGTGGATAGCAGTACCTGAGCCATAATCATTTTTCATGTTGTTATAAATACTCTTTTGAACTT
TAGCATCAATAGTTAGTTGAATATCTTTGCCATCTTTTTTCTTTTTCTCTATTAATGTATGTGCGATTGTATTGCTA
TTATCGTCAACGATTGTGACACGATAGCCATCTTCATGTTGGAGCTTTTTATCGTAAAGTTTTTCGAGTCCCTT
TTTACCAATAACTGCATCATCTTTATAGCCTTTATATTCTTTTTGTTTTAATTCTTCAGAGTTAATGGGACCAACA
TAACCTAATAGATGTGAAGTCGCTTTTTCTAGAGGATAGTTACGACTTTCTGTTTCATTAGTTGTAAGATGAAA
TTTTTTTGCGAAATCACTTAAATATTCATCCATTTTTTTAACGGTTTTAAGTGGAACGAAGGTATCATCTTGTAC
CCAATTTTGATCCATTTGTTGTTTGATATAGTCTTCAGAAATACTTAGTTCTTTAGCGATTGCTTTATAATCTTTT
TTAGATACATTCTTTGGAACGATGCCTATCTCATATGCTGTTCCTGTATTGGCCAATTCCACATTGTTTCGGTCT
AAAATTTTACCACGTTCTGATTTTAAATTTTCAATATGTATGCTTTGGTCTTTCTGCATTCCTGGAATAATGACG
CTATGATCCCAATCTAACTTCCACATACCATCTTCTTTAACAAAATTAAATTGAACGTTGCGATCAATGTTACCG
TAGTTTGTTTTAATTTTATATTGAGCATCTACTCGTTTTTTATTTTTAGATACTTTTTTTATTTTACGATCCTGAAT
GTTTATATCTTTAACGCCTAAACTATTATATATTTTTATCGGACGTTCAGTCATTTCTACTTCACCATTATCGCTTT
TAGAAATATAACTGCTATCTTTATAAACTTGTTTGAAATTTTTATCTTCAATTGCATCAATAGTATTATTAATTTCT
TTATCTTTTGAAGCATAAAAATATATACCAAACCCGACAACTACAACTATTAAAATAAGTGGAACAATTTTTATC
TTTTTCATCAATATCCTCCTTATATAAGACTACATTTGTAGTATATTACAAATGTAGTATTTATGTCAAAATAATGT
TATAATTTTTGTGATATGGAGGTGTAGAAGGTGTTATCATCTTTTTTAATGTTAAGTATAATCAGTTCATTGCTC
ACGATATGTGTAATTTTTTTAGTGAGAATGCTCTATATAAAATATACTCAAAATATTATGTCACATAAGATTTGGT
TATTAGTGCTCGTCTCCACGTTAATTCCATTAATACCATTTTACAAAATATCGAATTTTACATTTTCAAAAGATAT
GATGAATCGAAATGTATCTGACACGACTTCTTCGGTTAGTCATATGTTAGATGGTCAACAATCATCTGTTACGA
AAGACTTAGCAATTAATGTTAATCAGTTTGAGACCTCAAATATAACGTATATGATTCTTTTGATATGGGTATTTG
GTAGTTTGTTGTGCTTATTTTATATGATTAAGGCATTCCGACAAATTGATGTTATTAAAAGTTCGTCATTGGAAT
CGTCATATCTTAATGAACGACTTAAAGTATGTCAAAGTAAGATGCAGTTCTACAAAAAGCATATAACAATTAGT
TATAGTTCAAACATTGATAATCCGATGGTATTTGGTTTAGTGAAATCCCAAATTGTACTACCAACTGTCGTAGT
CGAAACCATGAATGACAAAGAAATTGAATATATTATTCTACATGAACTATCACATGTGAAAAGTCATGACTTAA
TATTCAACCAGCTTTATGTTGTTTTTAAAATGATATTCTGGTTTAATCCTGCACTATATATAAGTAAAACAATGAT
GGACAATGACTGTGAAAAAGTATGTGATAGAAACGTTTTAAAAATTTTGAATCGCCATGAACATATACGTTAT
GGTGAATCGATATTAAAATGCTCTATTTTAAAATCTCAGCACATAAATAATGTGGCAGCACAATATTTACTAGG
TTTTAATTCAAATATTAAAGAACGTGTTAAGTATATTGCACTTTATGATTCAATGCCTAAACCTAATCGAAACAA
GCGTATTGTTGCGTATATTGTATGTAGTATATCGCTTTTAATACAAGCACCGTTACTATCTGCACATGTTCAACA

AGACAAATATGAAACAAATGTATCATATAAAAAATTAAATCAACTAGCTCCGTATTTCAAAGGATTTGATGGAA
GTTTTGTGCTTTATAATGAACGGGAGCAAGCTTATTCTATTTATAATGAACCAGAAAGTAAACAACGATATTCA
CCTAATTCTACTTACAAAATTTATTTAGCGTTAATGGCATTCGACCAAAATTTACTCTCATTAAATCATACTGAA
CAACAATGGGATAAACATCAATATCCATTTAAAGAATGGAACCAAGATCAAAATTTAAATTCTTCAATGAAATA
TTCAGTAAATTGGTATTACGAAAATTTAAACAAACATTTAAGACAAGATGAGGTTAAATCTTATTTAGATCTAA
TTGAATATGGTAATGAAGAAATATCAGGGAATGAAAATTATTGGAATGAATCTTCATTAAAAATTTCTGCAATA
GAACAGGTTAATTTGTTGAAAAATATGAAACAACATAACATGCATTTTGATAATAAGGCTATTGAAAAAGTTG
AAAATAGTATGACTTTGAAACAAAAAGATACTTATAAATATGTAGGTAAAACTGGAACAGGAATCGTGAATCA
CAAAGAAGCAAATGGATGGTTCGTAGGTTATGTTGAAACGAAAGATAATACGTATTATTTTGCTACACATTTA
AAAGGCGAAGACAATGCGAATGGCGAAAAAGCACAACAAATTTCTGAGCGTATTTTAAAAGAAATGGAGT
TAATATAATGGATAATAAAACGTATGATATATCATCTGCAGAATGGGAAGTTATGAATATCATTTGGATGAAAAA
ATATGCAAGTGCGAATAATATAATAGAAGAAATACAAATGCAAAAGGACTGGAGTCCAAAAACCATTCGTAC
ACTTATAACGAGATTGTATAAAAAGGGATTTATAGATCGTAAAAAAGACAATAAAATTTTTCAATATTACTCTC
TTGTAGAAGAAAGTGATATAAAATATAAAACATCTAAAAACTTTATCAATAAAGTATACAAAGGCGGTTTCAAT
TCACTTGTCTTAAACTTTGTAGAAAAAGAAGATCTATCACAAGATGAAATAGAAGAATTGAGAAATATATTGA
ATAAAAAATAAAATTGTTGTGTTTACAACAATACATAGAAAACAGAGGAAACAATCAAGTCGTTGAATATTTC
CTCTGTTTTTTAGTTGAAAAAATTAACCGAAAGCCTGAATGCAAGTCTTGATTAAATCAATAATGCTTGTAATA
ACACCAGTGAAATCCATATGCATACCCTCTTTCTATTTAAGATACATTAAGTATAATATCAAACAAATAAAAAAT
GTTAAAAATTCCCTAATTGGCTATTTAGATTGCATAAATGTCAAAAATTTGAAAAACATACAACGACTTTGCAT
AAAAAATCGTCATATTGGAAATACGTAATTTATTGAAATAATAAAAAAAATAAAAGAACGAAGATGATAACCT
AAGTGAGGTTTTAAGTTGTTCTAAGGTTTAATTTAATTTATGTTAAAATAGTTGGTATAAAAATACATGATAAA
CTATAAACTAAATTCAAAATAACTTATGGGGTAGGCAATTATGGAAAATATTTTAAATATAAATGATAATGAAA
AAAGAGTGCTAAGGGAAATTTATAACCATCATAATATTTCGCGTACTCAAATATCTAAAAATCTTGAGATTAAT
AAGGCAACGATTTCTAGTATTTTGAATAAGTTAAAGTATAAATCTCTTGTTAATGAGGTTGGTGAGGGTGATA
GCACGAAGAGTGGTGGTAGAAAACCTATTCTTCTGAAGGTTAATCATCTTTATGGTTATTTTATTTCTTTGGAT
TTAACTTATAGTTCTGTTGAAGTGATGTACAATTATTTTGATGGTAATGTCATTAAGCATGAATCTTATGATTTAC
CTGATGAAAAGGTTAGTAGTATATTAAGCATAATAAAAAAACATATTGATATTCAGGAGAAACTTGATACTTAT
AACGGACTATTAGGTGTGTCTGTTTCTATACATGGAGTTGTGGATAATGAGCAGCATGTGACATATTTACCATT
CCATGAAACTGAAGGAATTTCAATTGCTAAGAAAATAAAAGAAATTACTAATGTTCCAGTCGTAGTTGAAAAT
GAAGCGAATCTTTCAGCGTTATATGAACGTAATTTTAATCATAATTTATCCTACAATAATCTTATTGTTTTAAGTA
TACATAAAGGTATTGGTGCTGGGCTTATTATTAATAATCAATTGTATCGTGGTGCAAATGGGGAAGCGGGTGA
AATTGGAAAAACACTTGTCTCAAAAGTTAGCGATAATGTGGAGATCTTTCATAAGATTGAAGATATTTTTTCA
CAAGAAGCTTTACTGCATAATTTAAGTAATCAACTAAATGAGAAGATGACGCTTAGCAAATTAATTCAATTTTA
TAATGAAAAAAATCCAGTCGTAGTTGAAGAAATGGAACAATTTATAAATAAAATTGCTGTTTTAATACATAATT
TAAACACCCAGTTTAATCCGAATGCAATTTACATTAACTGTCCATTGTTCAATGAAATGCCTGAAATATTAGAA
GCAATTAAGAACCAGTTCAAACAATATTCACGTAACGAAATTCAAATAAAGTTAACATCTAATGTCAAATTTG
CAACTTTGCTAGGTGGTACATTAGCAATTATCCAAAAAGTACTACAGATTAATGATATTTACTTAGATATAAAA
GCATAAAAAACTAATTCAAATGAATAATCAAAGTTCGTAATTGTCTTTATAAAAAAATCCCTCAATCCGAATTG
AATTTTCGGATTGAGGGATTTTTATAGTTCTATTGCAGAAGAAAACTATTTTAAAAATGCTGGTAAATGTTGAT
AGCCACCTCTAACGTTAACAATATTCGTAAATCCTTTATATTCTAATATTCCTACCGCTATTGAACTTCTAACACC
TGATTGACAATGTACATAAATTAGGTCATTTTTATCGAAAGGTATATCTTCATTTAAAAGTTTACCGTGAGGAA
TATGAATTGCTTGTTTTAAATGACCTTTACGCCATTCATCATCATTACGAACATCTAATACATTATGTTCTTCACC
AGTCATTTCAGAACTATGAATAGATGATGTGACGATATTTGTTTGTGGCAAACGGTAACTTTTTACATTTTCAA
AACCAATTAATTGTAAAGCATGAATAGCTTGTTGAACGGTAGATTTATCGCCAATTAATTCAATATCTTAGTCAT

AATCTAAATACCAACCAATTTGATTTATAAAAGTTTTATTAAAAGGAATATTGATAGTTCCATGCATATGACCAC
CATGGAATGCTTCTTTACTTCGAAGATCAAAAGAAGTTTGTGTATTGCTTGAACTAGGGTAAACATTATATGG
TTGGTACATTTGCATACCAAATTGATTTATTTTTTTCATTTGTGAAAAATGGTGTGGTGGAGCTGGCTGATTGA
GTGTTAAAGTTTCGATAAATGAAGTTTCATCTTTAACATTAAAAGCCCAGTTGTTTATTTTCTCATAACCCAAA
GTAGTTGTAGGTAATGAACCTAGCGCTTTACCACAAGGACTCCCTGCACCATGACCTGGCCAAATTTGAATAT
AGTCTGGTAATGTTGCAGCAAATTGTATGGACTGATACATTTGTTTTGCTCCGATTTTTGTAGAACCTTCAACA
TTTACAGCTTTTTCTAATAGATCTGGTCTACCTACATCACCAACAAAGATGAAGTCACCGCTAAATAATCCCAT
TGGTATACTGGAACCCCCACCTTCGTCAGTAAGTAAAAAACTAATACTCTCAGGGGGATGGCCTGGAGTGTG
TAAGACTTCTAATTTAATCTTTCCTAAATAGATAATATCTTGATGCTTAACGAAATGTGTTTGTTTAGGCATATTT
TTATAATTAAATTCATCTTTACCTTCATCAGATACGTATATACTTGCATTCAATTTATTTGCCACATCTCTAATACC
TGAAGCAAAATCAGCATGAATATGTGTTTCTGCAGCTTTAGTAATTGTGAATCCTTCTTTATCTGCAACTTTTA
AATATTTTGTTAAATCTCGTATAGGGTCAATAATCATTGCTTCTCCTGTACGTTGACATCCAATTAAATAAGATG
CTTGTGAAAAATTGTCTTCATAAAATTGTTTGAAAAACAAAAAAAACTCCTTTTTAAAATAGATTTTATTGATT
AGATAAATAAGTTATGATTTGCTTGCTCAGTATGTCCAATATAAGTACCTACGCCACCATAATCGACTTCATCTC
TTAATTCTTCTTTTGAAATTCCCATAACATCCATACTCATGGTACAAGCAATTAACTTTATATCTTGATCGATTGC
TTGATCGATAAGTGAGTATAAAGAATCAACATTTTTCTTGTTCATTACATAACGCATAATAATATTACCTAGTCC
AAACATATTCATTTTTGATAATGGCATATGTATTGGATCCTTAGGTAACATAAGGTCAAACATTTTGGAAATACC
TTTCTTTTTAACGCGAGTTGATTGCGCTTTTTTTAATGCGTTGAGGCCCCAAAAAGTAAAGAAAATAGTTACA
TCTTTACCTGCTGCTTTAGCGCCATTTGCGATGATCATTGCTGCTACTGCCTTATCTAACTCACCGCTAAATAAA
ACAATTGTTGTACCTGTAGCAGTGTCATTGATTTCAAATTCTTTTGGCTTTTCTTTTTGAATAATTGCATTAATT
ACATTTGCTTCTTCAGTAAGATTTACAAGGGTATTCCCTGTTTGTTTCGCCCAACTTTTAATATCACTATTGAAA
CCAGGATCTGTAACTGTTACCTCGATTTGCTCACCCGTTGAAATATTGTTAATTTCTTTACTGATATTAACAATA
GGTCCAGGGCACTGAAGACCTCTAAAATCAAATTGTTTACGATTCTCTTTGATTTCAATATCTTTTTCTATTAA
AGGAGCACTATTGAAGTTCTTTGCTTCATAATCTTTATATCCACCCTTTAAATTCACGACATCATAACCTTGTTT
GGCTAAATAATCGCAAGCTTTAGTGCTTCGGTTACCGCTTTTACAATGTATATAATACGTTTTGTTGCTATTCTT
ATTGAATGATTTAATCTCTTCTACTGGGTGTAAAGTTGAACCGTTAATGTGTCCTAATTCATATTCTTCTTTTGT
TCTAACATCAATCAATTGACCCATTTTTGCCAATTTTTCTAATTCTTCTTTGTTTAATGAATTAATGTGTACTTCT
TTGTATTGTTCCATACTTACCTCCTATAAATACCTATGAGGGTATAATAAAACGGATAGAATTATTTGCCAAATAC
CTATAGGGGTATTTGACAATTTTGTTTTAATTTATTATTATTAACTTAATCAATTTATGTGGAGGAAATGAATATG
ACTTATGATAAAAAAATGATTAATCGTATAAATAGAATACAAGGTCAATTAAATGGTGTCGTAAAAATGATGGA
AGAAGAAAAAGATTGCAAAGATATAATTACGCAACTTAGTGCATCTAAAGGTTCTATACAACGTTTAATGGG
GATTATAATTAGTGAAAATTTAATAGAATGCGTTAAAACAGCAGAAGAAAATAATGAAAGTTCTCAAGAATTA
ATTAATGAAGCAGTTAATTTATTAGTTAAAAGTAAATAATGGATATAGCAAATATGACTATTATGTTGCTAATTG
GCGTACTGGGTGGATTTATATCTGGATTAATAGGTATTGGGGGCGCAATTATTATTTACCCAGCTATTCTTATAT
TGCCACCATTAATAGGTATACCTGCGTATAGTGCATATATTGCTTCGGGACTTACCTCTAGTCAAGTATTTTTCA
GTACACTTAGTGGATCATTAAATGCAAGAAAACAACCAGCTTTCTCTCCTAAACTTGTTATATATATGGGAGG
GGGTATGTTGATTGGAAGCATGTTAGGGGCAATTTTAGCTAGTTTGTTTAATGCTACTTTTGTAAATACGGTAT
ATGTAATAATCGCCATACTTGCTTTAATATTGATGTTTATTAAAGTTAAACCTACTACACAAGAGACGAAATCTA
AACCTTTGCTATTTATTATAGTTGGATTTGGAATTGGTGTAATTTCGGGAATTGTGGGTGCAGGTGGAGCATT
TATCATCATTCCTGTATTATTAGCATTATTTAAATTACCAATGAATACGGTAGTGAACAATAGCATAGCAATTGCT
TTTATATCTTCAGTAGGGGCATTTTTTATAAAATTAATGCAAGGATATATACCAGTAGAAAGTGCAATTTTTTTG
ATAATTGAATGATGATCATTTTCTGAAAATATTATGTGGTCATATAATATAATGCCCATCATTTCACTAATCTCTTT
TATTCTCTGAGTTATTTTGATATCTCCTGGAGAAGGTGTCGGATCTTATCAAGAGTAAATTATGAAAAAAATGG
TTTAAATCAATGTTCTTACTTTAAGTACATGTGTTTAACGTAAATGATAATTAGTTAAATATAAAAAGGTTATCC

CAATCAATAGGATAACCTCTTTATACTTTACTTTATGGTTTGGATTCGCCACTTCAAAGAAGAAATGAAATCAG
AGCATTGAATGTTCCATTTTCAAACAATATATAAATACCTAGCCCAATGAATACAATTGGTACAATCCAACGTTC
ATATTTCTCAATTGTTTCCGATATAAAATCGAAGGAAGCTAGACGGTAACTGACATAGCACAAAACTCCAACC
ATAATTAGAAAGACAATAGTGACAATAAAGATTTCAGACATACTTAAGGTCGTGAAGTACGGTATATAAATGG
AAAAGTCATCCGCACTGGAAGCCAATACGATGAAAATCATCGTCAAAAATAACTGATTAAATTTTCCAGAGG
AGAATAAAGATAAAATGCTACTTTCATCTTCATCCTCTTCTCCTTTAATCCATATTTTCACGCCTAGGTAAAGTG
GTAAAAGTCCAAGTAGTCCGATAACCCATTGCTGAGGAATTAAATTTACAACCCCCTGTGCAACTAAAAGAC
TTGCTCCTATCACAATTGCAGTCCCTATATATTGTCCTATCCAAATATGTTTTACCTGACCTTTTTTTACTTGCGA
AAACAAAAGAATTAATATGACGAGATAATCAATTCCTGTTGCTACATATACCGCAGTAGCCGTCAGTATCGTCG
CGATCATTTTATCATCTCCAAATATTTTAGGGATAGGACTTTTCTTCAAAATGAAAAGTCCTTCCGTAAATTGC
ACACATATAGTACCTATTTATCCTTCACTCTCATCAGTCGCAAACTATTTAATGCTACCAAAATAGTGGCTCCCA
TATCGGAAAGAATCGCAATCCAAAGGGTTAGCCAGCCTGGAATAACCAATAGTAAGGCAATTATCTTAATTCC
GATGGCAAACGTGATGTTCGCTTTGATGATATTTAGCGTTTTCCTGCTAAGTCTTACTGCAAATGGAAGCTTA
CTTAAATCATCTCCCATTAATGCAATATCAGCTGTCTCGATGGCAGTATCTGTTCCAGCACCGCCCATTGCAAT
GCCAACAGTGGATGCAGCAAGTGCAGGAGCATCATTGACGCCATCGCCAATCATAGCTACATTACCATGCTC
GGCTTTCATTTTTTTAATATAGTCCAACTTATCCTGTGGCAACAATTCGGACTGAATATCAGAAACGCCTACAT
GAGCACCGATTGCTTCTGCGGTACCTTGATTATCACCTGTCAGCATAATTGTTTGCTTGATTCCTAACTGATGA
AGTTTTAGAATCACATTTTTACTTGTTTCGCGGACCTCATCTGCTACAGCAATCACGCCGAGGATTGTTTGGT
CCGTTCCAATAATCATGGCCGTTTTCCCTTGGTTTTGTAGAACCTTCACCTTATTTTCAAATGCTGGACTAAAA
TCGGAAACATTTAATTCTTTAAAAAGCCTTGGACTGCCAATATAATAGGTTGTTCCGTTTATAGTCCCTTGAAT
ACCTCGCCCCGTAATCGAAGTGAAGTCCTCCACTCGAACATCCGAATAAGAAATAGTATCTTGCTCCGCCTTC
TTCATAATTGCTGAAGCGAGTGGATGTTGGGAACGGTATTCTAACGCTGTAATGATGGAGAATAGCTCTTTTT
CTTCCACTTGATCATTTAATACTTTGAAATCTGTTACCACAGGGACACCTTTTGTCAGTGTTCCTGTTTTATCAA
ATGCGATTGCTTTAATGGCTCCTAATTCCTCTAGATAGACACCGCCTTTAATCAACACACCTTTTTTAGCTGCAT
TTCCAATTGCCGAGACAATCGAGATTGGAGTAGAAATAACTAATGCACACGGACATCCAACTACAAGTACCG
CTAATCCTTGATAAACCCAAGTATCCCAACTTCCACCAAAGAATAAAGGTGGAACGACTGCAACGAGCGCCG
CAATAACCATAATGATCGGCGTATAATATTTCGCAAATTTATCTACGAATGCTTGCGCTGGAGCGCGCTCCCCT
TGTGCTTCCTCAACCAGATGAATAATCTTGGAGATGGTTGTATCCTCTACGTATTTGGTGATTTTTACTTCAAG
TAGTCCCTCTTCGTTAAGCGTACCTGCAAATACTTCATCATCTACCGTTTTGGCAACAGGGACAGATTCTCCTG
TTATAGCAGCCTGGTTGACAGCCGACACCCCATTTATAATGATCCCATCCATGGCAATTTTCTCCCCTGGTTTG
ACGATCATAATATCACCCACGGCGATATCGTCCACATGGATCATTATTTCCTGACCATTCCGCCTAACAAGTGC
TTCTTTTGGGGCAATATCCATCAATGAACGAATGGACTGTCTTGCTCTATCCATAGAAAAACGTTCAAGTGCT
TCACTGATTGCAAAGAGAATGACAACAATGGATGCCTCTGCCCATTCACCAATGATGGCAGCTCCAATAACT
GCAACGGTCATCAGGGTTTTCATGTCGAAATCAAAGCGTATCAAATTTTGAAAACCAACTTTAAATAGTGAAT
ATCCGCCAATTACAATCGAACTTACAAATAACATGGACGTTACAAGGTTATCTTCTCCATTTACAAAGTGAGA
AAGGTAACCAAAAGCAATCAGTAATGTGGCAAACAGCAATGTGCTGTGTTTTTTATAAAACGGTATTTTCTCT
TCTTTAGGAGCCTTAGTGTCTTCTTTGACCGCTTGTATCGATGGATTCGCCAGTTTTTCAGGAATTACCTTAAG
ATTCTCGAAAGCACCTGCTTTTTCAAGCTCTTCAACCGATGCATTTCCATATATATCAATTTTGGAAGCGCCAA
AGTTCACTTTTGCATCCTGAACTCCAGCTAGTTGTTTTACATTTTTTTCAAACTTCCCAGCACAGTTTGCGCA
GGAAAATCCCTCCACACGGTAAACATTTTTACCTTCTGTTACCACTGGTTCGACCCGTCTTCTAGCCTTCTCTG
GTGCCACTTTAAGATTCTCGAAAGCACCAGCCTTTTCCAGATCTTCAACGGTTGCACTGCCAAAGACATCAA
TTTTGGAAGCTCCGAAATTGACTTTAGCATCATGCACCCCTGATAGTTCTTTTACATTTTTTTCAAACTTCCCA
GCACAATTCGCACACGAGAAACCCTCCACACGGTAAACCTGTTTATCTTCTGTTAATGTTTTTGCTGAACTAT
CCAATACTAGCAACCTCCCTTTGATGCAAGAAAGCTTTTTCTACAAGCTGTTTAACATGCTCATCATCTAGTGA

ATAATAGACTAATTTTCCTTCTTTACGGTATTTTGCTATACCTAAATTTTTCAATAATCTTAAATGATGGGATGCC
GTAGCCGTTGAAGATTCAATGATATTAGCTACATCACAAACACATAACTCTCCCTCTAAAGACAAAACATAAG
CAATTTTAACTCTTGTATCATCTGATAGAGCCTTAAAAACTTTCGCTACATCCATAGGATTCTGTTTAGCAAGG
TCTTTTTTAGCCCTGTTTACCTTATCTTCATGAATATAGGTAACTTCACACATATCTTTTGTCATAATTACCCTCCT
TATTCAAATGACTGTTTGTTTGATGATTATAATATATCCCACTATAAACAAATAGTCAAATGATTGTTTGAATGAT
ATATAATTAATATTAAAAAGGATTGGTTTCTAATGTTAGAAACCAATCCTTTCGGAGATTTTAACCAAATTTTAA
AGTATCTTAAACATAACTGCCCCGTTAGTTTAAGTGCATCCTTTCACAATCTGTCTACAGATTAATAATAAAAAC
TACTCTTTATTATACAGATCTCCATATAATTTTTGAATTTGGTTCTGTAATTTTTTATTTTCTTTTTCTAATTCCATA
ACTCTTCTTTTTAAGGTTTTTATAAGGATTTCCTCCGAACGAGAACTTTTCTTGGGTTTTGAGACTACATTTGA
TGTTATTTGACGCTCACGAAGGGATTCGATTCTTTGCCTAATATCGTGTTCCTTATAAAGCCATGATTTAGAAA
CATTAGCTTCCTTTGCTATTGAATTAAAATTAATAGCTTTACCTTCAATCGAAAATTTAGAAATCGCTTTGTCTA
CTTTTTCTCTTGTCTTTTGTGATTTCTGCTTCGCCAAACGTACAATTTCTGTTGTATTTCTAACTTGTTTATTCAT
TGATAATTACCCCGTTAAACTTCCAATGATTTGTTCTAAACGCTCTTTAACACGGCTATTAGTCTCTATTTGTCT
TTGCCATTGTTTATCCTTTGCTATGGTTAATAACTCTTCTGTACGCTCTAACTGTTCTTCGTGCTGTGGTAAGAA
TTGCTTACTGGTACAGAAGTGAGTGCAATCTAAGCATGCATTCGCATGTGGACAACCACCTGCTATTACTGGC
AATCTACAATAACCATTTGGAAGCACTTGTGCATTTATATTTTTCTTGAACCATTGAAGCTCTACATCATCGACT
TCACTATCATCATCTAGATTGAGCACATCTCCATTATTGGTAACCAGTTTTTCTTGAAATTTAGTAAATTCATTTT
TTAGAGTTTCATCAAAGATATGAGCGTATCTGCTTGTCATTTCTGGGCTTTCATGTCCCAAAAATTTCTGCACA
ATATGCTGGGGCACCCCGTTGTTAATCATTCTTGTTCCTACTGAATGGCGAAAGGCATGGGCATGGAATCTAT
AAATCTCGCCTAATTTATCCACTATATTTTGCTCATAAGCTAATTTATTTAATTCGCCTCTAAATGTTTCTTGTTTT
AATGGCGATCCATCTTTTCTTGGAAAGAGGTATTCACTATCTGGAAATTCCTCTGAAACTTTATCTTCCCGAAC
TTTAATAAGTAAAACTACCTCTTTAGATATTGGAACTATATGCTCCTTTTTCATTTTCCATTGATAATACTTTAAA
AAGTAATCTCCATCTTTGTCCTCTAATAAACAGCCTTTTTTCAAGGTGCACAATTCACTTATCCTCATTCCACAT
TCTTGAACAATCATAGTCATCGTAGCTATATATTCGGGTAATTTATCAAGATGACTGTTCAATTGCTCTAGAACG
AATTCGTCTATAAAGCGAGGTTTTGCTCTTGGTATTTTCGGATAGTCCTCGGGATAAATTAATAATTTGGAAG
GAACATCATCCCATTCTAGCCTATGAAGGGTACTAAATAGTCCTTCCAATATAGAGATCCTCCCAGTTATTGTAC
TAGGTTTTATCCCCATCATGTTTAGTTCACTTAAATATGCTTCAATTTCCACTCTCGTTAATTGGTGTACTCTCTG
AACGTGTTTAAACTTTATGTCCAGAAAATTAAAGAACTCTTTAAGTTTTTGGGCAATATCACTTACATAGGAA
AAGCTATCCATGTTCAATCTTAACTTACAATATCTTTTTACAAGCTGTTTAAAATATGTATTCCGAAACCCTTTA
AAGTTAATTGTATATTCATATTGTGTTGGGTTAACCTTATCATCTGGCAAAGGTAAGTTTCGTCTATCCCAAAC
GTCTTTATCCCACTCCTCTCCATCAAAATAAAAATCCTCATAAAACTCAATAAATTGTTTAAGATTAGTAACATA
GTAAGAATTAGCTTTTACAGGTGTTTTTTCTTGATTAGCAGTAATCTTATAATTAGTGGTGGTAATTCTAACAC
CCTGTTTTGTCAAATATGTTCTATACTCCGTCATTGCTTTATCAATAGGAACTTCAGTAATTGAAGTAATGCTGG
GATACTTTAAATCTAAAAAATCCAACATTTTATTAATTACTGTTCCTTTTCTAATCCAGACAGTTTTTGCATTCC
ATATTCCATTGTTTAAATGGTAAAAATAAAAATATTTCAATTCTGTTCTTAACCACAGATTTTTAACACGTTCAA
AACGAACCCAACGATTTCTTAAAGCAGGATTCTTACTTAATTCTATGGCAGAAGGATGTGGACATTTTCTTAT
ATCCCAACTATTATTAGCCCAAAACCCTTGCATTTCTTCATTCATTACAGCTATTTTTTTGCTGATCTCACTCTGA
CTGATAATTTTCCTTTTACTAGAAGCATTCATTTAGCTGGTATTCTTTATCTAGTAATAAATACAATGGATACGG
CTTATTCTCCACTTCTATCCTTTGAACCCTCACACGCGCCCCCCTCTTTAAACACTTTAAGTACTACAAATTATA
AACCTTGTTAATTTTTATTTAAAGTAAAATCTCCCTTTAACATCTCCTCCTGAACTACTTTAACTACTATTATTTAT
TATACTATGGTTAATATGTCTCCACTTGGGTGGTTATGGCCAATAATCATACCCGTTGCGTTACTTAATATGGCT
GTTTTAAATATTTCTCGAGGATGTATAACCGCAAAATTAATGGTTCCTATAGAGACGGTTTCAATATGTGTAGG
TTCATTTTTGCTATTCAAGCAAATTAAAATGAAGTGTTCTCGGTCAGCATTTCCTATGAAGTCACGCATAATAT
CAGCAGCGTCTTTTGGTTCTTCAATACGTCTTTTTAAATACCAAAGTGTATCTTCTTTAACCATTTGGACTTTG

ACAACATCTATTCTTTTTCTACTCATTATGGATAAGCTCTATACTTTCTATTAAGTCAGGGTGAACAATGGATGT
GATAGAATCATCATTCATATAAATGGAATATAGCTCATCTAATTCTGAATTATTTTTTTTGTTTGATGAAAAGGC
GATAATGCCTTTAATAACATCGATGTGTGGTTGATGCATTAAATATTGGTATACTTCTTTCCATGTTTGATTCATT
CTATTTAGTCTCCTTTAATGGTTTAATTGTCCATGCCTTTCTAGCGTATGGATTTAATGGATGCATGATTTCATTT
GTTTGAGGATTTGTTAATATACTGATTTTCTTACTGTGAGGTTTTAACATGTCTAATACTTCATTCACTCGATTT
TCCACAAGAGGTTTTTTTCCGTAGGAACCCCAAGCCAATAAAACACTATCGGCTTCTTTGACAGCTTTCATAA
TGTGAATATCCGTGTGCTTGTCATGGCTGTTTTCGATATGTTTTAAATTAATGGGGGTATCAATATTTGAAAAA
AGATTTATAAAATTAACGCCACCATATTCATCTTTTTCAGCTACTTTATTGACAATAAGTTGTGTTGTAAGGTCA
ACGTTGATAACACCATCATAATGAGGATACATCGTTATAATTGTAAGTACTTCTTTATCAATATCCCAAGTTTTCT
TGAGAAGATAACGATGCATTTTATCCTGACTAAATATCGCTTCAGTTTCTAATACACTTTTGATTGATTTCATTT
TATCTAACTCCTTTATTTAATATTCTTCAGGTAACAACATGACGTAATAAGAAAGGTCTACATCATCTTCTCGGA
TAATGTAGACCTTATTAATATTGATAGCTTTTGAATAATTTGCCTTATGAATTTTTACGTACTCTGGACGTTCTTG
CTTATGTTTGATATATAATTGACTATTATGCATATTGAACTGGAATATATGAAAGTAATCAATCTCTTCTAGTTCCT
TGAATTGTTCGTTTTCGCGTTGCGCTACAAGTTTCCATAACTGTTGCTGCAACATTGCTGGCAAGTAATTGGC
AATACTACGTGTAATATAACGTTTCATGCTCATTTCTCCTTTTCTATATCTTTCATTATTGTAATAACTTCTGTAAT
GACTGTGACGCTTATCTGAGCTATGTGTATCCATTTATTCATTATTATAATTCTCCTTTTCAAGTAAGTGTCTTTG
ATCTAGTAGTGGCATAACTGTGTTATGAAGATATAAGAAGTCCATATCGTTTGTTTCATCAATATAACCAATCGC
AATAAGTTGTTTTTGATAGTAGAGGATAAAGGGGTATATATGCCTTTCATGAAGTGTGTCAGAGTAGTAAGTA
TACGTATGAAGGATTTTGTTGGTAAGCGGACCAAGTCTGATATATTGATCAGGAAGGATATTATCTGCCACTT
CCTCGATAGATTCACATTCCCATTTTTCGTCCTTTGGCAGATTGAATAAGTTAGTTATATACTCTTTGAGTTGTT
TTTCTAGTGTCATGGTTATGACCTCCTATAATGTTTTTGATGTATACATGTAATACACCATATAGATAATATATGCT
TCAAAAAAAGTTTGTTTCGTTTATGAATCCCATTGTTATTAAGAAAATATCCCAAGCACTATTTGCGAGAAAG
GTTGATATGACAAGGAAATCCCGTTATCCCGCTTATTTCTCAGGAAATATAGCCATATATTTATATAATTTAGATT
TGTAGGTGTAATTGTGACTTACTTTCCCTTTTTATATTATTTATTTATAAATAATAATGGGATTTCGGGATTACGC
ATGCGTAATCCTTCTCGTACTTGTATTTATCAAATCCCATTGTCATCCCGTTGGTTTATTATTTAGGGATGTTCTT
TTGTCATATCAATATAAGCTTCACATTGAGTTGTACTTATATGTAAACTGAATTTATAAGTTAGAAAAGTGTTAT
TGTATGACAAAAGGACAACAAGTATAAATTTAGCTTGTTTAAGGAAAAAATAAAGGCGAAGTGCTCCTCAGC
ACTTCGCCTAAAATTTATTCAAGAGAACAATTTATATTTAATTGATTTATCATTTATAGTGCAGTAATACCACCA
TCTATAACAAATTCTGATCCTGTAGAATAAGAAGCATCGTCAGACCCTAAGAAACATACAAGGTTAGATACTT
CTTCGGGTTGCGCAACTCTTCGCATTGGAATTGTTTTTTCGAATTCTTTAACGGCTTCTTTAACATCGTCTTGT
TCTAACATTGGTGTTTTTATAACACCTGGGTGTACTGAATTAACTCGAATATTGTATGGAGACAATTCTTTAGC
CGCAGCTTTTGTCATACCTCTGACCGCAAATTTTGTATCTGTATAGCCAATTGCACCACCAACAAGACCATTCA
TTGAAGAAATATTGATGATTGACCCTTGTTTTTGACTTTTCATAATTTTTGATACAGTTTTGATGCCTAAAAATA
CAGATACTTGATTAATATTTACAATCTTCATATAATCTTCTAATGATATTTGGTCAATTGTTTTATTGTATGTGATT
CCTGCATTATTAACCAATACATCAATTCGATTCCATTTGTTCATAACTTCTTGAATGACATGATTCCAATCCGTTT
CTTTAGATACATCATGTTTGATAAATAAAACATCGTCTCCAAGAGAATTAGCTGTTTTTTGACCTAATTCATCAT
TAATATCAGTTATCACGACTTTTGCGCCTTCAGCTATCGCTTTTTTGGCATGAAGTTTTCCCATACCTTGTGCAC
CACCTGTAATAACGACGACTTTATCTTGTAATTTTCCCATTTTAAAACATCCTTCCTCTAAATTAATATTGTTCAT
AATTTTATTGTATACCTAACTCAATCAAGAAGCTAAAAAAGTCACTTATACTTATATATGAATATAGAGTGTTTG
AGATAAAAATAAAGACGAAGTGCTGAGGCGCACTTCGTCGATAGGGGTATTATGGAAGAGTTGTTTAGTGA
TTTCATGATTGAGTTTTAATGTGACATAGAATTGCTTTTTACGATGTTCGTCTTTACGAATATCTATTCGGTCAA
TGACTGTGAGATATAACGCTTTGAGTTGTGATTTATCCATGGATTCTACGTTTTGAAATATGCGACGTAAAACA
GCGGATAGTTGTTTCGTATTATATGATGTTTTCTCTTGATTTTGTTGTTGCTTCAGTTGATTAATTTGATTCGTA
ATGTCATTCAGTTGTGTTTCATATTTATGGATTGTTTCTTTGAGTATTGTTGTTAAGTCTGGATTATCTTCGATTG

TTTTAATCAAGTTGTCTAGTTTCGCATGGACTTCATCGTATTGTTGTTGTTTATAAGCGATATCATGGTTCAATG
CACCAATATCGACTTTATTCTCTTGATTGACACGTTCTAAGACTTTATTAATGACTTTATCACTTTTGACAATTTC
GAGTATTTGATTCATGACGTATTTCTCAATCACGTCAGCTCTAACGCTATTCGCAGAACATACTTTTGAGCCTT
TGTTTCGAAAGTTACTACAAGAATAATAGCGTATTCGCTTCTTGGTACCATCTTTCAATGTGTTCGTTGTGTTA
CTAGCTGCCATTGGTGCACCACATTGTGGGCAGTGAATAATACCTGTTAATAGATTGGTTCCTTTTCCATGAAC
TTGAGGTTTTTGACTAACTTGCTTCATACGTGAATGTACTTTATCCCATAGTTCTTGACTAATGATAGGTGAAT
GCTTCCCGTCAGCTACTACAGGATTATCATTAAGGCCTTTACGTCGTTTTTCATTCCAATCCTTATATTTCGCAA
ATTGTATTTTACCAATGTAGAAGGGATTGGAAAGAATATAAGCTATAGCTGATGTACTGAATGGATTACCTTTC
TTAGTGACATAGCCTTTATGATTAAGTGCGTTAGCCATTTTCCGATATCCATGACCTTTAGCATATGACTCAAAT
ATATATTTGACGATATTCGCTTCATGTTGGTTTATCATGAGTTCGTGTTTATTGTCTGGAATTTTCTCATAGCCA
AGTGGAATATTGCCTTGGTAATATCCTTCTTGTGCACGTCTGGTTTGACCCATGAATACGTTCTCGACAATGTT
ATTGCGTTCGAATTCAGAAAAGCTTGCAAGTATTTGTAACATAAGCTTACCGCTACTCGTATTAACTTCCATAC
GTTCTGATAGACTGAAAAATTCAACATTTTGTTTATGCAAATCTTCTACAATTTTGAGTAAGTCTGATGTATTAC
GTGCCAGTCGGTTTGTTTTGTACACCATGACACAATCGATATGACCTTCATTCGCATCCTTCAACATACGTTGT
AGTGCAGGACGGTTCATAGACTTTCCTGAGATACCACGATCAGCGTAAATATCTGTGACTTCAAAATGATGAA
ATTGACAGTATTCTCTAATCTGATTGATTTGACCATCAATACTATAACCTTCTGTACTTTGCATTTCTGTTGATAC
ACGGACATAAATACCGACACGTTTTTGTTTGAGTTGTTGCATAGCTGATTCATCCTTTCTTGTTTAGGCAGTTG
ATGATTGTACCGCTTGATTAACAATGTTTAGTGGCTCATCTTTTAAATAGATGCCGCTTAGTTCTTTATTTTTAG
TAATATGAATGACATCAATAAAGGGATATAACATGTTTAACGTGAAACTTTTTTGTATAACCTTTTGTAGTGATG
TTTGCAACTGAGTATCACTTATTGAGGATATGGTTTTGCGCTTTTGAAGCACTGATTGAGACTGATTCCTGAA
TGTTTCAGCATCAATCATACCTTTGGCAAGCTTATCTATCAGTTGTTCTTGCGTGAGTGTATATTTAGCTTCTAT
GTCCCTTTGTCTTTTGAGTCGTTGTTGAATTGTATGGTTGATTTTTGAATAGAGCTGTTGGTTCTGAAAGAAG
TCCTGACAAGTCGCTAAGACACTTGTTTCTAATACTTGCGCATTGATTCCTTTGAATGCACAGACAAAACGTG
ATGCATTCATATTTTGAGGACAAGTATAATAACGTAATATATGGTGCTTTTTTCTAATCGTCATATTAGTCAGGG
TTGATCCACAATATGGACATTTTATTTTCTTCTGAAGTTGATTATCTGATGGAGTCCGTTTGGTATGTTTTTGTG
ACCGTATTGCTTGAGCTTGTTCGTATATATTGGTCGAAACAATAGATGGGAACATGTTGTCATATTGTCCATATT
GATTGATAACGCGACCACAATAATTAGGATTCGTGATAATAGCGCGTACTTGGTAGGGTTTACGATTAATACAT
TTATCATCTTTTTCTAGGTATTGCGCAATTTTCTTATAACCATAGTCTTGTAGGTAGTAATGGAATATTGCCTTAA
CCGTTGGTGCTTCATCTTGATTAATTGTGAATGTGCCATTGCGATAGGTATAACCAAATGGTGCATGTGTCGTG
ATGAGTTTACCTTGTTTTGCCTTTTCTCTAAGTCCATTTTTGACTTGTTCACCAATATTATCGGACTCTAATTCT
GCGAGACTTATGAATATATTGAGCTTTAGGCGATCAAACGCTTTATCCATATCAAAATAGCCATCATGAATACT
TAGAATATGAACATGATATTTTTGACACAGTTTCATAAGTTTTAATGCATTTTTCAAATTACGATGTAATCGATT
TAAGCGATAACAACATAATACGTCACATTGTCTTTGTTGAATGCGTTCGATAACTTGTTGGTAACCCGTTCGAT
TATCCGTTCTGCCTGATTGCTTATCACTATAAAAGGTGATGTGTTGGATATTGTATTTTTTAGCGAGCGTTTCG
ATAGTTTGTTTCTGTGCGGTGAGTGATTGTTGCTTGGTTGTACTTTGGCGTAAATAACCAATAGCTTGTTTCAT
CGAGTTTCCTCCTTTCATCATGATAATATATATTTATGAACGAATTTATAGATGAGCCCAACACCTTACTGGTGT
TGGGCGATATGATTAGTCATCAGTGCGATTAATTTCTTCAATAACCAAATCGGCTAATAGTGTGATTAATTCGT
CCACATATATCACCTCGTTATTCATAGTGTTAACAACAGTTCTTTAGTTGCATATCAAAAGTGGTAAGGAAATC
GATATTTTTCCTTGAGCAAATTTCTTCTAAATGAATGTAATCCTCATAGTCTCCATAGGCTACAATGGTCGTAGG
TGTGATTACGTTATCTAAAATATCATTAACTAACTTGCGTTCATCTGAGTAAGTGTTAGTTATTTGGTGTCCCTC
TTGTTCGCAGCACTCCTTCACGGTATCTAAAGGTGTGCCTTCTAAGTAATAAAACCTTTCATCTTCAATTTCAT
GAGCTAGTACGATTGTATTCATAGTGCAATTCCTCTCTATAACTTAATATTTGTGCTACAACACCTTTAGACAGG
TGTTGTAGCGAATTTATTGTGTAACATATTGTAACCATTACAGGTCAATATCTCCGTTCTTATCCCATTCATTTGC
TTGTGCAATCATGTCTCCAAGTAAAGGATGTGGATGTTTAGAATCAAATGGTAAGGGCATATTGGATTCGCTT

TTATCAGCTAGACCAAAAATATGGGCGTAAGATTTATCAAGCTTAATGTGATAAAACACAATTGTTGTTGGTTT
GCCTGCTTTGTCTTTGACACTACGCTTGGTTGTTTTACGATTGGCATCTGAATGTATATAATCTTTATCTCGGAG
TGCATCGATAACATTATTTACGTCTTGATATTGATTTTCTTCCAACATATGTTTAAAAACATCTTTAAGCATTTTG
ACTTGAATACAATTATCCTTTAATTCAATGAGCCCATAATTTTCTACCATGGTTGATAACTTATTATCTTCAGAAA
ACTTCCCTCTATTTTGTGCTACAAATTGAATAATTGTTTCGATTGCTTTATCAGCAAGAGAGCGCTCACTTACT
GAATCTGAATGGTAATTGAGTAAATAATTTCTAATCGCATCCAAATCAATAGGTGTCGCAATGACACGTTCTAA
TATACGAGCTGATGTCGTAATGGTAGCGTAGCGCTTGAACATACGAATACCTGTATGACTCGTTTCATTTTCAA
GTTTCGTTTTAAACCATTGATGTTCAGTATGGAACCATTGTATGACTTCTTGTTCACGATTAAGTAGATATTGT
GCGACTAATGGCATCACATGACCGTAATTAGCTGATATTGCCTTTTTAATGGCATCTGCATTATCAGCACTTGT
CGTAAAAGCTTCTGATATTTCAATGGTACGAACATTTAATCCATCATTACGTGCACTATTATTGAAAATACTATG
TTCGGCTGTACTGATGACACTTGTCCCCCAATTTTTCAAGCTTTTAACACCACCATGTATATTTGCTCGTTGAC
GTCCTTGACCTTCAGCAATAGAATAGAGCAAACCAGTTGTACTTTTAAATGTTGCAGCTGATAGTTCATCTAAT
ACAATAGGTAAGCCAAAATTTTGACTCAAGTATCCTTCAAGTGCATTTCTCGTACCATTCCATGATCTAAACAG
TGTATTGTTACCTTTGGTAGGATTACCGGCTATTGATACTGCCAAAGCGGCCGCTGTGGATTTTCCCGTACTA
GAATTCCCAGTAAATGAGAAAATAGTACCTGTGAATTCAACCTCATATTTTGATTTTAAGAAAGCAGTCACTA
ATGATGAAGCACCAAATATCACAGCAAGTTCGAGTAACAAGTTTCCTTTTACTTCATCAAGATACATTTGCCA
CCAGCCTTCAAAAGTACCTTTTGGCCGTAAATCATATTGTGTATCGCATAGAATATCTTCTTTTAATGAAGATAG
CACCTTCTTAGATACATAAGGTTCATCTAAAGATATGATAGGATAGTTCTCAGTGTTAATGACACCTACACCTT
GATACATAGGTGATAGGGGTGTTTGAGCACGCATTTGTTGGAGAATGAAACCCAAGTCTCGTATATATTTTTC
ATTAACATTAAAGCCAAATTTGATAATGGTTGGAAGCTTTTGAGATGATAATATATCAGCTTCTTCAATTAATTC
TGCATGATTGAAGTCTGATAGAATAATCTTTTCGACTTTTGAGATAGGATCAATGAATCTGGCTGTTATGACAA
TTGGACTTGCGATAAACACATCTTGATTATCTGCATCTTTCTTTTTTGAAGGGATGGTTAAAAACCATCCCGCC
ATTGTCAATGTTGCATTTCCATACTTTAAAGGTAAGTGTGAAGTATTCATTAGAAGCCACTCCCTTTCTGATAG
TTATTATTGTGATGAGGATTAGGACCTACTTTACGGTAAATTAAATGACCGTTAGAATCCTTGCCAATAATAAC
GAATGGTACACGTGGTGCGTGCTTCACAAAATACGCAAACCAGCGACCAACATCCTGTTGAACCTTAGTTGA
ACAGATAACATGAGCCTTCATATTTAAATCGTGAAACGTAAACTGAGTGCCAACAGGTAAATTAAATGCGACC
CCCGTAGCGCGTTTTTTTAATAGATAAAATTCTTTAGTTTTTTCCAT
>Staphylococcus argenteus MSHR1132
ATGAAAATCACCATTTTAGCTGTAGGGAAACTAAAAGAGAAATATTGGAAGCAAGCCATAGCAGAATATGAA
AAACGTTTAGGCCCATACACCAAGATAGACATCATAGAAGTTCCAGACGAAAAAGCACCAGAAAATATGAG
CGACAAAGAAATTGAGCAAGTAAAAGAAAAAGAAGGCCAACGAATACTAGCCAAAATCAAACCACAATCA
ACAGTCATTACATTAGAAATACAAGGAAAGATGCTATCTTCCGAAGGATTGGCCCAAGAATTGAACCAACGC
ATGACCCAAGGGCAAAGCGACTTTGTATTCGTCATTGGCGGATCAAACGGCCTGCACAAGGACGTCTTACA
ACGTAGTAACTACGCGCTATCATTCAGTAAAATGACATTCCCACATCAAATGATGCGGGTTGTGTTAATTGAG
CAAGTGTATAGAGCGTTTAAGATTATGCGTGGAGAAGCATATCATAAATGATGCGGTTTTTTCAGCCGCTTCA
TAAAGGGATTTTGAATGTATCAGAACATATGAGGTTTATGTGAATTGCTGTTATGTTTTTAAGAAGCTTATCAT
AAGTAATGAGGTTCATGATTTTTGACATAGTTAGCCTCCGCAGTCTTTCATTTCAAGTAAATAATAGCGAAATA
TTCTTTATACTGAATACTTATAGTGAAGCAAAGTTCTAGCTTTGAGAAAATTCTTTCTGCAACTAAATATAGTA
AATTACGGTAAAATATAAATAAGTACATATTGAAGAAAATGAGACATAAATATATTTTATAATAGGAGGGAATTT
CAAATGATAGACAACTTTATGCAGGTCCTTAAATTAATTAAAGAGAAACGTACCAATAATGTAGTTAAAAAAT
CTGATTGGGATAAAGGTGATCTATATAAAACTTTAGTCCATGATAAGTTACCCAAGCAGTTAAAAGTGCATATA
AAAGAAGATAAATATTCAGTTGTAGGGAAGGTTGCTACTGGGAACTATAGTAAAGTTCCTTGGATTTCAATAT
ATGATGAGAATATAACAAAAGAAACAAAGGATGGATATTATTTGGTATATCTTTTTCATCCGGAAGGAGAAGG
CATATACTTATCTTTGAATCAAGGATGGTCAAAGATAAGTGATATGTTTCCGCGGGATAAAAATGCTGCAAAA

CAAAGAGCATTAACTTTATCTTCCGAACTCAATAAATATATTACATCAAATGAATTTAATACTGGAAGATTTTAT
TACGCAGAAAATAAAGATTCATCTTATGATTTAAAAAATGATTATCCATCAGGATATTCTCATGGATCAATAAGA
TTCAAATATTATGATTTGAATGAAGGATTCACAGAAGAAGATATGCTAGAGGATTTAAAGAAATTTTTAGAAC
TATTTAATGAATTAGCTTCAAAAGTTACAAAAACATCCTATGATAGCTTGGTCAATAGCATAGACGAAATACAG
GAAGACAGCGAAATTGAAGAAATTAGAACAGCACAAAAAGATAAGACACTCAAGGAAGTGGAAGCACCTA
AAGGAATAATTCCAAAATATAAAAAAGGTGTATCAAAGACTACTAAAAATGATTCAGAAATTGAAAAATCAA
ATAAAGAGAATAAATTAACCGGTAAAGTTGGAGAAAAATTAGCGCTAAATTACTTTAATGAGCTAATTGATAA
TAAAATAGACGAAGATAAGAAAGAACAGTTTAGGAATATTTTAAATGATAATCCAGGCTCTCAACACGGTCAT
GGCTATGATTTAGTAGCTTTTGATCCAACAAATACAGATAAAGCTGTAGAAAAATTTATTGAAATTAAAACATC
TACATCTTCTAGTATTGAGGAACCATTTTTTATGTCGCTAAATGAAATGTTTGCTATGAAAGAATATAAGCAGA
AATATTTAATATTAAGAATATTTAATGTTTCCGGTAAAGAACCACAATTTTATTTTATAGATCCATATGCAAATTA
TTCTGAATTTAAAGATGTAGATGATCTCATTGACAAAGTATTTAATGTAGAAGCTATTCAGTATAAAGTTTTTG
GCGAAAAAATGATTACTTGAACAAGAGCTAAAATAAAATTGTGATCTAATAAAAATAGAAACTGTAATTTAAAT
AAAACTTTCTAAATAAGCTAACTGATAAAAAATCAGTTTGTCCACAGTCTGAAACAAGATTCCTATATTCTTTA
GGAATCTTGTTTTTTCTATTTTTATGGTGATAAAGAGCAGATAAGATAATGTGTAATAATCACAAAAAAGTTAA
ATATTTTAAGGCTTGTTTAATTATTAATGATTTTATATATAAAGAGCAGTATAATAAAGTTGTTAATATATTATGAA
TAATATTCAAGTAATTTTATTGTTTTTTAATTTGTCGATATTTAAGTTGAGTTAAATTTAAAGGGTGTAATTTGTT
TTACAATGATGAAGATAATTAGTCTATCAAAATAAAGGGGTTGGGACTGTTATGAGTGATAATTTGTCATTATT
CATTGACTATATCAATGATAATATAATCTATGGTAGTGAAATCAAACGGGAGAAATTAGAGAATTTATTTAATCA
ATTTGCTATAAAAAAATGTTGAAAAGAACATTGTCTATGATGAACTGAAATCTTTAGATATTACAATCATTGAGT
CACAGGATTCATATAAAAATAAATTGAAGAGATTATTTTCGGTTCTGTTGCAAAGTAAAAAAATATAGCTAACC
ACTAATTTATCATGTCAGTGTTCGCTTAACTTGCTAGCATGATGCTAATTTCGTGGCATGGCGAAAATCCGTAG
ATCTGAAGAGACCTGCGGTTCTTTTTATATAGAGCGTAAATACATTCAATACCTTTTAAAGTATTCTTTGCTGTA
TTGATACTTTGATACCTTGTCTTTCTTACTTTAATATGACGGTGATCTTGCTCAATGAGGTTATTCAGATATTTC
GATGTACAATGACAGTCAGGTTTAAGTTTAAAAGCTTTAATTACTTTAGCCATTGCTACCTTCGTTGAAGGTG
CCTGATCTGTAATTACCTTTTGAGGTTTACCAAATTGTTTAATGAGACGTTTGATAAACGCATATGCTGAATGA
TTATCTCGTTGCTTACGCAACCAAATATCTAATGTATGTCCCTCTGCATCAATGGCACGATATAAATAGCTCCAT
TTTCCTTTTATTTTGATGTACGTCTCATCAATACGCCATTTGTAATAAGCTTTTTTATGCTTTTTCTTCCAAATTT
GATACAAAATTGGGGCATATTCTTGAACCCAACGGTAGACCGTTGAATGATGAACGTTTACACCACGTTCCCT
TAATATTTCAGATATATCACGATAACTCAATGTATATCTTAGATAGTAGCCAACGGCTACAGTGATAACATCCTT
GTTAAATTGTTTATATCTGAAATAGTTCATACAGAAGACTCCTTTTTGTTAAAATTATACTATAAATTCAACTTTG
CAACAGAACCGTATTATGGAATAGAGATGTTGGTAACATTTATACAGGATCATTATACTTAAGTTTAATTTCGTT
ATTACAGAACCACACATTCCAACCAGAAGAGAAAGTATGTCTATTTAGTTATGGTTCAGGAGCAGTAGGAGA
AATCTTTAGTGGTTCAATCGTTAAAGGATATGACAAAGCATTAGATAAAGAGAAACACTTAAATATGCTAGAA
TCTAGAGAGCAATTATCAGTCGAAGAATACGAAACATTCTTTAACAGATTTGATAATCAAGAATTTGATTTCG
AACGTGAATTGACACAAGATCCATATTCAAAAGTATACTTATACAGTATAGAAGACCATATCAGAACATATAAG
ATAGAGAAATAAACTAGTGGCCGATTGTGCTTGATGAGCTTGGGACATAAATCCTAACTCGAAATAAATAAGC
ATATCACTAAACTGATTTTTTAAAGTTTACAGTGATATGCTTATTTTTTTATCTTACGATTTTGTACGTGCATGCT
TGCCTAGGGGTATGGCTCGAGCCATTAGTCTCTCGCACATACTATTCCCTCAGGCGTCAGCACTTACAAAATC
GGTTGTAATTTTCATTTTTATACGCATTCTTACTGAGATTATACTAATAAGAGGAATAGTAAAAGCAATTCTAAG
TAAAATTGCAGATAAGAGGTTTGTTAAAAGCAGTTCTAAGTAAAATTGCAGATAAGAGGTTTGTTAAAAGCA
GTTCTCAGTAAAATTACAGATAAGAGGTACGTTAAAAGCAGTTCTAAGTAAAATTGCAGATAAGAGGTTTGT
TAAAAGCAGTTCTAAGTAAAATTGCAGATAAGAGGTACGTTAAAAGCAATTCCATGCAAAATTGCTGATAAG
GGGTAAGTTAAAAGCAGTTCTCAGTAAAATTGCAGATAAGAGGTACGTTAAAAGCAGTTCTAGGCAAAATT

GCAGATAAGAGGTGCGTTAAAAGCAGTTCTCAGTAAAATTGCTGATAAGGGGTAAGTTAAAAGCAATCCTA
AGTAAAATTGCAGATAAGGGGTACAGAAAAACTAGACTTGATTACAAAATGGAGCTTGGGACATAAATGATT
TTTTAAAAATGAGATGAGACGTAGATTAACTCCATAATCAATACGAATCTATCGACTTCTTTATTTATGATATTC
ATCTCTTTTTAATGGAAATAAAAGTGCGATTAATGTGATAATACAGTTACGTTAATTAAAAAAATAAAAATGCA
AGGAGAGGTAATATGCTAACTGTATATGGACATAGAGGATTACCTAGTAAAGCTCCGGAAAATACAATTGCAT
CATTTAAAGCTGCTTCAGAAGTAGAAGGTATAAACTGGTTGGAGTTAGATGTTGCAATTACAAAAGATGAAC
AACTGATTATCATTCATGATGATTATTTAGAACGGACTACAAATATGTCCGGGGAAATAACTGAATTGAATTAT
GATGAAATTAAAGATGCTTCTGCAGGATCTTGGTTTGGTGAAAAATTCAAAGATGAACATTTGCCAACTTTC
GATGATGTAGTAAAAATAGCAAATGAATATAATATGAATTTAAATGTAGAATTAAAAGGTATTACTGGACCGAA
TGGACTAGCACTTTCTAAAAGTATGGTTAAGCAAGTGGAAGAACAATTAACAAACTTAAATCAGAATCAAGA
AGTGCTCATTTCAAGCTTTAATGTTGTGCTTGTTAAACTTGCAGAAGAAATCATGCCACAATATAACAGAGCA
GTTATATTCCATACAACTTCGTTTCGTGAAGACTGGAGAACACTTTTAGATTACTGTAATGCTAAAATAGTAAA
CACTGAAGATGCCAAACTTACTAAAGCAAAAGTAAAAATGGTAAAAGAAGCGGGTTATGAATTGAACGTAT
GGACTGTAAACAAACCAGCACGTGCAAACCAACTTGCTAATTGGGGAGTTGATGGTATCTTTACAGACAATG
CAGATAAAATGGTGCATTTGTCTCAATAGAAAGTTAGAGGTGAGTCTTACGTTTCAGTGACGGTAGACTTAC
CTTTAACATGTTACATACTAAAAAATTAATTTGAATAAGAAAGAGAGACATATATGAAATACGATGATTTTATA
GTAGGAGAAACATTCAAAACAAAAAGCCTTCATATTACAGAAGAAGAAATTATCCAATTTGCAACAACTTTT
GATCCTCAATATATGCATATAGATAAAGAAAAAGCAGAACAAAGTAGATTTAAAGGTATCATTGCATCTGGCA
TGCATACACTTTCAATATCATTTAAATTATGGGTAGAAGAAGGTAAATACGGAGAAGAAGTTGTAGCAGGAA
CACAAATGAATAACGTTAAATTTATTAAACCTGTATACCCAGGTAATACATTGTACGTTATCGCTGAAATTACAA
ATAAGAAATCCATAAAAAAAGAAAATGGACTCGTTACAGTGTCACTTTCAACATACAATGAAAATGAAGAAA
TTGTATTTAAGGGAGAAGTAACAGCACTTATTAATAATTCATAATAAAACAGTGAAGCAACCATCGTTACGGA
TTGCTTCACTGTTTTGTTATTCATCTATATCGTATTTTTTATTACCGTTCTCATATAGCTCATCATACACTTTACCT
GAGATTTTGGCATTGTAGCTAGCCATTCCTTTATCTTGTACATCTTTAACATTAATAGCCATCATCATGTTTGGA
TTATCTTTATCATATGATATAAACCACCCAATTTGTCTGCCAGTTTCTCCTTGTTTCATTTTGAGTTCTGCAGTAC
CGGATTTGCCAATTAAGTTTGCATAAGATCTATAAATATCTTCTTTATGTGTTTTATTTACGACTTGTTGCATACC
ATCAGTTAATAGATTGATATTTTCTTTGGAAATAATATTTTTCTTCCAAACTTTGTTTTTCGTGTCTTTTAATAAG
TGAGGTGCGTTAATATTGCCATTATTTTCTAATGCGCTATAGATTGAAAGGATCTGTACTGGGTTAATCAGTATT
TCACCTTGTCCGTAACCTGAATCAGCTAATAATATTTCATTATCTAAATTTTTGTTTGAAATTTGAGCATTATAAA
ATGGATAATCACTTGGTATATCTTCACCAACACCTAGTTTTTTCATGCCTTTTTCAAATTTCTTACTGCCTAATTC
GAGTGCTACTCTAGCAAAGAAAATGTTATCTGATGATTCTATTGCTTGTTTTAAGTCGATATTACCATTTACCAC
TTCATATCTTGTAACGTTGTAACCACCCCAAGATTTATCTTTTTGCCAACCTTTACCATCGATTTTATAACTTGTT
TTATCGTCTAATGTTTTGTTATTTAACCCAATCATTGCTGTTAATATTTTTTGAGTTGAACCTGGTGAAGTTGTA
ATCTGGAACTTGTTGAGCAGAGGTTCTTTTTTATCTTCGGTTAATTTATTATATTCTTCGTTACTCATGCCATAC
ATAAATGGATAGACGTCATATGAAGGTGTGCTTACAAGTGCTAATAATTCACCTGTTTGAGGGTGGATAGCAG
TACCTGAGCCATAATCATTTTTCATGTTGTTATAAATACTCTTTTGAACTTTAGCATCAATAGTTAGTTGAATATC
TTTGCCATCTTTTTTCTTTTTCTCTATTAATGTATGTGCGATTGTATTGCTATTATCGTCAACGATTGTGACACGA
TAGCCATCTTCATGTTGGAGCTTTTTATCGTAAAGTTTTTCGAGTCCCTTTTTACCAATAACTGCATCATCTTTA
TAGCCTTTATATTCTTTTTGTTTTAATTCTTCAGAGTTAATGGGACCAACATAACCTAATAGATGTGAAGTCGCT
TTTCCTAGAGGATAGTTACGACTTTCTGTTTCATTAGTTGTAAGATGAAATTTTTTTGCGAAATCACTTAAATAT
TCATCCATTTTTTTAACGGTTTTAAGTGGAACGAAGGTATCATCTTGTACCCAATTTTGATCCATTTGTTGTTTG
ATATAGTCTTCAGAAATACTTAGTTCTTTAGCGATTGCTTTATAATCTTTTTTAGATACATTCTTTGGAACGATG
CCTATCTCATATGCTGTTCCTGTATTGGCCAATTCCACATTGTTTCGGTCTAAAATTTTACCACGTTCTGATTTTA
AATTTTCAATATGTATGCTTTGGTCTTTCTGCATTCCTGGAATAATGACGCTATGATCCCAATCTAACTTCCACA

TACCATCTTCTTTAACAAAATTAAATTGAACGTTGCGATCAATGTTACCGTAGTTTGTTTTAATTTTATATTGAG
CATCTACTCGTTTTTTATTTTTAGATACTTTTTTTATTTTACGATCCTGAATGTTTATATCTTTAACGCCTAAACTA
TTATATATTTTTATCGGACGTTCAGTCATTTCTACTTCACCATTATCGCTTTTAGAAATATAACTGCTATCTTTATA
AACTTGTTTGAAATTTTTATCTTCAATTGCATCAATAGTATTATTAATTTCTTTATCTTTTGAAGCATAAAAATAT
ATACCAAACCCGACAACTACAACTATTAAAATAAGTGGAACAATTTTTATCTTTTTCATCAATATACTCCTTATAT
AAGACTACATTTGTAGTATATTACAAATGTAGTATTTATGTCAAAATAATGTTATAATTTTTGTGATATGGAGGT
GTAGAAGGTGTTATCATCTTTTTTAATGTTAAGTATAATCAGTTCATTGCTCACGATATGTGTAATTTTTTTAGT
GAGAATGCTCTATATAAAATATACTCAAAATATTATGTCACATAAGATTTGGTTATTAGTGCTCGTCTCCACGTT
AATTCCATTAATACCATTTTACAAAATATCGAATTTTACATTTTCAAAAGATATGATGAATCGAAATGTATCTGA
CACGACTTCTTCGGTTAGTCATATGTTAGATGGTCAACAATCATCTGTTACGAAAGACTTAGCAATTAATGTTA
ATCAGTTTGAGACCTCAAATATAACGTATATGATTCTTTTGATATGGGTATTTGGTAGTTTGTTGTGCTTATTTT
ATATGATTAAGGCATTCCGACAAATTGATGTTATTAAAAGTTCGTCATTGGAATCGTCATATCTTAATGAACGA
CTTAAAGTATGTCAAAGTAAGATGCAGTTCTACAAAAAGCATATAACAATTAGTTATAGTTCAAACATTGATAA
TCCGATGGTATTTGGTTTAGTGAAATCCCAAATTGTACTACCAACTGTCGTAGTCGAAACCATGAATGACAAA
GAAATTGAATATATTATTCTACATGAACTATCACATGTGAAAAGTCATGACTTAATATTCAACCAGCTTTATGTT
GTTTTTAAAATGATATTCTGGTTTAATCCTGCACTATATATAAGTAAAACAATGATGGACAATGACTGTGAAAA
AGTATGTGATAGAAACGTTTTAAAAATTTTGAATCGCCATGAACATATACGTTATGGTGAATCGATATTAAAAT
GCTCTATTTTAAAATCTCAGCACATAAATAATGTGGCAGCACAATATTTACTAGGTTTTAATTCAAATATTAAAG
AACGTGTTAAGTATATTGCACTTTATGATTCAATGCCTAAACCTAATCGAAACAAGCGTATTGTTGCGTATATTG
TATGTAGTATATCGAGCTTCACATGAAACAGCTAAAGAAGCTTTGGGCGATAAAGAGTTAAGAGCCATTGCA
CATGAGTTAACTAAAACAGTTAAGGATAACATGAGTGTTGATTGGTCTAAACGAGACAGTGCTAAAGCTAAA
ATGAGAGTTCAAGTTAGACGCCTATTAAAGAAATATGGCTATCCACCAGATCTTCAAAAAATGGCTGTGGAA
CAAGTTGTAGAGCAAGCAGAATTAATGGCAAGTCAGCAATAAAAAAATAAATCATAATGAGTCCGGGACATA
AAGTTCTTGGATAAGTGAAAAAAGACAATTTCTATTGAAATAATATAGAAATTGTCTTTTTTATAAATTTTTTG
ATTATTTTCAGCTCGTTGAGCTACTACTTTTCTTATATTAAGTGCCATTAATACAAAACCAAGTTCTCTTTTGAC
TTTATTGAGTCCTCGGACAGACATCCGAGTGAAACCCAAAATAGCCTTCATAAATCCAAAAACAGGTTCCAC
ATCAATTTTTCTTTGACTGTAGATATTTTTTGTTTCTGGTTCTGAAAGCTTTTTGTTAATTTGGGATTTAAAATA
TTCCCAGTTATAATTCTTCATTATTTTTTTGTTTGTTTTTGAATTGAAGTTCATACATTGATTTTTCAGAGGACAT
TCTGAACAATCATCACATTCATATAATTTGAAGTCTCGCTTATAACCATACTTATCATGACGATAGGCATATCTTT
TAAAACCTAGCCGTTTATTATTCGGACAAATGAATTCGTCATTAATTTCGTCATAGTTCCAATTTTGAGTATTAA
AGATGTCACTTTTATATTTTTTAGTTTTATCTTTTATAAACATTCCATATGTTATGAGTGGCGTTCGATTAAAGTC
ATCTATAATTGCCTTATAATTTGATTCACTACCATAACCTGCATCAGCTACAATATATTCAGGTAAATGACCGTAG
GTCTCTTGAATTGAATTTAAAAATGGAATCATCGTTCTAGTATCCGTTGGATTTTGATACACATTATAAGATAAA
ACAAATTGGGAATTTGTTGCTATTTGTAAATTATACCCTGGCTTAAGTTGTCCATTTTTCATGTGATCTTCTTTC
ATTCTCATAAATGTCGCATCATAATCTGTCTTAGAATAACTATTTCTATCCTTTAAAATAGATTTTTGAAATTCGT
ATCGATACTTTCGCTCAAAATAATCATTGATTTGCTTTTTGTATTTTTTGATTTTAGTTCTTTTGAGACGTATTTG
TTTTCTTGTTTTAGTACATTTTTCATTGTTGATATGTTGGTTTAAATCTTCGATTTCTTTATCTAAGTGACTACCA
ATCAAATCTATTTCTTCTTTTGTTAATTCATTATCATGATCTTCTTTAATTTCCGGTATGATTTTATTGGTTACCAA
TTCATGGTAGAGGGCTTTAGAATCCTCATTCATCTTTGATTCATGGTTTTGAATACTCTTTTTCCATACAAATGT
ATATCGATTGGCATTTGCTTCAATTTTTGTACCATCAATAAAAATAGCTTTATCATCTATAAGATTTTGTTTTACA
CACTGACTGTAAAATTGAATAAATAAAGATTCTAATAAAGCATCTACTTTTGGATTTACTCTAAATTGATTAATT
GTTTTATAAGAAGGTTTTTGATTTTGTGATAGCCACATCATTCGGATGCTATCATTAAGCATTTTTTCTATTTTA
CGACCTGAGAATACAGATTGTGTGTAGGCATATAGAATCACTTTTAACATCATTTTAGGATGGTACGAAGTTG
CACCACGGTGATGTCTGAATTCGTCGAATTCATTGTCAGGAATTGTTTCAACAATATCATTTACAGTAAAACG

ATGTTGATTTGTTTTGTTTCCATATTGACCTCCATGTATTTGCTATGATTTCAAAATCCATTTTTGACGTGCCTTA
GGGTTGAGTGGATGCATAATTTCATTTGTTACTGGATTGATGAGCTTTTTTACTTTCTTTTTATGAGGTTTTAA
CATTTCCATCACTTGTTCGACACGGTCGATAACAACTGGTCGCTTCGCATAGGCACCATAAGCAAGAATCACT
GTGTCACTTTCACTAATCGCTTTCATCAAATGAATATCAGTGTGCTCATCGTATGGATTTTTGATATGTTTGAGG
TTTTCGGGTGTTCTAATATTAGAGAATAGATTTACAAGATATACAGCACCGTATCGTTCTGAATTGGCTAATTG
GTTGAGGATAAGAACAGTTGTGAGATCGAGTGATAATACACCGTCTAAATGAGGATACATCGTTATCACTGTG
CATGCAGCTTTCTTTTCATCCCATGTTTTCTTGAGTAAATAGCGGTGCTGTTCATCATCGCTAAATATGGCTTCT
GTGTGTATCGTACTTTTGATTGTATTCATCATCGTCACTTCCTTTAGTATTCTTCTGGTAAAAGCATCACATAATA
AAAAGCGTCCACGTCATCTTCACGAATGACGTAGACTTTCTTAGGTAATGCATTTTGATTTTTTTCATAGTTTG
TATAGTGATATTCCAATTTGTATGTGGGTTGTTCTTGTTCATGTGTGATTGAGAGTATATTCTCATCTTCTTGTAA
TTTAAAAATGTGTAGGTAATCTGTATGAGGCTGGTTATCTTTTTTTTCTACCATGTGCCAAAGTAAGATTTGAA
GGTCTAGTGGAATACTTTCATTAATTCCTCTTGTGATGTATCGATTGATTTTCATGCTATTTCCCTCCCTTCTGCT
TTTCTTTCATGATGTCGATGATTTCGTTGATAACTGTGACGGATAATTGAGCAACTATGATCCAATTATTCATGG
TCCTGACCTCCTTGTTTTAGTAAATGACGTTCATCAATAATGATATTTTGAGTATCTGTAAGGTACAGAAAGTC
CATGTCAAAATGGTCTAAGTATCCGACACTGATGAGTTGGTTATTGGCATACATTAGAAATGGATAGATACTTA
GCTCATGTAGTTCATCATTATAGTAGGTATAAGTCTCGAGTGTGAGATGTACCAGTGGAGAATCATTAATAAAA
CGTTCCGGTAGAATATTTTCTGCTGCTTCCTCCAGCGCTTCACATTCCCAAGCTTCGTTATTAGATAGTTGGAA
TAGGTGGGTTATATATTGTTTGAGTTCTTGAGTGATGGTTTTCATATTATTGCCTCCTAGATAGTGTAATAGTGA
TGTAGTTCATATACATCATTGAGATAATATATATTTGATTTGTCATTTATTACGAATCCCGGTGGGAATAAGAGA
AAATTCCATATAAAAACCCGCTACAAACGTTGGTATGCCAAGGAAATCCTGAAATTCCGCCTATTTTGACAAA
CAATCAACTCATTATTTATAAGTATTGATGATAGGGTTGTGTCTCTGCTTCCTTATATATATTATTTATTTATAAAA
AGTAACGGGATTTTGGGATTGTGCTTGCACAATCCTTCTGTTTCTTCGAATCTGCAAATCCCAATCATTTCCCG
ATAAAAAATCATTGTGGGATGTTCTTTAGCAATTTCAATATAAGCATTGTATAGTTATGAAAAAATTACGACAA
TAACTGTTTCATTAGATAAGTGTTATTGAAATTGATAAAGAGCAATTCTTGAAAATAGTTAGATAAAATAAGCG
AAAGAATATAGTGAAAATTATTGTTATAACAATGATTCTATTAGCTAAATAGTAAGATATAGTGTTTGGGGCAA
AAATAAAGACGAAGTGCTGAGATGCACTTCGTCGAGTTGTTTATTATTGAAAAGTTGTTTAATGATTTCGTTA
TTAAGTTTGAGTGTGACATAGAATTGTTTTTTATGATTACCATCTTTTTTAATATCAATGCGATCAATCACTGAT
AGATACAATGCTTTGAGTCGAGATTTTTCTATGTGCTTAATATCATGAAAGATGTGTTGTAATAGTTTACTGATT
TCTTTGGCATCAAATAAAGTCTTATCTTCATTTTGTTGATTTTTGAGTTGGTTGATTTGATTCGTAATGTCATTG
AGTTGCTTTTCATATTTTTGAATACTTGGTCTGATTACTGATGTTAAGTCCGGATTATCCTCGATGGTTTTAATC
AAGTTATTTATTTTGATTTGTACTTCATCATATTGTTGTTGCTTATAAGCAATATCGTGATGAAGTGCAGCGCCA
TCAACTTGATTTTCTTGATTGACGTGTGTTACTACGCGTTGAATGACTTTATCACTTTTGACTATTTCAAGTATT
TGCTTCATCACATAATCTTCAATCACATCAGCTCTTACACTGTTTGCCGAACATACTTTGGAACCCTTGTTCCG
AAAATTACTACATGAATAGTAACGAATACGTTTCTTAGTCCCGTCTTTAAGAGTATTCGTGGTATTGCTTGCTG
CCATAGGTGCGCCACATTGGGGACAGTGAATAATGCCTGTAAGCAGATTCGTTCCTTTGCCATGGACTTGGG
GTTTTTGACTGACTTGTTTCTTACGCATTTGTACTTTATCCCATAAATCTTGATTAATAATGGGGGAATGCTTAC
CTTCAGCTATCACTGGTTTATCATTCAGCCCTTTACGACGTTTTTCACTCCAATCTTTGTATTTCGCAAATTGAA
TTTTGCCGATATAGAAAGGGTTAGCTAAGATGTATGTGATTGAACTAATACTGAAAGGTTTCCCCTTTTTAGT
GACATATCCTTTGTGATTCAATGCATTGGCAATTTTACGATAGCCATGTCCTTTGGCATAGCACTCGAATATATA
TTTTACAATATTAGCTTCATGTTGGTTAATCATTAGCTCGTGTTTACTATCTGGTATTTTGTCATAACCTAGTGGT
AAATTGCCTTGATAATAGCCTTCTTGGGCACGTCTCGTTTGACCCATAAATACGTTCTCGACAATGTTATTACG
TTCGAATTCTGAGAAACTCGCAAGTATCTGTAACATGAGTTTACCAGAAGAAGTATTGACTTCCATACGCTCT
GACAAACTGAAAAATTCGACATTTTGTTTGTGTAAATCTTCGACAATTTTGAGAAGATCAGATGTATTACGAG
CTAATCGGTTTGTTTTGTATACCATAACACAGTCGATATTGCCTTCTTTTGCATCTTTCAACATACGTTGGAGCT

CAGGTCTGTTCATAGATTTACCTGAAATACCACGGTCAGCGTATATATCTTTAACTTCAAAATGATGGAAGTCA
CAGTATTCTTTGATTTGATTGATTTGTCCGTCGATACTATAACCTTCTGTGCTTTGCATTTCTGTTGATACACGT
ACATAGATACCGACACGTTTTGTTTTAAGTTGTTGCATTATGTTACATCCTTTCTTCATTTATGCAATCGATGAT
TGCATGGTTTGATTGACAATATTGAGTGGTTCATTTTTGAAATAGATTCCTATAAGATTTTTATCTTTCGTAATG
TGAATGGTTTCAATATAGGGGTACAATATGTTTAACGTGAAACGTTTTTGAATAATATTTTGAATGATGTGTTG
TATTTGATGCCCATTGATAGATGTAGTGCGTTGCGGTTGTTGACGTAATGATTGTGTTTGCTCTCTGAACGTTT
CTGCATCAATGATGCCTTGTGCCAACTTTTCTATCAGTTGTTCTTGAGTCAATGTGTGATGTTTTTCTATGTTTC
TTTGTCTTTTGATGCGTTTGTCAATCGCACTTTTAATTTTTGTGTAGATGCGTTGATTTTGATAAAAGTCTCGG
CACACTTCTAATACTTTATCTTCAAGTGTTTGTGCATTGATGCCTTTAAAATCACAGACAAAGCGTGAAGCATT
CATGTTTTTAGGACAGACGTAGTAACGTAATGTATGATTCTTTTTTCTAATGGTCATATTTGTAAGTGTTGCATT
ACAACATGGGCATTTGATTTTTTGTTTGAGTTGATTATCCGAAGGTGTCTGTTTGGTTTGTTTTTGCAATCGA
AGTCTCTGCGCTTGCTCATATATACTTGTGGAAACAATAGAAGGAAACATATTGTCGAATTGGCCATATTGATT
GTTGACACGACCACAATAATTAGGATTGATGATAATGTTACGAACTTGATAGGGTTGTCGATTGATATACGTGT
TATCTTCTTCTAATAACTGTGCAATTTTCTTATAACCATGACCTTTAATGTAATAATTGAATACAGCCTTTACCGT
TGGTGACTCATTTTGATTGATGATGAATGTTCCGTTGTGATATTCGTAACCAAAAGGTGCATGTGTTGTAATCA
ATCGACCTTGCTTTGCTTTTTCTTGAAGCCCATTTCTGACTTGTTCTCCAATGTTATCCGATTCAAGTTCGGCT
AAACTGATGAAGATATTAAGCTTGAGTCGGTCGAAAGCTTGATCCATATCAAAGTAACCATCATGTACGCTTA
AGATATGAACATGGTACGTTTGACATAATTTGATGAGTTTTAATGCATTTTTCAGATTACGATGCAACCTATTA
AGACGATAACAGCATAATATGTCACACTGTCCTTGTTGAATTAATTGTGTTATTTGTCGATACCCACTACGATTA
TCTTTGCGACCTGATTGTTTGTCGCTATAAAAGTTGATATGTTGAATATGATGTTTTTCGGCTATTGCTTCGATA
GCTTGTTTCTGTGCTGCAAGAGATTGTTGTTTCATCGTACTTTGACGTAAATAGCCTATGACTTGTTTCATATC
GGCTCCTCCTTTCACAGTGATAATATATATTTATGGATGAATTGATATATAAGCCCAACATCAATGAGATGTTGG
GCGTCCATATTAGTCATTTGTTTGATTGATTTCTTCAATTACCAAATCGGCTAATATCTCGATAAGTTCATCCATG
TTTTTCACTCCGTTATTTGTTCTATCTTCAATACGTCGATTATTCAGTTTGATGCTTCACAGTTGTATGATAAAG
ACAATTAGAAATCTTCGTGAACTCCTGAAGGGCCTATCCCTTCATTAGCGGATTTAAAAAGTTCTTTCGCAGC
TTTGTTATCATTTGACGGTGTCCAATTTTGAAGTAACGACTTATCTTTAGTTAATCCGAGGATAGATGCAAACT
CTACATCTAATTTTAGATGGTAAAATACAAGTGATTGTTTTTTACCGCTATTATCTTTGACACTTCTTTTAGTTGT
TTGGCGTCCACGGTCAGCTAATATGAAACCTTTATCTCTTAAGGCGTTGACAACATTATTAACATCTTGAAATT
GATGATTGTTTAGCATCTGTTTAAAAACGTTCGCAATTATTTTTACTTCGATATGGTCATCTTTTAATGAGATTA
ATCCATAGTTCTCAAACATATTTTTCAAAGCACCTTCATCTGAAAACTTACCTCTGTTTTGTGCCACAAATTGA
ATGATGACATCAATAGCTTTATCAGCTAATGAGCGTTCAGAGACTGTATGAGTATGATAATCAATAAAGTAGTC
TCTTATATTAGCGATATCAATATCTGTAGATAAAACACGACCTAATATTTTCGCAGATGTTGTAATGACTGCATA
ACGCTTAAACATACGAATACCTGTATTGTTTGTTTCATCTTTCAATTTAGCTTCAAACCAATCTACTTCCTTGTA
AAACCATTGAATAACTTCATCTTCACGATTTATAAGATATTTAGCTACTAACGGTAAAACATGACCATAGTTTAG
TGCTACAGCTTTTTTAATATTGTCAGCATTGGTCGCATTTGTAGTGAATTGTTCATTAATCTCGATGGTTCTTAC
ACGTAATCCATCGTTTTGAGCTGAATCATTAAAAATACTGTATTCTGACGTTGAAATGACAGAAGTACCCCAAT
TCTTAGGCGTTTTAACTTCTCCATGAACGTTTGAACGTTGACGACCTTGACCTTCAGCGATGGAATATAACAA
ACCCGTGGTATCTCTAAGTGTCGTAGATGAAAGCTCATCAAATACTATAGGAATGCCATAATTGTTACTCAAAT
AACCTTCAAGTGCGTTTCGTGTGGCATTCCAACTTCTAAAAAGAGTTTCATTACCTTTGGTAGGGTTACCAGC
GACTGATACAGCTAAAGCAGCTGCGGTTGACTTACCGGTTGAGGATTGACCTGTAAAACTAAAGAGAATTC
CTGCAAATTCGATTTCATGTTTATGCTTCAGAAAACTTGTCACTAAGGCAGAAATCCCAAATATGACTGCCAA
TTCTAAAAGAAGATGACCTTTAACCTCGTCAATATACATGTTAAACCAATTATCAAATGTACCTTTAGGAGTTA
AGTCATAAGCACTATCTACAATAGGGTATGAAGGTGAGGACTGATTAAATTGAATAGATTTGAGAACTTTATC
TAGCGATATCAAGTAGCCAAAAGGTGTCTCTAGTATACCTACACCCTCATATAATTCAGAAAGTGGTAATCTAT

CTCGCATCAATTGCAAGGCATAACTCAATGATCTAATATACTTCTCGTTAATACTGTAGCCATATTTAATTAAAG
AAGGTAAGTTTCGTGTTGTTAAGATATCAGACTCAAAAATGTCTTTTTTACCATTGTTGTTAGTAATAATCAAC
TTTTCAACACCAGTAATTGGATCAGAAAATCTTGCATTAACAATAATAGGGCTAGCCATTCTAACCACTTTTTC
ACTGTCACCATCTTTTTTAGGTGGTATCGTTTCTTTCCAACCGAAGGAATCTAAAGCAAATGGGCCAATTTCA
AACAATGTGTAACTCATTAGCAATCACCTCCTTTCGAAGGCGTGCTATTATTGCATGGGTTTATCCCTATTTTCT
GATATAATAAACTGCCATTTTTTGTTCCTACAATTTTAAATGGTAATCCTGGCGTGTACTTAATCCAGTAAGCAA
AACGACGTCCTACTTCACGTTGATCATTCGTGCTACAAACCACATTATATTTCTTTCTTAATTCACCATAAGTGA
ACGTACTTCCAACTGGTTGTTGAAAAGATTTAGCAATTATAAAATTATAAAGCTTTTCTTGCTGCTTAGATTTC
TTATTCATTTTAAAATTCCTCCAATAATTTTTAAGGAGTCAATCAATATCATATAGGTTAAACTATCTTCAATTGA
TTGCTATAATAAGCATAAATGAAGCATTATGATTAGACGCGGAACAAAAAATGGGGAGGGGGGGTATATATT
GATACCTACGCTAGAAGAAATTATTGATAAGTATGGAAACTTAGTTGATTATTTGAAATTTGATGTAACAGTTG
AGGTTTATGAAGATTTGTTGTTATTAAGAAGTTTAAGCGATTTAAATGAACATCAAAAGATGGATAGAGTTTC
ATTTATAAAAAAACATTTAAACTCAAATTTCGATTATTTATTAGATGATACTGAGTTTAGAAGTATCAATAAGAT
TTACAGTAATTTAAATATCATGACGCATATCAATAGTCAAAATAATAATTTTAAACGTAATCCATTTATAAATGAA
GAACAATTGAAATCACTACTCGCGATAAAGAAAGTAGATGCACATATGAGTTATGATTCAAATATTACTTCAAA
AGCATTGGCAGAAATAAACAAAACGCAAAAAAGATTAGTTACAAAGATAGACACACTCTATAATCAGTCAAA
AAAAGAAAATGAATATGTACGGTTAGGAGAAAAAATATCGTATACAAAATTGGAGAATCATTGGAAATATTTA
TATAATGAGATTCAGTTTTATAATCTTAATAATCAATTGATTAGTTATGTTGCATTAGAACAGGAATATGCTTGG
GCTTTTTTAAATGAACTATTTTATTTGATAGAATTGTATTTGAAGGCATTTAAAGGACAAAAGAAAACAGATT
ATGAGTTTGGGAAAAAATTAAACGAATTTATTCAATCATATGTCATTATTTTACTTATAGATATACGATTACCTGT
TGTTAGATTATATATTATTAGAGACATTGTAGATACATGTGAAGGAGAAAAAGATAATTACAAAAGAATTACAC
TGATAGAAGAATCTATTAAAAAGTATAGATACGTAAAAGATCAGATTAAAAGATTTAAAAATGGATTAGAAGA
ATCGTTATTAAATGCAACTTTATCTATTGAACAAAATATGTTGAAAGTTAAAATTGATTATTATAAAGCCTACTG
CTTTCCTAGAGAAAAAACAAAGCATAACAATATTGAGTACAATGTATCGTTGTTTTTTAAAGCATTAGATGCCT
TAAAGAAATAGTTTTTCACTGAAAATATTTATAGATATAATTAAAATTAATTAATATTAAGCCAGGATAATGTAG
TCTTAATCGTTCTGAAATACGAAAAATGTTGTGGAAATCGCGTGAAATAATCAGAGGAATCGTTTGAAATCAT
CGTAGTAATCGCGCGAAAATCACGTGAAATAATCAGAGGAATCGTTTGAAATCATCGTAGTAATCGCGCGAA
AATCACGTGAAATAATCAGAGGAATCGTTTGAAATCATCGTAGTAATCGCGCGAAAATCACGTGAAATAATCA
GAGGAATCGTTTGAAATCATCGTAGTAATCGCGCGAAAATCACGTGAAATAATCAGAGGAATCGTTTGAAAT
CATCGTAGTAATCGCGCGAAAATCGCGTGAAATAATCAGAGGAATCGTTTGAAATCATCGTAGTAATCGCGCG
AAAATCGCGTGAAATAATCAGAGGAATCGTTTGAAATCATCGTAGTAATCGCGCGAAAATCGCGTGAAATAC
TATGGTAGACGGTTTGAGTAAATTAATGGAGTATTTTAATATTTATGGTTTGGAGGAATTGCGTTGCATAGAG
AAAGTAAATATATAGAGTATAAGAAATCACGAAAAGGATTATCTAATGATATTTGGTCTACGTATAGTGCTTTT
GCAAATACTGAAGGTGGTACTATATATTTAGGAATTGAAGAAAAAAAGATCGAGGACAAAAAAGTCTTTGTT
TCAGTTGGTGTTGAAGATCCAGAGAAAATGATTGAAGATTTTTGGAATGCACTATATGGAAGAAGTAAAGTT
AGTCAAAATATTTTATCAAATAAAGATGTTAAAATTGTTAATATTGAAAATAAAGCGTGCATTGAAATTCATGT
ACCAGAAGCGCCTTATTCGAAGAAACCGATATATGTAGATAATAAAAAAGATTTAGTATATAAAAGAGTTGAT
GATGCTGATAGAATTGCGACTGAAGAAGAGTATAAATTCATGATTGTAAATTCTCAAGACGATATAGATACAG
AATTATTAGATAACTATGACATGTCTGATTTAAATCACGAATCTATCGAAAATTATAGGAAACTTCTATTAAAAA
ATACTAATGATGAGAGATATGCGAATATGAGCCAACTGGATTTAATGATAGATTTAGGAGCATATAGAAAAGA
TAGAAGTTCGAAAGACAAACAGTATAAAATGACTACAGCATGTTTATTATTCTTTGGTAAGTATAATGCGATTA
GTGATAGATTCCCAGGATTTCAATTAGATTATTTTAAGAAAACAAATTACCTAGATACTGATTGGAAAGATAG
AATATCAAGTGGAGATTTAGGTAATGAAGATTTAAACGTGTATAGTTTTTTTGAAAAAGTATTGATAAAATTAA
CTGATAACATTGAGGAATCATTTAGCCTAAATGATGGTTTGACTAGACAAAATTATGCAAGAGATTTAAAAGT

AGCAATTCGCGAAGCACTGGTTAATACATTAATGCATGCGTATTATGATACTAAGCAAAGTATTAAAATAGTTA
ATTGTGAAGATTTTATAGAGTTTTATAATCCGGGTAATATGAGAATAAATAAAGAAGATTTTATTCATGGAGGG
CATTCAAAGGACAGAAATAGTATATTATCGACGCTTTTCAGAAGAGTAGGATATTCAGAAAAAGCTGGATCT
GGAGGACCAAGGATATTCGATGTAGTTAATAGACATAAGCTTAAAACGCCTGAAATAGAATTAACGGACATG
GACACTAATGTAGTACTTTGGAAACAAGATTTAATGAAGGAGTTTGAAAAATATCCTGAGTTAGACAAAAAA
GTAATAAAGTATATTATTGACTATGGATCAATAAGTAAGGGTGAAGCCTTAAAAATGGAAAATATGACAGAAT
ATCAGTTTAGAAATATTTTAAAAAAACTAAAAGATGATAACTTGATAAAAAAAGAAGGTGAAGGTCCGGCTA
CTAAATATGTGTTAATAGAATCAAAAGAAGCTGATATATTGCGAACTAAAAAAGTAATTAAAAGTTTAGAGTC
TTTCTTTAGGAATAAATAAAAAAACAAGAGATAGGTGCGAAGTGTTTGGATTATACAAATGCTTCGCATCTTA
TATTTATTAATAAATATCATAGAAAACCGTATCATTAACCGATACGCAGAGATGCGGTTTTTAGACACTTCATA
AAGGGATTTTGAACGTATCAGAACATATGAGGTTTATAGGAATTGCTGTTATGTTTTTTGATCACATCAATAAA
CAAAAAAGGTATGTACTATGTAAAATATTTATTAAATGATATAAAGCGAGGGTATATAAATGATTTTTAAATAGA
TATTTATCCAATAATATAAAAAGGAACTATAAGCTATATCTAAAAGCTATATCTAACTACTTATAGTCCTTCTTCAT
TAGTATAAATATAATTATTAAGAAAGGTATATATCTTTGTAACCTTCGTTTACATTAATAATGTTGTGATAACCTTT
GTGTTCTAAAATACCAATAGCTATAGAACTTCTAATGCCAGACTGACAGTCTACATAAATAACATCGTTTCTATT
GAAAGGTAAATCTGTTTCTAAAAGTTTGCCGTGTGGCACATGAACCGCTTGGGATAAGTGGCCATTATTCCA
TTCATTATCATTACGTACATCTAAGACATGTGCTTCATTACCAGTTATGTCTTTACTATGAACAGATTGTGTTTG
AATTTGAGCTTGTGGTAACTGATATCCAGACACATTATCATATCCAATAAGTTGTAAAGTATGTTATGTGTTGCT
TTTGAAACAAGGTGATAGTCTCCAATCAAGTTAATTTCTTGATTATAGTTTAGATACCAGCCAATTTGATTGAT
GAAATTTTTATCATATGGAATATTGATTGTACCTTCAATATGTCCACCATGATAAGCCTCCTTACTGCGGAGATC
AAAAGTTAATCTGTTTGTACTTGTAGCTGGATAAACCGTATAAGGTTGATATAAATTCATACCGAATTGATTAAT
TTTTTTCATTTGTGCAAAATGATGTGGTGGTGCAGGTTGGTCAGAAATGAGTTTATCGATAAAGGTAGCTTCA
TTATTTTCAGAAAAAGCCCAGTTCGTTTGTTTTTCATAGCCAAGCGTAGATGTTGGAATAGCACCTAAAGATT
TACCACAAGGACTACCAGCGCCATGACCAGGCCAAATTTGAATGTAGTCTGGCAAGTCTTTAATACTTTCAAT
AGATTTAAACATTTGTTTTGCGCCTATTTCAGATAATCCTTCTACTTTAACAGCTTTTTCTAGTAAATCAGGTCT
ACCGATATCTCCTACAAAAATAAAATCACCACTGAATAGTCCCATTGGAACTTGTGCTCCAGCACCTTCGTCA
GTAAGTAAAAAACTTATACTTTCTGGCGTGTGACCAGGTGTATGAAGCACTTTTAATTTTATATTTCCTACATA
AATATCATCATTATGTTGAACAAAATGAGTGTGGTTAGGCATATTTTTATAACCTAACATGTCATCACTTTCGCC
CGATACATAAATACTAGCATTTAACTTTATAGCAACATCTCTAATTCCTGAAACAAAATTTGCATGTATATGTGTT
TCAGCTGCATGAGTAATGGTTAAACTCTCTTCATCGGCAACTCGAATATATGAAGATAAGTCACAAATAGGAT
CAATGATCATGGCTTCTCCAGTTTTTTGACAACCGATTAAATAAGATGCTTGAGATAAATGTTTATCATAAATT
GATGATAGTCAATTTGTTATCAAGGGTGATAATTATATTAACTATGGTAATTACATGGAATTAAAGTTATTAAAC
AAACTTGATGATGACCAATTAGATTTAGAATTTTTACAAGATAATGGGTTAAAAGGTAATGTTCAAGGTCCTA
TGACTTTAAAGGAACCTTTAAAAAGTTATATACAAAAGTCTTGAAAACGAATATTTTAAATTTAGATAATGAGT
TAGAAAACACTGAATATGTCGAAGGTAAACCATATGTACAGTACGGTATTAAATATGAAAGAAATCAGGCATT
AAGAAATGAAGCTATTAAAATTCATGGAACTACATGTAAAGTATGTGGATTTGATTTTAAAGCTAAGTATGGC
GATTTAGGTGAGGGTTTTATTGAAATTCATCATTTAAAACCAATGTTTTCAATAAAAAGAGAAATAAAAGTAA
ATCCACAAAAAGATTTAGTCCCACTATGTTCTAATTGCCATAAAATGATTCATAGAAATACTAAAAAACCTTTA
ACGATTAAAGAATTAACCAAAATAGTTAATTATAATAGCAAATAATTTAATATTTTATAAACTATCATTCAACCCT
CTTAATTTATTAGGAGGTTTTTTGTATTTATGCTTTCAAATGTGTGATATACTTTGTTTGTTGAAATATAGAGTAT
CTATAGATAGGGTGATTGAGTATGAAATTCACTGAAGTGGAAGTTATCGAACATCTTGTAAAGGCATATAAAG
AAGCAGGAAAGCCTACTTATCCTCATGAAAATTTATATCGAGGACGTAATCATAGTATTTCAGGTATTGGAGA
AGACTTGCTGGGTGCTTATTTGATTAGTAGATTGGAAGGTGTCCAAATATTTATTGATCAGCCTTTATCTATGA
TTGATAAATCTTTAAGTACAAGATATCCGGATTTATTAATTTGTGAAGATAATGAAATTAAAAATATACTAGAAG

TTAAAATGGACTTAGGATATCAAAGAAAAGATTTTATAGATTATTGCCGAAAGAAAGAAGAATGGATTTCAA
ATATCGTAGGAAAACAGTGTGTATTGTCTAGAAAGAGAGAAGACAAAATTCCTATGAATATAGCTGATGATAT
TAAATTTCATGTTGTGATTTACAGTGAAAACAATGGACCGAAGCGGTTTGATGAAGAAATCATGCCTATCGTT
AATGAAACATGTCCACATATTGAAGTATATGTCCTAACAAGCGGTCAACACCCTAATTTAGTAAATGTTAATCT
TGAAGGTATTAATATTAATAAAGATGAATTTGAAATATTAGTAAATGCGTTATAAAAAAATAGAGCATCCTCCA
CGTTATGGAGGTGCTCTGTTTTTTATTGAAAAGTATCAAGTTAATTAATTTAATATGCTTAATAAGTTCTACCTT
GACCTTTTTCTCTAGCTTCTGTTCGATCTCTTATGTACTCAGTACATACTGGATTTTCTGTTAATTCATCAACTGT
ATTAGTTTAATCAGAAGACGTGTCTACTTTGTAAGCTTCTAAGAACTTATTATATGATATAGCGTTTGAGTTTTG
TTGTTCTTCTATAAATTTCTGTAGTTATTTTTCAAAAACCGCATCATTAACTGATAAGCAGAGGCGTATCATAAA
T
>Staphylococcus argenteus strain RIVM_M020832
ATGAAAATCACCATTTTAGCTGTAGGGAAACTAAAAGAGAAATATTGGAAGCAAGCCATAGCAGAATATGAA
AAACGTTTAGGCCCATACACCAAGATAGACATCATAGAAGTTCCAGACGAAAAAGCACCAGAAAATATGAG
CGACAAAGAAATTGAGCAAGTAAAAGAAAAAGAAGGCCAACGAATATTAGCCAAAATCAAACCACAATCA
ACAGTCATTACATTAGAAATACAAGGAAAGATGCTATCTTCCGAAGGATTGGCCCAAGAATTGAACCAACGC
ATGACCCAAGGGCAAAGCGACTTTGTATTCGTCATTGGCGGATCAAACGGCCTGCACAAGGACGTCTTACA
ACGTAGTAACTACGCGCTATCATTCAGTAAAATGACATTCCCACATCAAATGATGCGGGTTGTGTTAATTGAG
CAAGTGTATAGAGCGTTTAAGATTATGCGTGGAGAAGCATATCATAAATGATGCGGTTTTTTCAGCCGCTTCA
TAAAGGGATTTTGAATGTATCAGAACATATGAGGTTTATGTGAATTGCTGTTATGTTTTTAAGAAGCTTATCAT
AAGTAATGAGGTTCATGATTTTTGACATAGTTAGCCTCCGCAGTCTTTCATTTCAAGTAAATAATAGCGAAATA
TTCTTTATACTGAATACTTATAGTGAAGCAAAGTTCTAGCTTTGAGAAAATTCTTTCTGCAACTAAATATAGTA
AATTACGGTAAAATATAAATAAGTACATATTGAAGAAAATGAGACATAATATATTTTATAATAGGAGGGAATTT
CAAATGATAGACAACTTTATGCAGGTCCTTAAATTAATTAAAGAGAAACGTACCAATAATGTAGTTAAAAAAT
CTGATTGGGATAAAGGTGATCTATATAAAACTTTAGTCCATGATAAGTTACCCAAGCAGTTAAAAGTGCATATA
AAAGAAGATAAATATTCAGTTGTAGGGAAGGTTGCTACTGGGAACTATAGTAAAGTTCCTTGGATTTCAATAT
ATGATGAGAATATAACAAAAGAAACAAAGGATGGATATTATTTGGTATATCTTTTTCATCCGGAAGGAGAAGG
CATATACTTATCTTTGAATCAAGGATGGTCAAAGATAAGTGATATGTTTCCGCGGGATAAAAATGCTGCAAAA
CAAAGAGCATTAACTTTATCTTCCGAACTCAATAAATATATTACATCAAATGAATTTAATACTGGAAGATTTTAT
TACGCAGAAAATAAAGATTCATCTTATGATTTAAAAAATGATTATCCATCAGGATATTCTCATGGATCAATAAGA
TTCAAATATTATGATTTGAATGAAGGATTCACAGAAGAAGATATGCTAGAGGATTTAAAGAAATTTTTAGAAC
TATTTAATGAATTAGCTTCAAAAGTTACAAAAACATCCTATGATAGCTTGGTCAATAGCATAGACGAAATACAG
GAAGACAGCGAAATTGAAGAAATTAGAACAGCACAAAAAGATAAGACACTCAAGGAAGTGGAAGCACCTA
AAGGAATAATTCCAAAATATAAAAAAGGTGTATCAAAGACTACTAAAAATGATTCAGAAATTGAAAAATCAA
ATAAAGAGAATAAATTAACCGGTAAAGTTGGAGAAAAATTAGCGCTAAATTACTTTAATGAGCTAATTGATAA
TAAAATAGACGAAGATAAGAAAGAACAGTTTAGGAATATTTTAAATGATAATCCAGGCTCTCAACACGGTCAT
GGCTATGATTTAGTAGCTTTTGATCCAACAAATACAGATAAAGCTGTAGAAAAATTTATTGAAATTAAAACATC
TACATCTTCTAGTATTGAGGAACCATTTTTTATGTCGCTAAATGAAATGTTTGCTATGAAAGAATATAAGCAGA
AATATTTAATATTAAGAATATTTAATGTTTCCGGTAAAGAATAAGTAGTTCAGTTTTGGAGTACAAAAAGTGAA
AATGTAAACCTAAATGAGTATCAAATTGCAACTGAATTTGATACTCGTTTTTTATTGTTAAATCAACGTTTGTTG
TTAATTTACACCACAAAATGTAAGTTAAAATGTATATAAAAATGCGAACGTGAAATAATTATTGTAGGAATGCA
AATTGGTGTGTTAAAATGGTTACAATTTACTAAGCAAAAAGGGGTGCATTTTCCTATGCAATTTCTTGAGCCA
AAAAATAAGAACGCTAAGTCTGTGGATTGGGAAATATCAGAGCAAGTAAGGGTGATAGTTAAGCAATACGC
TGAATATGCAGAGCGTACTGAAAGCGAAGCAGTGGATGAATTCTTATTGAACATTTTAGACGATAAGAAGTT
TATTGAATGGATTGCCAACAAGCGAAGTAATAAGCGGATTGTTGAAAAAAATGGGTATCAAAGATAGAGTGG

GTTGATAGACAATGACCAAGCTTAAAAGATTAGCCACGAAGGAAGATGAAATTGTAGATGTTAAAATTCCGA
TTTCAAACGAAGAATTAAAAGATAGGGCTAAACAATATGATTTATTAACTCCAAAACGATTTGCAACAAGGTA
TAACAAAATGTTGTTCTTGCCAACGTCTTTTAAATGGAACGGTTCTGAATATCCCATTCAATATAATTACTGCAT
CAATCCGTTTTGTTGTAATTTTGGGAAAGAACAGCACAAATTCAAAGATGTAAAAGGGAAACCAAGTCGAT
ATAAAATGACTGGTAGTTCCAAAGATAAAGGTCATAAAGGTATGTACTGCAATGACAATCCTATTGGCAGAG
GTGTATCGCAAAACTGTACAGTAACCCCTTTATCTAATTGGTCTGTTGTTGAAGAAATTAAACGCCTTATAGAA
ATCAATAGCATCCAAGATGTTGAGCCAGATTATCAATTTCATAAAGAAGGCTGCTCGGAAGAAGAATCAACC
CCATTCAATGAGCCGAAGCAATTTTACAAAAGGGGGAAAAGTAGGGGCAAATCTCAACGCTATCAATGTAA
GGCGTGTAAGAAGTTCACTAATGTTCTACCAAAAAGAGAAGAAACCACTACTTATCACCAACAAAAGAACA
CCATTCTTCCAATGTTCGCTAAGATGGTTGTTGGAAGGGTTTCAGTCAGTCGTACTTGTGATATTTTAGGAAT
TGGAGTTGGAACCTACTATCATAAGCTGGAGTGGCTGTATAGGCGTTGTTTGGAGTTTTTAGAACGATATGA
GACACAACCCCTTCAAACAAAGAAATTTAACGAGATGTGGCTTAATACGGACAAGATGCACTACTACCTAAA
TAACGTCCGTAAGAAAGGACAAGGTTCAAAGAAATATACGGGGTTTGAAGACCTTAATATGCAAACGTATAT
AGTTGTATCTGCTGAGGTCTTATCAAGATATGTGTTCCGTTCTGATGTTGCCTATGATTGGAATATTTCAATGG
ATGAATTAAATGAAGATACAAGAAAATTCAAAGAAGACCATCTCAATACTTTTAGTCGTAAAAATGATAGGTT
AGATTGGTCTTATTATCCACAAGAACCATCCGCTAACGATAGCGAAAATAGGAATGCTTATCTTCACGAATTA
GGGAAAATTACTAATAGGAGTAGATTTGTCGATGGTTTAAATGTTGATGCTCCTTATACTACAACTGCTCACTA
TTGGTTAATTAAACAAATGGTTAATGCCGACGAATGGCGAATGATTAGTGATGATGACTTCTCTATTAGAAAC
GCCTTTTATAGAGTATTCACGAAAGAATTAAGGTTGAGCGATGCACATCATTTTATTTGCCAAGTTAACAAGA
CGAAAAGCAGAAAACAATGCTTAAAAGAATTCGGACAAGCAAAAGCAGAACTGCTCGATTGGGGAGATAT
CAGAGGATTCAAAACAAAGTTTCTACGGACTTTGGCATCTCATTATCTAACTGAATTACTAACAAGTCATCAAT
TCCACGAAGAGGCTATTAGTAAAGATGGAGAAAGATATCGCAAATATGCCGATAACCCTATCAAACATCCATT
AGCCACTAAGGATAAAGGATTTTATTCAGTTGATTGTAGAACGGATTTATCTGCTCTGGAACCAAACGAAATC
GCAAAGATGCTATTAAATGTTAATGACCATTCGACCAATAGCTTTATTCAACAGATAAGACGTTATATATCATCT
CTTGAAAGACCATTAACAACAGCTCGTGGTGATAAGAAGAGCTATATTTACGCTAACTTCAATCCGAAGTATG
CTCAATTTGCGATTACAATTCTAAGAACTTACTACAACTTTTGTCGCCCTTTTAAATCAGCGGATAAAAAGGTT
TTGACACCTGCTCAAAGATTAGGGATTACAGATAAACAATTTGATTGGAAAGATATAATTTATTTCAAATGAC
AAAAAGAGCTTGGAGTAATCCCAAGCTCTTGTGCTTTATCTTTACTGTTCTTCTTCAATTAACGCACCCGTTAA
CTCAATAAGTTTTTTCTTTCATATACATGAAAATAGTAGTTATACGGATTTTTATCATTCTTTATCCCTTTTTGGG
CAAATACCTCATTCCATTCCTCATAATTCACTTCTGGAAAAAAAGTATCTCCTTCGAATTCATGATGTATTTTTG
TGATGTACATTTTCTCAACATAAGGGAAAAACAAATTATAAATCTGTTCTCCTCCGAAAATAAAAATTTCTTCT
TCGTTTTTACATAACTCAAAAACATCTTCTATTGAATGAACAATTTCACAACCATTAAAGGTAAACCCCTTATCT
CTCGTCAGAATAATATTTCTTCTGTCAGGTAAGGCTCTTCCGATTGATTCAAGGTTCTTCCTACCTAATATTATC
GGATGTCCCTTTGTAGTATTTTTAACATATTCCCAGTCCTTGGGAATCCTCCAAGGAATGTCATTCTCTTTGCC
AATCACTCTATTCTTATCCATCGCAGCAATCAAAGAAACTTTCATTTATAACACAGCCTTTTTAACTAATTTACC
ATCTAATTTCTTATTTCTATCAAAAAACCTTTTCTCATTATCCTGCCCGTTAGTTGAATAAGCAATTTTTTCTATA
AGTTATATTATCATAATACCACAAAATCACAAATTTTCATTCAATTATCATTTTCAATTGCATTTTATAAATCATTT
TGTATGTAATTTGGTATGTAAGTTAAAATGAAAAAAGTAGTCCATTTTGAAGTACATTTTGTCATTTTGTACTCC
AAAACTGAACTACTTAGGTAAAGAACCACAATTTTATTTTATAGATCCATATGCAAATTATTCTGAATTTAAAG
ATGTAGATGATCTCATTGACAAAGTATTTAATGTAGAAGCTATTCAGTATAAAGTTTTTGGCGAAAAATGATTA
CTTGAACAAGAGCTAAAATAAAATTGTGATCTAATAAAAATAGAAACTGTAATTTAAATAAAACTTTCTAAATA
AGCTAACTGATAAAAAATCAGTTTGTCCACAGTCTGAAACAAGATTCCTATATTCTTTAGGAATCTTGTTTTTT
CTATTTTTATGGTGATAAAGAGCAGATAAGATAATGTGTAATAATCACAAAAAAGTTAAATATTTTAAGGCTTG
TTTAATTATTAATGATTTTATATATAAAGAGCAGTATAATAAAGTTGTTAATATATTATGAATAATATTCAAGTAAT

TTTATTGTTTTTTAATTTGTCGATATTTAAGTTGAGTTAAATTTAAAGGGTGTAATTTGTTTTACAATGATGAAG
ATAATTAGTCTATCAAAATAAAGGGGTTGGGACTGTTATGAGTGATAATTTGTCATTATTCATTGACTATATCAA
TGATAATATAATCTATGGTAGTGAAATCAAACGGGAGAAATTAGAGAATTTATTTAATCAATTTGCTATAAAAA
ATGTTGAAAAGAACATTGTCTATGATGAACTGAAATCTTTAGATATTACAATCATTGAGTCACAGGATTCATAT
AAAAATAAATTGAAGAGATTATTTTCGGTTCTGTTGCAAAGTAAAAAAATATAGCTAACCACTAATTTATCATG
TCAGTGTTCGCTTAACTTGCTAGCATGATGCTAATTTCGTGGCATGGCGAAAATCCGTAGATCTGAAGAGACC
TGCGGTTCTTTTTATATAGAGCGTAAATACATTCAATACCTTTTAAAGTATTCTTTGCTGTATTGATACTTTGATA
CCTTGTCTTTCTTACTTTAATATGACGGTGATCTTGCTCAATGAGGTTATTCAGATATTTCGATGTACAATGACA
GTCAGGTTTAAGTTTAAAAGCTTTAATTACTTTAGCCATTGCTACCTTCGTTGAAGGTGCCTGATCTGTAATTA
CCTTTTGAGGTTTACCAAATTGTTTAATGAGACGTTTGATAAACGCATATGCTGAATGATTATCTCGTTGCTTA
CGCAACCAAATATCTAATGTATGTCCCTCTGCATCAATGGCACGATATAAATAGCTCCATTTTCCTTTTATTTTG
ATGTACGTCTCATCAATACGCCATTTGTAATAAGCTTTTTTATGCTTTTTCTTCCAAATTTGATACAAAATTGGG
GCATATTCTTGAACCCAACGGTAGACCGTTGAATGATGAACGTTTACACCACGTTCCCTTAATATTTCAGATAT
ATCACGATAACTCAATGTATATCTTAGATAGTAGCCAACGGCTACAGTGATAACATCCTTGTTAAATTGTTTATA
TCTGAAATAGTTCATACAGAAGACTCCTTTTTGTTAAAATTATACTATAAATTCAACTTTGCAACAGAACCGTA
TTATGGAATAGAGATGTTGGTAACATTTATACAGGATCATTATACTTAAGTTTAATTTCGTTATTACAGAACCAC
ACATTCCAACCAGAAGAGAAAGTATGTCTATTTAGTTATGGTTCAGGAGCAGTAGGAGAAATCTTTAGTGGT
TCAATCGTTAAAGGATATGACAAAGCATTAGATAAAGAGAAACACTTAAATATGCTAGAATCTAGAGAGCAAT
TATCAGTCGAAGAATACGAAACATTCTTTAACAGATTTGATAATCAAGAATTTGATTTCGAACGTGAATTGAC
ACAAGATCCATATTCAAAAGTATACTTATACAGTATAGAAGACCATATCAGAACATATAAGATAGAGAAATAAA
CTAGTGGCCGATTGTGCTTGATGAGCTTGGGACATAAATCCTAACTCGAAATAAATAAGCATATCACTAAACT
GATTTTTTAAAGTTTACAGTGATATGCTTATTTTTTTATCTTACGATTTTGTACGTGCATGCTTGCCTAGGGGTA
TGGCTCGAGCCATTAGTCTCTCGCACATACTATTCCCTCAGGCGTCAGCACTTACAAAATCGGTTGTAATTTTC
ATTTTTATACGCATTCTTACTGAGATTATACTAATAAGAGGAATAGTAAAAGCAATTCTAAGTAAAATTGCAGA
TAAGAGGTTTGTTAAAAGCAGTTCTAAGTAAAATTGCAGATAAGAGGTTTGTTAAAAGCAGTTCTAAGTAAA
ATTACAGATAAGAGGTACGTTAAAAGCAGTTCTAAGTAAAATTGCAGATAAGAGGTTTGTTAAAAGCAGTTC
TAAGTAAAATTGCAGATAAGAGGTACGTTAAAAGCAATTCCATGCAAAATTGCTGATAAGGGGTAAGTTAAA
AGCAGTTCTCAGTAAAATTGCAGATAAGAGGTACGTTAAAAGCAGTTCTAGGCAAAATTGCAGATAAGAGG
TGCGTTAAAAGCAGTTCTCAGTAAAATTGCTGATAAGGGGTAAGTTAAAAGCAATCCTAAGTAAAATTGCAG
ATAAGGGGTACAGAAAAACTAGACTTGATTACAAAATGGAGCTTGGGACATAAATGATTTTTTAAAAATGAG
ATGAGACGTAGATTAACTCCATAATCAATACGAATCTATCGACTTCTTTATTTATGATATTCATCTCTTTTTAATG
GAAATAAAAGTGCGATTAATGTGATAATACAGTTACGTTAATTAAAAAAATAAAAATGCAAGGAGAGGTAATA
TGCTAACTGTATATGGACATAGAGGATTACCTAGTAAAGCTCCGGAAAATACAATTGCATCATTTAAAGCTGCT
TCAGAAGTAGAAGGTATAAACTGGTTGGAGTTAGATGTTGCAATTACAAAAGATGAACAACTGATTATCATTC
ATGATGATTATTTAGAACGGACTACAAATATGTCCGGGGAAATAACTGAATTGAATTATGATGAAATTAAAGA
TGCTTCTGCAGGATCTTGGTTTGGTGAAAAATTCAAAGATGAACATTTGCCAACTTTCGATGATGTAGTAAAA
ATAGCAAATGAATATAATATGAATTTAAATGTAGAATTAAAAGGTATTACTGGACCGAATGGACTAGCACTTTC
TAAAAGTATGGTTAAGCAAGTGGAAGAACAATTAACAAACTTAAATCAGAATCAAGAAGTGCTCATTTCAAG
CTTTAATGTTGTGCTTGTTAAACTTGCAGAAGAAATCATGCCACAATATAACAGAGCAGTTATATTCCATACAA
CTTCGTTTCGTGAAGACTGGAGAACACTTTTAGATTACTGTAATGCTAAAATAGTAAACACTGAAGATGCCAA
ACTTACTAAAGCAAAAGTAAAAATGGTAAAAGAAGCGGGTTATGAATTGAACGTATGGACTGTAAACAAAC
CAGCACGTGCAAACCAACTTGCTAATTGGGGAGTTGATGGTATCTTTACAGACAATGCAGATAAAATGGTGC
ATTTGTCTCAATAGAAAGTTAGAGGTGAGTCTTACGTTTCAGTGACGGTAGACTTACCTTTAACATGTTACAT
ACTAAAAAATTAATTTGAATAAGAAAGAGAGACATATATGAAATACGATGATTTTATAGTAGGAGAAACATTC

AAAACAAAAAGCCTTCATATTACAGAAGAAGAAATTATCCAATTTGCAACAACTTTTGATCCTCAATATATGCA
TATAGATAAAGAAAAAGCAGAACAAAGTAGATTTAAAGGTATCATTGCATCTGGCATGCATACACTTTCAATA
TCATTTAAATTATGGGTAGAAGAAGGTAAATACGGAGAAGAAGTTGTAGCAGGAACACAAATGAATAACGT
TAAATTTATTAAACCTGTATACCCAGGTAATACATTGTACGTTATCGCTGAAATTACAAATAAGAAATCCATAAA
AAAAGAAAATGGACTCGTTACAGTGTCACTTTCAACATACAATGAAAATGAAGAAATTGTATTTAAGGGAGA
AGTAACAGCACTTATTAATAATTCATAATAAAACAGTGAAGCAACCATCGTTACGGATTGCTTCACTGTTTTGT
TATTCATCTATATCGTATTTTTTATTACCGTTCTCATATAGCTCATCATACACTTTACCTGAGATTTTGGCATTGTA
GCTAGCCATTCCTTTATCTTGTACATCTTTAACATTAATAGCCATCATCATGTTTGGATTATCTTTATCATATGATA
TAAACCACCCAATTTGTCTGCCAGTTTCTCCTTGTTTCATTTTGAGTTCTGCAGTACCGGATTTGCCAATTAAG
TTTGCATAAGATCTATAAATATCTTCTTTATGTGTTTTATTTACGACTTGTTGCATACCATCAGTTAATAGATTGA
TATTTTCTTTGGAAATAATATTTTTCTTCCAAACTTTGTTTTTCGTGTCTTTTAATAAGTGAGGTGCGTTAATATT
GCCATTATTTTCTAATGCGCTATAGATTGAAAGGATCTGTACTGGGTTAATCAGTATTTCACCTTGTCCGTAACC
TGAATCAGCTAATAATATTTCATTATCTAAATTTTTGTTTGAAATTTGAGCATTATAAAATGGATAATCACTTGGT
ATATCTTCACCAACACCTAGTTTTTTCATGCCTTTTTCAAATTTCTTACTGCCTAATTCGAGTGCTACTCTAGCA
AAGAAAATGTTATCTGATGATTCTATTGCTTGTTTTAAGTCGATATTACCATTTACCACTTCATATCTTGTAACGT
TGTAACCACCCCAAGATTTATCTTTTTGCCAACCTTTACCATCGATTTTATAACTTGTTTTATCGTCTAATGTTTT
GTTATTTAACCCAATCATTGCTGTTAATATTTTTTGAGTTGAACCTGGTGAAGTTGTAATCTGGAACTTGTTGA
GCAGAGGTTCTTTTTTATCTTCGGTTAATTTATTATATTCTTCGTTACTCATGCCATACATAAATGGATAGACGTC
ATATGAAGGTGTGCTTACAAGTGCTAATAATTCACCTGTTTGAGGGTGGATAGCAGTACCTGAGCCATAATCA
TTTTTCATGTTGTTATAAATACTCTTTTGAACTTTAGCATCAATAGTTAGTTGAATATCTTTGCCATCTTTTTTCT
TTTTCTCTATTAATGTATGTGCGATTGTATTGCTATTATCGTCAACGATTGTGACACGATAGCCATCTTCATGTTG
GAGCTTTTTATCGTAAAGTTTTTCGAGTCCCTTTTTACCAATAACTGCATCATCTTTATAGCCTTTATATTCTTTT
TGTTTTAATTCTTCAGAGTTAATGGGACCAACATAACCTAATAGATGTGAAGTCGCTTTTCCTAGAGGATAGT
TACGACTTTCTGTTTCATTAGTTGTAAGATGAAATTTTTTTGCGAAATCACTTAAATATTCATCCATTTTTTTAA
CGGTTTTAAGTGGAACGAAGGTATCATCTTGTACCCAATTTTGATCCATTTGTTGTTTGATATAGTCTTCAGAA
ATACTTAGTTCTTTAGCGATTGCTTTATAATCTTTTTTAGATACATTCTTTGGAACGATGCCTATCTCATATGCTG
TTCCTGTATTGGCCAATTCCACATTGTTTCGGTCTAAAATTTTACCACGTTCTGATTTTAAATTTTCAATATGTAT
GCTTTGGTCTTTCTGCATTCCTGGAATAATGACGCTATGATCCCAATCTAACTTCCACATACCATCTTCTTTAAC
AAAATTAAATTGAACGTTGCGATCAATGTTACCGTAGTTTGTTTTAATTTTATATTGAGCATCTACTCGTTTTTT
ATTTTTAGATACTTTTTTTATTTTACGATCCTGAATGTTTATATCTTTAACGCCTAAACTATTATATATTTTTATCG
GACGTTCAGTCATTTCTACTTCACCATTATCGCTTTTAGAAATATAACTGCTATCTTTATAAACTTGTTTGAAATT
TTTATCTTCAATTGCATCAATAGTATTATTAATTTCTTTATCTTTTGAAGCATAAAAATATATACCAAACCCGACA
ACTACAACTATTAAAATAAGTGGAACAATTTTTATCTTTTTCATCAATATACTCCTTATATAAGACTACATTTGTA
GTATATTACAAATGTAGTATTTATGTCAAAATAATGTTATAATTTTTGTGATATGGAGGTGTAGAAGGTGTTATC
ATCTTTTTTAATGTTAAGTATAATCAGTTCATTGCTCACGATATGTGTAATTTTTTTAGTGAGAATGCTCTATATA
AAATATACTCAAAATATTATGTCACATAAGATTTGGTTATTAGTGCTCGTCTCCACGTTAATTCCATTAATACCAT
TTTACAAAATATCGAATTTTACATTTTCAAAAGATATGATGAATCGAAATGTATCTGACACGACTTCTTCGGTTA
GTCATATGTTAGATGGTCAACAATCATCTGTTACGAAAGACTTAGCAATTAATGTTAATCAGTTTGAGACCTCA
AATATAACGTATATGATTCTTTTGATATGGGTATTTGGTAGTTTGTTGTGCTTATTTTATATGATTAAGGCATTCC
GACAAATTGATGTTATTAAAAGTTCGTCATTGGAATCGTCATATCTTAATGAACGACTTAAAGTATGTCAAAGT
AAGATGCAGTTCTACAAAAAGCATATAACAATTAGTTATAGTTCAAACATTGATAATCCGATGGTATTTGGTTT
AGTGAAATCCCAAATTGTACTACCAACTGTCGTAGTCGAAACCATGAATGACAAAGAAATTGAATATATTATTC
TACATGAACTATCACATGTGAAAAGTCATGACTTAATATTCAACCAGCTTTATGTTGTTTTTAAAATGATATTCT
GGTTTAATCCTGCACTATATATAAGTAAAACAATGATGGACAATGACTGTGAAAAAGTATGTGATAGAAACGT

TTTAAAAATTTTGAATCGCCATGAACATATACGTTATGGTGAATCGATATTAAAATGCTCTATTTTAAAATCTCA
GCACATAAATAATGTGGCAGCACAATATTTACTAGGTTTTAATTCAAATATTAAAGAACGTGTTAAGTATATTG
CACTTTATGATTCAATGCCTAAACCTAATCGAAACAAGCGTATTGTTGCGTATATTGTATGTAGTATATCGAGCT
TCACATGAAACAGCTAAAGAAGCTTTGGGCGATAAAGAGTTAAGAGCCATTGCACATGAGTTAACTAAAAC
AGTTAAGGATAACATGAGTGTTGATTGGTCTAAACGAGACAGTGCTAAAGCTAAAATGAGAGTTCAAGTTAG
ACGCCTATTAAAGAAATATGGCTATCCACCAGATCTTCAAAAAATGGCTGTGGAACAAGTTGTAGAGCAAGC
AGAATTAATGGCAAGTCAGCAATAAAAAAATAAATCATAATGAGTCCGGGACATAAAGTTCTTGGATAAGTG
AAAAAAGACAATTTCTATTGAAATAATATAGAAATTGTCTTTTTTATAAATTTTTTGATTATTTTCAGCTCGTTG
AGCTACTACTTTTCTTATATTAAGTGCCATTAATACAAAACCAAGTTCTCTTTTGACTTTATTGAGTCCTCGGAC
AGACATCCGAGTGAAACCCAAAATAGCCTTCATAAATCCAAAAACAGGTTCCACATCAATTTTTCTTTGACTG
TAGATATTTTTTGTTTCTGGTTCTGAAAGCTTTTTGTTAATTTGGGATTTAAAATATTCCCAGTTATAATTCTTC
ATTATTTTTTTGTTTGTTTTTGAATTGAAGTTCATACATTGATTTTTCAGAGGACATTCTGAACAATCATCACAT
TCATATAATTTGAAGTCTCGCTTATAACCATACTTATCATGACGATAGGCATATCTTTTAAAACCTAGCCGTTTAT
TATTCGGACAAATGAATTCGTCATTAATTTCGTCATAGTTCCAATTTTGAGTATTAAAGATGTCACTTTTATATTT
TTTAGTTTTATCTTTTATAAACATTCCATATGTTATGAGTGGCGTTCGATTAAAGTCATCTATAATTGCCTTATAAT
TTGATTCACTACCATAACCTGCATCAGCTACAATATATTCAGGTAAATGACCGTAGGTCTCTTGAATTGAATTTA
AAAATGGAATCATCGTTCTAGTATCCGTTGGATTTTGATACACATTATAAGATAAAACAAATTGGGAATTTGTC
GCTATTTGTAAATTATACCCTGGCTTAAGTTGTCCATTTTTCATGTGATCTTCTTTCATTCTCATAAATGTCGCAT
CATGATCTGTCTTAGAATAACTATTTCTATCCTTTAAAATAGATTTTTGAAATTCGTATCGATACTTTCGCTCAAA
ATAATCATTGATTTGCTTTTTGTATTTTTTGATTTTAGTTCTTTTGAGACGTATTTGTTTTCTTGTTTTAGTACAT
TTTTCATTGTTGATATGTTGGTTTAAATCTTCGATTTCTTTATCTAAGTGACTACCAATCAAATCTATTTCTTCTT
TTGTTAATTCATTATCATGATCTTTTTTAATTTCCGGTATGATTTTATTGGTTACCAATTCATGGTAGAGGGCTTT
AGAATCCTCATTCATCTTTGATTCATGGTTTTGAATACTCTTTTTCCATACAAATGTATATCGATTGGCATTTGCT
TCAATTTTTGTACCATCAATAAAAATAGCTTTATCATCTATAAGATTTTGTTTTACACACTGACTGTAAAATTGA
ATAAATAAAGATTCTAATAAAGCATCTACTTTTGGATTTACTCTAAATCGATTAATTGTTTTATAAGAAGGTTTT
TGATTTTGTGATAGCCACATCATTCGGATGCTATCATTAAGCATTTTTTCTATTTTACGACCTGAGAATACAGAT
TGTGTGTAGGCATATAGAATCACTTTTAACATCATTTTAGGATGGTACGAAGTTGCACCACGGTGATGTCTGA
ATTCGTCGAATTCATTGTCAGGAATTGTTTCAACAATATCATTTACAGTAAAACGATGTTGATTTGTTTTGTTTC
CATATTGACCTCCATGTATTTGCTATGATTTCAAAATCCATTTTTGACGTGCCTTAGGGTTGAGTGGATGCATA
ATTTCATTTGTTACTGGATTGATGAGCTTTTTTACTTTCTTTTTATGAGGTTTTAACATTTCCATCACTTGTTCG
ACACGGTCGATAACAACTGGTCGCTTCGCATAGGCACCATAAGCAAGAATCACTGTGTCACTTTCACTAATCG
CTTTCATCAAATGAATATCAGTGTGCTCATCGTATGGATTTTTGATATGTTTGAGGTTTTCGGGTGTTCTAATAT
TAGAGAATAGATTTACAAGATATACAGCACCGTATCGTTCTGAATTGGCTAATTGGTTGAGGATAAGAACAGT
TGTGAGATCGAGTGATAATACACCGTCTAAATGAGGATACATCGTTATCACTGTGCATGCAGCTTTCTTTTCAT
CCCATGTTTTCTTGAGTAAATAGCGGTGCTGTTCATCATCGCTAAATATGGCTTCTGTGTGTATCGTACTTTTGA
TTGTATTCATCATCGTCACTTCCTTTAGTATTCTTCTGGTAAAAGCATCACATAATAAAAAGCGTCCACGTCATC
TTCACGAATGACGTAGACTTTCTTAGGTAATGCATTTTGATTTTTTTCATAGTTTGTATAGTGATATTCCAATTT
GTATGTGGGTTGTTCTTGTTCATGTGTGATTGAGAGTATATTCTCATCTTCTTGTAATTTAAAAATGTGTAGGTA
ATCTGTATGAGGCTGGTTATCTTTTTTTTCTACCATGTGCCAAAGTAAGATTTGAAGGTCTAGTGGAATACTTT
CATTAATTCCTCTTGTGATGTATCGATTGATTTTCATGCTATTTCCCTCCCTTCTGCTTTTCTTTCATGATGTCGA
TGATTTCGTTGATAACTGTGACGGATAATTGAGCAACTATGATCCAATTATTCATGGTCCTGACCTCCTTGTTT
TAGTAAATGACGTTCATCAATAATGATATTTTGAGTATCTGTAAGGTACAGAAAGTCCATGTCAAAATGGTCTA
AGTATCCGACACTGATGAGTTGGTTATTGGCATACATTAGAAATGGATAGATACTTAGCTCATGTAGTTCATCA
TTATAGTAGGTATAAGTCTCGAGTGTGAGATGTACCAGTGGAGAATCATTAATAAAACGTTCCGGTAGAATAT

TTTCTGCTGCTTCCTCCAGCGCTTCACATTCCCAAGCTTCGTTATTAGATAGTTGGAATAGGTGGGTTATATAT
TGTTTGAGTTCTTGAGTGATGGTTTTCATATTATTGCCTCCTAGATAGTGTAATAGTGATGTAGTTCATATACAT
CATTGAGATAATATATATTTGATTTGTCATTTATTACGAATCCCGGTGGGAATAAGAGAAAATTCCATATAAAAA
CCCGCTACAAACGTTGGTATGCCAAGGAAATCCTGAAATTCCGCCTATTTTGACAAACAATCAACTCATTATTT
ATAAGTATTGATGATAGGGTTGTGTCTCTGCTTCCTTATATATATTATTTATTTATAAAAAGTAACGGGATTTTGG
GATTGTGCTTGCACAATCCTTCTGTTTCTTCGAATCTGCAAATCCCAATCATTTCCCGATAAAAAATCATTGTG
GGATGTTCTTTAGCAATTTCAATATAAGCATTGTATAGTTATGAAAAAATTACGACAATAACTGTTTCATTAGAT
AAGTGTTATTGAAATTGATAAAGAGCAATTCTTGAAAATAGTTAGATAAAATAAGCGAAAGAATATAGTGAAA
ATTATTGTTATAACAATGATTCTATTAGCTAAATAGTAAGATATAGTGTTTGGGGCAAAAATAAAGACGAAGTG
CTGAGATGCACTTCGTCGAGTTGTTTATTATTGAAAAGTTGTTTAATGATTTCGTTATTAAGTTTGAGTGTGAC
ATAGAATTGTTTTTTATGATTACCATCTTTTTTAATATCAATGCGATCAATCACTGATAGATACAATGCTTTGAGT
CGAGATTTTTCTATGTGCTTAATATCATGAAAGATGTGTTGTAATAGTTTACTGATTTCTTTGGCATCAAATAAA
GTCTTATCTTCATTTTGTTGATTTTTGAGTTGGTTGATTTGATTCGTAATGTCATTGAGTTGCTTTTCATATTTTT
GAATACTTGGTCTGATTACTGATGTTAAGTCCGGATTATCCTCGATGGTTTTAATCAAGTTATTTATTTTGATTT
GTACTTCATCATATTGTTGTTGCTTATAAGCAATATCGTGATGAAGTGCAGCGCCATCAACTTGATTTTCTTGAT
TGACGTGTGTTACTACGCGTTGAATGACTTTATCACTTTTGACTATTTCAAGTATTTGCTTCATCACATAATCTT
CAATCACATCAGCTCTTACACTGTTTGCCGAACATACTTTGGAACCCTTGTTCCGAAAATTACTACATGAATAG
TAACGAATACGTTTCTTAGTCCCGTCTTTAAGAGTATTCGTGGTATTGCTTGCTGCCATAGGTGCGCCACATTG
GGGACAGTGAATAATGCCTGTAAGCAGATTCGTTCCTTTGCCATGGACTTGGGGTTTTTGACTGACTTGTTT
CTTACGCATTTGTACTTTATCCCATAAATCTTGATTAATAATGGGGGAATGCTTACCTTCAGCTATCACTGGTTT
ATCATTCAGCCCTTTACGACGTTTTTCACTCCAATCTTTGTATTTCGCAAATTGAATTTTGCCGATATAGAAAG
GGTTAGCTAAGATGTATGTGATTGAACTAATACTGAAAGGTTTCCCCTTTTTAGTGACATATCCTTTGTGATTC
AATGCATTGGCAATTTTACGATAGCCATGTCCTTTGGCATAGCACTCGAATATATATTTTACAATATTAGCTTCA
TGTTGGTTAATCATTAGCTCGTGTTTACTATCTGGTATTTTGTCATAACCTAGTGGTAAATTGCCTTGATAATAG
CCTTCTTGGGCACGTCTCGTTTGACCCATAAATACGTTCTCGACAATGTTATTACGTTCGAATTCTGAGAAACT
CGCAAGTATCTGTAACATGAGTTTACCAGAAGAAGTATTGATTTCCATACGCTCTGACAAACTGAAAAATTCG
ACATTTTGTTTGTGTAAATCTTCGACAATTTTGAGAAGATCAGATGTATTACGAGCTAATCGGTTTGTTTTGTA
TACCATAACACAGTCGATATTGCCTTCTTTTGCATCTTTCAACATACGTTGGAGCTCAGGTCTGTTCATAGATT
TACCTGAAATACCACGGTCAGCGTATATATCTTTAACTTCAAAATGATGGAAGTCACAGTATTCTTTGATTTGA
TTGATTTGTCCGTCGATACTATAACCTTCTGTGCTTTGCATTTCTGTTGATACACGTACATAGATACCGACACGT
TTTGTTTTAAGTTGTTGCATTATGTTACATCCTTTCTTCATTTATGCAATCGATGATTGCATGGCTTGATTGACG
ATATTGAGTGGTTCATTTTTGAAATAGATTCCTATAAGATTTTTATCTTTCGTAATGTGAATGGTTTCAATATAG
GGGTACAATATGTTTAACGTGAAACGTTTTTGAATAATATTTTGAATGGTGTGTTGTATTTGATGCCCATTTATA
GATGTAGTGCGTTGCGGTTGTTGACGTAATGATTGCGTTTGTTCTCTGAACGTTTCTGCATCAATGATGCCTT
GTGCCAACTTTTCTATCAGTTGTTCTTGAGTCAATATGTGATGTTTTTCTATGTTTCTTTGTCTTTTGATGCGTT
TGTCAATCGCACCTTTAATTTTTGTGTAGATGCGTTGATTTTGATAAAAGTCTCGGCACACTTCTAATACTTTAT
CTTCAAGTGTTTGTGCATTGATGCCTTTAAAATCACAGACAAAGCGTGAAGCATTCATGTTTTTAGGACAGA
CGTAGTAACGTAATGTATGATTCTTTTTTCTAATGGTCATATTTGTAAGTGTTGCATTACAACATGGGCATTTGA
TTTTTTGTTTGAGTTGATTATCCGAAGGTGTCTGTTTGGTTTGTTTTTGCAATCGAAGTCTCTGCGCTTGCTCA
TATATACTTGTGGAAACAATAGAAGGAAACATATTGTCGAATTGGCCATATTGATTGTTGACACGACCACAAT
AATTAGGATTGATGATAATGTTACGAACTTGATAGGGTTGTCGATTGATATACGTGTTATCTTCTTCTAATAACT
GTGCAATTTTCTTATAACCATGACCTTTAATGTAATAATTGAATACAGCCTTTACCGTTGGTGACTCATTTTGAT
TGATGATGAATGTTCCGTTGTGATAATCGTAACCAAAGGGCGCATGGGTTGTAATCAATCGACCTTGCTTTGC
TTTTTCTTGAAGCCCATTTCTGACTTGTTCTCCAATGTTATCCGATTCAAGTTCGGCTAAACTGATGAAGATAT

TAAGCTTGAGTCGGTCGAAAGCTTGATCCATATCAAAGTAACCATCATGTACACTTAAGATATGAACACGATAT
GTTTGACATAATTTGATGAGTTTTAATGCATTTTTCAAATTACGATGCAACCTATTAAGACGATAACAGCATAA
TATGTCACATTGTCCTTGTTGAATTAATTGTGTCATTTGTCGATACCCACTACGATTATCTTTGCGTCCTGATTGT
TTGTCGCTATAAAAGTTGATATGTTGAATATGATGTTTTTCGGCTATTGCTTCGATAGCTTGTTTCTGTGCTGCA
AGAGATTGTTGTTTCATCGTACTTTGACGTAAATAGCCTATAACTTGTTTCATATCGGCTCCTCCTTTCACAGTG
ATAATATATATTTATAGATGAATTGATATATAAGCCCAACATCAATGAGATGTTGGGCGTCCATATTAGTCATTCG
TTTGATTGATTTCTTCAATTACCAAATCGGCTAATATCTCGATAAGTTCATCCATGTTTTTCACTCCGTTATTTGT
TCTATCTTCAATACGTCGATTATTCAGTTTGATGCTTCACGGTTGTATGATAAAGACAATCAGAAATCTTCGTG
AACTCCTGAAGGGCCTATCCCCTCATTAGCGGATTTAAAAAGTTCTTTCGCAGCTTTGTTATCATTTGCCGGT
GTCCAATTTTGAATTAACGACTTATCTTTAGTTAATCCCAGGATAGATGCAAATTTTTCATCTAATTTTAGGTGG
TAAAATACAAGTGATTGTTTTTTACCGCTATTATCTTTGACACTTCTTTTAGTTGTTTGGCGTCCACGATCAGCT
AATATGAAACCTTTATCTCTTAAGGCGTTGACAACATTATTAACATCTTGAAATTGATGATTGTTTAGCATCTGT
TTAAAAACGTTCGCAATTATTTTTACTTCGATATGGTCATCTTTTAATGAGATTAATCCATAGTTCTCAAACATAT
TTTTCAAAGCACCTTCATCTGAAAACTTACCTCTGTTTTGTGCCACAAATTGAATGATGACATCAATAGCTTTA
TCAGCTAATGAGCGTTCAGAGACTGTATGAGTATGATAATCAATAAAGTAGTCTCTTATATTAGCGATATCAATA
TCTGTAGATAAAACACGACCTAATATTTTCGCAGATGTTGTAATGACTGCATAACGCTTAAACATACGAATACC
TGTATTGTTTGTTTCATCTTTCAATTTAGCTTCAAACCAATCTACTTCCTTGTAAAACCATTGAATAACTTCATCT
TCACGATTTATAAGATATTTAGCTACTAACGGTAAAACATGACCATAGTTTAGTGCTACAGCTTTTTTAATATTG
TCAGCATTGGTCGCATTTGTAGTGAATTGTTCATTAATCTCGATGGTTCTTACACGTAATCCATCGTTTTGAGC
TGAATCATTAAAAATACTGTATTCTGACGTTGAAATGACAGAAGTACCCCAATTCTTAGGCGTTTTAACTTCTC
CATGAACGTTTGAACGTTGACGACCTTGACCTTCAGCGATGGAATATAACAAACCCGTGGTATCTCTAAGTGT
CGTAGATGAAAGCTCATCAAATACTATAGGAACGCCATAATTGTTACTCAAATAACCTTCAAGTGCGTTACGT
GTGGCATTCCAACTTCTAAAAAGAGTTTCATTACCTTTGGTAGGGTTACCAGCGACTGATACAGCTAAAGCA
GCTGCGGTTGACTTACCGGTTGAGGATTGACCTGTAAAACTAAAGAGAATTCCTGCAAATTCGATTTCATGT
TTATGCTTCAGAAAACTTGTCACTAAGGCAGAAATCCCAAATATGACTGCCAATTCTAAAAGAAGATGACCTT
TAACCTCGTCAATATACATGTTAAACCAATTATCAAATGTACCTTTAGGAGTTAAGTCATAAGCACTATCTACAA
TAGGGTATGAAGGTGAGGACTGATTAAATTGAACAGAGTTGAGAACTTTATCTAGCGATATCAAGTAGCCAA
AAGGTGTCTCTAGTATACCTACACCCTCATATAATTCAGAAAGTGGTAATCTATCTCGCATCAATTGCAAGGCA
TAACTCAATGATCTAATATGCTTCTCGTTAATACTGTATCCATATTTAATTAAAGAAGGTAAGTTTCGTGTTGTTA
AGATATCAGACTCAAAAATGTCTTTTTTACCATTGTTGTTAGTAATAATCAACTTTTCAACGCCAGTAACTGGA
TCAGAAAATCTTGCATTAACAATAATAGGGCTAGCCATTATAACCACTTTTTCTCTGTCACCATCTTTTTTAGGC
GGTTTCGTTTCTTTCCAACCGAAGGAATCTAAAGCAAATGGGCCAATTTCAAACAATGTGTAACTCATTAGCA
ATCACCTCCTTTCGAAGTGTTGCTGTTATAGTGTGGATTAGGACCTGTTTTAAGATAAACTAAGTGACCGTGA
GTATCCTTACCGATAATAATAAATGGAACACGTGGCGCCTGTTTTACAAAATATGCGAACCAACGTCCAACAT
TTTGTTGTACAGCTTTTGAACATTGTACATTTGCACGACTGTTCAAGTCATGAAATGTAAATTCTTCTCCTTCA
GGTAAATTGAATGAAATACCTAATATTTTCTTTTTTAACTGCTCGAATTTATCTGATTGATTGAAATACATTTAA
CTTCCTCCTACTAATGAACTAAGATAGGAAAATTAAATATGCGCACAATTAACTTTCTTAAGTTCATTTTAGCC
AAGAAGTTATCATTTGAAAGCTTGTAAAAATATGTTAGAAATTCCGTACTAAATTAAGATGGATTTTTGTTGAC
AAAATAAAAAAACGCTGATTTAACAGCGCTTTAAATAAAAATTAATCTGAAGTTATATAAAAGTAGTCAGAAGG
AGTGGTGTTATTATCATTATAATAATCTAGTGCTTTATTGATTTCTTTTATTAATTTATCATGATGTTCTTTTGTTAT
TTTCTTTGGATATTTGAGTTCTTTTTCTTCTAAGTCTTTTGGAGACACTAGGTCACTATTTAAAATTAATGATAA
CAGCATATTTTTAGGTGCCGAAGCTCTGGTGTCTCGATGAGTAATATATTTTGCTAAATTACTGTTTATACTTAC
TTTTTCATTTTCTAAAAAGTTTGTACTATTTTTCACATGAGCATCAGCAATTTTGGTACTTATTTCAGTGTTTAA
ATCGTCTATTTCATCTAAATAATATTCAAGAATCTGAAGTTTATCTTTAGAAGTGATACCTTTTTCAGTGAAAAT

CTTAATAATGATAGATTTGATTCCAATTTCTATATTACTGTCTACTAGAAAATAGAGATAATAATTAGTCAGAGC
ATGTTCATAATCTCTTTGATCAGTAATTTCATGATTGATGAAATAATATAAGTTCTCTTTGATACGATTAGAAGA
ATCTATATCTTTGTCATTGATACCGTTTTTAAAAAAGTGTAGATAAACTGAGTTGTTTGAGAGTATATCATATAA
TACAAATAAACTTTTTGGTTTAAATACATATGATTTTCCTGATTTAAAGTTTGCTATGTTTATTCCTAGAGCGTT
GCAGAATTTATTAAAATTAGTTGTAAAATTATTTTTTTCTTGAATAAACATGTCGCTGTCATTTAATTCTGAACT
ATGATTTTTAATCATATTGATATTTTCTTCAATCTTGTCACTATTTAAATTTTCCATGCAAAGTATACTATATCTTAC
TATATCACTTATATTTATGGAAAAATCTTTAGGAGAACGATTTAAAGTATCCTTTTTATTATCTTGATCTACAGCC
CTACATGAATCATCAAAAACTTCTTTGACATTTATTTTATCAATGTTCAAAATTATGTCTACTAATGCATTTTGAA
ATTTTTCATTATATATGTCTCTCTTATTCATAATATCACTACCTTTATCAATTATAATAATATAGTATATCTTAATATTT
GTAGAAGTACTTGATGAGAGGTTAGATAAAACACCACATTTCTCTGGTTAGTAAAAACATAACTTCTAAGACT
TTTTTTCATATACTAATAAATTATATTATAAATATTTTTGAACATAAAAGGAGTGTATAAAAATGTATGCAAAGAA
GGTGTTTTAGTATAGATTTTAAGGCATTCATTTTTAATATAAAATATTTATAATATTTAAAATTGAAAATTATTATC
CTTTCAAAATTTTTGCAACGAGTAATTTTACTTTAATTATATTATAAAGTAGAATTAATAGTAATTAAAGGAACG
TTTGTTCCTGTTTTTATATGTAAATACGAGAACGATTGAATAGATAAAGAAGGATAAAATTGACTTCGAAATAG
AATATTTAATTTCAAGAAGGAGAGTATTGGATGTTAAAGCATAAAATAATAAATTTAATACAAGAAAAGAGAG
AAGGGAGTTACTGGGATTTTAAAGCAGAATATCATAAAGATAAAGCAGAATTATTACATGATATAATTTGTTTA
TCAAATAACTTGTTGAATCAAGAAGCTTATTTAATTTTAGGAGTCGCAGATAACGGTCATATTCTGGGGGTTG
CAGGTGATTCAAATCGAAAAAACCAAGAAGAATTAATATCATTTATTACTGGAAAAAAATTTGCTGCGGGTA
GGCACCCCAAGATTTCATTAATGACATTTGAATATGAAGAAAAGGAAATCGATGTCATTATAATAAATCCTAAA
GGATATGTACCATACTATCTGGAGAAAGCAGAAACCGATCAAAAAAGTAAAAAAAACAAAACAGTCAATGC
AGGTAGCATTTATACTAGAGTTGAAGATAAAAATACCCCAATAGATTCTACTGCAAGTCCATTAGATACAGAA
ATATTATGGAAAATGCATTTTGGTTTATATCCTACTCCTATAAAGAGGTTACAGAATTATTTACTAACTCCTGAG
AAATGGATGCAGAACTCAACAGGTTATTTTTATAGTGAATCGCCAGAGTATATAGTATACAAAAATGAGGACA
TTGAAGAAAAAGAAAATTATTTTAATTTAGTAAGTCCATTCTATGCGTATAATCAAATCAATAGTAACACTTTAT
ACTCATATTACGAATTTAAATATCATAGTACAGTTTTATATGCTTGCCGGTGTATCTCTTTAGATTCAGGTATCTA
CACAACACCAGTACCTAAATCAGGTGAGATAAATTTTAATATGCATAGAGATGATACTATTTATTATCGTTATTT
CATTGAAGAGACGATGCTTTATAATATTCATCTTTTTATGTATAAGGGTGATTCGATGGAAGAGAAATTTGCAA
TGGAAAAATTTTTAGAGTGTGTTTTAGTTTATAAGAACGAAGAGGAAAAGGAACTTTTTGAAAATTATATATT
ACATAATTGGGATAAAGTTAATCAATCAATTAATGAAAATAATAAACGCGTTTTTGGAACTGAACATTTATCAC
AACTTAAAAAAGAAGATATCACTAAAAAAGTAAAAACAGTTAAAGTTTTAAAAGATAAACTTGAGAACTTTA
GAAAAATTATTGGTTAATAGGAGAAAATCATGAAAAAATCAACACTCATTGATAAATTTTTAGACTTACTTTCT
TCAAGAAGCTCACTCAGAGAAATTCAGAATAATTTTATTGATGCAGACATTATGAGAGATAGTTCGATAAATC
AAAAATATAACGGACAACGTAAATCGTTAGCGTGGGAATATATTAGTACTTTAAATTTAGAAGATGAAGCTGA
ATTTTCTAAATTACTTAATGTTTTAGAAACGTATTTGTTTCAATGGAATCTGTATATCCATGAAATTGATGAAGA
CGAAGAAATTAATAGATTAATAAAAATAATTAATGTATTAGGTTATAAATATAATAAAGAGACAGGAAAAATAA
CTAAAAATGAAAGTGAAGTTAATTTAAGTACTATTAAATCTTTAGCAATAAAATTTGATATTGAATATGTATTAA
AAGAATGTAATAGAATTGAAAAAGAGGCACTAACGGATCCAGAGGACGCAATTACATCTGCAAAATCGATG
GTTGAGAGTACTTTGAAGTATATTTTAGATTCTGAAGGAGAAAAATTTAATAATAATGAAACCTTGAGAGGTT
TATATAAAAAAGTAAGTAATATTATGAACCTTTCTCCTGGTGGACATAATGAAAATACTTTTAAAACAATTTTGA
GTGGAATGATAAATGTAATTAACGGTCTGGATGAAGTAAGGAATGAATATGGTGACGCACATGGTAAATCAA
AGAAAAATTATAGGCCTGAAACTAGACATGCTTTTCTAGCAATCAACGCAGCACGTACGATAACTGAATTTCT
TTTAGCTTCATATAAAAAGTAAAAGCTATTTGATGAATAGCAATCAAATATATTGGTTATAAAAAACCGCATCAT
CAACTGATAAGCAGAGGCGTATCATAAATGAAACTAAAAAATAGGTTGTATATAACTCACTTTGAAATTGATT
GAATATATAGTAACTTTAATAGAATGCAGCTATTGTGGCGGAGAATATGAGAATCAAAAAATGATTAATATAGT

TTGAAGAGAGTGAGCATAAATACTAGAAAAATGGAGGCTGGGACATTAAGTTCTTAGGCAATGTAAAAAAG
CTGATTTCTATTAATTATTTGATAGAAATCAGCTTTTTTTTAAATGTATTTGATAATATACAGCTCGTCGAGCTGC
TATTTTCCTTATATTAAGTGCCATTAATACAAAACCTAGCTCTCGTTTAACTTTATTTATTCCTCGAACTGACATT
CGAGTGAAACCCAAAATAGCCTTCATAAATCCAAAAACAGGCTCTACATCAATTTTTCTTTGACTATAGATTTT
TTTCGTTTCTGGTTCAGAAAGCTTTTGATTAATCTCTGCTTCAATTTCTGCAGGCGTTTTACGTGTTTTTCCTT
CTTTTGGCGGTTATAACAATTTAACAAGTCCTGCTTTAACCGCTTTAAAGAAGGCAATTTCATCATTGAGTTCT
TGGGCTGTAGGTTCAGTCGCACAAAGCGCAAAGGCTTTACCTAACTCTGTGACCGTTTTAATAAAACGTTGG
CGTTCTTATTCGCCTAAACCAATCACATAATCCATCGTATTCGATATTGTATAATAACGTTCTGATTTTTTATCTG
AATTAAATTTAGAATAATCAAGATTATATAACATATCTTGAATCACATCATACTTCAATAACATCAATTCAACCGC
TTTATCTGTATCTATTGTCGTTTGAGCTTGATCAGATTCTGTATATTCTTTAAGTGCTTCTTTTAAACTTTCGGCG
ATACCCACATAATCGACAATTAATCCACCCGGCTTATCTTTAAACACTCGATTAACTCGAGCAATTGCTTGCAT
CAAATTATGGCCTTTCATTGGTTTATCAATATACATCGTATGCATGGAAGGAACATCAAATCCTGTCAGCCGCA
TATCTCGAACAATCACGAGTTGTAATTCATCATTCACATCTTTCATATGTTTTTCTAATAAATTATGACGTTTTTT
AGGACCAATATGTCTTTGGAAAGAAGCAGGATCACTAGATGAGCCTGTCATTACTACTTTAATAACACCTTTA
TCATCATCATCCAAATGCCATTCCGGTTTTAGACGAATGATTTCATCGTATAAATCAACATCAATTCGACGACTC
ATCGTTACGATCATTCCTTTAGCTTTCATCGCTTGCTGACGTGTTTCAAAATGTTGGATGATGTCTTTGGCTAA
AGCTTCTACACGAGGTTTTGCGCCTGCTAAGGCTTCAATTCGTGACCATTTTGATTTTAAACGCTGTTTTACA
TCCTCTTTTTGATCTTCTGTAATGTCATTATATGCTTCGTCTAAGTCTAAATTTTGAGGTAAATTTAATGGAATTA
CGCGACTTTCATAGTAAATTTTAACTGTACTTCCATCAGCTACGGCTTGTGTCATATCATAAACATCGATATAGT
TCCCACAGACCATTTGCGTATTTTTATCCGTTGAAGCTACGGGTGTGCCCGTAAATCCAACAAATGTTGCATT
CGGTAAAGCATCTCTTAAATATTTAGCATAACCATATTTAATGCCTTCGCCTCTATCATCGTAATTTGCATTAAAA
CCATATTGTGTACGATGAGCCTCATCTGCCATTACAAACACATTTTTACGTTCTGTTAGGGACGCCATGGTCGT
TTCATTTTGTTCAGGTTCAAATTTTGCATTGTTGTAAATACAATACCATCCGACTCAACAGATAATAACGATTTT
AATTCTTTACGTGTTTCAGCTTGTTTTGGTGTTTGTCTTAATAATCCTTTACCAGAGCGACCTTTTGATTTAAC
AAACGTACTGTATAGTTGGTTATCTAAATCATTACGATCTGTTATGACAACTAAGGTAGGATTATTAAGCATTTG
AATTAATTTTCCAGAGAAAAAGACCATGGTTAAACTTTTACCAGACCCTTGGGTATGCCAAATGACGCCGCC
TTTACCATCACCTCGCCCAGATGAAGCTAATAAAGCTCTATCAACAGCTTTATTAACAGCATAGTATTGATGAT
ACGCTGCTAGAATTTTACTGATATGCCCTTTACCATTATCTTGGAATAATACAAAATATCGAATTAAATCAAGTA
AGGTTTCTGGATTTAACATCCCATGAATCAGTACGTCTAAGCTAGCTAAACTTGACGAAGATTCCGTTTCTCC
ATCTTTAGAACGCCAAGTCATAAAACGATCATAGTTCGCAGTTAGTGAACCGACTTTAGTATTAATGCCATCAC
TTGTAACAACCACTTCATTAAACGTAAATAATTGTGGAATACGCATCTTATACGTTTCTAATTGATGATAACCAT
CTTCGACGCCTACGGTTTCATTGGTTGAATTTTTAAGTTCAATCACAACGATAGGCAAGCCATTAATAAAGAG
GACAATATCGGGACGTTTTGTATAGTCTCCATTAACGACCGTGAATTGATTGACTGCTAAAAAATTATTGTTTT
GTGGATGTTCAAAATCAACGATTTTAACAATTTCTACAATCGATTATCCTTCATCATCATAATCTTCAACTTCAA
TACCATTGATTAAATTTTCATGAAAGGTAAGGTTATTTTCCAAAAGATTGATTGGGCGACTTCTCTAAAGTTA
GTTCATGTAGCGCTTTTTCAATAAAACGAGGATGAAGGAAGAAGCGTATCTCGTAATTTTACTAAATTATTAG
TTTCTTTATTAAGATTACTAATGTTATTGAAAAGAGTTGAATGAATTTGATTTGCTTTTGATAACATTGGATGAT
TTAAATTAGGTAAAGCAATTTTAAAATTTTCAAATATTTTCTTAGTGTAAAATTCTCTAGCAGAACCTGATGTTT
GGTTACTTACATTTTTATTATATTATAAATCAAAGGATTATTTATCTTTGAATTGAGAGTTCTAAATCTCCACATA
TTTTGATTTTGTAAAGATGGTAAATTAGATTCTGTAATAATACCTATATTACCTATAGATCCCCCAACTGTTACTA
ATAAAGTATCAAAAATTTCCAATGAATATTTATTATATTTTGGTATATTTATATAATCATTAGAAACAAATTTTAAA
TTATTCCTCTCAATTAAGCAGCTAGTTTTAGATATATTTAAAGTTCTTAAGACTGATAATTTACTACTGTAAATAT
ATTCACTAGATTTAAACGCGTATCCTGTTCTAATATCAGCTATATTTTTTAATGAATTAATATTCCAATTTAAAGG
TATTTCACCTAACTCACTATCAATCATTTCTCCACCATTAGATTTATAAGGAAGAGAAAGTTAAATTTATATTAA

GTTTTATATATGATTAATTATTAACGTATTAAATGTAGTATACTCTTAATATAAGTAAAATCATCATAATATTTGTCA
AAAAAAGTGACATCTCATATAGTAGCGCGGTGTTAGTTGTCACAATTTTTGACAGCCAAAATAATACTTGAAC
CCTTGTGGTTATGCTATCTATTAAAGTATCTGATCGATAACTACCCCGAAGAATAGGGGACGAGAACTTAATTG
CATTATCAAATGTATATGCTGGATTCCATGATCGATAACTACCCCGAAGAATAGGGGACGAGAACTGTACTTA
AGATTTCATCAACTTTCTTTTGTACTGATCGATAACTACCCCGAAGAATAGGGGACGAGAACCCGAATTTTGA
TTCTTTGTTTGTAAATAATGCTCTGATCGATAACTACCCCGAAGAATAGGGGACGAGAACTTAAATCTTTGATT
GCTCTTAGCTCTAGTTATGTATGATCGATAACTACCCCGAAGAATAGGGGACGAGAACCACGCTGTAGTGAA
GTATAGAAACGGCATGAGTACAATGATCGATAACTACCCCGAAGAATAGGGGACAGAGTGTAAATTTAATTA
CACTCTAAAATTCGTGAATTTTTAATGGAATACGCATGGATTAATTTTTAGGGGATGGAAAATGAAAGATGTT
ATTTATGTAGAAAACCATTACTTTGTTACCGTGAAAGAAAATAGTATTAAATTTAGAAATGTAATAGATAAAAG
TGAGAAATTTTATTTGTTTGAAGAAATAGAAGCGATTATTTTTGATCATTATAAAAGCTATTTTTCTCATAAGTT
AGTAATTAAATGTATTGAAAATGATATCGCTATTATTTTTTGTGATAAAAAGCACTCTCCATTAACGCAACTTAT
TTCTTCTTACGGTATGACTCATCGTCTTCAAAGGATTCAAAGTCAGTTTCAATTATCTGGGAGAACTAGAGAT
AGAATTTGGAAAAAGATTGTTGTAAATAAAATTATTAATCAATCAAAATGTTTAGAAAACAATTTACATAATGA
GAATGTGAAGTTATTAGTAAACTTAGCAAAAGATGTTAGTTCTGGAGATAAAAGTAATAAAGAAGCACAGGC
TGCAAGAATTTATTTTAAAGATTTATATGGTAAACAATTTAAACGTGGGCGGTATAATGATATTATTAATTCAGG
GTTAAATTATGGGTATTCTATACTTAGATCTTTTATTAAAAAAGAACTAGCTTTACATGGATTCGAAATGAGTTT
AGATATTAAACATCACTCAAAGGAAAATCCTTTTAATTTAGCTGACGATATTATTGAAGTTTTTCGTCCTTTCAT
TGATAATATAGTGTATGATATAGTTTTTAAAAATAATATAAATACTTTTGATATAAATGAAAAGAAACTATTATTA
AATGTTTTATATGAAAAGTGTATTATAGATAAAAAAGTAGTGAGATTACTTGATAGTATCAAAATAGTCGTACA
GTCGTTAATTAAATGTTACGACGAAAATACGCCTACGCCTTTATCTCTACCTAAAATGATTGAGGTGGGGAAC
TAATGTATTTATTAGTTAGTTTTGACTTACCTAGAGATACTAAATTTGAACGTAAGATGGCAAGTGTGTATCGT
ACTCGATTATTAGAACTCGGTTTTAGTATGAAACAGTTTAGTTTATACGAAAGGTATGTCAGCGATGTTCAGA
AAAAAGATAAGATTTTAGAAATTCTACAACAAGAAATTCCTGATACTGGAAGTATTACTCTATATGTCTTACCT
GATGAGGTAAATAATAGTCAGATTACCATATTAGGAAAAGAGGTTAAGGTTGTTGTGAGAAAAGAACCTAAG
CTAATTTTTCTATAAAATGGAGGTTACAATGGATAAAAAAACGACATTAATGTATGGTTCTTTACTACATGATAT
AGGTAAAATTATCTATCGGAGTAACGGTCATGCATTTGCAAGAGGAACGCATTCAAAGTTAGGACACAAATT
TTTGTCTCAATTTTCAGAATTTAAAGACAATGAACTAATCGATAGCGTTGCTTATCATCATTATAAAGAACTTG
CAAAAGCTAATTTAGCTAATGATAATACAGCTTATATTACCTATATTGCCGATAATATTGCGAGTGGTATTGATA
GACGAGATATTATAGAAGAGGGCGATGAAGAATACGAAAAACAACCATTTAATTTTGATAAGTATACACCCCT
ATATAGTGTGTTTAATATTGTGAATTCTGAAAAATTGAAACAAACAAGTGGGAAGTTTAAATTTTCTAATGAA
AGTAATATTGAGTATCCTAAAACTGAGAATATTCAATATTCAAGTGGAAATTATACAGCACTAATGAAAGATAT
GAGTTATGATTTAGAGCACAAATTAAGTATTAAAGAAGATACATTTCCTTCATTATTACAATGGACAGAAAGTC
TATGGCAATATGTGCCAAGTTCAACAAATAAAAATCAATTAATTGATATTTCTCTTTATGATCATAGTCGTATTAC
ATGTGCTATTGCCAGTTGTATATTTGATTATTTAAATGAAAATAATATACATAATTACAAAGATGAATTATTTACA
AAGTATGAAAATACCAAAGCATTTTATCAAAAAGAAGCTTTTTTACTACTTAGTATGGATATGAGTGGTATTCA
AGATTTTATTTACAACATAAGCGGTTCTAAAACATTAAAAAGTTTAAGATCTCGTAGTTTTTACTTAGAACTCA
TGCTTGAGGTAATTGTTGATCAATTACTAGAAAAATTAGAATTAACACGAGCAAATCTTTTATACACTGGTGG
CGGTCATGCATATTTATTAGTCTCTAATACAGATAAAGTGAAAGAAAAAATAAATCAATTTAATACTGAGTTAA
AAAATTGGTTTATGTTAGAATTCACAACCGATCTCTCATTATCAATTGCTTTTGAAAAATGTAGTGGCAGTGAT
TTAATGAATACCAATGGTAATTATAGAACTATTTGGCGTAATGTCAGCAGTAAACTTTCTGATATTAAAGCACA
TAAGTATTCTGCAGAAGATATATTAAAATTAAATCATTTTCATTCATATGGAGATCGAGAATGTAAAGAATGTTT
AAGAAGTGACATAGATATTAATGATGATGGATTATGCAGTATATGTGAAGGAATCATTAATATATCAAATGACTT
AAGAGATAAATCATTCTTTGTACTATCAGAAACTGGAAAATTAAAAATGCCATTCGATAAATTTATATCGGTTAT

CGATTATGAAGAAGCAGAAATATTAGCACAAAATAATAATCAGATTCGTATTTACAGTAAAAATAAACCTTATA
TTGGTGTAGGAATATCAACGAATTTATGGATGTGTGATTACGACTATGCTAGTCAAAATCAAGATATGAGAGA
AAAAAGTATTGGAAGTTATGTAGAAAGAGAAGAAGGTATTAAGCGTTTAGGCGTTGTACGTGCTGACATAG
ATAATCTTGGCGCTACATTTATATCAGGGATTCCAGAAAAATATAATTCAATTTCAAGAACAGCTACGTTGTCT
CGTCAATTATCATTATTTTTTAAATATGAATTAAATCATTTGTTAGAAAATTATCAAATTACAACTATATATTCAGG
TGGCGACGATTTATTTTTAATTGGCGCATGGGATGACATTATAGAAGCAAGCGTTTATATAAATGACAAGTTT
AAAAAGTTTACGCTTGGTAAATTAACAATGTCTGCTGGTGTTGGAATGTTCAGTGGCAAGTATCCAGTTTCTA
AAATGGCTTTTGAGACAGGACTACTTGAAGAAGCAGCTAAAACTGATGAAAAAAAATCAGATAGCACTTTGG
GTGCAAGAAAAAGTATATAACTGGGATGAGTTTAAAAAGTATATTTTAGAAGAAAAGCTTCTCGTTTTACAAC
AGGGATTTTTGCAAACAGATGAACACGGAAAAGCATTTATATATAAAATGCTAGCTTTATTAAGAAATAATGA
AGTAATTAATATAGCTCGATTAGCCTATTTATTAGCAAGAAGCAAAATGTCAGAGGAATTTACATCTAAAATTT
TTAATTGGGCTCAAAATGACAAAGATAAAGATCAATTAATTACAGCTTTAGAATATTATGTTTATCAAATAAGG
GAGGCTGATTAAGTATGATATTAGCTAAAACAAAAAGTGGTAAAACGATAGATTTAACTTTTGCACATGAGAT
TGTAAAAAAATAATGTGAAAAATGTTAAAGATCGAAGAGGCAGAGAAAAACAAGTTTTATTTAACGGGCTTAC
AACAAGCAAGTTAAGAAATTTAATGGAGCAGGTGAATCGACTTTATACTATTGCCTTTAATTCAAATGAAGAT
CAATTAAATGAAGAATTTATCGATGAATTAGAATACTTAAAAATTAAATTTTATTACGAGGCAGGTCGAGAAA
AAAGCGTTGATGAATTTTTGAAAAAAACATTGATGTTTCCAATTATTGATAGAGTGATACAAAAAGAATCAAA
AAAATTTTTCTTAGATTATTGTAAATATTTTGAAGCTTTAGTTGCATACGCTAAATATTATCAAAAGGAGGATTA
ATATGTATTCAAAAATTAAAATTTCAGGAACAATTGAAGTAGTTACTGGTTTACACATCGGTGGGGGCGGTG
AATCTAGTATGATTGGAGCAATTGATTCCCCTGTAGTTAGAGATTTACAAACTAAATTACCTATCATACCTGGC
AGTTCAATCAAAGGAAAAATGAGAAGTTTATTAGCAAAACATTTTGGCTTGAAAATGAAACAAGAGAATCAT
AATCAAGACGATGAACGTGTTTTAAGATTATTTGGTTCAAGTGAAAAAGGGAATATTCAAAGAGCTCGTCTA
CAAATTTCTGATGCATTCTTTTCTGAAAAAACGAAAGAGCATTTTGCGCAAAATGATATTGCTTATACAGAGA
CGAAATTTGAGAATGCAATTAATCGTTTAACTGCAGTTGCAAATCCAAGACAAATTGAGAGAGTAACAAGAG
GATCTGAGTTTGACTTTGTACTTATTTACAATGTCGATGAAGAGTCACAAGTTGAGGATGATTTTGAAAATAT
TGAAAAAGCGATTCACTTATTAGAGAATGACTATCTTGGTGGCGGCGGCACCAGAGGCAACGGACGTATCC
AATTTAATGATATAAATATCGAGACAGTTGTTGGAGAATACGACAGTACAAATCTTAAAATTAAGTAGGTGAA
AGACATTGACAACAAAAGTATTTAAACTTTCTTTTAAGACTCCTGTTCATTTTGGAAAAAAACGTTTGTCAGA
TGGGGAAATGACAATAACGTCTGATACTTTGTTTAGTGCTTTATTTATTGAAACGCTTCAATTGGGTAAAGAA
ACTGGTTGGTTATTAAACGATTTAATCATTAGTGATACATTTCCTTATGAAAATGAACTTTATTATCTTCCTAAAC
CTTTAATAAAAATAGAATCTAAAGAAGAAGGCAACCATAAAGCATTTAAAAAGTTAAAATATGTTCCGGTTCA
TCACTATAATCAATATTTAAATGGTGAGATAAGCGCTGAAGATGCGACAGATTTAAATGATATTTTTAATATTG
GGTATTTTTCTCTACAAACAAAGGTTTCATTATCAGCACAAGAAATTGATTCAAGTGCTGACAGTGAACCTTA
TTCAGTGGGTACATTTACTTTTGAACCTGAAGCTGGTTTATATTTTATTGCAAAAGGATCAGAAGAAACACTT
GACCATTTAAAAGATATTATGACTTCATTACAGTACTCGGGTTTAGGCGGTAAACGTAATGCAGGCTACGGAC
AATTTGAATATGAAATGGTAAATAATCAACAATTGTTTAAATTATTGAATCAAAAGGGAGAATATTCTATTCTTT
TATCAACGGCAATGGCTAAAGAAGAAGAGATAGAGAGTGCTTTAAAAGAAGCGAGATATATTTTAAATAAAC
GTTCTGGTTTTATACAATCAACGAATTATTCTGAAATTCTAGTTAAAAAAAGTGATTTCTATAGCTTTTCTTCGG
GTTCAGTTTTTAAAAATATCTTTAATGGCGACATTTTTAATGTTGGACATAATGGGAAACATCCAGTCTATCGC
TATGCAAAACCTTTATGGTTGGAGGTATAAGTATGACAATAAAAAATTATGAAGTTGTTATTAAAACTTTAGGT
CCAGTTCATATTGGTAGTGGTCAAGTTATGAAGAAGCAAGATTACATTTATGATTTTTATAATTCTAAAGTTTAT
ATGATTAATGGCAATAAACTAGTTAAATTTTTAAAAAGAAAAAATTTACTTGATACATATCAAAACTTTTTGAG
AAACCCACCAAAAAATCCAAGAGAAAATGGACTGAAAGACTATTTAGACGCTCAAAATGTTAAGCAAAGTG
AATGGAAAGCATTTGTGAGTTATTCTGAAAAGGTCAATCAAGGCAAGAAATATGGTAATATACGTCCTAAACC

GCTAAATGATTTACATCTAATGATAAGAGATGGGCAAAATAAAGTGTATCTTCCAGGTAGTTCAATTAAAGGC
GCTATCAAAATAGCCCTTGTATCAAAATATGATAATGAAAAAAATAAAGATATTTATAGCAAAATTAAAGTCAG
CGATTCAGAGCCTATCGATGAAAGTCATTTAGCGATTTATCAAAAAATAGACATTAATAAAAGTGAAAAACCA
ATGCCTCTATATAGAGAGTGTGTAGATGTAGATACTGAAATAAAATTTAAGTTAACCATCGAAGATGAAATTTA
TTCTATTAATGAAATTGAACAAAGTATCAGAGATTTTTACAAAAACTATTATGACAAATGGTTGGTCGGTTTTA
AAGAAACAAAAGGTGGAAGACGATTTGCATTAGAAGGTGGTATGCCCGATGTTCTAAACCAAAATATTTTAT
TCTTAGGAGCTGGCGCAGGATTTGTTAGTAAAACGACACATTATCAATTAAAAAGTCGAGAACAGGCGAAA
AGAGATTCTTTTAAAGAGTTAACTAAAAAATTCCGTAGAACTTATGGAAAAATGAAAGAAATACCTTCTAAC
GTACCTGTTGCTTTAAAAGGGACAACTAACCAAAGTCGTCGTACTTCATACCAGCAAGGTATGTGTAAAATAA
GTTTTCAAGAGTTAAATAATGAGGTGTTATAATGAAAGTACTATTTAGTCCAATAGGTAATTCAGATCCATGGA
GTAATGATAGAGATGGTGCAATGCTTCATATCGTACGTCATTATCAACCTGATATAGTAGTTTTATTTTTTACTG
AAAGTATCTGGGAAGGTAATAAAAATATACCTGGACGTAAAAACTTTGATTGGGAAAGTATTATTTTAAAAGT
ATCTCCAGGAACAAAAGTAAATATAAAAGTTGATAACATTAAATATGAAAATGATTTTGATAGCTATAAAGACT
TATTTCACTTTTATATAAATGAGATAAGAACTGAATATTCCGAGGCAGAAATTTTATTAAATGTTACAAGTGGC
ACGCCACAAATGGAATCTACTTTATGTTTAGAATACATTTCTAACCCTAATAATATGCAATGTATACAAGTTTCA
ACGCCTGCGCCTACTGAAGGGCCTAGACGTTCATTTGCGAAACCTGAAACTTTGATTGAAGATTTAAATAAA
GTTAATGATAATGAAAAAGTTGCTACTAATAGAAGCAAATCAATTGATATTATAAGTTTTAGAGAAGTTATGGT
GCGTTCACAGATAAAAGGTTTAGTAGATAATTATGATTATGAAGGTGCGCTTAATTTAGTAAGTAATCAAAAG
TCTTTTCGTAATGGAAAATTATTAAGAAAAAAATTATTAGCTTTAACAAATCAAATTAAAACACATGAAGTCTT
TCCAGAAATTAATGTGAAATATAATAATGCAGCTTTAAAAAAATCATTGTTTCATTATTTACTAGTAAATATGAG
ATATAATCGACTTGATGTGGCTGAAACATTAATTAGAGTGAAGTCTATTGCTGAGTTTATACTTAAAACATATCT
TGAAAATCATTGGACTCATTTAATGATTGAAGTTGACGGTAAACCATACTTAAATGCTGAAGATAATTTATCTT
TTATTTACAAATATAAATTATTATTAGAAAAAAGAAAACAGAATTTAGATACCTCAAGAATTTTAGGTCTTCCA
GCATTTATAGATATGCTTAGTATTTTAGAACCTAAGTCTAAATTATTAAAAGAAGTAAAAGCAGTGAATGATAT
CAATGGATTAAGAAATTCTATAGCTCACAATTTAGAAGTGTTACATCTAGATGAAAATAAAAATTATAAAAAAA
TTATGTTATCTGTTGAAGCTATAAAAAAATATGCTGAATATTTCATTTCCTGAGATAGATGAACAAGACTATAATT
ACTTTGAAGAAAAAAAATAAGGAATTTAAAGAATTATTATGATAAACAAAATTACAGTAGAATTAAACCTACCA
AATAGTATTCGTTTTCAATATTTAGGAAGTATTTTACATGGCGTGTTAATGGATTATCTTCCTAATGATATTGCTG
ATCAACTACATCATGAATTTGCTTATAGTCCATTGAAGCAAAGGATATATCATAAAAACAAAAAAGTAATATGG
GAAATTGTTTGTATGTCAGATAGATTATTTAATGAGATAGCCAAGCTATTCACTTCTAAAAATAGGTTGTTTTTA
AAATATTACCAAGTTTATATTGAGATTTATTCTTTTAATATTGAGAAGGTAAACGTTCAAAATATTATGAATCAA
CTCTTGCAAACAGAAGAATTGAATCGTTATGTAAGAATTAATATACAGACACCTATGTCTTTTAAATATCAGAG
TAGTTACATGATTTTTCCTGAGGTTAAACGTTTCTTTAGAAGTATTATGATACAATTTGATGCTTTTTTTGAAGA
ATATAAAATGTACGATAAAGAAACATTAGATTTTTTAGAAAAGAATATCAATATTGTTGACTATAAATTGAAAA
GCACACGGTTTAACTTAGAAAAAGTTAAAATTCCTTCATTTACAGGAGAAATAGTGTTTAAAATTAAAGGAC
CATTACCTTTTCTACAATTAACTCATTTTTTATTGAAGTTTGGTGAATTTTCTGGATCAGGTATGAAAACAAGC
TTAGGCATGGGGAAATATAGTATAATATAACTAGAATATAGCTGAAATTTAATTGTCAAAAAATGTGACATTCT
GCACTTATAGTACAAGTGATTTGTCACTATTTTTGACAGCAAATTTTATATTTGAACTATTGATTTAATAGGCTT
TAAAGAGCTATCTATTCGATAACTACCCCGAAGAAGAGGGGACGAGAACCTATAATAGTTACTGCTTTTGTAA
CCGTCCATATCATTCGATAACTACCCCGAAGAAGAGGGGACGAGAACAAATGCTTATCCATTCTAATCATATTT
TCAATTTGTTTATATTCGATAACTACCCCGAAGAAGAGGGGACGAGAACTGCCCACTTAATTAATTCATCTAG
TCTCATTTCTTTATTCGATAACTACCCCGAAGAAGAGGGGACGAGAACCATCAACTGACTTTTTAACTGTTTT
AGTGAATTCGTCTATTCGATAACTACCCCGAAGAAAAGGGGACGAGAACTTAAAGATCTCAACAATAGCGTC
CCATATTTTCTGTATTCGATAAATACCCCGGAGAACAGGGGGCGAAAACCTCATCTTGATGATCCCAATTGGC

CTGGTTAAAAAGAAATCGATAACCTTTCGTAGAATAGAGAATAAGTATTAAAAATATATTTGGTTTGATAAAAT
ATTCTGCAAAAAAGGGGACAAAAAAATCAAGATTTAAACTGTAATTCATTATCTTTGAAATTAATTGACATTC
AATTTCTAAATAAGCAGAGACAAAAATAACTTTCATACAAATAATTTCTTTGTGATTAAGTTGGAATTAGAAA
ACTAATACTAAACGAATGGAATGTATATTATTGAGGAATTGGATGCAGTGTGGTGCAATTTCGAGAACTACTA
CTTAAGAAAATAAACAATAAGAATTAAATCTGAGTCAAGATATAAAAACAAATATTTTTACCATAACTATATTAG
TAAATACTTTATTATTTAGTATACGGATGAAAGCGAAGTGACAGCTAGCAATGCTCCAGATATAGGTATTAGTA
TTATAAAAGAGCAAGATTACCATTTAGAAAATGGAACTTTTTAAAAAAATTGAAGATAAAGTAGATCATTCTG
TGAGTTTTTATGTTAATAAGTCTGAACATGAGGATGTAGTATGAATACGGTTTAGTAGAAACTGACGCCGGAG
CTGTAGCTAAGATTGACTATCAAAAATTAACTAAAGTATTTGTTGAAGAATAATAACTTTGAAAGTTTTTTAGC
AAAATCACTGATAGGGAGAGGCGTATCATAAGT
>Staphylococcus argenteus strain RIVM_M046968
ATGAAAATAACCATTTTAGCTGTAGGGAAACTAAAAGAGAAATATTGGAAGCAAGCCATGGCAGAATATGAA
AAACGTTTAGGCCCATACACCAAGATAGACATCATAGAAGTTCCAGACGAAAAAGCACCAGAAAATATGAG
CGACAAAGAAATTGAGCAAGTAAAAGAAAAAGAAGGCCAACGAATACTAGCCAAAATCAAACCACAATCA
ACAGTCATTACATTAGAAATACAAGGAAAGATGCTATCTTCCGAAGGATTAGCCCAAGAGTTGAACCAACGT
ATGACCCAAGGGCAAAGCGACTTTGTATTCGTCATTGGCGGATCAAACGGCCTGCACAAGGACGTCTTACA
ACGCAGTAACTACGCACTATCATTCAGCAAAATGACATTCCCACATCAGATGATGCGGGTTGTATTAATTGAG
CAAGTGTACAGAGCATTTAAGATTATGCGTGGAGAAGCATATCATAAATGATGCGGTTTTTTCAGCCGCTTCA
TAAAGGGATTTTGAATGTATCAGAACATATGAGGTTTATGTGAATTGCTGTTATGTTTTTAAGAAGCTTATCAT
AAGTAATGAGGTTCATGATTTTTGACATAGTTAGCCTCCGCAGTCTTTCATTTCAAGTAAATAATAGCGAAATA
TTCTTTATACTGAATACTTATAGTGAAGCAAAGTTCTAGCTTTGAGAAAATTCTTTCTGCAACTAAATATAGTA
AATTACGGTAAAATATAAATAAGTACATATTGAAGAAAATGAGACATAATATATTTTATAATAGGAGGGAATTT
CAAATGATAGACAACTTTATGCAGGTCCTTAAATTAATTAAAGAGAAACGTACCAATAATGTAGTTAAAAAAT
CTGATTGGGATAAAGGTGATCTATATAAAACTTTAGTCCATGATAAGTTACCCAAGCAGTTAAAAGTGCATATA
AAAGAAGATAAATATTCAGTTGTAGGGAAGGTTGCTACTGGGAACTATAGTAAAGTTCCTTGGATTTCAATAT
ATGATGAGAATATAACAAAAGAAACAAAGGATGGATATTATTTGGTATATCTTTTTCATCCGGAAGGAGAAGG
CATATACTTATCTTTGAATCAAGGATGGTCAAAGATAAGTGATATGTTTCCGCGGGATAAAAATGCTGCAAAA
CAAAGAGCATTAACTTTATCTTCCGAACTCAATAAATATATTACATCAAATGAATTTAATACTGGAAGATTTTAT
TACGCAGAAAATAAAGATTCATCTTATGATTTAAAAAATGATTATCCATCAGGATATTCTCATGGATCAATAAGA
TTCAAATATTATGATTTGAATGAAGGATTCACAGAAGAAGATATGCTAGAGGATTTAAAGAAATTTTTAGAAC
TATTTAATGAATTAGCTTCAAAAGTTACAAAAACATCCTATGATAGCTTGGTCAATAGCATAGACGAAATACAG
GAAGACAGCGAAATTGAAGAAATTAGAACAGCACAAAAAGATAAGACACTCAAGGAAGTGGAAGCACCTA
AAGGAATAATTCCAAAATATAAAAAAGGTGTATCAAAGACTACTAAAAATGATTCAGAAATTGAAAAATCAA
ATAAAGAGAATAAATTAACCGGTAAAGTTGGAGAAAAATTAGCGCTAAATTACTTTAATGAGCTAATTGATAA
TAAAATAGACGAAGATAAGAAAGAACAGTTTAGGAATATTTTAAATGATAATCCAGGCTCTCAACACGGTCAT
GGCTATGATTTAGTAGCTTTTGATCCAACAAATACAGATAAAGCTGTAGAAAAATTTATTGAAATTAAAACATC
TACATCTTCTAGTATTGAGGAACCATTTTTTATGTCGCTAAATGAAATGTTTGCTATGAAAGAATATAAGCAGA
AATATTTAATATTAAGAATATTTAATGTTTCCGGTAAAGAACCACAATTTTATTTTATAGATCCATATGCAAATTA
TTCTGAATTTAAAGATGTAGATGATCTCATTGACAAAGTATTTAATGTAGAAGCTATTCAGTATAAAGTTTTTG
GCGAAAAATGATTACTTGAACAAGAGCTAAAATAAAATTGTGATCTAATAAAAATAGAAACTGTAATTTAAAT
AAAACTTTCTAAATAAGCTAACTGATAAAAAATCAGTTTGTCCACAGTCTGAAACAAGATTCCTATATTCTTTA
GGAATCTTGTTTTTTCTATTTTTATGGTGATAAAGAGCAGATAAGATAATGTGTAATAATCACAAAAAAGTTAA
ATATTTTAAGGCTTGTTTAATTATTAATGATTTTATATATAAAGAGCAGTATAATAAAGTTGTTAATATATTATGAA
TAATATTCAAGTAATTTTATTGTTTTTTAATTTGTCGATATTTAAGTTGAGTTAAATTTAAAGGGTGTAATTTGTT

TTACAATGATGAAGATAATTAGTCTATCAAAATAAAGGGGTTGGGACTGTTATGAGTGATAATTTGTCATTATT
CATTGACTATATCAATGATAATATAATCTATGGTAGTGAAATCAAACGGGAGAAATTAGAGAATTTATTTAATCA
ATTTGCTATAAAAAATGTTGAAAAGAACATTGTCTATGATGAACTGAAATCTTTAGATATTACAATCATTGAGT
CACAGGATTCATATAAAAATAAATTGAAGAGATTATTTTCGGTTCTGTTGCAAAGTAAAAAAATATAGCTAACC
ACTAATTTATCATGTCAGTGTTCGCTTAACTTGCTAGCATGATGCTAATTTCGTGGCATGGCGAAAATCCGTAG
ATCTGAAGAGACCTGCGGTTCTTTTTATATAGAGCGTAAATACATTCAATACCTTTTAAAGTATTCTTTGCTGTA
TTGATACTTTGATACCTTGTCTTTCTTACTTTAATATGACGGTGATCTTGCTCAATGAGGTTATTCAGATATTTC
GATGTACAATGACAGTCAGGTTTAAGTTTAAAAGCTTTAATTACTTTAGCCATTGCTACCTTCGTTGAAGGTG
CCTGATCTGTAATTACCTTTTGAGGTTTACCAAATTGTTTAATGAGACGTTTGATAAACGCATATGCTGAATGA
TTATCTCGTTGCTTACGCAACCAAATATCTAATGTATGTCCCTCTGCATCAATGGCACGATATAAATAGCTCCAT
TTTCCTTTTATTTTGATGTACGTCTCATCAATACGCCATTTGTAATAAGCTTTTTTATGCTTTTTCTTCCAAATTT
GATACAAAATTGGGGCATATTCTTGAACCCAACGGTAGACCGTTGAATGATGAACGTTTACACCACGTTCCCT
TAATATTTCAGATATATCACGATAACTCAATGTATATCTTAGATAGTAGCCAACGGCTACAGTGATAACATCCTT
GTTAAATTGTTTATATCTGAAATAGTTCATACAGAAGACTCCTTTTTGTTAAAATTATACTATAAATTCAACTTTG
CAACAGAACCATATATGCATATAGATAAAGAAAAAGCAGAACAAAGTAGATTTAAAGGTATCATTGCATCTGG
CATGCATACACTTTCAATATCATTTAAATTATGGGTAGAAGAAGGTAAATACGGAGAAGAAGTTGTAGCAGG
AACACAAATGAATAACGTTAAATTTATTAAACCTGTATACCCAGGTAATACATTGTACGTTATCGCTGAAATTAC
AAATAAGAAATCCATAAAAAAAGAAAATGGACTCGTTACAGTGTCACTTTCAACATACAATGAAAATGAAGA
AATTGTATTTAAGGGAGAAGTAACAGCACTTATTAATAATTCATAATAAAACAGTGAAGCAACCATCGTTACG
GATTGCTTCACTGTTTTGTTATTCATCTATATCGTATTTTTTATTACCGTTCTCATATAGCTCATCATACACTTTAC
CTGAGATTTTGGCATTGTAGCTAGCCATTCCTTTATCTTGTACATCTTTAACATTAATAGCCATCATCATGTTTG
GATTATCTTTATCATATGATATAAACCACCCAATTTGTCTGCCAGTTTCTCCTTGTTTCATTTTGAGTTCTGCAGT
ACCGGATTTGCCAATTAAGTTTGCATAAGATCTATAAATATCTTCTTTATGTGTTTTATTTACGACTTGTTGCATA
CCATCAGTTAATAGATTGATATTTTCTTTGGAAATAATATTTTTCTTCCAAACTTTGTTTTTCGTGTCTTTTAATA
AGTGAGGTGCGTTAATATTGCCATTATTTTCTAATGCGCTATAGATTGAAAGGATCTGTACTGGGTTAATCAGT
ATTTCACCTTGTCCGTAACCTGAATCAGCTAATAATATTTCATTATCTAAATTTTTGTTTGAAATTTGAGCATTAT
AAAATGGATAATCACTTGGTATATCTTCACCAACACCTAGTTTTTTCATGCCTTTTTCAAATTTCTTACTGCCTA
ATTCGAGTGCTACTCTAGCAAAGAAAATGTTATCTGATGATTCTATTGCTTGTTTTAAGTCGATATTACCATTTA
CCACTTCATATCTTGTAACGTTGTAACCACCCCAAGATTTATCTTTTTGCCAACCTTTACCATCGATTTTATAACT
TGTTTTATCGTCTAATGTTTTGTTATTTAACCCAATCATTGCTGTTAATATTTTTTGAGTTGAACCTGGTGAAGT
TGTAATCTGGAACTTGTTGAGCAGAGGTTCTTTTTTATCTTCGGTTAATTTATTATATTCTTCGTTACTCATGCC
ATACATAAATGGATAGACGTCATATGAAGGTGTGCTTACAAGTGCTAATAATTCACCTGTTTGAGGGTGGATA
GCAGTACCTGAGCCATAATCATTTTTCATGTTGTTATAAATACTCTTTTGAACTTTAGCATCAATAGTTAGTTGA
ATATCTTTGCCATCTTTTTTCTTTTTCTCTATTAATGTATGTGCGATTGTATTGCTATTATCGTCAACGATTGTGAC
ACGATAGCCATCTTCATGTTGGAGCTTTTTATCGTAAAGTTTTTCGAGTCCCTTTTTACCAATAACTGCATCATC
TTTATAGCCTTTATATTCTTTTTGTTTTAATTCTTCAGAGTTAATGGGACCAACATAACCTAATAGATGTGAAGT
CGCTTTTCCTAGAGGATAGTTACGACTTTCTGTTTCATTAGTTGTAAGATGAAATTTTTTTGCGAAATCACTTA
AATATTCATCCATTTTTTTAACGGTTTTAAGTGGAACGAAGGTATCATCTTGTACCCAATTTTGATCCATTTGTT
GTTTGATATAGTCTTCAGAAATACTTAGTTCTTTAGCGATTGCTTTATAATCTTTTTTAGATACATTCTTTGGAA
CGATGCCTATCTCATATGCTGTTCCTGTATTGGCCAATTCCACATTGTTTCGGTCTAAAATTTTACCACGTTCTG
ATTTTAAATTTTCAATATGTATGCTTTGGTCTTTCTGCATTCCTGGAATAATGACGCTATGATCCCAATCTAACTT
CCACATACCATCTTCTTTAACAAAATTAAATTGAACGTTGCGATCAATGTTACCGTAGTTTGTTTTAATTTTATA
TTGAGCATCTACTCGTTTTTTATTTTTAGATACTTTTTTTATTTTACGATCCTGAATGTTTATATCTTTAACGCCTA
AACTATTATATATTTTTATCGGACGTTCAGTCATTTCTACTTCACCATTATCGCTTTTAGAAATATAACTGCTATCT

TTATAAACTTGTTTGAAATTTTTATCTTCAATTGCATCAATAGTATTATTAATTTCTTTATCTTTTGAAGCATAAA
AATATATACCAAACCCGACAACTACAACTATTAAAATAAGTGGAACAATTTTTATCTTTTTCATCAATATACTCC
TTATATAAGACTACATTTGTAGTATATTACAAATGTAGTATTTATGTCAAAATAATGTTATAATTTTTGTGATATGG
AGGTGTAGAAGGTGTTATCATCTTTTTTAATGTTAAGTATAATCAGTTCATTGCTCACGATATGTGTAATTTTTT
TAGTGAGAATGCTCTATATAAAATATACTCAAAATATTATGTCACATAAGATTTGGTTATTAGTGCTCGTCTCCA
CGTTAATTCCATTAATACCATTTTACAAAATATCGAATTTTACATTTTCAAAAGATATGATGAATCGAAATGTATC
TGACACGACTTCTTCGGTTAGTCATATGTTAGATGGTCAACAATCATCTGTTACGAAAGACTTAGCAATTAATG
TTAATCAGTTTGAGACCTCAAATATAACGTATATGATTCTTTTGATATGGGTATTTGGTAGTTTGTTGTGCTTAT
TTTATATGATTAAGGCATTCCGACAAATTGATGTTATTAAAAGTTCGTCATTGGAATCGTCATATCTTAATGAAC
GACTTAAAGTATGTCAAAGTAAGATGCAGTTCTACAAAAAGCATATAACAATTAGTTATAGTTCAAACATTGAT
AATCCGATGGTATTTGGTTTAGTGAAATCCCAAATTGTACTACCAACTGTCGTAGTCGAAACCATGAATGACA
AAGAAATTGAATATATTATTCTACATGAACTATCACATGTGAAAAGTCATGACTTAATATTCAACCAGCTTTATG
TTGTTTTTAAAATGATATTCTGGTTTAATCCTGCACTATATATAAGTAAAACAATGATGGACAATGACTGTGAA
AAAGTATGTGATAGAAACGTTTTAAAAATTTTGAATCGCCATGAACATATACGTTATGGTGAATCGATATTAAA
ATGCTCTATTTTAAAATCTCAGCACATAAATAATGTGGCAGCACAATATTTACTAGGTTTTAATTCAAATATTAA
AGAACGTGTTAAGTATATTGCACTTTATGATTCAATGCCTAAACCTAATCGAAACAAGCGTATTGTTGCGTATA
TTGTATGTAGTATATCGAGCTTCACATGAAACAGCTAAAGAAGCTTTGGGCGATAAAGAGTTAAGAGCCATT
GCACATGAGTTAACTAAAACAGTTAAGGATAACATGAGTGTTGATTGGTCTAAACGAGACAGTGCTAAAGCT
AAAATGAGAGTTCAAGTTAGACGCCTATTAAAGAAATATGGCTATCCACCAGATCTTCAAAAAATGGCTGTG
GAACAAGTTGTAGAGCAAGCAGAATTAATGGCAAGTCAGCAATAAAAAAATAAATCATAATGAGTCCGGGA
CATAAAGTTCTTGGATAAGTGAAAAAAGACAATTTCTATTGAAATAATATAGAAATTGTCTTTTTTATAAATTTT
TTGATTATTTTCAGCTCGTTGAGCTACTACTTTTCTTATATTAAGTGCCATTAATACAAAACCAAGTTCTCTTTT
GACTTTATTGAGTCCTCGGACAGACATCCGAGTGAAACCCAAAATAGCCTTCATAAATCCAAAAACAGGTTC
CACATCAATTTTTCTTTGACTGTAGATATTTTTTGTTTCTGGTTCTGAAAGCTTTTTGTTAATTTGGGATTTAAA
ATATTCCCAGTTATAATTCTTCATTATTTTTTTGTTTGTTTTTGAATTGAAGTTCATACATTGATTTTTCAGAGGA
CATTCTGAACAATCATCACATTCATATAATTTGAAGTCTCGCTTATAACCATACTTATCATGACGATAGGCATATC
TTTTAAAACCTAGCCGTTTATTATTCGGACAAATGAATTCGTCATTAATTTCGTCATAGTTCCAATTTTGAGTAT
TAAAGATGTCACTTTTATATTTTTTAGTTTTATCTTTTATAAACATTCCATATGTTATGAGTGGCGTTCGATTAAA
GTCATCTATAATTGCCTTATAATTTGATTCACTACCATAACCTGCATCAGCTACAATATATTCAGGTAAATGACCG
TAGGTCTCTTGAATTGAATTTAAAAATGGAATCATCGTTCTAGTATCCGTTGGATTTTGATACACATTATAAGAT
AAAACAAATTGGGAATTTGTTGCTATTTGTAAATTATACCCTGGCTTAAGTTGTCCATTTTTCATGTGATCTTCT
TTCATTCTCATAAATGTCGCATCATAATCTGTCTTAGAATAACTATTTCTATCCTTTAAAATAGATTTTTGAAATT
CGTATCGATACTTTCGCTCAAAATAATCATTGATTTGCTTTTTGTATTTTTTGATTTTAGTTCTTTTGAGACGTAT
TTGTTTTCTTGTTTTAGTACATTTTTCATTGTTGATATGTTGGTTTAAATCTTCGATTTCTTTATCTAAGTGACTA
CCAATCAAATCTATTTCTTCTTTTGTTAATTCATTATCATGATCTTCTTTAATTTCCGGTATGATTTTATTGGTTAC
CAATTCATGGTAGAGGGCTTTAGAATCCTCATTCATCTTTGATTCATGGTTTTGAATACTCTTTTTCCATACAAA
TGTATATCGATTGGCATTTGCTTCAATTTTTGTACCATCAATAAAAATAGCTTTATCATCTATAAGATTTTGTTTT
ACACACTGACTGTAAAATTGAATAAATAAAGATTCTAATAAAGCATCTACTTTTGGATTTACTCTAAATTGATTA
ATTGTTTTATAAGAAGGTTTTTGATTTTGTGATAGCCACATCATTCGGATGCTATCATTAAGCATTTTTTCTATT
TTACGACCTGAGAATACAGATTGTGTGTAGGCATATAGAATCACTTTTAACATCATTTTAGGATGGTACGAAG
TTGCACCACGGTGATGTCTGAATTCGTCGAATTCATTGTCAGGAATTGTTTCAACAATATCATTTACAGTAAAA
CGATGTTGATTTGTTTTGTTTCCATATTGACCTCCATGTATTTGCTATGATTTCAAAATCCATTTTTGACGTGCC
TTAGGGTTGAGTGGATGCATAATTTCATTTGTTACTGGATTGATGAGCTTTTTTACTTTCTTTTTATGAGGTTT
TAACATTTCCATCACTTGTTCGACACGGTCGATAACAACTGGTCGCTTCGCATAGGCACCATAAGCAAGAATC

ACTGTGTCACTTTCACTAATCGCTTTCATCAAATGAATATCAGTGTGCTCATCGTATGGATTTTTGATATGTTTG
AGGTTTTCGGGTGTTCTAATATTAGAGAATAGATTTACAAGATATACAGCACCGTATCGTTCTGAATTGGCTAA
TTGGTTGAGGATAAGAACAGTTGTGAGATCGAGTGATAATACACCGTCTAAATGAGGATACATCGTTATCACT
GTGCATGCAGCTTTCTTTTCATCCCATGTTTTCTTGAGTAAATAGCGGTGCTGTTCATCATCGCTAAATATGGC
TTCTTTGTGTATCGTACTTTTGATTGTATTCATCATCGTCACTTCCTTTAGTATTCTTCTGGTAAAAGCATCACAT
AATAAAAAGCGTCCACGTCATCTTCACGAATGACGTAGACTTTCTTAGGTAATGCATTTTGATTTTTTTCATAG
TTTGTATAGTGATATTCCAATTTGTATGTGGGTTGTTCTTGTTCATGTGTGATTGAGAGTATATTCTCATCTTCTT
GTAATTTAAAAATGTGTAGGTAATCTGTATGAGGCTGGTTATCTTTTTTTCTACCATGTGCCAAAGTAAGATTT
GAAGGTCTAGTGGAATACTTTCATTAATTCCTCTTGTGATGTATCGATTGATTTTCATGCTATTTCCCTCCCTTC
TGCTTTTCTTTCATGATGTCGATGATTTCGTTGATAACTGTGACGGATAATTGAGCAACTATGATCCAATTATTC
ATGGTCCTGACCTCCTTGTTTTAGTAAATGACGTTCATCAATAATGATATTTTGAGTATCTGTAAGGTACAGAA
AGTCCATGTCAAAATGGTCTAAGTATCCGACACTGATGAGTTGGTTATTGGCATACATTAGAAATGGATAGAT
ACTTAGCTCATGTAGTTCATCATTATAGTAGGTATAAGTCTCGAGTGTGAGATGTACCAGTGGAGAATCATTAA
TAAAACGTTCCGGTAGAATATTTTCTGCTGCTTCCTCCAGCGCTTCACATTCCCAAGCTTCGTTATTAGATAGT
TGGAATAGGTGGGTTATATATTGTTTGAGTTCTTGAGTGATGGTTTTCATATTATTGCCTCCTAGATAGTGTAAT
AGTGATGTAGTTCATATACATCATTGAGATAATATATATTTGATTTGTCATTTATTACGAATCCCGGTGGGAATAA
GAGAAAATTCCAATCAAAAACCCGTGATAAATGTTGGTGTAACAAGGAAATCCCGTAATCCCACTCATTTTGA
CGAACAATCAACTCATTATTTATAAGTATTGATGATAGGGTTGTGTCTCTGCTTCCTTATATATATTATTTATTTAT
AAAAAGTAACGGGATTTTGGGATTGTGCTTGCACAATCCTTCTGTTTCTTCGAATCTGCAAATCCCAATCATT
TCCCGATAAAAAATCATTGTGGGATGTTCTTTAGCAATTTCAATATAAGCATTGTATAGTTATGAAAAAATTAC
GACAATAACTGTTTCATTAGATAAGTGTTATTGAAATTGATAAAGAGCAATTCTTGAAAATAGTTAGATAAAAT
AAGCGAAAGAATATAGTGAAAATTATTGTTATAACAATGATTCTATTAGCTAAATAGTAAGATATAGTGTTTGG
GGCAAAAATAAAGACGAAGTGCTGAGATGCACTTCGTCGAGTTGTTTATTATTGAAAAGTTGTTTAATGATT
TCGTTATTAAGTTTGAGTGTGACATAGAATTGTTTTTTATGATTACCATCTTTTTTAATATCAATGCGATCAATCA
CTGATAGATACAATGCTTTGAGTCGAGATTTTTCTATGTGCTTAATATCATGAAAGATGTGTTGTAATAGTTTAC
TGATTTCTTTGGCATCAAATAAAGTCTTATCTTCATTTTGTTGATTTTTGAGTTGGTTGATTTGATTCGTAATGT
CATTGAGTTGCTTTTCATATTTTTGAATACTTGGTCTGATTACTGATGTTAAGTCCGGATTATCCTCGATGGTTT
TAATCAAGTTATTTATTTTGATTTGTACTTCATCATATTGTTGTTGCTTATAAGCAATATCGTGATGAAGTGCAGC
GCCATCAACTTGATTTTCTTGATTGACGTGTGTTACTACGCGTTGAATGACTTTATCACTTTTGACTATTTCAA
GTATTTGCTTCATCACATAATCTTCAATCACATCAGCTCTTACACTGTTTGCCGAACATACTTTGGAACCCTTGT
TCCGAAAATTACTACATGAATAGTAACGAATACGTTTCTTAGTCCCGTCTTTAAGAGTATTCGTGGTATTGCTT
GCTGCCATAGGTGCGCCACATTGGGGACAGTGAATAATGCCTGTAAGCAGATTCGTTCCTTTGCCATGGACT
TGGGGTTTTTGACTGACTTGTTTCTTACGCATTTGTACTTTATCCCATAAATCTTGATTAATAATGGGGGAATG
CTTACCTTCAGCTATCACTGGTTTATCATTCAGCCCTTTACGACGTTTTTCACTCCAATCTTTGTATTTCGCAAA
TTGAATTTTGCCGATATAGAAAGGGTTAGCTAAGATGTATGTGATTGAACTAATACTGAAAGGTTTCCCCTTT
TTAGTGACATATCCTTTGTGATTCAATGCATTGGCAATTTTACGATAGCCATGTCCTTTGGCATAGCACTCGAA
TATATATTTTACAATATTAGCTTCATGTTGGTTAATCATTAGCTCGTGTTTACTATCTGGTATTTTGTCATAACCTA
GTGGTAAATTGCCTTGATAATAGCCTTCTTGGGCACGTCTCGTTTGACCCATAAATACGTTCTCGACAATGTTA
TTACGTTCGAATTCTGAGAAACTCGCAAGTATCTGTAACATGAGTTTACCAGAAGAAGTATTGACTTCCATAC
GCTCTGACAAACTGAAAAATTCGACATTTTGTTTGTGTAAATCTTCGACAATTTTGAGAAGATCAGATGTATT
ACGAGCTAATCGGTTTGTTTTGTATACCATAACACAGTCGATATTGCCTTCTTTTGCATCTTTCAACATACGTTG
GAGCTCAGGTCTGTTCATAGATTTACCTGAAATACCACGGTCAGCGTATATATCTTTAACTTCAAAATGATGGA
AGTCACAGTATTCTTTGATTTGATTGATTTGTCCGTCGATACTATAACCTTCTGTGCTTTGCATTTCTGTTGATA
CACGTACATAGATACCGACACGTTTTGTTTTAAGTTGTTGCATTATGTTACATCCTTTCTTCATTTATGCAATCG

ATGATTGCATGGTTTGATTGACAATATTGAGTGGTTCATTTTTGAAATAGATTCCTATAAGATTTTTATCTTTCG
TAATGTGAATGGTTTCAATATAGGGGTACAATATGTTTAACGTGAAACGTTTTTGAATAATATTTTGAATGATG
TGTTGTATTTGATGCCCATTGATAGATGTAGTGCGTTGCGGTTGTTGACGTAATGATTGTGTTTGCTCTCTGAA
CGTTTCTGCATCAATGATGCCTTGTGCCAACTTTTCTATCAGTTGTTCTTGAGTCAATGTGTGATGTTTTTCTAT
GTTTCTTTGTCTTTTGATGCGTTTGTCAATCGCACTTTTAATTTTTGTGTAGATGCGTTGATTTTGATAAAAGTC
TCGGCACACTTCTAATACTTTATCTTCAAGTGTTTGTGCATTGATGCCTTTAAAATCACAGACAAAGCGTGAA
GCATTCATGTTTTTAGGACAGACGTAGTAACGTAATGTATGATTCTTTTTTCTAATGGTCATATTTGTAAGTGTT
GCATTACAACATGGGCATTTGATTTTTTGTTTGAGTTGATTATCCGAAGGTGTCTGTTTGGTTTGTTTTTGCAA
TCGAAGTCTCTGCGCTTGCTCATATATACTTGTGGAAACAATAGAAGGAAACATATTGTCGAATTGGCCATATT
GATTGTTGACACGACCACAATAATTAGGATTGATGATAATGTTACGAACTTGATAGGGTTGTCGATTGATATAC
GTGTTATCTTCTTCTAATAACTGTGCAATTTTCTTATAACCATGACCTTTAATGTAATAATTGAATACAGCCTTTA
CCGTTGGTGACTCATTTTGATTGATGATGAATGTTCCGTTGTGATATTCGTAACCAAAAGGTGCATGTGTTGTA
ATCAATCGACCTTGCTTTGCTTTTTCTTGAAGCCCATTTCTGACTTGTTCTCCAATGTTATCCGATTCAAGTTCG
GCTAAACTGATGAAGATATTAAGCTTGAGTCGGTCGAAAGCTTGATCCATATCAAAGTAACCATCATGTACGC
TTAAGATATGAACATGGTACGTTTGACATAATTTGATGAGTTTTAATGCATTTTTCAGATTACGATGCAACCTAT
TAAGACGATAACAGCATAATATGTCACACTGTCCTTGTTGAATTAATTGTGTTATTTGTCGATACCCACTACGAT
TATCTTTGCGACCTGATTGTTTGTCGCTATAAAAGTTGATATGTTGAATATGATGTTTTTCGGCTATTGCTTCGA
TAGCTTGTTTCTGTGCTGCAAGAGATTGTTGTTTCATCGTACTTTGACGTAAATAGCCTATGACTTGTTTCATAT
CGGCTCCTCCTTTCACAGTGATAATATATATTTATGGATGAATTGATATATAAGCCCAACATCAATGAGATGTTG
GGCGTCCATATTAGTCATTTGTTTGATTGATTTCTTCAATTACCAAATCGGCTAATATCTCGATAAGTTCATCCAT
GTTTTTCACTCCGTTATTTGTTCTATCTTCAATACGTCGATTATTCAGTTTGATGCTTCACAGTTGTATGATAAA
GACAATTAGAAATCTTCGTGAACTCCTGAAGGGCCTATCCCTTCATTAGCGGATTTAAAAAGTTCTTTCGCAG
CTTTGTTATCATTTGACGGTGTCCAATTTTGAAGTAACGACTTATCTTTAGTTAATCCGAGGATAGATGCAAAC
TCTACATCTAATTTTAGATGGTAAAATACAAGTGATTGTTTTTTACCGCTATTATCTTTGACACTTCTTTTAGTTG
TTTGGCGTCCACGGTCAGCTAATATGAAACCTTTATCTCTTAAGGCGTTGACAACATTATTAACATCTTGAAAT
TGATGATTGTTTAGCATCTGTTTAAAAACGTTCGCAATTATTTTTACTTCGATATGGTCATCTTTTAATGAGATT
AATCCATAGTTCTCAAACATATTTTTCAAAGCACCTTCATCTGAAAACTTACCTCTGTTTTGTGCCACAAATTG
AATGATGACATCAATAGCTTTATCAGCTAATGAGCGTTCAGAGACTGTATGAGTATGATAATCAATAAAGTAGT
CTCTTATATTAGCGATATCAATATCTGTAGATAAAACACGACCTAATATTTTCGCAGATGTTGTAATGACTGCAT
AACGCTTAAACATACGAATACCTGTATTGTTTGTTTCATCTTTCAATTTAGCTTCAAACCAATCTACTTCCTTGT
AAAACCATTGAATAACTTCATCTTCACGATTTATAAGATATTTAGCTACTAACGGTAAAACATGACCATAGTTTA
GTGCTACAGCTTTTTTAATATTGTCAGCATTGGTCGCATTTGTAGTGAATTGTTCATTAATCTCGATGGTTCTTA
CACGTAATCCATCGTTTTGAGCTGAATCATTAAAAATACTGTATTCTGACGTTGAAATGACAGAAGTACCCCA
ATTCTTAGGCGTTTTAACTTCTCCATGAACGTTTGAACGTTGACGACCTTGACCTTCAGCGATGGAATATAAC
AAACCCGTGGTATCTCTAAGTGTCGTAGATGAAAGCTCATCAAATACTATAGGAATGCCATAATTGTTACTCAA
ATAACCTTCAAGTGCGTTTCGTGTGGCATTCCAACTTCTAAAAAGAGTTTCATTACCTTTGGTAGGGTTACCA
GCGACTGATACAGCTAAAGCAGCTGCGGTTGACTTACCGGTTGAGGATTGACCTGTAAAACTAAAGAGAAT
TCCTGCAAATTCGATTTCATGTTTGTGCTTCAGAAAACTTGTCACTAAGGCAGAAATCCCAAATATGACTGCC
AATTCTAAAAGAAGATGACCTTTAACCTCGTCAATATACATGTTAAACCAATTATCAAATGTGCCCCTAGGTGC
TAAGTCATAAGTATTCTCACAAATGGCGTCAGATGGAGATTTATTATCAAATTCCGTAGTAGTATAGATTTCATT
TAACGATACAATAGGACCAAACGGTGTTTCCAGTATACCTACCCCTTCATATAAGTAGGAAATGGGTAATTGAT
TGCGCATTTGTTGCAACGCATAGCCTAAATCTTTTGTATATTTTTCATTAATACTAAATCCATATTTCATTAAAGA
GGGCAGTTTTTGTGTTGTTAAAATATCACTAGATTCAACAATTACTTTTTGATCCTCGTCTGTAATAATTACTTT
TTCAGTGTTAGTTTTAGGGTCAATAAATTTATTTTCGATAACGATAGGACCTGCGATTTCAACTTCAATAGGCA

TTCCTCCTTTTTCTTTGGGAGGCTTATCTTCATACCAACCTTTTTTTGGTACATAATACGGTAAAGGATTAAAC
GAAAAGTTAGTTTGAGCCATTAGCGAACACCTCCTTTCGAAGGGTTGCTGTTATTATATGGATTCGGTCCTGT
TTTAAGGTAAACTAAGTGTCCATGAGAATTTTTACCAATAATAACAAATGGAACACGTGGTGCATGTTTTACA
AAGTATGCGAACCAACGGCCTACCTCTCGTTGAACATCTCTAGAGCAAGTGATACCTGATTTTTGTGTTAAAT
CACTAATAGTAAATTCAGTACCTACTGCTAAATTAAATGCAATGGCTAAATCTGCATCTAGTAAATCTTTAAATT
CTTGACTTTTGTTAGTCATATTAAATCAACTCCTGTTCAATTAATGTGTAAGTGACTGAATGCTCAATCAACTGT
ACACATAAATTATTACAGGGATGAGAATTTAAAGTCATAGAGAGTTTTAAAGGTACTTTTTAGCACATAAAAG
CGATAAAGTAAAAAAGGAGAGATATAATGAATACGTGGAAAAATGATTTGTACGGGAGATTAGGAGATGTTA
TTGAACTTGAGATTGCAATACATCTAATGATTAGAAAATATGAACGCAATTCAATGGATAAATATATGAACATT
AATGAGTTTTATCATTATCTATTGCAAAAAGACATAGTTATTTATGATTATGAGACATTTCATCGATTCTATAAAA
CGAGTAATATTCATAATTACTTGCGAGTAAAGCTAAGACCAAAAAAAGCTTGGGAGGAAGAAAAGGTTATTC
ATCCAGAGTCTTTTAAAAGTATTTTAGCAATACGTTTAGGTGAAACAAAATTGATGGCTGGAACTAAATACAT
TGACTCCAATATTAAAAGCTTAAGCCAAACTTATAAACAACTAAATTCTAATATTGAACAGATTTTAGATAAAA
TAAGTAAGAATACATTTGACATAAAATTGGAACCAAAGTTGATTGCTTATAAAAATTTATATGAGTTATACAGT
TATTTTATGGACGAAATAGAAAACTATAGCAACGACTTTGAGCAATTACGAGCGCTTCTATATTTAGAATTAGA
TTACTCATTTGTACTCTATAATGAGTTACTAAGGATGATACATTTAATCAAAAAGTATAAACTAGCTGCTAATAG
TAAGATAATAGAAAAAGCAATAGGTGAAATTAGTATGAAGTTAGCAAATATAGAATTTCCATGGCTAAGATTA
GTAGTTCTAAGAGCTTTTATTGATAATATAAAAAAATATCATGATATAAATTCATTGATTAACGATATTAGTCATA
TTATCAATTATACGTTATTTGAAATCGAACAGTGGATTGAACGAGCAGAAATTGAAGAAGGAATTAGAGATT
TAATGAATGTGAAATATGGTAGGGTTCCTTATCTAGATGCAATTATAGAAAACAGATATAAAAGAAATAGTATT
AAAGAATATTTTATCTTACTCAAACATGAATTTGGAATTAGATGGTTAAGAGGTCGAGACTTTAATGATCAAG
ATAGAAATAGGTTTAATAGAGCTATCAAATATAAACCTTTGAATCTTATAGGATATAACTAATTAGAGGTGATAT
AACTATGATAACTGAAGAGATGCTTAAAAAAAATTGCAAATGTATTTAACGGGGACGATGAAAATAGTATTTAT
GAGTATAAAACAGGTTCAGATTTAGTGCGTTTTTTTAATCAGTACTTTAATAGAAAGGACACATACAAAAACC
CTTTTCCATCAAGGTGGAGGTATGTTGTTGATATATTACAGCAGTTGTTACAGACAAAAAAATTAGATGAATT
CTTTACTGTTATTTTAAGTATTCGTTATATCCAAACAGAATTACATCTTAGTGAAGTAGAGGCAGTGCAAAAAT
CTAATGATGCTTTGTTGTACTTTAATAAATTGTTACAATATGACGGTTATTATTTAGTATATAAGGATGATAAATT
TATACTAATGGAAAGGGATAAGGATTTAACATATTTAACAAGTGGCGGCTACGCTGATATTTATCTACAGAAAT
CAACTGGTCTTATCATAAAAAAAGTTAAGAAGTGAGTATTATAGCGATAAATCAATATGTAGTAGGTTTAAAAG
AGAGTTTGATATTACAAAGTCCTTATCATCAATGGAGTTAATTATTGATGTATACGAGTTTGATAATAGTCGTCT
ATCATATTCAATGGAAAAAGCCGATATGACATTAGAGCATTATATTAATAATTATGAAGTAGATTTAGAAATTAA
AATTAAAATAATTAGACTAATACTTTATACAATTTCTAATGTACATGAAAAAGGTATAATTCATAGAGATTTAAG
CCCTACAAATATATTTTTCACTAATGGGAATATAAAGGTTGCAGACTTTGGCTTAGGTAAAGATTTAAATGTTT
TGTATTCCAAGCAGACGCTAAATACTAATGCAGTGGGACAATTATTCTATTGTGCACCAGAGCAATTACTTGG
GCTCAAAGATAGTAGTAAGAGAAGTGATGTTTTTTCTTTGGGAAGAATTATTAATTTTATCATGACTAGGAGC
CCTAACAAAGTTTCACATATTTTTAGAACTGTGAGTGAAAAGTCTACACACGAGAGTTCAGAATATCGACATG
AAAATGCTCAAGATTTATTAAATCATTTTGAAAAGGCATTAAAATATCATAATGATAAAAATAAAAATTTAGAA
ATAAAGAATAAGATTAATCGAGGTGTGTTTGATGATGATGTAGAATTTTTTTATGCAGCTCTTTCAGAAAACG
AAATTTGTCAGGTGTTATTATCTTCGACCTCTATGGTTAGGAATACATTAATTGAGTTCATGAAGAAGAAGGAT
AGTTATGCAGAAGTAGCAATACAAAATATTAATAGTGAATATAAAAAAATATGTAAAAATTTTGAAGACTATGA
TCCATTCTCAAATTTTATGTATGAAATCCTGAAGGATAGATTTTCATTTAGGGTAAAAGAAATTGCAGCGATTA
TTCTCAATGAGATAGCATACTCTTTTAACAGATATCATGCTCAAGGATTAATTAAAGATATTATATCAATTGGAA
TTGAACCAATAATCGAAGATATTTTAAAAGGAGATAAATAATGGACAATAACTTTTTAGAAAGACTAAAAGAA
ATTTTAGAACCATATGACGATATAGAATCAAGTAAAGATGGTTTTGTATTTGGTGGATATATAGAATGGCCTAC

TTTAGATTATTACAAAGATTTTCAAGTTAGTAAGTATACCTAAAGAGTTCAGTTATACAGTTAATTTTTATATTTT
TGGATATATTAAAACTCCACCCAAATTATTTGTAACAATACTAGGGGAAGATTTAGAAGGAAATCAATTTAAA
GATGAAATTGAAATTAAAGTCCAAAGGTATAAATTTAGTTATGTTCCGGTTCAAGCAGAAGTTACTTTATATGC
TTGATAATTATATATTATAAGATTTTTATATTAATAAAATATAAATATTATTAGGATTTAATATTCATACTGTGCTAA
ACTTAAATAAAAAAATTAATAATTAATTTATAAGGAGATTAACAATGAAATTTTGTCCAGAGTGTGGTAATAAAT
TAATAGATGGTGCTAAATTTTGTCCAGAATGTGGTTATAAGATAGCTAATTTAAATAATGTTTCTAACATACGAG
ATGTACATATTGAAGAAGGATATAAAAATAGTGATAACTCTATTAAAATAAGTTCAGATGAATATATCAAAAAA
ATTCTAAGAGAATCTGACTTTACAAATATAGATATAACACCAAACATCTCTGAAAAAAAATTAGTAAATGCTTC
TGTAAGCATAGCTCAAAATACGGACCCCAATACATTAATAGCACTAATTGATACAAGCTTATTAACAAACGGTA
AGTCAGGTGTTGTATTTACAGGCTCTGAATTTTTTATAAAAAATAATTTTTCAGATTCTTTAAGAGTACCATTTA
AAGATTTGTTAAGTGCAAAACATAGTATAGATACAACATTTAACAAAAAAGGAATAGCAATAGAAGAAGAGA
AAGTAATTATTGAATATAAAGATGGAGCTACTGTAAATTTAAATGCAAACACTTATGGGAAAGATATACCGTAC
AAATTATTAGCTAATTTTTTAAATGAATTTGATAAAAATGTTGATAAAATATCTTCGAAAAATCAGGTTGTTCAA
TTAAATCAAATGAATGAAAAAATAATTAAATTATACTTCCGAATTATTATTGCTTATTTAAAAGATGACGATGGC
ATAATTGATAGTAAAGAATATAAAGAACTAATTATACTTATGACAAAAGTTAAAATTTCTAAGATTGTTGCAAA
AGAGTTAAGAGAATACAGATTTGAGCATAAAGAAAATTTAACTATAGATGAACTTATTGAGGAACTACAGTAT
GAGTTGAATGAAGCTGATTTATCTGATACTTCTGTAATACAATCTTTAGGTATGGACATGATATCTATGAACAGT
GATAAATTAGATGATTTAACTAATGATGTAGTATTAATGAGAAATTTAAATAGATTGAATTTCACAGATAAACA
AATTCAGTTTGCTGTTCATAAAATTAAAGCTGAAAAGAAAATACTTGAAGAAATAATGACGGACTCTCAAATT
AAAGAGACAGCAAAAGAGTTAGCAGCCATTGCAAGTGGAGCTGGAGTATCTTTAGGAGCTTTAGCAATTAC
TGGAGCAGTTGGCGGTCTGGGAATGGGTGTGACAAGTGGCTTATTCGCATTGGCCTTTACACTAAGTACAG
GGACATTATTTGTAGGTTTAGCAGCTATTACATCTGCAGGATTTGGTGTATATAAAGGAGTGAAATATTTCTCA
GGTACGGGTGAATTAGAAAAATATGGTATTAGAATACAAGCTTTACAAGATAATATTAAACAAATGAGAATTG
CTAATAATTATATAATTGAGGATATTAATTGGTTGTCTGAAAAGTTATCCCACTTTGCTGAAAAATTAAAACATT
CTAATGAAATGAGCAATGAATTATATGAAGAAATAAAAATACTTCTTTCACAGAATCAATTGTTAGCAAGTGC
AGGAACTTTAATTGAAGAAGAAGAACTAAATTCCGAGTATGAATTGTTGAAGACTAGTGTTCCTGCATCTCTT
AATCTTAGTAAATATAATGAATTACTGGCAAAAAATACAAATAAAGTTTATGCAGATGAAGTAATAAAAAAAG
CATATGTTGTGAGTGGAGACTTAGACGAACAAAATGATCTCTCCAGTATTGAATTAAAAGAAGATGTTACTTT
AGAAGAATTAGAAACAGTTAAAGCTATTTTAGAAGAAATTGGATACTTTGATACTAAAGCAGCAAGTATTGC
ACAAGGTAAATCATTAGCTAAAAAAGGTTTTTCAAACTTTAAAAAATCATTCCTAGGTGGGGAAGATTTTGA
ATAAGAAAACAAGAAATGAAAACAAAAAAATGAAGCATGCAGCTGAAAAAATTATTGTTGATAAAGAATTA
CGCGATAGAGGTAGAGATAATCTAGAAAAAGCATCATATAATATAGATACTATGCTTAATAATGTTGATCAACA
AATAGCTATGAAAAAGGAGTTACTTAGTCAGCTAAGACAAAGAAGAAATAACTCACATCAATTTAAAAAGAG
AGATTTATATCACGATAAAATATTAGAAAATAAGCTTAATTCTGCTCAAGAAATTTCTAAGAAAAATCTAAATTT
AATAGAGTTAGATAAAGTAACAACTATTGATATAGATCGAGAAGATTTTGATTCTATTCAGTATAATAGGGAGT
TTGCGCAGCTAAACAGTATAAACTTAGATAGTCCTTTTTTAACTTTATATAGCAAAACAGAACAAGTTAAAATA
GCGAATCAAGTTGTTCAACAATTTGATTTGCTAGAATTAGATGATATGGATTATCTTTTTGCTACCGCTGCAGG
AGTAATTGCAGGTTTTGTTGACGTTATGTATGGCGGGACAATAGCTAAAGGTAAAGATGCGAAAGGCTTACA
AAAAATTGTTGATAAAAGTACAGAAGAATTAGTGAAAAAATATGCTTTACATGAAAAAATAGCAGAGTTGAA
TAAGCAAAAGAAGAATACTAACTCACAAAAGTCAATAGATAACATTAATAAAAAAAATAAAAGAAATAAAAAG
AAATAAAACAAATATTAATCTAAAAAGTAGTATTAAGTATTTAGAAAACAATCATCTAGTTGGATACGATACTG
CACATTCGAAATATATAACAGAAATGATTGGAAAGATGTCGCCTGATAATCATCATTTATTATCAATAGCGCATG
AACCTAGTTTGTTAGGTTTAATTGTCGGAATAAAGGATCAACTTACAAACACTTCAACTTTTATGACTAAAGA
AGGTAAAATTATAAATGTAGTTTCACAAAATAAAAATAACGAATTAACTGGAAATATGTTTGAACAAATTATAC

AAGCAACAAATAATTGGTTTGGTCATATTATGAGTGATATATCAGGCTCTAGTTCTTCTAAAGGAAGAGGTAG
TGGTTTACCAGTCCCAGGTTGGTTCTCCTTGCAAAAATTACAATTTGGTAAAATACCATTAAATGGAAAAGAT
ATGACCATTGCTCAAGTTTCAGAATGGATGTTTAAAAATGGTTATGACATAAGAGCGTTTGCTTCAGAATCAA
TATCTGTTATTATATTCGAAACACTTATTAGAATTTACTGGTTTTATAAGCAATATTTTTATTACGGAAAAAGTTT
AAAAGAAAGTATTCCTATTGCAAACAGTCGAGAATTGTCTAGATTATTACTTATAGGCTCAGCTACATTTTCAT
CTATAGATATAGGTCATGGATTAATAAAAAGTACTTCTAAAGGTGGAGTGCACCCAATAGGCATTGCTACCTTT
ATAATGACTGTTAATAAGCCAGGCCTACTAGATTTAGGTTTTAGAAGTTATCAAAATGTGAGATTTGAATTACA
ACATAGAAAACATGTAGAAAAAAATAATTGAAACAGATATTTTAATGGAGTATAGAAGAATTACCATATCAAATT
CTATATTTTAGTGTTGAAAATAAAGTATTAATATTATAAGAAACCAATTTATACTTATGGGACAAGGCGATAAAT
TCGATATGAATTCTGTCTTGGTCCCTTTTATTTATCTTACGAAAATTTATACAAAAAACGATGCTTAAACTTTCG
ATTTATAAAACCGCATCATTAACCGATACGCAGAGGCGTATCATAAGT
>Staphylococcus arlettae strain SA283
ATGAAAATTACAATACTCGCAGTTGGTAAATTAAAAGAAAAATACTGGAAACAAGCACTTGCTGAATATGAA
AAGAGGTTAGGTGGCTACACTAAAATCGAACTCATCGAAGTTGCAGATGAAAAAGCACCGGAAAACCTGA
GCGACAAAGAGGTCGAACAAGTCAAAGCGAAAGAAGGTCAACGCCTACTTGCCAAAGTCAAACCACAATC
CACAGTGATTACCCTTGAAATTCGAGGCAAAATGTTAACATCCGAAGGTTTGGCACAAGAACTTCAAGGTA
GAATGACCCAAGGGCAAAGCGACTTTACTTTTATTATTGGCGGCTCACATGGTCTGCATCAAGACGTCTTAA
GTAGAAGCAACTACGCTCTTTCATTCAGTACCATGACCTTCCCACATCAAATGATGCGCGTCATGCTCATCGA
ACAAATCTACAGAGCCTTTAAGATCATGAAAGGCGAAGCATATCATAAGTGATGCGGTTTTTATTAATTAGTT
GGTTAAAAACGAAGTATGCAATATTAATTATTACTAAATTTTGATATATTTAAGGAAAGATTAAGTTTAGGGTG
GATGAATGAATGGCTTATCAGAGTGAATATGCATTAGAAAATGAGGTACTTCAACAACTTGAGAATTTAGGAT
ACGAACGAGTAAACATACATAATGTTGAACAATTACATGATAATTTTCGTACTATAATTAATGAACGTCATAAG
GATAAATTAAGTGGGAAACCGCTGACGGATCGTGAATTTGAACGTTTAATGACGGGAATTAACGGTAAATCT
GTATTTGATAGTGCAATGCAATTACGCGATCGCTATGTACTAAAGAGAGATGATGATACGGATCTTTATATAGA
TTTCATGAACTTAAAACAATGGTGTCAGAACAAATTCCAAGTAACCAATCAAATCAGTGTGAAAGATAAATAT
AAAAGTCGTTATGATGTGACGATTTTGATTAATGGTCTACCATTAGTTCAAATAGAATTGAAACGTAGTGGGG
TAGCGATTACGGAAGCATTTAATCAAATTGAGCGTTATCGTCGTCAAAACTATACGGGTTTATTCCGATACATA
CAATTATTTGTCGTTAGTAACAAAATGGAAACACGCTACTACGCCAATAGTGATCGTGAAATCTTTAAGGGAC
AAATGTTTTATTGGAGTAATGAGCAAAATGAGCGTATTAACTATCTTAAAGACTTTATTGAAGATTTCTTAGAA
CCATGTCACATTGCTAAGATGATTAGTCGTTATATGATCGTCAATGAAACAGATAAAATTTTAATGGCATTACG
TCCTTATCAAGTCTATGCGGTTGAGGCCATTTTAAATCGTGCGTTAGAAACAAATAACAATGGTTACATTTGG
CATACCACAGGTAGTGGTAAAACACTGACCTCTTTTAAAGCGAGTCAATTATTATCGCAAGAAGAAAATATTA
AAAAAGTGATTTTCTTGGTCGACCGTAAGGACTTAGATAATCAAACGTTAGCTGAATTTAATAAATTTCAAGA
AGACTCGGTTGATTTTACGGATAATACGCGTAAATTATTGAGACAGTTAGCCGATCCAACTTTACCTTTAATTG
TGACAACGATTCAAAAAATGGCGAATGCAGTGAAGTCCAATCATTCAGTAATGGAATCATATAAACAAGATA
AAGTGATTTTTATTATTGATGAGTGTCATCGTACGCAATTTGGTGACATGCATCGTTTAATTAAACAACATTTT
GAAAATGCACAATACTTTGGATTCACGGGCACACCACGTTTTGAAGAAAATAAGAGCCAAGATGGGCGTGC
TACAGCGGACATTTTTGATACATGTTTACATCACTATCTCATTAAAGATGCTATTAGGGACCATAATGTGCTTG
GATTTTCCGTTGAATACAATCAAACATTTAATGCACATGAAGATTTGGATGAAGAGTATATGTCTAAAATTAAT
ACGTCGGAAATTTGGATGGCAGATGAACGTATTGAAGCTGTTTGCAGACATTTAATTTCAAATTATCACAAG
AAAACAGATAACGGAAATTATACGTCAATGTTTGCCGTTCAAAGTATTCCAATGGCGATTAAATATTATGATAC
ATTCCAAAGACTAAAAAACGAAGGTGTTCATGATTTAAATGTTGCAACTATTTTTACGTATCAGGCTAATGAA
GATGCACAAGAGGATGACAATCATGTTCATTCAAGAGAAGTGCTCGATCGCATTATGAATGATTATAATCAAA
CATTTAAAACGAACTATAATACGGATAATTTCGAAGGCTACTTTTCTGACGTATCTAAGCGTATGAAAGAAGT

GGTTCGTGATGACAAGATTGATATTCTTATTGTAGTTAATATGTTTTTGACTGGTTTTGATAGTAAGAAACTGA
ATACCCTTTACGTTGATAAAAACTTGAAACATCATGATTTAATTCAAGCCTATTCAAGAACGAATCGCGTTGAA
AAAGAACGTAAACCTTACGGTAATATTGTATGTTATCGTGATTTAAAAAAACAAACGGATGAAGCCATTGAAA
TTTTCTCACAAACAGATAATACAGATACAGTATTAAGTCTGTCTTACGAAGAATATTTAGATAATTTCAGAGAT
ATCTTGCAAAACGTTTTTCAACTAGCGCCAACTCCTTTAGATGTCGATAAATTAGAGGCTGAGGATTTAAAAA
AAGAATTTGTCATTTCGTTCCGTGATTTATCGAATACTTTAATCAAATTAAAAACCTTTGATGAATTTCAATTTA
CGAAAAACGAGCTAGGCATTACAGAACAAACGTATGAAGATTATAAAGGAAAATACTTAAATATTTATGAAG
AAGTCGTGCGTGTTATAAAAGGAGATAATGATGACGCTGTTTCTGTCTTAAATGATATTGATTTCCAAGTTGA
ATTAATGCGTAATGATTTGATTAATGTGAAGTATATTATGGATCTCATTGGTCAAATTAATCTATCTGATGCAAA
AGCACGTGATGAAAAACGTCATCAAATTCATAAGTTATTAGATAAAGCCGATGATCAACAACTACGTTTGAAA
GCTGATCTGATTCGTAGCTTCTTAGATAAAGTTGTGCCGTCACTAAAAGAAGATTCCGATATTAATGAAGCCT
ATTATGAATTTGAAGACAAAGAAAAAACAAAAGAAATTGATGCATTTGCAGAACAAAAAGCCTTTTCTGCTA
TGTTACTTAACGAAGCTGTTAATGAATATGAATATAGTGGTAATATCGATCGTAAATCTATCGGTCAAAATATTT
CAGAGCCTTTTATGAAACGTAAGCGAAAAACAGATCAAATTATACAATTTATTGAAAATACTGTTGAAAAATA
TGGTATGATTGAATAATTTTTATAAAATCCTTGTATTTTTTCTTTACTACAAGGATTTTTTGTTATATACTCAATTT
GTAGTAATAAAAAAATACAAAAAAGAAGGTGAGTCAAATATCTCAATTATTTACGTTTGATAATGGTTCTTTAA
TCAATCGACTTGAGACCGTAGAAGCTTCTCAAGGCATAACATTACCTATCTACGATCAACGTATGATGGAGTT
CGATAGTGGAGAGTTTCAACCGACAACATATCAACCTAAGCAGGTAACATTAAGCACAACACATCAAGCCAA
GATGGTTCATACGGGCGATATCGTGATTAATATGATGACGAGTGAGTGTGTCATTGTGAGTCAACAACATCAT
GAAAGTATTCTACCTTATAATTATACGCATATTGAGATTGATACTACACACATCGATGCTAATTATTTTGTTTATT
GGATGAATGCTTCATCTCAAGCGAAGAGTCAGTTAAATCAATTTAAACAAGGGGGTTCATTAGTTAAAAAAT
TAACATTGAATCAACTTAAACAACTTAAGATGACGCCACCTCCATTAGAACAACAACAACGTATAGGAAAACT
TGATGAACGACGTAGACACTTAAAGTATTTACAAACGAAACGAACTTACTTGATGGACCAATTTTTATCAGAA
TATTTATTTAAGGAGGGCATTTAGATGTCAACAACAGAAAAACAAAGACAACAACAAGCCGAATTACAAAA
GAAATTATGGAGTATTGCGAATGATTTACGTGGAAATATGGATGCAAGCGAGTTTAGAAATTATATTTTAGGA
CTTATCTTTTATCGATTCTTATCAGAAAAAACAGAAGAAGAAGTAGCTGAGTTATTAAAAGAAGACAACATTT
CATATGCAGACGCTTGGGAAGATGAAGAATATCGTGAAGCGTTACAGCAAGAATTAATTAATTTAATTGGTTT
TGTGATTGAACCTCAAGATTTATTTAGTCACTTAATTCAGAAAATTGAAACTCAAACGTTTGAAATTGAAGAT
TTGCATAAAGCGATTAATAAAATCGAAGAATCAACACGTGGTGAAGACAGTGAAGAAGATTTTGATCACTTA
TTCGCAGATATGGATTTAAACTCGACACGTTTAGGTAATACCAATGCTGCTCGTACAAAACTTATTTCAAAAGT
CATGGTTAATTTAGCTACACTTCCGTTTGTGCATAGTGATATTGAAATTGATATGTTAGGGGATGCTTATGAATA
TTTAATTGGTCAATTTGCAGCCAATGCTGGTAAAAAGGCGGGAGAATTCTATACACCTCAACAAGTGTCTAA
AATTTTAGCTAAGATTGTCACAACGAATAAACCGAACTTAAAAAATGTATATGACCCAACATGTGGTTCTGGT
TCATTGTTGTTACGTGTTGGACGTGAAGCTGATGTTCGATTCTACTATGGTCAAGAATATAACAATACTACATT
CAACTTAGCACGAATGAATATGTTACTTCATGATGTGAACTATACACGTTTTAAAATTGATAATGATGATACCTT
AGAAAATCCTGCTTTTAGAGGAGAAAAATTTGATGCAGTAGTAGCTAATCCGCCTTATAGTGCGAAATGGAG
TGCCGATCCATCATTTTTAGATGACGAACGCTTTAGCGGCTATGGTAAATTGGCGCCTAAATCAAAAGCCGAC
TTCGCCTTTATTCAACACATGATTCATTATTTAGATGATAATGGCACAATGGCTGTAGTTTTACCACATGGCGTA
CTATTCCGAGGTGCAGCTGAAGGAACGATTCGTAAATATTTAATTGAAGAAAAAAATTATCTAGATGCGGTG
ATTGGATTACCAGCAAATCTATTCTTCGGTACATCGATACCAACAAGTATACTCGTCTTTAAAAAATGTCGTGA
AGCCGATGAAAATGTGTTGTTTATTGATGCGTCTCAATCATTTGAAAAAGGTAAGAACCAAAACCACTTAAC
AACTGAAGACGTTGAAAAAATCGTTGAAACGTATAAAAATCGTGAAGCATTAGATAAATATAGTTATGCGGC
AAGTTTAGAAGAGATTGCAGAAAATGATTACAACCTTAATATCCCAAGATATGTCGATACATTTGAAGAAGAA
GAACCTGTTGATTTGGAGCAAGTTCAAAAAGAATTAAATCAAATTGATGATGAAATTGTAGAAGTAGAGCAA

GAAATTAATGGTTATTTGAAAGAATTAGGAGTGTTGAACCATGACTAATGAAGTTGAAAATGTTCCAGAATTA
AGGTTTCCAGAGTTTAAAGGAGAATGGGAAAGTCAAAAATTAAAAAATAAAATTGTATCAATTGAAACAGG
TACAAACTTATTAGGATCAGAAGACGGTCAAGGAATTCCTTTAATAAAAATGGGTAATATCAAAAGAGGATAT
TTTTCATTTGATAAATTAGAAAAATTAAATAAGGATGCAAATATTGATAAAAAAAACATAGCTAAAAAAGGTG
ATTTTTTATTTAATACAAGAAATACTATGGAATTAGTAGGGAAAAGTGCTACATGGACCAAAAAAAATGCTGA
ATATGCATTTAACAGTAATATAGCTAGGTTGGTGTTTAATGATATAAATACATACTTTTTTAATTATTTATATAATA
CACCTATAATGATAAATAAGATTCGATCATTAGCAGTGGGAACAACAAGTGTAGCAGCTATTTATCCTAGAGA
CCTTAAGCATTTGAATTTCTATTTACCTACTTTAAGAGAACAGAAAAAAAATAGGTGATTTTTTCAGCAAACTC
GACCAACAAATTGAATTGGAAGAGAAGAAGTTAGAGTTATTAGAGCAACAAAAGCGTGGATATATGCAAAA
GCTTTTCTCGCAAGAATTGCGATTTAAAACGAAAGATAACGTTTATTACTCTGAATGGCAATTACATAAATTAG
GTGAGATATTTAAATATAGTCAAGGACAACAAATTTCTGTCGATAAACAATTTAGTGTTAAGAGTAATGAAAT
GAAACGTTTTATCAGAATAGTTGATATAACTGAGAATCCAGAACCAAGATTTGTTTTATATGAATCAGATACTG
GATTGGTAAATAAAGAAGATTTATTTATGATTCGTTATGGTCGTCCGGGCATTGTGTCTATAGGATTTGAGGG
GATAATTGCTAACAATTTATTTAAGTTAATTCCTAAAATAAAAATATCTACTGTTTATTTTTATTATTTATTATCTTA
TAACTACTATAGAATTAATTTATTAACAAATCAGTCTACAATGCCTGCGTTAAACTTTAAATCTTTAGATTTACTT
AAATTTAAAATACCTTCATACAAAGAACAAAATAAAATCGGCAATTTCTTTACTAAATTCGATGAACTGATTGA
AAAACAAAATCAAAAAATTGATGGATTGAAAAAACGTAAAAAAGGCTTTTTACAGAAGATGTTTATTTAGGT
TCTGATAACGGCCTATTATGTTAATTGAAATTAGAAAAAATAAAAATTAGAGCCTCCTTCATCGTAAAGTGAA
GGAGGCTTTAAATTTATAAATTTACTAAATTCTTAGACAGAAGATTAATTCGTTGTGCTTTCTAATATAGTCACA
TCAATTTTTTTTGAGTTGTTTTATTATATACCCAACGTATTAATAAAGCGATTGCAATGAATAAGCCGATGTAAC
CTACAATTGGAAAGACAATATTAATTAAATCGGCGAATCCCACAAAGCTTAGTATAAATGCTAAAACTAACGA
AGAAATTAATAATATAAAGTAACGTGGTGTACGAAAATCAGAAAAGCGAATTAAGAAAGGATAAATCATTCCT
ACACAACTATTAAAAATAACACCTACCATGACAGCAGACATAAGAACACCAAGCCAAGGATGAATATCTTTAG
CTAATAATAATGTAGGTATATCGACACTGTTAGCTTTTTCAACATTCGCAAGTAAACCTGCAATCATAATCAGTA
ATAGTATACTAAATACGATACCTCCATAAGCTGCACCTCGTCTGGCAATCTTATGACTTTCTGCATCTCCGCCTA
TAATAGTTAAAAAACTGAAGCTATTTCCTATAATAATACCACCATATAAAATAGCGTCCCACCACCAGGCATGA
GGAGTATTTGTTTTTGAAGGTTGAATATGCTGAGCTACCTCATTAAAAGGGACAGTAGGTTGAATAATATTAA
ATCCGGCAATAATAAACACAGCGATAACTAAAAAGGGTGTCACGATACCTAACACGCTAATGATTTTATTGAA
ATTTAAGAGTAGAATGAAAAATAATAATAAAACAATTATGAATGAACCTATCCATGTAGGAACATTAAATCCTT
GTTGTAATGCGGATCCACCACCAGCAATCATAACGACAGTTAATCCAAATAAAAAGAAAACAATTAAATAATC
AATAATGGTTCCTAATACTTTACCAAATAAGCTATGTAATGAAATATCGTATTGATTAGCATCTAAACGGTAACC
TACTTTTGAAGTTTGACGTATACCAAAGGTTACAATGATACCTGTAATCATTGCGGCTATATATGCATAATTTCC
ATGGTTTACAAAAAATTGTAATAACTCTTGGCCAGTAGTAAAACCAGCACCTACAATGAAACTTAAGTAAGCA
AGAGTAATTTTAATTGCGGTTTTGTTTTCTTTACTCACCAACATTCCTCCTTAAATAATACATACATATAGAATAA
CACAATTTGAATAGGTATTGAGATTATAAGAAAAAATAAAATTGAGCATCCCTCACCGTAAAAGTGAAGGATG
CTCTAGTTTTATTAGAATATAATTTTCATTTTGACAGATAATGGTTAATAATTGTCTGTAAATCATTATTAAGTAC
TGTGGTTATAGAGTCATCATTCATAAAATCGTCATAGATTTTATCAAGTATTTCTTCATCTTCAATCGCTGTAAA
ATGATATATTAGTCCTTTTATAATTGAAATATAGTTTTGCGATGCTAAGCAACTAGCAACTATTCGCCAGTCTGA
TTGTGTAGTGTTATGGTTCATAGATAATCCTCCTTTATTTCAGTGTCCATTTTTGTCGAGCCTTAGGATTGAGTG
GATGCATAATTTCATTTGTTGCCGGATTTATGAGCTTTTTAATTTTCTTTTTGTGAGGCTTCAACATTTCCATTA
CTTGTTCAACACGTTTGATAACAACTGGCCGCTTCGCATAAGCTCCATAAGCTAGAATCACTGTGTCACTTTC
ACTTATCGCTTTCATCAAGTGAATGTCTGTGTGCTCATCGTATGGCTCTTTAATATGTTTAAGGTTTTCTGGTGT
TTTAATATTTGAAAATAGATTTACAAGATATACAGCACCGTATCGTTCTGAATTCGCTAATTGATTGAGAATGA
GAACAGTTGTGAGATCAAGTGATAATACACCATCTAAATGAGGGTACATTGTTATGACGGTACATGCGGGTTT

CTTTTCATCCCATGTCTTTTTGAGTAAGTAGCGGTGTTGTTCATCATCGCTAAATATGGCTTCTGTGTGTATCGT
ACTTTTGATTGTATTCATCATCGTCACTCCTTTTAGTATTCTTCTGGTAAAAGCATCACATAATAAAAAGCGTCT
ACATCATCTTCTCGGATGACGTAGACTTTCTTAGGTAATGCATTTTGATTTTTCATATAGTTTGTATAGTGATATT
CCAATTTGTATGCAGGTTGTTCTTGTTCATGTATGATTGAGAGTATAGTCTCATCTTCTTGCAGTCTAAAAATGT
GTAGGTAATCTGTATCAGGTTGATTATCTCTTTCTTTTACCATATTCCAAAGTAAGATTTGAAGGTCTAGAGATA
GGTGTTCACTAATGCCTTTTGTGATGTATCGATTGATTTTCATACTATTTTCCTCCATTTTGCTTTTCTTTCATGA
TGTCAATTATTTCGTTAATGACTGTAACAGATATTTGTGCCACTTTGATCCAATTATTCATGGTCATTCCCTCCTT
CTTTTAGTAGATGACGTTCATCAATAATCGTATTTTTAGTATCTGTGAGGTAGAGAAAGTCCATATCAAAATGA
TCTAGATAACCAATACTGATGAGTTGATTATTAGCATACATTAGAAATGGATAGATACTTAGCTCATGTAGCTCA
TCATTATAGTAGGTATAAGTGTCAAGTGTAAGATGCGCAAGAGGAGAATGCTCTACAAAACGAGTAGGGAGT
ATATTTTCTGCTGCTTCCTCCAGTGCTTCACATTCCCATGTTTCATTGTTAGATAGTTGGAATAGACGAGTTATA
TATTGTTTGAGCTCTTGAGTGGTTATTTTCATATCATTGCCTCCTAGATAGTGTGATAGTGATGTAGTTGATATA
CATCATTGGGATAATATATATTTGATTTGTCATTTATTACGAATCCCGGTGGGAAAAGGAGAATATCCCAATCG
AAAATCATCACAATTGTTGATATGACAAGGAAATCCCGTTATCCCGCTCATTTCTCGGGAAATATAGCTATATAT
TTATATATGATTTGGATTTGTAGATGTCAGTACGACTTACTTTTTCTTTTTATATTATTTATTTATAAATAATAATG
GGATTTTGGGATTACGCTTGCGTAATCCTTCTGCTACATGAAGTTATTGAATCCCGCTGTTATCCCGTTGACAT
TTTATTTTGGGATGTTCTTTCGTCATATCAATATAAGCTTCATATCGAGTTATGACTGATTAGTTTGTTGAAATAT
TTGATAGATGCGTGTTATTGAATGACGAAAGGACAAGTATGTTTATATTATATTTTGAGAAAGAGGGTTTGAG
GTAAAAATAAAGACGAAGTGCTGAGACGCACTTCGTCGAGAGAAGAATGATTAAATTTGATTTTGTAGTTAT
TATAATTTTGATTCTAATTGGTGGACAAGATGAGTTAGCATTGTATTAATCGGTGTAGGTATATTAAATTCTTTT
CCATAAGCAGCTATTTGTCCATTAAGGTAGTCTATTTCAGTTAAGCGGCCATTTGAATAATCTTGATGCATTGA
TGGGTAATGAAGTCCTTGAGCTTCTTTTGGATAGGCTGATACAACTTTTTTAAAAAGCTCATTACGATCCAAA
TGAATGTCTTTTGCCTTTGCAACTGCGACAATTTCATCAATAATTGGCATAATCATTTCATCTGATTGTGCATAA
TTACCAAATTCGTAAATGGTTTTGTTTAATATCGTACATAGAGGATTTAAAACACTATTGAGTGTAGCTTTAGA
CCATATTGATTCGAATACATTGGAACTAATTTGTGCATTCAAGCCAGAGTCATTGAATATTTGAAGTATAGTATT
AGCTTGTTCATCTACTTCACCATCTGCTCTTTGTAGATATATAGATCCTTCGCCTTCGAGTAATAATTGACCTGG
CCCGCGCAGTCCAGCTGTCCACATAGTTACGCCAAGATAGATTTGTGATAGCGGAACAATTTGTGATAATCGC
TCTTCATGACCTAAGCCATTCATCATACTTAATATAGCTGTCCCACTATGAATACAGCCAGCTTCTTGTAAACGT
TGTAACATCTCTTCAGATTGCATAGCTTTGGTTAATATTATGATGAGATCAAATTCAGCTGAAACATCTTTGGTT
AATATTGCGGGAATGTTAATTGAGTAGTTATCTGTTTCTGTTTTAATGTTCATGCCATCTTGATTAATTTGATTA
ACATGCTCTTCCCAATTATCTATAAGTGTTACGTCATAACCAGCTTCTTTTATAGAAATACCAATTCTTCCACCC
ATTGCGCCTGCACCTGCTATGGCTATTTTGTACATAAAATACACTCCTTAGCTATTTTTAGATTTTATAATCAATA
TTACAATATAATTGAATTATATCATTTTAGACCTTTTAGTGAATTGTTGCGCAATTCTTTGATTTGAACCATCATT
CAGTTGGTTATCATATTATGGTTTTTAGTATTATTTGAAAATCCCATTGAAATACAGAAAATATCCCAATCGAAA
ATCATCACAATTATTGATATGACGAGGAAATCCCGTTATCCCGCTTATTTCTCGGGAAATATAGCTATATATTTAT
ATATGATTTGGATTTGTAGATGTCAGTACGACTTACTTTTTCTTTTTATATTATTTATTTATAAATAATAATGGGAT
TTCGGGATTACGCTTGCGTAATCCTTCTGTCACTTGAAGTTATCCAATCCCATTGCTATCCCGTTCATATTATATT
TTGGGATGTTCTTTCGTCATACAATATAAGCTTCACATTGAGTTGTGACTTGTTAGTATGTTGAAAAGGTTCGT
TAGATGGGTGTTATTGAATGACGAAAGGACAGTACATAGATATTAGATTTGAAAGTAGAGTGTTTGAGGCAA
AAACAAAGACGAAGTGCTGAGGAGCACTTCGTCTAGATTATTATTATTGAAAAGTTGTTTAATAATTTCATTAT
TAAGTTTTAGTGTAACGTAGAATTGCTTTTTATGATTCTCATGTTTACGAATGTCAATACGGTCAATAACCGTTA
GGTACAAAGCTTTGAGTTGTGATTTATCCATGGATTCTATATTTTGAAATATTCGTTGTAATAGGGCAGCGATT
TGTTTCGTATCATAAGATGGTTTCTCTTGATTTTGTTGGTGCTTGAGTTGATTCATTTGATTTGTAATGTCATTG
AGTTGTGTTTCATATTGATGAATGGTTGGTTTGAGTGCAGATGTTAAGTCTGGATTGTCTTCGATGGTTTGAA

TTAGATTTTTTAGTTTAGTGTTTATTTCATCAAATTGTTGTTGCTTATATGCGATATCATGGTTAAGTGCAGCCAT
ATCAATTTGACTATCTTGATTAACACGTTCGACAACTTGTTTGAGAACTTTATCGCTTTTGACAATTTCAAGTA
TTTGATCCATAACGTATTTCTCAATCACCTCAGCTCTAACACTATTCGCAGAACATACTTTTGAGCCTTTGTTTC
GAAAGTTACTACAAGAATAATAGCGTATTCGCTTCTTGGTACCATCTTTCAATGTGTTCGTTGTGTTACTAGCT
GCCATTGGTGCACCACATTCTGGGCAGTGAATAATACCTGTTAATAGATTGGTTCCTTTTCCATGAACTTGAG
GTTTTTGACTAACTTGCTTCATACGTGAATGTACTTTATCCCATAGTTCTTGACTAATGATAGGTGAATGCTTCC
CGTCAGCTACTACAGGATTATCATTAAGTCCTTTACGTCGCTTTTCATTCCAATCTTTATATTTCGCAAATTGTAT
TTTACCAATGTAAAAAGGATTGGAAAGGATGTATGCTATAGCTGATGTACTGAATGGATTACCTTTCTTTGTTA
CATAACCTTTATGATTAAGTGCATTAGCAATTTTACGATATCCATGTCCTTTCGCATATGATTCAAAGATGTATTT
AACGATATTGGCTTCATGTTGATTAATCATGAGTTCATGTTTATTATCTGGTATCTTACCATAGCCTAATGGCAA
ATTGCCTTGATAATAGCCTTCTTGAGCACGTCGGGTTTGTCCCATGAATACGTTCTCGACAATATTGTTTCTTT
CAAATTCAGAAAAGCTTGCAAGTATTTGTAACATGAGCTTACCGCTACTCGTATTGACTTCCATACGTTCAGA
CAAACTGAAAAATTCGACATTTTGTTTATGTAAATCTTCGACAATTTTCAGTAAGTCTGATGTATTACGTGCTA
GTCGGTTTGTTTTGTAGACCATAACAGAATCGATATGACCTTCGTTCGCATCTTTTAACAAACGTTGTAGTTCT
GGTCGGTTCATAGATTTTCCAGAAATACCTCTATCTGCGTATACATCTACAACACCAAAGTTATTGAAATCACA
ATATTCTCGAATTTGATTGATTTGTCCATCGATACTATAGCCTTCAGTACTTTGGATTTCCGTTGATACACGAAC
ATAGATACCGACACGTTTTTGTTTGAGTTGTTGCATGATTTTTTCATCCTTTCTTGTTAGACAATCGATGATTGC
GAGGTTTGGTTCACAATGTTCAATGGTTCATTTTTGAAATAAATCCCAACAAGGGCTTTGTTTTTAGTAATGT
GAATTTCATCAATATAGGGATGCAGCATGTTTAACGTGAAACTTTTCTGTATAACCTTTTGTAGTGATGCTTTT
ATCCGAATATCACTTATTGCTTGTATAGGCTTTTTCTGTAGGTTTATTGATTGAGATTGTTCTCTGAATGTTTCTA
CATCAATTGTTCCTTGTGCTAATTTTTCAATGAGCTTTTCATGAGTTAATGTGTTACTAGTATCATGCATCCTTT
GTTTTTTGAGCTGTTTCTCAATAGCATGTTTGATTTTCGAATAGAGCTGTTGGTTTCGAAAGAATTTTTGGCA
AGATTCTAAGACCTGTTTTTCCAGGTTTTCAGCGTTGATTCCTTTAAATTCACAAACAAAGCGAGCATTATTC
ATATTTTGCGGACAGACATAGTAACGCAATGAATGATCTGGTTTTCGGACAGTCATGTTTGTGAGTGTCGAAT
GACAATAGGGACATTTGATTTTTTGTTTGAGTTGATTTTCTGACGGCTTACGTTTTACTTGTTTCTGAGTTCG
GGTAACTTGAGCTTCTTCGTATATCGTTGTACTGACAATAGCTGGGAACATGTTTTCATATTGTCCGTATTGATT
GATAACACGGCCACAGTAATTAGGGTTAAGGATAATATTACGCACTTGATAGGGTTTTCGGTTGATTAACTTA
TTATCTTCTTCCAAATATTGTGCAATTTTTTTATAACCATAACCTTGAAGGTAATAATTGAACACAGCTTTTATT
GTTGGTGCTTTTACTGTGTCTATTGTGAAAGTACCATTATGATAGTGATACCCAAAGGGTGCATGTGTTGTAAT
CATTTTACCTTGTTTAGCTTTTTCTTTGATTCCATTTTTGACTTGTTCACCTATATTATCAGATTCTAGTTCGGCC
AAGCTGATGAAAATATTGAGTTTGAGCCGATCGAATGCTTTATCCATATCAAAATAGCCATCATGAACGCTTAA
GATATGGACATGGTATTTTTGACACAATTTCATGAGTTTTAATGCATTTTTAAGATTGCGATGAAGTCGGTTTA
ATCTATAACAACATAATACGTCACATTGTCCCTGTTGAATCAGTTCAGTAATTTGTTGATAACCATTCCGCTTAT
CAGTACGTCCTGATTGTTTATCGCTATAAAAGGTAATGTGTTGAATATTATGTTTTTCGGCTAATGCCTTGATGG
TTTGTTTTTGTGCTGCTAAGGATTGTTGCTTTGTAGTGCTCTGTCGTAAGTAACCAATTGCTTGTTTCATCTTG
TTTCCTCCTTCCACAGTGATAATATATATTTATGAACGAATTTACACATAAGCCCAACACCAATCAGGTGTTGG
GCGTTATTATTAGTCATCAGCGTGATTAATTTCTTCAATCACTAAATCAGCGAGTAATGTGATTAATTCGTCCAT
ATGGTTCAACCTCCAAATATCAAATTATATTGAATTGATAACATGTTGCATTGTTATTCGTTCTAACCGTGTACTT
GGGACAAAATGTTCAGTTATCTCTTTTTCATTTATGAATTCAGGGTACAAGTGATAAAATTTTAGTAGCTTCTG
TTCCGTTTCAGTTAATACATATTGTTGCATATGGTAGTATCTGTCGATATAATTAACGACAATAGGTTTTTGATG
ATCATCGACTTCTACTGAATCAATTGTGTAATTTAATAAGTTGACGTTTATTTTCATTTTTATGCCTCCGATTAAT
TTAATGAGCTACAACGCGCTATGTGCGTTGTAGCGATATTCATATTTAGAGTTCTAAATCGCCCTTTTCTTCCCA
TTCTCTACCGTCTTTAACAAATTTGTTGAGTAATTCCTTATTTGTTGTATTATTATCAAATTGCGGTGGTGTGTAT
GGTTCAGAATTTGAGCTTAAACCGAGAACAGATGCAAACTCACTAGGCAATATAAGATGGTAGAACAAGATC

GTTTTTGTTTTGCCTTGAGTATCTTTGACATTACGTTTGATAGCTATCCGATCGGAATCCATCTGGATATATCCT
TCTGCTCTTAAACTATCTATTACATTTTTTACATCTTGGTATTGGTTTTCTTCCAACATATGTTTAAAAACATCTT
TAAGCATCTTGACTTGAATGTGATCGTCTTTCAGCTCAATAAGGCCATAATTTTCAGTCATTATGGAGAGTTTT
TCATTTTCCGCAAACTTACTACGATTGCGTGCCACGAACTGAACTACACTATCAATCGCCTTGGCTCCCAAAC
TGCGCTCACTTATGGAATCTACATGATAACTAACTAGGTACTCTCTAACTGCGACCAAATCGATAGGGGTTGC
AATTACACGTTCTAATATTCGTGCTGATGTCGTTATTGTTGCATAGCGTTTGAACATACGAATACCAGTATTACT
CGTTTCATTTTTTAGTTGGTTTTTGAACCAATCATTTTCCGCATGAAACCATTTAATTACTTCGTCTTCACGTTT
TAAAAGATATTCAGCTACTAATGGCATTATATGGCCATAATTGACCGATGTAGCCCTTTTAATTGCATCAGCATT
GTCAGCACTTGTTGTAAAGGCTTCAGATATTTCAATCGTACGGACGTTCAAACCATCATTACGTGCACTATCAT
TTAAAATACTGTGTTCTGCGGTACTTATCACTGTGGTGCCCCAATTTTTGAGTGCTTTGACATTACCATCAATAT
TAGAGCGTTGTCGGCCTTGTCCCTCTGCCAAACTGTACAACAATCCAGTAGTGTCTTTGAAGGTTGCAGCTG
ACAATTCATCTAATACGATAGGCACACCAAAGTTACTACTTAAATATCCTTCAAGCGCGTTTCTAGTACCATTCC
ATGATCTAAACAGGGTATTACTACCTTTAGTAGGGTTGCCGGCTATTGAAACAGCTAAAGCAGCTGCAGTCG
ATTTACCTGTGCTTGAATTTCCCATAAATGAGAATATGGTTCCCGCAAACTCGACCTCATGTCTTGTTTTTAGG
AACGCAGTAACTAATGAAGAAGCAGCAAATACTACTGCAAGTTCTAGTAGTAAGTTCCCTTTAACTTGTTTTA
AGTACATCTCCCACCAGCCTTTGAAAGTACCTTTTGGTTGTAAATCATAGTGTGTTTCACAAATGATTTCATTA
GCTTGAGATTGCTCTATTTCTTTTGAAAAGTATGGTTTATCCAATGAAATGACCATACCTTCATCATCAGACTG
TAATACACCTACGCCGGTATATAATTTAGATAATGGAAGTGACTGGCGCATCGATTGTAACGCATAGCTTAATG
ATTTAATGTACCGTTCATTGATGTTGAAGCCGTACTTAATCAATCCAGGTAGCTTGAAAGATGTTAAGATATCT
GATGCTTCAATACGTTCAATATTTTTTCCATCTGTAATAATTAACTTCTCAACCCCTGTAGATGGGTCGAGAAAT
TTATTTTCAATAATGATGGGGCTAGACAATTGAATGATTTTTTCTACATCATTGTTACTTTTAGGTGGAATCACT
TCATAAAAGCCATTTGAATTTAAAAAATATGGATATTGCTTGAATATGTTGTTAGTCATTAGTTGTTACCACCTT
TCCAGTTAAGTTTGTGACGCATTGGATTAGGTCCTGTCTTACGATAAACTAAGTTCCCGTTGCTATTCTTACCA
ATAATGACAAATGGAACACTTGGCGTGTTCTTTACAAAGTATGCAAACCAGCGACCAACATCTTGTTGTACGT
CAGTTGAACATGTAACGTTGGACGCCAAATTTAAATCGTTAAACGTAAAATCCTTACCAATTGCTAAATTAAA
AGCAACACCACAAGCGCGTCTTTTTAACAAAAGAAAGCAATAACTTTTTTTGCTCATATAAAATTCCTCCTAA
TAATTAAAATCATCAAATTTGATGTAATTACATACTAATCCCTCACAAGTAGGCTTAGTTTCAAATTTTCAAGGG
GAATTTTAGGACATTGATATAAAGGGATTTAAAAGAAAAAATACCTATCGAATACATGTATTCGATAGGCAATT
AATTTATTAAAAATAAATATTTCTATGCTTTTTACTGTTTTTAAAACGTGATTCATAAAATTTACTAATAAGTTGA
GTCATTAATGTATCACGAAAATCGTTAGGATTAGGGGTCAAACCATATCTATTATAAAATGTGCTTATGTCATAG
TCAGCAAGTGTTGAAAGTAGTTCACTTTCAATAGTTGAAGTTAGCTTGCGATTCATTTGAATGGTTTCAGCAA
TTACCGATTTTAGATTTGAATTCCTAGCATAGCCATTAATTAAATTTTCTATATGTTGTGATTTTTGATCGTATTT
TTCAATCCAAGTTTTTAAGTCCTGTTTAAGATTACTTATTATATATAAAGATACTAGTATGTCTTGCTCTGTTGCT
TTTTGTATTATGACACTTACAGTTTGAATGAGTTCATCTAATAAATCTGAAATAGCGGTAAATAGGGTGAAATG
AAATGTATCTTCAATGGTTGTCTTATAATAGAGTTCATTATGACTGATTAGATTTTCACTCTCCATGAATTTATCA
ATTCTTTCTTTAGATTGAATAACGTCATCAACAGTTATTGTTTTTCTGCGCAAGCTTTCATTTGCAATACTGCGA
ATAAATTTAAAGTAAGGGTCATTTTTATCGTATAGATAAAGCGAAACACCAATAGCAGCTTGTCTGAATTGATC
GATATTGTCAGATTGTTCTAAAGAATCATTTTTATGAGTTGACGGTTTAATACTATTTGCAATGTCAAAATATTG
GGAGTCACTTTTTCGTTGTTTAATTAAGTTTTTATTAATTAATTTTATAAAGTAATTTGACTTATTTTGACTTAAT
ATGTCTTTTTCCAGATGGGAGTCCTGCTTTTCTTTATATACAAAATATTTAGTTAAGAAATCACTTATTAAAATG
TCGTTATATGATTCTGCAGTTAAGTCTTGATGGTTCTTTATAATTTCATTTTTAAGTTTGTTGAATTCTGAAGAT
GAATTTAATTTATCTTTGTTTTTATTCATTTTAAATTGAATATCCCATTTTTTAAGATTTATCTCACCCATTATTTG
TCTTTTTACATTTTCTGCAGTTTGTTTAGCAAATTCTGTTTTGTCTGAAATGGCAAGTAAGAATTCATCAAAGT
ATGATTCTATTGTTTTTAAATCTAAGAATAGGAGAACGGAAGAGCCTTCTTCAACGATTTCTTGTGATATATCA

TCAATATCGATTTTACTTTGACTATCTATAAAAAATGATAATTTATCATTTTTAAAAATTTCCGATTGATATATATT
TGGGACATTGTCAGTAAATATACCGTTTGAATCTGATTTTACTGTATGTTGTTCAATAATTTGATCTGGTTGATT
TACGAAGTAACTAATATAAACTGTTTCATTAGGTAATGATTGTCCTTTAATTAAGTTATATTCATTAAATGTGATT
CTTTCTGGTACAAATTCATTGCTGATTTTATGTATAAAATAGTTGAATTTCTGAAACCATAATTCTATTTTCCATT
TGTTCTTTATCATACTAATCATCCATTTCTATAAAATATTAATATTTTTGGGTGAATTTTGAATGGTATAACTATTA
TAAGATGGTAAGTGCTGTTTTAATATGTTTAGCCTTTTTTTGCAGTTTTTCTAAATCAGACTCTATGTCTTTTAT
AGCTTCCTCAATATCATTAATCATTGGTTTATTAGCTTTAAAAAAAGCAATTCCATGCTCATCTTTCTCGGGATG
GCTATTTAATCGGTATGGAGTTATGTTATGAGTATATTCATCTCTATACTTTACGGTACGAATAGGACTGTTTTTA
TTAAGTATATTTCCTAATTTTTTAAAAGTTTCATTGTCAGAAGCCTTTAATTTGCCTACTAATTCTCTTTTAAAAC
CTAATTCTAATTTAACTTCATAAGAATGGGTTAATTCTCTTATCAAGATATACATTAAATCGTGTATAGTATGTAA
AAAATGAAGAGTTATGTTGTGATAATAAAATACCCAATAATAATTTTTCTCTAGGTTATTTGTATATGGTAAATT
TTTATGTGCATACTTCAAACATACAAAAGAATTGCAAAAATATTCACAAGTATCATGTATTGAATTGATAGCGG
AGGGGACATTCGAACCTATTTGCTCAAATCCACTTACTAAAGTATTAACCTTTGAAAAATCAAATATATTGATC
TCAGTAAGTAATTTGTTATATTCAATGTCAAAATAGTTTTCGTCGATAACAAAAGGTTTCATAACATTAGATCCT
TTCAAATTAGTGATTAACTTTTGTTAATCTTGTTTTTAGTGCATTTAGTTAAGATTTTACTTATATTTATTATACTA
TAAGATAAATTTATCTTTAACTTAATTTACTTTATTTAAAAAAAGAGAGTTGAATTGAGATGACAATATATGTCA
TCTTTACTCAGAACATGATAAATGGGGCTTTTCAAACAAAGAAATTACGTTCTATACACATACAAAACGAATG
AAAATAAAAGGGGTTTCTAAAGAAAATCATCTTTTAGTTAGTATACCAAGTGAATTTAGTTATGCTACAAATTT
CTATCTATTTGGCTATATTGTTAACCCACCAAAATTGCTTGTAAAGATACGAGGTGAGACTCTTTCAGGAAATG
AGTTTACCGATGAAATTGAAATCCAACTCCAACTCCAAAGATTTAAACTGAGCTATAACCCTTTCAAAGCGGA
GATTACATTATTTGCTTAAAATAAAGAAATATTCATCAAAAAACCGCATCACCAAGCGATAAGCAGAAGCTTA
TCATAAGTAATGAGGTTCATGATTTTTGACATAGTTAGCCTCCGCAGTCTTTCATTTCAAGTAAATAATAGCGA
AATATTCTTTATACTGAATACTTATAGTGAAGCAAAGTTCTAGCTTTGAGAAAATTCTTTCTGCAACTAAATATA
GTAAATTACGGTAAAATATAAATAAGTACATATTGAAGAAAATGAGACATAATATATTTTATAATAGGAGGGAA
TTTCAAATGATAGACAACTTTATGCAGGTCCTTAAATTAATTAAAGAGAAACGTACCAATAATGTAGTTAAAA
AATCTGATTGGGATAAAGGTGATCTATATAAAACTTTAGTCCATGATAAGTTACCCAAGCAGTTAAAAGTGCAT
ATAAAAGAAGATAAATATTCAGTTGTAGGGAAGGTTGCTACTGGGAACTATAGTAAAGTTCCTTGGATTTCA
ATATATGATGAGAATATAACAAAAGAAACAAAGGATGGATATTATTTGGTATATCTTTTTCATCCGGAAGGAGA
AGGCATATACTTATCTTTGAATCAAGGATGGTCAAAGATAAGTGATATGTTTCCGCGGGATAAAAATGCTGCA
AAACAAAGAGCATTAACTTTATCTTCCGAACTCAATAAATATATTACATCAAATGAATTTAATACTGGAAGATTT
TATTACGCAGAAAATAAAGATTCATCTTATGATTTAAAAAATGATTATCCATCAGGATATTCTCATGGATCAATA
AGATTCAAATATTATGATTTGAATGAAGGATTCACAGAAGAAGATATGCTAGAGGATTTAAAGAAATTTTTAG
AACTATTTAATGAATTAGCTTCAAAAGTTACAAAAACATCCTATGATAGCTTGGTCAATAGCATAGACGAAATA
CAGGAAGACAGCGAAATTGAAGAAATTAGAACAGCACAAAAAGATAAGACACTCAAGGAAGTGGAAGCA
CCTAAAGGAATAATTCCAAAATATAAAAAAGGTGTATCAAAGACTACTAAAAATGATTCAGAAATTGAAAAAT
CAAATAAAGAGAATAAATTAACCGGTAAAGTTGGAGAAAAATTAGCGCTAAATTACTTTAATGAGCTAATTG
ATAATAAAATAGACGAAGATAAGAAAGAACAGTTTAGGAATATTTTAAATGATAATCCAGGCTCTCAACACGG
TCATGGCTATGATTTAGTAGCTTTTGATCCAACAAATACAGATAAAGCTGTAGAAAAAATTTATTGAAATTAAAA
CATCTACATCTTCTAGTATTGAGGAACCATTTTTTATGTCGCTAAATGAAATGTTTGCTATGAAAGAATATAAGC
AGAAATATTTAATATTAAGAATATTTAATGTTTCTGGTAAAGAACCACAATTTTATTTTATAGATCCATATGCAA
ATTATTCTGAATTTAAAGATGTAGATGATCTCATTGACAAAGTATTTAATGTAGAAGCTATTCAGTATAAAGTTT
TTGGCGAAAAAATGATTACTTGAACAAGAGCTAAAATAAAATTGTGATCTAATAAAAATAGAAACTGTAATTTA
AATAAAACTTTCTAAATAAGCTAACTGATAAAAAATCAGTTTGTCCACAGTCTGAAACAAGATTCCTATATTCT
TTAGGAATCTTGTTTTTTCTATTTTTATGGTGATAAAGAGCAGATAAGATAATGTGTAATAATCACAAAAAAGT

TAAATATTTTAAGGCTTGTTTAATTATTAATGATTTTATATATAAAGAGCAGTATAATAAAGTTGTTAATATATTAT
GAATAATATTCAAGTAATTTTATTGTTTTTTAATTTGTCGATATTTAAGTTGAGTTAAATTTAAAGGGTGTAATT
TGTTTTACAATGATGAAGATAATTAGTCTATCAAAATAAAGGGGTTGGGACTGTTATGAGTGATAATTTGTCAT
TATTCATTGACTATATCAATGATAATATAATCTATGGTAGTGAAATCAAACGGGAGAAATTAGAGAATTTATTTA
ATCAATTTGCTATAAAAAAATGTTGAAAAGAACATTGTCTATGATGAACTGAAATCTTTAGATATTACAATCATTG
AGTCACAGGATTCATATAAAAATAAATTGAAGAGATTATTTTCGGTTCTGTTGCAAAGTAAAAAAATATAGCT
AACCACTAATTTATCATGTCAGTGTTCGCTTAACTTGCTAGCATGATGCTAATTTCGTGGCATGGCGAAAATCC
GTAGATCTGAAGAGACCTGCGGTTCTTTTTATATAGAGCGTAAATACATTCAATACCTTTTAAAGTATTCTTTG
CTGTATTGATACTTTGATACCTTGTCTTTCTTACTTTAATATGACGGTGATCTTGCTCAATGAGGTTATTCAGAT
ATTTCGATGTACAATGACAGTCAGGTTTAAGTTTAAAAGCTTTAATTACTTTAGCCATTGCTACCTTCGTTGAA
GGTGCCTGATCTGTAATTACCTTTTGAGGTTTACCAAATTGTTTAATGAGACGTTTGATAAACGCATATGCTGA
ATGATTATCTCGTTGCTTACGCAACCAAATATCTAATGTATGTCCCTCTGCATCAATGGCACGATATAAATAGCT
CCATTTTCCTTTTATTTTGATGTACGTCTCATCAATACGCCATTTGTAATAAGCTTTTTTATGCTTTTTCTTCCAA
ATTTGATACAAAATTGGGGCATATTCTTGAACCCAACGGTAGACCGTTGAATGATGAACGTTTACACCACGTT
CCCTTAATATTTCAGATATATCACGATAACTCAATGTATATCTTAGATAGTAGCCAACGGCTACAGTGATAACAT
CCTTGTTAAATTGTTTATATCTGAAATAGTTCATACAGAAGACTCCTTTTTGTTAAAATTATACTATAAATTCAAC
TTTGCAACAGAACCGTATTATGGAATAGAGATGTTGGTAACATTTATACAGGATCATTATACTTAAGTTTAATTT
CGTTATTACAGAACCACACATTCCAACCAGAAGAGAAAGTATGTCTATTTAGTTATGGTTCAGGAGCAGTAG
GAGAAATCTTTAGTGGTTCAATCGTTAAAGGATATGACAAAGCATTAGATAAAGAGAAACACTTAAATATGCT
AGAATCTAGAGAGCAATTATCAGTCGAAGAATACGAAACATTCTTTAACAGATTTGATAATCAAGAATTTGAT
TTCGAACGTGAATTGACACAAGATCCATATTCAAAAGTATACTTATACAGTATAGAAGACCATATCAGAACATA
TAAGATAGAGAAATAAACTAGTGGCCGATTGTGCTTGATGAGCTTGGGACATAAATCCTAACTCGAAATAAAT
AAGCATATCACTAAACTGATTTTTTAAAGTTTACAGTGATATGCTTATTTTTTTATCTTACGATTTTGTACGTGC
ATGCTTGCCTAGGGGTATGGCTCGAGCCATTAGTCTCTCGCACATACTATTCCCTCAGGCGTCAGCACTTACA
AAATCGGTTGTAATTTTCATTTTTATACGCATTCTTACTGAGATTATACTAATAAGAGGAATAGTAAAAGCAATT
CTAAGTAAAATTGCAGATAAGAGGTTTGTTAAAAGCAGTTCTAAGTAAAATTGCAGATAAGAGGTTTGTTAA
AAGCAGTTCTCAGTAAAATTACAGATAAGAGGTACGTTAAAAGCAGTTCTAAGTAAAATTGCAGATAAGAGG
TTTGTTAAAAGCAGTTCTCAGTAAAATTGCAGATAAGAGGTACGTTAAAAGCAATTCCATGCAAAATTGCTG
ATAAGGGGTAAGTTAAAAGCAGTTCTCAGTAAAATTGCAGATAAGAGGTACGTTAAAAGCAGTTCTAGGCA
AAATTGCAGATAAGAGGTGCGTTAAAAGCAGTTCTCAGTAAAATTGCAGATAAGGGGTAAGTTAAAAGCAA
TCCTAAGTAAAATTGCAGATAAGGGGTACAGAAAAACTAGACTTGATTACAAAATGGAGCTTGGGACATAAA
TGATTTTTTAAAAATGAGATGAGACGTAGATTAACTCCATAATCAATACGAATCTATCGACTTCTTTATTTATGA
TATTCATCTCTTTTTAATGGAAATAAAAGTGCGATTAATGTGATAATACAGTTACGTTAATTAAAAAAATAAAAA
TGCAAGGAGAGGTAATATGCTAACTGTATATGGACATAGAGGATTACCTAGTAAAGCTCCGGAAAATACAATT
GCATCATTTAAAGCTGCTTCAGAAGTAGAAGGTATAAACTGGTTGGAGTTAGATGTTGCAATTACAAAAGAT
GAACAACTGATTATCATTCATGATGATTATTTAGAACGGACTACAAATATGTCCGGGGAAATAACTGAATTGA
ATTATGATGAAATTAAAGATGCTTCTGCAGGATCTTGGTTTGGTGAAAAATTCAAAGATGAACATTTGCCAAC
TTTCGATGATGTAGTAAAAATAGCAAATGAATATAATATGAATTTAAATGTAGAATTAAAAGGTATTACTGGAC
CGAATGGACTAGCACTTTCTAAAAGTATGGTTAAGCAAGTGGAAGAACAATTAACAAACTTAAATCAGAATC
AAGAAGTGCTCATTTCAAGCTTTAATGTTGTGCTTGTTAAACTTGCAGAAGAAATCATGCCACAATATAACAG
AGCAGTTATATTCCATACAACTTCGTTTCGTGAAGACTGGAGAACACTTTTAGATTACTGTAATGCTAAAATAG
TAAACACTGAAGATGCCAAACTTACTAAAGCAAAAGTAAAAATGGTAAAAGAAGCGGGTTATGAATTGAAC
GTATGGACTGTAAACAAACCAGCACGTGCAAACCAACTTGCTAATTGGGGAGTTGATGGTATCTTTACAGAC
AATGCAGATAAAATGGTGCATTTGTCTCAATAGAAAGTTAGAGGTGAGTCTTACGTTTCAGTGACGGTAGAC

TTACCTTTAACATGTTACATACTAAAAAATTAATTTGAATAAGAAAGAGAGACATATATGAAATACGATGATTT
TATAGTAGGAGAAACATTCAAAACAAAAAGCCTTCATATTACAGAAGAAGAAATTATCCAATTTGCAACAACT
TTTGATCCTCAATATATGCATATAGATAAAGAAAAAGCAGAACAAAGTAGATTTAAAGGTATCATTGCATCTGG
CATGCATACACTTTCAATATCATTTAAATTATGGGTAGAAGAAGGTAAATACGGAGAAGAAGTTGTAGCAGG
AACACAAATGAATAACGTTAAATTTATTAAACCTGTATACCCAGGTAATACATTGTACGTTATCGCTGAAATTAC
AAATAAGAAATCCATAAAAAAAGAAAATGGACTCGTTACAGTGTCACTTTCAACATACAATGAAAATGAAGA
AATTGTATTTAAGGGAGAAGTAACAGCACTTATTAATAATTCATAATAAAACAGTGAAGCAACCATCGTTACG
GATTGCTTCACTGTTTTGTTATTCATCTATATCGTATTTTTTATTACCGTTCTCATATAGCTCATCATACACTTTAC
CTGAGATTTTGGCATTGTAGCTAGCCATTCCTTTATCTTGTACATCTTTAACATTAATAGCCATCATCATGTTTG
GATTATCTTTATCATATGATATAAACCACCCAATTTGTCTGCCTGTTTCTCCTTGTTTCATTTTGAGTTCTGCAGT
ACCGGATTTGCCAATTAAGTTTGCATAAGATCTATAAATATCTTCTTTATGTGTTTTATTTACGACTTGTTGCATA
CCATCAGTTAATAGATTGATATTTTCTTTGGAAATAATATTTTTCTTCCAAACTTTGTTTTTCGTGTCTTTTAATA
AGTGAGGTGCGTTAATATTGCCATTATTTTCTAATGCGCTATAGATTGAAAGGATCTGTACTGGGTTAATCAGT
ATTTCACCTTGTCCGTAACCTGAATCAGCTAATAATATTTCATTATCTAAATTTTTGTTTGAAATTTGAGCATTAT
AAAATGGATAATCACTTGGTATATCTTCACCAACACCTAGTTTTTTCATGCCTTTTTCAAATTTCTTACTGCCTA
ATTCGAGTGCTACTCTAGCAAAGAAAATGTTATCTGATGATTCTATTGCTTGTTTTAAGTCGATATTACCATTTA
CCACTTCATATCTTGTAACGTTGTAACCACCCCAAGATTTATCTTTTTGCCAACCTTTACCATCGATTTTATAACT
TGTTTTATCGTCTAATGTTTTGTTATTTAACCCAATCATTGCTGTTAATATTTTTTGAGTTGAACCTGGTGAAGT
TGTAATCTGGAACTTGTTGAGCAGAGGTTCTTTTTTATCTTCGGTTAATTTATTATATTCTTCGTTACTCATGCC
ATACATAAATGGATAGACGTCATATGAAGGTGTGCTTACAAGTGCTAATAATTCACCTGTTTGAGGGTGGATA
GCAGTACCTGAGCCATAATCATTTTTCATGTTGTTATAAATACTCTTTTGAACTTTAGCATCAATAGTTAGTTGA
ATATCTTTGCCATCTTTTTTCTTTTTCTCTATTAATGTATGTGCGATTGTATTGCTATTATCGTCAACGATTGTGAC
ACGATAGCCATCTTCATGTTGGAGCTTTTTATCGTAAAGTTTTTCGAGTCCCTTTTTACCAATAACTGCATCATC
TTTATAGCCTTTATATTCTTTTTGTTTTAATTCTTCAGAGTTAATGGGACCAACATAACCTAATAGATGTGAAGT
CGCTTTTTCTAGAGGATAGTTACGACTTTCTGTTTCATTAGTTGTAAGATGAAATTTTTTTGCGAAATCACTTA
AATATTCATCCATTTTTTTAACGGTTTTAAGTGGAACGAAGGTATCATCTTGTACCCAATTTTGATCCATTTGTT
GTTTGATATAGTCTTCAGAAATACTTAGTTCTTTAGCGATTGCTTTATAATCTTTTTTAGATACATTCTTTGGAA
CGATGCCTATCTCATATGCTGTTCCTGTATTGGCCAATTCCACATTGTTTCGGTCTAAAATTTTACCACGTTCTG
ATTTTAAATTTTCAATATGTATGCTTTGGTCTTTCTGCATTCCTGGAATAATGACGCTATGATCCCAATCTAACTT
CCACATACCATCTTCTTTAACAAAATTAAATTGAACGTTGCGATCAATGTTACCGTAGTTTGTTTTAATTTTATA
TTGAGCATCTACTCGTTTTTTATTTTTAGATACTTTTTTTATTTTACGATCCTGAATGTTTATATCTTTAACGCCTA
AACTATTATATATATTTTTATCGGACGTTCAGTCATTTCTACTTCACCATTATCGCTTTTAGAAATATAACTGCTATCT
TTATAAACTTGTTTGAAATTTTTATCTTCAATTGCATCAATAGTATTATTAATTTCTTTATCTTTTGAAGCATAAA
AATATATACCAAACCCGACAACTACAACTATTAAAATAAGTGGAACAATTTTTATCTTTTTCATCAATATCCTCC
TTATATAAGACTACATTTGTAGTATATTACAAATGTAGTATTTATGTCAAAATAATGTTATAATTTTTGTGATATGG
AGGTGTAGAAGGTGTTATCATCTTTTTTAATGTTAAGTATAATCAGTTCATTGCTCACGATATGTGTAATTTTTT
TAGTGAGAATGCTCTATATAAAATATACTCAAAATATTATGTCACATAAGATTTGGTTATTAGTGCTCGTCTCCA
CGTTAATTCCATTAATACCATTTTACAAAATATCGAATTTTACATTTTCAAAAGATATGATGAATCGAAATGTATC
TGACACGACTTCTTCGGTTAGTCATATGTTAGATGGTCAACAATCATCTGTTACGAAAGACTTAGCAATTAATG
TTAATCAGTTTGAGACCTCAAATATAACGTATATGATTCTTTTGATATGGGTATTTGGTAGTTTGTTGTGCTTAT
TTTATATGATTAAGGCATTCCGACAAATTGATGTTATTAAAAGTTCGTCATTGGAATCGTCATATCTTAATGAAC
GACTTAAAGTATGTCAAAGTAAGATGCAGTTCTACAAAAAGCATATAACAATTAGTTATAGTTCAAACATTGAT
AATCCGATGGTATTTGGTTTAGTGAAATCCCAAATTGTACTACCAACTGTCGTAGTCGAAACCATGAATGACA
AAGAAATTGAATATATTATTCTACATGAACTATCACATGTGAAAAGTCATGACTTAATATTCAACCAGCTTTATG

TTGTTTTTAAAATGATATTCTGGTTTAATCCTGCACTATATATAAGTAAAACAATGATGGACAATGACTGTGAA
AAAGTATGTGATAGAAACGTTTTAAAAATTTTGAATCGCCATGAACATATACGTTATGGTGAATCGATATTAAA
ATGCTCTATTTTAAAATCTCAGCACATAAATAATGTGGCAGCACAATATTTACTAGGTTTTAATTCAAATATTAA
AGAACGTGTTAAGTATATTGCACTTTATGATTCAATGCCTAAACCTAATCGAAACAAGCGTATTGTTGCGTATA
TTGTATGTAGTATATCGCTTTTAATACAAGCACCGTTACTATCTGCACATGTTCAACAAGACAAATATGAAACA
AATGTATCATATAAAAAATTAAATCAACTAGCTCCGTATTTCAAAGGATTTGATGGAAGTTTTGTGCTTTATAAT
GAACGGGAGCAAGCTTATTCTATTTATAATGAACCAGAAAGTAAACAACGATATTCACCTAATTCTACTTACA
AAATTTATTTAGCGTTAATGGCATTCGACCAAAATTTACTCTCATTAAATCATACTGAACAACAATGGGATAAA
CATCAATATCCATTTAAAGAATGGAACCAAGATCAAAATTTAAATTCTTCAATGAAATATTCAGTAAATTGGTAT
TACGAAAATTTAAACAAACATTTAAGACAAGATGAGGTTAAATCTTATTTAGATCTAATTGAATATGGTAATGA
AGAAATATCAGGGAATGAAAATTATTGGAATGAATCTTCATTAAAAATTTCTGCAATAGAACAGGTTAATTTG
TTGAAAAATATGAAACAACATAACATGCATTTTGATAATAAGGCTATTGAAAAAGTTGAAAATAGTATGACTTT
GAAACAAAAAGATACTTATAAATATGTAGGTAAAACTGGAACAGGAATCGTGAATCACAAAGAAGCAAATG
GATGGTTCGTAGGTTATGTTGAAACGAAAGATAATACGTATTATTTTGCTACACATTTAAAAGGCGAAGACAA
TGCGAATGGCGAAAAAGCACAACAAATTTCTGAGCGTATTTTAAAAGAAATGGAGTTAATATAATGGATAAT
AAAACGTATGAAATATCATCTGCAGAATGGGAAGTTATGAATATCATTTGGATGAAAAAATATGCAAGTGCGA
ATAATATAATAGAAGAAATACAAATGCAAAAGGACTGGAGTCCAAAAACCATTCGTACACTTATAACGAGATT
GTATAAAAAGGGATTTATAGATCGTAAAAAAGACAATAAAATTTTTCAATATTACTCTCTTGTAGAAGAAAGT
GATATAAAATATAAAACATCTAAAAACTTTATCAATAAAGTATACAAAGGCGGTTTCAATTCACTTGTCTTAAA
CTTTGTAGAAAAAGAAGATCTATCACAAGATGAAATAGAAGAATTGAGAAATATATTGAATAAAAAATAAAAT
TGTTGTGTTTACAACAATACATAGAAAACAGAGGAAACAATCAAGTCGTTGAATATTTCCTCTGTTTTTTAGT
TGAAAAAATTAACCGAAAGCCTGAATGCAAGTCTTGATTAAATCAATAATGCTTGTAATAACACCAGTGAAAT
CCATATGCATACCCTCTTTCTATTTAAGATACATTAAGTATAATATCAAACAAATAAAAAATGTTAAAAATTCCCT
AATTGGCTATTTAGATTGCATAAATGTCAAAAATTTGAAAAACATACAACGACTTTGCATAAAAAATCGTCATA
TTGGAAATACGTAATTTATTGAAATAATAAAAAAATAAAAGAACGAAGATGATAACCTAAGTGAGGTTTTAAG
TTGTTCTAAGGTTTAATTTAATTTATGTTAAAATAGTTGGTATAAAAATACATGATAAACTATAAACTAAATTCA
AAATAACTTATGGGGTAGGCAATTATGGAAAATATTTTAAATATAAATGATAATGAAAAAAGAGTGCTAAGGG
AAATTTATAACCATCATAATATTTCGCGTACTCAAATATCTAAAAATCTTGAGATTAATAAGGCAACGATTTCTA
GTATTTTGAATAAGTTAAAGTATAAATCTCTTGTTAATGAGGTTGGTGAGGGTGATAGCACGAAGAGTGGTG
GTAGAAAACCTATTCTTCTGAAGGTTAATCATCTTTATGGTTATTTTATTTCTTTGGATTTAACTTATAGTTCTGT
TGAAGTGATGTACAATTATTTTGATGGTAATGTCATTAAGCATGAATCTTATGATTTACCTGATGAAAAGGTTA
GTAGTATATTAAGCATAATAAAAAAACATATTGATATTCAGGAGAAACTTGATACTTATAACGGACTATTAGGT
GTGTCTGTTTCTATACATGGAGTTGTGGATAATGAGCAGCATGTGACATATTTACCATTCCATGAAACTGAAG
GAATTTCAATTGCTAAGAAAATAAAAGAAATTACTAATGTTCCAGTCGTAGTTGAAAATGAAGCGAATCTTTC
AGCGTTATATGAACGTAATTTTAATCATAATTTATCCTACAATAATCTTATTGCTTTAAGTATACATAAAGGTATT
GGTGCTGGGCTTATTATTAATAATCAATTGTATCGTGGTGCAAATGGGGAAGCGGGTGAAATTGGAAAAACA
CTTGTCTCAAAAGTTAGCGATAATGTGGAGATCTTTCATAAGATTGAAGATATTTTTTCACAAGAAGCTTTACT
GCATAATTTAAGTAATCAACTAAATGAGAAGATGACGCTTAGCAAATTAATTCAATTTTATAATGAAAAAAATC
CAGTCGTAGTTGAAGAAATGGAACAATTTATAAATAAAATTGCTGTTTTAATACATAATTTAAACACCCAGTTT
AATCCGAATGCAATTTACATTAACTGTCCATTGTTCAATGAAATGCCTGAAATATTAGAAGCAATTAAGAACCA
GTTCAAACAATATTCACGTAACGAAATTCAAATAAAGTTAACATCTAATGTCAAATTTGCAACTTTGCTAGGTG
GTACATTAGCAATTATCCAAAAAGTACTACAGATTAATGATATTTACTTAGATATAAAAGCATAAAAAACTAATT
CAAATGAATAATCAAAGTTCGTAATTGTCTTTATAAAAAAATCCCTCAATCCGAATTGAATTTTCGGATTGAGG
GATTTTTATAGTTCTATTGCAGAAGAAAACTATTTTAAAAATGCTGGTAAATGTTGATAGCCACCTCTAACGTT

AACAATATTCGTAAATCCTTTATATTCTAATATTGGTTCTGTTGCAAAGTAAAAAAATATAGCTAACCACTAATT
TATCATGTCAGTGTTCGCTTAACTTGCTAGCATGATGCTAATTTCGTGGCATGGCGAAAATCCGTAGATCTGAA
GAGACCTGCGGTTCTTTTTATATAGAGCGTAAATACATTCAATACCTTTTAAAGTATTCTTTGCTGTATTGATAC
TTTGATACATTGTCTTTCTTACTTTAATATGACGGTGATCTTGCTCAATGAGGTTATTCAGATATTTCGATGTAC
AATGACAGTCAGGTTTAAGTTTAAAAGCTTTAATTACTTTAGCCATTGCTACCTTCTTTGAAGGTGCCTGATCT
GTAATTACCTTTTGAGGTTTACCAAATTGTTTAATGAGACGTTTGATAAACGCATATGCTGAATGATTATCTCG
TTGCTTACGCAACCAAATATCTAATGTATGTCCCTCTGCATCAATGGCACGATATAAATAACTCCATTTTCCTTTT
ATTTTGATGTACGTCTCATCAATACGCCATTTGTAATAAGCTTTTTTATGCTTTTTCTTCCAAATTTGATATAAAA
TTGGGGCATATTCTTGAACCCAACGGTAGACCGTTGAATGATGAACGTTTACACCACGTTCCCTTAATATTTC
AGATATATCACGATAACTCAATGCATATCTTAGATAGTAGCCAACGGCTACAGTGATAACATCCTTGTTAAATTG
TTTATATCTGAAATAGTTCATACAGAAGACTCCTTTTTGTTAAAATTATACTATAAATTCAACTTTGCAACAGAA
CCCAGTATTTGATAGATTATGATAATGCATCTAATGTACATGGATGGCAGTGGTCAGCATCAACTGGAACAGAT
GCAGTGCCTTATTTCAGAATGTTTAATCCTATACGTCAAAGTGAGCGTTTTGATGCGCAAGGGTATTTTATTAA
AACACAGCTAGAAATATTTAATGATGTGAGCAGTAAATACATTCATGATCCAACTAAATATAAAGACAAGTTAC
AAAAAATTTATCATATTGAAATTGGAAAAGATTATCCACAGACTATCGTGAATCATAAAAAATAGTAGAGATTAT
GTTATGGATAAATTTAAGCAATTTTGACATATCATCCAATAAAAAGGAGTGATTCATTTGTTGAAAAATGAAAT
ACATCGTATTTATTTAGACAAAAATAATAATGAGGTAACTATTAATAGATTTAATGTGACATATCACTATACTATA
TTAGAACAAATTAATGATTTGCCACAATATGGTTATGCAGTTTTAAACCATTTATATGCTAATCCTGGTTTAGAA
GCAGACTTCGAGCGTGTGTTCTTAAATAGAGATAAACATCTAGAGAATACTGAAGGGTTTGAAAATCTATTAT
TTCTAAAGCCACATAGTACACATGAACATCATGTTATCATTACGTTTTGGCAAGACGAAGCAGCTTACAAACA
TTGGCAAGAGTCTCAGGAATATAAAGCATCACATAAAAAATAGAGGAACCAAGCAAGGTGCTGATAAATCTAT
TGTAAACAGAAACCTATCATTTAATATTAGTTTAGAATTTAAATAACATAATTTTTGAATTAAATGATATTTCTGC
AAACATATAGGCGAGTTATTTAAATACTAACTTGCCTATTTTTTAAGCGATATAAAAATTTTAGCAAAATCACTG
ATAAGGGGAAGCGTATCACAAATAAAACTAAAAAATAAGTTGCGAACAACTTAGAGGGTGGTTTAGAGATG
GAAAGAGAAATAATAATTTCAATATGCTCAATTATTGTTGCGATTTTTGCATGTGCTGCAGCTATTATAACTGCC
ATATATACAAGAAAAGCATATATTACACAAAATAAAGGTTATATTGGTGTATATTGTGAAGGCTATTATATAGGT
AAGTTTGTCAAAAAAATTGTAATTAAAAATTATGGTAATTCCCCAGCAAAAATTAATAAGATAAGTGTAAAAG
GGATTGAAAATCAAGATTACTTAAAAAAATTTATTAATGCTCAAAAAGATAAAACATATATGCCTAATCAAATT
TCGACGTGCATAATTTTACCTGATAACTATGACTTAGTAATAGAATTGGAATACAGTTATATAAGTATGAATAGA
AATGAAATTCAATATTTGACTATTGATTTAAGCAATACGAATAAAGAATTATATACTATAGTTGAAAATGTAGGT
AGCAAATTTGAAAATGAAATACTTACAGCATTAAATCAATTATTTAGTAAAATTTAAAAAGGAGTTAAAAAGC
AATGTCATCAAATGAGATAGTAAGAAAATTATATTCAAAGGAAGATATTCGCATAGAATTAGAACTTACTCCAC
ATAAATTCAATAAAAAAATGGATACAATTTCTAACCTTTTTAAAATTGATATGAAAAATTTTCACAATTACAAA
GGCCAAGATATAAATAATCAATATACATTTAACGGCGTCGCAAAAGAATTAATAAAGGTGTTACTCAAAAGTG
TTGACTATTACCCAGTGGATATAAATTCCAAAGCTTTTAAACAAAATGGAAAATCTAAAAAAGATATGATTGA
AAATATAAATGCCTCGAGTTACATGGAATATACTTATCAGTTAATGAAATCTATCAATGAAATTAAATACAAAAG
ATTAATTGCAGACATACATACGAAAGACGTATATCAAAATACTAAAGCATGGTTAAGCATTGGTGAATCAATTA
ATAAAAAGGAACAAGAATTATACCAATATATGACACCTTTGCCCTTACATAAAAGAATTGAGCTACAAAATGA
AGTATTAAAATCTATAGATGAAACGATTTTTCAATTTATGGCAAAAGAACATAGAAACACCCAAATAAAAGAA
AATAATGAATTAGAAGAATACACAAATGCAATAAAAGAAGGTAAGTATCCCAAAAAGGATTACGAATTAAAC
CACCTATTATACAAGAAAAATGAAATACCATTAGATAGCTTAATTAAAGATGTATGGGACTATGATGAAACAGA
GTATATGGAGCTTGATTGGTTAATTGCAGATATGCTAAAACGTTCACAAAGAGCTGATAGTAATTTTATCGAA
AGTTTAGATAAGAAAAATAAATTGAGAAAAAAATATTCATAAAGAATCTATTAAAAGTATATCTAAGTTGATTGA
TCATGAATTGATTAAAAATAATAGAAAGTGGGACGTAGCAGAATTTTATAAAAATCAACAATTTTGGAACAAT

AGTACATTAAGACGTAGCTCTCAAAATTTTGGAATTTTATATTACAAAAAAAATAGGTTACATACAATGAACCA
AAGTAATATTATTAAAAAAATAAAAATTATCGAGGCTAATATAGAGAGTGCAAATAAACAGCCGAGCTATTTA
AATTTCTTTTTACAAGCTAAGTTTGACTTAGAGAGGTTAGAAAGTGAATTAATGAAATATAGCATTAATCCGA
AATATTTTAATAAGATAAATAGTGATTACGTAAGATATGCTATTGAAGATGTACATAATGCAATTATTAAAATTG
ACAGATATATAAGTAACGACAAAGTGAAGTTAAACTATTCAAATGCCCAAATGGTAGTGAGAAACGAAATAA
CAGAACAAGGAAGCTACTTTGTAACCCAGGCATTGAATACACTGTTAAAGGTAGAAGAGTCTGGCTACAGC
TTTGATAATTTCATGGTAGGACCTTTCGTAGAAGACTTTAAAAAAGCAATTAAAAGTCGTAGATTTGGTTCTA
AAACAGATTAAGATTGTTTTTTAGAGTTAAACATAAGAATAAACTTAAATAAAAAAATAAACCCAACAAACCT
TGTTATATCAATGTTTGTTGGGTTTATTTTTTTTGGTGATTTTTTAATAACTCAATTACATCTTAGGATAATATTG
CTAAATTATACATTGTCGAGGCCAAGACAAATAAAAAATTAAAGCAAAAGTTAGCGCGCAACTTTCTGCTATA
AAGGAGCAGATTTATATGGAGACAAGAGACAAACTGATGTCATTGACTCAAAGTGACAAAACACAGCAATG
GTTAATGGACAAATCATCTAACCAAACTGATGTTCAACAATTGCAGCAACAATTCAGCCAACTGCTAGATCAA
AAATATAATGCACTTTTAGCTGATGAACAAGTTAAGTTAGACCAATATGTGGAAGTACATCATAATTTAGAAGT
ATTAAAGGAAGAGATTGAATCAGAACCTATTACGCTTAATATCGATAAATTACCCGATATCAAATCAACAATGC
TTGAAAGAGCCAAGAATGATGAACATTCTGATAAAATCGAAAAGCTATTTGATAGGTTAGAACAGGCATTAA
ATGGTACGAATCGATTATATACGCAATTATCGTTGATTGGTACACGGACACATCGAATCACAACTAAAAATTTT
AATATCCAAGGCTTACCAAAATCAGTCCAACGTATAATTTTACCTTCACAATTCAAAAAGGTATATACAATCGA
TTTCAAGTCATTTGATCCTTCGGTTGTTGGATACATGACACAAGATTCTAAACTGATTGACTATTTGAATCATA
AAGAAGGTTTATATGACGCATTACTAGAAGATTTATCACTGTCCAAAAAGGAGAAAAAGTTCGTTAAACGTG
CATTTATTGGTTCGTTTCTATTTGGCGGCAACTTCGATAGCCCTAAATTCAAGTTGAAACATTATGTAAGTGAA
GTGCAATGGTTGGATGCGGTCAGCCAATTTACAAAAGTCATTGAACTTAAGAAACAAATCGAAGAGCATAA
AACCATGCCTATGCCTTACGGCATTGAACATGATATGAGCGCATTTCAAGGTAGCAGTATTATGGCATTTTATG
TACAAACTGTAGCGAGTTATATTTTCAAACACATTCTATTGGAAGTGTACAGAGCACAATGCGAACAAAAAA
CGTTCAAGATTATAGTGCCGATACACGATGCGATTATGATTGAATGTGAAGACGAAGAAATTGCACAAAATGT
GGGTCAGTTAATGAAAGATACGGCTAACCAGTTGTTCAATGGTGAATTTGCACATGTGACAGTGGAAGAAAT
AGGAGGCGTAGACCATGAATAATGATAGAGGACAAAGCCTACACATCCCAAGTAGTACACCAATCAAAGAA
AATAATATATATGTAGCTACGTTACATTCTGTGATCCAAACAGATTTCTCAGGTGAAATAAAGCACCAATTCAC
GTATGAAATTGAAGTGAACAATCAGGTTGTATATGCGAATCGTAATATTCCAACAAAACCGAGCGCTAATCAG
TTGTCAATTCATGATTGGCTTAAACGTCATAGCAACTATAACCTGAGTCATAAAACGTATGAACCTTACATTGA
CCGAAAGCATTTAATCCATATTGGTCAATATAATGGTAATTATTATGTACACGATGTAGCACCATTAAATGAAAC
TGGAGGCATATTATAATGACCAGTATATTCGAAATGTTAACTAAAATAATAAGTGTGGCTAAGGAGGCAATCG
ACCGTCAAGGTCTGATTGCTATCCTAACTATTCCTGTTAATAATGACAATGAAATAGAAGAAACAGCTCAAGG
TGAAACCGTGTATAACGAACTTATCGATCAGTTACGACTTAATATCCCAAAAGATACGGATTATCGACCTAACA
TCTATAGCTATTTTGGTATTAAGAAGAATCCTAATGACACCGTACTCATGGAAATGATGATAAAGGTTTTTCAT
ATCAAACGCTTTAATTCAGAACTGTTTGTTTTCAAAGCTAACGAGTGGCAAAAGATAAATGGAAATGAATTA
CAAGACTTGATATCTAAAATGATACAAGTATTGCTTGTAGATTATAAGCCTTCACTAAGCACTCTAAAAAATGT
CGTAGATGGATTGCAAAAATCAACAGATGTAGAAGAACTTGTTGAAAATGAGCACTATATTGGTTGTGGTGA
AAATATGTTCGATCTTAATACGTTTCAAGTCGTTAAAAATTCAATCGATATCTTTCCAAAAACACGATTGAATTT
ATCATTAAGTACAAATGATGTAATTACTGATAAGATACCGCCATATTTTAACCAATATATGTTACAACTTGCGAA
TTATGACGATGATTTACAATACTTTCTTTTCCAACATACAGCAGTGTTACTTACAGCTGATACTAAATACCGTAG
GGGTCTCATATTATATGGTGGAGCTAAGAATGGTAAATCTGTATATATTGAACTAGTTAAATCATTTTTCTATAG
TAAAGATATTGTGTCTAAGGCACTTAATGAGCTTGAAGGTCGCTTTGACAAAGAAAGTTTAATTGACAAAAG
TCTAATGGCAAGTCATGAAATTGGAAAATCTAGGATTCAAGAAAAGACTGTAAATGACTTCAAAAAGTTATT
ATCTGTAGAATCAATGCATGTTGATCGTAAAGGAAAAACTCAAGTGGAGGTCACTTTGGATTTGAAACTTGT

TTTTGGTACAAATGCGGTACTTAATTTTCCTGCTGAACATGCGAAAGCTTTGGAGCGTCGAATTAATGTTATT
CCATGTGAATATTATGTTGAAAAAGCGGATACTTCATTAATTGATAAACTCATGAGTGAGAAGAAAGAAATCT
TTCTTTACTTGATGTATGTGTATCAAAAAATTGTAGAAGCAGATATCGAATATCTTGAAAATAGTCGTGTTACT
GAAATTTCTCATGATTGGTTAAATTTTGGATATGAATTTGTTTCTAGTAAATCAGCAAGTATTGCACATCAGAA
AGCGTGTATTAATTTACTCAGAAAACTTATAGAAATCAAACCAGGATCACGAATCAAAGTATCCGAGTTAAAT
AAAGTTATTAATGAAGAAATAAAGGTGAGTTCTCAGGTTATTAAACAGTTAATTCAAGCAAACTTTGATACTC
AAACCAAACTATACAATGGCTACGATTATTGGATTGATTTAGGTTGGAAAGAAGCCGATAAAAAAGAGATTC
ATGATATTTCGAAAAAAGATAATATTATTTCATTAGATAAAAATGAAAATATAACAGACGATGAGACATTAGAT
GAAGAGAATTTGGACTTTGATTGGGAGGACTTTGACGATGAATAATGAACAAATTGAAGCATTTGTAGAAG
TGCTTGTGCCTATCATAGAAGAACGTATCAATAAAGGTAAGTAATCTAATTACGTACTACAGGCAGTTGCCTGT
AGTACTCATATGATTAAGTGGTAAAAGTGATAAAAATGAAACGAAATTATAAATATATATTATCCATATGATGTT
ACAAGACCGATGGTCTGTAGCAATAATCTAATAAAAGGAGCGGTATGATATGAAGGGTAAAATTGCACTTTAT
TCACGCGTTAGTACGTCAGAGCAGTCGGAGCATGGGTACTCAATCCATGAACAAGAACAAGTACTCATCAAA
GAGGTTGTGAACAACTATCCAGGTTATGACTATGAAACTTATATCGATTCTGGTATATCAGGTAAAAATATTGA
AGGTCGACCAGCAATGAAACGTCTATTACAAGATGTTAAGGATAATAAAATCGAAATGGTGTTAAGTTGGAA
ATTGAATCGTATCTCACGCTCAATGAGAGACGTTTTTAATATTATTCATGAATTCAAAGAGCATGGCGTAGGG
TATAAATCGATTTCTGAGAATATTGATACATCCAATGCATCTGGTGAAGTTTTAGTTACAATGTTTGGGTTAATA
GGATCTATAGAACGCCAGACTTTGATTTCCAATGTGAAACTTTCTATGAATGCTAAGGCACGGAGCGGAGAG
GCAATCACCGGTCGTGTTTTAGGCTACAAATTATCACTTAATCCATTGACACAGAAAAATGATTTAGTTATTGA
TGAAAATGAAGCTAATATTGTACGTGAAATTTTCGATTTATATTTGAATCATAATAAAGGGCTCAAAGCCATTA
CAACTGTACTTAATCAAAAGGGGTATCGTACTATTAATCAAAAGCCATTTTCAGTGTATGGTGTTAAATACATT
TTGAATAATCCAGTCTATAAAGGCTATGTCAGATTCAATAACCATCAAAACTGGGCTGTACAGCGAAGAAGTG
GCAAAAGTGATAAAAATGATGTGATATTGGTCAAAGGTAAACATAAAGCCATTATAAGTGAAGAGGTATTTG
ACCAAGTTCATGAAAAATTAGCTTCTAAAAGTTTTAAACCGGGCAGACCTATTGGTGGGGATTTTTACCTTC
GTAGCCTTATTAAATGTCCAGAATGCGGTAATAATATGGTATGTCGACGGACTTATTATAAAACCAAAAAGTCA
AAAGAACGGACAATCAAGCGTTATTACATTTGTTCCTTATTCAATCGTTCAGGGAGTTCTGCATGTCACAGTA
ATGCCATCAATGCTGAAGTCGTCGAGCGTGTAATTAATGTTCATTTGAATCGTATTCTGTCTCAACCAGATATT
ATCAAGCAGATTGCGTCAAATGTGATAGAAGAACTGAAACAAAAGCATAGTAACCAAACAGAAATTAAATAT
GACATTGATAGTTTAGAAAAACAAAAAGCTAAGCTTAAAACACAACAAGAACGATTGTTAGAATTGTTCTTA
GATGATCAAATGGATAGCGAAATGTTAAAAGCAAAACAAAGTCAGATGAATGGACAACTAAAGATGCTAGA
TAAGCAAATTAAAGAAGCGCAACAAGCTACTGAATCACAAGATGAAGTACCAAATTTTGATAAACTGAAAA
GCCGTTTAACCATGATGATTAGCCGATTTAGTGTCTACTTAAGGGAAGCTACACCCGAAGCTAAAAATCAACT
TATGAAAATGTTGATTGATTCTATTGAAATTACGACAGATAAACAAGTAAAACTTGTAAGGTATAAAATTGATG
AAAGTCTTATCCCTCAATCTTTGAAAAAAGATTGGGGGTCTTTTTTTATGCCTAAATTCCAATTCGAAATAGAC
GGTCGAAACAATTATTTCATCGACCAAATTACCACTTTTACCACTTAGTTCATAGTGACAAAAGTGAGAGAAA
TGAAATAAAAATCAAATATATATTATCTAAATGATGTATCACATGTATACATCAACCAAATACATTAGGAGGTCAT
AACAATGACATTAGAACTACAACTCAAACACTATATAACCAATCTATTCAACCTACCAAAGAATGAAAAGTGG
GAATGTGAATCTATCGAGGAAATCGCTGATGATATCCTACCTGACCAATATGTAAGGCTTGGTCCACTCAGTA
ATAAAATACTTCAAACCTATACCTACTACTCTGACACACTTTACGAAAGTAATATCTATCCTTTCATTCTCTACTA
TCAGAAACAGCTCATAGCCATTGGTTATATTGATGAAAACCACGATATGGATTTCTTATACCTACACAACACTA
TCATGCCCCTTTTGGATCAACGATACTTACTAACAGGAGAACAATAATATGCATAAATACATCAAAATTACACA
AATCGTCATTACAATTCTTAGTGAAATTATCATTTGGATGAAAGAATCAGAACGAAAGGAAATCTCTTATGAAT
AAATATATCACACGTGGTATTGCCCAAAACTTACCTACCACATTGCAACACCAATTATGGCAACTTGTAGCGCA
ACGTGAAAACGAACAGTCCAAGGAATTAGAAGCAATAGATTACTTTCATGTCTTCCAGTTCAACATGCACAA

TAATCAATTATATGTCAAACACAAACAAGAACGTCCTGAATATGTCAAAATTCATAAAGCAAATTATTCAAAAG
CTATCGATATCAATAAGGTCTACATTATCCGAGAAGATGATGTAGACCTTACTTATTTCGTCATGTTATTACCCG
AAGAATACTAATTAAGGAGAAAAACATATGGAAACAATCAAAAGTACATTAGAAACCGAAGCCATATTCAGT
GATGACCAACAACACCGCTATCTACTTAAGAAAACATGGGACAGTGAAAAACAAACAATCACAATCATCACA
ATGTATCCGCATTATGATGGCATTCTCAACATCGACCTAACAACTCAGCTCATTATGAACAAAGTTTCAGAAAT
GGATGCGTTTGGTGCCATCAATTTTGTGAATCTATACTCTAATATTACAACACCTATCAATCTCAAACATTTAGA
AAATGCCTATGATAAGCATACAGATATTCAAATTATGAAGGCAGTGAAAGAATCAGATGAAGTGATATTAGCA
TGGGGTGCTTACGCTAAAAAGCCCGTTGTTGAAGCACGCGTCAATGAAGTATTAGAGATGTTAAAACCACAT
AAAAAGAAAGTGAAACAACTCATGAACCCAGCAACCAATGAAATCATGCATCCCCTTAACCCGAAAGCACG
TCAAAAATGGATTTTGAAAGCGTAACAAATTTAAAAAGTAAAACTTGTGAAATATTTTTATATTGAATTTAAAT
TTTAGTATACAAGTTTTTCAAAATAAATTAAAATAGTTATATAGTAATTCAATTCTGTTTAAAACAACTGGTTCA
AGACCAAACATAACGCTTTATAAATAAAATTAATAATTACGAAAGGAATGGAGATTATGAGTAACATAATAAA
AACGTTAGGCAAAGGAATTGGGAAAGGTGTCGGTGCAGCAGCATATTATTCAGTTGCGACTGTTGGCGCTG
CCATCGGTGCGATATGGGAAACAACAGGCGATAGAGGTTTGATGAGAGATGCTTTAAGTGATAGTTCAAAC
CAAGACTTAATGGAAATATATGAGATACAGAAGAAAGATGATGGGGATTATGATACTTATATTGCGGCTAAAT
CCTTATTACAAGAGAGAGGATTTACTTATAATCACGAAACGAACAAATGGGAAAAATAA
>Staphylococcus aureus 04-02981
ATGAAAATCACCATTTTAGCTGTAGGGAAACTAAAAGAGAAATATTGGAAGCAAGCCATAGCAGAATATGAA
AAACGTTTAGGCCCATACACCAAGATAGACATCATAGAAGTTCCAGACGAAAAAGCACCAGAAAATATGAG
CGACAAAGAAATTGAGCAAGTAAAAGAAAAAGAAGGCCAACGAATACTAGCCAAAATTAAACCACAATCCA
CAGTCATTACATTAGAAATACAAGGAAAGATGCTATCTTCCGAAGGATTGGCCCAAGAATTGAACCAACGCA
TGACCCAAGGGCAAAGCGACTTTGTATTCGTCATTGGCGGATCAAACGGCCTGCACAAGGACGTCTTACAA
CGCAGTAACTACGCACTATCATTCAGCAAAATGACATTCCCACATCAAATGATGCGGGTTGTGTTAATTGAGC
AAGTGTATAGAGCATTTAAGATTATGCGTGGAGAAGCATATCATAAATGATGCGGTTTTTTCAGCCGCTTCAT
AAAGGGATTTTGAATGTATCAGAACATATGAGGTTTATGTGAATTGCTGTTATGTTTTTAAGAAGCTTATCATA
AGTAATGAGGTTCATGATTTTTGACATAGTTAGCCTCCGCAGTCTTTCATTTCAAGTAAATAATAGCGAAATAT
TCTTTATACTGAATACTTATAGTGAAGCAAAGTTCTAGCTTTGAGAAAATTCTTTCTGCAACTAAATATAGTAA
ATTACGGTAAAATATAAATAAGTACATATTGAAGAAAATGAGACATAATATATTTTATAATAGGAGGGAATTTC
AAATGATAGACAACTTTATGCAGGTCCTTAAATTAATTAAAGAGAAACGTACCAATAATGTAGTTAAAAAATC
TGATTGGGATAAAGGTGATCTATATAAAACTTTAGTCCATGATAAGTTACCCAAGCAGTTAAAAGTGCATATAA
AAGAAGATAAATATTCAGTTGTAGGGAAGGTTGCTACTGGGAACTATAGTAAAGTTCCTTGGATTTCAATATA
TGATGAGAATATAACAAAAGAAACAAAGGATGGATATTATTTGGTATATCTTTTTCATCCGGAAGGAGAAGG
CATATACTTATCTTTGAATCAAGGATGGTCAAAGATAAGTGATATGTTTCCGCGGGATAAAAATGCTGCAAAA
CAAAGAGCATTAACTTTATCTTCCGAACTCAATAAATATATTACATCAAATGAATTTAATACTGGAAGATTTTAT
TACGCAGAAAATAAAGATTCATCTTATGATTTAAAAAATGATTATCCATCAGGATATTCTCATGGATCAATAAGA
TTCAAATATTATGATTTGAATGAAGGATTCACAGAAGAAGATATGCTAGAGGATTTAAAGAAATTTTTAGAAC
TATTTAATGAATTAGCTTCAAAAGTTACAAAAACATCCTATGATAGCTTGGTCAATAGCATAGACGAAATACAG
GAAGACAGCGAAATTGAAGAAATTAGAACAGCACAAAAAGATAAGACACTCAAGGAAGTGGAAGCACCTA
AAGGAATAATTCCAAAATATAAAAAAGGTGTATCAAAGACTACTAAAAATGATTCAGAAATTGAAAAATCAA
ATAAAGAGAATAAATTAACCGGTAAAGTTGGAGAAAAATTAGCGCTAAATTACTTTAATGAGCTAATTGATAA
TAAAATAGACGAAGATAAGAAAGAACAGTTTAGGAATATTTTAAATGATAATCCAGGCTCTCAACACGGTCAT
GGCTATGATTTAGTAGCTTTTGATCCAACAAATACAGATAAAGCTGTAGAAAAATTTATTGAAATTAAAACATC
TACATCTTCTAGTATTGAGGAACCATTTTTTATGTCGCTAAATGAAATGTTTGCTATGAAAGAATATAAGCAGA
AATATTTAATATTAAGAATATTTAATGTTTCCGGTAAAGAACCACAATTTTATTTTATAGATCCATATGCAAATTA

TTCTGAATTTAAAGATGTAGATGATCTCATTGACAAAGTATTTAATGTAGAAGCTATTCAGTATAAAGTTTTTG
GCGAAAAATGATTACTTGAACAAGAGCTAAAATAAAATTGTGATCTAATAAAAATAGAAACTGTAATTTAAAT
AAAACTTTCTAAATAAGCTAACTGATAAAAAATCAGTTTGTCCACAGTCTGAAACAAGATTCCTATATTCTTTA
GGAATCTTGTTTTTTCTATTTTTATGGTGATAAAGAGCAGATAAGATAATGTGTAATAATCACAAAAAAGTTAA
ATATTTTAAGGCTTGTTTAATTATTAATGATTTTATATATAAAGAGCAGTATAATAAAGTTGTTAATATATTATGAA
TAATATTCAAGTAATTTTATTGTTTTTTAATTTGTCGATATTTAAGTTGAGTTAAATTTAAAGGGTGTAATTTGTT
TTACAATGATGAAGATAATTAGTCTATCAAAATAAAGGGGTTGGGACTGTTATGAGTGATAATTTGTCATTATT
CATTGACTATATCAATGATAATATAATCTATGGTAGTGAAATCAAACGGGAGAAATTAGAGAATTTATTTAATCA
ATTTGCTATAAAAAAATGTTGAAAAGAACATTGTCTATGATGAACTGAAATCTTTAGATATTACAATCATTGAGT
CACAGGATTCATATAAAAATAAATTGAAGAGATTATTTTCGGTTCTGTTGCAAAGTAAAAAAATATAGCTAACC
ACTAATTTATCATGTCAGTGTTCGCTTAACTTGCTAGCATGATGCTAATTTCGTGGCATGGCGAAAATCCGTAG
ATCTGAAGAGACCTGCGGTTCTTTTTATATAGAGCGTAAATACATTCAATACCTTTTAAAGTATTCTTTGCTGTA
TTGATACTTTGATACCTTGTCTTTCTTACTTTAATATGACGGTGATCTTGCTCAATGAGGTTATTCAGATATTTC
GATGTACAATGACAGTCAGGTTTAAGTTTAAAAGCTTTAATTACTTTAGCCATTGCTACCTTCGTTGAAGGTG
CCTGATCTGTAATTACCTTTTGAGGTTTACCAAATTGTTTAATGAGACGTTTGATAAACGCATATGCTGAATGA
TTATCTCGTTGCTTACGCAACCAAATATCTAATGTATGTCCCTCTGCATCAATGGCACGATATAAATAGCTCCAT
TTTCCTTTTATTTTGATGTACGTCTCATCAATACGCCATTTGTAATAAGCTTTTTTATGCTTTTTCTTCCAAATTT
GATACAAAATTGGGGCATATTCTTGAACCCAACGGTAGACCGTTGAATGATGAACGTTTACACCACGTTCCCT
TAATATTTCAGATATATCACGATAACTCAATGTATATCTTAGATAGTAGCCAACGGCTACAGTGATAACATCCTT
GTTAAATTGTTTATATCTGAAATAGTTCATACAGAAGACTCCTTTTTGTTAAAATTATACTATAAATTCAACTTTG
CAACAGAACCATCTAATCTTCAACAAACTGGCCCGTTTGTTGAACTACTCTTTAATAAAATAATTTTTCCGTTC
CCAATTCCACATTGCAATAATAGAAAATCCATCTTCATCGGCTTTTTCGTCATCATCTGTATGAATCAAATCGCC
TTCTTCTGTGTCATCAAGGTTTAATTTTTTATGTATTTCTTTTAACAAACCACCATAGGAGATTAACCTTTTACG
GTGTAAACCTTCCTCCAAATCAGACAAACGTTACAAATTCTTTTCTTCATCATCGGTCATAAAATCCGTATCCTT
TACAGGATATTTTGCAGTTTCGTCAATTGCCGATTGTATATCCGATTTATATTTATTTTTCGGTCGAATCATTTGA
ACTTTTACATTTGGATCATAGTCTAATTTCATTGCCTTTTTCCAAAATTGAATCCATTGTTTTTGATTCACGTAG
TTTTCTGTATTCTTAAAATAAGTTGGTTCCACACATACCAATACATGCATGTGCTGATTATAAGAATTATCTTTAT
TATTTATTGTCACTTCCGTTGCACGCATAAAACCAACAAGATTTTTATTAATTTTTTTATATTGCATCATTCGGC
GAAATCCTTGAGCCATATCTGACAAACTCTTATTTAATTCTTCGCCATCATAAACATTTTTAACTGTTAATGTGA
GAAACAACCAACGAACTGTTGGCTTTTGTTTAATAACTTCAGCAACAACCTTTTGTGACTGAATGCCATGTTT
CATTGCTCTCCTCCAGTTGCACATTGGACAAAGCCTGGATTTACAAAACCACACTCGATACAACTTTCTTTCG
CCTGTTTCACGATTTTGTTTATACTCTAATATTTCAGCACAATCTTTTACTCTTTCAGCCTTTTTAAATTCAAGAA
TATGCAGAAGTTCAAAGTAATCAACATTAGCGATTTTCTTTTCTCTCCATGGTCTCACTTTTCCACTTTTTGTCT
TGTCCACTAAAACCCTTGATTTTTCATCTGAATAAATGCTACTATTAGGACACATAATATTAAAAGAAACCCCC
ATCTATTTAGTTATTTGTTTAGTCACTTATAACTTTAACAGATGGGGTTTTTCTGTGCAACCAATTTTAAGGGTT
TTCAATACTTTAAAACACATACATACCAACACTTCAACGCACCTTTCAGCAACTAAAATAAAAATGACGTTATT
TCTATATGTATCAAGATAAGAAAGAACAAGTTCAAAACCATCAAAAAAAGACACCTTTTCAGGTGCTTTTTTT
ATTTTATAAACTCATTCCCTGATCTCGACTTCGTTCTTTTTTTACCTCTCGGTTATGAGTTAGTTCAAATTCGTTC
TTTTTAGGTTCTAAATCGTGTTTTTCTTGGAATTGTGCTGTTTTATCCTTTACCTTGTCTACAAACCCCTTAAAA
ACGTTTTTAAAGGCTTTTAAGCCGTCTGTACGTTCCTTAAGGAATTCCTTAGTGCTTTCATAGATTAAACTCAC
ATCACGCTTTAAATCGCTTATTTTAGACTTTAAAGACTTGTTTTCTTCAAGCAACTCATTATAATCATTTACATTT
TCATTAAATCGCTCTACAAGACCACTATATTTTTCTTTAACTTGCCCATGTTCTTTACTTAATTTTTTATATTCTCT
CGCCATATCAGTACTCATGAGATTTCTAACATGCTGTTTTAACCTATCGTTATCTCTCGCAGCAGTCACTAAGTT
TTTATAATCACGCTCCGATATAACAACATTTTTGGTTGGTTTCTTTTCTGTTTTCATTATTTCTTTTCCCAAACCA

AACATAGACTTTTCACCCGTTGGCACTTCAACACTTTTCATGTGTCGTTTCGCTGGTACTTCTAAATCTGATTT
AACTTTATCGCTATAAGCAGTCCATTCATCTTTTTTAACTGCTAAATTTTTTTCTAGAAAATCAATCTCTTTTTCC
AAAGTTTGTTTTTTAAATTTAGCTGTCTCAATATGTTTACGGTCAGAGCCACGTTCACCACGCTTCAACTCAA
AACCCTGTTTTTTCATATGCTCGGGGAATTTATCTTGTAGCCATAACAGTTCTTGACGATTAAACACATTTTTTC
CTTGCAGTTTTCCATCACGCATAGGCACAACACCTAAATGCATGTGAGGGGTTTGCTCATCATTATGAACTGT
TGCATAAGCAATATTTTGCTTGCCATATCGTTCGGAAAATAATTTATAACTTTCCTCAAAAAATCGTTTTTGTTC
TCCTGGATCCAGTTGCTCAAAAAAATCTCGGTCAGATGTTACTAGCAACTCATTTACAAGAACAGCATCTTTC
CTCGTTTTTCTTGTACCTGTTTTTTGTGATTCAATAATTTCTTTGACACGTTCGTTGTAATCAATATTTTTATCAT
TTTTCAAATCATAATTTTCACGTGTTCGCTCATGGTCAATATCATCATTCGTTCTACTTTTTCGCTCTCTTTGATT
ATGAAATTGCATGCCTTTTAGTCCAGCTGATTTCACTTTTTGCATTCTACAAACTGCATAACTCATATGTAAATC
GCTCCTTTTTAGGTGGCACAAATGTGAGGCATTTTCGCTCTTTCCGGCAACCACTTCCAAGTAAAGTATAACA
CACTATACTTTATATTCATAAAGTGTGTGCTCTGCGAGGCTGTCGGCAGTGCCGACCAAAACCATAAAACCTT
TAAGACCTTTCTTTTTTTTACGAGAAAAAAGAAACAAAAAAACCTGCCCTCTGCCACCTCAGCAAAGGGGG
GTTTTGCTCTCGTGCTCGTTTAAAAATCAGCAAGGGACAGGTAGTATTTTTTGAGAAGATCACTCAAAAAAT
CTCCACCTTTAAACCCTTGCCAATTTTTATTTTGTCCGTTTTGTCTAGCTTACCGAAAGCCAGACTCAGCAAG
AATAAAATTTTTATTGTCTTTCGGTTTTCTAGTGTAACGGACAAAACCACTCAAAATAAAAAAGATACAAGAG
AGGTCTCTCGTATCTTTTATTCAGCAATCGCGCCCGATTGCTGAACAGATTAATAATAGATTTTAGCTTTTTATT
TGTTGAAAAAAGCTAATCAAATTGTTGTCGGGATCAATTACTGCAAAGTCTCGTTCATCCCACCACTGATCTT
TTAATGATGTATTGGGGTGCAAAATGCCCAAAGGCTTAATATGTTGATATAATTCATCAATTCCCTCTACTTCAA
TGCGGCAACTAGCAGTACCAGCAATAAACGACTCCGCACCTGTACAAACCGGTGAATCATTACTACGAGAGC
GCCAGCCTTCATCACTTGCCTCCCATAGATGAATCCGAACCTCATTACACATTAGAACTGCGAATCCATCTTCA
TGGTGAACCAAAGTGAAACCTAGTTTATCGCAATAAAAACCTATACTCTTTTTAATATCCCCGACTGGCAATGC
CGGGATAGACTGTAACATTCTCACGCATAAAATCCCCTTTCATTTTCTAATGTAAATCTATTACCTTATTATTAAT
TCAATTCGCTCATAATTAATCCTTTTTCTTATTACGCAAAATGGCCCGATTTAAGCACACCCTTTATTCCGTTAA
TGCGCCATGACAGCCATGATAATTACTAATACTAGGAGAAGTTAATAAATACGTAACCAACATGATTAACAATT
ATTAGAGGTCATCGTTCAAAATGGTATGCGTTTTGACACATCCACTATATATCCGTGTCGTTCTGTCCACTCCTG
AATCCCATTCCAGAAATTCTCTAGCGATTCCAGAAGTTTCTCAGAGTCGGAAAGTTGACCAGACATTACGAA
CTGGCACAGATGGTCATAACCTGAAGGAAGATCTGATTGCTTAACTGCTTCAGTTAAGACCGAAGCGCTCGT
CGTATAACAGATGCGATGATGCAGACCAATCAACATGGCACCTGCCATTGCTACCTGTACAGTCAAGGATGG
TAGAAATGTTGTCGGTCCTTGCACACGAATATTACGCCATTTGCCTGCATATTCAAACAGCTCTTCTACGATAA
GGGCACAAATCGCATCGTGGAACGTTTGGGCTTCTACCGATTTAGCAGTTTGATACACTTTCTCTAAGTATCC
ACCTGAATCATAAATCGGCAAAATAGAGAAAAATTGACCATGTGTAAGAGGCCAATCTGATTCCACCTGAGA
TGCATAATCTAGTAGAATCTCTTCGCTATCAAAATTCACTTCCACCTTCCACTCACCGGTTGTCCATTCATGGCT
GAACTCTGCTTCCTCTGTTGACATGACACACATCATCTCAATATCCGAATAGGGCCCATCAGTCTGACGACCA
AGAGAGCCATAAACACCAATAGCCTTAACATCATCCCCATATTTATCCAATATTCGTTCCTTAATTTCATGAACA
ATCTTCATTCTTTCTTCTCTAGTCATTATTATTGGTCCATTCACTATTCTCATTCCCTTTTCAGATAATTTTAGATT
TGCTTTTCTAAATAAGAATATTTGGAGAGCACCGTTCTTATTCAGCTATTAATAACTCGTCTTCCTAAGCATCCT
TCAATCCTTTTAATAACAATTATAGCATCTAATCGGTTCTGTTGCAAAGTAAAAAAATATAGCTAACCACTAATT
TATCATGTCAGTGTTCGCTTAACTTGCTAGCATGATGCTAATTTCGTGGCATGGCGAAAATCCGTAGATCTGAA
GAGACCTGCGGTTCTTTTTATATAGAGCGTAAATACATTCAATACCTTTTAAAGTATTCTTTGCTGTATTGATAC
TTTGATACCTTGTCTTTCTTACTTTAATATGACGGTGATCTTGCTCAATGAGGTTATTCAGATATTTCGATGTAC
AATGACAGTCAGGTTTAAGTTTAAAAGCTTTAATTACTTTAGCCATTGCTACCTTCGTTGAAGGTGCCTGATC
TGTAATTACCTTTTGAGGTTTACCAAATTGTTTAATGAGACGTTTGATAAACGCATATGCTGAATGATTATCTC
GTTGCTTACGCAACCAAATATCTAATGTATGTCCCTCTGCATCAATGGCACGATATAAATAGCTCCATTTTCCTT

TTATTTTGATGTACGTCTCATCAATACGCCATTTGTAATAAGCTTTTTTATGCTTTTTCTTCCAAATTTGATACAA
AATTGGGGCATATTCTTGAACCCAACGGTAGACCGTTGAATGATGAACGTTTACACCACGTTCCCTTAATATT
TCAGATATATCACGATAACTCAATGTATATCTTAGATAGTAGCCAACGGCTACAGTGATAACATCCTTGTTAAAT
TGTTTATATCTGAAATAGTTCATACAGAAGACTCCTTTTTGTTAAAATTATACTATAAATTCAACTTTGCAACAG
AACCGAAAAACTAGACTTGATTACAAAATGGAGCTTGGGACATAAATGATTTTTTAAAAATGAGATGAGACG
TAGATTAACTCCATAATCAATACGAATCTATCGACTTCTTTATTTATGATATTCATCTCTTTTTAATGGAAATAAA
AGTGCGATTAATGTGATAATACAGTTACGTTAATTAAAAAAATAAAAATGCAAGGAGAGGTAATATGCTAACT
GTATATGGACATAGAGGATTACCTAGTAAAGCTCCGGAAAATACAATTGCATCATTTAAAGCTGCTTCAGAAG
TAGAAGGTATAAACTGGTTGGAGTTAGATGTTGCAATTACAAAAGATGAACAACTGATTATCATTCATGATGA
TTATTTAGAACGGACTACAAATATGTCCGGGGAAATAACTGAATTGAATTATGATGAAATTAAAGATGCTTCT
GCAGGATCTTGGTTTGGTGAAAAATTCAAAGATGAACATTTGCCAACTTTCGATGATGTAGTAAAAATAGCA
AATGAATATAATATGAATTTAAATGTAGAATTAAAAGGTATTACTGGACCGAATGGACTAGCACTTTCTAAAAG
TATGGTTAAGCAAGTGGAAGAACAATTAACAAACTTAAATCAGAATCAAGAAGTGCTCATTTCAAGCTTTAA
TGTTGTGCTTGTTAAACTTGCAGAAGAAATCATGCCACAATATAACAGAGCAGTTATATTCCATACAACTTCGT
TTCGTGAAGACTGGAGAACACTTTTAGATTACTGTAATGCTAAAATAGTAAACACTGAAGATGCCAAACTTAC
TAAAGCAAAAGTAAAAATGGTAAAAGAAGCGGGTTATGAATTGAACGTATGGACTGTAAACAAACCAGCAC
GTGCAAACCAACTTGCTAATTGGGGAGTTGATGGTATCTTTACAGACAATGCAGATAAAATGGTGCATTTGTC
TCAATAGAAAGTTAGAGGTGAGTCTTACGTTTCAGTGACGGTAGACTTACCTTTAACATGTTACATACTAAAA
AATTAATTTGAATAAGAAAGAGAGACATATATGAAATACGATGATTTTATAGTAGGAGAAACATTCAAAACAA
AAAGCCTTCATATTACAGAAGAAGAAATTATCCAATTTGCAACAACTTTTGATCCTCAATATATGCATATAGATA
AAGAAAAAGCAGAACAAAGTAGATTTAAAGGTATCATTGCATCTGGCATGCATACACTTTCAATATCATTTAA
ATTATGGGTAGAAGAAGGTAAATACGGAGAAGAAGTTGTAGCAGGAACACAAATGAATAACGTTAAATTTAT
TAAACCTGTATACCCAGGTAATACATTGTACGTTATCGCTGAAATTACAAATAAGAAATCCATAAAAAAAGAAA
ATGGACTCGTTACAGTGTCACTTTCAACATACAATGAAAATGAAGAAATTGTATTTAAGGGAGAAGTAACAG
CACTTATTAATAATTCATAATAAAACAGTGAAGCAACCATCGTTACGGATTGCTTCACTGTTTTGTTATTCATCT
ATATCGTATTTTTTATTACCGTTCTCATATAGCTCATCATACACTTTACCTGAGATTTTGGCATTGTAGCTAGCCA
TTCCTTTATCTTGTACATCTTTAACATTAATAGCCATCATCATGTTTGGATTATCTTTATCATATGATATAAACCAC
CCAATTTGTCTGCCAGTTTCTCCTTGTTTCATTTTGAGTTCTGCAGTACCGGATTTGCCAATTAAGTTTGCATA
AGATCTATAAATATCTTCTTTATGTGTTTTATTTACGACTTGTTGCATACCATCAGTTAATAGATTGATATTTTCTT
TGGAAATAATATTTTTCTTCCAAACTTTGTTTTTCGTGTCTTTTAATAAGTGAGGTGCGTTAATATTGCCATTAT
TTTCTAATGCGCTATAGATTGAAAGGATCTGTACTGGGTTAATCAGTATTTCACCTTGTCCGTAACCTGAATCA
GCTAATAATATTTCATTATCTAAATTTTTGTTTGAAATTTGAGCATTATAAAATGGATAATCACTTGGTATATCTT
CACCAACACCTAGTTTTTTCATGCCTTTTTCAAATTTCTTACTGCCTAATTCGAGTGCTACTCTAGCAAAGAAA
ATGTTATCTGATGATTCTATTGCTTGTTTTAAGTCGATATTACCATTTACCACTTCATATCTTGTAACGTTGTAAC
CACCCCAAGATTTATCTTTTTGCCAACCTTTACCATCGATTTTATAACTTGTTTTATCGTCTAATGTTTTGTTATT
TAACCCAATCATTGCTGTTAATATTTTTTGAGTTGAACCTGGTGAAGTTGTAATCTGGAACTTGTTGAGCAGA
GGTTCTTTTTTATCTTCGGTTAATTTATTATATTCTTCGTTACTCATGCCATACATAAATGGATAGACGTCATATG
AAGGTGTGCTTACAAGTGCTAATAATTCACCTGTTTGAGGGTGGATAGCAGTACCTGAGCCATAATCATTTTT
CATGTTGTTATAAATACTCTTTTGAACTTTAGCATCAATAGTTAGTTGAATATCTTTGCCATCTTTTTTCTTTTTC
TCTATTAATGTATGTGCGATTGTATTGCTATTATCGTCAACGATTGTGACACGATAGCCATCTTCATGTTGGAGC
TTTTTATCGTAAAGTTTTTCGAGTCCCTTTTTACCAATAACTGCATCATCTTTATAGCCTTTATATTCTTTTTGTTT
TAATTCTTCAGAGTTAATGGGACCAACATAACCTAATAGATGTGAAGTCGCTTTTCCTAGAGGATAGTTACGA
CTTTCTGTTTCATTAGTTGTAAGATGAAATTTTTTTGCGAAATCACTTAAATATTCATCCATTTTTTTAACGGTT
TTAAGTGGAACGAAGGTATCATCTTGTACCCAATTTTGATCCATTTGTTGTTTGATATAGTCTTCAGAAATACT

TAGTTCTTTAGCGATTGCTTTATAATCTTTTTTAGATACATTCTTTGGAACGATGCCTATCTCATATGCTGTTCCT
GTATTGGCCAATTCCACATTGTTTCGGTCTAAAATTTTACCACGTTCTGATTTTAAATTTTCAATATGTATGCTT
TGGTCTTTCTGCATTCCTGGAATAATGACGCTATGATCCCAATCTAACTTCCACATACCATCTTCTTTAACAAAA
TTAAATTGAACGTTGCGATCAATGTTACCGTAGTTTGTTTTAATTTTATATTGAGCATCTACTCGTTTTTTATTTT
TAGATACTTTTTTTATTTTACGATCCTGAATGTTTATATCTTTAACGCCTAAACTATTATATATTTTTATCGGACGT
TCAGTCATTTCTACTTCACCATTATCGCTTTTAGAAATATAACTGCTATCTTTATAAACTTGTTTGAAATTTTTAT
CTTCAATTGCATCAATAGTATTATTAATTTCTTTATCTTTTGAAGCATAAAAATATATACCAAACCCGACAACTAC
AACTATTAAAATAAGTGGAACAATTTTTATCTTTTTCATCAATATCCTCCTTATATAAGACTACATTTGTAGTATAT
TACAAATGTAGTATTTATGTCAAAATAATGTTATAATTTTTGTGATATGGAGGTGTAGAAGGTGTTATCATCTTT
TTTAATGTTAAGTATAATCAGTTCATTGCTCACGATATGTGTAATTTTTTTAGTGAGAATGCTCTATATAAAATAT
ACTCAAAATATTATGTCACATAAGATTTGGTTATTAGTGCTCGTCTCCACGTTAATTCCATTAATACCATTTTACA
AAATATCGAATTTTACATTTTCAAAAGATATGATGAATCGAAATGTATCTGACACGACTTCTTCGGTTAGTCAT
ATGTTAGATGGTCAACAATCATCTGTTACGAAAGACTTAGCAATTAATGTTAATCAGTTTGAGACCTCAAATAT
AACGTATATGATTCTTTTGATATGGGTATTTGGTAGTTTGTTGTGCTTATTTTATATGATTAAGGCATTCCGACA
AATTGATGTTATTAAAAGTTCGTCATTGGAATCGTCATATCTTAATGAACGACTTAAAGTATGTCAAAGTAAGA
TGCAGTTCTACAAAAAGCATATAACAATTAGTTATAGTTCAAACATTGATAATCCGATGGTATTTGGTTTAGTG
AAATCCCAAATTGTACTACCAACTGTCGTAGTCGAAACCATGAATGACAAAGAAATTGAATATATTATTCTACA
TGAACTATCACATGTGAAAAGTCATGACTTAATATTCAACCAGCTTTATGTTGTTTTTAAAATGATATTCTGGTT
TAATCCTGCACTATATATAAGTAAAACAATGATGGACAATGACTGTGAAAAAGTATGTGATAGAAACGTTTTAA
AAATTTTGAATCGCCATGAACATATACGTTATGGTGAATCGATATTAAAATGCTCTATTTTAAAATCTCAGCACA
TAAATAATGTGGCAGCACAATATTTACTAGGTTTTAATTCAAATATTAAAGAACGTGTTAAGTATATTGCACTTT
ATGATTCAATGCCTAAACCTAATCGAAACAAGCGTATTGTTGCGTATATTGTATGTAGTATATCGCTTTTAATAC
AAGCACCGTTACTATCTGCACATGTTCAACAAGACAAATATGAAACAAATGTATCATATAAAAAATTAAATCAA
CTAGCTCCGTATTTCAAAGGATTTGATGGAAGTTTTGTGCTTTATAATGAACGGGAGCAAGCTTATTCTATTTA
TAATGAACCAGAAAGTAAACAACGATATTCACCTAATTCTACTTACAAAATTTATTTAGCGTTAATGGCATTCG
ACCAAAATTTACTCTCATTAAATCATACTGAACAACAATGGGATAAACATCAATATCCATTTAAAGAATGGAAC
CAAGATCAAAATTTAAATTCTTCAATGAAATATTCAGTAAATTGGTATTACGAAAATTTAAACAAACATTTAAG
ACAAGATGAGGTTAAATCTTATTTAGATCTAATTGAATATGGTAATGAAGAAATATCAGGGAATGAAAATTATT
GGAATGAATCTTCATTAAAAATTTCTGCAATAGAACAGGTTAATTTGTTGAAAAATATGAAACAACATAACAT
GCATTTTGATAATAAGGCTATTGAAAAAGTTGAAAATAGTATGACTTTGAAACAAAAAGATACTTATAAATATG
TAGGTAAAACTGGAACAGGAATCGTGAATCACAAAGAAGCAAATGGATGGTTCGTAGGTTATGTTGAAACG
AAAGATAATACGTATTATTTTGCTACACATTTAAAAGGCGAAGACAATGCGAATGGCGAAAAAGCACAACAA
ATTTCTGAGCGTATTTTAAAAGAAATGGAATTAATATAATGGATAATAAAACGTATGAAATATCATCTGCAGAA
TGGGAAGTTATGAATATCATTTGGATGAAAAAATATGCAAGTGCGAATAATATAATAGAAGAAATACAAATGC
AAAAGGACTGGAGTCCAAAAACCATTCGTACACTTATAACGAGATTGTATAAAAAGGGATTTATAGATCGTAA
AAAAGACAATAAAATTTTTCAATATTACTCTCTTGTAGAAGAAAGTGATATAAAATATAAAACATCTAAAAACT
TTATCAATAAAGTATACAAAGGCGGTTTCAATTCACTTGTCTTAAACTTTGTAGAAAAAGAAGATCTATCACA
AGATGAAATAGAAGAATTGAGAAATATATTGAATAAAAAATAAAATTGTTGTGTTTACAACAATACATAGAAA
ACAGAGGAAACAATCAAGTCGTTGAATATTTCCTCTGTTTTTTAGTTGAAAAAATTAACCGAAAGCCTGAAT
GCAAGTCTTGATTAAATCAATAATGCTTGTAATAACACCAGTGAAATCCATATGCATACCCTCTTTCTATTTAAG
ATACATTAAGTATAATATCAAACAAATAAAAAATGTTAAAAATTCCCTAATTGGCTATTTAGATTGCATAAATGT
CAAAAATTTGAAAAACATACAACGACTTTGCATAAAAAATCGTCATATTGGAAATACGTAATTTATTGAAATAA
TAAAAAAAATAAAAGAACGAAGATGATAACCTAAGTGAGGTTTTAAGTTGTTCTAAGGTTTAATTTAATTTAT
GTTAAAATAGTTGGTATAAAAATACATGATAAACTATAAACTAAATTCAAAATAACTTATGGGGTAGGCAATTA

TGGAAAATAAAATATAAATGATAATGAAAAAAGAGTGCTAAGGGAAATTTATAACCATCATAATATTTCGCGTA
CTCAAATATCTAAAAATCTTGAGATTAATAAGGCAACGATTTCTAGTATTTTGAATAAGTTAAAGTATAAATCTC
TTGTTAATGAGGTTGGTGAGGGTGATAGCACGAAGAGTGGTGGTAGAAAACCTATTCTTCTGAAGGTTAATC
ATCTTTATGGTTATTTTATTTCTTTGGATTTAACTTATAGTTCTGTTGAAGTGATGTACAATTATTTTGATGGTAA
TGTCATTAAGCATGAATCTTATGATTTACCTGATGAAAAGGTTAGTAGTATATTAAGCATAATAAAAAAACATAT
TGATATTCAGGAGAAACTTGATACTTATAACGGACTATTAGGTGTGTCTGTTTCTATACATGGAGTTGTGGATA
ATGAGCAGCATGTGACATATTTACCATTCCATGAAACTGAAGGAATTTCAATTGCTAAGAAAATAAAAGAAAT
TACTAATGTTCCAGTCGTAGTTGAAAATGAAGCGAATCTTTCAGCGTTATATGAACGTAATTTTAATCATAATTT
ATCCTACAATAATCTTATTGCTTTAAGTATACATAAAGGTATTGGTGCTGGGCTTATTATTAATAATCAATTGTAT
CGTGGTGCAAATGGGGAAGCGGGTGAAATTGGAAAAACACTTGTCTCAAAAGTTAGCGATAATGTGGAGA
TCTTTCATAAGATTGAAGATATTTTTTCACAAGAAGCTTTACTGCATAATTTAAGTAATCAACTAAATGAGAAG
ATGACGCTTAGCAAATTAATTCAATTTTATAATGAAAAAAATCCAGTCGTAGTTGAAGAAATGGAACAATTTA
TAAATAAAATTGCTGTTTTAATACATAATTTAAACACCCAGTTTAATCCGAATGCAATTTACATTAACTGTCCAT
TGTTCAATGAAATGCCTGAAATATTAGAAGCAATTAAGAACCAGTTCAAACAATATTCACGTAACGAAATTCA
AATAAAGTTAACATCTAATGTCAAATTTGCAACTTTGCTAGGTGGTACATTAGCAATTATCCAAAAAGTACTAC
AGATTAATGATATTTACTTAGATATAAAAGCATAAAAAACTAATTCAAATGAATAATCAAAGTTCGTAATTGTCT
TTATAAAAAAATCCCTCAATCCGAATTGAATTTTCGGATTGAGGGATTTTTATAGTTCTATTGCAGAAGAAAAC
TATTTTAAAAATGCTGGTAAATGTTGATAGCCACCTCTAACGTTAACAATATTCGTAAATCCTTTATATTCTAATA
TTCCTACCGCTATTGAACTTCTAACACCTGATTGACAATGTACATAAATTAGGTCATTTTTATCGAAAGGTATAT
CTTCATTTAAAAGTTTACCGTGAGGAATATGAATTGCTTGTTTTAAATGACCTTTACGCCATTCATCATCATTAC
GAACATCTAATACATTATGTTCTTCACCAGTCATTTCAGAACTATGAATAGATGATGTGACGATATTTGTTTGTG
GCAAACGGTAACTTTTTACATTTTCAAAACCAATTAATTGTAAAGCATGAATAGCTTGTTGAACGGTAGATTT
ATCGCCAATTAATTCAATATCTTAGTCATAATCTAAATACCAACCAATTTGATTTATAAAAGTTTTATTAAAAGG
AATATTGATAGTTCCATGCATATGACCACCATGGAATGCTTCTTTACTTCGAAGATCAAAAGCAGTTTGTGTAT
TGCTTGAACTAGGGTAAACATTATATGGTTGGTACATTTGCATACCAAATTGATTTATTTTTTTCATTTGTGAAA
AATGGTGTGGTGGAGCTGGCTGATTGAGTGTTAAAGTTTCGATAAATGAAGTTTCATCTTTAACATTAAAAG
CCCAGTTGTTTATTTTCTCATAACCCAAAGTAGTTGTAGGTAATGAACCTAGCGCTTTACCACAAGGACTCCC
TGCACCATGACCTGGCCAAATTTGAATATAGTCTGGTAATGTTGCAGCAAATTGTATGGACTGATACATTTGTT
TTGCTCCGATTTTTGTAGAACCTTCAACATTTACAGCTTTTTCTAATAGATCTGGTCTACCTACATCACCAACAA
AGATGAAGTCACCGCTAAATAATCCCATTGGTATACTGGAACCCCCACCTTCGTCAGTAAGTAAAAAACTAAT
ACTCTCAGGGGGATGGCCTGGAGTGTGTAAGACTTCTAATTTAATCTTTCCTAAATAGATAATATCTTGATGCT
TAACGAAATGTGTTTGTTTAGGCATATTTTTATAATTAAATTCATCTTTACCTTCATCAGATACGTATATACTTGC
ATTCAATTTATTTGCCACATCTCTAATACCTGAAGCAAAATCAGCATGAATATGTGTTTCTGCAGCTTTAGTAAT
TGTGAATCCTTCTTTATCTGCAACTTTTAAATATTTTGTTAAATCTCGTATAGGGTCAATAATCATTGCTTCTCCT
GTACGTTGACATCCAATTAAATAAGATGCTTGTGAAAAATTGTCTTCATAAAATTGTTTGAAAAACAAAAAAA
ACTCCTTTTTAAAATAGATTTTATTGATTAGATAAATAAGTTATGATTTGCTTGCTCAGTATGTCCAATATAAGTA
CCTACGCCACCATAATCGACTTCATCTCTTAATTCTTCTTTTGAAATTCCCATAACATCCATACTCATGGTACAA
GCAATTAACTTTATATCTTGATCGATTGCTTGATCGATAAGTGAGTATAAAGAATCAACATTTTTCTTGTTCATT
ACATAACGCATCATAATATTACCTAGTCCAAACATATTCATTTTTGATAATGGCATATGTATTGGATCCTTAGGTA
ACATAAGGTCAAACATTTTGGAAATACCTTTCTTTTTAACGCGAGTTGATTGCGCTTTTTTTAATGCGTTGAG
GCCCCAAAAAGTAAAGAAAATAGTTACATCTTTACCTGCTGCTTTAGCGCCATTTGCGATGATCATTGCTGCT
ACTGCCTTATCTAACTCACCGCTAAATAAAACAATTGTTGTACCTGTAGCAGTGTCATTGATTTCAAATTCTTTT
GGCTTTTCTTTTTGAATAATTGCATTAATTACATTTGCTTCTTCAGTAAGATTTACAAGGGTATTCCCTGTTTGT
TTCGCCCAACTTTTAATATCACTATTGAAACCAGGATCTGTAACTGTTACCTCGATTTGCTCACCCGTTGAAAT

ATTGTTAATTTCTTTACTGATATTAACAATAGGTCCAGGGCACTGAAGACCTCTAAAATCAAATTGTTTACGAT
TCTCTTTGATTTCAATATCTTTTTCTATTAAAGGAGCACTATTGAAGTTCTTTGCTTCATAATCTTTATATCCACC
CTTTAAATTCACGACATCATAACCTTGTTTGGCTAAATAATCGCAAGCTTTAGTGCTTCGGTTACCGCTTTTAC
AATGTATATAATACGTTTTGTTGCTATTCTTATTGAATGATTTAATCTCTTCTACTGGGTGTAAAGTTGAACCGTT
AATGTGTCCTAATTCATATTCTTCTTTTGTTCTAACATCAATCAATTGACCCATTTTTGCCAATTTTTCTAATTCTT
CTTTGTTTAATGAATTAATGTGTACTTCTTTGTATTGTTCCATACTTACCTCCTATAAATACCTATGAGGGTATAA
TAAAACGGATAGAATCATTTGCCAAATACCTATATGGGTATTTGACAATTTTGTTTTAATTTATTATTATTAACTT
AATCAATTTATGTGGAGGAAATGAATATGACTTATGATAAAAAAATGATTAATCGTATAAATAGAATACAAGGT
CAATTAAATGGTGTCGTAAAAATGATGGAAGAAGAAAAAGATTGCAAAGATATAATTACGCAACTTAGTGCA
TCTAAAGGTTCTATACAACGTTTAATGGGGATTATAATTAGTGAAAATTTAATAGAATGCGTTAAAACAGCAG
AAGAAAATAATGAAAGTTCTCAAGAATTAATTAATGAAGCAGTTAATTTATTAGTTAAAAGTAAATAATGGATA
TAGCAAATATGACTATTATGTTGCTAATTGGCGTACTGGGTGGATTTATATCTGGATTAATAGGTATTGGGGGC
GCAATTATTATTTACCCAGCTATTCTTATATTGCCACCATTAATAGGTATACCTGCGTATAGTGCATATATTGCTTC
GGGACTTACCTCTAGTCAAGTATTTTTCAGTACACTTAGTGGATCATTAAATGCAAGAAAACAACCAGCTTTC
TCTCCTAAACTTGTTATATATATGGGAGGGGGTATGTTGATTGGAAGCATGTTAGGGGCAATTTTAGCTAGTT
TGTTTAATGCTACTTTTGTAAATACGGTATATGTAATAATCGCCATACTTGCTTTAATATTGATGTTTATTAAAGT
TAAACCTACTACACAAGAGACGAAATCTAAACCTTTGCTATTTATTATAGTTGGATTTGGAATTGGTGTAATTT
CGGGAATTGTGGGTGCAGGTGGAGCATTTATCATCATTCCTGTATTATTAGCATTATTTAAATTACCAATGAAT
ACGGTAGTGAACAATAGCATAGCAATTGCTTTTATATCTTCAGTAGGGGCATTTTTTATAAAATTAATGCAAGG
ATATATACCAGTAGAAAGTGCAATTTTTTTGATAATTGAATGATGATCATTTTCTGAAAATATTATGTGGTCATAT
AATATAATGCCCATCATTTCACTAATCTCTTTTATTCTCTGAGTTATTTTGATATCTCCTGGAGAAGGTGTTACAT
CTTATCAAGAGTAAATTACAAAAGAATCATTTAAATCAATACTTTCACTTTGAATACATGTATTTGAAGTGGAA
GGTACTTATTTCAAAAATAGTAAAACCTGTATCTTAAATTACTTAATAGTAACATAAGATACAGGCTGATTTTTT
ATTCATTGTTATTTATACTAAAGCACCCGATAGCTCTGAAAACAATCACAAATTCAACTTTTCAAAGCCACAGC
TTTAAGTTATTTTGTCCAGACAACCCCCATTGCCCAACCCATTTTATGGAATTGGCATCCAGGCAACAACTTTT
CATATAAATCGTAATAATTTTGTTCAGATAGGTACTTATCTGAAGCTAAATGCTCAAGCCATGATTTAGATGTGT
TGTGATTATAAATTCTAATCGCATTTTTTATTCCAAATTTGAAACAATGCGGTAAAAACTCTTGGATTGCCCCTA
ATTTATAAACATAGGTAGGCGGTGTCTCGACTTCAGATACATTATCAAGAATAACTATTCTTCCTTCCTCATTCA
ACAGTTCCTTCATTTGCTGTATTACGCTGGCTATATCATCCAAATGATGAAAGGTTGTTCGGCTTACAATAAAA
TCAAACTTCTCATTAAAATTAAGTTGTTCTGCATTCATATTCAGATAGACCGTATTTGTTAGTTGACGTTTAGAT
TTGGCAAGATCGAGCATTTGATTAGAAATATCAATCCCTACCACTTCATCATAATAACTTGCTAATTTCTCCACT
AACAAACCCGAGCCACATCCGATATCTAATGCTCTGCCTTTCTTTGGAGACATATTAGACACAAAGAATGAAT
AATCATTCAAAAGCTCATTCACGAAATCGTAATCTTCTGCAACCTTATCAAACTGTGATTCTATTGTATTCAAAA
AGATCCCCCATTCCTACTTTATCGACATTCTTTCATTACTTACCACTTTAGATGTTTTTTCGTTGGGGATAAAAC
TTCCCTTTAGACAATTTTATCCAAAGACAATACAACAGTGCAACTTTATTAAAGTCACTGTCCTTTATCGCAGC
CTTTACTTTTTAGTAAAGACAGTGGCTTCTCTTATCAAGTTTCAAAACATATTATTTTGAAGAAAACGTCCATC
TGAAGTGTCAAGTGCAAAATTACATATAAAGGTTTATTCTAAAATGAAAAGATGATACAATCATATTCAGTTAC
ATAAGGAGGTTTCAATTATGTGCACCAGTATCGCAGTAGTAGAAATTACTTTATCTCATTCATAATGAAAAAAA
TGGAAAGGAGATAAAAGTATGGGTACTTTTTCTATATTTGTTATTAATAAAGTTCGTTATCAACCAAATCAAAA
TTAATTGGTTATAATGAACGCTTAATGTCAGTTCATTATAACCAGTAAGGAGAAGGTTATAATGAACCAGTAA
GGAGAAGGTTATAATGAACCAGAAAAACCCTAAAGACACGCAAAATTTTATTACTTCTAAAAAGCATGTAAA
AGAAATATTGAATCACACGAATATCAGTAAACAAGACAACGTAATAGAAATCGGATCAGGAAAAGGACATTT
TACCAAAGAGCTAGTCAAAATGAGTCGATCAGTTACTGCTATAGAAATTGATGGAGGCTTATGTCAAGTGAC
TAAAGAAGCGGTAAACCCCTCTGAGAATATAAAAGTGATTCAAACGGATATTCTAAAATTTTCCTTCCCAAAA

CATATAAACTATAAGATATATGGTAATATTCCTTATAACATCAGTACGGATATTGTCAAAAGAATTACCTTTGAAA

GTCAGGCTAAATATAGCTATCTTATCGTTGAGAAGGGATTTGCGAAAAGATTGCAAAATCTGCAACGAGCTT

TGGGTTTACTATTAATGGTGGAGATGGATATAAAAATGCTCAAAAAAGTACCACCACTATATTTTCATCCTAAG

CCAAGTGTAGACTCTGTATTGATTGTTCTTGAACGACATCAACCATTGATTTCAAAGAAGGACTACAAAAAGT

ATCGATCTTTTGTTTATAAGTGGGTAAACCGTGAATATCGTGTTCTTTTCACTAAAAACCAATTCCGACAGGCT

TTGAAGCATGCAAATGTCACTAATATTAATAAACTATCGAAGGAACAATTTCTTTCTATTTTCAATAGTTACAAA

TTGTTTCACTAAATTAAAGTAATAAAGCGTTCTCTAATTTCACAAGAGGACGCTTTATTCTTCCCAAAAATTGT

TCAATATTTATCAATAAATCAGTAGTTTTAAAAGTAAGCACCTGTTATTGCAATAAAATTAGCCTAATTGAGAG

AAGTTTCTATAGAATTTTTCATATACTTAACGAGTGCTTTCACCTTTGAATATAGTCCTTCCCACTTATCATCACA

CTCTCCCCGATAGCCTTTTCTAGCTATATCCAGTAAAGTTACATGCTCTTTAGGTAAAAGAGGTATAGCCCATT

CTGCAGCGACATCTTTCGAGGTAATTTCACCAGTAGTCACTGTTTGCCACATTCGAGCTAGGGTTAAAATTAC

ATTACGCTCATCACCTTTTATCCCCTCAATTAGTTCTGGCAAAGAATCCTTAATTGCTCTTCGAATATCTGTCAA

AGGTACGGAGACAAGTATACTTGAAGAATCAGGACCAAATAGAGAAATACTATTCTTTCTTGCTTGTGCTAA

AACAATAGCCAAATCAGGATCATAGCTTGGTTCCTGAATTTGTCCATTCTCAAATTCACCCCTGAGCCACTCA

CCGTATATAAATTCTCTTTTTGGAGGATATTGCCAAGGGACAACTTCACTCCTATTTATAACCGTAACTTCAAG

TGGTCTAACAGAATCCGTATTTCCAATCTTTCCTGATATAGTCATTAGTCTTTCTGTTAGTTTTTTTCGAGTTAAT

TGAGGTAAACTATGATTCACGACGACTAGAACATCTACATCGCTGTTAATGCGTAAACCACCATTTACTGCTG

AACCAAATAGATATACTCCAACTATTGAACTTCCAAATAAATCTTTTACGATTTTTAATGTTTGAATCGCTTGAT

TTGGTATTTTTCCGTTAATCAAATTGCTCATGATTTCACCTCGTTGATTATGTTCATATAAAGTTTATATTGATAC

TCAATTTACTTACCCTAGATTGGACATATACTTAAATTACTGTTCAATAAAGCTGACCGTTAGCGTTTAAGTACA

TCCTTTCACAATTTGTCTACAGATTAATAATTATTCTTTATTATACAGATCTCCATATAATTTTTGAATTTGGTTCT

GTAATTTTTTATTTTCTTTTTCTAATTCCATTACTCTTCTTTTTAAGGTTTTAATAAGGATTTCCTCCGAACGAGA

ACTTTTCTTGGGTTTTGAGACTACATTTGCTGTTATTTGACGCTCACGAAGGGATTCGATTCTTTGCCTAATAT

CGTGTTCCTTATAAAGCCATGATTTAGAAACATTAGCTTCCTTTGCTATTGAATTAAAATTAATAACTTTACCTT

CAATCGAAAATTTAGAAATCGCTTTGTCTACTTTTTCCCTTGTCTTTTTTGATTTCTGCTTCGCCAAACGTACA

ATTTCTGTTGTATTTCTAACTTGTTTATCCATTGATAATTACCCCGTCAAACTTCCAATGATTTGTTCTAAACGCT

CTTTAACACGGCTATTAGTCTCTACTTGTCTTTGCCATTGTTTATCCTTAGCTATGGCTAATAACTCTTCTGTACG

CTCTAACTGTTCTTCGTGCTGTGGTAAGAATTGCTTACTGGTACAGAAGTGAGTGCAATCTAAGCATGCATTC

GCATGTGGACAACCACCTGCTACTACTGGCAATCTACAATAACCATTTGGAAGCACTTGTGCATTTATATTTTT

CTTGAACCATTGAAGCTCTACATCATCGACTTCATTATCTTCATCTAGATCAAGCACATCTCCATTATTGGTAAC

CAGTTTTTCCTGAAATTTAGTAAATTCATTTTTTAGAGTTTCATCAAAGATATGAGCGTATCTGCTTGTCATTTC

TGGGCTTTCATGCCCCAAAAATTTCTGCACAATATGCTGGGGCATCCCGTTGTTAATCATTCTTGTTCCTACTG

TATGGCGAAAGGCATGGGCATGGAATCTATAAATCTCACCTGATTTATCCACTATATTTTGCTCATAAGCTAATT

TATTTAACTCACCTCTAAATGTTTCTTGTTTTAATGGCGATCCATCTTTTCTTGGAAAGAGGTATTCACTATCTG

GAAATTCCTCTGAAACTTTATCTTCCCGAACTTTAATAAGTAAAGCTACCTCTTTAGATATTGGAACTATATGCT

CCTTTTTCATTTTCCATTGATAATACTTTAAAAAGAAATCTCCATCTTTGTCCTCTAATAGACAGCCTTTTTTCA

AGGTGCACAATTCACTTATCCTCATTCCACATTCTTGAACAATCATAGTCATCGTAGCTATATATTCGGGTAATT

TATCAAGATGACTGTTCAATTGCTCTAGGACGAATTCATCTATAAAGCGTGGTTTTGCTCTTGGTATTTTCGGA

TAGTCCTCAGAATAAATTAATATTTTGGAAGGAACATCATCCCATTCTAGCCTAAGAAGGGTACTAAATAGTCC

TTCCAATATAGAGATCCTCCCAGTTATTGTACTAGGTTTTATTCCCATCATGTTTAGTTCACTTAAATATGCTTCA

ATTTCCACTCTCGTTAATTGGTGTACTCTCTGAACTTGTTTAAATTTCATGTCCAGAAAATTAAAGAACTCTTTA

AGTCTTTGGGCAATATCACTTACATAGGAAAAGCTATCCACGTTCAATCTCAACTTACAATATCTTTTTACAAG

TTGTTTAAAATATGTATTCCGAAACCCTTTAAAGTTAATTGTATATTCATATTGTGTTGGGTTAACCTTATCATCT

GGCAAAGGTAAGTTACGTCTATCCCAAACGTCTTTATCCCACTCCTCTCCATCAAAATAAAAGTTCTCATAAAA

CTCCATAAATTGTTTTAGATTAGTAACATAGTAGGAATTAGCTTTTACAGGTGTTTTTTCTTGATTAGCAGTAAT
CTTATAATTAGTAGTGGTAATTCTAACACCCCGTTTTGTCAAATAAGTTCTATACTCCGTCATTGCTTTTTCAATA
GGAACTTCAGTAATTGAAGTAATGCTAGGATACTTTAAATCTAAGAAATCTAACATTTTATTAATTACTGTTCCT
TTTCTAATCCAGACAGTTTTTGCATTCCATATTCCATTGTTTAAATGGTAAAAATAAAAATATTTCAATTCTGTTC
TTAACCACAGATTTTTAACACGTTCAAAACGAACCCAACGATTCCTTAAAGCAGGATTCTTACTTAATTCTATG
GCAGAAGGATGTGGACATTTTCTTATATCCCAACTATTATTAGCCCAAAACCCCTGCATTTCTTCATTCATTACA
GCTATTTTTTTGCTAATCTCACTCTGACTAATAATTTTCCTTTTACTAGAAGCATTCATTTCTTATGCTCCTTTCT
CTCGAGGTATTTATTAAACTCATTTTTCATATCCTGATCTGAAAGATGAACATAGGTATTTAACGTTGTCTGAAC
ATGTGCGTGACCTAATCTCTTTTGAACGAACGCAACATCCCATCCTTCCCTAATTAGCTGCGTTGCGTGAGTG
TGGCGAAGCATATGTGATGTAAATTCTATTCCAGTCCTTTTAACTATTCTTCTAACTAGATCAAGAACACTTTG
GTACTTTAGTGGTTTCCCAAAATAGCCTTCTTTTAAGGAAATAAAAACATAATCATGCTCCAATTCCTCACTATA
CTCATATATCAAGTAATCTGTATAAAGTGACATAAGTTCTTTACTCACATGTATTGTTCTTTCCTTCCTTAATTTA
ATATAAGCTTCATTAACATTAACATCTCTAGGTGTTAAATGGATTTGATTGTCCCAAGTGACAATATCTTCAAGC
CTAAGCGATAACACTTCACCGATTCTTAAACCACCCTCATACATAAGCATTAAAATTAATTTATCTCTTTTCGTAT
GACAAGCATCAATAATTTGCTTAACTTCCTTTGATCTCAATGTTCTTATCTGTTTCTTTTTAACCCTTAACTTTAA
GACATTCTTTTGGTATCTACCCTTATTAACATGATGTAAAAATCCTTTGAAATTTCTTCCCTTTGCTTGTTTAAAT
ACATCAATTGATTTAAATTCTCCTAATCTACTTAAATAATCAAGAAAACTCATAACTACATTTAAAATTGTATTCA
CTGTCGTTTCTTCTCTTATGGCTTTTTTTGACTGAAGATCAATTACATTTGATGCTGAAGGATATCTCAACCAA
CCTACGAAGTCTGCTAACAACTCAAAGTTAATATCATTAAGAATAACACCTCTCTGTTCCATGAACTCGTACAG
CAACTTTAAATGATAGCAGTATGCCTTAATGGTATTAGGAGACTTACCAGTATTATCTAAGTATTTAATAAATTT
CATTACTGGTTCTATTAGCTGGTATTCTTTATCTAGTAATAAATACAATGGATACGGCTTATTCTCCACTTCTATC
CTTTGAACCTTCACATGTTCCCACCTCTTTAAATACTTTAACTACTACAAATTATAAACCTTGTTAATTTTTATTT
AAAGTAAAATATCCCTTTAATATCTCCTCTTAAACTACTTTAACTACTATTATTTATTATACTATGGTTAATACATCT
CCACTTGGATGATTATGACCGAGCATTATACTATTTGCGTTACTGAGTATCGCTGTTTTGAATATTTCTCTAGGG
TGAATCACCGTTTGGTTAATAGATCCAATCGATAGTGTTTGAATATGTGTAGGTTCATTTTTACTGTTCATACAT
ATGAGAATGAGATGCTCTCGGTCACTGTTTCCAATGAATGAACGCATGATTTCTGCCGCATCCTCAGGGTTTG
AAATACGATTTTTTAGATAACTTAATGTATCTGTTTTTATCATTTGTAGTGAAACAATATTGATTTCCTTCATCGT
TTACCTCCATATATAGGTTATGCTTTCAAAGTCCATTTTTGACGTGCTTTAGGGTTGAGTGGGTGCATGATTTC
ATTTGTGGCTGGATTAATGAGCTTTTTGACTTTCTTTTTATGAGGCTTCAACATTTCCATTACTTGTTCGACAC
GTTCTACAACAACTGGCCGCTTCGCATAAGCACCATAAGCTAGAATCACTGTGTCACTTTCGCTAATTGCTTT
CATCAAGTGGATGTCTGTGTGTTTATCATAAGGTTCTTTAATATGTTTAAGGTTCTCTGGTGTTTTAATATTAGA
GAATAGATTTACAAGATATACAGCACCGTATCGTTCTGAATTCGCTAATTGATTGAGGATAAGAACAGTTGTG
AGATCGAGTGATAATACACCATCTAAATGAGGGTACATCGTTATCACTGTACAAGCGGGTTTCTTTTCATCCCA
TGTTTTCTTGAGTAAATAGCGGTGTTTTTCATCCTTGCTAAATATAGCTTCTGTGTATATCGTATTTTTGATTGTA
TTCATATATATAATCACTTCCTTTAGTATTCTTCTGGTAAAAGCATCACATAATAAAAAGCGTCTACGCCATCTTC
ACGAATGACGTAGACTTTCTTAGGTAATGCATTTTGATTTTTTACATAGTTTGTATAGTGATATTCCAATTTGTA
TGCAGGTTGTTCTTGTTCATGTGTGATTGAGAGTATATTCTCATCTTCTTGCAGTTTAAAAATGTGTAGGTAAT
CTGTATGAGGTTGATTATCTCTTTCTTTTACCATATTCCAAAGTAAGATTTGAAGGTCTAGAGATAGGTATTCA
CTAATACCTCTTGTGATGTATCGATTGATTTCATGTTATTTTACCTCGTCTTGAATTTCTTTCATAATGATAATCG
CTTGGCTAATAATCGTAACAGATATTTGTGCCACTTTGATCCAGTTATTCATGGTGAGTCCCTCCTTCTCTTAGT
AAATGACGTTCATCGATAATCGTATTTTTAGTATCTGTGAGATATAAAAAGTCCATGTCAAAATGATTCAGATA
ACCAACGCTGATGAGTTGGTTATTAGCGTACATAAGAAATGGATAGATACTTAGGTCATGTAGTTCATTATTGT
AGTAGGTATAAGTTTCAAGTGTAAGATGTGCAAGTGGGGAATCATTAATAAAACGTTCCGGTAGAATATTTTC
TGCTGCTTCTTCTAACGCTTCGCATTCCCATGTTTCATTGTTAGATAGTTGGAATAGACGAGTTATATATTGTTT

GAGTTCTTGAGTGGTCGTTTTCATATCATTGCCTCCTAGATAGTGTTATAGTGATGTAGTTTATGTACATCATTG
GGATAATATATATTTGATTTGTCATTTATTACGCATCCCGGTGAGAATGAGAGAAAATTCCATATGAAAAACCG
CTTCAAACCTTGGTATGACAAGGAAATCCCGAAATTCCGCCTATTTTGACGAACAATCAACTCATTCTTTATAA
CTATTGATGTTAGGGTGGGGCTCTGCTTTCTTATATATTATTTATTTATAAAGAATAACGGGATTTTGGGATTGT
GCTTGCACAATCCTTCTGCTTCTTCGAATCTGCAAATCCCAATCATTTCCCGATAAAAAATCATTGTGGGATGT
TCTTTAGCAATTTCAATATAAGCATCGTGTAGTTATGAAAAAAATTACGACAATGACTGTTTCATTAGATAAGT
GTTATTGAAATTGATAAAGAGAATTTTAAAAATGGTTAGATAAAATAAATGAAAGAATATAATGAAAATTATTG
TTATAACAATGATTCTATTAGCTAAATAGTAAGATATAGTGTTTGGGGCAAAAACAAAGACGAAGTGCTGAGA
TGCACTTCGTCGAGTTGTTTATTATTGAAAAGTTGTTTTATGATTTCGTTATTAAGTTTGAGTGTGACATAGAA
TTGTTTTTTATGATTACCATCTTTTTTAATATCAATGCGATCAATCACTGATAGATACAATGCTTTGAGTCGAGA
TTTTTCTATGTGCTTAATATCATGAAAGATGTGTTGTAATAGTTTACTGATTTCTTTGGCATCAAATAAAGGCTT
ATCTTCATTTTGTTGATTTTTGAGTTGGTTGATTTGATTCGTAATGTCATTGAGTTGCTTTTCATATTTTTGAAT
ACTTGGTCTGATTACTGATGTTAAGTCCGGATTATCCTCGATGGTTTTAATCAAGTTATTTAGTTTGATTTGTAC
TTCATCATATTGTTGTTGCTTATAAGCAATATCGTGATGAAGTGCAGCGCCATCAACTTGATTTTCTTGATTGAC
GTGTGTTACTACGCGTTGAATGACTTTATCACTTTTGACTATTTCAAGTATTTGCTTCATCACATAATCTTCAAT
CACATCAGCTCTTACACTGTTTGCCGAACATACTTTGGAACCCTTGTTCCGAAAATTACTACATGAATAGTAAC
GAATACGTTTCTTAGTCCCGTCTTTAAGTGTATTCGTGGTATTGCTTGCTGCCATAGGTGCGCCACATTGGGG
ACAGTGAATAATGCCTGTAAGCAGATTCGTTCCTTTGCCATGGACTTGGGGTTTTTGACTGACTTGTTTTTTA
CGCATTTGTACTTTATCCCATAAATCTTGATTAATAATGGGGGAATGCTTACCTTCAGCTATCACTGGTTTATCA
TTAAGCCCTTTACGACGTTTTTCACTCCAATCTTTGTATTTCGCAAATTGAATTTTGCCGATATAGAATGGGTT
AGCTAATATATATGTGATAGAACTAATACTAAAAGGTTTACCCTTTTTAGTGACATAGCCTTTGTGATTTAATGC
ATTGGCTATTTTACGATAGCCATGGCCTTTGGCATAGGACTCGAATATATATTTCACAATATTAGCTTCATGTTG
ATTAATCATCAGTTCATGTTTACTATTAGGTATTTTGTCATAGCCCAGCGGCAAATTGCCTTGATAATAGCCTTC
TTGGGCACGTCTCGTTTGACCCATAAATACATTCTCGACAATGTTATTACGTTCGAATTCTGAGAAACTCGCA
AGTATTTGTAACATGAGCTTACCCGATGAAGTATTGACTTCCATACGCTCTGATAAACTGAAAAATTCGACATT
TTGTTTGTGTAAATCTTCGACAATTTTGAGAAGATCAGATGTATTACGAGCTAATCGGTTTGTTTTGTAGACCA
TAACACAGTCGATATAGCCTTCTTTCGCATCCTTCAATATACGTTGGAGCTCAGGTCGATTCATAGATTTACCT
GAAATACCACGGTCAGCGTATATATCTTTAACTTCAAAATGATGGAAGTCACAGTATTCTTTGATTTGATTGAT
TTGTCCGTCGATACTATAACCTTCTGTGCTTTGCATTTCTGTTGATACACGTACATAGATACCGACACGTTTTGT
TTTAAGTTGTTGCATTATGTTTCATCCTTTCTTCGTTTATGCAATCGATGATTGCATGGTTTGATTGACGATATT
GAGTGGTTCATTTTTGAAATAGATTCCTATAAGATTTTTATCTTTCGTAATGTGAATGGTTTCAATATAGGGGTA
CAATATGTTTAACGTGAAACGTTTTTGAATAATATTTTGAATGGTGTGTTGTATTTGATGTCCATTGATAGATGT
AGTGCGTTGCGGTTGTTGACGTAATGATTGCGTTTGTTCTCTGAACGTTTCTGCATCGATGATGCCTTGTGCC
AACTTTTCTATCAGTTGTTCTTGAGTCAATGTGTGATGTTTTTCTATGTTTCTTTGTCTTTTGATGCGTTTGTCA
ATCGCACCTTTAATTTTTGTGTAGATGCGTTGATTTTGATAAAAGTCTTTGCACACTTCTAATACTTTATCTTCA
AGTGTTTGTGCATTGATGCCTTTAAAATCACAGACAAAGCGAGAAGCATTCATGTTTTTAGGACAGACGTAG
TAACGTAATGTATGATTCTTTTTTCTAACGGTCATATTCGTAAGTGTTGTATTACAACATGGGCATTTGATTTTT
TGTTTTAGTTGGTTATCCGAAGATGTCTGTTTGATTTGTTTTTGCGATCGAAGTCTCTGCGCTTGCTCATATATA
CTTGTGGAAACAATAGAAGGAAACATATTGTCGAATTGGCCATATTGATTGTTGACACGACCACAATAATTAG
GATTGATGATAATGTTACGAACTTGATAGGGTTGTCGATTGATATACGTGTTATCTTCTTCTAATAACTGTGCAA
TTTTCTTATAACCATGACCTTTAATGTAATAATTGAATACAGCCTTTACCGTTGGTGACTCATTTTGATTGATGA
TGAATGCTCCGTTGTGATATTCGTAACCAAAGGGCGCATGGGTTGTAATCAATCGACCTTGCTTTGCTTTTTC
TTGAAGCCCATTTCTGACTTGTTCTCCAATGTTATCTGATTCAAGTTCGGCCAAGCTGATAAAAAATATTAAGCT
TGAATCGGTCGAAAGCTTGATCCATATCAAAGTAACCATCGTGTACGCTTAAGATATGAACATGGTATGTTTG

ACATAATTTGATGAGTTTTAATGCATTTTTCAGATTACGATGTAATCTATTAAGACGATAACAACATAATATGTC
ACATTGCCCTTGTTGAATTAATTGCGTCATTTGTCGATACCCACTACGATTATCTTTGCGTCCTGATTGTTTGTC
GCTATAAAAGTTGATATGTTGAATATGATGTTTTTCGGCTATTGCTTCGATAGCCTGTTTCTGTGCTGCAAGAG
ATTGTTGTTTCATCGTACTTTGACGTAAATAGCCTATGACTTGTTTCATATCGGCTCCTCCTTTCACAGTAATAA
TATATATTTATGGATGAATTGATATATAAGCCCAACATCAATAAGATGTTGGGCGTTCATATTAGTCATTCATTTG
ATTGATTTCTTCAATTACCAAATCGGCTAATATCTCGATAAGTTCATCCATGTTTTTCACTCCGTTATTTGTTCTA
TCTTCAATACGTCGATTATTCAGTTTGATGCTTCACGGTTGTATGATAAAGACAATCAGAAATCTTCGTGAACT
CCTGAAGGGCCTATCCCCTCATTAGCGGATTTAAAAAGTTCTTTCGCAGCTTTGTTATCATTTGCCGGTGTCC
AATTTTGAATTAACGACTTATCTTTAGTTAATCCCAGGATAGATGCAAACTCTACATCTAATTTTAGATGGTAAA
ATACAAGTGATTGTTTTTTACCGCTATTATCTTTGACACTTCTTTTAGTTGTTTGGCGTCCACGGTCAGCTAATA
TGAAACCTTTATCTCTTAAGGCGTTGACAACATTATTAACATCTTGAAATTGATGATTGTTTAGCATCTGTTTAA
AAACGTTTGCAATCATTTTTACTTCGATATGGTCATCTTTTAATGAGATTAATCCATAGTTCTCAAACATATTTTT
CAAAGCACCTTCATCTGAAAACTTACCTCTGTTTTGTGCTACAAATTGAATGATGACATCGATAGCTTTATCAG
CTAATGAGCGTTCAGAGATTGTATGACCATGATAATCAATAAAGTAGTCTCTTATTTTAGCGATATCAATATCTG
TAGCTAAAACACGACCTAATATTTTTGCAGATGTTGTAATGACTGCATAACGCTTAAACATACGATTGCCTGTG
TTGCTTTTATCATCTTTCAATTTAGCTTCAAACCAATCTACTTCCTTGTAAAACCATTGAATAACTTCATCTTCAC
GATTTATAAGATATTTAGCTACTAACGGTAAAACATGACCATAGTTTAGTGCTACAGCTTTTTTAATATTGTCAG
CATTGGTCGCATTTGTAGTGAATTGTTCATTAATCTCGATGGTTCTTACACGTAATCCATCGTTTTGAGCTGAA
TCATTAAAAATACTGTGTTCTGACGTTGAAATGACAGAAGTACCCCAATTCTTAGGCGTTTTAACTTCTCCATG
AACGTTTGAACGTTGACGACCTTGACCTTCAGTGATGGAGTACAATAACCCCGTTGTATCTCTAAAAGTTGCT
GATGAGAGTTCATCAAATACAATAGGTATACCAAAATTGTTACTCAAGTAACCTTCAAGTGCATTACGTGTGG
CATTCCAATTTCTAAAGAGAGTTTCATTACCTTTGGTAGGGTTACCAGCGACTGATACAGCTAAAGAAGCTGC
AGTTGACTTACCGGTTGAGGATTGACCTGTAAAACTAAAAATGATTCCGGCAAATTCGGTTTCATGTTTGTGC
TTCAGGAAACTCGTCACTAAGGCAGAAATACCAAATACGACTGCTAATTCTAAAAGAAGAGAACCTTTAACC
TCTTTTAGATACATGTTAAACCAATTATCAAATGTTCCCCTAGGTGCTAAGTCATAAGTATTCTCACAAATGGCG
TCAGATGGAGATTTATTATCAAATTCCGTAGTAGTATAGATTTCATTTAACGATACAATAGGACCAAACGGTGT
TTCCAGTATACCTACCCCTTCATATAAGTAGGAAATGGGTAATTGGTTGCGCATTTGTTGCAACGCATAACCTA
AATCTTTTGTATATTTTTCATTAATACTAAATCCATATTTCATTAAAGAGGGCAGTTTTTGTGTTGTTAAAATATC
ACTAGATTCAACAATTACTTTTTGATCCTCGTCTGTAATAATTACTTTTTCAGTGTTAGTTTTAGGGTCAATAAA
CTTATTTTCGATAACGATAGGACCTGCGATTTCAACTTCAGTAGGCATTCCTCCTTTTTCTTTGGGAGGCTTGT
CTTTATACCAACCTTTTTTTGATTTGTATCGTGGTGAAGGATTAAATGAAGGGTTAGTTTGAGTCATTAGCGA
ACACCTCCTTTCGAAGGGTTGCTGTTATAGTGTGGATTAGGACCTGTTTTAAGATAAACTAAGTGACCGTGA
GTATCCTTACCGATAATAATAAATGGAACACGTGGCGCATGTTTTACAAAATATGCGAACCAACGTCCAACATT
TTGTTGTACAGCTTTTGAACATTGTACATTTGCACGACTGTTCAAGTCATGAAATGTAAATTCTTCTCCTTCAG
GTAAATTGAATGAAATACCTAATATTTTCTTTTTTAACTGCTCGAATTTATCTGATTGATTGAAATACATTTAACT
TCCTCCTACTAATGAACTAAGATAGGAAAATTAAATATGCGCACAATTAACTTTCTTAAGTTCATTTTAGCCAA
GAAGTTATCATTTGAAAGCTTGTAAAAATATGTTAGAAATTCCGTACTAAATTAAGATGGATTTTTGTTGACAA
AATAAAAAAACGCTGATTTAACAGCGCTTTAAATAAAAATTAATCTGAAGTTATATAAAAGTAGTCAGAAGGAG
TGGTGTTATTATCATTATAATAATCTAGTGCTTTATTGATTTCTTTTATTAATTTATCATGATGTTCTTTTGTTATTT
TCTTTGGATATTTGAGTTCTTTTTCTTCTAAGTCTTTTGGAGACACTAGGTCACTATTTAAAATTAATGATAACA
GCATATTTTTAGGTGCCGAAGCTCTGGTGTCTCGATGAGTAATATATTTTGCTAAATTACTGTTTATACTTACTT
TTTCATTTTCTAAAAAGTTTGTACTATTTTTAACATGAGCATCAGCAATTTTGGTACTTATTTCAGTGTTTAAAT
CGTCTATTTCATCTAAATAATATTCAAGAATCTGAAGTTTATCTTTAGAAGTGATACCTTTTTCAGTGAAAATCT
TAATAATGATAGATTTGATTCCAATTTCTATATTACTGTCTACTAGAAAATAGAGATAATAATTAGTCAGAGCAT

GTTCATAATCTCTTTGATCAGTAATTTCATGATTGATGAAATAATATAAGTTCTCTTTGATACGATTAGAAGAAT
CTATATCTTTGTCATTGATACCGTTTTTAAAAAAGTGTAGATAAACTGAGTTGTTTGAGAGTATATCATATAATA
CAAATAAACTTTTTGGTTTAAATACATATGATTTTCCTGATTTAAAGTTTGCTATGTTTATTCCTAGAGCGTTGC
AGAATTTATTAAAATTAGTTGTAAAATTATTTTTTTCTTGAATAAACATGTCGTTGTCATTTAATTCTGAACTATG
ATTTTTAATCATATTGATATTTTCTTCAATCTTGTCACTACTTAAATTTTCCATGCAAAGTATACTATATCTTACTAT
ATCACTTATATTTATGGAAAAATCTTTAGGAGAACGATTTAAAGTATCCTTTTTATTATCTTGATCTACAGCCCT
ACATGAATCATCAAAAACTTCTTTGACATTTATTTTATCAATGTTCAAAATTATGTCTACTAAAGCATTTTGAAA
ATCATCGCTATATATGTCTTTATTATTCATAGTGTCACTACCTTTATTTTTAATAACACTATATATTATATCTTATAAA
CCTAATACCATGAAGAATAAAAAGAATGATTCAAATTTTATATTTAATCCATAGTTGGCTGCTATGGTTAGTGTA
CTAGTCTAGTTTTACTAGTATATAAAACAGTACTCAATTTTTATTTTTTCAAAATTATAAAAAAGAACACCTATC
ATCGATAGGCACTGAACCCCTAAAACGGGAACTTAATAAAAACACCATGTTCTAGGCTATTAATTCTCTGTATT
TTACAGGGGATAAGTAGCCTAGTTTTTGTTGAATTTGATTATTATTATAGTTTTTAATGTACTTTTCGACAATAT
CTATTACAATATGATTAGAGCTATTAAGCTGATTATTGATGTAAAGAGTTTCAGACTTTAGCGAGGAATGGAA
ACTTTCTATCGGGGCGTTATCGGCAGGTGTTCCCTTTCGGGACATACTTCTGATAATGCCTTTTTCTTCGCATA
ATTGATAATAAGCATAAGATGTATAAACGCTGCCTTGATCACTATGTAATATATACCCCTCAGGTATATCGATTTG
ATTTAATGTATCATTAACTAAACGTTGGTCTTGTTTATCATCTATTTTATACGCCACAATTTCTCCGTTATAAATAT
CCATTATCGAAGATAAATACAACATAGAATGATCAAATGGTAAATAAGTAATATCGGTTGTTAATACTTCTATGG
GACAATTCGCTTTAAATTATCTTTGTAATAAATTGTCTGTTTTATAATACGGTTTACCTATCCTTGTCGTCTTTTT
AGGTCTAACTCGGCAGTTCAAATGATGCTTCTGCATCATTCTCTGTACTCTCTTATGATTAATTGGTGATGTATA
ACATTGATTAATCAGTGCTGTAATCTTACGATAACCGTAGGTATAATGGTTAGCTTCACATAATTCAATAACTTT
TTGTGTTACAGTATCATTTTTATAGGTTTTGTTTTTCCATCGGTAATATGTTGATTTAGGTATATTTAATACTTCTA
GTATCAATTTGATTGAATAGTTTCCTTTTAATTGATCCACTAAATCTATGACTACTGTTGGTACCACTTCCTTTCC
AATGCCTTGTACTTTTTTAAAATATCCAATTCTATATCTTTTCTCTTATTTTCTAATTTTAATTGTTCTACTTCTGA
CAGCTCTTCTAATCCTTTACCGTAGGTATATTGTTTACCAACGTGTTGTGAAAATCTATAACTTTCCCCATTTCG
ATACCATCGCCACCAAGTTTCCACTTGTGTTCTATTTTTAATATTTAATTCTTTCATAATTTCTTTTGTTGAAAAT
CCTGCTGCTTTCATTTCAACTGCTTTATACTTTGTTTCTACTGAATAAGAAACTCTTTTCATAGAAAAAACACC
TCCGTATGATTCATTTTAATATGAATTCAACGAAAGTGTTTTTATATAATTCCCACTAATTGGGGTCAGTCTACT
ATATGATACGGTTTTTTAATTTAAAGTATCAATAATAATTGGATATAGAGGGAAAGAAGCTATAATGATATTTGC
TTACTAAGTGGATAAAGATATTAGAAAAACGAGAAGCAGGACAGTTATTAAATCGGTAGATATTAATCATAAG
TATTTAACTGAATTTTTACCTTTTGTCGAATATATAACTAAAGTTGAAGGTAGTAAAATATTTATCTAGTCGATAT
TAAAACAATTTATCGAAAACGATGGTTTTCCATATCCTCTTTAGTAGCAATTTTCTAACAATATATAATTAATTAT
ATACAAATTAAGAATAATTAAGAAGTATATATTAATTAGAGATAGTTTAAAAATTAGATTTCAATCAATTAATTA
AAACAGGTTAGACTATTAATTTATACTAACCTGTTAAAAGTAACAAAATTAAGAATATATTAAAACTTGTGTAAT
GAGTAAGCCTCCACCAAATATTAAAACAAACGCGACAACAGGCCATACGAAACGTAACCAGTGTGAGTAGC
GCACGTTTAACATTTGAAGTGTTGCCATTACAAGTCCAGTAGGCGCTAAGAACAACATTGCATACTGACCGA
ATTGATATGTTGTAACAATAACAAATCTTGGTATACCTACTGTATCAGCTAATGGCGCAAAGATAGGCATAGAT
AATACTGCTAATCCTGATGATGATGGTACGATAAATCCTAAACAGAAAAAGATAAAGAGCAGAACAATGATAA
ATAAAGGCCCACTCATATGTTGCACGATAGATGATGAAAAGTGCAAGATTGTGTCAGAAATCATTCCTTTATT
CAATACTAAGTTGATTCCACGAGCTAAACCAATGATTAAAGATACACCTACTAAACTTGAAGCGCCATTAACG
AATGCATCTACAGTGCCTTTTTCGCCTAAACCATATTGTCCTGTTCCAGCAATAAACATGATGACAATGGTAAA
GATTAAGAATGCAGATGCCATGACTGGGAACCACCATCCTTGTGTCATAACACCCCAAACCATAATAGGGAAT
GGTAGGACGAAAAGCGTAAGAATAATCTTTTTACGTAATGTAAACTCAGAAGAACCGTCATCATGGAGCACA
GACCACTGTTTTTCAAATGCTGCTTTGTCTTCATAAGAATAAGAGGATTTAGGATCTTTTTTAATTTTTTTACA
ATACCAGAATAAATAACTAATAACAAATATGGCACCGATGATACAAGCGCCTATTCTCCAATAAAGACCATCAG

TAAAAGTTGTTCCTGCTGCATTAGAAGCAATGACGACTGAGAATGGGTTGATTGTTGAGAATGTACTACCCA
CAGAGCTTGCTAAGAAAATTGCACCGAAAGTAAGACTTTAGAATCTATGGGAAAGATAATAAGGTTTAATAG
AAATACAAGAAGTGTAAATAGTAAGCATATAATTTATACATTTTAATATAATATAACAAGAAAGGTAAACACAA
AGATAATAAAAATAAAAATTTAATAAAGATAGAGAATTATTAAATTGTTTTTGATATAAAACAACTTAATAAAA
AATTTAATTTTAGGGGAGGATAATTATAATATAAAGAAACATAGAATGGAATTGCAATTGGGAAGTATACGAT
TTTAACTGCGAATCATATGTTCAACGACTTACAAATGCGTAAATCGATAACACATTCAAAAATATATAATCTGAA
AATTGATTTACTAAGAGAGGATTATTGTAGTTACAGGTGTTAAGTTGCTCCAATATGGAGTTGTCGTCTTGTTT
TATATTAAACAAGTTTTATTCAAATACATGACAATACGTAAAATAACACTAGAATTATCAATCACTAAAATTAAA
ACAAATGTATCTATTCAAACTTTTCTTTAGGGTTTACTTGCTGAATAATTTAAAAATGAAGTATTAGGTGTACAT
GCTTTCAGAATTGCTTTTGAAGAATATTAAAAGTATAATCTGAATACGATGACAGAATGAATAGGTAAACTATG
ACAAGAAGTCATCAAGGTTATCAAATAAATATATCCCAGCGGTTGAGACGACTCACTGCTGGGATTTTATTGT
TAGTTATTTATTCTAAGTGTTGCATTTGGTAGCCGATTCTAGGATGTGTGATGATTAATTTCTTTTCAGTGTTTG
GGTCTTGCGTATAATTCAACTTTTTACGTAAAGAGGCCATATGTACACGTAAGGCAGCCATTTCTGTATGATTG
ACATAGCCATAGAGTGATTTTAATAACACTTGATAGGTTAATACTTTGCCGACATGATGGCATAATATCGTTAG
GAGTTGGAATTCATTCGGTGTTAGATGTACGGACTGTTCATTGACAAGGACTGATTTCGCATCGAAATCAATG
GTTAATGGACCATTTGTGAAACGACTTTGAATTGTCTCAGTAGAACGTGACATACGTAATGCTACTCTGATAC
GTGCTCTTAACTCATCGATATTGAAAGGCTTAGTCATATAATCATTGGCACCGCGATCTAATACTTCGACAATG
GTTTGTTCCTCTGTTCGTGCACTAATCACAATAATAGGTGTGTCCACAAAGTCTCTGAATTGCTGAATGAGAG
ATAAACCATCAATATCTGGTAAGCCTAAATCTAATAAGATAATATCTGGTTGTTCTGTTCTTAGGCGAAAGTCC
GCTTCTTTCCCCGTCTTCGCCGTAACCACTTTATAATAATTCATAGTTAGCGCAACATCGATTAAATGTAAAATC
GCTTCATCATCTTCAACGACCAATAATGTTGTTTTCATCCATGGCTCCCTCCATTTCAATTGGATTAACAGTCAA
ATAAAAATAAAAAATACTGCCTTGTGGTGTATTCGGTTGATATTCTAATTCACTGTTGTGTTGTTTCAAGATGA
GTTGTACTAAATAGAGCCCTAATCCCAAACTATCTTTTTTATTGTCTTTAAAGTTGTCTCCTGAATAATAGGGAT
TAAAAATCAATTGACGTTCTTCTTCTGGAATACCTTTTCCGCAATCTATCATTTCGAATTTTATTTTGTTTGTTTC
ATGTTGAACGTGCAGTTTTATTTCAGAATGTGATTCTGCATGCTTTAAAGCATTATCGATAAGGTTGAATAGCA
CTTGCAGTATTAATTTACTGTCGATATTAATGAGTGAAGCGTCATCCTCATTTTCAATAATGACATGATTTGCTT
GGTGTCTGCGTATGAGGCCTTCTTCAAATTCTTCTAGAAGTTCTTCTACTAAATAAGGGGTGCGTTGTATTTG
AATGTCAGAGCTTTCTAACTTAGTCAAAGATAAAATATTTGTGACTAAGGTATGCAGATATTGTGCTTCGCCAT
AAGAAGCAGTTAAGAGTTCTGCTTGTTGTTGATCGTTTAAATGCTCGTTATGGTATTTCAGCATATCTAAGTTG
CCCATAATGGAAGTCAGTGGTGTTCTAATATCATGTGAAATTGAATGCAAGAAGTTTGAACGTGTGGCTTCTC
GTTCAGCTTTCAATATGGATTGTCTGGTTTGTTTTAATAGATCCACATTCTCTATTGCCAGGGTAATATCGTTCA
ACATAGAGTCTAATATTGAATTGTCATAGGTGTCGATGTAAGTTTCGTCGGTGAAGCGGATAGAAATCACACC
TTTGACTGGATTTGTGCCAATGGGAATACACAAAAATTTACTGCCAGGAAAGGTATCGGTTAATTTACCGGC
ACGGCTTTCATTTTCAATGACCCAGCTCAATGTCTCAGCATCATGTGTCTTATCTGAGCTTGAAATGCTTCTGT
TTCCAAATGAGTTGGAAGCAGCGACTTTCTTACTTTGAATTAAGAATACCGTGACATCCTGATTGAGTAGCTG
ATGAATCTGATCACCAGCAATATTTAATAAGCGTTCAATTGAATAAGATTCTTTAATGGACTGGTTAAATTGCA
GCATAATATTGGTCCGATATAACTGCCGTTCTGTTAAGGAATGCTGATGTTTTAAATTCTTTAAGATGGCACTC
GTAAAGATACTAGCAAAAATACTGGTCGCAAACGTAATCGGATATTCAAAGCGATACATTTCTAAAGTGAATC
TTGGCACCGTAAAGAAATAATTAAAAACAAACACATTTAAGATAGACGCAAAGAATCCGATTAAATAGGATT
GGGTCCAAATAGAAAGCACGATAATACCGATAAAGAACATCAGCAAGATAATGGCACTGGATTCGCTTTTAT
CTAAGTTGTAAACCCATATACCGAGCAAGACACAGATCGTTTGAATTACAAGCATCTTCATAATCTCTACAGTT
AAACGAGAGGATTTTTGAGCTTGCTTAGGTGTATTTGTAGGTTTGTCTGAATGAATATAATGAATCGGTACAA
TTTCTAACTTGAAATGGTGTGGTACGTGATTAATTTGTTCAATGAGTGACTGTTTAAAGTAATCTTTCCAACGT
GGCTGTTCTGACTGTCCAAGGACTAGCTTCGTCACAAAAGCGAGATCACACCAATCGGTTAACGCTTTCGCA

ATATCTTGTGCATACAACACTTTGATTTCTGCACCTAACGCTTTGGCGAGCATTAGATTTTTATGGACATAATG
ATCTTGTTTTCTGCTTTCTGACGGTGTCTCAAAGACATCAATATACACAGCTGTGAATTTAGCATGTTCTTTATA
GGCAGCACGTCTTGCTTCTCGTATGACCCGTTCGTTATAAATACTGCCGCTGATAGCTACCGCAATATGCGGT
GTAATGTCGGTATGTTTAGTTTTATATTGTTGTCCTTTTTGACTCATAATATCTGCGACAGTTCTGAGTGTAAGT
TCACGCAGCTCTGTCAGATTTTCATACGTAAAGAAATTAGAAAAGGCTGTTTCTAAGCGTTCTTTTTTATATAC
TTTTCCTGCTTTAAGGCGCTGAATCAACATATTTGGTGAAATATCCACAACTTCAAAGGCATCTGCTGACGTA
ATGAATTGGTCGGGCACACGTTCTGTAACTTGAATACCTGTCATTAACGCAATTTGTCCGCTTAGACTCTCGA
TATGTTGGATGTTGAGTGTTGTCCAGACATCGATACCATGCGATAGAATTTCTTCTATATCCTTATAACGTTTTA
AATGGCGCTCTTTTGAAATGTTTGTATGTGCTAGTTCATCAATTAAGACCACATCTGGATTAGCTTCTATGATTT
TAGAGACATCTATATAGTGAAAGGTGTGGCTGCCAAATTTACGGCTGGAGGTCGAAATTTCAGGCAATTGTT
GAACCAGTGCATTGGTTTCAGGGCGTTGATGGGGTTCGATATAACCAATTTTAATATCTGCACCTTCTTGATA
CTGATCAATACCATTTGATAACATTTCATACGTTTTACCTACCCCTGGGCTATAGCCTAAATAAATGGTAAGTTT
CCCTCTTTTTTTATATGTACTTTCCATGAGGCACCCCCTCTTAATCACATCATAATTAATATACAACATATTATCCA
TTTATCTTTCTGTTTTGTTAATCATCTTTATAGTTTCTTTATAACTTTCCTTAAATGTTAATACTTCGATTAGATAA
CTCTTGTTAAGATTTTGATGAAAGTAAGGAGGGAGCGCAATGATTACACTATTAGCTGTCGTTGTCATCGCAT
TAATTTTATTTTTATTTTACGCATTAATTTGGAGTGAAAAATTTTAACAGAGAAAGAGGGAAGCATCATGAGT
ATTGTGTTGTTTTTGATTGTATTTATCTTGCTCTCACTCATTGTGAGCCGATATTTATATTCAGTTGCTTTAAATG
TGCCATCTAAAATAGATGTTGTTTTTAATCCGATTGAGAAATTGATTTATCAACTGATTGGCACGAAATTAGAA
CACATGTCTGGGAAGACGTATATCAAACATTTTTTGTTGTTTAACGGATTGATGGGCGGATTGTCCTTTGTATT
ATTGCTTATTCAACAATGGCTGTTTTTGAATCCTAACCATAATTTAAATCAATCTGTATCGTTAGCCTTTAATACT
ATGGCATCTTTTTTGACCAATACTAACTTACAGCATTATGCAGGTGAAACAGATTTAAGTTATTTAACACAAAT
GTGTGTCATCACTTTCTTAATGTTCACGTCAGCAGCGTCAGGTTACGCCGTATGTATTGCGATGTTAAGACGT
TTGACTGGAATGACAGATGTGATTGGTAATTTCTATCAAGATATTACGCGTTTTATTGTACGGGTGCTCATACC
TTTCGCATTGATCATCAGTTTGTTTTTAATCAGTCAGGGCACACCGCAAACGCTTAAAGGTAATTTGGTGATT
GAGACATTATCAGGTGTGAAACAAACGATTGCATATGGACCGATGGCGTCTTTAGAATCTATTAAACATTTAG
GGACAAATGGTGGTGGTTTCTTAGGTGCGAACTCTTCTACACCTTTTGAAAATCCGACATACTGGTCTAATTA
CGCTGAAGCTTTAAGTATGATGTTGATTCCAGGTTCATTAGTCTTTCTATTCGGTAGAATGTTGAAAACTAAAC
TACAGATTCATCCGCATGCGATTATGATTTTCGTTGCGATGTTTGTAATGTTCATCGGCTTTTTAGTGACATGTC
TCTATTTTGAATTTGCGGGGAATCCAGTGTTGCATCACTTAGGTATTGCCGGTGGCAATATGGAAGGCAAAG
AAACACGTTTCGGTATTGGCTTATCCGCTTTATTTACAACCATTACGACCGCTTTTACTACAGGAACAGTTAAC
AATATGCACGATAGTCTTACACCGCTAGGCGGCATGGTTCCAATGGTATTAATGATGTTGAATGCAGTTTTTG
GCGGTGAAGGTGTTGGGCTGATGAACATGTTGATTTATGTCGTGTTAACGGTCTTTATCTGTAGTTTGATGAT
TGGGAAAACACCAAGTTATTTAGGAATGAAGATTGAAGGTAAAGAGATGAAACTCATTGCGCTTTCTTTCTT
AGTACATCCTTTACTTATTTTGGTTTTTTCAGCACTAGCTTTTATTGTGCCAGGGGCATCAGATGCGTTAACTA
ATCCGCAATTCCACGGTGTATCACAAGTGTTGTATGAGTTTACATCATCTTCAGCGAATAATGGCTCTGGTTTT
GAAGGATTAGGAGACAATACGGTATTTTGGAACATTTCAACAGGCATTGTGATGTTGCTTGCACGATATATTC
CAATCGTTTTACAAATTTTGATTGTATCTAGTTTGGTAAATAAAAAGACCTATCAGCAACATACTCAAGATGTA
CCGATTAATAATTTATTTTTCAGCAGTGTATTGATTATCTTTATTATTTTGTTGAGCGGCTTAACGTTCTTACCTG
ACTTAATGCTTGGACCAATAGGCGAACAGCTTTTGCTGCACGCATAGATAAAGGAGGATTAGAAAATGGCTG
AAACTACTAAAATATTTGAATCACATTTGGTCAAACAGGCTCTAAAAGACAGTGTATTGAAGCTCTATCCTGTT
TATATGATTAAAAATCCGATTATGTTTGTTGTAGAAGTGGGCATGCTGCTTGCCTTAGGATTAACCATTTATCC
GGATTTATTTCACCAAGAAAGTGTATCACGGCTATATGTGTTCAGTATCTTTATCATATTATTACTGACACTTGT
CTTTGCGAACTTCTCTGAAGCATTAGCTGAAGGTCGCGGTAAAGCACAAGCCAACGCTTTACGCCAAACAC
AAACTGAAATGAAGGCACGTCGTATTAAACAAGACGGCAGTTATGAAATGATTGACGCTAGTGACCTGAAA

AAAGGACATATCGTACGTGTCGCGACAGGTGAACAAATCCCAAATGACGGTAAAGTTATTAAGGGCCTCGC
AACAGTGGATGAATCTGCGATTACAGGTGAATCTGCACCTGTAATCAAAGAAAGCGGTGGAGATTTCGATAA
TGTAATTGGAGGAACTTCTGTAGCTTCAGACTGGTTAGAAGTTGAGATTACTTCAGAACCAGGTCATTCATTT
TTAGATAAAATGATTGGTTTGGTTGAAGGGGCTACAAGAAAGAAAACACCTAATGAAATTGCGTTATTTACT
TTATTGATGACATTAACGATTATCTTCTTGGTCGTTATTTTAACGATGTATCCATTGGCGAAATTCCTGAATTTC
AATTTATCCATTGCGATGCTGATTGCTTTGGCTGTGTGTTTAATTCCAACAACCATTGGGGGATTATTATCGGC
TATAGGGATTGCAGGGATGGATCGTGTGACACAGTTTAATATCTTGGCTAAAAGCGGACGTTCTGTAGAGAC
TTGTGGTGATGTGAATGTCTTGATTTTAGATAAAACAGGTACCATTACCTACGGCAACCGTATGGCAGATGCG
TTTATTCCGGTGAAATCATCAAGCTTTGAACGTTTAGTTAAAGCGGCCTATGAAAGTTCTATCGCAGATGACA
CACCAGAGGGACGTAGTATTGTGAAATTAGCTTATAAACAACATATCGACTTACCGCAAGAGGTCGGAGAAT
ATATTCCGTTTACTGCTGAAACACGTATGAGCGGTGTGAAATTTACGACACGTGAAGTATATAAAGGTGCACC
GAATAGTATGGTTAAGCGTGTGAAAGAAGCAGGGGGACATATTCCAGTTGATTTAGACGCTCTTGTCAAAG
GGGTGTCTAAAAAAGGTGGCACACCGCTGGTTGTGCTTGAAGATAATGAGATTTTAGGTGTTATTTATTTGA
AAGATGTCATTAAAGATGGACTCGTAGAACGTTTCCGTGAATTACGTGAGATGGGGATTGAAACGGTGATGT
GTACAGGAGATAACGAATTGACAGCTGCGACAATAGCGAAAGAAGCGGGTGTGGATCGCTTTGTGGCAGA
GTGTAAACCTGAAGATAAAATCAATGTGATTAGAGAAGAACAAGCGAAAGGTCATATTGTTGCGATGACGG
GTGACGGTACGAATGACGCGCCAGCTTTAGCAGAAGCTAATGTAGGTTTGGCAATGAACTCAGGAACCATG
AGTGCCAAAGAAGCGGCGAATTTAATTGATTTAGATTCTAATCCAACCAAACTGATGGAAGTCGTTCTAATTG
GGAAACAATTATTAATGACACGTGGCTCACTCACTACATTTAGTATTGCGAATGACATTGCGAAATACTTTGC
GATTTTACCAGCCATGTTTATGGCGGCTATGCCTGCGATGAATCATTTGAATATTATGCATCTGCATTCACCTGA
ATCAGCAGTATTATCTGCGTTAATCTTTAATGCGTTGATTATTGTATTATTGATTCCGATTGCGATGAAAGGCGT
GAAATTTAAAGGTGCCTCAACGCAAACCATATTGATGAAAAATATGTTAGTTTACGGCTTAGGCGGTATGATC
GTGCCATTTATCGGCATTAAGCTCATTGATCTCATCATCCAACTCTTTGTCTAAAAGGAGGACAAAACAATGC
AGACAATAAGAAAAAGTTTAGACTAGTACTGATTATGTTTGTTTTATGCGGATTTATCTTCCCGCTGACTGTCA
CAGCGCTTGGACAAGTATTATTTCCAGAACAAGCAAACGGCAGTTTAGTGAAACAAGATGGCAAAGTAATT
GGTTCAAAGCTCATTGGACAACAATGGACAGAACCTAAATATTTCCATGGACGTATCAGTGCAGTCAATTACA
ATATGAATGCGAATGAAGTGAAAGAAAGTGGCGGACCTGCTTCAGGCGGCTCAAACTACGGCAATTCAAAT
CCTGAATTGAAAAAAAGAGTTCAAGAGACTATTAAACAAGAAGGAAAAAAAATTTCAAGTGATGCGGTGAC
CGCTTCTGGCTCTGGTTTAGACCCAGATATTACGGTTTGACAAATGCGAAACAACAAGTAAAACGCATTGCG
AAAGAAAGAAACATAGATGCTTCAAAAATTAATCACCTTATTGATGAAAACAAACAAGCATCACCAATGGCA
GATGATTATGTTAATGTCTTAAAATTGAATATCACTTTAGATAAACTCTAAATAAACAGGGAGTGAGGTGAGA
CATCCATGTGGTTCATTAGCATTATTATTTTAATAGCATTCCTAATTATATTAATGATTGACGATTTAATTAAAACA
TGACAACTTCACGCAAAACAATATGAATTGGATGTAATAGAGAAGGGAGTACCAAGAAATACTCAGTTTATT
GAGGATAATTATTAGGGGAGATTAGAGAAGAATTCTCTATTCACGAAATTACTTTAACAGTTGTAATCATTGG
TTTCATCATATCGATTAATAGTACAGTCATCTTGAGATTTTCAACACACTATTATAGCTAGCTAAGATACTTTTAA
ATTATGACTTATTTTTCATCATTTACGATACATCATTCTCGATAACTTACTATTTGTTATGGTATTTATAGATTTAA
AGAAAGTGAAGGAGATTAAGCAATGTAACTTGGCTTAATCTCTTTTTGATTATATTTATTAAGGCAAAGCTTC
ATTTCCCTTAAATCAAGTATATCTTCTGAATAAGCAAATGAAATCGTGACGCCAACTACAGTGATAACTTCGCC
TAAAACACAATGAGTGTGGTGTTTTCACTTTTCATAAACTCCTCTAAGTTTGTGTTTTTAAACTGTGATGGATT
TGAATGTTTTTGAATTACGTTGTCGCGTCCTTGGGATTGCAATCCAAGAACGTCAGCATAATCTAAATCAATTT
TGATTTGGTAGAAGACAAGAGAAGTACTTTTGCCTGTATCATCTTTCGCATTTCTTTTTGTTGTCTTTCGATCC
CGATCGGAAACTAATTTTCCTGCATTATCCAAAGCATCAATAACATTTTTCGTGTCTTCGAATTGACTGTCTGT
TAACATCTTATCGAATACATTTCGGATAATTTTTACTTCGATGTAGTCATCTTTAAGTGAGAGGATACCAAAGTT
TTGAATCATATTAGCAAGACGTGTTTGTTGTGAAAACTTGCCCAGGTTTTCCGCAATAAACTGTACGATTGTA

TCCATCGCTTTATCTGCAAGAGAACGTTCTGACACCGTTTCATTATGATAGCTTAATAAATAGTTCTTTATGCTG
CTAACGTTTAACTGTAGGTCTAGTGATTGATTTAAAATATGGGCTGTTGTTGTTAGAGTTGCATAATGTTTAAT
CATTCGAATACCAGTGTTACTGGATTCAGTTGATAGTAATTGATAAAAAACACTGTCTTCTTTTTGAAAAAGG
GAAGTAACATCATTAGTGTTGTCTAATAGGAATTGAGCAATTATAGGTAAAACATGACCATTATTTTGACTAAC
CACTTTTTTTATAGTATCTGAGTTTTGAGCACTTGATGTGAATTTTTCAGACACCTCAATAGTACGAACACGCA
AACCATCATTTTTAGCTGGATTTTTGAAAATACTATGTTCAGAAGTTGAAATCACTGATGTATCCTAGTTTTTA
GGTGTTTTGACATCACCATTTATATTTGCTCTTTGACGTCCTTGTCCCTCTGCAAATGAATATAGAAGACCTGT
TGTATCATGAAATGTTGCCGCTGATAATTCATCTAATACTATGGGTACACCATAATTTCCGCTAAGATATCCTTCT
AGTGCATTTCTTGTTGCATTCCAACTCCGGAATAGGTTTTCAGTACCTTTTGTTGGGTTGCCAGCTACCGATG
CGGCTAACATAGCGGCTGTCGATTTTCCTGTTGATGATTGGCCAGTGAAAGAGAAAATAGTTCCTGAAAACT
CGACGTTATTATGATACTTAAGGAAAGCAGTGACTAATGAAGATACGCCAAACAACACATCTAGTTCTAAAGA
TAAGTTCCCATGGACTTCTTCTTTATACATTTGTAACCATTCTGATAAATTGCCTTTTGGTATTAAATCGTACTTG
TTATCACATATAATAGAAGTGCTATTAGTAATCTCATTAGAAAAATAAGGCTGGTCTAATGAAATTAATGGTCCA
AGTAATGTATGAAGCATTCCTACACCTGAGTATATTGTTGATAAAGGTGTGGTGCTTCTCATTTGTTGCAGTAC
GAACCCTAAATCTCTAATGTGCTTTTCATTAATGGTAAATCCATATTCAATTAAGGAGGGTAATTGCAAAGCAG
TAAGGATTGAAGCCTTTTCGACAAGCTCTATATTATTCAAATCCGAAAGAAATACTTTTTCTTTACGAGTCGCG
GGGTCTATAAAACGATCTGTGAAAGCAATCGGTCCAGATAACATTATTTCAAACTCCGTACCCCCATCTTTTTT
AGGAGGGATGGTTTTGTACCATCCCGAAGGTTTGAGCTTGAATTCGTCAGTTTGATATAGAATTTTTTCCATT
AACAGTGACCCCCTTGGTTGTAAGAGGAAATTATAATGATATGTCGGTACTTAAGTGGGTTAGTTCCTATAATA
ATGAAAGGGATGCTTGGTTTGTACTTTATCCACCAAGCAAAGCGTCTTCCTGCTTCACGTTGATCGTTGATGT
TGCACTGTTCTCTGAATAACTGTAATTATAAGGTTTAGTAATGGCAATTTTTTATTTAAACAAATTTTTACGAAT
GGATTGTCTGTTTATTAATTCAGCAACGTTTATTGAAGAAAAATAAGTGTCTTCTGATAATTGCAAAGGGTTT
AATTCAATGTAAACTGAATTAAGGGTAGGGTAGGTATGCCTGCTTTTTGGTTCTTTTAAGTTCAATCGATTAAT
AAAATTGTTGAACCTTTGTTCTAAGTCATACAAAAACATCAGTAATTCATTAGGAGAATGATTTATTATGCAAT
CCATATTTTCGTTTATGCTTGAAGTTATACATCTAAAAACAGTATTGGTTTCTTCGTATAAAAATAACTTATTAAT
AGAGGTAACATTGATTAAGACTGAGTATACGTATAATAAGAGTGACTCTGACATCTCATTTAGATAAATAAGAC
TGTGATCATTCTTATTTACGATTATTATATGTGATTGAATCAATTCAATAATGTCTAGAAGAAGACTTGAAAAGT
CGCTGTTAATCTTGGTCTTTGAAAAATCATCGTGACCATTTGTCTTATTACTTAGATGTAGTTGTTCGTATTTTA
TAGTTTGGCGGTTGAGGAATTAAGCATTTGATTATTATAAACTATCTCAAAAATTAAATTACCTCCTTTTTTCTT
CCTCGATGAAGGTTTATCTAATCTTCCAATATACAGGTTTTTGAAGTAAATTATAGATATGATAATCATTAATTCA
GTAAATGTATACTTATTAAGGGGTGGAGCTTTAGCTAACTTATCGATAGTACTGCCGATGCGTGCTTTGAAAC
TATTCAAATTGTCGTGACTGTCTATATCTAAATCTTCAAATTTACTAGCAAGGGTTTGCAAAGTAGAAATTACA
ACTTCTTCTTTTGTATAATATTTATTGAAAATATCATCTTCTTTATCATCGCTTTGGAGTTTTAAAGTGCCTGGTT
CTAAGATGTCAAATTTTTTGTTTCGATTGGTATTTTTTTAAAATGTGCCTTGTATTTTTGGGGTTGTTCCAAATG
TGTATTTGCTAGACTACCTGTAATTGATATTGCTTTATTTAGCATTTGGTGTGTATTAACTGTATCTGATTCTTCT
GGAAATTTAAATCTAAAAGTAAAATCTCTACTACCGAATGTACTTTTTACGGCTACAGTTTTGTACTTATGCTC
AGGCAATGTAATTAAACCATCTTGATGCAGGAGGAAAAGTGTGTCATCATTTAATTTCTCAATTGTATTAAATA
AGTAATTATGCATAGCCTCTTTATACATGGCGAATTGATAATTTAGATTATCCCATTCTATTAGTTGATTTAAAAT
ACAATATAGACCTTCATTCAATCGGTTTACGAATATCAATACAGCTTCTTCTCGTCCAACAAGGCTTATTAACTC
CTTCAAGTCATCCTTTAATTTTAATTTTTCTCCTTCAAAATTGTATTGAGTGAAGCCTATAGCCTTTTGAAAAGT
AACTTCCATGACAGAGTTGGTTTCAGAATATTCTAACGATTCATTTATTGCAAAAATCAAATTTTTTATAAATAT
GGAATTACTCATGGTTTGTAATTGCATATACTACCCAACTTTCTTATTTACTTTAAAACAAATATAAGCTAAAGT
AGTTATTATCATAAAGGGAACGTACGTTCTTATTTAGCTGATTTCTATGAAGAGAGGTTATGGATATTGAATGA
AGAAATCTTACGCGAAGTAGCAGATATTTTTATAGGTGATGACAGAGATAGCATTTATGATTATAAAACTGGG

AACGAATTAGTGAGGTTTTTAATCATTACTTTAATAAAGGGGACATATATCAGGCTCCGTTTCCATCTAGATG
GCTATATGTTGTGAAACATTTGCAAACTCTGATTCAGGAGAGAAAGATCAATCAATTTTTCACATTAATTTTAA
GCAATCACTATATTAAGTATGAACTGAAAATTGACGAGGTTGAAGCAGCAAAGCAGGCTGCTAAAGCACTTA
AGTTGTTCAACAAAAGATTAAATCATTATGGGTACTACATAACAGGAACTAACAATGCTAGATATTTTATGGAT
AAGGATGAAGATACGGAGTCTATTGGTTATGGAGGGTATGCGAATATTTATTTACAGAAGTCTACAGGTCTTG
CTGTAAAAAAATTGAAAGAGGAGTATCTTACTGATTCTTCAATTAAAAGTAGGTTTAAGAGAGAATTTGATCT
CACTAAATCTTTTGATACAAATCCATTGTTCATTAATGTGTTTGAATTTAATGAATCAGATTATTCATACACGATG
GAGTTAGCTGATGAAACTTTGAAAGATTACATTGAAAGCAAGACAATTAGTGAGCTAGAAAAAGTAAAGAT
AATAATGAAAATTTTAAAAGCGATGAGTCAAGCACATAGTGAAAATAAAATACACAGAGATATCAGTTCTAAA
AATGTATTAATGTTTAGAGGAAAAGTCAAAATATCAGACTTAGGATTAGGTAAAAACCTTGATGAAATTCATT
CGCATCAAACCTTTGATACAAACGGTGTAGGACAATATAAATACTGTGCACCGGAACAGATGTATAGTTTAAA
ACAAGCAGATAAACAATCTGATGTTTTTAGTTTAGGAAGATTGATAAATTTTATTATGACTGGAAATGTAGTTA
ACAACCATCACCTATTTAGAGGTGTATCTGATAAGGCTACGAACAGTAGTAAAGAATACAGATTTGAAGATGC
AAATGAAATGTTGAAAATGCTGCAGAGAATTTTAGAGTATCACAGTAGTGCAAAGCACGTCGAAAAATGTCA
AGAAAAGCTGAAAAGAGGAGTGTTTGATGATGAAAGCGAAGAATTTATTATGACACGAAGTGATGAACAAT
TATGTCAAATGGTTCTAAGTTCTAATAATAATGAGCAAGCGTGTTTAATTCGTTATATGCAAAAAAACGAATCT
TCAGCATGTGATTTAATAGAGAGTATTAATAGAAAGTATCAAGAGTTTTGTGGAAGGTTTGAAGACTACGATC
CTTTTGCTAAATTAGCATATATGATTTTATGTAATAACTTCAGTTATAGAGTGAATGAAACAGCAGCTAGAGTG
CTAAATTATGTTGCTTGGTCTGTAAATAGATTTTCGGCACAAGACTTAATTAAAGGTTTAATTAATAGAGGAGT
GGAGCCTTTGATTGAAGAAAAATTAAAGGACAATTAAAAAAACCTCATCATTAACTGATACGCAGAGGCGTA
TCATAAGT
>Staphylococcus aureus CA-347
ATGAAAATCACCATTTTAGCTGTAGGGAAACTAAAAGAGAAATATTGGAAGCAAGCCATAGCAGAATATGAA
AAACGTTTAGGCCCATACACCAAGATAGACATCATAGAAGTTCCAGACGAAAAAGCACCAGAAAATATGAG
CGACAAAGAAATTGAGCAAGTAAAAGAAAAAGAAGGCCAACGAATACTAGCCAAAATCAAACCACAATCC
ACAGTCATTACATTAGAAATACAAGGAAAGATGCTATCTTCCGAAGGATTGGCCCAAGAATTGAACCAACGC
ATGACCCAAGGGCAAAGCGACTTTGTATTCGTCATTGGCGGATCAAACGGCCTGCACAAGGACGTCTTACA
ACGCAGTAACTATGCACTATCATTTAGCAAAATGACATTCCCACATCAAATGATGCGGGTTGTGTTAATTGAAC
AAGTGTATAGAGCATTTAAGATTATGCGTGGAGAAGCATATCATAAATGATGCGGTTTTTTCAGCCGCTTCAT
AAAGGGATTTTGAATGTATCAGAACATATGAGGTTTATGTGAATTGCTGTTATGTTTTTAAGAAGCTTATCATA
AGTAATGAGGTTCATGATTTTTGACATAGTTAGCCTCCGCAGTCTTTCATTTCAAGTAAATAATAGCGAAATAT
TCTTTATACTGAATACTTATAGTGAAGCAAAGTTCTAGCTTTGAGAAAATTCTTTCTGCAACTAAATATAGTAA
ATTACGGTAAAATATAAATAAGTACATATTGAAGAAAATGAGACATAATATATTTTATAATAGGAGGGAATTTC
AAATGATAGACAACTTTATGCAGGTCCTTAAATTAATTAAAGAGAAACGTACCAATAATGTAGTTAAAAAATC
TGATTGGGATAAAGGTGATCTATATAAAACTTTAGTCCATGATAAGTTACCCAAGCAGTTAAAAGTGCATATAA
AAGAAGATAAATATTCAGTTGTAGGGAAGGTTGCTACTGGGAACTATAGTAAAGTTCCTTGGATTTCAATATA
TGATGAGAATATAACAAAAGAAACAAAGGATGGATATTATTTGGTATATCTTTTTCATCCGGAAGGAGAAGG
CATATACTTATCTTTGAATCAAGGATGGTCAAAGATAAGTGATATGTTTCCGCGGGATAAAAATGCTGCAAAA
CAAAGAGCATTAACTTTATCTTCCGAACTCAATAAATATATTACATCAAATGAATTTAATACTGGAAGATTTTAT
TACGCAGAAAATAAAGATTCATCTTATGATTTAAAAAATGATTATCCATCAGGATATTCTCATGGATCAATAAGA
TTCAAATATTATGATTTGAATGAAGGATTCACAGAAGAAGATATGCTAGAGGATTTAAAGAAATTTTTAGAAC
TATTTAATGAATTAGCTTCAAAAGTTACAAAAACATCCTATGATAGCTTGGTCAATAGCATAGACGAAATACAG
GAAGACAGCGAAATTGAAGAAATTAGAACAGCACAAAAAGATAAGACACTCAAGGAAGTGGAAGCACCTA
AAGGAATAATTCCAAAATATAAAAAAGGTGTATCAAAGACTACTAAAAATGATTCAGAAATTGAAAAATCAA

ATAAAGAGAATAAATTAACCGGTAAAGTTGGAGAAAAATTAGCGCTAAATTACTTTAATGAGCTAATTGATAA
TAAAATAGACGAAGATAAGAAAGAACAGTTTAGGAATATTTTAAATGATAATCCAGGCTCTCAACACGGTCAT
GGCTATGATTTAGTAGCTTTTGATCCAACAAATACAGATAAAGCTGTAGAAAAATTTATTGAAATTAAAACATC
TACATCTTCTAGTATTGAGGAACCATTTTTTATGTCGCTAAATGAAATGTTTGCTATGAAAGAATATAAGCAGA
AATATTTAATATTAAGAATATTTAATGTTTCCGGTAAAGAACCACAATTTTATTTTATAGATCCATATGCAAATTA
TTCTGAATTTAAAGATGTAGATGATCTCATTGACAAAGTATTTAATGTAGAAGCTATTCAGTATAAAGTTTTTG
GCGAAAAAATGATTACTTGAACAAGAGCTAAAATAAAATTGTGATCTAATAAAAATAGAAACTGTAATTTAAAT
AAAACTTTCTAAATAAGCTAACTGATAAAAAATCAGTTTGTCCACAGTCTGAAACAAGATTCCTATATTCTTTA
GGAATCTTGTTTTTTCTATTTTTATGGTGATAAAGAGCAGATAAGATAATGTGTAATAATCACAAAAAAGTTAA
ATATTTTAAGGCTTGTTTAATTATTAATGATTTTATATATAAAGAGCAGTATAATAAAGTTGTTAATATATTATGAA
TAATATTCAAGTAATTTTATTGTTTTTTAATTTGTCGATATTTAAGTTGAGTTAAATTTAAAGGGTGTAATTTGTT
TTACAATGATGAAGATAATTAGTCTATCAAAATAAAGGGGTTGGGACTGTTATGAGTGATAATTTGTCATTATT
CATTGACTATATCAATGATAATATAATCTATGGTAGTGAAATCAAACGGGAGAAATTAGAGAATTTATTTAATCA
ATTTGCTATAAAAAAATGTTGAAAAGAACATTGTCTATGATGAACTGAAATCTTTAGATATTACAATCATTGAGT
CACAGGATTCATATAAAAATAAATTGAAGAGATTATTTTCGGTTCTGTTGCAAAGTAAAAAAATATAGCTAACC
ACTAATTTATCATGTCAGTGTTCGCTTAACTTGCTAGCATGATGCTAATTTCGTGGCATGGCGAAAATCCGTAG
ATCTGAAGAGACCTGCGGTTCTTTTTATATAGAGCGTAAATACATTCAATACCTTTTAAAGTATTCTTTGCTGTA
TTGATACTTTGATACCTTGTCTTTCTTACTTTAATATGACGGTGATCTTGCTCAATGAGGTTATTCAGATATTTC
GATGTACAATGACAGTCAGGTTTAAGTTTAAAAGCTTTAATTACTTTAGCCATTGCTACCTTCGTTGAAGGTG
CCTGATCTGTAATTACCTTTTGAGGTTTACCAAATTGTTTAATGAGACGTTTGATAAACGCATATGCTGAATGA
TTATCTCGTTGCTTACGCAACCAAATATCTAATGTATGTCCCTCTGCATCAATGGCACGATATAAATAGCTCCAT
TTTCCTTTTATTTTGATGTACGTCTCATCAATACGCCATTTGTAATAAGCTTTTTTATGCTTTTTCTTCCAAATTT
GATACAAAATTGGGGCATATTCTTGAACCCAACGGTAGACCGTTGAATGATGAACGTTTACACCACGTTCCCT
TAATATTTCAGATATATCACGATAACTCAATGTATATCTTAGATAGTAGCCAACGGCTACAGTGATAACATCCTT
GTTAAATTGTTTATATCTGAAATAGTTCATACAGAAGACTCCTTTTTGTTAAAATTATACTATAAATTCAACTTTG
CAACAGAACCATCTAATCTTCAACAAACTGGCCCGTTTGTTGAACTACTCTTTAATAAAATAATTTTTCCGTTC
CCAATTCCACATTGCAATAATAGAAAATCCATCTTCATCGGCTTTTTCGTCATCATCTGTATGAATCAAATCGCC
TTCTTCTGTGTCATCAAGGTTTAATTTTTTATGTATTTCTTTTAACAAACCACCATAGGAGATTAACCTTTTACG
GTGTAAACCTTCCTCCAAATCAGACAAACGTTACAAATTCTTTTCTTCATCATCGGTCATAAAATCCGTATCCTT
TACAGGATATTTTGCAGTTTCGTCAATTGCCGATTGTATATCCGATTTATATTTATTTTTCGGTCGAATCATTTGA
ACTTTTACATTTGGATCATAGTCTAATTTCATTGCCTTTTTCCAAAATTGAATCCATTGTTTTTGATTCACGTAG
TTTTCTGTATTCTTAAAATAAGTTGGTTCCACACATACCAATACATGCATGTGCTGATTATAAGAATTATCTTTAT
TATTTATTGTCACTTCCGTTGCACGCATAAAACCAACAAGATTTTTATTAATTTTTTTATATTGCATCATTCGGC
GAAATCCTTGAGCCATATCTGACAAACTCTTATTTAATTCTTCGCCATCATAAACATTTTTAACTGTTAATGTGA
GAAACAACCAACGAACTGTTGGCTTTTGTTTAATAACTTCAGCAACAACCTTTTGTGACTGAATGCCATGTTT
CATTGCTCTCCTCCAGTTGCACATTGGACAAAGCCTGGATTTACAAAACCACACTCGATACAACTTTCTTTCG
CCTGTTTCACGATTTTGTTTATACTCTAATATTTCAGCACAATCTTTTACTCTTTCAGCCTTTTTAAATTCAAGAA
TATGCAGAAGTTCAAAGTAATCAACATTAGCGATTTTCTTTTCTCTCCATGGTCTCACTTTTCCACTTTTTGTCT
TGTCCACTAAAACCCTTGATTTTTCATCTGAATAAATGCTACTATTAGGACACATAATATTAAAAGAAACCCCC
ATCTATTTAGTTATTTGTTTAGTCACTTATAACTTTAACAGATGGGGTTTTTCTGTGCAACCAATTTTAAGGGTT
TTCAATACTTTAAAACACATACATACCAACACTTCAACGCACCTTTCAGCAACTAAAATAAAAATGACGTTATT
TCTATATGTATCAAGATAAGAAAGAACAAGTTCAAAACCATCAAAAAAAGACACCTTTTCAGGTGCTTTTTTT
ATTTTATAAACTCATTCCCTGATCTCGACTTCGTTCTTTTTTTACCTCTCGGTTATGAGTTAGTTCAAATTCGTTC
TTTTTAGGTTCTAAATCGTGTTTTTCTTGGAATTGTGCTGTTTTATCCTTTACCTTGTCTACAAACCCCTTAAAA

ACGTTTTTAAAGGCTTTTAAGCCGTCTGTACGTTCCTTAAGGAATTCCTTAGTGCTTTCATAGATTAAACTCAC
ATCACGCTTTAAATCGCTTATTTTAGACTTTAAAGACTTGTTTTCTTCAAGCAACTCATTATAATCATTTACATTT
TCATTAAATCGCTCTACAAGACCACTATATTTTTCTTTAACTTGCCCATGTTCTTTACTTAATTTTTTATATTCTCT
CGCCATATCAGTACTCATGAGATTTCTAACATGCTGTTTTAACCTATCGTTATCTCTCGCAGCAGTCACTAAGTT
TTTATAATCACGCTCCGATATAACAACATTTTTGGTTGGTTTCTTTTCTGTTTTCATTATTTCTTTTCCCAAACCA
AACATGGACTTTTCACCCGTTGGCACTTCAACACTTTTCATGTGTCGTTTCGCTGGTACTTCTAAATCTGATTT
AACTTTATCGCTATAAGCAGTCCATTCATCTTTTTTAACTGCTAAATTTTTTTCTAGAAAATCAATCTCTTTTTCC
AAAGTTTGTTTTTTAAATTTAGCTGTCTCAATATGTTTACGGTCAGAGCCACGTTCACCACGCTTCAACTCAA
AACCCTGTTTTTTCATATGCTCGGGGAATTTATCTTGTAGCCATAACAGTTCTTGACGATTAAACACATTTTTTC
CTTGCAGTTTTCCATCACGCATAGGCACAACACCTAAATGCATGTGAGGGGTTTGCTCATCATTATGAACTGT
TGCATAAGCAATATTTTGCTTGCCATATCGTTCGGAAAATAATTTATAACTTTCCTCAAAAAATCGTTTTTGTTC
TCCTGGATCCAGTTGCTCAAAAAAATCTCGGTCAGATGTTACTAGCAACTCATTTACAAGAACAGCATCTTTC
CTCGTTTTTCTTGTACCTGTTTTTTGTGATTCAATAATTTCTTTGACACGTTCGTTGTAATCAATATTTTTATCAT
TTTTCAAATCATAATTTTCACGTGTTCGCTCATGGTCAATATCATCATTCGTTCTACTTTTTCGCTCTCTTTGATT
ATGAAATTGCATGCCTTTTAGTCCAGCTGATTTCACTTTTTGCATTCTACAAACTGCATAACTCATATGTAAATC
GCTCCTTTTTAGGTGGCACAAATGTGAGGCATTTTCGCTCTTTCCGGCAACCACTTCCAAGTAAAGTATAACA
CATTATACTTTATATTCATAAAGTGTGTGCTCTGCGAGGCTGTCGGCAGTGCCGACCAAAACCATAAAACCTT
TAAGACCTTTCTTTTTTTTACGAGAAAAAAGAAACAAAAAAACCTGCCCTCTGCCACCTCAGCAAAGGGGG
GTTTTGCTCTCGTGCTCGTTTAAAAATCAGCAAGGGACAGGTAGTATTTTTTGAGAAGATCACTCAAAAAAT
CTCCACCTTTAAACCCTTGCCAATTTTTATTTTGTCCGTTTTGTCTAGCTTACCGAAAGCCAGACTCAGCAAG
AATAAAATTTTTATTGTCTTTCGGTTTTCTAGTGTAACGGACAAAACCACTCAAAATAAAAAAGATACAAGAG
AGGTCTCTCGTATCTTTTATTCAGCAATCGCGCCCGATTGCTGAACAGATTAATAATAGATTTTAGCTTTTTATT
TGTTGAAAAAAGCTAATCAAATTGTTGTCGGGATCAATTACTGCAAAGTCTCGTTCATCCCACCACTGATCTT
TTAATGATGTATTGGGGTGCAAAATGCCCAAAGGCTTAATATGTTGATATAATTCATCAATTCCCTCTACTTCAA
TGCGGCAACTAGCAGTACCAGCAATAAACGACTCCGCACCTGTACAAACCGGTGAATCATTACTACGAGAGC
GCCAGCCTTCATCACTTGCCTCCCATAGATGAATCCGAACCTCATTACACATTAGAACTGCGAATCCATCTTCA
TGGTGAACCAAAGTGAAACCTAGTTTATCGCAATAAAAACCTATACTCTTTTTAATATCCCCGACTGGCAATGC
CGGGATAGACTGTAACATTCTCACGCATAAAATCCCCTTTCATTTTCTAATGTAAATCTATTACCTTATTATTAAT
TCAATTCGCTCATAATTAATCCTTTTTCTTATTACGCAAAATGGCCCGATTTAAGCACACCCTTTATTCCGTTAA
TGCGCCATGACAGCCATGATAATTACTAATACTAGGAGAAGTTAATAAATACGTAACCAACATGATTAACAATT
ATTAGAGGTCATCGTTCAAAATGGTATGCGTTTTGACACATCCACTATATATCCGTGTCGTTCTGTCCACTCCTG
AATCCCATTCCAGAAATTCTCTAGCGATTCCAGAAGTTTCTCAGAGTCGGAAAGTTGACCAGACATTACGAA
CTGGCACAGATGGTCATAACCTGAAGGAAGATCTGATTGCTTAACTGCTTCAGTTAAGACCGAAGCGCTCGT
CGTATAACAGATGCGATGATGCAGACCAATCAACATGGCACCTGTCATTGCTACCTGTACAGTCAAGGATGGT
AGAAATGTTGTCGGTCCTTGCACACGAATATTACGCCATTTGCCTGCATATTCAAACAGCTCTTCTACGATAAG
GGCACAAATCGCATCGTGGAACGTTTGGGCTTCTACCGATTTAGCAGTTTGATACACTTTCTCTAAGTATCCA
CCTGAATCATAAATCGGCAAAATAGAGAAAAATTGACCATGTGTAAGCGGCCAATCTGATTCCACCTGAGAT
GCATAATCTAGTAGAATCTCTTCGCTATCAAAATTCACTTCCACCTTCCACTCACCGGTTGTCCATTCATGGCT
GAACTCTGCTTCCTCTGTTGACATGACACACATCATCTCAATATCCGAATAGGGCCCATCAGTCTGACGACCA
AGAGAGCCATAAACACCAATAGCCTTAACATCATCCCCATATTTATCCAATATTCGTTCCTTAATTTCATGAACA
ATCTTCATTCTTTCTTCTCTAGTCATTATTATTGGTCCATTCACTATTCTCATTCCCTTTTCAGATAATTTTAGATT
TGCTTTTCTAAATAAGAATATTTGGAGAGCACCGTTCTTATTCAGCTATTAATAACTCGTCTTCCTAAGCATCCT
TCAATCCTTTTAATAACAATTATAGCATCTAATCGGTTCTGTTGCAAAGTAAAAAAATATAGCTAACCACTAATT
TATCATGTCAGTGTTCGCTTAACTTGCTAGCATGATGCTAATTTCGTGGCATGGCGAAAATCCGTAGATCTGAA

GAGACCTGCGGTTCTTTTTATATAGAGCGTAAATACATTCAATACCTTTTAAAGTATTCTTTGCTGTATTGATAC
TTTGATACCTTGTCTTTCTTACTTTAATATGACGGTGATCTTGCTCAATGAGGTTATTCAGATATTTCGATGTAC
AATGACAGTCAGGTTTAAGTTTAAAAGCTTTAATTACTTTAGCCATTGCTACCTTCGTTGAAGGTGCCTGATC
TGTAATTACCTTTTGAGGTTTACCAAATTGTTTAATGAGACGTTTGATAAACGCATATGCTGAATGATTATCTC
GTTGCTTACGCAACCAAATATCTAATGTATGTCCCTCTGCATCAATGGCACGATATAAATAGCTCCATTTTCCTT
TTATTTTGATGTACGTCTCATCAATACGCCATTTGTAATAAGCTTTTTTATGCTTTTTCTTCCAAATTTGATACAA
AATTGGGGCATATTCTTGAACCCAACGGTAGACCGTTGAATGATGAACGTTTACACCACGTTCCCTTAATATT
TCAGATATATCACGATAACTCAATGTATATCTTAGATAGTAGCCAACGGCTACAGTGATAACATCCTTGTTAAAT
TGTTTATATCTGAAATAGTTCATACAGAAGACTCCTTTTTGTTAAAATTATACTATAAATTCAACTTTGCAACAG
AACCGAAAAACTAGACTTGATTACAAAATGGAGCTTGGGACATAAATGATTTTTTAAAAATGAGATGAGACG
TAGATTAACTCCATAATCAATACGAATCTATCGACTTCTTTATTTATGATATTCATCTCTTTTTAATGGAAATAAA
AGTGCGATTAATGTGATAATACAGTTACGTTAATTAAAAAAATAAAAATGCAAGGAGAGGTAATATGCTAACT
GTATATGGACATAGAGGATTACCTAGTAAAGCTCCGGAAAATACAATTGCATCATTTAAAGCTGCTTCAGAAG
TAGAAGGTATAAACTGGTTGGAGTTAGATGTTGCAATTACAAAAGATGAACAACTGATTATCATTCATGATGA
TTATTTAGAACGGACTACAAATATGTCCGGGGAAATAACTGAATTGAATTATGATGAAATTAAAGATGCTTCT
GCAGGATCTTGGTTTGGTGAAAAATTCAAAGATGAACATTTGCCAACTTTCGATGATGTAGTAAAAATAGCA
AATGAATATAATATGAATTTAAATGTAGAATTAAAAGGTATTACTGGACCGAATGGACTAGCACTTTCTAAAAG
TATGGTTAAGCAAGTGGAAGAACAATTAACAAACTTAAATCAGAATCAAGAAGTGCTCATTTCAAGCTTTAA
TGTTGTGCTTGTTAAACTTGCAGAAGAAATCATGCCACAATATAACAGAGCAGTTATATTCCATACAACTTCGT
TTCGTGAAGACTGGAGAACACTTTTAGATTACTGTAATGCTAAAATAGTAAACACTGAAGATGCCAAACTTAC
TAAAGCAAAAGTAAAAATGGTAAAAGAAGCGGGTTATGAATTGAACGTATGGACTGTAAACAAACCAGCAC
GTGCAAACCAACTTGCTAATTGGGGAGTTGATGGTATCTTTACAGACAATGCAGATAAAATGGTGCATTTGTC
TCAATAGAAAGTTAGAGGTGAGTCTTACGTTTCAGTGACGGTAGACTTACCTTTAACATGTTACATACTAAAA
AATTAATTTGAATAAGAAAGAGAGACATATATGAAATACGATGATTTTATAGTAGGAGAAACATTCAAAACAA
AAAGCCTTCATATTACAGAAGAAGAAATTATCCAATTTGCAACAACTTTTGATCCTCAATATATGCATATAGATA
AAGAAAAAGCAGAACAAAGTAGATTTAAAGGTATCATTGCATCTGGCATGCATACACTTTCAATATCATTTAA
ATTATGGGTAGAAGAAGGTAAATACGGAGAAGAAGTTGTAGCAGGAACACAAATGAATAACGTTAAATTTAT
TAAACCTGTATACCCAGGTAATACATTGTACGTTATCGCTGAAATTACAAATAAGAAATCCATAAAAAAAGAAA
ATGGACTCGTTACAGTGTCACTTTCAACATACAATGAAAATGAAGAAATTGTATTTAAGGGAGAAGTAACAG
CACTTATTAATAATTCATAATAAAACAGTGAAGCAACCATCGTTACGGATTGCTTCACTGTTTTGTTATTCATCT
ATATCGTATTTTTTATTACCGTTCTCATATAGCTCATCATACACTTTACCTGAGATTTTGGCATTGTAGCTAGCCA
TTCCTTTATCTTGTACATCTTTAACATTAATAGCCATCATCATGTTTGGATTATCTTTATCATATGATATAAACCAC
CCAATTTGTCTGCCAGTTTCTCCTTGTTTCATTTTGAGTTCTGCAGTACCGGATTTGCCAATTAAGTTTGCATA
AGATCTATAAATATCTTCTTTATGTGTTTTATTTACGACTTGTTGCATACCATCAGTTAATAGATTGATATTTTCTT
TGGAAATAATATTTTTCTTCCAAACTTTGTTTTTCGTGTCTTTTAATAAGTGAGGTGCGTTAATATTGCCATTAT
TTTCTAATGCGCTATAGATTGAAAGGATCTGTACTGGGTTAATCAGTATTTCACCTTGTCCGTAACCTGAATCA
GCTAATAATATTTCATTATCTAAATTTTTGTTTGAAATTTGAGCATTATAAAATGGATAATCACTTGGTATATCTT
CACCAACACCTAGTTTTTTCATGCCTTTTTCAAATTTCTTACTGCCTAATTCGAGTGCTACTCTAGCAAAGAAA
ATGTTATCTGATGATTCTATTGCTTGTTTTAAGTCGATATTACCATTTACCACTTCATATCTTGTAACGTTGTAAC
CACCCCAAGATTTATCTTTTTGCCAACCTTTACCATCGATTTTATAACTTGTTTTATCGTCTAATGTTTTGTTATT
TAACCCAATCATTGCTGTTAATATTTTTTGAGTTGAACCTGGTGAAGTTGTAATCTGGAACTTGTTGAGCAGA
GGTTCTTTTTTATCTTCGGTTAATTTATTATATTCTTCGTTACTCATGCCATACATAAATGGATAGACGTCATATG
AAGGTGTGCTTACAAGTGCTAATAATTCACCTGTTTGAGGGTGGATAGCAGTACCTGAGCCATAATCATTTTT
CATGTTGTTATAAATACTCTTTTGAACTTTAGCATCAATAGTTAGTTGAATATCTTTGCCATCTTTTTTCTTTTTC

TCTATTAATGTATGTGCGATTGTATTGCTATTATCGTCAACGATTGTGACACGATAGCCATCTTCATGTTGGAGC
TTTTTATCGTAAAGTTTTTCGAGTCCCTTTTTACCAATAACTGCATCATCTTTATAGCCTTTATATTCTTTTTGTTT
TAATTCTTCAGAGTTAATGGGACCAACATAACCTAATAGATGTGAAGTCGCTTTTCCTAGAGGATAGTTACGA
CTTTCTGTTTCATTAGTTGTAAGATGAAATTTTTTTGCGAAATCACTTAAATATTCATCCATTTTTTTAACGGTT
TTAAGTGGAACGAAGGTATCATCTTGTACCCAATTTTGATCCATTTGTTGTTTGATATAGTCTTCAGAAATACT
TAGTTCTTTAGCGATTGCTTTATAATCTTTTTTAGATACATTCTTTGGAACGATGCCTATCTCATATGCTGTTCCT
GTATTGGCCAATTCCACATTGTTTCGGTCTAAAATTTTACCACGTTCTGATTTTAAATTTTCAATATGTATGCTT
TGGTCTTTCTGCATTCCTGGAATAATGACGCTATGATCCCAATCTAACTTCCACATACCATCTTCTTTAACAAAA
TTAAATTGAACGTTGCGATCAATGTTACCGTAGTTTGTTTTAATTTTATATTGAGCATCTACTCGTTTTTTATTTT
TAGATACTTTTTTTATTTTACGATCCTGAATGTTTATATCTTTAACGCCTAAACTATTATATATTTTTATCGGACGT
TCAGTCATTTCTACTTCACCATTATCGCTTTTAGAAATATAACTGCTATCTTTATAAACTTGTTTGAAATTTTTAT
CTTCAATTGCATCAATAGTATTATTAATTTCTTTATCTTTTGAAGCATAAAAATATATACCAAACCCGACAACTAC
AACTATTAAAATAAGTGGAACAATTTTTATCTTTTTCATCAATATCCTCCTTATATAAGACTACATTTGTAGTATAT
TACAAATGTAGTATTTATGTCAAAATAATGTTATAATTTTTGTGATATGGAGGTGTAGAAGGTGTTATCATCTTT
TTTAATGTTAAGTATAATCAGTTCATTGCTCACGATATGTGTAATTTTTTTAGTGAGAATGCTCTATATAAAATAT
ACTCAAAATATTATGTCACATAAGATTTGGTTATTAGTGCTCGTCTCCACGTTAATTCCATTAATACCATTTTACA
AAATATCGAATTTTACATTTTCAAAAGATATGATGAATCGAAATGTATCTGACACGACTTCTTCGGTTAGTCAT
ATGTTAGATGGTCAACAATCATCTGTTACGAAAGACTTAGCAATTAATGTTAATCAGTTTGAGACCTCAAATAT
AACGTATATGATTCTTTTGATATGGGTATTTGGTAGTTTGTTGTGCTTATTTTATATGATTAAGGCATTCCGACA
AATTGATGTTATTAAAAGTTCGTCATTGGAATCGTCATATCTTAATGAACGACTTAAAGTATGTCAAAGTAAGA
TGCAGTTCTACAAAAAGCATATAACAATTAGTTATAGTTCAAACATTGATAATCCGATGGTATTTGGTTTAGTG
AAATCCCAAATTGTACTACCAACTGTCGTAGTCGAAACCATGAATGACAAAGAAATTGAATATATTATTCTACA
TGAACTATCACATGTGAAAAGTCATGACTTAATATTCAACCAGCTTTATGTTGTTTTTAAAATGATATTCTGGTT
TAATCCTGCACTATATATAAGTAAAACAATGATGGACAATGACTGTGAAAAAGTATGTGATAGAAACGTTTTAA
AAATTTTGAATCGCCATGAACATATACGTTATGGTGAATCGATATTAAAATGCTCTATTTTAAAATCTCAGCACA
TAAATAATGTGGCAGCACAATATTTACTAGGTTTTAATTCAAATATTAAAGAACGTGTTAAGTATATTGCACTTT
ATGATTCAATGCCTAAACCTAATCGAAACAAGCGTATTGTTGCGTATATTGTATGTAGTATATCGCTTTTAATAC
AAGCACCGTTACTATCTGCACATGTTCAACAAGACAAATATGAAACAAATGTATCATATAAAAAATTAAATCAA
CTAGCTCCGTATTTCAAAGGATTTGATGGAAGTTTTGTGCTTTATAATGAACGGGAGCAAGCTTATTCTATTTA
TAATGAACCAGAAAGTAAACAACGATATTCACCTAATTCTACTTACAAAATTTATTTAGCGTTAATGGCATTCG
ACCAAAATTTACTCTCATTAAATCATACTGAACAACAATGGGATAAACATCAATATCCATTTAAAGAATGGAAC
CAAGATCAAAATTTAAATTCTTCAATGAAATATTCAGTAAATTGGTATTACGAAAATTTAAACAAACATTTAAG
ACAAGATGAGGTTAAATCTTATTTAGATCTAATTGAATATGGTAATGAAGAAATATCAGGGAATGAAAATTATT
GGAATGAATCTTCATTAAAAATTTCTGCAATAGAACAGGTTAATTTGTTGAAAAATATGAAACAACATAACAT
GCATTTTGATAATAAGGCTATTGAAAAAGTTGAAAATAGTATGACTTTGAAACAAAAAGATACTTATAAATATG
TAGGTAAAACTGGAACAGGAATCGTGAATCACAAAGAAGCAAATGGATGGTTCGTAGGTTATGTTGAAACG
AAAGATAATACGTATTATTTTGCTACACATTTAAAAGGCGAAGACAATGCGAATGGCGAAAAAGCACAACAA
ATTTCTGAGCGTATTTTAAAAGAAATGGAGTTAATATAATGGATAATAAAACGTATGAAATATCATCTGCAGAA
TGGGAAGTTATGAATATCATTTGGATGAAAAAATATGCAAGTGCGAATAATATAATAGAAGAAATACAAATGC
AAAAGGACTGGAGTCCAAAAACCATTCGTACACTTATAACGAGATTGTATAAAAAGGGATTTATAGATCGTAA
AAAAGACAATAAAATTTTTCAATATTACTCTCTTGTAGAAGAAAGTGATATAAAATATAAAACATCTAAAAACT
TTATCAATAAAGTATACAAAGGCGGTTTCAATTCACTTGTCTTAAACTTTGTAGAAAAAGAAGATCTATCACA
AGATGAAATAGAAGAATTGAGAAATATATTGAATAAAAAATAAAATTGTTGTGTTTACAACAATACATAGAAA
ACAGAGGAAACAATCAAGTCGTTGAATATTTCCTCTGTTTTTTAGTTGAAAAAATTAACCGAAAGCCTGAAT

GCAAGTCTTGATTAAATCAATAATGCTTGTAATAACACCAGTGAAATCCATATGCATACCCTCTTTCTATTTAAG
ATACATTAAGTATAATATCAAACAAATAAAAAATGTTAAAAATTCCCTAATTGGCTATTTAGATTGCATAAATGT
CAAAAATTTGAAAAACATACAACGACTTTGCATAAAAAAATCGTCATATTGGAAATACGTAATTTATTGAAATAA
TAAAAAAAAATAAAAGAACGAAGATGATAACCTAAGTGAGGTTTTAAGTTGTTCTAAGGTTTAATTTAATTTAT
GTTAAAATAGTTGGTATAAAAATACATGATAAACTATAAACTAAATTCAAAATAACTTATGGGGTAGGCAATTA
TGGAAAATAAAATATAAATGATAATGAAAAAAGAGTGCTAAGGGAAATTTATAACCATCATAATATTTCGCGTA
CTCAAATATCTAAAAATCTTGAGATTAATAAGGCAACGATTTCTAGTATTTTGAATAAGTTAAAGTATAAATCTC
TTGTTAATGAGGTTGGTGAGGGTGATAGCACGAAGAGTGGTGGTAGAAAACCTATTCTTCTGAAGGTTAATC
ATCTTTATGGTTATTTTATTTCTTTGGATTTAACTTATAGTTCTGTTGAAGTGATGTACAATTATTTTGATGGTAA
TGTCATTAAGCATGAATCTTATGATTTACCTGATGAAAAGGTTAGTAGTATATTAAGCATAATAAAAAAACATAT
TGATATTCAGGAGAAACTTGATACTTATAACGGACTATTAGGTGTGTCTGTTTCTATACATGGAGTTGTGGATA
ATGAGCAGCATGTGACATATTTACCATTCCATGAAACTGAAGGAATTTCAATTGCTAAGAAAATAAAAGAAAT
TACTAATGTTCCAGTCGTAGTTGAAAATGAAGCGAATCTTTCAGCGTTATATGAACGTAATTTTAATCATAATTT
ATCCTACAATAATCTTATTGCTTTAAGTATACATAAAGGTATTGGTGCTGGGCTTATTATTAATAATCAATTGTAT
CGTGGTGCAAATGGGGAAGCGGGTGAAATTGGAAAAACACTTGTCTCAAAAGTTAGCGATAATGTGGAGA
TCTTTCATAAGATTGAAGATATTTTTTCACAAGAAGCTTTACTGCATAATTTAAGTAATCAACTAAATGAGAAG
ATGACGCTTAGCAAATTAATTCAATTTTATAATGAAAAAAATCCAGTCGTAGTTGAAGAAATGGAACAATTTA
TAAATAAAATTGCTGTTTTAATACATAATTTAAACACCCAGTTTAATCCGAATGCAATTTACATTAACTGTCCAT
TGTTCAATGAAATGCCTGAAATATTAGAAGCAATTAAGAACCAGTTCAAACAATATTCACGTAACGAAATTCA
AATAAAGTTAACATCTAATGTCAAATTTGCAACTTTGCTAGGTGGTACATTAGCAATTATCCAAAAAGTACTAC
AGATTAATGATATTTACTTAGATATAAAAGCATAAAAAACTAATTCAAATGAATAATCAAAGTTCGTAATTGTCT
TTATAAAAAAAATCCCTCAATCCGAATTGAATTTTCGGATTGAGGGATTTTTATAGTTCTATTGCAGAAGAAAAC
TATTTTAAAAATGCTGGTAAATGTTGATAGCCACCTCTAACGTTAACAATATTCGTAAATCCTTTATATTCTAATA
TTCCTACCGCTATTGAACTTCTAACACCTGATTGACAATGTACATAAATTAGGTCATTTTTATCGAAAGGTATAT
CTTCATTTAAAAGTTTACCGTGAGGAATATGAATTGCTTGTTTTAAATGACCTTTACGCCATTCATCATCATTAC
GAACATCTAATACATTATGTTCTTCACCAGTCATTTCAGAACTATGAATAGATGATGTGACGATATTTGTTTGTG
GCAAACGGTAACTTTTTACATTTTCAAAACCAATTAATTGTAAAGCATGAATAGCTTGTTGAACGGTAGATTT
ATCGCCAATTAATTCAATATCTTAGTCATAATCTAAATACCAACCAATTTGATTTATAAAAGTTTTATTAAAAGG
AATATTGATAGTTCCATGCATATGACCACCATGGAATGCTTCTTTACTTCGAAGATCAAAAGCAGTTTGTGTAT
TGCTTGAACTAGGGTAAACATTATATGGTTGGTACATTTGCATACCAAATTGATTTATTTTTTTCATTTGTGAAA
AATGGTGTGGTGGAGCTGGCTGATTGAGTGTTAAAGTTTCGATAAATGAAGTTTCATCTTTAACATTAAAAG
CCCAGTTGTTTATTTTCTCATAACCCAAAGTAGTTGTAGGTAATGAACCTAGCGCTTTACCACAAGGACTCCC
TGCACCATGACCTGGCCAAATTTGAATATAGTCTGGTAATGTTGCAGCAAATTGTATGGACTGATACATTTGTT
TTGCTCCGATTTTTGTAGAACCTTCAACATTTACAGCTTTTTCTAATAGATCTGGTCTACCTACATCACCAACAA
AGATGAAGTCACCGCTAAATAATCCCATTGGTATACTGGAACCCCCACCTTCGTCAGTAAGTAAAAAACTAAT
ACTCTCAGGGGGATGGCCTGGAGTGTGTAAGACTTCTAATTTAATCTTTCCTAAATAGATAATATCTTGATGCT
TAACGAAATGTGTTTGTTTAGGCATATTTTTATAATTAAATTCATCTTTACCTTCATCAGATACGTATATACTTGC
ATTCAATTTATTTGCCACATCTCTAATACCTGAAGCAAAATCAGCATGAATATGTGTTTCTGCAGCTTTAGTAAT
TGTGAATCCTTCTTTATCTGCAACTTTTAAATATTTTGTTAAATCTCGTATAGGGTCAATAATCATTGCTTCTCCT
GTACGTTGACATCCAATTAAATAAGATGCTTGTGAAAAATTGTCTTCATAAAATTGTTTGAAAAACAAAAAAA
ACTCCTTTTTAAAATAGATTTTATTGATTAGATAAATAAGTTATGATTTGCTTGCTCAGTATGTCCAATATAAGTA
CCTACGCCACCATAATCGACTTCATCTCTTAATTCTTCTTTTGAAATTCCCATAACATCCATACTCATGGTACAA
GCAATTAACTTTATATCTTGATCGATTGCTTGATCGATAAGTGAGTATAAAGAATCAACATTTTTCTTGTTCATT
ACATAACGCATCATAATATTACCTAGTCCAAACATATTCATTTTTGATAATGGCATATGTATTGGATCCTTAGGTA

ACATAAGGTCAAACATTTTGGAAATACCTTTCTTTTTAACGCGAGTTGATTGCGCTTTTTTTAATGCGTTGAG
GCCCCAAAAAGTAAAGAAAATAGTTACATCTTTACCTGCTGCTTTAGCGCCATTTGCGATGATCATTGCTGCT
ACTGCCTTATCTAACTCACCGCTAAATAAAACAATTGTTGTACCTGTAGCAGTGTCATTGATTTCAAATTCTTTT
GGCTTTTCTTTTTGAATAATTGCATTAATTACATTTGCTTCTTCAGTAAGATTTACAAGGGTATTCCCTGTTTGT
TTCGCCCAACTTTTAATATCACTATTGAAACCAGGATCTGTAACTGTTACCTCGATTTGCTCACCCGTTGAAAT
ATTGTTAATTTCTTTACTGATATTAACAATAGGTCCAGGGCACTGAAGACCTCTAAAATCAAATTGTTTACGAT
TCTCTTTGATTTCAATATCTTTTTCTATTAAAGGAGCACTATTGAAGTTCTTTGCTTCATAATCTTTATATCCACC
CTTTAAATTCACGACATCATAACCTTGTTTGGCTAAATAATCGCAAGCTTTAGTGCTTCGGTTACCGCTTTTAC
AATGTATATAATACGTTTTGTTGCTATTCTTATTGAATGATTTAATCTCTTCTACTGGGTGTAAAGTTGAACCGTT
AATGTGTCCTAATTCATATTCTTCTTTTGTTCTAACATCAATCAATTGACCCATTTTTGCCAATTTTTCTAATTCTT
CTTTGTTTAATGAATTAATGTGTACTTCTTTGTATTGTTCCATACTTACCTCCTATAAATACCTATGAGGGTATAA
TAAAACGGATAGAATCATTTGCCAAATACCTATAGGGGTATTTGACAATTTTGTTTTAATTTATTATTATTAACTT
AATCAATTTATGTGGAGGAAATGAATATGACTTATGATAAAAAAATGATTAATCGTATAAATAGAATACAAGGT
CAATTAAATGGTGTCGTAAAAATGATGGAAGAAGAAAAAGATTGCAAAGATATAATTACGCAACTTAGTGCA
TCTAAAGGTTCTATACAACGTTTAATGGGGATTATAATTAGTGAAAATTTAATAGAATGCGTTAAAACAGCAG
AAGAAAATAATGAAAGTTCTCAAGAATTAATTAATGAAGCAGTTAATTTATTAGTTAAAAGTAAATAATGGATA
TAGCAAATATGACTATTATGTTGCTAATTGGCGTACTGGGTGGATTTATATCTGGATTAATAGGTATTGGGGGC
GCAATTATTATTTACCCAGCTATTCTTATATTGCCACCATTAATAGGTATACCTGCGTATAGTGCATATATTGCTTC
GGGACTTACCTCTAGTCAAGTATTTTTCAGTACACTTAGTGGATCATTAAATGCAAGAAAACAACCAGCTTTC
TCTCCTAAACTTGTTATATATATGGGAGGGGGTATGTTGATTGGAAGCATGTTAGGGGCAATTTTAGCTAGTT
TGTTTAATGCTACTTTTGTAAATACGGTATATGTAATAATCGCCATACTTGCTTTAATATTGATGTTTATTAAAGT
TAAACCTACTACACAAGAGACGAAATCTAAACCTTTGCTATTTATTATAGTTGGATTTGGAATTGGTGTAATTT
CGGGAATTGTGGGTGCAGGTGGAGCATTTATCATCATTCCTGTATTATTAGCATTATTTAAATTACCAATGAAT
ACGGTAGTGAACAATAGCATAGCAATTGCTTTTATATCTTCAGTAGGGGCATTTTTTATAAAATTAATGCAAGG
ATATATACCAGTAGAAAGTGCAATTTTTTTGATAATTGAATGATGATCATTTTCTGAAAATATTATGTGGTCATAT
AATATAATGCCCATCATTTCACTAATCTCTTTTATTCTCTGAGTTATTTTGATATCTCCTGGAGAAGGTGTTACAT
CTTATCAAGAGTAAATTACAAAAGAATCATTTAAATCAATACTTTCACTTTGAATACATGTATTTGAAGTGGAA
GGTACTTATTTCAAAAATAGTAAAACCTGTATCTTAAATTACTTAATAGTAACATAAGATACAGGCTGATTTTTT
ATTCATTGTTATTTATACTAAAGCACCCGATAGCTCTGAAAACAATCACAAATTCAACTTTTCAAAGCCACAGC
TTTAAGTTATTTTGTCCAGACAACCCCCATTGCCCAACCCATTTTATGGAATTGGCATCCAGGCAACAACTTTT
CATATAAATCGTAATAATTTTGTTCAGATAGGTACTTATCTGAAGCTAAATGCTCAAGCCATGATTTAGATGTGT
TGTGATTATAAATTCTAATCGCATTTTTTATTCCAAATTTGAAACAATGCGGTAAAAACTCTTGGATTGCCCCTA
ATTTATAAACATAGGTAGGCGGTGTCTCGACTTCAGATACATTATCAAGAATAACTATTCTTCCTTCCTCATTCA
ACAGTTCCTTCATTTGCTGTATTACGCTGGCTATATCATCCAAATGATGAAAGGTTGTTCGGCTTACAATAAAA
TCAAACTTCTCATTAAAATTAAGTTGTTCTGCATTCATATTCAGATAGACCGTATTTGTTAGTTGACGTTTAGAT
TTGGCAAGATCGAGCATTTGATTAGAAATATCAATCCCTACCACTTCATCATAATAACTTGCTAATTTCTCCACT
AACAAACCCGAGCCACATCCGATATCTAATGCTCTGCCTTTCTTTGGAGACATATTAGACACAAAGAATGAAT
AATCATTCAAAAGCTCATTCACGAAATCGTAATCTTCTGCAACCTTATCAAACTGTGATTCTATTGTATTCAAAA
AGATCCCCCATTCCTACTTTATCGACATTCTTTCATTACTTACCACTTTAGATGTTTTTTCGTTGGGGATAAAAC
TTCCCTTTAGACAATTTTATCCAAAGACAATACAACAGTGCAACTTTATTAAAGTCACTGTCCTTTATCGCAGC
CTTTACTTTTTAGTAAAGACAGTGGCTTCTCTTATCAAGTTTCAAAACATATTATTTGAAGAAAACGTCCATC
TGAAGTGTCAAGTGCAAAATTACATATAAAGGTTTATTCTAAAATGAAAAGATGATACAATCATATTCAGTTAC
ATAAGGAGGTTTCAATTATGTGCACCAGTATCGCAGTAGTAGAAATTACTTTATCTCATTCATAATGAAAAAAA
TGGAAAGGAGATAAAAGTATGGGTACTTTTTCTATATTTGTTATTAATAAAGTTCGTTATCAACCAAATCAAAA

TTAATTGGTTATAATGAACGCTTAATGTCAGTTCATTATAACCAGTAAGGAGAAGGTTATAATGAACCAGTAA
GGAGAAGGTTATAATGAACCAGAAAAACCCTAAAGACACGCAAAATTTTATTACTTCTAAAAAGCATGTAAA
AGAAATATTGAATCACACGAATATCAGTAAACAAGACAACGTAATAGAAATCGGATCAGGAAAAGGACATTT
TACCAAAGAGCTAGTCAAAATGAGTCGATCAGTTACTGCTATAGAAATTGATGGAGGCTTATGTCAAGTGAC
TAAAGAAGCGGTAAACCCCTCTGAGAATATAAAAGTGATTCAAACGGATATTCTAAAATTTTCCTTCCCAAAA
CATATAAACTATAAGATATATGGTAATATTCCTTATAACATCAGTACGGATATTGTCAAAAGAATTACCTTTGAAA
GTCAGGCTAAATATAGCTATCTTATCGTTGAGAAGGGATTTGCGAAAAGATTGCAAAATCTGCAACGAGCTT
TGGGTTTACTATTAATGGTGGAGATGGATATAAAAATGCTCAAAAAAGTACCACCACTATATTTTCATCCTAAG
CCAAGTGTAGACTCTGTATTGATTGTTCTTGAACGACATCAACCATTGATTTCAAAGAAGGACTACAAAAAGT
ATCGATCTTTTGTTTATAAGTGGGTAAACCGTGAATATCGTGTTCTTTTCACTAAAAACCAATTCCGACAGGCT
TTGAAGCATGCAAATGTCACTAATATTAATAAACTATCGAAGGAACAATTTCTTTCTATTTTCAATAGTTACAAA
TTGTTTCACTAAATTAAAGTAATAAAGCGTTCTCTAATTTCACAAGAGGACGCTTTATTCTTCCCAAAAATTGT
TCAATATTTATCAATAAATCAGTAGTTTTAAAAGTAAGCACCTGTTATTGCAATAAAATTAGCCTAATTGAGAG
AAGTTTCTATAGAATTTTTCATATACTTAACGAGTGCTTTCACCTTTGAATATAGTCCTTCCCACTTATCATCACA
CTCTCCCCGATAGCCTTTTCTAGCTATATCCAGTAAAGTTACATGCTCTTTAGGTAAAAGAGGTATAGCCCATT
CTGCAGCGACATCTTTCGAGGTAATTTCACCAGTAGTCACTGTTTGCCACATTCGAGCTAGGGTTAAAATTAC
ATTACGCTCATCACCTTTTATCCCCTCAATTAGTTCTGGCAAAGAATCCTTAATTGCTCTTCGAATATCTGTCAA
AGGTACGGAGACAAGTATACTTGAAGAATCAGGACCAAATAGAGAAATACTATTCTTTCTTGCTTGTGCTAA
AACAATAGCCAAATCAGGATCATAGCTTGGTTCCTGAATTTGTCCATTCTCAAATTCACCCCTGAGCCACTCA
CCGTATATAAATTCTCTTTTTGGAGGATATTGCCAAGGGACAACTTCACTCCTATTTATAACCGTAACTTCAAG
TGGTCTAACAGAATCCGTATTTCCAATCTTTCCTGATATAGTCATTAGTCTTTCTGTTAGTTTTTTTCGAGTTAAT
TGAGGTAAACTATGATTCACGACGACTAGAACATCTACATCGCTGTTAATGCGTAAACCACCATTTACTGCTG
AACCAAATAGATATACTCCAACTATTGAACTTCCAAATAAATCTTTTACGATTTTTAATGTTTGAATCGCTTGAT
TTGGTATTTTTCCGTTAATCAAATTGCTCATGATTTCACCTCGTTGATTATGTTCATATAAAGTTTATATTGATAC
TCAATTTACTTACCCTAGATTGGACATATACTTAAATTACTGTTCAATAAAGCTGACCGTTAGCGTTTAAGTACA
TCCTTTCACAATTTGTCTACAGATTAATAATTATTCTTTATTATACAGATCTCCATATAATTTTTGAATTTGGTTCT
GTAATTTTTTATTTTCTTTTTCTAATTCCATTACTCTTCTTTTTAAGGTTTTAATAAGGATTTCCTCCGAACGAGA
ACTTTTCTTGGGTTTTGAGACTACATTTGCTGTTATTTGACGCTCACGAAGGGATTCGATTCTTTGCCTAATAT
CGTGTTCCTTATAAAGCCATGATTTAGAAACATTAGCTTCCTTTGCTATTGAATTAAAATTAATAACTTTACCTT
CAATCGAAAATTTAGAAATCGCTTTGTCTACTTTTTCCCTTGTCTTTTTTGATTTCTGCTTCGCCAAACGTACA
ATTTCTGTTGTATTTCTAACTTGTTTATCCATTGATAATTACCCCGTCAAACTTCCAATGATTTGTTCTAAACGCT
CTTTAACACGGCTATTAGTCTCTACTTGTCTTTGCCATTGTTTATCCTTAGCTATGGCTAATAACTCTTCTGTACG
CTCTAACTGTTCTTCGTGCTGTGGTAAGAATTGCTTACTGGTACAGAAGTGAGTGCAATCTAAGCATGCATTC
GCATGTGGACAACCACCTGCTACTACTGGCAATCTACAATAACCATTTGGAAGCACTTGTGCATTTATATTTTT
CTTGAACCATTGAAGCTCTACATCATCGACTTCATTATCTTCATCTAGATCAAGCACATCTCCATTATTGGTAAC
CAGTTTTTCCTGAAATTTAGTAAATTCATTTTTTAGAGTTTCATCAAAGATATGAGCGTATCTGCTTGTCATTTC
TGGGCTTTCATGCCCCAAAAATTTCTGCACAATATGCTGGGGCATCCCGTTGTTAATCATTCTTGTTCCTACTG
TATGGCGAAAGGCATGGGCATGGAATCTATAAATCTCACCTGATTTATCCACTATATTTTGCTCATAAGCTAATT
TATTTAACTCACCTCTAAATGTTTCTTGTTTTAATGGCGATCCATCTTTTCTTGGAAAGAGGTATTCACTATCTG
GAAATTCCTCTGAAACTTTATCTTCCCGAACTTTAATAAGTAAAGCTACCTCTTTAGATATTGGAACTATATGCT
CCTTTTTCATTTTCCATTGATAATACTTTAAAAAGAAATCTCCATCTTTGTCCTCTAATAGACAGCCTTTTTTCA
AGGTGCACAATTCACTTATCCTCATTCCACATTCTTGAACAATCATAGTCATCGTAGCTATATATTCGGGTAATT
TATCAAGATGACTGTTCAATTGCTCTAGGACGAATTCATCTATAAAGCGTGGTTTTGCTCTTGGTATTTTCGGA
TAGTCCTCAGAATAAATTAATATTTTGGAAGGAACATCATCCCATTCTAGCCTAAGAAGGGTACTAAATAGTCC

TTCCAATATAGAGATCCTCCCAGTTATTGTACTAGGTTTTATTCCCATCATGTTTAGTTCACTTAAATATGCTTCA
ATTTCCACTCTCGTTAATTGGTGTACTCTCTGAACTTGTTTAAATTTCATGTCCAGAAAATTAAAGAACTCTTTA
AGTCTTTGGGCAATATCACTTACATAGGAAAAGCTATCCACGTTCAATCTCAACTTACAATATCTTTTTACAAG
TTGTTTAAAATATGTATTCCGAAACCCTTTAAAGTTAATTGTATATTCATATTGTGTTGGGTTAACCTTATCATCT
GGCAAAGGTAAGTTACGTCTATCCCAAACGTCTTTATCCCACTCCTCTCCATCAAAATAAAAGTTCTCATAAAA
CTCCATAAATTGTTTTAGATTAGTAACATAGTAGGAATTAGCTTTTACAGGTGTTTTTTCTTGATTAGCAGTAAT
CTTATAATTAGTAGTGGTAATTCTAACACCCCGTTTTGTCAAATAAGTTCTATACTCCGTCATTGCTTTTTCAATA
GGAACTTCAGTAATTGAAGTAATGCTAGGATACTTTAAATCTAAGAAATCTAACATTTTATTAATTACTGTTCCT
TTTCTAATCCAGACAGTTTTTGCATTCCATATTCCATTGTTTAAATGGTAAAAATAAAAATATTTCAATTCTGTTC
TTAACCACAGATTTTTAACACGTTCAAAACGAACCCAACGATTCCTTAAAGCAGGATTCTTACTTAATTCTATG
GCAGAAGGATGTGGACATTTTCTTATATCCCAACTATTATTAGCCCAAAACCCCTGCATTTCTTCATTCATTACA
GCTATTTTTTTGCTAATCTCACTCTGACTAATAATTTTCCTTTTACTAGAAGCATTCATTTCTTATGCTCCTTTCT
CTCGAGGTATTTATTAAACTCATTTTTCATATCCTGATCTGAAAGATGAACATAGGTATTTAACGTTGTCTGAAC
ATGTGCGTGACCTAATCTCTTTTGAACGAACGCAACATCCCATCCTTCCCTAATTAGCTGCGTTGCGTGAGTG
TGGCGAAGCATATGTGATGTAAATTCTATTCCAGTCCTTTTAACTATTCTTCTAACTAGATCAAGAACACTTTG
GTACTTTAGTGGTTTCCCAAAATAGCCTTCTTTTAAGGAAATAAAAACATAATCATGCTCCAATTCCTCACTATA
CTCATATATCAAGTAATCTGTATAAAGTGACATAAGTTCTTTACTCACATGTATTGTTCTTTCCTTCCTTAATTTA
ATATAAGCTTCATTAACATTAACATCTCTAGGTGTTAAATGGATTTGATTGTCCCAAGTGACAATATCTTCAAGC
CTAAGCGATAACACTTCACCGATTCTTAAACCACCCTCATACATAAGCATTAAAATTAATTTATCTCTTTTCGTAT
GACAAGCATCAATAATTTGCTTAACTTCCTTTGATCTCAATGTTCTTATCTGTTTCTTTTTAACCCTTAACTTTAA
GACATTCTTTTGGTATCTACCCTTATTAACATGATGTAAAAATCCTTTGAAATTTCTTCCCTTTGCTTGTTTAAAT
ACATCAATTGATTTAAATTCTCCTAATCTACTTAAATAATCAAGAAAACTCATAACTACATTTAAAATTGTATTCA
CTGTCGTTTCTTCTCTTATGGCTTTTTTTGACTGAAGATCAATTACATTTGATGCTGAAGGATATCTCAACCAA
CCTACGAAGTCTGCTAACAACTCAAAGTTAATATCATTAAGAATAACACCTCTCTGTTCCATGAACTCGTACAG
CAACTTTAAATGATAGCAGTATGCCTTAATGGTATTAGGAGACTTACCAGTATTATCTAAGTATTTAATAAATTT
CATTACTGGTTCTATTAGCTGGTATTCTTTATCTAGTAATAAATACAATGGATACGGCTTATTCTCCACTTCTATC
CTTTGAACCTTCACATGTTCCCACCTCTTTAAATACTTTAACTACTACAAATTATAAACCTTGTTAATTTTTATTT
AAAGTAAAATATCCCTTTAATATCTCCTCTTAAACTACTTTAACTACTATTATTTATTATACTATGGTTAATACATCT
CCACTTGGATGATTATGACCGAGCATTATACTATTTGCGTTACTGAGTATCGCTGTTTTGAATATTTCTCTAGGG
TGAATCACCGTTTGGTTAATAGATCCAATCGATAGTGTTTGAATATGTGTAGGTTCATTTTTACTGTTCATACAT
ATGAGAATGAGATGCTCTCGGTCACTGTTTCCAATGAATGAACGCATGATTTCTGCCGCATCCTCAGGGTTTG
AAATACGATTTTTTAGATAACTTAATGTATCTGTTTTTATCATTTGTAGTGAAACAATATTGATTTCCTTCATCGT
TTACCTCCATATATAGGTTATGCTTTCAAAGTCCATTTTTGACGTGCTTTAGGGTTGAGTGGGTGCATGATTTC
ATTTGTGGCTGGATTAATGAGCTTTTTGACTTTCTTTTTATGAGGCTTCAACATTTCCATTACTTGTTCGACAC
GTTCTACAACAACTGGCCGCTTCGCATAAGCACCATAAGCTAGAATCACTGTGTCACTTTCGCTAATTGCTTT
CATCAAGTGGATGTCTGTGTGTTTATCATAAGGTTCTTTAATATGTTTAAGGTTCTCTGGTGTTTTAATATTAGA
GAATAGATTTACAAGATATACAGCACCGTATCGTTCTGAATTCGCTAATTGATTGAGGATAAGAACAGTTGTG
AGATCGAGTGATAATACACCATCTAAATGAGGGTACATCGTTATCACTGTACAAGCGGGTTTCTTTTCATCCCA
TGTTTTCTTGAGTAAATAGCGGTGTTTTTCATCCTTGCTAAATATAGCTTCTGTGTATATCGTATTTTTGATTGTA
TTCATATATATAATCACTTCCTTTAGTATTCTTCTGGTAAAAGCATCACATAATAAAAAGCGTCTACGCCATCTTC
ACGAATGACGTAGACTTTCTTAGGTAATGCATTTTGATTTTTTACATAGTTTGTATAGTGATATTCCAATTTGTA
TGCAGGTTGTTCTTGTTCATGTGTGATTGAGAGTATATTCTCATCTTCTTGCAGTTTAAAAATGTGTAGGTAAT
CTGTATGAGGTTGATTATCTCTTTCTTTTACCATATTCCAAAGTAAGATTTGAAGGTCTAGAGATAGGTATTCA
CTAATACCTCTTGTGATGTATCGATTGATTTTCATGTTATTTTACCTCGTCTTGAATTTCTTTCATAATGATAATCG

CTTGGCTAATAATCGTAACAGATATTTGTGCCACTTTGATCCAGTTATTCATGGTGAGTCCCTCCTTCTCTTAGT
AAATGACGTTCATCGATAATCGTATTTTTAGTATCTGTGAGATATAAAAAGTCCATGTCAAAATGATTCAGATA
ACCAACGCTGATGAGTTGGTTATTAGCGTACATAAGAAATGGATAGATACTTAGGTCATGTAGTTCATTATTGT
AGTAGGTATAAGTTTCAAGTGTAAGATGTGCAAGTGGGGAATCATTAATAAAACGTTCCGGTAGAATATTTTC
TGCTGCTTCTTCTAACGCTTCGCATTCCCATGTTTCATTGTTAGATAGTTGGAATAGACGAGTTATATATTGTTT
GAGTTCTTGAGTGGTCGTTTTCATATCATTGCCTCCTAGATAGTGTTATAGTGATGTAGTTTATGTACATCATTG
GGATAATATATATTTGATTTGTCATTTATTACGCATCCCGGTGAGAATGAGAGAAAATTCCATATGAAAAACCG
CTTCAAACCTTGGTATGACAAGGAAATCCCGAAATTCCGCCTATTTTGACGAACAATCAACTCATTCTTTATAA
CTATTGATGTTAGGGTGGGGCTCTGCTTTCTTATATATTATTTATTTATAAAGAATAACGGGATTTTGGGATTGT
GCTTGCACAATCCTTCTGCTTCTTCGAATCTGCAAATCCCAATCATTTCCCGATAAAAAATCATTGTGGGATGT
TCTTTAGCAATTTCAATATAAGCATCGTGTAGTTATGAAAAAAATTACGACAATGACTGTTTCATTAGATAAGT
GTTATTGAAATTGATAAAGAGAATTTTAAAAATGGTTAGATAAAATAAATGAAAGAATATAATGAAAATTATTG
TTATAACAATGATTCTATTAGCTAAATAGTAAGATATAGTGTTTGGGGCAAAAACAAAGACGAAGTGCTGAGA
TGCACTTCGTCGAGTTGTTTATTATTGAAAAGTTGTTTTATGATTTCGTTATTAAGTTTGAGTGTGACATAGAA
TTGTTTTTTATGATTACCATCTTTTTTAATATCAATGCGATCAATCACTGATAGATACAATGCTTTGAGTCGAGA
TTTTTCTATGTGCTTAATATCATGAAAGATGTGTTGTAATAGTTTACTAATTTCTTTGGCATCAAATAAAGGCTT
ATCTTCATTTTGTTGATTTTTGAGTTGGTTGATTTGATTCGTAATGTCATTGAGTTGCTTTTCATATTTTTGAAT
ACTTGGTCTGATTACTGATGTTAAGTCCGGATTATCCTCGATGGTTTTAATCAAGTTATTTAGTTTGATTTGTAC
TTCATCATATTGTTGTTGCTTATAAGCAACAACAATATGATGAAGTGCAGCGCCATCAACTTGATTTTCTTGAT
TGACGTGTGTTACTACGCGTTGAATGACTTTATCACTTTTGACTATTTCAAGTATTTGCTTCATCACATAATCTT
CAATCACATCAGCTCTTACACTGTTTGCCGAACATACTTTGGAACCCTTGTTCCGAAAATTACTACATGAATAG
TAACGAATACGTTTCTTAGTCCCGTCTTTAAGTGTATTCGTGGTATTGCTTGCTGCCATAGGTGCGCCACATTG
GGGACAGTGAATAATGCCTGTAAGCAGATTCGTTCCTTTGCCATGGACTTGGGGTTTTTGACTGACTTGTTT
TTTACGCATTTGTACTTTATCCCATAAATCTTGATTAATAATGGGGGAATGCTTACCTTCAGCTATCACTGGTTT
ATCATTAAGCCCTTTACGACGTTTTTCACTCCAATCTTTGTATTTCGCAAATTGAATTTTGCCGATATAGAATGG
GTTAGCTAATATATATGTGATAGAACTAATACTAAAAGGTTTACCCTTTTTAGTGACATAGCCTTTGTGATTTAA
TGCATTGGCTATTTTACGATAGCCATGGCCTTTGGCATAGGACTCGAATATATATTTCACAATATTAGCTTCATG
TTGATTAATCATCAGTTCATGTTTACTATTAGGTATTTTGTCATAGCCCAGCGGCAAATTGCCTTGATAATAGCC
TTCTTGGGCACGTCTCGTTTGACCCATAAATACATTCTCGACAATGTTATTACGTTCGAATTCTGAGAAACTCG
CAAGTATTTGTAACATGAGCTTACCAGATGAAGTATTGACTTCCATACGCTCTGATAAACTGAAAAATTCGAC
ATTTTGTTTGTGTAAATCTTCGACAATTTTGAGAAGATCAGATGTATTACGAGCTAATCGGTTTGTTTTGTAGA
CCATAACACAGTCGATATAGCCTTCTTTCGCATCCTTCAATATACGTTGGAGCTCAGGTCGATTCATAGATTTAC
CTGAAATACCACGGTCAGCGTATATATCTTTAACTTCAAAATGATGGAAGTCACAGTATTCTTTGATTTGATTG
ATTTGTCCGTCGATACTATAACCTTCTGTGCTTTGCATTTTTGTTGATACACGTACATAGATACCGACACGTTTT
GTTTTAAGTTGTTGCATTATGTTTCATCCTTTCTTCGTTTATGCAATCGATGATTGCATGGTTTGATTGACGATA
TTGAGTGGTTCATTTTTGAAATAGATTCCTATAAGATTTTTATCTTTCGTAATGTGAATGGTTTCAATATAGGGG
TACAATATGTTTAACGTGAAACGTTTTTGAATAATATTTTGAATGGTGTGTTGTATTTGATGTCCATTGATAGAT
GTAGTGCGTTGCGGTTGTTGACGTAATGATTGCGTTTGTTCTCTGAACGTTTCTGCATCGATGATGCCTTGTG
CCAACTTTTCTATCAGTTGTTCTTGAGTCAATGTGTGATGTTTTTCTATGTTTCTTTGTCTTTTGATGCGTTTGT
CAATCGCACCTTTAATTTTTGTGTAGATGCGTTGATTTTGATAAAAGTCTTTGCACACTTCTAATACTTTATCTT
CAAGTGTTTGTGCATTGATGCCTTTAAAATCACAGACAAAGCGAGAAGCATTCATGTTTTTAGGACAGACGT
AGTAACGTAATGTATGATTCTTTTTTCTAACGGTCATATTCGTAAGTGTTGTATTACAACATGGGCATTTGATTT
TTTGTTTTAGTTGGTTATCCGAAGATGTCTGTTTGATTTGTTTTTGCGATCGAAGTCTCTGCGCTTGCTCATAT
ATACTTGTGGAAACAATAGAAGGAAACATATTGTCGAATTGGCCATATTGATTGTTGACACGACCACAATAAT

TAGGATTGATGATAATGTTACGAACTTGATAGGGTTGTCGATTGATATACGTGTTATCTTCTTCTAATAACTGTG
CAATTTTCTTATAACCATGACCTTTAATGTAATAATTGAATACAGCCTTTACCGTTGGTGACTCATTTTGATTGA
TGATGAATGCTCCGTTGTGATATTCGTAACCAAAGGGCGCATGGGTTGTAATCAATCGACCTTGCTTTGCTTT
TTCTTGAAGCCCATTTCTGACTTGTTCTCCAATGTTATCTGATTCAAGTTCGGCCAAGCTGATAAAAATATTAA
GCTTGAATCGGTCGAAAGCTTGATCCATATCAAAGTAACCATCGTGTACGCTTAAGATATGAACATGGTATGT
TTGACATAATTTGATGAGTTTTAATGCATTTTTCAGATTACGATGTAATCTATTAAGACGATAACAACATAATAT
GTCACATTGCCCTTGTTGAATTAATTGCGTCATTTGTCGATACCCACTACGATTATCTTTGCGTCCTGATTGTTT
GTCGCTATAAAAGTTGATATGTTGAATATGATGTTTTTCGGCTATTGCTTCGATAGCCTGTTTCTGTGCTGCAA
GAGATTGTTGTTTCATCGTACTTTGACGTAAATAGCCTATGACTTGTTTCATATCGGCTCCTCCTTTCACAGTAA
TAATATATATTTATGGATGAATTGATATATAAGCCCAACATCAATAAGATGTTGGGCGTTCATATTAGTCATTCAT
TTGATTGATTTCTTCAATTACCAAATCGGCTAATATCTCGATAAGTTCATCCATGTTTTTCACTCCGTTATTTGTT
CTATCTTCAATACGTCGATTATTCAGTTTGATGCTTCACGGTTGTATGATAAAGACAATCAGAAATCTTCGTGA
ACTCCTGAAGGGCCTATCCCCTCATTAGCGGATTTAAAAAGTTCTTTCGCAGCTTTGTTATCATTTGCCGGTG
TCCAATTTTGAATTAACGACTTATCTTTAGTTAATCCCAGGATAGATGCAAACTCTACATCTAATTTTAGATGGT
AAAATACAAGTGATTGTTTTTTACCGCTATTATCTTTGACACTTCTTTTAGTTGTTTGGCGTCCACGGTCAGCT
AATATGAAACCTTTATCTCTTAAGGCGTTGACAACATTATTAACATCTTGAAATTGATGATTGTTTAGCATCTGT
TTAAAAACGTTTGCAATCATTTTTACTTCGATATGGTCATCTTTTAATGAGATTAATCCATAGTTCTCAAACATAT
TTTTCAAAGCACCTTCATCTGAAAACTTACCTCTGTTTTGTGCTACAAATTGAATGATGACATCGATAGCTTTA
TCAGCTAATGAGCGTTCAGAGATTGTATGACCATGATAATCAATAAAGTAGTCTCTTATTTTAGCGATATCAATA
TCTGTAGCTAAAACACGACCTAATATTTTTGCAGATGTTGTAATGACTGCATAACGCTTAAACATACGATTGCC
TGTGTTGCTTTTATCATCTTTCAATTTAGCTTCAAACCAATCTACTTCCTTGTAAAACCATTGAATAACTTCATCT
TCACGATTTATAAGATATTTAGCTACTAACGGTAAAACATGACCATAGTTTAGTGCTACAGCTTTTTTAATATTG
TCAGCATTGGTCGCATTTGTAGTGAATTGTTCATTAATCTCGATGGTTCTTACACGTAATCCATCGTTTTGAGC
TGAATCATTAAAAATACTGTGTTCTGACGTTGAAATGACAGAAGTACCCCAATTCTTAGGCGTTTTAACTTCTC
CATGAACGTTTGAACGTTGACGACCTTGACCTTCAGTGATGGAGTACAATAACCCCGTTGTATCTCTAAAAGT
TGCTGATGAGAGTTCATCAAATACAATAGGTATACCAAAATTGTTACTCAAGTAACCTTCAAGTGCATTACGTG
TGGCATTCCAATTTCTAAAGAGAGTTTCATTACCTTTGGTAGGGTTACCAGCGACTGATACAGCTAAAGAAG
CTGCAGTTGACTTACCGGTTGAGGATTGACCTGTAAAACTAAAAATGATTCCGGCAAATTCGGTTTCATGTTT
GTGCTTCAGGAAACTCGTCACTAAGGCAGAAATACCAAATACGACTGCTAATTCTAAAAGAAGAGAACCTTT
AACCTCTTTTAGATACATGTTAAACCAATTATCAAATGTACCTTTAGGAGTTAAGTCATAAGCATTATCACAAAT
GGCGTCAGATGGAGATTTATTATCAAATTCTTTAGTAGTATAGATTTCACTTAACGATACAATAGGACCAAACG
GTGTTTCCAGTATACCTACCCCTTCATATAAGTAGGAAATGGGTAATTGATTGCGCATTTGTTGCAACGCATAA
CCTAAATCTTTTGTATATTTTTCATTAATACTAAATCCATACTTCATTAAAGATGGCAGTTTTTGTGTTGTTAAAA
TATCACTAGATTCAACAATTTCTTTTTGATCCTCGTCTGTAATAATTACTTTTTCAGTGTTAGTTTTAGGGTCAA
TAAACTTATTTTCGATAACGATAGGACCTGCGATTTCAACTTCAGTAGGCATTCCTCCTTTTTCTTTGGGAGGC
TTGTCTTTATACCAACCTTTTTTTGATTTGTATCGTGGTGAAGGATTAAATGAAGGGTTAGTTTGAGTCATTAG
CGAACACCTCCTTTCGAAGGGTTGCTGTTATAGTGTGGATTAGGACCTGTTTTAAGATAAACTAAGTGACCG
TGAGTATCCTTACCGATAATAATAAATGGAACACGTGGCGCATGTTTTACAAAATATGCGAACCAACGTCCAA
CATTTTGTTGTACAGCTTTTGAACATTGTACATTTGCACGACTGTTCAAGTCATGAAATGTAAATTCTTCTCCT
TCAGGTAAATTGAATGAAATACCTAATATTTTCTTTTTTAACTGCTCGAATTTATCTGATTGATTGAAATACATTT
AACTTCCTCCTACTAATGAACTAAGATAGGAAAATTAAATATGCGCACAATTAACTTTCTTAAGTTCATTTTAG
CCAAGAAGTTATCATTTGAAAGCTTGTAAAAATATGTTAGAAATTCCGTACTAAATTAAGATGGATTTTTGTTG
ACAAAATAAAAAACGCTGATTTAACAGCGCTTTAAATAAAAATTAATCTGAAGTTATATAAAAGTAGTCAGAA
GGAGTGGTGTTATTATCATTATAATAATCTAGTGCTTTATTGATTTCTTTTATTAATTTATCATGATGTTCTTTTGT

TATTTTCTTTGGATATTTGAGTTCTTTTTCTTCTAAGTCTTTTGGAGACACTAGGTCACTATTTAAAATTAATGA
TAACAGCATATTTTTAGGTGCCGAAGCTCTGGTGTCTCGATGAGTAATATATTTTGCTAAATTACTGTTTATACT
TACTTTTTCATTTTCTAAAAAGTTTGTACTATTTTTAACATGAGCATCAGCAATTTTGGTACTTATTTCAGTGTT
TAAATCGTCTATTTCATCTAAATAATATTCAAGAATCTGAAGTTTATCTTTAGAAGTGATACCTTTTTCAGTGAA
AATCTTAATAATGATAGATTTGATTCCAATTTCTATATTACTGTCTACTAGAAAATAGAGATAATAATTAGTCAGA
GCATGTTCATAATCTCTTTGATCAGTAATTTCATGATTGATGAAATAATATAAGTTCTCTTTGATACGATTAGAA
GAATCTATATCTTTGTCATTGATACCGTTTTTAAAAAAGTGTAGATAAACTGAGTTGTTTGAGAGTATATCATAT
AATACAAATAAACTTTTTGGTTTAAATACATATGATTTTCCTGATTTAAAGTTTGCTATGTTTATTCCTAGAGCG
TTGCAGAATTTATTAAAATTAGTTGTAAAATTATTTTTTTCTTGAATAAACATGTCGTTGTCATTTAATTCTGAA
CTATGATTTTTAATCATATTGATATTTTCTTCAATCTTGTCACTACTTAAATTTTCCATGCAAAGTATACTATATCT
TACTATATCACTTATATTTATGGAAAAATCTTTAGGAGAACGATTTAAAGTATCCTTTTTATTATCTTGATCTACA
GCCCTACATGAATCATCAAAAACTTCTTTGACATTTATTTTATCAATGTTCAAAATTATGTCTACTAAAGCATTT
TGAAAATCATCGCTATATATGTCTTTATTATTCATAGTGTCACTACCTTTATTTTTAATAACACTATATATTATATCT
TATAAACCTAATACCATGAAGAATAAAAAGAATGATTCAAATTTTATATTTAATCCATAGTTGGCTGCTATGGTT
AGTGTACTAGTCTAGTTTTACTAGTATATAAAACAGTACTCAATTTTTATTTTTTCAAAATTATAAAAAAGAACA
CCTATCATCGATAGGCACTGAACCCCTAAAACGGGAACTTAATAAAAACACCATGTTCTAGGCTATTAATTCTC
TGTATTTTACAGGGGATAAGTAGCCTAGTTTTTGTTGAATTTGATTATTATTATAGTTTTTAATGTACTTTTCGA
CAATATCTATTACAATATGATTAGAGCTATTAAGCTGATTATTGATGTAAAGAGTTTCAGACTTTAGCGAGGAA
TGGAAACTTTCTATCGGGGCGTTATCGGCAGGTGTTCCCTTTCGGGACATACTTCTGATAATGCCTTTTTCTTC
GCATAATTGATAATAAGCATAAGATGTATAAACGCTGCCTTGATCACTATGTAATATATACCCCTCAGGTATATCG
ATTTGATTTAATGTATCATTAACTAAACGTTGGTCTTGTTTATCATCTATTTTATACGCCACAATTTCTCCGTTATA
AATATCCATTATCGAAGATAAATACAACATAGAATGATCAAATGGTAAATAAGTAATATCGGTTGTTAATACTTC
TATGGGACAATTCGCTTTAAATTATCTTTGTAATAAATTGTCTGTTTTATAATACGGTTTACCTATCCTTGTCGTC
TTTTTAGGTCTAACTCGGCAGTTCAAATGATGCTTCTGCATCATTCTCTGTACTCTCTTATGATTAATTGGTGAT
GTATAACATTGATTAATCAGTGCTGTAATCTTACGATAACCGTAGGTATAATGGTTAGCTTCACATAATTCAATA
ACTTTTTGTGTTACAGTATCATTTTTATAGGTTTTGTTTTTCCATCGGTAATATGTTGATTTAGGTATATTTAATA
CTTCTAGTATCAATTTGATTGAATAGTTTCCTTTTAATTGATCCACTAAATCTATGACTACTGTTGGTACCACTTC
CTTTCCAATGCCTTGTACTTTTTTAAAATATCCAATTCTATATCTTTTCTCTTATTTTCTAATTTTAATTGTTCTACT
TCTGACAGCTCTTCTAATCCTTTACCGTAGGTATATTGTTTACCAACGTGTTGTGAAAATCTATAACTTTCCCCA
TTTCGATACCATCGCCACCAAGTTTCCACTTGTGTTCTATTTTTAATATTTAATTCTTTCATAATTTCTTTTGTTG
AAAATCCTGCTGCTTTCATTTCAACTGCTTTATACTTTGTTTCTACTGAATAAGAAACTCTTTTCATAGAAAAA
ACACCTCCGTATGATTCATTTTAATATGAATTCAACGAAAGTGTTTTTATATAATTCCCACTAATTGGGGTCAGT
CTACTATATGATACGGTTTTTTAATTTAAAGTATCAATAATAATTGGATATAGAGGGAAAGAAGCTATAATGATA
TTTGCTTACTAAGTGGATAAAGATATTAGAAAAACGAGAAGCAGGACAGTTATTAAATCGGTAGATATTAATC
ATAAGTATTTAACTGAATTTTTACCTTTTGTCGAATATATAACTAAAGTTGAAGGTAGTAAAATATTTATCTAGT
CGATATTAAAACAATTTATCGAAAACGATGGTTTTCCATATCCTCTTTAGTAGCAATTTTCTAACAATATATAATT
AATTATATACAAATTAAGAATAATTAAGAAGTATATATTAATTAGAGATAGTTTAAAAATTAGATTTCAATCAATT
AATTAAAACAGGTTAGACTATTAATTTATACTAACCTGTTAAAAGTAACAAAATTAAGAATATATTAAAACTTGT
GTAATGAGTAAGCCTCCACCAAATATTAAAACAAACGCGACAACAGGCCATACGAAACGTAACCAGTGTGA
GTAGCGCACGTTTAACATTTGAAGTGTTGCCATTACAAGTCCAGTAGGCGCTAAGAACAACATTGCATACTG
ACCGAATTGATATGTTGTAACAATAACAAATCTTGGTATACCTACTGTATCAGCTAATGGCGCAAAGATAGGCA
TAGATAATACTGCTAATCCTGATGATGATGGTACGATAAATCCTAAACAGAAAAAGATAAAGAGCAGAACAAT
GATAAATAAAGGCCCACTCATATGTTGCACGATAGATGATGAAAAGTGCAAGATTGTGTCAGAAATCATTCCT
TTATTCAATACTAAGTTGATTCCACGAGCTAAACCAATGATTAAAGATACACCTACTAAACTTGAAGCGCCATT

AACGAATGCATCTACAGTGCCTTTTTCGCCTAAACCATATTGTCCTGTTCCAGCAATAAACATGATGACAATGG
TAAAGATTAAGAATGCAGATGCCATGACTGGGAACCACCATCCTTGTGTCATAACACCCCAAACCATAATAGG
GAATGGTAGGACGAAAAGCGTAAGAATAATCTTTTTACGTAATGTAAACTCAGAAGAACCGTCATCATGGAG
CACAGACCACTGTTTTTCAAATGCTGCTTTGTCTTCATAAGAATAAGAGGATTTAGGATCTTTTTTAATTTTTT
TACAATACCAGAATAAATAACTAATAACAAATATGGCACCGATGATACAAGCGCCTATTCTCCAATAAAGACCA
TCAGTAAAAGTTGTTCCTGCTGCATTAGAAGCAATGACGACTGAGAATGGGTTGATTGTTGAGAATGTACTA
CCCACAGAGCTTGCTAAGAAAATTGCACCGAAAGTAAGACTTTAGAATCTATGGGAAAGATAATAAGGTTTA
ATAGAAATACAAGAAGTGTAAATAGTAAGCATATAATTTATACATTTTAATATAATATAACAAGAAAGGTAAACA
CAAAGATAATAAAAATAAAAATTTAATAAAGATAGAGAATTATTAAATTGTTTTTGATATAAAACAACTTAATAA
AAAATTTAATTTTAGGGGAGGATAATTATAATATAAAGAAACATAGAATGGAATTGCAATTGGGAAGTATACG
ATTTTAACTGCGAATCATATGTTCAACGACTTACAAATGCGTAAATCGATAACACATTCAAAAATATATAATCTG
AAAATTGATTTACTAAGAGAGGATTATTGTAGTTACAGGTGTTAAGTTGCTCCAATATGGAGTTGTCGTCTTG
TTTTATATTAAACAAGTTTTATTCAAATACATGACAATACGTAAAATAACACTAGAATTATCAATCACTAAAATTA
AAACAAATGTATCTATTCAAACTTTTCTTTAGGGTTTACTTGCTGAATAATTTAAAAATGAAGTATTAGGTGTA
CATGCTTTCAGAATTGCTTTTGAAGAATATTAAAAGTATAATCTGAATACGATGACAGAATGAATAGGTAAACT
ATGACAAGAAGTCATCAAGGTTATCAAATAAATATATCCCAGCGGTTGAGACGACTCACTGCTGGGATTTTAT
TGTTAGTTATTTATTCTAAGTGTTGCATTTGGTAGCCGATTCTAGGATGTGTGATGATTAATTTCTTTTCAGTGT
TTGGGTCTTGCGTATAATTCAACTTTTTACGTAAAGAGGCCATATGTACACGTAAGGCAGCCATTTCTGTATGA
TTGACATAGCCATAGAGTGATTTTAATAACACTTGATAGGTTAATACTTTGCCGACATGATGGCATAATATCGTT
AGGAGTTGGAATTCATTCGGTGTTAGATGTACGGACTGTTCATTGACAAGGACTGATTTCGCATCGAAATCA
ATGGTTAATGGACCATTTGTGAAACGACTTTGAATTGTCTCAGTAGAACGTGACATACGTAATGCTACTCTGA
TACGTGCTCTTAACTCATCGATATTGAAAGGCTTAGTCATATAATCATTGGCACCGCGATCTAATACTTCGACA
ATGGTTTGTTCCTCTGTTCGTGCACTAATCACAATAATAGGTGTGTCCACAAAGTCTCTGAATTGCTGAATGA
GAGATAAACCATCAATATCTGGTAAGCCTAAATCTAATAAGATAATATCTGGTTGTTCTGTTCTTAGGCGAAAG
TCCGCTTCTTTCCCCGTCTTCGCCGTAACCACTTTATAATAATTCATAGTTAGCGCAACATCGATTAAATGTAAA
ATCGCTTCATCATCTTCAACGACCAATAATGTTGTTTTCATCCATGGCTCCCTCCATTTCAATTGGATTAACAGT
CAAATAAAAATAAAAAATACTGCCTTGTGGTGTATTCGGTTGATATTCTAATTCACTGTTGTGTTGTTTCAAGA
TGAGTTGTACTAAATAGAGCCCTAATCCCAAACTATCTTTTTTATTGTCTTTAAAGTTGTCTCCTGAATAATAGG
GATTAAAAATCAATTGACGTTCTTCTTCTGGAATACCTTTTCCGCAATCTATCATTTCGAATTTTATTTTGTTTG
TTTCATGTTGAACGTGCAGTTTTATTTCAGAATGTGATTCTGCATGCTTTAAAGCATTATCGATAAGGTTGAAT
AGCACTTGCAGTATTAATTTACTGTCGATATTAATGAGTGAAGCGTCATCCTCATTTTCAATAATGACATGATTT
GCTTGGTGTCTGCGTATGAGGCCTTCTTCAAATTCTTCTAGAAGTTCTTCTACTAAATAAGGGGTGCGTTGTA
TTTGAATGTCAGAGCTTTCTAACTTAGTCAAAGATAAAATATTTGTGACTAAGGTATGCAGATATTGTGCTTCG
CCATAAGAAGCAGTTAAGAGTTCTGCTTGTTGTTGATCGTTTAAATGCTCGTTATGGTATTTCAGCATATCTAA
GTTGCCCATAATGGAAGTCAGTGGTGTTCTAATATCATGTGAAATTGAATGCAAGAAGTTTGAACGTGTGGC
TTCTCGTTCAGCTTTCAATATGGATTGTCTGGTTTGTTTTAATAGATCCACATTCTCTATTGCCAGGGTAATATC
GTTCAACATAGAGTCTAATATTGAATTGTCATAGGTGTCGATGTAAGTTTCGTCGGTGAAGCGGATAGAAATC
ACACCTTTGACTGGATTTGTGCCAATGGGAATACACAAAAAATTTACTGCCAGGAAAGGTATCGGTTAATTTAC
CGGCACGGCTTTCATTTTCAATGACCCAGCTCAATGTCTCAGCATCATGTGTCTTATCTGAGCTTGAAATGCTT
CTGTTTCCAAATGAGTTGGAAGCAGCGACTTTCTTACTTTGAATTAAGAATACCGTGACATCCTGATTGAGTA
GCTGATGAATCTGATCACCAGCAATATTTAATAAGCGTTCAATTGAATAAGATTCTTTAATGGACTGGTTAAAT
TGCAGCATAATATTGGTCCGATATAACTGCCGTTCTGTTAAGGAATGCTGATGTTTTAAATTCTTTAAGATGGC
ACTCGTAAAGATACTAGCAAAAATACTGGTCGCAAACGTAATCGGATATTCAAAGCGATACATTTCTAAAGTG
AATCTTGGCACCGTAAAGAAATAATTAAAAACAAACACATTTAAGATAGACGCAAAGAATCCGATTAAATAG

GATTGGGTCCAAATAGAAAGCACGATAATACCGATAAAGAACATCAGCAAGATAATGGCACTGGATTCGCTT
TTATCTAAGTTGTAAACCCATATACCGAGCAAGACACAGATCGTTTGAATTACAAGCATCTTCATAATCTCTAC
AGTTAAACGAGAGGATTTTTGAGCTTGCTTAGGTGTATTTGTAGGTTTGTCTGAATGAATATAATGAATCGGT
ACAATTTCTAACTTGAAATGGTGTGGTACGTGATTAATTTGTTCAATGAGTGACTGTTTAAAGTAATCTTTCCA
ACGTGGCTGTTCTGACTGTCCAAGGACTAGCTTCGTCACAAAAGCGAGATCACACCAATCGGTTAACGCTTT
CGCAATATCTTGTGCATACAACACTTTGATTTCTGCACCTAACGCTTTGGCGAGCATTAGATTTTTATGGACAT
AATGATCTTGTTTTCTGCTTTCTGACGGTGTCTCAAAGACATCAATATACACAGCTGTGAATTTAGCATGTTCT
TTATAGGCAGCACGTCTTGCTTCTCGTATGACCCGTTCGTTATAAATACTGCCGCTGATAGCTACCGCAATATG
CGGTGTAATGTCGGTATGTTTAGTTTTATATTGTTGTCCTTTTTGACTCATAATATCTGCGACAGTTCTGAGTGT
AAGTTCACGCAGCTCTGTCAGATTTTCATACGTAAAGAAATTAGAAAAGGCTGTTTCTAAGCGTTCTTTTTTA
TATACTTTTCCTGCTTTAAGGCGCTGAATCAACATATTTGGTGAAATATCCACAACTTCAAAGGCATCTGCTGA
CGTAATGAATTGGTCGGGCACACGTTCTGTAACTTGAATACCTGTCATTAACGCAATTTGTCCGCTTAGACTC
TCGATATGTTGGATGTTGAGTGTTGTCCAGACATCGATACCATGCGATAGAATTTCTTCTATATCCTTATAACGT
TTTAAATGGCGCTCTTTTGAAATGTTTGTATGTGCTAGTTCATCAATTAAGACCACATCTGGATTAGCTTCTATG
ATTTTAGAGACATCTATATAGTGAAAGGTGTGGCTGCCAAATTTACGGCTGGAGGTCGAAATTTCAGGCAAT
TGTTGAACCAGTGCATTGGTTTCAGGGCGTTGATGGGGTTCGATATAACCAATTTTAATATCTGCACCTTCTT
GATACTGATCAATACCATTTGATAACATTTCATACGTTTTACCTACCCCTGGGCTATAGCCTAAATAAATGGTAA
GTTTCCCTCTTTTTTTATATGTACTTTCCATGAGGCACCCCCTCTTAATCACATCATAATTAATATACAACATATTA
TCCATTTATCTTTCTGTTTTGTTAATCATCTTTATAGTTTCTTTATAACTTTCCTTAAATGTTAATACTTCGATTAG
ATAACTCTTGTTAAGATTTTGATGAAAGTAAGGAGGGAGCGCAATGATTACACTATTAGCTGTCGTTGTCATC
GCATTAATTTTATTTTTATTTTACGCATTAATTTGGAGTGAAAAATTTTAACAGAGAAAGAGGGAAGCATCAT
GAGTATTGTGTTGTTTTTGATTGTATTTATCTTGCTCTCACTCATTGTGAGCCGATATTTATATTCAGTTGCTTTA
AATGTGCCATCTAAAATAGATGTTGTTTTTAATCCGATTGAGAAATTGATTTATCAACTGATTGGCACGAAATT
AGAACACATGTCTGGGAAGACGTATATCAAACATTTTTTGTTGTTTAACGGATTGATGGGCGGATTGTCCTTT
GTATTATTGCTTATTCAACAATGGCTGTTTTTGAATCCTAACCATAATTTAAATCAATCTGTATCGTTAGCCTTTA
ATACTATGGCATCTTTTTTGACCAATACTAACTTACAGCATTATGCAGGTGAAACAGATTTAAGTTATTTAACA
CAAATGTGTGTCATCACTTTCTTAATGTTCACGTCAGCAGCGTCAGGTTACGCCGTATGTATTGCGATGTTAAG
ACGTTTGACTGGAATGACAGATGTGATTGGTAATTTCTATCAAGATATTACGCGTTTTATTGTACGGGTGCTCA
TACCTTTCGCATTGATCATCAGTTTGTTTTTAATCAGTCAGGGCACACCGCAAACGCTTAAAGGTAATTTGGT
GATTGAGACATTATCAGGTGTGAAACAAACGATTGCATATGGACCGATGGCGTCTTTAGAATCTATTAAACAT
TTAGGGACAAATGGTGGTGGTTTCTTAGGTGCGAACTCTTCTACACCTTTTGAAAATCCGACATACTGGTCTA
ATTACGCTGAAGCTTTAAGTATGATGTTGATTCCAGGTTCATTAGTCTTTCTATTCGGTAGAATGTTGAAAACT
AAACTACAGATTCATCCGCATGCGATTATGATTTTCGTTGCGATGTTTGTAATGTTCATCGGCTTTTTAGTGAC
ATGTCTCTATTTTGAATTTGCGGGGAATCCAGTGTTGCATCACTTAGGTATTGCCGGTGGCAATATGGAAGGC
AAAGAAACACGTTTCGGTATTGGCTTATCCGCTTTATTTACAACCATTACGACCGCTTTTACTACAGGAACAG
TTAACAATATGCACGATAGTCTTACACCGCTAGGCGGCATGGTTCCAATGGTATTAATGATGTTGAATGCAGTT
TTTGGCGGTGAAGGTGTTGGGCTGATGAACATGTTGATTTATGTCATGTTAACGGTCTTTATCTGTAGTTTGA
TGATTGGGAAAACACCAAGTTATTTAGGAATGAAGATTGAAGGTAAAGAGATGAAACTCATTGCGCTTTCTT
TCTTAGTACATCCTTTACTTATTTTGGTTTTTTCAGCACTAGCTTTTATTGTGCCAGGGGCATCAGATGCGTTA
ACTAATCCGCAATTCCACGGTGTATCACAAGTGTTGTATGAGTTTACATCATCTTCAGCGAATAATGGCTCTGG
TTTTGAAGGATTAGGAGACAATACGGTATTTTGGAACATTTCAACAGGCATTGTGATGTTGCTTGCACGATAT
ATTCCAATCGTTTTACAAATTTTGATTGTATCTAGTTTGGTAAATAAAAAGACCTATCAGCAACATACTCAAGA
TGTACCGATTAATAATTTATTTTTCAGCAGTGTATTGATTATCTTTATTATTTTGTTGAGCGGCTTAACGTTCTTA
CCTGACTTAATGCTTGGACCAATAGGCGAACAGCTTTTGCTGCACGCATAGATAAAGGAGGATTAGAAAATG

GCTGAAACTACTAAAATATTTGAATCACATTTGGTCAAACAGGCTCTAAAAGACAGTGTATTGAAGCTCTATC
CTGTTTATATGATTAAAAATCCGATTATGTTTGTTGTAGAAGTGGGCATGCTGCTTGCCTTAGGATTAACCATT
TATCCGGATTTATTTCACCAAGAAAGTGTATCACGGCTATATGTGTTCAGTATCTTTATCATATTATTACTGACAC
TTGTCTTTGCGAACTTCTCTGAAGCATTAGCTGAAGGTCGCGGTAAAGCACAAGCCAACGCTTTACGCCAAA
CACAAACTGAAATGAAGGCACGTCGTATTAAACAAGACGGCAGTTATGAAATGATTGACGCTAGTGACCTG
AAAAAAGGACATATCGTACGTGTCGCGACAGGTGAACAAATCCCAAATGACGGTAAAGTTATTAAGGGCCT
CGCAACAGTGGATGAATCTGCGATTACAGGTGAATCTGCACCTGTAATCAAAGAAAGCGGTGGAGATTTCG
ATAATGTAATTGGAGGAACTTCTGTAGCTTCAGACTGGTTAGAAGTTGAGATTACTTCAGAACCAGGTCATTC
ATTTTTAGATAAAATGATTGGTTTGGTTGAAGGGGCTACAAGAAAGAAAACACCTAATGAAATTGCGTTATT
TACTTTATTGATGACATTAACGATTATCTTCTTGGTCGTTATTTTAACGATGTATCCATTGGCGAAATTCCTGAA
TTTCAATTTATCCATTGCGATGCTGATTGCTTTGGCTGTGTGTTTAATTCCAACAACCATTGGGGGATTATTATC
GGCTATAGGGATTGCAGGGATGGATCGTGTGACACAGTTTAATATCTTGGCTAAAAGCGGACGTTCTGTAGA
GACTTGTGGTGATGTGAATGTCTTGATTTTAGATAAAACAGGTACCATTACCTACGGCAACCGTATGGCAGAT
GCGTTTATTCCGGTGAAATCATCAAGCTTTGAACGTTTAGTTAAAGCGGCCTATGAAAGTTCTATCGCAGATG
ACACACCAGAGGGACGTAGTATTGTGAAATTAGCTTATAAACAACATATCGACTTACCGCAAGAGGTCGGAG
AATATATTCCGTTTACTGCTGAAACACGTATGAGCGGTGTGAAATTTACGACACGTGAAGTATATAAAGGTGC
ACCGAATAGTATGGTTAAGCGTGTGAAAGAAGCAGGGGGACATATTCCAGTTGATTTAGACGCTCTTGTCAA
AGGGGTGTCTAAAAAAGGTGGCACACCGCTGGTTGTGCTTGAAGATAATGAGATTTTAGGTGTTATTTATTT
GAAAGATGTCATTAAAGATGGACTCGTAGAACGTTTCCGTGAATTACGTGAGATGGGGATTGAAACGGTGA
TGTGTACAGGAGATAACGAATTGACAGCTGCGACAATAGCGAAAGAAGCGGGTGTGGATCGCTTTGTGGC
AGAGTGTAAACCTGAAGATAAAATCAATGTGATTAGAGAAGAACAAGCGAAAGGTCATATTGTTGCGATGA
CGGGTGACGGTACGAATGACGCGCCAGCTTTAGCAGAAGCTAATGTAGGTTTGGCAATGAACTCAGGAACC
ATGAGTGCCAAAGAAGCGGCGAATTTAATTGATTTAGATTCTAATCCAACCAAACTGATGGAAGTCGTTCTA
ATTGGGAAACAATTATTAATGACACGTGGCTCACTCACTACATTTAGTATTGCGAATGACATTGCGAAATACTT
TGCGATTTTACCAGCCATGTTTATGGCGGCTATGCCTGCGATGAATCATTTGAATATTATGCATCTGCATTCACC
TGAATCAGCAGTATTATCTGCGTTAATCTTTAATGCGTTGATTATTGTATTATTGATTCCGATTGCGATGAAAGG
CGTGAAATTTAAAGGTGCCTCAACGCAAACCATATTGATGAAAAATATGTTAGTTTACGGCTTAGGCGGTATG
ATCGTGCCATTTATCGGCATTAAGCTCATTGATCTCATCATCCAACTCTTTGTCTAAAAGGAGGACAAAACAAT
GCAGACAATAAGAAAAAGTTTAGGACTAGTACTGATTATGTTTGTTTTATGCGGATTTATCTTCCCGCTGACT
GTCACAGCGCTTGGACAAGTATTATTTCCAGAACAAGCAAACGGCAGTTTAGTGAAACAAGATGGCAAAGT
AATTGGTTCAAAGCTCATTGGACAACAATGGACAGAACCTAAATATTTCCATGGACGTATCAGTGCAGTCAAT
TACAATATGAATGCGAATGAAGTGAAAGAAAGTGGCGGACCTGCTTCAGGCGGCTCAAACTACGGCAATTC
AAATCCTGAATTGAAAAAAAGAGTTCAAGAGACTATTAAACAAGAAGGAAAAAAAATTTCAAGTGATGCGG
TGACCGCTTCTGGCTCTGGTTTAGACCCAGATATTACGGTTGACAATGCGAAACAACAAGTAAAACGCATTG
CGAAAGAAAGAAACATAGATGCTTCAAAAATTAATCACCTTATTGATGAAAACAAACAAGCATCACCAATGG
CAGATGATTATGTTAATGTCTTAAAATTGAATATCACTTTAGATAAACTCTAAATAAACAGGGAGTGAGGTGA
GACATCCATGTGGTTCATTAGCATTATTATTTTAATAGCATTCCTAATTATATTAATGATTGACGATTTAATTAAA
ACATGACAACTTCACGCAAAACAATATGAATTGGATGTAATAGAGAAGGGAGTACCAAGAAATACTCAGTTT
ATTGAGGATAATTATTAGGGGAGATTAGAGAAGAATTCTCTATTCACGAACATTACTTTAACAGTTGTAATCAT
TGGTTTCATCATATCGATTAATAGTACAGTCATCTTGAGATTTTCAACACACTATTATAGCTAGCTAAGATACTT
TTAAATTATGACTTATTTTTCATCATTTACGATACATCATTCTCGATAACTTACTATTTGTTATGGTATTTATAGATT
TAAAGAAAGTGAAGGAGATTAAGCAATGTAACTTGGCTTAATCTCTTTTTGATTATATTTATTAAGGCAAAGC
TTCATTTCCCTTAAATCAAGTATATCTTCTGAATAAGCAAATGAAATCGTGACGCCAACTACAGTGATAACTTC
GCCTAAAACACAATGAGTGTGGTGTTTTCACTTTTCATAAACTCCTCTAAGTTTGTGTTTTTAAACTGTGATG

GATTTGAATGTTTTTGAATTACGTTGTCGCGTCCTTGGGATTGCAATCCAAGAACGTCAGCATAATCTAAATC
AATTTTGATTTGGTAGAAGACAAGAGAAGTACTTTTGCCTGTATCATCTTTCGCATTTCTTTTTGTTGTCTTTC
GATCCCGATCGGAAACTAATTTTCCTGCATTATCCAAAGCATCAATAACATTTTTCGTGTCTTCGAATTGACTG
TCTGTTAACATCTTATCGAATACATTTCGGATAATTTTTACTTCGATGTAGTCATCTTTAAGTGAGAGGATACCA
AAGTTTTGAATCATATTAGCAAGACGTGTTTGTTGTGAAAACTTGCCCAGGTTTTCCGCAATAAACTGTACGA
TTGTATCCATCGCTTTATCTGCAAGAGAACGTTCTGACACCGTTTCATTATGATAGCTTAATAAATAGTTCTTTA
TGCTGCTAACGTTTAACTGTAGGTCTAGTGATTGATTTAAAATATGGGCTGTTGTTGTTAGAGTTGCATAATGT
TTAATCATTCGAATACCAGTGTTACTGGATTCAGTTGATAGTAATTGATAAAAAACACTGTCTTCTTTTTGAAA
AAGGGAAGTAACATCATTAGTGTTGTCTAATAGGAATTGAGCAATTATAGGTAAAACATGACCATTATTTTGA
CTAACCACTTTTTTTATAGTATCTGAGTTTTGAGCACTTGATGTGAATTTTTCAGACACCTCAATAGTACGAAC
ACGCAAACCATCATTTTTAGCTGGATTTTTGAAAATACTATGTTCAGAAGTTGAAATCACTGATGTATCCTAGT
TTTTAGGTGTTTTGACATCACCATTTATATTTGCTCTTTGACGTCCTTGTCCCTCTGCAAATGAATATAGAAGAC
CTGTTGTATCATGAAATGTTGCCGCTGATAATTCATCTAATACTATGGGTACACCATAATTTCCGCTAAGATATC
CTTCTAGTGCATTTCTTGTTGCATTCCAACTCCGGAATAGGTTTTCAGTACCTTTTGTTGGGTTGCCAGCTACC
GATGCGGCTAACATAGCGGCTGTCGATTTTCCTGTTGATGATTGGCCAGTGAAAGAGAAAATAGTTCCTGAA
AACTCGACGTTATTATGATACTTAAGGAAAGCAGTGACTAATGAAGATACGCCAAACAACACATCTAGTTCTA
AAGATAAGTTCCCATGGACTTCTTCTTTATACATTTGTAACCATCCTGATAAATTGCCTTTTGGTATTAAATCGT
ACTTGTTATCACATATAATAGAAGTGCTATTAGTAATCTCATTAGAAAAATAAGGCTGGTCTAATGAAATTAATG
GTCCAAGTAATGTATGAAGCATTCCTACACCTGAGTATATTGTTGATAAAGGTGTGGTGCTTCTCATTTGTTGC
AGTACGAACCCTAAATCTCTAATGTGCTTTTCATTAATGGTAAATCCATATTCAATTAAGGAGGGTAATTGCAA
AGCAGTAAGGATTGAAGCCTTTTCGACAAGCTCTATATTATTCAAATCCGAAAGAAATACTTTTTCTTTACGA
GTCGCGGGGTCTATAAAACGATCTGTGAAAGCAATCGGTCCAGATAACATTATTTCAAACTCCGTACCCCCAT
CTTTTTTAGGAGGGATGGTTTTGTACCATCCCGAAGGTTTGAGCTTGAATTCGTCAGTTTGATATAGAATTTT
TTCCATTAACAGTGACCCCCTTGGTTGTAAGAGGAAATTATAATGATATGTCGGTACTTAAGTGGGTTAGTTC
CTATAATAATGAAAGGGATGCTTGGTTTGTACTTTATCCACCAAGCAAAGCGTCTTCCTGCTTCACGTTGATC
GTTGATGTTGCACTGTTCTCTGAATAACTGTAATTATAAGGTTTAGTAATGGCAATTTTTTATTTAAACAAATTT
TTACGAATGGATTGTCTGTTTATTAATTCAGCAACGTTTATTGAAGAAAAATAAGTGTCTTCTGATAATTGCAA
AGGGTTTAATTCAATGTAAACTGAATTAAGGGTAGGGTAGGTATGCCTGTTTTTTGGTTCTTTTAAGTTCAAT
CGATTAATAAAATTGTTGAACCTTTGTTCTAAGTCATACAAAAACATCAGTAATTCATTAGGAGAATGATTTAT
TATGCAATCCATATTTTCGTTTATGCTTGAAGTTATACATCTAAAAACAGTATTGGTTTCTTCGTATAAAAATAAC
TTATTAATAGAGGTAACATTGATTAAGACTGAGTATACGTATAATAAGAGTGACTCTGACATCTCATTTAGATAA
ATAAGACTGTGATCATTCTTATTTACGATTATTATATGTGATTGAATCAATTCAATAATGTCTAGAAGAAGACTT
GAAAAGTCGCTGTTAATCTTGGTCTTTGAAAAATCATCGTGACCATTTGTCTTATTACTTAGATGTAGTTGTTC
GTATTTTATAGTTTGGCGGTTGAGGAATTAAGCATTTGATTATTATAAACTATCTCAAAAATTAAATTACCTCCT
TTTTTCTTCCTCGATGAAGGTTTATCTAATCTTCCAATATACAGGTTTTTGAAGTAAATTATAGATATGATAATCA
TTAATTCAGTAAATGTATACTTATTAAGGGGTGGAGCTTTAGCTAACTTATCGATAGTACTGCCGATGCGTGCT
TTGAAACTATTCAAATTGTCGTGACTGTCTATATCTAAATCTTCAAATTTACTAGCAAGGGTTTGCAAAGTAGA
AATTACAACTTCTTCTTTTGTATAATATTTATTGAAAATATCATCTTCTTTATCATCGCTTTGGAGTTTTAAAGTG
CCTGGTTCTAAGATGTCAAATTTTTTGTTTCGATTGGTATTTTTTTAAAATGTGCCTTGTATTTTTGGGGTTGTT
CCAAATGTGTATTTGCTAGACTACCTGTAATTGATATTGCTTTATTTAGCATTTGGTGTGTATTAACTGTATCTGA
TTCTTCTGGAAATTTAAATCTAAAAGTAAAATCTCTACTACCGAATGTACTTTTTACGGCTACAGTTTTGTACTT
ATGCTCAGGCAATGTAATTAAACCATCTTGATGCAGGAGGAAAAGTGTGTCATCATTTAATTTCTCAATTGTAT
TAAATAAGTAATTATGCATAGCCTCTTTATACATGGCGAATTGATAATTTAGATTATCCCATTCTATTAGTTGATT
TAAAATACAATATAGACCTTCATTCAATCGGTTTACGAATATCAATACAGCTTCTTCTCGTCCAACAAGGCTTAT

TAACTCCTTCAAGTCATCCTTTAATTTTAATTTTTCTCCTTCAAAATTGTATTGAGTGAAGCCTATAGCCTTTTG
AAAAGTAACTTCCATGACAGAGTTGGTTTCAGAATATTCTAACGATTCATTTATTGCAAAAATCAAATTTTTTA
TAAATATGGAATTACTCATGGTTTGTAATTGCATATACTACCCAACTTTCTTATTTACTTTAAAACAAATATAAGC
TAAAGTAGTTATTATCATAAAGGGAACGTACGTTCTTATTTAGCTGATTTCTATGAAGAGAGGTTATGGATATT
GAATGAAGAAATCTTACGCGAAGTAGCAGATATTTTTATAGGTGATGACAGAGATAGCATTTATGATTATAAA
ACTGGGAACGAATTAGTGAGGTTTTTTAATCATTACTTTAATAAAGGGGACATATATCAGGCTCCGTTTCCATC
TAGATGGCTATATGTTGTGAAACATTTGCAAACTCTGATTCAGGAGAGAAAGATCAATCAATTTTTCACATTA
ATTTTAAGCAATCACTATATTAAGTATGAACTGAAAATTGACGAGGTTGAAGCAGCAAAGCAGGCTGCTAAA
GCACTTAAGTTGTTCAACAAAAGATTAAATCATTATGGGTACTACATAACAGGAACTAACAATGCTAGATATTT
TATGGATAAGGATGAAGATACGGAGTCTATTGGTTATGGAGGGTATGCGAATATTTATTTACAGAAGTCTACA
GGTCTTGCTGTAAAAAAATTGAAAGAGGAGTATCTTACTGATTCTTCAATTAAAAGTAGGTTTAAGAGAGAA
TTTGATCTCACTAAATCTTTTGATACAAATCCATTGTTCATTAATGTGTTTGAATTTAATGAATCAGATTATTCAT
ACACGATGGAGTTAGCTGATGAAACTTTGAAAGATTACATTGAAAGCAAGACAATTAGTGAGCTAGAAAAA
GTAAAGATAATAATGAAAATTTTAAAAGCGATGAGTCAAGCACATAGTGAAAATAAAATACACAGAGATATCA
GTTCTAAAAATGTATTAATGTTTAGAGGAAAAGTCAAAATATCAGACTTAGGATTAGGTAAAAACCTTGATGA
AATTCATTCGCATCAAACCTTTGATACAAACGGTGTAGGACAATATAAATACTGTGCACCGGAACAGATGTAT
AGTTTAAAACAAGCAGATAAACAATCTGATGTTTTTAGTTTAGGAAGATTGATAAATTTTATTATGACTGGAA
ATGTAGTTAACAACCATCACCTATTTAGAGGTGTATCTGATAAGGCTACGAACAGTAGTAAAGAATACAGATT
TGAAGATGCAAATGAAATGTTGAAAATGCTGCAGAGAATTTTAGAGTATCACAGTAGTGCAAAGCACGTCG
AAAAATGTCAAGAAAAGCTGAAAAGAGGAGTGTTTGATGATGAAAGCGAAGAATTTATTATGACACGAAGT
GATGAACAATTATGTCAAATGGTTCTAAGTTCTAATAATAATGAGCAAGCGTGTTTAATTCGTTATATGCAAAA
AAACGAATCTTCAGCATGTGATTTAATAGAGAGTATTAATAGAAAGTATCAAGAGTTTTGTGGAAGGTTTGA
AGACTACGATCCTTTTGCTAAATTAGCATATATGATTTTATGTAATAACTTCAGTTATAGAGTGAATGAAACAGC
AGCTAGAGTGCTAAATTATGTTGCTTGGTCTGTAAATAGATTTTCGGCACAAGACTTAATTAAAGGTTTAATTA
ATAGAGGAGTGGAGCCTTTGATTGAAGAAAAATTAAAGGACAATTAAAAAAACCTCATCATTAACTGATACG
CAGAGGCGTATCATAAGT
>Staphylococcus aureus CN05
ATGAAAATCACCATTTTAGCTGTAGGGAAACTAAAAGAGAAATATTGGAAGCAAGCCATAGCAGAATATGAA
AAACGTTTAGGCCCATACACCAAGATAGACATCATAGAAGTTACAGACGAAAAAGCACCAGAAAATATGAG
CGACAAAGAAATCGAGCAAGTAAAAGAAAAAGAAGGCCAACGAATACTAGCCAAAATCAAACCACAATCC
ACAGTCATTACATTAGAAATACAAGGAAAGATGCTATCTTCCGAAGGATTGGCCCAAGAATTGAACCAACGC
ATGACCCAAGGGCAAAGCGACTTTGTATTCGTCATTGGCGGATCAAACGGCCTGCACAAGGACGTCTTACA
ACGTAGTAACTACGCACTATCATTCAGCAAAATGACATTTCCACATCAAATGATGCGGGTTGTGTTAATTGAA
CAAGTGTACAGAGCATTTAAGATTATGCGTGGAGAAGCATATCATAAATGATGCGGTTTTTTCAGCCGCTTCA
TAAAGGGATTTTGAATGTATCAGAACATATGAGGTTTATGTGAATTGCTGTTATGTTTTTAAGAAGCTTATCAT
AAGTAATGAGGTTCATGATTTTTGACATAGTTAGCCCCCGCAGTCTTTCATTTCAAGTAAATAATAGCGAAATA
TTCTTTATACTGAATACTTATAGTGAAGCAAAGTTCTAGCTTTGAGAAAATTCTTTCTGCAACTAAATATAGTA
AATTACGGTAAAATATAAATAAGTACATATTGAAGAAAATGAGACATAATATATTTTATAATAGGAGGGAATTT
CAAATGATAGACAACTTTATGCAGGTCCTTAAATTAATTAAAGAGAAACGTACCAATAATGTAGTTAAAAAAT
CTGATTGGGATAAAGGTGATCTATATAAAACTTTAGTCCATGATAAGTTACCCAAGCAGTTAAAAGTGCATATA
AAAGAAGATAAATATTCAGTTGTAGGGAAGGTTGCTACTGGGAACTATAGTAAAGTTCCTTGGATTTCAATAT
ATGATGAGAATATAACAAAAGAAACAAAGGATGGATATTATTTGGTATATCTTTTTCATCCGGAAGGAGAAGG
CATATACTTATCTTTGAATCAAGGATGGTCAAAGATAAGTGATATGTTTCCGCGGGATAAAAATGCTGCAAAA
CAAAGAGCATTAACTTTATCTTCCGAACTCAATAAATATATTACATCAAATGAATTTAATACTGGAAGATTTTAT

TACGCAGAAAATAAAGATTCATCTTATGATTTAAAAAATGATTATCCATCAGGATATTCTCATGGATCAATAAGA
TTCAAATATTATGATTTGAATGAAGGATTCACAGAAGAAGATATGCTAGAGGATTTAAAGAAATTTTTAGAAC
TATTTAATGAATTAGCTTCAAAAGTTACAAAAACATCCTATGATAGCTTGGTCAATAGCATAGACGAAATACAG
GAAGACAGCGAAATTGAAGAAATTAGAACAGCACAAAAAGATAAGACACTCAAGGAAGTGGAAGCACCTA
AAGGAATAATTCCAAAATATAAAAAAGGTGTATCAAAGACTACTAAAAATGATTCAGAAATTGAAAAATCAA
ATAAAGAGAATAAATTAACCGGTAAAGTTGGAGAAAAATTAGCGCTAAATTACTTTAATGAGCTAATTGATAA
TAAAATAGACGAAGATAAGAAAGAACAGTTTAGGAATATTTTAAATGATAATCCAGGCTCTCAACACGGTCAT
GGCTATGATTTAGTAGCTTTTGATCCAACAAATACAGATAAAGCTGTAGAAAAATTTATTGAAATTAAAACATC
TACATCTTCTAGTATTGAGGAACCATTTTTTATGTCGCTAAATGAAATGTTTGCTATGAAAGAATATAAGCAGA
AATATTTAATATTAAGAATATTTAATGTTTCCGGTAAAGAACCACAATTTTATTTTATAGATCCATATGCAAATTA
TTCTGAATTTAAAGATGTAGATGATCTCATTGACAAAGTATTTAATGTAGAAGCTATTCAGTATAAAGTTTTTG
GCGAAAAAATGATTACTTGAACAAGAGCTAAAATAAAATTGTGATCTAATAAAAAATAGAAACTGTAATTTAAAT
AAAACTTTCTAAATAAGCTAACTGATAAAAAATCAGTTTGTCCACAGTCTGAAACAAGATTCCTATATTCTTTA
GGAATCTTGTTTTTTCTATTTTTATGGTGATAAAGAGCAGATAAGATAATGTGTAATAATCACAAAAAAGTTAA
ATATTTTAAGGCTTGTTTAATTATTAATGATTTTATATATAAAGAGCAGTATAATAAAGTTGTTAATATATTATGAA
TAATATTCAAGTAATTTTATTGTTTTTTAATTTGTCGATATTTAAGTTGAGTTAAATTTAAAGGGTGTAATTTGTT
TTACAATGATGAAGATAATTAGTCTATCAAAATAAAGGGGTTGGGACTGTTATGAGTGATAATTTGTCATTATT
CATTGACTATATCAATGATAATATAATCTATGGTAGTGAAATCAAACGGGAGAAATTAGAGAATTTATTTAATCA
ATTTGCTATAAAAAAATGTTGAAAAGAACATTGTCTATGATGAACTGAAATCTTTAGATATTACAATCATTGAGT
CACAGGATTCATATAAAAATAAATTGAAGAGATTATTTTCGGTTCTGTTGCAAAGTAAAAAAATATAGCTAACC
ACTAATTTATCATGTCAGTGTTCGCTTAACTTGCTAGCATGATGCTAATTTCGTGGCATGGCGAAAATCCGTAG
ATCTGAAGAGACCTGCGGTTCTTTTTATATAGAGCGTAAATACATTCAATACCTTTTAAAGTATTCTTTGCTGTA
TTGATACTTTGATACCTTGTCTTTCTTACTTTAATATGACGGTGATCTTGCTCAATGAGGTTATTCAGATATTTC
GATGTACAATGACAGTCAGGTTTAAGTTTAAAAGCTTTAATTACTTTAGCCATTGCTACCTTCGTTGAAGGTG
CCTGATCTGTAATTACCTTTTGAGGTTTACCAAATTGTTTAATGAGACGTTTGATAAACGCATATGCTGAATGA
TTATCTCGTTGCTTACGCAACCAAATATCTAATGTATGTCCCTCTGCATCAATGGCACGATATAAATAGCTCCAT
TTTCCTTTTATTTTGATGTACGTCTCATCAATACGCCATTTGTAATAAGCTTTTTTATGCTTTTTCTTCCAAATTT
GATACAAAATTGGGGCATATTCTTGAACCCAACGGTAGACCGTTGAATGATGAACGTTTACACCACGTTCCCT
TAATATTTCAGATATATCACGATAACTCAATGTATATCTTAGATAGTAGCCAACGGCTACAGTGATAACATCCTT
GTTAAATTGTTTATATCTGAAATAGTTCATACAGAAGACTCCTTTTTGTTAAAATTATACTATAAATTCAACTTTG
CAACAGAACCGTATTATGGAATAGAGATGTTGGTAACATTTATACAGGATCATTATACTTAAGTTTAATTTCGTT
ATTACAGAACCACACATTCCAACCAGAAGAGAAAGTATGTCTATTTAGTTATGGTTCAGGAGCAGTAGGAGA
AATCTTTAGTGGTTCAATCGTTAAAGGATATGACAAAGCATTAGATAAAGAGAAACACTTAAATATGCTAGAA
TCTAGAGAGCAATTATCAGTCGAAGAATACGAAACATTCTTTAACAGATTTGATAATCAAGAATTTGATTTCG
AACGTGAATTGACACAAGATCCATATTCAAAAGTATACTTATACAGTATAGAAGACCATATCAGAACATATAAG
ATAGAGAAATAAACTAGTGGCCGATTGTGCTTGATGAGCTTGGGACATAAATCCTAACTCGAAATAAATAAGC
ATATCACTAAACTGATTTTTTAAAGTTTACAGTGATATGCTTATTTTTTTATCTTACGATTTTGTACGTGCATGCT
TGCCTAGGGGTATGGCTCGAGCCATTAGTCTCTCGCACATACTATTCCCTCAGGCGTCAGCACTTACAAAATC
GGTTGTAATTTTCATTTTTATACGCATTCTTACTGAGATTATACTAATAAGAGGAATAGTAAAAGCAATTCTAAG
TAAAATTGCAGATAAGAGGTTTGTTAAAAGCAGTTCTAAGTAAAATTGCAGATAAGAGGTTTGTTAAAAGCA
GTTCTCAGTAAAATTACAGATAAGAGGTACGTTAAAAGCAGTTCTAAGTAAAATTGCAGATAAGAGGTTTGT
TAAAAGCAGTTCTAAGTAAAATTGCAGATAAGAGGTACGTTAAAAGCAATTCCATGCAAAATTGCTGATAAG
GGGTAAGTTAAAAGCAGTTCTCAGTAAAATTGCAGATAAGAGGTACGTTAAAAGCAGTTCTAGGCAAAATT
GCAGATAAGAGGTGCGTTAAAAGCAGTTCTCAGTAAAATTGCTGATAAGGGGTAAGTTAAAAGCAATCCTA

AGTAAAATTGCAGATAAGGGGTACAGAAAAACTAGACTTGATTACAAAATGGAGCTTGGGACATAAATGATT
TTTTAAAAATGAGATGAGACGTAGATTAACTCCATAATCAATACGAATCTATCGACTTCTTTATTTATGATATTC
ATCTCTTTTTAATGGAAATAAAAGTGCGATTAATGTGATAATACAGTTACGTTAATTAAAAAAATAAAAATGCA
AGGAGAGGTAATATGCTAACTGTATATGGACATAGAGGATTACCTAGTAAAGCTCCGGAAAATACAATTGCAT
CATTTAAAGCTGCTTCAGAAGTAGAAGGTATAAACTGGTTGGAGTTAGATGTTGCAATTACAAAAGATGAAC
AACTGATTATCATTCATGATGATTATTTAGAACGGACTACAAATATGTCCGGGGAAATAACTGAATTGAATTAT
GATGAAATTAAAGATGCTTCTGCAGGATCTTGGTTTGGTGAAAAATTCAAAGATGAACATTTGCCAACTTTC
GATGATGTAGTAAAAATAGCAAATGAATATAATATGAATTTAAATGTAGAATTAAAAGGTATTACTGGACCGAA
TGGACTAGCACTTTCTAAAAGTATGGTTAAGCAAGTGGAAGAACAATTAACAAACTTAAATCAGAATCAAGA
AGTGCTCATTTCAAGCTTTAATGTTGTGCTTGTTAAACTTGCAGAAGAAATCATGCCACAATATAACAGAGCA
GTTATATTCCATACAACTTCGTTTCGTGAAGACTGGAGAACACTTTTAGATTACTGTAATGCTAAAATAGTAAA
CACTGAAGATGCCAAACTTACTAAAGCAAAAGTAAAAATGGTAAAAGAAGCGGGTTATGAATTGAACGTAT
GGACTGTAAACAAACCAGCACGTGCAAACCAACTTGCTAATTGGGGAGTTGATGGTATCTTTACAGACAATG
CAGATAAAATGGTGCATTTGTCTCAATAGAAAGTTAGAGGTGAGTCTTACGTTTCAGTGACGGTAGACTTAC
CTTTAACATGTTACATACTAAAAAATTAATTTGAATAAGAAAGAGAGACATATATGAAATACGATGATTTTATA
GTAGGAGAAACATTCAAAACAAAAAGCCTTCATATTACAGAAGAAGAAATTATCCAATTTGCAACAACTTTT
GATCCTCAATATATGCATATAGATAAAGAAAAAGCAGAACAAAGTAGATTTAAAGGTATCATTGCATCTGGCA
TGCATACACTTTCAATATCATTTAAATTATGGGTAGAAGAAGGTAAATACGGAGAAGAAGTTGTAGCAGGAA
CACAAATGAATAACGTTAAATTTATTAAACCTGTATACCCAGGTAATACATTGTACGTTATCGCTGAAATTACAA
ATAAGAAATCCATAAAAAAAGAAAATGGACTCGTTACAGTGTCACTTTCAACATACAATGAAAATGAAGAAA
TTGTATTTAAGGGAGAAGTAACAGCACTTATTAATAATTCATAATAAAACAGTGAAGCAACCATCGTTACGGA
TTGCTTCACTGTTTTGTTATTCATCTATATCGTATTTTTTATTACCGTTCTCATATAGCTCATCATACACTTTACCT
GAGATTTTGGCATTGTAGCTAGCCATTCCTTTATCTTGTACATCTTTAACATTAATAGCCATCATCATGTTTGGA
TTATCTTTATCATATGATATAAACCACCCAATTTGTCTGCCAGTTTCTCCTTGTTTCATTTTGAGTTCTGCAGTAC
CGGATTTGCCAATTAAGTTTGCATAAGATCTATAAATATCTTCTTTATGTGTTTTATTTACGACTTGTTGCATACC
ATCAGTTAATAGATTGATATTTTCTTTGGAAATAATATTTTTCTTCCAAACTTTGTTTTTCGTGTCTTTTAATAAG
TGAGGTGCGTTAATATTGCCATTATTTTCTAATGCGCTATAGATTGAAAGGATCTGTACTGGGTTAATCAGTATT
TCACCTTGTCCGTAACCTGAATCAGCTAATAATATTTCATTATCTAAATTTTTGTTTGAAATTTGAGCATTATAAA
ATGGATAATCACTTGGTATATCTTCACCAACACCTAGTTTTTTCATGCCTTTTTCAAATTTCTTACTGCCTAATTC
GAGTGCTACTCTAGCAAAGAAAATGTTATCTGATGATTCTATTGCTTGTTTTAAGTCGATATTACCATTTACCAC
TTCATATCTTGTAACGTTGTAACCACCCCAAGATTTATCTTTTTGCCAACCTTTACCATCGATTTTATAACTTGTT
TTATCGTCTAATGTTTTGTTATTTAACCCAATCATTGCTGTTAATATTTTTTGAGTTGAACCTGGTGAAGTTGTA
ATCTGGAACTTGTTGAGCAGAGGTTCTTTTTTATCTTCGGTTAATTTATTATATTCTTCGTTACTCATGCCATAC
ATAAATGGATAGACGTCATATGAAGGTGTGCTTACAAGTGCTAATAATTCACCTGTTTGAGGGTGGATAGCAG
TACCTGAGCCATAATCATTTTTCATGTTGTTATAAATACTCTTTTGAACTTTAGCATCAATAGTTAGTTGAATATC
TTTGCCATCTTTTTTCTTTTTCTCTATTAATGTATGTGCGATTGTATTGCTATTATCGTCAACGATTGTGACACGA
TAGCCATCTTCATGTTGGAGCTTTTTATCGTAAAGTTTTTCGAGTCCCTTTTTACCAATAACTGCATCATCTTTA
TAGCCTTTATATTCTTTTTGTTTTAATTCTTCAGAGTTAATGGGACCAACATAACCTAATAGATGTGAAGTCGCT
TTTCCTAGAGGATAGTTACGACTTTCTGTTTCATTAGTTGTAAGATGAAATTTTTTTGCGAAATCACTTAAATAT
TCATCCATTTTTTTAACGGTTTTAAGTGGAACGAAGGTATCATCTTGTACCCAATTTTGATCCATTTGTTGTTTG
ATATAGTCTTCAGAAATACTTAGTTCTTTAGCGATTGCTTTATAATCTTTTTTAGATACATTCTTTGGAACGATG
CCTATCTCATATGCTGTTCCTGTATTGGCCAATTCCACATTGTTTCGGTCTAAAATTTTACCACGTTCTGATTTTA
AATTTTCAATATGTATGCTTTGGTCTTTCTGCATTCCTGGAATAATGACGCTATGATCCCAATCTAACTTCCACA
TACCATCTTCTTTAACAAAATTAAATTGAACGTTGCGATCAATGTTACCGTAGTTTGTTTTAATTTTATATTGAG

CATCTACTCGTTTTTTATTTTTAGATACTTTTTTTATTTTACGATCCTGAATGTTTATATCTTTAACGCCTAAACTA
TTATATATTTTTATCGGACGTTCAGTCATTTCTACTTCACCATTATCGCTTTTAGAAATATAACTGCTATCTTTATA
AACTTGTTTGAAATTTTTATCTTCAATTGCATCAATAGTATTATTAATTTCTTTATCTTTTGAAGCATAAAAATAT
ATACCAAACCCGACAACTACAACTATTAAAATAAGTGGAACAATTTTTATCTTTTTCATCAATATACTCCTTATAT
AAGACTACATTTGTAGTATATTACAAATGTAGTATTTATGTCAAAATAATGTTATAATTTTTGTGATATGGAGGT
GTAGAAGGTGTTATCATCTTTTTTAATGTTAAGTATAATCAGTTCATTGCTCACGATATGTGTAATTTTTTTAGT
GAGAATGCTCTATATAAAATATACTCAAAATATTATGTCACATAAGATTTGGTTATTAGTGCTCGTCTCCACGTT
AATTCCATTAATACCATTTTACAAAATATCGAATTTTACATTTTCAAAAGATATGATGAATCGAAATGTATCTGA
CACGACTTCTTCGGTTAGTCATATGTTAGATGGTCAACAATCATCTGTTACGAAAGACTTAGCAATTAATGTTA
ATCAGTTTGAGACCTCAAATATAACGTATATGATTCTTTTGATATGGGTATTTGGTAGTTTGTTGTGCTTATTTT
ATATGATTAAGGCATTCCGACAAATTGATGTTATTAAAAGTTCGTCATTGGAATCGTCATATCTTAATGAACGA
CTTAAAGTATGTCAAAGTAAGATGCAGTTCTACAAAAAGCATATAACAATTAGTTATAGTTCAAACATTGATAA
TCCGATGGTATTTGGTTTAGTGAAATCCCAAATTGTACTACCAACTGTCGTAGTCGAAACCATGAATGACAAA
GAAATTGAATATATTATTCTACATGAACTATCACATGTGAAAAGTCATGACTTAATATTCAACCAGCTTTATGTT
GTTTTTAAAATGATATTCTGGTTTAATCCTGCACTATATATAAGTAAAACAATGATGGACAATGACTGTGAAAA
AGTATGTGATAGAAACGTTTTAAAAATTTTGAATCGCCATGAACATATACGTTATGGTGAATCGATATTAAAAT
GCTCTATTTTAAAATCTCAGCACATAAATAATGTGGCAGCACAATATTTACTAGGTTTTAATTCAAATATTAAAG
AACGTGTTAAGTATATTGCACTTTATGATTCAATGCCTAAACCTAATCGAAACAAGCGTATTGTTGCGTATATTG
TATGTAGTATATCGAGCTTCACATGAAACAGCTAAAGAAGCTTTGGGCGATAAAGAGTTAAGAGCCATTGCA
CATGAGTTAACTAAAACAGTTAAGGATAACATGAGTGTTGATTGGTCTAAACGAGACAGTGCTAAAGCTAAA
ATGAGAGTTCAAGTTAGACGCCTATTAAAGAAATATGGCTATCCACCAGATCTTCAAAAAATGGCTGTGGAA
CAAGTTGTAGAGCAAGCAGAATTAATGGCAAGTCAGCAATAAAAAAATAAATCATAATGAGTCCGGGACATA
AAGTTCTTGGATAAGTGAAAAAAGACAATTTCTATTGAAATAATATAGAAATTGTCTTTTTTATAAATTTTTTG
ATTATTTTCAGCTCGTTGAGCTACTACTTTTCTTATATTAAGTGCCATTAATACAAAACCAAGTTCTCTTTTGAC
TTTATTGAGTCCTCGGACAGACATCCGAGTGAAACCCAAAATAGCCTTCATAAATCCAAAAACAGGTTCCAC
ATCAATTTTTCTTTGACTGTAGATATTTTTTGTTTCTGGTTCTGAAAGCTTTTTGTTAATTTGGGATTTAAAATA
TTCCCAGTTATAATTCTTCATTATTTTTTTTGTTTGTTTTTGAATTGAAGTTCATACATTGATTTTTCAGAGGAC
ATTCTGAACAATCATCACATTCATATAATTTGAAGTCTCGCTTATAACCATACTTATCATGACGATAGGCATATCT
TTTAAAACCTAGCCGTTTATTATTCGGACAAATGAATTCGTCATTAATTTCGTCATAGTTCCAATTTTGAGTATT
AAAGATGTCACTTTTATATTTTTTAGTTTTATCTTTTATAAACATTCCATATGTTATGAGTGGCGTTCGATTAAAG
TCATCTATAATTGCCTTATAATTTGATTCACTACCATAACCTGCATCAGCTACAATATATTCAGGTAAATGACCGT
AGGTCTCTTGAATTGAATTTAAAAATGGAATCATCGTTCTAGTATCCGTTGGATTTTGATACACATTATAAGATA
AAACAAATTGGGAATTTGTTGCTATTTGTAAATTATACCCTGGCTTAAGTTGTCCATTTTTCATGTGATCTTCTT
TCATTCTCATAAATGTCGCATCATAATCTGTCTTAGAATAACTATTTCTATCCTTTAAAATAGATTTTTGAAATTC
GTATCGATACTTTCGCTCAAAATAATCATTGATTTGCTTTTTGTATTTTTTGATTTTAGTTCTTTTGAGACGTATT
TGTTTTCTTGTTTTAGTACATTTTTCATTGTTGATATGTTGGTTTAAATCTTCGATTTCTTTATCTAAGTGACTAC
CAATCAAATCTATTTCTTCTTTTGTTAATTCATTATCATGATCTTCTTTAATTTCCGGTATGATTTTATTGGTTACC
AATTCATGGTAGAGGGCTTTAGAATCCTCATTCATCTTTGATTCATGGTTTTGAATACTCTTTTTCCATACAAAT
GTATATCGATTGGCATTTGCTTCAATTTTTGTACCATCAATAAAAATAGCTTTATCATCTATAAGATTTTGTTTTA
CACACTGACTGTAAAATTGAATAAATAAAGATTCTAATAAAGCATCTACTTTTGGATTTACTCTAAATTGATTAA
TTGTTTTATAAGAAGGTTTTTGATTTTGTGATAGCCACATCATTCGGATGCTATCATTAAGCATTTTTTCTATTT
TACGACCTGAGAATACAGATTGTGTGTAGGCATATAGAATCACTTTTAACATCATTTTAGGATGGTACGAAGT
TGCACCACGGTGATGTCTGAATTCGTCGAATTCATTGTCAGGAATTGTTTCAACAATATCATTTACAGTAAAAC
GATGTTGATTTGTTTTGTTTCCATATTGACCTCCATGTATTTGCTATGATTTCAAAATCCATTTTTGACGTGCCTT

AGGGTTGAGTGGATGCATAATTTCATTTGTTACTGGATTGATGAGCTTTTTTACTTTCTTTTTATGAGGTTTTA
ACATTTCCATCACTTGTTCGACACGGTCGATAACAACTGGTCGCTTCGCATAGGCACCATAAGCAAGAATCAC
TGTGTCACTTTCACTAATCGCTTTCATCAAATGAATATCAGTGTGCTCATCGTATGGATTTTTGATATGTTTGAG
GTTTTCGGGTGTTCTAATATTAGAGAATAGATTTACAAGATATACAGCACCGTATCGTTCTGAATTGGCTAATT
GGTTGAGGATAAGAACAGTTGTGAGATCGAGTGATAATACACCGTCTAAATGAGGATACATCGTTATCACTGT
GCATGCAGCTTTCTTTTCATCCCATGTTTTCTTGAGTAAATAGCGGTGCTGTTCATCATCGCTAAATATGGCTT
CTGTGTGTATCGTACTTTTGATTGTATTCATCATCGTCACTTCCTTTAGTATTCTTCTGGTAAAAGCATCACATAA
TAAAAAGCGTCCACGTCATCTTCACGAATGACGTAGACTTTCTTAGGTAATGCATTTTGATTTTTTTCATAGTT
TGTATAGTGATATTCCAATTTGTATGTGGGTTGTTCTTGTTCATGTGTGATTGAGAGTATATTCTCATCTTCTTGT
AATTTAAAAATGTGTAGGTAATCTGTATGAGGCTGGTTATCTTTTTTTTCTACCATGTGCCAAAGTAAGATTTG
AAGGTCTAGTGGAATACTTTCATTAATTCCTCTTGTGATGTATCGATTGATTTTCATGCTATTTCCCTCCCTTCTG
CTTTTCTTTCATGATGTCGATGATTTCGTTGATAACTGTGACGGATAATTGAGCAACTATGATCCAATTATTCAT
GGTCCTGACCTCCTTGTTTTAGTAAATGACGTTCATCAATAATGATATTTTGAGTATCTGTAAGGTACAGAAAG
TCCATGTCAAAATGGTCTAAGTATCCGACACTGATGAGTTGGTTATTGGCATACATTAGAAATGGATAGATACT
TAGCTCATGTAGTTCATCATTATAGTAGGTATAAGTCTCGAGTGTGAGATGTACCAGTGGAGAATCATTAATAA
AACGTTCCGGTAGAATATTTTCTGCTGCTTCCTCCAGCGCTTCACATTCCCAAGCTTCGTTATTAGATAGTTGG
AATAGGTGGGTTATATATTGTTTGAGTTCTTGAGTGATGGTTTTCATATTATTGCCTCCTAGATAGTGTAATAGT
GATGTAGTTCATATACATCATTGAGATAATATATATTTGATTTGTCATTTATTACGAATCCCGGTGGGAATAAGA
GAAAATTCCATATAAAAACCCGCTACAAACGTTGGTATGCCAAGGAAATCCTGAAATTCCGCCTATTTTGACA
AACAATCAACTCATTATTTATAAGTATTGATGATAGGGTTGTGTCTCTGCTTCCTTATATATATTATTTATTTATAA
AAAGTAACGGGATTTTGGGATTGTGCTTGCACAATCCTTCTGTTTCTTCGAATCTGCAAATCCCAATCATTTCC
CGATAAAAAATCATTGTGGGATGTTCTTTAGCAATTTCAATATAAGCATTGTATAGTTATGAAAAAATTACGAC
AATAACTGTTTCATTAGATAAGTGTTATTGAAATTGATAAAGAGCAATTCTTGAAAATAGTTAGATAAAATAAG
CGAAAGAATATAGTGAAAATTATTGTTATAACAATGATTCTATTAGCTAAATAGTAAGATATAGTGTTTGGGGC
AAAAATAAAGACGAAGTGCTGAGATGCACTTCGTCGAGTTGTTTATTATTGAAAAGTTGTTTAATGATTTCGT
TATTAAGTTTGAGTGTGACATAGAATTGTTTTTTATGATTACCATCTTTTTTAATATCAATGCGATCAATCACTG
ATAGATACAATGCTTTGAGTCGAGATTTTTCTATGTGCTTAATATCATGAAAGATGTGTTGTAATAGTTTACTGA
TTTCTTTGGCATCAAATAAAGTCTTATCTTCATTTGTTGATTTTTGAGTTGGTTGATTTGATTCGTAATGTCAT
TGAGTTGCTTTTCATATTTTTGAATACTTGGTCTGATTACTGATGTTAAGTCCGGATTATCCTCGATGGTTTTAA
TCAAGTTATTTATTTTGATTTGTACTTCATCATATTGTTGTTGCTTATAAGCAATATCGTGATGAAGTGCAGCGC
CATCAACTTGATTTTCTTGATTGACGTGTGTTACTACGCGTTGAATGACTTTATCACTTTTGACTATTTCAAGTA
TTTGCTTCATCACATAATCTTCAATCACATCAGCTCTTACACTGTTTGCCGAACATACTTTGGAACCCTTGTTCC
GAAAATTACTACATGAATAGTAACGAATACGTTTCTTAGTCCCGTCTTTAAGAGTATTCGTGGTATTGCTTGCT
GCCATAGGTGCGCCACATTGGGGACAGTGAATAATGCCTGTAAGCAGATTCGTTCCTTTGCCATGGACTTGG
GGTTTTTGACTGACTTGTTTCTTACGCATTTGTACTTTATCCCATAAATCTTGATTAATAATGGGGGAATGCTTA
CCTTCAGCTATCACTGGTTTATCATTCAGCCCTTTACGACGTTTTTCACTCCAATCTTTGTATTTCGCAAATTGA
ATTTTGCCGATATAGAAAGGGTTAGCTAAGATGTATGTGATTGAACTAATACTGAAAGGTTTCCCCTTTTTAGT
GACATATCCTTTGTGATTCAATGCATTGGCAATTTTACGATAGCCATGTCCTTTGGCATAGCACTCGAATATATA
TTTTACAATATTAGCTTCATGTTGGTTAATCATTAGCTCGTGTTTACTATCTGGTATTTTGTCATAACCTAGTGGT
AAATTGCCTTGATAATAGCCTTCTTGGGCACGTCTCGTTTGACCCATAAATACGTTCTCGACAATGTTATTACG
TTCGAATTCTGAGAAACTCGCAAGTATCTGTAACATGAGTTTACCAGAAGAAGTATTGACTTCCATACGCTCT
GACAAACTGAAAAATTCGACATTTTGTTTGTGTAAATCTTCGACAATTTTGAGAAGATCAGATGTATTACGAG
CTAATCGGTTTGTTTTGTATACCATAACACAGTCGATATTGCCTTCTTTTGCATCTTTCAACATACGTTGGAGCT
CAGGTCTGTTCATAGATTTACCTGAAATACCACGGTCAGCGTATATATCTTTAACTTCAAAATGATGGAAGTCA

CAGTATTCTTTGATTTGATTGATTTGTCCGTCGATACTATAACCTTCTGTGCTTTGCATTTCTGTTGATACACGT
ACATAGATACCGACACGTTTTGTTTTAAGTTGTTGCATTATGTTACATCCTTTCTTCATTTATGCAATCGATGAT
TGCATGGTTTGATTGACAATATTGAGTGGTTCATTTTTGAAATAGATTCCTATAAGATTTTTATCTTTCGTAATG
TGAATGGTTTCAATATAGGGGTACAATATGTTTAACGTGAAACGTTTTTGAATAATATTTTGAATGATGTGTTG
TATTTGATGCCCATTGATAGATGTAGTGCGTTGCGGTTGTTGACGTAATGATTGTGTTTGCTCTCTGAACGTTT
CTGCATCAATGATGCCTTGTGCCAACTTTTCTATCAGTTGTTCTTGAGTCAATGTGTGATGTTTTTCTATGTTTC
TTTGTCTTTTGATGCGTTTGTCAATCGCACTTTTAATTTTTGTGTAGATGCGTTGATTTTGATAAAAGTCTCGG
CACACTTCTAATACTTTATCTTCAAGTGTTTGTGCATTGATGCCTTTAAAATCACAGACAAAGCGTGAAGCATT
CATGTTTTTAGGACAGACGTAGTAACGTAATGTATGATTCTTTTTTCTAATGGTCATATTTGTAAGTGTTGCATT
ACAACATGGGCATTTGATTTTTTGTTTGAGTTGATTATCCGAAGGTGTCTGTTTGGTTTGTTTTTGCAATCGA
AGTCTCTGCGCTTGCTCATATATACTTGTGGAAACAATAGAAGGAAACATATTGTCGAATTGGCCATATTGATT
GTTGACACGACCACAATAATTAGGATTGATGATAATGTTACGAACTTGATAGGGTTGTCGATTGATATACGTGT
TATCTTCTTCTAATAACTGTGCAATTTTCTTATAACCATGACCTTTAATGTAATAATTGAATACAGCCTTTACCGT
TGGTGACTCATTTTGATTGATGATGAATGTTCCGTTGTGATATTCGTAACCAAAAGGTGCATGTGTTGTAATCA
ATCGACCTTGCTTTGCTTTTTCTTGAAGCCCATTTCTGACTTGTTCTCCAATGTTATCCGATTCAAGTTCGGCT
AAACTGATGAAGATATTAAGCTTGAGTCGGTCGAAAGCTTGATCCATATCAAAGTAACCATCATGTACGCTTA
AGATATGAACATGGTACGTTTGACATAATTTGATGAGTTTTAATGCATTTTTCAGATTACGATGCAACCTATTA
AGACGATAACAGCATAATATGTCACACTGTCCTTGTTGAATTAATTGTGTTATTTGTCGATACCCACTACGATTA
TCTTTGCGACCTGATTGTTTGTCGCTATAAAAGTTGATATGTTGAATATGATGTTTTTCGGCTATTGCTTCGATA
GCTTGTTTCTGTGCTGCAAGAGATTGTTGTTTCATCGTACTTTGACGTAAATAGCCTATGACTTGTTTCATATC
GGCTCCTCCTTTCACAGTGATAATATATATTTATGGATGAATTGATATATAAGCCCAACATCAATGAGATGTTGG
GCGTCCATATTAGTCATTTGTTTGATTGATTTCTTCAATTACCAAATCGGCTAATATCTCGATAAGTTCATCCATG
TTTTTCACTCCGTTATTTGTTCTATCTTCAATACGTCGATTATTCAGTTTGATGCTTCACAGTTGTATGATAAAG
ACAATTAGAAATCTTCGTGAACTCCTGAAGGGCCTATCCCTTCATTAGCGGATTTAAAAAGTTCTTTCGCAGC
TTTGTTATCATTTGACGGTGTCCAATTTTGAAGTAACGACTTATCTTTAGTTAATCCGAGGATAGATGCAAACT
CTACATCTAATTTTAGATGGTAAAATACAAGTGATTGTTTTTTACCGCTATTATCTTTGACACTTCTTTTAGTTGT
TTGGCGTCCACGGTCAGCTAATATGAAACCTTTATCTCTTAAGGCGTTGACAACATTATTAACATCTTGAAATT
GATGATTGTTTAGCATCTGTTTAAAAACGTTCGCAATTATTTTTACTTCGATATGGTCATCTTTTAATGAGATTA
ATCCATAGTTCTCAAACATATTTTTCAAAGCACCTTCATCTGAAAACTTACCTCTGTTTTGTGCCACAAATTGA
ATGATGACATCAATAGCTTTATCAGCTAATGAGCGTTCAGAGACTGTATGAGTATGATAATCAATAAAGTAGTC
TCTTATATTAGCGATATCAATATCTGTAGATAAAACACGACCTAATATTTTCGCAGATGTTGTAATGACTGCATA
ACGCTTAAACATACGAATACCTGTATTGTTTGTTTCATCTTTCAATTTAGCTTCAAACCAATCTACTTCCTTGTA
AAACCATTGAATAACTTCATCTTCACGATTTATAAGATATTTAGCTACTAACGGTAAAACATGACCATAGTTTAG
TGCTACAGCTTTTTTAATATTGTCAGCATTGGTCGCATTTGTAGTGAATTGTTCATTAATCTCGATGGTTCTTAC
ACGTAATCCATCGTTTTGAGCTGAATCATTAAAAATACTGTATTCTGACGTTGAAATGACAGAAGTACCCCAAT
TCTTAGGCGTTTTAACTTCTCCATGAACGTTTGAACGTTGACGACCTTGACCTTCAGCGATGGAATATAACAA
ACCCGTGGTATCTCTAAGTGTCGTAGATGAAAGCTCATCAAATACTATAGGAATGCCATAATTGTTACTCAAAT
AACCTTCAAGTGCGTTTCGTGTGGCATTCCAACTTCTAAAAAGAGTTTCATTACCTTTGGTAGGGTTACCAGC
GACTGATACAGCTAAAGCAGCTGCGGTTGACTTACCGGTTGAGGATTGACCTGTAAAACTAAAGAGAATTC
CTGCAAATTCGATTTCATGTTTATGCTTCAGAAAACTTGTCACTAAGGCAGAAATCCCAAATATGACTGCCAA
TTCTAAAAGAAGATGACCTTTAACCTCGTCAATATACATGTTAAACCAATTATCAAATGTACCTTTAGGAGTTA
AGTCATAAGCACTATCTACAATAGGGTATGAAGGTGAGGACTGATTAAATTGAATAGATTTGAGAACTTTATC
TAGCGATATCAAGTAGCCAAAAGGTGTCTCTAGTATACCTACACCCTCATATAATTCAGAAAGTGGTAATCTAT
CTCGCATCAATTGCAAGGCATAACTCAATGATCTAATATACTTCTCGTTAATACTGTAGCCATATTTAATTAAAG

AAGGTAAGTTTCGTGTTGTTAAGATATCAGACTCAAAAATGTCTTTTTTACCATTGTTGTTAGTAATAATCAAC
TTTTCAACACCAGTAATTGGATCAGAAAATCTTGCATTAACAATAATAGGGCTAGCCATTCTAACCACTTTTTC
ACTGTCACCATCTTTTTTAGGTGGTATCGTTTCTTTCCAACCGAAGGAATCTAAAGCAAATGGGCCAATTTCA
AACAATGTGTAACTCATTAGCAATCACCTCCTTTCGAAGGCGTGCTATTATTGCATGGGTTTATCCCTATTTTCT
GATATAATAAACTGCCATTTTTTGTTCCTACAATTTTAAATGGTAATCCTGGCGTGTACTTAATCCAGTAAGCAA
AACGACGTCCTACTTCACGTTGATCATTCGTGCTACAAACCACATTATATTTCTTTCTTAATTCACCATAAGTGA
ACGTACTTCCAACTGGTTGTTGAAAAGATTTAGCAATTATAAAATTATAAAGCTTTTCTTGCTGCTTAGATTTC
TTATTCATTTTAAAATTCCTCCAATAATTTTTAAGGAGTCAATCAATATCATATAGGTTAAACTATCTTCAATTGA
TTGCTATAATAAGCATAAATGAAGCATTATGATTAGACGCGGAACAAAAAATGGGGAGGGGGGGGTATATAT
TGATACCTACGCTAGAAGAAATTATTGATAAGTATGGAAACTTAGTTGATTATTTGAAATTTGATGTAACAGTT
GAGGTTTATGAAGATTTGTTGTTATTAAGAAGTTTAAGCGATTTAAATGAACATCAAAAGATGGATAGAGTTT
CATTTATAAAAAAACATTTAAACTCAAATTTCGATTATTTATTAGATGATACTGAGTTTAGAAGTATCAATAAGA
TTTACAGTAATTTAAATATCATGACGCATATCAATAGTCAAAATAATAATTTTAAACGTAATCCATTTATAAATGA
AGAACAATTGAAATCACTACTCGCGATAAAGAAAGTAGATGCACATATGAGTTATGATTCAAATATTACTTCAA
AAGCATTGGCAGAAATAAACAAAACGCAAAAAAGATTAGTTACAAAGATAGACACACTCTATAATCAGTCAA
AAAAAGAAAATGAATATGTACGGTTAGGAGAAAAAATATCGTATACAAAATTGGAGAATCATTGGAAATATTT
ATATAATGAGATTCAGTTTTATAATCTTAATAATCAATTGATTAGTTATGTTGCATTAGAACAGGAATATGCTTG
GGCTTTTTTAAATGAACTATTTTATTTGATAGAATTGTATTTGAAGGCATTTAAAGGACAAAAGAAAACAGAT
TATGAGTTTGGGAAAAAATTAAACGAATTTATTCAATCATATGTCATTATTTTACTTATAGATATACGATTACCTG
TTGTTAGATTATATATTATTAGAGACATTGTAGATACATGTGAAGGAGAAAAAGATAATTACAAAAGAATTACA
CTGATAGAAGAATCTATTAAAAAGTATAGATACGTAAAAGATCAGATTAAAAGATTTAAAAATGGATTAGAAG
AATCGTTATTAAATGCAACTTTATCTATTGAACAAAATATGTTGAAAGTTAAAATTGATTATTATAAAGCCTACT
GCTTTCCTAGAGAAAAAACAAAGCATAACAATATTGAGTACAATGTATCGTTGTTTTTTAAAGCATTAGATGC
CTTAAAGAAATAGTTTTTCACTGAAAATATTTATAGATATAATTAAAATTAATTAATATTAAGCCAGGATAATGTA
GTCTTAATCGTTCTGAAATACGAAAAATGTTGTGGAAATCGCGTGAAATAATCAGAGGAATCGTTTGAAATC
ATCGTAGTAATCGCGCGAAAATCACGTGAAATAATCAGAGGAATCGTTTGAAATCATCGTAGTAATCGCGCGA
AAATCACGTGAAATAATCAGAGGAATCGTTTGAAATCATCGTAGTAATCGCGCGAAAATCACGTGAAATAATC
AGAGGAATCGTTTGAAATCATCGTAGTAATCGCGCGAAAATCACGTGAAATAATCAGAGGAATCGTTTGAAA
TCATCGTAGTAATCGCGCGAAAATCACGTGAAATAATCAGAGGAATCGTTTGAAATCATCGTAGTAATCGCGC
GAAAATCACGTGAAATAATCAGAGGAATCGTTTGAAATCATCGTAGTAATCGCGCGAAAATCGCGTGAAATA
ATCAGAGGAATCGTTTGAAATCATCGTAGTAATCGCGCGAAAATCGCGTGAAATAATCAGAGGAATCGTTTG
AAATCATCGTAGTAATCGCGCGAAAATCGCGTGAAATACTATGGTAGACGGTTTGAGTAAATTAATGGAGTAT
TTTAATATTTATGGTTTGGAGGAATTGCGTTGCATAGAGAAAGTAAATATATAGAGTATAAGAAATCACGAAA
AGGATTATCTAATGATATTTGGTCTACGTATAGTGCTTTTGCAAATACTGAAGGTGGTACTATATATTTAGGAAT
TGAAGAAAAAAAGATCGAGGACAAAAAAGTCTTTGTTTCAGTTGGTGTTGAAGATCCAGAGAAAATGATTG
AAGATTTTTGGAATGCACTATATGGAAGAAGTAAAGTTAGTCAAAATATTTTATCAAATAAAGATGTTAAAATT
GTTAATATTGAAAATAAAGCGTGCATTGAAATTCATGTACCAGAAGCGCCTTATTCGAAGAAACCGATATATG
TAGATAATAAAAAAGATTTAGTATATAAAAGAGTTGATGATGCTGATAGAATTGCGACTGAAGAAGAGTATAA
ATTCATGATTGTAAATTCTCAAGACGATATAGATACAGAATTATTAGATAACTATGACATGTCTGATTTAAATCA
CGAATCTATCGAAAATTATAGGAAACTTCTATTAAAAAATACTAATGATGAGAGATATGCGAATATGAGCCAAC
TGGATTTAATGATAGATTTAGGAGCATATAGAAAAGATAGAAGTTCGAAAGACAAACAGTATAAAATGACTA
CAGCATGTTTATTATTCTTTGGTAAGTATAATGCGATTAGTGATAGATTCCCAGGATTTCAATTAGATTATTTTA
AGAAAACAAATTACCTAGATACTGATTGGAAAGATAGAATATCAAGTGGAGATTTAGGTAATGAAGATTTAA
ACGTGTATAGTTTTTTTGAAAAAGTATTGATAAAATTAACTGATAACATTGAGGAATCATTTAGCCTAAATGAT

GGTTTGACTAGACAAAATTATGCAAGAGATTTAAAAGTAGCAATTCGCGAAGCACTGGTTAATACATTAATGC
ATGCGTATTATGATACTAAGCAAAGTATTAAAATAGTTAATTGTGAAGATTTTATAGAGTTTTATAATCCGGGTA
ATATGAGAATAAATAAAGAAGATTTTATTCATGGAGGGCATTCAAAGGACAGAAATAGTATATTATCGACGCT
TTTCAGAAGAGTAGGATATTCAGAAAAAGCTGGATCTGGAGGACCAAGGATATTCGATGTAGTTAATAGACA
TAAGCTTAAAACGCCTGAAATAGAATTAACGGACATGGACACTAATGTAGTACTTTGGAAACAAGATTTAATG
AAGGAGTTTGAAAAATATCCTGAGTTAGACAAAAAAGTAATAAAGTATATTATTGACTATGGATCAATAAGTA
AGGGTGAAGCCTTAAAAATGGAAAATATGACAGAATATCAGTTTAGAAATATTTTAAAAAAACTAAAAGATG
ATAACTTGATAAAAAAAGAAGGTGAAGGTCCGGCTACTAAATATGTGTTAATAGAATCAAAAGAAGCTGATA
TATTGCGAACTAAAAAAGTAATTAAAAGTTTAGAGTCTTTCTTTAGGAATAAATAAAAAAACAAGAGATAGG
TGCGAAGTGTTTGGATTATACAAATGCTTCGCATCTTATATTTATTAATAAATATCATAGAAAACCGTATCATTAA
CCGATACGCAGAGATGCGGTTTTTTAGACACTTCATAAAGGGATTTTGAACGTATCAGAACATATGAGGTTTA
TAGGAATTGCTGTTATGTTTTTTGATCACATCAATAAACAAAAAAGGTATGTACTATGTAAAATATTTATTAAAT
GATATAAAGCGAGGGTATATAAATGATTTTTAAATAGATATTTATCCAATAATATAAAAAGGAACTATAAGCTAT
ATCTAAAAGCTATATCTAACTACTTATAGTCCTTCTTCATTAGTATAAATATAATTATTAAGAAAGGTATATATCTT
TGTAACCTTCGTTTACATTAATAATGTTGTGATAACCTTTGTGTTCTAAAATACCAATAGCTATAGAACTTCTAAT
GCCAGACTGACAGTCTACATAAATAACATCGTTTCTATTGAAAGGTAAATCTGTTTCTAAAAGTTTGCCGTGT
GGCACATGAACCGCTTGGGATAAGTGGCCATTATTCCATTCATTATCATTACGTACATCTAAGACATGTGCTTC
ATTACCAGTTATGTCTTTACTATGAACAGATTGTGTTTGAATTTGAGCTTGTGGTAACTGATATCCAGACACAT
TATCATATCCAATAAGTTGTAAAGTATGTTATGTGTTGCTTTTGAAACAAGGTGATAGTCTCCAATCAAGTTAAT
TTCTTGATTATAGTTTAGATACCAGCCAATTTGATTGATGAAATTTTTATCATATGGAATATTGATTGTACCTTCA
ATATGTCCACCATGATAAGCCTCCTTACTGCGGAGATCAAAAGTTAATCTGTTTGTACTTGTAGCTGGATAAAC
CGTATAAGGTTGATATAAATTCATACCGAATTGATTAATTTTTTTCATTTGTGCAAAATGATGTGGTGGTGCAG
GTTGGTCAGAAATGAGTTTATCGATAAAGGTAGCTTCATTATTTTCAGAAAAAGCCCAGTTCGTTTGTTTTTC
ATAGCCAAGCGTAGATGTTGGAATAGCACCTAAAGATTTACCACAAGGACTACCAGCGCCATGACCAGGCCA
AATTTGAATGTAGTCTGGCAAGTCTTTAATACTTTCAATAGATTTAAACATTTGTTTTGCGCCTATTTCAGATAA
TCCTTCTACTTTAACAGCTTTTTCTAGTAAATCAGGTCTACCGATATCTCCTACAAAAATAAAATCACCACTGAA
TAGTCCCATTGGAACTTGTGCTCCAGCACCTTCGTCAGTAAGTAAAAAACTTATACTTTCTGGCGTGTGACCA
GGTGTATGAAGCACTTTTAATTTTATATTTCCTACATAAATATCATCATTATGTTGAACAAAATGAGTGTGGTTA
GGCATATTTTTATAACCTAACATGTCATCACTTTCGCCCGATACATAAATACTAGCATTTAACTTTATAGCAACAT
CTCTAATTCCTGAAACAAAATTTGCATGTATATGTGTTTCAGCTGCATGAGTAATGGTTAAACTCTCTTCATCG
GCAACTCGAATATATGAAGATAAGTCACAAATAGGATCAATGATCATGGCTTCTCCAGTTTTTTGACAACCGA
TTAAATAAGATGCTTGAGATAAATGTTTATCATAAATTGATGATAGTCAATTTGTTATCAAGGGTGATAATTATA
TTAACTATGGTAATTACATGGAATTAAAGTTATTAAACAAACTTGATGATGACCAATTAGATTTAGAATTTTTAC
AAGATAATGGGTTAAAAGGTAATGTTCAAGGTCCTATGACTTTAAAGGAACCTTTAAAAAGTTATATACAAAA
GTCTTGAAAACGAATATTTTAAATTTAGATAATGAGTTAGAAAACACTGAATATGTCGAAGGTAAACCATATG
TACAGTACGGTATTAAATATGAAAGAAATCAGGCATTAAGAAATGAAGCTATTAAAATTCATGGAACTACATG
TAAAGTATGTGGATTTGATTTTAAAGCTAAGTATGGCGATTTAGGTGAGGGTTTTATTGAAATTCATCATTTAA
AACCAATGTTTTCAATAAAAAGAGAAATAAAAGTAAATCCACAAAAAGATTTAGTCCCACTATGTTCTAATTG
CCATAAAATGATTCATAGAAATACTAAAAAACCTTTAACGATTAAAGAATTAACCAAAATAGTTAATTATAATA
GCAAATAATTTAATATTTTATAAACTATCATTCAACCCTCTTAATTTATTAGGAGGTTTTTTGTATTTATGCTTTC
AAATGTGTGATATACTTTGTTTGTTGAAATATAGAGTATCTATAGATAGGGTGATTGAGTATGAAATTCACTGA
AGTGGAAGTTATCGAACATCTTGTAAAGGCATATAAAGAAGCAGGAAAGCCTACTTATCCTCATGAAAATTTA
TATCGAGGACGTAATCATAGTATTTCAGGTATTGGAGAAGACTTGCTGGGTGCTTATTTGATTAGTAGATTGG
AAGGTGTCCAAATATTTATTGATCAGCCTTTATCTATGATTGATAAATCTTTAAGTACAAGATATCCGGATTTATT

AATTTGTGAAGATAATGAAATTAAAAATATACTAGAAGTTAAAATGGACTTAGGATATCAAAGAAAAGATTTT
ATAGATTATTGCCGAAAGAAAGAAGAATGGATTTCAAATATCGTAGGAAAACAGTGTGTATTGTCTAGAAAG
AGAGAAGACAAAATTCCTATGAATATAGCTGATGATATTAAATTTCATGTTGTGATTTACAGTGAAAACAATG
GACCGAAGCGGTTTGATGAAGAAATCATGCCTATCGTTAATGAAACATGTCCACATATTGAAGTATATGTCCTA
ACAAGCGGTCAACACCCTAATTTAGTAAATGTTAATCTTGAAGGTATTAATATTAATAAAGATGAATTTGAAAT
ATTAGTAAATGCGTTATAAAAAAATAGAGCATCCTCCACGTTATGGAGGTGCTCTGTTTTTTATTGAAAAGTAT
CAAGTTAATTAATTTAATATGCTTAATAAGTTCTACCTTGACCTTTTTCTCTAGCTTCTGTTCGATCTCTTATGTA
CTCAGTACATACTGGATTTTCTGTTAATTCATCAACTGTATTAGTTTAATCAGAAGACGTGTCTACTTTGTAAGC
TTCTAAGAACTTATTATATGATATAGCGTTTGAGTTTTGTTGTTCTTCTATAAATTTCTGTAGTTATTTTTCAAAA
ACCGCATCATTAACTGATAAGCAGAGGCGTATCATAAGT
>Staphylococcus aureus CN09
ATGAAAATCACCATTTTAGCTGTAGGGAAACTAAAAGAGAAATATTGGAAGCAAGCCATAGCAGAATATGAA
AAACGTTTAGGCCCATACACCAAGATAGACATCATAGAAGTTACAGACGAAAAAGCACCAGAAAATATGAG
CGACAAAGAAATCGAGCAAGTAAAAGAAAAAGAAGGCCAACGAATACTAGCCAAAATCAAACCACAATCC
ACAGTCATTACATTAGAAATACAAGGAAAGATGCTATCTTCCGAAGGATTGGCCCAAGAATTGAACCAACGC
ATGACCCAAGGGCAAAGCGACTTTGTATTCGTCATTGGCGGATCAAACGGCCTGCACAAGGACGTCTTACA
ACGTAGTAACTACGCACTATCATTCAGCAAAATGACATTTCCACATCAAATGATGCGGGTTGTGTTAATTGAA
CAAGTGTACAGAGCATTTAAGATTATGCGTGGAGAAGCATATCATAAATGATGCGGTTTTTTCAGCCGCTTCA
TAAAGGGATTTTGAATGTATCAGAACATATGAGGTTTATGTGAATTGCTGTTATGTTTTTAAGAAGCTTATCAT
AAGTAATGAGGTTCATGATTTTTGACATAGTTAGCCCCCGCAGTCTTTCATTTCAAGTAAATAATAGCGAAATA
TTCTTTATACTGAATACTTATAGTGAAGCAAAGTTCTAGCTTTGAGAAAATTCTTTCTGCAACTAAATATAGTA
AATTACGGTAAAATATAAATAAGTACATATTGAAGAAAATGAGACATAATATATTTTATAATAGGAGGGAATTT
CAAATGATAGACAACTTTATGCAGGTCCTTAAATTAATTAAAGAGAAACGTACCAATAATGTAGTTAAAAAAT
CTGATTGGGATAAAGGTGATCTATATAAAACTTTAGTCCATGATAAGTTACCCAAGCAGTTAAAAGTGCATATA
AAAGAAGATAAATATTCAGTTGTAGGGAAGGTTGCTACTGGGAACTATAGTAAAGTTCCTTGGATTTCAATAT
ATGATGAGAATATAACAAAAGAAACAAAGGATGGATATTATTTGGTATATCTTTTTCATCCGGAAGGAGAAGG
CATATACTTATCTTTGAATCAAGGATGGTCAAAGATAAGTGATATGTTTCCGCGGGATAAAAATGCTGCAAAA
CAAAGAGCATTAACTTTATCTTCCGAACTCAATAAATATATTACATCAAATGAATTTAATACTGGAAGATTTTAT
TACGCAGAAAATAAAGATTCATCTTATGATTTAAAAAATGATTATCCATCAGGATATTCTCATGGATCAATAAGA
TTCAAATATTATGATTTGAATGAAGGATTCACAGAAGAAGATATGCTAGAGGATTTAAAGAAATTTTTAGAAC
TATTTAATGAATTAGCTTCAAAAGTTACAAAAACATCCTATGATAGCTTGGTCAATAGCATAGACGAAATACAG
GAAGACAGCGAAATTGAAGAAATTAGAACAGCACAAAAAGATAAGACACTCAAGGAAGTGGAAGCACCTA
AAGGAATAATTCCAAAATATAAAAAAGGTGTATCAAAGACTACTAAAAATGATTCAGAAATTGAAAAATCAA
ATAAAGAGAATAAATTAACCGGTAAAGTTGGAGAAAAATTAGCGCTAAATTACTTTAATGAGCTAATTGATAA
TAAAATAGACGAAGATAAGAAAGAACAGTTTAGGAATATTTTAAATGATAATCCAGGCTCTCAACACGGTCAT
GGCTATGATTTAGTAGCTTTTGATCCAACAAATACAGATAAAGCTGTAGAAAAATTTATTGAAATTAAAACATC
TACATCTTCTAGTATTGAGGAACCATTTTTTATGTCGCTAAATGAAATGTTTGCTATGAAAGAATATAAGCAGA
AATATTTAATATTAAGAATATTTAATGTTTCCGGTAAAGAACCACAATTTTATTTTATAGATCCATATGCAAATTA
TTCTGAATTTAAAGATGTAGATGATCTCATTGACAAAGTATTTAATGTAGAAGCTATTCAGTATAAAGTTTTTG
GCGAAAAAATGATTACTTGAACAAGAGCTAAAATAAAATTGTGATCTAATAAAAATAGAAACTGTAATTTAAAT
AAAACTTTCTAAATAAGCTAACTGATAAAAAATCAGTTTGTCCACAGTCTGAAACAAGATTCCTATATTCTTTA
GGAATCTTGTTTTTTCTATTTTTATGGTGATAAAGAGCAGATAAGATAATGTGTAATAATCACAAAAAAGTTAA
ATATTTTAAGGCTTGTTTAATTATTAATGATTTTATATATAAAGAGCAGTATAATAAAGTTGTTAATATATTATGAA
TAATATTCAAGTAATTTTATTGTTTTTTAATTTGTCGATATTTAAGTTGAGTTAAATTTAAAGGGTGTAATTTGTT

TTACAATGATGAAGATAATTAGTCTATCAAAATAAAGGGGTTGGGACTGTTATGAGTGATAATTTGTCATTATT
CATTGACTATATCAATGATAATATAATCTATGGTAGTGAAATCAAACGGGAGAAATTAGAGAATTTATTTAATCA
ATTTGCTATAAAAAAATGTTGAAAAGAACATTGTCTATGATGAACTGAAATCTTTAGATATTACAATCATTGAGT
CACAGGATTCATATAAAAATAAATTGAAGAGATTATTTTCGGTTCTGTTGCAAAGTAAAAAAAATATAGCTAACC
ACTAATTTATCATGTCAGTGTTCGCTTAACTTGCTAGCATGATGCTAATTTCGTGGCATGGCGAAAATCCGTAG
ATCTGAAGAGACCTGCGGTTCTTTTTATATAGAGCGTAAATACATTCAATACCTTTTAAAGTATTCTTTGCTGTA
TTGATACTTTGATACCTTGTCTTTCTTACTTTAATATGACGGTGATCTTGCTCAATGAGGTTATTCAGATATTTC
GATGTACAATGACAGTCAGGTTTAAGTTTAAAAGCTTTAATTACTTTAGCCATTGCTACCTTCGTTGAAGGTG
CCTGATCTGTAATTACCTTTTGAGGTTTACCAAATTGTTTAATGAGACGTTTGATAAACGCATATGCTGAATGA
TTATCTCGTTGCTTACGCAACCAAATATCTAATGTATGTCCCTCTGCATCAATGGCACGATATAAATAGCTCCAT
TTTCCTTTTATTTTGATGTACGTCTCATCAATACGCCATTTGTAATAAGCTTTTTTATGCTTTTTCTTCCAAATTT
GATACAAAATTGGGGCATATTCTTGAACCCAACGGTAGACCGTTGAATGATGAACGTTTACACCACGTTCCCT
TAATATTTCAGATATATCACGATAACTCAATGTATATCTTAGATAGTAGCCAACGGCTACAGTGATAACATCCTT
GTTAAATTGTTTATATCTGAAATAGTTCATACAGAAGACTCCTTTTTGTTAAAATTATACTATAAATTCAACTTTG
CAACAGAACCGTATTATGGAATAGAGATGTTGGTAACATTTATACAGGATCATTATACTTAAGTTTAATTTCGTT
ATTACAGAACCACACATTCCAACCAGAAGAGAAAGTATGTCTATTTAGTTATGGTTCAGGAGCAGTAGGAGA
AATCTTTAGTGGTTCAATCGTTAAAGGATATGACAAAGCATTAGATAAAGAGAAACACTTAAATATGCTAGAA
TCTAGAGAGCAATTATCAGTCGAAGAATACGAAACATTCTTTAACAGATTTGATAATCAAGAATTTGATTTCG
AACGTGAATTGACACAAGATCCATATTCAAAAGTATACTTATACAGTATAGAAGACCATATCAGAACATATAAG
ATAGAGAAATAAACTAGTGGCCGATTGTGCTTGATGAGCTTGGGACATAAATCCTAACTCGAAATAAATAAGC
ATATCACTAAACTGATTTTTTAAAGTTTACAGTGATATGCTTATTTTTTTATCTTACGATTTTGTACGTGCATGCT
TGCCTAGGGGTATGGCTCGAGCCATTAGTCTCTCGCACATACTATTCCCTCAGGCGTCAGCACTTACAAAATC
GGTTGTAATTTTCATTTTTATACGCATTCTTACTGAGATTATACTAATAAGAGGAATAGTAAAAGCAATTCTAAG
TAAAATTGCAGATAAGAGGTTTGTTAAAAGCAGTTCTAAGTAAAATTGCAGATAAGAGGTTTGTTAAAAGCA
GTTCTCAGTAAAATTACAGATAAGAGGTACGTTAAAAGCAGTTCTAAGTAAAATTGCAGATAAGAGGTTTGT
TAAAAGCAGTTCTAAGTAAAATTGCAGATAAGAGGTACGTTAAAAGCAATTCCATGCAAAATTGCTGATAAG
GGGTAAGTTAAAAGCAGTTCTCAGTAAAATTGCAGATAAGAGGTACGTTAAAAGCAGTTCTAGGCAAAATT
GCAGATAAGAGGTGCGTTAAAAGCAGTTCTCAGTAAAATTGCTGATAAGGGGTAAGTTAAAAGCAATCCTA
AGTAAAATTGCAGATAAGGGGTACAGAAAAACTAGACTTGATTACAAAATGGAGCTTGGGACATAAATGATT
TTTTAAAAATGAGATGAGACGTAGATTAACTCCATAATCAATACGAATCTATCGACTTCTTTATTTATGATATTC
ATCTCTTTTTAATGGAAATAAAAGTGCGATTAATGTGATAATACAGTTACGTTAATTAAAAAAATAAAAATGCA
AGGAGAGGTAATATGCTAACTGTATATGGACATAGAGGATTACCTAGTAAAGCTCCGGAAAATACAATTGCAT
CATTTAAAGCTGCTTCAGAAGTAGAAGGTATAAACTGGTTGGAGTTAGATGTTGCAATTACAAAAGATGAAC
AACTGATTATCATTCATGATGATTATTTAGAACGGACTACAAATATGTCCGGGGAAATAACTGAATTGAATTAT
GATGAAATTAAAGATGCTTCTGCAGGATCTTGGTTTGGTGAAAAATTCAAAGATGAACATTTGCCAACTTTC
GATGATGTAGTAAAAATAGCAAATGAATATAATATGAATTTAAATGTAGAATTAAAAGGTATTACTGGACCGAA
TGGACTAGCACTTTCTAAAAGTATGGTTAAGCAAGTGGAAGAACAATTAACAAACTTAAATCAGAATCAAGA
AGTGCTCATTTCAAGCTTTAATGTTGTGCTTGTTAAACTTGCAGAAGAAATCATGCCACAATATAACAGAGCA
GTTATATTCCATACAACTTCGTTTCGTGAAGACTGGAGAACACTTTTAGATTACTGTAATGCTAAAATAGTAAA
CACTGAAGATGCCAAACTTACTAAAGCAAAAGTAAAAATGGTAAAAGAAGCGGGTTATGAATTGAACGTAT
GGACTGTAAACAAACCAGCACGTGCAAACCAACTTGCTAATTGGGGAGTTGATGGTATCTTTACAGACAATG
CAGATAAAATGGTGCATTTGTCTCAATAGAAAGTTAGAGGTGAGTCTTACGTTTCAGTGACGGTAGACTTAC
CTTTAACATGTTACATACTAAAAAATTAATTTGAATAAGAAAGAGAGACATATATGAAATACGATGATTTTATA
GTAGGAGAAACATTCAAAACAAAAAGCCTTCATATTACAGAAGAAGAAATTATCCAATTTGCAACAACTTTT

GATCCTCAATATATGCATATAGATAAAGAAAAAGCAGAACAAAGTAGATTTAAAGGTATCATTGCATCTGGCA
TGCATACACTTTCAATATCATTTAAATTATGGGTAGAAGAAGGTAAATACGGAGAAGAAGTTGTAGCAGGAA
CACAAATGAATAACGTTAAATTTATTAAACCTGTATACCCAGGTAATACATTGTACGTTATCGCTGAAATTACAA
ATAAGAAATCCATAAAAAAAGAAAATGGACTCGTTACAGTGTCACTTTCAACATACAATGAAAATGAAGAAA
TTGTATTTAAGGGAGAAGTAACAGCACTTATTAATAATTCATAATAAAACAGTGAAGCAACCATCGTTACGGA
TTGCTTCACTGTTTTGTTATTCATCTATATCGTATTTTTTATTACCGTTCTCATATAGCTCATCATACACTTTACCT
GAGATTTTGGCATTGTAGCTAGCCATTCCTTTATCTTGTACATCTTTAACATTAATAGCCATCATCATGTTTGGA
TTATCTTTATCATATGATATAAACCACCCAATTTGTCTGCCAGTTTCTCCTTGTTTCATTTTGAGTTCTGCAGTAC
CGGATTTGCCAATTAAGTTTGCATAAGATCTATAAATATCTTCTTTATGTGTTTTATTTACGACTTGTTGCATACC
ATCAGTTAATAGATTGATATTTTCTTTGGAAATAATATTTTTCTTCCAAACTTTGTTTTTCGTGTCTTTTAATAAG
TGAGGTGCGTTAATATTGCCATTATTTTCTAATGCGCTATAGATTGAAAGGATCTGTACTGGGTTAATCAGTATT
TCACCTTGTCCGTAACCTGAATCAGCTAATAATATTTCATTATCTAAATTTTTGTTTGAAATTTGAGCATTATAAA
ATGGATAATCACTTGGTATATCTTCACCAACACCTAGTTTTTTCATGCCTTTTTCAAATTTCTTACTGCCTAATTC
GAGTGCTACTCTAGCAAAGAAAATGTTATCTGATGATTCTATTGCTTGTTTTAAGTCGATATTACCATTTACCAC
TTCATATCTTGTAACGTTGTAACCACCCCAAGATTTATCTTTTTGCCAACCTTTACCATCGATTTTATAACTTGTT
TTATCGTCTAATGTTTTGTTATTTAACCCAATCATTGCTGTTAATATTTTTTGAGTTGAACCTGGTGAAGTTGTA
ATCTGGAACTTGTTGAGCAGAGGTTCTTTTTTATCTTCGGTTAATTTATTATATTCTTCGTTACTCATGCCATAC
ATAAATGGATAGACGTCATATGAAGGTGTGCTTACAAGTGCTAATAATTCACCTGTTTGAGGGTGGATAGCAG
TACCTGAGCCATAATCATTTTTCATGTTGTTATAAATACTCTTTTGAACTTTAGCATCAATAGTTAGTTGAATATC
TTTGCCATCTTTTTTCTTTTTCTCTATTAATGTATGTGCGATTGTATTGCTATTATCGTCAACGATTGTGACACGA
TAGCCATCTTCATGTTGGAGCTTTTTATCGTAAAGTTTTTCGAGTCCCTTTTTACCAATAACTGCATCATCTTTA
TAGCCTTTATATTCTTTTTGTTTTAATTCTTCAGAGTTAATGGGACCAACATAACCTAATAGATGTGAAGTCGCT
TTTCCTAGAGGATAGTTACGACTTTCTGTTTCATTAGTTGTAAGATGAAATTTTTTTGCGAAATCACTTAAATAT
TCATCCATTTTTTTAACGGTTTTAAGTGGAACGAAGGTATCATCTTGTACCCAATTTTGATCCATTTGTTGTTTG
ATATAGTCTTCAGAAATACTTAGTTCTTTAGCGATTGCTTTATAATCTTTTTTAGATACATTCTTTGGAACGATG
CCTATCTCATATGCTGTTCCTGTATTGGCCAATTCCACATTGTTTCGGTCTAAAATTTTACCACGTTCTGATTTTA
AATTTTCAATATGTATGCTTTGGTCTTTCTGCATTCCTGGAATAATGACGCTATGATCCCAATCTAACTTCCACA
TACCATCTTCTTTAACAAAATTAAATTGAACGTTGCGATCAATGTTACCGTAGTTTGTTTTAATTTTATATTGAG
CATCTACTCGTTTTTTATTTTTAGATACTTTTTTTATTTTACGATCCTGAATGTTTATATCTTTAACGCCTAAACTA
TTATATATTTTTATCGGACGTTCAGTCATTTCTACTTCACCATTATCGCTTTTAGAAATATAACTGCTATCTTTATA
AACTTGTTTGAAATTTTTATCTTCAATTGCATCAATAGTATTATTAATTTCTTTATCTTTTGAAGCATAAAAATAT
ATACCAAACCCGACAACTACAACTATTAAAATAAGTGGAACAATTTTTATCTTTTTCATCAATATACTCCTTATAT
AAGACTACATTTGTAGTATATTACAAATGTAGTATTTATGTCAAAATAATGTTATAATTTTTGTGATATGGAGGT
GTAGAAGGTGTTATCATCTTTTTTAATGTTAAGTATAATCAGTTCATTGCTCACGATATGTGTAATTTTTTTAGT
GAGAATGCTCTATATAAAATATACTCAAAATATTATGTCACATAAGATTTGGTTATTAGTGCTCGTCTCCACGTT
AATTCCATTAATACCATTTTACAAAATATCGAATTTTACATTTTCAAAAGATATGATGAATCGAAATGTATCTGA
CACGACTTCTTCGGTTAGTCATATGTTAGATGGTCAACAATCATCTGTTACGAAAGACTTAGCAATTAATGTTA
ATCAGTTTGAGACCTCAAATATAACGTATATGATTCTTTTGATATGGGTATTTGGTAGTTTGTTGTGCTTATTTT
ATATGATTAAGGCATTCCGACAAATTGATGTTATTAAAAGTTCGTCATTGGAATCGTCATATCTTAATGAACGA
CTTAAAGTATGTCAAAGTAAGATGCAGTTCTACAAAAAGCATATAACAATTAGTTATAGTTCAAACATTGATAA
TCCGATGGTATTTGGTTTAGTGAAATCCCAAATTGTACTACCAACTGTCGTAGTCGAAACCATGAATGACAAA
GAAATTGAATATATTATTCTACATGAACTATCACATGTGAAAAGTCATGACTTAATATTCAACCAGCTTTATGTT
GTTTTTAAAATGATATTCTGGTTTAATCCTGCACTATATATAAGTAAAACAATGATGGACAATGACTGTGAAAA
AGTATGTGATAGAAACGTTTTAAAAATTTTGAATCGCCATGAACATATACGTTATGGTGAATCGATATTAAAAT

GCTCTATTTTAAAATCTCAGCACATAAATAATGTGGCAGCACAATATTTACTAGGTTTTAATTCAAATATTAAAG
AACGTGTTAAGTATATTGCACTTTATGATTCAATGCCTAAACCTAATCGAAACAAGCGTATTGTTGCGTATATTG
TATGTAGTATATCGAGCTTCACATGAAACAGCTAAAGAAGCTTTGGGCGATAAAGAGTTAAGAGCCATTGCA
CATGAGTTAACTAAAACAGTTAAGGATAACATGAGTGTTGATTGGTCTAAACGAGACAGTGCTAAAGCTAAA
ATGAGAGTTCAAGTTAGACGCCTATTAAAGAAATATGGCTATCCACCAGATCTTCAAAAAATGGCTGTGGAA
CAAGTTGTAGAGCAAGCAGAATTAATGGCAAGTCAGCAATAAAAAAATAAATCATAATGAGTCCGGGACATA
AAGTTCTTGGATAAGTGAAAAAAGACAATTTCTATTGAAATAATATAGAAATTGTCTTTTTTATAAATTTTTTG
ATTATTTTCAGCTCGTTGAGCTACTACTTTTCTTATATTAAGTGCCATTAATACAAAACCAAGTTCTCTTTTGAC
TTTATTGAGTCCTCGGACAGACATCCGAGTGAAACCCAAAATAGCCTTCATAAATCCAAAAACAGGTTCCAC
ATCAATTTTTCTTTGACTGTAGATATTTTTTGTTTCTGGTTCTGAAAGCTTTTTGTTAATTTGGGATTTAAAATA
TTCCCAGTTATAATTCTTCATTATTTTTTTTGTTTGTTTTTGAATTGAAGTTCATACATTGATTTTTCAGAGGAC
ATTCTGAACAATCATCACATTCATATAATTTGAAGTCTCGCTTATAACCATACTTATCATGACGATAGGCATATCT
TTTAAAACCTAGCCGTTTATTATTCGGACAAATGAATTCGTCATTAATTTCGTCATAGTTCCAATTTTGAGTATT
AAAGATGTCACTTTTATATTTTTTAGTTTTATCTTTTATAAACATTCCATATGTTATGAGTGGCGTTCGATTAAAG
TCATCTATAATTGCCTTATAATTTGATTCACTACCATAACCTGCATCAGCTACAATATATTCAGGTAAATGACCGT
AGGTCTCTTGAATTGAATTTAAAAATGGAATCATCGTTCTAGTATCCGTTGGATTTTGATACACATTATAAGATA
AAACAAATTGGGAATTTGTTGCTATTTGTAAATTATACCCTGGCTTAAGTTGTCCATTTTTCATGTGATCTTCTT
TCATTCTCATAAATGTCGCATCATAATCTGTCTTAGAATAACTATTTCTATCCTTTAAAATAGATTTTTGAAATTC
GTATCGATACTTTCGCTCAAAATAATCATTGATTTGCTTTTTGTATTTTTTGATTTAGTTCTTTTGAGACGTATT
TGTTTTCTTGTTTTAGTACATTTTTCATTGTTGATATGTTGGTTTAAATCTTCGATTTCTTTATCTAAGTGACTAC
CAATCAAATCTATTTCTTCTTTTGTTAATTCATTATCATGATCTTCTTTAATTTCCGGTATGATTTTATTGGTTACC
AATTCATGGTAGAGGGCTTTAGAATCCTCATTCATCTTTGATTCATGGTTTTGAATACTCTTTTTCCATACAAAT
GTATATCGATTGGCATTTGCTTCAATTTTTGTACCATCAATAAAAATAGCTTTATCATCTATAAGATTTTGTTTTA
CACACTGACTGTAAAATTGAATAAATAAAGATTCTAATAAAGCATCTACTTTTGGATTTACTCTAAATTGATTAA
TTGTTTTATAAGAAGGTTTTTGATTTTGTGATAGCCACATCATTCGGATGCTATCATTAAGCATTTTTTCTATTT
TACGACCTGAGAATACAGATTGTGTGTAGGCATATAGAATCACTTTTAACATCATTTTAGGATGGTACGAAGT
TGCACCACGGTGATGTCTGAATTCGTCGAATTCATTGTCAGGAATTGTTTCAACAATATCATTTACAGTAAAAC
GATGTTGATTTGTTTTGTTTCCATATTGACCTCCATGTATTTGCTATGATTTCAAAATCCATTTTTGACGTGCCTT
AGGGTTGAGTGGATGCATAATTTCATTTGTTACTGGATTGATGAGCTTTTTTACTTTCTTTTTATGAGGTTTTA
ACATTTCCATCACTTGTTCGACACGGTCGATAACAACTGGTCGCTTCGCATAGGCACCATAAGCAAGAATCAC
TGTGTCACTTTCACTAATCGCTTTCATCAAATGAATATCAGTGTGCTCATCGTATGGATTTTTGATATGTTTGAG
GTTTTCGGGTGTTCTAATATTAGAGAATAGATTTACAAGATATACAGCACCGTATCGTTCTGAATTGGCTAATT
GGTTGAGGATAAGAACAGTTGTGAGATCGAGTGATAATACACCGTCTAAATGAGGATACATCGTTATCACTGT
GCATGCAGCTTTCTTTTCATCCCATGTTTTCTTGAGTAAATAGCGGTGCTGTTCATCATCGCTAAATATGGCTT
CTGTGTGTATCGTACTTTTGATTGTATTCATCATCGTCACTTCCTTTAGTATTCTTCTGGTAAAAGCATCACATAA
TAAAAAGCGTCCACGTCATCTTCACGAATGACGTAGACTTTCTTAGGTAATGCATTTTGATTTTTTTCATAGTT
TGTATAGTGATATTCCAATTTGTATGTGGGTTGTTCTTGTTCATGTGTGATTGAGAGTATATTCTCATCTTCTTGT
AATTTAAAAATGTGTAGGTAATCTGTATGAGGCTGGTTATCTTTTTTTTCTACCATGTGCCAAAGTAAGATTTG
AAGGTCTAGTGGAATACTTTCATTAATTCCTCTTGTGATGTATCGATTGATTTCATGCTATTTCCCTCCCTTCTG
CTTTTCTTTCATGATGTCGATGATTTCGTTGATAACTGTGACGGATAATTGAGCAACTATGATCCAATTATTCAT
GGTCCTGACCTCCTTGTTTTAGTAAATGACGTTCATCAATAATGATATTTTGAGTATCTGTAAGGTACAGAAAG
TCCATGTCAAAATGGTCTAAGTATCCGACACTGATGAGTTGGTTATTGGCATACATTAGAAATGGATAGATACT
TAGCTCATGTAGTTCATCATTATAGTAGGTATAAGTCTCGAGTGTGAGATGTACCAGTGGAGAATCATTAATAA
AACGTTCCGGTAGAATATTTTCTGCTGCTTCCTCCAGCGCTTCACATTCCCAAGCTTCGTTATTAGATAGTTGG

AATAGGTGGGTTATATATTGTTTGAGTTCTTGAGTGATGGTTTTCATATTATTGCCTCCTAGATAGTGTAATAGT
GATGTAGTTCATATACATCATTGAGATAATATATATTTGATTTGTCATTTATTACGAATCCCGGTGGGAATAAGA
GAAAATTCCATATAAAAACCCGCTACAAACGTTGGTATGCCAAGGAAATCCTGAAATTCCGCCTATTTTGACA
AACAATCAACTCATTATTTATAAGTATTGATGATAGGGTTGTGTCTCTGCTTCCTTATATATATTATTTATTTATAA
AAAGTAACGGGATTTTGGGATTGTGCTTGCACAATCCTTCTGTTTCTTCGAATCTGCAAATCCCAATCATTTCC
CGATAAAAAATCATTGTGGGATGTTCTTTAGCAATTTCAATATAAGCATTGTATAGTTATGAAAAAATTACGAC
AATAACTGTTTCATTAGATAAGTGTTATTGAAATTGATAAAGAGCAATTCTTGAAAATAGTTAGATAAAATAAG
CGAAAGAATATAGTGAAAATTATTGTTATAACAATGATTCTATTAGCTAAATAGTAAGATATAGTGTTTGGGGC
AAAAATAAAGACGAAGTGCTGAGATGCACTTCGTCGAGTTGTTTATTATTGAAAAGTTGTTTAATGATTTCGT
TATTAAGTTTGAGTGTGACATAGAATTGTTTTTTATGATTACCATCTTTTTTAATATCAATGCGATCAATCACTG
ATAGATACAATGCTTTGAGTCGAGATTTTTCTATGTGCTTAATATCATGAAAGATGTGTTGTAATAGTTTACTGA
TTTCTTTGGCATCAAATAAAGTCTTATCTTCATTTTGTTGATTTTTGAGTTGGTTGATTTGATTCGTAATGTCAT
TGAGTTGCTTTTCATATTTTTGAATACTTGGTCTGATTACTGATGTTAAGTCCGGATTATCCTCGATGGTTTTAA
TCAAGTTATTTATTTTGATTTGTACTTCATCATATTGTTGTTGCTTATAAGCAATATCGTGATGAAGTGCAGCGC
CATCAACTTGATTTTCTTGATTGACGTGTGTTACTACGCGTTGAATGACTTTATCACTTTTGACTATTTCAAGTA
TTTGCTTCATCACATAATCTTCAATCACATCAGCTCTTACACTGTTTGCCGAACATACTTTGGAACCCTTGTTCC
GAAAATTACTACATGAATAGTAACGAATACGTTTCTTAGTCCCGTCTTTAAGAGTATTCGTGGTATTGCTTGCT
GCCATAGGTGCGCCACATTGGGGACAGTGAATAATGCCTGTAAGCAGATTCGTTCCTTTGCCATGGACTTGG
GGTTTTTGACTGACTTGTTTCTTACGCATTTGTACTTTATCCCATAAATCTTGATTAATAATGGGGGAATGCTTA
CCTTCAGCTATCACTGGTTTATCATTCAGCCCTTTACGACGTTTTTCACTCCAATCTTTGTATTTCGCAAATTGA
ATTTTGCCGATATAGAAAGGGTTAGCTAAGATGTATGTGATTGAACTAATACTGAAAGGTTTCCCCTTTTTAGT
GACATATCCTTTGTGATTCAATGCATTGGCAATTTTACGATAGCCATGTCCTTTGGCATAGCACTCGAATATATA
TTTTACAATATTAGCTTCATGTTGGTTAATCATTAGCTCGTGTTTACTATCTGGTATTTTGTCATAACCTAGTGGT
AAATTGCCTTGATAATAGCCTTCTTGGGCACGTCTCGTTTGACCCATAAATACGTTCTCGACAATGTTATTACG
TTCGAATTCTGAGAAACTCGCAAGTATCTGTAACATGAGTTTACCAGAAGAAGTATTGACTTCCATACGCTCT
GACAAACTGAAAAAATTCGACATTTTGTTTGTGTAAATCTTCGACAATTTTGAGAAGATCAGATGTATTACGAG
CTAATCGGTTTGTTTTGTATACCATAACACAGTCGATATTGCCTTCTTTTGCATCTTTCAACATACGTTGGAGCT
CAGGTCTGTTCATAGATTTACCTGAAATACCACGGTCAGCGTATATATCTTTAACTTCAAAATGATGGAAGTCA
CAGTATTCTTTGATTTGATTGATTTGTCCGTCGATACTATAACCTTCTGTGCTTTGCATTTCTGTTGATACACGT
ACATAGATACCGACACGTTTTGTTTTAAGTTGTTGCATTATGTTACATCCTTTCTTCATTTATGCAATCGATGAT
TGCATGGTTTGATTGACAATATTGAGTGGTTCATTTTTGAAATAGATTCCTATAAGATTTTTATCTTTCGTAATG
TGAATGGTTTCAATATAGGGGTACAATATGTTTAACGTGAAACGTTTTTGAATAATATTTTGAATGATGTGTTG
TATTTGATGCCCATTGATAGATGTAGTGCGTTGCGGTTGTTGACGTAATGATTGTGTTTGCTCTCTGAACGTTT
CTGCATCAATGATGCCTTGTGCCAACTTTTCTATCAGTTGTTCTTGAGTCAATGTGTGATGTTTTTCTATGTTTC
TTTGTCTTTTGATGCGTTTGTCAATCGCACTTTTAATTTTTGTGTAGATGCGTTGATTTTGATAAAAGTCTCGG
CACACTTCTAATACTTTATCTTCAAGTGTTTGTGCATTGATGCCTTTAAAATCACAGACAAAGCGTGAAGCATT
CATGTTTTTAGGACAGACGTAGTAACGTAATGTATGATTCTTTTTTCTAATGGTCATATTTGTAAGTGTTGCATT
ACAACATGGGCATTTGATTTTTTGTTTGAGTTGATTATCCGAAGGTGTCTGTTTGGTTTGTTTTTGCAATCGA
AGTCTCTGCGCTTGCTCATATATACTTGTGGAAACAATAGAAGGAAACATATTGTCGAATTGGCCATATTGATT
GTTGACACGACCACAATAATTAGGATTGATGATAATGTTACGAACTTGATAGGGTTGTCGATTGATATACGTGT
TATCTTCTTCTAATAACTGTGCAATTTTCTTATAACCATGACCTTTAATGTAATAATTGAATACAGCCTTTACCGT
TGGTGACTCATTTTGATTGATGATGAATGTTCCGTTGTGATATTCGTAACCAAAAGGTGCATGTGTTGTAATCA
ATCGACCTTGCTTTGCTTTTTCTTGAAGCCCATTTCTGACTTGTTCTCCAATGTTATCCGATTCAAGTTCGGCT
AAACTGATGAAGATATTAAGCTTGAGTCGGTCGAAAGCTTGATCCATATCAAAGTAACCATCATGTACGCTTA

AGATATGAACATGGTACGTTTGACATAATTTGATGAGTTTTAATGCATTTTTCAGATTACGATGCAACCTATTA
AGACGATAACAGCATAATATGTCACACTGTCCTTGTTGAATTAATTGTGTTATTTGTCGATACCCACTACGATTA
TCTTTGCGACCTGATTGTTTGTCGCTATAAAAGTTGATATGTTGAATATGATGTTTTTCGGCTATTGCTTCGATA
GCTTGTTTCTGTGCTGCAAGAGATTGTTGTTTCATCGTACTTTGACGTAAATAGCCTATGACTTGTTTCATATC
GGCTCCTCCTTTCACAGTGATAATATATATTTATGGATGAATTGATATATAAGCCCAACATCAATGAGATGTTGG
GCGTCCATATTAGTCATTTGTTTGATTGATTTCTTCAATTACCAAATCGGCTAATATCTCGATAAGTTCATCCATG
TTTTTCACTCCGTTATTTGTTCTATCTTCAATACGTCGATTATTCAGTTTGATGCTTCACAGTTGTATGATAAAG
ACAATTAGAAATCTTCGTGAACTCCTGAAGGGCCTATCCCTTCATTAGCGGATTTAAAAAGTTCTTTCGCAGC
TTTGTTATCATTTGACGGTGTCCAATTTTGAAGTAACGACTTATCTTTAGTTAATCCGAGGATAGATGCAAACT
CTACATCTAATTTTAGATGGTAAAATACAAGTGATTGTTTTTTACCGCTATTATCTTTGACACTTCTTTTAGTTGT
TTGGCGTCCACGGTCAGCTAATATGAAACCTTTATCTCTTAAGGCGTTGACAACATTATTAACATCTTGAAATT
GATGATTGTTTAGCATCTGTTTAAAAACGTTCGCAATTATTTTTACTTCGATATGGTCATCTTTTAATGAGATTA
ATCCATAGTTCTCAAACATATTTTTCAAAGCACCTTCATCTGAAAACTTACCTCTGTTTTGTGCCACAAATTGA
ATGATGACATCAATAGCTTTATCAGCTAATGAGCGTTCAGAGACTGTATGAGTATGATAATCAATAAAGTAGTC
TCTTATATTAGCGATATCAATATCTGTAGATAAAACACGACCTAATATTTTCGCAGATGTTGTAATGACTGCATA
ACGCTTAAACATACGAATACCTGTATTGTTTGTTTCATCTTTCAATTTAGCTTCAAACCAATCTACTTCCTTGTA
AAACCATTGAATAACTTCATCTTCACGATTTATAAGATATTTAGCTACTAACGGTAAAACATGACCATAGTTTAG
TGCTACAGCTTTTTTAATATTGTCAGCATTGGTCGCATTTGTAGTGAATTGTTCATTAATCTCGATGGTTCTTAC
ACGTAATCCATCGTTTTGAGCTGAATCATTAAAAATACTGTATTCTGACGTTGAAATGACAGAAGTACCCCAAT
TCTTAGGCGTTTTAACTTCTCCATGAACGTTTGAACGTTGACGACCTTGACCTTCAGCGATGGAATATAACAA
ACCCGTGGTATCTCTAAGTGTCGTAGATGAAAGCTCATCAAATACTATAGGAATGCCATAATTGTTACTCAAAT
AACCTTCAAGTGCGTTTCGTGTGGCATTCCAACTTCTAAAAAGAGTTTCATTACCTTTGGTAGGGTTACCAGC
GACTGATACAGCTAAAGCAGCTGCGGTTGACTTACCGGTTGAGGATTGACCTGTAAAACTAAAGAGAATTC
CTGCAAATTCGATTTCATGTTTATGCTTCAGAAAACTTGTCACTAAGGCAGAAATCCCAAATATGACTGCCAA
TTCTAAAAGAAGATGACCTTTAACCTCGTCAATATACATGTTAAACCAATTATCAAATGTACCTTTAGGAGTTA
AGTCATAAGCACTATCTACAATAGGGTATGAAGGTGAGGACTGATTAAATTGAATAGATTTGAGAACTTTATC
TAGCGATATCAAGTAGCCAAAAGGTGTCTCTAGTATACCTACACCCTCATATAATTCAGAAAGTGGTAATCTAT
CTCGCATCAATTGCAAGGCATAACTCAATGATCTAATATACTTCTCGTTAATACTGTAGCCATATTTAATTAAAG
AAGGTAAGTTTCGTGTTGTTAAGATATCAGACTCAAAAATGTCTTTTTTACCATTGTTGTTAGTAATAATCAAC
TTTTCAACACCAGTAATTGGATCAGAAAATCTTGCATTAACAATAATAGGGCTAGCCATTCTAACCACTTTTTC
ACTGTCACCATCTTTTTTAGGTGGTATCGTTTCTTTCCAACCGAAGGAATCTAAAGCAAATGGGCCAATTTCA
AACAATGTGTAACTCATTAGCAATCACCTCCTTTCGAAGGCGTGCTATTATTGCATGGGTTTATCCCTATTTTCT
GATATAATAAACTGCCATTTTTTGTTCCTACAATTTTAAATGGTAATCCTGGCGTGTACTTAATCCAGTAAGCAA
AACGACGTCCTACTTCACGTTGATCATTCGTGCTACAAACCACATTATATTTCTTTCTTAATTCACCATAAGTGA
ACGTACTTCCAACTGGTTGTTGAAAAGATTTAGCAATTATAAAATTATAAAGCTTTTCTTGCTGCTTAGATTTC
TTATTCATTTTAAAATTCCTCCAATAATTTTTAAGGAGTCAATCAATATCATATAGGTTAAACTATCTTCAATTGA
TTGCTATAATAAGCATAAATGAAGCATTATGATTAGACGCGGAACAAAAAATGGGGAGGGGGGGGTATATAT
TGATACCTACGCTAGAAGAAATTATTGATAAGTATGGAAACTTAGTTGATTATTTGAAATTTGATGTAACAGTT
GAGGTTTATGAAGATTTGTTGTTATTAAGAAGTTTAAGCGATTTAAATGAACATCAAAAGATGGATAGAGTTT
CATTTATAAAAAAACATTTAAACTCAAATTTCGATTATTTATTAGATGATACTGAGTTTAGAAGTATCAATAAGA
TTTACAGTAATTTAAATATCATGACGCATATCAATAGTCAAAATAATAATTTTAAACGTAATCCATTTATAAATGA
AGAACAATTGAAATCACTACTCGCGATAAAGAAAGTAGATGCACATATGAGTTATGATTCAAATATTACTTCAA
AAGCATTGGCAGAAATAAACAAAACGCAAAAAAGATTAGTTACAAAGATAGACACACTCTATAATCAGTCAA
AAAAAGAAAATGAATATGTACGGTTAGGAGAAAAAATATCGTATACAAAATTGGAGAATCATTGGAAATATTT

ATATAATGAGATTCAGTTTTATAATCTTAATAATCAATTGATTAGTTATGTTGCATTAGAACAGGAATATGCTTG
GGCTTTTTTAAATGAACTATTTTATTTGATAGAATTGTATTTGAAGGCATTTAAAGGACAAAAGAAAACAGAT
TATGAGTTTGGGAAAAAATTAAACGAATTTATTCAATCATATGTCATTATTTTACTTATAGATATACGATTACCTG
TTGTTAGATTATATATTATTAGAGACATTGTAGATACATGTGAAGGAGAAAAAGATAATTACAAAAGAATTACA
CTGATAGAAGAATCTATTAAAAAGTATAGATACGTAAAAGATCAGATTAAAAGATTTAAAAATGGATTAGAAG
AATCGTTATTAAATGCAACTTTATCTATTGAACAAAATATGTTGAAAGTTAAAATTGATTATTATAAAGCCTACT
GCTTTCCTAGAGAAAAAACAAAGCATAACAATATTGAGTACAATGTATCGTTGTTTTTTAAAGCATTAGATGC
CTTAAAGAAATAGTTTTTCACTGAAAATATTTATAGATATAATTAAAATTAATTAATATTAAGCCAGGATAATGTA
GTCTTAATCGTTCTGAAATACGAAAAATGTTGTGGAAATCGCGTGAAATAATCAGAGGAATCGTTTGAAATC
ATCGTAGTAATCGCGCGAAAATCACGTGAAATAATCAGAGGAATCGTTTGAAATCATCGTAGTAATCGCGCGA
AAATCACGTGAAATAATCAGAGGAATCGTTTGAAATCATCGTAGTAATCGCGCGAAAATCACGTGAAATAATC
AGAGGAATCGTTTGAAATCATCGTAGTAATCGCGCGAAAATCACGTGAAATAATCAGAGGAATCGTTTGAAA
TCATCGTAGTAATCGCGCGAAAATCACGTGAAATAATCAGAGGAATCGTTTGAAATCATCGTAGTAATCGCGC
GAAAATCACGTGAAATAATCAGAGGAATCGTTTGAAATCATCGTAGTAATCGCGCGAAAATCGCGTGAAATA
ATCAGAGGAATCGTTTGAAATCATCGTAGTAATCGCGCGAAAATCGCGTGAAATAATCAGAGGAATCGTTTG
AAATCATCGTAGTAATCGCGCGAAAATCGCGTGAAATACTATGGTAGACGGTTTGAGTAAATTAATGGAGTAT
TTTAATATTTATGGTTTGGAGGAATTGCGTTGCATAGAGAAAGTAAATATATAGAGTATAAGAAATCACGAAA
AGGATTATCTAATGATATTTGGTCTACGTATAGTGCTTTTGCAAATACTGAAGGTGGTACTATATATTTAGGAAT
TGAAGAAAAAAAGATCGAGGACAAAAAAGTCTTTGTTTCAGTTGGTGTTGAAGATCCAGAGAAAATGATTG
AAGATTTTTGGAATGCACTATATGGAAGAAGTAAAGTTAGTCAAAATATTTTATCAAATAAAGATGTTAAAATT
GTTAATATTGAAAATAAAGCGTGCATTGAAATTCATGTACCAGAAGCGCCTTATTCGAAGAAACCGATATATG
TAGATAATAAAAAAGATTTAGTATATAAAAGAGTTGATGATGCTGATAGAATTGCGACTGAAGAAGAGTATAA
ATTCATGATTGTAAATTCTCAAGACGATATAGATACAGAATTATTAGATAACTATGACATGTCTGATTTAAATCA
CGAATCTATCGAAAATTATAGGAAACTTCTATTAAAAAATACTAATGATGAGAGATATGCGAATATGAGCCAAC
TGGATTTAATGATAGATTTAGGAGCATATAGAAAAGATAGAAGTTCGAAAGACAAACAGTATAAAATGACTA
CAGCATGTTTATTATTCTTTGGTAAGTATAATGCGATTAGTGATAGATTCCCAGGATTTCAATTAGATTATTTTA
AGAAAACAAATTACCTAGATACTGATTGGAAAGATAGAATATCAAGTGGAGATTTAGGTAATGAAGATTTAA
ACGTGTATAGTTTTTTTGAAAAAGTATTGATAAAATTAACTGATAACATTGAGGAATCATTTAGCCTAAATGAT
GGTTTGACTAGACAAAATTATGCAAGAGATTTAAAAGTAGCAATTCGCGAAGCACTGGTTAATACATTAATGC
ATGCGTATTATGATACTAAGCAAAGTATTAAAATAGTTAATTGTGAAGATTTTATAGAGTTTTATAATCCGGGTA
ATATGAGAATAAATAAAGAAGATTTTATTCATGGAGGGCATTCAAAGGACAGAAATAGTATATTATCGACGCT
TTTCAGAAGAGTAGGATATTCAGAAAAAGCTGGATCTGGAGGACCAAGGATATTCGATGTAGTTAATAGACA
TAAGCTTAAAACGCCTGAAATAGAATTAACGGACATGGACACTAATGTAGTACTTTGGAAACAAGATTTAATG
AAGGAGTTTGAAAAATATCCTGAGTTAGACAAAAAAGTAATAAAGTATATTATTGACTATGGATCAATAAGTA
AGGGTGAAGCCTTAAAAATGGAAAATATGACAGAATATCAGTTTAGAAATATTTTAAAAAAACTAAAAGATG
ATAACTTGATAAAAAAAGAAGGTGAAGGTCCGGCTACTAAATATGTGTTAATAGAATCAAAAGAAGCTGATA
TATTGCGAACTAAAAAAGTAATTAAAAGTTTAGAGTCTTTCTTTAGGAATAAATAAAAAAACAAGAGATAGG
TGCGAAGTGTTTGGATTATACAAATGCTTCGCATCTTATATTTATTAATAAATATCATAGAAAACCGTATCATTAA
CCGATACGCAGAGATGCGGTTTTTTAGACACTTCATAAAGGGATTTTGAACGTATCAGAACATATGAGGTTTA
TAGGAATTGCTGTTATGTTTTTTGATCACATCAATAAACAAAAAAGGTATGTACTATGTAAAATATTTATTAAAT
GATATAAAGCGAGGGTATATAAATGATTTTTAAATAGATATTTATCCAATAATATAAAAAGGAACTATAAGCTAT
ATCTAAAAGCTATATCTAACTACTTATAGTCCTTCTTCATTAGTATAAATATAATTATTAAGAAAGGTATATATCTT
TGTAACCTTCGTTTACATTAATAATGTTGTGATAACCTTTGTGTTCTAAAATACCAATAGCTATAGAACTTCTAAT
GCCAGACTGACAGTCTACATAAATAACATCGTTTCTATTGAAAGGTAAATCTGTTTCTAAAAGTTTGCCGTGT

GGCACATGAACCGCTTGGGATAAGTGGCCATTATTCCATTCATTATCATTACGTACATCTAAGACATGTGCTTC
ATTACCAGTTATGTCTTTACTATGAACAGATTGTGTTTGAATTTGAGCTTGTGGTAACTGATATCCAGACACAT
TATCATATCCAATAAGTTGTAAAGTATGTTATGTGTTGCTTTTGAAACAAGGTGATAGTCTCCAATCAAGTTAAT
TTCTTGATTATAGTTTAGATACCAGCCAATTTGATTGATGAAATTTTTATCATATGGAATATTGATTGTACCTTCA
ATATGTCCACCATGATAAGCCTCCTTACTGCGGAGATCAAAAGTTAATCTGTTTGTACTTGTAGCTGGATAAAC
CGTATAAGGTTGATATAAATTCATACCGAATTGATTAATTTTTTTCATTTGTGCAAAATGATGTGGTGGTGCAG
GTTGGTCAGAAATGAGTTTATCGATAAAGGTAGCTTCATTATTTTCAGAAAAAGCCCAGTTCGTTTGTTTTTC
ATAGCCAAGCGTAGATGTTGGAATAGCACCTAAAGATTTACCACAAGGACTACCAGCGCCATGACCAGGCCA
AATTTGAATGTAGTCTGGCAAGTCTTTAATACTTTCAATAGATTTAAACATTTGTTTTGCGCCTATTTCAGATAA
TCCTTCTACTTTAACAGCTTTTTCTAGTAAATCAGGTCTACCGATATCTCCTACAAAAATAAAATCACCACTGAA
TAGTCCCATTGGAACTTGTGCTCCAGCACCTTCGTCAGTAAGTAAAAAACTTATACTTTCTGGCGTGTGACCA
GGTGTATGAAGCACTTTTAATTTTATATTTCCTACATAAATATCATCATTATGTTGAACAAAATGAGTGTGGTTA
GGCATATTTTTATAACCTAACATGTCATCACTTTCGCCCGATACATAAATACTAGCATTTAACTTTATAGCAACAT
CTCTAATTCCTGAAACAAAATTTGCATGTATATGTGTTTCAGCTGCATGAGTAATGGTTAAACTCTCTTCATCG
GCAACTCGAATATATGAAGATAAGTCACAAATAGGATCAATGATCATGGCTTCTCCAGTTTTTTGACAACCGA
TTAAATAAGATGCTTGAGATAAATGTTTATCATAAATTGATGATAGTCAATTTGTTATCAAGGGTGATAATTATA
TTAACTATGGTAATTACATGGAATTAAAGTTATTAAACAAACTTGATGATGACCAATTAGATTTAGAATTTTTAC
AAGATAATGGGTTAAAAGGTAATGTTCAAGGTCCTATGACTTTAAAGGAACCTTTAAAAAGTTATATACAAAA
GTCTTGAAAACGAATATTTTAAATTTAGATAATGAGTTAGAAAACACTGAATATGTCGAAGGTAAACCATATG
TACAGTACGGTATTAAATATGAAAGAAATCAGGCATTAAGAAATGAAGCTATTAAAATTCATGGAACTACATG
TAAAGTATGTGGATTTGATTTTAAAGCTAAGTATGGCGATTTAGGTGAGGGTTTTATTGAAATTCATCATTTAA
AACCAATGTTTTCAATAAAAAGAGAAATAAAAGTAAATCCACAAAAAGATTTAGTCCCACTATGTTCTAATTG
CCATAAAATGATTCATAGAAATACTAAAAAACCTTTAACGATTAAAGAATTAACCAAAATAGTTAATTATAATA
GCAAATAATTTAATATTTTATAAACTATCATTCAACCCTCTTAATTTATTAGGAGGTTTTTTGTATTTATGCTTTC
AAATGTGTGATATACTTTGTTTGTTGAAATATAGAGTATCTATAGATAGGGTGATTGAGTATGAAATTCACTGA
AGTGGAAGTTATCGAACATCTTGTAAAGGCATATAAAGAAGCAGGAAAGCCTACTTATCCTCATGAAAATTTA
TATCGAGGACGTAATCATAGTATTTCAGGTATTGGAGAAGACTTGCTGGGTGCTTATTTGATTAGTAGATTGG
AAGGTGTCCAAATATTTATTGATCAGCCTTTATCTATGATTGATAAATCTTTAAGTACAAGATATCCGGATTTATT
AATTTGTGAAGATAATGAAATTAAAAATATACTAGAAGTTAAAATGGACTTAGGATATCAAAGAAAAGATTTT
ATAGATTATTGCCGAAAGAAAGAAGAATGGATTTCAAATATCGTAGGAAAACAGTGTGTATTGTCTAGAAAG
AGAGAAGACAAAATTCCTATGAATATAGCTGATGATATTAAATTTCATGTTGTGATTTACAGTGAAAACAATG
GACCGAAGCGGTTTGATGAAGAAATCATGCCTATCGTTAATGAAACATGTCCACATATTGAAGTATATGTCCTA
ACAAGCGGTCAACACCCTAATTTAGTAAATGTTAATCTTGAAGGTATTAATATTAATAAAGATGAATTTGAAAT
ATTAGTAAATGCGTTATAAAAAAATAGAGCATCCTCCACGTTATGGAGGTGCTCTGTTTTTTATTGAAAAGTAT
CAAGTTAATTAATTTAATATGCTTAATAAGTTCTACCTTGACCTTTTTCTCTAGCTTCTGTTCGATCTCTTATGTA
CTCAGTACATACTGGATTTTCTGTTAATTCATCAACTGTATTAGTTTAATCAGAAGACGTGTCTACTTTGTAAGC
TTCTAAGAACTTATTATATGATATAGCGTTTGAGTTTTGTTGTTCTTCTATAAATTTCTGTAGTTATTTTTCAAAA
ACCGCATCATTAACTGATAAGCAGAGGCGTATCATAAGT
>Staphylococcus aureus isolate HU-14
ATGAAAATCACCATTTTAGCTGTAGGGAAACTAAAAGAGAAATATTGGAAGCAAGCCATAGCAGAATATGAA
AAACGTTTAGGCCCATACACCAAGATAGACATCATAGAAGTTCCAGACGAAAAAGCACCAGAAAATATGAG
CGACAAAGAAATTGAGCAAGTAAAAGAAAAAGAAGGCCAACGAATACTAGCCAAAATTAAACCACAATCCA
CAGTCATTACATTAGAAATACAAGGAAAGATGCTATCTTCCGAAGGATTGGCCCAAGAATTGAACCAACGCA
TGACCCAAGGGCAAAGCGACTTTGTATTCGTCATTGGCGGATCAAACGGCCTGCACAAGGACGTCTTACAA

CGCAGTAACTACGCACTATCATTCAGCAAAATGACATTCCCACATCAAATGATGCGGGTTGTGTTAATTGAGC
AAGTGTATAGAGCATTTAAGATTATGCGTGGAGAAGCTTATCATAAGTAATGAGGTTCATGATTTTTGACATA
GTTAGCCTCCGCAGTCTTTCATTTCAAGTAAATAATAGCGAAATATTCTTTATACTGAATACTTATAGTGAAGCA
AAGTTCTAGCTTTGAGAAAATTCTTTCTGCAACTAAATATAGTAAATTACGGTAAAATATAAATAAGTACATATT
GAAGAAAATGAGACATAATATATTTTATAATAGGAGGGAATTTCAAATGATAGACAACTTTATGCAGGTCCTT
AAATTAATTAAAGAGAAACGTACCAATAATGTAGTTAAAAAATCTGATTGGGATAAAGGTGATCTATATAAAA
CTTTAGTCCATGATAAGTTACCCAAGCAGTTAAAAGTGCATATAAAAGAAGATAAATATTCAGTTGTAGGGAA
GGTTGCTACTGGGAACTATAGTAAAGTTCCTTGGATTTCAATATATGATGAGAATATAACAAAAGAAACAAAG
GATGGATATTATTTGGTATATCTTTTTCATCCGGAAGGAGAAGGCATATACTTATCTTTGAATCAAGGATGGTC
AAAGATAAGTGATATGTTTCCGCGGGATAAAAATGCTGCAAAACAAAGAGCATTAACTTTATCTTCCGAACTC
AATAAATATATTACATCAAATGAATTTAATACTGGAAGATTTTATTACGCAGAAAATAAAGATTCATCTTATGAT
TTAAAAAATGATTATCCATCAGGATATTCTCATGGATCAATAAGATTCAAATATTATGATTTGAATGAAGGATTC
ACAGAAGAAGATATGCTAGAGGATTTAAAGAAATTTTTAGAACTATTTAATGAATTAGCTTCAAAAGTTACAA
AAACATCCTATGATAGCTTGGTCAATAGCATAGACGAAATACAGGAAGACAGCGAAATTGAAGAAATTAGAA
CAGCACAAAAAGATAAGACACTCAAGGAAGTGGAAGCACCTAAAGGAATAATTCCAAAATATAAAAAAGGT
GTATCAAAGACTACTAAAAATGATTCAGAAATTGAAAAATCAAATAAAGAGAATAAATTAACCGGTAAAGTT
GGAGAAAAATTAGCGCTAAATTACTTTAATGAGCTAATTGATAATAAAATAGACGAAGATAAGAAAGAACAG
TTTAGGAATATTTTAAATGATAATCCAGGCTCTCAACACGGTCATGGCTATGATTTAGTAGCTTTTGATCCAAC
AAATACAGATAAAGCTGTAGAAAAATTTATTGAAATTAAAACATCTACATCTTCTAGTATTGAGGAACCATTTT
TTATGTCGCTAAATGAAATGTTTGCTATGAAAGAATATAAGCAGAAATATTTAATATTAAGAATATTTAATGTTT
CCGGTAAAGAACCACAATTTTATTTTATAGATCCATATGCAAATTATTCTGAATTTAAAGATGTAGATGATCTCA
TTGACAAAGTATTTAATGTAGAAGCTATTCAGTATAAAGTTTTTGGCGAAAAATGATTACTTGAACAAGAGCT
AAAATAAAATTGTGATCTAATAAAAATAGAAACTGTAATTTAAATAAAACTTTCTAAATAAGCTAACTGATAAA
AAATCAGTTTGTCCACAGTCTGAAACAAGATTCCTATATTCTTTAGGAATCTTGTTTTTTCTATTTTTATGGTGA
TAAAGAGCAGATAAGATAATGTGTAATAATCACAAAAAAGTTAAATATTTTAAGGCTTGTTTAATTATTAATGA
TTTTATATATAAAGAGCAGTATAATAAAGTTGTTAATATATTATGAATAATATTCAAGTAATTTTATTGTTTTTTAA
TTTGTCGATATTTAAGTTGAGTTAAATTTAAAGGGTGTAATTTGTTTTACAATGATGAAGATAATTAGTCTATCA
AAATAAAGGGGTTGGGACTGTTATGAGTGATAATTTGTCATTATTCATTGACTATATCAATGATAATATAATCTA
TGGTAGTGAAATCAAACGGGAGAAATTAGAGAATTTATTTAATCAATTTGCTATAAAAAAATGTTGAAAAGAA
CATTGTCTATGATGAACTGAAATCTTTAGATATTACAATCATTGAGTCACAGGATTCATATAAAAATAAATTGAA
GAGATTATTTTCGGTTCTGTTGCAAAGTAAAAAAATATAGCTAACCACTAATTTATCATGTCAGTGTTCGCTTA
ACTTGCTAGCATGATGCTAATTTCGTGGCATGGCGAAAATCCGTAGATCTGAAGAGACCTGCGGTTCTTTTTA
TATAGAGTGTAAATACATTCAATACCTTTTAAAGTATTCTTTGCTGTATTGATACTTTGATACCTTGTCTTTCTTA
CTTTAATATGACGGTGATCTTGCTCAATGAGGTTATTCAGATATTTCGATGTACAATGACAGTCAGGTTTAAGT
TTAAAAGCTTTAATTACTTTAGCCATTGCTACCTTCGTTGAAGGTGCCTGATCTGTAATTACCTTTTGAGGTTT
ACCAAATTGTTTAATGAGACGTTTGATAAACGCATATGCTGAATGATTATCTCGTTGCTTACGCAACCAAATAT
CTAATGTATGTCCCTCTGCATCAATGGCACGATATAAATAGCTCCATTTTCCTTTTATTTTGATGTACGTCTCATC
AATACGCCATTTGTAATAAGCTTTTTTATGCTTTTTCTTCCAAATTTGATACAAAATTGGGGCATATTCTTGAAC
CCAACGGTAGACCGTTGAATGATGAACGTTTACACCACGTTCCCTTAATATTTCAGATATATCACGATAACTCA
ATGTATATCTTAGATAGTAGCCAACGGCTACAGTGATAACATCCTTGTTAAATTGTTTATATCTGAAATAGTTCA
TACAGAAGACTCCTTTTTGTTAAAATTATACTATAAATTCAACTTTGCAACAGAACCGTATTATGGAATAGAGA
TGTTGGTAACATTTATACAGGATCATTATACTTAAGTTTAATTTCGTTATTACAGAACCACACATTCCAACCAGA
AGAGAAAGTATGTCTATTTAGTTATGGTTCAGGAGCAGTAGGAGAAATCTTTAGTGGTTCAATCGTTAAAGG
ATATGACAAAGCATTAGATAAAGAGAAACACTTAAATATGCTAGAATCTAGAGAGCAATTATCAGTCGAAGAA

TACGAAACATTCTTTAACAGATTTGATAATCAAGAATTTGATTTCGAACGTGAATTGACACAAGATCCATATTC
AAAAGTATACTTATACAGTATAGAAGACCATATCAGAACATATAAGATAGAGAAATAAACTAGTGGCCGATTG
TGCTTGATGAGCTTGGGACATAAATCCTAACTCGAAATAAATAAGCATATCACTAAACTGATTTTTTAAAGTTT
ACAGTGATATGCTTATTTTTTTATCTTACGATTTTGTACGTGCATGCTTGCCTAGGGGTATGGCTCGAGCCATT
AGTCTCTCGCACATACTATTCCCTCAGGCGTCAGCACTTACAAAATCGGTTGTAATTTTCATTTTTATACGCATT
CTTACTGAGATTATACTAATAAGAGGAATAGTAAAAGCAATTCTAAGTAAAATTGCAGATAAGAGGTTTGTTA
AAAGCAGTTCTAAGTAAAATTGCAGATAAGAGGTTTGTTAAAAGCAGTTCTCAGTAAAATTACAGATAAGAG
GTACGTTAAAAGCAGTTCTAAGTAAAATTGCAGATAAGAGGTTTGTTAAAAGCAGTTCTAAGTAAAATTGCA
GATAAGAGGTACGTTAAAAGCAATTCCATGCAAAATTGCTGATAAGGGGTAAGTTAAAAGCAGTTCTCAGTA
AAATTGCAGATAAGAGGTACGTTAAAAGCAGTTCTAGGCAAAATTGCAGATAAGAGGTGCGTTAAAAGCAG
TTCTCAGTAAAATTGCTGATAAGGGGTAAGTTAAAAGCAATCCTAAGTAAAATTGCAGATAAGGGGTACAGA
AAAACTAGACTTGATTACAAAATGGAGCTTGGGACATAAATGATTTTTTAAAAATGAGATGAGACGTAGATT
AACTCCATAATCAATACGAATCTATCGACTTCTTTATTTATGATATTCATCTCTTTTTAATGGAAATAAAAGTGCG
ATTAATGTGATAATACAGTTACGTTAATTAAAAAAATAAAAATGCAAGGAGAGGTAATATGCTAACTGTATATG
GACATAGAGGATTACCTAGTAAAGCTCCGGAAAATACAATTGCATCATTTAAAGCTGCTTCAGAAGTAGAAG
GTATAAACTGGTTGGAGTTAGATGTTGCAATTACAAAAGATGAACAACTGATTATCATTCATGATGATTATTTA
GAACGGACTACAAATATGTCCGGGGAAATAACTGAATTGAATTATGATGAAATTAAAGATGCTTCTGCAGGA
TCTTGGTTTGGTGAAAAATTCAAAGATGAACATTTGCCAACTTTCGATGATGTAGTAAAAATAGCAAATGAAT
ATAATATGAATTTAAATGTAGAATTAAAAGGTATTACTGGACCGAATGGACTAGCACTTTCTAAAAGTATGGTT
AAGCAAGTGGAAGAACAATTAACAAACTTAAATCAGAATCAAGAAGTGCTCATTTCAAGCTTTAATGTTGTG
CTTGTTAAACTTGCAGAAGAAATCATGCCACAATATAACAGAGCAGTTATATTCCATACAACTTCGTTTCGTGA
AGACTGGAGAACACTTTTAGATTACTGTAATGCTAAAATAGTAAACACTGAAGATGCCAAACTTACTAAAGC
AAAAGTAAAAATGGTAAAAGAAGCGGGTTATGAATTGAACGTATGGACTGTAAACAAACCAGCACGTGCAA
ACCAACTTGCTAATTGGGGAGTTGATGGTATCTTTACAGACAATGCAGATAAAATGGTGCATTTGTCTCAATA
GAAAGTTAGAGGTGAGTCTTACGTTTCAGTGACGGTAGACTTACCTTTAACATGTTACATACTAAAAAATTAA
TTTGAATAAGAAAGAGAGACATATATGAAATACGATGATTTTATAGTAGGAGAAACATTCAAAACAAAAAGC
CTTCATATTACAGAAGAAGAAATTATCCAATTTGCAACAACTTTTGATCCTCAATATATGCATATAGATAAAGAA
AAAGCAGAACAAAGTAGATTTAAAGGTATCATTGCATCTGGCATGCATACACTTTCAATATCATTTAAATTATG
GGTAGAAGAAGGTAAATACGGAGAAGAAGTTGTAGCAGGAACACAAATGAATAACGTTAAATTTATTAAAC
CTGTATACCCAGGTAATACATTGTACGTTATCGCTGAAATTACAAATAAGAAATCCATAAAAAAAGAAAATGG
ACTCGTTACAGTGTCACTTTCAACATACAATGAAAATGAAGAAATTGTATTTAAGGGAGAAGTAACAGCACT
TATTAATAATTCATAATAAAACAGTGAAGCAACCATCGTTACGGATTGCTTCACTGTTTTGTTATTCATCTATATC
GTATTTTTTATTACCGTTCTCATATAGCTCATCATACACTTTACCTGAGATTTTGGCATTGTAGCTAGCCATTCCT
TTATCTTGTACATCTTTAACATTAATAGCCATCATCATGTTTGGATTATCTTTATCATATGATATAAACCACCCAAT
TTGTCTGCCAGTTTCTCCTTGTTTCATTTTGAGTTCTGCAGTACCGGATTTGCCAATTAAGTTTGCATAAGATC
TATAAATATCTTCTTTATGTGTTTTATTTACGACTTGTTGCATACCATCAGTTAATAGATTGATATTTTCTTTGGA
AATAATATTTTTCTTCCAAACTTTGTTTTTCGTGTCTTTTAATAAGTGAGGTGCGTTAATATTGCCATTATTTTCT
AATGCGCTATAGATTGAAAGGATCTGTACTGGGTTAATCAGTATTTCACCTTGTCCGTAACCTGAATCAGCTAA
TAATATTTCATTATCTAAATTTTTGTTTGAAATTTGAGCATTATAAAATGGATAATCACTTGGTATATCTTCACCA
ACACCTAGTTTTTTCATGCCTTTTTCAAATTTCTTACTGCCTAATTCGAGTGCTACTCTAGCAAAGAAAATGTT
ATCTGATGATTCTATTGCTTGTTTTAAGTCGATATTACCATTTACCACTTCATATCTTGTAACGTTGTAACCACCC
CAAGATTTATCTTTTTGCCAACCTTTACCATCGATTTTATAACTTGTTTTATCGTCTAATGTTTTGTTATTTAACC
CAATCATTGCTGTTAATATTTTTTGAGTTGAACCTGGTGAAGTTGTAATCTGGAACTTGTTGAGCAGAGGTTC
TTTTTTATCTTCGGTTAATTTATTATATTCTTCGTTACTCATGCCATACATAAATGGATAGACGTCATATGAAGGT

GTGCTTACAAGTGCTAATAATTCACCTGTTTGAGGGTGGATAGCAGTACCTGAGCCATAATCATTTTTCATGTT
GTTATAAATACTCTTTTGAACTTTAGCATCAATAGTTAGTTGAATATCTTTGCCATCTTTTTTCTTTTTCTCTATTA
ATGTATGTGCGATTGTATTGCTATTATCGTCAACGATTGTGACACGATAGCCATCTTCATGTTGGAGCTTTTTAT
CGTAAAGTTTTTCGAGTCCCTTTTTACCAATAACTGCATCATCTTTATAGCCTTTATATTCTTTTTGTTTTAATTC
TTCAGAGTTAATGGGACCAACATAACCTAATAGATGTGAAGTCGCTTTTCCTAGAGGATAGTTACGACTTTCT
GTTTCATTAGTTGTAAGATGAAATTTTTTTGCGAAATCACTTAAATATTCATCCATTTTTTTAACGGTTTTAAGT
GGAACGAAGGTATCATCTTGTACCCAATTTTGATCCATTTGTTGTTTGATATAGTCTTCAGAAATACTTAGTTCT
TTAGCGATTGCTTTATAATCTTTTTTAGATACATTCTTTGGAACGATGCCTATCTCATATGCTGTTCCTGTATTGG
CCAATTCCACATTGTTTCGGTCTAAAATTTTACCACGTTTTGATTTTAAATTTTCAATATGTATGCTTTGGTCTTT
CTGCATTCCTGGAATAATGACGCTATGATCCCAATCTAACTTCCAAATACCATCTTCTTTAACAAAATTAAATTG
AACGTTGCGATCAATGTTACCGTAGTTTGTTTTAATTTTATATTGAGCATCTACTCGTTTTTTATTTTTAGATACT
TTTTTTATTTTACGATCCTGAATGTTTATATCTTTAACGCCTAAACTATTATATATTTTTATCGGACGTTCAGTCAT
TTCTACTTCACCATTATCGCTTTTAGAAATATAACTGCTATCTTTATAAACTTGTTTGAAATTTTTATCTTCAATT
GCATCAATAGTATTATTAATTTCTTTATCTTTTGAAGCATAAAAATATATACCAAACCCGACAACTACAACTATTA
AAATAAGTGGAACAATTTTTATCTTTTTCATCAATATCCTCCTTATATAAGACTACATTTGTAGTATATTACAAAT
GTAGTATTTATGTCAAAATAATGTTATAATTTTTGTGATATGGAGGTGTAGAAGGTGTTATCATCTTTTTTAATG
TTAAGTATAATCAGTTCATTGCTCACGATATGTGTAATTTTTTTAGTGAGAATGCTCTATATAAAATATACTCAAA
ATATTATGTCACATAAGATTTGGTTATTAGTGCTCGTCTCCACGTTAATTCCATTAATACCATTTTACAAAATATC
GAATTTTACATTTTCAAAAGATATGATGAATCGAAATGTATCTGACACGACTTCTTCGGTTAGTCATATGTTAG
ATGGTCAACAATCATCTGTTACGAAAGACTTAGCAATTAATGTTAATCAGTTTGAGACCTCAAATATAACGTAT
ATGATTCTTTTGATATGGGTATTTGGTAGTTTGTTGTGCTTATTTTATATGATTAAGGCATTCCGACAAATTGAT
GTTATTAAAAGTTCGTCATTGGAATCGTCATATCTTAATGAACGACTTAAAGTATGTCAAAGTAAGATGCAGTT
CTACAAAAAGCATATAACAATTAGTTATAGTTCAAACATTGATAATCCGATGGTATTTGGTTTAGTGAAATCCC
AAATTGTACTACCAACTGTCGTAGTCGAAACCATGAATGACAAAGAAATTGAATATATTATTCTACATGAACTA
TCACATGTGAAAAGTCATGACTTAATATTCAACCAGCTTTATGTTGTTTTTAAAATGATATTCTGGTTTAATCCT
GCACTATATATAAGTAAAACAATGATGGACAATGACTGTGAAAAAGTATGTGATAGAAACGTTTTAAAAATTT
TGAATCGCCATGAACATATACGTTATGGTGAATCGATATTAAAATGCTCTATTTTAAAATCTCAGCACATAAATA
ATGTGGCAGCACAATATTTACTAGGTTTTAATTCAAATATTAAAGAACGTGTTAAGTATATTGCACTTTATGATT
CAATGCCTAAACCTAATCGAAACAAGCGTATTGTTGCGTATATTGTATGTAGTATATCGAGCTTCACATGAAAC
AGCTAAAGAAGCTTTGGGCGATAAAGAGTTAAGAGCCATTGCACATGAGTTAACTAAAACAGTTAAGGATA
ACATGAGTGTTGATTGGTCTAAACGAGACAGTGCTAAAGCTAAAATGAGAGTTCAAGTTAGACGCCTATTAA
AGAAATATGGCTATCCACCAGATCTTCAAAAAATGGCTGTGGAACAAGTTGTAGAGCAAGCAGAATTAATGG
CAAGTCAGCAATAAAAAAATAAATCATAATGAGTCCGGGACATAAAGTTCTTGGATAAGTGAAAAAAGACAA
TTTCTATTGAAATAATATAGAAATTGTCTTTTTTATAAATTTTTTGATTATTTTCAGCTCGTTGAGCTACTACTTT
TCTTATATTAAGTGCCATTAATACAAAACCAAGTTCTCTTTTGACTTTATTGAGTCCTCGGACAGACATCCGAG
TGAAACCCAAAATAGCCTTCATAAATCCAAAAACAGGTTCCACATCAATTTTTCTTTGACTGTAGATATTTTTT
GTTTCTGGTTCTGAAAGCTTTTTGTTAATTTGGGATTTAAAATATTCCCAGTTATAATTCTTCATTATTTTTTTGT
TTGTTTTTGAATTGAAGTTCATACATTGATTTTTCAGAGGACATTCTGAACAATCATCACATTCATATAATTTGA
AGTCTCGCTTATAACCATACTTATCATGACGATAGGCATATCTTTTAAAACCTAGCCGTTTATTATTCGGACAAA
TGAATTCGTCATTAATTTCGTCATAGTTCCAATTTTGAGTATTAAAGATGTCACTTTTATATTTTTTAGTTTTATC
TTTTATAAACATTCCATATGTTATGAGTGGCGTTCGATTAAAGTCATCTATAATTGCCTTATAATTTGATTCACTA
CCATAACCTGCATCAGCTACAATATATTCAGGTAAATGACCGTAGGTCTCTTGAATTGAATTTAAAAATGGAAT
TATCGTTCTAGTATCCGTTGGATTTTGATACACATTATAAGATAAAACAAATTGGGAATTTGTTGCTATTTGTAA
ATTATACCCTGGCTTAAGTTGTCCATTTTTCATGTGATCTTCTTTCATTCTCATAAATGTCGCATCATAATCTGTC

TTAGAATAACTATTTCTATCCTTTAAAATAGATTTTTGAAATTCGTATCGATACTTTCGCTCAAAATAATCATTGA
TTTGCTTTTTGTATTTTTTGATTTTAGTTCTTTTGAGACGTATTTGTTTTCTTGTTTTAGTACATTTTTCATTGTT
GATATGTTGGTTTAAATCTTCGATTTCTTTATCTAAGTGACTACCAATCAAATCTATTTCTTCTTTTGTTAATTCA
TTATCATGATCTTCTTTAATTTCTGGTATGATTTTATTGGTTACCAATTCATGGTAGAGGGCTTTAGAATCCTCAT
TCATCTTTGATTCATGGTTTTGAATACTCTTTTTCCATACAAATGTATATCGATTGGCATTTGCTTCAATTTTTGT
ACCATCAATAAAAATAGCTTTATCATCTATAAGATTTTGTTTTACACACTGACTGTAAAATTGAATAAATAAAGA
TTCTAATAAAGCATCTACTTTTGGATTTACTCTAAATTGATTAATTGTTTTATAAGAAGGTTTTTGATTTTGTGA
TAGCCACATCATTCGGATGCTATCATTAAGCATTTTTTCTATTTTACGACCTGAGAATACAGATTGTGTGTAGG
CATATAGAATCACTTTTAACATCATTTTAGGATGGTACGAAGTTGCACCACGGTGATGTCTGAATTCGTCGAAT
TCATTGTCAGGAATTGTTTCAACAATATCATTTACAGTAAAACGATGTTGATTTGTTTTGTTTCCATATTGACCT
CCATGTATTTGCTATGATTTCAAAATCCATTTTTGACGTGCCTTAGGGTTGAGTGGATGCATAATTTCATTTGTT
ACTGGATTGATGAGCTTTTTTACTTTCTTTTTATGAGGTTTTAACATTTCCATCACTTGTTCGACACGGTCGAT
AACAACTGGTCGCTTCGCATAGGCACCATAAGCAAGAATCACTGTGTCACTTTCACTAATCGCTTTCATCAAA
TGAATATCAGTGTGCTCATCGTATGGATTTTTGATATGTTTGAGGTTTTCGGGTGTTCTAATATTTGAAAATAG
ATTTACAAGATATACAGCACCATATTGTTCAGTATTAGCTAATTGATTGAGGATAAGAACAGTTGTGAGATCGA
GTGATAATACACCATCTAAATGAGGGTACATCGTTATCACTGTACAAGCGGGTTTCTTTTCATCCCATGTTTTC
TTGAGTAAATAGCGGTGTTGTTCATCATCGCTAAATATGGCTTCTGTGTTTATCGTACTTTTGATTGTATTCATC
ATCGTCACTCCTTTTAGTATTCTTCTGGTAAAAGCATCACATAATAAAAAACATCTACATCATCTTCTCGGATGA
CGTAGACTTTCTTAGGTAATACATTTTGATTTTTTATATAGTTTGTATAGTGATATTCCAATTTGTACGTGGGTTG
TTCTTGCTCATGTATGATTGAAAGTATATTCTCATCTTCTTTCAGTCTAAAAATGTGTAGGTAATCTGTATCAGG
TTGATTATCTCTTTCTTTTACCATGTTCCAAAGTAAGATTTGAAGATCTAGAGATAGGTGTTCACTAATGCCTCT
TGTGATATATCGATTGATGTTCATACTATTTTCCTCCATTTTGCTTTTCTTTCATGATGTCAATCACTTCGTTAATG
ACTGTAACAGATATTTGTGCCACTTTGATCCAATTATTCATGGCGAGTCCCTCCTTCTTTTAGTAGATGACGTT
CATCGATAATCGTATTTTTAGTATCTGTGAGATATAAAAAGTCCATGTCAAAATGATCCAGATAACCGATACTGA
TGAGTTGGTTATTGGTGTACATTAGAAATGGATAGATACTTAGCTCATGTAGCTCATCATTATAGTAGGTATAA
GTGTCAAGTGTAAGATGCGCAAGTGGGGTATGGTCTACAAAGCGAGTAGGAAGTATATTTTCTGCTGCTTCT
TCTAACGCTTCACATTCCCATGTTTCATTGTTAGATAGTTGGAATAGACGAGTTATATATTGTTTGAGTTCTTGA
GTGGTTGTTTTCATATCATTGCCTCCTAGATAGTGTTAGCGATGTATTTCATATACATCACTGAGATAATATATAT
TTGATTTATCATTTATTACGAATCCCGGTGGGAATAAGAGAAAATCCCATATGAAAAACTGCTACAAACATTG
ATATGACAAGAAAATCCCGTTATCCCGCTCATTTGTTGGGAAATATAGCTATATATTTATATATCATTTGGATTTG
GTGGTGTTATTACGACTTACTTTTTCTTTTTATATTATTTATTTATAAATAATAACGGGATTTTGGGATTACGCTT
GCGTAATCCTTCTCCTACATGAAGTTAGCCAATCCCGTTACTATCCCACTTATATTTTGTTTTGGGATGTTCTTT
CGTCATATCAATATAAGCTTCACATTGAGCCATGATTTGTTAGCGTGTTGAATATATTTGTTAGATGAGTGTTAT
TGAATGACGAAAGGACAGTACATAGATATTATATTTGAAAGTAGAGTGTTTGAGGCAAAAACAAAGACGAA
GTGCTGAGAAGCACTTCGTCTAGATTATTATTATTGAAAAGTTGTTTAATAATTTCATTATTAAGTTTTAGTGTA
ACAAAGAATTGCTTTTTATGATTCTCATCTTTACGAATGTCAATACGGTCAATAACCGTTAGGTACAAAGCTTT
GAGCTGTGATTTATCCATGGATTCTATATTTTGAAATATTCGTTGTAATAAAGCAGCGATTTGTTTAGTATCATA
AAATGGTTTCTCTTGATTTTGTTGGTGCTTGAGTTGATTAATTTGGTTTGTGATATCATTCAGTTGTGTTTCATA
TTGATGAATGGTTGGTTTGAGTGCAGATGTTAAGTCTGGATTGTCTTCGATGGTTTGAATTAGATTTTTAAGT
TTAGTGCTAATTTCATCAAATTGTTGTTGTTTATAAGCAATATCATGGTTAAGTGCAGCTACATCGACTTGATTC
TCTTGATTGACACGTTCGACAACTTGTTTGAGAACTTTATCACTTTTGACAATTTCAAGTATTTGGTCCATAAC
ATATTTTTCAATGACATCGGCTCTAACACTATTCGCAGAACATACCTTTGAGCCTTTATTTCGAAAATTACTACA
CGAATAGTAACGAATACGTTTTTAGTGCCATCTTTAAGTGTGTTTGTGGTGTTACTCGCAGCCATAGGTGCAG
AACATTGGGGACACGAAATTATTCCAGTTAAAATATTGGTTCCTTTACCATGGACTTGTGGTTTTTCACTTACT

TGTTTCTTACGTGCTTGCACTTTATCCCATAATGATTGACTAATAATAGGCGTGTGTTTACCTTCAGCGATTACT
GGCTTATCGTTTAATCCTTTACGTCGTTTATCATTCCAATCTTTGTATTTCGCGAATTGAATTTTACCAATATAGA
ATGGGTTTGAGAGAATATAAGTAACAGCTGAAATACTAAATGGATTGCCTTTTTTAGTCACATAGCCTTTGTG
ATTGAATGCATTGGCTATTTTACGATAACCATGACCTTTGGCATAAGATTCAAAGATATATTTAACGATGTTAGC
TTCATGTTGATTAATCATTAATTCTTTTTTATTATCAGGTATGTTATTATATCCTAATGGAAGATTGCCTTGATAAT
AGCCCTCTAAAGCTCTTTGACGTTGTCCGGTGTAAATATTCTCTAAAATTGTATTTCTTTCGAATTCGGAAAAA
CTTGCAAGTATCTGGAGCATTAACTTGCCTGTTGAATTTTTGACTTCCATACGTTCAGACAAGCTAAAAAATT
CAACATTTTGGCGATGAAGTTCTTCGACTATTGTAAGTAAATCGGAAGTATTACGTGCCAAACGATTTGTTTT
ATAAACCATAACACAGTCTAATTTTCCATTTTTAGCATCATTTAACATGCGCTGTAATTCAGGACGGTTCATAG
ATTTTCCTGATATACCCCGATCTGCATATATATCAACAAGTTCATAGCCGTTAAATTGGCAATATTGCTCTATTTG
TGTAATTTGTCCCTCTATGCTATAACCTTCTACTTGTCTCTCTGTGGACACACGAATGTAGCCTCCTACAAGCTT
TTTCTTCATTTTATCCATTATAATCCATCCTTTCTTTAATTATACAATTGATAATTCCATAGTCTGCTTTACAATATT
CAGTGGTTCATTTTTGAAATAGATGCCAGCGAGGCTTTTATTTTTAGAAATATTAATTTCATCAATATAGGGGT
ACAACATGTTTAGCGTGAAACGTTGTTGAATGATGTTTTGGAAAGCTTTTCGAATTTGATACGTACTGATTGA
TGATATAGGTTTTGATTGCTGACGTAATGATTGCGTTTGTTCTCTGAACGTTTCTGCATCAATTTTGTCTTGGG
CTAATTTTTCTATCAGCTGCTCGTGATTGAGTGTAGTTTTAGTTTCTATATCTCTTTGTCTTTTGAGTCGTTGTT
GAATAGTATGGTTTATTTTTGAATAGAGCTGTTGATTTTGAAAGAAGTCCTGACAAGTCGCTAAAACACTTGT
TTCTAATTCTTGTGCGTTGATCCCTTTGAATTCACAGACAAAACGAGATGCATTCATATTTTGAGGACAAACAT
AATAACGTAATGTATGGTGCTTTTTTCTAATGGTCATATTAGTTAGTGTTGAATCACAATAAGGACATTTGATTT
TTTGTTTGAGTTGGTTTTCTGACGGCTTACGTTTTACTGGTTTCTGAGTTCGGGTAACTTGAGCTTCTTCGTA
TATCGTTGTACTGACAATAGCTGGGAACATGTTTTCATATTGTCCGTATTGATTGATAACACGGCCACAGTAAT
TAGGGTTAAGGATAATATTACGCACTTGATAGGGCTTACGATTAATGAATTTATCATCAGCTTCTAAGTATTGC
GCAATTTTTTTATAACCATAACCTTGAAGGTAATAATTGAACACAGCTTTTACTGTTGGTGCTTTTACTGTGTC
TATCGTGAAAGTACCATTATGATAGTGATACCCAAAGGGTGCATGTGTTGTAATCATTTTACCTTGTTTCGCTT
TTTCTTTGATTCCATTTTTGACTTGTTCGCCTATATTATCAGATTCTAGTTCGGCCAAGCTGATGAAAATATTGA
GTTTGAGCCGATCGAATGCTTTATCCATATCAAAATAGCCATCATGAACGCTTAAGATATGGACATGGTATTTT
TGACACAATTTCATGAGTTTTAATGCATTTTTAAGATTGCGATGAAGTCGGTTTAATCTGTAACAACATAATAC
ATCACATTGTCCTTGTTGAATCAGTTCAGTAATTTGTTGGTAACCGTTCCGCTTATCAGTGCGTCCTGATTGCT
TATCGCTATAAAAGGTAATGTATTGAATATTATGTTTTTTGGCTAATGCCTCGATGGTTTGTTTTTGTGCTGCTA
AGGATTGTTGCTTTGTAGTGCTCTGTCGTAAGTAACCTATTGCTTGTTTCATCGTATTTCCTCCTTCCAAAGTG
ATAATATATATTTATGAACGAATTTATAGATGAGCCCAACACCTGCTGGTGTTGGGCGTTATTATTAGTCATCAG
CATGATTAATTTCTTCAAAAACTAAATCAGCTAGTAATTCAATCAATTCATCCATTTTATTCACTCCTGTACGATT
TCATCTTTAAGTTATTAAAAATCAATTAAATCATCATTCTGTTTTTTCCATGATTCAAGTATCTTTTTGTTATCCA
AGTTAATTTCAGCTTTAATAGGTTCAGCATCTTTAGTTAGACCAAAAATAGACGCATATTCTGAATCTAGCTTT
AAATGGTAAAAGACAAGTGACTGTTTTTTGCCATTGCCATCTTTGACTGTTCTTTTTGTTGTAATTCGATCATG
GTCAGATTCGATAAATCCTTTGTCCCTTAGCGCATTAACAACATTATTAACATCTTGGAAGTGATGCTCTAACA
ACATATTTTTAAATACAGACGCAATGATTTTGACTTCGATATGATTATCTTTTAAGGCAATTAGTCCATAGTTCT
CGAACATATTCTTTAACGCTGTATCATCAGAAAATTTACCACGGTTTTGTGCTACAAATTGAGTAATGACTTCG
ATCGCTTTATCTGCCAGTGATCGTTCAGAGACTGTATGAGCATGATAATCAATAAAGTAGTCTCTGATTTTAGC
GATATCAATATCTGTAGCTAAAACACGACCTAATATTTTCGCAGATGTTGTAATGACTGCATAACGCTTAAACAT
ACGAATACCTGTATTGTTTGTTTCACTGTTCAATTTAGCTTCAAACCAATCTACTTCCTTGTAAAACCATTGAAT
AACTTCATCTTCACGATTTATAAGGTATTGAGCTACTAACGGTAAAACATGACCATGGTTTAGTGCCACAGCTT
TTTTGATATTGTCAGCATTGGTCGCATTTGTAGTAAATTGTTCATTAATCTCGATGGTTCTTACACGTAATCCAT
CGTTTTGAGCAGAATCAGTAAAGATACTGTATTCAGAGGTTGAAATCACAGAAGTGCCCCAATTCTTAGGCG

TTTTAACTTCTCCGTGTACATTGGAACGTTGACGCCCTTGACCTTCAGCGATAGAGTACAATAACCCCGTTGT
ATCTTTAAAAGTTGCTGATGAGAGTTCATCAAATACAATAGGTATACCAAAATTGTTACTCAAGTAACTTTCAA
GTGCATTACGTGTGGCATTCCAACTTCGAAAAAGTGTTTGATTACCTTTAGTTGGATTTCCAGCGACGGATAC
TGCTAAAGCTGCTGCTGTTGATTTACCAGTTGATGACTGGCCTGTAAAACTAAAGATAATTCCTGCAAATTCG
ATTTCACGTTTGTACTTCAGAAAGCTTGTCACTAAGGCAGAAATTCCAAATACGACCGCTAACTCTAGAAGA
AGATGACCTTTGACTTCGTTAATATACATATTTAACCAATCTTTAAATGTACCTTTAGGCTCTAATGGATAAGCA
CTATCGACAATAGGATCTAAAGATGATAATTGATCATATTGTTTAGATGTATAAATGGTATCTATCATTACAATAT
AACCGTGGGGTGTTTCTATGATGCCCGAGCCACCATATAAATCAGAAGTGGGCATTTCATCGCGCATTAATTG
TAGTGCATAGCTCAAATCTTTTATATAATTTTCATTGATACTGTGACCATATTTAATCAAGGAAGGTAACTTTCG
AGTTGTTAAAATATCAGAGTCAAATGTATGTTCTTTATCCTTACCGTTAGAAATAATTATTTTTTCTGTATTTTCT
AAGACATGCCAAAATTTAGCTTCAACAGCCATACAACTAGACATAGTCACTACTTTTTTCTCATCTCCATCTTT
TTTAGGTGGGATAGTTTTATGCCAACCCATTGTATCTAAATGGTATAGGCCTAGTCTAAAAATGTCATGATGAC
TCATTAGCGAACACCTCCTTTCGAAGGGTTGCTATCATTGAGTGGATTAGGTCCGACTTTCATATATACTAAAT
GACCATTGGTATTTTTACCGATAATAATAAATGGAACACGTGGCGCTTGTTTTACAAAATATGCGAACCAACG
TCCAACATTTTGTTGTACAGCTTTTGAGCATTGTACATTTGCACGACGGTTCAAGTCATGGAAAGTAAATGTT
TGTCCTTCTGGTAAATTAATTGAAATACCTAAAATTCTATTTTTTAATTTTTTAAAATCATCTTCTTGCTTGTAAG
ACATTTGAATTCCTCCTAAATATTATTAATTAATTCTATCTTCTGGCTATTTCAGAAGATTACATTGCTTATAATAG
AGGTGTACTGTTTTTATTGCTCGAATAGTATTGAGCAAAAGACGAACACATAAATGCAAGCCTTTTTGCTTGA
CAAATTAGAAAAGAGGTAAACAAAGTGTATGATTTTTTAGAATACTTACATCGTTATTGTATAAAATCAATTAA
TAATTGGGAGGCTGTTGCAGACATATCATGCAAAGTTGAACGTGGAAATGTTAGTACGGGCTATTATGTTAA
ACAAGATAGAATTGTAATAGATGATAGTGATATACCTCCAATTATTAAAATAAGTGGAAGTTATATAGCAGCAG
ACATATTAGTTACTAGATATTTAGATGGAATGGGTGAATTTATAAAGGTATCAAAATTAGATCAATCGAGCTATT
ACGAATGGTTTATGTTTGTTGAAGAAACGAGAAGAATATTTAGAGGTACAGAAATAGTTTATCAATTTTTAAA
ATATCTATTTACGCATACCTATGAACATAAAAATGAAGAAAAAAGAATAGAAAAACATATGAAAAACATTATCA
TAGATAATCAATTTAGAGGGCAGCATATCAATGATATCGTTCGTTTAGACATGAAAGATGATAATATGTTATTTT
ACAATTTTGATATATACGATACAAATGTTCTTTATAAAGAAATAGACGAGGAAAAATTACTTAGAATTTTAGAT
GAAATAAGTAATACTGACAATTACAAAGCGCTATTTAACATTATAAGTGAGAATCAAAAGAAATTTTGGAAAT
CAAATATTAAACCAATTGTAAGTGAATGTTCAGATGGTATAAATACAACCACATTTGATTTATATGCTTATGTAA
ATAAGTATATAAGTAATAAAATATTTGACTGTGATAATTATTTAATTACTAAAAATGAGGTTGTGGATTTCTATTT
TAATCATGGTAGTGACTTTAATAATTTCAAGATGGGACAGACAGGCACAAAGTCCAAATTTAAGCAGGGAAA
TAAACTTAAAATACACGAATATATCATTGCAGTAACTTATGTAAATTTATTAAATCATTCATATATGAAATATATCA
ATGCTGAAAGTTACTATGACCCTAGCAAAAAAATAATAAAAGGAATGAAAAATGATTATCATAAATTTACAGA
TTCATTCAGTGATTTTGTAGAAATGGTGGACGATATAACATGGAGAATCCGTGATTCGTTTGATTATATTGGAG
GATTAAAAAATAAAGAAATTAGCGAAGATGAGAACGAATATATCGAACAATTAATTCAATTTGTTATATTATCT
AAATTCGGCTATATGAATGTGGAAAATCATTTTAATTATATTGAGAACTTAAATACACTTAATAACGAATTAACG
ACTATATTAAGAAGTGAATTCGCGCACATTTCAAATGATATGTATAATAATTTATTTGGTAATTTATTCGATGTTG
TAAATACATGGAAAAAAGATTATTCAAATAATTTTTATAATAATACTCAGAATACTATACCTACAGAAAAATTAG
ATATAATATCTTCATTTATAAAACAATATGATAAACCAATTGAACGAGACGAACATGGCAAACACGTTAAAGCA
AAAGTGTTTGAAGGTATAAGTGATTTTCATAAGCGTGTAAAAAAAGGATTTGATAATAATGATGTGCTATTGC
ATTTGAGATTGGAAATGAGTGTACATGAAAATAAGTATGATTAACTACTAGTTTATCCAATAATATAAAAAGGA
CTATAAGCTATATCTAAATACTTATAGTCCTTCTTCATTAGTATAAATATTAAGAAAGTTGTATATCTTTATAACCT
TCATTTACATTAATAATGTTGTGATAACCTTTGTGTTCTAAAATACCAATAGCTATCGAACTTCTAATGCCAGAC
TGACAGTGTACATAAATAACATCGTTTTTATTGAAAGGTAAATCTGTTTCTAAAAGTTTGCCGTGTGGTACATG
AACCGCTTGAGATAAGTGGCCATTATTCCATTCATTATCATTACGTACATCTAATATATGTGATTCGTTACCTGTA

ATGTCTTCACTATGAATGGAACGTGTTTGAATCTTAGATTGCGGTAATTGATATCCAGCAATATCATCGTATCCA
ATGAGTTGTAAGGTGTGTGTTGCTTTTGAAACAAGGTGATAGTCTCCAATCAAGTTAATTTCTTGATCATAGT
TTAGATACCAGCCAATTTGATTGATGAAATTTTTATCATATGGAATATTGATTGTACCTTCAATATGTCCACCATG
ATAAGCCTCCTTACTGCGGAGATCAAAAGTTAATCTGTTTGTATTTGTAGCTGGATAAACCGTATAAGGTTGAT
ATAAATTCATACCGAATTGATTAATTTTTTTCATTTGTGCAAAATGATGTGGTGGTGCAGGTTGGTCAGAAAT
GAGTTTATCGATAAAGGTAGCTTCGTTATTTTCAGAAAAAGCCCAGTTCGTTTGTTTTTCATAGCCAAGAGTA
GATGTTGGAATAGCACCTAAAGATTTACCACAAGGACTACCAGCGCCATGGCCAGGCCAAATTTGAATGTAA
TCTGGCAAGTCTTTAATACTTTCAATCGATTTAAACATTTGTTTAGCGCCTATTTCAGATGATCCTTCTACTTTA
ACCGCTTTTTCTAGTAAATCAGGTCTACCGATATCTCCTACAAAAATAAAATCACCACTGAATAGTCCCATTGG
AACTTGTGCTCCAGCACCTTCGTCAGTAAGTAAAAAACTTATACTTTCTGGCGTGTGACCCGGTGTATGAAGC
ACTTTTAATTTTATATTTCCTACATAAATATCGTCATTATGTTGAACAAAATGAGTGTGGTTAGGCATATTTTTAT
AACCTAATGTGTCATCACTTTCACCCGATACATAAATATTAGCATTTAACTTTATAGCAACATCTCTAATTCCTGA
AGCAAAATCTGCATGTATATGTGTTTCAGCTGCATGAGTAATGGTTAAACCTTCTTCATCAGCAACTCGAATAT
ATGAAGATAAGTCACGAATAGGATCAATAATCATGGCTTCTCCAGTTTTTTGACAACCGATTAAATAAGATGC
TTGAGATAAATGTTTATCATAAAATTGTTTAAAAAACATAATATCATTCCTTTCTAATTATATAAATAAATTGTGAT
TAGCTTGTTCTGTATATCCAATATATGCACCTACGCCACCGTATTCTACATCATCACGTAGTTCTTCTTTTGTAAT
GCCCATCACATCCATACTCATTGTACAAGCGATAAGTTTAACACCTTGCTCAACTGCTTGATCAATAAGAGAA
GGTAAAGTATCGACATTTTTCTTTTTCATGACATAACGCATCATTAGATTGCCTAAACCAAACATATTCATTTTA
GAAATAGGCATGTTAATTGGTGAATTTGGAAGCATGAAGTCAAAAAGTTTTGCAATACCTTTTTTCTTAATAC
GTTGACTTTGAATTTTTTTAAGTGCATTTAAGCCCCAAAATGTGCAAAAAAATGGTTACATCTCTACCTGCAGC
TTTAGCACCATTAGCAATAATCATTGCTGCAACTGCTTTATCTAGCTCGCCGCTAAATAAAACAATTGTTGTTC
CACTTTTCGTGTGTGTCACTTCTATATTTTTATTTTCTTCTTTTTGAATAATAGCGCGAATTTCATTGCCAAAATC
GTTGAGTCTAACAAGCGTATGTCCAGTTTGTTTGACCCAACTTTTAATATCATTTAAAAATCCGTGATCAGTCA
CAACAACTTCTATTTGATCGCCAATAGCGATGTTTTTGATTTCTTTACTAATATTAACAATAGGTCCTGGACACT
GTAGATTACTATAATTGAATGTCTTACGATTATCTTTAATTTCTCTATCTTCTTTTACTTTAGATAAATTATCATTAT
GATGTTGTTGTTCGTATGCGGTATAACCTCCGTCTAAATTCACTACATCGTATCCGCGTTGTGCAAGAAATTGA
CTGGCTTTTCTACTTCTATTACCACTTTTACAATAGATATAGTAAGTTTTATTTTTGCTTTGTTTAAAAGTCTCTA
TATTTTCAACTGAATGTAATAAAGCGTTTTTAATGTGTCCTAATTCAAATTCTTCAGGATGTCGAACGTCAATTA
ATTGACCCTTAGAACTCAATTCTTGTAATTCTTTTTTAGTGAAATCATTAATGTGTTTCGTTTTATATTGTGACAT
AATTGCCTCCTTTTAAATACCTATGGGGGTATATTATAACGAATTAGATAGTTTGTCAAATACCTATGGGGGTAT
TTGACAAACTAAAATTTAATCAATATGATGTAATTATTAAAAAATGAATGTGGAGTGAAATATATTGGAATATAAT
AAAAAGATGATTAATCGTATTCATCGCATACAAGGACAGCTTAATGGAGTCATTAAAATGATGGAGGAAGAA
AAAAATTGTAAAGACGTCATTAGTCAATTAAGTGCATCTAAAAGTTCTATTCAACGTTTAATGGGTATTATTATT
AGCGAGAACTTAGTAGAATGCGTCAAAATGTCTGAAGAAAATAGTGAAGATTCTCAGGCACTAATTAATGAA
GCTGTTGAATTATTAATAAAAAGTAAATGATAAGTATAACAGTAATTATAGTAATGTTAGTTATTGGTATATTAG
GAGGATTTATTTCTGGATTAGTCGGTATAGGGGGAGCCATTGTTATTTACCCTGCTCTCTTATTATTACCACCAT
TATTTGGTTTACCTACTTATAGTGCTTATATTGCCTCAGGATTAACTTCAAGTCAGGTGTTTTTTAGTACATTGA
GTGGATCTTTAAAAGCATATAAAAATAAAAATTTTTCTAGAACGCTTATACTTAATATGGGAAGTGGAATGGTA
ATAGGTAGTATTCTAGGTGCGTTATTAGCAACTGTATTTAATAGTCAGTTTGTTAATGTAATTTATATAATTATTG
CTTTGTTAGCTCTTATTCTAATGTTTATTAAAGTCACACCATCTACTAGTCATATTAAGTTTAATCGCGTACTTTT
AATTACAATAGGTGGTATCATCGGTTTAGTATCTGGTATTGTAGGAGCTGGTGGAGCATTCATTATTATTCCAG
TACTACTTGTTATATTTAAATTACCAATGAATATGGTTGTTACAAATAGTATTGTAATTGCATTCATTTCTTCTATT
GGAGCATTTATTATTAAATTATTACAGGGTTATATTCCTATCAATAGTGCAATACCGTTAATTTTAGGTAGTATAC
TATTTACGCCTCTAGGCATGAAAATAGGGCAAAAAATACCTGATTCTATTCAAAAAGGGATTGTAAGTATTTTA

ATCGTCATTGCAATCATTAAGTTGATATTTTAAAAATATGGGAGTGGGGCAACATTTATTTTGAATTTGTAGTC
TCGCTCCCTAAATCAATTTATACGGTATTTAATTCAAAGGTTATTTATTAGAATCTAATCAATTTTACAAAATTTT
AACAAAAAAACTTTTTCTAAATTGATATATAATAGCGGGTATGGTAATTTTAAAATAGATACTCGTATCTAACTA
ATTTTTAGGGGTGGTTAATTGTATGAATAAAAATTCGAAGAAGAAGCTCGATTTTCTTCCAAACAAGCTTAAT
AAGTACTCAATTAGACGTTTCACTGTAGGGACAGCTTCGATTTTAGTAGGAGCTACTTTAATTTTCGGTGTTG
CAAATGATCAAGCAGAAGCCGCTGAGAATAACACAACTCAAAAGCAAGATGATAGTTCAGATGCAAGTAAA
GTAAAAGGTAATGTTCAAACTATTGAACAATCTTCTGCAAATTCAAATGAATCTGATATTCCTGAACAAGTTG
ATGTAACTAAAGATACAACTGAACAAGCATCAACAGAAGAAAAAGCAAATACAACTGAACAAGCATCAACA
GAAGAAAAAGCAGATACAACTGAACAAGCAACAACAGAAGAAGCGCCAAAAGCTGAAGGAACAGACAAA
GTAGAAACAGAAGAAGCGCCAAAAGCTGAAGAAACAGACAAAGCAACAGAAGAAGCACCAAAAACTGA
AGAAACAGACAAAGCAACAACAGAAGAAGCGCCAAAAGCTGAAGAAACAGACAAAGCAACAGAAGAAG
CACCAAAAACTGAAGAAACAGACAAAGCAACAACAGAAGAAGCGCCAGCAGCTGAAGAAACAAGCAAA
GCAGCAACAGAAGAAGCGCCAAAAGCTGAAGAAACAAGCAAAGCAGCAACAGAAGAAGCGCCAAAAGC
TGAAGAAACAGAAAAAACAGCAACAGAAGAAGCACCAAAAACTGAAGAAACAGACAAAGTAGAAACAG
AAGAAGCGCCAAAAGCTGAAGAAACAAGCAAAGCAGCAACAGAAAAAGCACCAAAAGCTGAAGAAACA
AACAAAGTAGAAACAGAAGAAGCGCCAGCAGCTGAAGAAACAAACAAAGCAGCAACAGAAGAAACACCA
GCAGTTGAAGACACAAATGCTAAGAGCAATTCAAATGCTCAACCATCAGAAACTGAGAGAACTCAAGTTGT
AGATACAGTTGCTAAAGATTTATATAAAAAATCTGAAGTTACAGAAGCAGAAAAAGCTGAAATTGAAAAAGT
ATTACCAAAAGATATTTCAAACTTATCTAATGAAGAAATTAAAAAAATAGCTTTAAGTGAAGTACTTAAAGAA
ACAGCTAACAAAGAAAACGCACAACCAAGAGCAACATTCCGTTCAGTAAGCAGCAATGCTAGAACAACAAA
TGTTAACTATTCAGCAACAGCATTAAGAGCAGCTGCACAAGACACAGTTACTAAAAAAGGAACTGGTAACTT
TACTGCGCATGGAGATATAATCCATAAAACTTATAAAGAAGAATTCCCTAATGAAGGCACGCTAACTGCATTC
AATACAAACTTCAATCCTAATACAGGAACTAAAGGCGCATTAGAATATAATGATAAAATAGATTTTAATAAAGA
CTTTACAATTACTGTTCCAGTAGCAAACAACAACCAAGGTAATACAACAGGAGCAGATGGCTGGGGCTTCAT
GTTTACTCAAGGGAATGGCCAAGACTTCTTAAACCAAGGTGGTATTTTAAGAGACAAAGGTATGGCAAATG
CATCTGGTTTTAAAATTGATACGGCATATAATAATGTTAATGGTAAAGTCGATAAACTCGATGCAGATAAAACA
AACAATCTAAGTCAAATTGGCGCAGCAAAAGTTGGTTACGGTACATTTGTTAAAAATGGTGCAGATGGTGTG
ACTAACCAAGTTGGTCAAAATGCCCTAAATACAAAAGATAAACCTGTAAATAAAATAATTTATGCAGATAATAC
AACTAATCATCTTGATGGTCAATTCCATGGCCAAAGATTAAATGATGTAGTATTAAATTATGATGCAGCAACAA
GTACAATAACTGCTACATATGCAGGAAAAACATGGAAAGCTACTACAGATGATTTAGGAATTGATAAATCACA
AAAATATAATTTCTTAATTACTTCAAGTCATATGCAAAATAGATATTCTAATGGAATTATGAGAACAAATCTTGA
AGGTGTAACAATTACAACGCCTCAAGCTGATTTAATTGATGATGTGGAAGTAACGAAACAACCAATTCCTCAT
AAAACTATTCGTGAGTTTGATCCAACTCTAGAACCAGGCTCACCTGATGTTATTGTACAAAAAGGTGAAGAT
GGAGAGAAAACAACAACTACACCAACTAAAGTTGACCCTGATACAGGAGATGTAGTTGAACGTGGTGAACC
AACAACAGAAGTAACAAAAAATCCAGTTGACGAGATTGTACACTTTGCACCTGAAGAAGTACCACAAGGTC
ATAAAGATGAGTTCGATCCAAACTTACCAATTGACGGTACAGAAGAAGTACCAGGTAAACCAGGCATCAAG
AATCCTGAAACAGGTGAAGTAGTAACACCACCAGTAGACGATGTCACAAAACATGGTCCAAAAGCAGGCGA
ACCAGAGGTTACTAAAGAAGAAATTCCATATGAAACTAAACGCGTATTAGATCCAACAATGGAACCAGGTAG
TCCTGATAAAGTAGCTCAAAAAGGTGAAAATGGTGAAAAAACAACAACAACACCAACTACAATTAATCCATT
AACGGGAGAAAAAGTAGGCGAAGGCGAACCAACAACGGAAGTAACGAAAGAACCAATAGACGAAATTGT
TAACTATGCACCTGAAATTATTCCTCATGGTACACGTGAAGAAATTGATCCAAACTTACCAGAAGGTGAAACT
AAAGTTATCCCAGGTAAAGATGGCTTGAAAGATCCTGAAACTGGAGAAATCATTGAAGAACCACAAGATGA
AGTAATCATCCATGGTGCTAAAGATGATTCAGATGCGGACAGCGATTCAGACGCAGATAGCGATTCTGATGC
AGACAGCGACTCAGACGCAGATAGCGACTCTGATGCGGACAGCGATTCAGACAGCGATAGCGATTCAGATT

CAGATAGCGACTCTGATGCGGACAGCGATTCAGACAGCGATAGCGATTCAGACGCAGATAGCGATTCTGAT
GCAGACAGCGACTCAGACGCAGATAGCGACTCAGATTCAGACGCAGATAGCGACTCAGATTCAGACAGCG
ACTCAGACGCAGACAGCGACTCAGATTCAGACAGCGATTCAGACGCAGACAGCGACTCAGACGCAGATAG
CGACTCAGATTCAGACAGCGATTCAGACGCAGATAGCGATTCAGATTCAGATAGTGACTCTGATGCGGACA
GCGACTCAGACGCAGATAGCGACTCTGATGCGGACAGCGACTCAGACGCAGATAGCGATTCTGATTCAGAC
AGCGATTCAGACGCAGATAGCGACTCAGACGCAGATAGCGATTCAGACGCAGATAGCGACTCAGACGCAG
ATAGCGATTCAGATTCAGATAGCGACTCTGATGCGGACAGCGATAGCGATTCAGATTCAGACAGCGACTCAG
ACGCAGATAGCGACTCAGACGCAGATAGCGATAGCGATTCTGATGCAGACAGCGACTCAGACGCAGATAGC
GACTCTGATGCGGACAGCGACTCAGACGCAGATAGCGACTCAGATTCAGACAGCGACTCAGACGCAGATA
GCGACTCTGATGCGGACAGCGACTCAGACGCAGATAGCGATTCTGATGCAGACAGCGACTCAGACAGCGAT
AGCGATTCTGATTCAGACAGCGATTCAGACGCAGATAGCGACTCAGATTCAGACAGCGATTCAGACGCAGA
TAGAGATCATAATGACAAAACAGATAAACCAAATAATAAAGAGTTACCAGATACTGGTAATGATGCTCAAAAT
AATGGCACATTATTTGGTTCACTATTCGCTGCGCTTGGAGGATTATTCTTAGTTGGCAGACGTCGTAAAAACA
AAAATAATGAAGAAAAATAATATTTAAACTTCATAATTTTGGGTAAAATTTAAAACCAGGCCTTACATGGCCT
GGTTTTATTTTAAATACTATGATATAAATTAATGGAAATAGGACAAATATATAAAAATAAGGAGGAAACAATGG
AATTTGAACATAAATTAGAGAAATTAATATCTGAAGTAAATAATAAGACTGAAATTAATAATTATGTTTTTTTTA
GTTTAGGTAAGTCAAGTGTTAAAGCACAGGTTAAGTTATTAAAAAAAACTAATTATCTCAAACAAGATATTTC
AAAATTGGCACTTAAATTTAAAAAGAAATCTGGGGAATTTCCTGAGTGGATTAAATTAGATATTGTAACCTCG
ACTGAAAAAATATTATTTAAAGAATTAAAAAAAACACTGATCAATACAAGAAGAAATTATGTAGATTTTGGTA
TAGCATTTGATAGTCAATGGAATTTTGCAGTATTACCTGAAGAAATTAACGCGAATGCTTTTGTTCGACCAGA
TAATACTACTAAAGAGTTATTTCTTTCGGAAAAAAACATCAATAATTATTTACGCAAATATACTACAAATAAAAA
GGCATTTTCTAGTGAGTTCTATAATGAAAAAGAAGTTATTAAATTCTATACACAAGGTTTCTTTATAGGTGATG
AAGAAGTACATGAACTATATAGCGAAGGCTATAAAAAAGGTTTAAGAAAAGTAAATGATTTAAATAATGAAAT
TGATCAATTAATTGAAAGTAGTACTAATTTCTTACAAAATATGTTGTTAGATAATGGTAAATATATTTATGGATAT
TTTCCTCATTTTGATAATGAAATTGGATTCTACAATGTTTTAAGACACTCTTCTTCTACTTATGCCTTAATTGAG
GGTCTGTCCTATTTAGGTAAAAGCTTACAACCTGTTGAAAAAGCAATTGATTATATCATTTTAAATCAACTGTT
TGAGATTGGTGATAAAGCCTATATCTTTGATGATACAGAGGAAGCAAATGAAATCAAATTAGGACAAAATGC
TTCATTTATATTCGCAGTTTGTGAATATTTAAAGCATGAAGATAATCCTAAATTTCTTGAGTCAGCTCAGAAAG
TGGCTAAAGGTATTCTTTCAATGATAGATGAAGATACATATGAGACAACTCACTTATTGAATTATCCTGATTTAA
GCGTAAAAGAGAAGTTTAGAATTATTTATTACGATGGTGAAGCAGCGCTTGCTTTATTGAGATTGTATCAAAA
GGATGAAAATGAACTATGGCTAAAAACTGTAGAAAATTTGATGGACCGTTTCATTGAGAAGAAATATTGGCA
ATATCATGATCATTGGTTAGGATATTGTACGAATGAATTAGTTCAAATTAATCCACAAGACAAATATTTTGAATT
TGGAATCAAAAATGTGAATAATTACTTAGATTATATTAAAAATCGTGAAACAACATTTCCAACATTCTTAGAAA
TGTTAATGGCCACATATAGATTAGTTCAAAAAGCGAAAGATACGGGTCGCGAGGAATTGGTAAACAATTTAA
TAGATGAACAATATTTGATAGATGTAATTAATATTAGAGCAGATTATCAAAGAGTTGGATTCTTTTATCCTGAA
ATTGCTATGTATTTTAAAAACCCATCAAGAATACTAGGAAGTTTCTTTATTAAACACCACGGTTATCGTGTTCG
AATTGATGATATCGAACATTATTTATCTGGATATGTACAATATCAGCTTGCATTTAATAGGTAAAATAAGTATTTG
AAATTTAAATTAATGACTTACAAAAAATGGTTGATTTACATAAAAACGAAATCAACCATTTTTAATTTGCTCTT
TAATAATTATTTTTCATATAACTAAAATTCTAATTTCATAAAGAATAACTGAAAAAACTAAACAACATGTGAGCT
ATTCATAACTTGAATTAATTGTAGAAAATCTATCTTCCCATAACTTCATAACAATTTTACTTGAAAAAGCTTTAG
AGTGCTTGATACATTCTTGAGAAAAAGTATCTCTTAAAGTCTTATCTAATAATAGGTTTCGCACTCGATTATATA
ATTCATCTTTATCATTTTTGTTAATTAAATAACCATTTTCATTATTTAATATTAATTCGGAAGGACCATACTTCGA
ATTATAGCCAACTGGAGGTATGCCTTCAGTTATAGTTTCTAATAAACCTAAATTAAATCCTTCCATATTACTAGTA
ATTAGAGACATATAAGCATCTTGAATCTCAGCACTTAAATTTCTTCTAAACCCTCGTAAAAACACATTGTTTTCT

AAATTATATTCAGTGATTAGTTGCTTGTATTTTTCTTCTTCTTTACCAAACCCATACAAATGTAGTTGGATGTTA
GGAAATTCTTTAATTAATTTAGAAACTAATTCAATTTGATGATTCAACTGTTTTTCAGGAGAATAACGTGCAAC
GGAAATAATTTTATTATTATTTATACTATGATTATTTCTTTTTAAATTAGTAAAATGTTCATCAATATATCCTACTG
GTATAGTATGAACAGGGATTTCATTATTTATTCGAGCTGATATATCCAATTGTTGCTGTTTAGTCGATACAATTAT
CCCAGAGTATCTATTTAGATTGTTAAAAACATGTTTATAAGTATTTTTGATGTCCGATTCATATACCATGTCAATA
TTCTTGACATGAGTACTATGCAAAACAGCTAAAACAGGTATTGTTTCACTAGTATTATTGAATATTGGTGCAGT
ATTTATATTTTTATCACTCAGGAAAATATCTCCATTTTTATACAATTTTTCAATTGCAAATGCTAGTAATTCCTGA
TCATTATTAAAATACTTAAGTTGAGAATCGGTGTTATAAATAATAAGTTGTGATTGTGAATTTTCAAGTTCTGG
ATTAAAAAATTTTTGGAACTTTATAGTACCTTCAGGAGTGTAATAATGTTCACAAACTACTTTTTGATTTGTTG
TTAAAGTTCTTGAACAACTTAAAAAGCCTCTTGAATCATATAGATCTCGACGTATTTTACGTTGATTTACATCA
AAATGATTTATGTAATCCAAATGTTGGTAATTACTATCATTAAAATGGACATACATTCTATAGTTGCTTTTTTCAT
ATATCTTTATATCATTGGTATTTTCAATAAATTTTAGTGTATAATTACACTCTTTAGTCCAATGAGTGATCCAGTC
CTTATGGATGCTTTTTATATTTATACTGTCTTGTAGCACATCATATAAGCTTAATATATCGTCATCTTCAATTTGAA
ATAGACTTGCATTAAAATGTAAACGAGGTGACCACGATGCAAAAATCAATTTACTAGTTATCGAGTGATTTTT
AAATAAGTTGTGTCTATTAATCATCGCTTTTTCAATTCCGGTTAATTTATTTCCTAACCTATTCCCTACTGAATAT
ATCATTTTAATTCAACCTCATTTTTTGTTATTTATTATTTAGTTTATTTTTAATTTCATTTAAGTTTGGGTAAGAG
GTAAATATAATAAATTTATCTTTATCTTTTATAATACCGCTATTATATTTCCTGCCAATAATTATTTCGTTAGTAAAT
TTAAATAGGGTATTTTTATATGCTTTTAAAATTTTAAATAAGTCGTCAACAGTGAAGTTTTGTCGTTCTCTCATA
GTATGACGTCCAGTTAAATTTATTGCTACAGAATTTGAAAGACTTAAATGTTCTATTTTTGAATTGATATATTCA
TCGCGCTTTGGACCGTTTTCAAATAAATACTTAGATAATTCATAAGAAGGTGTATACATAGGTGAATTAAGCAT
GGCTTTCATCAATTCGTAAGTTGTCGTTTCTTTTCCTTCATAATAAAGTTTGTTATTTGTTGCCCAGTTAGCAAT
AAAAATAGTATCTTCTGATACATTATCATCTATAGCTTTTAAATAATTAAAGATAGGGCCTAAGCCTTCAATTGT
ACCTGAATGTTCTCTGTTATGAGATTCAATGACATTATAATTTTCATCAATAATCATATAACTGCGACCGTTCAA
GGATTGACCATCAAATAAAGAAGTGTTCGAATTATTAATATTATATTTATTTAATTTCTCAGGTAATTCTTTTGCT
AATTTTGGAAATTCTGTACCACCTGCTGATGATTTTAATAGAGTTAATGAATCTGGTTTATTTAGGTACAATAA
ATCTTTTTCTAATAGATGTCTATTAGAATACCATGTTATATTTTTGCTTTTTACTCCTGTAACGACACTCTTTAAA
GCATCATCCATACATAGAATATAATCTGCATTACTATTTTCTAGAACATCAACTAATTGCAAATGCAATGATTTAG
AGTGCTTACCTAAGTCACTAATTTGCCCAATCGCAATAATTTTATTACCTTTAAAAAATTTTGTTTGAGTATTAA
ATGCTTTTATAGCATTAATCATTGCTGGTAATGAAGCGTTGTGTGTATCATCTATGTAAGTTAACTTTATAGTTA
GGTGTTTCAACTTCTTTTAGGTTTAAAACTTTTTCAAATGGTTTAAATGTACTTAAGTTCTCTAAGGCTCTTTC
TAGTGGTATATCTAAATGCGATAATGTAGCAAATGTGGCTAGAGAGTTTTCAACCATACCGTCACTTATTGAAT
TAATTCTATAAGTATATTTTTGTCCGTTAAAATCAATTGTTATAACTGTATAGCCTTTACTATATTGAATAGATTTA
GGACAAATTGTTGCGCTAGAGTCATGTGTACTATAAGTAATAACATTACTTGTATTTTGTTTGGCGCGTTCGAT
TAATATATCTGAATGTAATGTATCTTTATTAATTATTGCTACTCCCTCAGGAGTTAAACCATCAAAAATACTTGCT
TTCACTTCTACAATATTTAGAATATCTTTAAATGTAGACATATGAGCAGCACCTATACCAGTAACTATAGCAATAT
TTGGTTTTATTAAATATGATGAATTACCAACTGCATTTAATGCGTTTAATGATACTTCTAAAACAGCAAAATTTG
GTTGTCTAATCATTTTGCATAATAATAAATATACTGCGGATCTTATGTTATTATTAAATCTATTTTGAAGAGGATA
ATAATCTTTAAGACCAGCACTTATCAGCATTCTAGTTGAACTTTTTCCCATTGATCCTGTTATAGCAACAACAG
GGTGTTTATACTGTAGTCTCATTTCTTCAGCTAATTTAGTTAGTGTGTTTGCAGAATCATTGATAACTAATTGAG
GAGTTTTAAACTTCATGTCAGGAACATAGGTTTCAGTAATAATTAATCCTAAATCTTCCTTATCCTTCTTTATATA
TTCATTACCTTCTTTTGCGTTTGCATCTCTACCAAAACGTCTCCATGTATGCTTATTGGGCGATATGTAGGCTGT
GCTTTTATTATGAATAAAGGGGAACCAATTCTCAAAATTATTTATAACATTGTCCTCTTTACCTTCAGCATCAAG
GAGATTACCTCCTAAAATATTATGAAGTTCTTTAATTGTTAATGACATTTTGTACTCCTTTATAAAATGTAAAATT
GTGTTTAATTTCTAAATATATATATAGTATAGCATGTTGATATACTGAAATGATTTGAAAAATTTTTGAAACTTAA

TGACTTATATATTCATATTAGTTTAAAATTCGATACTATTCCATAATGATGGAATGTGATGTATATGATTAATCAAT
ATACATCACATTTCATTTAATGCAACTATTGCACAAGTTAATAGTATATTTAAAAATAGGTTAGTGAGATTGATT
AATTATTGATTATGTTGGTATCAATGAAAGCTTGGAAGAAGGGCTTAAAAATATAAAAACTTGGATAAATCAC
AATACAATCATAGACACAGATAAGTTCGTCAATCTGTTAATGAATTATGATGTGATAGAGTTAAGTGAGGATAA
ATGTTAACGTTTTATTAAAACAGTCACAGAATTAGATAGAGCATTTGCAATTTGTGCTACTGAACTTATAATCT
AAGAGCTCAATAGTGAAATTGTCTTTTTTAAAGTTGTTAAAGCATAAATTTGTTGAATTATTACAGAAAAAGA
TCGAAATTTAGTTACTGCTAATGGAACGGCAGCATTAGAGTTTGCCGAACTCATTTTAAAAGGACTTGAATTT
GATAGTAAAGAAAATATTGAAAGGCAAATCTATATGTATCAAAGAGGCTTCTATGAGTATTGTAATAAGTATGG
CAATCCTTATCAATAATTAAAAACCGCATCATTATCTGATACGCAGAGGCGTATCATAAGT
>Staphylococcus aureus JCSC6690
AAAAACTGCAAAAAAACTTGGTATAATAAGAGGGAACAGTGTGAACAAGTTAATAACTTGTGGATAACTGG
AAAGTTGATAACAATTTGGAGGACCAAACGACATGAAAATCACCATTTTAGCTGTAGGGAAACTAAAAGAG
AAATACTGGAAGCAAGCCATAGCAGAATATGAAAAACGTTTAGGCCCATACACCAAGATAGACATCATAGAA
GTTCCAGACGAAAAAGCACCAGAAAATATGAGCTACAAAGAAATTGAGCAAGTAAAAGAAAAAGAAGGCC
AACGAATACTAGCCAAAATCAAACCACAATCAACAGTCATTACATTAGAAATACAAGGAAAGATGCTATCTTC
CGAAGGATTGGCCCAAGAATTGAACCAACGCATGACCCAAGGGCAAAGCGACTTTGTATTCGTCATTGGCG
GATCAAACGGCCTGCACAAGGACGTCTTACAACGCAGTAACTACGCACTATCATTCAGCAAAATGACATTCC
CACATCAAATGATGCGGGTTGTGTTAATTGAACAAGTGTACAGAGCATTTAAGATTATGCGAGGAGAAGCGT
ACCACAAGTGATGCGGTTTTTATACATGTTAATTAAATTTAGTTAAATTTTCATAATTAAATGGCTAAAAAAATT
TACATTCCTTGTGTATTTATATTATAAAGTCTATTATCATAAAGATATACATTGATATTCGATACATAGATATGGAG
TGGTAATATGAGTACAAAAATACTCGATGAAATAAGCGAGATATTAAATGAGATGGAAAGATACTGGATAAAA
GGAAAATTAGTAAATCAATTGGTAATAGAAGATTTAAGAATAATGATAGCAAATTAATTTCTAAGCTTTTATCT
AATCAAACTATTAATGAAATATATGTTCAAGATGTTGATGGATACAAGTTATTTGATAAAGAAGCGTTTATTTC
AATGTTACGCTATAAAAATTATTGGCTGGATAGCTATACTAAATATGCAAATAAAATTGGTCTAACGACTGAAA
ATCAGTATTTAAATTATAACTCGGATGTAATATTAGATTTTCCATTTAAAGACTGCATACTTGAAGGTGCTGTTA
CGAAAGAAGATAGTATTTTAAAAAATGATGAGAAATTTTATAATCAAGTAATTGCTAGAGAAGAAATTGATAC
TTTATTATCAACTAAGGCTTTTACTAATATTAAGAAGTATAATGAAGATGGAAAACATGATGTGAATTATATCGA
AGATACCGATAATTTGATTATAAGGGGTAACAATCTAATCACATTACACAGTTTGAAAAAGAAATATGCAAATA
AAATCAAATTAATTTACATTGATCCTCCTTATAATACTGGTGGAGATAGCTTTAAGTACAATGATAGATTTAACA
GAGCCAGTTGGCTTACCTTTATGAAAAACAGATTAGAAGTGGCTAAAGAATTATTATCTGATGAAGGGAGTA
TTTTTGTTCAAATTGATTGGCATGAAGCACATTACTTAAAAATTTTATTAGATTCAATTTTTGAGGAAAAAAAT
TTAATAAATGAAATTATTTGGAGTTATTCATCAGGAGGTAATTACTACGAATTCGTTTCCTAAAAAGCATGACA
CAATCTTTTTGGTATAGTAAGTCAGATAATTACTCATTTTTATGCTGAAAATAAAATGGTAGGTGAAAGACGCG
GTAGAGATAAAAAAAATAATATGAAAAAAAATATAGATGAAAACGGAAGTATTTACTACTCAATCAAGTCTAA
TGGAAAAGAGTATAGATATTATGAAGATGATATAGTAGTTCCACAGGATGTTTGGGACATACCAATTTTACAG
CAAAAAGATCCGGAAAGATTAAGCTTTAACTCTCAAAAACCAGAACGTTTAATATCTAGAATTATTGGTGCCA
CTACAAACGAAAAAGATTTGATTCTCGACTTTTTTATGGGATCGGCAACTACTCAAGCAGTAGCTCATAAAAT
GAAACGACAATATATTGGGATTGAACAAATGAATTACATTAATACAATCTCAGTGCCTAGATTACAAAAAGTT
ATAAAAGGAGATCAAAGTGGCATTTCTAAAGATATAGAGTGGCAAGGTGGAGGCTCGTTTGTATATGCCGAA
TTAGCAAAAGAAAATCAAGAAATTGTAGATAAAATTATAAATAGTAATACAAAAGAAGAACTTAACGAACAA
ATTGATGCTCTATTAAGCAATGGTGTGCTTAATTATGAAGTAGACTTTAATGAGTTCATAAATACAAAAAAAGA
GTTCAATGAATTGAAGCTTGAAGAACAAAAAGAAGTATTAATTAGAGTCTTAGATAGTAACCAACTATATGTT
AATTACTCTGATATTGAGGATACTGCATATAATTTTACAGAAGATGAAATAGCATTTAACCATAGCTTCTACGG
AGGTGAGTAATTATGTCAGAAATGCTACATGATCGTATAGAAAACCAATTATTTTTACAATATCCGGATATACCT

AATTATATTATTGATAATTTAAATCATCGTTTAAGACCATATCAAGAGGAAGCCGTTCAAAGGCTAATGTATGTC
GGGGAACAAGATGAAGATAATTTATACAACAAGTTAATGTTTAATATGGCGACAGGTTCTGGTAAAACGCTC
GTTTTAGCTGCTTCAATATTATATGTATTTAAAGAAAAAGGTTATCAAAATTTTTTGTTTTTCGTTAACAGTACT
GCGGTTGTTAATAAAACATATGACAATCTAACTAATACAGCATCTAGTAAGTATTTATTTAATCCTGAAGGTATC
GTAATTGATGGTAAATCAATCAGTATTCAAGTAGTAGATAATTATCCCGTTTTACCGGATGAAAACACGATATAT
CTTAAATTAACTACCATTAATGCATTACATGATAAATTAAATTATCCAAGAGAAAATAGTATTACATATGAAGAT
TTAGCTCAATTTCCAATTGTATTATTAGCTGACGAAGCTCACCATTTGAATGTGAGTACAAAGAAAAAAAGAA
AAAAGAATAAAGAGCAAATTGAAGAAACTAATTGGGAAGTAACTGTAGATAGAATTATGAAGTTAAATCGTA
AAAATAAATTATTAGAATATACAGCTACAATACAGTTAGAAGACGATATTTTTGAAAAATATAAAGATAGAATT
TTATATCGCTATGATTTAAAGGAATTTATGCAACAAGGATATTCAAAAAATGTAACGCTTCTATATACAAGTGA
AGAAAACGAAAAAAAAATATTACATGCTTTGCTAATGAATGAATATAAAAAATATGTAGCTGAAAAAAATGAC
ATTATACTTAAACCTGTTATTATGTTTCAATCTACCTCTATAAAGGGATCACAGGAATTACATGAAATGATGTTA
AACCATTTGGAACAGTTGACTTTAGAAAAATTAGAAAAGATTGTTGAAGATGGTTATAAATTTTATAGTGGCC
AAAATAGCATTTGGAGTAAAGTCTTTAGTTTTTATAAACAATGTAGCCTAGCAAAAGTATTAAATGACTTAAA
GTGGGACTTTAATAAACAAACGACATTGAACGTAAATAATGATAAAGAAAAAGAGAAAAATTCTAAATTACT
AAACAACCTAGAAGATATAGATAATCCAATAAGAGCTATATTCGCAGTATATAAGCTTAATGAAGGTTGGGAT
GTTTTTAATCTTTTTGACATTGTGAAAGTTGATGAGAAGAAAAAAATTAATAAAGCTGCTACAAATGCTGAA
GCGCAACTGATTGGTCGTGGGGCTAGATACTATCCATTTACTTATAATAAAGAAAAATCCTATAAACGTAGATT
TGATAACGAATTTAGTGATTTAAAAGTTATTGAAACACTTCATTATCATACGATTAATAATTCTTCTTATATTAGC
AATTTAAAAAATTCTTTAAAAACAGCAAAAGTCCAAACAAACGAGGATAATAGTGAAGTGCACGAAGGCAA
AGTAAAAAGCAAATTTAAAAAGACAGAGCTTTTTAAGAATGGAAAATTATACATCAATAAAACAGTCCCGAC
AACTTCAGAAGATTATAAAAGTTTAGATGATTATAGTGCGACCAGAACATATCAAAAAGATTTATATAAAACG
AGTGAATCAAGTTTAATTCAAGATATTAAAAATATAGCGGAAAACCGTAAAGAAGTAAAAGTACTTCTCAATA
AACCTTTATTGCAAAAAGCATTAAGAAGTAATCCATTTTTTAGATATTCTAATTTAAAAGAGTACGTACCAAGT
ATTACGAGTATGCAGACATTTATTAAATCAAAAGATTTTCTAGGTAATTTAGATATAAGCGTAACTATTCCTAAA
GAAATGGATGTCAGTGATATAACACCTAAAATTAAATTATCTGTATTAAATGATTATTTGAGCAAACTGGAAAC
AAAAATAAAAAATAATTATCTAAAAGTTAAAGGTACAACGATTTTTGAAGGTATTAAGATATCAGAATTAATCG
ATGATTACGTTGTTGAAGTTAATAATATTAATAGAGATGTTTCGGATTTAGAGAGTCAGAAACAACCAAAAAG
TATGACCCAACATGATTGGTATATTTATGATAAAGCAATTGTAAATGGTTTAGAGAGTGATTTGATCGATTTAAT
TAATAATATGATGGAAGATTTACAAAAAAAAATATGATGAAGTTTATTTAATTCGTAATGAACGTAAAATTAAATT
TAGAGAAATCAATGGTGTTAGAGGATTTATGCCTGATTTCTTACTCTACTTAAAAGATGATAATTATACTTATCA
GGTGTTTATAGAACCTAAGGGACAACATTTATTATTAAATGATGAATGGAAAGAACAGTTTATGTTGTCTATTA
ATAGGAGAGAAGATATTGAAGTACTTGCTGAAAATGACAACGTTAGATTAGTTGGTTTATGTTTTTATTCTGA
TGATGCATTAAAACGTCAAGAATTTAAAGAACAATTTAATGACAAATTGTTAAATTAAATTTATTTATCATAAA
AAATTAAATTAACAAGAGCATCCCTCACCGTAAAGTGAAGGATGCTCTAGTTTTATTAGGATATAGTTTTCATT
TTGATAGATAATGGTTAATAATTGTCTGTAAATCATTATTAAGTACCTTGTAATACAATCATTATTCATAAAATCTT
CATAGATTTTATCTAGTATTTCTTCATCTTCAATCGCTGTGAAGTGATGTACTAGCCCTTTTAAGATTGAAGTAT
AATTTTGAGATGCTAAGCAACTAGCAACCATTCGCCAATCGATTGATGTGGTTTCATGGATCATAGATAATCCT
CCTTTATTTCAATGTCCAATTTTGTCTCGCTTTAGGGTTGAGTGGATGCATGATTTCATTCGTTGCGGGATTAA
TGAGCTTTTTGACTTTCTTTTTGTGAGACTTTAACATTTCCATCACTTGTGATACACGTTCTATAACAACTGGT
CGCTTCGCATAGGCGCCATAGGCTAGAAATGACTGTGTCACTTTCGCTTATCGCTTTCATCAAGTGAATGTCT
GTGTGTTTGTCATAAGGCTCTTTGATATGTTTAAGATTTTCAGGTGTTTTGATATTAGAGAATAGATTTACAAG
ATATACAGCGCCATATTGTTCAGAATTTGCTAATTGGTTGAGGATAAGAACTGTTGTAAGATCGAGTGATAATA
CGCCATCTAAATGAGGATACATCGTTATCACTGTACATGCGGGTTTGTTTTTATCCCAAGTCTTTTTGAGTAAG

TAACGATGTTGTTCATCGTCGCTAAAAATTGCTTCTGTGTGTATGGTGCTTTTGATTGTATTCATAGTATTCACT
TCCTTTAGTATTCTTCAGGTAAAAGCATGACGTAATAAGAAAGGTCTATATCATCTTCACGAATAATATAGACC
CTATTGATATTGATAGGATGTTTGACATTTGACTTATGAGTTTTCACATATGTTGGTCGTTCTTGCTTGTGTTTG
ATATATAGTCGATCATTGTGCATGTTGAACTGGAAAATATGAAAGTAATCTATTTCTTCTAGTTCCTTAGACTGT
TCGTTTTCACGTTGAGCTACAAGTTGCCATAATTGATGTTGTAAGGTGATAGGTAAGTTGTTATCGATACCGC
GGGTAATATATCTATTCATAAGCGTTTTCCTTTCGTTCTGATTCTTTCATCCAAATGATAATTTCACTAAGAATTG
TAATGACGAGTTGTGTAATTTTGATGTATTTATGCATATTATTGTCCTCCTGTTAGTAAGTATCGTTGATCCAAA
AGGGTCATGATAGTGTTGTGTAGATATAAGAAATCCATATCGTGGTTTTCATCAATATAACCAATGGCTATGAG
CTGTTTCTGATAGTAGAGAATGAAAGGGTAGATATTACTTTCGTGAAGTGTATCAGAGTAGTAGGTGTAGGTT
CGAAGTATTTTATTACTGAGTGGCCCAAGCCTTACATATTGGTCAGGTAAGATATGATCAGCGATTTCCTCGAT
AGATTCACATTCCCATTTTTCGTCCTTTGGTAGACTGAATAAGTTTGTTATATAGTACTTGAGTTGTTGTTCTAG
TGTCATCATTATGACCTCCAAATGTATTTGATTGACGTATGCATGTGATACATCATTTTGATAATATAAGTTTGAA
ATAATGTTTGTTTCGCTTAGAAATCCCACTGAAATACAGAAAATATCCCAATCGACAATCATCACAATTGTTGA
TATGACAAGGAAATCCCGTTATCCCGCTCATTTGTCGGGAAATATAGCTATATATTTGTATATCATTTGGATTAAT
AGGTATGGTTATGATTTACTTTTTCTTTTTATATTATTTATTTATAAATAATAACGGGATTTTGGGATTACGCTTG
CGTAATCCTTCTTATACATGAAGTTATCCAATCCCGTTACTATCCCGCTTGTATTTTATTTTGGGATGTTCTTTCG
TCATATCAATATAAGCTTCACATTGAGTCATGAGTAGTTAGCGTGTTGAAGATATTTGTTAGATAGGTGTTATT
GAATGACGAAAGGACACTGAGAAAATAAGTTGAAATACATATAATTTAACTTTGACTTTTGGTGTAATATAAA
GTGAAAGCCAAAGGAGTTGAAGCTAGTGGCAAGTTTTAGTAAATATGGTCATTATTCAGATGATGACTTGAA
GTATGATATGAATGGTTCCATTGATGGTTATCATTTATATGATGGAATATACATTACTGATAGAGTTTGGTTTTAC
ATGCAGGAAAAAGATATGGATTTTGAAGATGCTCTCAATGCATTAGGTTTGAATTATGATGAAGCTATTGCAG
ATGAGGATGATATCCCAAAACTAGACGAAAAAAGACAGCGTCTGATGGACGATCCATACAGGTTTCCATTTG
AATATGATGAAGATAAGTGATAGGCAAGGTGCATAAATGCATCTTGCTTTTTTTATTAGATTTTAAGGTAAAGA
GTTTGAGGCAAAAACAAAGACGAATTGCTGAGAAGCACTTCGTCGAGCTGAGTATTATTGAAAAGTTGTTT
AATAATCTCATTATTGAGCTTGAGTGTGACATAGAATTGCTTTTTATGATTCCCATCTTTACGAATATCGATACG
ATCAATGACCGTAAGGTATAATGCTTTGAGTTGTGATTTATCCATGGATTCTATGTTTTGAAATATTCGTTGTAA
TAGGAGAGCGATTTGTTTCGTATCATAAGATGGTTTCTCTTGATTTTGTTGCTGCTTTAGTTGATTCATTTGGT
TTGTAATGTCATTGAGTTGTGTTTCATATTGATGAATGGTTGGTTTGAGTGCAGATGTTAAGTCTGGATTGTCT
TCGATGGTTTGAACGAGATTTTTAAGTTTAGTGTTAATTTCATCAAATTGTTGTTGTTTATAAGCAATATCATG
GTTAAGTGCTGCTACATCTACATGATTCTCTTGATTGACGCGTTCGATAACTTGCTTGAGAACTTTATCACTTT
TGACAACTTCGAGTATTTGATCCATAACATATTTTTCTATGACATCAGCTCTAACACTATTGGCAGAACACACTT
TTGAACCTTTATTGCGAAAGTTACTACATGAATAATACCGAATGCGTTTTTTAGTACCGTCTTTGAGTGTATTG
GTTGTATTTGAAGCTGCATATGCCGCACCGCATTTCTCACAAAAAATTATTCCAGTTAACAGATTTGTCCCTTT
ACCATGTACCTGTGGCTTTTTGCTTACTTGCTTCTTACGTGCTTGTACTTTATTCCATAAATCTTGACTAATAATA
GGCGTGTGTTTGCCTTCAGCGATCACTGGCTTATCGTTTAATCCTTTACGTCGTTTATCATTCCAATCTTTGTAT
TTTGCGAATTGAATTTTACCAATATAGAATGGGTTTGAGAGAATATAAGTAACAGCTGAAATACTAAATGGAT
TACCTTTCTTAGTGACATAACCTTTATGATTGAGTGCATTGGCTATTTTACGATAACCATGACCTTTGGCATAAG
ATTCAAAGATATATTTAACGATGTTAGCTTCATGTTGATTAATCATTAATTCTTTTTTATTATCAGGTATGTTATTA
TATCCTAATGGAAGATTGCCTTGATAATAGCCCTCTAAAGCTCTTTGATGTTGCCCGTTGTAAATATTTTCTAAA
ATTGTATTTCTTTCAAATTCGGAAAAACTTGCAAGTATCTGGAGCATTAACTTGCCTGTTGAATTTTTGACTTC
CATGCGTTCAGACAAGCTAAAAAATTCAACATTTTGGCGATGAAGTTCTTCGACGATTGTAAGTAAATCGGA
AGTATTACGTGCCAAACGATTTGTTTTATAAACCATAACACAGTCTAATTTTCCATTTTTAGCATCATTTAACAT
GCGCTGTAATTCAGGACGGTTCATAGATTTTCCTGATATACCCCGATCTGCATATATATCAACGAGTTCATAGCC
GTTAAATTGGCAATATTGCTCTATTTGTGTAATTTGCCCCTCTATGCTATAGCCCATTACCTGTTTTTCAGTGGA

TACGCGGATGTACCCTCCGATTCTCTTCTTCTTCATTTCACTCATATTTTTTCATCCTTTCATCTATTAAGCAATC
GATGATTGCGTAGTTTGATTGACAATGTTTAGAGGTTCATTTTTGAAATAGATCCCCGCAAGGGTTTTGTTTT
TAGAAATATTAATTTCATCAATATAGGGGTACAACATGTTTAGCGTGAAACGTTGTTGAATGATGTTTTGGAA
AGCTTTTTGAATTTGATACGCACTGATTGATGATATAGGTTTTGATTGTTGATGTAATGATTGCGTTTGTTCTCT
GAACGTTTCTGCATCAATTTTGCCTTGGGCTAATTTTTCTATCAGCTGCTCGTGATTGAGTGTAGTTTTAGTTT
CTACATCTCTTTGTCTTTTGAGTCGTTGTTGAATAGTATGGTTTATTTTTGAATAGAGCTGTTGATTTTGAAAG
AAGTCCTGACAAATCGCTAAAACACTTGTTTCTAATTCTTGTGCGTTGATTCCTTTAAATTCACAAACAAAGC
GAGCATTATTCATATTTTGCGGACAGACATAGTAACGCAATGAATGATCTGTTTTTCGGACAGTCATGTTTGT
GAGTGTCGAATGACAATAGGGACATTTGATTTTTTGTTTGAGTTGGTTTTCTGACGGCTTATGTTTGACTTGT
TTCTGAGTTCGGGTAACTTGAGCTTCTTCGTATATCGTTGTACTGACAATAGCTGGGAACATGTTTTCATATTG
TCCGTATTGATTGATAACACGGCCACAGTAATTAGGGTTAAGGATAATATTACGCACTTGATAGGGCTTGCGA
TTAATGAATTTATCATCAGCTTCTAAGTATTGCGCAATTTTTTTATAACCATAACCTTGAAGGTAATAATTGAAC
ACAGCTTTTACTGTTGGTGCTTTTACTGTGTCTATCGTGAAAGTACCATTATGATAGTGATACCCAAAGGGTGC
ATGTGTCGTGATGAGTTTACCTTGTTTCGCTTTTTCTCTGATTCCATTTTTGACTTGTTCGCCTATATTATCAGAT
TCTAGTTCGGCCAAGCTGATGAAAATATTGAGTTTGAGCCGATCGAATGCTTTATCCATGTCGAAGTAGCCAT
CATGAACGCTTAAGATATGGACATGGTATTTTTGACACAATTTCATGAGTTTTAATGCATTTTTCAAATTACGA
TGAAGTCGATTTAGACGATAGCAACATAATACGTTACATTGTCCTTGTTGAATCAGTTCAGTAACTTGTTGATA
ACCATTTCGCTTATCAGTGCGTCCTGATTGCTTATCGCTATAAAAGGTAATGTGTTGAATACTGTGTTTTTCGG
CTAGTGCTTTGATGGTCTGTTTCTGTGCTGCTAGAGATTGTTGTTTGAGTGTACTTTGACGTAAGTAACCAAT
TGCTTGTTTCATCTTATTTCCTCCTTCCAAAGTGATAATATATATTTATGAACGAATTTATAGATAAGCCCAACAC
CTGGCTGGTGTTGGGCGTTATTATTAGTCATCAGCGTGATTAATTTCTTCAATCACTAAATCAGCAAGTAATGT
GATTAATTCGTCCATATTCTTCCTCCCAATTGACTTTGATAGTAGTAAAAGTGTTATACCAACTTTGGAAATATT
GTAATGAATTCAATTTTTCGCTTTGAGCAGATTTGCTCCAACTCAGGATAATCTTCATAAATGCCCCAAAAGA
CGATACGTTTAGGTTTGACGTGCTTCAGTTTAATATCATTTATTAATTGGTTATCGTCGTTATAATCTTTTGTGAT
TTTATAACCATGTTCTTTACAATATGCTTTTACAGTATCAATAGGTTCGTCTAAGTAGACTTCTCTTTCATCTGTA
ATATTATGTGCTAATGCAACTACATTCATAGTTAATACCTCCGGTTGATTTAATGAGCTACAACGCATAGAGCGT
TGTAGCGGTATAATATTCAAATTTATAGTTCTAAATCGTCACTTGCTTCTTTTTCTTTTGCTTGTTTAATAAAATT
ATTAAGCAAATCTGGATTTGTATTATTATCAAATTTCGATGGTGTGAAAGTTTCTGTATTGGAACTTAAGCCAA
AAATAGGGGCAAGTTCTTTATCCAATTTAAGATGATAGAACACAATGGTCTTTGATTTACCTTGAGTATCTTTG
ACACTGCGTTTGGTTGTTTTTCGATTAGGGTCAGATTGAATATAGCCTTTATCTCGCAAAGCATTGATAACATT
ATTGACATCTTGGTATTGGTTTTCTTCTAACATATGTTTAAAAACATCTTTCAGTATTTTGACTTGAATATGATCA
TCTTTCAGTTCAATAAGCCCGTAATTTTCGATCATATTTGAGAGTTTATTGTCTTCCGAGAATTTTCCTCTATTT
TGTGCCACAAATTGGGTGATAGTTTCGAGAGCTTTGGAAGACAGATCTCTTTCACTAACAGAATCTAAATGA
TAGTTAATGAGGTAGTCTCTAACTGCATTCAGATCGATAGGTGTCGCGATTACACGTTCTAATATTAGTGCTGA
AGTCGTAATGGTGGCGTAGCGCTTGAACATGCGGATACCTGTATTGCTCGTTTCATTTTCTAGTTGCGTTTTG
AACCATTGATGCTCAGTTCGGAACCATTGAATGACTTCTGCTTCACGATTAAGTAGGTACTCGGCGACTAATG
GCATCACATGACCGTAATTAGCTGATGTTGCCTTTTTAATTGCATCGGCATTATCAGCACTTCTCGTAAAAGCT
TCTGATATTTCAATGGTACGAACATTTAATCCATCATTACGTGCACTATTATTGAAAATACTATGTTCGGCTGTA
CTGATGACACTTGTTCCCCAATTTTTCAAGCTTTTAACACCACCATGTATATTAGCTCGTTGACGCCCTTGGCC
TTCAGCAATAGAATAGAGCAAACCAGTTGTACTTTTAAATGTTGCAGCTGATAGTTCATCTAACACAATAGGT
AAGCCAAAATTCTGACTCAAGTATCCTTCAAGTGCATTTCTCGTACCATTCCATGATCTAAACAGTGTATTGTT
ACCTTTGGTAGGGTTACCAGCTATTGATACTGCCAAAGCAGCCGCTGTGGATTTTCCGGTACTAGAATTCCCA
GTAAATGAGAAAATAGTACCTGTGAATTCAACCTCATATTTTGATTTTAAGAAAGCAGTCACTAATGAAGAAG
CACCAAATATCACAGCAAGCTCCAGTAACAAGTTTCCTTTTACTTCATCAAGATACATTTGCCACCAGCCTTCA

AATGTACCTTTAGGTTGTAAGTCATACTTTGTTTCACAAATGATTTCATCAGCCTGAGACTGCTCGATGTCTTT
TGAAAGATATGGTTCATCTAATGAAATGACAGTACCTTCATCAGTACTTAAGACACCTACACCTGTATACAATG
TAGAAATGGGCAGTGATTGACGCATTAATTGTAGTGCATTACCTAGCGATTTAATGTACTTTTCATTGATGCTG
AAGCTATACAGAACCAGTCTAGGTAATTTTTGTGTCGTTAGAATATCTGACGTTTCAATACGTTTAATATTTTTG
CCATCCGTGATAGTCAATTTCTCAATCCCAGTAGAGGGATCAAGAAATTTGTTTTCAATGATGATTGGACTAG
ATAAACGAACTACCTTATCTTCGTCGTTTTTGTTTTTTGCTCGAATAAGTTCATACCATCCAGTTGAATTTACCC
AATATGGATATTGCTTGAAGATGTTATGAGTCATAATTAATCTCTCCTTTGCTATATAAGCATTAAATTTGTGTTG
CTACATACATATTAAAGGAGAATTAAACGCAAATAAGTAGGTCGTCCATATACAAAAAAATTGGACGACCTAC
TTAGGTAATGCTATTCAAATTTTAAATTGTCCAAGGTTTCACTTAGAGCTTTTGCGATAGACCAACTAGATAAA
TCATTGACTTGTTTACTGACGGTTTCGTCTTTAATTTGAGTAGATTTTATTTCTTCAAATAACTGGAAAGCGTT
CGTTAATTCGGTAAAGTTTATTTTTGAAAGTGGCTCAATATCTTTATTAAATGAATATTTATCTAATAAAAGATG
AATTCGATACAAGTCATTTAAGAAGCTCTCTACTTGATTTGGACTTATACCATCATCAACATAGGATTGGCGAT
TAGAATAAAATAGGTTAGAAAATTTAGACAATGATAAATAAAAGTCTTTATAAATATTGAGAAACTTATCGTTT
GATATTAATGGATCGGGGCTATTTATTATATTCTCTATGCGCGATTCAAAATTTTGTAAATCAAAAGTGCTGTTT
TTATATTGATTTATAGTATTAATAATTCTTTGATAGTTAGAAAATAAGTCGTTTTCAATACTGAGAAAGTATCCAT
TAACGTTGAATGGGTATTTTACAATTTCTTTTTTTCTTAATTTTTCACGGATTTTCGATTCATCATTTTGTATATC
ATCAGTCACAATTTTATGATTGATTCTTTTATTCAAACTTGGAAGGTAGAGTTTGTTTAATATATCAATAATGAA
AAAATCTAATAATTTGTTATCAGTACGTTCACTATACTCATCTATAGTTGGCTCATTTGATATATATCTAGCTACTT
CATGATTTACATTTGAATACTTGGAATCATCAGGTTCTACTTGGTTGTATATAATAAAATTTTCGGGATTGTTTT
CTTTATTATATTGATGAGCTTTATATAATTGCTTGAAATTGAAGTCATTATCAAATGTAATATTATCTTCCATTAAT
GTTTCAAACAGTAACTCGTCAATTCTATGGGCTAATTTTCTATATAATTTTGAACTCTTATCTAAATCTAGAGAA
TTTGTATAATTAAAAAATGAAGACAATGCATCATTACACTTTGACTGAGCACTAAGCCATTCTAAAGTAAAAG
AATAGTTTTCAGATGCATAAATTTGCGCTTTTAATGCATCATTTTTAATATTATCAATTGAATCAGTGATATTAGT
AATATAGCTAATAAATTGACTATTATTTTTCTTTAATGCCTCCTCTCTTAAATCTATGTCATCAAATTTTTTAGCAG
ATGTTGGAACAGGAAAGTCTTCAATAGTATTTAGTAAAACTTTAAATAATTCTAATTCAAATAAGTTAAAGCTA
TAACTGCTTCTAGTTGCTGATGGATTGGATTTGTATCTAGTTATATCTAACTTATAAACTCGTTCGAGCTTATCC
ATACTATTCTTAAACATTTTTTCACTTAAGTTACATTCTTTCATTAAATCTTTTCCCGTAAAATATTTATAATTTTC
AAAATTCAAAGTTATTGTCCTTTCAAACCTTCTTCAATGGTTATGTTTACTTTAATTATATTATAAAGTAGAATTA
ATAGTAATAAAGGGAACATTTGTTCCTGTTTGAAGTATACTAATGAAAGTTATGTTTAAATCATTTAAAGAATT
GTATGAAGTGCTTGATAGTTATATAGGTATTGATTTGTGATTTTGGAGAAAATGATAACTTGAAGTATGATTTG
ATGTCAGAATTAGGTATGCACTTTGATAGATATTAAGAAAAGAAAGCTGTTTTTATTTGTGGAAACGTCTAGC
TATGTTTTGTATAAAAGACACTTATAATAATTGTAGTATGTTAACAACTGTTAAGCATCATAAGGTAGTGATTAG
AGGGTTCTGTTGCAAAGTAAAAAAATATAGCTAACCACTAATTTATCATGTCAGTGTTCGCTTAACTTGCTAGC
ATGATGCTAATTTCGTGGCATGGCGAAAATCCGTAGATCTGAAGAGATCTGCGGTTCTTTTTATATAGAGCGT
AAATACATTCAATACCTTTTAAAGTATTCTTTGCTGTATTGATACTTTGATACCTTGTCTTTCTTACTTTAATATGA
CGGTGATCTTGCTCAATGAGGTTATTCAGATATTTCGATGTGCAATGACAGTCAGGTTTAAGTTTAAAAGCTT
TAATTACTTTAGCCATTGCTACCTTCGTTGAAGGTGCCTGATCTGTAATTACCTTTTGAGGTTTACCAAATTGT
TTAATGAGACGTTTGATAAACGCATATGCTGAATGATTATCTCGTTGCTTACGCAACCAAATATCTAATGTATGT
CCCTCTGCATCAATGGCACGATATAAATAACTCCATTTTCCTTTTATTTTGATGTACGTCTCATCAATACGCCATT
TGTAATAAGCTTTTTTATGCTTTTTCTTCCAAATTTGATATAAAATTGGGGCATATTCTTGAACCCAACGGTAG
ACCGTTGAATGATGAACGTTTACACCACGTTCCCTTAATATTTCGGATATATCACGATAACTCAATGCATATCTT
AGATAGTAGCCAACGGCTACAGTGATAACATCCTTGTTAAATTGTTTATATCTGAAATAGTTCATACAGAAGAC
TCCTTTTTGTTAAAATTATACTATAAATTCAACTTTGCAACAGAACCGTATTATGGAATAGAGATGTTGGTAAC
ATTTATACAGGATCATTATACTTAAGTTTAATTTCGTTATTACAGAACCACACATTCCAACCAGAAGAGAAAGT

ATGTCTATTTAGTTATGGTTCAGGAGCAGTAGGAGAAATCTTTAGTGGTTCAATCGTTAAAGGATATGACAAA
GCATTAGATAAAGAGAAACACTTAAATATGCTAGAATCTAGAGAGCAATTATCAGTCGAAGAATACGAAACAT
TCTTTAACAGATTTGATAATCAAGAATTTGATTTCGAACGTGAATTGACACAAGATCCATATTCAAAAGTATAC
TTATACAGTATAGAAGACCATATCAGAACATATAAGATAGAGAAATAAACTAGTGGCCGATTGTGCTTGATGA
GCTTGGGACATAAATCCTAACTCGAAATAAATAAGCATATCACTAAACTGATTTTTTAAAGTTTACAGTGATAT
GCTTATTTTTTTATCTTACGATTTTGTACGTGCATGCTTGCCTAGGGGTATGGCTCGAGCCATTAGTCTCTCGC
ACATACTATTCCCTCAGGCGTCAGCACTTACAAAATCGGTTGTAATTTTCATTTTTATACGCATTCTTACTGAGA
TTATACTAATAAGAGGAATAGTAAAAGCAATTCTAAGTAAAATTGCAGATAAGAGGTTTGTTAAAAGCAGTTC
TAAGTAAAATTGCAGATAAGAGGTTTGTTAAAAGCAGTTCTCAGTAAAATTACAGATAAGAGGTTTGTTAAA
AGCAGTTCTAAGTAAAATTGCAGATAAGAGGTTTGTTAAAAGCAGTTCTAAGTAAAATTGCAGATAAGAGGT
ACGTTAAAAGCAATTCCATGCAAAATTGCTGATAAGGGGTAAGTTAAAAGCAGTTCTCAGTAAAATTGCAGA
TAAGAGGTAAGTTAAAAGCAGTTCTAGGCAAAATTGCAGATAAGAGGTGCGTTAAAAGCAGTTCTCAGTAA
AATTGCTGATAAGGGGTAAGTTAAAAGCAATCCTAAGTAAAATTGCAGATAAGAGGTAAGTTAAAAGCAATC
CTAAGTAAAATTGCAGATAAGGGGTACAGAAAAACTAGACTTGATTACAAAATGGAGCTTGGGACATAAATG
ATTTTTTAAAAATGAGATGAGACGTAGATTAACTCCATAATCAATACGAATCTATCGACTTCTTTATTTATGATAT
TCATCTCTTTTTAATGGAAATAAAAGTGCGATTAATGTGATAATACAGTTACGTTAATTAAAAAAATAAAAATG
CAAGGAGAGGTAATATGCTAACTGTATATGGACATAGAGGATTACCTAGTAAAGCTCCGGAAAATACAATTGC
ATCATTTAAAGCTGCTTCAGAAGTAGAAGGTATAAACTGGTTGGAGTTAGATGTTGCAATTACAAAAGATGA
ACAACTGATTATCATTCATGATGATTATTTAGAACGGACTACAAATATGTCCGGGGAAATAACTGAATTGAATT
ATGATGAAATTAAAGATGCTTCTGCAGGATCTTGGTTTGGTGAAAAATTCAAAGATGAACATTTGCCAACTTT
CGATGATGTAGTAAAAATAGCAAATGAATATAATATGAATTTAAATGTAGAATTAAAAGGTATTACTGGACCGA
ATGGACTAGCACTTTCTAAAAGTATGGTTAAGCAAGTGGAAGAACAATTAACAAACTTAAATCAGAATCAAG
AAGTGCTCATTTCAAGCTTTAATGTTGTGCTTGTTAAACTTGCAGAAGAAATCATGCCACAATATAACAGAGC
AGTTATATTCCATACAACTTCGTTTCGTGAAGACTGGAGAACACTTTTAGATTACTGTAATGCTAAAATAGTAA
ACACTGAAGATGCCAAACTTACTAAAGCAAAAGTAAAAATGGTAAAAGAAGCGGGTTATGAATTGAACGTA
TGGACTGTAAACAAACCAGCACGTGCAAACCAACTTGCTAATTGGGGAGTTGATGGTATCTTTACAGACAAT
GCAGATAAAATGGTGCATTTGTCTCAATAGAAAGTTAGAGGTGAGTCTTACGTTTCAGTGACGGTAGACTTA
CCTTTAACATGTTACATACTAAAAAATTAATTTGAATAAGAAAGAGAGACATATATGAAATACGATGATTTTATA
GTAGGAGAAACATTCAAAACAAAAAGCCTTCATATTACAGAAGAAGAAATTATCCAATTTGCAACAACTTTT
GATCCTCAATATATGCATATAGATAAAGAAAAAGCAGAACAAAGTAGATTTAAAGGTATCATTGCATCTGGCA
TGCATACACTTTCAATATCATTTAAATTATGGGTAGAAGAAGGTAAATACGGAGAAGAAGTTGTAGCAGGAA
CACAAATGAATAACGTTAAATTTATTAAACCTGTATACCCAGGTAATACATTGTACGTTATCGCTGAAATTACAA
ATAAGAAATCCATAAAAAAAGAAAATGGACTCGTTACAGTGTCACTTTCAACATACAATGAAAATGAAGAAA
TTGTATTTAAGGGAGAAGTAACAGCACTTATTAATAATTCATAATAAAACAGTGAAGCAACCATCGTTACGGA
TTGCTTCACTGTTTTGTTATTCATCTATATCGTATTTTTTATTACCGTTCTCATATAGCTCATCATACACTTTACCT
GAGATTTTGGCATTGTAGCTAGCCATTCCTTTATCTTGTACATCTTTAACATTAATAGCCATCATCATGTTTGGA
TTATCTTTATCATATGATATAAACCACCCAATTTGTCTGCCAGTTTCTCCTTGTTTCATTTTGAGTTCTGCAGTAC
CGGATTTGCCAATTAAGTTTGCATAAGATCTATAAATATCTTCTTTATGTGTTTTATTTACGACTTGTTGCATACC
ATCAGTTAATAGATTGATATTTTCTTTGGAAATAATATTTTTCTTCCAAACTTTGTTTTTCGTGTCTTTTAATAAG
TGAGGTGCGTTAATATTGCCATTATTTTCTAATGCGCTATAGATTGAAAGGATCTGTACTGGGTTAATCAGTATT
TCACCTTGTCCGTAACCTGAATCAGCTAATAATATTTCATTATCTAAATTTTTGTTTGAAATTTGAGCATTATAAA
ATGGATAATCACTTGGTATATCTTCACCAACACCTAGTTTTTTCATGCCTTTTTCAAATTTCTTACTGCCTAATTC
GAGTGCTACTCTAGCAAAGAAAATGTTATCTGATGATTCTATTGCTTGTTTTAAGTCGATATTACCATTTACCAC
TTCATATCTTGTAACGTTGTAACCACCCCAAGATTTATCTTTTTGCCAACCTTTACCATCGATTTTATAACTTGTT

TTATCGTCTAATGTTTTGTTATTTAACCCAATCATTGCTGTTAATATTTTTTGAGTTGAACCTGGTGAAGTTGTA
ATCTGGAACTTGTTGAGCAGAGGTTCTTTTTTATCTTCGGTTAATTTATTATATTCTTCGTTACTCATGCCATAC
ATAAATGGATAGACGTCATATGAAGGTGTGCTTACAAGTGCTAATAATTCACCTGTTTGAGGGTGGATAGCAG
TACCTGAGCCATAATCATTTTTCATGTTGTTATAAATACTCTTTTGAACTTTAGCATCAATAGTTAGTTGAATATC
TTTGCCATCTTTTTTCTTTTTCTCTATTAATGTATGTGCGATTGTATTGCTATTATCGTCAACGATTGTGACACGA
TAGCCATCTTCATGTTGGAGCTTTTTATCGTAAAGTTTTTCGAGTCCCTTTTTACCAATAACTGCATCATCTTTA
TAGCCTTTATATTCTTTTTGTTTTAATTCTTCAGAGTTAATGGGACCAACATAACCTAATAGATGTGAAGTCGCT
TTTCCTAGAGGATAGTTACGACTTTCTGTTTCATTAGTTGTAAGATGAAATTTTTTTGCGAAATCACTTAAATAT
TCATCCATTTTTTTAACGGTTTTAAGTGGAACGAAGGTATCATCTTGTACCCAATTTTGATCCATTTGTTGTTTG
ATATAGTCTTCAGAAATACTTAGTTCTTTAGCGATTGCTTTATAATCTTTTTTAGATACATTCTTTGGAACGATG
CCTATCTCATATGCTGTTCCTGTATTGGCCAATTCCACATTGTTTCGGTCTAAAATTTTACCACGTTCTGATTTTA
AATTTTCAATATGTATGCTTTGGTCTTTCTGCATTCCTGGAATAATGACGCTATGATCCCAATCTAACTTCCACA
TACCATCTTCTTTAACAAAATTAAATTGAACGTTGCGATCAATGTTACCGTAGTTTGTTTTAATTTTATATTGAG
CATCTACTCGTTTTTTATTTTTAGATACTTTTTTTATTTTACGATCCTGAATGTTTATATCTTTAACGCCTAAACTA
TTATATATTTTTATCGGACGTTCAGTCATTTCTACTTCACCATTATCGCTTTTAGAAATATAACTGCTATCTTTATA
AACTTGTTTGAAATTTTTATCTTCAATTGCATCAATAGTATTATTAATTTCTTTATCTTTTGAAGCATAAAAATAT
ATACCAAACCCGACAACTACAACTATTAAAATAAGTGGAACAATTTTTATCTTTTTCATCAATATCCTCCTTATAT
AAGACTACATTTGTAGTATATTACAAATGTAGTATTTATGTCAAAATAATGTTATAATTTTTGTGATATGGAGGT
GTAGAAGGTGTTATCATCTTTTTTAATGTTAAGTATAATCAGTTCATTGCTCACGATATGTGTAATTTTTTTAGT
GAGAATGCTCTATATAAAATATACGGTTCTGTTGCAAAGTTGAATTTATAGTATAATTATAACCAAAAGGAGTC
TTCTGTATGAACTATTTCAGATATAAACAATTTAACAAGGATGTTATCACTGTAGCCGTTGGCTACTATCTAAGA
TATGCATTGAGTTATCGTGATATATCTGAAATATTAAGGGAACGTGGTGTAAACGTTCATCATTCAACGGTCTA
CCGTTGGGTTCAAGAATATGCACCAATTTTATATCAAATTTGGAAGAAAAAGCATAAAAAAGCTTATTACAAA
TGGCGTATTGATGAGACGTACATCAAAATAAAAGGAAAATGGAGCTATTTATATCGTGCCATTGATACAGAGG
GACATACATTAGATATTTGGTTGCGTAAGCAACGAGATAATCATTCAGCATATGCGTTTATCAAACGTCTCATT
AAACAATTTGGTAAACCTCAAAAGGTAGTTACAGATCAGGCACCTTCAACGAAGGTAGCAATGGCTAAAATA
ATTAAAGCTTTTAAACTTAAACCTGACTGTCATTGTACATCGAAATATCTGAATAACCTCATTGAGCAAGATCA
CCGTCATATTAAAGTAAGAAAGACAAGATATCAAAGTATCAATACGGCAAAGAATACTTTAAAAGGTATTGAA
TGTATTTACGCTCTATATAAAAAGAACCGTAGGTCTCTTCAGATCTACGGATTTTCCCCATGCCACGAAATTAG
CATCATGCTAGCAAGTTAAGCAAACACTGACATGATAAATTAGTGGTTAGCTATATTTTTTTACTTTGCAACAG
AACCCCATTTTCACTCATTCTAATAGCCCATTCTTTAGCGTCTGATGGCTTAATACTGTCAATACTTCTTACACC
TAACTTGTCTTTCTTCAAAATATCCATAAGATATTTGCGTCCAGTTTCAGTGTTTTTTCTAACCTTTGGTCTTTG
AGCGTTCTGTTTTGCGTAAAGCTGGCAGAGTGTCATTTTCTTTCCTACAACATCAATACCATCATGAATGTCTT
TCTGTAACTCTGCGATTTTCTCTCTAAGTGAGATACAATCACGCTTTCCTGCTGGTACTCGGTCTGTAGCCACA
AGTTTCCACGAGTAAACAAATTGCGGTTCTCCAAATGAATCTATATATTTGTATAAGTATCTTCCGTCTTTTCGT
TGGCTCTCTCCAGTCTTTAAGATTCGACCTTTATTGTCACGTCTTTTTTCTGACATGGCATTTGCTCCTTTCCTT
TATGGAAAGAGCCTTGATACGACTTAATACTATTTTATCATATACAAGACCCTTTGGCGACGCTAGATTGCGTC
CAATGTATCTATAATTTTTTCAAATTGTTTTCGTTTAATCTGAATACGATTGCCATTCATAATCAGCCAATTTGCA
TTTTTATTTTCCTCTGCCAAGCGTCGTAGCTTGTTTTCGCCAATACGAAAATATTTTGACGCTTCTTCAATGGT
TAGGGTATAACGTTCCCAAATAGGAATGTCAGTCTGCTTCATAAAATCCTCCTTTCCAAATCACTTATTTGGAT
TTCATAAAAGTTGTTTTACCAGCAATCGAACAGCTTTAGCAAAGCTCACGGGAGTTCCACCCCTGCATGGTT
CTCATGTAGCCATACTCATTGCCTGCGACGGTTTTATCACGCTCGGACTATTGACTGTATGGGAGTATCATTAT
CACGATAAGAATGTCGTTGCAGGCAATCCTGCTAAAGATTGCTTCTCGGATCACTAACATGAATCGCTCGCTA
TCTTTATAAGATAGGTCATGGCGGTTAGTTCCGTTGGCTCTTTTCTTATCGAAACGTATTCGATTACTTTTATTC

AGTTTTCAAAGAACAATGGCTCGTTAGCCTATCAAAACACATTGAAAGCTCAATATGCTTTGGTGGAATAACA
AACCTCCCTGTTCGGGAAGCGTGGAATGGTTTAGCACGCTTCCACGAAAGGAGAGAGGATATTACTTAATTT
CAAATGACAAAATCTTTGTAATCAGTCTGGTTTCCATTCTTCCACGTAAGACTTCATCAACGACCATACTTTGA
TTGCCATATTCATCTTTCATAAGTCGTAGGGAACGCTTCGTTATGTACCCTCTGTAATGATGTAGAATCTGGTTA
ATCGCTTCGGTATCGCCATCTGTTGCCTTTACAATGAGAGGAAAGGGAATCATAGGATATTGTGTTTTCATTCT
TCAAATTCCTCCATAAACTTTTTAATTAAGGCTAGTCCACTGGTTCTATGCCGATAGACAGTAGAACGGTTCAA
TTTCAACAGGTCTGCAATTTCTGAATCGCTCATGTCCATAAAGTAAAACAGCAGTAGAATTTCACGTTTCTTG
TCTGGCAACTCACGTAATGCTTCACTCAACAAATCATTTTCAACGCCTACTGATAACCCATTGAGTGTAAAAAT
CTGAAAGTCAGTTGAATAGTTATCTGTTGTCGCAAACTGGCTAACAAGATAATCGCCAACATCCGAAAAGGA
CACCTCACGCTTTGCAATCCTTGAAAGATAAAGCATATAATTCTTTCGCTCGTCTTCCATAGCACGTTTACAGA
TATAGTCAAACTGATTTTCTATTGTGGTCTGAAAAGAAGATGGTTTCATGTTTCTCACCCCCTTTCTGTCTAGG
AAAGGAAGTGAGCCTTGCTCGTTTATCTCCTTTCACTCTTAGTCCCAATGTGAAAGGGGGATTTGTTGCATTA
CTGATAAATAAACTTTGTAAAAAAGTTCTGAATAGCCAAAAAAGCATATAAACAGATTTATTTCTCTGTTTACA
TGCTTCTGTTATTCTATCTATATGATTTATAAAACCACATTGGTGGACGTACTTATCTATTGCAGATAGACGACTT
TTTTTGACAAGAACCCAATGTAAGGAAATTTATTGTATATGATGTACTTCATGGCGACGTTGACCTCCAACAA
ACCGCCATTTGGAAGTAATATACAATATTTTAACAGCGTAAATAGCACTACCATATAACGGTTTTTTTATTGGC
GTTTAGTAGTGCTTTTTATTAAATATAAACCTATAAACCATATAACACGTTTTTCTATACCTGTTTTTAATTCAGT
AGGAACAATAAAATGTATAGAGGTGGTCTACTATGCGTAAAAAAGAAGATAAATATGATTTTAGAGCCTTTGG
TTTAGCCATTAAAGAAGCTCGATTGAAACGAGGTTTAACTCGTGAACAAGTGGGAGCATTGATTGAAATTG
ACCCACGGTACTTAACTAATATTGAAAATAAAGGGCAACACCCCAGCATACAAGTTCTTTATGACCTTGTATC
GTTACTTCATGTTTCCGTTGATGAATTTTTCTTACCTGCTAATAACTTGGTAAAAAGCACCCGACGATTACAGA
TAGAGAAATACATGGATAGCTTTACAGACAAAGAACTATCCTTAATGGAATCTTTAGCCAGCGGTATCAACGA
AGCAAGAAACATCGAAGACTAATTAAAAGAATCCATACATAACGGAAAGAGCCGATAAAATGAGATTGTATT
AATCTCATTTTATCGGCTCTGCGTCTTTGCGTCTGGCTCTGTAATCACAGTTACTTTGAACTGCTTTATTTCAAT
TAAATTTTCTTGTCTGCATTTCGGACAATAGAGGGGGAATTTTTTTAATTCAGTATCTTCCCTTATCTTTAATCG
TGTTTTATTTCCACATACAGGACACAATATCCACTTGTAGTTTATAATAACTATCTCCTCCTTTACACTTTAATTC
AAATCTTTATTAAAAAATATTTCATCTTATTTAACAAGAAACCATATTTATATAACAACATAAAATACACTAAGTT
ATTTTATTGAACATATATCGTACTTTATCTATCCGACTATTTGGACGACGGGGCTGGCAAACAGGTTCACCGGT
AGTAACATGGTACCCTTTTAACTCTGTTAAACAAACACTACGTCCATTTGTAAAGAAAGTTAAATCACTACGAT
ATTCTTGAATACACCGAGCAGGGATTTCTCCACTAAGAATGACCTCATTATTTTTCAATTGAGTGTCTACGATG
TTCGCACAATATTTAGGAGCATCGTTGTATGCTCGTGAAAGATATTCCTGTGGCGCATAAATTTTAAAACTAAG
ATATGGCTCTAACAATTCTGTTCCAGCTTTTTTTTAGACTTGTTCCATACAATAGGAGCAAGCATCCGAAAATC
TGCTGGAGTACTAACAGGGCTATAGTATAAACCGTACTTAAAACAGATTTTACAATCCGTCACATTCCAACCAT
ATAATCCTTGTTCGCAACCATAGCGTATCCCTTCCATAACTGCATTTTGAAATGATTGATTTAAGTATCCAAGAG
AAACCGAGCTCTCATACTGCATTCCACTTCCCAACGGAAGCGGTGATACAGATAAACCAATGGAAGCCCAGA
AAGGATTTGGCGGCACTTCGATGTGAATGGTATATTCTGCATTTTTTAACGGTCTCTCCATATAAATGACTGTA
GGCTCTTTTAGTTCTATCTCCACATGATACTTTTCTTGCAACAGTGCACTAATCACTTCCATTTGTACTTTCCCT
AAGAAAGAAAGTATAATTTCATGTGTCGTAGAATCCACGTAATATCGTAGAAGCGGATCACTATCTGAGATTT
CCAAAAGGGCATCAAGCAACATTTCTCTCTGTTCAGGTTTACTCGGTTCAACAGTTGTTTGTAGTAGAGGGT
GCGGATTTTCAATCTTTTTTCTCTGTGGCAATAGTTTTGTATCTCCAAGAACACTATTTAACTTCAAAAACTCA
TTTTGCAAAATAACAATTTCTCCAGAATAAGCTCTATCAATCTTACATAATTCACCATTTATTGAAGTATACATTT
CTGTAACTTTTATTTTTTCTTTTTCTGATACTCTAACCGAATCTCGTAAATGTAGTACTCCACTATAAAGGCGTAT
ATATGCAAGACGTTGTCTTTTTTTTGTATATTCAATTTTGAAAACATTTCCGCAAAGTTCAGACGGACCTCGAT
GTGTTGATGAATAAAATTTATTAGTAATAACTTCTATAAGGTTATCAATCCCTATATTACTTTTTGCACTTCCATG

ATAAAGAGGGAACAGAGAACAATTCTGAAATCTTATGCTTTCCTCTTGTTCGAGTTCCAATGCTTCTAATGAT
TTACCGGACATATATTTCTCTAAAAGGTCATCGTTTCCCTCTATTACCGTATCCCATTGTTCAGATTCGGTAAAG
TTCGTCACACACATATTAGGATACAGTTCTACCTTCTGTTTGATTACAATTTCGGCAGAAAGTTTCTCTTTAATA
TCCTGATAAACCGTTGATAAATCAATTCCATTTTGGTCAATCTTATTGATAAAAAAGATTGTGGGAATCCCCAT
TTTCCTAAGTGCATGAAATAATATACGAGTTTGTGCTTGTACGCCATCTTTTGCAGAAATCAGTAGAATTGCCC
CATCTAAAACTGATAATGAACGGTATACT
>Staphylococcus aureus JCSC6945, SCCmec
AACTGCAAAAAATATTGGTATAATAAGAGGGAACAGTGTGAACAAGTTAATAACTTGTGGATAACTGGAAAG
TTGATAACAATTTGGAGGACCAAACGACATGAAAATCACCATTTTAGCTGTAGGGAAACTAAAAGAGAAATA
TTGGAAGCAAGCCATAGCAGAATATGAAAAACGTTTAGGCCCATACACCAAGATAGACATCATAGAAGTTCC
AGACGAAAAAGCACCAGAAAATATGAGCGACAAAGAAATTGAGCAAGTAAAAGAAAAAGAAGGCCAACG
AATACTAGCCAAAATCAAACCACAATCCACAGTCATTACATTAGAAATACAAGGAAAGATGCTATCTTCCGAA
GGATTGGCCCAAGAGTTGAACCAACGCATGACCCAAGGGCAAAGCGACTTTGTATTCGTCATTGGCGGATC
AAACGGCCTGCACAAGGATGTCTTACAACGCAGTAACTACGCACTATCATTCAGCAAAATGACATTTCCACA
CCAAATGATGCGGGTTGTGTTAATTGAGCAAGTGTATAGAGCATTTAAGATTATGCGTGGAGAAGCGTACCA
CAAATAAAACTAAAAAATAGATTGCGTAGCACATATTATGAAATAATTCATTAGATAAAGGAGAAATTGTTAAT
GACTATGTTTCGTGAGGCATTAATATGGCTAGTACTCCTAGTATTTAATTTAATAAACACGTTCTTAGTTATTATA
GGGAAAACACAATTATTTAAAGTTCCACTATGGAGTACGTGGCTATTATGGGGAATTATTACGATCATTATACT
AGGTATTTTATTCTTTAGAAAATATCTACAAAAAACGTATTCTCTAACTAATATAAATTCCGATAAAAAGTTTAA
AGACGGTGAGTTCTTTGTACAAATCCCTTTATACATCATTGAGAATCAAAGCAATGTTATATACGGTAACGAG
ACAATAACGTATAAACCTGTTTTTGTTAATATATTTCATAAATTATTGAGTCTCTTTGGTGTTCAAACAAAATATA
GTGTATATATGAATTCTAGTGAGAACAATGTAAAAGTAATTCGTAAACATGTGGTAGCGAATAAACATCAATAT
ACGATGTATTTGAATGATGAAGAAGTAGGCATACTTGAGATGAAACAGTTCTTCAAAAGTGGGGGAAAGCA
ACAAATTCCTTATACGTTTAATTACAAATCTGAGTTATTTGATGTAAGCAATCCGTTTTTTAGTAATGAAACCAA
AATTACATTTGAGAATGAAGTATTATTAACCGCAAAGCGTAGTTTTTTAGATATTTCAAAAAGTAAACTGACTA
AAAAACGTGGGGAAAAACACAATATACACATTCACAGTACTAGAGTAGAGAAAGAAATATTAATAGCCATTT
ACTTACAATGCATGATAAACAAGCAAACACAATAAATGAAGTATAGGTGTAGTATAAATGAATCAAAATAATAA
TTGATTTAACCATTAACGAATAAAGATTTTAGTACAAATATACCCTATTATCATAACTGCTAAAAAAGATAGTGA
AGGCAACAAAACAAACCATATTGACATACATCTCTCTCCTTACTCACCATGTTTTTTATTTAAAATCTAAACATA
TGATTAAAAGTGCTAGATGATTTTAAATACAGTTTAACAAAAATAATTTAAAATGAAATAACTCACAATTGATA
TATTAAAATTTGTATAAAAGGTTTAAAGATAAAGTAAGTTATATATCACTCACTTGGAAATGAATTGACTAAGA
ATGTTACAATGCAAATGAGTAGAGGAGAGTGTTTATGAAAAAGATAATATTATTAGCTAGTCTATTGGGTTTG
TCTTTTATGTTAGTAGCTTGTGGAACATCGGGAGAACAGAAAGAACCTTCTAAAGATAGTCAAAAGTCAGAG
AAATATACATATGAGTATTATGAAGTGCTAAATAATGGTTCAGAAGATACACCCAATGTGGAAATAAAATATAA
AGATAAAAATGGTAAATCACATTTACAAAAGACGGATTTAGAACACGTTTATGAACATATTTTAAATGATGGA
AATAAAAAGCCTTATATAATTAAAGATGGCAAAAAGATTCATGTCTATCGTCCACCATATATGATTTATGGCGAT
GACGATATTGAAGGACAAGTTACTGCTAAAGATGAAGTGTCTAAAGAGTAAGAGGTGAGTTGGTGAAGCAT
AAAGTATTAAAAATGATAAGTATAATGATAATCTTTAATGTGACCTCGCTTTGTATATTGTCTACAAACTTAGAA
GCTGTAACCTCAAGCACTAGTAGCTCAAGTTCGAGTAGTAGTTCGTCATCTTCTGCGGTTAGAAGTTCAACGT
CTTCAAGTTCGTCAATGAGTCGTTCAAGTACGATGAATGCTTCACGAGTTGCAAAACAATCAAGTCAGCGTG
CAGCTCAACAAGCTAGTCGAACAAACAGTATTACATATAATAATAAGGCATCAAAATTTAAACAAGACACTGG
TAAAATTCAATCTCAACAAGCACGATCAATGGTTTCTACAAGACATAGATATAGACCAGGGGCCTCATATGCG
TCACAATTTATGGCTACTTCATATTATAATAATTGGTTATATTTTTATTTTATTACATCCGGTATGTATAAAAATAA
AACCAATCATGTTGAATACCAAAAAAACATGTTGAAACAACAAATGAAAGCGCATGAGAAATTATATACCATT

ACCGTTCAAACTAATAAAGGCAAACGTTTAGTTGTTGTCCCGAAAAAGCAATACGATGAAATTAAAAACGGT
GAGAAAGTGAAAATTAAAAATAGTGCATTACAATAACTCACATTGAGTAGTCCAACGTTCTTAATAGAAGGG
AGTTTTAGTAAAGTTTGTTCATCATTTTAAACTCTGTAATAGTCTAAAATGACATATTAAAAAAAGCGTCCCAA
TTATATTGAGACGCTTAAAGTTTATCTAGCATTAAATGATTCAATGGCTGCTTGGATTTCATCTCTAACACGCTG
GAATTCTGACTAAGGTTTGCCAGCAGGATCGTCAAATCCCCAATGTTCCTTCTTTACATTAGGCGGCAAAACA
GGACAGTTTTGATCAGCATCACTACATAGAGTTACTACTAAATCGGATTGGTTTAGTATAGTGTTGTCAATTAA
ATTAGAAGTATGGTTTGAGATATCAATTCCAACTTCTTTCATCGCTTCGATTGCTTTCGGATTGACACCGTGTG
CCTCAATACCGCCAGAATATACTTGCCATTCATCACCTAAGATGTTCTTAGCCCAACCTTCAGCCATTTGGCTA
CGGCATGAATTACCTGTACATATAAAATAAATTATTTTCTTTGTCATCATTGACACCTCTTTTTTAAAATATAATA
AGTGTAACGTATAAACCAACTAATGTGATAAACAGAACAGGAATCGTAATAACAATACCCGTTTTAAAATATGT
GCCCCACGAAATTTTTACTCCCTTTTGTGTTAAAACGTGCAACCATAGTAATGTTGCTAAGGAGCCGATAGGT
GTGATTTTAGGTCCTAAATCTGCACCAATGATATTGGCATAAATCATGCCTTCTTTAATTAAACCGGTAGTTGC
AGATTGTCCAATAGCAATGGCATCAATTAATACGGTAGGCATATTATTCATAATGGCTGAAAGAAATGCGGATA
TAAAGCCCATACCTATCACACTACTAAACAATCCATAGTTTGATATGCTTGATAGAATATTAGCTAATAATGCAG
TAATACCCACATTTTTCAGACCAAATACAACTAAATACATACCTATAGAAAAGAGCACAATATTCCAAGGTGCA
CCTTTGATCACTTGTTTCGTGTTTACAACGCTTGATTGATAGGCCAATACAGTAAATATAAACGCAATTGAACA
AGCAATGATCGATACTGGAATTTGAATAAATTCACTAACTATATAACCAATGAGTAAGATTGATAAAACAATCC
ATGAAAACTTGAATAATTTTGGATCTTTAATGGCTTGATCAGGTGTTTTAAGGTTTTTAATTTCAAAATGGTTA
GGAATAACTTTTCTGAAATATAGCCATAATACAACAATACTTGCAAGTAAAGAGAATAGATTGGGAATCATCAT
GCGACTTACATATTCAATAAATCCAATATGGAAGTAGTCAGCAGAAACGATATTAACCAAATTACTTACAATTA
ATGGAAGAGACGTAGTATCTGCAATGAATCCACTTGCAATGATAAAAGGAAAAATGACTTTTTGATTAAACC
CAAGATTTCTTACCATAGCGAGCACGATTGGAGTGAGAATTAATGCAGCTCCATCATTCGCAAAGAAAGCTG
CTACAATAGCGCCTAACAACATAATGTAGACAAACATCTTAAACCCATGACCATTTGAAGCTCTGACCATATGT
ATAGCCGCCCATTCGAAAAATCCAATTTCATCTAAAATTAATGAAATGAGTATAACAGCGACAAAGGTTAACG
TCGCATTCCAAACAATTCCAGTAACTTCAATAACATCCGAGAGACTGACAACGCCTGTAATAATAGCGATGAT
AGCACCAAACAATGCTGTAATACCAATATCTAACCCCTTAGGTTGCCAAATAACAAACATGAGAGTTAAAAGA
AAAATAATAATCGCTAGAATAGTCATTATGTACATTCACCTGTCTTTATCTTTTTACACATACATTGTTGAGTAG
GAGAGCTAATATAAGTTAATTCATTGTTGATTGAATCTAAGGACTTATGATTAAGTCGGTACATGCGCTTAGTA
CCGTCTTTTCGTGTAGATACTAATTCATTATCTACTAGAATTTTCATATGATGACTTAATGTAGGTTGTGAAAATT
GAAAATACGCTAATAAATCACAAGCACATAATTCACCGCAAGATAATAAGTCTAATATTTCTAATCGACTTGGA
TCCGATAAAATTTTTAATTGTAATGATAGTTCCTTATAAGACATATGGATGTCATCCCTCCTTTATTATAGATTATC
ATCTATATAGACAAATATCTATATAGATAATATTTTTTGCGGTTCTGTTGCAAAGTTGAATTTATAGTATAATTTTA
ACAAAAAGGAGTCTTCTGTATGAACTATTTCAGATATAAACAATTTAACAAGGATGTTATCACTGTAGCCGTT
GGCTACTATCTAAGATATACATTGAGTTATCGTGATATATCTGAAATATTAAGGGAACGTGGTGTAAACGTTCA
TCATTCAACGGTCTACCGTTGGGTTCAAGAATATGCCCCAATTTTGTATCAAATTTGGAAGAAAAAGCATAAA
AAAGCTTATTACAAATGGCGTATTGATGAGACGTACATCAAAATAAAAGGAAAATGGAGCTATTTATATCGTG
CCATTGATGCAGAGGGACATACATTAGATATTTGGTTGCGTAAGCAACGAGATAATCATTCAGCATATGCGTT
TATCAAACGTCTCATTAAACAATTTGGTAAACCTCAAAAGGTAATTACAGATCAGGCACCTTCAACGAAGGTA
GCAATGGCTAAAGTAATTAAAGCTTTTAAACTTAAACCTGACTGTCATTGTACATCGAAATATCTGAATAACCT
CATTGAGCAAGATCACCGTCATATTAAAGTAAGAAAGACAAGGTATCAAAGTATCAATACAGCAAAGAATAC
TTTAAAAGGTATTGAATGTATTTACGCTCTATATAAAAAGAACCGCAGGTCTCTTCAGATCTACGGATTTTCGC
CATGCCACGAAATTAGCATCATGCTAGCAAGTTAAGCGAACACTGACATGATAAATTAGTGGTTAGCTATATTT
TTTTACTTTGCAACAGAACCAAAAAAGATGATAACACCTTCTACACCTCCATATCACAAAAATTATAACATTAT
TTTGACATAAATACTACATTTGTAATATACTACAAATGTAGTCTTATATAAGGAGGATATTGATGAAAAAGATAA

AAATTGTTCCACTTATTTTAATAGTTGTAGTTGTCGGGTTTGGTATATATTTTTATGCTTCAAAAGATAAAGAAA
TTAATAATACTATTGATGCAATTGAAGATAAAAATTTCAAACAAGTTTATAAAGATAGCAGTTATATTTCTAAAA
GCGATAATGGTGAAGTAGAAATGACTGAACGTCCGATAAAAATATATAATAGTTTAGGCGTTAAAGATATAAA
CATTCAGGATCGTAAAATAAAAAAAGTATCTAAAAATAAAAAACGAGTAGATGCTCAATATAAAATTAAAACA
AACTACGGTAACATTGATCGCAACGTTCAATTTAATTTTGTTAAAGAAGATGGTATGTGGAAGTTAGATTGGG
ATCATAGCGTCATTATTCCAGGAATGCAGAAAGACCAAAGCATACATATTGAAAATTTAAAATCAGAACGTGG
TAAAATTTTAGACCGAAACAATGTGGAATTGGCCAATACAGGAACAGCATATGAGATAGGCATCGTTCCAAA
GAATGTATCTAAAAAAGATTATAAAGCAATCGCTAAAGAACTAAGTATTTCTGAAGACTATATCAAACAACAA
ATGGATCAAAATTGGGTACAAGATGATACCTTCGTTCCACTTAAAACCGTTAAAAAAATGGATGAATATTTAA
GTGATTTCGCAAAAAAATTTCATCTTACAACTAATGAAACAGAAAGTCGTAACTATCCTCTAGAAAAAGCGAC
TTCACATCTATTAGGTTATGTTGGTCCCATTAACTCTGAAGAATTAAAACAAAAAGAATATAAAGGCTATAAAG
ATGATGCAGTTATTGGTAAAAAGGGACTCGAAAAACTTTACGATAAAAAGCTCCAACATGAAGATGGCTATC
GTGTCACAATCGTTGACGATAATAGCAATACAATCGCACATACATTAATAGAGAAAAAGAAAAAAGATGGCA
AAGATATTCAACTAACTATTGATGCTAAAGTTCAAAAGAGTATTTATAACAACATGAAAAATGATTATGGCTCA
GGTACTGCTATCCACCCTCAAACAGGTGAATTATTAGCACTTGTAAGCACACCTTCATATGACGTCTATCCATT
TATGTATGGCATGAGTAACGAAGAATATAATAAATTAACCGAAGATAAAAAAGAACCTCTGCTCAACAAGTTC
CAGATTACAACTTCACCAGGTTCAACTCAAAAAATATTAACAGCAATGATTGGGTTAAATAACAAAACATTAG
ACGATAAAACAAGTTATAAAATCGATGGTAAAGGTTGGCAAAAAGATAAATCTTGGGGTGGTTACAACGTTA
CAAGATATGAAGTGGTAAATGGTAATATCGACTTAAAACAAGCAATAGAATCATCAGATAACATTTTCTTTGCT
AGAGTAGCACTCGAATTAGGCAGTAAGAAATTTGAAAAAGGCATGAAAAAACTAGGTGTTGGTGAAGATAT
ACCAAGTGATTATCCATTTTATAATGCTCAAATTTCAAACAAAAATTTAGATAATGAAATATTATTAGCTGATTC
AGGTTACGGACAAGGTGAAATACTGATTAACCCAGTACAGATCCTTTCAATCTATAGCGCATTAGAAAATAAT
GGCAATATTAACGCACCTCACTTATTAAAAGACACGAAAAACAAAGTTTGGAAGAAAAATATTATTTCCAAA
GAAAATATCAATCTATTAACTGATGGTATGCAACAAGTCGTAAATAAAACACATAAAGAAGATATTTATAGATC
TTATGCAAACTTAATTGGCAAATCCGGTACTGCAGAACTCAAAATGAAACAAGGAGAAACAGGCAGACAAA
TTGGGTGGTTTATATCATATGATAAAGATAATCCAAACATGATGATGGCTATTAATGTTAAAGATGTACAAGATA
AAGGAATGGCTAGCTACAATGCCAAAATCTCAGGTAAAGTGTATGATGAGCTATATGAGAACGGTAATAAAA
AATACGATATAGATGAATAACAAAACAGTGAAGCAATCCGTAACGATGGTTGCTTCACTGTTTTATTATGAATT
ATTAATAAGTGCTGTTACTTCTCCCTTAAATACAATTTCTTCATTTTCATTGTATGTTGAAAGTGACACTGTAAC
GAGTCCATTTTCTTTTTTTATGGATTTCTTATTTGTAATTTCAGCGATAACGTACAATGTATTACCTGGGTATAC
AGGTTTAATAAATTTAACGTTATTCATTTGTGTTCCTGCTACAACTTCTTCTCCGTATTTACCTTCTTCTACCCAT
AATTTAAATGATATTGAAAGTGTATGCATGCCAGATGCAATGATACCTTTAAATCTACTTTGTTCTGCTTTTTCT
TTATCTATATGCATATATTGAGGATCAAAAGTTGTTGCAAATTGGATAATTTCTTCTTCTGTAATATGAAGGCTT
TTTGTTTTGAATGTTTCTCCTACTATAAAATCATCGTATTTCATATATGTCTCTCTTTCTTATTCAAATTAATTTTTT
AGTATGTAACATGTTAAAGGTAAGTCTACCGTCACTGAAACGTAAGACTCACCTCTAACTTTCTATTGAGACA
AATGCACCATTTTATCTGCATTGTCTGTAAAGATACCATCAACTCCCCAATTAGCAAGTTGGTTTGCACGTGCT
GGTTTGTTTACAGTCCATACGTTCAATTCATAACCCGCTTCTTTTACCATTTTTACTTTTGCTTTAGTAAGTTTG
GCATCTTCAGTGTTTACTATTTTAGCATTACAGTAATCTAAAAGTGTTCTCCAGTCTTCACGAAACGAAGTTGT
ATGGAATATAACTGCTCTGTTATATTGTGGCATGATTTCTTCTGCAAGTTTAACAAGCACAACATTAAAGCTTG
AAATGAGCACTTCTTGATTCTGATTTAAGTTTGTTAATTGTTCTTCCACTTGCTTAACCATACTTTTAGAAAGT
GCTAGTCCATTCGGTCCAGTAATACCTTTTAATTCTACATTTAAATTCATATTATATTCATTTGCTATTTTTACTAC
ATCATCGAAAGTTGGCAAATGTTCATCTTTGAATTTTTCACCAAACCAAGATCCTGCAGAAGCATCTTTAATTT
CATCATAATTCAATTCAGTTATTTCCCCGGACATATTTGTAGTCCGTTCTAAATAATCATCATGAATGATAATCAG
TTGTTCATCTTTTGTAATTGCAACATCTAACTCCAACCAGTTTATACCTTCTACTTCTGAAGCAGCTTTAAATGA

TGCAATTGTATTTTCCGGAGCTTTACTAGGTAATCCTCTATGTCCATATACAGTTAGCATATTACCTCTCCTTGCA
TTTTTATTTTTTTAATTAACGTAACTGTGTTATCACATTAATCGCACTTTTATTTCCATTAAAAAGAGATGAATAT
CATAAATAAAGAAGTCGATAGATTCGTATTGATTATGGAGTTAATCTACGTCTCATCTCATTTTTAAAAAATCAT
TTATGTCCCAAGCTCCATTTTGTAATCAAGTCTAGTTTTTCTGTACCCCTTATCTGCAATTTTACTTAGGATTGC
TTTTAACTTACCTCTTATCTGCAATTTTACTTTGGATTGCTTTTAACTTACCCCTTATCAGCAATTTTACTGAGA
ACTGCTTTTAACGCACCTCTTATCTGCAATTTTGCCTAGAACTGCTTTTAACGTACCTCTTATCTGCAATTTTAC
TGAGAACTGCTTTTAACTTACCCCTTATCAGCAATTTTGCATGGAATTGCTTTTAACGTACCTCTTATCTGCAAT
TTTACTTAGAACTGCTTTTAACAAACCTCTTATCTGCAATTTTACTGAGAACTGCTTTTAACTTACCCCTTATCA
GCAATTTTGCATGGAATTGCTTTTAACGTACCTCTTATCTGCAATTTTACTTAGAACTGCTTTTAACAAACCTCT
TATCTGCAATTTTACTTAGAACTGCTTTTAACGTACCTCTTATCTGTAATTTTACTGAGAACTGCTTTTAACAAA
CCTCTTATCTGCAATTTTACTTAGAACTGCTTTTAACAAACCTCTTATCTGCAATTTTACTTAGAATTGCTTTTAC
TATTCCTCTTATTAGTATAATCTCAGTAAGAATGCGTATAAAAATGAAAATTACAACCGATTTTGTAAGTGCTGA
CGCCTGAGGGAATAGTATGTGCGAGAGACTAATGGCTCGAGCCATACCCCTAGGCAAGCATGCACGTACAA
AATCGTAAGATAAAAAAATAAGCATATCACTGTAAACTTTAAAAAATCAGTTTAGTGATATGCTTATTTATTTCG
AGTTAGGATTTATGTCCCAAGCTCATCAAGCACAATCGGCCACTAGTTTATTTCTCTATCTTATATGTTCTGATA
TGGTCTTCTATACTGTATAAGTATACTTTTGAATATGGATCTTGTGTCAATTCACGTTCGAAATCAAATTCTTGA
TTATCAAATCTGTTAAAGAATGTTTCGTATTCTTCGACTGATAATTGCTCTCTAGATTCTAGCATATTTAAGTGTT
TCTCTTTATCTAATGCTTTGTCATATCCTTTAACGATTGAACCACTAAAGATTTCTCCTACTGCTCCTGAACCATA
ACTAAATAGACATACTTTCTCTTCTGGTTGGAATGTGTGGTTCTGTAATAACGAAATTAAACTTAAGTATAATG
ATCCTGTATAAATGTTACCAACATCTCTATTCCATAATACGGTTCTGTTGCAAAGTTGAATTTATAGTATAATTTT
AACAAAAAGGAGTCTTCTGTATGAACTATTTCAGATATAAACAATTTAACAAGGATGTTATCACTGTAGCCGT
TGGCTACTATCTAAGATATACATTGAGTTATCGTGATATATCTGAAATATTAAGGGAACGTGGTGTAAACGTTC
ATCATTCAACGGTCTACCGTTGGGTTCAAGAATATGCCCCAATTTTGTATCAAATTTGGAAGAAAAAGCATAA
AAAAGCTTATTACAAATGGCGTATTGATGAGACGTACATCAAAATAAAAGGAAAATGGAGCTATTTATATCGT
GCCATTGATGCAGAGGGACATACATTAGATATTTGGTTGCGTAAGCAACGAGATAATCATTCAGCATATGCGT
TTATCAAACGTCTCATTAAACAATTTGGTAAACCTCAAAAGGTAATTACAGATCAGGCACCTTCAACGAAGGT
AGCAATGGCTAAAGTAATTAAAGCTTTTAAACTTAAACCTGACTGTCATTGTACATCGAAATATCTGAATAACC
TCATTGAGCAAGATCACCGTCATATTAAAGTAAGAAAGACAAGGTATCAAAGTATCAATACAGCAAAGAATAC
TTTAAAAGGTATTGAATGTATTTACGCTCTATATAAAAAGAACCGCAGGTCTCTTCAGATCTACGGATTTTCGC
CATGCCACGAAATTAGCATCATGCTAGCAAGTTAAGCGAACACTGACATGATAAATTAGTGGTTAGCTATATTT
TTTTACTTTGCAACAGAACCAAATGAAGACTATTTAGAAAATGAATATAAGCTTAAAAGTTTTGAAGAATACG
CTGAATATTATACTAGTAAAACTAATAATAAAGAAAAAGTTATGAGAAACACTTATCAATTAAATGAGCTATTAC
ATAGTGTTGGAATAAGAGAAAAAATTAAGAAGTCAATTTGTTGGAACTTGTTTATTATCTTTAAAATATAGTAAG
GTTGATCTAATAAATCCAACTCTTAATACAGATATAATACGAAGTGAGATGAAAAGAATTTTAAGTACGTTATT
ACAAAATGATTTGCATAGAGCTTCTAAAGTATCATTACTAGATAAAATATTTTGGATAGCCAAGATATACGAAC
TTTAGAATCGTCTTCTTTTAGAAAAATATTAAATTATATTAATATAAATATACTTCCATATATAAATGATAAATCTAC
TGCTGGTCAGGATTTATTAAATTTATTTTTTATTACCTTCAATAAATATGTTGGTAAAGAAGATAAAAACCAAG
CATTTACGCCAGATCATATTACTGATTTTATGGTTAAAGTAACAGAAGTTAATAGACATTCTAAGGTTTTAGAT
CCATGTTGTGGTAGTGGTTCTTTTTTAGTGAGAGCTATGACTCAAGCTTTAGATGATTGCGCAACCGCTGAA
GAACAAGATGAAGTAAAAAGAAACAATATTTATGGTATTGAATATGATGAAAATATTTATGGTTTAGCTACTAC
TAATATGCTAATTCACGGAGATGGAAATACAAATATTTTTCAAGATAGTTGTTTTCAATTAAATGACCAAATTG
CAAAGTGGGGCATCGATGTAGTACTAATGAACCCCCCATATAATGCTACTAAAAGTTACATGCCTAAAGAATA
CACTGATAAATGGACATCTAATAAAGGTCAGGATCCTTCTAAAGGATTTTATTATGTAAAAAAAACAATAGAA
GCTGTGAAAACTGGAAAAAATGGCCGTATTATTGCCTATGGCTTGTGCTATTGGTAATAATAAGGAAATAAAAA

AATTAAAGAAGAAATACTAAGAGAAAATACATTAGAAGCGGTTTTTTCTTTACCTGATGAAATATTTTATCCTG
GAGCGTCTTCAGTTGCTTGTTGTATGGTCTTCACATTAGGAAAACGTCATGATTCAACAAAACCTACATTTTT
TGGATATTATAAAAATGATGGATTTGTAAAGAAGAAATACTTAGGAAGAATTGAGAAAGTTAATGAAGATGG
ATTAGGAGCATGGACAGAAATTCAAAAAAAGTGGCTTGATTTATATTTTAATAAGGTTGAAAAGCCTGGATT
ATCAGCTTTAAAGTCGGTAACTGGAAATGATGAATGGTTAGCAGAAGCATATATGAAAAGTGACTATAGTAAT
TTAAAACAAGATGACTTTCAAATTACTGTAAATAACTATTTAGCTCATTTAGTTCAAAAGGGTGATTTACATGA
ATCTGAGTAATAAAATTTGGAAAGAATTTTCATTAAATAACTTATTTGATATTAAACCAGGTATTTATCACTATC
CTGAAGAATATGATGAAGGAGAAACTGCTTATGTTTCCGCATCTAATACTAATAATGGAATTAAACAAAGAAT
CAATTTAAAGCCGGATTTTAAAGGAAACTGCATTGTAACAGGTAAAGTAGGATGTACAGCATTTTATCAATAC
GAAGATTTTTGTGCAACAAGTGATGTAAATATTTTAACTCCTCAAAACTTTCAAATGAATGAAGACATCGGAT
TGTTTTTTGTCACTATTATCAATTTTAGTGAAAATTATAAATGGAATTATGGTAGACAGTGTCGAGTAAGTAATA
GTAAAAAAATTATTATAAATCTTCCGGTGGTTAGTAATAATGAGAGAGCAATTATTGATAAGAAAAAAATTTTT
TCTGAAGAAGGATATATTCCGGATTTTGAGTTTATGAAAAATTACATAAAATCGCTTAAAGGGAAACCTATCA
CAACTAAAAACAGTCGAAAAAATAAATTATCAATTTTTAATAGAAAATGGGAAGAATTCAAATTAGGAAATCT
ATTTTCTGAGATTTATAAAGGAAAACCCCATATAAAAGGCGAATTATATTTAACAAATTCAACTGATGCTCAAG
GAATTAATTTTATTAGTAGAACAGATACAAATAATGGCTGTGATGCATATGTTTTAGATAAAAATTTAAAAAAT
ATAGAGCATAGTAATGCAATAACTATAGGTGACACGACTGCAACAGTATTTTATCAAAATCAAAGATTTGTTAC
TGGAGACCATATTGTTGTTTGTAGGGCTAAATGGATGAATAAATATACTGCATTATTTATGAAAACCATTATCG
ATACAGAAAAGTATAAATATAGTTATGGAAGAGCTTTCAAAATGAATCTAATTAAAAATACAGTTATTAAATTA
CCGGCAAAAAACGATAGCGAACCAGATTGGGAATTAATAGAAGATTACGTAAAATCTTTACCTTTTGCAGAT
AGAATATAGTGAAAGCTTTAAGTCTATAATCCCTTCCAAATTGAATATACTCATTGGAATAATAATATGAAAACT
CAATTACAAGAGCCTGAGACGTAAGTTCTAGAAAAATAAGCAGTAAAAAGTTTACTATAATTTCATTTCTCCG
CACATTAATTTATGATACTTCGTATTATTAGTTCGCTTTCTTATCGAGACTAGTTTCATTCTAGTCTAATACTTCA
AGAATCTCGTTAATTTACTAAAGTCATTATGCTCAGTTTAATGCTTTAGATGACAAACTAGAGTACAAGAGGAT
AGACAAATTAGCTTTGAGCTAAAAATTCACGACTAATACTAATATAGCATTACTATATTAATTGAAATGTAATAT
CGTGGAATATGTAACGAAGAAAAAACAAAAATTAAATATAAGAGCATCCCTCACCGCTAAGTGAGGGATGCT
CTATTTTTATTAGAATATAGTTTTCATTTTGATAGATAATGTTTAATAATTGTCTGTAAATCATTGTTAAGTACCGT
TGTAATAGAGTCATCATTCATAAAATCATCATAGACTTTATCCAATATTTCTTCATCTTCAATTGCTGCGAAATGA
CTTATTAGACCTTTTACTATTGATACATAATCTTGTGATGCTAAACAGCTAGCAACCGTTCTCCAATCTGTTTGT
TTAGTGTCATGGTTCATAGATAATCCTCCTTTATTTCAATGTCCATTTTTGACGTGCTTTAGGATTAAGTGGATG
CATAATTTCATTTGTGGCTGGATTAATGAGCTTTTTAATTTTCTTTTTGTGAGGCTTCAACATTTCCATCACTTG
CTCAACACGTTCAACGACAACTGGCCGCTTCGCATAAGCACCATAAGCAAGAATTACTGTGTCACTCTCACTT
ATCGCTTTCATCAAGTGGATGTCTGTATGTTTATCAAAAGGTTCTTTGATATGTTTAAGGTTCTCTGGAGTTTT
AATATTAGAGAATAGATTTACAAGATATACTGCGCCATATTGTTCAGAATTCGCTAATTGGTTAAGAATTAGAA
CAGTGGTAAGATCGAGTGATAATACGCCATCTAAATGAGGATACATCGTTATCACTGTACATGCGGGTTTGTT
TTTATCCCAAGTCTTTTTGAGTAAGTAACGATGTTGTTCATCGTCGCTAAAAATTGCTTCTGTGTGTATGGTGC
TTTTGACCGTATTCATAGTATTCACTTCCTTTAGTATTCTTCAGGTAACAACATGACGTAATAAGAAAGGTCTAC
ATCATCTTCTCGGATAATGTAGACCTTATTGATATCGATAGACTGTTTGACATTTGCCTTATGCGTTTTGACATA
CTCAGGACGTTCTTGTTTGTGTTTGATATATAGTTGGTTATTGTGCATATTGAATTGGAATATATGAAAATAATC
CAATGATTCCTTGCCTTTTGATTGTGTTTGTTCTCGTCGTGCTACGAGTTGCCATAATTGTTTTTGTAAGATGAT
AGGTAAGCTATTGGCAATACCACGAGTGATATATCTATTCATAAGCGTTTTCCTTTCGTTCTGATTCTTTCATCC
AAATGATAATTTCACTAAGAATTGTAATGACGAGTTGTGTAATTTTGATGTATTTATGCATATTATTGTCCTCCT
GTTAGTAAGTATCGTTGATCCAAAAGGGGCATGATAGTGTTGTGTAGGTATAAGAAATCCATATCGTGGTTTT
CATCAATATAACCAATGGCTATGAGCTGTTTCTGATAGTAGAGAATGAAAGGATAGATATTACTTTTGTGAAGT

GTATCAGAGTAGTAGGTGTAGGTTTGAAGTGTTTTATTACTGAGTGGTCCAAGCCTTACATATTGGTCAGGTA
AGATATGATCAGCGATTTCCTCGATAGATTCACATTCCCACTTTTCATCTTTTGGCAGTTCGAATAAGTTGGTT
ATATAGTGCTTGAGTTGTTGTTCTAGTGTCATGGTTATGACCTCCTAATGTATTTGATTGATGTATGTACGTGAT
ACATCTTATTGATAATATAAGTTTGAAATAATGTTTGTTTCGCTTAGAAATCCCACTGAAATACAGAAAAGATC
CCAATAGAAAGTCGTCACAATTGTTGATATAACAAGGAAATCCCGTTATCCCGCTCATTTCTCGGGAAATATAG
CTATATATTTGTATATCATTTGGATTTGTAGATGTCAGTACGACTTACTTTTTCTTTTTATATTATTTATTTATAAAT
AATAATGGGATTTCGGGATTACGCTTGCGTAATCCTTTTCTTATATAGATTTATCAAATCCCATTATCATCCCGTT
CATTTTTTATTTTGGGATGTTCTTTCGTCATCCAATATAAGCATCCGTGTTCGTTAGGCTAGCTTCATCTATGGA
TAGAATATATGAGATGTGTGTTATTGGATGACGAAAGGACAGTATCCATTATGAGATAGGCAAATAGAGTGTT
TGAGATAATAATAAAGACGAAGTGCTGAGTTGCACTTCGTCGAGAGGGGTATTATGGAAGAGTTGTTTAATG
ATTTCATGATTGAGTTTTAATGTGACGTAGAACTGTTTTTTATGATGTTCGTCTTTACGAATATCAATGTGGTCT
ATGACCGTAAGGTATAGTGCTTTGAGTTGTGCTTTATCCATGGTTTCGATATTTTGAATATATGACGTAATATAG
TTGCAATCTCATTAGCGTCATATATTGTTTTGGGAGCACTTTGTTGTTGCTTAAGTTGATTGATTTGATTTGTG
ATGTCATTGAGTTGTTTTTCATACTGATGAATCGTATTTTTAAGCACAGATGTTAAGTCTGGATTATCTTCAAGT
GTTTTGATCAAGTTATCTAATTTGATTTGGACCTCATCATATTGTTGTTGCTTATATGCGATATCATGATTGAGTG
CAGACGTATCAATTTGATGTTCTTGATTGACACGCTCAACGACTTGTTTGATAACTTTGTCACTTTTAACGATT
TCGAGTATTTGATCCATAACATATTTTTCAATTACATCGGCTCTGACACTATTCGCAGAACATACTTTTGACCCT
TTATTACGAAAGTTACTACACGAATAGTAGCGAATGCGTTTTTTAGTACCATCTTTAAGTGTATTAACTGTTGT
GCTCGCAGACATTGAAGCAGAACATTGGGGACACGAAATTAATCCAGTTAAAATATTGGTTCCTTTACCATG
AACTTGTGGTTTTTCACTTACTTGTTTTTTACGTGCTTGGACTTTATCCCATAATGATTGGCTAATAATAGGGG
TGTGTTTGCCTTCGGCGATTACTGGTTTATCATTCAAGCCTTTACGACGTTTATCATTCCAATCTTTGTATTTCG
CGAATTGGATTTTACCAATATAGAATGGGTTTGAGAGAATATAAGTAACAGCTGAAATACTAAAAGGATTACC
TTTCTTAGTGACATAACCTTTATGATTGAGTGCGTTGGCAATTTTACGATAACCATGACCTTTGGCGTAAGATT
CAAAAATATATTTCACAATATTAGCTTCATGCTGATTAATCATCAAATCTTTTTTATTATCTGGTATGTTATTATAT
CCTAAAGGAAGATTACCTTGGTAATAGCCTTCTAAGGCTCTTTGATGTTGTCCGGTGTAAATATTCTCTAAAAT
GGTATTTCTTTCAAATTCGGAAAAACTTGCAAGTATATTTAATAATAATTTACCTGTCGAATTTTTGACTTCCAT
GCGTTCAGACAAGCTGAAAAATTCAACATTTTGACGATGTAGTTCTTCGACGATTGTCAGTAAGTCAGAAGT
ATTACGTGCTAAACGATTTATTTTATAAACAATCACACAGTCTAACTTTCCATTTTTTGCGTCTTGTAACATGCG
CTGTAGTGCAGGGCGATTCATAGATTTTCCAGAAATACCCCGATCTGCGTATATATCAACGAGGTCATAACCAT
TAAATTGGCAAAATTGCTCTATTTGTGTAATTTGACCATCTATACTATAACCTTCCACTTGTCTCTCTGTGGACA
CGCGAATGTACGCTCCGATTCTCTTCTTCTTCATTTCATTCATATTTTTTCATCCTTTCATCTATTAAGCAATCGA
TGATTGCGTCGTTTGATTGACAATGTTTAGAGGTTCATTTTTGAAATAGATGCCAGCGAGGCTTTTATTTTTA
GAAATATTAATTTCATCAATATAGGGGTACAACATGTTTAGCGTGAAACGTTGTTGAATGATGTTTTGGAAAG
CTTTTCGAATTTGATACGCACTGATTGATGATATAGGTTTTGATTGTTGATGTAATGATTGCGTTTGTTCTCTGA
ACGTTTCTGCATCAATTTTGCCTTGGGCTAATTTTTCTATCAGCTGCTCGTGATTGAGTGTAGTTTTAGTTTCTA
TATCTCTTTGTCTTTTGAGTCGTTGTTGAATAGTATGGTTTATTTTTGAATAGAGCTGTTGATTTTGAAAGAAG
TCCTGACAAGTCGCTAAAACACTTGTTTCTAATTCTTGTGCGTTGATTCCTTTAAATTCACAAACAAAGCGAG
CGTTATTCATATTTTGAGGACAGACATAGTAACGCAATGAATGATTTGGTTTTCGGACGGTCATGTTTGTGAG
TGTCGAATGGCAATAGGGACATTTGATTTTTTGTTTGAGTTGATTTTCTGACGGCTTACGTTTGACTTGTTTC
TGAGTTCGGATAACTTGAGCTTCTTCGTATATCGTTGTACTGACAATAGCTGGGAACATGTTTTCATATTGTCC
GTATTGATTGATAACACGGCCACAGTAATTAGGGTTAAGGATAATATTACGCACTTGATAGGGCTTACGATTA
ATGAAGTTATCATCAGCTTCTAAGTATTGCGCAATTTTTTTATAACCATAACCTTGAAGGTAATAATTGAACAC
AGCTTTTACTGTTGGTGCTTTTACTGTGTCTATCGTGAAAGTACCATTATGATAGTGATACCCAAAGGGTGCAT
GTGTTGTAATCATTTTACCTTGTTTCGCTTTTTCTTTGATTCCATTTTTGACTTGTTCGCCTATATTATCAGATTC

TAGTTCGGCCAAGCTGATGAAAATATTGAGTTTGAGCCGATCGAATGCTTTATCCATGTCGAAGTAGCCATCA
TGAACGCTTAAGATATGAACATGATACGTTTGACATAATTTCATGAGCTTTAATGCATTTTTCAAATTACGATG
AAGTCGATTTAGACGATAGCAACATAATACGTCACATTGTCCCTGTTGAATCAGTTCAGTAATTTGTTGATAAC
CATTCCGCTTATTAGTACGTCCTGATTGTTTATCGCTATAAAAGGTAATGTGTTGAATATTATGTTTTTCGGCTA
ATACCTTGATGGTTTGTTTTTGAGCTGCTAAGGATTGTTGCTTTGTAGTGCTCTGTCGTAAGTAACCAATTGCT
TGTTTCATCTTGTTTCCTCCTTTCGAAGTGATAATATATATTTATGAACGAATTTATAGATGAGCCCAACACCTG
CCTGGTGTTGGGCGTTATTATTAGTCATCAGCATGATTAATTTCTTCAATCACTAAATCAGCGAGTAATGTGAT
TAATTCGTCCATATGGTTCAACCTCCAAATATCAAATTATATTGAATTGATAACATGTTGCATTGTTATTCGTTCA
CTCCGTGTACTTGGAACAATATGTTCAGTTATCTCTTTTTCATTTATGAATTCAGGGTACAAGTGATAAAATTTT
AGTAGCTTCTGTTCCGTTTCAGTTAATACATATTGTTGCATATGGTAGTATCTGTCGATATAATTAACGACAATA
GGTTTTTGATGATCATCGACTTCTACCGAATCAATTGTGTAATTTAATAAGTTGACTTTTGTTTTCATTTTTATG
CCTCCGATTAATTTAATGAGCTACAACGCACATAGCGCGTTGTAGCGATATTCATATTTAGAGTTCTAAATCGC
CCTTTTCTTCCCATTCTCTACCGTCTTTAACAAATTTGTTGAGTAATTCCTTATTTGTTGTATTATTATCAAATTG
CGGTGGTGTGAATGGTTCAGAATTTGAGCTTAAACCGAGAACAGGTGCAAACTCACTAGGCAATACAAGAT
GGTAGAACAAGATCGTTTTTGTTTTGCCTTGAGTATCTTTGACATTACGTTTGATAGCTATCCGATCGGAATCC
ATCTGGATATATCCTTCTGCTCTTAAACTATCTATTACATTTTTTACATCTTGGTATTGGTTTTCTTCTAACATATG
TTTAAAAACATCTTTAAGCATCTTGACTTGAATGTGATCGTCTTTCAGCTTAATAAGGCCATAATTTTCAGTCAT
TATGGAGAGTTTTTCATTTTCCGCAAACTTACTACGATTGCGTGCCACGAACTGAACTACACTATCAATCGCCT
TGGCTCCCAAACTGCGCTCACTTACGGAATCTACATGATAACTAACTAGATATTCTCTAACTGCATCCAAATCG
ATAGGAGTTGCGATTACACGTTCTAATATTCGTGCTGAAGTCGTAATGGTGGCATAGCGCTTGAACATACGTA
TACCTACATTGTTTGTTTCATTGTTAAGCTGTACTTTGAACCAATCGTGTTCTTTATAAAACCACTTGATTACTT
CAGATTCACGATTTAGAAGGTATTCAGCTACTAACGGCATTACGTGTCCATAGTTTTTTGATGTTCCTTTTTTA
ATGGCGTCTGCATTGTCAGCACTTGTTGTAAAGACATCAGAAATTTCGATGCATCTTGCTCTCAAACCATCATT
TGCACTAGCATCTTTAAAAATCGTGTGTTCTGCTGTACTAATCACACTAGATCCAAAATGGTGAGGTTTTTTTA
CATTTCCCTCTCGATTAGCACGTAGGCGACCTTGACCTTCTGCAAGCGAATAAAGCAAACCATTGGTATCTTT
AAAGGTCGCTGCTGATAATTCGTCTAATACAATAGGAACACCAAAGTTGTTACTTAAGTATCCTTCAATTGCAT
TACGGGTGCCGTTCCAACCTCTGAATAATGTTTGGTCACCTTTAGATGGATTACCTGCTATTGAAACTGCTAAT
GCCGCTGCAGTCGATTTACCCGTACTAGAGTTCCCCATAAATGAAAATATCGTGCCAAAATATTCGACTTCATG
CTTTGTTTTTAAGAACGCAGTCACTAATGAAGAAACACCAAATATCACAGCAAGCTCCAGTAACAAGTTTCC
TTTTACTTCATCAAGATACATTTGCCACCAGCCTTCAAATGTACCTTTAGGTTGTAAGTCATACTTTGTTTCACA
AATGATTTCATCAGCCTGAGACTGCTCGATGTCTTTTGAAAGATATGGTTCATCTAATGAAATGACAGTACCTT
CATCAGTACGTAAGACACCTACACCTGTATACAATGTAGAAATGCGTAGTGATTGACGCATTAATTGTAGTGC
ATTACCTAGCGATTTAATGTACTTTTCATTGATGCTGAACCCAAACATAACCAGTCTAGGTAACTTTTGTGTCG
TTAGAATATCTGACGTTTCAATACGTTCAATATTTTTGCCATCCGTGATAGTCAATTTCTCAACCCCTGTAGTAG
GGTCGAGAAATTTGTTTTTTATGATGATTGGACTAGATAAACGAATTATCTTATCTTCGTCATTTTTGTTTTTTG
CTCGAATAAGTTCATACCATGCAGTTGAATCTAACCAGTATGGATATTGCTTGAATATGTTGTTCGTCATTAGTT
GTTACCGCCTTTCCAATTACGCTTTTGACGCTTTGGATTCGGGCCTGTTTTCAGATAAACTGAGTTCCATTTAC
TATTCTTACTAATAATGATAAATGGAACACTTGGTGTGTGCTTTACAAAATATTCACCTTCGTACTTCATAATTAT
TTACCTCCGAATGTATTAATTTGTGTATCGGCCGAATACATATATAACTATATAGGTAATAGTGTGAGGAGTAAG
TCTGTAAAATAACCTTTAAAATTCCAGACAAAAATTATGAAGAAATATATTGACAAAAAAGAAGTGCCGATGA
AAAATCGGCACTTCTATAATGATGGTTACTATTTTGACGTGATATAAAAATATTCAGAATCATCAAGGTTATTTT
TATCGTTGTTGTTCTTTAAATGATAATTTAAAGCTTTTTTTAATTCAGAAATAAGTTTGTTGCGATTTTCTTTGG
TGTACTCTGGTGGATCTACTAGCTCGCTTTGACTTAATAATCTAGTTACTAAGGATTTAGGGGCAGATGCTTGA
GTTTCTTTTTTAGTTAAATACTTTGCAAGGTTTACGTCAATTTGAACACGGCATTCTTGTTCTAAATAGTCACT

ATAGCTATAAGATTGGGTTGCAGCTAATCTTGCATTAACTTCGTCCTTGAGTTCATCTAATTCATCCATATAGTA
ATTAATTATATCTATTTTTTGAGCACTGCTAACATTTGGTGCTGTTATAATATCGATAACCTTAGATTTTATACCA
ACTTCGATATTTGAATCGATTAAGAAATATAAATAATAATTTGTCATAGCAGTTTTATAATCAATAGGATTAGTA
ATTTCATTTTTTATAAAATAATATATGTTATCATTAATTTTATTTGTAGATTCTAAGTCTTTTTTATTAACGCCATTT
TTTAAAAAATGGATATAATCAGAGTTATTCGACAATATATCATGTATAACAAGTTTACTATCATATTTAAATGCAT
AGAATTTTCCAATTTTAAAATTTTCAGAATCAATACCAAGTGCAAGACAAAACTTTTTGAAATTTGCTGAAAA
GTTAGTTTTAGTTGGCACAAATAAATCATGATCTTCCCACTTTTTTGGTTTGTTTTCCTCATCTACATTATTGCA
TCCATCAAGAAGATATAATTCATATTTAACTATGTCATTTAAAATCATTGGATATTTTTCCTTGTTACGAGTGTAG
CGTTTACTATTTAATTCCTCATAAGACTTACTTGCTTGACTCATCACTTCTTTTACGTCTTTCGTATCTATATTTTC
AATTATTGATTGTAACTCACGTCCCCAATTGCTATACATTAAATCACTCATTTCTATTTTTTAAATAATTATATCTT
AAGAATTGTGAATATATAGAGAGAACATAGGTTCTTGATTTGTTATAATTATTTGTATTAAGTAATTTCGTTATA
AAGTGGATTGAACAATATATTGCAAGTCTTTTTATTACAGATAATTACCGTAATAAAGGTATAGGAAGGTTTTT
TATTGGAAAGATTAAAAAGCGATTATAATGTACTTAATTTACATGTAAATGAGAAGAATGAAGGAGCCATTCG
TTTTTATACAAAACATCAATTTGAAATACGATCTAAGGAATTTGAAGTAGAAACTCAAGAATATGAATATTTTA
TGAAATGGGATAGTAATTGAAATTACAAAAGTACCAATAAGTAATTTTATGTAAAAAGATAACAAATAATATAC
TCTATTAAGTCTTACAACTTGAGAGAGCTTTTTGTATATATTAAGCATTTATGCTACTAGTTGATATTAGATGTAA
AGTATTTATAGTAGTAATATATCGTTTTATCTAGCTGTACGAAAAATCAATTACAAAAAACCCCATCAACAACCG
ATAAGCAGAACTCGTCAAGAGATGTCAGATAAGATAGTTTTAGTACTCTGTAATTTTAGGTATAAGGTATATTA
TAACCGTTTTAGCCTATCCATCCATCGCCCACTCATTCTATTCTAGCTTATCCACCTATCGCCTTTACATACACAC
TTCATCTTTAAAATCTAAAATTTCCTTATGCTTATTCATTAACTATTCCTCCATACTCTTCTCATCGTTCTTTTAATT
CACACACTCCCTTTTCCCTCGTCACTCAGTCATGGCAGTTTGTAATTTTTGTTCAATTAAGTCGATATGTGTACT
GATCAAATTCCGTTGTTCATTCAAGAATTCTAGGTGTGCGCGTAACACGCTTGGGACATCTATTTGGTTTTGA
TAAAGCATGTCTGTGTTTTTTCGGATTTCTTTTAATGGCATATTCAAATTGCTTAAGCACTGGATAAATTTAATC
ATTTCGATATCGTCGTCTGTGTATAAACGCTTGTTATTGCTATCTCTGTCGATGTGTTTTAAGATACTCTTTTTCT
CGTAATAACGCAGTTTGCTTTTAGGTATATTGCAATAATCTGAGACATATTCAATGTAATATTGTTTCATGTAAG
CATATCCTTTCTCTTAAACCAAGGTTTAACTGATATTGTATTCTATATAAACATGAATGAGGAGGAAAGACAAT
GAAAAAGGCTTTAATTGTTGTAACAAACATTGCGAAATACGATAATTTAGAAAGACCTACAGGTGCGTGGTT
CTCAGAGGTTACGCACTTTGCGAAAGATTTCTATGACGCAGGTTATGATGTTGATTTTGTAAGTCCGAATGGT
GGGTATGTGCCTCTTGATCCTATCAGTCTTAGCCCTGAAATGATGGGAGCCGAGGACTGGGAATACTACACG
GATCATGATTATATGAATAAATTTGGACAAACATTATCTCCAAAAGAGGTTAATCCTAGCGACTATCAAGCCAT
TTACTTTGCAGGTGGACATGGTGCGATTTGGGATTTAAGAAATAACAAAGAACTAAACGACATTGCGCTAAG
TATTTACAATAACCAAGGCGTTCTCTCTTCTGTATGTCATGGTGCGGCTGGTCTACTCGATATTAAAGAAAATG
GCGATTATCTCGTTCGTCATAAAGATGTGACTGGTTTTACAAATAGTGAAGAACAAGAAAATGGTACAACTG
AATACATGCCATATTTATTAGAAGACGAATTTATTAGTAACGGTGCACATTTTAAAAAAGAGGCTGACTGGAG
TAATTTTGCAGTCGTAGATGGTCGCATTGTCACAGGTCAAAATCCCCAATCAGGACATGCAGTTGCTGAAAAT
GCTTTAAAAATCTTAGCTAATCAATAATTTTATAAAGGAGCGATACACATGAAATCATTAATTATCGGTGCTAAT
GGTGGCGTCGGTCAACATCTCGTACGTAAACTGAAAGCAAGAGATGTTGATTTTACTGCCGGTGTTAGGAA
AGAAGAACAAGTTGAAGCTTTAAAAGCAGATGGTATCGATGCAACTTACATTGATGTCGCAAAACAATCTAT
TGATGAATTAATAGAATTATTTAAATCGTATGATCAAATCCTTTTTTCCGTCGGTTCTGGTGGGAGCACAGGTG
ACGATCAAACAATCATTGTAGATTTAGACGGTGCAGTGAAAGCAATTAAAGCAAGTGAACAGATCGATCATC
AACACTTTATTATGGTATCAACGTACGACTCACGCCGAGAAGCGTTTGATGCGTCAGGCGACTTGAAACCAT
ACACCATTGCTAAACATTATGCGGATGACTACTTAAGACATGCAAATTTAAAATATACCATCGTGCATCCAGGC
GCTTTAACAAACGAACATGAAACGCAACAATTCAATATGAGTGCGCAATTTGAAAATGTACAAAATCCGTCTA
TTACAAGAGAAGATGTAGCAGAAGTGCTTGTTTCTGTATTAACTGATGAAGTATTACAAGGTCACGAATTCCA

AATCATCAATGGTGATTTGTCATTATTAGACGCAACGACTAAATATCTGGAGGAATAATAAATGAATAATCAAC
AAGTTGTACTTGCAAAACGACCACAAAGTATCCCTCAAGACGATGTATTTAGATTTGAAACAATAGAAACTC
GAGAACCACATGCAGGTGAGGTTCAAGTAGAATCCATTTATGTATCTGTAGATCCTTACATGAGAGGCAGAA
TGAATGATACAAAAAGTTATGTTCAACCTTTCCAAGTGAATGAGCCATTACAAGGTCATATTGTTGGAAAAGT
CACACAATCGAACGATGAACGTCTATCTGTCGGAGATTATGTCACAGGCATATTACCATGGAAAAAGATAAAT
ACAGTGAATGGAGACGATGTGACCCCTGTGCCATCAAAAGATGTACCATTACATTTATATTTGAGTGTTTTAG
GCATGCCGGGAATGACAGCCTATACAGGGTTGCTTCAAATTGGTCAGCCACAATCTGGCGAGACGGTTGTT
GTGTCAGCTGCATCAGGTGCAGTAGGCTCTGTCGTAGGACAAATTGCTAAGATTAAAGGCGCAAAAGTTGT
CGGTATTGCTGGTGGTAAGCAGAAAACAACATATTTAACAGATGAATTAGGATTTGATGCGGCCATTGACTAT
AAACAAGATGATTTCGTACAGCAACTCGAAGCGGCTGTACCAGATGGTATTGATGTGTATTTTGAAAATGTTG
GCGGCGCAATTTCTGATGAAGTGTTTAAATACTTAAATCGATTTGCACGCGTTCCGGTATGTGGTGCAATTTC
AGCATATAATAATGAAAAAGACGATATTGGACCACGTATCCAAGGAACGTTGATTAAAAATCAAGCATTGATG
CAAGGTTTTGTAGTAGCACAATTCGCTGATCATTTTAAAGAAGCAAGCGAACAACTCGCACAATGGGTGTCT
GAAGGTAAAATTAAATTTGAAGTGACGATAGATGAAGGTTTTGACAATTTACCTTCTGCATTTAGAAAGTTAT
TCACAGGAGAGAATTTTGGTAAACAAGTTGTCAAAGTCGCTGAAGAATAGAGAAGACTATGAAAAATATCAT
CGTAAGATTCATATTTAGTTTACTGTTTTATAAAAGTGGTGTTTCACACTTTAAAAATAAAGATATGTTTTTAAA
AATCGTGCCGTCCTATTTACCTTTCAAACACGCAATAGTTAAGTGTTCGGGCATATTAGAATTTATAATTGTTTT
TTATGTACTCGGTGCTAAAAATAGATCACGCGTAAGAAAAGTAGTACAAGGATTCCTTTGGCTCGTATTTCCT
GCAAATATTTACGCTGCACGTAAGAACATCTCTTATAAAGATGAAAGAGATAGAACAGATAGTGTTAAGCAG
CATGTCATTCGTTTACCATTACAATTTGTCATGGTCGCACTTGCGAAACTATTATAAGACTTATCATAGCCACGC
CTGGAACATTACTTTTGTTCCAGGCGATTTTTTAATATTTACACTTAAAATCGACGTATATCAAATGATTCTCTT
GACAACGCTTATGTGCTCTTTGTTTAAATAAGCTCATATCAGACATTTACTTTTCAATTTTTCTATAGTGATAAG
CTTCTAAATTAAGAAAAGCATAAGTTAATTTGCGTGTATGAGTATAAGAATAAATAAAATAATTATATTTTCATA
TAATTATTATGTGGCATTGTACCATTAATGTAATAATGTGGCCTGTAAGACAAATATGAGGAGGGTTCTATAAG
GTGCTTCTTATTATTGAAATTGTTAAAAAAACAAAATGCAATGAACTGACAAATTAAAATCGCTTTAAGTCGT
ATTCTCAAAGAATCTATAAATCACTCGTATATACACAGCCCCTTACTATCAGTTAAAGTGTCACTTTTCGGTTCT
AATATTTATTATTCATAAGATATGCAATATATGATACAATTCATTCAAATGTATATTTGAATGAGGTGCTCTATGGT
TCAGACTATTATAACTGCGGCAATACTTTATATTGCAACTGCGGTAGATTTATTAGTGATTTTATTAATATTTTTT
GCTAGAGCAAAGACTAGAAAAGAATATAGGGATATTTATATAGGTCAATATCTAGGATCGATGATCCTCATATT
AGTCAGTTTATTTTTAGCTTTTGTGTTAAATTATGTTCCAGAGAAATGGATATTAGGTTTATTAGGTTTGATCCC
CATCTACTTAGGTATAAAAGTTGCTATTTATGATGATTGTGAAGGTGAAAAAAGAGCAAAAAAAGAATTAAA
CGAAAAAGGTTTGTCCAAATTAGTAGGGACTGTTTCTTTAGTGACAATTGCAAGTTGTGGTGCAGATAATATT
GGTTTATTTGTCCCTTATTTTGTAGCATTGGATATTATTGAATTACTGACTACCTTAATTGTATTTTTAGTTTTAAT
TTTCTTTTTAGTATTCACAGCACAAAAACTAGCTAGAATTCCTGGTGTGGGTGAAATTGTTGAGAAATTCAGT
CGTTGGATTATGGCTGTTATTTACATAGCATTGGGCTTATTTATTATTATTGAAAATGAAACCATACAAACGATA
TTAGGATTTATATTATAAATTAAGGTGTGACTTAATATGAGTAACAATAAAATTTGTGACGTCATTTGTGTTCAT
GAGGAAAAAGTGAATTATGCTTTAAGATTTTTAAATGAAGAAAAAACTCAACGACTTATCAATACATTATTAA
AGATAAGTGATGAAAATAAGCTGAAAATTATATTAGCTCTAATTAAAGAGAAAGAATTATGTGTTTGTGACTT
ATCTATAACATTAGGTTTAAGTATAGCTTCAACATCGTATCATTTGAGAGCCCTTTATAAAAGAGACGTGCTGA
ATTTTTACAAGGATGGGAAAATGGTATACTATTATATCAAAGATATAGAAATGAAATCATTATTAATAAAAAGTA
TGACTTAAATATAAAGAAACGCTCACATTAAACGAGCGTTTCTTTGAATTCTTGTATTCTTTTTCCAATTTCATC
TCTAACACGTTAGAATTCTGACCATTCTTTTCTTGATGGCTCATCATAGCCCCAGTAATCTTTTTTTGACGTTAG
GTGATATGATGATCTCTGGTGCGGCGGACTGCGCTTTATTGCTTTGCCATAATAATATCTTGAACACGTCTCAC
GGTGTTCAGGATTAATGTTTATTTACTTTAATTTTAAAATAATAAATATCTCCTTTAGAAAAATCAAATGATTATT

ATCTAACATCCTCTAAATTTTTATTATAATCATAATTTTAAAAAATTTTCAATAAGCTCTGAATCTGAGATTCTAA
ATTAATATATATATAATATAATAGAAACTTCAATATTTCGTTTAGAGTATATAAAAAATGACGATTAATGAAATTCC
TCCAATTAGAAAAGACAATAGCGATACAAATGAATTAAAATGTATACAATGAGAAAAAACAAAAATTAATTTA
GTAAAACTAATAGACAATTATAGATACTTGATTACCTTAGTGCTAAATTATCATATATTATACTAATGTATTTCAAT
AAATACTTTATGATAAATTTGAAAAGTTACAATAATTATCATTGTAATTGAAATGACAATGATATACGGTGTATA
ACTATTTTCTCCTTGTAATCCTACAAGTGGTGTTGTAATAGCACCAATTAAATATTGCATTAAACCTACTAAGCT
AGAAGCACTTCCGATACCTCTCGTTTGACCTTCTATTGCTATTGCGTATCCTAAAGTACCTATACCGGCGACTG
GTCCAACTATAAGAATAAATGATGGTAATAAAATCCATAAAGTAAGGTGATTGGTTAACGCAATTATCGTAATA
ACTGTCCCTACTAATTGGATAGCTGAATATATACGAAAGAGTGTATGTTGATTCATGTGTTCAACGAGACGCCC
TGTTATTTGGGCAGTGAAAATTAATCCTAGACCCGTCAAAGCAAAAATGTAGCTATATTGTTGGGCACTGAGA
CCGTATATATTTTGCATAATAAACGGAGAAGCTGCCATATATCCAAAGAACATAGCAAATGTAAAACCTTGAAT
AATCATTGGAATGACAAATGATTGACGTGTTAATAATAATTTAAAATCTTCAGCTATAATTTTAAAATGAGCCT
GTCTACGATTTTGAGGAGGTAAAGTTTCATTGATTTTTAAATGTGTACCTATAAGCATTAAACTACTAAGTAAT
GCTAAAATCCAGAAAAGTATACGGAAATTTAAAAAATTTAAAATTATACCACCAAACATTGGTGCTAACACTG
GTGCTGCTCCATTGACAAGCATTAACATTGCTAAAAACTGCGTGAGCTCTTTCCCTTTATATAAATCTGTGGCA
ATAGCTCTTGATAAGACGATACCAGCTCCACCAGCAAATCCTTGTATGAAACGAAATATAAGAACGATTGTAA
TATGCGGTGCAATTGCAATAACGATTGAAGATAAAATATAAACAATTAAACTAAATATTAAAGGATTTTTTCGA
CCAAACGTATCAGATAGTGTTCCAATAGTAATATTACCAAAAGCCAGGCCAATCATAAAAAAATGTAAGTGTCAT
TTGAACTTGAGCAGTACTTGTATTTAGCATAGATTTAACTTCAGGCAATGCTGGTAGATACATATCAATTGATA
GTGGACCAAACGCAGCCAGACCAGCCATAATTAGAATAAATAAAGGGGATATATTTTTTTCTTCATTATAAATT
TCCTTTCTGGTTCTATTTTAAGAATTTGTCAATAAAAGAACACGAAAGTCAAAATAAAGACTTTCGTGTTCTTT
TAAAGATTACTACTTTTTAGAGTCTAAAATTTTAATCATATATTCTGTTGTAACAACTTGTCCCATAAATGGAAA
TGTTTGTTCAATTGAAAATTGATGTAATGATGCTTCAGGTGCAGACATCATGTCAGAAACAAAATATTGATTAT
ATGCATTTTGATAAGCATCACGCGCAGTAGTGTCAACACCCATATGTGTAGAAATGCCACCAATGACGATATTT
TGAATACCTCGACGTCGTAGTTGTAAATCTAAGTCTGTGCCAAAAAAGCCACTAAATCCTCGTTTACTTACAA
CATAATCCGTTGATTTTACCCCAAGTTCATCAACTATTTCAGCAAAATCAGGATTCGGACTACCTCCTGGCAAG
GATTTCATCGCATTAGGCTTTAGAGCATCTTTACCGTCAATAAAATCAACCCTAATAAATGCTATAAAACCTTCA
TTCTTTCTAAATAATTCCACCATTTTTGCAGCATTATTTAATACTGTTGAGACACTATGCGGAGCTGTTTCAGCA
GTATTCGCAATACCTTTTTGTAAGTCAACTAAAACTAAAGCTGTATTCTTGAAATCCATTTTTATCTTCCTTTCG
TTATCAAAGTTTTGAGGACTGAATACCTCGAACAAAAATTTTCAAAATACGATCTACAAATTGTTTGGAGGGA
ATCTTTTTAACATAATGTTGATACTCAAATACATCTGCTGAAAAAGAATTCAGTAAAATATCAGCATGAAAATC
AGGATCATTGACAATATCCATTTTTGTTATACATGACATAACTTCTGATTTAAGTTTTACATAGAAGGGTGTCTG
CATAAGTACACGATTTTTTTTGTCTGAACGTTCAATTTCTTTAATTAAACTTACATTTTTCTCCATAATTTCAATA
AGAGATAAAAATATAAAACGAAGTAATTCTTGCTGATCAGTTTGAATTTTCTTTTTATAATCAATCTGATTAAAC
ATGTGTTCAATTTCTTGACTCAACAGTGCAGAACAAATATCGGACTTATTGGAAAAGTGACGATATAATGTTC
CTTTACCAATTTGAGCGGCTTCCGCTATTTTTTTCATACTTATCGAATCTACACCATACATATTGAAAAGTTCAT
TAGCTGTTTTTATAATAAGCTTTTCATTTTTAATAGCATCACTTCGCTTTGACATATAAACCTCACCTTCAAATTA
TTTGTTTCATACATATTTAATAAAACCATTATAAGTGAGACGGGTCTCCGTTTCAAGCGTTTAATCTAAAAAATA
TTTATTCTAATATACTTTTAAAAAAGTGGGTTTTAAAGATAGTGGGTGAATCAATTACTATTCTTTAATTTACTT
TGATATTAGTATCATCTATTAAAACAAATCAATTTTTGCTAAGTTATAATTACTAGCTTTATTCATTTCTTCTAATA
CAATACTTTTAGTAAGAAGTAAAATTACGAAAACGATTACAAAAGCGATAAAATATGGCTCTCTGTCAAATTG
GACTGATGGCCTCAATTATAGAGATTAAGCAACGAAACATTGCTTAATCTCTTTTTTGGTCTATATGCGTAAAG
TTTAGAACACAAAATTATGCGATTGCGCAGGTGATTGTAATTTCTATAACCATAAGATACCCGTTTAATTAATTT
AATTTTATTGTTAATGCCTTCAATTGGACCGTTGGTCAGATTAGTATAAGTGAGGGTGTTTTTGATGAAATCA

GTGTACTTGCGTAAAGTCCCTACAACTGGCTTAAATTTTTTATGAGTCAGTGATAAGTCAATCATAGATAAAGT
TGTCACTAATGCCGAGAAGTCATTCGTTTTAATACAGGTGCGTAGTTGATTTACATAGTGGTAAGTACGTTCTA
ATGTTGAGTTTAAAGCTAAAATATGCTTGACGACACCTTTTTCGGTCTTCCATTCTTTAAACAATCTCATTTTTT
TATATTCAAAGGATTCTAAGTTTTCCGTAGGTTTTAAGACCAGTTTCCAATAACGTTTACACTTATTGTAATCTG
GGCGATGTGCGTTTTTTAGGTCATTCATGGCACTGACACGTGCCATATTAAGCGCACGATGAATAGATTGTAC
AATATGGAAGCGATCAATAATAATATGTGCATTAGGAAACATATTACGAATCAAATTTATATAAGGTGGATACAT
ATCAATCGTAATGGTTTTTACTTTTTGACGTTCTTTTAAAGGAAACCGATTAAAATATCGTTTTAACGAAAATA
AACGGTGATCTTCTACGATATCTACAATAGGATGCGTCAACGCATCGGCGTAAATAAAATTCATACTCCCTGAT
ACATTTTTAACTCTTTTAAATTCATCCATCATAAGGTGTTCGGGCGAGGATTGATTAAGGCGTTTGATGTATTT
GATTCGCAATCATATCAATGTATCTAGAGACCGTGGTAGGTGATACAGATGTATCACGGGCGATGGTTTTCTCT
GAACGGACTTCTGTGGCTTTATCGACAATCGCTAGCTTGGTCGAGTTAGCAATATAGCAGTACATATCAACAA
CATTTGTTTTAGCAGTAAAGTGAGACTCACAACATTTACAATAAAAGCGTTGCTTCGCTAATACTAAATAAACT
GGTCTTTCATTCATTTTATTAAGCGTAATGCATGATTGTTTCTTGCCATTTTTAACGATTGAATAATCCTTATTTT
TATACCCACATAATGCCCACCTTGGAGGTGTATAGGATAGTTCACCGTAAAAGAATAAACAGGTCATTCCTTTA
TAGACTTTTTCATCTAATGTGTCTTCAAATTTAATATTTTTATCTTTAATTCTAAGTATTTTTGATATACAATCAGT
CATAGGCGCAATATCTCTCCTCATTGTTGGTTTAAGGCACTTACAATTATAGAGATATTTGCGCCATTTTTATAT
TCTTAAAGACATAAAAAAAATTGAACTGATTTTCATCAGTCCAATTAAGTATAGAACCTAAAAATATGTATATTAA
TTGATAAAGCGATGATATTATGTTAAACAATCAGCAGTTATTAACAAAGTTAAAGGGGATTATGAATTCGGAG
ATTTAGAACTTCAAGATATACAAAGTGAGTAAGTTATTGTTAAAGTGGTAGTTTTATATATTTATCACTCTGACG
AAGCAATGAGAATTGGTGATGTAGAATTTTCTTTTCCAGCACTATTAGGACATGAAGGAGCAGGAATTATCG
AAAAGGTAGGTGAGCAAGCTACAAATTTTAAAGTTGGAGATCAAGTTTTAATGGCTTACAATACTTGGGGA
GAATGTGAAAACTGCAATTCAGAGAATCCTTCTTCTTGTATAAATTGGACTACATTAAATATGAGTGGGGCTA
GAATAGACGGATCATACACATTCAAAAAGCAAGATGGAACAGCAGTATCTAATTTCTTTACGTAAAGCTCTTT
TTCTACACATACAATTACAAATTATAGAAACTTAGTTAAAATAGATAATGATATTGATTTAAGATTAGTTGTCCC
TTTAGGATGTGGATTCTTAACTGGAGCTTGAACAATTGTAAATGGCTTAAAACCTAAAGTTGGAGATAGTATA
GTTGTATTTGGTACTGGTGCGGTAAGTTCAGCAGCATTAATGTTAGCAAAAGCACAAGGCTGTACAAAAGTT
ATTGCTGTAGAGATTCATGGTCGCAGATTAAGTACTGCAAAGGAATTAGGTGCCACGCATGTGATTAATATTT
CTAATGAAGATTTAATTGAAGAAGTAAATAAAATAACTAATGGCAAAGGGGTGAGTTTTTCTGTTGACAAAA
TAGGAGTTTCAACTGTTATGTAGTCTGCTATAGATGTTCTTGGTACGCAAGGAGTAATGGCCTCTATAGCTGTA
ACACAAAATAAATTAGAAATGAATAGCTTTACAGATTTAGTACTCCAAAATAAGTCCATTAAGGTGTATTGATG
AGTGATGCGATTGCTCAATTTGAAATATCTCAATTAATTGAATTATATAAGCAAGGACAATTTGATTTTAATAAA
TTAGTTAAATTTTATGACTTCGAATATATTAACCAAGCAATTTTAGATTCAAATTCTGGAAAAGTTATTAAACCG
GTATTATTAATATCTATATCCAAAGTAATTCCAACTTTTTAAATTCAATACTTAGATTCTCAATTTTATATGTGATT
TCTAAATAAAAAGCATTTAAAGATTACAAAAAAGAGCAGAACAGATAGCTAATTACTAGTTATCCGTTCTGCT
CTTATATATATCTTTACCTCTTATGAGCCCAACTTTATATGAACTTACTTTCGTATATTTTCGTAGTTAATTTACAT
GGAATTTAAAGATTGTTTATGTTCTATATGTCTTTTCAACAGCCTTTATTGTTAATCAAGTATATAAGATTAAAA
CAAGCATTTTTAATCTCATAACTACAATTAACACAGCTCAAATAATAATTTCATTTCTTATAAATTGCTAACATGA
ATAAGTAGTAGTAGAAATATAAAATTAAATATTAAAGAGCTGAATTGCATTACCTCTTAAAATACCGTCAATTGT
GTCTGGATCAATATCACTGTTTTTAATATAGTCTACACCACGTGTATATTTCTCATCTTGGAAGTAAGGGAAGT
CTGACCCCATCATGAGCTTATCCTTACCGAATGTATCAATGGTATTGATTAAAGAAGGTTCATGGAAATTAGCA
GTATCATACCAAAATTGACGATTTAATACTTCATATGGATCAATGTTAAAGGCGTTCCAGTCTTCGTAATTGTCT
TTAATACGAGTCATGAAGAATGGTAAAGCACCACCTAAATGTGAAATATGGAATTTGATATTTGGATATTTTTG
TGGAATCTCGTTTTTAATTAATTGTAACGTAATGAATGTAGATTCTAATGGTGCGCCAATCACCCATTCTAATTG
ATAGTCGTTCACGAGTGGACTTTGGGCACCACAACCAGTTGGATGAATATAAAGTGTTGCATTTAATTCGTTC

ATAGCTTCGAAGAAAGGTTCGAATTGTTTGTCGGCAACTGACACTTTATCTTTCACAATAGTTGGAATCGCAA
TACCTACAAAGGCATCTTTTTTAAGTAGTTCTTGCGCTTCTTTAATAGCTTGATCGACATATGGAAGAGATACT
GCACCATATGCTAAGAAACGGTCTGGATATTGTTGAATCAATGATTCATATAAATCGTTAATCTCTTGTGCACTT
TGATGTGCTTCTTCTTTTGTACCCCATTGTGGTGATTGTGGTGTGGCAGAGATGATTTGCATATCAACGCCAG
CATCGTCCATCATTTTAAAGCGCTTGTCTAGATCTTCTTTAGAGGCAGATTGATTAATTCCTTTAGCAACCTCT
GTACCTTGACTACCTAATTTGCCTAATTTCTCTAAATAGTCTTCGCTCCATAAATGTGCATGTGTATCAATCGCTT
TTGCATTTGACATATGATTGTTCTCCTTTAATTAAATGACTATATTAAATATTTATCCAGTTTTAAAAAATATAATC
TTTTATTGACGACGATTTAAACTTAAAACTTCGATTAAGCTAAGAACATTTAACATAAAGAATTTAGAAGCGG
CTTTGGCACCTTGGTGACCATGTCCTGCTTTATAGTGATTGTAGATTGCTGTACCCATCACTATGTTAATCATTA
GAGTGCCCACAACGACTAATTTTTGGCCAAGTTTGCCTAAAATTGAACTAAATAAAAAGATAGAACCTACAA
ATTCGAATAATCCTGCTAATCGCATTGAACGACGTGATAATCCGAATCCTTCTTTAAATTGTTGTGCCATAGCAT
CGTCATTTTTTACTTTTGGTAAACTACTTTTTAAAATTTCTTTTCCTACATATGCGTTAATAACATGTCTTAGTAA
CATGCTTAACACCTCCTATTAAATAATATTAAAGATTAGTGTGTTGTATTTGATTGTCTTTTTTCTGAGAAGAGT
GCAATGAATGCACCTATCAGTGCTACAAAGGCAACGATATAAAATGTCATATCTAGACCATGGATAAGGCCGG
CTGCACCGTGTAATGGTGAAAGGTGACCGAAACCAGTCATGAGTGTTACAAGTATACCTGTACCGATAGCTG
CTGAAATTTGGCGAATTGTATTATTCATTGCCGTTCCATGCGCAATCAAGGATACAGGTAAAGCGTTTAATGC
TTGTGTAGTCATTGGTGTCATAATCATAGAATTACCTAACATCAAGATTGAGAATGTCACAATGACATACAATG
CTGATGTTTGTGGATTGAACTGTGCCATGAATAAGGCACCTATCATAATCAATAACATGCCAGTGATACTTAAA
CTTCGGCCGCCAACACGGTCGTATAATTTACCAGTAACAGGCGACAACAAGCCCATGACGAGTCCACCAGG
TAATAAGATGAGACCAGATTCTAAAGGTGACCAATGCATCATGGTTTGCATGTATATAGGTAAAATAGTCAAAT
TACCGATAAAGAGTACAAACATTAATACAATTAAAGTCATCGAAATGGTGAAACTACGGTATTTAAATACTCTA
AATTCGAGTAACGGTGAAGGTAGTTTCAACTGACGACGTATAAATAGCGCAAGTATAATGATGGAAATGATG
ATCGTCACGTAAACGGATGGATGTGACCAACCCAGGTTACCGGCTGAACTAAAGCCATAAAGTAAACCACCA
AATCCTAATGTAGACATGATGACTGACAAGATGTCTAACGAAGGTTGTTGTGTCTCGGTAATATTTTTCAAATA
TAAAAAGCCGAAAATAGCGTCAACCACACTAATTAAGAGCACGACTAAAAATAGATAGCGCCAATCGAATAG
GTGGATGAACCAACCTGCTGCTGTAGGACCAATCGCAGGTGCAAAGCCTATGACAAGACCGAAGATACCCA
TTGCCATACCACGTTTTTCAACAGGAAAAATAATGAATAATAGTGTTTGAGATAATGGCATTAAAATACCTGCA
CCTAACGCTTGAATACTACGACCCACTAATAATAATGGGAAATTAACACCCAACATACAGATTAAAGAACCAA
GTATTAAACAGGTCGCAGCCGTAAAGAATAGTGTACGTAATGAAAATCTCTCAATTAAGTAGGCTGTGACAG
GAATCATAATACCGTTCACTAACATAAAGATGGTCGTTAACCACTGTGCCGTACTACTTGATATCGCAAAGTCC
TTCATGACCTCTGGCAATATCGTTGTAATTAACGTTTGATTTAAGACACCAATAAAAGCACCAATCATCATGAC
CGCAATCATTAAATTACGTTGTTTTATATTTAAATGTGATGGTTGTGTCTGAGTCATAATTTCACCTCTTTTCTTA
AATGGAATCAACTATCACTTATTTTATTCGGAATGCGTTCCGTTTGTCAAATTTATGAATTCAATATAATATAGAT
AGTGTTAAAAAACGAAAAAAGTAAAAAGGAGATGCTTAGTAATGAATATTGAAGGTCTTGAAAAAATATTTAA
ATATAGATCCAAATAAATATAATGAACCAAGCTTGGAAGCATTAAATTATTATTTAAAACGATATATGTTAACAG
TGCCTTTTGAAAATATAGATGTTCAAAATGGTGTGAGAATATCAGTAGAAGTAGATGATATCTATGAAAAAATC
GTTAATCACCAACGTGGTGGTTTCTGTTATGAAATGAATCACTTCTTCAAAGCGTACTTAGAAGCAAAAGGC
TTCACAGCAAATATGGTATCTGCAACTATTCATACACCAGGGGGCGGACGTAGTCTTAAAGGCTCTCATATGT
CATTAATCGTGCCGATTGATAGTGTCAATTATGTCGCTGATGTCGGTTATGGTGACTTGCCAATAAGTGCTATG
CCAATTAATAATCAAGATAGTGATGCAATCATTGAGGATATTAACGGAGAATATCGTGCCATTTATGTAAATGA
TAACTTATTCTACATTCAAAAATGGAAAGACAATGAGTGGGATACAGAATACGAAGCTGAGTTAGAACCTAG
AGACATTCATGATTTTGATTATAATATCGAATACAATCAAACTAATCCTAATTCTACATTCGTTAAACGTTTGCTC
GTTACAATGCCTAAATCATATGGTCGTGCGACAATGTCTCAAAATAATTTAACGTTAACGAAACAACGAGATA
AAGAAAAATATGATGTGACAAGTGAAAATTATCGTCAATTTTTAAAAGAAGAATTCAATTTAGATGTTAAAAT

TAATAGATTAGAACCATAAAAGGGAGCACATCGTTTGGAAGGGGATAAGATGACCAATCAACGGAAAAAAC
GTAGTGATGCAACGCATAACAAAGCAATCATCTTACAGACAACGACCCAATTATTAGCACAGGGTGAAGATA
TTAGTGAGATGAATATGTCGGAAATCGCGAAGAAAGCTGGCGTGGGTGTAGGCACTTTATATCGTCACTTTG
AAAGTAAATCGTTACTATGCCAAGCGATGATGGATGAAAAGGTTCATGATATGTTTGATGAAATGGATACGTT
CCTTCATCAGCATCAAGATGCGTCTGTGAGGGATAAAATATATGGTGTTTTATCTATTTATTTAGATTTAAAAGA
GGCAAACTTTAATGTCCTAAATTTTATTGAGAAATCAAATTCTCAACATCAGTCAATGATTAATATTCCGTTTTA
TGAACAACTGAAAGAATTGATTAAAGACCAGTTCACTAGTCAGCAACAGACCCAAGATTTAGAGTTTAAGAT
TAATTTGATGCTAAACTCATTTTCATCAGATTTCTATTACTTTGCCAAACACGATCAACAACTGACAAAAGACC
AATTTTTAAGTAGACTATTAGATATATTTATAGGATAGATAATAGCTTGAAAGTTGGACAGAATTAAGAAAGGA
GCAAATGTAAATAAACACTAGTTCATTAAATAGCCCCTTAAAATTTATTGTAGCCTGAGATATTAATTTATGTCC
CAGGCTACAATTTTTATAAGTCATATGATAGATTGTTAAACTTTGCGATAACGCTTAAGTCCGATGATATTTAAA
ATGATAAATAAAATAATAAAGATAAGAAGAATAAGAACATCTTACCTGAACCACTAGGTCCTATTAGTCCCAAA
AGTTCGCCTTCGTTAATGTCTAGACTGATTTGGTCTAAAACTTTATGTTTATCATAGGATTTTACTGCGTCTTTA
AGTGAAGCAATCGTCTGCATCATGCTGTGTACCTCCTTATTTGACACTCTGCTTAATTACTGTATATTCAATATA
AATCAATTTTCGACAATTGCATGCATCAATTGAAATAATCAGACACATGAGATATAAATGTTCAATAATGTTATG
GGGGCGACACAAATGAACGAAGAAACCATGGATAAACGTGTGTATAAAATATTAACCAAAATTGAAAAAAA
GTATGATTACACTACTAAATGTTAAATCTTATGATGACATTAGTATTAAAGATATTTGTGATGAAAGTGGCATTA
GTAGGGGAACATTTTATCAACACTATAGAGATAAAGATGACTTTTTATTTCAATATCAAAAAGCCATGATGAA
AAAAGGGAAGCGTCAATTGACACAAATTCAATTTGAGGAACGACGTCAATTCTTTGAACACGCGTTAAACT
CTTGGATTAATGAAGGAGAATTACTACTTTTATTGTTAAGAGATAATGGCGTTTATATTGTCCACCAAGCCATG
AAAAAGAATTTACAACAAAACATTGAAGTTCGTTTAGTGCCTATTATGAATACTTAGGCACTTACAAATAAGG
AAAAATAATTTTTAATTATATGACACTGGCCCCCAAATAATGGGAATAATATAAAAACACTTTCGTTGAATTCAT
TTAAAATGAATCATACGGAGGTGTTTTTTCTATGAAAAGAGTGTCTTATTCATTAAAAACAAGTATAAAACTAT
TGAGATGAAAAAATCTGGTTATTCGATAAAAAAATTATAGAAACATTAAATATTAGAAATAGAACACAGGTGG
AAACATGGTGGAGATGGTATCGAAATGGGGAAACTTATAGATTTTCTCAACTAGTCGGTAAGCAATATACTTA
TGGTAAAGAACTTGAAGAACTCTCAGAAGTTGAAGAATTAAAATTAGAGAATAATAGAAAAGATATTGAAAT
AGATATTTTAAAAAAGTAAAAAGAATTGGAAAGGAAGGGGTACCAAAAGTAATCGTAAAATTAGTTGATCA
ATTAAAGCATAAGTATTCAGTAAAAATAATACTTGATGTTTTAGAAATACCTGAACTGACATATTATAGATGGG
AACACAAGAACTATAAAAATGATGACTTGAAGCAAAAAATCATTCAATTGTGTAAAGACAACCAATACATCTA
TGGTTATCGAAAAATTACAACCTTGATTAATCAATTGTCTTCAGTAGCAGTTAATCATAAAAGAATTCAGAGA
ATCATGCAGAAGAATGATTTAAATTGTCGAGTTAGACCTAAAAAGACTAAAAGCATTGGTAAACCTTGTTATA
AAACAGATAATTTACTACAAAGACAATTTAAAGCAAGTCAACCAATGGAAGTATTAACAACAAATATTACTTA
TTTATCTTTCGGTAACCCCATGTTGTATTTATCTTCAATTATGGATCTTTATAACGGAGAAATTGTTGCATATAAA
ATAAATAACAAACAGGATCAAAGCTTAGTAAATGATACATTGAATCAAATTGTAATTGATATTGTTGAAAATTA
CATTAAAAACTATAATAATAATCGAATTCAACAAAAGCTAGGCTACTTAGCCCCTGTAAAATACAGAGAATTAG
CAGCCTAGAATATTGTTTTTTTATTAAGTTCCCACTTTAGGGGTTCAGTACCTCTAATTACTTATAGTCTTTCTTA
ATTAGTATAAATATTAAGAAAGGTGTATATCTTTGTAACCTTCATTTACATTAATAATGTTGTGATAACCTTTGTG
TTCTAAAATACCAATAGCTATAGAACTTCTAATGCCAGATTGACAGTGTACATAAATATCATCATTTTTATTGAA
AGGTAAATCTGTGTCTAAAAGTTTGCCGTGTGGTACATGAACCGCTTGAGATAAGTGGCCATTATTCCATTCA
TTATCATTACGTACATCTAAGACATGTGCTTCATTACCAGTTATGTCTTTACTATGAACAGATTGTGTTTGAATTT
GAGAATGTGGTAACTGGTATCCAGACACATTATCATATCCAATAAGTTGTAAAGTATGTGTTGCTTTTGAAACA
AGGTAATAGTCTCCAATCAAGTTAATTTCTTGATCATAGTTTAGATACCAGCCAATTTGATTGATGAAATTTTTA
TCATATGGAATATTGATTGTACCTTCAATATGTCCCCCATGATAAGCCTCTTTACTGCGGAGATCAAAAGTTAAT
CTGTTTGTACTTGTAGCTGGATAAACTGTATAAGGTTGATATAAATTCATACCGAATTGATTAATTTTTTTCATT

TGTGCAAAATGATGTGGTGGTGCAGGTTGGTCAGAAATGAGTTTATCGATAAAGGTAGCTTCATTATTTTCA
GAAAAAGCCCAGTTTGTTTGTTTTTCATAGCCAAGCGTAGATGTTGGAATAGCACCTAAAGATTTACCACAA
GGACTACCAGCGCCATGACCAGGCCAAATTTGAATGTAGTCTGGCAAGTCTTTAATACTTTCAATAGATTTAA
ACATTTGTTTAGCGCCTATTTCAGATGATCCTTCTACTTTAACAGCTTTTTCTAGTAAATCAGGTCTACCGATAT
CTCCTACAAAAATAAAATCACCACTGAATAGTCCCATTGGAACTTGTGCTCCAGCACCTTCATCAGTAAGTAA
AAAACTTATACTTTCTGGCGTGTGACCAGGTGTATGAAGCACTTTTAATTTTATATTTCCTACATAAATATCATC
ATTATGTTGAACAAAATGAGTCTGGTTAGGCATATTTTTATAACCTAACGTGTCATCACTTTCACCCGATACATA
AATACTAGCATTTAACTTTATAGCAACATCTCTAATTCCTGAAGCAAAATCTGCATGTATATGTGTTTCAGCTGC
ATGAGTAATGGTTAAACCTTCTTCATCAGCAACTCGAATATATGAAGATAAGTCACGAATAGGATCAATAATCA
TGGCTTCTCCAGTTTTTTGACAACCGATTAAATAAGATGCTTGAGATAAGTGTTTATCATAAAATTGTTTAAAA
AACATAATATCATTCCTTTTTAATTATATAAATAAATTGTGATTAGCTTGTTCTGTATATCCTATATATGCACCTAC
GCCACCGTATTCTACATCATCGCGTAGTTCTTCTTTTGAAATGCCCATAACATCCATGCTCATTGTACAAGCAAT
AAGTTTAACACCTTGTTCAACTGCTTGATTGATAAGAGAAGGTAAATCATCGACATTTTTCTTTTTCATGACAT
AACGCATCATTAAATTTCCTACACCAAACATATTCATTTTTGAAATAGGCATGTTAATTGGTGAATTTGGAAGC
ATGAAGTCAAAAAGTTTTGCGATACCTTTTTTCTTAATGCGTTGATTTTGAATTTTTTTAAGTGCATTTAAGCC
CCAAAATGTGCAAAAAAATGGTTACATCTCTACCTGCGGCTTTAGCACCATTAGCAATAATCATTGCTGCAACT
GCTTTATCTAGCTCACCGCTAAATAAAACAATCGTTGTTCCATTTTTCGTGTGTGTCACTTCTATATTTTTATTTT
CTTCTTTTTGAATAATAGCGCGAATTTCATTACCAGAATCATTGAGTCTAACAAGTGTATGTCCAGTTTGTTTG
ATCCAGCTTTTAATATCATTTAAAAATCCATGATCAGTCACAACAACTTCTATTTGATCACCAATAGCGATGTTT
TTGATTTCTTTACTAATATTAACAATAGGTCCTGGACATTGTAGATTACTATAATTGAACATCTTACGATTATCTT
TGATTTGAATTTCTTTATCTTTTTTTAATTTAAATGATTCATTATTATGTTGTTGTTCGTATGCGGTATAACCTCCA
TCTAAATTCACCACATCGTATCCACGTTGTGTAAGAAATTGACTTGCTTTTCTACTTCTATTACCACTTTTGCAA
TAAACATAGTAAGTTTTATTTTTACTTTGTTTAAAAGTCTCTATATTTTCAACTGAATGTAATAAAGCATTTTTAA
CGTGTCCTAATTCAAATTCTTCAGGCTGTCGAACGTCAATTAATTGACCATTAGAACCCAATTCTTGTAATTCT
TTTTTAGTGAAATCATTAATGTGTTTCGTTTTATATTGTGACATAATAGCCTCCTTTTAAATACCTGTAGGGGTAT
ATTATAATGAATTAGATAGTTTGTCAAATACCTATGGGGGTATTTGACAAAATAAAATTTAATCAATATGATGTA
ATTATTAAAAAATTAATTTGGAGTGAAATATATTGGAATATAATAAAAAGATGATTAATCGTATTCATCGCATAC
AAGGACAGCTTAATGGTGTGATTAAAATGATGGAGGAAGAAAAAAGTTGTAAAGATGTCATTAGTCAATTAA
GTGCATCTAAAAGTTCTATTCAACGTTTAATGGGTATTATCATTAGTGAGAACTTAGTAGAATGTGTCAAAATG
TCTGAAGAAAATAGTGAAGATTCTCAGGCACTAATTAATGAAGCAGTTGAATTATTAATAAAAAGTAAATGAT
AAGTGTAACAGCAATTATAGTAATGTTAGCTATTGGTATCTTAGGAGGATTTATTTCTGGATTAGTTGGTATAG
GAGGAGCCATTGTTATTTACCCTGCTCTCTTATTATTACCACCTTTATTTGGTTTACCTACTTATAGTGCTTATAT
TGCTTCAGGATTAACTTCAAGTCAGGTGTTTTTTAGTACATTGAGTGGATCTTTAAAAGCATATAAAAATAAA
AATTTTTCTAGAACGCTTATACTTAATATGGGAAGTGGAATGGTAATAGGTAGTATTCTAGGTGCATTATTAGC
AACTGTATTTAATAGTCATTTTGTTAATGTAATTTATATAATCATTGCTTTGTTAGCTCTTATTCTAATGTTTATTA
AAATCACACCATCTACTAGTCATATTAAGTTTAATCGCGTACTTTTAATTACAATAGGTAGTATCATCGGTTTGG
TATCTGGTATTGTAGGAGCTGGTGGAGCATTTATTATTATTCCAGTACTACTTGTTATATTTAAATTACCAATGA
ATATGGTTGTTACAAATAGTATTGTAATTGCATTTATTTCTTCTATTGGAGCATTTATTATTAAATTATTACAGGG
TTATATTCCTATCAATAGTGCAATACCGTTAATTTTAGGTAGTATACTATTTACGCCTCTAGGTATGAAAATAGG
GCAAAAAAATACCTGATTCTATTCAAAAAGTGATTGTAAGTGTTTTAATCGTCTTTGCAATCATTAAGTTGATTT
TTTAAGAACATTATATGATAGAAAATCTATTAAAAAGCGTCTCAATCATATTGAGACGCTTGAAGTTTATCTAG
CTTTAAATGATTCAATGGAGGTTTTGATTTCATCTCTAACACGTTGAAATTCTGACCAAGGTTTTCCTGCAGG
ATCATCAAATCCCCAATGTTCCTTTTTTACATTAGGCGGCAATATAGGACAGTTTTGATCAGCATCACTACATA
GAGTTACTACTAAATCGGATTGGTTTAGTATATTGTTGTCAATTAAATTAGAAGTATGGTTTGATATATCAATTC

CAACTTCTTTCATAGCTTCGATTGCTTTCGGATTGACACCGTGTGCCTCAATACCTCCAGAATATACTTGCCAT
TCATCACCTAAGATTTTTTTACCCCAGCCTTCAGCCATTTGGCTACGGCATGAATTACCTGTACATATAAAATAA
ATTATTTTCTTTGTCATCATTGACACCTCTTTTTTAAAATATAATAAGTGTAACGTACAAACCAACTAATGTAATA
AACAGAACAGGAATCGTAATAACAATACCCGTTTTAAAATATGTGCCCCACGAAATTTTTACTCCTTTTTGTGT
TAAAACATGTAGCCATAGTAATGTTGCTAAGGAGCCGATAGGTGTGATTTTAGGTCCTAAATCTGCGCCAATG
ATATTAGCATAAATCATGCCTTCTTTGATTAAACCGGTAGTTGCAGATTGTCCAATAGCAATTGCATCAATTAAT
ACGGTAGGCATATTATTCATAATGGCTGAAAGGAATGCGGATATAAAGCCCATACCTATAACACTACTAAACAA
TCCATAGTTTGATATGCTTGATAGAATATTAGCTAATAGTGCAGTAATACCTACATTTTTCAGACCAAATACAAC
TAAATACATTCCTATAGAAAAGAGCACAATATTCCATGGTGCACCTTTGATAACTTGTTTTGTATTTACTACTCT
CGATTTATAGGCCAATACAGTAAATATAAACGCAATCAAACAAGCAATTAACGATACTGGAATTTGAATAAATT
CACTAACTATATAACCGATGAGTAAGATTGATAAAACAATCCATGAAAACTTGAATAATTTTGGATCTTTAATG
GCTTGATTAGGTGCTTTAAGATTTTTAATTTCAAAATGGTTAGGAATAACTTTTCTGAAATATAGCCATAATAC
AACGATACTTGCAAGTAAAGAGAATAGATTGGGAATGATCATGCGGCTTACATATTCAATAAATCCAATATGG
AAGTAGTCAGCAGAAACGATATTAACCAAATTACTTACAATTAGTGGAAGAGACGTAGTATCTGCAATGAATC
CACTTGCAATGATAAAAGGAAAAATGACTTTTTGATTAAACCCTAGATTTCTTACCATAGCTAGCACGATTGG
AGTGAGAATTAATGCAGCTCCATCGTTCGCAAAGAAAGCTGCTACAATAGCGCCTAACAACATAATGTAAAC
AAACATTTTAAATCCATGACCATTTGAAGCTCTAACCATATGTATAGCTGCCCATTCGAAAAATCCAATTTCATC
TAAAATTAATGAAATGAGTATAACAGCGACAAAGGTTAACGTAGCATTCCAAACTATCCCTGTAACTTCAACA
ACATCTGAAAGACTAACAACACCTGTAATAATAGCAATGATAGCACCAAACAATGCTGTAATACCAATATCTAA
CCCCTTAGGTTGCCAAATAACAAACATGAGAGTTAAAAGAAAAATAATAATCGCTAGAATAGTCATTATGTAC
ATTCACCTGTCTTTATCTTTTTACACATACATTGTTGAGTAGGAGAGCTAATATAAGTTAATTCATTGTTGATTG
AATCTAAGGACTTATGATTAAGTCGGTACATGCGCTTAGTACCGTCTTTTCGTGTAGATACTAATTCATTATCTA
CTAGAATTTTCATATGATGACTTAATGTAGGTTGTGAAAATTGAAAATACGCTAATAAATCACAAGCACATAAT
TCACCGCAAGATAATAAGTCTAATATTTCTAATCGACTTGGATCCGATAAAATTTTTAATTGTAATGATAGTTCC
TTATAAGACATATGGATGTCATCCCTCCTTTATTATAGATTATCATCTATATAGACAAATATCTATATAGATAATACT
TTTTGCAATAATACATTTAATTAAAACCTTGTTTTAAACATCGATGACAAGGTCTAATGTAGGACGTGGAGAC
ATCATTTTCGGAATGATATAAATAAATCCATAAATGGAGATAAATCGAAAATATTTATACCCCTAGGTGGTATGT
GTTATAGTATAAGTATAGCTAATATAATATTTTCATAAATAGGAGGGGTTAATTTGAATAATAATGGTGAAGAGC
ATAATCATCAAAATCACATGAATCATTCCAATCACATGCATCATGATAACCATGCCTCACATCATCATAGTGGCC
ATGCACATCATCATGGAAATTTTAAAGTTAAGTTTTTTGTTTCATTAATTTTTGCAATACCTATCATTCTTTTATC
GCCAATGATGGGTGTTAACTTACCTTTTCAATTCACATTTCCAGGTTCTGAATGGGTAGTGTTAATATTAAGTA
CAATTTTATTCTTTTATGGTGGTAAACCGTTCTTGTCTGGTGGTAAAGATGAAATTGCTACAAAAAAACCAGG
CATGATGACCTTAGTTGCCCTAGGTATTTCAGTAGCTTATATTTATAGCTTGTATGCTTTTTATATGAATAACTTT
AGTAGTGCAACTGGTCATACAATGGACTTTTTTTGGGAATTAGCAACCTTGATTTTAATTATGCTATTAGGACA
TTGGATAGAAATGAATGCTGTCGGAAATGCTGGAGATGCTTTAAAGAAAATGGCAGAACTGTTACCTAATAG
TGCTATTAAAGTTATGGATAATGGCCAACGCGAAGAAGTTAAAATATCAGACATCATGACTGATGATATCGTC
GAAGTAAAAGCCGGAGAAAGCATTCCGACAGATGGTATTATCGTTCAAGGACAAACATCTATAGATGAATCC
CTAGTCACTGGAGAATCTAAAAAAGTACAAAAAAATCAAAATGACAACGTCATCGGGGGTTCTATTAATGGG
TCTGGAACAATACAAGTCAAGGTTACAGCTGTGGGAGAAGATGGATATCTTTCTCAAGTTATGGGACTTGTT
AATCAAGCACAAAATGATAAATCTAGTGCTGAATTGTTATCTGATAAAGTAGCGGGTTATTTATTCTACTTTGC
TGTAATTGTTGGCGTGATTTCGTTTATTGTCTGGATGCTCATTCAAAATGATGTTGATTTTGCATTAGAACGTC
TTGTAACTGTGTTAGTCATTGCTTGTCCACATGCTTTAGGCTTGGCAATACCTTTAGTCACTGCACGTTCTACT
TCAATTGGTGCACATAATGGTTTAATTATTAAAAATAGAGAGTCTGTAGAAATAGCTCAACATATCGATTATGT
AATGATGGATAAAACTGGTACTTTAACTGAGGGTAACTTTTCTGTGAATCATTATGAGAGCTTTAAAAATGAT

TTGAGTAATGATACAATATTAAGCCTTTTCGCCTCATTAGAAAGTCAATCTAATCACCCATTAGCTATAAGTATT
GTTGATTTTGCGAAAAGTAAAAATGTTTCATTTACTAATCCACAAGACGTTAATAATATTCCAGGTGTCGGATT
AGAAGGTCTAATTGATAATAAAACATATAAAATAACAAATGTCTCTTATCTTGATAAACATAAACTTAATTATGA
CGATGACTTGTTTACTAAATTAGCTCAACAAGGTAATTCAATCAGCTATTTAATTGAGGATCAACAAGTCATTG
GCATGATTGCTCAAGGAGATCAAATTAAAGAAAGCTCAAAACAAATGGTAGCTGATTTACTATCAAGAAATAT
TACACCAGTCATGCTTACAGGTGACAATAATGAAGTGGCACACGCTGTCGCAAAAGAATTAGGTATTAGTGA
TGTCCATGCACAACTCATGCCAGAAGATAAGGAAAGCATTATAAAAGATTATCAAAGTAACGGTAATAAAGTC
ATGATGGTCGGAGACGGTATCAACGATGCGCCGAGCCTTATAAGAGCCGATATTGGTATAGCAATTGGTGCA
GGCACAGATGTTGCAGTGGATTCAGGTGATATCATACTTGTTAAAAGTAATCCATCAGATATCATTCATTTCTT
GACCCTTTCAAATAATACTATGAGAAAAATGGTTCAAAACTTATGGTGGGGTGCAGGTTATAATATTGTTGCT
GTACCTTTAGCAGCTGGCGCATTAGCTTTTGTCGGGTTAATATTATCACCAGCTGTAGGAGCAATATTAATGTC
TTTAAGTACAGTTATAGTAGCGATTAATGCTTTTACATTAAAATTAAAATAAAAGAGGTAAACCTTATGTATAAT
AAAGTTTTTGCAATTTTAATTATAATTTTTTCCATAATAATTATTGCGTCTAATGATACTTTCGCAGAAAGTAAG
AATGACATGATGAACATGAAAGAAGATAAGAAAAATACAATGGATATGAAAAATATGAAACATCATGACGAA
AGAAAGAAATTAAATTCTTCACAAGGAAAAAATGAAATAATATTTCCTGAAGTTGCAGAGTCAAAAAAAGAT
AACAATGGTTATAAAAATTATACATTAAAAGCTCAGGAAGGAAAGACAGAGTTTTACAAAAATAATTTTTCTA
ATACTCTAGGCTACAATGGAAATTTACTTGGACCAACTTTAAAATTAAAAAAAGGAGATAAAGTTAAAATTAA
GTTAATAAATAACTTAGATGAAAATACAACATTTCATTGGCATGGATTAGAAATAAATGGAAAAGTGGATGGA
GGGCCTTCTCAGGTTATAAAACCAGGAAAAGAAAAAACTATAAAATTTGAGGTTAATCAAGATTCTGCTACG
TTATGGTATCACCCCCACCCCTCTCCAAATACAGCTAAACAAGTTTATAATGGCTTATCAGGATTATTATATATAG
AAGATAGTAAAAAGAATAATTATCCTAGTAATTATGGAAAAAATGATTTGCCTATAATAATCCAAGATAAAACA
TTTGTATCTAAAAAATTAAATTATTCAAAAACGAAAGACGAAGATGGCACTCAAGGTGATACTGTTCTTGTGA
ACGGAATAGTAAACCCCAAACTGACAGCAAAAGAAGAGAAAATACGTTTGAGACTTTTAAATGGTTCTAAT
GCTCGAGATTTAAATCTTAAGCTAAGTAATAATCAAAGTTTTGAATATATTGCTTCAGATGGCGGTCAATTAAA
AAACGCTAAAAAATTAAAAGAAATTAATTTAGCTCCTTCAGAAAGAAAAGAAATAGTAATAGATTTATCTAAA
ATGAAAGGCGAGAAAATCAGTCTGGTTGATAATGATAAAACTGTAATTTTACCGATTAGTAACAAAGAGAAA
AGTTCTAACAAAGGTAATACACCAAAAGTAAGTAAAAAAATAAAATTAGAAGGTATGAATGATCATGTTACCA
TTAATGGTAATAAATTCGATCCTAACAGAATAGATTTTACACAAAAGTTAAACCAGAAAGAAGTATGGGAAAT
TGAAAATGTCAAAGATAAAATGGGTGGTATGAAACATCCTTTCCACATCCATGGAACGCAATTTAAAGTTTTA
TCTGTGGATGGGGAGAAACCTCCAAAAGATATGAGGGGTAAAAAGGATGTTATATCTTTGGAACCTGGACA
AAAAGCTAAAATAGAGGTTGTATTTAAAAATACTGGAACATACATGTTTCACTGTCATATACTTGAGCATGAA
GAGAATGGAATGATGGGTCAAATAAAAGTAACAAACTAATCAATAGGGAGATTTTAATATGATGAAAAAAGA
TAAAGACACTAATGACCAAAAAAGTGAGAGCCATATGAAGCATAATGATGAAAGTAAAGTTCCTGAAGATAT
GACATCGACTAATGAGGGTGAATTTAAAGTAGGAGATAAAGTAACGATTACAGCAGGGCATATGCCAGGTAT
GAAAGGTGCAGAAGCTACTGTAAAAGGTGCGTATAAAACATATGCTTATGTTGTAAGTTATAAACCCACAAAT
GGAAATGAAAAAGTAAACAATCATAAATGGGTCGTAAACGAAGAGATCAAAGATGCACCTAAAGATGGATT
TAGTAAAGACGATACTGTTAAATTAGAAGCAAATCATATGTCTGGTATGAAAGGTGCTACAGCCAATATTGATA
ACGTGAAAAAGACTACTGTTTACGTGGTTGATTACAAATCCAAAGATAATGGTAAAATCATTAAAAATCATAA
ATGGATGACAGGAAATGAGCTGAAAGCACGATAAAAATCTAGTTCTAGATTGAGAAATAAATAGATATAAAA
ATATCCTCCTTAATCAATAATTTAAATAACTTATTATTGTTAAGGAGGATATTTTTTAGTGTGTAAATTAAAAAGA
ATTTTAGAAGAATAACATTTATCAAAAAACTGTTCATTACCTTATTAATTGAAATTATATAATTAAAAACCGCAT
CATTAACCGATAAGCAGAGGCTTATCATAAATGATGAGGTTAAATACTAAAAATAATATTAAATACATTAGTAA
CATCTCAGACATAATTTTGATTATTAGAAATAAATTTAAAGCCCACAGTTAGCAGTTGAT

>Staphylococcus aureus LAC

ATGAAAATCACCATTTTAGCTGTAGGGAAACTAAAAGAGAAATATTGGAAGCAAGCCATAGCAGAATATGAA
AAACGTTTAGGCCCATACACCAAGATAGACATCATAGAAGTTCCAGACGAAAAAGCACCAGAAAATATGAGT
GACAAAGAAATTGAGCAAGTAAAAGAAAAAGAAGGCCAACGAATACTAGCCAAAATCAAACCACAATCCA
CAGTCATTACATTAGAAATACAAGGAAAGATGCTATCTTCCGAAGGATTGGCCCAAGAATTGAACCAACGCA
TGACCCAAGGGCAAAGCGACTTTGTTTTCGTCATTGGCGGATCAAACGGCCTGCACAAGGACGTCTTACAA
CGCAGTAACTACGCACTATCATTCAGCAAAATGACATTCCCACATCAAATGATGCGGGTTGTGTTAATTGAAC
AAGTGTACAGAGCATTTAAGATTATGCGAGGAGAAGCATATCATAAATGATGCGGTTTTTTCAGCCGCTTCAT
AAAGGGATTTTGAATGTATCAGAACATATGAGGTTTATGTGAATTGCTGTTATGTTTTTAAGAAGCTTATCATA
AGTAATGAGGTTCATGATTTTTGACATAGTTAGCCTCCGCAGTCTTTCATTTCAAGTAAATAATAGCGAAATAT
TCTTTATACTGAATACTTATAGTGAAGCAAAGTTCTAGCTTTGAGAAAATTCTTTCTGCAACTAAATATAGTAA
ATTACGGTAAAATATAAATAAGTACATATTGAAGAAAATGAGACATAATATATTTTATAATAGGAGGGAATTTC
AAATGATAGACAACTTTATGCAGGTCCTTAAATTAATTAAAGAGAAACGTACCAATAATGTAGTTAAAAAATC
TGATTGGGATAAAGGTGATCTATATAAAACTTTAGTCCATGATAAGTTACCCAAGCAGTTAAAAGTGCATATAA
AAGAAGATAAATATTCAGTTGTAGGGAAGGTTGCTACTGGGAACTATAGTAAAGTTCCTTGGATTTCAATATA
TGATGAGAATATAACAAAAGAAACAAAGGATGGATATTATTTGGTATATCTTTTTCATCCGGAAGGAGAAGG
CATATACTTATCTTTGAATCAAGGATGGTCAAAGATAAGTGATATGTTTCCGCGGGATAAAAATGCTGCAAAA
CAAAGAGCATTAACTTTATCTTCCGAACTCAATAAATATATTACATCAAATGAATTTAATACTGGAAGATTTTAT
TACGCAGAAAATAAAGATTCATCTTATGATTTAAAAAATGATTATCCATCAGGATATTCTCATGGATCAATAAGA
TTCAAATATTATGATTTGAATGAAGGATTCACAGAAGAAGATATGCTAGAGGATTTAAAGAAATTTTTAGAAC
TATTTAATGAATTAGCTTCAAAAGTTACAAAAACATCCTATGATAGCTTGGTCAATAGCATAGACGAAATACAG
GAAGACAGCGAAATTGAAGAAATTAGAACAGCACAAAAAGATAAGACACTCAAGGAAGTGGAAGCACCTA
AAGGAATAATTCCAAAATATAAAAAAGGTGTATCAAAGACTACTAAAAATGATTCAGAAATTGAAAAATCAA
ATAAAGAGAATAAATTAACCGGTAAAGTTGGAGAAAAATTAGCGCTAAATTACTTTAATGAGCTAATTGATAA
TAAAATAGACGAAGATAAGAAAGAACAGTTTAGGAATATTTTAAATGATAATCCAGGCTCTCAACACGGTCAT
GGCTATGATTTAGTAGCTTTTGATCCAACAAATACAGATAAAGCTGTAGAAAAATTTATTGAAATTAAAACATC
TACATCTTCTAGTATTGAGGAACCATTTTTTATGTCGCTAAATGAAATGTTTGCTATGAAAGAATATAAGCAGA
AATATTTAATATTAAGAATATTTAATGTTTCCGGTAAAGAACCACAATTTTATTTTATAGATCCATATGCAAATTA
TTCTGAATTTAAAGATGTAGATGATCTCATTGACAAAGTATTTAATGTAGAAGCTATTCAGTATAAAGTTTTTG
GCGAAAAAATGATTACTTGAACAAGAGCTAAAATAAAATTGTGATCTAATAAAAATAGAAGGTTCTGTTGCAA
AGTAAAAAAAATATAGCTAACCACTAATTTATCATGTCAGTGTTCGCTTAACTTGCTAGCATGATGCTAATTTCGT
GGCATGGCGAAAATCCGTAGATCTGAAGAGACCTGCGGTTCTTTTTATATAGAGCGTAAATACATTCAATACC
TTTTAAAGTATTCTTTGCTGTATTGATACTTTGATACCTTGTCTTTCTTACTTTAATATGACGGTGATCTTGCTCA
ATGAGGTTATTCAGATATTTCGATGTACAATGACAGTCAGGTTTAAGTTTAAAAGCTTTAATTACTTTAGCCAT
TGCTACCTTCGTTGAAGGTGCCTGATCTGTAATTACCTTTTGAGGTTTACCAAATTGTTTAATGAGACGTTTG
ATAAACGCATATGCTGAATGATTATCTCGTTGCTTACGCAACCAAATATCTAATGTATGTCCCTCTGCATCAATG
GCACGATATAAATAGCTCCATTTTCCTTTTATTTTGATGTACGTCTCATCAATACGCCATTTGTAATAAGCTTTTT
TATGCTTTTTCTTCCAAATTTGATACAAAATTGGGGCATATTCTTGAACCCAACGGTAGACCGTTGAATGATG
AACGTTTACACCACGTTCCCTTAATATTTCAGATATATCACGATAACTCAATGTATATCTTAGATAGTAGCCAAC
GGCTACAGTGATAACATCCTTGTTAAATTGTTTATATCTGAAATAGTTCATACAGAAGACTCCTTTTTGTTAAA
ATTATACTATAAATTCAACTTTGCAACAGAACCGTATTATGGAATAGAGATGTTGGTAACATTTATACAGGATC
ATTATACTTAAGTTTAATTTCGTTATTACAGAACCACACATTCCAACCAGAAGAGAAAGTATGTCTATTTAGTT
ATGGTTCAGGAGCAGTAGGAGAAATCTTTAGTGGTTCAATCGTTAAAGGATATGACAAAGCATTAGATAAAG
AGAAACACTTAAATATGCTAGAATCTAGAGAGCAATTATCAGTCGAAGAATACGAAACATTCTTTAACAGATT
TGATAATCAAGAATTTGATTTCGAACGTGAATTGACACAAGATCCATATTCAAAAGTATACTTATACAGTATAG

AAGACCATATCAGAACATATAAGATAGAGAAATAAACTAGTGGCCGATTGTGCTTGATGAGCTTGGGACATA
AATCCTAACTCGAAATAAATAAGCATATCACTAAACTGATTTTTAAAGTTTACAGTGATATGCTTATTTTTTTAT
CTTACGATTTTGTACGTGCATGCTTGCCTAGGGGTATGGCTCGAGCCATTAGTCTCTCGCACATACTATTCCCT
CAGGCGTCAGCACTTACAAAATCGGTTGTAATTTTCATTTTTATACGCATTCTTACTGAGATTATACTAATAAGA
GGAATAGTAAAAGCAATTCTAAGTAAAATTGCAGATAAGAGGTTTGTTAAAAGCAGTTCTCAGTAAAATTAC
AGATAAGAGGTACGTTAAAAGCAGTTCTAAGTAAAATTGCAGATAAGAGGTTTGTTAAAAGCAGTTCTAAGT
AAAATTGCAGATAAGAGGTACGTTAAAAGCAATTCCATGCAAAATTGCTGATAAGGGGTAAGTTAAAAGCA
GTTCTCAGTAAAATTGCAGATAAGAGGTACGTTAAAAGCAGTTCTAGGCAAAATTGCAGATAAGAGGTGCG
TTAAAAGCAGTTCTCAGTAAAATTGCTGATAAGGGGTAAGTTAAAAGCAATCCTAAGTAAAATTGCAGATAA
GGGGTACAGAAAAACTAGACTTGATTACAAAATGGAGCTTGGGACATAAATGATTTTTTAAAAATGAGATGA
GACGTAGATTAACTCCATAATCAATACGAATCTATCGACTTCTTTATTTATGATATTCATCTCTTTTTAATGGAAA
TAAAAGTGCGATTAATGTGATAATACAGTTACGTTAATTAAAAAAATAAAAATGCAAGGAGAGGTAATATGCT
AACTGTATATGGACATAGAGGATTACCTAGTAAAGCTCCGGAAAATACAATTGCATCATTTAAAGCTGCTTCA
GAAGTAGAAGGTATAAACTGGTTGGAGTTAGATGTTGCAATTACAAAAGATGAACAACTGATTATCATTCATG
ATGATTATTTAGAACGGACTACAAATATGTCCGGGGAAATAACTGAATTGAATTATGATGAAATTAAAGATGC
TTCTGCAGGATCTTGGTTTGGTGAAAAATTCAAAGATGAACATTTGCCAACTTTCGATGATGTAGTAAAAATA
GCAAATGAATATAATATGAATTTAAATGTAGAATTAAAAGGTATTACTGGACCGAATGGACTAGCACTTTCTAA
AAGTATGGTTAAGCAAGTGGAAGAACAATTAACAAACTTAAATCAGAATCAAGAAGTGCTCATTTCAAGCTT
TAATGTTGTGCTTGTTAAACTTGCAGAAGAAATCATGCCACAATATAACAGAGCAGTTATATTCCATACAACTT
CGTTTCGTGAAGACTGGAGAACACTTTTAGATTACTGTAATGCTAAAATAGTAAACACTGAAGATGCCAAAC
TTACTAAAGCAAAAGTAAAAATGGTAAAAGAAGCGGGTTATGAATTGAACGTATGGACTGTAAACAAACCA
GCACGTGCAAACCAACTTGCTAATTGGGGAGTTGATGGTATCTTTACAGACAATGCAGATAAAATGGTGCAT
TTGTCTCAATAGAAAGTTAGAGGTGAGTCTTACGTTTCAGTGACGGTAGACTTACCTTTAACATGTTACATAC
TAAAAAATTAATTTGAATAAGAAAGAGAGACATATATGAAATACGATGATTTTATAGTAGGAGAAACATTCAA
AACAAAAAGCCTTCATATTACAGAAGAAGAAATTATCCAATTTGCAACAACTTTTGATCCTCAATATATGCATA
TAGATAAAGAAAAAGCAGAACAAAGTAGATTTAAAGGTATCATTGCATCTGGCATGCATACACTTTCAATATC
ATTTAAATTATGGGTAGAAGAAGGTAAATACGGAGAAGAAGTTGTAGCAGGAACACAAATGAATAACGTTA
AATTTATTAAACCTGTATACCCAGGTAATACATTGTACGTTATCGCTGAAATTACAAATAAGAAATCCATAAAAA
AAGAAAATGGACTCGTTACAGTGTCACTTTCAACATACAATGAAAATGAAGAAATTGTATTTAAGGGAGAAG
TAACAGCACTTATTAATAATTCATAATAAAACAGTGAAGCAACCATCGTTACGGATTGCTTCACTGTTTTGTTA
TTCATCTATATCGTATTTTTTATTACCGTTCTCATATAGCTCATCATACACTTTACCTGAGATTTTGGCATTGTAGC
TAGCCATTCCTTTATCTTGTACATCTTTAACATTAATAGCCATCATCATGTTTGGATTATCTTTATCATATGATATA
AACCACCCAATTTGTCTGCCAGTTTCTCCTTGTTTCATTTTGAGTTCTGCAGTACCGGATTTGCCAATTAAGTT
TGCATAAGATCTATAAATATCTTCTTTATGTGTTTTATTTACGACTTGTTGCATACCATCAGTTAATAGATTGATAT
TTTCTTTGGAAATAATATTTTTCTTCCAAACTTTGTTTTTCGTGTCTTTTAATAAGTGAGGTGCGTTAATATTGC
CATTATTTTCTAATGCGCTATAGATTGAAAGGATCTGTACTGGGTTAATCAGTATTTCACCTTGTCCGTAACCTG
AATCAGCTAATAATATTTCATTATCTAAATTTTTGTTTGAAATTTGAGCATTATAAAATGGATAATCACTTGGTAT
ATCTTCACCAACACCTAGTTTTTTCATGCCTTTTTCAAATTTCTTACTGCCTAATTCGAGTGCTACTCTAGCAAA
GAAAATGTTATCTGATGATTCTATTGCTTGTTTTAAGTCGATATTACCATTTACCACTTCATATCTTGTAACGTTG
TAACCACCCCAAGATTTATCTTTTTGCCAACCTTTACCATCGATTTTATAACTTGTTTTATCGTCTAATGTTTTGT
TATTTAACCCAATCATTGCTGTTAATATTTTTTGAGTTGAACCTGGTGAAGTTGTAATCTGGAACTTGTTGAGC
AGAGGTTCTTTTTTATCTTCGGTTAATTTATTATATTCTTCGTTACTCATGCCATACATAAATGGATAGACGTCAT
ATGAAGGTGTGCTTACAAGTGCTAATAATTCACCTGTTTGAGGGTGGATAGCAGTACCTGAGCCATAATCATT
TTTCATGTTGTTATAAATACTCTTTTGAACTTTAGCATCAATAGTTAGTTGAATATCTTTGCCATCTTTTTTCTTT

TTCTCTATTAATGTATGTGCGATTGTATTGCTATTATCGTCAACGATTGTGACACGATAGCCATCTTCATGTTGG
AGCTTTTTATCGTAAAGTTTTTCGAGTCCCTTTTTACCAATAACTGCATCATCTTTATAGCCTTTATATTCTTTTT
GTTTTAATTCTTCAGAGTTAATGGGACCAACATAACCTAATAGATGTGAAGTCGCTTTTCCTAGAGGATAGTT
ACGACTTTCTGTTTCATTAGTTGTAAGATGAAATTTTTTTGCGAAATCACTTAAATATTCATCCATTTTTTTAAC
GGTTTTAAGTGGAACGAAGGTATCATCTTGTACCCAATTTTGATCCATTTGTTGTTTGATATAGTCTTCAGAAA
TACTTAGTTCTTTAGCGATTGCTTTATAATCTTTTTTAGATACATTCTTTGGAACGATGCCTATCTCATATGCTGT
TCCTGTATTGGCCAATTCCACATTGTTTCGGTCTAAAATTTTACCACGTTCTGATTTTAAATTTTCAATATGTATG
CTTTGGTCTTTCTGCATTCCTGGAATAATGACGCTATGATCCCAATCTAACTTCCACATACCATCTTCTTTAACA
AAATTAAATTGAACGTTGCGATCAATGTTACCGTAGTTTGTTTTAATTTTATATTGAGCATCTACTCGTTTTTTA
TTTTTAGATACTTTTTTTATTTTACGATCCTGAATGTTTATATCTTTAACGCCTAAACTATTATATATTTTTATCGG
ACGTTCAGTCATTTCTACTTCACCATTATCGCTTTTAGAAATATAACTGCTATCTTTATAAACTTGTTTGAAATTT
TTATCTTCAATTGCATCAATAGTATTATTAATTTCTTTATCTTTTGAAGCATAAAAATATATACCAAACCCGACAA
CTACAACTATTAAAATAAGTGGAACAATTTTTATCTTTTTCATCAATATACTCCTTATATAAGACTACATTTGTAG
TATATTACAAATGTAGTATTTATGTCAAAATAATGTTATAATTTTTGTGATATGGAGGTGTAGAAGGTGTTATCAT
CTTTTTTAATGTTAAGTATAATCAGTTCATTGCTCACGATATGTGTAATTTTTTTTAGTGAGAATGCTCTATATAA
AATATACTCAAAATATTATGTCACATAAGATTTGGTTATTAGTGCTCGTCTCCACGTTAATTCCATTAATACCATT
TTACAAAATATCGAATTTTACATTTTCAAAAGATATGATGAATCGAAATGTATCTGACACGACTTCTTCGGTTA
GTCATATGTTAGATGGTCAACAATCATCTGTTACGAAAGACTTAGCAATTAATGTTAATCAGTTTGAGACCTCA
AATATAACGTATATGATTCTTTTGATATGGGTATTTGGTAGTTTGTTGTGCTTATTTTATATGATTAAGGCATTCC
GACAAATTGATGTTATTAAAAGTTCGTCATTGGAATCGTCATATCTTAATGAACGACTTAAAGTATGTCAAAGT
AAGATGCAGTTCTACAAAAAGCATATAACAATTAGTTATAGTTCAAACATTGATAATCCGATGGTATTTGGTTT
AGTGAAATCCCAAATTGTACTACCAACTGTCGTAGTCGAAACCATGAATGACAAAGAAATTGAATATATTATTC
TACATGAACTATCACATGTGAAAAGTCATGACTTAATATTCAACCAGCTTTATGTTGTTTTTAAAATGATATTCT
GGTTTAATCCTGCACTATATATAAGTAAAACAATGATGGACAATGACTGTGAAAAAGTATGTGATAGAAACGT
TTTAAAAATTTTGAATCGCCATGAACATATACGTTATGGTGAATCGATATTAAAATGCTCTATTTTAAAATCTCA
GCACATAAATAATGTGGCAGCACAATATTTACTAGGTTTTAATTCAAATATTAAAGAACGTGTTAAGTATATTG
CACTTTATGATTCAATGCCTAAACCTAATCGAAACAAGCGTATTGTTGCGTATATTGTATGTAGTATATCGAGCT
TCACATGAAACAGCTAAAGAAGCTTTGGGCGATAAAGAGTTAAGAGCCATTGCACATGAGTTAACTAAAAC
AGTTAAGGATAACATGAGTGTTGATTGGTCTAAACGAGACAGTGCTAAAGCTAAAATGAGAGTTCAAGTTAG
ACGCCTATTAAAGAAATATGGCTATCCACCAGATCTTCAAAAAATGGCTGTGGAACAAGTTGTAGAGCAAGC
AGAATTAATGGCAAGTCAGCAATAAAAAAATAAATCATAATGAGTCCGGGACATAAAGTTCTTGGATAAGTG
AAAAAAGACAATTTCTATTGAAATAATATAGAAATTGTCTTTTTTATAAATTTTTTGATTATTTTCAGCTCGTTG
AGCTACTACTTTTCTTATATTAAGTGCCATTAATACAAAACCAAGTTCTCTTTTGACTTTATTGAGTCCTCGGAC
AGACATCCGAGTGAAACCCAAAATAGCCTTCATAAATCCAAAAACAGGTTCCACATCAATTTTTCTTTGACTG
TAGATATTTTTTGTTTCTGGTTCTGAAAGCTTTTTGTTAATTTGGGATTTAAAATATTCCCAGTTATAATTCTTC
ATTATTTTTTTGTTTGTTTTTGAATTGAAGTTCATACATTGATTTTTCAGAGGACATTCTGAACAATCATCACAT
TCATATAATTTGAAGTCTCGCTTATAACCATACTTATCATGACGATAGGCATATCTTTTAAAACCTAGCCGTTTAT
TATTCGGACAAATGAATTCGTCATTAATTTCGTCATAGTTCCAATTTTGAGTATTAAAGATGTCACTTTTATATTT
TTTAGTTTTATCTTTTATAAACATTCCATATGTTATGAGTGGCGTTCGATTAAAGTCATCTATAATTGCCTTATAAT
TTGATTCACTACCATAACCTGCATCAGCTACAATATATTCAGGTAAATGACCGTAGGTCTCTTGAATTGAATTTA
AAAATGGAATCATCGTTCTAGTATCCGTTGGATTTTGATACACATTATAAGATAAAACAAATTGGGAATTTGTT
GCTATTTGTAAATTATACCCTGGCTTAAGTTGTCCATTTTTCATGTGATCTTCTTTCATTCTCATAAATGTCGCAT
CATAATCTGTCTTAGAATAACTATTTCTATCCTTTAAAATAGATTTTTGAAATTCGTATCGATACTTTCGCTCAAA
ATAATCATTGATTTGCTTTTTGTATTTTTTGATTTTAGTTCTTTTGAGACGTATTTGTTTTCTTGTTTTAGTACAT

TTTTCATTGTTGATATGTTGGTTTAAATCTTCGATTTCTTTATCTAAGTGACTACCAATCAAATCTATTTCTTCTT
TTGTTAATTCATTATCATGATCTTCTTTAATTTCCGGTATGATTTTATTGGTTACCAATTCATGGTAGAGGGCTTT
AGAATCCTCATTCATCTTTGATTCATGGTTTTGAATACTCTTTTTCCATACAAATGTATATCGATTGGCATTTGCT
TCAATTTTTGTACCATCAATAAAAATAGCTTTATCATCTATAAGATTTTGTTTTACACACTGACTGTAAAATTGA
ATAAATAAAGATTCTAATAAAGCATCTACTTTTGGATTTACTCTAAATTGATTAATTGTTTTATAAGAAGGTTTT
TGATTTTGTGATAGCCACATCATTCGGATGCTATCATTAAGCATTTTTTCTATTTTACGACCTGAGAATACAGAT
TGTGTGTAGGCATATAGAATCACTTTTAACATCATTTTAGGATGGTACGAAGTTGCACCACGGTGATGTCTGA
ATTCGTCGAATTCATTGTCAGGAATTGTTTCAACAATATCATTTACAGTAAAACGATGTTGATTTGTTTTGTTTC
CATATTGACCTCCATGTATTTGCTATGATTTCAAAATCCATTTTTGACGTGCCTTAGGGTTGAGTGGATGCATA
ATTTCATTTGTTACTGGATTGATGAGCTTTTTTACTTTCTTTTTATGAGGTTTTAACATTTCCATCACTTGTTCG
ACACGGTCGATAACAACTGGTCGCTTCGCATAGGCACCATAAGCAAGAATCACTGTGTCACTTTCACTAATCG
CTTTCATCAAATGAATATCAGTGTGCTCATCGTATGGATTTTTGATATGTTTGAGGTTTTCGGGTGTTCTAATAT
TAGAGAATAGATTTACAAGATATACAGCACCGTATCGTTCTGAATTGGCTAATTGGTTGAGGATAAGAACAGT
TGTGAGATCGAGTGATAATACACCGTCTAAATGAGGATACATCGTTATCACTGTGCATGCAGCTTTCTTTTCAT
CCCATGTTTTCTTGAGTAAATAGCGGTGCTGTTCATCATCGCTAAATATGGCTTCTGTGTGTATCGTACTTTTGA
TTGTATTCATCATCGTCACTTCCTTTAGTATTCTTCTGGTAAAAGCATCACATAATAAAAAGCGTCCACGTCATC
TTCACGAATGACGTAGACTTTCTTAGGTAATGCATTTTGATTTTTTTCATAGTTTGTATAGTGATATTCCAATTT
GTATGTGGGTTGTTCTTGTTCATGTGTGATTGAGAGTATATTCTCATCTTCTTGTAATTTAAAAATGTGTAGGTA
ATCTGTATGAGGCTGGTTATCTTTTTTTTCTACCATGTGCCAAAGTAAGATTTGAAGGTCTAGTGGAATACTTT
CATTAATTCCTCTTGTGATGTATCGATTGATTTTCATGCTATTTCCCTCCCTTCTGCTTTTCTTTCATGATGTCGA
TGATTTCGTTGATAACTGTGACGGATAATTGAGCAACTATGATCCAATTATTCATGGTCCTGACCTCCTTGTTT
TAGTAAATGACGTTCATCAATAATGATATTTTGAGTATCTGTAAGGTACAGAAAGTCCATGTCAAAATGGTCTA
AGTATCCGACACTGATGAGTTGGTTATTGGCATACATTAGAAATGGATAGATACTTAGCTCATGTAGTTCATCA
TTATAGTAGGTATAAGTCTCGAGTGTGAGATGTACCAGTGGAGAATCATTAATAAAACGTTCCGGTAGAATAT
TTTCTGCTGCTTCCTCCAGCGCTTCACATTCCCAAGCTTCGTTATTAGATAGTTGGAATAGGTGGGTTATATAT
TGTTTGAGTTCTTGAGTGATGGTTTTCATATTATTGCCTCCTAGATAGTGTAATAGTGATGTAGTTCATATACAT
CATTGAGATAATATATATTTGATTTGTCATTTATTACGAATCCCGGTGGGAATAAGAGAAAATTCCATATAAAAA
CCCGCTACAAACGTTGGTATGCCAAGGAAATCCTGAAATTCCGCCTATTTTGACAAACAATCAACTCATTATTT
ATAAGTATTGATGATAGGGTTGTGTCTCTGCTTCCTTATATATATTATTTATTTATAAAAAGTAACGGGATTTTGG
GATTGTGCTTGCACAATCCTTCTGTTTCTTCGAATCTGCAAATCCCAATCATTTCCCGATAAAAAATCATTGTG
GGATGTTCTTTAGCAATTTCAATATAAGCATTGTATAGTTATGAAAAAATTACGACAATAACTGTTTCATTAGAT
AAGTGTTATTGAAATTGATAAAGAGCAATTCTTGAAAATAGTTAGATAAAATAAGCGAAAGAATATAGTGAAA
ATTATTGTTATAACAATGATTCTATTAGCTAAATAGTAAGATATAGTGTTTGGGGCAAAAATAAAGACGAAGTG
CTGAGATGCACTTCGTCGAGTTGTTTATTATTGAAAAGTTGTTTAATGATTTCGTTATTAAGTTTGAGTGTGAC
ATAGAATTGTTTTTTATGATTACCATCTTTTTTAATATCAATGCGATCAATCACTGATAGATACAATGCTTTGAGT
CGAGATTTTTCTATGTGCTTAATATCATGAAAGATGTGTTGTAATAGTTTACTGATTTCTTTGGCATCAAATAAA
GTCTTATCTTCATTTTGTTGATTTTTGAGTTGGTTGATTTGATTCGTAATGTCATTGAGTTGCTTTTCATATTTTT
GAATACTTGGTCTGATTACTGATGTTAAGTCCGGATTATCCTCGATGGTTTTAATCAAGTTATTTATTTTGATTT
GTACTTCATCATATTGTTGTTGCTTATAAGCAATATCGTGATGAAGTGCAGCGCCATCAACTTGATTTTCTTGAT
TGACGTGTGTTACTACGCGTTGAATGACTTTATCACTTTTGACTATTTCAAGTATTTGCTTCATCACATAATCTT
CAATCACATCAGCTCTTACACTGTTTGCCGAACATACTTTGGAACCCTTGTTCCGAAAATTACTACATGAATAG
TAACGAATACGTTTCTTAGTCCCGTCTTTAAGAGTATTCGTGGTATTGCTTGCTGCCATAGGTGCGCCACATTG
GGGACAGTGAATAATGCCTGTAAGCAGATTCGTTCCTTTGCCATGGACTTGGGGTTTTTGACTGACTTGTTT
CTTACGCATTTGTACTTTATCCCATAAATCTTGATTAATAATGGGGGAATGCTTACCTTCAGCTATCACTGGTTT

ATCATTCAGCCCTTTACGACGTTTTTCACTCCAATCTTTGTATTTCGCAAATTGAATTTTGCCGATATAGAAAG
GGTTAGCTAAGATGTATGTGATTGAACTAATACTGAAAGGTTTCCCCTTTTTAGTGACATATCCTTTGTGATTC
AATGCATTGGCAATTTTACGATAGCCATGTCCTTTGGCATAGCACTCGAATATATATTTTACAATATTAGCTTCA
TGTTGGTTAATCATTAGCTCGTGTTTACTATCTGGTATTTTGTCATAACCTAGTGGTAAATTGCCTTGATAATAG
CCTTCTTGGGCACGTCTCGTTTGACCCATAAATACGTTCTCGACAATGTTATTACGTTCGAATTCTGAGAAACT
CGCAAGTATCTGTAACATGAGTTTACCAGAAGAAGTATTGACTTCCATACGCTCTGACAAACTGAAAAATTCG
ACATTTTGTTTGTGTAAATCTTCGACAATTTTGAGAAGATCAGATGTATTACGAGCTAATCGGTTTGTTTTGTA
TACCATAACACAGTCGATATTGCCTTCTTTTGCATCTTTCAACATACGTTGGAGCTCAGGTCTGTTCATAGATT
TACCTGAAATACCACGGTCAGCGTATATATCTTTAACTTCAAAATGATGGAAGTCACAGTATTCTTTGATTTGA
TTGATTTGTCCGTCGATACTATAACCTTCTGTGCTTTGCATTTCTGTTGATACACGTACATAGATACCGACACGT
TTTGTTTTAAGTTGTTGCATTATGTTACATCCTTTCTTCATTTATGCAATCGATGATTGCATGGTTTGATTGACA
ATATTGAGTGGTTCATTTTTGAAATAGATTCCTATAAGATTTTTATCTTTCGTAATGTGAATGGTTTCAATATAG
GGGTACAATATGTTTAACGTGAAACGTTTTTGAATAATATTTTGAATGATGTGTTGTATTTGATGCCCATTGATA
GATGTAGTGCGTTGCGGTTGTTGACGTAATGATTGTGTTTGCTCTCTGAACGTTTCTGCATCAATGATGCCTT
GTGCCAACTTTTCTATCAGTTGTTCTTGAGTCAATGTGTGATGTTTTTCTATGTTTCTTTGTCTTTTGATGCGTT
TGTCAATCGCACTTTTAATTTTTGTGTAGATGCGTTGATTTTGATAAAAGTCTCGGCACACTTCTAATACTTTAT
CTTCAAGTGTTTGTGCATTGATGCCTTTAAAATCACAGACAAAGCGTGAAGCATTCATGTTTTTAGGACAGA
CGTAGTAACGTAATGTATGATTCTTTTTTCTAATGGTCATATTTGTAAGTGTTGCATTACAACATGGGCATTTGA
TTTTTTGTTTGAGTTGATTATCCGAAGGTGTCTGTTTGGTTTGTTTTTGCAATCGAAGTCTCTGCGCTTGCTCA
TATATACTTGTGGAAACAATAGAAGGAAACATATTGTCGAATTGGCCATATTGATTGTTGACACGACCACAAT
AATTAGGATTGATGATAATGTTACGAACTTGATAGGGTTGTCGATTGATATACGTGTTATCTTCTTCTAATAACT
GTGCAATTTTCTTATAACCATGACCTTTAATGTAATAATTGAATACAGCCTTTACCGTTGGTGACTCATTTTGAT
TGATGATGAATGTTCCGTTGTGATATTCGTAACCAAAAGGTGCATGTGTTGTAATCAATCGACCTTGCTTTGCT
TTTTCTTGAAGCCCATTTCTGACTTGTTCTCCAATGTTATCCGATTCAAGTTCGGCTAAACTGATGAAGATATT
AAGCTTGAGTCGGTCGAAAGCTTGATCCATATCAAAGTAACCATCATGTACGCTTAAGATATGAACATGGTAC
GTTTGACATAATTTGATGAGTTTTAATGCATTTTTCAGATTACGATGCAACCTATTAAGACGATAACAGCATAA
TATGTCACACTGTCCTTGTTGAATTAATTGTGTTATTTGTCGATACCCACTACGATTATCTTTGCGACCTGATTG
TTTGTCGCTATAAAAGTTGATATGTTGAATATGATGTTTTTCGGCTATTGCTTCGATAGCTTGTTTCTGTGCTGC
AAGAGATTGTTGTTTCATCGTACTTTGACGTAAATAGCCTATGACTTGTTTCATATCGGCTCCTCCTTTCACAG
TGATAATATATATTTATGGATGAATTGATATATAAGCCCAACATCAATGAGATGTTGGGCGTCCATATTAGTCATT
TGTTTGATTGATTTCTTCAATTACCAAATCGGCTAATATCTCGATAAGTTCATCCATGTTTTTCACTCCGTTATTT
GTTCTATCTTCAATACGTCGATTATTCAGTTTGATGCTTCACAGTTGTATGATAAAGACAATTAGAAATCTTCGT
GAACTCCTGAAGGGCCTATCCCTTCATTAGCGGATTTAAAAAGTTCTTTCGCAGCTTTGTTATCATTTGACGG
TGTCCAATTTTGAAGTAACGACTTATCTTTAGTTAATCCGAGGATAGATGCAAACTCTACATCTAATTTTAGAT
GGTAAAATACAAGTGATTGTTTTTTACCGCTATTATCTTTGACACTTCTTTTAGTTGTTTGGCGTCCACGGTCA
GCTAATATGAAACCTTTATCTCTTAAGGCGTTGACAACATTATTAACATCTTGAAATTGATGATTGTTTAGCATC
TGTTTAAAAACGTTCGCAATTATTTTTACTTCGATATGGTCATCTTTTAATGAGATTAATCCATAGTTCTCAAAC
ATATTTTTCAAAGCACCTTCATCTGAAAACTTACCTCTGTTTTGTGCCACAAATTGAATGATGACATCAATAGC
TTTATCAGCTAATGAGCGTTCAGAGACTGTATGAGTATGATAATCAATAAAGTAGTCTCTTATATTAGCGATATC
AATATCTGTAGATAAAACACGACCTAATATTTTCGCAGATGTTGTAATGACTGCATAACGCTTAAACATACGAA
TACCTGTATTGTTTGTTTCATCTTTCAATTTAGCTTCAAACCAATCTACTTCCTTGTAAAACCATTGAATAACTTC
ATCTTCACGATTTATAAGATATTTAGCTACTAACGGTAAAACATGACCATAGTTTAGTGCTACAGCTTTTTTAAT
ATTGTCAGCATTGGTCGCATTTGTAGTGAATTGTTCATTAATCTCGATGGTTCTTACACGTAATCCATCGTTTTG
AGCTGAATCATTAAAAATACTGTATTCTGACGTTGAAATGACAGAAGTACCCCAATTCTTAGGCGTTTTAACT

TCTCCATGAACGTTTGAACGTTGACGACCTTGACCTTCAGCGATGGAATATAACAAACCCGTGGTATCTCTAA
GTGTCGTAGATGAAAGCTCATCAAATACTATAGGAATGCCATAATTGTTACTCAAATAACCTTCAAGTGCGTTT
CGTGTGGCATTCCAACTTCTAAAAAGAGTTTCATTACCTTTGGTAGGGTTACCAGCGACTGATACAGCTAAA
GCAGCTGCGGTTGACTTACCGGTTGAGGATTGACCTGTAAAACTAAAGAGAATTCCTGCAAATTCGATTTCA
TGTTTATGCTTCAGAAAACTTGTCACTAAGGCAGAAATCCCAAATATGACTGCCAATTCTAAAAGAAGATGAC
CTTTAACCTCGTCAATATACATGTTAAACCAATTATCAAATGTACCTTTAGGAGTTAAGTCATAAGCACTATCTA
CAATAGGGTATGAAGGTGAGGACTGATTAAATTGAATAGATTTGAGAACTTTATCTAGCGATATCAAGTAGCC
AAAAGGTGTCTCTAGTATACCTACACCCTCATATAATTCAGAAAGTGGTAATCTATCTCGCATCAATTGCAAGG
CATAACTCAATGATCTAATATACTTCTCGTTAATACTGTAGCCATATTTAATTAAAGAAGGTAAGTTTCGTGTTG
TTAAGATATCAGACTCAAAAATGTCTTTTTTACCATTGTTGTTAGTAATAATCAACTTTTCAACACCAGTAATTG
GATCAGAAAATCTTGCATTAACAATAATAGGGCTAGCCATTCTAACCACTTTTTCACTGTCACCATCTTTTTTA
GGTGGTATCGTTTCTTTCCAACCGAAGGAATCTAAAGCAAATGGGCCAATTTCAAACAATGTGTAACTCATTA
GCAATCACCTCCTTTCGAAGGCGTGCTATTATTGCATGGGTTTATCCCTATTTTCTGATATAATAAACTGCCATT
TTTTGTTCCTACAATTTTAAATGGTAATCCTGGCGTGTACTTAATCCAGTAAGCAAAACGACGTCCTACTTCAC
GTTGATCATTCGTGCTACAAACCACATTATATTTCTTTCTTAATTCACCATAAGTGAACGTACTTCCAACTGGTT
GTTGAAAAGATTTAGCAATTATAAAATTATAAAGCTTTTCTTGCTGCTTAGATTTCTTATTCATTTTAAAATTCC
TCCAATAATTTTTAAGGAGTCAATCAATATCATATAGGTTAAACTATCTTCAATTGATTGCTATAATAAGCATAAA
TGAAGCATTATGATTAGACGCGGAACAAAAAATGGGGAGGGGGGGTATATATTGATACCTACGCTAGAAGA
AATTATTGATAAGTATGGAAACTTAGTTGATTATTTGAAATTTGATGTAACAGTTGAGGTTTATGAAGATTTGT
TGTTATTAAGAAGTTTAAGCGATTTAAATGAACATCAAAAGATGGATAGAGTTTCATTTATAAAAAAACATTTA
AACTCAAATTTCGATTATTTATTAGATGATACTGAGTTTAGAAGTATCAATAAGATTTACAGTAATTTAAATATC
ATGACGCATATCAATAGTCAAAATAATAATTTTAAACGTAATCCATTTATAAATGAAGAACAATTGAAATCACTA
CTCGCGATAAAGAAAGTAGATGCACATATGAGTTATGATTCAAATATTACTTCAAAAGCATTGGCAGAAATAA
ACAAAACGCAAAAAAGATTAGTTACAAAGATAGACACACTCTATAATCAGTCAAAAAAAGAAAATGAATATG
TACGGTTAGGAGAAAAAATATCGTATACAAAATTGGAGAATCATTGGAAATATTTATATAATGAGATTCAGTTT
TATAATCTTAATAATCAATTGATTAGTTATGTTGCATTAGAACAGGAATATGCTTGGGCTTTTTTAAATGAACTA
TTTTATTTGATAGAATTGTATTTGAAGGCATTTAAAGGACAAAAGAAAACAGATTATGAGTTTGGGAAAAAA
TTAAACGAATTTATTCAATCATATGTCATTATTTTACTTATAGATATACGATTACCTGTTGTTAGATTATATATTATT
AGAGACATTGTAGATACATGTGAAGGAGAAAAAGATAATTACAAAAGAATTACACTGATAGAAGAATCTATT
AAAAAGTATAGATACGTAAAAGATCAGATTAAAAGATTTAAAAATGGATTAGAAGAATCGTTATTAAATGCAA
CTTTATCTATTGAACAAAATATGTTGAAAGTTAAAATTGATTATTATAAAGCCTACTGCTTTCCTAGAGAAAAA
ACAAAGCATAACAATATTGAGTACAATGTATCGTTGTTTTTTAAAGCATTAGATGCCTTAAAGAAATAGTTTTT
CACTGAAAATATTTATAGATATAATTAAAATTAATTAATATTAAGCCAGGATAATGTAGTCTTAATCGTTCTGAAA
TACGAAAAATGTTGTGGAAATCGCGTGAAATAATCAGAGGAATCGTTTGAAATCATCGTAGTAATCGCGCGA
AAATCACGTGAAATAATCAGAGGAATCGTTTGAAATCATCGTAGTAATCGCGCGAAAATCACGTGAAATAATC
AGAGGAATCGTTTGAAATCATCGTAGTAATCGCGCGAAAATCACGTGAAATAATCAGAGGAATCGTTTGAAA
TCATCGTAGTAATCGCGCGAAAATCACGTGAAATAATCAGAGGAATCGTTTGAAATCATCGTAGTAATCGCGC
GAAAATCACGTGAAATAATCAGAGGAATCGTTTGAAATCATCGTAGTAATCGCGCGAAAATCACGTGAAATA
ATCAGAGGAATCGTTTGAAATCATCGTAGTAATCGCGCGAAAATCGCGTGAAATAATCAGAGGAATCGTTTG
AAATCATCGTAGTAATCGCGCGAAAATCGCGTGAAATAATCAGAGGAATCGTTTGAAATCATCGTAGTAATCG
CGCGAAAATCGCGTGAAATACTATGGTAGACGGTTTGAGTAAATTAATGGAGTATTTTAATATTTATGGTTTG
GAGGAATTGCGTTGCATAGAGAAAGTAAATATATAGAGTATAAGAAATCACGAAAAGGATTATCTAATGATAT
TTGGTCTACGTATAGTGCTTTTGCAAATACTGAAGGTGGTACTATATATTTAGGAATTGAAGAAAAAAAGATC
GAGGACAAAAAAGTCTTTGTTTCAGTTGGTGTTGAAGATCCAGAGAAAATGATTGAAGATTTTTGGAATGC

ACTATATGGAAGAAGTAAAGTTAGTCAAAATATTTTATCAAATAAAGATGTTAAAATTGTTAATATTGAAAATA
AAGCGTGCATTGAAATTCATGTACCAGAAGCGCCTTATTCGAAGAAACCGATATATGTAGATAATAAAAAAGA
TTTAGTATATAAAAGAGTTGATGATGCTGATAGAATTGCGACTGAAGAAGAGTATAAATTCATGATTGTAAATT
CTCAAGACGATATAGATACAGAATTATTAGATAACTATGACATGTCTGATTTAAATCACGAATCTATCGAAAATT
ATAGGAAACTTCTATTAAAAAATACTAATGATGAGAGATATGCGAATATGAGCCAACTGGATTTAATGATAGAT
TTAGGAGCATATAGAAAAGATAGAAGTTCGAAAGACAAACAGTATAAAATGACTACAGCATGTTTATTATTCT
TTGGTAAGTATAATGCGATTAGTGATAGATTCCCAGGATTTCAATTAGATTATTTTAAGAAAACAAATTACCTA
GATACTGATTGGAAAGATAGAATATCAAGTGGAGATTTAGGTAATGAAGATTTAAACGTGTATAGTTTTTTTG
AAAAAGTATTGATAAAATTAACTGATAACATTGAGGAATCATTTAGCCTAAATGATGGTTTGACTAGACAAAA
TTATGCAAGAGATTTAAAAGTAGCAATTCGCGAAGCACTGGTTAATACATTAATGCATGCGTATTATGATACTA
AGCAAAGTATTAAAATAGTTAATTGTGAAGATTTTATAGAGTTTTATAATCCGGGTAATATGAGAATAAATAAA
GAAGATTTTATTCATGGAGGGCATTCAAAGGACAGAAATAGTATATTATCGACGCTTTTCAGAAGAGTAGGA
TATTCAGAAAAAGCTGGATCTGGAGGACCAAGGATATTCGATGTAGTTAATAGACATAAGCTTAAAACGCCT
GAAATAGAATTAACGGACATGGACACTAATGTAGTACTTTGGAAACAAGATTTAATGAAGGAGTTTGAAAAA
TATCCTGAGTTAGACAAAAAAGTAATAAAGTATATTATTGACTATGGATCAATAAGTAAGGGTGAAGCCTTAA
AAATGGAAAATATGACAGAATATCAGTTTAGAAATATTTTAAAAAAACTAAAAGATGATAACTTGATAAAAAA
AGAAGGTGAAGGTCCGGCTACTAAATATGTGTTAATAGAATCAAAAGAAGCTGATATATTGCGAACTAAAAA
AGTAATTAAAAGTTTAGAGTCTTTCTTTAGGAATAAATAAAAAAACAAGAGATAGGTGCGAAGTGTTTGGAT
TATACAAATGCTTCGCATCTTATATTTATTAATAAATATCATAGAAAACCGTATCATTAACCGATACGCAGAGATG
CGGTTTTTTAGACACTTCATAAAGGGATTTTGAACGTATCAGAACATATGAGGTTTATAGGAATTGCTGTTAT
GTTTTTTGATCACATCAATAAACAAAAAAGGTATGTACTATGTAAAATATTTATTAAATGATATAAAGCGAGGG
TATATAAATGATTTTTAAATAGATATTTATCCAATAATATAAAAAGGAACTATAAGCTATATCTAAAAGCTATATCT
AACTACTTATAGTCCTTCTTCATTAGTATAAATATAATTATTAAGAAAGGTATATATCTTTGTAACCTTCGTTTACA
TTAATAATGTTGTGATAACCTTTGTGTTCTAAAATACCAATAGCTATAGAACTTCTAATGCCAGACTGACAGTCT
ACATAAATAACATCGTTTCTATTGAAAGGTAAATCTGTTTCTAAAAGTTTGCCGTGTGGCACATGAACCGCTT
GGGATAAGTGGCCATTATTCCATTCATTATCATTACGTACATCTAAGACATGTGCTTCATTACCAGTTATGTCTT
TACTATGAACAGATTGTGTTTGAATTTGAGCTTGTGGTAACTGATATCCAGACACATTATCATATCCAATAAGTT
GTAAAGTATGTTATGTGTTGCTTTTGAAACAAGGTGATAGTCTCCAATCAAGTTAATTTCTTGATTATAGTTTA
GATACCAGCCAATTTGATTGATGAAATTTTTATCATATGGAATATTGATTGTACCTTCAATATGTCCACCATGATA
AGCCTCCTTACTGCGGAGATCAAAAGTTAATCTGTTTGTACTTGTAGCTGGATAAACCGTATAAGGTTGATATA
AATTCATACCGAATTGATTAATTTTTTTCATTTGTGCAAAATGATGTGGTGGTGCAGGTTGGTCAGAAATGAG
TTTATCGATAAAGGTAGCTTCATTATTTTCAGAAAAAGCCCAGTTCGTTTGTTTTTCATAGCCAAGCGTAGATG
TTGGAATAGCACCTAAAGATTTACCACAAGGACTACCAGCGCCATGACCAGGCCAAATTTGAATGTAGTCTG
GCAAGTCTTTAATACTTTCAATAGATTTAAACATTTGTTTTGCGCCTATTTCAGATAATCCTTCTACTTTAACAG
CTTTTTCTAGTAAATCAGGTCTACCGATATCTCCTACAAAAATAAAATCACCACTGAATAGTCCCATTGGAACT
TGTGCTCCAGCACCTTCGTCAGTAAGTAAAAAACTTATACTTTCTGGCGTGTGACCAGGTGTATGAAGCACTT
TTAATTTTATATTTCCTACATAAATATCATCATTATGTTGAACAAAATGAGTGTGGTTAGGCATATTTTTATAACC
TAACATGTCATCACTTTCGCCCGATACATAAATACTAGCATTTAACTTTATAGCAACATCTCTAATTCCTGAAAC
AAAATTTGCATGTATATGTGTTTCAGCTGCATGAGTAATGGTTAAACTCTCTTCATCGGCAACTCGAATATATG
AAGATAAGTCACAAATAGGATCAATGATCATGGCTTCTCCAGTTTTTTGACAACCGATTAAATAAGATGCTTG
AGATAAATGTTTATCATAAATTGATGATAGTCAATTTGTTATCAAGGGTGATAATTATATTAACTATGGTAATTAC
ATGGAATTAAAGTTATTAAACAAACTTGATGATGACCAATTAGATTTAGAATTTTTACAAGATAATGGGTTAAA
AGGTAATGTTCAAGGTCCTATGACTTTAAAGGAACCTTTAAAAAGTTATATACAAAAGTCTTGAAAACGAATA
TTTTAAATTTAGATAATGAGTTAGAAAACACTGAATATGTCGAAGGTAAACCATATGTACAGTACGGTATTAAA

TATGAAAGAAATCAGGCATTAAGAAATGAAGCTATTAAAATTCATGGAACTACATGTAAAGTATGTGGATTTG
ATTTTAAAGCTAAGTATGGCGATTTAGGTGAGGGTTTTATTGAAATTCATCATTTAAAACCAATGTTTTCAATA
AAAAGAGAAATAAAAGTAAATCCACAAAAAGATTTAGTCCCACTATGTTCTAATTGCCATAAAATGATTCATA
GAAATACTAAAAAACCTTTAACGATTAAAGAATTAACCAAAATAGTTAATTATAATAGCAAATAATTTAATATTT
TATAAACTATCATTCAACCCTCTTAATTTATTAGGAGGTTTTTTGTATTTATGCTTTCAAATGTGTGATATACTTT
GTTTGTTGAAATATAGAGTATCTATAGATAGGGTGATTGAGTATGAAATTCACTGAAGTGGAAGTTATCGAAC
ATCTTGTAAAGGCATATAAAGAAGCAGGAAAGCCTACTTATCCTCATGAAAATTTATATCGAGGACGTAATCAT
AGTATTTCAGGTATTGGAGAAGACTTGCTGGGTGCTTATTTGATTAGTAGATTGGAAGGTGTCCAAATATTTA
TTGATCAGCCTTTATCTATGATTGATAAATCTTTAAGTACAAGATATCCGGATTTATTAATTTGTGAAGATAATG
AAATTAAAAATATACTAGAAGTTAAAATGGACTTAGGATATCAAAGAAAAGATTTTATAGATTATTGCCGAAA
GAAAGAAGAATGGATTTCAAATATCGTAGGAAAACAGTGTGTATTGTCTAGAAAGAGAGAAGACAAAATTC
CTATGAATATAGCTGATGATATTAAATTTCATGTTGTGATTTACAGTGAAAACAATGGACCGAAGCGGTTTGAT
GAAGAAATCATGCCTATCGTTAATGAAACATGTCCACATATTGAAGTATATGTCCTAACAAGCGGTCAACACCC
TAATTTAGTAAATGTTAATCTTGAAGGTATTAATATTAATAAAGATGAATTTGAAATATTAGTAAATGCGTTATAA
AAAAATAGAGCATCCTCCACGTTATGGAGGTGCTCTGTTTTTTATTGAAAAGTATCAAGTTAATTAATTTAATA
TGCTTAATAAGTTCTACCTTGACCTTTTTCTCTAGCTTCTGTTCGATCTCTTATGTACTCAGTACATACTGGATTT
TCTGTTAATTCATCAACTGTATTAGTTTAATCAGAAGACGTGTCTACTTTGTAAGCTTCTAAGAACTTATTATAT
GATATAGCGTTTGAGTTTTGTTGTTCTTCTATAAATTTCTGTAGTTATTTTTCAAAAACCGCATCATTAACTGAT
AAGCAGAAGCGTATCACAAATAAAACTAAAAAATAGATTGTGTATAATATAAAAGGAAGGGATTTATATTAAA
ATTTTGAATTCAAAAATTATTGAAAGGGAAGCTACCTTAGAAATTGAATCTATGGCCACTAACTGCTTTTTTAT
ATCACCACTTATTAAAAAATGTAAGAATATTCCTCAGTGCTAAGTGAAGGAGGTTCTAGTTTTATTTAATATTC
ATATTGATAGGTAATGGTTAATAATTGCCTGTAAATTATTGTTAAATATCGTTGTTATAGTATCATCATTCATAAA
ATTTTTATAGATTTTATCAAGTATTTCCTCATCTTCAATAGCTGTAAAGCGATGTACTAGTCTTTTTATGAGCTAT
ATATAATTTTGCGAAGCTAAACTGTTTGTAATTATTCACCAATCAGGTTATGTAGTGTCACGGTTCATAGGTAAT
TCTCTTTTTAAAATCATCTAATATTTTATATAATTCATATAAGTGTAGTATTAACGTTTTCTACTTTATCTATAAAAT
ATTTAGAATTACATTAGAATTTTAAATTATACGGCTGGAAATTCAATGCTGTAAAAATAGACAGATTTTAAAAA
AGTAGTAATAAAAAGTCTAAGTTTTATCTTTGTAATAAACTTCGACTATATTTAAAAATAAATATGAATGTCTTT
TAATTTCTATAATATCAAAGCTATTATATGATTTGAAATTTTGTAACTTTTTCTCTAATTTGGAAGTAGATAATGA
CTTAAAGGTTTTAACTTTTACCCAACGACTTGGCATTTTATCACTCCTATGTAATGTATTACTTCAATATACCCTT
AAATTTTTATAATTAATTATATTGTAATATTATTTTATTTTATAAAAATAGTTTAATAAAATGTTTAATTTTTATAAA
AAATAATTAAATATTATAGACATTACGATAGTTTAATTAGAAAATATATTTAAGAATTATGAATTTAGTTCTTATTA
ATGTTAAAGATCATCAATTATTTTGTCACATTATCTTGAGAGGATTACCTTATGAGGTAGCCTTTTATTTGTCAC
CTAATATTGCTCTAAGGCTAATAATACATATATAAAATAAATAATTATCAGAATATATTTAGAAATTGATATTTAAT
AATTAAATAAAAGTATGCTAAATATTTATAATTTACAAGTGAGCTGATAGAAAGATGAGTATAAGCGAATATAA
AAATAGATACTTAGAGTCACTATATAACACCTTTAAAACTGAGTTAGAAAACGAACGCACTCGCTATTTTATAT
TAGATACCAACTATACAAATAGTGATGATACTGAAAAGGAGTTTACTTGGGATGTAAAACAGAATAATAAGAT
CAGAGAAGGCGATTTGTTCATTTTTAGACGGCCAACTAATTTATCTCAAATTGCTCAACAATTTTATTTCTTTG
GAGCTGGGAAAATAGAAAAAATTGAGCGAAAAAATAATGTAGCAACCGCTTACATTTCAAAACCCTTACTTT
TTGTGGATCGTGTTTTAAAAGATAATATTGAAAATTTAAAATGGGAGTTCAAAGAAAGAATTAAAGAAGAGT
GGGAGCAGTTTTTCTTTAAAAATAAAATCAGTAATATTACAAAAAATGATTTTTTAAAACTACTAAGTTTGAGT
AGAAATGTAGTGGAAATAGAAAGTAATTATATTAATAATGATATTTCGCTAACTTTTAAACAAATGGCTGATAT
GGCAACTCGAATAAAGAAAAATATTTACTACGTAGGGAATCATGAAGCATTTGTTCAAGCTAAGGGAAGTGC
TCATTTTGAGTTTTCAAAAAGGATTAAACAAAATTATAGTTATAGATGTGCAATTACCGGGATTAAAACTAAA
GACTTTTTAGTCGTTACACATATTATTCCTTGGCATGAAAATGAGTTTATTAGACTAGATCCTTCAAATGGTATT

TGTTTGTCTTTATTTTTAGCAAAAGCATTTAAGAAAGGCTTTATCACTTTTTCTAACAGTTATAGAGTTGTTTTA
TCTAAAGAAGCAGAGAAAGATGCTGCTTTATATGAAGAATTAAAGATTTATGAAAATCAAAAAATCGAACTA
CCAGATTGTCAGAAACCTAATTTGAAATATTTAGATTGGCATAGAGAACATATTTTTAAAAATTAATTTTGGGA
GAGGTTGTATGACAGATATTAGTCAATTAGTAGAGGTGCCATACATCTTTCATTAGTAGATTGAAAAAGAGCA
CTTGAAACAGATAATTTGTATTATTTAGCCTATGGATTAACAACTTTAGATAATTGTTTTAAATATTACCAATTAA
TTACACAAAATGAACATTTTTGCTATTCTCAACAAGAAGAAAGAGAAAGTTATCAGCAAAAATTTCAAAGTAT
GATTAATGGTTTAAAAATTAAAATTGGCATACAAAGTTCAAATGATGATGCGCAATAAGCTACAAAGTTTGAT
TTTTTAATTGAACGTTATATGTTTTTAAAAGAAGAAAGAGCTTCGAATTAATATCTTAATGGAGGAAGATTAGT
AAATGGCTAGAACGTATAATGTGATTAAATATGTCATTAAGAAAAATAAGGATGATCTTAAAATACATGGATTA
AGATTATATGAATTTAATATAAAAGATGAAAAAGCTATAAATATTAGCCAACCCATTTTTGTGAACAGAGCAAC
ATTGATTGAGAAAATACAAAATGGTGAAGCATTTACGCACGCCTTTAGAGTAAATCACAATACTTATTATGCA
GGCCAATTAATTTCTTTATCATTGAATAAAAATGGTTATATTAATAACAACTTTGAGGCCTCACGAGATATTATT
AGAAACATTGAAGTAAGTATTTTAGACTATGAATAAAGGTAATACTGATAGAATACATTACAATTAAAAAAGG
GAGTATAGGACTCCCTTAATAAGTTGATATATAAGTGACTTATTTTTTTAATAGAATTTTAATTCCTCTGTTAATG
TTCAAGATATATAATATTAAAGCAATTAAAAAGAATATAATAGTAATAATTAAAAGAATGCTAAATATTAATAATT
GATGATCCAAAGGTTTATCAAAGACTTCTTTAGAAAAAATAAATGCCAATCTACCTATAAAAAAACTAACCCA
AGTCATAATATGATTAATGAATGCTTTTTTGCCATGTTCTGATGTTGGTTCATCAGCAAGTATCCATACAAATAA
AGGTAAAATGACAGGTGCGAAAAAGATACTTAAATAACTCAATGCTGATAAAACGTAACTATTGTCATTTTTA
TGCATATTACTATTTGTATTTTCCACTTTTTTCACCTTCTTTACTTTTCTTTACTATTATAAAAACATTAATAGCGA
GCTATTACAACAGTTATAAGAGAAGCTTACGATATTGTAAGTATGTGTTGTTATAATCAATATAAAATTAAGAAA
GACCTGAGAATTTATATTATCATCCACAATCGAAGTGAGTAGCGAAATTTATTGGAAAAGGTACTTATATTGAA
GATATTGTTAATGGTTCATTTTTAAAAAGCCAAGACGATTTGAAGTTAAAAGCTTCTGACTTCACTGTCGAAG
ATAACTATAACTACGGTGTACTTATATTTCCAGAGAATCTTTCTCTTACAGATAATAATAAGAAATTGGAATCTT
TAATAAAAACTATACTATTTACAGGTAAACGATATCAATATACTTATTTTAAAATTTGATCTTAATAGATAAGGAT
ATAATGATTTAAACAATTTAAGAGATATTATTTTCACAAAAAAAGAATGCTTTTTGTTAGAGGCACTGAACCCT
TAAAATGGGAACTTAATAAAAACACTATGTTCTAGGCTGCTAATTCTCTGTATTTTACAGGGGATAAGTAGCCT
AGTTTTTGTTGAATTCGATTATTATTATAGTTTTTAATGTATTTTCGACAATATCTATTACAATATGATTAGAGTTA
TTAAGCTGATTATTGATGTAAAAAGTTTCAGACTTTAGCGAGGAATGGAAACTTTCTATCGGGGCGTTATCGG
CAGGTGTTCCCTTTCGGGACATACTTCTGATAATGCCTTTTTCTTCGCATAATTGATAATAAGCATAAGATGTAT
AAACGCTGCCTTGATCACTATGTAATATACACCCCTCAGGTATATCGATTTGATTTAATGTATCATTAACTAAAC
GTTGGTCTTGTTTATCATCTATTTTATACGCCACAATTTCTCCGTTATAAATGTCCATTATCGAAGATAAATACAA
CATAGAATGACCAAATGGTAAATAAGTAATATCGGTTGTTAATACTTCCATGGGACAACTCGCTTTAAATTGTC
TTTGTAATAAATTGTCCGTTTTATAATACGGTTTACCTATTCTTGTCGTCTTTTTAGGTCTAACTCGGCAGTTCA
AATGATGCTTCTGCATCACTCTCTGTACTCTCTTATGATTAATTGGTGATGTATAACATTGATTAATCAATGCTGT
AATCTTACGATAACCGTAGGTATATTGTTTACCAACATGTTGTGAAAATCTATAACTTTCCCCATTTCGATACCA
TCGCCACCAATTTTTCACTTGTGTTCTATTTCTAATATTTAATTCTTTCATAATTTCTTTTGTTGGAAATCCTGCT
GCTTTCATTTCAACTGCTTTATACTTTGTTTCTACTGAATAAGAAGCTCTTTTCATAGAAAAAAACACCTCCGT
ATGATTCATTTTAATATGAATTCAACGAAAGTGTTTTTATATAATTCCCACTATTGGGGTCAGTCTAAAATTAAA
ATTGATAACTTTTTTAATAAAATAGATACACTAATTTTAAAACAAGGTAAAAAAATAGAATTATTAAAACAACG
CAAACAAGGCTTACTACAAAAAATGTTTGTGTAATTCTGATAACGTCCTATTATGTTAATTGAAAATAGACAAA
AATAAATTGAGAGCATCCCTTACCGCAAAGTGAAGGATGCTCTATTTTTGTTGAAATTAATATAAGCTTCATTC
TGATAGATAGTCATTAATAATTGTTTGCAAATCGTTATTAAGTACCGTTGTTACAGCGTCATCATTCATAAAATC
CTCGTAGATTTTATCAAGAATTTATTCCTCTTACAGACATTCGCGAGAAGTCCGTTTTAATTTTATTAGAAGTA
ATTCAGGTTTGAACCTACCTAAATGAATATATGAGTTATCTTTTATACTACAAAATATATTCAGATTTCAATAATG

ACATAAAATAGGCATCTTTATATTTACCTTTAGTGTAGAATTGCTCTTTAAGTAATCCTTCTGTTTTAAATCCTTG
TGACTCGTATATATGAATAGCTTTTTTGTTATCTGCATCAACATATAGATAGATCTTGTGCATATTTAAAATATTG
AATGCATAAATTATCGCTTTTTCGAATGCGAATTTTGCATAACCTTTACCACTGAACTCAGGTTTAATAATTATT
TGTATTTCACAATTACGATGGATGTAATTAATTTCTACTAATTCAACAATACCTACGACTTGATTTTCATCTTCAA
CAATAAAACGTCTTTCCGATTCATCTAATAAATGCTTATCAAATAAATGTTGAAGTTCCGTTAAGGATTCATAA
GGCTCTTCAAACCAATAAGACATAATAGAATATTCATTATTTAATTCATGAACAAAAAGTAAATCACTATACTCT
AATGCTCTTAGTTTCATAATCCCACTCCCAAAATTTTCTTATATATTTGCATTATAAATATAAATAACGAATAAGT
CATTATTCACTATGAATAAATCTATTTTAACAATTCACTACATACTAATTCTCATTTTCTTATTATTCTCTAATATCT
CTGATTTATTACTCAGTGAAAGATGCCCTATTTTTATCAGTATATAGTTTTTATTTTGATAGATATTGATTAATAAT
CGTCTGTAAATCATTGTTAAGTAAGGTTGTGATAGCGTCGTTATTCATAAAATCTTCATAAATTTTATCAAGTAT
TTCTTTAATATATTTTTTAATCTTCACACCTTAAATTCACTTGAAAACCAAATCACTTCTAAATTATGATTTTACAT
AATAAATTTTTAAAAATTGGTATTATATAAAATAATCTCAATAATCAGAATTTTTTGTAAATAATAATATTAAAGT
GTAAAATAACAGATAAGAATAGACTTAATGTATATAAATTAAAGGAGTGTTGGAAGTGAAAACTAGAGCAGC
AGTTTTACATGAAATGGGGGCTAAAAAGCCCTATAAAGAATCTAAACCTTTAAAAATTGAATACTTAGAACTT
GATAATCCTAGCGAACACGAAGTATTAATTAAAATTCATGCAGCTGGATTATGTCACTCTGATTTGTCAGTAAT
TAATGGTAACAGACCTAGACCTTTACCTATGGCACTTGGTCATGAAGCTTCCGGTGAAGTAATTAAAGTTGGA
AAAGCTGTTACAAGAGTTAGCGAAGGAGATCATGTGGTATGCACATTTATTCCCAGTTGTGGAAAATGTATCC
CATGTAAAGAAGGACGTCCTGCATTATGTGAAAACGGAGCAATATCTAATGAAAAAGGCGAAATGTTAGAG
GGAGGGATGCGTTTATCTAATGATGAAGGAAAAGTATATCATCACTTGGGGATATCAGGTTTTGCTGAATATT
CTGTTGTCTCTGAAAACTCTATAGTTAAAATTGATAAAAAAATACCTTTTGAACGTGCAGCTGCATTTGGTTGT
GCTATTATCACAGGTATCGGTGCTGTGGTGAATACAGCCCAAATTCGTTCTGGTAGTAATGTAGCGGTTGTTG
GTTTAGGAGGTATTGGATTGAATGCTATTATTGGAGCTAAACTAGCGGGAGCCAATGAAATTATTGCTTTAGA
TATTAACGAAGATAAATTTGAATTAGCAAAGCAATTTGGGGCTACAGCAACATTTAATTCAAGCGATAAAGAT
ATCGATGAGCAAATTAAAGAATATATTCCTGGCGGAGTAGAATATGCTTTTGAAACAGCGGGTGTAGTGCCA
GCTATGAAAGTTGCTTATCAGATTACTAAACGAGGGGGGACAACTGTAACAACAGGACTGCCTAATCCTAAA
GATAATTTTTCTTTCCCTCAAGTTACTTTAGCGGCTGAAGAACGTACCATTAAAGGATCATATGTAGGAAGTT
GTGTACCTGACAGAGATATACCAAGGTTCGTTAATCTATACAACCAAGGACGTTTAAATATTGATTCACTCATC
AGTGAGGTTATCACTTTAGATGAAATTAATGAGGGATTTGATCGTTTGTCTAATGGTGAAGTAGGTAGAATTA
TAATGAAGATGCATTGAATATAATAGAATTCAAGTCGTTCCTCCTCTTGATTTCTATGAAAAGAATAACAATTT
GAGTAGGAAGATTACTGACAAATATTACATTTGCATTGTGACTGCCAATCTAATTGTATTTTTACATTTCATATC
TATATATTAGAAGATAAATGTTTTAAAACTAATTACTAATAGTTTTTTTAGATAAAAAAAGATATAGGAGCATCC
TTCACCGCAAAAGTGAAGGATGCTCTAGTTTTATTAGAATATAGTTTTCATTTTGATAGATAATGATTAATAATT
GTTTGTAAATCATTGTTAAGTACCGTTGTAATAGAGTCATCATTTATAAACTCTTCATAGATTTTATCAAGTATTT
CCTCGTCATCGATGGCTGTGAAATGATGCACCAGACCTTTTACTATTGAAATGTAATTTTGTGATGCTAAACAA
CTAGCAACCGTTCGCCAATTGGATTGTATAGTGTTGTCATGGTTCATAGATAATACTCCTTTACTTTAGTGTCCA
TTTTTGACGTGCTTTAGGGTTGAGTGGATGCATAACTTCGTTCGTTACTGGATTTATGAGCTTTTTTACTTTCT
TTTTATGAGGTTTTAACATTTCCATCACTTGTTCGACACGGTCGATAACAACTGGTCGCTTCGCATAGGCACC
ATAAGCAAGAATGACTGTGTCACTTTCACTAATTGCTTTCATTAAGTGTATATCTGTGTGCTCATCATAAGGCTC
TTTGATATGTTTAAGGTTCTCTGGTGTTTTAATATTAGAGAAAAGATTAACGAGATAAACAGCGCCATATTGCT
CAGAATTAGCTAATTGATTGAGGATGAGAACCGTAGTAAGATCGAGTGATAATACACCATCTAAATGAAGATA
CATCGTTATCACAGTACAAGCCGGTTTCTTGTCATCCCATATCTTTTTGAGTAAGTAGCGATGTTGTTCATCATC
GCTAAATATCGCTTCTGTGTGTATCGTACTTTTGATTGTATTCATATATCGTCACTTCCTTTAGTATTCTTCTGGC
AAAAGCATCACATAATAAAAAGCGTCTACATCATCTTCTCGGATGACGTAGACTTTCTTAGGTAATGCATTTTG
ATTTTTTATATAGTTTGTATAGTGATATTCCAATTTGTATGCGGGTTGTTCTTGTTCATGTGTAATTGACAACATA

TTATCATCTTCTTGTAGTTTGAAAATGTGTAGGTAATCTGTATTAAGTTGATTGTCTCGATCTTTTACCATGTTCC
AAAGTAAGATTTGAAGGTCTAGAGATAGTTGTTCACTAATGCCTCTTGTGATGTATCGATTGATTTTCATACTA
TTTTCCTCCATTTTGCTTTTCTTTCATGATGTCAATCACTTCGTTAATGACTGTAACAGATATTTGTGCCACTTT
GATCCAATTATTCATGGTTATTCCCTCCTTCTTTTAGTAAATGACGTTCATCGATAATCGTATTTTTAGTATCTGT
GAGGTATAGAAAGTCCATATCAAAATGATCCAGATAACCAATACTGATGAGTTGGTTATTAGAATACATTAGAA
ATGGATAGATACTTAGTTCATGTAGCTCATTATTATAGTAGGTATAAGTTTCAAGTATAAGATGTGCAAGTGGG
GAATCATTAATAAAACGTTTGGGTAGAATATTTTCTGCCGCTTCCTCCAGTGTTTCACATTCCCATGTTTCATTG
TTAGATAATTGGAATAAGCGAGTCATATATTGTTTGAGTTCTTGAGTAGTTGTTTTCATATCATTGCCTCCCTAG
ATAGTGTGATAGTGATGTACTTCATATACATCATTGAGATAATATATATTTGATTTGTCATTTATTACTGATCCCGT
TGACAATATGAGAAAATTCCATATAAAAAACCGCTACAAACCTTGGTATGACAAGGAAATCCCGAAATTCCGC
CTATTTTGACGAACAATCAACTCATTATTTATAAGTATTGATGATAGGGTGGGGTCTCTGCTTCCTTATATATATT
ATTTATTTATAAAGAATAACGGGATTTTGGGATTGTGCTTGCACAATCCTTCTGCTTCTTCTAATCTGCAAATCC
CATTCCTTTCCCGATAAAAAATCATCGTGGGATGTTCTTTAGCAATTTCAATATAAGCCTCGTGTAGTTATGAA
AAAATTACGACAATGACTGTTTCATTAGATAAGTGTTATTGAAATTGATAAAGAGAATTCTCAAAAATTTTTAG
AAAAAGAAAGAGGAAGTATGTAGATATAAAATAGATTTATTAAATAGTAAGGTATAATGTTTGCGGTAAAAAT
AAAGACGAAGTGCTGGAATACACTTCGTCGATAATAAGGTAGTTGAAGTTTCGATTATTTTAAATTGATCATT
TTAAGCCTACAAATCCCTCTAATGTTTCACCAGATTTGTACACTTTTGTCGCCTTCTTTTTCCTAAATAACGTAA
ATGCCAAGGTTCATATTGATATCCTGTGATGCTTTCTTTGTTTTTAGGATATCTTATAATAAATCCATAATTATGG
GCATTCTTAGCTATCCAGCGTCCTTCCTCAGTTTTACCAAAACTAGCATATAGATTTTCACAACAAGTTGGTAA
ACAATATACCTACGGTAAAGGATTAGAAGAGCTGTCAGAAGTAGAACAATTAAAATTAGAAAATAAGAGAAA
AGATATAGAATTGGATATTTTAAAAAAGTACAAGGTATTGGAAAGGAAGTGGTACCAACAGTAGTCATAGAT
TTAGTGGATCAATTAAAAGTAAAATATTCAATCAAATTGCTACTAAAAGTATTAAACATACCTAAATCAACATAT
TACCGATGGAAAAACAAAACCCATAAAAATGATACCGTAACACAAAAAGTTATTGAATTATGTAAAGCTAACC
ACTATACCTACGGTTATCGTAAGATTACAGCATTGATTAATCAATGTTATACATCACCAATTAATCATAAGAGAG
TACAGAGAATGATGCAGAAGCATCATTTGAACTGCCGAGTTAGACCTAAAAAGATGACAAAAATAGGTAAA
CCGTATTATAAAACGGACAATTTATTACAAAGACAATTTAAAGCGAGTTGTCCAATGGAAGTATTAACAACCG
ATATTACTTATTTACCATTTGGTCATTCTATGTTGTATTTATCTTCGATAATGGATATTTATAACGGAGAAATTGT
GGCGTATAAAATAGATGATAAACAAGACCAAAGTTTAGTTAATGATACATTAAATCAAATCGATATACCTGAGG
GTTGTATATTACATAGTGATCAAGGCAGCGTTTATACATCTTATGCTTATTATCAATTGTGCGAAGAAAAAGGC
ATTATCAGAAGTATGTCCCGAAAGGGAACACCTGCCGATAACGCCCCGATAGAAAGTTTCCATTCCTCGCTAA
AGTCTGAAACTTTTTACATCAATAATGAGCTTAATCACTCTAATCACATTGTAATAGATATTGTCGAAAAGTACA
TTAAAAACAATAATAATAATAATAATCGAATTCAACAAAAACTAGGCTACTTATCCCCTGTAAAATACAGA
GAATTAATAGCCTAGAACATGGTGTTTTTATTAAGTTCCCGTTTTAAGGGTTCAGTGCCCTAGGATTATAGGCT
CTTTTGTTTATAAAGGTAATTGAACTAAAGTATTATAATTTCAATTCTTAATTAATGTTCCTATTTTACCGTCTAA
AGCGTCCCCTAATCCTGCTAGAGATGTAATCAAAACACTGCCTTTAGTATTTTTTTCAAGAAATTGAAGTGCA
GCTTCAACTTTTGGAAGCATACTTCCTTTAGCAAATTGACCATCAGAGATATGTTTTTTCATTTCATCCACAGA
CACTTCATCGAGACCTCTTTGGTTTTCTTTTCCATAGTTAATGTAAACATGGTCCACAGCAGTTAATATGATTA
ATTGATCAGATTGTAAATGTGCTGCTAATAAAGCACTCGTTTTATCTTTATCAATAACTGCATCAACACCTGTAT
AAACTTCATTTTCCTTAATTACTGGAATTCCACCACCGCCGGCAGCGATAACTAGTGTTCCATGAGTGATTAAT
GTTTCTATACTATCTAACTCAACTATACTTATAGGTTGTGGGGAAGGGACAACGCGACGATAGCCGCGTCCAG
AATCTTCTACAAAAGTATAACCTTTTTCTTTCGTAAATTTATCAGCCTGCTCTTTTGTATAAAATAATCCAATTG
GTTTAGTAGGGTTATTGAAAGCAGAATCATCGCTTGCAACTTGAACTTGTGTTACTAGCGTAACAACTTGTTT
ATCTATGCCCATTGAATGAAGTTCGTTTTGTAAACTTTCTTGCATCTGATAGCCAATATAAGCTTGACTCATAGC
ACCACATTCAGGGAAAGGAAAAGGAGGACCTTGTTTGTGTTCCGCCGCATAATTTAAACCTAAGTTAATACT

TCCAACTTGTGGACCATTACCATGACTAATTACAATTTCGTATCCTTTATCGATTAAACTTACTAGAGATTTAGA
TGTACTTTTTAATAAATCTAATTGTTCTTTAGGCGATTGTCCTAAAGCGTTACCACCCAAAGCTACGACGATTT
TAGACATATTTATATCCTCCTTTCATTTACTCTCCTAATGTTGCTACCATGACTGCTTTTATTGTATGTGCTCTATT
TTCTGCTTCTTGGAAAACAACTGATTGTTCACTTTCAAATACTTCATTTGTTACTTCCATTTCAGTTAGACCATA
TTTTTCTTGAATTTGTTTACCGATTATTGTTTCAGTATCATGGAATGATGGTAAGCAATGTTCAAAAATTGTATG
TGGATTACCTGTTTTTTTCATTAATTCTTTAGTTACACGGTATGGTTCTAATAATTTGATACGTTTTTCCCAAACT
TCATCAGGTTCACCCATAGATACCCAAACATCTGTGTAAATTACATCAGATCCTTTGACACCTTCATCAATATCA
TCAGTTATAAGGATTTCACCACCGTTTTTGTCAGCTATATCATTACACCGATTTAATAATTCATCAGTTGGATTT
AGTTCTTTAGGACATACAAGATGGAAAGTCATGCCCATGATTGCTGCTCCTTGCATTAAGGCATTTGCAACGT
TATTACGTCCATCTCCAACATATGTGAAGTTAATTTCATTATATGGTTTTTTCAATACTTCTTTAGCTGTTAAAAA
ATCAGCAAGTACTTGTGTAGGATGATCTTCATCTGTTAAACCATTCCATACGGGAACGCCAGAATATTTTGCC
AAATCCTCAACTACTCTTTGTGAGAATCCACGATATTCTATGCCATCATACATTCCACCTAAAACACGAGCAGT
ATCTTTGGTAGACTCTTTTTTACCCATTTGAGAACCTGTTGGCCCAAGGTATGTTACATGTGCACCTTGATCAT
AAGCCGCTGTTTCAAATGCACATCGAGTGCGTGTTGAATCTTTTTCAAAAAGTAGAGCGATATTTTTACCTTT
CATTTTTTGTTGTTCTATTCCTGCATATTTTGCGCGTTTAAGATCTTCAGATAAATTAAGTAAAAATTCCATTTCT
TTTTGTGTGAAGTCTAACAAAGTTAAAAAGTTTCTATTTCTTAAATTTTTCATTTTGAATATCTCCTTTTCAATT
AATATTTTATTCATGTTATTTTTTTATAATCCAATTTCTTTTATCTTGATATATAATATTTTCTATTTTTAATTCTCTG
ATTATTTCACCAGTAGTTTTTCTGTTAAGTCCAGATAAATTACTTATTAATTGAACAGTCATAATTTGTTTTAATT
CATAGAATTCTCCATTATCATCCCCAATTGAAAGGCATAAAATTTGTAGTATTCTTTCAATTCTTTCTTTAGCTG
AATTAGCTCTTAACGCCATAATATATTCTACTTGAAATTGAATAGTCTCATTAAGTTTCTTGAAGAGACTTTCAA
ATATTTCATTATGCTTTCTACATAAATACTCAAGTAAATCTTTCGGTAAAGTTAATATTTTACAATCTGTCAAAGC
TGTACATATTTCATATGGTGCAGGGGTTTCATTAAATATGAAGTTCATTGGAAATATATTTTCGTCTTTACTTAAT
CTTAAATAATTGTCACCAGTAATATTAGAAGATTCATGTAAAATACAACCATTTACTAAAAAGTATACAAATTTT
ATTTGATCAGTTGAATGATATATGACTTGTCCTTTTTTATATTGATAAAGTGTTAAATCCTCTTTATAAGGTCTAA
CAATACTAACAGGAATATTTAAGTATGATGCTAATTGTTTAAGATTATTATCAAATTCATATTCTGAATTTTTAAT
ATAAATATTTTCTTCATACATAACTATAAACCCCTTAAAAATTACTTAGTTTAAATCATAACTTAAAATACAGAAA
TATTACCTGTAATCAAACGAACTATTCCTATTATTGCAAGTACAATGATGAATATGAATAACGTATAATCCAATTT
TGTCAAATGTTTGTTATTATCCCTTTGTACGTAGCTGTATACGAGTAATCCAGGTATATATAACAACATCGTTAAT
AGTAAATAATCTAATCCAGCTGCATAAACCAACCAAATTGTGTAAATAGATGCAATAATTCCTATTATCCATTGT
TTTAAATTAGCTTTAGATTTATTTTGAATAGTATATTTAACCTGGTAAAAAGCACTGAGTGTATATGGAATTAAG
ATTGCACTTGATGCAAGTGAAAACGCAAACTGATAGGCACTATCTGTAAACAACATACTAATTAAAAATAACT
GAACTAATATATTAGTAATAATTAAAGCGTTGACCGGAGCTTTATTCTTATTTTCTTTAGCAAACCATTTCGGG
AAAAGTCCATCTTTAGCTACAATGAATGGTAATTCACCAGCTAGTAATGTCCATCCTAACCAAGCTCCTAAAAC
AGAGATAATTAAGCCTATATTAACTAACACTGAACCCCAATGACCTACAATATGTTCTAATACTTGTGCCATTGA
TGGATTAGCAAGTTTTGAAATTTGGTTCTGCTGAATGACACCTTGGGCTAGTACAGTCATTAAGAAATAAATG
ACTAGCACAGAAATCAAACCAATAACGGTAGCAGTTCCTACATCCTTTTTAGACTTTGCACGTCCAGAAAAG
ACAACGGCTCCTTCAATCCCTGTGAATACCCATACAGTTACTAACATAGTACTTTTTACTTGTGCCATTGTATCT
CCCCAACTAAAAACGCCAACACTTCCACTAGTCATACCATAAAAACCGGATTTAAAAGTACTGAAGTTGAATA
CAACTATCATGCATATAATAACTAGAAATATAGGTATTAATTTAGCTACTGTAACAATACTATTTATAAACGCTGC
AGTTTCTACACCTCTAAGTATTAAAAAATGTACACCCCATAATAAAATTGATGCTATGATAATACTTGGAAGTGT
GTTACCTCCTTTAAATATAGGGAAAAAGTTACCCACAGCTGACATTAATAGGGTTGCATAAGCCACATTACCT
AGAAATGCTGCAAACCAATATCCCCAAGCACTTGAAAAACCAATAAAATCTCCAAACCCTGTTTGAGCATAA
CTATAAATTCCTCCATCAAGATCTGGTCGCTCATTTGTTAAATTTTGAAATACGAAAGCAAGAGAAATCATACC
AATAGCAGTTATTATCCAACCGATAATTATTGCAAGTCCACCAGCTTGGCCACCCATATCTGAGATGATATTGA

ATGCACCACCGCCTATCATAGAGCCTATGACTAAACCAATTAAGGAAGTTTTACCTAATTTATTTTCATCCATAT
TAATCTCCCCTAATAAAGGTGGTAAAAATCTTGTTATTTGATTTAATGAATGAATTACTTGTGACTTTTACCACC
TTTCAAATTTAAATATCTTCTCTAAATAACGGCTGACTCATACATCTTGGGCCTCCGCGTCCACGTACAAGTTC
ACTACCAGTAATTTCAATCACTTTAATTCCTTTGTCGCGTAAAAGTTGGTTTGATACATAGTTGCGATCGTATGT
CACCACAACCCCTGGTCGAATACATAATGTGTTTGAGCCATCATTCCATTGTTCACGTGCACCATCAATAACGT
CGCCATTACCTGTTGGAATAAAGTCCACTTTTTCTACTTCTAAAACTTCAGCAAGTGTTTCACGTAACTTGCTA
GAACGAGTAATTTTTATATCGTCCTTACCATCATTTTGTTCTATGGTAAATATATTCATATTATTTTCTTCTTTAAA
TATTGCTGCATGTACTGTAAACTTATCGTAGTCAATCATAGTTAGTACTGTATCTAGGTGCATAAATGTACGTGT
ATTAGGTATTTCAATAGCTACGATTTTTTTAAAACTTGTGTTTGCATCTTTGAAAATATTACGTGCTAACTTTTC
TATTGCTTGAGCTGATGTACGTTCTGATATACCAATAGCTAAAACATCTTTCGATAATACTAATTCATCTCCACCT
TCAATATTAAATGGTGAGTTACGATCTAACCATACTGGTACATCTTTATCTTTAAATCTTGGATGATGTTTCAGT
ATATATGTCATAAAAATAGATTCTCTACGTCGTGCTCTCCAATACATTCTGTTAATTGTCATTCCTCTACCAATTG
AAGCTTGGGGATCTCTTGTAAAATAAAGGTTGGGCATTGGATCTAAGTAAAATGGATATCTATCATCCATATAT
TCTACTAAATGGGTTGTTTCAAGTTGAATTTCTTCTTTACGTACGCCAGCCATGATTTTATTTACAAGTTCTTG
GTCAGATAACTTTGAAAAGAATTCTTTAATTTCAGTTTCATGACCTAATATTGTCTTTTTAGATTCTGTTAATAT
GTCGTTTATGAAGTTCTCGCGTACTTCTGGCTCAGTAATAGATTCTGCTGCAAGTTTTTCTAAATAAACTACTT
CGATTCCTTCATCTCTCAAAGTTTGAGCAAATTTGTCATGCTCTTCTTGTGCAACTTTTAAGTAGGGAATATCA
TCGAATAATAAACCACTTAAATGATCAGGTACTAAATTTTCTAATTCTTTTCCTGGTCTTTTTAACAACACAGTT
TTCAATTTGCCTATTTCACTATTTACTTGAATGGGTCCTTGTACCATTTCAAATTCCTCCTTTGCTTTATTACATT
CACATTATAAAACGCTTTCAAAAAACATCGTGTGATTTAATTCACAATTGTTTAGGGAGGGTTTTCACATATAT
AGATTTATTCAATGCAAAATTTTGTATGAATATGTATTTTGTACTATATAATAGTGAATAATTTGAAAAATCTCAT
TGTTAAAACCTTATCTTATTCAAGCAAAAAGATTTTTTACTCATTTTTTTGTATTAGTAATCATTCCTATTAATATA
ATCATTTAATTTTTAAGGGAATAATTGATAACAAACAAATTCTGCTATTTCATTTGAAGAAGTCAAAATCATCA
AAGTATCGTTACCTCCAATAATTCCTAATATTTCTTTCATTTGTAATTGATCTATGTAATAACTTATACTTTGAGCA
AAGCCAGGAGATGTTTTTATTAAGACATAGTTATTTAGCGTTATAAATTCAATAATCTCATCACTAAATATTTCT
AATTGTTTTTTTGCACTTAATTGATTTGTTTGATTTATTTTCTTGTAAATATACTTTTTATTTTCAACAGGGATTT
TGTAAATTTCTAATTCTTGTAAGTCACGAGAAATAGTTGTCAAGCTATAGTAAACTCCAAAATGTCTTGCCATG
TAATCCACTATTTGTTGTTTTTTATTAAACTGATTCTGTTGTATAACAGTTAAGATAAGATTTAAACGTTTTTCTT
TTTTCATTTTTATTACCCCCTTTATTATTTTTGATTATGAAATAGATTTTAAATAACTGTATAAAAATCACTTATAA
ATCTCAATTTACAAATAATTTTGAAATAGTGGATGCTTTAATTATAAGTAGATTATTTTATGCCTAAAAGGAGAG
ATTAATTAGAATAGATTATAGTCATCATAATTATAAAATCAAAATAATTTGCGTAATAATAGCGAAATGTGTAAAT
TGTAAATGTCAGGTAATAACCACAATATCTCATAAAAGAAGTTTGGAAAAAGGCATTGAAATAAAAAGTAGT
TTATAAGTTGAAGATATTTAGGTATGTATTAGAGCGCTTTATAAATAAATAGGTTTATACAAAGAATTACAAAA
ATAATATAAAAAATAGAATCTAATACATGATAAGGAATATATTTGAATAATTTGAATATGAGAATAAGCATACATA
TTTAGGGTATATATAAGTAATAGACATCTAAATAATAAGTAATAGGAGGCTGGTGTAGATGTTCAAAAATATATT
ATTACCCTATGATTTCGAAAATGATTTTAGTGCTATCCCTGACTATTTAGAAAAAGTCACCGATGAAGATTCAG
TTGTTGTAATTTATCACGTTGTAACAGAAAATGATCTTGCAATTAGTGTCAAGTATTATAATAAGCATAAAGAA
GATATTATTAGAGAAAAAGAGAAAAAACTCACTCCATTTTTACGTGAATTAGAAAAAAGAGATATTCAATATA
AAATAGATGTAGATTTTGGGCATATTAAAGATACAATCTTAGAAAAAATTACTTCTGGAGATATAAATAATGGT
GAATTTGATTTAGTAATTATGAGTAATCATAGAGTCGATTTGAATATTAAACATGTTTTAGGAGATGTTACACAT
AAGATTGCTAAAAGAAGTTCTGTCCCAGTACTAATTGTTAAATAAACATAAGAAGTAAGAAATTTATTAATTCA
AAAAGCCTAAATACTTTCTCACAAATCGAGAGAGTATTTAGGCTTTTTTATTTTTTTAATAAACGTAATGAATT
TAATATAACTAATATTGCTGCACCTGTATCACTTAGAACAGCTAACCAAAGTGTTAGTAATCCAGGGAATACTA
ACACAAAGGCAATTAATTTAATAATTATAGCAAAATATAGGTTCTGTTGCAAAGTTGAATCTATAGTATAATTTT

AACAAAAAGGAGTCTTCTGTATGAACTATTTCAGATATAAACAATTTAACAAGGATGTTATCACTGTAGCCGT
TGGCTACTATCTAAGATATGCATTGAGTTATCGTGATATATCTGAAATATTAAGGGAACGTGGTGTAAACGTTC
ATCATTCAACGGTCTACCGTTGGGTTCAAGAATATGCCCCAATTTTATATCAAATTTGGAAGAAAAAGCATAA
AAAGCTAATTACAAATGGCGTATTGATGAGACGTACATCAAAATAAAAGGAAAATGGAGCTATTTATATCGTG
CCATTGATGCAGAGGGATATACATTAGATATTTGGTTGCGTAAGCAACGAGATAATCATTCAGCATATGCGTTT
ATCAAACGTCTCATTAAACAATTTGGTAAACCTCAAAAGGTAATTACAGATCAGGCACCTTCAACGAAGGTA
GCAATGGCTAAAGTAATTAAAGCTTTTAAACTTAAACCTGACTGTTATTGTACATCGAAATATCTGAATAACCT
CATTGAGCAAGATCACTGTCATATTAAAATAAGAAAGACAAGGTATCAAAATATCAATACAGCAAAGAATACT
TTAAAAGGTATTGAATGTATTTACGCTCTATATAAAAAGAACCGCAGGTCTCTTCAGATCTACGGATTTTCGCC
ATGCCACGAAATTAGCATCATGCTAGCAAGTTAAGCGAACACTGACATGATAAATTAGTGGTTAGCTATATTTT
TTTACTTTGCAACAGAACCTATAAAAAACATGGGCTTAAAGGGTTAATTTATATAGTATTACTGCAAACATTGA
TTTGGGTCAAAAATTAGGGGTATTAAAAAAATGAATGAATCATAATTTTATCAAGCTGATTGGAGAGGTTAAA
ATGCATTATATAAAATTTATTGAGTCAAAAGATAATACAAAACTTTATATGAAAGTGAATGATATTCAAGATGCA
AAAGCGAATATCATTATAGCTCATGGTGTGGCAGAACATTTAGATCGTTATGATGAGATAACAGCATATTTAAA
TGAAGCGGGTTTTAGTGTTATTAGATATGATCAAAGAGGGCATGGTCGTTCTGAAGGCAAGCGTGCCTTTTA
TAGCAATAGTAATGAAATTGTCGAAGATTTAGATGCGATAATAAATTATGTGAAGTCAAACTTTGAAGGTAAA
GTTTACTTAATCGGTCATAGTATGGGTGGTTATACAGTCACTTTATATGGAACGAAACATCCAAATACAGTGAA
TGGTATTATAACTTCTGGAGCATTAACACGTTATAATAATAAACTATTTGGCAATCCTGATAGAAACATATCACC
TGATACTTATATAGAAAACAATTTAAGTGAGGGGGTATGTTCTGATTTAGAGGTAATGGAAAAATATAAACTT
GATGATTTGAATGCGAAACAAATCTCTATGGGGCTCGTCTTTTCAATAATGGATGGTGTTAGGTATTTGAAAG
ACAATGCTCAACAATTTACAGATAATATTTTGATATTGCATGGCAAGGAAGATGGGCTAGTAAGCTATGTAGA
TTCTTTACAGCTTTATCAAGAAATAGGATCAGCACATAAATCATTACACATCTATGATCGTTTGGAGCATGAAA
TATTTAATGAAAGTTCTTATAATAGAACTATTTTTAACGAAGTTATTGAATGGCTTGAAACGGAATTAACTTATA
ACTAAAACAGTATAGTTCCGTGTATTTGATTATAAGAAATTATGAGGATATTAAACATACTAAGATTAGCTATGA
AGAAATCTATGACGATAGATTTTTTCATAGCTATTTTTTATAGTTATAGAGAGGAGTAGACTGTCCAGACTCTT
GGATTTTAAATCCGTAAAAAAAACAAGTCAGCTTTACTCTCACCTTTTGAAATTCGTTTGTAGTATGTTGGGT
TCTTGAAACCGTGTATAGGAAAATGAAATGAGAAAGGTTAAGTAAAGTTTTTAGCTTCTCAACTATTCAAAG
GAGGTTTTTTTATCGATTACTTAGGTGTTGATATTAGTAAAAGGAGTAGTGTAGTTGCACATTATAAAAATGG
AAAATTCCAAAAAGAGTTTTTCATCCAAAATAATAAAAATGGCTACAATTATTTACTCAAGTATTTGAATGACT
TAGACCACCCACAACTCATTTTTGAATCTACAGGTATCTATTCAAGAGGTATGGAACGATTTTGTTGTGTAAAT
CAAATTAACTATATTCAAATGAATCCGTTAGAAGCCAAATTTAAAACGAGCGCTCTAAGATCATGGAAAACTG
ATCAGGCAGATGCTCATAAGCTTGCTTGTTTAGGACCGACGCTCAAACAAACAGGCAGCTTACCTATACATG
AGTTAATATTCTTTGAATTAAGAGAACGTGCCCGTTTTCATCTAGAAATCGAGAATGAACAAAATCGACTTAA
ATTTCAGATTCTTGAATTACTCCATCAAACATTCCCTGGTTTAGAAAGATTATTTAGTAGTCGATATTCAATCAT
TGCACTCAACATCGCAGAAATTTTTACTCATCCAGACGTGGTTCTTGATATCGACAAGGATGTACTTATTACAC
ATATATTCAATTCTACAGATAAGGGAATGTCAATGGATAAAGCTACAAAATATGCACTTCAATTAAGAGTGATT
GCTCAAGAAAGCTATCCTAATGTCGATAGACATTCCTTTCTAGTCGAAAAATTACGCTTACTTATTCAACAATT
AAAACAATCTATTCATCATCTCAAACAATTAGATGATGCCATGATTCAATTAGCACAACAACTCGATTATTTTGA
AAATATTCATTCGATACCTGGTATTGGTAAGCTAAGCACAGCTATGATTATTGGGGAGATTGGTGATATTAAGC
GATTTAAATCAAATAAACAACTCAACGCTTTTGTAGGCATTGATATCAAACGATATCAATCAGGTCATACACAC
TGTAGAGATACCATCAACAAGCGTGGTAATAAAAAAGCGAGAAAACTTTTATTTTGGGTGATTATGAATATAA
TAAGAGGGCAGCATCATTATGACAATCATGTCGTCGATTATTACTACAAACTAAGAAAGCAGCCTAATGAGAA
ACCTCATAAGACTGCCATCATTGCTTGTATAAATCGATTATTAAAAACGATTCATTATCTGGTAATGAATCATAA
ATTGTACGATTATCAAATGTCACCACATTAGCCAAACGTACAATTAAATATATTTTAATACCTTATTCAAAAAATT

AAAATGAACGGTTTAGTTAAGTAATGCTTATTTTAATTATAAGTACTTGACTAATCGTAAGAAAGAGCCTAGG
ACATAAATCAATGTCTCGCGACCACAGCTTAATTTTTGGTGTTCATTCATGACTTTAAAAAATCCTTATTGCATA
AATGTACATAGTGTAGTACTATTCAAAACGTAATTATTACGATTTGAATTAAGCGAGGAGAATGAAATGACTAA
GACTTATGACGTTTGGTGGCAAAAAGGTCAAGAATCAGATGATGATATGGCACGAGACCATCAAGAAGCTT
GGGAGAGAACAATAAAAATGCTTGATACATCTGACATCGAAGGGAAAACGATTTTAGATGTGGGATGTAATC
AAGGCGGATTTTTACGACAGTTATACGATACAACACCGTTTAAAAAAGGTGTTGGCATAGATTTAGCACGTT
TATCTTTGGAAAAGGCAGAGACATTAAAAGGACAACGTCCACTTACATACTATTTAACAGATAAACCGCAAG
AAACGAAGCACGTGTTTGATACGGCAGTAAGTACGTCTGTCTTGTACTTAATAGAAGATATTCCGCAACATGC
AAAAGATTTAAAAGAGGTATTGAAACCAGGCGGTGTTTATTACGCTTCATTCGCGGATTTAACTAATAACCCA
AGTCGTCAGTTTATGGATGACACGATTAATCAATATGGTGCAACACCTTCTCAGAATCACTCTCTAAAACATAT
CGTTGATAGCTTTGTGGATGCAGGATTTGAAGTTGCAGTAATGAAAGAGCATGTACCTGACGTGATTGATTT
AACACATTATAGCGATTTTTATTTATCACCGAATGATTATTTACAAACACTATATGAAGAATCGTTTTTAATAAA
AGCAAGTGTGAAAGAAGGTACTGAGAAATGAGGAAATGTGTATTAATGACGGTAGCAGCAAGTGCTACGCT
CTTATTGGCAGGTTGTGGCAATGGTCAAAAAGAAGATAAAGATGTTACGGTATCGCTACCTACTGAAGCAAA
GGCGGATAAACTTGACGCGCAAGGCTATGATGCAGCGATGCCCGTTTATAGTGCAGTGTATGATGCATTAGT
TAAATATGATAAAGATAAGGGTATTAAAGCAGGTTTAGCAGATAAATGGAGCGTTGATGAATCAGGGAAAGT
TTATGAATTCCATTTGAAAAAGAATGTTAAATTCTCAGATGGTTCAGCATTAGATGCTAAGGACGTGAAATTC
TCGATTGATCGTGCGAAAGCGATGAACAAAGATTCGACTGTAGAAACGTTAAAAAAATTAGATAAGGTCGTT
GTTAAAAATGAGCACGTGGTCCAAATTAGATTGAAATCTCCTTCAAATCAAGTGTTAAATGAATTAACACAAG
TGAGACCGTTGCGTATTATGAGTCCACATTCAGTAGAAGATGGTAAAGTAAACGGTAAATTTGAAAAAGCGA
TTGGAACAGGTGCATTTGTTGTTGATAAAACTGGTAAAGAAAAAACGACAATGAAGCCAAATAAATATTTTG
ACAACGGTCACCCAGTCAATTATCATCTTGCATTCCAAACGATTGAAGATGGGGACTCAAGAAATTCTGCAG
TACAAAGTGGTTCTGTAGATATTTCTGGTGGTGCTTTAGGTATGCTCTCAGACGAACAAATCAAACAAGATAA
GAAAAATAAGAACTTAACGATTGAAGATAGACCTAGCACAGTAAGTCACTTTATGGCATTTAACCCTAAAAAT
GATGTATTAAATCAACGCACAATTCGTGAAGCGATAAGTAAGAGCATCGATGCGAAAGACATTGCGGGCAA
ATCTGTAAATGGTCTGTTCCAGAAGAACGTACAATTTGTGACTAAAAATAATCAACAGCCACACGATTATGAT
ATGAAAGCGGCTGAAAGGTTACTTAAATCAGAAGGATATCATAAAAACGATGACGGCATCTTTGAAAAGAAT
GGCAAACCTTTATCATTTAACTTAGTCATTCAAACTGCAGAGTTCCCAAATTGGAAAGATAAAGCTGAAAAA
GTGCAACGTCAGCTTAAACAAGCCGGTATTAAGTTAAATGTGAAAACGTTAGATTCACAATCATACTATGATA
CATTATGGACGAAAAAAGACTATGATTTGATTTTCTATAGAACGTATTCAGATGCATTAATGCCTTACAACTTT
ATAAGTTCAGTGTTTAAAAATAATGATGGTCAACCAGGGGTGTTAGCTGATGATGAAACATTAACGAAACAG
CTAGACGATTTCCCATCAACCGTATCAAAAGAAGACCAACAGTGTTCATTTGATGACATATTTAAACACTTTA
ATCAACAATACTATGGTGTGCCAATTGCTTATCCAAATGAGACGTTTGTAGTGAGTGATAAAGTAAAACAATT
CAAATTCTCTGGACTTACGGATGCACCAATTGATTATAAAGCGTTGAAAGTTAATGAATAGCAATGCTCAAAC
GTACAATTAAATTCATACTTTATTTAATCGTAAGTTCGTTTATTATCTTCATTTTAGTTGAGAAGACATCTGGTAA
TCCAGCGATTCTGTATCTACAACGTCATGGTTATACGTCGATTACGCAAGACAATATTGAAGCGGCACAACAT
CAACTTGGCTTAGGACAACATGTGTTACTAAGATATATCGATTGGGTTGGACATGCACTCACGGGCAACTTA
GGATACGGCTTTAGTACGAACGAAGCAGTTACCGCTATGATAATGGAAGCCATCGTGCCGACGCTTGTGCTA
ATCATTGTCTCTAGTTGTATCATGTTGCCATTTGGCTATATTGTTGGTTACTTCGTTGGGACGCGTCCGCATACA
CGTTACGCTAATGGAATTCGTGGATTCGCCCAAGTGATGACCTCAATGCCAGAATACTGGTTAGCTATTTTATT
CATTTATTATTTAGGCGTACGTTGGCAATTGTTACCATTTGTAGGTAGTGATTCATGGCAACACTTTGTGCTGC
CAATCTTCACAATTGTTGTTATAGAAGGGTGTCATATCTTATTGATGACAGCACATCTGATTACACAAACGTTA
GATCAAGATGCGTATCAACTGGCGCAGTTAAGACATTTTTCGTTAAAAGCGCGTATCATCGTACAAATTAAAG
AGATATTTGCACCACTAATGACGATTTCAATTAACAGTATCATTCATTTAATTGGAAAAGCCGTAATACTAGAA

GTCATCTTCAGCATGTCTGGTATAGGTAAATTGTTGATTAATGCTATTAACCAACGAGATTATCCACTGATTCA
GGGCATTGTCATCTTTATCATTGTCTTTATTATGCTAATGAATTATTTAGGCGATGTGATTATTTTGAAGAATGA
ACCTAGACTTCGACGACGTCATACCCAGCAGTCAGGCAATGAGAAAAGAGGTACGATGTGATGAAAAAATA
TCAAACGTACATCGCAATAGGTTCACTATTGAGTTTGATGGTTGTATTAATTACGTATGGTTTAATGCAAGACA
CGCAACATTTGAACCCACTTGAGTCACCTAATGGACAACATTGGTTGGGTACCGATCAATTAGGCAGAGACT
TCTTAGTAAGACTGATTGTCGGTAGTCTTGTCACATTGAGTTTAACAGGCATAGTGATTCTATTAAGCGTTTGT
ATGGGACTTATCTTTGGCTTAATTGCAGGCATAGAAAGACGATGGTTAGATCAAATCATCATGTTTGTTGCCG
ATATGTTGCTGGCTATTCCGTCATTTATTATCGCATTAGTCATCTTAAGTTTAGTAAGTAACTCCATGATAGGTTT
GATACTTGCTTTAACGATTGGATGGATAGGACGTTATTTACGTTACTTCAGAAATTTAACGCGAGATATTCAAA
AACGTCCATTTGTTCAATATGCACGATTGAGTGGGAACTCAACATTCAAAACGACAGTAACACATGTGATTCC
ACATTTATTAAGTAGTATATTCGCTTTGGTAACGGCTGACTTTGGCAAAATGATGCTCAGCATATCTGGACTTG
CTTTTCTAGGACTAGGTATTAAACCGCCGACGCCTGAGTTAGGAACAATTCTTTTTGATGGGAAAAGTTATTT
CAACGGCGCACCGTGGCTCTTCTTCTTCCCTGGTGTATTGTTAGGAGGTTTCGCCTTATTATGTCAAATTATCA
ACAAAAAAATAACGCAGTAAATACGGTAGTCAACGTCAATCAATTATCGATTTTAGATCAAGAGAAAGTATTG
TTAAAGGATGTTGATTTGACAGTAACTAAAGGTGCATTTCATTGCATTATAGGTGAAAGTGGCAGTGGGAAA
TCACTGTTAACAAGAACAATACTTGGAATGAAACAATCACAATTATGTTATCAAGGAGATATTGACATCGATTT
AACTCAAACAGATGCAGTGTTTCAAGATGTTCAAAGTAATATGTTTCAAAATATAACATTAGCTAAGCATTTCC
AATACATTTATGAAGCCAATCGCACACATCTCACTAAACAGCGTATTAAGGAAGATGTCTTACAGATGATGCA
ATTACTTGGTTTAAGACAAGGGGAACAATTGCTTGAGCGTTATCCCTTCGAACTTAGTGGAGGTATGGCACA
ACGTGTCGCCTTTATAATGTCATTAATTAGACGTCCGAACTACTTATTTTTAGATGAACCAACGAGTGCACTTG
ATCAAGAAAATATTAAAAAGTTTATGCATTACCTTCTTAGGGCACAGGAGCGCTACCAAATGACCATTGTTTT
TATCACACATGATATTAACTTAGTGAAAGATTGTGCCACACATATTAGTATTATGCAGCAAGGTAAATTGATAG
AAAATGGTGAGGCCGCGTCGATCTTAACTAAGCCGACACATAATTACACGAAAAAATTAATTACGATTGCAC
ATCGGAGACAACCTTATGCTTAAAATAGAGAGATTAACCAAATATATAGACACGCAACTGATATTTAAAGAGA
TATCATGTACAATTAACGACCAGCACTTACTCATAAGTGGGGAGAGTGGTTGTGGTAAATCCACATTAGCCAA
GATTATCGCTGGCTTAGATACGGATTATCAGGGCGAATTATATCTTAATGGGCGCTTACGTGAATCTTATACGT
CTAAAGAGTGGATGAAGCACATCCAATATGTACCTCAATATCAACGTGATACTTTAAATCAGCGTAAAACGGT
ATTAGCTACATTATTAGAACCACTTAAGAATTATAAGGTAAATAAACAGCGTTATACATCAAGCATTGAAGCAG
TGCTTGATCAGTGTAATTTACCACACGATATACTTAATCATAAAGTTTCGACATTAAGTGGTGGCCAATTTCAA
CGCGTCTGGATAGCTAAAGCTTTAATATTAGAACCAGAGATTCTCATATTGGATGAAGCTACAACCAACTTAG
ATGTCATTAATGAAGAAGCTATACTTCAAATGTTGATTTCCTTAAAGATGACACAATTAATCATTATTTCACATG
ATACATACGTCTTAAGCCAATTTGAAGGAATTCAGTTACAGCTAAATAAATTGAATAATTAAGATCACAAATCT
TAATATGGTGAATATTTAATGGTACCTAAAAAATAAAATTTAAACTACAATGTCTAAATCCATATGTTGTTTCATT
AGAGGATTTAAAAATGATTATAACACTAAAAGATTTCAAATTATTATATTTAATATAAATTTACATATGATAAACG
AATAACAATTCCAATATAAATTATTTTTTGATTATTTTTATTTATAACTATATATTTTATATGAAAAATATAAGTTAT
AATAATAAGTTTAATATTGCCTCGTGGTTCTGAGCTTGAACTTATCTCTAAATCATTTTGAGCTACTTATCTATCA
ATTCATATATTCTATAACAATATTTGTGACATCACGTGCTATTTCATGAAGTGATTTTACGATATCACCTTCTTTA
GAAAAAATATATTTCAGTAGGCACCGACGTATACAGAATCATTTGAGTATTAAAAATAAAAACTAGAAAAAGA
AACCGCATCATTAACTGATACGCAGAATCATATTATAAATAAAACTAAAAATGAGGTTGTATATAACTCACTCTG
AAATTGATTGAATATATAGTATCTTTAATAAAATGCAGCTATTGTGGCGTAGAATTTGAGAATCAAAAAATGAT
TAATATAGTTTGAAGAGACTGAGCATAAATACTAGAAAAATGGCCAGTAAATGAGTTTACTATAAACTCATTTA
CTGGCTTCTCTAATAATTATCAAGACAATTTGCGTTTCTAGGCATACTTTGAAATGCGCTATTTTCTTTAAGAAT
ATTAATATATGACTTGTGTTGGTAATGATAACCTTGGTTAGAGTGTATTGTCATGTGATAGTTAAGATTAGATCT
TTTAATTAAAACATTGTTTTAAATATCGATGACAAGGTCTAATGTAGGACGTGTAGATATAAGGTTCGGAATTA

TATAAATCCATAAAAGGAGATAAATCGAAAACCTTTATACCCTGGGTGGGTATATGTTATAGTATAAGTAGCTT
TACTATAACATTTTCATTAGGAGGGGTTAATTTGAATAATAATGGTGAAGAGCATAATCATCAAAATCACATGA
ATCATTCCAATCAAATGCATCATGATAACCATGCCTCACATGATCATCATAGTGGCCATGCACATCATCATGGAA
ATTTTAAAGTTAAGTTTTTTGTTTCATTAATTTTTGCAATACCTATCATTCTTTTATCGCCACTGATGGGTGTTAA
CTTACCTTTTCAATTCACATTTCCAGGTTCTGAATGGGTAGTGTTAATATTAAGTACAATTTTATTCTTTTATGG
TGGTAAACCGTTCTTGTCTGGTGGTAAAGATGAAATTGCTACAAAAAAACCAGGCATGATGACCTTAGTTGC
CCTAGGTATTTCAGTAGCTTATATTTATAGCTTGTATGCTTTTTATATGAATAACTTTAGTAGTGCAACTGGTCAT
ACAATGGACTTTTTTTGGGAATTAGCAACCTTAATTTTAATTATGCTATTAGGACATTGGATAGAAATGAATGC
TGTCGGAAATGCTGGAGATGCTTTAAAGAAAATGGCAGAACTGTTACCTAATAGTGCTATTAAAGTTATGGAT
AATGGCCAACGCGAAGAAGTTAAAATATCAGACATCATGACTGATGATATCGTCGAAGTAAAAGCCGGAGA
AAGCATTCCAACAGATGGTATTATCGTTCAAGGACAAACATCTATAGATGAATCCCTAGTCACTGGAGAATCT
AAAAAAGTACAAAAAAATCAAAATGACAACGTCATCGGGGGTTCTATTAATGGGTCTGGAACAATACAAGTC
AAGGTTACAGCTGTGGGAGAAGATGGATATCTTTCTCAAGTTATGGGACTTGTTAATCAAGCACAAAATGAT
AAATCTAGTGCTGAATTGTTATCTGATAAAGTAGCGGGTTATTTATTCTACTTTGCTGTAAGTGTTGGCGTGAT
TTCTTTTATTGTCTGGATGCTCATTCAAAATGATGTTGATTTTGCATTAGAACGTCTTGTAACTGTGTTAGTCAT
TGCTTGTCCACATGCTTTAGGCTTGGCAATACCTTTAGTCACTGCACGTTCTACTTCAATTGGTGCACATAATG
GTTTAATTATTAAAAATAGAGAGTCTGTAGAAATAGCTCAACATATCGATTATGTAATGATGGACAAAACTGGT
ACTTTAACTGAGGGTAACTTTTCTGTGAATCATTATGAGAGCTTTAAAAATGATTTGAGTAATGATACAATATT
AAGCCTTTTCGCCTCATTAGAAAGTCAATCTAATCACCCATTAGCTATAAGTATTGTTGATTTTGCGAAAAGTA
AAAATGTTTCATTTACTAACCCACAAGACGTTAATAATATTCCAGGTGTCGGATTAGAAGGTCTAATTGATAAT
AAAACATATAAAATAACAAATGTCTCTTATCTTGATAAACATAAACTTAATTATGACGATGACTTGTTTACTAAA
TTAGCTCAACAAGGTAATTCAATCAGTTATTTAATTGAGGATCAACAAGTCATTGGCATGATTGCTCAAGGAG
ATCAAATTAAAGAAAGCTCAAAACAAATGGTAGCTGATTTACTATCAAGAAATATTACACCAGTCATGCTTAC
AGGTGACAATAATGAAGTGGCACACGCTGTCGCAAAAGAATTAGGTATTAGTGATGTCCACGCACAACTCAT
GCCAGAAGATAAGGAAAGCATTATAAAAGATTATCAAAGTGACGGTAATAAAGTCATGATGGTCGGAGACG
GTATCAACGATGCGCCGAGTCTTATAAGAGCGGATATTGGTATAGCAATTGGTGCAGGTACAGATGTTGCAG
TGGATTCAGGTGATATCATACTTGTTAAAAGTAATCCATCAGATATCATTCATTTCTTGACCCTTTCAAATAATA
CTATGAGAAAAATGGTGCAAAACTTATGGTGGGGTGCAGGTTATAATATTGTTGCTGTACCTTTAGCAGCTG
GTATTTTAGCATTTATTGGCTTGATTTTATCACCTGCAATAGGTGCTATTTTAATGTCTTTAAGTACAATTATCGT
TGCAATTAATGCCTTTACATTAAAATTAAAATAAAAGATAGGAGTTTTATTATGATTAAAAAATTATTTTTTATG
ATATTAGGATCATTACTAATATTATCAGCTTGCTCCAATAATGATGAAAAAGATAAAGACACTAATGACCAAAA
AAGTGAGAGCCATATGAAGCATAATGATGAAAGTAAAGTTCCAGAAGATATGAAATCGACTAATGAGGGTGA
ATTTAAAGTGGGAGATAAAGTAACGATTACAGCAGGGCATATGCCAGGTATGAAAGGTGCAGAAGCTACTG
TAAAAGGTGCGTATAAAACATATGCCTATGTTGTAAGTTATAAACCCACAAATGGAAATGAAAAAGTAAACAA
TCATAAATGGGTCGTAAACGAAGAGATTAAAGATGCACCTAAAGATGGATTTAGTAAGGGCGATACTGTTAA
ATTAGAAGCAAGTCATATGTCTGGTATGAAAGGTGCTACAGCCAATATAGATAACGTGAAAAAGACTACTGTT
TACGTAGTTGATTACAAATCCAAAGATAATGGTAAAATCATTAAAAATCATAAATGGATGACAGGAAATGAGC
TGAAAGCACGATAAAAATCTAGTTCTAAATTGAGAAATAAATAGATATAAAAATATCCTCCTTAATCAATAATTT
AAATAACTTATTATTGTTAAGGAGGATATTTTTTAGTGTGTAAATTAAAAAGAATTTTAGAAGAATAACATTTAT
CAAAAAACTGTTCATTACCTTATTAATTGAAATTATATAATTAAAAACCGCATCATTAACCGATACGCAGAGGC
GTATCATAAGT
>Staphylococcus aureus PM1
GTATTATGGAATAGAGATGTTGGTAACATTTATACAGGATCATTATACTTAAGTTTAATTTCGTTATTACAGAAC
CACACATTCCAACCAGAAGAGAAAGTATGTCTATTTAGTTATGGTTCAGGAGCAGTAGGAGAAATCTTTAGT

GGTTCAATCGTTAAAGGATATGACAAAGCATTAGATAAAGAGAAACACTTAAATATGCTAGAATCTAGAGAG
CAATTATCAGTCGAAGAATACGAAACATTCTTTAACAGATTTGATAATCAAGAATTTGATTTCGAACGTGAATT
GACACAAGATCCATATTCAAAAGTATACTTATACAGTATAGAAGACCATATCAGAACATATAAGATAGAGAAAT
AAACTAGTGGCCGATTGTGCTTGATGAGCTTGGGACATAAATCCTAACTCGAAATAAATAAGCATATCACTAA
ACTGATTTTTTAAAGTTTACAGTGATATGCTTATTTTTTTATCTTACGATTTTGTACGTGCATGCTTGCCTAGGG
GTATGGCTCGAGCCATTAGTCTCTCGCACATACTATTCCCTCAGGCGTCAGCACTTACAAAATCGGTTGTAATT
TTCATTTTTATACGCATTCTTACTGAGATTATACTAATAAGAGGAATAGTAAAAGCAATTCTAAGTAAAATTGCA
GATAAGAGGTTTGTTAAAAGCAGTTCTAAGTAAAATTGCAGATAAGAGGTTTGTTAAAAGCAGTTCTAAGTA
AAATTGCAGATAAGAGGTTTGTTAAAAGCAGTTCTCAGTAAAATTACAGATAAGAGGTTTGTTAAAAGCAGT
TCTAAGTAAAATTGCAGATAAGAGGTTTGTTAAAAGCAGTTCTAAGTAAAATTGCAGATAAGAGGTACGTTA
AAAGCAATTCCATGCAAAATTGCTGATAAGGGGTAAGTTAAAAGCAGTTCTCAGTAAAATTGCAGATAAGAG
GTACGTTAAAAGCAGTTCTAGGCAAAATTGCAGATAAGAGGTGCGTTAAAAGCAGTTCTAAGTAAAATTGCT
GATAAGGGGTAAGTTAAAAGCAATCCTAAGTAAAATTGCAGATAAGAGGTAAGTTAAAAGCAATCCTAAGTA
AAATTGCAGATAAGGGGTACAGAAAAACTAGACTTGATTACAAAATGGAGCTTGGGACATAAATGATTTTTT
AAAAATGAGATGAGACGTAGATTAACTCCATAATCAATACGAATCTATCGACTTCTTTATTTATGATATTCATCT
CTTTTTAATGGAAATAAAAGTGCGATTAATGTGATAATACAGTTACGTTAATTAAAAAAATAAAAATGCAAGG
AGAGGTAATATGCTAACTGTATATGGACATAGAGGATTACCTAGTAAAGCTCCGGAAAATACAATTGCATCATT
TAAAGCTGCTTCAGAAGTAGAAGGTATAAACTGGTTGGAGTTAGATGTTGCAATTACAAAAGATGAACAACT
GATTATCATTCATGATGATTATTTAGAACGGACTACAAATATGTCCGGGGAAATAACTGAATTGAATTATGATG
AAATTAAAGATGCTTCTGCAGGATCTTGGTTTGGTGAAAAATTCAAAGATGAACATTTGCCAACTTTCGATG
ATGTAGTAAAAATAGCAAATGAATATAATATGAATTTAAATGTAGAATTAAAAGGTATTACTGGACCGAATGGA
CTAGCACTTTCTAAAAGTATGGTTAAGCAAGTGGAAGAACAATTAACAAACTTAAATCAGAATCAAGAAGTG
CTCATTTCAAGCTTTAATGTTGTGCTTGTTAAACTTGCAGAAGAAATCATGCCACAATATAACAGAGCAGTTAT
ATTCCATACAACTTCGTTTCGTGAAGACTGGAGAACACTTTTAGATTACTGTAATGCTAAAATAGTAAACACT
GAAGATGCCAAACTTACTAAAGCAAAAGTAAAAATGGTAAAAGAAGCGGGTTATGAATTGAACGTATGGAC
TGTAAACAAACCAGCACGTGCAAACCAACTTGCTAATTGGGGAGTTGATGGTATCTTTACAGACAATGCAGA
TAAAATGGTGCATTTGTCTCAATAGAAAGTTAGAGGTGAGTCTTACGTTTCAGTGACGGTAGACTTACCTTTA
ACATGTTACATACTAAAAAATTAATTTGAATAAGAAAGAGAGACATATATGAAATACGATGATTTTATAGTAGG
AGAAACATTCAAAACAAAAAGCCTTCATATTACAGAAGAAGAAATTATCCAATTTGCAACAACTTTTGATCCT
CAATATATGCATATAGATAAAGAAAAAGCAGAACAAAGTAGATTTAAAGGTATCATTGCATCTGGCATGCATA
CACTTTCAATATCATTTAAATTATGGGTAGAAGAAGGTAAATACGGAGAAGAAGTTGTAGCAGGAACACAAA
TGAATAACGTTAAATTTATTAAACCTGTATACCCAGGTAATACATTGTACGTTATCGCTGAAATTACAAATAAGA
AATCCATAAAAAAAGAAAATGGACTCGTTACAGTGTCACTTTCAACATACAATGAAAATGAAGAAATTGTATT
TAAGGGAGAAGTAACAGCACTTATTAATAATTCATAATAAAACAGTGAAGCAACCATCGTTACGGATTGCTTC
ACTGTTTTGTTATTCATCTATATCGTATTTTTTATTACCGTTCTCATATAGCTCATCATACACTTTACCTGAGATTT
TGGCATTGTAGCTAGCCATTCCTTTATCTTGTACATCTTTAACATTAATAGCCATCATCATGTTTGGATTATCTTT
ATCATATGATATAAACCACCCAATTTGTCTGCCAGTTTCTCCTTGTTTCATTTTGAGTTCTGCAGTACCGGATTT
GCCAATTAAGTTTGCATAAGATCTATAAATATCTTCTTTATGTGTTTTATTTACGACTTGTTGCATACCATCAGTT
AATAGATTGATATTTTCTTTGGAAATAATATTTTTCTTCCAAACTTTGTTTTTCGTGTCTTTTAATAAGTGAGGT
GCGTTAATATTGCCATTATTTTCTAATGCGCTATAGATTGAAAGGATCTGTACTGGGTTAATCAGTATTTCACCT
TGTCCGTAACCTGAATCAGCTAATAATATTTCATTATCTAAATTTTTGTTTGAAATTTGAGCATTATAAAATGGA
TAATCACTTGGTATATCTTCACCAACACCTAGTTTTTTCATGCCTTTTTCAAATTTCTTACTGCCTAATTCGAGT
GCTACTCTAGCAAAGAAAATGTTATCTGATGATTCTATTGCTTGTTTTAAGTCGATATTACCATTTACCACTTCA
TATCTTGTAACGTTGTAACCACCCCAAGATTTATCTTTTTGCCAACCTTTACCATCGATTTTATAACTTGTTTTAT

CGTCTAATGTTTTGTTATTTAACCCAATCATTGCTGTTAATATTTTTTGAGTTGAACCTGGTGAAGTTGTAATCT
GGAACTTGTTGAGCAGAGGTTCTTTTTTATCTTCGGTTAATTTATTATATTCTTCGTTACTCATGCCATACATAA
ATGGATAGACGTCATATGAAGGTGTGCTTACAAGTGCTAATAATTCACCTGTTTGAGGGTGGATAGCAGTACC
TGAGCCATAATCATTTTTCATGTTGTTATAAATACTCTTTTGAACTTTAGCATCAATAGTTAGTTGAATATCTTTG
CCATCTTTTTTCTTTTTCTCTATTAATGTATGTGCGATTGTATTGCTATTATCGTCAACGATTGTGACACGATAGC
CATCTTCATGTTGGAGCTTTTTATCGTAAAGTTTTTCGAGTCCCTTTTTACCAATAACTGCATCATCTTTATAGC
CTTTATATTCTTTTTGTTTTAATTCTTCAGAGTTAATGGGACCAACATAACCTAATAGATGTGAAGTCGCTTTTC
CTAGAGGATAGTTACGACTTTCTGTTTCATTAGTTGTAAGATGAAATTTTTTTGCGAAATCTCTTAAATATTCAT
CCATTTTTTTAACGGTTTTAAGTGGAACGAAGGTATCATCTTGTACCCAATTTTGATCCATTTGTTGTTTGATAT
AGTCTTCAGAAATACTTAGTTCTTTAGCGATTGCTTTATAATCTTTTTTAGATACATTCTTTGGAACGATGCCTA
TCTCATATGCTGTTCCTGTATTGGCCAATTCCACATTGTTTCGGTCTAAAATTTTACCACGTTCTGATTTTAAAT
TTTCAATATGTATGCTTTGGTCTTTCTGCATTCCTGGAATAATGACGCTATGATCCCAATCTAACTTCCACATAC
CATCTTCTTTAACAAAATTAAATTGAACGTTGCGATCAATGTTACCGTAGTTTGTTTTAATTTTATATTGAGCAT
CTACTCGTTTTTTATTTTTAGATACTTTTTTTATTTTACGATCCTGAATGTTTATATCTTTAACGCCTAAACTATTA
TATATTTTTATCGGACGTTCAGTCATTTCTACTTCACCATTATCGCTTTTAGAAATATAACTGCTATCTTTATAAAC
TTGTTTGAAATTTTTATCTTCAATTGCATCAATAGTATTATTAATTTCTTTATCTTTTGAAGCATAAAAATATATAC
CAAACCCGACAACTACAACTATTAAAATAAGTGGAACAATTTTTATCTTTTTCATCAATATCCTCCTTATATAAG
ACTACATTTGTAATATACTACAAATGTAGTATTTATGTCAAAATAATGTTATAATTTTTGTGATATGGAGGTGTAG
AAGGTGTTATCATCTTTTTTAATGTTAAGTATAATCAGTTCATTGCTCACGATATGTGTAATTTTTTTAGTGAGA
ATGCTCTATATAAAATATACGGTTCTGTTGCAAAGTTGAATTTATAGTATAATTATAACCAAAAGGAGTCTTCTG
TATGAACTATTTCAGATATAAACAATTTAACAAGGATGTTATCACTGTAGCCGTTGGCTACTATCTAAGATATGC
ATTGAGTTATCGTGATATGTCTGAAATATTAAGGGAACGTGGTGTAAACGTTCATCATTTAACGGTCTAGCGTT
GAGTTCAAGAATATGCCCCGATTTTATATCAAATTTGGAAGAAAAAGCATAAAAAAGCTTATTACAAATGGCG
TATTGATGAGACGTACATCAAAATAAAAGGAAAATGGAACTATTTATATCGTGCCATTGATACAGAGGGACAT
ACATTAGATATTTGGTTGCGTAAGCAACGAGATAATCATTCAGCATATGTATTTATCAAACGTCTCATTAAACA
ATTTGGTAAACCTCAAAAGGTAATTACAGATTAGGCACCTTCAACGAAGGTCGCAATGGCTAAAGTCATTAA
AGCTTTTAAACTTAAACCTGACTGTCATTGTACATCGAAATATCTGAATAACCTCATTGAGCAAGATCACCGTC
ATATTAAAGTAAGAAAGACAAGATATCAAAGTATCAATACGGCAAAGAATACTTTAAAAGGTATTGAATGTAT
TTACGGTCTATATAAAAAGAACCGCAGGTCTCTTCAGATCTGCGGATTTTCGCCATGCCATGAAATTAGCATC
ATGCTAACAAGTTAACACAAAGTATTATTTTAAAATTGAGATTAGACATTTATTTTTCAACTTTGCAGCAGAAC
CTAATTTTTTCTCTTTGTAAATAAATGCAAAGAGATACCATAAGGATCTCTTACATAACCATAACCTTCAGTATA
GAATTCTGGACTAAATGTTTTCAATACCTCACTGCCTTTTTCTATTAACTGGTCATATACATGTTTAGTTTCTTCT
ACTTGGTCAAAAGTGAGACAAAGAGATATATTATTACCTTGTGTTATGGGCAAACCTTCAGTGTCATCTGCGA
TCATAATTTTTATATCTCCAAATTGAAGTACACATTGATCAATTTTATTTAAATCATTTTCGTCAATATTAAGTTTC
TTATCTATAGGTCTATCTTTAATACGTTGAATATACAGTGTTTTAGCGCCAAACAGCTCTTCATACAACTTTTTTA
AACCCTCTGCATTTTGAGTGATTAAAAAATATGGACTTACTTGAAATTTCATGTTTTTTCCTCCTTAGATTTGTT
ATAAATAGAGTATATACTTAATTAGTGTCATCTATTGACACTAAAAGGAGAATAATAATGAAAAAATCTGTTAG
ATTATATAATATGATTGAATATTGTAATGAAAATAGGAACTTCAAATTAAATGATTTAATGTCAGAATTTAATATT
TCTCGTAGTACCGCTTTAAGGGATATAAAAGAAATTGAAGCATTAGGAGTACCTTTATATAGTAATCCAGGGA
AAAATGGTGGTTATACGATCATAGGTAATCGAGACCAAACGAAAATAGCAATCTCAGATGAAGAGTTGAAAG
CTTTAGTATTTACACTTTCGAGTATTTCAAATGTGAGTAATCTACCTTTTCAAACAGAATATCAAGAAATATTAA
AAAAATTATATAATAACTCGAATAAAAAAGAGTTAATAAACCAATATAATGATCTATTTCAATATTTTAATGAAG
ATAAGTATCAGTTCAAAAGTTATAAGTTATTTAATGAAATCATTAGATTGATAATTGAGAATAAGTCTTTTGAAA
CCTGTTATTCACAAAACTATATTAAAGAACAATATAAAGGTATTGGCATTATGTATAAAAATCATCAATGGTATT

TTGTTGTAGCTAATATAGAATCAAAGTTAGTGAATCTATTAAATATTTCGAAAATAAAAGAACTATATGAAATG
GGAGAGACTCAAGAGTGTAATGATATAACTATGCAGAATTTTCAACAGTTCATGGTTAAAAATGAAACAGCT
ATTGATATTCTTATTAGAAGCAATGTTATGGGATTGAATATCTTGAAAGGCTACCTGTGGAGTGACTATATGAT
TGAAAATATTGACGAAGAGACATATTTATTTAAATCAAAAGTGAACGCGAAAGATATAGATTTTATAGCTAAG
TTAATTGTCACAGGTGGTGTCAATGTAAAAGTAGAGACCCCTAATAGTTTGAAAAATGCTGTTAAAGTTGAAT
TAACTAAAATAATAAACATGTATTAATAGTAATTTAATCATAATTGTAATAACAAAAAGATGACAACATTAAACA
ATTTAAATTTATACAAAAAAGCATTACCAAATTTAAAATTGGGTAGTGGTAGTGCTACAGCATACATAAATAGT
TATAATTAAATAAAGATGGGTCAATGAAGGTGAATTAAGTGAATAAAAATATTACAGCTCTAAAATCTACTGAA
AATACTACATATATGCAGTATACGGTTCATATCGATGATCAAATTATAAATAATATAAAAGAGTGTACCAAAAAG
TTTAAAATTTTGCCTATGGAAGATAAAATTCCATTATCACCGTTGTTACAACCAGAATATGCAGGAGAGGTAC
AAGATTTTATTAGTACATATGAGCAGTTTATGATTAATTTTGGTAAAGTAATATTGGATAGTCAAGGCATAAAA
ATACAGTTTGAAAGTGAATCATTAAGTAGTATTCAACGAGGCATTCAAGAACATTGTTACTTAAATGAGCGAA
CAAATGACATTGATGTGACTAAAGAATGGTATTTATGCAAATTTTCAAATCAAATATTAGAAGAGGATAAAAA
TCAATTGTATAATGCTTTAAAGCAACTCATGAATGATTCTAAAAATAAAAAGCAAGCTTTTTGGACTGACCCC
AATTAGTGGGAATTATATAAAAACACTTTCGTTGAATTCATAATAAAATGAATCATGCGGAGGTGTTTTTTCTA
TGAAAAGAGTTTCTTATTCAGTAGAAACAAAGTATAAAGCAGTTGAAATGAAAATAGCTGGTTTTTCGACAA
AAGAAATTATGAAAGAATTAAATATTAAAAATAGAACACAAGTGGAAACTTGGTGGCGATGGTATCGAAATG
AGGAAAGTTATAGATTTTCACAACACGTTGGGAAACAATATACCTACGGTAAAGGATTAGAAGAATTGTCAG
AAATAGAACGATTAAAATTAGAAAATAAGAGAAAAGATATAGAATTGGATATTTTAAAAAAGTACAAGGCAT
TGGAAAGGAAGTGGTACCAGCAGTAGTCATAGATTTAGTGGATCAATTAAAAGTAAAATATTCAATCAAATTG
ATACTAGAAGTATTAAACATACCTAAATCAACATATTACCGATGGAAAAACAAAACCTATAAAAATGATACTGT
AACACAAAAAGTTATTGAATTATGTGAAGCTAACCACTATACCTACGGTTATCGTAAGATTACAGCACTGATTA
ATCAATGTTATACATCACCAATTAATCATAAGAGAGTACAGAGAATTATGCAGAAGCATCATTTGAACTGCCG
AGTTAGACCTAAAAAGACGACAAGGATAGGTAAACCGTATTATAAAACTGACAATTTATTACAAAGACAATTT
AAAGCGAGTTGTCCCATGGAAGTATTAACAACCGATATTACTTATTTACCATTTGGTCATTCTATGTTGTATTTA
TCTTCGATAATGGATATTTATAACGGAGAAATTGTGGCGTATAAAATAGATGATAAACAAGACCAAAGTTTAG
TTAATGATACATTAAATCAAATCGATATACCCGAGGGGTGTATATTACATAGTGATCAAGGCAGCGTTTATACAT
CTTATGCTTATTATCAATTATGCGAAGAAAAAGGCATTATCAGAAGTATGTCCCGAAAGGGAACACCTGCTGA
TAACGCCCCGATAGAAAGTTTTCATTCCTCGCTAAAGTCTGAAACTTTTTACATCAATAATCAGCTTAATAGCT
CTAATCATATTGTAATAGATATTGTCGAAAAATACATTAAAAACTATAATAATAATCGAATTCAACAAAAACTAG
GCTACTTATCTCCTGTAAAATACAGAGAATTAGCAGCCTAGAACATAGTGTTTTTATTAAGTTCCCGTTTAAGG
GGGTCAGTACCTTTTCAAGAGGTGTTTAAGGTTCATATTGATATTTATCTCTACAATAAAGAAAAATCTGAATA
TAAGTATCAAGTATCTAGTTATTTCAATCTTGTAAAAGAAAATCCTAAAATAACTTATAAAAAAAGACATTTACA
AGAGAAACAAGGTGTCAAGGGTACAACTTTCACGAACAAATTAAAATTATTGAACAAAATGTATGGTGTAGA
TGTTGCAAAATATCAGCCATTTTATAATTCTAATAATCCTGAATATGAAAGAGGTCAATTTGGTGAAAGATACA
TTTCTCAAAGATCTAACTATGAATTTAATAGACTTCAATATCAAATTATAGATATGTTATCGAAAATACTTGATAA
GCATCCATTACCAAAGTCAGATAATAATTATAAACATATTCCAACTATTGAAAAAGCAATATTAAGTGGGGATT
CTCATAGTTTTTATGAGTATTTTGAAGATATAATGAAAGAAATTATAAGTATGGAAAATTCTTCTTTAAAGGAA
AAATTATTGACTGATTTTACATATCAATCTCAATGTAGATGGTACTCTGAAAGTGAAAAATTAAAATTGCAACT
TGAAAGTTTTATGCATAAAGTTTTGGAGAGCAATTATTATGAGGGTAACAAATTATATAGAATGTTGTCTCATG
CAATTGAAGAAACAATCAATGAGGCTGATGAAGATAAGGTTCATTTCAATTATTTTAAAGATTATTTTTTGAC
AGACGGTGGGGTGAAGAATTGGGAGCAGATTAGCGAGAAAATTACTGAATTCAATGGTAAAGTTATTAACG
ATATCCAAAATGAATATAATAAGATTCAATTTAATAACGCAAATCGAAATCAGAAACTAAACTTTAATTATTTAT
ACCATTGCTTTGAGTTTAGTAATAACTTAGTAAAAGCAAGGGTAAATGGAAATAGAGGTTACATTTTATATTTT

GTAGATAAATATAAAATAAAAATGCCCTTTTTGGATCAATTATTAATCAATTATTTGAATCAAGAAATAGGTCAG
GAAAGTATTAATTATAATATGCAAACATTGTTTGAAAAAGAAAGATATGATAGAAGTAGTACCATTGAAAAAT
TAGTAGCAACAAGCAAATTTAAGTATGAAAAAGATGATTCAGATTTATTCAAACAACTTTTCAATGATGTTGA
AAATTCAATAGACAGATTAGGTATTTACTTACTAAATAATGGTATAAATTCGAATGATGAAAATGCAAGATATTA
TAGATCGTTTTTAAAGGAACTTAGTAGAATAAAAAGTAAATTAACGCCATTTTCTCTTGAAATAAGTAAGTCTA
GCGGAAGAGAGCAACATTATCCTGATGATGCTATTGATGATAAAGATGAGAGAAGAAAAAATAAAGAAGAA
ACATATCATGCTTTTGATGATAAAAGCGATATTGACTCCAAATTAAAGAATAAAATAAATGTTTCTATCGATAAT
TTATTGTCAGTTAAAATTAAGAGCAATTTGTATGATATTAGTCCAAATACAAAAAAACAATAACTATAATGAGC
CAACTCAAGAATGAGTTGGCTTTTTAATTGTAATTTTACGCTTCCATTACAGGAATCAATGTTATGGAAGCCAT
TAAGCAATTAATTTGAGTCATTTATAGTATAGGTACAACGTTGTGATGTTCACATGATAATCAGCCCGGGAGAT
TATTGTGTAATAACTTATAGAAGGAGCAAATCACCATGTATCAAGCAAATATACGTGATTTAATTACAAAATTG
CCTCAAAGCAATAAAACAGAACACTTTTTAATGAACAAATTTTCAAATCAAGATAAAGTTCAGCAGCTACAA
AGACAAATTAGCCAACAGCTAGATCAACAATATAATGAGCTTTTGGCTAATGAAAAAGCTAAGCTAGACCAA
TACGTGGAAGTACACCATAATTTAGAACCATTAAAGAAAGAGATTGAATCAGAATCTATTAACCTTGATACCG
ATAAATTACCTGATATCAAAGCGACAATGCTTGAAAAGGCTAAGAACGATGAACATTTTGATAAAATCGAACA
GCTATTTGATAGATTAGATCAGTCATTAAATGGTACGAATCGATTATATACGCAATTATCGTTGATTGGCACACG
AACACATCGGATCACAACGAAAAGATTTAATGTTCAAGGCTTGCCTAAATTAGTCCAACAAATGATTTTACCT
TCGCAATTTAAAAAGGTTTATACAATAGATTTTAAATCATTCGAACCATCAGTTGCTGCGTATATGACACAAGA
CGAACAACTGATTGACTACTTGAATCATGAAGAAGGGTTATACGATGCATTACTGAGAGACTTATCTTTGTCA
AAAGAGAAGCGCGTGAGTGTGAAACGTGCATTTATAGGGTCATTTCTTTTTGGCGGTCGTTATAGTAGCTCT
AAATTCAAAATCAATCAAGAGGTTAGTGAAATTAACTGGCTACAAGTAATGAGCAAATTCAAGAAGGTCATT
GAATTTAAGGAGCAAGTCGAAAAATATAAAACAATGCCTACGCCTTACGGCATTGAACATGATATGAGCGCA
TTTCAAGGTAGTAGTATTATGGCAATTTATGTACAAACGGTAGCAAGCTATATTTTCAAGCACATTTTGTTGGA
AGTGTACAAAGCACAGTGCGAAAAAAAAACGTTCAAGATTATAGTGCCGATACACGATGCGATTATGATTGA
ATGTAATGATAAGGGGATTGCACAAAATGTAGCACAGCTCATGAAAGATACAGCTAATCAACTGTTTAATGGT
GAATTTGCACATGTGACAGTGGAAGCTTTAGGAGGTATAGACAATGAATAATGATAGAGGAAAAAGTCTTCA
AATTCCCCAAAGTACATTGTTAAAAGAAGGATCTATATACGTTGCTACGTTACATTCTGTGTACGAGAAGAAC
TTCTCAGGTGATATTAAACATCAGTTTACGTATGAAGTAGAACTTAACCAAGAAACGCACTATGTAAATCGCA
ATATTACCGTAAAATCTATGAGTCACCAATTATCAATTGCTGATTGGATTAAACGTCACAGTAACTATAACGTAA
ACCACATTAATTATGATCCATACATTGATCGAAAACATTTGGTCCTTGTAGGGCAATATAACGGAAATTATTATA
TTCAAGATGTAGCACCATTAGATGAATTTGGAGGGGTATTGTAATGAATCATATTTTAGAAATGTTAATAAAAT
TATTAAAAGTGGGTATGGAGGCAATCGACCGTAAAGGTCTGATTGCCATCCTAACAAGTAGTATTGGAAATG
ATGAAATGGATGATTCTGAACAAGCTGTAATGGTGTATAACGAGCTTATCGATAAGCTACAGCTTAACATTCCT
AAAGATGTCGACTATAGACCTAACATATACAGTTATTTTGGTATTCAAAAAAAGCCAAATGACACAATATTAGT
GGAAATGATGATATGTATTTTTCATATCAAGCGTTTTGATTCAGAGTTGTTTGTTTTCAAAGATAAAGGTTGGC
AAAAGGTAAGTGAAGATGAATTGCAAGGGTTGATATCTAAAATGATACAAGTGTTGCTAGTTGATTATAAACC
TTCACTAAGTACTTTGAAAAACGTAGTAGATGGCATACAGAAATCAACGGACATAGAAAAACTTGTTGAGCA
TAGACAGTACATTGGTTGTGGACGAAATATGTTCAATCTAAAGACCTTTAAAGTGGTTGATAATGACCTTGAA
ATATTCCCTAAAACACGCTTAGATTTGGAATTAGATATAAATGACACGATTACGGACAAGATACCCCCGAATTT
CAAACAATATATGTTAGAGTTGGCGAATTATGACCATGATTTACAATATTTCCTTTTCCAACATATGGCAGTGTT
ATTGACGGCAGATACTAAACTACGTCGTGGACTTTTTTTGTATGGAACTGCAAAAAATGGGAAATCGGTCTAT
ATTAAATTAGTTAAGTCATTCTTTTATAGTAATGATATCGTATCTAAAACACTTAATGAACTTGGCGGGCGTTTC
GATAAGGAAAGTCTAATTGGTAAACGAATTATGGCAAGTGATGAAGTGGGGAAAGCTAATATTGATGAAGC
AACTGTGAATGATTTCAAAAAATTACTATCTGTTGAACCAATTCATGCTGACCGTAAAGGAAGAACGCAAGT

AGAAGTTACTTTAGATTTAAAACTCATTTTTAATACGAATGCTGTACTCAATTTTCCATCATCACATGCAAAAG
CATTAGAGCGTAGAATTGCTGTTATTCCATGTGAATATTATGTTGAAAAAGCTGACCCTGACTTAATTGAAAA
GTTACAGGATGAAAAGAAAGAAATCTTTCTTTACTTGATGTATGTGTATAAGCAAATTGTAAAAAATGATATC
GAGTACCTCCAAAATGATCGTGTTACTGAAATTTCTCATGATTGGTTAAATTTTGGATATGAATTTGTTTCTAG
TAAATCAGCAAATATTGCACATCAGAAAGCGTGTATTAATTTACTCAGAAAACTTATAGAAATCAAACCAGGG
TCACGTATCAAAGTGTCTAGGCTAAATGAGGTTATTAGAGATGAAATTAAAGTAAGCTCTCAAGTTATTAATG
ATTTGGTTCAAGCTAACTTTAATGTACAAAGTAGACTAAATAATGGTTATAAGTATTGGGTCGATTTAGGATGG
AAAGAAACTGATAAAAAAGATGACATGATTTCATTCGATAAAAATGAGAATGTAACAGATGATGAATTCTTAT
ACGAAGATGATTTGAACTTAGGTTGGGAGGACTTTGACGATGAATAATGAACAAATTGAAGCATTTGTAGA
AGTGCTTGTGCCTATCATAGAAGAACGTATCAATAAAGGTAAGTAATCTAATTACGTACTACAGGCAGTTGCC
TGTAGTACTCATATGATTAAGTGGTAAAAGTGATAAAAATGAAACGAAATTATAAATATATATTATCTATATGTTG
TTACAAGACCGATGGTCTGTAGCAATAATCTAATAAAAGGAGCGGTATGATATGAAGGGTAAAATTGCACTTT
ATTCACGTGTTAGTACGTCTGAGCAGTCAGAACATGGTTATTCTGAAAAGGAGCAGGAACAACTACTCATCA
AAGAAGTTATGAAAAATTTCCCAGGTTATGACTATGAGACATATACTGACTCAGGCATTTCAGGTAAAAATAT
TGAAGGTCGTCCGGCAATGAAACGTCTATTACAAGATGTTAAGGATAATAAAATCGAAATGGTATTAAGTTGG
AAATTGAATCGTATTTCTCGCTCAATGAGAGACGTGTTTAATATTATTCATGAATTCAAAGAACATGACGTAGG
GTATAAATCGATTTCTGAGAATATTGATACATCCAATGCTTCTGGAGAAGTACTCGTTACAATGTTTGGACTAA
TAGGATCTATAGAACGCCAGACTTTGATTTCCAATGTGAAACTTTCTATGAATGCTAAGGCAAGGAGCGGAG
AGGCAATCACCGGTCGTGTTTTAGGCTACAAATTATCACTTAATCCATTGACACAGAAAAATGATTTAGTTATT
GATGAAAATGAAGCTCATATTGTACGGGAAATCTTTGATTTATATTTGAATCACAATAAAGGATTTAAAGCAAT
CACGACAATTCTAAATCAAAAAGGATATCGTACCATTAATCAAAAACCATTTTCAGTGTTTGGCGTGAAATAC
ATTTTGAATAATCCAGTCTATAAAGGCTATGTCAGATTCAATAATCATCAAAATTGGGCTGTTCAGCGAAGAA
GTGGTAAAAGTGATAAAAATGATGTGATATTGGTCAAAGGTAAGCATGAAGCCATTATAAGTGAAGATGTATT
TGATCAAGTTCATGAGAAACTAGCTTCTAAAAGTTTTAAACCGGGTCGACCTATTGGTGGAGATTTCTACTTA
CGTGGCCTTATTAAATGCCCAGAATGCGGAAATAATATGGTATGTCGACGGACGTATTATAAAACGAAAAAGT
CCAAAGAACGCACAATCAAACGCTATTACATTTGTTCATTATTCAATCGCTCAGGAAGTTCTGCCTGTCATAGT
AATGCGATTAATGCTGAAGTCGTCGAACGCGTAATCAATGTTCATTTGAATCGTATTCTTTCACAACCTAATGT
TATTAAGCAGATTGCGTCAAGTGTGATAGAAGAACTGAAACAAAAGCATAGTAAACAAACAGAAATAAAATA
TGATATTGATAGTCTAGAAAAACAAAAAGCAAAAGTTAAAACACAACAAGAACGATTATTGGAATTGTTCTT
AGATGATGAAATGGATAGCGAAATGTTAAAAGCTAAACAAAGTGAAATGAATCAACAGTTAGAAGTATTAGA
CCAACAAATTAAAGAAGCAAAACAAGCAAATCAATCACAGGATGATATACCCAATTTTGATAAGTTAAAAGC
ACGACTCATTTTGATGATAACACGATTCAGTGTGTACTTAAGAAAGGCTACACCCGAAGCTAAAAATCAACTT
ATGAAAATGTTAATTGATTCAATTGAAATTACGACAGATAAACAAGTAAAACTTGTAAGGTATAAAATTGATG
AAAGTCTTATCCCTCAATCTTTGAAAAAAGATTGGGGGTCTTTTTTTATACCTAAATTTAACTTTGTGATAAAT
GTCACAAAGAAAAATAGGATTGAAAATTTATCACTTTTACCACTTTTTTAGAGTGACAAAAGTGGAGGAGTT
TTGAAATATTTTATAAATATATATTTTATTTATGGAGTACACATTATTAATTAAGGAGGTCATTATAATGACGCTA
AGCAAACAACTTAAAACGTATATCACTGAACGATTTAAATTAAATTATCAAGAAACTTGGGCTTGTGAAACCA
TAGATGCGGTGGCTGAAGATGTATTACCTGAAAAATATATTAAAAATAGTCCACTTGAACATAAAATTTTAAAT
ACTTTTACCTATTACAATGATGAATTACATGAAATCAGCATTTACCCTTTTTTATGTTATCTAGATAAGGAATTAG
TAGCAATAGGTTATTTAGATAATTTTGATTTAGACTTTATATTTTTAAATGACACTCATCAAGTCATTATTGATGA
ACGCTACTTGTTACAAAAAGGGGGCGAGTAATTATGAATTGGATCAAGGTCGCTCAACTATCTGTCACAGTT
ATCAATGAAGTCATTGAGATTATGAAAGAAAAACAAAATGGAGGGGAATAGTATGAAAATCAATCGATACAT
TACAAGAGGTGTTAGTGAACATTTATCTCTAGACCTTCAAATCTTACTTTGGAACATGGTAAAAGATCGAGAC
AATCAACTTCATACAGATTACCTACACATTTTTAAACTACAAGAAGATGATAATATACTCTCAATCACACATGAA

CAAGAACAACCCGCATACAAATTGGAATATCACTATACAAACTATGTAAAAAATCAAAATGCATTACCTAAGA
AAGTCTACGTCATCCGAGAAGATGATGTAGACGCTTTTTATTATGTGATGCTTTTACCAGAAGAATACTAAAA
GGAGTGACAATGATGAATACAATCAAAAATACAATACACACAGAAGCGATATTTAGCGATGATGAACAACAC
CGCTACTTACTCAAAAAGACTTGGGATGAAAAGAAACCTGCATGTACGGTAATCACCATGTACCCTCATCTAG
ATGGCGTATTATCACTCGATCTTACAACTGTTCTTATCCTCAATCAATTAGCGAATTCTGAACAATATGGCGCTG
TATATCTTGTAAATCTATTTTCTAATATTAAAACCCCAGAGAACCTTAAACATATTAAAGAACCTTATGATAAAC
ACACAGACATTCATTTAATGAAAGCGATAAGTGAAAGTGACACAGTGATTTTAGCCTATGGTGCTTATGCGA
AGCGACCCGTTGTTGTCGAACGCGTTGAGCAAGTGATGGAAATGTTAAAACCTCATAAAAAGAAAGTAAAA
AAGCTCATAAATCCGGCAACGAATGAAATTATGCATCCGCTTAATCCTAAAGCGCGTCAAAAATGGACATTGA
AATAAAGGAGGATTATCTATGAACCATGAAACTAAACAATCAGATTGGCGAACGGTTGCTAGTTGTTTAGCAT
CACAAAATTATATATCGATTGTAAAAGGTTTAGTACATCATTTTACAGCGATCGAAGATGAGGAAATTCTTGAT
AAAATCTATGATGATTTTATGAATGATGACTCTATAACAACGGTACTTAACAATGATTTACAGACAATCATTAAC
CATTATCTATCAAAATAAAGACTATACTAATTACAATAAAAACAGAGCATCCTTCACTTTTATGGTGAGGGATG
CTCTTTTAATTTATTTTTTTATTGATTATTCGCCATAAGTTCTGCTTGCTCTACAACCTGCTCTACAGCCATTTTT
TGTAAATCTGGTGGATAGCCATATTTTTTAAGCAATCGTCTAACAGCTACGCGCATTTTAGCTTTTGCGCTATC
ACGTTTAGACCAATCAACACCCATGTTTTCTTTCACTGTTTTAGTTAGCTCATGAGCAATCGCACGTAGTTCTT
TATCTCCCATGGCTTCTTTTGCTGTTTCATGTGAAGCTAAAGCATCGTAAAACGCAATCTCATCTGAATTCAGG
CCTAATTCATTTCCTCGTTGTTGTTCTTGTTTAATATCTTTAGCGAGTTGAATAAGTTCTTCAATCACTTTAGAT
GTTTCAATGGAACGACTATTATATTTATTAATCGAATTTCCTAACATCTCAGAGAAACGCTTAGATACCGTTGC
ATTCGTTTTCATTAATGATTTAACTTGTCCTTTGAGTAATCGATTTAATAATTCTACCGCCACATTTTTCTGTTTC
AATCCTTCGACATCTTTTAAAAAGTCATCAGATAAGATTGATAAATCGGGTTGTTCAAGACCTAGCGTTTGAT
AAACATCAATGACATCTTCAGTCACAACAGATTGTGACACAAGTTGATTAATCTCTGCTTCAACTTCTGCAGG
TGTTTTACGTGTTTTTCCTTCTTTTGGCGGTTGTAACAATTTAACAAGTCCTGCTTTAACTGCTTTAAAGAAG
GCAATCTCATCGTTGAGTTCTTGGGCTGTAGGTTCAGTCGCACAAAGAGCAAAGGCTTTCCCTAATTCTGTG
ACTGTTTTAATAAAACGTTGACGTTCATCTTCACCTAAACCAATTACATAATCCATCGTATTCGATATTGTATAAT
AACGTTCAGATTTTTTATCTGAATTAAATTTAGAGTAATCAAGATTATATAACATATCTTGAATTACATCATACTT
CAATAACATCAATTCAACCGCTTTATCCGTATCTATTGCCGTTTGAGCTTGATCAGATTCTGTATATTCTTTAAGT
GCCTCTTTTAAACTTTCGGCAATCCCCACATAATCGACAATTAAACCACCCGGCTTATCTTTAAACACTCGATT
AACTCGAGCAATTGCTTGCATCAAATTATGCCCTTTCATCGGTTTATCAATATACATCGTATGCATAGAAGGAA
CATCAAACCCTGTCAGCCACATATCTCGAACAATCACGAGTTGTAACTCATCATCCACATCTTTCATACGTTTTT
CTAATAAATTACGGCGTTTTTTAGGACCAATGTGTCTTTGGAAAGAAGTTGGGTCACTAGATGAGCCTGTCAT
TACCACTTTAATGACTCCTTTATCATCATCATCTGAATGCCATTCTGGTTTTAGACGAATGATTTCATCATATAAA
TCAACAGCAATTCGACGACTCATCGTTACAATCATTCCTTTGCCTTTCATCGCTTGTTGACGTGTTTCAAAGTG
TTGGATGATATCTTTGGCTAGGGCTTCTACACGAGGTTTTGCGCCTGCTAAAGCTTCAATACGGGACCATTTT
GATTTTAAACGCTGTTTTACATCCTCTTCTTGATCTTCAGTAATGTCATTATACGCTTCATCTAAATCTAAACTTT
GAGGTAGATTTAATGGAATTACACGACTTTCATAGTAAATTTTAACGGTGCTTCCATCAGCGACGGCTTGTGT
CATATCATAAACATCGATATAGTTTCCGAAAACCATTTGCGTATTTTTATCCGTTGAAGCTACGGGTGTGCCTG
TAAATCCTACGAATGTTGCATTCGGTAAAGCATCTCTTAAATATTTGGCATAACCATATTTAATGCCTTCACCTT
TATCATCGTATTTTGCGTTAAAGCCATATTGTGTACGATGAGCTTCGTCTGCCATGACAATCACATTTTTTCGTT
CTGTTAGGGCAGCCATGGTCGTTTCGTTTTGTTCAGGTTCAAATTTTTGCATCGTTGTAAATACAATACCACCC
GACTCAACAGATAATAATGATTTTAATTCTTTACGTGTTTCAGCTTGTTTTGGTGTTTGTCTTAATAATCCTTTA
CCAGAACGTCCTTTTGATTTAACAAACGTACTGTACAGTTGGTTATCTAAATCATTACGATCCGTTACGACGAC
TAAGGTAGGGTTATTCAGCATTTGAATTAATTTTCCAGAGAAAAAGACCATGGTTAAACTTTTACCGGACCCT
TGGGTATGCCAAATAACGCCGCCTTTACCATCACCTTGCTCAGATGAAGCTAATAAAGCTCTGTCAACGGCTT

TATTAACGGCATAATATTGATGATACGCTGCTAGAATTTTACTGATATGTCCTTTGCCATCATCTTGAAATAAAA
CAAAATATCGAATTAAATCAAGTAATGTTTCTGGATTTAACATGCCATGAATGAGTACGTCTAAGCTAGCTAAA
CCTGGTGATGATTCAGTTTGACCATCTTTACTGCGCCAAGTCATAAACCGATCATAATTTGCGGTTAGAGAGC
CAGCCTTTGTATTAATACCATCACTTGTAACAAGCACTTCGTTAAACGTAAATAATTGTGGAATACGCATCTTAT
ACGTTTCTAATTGATGATAACCGTCTTCGACGCCCACGGTTTCATTTGTTGAATTTTTAAGTTCAATCACTACG
ATAGGCAAGCCATTGATAAAGAGTACAATATCGGGACGTTTTTTATAGTCTCCATTAACGATAGTTAATTGATT
GACTGCTAAAAAATCATTGTTTTGTGGATATTCAAAATCAATAATTTTAACAATTTCTACAATCGATTGTCCTTC
ATCATCATAGTCTTCGATTTCAATACCATTAATCAAATTTTCATGAAAGGTAAGGTTATTTTCTAAAAGATTGGG
CGACTTCTCTAAAGTTAGTTCATGTAGCGCTTTTTCAATAAAACGAGGATGGATATCTGAGTTGATTTTTCTTA
ATGCTTTCTCTAAACGTTCATGAAGGACAACATCTTTATCACTTTTACGTTCTGGTGTTAGACTTGTCATACTA
ATCTCATTACCTTTTTTATAGTCATAGCCTAGTGATTGTAGCCATTCTAATGCGACTTGTTCTAAATCATCTTCAT
TAAATTGAAAGTTCATCACTATTCACCTCAATATCATCAGATATTTCTAGTTCTCCAGACATTAGTTTAGGAAGA
AGAGTATCTCGTAATTGAGTTAATTTACCAATTTCTGATCTTAATATTTTCATTTCTTCCATATATGGAGTGATTA
TTTCAGAATATTTTTTTACAATACTATCTTCAATTGCTAATTTATAATTAACAGCTATTGTTGGTTTTACTCTTTGT
CTACTATTTGTTGAACCGGTAGTATTAGCTTTTAAATAATCTATAAATTGAGAATTAAGACATATATTATATATGA
ATGAATTAATTTTATTATTAGGAGATTCCATTACAACAAATTCTGATGAAGCAACATTTAATTTTTTATTATCTAT
TACAGGTAACCAAATCCTTTTAGTATCAGGATTCATTTTTGAAAATAATACGCAATTGTTATTAATAATCCATTT
ATTACTTTTTATTTTATTGACTTCTTCCTCTATAGCTTGTTCTTCATTATCATAAGCAGGTAAACTAAAATGTTTA
ACTGTTACTTCTTCACTCTTTTTAGGATTAAAAGTTTCCTTTTTATGACTTGCTATATCTTTTAATTTATAGATTTT
CCAATTACTTGGTATCTTACCTAACTCACTATCAATCATTTCTCCTCCACTAGATTTATAAGGATTACCATCTTCA
TCTGGAAATTCAAAATCTACAAACCAATGCTTAAATAGTGTTTGTGAAAGTTCTTTAAGGTTTGCTATCATTTT
TTGGTTAGTTTCTATTTTTCTTGTGATTTGATTTAAAAATTTAGAAATGCTTTTTTGTTTTATTAAAGAAAAATT
AGGAATTTTGACAGTTTCAATAAGTTTAGAATTTATATTAGCTTGATTAGCACTCCCTGTAGAATTTCTAACTA
AATACTCAGTTATTTTAGGTTGTAGAAAATAATAAAATAAAAAATCTAAGTCTGCACTATTACTATATTTTAATTC
GAATTTTCCCACACGTTGATTTTGTAATGCTGGATAATCAAATTCAATTCTTCCAACTTTTCCTACTGCAGAAG
ACATTTGATTCACGCCTGATCCAGTTAATGATATTAATATATCATTTTTCTTTAATGAAAAACCTTTAGTTTTTTC
ATATGTTTTAATAGAGACTTTTTGAGAGTCTTTTGTATCAACAATGGGTGAAATTATATTCTTAATTTTAATAAC
CGGTATACCTTTTTCTTGAAATTCTTTTGATTTAAAAGCATATCCATTTTTAATTTTAGCTACATCACCGAAAAA
AATTGTATTATACTCCATATCCTAACCCCTCCAATGATTTTCGGATTTGGTCTTCAAGTTCTTTTGATTTTGCGA
ATTGTTCACTTAATTCAGAAGTAATACGTTCCATTTTTTGTTCAAAAGGTTCTTCATCTTCTTCAACGTCAGCTA
AACCAACATATCGTCCAGGTGTTAAAATGTATTCATTATTCTTAACTTCTTCTAAATTAGCTACTTTACAAAAAC
CAGCTATATCTTCATAAGGTTTATCGTTTGTACCTCTCCACGCATGATACGTCTGTGCTACTTTTTGAATATCTTC
ATCTGAAAATTCTTTTAATGTTCTAGATACCATATGGCCGATTTCACGAGCATCGATAAATAAAATTTCATTTTC
ACGCTCTTTTTTACCATTTTGACCTTTATTATTACTAATGAACCAAAGACATACAGGTATTTGGGTTGAGTAAA
AGAGTTGTCCAGGTAAAGTAACAATACATTCAACTAAATCTTGTTCAATTAGATTTTTACGAATTTCTAATTCAT
CTTTTCCGCTTGTAGACATTGATCCGTTGGCTAATACGAATCCTGCTGTACCATTAGGTGCTAATTTTGAAATC
ATATGTTCAATCCATGCATAGTTAGCATTACCTTTTGGTGGAATACCAAATTGCCAACGGTAATCATCAAGTAA
TCGTTCTTGACCCCAATCACTTGCGTTAAAAGGAGGATTGGCTAATATGTAATCTGCTTTTAATCCTTTATGTA
AATCATTATGGAATGTATCAGCATTTCGTTCACCTAAGTCATTATCAATACCACGAATCGCTAAGTTCATTTTTG
CTAATTTCCAAGTCGTAGGGTTAGATTCTTGTCCGTAAATCGCAATATCGTCTAATCGACCTTGATGTTTCTCA
ACGAAGCGTTCACTTTGTACAAACATCCCACCTGAACCGCAACACGGATCGTAAATACGGCCTTTATAAGGC
TCAATCATCTCAACTAATAACTTTACAATTGATGACGGGGTATAGAATTCTCCAGCATTTTTACCTTCGGCACT
TGCGAATTTAGCAATAAAGTACTCATACACACGACCTAACACATCTTGTTTACGACTCTCTGTGTCGCCAACTT
TAAATGTAAATAAATCAATAATATCGCCTAATTTTTCTTTATCTAATGCAGGTCTTGCATATTCTTTAGGTAATAC

ACCTTTGAGTGATTCATTTTCATTTTCAATTGCAATCATTGCTTTATCAATAATTTGTCCAATTTCTGGTTTTTTC
GCATTATCATTGATATATTGCCATCTTGCTTCTTTTGGCACCCAGAAAATGTTTTCTGCTAAGTATTCATCTTGG
TCCTCTTCATCTGCATATGGATCCTGCTTCAATTCTTCATACTTTTCTTCAAAAGAATCTGATACATATTTTAAAA
AGATTAAACCTAGTGCTACATTTTTATACTCAGCAGCATCCATACTTCCTCTCAATTTATCGGCAGCTTGCCATA
ATTTTTCTTCAAAACCTATAGTGGCCATTAATAACACCTCTTAAAATTAATGTTAAATCAACTATAGCAAAAAAT
AGAAGATGATTGTGTAGTGTTTTATTAATATTTAATTTATCTAGACTATCAAAAAAATATAGATTATTATTATCTTA
AATTAATAAACAGGAGTGAATTAAAATGAATAAAAAATATAAATTAAAACGATACGATAAAAGATTTAGAGGT
GGATGGTGTTACAAATGTGAAGATTCATATTCTTACCTTTGTGAAGATTGTGGTTATTGTAGATATTGTTGCCA
TTGTTAATACAAGACTAGATAAATTGGGTATCTTTAACTCAATTAATAAAAAATTTAAATGTAGTCAATAGCTTG
CTAACTTTATTTGATGTAATATAATTTTGGCTTTTAGCATTCAGTTAAAAAGAAGTAAATAATTGACAGATTTTT
TAGAGAACTGGAGAAAAAAGATGTACATTTCTAGTGTTAAAATTAAGAATTTTAGAAATTTTGATAATATCGA
TATTGATTTCCATGAGGGAGTTAATGTATTAATCGGACATAATAATTCGGGGAAATCAAATTTATTACGTGCTT
TATCTTTAATTTTTGACGGTTCAGTTAGAAAGCAACTGTCAGTGGAAGATTTTAATAACTCTTTAACAAAAGA
ATCTTTAAAAAAAGAGGCTCCAAAAATAGTAATTTCAGTACATATAACACAATCTGAAAACGAAAGACTGATG
TCTGATGAGTTAATTACGGTAAGTAATTGGCTGACTAAATTAGAAGAACCTTATGAAGCTAGAATACAATATG
AATTCTTTTTACCAAAAGATGAGGAAAAAAAATTACATAGATTTAGTAAAAAATATAGATGAAAAAGAAGAAA
TATGGAATTTAATTAGAAGCCAGTTTATTAGGTTGTATGTCAATAAAATCTGGGTGGGTAATCCAGAACATCA
GATTCCTATAGATAATGATAGTTTAAATAAATTTGATTTCCAATTTTTAGACGCAATAAGAGATGTAGAGAGAG
ATATGTTTAGTGGTAAAAATACATTATTAAAAAGTGTAATAGATTTTTTTATAGATTACGATATAAAATCCGATG
AAACAATCACAGAAGAAGAACAGAAAGAAAAATTAGAAGAAAGAAGAAAAGAATTTTCTGATAATTCTAGT
GATTTGATTGAAACTATTCATAAAAGATTAGATAGTGGGAATCAAAAAATTTTGTCTTATACAAATGGCATAGG
AGCATCTTATGATAAATCAACACCGGATTTTAAAGGTAATCTTACAGAAAGTGAAATTTATACTGTATTGCAAT
TGATAATTAAACATGAAACTGGAATGACTTTACCTATAACTCATAACGGGTTGGGTTACAATAATTTAATTTTC
ATGGCCTTGCTATTGTCTAAAATGCAAGCTGATTCTGATGGAAATTTTCTAGGTAGTAATGCAAAAGTATTTCC
GATACTAGCTATTGAAGAACCAGAGGCTCATCTACATCCTACTATGCAAAATGAATTTATTAAATTTTTAAAAA
ATAACATTAGAGAAAAAAAAGGTAAAGCAGATTTTCATAACTACTCATTCAACACATATTTCTTCTTCTACAAAT
ATTGATGATATTATTTGTCTTTACACAGATGATAGTAAAACTAATGTTAGTTATCCTGGAAAAGTTTTTGATAAG
AATAATCAAAGTAAGAAGTACGTACAACGGTTTTTAGATGCCACTAAGTCAAATATGCTATTTGCAGAAAAGG
TAATATTTGTGGAAGGTATAGCAGAACAATTACTATTACATATATTTGCAGACTATTTAAATAAACCGTTAGAAA
AGAATCATGTTGCAGTAATAAATGTAGGCGGGAGATATTTTAATCATTTTTTATCTTTGTTTGATAGTAATAATA
AATATGCAATTAATCGTAAGGTTTCTTGTATAACAGATTTAGATCCAATGCGAAAAGAAAAAAATGAAAATGG
ACAAAAAAATAATTTTAAAGCATGCTATCCTTTTGAATATGAAATGGATTTAGATAAATTTGATTATTCAACTAA
TAATTCTCTAGATAAATATCTAAATAATGAGCACAGTAATATACGAGTATTTGTTCAACCTAATAAATATGGAAA
AACATTTGAGTACCAACTTATGTTTGACAATCCTTCATTAAAACTGTTACTTACAGACTCGATTTCTAATAGTCA
AGAGCTAGCAGAATTAATGGATCACTATGAAGAAAATGAATCTTTACAAAAATTAATTGATACATTATCACCAT
CTAATGAAAATCAGAGAATAATAAAATCTTTAAAAGACACAAATGACAGTTGGAATGAAGATAATAAAAAGA
AAGCATTAATAGCATCAAGATACCTAAATTCCATAGGAAAAGGAGAAAATGCTCTAGAGCTAGCTTCAGTATT
AAAAGATAATTTAGAGTTAAAAGGCCAGAGTGACTATGAAGATTTTGTAGTACCAAAATATATAGAAGGTGC
AATTCGATGGGTGTGTGAATAATATGATTATTAATGCTCAAACGAAATTAAATATAGAACAAGATTTTAAAATA
CAGGCTGGTCCTGGTGCTGGAAAAACAGAATTTTTGGTGAATCATATCAAAAATGTAATACAAACTTCTGAA
AAGCTCGAACGTATGAAGAAAATAGCGTGTATTACTTATACGAATACGGCTGCTCAGACAGTTTTACAAAGAT
TAGGAAAAAGTGTTTCAAATAGAGTAGATATATCTACAATACATAGCTTTTTGTATAGAAACGTAGTTAAACCA
TATTGTTCTTTTCTACCCAAAGAATACGGAATTTGTGTGCAAAAATTAAAAGGACATAGTGATCCAATTGTGAT
TAACAAATATGTAAATGCATGGTTAAAAACTGAATATTTTACGAAATTAAAACCGCCTAGTAATAGAAATCAAC

TACTAAAAATGCCAGTACTAAAGAAAGCTTTGCAAAACTGGCTACTATCTATGAAACTTTCTTACGAAAATAA
TGATGTGCAATTTATTTGTGATAATACCAAAGCTCAAGCAATTGATAAAAAAAGTAAAAAAAGAATGGGAATA
AATAAATCAAACTTAAATATACTAGAAAAGCAATTATTAAATTATAAAAAAATATATTGGGAGAAAGGAATTAT
CGATCATGATGATGTTTTGTTTTTTTCATATGTATTAATTAGAGATTACCCATTCATTCTCACTATTTTATGTGCTA
AATATCCCTACTTTTTTATAGATGAATTTCAAGATACTAGTCAATTACAAGATTTTATTATAGATAAAATAAGACA
GAAAGGCTGCATAGTGGGGGTTATTGGCGATAAGGCACAGGCAATTTACAGCTTTCAAGGTGCACGGGTTT
CCTTATTTGAGAAGTTTAAAGTTGATTTAATAAATTCTCACACTATCTTAGAAAATCATAGAAGTTCATATCAAA
TAGTTACTTTTTTAAATAGTATCAGAGAAGATATTACTCAAACAGTATATAGTGGTATTGAAAATGATAAAGTTA
CAATTTTGATTGGAAATCGGATTCGCAGTTATACTAAGGTAGTTAATATTTGTAACAAGGAGTCACTTGTTACC
TTGTCAAGAGATAATATTACTTCTAATGCAATGAGAATAAAGTTGGAAGGAAATAATTTTGATAAAAAGTTATT
GGAAAAATTTCAAGAAATTGATAGTAATAGTAAAAGAAGGAATACTATTATTTCGCTTATAAAAGCTATTGAAT
TAGCTAAGAATACTAAATATAAAGATGCCTTAAAAATAGTCGAATCGATTTATAAAGATGAAAATAATCCTAAA
AAAATAGCTCTTAATTCTCTAATAAATATGATTAATAAATATGATAAATACGGTAATGGTACATTAATGAATTTCT
ACAATACTTTATGTAAAACTTTAGATATTAAACTTTCAGGGTTTAGAAAGGGGCAAGTAAAAGATTTTTACGA
AAACACTTTATATAAAAATATGGTGATATGTACAAATATCGTTGAAGACACAAGTAATCATATTACTATCCACAA
AGCGAAAGGCGCAGAATTCGAAAATGTATTTGTTATAGGTAATAAAGACACTTTAAGTCTACTTTTGAAACCA
GATTTAATGAACGATGAAGAGCATCGTATATTCTATGTAGCTATGAGTCGAGCAAAGAAGAAACTTTTTTTTA
CAATTTGATTATCTTGATAATAATGAACAAAAGAAAATTAAGGAACGTTATGATGTAAGTATTATACAATAACC
TAATCTAAGAATATTTAAATCCAATATATTTCTTGATTATAATTCAATAATAGATAAAATCTATAAACGATTAAAAA
GAAAATATATTTGCTAAGACTTATAAAAATGAAGAGATAATAGATTTTCCTAAATTACAGTTAAAAGAAGTAAA
TAGATTGAACACTTTGAAAGATTCTAATCCAAAATTTGAACCACTAAAATTGAAAGATGAAGAAGATGCAAG
ATGTAGCCATCAACGCTGATCACAAAGTATTTTTCTTAATGATTATTTTTAATGTTATCATGCTCGTTTCTTCTAT
TATTCAAATGATTTTAGGAAAAGGCAGACGTATCACACCAGCAAAACCTACTAAAGATGCTAAGTTACACTTA
GATAATCAATCCTCATAAAGAGAAAAAAGAACCCCTAAAAAGTTCAACTTTTTGGGGGTTACTTACTATTAAG
AAGTATTTTTGATTAAGCATCTTTCATATCGTAATCACCAATATACTGATCAAGGCGATTTATTGTTTCTCTGATA
TCATCATGTGTCGTTAATGGGTTCGTTGTACATAAACGAATGACACGTTGGTTATTCAATACTGTGGTATAAGC
AATGGCATAACCAGAATCTGCAATTTTTTGCGCAGCCATGCTATTGAGTTGATTATTTTGTGCTTCAGTTAAAT
CACTGTTTTCATATCTGAAGTTCACAATGGAAAGATTAGCGTGTGAGATAATACGCCAGTTATCGAGACCTGA
CACGTATTCTTCTGCATATTCTGCTAAACCTTCGCCGTATTTAATGCGCTCGATAATTTCATCTTCACCAATGAC
TTGCAGAGTAATCCATAATTTTAAAGCACGTGCAGGGCGTGTAAGTTCAATGCCTAACATTTCTGGATCAATC
ACATCATCATCTGAAGCAATATCATCCAAGTACTCAGCATCTTCGCCAAAGCTTTGCAGCAGATGATTCTTTTC
TTTCACGATTACCATAGCGCAACTATATGTTTGGAACAGTAATTTGTGAGCATCCCAACTGGCACTATCTGAAC
GTTCAATGCCTTTAAATAAGTGTTGTGCTTTATCGGATAAGATATGTGATAGTCCATAAGCACCGTCTACATGG
AGCCATAGATTGTAGTGATCACATGTATCTCCAAGTGTGGTGAAATCATCGACAGAACCTGTATTAGTCGTGC
CTGCAGTCGCAATCACCATAGCAGGTTTGTAACCTTCTTCAATATCTTTATCAATTGCGGCTTTTAAGCTATCT
GTATTCATTGTAAAGTCATCGTTGTACTCAATTCTGCGGATATTATTTTTAAGGAAGCCTGCAACATGCAAGGC
TTTGCCTACAGAATGATGCGTTTGAGATGTAAGATAAACGGTTGCTTTTTTAATATCTTCCATTTCTACCTGTG
CATCTCTAGCCGCAACGATAGCTGTTAAATTGGCCATTGAACCGCCAGATACAAAGACACCACCGGCCGGTT
TGATCTCATAACCGATTTTTCCTACCAAGTAGTTAATTAAATTACGCTCAATATTAATTGGCAGCGTCGCATTGG
CAAAATTAGAGGCATGGATATTATTTGCTGTTGTTAAAATGTCTCCAAGCCATGATAGACGTGATGCTGGACC
TGGTATAAATGAGAATGAACGTGGATGGTTCGGACGATATAAATAATTCAGTACTTCATTATTTAAATCTTTGA
GTACTTCATAGATATCTCGCCCTTTATTCGGCACTTCCATCTTTTCATATTTCTCTCTCAATTCGAGCGGTGCCT
GTTGTGTCGCAGGTAAGTCTCCAATATCTCTAGATAAATATTTATTAACTTCATCTCTGATAATCGTCTCTAAGTC
GATATTGTTCTCATTGAATTCCATTTCCATACTCTCTCCTTCAATTATATTTTTAATAAAAGACAGACGTATTAAT

TACGACGTTTTACTACAATATAAATGATAGCAGATATTAATAAAACCACGCTAACCATAATGACGAAACCCAGA
AGGTGTCGCCCCAATGAATAACGTGCTGTGCTTTAACACTCGGTGTATGTGCGCCTTTCAGAAATTGACGAG
GGTAATCGACTTGCAGATTATCATCTGTGATTTTCAATTTGTATTGATGCTTGTCTTTTGGAATTCGATAACTAC
CCCGTAGAACAGGGGACGAGGACCTCATCTTGATGATCCCAATTGGCTTGGTTAAAAAGAAATCGATAATCC
TCGTAGAATAGAGAAAAAGTATTAAAAATATATTTGATTTGATAAAATATTTTGCAAAAAAGAGAAACAAAAA
TAACTTTCATATAAATAATTTCTTTGCGATTAAGTTGGAATTAGAAATACTAAATACAAATAACAGCGAATGGA
ATGCATATTATTGAGGAATTGGATGTGTAGTGCAATTTCGAGAGTTACTACTTAACTTAAGAAAATAAAAAGA
AGAAAACAAACAATAAGAATTAAATCTGAAGCAAGATATAAAAGCAAATATTTTTGCTACAATTATATTAGTAA
ATACTTTATTAATTAGCATACGACTGAAAGCGAAGTAACAGCTAATAATGTTCCAGATATAGACATTAGTGTTAT
GAAAGAACAATATTACCATCTAGAAAATGTAATTTTTAAAAACCTGAAGATAAAGTAGACTATTCTGTGAGTT
TTCATGTTAAGAATTCTGAACTTGGGGATGTAGTATAAATATGGTTTAGTAGAAAATGACGCTGGAGCTGTAG
CTGAGATTGACTATCAAGAATTAACTAAAGTAGATTATTACCCATCAACAAAAATCCAAGATTTAACACTATCT
AATAATAGTGCGAATAATGAAGTTTAAAAGTGAAAAACTGATAATAGCGAATCAAACTAAAGTAATCAATTAC
AGAATTCAAGTACTACTCAAGCCTCAAACAAATATTGAAACTCAAACACAAGTTAAGAATAATACACTGTCAG
AAACAGATGAACAGTCTAATGCCGATTTAGTCCAGTAATAGCCTCTGTATTATTAGCCGCACGATTATTATCAA
TAATTAAATAAAGTTAATATTAAATAATAAAAATCCTTCAAATTATTTGTTGAAAAAATAATAACTTTGAAGGTT
TTTTAGCAAAATCACTGATAGGGAGAAGCGTATCATAAATGATGGGGTTTTAAGTACGATTTAATAAACTAAA
ATAAGATAGGGGAATTAAGAAATGGGAATTTATCATTTAAATAAAGATAAAGATGTATTGACAGATTTAAAGT
CAAATGAAAAACAAGAGCAGGTTGCTACATTTATAAATAAACATCTTTCAGCAAATAATTTAACGATATTTATC
GGTTCAGGATGCTCGACAGGTGCTGTTCCTTTAATGTCTACTACAATGAAAAACATATTAGAAGAAAATGAG
AGTGTCTTAAATTATGTAAAAAAATTTCTGAATTCTAAAGGGATTAAAGAATTTATAAAGTATGTAGAAGAAC
AAGAACAAGAAAAAAATACAAGAGAAAGAAAGAAAAGCGTTACATACAATAATGGATCAATTAGAGGCTGA
AAATTTTAAAAATTTAGAAGAATATAGTGGTTGGTTAGATATGCAAGATAGTGAATATAAAGAAGAAATATTG
AATTTTTTAGATTGTTATTATCTAAATTATAGTAATATTGAGGAATTACTAAATTGGATACAAAATGGACTTCATT
ATGACAATAATAATGGAGATTTGAAAGACGTGTTTACTACTTTAAAAAGTGAGTTTATAAAAACTATTCCTAAA
GTGGGTGATAAAGAATACAGTACAGAAACTTATGAAATATATAAGGACTTTTATCGTTATGTATTTGATAAAAG
AACTGAACAAAAATCTAAAGTATCAATTTTCACTACAAATTATGATTTGTTTAATGAATATGCACTTGAGAATA
ATAATATTATCTATAGTACGGGGATTCAAAATACAATACTTAAAAAAATTTGACATTAATCAATTTAAATACAGAG
TTGTAGATGATACAAACAGGTATAAAGAAAAATGGCAACCAGTATCTAAAGAGGCAAATTTATATAAAATACA
TGGTTCGATAAATTGGAAGTCCAATGAAGAAGGGGAATTGCAACAAATCGATTTTAATGATGAAGATGATCA
GGTGGTCATTTATCCAACCATGTTGAAACACAAGGAAACAGCACAAGCACCGTATTCAGAATTGTTCAGAGA
ATTCTCTAATTGTTTACAGATAAAAGATACTACTTTAATTATTATAGGGTATGGATTCCCAGATGAACATATTAAT
AATATAATAGCTCAGAATTTAAAGAATCAAGATTTTAATTTAATAATATTTGGTGATGTTAAAGAAGAAAACGT
GAAGAATTTCTATGATAATTTTAAGAATTTTAATTTACATCTTATCGGAGGTAATTCAAGTAAAGCAGAGCAAA
AAGCACATTATTTCCAGTTTATCGTAGAAAATTTTCTGAAAAATCAAAGGAGAAGATAAAATAAATGCATAGT
ATTGGAAAAGTCACAAGTGTTACATTTGAAAAGTTAATATTCGAAGTTAGTGATTTTGAAAAACTAAATTATA
ATTTGTTAGGTCAAATTTACATAGCTAAAGGTGTAATAGACTATGTAACAATAAAAAATGAATATAGTGAAAAA
TTTATATATCAAGTTGTAAAAGTAGAAGATAAAGAAATTCCATTATCTTCTGAAGAGCACTCTAAATTTAAATAT
CATGGTAGGTTTGAGTGTGTACCAGTAGGAATGATTAAACATGGAAAAATTGAGTTTAATTTAAAGAAATATC
CTTTTTTACAAGATAAAGTTTATTTAACAAGTCAAGAAGAAATGGAAATGGTATTTTCACATTTTCATAATGGA
AATGATATAACTATTGGACTCATTGATGACCAATATCCAGCTTATTTTAATACTGCTAAATTATTAACGAATCATA
CAGCTATAATTGGAAATACTGGATCTGGCAAATCTACAACAGTACGTCAGATTATTTCAAAAATCAACAATCTA
AACACCCAAAATTTACACTTTCACATATTCGATGTCCATGATGAATATAAAGATATAAATGGAGTGAAAATAGT
AGATGTAATAAATGACTTTAAAATTAATATTAAAAATTTAGAGATGCAAGACTGGATAAATTTGATTAAACCAT

CGGAACTAGTTCAATTACCTATTTTACAAATGGGATTAAAATATGCCAATGCTATTGAAAATAAAATTATAGAG
GAAGAATGGTTGAAGTGTTATATAGCGCTATCATTATATAGAAATCAGCAAACAGATGCTGTTACAAAGCGAA
CTAAGATTTTAAGCATATTAGATGGAACAAATATTGACACAGAAAAATATGATTCTAAATATGGAAATATGGAC
AGCAATACTGAAAAAAAATTTATCGAGAGTTTGAAAAATGTAGTAGATAATGGTGGTAATATATTTACTTTAA
GCGAAGTAATTGAAAAAGCAAAATATAATGTATCGAGTTTTAATAAATTATTAGAAGGATTAAACTATGTATTT
TTACTTGAAGAAAGTAAAGGAAATAATCAAGCAAGATCATATTCAGCAACACTTGAAACGCGTATAAAAAAT
GTTCAAACACGATTTTCAAATTTATTTGGAAATAATGATACTGAACTGGAAGATAAATCAATTGTTTATTCAGT
TTCAGAATTAGATGATGATTTGTTATTATTTTTCACAACATTTATTTTAAAAAAAGAATTTGAAAAAAACAAAA
AAAGAAATTAGAAGATCGTTCAGTGAACGTTTTTATATTTGAAGAAGCTCATAGATATATATCGAAATTTAAAG
AAAGTAGCCAATTTAATGAAGTAGAAGCATTCAAAAAGATAGCTAGAGAAGGAAGGAAATTCGGTTGTTTT
TTAATGTTATCTAGTCAAAGGCCAAGTGAGCTGTCTTCTACTGTCTTATCACAATGCAATAATTATATAGTCCAT
AGGGTAAAAAATAATGTAGATTTAGAATATTTACTAAATTCCATTCCCTACATAAATAAATTTCAATTAAATCGT
TTTTCTTATTTACCTACTGGAACAGCTTATATAGTAGGAGAATTATTTCCAATCCCTGTAGAAATAGAAATATTT
GAAGAGTTTTCGAAAAACAGCACGATTACACCTGAAATAGTATATAGATCTTGAATAATAACTACGCTCATTTT
GGATGATTATGTGTTATACGAAATTAATAAACATAACTACGCGTTGATAACAAAAATAAATGACTTTAAAATTA
CTACTTGACATATTTTATAAAGACTAGAGGTTAAAATTTTTTTACTCTCTTAATGAGCAGGAATTTATGGCCAA
ATTAATACATTTACTTTCAATTTTATATAGACTTAGTTCAGATAAGAAATTTACTGTGAAACAAATTTCAGACAC
CTGTCATAATGGTGGGAAATCTTTATATAGTGCTATTACAAATTTGAAAAAATAATCACATTTGGAGCAAGTAA
TTGATATAGAAAAGCATGCCGCTAGTCTTGAAATAGCAAATGAAGAGTTGGATAAAATCTAAAGTAAGTTATA
ATAAAAAAGCAATTAATATAAATGTATCATTAATTATATAATACAGAAAACGTTGTTTTTTGACATTGAAATATTT
GTATATACTTTGATGAGAAATAGTTGTGTGAAAGTTACGAAATAGAAATATATCCCAAAAAGCGTATGAGAAC
TAAGAAAGGAAGTCTCGAATAATGAAAACACCTATTTATTATACGATTGTAAATAAAGGCAATAATATTGAAA
ATACAATATTTCAGACACCACCAAATACAGTTAGTGACTTTGACCATTTGGATGTTATGCGTTACTTACCATCA
ATTGTTGATATTAGTGAGATTTGTTTATTAGCCGAAGCAAAAAAAATACCAGAGTTTAAAAAATCGCTAGACT
TCATTCTTAATAAATTTGAAACATATACATTTAATATTGTTAATGATTTAATTGAAATAACACTAACGAAAGACG
CATTATTAAATTATCAAAAGTACTTGTTACATCAATCAATCATAATAAGTAAATCATTAGCTTTAAAAAATAGTAA
CAATGGTTTATGTACACAAGAATTAAAATCCCAATTATATGGATATGAAAAGCTTCATTATGAACCAGCCTTTC
ATATATTAGATAACGATTTAATTCCTAATATTAATGAAGAGCAAAGTGAATTGATACCCCTTGAGCATGTTGTTT
TATTATCTGATGGGATTAGTTATTTAATGGATTATTTTGACAAAAATCCTGAAGCAACTTTCACATTATACATTG
TGAAAGATTACACAAGTTATTACGAGGTAGAATTATAAAAGCATTTTTATTAATTAAAATATTTAGGCGAGCTA
AATGCATAGATTGTGGCAAGTATTGTATTAACCATATTGGTCAACATTAAATTGGGAATGTACTAAACCTAACA
CACAAAGTTATGTGAATAAAAAAGATGATCTTACTTGATTTACATTTCCTGAGTTAAATAATGAAATATAGAGC
AACTTTTCAAGAGGCATTAAAGTATCTTGAAAAGTTGCTTATTAAATAATAATTGCTCAAATAAAAAAACATAT
AAATGCTCTTACCAAAAAAATATTCGTAACTAAAATATTTCAAAGTAATTAAAAAGTGTGATTACCCAAAAATC
CATTTTTTATTCAGATAGCCTTAATATTGCTCCTAAATGTTGCTGTGCAGTAGTAAATAATACGAATGTAATTAAT
TCAATGATTTCTTCATCTGTCATCGTTTCTTTTAATAAACTTATACTTGATTCGGGTATGTTTTGTTTAGATTTTA
AATAAACTTCAACAAAGCCAACACAAAGTAGAGATTTTTCGTTTAGAAATTTATTTGTAGGACTACCTTTTGC
TTTGCAATATTGACATCCGTTTTGCTGGGCCAGTAACCTTCTTAACTCCTCTTTTAATTCTTTACTTATTGCCCC
ATCGCTACTTAAAAAATCGGATAAATTATTCCAGTATTTTAATAAGTTTTTATTATGACCTAATAGCTTTTCAAAT
GGTGTGTTTCCAATATTACTGTATTCTAATAATGTCATATCCTCATCTCCTATCTTAATGATATACATGATAAAATT
GTATGGAAATAAGATTTTGAAAGTTTATATCATTTAATACTTTGAAAAGAGAGGATTTTCTATGAATGACTTAA
TAAATGAAAAAATAATCAATGTTTTGTTAAATAATAGTAGAATTACTATTAATGAATTAAGTAAACAAGTGAAC
TTATCTGCTCCAGCTGTAAGAGAAAGGGTAAATAAACTTGAAGATCAAGGTATTATAAAAGGTTATACAATTA
ACATAGATTATAAGGAATTAGGTTACGATGTAGAAATATTAATTGAGTTAACTATAAAGAATAATAGATACAAA

GATTTTAAAGAATTTATTTCAAAACAAGATAATGTTGAGTTTTGTTATCGTGTATCAGGAGAGGCATGTTTTGT
ATTTAAGGTTCGGTTGGAAAATATGGATGAAGTGGAAACATTTGTTGATACCATACAACCATATGGGCAGAC
AAAATGTCAGTTTATCTTTTCATCAGTTGTCTAAACTACATGGAAAAAGTTTAATAAGCATAGGAAAGATTTTT
AACAGAGAGAAGTGACTTACTTTGGAATATAAATCTTACAACAAAATGAGTAATAATATTTTATCTATCTTGTT
GAGTGAAAAAAAATTTCGATAATAAAATGGTAGTCTCAACAGGTAGTTACATTTTGTCTGAGTTGCCATGTATT
GTTGCCTATAAAAATAATGATATCGTGGGGCTACTAACGTATAAAGTTTATGATGAGTATATTGAAATCATTTCT
TTAGATAGTTTTGTAGAGAACAAGGGTATTGGTAGTCATTTACTTAATTATGCTGAAATAATTGCCTCAGATAT
GAGTAAAAGAAGTATCCGTGTTATTACTACAAATGAAAATATTAAGGCACTCTATTTCTATCAAAAAAATAAAT
ATAGAATCACAGACGTTATTTTTGATGCTGTAACAGAAGCTAGGAAAATAAAGCCGTCGATTCCTGCAATTGA
TAAAAATGGCATTGAAATTAGAGATGAAATTGTTTTGAGGAAATGTTTGTAATGTATAAATATACTCAGCTTTA
AATCACCCTAGTTCATACCTAAAAGACTTTCACACAAACAAAAAAGGAGGAACATTAAATTCCTCCTCAAACT
TTATTACTCATACTATAATTCAATTTTAACGTCTTCGTCCATTTGGGCTTCAAATTCATCTAGTAGTGCTCGTGCT
TCTGCAATTGATTGTGTGTTCATCAATTGATGTCGAAGTTCACTAGCGCCTCTTATGCCACGCACATAGATTTT
AAAGAATCTACGCAAGCTCTTGAATTGTCGTATTTCATCTTTTTCATATTTGTTAAACAATGATAAATGCAATCT
CAACAGATCTAATAGTTCTTTGCTTGTGTGTTCGCGTGGTTCTTTTTCAAAAGCGAATGGATTGTGGAAAATG
CCTCTACCAATCATGACGCCATCAATGCCATATTTTTCTGCAAGTTCAAGTCCTGTTTTTCTATCGGGAATATCA
CCGTTAATTGTTAACAATGTGTTTGGTGCAATTTCGTCACGTAAATTTTTAATAGCTTCGATTAATTCCCAATGT
GCATCTACTTTACTCATTTCTTTACGTGTACGAAGATGAATAGATAAATTTGCAATGTCTTGTTCGAAGACATG
CTTCAACCAATCTTTCCATTCATCGATTTCATAGTAGCCAAGGCGTGTTTTAACACTTACTGGAAGCCCGCCTG
CTTTAGTTGCTTGAATAATTTCGGCAGCGACGTCAGGTCTTAAGATTAAGCCGGAACCCTTACCCTTTTTAGC
AACATTTGCTACAGGACATCCCATATTTAAGTCAATGCCTTTAAAGCCCATTTTAGCTAATTGAATACTCGTTTC
ACGGAACTGTTCTGGCTTATCTCCCCATATGTGAGCGACCATCGGCTGTTCATCTTCACTAAAAGTTAAGCGT
CCGCGCACACTATGTATGCCTTCAGGGTGACAAAAGCTCTCGGTATTTGTAAATTCAGTGAAAAACACATCC
GGTCTAGCTGCTTCACTTACAACGTGTCGAAAGACGATATCTGTAACGTCTTCCATTGGCGCCAAAATAAAAA
ATGGACGTGGTAATTCACTCCAAAAATTTTCCTTCATAATATATTTATACCCTCTTTATAATTAGTATCTCGATTTT
TTATGCATGATGATATTACCACAAAAGACGAACTTATACAAAAGGAATTTTAGTAGATACAACCATTCGAAAG
GGAAGTCTACGAGTAGTCTAAAATGAATGTTGTGTAAGTTGATTAATATACAAATCAAGGATTATCGCGTTAA
ATTGTTCATTATTAATGATACACTGCTTATTATTATGATTCAGAATTTTCTTTAGCTACATATACAGTAATGTGATT
TATACGTTATCTTATAACAAAGACAAATTTATAAAGGTGATATTATGGAAGATTTAAAGGAATCTTTAAAAAGT
TTAGGTTGGTGGGATTTATTTTTTGCGATACCTATGTTTCTGCTATTCGCATACCTTCCAAACTATAATTTTATAA
CGATATTTCTTAACATTGTTATCATTATTTTCTTTTCCATAGGTTTGATTTTAACTACGCATATAATTATAGATAAA
ATTAAGAGCAACACGAAATGAATCATTAATACGAATGTGATTAAACATAAAACTGAAGGAGCGATTACAATG
GCGACTGAGAAAGATGTAAATGATTTATTTTTAAATCATGTGAATTCAAATGCCGTTAAAACTAGAAAGATGA
TGGGAGAATATATTGTTTATTATGATGGTGTGGTTATAGGTGGTTTGTATGATAATAGACTATTGGTCAAGGCG
ACTAAAAGTGCACGTCATCAATTTCAAGATAATACATTAGTATCGCCGTATCCTGGTGCTAAAGAAATGATATT
AATTCCAGACATTACCGAAGTAACAAATCTCACTGATTTATTTGAGCTCATAAAAAATGATTTGCAAAAGTGA
AGTTCGGATGGAGTAAACTAAGCACATCGATAAATGGTCGCTTGTTAAAGAAGAGTGACGGTCACTCTTCTT
TATGTGCATATTTTATTTTGTCTGTTTTGTTAACGAGCAGCAGTGTAACAAATATGAGTAAGGATAAAATGAGT
ATAATATAGAAACCGAATTTATCATTAATTTCATTAATCCATCTTCCTAAAAATGGAGCAATGAAACTTTGCAGT
AACAATGAAATTGACGTCCATATCGTAAATGAGCGACCGACATATTTATCTGAAACAGTGTTCATTATAGCTGT
ATTCATATAAATTCTGATTGATGAAATTGAGTAGCCTAGTATAAATGATCCTATGAATAAGTAAAATGCTGAGTT
TATCCAAATAAATAGTGCTGAATTTATGACTAATATGAAATATAACAAAAATATCAATGCTTTAGTTGAGATTTT
TTTCGAAAGAATAGCTGAAATTAAACCTGCACATAATCCTCCAATGCCATATAACATATCTGAAAAACCAAATT
GTACAGACGAAAGTTTTAAAACATTATATACATATCCTGGTAATGATATGTTAAAGATCATTGTAAACACCATTG

GTATGATTGAAATAACTCCAAAAATAAATATCATCATGTTGTCTTTTAAAAATTTCCATCCTAATAAATATTCTTG
CAATAAGCTATTTGTTGATTCTTCCTCTGAATGAGTTGGTTTATCTACATGCAATCTAAATAACATAAAAATGCT
GATTAAAAACATCATTATAGTCATCGCTATAATTAGAGTGAATCCATTTATTTCATATAATATTCCTGATAATCCAC
CTGCAATAAACATACCTGTTTGCAAACTAATCTCTAATAGAGAATTTGCATCTGTATATTGATCTGGTTTTAAAA
TCTGTTTAACTAAACTTCTAGATGTTGCCATATACGTAGTCCAACCTATCCCATTAACAATCGCAAATCCAATCA
CTAAATAGGTCTCAAAGCCTATCATTAAGAGTGCTATGACAATGAGTAAATATAATATTACCTGAAGAAGATAG
GTGATTAAAATAATATTTCGTCTATTATATTTATCTGCTAGTCCACCTATTATAGGAGAAGCTAAGAAACCGGAT
AATACATTTAAAGCTAACATAATACCTAATAGTTGGGAGTCGTTAGTTTTGTCGATTAAATACCAATTAGCCCC
AACTGTACTAATTCCAACTCCGAATGCTGAAATTATTTCTGCTAAAAAGAACAATCTGAAGTTTTTAATTTTCA
ACAAATCAGTCATAAATATCACCTTTTTTCAATGCAAGATTTATATTTGCGAGCTCTTCTATAAATTGAGAGTCG
TTTAAAATATCGAATGGTTTATTGCCTTCGCCGATCAAAAATCTATAGTTAGTAATATTCATAAACTCAAATATTA
ATTTGAATTGGTTGACTAAAGGTTGTGCTTTGGTATGCGGATTGTCGCCACCTATGATTAAAATCAAATACTTT
TTTTGTGACATGATTTCTTTGAAGTTATCTATTTGCGTATCTCGCAATGACTCGGTCCATCTATCAATGAAGAGT
TTTAAAGACGCACTCATGGAGTACCAATAGAGTGGTGTTGAAAAAATAATAATATCTGATTCTAAAACTTTGA
TTAAAATTTGTTCGTAATCATCATTATGAAAGTTGGCATCTTTGCTATGACGATTATCTGTAACCTTTTCAATGT
TACTTTGATATAAATTCACAAAGTTGACATTTAAATTCTTCAATAGATTCTCTACTGCGATAGCTGAATTGCCAT
CTTTTCTACTACTTCCAAATAAACAAGTAATCATAGTCATAACTCCTTTCGATTTACCTTTATAATAATATTTAATA
TATTATTATTAAATCAGAATTCTTAGAATGCAGAATTCGATAAAAGTGAATCCTAAAGGAGGTACTACTTTGAA
CTTAAACATTTTAAAATCATTTTTAGTAACTAGTGAAACAAAGAACCTTACTAAAGCATCAGAATTACTTAACT
ATTCACAGTCAACTGTATCTACACATATTGAAAAATTAGAAAAGCAATTAGATGTTAAATTATTTTATAGAAAA
AAATATGGTATGGAACTAACGGAAGAAGGCTTAGCATACGTTAAATACGCTAAAGTGATTTTAGATAGTAATA
GCGAATACGAGAGAGAAATAAAAGGACTTTACAATAAGAAGGTAAATATAAGTATTAACATGCAAGAAAGTC
AGTATTTGTATCGCTACTATAATAAGATTAGTGAATGGTTAGCTGAACACCCATATGTAAACTTAAAGTTTAAAT
CCGCACATTCTAATTTCTATATTAAAGAAGAAATTGCTAATTTTAAATCGGATATTAGCCTTATCACAGACGAA
AAGATTATTAATAGTAACTTAACTGCTATTCCTATAACT
>Staphylococcus aureus strain 0213-M-4A
ATGCAAATGGCTAGAAAAGTTGTTGTAGTTGATGATGAAAAACCGATTGCTGATATTTTAGAATTTAATTTAA
AAAAAGAAGGATACGATGTGTACTGTGCATACGATGGTAATGATGCAGTCGACTTAATTTATGAAGAAGAAC
CAGACATCGTATTGCTAGATATCATGTTACCTGGTCGTGATGGTATGGAAGTATGTCGTGAAGTGCGCAAAAA
ATACGAAATGCCAATTATAATGCTTACTGCTAAAGATTCAGAAATTGATAAAGTGCTTGGTTTAGAACTAGGT
GCAGATGACTATGTAACGAAACCGTTTAGTACGCGTGAATTAATCGCACGTGTGAAAGCGAACTTACGTCGT
CATTACTCACAACCAGCACAAGACACTGGAAATGTAACGAATGAAATCACAATTAAAGATATTGTGATTTATC
CAGACGCATATTCTATTAAAAAGCGTGGCGAAGATATTGAATTAACACATCGTGAATTTGAATTGTTCCATTAT
TTATCAAAACATATGGGACAAGTAATGACACGTGAACATTTATTACAAACAGTATGGGGCTATGATTACTTTG
GCGATGTACGTACGGTCGATGTAACGATTCGTCGTTTACGTGAAAAGATTGAAGATGATCCGTCACATCCTGA
ATACATTGTGACGCGTAGAGGCGTTGGATATTTCCTCCAACAACATGAGTAGAGGTCGAAACGAATGAAGTG
GCTAAAACAACTACAATCCCTTCATACTAAACTTGTAATTGTTTATGTATTACTGATTATCATTGGTATGCAAATT
ATCGGGCTGTATTTTACAAATAACCTTGAAAAAGAGCTGCTTGATAATTTTAAGAAGAATATTACGCAGTACG
CTAAACAATTAGAAATTAGTATTGAAAAAGTATATGACGAAAAGGGCTCCGTAAATGCACAAAAAGATATTCA
AAATTTATTAAGTGAGTATGCCAACCGTCAAGAAATTGGAGAAATTCGTTTTATAGATAAAGACCAAATTATT
ATTGCGACGACGAAGCAGTCTAACCGTAGTCTAATCAATCAAAAAGCGAATGATAGTTCTGTCCAAAAAGCA
CTATCACTAGGACAATCAAACGATCATTTAATTTTAAAAGATTATGGCGGTGGTAAGGACCGTGTCTGGGTAT
ATAATATCCCAGTTAAAGTCGATAAAAAGGTAATTGGTAATATTTATATCGAATCAAAAATTAATGACGTTTATA
ACCAATTAAATAATATAAATCAAATATTCATTGTTGGTACAGCTATTTCATTATTAATCACAGTCATCCTAGGATT

CTTTATAGCGCGAACGATTACCAAACCAATCACCGATATGCGTAACCAGACGGTTGAAATGTCCAGAGGTAA
CTATACGCAACGTGTGAAGATTTATGGTAATGATGAAATTGGCGAATTAGCTTTAGCATTTAATAACTTGTCTA
AACGTGTACAAGAAGCGCAGGCTAATACTGAAAGTGAGAAACGTAGACTGGACTCAGTTATCACCCATATGA
GTGATGGTATTATCGCAACAGACCGCCGTGGACGTATTCGTATCGTCAATGATATGGCACTTAAGATGCTTGG
TATGGCGAAAGAAGACATCATCGGTTATTACATGTTAAGTGTATTAAGTCTTGAAGATGAATTTAAACTTGAA
GAAATTCAAGAGAATAATGATAGTTTCTTATTAGATTTAAATGAAGAAGAAGGTCTAATCGCACGTGTTAACT
TTAGTACGATTGTGCAGGAAACAGGATTTGTAACTGGTTATATCGCTGTGTTACATGACGTTACTGAACAACA
ACAAGTTGAACGTGAGCGTCGTGAATTTGTTGCCAATGTATCACATGAGTTACGTACACCTTTAACTTCTATG
AATAGTTACATTGAAGCACTTGAAGAAGGTGCATGGAAAGATGAGGAACTTGCGCCACAATTTTTATCTGTT
ACCCGTGAAGAAACAGAACGAATGATTCGACTGGTCAATGACTTGCTACAGTTATCTAAAATGGATAATGAG
TCTGATCAAATCAACAAAGAAATTATCGACTTTAACATGTTCATTAATAAAATTATTAATCGACATGAAATGTCT
GCGAAAGATACAACATTTATTCGAGATATTCCGAAAAAGACGATTTTCACAGAATTTGATCCTGATAAAATGA
CGCAAGTATTTGATAATGTCATTACAAATGCGATGAAATATTCTAGAGGCGATAAACGTGTCGAGTTCCACGT
GAAACAAAATCCACTTTATAATCGAATGACGATTCGTATTAAAGATAATGGCATCGGTATTCCTATCAATAAAG
TCGATAAGATATTCGACCGATTCTATCGTGTAGATAAGGCACGTACGCGTAAAATGGGTGGTACTGGATTAGG
ACTAGCCATTTCGAAAGAGATTGTGGAAGCGCACAATGGTCGTATATGGGCAAACAGTGTAGAAGGTCAAG
GTACATCTATCTTTATCACACTTCCATGTGAAGTCATTGAAGACGGTGATTGGGATGAATAATAAGGAACATAT
TAAATCTGTCATTTTAGCGCTACTCGTCTTGATGAGTGTCGTATTGACATATATGGTATGGAACTTTTCTCCTGA
TATTGCAAATGTCGACAATACAGATAGTAAGAAGAGTGAAACGAAACCTTTAACGACACCTATGACAGCCAA
AATGGATACAACTATTACGCCATTTCAGATTATTCATTCGAAAAATGATCATCCAGAAGGTACGATTGCGACG
GTATCTAATGTGAATAAGCTGACGAAACCTTTGAAAAATAAAGAAGTGAAGTCCGTGGAACATGTTCGTCGT
GATCATAACTTGATGATTCCTGATTTGAGCAGTGATTTTACATTATTCGATTTTACGTATGATTTACCGTTATCA
ACATATCTTGGTCAAGTACTGAACATGAATGCGAAAGTACCAAATCATTTCAATTTCAATCGTTTGGTCATAGA
TCATGATGCTGATGATAATATCGTGCTTTATGCTATAAGCAAAGATCGCCACGATTACGTAAAATTGACAACTA
CAACGAAAAATGATCATTTTTTAGATGCATTAGCAATAGTGAAAAAAGATATGCAACCTTACACAGATATCATC
ACAAACAAAGATACAATTGATCGTACGACGCATGTTTTTGCACCAAGTAAACCTGAGAAGTTAAAAACATAT
CGCATGGTATTTAACACGATTAGTGTTGAGAAAATGAATGCTATACTATTTGACGATTCAACCATCGTTCGTAG
TTCAAAGAGTGGTGTTACAACCTACAACAATAATACAGGTGTCGCAAACTATAACGATAAAAATGAAAAATAC
CATTATAAAAACTTGTCCGAAGATGAAGCAAGTTCAAGCAAAATGGAAGAAACGATCCCAGGAACCTTTGA
TTTTATTAATGGTCATGGTGGTTTCTTAAACGAAGACTTCAGATTGTTTAGTACGAATAATCAGTCAGGCGAG
TTAACATATCAACGTTTCCTTAATGGTTATCCAACGTTTAATAAAGAAGGCGCTAATCAAATTCAAGTCACTTG
GGGTGAAAAGGGCGTCTTTGATTATCGTCGTTCGTTATTGCGCACCGATGTTGTGTTAAATAGTGAGGATAAT
AAATCGTTGCCGAAATTAGAGTCTGTGCGTTCAAGCTTAGCGAACAATAGTGATATTAATTTTGAAAAAGTAA
CGAATATCGCTATCGGTTACGAAATGCAAGATAATCCGGATCATAATCACATTGAAGTGCAGATTAATAGTGA
ACTCGTACCACGCTGGTATGTAGAATATGATGGCGAATGGTATGTTTATAACGATGGGAGGCTTGAATAAATG
AACTGGAAACTGACGAAGACACTTTTCATTTTCGTGTTTATTCTTGTCAACATCGTGTTAGTATCGATTTATGT
TAATAAAGTCAATCGCTCACACATTAATGAAGTTGAGAGTAATAATGAAGTTAATTTTCAGCAAGAAGAAATT
AAAGTACCGGCTAGTATTTTAAATAAATCAGTTAAAGGCATACAATTAGAACAAATTACGGGGCGTTCAAAA
GACTTTAGTTCTAAAGCTAAGGGTGATTCGGATTTGACCACATCAGACGGTGGAAAATTATTGAATGCGAAC
ATTAGTCAATCGGTAAAGGTCAGTGACAATAACTTAAAAGATTTGAAAGATTATGTTAACAAACGTGTGTTCA
AAGGTTCAGAATATCAATTAAGTGAGATTAGTTCTGGTTCTGTAAAATACGAACAAACGTATGATAATTTCCC
GATTTTGAATAATAGTAAAGCGATGTTGAACTTTAATATAGAAGATAACAAAGCGACTAGTTATAAACAATCA
ATGATGGATGACATTAAGCCCACAGATGGTGCAGATAAGAAGCATCAAGTGATTGGTGTGAGAAAAGCAAT
CGAGGCATTATATTATAATCGTTACTTGAAAAAAGGTGATGAAGTCATTAATGCTAGACTTGGTTACTACTCAG

TCGTGAACGAAACGAATGTTCAATTGTTACAACCAAACTGGGAAATTAAAGTGAAGCATGACGGTAAGGAC
AAAACGAATACTTACTATGTCGAAGCGACAAATAATAACCCTAAAATTATTAATCATTAATATGAATCGTAATAA
GCTAGCATTGCAAGCTCATCATATGTGAGAAGCGGTGTTAGCTTTTTGCTGGTATGGTTTATTATGGCTGACG
TTTTTGTGTCCTAACATGCGCATTTATTCATATATTAAGTAGAACCGCATTGTAAAATTAACGTAACTGCTATTTT
AAAAACTTTAGTATTTGTCTAATCATTGTTATAATAATTAGGACATTCATTGCACGTGATTATCAAAATTTAATTA
TAAGAGACCGGTCGATGAACTAAAGTTACATAATAGGAAAGATATACAAAACAGCTAATATACTGATAGTTTC
TGTAGGGGAAATCGTATATTTGCACTGATGTATATTGCAGTCATTTAGAGAGAGTGACTGCATAAATAGAAAG
GATGAGCCGCTTGATACGCATGAGTGTATTAGCAAGTGGTAGTACAGGTAACGCCACTTTTGTAGAAAATGA
AAAAGGTAGTCTATTAGTTGATGTTGGTTTGACTGGCAAGAAAATGGAAGAATTGTTTAGTCAAATTGACCG
TAATATTAAAGATTTAAATGGTATTTTAGTAACCCATGAACATATTGATCATATTAAAGGATTAGGTGTTTTGGC
GCGTAAATATCAATTGCCAATTTATGCGAATGAAAAGACTTGGCAGGCAATTGAAAAGAAAGATAGTCGCAT
TCCTATGGATCAGAAATTTATTTTTAATCCTTATGAAACGAAATCTATTGCAGGTTTCGATGTTGAATCGTTTAA
CGTGTCACATGATGCGATAGATCCGCAATTTTATATTTTCCATAATAACTATAAGAAGTTTACGATTTTAACGGA
TACGGGTTACGTGTCTGATCGTATGAAAGGTATGATACGTGGCAGCGATGCGTTTATTTTTGAGAGTAATCAT
GACGTCGATATGTTGAGAATGTGTCGTTATCCATGGAAGACGAAACAACGTATTTTAGGCGATATGGGTCATG
TATCTAATGAGGATGCGGGTCATGCGATGACAGACGTGATTACAGGTAACACGAAACGTATTTACTTATCGCA
TTTATCACAAGATAATAACATGAAAGATTTGGCGCGTATGAGTGTTGGCCAAGTATTGAACGAACACGATATT
GATACGGAAAAAGAAGTATTGCTATGTGATACGGATAAAGCTATTCCAACGCCAATATATACAATATAAATGAG
AGTCACCCGATAAAGTTCCGCACTGCTGTGAAACGACTTTATCGGGTTTTTGTTTATGTTGTTGGTGGGAAAT
GGCTGTTGTTGAGATTAAGGTTCTAGTTGAAATGTAAAAAAATAATTCGATATTAAATGTAATTTATAAATAATTT
ACATAAAATCAATCATTTTAATATAAGGATTATGATAATATATTGGTGTATGACAGTTAATGGAGGGAACGAAA
TGAAAGCTTTATTACTTAAAACAAGTGTATGGCTCGTTTTGCTTTTTAGTGCAATGGGATTATGGCAAGTCTC
GAGCGCGGCTGAGCAGCATACACCAATGAAAGCACATGCAGTAACAGTGATAGACAAAGCAACAACAGAT
AAGCAACAAGTAACGCCAACAAAGGAAGCAGCGGCTCATCAATCTGGTGAAGAAGCGGCAACCAACGTAT
CAGCATCAGTACAGGGAACAGCTGATGATACAAACAACAAAGTAACATCCAACGCACCATCTAACAAACCAT
CTACAGCAGTTTCAACAACAGTAAACGAAACGCGCGACGTAGATACACAACAAGCCTCAACACAAAAACCA
ACTCAATCAGCGACATTCACATTATCAAATGCTAAAACAGCATCACTTTCACCACGAATGTTTGCTGCTAATGC
ACCACAAACAACTACACATAAAATATTACATACAAATGATATCCATGGCCGACTAGCTGAAGAAAAAGGGCGT
GTCATCGGTATGGCTAAATTAAAAACAGTAAAAGAACAAGAAAAGCCAGATTTAATGTTAGACGCAGGAGA
CGCCTTCCAAGGTTTACCACTTTCAAACCAGTCTAAAGGTGAAGAAATGGCTAAAGCAATGAATGCAGTAG
GTTATGATGCTATGGCAGTCGGTAACCACGAATTTGACTTTGGATACGACCAGTTGAAAAAGTTAGAGGGTA
TGTTAGACTTCCCGATGCTAAGTACTAACGTTTATAAAGATGGAAAACGCGCATTTAAACCTTCAACGATTGT
AACGAAAAAGGGTATTCGTTATGGAATTATTGGCGTAACGACACCAGAAACAAAGACGAAAACAAGACCTG
AAGGCATTAAAGGTGTTGAATTTAGAGATCCATTACAAAGTGTGACAGCAGAAATGATGCGTATTTATAAAG
ACGTAGATACATTTGTTGTTATATCACATTTAGGGATTGATCCTTCAACACAAGAAACATGGCGTGGTGATTAC
TTAGTGAAACAATTAAGTCAAAATCCACAATTGAAAAAACGTATTACAGTCATTGATGGTCATTCACATACCG
TACTTCAAAATGGTCAAATTTATAACGATGATGCATTAGCACAAACAGGTACAGCACTTGCGAATATCGGTAA
GATTACATTTAATTACCGCAATGGAGAGGTATCAAATATTAAACCATCATTGATTAATGTTAAAGACGTTGAAA
ATGTAACACCGAACAAAGCATTAGCTGAACAAATTAATCAAGCTGATCAAACATTTAGAGCACAAACAGCAG
AGGTTATTATTCCAAATAATACGATCGATTTCAAAGGAGAAAGAGATGACGTTAGAACGCGTGAAACAAATT
TAGGAAACGCAATTGCAGATGCTATGGAAGCGTATGGCGTTAAGAATTTCTCTAAAAAGACTGACTTTGCCG
TGACAAATGGTGGAGGTATTCGTGCCTCTATCGCAAAAGGTAAGGTGACACGCTATGATTTAATCTCAGTATT
ACCATTTGGAAATACGATTGCGCAAATTGATGTAAAAGGTTCAGACGTCTGGACAGCTTTTGAACATAGTTT
AGGCGCACCAACAACACAAAAAGATGGTAAGACAGTATTAACAGCGAATGGTGGTTTACTACATATCTCTGA

TTCAATTCGTGTTTACTATGATATGAATAAACCATCTGGCAAACGAATTAACGCTATTCAAATTTTAAATAAAGA
GACAGGTAAGTTTGAAAATATTGATTTAAAACGTGTATATCACGTAACGATGAATGACTTCACAGCATCAGGT
GGCGACGGATATAGTATGTTCGGTGGCCCTAGAGAAGAAGGTATTTCATTAGATCAAGTACTAGCAAGTTATT
TAAAAACAGCTAACTTAGCTAAGTATGATACGACAAAACCACAACGTATGTTATTAGGTAAACCAGCAGTAAG
TGAACAACCAGCTAAAGGACAACAAGGTAGCAAAGGTAGTAAGTCTGGTAAAGATGCACAACCAATTGGTA
AAGACAAAGTGATGGATCCAGCGAAACAACCAGCGCCAAGTAAAGTTGTGTTGTTGCCAACGAATAGAGG
AACTGTTAGTAGTGGTAGAGAAGGTTCTGATCGTGCATTGGAAGGAACTGCTGTATCAAGTAAGAGTGGGA
AACAATTGGCTAGCATGTCAGCGCCTAAAGGTAGCACACATGAGAAACAGTTACCAAAAACTGGAACTGAT
CAAAGTTCAAGCCCAGCAGCGATGTTTGTATTAGTAGTAGGTATAGGTTTAATCGCGACTGTACGACGTAGA
AAAGCTAGCTAAAATATATTGAAAACCATACTACTGTATTTCTTAAATAAGAGGTACGGTAGTGTTTTTTATGG
AAAAAGCGATAACCGTTGATAAATATGGGATATAAAAACGGGGATAAGTAATAAGACATCAAGGTATTTATCC
ACAGAAATGGGGATAGTTATCCAGAATTGTGTACAATTTAAAGAGAAATACCCACAATGCCCACAGAGTTATC
CACAAATACACAGGTTATACACTAAAAAACGGGCATAAATGTCAGGAAAATATCAAAAACTGCAAAAAATAT
TGGTATAATAAGAGGGAACAGTGTGAACAAGTTAATAACTTGTGGATAACTGGAAAGTTGATAACAATTTGG
AGGACCAAACGACATGAAAATCACCATTTTAGCTGTAGGGAAACTAAAAGAGAAATATTGGAAGCAAGCCA
TAGCAGAATATGAAAAACGTTTAGGCCCATACACCAAGATAGACATCATAGAAGTTCCAGACGAAAAAGCAC
CAGAAAATATGAGCGACAAAGAAATTGAGCAAGTAAAAGAAAAAGAAGGCCAACGAATACTAGCCAAAAT
CAAACCACAATCCACAGTCATTACATTAGAAATACAAGGAAAGATGCTATCTTCCGAAGGATTGGCCCAAGA
GTTGAACCAACGCATGACCCAAGGGCAAAGCGACTTTGTATTCGTCATTGGCGGATCAAACGGCCTGCACA
AGGATGTCTTACAACGCAGTAACTACGCACTATCATTCAGCAAAATGACATTTCCACACCAAATGATGCGGGT
TGTGTTAATTGAGCAAGTGTATAGAGCATTTAAGATTATGCGTGGAGAAGCGTATCACAAATAAAACTAAAAA
ATAGATTGTGTATAATATAAAAGGAAGGGATTTATATTAAAATTTTGAATTCAAAAATTATTGAAAGGGAAGCT
ACCTTAGAAATTGAATCTATGGCCACTAATACATTGAAAATAAACCCAGACATTAATTCTTACTATACAGAAAT
GTCTTTCGATGGAGAATTGGAAGTGTATGATCCTGAAAATTTGAATAAAAAATTTCGTTGGAAAAATACAAG
TTCAAGTTAAAGGAAAAGAAGTAGCTAAAAGAGGAGGTAAGATTATTCGTCGAAGTAATGGGTTCTGTTGC
AAAGTAAAAAAATATAGCTAACCACTAATTTATCATGTCAGTGTTCGCTTAACTTGCTAGCATGATGCTAATTTC
GTGGCATGGCGAAAATCCGTAGATCTGAAGAGACCTGCGGTTCTTTTTATATAGAGCGTAAATACATTTAATA
CCTTTTAAAGTATTCTTTGCTGTATTGATACTTTGATACCTTGTCTTTCTTACTTTAATATGACGGTGATCTTGCT
CAATGAGGTTATTCAGATATTTCGATGTACAATGACAGTCAGGTTTAAGTTTAAAACCTTTAATTACTTTAGCC
ATTGCTACCTTCGTTGAAGGTGCCTGATCTGTAATTACCTTTTGAGGTTTACCAAATTGTTTAATGAGACGTTT
GATAAACGCATATGCTGAATGATTATCTCGTTGCTTACGCAACCAAATATCTAATGTATATCCCTCTGCATCAAT
GGCACGATATAAATAGCTCCATTTTCCTTTTATTTTGATGTACGTCTCATCAATACGCCATTTGTAATAAGCTTTT
TTATGCTTTTTCTTCCAAATTTGATACAAAATTGGGGCATATTCTTGAACCCAACGGTAGACCGTTGAATGATG
AACGTTTACACCACGTTCCCTTAATATTTCAGATATATCACGATAACTCAATGTATATCTTAGATAGTAGCCAAC
GGCTACAGTGATAACATCCTTGTTAAATTGTTTATATCTGAAATAGTTCATACAGAAGACTCCTTTTTGTTAAA
ATTATACTATAAATTCAACTTTGCAACAGAACCGTATTATGGAATAGAGATGTTGGTAACATTTATACAGGATC
ATTATACTTAAGTTTAATTTCGTTATTACAGAACCACACATTCCAACCAGAAGAGAAAGTATGTCTATTTAGTT
ATGGTTCAGGAGCAGTAGGAGAAATCTTTAGTGGTTCAATCGTTAAAGGATATGACAAAGCATTAGATAAAG
AGAAACACTTAAATATGCTAGAATCTAGAGAGCAATTATCAGTCGAAGAATACGAAACATTCTTTAACAGATT
TGATAATCAAGAATTTGATTTCGAACGTGAATTGACACAAGATCCATATTCAAAAGTATACTTATACAGTATAG
AAGACCATATCAGAACATATAAGATAGAGAAATAAACTAGTGGCCGATTGTGCTTGATGAGCTTGGGACATA
AATCCTAACTCGAAATAAATAAGCATATCACTAAACTGATTTTTTAAAGTTTACAGTGATATGCTTATTTTTTTAT
CTTACGATTTTGTACGTGCATGCTTGCCTAGGGGTATGGCTCGAGCCATTAGTCTCTCGCACATACTATTCCCT
CAGGCGTCAGCACTTACAAAATCGGTTGTAATTTTCATTTTTATACGCATTCTTACTGAGATTATACTAATAAGA

GGAATAGTAAAAGCAATTCTAAGTAAAATTGCAGATAAGAGGTTTGTTAAAAGCAGTTCTAAGTAAAATTGC
AGATAAGAGGTTTGTTAAAAGCAGTTCTCAGTAAAATTACAGATAAGAGGTACGTTAAAAGCAGTTCTAAGT
AAAATTGCAGATAAGAGGTTTGTTAAAAGCAGTTCTAAGTAAAATTGCAGATAAGAGGTACGTTAAAAGCA
ATTCCATGCAAAATTGCTGATAAGGGGTAAGTTAAAAGCAGTTCTCAGTAAAATTGCAGATAAGAGGTACGT
TAAAAGCAGTTCTAGGCAAAATTGCAGATAAGAGGTGCGTTAAAAGCAGTTCTCAGTAAAATTGCTGATAAG
GGGTAAGTTAAAAGCAATCCTAAGTAAAATTGCAGATAAGAGGTAAGTTAAAAGCAATCCTAAGTAAAATTG
CAGATAAGGGGTACAGAAAAACTAGACTTGATTACAAAATGGAGCTTGGGACATAAATGATTTTTTAAAAAT
GAGATGAGACGTAGATTAACTCCATAATCAATACGAATCTATCGACTTCTTTATTTATGATATTCATCTCTTTTTA
ATGGAAATAAAAGTGCGATTAATGTGATAATACAGTTACGTTAATTAAAAAAATAAAAATGCAAGGAGAGGT
AATATGCTAACTGTATATGGACATAGAGGATTACCTAGTAAAGCTCCGGAAAATACAATTGCATCATTTAAAGC
TGCTTCAGAAGTAGAAGGTATAAACTGGTTGGAGTTAGATGTTGCAATTACAAAAGATGAACAACTGATTAT
CATTCATGATGATTATTTAGAACGGACTACAAATATGTCCGGGGAAATAACTGAATTGAATTATGATGAAATTA
AAGATGCTTCTGCAGGATCTTGGTTTGGTGAAAAATTCAAAGATGAACATTTGCCAACTTTCGATGATGTAG
TAAAAATAGCAAATGAATATAATATGAATTTAAATGTAGAATTAAAAGGTATTACTGGACCGAATGGACTAGCA
CTTTCTAAAAGTATGGTTAAGCAAGTGGAAGAACAATTAACAAACTTAAATCAGAATCAAGAAGTGCTCATT
TCAAGCTTTAATGTTGTGCTTGTTAAACTTGCAGAAGAAATCATGCCACAATATAACAGAGCAGTTATATTCCA
TACAACTTCGTTTCGTGAAGACTGGAGAACACTTTTAGATTACTGTAATGCTAAAATAGTAAACACTGAAGAT
GCCAAACTTACTAAAGCAAAAGTAAAAATGGTAAAAGAAGCGGGTTATGAATTGAACGTATGGACTGTAAA
CAAACCAGCACGTGCAAACCAACTTGCTAATTGGGGAGTTGATGGTATCTTTACAGACAATGCAGATAAAAT
GGTGCATTTGTCTCAATAGAAAGTTAGAGGTGAGTCTTACGTTTCAGTGACGGTAGACTTACCTTTAACATGT
TACATACTAAAAAATTAATTTGAATAAGAAAGAGAGACATATATGAAATACGATGATTTTATAGTAGGAGAAA
CATTCAAAACAAAAAGCCTTCATATTACAGAAGAAGAAATTATCCAATTTGCAACAACTTTTGATCCTCAATAT
ATGCATATAGATAAAGAAAAAGCAGAACAAAGTAGATTTAAAGGTATCATTGCATCTGGCATGCATACACTTT
CAATATCATTTAAATTATGGGTAGAAGAAGGTAAATACGGAGAAGAAGTTGTAGCAGGAACACAAATGAATA
ACGTTAAATTTATTAAACCTGTATACCCAGGTAATACATTGTACGTTATCGCTGAAATTACAAATAAGAAATCCA
TAAAAAAAGAAAATGGACTCGTTACAGTGTCACTTTCAACATACAATGAAAATGAAGAAATTGTATTTAAGG
GAGAAGTAACAGCACTTATTAATAATTCATAATAAAACAGTGAAGCAACCATCGTTACGGATTGCTTCACTGT
TTTGTTATTCATCTATATCGTATTTTTTATTACCGTTCTCATATAGCTCATCATACACTTTACCTGAGATTTTGGCA
TTGTAGCTAGCCATTCCTTTATCTTGTACATCTTTAACATTAATAGCCATCATCATGTTTGGATTATCTTTATCATA
TGATATAAACCACCCAATTTGTCTGCCAGTTTCTCCTTGTTTCATTTTGAGTTCTGCAGTACCGGATTTGCCAA
TTAAGTTTGCATAAGATCTATAAATATCTTCTTTATGTGTTTTATTTACGACTTGTTGCATACCATCAGTTAATAG
ATTGATATTTTCTTTGGAAATAATATTTTTCTTCCAAACTTTGTTTTTCGTGTCTTTTAATAAGTGAGGTGCGTT
AATATTGCCATTATTTTCTAATGCGCTATAGATTGAAAGGATCTGTACTGGGTTAATCAGTATTTCACCTTGTCC
GTAACCTGAATCAGCTAATAATATTTCATTATCTAAATTTTTGTTTGAAATTTGAGCATTATAAAATGGATAATCA
CTTGGTATATCTTCACCAACACCTAGTTTTTTCATGCCTTTTTCAAATTTCTTACTGCCTAATTCGAGTGCTACT
CTAGCAAAGAAAATGTTATCTGATGATTCTATTGCTTGTTTTAAGTCGATATTACCATTTACCACTTCATATCTTG
TAACGTTGTAACCACCCCAAGATTTATCTTTTTGCCAACCTTTACCATCGATTTTATAACTTGTTTTATCGTCTA
ATGTTTTGTTATTTAACCCAATCATTGCTGTTAATATTTTTTGAGTTGAACCTGGTGAAGTTGTAATCTGGAAC
TTGTTGAGCAGAGGTTCTTTTTTATCTTCGGTTAATTTATTATATTCTTCGTTACTCATGCCATACATAAATGGAT
AGACGTCATATGAAGGTGTGCTTACAAGTGCTAATAATTCACCTGTTTGAGGGTGGATAGCAGTACCTGAGC
CATAATCATTTTTCATGTTGTTATAAATACTCTTTTGAACTTTAGCATCAATAGTTAGTTGAATATCTTTGCCATC
TTTTTTCTTTTTCTCTATTAATGTATGTGCGATTGTATTGCTATTATCGTCAACGATTGTGACACGATAGCCATCT
TCATGTTGGAGCTTTTTATCGTAAAGTTTTTCGAGTCCCTTTTTACCAATAACTGCATCATCTTTATAGCCTTTA
TATTCTTTTTGTTTTAATTCTTCAGAGTTAATGGGACCAACATAACCTAATAGATGTGAAGTCGCTTTTCCTAG

AGGATAGTTACGACTTTCTGTTTCATTAGTTGTAAGATGAAATTTTTTTGCGAAATCACTTAAATATTCATCCAT
TTTTTTAACGGTTTTAAGTGGAACGAAGGTATCATCTTGTACCCAATTTTGATCCATTTGTTGTTTGATATAGTC
TTCAGAAATACTTAGTTCTTTAGCGATTGCTTTATAATCTTTTTTAGATACATTCTTTGGAACGATGCCTATCTC
ATATGCTGTTCCTGTATTGGCCAATTCCACATTGTTTCGGTCTAAAATTTTACCACGTTCTGATTTTAAATTTTC
AATATGTATGCTTTGGTCTTTCTGCATTCCTGGAATAATGACGCTATGATCCCAATCTAACTTCCACATACCATC
TTCTTTAACAAAATTAAATTGAACGTTGCGATCAATGTTACCGTAGTTTGTTTTAATTTTATATTGAGCATCTAC
TCGTTTTTTATTTTTAGATACTTTTTTTATTTTACGATCCTGAATGTTTATATCTTTAACGCCTAAACTATTATATAT
TTTTATCGGACGTTCAGTCATTTCTACTTCACCATTATCGCTTTTAGAAATATAACTGCTATCTTTATAAACTTGT
TTGAAATTTTTATCTTCAATTGCATCAATAGTATTATTAATTTCTTTATCTTTTGAAGCATAAAAATATATACCAAA
CCCGACAACTACAACTATTAAAATAAGTGGAACAATTTTTATCTTTTTCATCAATATCCTCCTTATATAAGACTA
CATTTGTAATATATTACAAATGTAGTATTTATGTCAAAATAATGTTATAATTTTTGTGATATGGAGGTGTAGAAG
GTGTTATCATCTTTTTTAATGTGGTTCTGTTGCAAAGTTGAATTTATAGTATAATTATAACCAAAAGGAGTCTTC
TGTATGAACTATTTCAGATATAAACAATTTAACAAGGATGTTATCACTGTAGCCGTTGGCTACTATCTAAGATAT
ACATTGAGTTATCGTGATATATCTGAAATATTAAGGGAACGTGGTGTAAACGTTCATCATTCAACGGTCTACCG
TTGGGTTCAAGAATATGCCCCAATTTTGTATCAAATTTGGAAGAAAAAGCATAAAAAAGCTTATTACAAATGG
CGTATTGATGAGACGTACATCAAAATAAAAGGAAAATGGAGCTATTTATATCGTGCCATTGATGCAGAGGGAT
ATACATTAGATATTTGGTTGCGTAAGCAACGAGATAATCATTCAGCATATGCGTTTATCAAACGTCTCATTAAA
CAATTTGGTAAACCTCAAAAGGTAATTACAGATCAGGCACCTTCAACGAAGGTAGCAATGGCTAAAGTAATT
AAAGGTTTTAAACTTAAACCTGACTGTCATTGTACATCGAAATATCTGAATAACCTCATTGAGCAAGATCACC
GTCATATTAAAGTAAGAAAGACAAGGTATCAAAGTATCAATACAGCAAAGAATACTTTAAAAGGTATTAAATG
TATTTACGCTCTATATAAAAAGAACCGTAGGTCTCTTCAGATCTACGGATTTTCCCCATGCCACGAAATTAGCA
TCATGCTAGCAAGTTAAGCAAACACTGACATGATAAATTAGTGGTTAGCTATATTTTTTTACTTTGCAACAGAA
CCTAATATACTCTATCAAGTCCCACAACTTGAGAGAGCTTTTTGTATATATTAAGCATTTATGCTACTAGTTGAT
ATTAGATGTAAAGTATTTATAGTAGTAATATATCGTTTTATCTAGCTGTACGAAAAATCAATTACAAAAAACCCC
ATCAACAACCGATAAGCAGAACTCGTCAAGAGATGTCAGATAAGATAGTTTTAGTACTCTGTAATTTTAGGTA
TAAGGTATATTATAACCGTTTTAGCCTATCCATCCATCGCCCACTCATTCTATTCTAGCTTATCCACCCGACGCAA
TTTTCTGACTCATTCCAGCTTTGCCTATCCACCTATCGCCTTTACATACACACTTCATCTTTAAAATCTAAAATTT
CCTTATGCTTATTCATTAACTATTCCTCCATACTCTTCTCATCGTTCTTTTAATTCACACACTCCCTTTTCCCTCGT
CACTTAGTCATGGCAGTTTGTAATTTTTGTTCAATTAAGTCGATATGTGTACTGATCAAGTTCCGTTGTTCATTC
AAGAATTCTAGGTGTGCGCGTAACACGCTTGGGACATCTATTTGGTTTTGATAAAGCATGTCTGTGTTTTTTC
GGATTTCTTTTAATGGCATATTCAAATTGCTTAAGCACTGGATAAATTTAATCATTTCGATATCGTCGTCTGTGT
ATAAACGCTTGTTATTGCTATCTCTGTCGATGTGTTTTAAGATACTCTTTTTCTCGTAATAACGCAGTTTGCTTT
TAGGTATATTGCAATAATCTGAGACATATTCAATGTAATATTGTTTCATGTAAGCATATCCTTTCTCTTAAACCAA
GGTTTAACTGATATTGTATTCTATATAAACATGAATGAGGAGGAAAGACAATGAAAAAGGCTTTAATTGTTGT
AACAAACATTGCGAAATACGATAATTTAGAAAGACCTACAGGTGCGTGGTTCTCAGAGGTTACGCACTTTGC
GAAAGATTTCTATGACGCAGGTTATGATGTTGATTTTGTAAGTCCGAACGGTGGGTATGTGCCTCTTGATCCT
ATCAGTCTTAGCCCTGAAATGATGGGAGCCGAGGACTGGGAATACTACACGGATCATGATTATATGAATAAAT
TTGGACAAACATTATCTCCAAAAGAGGTTAATCCTAGCGACTATCAAGCCATTTACTTTGCAGGTGGACATGG
TGCGATTTGGGATTTAAGAAATAACAAAGAACTAAACGACATTGCGCTAAGTATTTACAATAACCAAGGCGT
TCTCTCTTCTGTATGTCATGGTGCGGCTGGTCTACTCGATATTAAAGAAAATGGCGATTATCTCGTTCGTCATA
AAGATGTGACTGGTTTTACAAATAGTGAAGAACAAGCAAATGGTACAACTGAATACATGCCATATTTATTAGA
AGACGAATTTATTAGTAACGGTGCACATTTTAAAAAAGAGGCTGACTGGAGTAATTTTGCAGTCGTAGATGG
TCGCATTGTCACAGGTCAAAATCCCCAATCAGGACATGCAGTTGCTGAAAATGCTTTAAAAATCTTAGCTAAT
CAATAATTTTATAAAGGAGCGATACACATGAAATCATTAATTATCGGTGCTAATGGTGGCGTCGGTCAACATCT

CGTACGTAAACTGAAAGCAAGAGATGTTGATTTTACTGCCGGTGTTAGGAAAGAAGAACAAGTTGAAGCTT
TAAAAGCAGATGGTATCGATGCAACTTACATTGATGTCGCAAAACAATCTATTGATGAATTAATAGAATTATTT
AAATCGTATGATCAAATCCTTTTTTCCGTCGGTTCTGGTGGGAGCACAGGTGACGATCAAACAATCATTGTAG
ATTTAGACGGTGCAGTGAAAGCAATTAAAGCAAGTGAACAGATCGATCATCAACACTTTATTATGGTATCAAC
GTACGACTCACGCCGAGAAGCGTTTGATGCGTCAGGCGACTTGAAACCATACACCATTGCTAAACATTATGC
GGATGACTACTTAAGACATGCAAATTTAAAATATACCATCGTGCATCCAGGCGCTTTAACAAACGAACATGAA
ACGCAACAATTCAATATGAGTGCGCAATTTGAAAATGTACAAAATCCGTCTATTACAAGAGAAGATGTAGCA
GAAGTGCTTGTTTCTGTATTAACTGATGAAGTATTACAAGGTCACGAATTCCAAATCATCAATGGTGATTTGTC
ATTATTAGACGCAACGACTAAATATCTGGAGGAATAATAAATGAATAATCAACAAGTTGTACTTGCAAAACGA
CCACAAAGTATCCCTCAAGACGATGTATTTAGATTTGAAACAATAGAAACTCGAGAACCACATGCAGGTGAG
GTTCAAGTAGAATCCATTTATGTATCTGTAGATCCTTACATGAGAGGCAGAATGAATGATACAAAAAGTTATGT
TCAACCTTTCCAAGTGAATGAGCCATTACAAGGTCATATTGTTGGAAAAGTCACACAATCGAACGATGAACG
TCTATCTGTCGGCGATTATGTCACAGGCATATTACCATGGAAAAAGATAAATACAGTGAATGGAGACGATGTG
ACCCCTGTGCCATCAAAAGATGTACCATTACATTTATATTTGAGTGTTTTAGGCATGCCGGGAATGACAGCCT
ATACAGGATTGCTTCAAATTGGTCAGCCACAATCTGGCGAGACGGTTGTCGTGTCAGCTGCATCAGGTGCAG
TAGGCTCTGTCGTAGGACAAATTGCTAAGATTAAAGGCGCAAAAGTTGTCGGTATTGCTGGTGGTAAGCAG
AAAACAACATATTTAACAGATGAATTAGGATTTGATGCGGCCATTGACTATAAACAAGATGATTTCGCACAGC
AACTCGAAGCGGCTGTACCAGATGGTATTGATGTGTATTTTGAAAATGTTGGCGGCGCAATTTCTGATGAAG
TATTTAAATACTTAAATCGATTTGCACGCGTTCCGGTATGTGGTGCAATTTCAGCATATAATAATGAAAAAGAC
GATATTGGACCACGTATCCAAGGAACGTTGATTAAAAATCAAGCATTAATGCAAGGTTTTGTAGTAGCACAAT
TCGCTGATCATTTTAAAGAAGCAAGCGAACAACTCGCACAATGGGTGTCTGAAGGTAAAATTAAATTTGAAG
TGACGATAGATGAAGGTTTTGACAATTTACCTTCTGCATTTAGAAAGTTATTCACAGGAGAGAATTTTGGTAA
ACAAGTTGTCAAAGTCGCTGAAGAATAGAGAAGACTATGAAAAATATCATCGTAAGATTCATATTTAGTTTAC
TGTTTTATAAAAGTGGTGTTTCACACTTTAAAAATAAAGATATGTTTTTAAAAATCGTGCCGTCCTATTTACCTT
TCAAACACGCAATAGTTAAGTGTTCGGGCATATTAGAATTTATAATTGTTTTTTATGTACTCGGTGCTAAAAAT
AGATCACGCGTAAGAAAAGTAGTACAAGGATTCCTTTGGCGCGTATTTCCTGCAAATATTTACGCTGCACGTA
AGAACATCTCTTATAAAGATGAAAGAGATAGAACAGATAGTGTTAAGCAGCATGTCATTCGTTTACCATTACA
ATTTGTCATGGTCGCACTTGCGAAACTATTATAAGACTTATCATAGCCACGCCTGGAACATTACTTTTGTTCCA
GGCGATTTTTTAATATTTACACTTAAAATCGACGTATATCAAATGATTCTCTTGACAACGCTTATGTGCTCTTTG
TTTAAATAAGCTCATATCAGACATTTACTTTTCAATTTTTCTATAGTGATAAGCTTCTAAATTAAGAAAAGCATA
AGTTAATTTGCGTGTATGAGTGTAAGAATAAATAAAATAATTATATTTTCATATAATTATTATGTGGCATTGTACC
ATTAATGTAATAATGTGGCCTGTAAGACAAATATGAGGAGGGTTCTATAAGGTGCTTCTTATTATTGAAATTGT
TAAAAAAACAAAATGCAATGAACTGACAAATTAAAATCGCTTTAAGTCGTATTCTCAAAGAATCTATAAATCA
CTCGTATATACACAGCCCCTTACTATCAGTTAAAGTTTCGCTTTTCGATTCGTAATAATTATTCATAAAGTATACA
ATATATGATACAATTCATTCAAATATATATTTGAATGAGGTGCTCTATGGTTCAGACTATTGTAACTGCGGCAAT
ACTTTACATTGCTACTGCAGTAGACTTATTAGTAATACTATTAATATTTTTTGCAAGAGCAAAGACTAGAAAAG
AATATAGGGATATTTATATAGGTCAATATTTAGGATCGATTATCCTCATATTAGTCAGTTTATTTTTAGCTTTTGT
GTTAAATTATGTTCCAGAGAAATGGATATTAGGTTTATTAGGTTTGATCCCCATCTACTTAGGTATAAAAGTTG
CTATTTATGATGATTGTGAAGGCGAAAAAAGAGCAAAAAAAGAATTAAACGAAAAAGGTTTGTCCAAATTA
GTAGGGACTGTTTCTTTAGTGACAATTGCAAGTTGTGGTGCAGATAATATTGGTTTATTTGTCCCTTATTTTGT
AACATTGGATATTGTTGAATTACTGACTACCTTAATTGTATTTTTAGTTTTAATTTTCTTTTTAGTATTCACAGCA
CAAAAACTAGCTAGAATTCCTGGTGTGGGTGAAATTGTTGAGAAGTTCAGTCGTTGGATTATGGCTGTTATC
TACATAGCATTGGGCTTATTTATTATTATTGAAAATGAAACCATACAAACAATATTAGGATTTATATTATAAATTA
AGGTGTGACTTAATATGAGTAACAATAAAATTTGTGACGTCATTTGTGTTCATGAGGAAAAAGTGAATTATGC

TTTAAGATTTTTAAAAGAAGAAAAAACTCAACGGCTTATCAATACATTATTAAAGATAAGTGATGAGAATAAG
CTGAAAATTATATTAGCTCTAATTAAAGAGAAAGAATTATGTGTTTGTGACTTATCTATAACATTAGGTTTGAGT
ATAGCTTCAACATCACATCATTTGAGAGCGCTGTATAAAAGAGACGTGCTGGATTTTTACAAGGATGGGAAA
ATAGTATACTATTATATCAAAGATATAGAAATGAAATCATTATTAATAAAATGTATGACTTAAATATAAAGAAACG
CTCACATTAAACGAGCGTTTCTTTGAATTCTTGTATTCTTTTTCCAATTTCATCTCTAACACGTTGAAATTCTGA
CCACTCTTTACCTGCTGGATCATCAAAACCCCAGTGTTCTTTTTTAACATTAGGTGGTAATATAGGGCAATTAT
CGTCTGCATCACTACATAATGTTACGACCAAAGCTGACTGTTCTAAGATATCACTATCAATCAAATCTGATGTAT
GGTTTGAGATATCAATATCCACTTCTTTCATAGCTTCTATTGCTTTAGGATTAACACCATGCGTTTCTATACCAG
CAGAATAGACATTCCAATCTTCACCCAATATTTCCTTTCCCCAACCTTCAGCCATTTGGCTACGACAAGAGTTA
CCACTACATATAAAATAAATTGTTTTCTTATCCATAATTAAAGCCTCTTTTCTTAAAATATGATTAGTGTAAGGTA
TAAACCTAAGAGTGTTACAAATAGGACTGGAATAGTAATGATAATTCCAGTTTTAAAGTATGTTCCCCACGAA
ATCTTCACACCTTTTTGTGTTAAGACATGTAGCCACAATAATGTTGCTAAAGAACCAATTGGCGTAATTTTAGG
TCCTAAATCAGAACCTATGACATTCGCATAAATCATACCTTCTTTTAATATACCTGTAGCACTAGATTGACCAAT
AGCTATTGCGTCTATTAAAACAGTAGGCATATTATTCATAATTGATGATAAAAAAGCTGAAATAAAGCCCATGC
CCATAATACTGCTGAATAATCCATGGCTTGAAATATTGGATAATACATCAGCTAAAATTGTTGTGATTTCTACGT
TTTTTAAACCAAATACAACTAAGTACATACCAATAGAAAATAGAACGATATTCCATGGCGCACCTTTAATCACT
TGTTTTGTATGAACTGCTTTTGATTTATGAGCTAATATTACAAAGATAAAAGCAATAATACCAGCAATAATTGAT
ACAGGAATTTGGATAAACTCGCTAACAAGATATCCAACGAGTAGTACTGCTAATACTATCCATGAAAGCTTAA
ATAACTTAGAATCTTTAATTACACTTTTAGGATCTGATAGATTTTCTGTATCGAACGTTTTAGGTATGGATTTTC
TGAAATATAGCCATAATACAACAATACTTGCAAGTAAAGAGAATAGATTGGGAATCATCATGCGACTTACATAT
TCAATAAATCCAATATGGAAGTAGTCAGCAGAAACGATATTAACCAAATTACTTACAATTAGTGGAAGAGACG
TAGTATCTGCAATGAATCCACTTGCAATGATAAAAGGAAAAATGACTTTTTGATTAAACCCTAAATTTCTTACC
ATAGCGAGCACGATTGGAGTGAGAATTAATGCAGCTCCATCGTTCGCAAAGAAAGCTGCTACAATAGCGCCT
AACAACATAATGTAGACAAACATTTTAAACCCATGACCATTTGAAGCTCTGACCATATGTATAGCTGCCCATTC
GAAAAATCCAATTTCATCTAAAATTAATGAAATGAGTATAACAGCGACAAAGGTTAACGTCGCATTCCAAACA
ATCCCTGTAACTTCAATAACATCCGAGAGACTGACAACGCCTGTAATAATAGCGATGATAGCACCAAACAATG
CTGTAATACCAATATCTAACCCCTTAGGTTGCCAAATAACAAACATGAGAGTTAAAAGAAAAATAATAATCGCT
AGAATAGTCATTATGTACATTCACCTGTCTTTATCTTTTTACACATACATTGTTGAGTAGGAGAGCTAATATAAG
TTAATTTATTGTTGATTGAATCTAAGGACTTATGATTAAGTCGGTACATGCGCTTAGTACCGTCTTTTCGTGTAG
ATACTAATTCATTATCTACTAGAATTTTCATATGATGACTTAATGTAGGTTGTGAAAATTGAAAATACGCTAATA
AATCACAAGCACATAATTCACCGCAAGATAATAAGTCTAATATTTCTAATCGACTTGGATCCGATAAAATTTTTA
ATTGTAATGATAGTTCCTTATAAGACATATGGATGTCATCCCTCCTTTATTATAGATTATCATCTATATAGACAAAT
ATCTATATAGATAATACTTTTTGCAATAATACATTTAATTAAAACCTTGTTTTAAACATCGATGACAAGGTCTAAT
GTAGGACGTGGAGACATCATTTTCGGAATGATATAAATAAATCCATAAATGGAGATAAATCGAAAATATTTATA
CCCCTAGGGGGTATGTGTTATAGTATAAGTATAGCTAATATAATATTTTCATAAATAGGAGGGGTTAATTTGAAT
AATAATGGTGAAGAGCATAATCATCAAAATCACATGAATCATTCCAATCACATGCATCATGATAACCATGCCTC
ACATCATCATAGTGGCCATGCACATCATCATGGAAATTTTAAAGTTAAGTTTTTTGTTTCATTAATTTTTGCAAT
ACCTATCATTCTTTTATCGCCAATGATGGGTGTTAACTTACCTTTTCAATTCACATTTCCAGGTTCTGAATGGGT
AGTGTTAATATTAAGTACAATTTTATTCTTTTATGGTGGTAAACCGTTCTTGTCTGGTGGTAAAGATGAAATTG
CTACAAAAAAACCAGGCATGATGACCTTAGTTGCCCTAGGTATTTCAGTAGCTTATATTTATAGCTTGTATGCT
TTTTATATGAATAACTTTAGTAGTGCAACTGGTCATACAATGGACTTTTTTTGGGAATTAGCAACCTTAATTTTA
ATTATGCTATTAGGACATTGGATAGAAATGAATGCTGTCGGAAATGCTGGAGATGCTTTAAAGAAAATGGCA
GAACTGTTACCTAATAGTGCTATTAAAGTTATGGATAATGGCCAACGCGAAGAAGTTAAAATATCAGACATCA
TGACTGATGATATCGTCGAAGTAAAAGCCGGAGAAAGCATTCCGACAGATGGTATTATCGTTCAAGGACAAA

CATCTATAGATGAATCCCTAGTCACTGGAGAATCTAAAAAAGTACAAAAAAATCAAAATGACAACGTCATCGG
GGGTTCTATTAATGGGTCTGGAACAATACAAGTCAAGGTTACAGCTGTGGGAGAAGATGGATATCTTTCTCA
AGTTATGGGACTTGTTAATCAAGCACAAAATGATAAATCTAGTGCTGAATTGTTATCTGATAAAGTAGCAGGT
TATTTATTCTACTTTGCTGTAATTGTTGGCGTGATTTCTTTTATTGTCTGGATGCTCATTCAAAATGATGTTGATT
TTGCATTAGAACGTCTTGTAACTGTGTTAGTCATTGCTTGTCCACATGCTTTAGGCTTGGCAATACCTTTAGTC
ACTGCACGTTCTACTTCAATTGGTGCACATAATGGTTTAATTATTAAAAATAGAGAGTCTGTAGAAATAGCTCA
ACATATCGATTATGTAATGATGGATAAAACTGGTACTTTAACTGAGGGTAACTTTTCTGTGAATCATTATGAGA
GCTTTAAAAATGATTTGAGTAATGATACAATATTAAGCCTTTTCGCCTCATTAGAAAGTCAATCTAATCACCCAT
TAGCTATAAGTATTGTTGATTTTGCGAAAAGTAAAAATGTTTCATTTACTAATCCACAAGACGTTAATAATATTC
CAGGTGTCGGATTAGAAGGTCTAATTGATAATAAAACATATAAAATAACAAATGTCTCTTATCTTGATAAACAT
AAACTTAATTATGACGATGACTTATTTACTAAATTAGCTCAACAAGGTAATTCAATCAGCTATTTAATTGAGGAT
CAACAAGTCATTGGCATGATTGCTCAAGGAGATCAAATTAAAGAAAGCTCAAAACAAATGGTAGCTGATTTA
CTATCAAGAAATATTACACCAGTCATGCTTACAGGTGACAATAATGAAGTGGCACACGCTGTCGCAAAAGAA
TTAGGTATTAGTGATGTCCACGCACAACTCATGCCAGAAGATAAGGAAAGCATTATAAAAGATTATCAAAGTG
ACGGTAATAAAGTCATGATGGTCGGAGACGGTATCAACGATGCGCCGAGTCTTATAAGAGCCGATATTGGTA
TAGCAATTGGTGCAGGCACAGATGTTGCAGTAGATTCAGGTGATATCATACTTGTTAAAAGTAATCCATCAGA
TATTATTCATTTCTTGACCCTTTCAAATAATACTATGAGAAAAATGGTGCAAAACTTATGGTGGGGTGCAGGTT
ATAATATTGTTGCTGTACCTTTAGCAGCTGGTATTTTAGCATTTATCGGCTTGATTTTATCACCAGCTGTAGGAG
CAATATTAATGTCTTTAAGTACAGTTATAGTAGCGATTAATGCCTTTACATTAAAATTAAAATAAAAGATAGGAG
TTTTATTATGATTAAAAAATTATTTTTTATGATATTAGGATCATTACTAATATTATCAGCTTGCTCCAATAATGATG
AAAAAGATAAAGACACTAATGACCAAAAAAGTGAGAGCCATATGAAGCATAATGATGAAAGTAAAGTTCCA
GAAGATATGACATCGACTAATGAGGGTGAATTTAAAGTGGGAGATAAAGTAACGATTACAGCAGGGCATATG
CCAGGTATGAAAGGTGCAGAAGCTACTGTAAAAGGTGCGTATAAAACATATGCTTATGTTGTAAGTTATAAAC
CCACAAATGGAAATGAAAAAGTAAGCAATCATAAATGGGTCGTAAACGAAGAGATCAAAGATGCACCTAAA
GATGGATTTAGTAAGGGCGATACTGTTAAATTAGAAGCAAGTCATATGTCTGGTATGAAAGGTGCTACAGCC
AATATAGATAACGTGAAAAAGACGACTGTTTACGTAGTTGATTACAAATCCAAAGATAATGGTAAAATCATTA
AAAATCATAAATGGATGACAGGAAATGAATTGAAAGCACGATAAAAATCTAGTTCTAGATTGAGAAATAAATA
GATATAAAGATATCCTCCTTAATCAATAATTTAAATAACTTATTATTGTTAAGGAGGATATTTTTTAGTGTGTAAA
TTAAAAAGAATTTTAGAAGAATAACATTTATCAAAAAACTGTTCATTACCTTCTTAAATGAAATTATATAATTAA
AAACCGCATCATTAACCGATACGCAGAAGCGTATCACAAATAAAACTAAAAAATAGGTTGTGAAAAACGGAA
ATATTTATTGATAAAAAGCCACAATCTTATGTTAGGATTGTGGCGCTAAAATTTAATACTGTAAGTTTTAGTTTT
TATAAATGTCATTATAAATTTCAAATGCTTCATTAATTTCTTCATTTAATGCGGTTTTCAATGAAGTATCTATATAA
TTAGGTCCAAATTCATAGAATAACAAACCATGTTCAACAGATAAAATATCAGTTTTAGAGGTAGAACTAGTGT
CTTTAGGGTATAAAATAATAACCTTTGTAATAACCGTATGGTTATTTTTTCTAATATGTTTAGCACCTGCTAAATG
TAATAAAGATCTTGGAGAATCTCTGTAAGCAAGTAATTGTTTAAGAGAACCCTTTTTATCATTGGTTATATTTTT
CAGTTTTCTGTACTTAGCATCTATGGGTATACTTTTTAAATATATATCCTCTACAAAAATATCAATTCTAATATCAG
GTTTATTTTTTTTGTTCGCAGTATAAAGTGGATTTATTAAATCAGTTTCAGAACTTTTATATTTAAGTAGTGCAT
TATAATGGATATTTAAAGTAATTTCTGCTTTTTTCATAGTTACATAAGTACCTTCATCGAGATCATTTTGGATGTC
ACCGTTAAAAATCCAACCTTTTACAGGAATAAAGCCACTACTTTTTAATATTTCAATTATATTGATATAACACCA
GATTTCATATAATTCATCTGTCTTCTTCCATGAATTTAATACTTGATCTGAAAAAATGAAATCAAAATTACTTTTA
TTAAATTCTACATACCATGTATATAATAAATTATAATTTTTATTCATAAGAGAAGACTGAGTTGGCATTATATTGT
TTTTATAAGAAACTTGATTATACCATTCAGTACTTCTTAATTTTATTATTAAATTTTTAATTATAGTTATATTTTTTT
TAGATTGCTTTAAATTGTACTTAGTAATTTTGTTTTTTTTAATTTCATTACTATATTGCAAATTGCATTTAGATGTC
TCATTTTTAATTTTTAAAATAATTTCTTTATCAATTGAGTTTATTTCTTTTAATAAAAACTCTACTACAAATTTCAA

CCACATATTTTCTGGAATGTTAAAATTAACAAGTCTTTTATAAGTGTACAAGTATTGTTTTTTATTTGGATATTTA
GATGAATATTTAAAAGTATTTTTATCGACAGGTGGTTGTGTATTTTTAGATGCCCAATGATGTTTTTTTTGAATC
GTTAGTCTAGGATTTGCTATTAATTGATTTATAGAATTAATAATTTTTATATAGTATTCATTAATGAAATTAATTTT
GTTGTGTAAATTATTATCTTTTTTACTTTTAATAACTTTAACGTTATTTTTATTGATAAATGAATTTGCTAAACCAT
TTAAGTAATTATTTACGTCTTCGTATAAATATTTCCATTCTTCCTCAGTAAAATCTTTGGGAACTATTTTAATAAT
TGCATACTCAGTTTTTTCATTGACCAAAGTTAATAAATAATAACCAGGAACTAGTACATTTTGATCATTAGTACT
AGTGTAATTAATTATATCTAGTGTAGCGTTTGAAAGATAACTTTCTCCCTTTTCATCAATTTTTAATACATCATCT
ACGATAATATCAAAACCATTGAAATATAGTTTTTCATTATAATTAGTAGATGTATGGCGAAACTTCATAGAGTGA
TATTCTTTGAAGGATAATACGTCTTTTTCAAAATTTATATCTTTTATTGATTCTTTGAATACAATATTAAAAACAT
TTTCTCGGTTTCCATTACTAACAATTATATCTAAGTTAGAATGTAAATCCATTTAAATATAACTCCTTAGATTTATT
TAAAATTACCTTTTTAGAATTTGTGAAATCAGAGATACTTTTATATTTATCCATCAATGCTAATAAATTAGAAGA
AGTTACGGAATTATTATTAGGATTATATGAACCTATTAATAATTCAAGTTCTTCTCTTGAACCTCTGATTTTTGAT
AAAATTCTCTGGATAACCTGAAAGTCTAAACCTTCTCCATAACTTAAAATTCCTTCATAATTAGGAATGTTTTTT
AAGTAATAATCAATATGTTTTATGACTCTAGGTCCTATACCAGATTGATTGTTTATATTTAATAGAGTATTGTGCA
TTTCTTGTAAGAAATCAATAGTATCATTATCTAGATCAATAGTTTTACTATTTTTAAAGAAACTTTCAATATATTC
TTGTTCGAAGGAAATACCTTCTGTCGTTTTGATTTTTTCAATGTTAGTAATTTCAACTAACTGTTTAAAGGGCA
TTACTTCTAAAGTGATTAAATCTGCTCTATCTAGTACTTTATTTGAAAAATGATAAGTCGATTCGTCTATATTAAC
TGTGCCTACAAATCTAACATTTTTTCCAATAGTAATTTCATGATTATATTTAGAGGAATTATATAATTTGCCACCT
AATTCTTTATTATATAATTGTAGTTTACGATTTTCTACATCTGATTCTAGTAAAGATAAAAATTGAGAAAAGTAA
TGTTCTATACGAGCTAAATTCATTTCATCAAAACAAATTATAAATAATTTGTCAGGATTAAGTTTTGCTTGAATA
AGGGTTTCTATTAAACCATGATCATCAGGTCTATAAATCATATTTAATGTATCTGCATAACCAATAATGTCTGAAT
CTTCAGACCAACTTGGACTGACCGGTATAAATTTAAAATTATCTTTTAGACCTAAAGAATTTGCATAGGCTTTA
ATCAATTTTGACTTTCCTGTTCCACTCATCCCTGATAAAATAACTAATTTTGAAGTCTTCATTGCAGTGTGAAA
ATTCAAAATATCTTTTGGGCTATAAACTAAATTTTCATTAGAAAGTTTATTCATGAAGTGTTTTAAAAATTTTCT
TTCTAGTTCATCATTTTCAGCCTTTTTATCAGTTTCTAGTTCAATAATATTCTCAGTGTCTTTTAAAATCTTATCTT
CAATTTTTTGAATTAAGGCAGTTGTTATAAATGCTATATTATTTTTATTTATAATTAAACTTTTGTAATCTTCTTCT
TCTAATTTGAAAAAGTGTATTTTTTTATCGAAATTAAATGAAATCCCACTGTCTTCATTAAGATCAAATGATTTG
ATTTCTCCTATTGCGAATATGTCGTTATCATCATCTTCATAAAAAATTATTTCAGGAGTATCATCTAGTTCTTTCG
ATATATGTAAATTGTTTCTAAATTTTAAATTTTTAGATAAATCGTTTAAAAAACTATCTATATCTTTTGATGCATTA
ATTTTATTGAATATTGGCACTGGGTGTAATACATCCGTGTACTCGATTTTTGATGGTCGAGGTAATATTTGTTTA
ATATGTCCATTATAAAAATATTTGTTAAGAATTGTGTAGTCTTCCTTTTGTTCTACATTTAAGCAAATAAATTTAT
CATGTATAAAATTATAAAAGTCATTTATTCTTTCCGTTTGAGATTTGCTTGTGTCATCTTTATATGAATCATATAA
ACTAGTCGAAAAGCAAGCTATATACTTAGTTTTGTTATCTAAATTTGGATAATCATAAGGTGTTGTTGATATTAA
TTTTATATAAAATTGAATGAAATCATTAGAGTTTATGAAAATATTTTCTTTATTATAGGCTTTACTAATCTCCTCTT
CTGTAGCAAGAATACCAATAAGGTCAACTTTTTCCCAATTATTTTTCATTTTTTCATTCTCCTTTTATTATTTTTA
TATATTCATCTTTATTATTTATAAAAATTGCGTGTGTTTGTTTTTTAATTTTTCGAGATGAGTATGAAGTAACACC
GAATCTTACCACATAGAATTTTGAATTTTCATTATTATAATTATTTATAAAGTTATCGATTTCATCTTCGTTATAAA
AACATATATCTTCAGTACTTTCTAATTTCCATAAATCTGGAGCTCCAATTATATTGATTTTAGTATCATTAATATTT
TCAGAATGAAGGGAATTATTATCTTTTGGGTAAGGTTGTTCTTTTAATTGCTCTAATTCGATTATTTTATTTTGT
AAGACTTCTTTTTTCTCATTTAATTCGTCATTTTTAACTTTAATTGCTTCATTTAATTTTATAGTTAAGTCTTCGTA
TTTTTTGATTTTTTTTTCTGCCTCTTTATTCAATTTAAGTAGTTCATCTATTTTTTTATCTTTAATTTGTAACTTTG
ATTCTAAAGACTGTATTATATTTCTTAAAACGAATTCTTTATCTTTATTTTTATCCGAATTTTCATATTTTATTGGT
GAATTATCTATAACAAAATTACTTATAATTTTTTCAGTTAATTCACCATATTTGTCATTTTCAAATATAGTAATTAA
ACCATTAAAATCTTTATTATATTTTTCAGAAATTTTATCAAATGATAATAAATAATCTAAGTTGTGTTCATTTTTTA

TGTTACTTTTAATGATATTTGTAATTTTATTAATATTTTGTGTAGTTGCAATTTGATTGGAAATATATTTTTCATTT
TGTTCAAGTAAATCAATAGTTATATTTGAAAAGCCTTTAATTTTTAAATCATAATTTTTAATTATTTTAAATTTATC
TTCAGTATCTTGAATTTTAAATAATTCTTTTATAGCTTTTGATTTTTGCATTGCTTTGCTCCTTTCCTAAAAGTAA
GTAATTAGAAAATCAATTATACAAATACTCCTTTCAAAGATATATATTTTCTATATGTTCTTTATTAATAATATCTCT
ATAAATGAAAATTATCTACATTTTTTCAACCATTAGTATACAATCAAAATAGTAAAACCTATACAGCAGTTGTAT
AAAATAAATAATAAGCGTTTTAATTTATATAAATATATTGCTCTTACAAAAAAATTAATAGAAAGTTGAGATTAT
GACGAAAAATACTTTTAGAGAAAGAATTCAAAAAACATATACAAAAAAAGATCTTGAGAAAGCTTTAGGTCT
TAAAAACAAAGCTCAATTGCAGAAACGAATTGATAAATTAGTTGATATTTATAAGGTTGATATGAAGAAATTT
CAAAAAAATTCTGGCGAAAGTGAAAGAGCGGCTTATAGTTTCAATGGGATTGCTTTTGATATATTATGTGTCT
TATTAAATAATATGACAGATGATATGCTCCCTAAAGCAATAACAGATGAAGACGTAAAGTTAAGAAAAAATGC
GATAGAGCATATCGATATTGACCAATATGCATATTTTATTAAAAGCATTAAAAATGATATAGATAAAATTGAATT
TAAGCCATTAAAGTTACATATACATGCACAAAAAGCATATCAAGATGTAAAAAAAGTATTTGATTCAGGTAATA
AAATAAATGAAAAGTTGCAATTAGTTTCAGAAGTAATGAATCAATTACCTATTGATAAACAAGTTGAATTAAG
TGATAAAATCGCATTTGCTATACATGAAACTATTTATTCTACATTCGCAGAGATCAAGTATAATGAAAGGATTG
CTGAGCATAATCAAACAAGTGAAAACTCAAGTCAGTATGATTTTAGATTTAACTATTTCGTAAATCAATTAACT
GATAATGTAATAACAGAAGATATAAATGAAGAAAAAGCCTTTATATATGATAATGATGAACAATTAGATTGGTT
ATTGGTTAACATACTAAATAAAGTTCAACGGGCTGCTGATGGTTGGGGGATGAGAAACGCTAAGCCTCATCA
TAGAACACAAAAGTTTAGAGATATTGAACATTATTTCCTTAGAGATGTATCGAAAATGATAGATAAAGAGCTT
AACAAAAAGGAAAACTTAAATAAACAATCTAAAAAATATGACTTCGATAATAAGATAAAGGTCATTAATGACA
ATATTGAGTTTTTTAATGAAGATGAACAAATTAGAAAGCATTTACTAGAGTTACGTAAACGATTAGACTTTCAT
TCTGCAGCAGTATTAAATCATAAAAATCATTCACACATAGGCAATGAAATGTTCCAATATTTTATGGATGATATG
CAACAAGTTGTAACATTGATAGACCGATACATTAATAAGACTGAACATTATAATGATATGAAAAATGGCTATAT
TAATGAATATAAATCATTTTATAATGCATATAATAATGAAATTGAAGTGACTAAGACGTACGAAAATAACATCCC
AATGGCAATGTTAATGAAACAATTTAAGAATCAACTGTTAAAAAGAAGGAATAACCGAAAAAGCTCTAATTG
AAGTTAAGTAGAAAATTAATAAATAATTGGTAAGCTCAATCCCCCATATAAAGGGATTGAGCTTATTTATTTGG
TGGAATTTTTTTTGAAATTTTTTGCCACTTCATCAATGACCTCTATTTAGTATTAGGATTGTCTTATCAAAACGT
TTTGAACAAATTTTAAGTTGAAAGTGAGCGCGCCACTTTCAACCATAAAGGAGAAGATTTACATGGAGACA
AGAGACAAACTGATGTCATTGACTCAAAGTGACAAAACACAGCAATGGCTAATGGACAAGTCATCTAACCA
AGATGACATTCAACAATTGCAGCAACAATTCAGCCAACAGTTAGATCAAAAATATAATGCACTTTTAGCTGGT
GAACAAGCTAAATTAGATCAATACGTGGAAGTACATCAAGGATTGGAAGCATTAAAGGAAGAGATTGAATC
AGAATCCATTAAACTTAATATCGATAAATTACCCGATATCAAAGCAACAATGCTTGAAAGAGCCAAGAATGAT
GAACATTCTGATAAGATCGAAAAGCTATTTGATAGGTTAGAACAGGCATTAAATGGTACGAATCGATTATATA
CGCAATTATCGTTGATTGGTACACGAACACATCGAATTACAACTAAAAATTTTAATCTTCAAGGCTTACCAAA
ATCCGTCCAACGTATGATTTTACCTTCACAATTCAAAAAGGTATATACAATCGATTTCAAGTCATTTGACCCTTC
AGTTGTTGGATACATGACGCAAGATTCTAAACTGATTGACTATTTGAATCATAAAGAAGGTTTATATGACGCAT
TATTAGAAGATTTATCACTGTCCAAAAAGGAGAAAAAGTTCGTTAAACGTACATTTATTGGTTCGTTTTTATTT
GGCGGCAACTTCGATAGCCCCAAATTCAAGTTGAAACATTATGTAAGTGAAGAGCAATGGTTGGATACGGTC
AGCCAATTTACAAAAGCCAATGAACTTAAGAAACAAATCGAAGAGCATAAAAGCATGCCGATGCCCTACGG
CATTGAACATGATATGAGTGCATTTCAAGGTAGTAGTATTATGGCAATTTACGTACAAACGGTAGCGAGCTATA
TTTTCAAGCACATTTTGTTGGAAGTGTACAAAGCACAGTGCGAACAAAAAACGTTCAAGATTATAGTGCCGA
TACACGATGCGATTATGATTGAATGTGATGATGAAGTAACTGCACGTGATGTTGCTCAGCTAATGAAAAGTAC
AGCTAACCAGTTGTTCAATGATGAATTTGCACATGTGACAGTGGAAGAACTGGGAGGTGAGGGCCATGAAT
AATGATAGAGGACAAAGTTTACAAATACCAAGTAGTACATCACTCAAAGAAAATTATATATATGTAGCTACGTT
ATATTCTGTGAGAGAAACAAATTTCTCAGGTGATGTGAAACATCAATTCACATATGAAATTGAAGTGAATAAT

CAGATCGTATATGTGAATCGTAATATCTCAACACAAAAGACAGCTAAGCAGTTGTCAATCAATGATTGGCTTA
AACGTCACAGTAATTATAGCTCCACTCATGAGAATTATGACCCTTACATTGATAGAAAGCATTTAATCCATGTA
GGTTACTTTAACGGTAATTACTATGTACAAGATGTAGCATCATTAAATAAAGATGGAGGATTATTATAATGAATC
ATATATTACAAATGTTATCTAAGCTATTAAGTGTGGCTAAGGAGGCAATCGACCGTCAAGGTCTGATTGCTATC
CTAACCATTAGTGTTGGTAATGACGATGAAATAGAAGAATCGGCACAGGGTGAAACAGTTTATAACGAACTT
GTCGATAAGTTACAACTTAACATCCCTAAAGATAGGGATTATAGACCTAACATCTATAGCTATTTTGGTATTAAG
AAGAAACCAAGTGACACAATATTAATAGATATGATGATAAAAGTTTTTCATATCAAACGCTTTAATTCAGAACT
GTATATATTCAAAGTTAATGGTTGGCAAAAGCTAAATGAAGACGAATTACAAGGATTTGTATCAAAAATGATA
CAAGTACTGTTAATCGGTTATACACCTACGCAAAGTGCACTGAAAAATGTGGTTGAAGGTCTACAGAAATCG
TCAGATATAGAAGAACTTAATGAGGATAAAAATTATATTGGTTGTGGGAGAAACATGTTTAGTTTAAAGACAT
TTAAAGTGGTTGAAAACGATATAAAAATCTTCCCTAAAACACGTTTAAATCTGATGTTAGACAAAAGCGATAT
CATAACAGATAAAGTACCTTCGCATTTCAACCAATATATGCTAGAACTTGCTAATTTGGATTCTGATTTACAATA
CTTTCTTTTCCAACATACAGCGGTGTTACTCACTGCAGATACTAAACTACGTCGTGGACTTATTTTGTATGGAA
CTGCAAAAAATGGGAAATCGGTCTATATCAAATTAGTTAAGTCATTCTTTTATAGTAATGATATCGTATCTAAAA
CACTTAATGAACTTGGCGGGCGTTTCGATAAGGAAAGTCTAATTGGTAAACGAATTATGGCAAGTGATGAAG
TAGGGAAAGCTAATGTTGATGAAGCAACTGTGAATGATTTCAAAAAATTACTATCTGTTGAACCAATTCATGC
TGACCGTAAAGGAAGAACACAAGTAGAAGTCACACTAGATTTAAAACTTATTTTTAATACGAATGCTGTACTC
AATTTTCCATCATCACATGCAAAAGCATTAGAGCGTAGAATTGCTGTTATTCCATGTGAATATTATGTTGAAAA
ATCTGACCCTGACTTAATTGAAAAGTTACAGGATGAAAAGAAAGAAATCTTTCTTTACTTGATGTATGTGTAT
AAGCAAATTGTAAAAAATGATATCGAGTACCTCCAAAATGATCGTGTTACTGAAATTTCTCATGATTGGTTGA
ATTTTGGATATGAATTTGTTTCTAGCAGGTCCGTAAGTAATGCAAATCAGAAAGCATGTATTGATTTACTCCGA
AAACTTATAGCAATTAAACCAGGGTCACGTATCAAAGTGTCTAGGCTAAATGAGGTTATTAGAGATGAAATTA
AAGTAAGCTCTCAAGTTATTAATGATTTGGTTCAAGCTAATTTTAATGTACAAAGTAGACTAAATAATGGTTAT
AAGTATTGGGTCGATTTAGGATGGAAAGAAACTGATAAAAAAGATGACATGATTTCATTCGATGAAAATGAG
AATGTAACAGATGATGAGTTCTTATACGAAGATGATTTGAACTTAGGTTGGGAGGACTTTGACGATGAATAAT
GAACAAATTGAAGCATTTGTAGAAGTGCTTGTACCTATCATAGAAGAACGTATCAATAAAGGTAATTAAGGCT
AATTACGTACTACAGGCAACTGCCTGTAGTACTCATATGATTAAGTGGTAAAAGTGATAAAAATGAAACGAAA
TTATAAATATATATTATCTATATGTTGTTACAAGACCGATAGTCTGTAGCAATAATCTAATAAAAGGAGAGCGAT
ATGATATGAAGGGTAAAATTGCACTTTATTCACGTGTTAGTACGTCTGAGCAGTCAGAACATGGTTATTCTGA
AAAGGAGCAGGAACAAGTACTCATCAAAGAAGTTGTGAAAAATTTCCCAGGTTATGACTATGAGACATATAC
TGACTCAGGCATTTCAGGTAAAAATATTGAAGGTCGTCCGGCAATGAAACGTCTATTACAAGATGTTAAGGA
TAATAAAATCGAAATGGTGTTAAGTTGGAAATTGAATCGTATCTCACGATCAATGAGAGACGTGTTTAATATTA
TTCATGAATTCAAAGAACATGGCGTAGGGTATAAATCGATTTCTGAGAATATTGATACATCCAATGCTTCTGGA
GAAGTACTCGTTACAATGTTTGGGTTAATAGGATCTATAGAACGCCAGACTTTGATTTCCAATGTGAAACTTT
CTATGAATGCTAAGGCACGGAGCGGAGAGGCAATCACAGGTCGTGTTTTAGGCTACAAATTATCACTTAATC
CATTGACACAGAAAAATGATTTAGTTATTGATGAAAATGAAGCTCATATTGTACGGGAAATCTTTGATTTATAT
TTGAATCACAATAAAGGACTTAAAGCAATCACGACAGTTCTAAATCAAAAAGGATATCGCACCATTAATAAAA
AACCATTTTCAGTGTTTGGCGTGAAATATATTTTGAATAATCCAGTCTATAAAGGCTATGTCAGATTCAATAAC
CATCAAAATTGGGCTGTTCAGCGAAGAAGTGGTAAAAGTGATAAAAATGATGTGATATTGGTCAAAGGTAA
GCATGAAGCCATTATAAGTGAAGATGTATTTGATCAAGTTCATGAGAAACTAGCTTCTAAAAGTTTTAAACCG
GGTCGACCTATTGGTGGAGATTTCTACTTACGTGGCCTTATTAAATGCCCAGAATGCGGAAATAATATGGTAT
GTCGACGGACGTATTATAAAACGAAAAGATCCAAAGAACGGACAATCAAACGCTATTACATTTGTTCATTATT
CAACCGCTCAGGAAGTTCTGCCTGTCATAGTAATGCGATTAATGCTGAAGTCGTCGAACGCGTAATCAATGTC
CATTTGAATCGTATTCTTTCACAACCTAATGTTATTAAGCAGATTGCGTCAAGTGTGATAGAAGAACTGAAAC

AAAAGCATAGTAAACAAACAGAAATAAAATATGATATTGATAGTCTAGAAAAACAAAAAGCAAAAGTTAAAA
CACAACAAGAACGATTATTGGAATTGTTCTTAGATGATGAAATGGATAGCGAAATGTTAAAAGCTAAACAAA
GTGAAATGAATCAACAGTTAGAAGTATTAGACCAACAAATTAAAGAAGCAAAACAAGCAAATCAATCACAG
GATGATATACCCAATTTTGATAAGTTAAAAGCACGACTCATTTTGATGATAACACGATTCAGTGTGTACTTAAG
AAAGGCTACACCCGAAGCTAAAAATCAACTTATGAAAATGTTAATTGATTCAATTGAAATTACGACAGATAAA
CAAGTAAAACTTGTAAGGTATAAAATTGATGAAAGTCTTATCCCTCAATCTTTGAAAAAAGATTGGGGGTCTT
TTTTTATGCCAAAAATTCATTTTGTAATTGATGGTTCACAGAAATATTCTATTGACCAAATTTCCACTTTTGCCA
CTTAATCATATTAGTGATAGAAGTGAGGGAAATGAACTGAATATCAGATATATATTATAAAAATGATGTGATAA
ATGTATGCATCAATCAAATACACTAGGAGGTCATAATTATGACATTGGAACAACAAGTCAAAAACTATATTACG
AATCTATTCAAATTACCCAAAGACGAGATGTGGGACTGTGAAGCAATTGATGAAGTTGCTGATAATATCATAC
CTGATCAATATGTAAGACTCGGGTCAAAGAAGTATTGGATATGTTAGAAACAAGCAATAAAAAGGTTAGCAT
ACTGACAAATCCTCAAACAAATGAGATGATGCACCCGTTGAATCTCTTTGCCAGAAAAGCATGGACAATCAA
ATCATTAAAGGAAATGAAAATAAATAATTTATGACACTTTCTTATTAGTTAGTTATTTTAAAAAGTCTTTATTATC
AAAATAAATATAGAGAAGTTTTGTAAATTTGATATATAATGATAACAAACGAAGTGTAAATGCATCGCTTATTAT
ATCATTAGTGATATTATTGGTAATTTTATTAGCAGTAGGATTAGATATAGCAAGTCATATGTATCAGGTTGATTG
AAGATATGTAGACCTCTAAAAAGTCTAGCAGTAGTTTATTATATCGTCGTAGTTTGGGGTTACTAATTTAAAAT
AATAAGGGGAGATAAAATGAAAAATTTATTAATGGTCTTAGCATCAGTAGCTTTATTAGCAGTTATCTTAGCG
GCAGGAGTATTTGGATATAAGAGTTTTACAGATATTAATAATAGTAAAGAAGATAAGAACGTGGAGGAAGTA
AAATCAAATAAAGAAAAAGAGTCTAAAAAAGAAGCCAAAAAAGAGCAAAAAAATGAAGAACAGAATGTTG
AAGAATCAACTGAATTAGCACAAGAACAAACAATAGAAGAACAAACAATAGAAGAACAAACTGTAGAGGA
AAATAAAGAACTAGATGTAAATGCGGAAATTGCTAAAGCAGATAAAGATGGTGACGGTGTTGCAACAAGAG
ATGAAATGACACCAGAATTATGGGAATTAACCAGACAAGGAAAGTTCCAACCAACTTCTCGTGAAATATATTA
TCAAGATCCAAACGCAAGTGAAAAAGATGATGAGCCAGTAACAGATAATGAAGTTAACAATATTGATGATGA
AGAAGAACGTGGAGATGTAGGTATGGACATGGAGCAACAACAAAGAAACATTGATGCTCTTAAGGAATCAG
AATAAAATAATAAGGAAAGTAATTATTTTAAATATCAATGATGATAAACATACTAAATGCCCTTTATGAAAAATA
TTATGGATGCCGACTTGATTTTATTAGCTTGAAGAACAAATCAAAACGTCTCAACAATCTAATCTAGGAGAAA
CAAAAGTACCAGACTTTGATATAAGTTGAAGAGTCTCCTAAATTTGATAATAAGTCGTTTTAGTGTTTATTTAA
GAGAAGCAAATCCTGAGGCAAAAAATCAGCTTATGAAGTTGTTGATTGATTCTGTTGAAATTACTACAGATA
AAAAAGTGAAGCTCATAAGGTATAAAATTGATGAAAGTCTTATCCCTCAATCTTTGAAAAAAGATTGGGGGT
CTTTTTTTATGCCTAAATTTAATTTTGTGATAAATGTCGCAAATGAAAATAGGAAAATTTATCACTTTTACCACT
TTTTTAGAGTGACAAAAGTGGAGGAGTTTTGAAATATTTTATAAATATATATTTTATTTATGGAGTACAAATTAT
TAATTAGGGAGGTCATTATAATGACGCTAAGCAAACAACTTAAAACGTATATTACGGAACGATTTAAATTAAAT
TATCAAGAAACTTGGGCTTGTGAAACCGTAGATGCGGTGGCTGAAGATGTATTACCTGAAAAATATATTAAAA
ATAGTCCACTTGAAAATAAAATTTTAAATACTTTTACCTATTACAATGATGAATTACATGAAATCAGCATTTACC
CTTTTTTATGTTATCTAGATAAGGAATTAGTAGCAATAGGTTATTTAGATAATTTTGATTTAGACTTTATATTTTT
AAATGACACTCATCAAGTCATTATTGATGAACGCTACTTGTTACAAAAAGGGGGCGAGTAATTATGAACTGG
ATCAAGGTCGCTCAACTATCTGTCACAGTTATCAATGAATTAATTGAGATTATGAAAGAAAAACAAAATGGAG
GGGAATAGTATGAACATCAATCGATACATTACACGAGGGATTAGTGAACACATATCTCTAGACCTTCAAATTTT
ACTTTGGAATATGGTAAAAGAACGGGACAATCAACCTCATACAGATTATCTACACATTTTTAGACTGCAGGAA
GATGAGAATATACTCTCAATCACACATGAACAAGAACAACCCGCATACAAATTGGAATATCACTATATAAACTA
TGTAAAAAAATCAAAAAGCAATACCTAAGAAAGTCTACGTCATCCGAGAAGATGATGTAGACGTTTTTTATTAT
GTCATGCTTTTACCTGAAGAATATTAAAAGGAGTGAAGATGATGAATACAATCAAAAGTACGATACACACAG
AAGCGATTTTTAGCGATGATGAACAACACCGCTACTTACTCAAGAAAATTTGGGATGAAAAGAAACCTGTTT
GTACGGTGATAACCATGTATCCTCATTTAGATGGCGTATTATCACTCGATCTTACGACTGTTCTTATCCTCAATC

AATTAGCGAACTCTGAACAATATGGCGCTGTATATCTTGTAAATCTATTCTCTAATATTAAAACCCCAGAGAATC
TTAAACATATTAAAGAACCTTATGATAAACACAGCGACATTCATTTAATGAAAGCGATAAGTGAAAGTGACGC
AGTGATTTTAGCCTATGGTGCTTATGCGAAGCGTCCCGTTGTTGTCGAACGCGTTGAGCAAGTGATGGAAAT
GTTAAAACCTCATAAAAAGAAAGTAAAAAAGCTCATAAATCCAGCAACGAATGAAATTATGCATCCGCTTAAT
CCTAAAGCGCGTCAAAAATGGACATTGAAATAAAGGAGGATTATCTATGAACCATGAAACTAAACAATCAGA
TTGGCGAACGGTTGCTAATTGTTTAGAATCGAAAAATTATATATCGATTGTAAAAGGATTAGTACATCATTTCA
CAGCGATTGAAGATGAAGAAATACTTGATAAAATCTATGAAGATTTTATGAATGATGACTCTATTACAACGGT
CCTTAACAATGATTTACAAGATATTATTAATTATTACCTATCAAAATAAAAATATATTACTTTTAATAAAATTAGA
GCATCCTTCACTTTGCGGTGAGGGATGCTCTCTATTTAATTTTTTTGCTATTTTCCATTATATATATTTAATTTTAT
TTGAAATGTATTGTACAATTTAAAATAATGGAGGGACATTTAATTTATGAAAAAATATATAATTAGTTTAGTACT
TTTAACTTCTTTTTTACTATCTGCTTGTACAATTAATATTGAGCGCGATGAGGAAAATAGTGAGAAAAATAAAC
AGAAAGAAAACCCTCATACTGATTCAAACAATACAAGTTCGAATGAATCAGATACTTCTAAACAATCATCTCA
AGAAAATATTATAAATCAAACAGAACAAAATCAACCAACTGAGAATAATTCAAGTTCACAACAAGATCTGCA
ACTAATAACAGAAAATGAAGCTATTCAAAAAATAAAAGATGAGTTTCCACCTATACGAACAGGAAATGACTAT
CGTATAGATAGTACTAGAACTGATAATAATGTGTACGCTATCAAATTTACTTCTCAAGATGCAGAAGGTTATCCT
ATGAAAGCTGCTGTCACTATTGATAAGCGAACAGGAGAATTTATTGATTATATTGATGATAGAAGCGATGAAG
ATAAAGAGCGTCATGTCCAACATGCTAAAGAAAGTACTCTTTATAAAGGTCCATACGATGCTTTCAGAAAAGA
TTTTAGTCATAAAATAAATGAATGAAGTATATATGAATTGTCATCTTTAATATAAGAGCATCCTTCACTTTGCGG
TGAGGGATGCTCTTTTAGTTTATTTTTTTACTGAGTACTCGCCATAAGCTCTGCTTGTTCTACGACCTGCTCTA
CAGCCATTTTTTGTAAATCTGGTGGATAGCCATATTTCTTAAGCAATCGTCTCACAGCTACGCGCATTTTAGCT
TTTGCGCTATCACGTTTAGACCAATCAACACCCATGTTTTCTTTCACTGTTTTAGTTAGCTCATGAGCAATCGC
ACGTAGTTCTTTATCTCCCATGGCTTCTTTTGCTGTTTCATGTGAAGCTAAAGCATCGTAAAACGCAATCTCAT
CTGAATTCAGGCCTAATTCTTTTCCTCGTTGTTGTTCTTGTTTGATATCTTTAGCGAGTTGAATAAGTTCTTCAA
TCACTTTAGATGTTTCAATGGAACGACTATTATATTTATTAATCGAGTTTCCTAACATTTCAGAGAAACGCTTAG
ATACTGTCGCGTTCGTTTTCATTAATGATTTAACTTGCCCTTTGAGTAATCGATTTAATAATTCTACCGCAACAT
TTTTTTGTTTCAATCCTTCAACATCTTTTAGGAAGTCATCTGATAGGATTGATAAATCGGGTTGTTCAAGACCT
AGCGTTTGGTAAACATCAATGACATCTTCAGTCACAACAGATTGTGACACAAGTTGATTAATCTCTGCTTCAA
CTTCTGCAGGCGTTTTACGTGTTTTCCCTTCTTTTGGTGGTTGTAATAATTTAACAAGCCCTGCTTTAACTGCT
TTAAAGAAAGCAATTTCATCATTGAGTTCTTGGGCTGTTGGTTCAGTCGCACAAAGAGCAAAAGCTTTCCCT
AACTCTGTGACCGTTTTAATAAAACGCTGGCGTTCATCTTCGCCTAAACCAATCACGTAATCCATCGTATCTGA
AATCGCATAATAACGTTCAGACTTTTTCTCTGAATTAAATTTAGAATAATCAAGATTATATAACATATCTTGAATC
ACATCATATTTCAATAACATCAATTCAACCGCTTTATCTGTATCTATCGCCGTTTGTGCTTGATCAGATTCTGTAT
ATTCTTTAAGTGCTTCTTTTAAACTTTCAGCGATACCCACATAATCGACAATCAATCCACCCGGTTTATCTTTAA
ACACTCGATTGACACGAGCAATCGCTTGCATTAAATTATGACCTTTCATCGGTTTATCGATATACATTGTATGCA
TGGAAGGTACATCAAATCCTGTGAGCCACATATCTCGAACAATCACGAGTTGTAATTCATCATTCACATCTTTC
ATACGTTTTTCTAATAAATTACGACGTTTTTTAGGACCAATATGTCTTTGGAAAGAAGTTGGGTCACTAGATG
AGCCTGTCATCACCACTTTAATGACCCCTTTATCATCATCATCTGAATGCCATTCTGGTTTTAGACGAATGATTT
CATCATATAAATCAACAGCAATTCGACGACTCATGGTTACGATCATTCCTTTACCTTTCATCGCTTGCTGACGT
GTTTCAAAATGTTGGATGATATCTTTGGCTAGGGCTTCGATACGAGGTTTTGCACCTGCTAAAGCTTCAATAC
GGGACCATTTTGATTTTAAACGCTGTTTTACATCCTCTTCTTGATCTTCAGTAATGTCATTATACGCTTCATCTA
AATCTAAATTTTGGGGTAGATTTAATGGAATTACACGACTTTCATAGTAAATTTTAACGGTACTTCCATCAGCT
ACGGCTTGTGTCATATCATAAACATCGATATAGTTTCCGAAAACCATTTGCGTATTTTTATCCGTTGAAGCTAC
GGGTGTGCCTGTAAATCCTACGAATGTTGCATTCGGTAAAGCATCTCTTAAATATTTGGCATAACCATATTTAA
TGCCTTCACCTTTATCATCGTATTTCGCGTTAAAGCCATATTGTGTACGATGCGCTTCATCTGCCATAACAATCA

CATTTTTACGTTCAGTTAGGGCAGCCATGGTCGTTTCATTTTGTTCAGGTTCAAATTTTTGCATTGTTGTAAAT
ACAATACCACCCGACTCAACAGATAATAACGATTTTAATTCTTTACGTGTTTCAGCTTGTTTTGGTGTTTGTCT
TAATAATCCTTTACCAGAGCGACCTTTTGATTTAACAAACGTACTGTATAGTTGGTTATCTAAATCATTACGGTC
TGTTACGACAACTAAGGTAGGATTATTCAGCATTTGAATTAACTTTCCAGAGAAAAAGACCATGGTTAAACTT
TTACCAGACCCTTGGGTATGCCAAATAACGCCGCCTTTACCATCACCCGGCCCAGATGAAGCTAATAAAGCTC
TATCAACAGCTTTATTAACAGCATAGTATTGATGATATGCTGCTAGAATTTTACTGATATGCCCTTTACCATCATC
TTGGAATAATACAAAATATCGAATTAAATCAAGTAGAGTATTAGGATTTAACATCCCATGAATCAGTACGTCTA
AGCTAGCTAAACTTGACGAAGATTCTGTTTCTCCATCTTTAGAACGCCAAGTCATAAAACGATCATAGTTCGC
AGTTAGTGAACCGGCTTTAGTATTAATACCATCACTTGTAACAAGCACTTCATTAAATGTAAATAATTGTGGAA
TACGCATCTTATACGTTTCTAATTGATGATAACCATCTTCGACGCCTACGGTTTCATTGGTTGAATTTTTAAGTT
CGATCACAACGATAGGCAAGCCATTGATAAAGAGGACAATATCGGGACGTTTTGTATAGTCTCCGTTAATGAC
AGTGAATTGATTGACTGCTAAAAAATCATTGTTTTGTGGATGTTCAAAATCAACGATTTTAACAATTTCTACTA
CCGATTGTCCTTCGTCATCATAGTCTTCGATTTCAATACCATTGATCAAATTTTCATGAAAGGTAAGGTTATTTT
CTAAAAGATTGGGCGACTTCTCTAAAGTTAGTTCATGTATGGCTTTTTCGATAAAACGATGATGAATATCTGA
ATTAATCTTTCTTAATGATTTTTCTAATCGTTCATGAAGGACAACATCTTTATCACTTTTACGTTCTGGCGCTAG
ACCTGTCATACTAATCTCATTACCTTTTTTGTAGTCATAGCCCAGTGATTGTAGCCATTCTAATGCGACTTGTTC
TAAATCATCTTCACTAAATTGAAAGCTCATCTTCATTCACCTCGATATCATCAGGTATCTCTATTTCCCCCGACAT
TAATTTTGGTAGTAAAGTATCTCTTAATTGTGTTAGTTTTTGGTTTTCTATATTTTTTTGGTCCATCATTTTTAAA
ATATGATTATTTTCCTTTTGATATGTTGATAGCAAAGTTTCATTAGTTGGCATTGCTACTTTAGTGTTCTTAATAT
CTTTTTGAGATAATAGAGGTTGTGTAGAACCTCTATTCAATGATATATAGTCTATACTTTTAAGTACTTGATATAT
AATGCTTTCAAAATCACTGATTATTACAAAAGTATTATCTGATGGCCATGTTCTTGTAGAAAATCTTTGGATTAC
ACCATGAGTTCCTACTCTTCCAATTATAATAATTTTTTCATTATATAAATAATCATTGGTATAACCCATTATTTTAC
TAGCACCAATTATTGGCACCACATTTTCTATATCTTTTTTATCAACTTTATTTTTAGGTCTTTTACCACTTTTTATT
TTTATATAATTTCCTAATTCATCAACTTTCCAACCGCTAGGTATCTCACCTAGTTCACTATCAATCATCTCGCCAC
CACTTGATTTGTACGGATTTCCATTTTCATCTGGGAATTCAAAATCAACAAACCAACGTTTGAACAGTGTTTG
TGATAGTTCCTCTAGGTTTGCTATGATTCTTTTATTAAGTTCTATTTTTTTATCTAAAATTTTTAAAATAGAACTT
ATAATAGTTTCTTTTCTTTTGCTTTTAGGAGAATAAATTTCTAAATTATCTAGTATAGGTTTTGTGAGATATTTAG
TAGCACTACCTATTGATTGCGAACGTAAAAAATTCAATTTAAATTTAAGATAATAATATAAATAATCAATATCTAT
GACGTCAAATTTAGGTTTTATAATATATGTCCTTTGATAAGCGTCAAATTTTCCATTGAATCTATTTATATGGAA
AATTCCATTAGCATTATTGCCAGCAAGTAAGATTACATCATCATCATATGAATAAGAATTAATTGTTAAAGGATT
AGGAGCACAAGTGAAAAATTTATATTTGCCATTTTCAATCGCAGCATTAGAATTAAGTCGACCTGTCTTATAAT
CGCTTATTTCAGATAAAAGATATTTTTTAAACTCCATAGCCTAATCCCTCCAATGATTTGCGGATTTGGTTTTCA
AGTTCTTTTGATTTCGCAAATTGTTCGCTTAATTCAGAAGTAATGCGTTCCATTTTTTGCTCAAACGGTTCTTT
ATCTTCTTCAACATCAGTTAAACCTACATAACGTCCTGGCGTTAAAATATATTCATTATTCTTAATTTCTTCAAGG
TTAGCTACTTTACAAAAACCAGCTATATCTTCATAGGATTTGTCGTTTGTACCTTTCCACGCATGATACGTTTGT
GCTACTTTTTGAATATCTTCATCTGAAAATTCTTTTAATGTTCTAGATACCATATGACCGATTTCACGAGCATCA
ATAAATAAAATTTCATTTTTACGTTCTTTTTTACCATTTTGACCTTTATTATTACTAATGAACCAAAGACATACAG
GTATTTGTGTTGAATAAAAGAGTTGACCTGGTAAGGTAACAATACATTCCACTAAATCTTGTTCGATAAGATTT
TTTCGGATTTCTAATTCATCTTTTCCACTTGTAGACATTGAACCGTTGGCTAATACAAATCCTGCTGTACCATTA
GGTGCTAATTTTGAAATCATATGTTCAATCCATGCATAGTTGGCATTACCTTTTGGCGGAATACCAAATTGCCA
ACGGTAATCATCAAGTAATCGTTCTTGACCCCAATCACTTGCGTTAAAAGGAGGATTGGCTAATATGTAATCT
GCTTTTAATCCTTTATGTAAATCGTTATGGAATGTATCAGCATTTCGTTCACCTAAGTCATTATCAATACCACGA
ATCGCTAAGTTCATTTTAGCTAATTTCCAAGTTGTAGGATTAGATTCTTGCCCATAAATCGCAATATCGTCTAAT
CGACCTTGATGTCTTTCAACGAAGCGTTCACTTTGTACAAACATCCCACCTGAACCACAGCATGGATCGTAG

ATACGACCTTTATAAGGTTCAATCATCTCAACCAATAATTTTACAATTGATGAGGGGGTATAGAACTCTCCGGC
ATTTTTCCCTTCAGCGCTTGCAAACTTAGCAATAAAATACTCATACACTCGGCCTAATACATCTTGCTTACGAC
TTTCAGTATCGCCTACCTTAAAAGTAAATAAATCAATAATATCGCCTAATTTTTCTTTATCTAACGCAGGGCGCG
CATATTCTTTAGGTAACACACCTTTTAATGATTCATTTTCTTTTTCAATCGCAATCATGGCTTTATCAATAATTTG
TCCAATTTCTGGTTTTTTCGCATTATCATTAATATATTGCCATCTTGCTTCTTTTGGCACCCAGAAAATATTTTCT
GCTAAATATTCATCTTGATCCTCTTCATCAGCATAAGGATCTTGCTTCAATTCTTCATACTTTTCTTCAAAAGAA
TCTGATACATATTTTAAAAAGATTAAACCTAATGCTACATTTTTATACTCAGCAGCATCCATGCTTCCTCTTAATT
TATCGGCAGCTTGCCATAATTTTTCTTCAAAACCTATCGTTGCCATTAATAACACCTCTTTGATTACTATTAGTTT
CAATATATCAAAAAATAAATACTTATTGTGTAGGTTTTTTGCTAATATTTTAATATGAATAATCTTTTTTGTGAAA
ATAAAATATTTATGGGAGTGTTTTTATTGGATAAAATTCGAATAAAAGAAGAATTCAATATGTTAAAAAAAGA
AAAGTATGTGAATGAGTGTTTTAAAAGAAAAGGGAATAGTAAATTAATAACTTTCAAATACTTATTGCCATTTA
AAATACCTATAAGTGAATTTGAAAATATTGATTTTATAAATGAAGAAAACACATTTATGATTTTCAACCACTTAG
AAGATAATGTGTTGGAAAATGATAATATCAAACGAGATAGAAAAACCTATGTTGAAATAACTTCAATAATCAA
GCACAATCAGTTTAAAAAATTGAAATCAAATTCTACTTCTAGGGAATCAAAGAAAAGTAATAGTCTAACGGA
AATTTTTAATAAGCAATTTAATTGTCTAAATAATCTTATTAAAATTATTAGCGTTAAATATCAGTATCATAATATAT
ACCAACTATCATTAGGGGATATATTAAGTATTCCATATTATGTAATATATACTATAGATGGAAATATAAAAGAGAT
GAGTATTTTCATGATTGACATGCCTGGAAAGATAGAGGAAGATCAGTATTCATCTATCAAAAAAAGTGATTTA
TTAAATATTAAAAAAAATTATAATATTTTCAATAATCATCCTAGCAATATTTATGTTTTGGCTATGAGAAAAGGT
GAACGTTCTTTTTATAAATCTGACTATAATTTAGCAATTGTACAAATTCAGACTGCATTAGAAGTTTTTATCACA
AATTTTTTAGAAAGATATTATAAATTAGATGAAAATTTAAGTGATGAAGAGATAAAAAAGAAGCTTGGATGTG
GGTATGCTAATGTTGTTAATGACCATTTATTAAAGACAATAGATAATCTAAATTTGGATAATTCTAATGAAATTA
AAAATTGTGTAAAAAAATATATGAAAGATTACTATGATATGAGAAATAAAATTGTTCATACAGGCGCTACTTAT
AAAAAAGAAGATGCAATAGAATTTAAAGAGATAGTAGCTGATATTATTAGATTAATAACGTTTAGTATGAAGA
ATACGAGTGATAGTGACTTTTCTAAAGAATTTAATATGTATAATATTATTAATAAAAAAAATAGATATTAATGAGAT
AAAGAACAAATATAAAAAAGATACTCATGAATCCAAATAAAGCAGAAATTGAAAACTTTGAGGGTATTTTTA
CAAACAACGTTATTGATAGAAAAGAAATTACCTCTTAGAAGTCGAACCAACAATATGAATTATTGTAAATAAA
ATAAACAGTAAACGAAATTAACACTCATTTACTGTTTGTTTTTTAGGGTAATATGTCTCACATTCATATATAGAT
TATAGAGAGAAAAATAAATTTAAAAGATTAAGTTAAGAAATGTAATACTTTTTTTGTTGTTATTAAAATGTATCC
AATTAATTTGAAAAATAATAAGTCTATCTCTTATTATTTGATTCAATAATAGAATCTAACTCATTTTCAAAATTAG
ATAAATTAATTTTCCTGTTTAGTTCTTCAATAACTTGATCGATCGTTTTTAAACTATTATTAGAATTAGCCGGTTT
TATATTTTGCTTAGAGATAGATCCATTTTGAGGATTGACAAATATATAGATTGATGGTTCAATTTGAGATTTAAA
GTCTATATTTTTTTCTTCGCAAATTTCTTTCAATAAATTTTTCCAATTATATCCTTTCTCTGTATCATAAGATCTTA
ATATAACAATTGTATGTTGGTCTTGAATAATTTTTTCAATAATAGCATTAGCACTAATTTTACTACTTTCTAACTC
CATTTGATTAGGTTTTTTTTCTGTTCTATATGGGATGAGTTCGATATTTGTTATTGGCATTTTTAGTAGTGCTTC
CTCAAATTTTTGCTGAATTACTTCACTTCCAACATTAACTCTTGCTTTCTCCATTTTTTCAAAAATATTTTTATAA
TGATTTTTAGCTAATTCAAAGCTATTAAATACTCCTTTAAAGTACATAGAAAAATAATAACCAATGTTTTTCATC
TTTTTTTGTTCCATATTTTCGAAAAATAAATTATCATTATTATAAATTTTATATAATTTTTTCAATTCTTGAGAGAG
GACATTTTCTTTGCTTAAAATATGATTATAATAAAAATCATCCTTGTTTAATTCTGTATTTATTTTTTTTTCATATA
CTTCTTTTGAAGGAAAAGAAATTTCTTTAAGTTCTTTTTTTTCACTTTTAAGTTCTTTTTCTATATATTCACCAA
CAGTATTACAGTTTTCTGCCTCATCTTGTTTCATATTTATTCCTGGATTAAGTAAACCTATTATAAATTTTGAATT
ATCAATATCTCCTAAAATATGGTTAGGAATCCCCCATTTCATAGATTTTGAAATATTAATTTTTTTCAAACTATCT
TTATCTTTTTCATTAATCCATGAAAAAGATTCTTCTTTATTAGGATTTTCAAAATAATTATGATAAAGTTCAGATT
TTTGTTTGATTTCATTTTTATTCCAAAAATTCTCAAAGTTTTTCCAAATATTTTCACTCATAATTTCAATCTCCTT
AAAATTTTATTGTTTAGTAAAAATAGAACTAATTGTGAAGAAGTTATAATTGTAAAAGTGTCTACTATTTAAGG

TATATAATTGTTATACTCGTTTAATATAAGAATATGATATCCATTATTATTATTTTAACTCTCTACTGATGGATTCAA
GGTTATACATCTCTTCTATATCAATTTTGGAAGAAGATGTATGAATGAACCAAATTTTAAAAGTAAATAAACTA
AGAATGCAACGAATAAATAACTAAGTAACGATGGTAAAATACTAAATTTATCTTTTAGAACATTATTAAATACA
GTCTTACTAAGAGAGAAACCATACTTACATAGTTTAATTGATAATATCAAATAAATAGGTATGAATATTATGCGC
AATAAACCTATAAATAGATCTAAAATACTGTGTAATATTGAGGCAGATTCGGAATCACCATGTATTGCTGTTATT
ATAATAATAATCATTAAAACTACAAATGTAATTAAAGCATTAATGATACATCCAGCCTGTGTTTCCTTATATATAG
GACGTCCATTATTATCCCAACCTGCAAATCTGCCTTCATCATAACTTCTTTCTTCTGGCGGCTTTGGCCATTCAA
GTCTACTCATTATTAATTCTCCTATTCAAATTTATCATTAGTAAAAATAAATTTAGTTATATTCACTATAATATTGA
GGGATAACATTTTTTACTTAAAATATTATAAGTAATATATAATATAAACTATAAAAAAGACATAATATGTCTGTTT
TTTATAAATTTACTCTTTTGATACTGAACTTATTTATTACTCATATTTATTTCTTTATTACGAAATTTTTGCAAGTA
GTCGTATCATATTGATTAAATATTTCTAACATTCTTTCTAAAAAACCATCTACATAAGTTTGTGGTGCAAGTATTT
GCGCATAGGGTGCCTTTTGATAAGCTATGTAGAAAGCATCTAGCTTAGTACATGCTATTTCAAAAATAAGCCA
ATCGTTTTCTTTAGCTACGAATTTTGCTTCTTCTTCATTTAATGAACGTTTATATTGCACATCATATAATTGAATT
AATTCATTACTTATATTTTTATGTACTCTTACTGTTACTAATTCATTAACCTCAGATTTTTTCTTTGAATAATTTTC
ATCAATATATATATCTGATAAATCACGAACTTTGATATCATAAAATTTGCCCCCAAGGTCATAGGTAAACCAATA
GTCATAATTCATATACATTAATGAATGAGGTGTAGCAAAAATATATTTATTTGTGCTTTTTTGTTTAAATCCAATC
ATTTTCTCTTTAATTAATGCCTTTTGAAGCATCATTAATTCTTGGCCAGGAAAATTACCGTCATAAGCTTTGTCT
CTTATAGAAAAGTGATTTAATACTGACATTAATGTTGTTCTATCTTCTGCTCTTCCATTTGAAATGAGTTGTTTA
AATAATTTATCGACATTGCTATGTATTTTAGGAGTTAAACTTTTAATGCTAAGTAATAACCCTAAAGTAGCATAA
CTATCATAATTAAATGAATTATTCTCTAATTGATAATAAGCATTATCATTTTTACCTATTAGTCTTATTTGTGTATTA
CTGTCATACCATGGTAATTCTGGATCAATAAAAAAACGATTGATAGAGTCCATATCTCTACTTATTGTACGAGAT
GTTCTCATTGTCTTTCTTATTAATTCAGAGCGGTTTGCCTTTTTTCCATTAATCATTAATAGAAAAAAATATAATA
GACGTTTTTCTTTTTCCATAACTTTTCACCTCTATAAATAAAGATACATTTATAAAAAGACATATATTGTCCATAT
AAAATGTTTTACTAAATATTATTGATAGACAATCTAAATATATGTAAGTATTAAGACTTTGTTTATCTGTTTGATT
AAAAATGAATAGAGTGATTAATATGAATAAAAAAATTATGGAATGAATTTGAAAAACTTTGAGATAAAAGAAA
AACAGAAAGAAAATTAAATAATAAATCACGGATGATTCATTGATATATATCTAATCATGTTTTAATAATCAATAG
TAATTGATGGTATACGTGAGATATTGAAATATTTTTAGATGATGTTATTGCTAATAATAATCAAATGGAGGTTTT
TAATTATGAAAGTAACTACAAATAATTTAAATTTAATAGAGTATAAAATTACAAAGCAATGTAAAGAAGTGTTT
GAGAATGTACTTAATTTGATTGCGAAAAAAAAGCGAAAGATTATTGAAACACAAGATCTTCTTCTAGCCTTTG
CTTCTACTGCGGATACTGGAGCGGCCTATTCACTAGGTCGCTTTTCCATAACTAAAAAGAAATTAGAAGATGA
AATTAAACAAGCAAAATTAATGGATAAACGTTATATAGAAATAAATGAAGATGAATTTGAACAAAATGATTTAT
TTATTTCTCAAAAAAAGAAAAGAAAAATTCAAGAATATATAAAAACAAACCAGGTACAATTTATATATGAATCA
TCAACAGACCAAGCGATAAAGTATATGTCAGAATACCCTATTTCAAACAATGTAAAAGATATAATTGATTTTGC
GGAAGAGATGAGATTTCAAAACAATCCAAGCGGTGGGATAGATACCTATTGGATTATCATGGGAATGTCTCA
AGATGAAGATTGTAATGCTTATCATGTTTTAAAAAAATTAATGTTAAAATATGACAATATATTTGATGGCGATAA
AATGTCAGAGCGTTTTCAAAACCGTAGCTATTTGGCAAATAATTATTATGATGGAAGAAATGAAGAAGAGCA
ACGTAAGAATGCTACAATAAGAAATCGAATTTCTAATAAATTAGCAGACCCTAGTTATTCAATATTAGAAGATA
TTACTACAGATTTAACTGAAAAAGCGCGTAATAAAGAATTAATGCCTGCTATTAAGAGAGAGAGAGAAATTC
ATCATATGGAGATAGCTTTATCTCGTCGTGATAAAAATAATGTTAGTTTAATAGGGAAAGGTGGGGTAGGTAA
ATCAGCTATAGTTGATGGTTTAGCCTTAAAAATAGTTAATAATGTAATACCATCATTAAAAAATAAGAAAATATT
ACAATTTAGTGTTAATGATTTAATTTCTGTAATTCAAGGAGAAACTTTTAAAGGTATTCAAAGATTTATGTCTG
AAATGAAAAGAGAGAAAGATGTTATTTTATTTGTTGATGAAATCCACATGTTGGGTAAAAGTAAAGGATTAA
CAGATATTTTGAAACCAGCAATGGCTAGAAGTGATTTTCGTATAATTGGAGCAACTACTCCTAGAGAATGGCA
AAGTTATATATCATCTGACACGGCATTAACTAGACGTTTTGAAATTATTAAAATAGATGAGCCTAGTGTAGAAG

ATACCGTAGAAATAATAAATCAAGTTATTCCTATTTATGAAAATTTTCACCATGTTAATTTTGAAAAAGAAACG
ATTAAACTTGCCTCACAACTAGGAAAAAAGTATTTTCCGAAAGAACAGCTACCAGACTTAGCGTTTACTATTT
TAGATAATGCAGGAGCAATTTGTCGAATTGAACAAGGACAGACTACCAATTTACAAACTCCTTATTTAGATAA
AATGAATCGATTAAAAGAAGACTTAAAACAAGCTCAAGTAAAAGAATTTAATGATAAACAGGTTGAAAAATT
ACGTCGAGAAATACAACAACTTGAAAAAAACTATTCACGTAAGATGAGCAATATAGAAAAAATTACATTTGAT
TACTTAGTTACTAAAGAGCATATAAAAAAAGCAATTGAACAAAGATTGGGTGAAGGATTTGAAGTAGTCGAA
TTAAAAGAAGATGAAGATTATTCTGATATTGAGATTGATCGCCTAAGACAATTAAAAGATACTATGAAATCAC
AAATTATTGGGCAAGATGAAGCTATTGAGACTATAGCAAATGCAGTAATAAGAAAGAAATTAGGCTTTAAGC
AATCAAATCGTCCTGTGGGCGTATTTATGCTTTTAGGTACTACAGGAGTAGGTAAAACAGAAACAGCCAAAA
TATTAAATAAAACTTTATATAGAGATGAGCAGAATATCATTCGATATGATATGTCAGAATATCAAAAGGAGCAT
GAAGTTTCTAAATTAATTGGTCCACCACCAGGCTACATTGGTTTTGGTCAAGACGGAGACTTAGTAAAGACT
GTGCTTGAACATCCTCGTGCAGTTATTTTGTTTGACGAAATAGAAAAAGCTCATCCGAAAATATTTGATGTATT
ATTACAAGTATTTGATGATGGTAGATTAACTAACTCATTAGGTGAAACAGCAGATTTTAGTGAAAGTATTATTT
TATTGACCTCAAATATTGGAGCTAGTGATATTCAGAATCGAAAAGTAGTAGGTTTAAATCAAACTAATAAAAAT
GGAACAGACTTTGAAATGATAGATGAAAGTATTAGAGAAGCTTTAAAACAATATTTTAGACCTGAATTTTTAA
ATCGTATTGATGAGATGATTACTTTTAAACCACTTAACCAACAAGAAATTTTTGAAATCACACATCTATTGATTA
AGAAAGAAGTAGATTTAATTGAATCTATGGGATATAATATTGAATTTTCAAATGAAGCGATTAGATTGATTGCT
AATTTGTGTTATGAACCTAAAAACGGAGCTAGACCAATAAAACGTGGCATTTCTAAGTTACTAGAGGATCGTC
TTTCGGAAGAAATAATAAATGGTACCTTAAAAAAAGGAAATACTATAAAAGTAACTGAATATAATAATGAACT
ATTGATAGATTATATTTAGTTACAAAAAAGACATAAAATTTATAACTATAAACTAATGAGCCTGGGATATAATTC
CTAGGCTTTATTATTATAGTATTATTTAGCAAAATCACTGATAAGGAGAGGCTTATCATAAAT
>Staphylococcus aureus strain 07-059
ATGAAAATCACCATTTTAGCTGTAGGGAAACTAAAAGAGAAATATTGGAAGCAAGCCATAGCAGAATATGAA
AAACGTTTAGGCCCATACACCAAGATAGACATCATAGAAGTTACAGACGAAAAAGCACCAGAAAATATGAG
CGACAAAGAAATCGAGCAAGTAAAAGAAAAAGAAGGCCAACGAATACTAGCCAAAATCAAACCACAATCC
ACAGTCATTACATTAGAAATACAAGGAAAGATGCTATCTTCCGAAGGATTGGCCCAAGAATTGAACCAACGC
ATGACCCAAGGGCAAAGCGACTTTGTATTCGTCATTGGCGGATCAAACGGCCTGCACAAGGACGTCTTACA
ACGTAGTAACTACGCACTATCATTCAGCAAAATGACATTTCCACATCAAATGATGCGGGTTGTGTTAATTGAA
CAAGTGTACAGAGCATTTAAGATTATGCGTGGAGAAGCATATCATAAATGATGCGGTTTTTTCAGCCGCTTCA
TAAAGGGATTTTGAATGTATCAGAACATATGAGGTTTATGTGAATTGCTGTTATGTTTTTAAGAAGCTTATCAT
AAGTAATGAGGTTCATGATTTTTGACATAGTTAGCCTCCGCAGTCTTTCATTTCAAGTAAATAATAGCGAAATA
TTCTTTATACTGAATACTTATAGTGAAGCAAAGTTCTAGCTTTGAGAAAATTCTTTCTGCAACTAAATATAGTA
AATTACGGTAAAATATAAATAAGTACATATTGAAGAAAATGAGACATAATATATTTTATAATAGGAGGGAATTT
CAAATGATAGACAACTTTATGCAGGTCCTTAAATTAATTAAAGAGAAACGTACCAATAATGTAGTTAAAAAAT
CTGATTGGGATAAAGGTGATCTATATAAAACTTTAGTCCATGATAAGTTACCCAAGCAGTTAAAAGTGCATATA
AAAGAAGATAAATATTCAGTTGTAGGGAAGGTTGCTACTGGGAACTATAGTAAAGTTCCTTGGATTTCAATAT
ATGATGAGAATATAACAAAAGAAACAAAGGATGGATATTATTTGGTATATCTTTTTCATCCGGAAGGAGAAGG
CATATACTTATCTTTGAATCAAGGATGGTCAAAGATAAGTGATATGTTTCCGCGGGATAAAAATGCTGCAAAA
CAAAGAGCATTAACTTTATCTTCCGAACTCAATAAATATATTACATCAAATGAATTTAATACTGGAAGATTTTAT
TACGCAGAAAATAAAGATTCATCTTATGATTTAAAAAATGATTATCCATCAGGATATTCTCATGGATCAATAAGA
TTCAAATATTATGATTTGAATGAAGGATTCACAGAAGAAGATATGCTAGAGGATTTAAAGAAATTTTTAGAAC
TATTTAATGAATTAGCTTCAAAAGTTACAAAAACATCCTATGATAGCTTGGTCAATAGCATAGACGAAATACAG
GAAGACAGCGAAATTGAAGAAATTAGAACAGCACAAAAAGATAAGACACTCAAGGAAGTGGAAGCACCTA
AAGGAATAATTCCAAAATATAAAAAAGGTGTATCAAAGACTACTAAAAATGATTCAGAAATTGAAAAAATCAA

ATAAAGAGAATAAATTAACCGGTAAAGTTGGAGAAAAATTAGCGCTAAATTACTTTAATGAGCTAATTGATAA
TAAAATAGACGAAGATAAGAAAGAACAGTTTAGGAATATTTTAAATGATAATCCAGGCTCTCAACACGGTCAT
GGCTATGATTTAGTAGCTTTTGATCCAACAAATACAGATAAAGCTGTAGAAAAATTTATTGAAATTAAAACATC
TACATCTTCTAGTATTGAGGAACCATTTTTTATGTCGCTAAATGAAATGTTTGCTATGAAAGAATATAAGCAGA
AATATTTAATATTAAGAATATTTAATGTTTCCGGTAAAGAACCACAATTTTATTTTATAGATCCATATGCAAATTA
TTCTGAATTTAAAGATGTAGATGATCTCATTGACAAAGTATTTAATGTAGAAGCTATTCAGTATAAAGTTTTTG
GCGAAAAAATGATTACTTGAACAAGAGCTAAAATAAAATTGTGATCTAATAAAAATAGAAACTGTAATTTAAAT
AAAACTTTCTAAATAAGCTAACTGATAAAAAATCAGTTTGTCCACAGTCTGAAACAAGATTCCTATATTCTTTA
GGAATCTTGTTTTTTCTATTTTTATGGTGATAAAGAGCAGATAAGATAATGTGTAATAATCACAAAAAAGTTAA
ATATTTTAAGGCTTGTTTAATTATTAATGATTTTATATATAAAGAGCAGTATAATAAAGTTGTTAATATATTATGAA
TAATATTCAAGTAATTTTATTGTTTTTTAATTTGTCGATATTTAAGTTGAGTTAAATTTAAAGGGTGTAATTTGTT
TTACAATGATGAAGATAATTAGTCTATCAAAATAAAGGGGTTGGGACTGTTATGAGTGATAATTTGTCATTATT
CATTGACTATATCAATGATAATATAATCTATGGTAGTGAAATCAAACGGGAGAAATTAGAGAATTTATTTAATCA
ATTTGCTATAAAAAAATGTTGAAAAGAACATTGTCTATGATGAACTGAAATCTTTAGATATTACAATCATTGAGT
CACAGGATTCATATAAAAATAAATTGAAGAGATTATTTTCGGTTCTGTTGCAAAGTAAAAAAATATAGCTAACC
ACTAATTTATCATGTCAGTGTTCGCTTAACTTGCTAGCATGATGCTAATTTCGTGGCATGGCGAAAATCCGTAG
ATCTGAAGAGACCTGCGGTTCTTTTTATATAGAGCGTAAATACATTCAATACCTTTTAAAGTATTCTTTGCTGTA
TTGATACTTTGATACCTTGTCTTTCTTACTTTAATATGACGGTGATCTTGCTCAATGAGGTTATTCAGATATTTC
GATGTACAATGACAGTCAGGTTTAAGTTTAAAAGCTTTAATTACTTTAGCCATTGCTACCTTCGTTGAAGGTG
CCTGATCTGTAATTACCTTTTGAGGTTTACCAAATTGTTTAATGAGACGTTTGATAAACGCATATGCTGAATGA
TTATCTCGTTGCTTACGCAACCAAATATCTAATGTATGTCCCTCTGCATCAATGGCACGATATAAATAGCTCCAT
TTTCCTTTTATTTTGATGTACGTCTCATCAATACGCCATTTGTAATAAGCTTTTTTATGCTTTTTCTTCCAAATTT
GATACAAAATTGGGGCATATTCTTGAACCCAACGGTAGACCGTTGAATGATGAACGTTTACACCACGTTCCCT
TAATATTTCAGATATATCACGATAACTCAATGTATATCTTAGATAGTAGCCAACGGCTACAGTGATAACATCCTT
GTTAAATTGTTTATATCTGAAATAGTTCATACAGAAGACTCCTTTTTGTTAAAATTATACTATAAATTCAACTTTG
CAACAGAACCATCTAATCTTCAACAAACTGGCTCGTTTGTTGAACTACTCTTTAATAAAATAATTTTTCCGTTC
CCAATTCCACATTGCAATAATAGAAAATCCATCTTCATCGGCTTTTTCGTCATCATCTGTATGAATCAAATCGCC
TTCTTCTGTGTCATCAAGGTTTAATTTTTTATGTATTTCTTTTAACAAACCACCATAGGAGATTAACCTTTTACG
GTGTAAACCTTCCTCCAAATCAGACAAACGTTACAAATTCTTTTCTTCATCATCGGTCATAAAATCCGTATCCTT
TACAGGATATTTTGCAGTTTCGTCAATTGCCGATTGTATATCCGATTTATATTTATTTTTCGGTCGAATCATTTGA
ACTTTTACATTTGGATCATAGTCTAATTTCATTGCCTTTTTCCAAAATTGAATCCATTGTTTTTGATTCACGTAG
TTTTCTGTATTCTTAAAATAAGTTGGTTCCACACATACCAATACATGCATGTGCTGATTATAAGAATTATCTTTAT
TATTTATTGTCACTTCCGTTGCACGCATAAAACCAACAAGATTTTTATTAATTTTTTTATATTGCATCATTCGGC
GAAATCCTTGAGCCATATCTGACAAACTCTTATTTAATTCTTCGCCATCATAAACATTTTTAACTGTTAATGTGA
GAAACAACCAACGAACTGTTGGCTTTTGTTTAATAACTTCAGCAACAACCTTTTGTGACTGAATGCCATGTTT
CATTGCTCTCCTCCAGTTGCACATTGGACAAAGCCTGGATTTACAAAACCACACTCGATACAACTTTCTTTCG
CCTGTTTCACGATTTTGTTTATACTCTAATATTTCAGCACAATCTTTTACTCTTTCAGCCTTTTTAAATTCAAGAA
TATGCAGAAGTTCAAAGTAATCAACATTAGCGATTTTCTTTTCTCTCCATGGTCTCACTTTTCCACTTTTTGTCT
TGTCCACTAAAACCCTTGATTTTTCATCTGAATAAATGCTACTATTAGGACACATAATATTAAAAGAAACCCCC
ATCTATTTAGTTATTTGTTTAGTCACTTATAACTTTAACAGATGGGGTTTTTCTGTGCAACCAATTTTAAGGGTT
TTCAATACTTTAAAACACATACATACCAACACTTCAACGCACCTTTCAGCAACTAAAATAAAAATGACGTTATT
TCTATATGTATCAAGATAAGAAAGAACAAGTTCAAAACCATCAAAAAAAGACACCTTTTCAGGTGCTTTTTTT
ATTTTATAAACTCATTCCCTGATCTCGACTTCGTTCTTTTTTTACCTCTCGGTTATGAGTTAGTTCAAATTCGTTC
TTTTTAGGTTCTAAATCGTGTTTTTCTTGGAATTGTGCTGTTTTATCCTTTACCTTGTCTACAAACCCCTTAAAA

ACGTTTTTAAAGGCTTTTAAGCCGTCTGTACGTTCCTTAAGGAATTCCTTAGTGCTTTCATAGATTAAACTCAC
ATCACGCTTTAAATCGCTTATTTTAGACTTTAAAGACTTGTTTTCTTCAAGCAACTCATTATAATCATTTACATTT
TCATTAAATCGCTCTACAAGACCACTATATTTTTCTTTAACTTGCCCATGTTCTTTACTTAATTTTTTATATTCTCT
CGCCATATCAGTACTCATGAGATTTCTAACATGCTGTTTTAACCTATCGTTATCTCTCGCAGCAGTCACTAAGTT
TTTATAATCACGCTCCGATATAACAACATTTTTGGTTGGTTTCTTTTCTGTTTTCATTATTTCTTTTCCCAAACCA
AACATAGACTTTTCACCCGTTGGCACTTCAACACTTTTCATGTGTCGTTTCGCTGGTACTTCTAAATCTGATTT
AACTTTATCGCTATAAGCAGTCCATTCATCTTTTTTAACTGCTAAATTTTTTTCTAGAAAATCAATCTCTTTTTCC
AAAGTTTGTTTTTTAAATTTAGCTGTCTCAATATGTTTACGGTCAGAGCCACGTTCACCACGCTTCAACTCAA
AACCCTGTTTTTTCATATGCTCGGGGAATTTATCTTGTAGCCATAACAGTTCTTGACGATTAAACACATTTTTTC
CTTGCAGTTTTCCATCACGCATAGGCACAACACCTAAATGCATGTGAGGGGTTTGCTCATCATTATGAACTGT
TGCATAAGCAATATTTTGCTTGCCATATCGTTCGGAAAATAATTTATAACTTTCCTCAAAAAATCGTTTTTGTTC
TCCTGGATCCAGTTGCTCAAAAAAATCTCGGTCAGATGTTACTAGCAACTCATTTACAAGAACAGCATCTTTC
CTCGTTTTTCTTGTACCTGTTTTTTGTGATTCAATAATTTCTTTGACACGTTCGTTGTAATCAATATTTTTATCAT
TTTTCAAATCATAATTTTCACGTGTTCGCTCATGGTCAATATCATCATTCGTTCTACTTTTTCGCTCTCTTTGATT
ATGAAATTGCATGCCTTTTAGTCCAGCTGATTTCACTTTTTGCATTCTACAAACTGCATAACTCATATGTAAATC
GCTCCTTTTTAGGTGGCACAAATGTGAGGCATTTTCGCTCTTTCCGGCAACCACTTCCAAGTAAAGTATAACA
CACTATACTTTATATTCATAAAGTGTGTGCTCTGCGAGGCTGTCGGCAGTGCCGACCAAAACCATAAAACCTT
TAAGACCTTTCTTTTTTTTACGAGAAAAAAGAAACAAAAAAACCTGCCCTCTGCCACCTCAGCAAAGGGGG
GTTTTGCTCTCGTGCTCGTTTAAAAATCAGCAAGGGACAGGTAGTATTTTTTGAGAAGATCACTCAAAAAAT
CTCCACCTTTAAACCCTTGCCAATTTTTATTTTGTCCGTTTTGTCTAGCTTACCGAAAGCCAGACTCAGCAAG
AATAAAATTTTTATTGTCTTTCGGTTTTCTAGTGTAACGGACAAAACCACTCAAAATAAAAAAGATACAAGAG
AGGTCTCTCGTATCTTTTATTCAGCAATCGCGCCCGATTGCTGAACAGATTAATAATAGATTTTAGCTTTTTATT
TGTTGAAAAAAGCTAATCAAATTGTTGTCGGGATCAATTACTGCAAAGTCTCGTTCATCCCACCACTGATCTT
TTAATGATGTATTGGGGTGCAAAATGCCCAAAGGCTTAATATGTTGATATAATTCATCAATTCCCTCTACTTCAA
TGCGGCAACTAGCAGTACCAGCAATAAACGACTCCGCACCTGTACAAACCGGTGAATCATTACTACGAGAGC
GCCAGCCTTCATCACTTGCCTCCCATAGATGAATCCGAACCTCATTACACATTAGAACTGCGAATCCATCTTCA
TGGTGAACCAAAGTGAAACCTAGTTTATCGCAATAAAAACCTATACTCTTTTTAATATCCCCGACTGGCAATGC
CGGGATAGACTGTAACATTCTCACGCATAAAATCCCCTTTCATTTTCTAATGTAAATCTATTACCTTATTATTAAT
TCAATTCGCTCATAATTAATCCTTTTTCTTATTACGCAAAATGGCCCGATTTAAGCACACCCTTTATTCCGTTAA
TGCGCCATGACAGCCATGATAATTACTAATACTAGGAGAAGTTAATAAATACGTAACCAACATGATTAACAATT
ATTAGAGGTCATCGTTCAAAATGGTATGCGTTTTGACACATCCACTATATATCCGTGTCGTTCTGTCCACTCCTG
AATCCCATTCCAGAAATTCTCTAGCGATTCCAGAAGTTTCTCAGAGTCGGAAAGTTGACCAGACATTACGAA
CTGGCACAGATGGTCATAACCTGAAGGAAGATCTGATTGCTTAACTGCTTCAGTTAAGACCGAAGCGCTCGT
CGTATAACAGATGCGATGATGCAGACCAATCAACATGGCACCTGCCATTGCTACCTGTACAGTCAAGGATGG
TAGAAATGTTGTCGGTCCTTGCACACGAATATTACGCCATTTGCCTGCATATTCAAACAGCTCTTCTACGATAA
GGGCACAAATCGCATCGTGGAACGTTTGGGCTTCTACCGATTTAGCAGTTTGATACACTTTCTCTAAGTATCC
ACCTGAATCATAAATCGGCAAAATAGAGAAAAATTGACCATGTGTAAGAGGCCAATCTGATTCCACCTGAGA
TGCATAATCTAGTAGAATCTCTTCGCTATCAAAATTCACTTCCACCTTCCACTCACCGGTTGTCCATTCATGGCT
GAACTCTGCTTCCTCTGTTGACATGACACACATCATCTCAATATCCGAATAGGGCCCATCAGTCTGACGACCA
AGAGAGCCATAAACACCAATAGCCTTAACATCATCCCCATATTTATCCAATATTCGTTCCTTAATTTCATGAACA
ATCTTCATTCTTTCTTCTCTAGTCATTATTATTGGTCCATTCACTATTCTCATTCCCTTTTCAGATAATTTTAGATT
TGCTTTTCTAAATAAGAATATTTGGAGAGCACCGTTCTTATTCAGCTATTAATAACTCGTCTTCCTAAGCATCCT
TCAATCCTTTTAATAACAATTATAGCATCTAATCGGTTCTGTTGCAAAGTAAAAAAATATAGCTAACCACTAATT
TATCATGTCAGTGTTCGCTTAACTTGCTAGCATGATGCTAATTTCGTGGCATGGCGAAAATCCGTAGATCTGAA

GAGACCTGCGGTTCTTTTTATATAGAGCGTAAATACATTCAATACCTTTTAAAGTATTCTTTGCTGTATTGATAC
TTTGATACCTTGTCTTTCTTACTTTAATATGACGGTGATCTTGCTCAATGAGGTTATTCAGATATTTCGATGTAC
AATGACAGTCAGGTTTAAGTTTAAAAGCTTTAATTACTTTAGCCATTGCTACCTTCGTTGAAGGTGCCTGATC
TGTAATTACCTTTTGAGGTTTACCAAATTGTTTAATGAGACGTTTGATAAACGCATATGCTGAATGATTATCTC
GTTGCTTACGCAACCAAATATCTAATGTATGTCCCTCTGCATCAATGGCACGATATAAATAGCTCCATTTTCCTT
TTATTTTGATGTACGTCTCATCAATACGCCATTTGTAATAAGCTTTTTTATGCTTTTTCTTCCAAATTTGATACAA
AATTGGGGCATATTCTTGAACCCAACGGTAGACCGTTGAATGATGAACGTTTACACCACGTTCCCTTAATATT
TCAGATATATCACGATAACTCAATGTATATCTTAGATAGTAGCCAACGGCTACAGTGATAACATCCTTGTTAAAT
TGTTTATATCTGAAATAGTTCATACAGAAGACTCCTTTTTGTTAAAATTATACTATAAATTCAACTTTGCAACAG
AACCGAAAAACTAGACTTGATTACAAAATGGAGCTTGGGACATAAATGATTTTTTAAAAATGAGATGAGACG
TAGATTAACTCCATAATCAATACGAATCTATCGACTTCTTTATTTATGATATTCATCTCTTTTTAATGGAAATAAA
AGTGCGATTAATGTGATAATACAGTTACGTTAATTAAAAAAATAAAAATGCAAGGAGAGGTAATATGCTAACT
GTATATGGACATAGAGGATTACCTAGTAAAGCTCCGGAAAATACAATTGCATCATTTAAAGCTGCTTCAGAAG
TAGAAGGTATAAACTGGTTGGAGTTAGATGTTGCAATTACAAAAGATGAACAACTGATTATCATTCATGATGA
TTATTTAGAACGGACTACAAATATGTCCGGGGAAATAACTGAATTGAATTATGATGAAATTAAAGATGCTTCT
GCAGGATCTTGGTTTGGTGAAAAATTCAAAGATGAACATTTGCCAACTTTCGATGATGTAGTAAAAATAGCA
AATGAATATAATATGAATTTAAATGTAGAATTAAAAGGTATTACTGGACCGAATGGACTAGCACTTTCTAAAAG
TATGGTTAAGCAAGTGGAAGAACAATTAACAAACTTAAATCAGAATCAAGAAGTGCTCATTTCAAGCTTTAA
TGTTGTGCTTGTTAAACTTGCAGAAGAAATCATGCCACAATATAACAGAGCAGTTATATTCCATACAACTTCGT
TTCGTGAAGACTGGAGAACACTTTTAGATTACTGTAATGCTAAAATAGTAAACACTGAAGATGCCAAACTTAC
TAAAGCAAAAGTAAAAATGGTAAAAGAAGCGGGTTATGAATTGAACGTATGGACTGTAAACAAACCAGCAC
GTGCAAACCAACTTGCTAATTGGGGAGTTGATGGTATCTTTACAGACAATGCAGATAAAATGGTGCATTTGTC
TCAATAGAAAGTTAGAGGTGAGTCTTACGTTTCAGTGACGGTAGACTTACCTTTAACATGTTACATACTAAAA
AATTAATTTGAATAAGAAAGAGAGACATATATGAAATACGATGATTTTATAGTAGGAGAAACATTCAAAACAA
AAAGCCTTCATATTACAGAAGAAGAAATTATCCAATTTGCAACAACTTTTGATCCTCAATATATGCATATAGATA
AAGAAAAAGCAGAACAAAGTAGATTTAAAGGTATCATTGCATCTGGCATGCATACACTTTCAATATCATTTAA
ATTATGGGTAGAAGAAGGTAAATACGGAGAAGAAGTTGTAGCAGGAACACAAATGAATAACGTTAAATTTAT
TAAACCTGTATACCCAGGTAATACATTGTACGTTATCGCTGAAATTACAAATAAGAAATCCATAAAAAAAGAAA
ATGGACTCGTTACAGTGTCACTTTCAACATACAATGAAAATGAAGAAATTGTATTTAAGGGAGAAGTAACAG
CACTTATTAATAATTCATAATAAAACAGTGAAGCAACCATCGTTACGGATTGCTTCACTGTTTTGTTATTCATCT
ATATCGTATTTTTTATTACCGTTCTCATATAGCTCATCATACACTTTACCTGAGATTTTGGCATTGTAGCTAGCCA
TTCCTTTATCTTGTACATCTTTAACATTAATAGCCATCATCATGTTTGGATTATCTTTATCATATGATATAAACCAC
CCAATTTGTCTGCCAGTTTCTCCTTGTTTCATTTTGAGTTCTGCAGTACCGGATTTGCCAATTAAGTTTGCATA
AGATCTATAAATATCTTCTTTATGTGTTTTATTTACGACTTGTTGCATACCATCAGTTAATAGATTGATATTTTCTT
TGGAAATAATATTTTTCTTCCAAACTTTGTTTTTCGTGTCTTTTAATAAGTGAGGTGCGTTAATATTGCCATTAT
TTTCTAATGCGCTATAGATTGAAAGGATCTGTACTGGGTTAATCAGTATTTCACCTTGTCCGTAACCTGAATCA
GCTAATAATATTTCATTATCTAAATTTTTGTTTGAAATTTGAGCATTATAAAATGGATAATCACTTGGTATATCTT
CACCAACACCTAGTTTTTTCATGCCTTTTTCAAATTTCTTACTGCCTAATTCGAGTGCTACTCTAGCAAAGAAA
ATGTTATCTGATGATTCTATTGCTTGTTTTAAGTCGATATTACCATTTACCACTTCATATCTTGTAACGTTGTAAC
CACCCCAAGATTTATCTTTTTGCCAACCTTTACCATCGATTTTATAACTTGTTTTATCGTCTAATGTTTTGTTATT
TAACCCAATCATTGCTGTTAATATTTTTTGAGTTGAACCTGGTGAAGTTGTAATCTGGAACTTGTTGAGCAGA
GGTTCTTTTTTATCTTCGGTTAATTTATTATATTCTTCGTTACTCATGCCATACATAAATGGATAGACGTCATATG
AAGGTGTGCTTACAAGTGCTAATAATTCACCTGTTTGAGGGTGGATAGCAGTACCTGAGCCATAATCATTTTT
CATGTTGTTATAAATACTCTTTTGAACTTTAGCATCAATAGTTAGTTGAATATCTTTGCCATCTTTTTTCTTTTTC

TCTATTAATGTATGTGCGATTGTATTGCTATTATCGTCAACGATTGTGACACGATAGCCATCTTCATGTTGGAGC
TTTTTATCGTAAAGTTTTTCGAGTCCCTTTTTACCAATAACTGCATCATCTTTATAGCCTTTATATTCTTTTTGTTT
TAATTCTTCAGAGTTAATGGGACCAACATAACCTAATAGATGTGAAGTCGCTTTTCCTAGAGGATAGTTACGA
CTTTCTGTTTCATTAGTTGTAAGATGAAATTTTTTTGCGAAATCACTTAAATATTCATCCATTTTTTTAACGGTT
TTAAGTGGAACGAAGGTATCATCTTGTACCCAATTTTGATCCATTTGTTGTTTGATATAGTCTTCAGAAATACT
TAGTTCTTTAGCGATTGCTTTATAATCTTTTTTAGATACATTCTTTGGAACGATGCCTATCTCATATGCTGTTCCT
GTATTGGCCAATTCCACATTGTTTCGGTCTAAAATTTTACCACGTTCTGATTTTAAATTTTCAATATGTATGCTT
TGGTCTTTCTGCATTCCTGGAATAATGACGCTATGATCCCAATCTAACTTCCACATACCATCTTCTTTAACAAAA
TTAAATTGAACGTTGCGATCAATGTTACCGTAGTTTGTTTTAATTTTATATTGAGCATCTACTCGTTTTTTATTTT
TAGATACTTTTTTTATTTTACGATCCTGAATGTTTATATCTTTAACGCCTAAACTATTATATATTTTTATCGGACGT
TCAGTCATTTCTACTTCACCATTATCGCTTTTAGAAATATAACTGCTATCTTTATAAACTTGTTTGAAATTTTTAT
CTTCAATTGCATCAATAGTATTATTAATTTCTTTATCTTTTGAAGCATAAAAATATATACCAAACCCGACAACTAC
AACTATTAAAATAAGTGGAACAATTTTTATCTTTTTCATCAATATACTCCTTATATAAGACTACATTTGTAGTATAT
TACAAATGTAGTATTTATGTCAAAATAATGTTATAATTTTTGTGATATGGAGGTGTAGAAGGTGTTATCATCTTT
TTTAATGTTAAGTATAATCAGTTCATTGCTCACGATATGTGTAATTTTTTAGTGAGAATGCTCTATATAAAATATA
CTCAAAATATTATGTCACATAAGATTTGGTTATTAGTGCTCGTCTCCACGTTAATTCCATTAATACCATTTTACAA
AATATCGAATTTTACATTTTCAAAAGATATGATGAATCGAAATGTATCTGACACGACTTCTTCGGTTAGTCATAT
GTTAGATGGTCAACAATCATCTGTTACGAAAGACTTAGCAATTAATGTTAATCAGTTTGAGACCTCAAATATAA
CGTATATGATTCTTTTGATATGGGTATTTGGTAGTTTGTTGTGCTTATTTTATATGATTAAGGCATTCCGACAAAT
TGATGTTATTAAAAGTTCGTCATTGGAATCGTCATATCTTAATGAACGACTTAAAGTATGTCAAAGTAAGATGC
AGTTCTACAAAAAGCATATAACAATTAGTTATAGTTCAAACATTGATAATCCGATGGTATTTGGTTTAGTGAAA
TCCCAAATTGTACTACCAACTGTCGTAGTCGAAACCATGAATGACAAAGAAATTGAATATATTATTCTACATGA
ACTATCACATGTGAAAAGTCATGACTTAATATTCAACCAGCTTTATGTTGTTTTTAAAATGATATTCTGGTTTAA
TCCTGCACTATATATAAGTAAAACAATGATGGACAATGACTGTGAAAAAGTATGTGATAGAAACGTTTTAAAA
ATTTTGAATCGCCATGAACATATACGTTATGGTGAATCGATATTAAAATGCTCTATTTTAAAATCTCAGCACATA
AATAATGTGGCAGCACAATATTTACTAGGTTTTAATTCAAATATTAAAGAACGTGTTAAGTATATTGCACTTTAT
GATTCAATGCCTAAACCTAATCGAAACAAGCGTATTGTTGCGTATATTGTATGTAGTATATCGAGCTTCACATG
AAACAGCTAAAGAAGCTTTGGGCGATAAAGAGTTAAGAGCCATTGCACATGAGTTAACTAAAACAGTTAAG
GATAACATGAGTGTTGATTGGTCTAAACGAGACAGTGCTAAAGCTAAAATGAGAGTTCAAGTTAGACGCCTA
TTAAAGAAATATGGCTATCCACCAGATCTTCAAAAAATGGCTGTGGAACAAGTTGTAGAGCAAGCAGAATTA
ATGGCAAGTCAGCAATAAAAAAATAAATCATAATGAGTCCGGGACATAAAGTTCTTGGATAAGTGAAAAAAG
ACAATTTCTATTGAAATAATATAGAAATTGTCTTTTTTATAAATTTTTTGATTATTTTCAGCTCGTTGAGCTACTA
CTTTTCTTATATTAAGTGCCATTAATACAAAACCAAGTTCTCTTTTGACTTTATTGAGTCCTCGGACAGACATCC
GAGTGAAACCCAAAATAGCCTTCATAAATCCAAAAACAGGTTCCACATCAATTTTTCTTTGACTGTAGATATTT
TTTGTTTCTGGTTCTGAAAGCTTTTTGTTAATTTGGGATTTAAAATATTCCCAGTTATAATTCTTCATTATTTTTT
TGTTTTGTTTTTGAATTGAAGTTCATACATTGATTTTTCAGAGGACATTCTGAACAATCATCACATTCATATAAT
TTGAAGTCTCGCTTATAACCATACTTATCATGACGATAGGCATATCTTTTAAAACCTAGCCGTTTATTATTCGGA
CAAATGAATTCGTCATTAATTTCGTCATAGTTCCAATTTTGAGTATTAAAGATGTCACTTTTATATTTTTTAGTTT
TATCTTTTATAAACATTCCATATGTTATGAGTGGCGTTCGATTAAAGTCATCTATAATTGCCTTATAATTTGATTC
ACTACCATAACCTGCATCAGCTACAATATATTCAGGTAAATGACCGTAGGTCTCTTGAATTGAATTTAAAAATG
GAATCATCGTTCTAGTATCCGTTGGATTTTGATACACATTATAAGATAAAACAAATTGGGAATTTGTTGCTATTT
GTAAATTATACCCTGGCTTAAGTTGTCCATTTTTCATGTGATCTTCTTTCATTCTCATAAATGTCGCATCATAATC
TGTCTTAGAATAACTATTTCTATCCTTTAAAATAGATTTTTGAAATTCGTATCGATACTTTCGCTCAAAATAATCA
TTGATTTGCTTTTTGTATTTTTTGATTTTAGTTCTTTTGAGACGTATTTGTTTTCTTGTTTTAGTACATTTTTCAT

TGTTGATATGTTGGTTTAAATCTTCGATTTCTTTATCTAAGTGACTACCAATCAAATCTATTTCTTCTTTTGTTAA
TTCATTATCATGATCTTCTTTAATTTCCGGTATGATTTTATTGGTTACCAATTCATGGTAGAGGGCTTTAGAATC
CTCATTCATCTTTGATTCATGGTTTTGAATACTCTTTTTCCATACAAATGTATATCGATTGGCATTTGCTTCAATT
TTTGTACCATCAATAAAAATAGCTTTATCATCTATAAGATTTTGTTTTACACACTGACTGTAAAATTGAATAAAT
AAAGATTCTAATAAAGCATCTACTTTTGGATTTACTCTAAATTGATTAATTGTTTTATAAGAAGGTTTTTGATTT
TGTGATAGCCACATCATTCGGATGCTATCATTAAGCATTTTTTCTATTTTACGACCTGAGAATACAGATTGTGTG
TAGGCATATAGAATCACTTTTAACATCATTTTAGGATGGTACGAAGTTGCACCACGGTGATGTCTGAATTCGTC
GAATTCATTGTCAGGAATTGTTTCAACAATATCATTTACAGTAAAACGATGTTGATTTGTTTTGTTTCCATATTG
ACCTCCATGTATTTGCTATGATTTCAAAATCCATTTTTGACGTGCCTTAGGGTTGAGTGGATGCATAATTTCATT
TGTTACTGGATTGATGAGCTTTTTTACTTTCTTTTTATGAGGTTTTAACATTTCCATCACTTGTTCGACACGGTC
GATAACAACTGGTCGCTTCGCATAGGCACCATAAGCAAGAATCACTGTGTCACTTTCACTAATCGCTTTCATC
AAATGAATATCAGTGTGCTCATCGTATGGATTTTTGATATGTTTGAGGTTTTCGGGTGTTCTAATATTAGAGAA
TAGATTTACAAGATATACAGCACCGTATCGTTCTGAATTGGCTAATTGGTTGAGGATAAGAACAGTTGTGAGA
TCGAGTGATAATACACCGTCTAAATGAGGATACATCGTTATCACTGTGCATGCAGCTTTCTTTTCATCCCATGTT
TTCTTGAGTAAATAGCGGTGCTGTTCATCATCGCTAAATATGGCTTCTGTGTGTATCGTACTTTTGATTGTATTC
ATCATCGTCACTTCCTTTAGTATTCTTCTGGTAAAAGCATCACATAATAAAAAGCGTCCACGTCATCTTCACGA
ATGACGTAGACTTTCTTAGGTAATGCATTTTGATTTTTTTCATAGTTTGTATAGTGATATTCCAATTTGTATGTG
GGTTGTTCTTGTTCATGTGTGATTGAGAGTATATTCTCATCTTCTTGTAATTTAAAAATGTGTAGGTAATCTGTA
TGAGGCTGGTTATCTTTTTTTTCTACCATGTGCCAAAGTAAGATTTGAAGGTCTAGTGGAATACTTTCATTAAT
TCCTCTTGTGATGTATCGATTGATTTTCATGCTATTTCCCTCCCTTCTGCTTTTCTTTCATGATGTCGATGATTTC
GTTGATAACTGTGACGGATAATTGAGCAACTATGATCCAATTATTCATGGTCCTGACCTCCTTGTTTTAGTAAA
TGACGTTCATCAATAATGATATTTTGAGTATCTGTAAGGTACAGAAAGTCCATGTCAAAATGGTCTAAGTATCC
GACACTGATGAGTTGGTTATTGGCATACATTAGAAATGGATAGATACTTAGCTCATGTAGTTCATCATTATAGT
AGGTATAAGTCTCGAGTGTGAGATGTACCAGTGGAGAATCATTAATAAAACGTTCCGGTAGAATATTTTCTGC
TGCTTCCTCCAGCGCTTCACATTCCCAAGCTTCGTTATTAGATAGTTGGAATAGGTGGGTTATATATTGTTTGA
GTTCTTGAGTGATGGTTTTCATATTATTGCCTCCTAGATAGTGTAATAGTGATGTAGTTCATATACATCATTGAG
ATAATATATATTTGATTTGTCATTTATTACGAATCCCGGTGGGAATAAGAGAAAATTCCATATAAAAACCCGCTA
CAAACGTTGGTATGCCAAGGAAATCCTGAAATTCCGCCTATTTTGACAAACAATCAACTCATTATTTATAAGTA
TTGATGATAGGGTTGTGTCTCTGCTTCCTTATATATATTATTTATTTATAAAAAGTAACGGGATTTTGGGATTGT
GCTTGCACAATCCTTCTGTTTCTTCGAATCTGCAAATCCCAATCATTTCCCGATAAAAAATCATTGTGGGATGT
TCTTTAGCAATTTCAATATAAGCATTGTATAGTTATGAAAAAATTACGACAATAACTGTTTCATTAGATAAGTGT
TATTGAAATTGATAAAGAGCAATTCTTGAAAATAGTTAGATAAAATAAGCGAAAGAATATAGTGAAAATTATT
GTTATAACAATGATTCTATTAGCTAAATAGTAAGATATAGTGTTTGGGGCAAAAATAAAGACGAAGTGCTGAG
ATGCACTTCGTCGAGTTGTTTATTATTGAAAAGTTGTTTAATGATTTCGTTATTAAGTTTGAGTGTGACATAGA
ATTGTTTTTTATGATTACCATCTTTTTTAATATCAATGCGATCAATCACTGATAGATACAATGCTTTGAGTCGAG
ATTTTTCTATGTGCTTAATATCATGAAAGATGTGTTGTAATAGTTTACTGATTTCTTTGGCATCAAATAAAGTCT
TATCTTCATTTTGTTGATTTTTGAGTTGGTTGATTTGATTCGTAATGTCATTGAGTTGCTTTTCATATTTTTGAAT
ACTTGGTCTGATTACTGATGTTAAGTCCGGATTATCCTCGATGGTTTTAATCAAGTTATTTATTTTGATTTGTAC
TTCATCATATTGTTGTTGCTTATAAGCAATATCGTGATGAAGTGCAGCGCCATCAACTTGATTTTCTTGATTGAC
GTGTGTTACTACGCGTTGAATGACTTTATCACTTTTGACTATTTCAAGTATTTGCTTCATCACATAATCTTCAAT
CACATCAGCTCTTACACTGTTTGCCGAACATACTTTGGAACCCTTGTTCCGAAAATTACTACATGAATAGTAAC
GAATACGTTTCTTAGTCCCGTCTTTAAGAGTATTCGTGGTATTGCTTGCTGCCATAGGTGCGCCACATTGGGG
ACAGTGAATAATGCCTGTAAGCAGATTCGTTCCTTTGCCATGGACTTGGGGTTTTTGACTGACTTGTTTCTTA
CGCATTTGTACTTTATCCCATAAATCTTGATTAATAATGGGGGAATGCTTACCTTCAGCTATCACTGGTTTATCA

TTCAGCCCTTTACGACGTTTTTCACTCCAATCTTTGTATTTCGCAAATTGAATTTTGCCGATATAGAAAGGGTT
AGCTAAGATGTATGTGATTGAACTAATACTGAAAGGTTTCCCCTTTTTAGTGACATATCCTTTGTGATTCAATG
CATTGGCAATTTTACGATAGCCATGTCCTTTGGCATAGCACTCGAATATATATTTTACAATATTAGCTTCATGTT
GGTTAATCATTAGCTCGTGTTTACTATCTGGTATTTTGTCATAACCTAGTGGTAAATTGCCTTGATAATAGCCTT
CTTGGGCACGTCTCGTTTGACCCATAAATACGTTCTCGACAATGTTATTACGTTCGAATTCTGAGAAACTCGC
AAGTATCTGTAACATGAGTTTACCAGAAGAAGTATTGACTTCCATACGCTCTGACAAACTGAAAAATTCGACA
TTTTGTTTGTGTAAATCTTCGACAATTTTGAGAAGATCAGATGTATTACGAGCTAATCGGTTTGTTTTGTATAC
CATAACACAGTCGATATTGCCTTCTTTTGCATCTTTCAACATACGTTGGAGCTCAGGTCTGTTCATAGATTTAC
CTGAAATACCACGGTCAGCGTATATATCTTTAACTTCAAAATGATGGAAGTCACAGTATTCTTTGATTTGATTG
ATTTGTCCGTCGATACTATAACCTTCTGTGCTTTGCATTTCTGTTGATACACGTACATAGATACCGACACGTTTT
GTTTTAAGTTGTTGCATTATGTTTCATCCTTTCTTCGTTTATGCAATTGATGATTGCATGGTTTGATTGACGATA
TTGAGTGGTTCATTTTTGAAATAGATTCCTATAAGATTTTTATCTTTCGCAATGTGAATGGTTTCAATATAGGG
GTACAATATGTTTAACGTGAAACGTTTTTGAATAATATTTTGAATGGTGTGTTGTATTTGATGCTCACTGATGG
ATGTGATGCGTTGTGGTTGTTGATGTAATGATTGCATTTGTTTTCTAAACGTTTCTGCATCGATGATGCCTTGT
GCCAACTTTTCTATCAGTTGTTCTTGAGTCAATGTGTGATGCTTTTCTATGTTTCTTTGTCTTTTGATGCGTTTG
TCAATCGCATTTTTAATTTTTACGTAGATATGTTGGTCTTGATAAAAGTCTTGGCACACTTTTAATACTTTATCTT
CCAATGTCTGTGCATTGATGCCTTTGAAATCACAGACAAAGCGTGAAGCATTCATATTTTTAGGACAGACGTA
GTAACGTAATATATGATTCTTTTTTCTAACGGTCATATTTGTAAGTGTTGCATTACAACATGGGCATTTGATTTT
TTGTTTGAGTTGATTATCCGAAGATGTCTGTTTGGTTTGTTTTTGCGATCGAAGTCTCTGCGCTTGCTCATATA
TACTTGTGGAAACAATAGAAGGAAACATATTGTCGAATTGGCCATATTGATTGTTGACACGACCACAATAATT
AGGATTGATGATAATGTTACGAACTTGATAGGGTTGTCGATTGATATACGTGTTATCTTCTTCTAATAACTGTGC
AATTTTCTTATAACCATGGCCTTTAATGTAATAATTGAATACAGCCTTTACCGTTGGTGACTCATTTTGATTGAT
GATGAATGTTCCGTTGTGATAATCGTAACCAAAAGGTGCATGTGTTGTAATCAATCGACCTTGCTTTGCTTTTT
CTTGAAGCCCATTTCTGACTTGTTCTCCAATGTTATCCGATTCAAGTTCGGCTAAACTGATGAAGATATTAAGC
TTGAGTCGGTCGAAAGCTTGATCCATATCAAAGTAACCATCATGTACGCTTAAGATATGAACATGGTACGTTT
GACATAATTTGATGAGTTTTAATGCATTTTTCAGATTACGATGCAACCTATTAAGACGATAACAGCATAATATGT
CACATTGTCCTTGTTGAATTAATTGTGTTAATTGTCGATACCCACTACGATTATCTTTGCGTCCTGATTGTTTGT
CGCTATAAAAGTTGATATGTTGAATATGATGTTTTTCGGCTATTGCTTCGATAGCTTGTTTCTGTGCTGCAAGA
GATTGTTGTTTCATCGTACTTTGACGTAAATAGCCTATGACTTGTTTCATATCGGCTCCTCCTTTCACAGTGATA
ATATATATTTATGGATGAATTGATATATAAGCCCAACATCAATGAGATGTTGGGCGTCCATATTAGTCATTCGTTT
GATTGATTTCTTCAATTACTAAATCGGCTAATATCTCGATAAGTTCATCCATGTTTTTCACTCCGTTATTTGTTCT
ATCTTCAATACGTCGATTATTCAGTTTGATGCTTCACAGTTGTATGATAAAGACAATTAGAAATCTTCGTGAAC
TCCTGAAGGGCCTATCCCTTCATTAGCGGATTTAAAAAGTTCTTTCGCAGCTTTGTTATCATTTGACGGTGTCC
AATTTTGAAGTAACGACTTATCTTTAGTTAATCCGAGGATAGATGCAAACTCTACATCTAATTTTAGATGGTAA
AATACAAGTGATTGTTTTTTACCGCTATTATCTTTGACACTTCTTTTAGTTGTTTGGCGTCCACGGTCAGCTAA
TATGAAACCTTTATCTCTTAAGGCGTTGACAACATTATTAACATCTTGAAATTGATGATTGTTTAGCATCTGTTT
AAAAACGTTCGCAATTATTTTTACTTCGATATGGTCATCTTTTAATGAGATTAATCCATAGTTCTCAAACATATTT
TTCAAAGCACCTTCATCTGAAAACTTACCTCTGTTTTGTGCCACAAATTGAATGATGACATCAATAGCTTTATC
AGCTAATGAGCGTTCAGAGACTGTATGAGTATGATAATCAATAAAGTAGTCTCTTATATTAGCGATATCAATATC
TGTAGATAAAACACGACCTAATATTTTCGCAGATGTTGTAATGACTGCATAACGCTTAAACATACGAATACCTG
TATTGTTTGTTTCGTCTTTCAATTTAGCTTCAAACCAATCTACTTCCTTGTAAAACCATTGAATAACTTCATCTTC
ACGATTTATAAGATATTTAGCTACTAACGGTAAAACATGACCATAGTTTAGTGCTACAGCTTTTTTAATATTGTC
AGCATTGGTCGCATTTGTAGTGAATTGTTCATTAATCTCGATGGTTCTTACACGTAATCCATCGTTTTGAGCTG
AATCATTAAAAATACTGTATTCTGACGTTGAAATGACAGAAGTACCCCAATTCTTAGGCGTTTTAACTTCTCCA

TGAACGTTTGAACGTTGACGACCTTGACCTTCAGTGATGGAGTACAATAACCCCGTTGTATCTCTAAAAGTTG
CTGATGAGAGTTCATCAAATACAATAGGTATACCAAAATTGTTACTCAAGTAACCTTCAAGTGCATTACGTGTG
GCATTCCAACTTCTAAAGAGAGTTTCATTACCTTTGGTGGGATTACCAGCGACTGATACAGCTAAAGAAGCT
GCAGTTGACTTACCGGTTGAGGATTGACCTGTAAAACTAAAAATGATTCCGGCAAATTCGGTTTCATGTTTG
TGCTTCAGGAAACTCGTCACTAAGGCAGAAATACCAAATACGACTGCTAATTCTAAAAGAAGAGAACCTTTA
ACCTCTTTTAGATACATGTTATACCAATTATCAAATGTACCTTTAGGAGTTAAGTCATAAGCATTATCACAAATG
GCGTCAGATGGAGATTTATTATCAAATTCTTTAGTAGTATAGATTTCACTTAACGATACAATAGGACCAAACGG
TGTTTCCAGTATACCTACCCCTTCATATAAGTAGGAAATGGGTAATTGATTGCGCATTTGTTGCATCGCATAAC
CTAAATCTTTTGTATATTTTTCATTAATACTAAATCCATACTTCATTAAAGATGGCAATTTTTGTGTCGTTAAAAT
ATCACTAGATTCAACAATTTCTTTTTGATCTTCGTCTGTGATAATTAGTTTTTCAGTATTAGTTTTAGGGTCAAT
AAACTTATTTTCGATAACGATAGGACCTGCGATTTCAACTTCAGTAGGCATTCCTCCTTTTTCTTTTGGGGGC
GTGTCTTTATACCAACCTTTTTTTGATTTGTATCGTGGTGAAGGATTAAATGAAAGGTTAGTTTGAGCCATTAG
CAATCACCTCCTTTCGAAGAGTTGCTGTTATGGTGTGGATTAGGACCTGTTTTAAGATAAACTAAGTGACCGT
GAGTATCCTTACCGATAATAATAAATGGAACACGTGGCGCATGTTTTACAAAATATGCGAACCAACGTCCAAC
ATTTTGTTGTACAGCTTTTGAACATTGTACATTTGCACGACTGTTCAAGTCATGAAATGTAAATTCTTCTCCTT
CAGGTAAATTGAATGAAATACCTAATATTTTCTTTTTTAACTGCTCGAATTTATCTGATTGATTGAAATACATTT
AACTTCCTCCTACTAATGAACTAAGATAGGAAAATTAAATATGCGCACAATTAACTTTCTTAAGTTCATTTTAG
CCAAGAAGTTATCATTTGAAAGCTTGTAAAAATATGTTAGAAATTCCGTACTAAATTAAGATGGATTTTTGTTG
ACAAAATAAAAAACGCTGATTTAACAGCGCTTTAAATAAAAATTAATCTGAAGTTATATAAAAGTAGTCAGAA
GGAGTGGTGTTATTATCATTATAATAATCTAGTGCTTTATTGATTTCTTTTATTAATTTATCATGATGTTCTTTTGT
TATTTTCTTTGGATATTTGAGTTCTTTTTCTTCTAAGTCTTTTGGAGACACTAGGTCACTATTTAAAATTAATGA
TAACAGCATATTTTTAGGTGCCGAAGCTCTGGTGTCTCGATGAGTAATATATTTTGCTAAATTACTGTTTATACT
TACTTTTTCATTTTCTAAAAAGTTTGTACTATTTTTCACATGAGCATCAGCAATTTTGGTACTTATTTCAGTGTT
TAAATCGTCTATTTCATCTAAATAATATTCAAGAATCTGAAGTTTATCTTTAGAAGTGATACCTTTTTCAGTGAA
AATCTTAATAATGATAGATTTGATTCCAATTTCTATATTACTGTCTACTAGAAAATAGAGATAATAATTAGTCAGA
GCATGTTCATAATCTCTTTGATCAGTAATTTCATGATTGATGAAATAATATAAGTTCTCTTTGATACGATTAGAA
GAATCTATATCTTTGTCATTGATACCGTTTTTAAAAAAGTGTAGATAAACTGAGTTGTTTGAGAGTATATCATAT
AATACAAATAAACTTTTTGGTTTAAATACATATGATTTTCCTGATTTAAAGTTTGCTATGTTTATTCCTAGAGCG
TTGCAGAATTTATTAAAATTAGTTGTAAAATTATTTTTTTCTTGAATAAACATGTCGTTGTCATTTAATTCTGAA
CTATGATTTTTAATCATATTGATATTTTCTTCAATCTTGTCACTATTTAAATTTTCCATGCAAAGTATACTATATCTT
ACTATATCACTTATATTTATGGAAAAATCTTTAGGAGAACGATTTAAAGTATCCTTTTTATTATCTTGATCTACAG
CCCTACATGAATCATCAAAAACTTCTTTGACATTTATTTTATCAATGTTCAAAATTATGTCTACTAATGCATTTTG
AAATTTTTCATTATATATGTCTCTCTTATTCATAATATCACTACCTTTATCAATTATAATAATATATTATATCTTAATAT
TTGTAGAAGTACTTGATGAGAGGTTAGATAAAACACCACATTTCTCTGGTTAGTAAAAACATAACTTCTAAGA
CTTTTTTTCATATACTAATAAATTATATTATAAATATTTTTGAACATAAAAGGAGTGTATAAAAATGTATGCAAAG
AAGGTGTTTTAGTATAGATTTTAAGGCATTCATTTTTAATATAAAATATTTATAATATTTAAAATTGAAAATGATT
ATCCTTTCAAAATTTTTGCAACGAGTAATTTTACTTTAGTTATATTATAAAGTAGAATTAATAGTAATTAAAGGA
ACGTTTGTTCCTGTTTTTATATGTAAATACGAGAACGATTGAATAGATAAAGAAGGATAAAATTGACTTCGAA
ATAGAATATTTAATTTCAAGAAGGAGAGTATTGGATGTTAAAGCATAAAATAATAAATTTAATACAAGAAAAG
AGAGAAGGGAGTTACTGGGATTTTAAAGCAGAATATCATAAAGATAAAGCAGAATTATTACATGATATAATTT
GTTTATCAAATAACTTGTTGAATCAAGAAGCTTATTTAATTTTAGGAGTCGCAGATAACGGTCATATTCTGGG
GGTTGCAGGTGATTCAAATCGAAAAAACCAAGAAGAATTAATATCATTTATTACTGGAAAAAAATTTGCTGC
GGGTAGGCACCCCAAGATTTCATTAATGACATTTGAATATGAAGAAAAGGAAATCGATGTCATTATAATAAAT
CCTAAAGGATATGTACCATACTATCTGGAGAAAGCAGAAACCGATCAAAAAAAGTAAAAAAAACAAAACAG

TCAATGCAGGTAGCATTTATACAAGAGTTGAAGATAAAAATACCCCAATAGATTCTACTGCAAGTCCATTAGAT
ACAGAAATATTATGGAAAATGCATTTTGGTTTATATCCTACTCCTATAAAGAGGTTACAGAATTATTTACTAACT
CCTGAGAAATGGATGCAGAACTCAACAGGTTATTTTCATAGTGAATCGCCAGAGTATATAGTATACAAAAATG
AGGACATTGAAGAAAAAGAAAATTATTTTAATTTAGTAAGTCCATTTTATGCGTATAATCAAATCAATAGTAAC
ACTTTATACTCATATTACGAATTTAAATATCATAGTACAGTTTTATATGGTTGCCGGTGTATCTCTTTAGATTCAG
GTATCTACACAACACCAGTACCTGAACTAGGTGAGATAAATTTTAATATGCATAGAGATGATACTATTTATTATC
GTTATTTCATTGAAGAGACGATGCTTTATAATATTCATCTTTTTATGTATAAGGGTGATTCGATGGAAGAGAAA
TTTGCAATGGATAAATTTTTAGAATGTGTTTTGGTTTATAAAAGTGATGTAGAAAAGGAACTTTTTGAAAATT
ATATATTAGATAATTGGGATAAAGTTAATCAGTCAATTAATGAAAATAATAAACGTGTATTTGGAACTGAACAT
TTGTCACAACTTGAAAAAGAAGATATCACTAAAAAAGTAAAAACAGTTAAAGTTTTAAAAGATGAACTTGAG
AACTTTAGAACTTGAGAAAAATTATTGGTTAATAGGAGAAAATCATGAAAAAATCAACACTCATTGATAAATT
TTTAGACTTACTTTCTTCAAGAAGCTCACTCAGAGAAATTCAGAATAATTTTATTGATGCAAACATTATGAGA
GATAGTTCTATAAATCAAAAATATAACGGACAACGTAAATCGTTAGCGTGGGAATATATTAGTACTTTAAATTT
AGAAGATGAAGCTGAATTTTCTAAATTACTTAATGTTATAGAAACGTATTTGTTTCAATGGAATCTGTATATCCA
TGAAGTTGATGAAGACGAAGAAATTAATAGATTAATAAAAATAGTTAATGTATTAGGTTATGAATATAATAAAG
ATACAGGAAAAATAACTAAAAATGAAAGAGAAGTTAATTTAAGTACTATTAAATCTTTGGCAATAAAATTTGA
TATTGAATATGTATTAAAAGAATGTAATAGAATTGAAAAAGAGGCACTAACGGATCCAGAGGACGCAATTAC
ATCTGCAAAATCGATGGTTGAGAGTACTTTGAAGCATATTTTAGATTCTGAAGGAGAAAAATTTAATAATAAT
GAAACCTTGAGAGGTTTATATAAAAAAGTAAGTAATATTATGAATCTTTCTCCTGGTGGACATAATGAAAATAC
TTTTAAAACAATTTTGAGTGGAATGATAAATGTAATTAACGGTCTGGACGAAGTAAGGAATGAATATGGTGA
CGCACATGGTAAATCAAAGAAAAATTATAGGCCTGAAACTAGACATGCTTTTCTAGCAATCAACGCAGCACG
TACGATAACTGAATTTCTTTTAGCTTCATATAAAAAGTAAAAGCTATTTGATGAATAGCAATCAAATATATTGGT
TATAAAAAACCGCATCATCAACTGATAAGCAGAGGCGTATCATAAGT
>Staphylococcus aureus strain 110900
CTAAAAATCGGGCATAAATGTCAGGAAAATATCAAAAACTGCAAAAAATATTGGTATAATAAGAGGGAACAG
TGTGAACAAGTTAATAACTTGTGGATAACAATTTGGAGGACCAAACGACATGAAAATCACCATTTTAGCTGT
AGGGAAACTAAAAGAGAAATATTGGAAGCAAGCCATAGCAGAATATGAAAAACGTTTAGGCCCATACACCA
AGATAGACATCATAGAAGTTCCAGACGAAAAAGCACCAGAAAATATGAGCGACAAAGAAATTGAGCAAGTA
AAAGAAAAAGAAGGCCAACGAATACTAGCCAAAATTAAACCACAATCCACAGTCATTACATTAGAAATACAA
GGAAAGATGCTATCTTCCGAAGGATTGGCCCAAGAATTGAACCAACGCATGACCCAAGGGCAAAGCGACTT
TGTATTCGTCATTGGCGGATCAAACGGCCTGCACAAGGACGTCTTACAACGCAGTAACTACGCACTATCATTC
AGCAAAATGACATTCCCACATCAAATGATGCGGGTTGTGTTAATTGAGCAAGTGTATAGAGCATTTAAGATTA
TGCGTGGAGAAGCGTATCACAAATAAAACTAAAAAATAGATTGTGCATAATATAAAAGGAGCGGATTTATATT
AAAACTTTGAATTCAAAAATTATTGAAAGGGAAGCTACCTTAGAAATTGAATCTATGGCAACTAATACATTGA
AAATAAACCCGGATATTAATTCAAACGATACAAAAATGTCTTTCGATGGAGAATTGGAAGTGTATGATTCTGA
AAATTTGAGTAAAAAAAAATTTCGTTGGAAAAATACAAGTTCAAGTTAAAGGAAAGGAAGTAGCTAAAAGAG
GAGGTAAGGTTATTCATCGAAGTAATGTCAAAATGAATGATTTAAAGGCATACCAACGAGAAGGTGGTGTGT
ATTACTTTGTCGTGTATTTAATCGTTGAGAATAAAAAAGTTGTTGAGAAGCAGGTTTATGGTAAACAATTACA
TCAATTAGATTTACAATTTTTACTGCAAAAAAAGCAGAAAAGCGTCACTATAAAAATGTATGAAATTGAAAAT
GAAAAAATTTTATATAATAATTGCGTAAAATATATAAATGAAAAGAGATTACAAAACCAAGTAGGTCAAGTTA
AAGTAAAGAACATAGAAAAAGCTTTATCATATATAGCTACACCTGAAAATATTGTGATGGATCATAGAGGTTTA
CCATTAAATGATTTTTATGGCTATATAAAAATTAATTCATCTGAATTAGATGTAACTATACCAGATGGAGTATTAA
GTATGGAAAAAGTAAAAAGAGTGAACAAAAAGCAGATAATAAAAGAAGGCAAGTTATTATTTGAAGGTATG
GTAGGTATAGAAACTTCAAAGGAATCCATCTCTATAACTATAGATGATATTTTCAAAATTCAAACATTTGAAAG

TGATAACAAAAGTACATATACAATGTTACCATTTAAAAAATTAAATATAGCCGAACAATCTTTTAATGTTATAAA
CGAATTGTCGAAAGGCGGCGAATTCTTTTTGGATCAAATCCAATTAGTAATCCAACCTTTTGAAATTAATATTA
TTGAAATAAAAGAAACAATAAATAAGTTGAATATTAAATTATCAGAGTATAGTAACTTGCTTTCGTTTGATGTG
AGTCTAAAATCTACAGAATTTGATAAGCAGATGAATGAGATAAAAGGTTTATTAGAATTATTGGAATATAAAA
ATTTTAAAGATTTTAAAATGCATAATAATGGATACTATAAAATGAAGTTTTGTGGAAAATTTATATTATTATTTAA
AGACAATACATCATTGTATAATGTCTACTCTAATGACTTTGTAGATAGATTTGAGGCTGTTACAAAAGAAAGA
GTTGTACAGATGCCAATTGTTTACACATTAACAAGAGATATGATTGTAGATGTACTGAATTTTGATATAAATGT
TATTAAAGAATGTATTGAATCAGATAAAATTGCTATTCAATCTGACATTAAATGGGAGAAATTAAATAACTTTG
CATTAGAATTAATCGCAGCCTATGATGAAACTCAAAGAACCGATTTATTAGAATTAGCTGAATATGTATTAAAT
AATTTGCTGAATTTTGATAATGATAAAATATTTATGAACTTAATCAACAAAGCTCAAATTATAAAGAGAAGAAG
AAATGATGTAGATGAAAAATTGTTAATTAAGTTATTCAAATTGAGAGATAAATTAATAGACATTGATAAAGAAA
TAGCAACATTGTATATCAACGTAGTATCAGGGAGTAAGCAAGAGGCAAAAATAAGATATGAAACCCTCAATG
CTGCAGATTTGGAAATATTTAATGCTTACCCGATTTTTAATTTATATAAAAAATTAATAGAAAGTTGAGATTATG
ACGAAAAATACTTTTAGAGAAAGAATTCAAAAAACATATACAAAAAAAGATCTTGAGAAAGCTTTAGGTCTT
AAAAACAAAGCTCAATTGCAGAAACGAATTGATAAATTAGTTGATATTTATAAGGTTGATATGAAGAAATTTC
AAAAAAATTCTGGCGAAAGTGAAAGAGCTGCTTATAGTTTCAATGGGATTGCTTTTGATATACTATGTGTCTT
ATTAAATAATATGACAGATGATATGCTCCCTAAAGCAATAACAGATGAAGACGTAAAGTTAAGAAAAAATGCG
ATAGAGCATATCGATATTGACCAATATGCACATTTTATTAAAAGCATTAAAAATGATATAGATAAAATTGAATTT
AAGCCATTAAAGTTACATATACATGCACAAAAAGCATATCAAGATGTAAAAAAAGTATTTGATTCAGGTAATA
AAATAAATGAAAAGTTGCAATTAGTTTCAGAAGTAATGAATCAATTACCTATTGATAAACAAGTTGAATTAAG
TGATAAAATCGCATTTGCTATACATGAAACTATTTATTCTACATTCGCAGAGATCAAGTATAATGAAAGGATTG
CTGAGCATAATCAAACAAGTGAAAACTCAAGTCAGTATGATTTTAGATTTAACTATTTCGTAAATCAATTAACT
GATAATGTAATAACAGAAGATATAAATGAAGAAAAAGCCTTTATATATGATAATGATGAACAATTAGATTGGTT
ATTGGTTAACATACTAAATAAAGTTCAACGGGCTGCTGATGGTTGGGGGATGAGAAACGCTAAGCCTCATCA
TAGAACACAAAAGTTTAGAGATATTGAACATTATTTCCTTAGAGACGTATCGAAAATGATTGATAAAGAGCTT
AACAAAAAGGAAAACTTAAATAAACAATCTAAAAAATATGACTTCGATAATAAGATAAAGGTCATTAATGACA
ATATTGAGTTTTTTAATGAGGATGAACAAATTAGAAAGCATTTATTAGAGTTACGTAAACGATTAGACTTTCAT
TCTGCAGCAGTATTAAATCAGAAAAATCATTCACACATAGGCAATGAAATGTTCCAATATTTTATGGATGATAT
GCAACAAGTTGTAACATTGATAGACCGATACATTAATAAGACTGAACATTATAATGATATGAAAAATGGCTATA
TTAATGAATATAAATCATTTTATAATGCATATAATAATGAAATTGAAGTGACTAAGACGTACGAAAATAACATCC
CAATGGCAATGTTAATGAAACAATTTAAGAATCAACTGTTAAAAAGAAGGAATAACCGAAAAAGCTCTAATT
GAAGTTAAGTAGAAAATTAATAAATAATTGGTAAGCTCAATCCCTCATATAAAGGGATTGAGCTTATTTATTTG
GTGGAATTTTTTTTGAAATTTTTTGCCACTTCATCAATGACCTCTATTTAGTATTAGGATTGTCTTATCAAAACG
TTTTGAACAAATTTTAAGTTGAAAGTGAGCGCGCCACTTTCAACCATAAAGGAGAAGATTTACATGGAGAC
AAGAGACAAACTGATGTCATTGACTCAAAGTGACAAAACACAGCAATGGCTAATGGACAAGTCATCTAACC
AAGATGACATTCAACAATTGCAGCAACAATTCAGCCAGCAGTTAGATCAAAAATATAATGCACTTTTAGCTGA
CAATCAAGCTAAGTTAGACCAATATGTGGAAGTACATCAAGGATTGGAAGCATTAAAGGAAGAGATTGAATC
AGAATCCATTAAACTTAATATAGATAAATTACCCGATATCAAAGCAACAATGCTTGAAAGAGCCAAGAATGAT
GAACATTCTGATAAAATCGAAAAGCTATTTGATAGGTTAGAACAGGCATTAAATGGTACGAATCGATTATATAC
GCAATTATCGTTGATTGGTACACGAACACATCGAATTACAACTAAAAATTTTAATCTTCAAGGCTTACCTAAAG
CAGTCCAACATACGATTTTACCTTCAAAATTTAAGAAGGTGTATACAGTCGATTTTAAATCGTTTGAACCATCA
GTTGCAGCGTACATGACTCAAGATTCTAAACTGATTGACTATTTGAATCATAAAGAAGGTTTATATGACGCATT
ATTAGAAGATTTATCACTGTCCAAAAAGGAGAAAAAGTTCGTTAAACGTACATTTATTGGTTCGTTTTTATTT
GGCGGCAACTTCGATAGCCCCAAATTCAAGTTGAAACATTATGTAAGTGAAGAGCAATGGTTGGATACGGTC

AGCCAATTTACAAAAGTCAATGAACTTAAGAAACAAATCGAAGAGCATAAAACCATGCCGATGCCCTACGGC
ATTGAACATGATATGAGCGCATTTCAAGGTAGCAGTATTATGGCAATCTACGTACAAACTGTAGCGAGTTATAT
TTTCAAGCACATTCTGCTAAAAGTGTACAAAGCACAGTGCGATCAAAAAACGTTCAAGATTATAGTACCTATA
CACGATGCGATTATGATTGAATGTGAAGATGAAGAAATTGCACAAAATGTGGGTCAGTTAATGAAAGATACG
GCTAACCAGTTGTTCAATGGTGAATTTGCACATGTGACAGTGGAAGAAATAGGAGGCGTAGACCATGAATA
ATGATAGAGGACAAAGCCTACACATCCCAAGTAGTACACCAATCAAAGAAAATAATATATATGTAGCTACGTTA
CATTCTGTGATCCAAACAGATTTCTCAGGTGAAATAAAGCACCAATTCACGTATGAAATTGAAGTGAACAATC
AGATTGTATATGCGAATCGTAATATTCCAACAAAACCGAGCGCTAATCAGTTGTCAATTCATGATTGGCTGAA
ACGTCATAGCAACTATAGCGCAAGTCATGAAAACTATGAGCCTTATATTGATCAGAAACATTTAATTCTATTAG
GTCAATATAACGGTAACTATTATGTACAAGATGTAGCATCGTTAGATGCGTTTGGAGGCGTATTATCATGAATC
ATATATTACAAATGTTATCTAAGCTATTAAGTGTGGCCAAGGAGGCAATCGACCGTCAAGGTCTGATTGCTATC
CTAACTATTCCTGTTAATAATAACGATGAAATAGAAGAAACGGCTTAAGGTGAAACCGTGTATAACGAACTTA
TCGATCAGTTACGACTTAATATCCCAAAAGATACGGATTATCAACCTAACATCTATAGTTATTTTGGTATTAAGA
AGAATCCTAATGACACCGTACTCATGGAAATGATGATAAAGGTTTTTCATATCAAACGCTTTAATTCAGAACTG
TTTATTTTCAAAGTTAACGGGTGGCAAAAGATAAATGGAGATGAATTACAAGGGTTGATATCTAAAATGATAC
AAGTATTGCTTGTAGATTATAAGCCTTCACTAAGCACTCTAAAAAAATGTCGTAGATGGATTGCAAAAATCAAC
AGATGTAGAAGAACTTGTTGAGAATGAGCGCTATATTGGTTGTGGTGAAAATATGTTCGATCTTAATACGTTT
CAAGTCGTTAAAAATTCAATCGATATCTTTCCAAAAACACGATTGAATTTATCATTAAGTACAAATGATGTAATT
ACTGATAAGATACCGCCTTATTTTAAGCAATATATGTTACAACTTGCGAATTATGACGATGATTTACAATACTTT
CTTTTCCAACATACAGCAGTATTACTTACAGCTGATACTAAATACCGTAGGGGTCTCATATTATATGGTGGAGC
TAAGAATGGTAAATCTGTATATATTGAACTAGTTAAATCATTTTTCTATAGTAAAGATATTGTGTCTAAGCCACT
TAATGAGCTTGAAGGTCGTTTTGACAAAGAAAGTTTAATTGACAAAAGTCTAATGGCAAGTCATGAAATTGG
GCAATCTAGGATTCAAGAAAAGATCGTAAATGACTTCAAAAAGTTATTATCTGTAGAATCAATGCATGTTGAT
CGTAAAGGAAAAACTCAAGTGGAAGTCATTTTGGATTTGAAACTTATTTTTAGTACAAATGCGATACTTAATT
TTCCTCCTGAACATGCGAAAGCTTTGGAGCGTCGAATTAATATTATTCCATGTGAGTATTATGTTGAAAAAGC
GGACACTTCATTAATTGATAAGCTCCAGAGTGAGAAGAAAGAAATCTTTCTTTACTTGATGTATGTGTATCAA
CAGATTGTAAAAGCAGATATCGAGTATCTTGAAAATAGCCGTGTCACTGAAATTACTCACGATTGGTTAAATT
TTGGATATGAATTTGTTTCTAGCAGGTCCGTAAGTAATGCAAATCAGAAAGCATGTATTAATTTACTCAGAAA
ACTTATAGAAATCAAATCAGGATCACGAATCAAAGTATCCGAGTTAAATAAAGTTATTAATGAAGAAATAAAG
GTAAGTTCTCAGGTTATTAAACAGTTAATTCAAGCAAACTTTGATACTCAAACCAAACTATACAATGGCTACG
ATTATTGGATTGATTTAGGTTGGAAAGAAGCCAATAAAAAAGAGATTCATGATATTTCGGAAAAAGATAATAT
TATTTCATTAGATAAAAATGAAAATATAACAGACGATGAGGCATTAGATGAAGAGAATTTGGACTTTGATTGG
GAGGACTTTGACGATGAATAATGAACAAATTGAAGCATTTGTAGAAGTGCTTGTACCTATCATAGAAGAACG
TATCAATAAAGGTAATTAAGGCTAATTACGTACTACAGGCAGTTGCCTGTAGTACTCATATGATTAAGTGGTAA
AAGTGATAAAAATGAAACGAAATTATAAATATATATTATCTATATGTTGTTACAAGACCGATAGTCTGTAGCAAT
AATCTAATAAAAGGAGCGGTATGATATGAAGGGTAAAATTGCACTTTATTCACGCGTTAGTACGTCAGAGCA
GTCGGAGCATGGGTACTCAATCCATGAGCAGGAACAAGTACTCATCAAAGAAGTTGTGAAAAATTTCCCAG
GTTATGACTATGAGACATATACTGACTCAGGCATTTCAGGTAAAAATATTGAAGGTCGTCCGGCAATGAAACG
TCTATTACAAGATGTTAAGGATAATAAAATCGAAATGGTGTTAAGTTGGAAATTGAATCGTATCTCACGATCAA
TGAGAGACGTGTTTAATATTATTCATGAATTCAAAGAACATGATGTAGGGTATAAATCGATTTCTGAGAATATT
GATACATCCAATGCTTCTGGAGAAGTACTCGTTACAATGTTTGGGTTAATAGGATCTATAGAACGCCAGACTT
TGATTTCGAATGTGAAACTTTCTATGAATGCTAAGGCAAGGAGCGGAGAGGCAATCACCGGTCGTGTTTTAG
GCTACAAATTATCACTTAATCCACTTACACAGAAAAATGATTTGGTTATCGATGAAAATGAAGCTAATATTGTA
CGTGAAATTTTCGATTTATATTTGAATCATAATAAAGGCCTCAAAGCCATTACAACCGTACTTAATCAAAAGGG

GTATCGTACTATTAATCAAAAGCCATTTTCAGTGTATGGTGTTAAATACATTTTGAATAATCCAGTCTATAAAGG
CTATGTCAGATTCAATAACCATCAAAACTGGGCTGTACAGCGAAGAAGTGGCAAAAGTGATAAAAATGATGT
GATATTGGTCAAAGGTAAACATGAAGCCATTATAAGTGAAGAGGTATTTGATAAAGTTCATGAAAAATTAGCT
TCTAAAAGTTTTAAACCGGGCAGACCTATTGGTGGAGATTTCTACTTACGTGGCCTTATTAAATGCCCAGAAT
GCGGAAATAATATGGTATGTCGACGGACGTATTATAAAACGAAAAAGTCCAAAGAACGGACAATCAAGCGTT
ATTACATTTGTTCCTTATTCAATCGTTCAGGGAGTTCTGCATGTCACAGTAATTCCATCAATGCTGAAGTCGTC
GAGCGTGTAATTAATGTTCATTTGAATCGTATTCTGTCTCAACCAGATATTATCAAGCAGATTGCGTCAAATGT
GATAGAAGAACTGAAACAAAAGCATAGTAACCAAACAGAAATTAAATATGACATTGATAGTTTAGAAAAACA
AAAAGCTAAGCTTAAAACACAACAAGAACGATTGTTAGAATTGTTCTTAGATGATCAGATGGATAGCGAAAT
GTTAAAAGCTAAACAAAGTCAAATGAATCAACAGTTAGAAGTATTAGATCAACAAATTAAAGAAGCGCAACA
AGCAAATCAATCACAGGATGAAATACCTAATTTTGATAAATTAAAAGGACGACTCATTTTGATGATAACACGA
TTCAGCGTGTACTTAAGAAAGGCTACACCCGAAGCTAAAAATCAACTTATGAAAATGTTAATTGATTCAATTG
AAATTACGACAGATAAACAAGTAAAACTTGTAAGGTATAAAATTGACGAAAGTCTTATCCCTCAATCTTTGAA
AAAAGATTGGGGGTCTTTTTTTATGCCCAAATTCCAATTTGAAATATATGGTCAAAATGATTATTTCATCGACC
AAATTACCACTTTTACCACTTAGTTATTAGTGACAAAAGTGAGCGAAATGAAATAAAAATCAAATATATATTAT
CAAAATGATGTATCACATGCATACATCAATCAAATACATTAGGAGGTCATAACCATGACACTAGAACAACAACT
CAAGCACTATATAACCAACTTATTCAATCTGCCAAGGGACGAAGTGTGGCACTGCGAATCTATCGAGGAAAT
CGCTGATGATATCTTACCCAATCAATATGTAAGACTTGGCCCACTCAGTAATAAAACACTTCAGACTAATACCT
ACTACTCTGACACACTTCATGAAAGTAATATCTATCCTTTCATTCTCTACTATCAGAAACAACTCATAGCCATCG
GTTATATCGACGAAAATCACGATATGGATTTCTTATACCTACACAACACTATCATGCCTCTTTTGGATCAACGAT
ACTTACTAACAGGAGGACAATAAAATGCATAAATACATCAAAATTACACAATTAGTCATTACAATACTAAGTGA
AATCATCATTTGGATGAAAGAGTCAGAACGAAAGGAAGTCTCTTATGAATAGATATATCACCCGGGGTATCGC
CAACAACTTACCTAATATCTTACAACACCAATTATGGCAACTCGTATCTGAGCGAGAACAAGAACAAACCAAA
GATAATACTTCAGTAGATTATTTTCATATATTCCAGTTCAATAAGCATCGCAATCAATTATATATCAAACACAAAC
AAGAACGACCTGAATATGCGAAAATCCATAAAGCTAATTATTCAAAAGCAATCAATATCAATAAGGTCTACATT
ATTCGAGAAGATGATGTAGACCTTTCTTATTATGTCATGTTATTACCTGAAGAATACTAGAGGAATGAAAATTA
TATGGAAACAATCAAAAGTACATTAAAAACAGAAGCCATATTCAGTGATGACAAACAACATCGCTATCTACTT
AAGAAAACATGGAATAGTGAAAAACAATCAATCACAATCATTACAATGTATCCGCATTATGATGGCATTCTCA
ATATTGACCTAACGACCCAACTCATCATGAACAAAGTTTCAGAAATGGATGCATTTGGTTCAATCAATTTTGT
GAATCTATACTCTAATATTACAACCCCTATCAATCTCAAACATTTAGAAAATGCGTATGATAAGCATACAGATATT
CAAATTATGAAGGCAGTGAAAGAGTCAGATGAAGTGATATTAGCTTGGGGCGCTTACGCTAAAAAGCCCGG
TGTTGAAGCACGTGTTAATGAAGTATTAGAGATGTTGAAACCACATAAAAAGAAAGTAAAACGACTCATGAA
TCCAGAAACCAATGAAATCATGCATCCCCTTAATTCGAAAGCACGTCAAAAATGGATATTAAAAGTATAGCAA
CTAAAATGTTTATTCACTATCCATGAAAATAATTGAATTTCATATATATCTTTTAATCTACAATATGATTAAAAGAT
ATATTATTTGGTTCTGTTGCAAAGTAAAAAAATATAGCTAACCACTAATTTATCATGTCAGTGTTCGCTTAACTT
GCTAGCATGATGCTAATTTCATGGCATGGCGAAAATCCGCAGATCTGAAGAGACCTGCGGTTCTTTTTATATA
GAGCGTAAATACATTCAATACCTTTTAAAGTATTCTTTGCTGTATTGATACTTTGATACCTTGTCTTTCTTACTTT
AATATGACGGTGATCTTGCTCAATGAGGTTATTCAGATATTTCGATGTACAATGACAGTCAGGTTTAAGTTTAA
AAGCTTTAATTACTTTAGCCATTGCTACCTTCGTTGAAGGTGCCTGATCTGTAATTACCTTTTGAGGTTTACCA
AATTGTTTAATGAGACGTTTGATAAACGCATATGCTGAATGATTATCTCGTTGCTTACGCAACCAAATATCTAAT
GTATGTCCCTCTGCATCAATGGCACGATATAAATAGCTCCATTTTCCTTTTATTTTGATGTACGTCTCATCAATAC
GCCATTTGTAATAAGCTTTTTTATGCTTTTTCTTCCAAATTTGATACAAAATTGGGGCATATTCTTGAACCCAAC
GGTAGACCGTTGAATGATGAACGTTTACACCACGTTCCCTTAATATTTCAGATATATCACGATAACTCAATGTAT
ATCTTAGATAGTAGCCAACGGCTACAGTGATAACATCCTTGTTAAATTGTTTATATCTGAAATAGTTCATACAGA

AGACTCCTTTTTGTTAAAATTATACTATAAATTCAACTTTGCAACAGAACCGTATTATGGAATAGAGATGTTGG
TAACATTTATACAGGATCATTATACTTAAGTTTAATTTCGTTATTACAGAACCACACATTCCAACCAGAAGAGA
AAGTATGTCTATTTAGTTATGGTTCAGGAGCAGTAGGAGAAATCTTTAGTGGTTCAATCGTTAAAGGATATGA
CAAAGCATTAGATAAAGAGAAACACTTAAATATGCTAGAATCTAGAGAGCAATTATCAGTCGAAGAATACGA
AACATTCTTTAACAGATTTGATAATCAAGAATTTGATTTCGAACGTGAATTGACACAAGATCCATATTCAAAA
GTATACTTATACAGTATAGAAGACCATATCAGAACATATAAGATAGAGAAATAAACTAGTGGCCGATTGTGCTT
GATGAGCTTGGGACATAAATCCTAACTCGAAATAAATAAGCATATCACTAAACTGATTTTTTAAAGTTTACAGT
GATATGCTTATTTTTTTATCTTACGATTTTGTACGTGCATGCTTGCCTAGGGGTATGGCTCGAGCCATTAGTCTC
TCGCACATACTATTCCCTCAGGCGTCAGCACTTACAAAATCGGTTGTAATTTTCATTTTTATACGCATTCTTACT
GAGATTATACTAATAAGAGGAATAGTAAAAGCAATTCTAAGTAAAATTGCAGATAAGAGGTTTGTTAAAAGC
AGTTCTAAGTAAAATTGCAGATAAGAGGTTTGTTAAAAGCAGTTCTCAGTAAAATTACAGATAAGAGGTACG
TTAAAAGCAGTTCTAAGTAAAATTGCAGATAAGAGGTTTGTTAAAAGCAGTTCTAAGTAAAATTGCAGATAA
GAGGTACGTTAAAAGCAATTCCATGCAAAATTGCTGATAAGGGGTAAGTTAAAAGCAGTTCTCAGTAAAATT
GCAGATAAGAGGTACGTTAAAAGCAGTTCTAGGCAAAATTGCAGATAAGAGGTGCGTTAAAAGCAGTTCTC
AGTAAAATTGCTGATAAGGGGTAAGTTAAAAGCAATCCTAAGTAAAATTGCAGATAAGAGGTAAGTTAAAAG
CAATCCTAAGTAAAATTGCAGATAAGGGGTACAGAAAAACTAGACTTGATTACAAAATGGAGCTTGGGACAT
AAATGATTTTTTAAAAATGAGATGAGACGTAGATTAACTCCATAATCAATACGAATCTATCGACTTCTTTATTTA
TGATATTCATCTCTTTTTAATGGAAATAAAAGTGCGATTAATGTGATAATACAGTTACGTTAATTAAAAAAATAA
AAATGCAAGGAGAGGTAATATGCTAACTGTATATGGACATAGAGGATTACCTAGTAAAGCTCCGGAAAATAC
AATTGCATCATTTAAAGCTGCTTCAGAAGTAGAAGGTATAAACTGGTTGGAGTTAGATGTTGCAATTACAAA
AGATGAACAACTGATTATCATTCATGATGATTATTTAGAACGGACTACAAATATGTCCGGGGAAATAACTGAAT
TGAATTATGATGAAATTAAAGATGCTTCTGCAGGATCTTGGTTTGGTGAAAAAATTCAAAGATGAACATTTGCC
AACTTTCGATGATGTAGTAAAAATAGCAAATGAATATAATATGAATTTAAATGTAGAATTAAAAGGTATTACTG
GACCGAATGGACTAGCACTTTCTAAAAGTATGGTTAAGCAAGTGGAAGAACAATTAACAAACTTAAATCAGA
ATCAAGAAGTGCTCATTTCAAGCTTTAATGTTGTGCTTGTTAAACTTGCAGAAGAAATCATGCCACAATATAA
CAGAGCAGTTATATTCCATACAACTTCGTTTCGTGAAGACTGGAGAACACTTTTAGATTACTGTAATGCTAAA
ATAGTAAACACTGAAGATGCCAAACTTACTAAAGCAAAAGTAAAAATGGTAAAAGAAGCGGGTTATGAATT
GAACGTATGGACTGTAAACAAACCAGCACGTGCAAACCAACTTGCTAATTGGGGAGTTGATGGTATCTTTAC
AGACAATGCAGATAAAATGGTGCATTTGTCTCAATAGAAAGTTAGAGGTGAGTCTTACGTTTCAGTGACGGT
AGACTTACCTTTAACATGTTACATACTAAAAAATTAATTTGAATAAGAAAGAGAGACATATATGAAATACGATG
ATTTTATAGTAGGAGAAACATTCAAAACAAAAAGCCTTCATATTACAGAAGAAGAAATTATCCAATTTGCAAC
AACTTTTGATCCTCAATATATGCATATAGATAAAGAAAAAGCAGAACAAAGTAGATTTAAAGGTATCATTGCAT
CTGGCATGCATACACTTTCAATATCATTTAAATTATGGGTAGAAGAAGGTAAATACGGAGAAGAAGTTGTAGC
AGGAACACAAATGAATAACGTTAAATTTATTAAACCTGTATACCCAGGTAATACATTGTACGTTATCGCTGAAA
TTACAAATAAGAAATCCATAAAAAAAGAAAATGGACTCGTTACAGTGTCACTTTCAACATACAATGAAAATGA
AGAAATTGTATTTAAGGGAGAAGTAACAGCACTTATTAATAATTCATAATAAAACAGTGAAGCAACCATCGTT
ACGGATTGCTTCACTGTTTTGTTATTCATCTATATCGTATTTTTTATTACCGTTCTCATATAGCTCATCATACACTT
TACCTGAGATTTTGGCATTGTAGCTAGCCATTCCTTTATCTTGTACATCTTTAACATTAATAGCCATCATCATGTT
TGGATTATCTTTATCATATGATATAAACCACCCAATTTGTCTGCCAGTTTCTCCTTGTTTCATTTTGAGTTCTGCA
GTACCGGATTTGCCAATTAAGTTTGCATAAGATCTATAAATATCTTCTTTATGTGTTTTATTTACGACTTGTTGC
ATACCATCAGTTAATAGATTGATATTTTCTTTGGAAATAATATTTTTCTTCCAAACTTTGTTTTTCGTGTCTTTTA
ATAAGTGAGGTGCGTTAATATTGCCATTATTTTCTAATGCGCTATAGATTGAAAGGATCTGTACTGGGTTAATC
AGTATTTCACCTTGTCCGTAACCTGAATCAGCTAATAATATTTCATTATCTAAATTTTTGTTTGAAATTTGAGCAT
TATAAAATGGATAATCACTTGGTATATCTTCACCAACACCTAGTTTTTTCATGCCTTTTTCAAATTTCTTACTGCC

TAATTCGAGTGCTACTCTAGCAAAGAAAATGTTATCTGATGATTCTATTGCTTGTTTTAAGTCGATATTACCATT
TACCACTTCATATCTTGTAACGTTGTAACCACCCCAAGATTTATCTTTTTGCCAACCTTTACCATCGATTTTATAA
CTTGTTTTATCGTCTAATGTTTTGTTATTTAACCCAATCATTGCTGTTAATATTTTTTGAGTTGAACCTGGTGAA
GTTGTAATCTGGAACTTGTTGAGCAGAGGTTCTTTTTTATCTTCGGTTAATTTATTATATTCTTCGTTACTCATG
CCATACATAAATGGATAGACGTCATATGAAGGTGTGCTTACAAGTGCTAATAATTCACCTGTTTGAGGGTGGA
TAGCAGTACCTGAGCCATAATCATTTTTCATGTTGTTATAAATACTCTTTTGAACTTTAGCATCAATAGTTAGTT
GAATATCTTTGCCATCTTTTTTCTTTTTCTCTATTAATGTATGTGCGATTGTATTGCTATTATCGTCAACGATTGT
GACACGATAGCCATCTTCATGTTGGAGCTTTTTATCGTAAAGTTTTTCGAGTCCCTTTTTACCAATAACTGCAT
CATCTTTATAGCCTTTATATTCTTTTTGTTTTAATTCTTCAGAGTTAATGGGACCAACATAACCTAATAGATGTG
AAGTCGCTTTTCCTAGAGGATAGTTACGACTTTCTGTTTCATTAGTTGTAAGATGAAATTTTTTTGCGAAATCT
CTTAAATATTCATCCATTTTTTTAACGGTTTTAAGTGGAACGAAGGTATCATCTTGTACCCAATTTTGATCCATT
TGTTGTTTGATATAGTCTTCAGAAATACTTAGTTCTTTAGCGATTGCTTTATAATCTTTTTTAGATACATTCTTTG
GAACGATGCCTATCTCATATGCTGTTCCTGTATTGGCCAATTCCACATTGTTTCGGTCTAAAATTTTACCACGTT
CTGATTTTAAATTTTCAATATGTATGCTTTGGTCTTTCTGCATTCCTGGAATAATGACGCTATGATCCCAATCTA
ACTTCCACATACCATCTTCTTTAACAAAATTAAATTGAACGTTGCGATCAATGTTACCGTAGTTTGTTTTAATTT
TATATTGAGCATCTACTCGTTTTTTATTTTTAGATACTTTTTTTATTTTACGATCCTGAATGTTTATATCTTTAACG
CCTAAACTATTATATATTTTTATCGGACGTTCAGTCATTTCTACTTCACCATTATCGCTTTTAGAAATATAACTGC
TATCTTTATAAACTTGTTTGAAATTTTTATCTTCAATTGCATCAATAGTATTATTAATTTCTTTATCTTTTGAAGCA
TAAAAATATATACCAAACCCGACAACTACAACTATTAAAATAAGTGGAACAATTTTTATCTTTTTCATCAATATC
CTCCTTATATAAGACTACATTTGTAATATATTACAAATGTAGTATTTATGTCAAAATAATGTTATAATTTTTGTGAT
ATGGAGGTGTAGAAGGTGTTATCATCTTTTTTAATGTTAAGTATAATCAGTTCATTGCTCACGATATGTGTAATT
TTTTTAGTGAGAATGCTCTATATAAAATATACGGTTCTGTTGCAAAGTTGAATTTATAGTATAATTATAACCAAA
AGGAGTCTTCTGTATGAACTATTTCAGATATAAACAATTTAACAAGGATGTTATCACTGTAGCCGTTGGCTACT
ATCTAAGATATGCATTGAGTTATCGTGATATGTCTGAAATATTAAGGGAACGTGGTGTAAACGTTCATCATTTA
ACGGTCTAGCGTTGAGTTCAAGAATATGCCCCGATTTTATATCAAATTTGGAAGAAAAAGCATAAAAAAGCT
TATTACAAATGGCGTATTGATGAGACGTACATCAAAATAAAAGGAAAATGGAACTATTTATATCGTGCCATTGA
TACAGAGGGACATACATTAGATATTTGGTTGCGTAAGCAACGAGATAATCATTCAGCATATGTATTTATCAAAC
GTCTCATTAAACAATTTGGTAAACCTCAAAAGGTAATTACAGATTAGGCACCTTCAACGAAGGTCGCAATGG
CTAAAGTCATTAAAGCTTTTAAACTTAAACCTGACTGTCATTGTACATCGAAATATCTGAATAACCTCATTGAG
CAAGATCACCGTCATATTAAAGTAAGAAAGACAAGATATCAAAGTATCAATACGGCAAAGAATACTTTAAAAG
GTATTGAATGTATTTACGGTCTATATAAAAAGAACCGCAGGTCTCTTCAGATCTGCGGATTTTCGCCATGCCAT
GAAATTAGCATCATGCTAACAAGTTAACACAAAGTATTATTTTAAAATTGAGATTAGACATTTATTTTTCAACT
TTGCAGCAGAACCTAATTTTTTCTCTTTGTAAATAAATGCAAAGAGATACCATAAGGATCTCTTACATAACCAT
AACCTTCAGTATAGAATTCTGGACTAAATGTTTTCAATACCTCACTGCCTTTTTCTATTAACTGGTCATATACAT
GTTTAGTTTCTTCTACTTGGTCAAAAGTGAGACAAAGAGATATATTATTACCTTGTGTTATGGGCAAACCTTCA
GTGTCATCTGCGATCATAATTTTTATATCTCCAAATTGAAGTACACATTGATCAATTTTATTTAAATCATTTTCGT
CAATATTAAGTTTCTTATCTATAGGTCTATCTTTAATACGTTGAATATACAGTGTTTTAGCGCCAAACAGCTCTTC
ATACAACTTTTTTAAACCCTCTGCATTTTGAGTGATTAAAAAATATGGACTTACTTGAAATTTCATGTTTTTTCC
TCCTTAGATTTGTTATAAATAGAGTATATACTTAATTAGTGTCATCTATTGACACTAAAAGGAGAATAATAATGA
AAAAATCTGTTAGATTATATAATATGATTGAATATTGTAATGAAAATAGGAACTTCAAATTAAATGATTTAATGT
CAGAATTTAATATTTCTCGTAGTACCGCTTTAAGGGATATAAAAGAAATTGAAGCATTAGGAGTACCTTTATAT
AGTAATCCAGGGAAAAATGGTGGTTATACGATCATAGGTAATCGAGACCAAACGAAAATAGCAATCTCAGAT
GAAGAGTTGAAAGCTTTAGTATTTACACTTTCGAGTATTTCAAATGTGAGTAATCTACCTTTTCAAACAGAAT
ATCAAGAAATATTAAAAAAATTATATAATAACTCGAATAAAAAAGAGTTAATAAACCAATATAATGATCTATTTC

AATATTTTAATGAAGATAAGTATCAGTTCAAAAGTTATAAGTTATTTAATGAAATCATTAGATTGATAATTGAGA
ATAAGTCTTTTGAAACCTGTTATTCACAAAACTATATTAAAGAACAATATAAAGGTATTGGCATTATGTATAAAA
ATCATCAATGGTATTTTGTTGTAGCTAATATAGAATCAAAGTTAGTGAATCTATTAAATATTTCGAAAATAAAAG
AACTATATGAAATGGGAGAGACTCAAGAGTGTAATGATATAACTATGCAGAATTTTCAACAGTTCATGGTTAA
AAATGAAACAGCTATTGATATTCTTATTAGAAGCAATGTTATGGGATTGAATATCTTGAAAGGCTACCTGTGG
AGTGACTATATGATTGAAAATATTGACGAAGAGACATATTTATTTAAATCAAAAGTGAACGCGAAAGATATAG
ATTTTATAGCTAAGTTAATTGTCACAGGTGGTGTCAATGTAAAAGTAGAGACCCCTAATAGTTTGAAAAATGC
TGTTAAAGTTGAATTAACTAAAATAATAAACATGTATTAATAGTAATTTAATCATAATTGTAATAACAAAAAGAT
GACAACATTAAACAATTTAAATTTATACAAAAAAGCATTACCAAATTTAAAATTGGGTAGTGGTAGTGCTACA
GCATACATAAATAGTTATAATTAAATAAAGATGGGTCAATGAAGGTGAATTAAGTGAATAAAAATATTACAGCT
CTAAAATCTACTGAAAATACTACATATATGCAGTATACGGTTCATATCGATGATCAAATTATAAATAATATAAAAG
AGTGTACCAAAAAGTTTAAAATTTTGCCTATGGAAGATAAAATTCCATTATCACCGTTGTTACAACCAGAATAT
GCAGGAGAGGTACAAGATTTTATTAGTACATATGAGCAGTTTATGATTAATTTTGGTAAAGTAATATTGGATAG
TCAAGGCATAAAAATACAGTTTGAAAGTGAATCATTAAGTAGTATTCAACGAGGCATTCAAGAACATTGTTAC
TTAAATGAGCGAACAAATGACATTGATGTGACTAAAGAATGGTATTTATGCAAATTTTCAAATCAAATATTAGA
AGAGGATAAAAATCAATTGTATAATGCTTTAAAGCAACTCATGAATGATTCTAAAAATAAAAAGCAAGCTTTT
CAAGAGGTGTTTAAGGTTCATATTGATATTTATCTCTACAATAAAGAAAAATCTGAATATAAGTATCAAGTATCT
AGTTATTTCAATCTTGTAAAAGAAAATCCTAAAATAACTTATAAAAAAAGACATTTACAAGAGAAACAAGGTG
TCAAGGGTACAACTTTCACGAACAAATTAAAATTATTGAACAAAATGTATGGTGTAGATGTTGCAAAATATCA
GCCATTTTATAATTCTAATAATCCTGAATATGAAAGAGGTCAATTTGGTGAAAGATACATTTCTCAAAGATCTA
ACTATGAATTTAATAGACTTCAATATCAAATTATAGATATGTTATCGAAAATACTTGATAAGCATCCATTACCAAA
GTCAGATAATAATTATAAACATATTCCAACTATTGAAAAAGCAATATTAAGTGGGGATTCTCATAGTTTTTATGA
GTATTTTGAAGATATAATGAAAGAAATTATAAGTATGGAAAATTCTTCTTTAAAGGAAAAATTATTGACTGATT
TTACATATCAATCTCAATGTAGATGGTACTCTGAAAGTGAAAAATTAAAATTGCAACTTGAAAGTTTTATGCAT
AAAGTTTTGGAGAGCAATTATTATGAGGGTAACAAATTATATAGAATGTTGTCTCATGCAATTGAAGAAACAA
TCAATGAGGCTGATGAAGATAAGGTTCATTTCAATTATTTTAAAGATTATTTTTTGACAGACGGTGGGGTGAA
GAATTGGGAGCAGATTAGCGAGAAAATTACTGAATTCAATGGTAAAGTTATTAACGATATCCAAAATGAATAT
AATAAGATTCAATTTAATAACGCAAATCGAAATCAGAAACTAAACTTTAATTATTTATACCATTGCTTTGAGTTT
AGTAATAACTTAGTAAAAGCAAGGGTAAATGGAAATAGAGGTTACATTTTATATTTTGTAGATAAATATAAAAT
AAAAATGCCCTTTTTGGATCAATTATTAATCAATTATTTGAATCAAGAAATAGGTCAGGAAAGTATTAATTATA
ATATGCAAACATTGTTTGAAAAAGAAAGATATGATAGAAGTAGTACCATTGAAAAATTAGTAGCAACAAGCA
AATTTAAGTATGAAAAAGATGATTCAGATTTATTCAAACAACTTTTCAATGATGTTGAAAATTCAATAGACAG
ATTAGGTATTTACTTACTAAATAATGGTATAAATTCGAATGATGAAAATGCAAGATATTATAGATCGTTTTTAAA
GGAACTTAGTAGAATAAAAAGTAAATTAACGCCATTTTCTCTTGAAATAAGTAAGTCTAGCGGAAGAGAGCA
ACATTATCCTGATGATGCTATTGATGATAAAGATGAGAGAAGAAAAAATAAAGAAGAAACATATCATGCTTTT
GATGATAAAAGCGATATTGACTCCAAATTAAAGAATAAAATAAATGTTTCTATCGATAATTTATTGTCAGTTAAA
ATTAAGAGCAATTTGTATGATATTAGTCCAAATACAAAAAAACAATAACTATAATGAGCCAACTCAAGAATGA
GTTGGCTTTTTAATTGTAATTTTACGCTTCCATTACAGGAATCAATGTTATGGAAGCCATTAAGCAATTAATTT
GAGTCATTTATAGTATAGGTACAACGTTGTGATGTTCACATGATAATCAGCCCGGGAGATTATTGTGTAATAAC
TTATAGAAGGAGCAAATCACCATGTATCAAGCAAATATACGTGATTTAATTACAAAATTGCCTCAAAGCAATAA
AACAGAACACTTTTTAATGAACAAATTTTCAAATCAAGATAAAGTTCAGCAGCTACAAAGACAAATTAGCCA
ACAGCTAGATCAACAATATAATGAGCTTTTGGCTAATGAAAAAGCTAAGCTAGACCAATACGTGGAAGTACA
CCATAATTTAGAACCATTAAAGAAAGAGATTGAATCAGAATCTATTAACCTTGATACCGATAAATTACCTGATA
TCAAAGCGACAATGCTTGAAAAGGCTAAGAACGATGAACATTTTGATAAAATCGAACAGCTATTTGATAGAT

TAGATCAGTCATTAAATGGTACGAATCGATTATATACGCAATTATCGTTGATTGGCACACGAACACATCGGATC
ACAACGAAAAGATTTAATGTTCAAGGCTTGCCTAAATTAGTCCAACAAATGATTTTACCTTCGCAATTTAAAA
AGGTTTATACAATAGATTTTAAATCATTCGAACCATCAGTTGCTGCGTATATGACACAAGACGAACAACTGATT
GACTACTTGAATCATGAAGAAGGGTTATACGATGCATTACTGAGAGACTTATCTTTGTCAAAAGAGAAGCGC
GTGAGTGTGAAACGTGCATTTATAGGGTCATTTCTTTTTGGCGGTCGTTATAGTAGCTCTAAATTCAAAATCA
ATCAAGAGGTTAGTGAAATTAACTGGCTACAAGTAATGAGCAAATTCAAGAAGGTCATTGAATTTAAGGAGC
AAGTCGAAAAATATAAAACAATGCCTACGCCTTACGGCATTGAACATGATATGAGCGCATTTCAAGGTAGTAG
TATTATGGCAATTTATGTACAAACGGTAGCAAGCTATATTTTCAAGCACATTTTGTTGGAAGTGTACAAAGCA
CAGTGCGAAAAAAAAACGTTCAAGATTATAGTGCCGATACACGATGCGATTATGATTGAATGTAATGATAAGG
GGATTGCACAAAATGTAGCACAGCTCATGAAAGATACAGCTAATCAACTGTTTAATGGTGAATTTGCACATGT
GACAGTGGAAGCTTTAGGAGGTATAGACAATGAATAATGATAGAGGAAAAAGTCTTCAAATTCCCCAAAGT
ACATTGTTAAAAGAAGGATCTATATACGTTGCTACGTTACATTCTGTGTACGAGAAGAACTTCTCAGGTGATAT
TAAACATCAGTTTACGTATGAAGTAGAACTTAACCAAGAAACGCACTATGTAAATCGCAATATTACCGTAAAAT
CTATGAGTCACCAATTATCAATTGCTGATTGGATTAAACGTCACAGTAACTATAACGTAAACCACATTAATTATG
ATCCATACATTGATCGAAAACATTTGGTCCTTGTAGGGCAATATAACGGAAATTATTATATTCAAGATGTAGCA
CCATTAGATGAATTTGGAGGGGTAATGTAATGAATCATATTTTAGAAATGTTAATAAAATTATTAAAAGTGGGT
ATGGAGGCAATCGACCGTAAAGGTCTGATTGCCATCCTAACAAGTAGTATTGGAAATGATGAAATGGATGAT
TCTGAACAAGCTGTAATGGTGTATAACGAGCTTATCGATAAGCTACAGCTTAACATTCCTAAAGATGTCGACT
ATAGACCTAACATATACAGTTATTTTGGTATTCAAAAAAAGCCAAATGACACAATATTAGTGGAAATGATGATA
TGTATTTTTCATATCAAGCGTTTTGATTCAGAGTTGTTTGTTTTCAAAGATAAAGGTTGGCAAAAGGTAAGTG
AAGATGAATTGCAAGGGTTGATATCTAAAATGATACAAGTGTTGCTAGTTGATTATAAACCTTCACTAAGTACT
TTGAAAAACGTAGTAGATGGCATACAGAAATCAACGGACATAGAAAAACTTGTTGAGCATAGACAGTACATT
GGTTGTGGACGAAATATGTTCAATCTAAAGACCTTTAAAGTGGTTGATAATGACCTTGAAATATTCCCTAAAA
CACGCTTAGATTTGGAATTAGATATAAATGACACGATTACGGACAAGATACCCCCGAATTTCAAACAATATATG
TTAGAGTTGGCGAATTATGACCATGATTTACAATATTTCCTTTTCCAACATATGGCAGTGTTATTGACGGCAGA
TACTAAACTACGTCGTGGACTTTTTTTGTATGGAACTGCAAAAAATGGGAAATCGGTCTATATTAAATTAGTTA
AGTCATTCTTTTATAGTAATGATATCGTATCTAAAACACTTAATGAACTTGGCGGGCGTTTCGATAAGGAAAGT
CTAATTGGTAAACGAATTATGGCAAGTGATGAAGTGGGGAAAGCTAATATTGATGAAGCAACTGTGAATGAT
TTCAAAAAATTACTATCTGTTGAACCAATTCATGCTGACCGTAAAGGAAGAACGCAAGTAGAAGTTACTTTA
GATTTAAAACTCATTTTTAATACGAATGCTGTACTCAATTTTCCATCATCACATGCAAAAGCATTAGAGCGTAG
AATTGCTGTTATTCCATGTGAATATTATGTTGAAAAAGCTGACCCTGACTTAATTGAAAAGTTACAGGATGAA
AAGAAAGAAATCTTTCTTTACTTGATGTATGTGTATAAGCAAATTGTAAAAAATGATATCGAGTACCTCCAAAA
TGATCGTGTTACTGAAATTTCTCATGATTGGTTAAATTTTGGATATGAATTTGTTTCTAGTAAATCAGCAAATAT
TGCACATCAGAAAGCGTGTATTAATTTACTCAGAAAACTTATAGAAATCAAACCAGGGTCACGTATCAAAGTG
TCTAGGCTAAATGAGGTTATTAGAGATGAAATTAAAGTAAGCTCTCAAGTTATTAATGATTTGGTTCAAGCTA
ACTTTAATGTACAAAGTAGACTAAATAATGGTTATAAGTATTGGGTCGATTTAGGATGGAAAGAAACTGATAA
AAAAGATGACATGATTTCATTCGATAAAAATGAGAATGTAACAGATGATGAATTCTTATACGAAGATGATTTG
AACTTAGGTTGGGAGGACTTTGACGATGAATAATGAACAAATTGAAGCATTTGTAGAAGTGCTTGTGCCTAT
CATAGAAGAACGTATCAATAAAGGTAAGTAATCTAATTACGTACTACAGGCAGTTGCCTGTAGTACTCATATGA
TTAAGTGGTAAAAGTGATAAAAATGAAACGAAATTATAAATATATATTATCTATATGTTGTTACAAGACCGATG
GTCTGTAGCAATAATCTAATAAAAGGAGCGGTATGATATGAAGGGTAAAATTGCACTTTATTCACGTGTTAGT
ACGTCTGAGCAGTCAGAACATGGTTATTCTGAAAAGGAGCAGGAACAACTACTCATCAAAGAAGTTATGAA
AAATTTCCCAGGTTATGACTATGAGACATATACTGACTCAGGCATTTCAGGTAAAAATATTGAAGGTCGTCCG
GCAATGAAACGTCTATTACAAGATGTTAAGGATAATAAAATCGAAATGGTATTAAGTTGGAAATTGAATCGTA

TTTCTCGCTCAATGAGAGACGTGTTTAATATTATTCATGAATTCAAAGAACATGACGTAGGGTATAAATCGATT
TCTGAGAATATTGATACATCCAATGCTTCTGGAGAAGTACTCGTTACAATGTTTGGACTAATAGGATCTATAGA
ACGCCAGACTTTGATTTCCAATGTGAAACTTTCTATGAATGCTAAGGCAAGGAGCGGAGAGGCAATCACCG
GTCGTGTTTTAGGCTACAAATTATCACTTAATCCATTGACACAGAAAAATGATTTAGTTATTGATGAAAATGAA
GCTCATATTGTACGGGAAATCTTTGATTTATATTTGAATCACAATAAAGGATTTAAAGCAATCACGACAATTCT
AAATCAAAAAGGATATCGTACCATTAATCAAAAACCATTTTCAGTGTTTGGCGTGAAATACATTTTGAATAATC
CAGTCTATAAAGGCTATGTCAGATTCAATAATCATCAAAATTGGGCTGTTCAGCGAAGAAGTGGTAAAAGTG
ATAAAAATGATGTGATATTGGTCAAAGGTAAGCATGAAGCCATTATAAGTGAAGATGTATTTGATCAAGTTCA
TGAGAAACTAGCTTCTAAAAGTTTTAAACCGGGTCGACCTATTGGTGGAGATTTCTACTTACGTGGCCTTATT
AAATGCCCAGAATGCGGAAATAATATGGTATGTCGACGGACGTATTATAAAACGAAAAAGTCCAAAGAACGC
ACAATCAAACGCTATTACATTTGTTCATTATTCAATCGCTCAGGAAGTTCTGCCTGTCATAGTAATGCGATTAAT
GCTGAAGTCGTCGAACGCGTAATCAATGTTCATTTGAATCGTATTCTTTCACAACCTAATGTTATTAAGCAGAT
TGCGTCAAGTGTGATAGAAGAACTGAAACAAAAGCATAGTAAACAAACAGAAATAAAATATGATATTGATAG
TCTAGAAAAACAAAAAGCAAAAGTTAAAACACAACAAGAACGATTATTGGAATTGTTCTTAGATGATGAAAT
GGATAGCGAAATGTTAAAAGCTAAACAAAGTGAAATGAATCAACAGTTAGAAGTATTAGACCAACAAATTAA
AGAAGCAAAACAAGCAAATCAATCACAGGATGATATACCCAATTTTGATAAGTTAAAAGCACGACTCATTTT
GATGATAACACGATTCAGTGTGTACTTAAGAAAGGCTACACCCGAAGCTAAAAATCAACTTATGAAAATGTTA
ATTGATTCAATTGAAATTACGACAGATAAACAAGTAAAACTTGTAAGGTATAAAATTGATGAAAGTCTTATCCC
TCAATCTTTGAAAAAAGATTGGGGGTCTTTTTTTATACCTAAATTTAACTTTGTGATAAATGTCACAAAGAAA
AATAGGATTGAAAATTTATCACTTTTACCACTTTTTTAGAGTGACAAAAGTGGAGGAGTTTTGAAATATTTTA
TAAATATATATTTTATTTATGGAGTACACATTATTAATTAAGGAGGTCATTATAATGACGCTAAGCAAACAACTT
AAAACGTATATCACTGAACGATTTAAATTAAATTATCAAGAAACTTGGGCTTGTGAAACCATAGATGCGGTGG
CTGAAGATGTATTACCTGAAAAATATATTAAAAATAGTCCACTTGAACATAAAATTTTAAATACTTTTACCTATT
ACAATGATGAATTACATGAAATCAGCATTTACCCTTTTTTATGTTATCTAGATAAGGAATTAGTAGCAATAGGTT
ATTTAGATAATTTTGATTTAGACTTTATATTTTTAAATGACACTCATCAAATTATTATTGATGAACGCTACTTGTT
ACAAAAAGGGGGCGAGTAATTATGAACTGGATCAAGGTTGCTCAGCTATCTGTTACAGTCATTAACGAAGTG
ATTGAGATCATGAAAGAAAAGCAGAATGGAGGAAAATAGTATGAACATCAATCGATACATCACGAGAGGCA
TTAGTGAACAACTATCTCTAGACCTTCAAATCTTACTTTGGCACATGGTAGAAGAAAAAGATAACCAGCCTCA
TACCGATTACCTACACATTTTCAAACTACAAGAAGATGATAATATGTTGTCAATTACACATGAACAAGAACAGC
CCGCATACAAGTTAGAATATCACTATATAAACTATGAAAAAAATCAAAATGCATTACCTAAGAAAGTCTACGTC
ATTCGTGAAGATGATGTAGACGTTTTTTATTATGTGATGCTTTTACCAGAAGAATACTAAAAGGAGTGACGTT
GATGAATACAATCAAAAGTACGATACACACAGAAGCTATATTTAGCGATGATGAACAACACCGCTACTTACTC
AAAAAGACTTGGGATGAAAAGAAACCTGTTTGTACAGTGATAACGATGTATCCTCATTTAGATGGCGTATTAT
CACTCGATCTTACTACTGTTCTTATCCTCAACCAATTAGCGAATTCTGAACAATATGGCGCTGTATATCTAGTGA
ATCTATTCTCTAATATTAAAACACCAGAGAATCTTAAACATATTAAAGAACCTTATGATAAACACACAGACATCC
ATTTGATGAAAGCAATTAGTGAGAGTGATACAGTAATTCTAGCTTATGGCGCCTATGCAAAGCGGCCTGTTGT
CGTCGAACGTGTTGAGCAAGTGATGGAAATGTTAAAACCTCATAAAAAGAAAGTCAAAAAACTCATAAACC
CAGCAACAAATGACATTATGCATCCACTTAACCCTAAAGCACGTCAAAAATGGATTTTGAAATAAAAGGAGG
ATTATCTATGAACCATGAAACTACACACTCAGACTGGCGAACGGTTGCTAATTGTTTAGCATCACAAAATTATA
TATCGATCGTAAAAGGATTAGTACATCATTTCACAGCGATTGAAGATGAAGAAATACTAGATAAAATCTATGAA
GATTTTATGAATAATGGCTCTATTACAACGGTACTTAACAATGATTTACAAGATATTATTAATTATTACCTATCAA
AATAAAAATATATTACTTTTAATAAAATTAGAGCATCCTTCACTTTGCGGTGAGAGATGCTCTTTTAGTTTATTT
TTTTATTGATTACTCGCCATAAGTTCTGCTTGCTCTACAGCCATTTTTATAAATCTGGTGGATAGCTATATTTTTT
AAGTAATCGTCTAACAGCTACGCGCATTTTAGCTTTTGCGCTATCACGTTTAGACCAATCACCCCCATATTTTC

TTTCACTTTTTTAGTTAGCTCATGAGCAATCGCACGTAGTTCTTCATCTTCCATTCCTTTTTTTCGCTGTTTCAT
GTGAAACTAAAGCATCGTAAAAATGCGATTTCATCTGAATTTAAACCTAATTCATTTCCTCGTTGTTGTTCTTG
TTTAATATCTTTGGCGAGTTGAATGAGTTCTTCAATCACTTTTGATGTTTCAATGGAACGGCTATTATATTTATT
AATCGAATTTCTTAACATTTCAGAGAAACGTTTAGATACCGTCGCATTCGTTTTCATTAATGATTTGACTTGTC
CCTTGAGTAATCGATTTAATAATTCTACCGCCACATTTTTCTGTTTCAATCCTTCTACATCTTTTAAAAAGTCATC
AGATAGGATTGATAAATCGGGTTGTTCAAGACCTAAAGTTTAATAAATGTCAAGTCAATGAAATCTTCAGTTA
ATAGTGTATATTTGATTATTATTAGTTTAAATATATTAAAATTAAATACTTATTATATATGGTTCTTTACTATTTTAG
AAGTATATTTTTTATTTTCCTGAACAGTAGTTATTAAATTTATCTCTTATTTTAAATTAGAAAGGAAGAAAAAGT
TAAATTTATATTAAGTTTTATATATGATTAATTATTAACGTATTAAATGTAGTATACTCTTAATATATAGAAAATTAT
CATAATATTTGTCAAAAAAAGTGACATTTCATATAGTAGCATGGTGTTAGTTGTCACAGTTTTTGACAGCCAAA
ATAACACTTGAACCTTTGTGGTTATGCTATTTATTAAAGTATCTGATCGATAACTACCCCGAATAACAGGGGAC
GAGAATTCTATAAGTTCATTAATTCCGATACCTAGATTATCTGATCGATAACTACCCCGAATAACAGGGGACGA
GAATTTTTTTCCACCCTTTCAGATCATCTATGATCTTGGATCGATAACTACCCCGAATAACAGGGGACGAGAA
TAATTTTCTAATTCTATAAGTTCATTAATTCCGATGATCGATAACTACCCCGAATAACAGGGGACGAGAATTATA
CTATTTACATAATTTTTTATGTGTCTGTCTACGATCGATAACTACCCCGAATAACAGGGGACGAGAATTAATAG
TGTTGTTCTCTATTAAAAGATACAATCCTGTGATCGATAACTACCCCGAATAACAGGGGACGAGAATTAGAAT
GTTATTATCTAAGTGGTCGATGTATTCCGATCGATAACTACCCCGAATAACAGGGGACGAGAATTAGACCATT
TACCTCATTATATTTATAGTCTTTATTAGATCGATAACTACCCCGAATAACAGGGGACGAGAATTTTTCTTTAAC
TGTTTTTACTGCCCATTTAATAGTGATCGATAACTACCCCGAATAACAGGGGACGAGAATATAAACCCGTTCA
ATTCGTTATCTTTAAATTCTTGGATCGATAACTACCCCGAATAACAGGGGACGAGAATAAGTTAACGGCATTA
CCTAATAAAAATATTTTAGGGATCGATAACTACCCCGAATAACAGGGGACGAGAACTCATCTTTCATGTCACT
GATTAATTCATTTGTAGATCGATAACTACCCCGAATAACAGGGGACGAGAACGGTAATAGTTGCTCAATAGGT
AATAAAACGTCGGTGATCGATAACTATCCCGAATAACAGGGGACAGAGTGTAAATTTAATTACACTCTAAAAT
TTGTAAATTTTTAATGGAATACGCATTGATTAATTTTTAGGGGATGAAAAATGAAAGATGTTATTTATGTAGAA
AATCATTACTTTGTTACCGTGAAAGAAAATAGTATTAAATTTAGAAATGTAATAGATAAAAGTGAGAAATTTTA
TTTGTTTGAAGAAATAGAAGCGATTATTTTTGATCATTATAAAAGCTATTTTTCTCATAAATTAGTAATTAAATG
TATAGAAAATGATATCGCTATTATTTTTTGTGATAAAAAGCACTCTCCATTAACGCAACTTATTTCTTCTTATGGT
ATGACTCATCGTCTTCAAAGGATTCAAAGTCAGTTTCAATTATCTGGGAGAACTAGAGATAGAATTTGGAAA
AAGATTGTTGTAAATAAAATTATTAATCAATCAAAATGTTTAGAAAACAATTTACATAATGAGAATGTGAAGTT
ATTAGTAAACTTAGCAAAAGATGTTAGTTCTGGAGATAAAAGTAATAAAGAAGCACAGGCTGCAAGAATTTA
TTTTAAAGATTTATACGGTAAACAATTTAAACGTGGACGGTACAATGATATTATTAACTCAGGGTTGAATTATG
GGTATTCGATACTTAGATCTTTTATAAAAAAAGAACTAGCTTTACATGGATTCGAAATGAGTTTAGGCATTAAT
CATCGTTCGAAAGAAAATCCATTTAATTTAGCAGATGATATTATTGAAGTTTTTCGTCCTTTTGTAGATAATATC
GTGTACGAGATAGTTTTTAAGAAAAATATTAATACATTTGATGTAAATGAAAAGAAATTATTGTTAAATGTTTT
GTATGAAAAGTGCATTATAGATAAAAAAGTGGTGAGGTTACTTGATAGTGTGAAGATAGTTATTCAATCACTT
ATTAGATGCTATGAAGAAAACACCCCTACTTATTTATTACTACCTAAAATGATTGAGGTGGGGAACTAATGTAT
TTATTAGTTAGTTTTGACTTACCTAGAGATACTAAATTTGAACGTAGAGTCGCAAGTAAGTATCGTACTCGATT
ATTAGAACTCGGTTTTAGTATGAAGCAGTTTAGTTTATACGAGAGGTATGTCAGCGATGTTCAAAAAAAAGAT
AAGATTTTAGAAATTCTGCAACAAGAAATTCCTGATACTGGAAGTATTACACTATATGTCTTACCTGATGAGGT
AAATAATAGTCAGATTACCATATTAGGAAAAGAGGTTAAGGTTGTTGTGCGAAAAGAACCTAAGCTAATTTTT
CTATAAAGTGGAGGTTACAATGGATAAAAAAACGACATTAATGTATGGTTCTTTACTACATGATATAGGTAAAA
TTATCTATCGGAGTAATGATCATGCATTTGCAAGAGGAACGCATTCAAAGTTAGGATACAATTTTTTATCTCAA
TTTTCAGAATTTAAAGATAACGAAGTGCTCGATAGTATTGCTTATCATCATTATAAAGAACTTGCAAAAGCTAA
TTTAGCTAATGATAATACAGCTTATATTACCTATATTGCCGATAATATTGCGAGTGGTATTGATAGACGAGATGT

TATAGAAGAGGGCGACGAAGAATACGAAAAACAAGCATTTAATTTTGATAAGTATACACCTCTATATAGTGTG
TTTAATATTGTGAATTCTGAAAAATTGAAACAAATAAGTGGAAAGTTTAAATTTTCTAATGAAAGTAATATTGA
GTATCCTAAAACTGAGAACATTCAATATTCAAGTGGAAATTATACAACACTAATGAAAGATATGAGTTATGATT
TAGAGCACAAATTAAGTATTAAAGAAGATACATTTCCTTCATTATTACAATGGACAGAAAGTCTATGGCAATAT
GTGCCAAGTTCGACAAATAAAAATCAATTAATTGATATTTCTCTTTATGATCATAGTCGTATTACATGTGCTATT
GCTAGTTGTATATTTGATTATTTAAATGAAAATAATATACATAATTACAAAGATGAATTGTTTACAAAGTATGAA
AATACCAAAGAATTTTATCAAAAAGAAGCTTTTTTACTACTTAGTATGGATATGAGTGGTATTCAAGATTTTAT
TTACAACATAAGTGGTTCTAAAGCATTAAAAAGTTTAAGATCTCGTAGTTTTTACTTAGAAATCATGCTTGAG
GTAATCGTTGATCAATTACTAGAAAAATTAGAATTAACACGAGCAAATCTTTTATACACTGGTGGAGGCCATG
CATATTTATTAGTCTCTAATACAGATAAAGGGAAAGAAAAAATAAATCAATTTAATACTGAATTAAAAAATTGG
TTTATGTCAGAATTCACTACAGATCTCTCATTATCAATTGCTTTTGAAAAATGTAGTGGTAATGACTTAATGAAT
ACTAGTGGTAATTATAGAAATATTTGGCGTAATGTCAGCAGTAAGCTTTCTGATATTAAAGCACATAAGTATTC
TTCAGAAGATATATTAAAATTAAATCATTTTCATTCATATGGGGATCGAGAATGTAAAGAATGTTTAAGAAGTG
ACATAGATATTAATGATGATGGATTATGCAGTATATGTGAAGGAATCATTAATATATCAAATGACTTAAGAGATA
AATCATTCTTTGTACTGTCAGAAACTGGAAAATTAAAAATGCCATTCGATAAATTTATATCGGTTATCGATTATG
AAGAAGCAGAAAGGTTAGCACAAAATAATAATCACATTCGTATTTACAGTAAAAATAAACCTTATATTGGTGT
AGGAATATCAACGAATTTATGGATGTGTGACTACGACTATGCTAGTCAAAATAAAGATATGAGAGAAAAAGG
TATTGGAAGTTATGTAGAAAGAGAAGAAGGCATTAAGCGTTTAGGCGTTGTACGTGCCGACATTGATAATCT
TGGCGCTACATTTATATCAGGAATTCCAGAAAAATATAATTCAATTTCAAGAACAGCTACATTGTCTCGTCAAT
TATCATTATTTTTTAAATATGAATTAAATCATTTATTAGAAAATTATCAAATTACAGCTATATATTCAGGTGGCGA
CGATTTATTTTTGATTGGCGCATGGGATGACATTATAGAAGTAAGCGTTTATATAAATGAAAAGTTTAAAGATT
TTACTCTTGGTAAATTAACAATGTCTGCTGGTGTTGGGATGTTCAGTGGTAAGTATCCAATTTCTAAAATGGCT
TTTGAGACAGGTCTACTTGAAGAAGCAGCTAAAACTGATGAAAAAAATCAGATAGCACTTTGGGTGCAAGA
AAAAGTATATAACTGGGATGAGTTTAAAAAGTATATCTTAGAAGAAAAACTTCTCGTTTTACAACATGGGTTT
TCACAAACAGATGAACACGGAAAAGCCTTTATTTATAAAATGTTAGCTTTACTGCGAAATAATGAAACTATTA
ATATTGCTCGCTTAGCCTATTTATTAGCAAGAAGTAAAATGACAGAGGAATTTACATCTAAAATTTTTAATTGG
GCTCAAAACGACAAAGATAAAAATCAATTAATTACAGCTTTAGAGTATTATGTTTATCAAATAAGGGAGGTTG
ATTGAGTATGATATTAGCTAAAACAAAAAGTGGGAAAAAAATAGATTTAACTTTTGCACATGAAGTAGTAAA
AAGTAATGTAAAGACAGTTAAAGATAAAAGAGGTAAAGAAAATCAAGTTTTATTTAACGGTCTTACAACAAG
CAAGTTAAGAAATTTGATGGAGCAAGTGAACCGACTTTATACTATTGCATTTAATTCGACTGAAGATCAATTG
AATGAAGAATTTATCGATGAATTAGAGTATTTAAAAATTAAATTTTATTATGAAGCAGGTCGAGAAAAAAGTG
TTGATGAATTCTTGAAAAAAACATTGATGTTCCCAATTATTGATAGAGTGATACAAAAAGAATCAAAAAAATT
TTTCTTAGATTACTGTAAATACTTTGAAGCTCTAGTTGCATACGCTAAATATTATCAAAAGGAGGATTAATATGT
ATTCAAAAATTAAAATTTCAGGAACAATTGAAGTAGTTACTGGTTTACACATCGGCGGGGGCGGTGAATCTA
GTATGATTGGCGCAATTGATTCCCCTGTAGTTAGAGATTTACAAACTAAATTACCTATCATACCTGGCAGTTCA
ATCAAAGGAAAAATGAGAAGTTTATTAGCAAAGCATTTTGGCTTGAAAATGAAACAAGAGAATCATAACCA
AGACGATGAAAGTGTTTTAAGATTATTCGGTTCAAGTGAAAAAGGAAATATTCAAAGAGCTCGTCTACAAAT
TTCTGATGCATTCTTTTCTGAAAAGACGAAAGAGCATTTTGCACAAAATGATATTGCTTATACAGAAACGAAA
TTTGAGAATACAATTAATCGTTTAACTGCAGTTGCAAATCCAAGACAAATTGAGAGAGTAACAAGAGGATCT
GAGTTTGACTTTGTATTTATTTACAATGTCGATGAAGAATCACAAGTTGAGAATGATTTTGAGAATATTGAAA
AAGCAATTCACTTATTAGAGAATGACTACCTTGGTGGCGGCGGTACCAGAGGTAACGGACGTATCCAATTTA
ATGATATAACTATCGAGACGGTTGTTGGAGAATACGACAGTACAAATCTTAAAATTAAGTAGGTGAAAGACAT
TGACAACAAAAGTATTTAAACTTTCTTTTAAGACTCCTGTTCATTTTGGGAAAAAAAGGTTGTCAGATGGGG
AAATGACAATAACTTCTGATACTTTGTTTAGTGCGTTATTTATTGAGGCGCTTCAATTGGGTATAGAAACTGAT

TGGTTATTAAACGATTTAATCATTAGTGATACATTTCCTTATGAAAATGAAATTTATTATCTTCCTAAACCTTTGA
TAAAAATTGAATCTAAAGAAGAAGGCAACCATAAAGCATTTAAAAAGTTAAAATATGTTCCGGTTCATCACTA
TAATCAATATTTAAATGGTGAGTTAAGCGCTGAAGATGCGACAGATTTAAATGATATTTTTAGTATTGGGCATT
TTTCTCTACAAACAAAGGTTTCATTATCAGCACAAGAAATTGATTCAAGTGCTGACAGTGAACCTTATTCAGT
GGGAACATTTACTTTTGAACCTGAAGCTGGTTTATATTTTATTGCAAAAGGATCAGAAGAAACCCTTGACCA
ATTAAAAGATATTATGACTTCATTACAGTACTCAGGTTTAGGTGGTAAACGTAATGCAGGATATGGACAATTTG
AATATGAAATAATAAATAATCAACAATTATTTAAGTTATTGAATCAAAATGGAGAACATTCTATTCTTTTATCAA
CGGCAATGGCTAAAGAAGATGAGATAAAGAGTGCTTTAAAAGAGGCAAGATATATTTTGAATAAACGTTCTG
GTTTCATACAATCAACAAATTATTCTGAAATGCTAGTTAAAAAAAGTGATTTCTATAGCTTTTCTTCTGGTTCA
GTTTTTAAAAATATCTTTAACGGTGACGTTTTTAATGTTGGACATAATGGTAAACACCCAGTCTATCGTTATGC
GAAGCCTTTATGGTTGGAGGTATAAGTATGACAATAAAAAATTACGAAGTTGTTATTAAAACTTTAGGTCCAG
TTCATATTGGTAGTGGTCAAGTTATGAAGAAACAAGATTACATTTATGATTTCTACAATTCTAAAGTTTATATGA
TTAATGGAAATAAACTGGTTAAGTTTTTAAAAAGAAAAAATATACTCGATACATATCAAAACTTTTTGAGATAC
CCACCAAAAAATCCAAGAGAAAATGGACTAAAAGACTATTTAGACGCTCAAAATGTTAAGCAAAGTGAATG
GAAAGCATTTGTGAGTTATTCTGAAAAAGTTAATCAAGGTAAGAAATATGGAAATATACGGCCTAAACCGCTA
AATGATTTACATTTAATGGTAAGAGATGGACAAAATAAGGTGTATCTTCCGGGAAGTTCAATCAAGGGAGCT
ATTAAAACAGCACTTGTTTCAAAATATAATAATGAAAAAAATACAGATGTATATAGCAAAATTAAAGTCAGCG
ATTCAGAGCCTATTGATGAAAGACATTTAGCAATTTATCAAAAAATAGATATTAATAAAAGCGAAAAACCAAT
GCCTTTATATAGAGAATGTGTAGATGTAGATACTGAAATAAAATTTAAATTAACCATAGAAGATGAAATTTATTC
TATTAATGAAATTGAACAAAGCATCCAAGATTTTTACAAAAACTATTATGACAAATGGTTGGTCGGTTTCAAA
GAAACAAAAGGTGGAAGACGATTTGCATTAGAAGGCGGTATGCCCGATGTTCTAAACCAAAATATTTTATTC
TTAGGAGCTGGCGCAGGATTTGTTAGTAAAACGACACATTATCAATTAAAAAGTCGAGAACAAGCAAAACG
AGATTCTTTTGATATTTTAACTAAAAAATTTCGTAGAACTTATGGGAAAATGAAAGAAATGCCTTCTAACGTAC
CAGTTGCTCTAAAGGGTACAACTAATCAAAGTCTTCATGTTTCATATCAACAAGGTATGTGTAAGATTAGTTTT
CAAGAGTTAAATAATGAGGTGCTATAATGAAAGTACTATTTAGTCCAATAGGTAACTCAGATCCATGGAGTAA
TGATAGAGATGGTGCAATGCTTCATATTGTGCGTCATTATAAACCTGATGTAGTCGTTTTATTTTTTACTGAAA
GCATTTGGAATGGTAATAGAAATATACCTGGACGTAAAAACTTCGATTGGGAAAACATTGTTTCAAAAGTATC
ATCGAGAACAAAAGTGGATATAAAAGTGGATAGTATTAAATATGAAAATGATTTTGATAGCTATAAAGATATAT
TTCACTTTTATATAAATGAGATTAGAACTAAATATTCTGATGCAGAAATTTTATTAAATGTTACAAGCGGAACA
CCACAAATGGAGTCAACTTTGTGTTTAGAATATATTTCTAATCCTCATAATATGAAATGTATACAAGTTTCAACA
CCTGCTCCTATTGAAGGACCTAAACGTTCATTTGCGAAACTTGAAACTGTGACTGAAGATTTAAATAAAGTTA
ATGCTAATGAAAAAATGGCTTCTAATAGAAGTAAATCAATCAATATTATTAGTTTTAGAGAAGTGATGGTGCG
CTCTCAAATAAAAAGTTTAGTGAATAATTATGATTATGAAGGTGCACTGAATTTAGTAAGTGATCAAAAGTCTT
TTCGTAATGGTAAACTATTAAGGAAAAGATTATTGGAATTAACAAATCAGATTAAAACGCATGAAGTTTTTCC
AGAAATTAATGATAAGTACAGAAGTGTTGCTTTAAAAAAATCATTATTCCATTATTTATTATTAAATATGCGATAT
AATCGTCTTGATGTAGCTGAAACGTTAATAAGAGTGAAATCTATTGCTGAGTTTATACTTAAAACATATATTGT
GGGTCATTGGCCTACTCTAATAATTGAAAAAGATGATAAACCTTATTTAAATGCTGAAGATAATTTATCTTTTAT
TTATAAATATAAATTACTATTAGAAAAAAGAAGACAGAATTTGGATGTTTCAAGGATTCTAGGTCTACCTGCGT
TTATAGATATACTTACAGTATTAGAACCTAACTCTAAACTATTAAAAGAAGTTAATGCTGTAAACGATATAAATG
GTTTAAGAAATTCTATAGCTCACAATTTGGAAACATTAGATTTGGATAAAAATAAAAATTATAAGAAAATAATG
TTATCTGTTGAAGCAATAAAGAATATGTTGCATATCTCATTTCCCGAGATAGAGGAAAAAGACTATAATTATTT
CGAAAGAAAAAACAAGGAATTTAGAGAATTATTATGATAAATAAAATTACAGTAGAATTAGACTTACCAGATA
GTATTCGTTTTCAATATTTAGGAAGTATTTTACATGGTGTGTTAATGGACTATCTTCCTAATGATATTGCTGATCA
GCTACATCATGAATTTGCTTATAGTCCATTGAAGCAAAGAATATATCATAAAAACAAAAAAGTAATTTGGGAA

ATTGTTTGTATGTCAGATCGATTATTTAATGAGATAGCCGAGCTATTCACTTCTAAAAACAGGCTGTTTTTAAA
ATATTACCAAGTTTATATTGAGATTTATTCTTTTAATATTGAGAAAGTAAACGTTCAAAATATTATGAATCAACTC
TTAGAAACAGAGGAATTGAATCGATATGTAAGAATTAATATACAGACACCTATGTCTTTTAAATATCAGAGTAA
TTACATGATTTTTCCTGAGGTTAAACGTTTCTTTAGAAGTATTATGATACAATTTGATGCTTTTTTCGAAGAATA
TAAAATGTACGATAAAGAAACATTAGATTTTTTGGAAAAAAATATTAATATTGTTGACTATAAATTGAAAAGTA
CACGATTTAACTTAGAAAAAGTTAAAATCCCTTCATTTACGGGAGAAATAGTGTTTAAAATTAAAGGACCCTT
ACCTTTTCTACAACTAACTCATTTTTTATTGAAGTTTGGCGAATTTTCTGGATCAGGTATGAAAACAAGCTTAG
GCATGGGGAAATATAGTATAATATAACTAGAGTATAGCTGAAATTTAGTTGTCAAAAAATGTGACATTCTGCAC
TTATAGTACAAGTGATTTGTCACTATTTTTGACAGTAAATTTTGCCTTTGAACTATTGATTTGATAGGCTTTAAA
GAGCTATCTAAATTCGATAACTACCCCGTAGAAGAGGGGACGAGAACTCTTCTAAGACGCGATATGATTCTA
ATTGGTCTTCATTCGATAACTACCCCCGTAGAAGAGGGGACGAGAACTGATATACTCCTTTACCATGTATTAAT
TCTGGACCACTATTCGATAACTACCCCGTAGAAGAGGGGACGAGGATCTCATCTTGATGATCCCAATTGGCTT
GGTTAAAAAGAAATCGATAATCCTCGTAGAATAGAGAAAAAGTATTAAAAATATATTTGATTTGATAAAATATT
CTGCAAAAAAGAGGAACAAAAAATCAAGATTTAAACTGTAATTCATTATTATTGAAATTAATCGACATTCAAT
TTCTAAATAAACAGAAACAAAAATAACTTTCATATAAATAATTTCTTTGCGATTAAGTTGGAATTAGAAATACT
AAATACAAATAACAGCGAATGAAATGCATATTATTGAGGAATTGGATGTGTAGTGCAATTTCGAGAGTTACTA
CTTAAGAAAATAAAAAGAAGAAAACAAACAATAAGAATTAAATCTGAAGCAAGATATAAAAGCAAATATTTT
TGATACAACCACATTAGTAAATACTTTATTAATTAGTATACGACTGAAAGCGAAGTAACAGCTAACAATGCTCC
AGATATAGACATTAGTGTTACGAAAGAATAATATTACCATCTAGAAAATGTAATTTTTTAAAAATCTGAAGATA
AAGTAGATTACTTTGTGAGTTTTCATGTTAAGAATTCTGAACTTGGAGACGTAGTATAAATATAGCTTAGTAGA
AAATGCCACCGGAGCTGTAGCTGAGATTGACTATCAAGAATTAACTAAATTAGGTTATTACCCATCAACAAAA
ATCCAAGATTTAACACTGTCTAATAATAGTGCGAATAATGAAGTTTAAAAGTAAACAACTGGTAATAGCGAAT
CAAACTAAAGTAATCAATTACAGAATTCAAGTACTACTCAAGCCTCAAGCAAATATTGAAACTCAAACACAAG
TTAAGAATAATACACTGCCTGAAACAGATGAACAGTCTAATGCCGATTGAGTCCAATAATAGCCTCTGTATTAT
TAGCCACACGATTATTATCAATATTTAAATAAAGTTAATATTAAATAATAAAAATCCTTCAAATTATTTGTTGAAA
AATTAATAACTTTGAAGGTTTTTAGCAAAATCACTGATAGGGAGAAGCATATCATAAAT
>Staphylococcus aureus strain 12-03119
ACTTATGATACGCCTCCCCTTGTTAGTGATATTGCTAAAAATACCAAAATAAAATTGAATTTATTTTGGTATCAA
TACTTTATTTTAGTTTTTTGAGTCTTAGATCACCTGTGGTTTCGGCTCCAGTCCAATACTTTCCAGTCAATTCAT
TTTCTTCAACTATTAATCTTGTTGTTCCTCGATTAATAGGATTTTTCTTTTCGAATTCGGCTTTTGGATTTGTTAT
ATAAGTATAGTACAATATATTTTTACCATGTTCTTCAATAATATCTGCTGTAATACTATTACTAGTAATTTCATCGC
TTCTGAATTCAATATTTACTGTTAAAAAAGTTTGTTTTATTTCCACACTTACCTTTTTAATTCCACCTTCTCCATA
AAAATGTCTGAGTGTTGCCTCATAATCTCCTGATATATCAGCTTGCTTATTAAATATACGAAACAATTTCCACTT
CCACATAAACCTTTCATACCCTATAGTAATAAATGTTGATATTGTTACAGTATAACTTATGATATCTATCCATCCTA
TTTGCCATACCGTACATGATACTATAATTAGTAACAAGAAAAAGAGTCCTGTAAACCATCCTGTTATTTTAATCA
AATGATTTATTTTGTCATTCATTTTTTTCTCACCACTATATTCAAAATAATTCTTTACATCCAGAATAAAATTTTTA
TAAACATTATTTTTTTCTTGAGAATCGCATAAATATAGAATGTCATTAGATAAAGTCCGTATAATTGTGTAAAAG
TAAAAAGGCCATATAACAGTCCTTTTACGGTACAATGTTTTTAACGACAAAAACATACCCAGGAGGACTTTTA
CATGACCCAAGTACATTTTACACTGAAAAGCGAAGAGATTCAAAGCATTATTGAATATTCTGTAAAGGATGAC
GTTTCTAAAAATATTTTAACAACGGTATTTAATCAACTAATGGAAAATCAACGAACAGAATATATTCAAGCAAA
AGAATATGAACGAACAGAAAACCGACAAAGTCAACGAAATGGCTATTATGAGCGCAGCTTTACGACACGTG
TAGGCACGCTAGAATTAAAAGTACCCAGAACACGTGATGGCCATTTTTCACCCACAGTGTTTGAACGTTATC
AACGAAACGAAAAAGCCCTCATGGTTTCAATGTTGGAAATGTATGTATCAGGCGTTTCAACTCGTAAAGTATC
AAAAATTGTGGAAGAACTTTGTGGTAAATCCGTCTCTAAGTCCTTCGTTTCTAGCTTAACAGAACAGCTAGA

ACCTATGGTTAACGAGTGGCAGAATCGTTTATTATCAGAAAAAAATTATCCTTACTTAATGACCGATGTACTCT
ATATAAAAGTACGAGAAGAAAATCGAGTACTCTCAAAAAGCTGTCATATAGCGATTGGAATAACCAAAGATG
GCGACCGTGAAATTATCGGCTTCATGATTCAAAGTGGCGAAAGCGAAGAGACCTGGACAACATTTTTTGAA
TACCTAAAAGAACGCGGTTTACAAGGTACGGAACTCGTTATTTCTGATGCGCACAAAGGATTAGTCTCTGCC
ATTAGAAAATCCTTCACCAACGTAAGTTGGCAAAGATGCCAAGTTCACTTCCTAAGAAATATCTTTACCACCA
TTCCTAAAAAAAATTCAAAATCTTTCAGAGAAGCTGTTAAAGGAATTTTTAAGTTCACAGATATTAACTTAGC
GCGTGAGGCTAAAAATCGATTGATTCATGATTATATCGATCAACCAAAATATTCAAAAGCTTGCGCATCATTG
GATGATGGATTCGAAGACGCCTTTCAATATACCGTACAAGGAAATTCCCACAATCGACTAAAGAGTACCAATC
TAATTGAACGACTGAATCAAGAAGTACGCAGAAGAGAAAAGATTATTCGCATCTTCCCCAATCAAACATCAG
CCAATCGCTTAATTGGAGCCGTTCTTATGGACCTACATGATGAATGGATTTATTCTTCAAGAAAATACATCAAT
TTTGATAAGTAGAAATGGTAAAAACATTGTATAGCATTTTACACAGGAGTCTGGACTTGACTCACTTCCTTTAT
TATTTTTCATTTTTTTAAACTATTTAATACTAATGTCTTTTATAATAGCTTTTCATATATTATATAATCAATCTTTATA
AGTCCTTTTATAAATTTCTTTTCTACCATTTTCGATAAATTCCTGTTTAATATTTTTAATTCCATAAACAATAGTTT
CAATAGGATAATATTCTTCAACTATATCTTGATATTCTTTTGCTTTCTCAATATCTATATTTCCATACATTCTTAATA
TATCTTCTCCAAAATTTGTTCCTATTTCTTCTTCACTATCTTCAAGTAAGTATATAAAATCACAATATTCATCTATA
ATTCCAGAATCTCCAAAATCAATTATTCCAGTTAATCTATTATTGCCATCTAACAATAGATGATTACAACTAAAAT
CATTATGGCATAAACACTTTTTACCCTCAAAAACTGTTGTTGCATTTAGTCTTTCCATAAAACTTTCTATATAATC
TTTTTCTATATCAGTTAAATCATTATAAATAGTTTCACGCAACAATATATACTCTTCTAATACATTTTGTTTATTATC
AATAGTACATTCACTAATATCTGTATAATCTAAACCGTGCATTTGTCTTAAAAAACTGGCAATATCTCGTTTTAA
CAAATTTTGTTCTTCTTCTGACATAGTAGAATAAATTTCTGGTGTTAAAAAAGTTCCTTTAATTTCTTTATAACC
TAGTATAGATAATTCATCACTAATATACGAATATTCAATATTAGGAATTTTTACATTAGTTTCTAAATTTGTATTTA
AAAAATTATATATTGCTTTTTCTTTTGCATAACCTTTTTTCTTATTAGTACTAAATTTTGTTTTAAAAATGTATTCA
TTATTAACTAAATATGCCACACTATCATAACCACTACCGATTATTTCAATACTATCTACTTTGAAATTATCAAAGT
AATGCTCAATTAAATATTTCATTGCCTTAACATTTGTGGCATTATCATCATATCTATATTCCATTAAATAACAATCT
TCTTTTTTGCCCTCGTGTAATTCATGTTCTGGCAAATCTTCAATAATTCTAAAACCAGATTTTTGGTATGCCCTT
ATTGCTCTTGGATTATTTTTATGAGGGTCTAAAATAACTGCATTAGCATTTCTTTCTTTTTTCAAAAATTCAAAA
ATCAATTTAATATATCTTGTACCAATTCCTTTACTCCAATAATTTGGCTCTCCTATAAATTGATCCATACCATAGAC
TATCTCATCAGTTTTTGGATAATGATAATCAGTATATAACTCATCATACATTTTATATATTTGTCCATATCCAATAG
GAACATTGTTATATTCAATAATTACTCTAAAAACTTCATCTTCCCAAGGCTCTGTATAATGTTTTTTTAATGATTC
TAATGTATATTTTTTATCTCTACCACCATAAAATTCTAATACTCTTTCATCAGTTAACCATTTTAACATCAAAGGA
AAATCATCATCTATTAAAGTTCTTATACATATTTCATTTTCAACTATATTCATTTATTTATCACCTTTTTCATAATCA
TATACATATACTATTTCATCTTTATAATCATTTTTACCACCTAATTTTTCATATACATGGCAAGCTCTAGGATTACC
TTTATCAGTTATTAAAAACATTTCAGAACAACCAATCTCTTTAGAATATTCCTTAATAAAAGATAATAATTTTGA
ACCATAACCTTTGTCTTGATAGTTAGGTAACATTCCTATTGAGTGTAAATAAAACATTGTTTTTCCATCAGGTCT
TAAAAGTGTATAGCAATATGCAAATCCTATAATTTTATTATTTTCTTTAGCTATAAACCCAAATGAACTGGTATCA
TTAAGAAATCCTTTTAAATTATCAATATCAAAAACCATATTATCATCAATTAAAACTTCTTCCATAAACTCAGTCA
AGTCCAGACTCCTGTGTAAAATGCTATACAATGTTTTTACCATTTCTACTTATCAAAATTGATGTATTTTCTTGA
AGAATAAATCCATTCATCATGTAGGTCCATAAGAACGGCTCCAATTAAGCGATTGGCTGATGTTTGATTGGGG
AAGATGCGAATAATCTTTTCTCTTCTGCGTACTTCTTGATTCAGTCGTTCAATTAGATTGGTACTCTTTAGTCGA
TTGTGGGAATTTCCTTGTACGGTATATTGAAAGGCGTCTTCGAATCCATCATCCAATGATGCGCAAGCTTTTG
AATATTTTGGTTGATCGATATAATCATGAATCAATCGATTTTTAGCCTCACGCGCTAAGTTAATATCTGTGAACT
TAAAAATTCCTTTAACAGCTTCTCTGAAAGATTTTGAAATTTTTTTAGGAATGGTGGTAAAGATATTTCTTAGG
AAGTGAACTTGGCATCTTTGCCAACTTACGTTGGTGAAGGATTTTCTAATGGCAGAGACTAATCCTTTGTGC
GCATCAGAAATAACGAGTTCCGTACCTTGTAAACCGCGTTCTTTTAGGTATTCAAAAAATGTTGTCCAGGTCT

CTTCGCTTTCGCCACTTTGAATCATGAAGCCGATAATTTCACGGTCGCCATCTTTGGTTATTCCAATCGCTATAT
GACAGCTTTTTGAGAGTACTCGATTTTCTTCTCGTACTTTTATATAGAGTACATCGGTCATTAAGTAAGGATAA
TTTTTTTCTGATAATAAACGATTCTGCCACTCGTTAACCATAGGTTCTAGCTGTTCTGTTAAGCTAGAAACGAA
GGACTTAGAGACGGATTTACCACAAAGTTCTTCCACAATTTTTGATACTTTACGAGTTGAAACGCCTGATACA
TACATTTCCAACATTGAAGCCATGAGGGCTTTTTCGTTTCGTTGATAACGTTCAAACACTGTGGGTGAAAAAT
GGCCATCACGTGTTCTGGGTACTTTTAATTCTAGCGTGCCTACACGTGTCGTAAAGCTGCGCTCATAATAGCC
ATTTCGTTGACTTTGTCGGTTTTCTGTTCGTTCATATTCTTTTGCTTGAATATATTCTGTTCGTTGATTTTCCATT
AGTTGATTAAATACCGTTGTTAAAATATTTTTAGAAACGTCATCCTTTACAGAATATTCAATAATGCTTTGAATC
TCTTCGCTTTTCAGTGTAAAATGTACTTGGGTCATGTAAAAGTCCTCCTGGGTATGTTTTTGTCGTTAAAAACA
TTGTACCGTAAAAGGACTGTTATATGGCCTTTTTACTTTTACACAATTATACGGACTTTATCTGTCATTAGGTTC
TTTAAATTCACTTAATTTATCTATATTATCTATTAAAAATGCAATTAATGCATTAAATGTATCACCTAACATTTCAT
CATTGCTATATTTGTAATCCGGTATGTTCCAAAACAAACCTTCAACTCCAAATGAAGAAGTTTGTTCCGCGTTT
CTATTTTTACTATCTATTAATTGATATCTTATTTCCTTTATTATTCTAACCATCTTTTTATATTTATAATTTGTATTGTT
ATTTTTTATAACACTATTATTTATATGCTGTTCCGGATAATTTATAATTCGTTCACCTTTATCTGAATAAATTGTGA
TTCCTCCAATGAAATTATTTGGATCATCCATATAATCATTACTATAATCTCTATATCTAAAACAAGGTACACAATC
TGTTTCTTTACGGTAAGTATTGCCATTGATTCTAATTGCTTTATTACCTCTTCTTACCTCACTTCTTCCAAATTTT
TCAATCAAAGCTTCTTCTACTTCATCTTTAAAATAATACGGAGGCTTATTACTAGAAATAAATTTATAATTTTCTC
TAGTTTTACCTTCTCTATATTTATCAAAAAACTCACTTTCTTTTACCACTGCAATATCAACGTCACTATTTTGTCT
TACATTGGTATTTGTTGCATACGAACCTTTCACAAATATACTTAGTTCATAATCTTTCGATGAATTAGTCATTTTA
ATTTGATATGATAGCGTATCTTCATTGTTTCTATGTATACCCTTTTTTATTTCATATCCTAATGACTCCAGAGATTC
TTGAATAATTCTTATTGCATTTTCACACTTTTCTTTTTCAGATTCTGACAATGGTTTAGAATATAATTTTAATTGT
TCTTCAGTAAATAACAAATTTGCTCCCCCATTCATACTCAGTTTAACTATATAATAATCTGTACTTTTATAATTAGC
TACTTAATATTACTTAAATAATACAACAAACAATATTTTTCACAAAGAAAATCACAAGAAAAGTCACATAAAGA
TTTAATATTTGCTATATTTGTATAAAGGATAATATAGAGGTGTAATATAAATGGCGACAATCGGATTTGAAGAAA
AATTATGGCAAGCTGCCGATAAATTAAGAGGAAGTATGGATGCTGCTGAATATAAGAACGTAGCATTAGGTTT
AATCTTTTTAAAATATGTATCAGATTCTTTTGAAGAAAAGTATGAAGAATTGAAGCAAGATCCTTATGCTGATG
AAGAGGATCAAGATGAATATTTAGCAGAAAATATTTTCTGGGTGCCAAAAGAAGCAAGATGGCAATATATTA
ATGATAATGCGAAAAAACCAGAAATTGGACAAATTATTGATAAAGCCATGATTGCGATTGAAAAAGAAAATG
AATCATTAAAAGGTGTGTTACCTAAAGAATATGCGCGCCCTGCGTTAGATAAAGAAAAATTAGGCGATATTAT
TGATTTATTTACTTTTAAGGTAGGCGATACTGAAAGTCGTAAGCAAGATGTATTAGGCCGAGTGTATGAGTAT
TTTATTGCTAAGTTTGCAAGCGCTGAAGGGAAAAATGCCGGAGAGTTCTATACCCCCTCATCAATTGTAAAAT
TATTGGTTGAGATGATTGAACCTTATAAAGGTCGTATCTACGATCCATGCTGTGGTTCAGGTGGGATGTTTGT
ACAAAGTGAACGCTTCGTTGAAAGACATCAAGGTCGATTAGACGATATTGCGATTTATGGGCAAGAATCTAA
TCCTACAACTTGGAAATTAGCTAAAATGAACTTAGCGATTCGTGGTATTGATAATGACTTAGGTGAACGAAAT
GCTGATACATTCCATAACGATTTACATAAAGGATTAAAAGCAGATTACATATTAGCCAATCCTCCTTTTAACGC
AAGTGATTGGGGTCAAGAACGATTACTTGATGATTACCGTTGGCAATTTGGTATTCCGCCAAAAGGTAATGC
CAACTATGCATGGATTGAACATATGATTTCAAAATTAGCACCTAATGGTACAGCAGGATTTGTATTAGCCAACG
GTTCAATGTCTACAAGTGGAAAAGATGAATTAGAAATCCGAAAAAATCTTATCGAACAAGATTTAGTGGAAT
GTATTGTTACCTTACCAGGTCAACTCTTTTATTCAACACAAATACCTGTATGTCTTTGGTTCATTAGTAATAATA
AAGGTCAAAATGGTAAAAAAGAACGTAAAAATGAAATTTTATTTATTGATGCTCGTGAAATCGGTCATATGGT
ATCTAGAACATTAAAAGAATTTTCAGATGAAGATATTCAAAAAGTAGCACAAACGTATCATGCGTGGAAAGG
TACAAACGACAAATCCTATGAAGATATAGCTGGTTTTTGTAAAGTAGCTAACCTTGAAGAAATTAAGAATAAT
GAATATATTTTAACGCCAGGACGTTATGTAGGTTTAACTGATGTTGAAGAAGATAAAGAACCGTTTGAGCAA
AAAATGGAACGCATTACTTCTGAATTAAGCGAACAATTTGCGAAATCAAAAGAACTTGAAAACCAAATCCGC

AAATCATTGGAGGGATTAGGCTATGGAGTTTAAAAAATATCTTTTATCTGAAATAAGCGATTATAAGACAGGT
CGACTTAATTCTAATGCTGCGATTGAAAATGGCAAATATAAATTTTTCACTTGTGCTCCTAATCCTTTAACAATT
AATTCTTATTCATATGATGATGATGTAATCTTACTTGCTGGCAATAATGCTAATGGAATTTTCCATATAAATAGAT
TCAATGGAAAATTTGACGCTTATCAAAGGACATATATTATAAAACCTAAATTTGACGTCATAGATATTGATTATT
TATATTATTATCTTAAATTTAAATTGAATTTTTTACGTTCGCAATCAATAGGTAGTGCTACTAAATATCTCACAAA
ACCTATACTAGATAATTTAGAAATTTATTCTCCTAAAAGCAAAAGAAAAGAAACTATTATAAGTTCTATTTTAAA
AATTTTAGATAAAAAAATAGAACTTAATAAAAGAATCATAGCAAACCTAGAGGAACTATCACAAACACTGTTC
AAACGTTGGTTTGTTGATTTTGAATTCCCAGATGAAAATGGAAATCCGTACAAATCAAGTGGTGGCGAGATG
ATTGATAGTGAACTAGGTGAGATACCTAGCGGTTGGAAAGTTGATGAATTAGGAAATTATATAAAAATAAAAA
GTGGTAAAAGACCTAAAAATAAAGTTGATAAAAAAGATATAGAAAATGTGGTGCCAATAATTGGTGCTAGTA
AAATAATGGGTTATACCAATGATTATTTATATAATGAAAAAATTATTATAATTGGAAGAGTAGGAACTCATGGT
GTAATCCAAAGATTTTCTACAAGAACATGGCCATCAGATAATACTTTTGTAATAATCAGTGATTTTGAAAGCAT
TATATATCAAGTACTTAAAAGTATAGACTATATATCATTGAATAGAGGTTCTACACAACCTCTATTATCTCAAAAA
GATATTAAGAACACTAAAGTAGCAATGCCAACTAATGAAACTTTGCTATCAACATATCAAAAGGAAAATAATC
ATATTTTAAAAATGATGGACCAAAAAAATATAGAAAACCAAAAACTAACACAATTAAGAGATACTTTACTACC
AAAATTAATGTCGGGGGAAATAGAGATACCTGATGATATCGAGGTGAATGAAGATGAGCTTTCAATTTAGTG
AAGATGATTTAGAACAAGTCGCATTAGAATGGCTACAATCACTGGGCTATGACTACAAAAAAGGTAATGAGA
TTAGTATGACAGGTCTAGCGCCAGAACGTAAAAGTGATAAAGATGTTGTCCTTCATGAACGATTAGAAAAAT
CATTAAGAAAGATTAATTCAGATATTCATCATCGTTTTATCGAAAAAGCCATACATGAACTAACTTTAGAGAAG
TCGCCCAATCTTTTAGAAAATAACCTTACCTTTCATGAAAATTTGATCAATGGTATTGAAATCGAAGACTATGA
TGACGAAGGACAATCGGTAGTAGAAATTGTTAAAATCGTTGATTTTGAACATCCACAAAACAATGATTTTTTA
GCAGTCAATCAATTCACTGTCATTAACGGAGACTATACAAAACGTCCCGATATTGTCCTCTTTATCAATGGCTT
GCCTATCGTTGTGATCGAACTTAAAAATTCAACCAATGAAACCGTAGGCGTCGAAGATGGTTATCATCAATTA
GAAACGTATAAGATGCGTATTCCACAATTATTTACATTTAATGAAGTGCTTGTTACAAGTGATGGTATTAATACT
AAAGCCAGTTCACTAACTGCGAACTATGATCGTTTTATGACTTGGCGTTCTAAAGATGGAGAAACAGAATCT
TCGTCAAGTTTAGCTAGCTTAGACGTACTGATTCATGGGATGTTAAATCCTAATACTCTACTTGATTTAATTCGA
TATTTTGTATTATTCCAAGATGATGGTAAAGGGCATATCAGTAAAATTCTAGCAGCATATCATCAATACTATGCT
GTTAATAAAGCTGTTGATAGAGCTTTATTAGCTTCATCTGGGCCGGGTGATGGTAAAGGCGGCGTTATTTGG
CATACCCAAGGGTCTGGTAAAAGTTTAACCATGGTCTTTTTCTCTGGAAAATTAATTCAAATGCTGAATAATCC
TACCTTAGTCGTTGTTACAGATCGTAATGATTTAGATAACCAACTATACAGTACATTTGTTAAATCAAAAGGAC
GTTCTGGTAAAGGATTATTAAGACAAACGCCAAAACAAGCTGAAACACGTAAAGAATTAAAATCGTTATTAT
CTGTTGAGTCGGGTGGTATTGTATTTACAACAATGCAAAAATTTGAACCTGAACAAAATGAAACGACCATGG
CTGCCCTAACAGAACGTAAAAATGTGATTGTTATGGCAGATGAGGCTCATCGTACACAATATGGCTTTAATGC
AAAGTATAATGATAAAGGTGAAGGCATTAAATATGGTTATGCTAAATATTTAAGAGATGCTTTACCGAATGCAA
CATTCGTCGGATTTACGGGCACACCCGTAGCTTCAACGGATAAAAATACACAAATGGTTTTCGGAAACTATAT
CGATGTTTATGACATGACACAAGCTGTAGCTGATGGAAGTACAGTTAAAATTTACTATGAAAGTCGTGTAATT
CCATTAAATCTACCCCAAAATTTAGATTTAGATGAAGCATATAATGACATTACAGAAGACCAAGAAGAGGATG
TAAAACAGCGTTTAAAATCAAAATGGTCACGAATTGAAGCCTTAGCAGGTGCAAAACCTCGTATCGAAGCCC
TAGCCAAAGATATCATCCAACACTTTGAAACACGTCAACAAGCGATGAAAGGCAAAGGAATGATTGTAACG
ATGAGTCGTCGAATTGCTGTTGATTTATATGATGAAATCATTCGTCTAAAACCAGAATGGCATTCAGATGATGA
TGATAAAGGGGTCATTAAAGTGGTAATGACGGGCTCCTCTAGTGACCCAGCTTCTTTCCAAAGACATATTGG
TCCTAAAAAACGTCGTAATTTATTAGAAAAACGTATGAAAGATGTGAATGATGAATTACAACTCGTGATTGTT
CGAGATATGTGGCTGACAGGGTTTGATGTTCCTTCTATGCATACGATGTATATTGATAAACCAATGAAAGGCC
ATAATTTGATGCAAGCAATTGCCCGAGTTAATCGAGTGTTTAAAGATAAACCGGGTGGATTGATTGTCGATTA

TGTGGGTATTGCCGAAAGTTTGAAAGAAGCACTTAAAGAGTATACAGAATCTGATCAAGCTCAAACAGCGAT
AGATACAGATAAAGCGGTTGAATTGATGTTATTGAAGTATGATGTAATTCAAGATATGTTATATAATCTTGATTA
TTCTAAATTTAATTCAAATAAAAAATTGGAACGTTATTATACAATATCGAATACGATGGATTATGTGATTGGTTT
AGGTGAAGATGAACGTCAACGTTTTATTAAAACGGTCACAGAGTTAGGTAAAGCCTTTGCTCTTTGTGCGAC
TGAACCAACAGCCCAAGAACTCAATGATGAAATTGCCTTCTTTAAAGCGGTTAAAGCAGGACTTGTTAAATT
GTTACAACCGCCAAAAGAAGGAAAAATACGTAAAACACCTGCAGAAGTTGAAGCAGAGATTAATCAACTTG
TGTCACAATCTGTTGTGACTGAAGATGTCATTGATGTTTATCAAACGCTAGGTCTTGAACAACCCGATTTATCA
ATCCTATCAGATGATTTCTTAAAAGACGTGGAAGGATTGAAACAAAAAAATGTTGCAGTAGAATTATTAAATC
GATTACTCAAAGGGCAAGTCAAATCATTAATGAAAACGAACGCGACAGTATCTAAGCGTTTCTCTGAAATGT
TAGGAAACTCGATTAATAAATATAATAGTCGTTCCATTGAAACATCTAAAGTGATTGAAGAACTTATTCAACTC
GCTAAAGATATCAAACAAGAACAACAACGAGGAAAAGAATTAGGCCTGAATTCAGATGAGATTGCGTTTTA
CGATGCTTTAGCTTCACATGAAACAGCAAAAGAAGCCATGGGAGATAAAGAACTACGTGCGATTGCTCATG
AGCTAACTAAAACAGTGAAAGAAAACATGGGTGTTGATTGGTCTAAACGTGATAGCGCAAAAGCTAAAATG
CGCGTAGCTGTTAGAAGATTGCTTAAAAAATATGGCTATCCACCAGATTTACAAAAAATGGCTGTAGAGCAA
GTCGTAGAACAAGCAGAGCTTATGGCGAGTAACCAATAAAAAAATAAACTAAAAGAGCATCCCTCACCGCA
AAGTGAAGGATGCTCTATTTTTATTGTAAGTAATATAATGTTCATTTTGATAGGTAATGATTAATAATTATCTGTA
AATCATTGTTAAGTACCGTTGTTATAGAGTCATCATTCATAAAATCATCATAGATTTTATCAAGTATTTCTTCATC
TTCAATCGCTGTGAAATGATGTACTAATCCTTTTACGATCGATGTATAATTTTGCGATTCTAAACAATTAGCAAC
CGTATGCCAATCTGATTGTTTAGTTTCATGGTTCATAGATAATCCTCCTTTATTTCAATGTCCATTTTTGACGCG
CTTTAGGATTAAGCGGATGCATAATATCATTCGTTGCTGGATTTATGAGCCTTTTTACTTTCTTTTTATGAGGTT
TTAACATTTCCATCACTTGCTCAACACGTTCGACAACAACGGGACGCTTTGCATAAACACCATAGGCTAAAAT
CACTGTGTCACTTTCACTTATCGCTTTCATTAAGTGTATATCTGTGTGCTCATCATAAGGTTCTTTAATATGTTTA
AGGTTCTCTGGTGTTTTAATATTAGAGAATAGATTTACAAGATATACAGAACCATATTGTTCAGAATTCGCTAA
TTGGTTAAGAATAAGAACAGTTGTGAGATCGAGTGATAATACACCATCTAAATGAGGGTACATCGTTATCACT
GTACAAGCAGGTTTCTTTTCATCCCATGTTTTCTTGAGTAAATAGCGGTGTTTTTCATCCTTGCTAAATATAGC
TTCTGTGTATATCGTATTTTTGATTGTATTCATATATATAATCACTTCCTTTAGTATTCTTCTGGTAAAAGCATCAC
ATAATAAAAAGCGTCTACGCCATCTTCACGAATGACGTAGACTTTCTTAGGTAATGCATTTTGATTTTTTTCAT
AGTTTGTATAGTGATATTCTAACTTGTATGCGGGCTGTTCTTGTTCATGTTTAATTGACAACATATTATCATCTTC
TTGTAGTTTGAAAATGTGTAGGTAATCGGTATGAGGCTGGTTATCTTTTTCTTCTACCATGTGCCAAAGTAAG
ATTTGAAGGTCTAGTGGAATACTTTCATTAATTCCTCTTGTGATGTATCGATTGATGTTCATACTATTTTCCTCCA
CTCTGTTTTTCTTTCATGATCTCAATGACTTCATTGATAACTGTGACAGATAGTTGAGCGACCTTGATCCAATT
CATAATTACTGGCCCCCTTTTTGTAACAAGTAGCGTTCATCAATAATAACTTGATGAGTGTCATTTAAAAATATA
AAGTCTAAATCAAAATTATCTAAATAACCTATTGCTACTAATTCCTTATCTAGATAACATAAAAAAGGGTAAATG
CTGATTTCATGTAATTCATCATTGTAATAGGTAAAAGTATTTAAAATTTTATGTTCAAGTGGACTATTTTTAATAT
ATTTTTCAGGTAATACATCTTCAGCCACCGCATCTATGGTTTCACAAGCCCAAGTTTCTTGATAATTTAATTTAA
ATCGTTCAGTGATATACGTTTTAAGTTGTTTGCTTAGCGTCATTATAATGACCTCCTTAATTAATAATGTGTACTC
CATAAATAAAATATATATTTATAAAATATTTCAAAACTCCTCCACTTTTGTCACTCTAAAAAAGTGGTAAAAGTG
ATAAATTTTCAATCCTATTTTTCTTTGTGACATTTATCACAAAGTTAAATTTAGGTATAAAAAAAGACCCCCAAT
CTTTTTTCAAAGATTGAGGGATAAGACTTTCGTCAATTTTATACCTTACGAGTTTCACTTGTTTATCTTTCGTAA
TTTCAATTGAATCAATTAACATTTTCATAAGTTGATTTTTAGCTTCGGGTGTAGCCTTTCTTAAGTACACACTG
AATCGTGTTATCATCAAAATGAGTCGTGCTTTTAACTTATCAAAATTGGGTATATCATCCTGTGATTGATTTGCT
TGTTTTGCTTCTTTAATTTGTTGGTCTAATACTTCTAACTGTTGATTCATTTCACTTTGTTTAGCTTTTAACATTT
CGCTATCCATTTCATCATCTAAGAACAATTCCAATAATCGTTCTTGTTGTGTTTTAACTTTTGCTTTTTGTTTTTC
TAGACTATTAATATCATATTTTATTTCTGTTTGTTTACTATGCTTTTGTTTCAGTTCTTCTATCACACTTGACGCAA

TCTGCTTAATAACATTAGGTTGTGAAAGAATACGATTCAAATGAACATTGATTACGCGTTCGACGACTTCAGC
ATTAATCGCATTACTATGACAGGCAGAACTTCCTGAGCGATTGAATAATGAACAAATGTAATAGCGTTTGATT
GTGCGTTCTTTGGACTTTTTCGTTTTATAATACGTCCGTCGACATACCATATTATTTCCGCATTCTGGGCATTTA
ATAAGGCCACGTAAGTAGAAATCTCCACCAATAGGTCGACCCGGTTTAAAACTTTTAGAAGCTAGTTTCTCAT
GAACTTGATCAAATACATCTTCACTTATAATGGCTTCATGCTTACCTTTGACCAATATCACATCATTTTTATCACT
TTTACCACTTCTTCGCTGAACAGCCCAATTTTGATGATTATTGAATCTGACATAGCCTTTATAGACTGGATTATT
CAAAATGTATTTCACGCCAAACACTGAAAATGGTTTTTGATTAATGGTACGATATCCTTTTTGATTTAGAATTG
TCGTGATTGCTTTAAATCCTTTATTGTGATTCAAATATAAATCAAAGATTTCCCGTACAATATGAGCTTCATTTTC
ATCAATAACTAAATCATTTTTCTGTGTCAATGGATTAAGTGATAATTTGTAGCCTAAAACACGACCGGTGATTG
CCTCTCCGCTCCTTGCCTTAGCATTCATAGAAAGTTTCACATTGGAAATCAAAGTCTGGCGTTCTATAGATCCT
ATTAGTCCAAACATTGTAACGAGTACTTCTCCAGAAGCATTGGATGTATCAATATTCTCAGAAATCGATTTATA
CCCTACGTCATGTTCTTTGAATTCATGAATAATATTAAACACGTCTCTCATTGAGCGAGAAATACGATTCAATTT
CCAACTTAATACCATTTCGATTTTATTATCCTTAACATCTTGTAATAGACGTTTCATTGCCGGACGACCTTCAAT
ATTTTTACCTGAAATGCCTGAGTCAGTATATGTCTCATAGTCATAACCTGGGAAATTTTTCATAACTTCTTTGAT
GAGTAGTTGTTCCTGCTCCTTTTCAGAATAACCATGTTCTGACTGCTCAGACGTACTAACACGTGAATAAAGT
GCAATTTTACCCTTCATATCATACCGCTCCTTTTATTAGATTATTGCTACAGACCATCGGTCTTGTAACAACATAT
AGATAATATATATTTATAATTTCGTTTCATTTTTATCACTTTTACCACTTAATCATATGAGTACTACAGGCAACTGC
CTGTAGTACGTAATTAGATTACTTACCTTTATTGATACGTTCTTCTATGATAGGCACAAGCACTTCTACAAATGC
TTCAATTTGTTCATTATTCATCGTCAAAGTCCTCCCAACCTAAGTTCAAATCATCTTCGTATAAGAATTCATCATC
TGTTACATTCTCATTTTTATCGAATGAAATCATGTCATCTTTTTTATCAGTTTCTTTCCATCCTAAATCGACCCAA
TACTTATAACCATTATTTAGTCTACTTTGTACATTAAAGTTAGCTTGAACCAAATCATTAATAACTTGAGAGCTT
ACTTTAATTTCATCTCTAATAACCTCATTTAGCCTAGACACTTTGATACGTGACCCTGGTTTGATTTCTATAAGT
TTTCTGAGTAAATTAATACACGCTTTCTGATGTGCAATATTTGCTGATTTACTAGAAACAAATTCATATCCAAAA
TTTAACCAATCATGAGAAATTTCAGTAACACGATCATTTTGGAGGTACTCGATATCATTTTTTACAATTTGCTTA
TACACATACATCAAGTAAAGAAAGATTTCTTTCTTTTCATCCTGTAACTTTTCAATTAAGTCAGGGTCAGCTTT
TTCAACATAATATTCACATGGAATAACAGCAATTCTACGCTCTAATGCTTTTGCATGTGATGATGGAAAATTGA
GTACAGCATTCGTATTAAAAATGAGTTTTAAATCTAAAGTAACTTCTACTTGCGTTCTTCCTTTACGGTCAGCA
TGAATTGGTTCAACAGATAGTAATTTTTTGAAATCATTCACAGTTGCTTCATCAATATTAGCTTTCCCCACTTCA
TCACTTGCCATAATTCGTTTACCAATTAGACTTTCCTTATCGAAACGCCCGCCAAGTTCATTAAGTGTTTTAGA
TACGATATCATTACTATAAAAGAATGACTTAACTAATTTAATATAGACCGATTTCCCATTTTTTGCAGTTCCATAC
AAAAAAAGTCCACGACGTAGTTTAGTATCTGCCGTCAATAACACTGCCATATGTTGGAAAAGGAAATATTGTA
AATCATGGTCATAATTCGCCAACTCTAACATATATTGTTTGAAATTCGGGGGTATCTTGTCCGTAATCGTGTCAT
TTATATCTAATTCCAAATCTAAGCGTGTTTTAGGGAATATTTCAAGGTCATTATCAACCACTTTAAAGGTCTTTA
GATTGAACATATTTCGTCCACAACCAATGTACTGTCTATGCTCAACAAGTTTTTCTATGTCCGTTGATTTCTGTA
TGCCATCTACTACGTTTTTCAAAGTACTTAGTGAAGGTTTATAATCAACTAGCAACACTTGTATCATTTTAGATA
TCAACCCTTGCAATTCATCTTCACTTACCTTTTGCCAACCTTTATCTTTGAAAACAAACAACTCTGAATCAAAA
CGCTTGATATGAAAAATACATATCATCATTTCCACTAATATTGTGTCATTTGGCTTTTTTTGAATACCAAAATAAC
TGTATATGTTAGGTCTATAGTCGACATCTTTAGGAATGTTAAGCTGTAGCTTATCGATAAGCTCGTTATACACCA
TTACAGCTTGTTCAGAATCATCCATTTCATCATTTCCAATACTACTTGTTAGGATGGCAATCAGACCTTTACGG
TCGATTGCCTCCATACCCACTTTTAATAATTTTATTAACATTTCTAAAATATGATTCATTACAATACCCCTCCAAAT
TCATCTAATGGTGCTACATCTTGAATATAATAATTTCCGTTATATTGCCCTACAAGGACCAAATGTTTTCGATCA
ATGTATGGATCATAATTAATGTGGTTTACGTTATAGTTACTGTGACGTTTAATCCAATCAGCAATTGATAATTGG
TGACTCATAGATTTTACGGTAATATTGCGATTTACATAGTGCGTTTCTTGGTTAAGTTCTACTTCATACGTAAAC
TGATGTTTAATATCACCTGAGAAGTTCTTCTCGTACACAGAATGTAACGTAGCAACGTATATAGATCCTTCTTTT

AACAATGTACTTTGGGGAATTTGAAGACTTTTTCCTCTATCATTATTCATTGTCTATACCTCCTAAAGCTTCCAC
TGTCACATGTGCAAATTCACCATTAAACAGTTGATTAGCTGTATCTTTCATGAGCTGTGCTACATTTTGTGCAA
TCCCCTTATCATTACATTCAATCATAATCGCATCGTGTATCGGCACTATAATCTTGAACGTTTTTTTTTCGCACTG
TGCTTTGTACACTTCCAACAAAATGTGCTTGAAAATATAGCTTGCTACCGTTTGTACATAAATTGCCATAATACT
ACTACCTTGAAATGCGCTCATATCATGTTCAATGCCGTAAGGCGTAGGCATTGTTTTATATTTTTCGACTTGCT
CCTTAAATTCAATGACCTTCTTGAATTTGCTCATTACTTGTAGCCAGTTAATTTCACTAACCTCTTGATTGATTT
TGAATTTAGAGCTACTATAACGACCGCCAAAAAGAAATGACCCTATAAATGCACGTTTCACACTCACGCGCTT
CTCTTTTGACAAAGATAAGTCTCTCAGTAATGCATCGTATAACCCTTCTTCATGATTCAAGTAGTCAATCAGTT
GTTCGTCTTGTGTCATATACGCAGCAACTGATGGTTCGAATGATTTAAAATCTATTGTATAAACCTTTTTAAATT
GCGAAGGTAAAATCATTTGTTGGACTAATTTAGGCAAGCCTTGAACATTAAATCTTTTCGTTGTGATCCGATG
TGTTCGTGTGCCAATCAACGATAATTGCGTATATAATCGATTCGTACCATTTAATGACTGATCTAATCTATCAAAT
AGCTGTTCGATTTTATCAAAATGTTCATCGTTCTTAGCCTTTTCAAGCATTGTCGCTTTGATATCAGGTAATTTA
TCGGTATCAAGGTTAATAGATTCTGATTCAATCTCTTTCTTTAATGGTTCTAAATTATGGTGTACTTCCACGTATT
GGTCTAGCTTAGCTTTTTCATTAGCCAAAAGCTCATTATATTGTTGATCTAGCTGTTGGCTAATTTGTCTTTGTA
GCTGCTGAACTTTATCTTGATTTGAAAATTTGTTCATTAAAAAGTGTTCTGTTTTATTGCTTTGAGGCAATTTT
GTAATTAAATCACGTATATTTGCTTGATACATGGTGATTTGCTCCTTCTATAAGTTATTACACAATAATCTCCCGG
GCTGATTATCATGTGAACATCACAACGTTGTACCTATACTATAAATGACTCAAATTAATTGCTTAATGGCTTCCA
TAACATTGATTCCTGTAATGGAAGCGTAAAATTACAATTAAAAAGCCAACTCATTCTTGAGTTGGCTCATTATA
GTTATTGTTTTTTTGTATTTGGACTAATATCATACAAATTGCTCTTAATTTTAACTGACAATAAATTATCGATAGA
AACATTTATTTTATTCTTTAATTTGGAGTCAATATCGCTTTTATCATCAAAAGCATGATATGTTTCTTCTTTATTTT
TTCTTCTCTCATCTTTATCATCAATAGCATCATCAGGATAATGTTGCTCTCTTCCGCTAGACTTACTTATTTCAAG
AGAAAATGGCGTTAATTTACTTTTTATTCTACTAAGTTCCTTTAAAAACGATCTATAATATCTTGCATTTTCATCA
TTCGAATTTATACCATTATTTAGTAAGTAAATACCTAATCTGTCTATTGAATTTTCAACATCATTGAAAAGTTGTT
TGAATAAATCTGAATCATCTTTTTCATACTTAAATTTGCTTGTTGCTACTAATTTTTCAATGGTACTACTTCTATC
ATATCTTTCTTTTTCAAACAATGTTTGCATATTATAATTAATACTTTCCTGACCTATTTCTTGATTCAAATAATTGA
TTAATAATTGATCCAAAAAGGGCATTTTTATTTTATATTTATCTACAAAATATAAAATGTAACCTCTATTTCCATTT
ACCCTTGCTTTTACTAAGTTATTACTAAACTCAAAGCAATGGTATAAATAATTAAAGTTTAGTTTCTGATTTCGA
TTTGCGTTATTAAATTGAATCTTATTATATTCATTTTGGATATCGTTAATAACTTTACCATTGAATTCAGTAATTTT
CTCGCTAATCTGCTCCCAATTCTTCACCCCACCGTCTGTCAAAAAATAATCTTTAAAATAATTGAAATGAACCT
TATCTTCATCAGCCTCATTGATTGTTTCTTCAATTGCATGAGACAACATTCTATATAATTTGTTACCCTCATAATA
ATTGCTCTCCAAAACTTTATGCATAAAACTTTCAAGTTGCAATTTTAATTTTTCACTTTCAGAGTACCATCTACA
TTGAGATTGATATGTAAAATCAGTCAATAATTTTTCCTTTAAAGAAGAATTTTCCATACTTATAATTTCTTTCATT
ATATCTTCAAAATACTCATAAAAACTATGAGAATCCCCACTTAATATTGCTTTTTCAATAGTTGGAATATGTTTAT
AATTATTATCTGACTTTGGTAATGGATGCTTATCAAGTATTTTCGATAACATATCTATAATTTGATATTGAAGTCT
ATTAAATTCATAGTTAGATCTTTGAGAAATGTATCTTTCACCAAATTGACCTCTTTCATATTCAGGATTATTAGA
ATTATAAAATGGCTGATATTTTGCAACATCTACACCATACATTTTGTTCAATAATTTTAATTTGTTCGTGAAAGT
TGTACCCTTGACACCTTGTTTCTCTTGTAAATGTCTTTTTTTATAAGTTATTTTAGGATTTTCTTTTACAAGATTG
AAATAACTAGATACTTGATACTTATATTCAGATTTTTCTTTATTGTAGAGATAAATATCAATATGAACCTTAAACA
CCTCTTGAAAAGCTTGCTTTTTATTTTTAGAATCATTCATGAGTTGCTTTAAAGCATTATACAATTGATTTTTAT
CCTCTTCTAATGTTTGATTTGAAAATTTGCATAAATACCATTCTTTAGTCACATCAATGTCATTTGTTCGCTCATT
TAAGTAACAATGTTCTTGAATGCCTCGTTGAATACTACTTAATGATTCACTTTCAAACTGTATTTTTATGCCTTG
ACTATCCAATATTACTTTACCAAAATTAATCATAAACTGCTCATATGTACTAATAAAATCTTGTACCTCTCCTGCA
TATTCTGGTTGTAACAACGGTGATAATGGAATTTTATCTTCCATAGGCAAAATTTTAAACTTTTTGGTACACTC
TTTTATATTATTTATAATTTGATCATCGATATGAACCGTATACTGCATATATGTAGTATTTTCAGTAGATTTTAGAG

CTGTAATATTTTTATTCACTTAATTCACCTTCATTGACCCATCTTTATTTAATTATAACTATTTATGTATGCTGTAGC
ACTACCACTACCCAATTTTAAATTTGGTAATGCTTTTTTGTATAAATTTAAATTGTTTAATGTTGTCATCTTTTTG
TTATTACAATTATGATTAAATTACTATTAATACATGTTTATTATTTTAGTTAATTCAACTTTAACAGCATTTTTCAA
ACTATTAGGGGTCTCTACTTTTACATTGACACCACCTGTGACAATTAACTTAGCTATAAAATCTATATCTTTCGC
GTTCACTTTTGATTTAAATAAATATGTCTCTTCGTCAATATTTTCAATCATATAGTCACTCCACAGGTAGCCTTTC
AAGATATTCAATCCCATAACATTGCTTCTAATAAGAATATCAATAGCTGTTTCATTTTTAACCATGAACTGTTGA
AAATTCTGCATAGTTATATCATTACACTCTTGAGTCTCTCCCATTTCATATAGTTCTTTTATTTTCGAAATATTTAA
TAGATTCACTAACTTTGATTCTATATTAGCTACAACAAAATACCATTGATGATTTTTATACATAATGCCAATACCT
TTATATTGTTCTTTAATATAGTTTTGTGAATAACAGGTTTCAAAAGACTTATTCTCAATTATCAATCTAATGATTT
CATTAAATAACTTATAACTTTTGAACTGATACTTATCTTCATTAAAATATTGAAATAGATCATTATATTGGTTTATT
AACTCTTTTTTATTCGAGTTATTATATAATTTTTTTAATATTTCTTGATATTCTGTTTGAAAAGGTAGATTACTCAC
ATTTGAAATACTCGAAAGTGTAAATACTAAAGCTTTCAACTCTTCATCTGAGATTGCTATTTTCGTTTGGTCTC
GATTACCTATGATCGTATAACCACCATTTTTCCCTGGATTACTATATAAAGGTACTCCTAATGCTTCAATTTCTTT
TATATCCCTTAAAGCGGTACTACGAGAAATATTAAATTCTGACATTAAATCATTTAATTTGAAGTTCCTATTTTC
ATTACAATATTCAATCATATTATATAATCTAACAGATTTTTTCATTATTATTCTCCTTTTAGTGTCAATAGATGACA
CTAATTAAGTATATACTCTATTTATAACAAATCTAAGGAGGAAAAAACATGAAATTTCAAGTAAGTCCATATTTT
TTAATCACTCAAAATGCAGAGGGTTTAAAAAAGTTGTATGAAGAGCTGTTTGGCGCTAAAACACTGTATATTC
AACGTATTAAAGATAGACCTATAGATAAGCAACTTAATATTGACGAAAATGATTTAAATAAAATTGATCAATGT
GTACTTCAATTTGGAGATATAAAAATTATGATCGCAGATGACACTGAAGGTTTGCCCATAACACAAGGTAATA
ATATATCTCTTTGTCTCACTTTTGACCAAGTAGAAGAAACTAAACATGTATATGACCAGTTAATAGAAAAAGGC
AGTGAGGTATTGAAAACATTTAGTCCAGAATTCTATACTGAAGGTTATGGTTATGTAAGAGATCCTTATGGTAT
CTCTTTGCATTTATTTACAAAGAGAAAAAATTAGGTTCTGCTGCAAAGTTGAAAAATAAATGTCTAATCTCAAT
TTTAAAATAATACTTTGTGTTAACTTGTTAGCATGATGCTAATTTCATGGCATGGCGAAAATCCGCAGATCTGA
AGAGACCTGCGGTTCTTTTTATATAGACCGTAAATACATTCAATACCTTTTAAAGTATTCTTTGCCGTATTGATA
CTTTGATATCTTGTCTTTCTTACTTTAATATGACGGTGATCTTGCTCAATGAGGTTATTCAGATATTTCGATGTAC
AATGACAGTCAGGTTTAAGTTTAAAAGCTTTAATGACTTTAGCCATTGCGACCTTCGTTGAAGGTGCCTAATC
TGTAATTACCTTTTGAGGTTTACCAAATTGTTTAATGAGACGTTTGATAAATACATATGCTGAATGATTATCTCG
TTGCTTACGCAACCAAATATCTAATGTATGTCCCTCTGTATCAATGGCACGATATAAATAGTTCCATTTTCCTTTT
ATTTTGATGTACGTCTCATCAATACGCCATTTGTAATAAGCTTTTTTATGCTTTTTCTTCCAAATTTGATATAAAA
TCGGGGCATATTCTTGAACTCAACGCTAGACCGTTAAATGATGAACGTTTACACCACGTTCCCTTAATATTTCA
GACATATCACGATAACTCAATGCATATCTTAGATAGTAGCCAACGGCTACAGTGATAACATCCTTGTTAAATTG
TTTATATCTGAAATAGTTCATACAGAAGACTCCTTTTTGTTAAAATTATACTATAAATTCAACTTTGCAACAGAA
CCGTATTATGGAATAGAGATGTTGGTAACATTTATACAGGATCATTATACTTAAGTTTAATTTCGTTATTACAGA
ACCACACATTCCAACCAGAAGAGAAAGTATGTCTATTTAGTTATGGTTCAGGAGCAGTAGGAGAAATCTTTA
GTGGTTCAATCGTTAAAGGATATGACAAAGCATTAGATAAAGAGAAACACTTAAATATGCTAGAATCTAGAG
AGCAATTATCAGTCGAAGAATACGAAACATTCTTTAACAGATTTGATAATCAAGAATTTGATTTCGAACGTGA
ATTGACACAAGATCCATATTCAAAAGTATACTTATACAGTATAGAAGACCATATCAGAACATATAAGATAGAGA
AATAAACTAGTGGCCGATTGTGCTTGATGAGCTTGGGACATAAATCCTAACTCGAAATAAATAAGCATATCAC
TAAACTGATTTTTTAAAGTTTACAGTGATATGCTTATTTTTTTATCTTACGATTTTGTACGTGCATGCTTGCCTA
GGGGTATGGCTCGAGCCATTAGTCTCTCGCACATACTATTCCCTCAGGCGTCAGCACTTACAAAATCGGTTGT
AATTTTCATTTTTATACGCATTCTTACTGAGATTATACTAATAAGAGGAATAGTAAAAGCAATTCTAAGTAAAAT
TGCAGATAAGAGGTTTGTTAAAAGCAGTTCTCAGTAAAATTACAGATAAGAGGTACGTTAAAAGCAGTTCTA
AGTAAAATTGCAGATAAGAGGTTTGTTAAAAGCAGTTCTAAGTAAAATTGCAGATAAGAGGTACGTTAAAAG
CAATTCCATGCAAAATTGCTGATAAGGGGTAAGTTAAAAGCAGTTCTCAGTAAAATTGCAGATAAGAGGTAC

GTTAAAAGCAGTTCTAGGCAAAATTGCAGATAAGAGGTGCGTTAAAAGCAGTTCTCAGTAAAATTGCTGATA
AGGGGTAAGTTAAAAGCAATCCTAAGTAAAATTGCAGATAAGAGGTAAGTTAAAAGCAATCCTAAGTAAAAT
TGCAGATAAGGGGTACAGAAAAACTAGACTTGATTACAAAATGGAGCTTGGGACATAAATGATTTTTTAAAA
ATGAGATGAGACGTAGATTAACTCCATAATCAATACGAATCTATCGACTTCTTTATTTATGATATTCATCTCTTTT
TAATGGAAATAAAAGTGCGATTAATGTGATAATACAGTTACGTTAATTAAAAAAATAAAAATGCAAGGAGAG
GTAATATGCTAACTGTATATGGACATAGAGGATTACCTAGTAAAGCTCCGGAAAATACAATTGCATCATTTAAA
GCTGCTTCAGAAGTAGAAGGTATAAACTGGTTGGAGTTAGATGTTGCAATTACAAAAGATGAACAACTGATT
ATCATTCATGATGATTATTTAGAACGGACTACAAATATGTCCGGGGAAATAACTGAATTGAATTATGATGAAAT
TAAAGATGCTTCTGCAGGATCTTGGTTTGGTGAAAAATTCAAAGATGAACATTTGCCAACTTTCGATGATGTA
GTAAAAATAGCAAATGAATATAATATGAATTTAAATGTAGAATTAAAAGGTATTACTGGACCGAATGGACTAG
CACTTTCTAAAAGTATGGTTAAGCAAGTGGAAGAACAATTAACAAACTTAAATCAGAATCAAGAAGTGCTCA
TTTCAAGCTTTAATGTTGTGCTTGTTAAACTTGCAGAAGAAATCATGCCACAATATAACAGAGCAGTTATATTC
CATACAACTTCGTTTCGTGAAGACTGGAGAACACTTTTAGATTACTGTAATGCTAAAATAGTAAACACTGAAG
ATGCCAAACTTACTAAAGCAAAAGTAAAAATGGTAAAAGAAGCGGGTTATGAATTGAACGTATGGACTGTAA
ACAAACCAGCACGTGCAAACCAACTTGCTAATTGGGGAGTTGATGGTATCTTTACAGACAATGCAGATAAAA
TGGTGCATTTGTCTCAATAGAAAGTTAGAGGTGAGTCTTACGTTTCAGTGACGGTAGACTTACCTTTAACATG
TTACATACTAAAAAATTAATTTGAATAAGAAAGAGAGACATATATGAAATACGATGATTTTATAGTAGGAGAAA
CATTCAAAACAAAAAGCCTTCATATTACAGAAGAAGAAATTATCCAATTTGCAACAACTTTTGATCCTCAATAT
ATGCATATAGATAAAGAAAAAGCAGAACAAAGTAGATTTAAAGGTATCATTGCATCTGGCATGCATACACTTT
CAATATCATTTAAATTATGGGTAGAAGAAGGTAAATACGGAGAAGAAGTTGTAGCAGGAACACAAATGAATA
ACGTTAAATTTATTAAACCTGTATACCCAGGTAATACATTGTACGTTATCGCTGAAATTACAAATAAGAAATCCA
TAAAAAAAGAAAATGGACTCGTTACAGTGTCACTTTCAACATACAATGAAAATGAAGAAATTGTATTTAAGG
GAGAAGTAACAGCACTTATTAATAATTCATAATAAAACAGTGAAGCAACCATCGTTACGGATTGCTTCACTGT
TTTGTTATTCATCTATATCGTATTTTTTATTACCGTTCTCATATAGCTCATCATACACTTTACCTGAGATTTTGGCA
TTGTAGCTAGCCATTCCTTTATCTTGTACATCTTTAACATTAATAGCCATCATCATGTTTGGATTATCTTTATCATA
TGATATAAACCACCCAATTTGTCTGCCAGTTTCTCCTTGTTTCATTTTGAGTTCTGCAGTACCGGATTTGCCAA
TTAAGTTTGCATAAGATCTATAAATATCTTCTTTATGTGTTTTATTTACGACTTGTTGCATACCATCAGTTAATAG
ATTGATATTTTCTTTGGAAATAATATTTTTCTTCCAAACTTTGTTTTTCGTGTCTTTTAATAAGTGAGGTGCGTT
AATATTGCCATTATTTTCTAATGCGCTATAGATTGAAAGGATCTGTACTGGGTTAATCAGTATTTCACCTTGTCC
GTAACCTGAATCAGCTAATAATATTTCATTATCTAAATTTTTGTTTGAAATTTGAGCATTATAAAATGGATAATCA
CTTGGTATATCTTCACCAACACCTAGTTTTTTCATGCCTTTTTCAAATTTCTTACTGCCTAATTCGAGTGCTACT
CTAGCAAAGAAAATGTTATCTGATGATTCTATTGCTTGTTTTAAGTCGATATTACCATTTACCACTTCATATCTTG
TAACGTTGTAACCACCCCAAGATTTATCTTTTTGCCAACCTTTACCATCGATTTTATAACTTGTTTTATCGTCTA
ATGTTTTGTTATTTAACCCAATCATTGCTGTTAATATTTTTTGAGTTGAACCTGGTGAAGTTGTAATCTGGAAC
TTGTTGAGCAGAGGTTCTTTTTTATCTTCGGTTAATTTATTATATTCTTCGTTACTCATGCCATACATAAATGGAT
AGACGTCATATGAAGGTGTGCTTACAAGTGCTAATAATTCACCTGTTTGAGGGTGGATAGCAGTACCTGAGC
CATAATCATTTTTCATGTTGTTATAAATACTCTTTTGAACTTTAGCATCAATAGTTAGTTGAATATCTTTGCCATC
TTTTTTCTTTTTCTCTATTAATGTATGTGCGATTGTATTGCTATTATCGTCAACGATTGTGACACGATAGCCATCT
TCATGTTGGAGCTTTTTATCGTAAAGTTTTTCGAGTCCCTTTTTACCAATAACTGCATCATCTTTATAGCCTTTA
TATTCTTTTTGTTTTAATTCTTCAGAGTTAATGGGACCAACATAACCTAATAGATGTGAAGTCGCTTTTCCTAG
AGGATAGTTACGACTTTCTGTTTCATTAGTTGTAAGATGAAATTTTTTTGCGAAATCTCTTAAATATTCATCCAT
TTTTTTAACGGTTTTAAGTGGAACGAAGGTATCATCTTGTACCCAATTTTGATCCATTTGTTGTTTGATATAGTC
TTCAGAAATACTTAGTTCTTTAGCGATTGCTTTATAATCTTTTTTAGATACATTCTTTGGAACGATGCCTATCTC
ATATGCTGTTCCTGTATTGGCCAATTCCACATTGTTTCGGTCTAAAATTTTACCACGTTCTGATTTTAAATTTTC

AATATGTATGCTTTGGTCTTTCTGCATTCCTGGAATAATGACGCTATGATCCCAATCTAACTTCCACATACCATC
TTCTTTAACAAAATTAAATTGAACGTTGCGATCAATGTTACCGTAGTTTGTTTTAATTTTATATTGAGCATCTAC
TCGTTTTTTATTTTTAGATACTTTTTTTATTTTACGATCCTGAATGTTTATATCTTTAACGCCTAAACTATTATATAT
TTTTATCGGACGTTCAGTCATTTCTACTTCACCATTATCGCTTTTAGAAATATAACTGCTATCTTTATAAACTTGT
TTGAAATTTTTATCTTCAATTGCATCAATAGTATTATTAATTTCTTTATCTTTTGAAGCATAAAAATATATACCAAA
CCCGACAACTACAACTATTAAAATAAGTGGAACAATTTTTATCTTTTTCATCAATATCCTCCTTATATAAGACTA
CATTTGTAATATATTACAAATGTAGTATTTATGTCAAAATAATGTTATAATTTTTGTGATATGGAGGTGTAGAAG
GTGTTATCATCTTTTTTAATGTTAAGTATAATCAGTTCATTGCTCACGATATGTGTAATTTTTTTAGTGAGAATGC
TCTATATAAAATATCCGGTTCTGTTGCAAAGTTGAATTTATAGTATAATTTTAACAAAAAGGAGTCTTCTGTATG
AACTATTTCAGATATAAACAATTTAACAAGGATGTTATCACTGTAGCCGTTGGCTACTATCTAAGATATGCATTG
AGTTATCGTGATATATCTGAAATATTAAGGGAACGTGGTGTAAACGTTCATCATTCAACGGTCTACCGTTGGAT
TCAAGAATATGCCACAATTTTATATCAAATTTGGAAGAAAAAGCATAAAAAAGCTAATTACAAATGGCGTATT
GATGAGACGTACATCAAAATAAAAGGAAAATGGAGCTATTTATATCGTGCCATTGATGCAGAGGGATATACAT
TAGATATTTGGTTGCGTAAGCAACGAGATAATCATTCAGCATATGCGTTTATCAAACGTCTCATTAAACAATTT
GGTAAACCTCAAAAGGTAATTACAGATCAGGCACCTTCAACGAAGGTAGCAATGGCTAAAGTAATTAAAGG
TTTTAAACTTAAACCTGACTGTCATTGTACATCGAAATATCTGAATAACCTCATTGAGCAAGATCACCGTCATAT
TAAAGTAAGAAAGACAAGGTATCAAAGTATCAATACAGCAAAGAATACTTTAAAAGGTATTAAATGTATTTAC
GCTCTATATAAAAAGAACCGCAGGTCTCTTCAGATCTACGGATTTTCGCCATGCCACGAAATTAGCATCATGC
TAGCAAGTTAAGCGAACACTGACATGATAAATTAGTGGTTAGCTATATTTTTTTACTTTGCAACAGAACCCATT
ACTTCGACGAATAATCTTACCTCCTCTTTTAGCTACTTCTTTTCCTTTAACTTGAACTTGTATTTTTCCAACGAA
ATTTTTTATTCAAATTTTCAGGATCATACACTTCCAATTCTCCATCGAAAGACATTTCTGTATAGTAAGAATTAA
TGTCTGGGTTTATTTTCAATGTATTAGTGGCCATAGATTCAATTTCTAAGGTAGCTTCCCTTTCAATAATTTTTG
AATTCAAAATTTTAATATAAATCCCTTCCTTTTATATTATACACAATCTATTTTTTAGTTTTATTTGTGATACGCTT
CTCCTCGCATAATCTTAAATGCTCTATACACTTGTTCAATTAACACAACCCGCATCATTTGATGTGGGAATGTCA
TTTTGCTGAATGATAGTGCGTAGTTACTGCGTTGTAAGACGTCCTTGTGCAGGCCGTTTGATCCGCCAATAAC
GAATACAAAGTCGCTTTGCCCTTGGGTCATGCGTTGGTTCAATTCTTGGGCCAATCCTTCGGAAGATAGCATC
TTTCCTTGTATTTCTAATGTAATGACTGTGGATTGTGGTTTGATTTTGGCTAGTATTCGTTGGCCTTCTTTTTCT
TTTACTTGCTCGATTTCTTTGTCGCTCATATTTTCTGGTGCTTTTTCGTCTGGAACTTCTATGATGTCTATCTTGG
TGTATGGGCCTAAACGTTTTTCATATTCTGCTATGGCTTGCTTCCAGTATTTCTCTTTTAGTTTCCCTACAGCTA
AAATGGTGATTTTCATGTCGTTTGGTCCTCCAAATTGTTATCAACTTTCCAGTTATCCACAAGTTATTAACTTGT
TCACACTGTTCCCTCTTATTATACCAATATTTTTTGCAGTTTTTGATATTTTCCTGACATTTATGCCCGATTTTTA
GTGTATAACCTGTGTATTTGTGGATAACTCTGTGGGCATTGTGGGTATTTCTCTTTAAATTGTACACAATCCTG
GATAACTATCCCCATTTCTGTGGATAAACACCTTGATGTCTTATTACTTATCCCCGTTTTTATATCCCATATTTATC
AACGGTTATCGCTTTTTTTCATAAAAAAACACTACCGTACCTCTTATTTAAGAAATACAGTAGTATTGTTTTCA
ATATATTTTAGCTAGCTTTTCTACGTCGTACAGTCGCAATTAAACCTATACCTGCTACTAATACAAACATCGCTG
CTGGGCTTGAACTTTGATCAGTTCCAGTTTTTGGTAACTGTTTCTCATGTGCGCTACCTTTAGGCGCTGACTT
GTTAGCCAATTGTTTCCCACTCTTACTTGATACAGCAGTTCCTTCCAATGCGCGATCAGAACCTTCTCTACCAC
TACTAACAGTTCCTCTATACGCTGGTAACAACACAACTTTACCTGAAGCTGTTTGTTTCGCTGGATCCATCACT
TTGTCGTCACCAATTGGTTGTGCATCTTTACCAGACTCACTACCTTTGCTACCTTGTTGTCCTTTAGCTGGTTG
TTCACTTACTGCTGGTTTACCTAATAACATACGTTGTGGTTCTGTCGTATCATACTTAGCTAAGTTAGCTGTTTT
TAAATAACTTGCTAGTACTTGATCTAATGAAATACCTTCTTCTCTAGGGCCACCGAACATACTATATCCGTCGCC
ACCTGATGCTGTGAAGTCATTCATCGTTACGTGATATACACGTTTTAAATCAATATTTTCAAACTTACCTGTCTC
TTTATTTAAAATTTGAATAGCGTTAATTCGTTTGCCAGATGGTTTATTCATATCATAGTAAACACGGATTGAATC
AGAGATATGTAGTAAACCACCATTCGCTGTTAATACTGTCTTACCATCTTTTTGTGTTGTTGGTGCGCCTAAAC

TATGTTCAAAAGCTGTCCAGACGTCTGAACCTTTTACATCAATTTGCGCAATCGTATTTCCAAATGGTAATACT
GAGATTAAATCATAGCGTGTCACCTTACCTTTTGCGATAGAGGCACGAATACCTCCACCATTTGTCACGGCAA
AGTCAGTCTTTTTAGAGAAATTCTTAACGCCATACGCTTCCATAGCATCTGCAATCGCGTTTCCTAAATTTGTT
TCACGCGTTCTAACGTCATCTCTTTCTCCTTTGAAATCAATGGTATTGTTTGGAATAATTACCTCTGCAGTTTGT
GCTCTAAATGTTTGATCAGCTTGATTAATTTGTTCAGCTAATGCTTTGTTCGGTGTCACATTTTCAACGTCTTTA
ACATTAATCAATGACGGCTTAATATTCGATACCTCTCCATTGCGGTAATTAAATGTAATCTTACCGATATTCGCA
AGTGCTGTACCTGTTTGTGCTAATGCATCATTGTTATAAATTTGACCATTTTGAAGTACGGTATGTGAATGACC
ATCAATAACTGTAATACGTTTCTTCAATTGTGGATTTTGACTTAATTGTTTCACTAAGTAATCACCACGCCATGT
TTCTTGCGTTGAAGGATCAATCCCTAAATGTGATATAACAACAAATGTATCTACGTCTTTATAAATACGCATCAT
TTCTGCTGTCACACTTTGTAATGGATCTCTAAATTCAACACCTTTAATGCCTTCAGGTCTTGTTTTCGTCTTTGT
TTCTGGTGTCGTTACGCCAATAATTCCAAAACGAATACCATTTTTTGTTACAATCGTTGATGGTTTAAACGCGC
GTTTCCCATCTTTATAAACGTTAGTGCTTAGCATCGGGAAGTCTAACATACCCTCTAACTTTTTCAACTGGTCG
TATCCAAAGTCAAATTCGTGGTTACCGACTGCCATAGCATCATAACCTACTGCATTCATTGCTTTAGCCATTTCT
TCACCTTTAGACTGGTTTGAAAGTGGTAAACCTTGGAAGGCGTCTCCTGCATCTAACATTAAATCAGGCTTTT
CTTGTTCTTTTACTGTTTTTAATTTAGCCATACCGATGACACGCCCTTTTTCTTCGGCTAGTCGGCCATGGATAT
CATTTGTATGTAATATTTTATGTGTTGTTGTTTGTGGTACATTGGCAGCAAACATTCGTGGTGAAAGTGATGCT
GTTTTAGCATTTGATAATGTGAATGTTGCTGTGTGAGTTGGTTTTTGTGTTGAGGCTTGTTGTGTATCTACATC
GCGCGTTTCGTTTACTGTTGTTGAAACTGCTGTAGATGGTTTATTAGATGGTTCGTTGGATGTTACTTTGTTGT
TTGTATCATCAGCTGTTCCCTGCGCTGATGCTGATACGTTGGTTGCCGCTTCTTCACCATAATAATGAGCCGCT
TCCTTTGTTGGCGTTACTAGTTGCTTATCTGTTGCTGCTTGGTCTATCGTTGTTACTGCATGTGCTTTCATTGGT
GTATGCTGCTCAGCCGCGTTCGAGACATGGTATAATCCCATTACACTAAAAAGCAAAACGAGCCATACACTTG
TTTTAAGTAATAAAGCTTTCATTTTGTTCCCTCCATTAACTGTCATACACCAATATATTATCATAATCCTTATATTA
AAATGATTGATTTTATGTAAATTATTTATAAATTACATTTAATATCGAATTATTTTCCACATTTCAACTAGACCCCT
TAATCTCAACAACAGCCATTTTCCACCAACAACATAAAAAAGCACCCGATAAAGTCGTCTCACAGCAGTGCC
GAACTTTATAGGGTACTCTCATTTATATTGTATATATTGGCGTTGGAATAGCTTTATCCGTATCACATAGCAATAC
TTCTTTTTCCGTATCAATATCGTGTTCGTTCAATACTTGACCAACACTCATACGCGCCAAATCTTTCATATTATTA
TCTTGTGATAAATGTGATAAGTAAATACGTTTCGTGTTACCTGTAATCACGTCTGTCATTGCATGACCCGCATCC
TCATTAGATACATGACCCATATCGCCTAAAATACGTTGTTTCGTCTTCCATGGATAACGACACATTCTCAACATA
TCGACGTCATGATTACTCTCAAAAATAAACGCATCGCTGCCACGTATCATACCTTTCATACGATCAGACACGTA
ACCCGTATCCGTTAAAATCGTAAACTTCTTATAGTTATTATGGAAAATATAAAATTGCGGATCTATTGCATCATG
TGACACGTTAAACGATTCAACATCGAAACCTGCAATAGATTTCGTTTCATAAGGATTAAAAATGAATTTCTGAT
CCATAGGGATGCGACTATCTTTCTTTTCAATTGCCTGCCAAGTCTTTTCATTCGCATAAATTGGCAATTGATATT
TACGCGCCAAAACACCTAATCCTTTAATATGATCAATATGTTCATGGGTTACTAAAATACCATTTAAATCTTGAA
TATTACGGTCAATTTGACTAAACAATTCTTCCATTTTCTTGCCAGTCAAACCAACATCAACTAATAGACTACCTT
TTTCATTTTCTACAAAAGTGGCGTTACCTGTACTACCACTTGCTAATACACTCATGCGTATCAAGCGGCTCATC
CTTTCTATTTAAGCAGTCAATCTCTCTATATGACTACAATATACATCAGTACAAATATACGATTTTCCCTACAGAA
ACTATCAGTATATTAGCTGTTTTGTATACCTTTCCTATTATGTAACTTTAGTTCATCGACCGGTCTCTTATAATTAA
ATTTTGATAATCACGTGCAATGAATTTCTTAATTATTATAACAATGATTAGACAAATACTAAAGTTTTTAAAATA
GCAGTTACGTTAATTTTACAATGCGGTTCTACTTAAAATATGAATAAATGCGCACGTTAGAGACGCAGAAATG
TCGCCTGTCAGCCATAATAAACCGTACCAGCAAAAAGCTAGCACCGCTTCTCACATATGATGAGCTTGCAATG
CTAGCTTATTACGATTCATATTAATGATTAATAATTTTAGGGTTATTATTTGTCGCTTCGACATAGTAAGTATTCG
TTTTGTCCTTACCGTCATGCTTCACTTTAATTTCCCAGTTTGGTTGTAACAATTGAACATTCGTTTCATTCACGA
CTGAGTAGTAACCGAGTCTAGCATTAATGACTTCATCACCTTTTTTCAAGTAACGATTATAATATAATGCCTCGA
TTGCTTTTCTCACACTAATAACTTGATGCTTCTTATCTGCACCATCAGTCGGCTTAATGTCATCCATCATTGACT

GTTTATAACTAGTTGCTTTGTTATCTTCTATTTAAAGTTCAACATCGCTTTGCTGTTATTTAAAATTGGAAAAT
CATCATACGTTTGTTCATATTTTACAGAATCTGAACTAATCTCGCTTAATTGATATTCAGCACCTTTAAATACGC
GTTTGTTAACGTAATCTTTTAAATCTTTTAAGTTATTATCACTAACTTTTACCGATTGGCTGATGTTCGCATTTAA
TAATTTTCCACTATCTGATGTGGATAAATCAGAATCACCTTTAGCTTTTGAACTAAAGTCTTTTGATCGTCCAG
TAATTTGTTCTAATTGTATGCCTTTAACAGATTTATTTAAAATACTGGCTGGCACCCTAATTTCTTCTTGTTGAA
AATTCACTTCATTGTTACTCTCGACTTCATTAATGTGTGAGCGATTGACTTTATTAACATAAATCGATACTAACA
CGATGTTGACAAGAATAAACACGAAAATGAAAAGTGTCTTTGTCAGTTTCCAGTTCATTTATTCAAGCCTCCC
ATCGTTATAAACATACCATTCGCCATCATATTCTACATACCAACGCGGTACGAGTTCACTGTTAATCTGCACTTC
AATGTGATTATGATCTGAGTTATCCTGCATTTCGTAACCGATAGCGATGTTTGTTACTTTTTCAAAATTAATATC
ACTATTGTTCGCTAAGCTTGAACGTACAGACTCTAATTTCGGCAACGTTTTATTATCCTCACTATTTAACACAA
CGTCGGTGCGCAATAACGAACGACGATAGTCAAAGACGCCTTTTTCACCCCAAGTGACTTGAATTTGATTAG
AGCCTTCTTTATTAAACGTTGGATAACCGTTAAGGAAACGTTGATATGTTAACTCGCCTGACTGATTATTCGTA
CTAAACAATCTGAAGTCTTCGTTTAAGAAACCACCATGACCATTAATAAAATCAAAGGTTCCTGGAATCGTTT
CTTCCATTTTGCTGGAACTTGCTTCATCTTCGGACAGGTTTTTATAATGGTATTTTTCATTTTTATCGTTATAGTT
TGCGACACCTGTATTATTGTTATAAGTAGTAACACCACTCTTTGAACTACGAACGATGGTTGAATCGTCAAATA
GTATAGCATTCATTTTCTCAACACTAATCGTGTTAAATACCATGCGATATGTTTTTAACTTTTCAGGTTTACTTG
GTGCAAAAACATGCGTCGTACGATCAATTGTATCTTTGTTTGTGATGATATCTGTGTATGGTTGCATATCTTTTT
TCACTGCTGCTAATGCATCTAAAAAATGATCATTTTTCGTTGTAGTTGTTAATTTTACGTAATCGTGGCGATCTT
TGCTTATAGCATAAAGCACGATATTATCATCAGCATCATGATCTATGACCAAACGATTGAAATTGAAATGATTT
GGTACTTTCGCATTCATGTTCAGTACTTGACCAAGATATGTTGATAACGGTAAATCATACGTAAAATCGAATAA
TGTAAAATCACTGCTCAAATCAGGAATCATCAAGTTATGATCACGACGAACATGTTCCACGGACTTCACTTCT
TTATTTTTTAAAGGTTTCGTCAGCTTATTCACATTAGAAACCGTCGCGATCGTACCTTCTGGATGATCATTTTTC
GAATGAATAATCTGAAATGGCGTAATAGTTGTATCCATTTTGGCTGTCATAGGTGTCGTTAAAGGTTTCGTTTC
ACTCTTCTTACTATCTGTATTGTCGACATTTGCAATATCAGGAGAAAAGTTCCATACCATATATGTCAATACGAC
ACTCATCAAGACAAGTAGCGCTAAAATGACAGATTTAATATGCTCCTTATTATTCATCCCAATCACCGTCTTCA
ATGACTTCACATGGAAGTGTGATAAAGATAGATGTACCTTGACCTTCTACACTGTTTGCCCAAATACGACCATT
GTGCGCTTCCACAATCTCTTTCGAAATGGCTAGTCCTAATCCAGTACCACCCATTTTACGCGTACGCGCCTTAT
CTACACGATAGAATCGGTCGAATATCTTATCGACTTTATTGATAGGAATACCGATGCCATTATCTTTAATACGAA
TCGTCATTCGATTATAAAGTGGATTTTGTTTCACGTGGAACTCGACACGTTTATCGCCTCTAGAATATTTCATC
GCATTTGTAATGACATTATCAAATACTTGCGTCATTTTATCAGGATCAAATTCTGTGAAAATCGTCTTTTTCGGA
ATATCTCGAATAAATGTTGTATCTTTCGCAGACATTTCATGTCGATTAATAATTTTATTAATGAACATGTTAAAGT
CGATAATTTCTTTGTTGATTTGATCAGACTCATTATCCATTTTAGATAACTGTAGCAAGTCATTGACCAGTCGA
ATCATTCGTTCTGTTTCTTCACGGGTAACAGATAAAAATTGTGGCGCAAGTTCCTCATCTTTCCATGCACCTTC
TTCAAGTGCTTCAATGTAACTATTCATAGAAGTTAAAGGTGTACGTAACTCATGTGATACATTGGCAACAAATT
CACGACGTTCACGTTCAACTTGTTGTTGTTCAGTAACGTCATGTAACACAGCGATATAACCAGTTACAAATCC
TGTTTCCTGCACAATCGTACTAAAGTTAACACGTGCGATTAGACCTTCTTCTTCATTTAAATCTAATAAGAAAC
TATCATTATTCTCTTGAATTTCTTCAAGTTTAAATTCATCTTCAAGACTTAATACACTTAACATGTAATATCCAAT
GATGTCTTCTTTCGCCATACCAAGCATCTTAAGTGCCATATCATTGACGATACGAATACGTCCACGGCGGTCTG
TTGCAATAATACCATCACTCATATGGGTGATAACTGAGTCCAGTCTACGTTTCTCACTTTCAGTATTAGCCTGC
GCTTCTTGTACACGTTTAGACAAGTTATTAAATGCTAAAGCTAATTCGCCAATTTCATCATTACCATAAATCTTC
ACACGTTGCGTATAGTTACCTCTGGACATTTCAACCGTCTGGTTACGCATATCGGTGATTGGTTTGGTAATCGT
TCGCGCTATAAAGAATCCTAGGATGACTGTGATTAATAATGAAATAGCTGTACCAACAATGAATATTTGATTTA
TATTATTTAATTGGTTATAAACGTCATTAATTTTTGATTCGATATAAATATTACCAATTACCTTTTTATCGACTTTA
ACTGGGATATTATATACCCAGACACGGTCCTTACCACCGCCATAATCTTTTAAAATTAAATGATCGTTTGATTGT

CCTAGTGATAGTGCTTTTTGGACAGAACTATCATTCGCTTTTTGATTGATTAGACTCCGGTTAGACTGCTTCGT
CGTCGCAATAATAATTTGGTCTTTATCTATAAAACGAATTTCTCCAATTTCTTGACGGTTGGCATACTCACTTAA
TAAATTTTGAATATCTTTTTGTGCATTTACGGAGCCCTTTTCGTCATATACTTTTTCAATACTAATTTCTAATTGT
TTCGCGTACTGCGTAATATTCTTCTTAAAATTATCAAGCAGCTCTTTTTCAAGGTTATTTGTAAAATACAGCCCG
ATAATTTGCATACCAATGATAATCAGTAATACATAAACAATTACAAGTTTAGTATGAAGGGATTGTAGTTGTTTT
AGCCACTTCATTCGTTTCGACCTCTACTCATGTTGTTGGAGGAAATATCCAACGCCTCTACGCGTCACAATATA
TTCAGGGTGTGACGGATCATCTTCAATCTTTTCACGTAAACGACGAATCGTTACATCGACCGTACGTACATCG
CCAAAGTAATCATAGCCCCATACTGTTTGTAATAAATGTTCACGTGTCATTACTTGTCCCATATGTTTTGATAAA
TAATGGAACAATTCAAATTCACGATGTGTTAATTCAATATCTTCGCCACGTTTTTTAATAGAATATGCGTCTGG
ATAAATCACAATATCTTTAATTGTGATTTCATTCGTTACATTTCCAGTGTCTTGTGCTGGTTGTGAGTAATGACG
ACGTAAGTTCGCTTTCACACGTGCGATTAATTCACGCGTACTAAACGGTTTCGTTACATAGTCATCTGCACCTA
GTTCTAAACCAAGCACTTTATCAATTTCTGAATCTTTAGCAGTAAGCATTATAATTGGCATTTCGTATTTTTTGC
GCACTTCACGACATACTTCCATACCATCACGACCAGGTAACATGATATCTAATAATACGATGTCTGGTTCTTCTT
CATAAATTAAGTCGACTGCATCATTACCATCGTATGCACAGTACACATCGTATCCTTCTTTTTTTAAGTTAAATT
CTAAAATATCAGCAATCGGTTTTTCATCATCAACTACAACAACTTTTCTAGCCATTTGCATAAACCTCTTTTCTT
AAATCATTAATATTATTTTATCATTTTTTGTTCTTTATCGTTTATGTTTGTGTAAAAAATCACAGGATGTCGCTTA
CTATTTTTGTTATTGTGCGCATTTTCCGTCTTATAACGTATAGTAACCTATTATATAATTTACCATTTATAAGCTATT
AATTCTATTTTCCAATATAAACTTATATATTAATATTTTTTATTAGTTTCTCACGGCGGATTGTGACAATGATATTT
AACACAATGATGAGGTCTCTATTCCAATAATAAAATTAGGGTAATGGCAACTTCTAGTTGCACGTCATTGCTCA
GAAAATAAATATCATTATTCGTTACTTTAACAATTGATGTTATGGAGGATTGGTGGCATATCACAACGAAGAAA
TATGTTGACAGTAGCATATGTTTGTACGTCTAATAAAGATTTTATTGTCTACGTTTATCTTTCATATCCAACCACT
AAGTTACTTGAGGTTCATACATTATAAAGAAAATGGACGAACAGATAAATCAATATGATTATCATTCGTCCATA
TATCTTTTATTAATATAATATTAAACGCAAAAAAATGCCTATCCTCTTGGATAGGCATTTGTACGGTCTCGACGG
GAATCGAACCCGCGATCTCCTGCGTGACAGGCAGGCGTGTTAACCGCTACACTACGAGACCTAAAAAATGG
TGACTCCTACGGGACTCGAACCCGTGTTACCGCCGTGAAAGGGCGGTGTCTTAACCGCTTGACCAAGGAGC
CGTTCTTAAGCACAAGATAGATTATAGCACAGCCAATCTTGCCATGACAAGATATTTTTTACATAATTTTGCTAT
TATTTTTTGAAGAATGTTATAAGTGAAAAATTGCATTTTATTGGCAGTCTATTTATTATAATAATTACGCTATTTT
TGAAATTTTTCTATTTTAGGTACTTTCATTTACACTTACGTCTACTCTATTTTAATAGCTATTTTCGACATCAGAC
TTATTACATTCAAGTATTTCTTCTTAATTTCGTTAAATATCATCTAATCTTTTAAATTATGACTAGCAAATATCACC
TTTACGTTAGTAATTTTTATCTAAATAATCGAATCGATACAGAAGCAAACAAAGCAAAAGCCTCCTACCAATAA
AGATAGAAGGCTCATGTATTTATAATCATTGCGTATGACTTATATAAAGTTCTACCACAATTCTTTTAATAGGTTT
GTTTGTTCTCTATCTGGACCAACTGAGAAGATAGAAATTTGTACATTACATAATTCTGAAATACGCTCTAAATAT
TTACGTGCATTTTCAGGTAATTCTTCTAAAGTACGCACACTTGTTACGTCTTCTGTCCAACCTGGTAACTCTTC
AAAGATTGGTTTACAACGTTTTAATTGATCTAAGTTTGCTGGGTACTCAGTAATTTCTTTACCGTCTAATTCATA
AGCTGTACAGATTTTCACTGTGTCTAGGCCTGTTAAAACATCGATTGAGTTAATAGATAAATCTGTAATACCAC
TTACACGACGAGAGTGACGTAATACAACTGAATCAAACCAACCTACACGACGTGGACGTCCTGTTGTTGTAC
CGTATTCACGACCAACCTCTCTAATATGATGTCCATCTTCATCGAATAATTCAGTAGGGAATGGACCATCACCA
ACACGTGATGTATAAGCTTTACATACACCAATTACCTTTGAAACGAATGTAGGACCTACACCTGTACCAACAG
TAACGTTACCTGCAATTGGATTACTTGATGTAACGAATGGGTATGTACCATGGTCGATATCTAACATTACACCTT
GCGCACCTTCGAAAAGTACCTTTTCATCTGCTACAAATGCATCGTCTAAGATTTTTGATGTGTCTGTTACAAAT
TCTTTTAAACGTTGACCTGCAGCATAGTATTCTTCAAAGATATCATCAAATGATGGACATGTTTCGTTAAACAT
ACCTTTGAAATATGCTTGTTTATATTCAATGTTTGATTTTAATAATCTTTCGAATGTTTCTTTTTCAAGTAAATCT
GCCATACGAATACCGATACGTTGAACTTTGTCTACATATGCTGGACCGATACCTTTTTTAGTTGTACCAATCTTA
TTGTCACCGCGTAAACGTTCTTCATATTCATCTTGTGCTAAGTGATATGGTAAAATCACTTGTGCACGATTAGA

TATACGTAAATTACTTGTAGGAATGCCACGTTCATTTAATCCGTCTAATTCTTTCAATAGTGCAACTGGATCAAC
AACGACACCGTTACCGATTACCGCTAATTTGTCTTTGTAAAAGATACCAGATGGTACTAAATGTAATTTATATG
TTTCTCCGCCAAATTGAATGGTATGGCCTGCATTATTACCACCTGAAAAACGCGCGATAACATCTGACTGTTCT
GCCAAGAAATCCGTTATTTTTCCTTTTCCTTCGTCTCCCCATTGTGTCCCAACTACTACGATTGATGACATGTG
AGCACCTCCAAGTTTTTCTCATTTAACCATTTGATATTCTACCAATATCGAGACCATAATGCAAATAAAAATCGA
ACATTGATTTTCGACCTTTTTAAAAGAAAAATCATTTTAACAAATCGTCTTCAACGTCCTAAAAACCCTTTAAC
AGAGGCGTTGACAGTACTAATTTATCACTTTCAATACACAAACTTAACATATATCAACCCGTTCAGAATAAAAT
AAAAACCGTACAATTTTATCATCATAATGATTATTGTACGGAAAAACTTTTTTTACATCATATCTGCATGTGCAT
AATCGATATCGGTAAATTTATTATATTGTTTCATAAAGTGTAACTTAACTGTGCCTGTTGGACCGTTACGTTGCT
TAGCAATGATAATTTCAATTTCACCGTTTTCATCATTCGTTTGTGGCTCGAAACCACCATCATCGTCATCATCTT
CATCGCCACCACGGTTATAGTAATCATCACGGTATAAGAACGCAACGATATCAGCATCTTGCTCAATCGAACC
AGATTCACGAATATCACTCATCATTGGACGTTTATCTTGTCGTTGTTCAACACCACGAGATAACTGACTTAATG
CGATAACTGGACATTCTAATTCACGGGCTAATGCTTTTAATGTACGAGAGATTTCAGAAACTTCCTGTTGTCT
GTTATCGGACGCACGTGAACCACTACCTTGAATCAACTGTAAGTAGTCAATCACAATCATGTCTAAGCCATGT
TCTTGCTTTAATCGACGACATTTAGAACGTAAATCATTAATTCGAATACCCGGTGTATCATCAATAAAAATCTTC
GTACGTGATAATTTACCTACCGCTATAGTAAAACGACTCCAATCTTCCTCAGTCATAGTACCCGTTCTTAAGCG
GTTTGAGTCAACATTACCAGAACTACAAATCATACGTGTGGCTAACTGATCAGCACCCATCTCTAGTGAGAAA
ATACCAACTGTATACATATCTTCATGCGTTGCAACTTTTTGTGCAATATTAAGTGCGAACGCAGTCTTACCTACA
GATGGACGCGCTGCAAGGATAATTAAATCATTTCGGTTGAACCCTGCTGTCATTTGGTCTAAATCTCGATACC
CTGTAGGTATACCTGGTGTTTGACCACTATTTTGATCAAGCTCTTCAGCTGTTTCATACACTTGTCCTAAGACG
TCTCGAATGTCTTTAAAGCCATCGCTTTCACGAGAAGATGACAGCTCTAAAATTCGACGTTCTGCATCACTTA
AAATCGCATCTAGTTCAAGTTCATCATTATATCCATCATTGGCAATACTATCTGCAGTTTGAATCAATCTACGTTT
TAATGCATGCTTAGAAACGATATCAGTATAATACTGAACATTTCGCGTCGTTGGTACATTTGTAGATAACTCTG
CAAGATATTGCGGGCCACCCGCTTCATTCAACGTACCTTCCGTCGATAATTGATCCATCAATGTTACAACATCA
ATTTCTTTATTATCTTCATTTAAGTGCATCATTGCACGGAAAATATGTTGATGGGCACCCCTATAAAACGACTCA
GGAAGCAAAACTTCCTGAGTAGTATTAATCAATTCTGGATCTATAATAATTGAACCTAAGACAGACTGTTCAG
CTTCATTGTTATGTGGCATTTGATTTTGCTCATACATTCTATCCATGAATGGTTACACCTCTTATTTCAATCCAAC
TTTATTGTTCAACTGTGTGTACGCGAATTGTACCTTCAACTTCTTTATCTAATTTAACAGGTACATTCGTATATCC
TAGGGAATGAATTCCATTTGGTAAATCCATTTTACGTTTATCAATTTTAATATCATGTTGTGCTTTTAGTGCTTC
GGCAATTTGTTTTGTACTTACTGATCCAAACAATTTACCACCTTCACCAGTTTTTGCTGATACTTCAACTTCAA
TGTTTGATAACGTTTCTTTTAATGCTTTAGCATCTTCAATTTCTTGTTGGCGTTCTTGTTTTGCACGTTTTTTCT
GTAACTCTAATTGTTTAAGGTTACCTGGTGTTGCTTCTACAGCATAATTCTTTTTCAATAAGAAGTTATTTGCAT
AACCTACTGGTACTTCTTTAACTTCACCTTTTTTACCTTTACCTTTAACATCTTGTGTAAAAATTACTTTCATGCA
TCTTCACTCCTACTTAATTGTTCTGTAATTGCTTGTTGTAATTGTGCTATCGCCTCTTCGACTGTCACACCTTTA
AGTTGTGTTGCAGCATTGGTTAAATGTCCACCGCCACCAAGTGCTTCCATTGTTAACTGAACATTTACTGAAC
CGAGTGAACGCGCAGATATACCAATCAGATTATCTTCACGTCTCGCAACAACATATGATGCTTCAATACCTTCT
AAACTTAACAGTTCATCTGCTGCTTGTGCAACTGTTACTGGATGATAAATTTTATCGTCTGAACCATGCGCAAT
GGCTATGCCATTATCTTCAACTTTTACAGTTCGAATTAATTCAGATCGATTAATGTAAGTATCCACATCATCTTTT
AAGAAATGTTGCGTTAAAATCGTATCTGCACCATGTGCACGTAAATAACTCGCTGCATCGAATGTTCTTGATCC
TGTTCGTAATGTAAAGTTTCTTGTATCTACAATAATACCTGCATACATCACTGTTGATTCAAGACGTGTTAAACG
TTGTTCTGTTGGTTGATATTCCAGTAACTCTGTTACCAATTCAGCTGTTGAACTTGCGTATGGTTCCATATATAT
CAACAATGGATTAGAGATGAAGCTTTCACCACGTCTATGATGATCGATAACAACTTTACGGTTTGCTTTATTTA
AGACATTTTCATCTAAAACCAGTTCCGGTTTATGCGTATCAACAATCACTACGGTTGTCTTAGATGTCATCATAT
CCCAAGCATCATCTGATGTTATAAATCGCTCTCTTAACTCTGGCTTTTTATCAATTTCGTTCATCACGCGTCGTA

ATGTTGGATCAATGTCAGTCTCATTTAATACGATGTATGCTTCTAAATTATTCATCATTGCAAATCTAGACACAC
CGATTGCTGCACCAATTGCATCTAAGTCAGGACGTTTATGTCCCATGATAATGACTTTGTCACCCTCTGCAAG
GATATCTTTTAACGCATGTGAGATCACTCGTGCTCTTACACGAGTACGTTTCTCCATCGGGTCAGTCTTACCGC
CATAGAAACGCACATTACCATTAATACTTTTAATTGCAACTTGGTCGCCACCGCGCCCTAATGCTAAGTCTAGG
CCTGATTGTGATAATTCACCTAAATCGATTAAATTTTCAGTACCTTCACCAACACCAATACTTAATGTTAATTGG
GCACGATAACCAACACTTTTTTCACGTAATTGACTCAAGATATCAAATTTAGATTCTTCTAAGTCAGCTAATATT
TTTTGATTTAAATAGGCTACGAATTGATCGGAACTATACCTTTTAAAGAATATATTATACTCAGTTGCCCATCGA
CTAATAACACGCGTTACCATTGAATTGATTTCCGAACGCTGCGTATCATTCATATTTTGAGTAATCTCATCGTAG
TTATCTAAAAAATAATGTCGCAATGATTGGTTTAGAATTTTCATATAGTTCATTTGTTTGTACTTGTTCAGTTATAT
CAAAGAAATAGAGGCAATGATCATTCTCAGAATAACGTACTTGGAAATGATACTGATTATATTCTATTTCAACG
GATTTCACTCTATCTAATTGCTTTAAAATGTTTGGAAATACTTCATTTACAGATTCAGAAATGACATTTGCTTCC
ATATGATTTGTCATAAATTGGTTAACCCATTCGATGTGATCATTTTCATCTAAAACAATGATACCAATTGGTAAA
TGTTTGATTGCTTTATTATTTGTTGTTGAAATTTGAGCACTCAAACCATCTACATAACTATCCATTTTCATTAAA
GCTTGTCTGAATAAAATGATGCTAACAATAATCATCACGACAAGAACGATAGATGCAATTAATGCTATAAGACT
ATTAAAGATAAACCATACACCCATTAAAACAATTGCTGTGATGATCATGATGACAAATGGTATTAGTAAAGCTT
TCTTAGTGGACTGCCGATTCATTATTCCACCTCTATTCACTTTTTAGAATTATTTTTCATGATTCGCTTCAAATTC
AAACTTAAATCGATAACACCAAGTAGTCCTACAATATGTGTCGTAGGTGTCAGTATTGTACCGATAATCAATAG
TAAAATCGTTACTGCATTCGGCAAACCTTTCGCTTTACCAAAGAAATGAATAACACTTAAACCTTGAATATACA
TTACTAATGATAACACAAGTTGGAAGTTTAAAAGAATGCTCTGGAACACACTCGGTTGACCTGTAAATAATAA
ACATATGATAACAATAATGTATATCCATAATAAAATACCGCTCATTTGCCACGCGAAAAGTGGCTTAAATACAG
GTGTAGCGATTTTAAATTTTCGTAAAACCGGAAATGTAACGATTAAGTTAATTAAGACAATTAAAAATGTAAT
GATAATGATGAAACCTGGTAATTGAACGGTCGCTTGTCTAAACCCTTCTTCTAATATTTGGGTCATATTCGCAT
CGGCACCGCTCATCGTAATCGCTTCATGTAATGTTTGCTTGAAAGGTTTTACTATGCTCGCTGATGGTGGAAT
CCTTCCGAATGTTTGTAGTAACATAAAAGCGATTAATGAAATTAAGCTCATCGCTACTGTTGTTACGTATAACA
TTCTTTCTTTAGACGTTCTTTCTTTGAGCAATTGACCAATAATTAAACTTGCAATTAAGACTAATATGATGGCA
CTTAAAACGAAAGTATTACCTAAAACAGTTGTTATAATTACTGTAATAAGTGCACTAACCCCGAAAGATTGTAT
TGATTTATTCCATAAAACGATACCTGGTATGGTTGCAAATAATGCAAATATTAGTCCCAAACCAGGCACTAGAT
ATAAAGCTAAAGCGACAAATACCAACGTAATCGTTGCAATTATTGTTGCTTTAGGTTGTATTTTTGAAAACAC
ATAAGCCACTCCCATATTTTTAACTATAGCTATTATTTTAACCTCTTTAATGGAAATTAACAATTTATAGATTGTAT
GCTTCTATTTCATTTAATTGAATAATAACTTTCATGTTTTATAAGTAATTAACATACTCATTTGAATCGCTTTTGT
GTGCTTTCATTTTCAACATGATTATTTAATCCCACTACATAGCAATCAAGCTTGATTTAGATTTACAATTCATTTC
CACTCTCATGTACTCTAGATGTCTTTGAATATGATAACTGTGATTTAGTAGCTTCATTCTTTAAAAATATATACAT
TATTACTTACGCTTAAAATGCTTTAAATTTAAGAAATGATATAAGTTAGGTGCCCAGGTACTAAAGTTTAGTAG
GAATCCATCATGTCCAACATTATCAGGCACGAAGAAATGACGATGATATTTAAAACGTTCACCTAATGCACGA
ACTTGATCGTCCGGATATAGCAAATCATCTATGAACCCCATCGTTAAGACTTTCGTTTCTAAATTTTTAAAAATA
TGCGTTACGTCTGTGCGACCTCGGTCAATGTTGTGACTATCCAATACATCTAGCAGTGTCAGATAACAATTCAA
ATCAAAATGTTCTTTAAATTTATTACCTTGATGTTGTTGGTATGCGACTACTTCATCCGGCGTGAAACGTTCATC
ATAACTTTTTGATGATCGATATGTCAAAAAAACCTAATTGGCGTGCAATACTTAGACCTTCCTTACCACCAAGAT
GAATGGCTTGCCTTGCAATTTCATTGAAAGCTCTACTATAAGATGATGTTCGACTTGTTGCAGCAAGGATAAT
GGCTTTATCTACTTCAAACTGTTGATTGTAAAGTAGTTCCATTGCTTGCATACCTCCAAGACTTCCCCCTATTAA
AATATTAATCTTATCATAACCAAGGGCTTGTATACCTCGTTCATTCGCTCTGACTATATCTCTTAATGTTAATTTTT
TAGGAAAATGAGGGTCGTTTAAAGGTGAACTTGAACCGAAAGGACTACCAATAACATCAAATGTTAAAAATT
GATAATCGTGAATGGGTATATATCCCCCATCAATAATTTCTCGCCACCAACCCGGATAATCATCTGTTCCATATG
TTAAATGATTGCCAGTTAATGCATGACAAACTACAACTAATGGTTGTCCATGATAACCAACATGCTCATATCTC

AAACGCAAGTTATCTATGACTTCCCCAGATTCTGTAATAAATTTCCCTAGATTTAAAGTATCTACTGTGTAATTT
GTCATTGTTCTTTCCTCCTTAAACAAAAAAACTTCTCACCCTATTGAAAAGTAAGAAGTCTTTATACTTATCATT
CGAGTAACTCGTTGGTTTTAGCACCGTGCTATAAAGTCGGTTGCTGAAGTATCACAGGGCCAAGTCCCTCAA
CTTCTCACAATAAGAAATATGAAATTGTTATGTGTTAGTTGAGATTCAGTGATGAATTACTTTTATCATTTAAAA
TATTGTTATCATTGTCATACGTTAGCCAAATCGCTTTCGTGTATACGATTCCCAGTCATACCATAGACGATTTGT
ATATCAGAATTTTCTGATTACTAACAGTTTACCTAAGTTTAAATATCTGTTCAATGATTTTCAGTTATTTTTAAAA
GAAAAATCGTAATGTTGCCATGATAACAATCCCACTAATAATTGTAATAGTTAAACTACGCGTGATTATAGATAA
AATAACCGTCGGAATGAGCGCGATAATGTAAGGGATGTTTAATGTATACCCCTCACCATGAGGCGTCTGTTGA
ATAATGCTGTCAATGACAAGTGCCGTAAATAGTGTGATTGGGATAAATGATAGCCATCGAACCACAACATCAG
GCAATTGCACTTTTGAAATCATGATAAAAGGTATAATTCGAATTAATAGCGTTACGATACCACACAATAAAATA
AGTATTAACATGTTCATATGAGTTATCAT
>Staphylococcus aureus strain 16445
ATGAAAATCACCATTTTAGCTGTAGGGAAACTAAAAGAGAAATATTGGAAGCAAGCCATAGCAGAATATGAA
AAACGTTTAGGCCCATACACCAAGATAGACATCATAGAAGTTCCAGACGAAAAAGCACCAGAAAATATGAGT
GACAAAGAAATTGAGCAAGTAAAAGAAAAAGAAGGCCAACGAATACTAGCCAAAATCAAACCACAATCCA
CAGTCATTACATTAGAAATACAAGGAAAGATGCTATCTTCCGAAGGATTGGCCCAAGAATTGAACCAACGCA
TGACCCAAGGGCAAAGCGACTTTGTTTTCGTCATTGGCGGATCAAACGGCCTGCACAAGGACGTCTTACAA
CGCAGTAACTACGCACTATCATTCAGCAAAATGACATTCCCACATCAAATGATGCGGGTTGTGTTAATTGAAC
AAGTGTACAGAGCATTTAAGATTATGCGAGGAGAAGCATATCATAAGTGATGCGGTTTTTATTAATTAGTTGC
TAAAAAATGAAGTATGCAATATTAATTATTATTAAATTTTGATATATTTAAAGAAAGATTAAGTTTAGGGTGAAT
GAATGGCTTATCAAAGTGAATATGCATTAGAAAATGAAGTACTTCAACAACTTGAGGAATTGAACTATGAAA
GAGTAAATATACATAATATTAAATTAGAAATTAATGAATATCTCAAAGAACTAGGAGTGTTGAAAAATGAATAA
GCAGACAAATACTCCAGAACTAAGATTTCCAGAGTTTGATGAGGAATGGAAAAAAAGGAAATTAGGTGAA
GTAGTAAATTATAAAAATGGTGGTTCATTTGAAAGTTTAGTGAAAAACCATGGTGTATATAAACTCATAACTCT
TAAATCTGTTAATACAGAAGGAAAGTTGTGTAATTCTGGAAAATATATCGATGATAAATGTGTTGAAACATTGT
GTAATGATACTTTAGTAATGATACTGAGCGAGCAAGCACCAGGACTAGTTGGAATGACTGCAATTATACCTAA
TAATAATGAGTATGTACTAAATCAACGAGTAGCAGCACTAGTGCCTAAACAATTTATAGATAGTCAATTTCTATC
TAAGTTAATTAATAGAAACCAGAAATATTTCAGTGTGAGATCTGCTGGAACAAAAGTGAAAAATATTTCTAAA
GGACATGTAGAAAACTTTAATTTTTTATCTCCTAATTACACTGAACAACAAAAAATAGGTAATTTCTTCAGCAA
ACTCGACCGCCAGATTGAGTTAGAAGAAGAGAAACTTGAACTCTTAGAGCAACAAAAGCGTGGATATATTC
AGAAGATTTTTTCTCAAGATTTAAGATTTAAAGATGAAAATGGAAACAGTTATCCTGATTGGTCTATTAAAAA
GATTGAAGATATTTCTAAAGTTAATAAAGGGTTTACTCCAAATACAAAAAATGATAAATACTGGGATGAATTA
AATGAAAATTGGTTATCTATAGCAGGTATGACACAGAAATATTTGTATAAAGGAAATAAAGGAATTACTGAAA
AAGGTGCATCAAAGCATGTAAAAGTAGATAAAGATACTCTAATAATGAGTTTTAAATTGACTTTAGGTAAGTT
AGCTATAGTAAAAGAGCCTATCTATACAAATGAAGCTATATGCCATTTCGTATGGAAAGAAAGTAATGTTAATA
CTGAGTATATGTACTACTATTTAAATTCTATAAATATAAGTACTTTTGGTGCACAGGCAGTTAAAGGAGTAACAT
TAAATAACGATGCAATTAATAGTATTATAGTAAAGTTACCAGTGATACAAGAACAAAATAAAATAGCATACTTT
TTCAATAAATTAGATAAATTAATTGAAAAACAATCTTCTAAAGTAGAATTATTAAAACAACGCAAACAAGGATT
TTTACAGAAAATGTTTGTTTAATTCTTATAAAGTTCTATTATGTAAAATATTAAATAGAGATAACATTATGAAAG
CGAGCCCAAGACATAAAGTTTTTGAATAAATAAAAAAGATAATTTCTATCAAATTAATATAGAAATTGTCTTTT
TTATAAATTTTTTGATTATTTTTAGCTGATTGAGCTGTTACTTTTCTTATAATAAGTGCTATTAGCACAAATCCTA
GTTCTCTTTTGGCTTTGTTTATTCCTCTTACGGACATTCGAGTGAAACCCATTTTAATTTTATTAGAAGTAATTT
AGGTTTGAACCCACCTAAATAAATATATGAGTTATTTTTTTATGCTACAAAATATATTCAGATTTCAATAATGAC
ATAAAATAGGCATCTTTATATTTACCTTTAGTGTAGAATTGCTCTTTGAGTAATCCTTCTGTTTTAAATCCTTGTG

ACTCGTATATATGCACAGCTTTTTGTTATCTGTATCAACATATAGATAAATTTTGTGCATGTTTAATATATCGAAT
GCATAATTTATCGCTTTTTCGAATGCGAATTTTGCATAACCTTTACCACTGAACTCAGGTTTAATAATTATTTGT
ATTTCACAATTACGATGGATGTAATTAATTTCTACTAATTCAACAATACCTACGACTTGATTTTCATCTTCAACA
ATAAAACGTCTCTCTGATTCATCTAATAAATGCTTATCAAATAAATATTGAAGTTCCGTTAAGGATTCATATGGT
TCTTCAAACCAATAAGACATAATAGAATATTCATTATTTAATTCATGAACAAAAAGTAAATCACTATACTCTAAT
GCTCTTAGTTTCATAATTCCACTCCCAAAATTTTCTCATATATTTGCATTATAAATATAAATAACGAATAAGTCAT
CATTCACTGTGAATACTCTATTTTAACAATTCACCACATACTAATTCTCATTTTCTTGTTATTCTCGATTTATTACT
CTTACTATGAAACCTATAAAATTCTCACATTTGTTTGTATTAAGAATAAATACGTCGATAGTAACAATAAAAAAA
TAAATAATAAAGCATCCCTCACCGTAAAAGTGAAGGATGCTCTAGTTTTATTGAAATATACATTTCATTTTGTTA
AATAATTATTAATAATATTTTGAAAATCATTATTACGTGAAATCTTCATAGATTTTATCAAGTATTTCTTTGCCTTC
AATTGCTGTGAAGTGATGTACCAATCTATTTTTACAATCATATGTAATTTTGTGACGCTAGGTAATTAGTAATTG
TTCGTCAGTCTGATTGTATAGTATCAAGTTTCATAGATAATACTCTTTGATTTTAATGTCCACTTTGACGTGCTT
TAAGATTGAGTATATACATAATGTCATTGTGGAATGTTAAAAATCCTACAAATGTTTATTCATCTGCAGGATTTT
TAAATCTCCAAGAATAAAAATCATCATAGGACAACTGGATTATTGTTTGGATAAATAACGTAAACAATAATTAG
GTACTATTATTTATTTTTGTTTATTCTTTTTCCTAACAAAATAAAGAAAAGAATAAACGCAATTGTTAAAAATAT
GTGTCCTAAACCAGCAATACCAGCAATAGCAGGACTTACACTTAGATCTTTAATGGTAGAAATACCGTTCACG
AATTGCATTGCCACAGTAACAAGCACACCTAAATGGTATATATAAAAGAAACTGTTAAACAGCTTTGTATGAG
TTGTTAATTTGAATTGGCCCTCGATAATCATGAAAATTAAGAACATAATTGTACCTAGTACTAATAAATGTGTAT
GTGTAACATTTAATTGAGAAAAAACCGCTAAAATCTTCCGCTTTTGTCATTTCTCTATAAAATAGACCACTTAATA
ACCCTAATAGTGTATAGAGCGCTGAACTATACATTAATCTTTTCATTTTAATTCCCCCTATTTTTAATTACGAGAT
AAGTATAGCGGTAGTTTATGAACTGAGTATGAACTTACAACAAAAAAAATTAATGAAGTACTTTACAATAAACT
CAATTTATTAGATGGTGGAGGGACGAAAAAGGATTTTAGAAAAATAAATTAATATATTTTTATTTTGATAAGTA
ATAATTAATAATATCTTGGAAATCATTGTTAAGTATTGTTGTAATACAATCGTCATTCATAAAATCTTCATAGATT
TTATCAAGAATTTCTTCATCTTCGATAGATGTGAAATGATTAGCTAACCCTTTTATAATTTAAGTGTAATTTGTG
AATCTAAACAACTAGCAACTGTTCGCCAATCTGATTGTGTAGTGTCATGGTTCATAGATAATCCTCCCTTTATTT
TAATGTCCATTTTTGACGTGCTTTAGGGTTAAGTGGATGCATAACTTCGTTCGTTACTGGATTAATGAGTTTTT
TGACTTTCTTTTTATGAGGTTTTAACATTTCCATCACTTGCTCAACACGTTCGACGACAACAGGCCGCTTCGC
ATAAGCTCCATAGGCTAGAATCACTGTGTCACTTTCACTAATCGCTTTCATTAAGTGAATGTCTGTGTGTTTGT
CATAAGGCTCTTTAATATGTTTAAGGTTTTCAGGTGTTTTAATATTAGAGAATAGATTTACTAGATATACAGCAC
CGTATTGTTCAGAATTAGCTAATTGGTTGAGAATGAGAACAGTGGTAAGATCGAGTGATAATACGCCATCTAA
ATGTGGATACATCGTTATCACTGTACAAGAGGGTTTCTTTTCATCCCATATTTTCTTGAGTAAGTAGCGGTGTT
GTTCATCGTCGCTAAATATAGCTTCTGTGTGTATCGTACTTTTGATTGTATTCATATATCGTTACCCCTTTTAATAT
TCTTCTGGCAAAAGCATCACATAATAAAAAGCGTCTACGTCATCTTCACGAATGACGTAGACTTTCTTAGGTA
ATGCATTTTGATTTTTTACATAGTTTGTATAGTGATATTCCAATTTGTATGCAGGTTGTTCTTGTTCATGTGTGAT
TGAGAGTATATTCTCATCTTCTTGTAGTCTAAAAATGTGTAGATAATCTGTATGAGATTGATTATCTCTTTCTTTT
ACCATATTCCAAAGTAGGATTTGAAGGTCTAGAGATAGTTGTTCACTTATGCCTCTTGTGATGTATCGATTGAT
TTTCATGTTATTTTACCTCGTCTTGAATTTCTTTCATAATGATAATTGCTTGGCTAATAATCGTAACAGATATTTG
TGCCACTTTGATCCAATTATTCATGGTTATTCTCTCCTTGTTTTAGTAAATGACGTTCATCGATAATCGTATTTTT
AGTATCTGTGAGGTATAGAAAGTCCATATCAAAATGATCCAAATAACCAATGCTGATGAGTTGGTCATTGGCG
TACATAAGAAATGGATAGATACTTAGCTCATGTAGCTCATCATTGTAGTAGGTATAAGTGTTGAGTGTGAGATG
CGCAAGTAGAGAATTATTAATAAAACGTTCCGGTAGAATATTTTCTGCTGCTTCCTCAAGTGCTTCACATTCCC
ATGTTTCGTTGTTAGATAGTTGGAAGAGACGAGTTATATATTGTTTGAGTTCTTGAGTGGTTGTTTTCATATCA
TTGCCTCCTAGATAGTGTGATAGTGATGTAGTTGATATACATCATTGGGATAATATATATTTGATTTATTATTTATT
ACGAATCCCGGTGGGAATAAGAGAAAATTCCATATAAAAACCCGTGATAAATGTTGGTGTAACAAGGAAATC

CCGGAATCCCACTCATTTTGACGAACAATCAACTCATTATTTATAAGTATTGATGATAGGGTTGTGTCTCTGCT
TCCTTATATATATTATTTATTTATAAAAAATAACGGGATTTTGGGATTGTGCTTGCACAATCCTTCTGCTTCTTCG
AATCTGCAAATCCCATTCCTTTCCCGGTAAAAAATCATTGTGGGATGTTCTTTAGCAATTTCAATATAAGCATT
GTGTAGTCATGAAAAAAATGACGGCAATGACTGTTTCATTAGATAAGTGTTATTGAAATTGATAAAGAGAATT
CTAAAAATGGTTAGATAAAATAAGTGAAAGAATAGCAGTGTAGTTATTGTTTATTCAATAGTTATATATAAAGT
TTGTGGCAAAAATAAAGACGAAGTGCTAGGGAGCACTTCGTCGAGTGGATGGTTATTAAATAGTTGTTTGAT
TATATCATTATTTAACTTGAGCGTAACATAAAATTGCTTCTTATGATGCTCATCTTTTCTTATGTCAATGCGATCG
ATGATTGTTAGATATAGTGATTTCAACTGAGATTTCTCTAATTTGTCTATATCTTTGAATATTGCTTGTAGTACAT
TCGCAATCATATCAGCATCATAATGTGGAGCTTCTGCTTGTTTATCTTGTTCTAGTTGATAGATTTGATTATTTAT
TTGATTCAATTCATCTTGGTAGCTAAGTATTGTTGGTTTTAATACGCTATCTAAGTCAGGTGAGTCTTCAATAGT
TTTCGTTAATGTATGCATTTTTGCTTTTATTTCTTCACATTGTGACTGTTTATAGGCAATATCATGGTTCAAAGA
AGAGACGTCTATTTGACTTTTTTCATTTACCTTTTCAACCAATTGCTTCAATACCTTTTTGCTTTTGATAATTTC
CAATATCTGATCCATAACATATTTTTCTAGTACATCTGCTCTAACGCTATTGGCAGAACAAACTTTTGAACCTTT
ATTTCTAAAATTGCTACATGAGTAATATCTGATTCTTTTCTTAGTGCCATCTTTTAATGTATTAGTTGTATTACTT
GCCGCCATTGCTGCACCACATTTCGGACATTTTACAATCCCAGTCAGTAGGTTTGTTCCTTTGCCGTGAACTT
GTGGTTTCTTGCGACTCTCTTGACGTTTAAACTGTACTTTATCCCATAGGTTTCTATCAATAATAGGCGCATGTT
TACCATCAGCGATAATCGGTTCCTCATTGAGTCCTTTTCGTCTTTTATCGCTCCAGTGTCTATACTTCGCAAACT
GTATCTTTCCAATGTAAAAAGGGTTTGAGATGATGTAGGTAATGGACGAAATACTAAAAGGTTTCCCTTTCTT
AGTCACATAACCTTTATGATTCAATGCGTTCGCAATCTTACGATAACCATGACCTTTAGCGTATGAATCAAAAA
TATATTTAACAATATTCGCTTCATGTTGATTGATCATGAGCTCTTTTTTACTGTCAGGTACTTTATCGTAGCCTAG
AGGTAAATTACCTTGATAATAACCTTCAATAGCACGTTGTCTTTGGCCATTGTAGACATTCTCTACAATCGTATT
ACGTTCAAATTCTGCGAAGCTGGCTAAAATTTGGAGCATCAATTTACCTGTTGAACTGGCAATTTCTATTTTTT
CAGTTAGACTAAAAAATTCGACATTAATCTTATACAATTCCTCGACAATATTTAACAAATCTGAGGTATTTCTAG
CTAAACGATTTGTTTTGTAGACCATAATACAATCTAATTTACCTTCGTTGGCATCTTTTAACATACGTTGTAATT
CTGGACGTTGCATTGTTTTACCTGATATACCACGATCGGTGTATTCATTGACGACTTCATAGCCTTGAAATTGA
CAATACTCTGTAAGTTGATTCAATTGACCTTGAATACTGTAACCATCCGTTTGCATTTCAGTCGATACACGTGC
ATATAATCCAATACGTTTCTTTTTGAGTTGTTTCATATTACTTCATTCCTTTCAGGAGTTATTAAATGTGATTGTT
CAACGATATTGAGTGGACTGTTTTTAAAGTAGATTCCTTGTAATTGTTTAGTTTGAGTTATTTTGATGCAATCG
ATAAAAGGTGCTATATCCTGTAAAGTTATTTTATTTTTTATGACATATTTTAATTTGTCATCGATTTGGTAGGTTG
AATAAGTAGAGTAATTCTCTTCACTTTTACAACTAGCAGATAAACGTTTGAACGTTTTTACATCAATACGATTT
TGAGCTAGCTTTTCGATAAGTTGTTCTTGTGTTAGATGGGTTTCTTTATGCTTCATTTGTTGCTGTTTTAGGAC
TTTGAGTATTGTGTTATTCAATCGTTTATGAAACGATTGTTCTTCAAAATACTTTTTACAAGTGTCGAGCACTT
CACTTTCAAGTTCTGGTGCATTGATACTTTTAAATGGGCATGTACGATAAGCGTCATTCATATTTTTAGGACAA
ACATAGTAACGTAGAGAATAGTTCTCTTTTTTTATCGTTAAGTTTGTTAATGTTGATTGACAGTAAGGACATTT
GATTCGTCGCTTTAGCTTATTTCTAGAATTGGATCGATTGAGTTGTTTATGAATACGACGCTCTTGTGCTTCTT
CAAATGTATCAATATCAATAATAGGTGGAACGATATCATTAAACGTGCCATATTTATTGATGACACGACCGCAAT
AGTTAGGGTTCAAGAGAATATTTCTAACTTGATAGGGCTTACGAGGAATAAGGTTAGGATTACTATCCAAATG
TTGGGAAATCTTTTTGTAGCCTAGACCTTGTAAGTACCAGCGATAAACCGATTTAACTGTATACGCTTCTTCTT
CTTGTACAACAAAACAACCTTTTCTATAACGATAGCCAAACGGAGCATGAGTTGTGATTAGCTTACCTTGTTT
GGCTTTTTCTCTAATCCCATTTTTTGTTTGTTCGCTGATATTGTTTGATTCCATTTCCGCTAGACTCATAAGTATG
TTCAAACGAAAGCAATCAAACTCTTTAGACAAATCAAAATATCCATCGTTAACACTGATGATTGTGATGTGAT
GCTTTTTACAAATTTCAAAGAATTGTATGGCATTTTTCAAATTACGATGTAGTCGGTTCAAGCGATAGCAACA
CAATACTTTACATTTTCCAGACGTAATTATTTCTACCATTTTTTGATAACCTGAACGTTTTGTATGTCGACCTGT
TTTCTTATCATCATAAAACGCCACATTAGACCATCCATATTGCTTAGCGGTATCCATAATGAGCGATTTTTGAGT

AGCTAAGCTTTGTTGTTTGAGTGTACTTTGACGTACATAAGCAATCGCTTCTTCCATGTTATACACCTCCAAAA
AGATAATATATATTTGTGAGTAAATTAGAATGAAAGGTCCAACGTGCTTTTAACACGTTGGACCGTCGTGATT
AGTTATCGTTCTGTAGCTCTTCAACGACTAAATCAGCTAGTAATTCAATCAATTCATCCATTTTATCTACTCCTGT
ACGATTTCATCTTTAAGTTATTAAAAATCAATTAAATCATCGTTCTGTTTTTTCCATGATTCAAGTATCTTTTTGT
TATCCAAGTTAATTTCAGCTTTAATAGGTTCAGCATCTTTAGTTAGACCAAAAATAGACGCATATTTTGAATCTA
GCTTTAAATGGTAAAAGACAAGTGACTGTTTCTTGCCATAGTCATCTTTAACTGCTCTTTTCGTAGTTATTCGA
TCACGGTCAGATTCGATAAATCCTTTATCCCTTAGTGCATTCACAACATTGTTGACATCTTGGAAATGATGCTC
TATCAACATATTCTTAAATACAGATGCAATGATTTTAACTTCGATATAATCATCTTTTAAGGCAATTAGTCCATAG
TTCTCAATCATATTCTTTAATGCTGTATCATCAGAAAACTTACCACGGTTTTGTGCTACAAATTGCGTAATGACT
TCAATCGCTTTATCAGCCAGTGATCGTTCAGAGACTGTATGAGCATGATAATCAATAAAGTAGTCTCTTATTTT
AGCGATATCAATATCTGTAGATAAAACATGACTAAGAATCTTGGCCGAAGTAGTGATAGCCGCATAGCGCTTA
AACATACGAATACCAGTATTATTTGTTTCATCCTTCAATTTATCTTTAAACCAATGATGTAATTCAAAAACGGAT
CAAAAAACAAAAGGCCCTAGAAACTAGGAGCACACTTACACAAGAACAATTGATAGATAAATTAGCTAAAG
GAGCCATCGATGCAGAAACGTTCAGAAAACAATCTCAATCGTTACTTCAACAATCAAAACCAACACTATCAAT
AAATGAGCAACAAATTCAAAGGTCTTTTGAAAATGTAATTCAACAACACTTCACGTTAAGCATGTTATACCGA
TATATTGATGAAATTCATATTTCTAAAAACAAAAGCCTTGTGGGAATCTATTTCAAAAATGAACCGCTAAACAT
TGTAAACCAAGCTACGCAATCATCGATTGCTTAATTAATGAAAGGATGAAAAATATGAAAGAAATGAAAAGA
AAATCTGTAGGATGTTATGTTAGAGTTTCAACAATTTCTCAAGACATTGATAAATTTAGTATTAATGGTCAAAT
TACACAAATAAAGGAATATTGCCAACAGGGAAATTATGAATTGCTATACTGATAATTAAAAGTACCCCCGATC
GAACGAAAATATCTTTTTGATGACTATTAGTGATAATTGTAATTTTATCCATTAATAGATGAATGGATTGGAATA
ATACTAAGCCTATTGCTATTCCACCGAGTAAAATTACCCACCCGCCTAATTCAATACTTTCCGGAATCATTTCAA
AAAATAATAAGCCAATTATAAGGCCTGCACATAATGAATAAATAAAGCCATGACCACTTTGAAAACCTTCCATT
ATCCAAGCGAGTCCACCGCCAACACCAATTCCAAGTGCGGAAGCTAACGCCCGATCATCCATACTGCACTCA
TCCCACTATTCTTAAAAAGGCTTTGTTTATTTTACTCTAAAAAATCAAAACAAAATATATGAATTCATATTAAATA
TTATTTGACTCTTTATTCAGATGGTTATATCATATATATGAACATATATTAATACATGTAGATAAATAGTGATTATTA
TATATAAGGTTTTGAATTGAAACCTAAAGTGAGGGAAGGATAATGGAAGAAAAAAAGGAATTAGAGGAAGT
AAATAATAAGGACTTAGATGATGAAACATTATTTGTCGTATCGCAAACATTTAAAGCGTTAGGTGATCCTACG
AGAATCCGGATTCTCCATTTGCTCTTTTATAAGGAGTATTCGGTAAACGGTATTGCTGAAACGCTACATCTTAG
ACAATCAACAGTTTCCCATCAATTGCGGTTCTTGAAAAATTTACGGTTAGTAAAATTCCGAAGGGAAGGCAC
AACATTGTTTTATTCCCATGATGATGAACATACTATGAATATGCTAAAACAGGCGATCGATCACGCCTGTCATC
ACTAGTATTCCGTAAACCAGTAATGAGTGTTAATAATAATTCACGTCATTAATATTGTTATATGATCATATAAAAA
TATTATAGGCATATGAAAAGGGGGTTTTGCAATGGATATGGAAAACAAAAAAACAGAATGGAAAGCGTTGT
ATGATATCTCGAAGGAAAGTGAGATGGGTGTAGCAGAGAGGGTTAGTGAATACGGTTGATAAATTCATAGG
ATGTATTATTGGATACAAAAAGTTTGGATGTGGTCAATTAACGTCCTGGTTTAGTAAAGCTTACTGCGAATATA
TTGGAACCCGATATATCATATTTATTTAGAAAGGTGATAGAAATGAAAAATATTCAAGAGCAACAAGCACACG
AAAGTCATAGCCACGATCATAGTCATGATCATGATCACGGAAAAATGCCAATTATTTCATATTTTATTGGCTTA
GTGTTGGCTATAATTGGGCTTTTTTTAAGTGATGCAAATTTATTAATACAAAACATCTTATTTTCAATTGCCACA
ATCACAGCCGGCTACCATGTAATTATTCTCGAAGGAATTGGAGAGACAGTTGAAAATACTAAATTAAAGGGA
AAATTCACTCCTAATTCTCATATTCTAATGGGATTAGCTGCAATCGGGGCTTCTCTGATAGGGAGTTTTTGGG
AAGGAACCCTTTTGATACTTATTTTTTCCGGCGCTCATTTTCTTGAAGATTACGCTGAAGGAAAAAGTAAAA
GAGAAATTACTAAGCTACTCGAAATGAACCCAACGACAGCTAAATTAATCCTACCTGATGGAAACACAAAAA
TTGTTGATGTCAGTGAATTAAAAGTTGGAGATCAACTCCAAGTGCTGAACGGTGATCAAGTTCCAATTGATG
GGATTATTTTATCCGGTACTACCTCAATTGATGAATCTTCTATTAATGGAGAAAGTATACCGAAAGAGAAGTCT
AAGGGTGACGAAGTTTTTGGAAGTACGATTAATGGAACAGGTACTTTTACTATGGAAGTCACTAAGGAAAA

CAAGGATACTGTATTCTCTAAAATTTTACAATTAGTTAGTCAAAACCAAGATAATCAAACAAAAGCTGCCAGT
ATCATTCAAAAATTCGAGCCTAAATATGTTAATATAGTTTTAATCGCAATACCATTAGTAATGTTACTTGCTCCTT
TTCTATTTGATTGGACATGGTCGCAAAGTGTATACAGGGGATTAGTGCTTTTAGTCGCAGCTTCACCGTGTGC
TTTGGCAGCAGCTACTGTATCTGTAACATTGTCTACAACATCTAACCTAGCTAAAAAAGGCGTGCTTTCAAAA
GGAAGTACTTACCTATCACAATTAGCGGATATAGATGCAATTGCCTTCGATAAAACAGGAACCCTTACGAACG
GAGAACCTAAAGTAACAAATTACTATTTCACTCATTCTGTGAACGAAGAAAATATTATTGATATTATAGTCGCC
CTTGAAAAGGAATCCAATCACCCACTCGCTAATGCTATTTTAGAAAAATTTGAAGTTAAAAATAAAATAGACA
TCGAAGTTACTAATCAAATTGGAAAAGGTCTGACAGGAGATTATAATGGAAAAAATTATCGTATTGGTAAGCC
TACTTCTTTTGAAAGTGCTTCTGAAGAGTATACCCAGTTCAATCATGATTGGGCATCAGAAGGAAAGACGGT
TGTATACGTAGCAGAAAATGAAGAAGTTATTGGGATTATAGCTCTAATGGATATTCCGAATGAGCATGCTAAA
GAAACAATTAATTACTTTAAGAAACTTGGTATCCACACGACTTTAATTACTGGTGATTCGGAAATGACGGGAA
AAGCTGTAGGCGAACAATTGGGAATAGACGAAGTTATTGCTAATGTAATGCCTGAAGATAAATCCAGAATTAT
AGAAGAACAAAAAGAAAAATTTGGAGTTACTGCCATGGTTGGAGATGGTGTGAACGATGCACCGGCCCTT
GTTAATGCTGATGTTGGTATAGCTATGGGGGGCGGTACTGATGTGGCAGTAGAAGTATCTGATTTGGTTTTAA
TGCAGAACAATTTATCTAAATTAGTACAGTCTCATAAAATTTCCTCAAATATGGGTCGTGTTATTAGGCAAAAT
ATTATTTTTTCAATGGCAGTTGTTGCCTTTTTAGTTGTCGTTAGTTTGTTAGGATTAACTGATATTACAATCAGT
GTAATTGTTCATGAAGGAAGTACTTTAGTTGTTATACTAAATGGACTTCGATTATTAAGATCTAAATAATGAAC
GAATCGATTGACATGAATGAACTTTGAAGTGTGGATTCTACAATGTTCCCATAACATTGGACACTAAAAAACA
GAGCAATCTAATAAAGATGTTATGAGTAAAAACAATGCCTTGCATACCGTTTTTATCATACGGGCGGTCAAAT
AAGCAATTAAAGAGCATGGGAAGCATAAAATCATAAACAGTGACTTAAGGCAGCCAGTTTACATTCAAAAGT
TAAATTGACTGTATTAAAAGTTTCGAAACGATTAATATCCGTATGGGGGCAAGCGTCGAGCCAAAGATAATGC
CAGGACAGAGCGTCTTTTTCCGTTCTTTTAAGTGGGAAAGGTTTTACCTTCTCTATGCCAAGACAGTCCCAG
AGCTAAAAGGGCCAGTTGCTCTTTTTGGGCGTTTGTGAACGGTAATCGCAGGAAGGGGATTTTCCCAACCG
CACCTGGTTTTCCATCACAATAAAGTTGGCGGCATTTGTTCTGATATTCTTGGGAATACTTACACTATGAATGG
CCGTATTAAGTGATACAGGAGCTGCTATTATTGTAATACTGAATGCTCTCCGCCTTTTGAGGGTAAAAGAATA
AAAGTAAGGATAACTAGGTAAAGCTGTTCAATCAAAAATTGAACAGCTTATTTTTCATCAAAATCAAAAACGT
TTATTATAATACCTACACTTGTTCTGTTAAATGTACCGATTTTTTAACCTTATTGTATCAGTAATATCTTGAAACG
AAGTAAGCGACTAAAATTTCTCTTTTATCATACGTTTATAAAAATACACTTTTAAGAACGGTTTGAAAATTTTT
GAAATAGATCAAAATAATCTTCTGGTTAAAAAACCTGTAGATATGATTTCTCTCTTAAATTTTTGTTTGATTAGA
TTAGACCCTAATAAGACGCATTCAATATGTCTACACGTGAATTTAGTCTTTGAAAATGTAAGGACCATTATTATT
ATAAAAACCCCAGTATAAAACGATACGCTGAAGCTTATCATAAGTAATGAGGTTCATGATTTTTGACATAGTTA
GCCTCCGCAGTCTTTCATTTCAAGTAAATAATAGCGAAATATTCTTTATACTGAATACTTATAGTGAAGCAAAG
TTCTAGCTTTGAGAAAATTCTTTCTGCAACTAAATATAGTAAATTACGGTAAAATATAAATAAGTACATATTGAA
GAAAATGAGACATAATATATTTTATAATAGGAGGGAATTTCAAATGATAGACAACTTTATGCAGGTCCTTAAAT
TAATTAAAGAGAAACGTACCAATAATGTAGTTAAAAAATTTGATTGGGATAAAGGTGATCTATATAAAACTTTA
GTCCATGATAAGTTACCCAAGCAGTTAAAAGTGCATATAAAAGAAGATAAATATTCAGTTGTAGGGAAGGTT
GCTACTGGGAACTATAGTAAAGTTCCTTGGATTTCAATATATGATGAGAATATAACAAAAGAAACAAAGGATG
GATATTATTTGGTATATCTTTTTCATCCGGAAGGAGAAGGCATATACTTATCTTTGAATCAAGGATGGTCAAAG
ATAAGTGATATGTTTCCGCGGGATAAAAATGCTGCAAAACAAAGAGCATTAACTTTATCTTCCGAACTCAATA
AATATATTACATCAAATGAATTTAATACTGGAAGATTTTATTACGCAGAAAATAAAGATTCATCTTATGATTTAA
AAAATGATTATCCATCAGGATATTCTCATGGATCAATAAGATTCAAATATTATGATTTGAATGAAGGATTCACA
GAAGAAGATATGCTAGAGGATTTAAAGAAATTTTTAGAACTATTTAATGAATTAGCTTCAAAAGTTACAAAAA
CATCCTATGATAGCTTGGTCAATAGCATAGACGAAATACAGGAAGACAGCGAAATTGAAGAAATTAGAACAG
CACAAAAAGATAAGACACTCAAGGAAGTGGAAGCACCTAAAGGAATAATTCCAAAATATAAAAAAGGTGTA

TCAAAGACTACTAAAAATGATTCAGAAATTGAAAAATCAAATAAAGAGAATAAATTAACCGGTAAAGTTGGA
GAAAAATTAGCGCTAAATTACTTTAATGAGCTAATTGATAATAAAATAGACGAAGATAAGAAAGAACAGTTTA
GGAATATTTTAAATGATAATCCAGGCTCTCAACACGGTCATGGCTATGATTTAGTAGCTTTTGATCCAACAAAT
ACAGATAAAGCTGTAGAAAAATTTATTGAAATTAAAACATCTACATCTTCTAGTATTGAGGAACCATTTTTTAT
GTCGCTAAATGAAATGTTTGCTATGAAAGAATATAAGCAGAAATATTTAATATTAAGAATATTTAATGTTTCCG
GTAAAGAACCACAATTTTATTTTATAGATCCATATGCAAATTATTCTGAATTTAAAGATGTAGATGATCTCATTG
ACAAAGTATTTAATGTAGAAGCTATTCAGTATAAAGTTTTTGGCGAAAAATGATTACTTGAACAAGAGCTAAA
ATAAAATTGTGATCTAATAAAAATAGAAACTGTAATTTAAATAAAACTTTCTAAATAAGCTAACTGATAAAAAA
TCAGTTTGTCCACAGTCTGAAACAAGATTCCTATATTCTTTAGGAATCTTGTTTTTTCTATTTTTATGGTGATAA
AGAGCAGATAAGATAATGTGTAATAATCACAAAAAAGTTAAATATTTTAAGGCTTGTTTAATTATTAATGATTT
TATATATAAAGAGCAGTATAATAAAGTTGTTAATATATTATGAATAATATTCAAGTAATTTTATTGTTTTTTAATTT
GTCGATATTTAAGTTGAGTTAAATTTAAAGGGTGTAATTTGTTTTACAATGATGAAGATAATTAGTCTATCAAA
ATAAAGGGGTTGGGACTGTTATGAGTGATAATTTGTCATTATTCATTGACTATATCAATGATAATATAATCTATG
GTAGTGAAATCAAACGGGAGAAATTAGAGAATTTATTTAATCAATTTGCTATAAAAAATGTTGAAAAGAACAT
TGTCTATGATGAACTGAAATCTTTAGATATTACAATCATTGAGTCACAGGATTCATATAAAAATAAATTGAAGA
GATTATTTTCGGTTCTGTTGCAAAGTAAAAAAATATAGCTAACCACTAATTTATCATGTCAGTGTTCGCTTAACT
TGCTAGCATGATGCTAATTTCGTGGCATGGCGAAAATCCGTAGATCTGAAGAGACCTGCGGTTCTTTTTATAT
AGAGCGTAAATACATTCAATACCTTTTAAAGTATTCTTTGCTGTATTGATACTTTGATACCTTGTCTTTCTTACTT
TAATATGACGGTGATCTTGCTCAATGAGGTTATTCAGATATTTCGATGTACAATGACAGTCAGGTTTAAGTTTA
AAAGCTTTAATTACTTTAGCCATTGCTACCTTCGTTGAAGGTGCCTGATCTGTAATTACCTTTTGAGGTTTACC
AAATTGTTTAATGAGACGTTTGATAAACGCATATGCTGAATGATTATCTCGTTGCTTACGCAACCAAATATCTA
ATGTATGTCCCTCTGCATCAATGGCACGATATAAATAGCTCCATTTTCCTTTTATTTTGATGTACGTCTCATCAAT
ACGCCATTTGTAATAAGCTTTTTTATGCTTTTTCTTCCAAATTTGATACAAAATTGGGGCATATTCTTGAACCCA
ACGGTAGACCGTTGAATGATGAACGTTTACACCACGTTCCCTTAATATTTCAGATATATCACGATAACTCAATG
TATATCTTAGATAGTAGCCAACGGCTACAGTGATAACATCCTTGTTAAATTGTTTATATCTGAAATAGTTCATAC
AGAAGACTCCTTTTTGTTAAAATTATACTATAAATTCAACTTTGCAACAGAACCGTATTATGGAATAGAGATGT
TGGTAACATTTATACAGGATCATTATACTTAAGTTTAATTTCGTTATTACAGAACCACACATTCCAACCAGAAG
AGAAAGTATGTCTATTTAGTTATGGTTCAGGAGCAGTAGGAGAAATCTTTAGTGGTTCAATCGTTAAAGGATA
TGACAAAGCATTAGATAAAGAGAAACACTTAAATATGCTAGAATCTAGAGAGCAATTATCAGTCGAAGAATA
CGAAACATTCTTTAACAGATTTGATAATCAAGAATTTGATTTCGAACGTGAATTGACACAAGATCCATATTCAA
AAGTATACTTATACAGTATAGAAGACCATATCAGAACATATAAGATAGAGAAATAAACTAGTGGCCGATTGTGC
TTGATGAGCTTGGGACATAAATCCTAACTCGAAATAAATAAGCATATCACTAAACTGATTTTTTAAAGTTTACA
GTGATATGCTTATTTTTTTATCTTACGATTTTGTACGTGCATGCTTGCCTAGGGGTATGGCTCGAGCCATTAGTC
TCTCGCACATACTATTCCCTCAGGCGTCAGCACTTACAAAATCGGTTGTAATTTTCATTTTTATACGCATTCTTA
CTGAGATTATACTAATAAGAGGAATAGTAAAAGCAATTCTAAGTAAAATTGCAGATAAGAGGTTTGTTAAAAG
CAGTTCTCAGTAAAATTGCAGATAAGAGGTTTGTTAAAAGCAGTTCTCAGTAAAATTACAGATAAGAGGTAC
GTTAAAAGCAGTTCTAAGTAAAATTGCAGATAAGAGGTTTGTTAAAAGCAGTTCTAAGTAAAATTGCAGATA
AGAGGTACGTTAAAAGCAATTCCATGCAAAATTGCTGATAAGGGGTAAGTTAAAAGCAGTTCTCAGTAAAAT
TGCAGATAAGAGGTACGTTAAAAGCAGTTCTAGGCAAAATTGCAGATAAGAGGTGCGTTAAAAGCAGTTCT
CAGTAAAATTGCTGATAAGGGGTAAGTTAAAAGCAATCCTAAGTAAAATTGCAGATAAGGGGTACAGAAAA
ACTAGACTTGATTACAAAATGGAGCTTGGGACATAAATGATTTTTTAAAAATGAGATGAGACGTAGATTAACT
CCATAATCAATACGAATCTATCGACTTCTTTATTTATGATATTCATCTCTTTTTAATGGAAATAAAAGTGCGATTA
ATGTGATAATACAGTTACGTTAATTAAAAAAATAAAAATGCAAGGAGAGGTAATATGCTAACTGTATATGGAC
ATAGAGGATTACCTAGTAAAGCTCCGGAAAATACAATTGCATCATTTAAAGCTGCTTCAGAAGTAGAAGGTAT

AAACTGGTTGGAGTTAGATGTTGCAATTACAAAAGATGAACAACTGATTATCATTCATGATGATTATTTAGAA
CGGACTACAAATATGTCCGGGGAAATAACTGAATTGAATTATGATGAAATTAAAGATGCTTCTGCAGGATCTT
GGTTTGGTGAAAAATTCAAAGATGAACATTTGCCAACTTTCGATGATGTAGTAAAAATAGCAAATGAATATAA
TATGAATTTAAATGTAGAATTAAAAGGTATTACTGGACCGAATGGACTAGCACTTTCTAAAAGTATGGTTAAG
CAAGTGGAAGAACAATTAACAAACTTAAATCAGAATCAAGAAGTGCTCATTTCAAGCTTTAATGTTGTGCTT
GTTAAACTTGCAGAAGAAATCATGCCACAATATAACAGAGCAGTTATATTCCATACAACTTCGTTTCGTGAAG
ACTGGAGAACACTTTTAGATTACTGTAATGCTAAAATAGTAAACACTGAAGATGCCAAACTTACTAAAGCAAA
AGTAAAAATGGTAAAAGAAGCGGGTTATGAATTGAACGTATGGACTGTAAACAAACCAGCACGTGCAAACC
AACTTGCTAATTGGGGAGTTGATGGTATCTTTACAGACAATGCAGATAAAATGGTGCATTTGTCTCAATAGAA
AGTTAGAGGTGAGTCTTACGTTTCAGTGACGGTAGACTTACCTTTAACATGTTACATACTAAAAAATTAATTT
GAATAAGAAAGAGAGACATATATGAAATACGATGATTTTATAGTAGGAGAAACATTCAAAACAAAAAGCCTT
CATATTACAGAAGAAGAAATTATCCAATTTGCAACAACTTTTGATCCTCAATATATGCATATAGATAAAGAAAA
AGCAGAACAAAGTAGATTTAAAGGTATCATTGCATCTGGCATGCATACACTTTCAATATCATTTAAATTATGGG
TAGAAGAAGGTAAATACGGAGAAGAAGTTGTAGCAGGAACACAAATGAATAACGTTAAATTTATTAAACCT
GTATACCCAGGTAATACATTGTACGTTATCGCTGAAATTACAAATAAGAAATCCATAAAAAAAGAAAATGGAC
TCGTTACAGTGTCACTTTCAACATACAATGAAAATGAAGAAATTGTATTTAAGGGAGAAGTAACAGCACTTAT
TAATAATTCATAATAAAACAGTGAAGCAACCATCGTTACGGATTGCTTCACTGTTTTGTTATTCATCTATATCGT
ATTTTTTATTACCGTTCTCATATAGCTCATCATACACTTTACCTGAGATTTTGGCATTGTAGCTAGCCATTCCTTT
ATCTTGTACATCTTTAACATTAATAGCCATCATCATGTTTGGATTATCTTTATCATATGATATAAACCACCCAATTT
GTCTGCCAGTTTCTCCTTGTTTCATTTTGAGTTCTGCAGTACCGGATTTGCCAATTAAGTTTGCATAAGATCTA
TAAATATCTTCTTTATGTGTTTTATTTACGACTTGTTGCATACCATCAGTTAATAGATTGATATTTTCTTTGGAAA
TAATATTTTTCTTCCAAACTTTGTTTTTCGTGTCTTTTAATAAGTGAGGTGCGTTAATATTGCCATTATTTTCTAA
TGCGCTATAGATTGAAAGGATCTGTACTGGGTTAATCAGTATTTCACCTTGTCCGTAACCTGAATCAGCTAATA
ATATTTCATTATCTAAATTTTTGTTTGAAATTTGAGCATTATAAAATGGATAATCACTTGGTATATCTTCACCAAC
ACCTAGTTTTTTCATGCCTTTTTCAAATTTCTTACTGCCTAATTCGAGTGCTACTCTAGCAAAGAAAATGTTATC
TGATGATTCTATTGCTTGTTTTAAGTCGATATTACCATTTACCACTTCATATCTTGTAACGTTGTAACCACCCCAA
GATTTATCTTTTTGCCAACCTTTACCATCGATTTTATAACTTGTTTTATCGTCTAATGTTTTGTTATTTAACCCAAT
CATTGCTGTTAATATTTTTTGAGTTGAACCTGGTGAAGTTGTAATCTGGAACTTGTTGAGCAGAGGTTCTTTT
TTATCTTCGGTTAATTTATTATATTCTTCGTTACTCATGCCATACATAAATGGATAGACGTCATATGAAGGTGTGC
TTACAAGTGCTAATAATTCACCTGTTTGAGGGTGGATAGCAGTACCTGAGCCATAATCATTTTTCATGTTGTTA
TAAATACTCTTTTGAACTTTAGCATCAATAGTTAGTTGAATATCTTTGCCATCTTTTTTCTTTTTCTCTATTAATGT
ATGTGCGATTGTATTGCTATTATCGTCAACGATTGTGACACGATAGCCATCTTCATGTTGGAGCTTTTTATCGTA
AAGTTTTTCGAGTCCCTTTTTACCAATAACTGCATCATCTTTATAGCCTTTATATTCTTTTTGTTTTAATTCTTCA
GAGTTAATGGGACCAACATAACCTAATAGATGTGAAGTCGCTTTTCCTAGAGGATAGTTACGACTTTCTGTTT
CATTAGTTGTAAGATGAAATTTTTTTGCGAAATCACTTAAATATTCATCCATTTTTTTAACGGTTTTAAGTGGA
ACGAAGGTATCATCTTGTACCCAATTTTGATCCATTTGTTGTTTGATATAGTCTTCAGAAATACTTAGTTCTTTA
GCGATTGCTTTATAATCTTTTTTAGATACATTCTTTGGAACGATGCCTATCTCATATGCTGTTCCTGTATTGGCC
AATTCCACATTGTTTCGGTCTAAAATTTTACCACGTTCTGATTTTAAATTTTCAATATGTATGCTTTGGTCTTTCT
GCATTCCTGGAATAATGACGCTATGATCCCAATCTAACTTCCACATACCATCTTCTTTAACAAAATTAAATTGAA
CGTTGCGATCAATGTTACCGTAGTTTGTTTTAATTTTATATTGAGCATCTACTCGTTTTTTATTTTTAGATACTTT
TTTTATTTTACGATCCTGAATGTTTATATCTTTAACGCCTAAACTATTATATATTTTTATCGGACGTTCAGTCATTT
CTACTTCACCATTATCGCTTTTAGAAATATAACTGCTATCTTTATAAACTTGTTTGAAATTTTTATCTTCAATTGC
ATCAATAGTATTATTAATTTCTTTATCTTTTGAAGCATAAAAATATATACCAAACCCGACAACTACAACTATTAAA
ATAAGTGGAACAATTTTTATCTTTTTCATCAATATACTCCTTATATAAGACTACATTTGTAGTATATTACAAATGTA

GTATTTATGTCAAAATAATGTTATAATTTTTGTGATATGGAGGTGTAGAAGGTGTTATCATCTTTTTTAATGTTA
AGTATAATCAGTTCATTGCTCACGATATGTGTAATTTTTTTAGTGAGAATGCTCTATATAAAATATACTCAAAATA
TTATGTCACATAAGATTTGGTTATTAGTGCTCGTCTCCACGTTAATTCCATTAATACCATTTTACAAAATATCGAA
TTTTACATTTTCAAAAGATATGATGAATCGAAATGTATCTGACACGACTTCTTCGGTTAGTCATATGTTAGATG
GTCAACAATCATCTGTTACGAAAGACTTAGCAATTAATGTTAATCAGTTTGAGACCTCAAATATAACGTATATG
ATTCTTTTGATATGGGTATTTGGTAGTTTGTTGTGCTTATTTTATATGATTAAGGCATTCCGACAAATTGATGTT
ATTAAAAGTTCGTCATTGGAATCGTCATATCTTAATGAACGACTTAAAGTATGTCAAAGTAAGATGCAGTTCTA
CAAAAAGCATATAACAATTAGTTATAGTTCAAACATTGATAATCCGATGGTATTTGGTTTAGTGAAATCCCAAA
TTGTACTACCAACTGTCGTAGTCGAAACCATGAATGACAAAGAAATTGAATATATTATTCTACATGAACTATCA
CATGTGAAAAGTCATGACTTAATATTCAACCAGCTTTATGTTGTTTTTAAAATGATATTCTGGTTTAATCCTGCA
CTATATATAAGTAAAACAATGATGGACAATGACTGTGAAAAAGTATGTGATAGAAACGTTTTAAAAATTTTGA
ATCGCCATGAACATATACGTTATGGTGAATCGATATTAAAATGCTCTATTTTAAAATCTCAGCACATAAATAATG
TGGCAGCACAATATTTACTAGGTTTTAATTCAAATATTAAAGAACGTGTTAAGTATATTGCACTTTATGATTCAA
TGCCTAAACCTAATCGAAACAAGCGTATTGTTGCGTATATTGTATGTAGTATATCGAGCTTCACATGAAACAGC
TAAAGAAGCTTTGGGCGATAAAGAGTTAAGAGCCATTGCACATGAGTTAACTAAAACAGTTAAGGATAACAT
GAGTGTTGATTGGTCTAAACGAGACAGTGCTAAAGCTAAATGAGAGTTCAAGTTAGACGCCTATTAAAGAA
ATATGGCTATCCACCAGATCTTCAAAAAATGGCTGTGGAACAAGTTGTAGAGCAAGCAGAATTAATGGCAAG
TCAGCAATAAAAAAATAAATCATAATGAGTCCGGGACATAAAGTTCTTGGATAAGTGAAAAAAGACAATTTC
TATTGAAATAATATAGAAATTGTCTTTTTTATAAATTTTTTGATTATTTTCAGCTCGTTGAGCTACTACTTTTCTT
ATATTAAGTGCCATTAATACAAAACCAAGTTCTCTTTTGACTTTATTGAGTCCTCGGACAGACATCCGAGTGA
AACCCAAAATAGCCTTCATAAATCCAAAAACAGGTTCCACATCAATTTTTCTTTGACTGTAGATATTTTTTGTT
TCTGGTTCTGAAAGCTTTTTGTTAATTTGGGATTTAAAATATTCCCAGTTATAATTCTTCATTATTTTTTTGTTTG
TTTTTGAATTGAAGTTCATACATTGATTTTTCAGAGGACATTCTGAACAATCATCACATTCATATAATTTGAAGT
CTCGCTTATAACCATACTTATCATGACGATAGGCATATCTTTTAAAACCTAGCCGTTTATTATTCGGACAAATGA
ATTCGTCATTAATTTCGTCATAGTTCCAATTTTGAGTATTAAAGATGTCACTTTTATATTTTTTAGTTTTATCTTTT
ATAAACATTCCATATGTTATGAGTGGCGTTCGATTAAAGTCATCTATAATTGCCTTATAATTTGATTCACTACCAT
AACCTGCATCAGCTACAATATATTCAGGTAAATGACCGTAGGTCTCTTGAATTGAATTTAAAAATGGAATCATC
GTTCTAGTATCCGTTGGATTTTGATACACATTATAAGATAAAACAAATTGGGAATTTGTCGCTATTTGTAAATTA
TACCCTGGCTTAAGTTGTCCATTTTTCATGTGATCTTCTTTCATTCTCATAAATGTCGCATCATGATCTGTCTTAG
AATAACTATTTCTATCCTTTAAAATAGATTTTTGAAATTCGTATCGATACTTTCGCTCAAAATAATCATTGATTTG
CTTTTTGTATTTTTTTGATTTTAGTTCTTTTGAGACGTATTTGTTTTCTTGTTTTAGTACATTTTTCATTGTTGATA
TGTTGGTTTAAATCTTCGATTTCTTTATCTAAGTGACTACCAATCAAATCTATTTCTTCTTTTGTTAATTCATTATC
ATGATCTTCTTTAATTTCCGGTATGATTTTATTGGTTACCAATTCATGGTAGAGGGCTTTAGAATCCTCATTCAT
CTTTGATTCATGGTTTTGAATACTCTTTTTCCATACAAATGTATATCGATTGGCATTTGCTTCAATTTTTGTACCA
TCAATAAAAAATAGCTTTATCATCTATAAGATTTTGTTTTACACACTGACTGTAAAATTGAATAAATAAAGATTCT
AATAAAGCATCTACTTTTGGATTTACTCTAAATTGATTAATTGTTTTATAAGAAGGTTTTTGATTTTGTGATAGC
CACATCATTCGGATGCTATCATTAAGCATTTTTTCTATTTTACGACCTGAGAATACAGATTGTGTGTAGGCATAT
AGAATCACTTTTAACATCATTTTAGGATGGTACGAAGTTGCACCACGGTGATGTCTGAATTCGTCGAATTCAT
TGTCAGGAATTGTTTCAACAATATCATTTACAGTAAAACGATGTTGATTTGTTTTGTTTCCATATTGACCTCCAT
GTATTTGCTATGATTTCAAAATCCATTTTTGACGTGCCTTAGGGTTGAGTGGATGCATAATTTCATTTGTTACT
GGATTGATGAGCTTTTTTACTTTCTTTTTATGAGGTTTTAACATTTCCATCACTTGTTCGACACGGTCGATAAC
AACTGGTCGCTTCGCATAGGCACCATAAGCAAGAATCACTGTGTCACTTTCACTAATCGCTTTCATCAAATGA
ATATCAGTGTGCTCATCGTATGGATTTTTGATATGTTTGAGGTTTTCGGGTGTTCTAATATTAGAGAATAGATTT
ACAAGATATACAGCACCGTATCGTTCTGAATTGGCTAATTGGTTGAGGATAAGAACAGTTGTGAGATCGAGT

GATAATACACCGTCTAAATGAGGATACATCGTTATCACTGTGCATGCAGCTTTCTTTTCATCCCATGTTTTCTTG
AGTAAATAGCGGTGCTGTTCATCATCGCTAAATATGGCTTCTGTGTGTATCGTACTTTTGATTGTATTCATCATC
GTCACTTCCTTTAGTATTCTTCTGGTAAAAGCATCACATAATAAAAAGCGTCCACGTCATCTTCACGAATGACG
TAGACTTTCTTAGGTAATGCATTTTGATTTTTTTCATAGTTTGTATAGTGATATTCCAATTTGTATGTGGGTTGTT
CTTGTTCATGTGTGATTGAGAGTATATTCTCATCTTCTTGTAATTTAAAAATGTGTAGGTAATCTGTATGAGGCT
GGTTATCTTTTTTTTCTACCATGTGCCAAAGTAAGATTTGAAGGTCTAGTGGAATACTTTCATTAATTCCTCTT
GTGATGTATCGATTGATTTTCATGCTATTTCCCTCCCTTCTGCTTTTCTTTCATGATGTCGATGATTTCGTTGATA
ACTGTGACGGATAATTGAGCAACTATGATCCAATTATTCATGGTCCTGACCTCCTTGTTTTAGTAAATGACGTT
CATCAATAATGATATTTTGAGTATCTGTAAGGTACAGAAAGTCCATGTCAAAATGGTCTAAGTATCCGACACTG
ATGAGTTGGTTATTGGCATACATTAGAAATGGATAGATACTTAGCTCATGTAGTTCATCATTATAGTAGGTATAA
GTCTCGAGTGTGAGATGTACCAGTGGAGAATCATTAATAAAACGTTCCGGTAGAATATTTTCTGCTGCTTCCT
CCAGCGCTTCACATTCCCAAGCTTCGTTATTAGATAGTTGGAATAGGTGGGTTATATATTGTTTGAGTTCTTGA
GTGATGGTTTTCATATTATTGCCTCCTAGATAGTGTAATAGTGATGTAGTTCATATACATCATTGAGATAATATAT
ATTTGATTTGTCATTTATTACGAATCCCGGTGGGAATAAGAGAAAATTCCATATAAAAACCCGCTACAAACGT
TGGTATGCCAAGGAAATCCTGAAATTCCGCCTATTTTGACAAACAATCAACTCATTATTTATAAGTATTGATGA
TAGGGTTGTGTCTCTGCTTCCTTATATATATTATTTATTTATAAAAAGTAACGGGATTTTGGGATTGTGCTTGCA
CAATCCTTCTGTTTCTTCGAATCTGCAAATCCCAATCATTTCCCGATAAAAAATCATTGTGGGATGTTCTTTAGC
AATTTCAATATAAGCATTGTATAGTTATGAAAAAATTACGACAATAACTGTTTCATTAGATAAGTGTTATTGAAA
TTGATAAAGAGCAATTCTTGAAAATAGTTAGATAAAATAAGCGAAAGAATATAGTGAAAATTATTGTTATAAC
AATGATTCTATTAGCTAAATAGTAAGATATAGTGTTTGGGGCAAAAATAAAGACGAAGTGCTGAGATGCACTT
CGTCGAGTTGTTTATTATTGAAAAGTTGTTTAATGATTTCGTTATTAAGTTTGAGTGTGACATAGAATTGTTTT
TTATGATTACCATCTTTTTTAATATCAATGCGATCAATCACTGATAGATACAATGCTTTGAGTCGAGATTTTTCTA
TGTGCTTAATATCATGAAAGATGTGTTGTAATAGTTTACTGATTTCTTTGGCATCAAATAAAGTCTTATCTTCAT
TTTGTTGATTTTTGAGTTGGTTGATTTGATTCGTAATGTCATTGAGTTGCTTTTCATATTTTTGAATACTTGGTC
TGATTACTGATGTTAAGTCCGGATTATCCTCGATGGTTTTAATCAAGTTATTTATTTTGATTTGTACTTCATCATA
TTGTTGTTGCTTATAAGCAATATCGTGATGAAGTGCAGCGCCATCAACTTGATTTTCTTGATTGACGTGTGTTA
CTACGCGTTGAATGACTTTATCACTTTTGACTATTTCAAGTATTTGCTTCATCACATAATCTTCAATCACATCAG
CTCTTACACTGTTTGCCGAACATACTTTGGAACCCTTGTTCCGAAAATTACTACATGAATAGTAACGAATACGT
TTCTTAGTCCCGTCTTTAAGAGTATTCGTGGTATTGCTTGCTGCCATAGGTGCGCCACATTGGGGACAGTGAA
TAATGCCTGTAAGCAGATTCGTTCCTTTGCCATGGACTTGGGGTTTTTGACTGACTTGTTTCTTACGCATTTGT
ACTTTATCCCATAAATCTTGATTAATAATGGGGGAATGCTTACCTTCAGCTATCACTGGTTTATCATTCAGCCCT
TTACGACGTTTTTCACTCCAATCTTTGTATTTCGCAAATTGAATTTTGCCGATATAGAAAGGGTTAGCTAAGAT
GTATGTGATTGAACTAATACTGAAAGGTTTCCCCTTTTTAGTGACATATCCTTTGTGATTCAATGCATTGGCAA
TTTTACGATAGCCATGTCCTTTGGCATAGCACTCGAATATATATTTTACAATATTAGCTTCATGTTGGTTAATCAT
TAGCTCGTGTTTACTATCTGGTATTTTGTCATAACCTAGTGGTAAATTGCCTTGATAATAGCCTTCTTGGGCAC
GTCTCGTTTGACCCATAAATACGTTCTCGACAATGTTATTACGTTCGAATTCTGAGAAACTCGCAAGTATCTGT
AACATGAGTTTACCAGAAGAAGTATTGACTTCCATACGCTCTGACAAACTGAAAAATTCGACATTTTGTTTGT
GTAAATCTTCGACAATTTTGAGAAGATCAGATGTATTACGAGCTAATCGGTTTGTTTTGTATACCATAACACAG
TCGATATTGCCTTCTTTTGCATCTTTCAACATACGTTGGAGCTCAGGTCTGTTCATAGATTTACCTGAAATACC
ACGGTCAGCGTATATATCTTTAACTTCAAAATGATGGAAGTCACAGTATTCTTTGATTTGATTGATTTGTCCGT
CGATACTATAACCTTCTGTGCTTTGCATTTCTGTTGATACACGTACATAGATACCGACACGTTTTGTTTTAAGTT
GTTGCATTATGTTACATCCTTTCTTCATTTATGCAATCGATGATTGCATGGTTTGATTGACGATATTGAGTGGTT
CATTTTTGAAATAGATTCCTATAAGATTTTTATCTTTCGTAATGTGAATGGTTTCAATATAGGGGTACAATATGT
TTAACGTGAAACGTTTTTGAATAATATTTTGAATGGTGTGTTGTATTTGATGTCCATTGATAGATGTAGTGCGT

TGAGGTTGTTGACGTAATGATTGCGTTTGTTCTCTGAACGTTTCTGCATCGATGATGCCTTGTGCCAACTTTT
CTATCAGTTGTTCTTGAGTCAATGTGTGATGTTTTTCTATGTTTCTTTGTCTTTTGATGCGTTTGTCAATCGCAC
CTTTAATTTTTGTGTAGATGCGTTGATTTTGATAAAAGTCTCGGCACACTTCTAATACTTTATCTTCAAATGTTT
GTGCATTGATGCCTTTGAAATCACATACAAAGCGTGAAGCATTCATGTTTTTAGGACAGACGTAGTAACGTAA
TATATGATTCTTTTTTCTAACGGTCATATTTGTAAGTGTTGCATTACAACATGGGCAATTGATTTTTTGTTTGAG
TTGATTATCCGAAGATGTCTGTTTGGTTTGTTTTTGCAATCGAAGTCTCTGCGCTTGCTCATATATACTTGTGG
AAACAATAGAAGGAAACATATTGTCGAATTGGCCATATTGATTGTTGACACGACCACAATAATTAGGATTGAT
GATAATGTTACGAACTTGATAGGGTTGTCGATTGATATACGTGTTATCTTCTTCTAATAACTGTGCAATTTTCTT
ATAACCATGACCTTTAATGTAATAATTGAATACAGCCTTTACCGTTGGTGACTCATTTTGATTGATGATGAATGT
TCCGTTGTGATATTCGTAACCAAAAGGTGCATGTGTTGTAATCAATCGACCTTGCTTTGCTTTTTCTTGAAGCC
CATTTCTGACTTGTTCTCCAATGTTATCCGATTCAAGTTCGGCTAAACTGATGAAGATATTAAGCTTGAGTCGG
TCGAAAGCTTGATCCATATCAAAGTAACCATCATGTACGCTTAAGATATGAACATGGTACGTTTGACATAATTT
GATGAGTTTTAATGCATTTTTCAGATTACGATGCAACCTATTAAGACGATAACAGCATAATATGTCACACTGTC
CTTGTTGAATTAATTGTGTTATTTGTCGATACCCACTACGATTATCTTTGCGACCTGATTGTTTGTCGCTATAAA
AGTTGATATGTTGAATATGATGTTTTTCGGCTATTGCTTCGATAGCTTGTTTCTGTGCTGCAAGAGATTGTTGT
TTCATCGTACTTTGACGTAAATAGCCTATGACTTGTTTCATATCGGCTCCTCCTTTCACAGTGATAATATATATTT
ATGGATGAATTGATATATAAGCCCAACATCAATGAGATGTTGGGCGTCCATATTAGTCATTTGTTTGATTGATTT
CTTCAATTACCAAATCGGCTAATATCTCGATAAGTTCATCCATGTTTTTCACTCCGTTATTTGTTCTATCTTCAAT
ACGTCGATTATTCAGTTTGATGCTTCACAGTTGTATGATAAAGACAATTAGAAATCTTCGTGAACTCCTGAAG
GGCCTATCCCTTCATTAGCGGATTTAAAAAGTTCTTTCGCAGCTTTGTTATCATTTGACGGTGTCCAATTTTGA
AGTAACGACTTATCTTTAGTTAATCCGAGGATAGATGCAAACTCTACATCTAATTTTAGATGGTAAAATACAAG
TGATTGTTTTTTACCGCTATTATCTTTGACACTTCTTTTAGTTGTTTGGCGTCCACGGTCAGCTAATATGAAACC
TTTATCTCTTAAGGCGTTGACAACATTATTAACATCTTGAAATTGATGATTGTTTAGCATCTGTTTAAAAACGTT
CGCAATTATTTTTACTTCGATATGGTCATCTTTTAATGAGATTAATCCATAGTTCTCAAACATATTTTTCAAAGCA
CCTTCATCTGAAAACTTACCTCTGTTTTGTGCCACAAATTGAATGATGACATCAATAGCTTTATCAGCTAATGA
GCGTTCAGAGACTGTATGAGTATGATAATCAATAAAGTAGTCTCTTATATTAGCGATATCAATATCTGTAGATAA
AACACGACCTAATATTTTCGCAGATGTTGTAATGACTGCATAACGCTTAAACATACGAATACCTGTATTGTTTG
TTTCATCTTTCAATTTAGCTTCAAACCAATCTACTTCCTTGTAAAACCATTGAATAACTTCATCTTCACGATTTAT
AAGATATTTAGCTACTAACGGTAAAACATGACCATAGTTTAGTGCTACAGCTTTTTTAATATTGTCAGCATTGG
TCGCATTTGTAGTGAATTGTTCATTAATCTCGATGGTTCTTACACGTAATCCATCGTTTTGAGCTGAATCATTAA
AAATACTGTATTCTGACGTTGAAATGACAGAAGTACCCCAATTCTTAGGCGTTTTAACTTCTCCATGAACGTTT
GAACGTTGACGACCTTGACCTTCAGCGATGGAATATAACAAACCCGTGGTATCTCTAAGTGTCGTAGATGAA
AGCTCATCAAATACTATAGGAATGCCATAATTGTTACTCAAATAACCTTCAAGTGCGTTTCGTGTGGCATTCCA
ACTTCTAAAAAGAGTTTCATTACCTTTGGTAGGGTTACCAGCGACTGATACAGCTAAAGCAGCTGCGGTTGA
CTTACCGGTTGAGGATTGACCTGTAAAACTAAAGAGAATTCCTGCAAATTCGATTTCATGTTTATGCTTCAGA
AAACTTGTCACTAAGGCAGAAATCCCAAATATGACTGCCAATTCTAAAAGAAGATGACCTTTAACCTCGTCAA
TATACATGTTAAACCAATTATCAAATGTTCCCCTAGGTGCTAAGTCATAAGTATTCTCACAAATGGCGTCAGAT
GGAGATTTATTATCAAATTCCGTAGTAGTATAGATTTCATTTAACGATACAATAGGACCAAACGGTGTTTCCAG
TATACCTACCCCTTCATATAAGTAGGAAATGGGTAATTGGTTGCGCATTTGTTGCAACGCATAACCTAAATCTT
TTGTATATTTTTCATTAATACTAAATCCATATTTCATTAAAGAGGGCAGTTTTTGTGTTGTTAAAATATCACTAGA
TTCAACAATTACTTTTTGATCCTCGTCTGTAATAATTACTTTTTCAGTGTTAGTTTTAGGGTCAATAAACTTATTT
TCGATAACGATAGGACCTGCGATTTCAACTTCAGTAGGCATTCCTCCTTTTTCTTTGGGAGGCTTGTCTTTATA
CCAACCTTTTTTTGATTTGTATCGTGGTGAAGGATTAAATGAAGGGTTAGTTTGAGTCATTAGCGAACACCTC
CTTTCGAAGGGTTGCTGTTATGGTGTGGATTAGGACCTGTTTTAAGATAAACTAAATGACCGTGAGTATCCTT

ACCGATAATAATAAATGGAACACGTGGCGCATGTTTTACAAAATATGCGAACCAACGTCCAACATTTTGTTGT
ACTTCTTTAGAACACGTTACATTTGCACGACTGTTCAAGTCATGAAATGTAAATTCTTCTCCTTCAGGTAAATT
GAATGCAATACCTAAAACCTTGCGTTTTAACAATAAAAAGTGGAGACTCTTTTTATTCATTTGGATCATCCTTT
GTACATTAGTCATCATTTAAATGATGCACTTGTATCTTATATCTTTACTGAGATATAAAGTTCTAAATTTGAGAG
GAAAATATTAGGACATTGGTATTACACCTTTTGATAAGTAAAAAAACAACGATTTTTTTAGACTGACCCCAAT
TAGTGGGAATTATATAAAAACACTTTCGTTGAATTCATATTAAAATGAATCATACGGGGGGTGTTTTTTTCTAT
GAAAAGAGTTTCTTATTCAGTAGAAACAAAGTATAAAGCAGTTGAAATGAAAGCAGCAGGATTTTCAACAA
AAGAAATTATGAAAGAATTAAATATTAGAAATAGAACACAAGTGAAAACTTGGTGGCGATGGTATCGAAATG
GGGAAAGTTATAGATTTTCACAACACGTTGGTAAACAATATACCTACGGTAAAGGATTAGAAGAGCTGTTAG
AAGTAGAACAATTAAAATTAGAAAATAAGAGAAAAGATATAGAATTGGATATTTTAAAAAAGTACAAGGCAT
TGGAGAGGAAGTGGTACCAACAGTAGTCGTAGATTTAGTGGATCAATTAAAAGTAAAATATTCAATCAAATT
GATACTAGAAGTATTAAACATACCTAAATCAACATATTACCGATGGAAAAACAAAACCCATAAAAATGATACCG
TAACACAAAAAGTTATTGAATTATGTAAAGCTAACCACTATACCTACGGTTATCGTAAGATTACAGCATTGATT
AATCAATGTTATACATCACCAATTAATCATAAGAGAGTACAGAGAATGATGCAGAAGCATCATTTGAACTGCC
GAGTTACCTAAAAAGACGACAAGAATAGGTAAACCGTATTATAAAACGGACAATTTATTACAAAGACAATTTA
AAGCGAGTTGTCCAATGGAAGTATTAACAACCGATATTACTTATTTACCATTTGGTCATTCTATGTTGTATTTAT
CTTCGATAATGGATATTTATAACGGAGAAATTGTGGCGTATAAAATAGATGATAAACAAGACCAAAGTTTAGT
TAATGATACATTAAATCAAATCGATATACCTGAGGGTTGTATATTACATAGTGATCAAGGCAGCGTTTATACATC
TTATGCTTATTATCAATTGTACGAAGAAAAAGGCATTATCAGAAGTATGTCCCGAAAGGGAACACCCGCCGAT
AACGCCCCGATAGAAAGTTTCCATTCCTCGCTAAAGTCTGAAACTTTTTACATCAATAATGAGCTTAATCGCTC
TAATCATATTGTAATAGATATTGTCGAAAAGTACATTAAAAACTATAATAATAATCGAATTCAACAAAAACTAGG
CTACTTATCCCCTGTGAAATACAGAGAATTAATAGCCTAGAACATGGTGTTTTTATTAAGTTCCCGTTTTAAGG
GTTCAGTGCCTTTAATCGTTGGTTTTTTTTATTGAATTAATAATATAAATTTGGTCCATCGTTAATATCTTCTAAA
CGTGTCTTGTAAAACTTACTGATTATTTGAATCATTAGTTTATCGAGAAAATCATTAGGATTATTATTAAAGTTG
TCTCTTTTATAAAAGTTGCTAATATTATATTTTTTTAGATCGGTGAGTAAAGACCTTTCTACTTTTTCTGTCATTT
TTATATCATTCATAATAATATTATGAATTAAATCTTTTAATCTAGTATTTCTTACATAACCATCAATAAGACTTTTAA
AACGGGTTAATGATCGTTCGTGTTTGTGTGCTAATTTATTCAAATCATTTTTTAAATTAGAAATACTATATAAAG
CGACTAATACATCGTATTGTGTCGCACTTTGAATGATTTTACTTGTTACTAAAACTAATTCATCCATTAAATCGG
AAATGGCAGTGAATAATGCAAAGTGGAACGTATCTTCAATAGTTGTTAAATAATATAATTCATTGTCGCTAATT
AGATCTTGTTTTTTCATAAATTCATTTATAGTGTTTTGAGACTCAATAAGTCCATCTATTGTTAAATTAACTTTAC
GTGACTTTTCTGTCAGAATACTACGGATGAATTTAAAGTATGTATCATTTTGATCATATAAGTTGAGAGAAATG
CCGATAACAGCTTGCCTAAATTGGTCTAACTCATCAGATTGATTTAAGCTATACTTGTTATGAATCGAAGGTTT
GATACTATTAGCAATTTCAAAGTATTGAGAATTACTTGTTCGTTCTTTGATTAAACCTTCCGTCACTAATTGTTT
AAAGTAGTTAGACTTATTTAAGTTTGAGATATTTTTCTTAACATAAGAGTCTTGTTTTTCTTTATAAATGAGATA
TTTCGTTAAAAAATCACTTATTAATACATCGTCAAAATTTTCTTTAGATAAATCTTGATGAGTTTCTAATATTGTA
TCTTTCAGATTTATAAATGATTCCAAAGAAGATACATCGTCAATAGCTTTTTCATAATCTATTCTATAGTTAGCTT
TTTTTAATTCAATATTTTCTCGAATCTGTTTTATTGTATTGTTAGCTATCTTTTTAGCAAAATCTGTTGTTTTATTG
ATTGTGGTTAAGAACTCGGTGAAATATGTATCTAGAGTTGTTGATTCTAACACATCTAGAGCATCTACACCTTG
GTCAATGATTTCATTTGAAAGTTCATCTATGTCCACCATACTTTGACTATCAATAGAATACGTCAGATTATCTTTA
GTAAAGCCACTAGGAATTTTATCGATAAATATACCCTCTTCACTACATTTCACAGTGTGTTGTTCAATATTTTGA
TTAGAGGTATTTACAAAGTAGCTTATATATATAGTTTCATTAGGTTGTGCTTCTCCCACTACATAATTTGTTCTCA
TTTCTTCAGGTAAAATATGTTGTGCATATTCTTTTAGTATAAATTCGGAATCAATTTTGTGTATAAAATAACTGA
AGAATTGAAACCACAGCTCTATTTTCCACTTATTTTTAATCATGATAATCACTCTCTTTCAATATTTGAATTAAGT
ATATCAAATCAATATTAATTGATATGATAGTTCTTTTACTAATTATTTTTATAAGATAAAATAGATAAAAAAGGAA

CAAATGTTCTCTTTGGGTATATCATATGATTAGAGGAGATCGATTCATTTAATGAATTATTTTATTTAATAGAATT
ATATTTGAAGGCATTTAAAGGACAAAAGAAAACAGGCTATGAATTTGAAGAAAGATTGAACACATTTATTCA
ATCATATGTCATTATTTTACTTATAGATATAAGGTTACCGACTGTTAGGTTATATATTATTAGGCATATTGTGGATA
CATGCGAAAAAGAAGTGGACAATCAAAAAAGAATTATACTAATAGAGAAATCTATTAAAAAGTACAGATATG
TAAAAGAACAGATTAAAAGATTTGGAAATGCGTTGAAAGAAGAATTATCTAACGCGACTTTGTCTATTGAAT
AGAATATGTTAGAAAATATCATTGATTATTATAAGGATTACTTTTATCCTAGAGAAAAAATAAAGCATAATAATG
TTGAGTGCAATGTATCATTGTTTTTTAAAGCTGTAGATGCTTTTAAGAAATAGTTTTTCACTGACAATATTTATG
GATATAATTAAATTTAAGCAATGTTAATTTATTCCAGTCTTAGTAGTTTTTGAATTTCACAAACACAATCTATGA
AGTGAGTTAATATTAAACGATTTTAATAGGAGAGGGTTAGTATGGCTAGTTATTTATTTTTTCATCCTAAACCA
GCGTGTGATACTTATGGGGATATGAATATTTATCACGACAAATTTGGAAATAATGAGGACCCATACGTATGGA
GTGAACGGTTTTTGCATAGCTTTTGTAAAATAACGGATTATGCATATAGTAAATCTACTCAAAAGGACATTATT
TTTTGGATATCAATAAATAAAGAAGGCAATAATTTAAAATATTTATGTGATTTAGTATTTAAAATAGAAAAGTG
GGATTTTTGGTATAAAACTTTTAGTGAACAAAAAGATGCCATAGCTACAAATAAAGAATTAACGATAAATGAC
GCAGTAGTAGAAGGCGATGAAGAAGCATATGAATATCATTATTCTTGGATCAACAGAGGGGAACACAAGTG
GGAACCAACTTATCGAAGGCGAAGACTCACATTAAAAGCAGATCCCGTCTTGAGTTTTCAGCCTCAAAATCG
ACAAGGTAATTTACTAGATGTCACGGAATTACTAAAAACAATTGTGAATTTTAATGTTGAAAAATCACCTGCA
AAAAGTGGGACTTCTTATAAAGCTTTCGAACTTGAAGAAGAGCAGGCAAGCAAACTATATGAGGAAATAAA
ACGATTATCTTTTATTCGATTAAAGGGAAGGGACTTAAAAAACTTAAGAAGAAACTTTTCTTGATATATCTTAA
TAGTTGTTGTTATTAAAACCATAAAGAAAGCTACCATTTTTAAGATACAAGCATTGATATGTACAATCTGAATG
GAGTGTAAAAAGTAAGTGATGTTGTGATGGACATAAAGTTGATACTCTATTTATACGAAAAATATTGATAAAG
ATTAATTATGATAAACATGTAGTAATAGAAAATGAATTCTATAGTTCAGGACTTAGCAACTAATATTATGGTAAT
GGGAAGAATAAACAAATTAATTTTTATAAAAAATATATAAATTAAAACTTAGAACAGTTAGAAACAGAATTATT
TGATTATGTAAATTGGTACAACAATTTTAGACCACATTCTTCGTTACAGTATTTAACGCCAATGGCGTATAAAG
ATATACACATGAAAAGTGTCTAAAAAACTGTTGACATTCCACTCTATATTTGATTGTAGTTGTTTATAGATAGTA
AGATCATTCATTATACATGGATTTTTGTCCGATTTCTTGACACAATTCCAGACAAACTAATAAACTAATAAAATA
AAAAAATAAAGGCGCGAAAACAGGAATTATTAAATAAAATATTAATTTAATAAGGGGAACAAATGAAAAATA
TAGATATCGCAATTTATGATATAGATAAAGTTATTTGTAAAAGTATTGAGAATAACTCATCAGATTTAGGTTATT
TATCACAAAGTATCTTATCACATTTAAGAAACTACGTAGAACATATTGGTATGAAATATTATTTCGAAAGTGTAA
ATGAAGATATGACTAATAGTTCTTCAAATATCTATAATGAAATACAAATGGGAATAGGTTATTTTAGGAAGCAT
TACAAATTGAATTGGCTTAAAAATTTTCATGAATTACTTCAGCAATCTGTTTCTCATTATACATTTGATGAAGAT
AGATCCGAACGGTTATTTATAAAGTATTATGACTTAATGTTAGAATTAAAAGAAAAGTTAAAGAAAGATTTTG
AAATGGATTTATTGCAAAATTTATATAAAGTACCGCTGAATATTGATAGTGATTTAAAAGGATATTATCATACAA
TATCTCAATTAATAGGTAATAAATACACTGATAATGAATATGAAGTAGATAATTCAAGGTATTATATTGAAAAAA
CAAAACCATTTGTTATTGAAAATAAAGTATATTATGAAATTACATTTAGAAATGCAAATGATAAAACTAGTAAAT
ACGAAAGGCTGATTGGTTATTCAAAACATAGAATAAACACAAATTATGCTGTAAAATTCGCATTAGAAATGCA
AACAATAAGTTATTTAGGGATTAAAGCTAACGTAATAATAATAAATGATTACGAAATATCTATAAGACCTTGTG
AATTTAATAATTTTGCTAAAATATTAGATTACGATTTAAAATTACAATCAAATCATAATGAATATAAAAAATTAAT
GAAATTGTTAAAAGAATTTAATTTACATTTACTTGATATTGTTTTGTTAGATGACGATGAATTTAATGAATTGG
AGTGTTCAGTGAATAGCGAATCAGCAGTTATTAATATTTTTAATGTGTTGAGACTAGCAAGAAGATATATTTTA
ACAAATAAATCAGGCGCAAATGTTCTTAGGTATTTATTATTTGTTTTTAACAATAGGATAATTCGTTTACAGTTA
CCTTATAATAGGAATAAATGTTATAAATTATCTAACCTTATATTGGATTATAAATGTGTTCCATTTGATCAAATGC
CATTTACAGCCTCTTTAAAAGGCCATAATCCTAATATCTATACTTTGTTACAATGTATAGAATACAAAGGGAGA
GAATATGAATTATTAGTCAGGAAGATTCAAAAGAATACTTTGAAGAATAAGAAAGTTTATACAAGCAAAGAA
GAAATTGAGCAGTATGGTGTTGTAAACGAACTAATCGATAAATATAATAATAATTTGTATTATAAGCATCGACC

GAATAGGGAAATACATTCATTTGGTGATAAATATTATTTATATGAAAATGAGCAGAGTATTATGAGTATAATAAA
GTCAATTAAACTATTGTCAAATGAAAGCGTAGAGGGCTATTCAAATTCAGTAGAGTTTTGGCTAAATAATGAA
TATACATCGTTGGATTGTAAAGAAAAGAAGGAAATACTCTTAAGAATGTTTTCAAATAGCAAAATATCGATGG
TATACGGAGCTGCTGGAACAGGGAAATCAACTTTAATAAACCATATTTGCAATTTTTTCTATGATAAAGATGTT
ATCGTCATAGCAAATACTAATACTGCAGTAGACAATATTAAAAGAAAAATCAAATTATCTAACATTAAAACGTC
TACTATTTCTAAATTTTTATATAATGATAAAGAAAAGTATGACTTGTTAATAATCGATGAGGCAGGTACAGTTAG
TAATAAGGACATGAATCGAATTCTTGAAAACAAGCAATTTGAATTATTATTAATTGTCGGTGATAATTATCAAA
TCGAATCGATAGATTTTGGAAATTGGTTCGAAATTGCCAAAGATGTTTTGTCAAAAAATATAATCAACGAACT
AACTGATATGTATCGAACTAAAAATGATGATTTACTTTACTTTTGGAAATCTGTTAGAGAAAAAAAGAGTAAT
TTAAATGAAATTATTAATATGAATAAATATTCTACAAGATTAGATGAAAGTATATTTAATGAATTCAATAAAGATG
AAATTATTCTTTGTTTAAATTACGATGGTATATATGGTATCAATAACATTAATAGATTATTACAAGCAAATAATAA
AAATGATTCTGTAATTTGGGGTGTGAAAGAATATAAAGTTGGTGATCCTATTCTATTTAATGAAACTAATAAGT
ACTCACCAATACTTTTTAATAATTTAAAAGGGTCAATAATTGAAATACATGTTTTTGAAGAATATATATTGTTTG
ATTTAGAAATAAATAAAGTTATAAATGAACTTGATATTATTTCGTTAGAAATTGATTTAATAAGTTCTTCTGAAA
ATAGTTCGGTGATTAGGATTAGAGTAGAAAAAAGTGATGGGTTGAATGATGATGATAATGATTCATCTGATAG
TATAGTTCCTTTTCAAGTGAGTTATGCAATTTCAATACATAAAGCTCAAGGGTTAGAGTTTAATTCAGTAAAAA
TTGTTATATCTGATGATTTAGACGAACAAATCACTAACAATATTTTTTATACTGCAATTACACGAGCTAGAGAA
AATTTAAAGATTTATTGGTCACCACGAACTGAAAAGAAAATTATTGATAATATAATTTCTAAAAGAAACTTGAA
GGATCTTTCTATATTAAAATCTCGAATAAAAAAACAAAATACTTAGAAATTTTCATACGACGCTTATATAGAACC
ATAAAAATCGTTTGACTAGTACCAGTGATTTTATTTAATTTTTCTCAACGACTCTACTTCTAAAAATATTTTAAAT
GACTTTGATACAGCACGAATTAAAATCGCATAAGCGATGACTTTTCCACTTAATTTCTTATGCCATTCTACGGA
AGTCATTTGAGAACAAAGTATTAATGATTTTTCACGGCTTCTTATTTCGAATACCTCCATGAGATACTATTGTTC
TTGTTCAGTTGTATACGGCAATAAAAAATCATCATTTACTAAAATATCCGTTCTGCTAATCACTTTTAATAGTTTA
TCTATGCTATTATAATCAGCTTGTTGTAGCTTACTTAATAAATCTGTCAATCTATAGAATAATACACTATACCGATT
ATCAATCGCATGATTTACTAAAGTAAAAGCGATGTAACTTTTACCATTTCCGGTGACACCTTTTTAAACCGCCA
TTTACATAAATACTATCTTTAAATATAACTTGATTATTTGAAAGTATGTGAAAGTAAATTAAGGATTGGTACGTT
TTGGTTAATTAAATATCTACCTAAATATTCCTCTAATTGACTTGTTGCATGTTCATTCAGCAAAACAGGAATAAT
CCATGCTGTATATAAATAATCATCTAGAAATTCTCTACATGTAGAATATTGTTTTTTTAATTCATCGTCTTTTATAT
ACAATGAAGATAATATTTTTCCGGTACCTTTATCTTCATCCATCTTCTTAATAAGTCACCTTCTTGATTTTTAACT
AATTTATATTCATTACCCTTAAATTCATATTTGAAATGTGATTTTCCAACTACGCAAACCATGCCATCTTTTCTTT
GAGTAACATAGATGTAATTTATTATTGGGTTATTTCCATTAGCTCTATTATTATTTGTTAATGTGTTACTTATATTA
GACAATAAATCCTCAAAATATGTAATAATATATTCATCTAAGGGTTTATTGTTATTACTAATATTACAATTTTGTTT
GATAGCATAAAAAGCTTCTAGGTTTAATTCAAATGGTTCCGCGCTAACGATTTTGCTATCTTTGTTGTCTATAAT
AAATTCGATTAGTAATTTCTTAATCATTTCGTGATGTTTTCTAGGCTTATTGTAATGACAGGTTTTATAAAGCTG
TTTATTAATTCTATTAAACATTTCTATTTGCAAGTCAGCAAGAATAGTTCTACAGTCTTCATAGATATCATGTACT
GGGTATGGTTTATTAGGAAACATGCTATATATTTCTTTATATGTTGTATTGAACACGTTAATTATACCTCCTACTA
TCTATTAATACTATTGTAATTATCAGATATAACTAAGTAAATGAATCGTCTACACTTAATTGGACAAATTCTATGA
GAATAGATATTGTTAATTTAAGAAAGAAATTACGAATGAACGCATTCGCAAAAATTGCATTTAAATAATGAAAT
ATAGAGTAACTTGAGAAAGTTGTTTCATCTAAAATATATAAACAGTAGAGGGGAGAACTTATGCACTGGAAA
GAAAATAGAATAAAATCAGCCAAAAATGACACGAATCCGATGGTAATCAAAGAATTAAAAGGTAGTTATGTG
GTTTTTGGAGATGTTCAGTTTCTTCCAGGTTATTGTGTATTACTTCCTAAAAGAGAGGTAAGATTATTGAATG
ATCTTACTTTAGAAGAACGACAAGATTACTTATTAGATATGAGCTTTGTTGGTGATGCTATGATGAAAGCATTG
AAACCTACAAGAGTAAATTATGAAATACTAGGTAATAAAAATCACTTTCTTCATGCACATTTATTCCAAAGATAT
GAATGGGAAGATGAATCTGTTAGGTATATGCCAGTGTGGTGTATGATGCTTCTAATTGGTCTAATGAAGAAAC

GTCTTACGATTCTGATAAACATGATGAAATTAGAAATAAGATAAAGAACGAACTTGAACAGTTGTATAACATAT
AAAACCAAAGTCGATATCATCATTTTGATATCGACTTTAATTATAAAAAACCGCACTCTTAACCGATACGCAGA
GGCGTATCATAAGT
>Staphylococcus aureus strain 25b_MRSA
ATGAAAATCACCATTTTAGCTGTAGGGAAACTAAAAGAGAAATATTGGAAGCAAGCCATAGCAGAATATGAA
AAACGTTTAGGCCCATACACCAAGATAGACATCATAGAAGTTCCAGACGAAAAAGCACCAGAAAATATGAGT
GACAAAGAAATTGAGCAAGTAAAAGAAAAAGAAGGCCAACGAATACTAGCCAAAATCAAACCACAATCCA
CAGTCATTACATTAGAAATACAAGGAAAGATGCTATCTTCCGAAGGATTGGCCCAAGAATTGAACCAACGCA
TGACCCAAGGGCAAAGCGACTTTGTTTTCGTCATTGGCGGATCAAACGGCCTGCACAAGGACGTCTTACAA
CGCAGTAACTACGCACTATCATTCAGCAAAATGACATTCCCACATCAAATGATGCGGGTTGTGTTAATTGAAC
AAGTGTACAGAGCATTTAAGATTATGCGAGGAGAAGCATATCATAAATGATGCGGTTTTTTCAGCCGCTTCAT
AAAGGGATTTTGAATGTATCAGAACATATGAGGTTTATGTGAATTGCTGTTATGTTTTTAAGAAGCTTATCATA
AGTAATGAGGTTCATGATTTTTGACATAGTTAGCCTCCGCAGTCTTTCATTTCAAGTAAATAATAGCGAAATAT
TCTTTATACTGAATACTTATAGTGAAGCAAAGTTCTAGCTTTGAGAAAATTCTTTCTGCAACTAAATATAGTAA
ATTACGGTAAAATATAAATAAGTACATATTGAAGAAAATGAGACATAATATATTTTATAATAGGAGGGAATTTC
AAATGATAGACAACTTTATGCAGGTCCTTAAATTAATTAAAGAGAAACGTACCAATAATGTAGTTAAAAAATC
TGATTGGGATAAAGGTGATCTATATAAAACTTTAGTCCATGATAAGTTACCCAAGCAGTTAAAAGTGCATATAA
AAGAAGATAAATATTCAGTTGTAGGGAAGGTTGCTACTGGGAACTATAGTAAAGTTCCTTGGATTTCAATATA
TGATGAGAATATAACAAAAGAAACAAAGGATGGATATTATTTGGTATATCTTTTTCATCCGGAAGGAGAAGG
CATATACTTATCTTTGAATCAAGGATGGTCAAAGATAAGTGATATGTTTCCGCGGGATAAAAATGCTGCAAAA
CAAAGAGCATTAACTTTATCTTCCGAACTCAATAAATATATTACATCAAATGAATTTAATACTGGAAGATTTTAT
TACGCAGAAAATAAAGATTCATCTTATGATTTAAAAAATGATTATCCATCAGGATATTCTCATGGATCAATAAGA
TTCAAATATTATGATTTGAATGAAGGATTCACAGAAGAAGATATGCTAGAGGATTTAAAGAAATTTTTAGAAC
TATTTAATGAATTAGCTTCAAAAGTTACAAAAACATCCTATGATAGCTTGGTCAATAGCATAGACGAAATACAG
GAAGACAGCGAAATTGAAGAAATTAGAACAGCACAAAAAGATAAGACACTCAAGGAAGTGGAAGCACCTA
AAGGAATAATTCCAAAATATAAAAAAGGTGTATCAAAGACTACTAAAAATGATTCAGAAATTGAAAAATCAA
ATAAAGAGAATAAATTAACCGGTAAAGTTGGAGAAAAATTAGCGCTAAATTACTTTAATGAGCTAATTGATAA
TAAAATAGACGAAGATAAGAAAGAACAGTTTAGGAATATTTTAAATGATAATCCAGGCTCTCAACACGGTCAT
GGCTATGATTTAGTAGCTTTTGATCCAACAAATACAGATAAAGCTGTAGAAAAATTTATTGAAATTAAAACATC
TACATCTTCTAGTATTGAGGAACCATTTTTTATGTCGCTAAATGAAATGTTTGCTATGAAAGAATATAAGCAGA
AATATTTAATATTAAGAATATTTAATGTTTCCGGTAAAGAACCACAATTTTATTTTATAGATCCATATGCAAATTA
TTCTGAATTTAAAGATGTAGATGATCTCATTGACAAAGTATTTAATGTAGAAGCTATTCAGTATAAAGTTTTTG
GCGAAAAAATGATTACTTGAACAAGAGCTAAAATAAAATTGTGATCTAATAAAAATAGAAGGTTCTGTTGCAA
AGTAAAAAAATATAGCTAAYCACTAATWTATCATGTCAGTGTTCGYTTAACTTGCTAGCATGATGCTAATTTCG
TGGCATGGCGAAAATCCGTAGATCTGAAGAGACCTGCGGTTCTTTTTATATAGAGCGTAAATACAYTCAATAC
CTTTTAAAGTATTCTTTGCTGTATTGATACTTTGATACCTTGTCTTTCTTACTTTAATATGACGGTGATCTTGCTC
AATGAGGTTATTCAGATATTTCGATGTACAATGRCAGTCAGGTTTAAGTTTAAAAGCTTTAATTACTTTARCCA
TTGCTACCTTCGTTGAAGGTGCCTGATCTGTAATTACCTTTTGAGGTTTACCAAATTGTTTAATGAGACGTTTG
ATAAACGCATATGCTGAATGATTATCTCGTTGCTTACGCAACCAAATATCTAATGTATGTCCCTCTGYATCAATG
GCACGATATAAATAGCTCCATTTTCCTTTTATTTTGATRTACGTCTCATCAATACGCCATTTGTAATAAGCTTTTT
TATGCTTTTTCTTCCAAATTTGATATAAAATTGGGGCATATTCTTGAACCCAACGGTAGAYCGTTGAATGATGA
ACGTTTACACCACGTTCYCTTAATATTTCAGATATATCACGATARCTCAATGCATATCTTAGATAGTAGCCAACG
GCTACAGTGATAACATCCTTGTTAAATTGTTTATATCTGAAATAGTTCATACAGAAGACTCCTTTTTGTTAAAAT
TATAYTATAAATTCAACTTTGCAACAGAACCGTATTATGGAATAGAGATGTTGGTAACATTTATACAGGATCATT

ATACTTAAGTTTAATTTCGTTATTACAGAACCACACATTCCAACCAGAAGAGAAAGTATGTCTATTTAGTTATG
GTTCAGGAGCAGTAGGAGAAATCTTTAGTGGTTCAATCGTTAAAGGATATGACAAAGCATTAGATAAAGAG
AAACACTTAAATATGCTAGAATCTAGAGAGCAATTATCAGTCGAAGAATACGAAACATTCTTTAACAGATTTG
ATAATCAAGAATTTGATTTCGAACGTGAATTGACACAAGATCCATATTCAAAAGTATACTTATACAGTATAGAA
GACCATATCAGAACATATAAGATAGAGAAATAAACTAGTGGCCGATTGTGCTTGATGAGCTTGGGACATAAAT
CCTAACTCGAAATAAATAAGCATATCACTAAACTGATTTTTTAAAGTTTACAGTGATATGCTTATTTTTTTATCTT
ACGATTTTGTACGTGCATGCTTGCCTAGGGGTATGGCTCGAGCCATTAGTCTCTCGCACATACTATTCCCTCAG
GCGTCAGCACTTACAAAATCGGTTGTAATTTTCATTTTTATACGCATTCTTACTGAGATTATACTAATAAGAGG
AATAGTAAAAGCAATTCTAAGTAAAATTGCAGATAAGAGGTTTGTTAAAAGCAGTTCTCAGTAAAATTACAG
ATAAGAGGTACGTTAAAAGCAGTTCTAAGTAAAATTGCAGATAAGAGGTTTGTTAAAAGCAGTTCTAAGTAA
AATTGCAGATAAGAGGTACGTTAAAAGCAATTCCATGCAAAATTGCTGATAAGGGGTAAGTTAAAAGCAGTT
CTCAGTAAAATTGCAGATAAGAGGTACGTTAAAAGCAGTTCTAGGCAAAATTGCAGATAAGAGGTGCGTTA
AAAGCAGTTCTCAGTAAAATTGCTGATAAGGGGTAAGTTAAAAGCAATCCTAAGTAAAATTGCAGATAAGGG
GTACAGAAAAACTAGACTTGATTACAAAATGGAGCTTGGGACATAAATGATTTTTTAAAAATGAGATGAGAC
GTAGATTAACTCCATAATCAATACGAATCTATCGACTTCTTTATTTATGATATTCATCTCTTTTTAATGGAAATAA
AAGTGCGATTAATGTGATAATACAGTTACGTTAATTAAAAAAATAAAAATGCAAGGAGAGGTAATATGCTAAC
TGTATATGGACATAGAGGATTACCTAGTAAAGCTCCGGAAAATACAATTGCATCATTTAAAGCTGCTTCAGAA
GTAGAAGGTATAAACTGGTTGGAGTTAGATGTTGCAATTACAAAAGATGAACAACTGATTATCATTCATGATG
ATTATTTAGAACGGACTACAAATATGTCCGGGGAAATAACTGAATTGAATTATGATGAAATTAAAGATGCTTCT
GCAGGATCTTGGTTTGGTGAAAAATTCAAAGATGAACATTTGCCAACTTTCGATGATGTAGTAAAAATAGCA
AATGAATATAATATGAATTTAAATGTAGAATTAAAAGGTATTACTGGACCGAATGGACTAGCACTTTCTAAAAG
TATGGTTAAGCAAGTGGAAGAACAATTAACAAACTTAAATCAGAATCAAGAAGTGCTCATTTCAAGCTTTAA
TGTTGTGCTTGTTAAACTTGCAGAAGAAATCATGCCACAATATAACAGAGCAGTTATATTCCATACAACTTCGT
TTCGTGAAGACTGGAGAACACTTTTAGATTACTGTAATGCTAAAATAGTAAACACTGAAGATGCCAAACTTAC
TAAAGCAAAAGTAAAAATGGTAAAAGAAGCGGGTTATGAATTGAACGTATGGACTGTAAACAAACCAGCAC
GTGCAAACCAACTTGCTAATTGGGGAGTTGATGGTATCTTTACAGACAATGCAGATAAAATGGTGCATTTGTC
TCAATAGAAAGTTAGAGGTGAGTCTTACGTTTCAGTGACGGTAGACTTACCTTTAACATGTTACATACTAAAA
AATTAATTTGAATAAGAAAGAGAGACATATATGAAATACGATGATTTTATAGTAGGAGAAACATTCAAAACAA
AAAGCCTTCATATTACAGAAGAAGAAATTATCCAATTTGCAACAACTTTTGATCCTCAATATATGCATATAGATA
AAGAAAAAGCAGAACAAAGTAGATTTAAAGGTATCATTGCATCTGGCATGCATACACTTTCAATATCATTTAA
ATTATGGGTAGAAGAAGGTAAATACGGAGAAGAAGTTGTAGCAGGAACACAAATGAATAACGTTAAATTTAT
TAAACCTGTATACCCAGGTAATACATTGTACGTTATCGCTGAAATTACAAATAAGAAATCCATAAAAAAAGAAA
ATGGACTCGTTACAGTGTCACTTTCAACATACAATGAAAATGAAGAAATTGTATTTAAGGGAGAAGTAACAG
CACTTATTAATAATTCATAATAAAACAGTGAAGCAACCATCGTTACGGATTGCTTCACTGTTTTGTTATTCATCT
ATATCGTATTTTTTATTACCGTTCTCATATAGCTCATCATACACTTTACCTGAGATTTTGGCATTGTAGCTAGCCA
TTCCTTTATCTTGTACATCTTTAACATTAATAGCCATCATCATGTTTGGATTATCTTTATCATATGATATAAACCAC
CCAATTTGTCTGCCAGTTTCTCCTTGTTTCATTTTGAGTTCTGCAGTACCGGATTTGCCAATTAAGTTTGCATA
AGATCTATAAATATCTTCTTTATGTGTTTTATTTACGACTTGTTGCATACCATCAGTTAATAGATTGATATTTTCTT
TGGAAATAATATTTTTCTTCCAAACTTTGTTTTTCGTGTCTTTTAATAAGTGAGGTGCGTTAATATTGCCATTAT
TTTCTAATGCGCTATAGATTGAAAGGATCTGTACTGGGTTAATCAGTATTTCACCTTGTCCGTAACCTGAATCA
GCTAATAATATTTCATTATCTAAATTTTTGTTTGAAATTTGAGCATTATAAAATGGATAATCACTTGGTATATCTT
CACCAACACCTAGTTTTTTCATGCCTTTTTCAAATTTCTTACTGCCTAATTCGAGTGCTACTCTAGCAAAGAAA
ATGTTATCTGATGATTCTATTGCTTGTTTTAAGTCGATATTACCATTTACCACTTCATATCTTGTAACGTTGTAAC
CACCCCAAGATTTATCTTTTTGCCAACCTTTACCATCGATTTTATAACTTGTTTTATCGTCTAATGTTTTGTTATT

TAACCCAATCATTGCTGTTAATATTTTTTGAGTTGAACCTGGTGAAGTTGTAATCTGGAACTTGTTGAGCAGA
GGTTCTTTTTTATCTTCGGTTAATTTATTATATTCTTCGTTACTCATGCCATACATAAATGGATAGACGTCATATG
AAGGTGTGCTTACAAGTGCTAATAATTCACCTGTTTGAGGGTGGATAGCAGTACCTGAGCCATAATCATTTTT
CATGTTGTTATAAATACTCTTTTGAACTTTAGCATCAATAGTTAGTTGAATATCTTTGCCATCTTTTTTCTTTTTC
TCTATTAATGTATGTGCGATTGTATTGCTATTATCGTCAACGATTGTGACACGATAGCCATCTTCATGTTGGAGC
TTTTTATCGTAAAGTTTTTCGAGTCCCTTTTTACCAATAACTGCATCATCTTTATAGCCTTTATATTCTTTTTGTTT
TAATTCTTCAGAGTTAATGGGACCAACATAACCTAATAGATGTGAAGTCGCTTTTCCTAGAGGATAGTTACGA
CTTTCTGTTTCATTAGTTGTAAGATGAAATTTTTTTGCGAAATCACTTAAATATTCATCCATTTTTTTAACGGTT
TTAAGTGGAACGAAGGTATCATCTTGTACCCAATTTTGATCCATTTGTTGTTTGATATAGTCTTCAGAAATACT
TAGTTCTTTAGCGATTGCTTTATAATCTTTTTTAGATACATTCTTTGGAACGATGCCTATCTCATATGCTGTTCCT
GTATTGGCCAATTCCACATTGTTTCGGTCTAAAATTTTACCACGTTCTGATTTTAAATTTTCAATATGTATGCTT
TGGTCTTTCTGCATTCCTGGAATAATGACGCTATGATCCCAATCTAACTTCCACATACCATCTTCTTTAACAAAA
TTAAATTGAACGTTGCGATCAATGTTACCGTAGTTTGTTTTAATTTTATATTGAGCATCTACTCGTTTTTTATTTT
TAGATACTTTTTTTATTTTACGATCCTGAATGTTTATATCTTTAACGCCTAAACTATTATATATTTTTATCGGACGT
TCAGTCATTTCTACTTCACCATTATCGCTTTTAGAAATATAACTGCTATCTTTATAAACTTGTTTGAAATTTTTAT
CTTCAATTGCATCAATAGTATTATTAATTTCTTTATCTTTTGAAGCATAAAAATATATACCAAACCCGACAACTAC
AACTATTAAAATAAGTGGAACAATTTTTATCTTTTTCATCAATATACTCCTTATATAAGACTACATTTGTAGTATAT
TACAAATGTAGTATTTATGTCAAAATAATGTTATAATTTTTGTGATATGGAGGTGTAGAAGGTGTTATCATCTTT
TTTAATGTTAAGTATAATCAGTTCATTGCTCACGATATGTGTAATTTTTTTAGTGAGAATGCTCTATATAAAATAT
ACTCAAAATATTATGTCACATAAGATTTGGTTATTAGTGCTCGTCTCCACGTTAATTCCATTAATACCATTTTACA
AAATATCGAATTTTACATTTTCAAAAGATATGATGAATCGAAATGTATCTGACACGACTTCTTCGGTTAGTCAT
ATGTTAGATGGTCAACAATCATCTGTTACGAAAGACTTAGCAATTAATGTTAATCAGTTTGAGACCTCAAATAT
AACGTATATGATTCTTTTGATATGGGTATTTGGTAGTTTGTTGTGCTTATTTTATATGATTAAGGCATTCCGACA
AATTGATGTTATTAAAAGTTCGTCATTGGAATCGTCATATCTTAATGAACGACTTAAAGTATGTCAAAGTAAGA
TGCAGTTCTACAAAAAGCATATAACAATTAGTTATAGTTCAAACATTGATAATCCGATGGTATTTGGTTTAGTG
AAATCCCAAATTGTACTACCAACTGTCGTAGTCGAAACCATGAATGACAAAGAAATTGAATATATTATTCTACA
TGAACTATCACATGTGAAAAGTCATGACTTAATATTCAACCAGCTTTATGTTGTTTTTAAAATGATATTCTGGTT
TAATCCTGCACTATATATAAGTAAAACAATGATGGACAATGACTGTGAAAAAGTATGTGATAGAAACGTTTTAA
AAATTTTGAATCGCCATGAACATATACGTTATGGTGAATCGATATTAAAATGCTCTATTTTAAAATCTCAGCACA
TAAATAATGTGGCAGCACAATATTTACTAGGTTTTAATTCAAATATTAAAGAACGTGTTAAGTATATTGCACTTT
ATGATTCAATGCCTAAACCTAATCGAAACAAGCGTATTGTTGCGTATATTGTATGTAGTATATCGAGCTTCACAT
GAAACAGCTAAAGAAGCTTTGGGCGATAAAGAGTTAAGAGCCATTGCACATGAGTTAACTAAAACAGTTAA
GGATAACATGAGTGTTGATTGGTCTAAACGAGACAGTGCTAAAGCTAAAATGAGAGTTCAAGTTAGACGCC
TATTAAAGAAATATGGCTATCCACCAGATCTTCAAAAAATGGCTGTGGAACAAGTTGTAGAGCAAGCAGAAT
TAATGGCAAGTCAGCAATAAAAAAATAAATCATAATGAGTCCGGGACATAAAGTTCTTGGATAAGTGAAAAA
AGACAATTTCTATTGAAATAATATAGAAATTGTCTTTTTTATAAATTTTTTGATTATTTTCAGCTCGTTGAGCTAC
TACTTTTCTTATATTAAGTGCCATTAATACAAAACCAAGTTCTCTTTTGACTTTATTGAGTCCTCGGACAGACAT
CCGAGTGAAACCCAAAATAGCCTTCATAAATCCAAAAACAGGTTCCACATCAATTTTTCTTTGACTGTAGATA
TTTTTTGTTTCTGGTTCTGAAAGCTTTTTGTTAATTTGGGATTTAAAATATTCCCAGTTATAATTCTTCATTATTT
TTTTGTTTGTTTTTGAATTGAAGTTCATACATTGATTTTTCAGAGGACATTCTGAACAATCATCACATTCATATA
ATTTGAAGTCTCGCTTATAACCATACTTATCATGACGATAGGCATATCTTTTAAAACCTAGCCGTTTATTATTCG
GACAAATGAATTCGTCATTAATTTCGTCATAGTTCCAATTTTGAGTATTAAAGATGTCACTTTTATATTTTTTAG
TTTTATCTTTTATAAACATTCCATATGTTATGAGTGGCGTTCGATTAAAGTCATCTATAATTGCCTTATAATTTGAT
TCACTACCATAACCTGCATCAGCTACAATATATTCAGGTAAATGACCGTAGGTCTCTTGAATTGAATTTAAAAA

TGGAATCATCGTTCTAGTATCCGTTGGATTTTGATACACATTATAAGATAAAACAAATTGGGAATTTGTTGCTA
TTTGTAAATTATACCCTGGCTTAAGTTGTCCATTTTTCATGTGATCTTCTTTCATTCTCATAAATGTCGCATCATA
ATCTGTCTTAGAATAACTATTTCTATCCTTTAAAATAGATTTTTGAAATTCGTATCGATACTTTCGCTCAAAATAA
TCATTGATTTGCTTTTTGTATTTTTTGATTTTAGTTCTTTTGAGACGTATTTGTTTTCTTGTTTTAGTACATTTTT
CATTGTTGATATGTTGGTTTAAATCTTCGATTTCTTTATCTAAGTGACTACCAATCAAATCTATTTCTTCTTTTGT
TAATTCATTATCATGATCTTCTTTAATTTCCGGTATGATTTTATTGGTTACCAATTCATGGTAGAGGGCTTTAGA
ATCCTCATTCATCTTTGATTCATGGTTTTGAATACTCTTTTTCCATACAAATGTATATCGATTGGCATTTGCTTCA
ATTTTTGTACCATCAATAAAAAATAGCTTTATCATCTATAAGATTTTGTTTTACACACTGACTGTAAAATTGAATA
AATAAAGATTCTAATAAAGCATCTACTTTTGGATTTACTCTAAATTGATTAATTGTTTTATAAGAAGGTTTTTGA
TTTTGTGATAGCCACATCATTCGGATGCTATCATTAAGCATTTTTTCTATTTTACGACCTGAGAATACAGATTGT
GTGTAGGCATATAGAATCACTTTTAACATCATTTTAGGATGGTACGAAGTTGCACCACGGTGATGTCTGAATT
CGTCGAATTCATTGTCAGGAATTGTTTCAACAATATCATTTACAGTAAAACGATGTTGATTTGTTTTGTTTCCA
TATTGACCTCCATGTATTTGCTATGATTTCAAAATCCATTTTTGACGTGCCTTAGGGTTGAGTGGATGCATAATT
TCATTTGTTACTGGATTGATGAGCTTTTTTACTTTCTTTTTATGAGGTTTTAACATTTCCATCACTTGTTCGACA
CGGTCGATAACAACTGGTCGCTTCGCATAGGCACCATAAGCAAGAATCACTGTGTCACTTTCACTAATCGCTT
TCATCAAATGAATATCAGTGTGCTCATCGTATGGATTTTTGATATGTTTGAGGTTTTCGGGTGTTCTAATATTAG
AGAATAGATTTACAAGATATACAGCACCGTATCGTTCTGAATTGGCTAATTGGTTGAGGATAAGAACAGTTGT
GAGATCGAGTGATAATACACCGTCTAAATGAGGATACATCGTTATCACTGTGCATGCAGCTTTCTTTTCATCCC
ATGTTTTCTTGAGTAAATAGCGGTGCTGTTCATCATCGCTAAATATGGCTTCTGTGTGTATCGTACTTTTGATTG
TATTCATCATCGTCACTTCCTTTAGTATTCTTCTGGTAAAAGCATCACATAATAAAAAGCGTCCACGTCATCTTC
ACGAATGACGTAGACTTTCTTAGGTAATGCATTTTGATTTTTTTCATAGTTTGTATAGTGATATTCCAATTTGTA
TGTGGGTTGTTCTTGTTCATGTGTGATTGAGAGTATATTCTCATCTTCTTGTAATTTAAAAATGTGTAGGTAATC
TGTATGAGGCTGGTTATCTTTTTTTTCTACCATGTGCCAAAGTAAGATTTGAAGGTCTAGTGGAATACTTTCAT
TAATTCCTCTTGTGATGTATCGATTGATTTTCATGCTATTTCCCTCCCTTCTGCTTTTCTTTCATGATGTCGATGA
TTTCGTTGATAACTGTGACGGATAATTGAGCAACTATGATCCAATTATTCATGGTCCTGACCTCCTTGTTTTAG
TAAATGACGTTCATCAATAATGATATTTTGAGTATCTGTAAGGTACAGAAAGTCCATGTCAAAATGGTCTAAGT
ATCCGACACTGATGAGTTGGTTATTGGCATACATTAGAAATGGATAGATACTTAGCTCATGTAGTTCATCATTAT
AGTAGGTATAAGTCTCGAGTGTGAGATGTACCAGTGGAGAATCATTAATAAAACGTTCCGGTAGAATATTTTC
TGCTGCTTCCTCCAGCGCTTCACATTCCCAAGCTTCGTTATTAGATAGTTGGAATAGGTGGGTTATATATTGTT
TGAGTTCTTGAGTGATGGTTTTCATATTATTGCCTCCTAGATAGTGTAATAGTGATGTAGTTCATATACATCATT
GAGATAATATATATTTGATTTGTCATTTATTACGAATCCCGGTGGGAATAAGAGAAAATTCCATATAAAAACCC
GCTACAAACGTTGGTATGCCAAGGAAATCCTGAAATTCCGCCTATTTTGACAAACAATCAACTCATTATTTATA
AGTATTGATGATAGGGTTGTGTCTCTGCTTCCTTATATATATTATTTATTTATAAAAAGTAACGGGATTTTGGGA
TTGTGCTTGCACAATCCTTCTGTTTCTTCGAATCTGCAAATCCCAATCATTTCCCGATAAAAAATCATTGTGGG
ATGTTCTTTAGCAATTTCAATATAAGCATTGTATAGTTATGAAAAAATTACGACAATAACTGTTTCATTAGATAA
GTGTTATTGAAATTGATAAAGAGCAATTCTTGAAAATAGTTAGATAAAATAAGCGAAAGAATATAGTGAAAAT
TATTGTTATAACAATGATTCTATTAGCTAAATAGTAAGATATAGTGTTTGGGGCAAAAATAAAGACGAAGTGCT
GAGATGCACTTCGTCGAGTTGTTTATTATTGAAAAGTTGTTTAATGATTTCGTTATTAAGTTTGAGTGTGACAT
AGAATTGTTTTTTATGATTACCATCTTTTTTAATATCAATGCGATCAATCACTGATAGATACAATGCTTTGAGTC
GAGATTTTTCTATGTGCTTAATATCATGAAAGATGTGTTGTAATAGTTTACTGATTTCTTTGGCATCAAATAAAG
TCTTATCTTCATTTTGTTGATTTTTGAGTTGGTTGATTTGATTCGTAATGTCATTGAGTTGCTTTTCATATTTTTG
AATACTTGGTCTGATTACTGATGTTAAGTCCGGATTATCCTCGATGGTTTTAATCAAGTTATTTATTTTGATTTG
TACTTCATCATATTGTTGTTGCTTATAAGCAATATCGTGATGAAGTGCAGCGCCATCAACTTGATTTTCTTGATT
GACGTGTGTTACTACGCGTTGAATGACTTTATCACTTTTGACTATTTCAAGTATTTGCTTCATCACATAATCTTC

AATCACATCAGCTCTTACACTGTTTGCCGAACATACTTTGGAACCCTTGTTCCGAAAATTACTACATGAATAGT
AACGAATACGTTTCTTAGTCCCGTCTTTAAGAGTATTCGTGGTATTGCTTGCTGCCATAGGTGCGCCACATTG
GGGACAGTGAATAATGCCTGTAAGCAGATTCGTTCCTTTGCCATGGACTTGGGGTTTTTGACTGACTTGTTT
CTTACGCATTTGTACTTTATCCCATAAATCTTGATTAATAATGGGGGAATGCTTACCTTCAGCTATCACTGGTTT
ATCATTCAGCCCTTTACGACGTTTTTCACTCCAATCTTTGTATTTCGCAAATTGAATTTTGCCGATATAGAAAG
GGTTAGCTAAGATGTATGTGATTGAACTAATACTGAAAGGTTTCCCCTTTTTAGTGACATATCCTTTGTGATTC
AATGCATTGGCAATTTTACGATAGCCATGTCCTTTGGCATAGCACTCGAATATATATTTTACAATATTAGCTTCA
TGTTGGTTAATCATTAGCTCGTGTTTACTATCTGGTATTTTGTCATAACCTAGTGGTAAATTGCCTTGATAATAG
CCTTCTTGGGCACGTCTCGTTTGACCCATAAATACGTTCTCGACAATGTTATTACGTTCGAATTCTGAGAAACT
CGCAAGTATCTGTAACATGAGTTTACCAGAAGAAGTATTGACTTCCATACGCTCTGACAAACTGAAAAATTCG
ACATTTTGTTTGTGTAAATCTTCGACAATTTTGAGAAGATCAGATGTATTACGAGCTAATCGGTTTGTTTTGTA
TACCATAACACAGTCGATATTGCCTTCTTTTGCATCTTTCAACATACGTTGGAGCTCAGGTCTGTTCATAGATT
TACCTGAAATACCACGGTCAGCGTATATATCTTTAACTTCAAAATGATGGAAGTCACAGTATTCTTTGATTTGA
TTGATTTGTCCGTCGATACTATAACCTTCTGTGCTTTGCATTTCTGTTGATACACGTACATAGATACCGACACGT
TTTGTTTTAAGTTGTTGCATTATGTTACATCCTTTCTTCATTTATGCAATCGATGATTGCATGGTTTGATTGACA
ATATTGAGTGGTTCATTTTTGAAATAGATTCCTATAAGATTTTTATCTTTCGTAATGTGAATGGTTTCAATATAG
GGGTACAATATGTTTAACGTGAAACGTTTTTGAATAATATTTTGAATGATGTGTTGTATTTGATGCCCATTGATA
GATGTAGTGCGTTGCGGTTGTTGACGTAATGATTGTGTTTGCTCTCTGAACGTTTCTGCATCAATGATGCCTT
GTGCCAACTTTTCTATCAGTTGTTCTTGAGTCAATGTGTGATGTTTTTCTATGTTTCTTTGTCTTTTGATGCGTT
TGTCAATCGCACTTTTAATTTTTGTGTAGATGCGTTGATTTTGATAAAAGTCTCGGCACACTTCTAATACTTTAT
CTTCAAGTGTTTGTGCATTGATGCCTTTAAAATCACAGACAAAGCGTGAAGCATTCATGTTTTTAGGACAGA
CGTAGTAACGTAATGTATGATTCTTTTTTCTAATGGTCATATTTGTAAGTGTTGCATTACAACATGGGCATTTGA
TTTTTTGTTTGAGTTGATTATCCGAAGGTGTCTGTTTGGTTTGTTTTTGCAATCGAAGTCTCTGCGCTTGCTCA
TATATACTTGTGGAAACAATAGAAGGAAACATATTGTCGAATTGGCCATATTGATTGTTGACACGACCACAAT
AATTAGGATTGATGATAATGTTACGAACTTGATAGGGTTGTCGATTGATATACGTGTTATCTTCTTCTAATAACT
GTGCAATTTTCTTATAACCATGACCTTTAATGTAATAATTGAATACAGCCTTTACCGTTGGTGACTCATTTTGAT
TGATGATGAATGTTCCGTTGTGATATTCGTAACCAAAAGGTGCATGTGTTGTAATCAATCGACCTTGCTTTGCT
TTTTCTTGAAGCCCATTTCTGACTTGTTCTCCAATGTTATCCGATTCAAGTTCGGCTAAACTGATGAAGATATT
AAGCTTGAGTCGGTCGAAAGCTTGATCCATATCAAAGTAACCATCATGTACGCTTAAGATATGAACATGGTAC
GTTTGACATAATTTGATGAGTTTTAATGCATTTTTCAGATTACGATGCAACCTATTAAGACGATAACAGCATAA
TATGTCACACTGTCCTTGTTGAATTAATTGTGTTATTTGTCGATACCCACTACGATTATCTTTGCGACCTGATTG
TTTGTCGCTATAAAAGTTGATATGTTGAATATGATGTTTTTCGGCTATTGCTTCGATAGCTTGTTTCTGTGCTGC
AAGAGATTGTTGTTTCATCGTACTTTGACGTAAATAGCCTATGACTTGTTTCATATCGGCTCCTCCTTTCACAG
TGATAATATATATTTATGGATGAATTGATATATAAGCCCAACATCAATGAGATGTTGGGCGTCCATATTAGTCATT
TGTTTGATTGATTTCTTCAATTACCAAATCGGCTAATATCTCGATAAGTTCATCCATGTTTTTCACTCCGTTATTT
GTTCTATCTTCAATACGTCGATTATTCAGTTTGATGCTTCACAGTTGTATGATAAAGACAATTAGAAATCTTCGT
GAACTCCTGAAGGGCCTATCCCTTCATTAGCGGATTTAAAAAGTTCTTTCGCAGCTTTGTTATCATTTGACGG
TGTCCAATTTTGAAGTAACGACTTATCTTTAGTTAATCCGAGGATAGATGCAAACTCTACATCTAATTTTAGAT
GGTAAAATACAAGTGATTGTTTTTTACCGCTATTATCTTTGACACTTCTTTTAGTTGTTTGGCGTCCACGGTCA
GCTAATATGAAACCTTTATCTCTTAAGGCGTTGACAACATTATTAACATCTTGAAATTGATGATTGTTTAGCATC
TGTTTAAAAACGTTCGCAATTATTTTTACTTCGATATGGTCATCTTTTAATGAGATTAATCCATAGTTCTCAAAC
ATATTTTTCAAAGCACCTTCATCTGAAAACTTACCTCTGTTTTGTGCCACAAATTGAATGATGACATCAATAGC
TTTATCAGCTAATGAGCGTTCAGAGACTGTATGAGTATGATAATCAATAAAGTAGTCTCTTATATTAGCGATATC
AATATCTGTAGATAAAACACGACCTAATATTTTCGCAGATGTTGTAATGACTGCATAACGCTTAAACATACGAA

TACCTGTATTGTTTGTTTCATCTTTCAATTTAGCTTCAAACCAATCTACTTCCTTGTAAAACCATTGAATAACTTC
ATCTTCACGATTTATAAGATATTTAGCTACTAACGGTAAAACATGACCATAGTTTAGTGCTACAGCTTTTTTAAT
ATTGTCAGCATTGGTCGCATTTGTAGTGAATTGTTCATTAATCTCGATGGTTCTTACACGTAATCCATCGTTTTG
AGCTGAATCATTAAAAATACTGTATTCTGACGTTGAAATGACAGAAGTACCCCAATTCTTAGGCGTTTTAACT
TCTCCATGAACGTTTGAACGTTGACGACCTTGACCTTCAGCGATGGAATATAACAAACCCGTGGTATCTCTAA
GTGTCGTAGATGAAAGCTCATCAAATACTATAGGAATGCCATAATTGTTACTCAAATAACCTTCAAGTGCGTTT
CGTGTGGCATTCCAACTTCTAAAAAGAGTTTCATTACCTTTGGTAGGGTTACCAGCGACTGATACAGCTAAA
GCAGCTGCGGTTGACTTACCGGTTGAGGATTGACCTGTAAAACTAAAGAGAATTCCTGCAAATTCGATTTCA
TGTTTATGCTTCAGAAAACTTGTCACTAAGGCAGAAATCCCAAATATGACTGCCAATTCTAAAAGAAGATGAC
CTTTAACCTCGTCAATATACATGTTAAACCAATTATCAAATGTACCTTTAGGAGTTAAGTCATAAGCACTATCTA
CAATAGGGTATGAAGGTGAGGACTGATTAAATTGAATAGATTTGAGAACTTTATCTAGCGATATCAAGTAGCC
AAAAGGTGTCTCTAGTATACCTACACCCTCATATAATTCAGAAAGTGGTAATCTATCTCGCATCAATTGCAAGG
CATAACTCAATGATCTAATATACTTCTCGTTAATACTGTAGCCATATTTAATTAAAGAAGGTAAGTTTCGTGTTG
TTAAGATATCAGACTCAAAAATGTCTTTTTTACCATTGTTGTTAGTAATAATCAACTTTTCAACACCAGTAATTG
GATCAGAAAATCTTGCATTAACAATAATAGGGCTAGCCATTCTAACCACTTTTTCACTGTCACCATCTTTTTTA
GGTGGTATCGTTTCTTTCCAACCGAAGGAATCTAAAGCAAATGGGCCAATTTCAAACAATGTGTAACTCATTA
GCAATCACCTCCTTTCGAAGGCGTGCTATTATTGCATGGGTTTATCCCTATTTTCTGATATAATAAACTGCCATT
TTTTGTTCCTACAATTTTAAATGGTAATCCTGGCGTGTACTTAATCCAGTAAGCAAAACGACGTCCTACTTCAC
GTTGATCATTCGTGCTACAAACCACATTATATTTCTTTCTTAATTCACCATAAGTGAACGTACTTCCAACTGGTT
GTTGAAAAGATTTAGCAATTATAAAATTATAAAGCTTTTCTTGCTGCTTAGATTTCTTATTCATTTTAAAATTCC
TCCAATAATTTTTAAGGAGTCAATCAATATCATATAGGTTAAACTATCTTCAATTGATTGCTATAATAAGCATAAA
TGAAGCATTATGATTAGACGCGGAACAAAAAATGGGGAGGGGGGGTATATATTGATACCTACGCTAGAAGA
AATTATTGATAAGTATGGAAACTTAGTTGATTATTTGAAATTTGATGTAACAGTTGAGGTTTATGAAGATTTGT
TGTTATTAAGAAGTTTAAGCGATTTAAATGAACATCAAAAGATGGATAGAGTTTCATTTATAAAAAAACATTTA
AACTCAAATTTCGATTATTTATTAGATGATACTGAGTTTAGAAGTATCAATAAGATTTACAGTAATTTAAATATC
ATGACGCATATCAATAGTCAAAATAATAATTTTAAACGTAATCCATTTATAAATGAAGAACAATTGAAATCACTA
CTCGCGATAAAGAAAGTAGATGCACATATGAGTTATGATTCAAATATTACTTCAAAAGCATTGGCAGAAATAA
ACAAAACGCAAAAAAGATTAGTTACAAAGATAGACACACTCTATAATCAGTCAAAAAAAGAAAATGAATATG
TACGGTTAGGAGAAAAAATATCGTATACAAAATTGGAGAATCATTGGAAATATTTATATAATGAGATTCAGTTT
TATAATCTTAATAATCAATTGATTAGTTATGTTGCATTAGAACAGGAATATGCTTGGGCTTTTTTAAATGAACTA
TTTTATTTGATAGAATTGTATTTGAAGGCATTTAAAGGACAAAAGAAAACAGATTATGAGTTTGGGAAAAAA
TTAAACGAATTTATTCAATCATATGTCATTATTTTACTTATAGATATACGATTACCTGTTGTTAGATTATATATTATT
AGAGACATTGTAGATACATGTGAAGGAGAAAAAGATAATTACAAAAGAATTACACTGATAGAAGAATCTATT
AAAAAGTATAGATACGTAAAAGATCAGATTAAAAGATTTAAAAATGGATTAGAAGAATCGTTATTAAATGCAA
CTTTATCTATTGAACAAAATATGTTGAAAGTTAAAATTGATTATTATAAAGCCTACTGCTTTCCTAGAGAAAAA
ACAAAGCATAACAATATTGAGTACAATGTATCGTTGTTTTTTAAAGCATTAGATGCCTTAAAGAAATAGTTTTT
CACTGAAAATATTTATAGATATAATTAAAATTAATTAATATTAAGCCAGGATAATGTAGTCTTAATCGTTCTGAAA
TACGAAAAATGTTGTGGAAATCGCGTGAAATAATCAGAGGAATCGTTTGAAATCATCGTAGTAATCGCGCGA
AAATCACGTGAAATAATCAGAGGAATCGTTTGAAATCATCGTAGTAATCGCGCGAAAATCACGTGAAATAATC
AGAGGAATCGTTTGAAATCATCGTAGTAATCGCGCGAAAATCACGTGAAATAATCAGAGGAATCGTTTGAAA
TCATCGTAGTAATCGCGCGAAAATCACGTGAAATAATCAGAGGAATCGTTTGAAATCATCGTAGTAATCGCGC
GAAAATCACGTGAAATAATCAGAGGAATCGTTTGAAATCATCGTAGTAATCGCGCGAAAATCACGTGAAATA
ATCAGAGGAATCGTTTGAAATCATCGTAGTAATCGCGCGAAAATCGCGTGAAATAATCAGAGGAATCGTTTG
AAATCATCGTAGTAATCGCGCGAAAATCGCGTGAAATAATCAGAGGAATCGTTTGAAATCATCGTAGTAATCG

CGCGAAAATCGCGTGAAATAATCAGAGGAATCGTTTGAAATCATCGTAGTAATCGCGCGAAAATCGCGTGAA
ATACTATGGTAGACGGTTTGAGTAAATTAATGGAGTATTTTAATATTTATGGTTTGGAGGAATTGCGTTGCATA
GAGAAAGTAAATATATAGAGTATAAGAAATCACGAAAAGGATTATCTAATGATATTTGGTCTACGTATAGTGCT
TTTGCAAATACTGAAGGTGGTACTATATATTTAGGAATTGAAGAAAAAAAGATCGAGGACAAAAAAGTCTTT
GTTTCAGTTGGTGTTGAAGATCCAGAGAAAATGATTGAAGATTTTTGGAATGCACTATATGGAAGAAGTAAA
GTTAGTCAAAATATTTTATCAAATAAAGATGTTAAAATTGTTAATATTGAAAATAAAGCGTGCATTGAAATTCAT
GTACCAGAAGCGCCTTATTCGAAGAAACCGATATATGTAGATAATAAAAAAGATTTAGTATATAAAAGAGTTG
ATGATGCTGATAGAATTGCGACTGAAGAAGAGTATAAATTCATGATTGTAAATTCTCAAGACGATATAGATACA
GAATTATTAGATAACTATGACATGTCTGATTTAAATCACGAATCTATCGAAAATTATAGGAAACTTCTATTAAAA
AATACTAATGATGAGAGATATGCGAATATGAGCCAACTGGATTTAATGATAGATTTAGGAGCATATAGAAAAG
ATAGAAGTTCGAAAGACAAACAGTATAAAATGACTACAGCATGTTTATTATTCTTTGGTAAGTATAATGCGATT
AGTGATAGATTCCCAGGATTTCAATTAGATTATTTTAAGAAAACAAATTACCTAGATACTGATTGGAAAGATA
GAATATCAAGTGGAGATTTAGGTAATGAAGATTTAAACGTGTATAGTTTTTTTGAAAAAGTATTGATAAAATTA
ACTGATAACATTGAGGAATCATTTAGCCTAAATGATGGTTTGACTAGACAAAATTATGCAAGAGATTTAAAAG
TAGCAATTCGCGAAGCACTGGTTAATACATTAATGCATGCGTATTATGATACTAAGCAAAGTATTAAAATAGTT
AATTGTGAAGATTTTATAGAGTTTTATAATCCGGGTAATATGAGAATAAATAAAGAAGATTTTATTCATGGAGG
GCATTCAAAGGACAGAAATAGTATATTATCGACGCTTTTCAGAAGAGTAGGATATTCAGAAAAAGCTGGATC
TGGAGGACCAAGGATATTCGATGTAGTTAATAGACATAAGCTTAAAACGCCTGAAATAGAATTAACGGACAT
GGACACTAATGTAGTACTTTGGAAACAAGATTTAATGAAGGAGTTTGAAAAATATCCTGAGTTAGACAAAAA
AGTAATAAAGTATATTATTGACTATGGATCAATAAGTAAGGGTGAAGCCTTAAAAATGGAAAATATGACAGAA
TATCAGTTTAGAAATATTTTAAAAAAACTAAAAGATGATAACTTGATAAAAAAAGAAGGTGAAGGTCCGGCT
ACTAAATATGTGTTAATAGAATCAAAAGAAGCTGATATATTGCGAACTAAAAAAGTAATTAAAAGTTTAGAGT
CTTTCTTTAGGAATAAATAAAAAAACAAGAGATAGGTGCGAAGTGTTTGGATTATACAAATGCTTCGCATCTT
ATATTTATTAATAAATATCATAGAAAACCGTATCATTAACCGATACGCAGAGATGCGGTTTTTTAGACACTTCAT
AAAGGGATTTTGAACGTATCAGAACATATGAGGTTTATAGGAATTGCTGTTATGTTTTTTGATCACATCAATAA
ACAAAAAAGGTATGTACTATGTAAAATATTTATTAAATGATATAAAGCGAGGGTATATAAATGATTTTTAAATAG
ATATTTATCCAATAATATAAAAAGGAACTATAAGCTATATCTAAAAGCTATATCTAACTACTTATAGTCCTTCTTCA
TTAGTATAAATATAATTATTAAGAAAGGTATATATCTTTGTAACCTTCGTTTACATTAATAATGTTGTGATAACCTT
TGTGTTCTAAAATACCAATAGCTATAGAACTTCTAATGCCAGACTGACAGTCTACATAAATAACATCGTTTCTAT
TGAAAGGTAAATCTGTTTCTAAAAGTTTGCCGTGTGGCACATGAACCGCTTGGGATAAGTGGCCATTATTCC
ATTCATTATCATTACGTACATCTAAGACATGTGCTTCATTACCAGTTATGTCTTTACTATGAACAGATTGTGTTTG
AATTTGAGCTTGTGGTAACTGATATCCAGACACATTATCATATCCAATAAGTTGTAAAGTATGTTATGTGTTGCT
TTTGAAACAAGGTGATAGTCTCCAATCAAGTTAATTTCTTGATTATAGTTTAGATACCAGCCAATTTGATTGAT
GAAATTTTTATCATATGGAATATTGATTGTACCTTCAATATGTCCACCATGATAAGCCTCCTTACTGCGGAGATC
AAAAGTTAATCTGTTTGTACTTGTAGCTGGATAAACCGTATAAGGTTGATATAAATTCATACCGAATTGATTAAT
TTTTTTCATTTGTGCAAAATGATGTGGTGGTGCAGGTTGGTCAGAAATGAGTTTATCGATAAAGGTAGCTTCA
TTATTTTCAGAAAAAGCCCAGTTCGTTTGTTTTTCATAGCCAAGCGTAGATGTTGGAATAGCACCTAAAGATT
TACCACAAGGACTACCAGCGCCATGACCAGGCCAAATTTGAATGTAGTCTGGCAAGTCTTTAATACTTTCAAT
AGATTTAAACATTTGTTTTGCGCCTATTTCAGATAATCCTTCTACTTTAACAGCTTTTTCTAGTAAATCAGGTCT
ACCGATATCTCCTACAAAAATAAAATCACCACTGAATAGTCCCATTGGAACTTGTGCTCCAGCACCTTCGTCA
GTAAGTAAAAAACTTATACTTTCTGGCGTGTGACCAGGTGTATGAAGCACTTTTAATTTTATATTTCCTACATA
AATATCATCATTATGTTGAACAAAATGAGTGTGGTTAGGCATATTTTTATAACCTAACATGTCATCACTTTCGCC
CGATACATAAATACTAGCATTTAACTTTATAGCAACATCTCTAATTCCTGAAACAAAATTTGCATGTATATGTGTT
TCAGCTGCATGAGTAATGGTTAAACTCTCTTCATCGGCAACTCGAATATATGAAGATAAGTCACAAATAGGAT

CAATGATCATGGCTTCTCCAGTTTTTTGACAACCGATTAAATAAGATGCTTGAGATAAATGTTTATCATAAATT
GATGATAGTCAATTTGTTATCAAGGGTGATAATTATATTAACTATGGTAATTACATGGAATTAAAGTTATTAAAC
AAACTTGATGATGACCAATTAGATTTAGAATTTTTACAAGATAATGGGTTAAAAGGTAATGTTCAAGGTCCTA
TGACTTTAAAGGAACCTTTAAAAAGTTATATACAAAAGTCTTGAAAACGAATATTTTAAATTTAGATAATGAGT
TAGAAAACACTGAATATGTCGAAGGTAAACCATATGTACAGTACGGTATTAAATATGAAAGAAATCAGGCATT
AAGAAATGAAGCTATTAAAATTCATGGAACTACATGTAAAGTATGTGGATTTGATTTTAAAGCTAAGTATGGC
GATTTAGGTGAGGGTTTTATTGAAATTCATCATTTAAAACCAATGTTTTCAATAAAAAGAGAAATAAAAGTAA
ATCCACAAAAAGATTTAGTCCCACTATGTTCTAATTGCCATAAAATGATTCATAGAAATACTAAAAAACCTTTA
ACGATTAAAGAATTAACCAAAATAGTTAATTATAATAGCAAATAATTTAATATTTTATAAACTATCATTCAACCCT
CTTAATTTATTAGGAGGTTTTTTGTATTTATGCTTTCAAATGTGTGATATACTTTGTTTGTTGAAATATAGAGTAT
CTATAGATAGGGTGATTGAGTATGAAATTCACTGAAGTGGAAGTTATCGAACATCTTGTAAAGGCATATAAAG
AAGCAGGAAAGCCTACTTATCCTCATGAAAATTTATATCGAGGACGTAATCATAGTATTTCAGGTATTGGAGA
AGACTTGCTGGGTGCTTATTTGATTAGTAGATTGGAAGGTGTCCAAATATTTATTGATCAGCCTTTATCTATGA
TTGATAAATCTTTAAGTACAAGATATCCGGATTTATTAATTTGTGAAGATAATGAAATTAAAAATATACTAGAAG
TTAAAATGGACTTAGGATATCAAAGAAAAGATTTTATAGATTATTGCCGAAAGAAAGAAGAATGGATTTCAA
ATATCGTAGGAAAACAGTGTGTATTGTCTAGAAAGAGAGAAGACAAAATTCCTATGAATATAGCTGATGATAT
TAAATTTCATGTTGTGATTTACAGTGAAAACAATGGACCGAAGCGGTTTGATGAAGAAATCATGCCTATCGTT
AATGAAACATGTCCACATATTGAAGTATATGTCCTAACAAGCGGTCAACACCCTAATTTAGTAAATGTTAATCT
TGAAGGTATTAATATTAATAAAGATGAATTTGAAATATTAGTAAATGCGTTATAAAAAAATAGAGCATCCTCCA
CGTTATGGAGGTGCTCTGTTTTTTATTGAAAAGTATCAAGTTAATTAATTTAATATGCTTAATAAGTTCTACCTT
GACCTTTTTCTCTAGCTTCTGTTCGATCTCTTATGTACTCAGTACATACTGGATTTTCTGTTAATTCATCAACTGT
ATTAGTTTAATCAGAAGACGTGTCTACTTTGTAAGCTTCTAAGAACTTATTATATGATATAGCGTTTGAGTTTTG
TTGTTCTTCTATAAATTTCTGTAGTTATTTTTCAAAAACCGCATCATTAACTGATAAGCAGAAGCGTATCACAA
ATAAAACTAAAAAATAGATTGTGTATAATATAAAAGGAAGGGATTTATATTAAAATTTTGAATTCAAAAATTATT
GAAAGGGAAGCTACCTTAGAAATTGAATCTATGGCCACTAACTGCTTTTTTATATCACCACTTATTAAAAAATG
TAAGAATATTCCTCAGTGCTAAGTGAAGGAGGTTCTAGTTTTATTTAATATTCATATTGATAGGTAATGGTTAAT
AATTGCCTGTAAATTATTGTTAAATATCGTTGTTATAGTATCATCATTCATAAAATTTTTATAGATTTTATCAAGTA
TTTCCTCATCTTCAATAGCTGTAAAGCGATGTACTAGTCTTTTTATGAGCTATATATAATTTTGCGAAGCTAAAC
TGTTTGTAATTATTCACCAATCAGGTTATGTAGTGTCACGGTTCATAGGTAATTCTCTTTTTAAAATCATCTAATA
TTTTATATAATTCATATAAGTGTAGTATTAACGTTTTCTACTTTATCTATAAAATATTTAGAATTACATTAGAATTTT
AAATTATACGGCTGGAAATTCAATGCTGTAAAAATAGACAGATTTTAAAAAAGTAGTAATAAAAAGTCTAAGT
TTTATCTTTGTAATAAACTTCGACTATATTTAAAAATAAATATGAATGTCTTTTAATTTCTATAATATCAAAGCTAT
TATATGATTTGAAATTTTGTAACTTTTTCTCTAATTTGGAAGTAGATAATGACTTAAAGGTTTTAACTTTTACCC
AACGACTTGGCATTTTATCACTCCTATGTAATGTATTACTTCAATATACCCTTAAATTTTTATAATTAATTATATTG
TAATATTATTTTATTTTATAAAAATAGTTTAATAAAATGTTTAATTTTTATAAAAAATAATTAAATATTATAGACATT
ACGATAGTTTAATTAGAAAATATATTTAAGAATTATGAATTTAGTTCTTATTAATGTTAAAGATCATCAATTATTT
TGTCACATTATCTTGAGAGGATTACCTTATGAGGTAGCCTTTTATTTGTCACCTAATATTGCTCTAAGGCTAATA
ATACATATATAAAATAAATAATTATCAGAATATATTTAGAAATTGATATTTAATAATTAAATAAAAGTATGCTAAAT
ATTTATAATTTACAAGTGAGCTGATAGAAAGATGAGTATAAGCGAATATAAAAATAGATACTTAGAGTCACTAT
ATAACACCTTTAAAACTGAGTTAGAAAACGAACGCACTCGCTATTTTATATTAGATACCAACTATACAAATAGT
GATGATACTGAAAAGGAGTTTACTTGGGATGTAAAACAGAATAATAAGATCAGAGAAGGCGATTTGTTCATT
TTTAGACGGCCAACTAATTTATCTCAAATTGCTCAACAATTTTATTTCTTTGGAGCTGGGAAAATAGAAAAAA
TTGAGCGAAAAAATAATGTAGCAACCGCTTACATTTCAAAACCCTTACTTTTTGTGGATCGTGTTTTAAAAGA
TAATATTGAAAATTTAAAATGGGAGTTCAAAGAAAGAATTAAAGAAGAGTGGGAGCAGTTTTTCTTTAAAA

ATAAAATCAGTAATATTACAAAAAATGATTTTTTAAAACTACTAAGTTTGAGTAGAAATGTAGTGGAAATAGA
AAGTAATTATATTAATAATGATATTTCGCTAACTTTTAAACAAATGGCTGATATGGCAACTCGAATAAAGAAAA
ATATTTACTACGTAGGGAATCATGAAGCATTTGTTCAAGCTAAGGGAAGTGCTCATTTTGAGTTTTCAAAAAG
GATTAAACAAAATTATAGTTATAGATGTGCAATTACCGGGATTAAAACTAAAGACTTTTTAGTCGTTACACATA
TTATTCCTTGGCATGAAAATGAGTTTATTAGACTAGATCCTTCAAATGGTATTTGTTTGTCTTTATTTTTAGCAA
AAGCATTTAAGAAAGGCTTTATCACTTTTTCTAACAGTTATAGAGTTGTTTTATCTAAAGAAGCAGAGAAAGA
TGCTGCTTTATATGAAGAATTAAAGATTTATGAAAATCAAAAAATCGAACTACCAGATTGTCAGAAACCTAATT
TGAAATATTTAGATTGGCATAGAGAACATATTTTTAAAAATTAATTTTGGGAGAGGTTGTATGACAGATATTAG
TCAATTAGTAGAGGTGCCATACATCTTTCATTAGTAGATTGAAAAAGAGCACTTGAAACAGATAATTTGTATTA
TTTAGCCTATGGATTAACAACTTTAGATAATTGTTTTAAATATTACCAATTAATTACACAAAATGAACATTTTTG
CTATTCTCAACAAGAAGAAAGAGAAAGTTATCAGCAAAAATTTCAAAGTATGATTAATGGTTTAAAAATTAAA
ATTGGCATACAAAGTTCAAATGATGATGCGCAATAAGCTACAAAGTTTGATTTTTTAATTGAACGTTATATGTT
TTTAAAAGAAGAAAGAGCTTCGAATTAATATCTTAATGGAGGAAGATTAGTAAATGGCTAGAACGTATAATGT
GATTAAATATGTCATTAAGAAAAATAAGGATGATCTTAAAATACATGGATTAAGATTATATGAATTTAATATAAA
AGATGAAAAAGCTATAAATATTAGCCAACCCATTTTTGTGAACAGAGCAACATTGATTGAGAAAATACAAAAT
GGTGAAGCATTTACGCACGCCTTTAGAGTAAATCACAATACTTATTATGCAGGCCAATTAATTTCTTTATCATT
GAATAAAAATGGTTATATTAATAACAACTTTGAGGCCTCACGAGATATTATTAGAAACATTGAAGTAAGTATTT
TAGACTATGAATAAAGGTAATACTGATAGAATACATTACAATTAAAAAAGGGAGTATAGGACTCCCTTAATAA
GTTGATATATAAGTGACTTATTTTTTTAATAGAATTTTAATTCCTCTGTTAATGTTCAAGATATATAATATTAAAGC
AATTAAAAAGAATATAATAGTAATAATTAAAAGAATGCTAAATATTAATAATTGATGATCCAAAGGTTTATCAAA
GACTTCTTTAGAAAAAATAAATGCCAATCTACCTATAAAAAAACTAACCCAAGTCATAATATGATTAATGAATG
CTTTTTTGCCATGTTCTGATGTTGGTTCATCAGCAAGTATCCATACAAATAAAGGTAAAATGACAGGTGCGAA
AAAGATACTTAAATAACTCAATGCTGATAAAACGTAACTATTGTCATTTTTATGCATATTACTATTTGTATTTTCC
ACTTTTTTCACCTTCTTTACTTTTCTTTACTATTATAAAAACATTAATAGCGAGCTATTACAACAGTTATAAGAG
AAGCTTACGATATTGTAAGTATGTGTTGTTATAATCAATATAAAATTAAGAAAGACCTGAGAATTTATATTATCA
TCCACAATCGAAGTGAGTAGCGAAATTTATTGGAAAAGGTACTTATATTGAAGATATTGTTAATGGTTCATTTT
TAAAAAGCCAAGACGATTTGAAGTTAAAAGCTTCTGACTTCACTGTCGAAGATAACTATAACTACGGTGTACT
TATATTTCCAGAGAATCTTTCTCTTACAGATAATAATAAGAAATTGGAATCTTTAATAAAAACTATACTATTTACA
GGTAAACGATATCAATATACTTATTTTAAAATTTGATCTTAATAGATAAGGATATAATGATTTAAACAATTTAAG
AGATATTATTTTCACAAAAAAAGAATGCTTTTTGTTAGAGGCACTGAACCCTTAAAATGGGAACTTAATAAAA
ACACTATGTTCTAGGCTGCTAATTCTCTGTATTTTACAGGGGATAAGTAGCCTAGTTTTTGTTGAATTCGATTAT
TATTATAGTTTTTAATGTATTTTCGACAATATCTATTACAATATGATTAGAGTTATTAAGCTGATTATTGATGTAAA
AAGTTTCAGACTTTAGCGAGGAATGGAAACTTTCTATCGGGGCGTTATCGGCAGGTGTTCCCTTTCGGGAC
ATACTTCTGATAATGCCTTTTTCTTCGCATAATTGATAATAAGCATAAGATGTATAAACGCTGCCTTGATCACTAT
GTAATATACACCCCTCAGGTATATCGATTTGATTTAATGTATCATTAACTAAACGTTGGTCTTGTTTATCATCTAT
TTTATACGCCACAATTTCTCCGTTATAAATGTCCATTATCGAAGATAAATACAACATAGAATGACCAAATGGTAA
ATAAGTAATATCGGTTGTTAATACTTCCATGGGACAACTCGCTTTAAATTGTCTTTGTAATAAATTGTCCGTTTT
ATAATACGGTTTACCTATTCTTGTCGTCTTTTTAGGTCTAACTCGGCAGTTCAAATGATGCTTCTGCATCACTCT
CTGTACTCTCTTATGATTAATTGGTGATGTATAACATTGATTAATCAATGCTGTAATCTTACGATAACCGTAGGTA
TATTGTTTACCAACATGTTGTGAAAATCTATAACTTTCCCCATTTCGATACCATCGCCACCAATTTTTCACTTGT
GTTCTATTTCTAATATTTAATTCTTTCATAATTTCTTTTGTTGGAAATCCTGCTGCTTTCATTTCAACTGCTTTATA
CTTTGTTTCTACTGAATAAGAAGCTCTTTTCATAGAAAAAAAACACCTCCGTATGATTCATTTTAATATGAATTC
AACGAAAGTGTTTTTATATAATTCCCACTATTGGGGTCAGTCTAAAATTAAAATTGATAACTTTTTTAATAAAAT
AGATACACTAATTTTAAAACAAGGTAAAAAAATAGAATTATTAAAACAACGCAAACAAGGCTTACTACAAAA

AATGTTTGTGTAATTCTGATAACGTCCTATTATGTTAATTGAAAATAGACAAAAATAAATTGAGAGCATCCCTT
ACCGCAAAGTGAAGGATGCTCTATTTTTGTTGAAATTAATATAAGCTTCATTCTGATAGATAGTCATTAATAATT
GTTTGCAAATCGTTATTAAGTACCGTTGTTACAGCGTCATCATTCATAAAATCCTCGTAGATTTTATCAAGAATT
TATTCCTCTTACAGACATTCGCGAGAAGTCCGTTTTAATTTTATTAGAAGTAATTCAGGTTTGAACCTACCTAA
ATGAATATATGAGTTATCTTTTATACTACAAAATATATTCAGATTTCAATAATGACATAAAATAGGCATCTTTATAT
TTACCTTTAGTGTAGAATTGCTCTTTAAGTAATCCTTCTGTTTTAAATCCTTGTGACTCGTATATATGAATAGCTT
TTTTGTTATCTGCATCAACATATAGATAGATCTTGTGCATATTTAAAATATTGAATGCATAAATTATCGCTTTTTC
GAATGCGAATTTTGCATAACCTTTACCACTGAACTCAGGTTTAATAATTATTTGTATTTCACAATTACGATGGAT
GTAATTAATTTCTACTAATTCAACAATACCTACGACTTGATTTTCATCTTCAACAATAAAACGTCTTTCCGATTC
ATCTAATAAATGCTTATCAAATAAATGTTGAAGTTCCGTTAAGGATTCATAAGGCTCTTCAAACCAATAAGACA
TAATAGAATATTCATTATTTAATTCATGAACAAAAAGTAAATCACTATACTCTAATGCTCTTAGTTTCATAATCCC
ACTCCCAAAATTTTCTTATATATTTGCATTATAAATATAAATAACGAATAAGTCATTATTCACTATGAATAAATCTA
TTTTAACAATTCACTACATACTAATTCTCATTTTCTTATTATTCTCTAATATCTCTGATTTATTACTCAGTGAAAGA
TGCCCTATTTTTATCAGTATATAGTTTTTATTTTGATAGATATTGATTAATAATCGTCTGTAAATCATTGTTAAGTA
AGGTTGTGATAGCGTCGTTATTCATAAAATCTTCATAAATTTTATCAAGTATTTCTTTAATATATTTTTTAATCTTC
ACACCTTAAATTCACTTGAAAACCAAATCACTTCTAAATTATGATTTTACATAATAAATTTTTAAAAATTGGTAT
TATATAAAATAATCTCAATAATCAGAATTTTTTGTAAATAATAATATTAAAGTGTAAAATAACAGATAAGAATAG
ACTTAATGTATATAAATTAAAGGAGTGTTGGAAGTGAAAACTAGAGCAGCAGTTTTACATGAAATGGGGGCT
AAAAAGCCCTATAAAGAATCTAAACCTTTAAAAATTGAATACTTAGAACTTGATAATCCTAGCGAACACGAAG
TATTAATTAAAATTCATGCAGCTGGATTATGTCACTCTGATTTGTCAGTAATTAATGGTAACAGACCTAGACCTT
TACCTATGGCACTTGGTCATGAAGCTTCCGGTGAAGTAATTAAAGTTGGAAAAGCTGTTACAAGAGTTAGCG
AAGGAGATCATGTGGTATGCACATTTATTCCCAGTTGTGGAAAATGTATCCCATGTAAAGAAGGACGTCCTGC
ATTATGTGAAAACGGAGCAATATCTAATGAAAAAGGCGAAATGTTAGAGGGAGGGATGCGTTTATCTAATGA
TGAAGGAAAAGTATATCATCACTTGGGGATATCAGGTTTTGCTGAATATTCTGTTGTCTCTGAAAACTCTATAG
TTAAAATTGATAAAAAAATACCTTTTGAACGTGCAGCTGCATTTGGTTGTGCTATTATCACAGGTATCGGTGCT
GTGGTGAATACAGCCCAAATTCGTTCTGGTAGTAATGTAGCGGTTGTTGGTTTAGGAGGTATTGGATTGAAT
GCTATTATTGGAGCTAAACTAGCGGGAGCCAATGAAATTATTGCTTTAGATATTAACGAAGATAAATTTGAATT
AGCAAAGCAATTTGGGGCTACAGCAACATTTAATTCAAGCGATAAAGATATCGATGAGCAAATTAAAGAATA
TATTCCTGGCGGAGTAGAATATGCTTTTGAAACAGCGGGTGTAGTGCCAGCTATGAAAGTTGCTTATCAGATT
ACTAAACGAGGGGGGACAACTGTAACAACAGGACTGCCTAATCCTAAAGATAATTTTTCTTTCCCTCAAGTT
ACTTTAGCGGCTGAAGAACGTACCATTAAAGGATCATATGTAGGAAGTTGTGTACCTGACAGAGATATACCA
AGGTTCGTTAATCTATACAACCAAGGACGTTTAAATATTGATTCACTCATCAGTGAGGTTATCACTTTAGATGA
AATTAATGAGGGATTTGATCGTTTGTCTAATGGTGAAGTAGGTAGAATTATAATGAAGATGCATTGAATATAAT
AGAATTCAAGTCGTTCCTCCTCTTGATTTCTATGAAAAGAATAACAATTTGAGTAGGAAGATTACTGACAAAT
ATTACATTTGCATTGTGACTGCCAATCTAATTGTATTTTTACATTTCATATCTATATATTAGAAGATAAATGTTTTA
AAACTAATTACTAATAGTTTTTTTAGATAAAAAAAGATATAGGAGCATCCTTCACCGCAAAAGTGAAGGATGC
TCTAGTTTTATTAGAATATAGTTTTCATTTTGATAGATAATGATTAATAATTGTTTGTAAATCATTGTTAAGTACC
GTTGTAATAGAGTCATCATTTATAAACTCTTCATAGATTTTATCAAGTATTTCCTCGTCATCGATGGCTGTGAAA
TGATGCACCAGACCTTTTACTATTGAAATGTAATTTTGTGATGCTAAACAACTAGCAACCGTTCGCCAATTGG
ATTGTATAGTGTTGTCATGGTTCATAGATAATACTCCTTTACTTTAGTGTCCATTTTTGACGTGCTTTAGGGTTG
AGTGGATGCATAACTTCGTTCGTTACTGGATTTATGAGCTTTTTTACTTTCTTTTTATGAGGTTTTAACATTTCC
ATCACTTGTTCGACACGGTCGATAACAACTGGTCGCTTCGCATAGGCACCATAAGCAAGAATGACTGTGTCA
CTTTCACTAATTGCTTTCATTAAGTGTATATCTGTGTGCTCATCATAAGGCTCTTTGATATGTTTAAGGTTCTCTG
GTGTTTTAATATTAGAGAAAAGATTAACGAGATAAACAGCGCCATATTGCTCAGAATTAGCTAATTGATTGAG

GATGAGAACCGTAGTAAGATCGAGTGATAATACACCATCTAAATGAAGATACATCGTTATCACAGTACAAGCC
GGTTTCTTGTCATCCCATATCTTTTTGAGTAAGTAGCGATGTTGTTCATCATCGCTAAATATCGCTTCTGTGTGT
ATCGTACTTTTGATTGTATTCATATATCGTCACTTCCTTTAGTATTCTTCTGGCAAAAGCATCACATAATAAAAA
GCGTCTACATCATCTTCTCGGATGACGTAGACTTTCTTAGGTAATGCATTTTGATTTTTTATATAGTTTGTATAG
TGATATTCCAATTTGTATGCGGGTTGTTCTTGTTCATGTGTAATTGACAACATATTATCATCTTCTTGTAGTTTGA
AAATGTGTAGGTAATCTGTATTAAGTTGATTGTCTCGATCTTTTACCATGTTCCAAAGTAAGATTTGAAGGTCT
AGAGATAGTTGTTCACTAATGCCTCTTGTGATGTATCGATTGATTTTCATACTATTTTCCTCCATTTTGCTTTTCT
TTCATGATGTCAATCACTTCGTTAATGACTGTAACAGATATTTGTGCCACTTTGATCCAATTATTCATGGTTATT
CCCTCCTTCTTTTAGTAAATGACGTTCATCGATAATCGTATTTTTAGTATCTGTGAGGTATAGAAAGTCCATATC
AAAATGATCCAGATAACCAATACTGATGAGTTGGTTATTAGAATACATTAGAAATGGATAGATACTTAGTTCAT
GTAGCTCATTATTATAGTAGGTATAAGTTTCAAGTATAAGATGTGCAAGTGGGGAATCATTAATAAAACGTTTG
GGTAGAATATTTTCTGCCGCTTCCTCCAGTGTTTCACATTCCCATGTTTCATTGTTAGATAATTGGAATAAGCG
AGTCATATATTGTTTGAGTTCTTGAGTAGTTGTTTTCATATCATTGCCTCCCTAGATAGTGTGATAGTGATGTAC
TTCATATACATCATTGAGATAATATATATTTGATTTGTCATTTATTACTGATCCCGTTGACAATATGAGAAAATTC
CATATAAAAAACCGCTACAAACCTTGGTATGACAAGGAAATCCCGAAATTCCGCCTATTTTGACGAACAATCA
ACTCATTATTTATAAGTATTGATGATAGGGTGGGGTCTCTGCTTCCTTATATATATTATTTATTTATAAAGAATAAC
GGGATTTTGGGATTGTGCTTGCACAATCCTTCTGCTTCTTCTAATCTGCAAATCCCATTCCTTTCCCGATAAAA
AATCATCGTGGGATGTTCTTTAGCAATTTCAATATAAGCCTCGTGTAGTTATGAAAAAATTACGACAATGACTG
TTTCATTAGATAAGTGTTATTGAAATTGATAAAGAGAATTCTCAAAAATTTTTAGAAAAAGAAAGAGGAAGT
ATGTAGATATAAAATAGATTTATTAAATAGTAAGGTATAATGTTTGCGGTAAAAATAAAGACGAAGTGCTGGA
ATACACTTCGTCGATAATAAGGTAGTTGAAGTTTCGATTATTTTAAATTGATCATTTTAAGCCTACAAATCCCTC
TAATGTTTCACCAGATTTGTACACTTTTGTCGCCTTCTTTTTCCTAAATAACGTAAATGCCAAGGTTCATATTGA
TATCCTGTGATGCTTTCTTTGTTTTTAGGATATCTTATAATAAATCCATAATTATGGGCATTCTTAGCTATCCAGC
GTCCTTCCTCAGTTTTACCAAAACTAGCATATAGATTTTCACAACAAGTTGGTAAACAATATACCTACGGTAAA
GGATTAGAAGAGCTGTCAGAAGTAGAACAATTAAAATTAGAAAATAAGAGAAAAGATATAGAATTGGATATT
TTAAAAAAGTACAAGGTATTGGAAAGGAAGTGGTACCAACAGTAGTCATAGATTTAGTGGATCAATTAAAAG
TAAAATATTCAATCAAATTGCTACTAAAAGTATTAAACATACCTAAATCAACATATTACCGATGGAAAAACAAA
ACCCATAAAAATGATACCGTAACACAAAAAGTTATTGAATTATGTAAAGCTAACCACTATACCTACGGTTATCG
TAAGATTACAGCATTGATTAATCAATGTTATACATCACCAATTAATCATAAGAGAGTACAGAGAATGATGCAGA
AGCATCATTTGAACTGCCGAGTTAGACCTAAAAAGATGACAAAAATAGGTAAACCGTATTATAAAACGGACA
ATTTATTACAAAGACAATTTAAAGCGAGTTGTCCAATGGAAGTATTAACAACCGATATTACTTATTTACCATTT
GGTCATTCTATGTTGTATTTATCTTCGATAATGGATATTTATAACGGAGAAATTGTGGCGTATAAAATAGATGAT
AAACAAGACCAAAGTTTAGTTAATGATACATTAAATCAAATCGATATACCTGAGGGTTGTATATTACATAGTGA
TCAAGGCAGCGTTTATACATCTTATGCTTATTATCAATTGTGCGAAGAAAAAGGCATTATCAGAAGTATGTCCC
GAAAGGGAACACCTGCCGATAACGCCCCGATAGAAAGTTTCCATTCCTCGCTAAAGTCTGAAACTTTTTACA
TCAATAATGAGCTTAATCACTCTAATCACATTGTAATAGATATTGTCGAAAAGTACATTAAAAACAATAATAATA
ATAATAATAATCGAATTCAACAAAAACTAGGCTACTTATCCCCTGTAAAATACAGAGAATTAATAGCCTAGAAC
ATGGTGTTTTTATTAAGTTCCCGTTTTAAGGGTTCAGTGCCCTAGGATTATAGGCTCTTTTGTTTATAAAGGTA
ATTGAACTAAAGTATTATAATTTCAATTCTTAATTAATGTTCCTATTTTACCGTCTAAAGCGTCCCCTAATCCTGC
TAGAGATGTAATCAAAACACTGCCTTTAGTATTTTTTTCAAGAAATTGAAGTGCAGCTTCAACTTTTGGAAGC
ATACTTCCTTTAGCAAATTGACCATCAGAGATATGTTTTTTCATTTCATCCACAGACACTTCATCGAGACCTCTT
TGGTTTTCTTTTCCATAGTTAATGTAAACATGGTCCACAGCAGTTAATATGATTAATTGATCAGATTGTAAATGT
GCTGCTAATAAAGCACTCGTTTTATCTTTATCAATAACTGCATCAACACCTGTATAAACTTCATTTTCCTTAATTA
CTGGAATTCCACCACCGCCGGCAGCGATAACTAGTGTTCCATGAGTGATTAATGTTTCTATACTATCTAACTCA

ACTATACTTATAGGTTGTGGGGAAGGGACAACGCGACGATAGCCGCGTCCAGAATCTTCTACAAAAGTATAA
CCTTTTTCTTTCGTAAATTTATCAGCCTGCTCTTTTGTATAAAATAATCCAATTGGTTTAGTAGGGTTATTGAAA
GCAGAATCATCGCTTGCAACTTGAACTTGTGTTACTAGCGTAACAACTTGTTTATCTATGCCCATTGAATGAA
GTTCGTTTTGTAAACTTTCTTGCATCTGATAGCCAATATAAGCTTGACTCATAGCACCACATTCAGGGAAAGG
AAAAGGAGGACCTTGTTTGTGTTCCGCCGCATAATTTAAACCTAAGTTAATACTTCCAACTTGTGGACCATTA
CCATGACTAATTACAATTTCGTATCCTTTATCGATTAAACTTACTAGAGATTTAGATGTACTTTTTAATAAATCTA
ATTGTTCTTTAGGCGATTGTCCTAAAGCGTTACCACCCAAAGCTACGACGATTTTAGACATATTTATATCCTCCT
TTCATTTACTCTCCTAATGTTGCTACCATGACTGCTTTTATTGTATGTGCTCTATTTTCTGCTTCTTGGAAAACAA
CTGATTGTTCACTTTCAAATACTTCATTTGTTACTTCCATTTCAGTTAGACCATATTTTTCTTGAATTTGTTTACC
GATTATTGTTTCAGTATCATGGAATGATGGTAAGCAATGTTCAAAAATTGTATGTGGATTACCTGTTTTTTTCAT
TAATTCTTTAGTTACACGGTATGGTTCTAATAATTTGATACGTTTTTCCCAAACTTCATCAGGTTCACCCATAGA
TACCCAAACATCTGTGTAAATTACATCAGATCCTTTGACACCTTCATCAATATCATCAGTTATAAGGATTTCACC
ACCGTTTTTGTCAGCTATATCATTACACCGATTTAATAATTCATCAGTTGGATTTAGTTCTTTAGGACATACAAG
ATGGAAAGTCATGCCCATGATTGCTGCTCCTTGCATTAAGGCATTTGCAACGTTATTACGTCCATCTCCAACAT
ATGTGAAGTTAATTTCATTATATGGTTTTTTCAATACTTCTTTAGCTGTTAAAAAATCAGCAAGTACTTGTGTAG
GATGATCTTCATCTGTTAAACCATTCCATACGGGAACGCCAGAATATTTGCCAAATCCTCAACTACTCTTTGT
GAGAATCCACGATATTCTATGCCATCATACATTCCACCTAAAACACGAGCAGTATCTTTGGTAGACTCTTTTTT
ACCCATTTGAGAACCTGTTGGCCCAAGGTATGTTACATGTGCACCTTGATCATAAGCCGCTGTTTCAAATGCA
CATCGAGTGCGTGTTGAATCTTTTTCAAAAAGTAGAGCGATATTTTTACCTTTCATTTTTTGTTGTTCTATTCCT
GCATATTTTGCGCGTTTAAGATCTTCAGATAAATTAAGTAAAAATTCCATTTCTTTTTGTGTGAAGTCTAACAA
AGTTAAAAAGTTTCTATTTCTTAAATTTTTCATTTTGAATATCTCCTTTTCAATTAATATTTTATTCATGTTATTTT
TTTATAATCCAATTTCTTTTATCTTGATATATAATATTTTCTATTTTTAATTCTCTGATTATTTCACCAGTAGTTTTT
CTGTTAAGTCCAGATAAATTACTTATTAATTGAACAGTCATAATTTGTTTTAATTCATAGAATTCTCCATTATCAT
CCCCAATTGAAAGGCATAAAATTTGTAGTATTCTTTCAATTCTTTCTTTAGCTGAATTAGCTCTTAACGCCATAA
TATATTCTACTTGAAATTGAATAGTCTCATTAAGTTTCTTGAAGAGACTTTCAAATATTTCATTATGCTTTCTACA
TAAATACTCAAGTAAATCTTTCGGTAAAGTTAATATTTTACAATCTGTCAAAGCTGTACATATTTCATATGGTGC
AGGGGTTTCATTAAATATGAAGTTCATTGGAAATATATTTTCGTCTTTACTTAATCTTAAATAATTGTCACCAGT
AATATTAGAAGATTCATGTAAAATACAACCATTTACTAAAAAGTATACAAATTTTATTTGATCAGTTGAATGATA
TATGACTTGTCCTTTTTTATATTGATAAAGTGTTAAATCCTCTTTATAAGGTCTAACAATACTAACAGGAATATTT
AAGTATGATGCTAATTGTTTAAGATTATTATCAAATTCATATTCTGAATTTTTAATATAAATATTTTCTTCATACAT
AACTATAAACCCCTTAAAAATTACTTAGTTTAAATCATAACTTAAAATACAGAAATATTACCTGTAATCAAACGA
ACTATTCCTATTATTGCAAGTACAATGATGAATATGAATAACGTATAATCCAATTTTGTCAAATGTTTGTTATTAT
CCCTTTGTACGTAGCTGTATACGAGTAATCCAGGTATATATAACAACATCGTTAATAGTAAATAATCTAATCCAG
CTGCATAAACCAACCAAATTGTGTAAATAGATGCAATAATTCCTATTATCCATTGTTTTAAATTAGCTTTAGATT
TATTTTGAATAGTATATTTAACCTGGTAAAAAGCACTGAGTGTATATGGAATTAAGATTGCACTTGATGCAAGT
GAAAACGCAAACTGATAGGCACTATCTGTAAACAACATACTAATTAAAAATAACTGAACTAATATATTAGTAAT
AATTAAAGCGTTGACCGGAGCTTTATTCTTATTTTCTTTAGCAAACCATTTCGGGAAAAGTCCATCTTTAGCTA
CAATGAATGGTAATTCACCAGCTAGTAATGTCCATCCTAACCAAGCTCCTAAAACAGAGATAATTAAGCCTATA
TTAACTAACACTGAACCCCAATGACCTACAATATGTTCTAATACTTGTGCCATTGATGGATTAGCAAGTTTTGA
AATTTGGTTCTGCTGAATGACACCTTGGGCTAGTACAGTCATTAAGAAATAAATGACTAGCACAGAAATCAA
ACCAATAACGGTAGCAGTTCCTACATCCTTTTTAGACTTTGCACGTCCAGAAAAGACAACGGCTCCTTCAATC
CCTGTGAATACCCATACAGTTACTAACATAGTACTTTTTACTTGTGCCATTGTATCTCCCCAACTAAAAACGCCA
ACACTTCCACTAGTCATACCATAAAAACCGGATTTAAAAGTACTGAAGTTGAATACAACTATCATGCATATAAT
AACTAGAAATATAGGTATTAATTTAGCTACTGTAACAATACTATTTATAAACGCTGCAGTTTCTACACCTCTAAG

TATTAAAAAATGTACACCCCATAATAAAATTGATGCTATGATAATACTTGGAAGTGTGTTACCTCCTTTAAATAT
AGGGAAAAAGTTACCCACAGCTGACATTAATAGGGTTGCATAAGCCACATTACCTAGAAATGCTGCAAACCA
ATATCCCCAAGCACTTGAAAAACCAATAAAATCTCCAAACCCTGTTTGAGCATAACTATAAATTCCTCCATCAA
GATCTGGTCGCTCATTTGTTAAATTTTGAAATACGAAAGCAAGAGAAATCATACCAATAGCAGTTATTATCCA
ACCGATAATTATTGCAAGTCCACCAGCTTGGCCACCCATATCTGAGATGATATTGAATGCACCACCGCCTATCA
TAGAGCCTATGACTAAACCAATTAAGGAAGTTTTACCTAATTTATTTTCATCCATATTAATCTCCCCTAATAAAG
GTGGTAAAAATCTTGTTATTTGATTTAATGAATGAATTACTTGTGACTTTTACCACCTTTCAAATTTAAATATCT
TCTCTAAATAACGGCTGACTCATACATCTTGGGCCTCCGCGTCCACGTACAAGTTCACTACCAGTAATTTCAAT
CACTTTAATTCCTTTGTCGCGTAAAAGTTGGTTTGATACATAGTTGCGATCGTATGTCACCACAACCCCTGGTC
GAATACATAATGTGTTTGAGCCATCATTCCATTGTTCACGTGCACCATCAATAACGTCGCCATTACCTGTTGGA
ATAAAGTCCACTTTTTCTACTTCTAAAACTTCAGCAAGTGTTTCACGTAACTTGCTAGAACGAGTAATTTTTAT
ATCGTCCTTACCATCATTTTGTTCTATGGTAAATATATTCATATTATTTTCTTCTTTAAATATTGCTGCATGTACTGT
AAACTTATCGTAGTCAATCATAGTTAGTACTGTATCTAGGTGCATAAATGTACGTGTATTAGGTATTTCAATAGC
TACGATTTTTTTAAAACTTGTGTTTGCATCTTTGAAAATATTACGTGCTAACTTTTCTATTGCTTGAGCTGATGT
ACGTTCTGATATACCAATAGCTAAAACATCTTTCGATAATACTAATTCATCTCCACCTTCAATATTAAATGGTGA
GTTACGATCTAACCATACTGGTACATCTTTATCTTTAAATCTTGGATGATGTTTCAGTATATATGTCATAAAAATA
GATTCTCTACGTCGTGCTCTCCAATACATTCTGTTAATTGTCATTCCTCTACCAATTGAAGCTTGGGGATCTCTT
GTAAAATAAAGGTTGGGCATTGGATCTAAGTAAAATGGATATCTATCATCCATATATTCTACTAAATGGGTTGTT
TCAAGTTGAATTTCTTCTTTACGTACGCCAGCCATGATTTTATTTACAAGTTCTTGGTCAGATAACTTTGAAAA
GAATTCTTTAATTTCAGTTTCATGACCTAATATTGTCTTTTTAGATTCTGTTAATATGTCGTTTATGAAGTTCTCG
CGTACTTCTGGCTCAGTAATAGATTCTGCTGCAAGTTTTTCTAAATAAACTACTTCGATTCCTTCATCTCTCAAA
GTTTGAGCAAATTTGTCATGCTCTTCTTGTGCAACTTTTAAGTAGGGAATATCATCGAATAATAAACCACTTAA
ATGATCAGGTACTAAATTTTCTAATTCTTTTCCTGGTCTTTTTAACAACACAGTTTTCAATTTGCCTATTTCACT
ATTTACTTGAATGGGTCCTTGTACCATTTCAAATTCCTCCTTTGCTTTATTACATTCACATTATAAAACGCTTTCA
AAAAACATCGTGTGATTTAATTCACAATTGTTTAGGGAGGGTTTTCACATATATAGATTTATTCAATGCAAAAT
TTTGTATGAATATGTATTTTGTACTATATAATAGTGAATAATTTGAAAAATCTCATTGTTAAAACCTTATCTTATTC
AAGCAAAAAGATTTTTTACTCATTTTTTTGTATTAGTAATCATTCCTATTAATATAATCATTTAATTTTTAAGGGA
ATAATTGATAACAAACAAATTCTGCTATTTCATTTGAAGAAGTCAAAATCATCAAAGTATCGTTACCTCCAATA
ATTCCTAATATTTCTTTCATTTGTAATTGATCTATGTAATAACTTATACTTTGAGCAAAGCCAGGAGATGTTTTTA
TTAAGACATAGTTATTTAGCGTTATAAATTCAATAATCTCATCACTAAATATTTCTAATTGTTTTTTTGCACTTAAT
TGATTTGTTTGATTTATTTTCTTGTAAATATACTTTTTATTTTCAACAGGGATTTTGTAAATTTCTAATTCTTGTA
AGTCACGAGAAATAGTTGTCAAGCTATAGTAAACTCCAAAATGTCTTGCCATGTAATCCACTATTTGTTGTTTT
TTATTAAACTGATTCTGTTGTATAACAGTTAAGATAAGATTTAAACGTTTTTCTTTTTTCATTTTTATTACCCCCT
TTATTATTTTTGATTATGAAATAGATTTAAATAACTGTATAAAAATCACTTATAAATCTCAATTTACAAATAATTT
TGAAATAGTGGATGCTTTAATTATAAGTAGATTATTTTATGCCTAAAAGGAGAGATTAATTAGAATAGATTATA
GTCATCATAATTATAAAATCAAAATAATTTGCGTAATAATAGCGAAATGTGTAAATTGTAAATGTCAGGTAATAA
CCACAATATCTCATAAAAGAAGTTTGGAAAAAGGCATTGAAATAAAAAGTAGTTTATAAGTTGAAGATATTTA
GGTATGTATTAGAGCGCTTTATAAATAAATAGGTTTATACAAAGAATTACAAAAATAATATAAAAAATAGAATCT
AATACATGATAAGGAATATATTTGAATAATTTGAATATGAGAATAAGCATACATATTTAGGGTATATATAAGTAAT
AGACATCTAAATAATAAGTAATAGGAGGCTGGTGTAGATGTTCAAAAATATATTATTACCCTATGATTTCGAAA
ATGATTTTAGTGCTATCCCTGACTATTTAGAAAAAGTCACCGATGAAGATTCAGTTGTTGTAATTTATCACGTT
GTAACAGAAAATGATCTTGCAATTAGTGTCAAGTATTATAATAAGCATAAAGAAGATATTATTAGAGAAAAAG
AGAAAAAACTCACTCCATTTTTACGTGAATTAGAAAAAAGAGATATTCAATATAAAATAGATGTAGATTTTGG
GCATATTAAAGATACAATCTTAGAAAAAATTACTTCTGGAGATATAAATAATGGTGAATTTGATTTAGTAATTAT

GAGTAATCATAGAGTCGATTTGAATATTAAACATGTTTTAGGAGATGTTACACATAAGATTGCTAAAAGAAGT
TCTGTCCCAGTACTAATTGTTAAATAAACATAAGAAGTAAGAAATTTATTAATTCAAAAAGCCTAAATACTTTC
TCACAAATCGAGAGAGTATTTAGGCTTTTTTATTTTTTTAATAAACGTAATGAATTTAATATAACTAATATTGCT
GCACCTGTATCACTTAGAACAGCTAACCAAAGTGTTAGTAATCCAGGGAATACTAACACAAAGGCAATTAATT
TAATAATTATAGCAAAATATAGGTTCTGTTGCAAAGTTGAATTTATAGTATAATTTTAACAAAAAGGAGTCTTCT
GTATGAACTATTTCAGATATAAACAATTTAACAAGGATGTTATCACTGTAGCYGTTGGCTACTATCTAAGATATG
CATTGAGYTATCGTGATATATCTGAAATATTAAGRGAACGTGGTGTAAACGTTCATCATTCAACGRTCTACCGT
TGGGTTCAAGAATATGCCCCAATTTTATATCAAATTTGGAAGAAAAAGCATAAAAAAGCTWATTACAAATGG
CGTATTGATGAGACGTAYATCAAAATAAAAGGAAAATGGAGCTATTTATATCGTGCCATTGATGCAGAGGGAY
ATACATTAGATATTTGGTTGCGTAAGCAACGAGATAATCATTCAGCATATGCGTTTATCAAACGTCTCATTAAA
CAATTTGGTAAACCTCAAAAGGTAATTACAGATCAGGCACCTTCAACGAAGGTAGCAATGGYTAAAGTAATT
AAAGCTTTTAAACTTAAACCTGACTGYYATTGTACATCGAAATATCTGAATAACCTCATTGAGCAAGATCACTG
TCATATTAAAATAAGAAAGACAAGGTATCAAARTATCAATACAGCAAAGAATACTTTAAAAGGTATTGARTGT
ATTTACGCTCTATATAAAAAGAACCGCAGGTCTCTTCAGATCTACGGATTTTCGCCATGCCACGAAATTAGCAT
CATGCTAGCAAGTTAARCGAACACTGACATGATAWATTAGTGRTTAGCTATATTTTTTTACTTTGCAACAGAA
CCTATAAAAAACATGGGCTTAAAGGGTTAATTTATATAGTATTACTGCAAACATTGATTTGGGTCAAAAATTAG
GGGTATTAAAAAAATGAATGAATCATAATTTTATCAAGCTGATTGGAGAGGTTAAAATGCATTATATAAAATTT
ATTGAGTCAAAAGATAATACAAAACTTTATATGAAAGTGAATGATATTCAAGATGCAAAAGCGAATATCATTAT
AGCTCATGGTGTGGCAGAACATTTAGATCGTTATGATGAGATAACAGCATATTTAAATGAAGCGGGTTTTAGT
GTTATTAGATATGATCAAAGAGGGCATGGTCGTTCTGAAGGCAAGCGTGCCTTTTATAGCAATAGTAATGAAA
TTGTCGAAGATTTAGATGCGATAATAAATTATGTGAAGTCAAACTTTGAAGGTAAAGTTTACTTAATCGGTCAT
AGTATGGGTGGTTATACAGTCACTTTATATGGAACGAAACATCCAAATACAGTGAATGGTATTATAACTTCTGG
AGCATTAACACGTTATAATAATAAACTATTTGGCAATCCTGATAGAAACATATCACCTGATACTTATATAGAAAA
CAATTTAAGTGAGGGGGTATGTTCTGATTTAGAGGTAATGGAAAAATATAAACTTGATGATTTGAATGCGAA
ACAAATCTCTATGGGGCTCGTCTTTTCAATAATGGATGGTGTTAGGTATTTGAAAGACAATGCTCAACAATTT
ACAGATAATATTTTGATATTGCATGGCAAGGAAGATGGGCTAGTAAGCTATGTAGATTCTTTACAGCTTTATCA
AGAAATAGGATCAGCACATAAATCATTACACATCTATGATCGTTTGGAGCATGAAATATTTAATGAAAGTTCTT
ATAATAGAACTATTTTTAACGAAGTTATTGAATGGCTTGAAACGGAATTAACTTATAACTAAAACAGTATAGTT
CCGTGTATTTGATTATAAGAAATTATGAGGATATTAAACATACTAAGATTAGCTATGAAGAAATCTATGACGATA
GATTTTTTCATAGCTATTTTTTATAGTTATAGAGAGGAGTAGACTGTCCAGACTCTTGGATTTTAAATCCGTAA
AAAAAACAAGTCAGCTTTACTCTCACCTTTTGAAATTCGTTTGTAGTATGTTGGGTTCTTGAAACCGTGTATA
GGAAAATGAAATGAGAAAGGTTAAGTAAAGTTTTTAGCTTCTCAACTATTCAAAGGAGGTTTTTTTATCGAT
TACTTAGGTGTTGATATTAGTAAAAGGAGTAGTGTAGTTGCACATTATAAAAATGGAAAATTCCAAAAAGAGT
TTTTCATCCAAAATAATAAAAATGGCTACAATTATTTACTCAAGTATTTGAATGACTTAGACCACCCACAACTC
ATTTTTGAATCTACAGGTATCTATTCAAGAGGTATGGAACGATTTTGTTGTGTAAATCAAATTAACTATATTCAA
ATGAATCCGTTAGAAGCCAAATTTAAAACGAGCGCTCTAAGATCATGGAAAACTGATCAGGCAGATGCTCAT
AAGCTTGCTTGTTTAGGACCGACGCTCAAACAAACAGGCAGCTTACCTATACATGAGTTAATATTCTTTGAAT
TAAGAGAACGTGCCCGTTTTCATCTAGAAATCGAGAATGAACAAAATCGACTTAAATTTCAGATTCTTGAATT
ACTCCATCAAACATTCCCTGGTTTAGAAAGATTATTTAGTAGTCGATATTCAATCATTGCACTCAACATCGCAG
AAATTTTTACTCATCCAGACGTGGTTCTTGATATCGACAAGGATGTACTTATTACACATATATTCAATTCTACAG
ATAAGGGAATGTCAATGGATAAAGCTACAAAATATGCACTTCAATTAAGAGTGATTGCTCAAGAAAGCTATCC
TAATGTCGATAGACATTCCTTTCTAGTCGAAAAATTACGCTTACTTATTCAACAATTAAAACAATCTATTCATCA
TCTCAAACAATTAGATGATGCCATGATTCAATTAGCACAACAACTCGATTATTTTGAAAATATTCATTCGATACC
TGGTATTGGTAAGCTAAGCACAGCTATGATTATTGGGGAGATTGGTGATATTAAGCGATTTAAATCAAATAAA

CAACTCAACGCTTTTGTAGGCATTGATATCAAACGATATCAATCAGGTCATACACACTGTAGAGATACCATCAA
CAAGCGTGGTAATAAAAAAGCGAGAAAACTTTTATTTTGGGTGATTATGAATATAATAAGAGGGCAGCATCA
TTATGACAATCATGTCGTCGATTATTACTACAAACTAAGAAAGCAGCCTAATGAGAAACCTCATAAGACTGCC
ATCATTGCTTGTATAAATCGATTATTAAAAACGATTCATTATCTGGTAATGAATCATAAATTGTACGATTATCAAA
TGTCACCACATTAGCCAAACGTACAATTAAATATATTTTAATACCTTATTCAAAAAATTAAAATGAACGGTTTAG
TTAAGTAATGCTTATTTTAATTATAAGTACTTGACTAATCGTAAGAAAGAGCCTAGGACATAAATCAATGTCTC
GCGACCACAGCTTAATTTTTGGTGTTCATTCATGACTTTAAAAAATCCTTATTGCATAAATGTACATAGTGTAG
TACTATTCAAAACGTAATTATTACGATTTGAATTAAGCGAGGAGAATGAAATGACTAAGACTTATGACGTTTG
GTGGCAAAAAGGTCAAGAATCAGATGATGATATGGCACGAGACCATCAAGAAGCTTGGGAGAGAACAATA
AAAATGCTTGATACATCTGACATCGAAGGGAAAACGATTTTAGATGTGGGATGTAATCAAGGCGGATTTTTA
CGACAGTTATACGATACAACACCGTTTAAAAAAGGTGTTGGCATAGATTTAGCACGTTTATCTTTGGAAAAG
GCAGAGACATTAAAAGGACAACGTCCACTTACATACTATTTAACAGATAAACCGCAAGAAACGAAGCACGT
GTTTGATACGGCAGTAAGTACGTCTGTCTTGTACTTAATAGAAGATATTCCGCAACATGCAAAAGATTTAAAA
GAGGTATTGAAACCAGGCGGTGTTTATTACGCTTCATTCGCGGATTTAACTAATAACCCAAGTCGTCAGTTTA
TGGATGACACGATTAATCAATATGGTGCAACACCTTCTCAGAATCACTCTCTAAAACATATCGTTGATAGCTTT
GTGGATGCAGGATTTGAAGTTGCAGTAATGAAAGAGCATGTACCTGACGTGATTGATTTAACACATTATAGC
GATTTTTATTTATCACCGAATGATTATTTACAAACACTATATGAAGAATCGTTTTTAATAAAAGCAAGTGTGAA
AGAAGGTACTGAGAAATGAGGAAATGTGTATTAATGACGGTAGCAGCAAGTGCTACGCTCTTATTGGCAGG
TTGTGGCAATGGTCAAAAAGAAGATAAAGATGTTACGGTATCGCTACCTACTGAAGCAAAGGCGGATAAACT
TGACGCGCAAGGCTATGATGCAGCGATGCCCGTTTATAGTGCAGTGTATGATGCATTAGTTAAATATGATAAA
GATAAGGGTATTAAAGCAGGTTTAGCAGATAAATGGAGCGTTGATGAATCAGGGAAAGTTTATGAATTCCAT
TTGAAAAAGAATGTTAAATTCTCAGATGGTTCAGCATTAGATGCTAAGGACGTGAAATTCTCGATTGATCGTG
CGAAAGCGATGAACAAAGATTCGACTGTAGAAACGTTAAAAAAATTAGATAAGGTCGTTGTTAAAAATGAG
CACGTGGTCCAAATTAGATTGAAATCTCCTTCAAATCAAGTGTTAAATGAATTAACACAAGTGAGACCGTTGC
GTATTATGAGTCCACATTCAGTAGAAGATGGTAAAGTAAACGGTAAATTTGAAAAAGCGATTGGAACAGGTG
CATTTGTTGTTGATAAAACTGGTAAAGAAAAAACGACAATGAAGCCAAATAAATATTTTGACAACGGTCACC
CAGTCAATTATCATCTTGCATTCCAAACGATTGAAGATGGGGACTCAAGAAATTCTGCAGTACAAAGTGGTT
CTGTAGATATTTCTGGTGGTGCTTTAGGTATGCTCTCAGACGAACAAATCAAACAAGATAAGAAAAATAAGA
ACTTAACGATTGAAGATAGACCTAGCACAGTAAGTCACTTTATGGCATTTAACCCTAAAAATGATGTATTAAAT
CAACGCACAATTCGTGAAGCGATAAGTAAGAGCATCGATGCGAAAGACATTGCGGGCAAATCTGTAAATGG
TCTGTTCCAGAAGAACGTACAATTTGTGACTAAAAATAATCAACAGCCACACGATTATGATATGAAAGCGGCT
GAAAGGTTACTTAAATCAGAAGGATATCATAAAAACGATGACGGCATCTTTGAAAAGAATGGCAAACCTTTA
TCATTTAACTTAGTCATTCAAACTGCAGAGTTCCCAAATTGGAAAGATAAAGCTGAAAAAGTGCAACGTCAG
CTTAAACAAGCCGGTATTAAGTTAAATGTGAAAACGTTAGATTCACAATCATACTATGATACATTATGGACGAA
AAAAGACTATGATTTGATTTTCTATAGAACGTATTCAGATGCATTAATGCCTTACAACTTTATAAGTTCAGTGTT
TAAAAATAATGATGGTCAACCAGGGGTGTTAGCTGATGATGAAACATTAACGAAACAGCTAGACGATTTCCC
ATCAACCGTATCAAAAGAAGACCAACAGTGTTCATTTGATGACATATTTAAACACTTTAATCAACAATACTATG
GTGTGCCAATTGCTTATCCAAATGAGACGTTTGTAGTGAGTGATAAAGTAAAACAATTCAAATTCTCTGGACT
TACGGATGCACCAATTGATTATAAAGCGTTGAAAGTTAATGAATAGCAATGCTCAAACGTACAATTAAATTCAT
ACTTTATTTAATCGTAAGTTCGTTTATTATCTTCATTTTAGTTGAGAAGACATCTGGTAATCCAGCGATTCTGTA
TCTACAACGTCATGGTTATACGTCGATTACGCAAGACAATATTGAAGCGGCACAACATCAACTTGGCTTAGGA
CAACATGTGTTACTAAGATATATCGATTGGGTTGGACATGCACTCACGGGCAACTTAGGATACGGCTTTAGTA
CGAACGAAGCAGTTACCGCTATGATAATGGAAGCCATCGTGCCGACGCTTGTGCTAATCATTGTCTCTAGTTG
TATCATGTTGCCATTTGGCTATATTGTTGGTTACTTCGTTGGGACGCGTCCGCATACACGTTACGCTAATGGAA

TTCGTGGATTCGCCCAAGTGATGACCTCAATGCCAGAATACTGGTTAGCTATTTTATTCATTTATTATTTAGGC
GTACGTTGGCAATTGTTACCATTTGTAGGTAGTGATTCATGGCAACACTTTGTGCTGCCAATCTTCACAATTGT
TGTTATAGAAGGGTGTCATATCTTATTGATGACAGCACATCTGATTACACAAACGTTAGATCAAGATGCGTATC
AACTGGCGCAGTTAAGACATTTTTCGTTAAAAGCGCGTATCATCGTACAAATTAAAGAGATATTTGCACCACT
AATGACGATTTCAATTAACAGTATCATTCATTTAATTGGAAAAGCCGTAATACTAGAAGTCATCTTCAGCATGT
CTGGTATAGGTAAATTGTTGATTAATGCTATTAACCAACGAGATTATCCACTGATTCAGGGCATTGTCATCTTTA
TCATTGTCTTTATTATGCTAATGAATTATTTAGGCGATGTGATTATTTTGAAGAATGAACCTAGACTTCGACGAC
GTCATACCCAGCAGTCAGGCAATGAGAAAAGAGGTACGATGTGATGAAAAAATATCAAACGTACATCGCAAT
AGGTTCACTATTGAGTTTGATGGTTGTATTAATTACGTATGGTTTAATGCAAGACACGCAACATTTGAACCCA
CTTGAGTCACCTAATGGACAACATTGGTTGGGTACCGATCAATTAGGCAGAGACTTCTTAGTAAGACTGATT
GTCGGTAGTCTTGTCACATTGAGTTTAACAGGCATAGTGATTCTATTAAGCGTTTGTATGGGACTTATCTTTGG
CTTAATTGCAGGCATAGAAAGACGATGGTTAGATCAAATCATCATGTTTGTTGCCGATATGTTGCTGGCTATTC
CGTCATTTATTATCGCATTAGTCATCTTAAGTTTAGTAAGTAACTCCATGATAGGTTTGATACTTGCTTTAACGA
TTGGATGGATAGGACGTTATTTACGTTACTTCAGAAATTTAACGCGAGATATTCAAAAACGTCCATTTGTTCA
ATATGCACGATTGAGTGGGAACTCAACATTCAAAACGACAGTAACACATGTGATTCCACATTTATTAAGTAGT
ATATTCGCTTTGGTAACGGCTGACTTTGGCAAAATGATGCTCAGCATATCTGGACTTGCTTTTCTAGGACTAG
GTATTAAACCGCCGACGCCTGAGTTAGGAACAATTCTTTTTGATGGGAAAAGTTATTTCAACGGCGCACCGT
GGCTCTTCTTCTTCCCTGGTGTATTGTTAGGAGGTTTCGCCTTATTATGTCAAATTATCAACAAAAAAATAACG
CAGTAAATACGGTAGTCAACGTCAATCAATTATCGATTTTAGATCAAGAGAAAGTATTGTTAAAGGATGTTGA
TTTGACAGTAACTAAAGGTGCATTTCATTGCATTATAGGTGAAAGTGGCAGTGGGAAATCACTGTTAACAAG
AACAATACTTGGAATGAAACAATCACAATTATGTTATCAAGGAGATATTGACATCGATTTAACTCAAACAGAT
GCAGTGTTTCAAGATGTTCAAAGTAATATGTTTCAAAATATAACATTAGCTAAGCATTTCCAATACATTTATGA
AGCCAATCGCACACATCTCACTAAACAGCGTATTAAGGAAGATGTCTTACAGATGATGCAATTACTTGGTTTA
AGACAAGGGGAACAATTGCTTGAGCGTTATCCCTTCGAACTTAGTGGAGGTATGGCACAACGTGTCGCCTT
TATAATGTCATTAATTAGACGTCCGAACTACTTATTTTTAGATGAACCAACGAGTGCACTTGATCAAGAAAATA
TTAAAAAGTTTATGCATTACCTTCTTAGGGCACAGGAGCGCTACCAAATGACCATTGTTTTTATCACACATGAT
ATTAACTTAGTGAAAGATTGTGCCACACATATTAGTATTATGCAGCAAGGTAAATTGATAGAAAATGGTGAGG
CCGCGTCGATCTTAACTAAGCCGACACATAATTACACGAAAAAATTAATTACGATTGCACATCGGAGACAACC
TTATGCTTAAAATAGAGAGATTAACCAAATATATAGACACGCAACTGATATTTAAAGAGATATCATGTACAATTA
ACGACCAGCACTTACTCATAAGTGGGGAGAGTGGTTGTGGTAAATCCACATTAGCCAAGATTATCGCTGGCT
TAGATACGGATTATCAGGGCGAATTATATCTTAATGGGCGCTTACGTGAATCTTATACGTCTAAAGAGTGGATG
AAGCACATCCAATATGTACCTCAATATCAACGTGATACTTTAAATCAGCGTAAAACGGTATTAGCTACATTATTA
GAACCACTTAAGAATTATAAGGTAAATAAACAGCGTTATACATCAAGCATTGAAGCAGTGCTTGATCAGTGTA
ATTTACCACACGATATACTTAATCATAAAGTTTCGACATTAAGTGGTGGCCAATTTCAACGCGTCTGGATAGCT
AAAGCTTTAATATTAGAACCAGAGATTCTCATATTGGATGAAGCTACAACCAACTTAGATGTCATTAATGAAG
AAGCTATACTTCAAATGTTGATTTCCTTAAAGATGACACAATTAATCATTATTTCACATGATACATACGTCTTAA
GCCAATTTGAAGGAATTCAGTTACAGCTAAATAAATTGAATAATTAAGATCACAAATCTTAATATGGTGAATAT
TTAATGGTACCTAAAAAATAAAATTTAAACTACAATGTCTAAATCCATATGTTGTTTCATTAGAGGATTTAAAAA
TGATTATAACACTAAAAGATTTCAAATTATTATATTTAATATAAATTTACATATGATAAACGAATAACAATTCCAA
TATAAATTATTTTTTGATTATTTTTATTTATAACTATATATTTTATATGAAAAATATAAGTTATAATAATAAGTTTAAT
ATTGCCTCGTGGTTCTGAGCTTGAACTTATCTCTAAATCATTTTGAGCTACTTATCTATCAATTCATATATTCTAT
AACAATATTTGTGACATCACGTGCTATTTCATGAAGTGATTTTACGATATCACCTTCTTTAGAAAAAATATATTT
CAGTAGGCACCGACGTATACAGAATCATTTGAGTATTAAAAATAAAAACTAGAAAAAGAAACCGCATCATTA
ACTGATACGCAGAATCATATTATAAATAAAACTAAAAATGAGGTTGTATATAACTCACTCTGAAATTGATTGAAT

ATATAGTATCTTTAATAAAATGCAGCTATTGTGGCGTAGAATTTGAGAATCAAAAAATGATTAATATAGTTTGA
AGAGACTGAGCATAAATACTAGAAAAATGGCCAGTAAATGAGTTTACTATAAACTCATTTACTGGCTTCTCTA
ATAATTATCAAGACAATTTGCGTTTCTAGGCATACTTTGAAATGCGCTATTTTCTTTAAGAATATTAATATATGAC
TTGTGTTGGTAATGATAACCTTGGTTAGAGTGTATTGTCATGTGATAGTTAAGATTAGATCTTTTAATTAAAAC
ATTGTTTTAAATATCGATGACAAGGTCTAATGTAGGACGTGTAGATATAAGGTTCGGAATTATATAAATCCATA
AAAGGAGATAAATCGAAAACCTTTATACCCTGGGTGGGTATATGTTATAGTATAAGTAGCTTTACTATAACATT
TTCATTAGGAGGGGTTAATTTGAATAATAATGGTGAAGAGCATAATCATCAAAATCACATGAATCATTCCAATC
AAATGCATCATGATAACCATGCCTCACATGATCATCATAGTGGCCATGCACATCATCATGGAAATTTTAAAGTT
AAGTTTTTTGTTTCATTAATTTTTGCAATACCTATCATTCTTTTATCGCCACTGATGGGTGTTAACTTACCTTTTC
AATTCACATTTCCAGGTTCTGAATGGGTAGTGTTAATATTAAGTACAATTTTATTCTTTTATGGTGGTAAACCGT
TCTTGTCTGGTGGTAAAGATGAAATTGCTACAAAAAAACCAGGCATGATGACCTTAGTTGCCCTAGGTATTTC
AGTAGCTTATATTTATAGCTTGTATGCTTTTTATATGAATAACTTTAGTAGTGCAACTGGTCATACAATGGACTT
TTTTTGGGAATTAGCAACCTTAATTTTAATTATGCTATTAGGACATTGGATAGAAATGAATGCTGTCGGAAATG
CTGGAGATGCTTTAAAGAAAATGGCAGAACTGTTACCTAATAGTGCTATTAAAGTTATGGATAATGGCCAACG
CGAAGAAGTTAAAATATCAGACATCATGACTGATGATATCGTCGAAGTAAAAGCCGGAGAAAGCATTCCAAC
AGATGGTATTATCGTTCAAGGACAAACATCTATAGATGAATCCCTAGTCACTGGAGAATCTAAAAAAGTACAA
AAAAATCAAAATGACAACGTCATCGGGGGTTCTATTAATGGGTCTGGAACAATACAAGTCAAGGTTACAGCT
GTGGGAGAAGATGGATATCTTTCTCAAGTTATGGGACTTGTTAATCAAGCACAAAATGATAAATCTAGTGCTG
AATTGTTATCTGATAAAGTAGCGGGTTATTTATTCTACTTTGCTGTAAGTGTTGGCGTGATTTCTTTTATTGTCT
GGATGCTCATTCAAAATGATGTTGATTTTGCATTAGAACGTCTTGTAACTGTGTTAGTCATTGCTTGTCCACAT
GCTTTAGGCTTGGCAATACCTTTAGTCACTGCACGTTCTACTTCAATTGGTGCACATAATGGTTTAATTATTAA
AAATAGAGAGTCTGTAGAAATAGCTCAACATATCGATTATGTAATGATGGACAAAACTGGTACTTTAACTGAG
GGTAACTTTTCTGTGAATCATTATGAGAGCTTTAAAAATGATTTGAGTAATGATACAATATTAAGCCTTTTCGC
CTCATTAGAAAGTCAATCTAATCACCCATTAGCTATAAGTATTGTTGATTTTGCGAAAAGTAAAAATGTTTCAT
TTACTAACCCACAAGACGTTAATAATATTCCAGGTGTCGGATTAGAAGGTCTAATTGATAATAAAACATATAAA
ATAACAAATGTCTCTTATCTTGATAAACATAAACTTAATTATGACGATGACTTGTTTACTAAATTAGCTCAACAA
GGTAATTCAATCAGTTATTTAATTGAGGATCAACAAGTCATTGGCATGATTGCTCAAGGAGATCAAATTAAAG
AAAGCTCAAAACAAATGGTAGCTGATTTACTATCAAGAAATATTACACCAGTCATGCTTACAGGTGACAATAA
TGAAGTGGCACACGCTGTCGCAAAAGAATTAGGTATTAGTGATGTCCACGCACAACTCATGCCAGAAGATAA
GGAAAGCATTATAAAAGATTATCAAAGTGACGGTAATAAAGTCATGATGGTCGGAGACGGTATCAACGATGC
GCCGAGTCTTATAAGAGCGGATATTGGTATAGCAATTGGTGCAGGTACAGATGTTGCAGTGGATTCAGGTGA
TATCATACTTGTTAAAAGTAATCCATCAGATATCATTCATTTCTTGACCCTTTCAAATAATACTATGAGAAAAATG
GTGCAAAACTTATGGTGGGGTGCAGGTTATAATATTGTTGCTGTACCTTTAGCAGCTGGTATTTTAGCATTTAT
TGGCTTGATTTTATCACCTGCAATAGGTGCTATTTTAATGTCTTTAAGTACAATTATCGTTGCAATTAATGCCTT
TACATTAAAATTAAAATAAAAGATAGGAGTTTTATTATGATTAAAAAATTATTTTTTATGATATTAGGATCATTAC
TAATATTATCAGCTTGCTCCAATAATGATGAAAAAGATAAAGACACTAATGACCAAAAAAGTGAGAGCCATAT
GAAGCATAATGATGAAAGTAAAGTTCCAGAAGATATGAAATCGACTAATGAGGGTGAATTTAAAGTGGGAG
ATAAAGTAACGATTACAGCAGGGCATATGCCAGGTATGAAAGGTGCAGAAGCTACTGTAAAAGGTGCGTATA
AAACATATGCCTATGTTGTAAGTTATAAACCCACAAATGGAAATGAAAAAGTAAACAATCATAAATGGGTCGT
AAACGAAGAGATTAAAGATGCACCTAAAGATGGATTTAGTAAGGGCGATACTGTTAAATTAGAAGCAAGTC
ATATGTCTGGTATGAAAGGTGCTACAGCCAATATAGATAACGTGAAAAAGACTACTGTTTACGTAGTTGATTA
CAAATCCAAAGATAATGGTAAAATCATTAAAAATCATAAATGGATGACAGGAAATGAGCTGAAAGCACGATA
AAAATCTAGTTCTAAATTGAGAAATAAATAGATATAAAAATATCCTCCTTAATCAATAATTTAAATAACTTATTAT
TGTTAAGGAGGATATTTTTTAGTGTGTAAATTAAAAAGAATTTTAGAAGAATAACATTTATCAAAAAACTGTT

CATTACCTTATTAATTGAAATTATATAATTAAAAACCGCATCATTAACCGATACGCAGAGGCGTATCATAAGT

>Staphylococcus aureus strain 515798

ATGAAAATCACCATTTTAGCTGTAGGGAAACTAAAAGAGAAATATTGGAAGCAAGCCATAGCAGAATATGAA
AAACGTTTAGGCCCATACACCAAGATAGACATCATAGAAGTTACAGACGAAAAAGCACCAGAAAATATGAG
CGACAAAGAAATCGAGCAAGTAAAAGAAAAAGAAGGCCAACGAATACTAGCCAAAATCAAACCACAATCC
ACAGTCATTACATTAGAAATACAAGGAAAGATGCTATCTTCCGAAGGATTGGCCCAAGAATTGAACCAACGC
ATGACCCAAGGGCAAAGCGACTTTGTATTCGTCATTGGCGGATCAAACGGCCTGCACAAGGACGTCTTACA
ACGTAGTAACTACGCACTATCATTCAGCAAAATGACATTTCCACATCAAATGATGCGGGTTGTGTTAATTGAA
CAAGTGTACAGAGCATTTAAGATTATGCGTGGAGAAGCATATCATAAATGATGCGGTTTTTTCAGCCGCTTCA
TAAAGGGATTTTGAATGTATCAGAACATATGAGGTTTATGTGAATTGCTGTTATGTTTTTAAGAAGCTTATCAT
AAGTAATGAGGTTCATGATTTTGACATAGTTAGCCTCCGCAGTCTTTCATTTCAAGTAAATAATAGCGAAATAT
TCTTTATACTGAATACTTATAGTGAAGCAAAGTTCTAGCTTTGAGAAAATTCTTTCTGCAACTAAATATAGTAA
ATTACGGTAAAATATAAATAAGTACATATTGAAGAAATGAGACATAATATATTTATAATAGGAGGGAATTTCAA
ATGATAGACAACTTTATGCAGGTCCTTAAATTAATTAAAGAGAAACGTACCAATAATGTAGTTAAAAATCTGAT
TGGGATAAAGGTGATCTATATAAAACTTTAGTCCATGATAAGTTACCCAAGCAGTTAAAAGTGCATATAAAAG
AAGATAAATATTCAGTTGTAGGGAAGGTTGCTACTGGGAACTATAGTAAAGTTCCTTGGATTTCAATATATGAT
GAGAATATAACAAAAGAAACAAAGGATGGATATTATTTGGTAGGTTCTGTTGCAAAGTAAAAAAATATAGCT
AACCACTAATTTATCATGTCAGTGTTCGCTTAACTTGCTAGCATGATGCTAATTTCGTGGCATGGCGAAAATCC
GTAGATCTGAAGAGACCTGCGGTTCTTTTTATATAGAGCGTAAATACATTCAATACCTTTTAAAGTATTCTTTG
CTGTATTGATACTTTGATACCTTGTCTTTCTTACTTTAATATGACGGTGATCTTGCTCAATGAGGTTATTCAGAT
ATTTCGATGTACAATGACAGTCAGGTTTAAGTTTAAAAGCTTTAATTACTTTAGCCATTGCTACCTTCGTTGAA
GGTGCCTGATCTGTAATTACCTTTTGAGGTTTACCAAATTGTTTAATGAGACGTTTGATAAACGCATATGCTGA
ATGATTATCTCGTTGCTTACGCAACCAAATATCTAATGTATGTCCCTCTGCATCAATGGCACGATATAAATAGCT
CCATTTTCCTTTTATTTTGATGTACGTCTCATCAATACGCCATTTGTAATAAGCTTTTTTATGCTTTTTCTTCCAA
ATTTGATACAAAATTGGGGCATATTCTTGAACCCAACGGTAGACCGTTGAATGATGAACGTTTACACCACGTT
CCCTTAATATTTCAGATATATCACGATAACTCAATGTATATCTTAGATAGTAGCCAACGGCTACAGTGATAACAT
CCTTGTTAAATTGTTTATATCTGAAATAGTTCATACAGAAGACTCCTTTTTGTTAAAATTATACTATAAATTCAAC
TTTGCAACAGAACCGTATTATGGAATAGAGATGTTGGTAACATTTATACAGGATCATTATACTTAAGTTTAATTT
CGTTATTACAGAACCACACATTCCAACCAGAAGAGAAAGTATGTCTATTTAGTTATGGTTCAGGAGCAGTAG
GAGAAATCTTTAGTGGTTCAATCGTTAAAGGATATGACAAAGCATTAGATAAAGAGAAACACTTAAATATGCT
AGAATCTAGAGAGCAATTATCAGTCGAAGAATACGAAACATTCTTTAACAGATTTGATAATCAAGAATTTGAT
TTCGAACGTGAATTGACACAAGATCCATATTCAAAAGTATACTTATACAGTATAGAAGACCATATCAGAACATA
TAAGATAGAGAAATAAACTAGTGGCCGATTGTGCTTGATGAGCTTGGGACATAAATCCTAACTCGAAATAAAT
AAGCATATCACTAAACTGATTTTTTAAAGTTTACAGTGATATGCTTATTTTTTATCTTACGATTTTGTACGTGCAT
GCTTGCCTAGGGGTATGGCTCGAGCCATTAGTCTCTCGCACATACTATTCCCTCAGGCGTCAGCACTTACAAA
ATCGGTTGTAATTTTCATTTTTATACGCATTCTTACTGAGATTATACTAATAAGAGGAATAGTAAAAGCAATTCT
AAGTAAAATTGCAGATAAGAGGTTTGTTAAAAGCAGTTCTAAGTAAAATTGCAGATAAGAGGTTTGTTAAAA
GCAGTTCTCAGTAAAATTACAGATAAGAGGTACGTTAAAAGCAGTTCTAAGTAAAATTGCAGATAAGAGGTT
TGTTAAAAGCAGTTCTAAGTAAAATTGCAGATAAGAGGTACGTTAAAAGCAATTCCATGCAAAATTGCTGATA
AGGGGTAAGTTAAAAGCAGTTCTCAGTAAAATTGCAGATAAGAGGTACGTTAAAAGCAGTTCTAGGCAAAA
TTGCAGATAAGAGGTGCGTTAAAAGCAGTTCTCAGTAAAATTGCTGATAAGGGGTAAGTTAAAAGCAATCCT
AAGTAAAATTGCAGATAAGGGGTACAGAAAAACTAGACTTGATTACAAAATGGAGCTTGGGACATAAATGA
TTTTTTAAAAATGAGATGAGACGTAGATTAACTCCATAATCAATACGAATCTATCGACTTCTTTATTTATGATATT
CATCTCTTTTTAATGGAAATAAAAGTGCGATTAATGTGATAATACAGTTACGTTAATTAAAAAAATAAAAATGC

AAGGAGAGGTAATATGCTAACTGTATATGGACATAGAGGATTACCTAGTAAAGCTCCGGAAAATACAATTGCA
TCATTTAAAGCTGCTTCAGAAGTAGAAGGTATAAACTGGTTGGAGTTAGATGTTGCAATTACAAAAGATGAA
CAACTGATTATCATTCATGATGATTATTTAGAACGGACTACAAATATGTCCGGGGAAATAACTGAATTGAATTA
TGATGAAATTAAAGATGCTTCTGCAGGATCTTGGTTTGGTGAAAAATTCAAAGATGAACATTTGCCAACTTTC
GATGATGTAGTAAAAATAGCAAATGAATATAATATGAATTTAAATGTAGAATTAAAAGGTATTACTGGACCGAA
TGGACTAGCACTTTCTAAAAGTATGGTTAAGCAAGTGGAAGAACAATTAACAAACTTAAATCAGAATCAAGA
AGTGCTCATTTCAAGCTTTAATGTTGTGCTTGTTAAACTTGCAGAAGAAATCATGCCACAATATAACAGAGCA
GTTATATTCCATACAACTTCGTTTCGTGAAGACTGGAGAACACTTTTAGATTACTGTAATGCTAAAATAGTAAA
CACTGAAGATGCCAAACTTACTAAAGCAAAAGTAAAAATGGTAAAAGAAGCGGGTTATGAATTGAACGTAT
GGACTGTAAACAAACCAGCACGTGCAAACCAACTTGCTAATTGGGGAGTTGATGGTATCTTTACAGACAATG
CAGATAAAATGGTGCATTTGTCTCAATAGAAAGTTAGAGGTGAGTCTTACGTTTCAGTGACGGTAGACTTAC
CTTTAACATGTTACATACTAAAAAATTAATTTGAATAAGAAAGAGAGACATATATGAAATACGATGATTTTATA
GTAGGAGAAACATTCAAAACAAAAAGCCTTCATATTACAGAAGAAGAAATTATCCAATTTGCAACAACTTTT
GATCCTCAATATATGCATATAGATAAAGAAAAAGCAGAACAAAGTAGATTTAAAGGTATCATTGCATCTGGCA
TGCATACACTTTCAATATCATTTAAATTATGGGTAGAAGAAGGTAAATACGGAGAAGAAGTTGTAGCAGGAA
CACAAATGAATAACGTTAAATTTATTAAACCTGTATACCCAGGTAATACATTGTACGTTATCGCTGAAATTACAA
ATAAGAAATCCATAAAAAAAGAAAATGGACTCGTTACAGTGTCACTTTCAACATACAATGAAAATGAAGAAA
TTGTATTTAAGGGAGAAGTAACAGCACTTATTAATAATTCATAATAAAACAGTGAAGCAACCATCGTTACGGA
TTGCTTCACTGTTTTGTTATTCATCTATATCGTATTTTTTATTACCGTTCTCATATAGCTCATCATACACTTTACCT
GAGATTTTGGCATTGTAGCTAGCCATTCCTTTATCTTGTACATCTTTAACATTAATAGCCATCATCATGTTTGGA
TTATCTTTATCATATGATATAAACCACCCAATTTGTCTGCCAGTTTCTCCTTGTTTCATTTTGAGTTCTGCAGTAC
CGGATTTGCCAATTAAGTTTGCATAAGATCTATAAATATCTTCTTTATGTGTTTTATTTACGACTTGTTGCATACC
ATCAGTTAATAGATTGATATTTTCTTTGGAAATAATATTTTTCTTCCAAACTTTGTTTTTCGTGTCTTTTAATAAG
TGAGGTGCGTTAATATTGCCATTATTTTCTAATGCGCTATAGATTGAAAGGATCTGTACTGGGTTAATCAGTATT
TCACCTTGTCCGTAACCTGAATCAGCTAATAATATTTCATTATCTAAATTTTTGTTTGAAATTTGAGCATTATAAA
ATGGATAATCACTTGGTATATCTTCACCAACACCTAGTTTTTTCATGCCTTTTTCAAATTTCTTACTGCCTAATTC
GAGTGCTACTCTAGCAAAGAAAATGTTATCTGATGATTCTATTGCTTGTTTTAAGTCGATATTACCATTTACCAC
TTCATATCTTGTAACGTTGTAACCACCCCAAGATTTATCTTTTTGCCAACCTTTACCATCGATTTTATAACTTGTT
TTATCGTCTAATGTTTTGTTATTTAACCCAATCATTGCTGTTAATATTTTTTGAGTTGAACCTGGTGAAGTTGTA
ATCTGGAACTTGTTGAGCAGAGGTTCTTTTTTATCTTCGGTTAATTTATTATATTCTTCGTTACTCATGCCATAC
ATAAATGGATAGACGTCATATGAAGGTGTGCTTACAAGTGCTAATAATTCACCTGTTTGAGGGTGGATAGCAG
TACCTGAGCCATAATCATTTTTCATGTTGTTATAAATACTCTTTTGAACTTTAGCATCAATAGTTAGTTGAATATC
TTTGCCATCTTTTTTCTTTTTCTCTATTAATGTATGTGCGATTGTATTGCTATTATCGTCAACGATTGTGACACGA
TAGCCATCTTCATGTTGGAGCTTTTTATCGTAAAGTTTTTCGAGTCCCTTTTTACCAATAACTGCATCATCTTTA
TAGCCTTTATATTCTTTTTGTTTTAATTCTTCAGAGTTAATGGGACCAACATAACCTAATAGATGTGAAGTCGCT
TTTCCTAGAGGATAGTTACGACTTTCTGTTTCATTAGTTGTAAGATGAAATTTTTTTGCGAAATCACTTAAATAT
TCATCCATTTTTTTAACGGTTTTAAGTGGAACGAAGGTATCATCTTGTACCCAATTTTGATCCATTTGTTGTTTG
ATATAGTCTTCAGAAATACTTAGTTCTTTAGCGATTGCTTTATAATCTTTTTTAGATACATTCTTTGGAACGATG
CCTATCTCATATGCTGTTCCTGTATTGGCCAATTCCACATTGTTTCGGTCTAAAATTTTACCACGTTCTGATTTTA
AATTTTCAATATGTATGCTTTGGTCTTTCTGCATTCCTGGAATAATGACGCTATGATCCCAATCTAACTTCCACA
TACCATCTTCTTTAACAAAATTAAATTGAACGTTGCGATCAATGTTACCGTAGTTTGTTTTAATTTTATATTGAG
CATCTACTCGTTTTTTATTTTTAGATACTTTTTTTATTTTACGATCCTGAATGTTTATATCTTTAACGCCTAAACTA
TTATATATTTTTATCGGACGTTCAGTCATTTCTACTTCACCATTATCGCTTTTAGAAATATAACTGCTATCTTTATA
AACTTGTTTGAAATTTTTATCTTCAATTGCATCAATAGTATTATTAATTTCTTTATCTTTTGAAGCATAAAAATAT

ATACCAAACCCGACAACTACAACTATTAAAATAAGTGGAACAATTTTTATCTTTTTCATCAATATACTCCTTATAT
AAGACTACATTTGTAGTATATTACAAATGTAGTATTTATGTCAAAATAATGTTATAATTTTTGTGATATGGAGGT
GTAGAAGGTGTTATCATCTTTTTTAATGTTAAGTATAATCAGTTCATTGCTCACGATATGTGTAATTTTTTTAGT
GAGAATGCTCTATATAAAATATACTCAAAATATTATGTCACATAAGATTTGGTTATTAGTGCTCGTCTCCACGTT
AATTCCATTAATACCATTTTACAAAATATCGAATTTTACATTTTCAAAAGATATGATGAATCGAAATGTATCTGA
CACGACTTCTTCGGTTAGTCATATGTTAGATGGTCAACAATCATCTGTTACGAAAGACTTAGCAATTAATGTTA
ATCAGTTTGAGACCTCAAATATAACGTATATGATTCTTTTGATATGGGTATTTGGTAGTTTGTTGTGCTTATTTT
ATATGATTAAGGCATTCCGACAAATTGATGTTATTAAAAGTTCGTCATTGGAATCGTCATATCTTAATGAACGA
CTTAAAGTATGTCAAAGTAAGATGCAGTTCTACAAAAAGCATATAACAATTAGTTATAGTTCAAACATTGATAA
TCCGATGGTATTTGGTTTAGTGAAATCCCAAATTGTACTACCAACTGTCGTAGTCGAAACCATGAATGACAAA
GAAATTGAATATATTATTCTACATGAACTATCACATGTGAAAAGTCATGACTTAATATTCAACCAGCTTTATGTT
GTTTTTAAAATGATATTCTGGTTTAATCCTGCACTATATATAAGTAAAACAATGATGGACAATGACTGTGAAAA
AGTATGTGATAGAAACGTTTTAAAAATTTTGAATCGCCATGAACATATACGTTATGGTGAATCGATATTAAAAT
GCTCTATTTTAAAATCTCAGCACATAAATAATGTGGCAGCACAATATTTACTAGGTTTTAATTCAAATATTAAAG
AACGTGTTAAGTATATTGCACTTTATGATTCAATGCCTAAACCTAATCGAAACAAGCGTATTGTTGCGTATATTG
TATGTAGTATATCGAGCTTCACATGAAACAGCTAAAGAAGCTTTGGGCGATAAAGAGTTAAGAGCCATTGCA
CATGAGTTAACTAAAACAGTTAAGGATAACATGAGTGTTGATTGGTCTAAACGAGACAGTGCTAAAGCTAAA
ATGAGAGTTCAAGTTAGACGCCTATTAAAGAAATATGGCTATCCACCAGATCTTCAAAAAATGGCTGTGGAA
CAAGTTGTAGAGCAAGCAGAATTAATGGCAAGTCAGCAATAAAAAAATAAATCATAATGAGTCCGGGACATA
AAGTTCTTGGATAAGTGAAAAAAGACAATTTCTATTGAAATAATATAGAAATTGTCTTTTTTATAAATTTTTTG
ATTATTTTCAGCTCGTTGAGCTACTACTTTTCTTATATTAAGTGCCATTAATACAAAACCAAGTTCTCTTTTGAC
TTTATTGAGTCCTCGGACAGACATCCGAGTGAAACCCAAAATAGCCTTCATAAATCCAAAAACAGGTTCCAC
ATCAATTTTTCTTTGACTGTAGATATTTTTTGTTTCTGGTTCTGAAAGCTTTTTGTTAATTTGGGATTTAAAATA
TTCCCAGTTATAATTCTTCATTATTTTTTTGTTTTGTTTTTGAATTGAAGTTCATACATTGATTTTTCAGAGGAC
ATTCTGAACAATCATCACATTCATATAATTTGAAGTCTCGCTTATAACCATACTTATCATGACGATAGGCATATCT
TTTAAAACCTAGCCGTTTATTATTCGGACAAATGAATTCGTCATTAATTTCGTCATAGTTCCAATTTTGAGTATT
AAAGATGTCACTTTTATATTTTTTAGTTTTATCTTTTATAAACATTCCATATGTTATGAGTGGCGTTCGATTAAAG
TCATCTATAATTGCCTTATAATTTGATTCACTACCATAACCTGCATCAGCTACAATATATTCAGGTAAATGACCGT
AGGTCTCTTGAATTGAATTTAAAAATGGAATCATCGTTCTAGTATCCGTTGGATTTTGATACACATTATAAGATA
AAACAAATTGGGAATTTGTTGCTATTTGTAAATTATACCCTGGCTTAAGTTGTCCATTTTTCATGTGATCTTCTT
TCATTCTCATAAATGTCGCATCATAATCTGTCTTAGAATAACTATTTCTATCCTTTAAAATAGATTTTTGAAATTC
GTATCGATACTTTCGCTCAAAATAATCATTGATTTGCTTTTTTGTATTTTTTGATTTTAGTTTCTTTTGAGACGTA
TTTGTTTTCTTGTTTTAGTACATTTTTCATTGTTGATATGTTGGTTTAAATCTTCGATTTCTTTATCTAAGTGACT
ACCAATCAAATCTATTTCTTCTTTTGTTAATTCATTATCATGATCTTCTTTAATTTCCGGTATGATTTTATTGGTTA
CCAATTCATGGTAGAGGGCTTTAGAATCCTCATTCATCTTTGATTCATGGTTTTGAATACTCTTTTTCCATACAA
ATGTATATCGATTGGCATTTGCTTCAATTTTTGTACCATCAATAAAAATAGCTTTATCATCTATAAGATTTTGTTT
TACACACTGACTGTAAAATTGAATAAATAAAGATTCTAATAAAGCATCTACTTTTGGATTTACTCTAAATTGATT
AATTGTTTTATAAGAAGGTTTTTGATTTTGTGATAGCCACATCATTCGGATGCTATCATTAAGCATTTTTTCTAT
TTTACGACCTGAGAATACAGATTGTGTGTAGGCATATAGAATCACTTTTAACATCATTTTAGGATGGTACGAA
GTTGCACCACGGTGATGTCTGAATTCGTCGAATTCATTGTCAGGAATTGTTTCAACAATATCATTTACAGTAAA
ACGATGTTGATTTGTTTTGTTTCCATATTGACCTCCATGTATTTGCTATGATTTCAAAATCCATTTTTGACGTGC
CTTAGGGTTGAGTGGATGCATAATTTCATTTGTTACTGGATTGATGAGCTTTTTTACTTTCTTTTTATGAGGTT
TTAACATTTCCATCACTTGTTCGACACGGTCGATAACAACTGGTCGCTTCGCATAGGCACCATAAGCAAGAAT
CACTGTGTCACTTTCACTAATCGCTTTCATCAAATGAATATCAGTGTGCTCATCGTATGGATTTTTGATATGTTT

GAGGTTTTCGGGTGTTCTAATATTAGAGAATAGATTTACAAGATATACAGCACCGTATCGTTCTGAATTGGCTA
ATTGGTTGAGGATAAGAACAGTTGTGAGATCGAGTGATAATACACCGTCTAAATGAGGATACATCGTTATCAC
TGTGCATGCAGCTTTCTTTTCATCCCATGTTTTCTTGAGTAAATAGCGGTGCTGTTCATCATCGCTAAATATGG
CTTCTGTGTGTATCGTACTTTTGATTGTATTCATCATCGTCACTTCCTTTAGTATTCTTCTGGTAAAAGCATCACA
TAATAAAAAGCGTCCACGTCATCTTCACGAATGACGTAGACTTTCTTAGGTAATGCATTTTGATTTTTTTCATA
GTTTGTATAGTGATATTCCAATTTGTATGTGGGTTGTTCTTGTTCATGTGTGATTGAGAGTATATTCTCATCTTCT
TGTAATTTAAAAATGTGTAGGTAATCTGTATGAGGCTGGTTATCTTTTTTTTCTACCATGTGCCAAAGTAAGAT
TTGAAGGTCTAGTGGAATACTTTCATTAATTCCTCTTGTGATGTATCGATTGATTTTCATGCTATTTCCCTCCCTT
CTGCTTTTCTTTCATGATGTCGATGATTTCGTTGATAACTGTGACGGATAATTGAGCAACTATGATCCAATTATT
CATGGTCCTGACCTCCTTGTTTTAGTAAATGACGTTCATCAATAATGATATTTTGAGTATCTGTAAGGTACAGA
AAGTCCATGTCAAAATGGTCTAAGTATCCGACACTGATGAGTTGGTTATTGGCATACATTAGAAATGGATAGA
TACTTAGCTCATGTAGTTCATCATTATAGTAGGTATAAGTCTCGAGTGTGAGATGTACCAGTGGAGAATCATTA
ATAAAACGTTCCGGTAGAATATTTTCTGCTGCTTCCTCCAGCGCTTCACATTCCCAAGCTTCGTTATTAGATAG
TTGGAATAGGTGGGTTATATATTGTTTGAGTTCTTGAGTGATGGTTTTCATATTATTGCCTCCTAGATAGTGTAA
TAGTGATGTAGTTCATATACATCATTGAGATAATATATATTTGATTTGTCATTTATTACGAATCCCGGTGGGAATA
AGAGAAAATTCCATATAAAAACCCGCTACAAACGTTGGTATGCCAAGGAAATCCTGAAATTCCGCCTATTTTG
ACAAACAATCAACTCATTATTTATAAGTATTGATGATAGGGTTGTGTCTCTGCTTCCTTATATATATTATTTATTTA
TAAAAAGTAACGGGATTTTGGGATTGTGCTTGCACAATCCTTCTGTTTCTTCGAATCTGCAAATCCCAATCATT
TCCCGATAAAAAATCATTGTGGGATGTTCTTTAGCAATTTCAATATAAGCATTGTATAGTTATGAAAAAATTAC
GACAATAACTGTTTCATTAGATAAGTGTTATTGAAATTGATAAAGAGCAATTCTTGAAAATAGTTAGATAAAAT
AAGCGAAAGAATATAGTGAAAATTATTGTTATAACAATGATTCTATTAGCTAAATAGTAAGATATAGTGTTTGG
GGCAAAAATAAAGACGAAGTGCTGAGATGCACTTCGTCGAGTTGTTTATTATTGAAAAGTTGTTTAATGATT
TCGTTATTAAGTTTGAGTGTGACATAGAATTGTTTTTTATGATTACCATCTTTTTTAATATCAATGCGATCAATCA
CTGATAGATACAATGCTTTGAGTCGAGATTTTTCTATGTGCTTAATATCATGAAAGATGTGTTGTAATAGTTTAC
TGATTTCTTTGGCATCAAATAAAGTCTTATCTTCATTTTGTTGATTTTTGAGTTGGTTGATTTGATTCGTAATGT
CATTGAGTTGCTTTTCATATTTTTGAATACTTGGTCTGATTACTGATGTTAAGTCCGGATTATCCTCGATGGTTT
TAATCAAGTTATTTATTTTGATTTGTACTTCATCATATTGTTGTTGCTTATAAGCAATATCGTGATGAAGTGCAGC
GCCATCAACTTGATTTTCTTGATTGACGTGTGTTACTACGCGTTGAATGACTTTATCACTTTTGACTATTTCAA
GTATTTGCTTCATCACATAATCTTCAATCACATCAGCTCTTACACTGTTTGCCGAACATACTTTGGAACCCTTGT
TCCGAAAATTACTACATGAATAGTAACGAATACGTTTCTTAGTCCCGTCTTTAAGAGTATTCGTGGTATTGCTT
GCTGCCATAGGTGCGCCACATTGGGGACAGTGAATAATGCCTGTAAGCAGATTCGTTCCTTTGCCATGGACT
TGGGGTTTTTGACTGACTTGTTTCTTACGCATTTGTACTTTATCCCATAAATCTTGATTAATAATGGGGGAATG
CTTACCTTCAGCTATCACTGGTTTATCATTCAGCCCTTTACGACGTTTTTCACTCCAATCTTTGTATTTCGCAAA
TTGAATTTTGCCGATATAGAAAGGGTTAGCTAAGATGTATGTGATTGAACTAATACTGAAAGGTTTCCCCTTT
TTAGTGACATATCCTTTGTGATTCAATGCATTGGCAATTTTACGATAGCCATGTCCTTTGGCATAGCACTCGAA
TATATATTTTACAATATTAGCTTCATGTTGGTTAATCATTAGCTCGTGTTTACTATCTGGTATTTTGTCATAACCTA
GTGGTAAATTGCCTTGATAATAGCCTTCTTGGGCACGTCTCGTTTGACCCATAAATACGTTCTCGACAATGTTA
TTACGTTCGAATTCTGAGAAACTCGCAAGTATCTGTAACATGAGTTTACCAGAAGAAGTATTGACTTCCATAC
GCTCTGACAAACTGAAAAATTCGACATTTTGTTTGTGTAAATCTTCGACAATTTTGAGAAGATCAGATGTATT
ACGAGCTAATCGGTTTGTTTTGTATACCATAACACAGTCGATATTGCCTTCTTTTGCATCTTTCAACATACGTTG
GAGCTCAGGTCTGTTCATAGATTTACCTGAAATACCACGGTCAGCGTATATATCTTTAACTTCAAAATGATGGA
AGTCACAGTATTCTTTGATTTGATTGATTTGTCCGTCGATACTATAACCTTCTGTGCTTTGCATTTCTGTTGATA
CACGTACATAGATACCGACACGTTTTGTTTTAAGTTGTTGCATTATGTTACATCCTTTCTTCATTTATGCAATCG
ATGATTGCATGGTTTGATTGACAATATTGAGTGGTTCATTTTTGAAATAGATTCCTATAAGATTTTTATCTTTCG

TAATGTGAATGGTTTCAATATAGGGGTACAATATGTTTAACGTGAAACGTTTTTGAATAATATTTTGAATGATG
TGTTGTATTTGATGCCCATTGATAGATGTAGTGCGTTGCGGTTGTTGACGTAATGATTGTGTTTGCTCTCTGAA
CGTTTCTGCATCAATGATGCCTTGTGCCAACTTTTCTATCAGTTGTTCTTGAGTCAATGTGTGATGTTTTTCTAT
GTTTCTTTGTCTTTTGATGCGTTTGTCAATCGCACTTTTTAATTTTTGTGTAGATGCGTTGATTTTGATAAAAGT
CTCGGCACACTTCTAATACTTTATCTTCAAGTGTTTGTGCATTGATGCCTTTAAAATCACAGACAAAGCGTGA
AGCATTCATGTTTTTAGGACAGACGTAGTAACGTAATGTATGATTCTTTTTTCTAATGGTCATATTTGTAAGTGT
TGCATTACAACATGGGCATTTGATTTTTTGTTTGAGTTGATTATCCGAAGGTGTCTGTTTGGTTTGTTTTTGCA
ATCGAAGTCTCTGCGCTTGCTCATATATACTTGTGGAAACAATAGAAGGAAACATATTGTCGAATTGGCCATAT
TGATTGTTGACACGACCACAATAATTAGGATTGATGATAATGTTACGAACTTGATAGGGTTGTCGATTGATATA
CGTGTTATCTTCTTCTAATAACTGTGCAATTTTCTTATAACCATGACCTTTAATGTAATAATTGAATACAGCCTTT
ACCGTTGGTGACTCATTTTGATTGATGATGAATGTTCCGTTGTGATATTCGTAACCAAAAGGTGCATGTGTTG
TAATCAATCGACCTTGCTTTGCTTTTTCTTGAAGCCCATTTCTGACTTGTTCTCCAATGTTATCCGATTCAAGTT
CGGCTAAACTGATGAAGATATTAAGCTTGAGTCGGTCGAAAGCTTGATCCATATCAAAGTAACCATCATGTAC
GCTTAAGATATGAACATGGTACGTTTGACATAATTTGATGAGTTTTAATGCATTTTTCAGATTACGATGCAACC
TATTAAGACGATAACAGCATAATATGTCACACTGTCCTTGTTGAATTAATTGTGTTATTTGTCGATACCCACTAC
GATTATCTTTGCGACCTGATTGTTTGTCGCTATAAAAGTTGATATGTTGAATATGATGTTTTTCGGCTATTGCTT
CGATAGCTTGTTTCTGTGCTGCAAGAGATTGTTGTTTCATCGTACTTTGACGTAAATAGCCTATGACTTGTTTC
ATATCGGCTCCTCCTTTCACAGTGATAATATATATTTATGGATGAATTGATATATAAGCCCAACATCAATGAGATG
TTGGGCGTCCATATTAGTCATTTGTTTGATTGATTTCTTCAATTACCAAATCGGCTAATATCTCGATAAGTTCAT
CCATGTTTTTCACTCCGTTATTTGTTCTATCTTCAATACGTCGATTATTCAGTTTGATGCTTCACAGTTGTATGAT
AAAGACAATTAGAAATCTTCGTGAACTCCTGAAGGGCCTATCCCTTCATTAGCGGATTTAAAAAGTTCTTTCG
CAGCTTTGTTATCATTTGACGGTGTCCAATTTTGAAGTAACGACTTATCTTTAGTTAATCCGAGGATAGATGCA
AACTCTACATCTAATTTTAGATGGTAAAATACAAGTGATTGTTTTTTACCGCTATTATCTTTGACACTTCTTTTA
GTTGTTTGGCGTCCACGGTCAGCTAATATGAAACCTTTATCTCTTAAGGCGTTGACAACATTATTAACATCTTG
AAATTGATGATTGTTTAGCATCTGTTTAAAAACGTTCGCAATTATTTTTACTTCGATATGGTCATCTTTTAATGA
GATTAATCCATAGTTCTCAAACATATTTTTCAAAGCACCTTCATCTGAAAACTTACCTCTGTTTTGTGCCACAA
ATTGAATGATGACATCAATAGCTTTATCAGCTAATGAGCGTTCAGAGACTGTATGAGTATGATAATCAATAAAG
TAGTCTCTTATATTAGCGATATCAATATCTGTAGATAAAACACGACCTAATATTTTCGCAGATGTTGTAATGACT
GCATAACGCTTAAACATACGAATACCTGTATTGTTTGTTTCATCTTTCAATTTAGCTTCAAACCAATCTACTTCC
TTGTAAAACCATTGAATAACTTCATCTTCACGATTTATAAGATATTTAGCTACTAACGGTAAAACATGACCATAG
TTTAGTGCTACAGCTTTTTTAATATTGTCAGCATTGGTCGCATTTGTAGTGAATTGTTCATTAATCTCGATGGTT
CTTACACGTAATCCATCGTTTTGAGCTGAATCATTAAAAATACTGTATTCTGACGTTGAAATGACAGAAGTACC
CCAATTCTTAGGCGTTTTAACTTCTCCATGAACGTTTGAACGTTGACGACCTTGACCTTCAGCGATGGAATAT
AACAAACCCGTGGTATCTCTAAGTGTCGTAGATGAAAGCTCATCAAATACTATAGGAATGCCATAATTGTTACT
CAAATAACCTTCAAGTGCGTTTCGTGTGGCATTCCAACTTCTAAAAAGAGTTTCATTACCTTTGGTAGGGTTA
CCAGCGACTGATACAGCTAAAGCAGCTGCGGTTGACTTACCGGTTGAGGATTGACCTGTAAAACTAAAGAG
AATTCCTGCAAATTCGATTTCATGTTTATGCTTCAGAAAACTTGTCACTAAGGCAGAAATCCCAAATATGACTG
CCAATTCTAAAAGAAGATGACCTTTAACCTCGTCAATATACATGTTAAACCAATTATCAAATGTACCTTTAGGA
GTTAAGTCATAAGCACTATCTACAATAGGGTATGAAGGTGAGGACTGATTAAATTGAATAGATTTGAGAACTT
TATCTAGCGATATCAAGTAGCCAAAAGGTGTCTCTAGTATACCTACACCCTCATATAATTCAGAAAGTGGTAAT
CTATCTCGCATCAATTGCAAGGCATAACTCAATGATCTAATATACTTCTCGTTAATACTGTAGCCATATTTAATTA
AAGAAGGTAAGTTTCGTGTTGTTAAGATATCAGACTCAAAAATGTCTTTTTTACCATTGTTGTTAGTAATAATC
AACTTTTCAACACCAGTAATTGGATCAGAAAATCTTGCATTAACAATAATAGGGCTAGCCATTCTAACCACTTT
TTCACTGTCACCATCTTTTTTAGGTGGTATCGTTTCTTTCCAACCGAAGGAATCTAAAGCAAATGGGCCAATT

TCAAACAATGTGTAACTCATTAGCAATCACCTCCTTTCGAAGGCGTGCTATTATTGCATGGGTTTATCCCTATTT
TCTGATATAATAAACTGCCATTTTTTGTTCCTACAATTTTAAATGGTAATCCTGGCGTGTACTTAATCCAGTAAG
CAAAACGACGTCCTACTTCACGTTGATCATTCGTGCTACAAACCACATTATATTTCTTTCTTAATTCACCATAAG
TGAACGTACTTCCAACTGGTTGTTGAAAAGATTTAGCAATTATAAAATTATAAAGCTTTTCTTGCTGCTTAGAT
TTCTTATTCATTTTAAAATTCCTCCAATAATTTTTAAGGAGTCAATCAATATCATATAGGTTAAACTATCTTCAAT
TGATTGCTATAATAAGCATAAATGAAGCATTATGATTAGACGCGGAACAAAAAATGGGGAGGGGGGGTATAT
ATTGATACCTACGCTAGAAGAAATTATTGATAAGTATGGAAACTTAGTTGATTATTTGAAATTTGATGTAACAG
TTGAGGTTTATGAAGATTTGTTGTTATTAAGAAGTTTAAGCGATTTAAATGAACATCAAAAGATGGATAGAGT
TTCATTTATAAAAAAACATTTAAACTCAAATTTCGATTATTTATTAGATGATACTGAGTTTAGAAGTATCAATAA
GATTTACAGTAATTTAAATATCATGACGCATATCAATAGTCAAAATAATAATTTTAAACGTAATCCATTTATAAAT
GAAGAACAATTGAAATCACTACTCGCGATAAAGAAAGTAGATGCACATATGAGTTATGATTCAAATATTACTT
CAAAAGCATTGGCAGAAATAAACAAAAACGCAAAAAAGATTAGTTACAAAGATAGACACACTCTATAATCAG
TCAAAAAAAGAAAATGAATATGTACGGTTAGGAGAAAAAATATCGTATACAAAATTGGAGAATCATTGGAAA
TATTTATATAATGAGATTCAGTTTTATAATCTTAATAATCAATTGATTAGTTATGTTGCATTAGAACAGGAATATG
CTTGGGCTTTTTTAAATGAACTATTTTATTTGATAGAATTGTATTTGAAGGCATTTAAAGGACAAAAGAAAAC
AGATTATGAGTTTGGGAAAAAATTAAACGAATTTATTCAATCATATGTCATTATTTTACTTATAGATATACGATTA
CCTGTTGTTAGATTATATATTATTAGAGACATTGTAGATACATGTGAAGGAGAAAAAGATAATTACAAAAGAAT
TACACTGATAGAAGAATCTATTAAAAAGTATAGATACGTAAAAGATCAGATTAAAAGATTTAAAAATGGATTA
GAAGAATCGTTATTAAATGCAACTTTATCTATTGAACAAAATATGTTGAAAGTTAAAATTGATTATTATAAAGC
CTACTGCTTTCCTAGAGAAAAAACAAAGCATAACAATATTGAGTACAATGTATCGTTGTTTTTTAAAGCATTAG
ATGCCTTAAAGAAATAGTTTTTCACTGAAAATATTTATAGATATAATTAAAATTAATTAATATTAAGCCAGGATA
ATGTAGTCTTAATCGTTCTGAAATACGAAAAATGTTGTGGAAATCGCGTGAAATAATCAGAGGAATCGTTTGA
AATCATCGTAGTAATCGCGCGAAAATCACGTGAAATAATCAGAGGAATCGTTTGAAATCATCGTAGTAATCGC
GCGAAAATCACGTGAAATAATCAGAGGAATCGTTTGAAATCATCGTAGTAATCGCGCGAAAATCGCGTGAAA
TAATCAGAGGAATCGTTTGAAATCATCGTAGTAATCGCGCGAAAATCGCGTGAAATAATCAGAGGAATCGTT
TGAAATCATCGTAGTAATCGCGCGAAAATCGCGTGAAATACTATGGTAGACGGTTTGAGTAAATTAATGGAGT
ATTTTAATATTTATGGTTTGGAGGAATTGCGTTGCATAGAGAAAGTAAATATATAGAGTATAAGAAATCACGAA
AAGGATTATCTAATGATATTTGGTCTACGTATAGTGCTTTTGCAAATACTGAAGGTGGTACTATATATTTAGGAA
TTGAAGAAAAAAAGATCGAGGACAAAAAAGTCTTTGTTTCAGTTGGTGTTGAAGATCCAGAGAAAATGATT
GAAGATTTTTGGAATGCACTATATGGAAGAAGTAAAGTTAGTCAAAATATTTTATCAAATAAAGATGTTAAAA
TTGTTAATATTGAAAATAAAGCGTGCATTGAAATTCATGTACCAGAAGCGCCTTATTCGAAGAAACCGATATAT
GTAGATAATAAAAAAGATTTAGTATATAAAAGAGTTGATGATGCTGATAGAATTGCGACTGAAGAAGAGTATA
AATTCATGATTGTAAATTCTCAAGACGATATAGATACAGAATTATTAGATAACTATGACATGTCTGATTTAAATC
ACGAATCTATCGAAAATTATAGGAAACTTCTATTAAAAAATACTAATGATGAGAGATATGCGAATATGAGCAAC
TGGATTTAATGATAGATTTAGGAGCATATAGAAAAGATAGAAGTTCGAAAGACAAACAGTATAAAATGACTA
CAGCATGTTTATTATTCTTTGGTAAGTATAATGCGATTAGTGATAGATTCCCAGGATTTCAATTAGATTATTTTA
AGAAAACAAATTACCTAGATACTGATTGGAAAGATAGAATATCAAGTGGAGATTTAGGTAATGAAGATTTAA
ACGTGTATAGTTTTTTTGAAAAAGTATTGATAAAATTAACTGATAACATTGAGGAATCATTTAGCCTAAATGAT
GGTTTGACTAGACAAAATTATGCAAGAGATTTAAAAGTAGCAATTCGCGAAGCACTGGTTAATACATTAATGC
ATGCGTATTATGATACTAAGCAAAGTATTAAAATAGTTAATTGTGAAGATTTTATAGAGTTTTATAATCCGGGTA
ATATGAGAATAAATAAAGAAGATTTTATTCATGGAGGGCATTCAAAGGACAGAAATAGTATATTATCGACGCT
TTTCAGAAGAGTAGGATATTCAGAAAAAGCTGGATCTGGAGGACCAAGGATATTCGATGTAGTTAATAGACA
TAAGCTTAAAACGCCTGAAATAGAATTAACGGACATGGACACTAATGTAGTACTTTGGAAACAAGATTTAATG
AAGGAGTTTGAAAAAATATCCTGAGTTAGACAAAAAAGTAATAAAGTATATTATTGACTATGGATCAATAAGTA

AGGGTGAAGCCTTAAAAATGGAAAATATGACAGAATATCAGTTTAGAAATATTTTAAAAAAACTAAAAGATG
ATAACTTGATAAAAAAAGAAGGTGAAGGTCCGGCTACTAAATATGTGTTAATAGAATCAAAAGAAGCTGATA
TATTGCGAACTAAAAAAGTAATTAAAAGTTTAGAGTCTTTCTTTAGGAATAAATAAAAAAACAAGAGATAGG
TGCGAAGTGTTTGGATTATACAAATGCTTCGCATCTTATATTTATTAATAAATATCATAGAAAACCGTATCATTAA
CCGATACGCAGAGATGCGGTTTTTTAGACACTTCATAAAGGGATTTTGAACGTATCAGAACATATGAGGTTTA
TAGGAATTGCTGTTATGTTTTTTGATCACATCAATAAACAAAAAAGGTATGTACTATGTAAAATATTTATTAAAT
GATATAAAGCGAGGGTATATAAATGATTTTTAAATAGATATTTATCCAATAATATAAAAAGGAACTATAAGCTAT
ATCTAAAAGCTATATCTAACTACTTATAGTCCTTCTTCATTAGTATAAATATAATTATTAAGAAAGGTATATATCTT
TGTAACCTTCGTTTACATTAATAATGTTGTGATAACCTTTGTGTTCTAAAATACCAATAGCTATAGAACTTCTAAT
GCCAGACTGACAGTCTACATAAATAACATCGTTTCTATTGAAAGGTAAATCTGTTTCTAAAAGTTTGCCGTGT
GGCACATGAACCGCTTGGGATAAGTGGCCATTATTCCATTCATTATCATTACGTACATCTAAGACATGTGCTTC
ATTACCAGTTATGTCTTTACTATGAACAGATTGTGTTTGAATTTGAGCTTGTGGTAACTGATATCCAGACACAT
TATCATATCCAATAAGTTGTAAAGTATGTTATGTGTTGCTTTTGAAACAAGGTGATAGTCTCCAATCAAGTTAAT
TTCTTGATTATAGTTTAGATACCAGCCAATTTGATTGATGAAATTTTTATCATATGGAATATTGATTGTACCTTCA
ATATGTCCACCATGATAAGCCTCCTTACTGCGGAGATCAAAAGTTAATCTGTTTGTACTTGTAGCTGGATAAAC
CGTATAAGGTTGATATAAATTCATACCGAATTGATTAATTTTTTTCATTTGTGCAAAATGATGTGGTGGTGCAG
GTTGGTCAGAAATGAGTTTATCGATAAAGGTAGCTTCATTATTTTCAGAAAAAGCCCAGTTCGTTTGTTTTTC
ATAGCCAAGCGTAGATGTTGGAATAGCACCTAAAGATTTACCACAAGGACTACCAGCGCCATGACCAGGCCA
AATTTGAATGTAGTCTGGCAAGTCTTTAATACTTTCAATAGATTTAAACATTTGTTTTGCGCCTATTTCAGATAA
TCCTTCTACTTTAACAGCTTTTTCTAGTAAATCAGGTCTACCGATATCTCCTACAAAAATAAAATCACCACTGAA
TAGTCCCATTGGAACTTGTGCTCCAGCACCTTCGTCAGTAAGTAAAAAACTTATACTTTCTGGCGTGTGACCA
GGTGTATGAAGCACTTTTAATTTTATATTTCCTACATAAATATCATCATTATGTTGAACAAAATGAGTGTGGTTA
GGCATATTTTTATAACCTAACATGTCATCACTTTCGCCCGATACATAAATACTAGCATTTAACTTTATAGCAACAT
CTCTAATTCCTGAAACAAAATTTGCATGTATATGTGTTTCAGCTGCATGAGTAATGGTTAAACTCTCTTCATCG
GCAACTCGAATATATGAAGATAAGTCACAAATAGGATCAATGATCATGGCTTCTCCAGTTTTTTGACAACCGA
TTAAATAAGATGCTTGAGATAAATGTTTATCATAAATTGATGATAGTCAATTTGTTATCAAGGGTGATAATTATA
TTAACTATGGTAATTACATGGAATTAAAGTTATTAAACAAACTTGATGATGACCAATTAGATTTAGAATTTTTAC
AAGATAATGGGTTAAAAGGTAATGTTCAAGGTCCTATGACTTTAAAGGAACCTTTAAAAAGTTATATACAAAA
GTCTTGAAAACGAATATTTTAAATTTAGATAATGAGTTAGAAAACACTGAATATGTCGAAGGTAAACCATATG
TACAGTACGGTATTAAATATGAAAGAAATCAGGCATTAAGAAATGAAGCTATTAAAATTCATGGAACTACATG
TAAAGTATGTGGATTTGATTTTAAAGCTAAGTATGGCGATTTAGGTGAGGGTTTTATTGAAATTCATCATTTAA
AACCAATGTTTTCAATAAAAAGAGAAATAAAAGTAAATCCACAAAAAGATTTAGTCCCACTATGTTCTAATTG
CCATAAAATGATTCATAGAAATACTAAAAAACCTTTAACGATTAAAGAATTAACCAAAATAGTTAATTATAATA
GCAAATAATTTAATATTTTATAAACTATCATTCAACCCTCTTAATTTATTAGGAGGTTTTTTGTATTTATGCTTTC
AAATGTGTGATATACTTTGTTTGTTGAAATATAGAGTATCTATAGATAGGGTGATTGAGTATGAAATTCACTGA
AGTGGAAGTTATCGAACATCTTGTAAAGGCATATAAAGAAGCAGGAAAGCCTACTTATCCTCATGAAAATTTA
TATCGAGGACGTAATCATAGTATTTCAGGTATTGGAGAAGACTTGCTGGGTGCTTATTTGATTAGTAGATTGG
AAGGTGTCCAAATATTTATTGATCAGCCTTTATCTATGATTGATAAATCTTTAAGTACAAGATATCCGGATTTATT
AATTTGTGAAGATAATGAAATTAAAAATATACTAGAAGTTAAAATGGACTTAGGATATCAAAGAAAAGATTTT
ATAGATTATTGCCGAAAGAAAGAAGAATGGATTTCAAATATCGTAGGAAAACAGTGTGTATTGTCTAGAAAG
AGAGAAGACAAAATTCCTATGAATATAGCTGATGATATTAAATTTCATGTTGTGATTTACAGTGAAAACAATG
GACCGAAGCGGTTTGATGAAGAAATCATGCCTATCGTTAATGAAACATGTCCACATATTGAAGTATATGTCCTA
ACAAGCGGTCAACACCCTAATTTAGTAAATGTTAATCTTGAAGGTATTAATATTAATAAAGATGAATTTGAAAT
ATTAGTAAATGCGTTATAAAAAATAGAGCATCCTCCACGTTATGGAGGTGCTCTGTTTTTTATTGAAAAGTATC

AAGTTAATTAATTTAATATGCTTAATAAGTTCTACCTTGACCTTTTTCTCTAGCTTCTGTTCGATCTCTTATGTAC
TCAGTACATACTGGATTTTCTGTTAATTCATCAACTGTATTAGTTTAATCAGAAGACGTGTCTACTTTGTAAGCT
TCTAAGAACTTATTATATGATATAGCGTTTGAGTTTTGTTGTTCTTCTATAAATTTCTGTAGTTATTTTTCAAAAA
CCGCATCATTAACTGATAAGCAGAAGCTTATCATAAAT
>Staphylococcus aureus strain 59731
ATGAAAATCACCATTTTAGCTGTAGGGAAACTAAAAGAGAAATATTGGAAGCAAGCCATAGCAGAATATGAA
AAACGTTTAGGCCCATACACCAAGATAGACATCATAGAAGTTACAGACGAAAAAGCACCAGAAAATATGAG
CGACAAAGAAATCGAGCAAGTAAAAGAAAAAGAAGGCCAACGAATACTAGCCAAAATCAAACCACAATCC
ACAGTCATTACATTAGAAATACAAGGAAAGATGCTATCTTCCGAAGGATTGGCCCAAGAATTGAACCAACGC
ATGACCCAAGGGCAAAGCGACTTTGTATTCGTCATTGGCGGATCAAACGGCCTGCACAAGGACGTCTTACA
ACGTAGTAACTACGCACTATCATTCAGCAAAATGACATTTCCACATCAAATGATGCGGGTTGTGTTAATTGAA
CAAGTGTACAGAGCATTTAAGATTATGCGTGGAGAAGCATATCATAAATGATGCGGTTTTTTCAGCCGCTTCA
TAAAGGGATTTTGAATGTATCAGAACATATGAGGTTTATGTGAATTGCTGTTATGTTTTTAAGAAGCTTATCAT
AAGTAATGAGGTTCATGATTTTTGACATAGTTAGCCCCCGCAGTCTTTCATTTCAAGTAAATAATAGCGAAATA
TTCTTTATACTGAATACTTATAGTGAAGCAAAGTTCTAGCTTTGAGAAAATTCTTTCTGCAACTAAATATAGTA
AATTACGGTAAAATATAAATAAGTACATATTGAAGAAAATGAGACATAATATATTTTATAATAGGAGGGAATTT
CAAATGATAGACAACTTTATGCAGGTCCTTAAATTAATTAAAGAGAAACGTACCAATAATGTAGTTAAAAAAT
CTGATTGGGATAAAGGTGATCTATATAAAACTTTAGTCCATGATAAGTTACCCAAGCAGTTAAAAGTGCATATA
AAAGAAGATAAATATTCAGTTGTAGGGAAGGTTGCTACTGGGAACTATAGTAAAGTTCCTTGGATTTCAATAT
ATGATGAGAATATAACAAAAGAAACAAAGGATGGATATTATTTGGTATATCTTTTTCATCCGGAAGGAGAAGG
CATATACTTATCTTTGAATCAAGGATGGTCAAAGATAAGTGATATGTTTCCGCGGGATAAAAATGCTGCAAAA
CAAAGAGCATTAACTTTATCTTCCGAACTCAATAAATATATTACATCAAATGAATTTAATACTGGAAGATTTTAT
TACGCAGAAAATAAAGATTCATCTTATGATTTAAAAAATGATTATCCATCAGGATATTCTCATGGATCAATAAGA
TTCAAATATTATGATTTGAATGAAGGATTCACAGAAGAAGATATGCTAGAGGATTTAAAGAAATTTTTAGAAC
TATTTAATGAATTAGCTTCAAAAGTTACAAAAACATCCTATGATAGCTTGGTCAATAGCATAGACGAAATACAG
GAAGACAGCGAAATTGAAGAAATTAGAACAGCACAAAAAGATAAGACACTCAAGGAAGTGGAAGCACCTA
AAGGAATAATTCCAAAATATAAAAAAGGTGTATCAAAGACTACTAAAAATGATTCAGAAATTGAAAAATCAA
ATAAAGAGAATAAATTAACCGGTAAAGTTGGAGAAAAATTAGCGCTAAATTACTTTAATGAGCTAATTGATAA
TAAAATAGACGAAGATAAGAAAGAACAGTTTAGGAATATTTTAAATGATAATCCAGGCTCTCAACACGGTCAT
GGCTATGATTTAGTAGCTTTTGATCCAACAAATACAGATAAAGCTGTAGAAAAATTTATTGAAATTAAAACATC
TACATCTTCTAGTATTGAGGAACCATTTTTTATGTCGCTAAATGAAATGTTTGCTATGAAAGAATATAAGCAGA
AATATTTAATATTAAGAATATTTAATGTTTCCGGTAAAGAACCACAATTTTATTTTATAGATCCATATGCAAATTA
TTCTGAATTTAAAGATGTAGATGATCTCATTGACAAAGTATTTAATGTAGAAGCTATTCAGTATAAAGTTTTTG
GCGAAAAAATGATTACTTGAACAAGAGCTAAAATAAAATTGTGATCTAATAAAAATAGAAACTGTAATTTAAAT
AAAACTTTCTAAATAAGCTAACTGATAAAAAATCAGTTTGTCCACAGTCTGAAACAAGATTCCTATATTCTTTA
GGAATCTTGTTTTTTCTATTTTTATGGTGATAAAGAGCAGATAAGATAATGTGTAATAATCACAAAAAAGTTAA
ATATTTTAAGGCTTGTTTAATTATTAATGATTTTATATATAAAGAGCAGTATAATAAAGTTGTTAATATATTATGAA
TAATATTCAAGTAATTTTATTGTTTTTTAATTTGTCGATATTTAAGTTGAGTTAAATTTAAAGGGTGTAATTTGTT
TTACAATGATGAAGATAATTAGTCTATCAAAATAAAGGGGTTGGGACTGTTATGAGTGATAATTTGTCATTATT
CATTGACTATATCAATGATAATATAATCTATGGTAGTGAAATCAAACGGGAGAAATTAGAGAATTTATTTAATCA
ATTTGCTATAAAAAAATGTTGAAAAGAACATTGTCTATGATGAACTGAAATCTTTAGATATTACAATCATTGAGT
CACAGGATTCATATAAAAATAAATTGAAGAGATTATTTTCGGTTCTGTTGCAAAGTAAAAAAATATAGCTAACC
ACTAATTTATCATGTCAGTGTTCGCTTAACTTGCTAGCATGATGCTAATTTCGTGGCATGGCGAAAATCCGTAG
ATCTGAAGAGACCTGCGGTTCTTTTTATATAGAGCGTAAATACATTCAATACCTTTTAAAGTATTCTTTGCTGTA

TTGATACTTTGATACCTTGTCTTTCTTACTTTAATATGACGGTGATCTTGCTCAATGAGGTTATTCAGATATTTC
GATGTACAATGACAGTCAGGTTTAAGTTTAAAAGCTTTAATTACTTTAGCCATTGCTACCTTCGTTGAAGGTG
CCTGATCTGTAATTACCTTTTGAGGTTTACCAAATTGTTTAATGAGACGTTTGATAAACGCATATGCTGAATGA
TTATCTCGTTGCTTACGCAACCAAATATCTAATGTATGTCCCTCTGCATCAATGGCACGATATAAATAGCTCCAT
TTTCCTTTTATTTTGATGTACGTCTCATCAATACGCCATTTGTAATAAGCTTTTTTATGCTTTTTCTTCCAAATTT
GATACAAAATTGGGGCATATTCTTGAACCCAACGGTAGACCGTTGAATGATGAACGTTTACACCACGTTCCCT
TAATATTTCAGATATATCACGATAACTCAATGTATATCTTAGATAGTAGCCAACGGCTACAGTGATAACATCCTT
GTTAAATTGTTTATATCTGAAATAGTTCATACAGAAGACTCCTTTTTGTTAAAATTATACTATAAATTCAACTTTG
CAACAGAACCGTATTATGGAATAGAGATGTTGGTAACATTTATACAGGATCATTATACTTAAGTTTAATTTCGTT
ATTACAGAACCACACATTCCAACCAGAAGAGAAAGTATGTCTATTTAGTTATGGTTCAGGAGCAGTAGGAGA
AATCTTTAGTGGTTCAATCGTTAAAGGATATGACAAAGCATTAGATAAAGAGAAACACTTAAATATGCTAGAA
TCTAGAGAGCAATTATCAGTCGAAGAATACGAAACATTCTTTAACAGATTTGATAATCAAGAATTTGATTTCG
AACGTGAATTGACACAAGATCCATATTCAAAAGTATACTTATACAGTATAGAAGACCATATCAGAACATATAAG
ATAGAGAAATAAACTAGTGGCCGATTGTGCTTGATGAGCTTGGGACATAAATCCTAACTCGAAATAAATAAGC
ATATCACTAAACTGATTTTTTAAAGTTTACAGTGATATGCTTATTTTTTTATCTTACGATTTTGTACGTGCATGCT
TGCCTAGGGGTATGGCTCGAGCCATTAGTCTCTCGCACATACTATTCCCTCAGGCGTCAGCACTTACAAAATC
GGTTGTAATTTTCATTTTTATACGCATTCTTACTGAGATTATACTAATAAGAGGAATAGTAAAAGCAATTCTAAG
TAAAATTGCAGATAAGAGGTTTGTTAAAAGCAGTTCTAAGTAAAATTGCAGATAAGAGGTTTGTTAAAAGCA
GTTCTCAGTAAAATTACAGATAAGAGGTACGTTAAAAGCAGTTCTAAGTAAAATTGCAGATAAGAGGTTTGT
TAAAAGCAGTTCTAAGTAAAATTGCAGATAAGAGGTACGTTAAAAGCAATTCCATGCAAAATTGCTGATAAG
GGGTAAGTTAAAAGCAGTTCTCAGTAAAATTGCAGATAAGAGGTACGTTAAAAGCAGTTCTAGGCAAAATT
GCAGATAAGAGGTGCGTTAAAAGCAGTTCTCAGTAAAATTGCTGATAAGGGGTAAGTTAAAAGCAATCCTA
AGTAAAATTGCAGATAAGGGGTACAGAAAAACTAGACTTGATTACAAAATGGAGCTTGGGACATAAATGATT
TTTTAAAAATGAGATGAGACGTAGATTAACTCCATAATCAATACGAATCTATCGACTTCTTTATTTATGATATTC
ATCTCTTTTTAATGGAAATAAAAGTGCGATTAATGTGATAATACAGTTACGTTAATTAAAAAAATAAAAATGCA
AGGAGAGGTAATATGCTAACTGTATATGGACATAGAGGATTACCTAGTAAAGCTCCGGAAAATACAATTGCAT
CATTTAAAGCTGCTTCAGAAGTAGAAGGTATAAACTGGTTGGAGTTAGATGTTGCAATTACAAAAGATGAAC
AACTGATTATCATTCATGATGATTATTTAGAACGGACTACAAATATGTCCGGGGAAATAACTGAATTGAATTAT
GATGAAATTAAAGATGCTTCTGCAGGATCTTGGTTTGGTGAAAAATTCAAAGATGAACATTTGCCAACTTTC
GATGATGTAGTAAAAATAGCAAATGAATATAATATGAATTTAAATGTAGAATTAAAAGGTATTACTGGACCGAA
TGGACTAGCACTTTCTAAAAGTATGGTTAAGCAAGTGGAAGAACAATTAACAAACTTAAATCAGAATCAAGA
AGTGCTCATTTCAAGCTTTAATGTTGTGCTTGTTAAACTTGCAGAAGAAATCATGCCACAATATAACAGAGCA
GTTATATTCCATACAACTTCGTTTCGTGAAGACTGGAGAACACTTTTAGATTACTGTAATGCTAAAATAGTAAA
CACTGAAGATGCCAAACTTACTAAAGCAAAAGTAAAAATGGTAAAAGAAGCGGGTTATGAATTGAACGTAT
GGACTGTAAACAAACCAGCACGTGCAAACCAACTTGCTAATTGGGGAGTTGATGGTATCTTTACAGACAATG
CAGATAAAATGGTGCATTTGTCTCAATAGAAAGTTAGAGGTGAGTCTTACGTTTCAGTGACGGTAGACTTAC
CTTTAACATGTTACATACTAAAAAATTAATTTGAATAAGAAAGAGAGACATATATGAAATACGATGATTTTATA
GTAGGAGAAACATTCAAAACAAAAAGCCTTCATATTACAGAAGAAGAAATTATCCAATTTGCAACAACTTTT
GATCCTCAATATATGCATATAGATAAAGAAAAAGCAGAACAAAGTAGATTTAAAGGTATCATTGCATCTGGCA
TGCATACACTTTCAATATCATTTAAATTATGGGTAGAAGAAGGTAAATACGGAGAAGAAGTTGTAGCAGGAA
CACAAATGAATAACGTTAAATTTATTAAACCTGTATACCCAGGTAATACATTGTACGTTATCGCTGAAATTACAA
ATAAGAAATCCATAAAAAAAGAAAATGGACTCGTTACAGTGTCACTTTCAACATACAATGAAAATGAAGAAA
TTGTATTTAAGGGAGAAGTAACAGCACTTATTAATAATTCATAATAAAACAGTGAAGCAACCATCGTTACGGA
TTGCTTCACTGTTTTGTTATTCATCTATATCGTATTTTTTATTACCGTTCTCATATAGCTCATCATACACTTTACCT

GAGATTTTGGCATTGTAGCTAGCCATTCCTTTATCTTGTACATCTTTAACATTAATAGCCATCATCATGTTTGGA
TTATCTTTATCATATGATATAAACCACCCAATTTGTCTGCCAGTTTCTCCTTGTTTCATTTTGAGTTCTGCAGTAC
CGGATTTGCCAATTAAGTTTGCATAAGATCTATAAATATCTTCTTTATGTGTTTTATTTACGACTTGTTGCATACC
ATCAGTTAATAGATTGATATTTTCTTTGGAAATAATATTTTTCTTCCAAACTTTGTTTTTCGTGTCTTTTAATAAG
TGAGGTGCGTTAATATTGCCATTATTTTCTAATGCGCTATAGATTGAAAGGATCTGTACTGGGTTAATCAGTATT
TCACCTTGTCCGTAACCTGAATCAGCTAATAATATTTCATTATCTAAATTTTTGTTTGAAATTTGAGCATTATAAA
ATGGATAATCACTTGGTATATCTTCACCAACACCTAGTTTTTTCATGCCTTTTTCAAATTTCTTACTGCCTAATTC
GAGTGCTACTCTAGCAAAGAAAATGTTATCTGATGATTCTATTGCTTGTTTTAAGTCGATATTACCATTTACCAC
TTCATATCTTGTAACGTTGTAACCACCCCAAGATTTATCTTTTTGCCAACCTTTACCATCGATTTTATAACTTGTT
TTATCGTCTAATGTTTTGTTATTTAACCCAATCATTGCTGTTAATATTTTTTGAGTTGAACCTGGTGAAGTTGTA
ATCTGGAACTTGTTGAGCAGAGGTTCTTTTTTATCTTCGGTTAATTTATTATATTCTTCGTTACTCATGCCATAC
ATAAATGGATAGACGTCATATGAAGGTGTGCTTACAAGTGCTAATAATTCACCTGTTTGAGGGTGGATAGCAG
TACCTGAGCCATAATCATTTTTCATGTTGTTATAAATACTCTTTTGAACTTTAGCATCAATAGTTAGTTGAATATC
TTTGCCATCTTTTTTCTTTTTCTCTATTAATGTATGTGCGATTGTATTGCTATTATCGTCAACGATTGTGACACGA
TAGCCATCTTCATGTTGGAGCTTTTTATCGTAAAGTTTTTCGAGTCCCTTTTTACCAATAACTGCATCATCTTTA
TAGCCTTTATATTCTTTTTGTTTTAATTCTTCAGAGTTAATGGGACCAACATAACCTAATAGATGTGAAGTCGCT
TTTCCTAGAGGATAGTTACGACTTTCTGTTTCATTAGTTGTAAGATGAAATTTTTTTGCGAAATCACTTAAATAT
TCATCCATTTTTTTAACGGTTTTAAGTGGAACGAAGGTATCATCTTGTACCCAATTTTGATCCATTTGTTGTTTG
ATATAGTCTTCAGAAATACTTAGTTCTTTAGCGATTGCTTTATAATCTTTTTTAGATACATTCTTTGGAACGATG
CCTATCTCATATGCTGTTCCTGTATTGGCCAATTCCACATTGTTTCGGTCTAAAATTTTACCACGTTCTGATTTTA
AATTTTCAATATGTATGCTTTGGTCTTTCTGCATTCCTGGAATAATGACGCTATGATCCCAATCTAACTTCCACA
TACCATCTTCTTTAACAAAATTAAATTGAACGTTGCGATCAATGTTACCGTAGTTTGTTTTAATTTTATATTGAG
CATCTACTCGTTTTTTATTTTTAGATACTTTTTTTATTTTACGATCCTGAATGTTTATATCTTTAACGCCTAAACTA
TTATATATTTTTATCGGACGTTCAGTCATTTCTACTTCACCATTATCGCTTTTAGAAATATAACTGCTATCTTTATA
AACTTGTTTGAAATTTTTATCTTCAATTGCATCAATAGTATTATTAATTTCTTTATCTTTTGAAGCATAAAAATAT
ATACCAAACCCGACAACTACAACTATTAAAATAAGTGGAACAATTTTTATCTTTTTCATCAATATACTCCTTATAT
AAGACTACATTTGTAGTATATTACAAATGTAGTATTTATGTCAAAATAATGTTATAATTTTTGTGATATGGAGGT
GTAGAAGGTGTTATCATCTTTTTTAATGTTAAGTATAATCAGTTCATTGCTCACGATATGTGTAATTTTTTTAGT
GAGAATGCTCTATATAAAATATACTCAAAATATTATGTCACATAAGATTTGGTTATTAGTGCTCGTCTCCACGTT
AATTCCATTAATACCATTTTACAAAATATCGAATTTTACATTTTCAAAAGATATGATGAATCGAAATGTATCTGA
CACGACTTCTTCGGTTAGTCATATGTTAGATGGTCAACAATCATCTGTTACGAAAGACTTAGCAATTAATGTTA
ATCAGTTTGAGACCTCAAATATAACGTATATGATTCTTTTGATATGGGTATTTGGTAGTTTGTTGTGCTTATTTT
ATATGATTAAGGCATTCCGACAAATTGATGTTATTAAAAGTTCGTCATTGGAATCGTCATATCTTAATGAACGA
CTTAAAGTATGTCAAAGTAAGATGCAGTTCTACAAAAAGCATATAACAATTAGTTATAGTTCAAACATTGATAA
TCCGATGGTATTTGGTTTAGTGAAATCCCAAATTGTACTACCAACTGTCGTAGTCGAAACCATGAATGACAAA
GAAATTGAATATATTATTCTACATGAACTATCACATGTGAAAAGTCATGACTTAATATTCAACCAGCTTTATGTT
GTTTTTAAAATGATATTCTGGTTTAATCCTGCACTATATATAAGTAAAACAATGATGGACAATGACTGTGAAAA
AGTATGTGATAGAAACGTTTTAAAAATTTTGAATCGCCATGAACATATACGTTATGGTGAATCGATATTAAAAT
GCTCTATTTTAAAATCTCAGCACATAAATAATGTGGCAGCACAATATTTACTAGGTTTTAATTCAAATATTAAAG
AACGTGTTAAGTATATTGCACTTTATGATTCAATGCCTAAACCTAATCGAAACAAGCGTATTGTTGCGTATATTG
TATGTAGTATATCGAGCTTCACATGAAACAGCTAAAGAAGCTTTGGGCGATAAAGAGTTAAGAGCCATTGCA
CATGAGTTAACTAAAACAGTTAAGGATAACATGAGTGTTGATTGGTCTAAACGAGACAGTGCTAAAGCTAAA
ATGAGAGTTCAAGTTAGACGCCTATTAAAGAAATATGGCTATCCACCAGATCTTCAAAAAATGGCTGTGGAA
CAAGTTGTAGAGCAAGCAGAATTAATGGCAAGTCAGCAATAAAAAAATAAATCATAATGAGTCCGGGACATA

AAGTTCTTGGATAAGTGAAAAAAGACAATTTCTATTGAAATAATATAGAAATTGTCTTTTTATAAATTTTTTG
ATTATTTTCAGCTCGTTGAGCTACTACTTTTCTTATATTAAGTGCCATTAATACAAAACCAAGTTCTCTTTTGAC
TTTATTGAGTCCTCGGACAGACATCCGAGTGAAACCCAAAATAGCCTTCATAAATCCAAAAACAGGTTCCAC
ATCAATTTTTCTTTGACTGTAGATATTTTTTGTTTCTGGTTCTGAAAGCTTTTTGTTAATTTGGGATTTAAAATA
TTCCCAGTTATAATTCTTCATTATTTTTTTTGTTTGTTTTTGAATTGAAGTTCATACATTGATTTTTCAGAGGAC
ATTCTGAACAATCATCACATTCATATAATTTGAAGTCTCGCTTATAACCATACTTATCATGACGATAGGCATATCT
TTTAAAACCTAGCCGTTTATTATTCGGACAAATGAATTCGTCATTAATTTCGTCATAGTTCCAATTTTGAGTATT
AAAGATGTCACTTTTATATTTTTTAGTTTTATCTTTTATAAACATTCCATATGTTATGAGTGGCGTTCGATTAAAG
TCATCTATAATTGCCTTATAATTTGATTCACTACCATAACCTGCATCAGCTACAATATATTCAGGTAAATGACCGT
AGGTCTCTTGAATTGAATTTAAAAATGGAATCATCGTTCTAGTATCCGTTGGATTTTGATACACATTATAAGATA
AAACAAATTGGGAATTTGTTGCTATTTGTAAATTATACCCTGGCTTAAGTTGTCCATTTTTCATGTGATCTTCTT
TCATTCTCATAAATGTCGCATCATAATCTGTCTTAGAATAACTATTTCTATCCTTTAAAATAGATTTTTGAAATTC
GTATCGATACTTTCGCTCAAAATAATCATTGATTTGCTTTTTGTATTTTTTGATTTTAGTTCTTTTGAGACGTATT
TGTTTTCTTGTTTTAGTACATTTTTCATTGTTGATATGTTGGTTTAAATCTTCGATTTCTTTATCTAAGTGACTAC
CAATCAAATCTATTTCTTCTTTTGTTAATTCATTATCATGATCTTCTTTAATTTCCGGTATGATTTTATTGGTTACC
AATTCATGGTAGAGGGCTTTAGAATCCTCATTCATCTTTGATTCATGGTTTTGAATACTCTTTTTCCATACAAAT
GTATATCGATTGGCATTTGCTTCAATTTTTGTACCATCAATAAAAATAGCTTTATCATCTATAAGATTTTGTTTTA
CACACTGACTGTAAAATTGAATAAATAAAGATTCTAATAAAGCATCTACTTTTGGATTTACTCTAAATTGATTAA
TTGTTTTATAAGAAGGTTTTTGATTTTGTGATAGCCACATCATTCGGATGCTATCATTAAGCATTTTTTCTATTT
TACGACCTGAGAATACAGATTGTGTGTAGGCATATAGAATCACTTTTAACATCATTTTAGGATGGTACGAAGT
TGCACCACGGTGATGTCTGAATTCGTCGAATTCATTGTCAGGAATTGTTTCAACAATATCATTTACAGTAAAAC
GATGTTGATTTGTTTTGTTTCCATATTGACCTCCATGTATTTGCTATGATTTCAAAATCCATTTTTGACGTGCCTT
AGGGTTGAGTGGATGCATAATTTCATTTGTTACTGGATTGATGAGCTTTTTTACTTTCTTTTTATGAGGTTTTA
ACATTTCCATCACTTGTTCGACACGGTCGATAACAACTGGTCGCTTCGCATAGGCACCATAAGCAAGAATCAC
TGTGTCACTTTCACTAATCGCTTTCATCAAATGAATATCAGTGTGCTCATCGTATGGATTTTTGATATGTTTGAG
GTTTTCGGGTGTTCTAATATTAGAGAATAGATTTACAAGATATACAGCACCGTATCGTTCTGAATTGGCTAATT
GGTTGAGGATAAGAACAGTTGTGAGATCGAGTGATAATACACCGTCTAAATGAGGATACATCGTTATCACTGT
GCATGCAGCTTTCTTTTCATCCCATGTTTTCTTGAGTAAATAGCGGTGCTGTTCATCATCGCTAAATATGGCTT
CTGTGTGTATCGTACTTTTGATTGTATTCATCATCGTCACTTCCTTTAGTATTCTTCTGGTAAAAGCATCACATAA
TAAAAAGCGTCCACGTCATCTTCACGAATGACGTAGACTTTCTTAGGTAATGCATTTTGATTTTTTTCATAGTT
TGTATAGTGATATTCCAATTTGTATGTGGGTTGTTCTTGTTCATGTGTGATTGAGAGTATATTCTCATCTTCTTGT
AATTTAAAAATGTGTAGGTAATCTGTATGAGGCTGGTTATCTTTTTTTTCTACCATGTGCCAAAGTAAGATTTG
AAGGTCTAGTGGAATACTTTCATTAATTCCTCTTGTGATGTATCGATTGATTTTCATGCTATTTCCCTCCCTTCTG
CTTTTCTTTCATGATGTCGATGATTTCGTTGATAACTGTGACGGATAATTGAGCAACTATGATCCAATTATTCAT
GGTCCTGACCTCCTTGTTTTAGTAAATGACGTTCATCAATAATGATATTTTGAGTATCTGTAAGGTACAGAAAG
TCCATGTCAAAATGGTCTAAGTATCCGACACTGATGAGTTGGTTATTGGCATACATTAGAAATGGATAGATACT
TAGCTCATGTAGTTCATCATTATAGTAGGTATAAGTCTCGAGTGTGAGATGTACCAGTGGAGAATCATTAATAA
AACGTTCCGGTAGAATATTTTCTGCTGCTTCCTCCAGCGCTTCACATTCCCAAGCTTCGTTATTAGATAGTTGG
AATAGGTGGGTTATATATTGTTTGAGTTCTTGAGTGATGGTTTTCATATTATTGCCTCCTAGATAGTGTAATAGT
GATGTAGTTCATATACATCATTGAGATAATATATATTTGATTTGTCATTTATTACGAATCCCGGTGGGAATAAGA
GAAAATTCCATATAAAAACCCGCTACAAACGTTGGTATGCCAAGGAAATCCTGAAATTCCGCCTATTTTGACA
AACAATCAACTCATTATTTATAAGTATTGATGATAGGGTTGTGTCTCTGCTTCCTTATATATATTATTTATTTATAA
AAAGTAACGGGATTTTGGGATTGTGCTTGCACAATCCTTCTGTTTCTTCGAATCTGCAAATCCCAATCATTTCC
CGATAAAAAATCATTGTGGGATGTTCTTTAGCAATTTCAATATAAGCATTGTATAGTTATGAAAAAATTACGAC

AATAACTGTTTCATTAGATAAGTGTTATTGAAATTGATAAAGAGCAATTCTTGAAAATAGTTAGATAAAATAAG
CGAAAGAATATAGTGAAAATTATTGTTATAACAATGATTCTATTAGCTAAATAGTAAGATATAGTGTTTGGGGC
AAAAATAAAGACGAAGTGCTGAGATGCACTTCGTCGAGTTGTTTATTATTGAAAAGTTGTTTAATGATTTCGT
TATTAAGTTTGAGTGTGACATAGAATTGTTTTTTATGATTACCATCTTTTTTAATATCAATGCGATCAATCACTG
ATAGATACAATGCTTTGAGTCGAGATTTTTCTATGTGCTTAATATCATGAAAGATGTGTTGTAATAGTTTACTGA
TTTCTTTGGCATCAAATAAAGTCTTATCTTCATTTTGTTGATTTTTGAGTTGGTTGATTTGATTCGTAATGTCAT
TGAGTTGCTTTTCATATTTTTGAATACTTGGTCTGATTACTGATGTTAAGTCCGGATTATCCTCGATGGTTTTAA
TCAAGTTATTTATTTTGATTTGTACTTCATCATATTGTTGTTGCTTATAAGCAATATCGTGATGAAGTGCAGCGC
CATCAACTTGATTTTCTTGATTGACGTGTGTTACTACGCGTTGAATGACTTTATCACTTTTGACTATTTCAAGTA
TTTGCTTCATCACATAATCTTCAATCACATCAGCTCTTACACTGTTGCCGAACATACTTTGGAACCCTTGTTCC
GAAAATTACTACATGAATAGTAACGAATACGTTTCTTAGTCCCGTCTTTAAGAGTATTCGTGGTATTGCTTGCT
GCCATAGGTGCGCCACATTGGGGACAGTGAATAATGCCTGTAAGCAGATTCGTTCCTTTGCCATGGACTTGG
GGTTTTTGACTGACTTGTTTCTTACGCATTTGTACTTTATCCCATAAATCTTGATTAATAATGGGGGAATGCTTA
CCTTCAGCTATCACTGGTTTATCATTCAGCCCTTTACGACGTTTTTCACTCCAATCTTTGTATTTCGCAAATTGA
ATTTTGCCGATATAGAAAGGGTTAGCTAAGATGTATGTGATTGAACTAATACTGAAAGGTTTCCCCTTTTTAGT
GACATATCCTTTGTGATTCAATGCATTGGCAATTTTACGATAGCCATGTCCTTTGGCATAGCACTCGAATATATA
TTTTACAATATTAGCTTCATGTTGGTTAATCATTAGCTCGTGTTTACTATCTGGTATTTTGTCATAACCTAGTGGT
AAATTGCCTTGATAATAGCCTTCTTGGGCACGTCTCGTTTGACCCATAAATACGTTCTCGACAATGTTATTACG
TTCGAATTCTGAGAAACTCGCAAGTATCTGTAACATGAGTTTACCAGAAGAAGTATTGACTTCCATACGCTCT
GACAAACTGAAAAAATTCGACATTTTGTTTGTGTAAATCTTCGACAATTTTGAGAAGATCAGATGTATTACGAG
CTAATCGGTTTGTTTTGTATACCATAACACAGTCGATATTGCCTTCTTTTGCATCTTTCAACATACGTTGGAGCT
CAGGTCTGTTCATAGATTTACCTGAAATACCACGGTCAGCGTATATATCTTTAACTTCAAAATGATGGAAGTCA
CAGTATTCTTTGATTTGATTGATTTGTCCGTCGATACTATAACCTTCTGTGCTTTGCATTTCTGTTGATACACGT
ACATAGATACCGACACGTTTTGTTTTAAGTTGTTGCATTATGTTACATCCTTTCTTCATTTATGCAATCGATGAT
TGCATGGTTTGATTGACAATATTGAGTGGTTCATTTTTGAAATAGATTCCTATAAGATTTTTATCTTTCGTAATG
TGAATGGTTTCAATATAGGGGTACAATATGTTTAACGTGAAACGTTTTTGAATAATATTTTGAATGATGTGTTG
TATTTGATGCCCATTGATAGATGTAGTGCGTTGCGGTTGTTGACGTAATGATTGTGTTTGCTCTCTGAACGTTT
CTGCATCAATGATGCCTTGTGCCAACTTTTCTATCAGTTGTTCTTGAGTCAATGTGTGATGTTTTTCTATGTTTC
TTTGTCTTTTGATGCGTTTGTCAATCGCACTTTTAATTTTTGTGTAGATGCGTTGATTTTGATAAAAGTCTCGG
CACACTTCTAATACTTTATCTTCAAGTGTTTGTGCATTGATGCCTTTAAAATCACAGACAAAGCGTGAAGCATT
CATGTTTTTAGGACAGACGTAGTAACGTAATGTATGATTCTTTTTTCTAATGGTCATATTTGTAAGTGTTGCATT
ACAACATGGGCATTTGATTTTTTGTTTGAGTTGATTATCCGAAGGTGTCTGTTTGGTTTGTTTTTGCAATCGA
AGTCTCTGCGCTTGCTCATATATACTTGTGGAAACAATAGAAGGAAACATATTGTCGAATTGGCCATATTGATT
GTTGACACGACCACAATAATTAGGATTGATGATAATGTTACGAACTTGATAGGGTTGTCGATTGATATACGTGT
TATCTTCTTCTAATAACTGTGCAATTTTCTTATAACCATGACCTTTAATGTAATAATTGAATACAGCCTTTACCGT
TGGTGACTCATTTTGATTGATGATGAATGTTCCGTTGTGATATTCGTAACCAAAAGGTGCATGTGTTGTAATCA
ATCGACCTTGCTTTGCTTTTTCTTGAAGCCCATTTCTGACTTGTTCTCCAATGTTATCCGATTCAAGTTCGGCT
AAACTGATGAAGATATTAAGCTTGAGTCGGTCGAAAGCTTGATCCATATCAAAGTAACCATCATGTACGCTTA
AGATATGAACATGGTACGTTTGACATAATTTGATGAGTTTTAATGCATTTTTCAGATTACGATGCAACCTATTA
AGACGATAACAGCATAATATGTCACACTGTCCTTGTTGAATTAATTGTGTTATTTGTCGATACCCACTACGATTA
TCTTTGCGACCTGATTGTTTGTCGCTATAAAAGTTGATATGTTGAATATGATGTTTTTCGGCTATTGCTTCGATA
GCTTGTTTCTGTGCTGCAAGAGATTGTTGTTTCATCGTACTTTGACGTAAATAGCCTATGACTTGTTTCATATC
GGCTCCTCCTTTCACAGTGATAATATATATTTATGGATGAATTGATATATAAGCCCAACATCAATGAGATGTTGG
GCGTCCATATTAGTCATTTGTTTGATTGATTTCTTCAATTACCAAATCGGCTAATATCTCGATAAGTTCATCCATG

TTTTTCACTCCGTTATTTGTTCTATCTTCAATACGTCGATTATTCAGTTTGATGCTTCACAGTTGTATGATAAAG
ACAATTAGAAATCTTCGTGAACTCCTGAAGGGCCTATCCCTTCATTAGCGGATTTAAAAAGTTCTTTCGCAGC
TTTGTTATCATTTGACGGTGTCCAATTTTGAAGTAACGACTTATCTTTAGTTAATCCGAGGATAGATGCAAACT
CTACATCTAATTTTAGATGGTAAAATACAAGTGATTGTTTTTTACCGCTATTATCTTTGACACTTCTTTTAGTTGT
TTGGCGTCCACGGTCAGCTAATATGAAACCTTTATCTCTTAAGGCGTTGACAACATTATTAACATCTTGAAATT
GATGATTGTTTAGCATCTGTTTAAAAACGTTCGCAATTATTTTTACTTCGATATGGTCATCTTTTAATGAGATTA
ATCCATAGTTCTCAAACATATTTTTCAAAGCACCTTCATCTGAAAACTTACCTCTGTTTTGTGCCACAAATTGA
ATGATGACATCAATAGCTTTATCAGCTAATGAGCGTTCAGAGACTGTATGAGTATGATAATCAATAAAGTAGTC
TCTTATATTAGCGATATCAATATCTGTAGATAAAACACGACCTAATATTTTCGCAGATGTTGTAATGACTGCATA
ACGCTTAAACATACGAATACCTGTATTGTTTGTTTCATCTTTCAATTTAGCTTCAAACCAATCTACTTCCTTGTA
AAACCATTGAATAACTTCATCTTCACGATTTATAAGATATTTAGCTACTAACGGTAAAACATGACCATAGTTTAG
TGCTACAGCTTTTTTAATATTGTCAGCATTGGTCGCATTTGTAGTGAATTGTTCATTAATCTCGATGGTTCTTAC
ACGTAATCCATCGTTTTGAGCTGAATCATTAAAAATACTGTATTCTGACGTTGAAATGACAGAAGTACCCCAAT
TCTTAGGCGTTTTAACTTCTCCATGAACGTTTGAACGTTGACGACCTTGACCTTCAGCGATGGAATATAACAA
ACCCGTGGTATCTCTAAGTGTCGTAGATGAAAGCTCATCAAATACTATAGGAATGCCATAATTGTTACTCAAAT
AACCTTCAAGTGCGTTTCGTGTGGCATTCCAACTTCTAAAAAGAGTTTCATTACCTTTGGTAGGGTTACCAGC
GACTGATACAGCTAAAGCAGCTGCGGTTGACTTACCGGTTGAGGATTGACCTGTAAAACTAAAGAGAATTC
CTGCAAATTCGATTTCATGTTTATGCTTCAGAAAACTTGTCACTAAGGCAGAAATCCCAAATATGACTGCCAA
TTCTAAAAGAAGATGACCTTTAACCTCGTCAATATACATGTTAAACCAATTATCAAATGTACCTTTAGGAGTTA
AGTCATAAGCACTATCTACAATAGGGTATGAAGGTGAGGACTGATTAAATTGAATAGATTTGAGAACTTTATC
TAGCGATATCAAGTAGCCAAAAGGTGTCTCTAGTATACCTACACCCTCATATAATTCAGAAAGTGGTAATCTAT
CTCGCATCAATTGCAAGGCATAACTCAATGATCTAATATACTTCTCGTTAATACTGTAGCCATATTTAATTAAAG
AAGGTAAGTTTCGTGTTGTTAAGATATCAGACTCAAAAATGTCTTTTTTACCATTGTTGTTAGTAATAATCAAC
TTTTCAACACCAGTAATTGGATCAGAAAATCTTGCATTAACAATAATAGGGCTAGCCATTCTAACCACTTTTTC
ACTGTCACCATCTTTTTTAGGTGGTATCGTTTCTTTCCAACCGAAGGAATCTAAAGTAAATGGGCCAATTTCA
AACAATGTGTAACTCATTAGCAATCACCTCCTTTCGAAGGCGTGCTATTATTGCATGGGTTTATCCCTATTTTCT
GATATAATAAACTGCCATTTTTTGTTCCTACAATTTTAAATGGTAATCCTGGCGTGTACTTAATCCAGTAAGCAA
AACGACGTCCTACTTCACGTTGATCATTCGTGCTACAAACCACATTATATTTCTTTCTTAATTCACCATAAGTGA
ACGTACTTCCAACTGGTTGTTGAAAAGATTTAGCAATTATAAAATTATAAAGCTTTTCTTGCTGCTTAGATTTC
TTATTCATTTTAAAATTCCTCCAATAATTTTTAAGGAGTCAATCAATATCATATAGGTTAAACTATCTTCAATTGA
TTGCTATAATAAGCATAAATGAAGCATTATGATTAGACGCGGAACAAAAAATGGGGAGGGGGGGTATATATT
GATACCTACGCTAGAAGAAATTATTGATAAGTATGGAAACTTAGTTGATTATTTGAAATTTGATGTAACAGTTG
AGGTTTATGAAGATTTGTTGTTATTAAGAAGTTTAAGCGATTTAAATGAACATCAAAAGATGGATAGAGTTTC
ATTTATAAAAAAACATTTAAACTCAAATTTCGATTATTTATTAGATGATACTGAGTTTAGAAGTATCAATAAGAT
TTACAGTAATTTAAATATCATGACGCATATCAATAGTCAAAATAATAATTTTAAACGTAATCCATTTATAAATGAA
GAACAATTGAAATCACTACTCGCGATAAAGAAAGTAGATGCACATATGAGTTATGATTCAAATATTACTTCAAA
AGCATTGGCAGAAATAAACAAAACGCAAAAAAGATTAGTTACAAAGATAGACACACTCTATAATCAGTCAAA
AAAAGAAAATGAATATGTACGGTTAGGAGAAAAAATATCGTATACAAAATTGGAGAATCATTGGAAATATTTA
TATAATGAGATTCAGTTTTATAATCTTAATAATCAATTGATTAGTTATGTTGCATTAGAACAGGAATATGCTTGG
GCTTTTTTAAATGAACTATTTTATTTGATAGAATTGTATTTGAAGGCATTTAAAGGACAAAAGAAAACAGATT
ATGAGTTTGGGAAAAAATTAAACGAATTTATTCAATCATATGTCATTATTTTACTTATAGATATACGATTACCTGT
TGTTAGATTATATATTATTAGAGACATTGTAGATACATGTGAAGGAGAAAAAGATAATTACAAAAGAATTACAC
TGATAGAAGAATCTATTAAAAAGTATAGATACGTAAAAGATCAGATTAAAAGATTTAAAAATGGATTAGAAGA
ATCGTTATTAAATGCAACTTTATCTATTGAACAAAATATGTTGAAAGTTAAAATTGATTATTATAAAGCCTACTG

CTTTCCTAGAGAAAAAACAAAGCATAACAATATTGAGTACAATGTATCGTTGTTTTTAAAGCATTAGATGCCT
TAAAGAAATAGTTTTTCACTGAAAATATTTATAGATATAATTAAAATTAATTAATATTAAGCCAGGATAATGTAG
TCTTAATCGTTCTGAAATACGAAAAATGTTGTGGAAATCGCGTGAAATAATCAGAGGAATCGTTTGAAATCAT
CGTAGTAATCGCGCGAAAATCACGTGAAATAATCAGAGGAATCGTTTGAAATCATCGTAGTAATCGCGCGAA
AATCACGTGAAATAATCAGAGGAATCGTTTGAAATCATCGTAGTAATCGCGCGAAAATCACGTGAAATAATCA
GAGGAATCGTTTGAAATCATCGTAGTAATCGCGCGAAAATCACGTGAAATAATCAGAGGAATCGTTTGAAAT
CATCGTAGTAATCGCGCGAAAATCGCGTGAAATAATCAGAGGAATCGTTTGAAATCATCGTAGTAATCGCGCG
AAAATCGCGTGAAATAATCAGAGGAATCGTTTGAAATCATCGTAGTAATCGCGCGAAAATCGCGTGAAATAC
TATGGTAGACGGTTTGAGTAAATTAATGGAGTATTTTAATATTTATGGTTTGGAGGAATTGCGTTGCATAGAG
AAAGTAAATATATAGAGTATAAGAAATCACGAAAAGGATTATCTAATGATATTTGGTCTACGTATAGTGCTTTT
GCAAATACTGAAGGTGGTACTATATATTTAGGAATTGAAGAAAAAAAGATCGAGGACAAAAAAGTCTTTGTT
TCAGTTGGTGTTGAAGATCCAGAGAAAATGATTGAAGATTTTTGGAATGCACTATATGGAAGAAGTAAAGTT
AGTCAAAATATTTTATCAAATAAAGATGTTAAAATTGTTAATATTGAAAATAAAGCGTGCATTGAAATTCATGT
ACCAGAAGCGCCTTATTCGAAGAAACCGATATATGTAGATAATAAAAAAGATTTAGTATATAAAAGAGTTGAT
GATGCTGATAGAATTGCGACTGAAGAAGAGTATAAATTCATGATTGTAAATTCTCAAGACGATATAGATACAG
AATTATTAGATAACTATGACATGTCTGATTTAAATCACGAATCTATCGAAAATTATAGGAAACTTCTATTAAAAA
ATACTAATGATGAGAGATATGCGAATATGAGCCAACTGGATTTAATGATAGATTTAGGAGCATATAGAAAAGA
TAGAAGTTCGAAAGACAAACAGTATAAAATGACTACAGCATGTTTATTATTCTTTGGTAAGTATAATGCGATTA
GTGATAGATTCCCAGGATTTCAATTAGATTATTTTAAGAAAACAAATTACCTAGATACTGATTGGAAAGATAG
AATATCAAGTGGAGATTTAGGTAATGAAGATTTAAACGTGTATAGTTTTTTTGAAAAAGTATTGATAAAATTAA
CTGATAACATTGAGGAATCATTTAGCCTAAATGATGGTTTGACTAGACAAAATTATGCAAGAGATTTAAAAGT
AGCAATTCGCGAAGCACTGGTTAATACATTAATGCATGCGTATTATGATACTAAGCAAAGTATTAAAATAGTTA
ATTGTGAAGATTTTATAGAGTTTTATAATCCGGGTAATATGAGAATAAATAAAGAAGATTTTATTCATGGAGGG
CATTCAAAGGACAGAAATAGTATATTATCGACGCTTTTCAGAAGAGTAGGATATTCAGAAAAAGCTGGATCT
GGAGGACCAAGGATATTCGATGTAGTTAATAGACATAAGCTTAAAACGCCTGAAATAGAATTAACGGACATG
GACACTAATGTAGTACTTTGGAAACAAGATTTAATGAAGGAGTTTGAAAAATATCCTGAGTTAGACAAAAAA
GTAATAAAGTATATTATTGACTATGGATCAATAAGTAAGGGTGAAGCCTTAAAAATGGAAAATATGACAGAAT
ATCAGTTTAGAAATATTTTAAAAAAACTAAAAGATGATAACTTGATAAAAAAAGAAGGTGAAGGTCCGGCTA
CTAAATATGTGTTAATAGAATCAAAAGAAGCTGATATATTGCGAACTAAAAAAGTAATTAAAAGTTTAGAGTC
TTTCTTTAGGAATAAATAAAAAAACAAGAGATAGGTGCGAAGTGTTTGGATTATACAAATGCTTCGCATCTTA
TATTTATTAATAAATATCATAGAAAACCGTATCATTAACCGATACGCAGAGATGCGGTTTTTTAGACACTTCATA
AAGGGATTTTGAACGTATCAGAACATATGAGGTTTATAGGAATTGCTGTTATGTTTTTTGATCACATCAATAAA
CAAAAAAGGTATGTACTATGTAAAATATTTATTAAATGATATAAAGCGAGGGTATATAAATGATTTTTAAATAGA
TATTTATCCAATAATATAAAAAGGAACTATAAGCTATATCTAAAAGCTATATCTAACTACTTATAGTCCTTCTTCAT
TAGTATAAATATAATTATTAAGAAAGGTATATATCTTTGTAACCTTCGTTTACATTAATAATGTTGTGATAACCTTT
GTGTTCTAAAATACCAATAGCTATAGAACTTCTAATGCCAGACTGACAGTCTACATAAATAACATCGTTTCTATT
GAAAGGTAAATCTGTTTCTAAAAGTTTGCCGTGTGGCACATGAACCGCTTGGGATAAGTGGCCATTATTCCA
TTCATTATCATTACGTACATCTAAGACATGTGCTTCATTACCAGTTATGTCTTTACTATGAACAGATTGTGTTTG
AATTTGAGCTTGTGGTAACTGATATCCAGACACATTATCATATCCAATAAGTTGTAAAGTATGTTATGTGTTGCT
TTTGAAACAAGGTGATAGTCTCCAATCAAGTTAATTTCTTGATTATAGTTTAGATACCAGCCAATTTGATTGAT
GAAATTTTTATCATATGGAATATTGATTGTACCTTCAATATGTCCACCATGATAAGCCTCCTTACTGCGGAGATC
AAAAGTTAATCTGTTTGTACTTGTAGCTGGATAAACCGTATAAGGTTGATATAAATTCATACCGAATTGATTAAT
TTTTTTCATTTGTGCAAAATGATGTGGTGGTGCAGGTTGGTCAGAAATGAGTTTATCGATAAAGGTAGCTTCA
TTATTTTCAGAAAAAGCCCAGTTCGTTTGTTTTTCATAGCCAAGCGTAGATGTTGGAATAGCACCTAAAGATT

TACCACAAGGACTACCAGCGCCATGACCAGGCCAAATTTGAATGTAGTCTGGCAAGTCTTTAATACTTTCAAT
AGATTTAAACATTTGTTTTGCGCCTATTTCAGATAATCCTTCTACTTTAACAGCTTTTTCTAGTAAATCAGGTCT
ACCGATATCTCCTACAAAAATAAAATCACCACTGAATAGTCCCATTGGAACTTGTGCTCCAGCACCTTCGTCA
GTAAGTAAAAAACTTATACTTTCTGGCGTGTGACCAGGTGTATGAAGCACTTTTAATTTTATATTTCCTACATA
AATATCATCATTATGTTGAACAAAATGAGTGTGGTTAGGCATATTTTTATAACCTAACATGTCATCACTTTCGCC
CGATACATAAATACTAGCATTTAACTTTATAGCAACATCTCTAATTCCTGAAACAAAATTTGCATGTATATGTGTT
TCAGCTGCATGAGTAATGGTTAAACTCTCTTCATCGGCAACTCGAATATATGAAGATAAGTCACAAATAGGAT
CAATGATCATGGCTTCTCCAGTTTTTTGACAACCGATTAAATAAGATGCTTGAGATAAATGTTTATCATAAATT
GATGATAGTCAATTTGTTATCAAGGGTGATAATTATATTAACTATGGTAATTACATGGAATTAAAGTTATTAAAC
AAACTTGATGATGACCAATTAGATTTAGAATTTTTACAAGATAATGGGTTAAAAGGTAATGTTCAAGGTCCTA
TGACTTTAAAGGAACCTTTAAAAAGTTATATACAAAAGTCTTGAAAACGAATATTTTAAATTTAGATAATGAGT
TAGAAAACACTGAATATGTCGAAGGTAAACCATATGTACAGTACGGTATTAAATATGAAAGAAATCAGGCATT
AAGAAATGAAGCTATTAAAATTCATGGAACTACATGTAAAGTATGTGGATTTGATTTTAAAGCTAAGTATGGC
GATTTAGGTGAGGGTTTTATTGAAATTCATCATTTAAAACCAATGTTTTCAATAAAAAGAGAAATAAAAGTAA
ATCCACAAAAAGATTTAGTCCCACTATGTTCTAATTGCCATAAAATGATTCATAGAAATACTAAAAAACCTTTA
ACGATTAAAGAATTAACCAAAATAGTTAATTATAATAGCAAATAATTTAATATTTTATAAACTATCATTCAACCCT
CTTAATTTATTAGGAGGTTTTTTGTATTTATGCTTTCAAATGTGTGATATACTTTGTTTGTTGAAATATAGAGTAT
CTATAGATAGGGTGATTGAGTATGAAATTCACTGAAGTGGAAGTTATCGAACATCTTGTAAAGGCATATAAAG
AAGCAGGAAAGCCTACTTATCCTCATGAAAATTTATATCGAGGACGTAATCATAGTATTTCAGGTATTGGAGA
AGACTTGCTGGGTGCTTATTTGATTAGTAGATTGGAAGGTGTCCAAATATTTATTGATCAGCCTTTATCTATGA
TTGATAAATCTTTAAGTACAAGATATCCGGATTTATTAATTTGTGAAGATAATGAAATTAAAAATATACTAGAAG
TTAAAATGGACTTAGGATATCAAAGAAAAGATTTTATAGATTATTGCCGAAAGAAAGAAGAATGGATTTCAA
ATATCGTAGGAAAACAGTGTGTATTGTCTAGAAAGAGAGAAGACAAAATTCCTATGAATATAGCTGATGATAT
TAAATTTCATGTTGTGATTTACAGTGAAAACAATGGACCGAAGCGGTTTGATGAAGAAATCATGCCTATCGTT
AATGAAACATGTCCACATATTGAAGTATATGTCCTAACAAGCGGTCAACACCCTAATTTAGTAAATGTTAATCT
TGAAGGTATTAATATTAATAAAGATGAATTTGAAATATTAGTAAATGCGTTATAAAAAAATAGAGCATCCTCCA
CGTTATGGAGGTGCTCTGTTTTTTATTGAAAAGTATCAAGTTAATTAATTTAATATGCTTAATAAGTTCTACCTT
GACCTTTTTCTCTAGCTTCTGTTCGATCTCTTATGTACTCAGTACATACTGGATTTTCTGTTAATTCATCAACTGT
ATTAGTTTAATCAGAAGACGTGTCTACTTTGTAAGCTTCTAAGAACTTATTATATGATATAGCGTTTGAGTTTTG
TTGTTCTTCTATAAATTTCTGTAGTTATTTTTCAAAAACCGCATCATTAACTGATAAGCAGAGGCGTATCATAAG
T
>Staphylococcus aureus strain 628
ATGAAAATCACCATTTTAGCTGTAGGGAAACTAAAAGAGAAATATTGGAAGCAAGCCATAGCAGAATATGAA
AAACGTTTAGGCCCATACACCAAGATAGACATCATAGAAGTTCCAGACGAAAAAGCACCAGAAAATATGAG
CGACAAAGAAATTGAGCAAGTAAAAGAAAAAGAAGGCCAACGAATACTAGCCAAAATTAAACCACAATCCA
CAGTCATTACATTAGAAATACAAGGAAAGATGCTATCTTCCGAAGGATTGGCCCAAGAATTGAACCAACGCA
TGACCCAAGGGCAAAGCGACTTTGTATTCGTCATTGGCGGATCAAACGGCCTGCACAAGGACGTCTTACAA
CGCAGTAACTACGCACTATCATTCAGCAAAATGACATTCCCACATCAAATGATGCGGGTTGTGTTAATTGAGC
AAGTGTATAGAGCATTTAAGATTATGCGTGGAGAAGCATATCATAAATGATGCGGTTTTTTCAGCCGCTTCAT
AAAGGGATTTTGAATGTATCAGAACATATGAGGTTTATGTGAATTGCTGTTATGTTTTTAAGAAGCTTATCATA
AGTAATGAGGTTCATGATTTTTGACATAGTTAGCCTCCGCAGTCTTTCATTTCAAGTAAATAATAGCGAAATAT
TCTTTATACTGAATACTTATAGTGAAGCAAAGTTCTAGCTTTGAGAAAATTCTTTCTGCAACTAAATATAGTAA
ATTACGGTAAAATATAAATAAGTACATATTGAAGAAAATGAGACATAATATATTTTATAATAGGAGGGAATTTC
AAATGATAGACAACTTTATGCAGGTCCTTAAATTAATTAAAGAGAAACGTACCAATAATGTAGTTAAAAAATC

TGATTGGGATAAAGGTGATCTATATAAAACTTTAGTCCATGATAAGTTACCCAAGCAGTTAAAAGTGCATATAA
AAGAAGATAAATATTCAGTTGTAGGGAAGGTTGCTACTGGGAACTATAGTAAAGTTCCTTGGATTTCAATATA
TGATGAGAATATAACAAAAGAAACAAAGGATGGATATTATTTGGTATATCTTTTTCATCCGGAAGGAGAAGG
CATATACTTATCTTTGAATCAAGGATGGTCAAAGATAAGTGATATGTTTCCGCGGGATAAAAATGCTGCAAAA
CAAAGAGCATTAACTTTATCTTCCGAACTCAATAAATATATTACATCAAATGAATTTAATACTGGAAGATTTTAT
TACGCAGAAAATAAAGATTCATCTTATGATTTAAAAAATGATTATCCATCAGGATATTCTCATGGATCAATAAGA
TTCAAATATTATGATTTGAATGAAGGATTCACAGAAGAAGATATGCTAGAGGATTTAAAGAAATTTTTAGAAC
TATTTAATGAATTAGCTTCAAAAGTTACAAAAACATCCTATGATAGCTTGGTCAATAGCATAGACGAAATACAG
GAAGACAGCGAAATTGAAGAAATTAGAACAGCACAAAAAGATAAGACACTCAAGGAAGTGGAAGCACCTA
AAGGAATAATTCCAAAATATAAAAAAGGTGTATCAAAGACTACTAAAAATGATTCAGAAATTGAAAAATCAA
ATAAAGAGAATAAATTAACCGGTAAAGTTGGAGAAAAATTAGCGCTAAATTACTTTAATGAGCTAATTGATAA
TAAAATAGACGAAGATAAGAAAGAACAGTTTAGGAATATTTTAAATGATAATCCAGGCTCTCAACACGGTCAT
GGCTATGATTTAGTAGCTTTTGATCCAACAAATACAGATAAAGCTGTAGAAAAATTTATTGAAATTAAAACATC
TACATCTTCTAGTATTGAGGAACCATTTTTTATGTCGCTAAATGAAATGTTTGCTATGAAAGAATATAAGCAGA
AATATTTAATATTAAGAATATTTAATGTTTCCGGTAAAGAACCACAATTTTATTTTATAGATCCATATGCAAATTA
TTCTGAATTTAAAGATGTAGATGATCTCATTGACAAAGTATTTAATGTAGAAGCTATTCAGTATAAAGTTTTTG
GCGAAAAATGATTACTTGAACAAGAGCTAAAATAAAATTGTGATCTAATAAAAATAGAAACTGTAATTTAAAT
AAAACTTTCTAAATAAGCTAACTGATAAAAAATCAGTTTGTCCACAGTCTGAAACAAGATTCCTATATTCTTTA
GGAATCTTGTTTTTTCTATTTTTATGGTGATAAAGAGCAGATAAGATAATGTGTAATAATCACAAAAAAGTTAA
ATATTTTAAGGCTTGTTTAATTATTAATGATTTTATATATAAAGAGCAGTATAATAAAGTTGTTAATATATTATGAA
TAATATTCAAGTAATTTTATTGTTTTTTAATTTGTCGATATTTAAGTTGAGTTAAATTTAAAGGGTGTAATTTGTT
TTACAATGATGAAGATAATTAGTCTATCAAAATAAAGGGGTTGGGACTGTTATGAGTGATAATTTGTCATTATT
CATTGACTATATCAATGATAATATAATCTATGGTAGTGAAATCAAACGGGAGAAATTAGAGAATTTATTTAATCA
ATTTGCTATAAAAAATGTTGAAAAGAACATTGTCTATGATGAACTGAAATCTTTAGATATTACAATCATTGAGT
CACAGGATTCATATAAAAATAAATTGAAGAGATTATTTTCGGTTCTGTTGCAAAGTAAAAAAATATAGCTAACC
ACTAATTTATCATGTCAGTGTTCGCTTAACTTGCTAGCATGATGCTAATTTCGTGGCATGGCGAAAATCCGTAG
ATCTGAAGAGACCTGCGGTTCTTTTTATATAGAGCGTAAATACATTCAATACCTTTTAAAGTATTCTTTGCTGTA
TTGATACTTTGATACCTTGTCTTTCTTACTTTAATATGACGGTGATCTTGCTCAATGAGGTTATTCAGATATTTC
GATGTACAATGACAGTCAGGTTTAAGTTTAAAAGCTTTAATTACTTTAGCCATTGCTACCTTCGTTGAAGGTG
CCTGATCTGTAATTACCTTTTGAGGTTTACCAAATTGTTTAATGAGACGTTTGATAAACGCATATGCTGAATGA
TTATCTCGTTGCTTACGCAACCAAATATCTAATGTATGTCCCTCTGCATCAATGGCACGATATAAATAGCTCCAT
TTTCCTTTTATTTTGATGTACGTCTCATCAATACGCCATTTGTAATAAGCTTTTTTATGCTTTTTCTTCCAAATTT
GATACAAAATTGGGGCATATTCTTGAACCCAACGGTAGACCGTTGAATGATGAACGTTTACACCACGTTCCCT
TAATATTTCAGATATATCACGATAACTCAATGTATATCTTAGATAGTAGCCAACGGCTACAGTGATAACATCCTT
GTTAAATTGTTTATATCTGAAATAGTTCATACAGAAGACTCCTTTTTGTTAAAATTATACTATAAATTCAACTTTG
CAACAGAACCGTATTATGGAATAGAGATGTTGGTAACATTTATACAGGATCATTATACTTAAGTTTAATTTCGTT
ATTACAGAACCACACATTCCAACCAGAAGAGAAAGTATGTCTATTTAGTTATGGTTCAGGAGCAGTAGGAGA
AATCTTTAGTGGTTCAATCGTTAAAGGATATGACAAAGCATTAGATAAAGAGAAACACTTAAATATGCTAGAA
TCTAGAGAGCAATTATCAGTCGAAGAATACGAAACATTCTTTAACAGATTTGATAATCAAGAATTTGATTTCG
AACGTGAATTGACACAAGATCCATATTCAAAAGTATACTTATACAGTATAGAAGACCATATCAGAACATATAAG
ATAGAGAAATAAACTAGTGGCCGATTGTGCTTGATGAGCTTGGGACATAAATCCTAACTCGAAATAAATAAGC
ATATCACTAAACTGATTTTTTAAAGTTTACAGTGATATGCTTATTTTTTTATCTTACGATTTTGTACGTGCATGCT
TGCCTAGGGGTATGGCTCGAGCCATTAGTCTCTCGCACATACTATTCCCTCAGGCGTCAGCACTTACAAAATC
GGTTGTAATTTTCATTTTTATACGCATTCTTACTGAGATTATACTAATAAGAGGAATAGTAAAAGCAATTCTAAG

TAAAATTGCAGATAAGAGGTTTGTTAAAAGCAGTTCTAAGTAAAATTGCAGATAAGAGGTTTGTTAAAAGCA
GTTCTAAGTAAAATTGCAGATAAGAGGTACGTTAAAAGCAATTCCATGCAAAATTGCTGATAAGGGGTAAGT
TAAAAGCAGTTCTCAGTAAAATTGCAGATAAGAGGTACGTTAAAAGCAGTTCTAGGCAAAATTGCAGATAAG
AGGTGCGTTAAAAGCAGTTCTCAGTAAAATTGCTGATAAGGGGTAAGTTAAAAGCAATCCTAAGTAAAATTG
CAGATAAGGGGTACAGAAAAACTAGACTTGATTACAAAATGGAGCTTGGGACATAAATGATTTTTTAAAAAT
GAGATGAGACGTAGATTAACTCCATAATCAATACGAATCTATCGACTTCTTTATTTATGATATTCATCTCTTTTTA
ATGGAAATAAAAGTGCGATTAATGTGATAATACAGTTACGTTAATTAAAAAAATAAAAATGCAAGGAGAGGT
AATATGCTAACTGTATATGGACATAGAGGATTACCTAGTAAAGCTCCGGAAAATACAATTGCATCATTTAAAGC
TGCTTCAGAAGTAGAAGGTATAAACTGGTTGGAGTTAGATGTTGCAATTACAAAAGATGAACAACTGATTAT
CATTCATGATGATTATTTAGAACGGACTACAAATATGTCCGGGGAAATAACTGAATTGAATTATGATGAAATTA
AAGATGCTTCTGCAGGATCTTGGTTTGGTGAAAAATTCAAAGATGAACATTTGCCAACTTTCGATGATGTAG
TAAAAATAGCAAATGAATATAATATGAATTTAAATGTAGAATTAAAAGGTATTACTGGACCGAATGGACTAGCA
CTTTCTAAAAGTATGGTTAAGCAAGTGGAAGAACAATTAACAAACTTAAATCAGAATCAAGAAGTGCTCATT
TCAAGCTTTAATGTTGTGCTTGTTAAACTTGCAGAAGAAATCATGCCACAATATAACAGAGCAGTTATATTCCA
TACAACTTCGTTTCGTGAAGACTGGAGAACACTTTTAGATTACTGTAATGCTAAAATAGTAAACACTGAAGAT
GCCAAACTTACTAAAGCAAAAGTAAAAATGGTAAAAGAAGCGGGTTATGAATTGAACGTATGGACTGTAAA
CAAACCAGCACGTGCAAACCAACTTGCTAATTGGGGAGTTGATGGTATCTTTACAGACAATGCAGATAAAAT
GGTGCATTTGTCTCAATAGAAAGTTAGAGGTGAGTCTTACGTTTCAGTGACGGTAGACTTACCTTTAACATGT
TACATACTAAAAAATTAATTTGAATAAGAAAGAGAGACATATATGAAATACGATGATTTTATAGTAGGAGAAA
CATTCAAAACAAAAAGCCTTCATATTACAGAAGAAGAAATTATCCAATTTGCAACAACTTTTGATCCTCAATAT
ATGCATATAGATAAAGAAAAAGCAGAACAAAGTAGATTTAAAGGTATCATTGCATCTGGCATGCATACACTTT
CAATATCATTTAAATTATGGGTAGAAGAAGGTAAATACGGAGAAGAAGTTGTAGCAGGAACACAAATGAATA
ACGTTAAATTTATTAAACCTGTATACCCAGGTAATACATTGTACGTTATCGCTGAAATTACAAATAAGAAATCCA
TAAAAAAAGAAAATGGACTCGTTACAGTGTCACTTTCAACATACAATGAAAATGAAGAAATTGTATTTAAGG
GAGAAGTAACAGCACTTATTAATAATTCATAATAAAACAGTGAAGCAACCATCGTTACGGATTGCTTCACTGT
TTTGTTATTCATCTATATCGTATTTTTTATTACCGTTCTCATATAGCTCATCATACACTTTACCTGAGATTTTGGCA
TTGTAGCTAGCCATTCCTTTATCTTGTACATCTTTAACATTAATAGCCATCATCATGTTTGGATTATCTTTATCATA
TGATATAAACCACCCAATTTGTCTGCCAGTTTCTCCTTGTTTCATTTTGAGTTCTGCAGTACCGGATTTGCCAA
TTAAGTTTGCATAAGATCTATAAATATCTTCTTTATGTGTTTTATTTACGACTTGTTGCATACCATCAGTTAATAG
ATTGATATTTTCTTTGGAAATAATATTTTTCTTCCAAACTTTGTTTTTCGTGTCTTTTAATAAGTGAGGTGCGTT
AATATTGCCATTATTTTCTAATGCGCTATAGATTGAAAGGATCTGTACTGGGTTAATCAGTATTTCACCTTGTCC
GTAACCTGAATCAGCTAATAATATTTCATTATCTAAATTTTTGTTTGAAATTTGAGCATTATAAAATGGATAATCA
CTTGGTATATCTTCACCAACACCTAGTTTTTTCATGCCTTTTTCAAATTTCTTACTGCCTAATTCGAGTGCTACT
CTAGCAAAGAAAATGTTATCTGATGATTCTATTGCTTGTTTTAAGTCGATATTACCATTTACCACTTCATATCTTG
TAACGTTGTAACCACCCCAAGATTTATCTTTTTGCCAACCTTTACCATCGATTTTATAACTTGTTTTATCGTCTA
ATGTTTTGTTATTTAACCCAATCATTGCTGTTAATATTTTTTGAGTTGAACCTGGTGAAGTTGTAATCTGGAAC
TTGTTGAGCAGAGGTTCTTTTTTATCTTCGGTTAATTTATTATATTCTTCGTTACTCATGCCATACATAAATGGAT
AGACGTCATATGAAGGTGTGCTTACAAGTGCTAATAATTCACCTGTTTGAGGGTGGATAGCAGTACCTGAGC
CATAATCATTTTTCATGTTGTTATAAATACTCTTTTGAACTTTAGCATCAATAGTTAGTTGAATATCTTTGCCATC
TTTTTTCTTTTTCTCTATTAATGTATGTGCGATTGTATTGCTATTATCGTCAACGATTGTGACACGATAGCCATCT
TCATGTTGGAGCTTTTTATCGTAAAGTTTTTCGAGTCCCTTTTTACCAATAACTGCATCATCTTTATAGCCTTTA
TATTCTTTTTGTTTTAATTCTTCAGAGTTAATGGGACCAACATAACCTAATAGATGTGAAGTCGCTTTTCCTAG
AGGATAGTTACGACTTTCTGTTTCATTAGTTGTAAGATGAAATTTTTTTGCGAAATCACTTAAATATTCATCCAT
TTTTTTAACGGTTTTAAGTGGAACGAAGGTATCATCTTGTACCCAATTTTGATCCATTTGTTGTTTGATATAGTC

TTCAGAAATACTTAGTTCTTTAGCGATTGCTTTATAATCTTTTTTAGATACATTCTTTGGAACGATGCCTATCTC
ATATGCTGTTCCTGTATTGGCCAATTCCACATTGTTTCGGTCTAAAATTTTACCACGTTCTGATTTTAAATTTTC
AATATGTATGCTTTGGTCTTTCTGCATTCCTGGAATAATGACGCTATGATCCCAATCTAACTTCCACATACCATC
TTCTTTAACAAAATTAAATTGAACGTTGCGATCAATGTTACCGTAGTTTGTTTTAATTTTATATTGAGCATCTAC
TCGTTTTTTATTTTTAGATACTTTTTTTATTTTACGATCCTGAATGTTTATATCTTTAACGCCTAAACTATTATATAT
TTTTATCGGACGTTCAGTCATTTCTACTTCACCATTATCGCTTTTAGAAATATAACTGCTATCTTTATAAACTTGT
TTGAAATTTTTATCTTCAATTGCATCAATAGTATTATTAATTTCTTTATCTTTTGAAGCATAAAAATATATACCAAA
CCCGACAACTACAACTATTAAAATAAGTGGAACAATTTTTATCTTTTTCATCAATATCCTCCTTATATAAGACTA
CATTTGTAGTATATTACAAATGTAGTATTTATGTCAAAATAATGTTATAATTTTTGTGATATGGAGGTGTAGAAG
GTGTTATCATCTTTTTTAATGTTAAGTATAATCAGTTCATTGCTCACGATATGTGTAATTTTTTTAGTGAGAATGC
TCTATATAAAATATACTCAAAATATTATGTCACATAAGATTTGGTTATTAGTGCTCGTCTCCACGTTAATTCCATT
AATACCATTTTACAAAATATCGAATTTTACATTTTCAAAAGATATGATGAATCGAAATGTATCTGACACGACTTC
TTCGGTTAGTCATATGTTAGATGGTCAACAATCATCTGTTACGAAAGACTTAGCAATTAATGTTAATCAGTTTG
AGACCTCAAATATAACGTATATGATTCTTTTGATATGGGTATTTGGTAGTTTGTTGTGCTTATTTTATATGATTAA
GGCATTCCGACAAATTGATGTTATTAAAAGTTCGTCATTGGAATCGTCATATCTTAATGAACGACTTAAAGTAT
GTCAAAGTAAGATGCAGTTCTACAAAAAGCATATAACAATTAGTTATAGTTCAAACATTGATAATCCGATGGTA
TTTGGTTTAGTGAAATCCCAAATTGTACTACCAACTGTCGTAGTCGAAACCATGAATGACAAAGAAATTGAAT
ATATTATTCTACATGAACTATCACATGTGAAAAGTCATGACTTAATATTCAACCAGCTTTATGTTGTTTTTAAAAT
GATATTCTGGTTTAATCCTGCACTATATATAAGTAAAACAATGATGGACAATGACTGTGAAAAAGTATGTGATA
GAAACGTTTTAAAAATTTTGAATCGCCATGAACATATACGTTATGGTGAATCGATATTAAAATGCTCTATTTTAA
AATCTCAGCACATAAATAATGTGGCAGCACAATATTTACTAGGTTTTAATTCAAATATTAAAGAACGTGTTAAG
TATATTGCACTTTATGATTCAATGCCTAAACCTAATCGAAACAAGCGTATTGTTGCGTATATTGTATGTAGTATAT
CGAGCTTCACATGAAACAGCTAAAGAAGCTTTGGGCGATAAAGAGTTAAGAGCCATTGCACATGAGTTAAC
TAAAACAGTTAAGGATAACATGAGTGTTGATTGGTCTAAACGAGACAGTGCTAAAGCTAAAATGAGAGTTCA
AGTTAGACGCCTATTAAAGAAATATGGCTATCCACCAGATCTTCAAAAAATGGCTGTGGAACAAGTTGTAGA
GCAAGCAGAATTAATGGCAAGTCAGCAATAAAAAAATAAATCATAATGAGTCCGGGACATAAAGTTCTTGGA
TAAGTGAAAAAAGACAATTTCTATTGAAATAATATAGAAATTGTCTTTTTTATAAATTTTTTGATTATTTTCAGC
TCGTTGAGCTACTACTTTTCTTATATTAAGTGCCATTAATACAAAACCAAGTTCTCTTTTGACTTTATTGAGTCC
TCGGACAGACATCCGAGTGAAACCCAAAATAGCCTTCATAAATCCAAAAACAGGTTCCACATCAATTTTTCTT
TGATTGTAGATATTTTTTGTTTCTGGTTCTGAAAGCTTTTTGTTAATTTGGGATTTAAAATATTCCCAGTTATAA
TTCTTCATTATTTTTTTGTTTGTTTTTGAATTGAAGTTCATACATTGATTTTTCAGAGGACATTCTGAACAATCA
TCACATTCATATAATTTGAAGTCTCGCTTATAACCATACTTATCATGACGATAGGCATATCTTTTAAAACCTAGCC
GTTTATTATTCGGACAAATGAATTCGTCATTAATTTCGTCATAGTTCCAATTTTGAGTATTAAAGATGTCACTTT
TATATTTTTTAGTTTTATCTTTTATAAACATTCCATATGTTATGAGTGGCGTTCGATTAAAGTCATCTATAATTGCC
TTATAATTTGATTCACTACCATAACCTGCATCAGCTACAATATATTCAGGTAAATGACCGTAGGTCTCTTGAATT
GAATTTAAAAATGGAATCATCGTTCTAGTATCCGTTGGATTTTGATACACATTATAAGATAAAACAAATTGGGA
ATTTGTTGCTATTTGTAAATTATACCCTGGCTTAAGTTGTCCATTTTTCATGTGATCTTCTTTCATTCTCATAAAT
GTCGCATCATAATCTGTCTTAGAATAACTATTTCTATCCTTTAAAATAGATTTTTGAAATTCGTATCGATACTTTC
GCTCAAAATAATCATTGATTTGCTTTTTGTATTTTTTGATTTTAGTTCTTTTGAGACGTATTTGTTTTCTTGTTTT
AGTACATTTTTCATTGTTGATATGTTGGTTTAAATCTTCGATTTCTTTATCTAAGTGACTACCAATCAAATCTATT
TCTTCTTTTGTTAATTCATTATCATGATCTTCTTTAATTTCCGGTATGATTTTATTGGTTACCAATTCATGGTAGA
GGGCTTTAGAATCCTCATTCATCTTTGATTCATGGTTTTGAATACTCTTTTTCCATACAAATGTATATCGATTGG
CATTTGCTTCAATTTTTGTACCATCAATAAAAATAGCTTTATCATCTATAAGATTTTGTTTTACACACTGACTGTA
AAATTGAATAAATAAAGATTCTAATAAAGCATCTACTTTTGGATTTACTCTAAATTGATTAATTGTTTTATAAGA

AGGTTTTTGATTTTGTGATAGCCACATCATTCGGATGCTATCATTAAGCATTTTTTCTATTTTACGACCTGAGAA
TACAGATTGTGTGTAGGCATATAGAATCACTTTTAACATCATTTTAGGATGGTACGAAGTTGCACCACGGTGA
TGTCTGAATTCGTCGAATTCATTGTCAGGAATTGTTTCAACAATATCATTTACAGTAAAACGATGTTGATTTGT
TTTGTTTCCATATTGACCTCCATGTATTTGCTATGATTTCAAAATCCATTTTTGACGTGCCTTAGGGTTGAGTGG
ATGCATAATTTCATTTGTTACTGGATTGATGAGCTTTTTTACTTTCTTTTTATGAGGTTTTAACATTTCCATCACT
TGTTCGACACGGTCGATAACAACTGGTCGCTTCGCATAGGCACCATAAGCAAGAATCACTGTGTCACTTTCA
CTAATCGCTTTCATCAAATGAATATCAGTGTGCTCATCGTATGGATTTTTGATATGTTTGAGGTTTTCGGGTGTT
CTAATATTTGAAAATAGATTTACAAGATATACAGCACCATATTGTTCAGTATTAGCTAATTGATTGAGGATAAGA
ACAGTTGTGAGATCGAGTGATAATACACCATCTAAATGAGGGTACATCGTTATCACTGTACAAGCGGGTTTCT
TTTCATCCCATGTTTTCTTGAGTAAATAGCGGTGTTGTTCATCATCGCTAAATATGGCTTCTGTGTTTATCGTAC
TTTTGATTGTATTCATCATCGTCACTCCTTTTAGTATTCTTCTGGTAAAAGCATCACATAATAAAAAACATCTAC
ATCATCTTCTCGGATGACGTAGACTTTCTTAGGTAATGCATTTTGATTTTTTATATAGTTTGTATAGTGATATTCC
AATTTGTACGTGGGTTGTTCTTGCTCATGTATGATTGAAAGTATATTCTCATCTTCTTTCAGTCTAAAAATGTGT
AGGTAATCTGTATCAGGTTGATTATCTCTTTCTTTTACCATGTTCCAAAGTAAGATTTGAAGATCTAGAGATAG
GTGTTCACTAATGCCTCTTGTGATATATCGATTGATGTTCATACTATTTTCCTCCATTTTGCTTTTCTTTCATGAT
GTCAATCACTTCGTTAATGACTGTAACAGATATTTGTGCCACTTTGATCCAATTATTCATGGCGAGTCCCTCCTT
CTTTTAGTAGATGACGTTCATCGATAATCGTATTTTTAGTATCTGTGAGATATAAAAAGTCCATGTCAAAATGAT
CCAGATAACCGATACTGATGAGTTGGTTATTGGTGTACATTAGAAATGGATAGATACTTAGCTCATGTAGCTCA
TCATTATAGTAGGTATAAGTGTCAAGTGTAAGATGCGCAAGTGGGGTATGGTCTACAAAGCGAGTAGGAAGT
ATATTTTCTGCTGCTTCTTCTAACGCTTCACATTCCCATGTTTCATTGTTAGATAGTTGGAATAGACGAGTTATA
TATTGTTTGAGTTCTTGAGTGGTTGTTTTCATATCATTGCCTCCTAGATAGTGTTAGCGATGTATTTCATATACAT
CACTGAGATAATATATATTTGATTTATCATTTATTACGAATCCCGGTGGGAATAAGAGAAAATCCCATATGAAAA
ACTGCTACAAACATTGATATGACAAGAAAATCCCGTTATCCCGCTCATTTGTTGGGAAATATAGCTATATATTTA
TATATCATTTGGATTTGGTGGTGTTATTACGACTTACTTTTTCTTTTTATATTATTTATTTATAAATAATAACGGGA
TTTTGGGATTACGCTTGCGTAATCCTTCTCCTACATGAAGTTAGCCAATCCCGTTACTATCCCACTTATATTTTG
TTTTGGGATGTTCTTTCGTCATATCAATATAAGCTTCACATTGAGCCATGATTTGTTAGCGTGTTGAATATATTT
GTTAGATGAGTGTTATTGAATGACGAAAGGACAGTACATAGATATTATATTTGAAAGTAGAGTGTTTGAGGCA
AAAACAAAGACGAAGTGCTGAGAAGCACTTCGTCTAGATTATTATTATTGAAAAGTTGTTTAATAATTTCATT
ATTAAGTTTTAGTGTAACAAAGAATTGCTTTTTATGATTCTCATCTTTACGAATGTCAATACGGTCAATAACCGT
TAGGTACAAAGCTTTGAGCTGTGATTTATCCATGGATTCTATATTTTGAAATATTCGTTGTAATAAAGCAGCGA
TTTGTTTAGTATCATAAAATGGTTTCTCTTGATTTTGTTGGTGCTTGAGTTGATTAATTTGGTTTGTGATATCAT
TCAGTTGTGTTTCATATTGATGAATGGTTGGTTTGAGTGCAGATGTTAAGTCTGGATTGTCTTCGATGGTTTG
AATTAGATTTTTAAGTTTAGTGCTAATTTCATCAAATTGTTGTTGTTTATAAGCAATATCATGGTTAAGTGCAGC
TACATCGACTTGATTCTCTTGATTGACACGTTCGACAACTTGTTTGAGAACTTTATCACTTTTGACAATTTCAA
GTATTTGGTCCATAACATATTTTTCAATGACATCGGCTCTAACACTATTCGCAGAACATACCTTTGAGCCTTTAT
TTCGAAAATTACTACACGAATAGTAACGAATACGTTTTTAGTGCCATCTTTAAGTGTGTTTGTGGTGTTACTCG
CAGCCATAGGTGCAGAACATTGGGGACACGAAATTATTCCAGTTAAAATATTGGTTCCTTTACCATGGACTTG
TGGTTTTTCACTTACTTGTTTCTTACGTGCTTGCACTTTATCCCATAATGATTGACTAATAATAGGCGTGTGTTT
ACCTTCAGCGATTACTGGCTTATCGTTTAATCCTTTACGTCGTTTATCATTCCAATCTTTGTATTTCGCGAATTG
AATTTTACCAATATAGAATGGGTTTGAGAGAATATAAGTAACAGCTGAAATACTAAATGGATTGCCTTTTTTAG
TCACATAGCCTTTGTGATTGAATGCATTGGCTATTTTACGATAACCATGACCTTTGGCATAAGATTCAAAGATA
TATTTAACGATGTTAGCTTCATGTTGATTAATCATTAATTCTTTTTTATTATCAGGTATGTTATTATATCCTAATGG
AAGATTGCCTTGATAATAGCCCTCTAAAGCTCTTTGACGTTGTCCGGTGTAAATATTCTCTAAAATTGTATTTCT
TTCGAATTCGGAAAAACTTGCAAGTATCTGGAGCATTAACTTGCCTGTTGAATTTTTGACTTCCATACGTTCA

GACAAGCTAAAAAATTCAACATTTTGGCGATGAAGTTCTTCGACTATTGTAAGTAAATCGGAAGTATTACGTG
CCAAACGATTTGTTTTATAAACCATAACACAGTCTAATTTTCCATTTTTAGCATCATTTAACATGCGCTGTAATT
CAGGACGGTTCATAGATTTTCCTGATATACCCCGATCTGCATATATATCAACAAGTTCATAGCCGTTAAATTGGC
AATATTGCTCTATTTGTGTAATTTGTCCCTCTATGCTATAACCTTCTACTTGTCTCTCTGTGGACACACGAATGTA
GCCTCCTACAAGCTTTTTCTTCATTTTATCCATTATAATCCATCCTTTCTTTAATTATACAATTGATAATTCCATAG
TCTGCTTTACAATATTCAGTGGTTCATTTTTGAAATAGATGCCAGCGAGGCTTTTATTTTTAGAAATATTAATTT
CATCAATATAGGGGTACAACATGTTTAGCGTGAAACGTTGTTGAATGATGTTTTGGAAAGCTTTTCGAATTTG
ATACGTACTGATTGATGATATAGGTTTTGATTGCTGACGTAATGATTGCGTTTGTTCTCTGAACGTTTCTGCATC
AATTTTGTCTTGGGCTAATTTTTCTATCAGCTGCTCGTGATTGAGTGTAGTTTTAGTTTCTATATCTCTTTGTCTT
TTGAGTCGTTGTTGAATAGTATGGTTTATTTTTGAATAGAGCTGTTGATTTTGAAAGAAGTCCTGACAAGTCG
CTAAAACACTTGTTTCTAATTCTTGTGCGTTGATCCCTTTGAATTCACAGACAAAACGAGATGCATTCATATTT
TGAGGACAAACATAATAACGTAATGTATGGTGCTTTTTTCTAATGGTCATATTAGTTAGTGTTGAATCACAATA
AGGACATTTGATTTTTTGTTTGAGTTGGTTTTCTGACGGCTTACGTTTTACTGGTTTCTGAGTTCGGGTAACT
TGAGCTTCTTCGTATATCGTTGTACTGACAATAGCTGGGAACATGTTTTCATATTGTCCGTATTGATTGATAACA
CGGCCACAGTAATTAGGGTTAAGGATAATATTACGCACTTGATAGGGCTTACGATTAATGAATTTATCATCAGC
TTCTAAGTATTGCGCAATTTTTTTATAACCATAACCTTGAAGGTAATAATTGAACACAGCTTTTACTGTTGGTG
CTTTTACTGTGTCTATCGTGAAAGTACCATTATGATAGTGATACCCAAAGGGTGCATGTGTTGTAATCATTTTAC
CTTGTTTCGCTTTTTCTTTGATTCCATTTTTGACTTGTTCGCCTATATTATCAGATTCTAGTTCGGCCAAGCTGA
TGAAAATATTGAGTTTGAGCCGATCGAATGCTTTATCCATATCAAAATAGCCATCATGAACGCTTAAGATATGG
ACATGGTATTTTTGACACAATTTCATGAGTTTTAATGCATTTTTAAGATTGCGATGAAGTCGGTTTAATCTGTA
ACAACATAATACATCACATTGTCCTTGTTGAATCAGTTCAGTAATTTGTTGGTAACCGTTCCGCTTATCAGTGC
GTCCTGATTGCTTATCGCTATAAAAGGTAATGTATTGAATATTATGTTTTTTGGCTAATGCCTCGATGGTTTGTT
TTTGTGCTGCTAAGGATTGTTGCTTTGTAGTGCTCTGTCGTAAGTAACCTATTGCTTGTTTCATCGTATTTCCTC
CTTCCAAAGTGATAATATATATTTATGAACGAATTTATAGATGAGCCCAACACCTGCTGGTGTTGGGCGTTATT
ATTAGTCATCAGCATGATTAATTTCTTCAAAAACTAAATCAGCTAGTAATTCAATCAATTCATCCATTTTATTCAC
TCCTGTACGATTTCATCTTTAAGTTATTAAAAATCAATTAAATCATCATTCTGTTTTTTCCATGATTCAAGTATCT
TTTTGTTATCCAAGTTAATTTCAGCTTTAATAGGTTCAGCATCTTTAGTTAGACCAAAAATAGACGCATATTCT
GAATCTAGCTTTAAATGGTAAAAGACAAGTGACTGTTTTTTGCCATTGCCATCTTTGACTGTTCTTTTTGTTGT
AATTCGATCATGGTCAGATTCGATAAATCCTTTATCCCTTAGCGCATTAACAACATTATTAACATCTTGGAAGTG
ATGCTCTAACAACATATTTTTAAATACAGACGCAATGATTTTGACTTCGATATGATTATCTTTTAAGGCAATTAG
TCCATAGTTCTCGAACATATTCTTTAACGCTGTATCATCAGAAAATTTACCACGGTTTTGTGCTACAAATTGAG
TAATGACTTCGATCGCTTTATCTGCCAGTGATCGTTCAGAGACTGTATGAGCATGATAATCAATAAAGTAGTCT
CTGATTTTAGCGATATCAATATCTGTAGCTAAAACACGACCTAATATTTTCGCAGATGTTGTAATGACTGCATAA
CGCTTAAACATACGAATACCTGTATTGTTTGTTTCACTGTTCAATTTAGCTTCAAACCAATCTACTTCCTTGTAA
AACCATTGAATAACTTCATCTTCACGATTTATAAGGTATTGAGCTACTAACGGTAAAACATGACCATGGTTTAG
TGCCACAGCTTTTTTGATATTGTCAGCATTGGTCGCATTTGTAGTAAATTGTTCATTAATCTCGATGGTTCTTAC
ACGTAATCCATCGTTTTGAGCAGAATCAGTAAAGATACTGTATTCAGAGGTTGAAATCACAGAAGTGCCCCA
ATTCTTAGGCGTTTTAACTTCTCCGTGTACATTGGAACGTTGACGCCCTTGACCTTCAGCGATAGAGTACAAT
AACCCCGTTGTATCTTTAAAAGTTGCTGATGAGAGTTCATCAAATACAATAGGTATACCAAAATTGTTACTCAA
GTAACTTTCAAGTGCATTACGTGTGGCATTCCAACTTCGAAAAAGTGTTTGATTACCTTTAGTTGGATTTCCA
GCGACGGATACTGCTAAAGCTGCTGCTGTTGATTTACCAGTTGATGACTGGCCTGTAAAACTAAAGATAATTC
CTGCAAATTCGATTTCACGTTTGTACTTCAGAAAGCTTGTCACTAAGGCAGAAATTCCAAATACGACCGCTAA
CTCTAGAAGAAGATGACCTTTGACTTCGTTAATATACATATTTAACCAATCTTTAAATGTACCTTTAGGCTCTAA
TGGATAAGCACTATCGACAATAGGATCTAAAGATGATAATTGATCATATTGTTTAGATGTATAAATGGTATCTAT

CATTACAATATAACCGTGGGGTGTTTCTATGATGCCCGAGCCATCATATAAATCAGAAGTGGGCATTTCATCGC
GCATTAATTGTAGTGCATAGCTCAAATCTTTTATATAATTTTCATTGATACTGTGACCATATTTAATCAAGGAAG
GTAACTTTCGAGTTGTTAAAATATCAGAGTCAAATGTATGTTCTTTATCCTTACCGTTAGAAATAATTATTTTTT
CTGTATTTTCTAAGACATGCCAAAATTTAGCTTCAACAGCCATACAACTAGACATAGTCACTACTTTTTTCTCAT
CTCCATCTTTTTTAGGTGGGATAGTTTTATGCCAACCCATTGTATCTAAATGGTATAGGCCTAGTCTAAAAATGT
CATGATGACTCATTAGCGAACACCTCCTTTCGAAGGGTTGCTATCATTGAGTGGATTAGGTCCGACTTTCATA
TATACTAAATGACCATTGGTATTTTTACCGATAATAATAAATGGAACACGTGGCGCTTGTTTTACAAAATATGC
GAACCAACGTCCAACATTTTGTTGTACAGCTTTTGAGCATTGTACATTTGCACGACGGTTCAAGTCATGGAA
AGTAAATGTTTGTCCTTCTGGTAAATTAATTGAAATACCTAAAATTCTATTTTTTAATTTTTTAAAATCATCTTCT
TGCTTGTAAGACATTTGAATTCCTCCTAAATATTATTAATTAATTCTATCTTCTGGCTATTTCAGAAGATTACATT
GCTTATAATAGAGGTGTACTGTTTTTATTGCTCGAATAGTATTGAGCAAAAGACGAACACATAAATGCAAGCC
TTTTTGCTTGACAAATTAGAAAAGAGGTAAACAAAGTGTATGATTTTTTAGAATACTTACATCGTTATTGTATA
AAATCAATTAATAATTGGGAGGCTGTTGCAGACATATCATGCAAAGTTGAACGTGGAAATGTTAGTACGGGC
TATTATGTTAAACAAGATAGAATTGTAATAGATGATAGTGATATACCTCCAATTATTAAAATAAGTGGAAGTTAT
ATAGCAGCAGACATATTAGTTACTAGATATTTAGATGGAATGGGTGAATTTATAAAGGTATCAAAATTAGATCA
ATCGAGCTATTACGAATGGTTTATGTTTGTTGAAGAAACGAGAAGAATATTTAGAGGTACAGAAATAGTTTAT
CAATTTTTAAAATATCTATTTACGCATACCTATGAACATAAAAATGAAGAAAAAAGAATAGAAAAACATATGAA
AAACATTATCATAGATAATCAATTTAGAGGGCAGCATATCAATGATATCGTTCGTTTAGACATGAAAGATGATA
ATATGTTATTTTACAATTTTGATATATACGATACAAATGTTCTTTATAAAGAAATAGACGAGGAAAAATTACTTA
GAATTTTAGATGAAATAAGTAATACTGACAATTACAAAGCGCTATTTAACATTATAAGTGAGAATCAAAAGAA
ATTTTGGAAATCAAATATTAAACCAATTGTAAGTGAATGTTCAGATGGTATAAATACAACCACATTTGATTTATA
TGCTTATGTAAATAAGTATATAAGTAATAAAATATTTGACTGTGATAATTATTTAATTACTAAAAATGAGGTTGTG
GATTTCTATTTTAATCATGGTAGTGACTTTAATAATTTCAAGATGGGACAGACAGGCACAAAGTCCAAATTTA
AGCAGGGAAATAAACTTAAAATACACGAATATATCATTGCAGTAACTTATGTAAATTTATTAAATCATTCATATA
TGAAATATATCAATGCTGAAAGTTACTATGACCCTAGCAAAAAAATAATAAAAGGAATGAAAAATGATTATCAT
AAATTTACAGATTCATTCAGTGATTTTGTAGAAATGGTGGACGATATAACATGGAGAATCCGTGATTCGTTTG
ATTATATTGGAGGATTAAAAAATAAAGAAATTAGCGAAGATGAGAACGAATATATCGAACAATTAATTCAATT
TGTTATATTATCTAAATTCGGCTATATGAATGTGGAAAATCATTTTAATTATATTGAGAACTTAAATACACTTAAT
AACGAATTAACGACTATATTAAGAAGTGAATTCGCGCACATTTCAAATGATATGTATAATAATTTATTTGGTAAT
TTATTCGATGTTGTAAATACATGGAAAAAAGATTATTCAAATAATTTTTATAATAATACTCAGAATACTATACCTA
CAGAAAAATTAGATATAATATCTTCATTTATAAAACAATATGATAAACCAATTGAACGAGACGAACATGGCAAA
CACGTTAAAGCAAAAGTGTTTGAAGGTATAAGTGATTTTCATAAGCGTGTAAAAAAAGGATTTGATAATAAT
GATGTGCTATTGCATTTGAGATTGGAAATGAGTGTACATGAAAATAAGTATGATTAACTACTAGTTTATCCAAT
AATATAAAAAGGACTATAAGCTATATCTAAATACTTATAGTCCTTCTTCATTAGTATAAATATTAAGAAAGTTGTA
TATCTTTATAACCTTCATTTACATTAATAATGTTGTGATAACCTTTGTGTTCTAAAATACCAATAGCTATCGAACT
TCTAATGCCAGACTGACAGTGTACATAAATAACATCGTTTTTATTGAAAGGTAAATCTGTTTCTAAAAGTTTGC
CGTGTGGTACATGAACCGCTTGAGATAAGTGGCCATTATTCCATTCATTATCATTACGTACATCTAATATATGTG
ATTCGTTACCTGTAATGTCTTCACTATGAATGGAACGTGTTTGAATCTTAGATTGCGGTAATTGATATCCAGCA
ATATCATCGTATCCAATGAGTTGTAAGGTGTGTGTTGCTTTTGAAACAAGGTGATAGTCTCCAATCAAGTTAAT
TTCTTGATCATAGTTTAGATACCAGCCAATTTGATTGATGAAATTTTTATCATATGGAATATTGATTGTACCTTCA
ATATGTCCACCATGATAAGCCTCCTTACTGCGGAGATCAAAAGTTAATCTGTTTGTATTTGTAGCTGGATAAAC
CGTATAAGGTTGATATAAATTCATACCGAATTGATTAATTTTTTTCATTTGTGCAAAATGATGTGGTGGTGCAG
GTTGGTCAGAAATGAGTTTATCGATAAAGGTAGCTTCGTTATTTTCAGAAAAAGCCCAGTTCGTTTGTTTTTC
ATAGCCAAGAGTAGATGTTGGAATAGCACCTAAAGATTTACCACAAGGACTACCAGCGCCATGGCCAGGCC

AAATTTGAATGTAATCTGGCAAGTCTTTAATACTTTCAATCGATTTAAACATTTGTTTAGCGCCTATTTCAGATG
ATCCTTCTACTTTAACCGCTTTTTCTAGTAAATCAGGTCTACCGATATCTCCTACAAAAATAAAATCACCACTGA
ATAGTCCCATTGGAACTTGTGCTCCAGCACCTTCGTCAGTAAGTAAAAAACTTATACTTTCTGGCGTGTGACC
CGGTGTATGAAGCACTTTTAATTTTATATTTCCTACATAAATATCGTCATTATGTTGAACAAAATGAGTGTGGTT
AGGCATATTTTTATAACCTAATGTGTCATCACTTTCACCCGATACATAAATATTAGCATTTAACTTTATAGCAACA
TCTCTAATTCCTGAAGCAAAATCTGCATGTATATGTGTTTCAGCTGCATGAGTAATGGTTAAACCTTCTTCATCA
GCAACTCGAATATATGAAGATAAGTCACGAATAGGATCAATAATCATGGCTTCTCCAGTTTTTTGACAACCGA
TTAAATAAGATGCTTGAGATAAATGTTTATCATAAAATTGTTTAAAAAACATAATATCATTCCTTTCTAATTATAT
AAATAAATTGTGATTAGCTTGTTCTGTATATCCAATATATGCACCTACGCCACCGTATTCTACATCATCACGTAGT
TCTTCTTTTGTAATGCCCATCACATCCATACTCATTGTACAAGCGATAAGTTTAACACCTTGCTCAACTGCTTGA
TCAATAAGAGAAGGTAAAGTATCGACATTTTTCTTTTTCATGACATAACGCATCATTAGATTGCCTAAACCAAA
CATATTCATTTTAGAAATAGGCATGTTAATTGGTGAATTTGGAAGCATGAAGTCAAAAAGTTTTGCAATACCT
TTTTTCTTAATACGTTGACTTTGAATTTTTTTAAGTGCATTTAAGCCCCAAAATGTGCAAAAAATGGTTACATC
TCTACCTGCAGCTTTAGCACCATTAGCAATAATCATTGCTGCAACTGCTTTATCTAGCTCGCCGCTAAATAAAA
CAATTGTTGTTCCACTTTTCGTGTGTGTCACTTCTATATTTTTATTTTCTTCTTTTTGAATAATAGCGCGAATTTC
ATTGCCAAAATCGTTGAGTCTAACAAGCGTATGTCCAGTTTGTTTGACCCAACTTTTAATATCATTTAAAAATC
CGTGATCAGTCACAACAACTTCTATTTGATCGCCAATAGCGATGTTTTTGATTTCTTTACTAATATTAACAATAG
GTCCTGGACACTGTAGATTACTATAATTGAATGTCTTACGATTATCTTTAATTTCTCTATCTTCTTTTACTTTAGA
TAAATTATCATTATGATGTTGTTGTTCGTATGCGGTATAACCTCCGTCTAAATTCACTACATCGTATCCGCGTTGT
GCAAGAAATTGACTGGCTTTTCTACTTCTATTACCACTTTTACAATAGATATAGTAAGTTTTATTTTTGCTTTGT
TTAAAAGTCTCTATATTTTCAACTGAATGTAATAAAGCGTTTTTAATGTGTCCTAATTCAAATTCTTCAGGATGT
CGAACGTCAATTAATTGACCCTTAGAACTCAATTCTTGTAATTCTTTTTTAGTGAAATCATTAATGTGTTTCGTT
TTATATTGTGACATAATTGCCTCCTTTTAAATACCTATGGGGGTATATTATAACGAATTAGATAGTTTGTCAAATA
CCTATGGGGGTATTTGACAAACTAAAATTTAATCAATATGATGTAATTATTAAAAATGAATGTGGAGTGAAATA
TATTGGAATATAATAAAAAGATGATTAATCGTATTCATCGCATACAAGGACAGCTTAATGGAGTCATTAAAATG
ATGGAGGAAGAAAAAAATTGTAAAGACGTCATTAGTCAATTAAGTGCATCTAAAAGTTCTATTCAACGTTTAA
TGGGTATTATTATTAGCGAGAACTTAGTAGAATGCGTCAAAATGTCTGAAGAAAATAGTGAAGATTCTCAGG
CACTAATTAATGAAGCTGTTGAATTATTAATAAAAAGTAAATGATAAGTATAACAGTAATTATAGTAATGTTAGT
TATTGGTATATTAGGAGGATTTATTTCTGGATTAGTCGGTATAGGGGGAGCCATTGTTATTTACCCTGCTCTCTT
ATTATTACCACCATTATTTGGTTTACCTACTTATAGTGCTTATATTGCCTCAGGATTAACTTCAAGTCAGGTGTTT
TTTAGTACATTGAGTGGATCTTTAAAAGCATATAAAAATAAAAATTTTTCTAGAACGCTTATACTTAATATGGG
AAGTGGAATGGTAATAGGTAGTATTCTAGGTGCGTTATTAGCAACTGTATTTAATAGTCAGTTTGTTAATGTAA
TTTATATAATTATTGCTTTGTTAGCTCTTATTCTAATGTTTATTAAAGTCACACCATCTACTAGTCATATTAAGTTT
AATCGCGTACTTTTAATTACAATAGGTGGTATCATCGGTTTAGTATCTGGTATTGTAGGAGCTGGTGGAGCATT
CATTATTATTCCAGTACTACTTGTTATATTTAAATTACCAATGAATATGGTTGTTACAAATAGTATTGTAATTGCAT
TCATTTCTTCTATTGGAGCATTTATTATTAAATTATTACAGGGTTATATTCCTATCAATAGTGCAATACCGTTAATT
TTAGGTAGTATACTATTTACGCCTCTAGGCATGAAAATAGGGCAAAAAATACCTGATTCTATTCAAAAAGGGA
TTGTAAGTATTTTAATCGTCATTGCAATCATTAAGTTGATATTTTAAAAATATGGGAGTGGGGCAACATTTATTT
TGAATTTGTAGTCTCGCTCCCTAAATCAATTTATACGGTATTTAATTCAAAGGTTATTTATTAGAATCTAATCAAT
TTTACAAAATTTTAACAAAAAAAACTTTTTCTAAATTGATATATAATAGCGGGTATGGTAATTTTAAAATAGATA
CTCGTATCTAACTAATTTTTAGGGGTGGTTAATTGTATGAATAAAAATTCGAAGAAGAAGCTCGATTTTCTTCC
AAACAAGCTTAATAAGTACTCAATTAGACGTTTCACTGTAGGGACAGCTTCGATTTTAGTAGGAGCTACTTTA
ATTTTCGGTGTTGCAAATGATCAAGCAGAAGCCGCTGAGAATAACACAACTCAAAAGCAAGATGATAGTTCA
GATGCAAGTAAAGTAAAAGGTAATGTTCAAACTATTGAACAATCTTCTGCAAATTCAAATGAATCTGATATTCC

TGAACAAGTTGATGTAACTAAAGATACAACTGAACAAACATCAACAGAAGAAAAAGCAAATACAACTGAAC
AAGCATCAACAGAAGAAAAAGCAGATACAACTGAACAAGCAACAACAGAAGAAGCGCCAAAAGCTGAAG
GAACAGACAAAGTAGAAACAGAAGAAGCGCCAAAAGCTGAAGAAACAGACAAAGCAACAGAAGAAGCA
CCAAAAACTGAAGAAACAGACAAAGCAACAACAGAAGAAGCGCCAAAAGCTGAAGAAACAGACAAAGC
AACAGAAGAAGCACCAAAAACTGAAGAAACAGACAAAGCAACAACAGAAGAAGCGCCAGCAGCTGAAG
AAACAAGCAAAGCAGCAACAGAAGAAGCGCCAAAAGCTGAAGAAACAAGCAAAGCAGCAACAGAAGAA
GCGCCAAAAGCTGAAGAAACAGAAAAAACAGCAACAGAAGAAGCACCAAAAACTGAAGAAACAGACAA
AGTAGAAACAGAAGAAGCGCCAAAAGCTGAAGAAACAAGCAAAGCAGCAACAGAAAAAGCACCAAAAG
CTGAAGAAACAAACAAAGTAGAAACAGAAGAAGCGCCAGCAGCTGAAGAAACAAACAAAGCAGCAACAG
AAGAAACACCAGCAGTTGAAGACACAAATGCTAAGAGCAATTCAAATGCTCAACCATCAGAAACTGAGAGA
ACTCAAGTTGTAGATACAGTTGCTAAAGATTTATATAAAAAATCTGAAGTTACAGAAGCAGAAAAAGCTGAA
ATTGAAAAAGTATTACCAAAAGATATTTCAAACTTATCTAATGAAGAAATTAAAAAAATAGCTTTAAGTGAAG
TACTTAAAGAAACAGCTAACAAAGAAAACGCACAACCAAGAGCAACATTCCGTTCAGTAAGCAGCAATGCT
AGAACAACAAATGTTAACTATTCAGCAACAGCATTAAGAGCAGCTGCACAAGACACAGTTACTAAAAAAGG
AACTGGTAACTTTACTGCGCATGGAGATATAATCCATAAAACTTATAAAGAAGAATTCCCTAATGAAGGCACG
CTAACTGCATTCAATACAAACTTCAATCCTAATACAGGAACTAAAGGCGCATTAGAATATAATGATAAAATAGA
TTTTAATAAAGACTTTACAATTACTGTTCCAGTAGCAAACAACAACCAAGGTAATACAACAGGAGCAGATGG
CTGGGGCTTCATGTTTACTCAAGGGAATGGCCAAGACTTCTTAAACCAAGGTGGTATTTTAAGAGACAAAG
GTATGGCAAATGCATCTGGTTTTAAAATTGATACGGCATATAATAATGTTAATGGTAAAGTCGATAAACTCGAT
GCAGATAAAACAAACAATCTAAGTCAAATTGGCGCAGCAAAAGTTGGTTACGGTACATTTGTTAAAAATGGT
GCAGATGGTGTGACTAACCAAGTTGGTCAAAATGCCCTAAATACAAAAGATAAACCTGTAAATAAAATAATTT
ATGCAGATAATACAACTAATCATCTTGATGGTCAATTCCATGGCCAAAGATTAAATGATGTAGTATTAAATTATG
ATGCAGCAACAAGTACAATAACTGCTACATATGCAGGAAAAACATGGAAAGCTACTACAGATGATTTAGGAA
TTGATAAATCACAAAAATATAATTTCTTAATTACTTCAAGTCATATGCAAAATAGATATTCTAATGGAATTATGA
GAACAAATCTTGAAGGTGTAACAATTACAACGCCTCAAGCTGATTTAATTGATGATGTGGAAGTAACGAAAC
AACCAATTCCTCATAAAACTATTCGTGAGTTTGATCCAACTCTAGAACCAGGCTCACCTGATGTTATTGTACAA
AAAGGTGAAGATGGAGAGAAAACAACAACTACACCAACTAAAGTTGACCCTGATACAGGAGATGTAGTTG
AACGTGGTGAACCAACAACAGAAGTTACAAAAAATCCAGTTGACGAGATTGTACACTTTGCACCTGAAGAA
GTACCACAAGGTCATAAAGATGAGTTCGATCCAAACTTACCAATTGACGGTACAGAAGAAGTACCAGGTAAA
CCAGGCATCAAGAATCCTGAAACAGGTGAAGTAGTAACACCTCCGGTTGACGATGTCACAAAACATGGTCC
AAAAGCAGGCGAACCAGAGGTTACTAAAGAAGAAATACCATTCGAGAAAAAACGTGAGTTCAATCCAGAC
TTAAAACCAGGTGAAGAGAAAGTAACGCAAGAAGGACAAACTGGAGAGAAAACAACAACAACGCCAACA
ACAATTAATCCATTAACGGGAGAAAAAGTAGGCGAAGGTGAACCAACAACAGAAGTAACAAAAGAACCAG
TAGATGAAATCACACAATTCGGTGGAGAAGAAGTACCACAAGGTCATAAAGATGAGTTCGATCCAAACTTAC
CAATTGACGGTACAGAAGAAGTACCAGGTAAACCAGGCATCAAGAATCCTGAAACAGGTGAAGTAGTAACA
CCACCAGTAGACGATGTCACAAAACATGGTCCAAAAGCAGGCGAACCAGAGGTTACTAAAGAAGAAATACC
ATTCGAGAAAAAACGTGAGTTCAATCCAGACTTAAAACCAGGTGAAGAGAAAGTAACGCAAGAAGGACAA
ACTGGAGAGAAAACAACAACAACGCCAACAACAATTAATCCATTAACGGGAGAAAAAGTAGGCGAAGGTG
AACCAACAACAGAAGTAACAAAAGAACCAGTAGATGAAATCACACAATTCGGTGGAGAAGAAGTACCACA
AGGTCATAAAGATGAGTTCGATCCAAACTTACCAATTGACGGTACAGAAGAAGTACCAGGTAAACCAGGCA
TCAAGAATCCTGAAACAGGTGAAGTAGTAACACCACCAGTAGACGATGTCACAAAACATGGTCCAAAAGCA
GGCGAACCAGAGGTTACTAAAGAAGAAATTCCATATGAAACTAAACGCGTATTAGATCCAACAATGGAACCA
GGTAGTCCTGATAAAGTAGCTCAAAAAGGTGAAAATGGTGAAAAAACAACAACAACACCAACTACAATTAA
TCCATTAACGGGAGAAAAAGTAGGCGAAGGCGAACCAACAACGGAAGTAACGAAAGAACCAATAGACGA

AATTGTTAACTATGCACCTGAAATTATTCCTCATGGTACACGTGAAGAAATTGATCCAAACTTACCAGAAGGT
GAAACTAAAGTTATCCCAGGTAAAGATGGCTTGAAAGATCCTGAAACTGGAGAAATCATTGAAGAACCACA
AGATGAAGTAATCATCCATGGTGCTAAAGATGATTCAGATGCGGACAGCGATTCAGACAGCGATAGCGATTC
AGACGCAGATAGCGATTCTGATGCAGACAGCGACTCAGACGCAGATAGCGACTCAGATTCAGACGCAGATA
GCGACTCAGATTCAGACAGCGACTCAGACGCAGACAGCGACTCAGATTCAGACAGCGATTCAGACGCAGA
CAGCGACTCAGACGCAGATAGCGACTCAGATTCAGACAGCGATTCAGACGCAGATAGCGATTCAGATTCAG
ATAGTGACTCTGATGCGGACAGCGACTCAGACGCAGATAGCGACTCTGATGCGGACAGCGATTCAGACGCA
GATAGCGATTCAGATTCAGATAGCGACTCTGATGCGGACAGCGACTCAGATTCAGACAGCGATTCAGACGC
AGATAGCGACTCAGATTCAGACGCAGATAGCGATTCTGATTCAGACAGCGACTCAGACAGCGATAGCGATTC
TGATTCAGACAGCGACTCAGACAGCGATAGCGATTCAGACGCAGATAGTGACTCTGATGCGGACAGCGACT
CAGACGCAGATAGCGACTCTGATGCGGACAGCGACTCAGACGCAGATAGCGACTCAGATTCAGACAGCGAT
AGCGATTCTGATGCAGACAGCGACTCAGACGCAGATAGCGACTCTGATGCGGACAGCGATTCAGACGCAG
ATAGCGACTCAGATTCAGACAGCGATTCAGACGCAGATAGCGACTCAGATTCAGACGCAGATAGCGATTCTG
ATTCAGACAGCGACTCAGACGCAGATAGCGACTCTGATGCGGACAGCGACTCAGACGCAGATAGCGATTCT
GATGCAGACAGCGACTCAGACAGCGATAGCGATTCTGATTCAGACAGCGATTCAGACGCAGATAGCGACTC
TGATGCGGACAGCGATTCAGACGCAGATAGAGATCATAATGACAAAACAGATAAACCAAATAATAAAGAGTT
ACCAGATACTGGTAATGATGCTCAAAATAATGGCACATTATTTGGTTCACTATTCGCTGCGCTTGGAGGATTAT
TCTTAGTTGGCAGACGTCGTAAAAACAAAAATAATGAAGAAAAATAATATTTAAACTTCATAATTTTGGGTAA
AATTTAAAACCAGGCCTTACATGGCCTGGTTTTATTTTAAATACTATGATATAAATTAATGGAAATAGGACAAA
TATATAAAAATAAGGAGGAAACAATGGAATTTGAACATAAATTAGAGAAATTAATATCTGAAGTAAATAATAA
GACTGAAATTAATAATTATGTTTTTTTTAGTTTAGGTAAGTCAAGTGTTAAAGCACAGGTTAAGTTATTAAAAA
AAACTAATTATCTCAAACAAGATATTTCAAAATTGGCACTTAAATTTAAAAAGAAATCTGGGGAATTTCCTGA
GTGGATTAAATTAGATATTGTAACCTCGACTGAAAAAATATTATTTAAAGAATTAAAAAAAACACTGATCAATA
CAAGAAGAAATTATGTAGATTTTGGTATAGCATTTGATAGTCAATGGAATTTTGCAGTATTACCTGAAGAAATT
AACGCGAATGCTTTTGTTCGACCAGATAATACTACTAAAGAGTTATTTCTTTCGGAAAAAAACATCAATAATTA
TTTACGCAAATATACTACAAATAAAAAGGCATTTTCTAGTGAGTTCTATAATGAAAAAGAAGTTATTAAATTCT
ATACACAAGGTTTCTTTATAGGTGATGAAGAAGTACATGAACTATATAGCGAAGGCTATAAAAAAGGTTTAAG
AAAAGTAAATGATTTAAATAATGAAATTGATCAATTAATTGAAAGTAGTACTAATTTCTTACAAAATATGTTGTT
AGATAATGGTAAATATATTTATGGATATTTTCCTCATTTTGATAATGAAATTGGATTCTACAATGTTTTAAGACAC
TCTTCTTCTACTTATGCCTTAATTGAGGGTCTGTCCTATTTAGGTAAAAGCTTACAACCTGTTGAAAAAGCAAT
TGATTATATCATTTTAAATCAACTGTTTGAGATTGGTGATAAAGCCTATATCTTTGATGATACAGAGGAAGCAA
ATGAAATCAAATTAGGACAAAATGCTTCATTTATATTCGCAGTTTGTGAATATTTAAAGCATGAAGATAATCCT
AAATTTCTTGAGTCAGCTCAGAAAGTGGCTAAAGGTATTCTTTCAATGATAGATGAAGATACATATGAGACAA
CTCACTTATTGAATTATCCTGATTTAAGCGTAAAAGAGAAGTTTAGAATTATTTATTACGATGGTGAAGCAGC
GCTTGCTTTATTGAGATTGTATCAAAAGGATGAAAATGAACTATGGCTAAAAACTGTAGAAAATTTGATGGAC
CGTTTCATTGAGAAGAAATATTGGCAATATCATGATCATTGGTTAGGATATTGTACGAATGAATTAGTTCAAAT
TAATCCACAAGACAAATATTTTGAATTTGGAATCAAAAATGTGAATAATTACTTAGATTATATTAAAAATCGTG
AAACAACATTTCCAACATTCTTAGAAATGTTAATGGCCACATATAGATTAGTTCAAAAAGCGAAAGATACGGG
TCGCGAGGAATTGGTAAACAATTTAATAGATGAACAATATTTGATAGATGTAATTAATATTAGAGCAGATTATC
AAAGAGTTGGATTCTTTTATCCTGAAATTGCTATGTATTTTAAAAACCCATCAAGAATACTAGGAAGTTTCTTT
ATTAAACACCACGGTTATCGTGTTCGAATTGATGATATCGAACATTATTTATCTGGATATGTACAATATCAGCTT
GCATTTAATAGGTAAAATAAGTATTTGAAATTTAAATTAATGACTTACAAAAAATGGTTGATTTACATAAAAAC
GAAATCAACCATTTTTAATTTGCTCTTTAATAATTATTTTTCATATAACTAAAATTCTAATTTCATAAAGAATAAC
TGAAAAAACTAAACAACATGTGAGCTATTCATAACTTGAATTAATTGTAGAAAATCTATCTTCCCATAACTTCAT

AACAATTTTACTTGAAAAAGCTTTAGAGTGCTTGATACATTCTTGAGAAAAAGTATCTCTTAAAGTCTTATCTA
ATAATAGGTTTCGCACTCGATTATATAATTCATCTTTATCATTTTTGTTAATTAAATAACCATTTTCATTATTTAATA
TTAATTCGGAAGGACCATACTTCGAATTATAGCCAACTGGAGGTATGCCTTCAGTTATAGTTTCTAATAAACCT
AAATTAAATCCTTCCATATTACTAGTAATTAGAGACATATAAGCATCTTGAATCTCAGCACTTAAATTTCTTCTAA
ACCCTCGTAAAAACACATTGTTTTCTAAATTATATTCAGTGATTAGTTGCTTGTATTTTTCTTCTTCTTTACCAAA
CCCATACAAATGTAGTTGGATGTTAGGAAATTCTTTAATTAATTTAGAAACTAATTCAATTTGATGATTCAACT
GTTTTTCAGGAGAATAACGTGCAACGGAAATAATTTTATTATTATTTATACTATGATTATTTCTTTTTAAATTAGT
AAAATGTTCATCAATATATCCTACTGGTATAGTATGAACAGGGATTTCATTATTTATTCGAGCTGATATATCCAAT
TGTTGCTGTTTAGTCGATACAATTATCCCAGAGTATCTATTTAGATTGTTAAAAACATGTTTATAAGTATTTTTG
ATGTCCGATTCATATACCATGTCAATATTCTTGACATGAGTACTATGCAAAACAGCTAAAACAGGTATTGTTTCA
CTAGTATTATTGAATATTGGTGCAGTATTTATATTTTTATCACTCAGGAAAATATCTCCATTTTTATACAATTTTTC
AATTGCAAATGCTAGTAATTCCTGATCATTATTAAAATACTTAAGTTGAGAATCGGTGTTATAAATAATAAGTTG
TGATTGTGAATTTTCAAGTTCTGGATTAAAAAATTTTTGGAACTTTATAGTACCTTCAGGAGTGTAATAATGTT
CACAAACTACTTTTTGATTTGTTGTTAAAGTTCTTGAACAACTTAAAAAGCCTCTTGAATCATATAGATCTCGA
CGTATTTTACGTTGATTTACATCAAAATGATTTATGTAATCCAAATGTTGGTAATTACTATCATTAAAATGGACAT
ACATTCTATAGTTGCTTTTTTCATATATCTTTATATCATTGGTATTTTCAATAAATTTTAGTGTATAATTACACTCTT
TAGTCCAATGAGTGATCCAGTCCTTATGGATGCTTTTTATATTTATACTGTCTTGTAGCACATCATATAAGCTTAA
TATATCGTCATCTTCAATTTGAAATAGACTTGCATTAAAATGTAAACGAGGTGACCACGATGCAAAAATCAATT
TACTAGTTATCGAGTGATTTTTAAATAAGTTGTGTCTATTAATCATCGCTTTTTCAATTCCGGTTAATTTATTTCC
TAACCTATTCCCTACTGAATATATCATTTTAATTCAACCTCATTTTTTGTTATTTATTATTTAGTTTATTTTTAATTT
CATTTAAGTTTGGGTAAGAGGTAAATATAATAAATTTATCTTTATCTTTTATAATACCGCTATTATATTTCCTGCC
AATAATTATTTCGTTAGTAAATTTAAATAGGGTATTTTTATATGCTTTTAAAATTTTAAATAAGTCGTCAACAGT
GAAGTTTTGTCGTTCTCTCATAGTATGACGTCCAGTTAAATTTATTGCTACAGAATTTGAAAGACTTAAATGTT
CTATTTTTGAATTGATATATTCATCGCGCTTTGGACCGTTTTCAAATAAATACTTAGATAATTCATAAGAAGGTG
TATACATAGGTGAATTAAGCATGGCTTTCATCAATTCGTAAGTTGTCGTTTCTTTTCCTTCATAATAAAGTTTGT
TATTTGTTGCCCAGTTAGCAATAAAAAATAGTATCTTCTGATACATTATCATCTATAGCTTTTAAATAATTAAAGAT
AGGGCCTAAGCCTTCAATTGTACCTGAATGTTCTCTGTTATGAGATTCAATGACATTATAATTTTCATCAATAAT
CATATAACTGCGACCGTTCAAGGATTGACCATCAAATAAAGAAGTGTTCGAATTATTAATATTATATTTATTTAA
TTTCTCAGGTAATTCTTTTGCTAATTTTGGAAATTCTGTACCACCTGCTGATGATTTTAATAGAGTTAATGAATC
TGGTTTATTTAGGTACAATAAATCTTTTTCTAATAGATGTCTATTAGAATACCATGTTATATTTTTGCTTTTTACTC
CTGTAACGACACTCTTTAAAGCATCATCCATACATAGAATATAATCTGCATTACTATTTTCTAGAACATCAACTA
ATTGCAAATGCAATGATTTAGAGTGCTTACCTAAGTCACTAATTTGCCCAATCGCAATAATTTTATTACCTTTAA
AAAATTTTGTTTGAGTATTAAATGCTTTTATAGCATTAATCATTGCTGGTAATGAAGCGTTGTGTGTATCATCTA
TGTAAGTTAACTTTATAGTTAGGTGTTTCAACTTCTTTTAGGTTTAAAACTTTTTCAAATGGTTTAAATGTACTT
AAGTTCTCTAAGGCTCTTTCTAGTGGTATATCTAAATGCGATAATGTAGCAAATGTGGCTAGAGAGTTTTCAAC
CATACCGTCACTTATTGAATTAATTCTATAAGTATATTTTTGTCCGTTAAAATCAATTGTTATAACTGTATAGCCTT
TACTATATTGAATAGATTTAGGACAAATTGTTGCGCTAGAGTCATGTGTACTATAAGTAATAACATTACTTGTAT
TTTGTTTGGCGCGTTCGATTAATATATCTGAATGTAATGTATCTTTATTAATTATTGCTACTCCCTCAGGAGTTAA
ACCATCAAAAATACTTGCTTTCACTTCTACAATATTTAGAATATCTTTAAATGTAGACATATGAGCAGCACCTAT
ACCAGTAACTATAGCAATATTTGGTTTTATTAAATATGATGAATTACCAACTGCATTTAATGCGTTTAATGATACT
TCTAAAACAGCAAAATTTGGTTGTCTAATCATTTTGCATAATAATAAATATACTGCGGATCTTATGTTATTATTAA
ATCTATTTTGAAGAGGATAATAATCTTTAAGACCAGCACTTATCAGCATTCTAGTTGAACTTTTTCCCATTGATC
CTGTTATAGCAACAACAGGGTGTTTATACTGTAGTCTCATTTCTTCAGCTAATTTAGTTAGTGTGTTTGCAGAA
TCATTGATAACTAATTGAGGAGTTTTAAACTTCATGTCAGGAACATAGGTTTCAGTAATAATTAATCCTAAATC

TTCCTTATCCTTCTTTATATATTCATTACCTTCTTTTGCGTTTGCATCTCTACCAAAACGTCTCCATGTATGCTTAT
TGGGCGATATGTAGGCTGTGCTTTTATTATGAATAAAGGGGAACCAATTCTCAAAATTATTTATAACATTGTCC
TCTTTACCTTCAGCATCAAGGAGATTACCTCCTAAAATATTATGAAGTTCTTTAATTGTTAATGACATTTTGTAC
TCCTTTATAAAATGTAAAATTGTGTTTAATTTCTAAATATATATATAGTATAGCATGTTGATATACTGAAATGATTT
GAAAAATTTTTGAAACTTAATGACTTATATATTCATATTAGTTTAAAATTCGATACTATTCCATAATGATGGAATG
TGATGTATATGATTAATCAATATACATCACATTTCATTTAATGCAACTATTGCACAAGTTAATAGTATATTTAAAA
ATAGGTTAGTGAGATTGATTAATTATTGATTATGTTGGTATCAATGAAAGCTTGGAAGAAGGGCTTAAAAATA
TAAAAACTTGGATAAATCACAATACAATCATAGACACAGATAAGTTCGTCAATCTGTTAATGAATTATGATGTG
ATAGAGTTAAGTGAGGATAAATGTTAACGTTTTATTAAAACAGTCACAGAATTAGATAGAGCATTTGCAATTT
GTGCTACTGAACTTATAATCTAAGAGCTCAATAGTGAAATTGTCTTTTTTAAAGTTGTTAAAGCATAAATTTGT
TGAATTATTACAGAAAAAGATCGAAATTTAGTTACTGCTAATGGAACGGCAGCATTAGAGTTTGCCGAACTC
ATTTTAAAAGGACTTGAATTTGATAGTAAAGAAAATATTGAAAGGCAAATCTATATGTATCAAAGAGGCTTCT
ATGAGTATTGTAATAAGTATGGCAATCCTTATCAATAATTAAAAACCGCATCATTATCTGATACGCAGAGGCGT
ATCATAAGT
>Staphylococcus aureus strain AR_0226
ATGAAAATCACCATTTTAGCTGTAGGGAAACTAAAAGAGAAATATTGGAAGCAAGCCATAGCAGAATATGAA
AAACGTTTAGGCCCATACACCAAGATAGACATCATAGAAGTTCCAGACGAAAAAGCACCAGAAAATATGAGT
GACAAAGAAATTGAGCAAGTAAAAGAAAAAGAAGGCCAACGAATACTAGCCAAAATCAAACCACAATCCA
CAGTCATTACATTAGAAATACAAGGAAAGATGCTATCTTCCGAAGGATTGGCCCAAGAATTGAACCAACGCA
TGACCCAAGGGCAAAGCGACTTTGTTTTCGTCATTGGCGGATCAAACGGCCTGCACAAGGACGTCTTACAA
CGCAGTAACTACGCACTATCATTCAGCAAAATGACATTCCCACATCAAATGATGCGGGTTGTGTTAATTGAAC
AAGTGTACAGAGCATTTAAGATTATGCGAGGAGAAGCATATCATAAATGATGCGGTTTTTTCAGCCGCTTCAT
AAAGGGATTTTGAATGTATCAGAACATATGAGGTTTATGTGAATTGCTGTTATGTTTTTAAGAAGCTTATCATA
AGTAATGAGGTTCATGATTTTTGACATAGTTAGCCTCCGCAGTCTTTCATTTCAAGTAAATAATAGCGAAATAT
TCTTTATACTGAATACTTATAGTGAAGCAAAGTTCTAGCTTTGAGAAAATTCTTTCTGCAACTAAATATAGTAA
ATTACGGTAAAATATAAATAAGTACATATTGAAGAAAATGAGACATAATATATTTTATAATAGGAGGGAATTTC
AAATGATAGACAACTTTATGCAGGTCCTTAAATTAATTAAAGAGAAACGTACCAATAATGTAGTTAAAAAATC
TGATTGGGATAAAGGTGATCTATATAAAACTTTAGTCCATGATAAGTTACCCAAGCAGTTAAAAGTGCATATAA
AAGAAGATAAATATTCAGTTGTAGGGAAGGTTGCTACTGGGAACTATAGTAAAGTTCCTTGGATTTCAATATA
TGATGAGAATATAACAAAAGAAACAAAGGATGGATATTATTTGGTATATCTTTTTCATCCGGAAGGAGAAGG
CATATACTTATCTTTGAATCAAGGATGGTCAAAGATAAGTGATATGTTTCCGCGGGATAAAAATGCTGCAAAA
CAAAGAGCATTAACTTTATCTTCCGAACTCAATAAATATATTACATCAAATGAATTTAATACTGGAAGATTTTAT
TACGCAGAAAATAAAGATTCATCTTATGATTTAAAAAATGATTATCCATCAGGATATTCTCATGGATCAATAAGA
TTCAAATATTATGATTTGAATGAAGGATTCACAGAAGAAGATATGCTAGAGGATTTAAAGAAATTTTTAGAAC
TATTTAATGAATTAGCTTCAAAAGTTACAAAAACATCCTATGATAGCTTGGTCAATAGCATAGACGAAATACAG
GAAGACAGCGAAATTGAAGAAATTAGAACAGCACAAAAAGATAAGACACTCAAGGAAGTGGAAGCACCTA
AAGGAATAATTCCAAAATATAAAAAAGGTGTATCAAAGACTACTAAAAATGATTCAGAAATTGAAAAATCAA
ATAAAGAGAATAAATTAACCGGTAAAGTTGGAGAAAAATTAGCGCTAAATTACTTTAATGAGCTAATTGATAA
TAAAATAGACGAAGATAAGAAAGAACAGTTTAGGAATATTTTAAATGATAATCCAGGCTCTCAACACGGTCAT
GGCTATGATTTAGTAGCTTTTGATCCAACAAATACAGATAAAGCTGTAGAAAAATTTATTGAAATTAAAACATC
TACATCTTCTAGTATTGAGGAACCATTTTTTATGTCGCTAAATGAATGAAATGTTTGCTATGAAAGAATATAAG
CAGAAATATTTAATATTAAGAATATTTAATGTTTCCGGTAAAGAACCACAATTTTATTTTATAGATCCATATGCA
AATTATTCTGAATTTAAAGATGTAGATGATCTCATTGACAAAGTATTTAATGTAGAAGCTATTCAGTATAAAGTT
TTTGGCGAAAAAATGATTACTTGAACAAGAGCTAAAATAAAATTGTGATCTAATAAAAATAGAAGGTTCTGTTG

CAAAGTAAAAAAATATAGCTAACCACTAATTTATCATGTCAGTGTTCGCTTAACTTGCTAGCATGATGCTAATT
TCGTGGCATGGCGAAAATCCGTAGATCTGAAGAGACCTGCGGTTCTTTTTATATAGAGCGTAAATACATTCAA
TACCTTTTAAAGTATTCTTTGCTGTATTGATACTTTGATACCTTGTCTTTCTTACTTTAATATGACGGTGATCTTG
CTCAATGAGGTTATTCAGATATTTCGATGTACAATGACAGTCAGGTTTAAGTTTAAAAGCTTTAATTACTTTAG
CCATTGCTACCTTCGTTGAAGGTGCCTGATCTGTAATTACCTTTTGAGGTTTACCAAATTGTTTAATGAGACGT
TTGATAAACGCATATGCTGAATGATTATCTCGTTGCTTACGCAACCAAATATCTAATGTATGTCCCTCTGCATCA
ATGGCACGATATAAATAGCTCCATTTTCCTTTTATTTTGATGTACGTCTCATCAATACGCCATTTGTAATAAGCTT
TTTTATGCTTTTTCTTCCAAATTTGATACAAAATTGGGGCATATTCTTGAACCCAACGGTAGACCGTTGAATGA
TGAACGTTTACACCACGTTCCCTTAATATTTCAGATATATCACGATAACTCAATGTATATCTTAGATAGTAGCCA
ACGGCTACAGTGATAACATCCTTGTTAAATTGTTTATATCTGAAATAGTTCATACAGAAGACTCCTTTTTGTTAA
AATTATACTATAAATTCAACTTTGCAACAGAACCTATATTTTGCTATAATTATTAAATTAATTGCCTTTGTGTTAG
TATTCCCTGGATTACTAACACTTTGGTTAGCTGTTCTAAGTGATACAGGTGCAGCAATATTAGTTATATTAAATT
CATTACGTTTATTAAAAAAATAAAAAAGCCTAAATACTCTCTCGATTTGTGAGAAAGTATTTAGGCTTTTTGAA
TTAATAAATTTCTTACTTCTTATGTTTATTTAACAATTAGTACTGGGACAGAACTTCTTTTAGCAATCTTATGTGT
AACATCTCCTAAAACATGTTTAATATTCAAATCGACTCTATGATTACTCATAATTACTAAATCAAATTCACCATTA
TTTATATCTCCAGAAGTAATTTTTTCTAAGATTGTATCTTTAATATGCCCAAAATCTACATCTATTTTATATTGAAT
ATCTCTTTTTTCTAATTCACGTAAAAATGGAGTGAGTTTTTTCTCTTTTTCTCTAATAATATCTTCTTTATGCTTAT
TATAATACTTGACACTAATTGCAAGATCATTTTCTGTTACAACGTGATAAATTACAACAACTGAATCTTCATCGG
TGACTTTTTCTAAATAGTCAGGGATAGCACTAAAATCATTTTCGAAATCATAGGGTAATAATATATTTTTGAACA
TCTACACCAGCCTCCTATTACTTATTATTTAGATGTCTATTACTTATATATACCCTAAATATGTATGCTTATTCTCAT
ATTCAAATTATTCAAATATATTCCTTATCATGTATTAGATTCTATTTTTTATATTATTTTTGTAATTCTTTGTATAAAC
CTATTTATTTATAAAGCGCTCTAATACATACCTAAATATCTTCAACTTATAAACTACTTTTTATTTCAATGCCTTTT
TCCAAACTTCTTTTATGAGATATTGTGGTTATTACCTGACATTTACAATTTACACATTTCGCTATTATTACGCAAA
TTATTTTGATTTTATAATTATGATGACTATAATCTATTCTAATTAATCTCTCCTTTTAGGCATAAAATAATCTACTTA
TAATTAAAGCATCCACTATTTCAAAATTATTTGTAAATTGAGATTTATAAGTGATTTTTATACAGTTATTTAAAAT
CTATTTCATAATCAAAAATAATAAAGGGGGTAATAAAAATGAAAAAAGAAAAACGTTTAAATCTTATCTTAACT
GTTATACAACAGAATCAGTTTAATAAAAAACAACAAATAGTGGATTACATGGCAAGACATTTTGGAGTTTACT
ATAGCTTGACAACTATTTCTCGTGACTTACAAGAATTAGAAATTTACAAAATCCCTGTTGAAAATAAAAAGTAT
ATTTACAAGAAAATAAATCAAACAAATCAATTAAGTGCAAAAAAACAATTAGAAATATTTAGTGATGAGATTA
TTGAATTTATAACGCTAAATAACTATGTCTTAATAAAAACATCTCCTGGCTTTGCTCAAAGTATAAGTTATTACA
TAGATCAATTACAAATGAAAGAAATATTAGGAATTATTGGAGGTAACGATACTTTGATGATTTTGACTTCTTCA
AATGAAATAGCAGAATTTGTTTGTTATCAATTATTCCCTTAAAAATTAAATGATTATATTAATAGGAATGATTACT
AATACAAAAAAATGAGTAAAAAATCTTTTTGCTTGAATAAGATAAGGTTTTAACAATGAGATTTTTCAAATTAT
TCACTATTATATCGTACAAAATACATATTCATACAAAATTTTGCATTGAATAAATCTATATATGTGAAAACCCTCC
CTAAACAATTGTGAATTAAATCACACGATGTTTTTTGAAAGCGTTTTATAATGTGAATGTAATAAAGCAAAGG
AGGAATTTGAAATGGTACAAGGACCCATTCAAGTAAATAGTGAAATAGGCAAATTGAAAACTGTGTTGTTAA
AAAGACCAGGAAAAGAATTAGAAAATTTAGTACCTGATCATTTAAGTGGTTTATTATTCGATGATATTCCCTAC
TTAAAAGTTGCACAAGAAGAGCATGACAAATTTGCTCAAACTTTGAGAGATGAAGGAATCGAAGTAGTTTA
TTTAGAAAAACTTGCAGCAGAATCTATTACTGAGCCAGAAGTACGCGAGAACTTCATAAACGACATATTAAC
AGAATCTAAAAAGACAATATTAGGTCATGAAACTGAAATTAAAGAATTCTTTTCAAAGTTATCTGACCAAGAA
CTTGTAAATAAAATCATGGCTGGCGTACGTAAAGAAGAAATTCAACTTGAAACAACCCATTTAGTAGAATATA
TGGATGATAGATATCCATTTTACTTAGATCCAATGCCCAACCTTTATTTTACAAGAGATCCCCAAGCTTCAATTG
GTAGAGGAATGACAATTAACAGAATGTATTGGAGAGCACGACGTAGAGAATCTATTTTTATGACATATATACT
GAAACATCATCCAAGATTTAAAGATAAAGATGTACCAGTATGGTTAGATCGTAACTCACCATTTAATATTGAAG

GTGGAGATGAATTAGTATTATCGAAAGATGTTTTAGCTATTGGTATATCAGAACGTACATCAGCTCAAGCAATA
GAAAAGTTAGCACGTAATATTTTCAAAGATGCAAACACAAGTTTTAAAAAAATCGTAGCTATTGAAATACCTA
ATACACGTACATTTATGCACCTAGATACAGTACTAACTATGATTGACTACGATAAGTTTACAGTACATGCAGCA
ATATTTAAAGAAGAAAATAATATGAATATATTTACCATAGAACAAAATGATGGTAAGGACGATATAAAAATTAC
TCGTTCTAGCAAGTTACGTGAAACACTTGCTGAAGTTTTAGAAGTAGAAAAAGTGGACTTTATTCCAACAGG
TAATGGCGACGTTATTGATGGTGCACGTGAACAATGGAATGATGGCTCAAACACATTATGTATTCGACCAGG
GGTTGTGGTGACATACGATCGCAACTATGTATCAAACCAACTTTTACGCGACAAAGGAATTAAAGTGATTGA
AATTACTGGTAGTGAACTTGTACGTGGACGCGGAGGCCCAAGATGTATGAGTCAGCCGTTATTTAGAGAAG
ATATTTAAATTTGAAAGGTGGTAAAAGTCACAAGTAATTCATTCATTAAATCAAATAACAAGATTTTTACCACC
TTTATTAGGGGAGATTAATATGGATGAAAATAAATTAGGTAAAACTTCCTTAATTGGTTTAGTCATAGGCTCTA
TGATAGGCGGTGGTGCATTCAATATCATCTCAGATATGGGTGGCCAAGCTGGTGGACTTGCAATAATTATCGG
TTGGATAATAACTGCTATTGGTATGATTTCTCTTGCTTTCGTATTTCAAAATTTAACAAATGAGCGACCAGATCT
TGATGGAGGAATTTATAGTTATGCTCAAACAGGGTTTGGAGATTTTATTGGTTTTTCAAGTGCTTGGGGATAT
TGGTTTGCAGCATTTCTAGGTAATGTGGCTTATGCAACCCTATTAATGTCAGCTGTGGGTAACTTTTTCCCTAT
ATTTAAAGGAGGTAACACACTTCCAAGTATTATCATAGCATCAATTTTATTATGGGGTGTACATTTTTTAATACT
TAGAGGTGTAGAAACTGCAGCGTTTATAAATAGTATTGTTACAGTAGCTAAATTAATACCTATATTTCTAGTTAT
TATATGCATGATAGTTGTATTCAACTTCAGTACTTTTAAATCCGGTTTTTATGGTATGACTAGTGGAAGTGTTGG
CGTTTTTAGTTGGGGAGATACAATGGCACAAGTAAAAAGTACTATGTTAGTAACTGTATGGGTATTCACAGG
GATTGAAGGAGCCGTTGTCTTTTCTGGACGTGCAAAGTCTAAAAAGGATGTAGGAACTGCTACCGTTATTGG
TTTGATTTCTGTGCTAGTCATTTATTTCTTAATGACTGTACTAGCCCAAGGTGTCATTCAGCAGAACCAAATTT
CAAAACTTGCTAATCCATCAATGGCACAAGTATTAGAACATATTGTAGGTCATTGGGGTTCAGTGTTAGTTAAT
ATAGGCTTAATTATCTCTGTTTTAGGAGCTTGGTTAGGATGGACATTACTAGCTGGTGAATTACCATTCATTGT
AGCTAAAGATGGACTTTTCCCGAAATGGTTTGCTAAAGAAAATAAGAATAAAGCTCCGGTCAACGCTTTAAT
TATTACTAATATATTAGTTCAGTTATTTTTAATTAGTATGTTGTTTACAGATAGTGCCTATCAGTTTGCGTTTTCA
CTTGCATCAAGTGCAATCTTAATTCCATATACACTCAGTGCTTTTTACCAGGTTAAATATACTATTCAAAATAAA
TCTAAAGCTAATTTAAAACAATGGATAATAGGAATTATTGCATCTATTTACACAATTTGGTTGGTTTATGCAGCT
GGATTAGATTATTTACTATTAACGATGTTGTTATATATACCTGGATTACTCGTATACAGCTACGTACAAAGGGAT
AATAACAAACATTTGACAAAATTGGATTATACGTTATTCATATTCATCATTGTACTTGCAATAATAGGAATAGTT
CGTTTGATTACAGGTAATATTTCTGTATTTTAAGTTATGATTTAAACTAAGTAATTTTTAAGGGGTTTATAGTTAT
GTATGAAGAAAATATTTATATTAAAAATTCAGAATATGAATTTGATAATAATCTTAAACAATTAGCATCATACTTA
AATATTCCTGTTAGTATTGTTAGACCTTATAAAGAGGATTTAACACTTTATCAATATAAAAAAGGACAAGTCAT
ATATCATTCAACTGATCAAATAAAATTTGTATACTTTTTAGTAAATGGTTGTATTTTACATGAATCTTCTAATATTA
CTGGTGACAATTATTTAAGATTAAGTAAAGACGAAAATATATTTCCAATGAACTTCATATTTAATGAAACCCCT
GCACCATATGAAATATGTACAGCTTTGACAGATTGTAAAATATTAACTTTACCGAAAGATTTACTTGAGTATTT
ATGTAGAAAGCATAATGAAATATTTGAAAGTCTCTTCAAGAAACTTAATGAGACTATTCAATTTCAAGTAGAA
TATATTATGGCGTTAAGAGCTAATTCAGCTAAAGAAAGAATTGAAAGAATACTACAAATTTTATGCCTTTCAAT
TGGGGATGATAATGGAGAATTCTATGAATTAAAACAAATTATGACTGTTCAATTAATAAGTAATTTATCTGGAC
TTAACAGAAAAACTACTGGTGAAATAATCAGAGAATTAAAAATAGAAAATATTATATATCAAGATAAAAGAAA
TTGGATTATAAAAAAATAACATGAATAAAATATTAATTGAAAAGGAGATATTCAAAATGAAAAATTTAAGAAA
TAGAAACTTTTTAACTTTGTTAGACTTCACACAAAAAGAAATGGAATTTTTACTTAATTTATCTGAAGATCTTA
AACGCGCAAAATATGCAGGAATAGAACAACAAAAAATGAAAGGTAAAAATATCGCTCTACTTTTTGAAAAAG
ATTCAACACGCACTCGATGTGCATTTGAAACAGCGGCTTATGATCAAGGTGCACATGTAACATACCTTGGGCC
AACAGGTTCTCAAATGGGTAAAAAAGAGTCTACCAAAGATACTGCTCGTGTTTTAGGTGGAATGTATGATGG
CATAGAATATCGTGGATTCTCACAAAGAGTAGTTGAGGATTTGGCAAAATATTCTGGCGTTCCCGTATGGAAT

GGTTTAACAGATGAAGATCATCCTACACAAGTACTTGCTGATTTTTTAACAGCTAAAGAAGTATTGAAAAAAC
CATATAATGAAATTAACTTCACATATGTTGGAGATGGACGTAATAACGTTGCAAATGCCTTAATGCAAGGAGC
AGCAATCATGGGCATGACTTTCCATCTTGTATGTCCTAAAGAACTAAATCCAACTGATGAATTATTAAATCGGT
GTAATGATATAGCTGACAAAAACGGTGGTGAAATCCTTATAACTGATGATATTGATGAAGGTGTCAAAGGATC
TGATGTAATTTACACAGATGTTTGGGTATCTATGGGTGAACCTGATGAAGTTTGGGAAAAACGTATCAAATTA
TTAGAACCATACCGTGTAACTAAAGAATTAATGAAAAAAACAGGTAATCCACATACAATTTTTGAACATTGCT
TACCATCATTCCATGATACTGAAACAATAATCGGTAAACAAATTCAAGAAAAATATGGTCTAACTGAAATGGA
AGTAACAAATGAAGTATTTGAAAGTGAACAATCAGTTGTTTTCCAAGAAGCAGAAAATAGAGCACATACAAT
AAAAGCAGTCATGGTAGCAACATTAGGAGAGTAAATGAAAGGAGGATATAAATATGTCTAAAATCGTCGTAG
CTTTGGGTGGTAACGCTTTAGGACAATCGCCTAAAGAACAATTAGATTTATTAAAAAGTACATCTAAATCTCTA
GTAAGTTTAATCGATAAAGGATACGAAATTGTAATTAGTCATGGTAATGGTCCACAAGTTGGAAGTATTAACT
TAGGTTTAAATTATGCGGCGGAACACAAACAAGGTCCTCCTTTTCCTTTCCCTGAATGTGGTGCTATGAGTCA
AGCTTATATTGGCTATCAGATGCAAGAAAGTTTACAAAACGAACTTCATTCAATGGGCATAGATAAACAAGTT
GTTACGCTAGTAACACAAGTTCAAGTTGCAAGCGATGATTCTGCTTTCAATAACCCTACTAAACCAATTGGAT
TATTTTATACAAAAGAGCAGGCTGATAAATTTACGAAAGAAAAAGGTTATACTTTTGTAGAAGATTCTGGACG
CGGCTATCGTCGCGTTGTCCCTTCCCCACAACCTATAAGTATAGTTGAGTTAGATAGTATAGAAACATTAATCA
CTCATGGAACACTAGTTATCGCTGCCGGCGGTGGTGGAATTCCAGTAATTAAGGAAAATGAAGTTTATACAG
GTGTTGATGCAGTTATTGATAAAGATAAAACGAGTGCTTTATTAGCAGCACATTTACAATCTGATCAATTAATC
ATATTAACTGCTGTGGACCATGTTTACATTAACTATGGAAAAGAAAACCAAAGAGGTCTCGATGAAGTGTCT
GTGGATGAAATGAAAAAACATATCTCTGATGGTCAATTTGCTAAAGGAAGTATGCTTCCAAAAGTTGAAGCT
GCACTTCAATTTCTTGAAAAAAATACTAAAGGCAGTGTTTTGATTACATCTCTAGCAGGATTAGGGGACGCTT
TAGACGGTAAAATAGGAACATTAATTAAGAATTGAAATTATAATACTTTAGTTCAATTACCTTTATAAACAAAA
GAGCCTATAATCCTAGGGCACTGAACCCTTAAAACGGGAACTTAATAAAAACACCATGTTCTAGGCTATTAAT
TCTCTGTATTTTACAGGGGATAAGTAGCCTAGTTTTTGTTGAATTCGATTATTATTATTATTATTGTTTTTAAT
GTACTTTTCGACAATATCTATTACAATGTGATTAGAGTGATTAAGCTCATTATTGATGTAAAAAGTTTCAGACTT
TAGCGAGGAATGGAAACTTTCTATCGGGGCGTTATCGGCAGGTGTTCCCTTTCGGGACATACTTCTGATAAT
GCCTTTTTCTTCGCACAATTGATAATAAGCATAAGATGTATAAACGCTGCCTTGATCACTATGTAATATACAACC
CTCAGGTATATCGATTTGATTTAATGTATCATTAACTAAACTTTGGTCTTGTTTATCATCTATTTTATACGCCACA
ATTTCTCCGTTATAAATATCCATTATCGAAGATAAATACAACATAGAATGACCAAATGGTAAATAAGTAATATCG
GTTGTTAATACTTCCATTGGACAACTCGCTTTAAATTGTCTTTGTAATAAATTGTCCGTTTTATAATACGGTTTA
CCTATTTTTGTCATCTTTTTAGGTCTAACTCGGCAGTTCAAATGATGCTTCTGCATCATTCTCTGTACTCTCTTAT
GATTAATTGGTGATGTATAACATTGATTAATCAATGCTGTAATCTTACGATAACCGTAGGTATAGTGGTTAGCTT
TACATAATTCAATAACTTTTTGTGTTACGGTATCATTTTTATGGGTTTTGTTTTTCCATCGGTAATATGTTGATTT
AGGTATGTTTAATACTTTTAGTAGCAATTTGATTGAATATTTTACTTTTAATTGATCCACTAAATCTATGACTACT
GTTGGTACCACTTCCTTTCCAATACCTTGTACTTTTTTAAAATATCCAATTCTATATCTTTTCTCTTATTTTCTAAT
TTTAATTGTTCTACTTCTGACAGCTCTTCTAATCCTTTACCGTAGGTATATTGTTTACCAACTTGTTGTGAAAAT
CTATATGCTAGTTTTGGTAAAACTGAGGAAGGACGCTGGATAGCTAAGAATGCCCATAATTATGGATTTATTAT
AAGATATCCTAAAAACAAAGAAAGCATCACAGGATATCAATATGAACCTTGGCATTTACGTTATTTAGGAAAA
AGAAGGCGACAAAAGTGTACAAATCTGGTGAAACATTAGAGGGATTTGTAGGCTTAAAATGATCAATTTAAA
ATAATCGAAACTTCAACTACCTTATTATCGACGAAGTGTATTCCAGCACTTCGTCTTTATTTTTACCGCAAACAT
TATACCTTACTATTTAATAAATCTATTTTATATCTACATACTTCCTCTTTCTTTTTCTAAAAATTTTTGAGAATTCTC
TTTATCAATTTCAATAACACTTATCTAATGAAACAGTCATTGTCGTAATTTTTTCATAACTACACGAGGCTTATAT
TGAAATTGCTAAAGAACATCCCACGATGATTTTTTATCGGGAAAGGAATGGGATTTGCAGATTAGAAGAAGC
AGAAGGATTGTGCAAGCACAATCCCAAAATCCCGTTATTCTTTATAAATAAATAATATATATAAGGAAGCAGAG

ACCCCACCCTATCATCAATACTTATAAATAATGAGTTGATTGTTCGTCAAAATAGGCGGAATTTCGGGATTTCC
TTGTCATACCAAGGTTTGTAGCGGTTTTTTATATGGAATTTTCTCATATTGTCAACGGGATCAGTAATAAATGA
CAAATCAAATATATATTATCTCAATGATGTATATGAAGTACATCACTATCACACTATCTAGGGAGGCAATGATATG
AAAACAACTACTCAAGAACTCAAACAATATATGACTCGCTTATTCCAATTATCTAACAATGAAACATGGGAATG
TGAAACACTGGAGGAAGCGGCAGAAAATATTCTACCCAAACGTTTTATTAATGATTCCCCACTTGCACATCTT
ATACTTGAAACTTATACCTACTATAATAATGAGCTACATGAACTAAGTATCTATCCATTTCTAATGTATTCTAATAA
CCAACTCATCAGTATTGGTTATCTGGATCATTTTGATATGGACTTTCTATACCTCACAGATACTAAAAATACGAT
TATCGATGAACGTCATTTACTAAAAGAAGGAGGGAATAACCATGAATAATTGGATCAAAGTGGCACAAATATC
TGTTACAGTCATTAACGAAGTGATTGACATCATGAAAGAAAAGCAAAATGGAGGAAAATAGTATGAAAATCA
ATCGATACATCACAAGAGGCATTAGTGAACAACTATCTCTAGACCTTCAAATCTTACTTTGGAACATGGTAAA
AGATCGAGACAATCAACTTAATACAGATTACCTACACATTTTCAAACTACAAGAAGATGATAATATGTTGTCAA
TTACACATGAACAAGAACAACCCGCATACAAATTGGAATATCACTATACAAACTATATAAAAAATCAAAATGCA
TTACCTAAGAAAGTCTACGTCATCCGAGAAGATGATGTAGACGCTTTTTATTATGTGATGCTTTTGCCAGAAG
AATACTAAAGGAAGTGACGATATATGAATACAATCAAAAGTACGATACACACAGAAGCGATATTTAGCGATGA
TGAACAACATCGCTACTTACTCAAAAAGATATGGGATGACAAGAAACCGGCTTGTACTGTGATAACGATGTAT
CTTCATTTAGATGGTGTATTATCACTCGATCTTACTACGGTTCTCATCCTCAATCAATTAGCTAATTCTGAGCAAT
ATGGCGCTGTTTATCTCGTTAATCTTTTCTCTAATATTAAAACACCAGAGAACCTTAAACATATCAAAGAGCCT
TATGATGAGCACACAGATATACACTTAATGAAAGCAATTAGTGAAAGTGACACAGTCATTCTTGCTTATGGTG
CCTATGCGAAGCGACCAGTTGTTATCGACCGTGTCGAACAAGTGATGGAAATGTTAAAACCTCATAAAAAGA
AAGTAAAAAAGCTCATAAATCCAGTAACGAACGAAGTTATGCATCCACTCAACCCTAAAGCACGTCAAAAAT
GGACACTAAAGTAAAGGAGTATTATCTATGAACCATGACAACACTATACAATCCAATTGGCGAACGGTTGCTA
GTTGTTTAGCATCACAAAATTACATTTCAATAGTAAAAGGTCTGGTGCATCATTTCACAGCCATCGATGACGA
GGAAATACTTGATAAAATCTATGAAGAGTTTATAAATGATGACTCTATTACAACGGTACTTAACAATGATTTAC
AAACAATTATTAATCATTATCTATCAAAATGAAAACTATATTCTAATAAAACTAGAGCATCCTTCACTTTTGCGG
TGAAGGATGCTCCTATATCTTTTTTTATCTAAAAAAACTATTAGTAATTAGTTTTAAAACATTTATCTTCTAATAT
ATAGATATGAAATGTAAAAATACAATTAGATTGGCAGTCACAATGCAAATGTAATATTTGTCAGTAATCTTCCTA
CTCAAATTGTTATTCTTTTCATAGAAATCAAGAGGAGGAACGACTTGAATTCTATTATATTCAATGCATCTTCAT
TATAATTCTACCTACTTCACCATTAGACAAACGATCAAATCCCTCATTAATTTCATCTAAAGTGATAACCTCACT
GATGAGTGAATCAATATTTAAACGTCCTTGGTTGTATAGATTAACGAACCTTGGTATATCTCTGTCAGGTACAC
AACTTCCTACATATGATCCTTTAATGGTACGTTCTTCAGCCGCTAAAGTAACTTGAGGGAAAGAAAAATTATCT
TTAGGATTAGGCAGTCCTGTTGTTACAGTTGTCCCCCCTCGTTTAGTAATCTGATAAGCAACTTTCATAGCTGG
CACTACACCCGCTGTTTCAAAAGCATATTCTACTCCGCCAGGAATATATTCTTTAATTTGCTCATCGATATCTTT
ATCGCTTGAATTAAATGTTGCTGTAGCCCCAAATTGCTTTGCTAATTCAAATTTATCTTCGTTAATATCTAAAGC
AATAATTTCATTGGCTCCCGCTAGTTTAGCTCCAATAATAGCATTCAATCCAATACCTCCTAAACCAACAACCG
CTACATTACTACCAGAACGAATTTGGGCTGTATTCACCACAGCACCGATACCTGTGATAATAGCACAACCAAA
TGCAGCTGCACGTTCAAAAGGTATTTTTTTATCAATTTTAACTATAGAGTTTTCAGAGACAACAGAATATTCA
GCAAAACCTGATATCCCCAAGTGATGATATACTTTTCCTTCATCATTAGATAAACGCATCCCTCCCTCTAACATT
TCGCCTTTTTCATTAGATATTGCTCCGTTTTCACATAATGCAGGACGTCCTTCTTTACATGGGATACATTTTCCA
CAACTGGGAATAAATGTGCATACCACATGATCTCCTTCGCTAACTCTTGTAACAGCTTTTCCAACTTTAATTAC
TTCACCGGAAGCTTCATGACCAAGTGCCATAGGTAAAGGTCTAGGTCTGTTACCATTAATTACTGACAAATCA
GAGTGACATAATCCAGCTGCATGAATTTTAATTAATACTTCGTGTTCGCTAGGATTATCAAGTTCTAAGTATTCA
ATTTTTAAAGGTTTAGATTCTTTATAGGGCTTTTTAGCCCCCATTTCATGTAAAACTGCTGCTCTAGTTTTCACT
TCCAACACTCCTTTAATTTATATACATTAAGTCTATTCTTATCTGTTATTTTACACTTTAATATTATTATTTACAAAA
AATTCTGATTATTGAGATTATTTTATATAATACCAATTTTTAAAAATTTATTATGTAAAATCATAATTTAGAAGTGA

TTTGGTTTTCAAGTGAATTTAAGGTGTGAAGATTAAAAAATATATTAAAGAAATACTTGATAAAATTTATGAAG
ATTTTATGAATAACGACGCTATCACAACCTTACTTAACAATGATTTACAGACGATTATTAATCAATATCTATCAA
AATAAAAACTATATACTGATAAAAATAGGGCATCTTTCACTGAGTAATAAATCAGAGATATTAGAGAATAATAA
GAAAATGAGAATTAGTATGTAGTGAATTGTTAAAATAGATTTATTCATAGTGAATAATGACTTATTCGTTATTTA
TATTTATAATGCAAATATATAAGAAAATTTTGGGAGTGGGATTATGAAACTAAGAGCATTAGAGTATAGTGATT
TACTTTTTGTTCATGAATTAAATAATGAATATTCTATTATGTCTTATTGGTTTGAAGAGCCTTATGAATCCTTAAC
GGAACTTCAACATTTATTTGATAAGCATTTATTAGATGAATCGGAAAGACGTTTTATTGTTGAAGATGAAAAT
CAAGTCGTAGGTATTGTTGAATTAGTAGAAATTAATTACATCCATCGTAATTGTGAAATACAAATAATTATTAAA
CCTGAGTTCAGTGGTAAAGGTTATGCAAAATTCGCATTCGAAAAAGCGATAATTTATGCATTCAATATTTTAAA
TATGCACAAGATCTATCTATATGTTGATGCAGATAACAAAAAAGCTATTCATATATACGAGTCACAAGGATTTAA
AACAGAAGGATTACTTAAAGAGCAATTCTACACTAAAGGTAAATATAAAGATGCCTATTTTATGTCATTATTGA
AATCTGAATATATTTTGTAGTATAAAAGATAACTCATATATTCATTTAGGTAGGTTCAAACCTGAATTACTTCTAA
TAAAATTAAAACGGACTTCTCGCGAATGTCTGTAAGAGGAATAAATTCTTGATAAAATCTACGAGGATTTTAT
GAATGATGACGCTGTAACAACGGTACTTAATAACGATTTGCAAACAATTATTAATGACTATCTATCAGAATGAA
GCTTATATTAATTTCAACAAAAATAGAGCATCCTTCACTTTGCGGTAAGGGATGCTCTCAATTTATTTTTGTCTA
TTTTCAATTAACATAATAGGACGTTATCAGAATTACACAAACATTTTTTGTAGTAAGCCTTGTTTGCGTTGTTT
TAATAATTCTATTTTTTTACCTTGTTTTAAAATTAGTGTATCTATTTTATTAAAAAAGTTATCAATTTTAATTTTAG
ACTGACCCCAATAGTGGGAATTATATAAAAACACTTTCGTTGAATTCATATTAAAATGAATCATACGGAGGTGT
TTTTTTTCTATGAAAAGAGCTTCTTATTCAGTAGAAACAAAGTATAAAGCAGTTGAAATGAAAGCAGCAGGA
TTTCCAACAAAAGAAATTATGAAAGAATTAAATATTAGAAATAGAACACAAGTGAAAAATTGGTGGCGATGG
TATCGAAATGGGGAAAGTTATAGATTTTCACAACATGTTGGTAAACAATATACCTACGGTTATCGTAAGATTAC
AGCATTGATTAATCAATGTTATACATCACCAATTAATCATAAGAGAGTACAGAGAGTGATGCAGAAGCATCATT
TGAACTGCCGAGTTAGACCTAAAAAGACGACAAGAATAGGTAAACCGTATTATAAAACGGACAATTTATTAC
AAAGACAATTTAAAGCGAGTTGTCCCATGGAAGTATTAACAACCGATATTACTTATTTACCATTTGGTCATTCT
ATGTTGTATTTATCTTCGATAATGGACATTTATAACGGAGAAATTGTGGCGTATAAAATAGATGATAAACAAGA
CCAACGTTTAGTTAATGATACATTAAATCAAATCGATATACCTGAGGGGTGTATATTACATAGTGATCAAGGCA
GCGTTTATACATCTTATGCTTATTATCAATTATGCGAAGAAAAAGGCATTATCAGAAGTATGTCCCGAAAGGGA
ACACCTGCCGATAACGCCCCGATAGAAAGTTTCCATTCCTCGCTAAAGTCTGAAACTTTTTACATCAATAATCA
GCTTAATAACTCTAATCATATTGTAATAGATATTGTCGAAAATACATTAAAAACTATAATAATAATCGAATTCAAC
AAAAACTAGGCTACTTATCCCCTGTAAAATACAGAGAATTAGCAGCCTAGAACATAGTGTTTTTATTAAGTTCC
CATTTTAAGGGTTCAGTGCCTCTAACAAAAAGCATTCTTTTTTTGTGAAAATAATATCTCTTAAATTGTTTAAAT
CATTATATCCTTATCTATTAAGATCAAATTTTAAAATAAGTATATTGATATCGTTTACCTGTAAATAGTATAGTTTT
TATTAAAGATTCCAATTTCTTATTATTATCTGTAAGAGAAAGATTCTCTGGAAATATAAGTACACCGTAGTTATA
GTTATCTTCGACAGTGAAGTCAGAAGCTTTTAACTTCAAATCGTCTTGGCTTTTTAAAAATGAACCATTAACA
ATATCTTCAATATAAGTACCTTTTCCAATAAATTTCGCTACTCACTTCGATTGTGGATGATAATATAAATTCTCAG
GTCTTTCTTAATTTTATATTGATTATAACAACACATACTTACAATATCGTAAGCTTCTCTTATAACTGTTGTAATAG
CTCGCTATTAATGTTTTTATAATAGTAAAGAAAAGTAAAGAAGGTGAAAAAAGTGGAAAATACAAATAGTAAT
ATGCATAAAAATGACAATAGTTACGTTTTATCAGCATTGAGTTATTTAAGTATCTTTTTCGCACCTGTCATTTTA
CCTTTATTTGTATGGATACTTGCTGATGAACCAACATCAGAACATGGCAAAAAAGCATTCATTAATCATATTAT
GACTTGGGTTAGTTTTTTTATAGGTAGATTGGCATTTATTTTTTCTAAAGAAGTCTTTGATAAACCTTTGGATC
ATCAATTATTAATATTTAGCATTCTTTTAATTATTACTATTATATTCTTTTTAATTGCTTTAATATTATATATCTTGAA
CATTAACAGAGGAATTAAAATTCTATTAAAAAAATAAGTCACTTATATATCAACTTATTAAGGGAGTCCTATACT
CCCTTTTTTAATTGTAATGTATTCTATCAGTATTACCTTTATTCATAGTCTAAAATACTTACTTCAATGTTTCTAAT
AATATCTCGTGAGGCCTCAAAGTTGTTATTAATATAACCATTTTTATTCAATGATAAAGAAATTAATTGGCCTGC

ATAATAAGTATTGTGATTTACTCTAAAGGCGTGCGTAAATGCTTCACCATTTTGTATTTTCTCAATCAATGTTGC
TCTGTTCACAAAAATGGGTTGGCTAATATTTATAGCTTTTTCATCTTTTATATTAAATTCATATAATCTTAATCCAT
GTATTTTAAGATCATCCTTATTTTTCTTAATGACATATTTAATCACATTATACGTTCTAGCCATTTACTAATCTTCC
TCCATTAAGATATTAATTCGAAGCTCTTTCTTCTTTTAAAAACATATAACGTTCAATTAAAAAATCAAACTTTGT
AGCTTATTGCGCATCATCATTTGAACTTTGTATGCCAATTTTAATTTTTAAACCATTAATCATACTTTGAAATTTT
TGCTGATAACTTTCTCTTTCTTCTTGTTGAGAATAGCAAAAATGTTCATTTTGTGTAATTAATTGGTAATATTTA
AAACAATTATCTAAAGTTGTTAATCCATAGGCTAAATAATACAAATTATCTGTTTCAAGTGCTCTTTTTCAATCT
ACTAATGAAAGATGTATGGCACCTCTACTAATTGACTAATATCTGTCATACAACCTCTCCCAAAATTAATTTTTA
AAAATATGTTCTCTATGCCAATCTAAATATTTCAAATTAGGTTTCTGACAATCTGGTAGTTCGATTTTTTGATTT
TCATAAATCTTTAATTCTTCATATAAAGCAGCATCTTTCTCTGCTTCTTTAGATAAAACAACTCTATAACTGTTAG
AAAAAGTGATAAAGCCTTTCTTAAATGCTTTTGCTAAAAATAAAGACAAACAAATACCATTTGAAGGATCTAG
TCTAATAAACTCATTTTCATGCCAAGGAATAATATGTGTAACGACTAAAAAGTCTTTAGTTTTAATCCCGGTAAT
TGCACATCTATAACTATAATTTTGTTTAATCCTTTTTGAAAACTCAAAATGAGCACTTCCCTTAGCTTGAACAA
ATGCTTCATGATTCCCTACGTAGTAAATATTTTTCTTTATTCGAGTTGCCATATCAGCCATTTGTTTAAAAGTTA
GCGAAATATCATTATTAATATAATTACTTTCTATTTCCACTACATTTCTACTCAAACTTAGTAGTTTTAAAAAATC
ATTTTTTGTAATATTACTGATTTTATTTTTAAAGAAAAACTGCTCCCACTCTTCTTTAATTCTTTCTTTGAACTCC
CATTTTAAATTTTCAATATTATCTTTTAAAACACGATCCACAAAAAGTAAGGGTTTTGAAATGTAAGCGGTTGC
TACATTATTTTTTCGCTCAATTTTTTCTATTTTCCCAGCTCCAAAGAAATAAAATTGTTGAGCAATTTGAGATAA
ATTAGTTGGCCGTCTAAAAATGAACAAATCGCCTTCTCTGATCTTATTATTCTGTTTTACATCCCAAGTAAACTC
CTTTTCAGTATCATCACTATTTGTATAGTTGGTATCTAATATAAAATAGCGAGTGCGTTCGTTTTCTAACTCAGT
TTTAAAGGTGTTATATAGTGACTCTAAGTATCTATTTTTATATTCGCTTATACTCATCTTTCTATCAGCTCACTTGT
AAATTATAAATATTTAGCATACTTTTATTTAATTATTAAATATCAATTTCTAAATATATTCTGATAATTATTTATTTTA
TATATGTATTATTAGCCTTAGAGCAATATTAGGTGACAAATAAAAGGCTACCTCATAAGGTAATCCTCTCAAGAT
AATGTGACAAAATAATTGATGATCTTTAACATTAATAAGAACTAAATTCATAATTCTTAAATATATTTTCTAATTA
AACTATCGTAATGTCTATAATATTTAATTATTTTTTATAAAAATTAAACATTTTATTAAACTATTTTTATAAAATAAA
ATAATATTACAATATAATTAATTATAAAAATTTAAGGGTATATTGAAGTAATACATTACATAGGAGTGATAAAATG
CCAAGTCGTTGGGTAAAAGTTAAAACCTTTAAGTCATTATCTACTTCCAAATTAGAGAAAAAGTTACAAAATT
TCAAATCATATAATAGCTTTGATATTATAGAAATTAAAAGACATTCATATTTATTTTTAAATATAGTCGAAGTTTAT
TACAAAGATAAAACTTAGACTTTTTATTACTACTTTTTTAAAATCTGTCTATTTTTACAGCATTGAATTTCCAGC
CGTATAATTTAAAATTCTAATGTAATTCTAAATATTTTATAGATAAAGTAGAAAACGTTAATACTACACTTATATG
AATTATATAAAATATTAGATGATTTTAAAAAGAGAATTACCTATGAACCGTGACACTACATAACCTGATTGGTG
AATAATTACAAACAGTTTAGCTTCGCAAAATTATATATAGCTCATAAAAAGACTAGTACATCGCTTTACAGCTAT
TGAAGATGAGGAAATACTTGATAAAATCTATAAAAATTTTATGAATGATGATACTATAACAACGATATTTAACA
ATAATTTACAGGCAATTATTAACCATTACCTATCAATATGAATATTAAATAAAACTAGAACCTCCTTCACTTAGCA
CTGAGGAATATTCTTACATTTTTTAATAAGTGGTGATATAAAAAAGCAGTTAGTGGCCATAGATTCAATTTCTA
AGGTAGCTTCCCTTTCAATAATTTTTGAATTCAAAATTTTAATATAAATCCCTTCCTTTTATATTATACACAATCTA
TTTTTTAGTTTTATTTGTGATACGCTTCTGCTTATCAGTTAATGATGCGGTTTTTGAAAAATAACTACAGAAATT
TATAGAAGAACAACAAAACTCAAACGCTATATCATATAATAAGTTCTTAGAAGCTTACAAAGTAGACACGTCT
TCTGATTAAACTAATACAGTTGATGAATTAACAGAAAATCCAGTATGTACTGAGTACATAAGAGATCGAACAG
AAGCTAGAGAAAAAGGTCAAGGTAGAACTTATTAAGCATATTAAATTAATTAACTTGATACTTTTCAATAAAA
AACAGAGCACCTCCATAACGTGGAGGATGCTCTATTTTTTTATAACGCATTTACTAATATTTCAAATTCATCTTT
ATTAATATTAATACCTTCAAGATTAACATTTACTAAATTAGGGTGTTGACCGCTTGTTAGGACATATACTTCAAT
ATGTGGACATGTTTCATTAACGATAGGCATGATTTCTTCATCAAACCGCTTCGGTCCATTGTTTTCACTGTAAA
TCACAACATGAAATTTAATATCATCAGCTATATTCATAGGAATTTTGTCTTCTCTCTTTCTAGACAATACACACTG

TTTTCCTACGATATTTGAAATCCATTCTTCTTTCTTTCGGCAATAATCTATAAAATCTTTTCTTTGATATCCTAAGT
CCATTTTAACTTCTAGTATATTTTTAATTTCATTATCTTCACAAATTAATAAATCCGGATATCTTGTACTTAAAGAT
TTATCAATCATAGATAAAGGCTGATCAATAAATATTTGGACACCTTCCAATCTACTAATCAAATAAGCACCCAG
CAAGTCTTCTCCAATACCTGAAATACTATGATTACGTCCTCGATATAAATTTTCATGAGGATAAGTAGGCTTTCC
TGCTTCTTTATATGCCTTTACAAGATGTTCGATAACTTCCACTTCAGTGAATTTCATACTCAATCACCCTATCTAT
AGATACTCTATATTTCAACAAACAAAGTATATCACACATTTGAAAGCATAAATACAAAAAACCTCCTAATAAAT
TAAGAGGGTTGAATGATAGTTTATAAAATATTAAATTATTTGCTATTATAATTAACTATTTTGGTTAATTCTTTAA
TCGTTAAAGGTTTTTTAGTATTTCTATGAATCATTTTATGGCAATTAGAACATAGTGGGACTAAATCTTTTTGTG
GATTTACTTTTATTTCTCTTTTTATTGAAAACATTGGTTTTAAATGATGAATTTCAATAAAACCCTCACCTAAAT
CGCCATACTTAGCTTTAAAATCAAATCCACATACTTTACATGTAGTTCCATGAATTTTAATAGCTTCATTTCTTAA
TGCCTGATTTCTTTCATATTTAATACCGTACTGTACATATGGTTTACCTTCGACATATTCAGTGTTTTCTAACTCA
TTATCTAAATTTAAAATATTCGTTTTCAAGACTTTTGTATATAACTTTTTAAAGGTTCCTTTAAAGTCATAGGAC
CTTGAACATTACCTTTTAACCCATTATCTTGTAAAAATTCTAAATCTAATTGGTCATCATCAAGTTTGTTTAATAA
CTTTAATTCCATGTAATTACCATAGTTAATATAATTATCACCCTTGATAACAAATTGACTATCATCAATTTATGATA
AACATTTATCTCAAGCATCTTATTTAATCGGTTGTCAAAAAACTGGAGAAGCCATGATCATTGATCCTATTTGT
GACTTATCTTCATATATTCGAGTTGCCGATGAAGAGAGTTTAACCATTACTCATGCAGCTGAAACACATATACA
TGCAAATTTTGTTTCAGGAATTAGAGATGTTGCTATAAAGTTAAATGCTAGTATTTATGTATCGGGCGAAAGT
GATGACATGTTAGGTTATAAAAATATGCCTAACCACACTCATTTTGTTCAACATAATGATGATATTTATGTAGGA
AATATAAAATTAAAAGTGCTTCATACACCTGGTCACACGCCAGAAAGTATAAGTTTTTTACTTACTGACGAAG
GTGCTGGAGCACAAGTTCCAATGGGACTATTCAGTGGTGATTTTATTTTTGTAGGAGATATCGGTAGACCTG
ATTTACTAGAAAAAGCTGTTAAAGTAGAAGGATTATCTGAAATAGGCGCAAAACAAATGTTTAAATCTATTGA
AAGTATTAAAGACTTGCCAGACTACATTCAAATTTGGCCTGGTCATGGCGCTGGTAGTCCTTGTGGTAAATCT
TTAGGTGCTATTCCAACATCTACGCTTGGCTATGAAAAACAAACGAACTGGGCTTTTTCTGAAAATAATGAAG
CTACCTTTATCGATAAACTCATTTCTGACCAACCTGCACCACCACATCATTTTGCACAAATGAAAAAAATTAAT
CAATTCGGTATGAATTTATATCAACCTTATACGGTTTATCCAGCTACAAGTACAAACAGATTAACTTTTGATCTC
CGCAGTAAGGAGGCTTATCATGGTGGACATATTGAAGGTACAATCAATATTCCATATGATAAAAATTTCATCAA
TCAAATTGGCTGGTATCTAAACTATAATCAAGAAATTAACTTGATTGGAGACTATCACCTTGTTTCAAAAGCAA
CACATAACATACTTTACAACTTATTGGATATGATAATGTGTCTGGATATCAGTTACCACAAGCTCAAATTCAAAC
ACAATCTGTTCATAGTAAAGACATAACTGGTAATGAAGCACATGTCTTAGATGTACGTAATGATAATGAATGGA
ATAATGGCCACTTATCCCAAGCGGTTCATGTGCCACACGGCAAACTTTTAGAAACAGATTTACCTTTCAATAG
AAACGATGTTATTTATGTAGACTGTCAGTCTGGCATTAGAAGTTCTATAGCTATTGGTATTTTAGAACACAAAG
GTTATCACAACATTATTAATGTAAACGAAGGTTACAAAGATATATACCTTTCTTAATAATTATATTTATACTAATG
AAGAAGGACTATAAGTAGTTAGATATAGCTTTTAGATATAGCTTATAGTTCCTTTTTATATTATTGGATAAATATC
TATTTAAAAATCATTTATATACCCTCGCTTTATATCATTTAATAAATATTTTACATAGTACATACCTTTTTTGTTTAT
TGATGTGATCAAAAAACATAACAGCAATTCCTATAAACCTCATATGTTCTGATACGTTCAAAATCCCTTTATGAA
GTGTCTAAAAAACCGCATCTCTGCGTATCGGTTAATGATACGGTTTTCTATGATATTTATTAATAAATATAAGAT
GCGAAGCATTTGTATAATCCAAACACTTCGCACCTATCTCTTGTTTTTTTATTTATTCCTAAAGAAAGACTCTAA
ACTTTTAATTACTTTTTTAGTTCGCAATATATCAGCTTCTTTTGATTCTATTAACACATATTTAGTAGCCGGACCT
TCACCTTCTTTTTTTATCAAGTTATCATCTTTTAGTTTTTTTAAAATATTTCTAAACTGATATTCTGTCATATTTTC
CATTTTTAAGGCTTCACCCTTACTTATTGATCCATAGTCAATAATATACTTTATTACTTTTTTGTCTAACTCAGGA
TATTTTTCAAACTCCTTCATTAAATCTTGTTTCCAAAGTACTACATTAGTGTCCATGTCCGTTAATTCTATTTCAG
GCGTTTTAAGCTTATGTCTATTAACTACATCGAATATCCTTGGTCCTCCAGATCCAGCTTTTTCTGAATATCCTA
CTCTTCTGAAAAGCGTCGATAATATACTATTTCTGTCCTTTGAATGCCCTCCATGAATAAAATCTTCTTTATTTAT
TCTCATATTACCCGGATTATAAAACTCTATAAAATCTTCACAATTAACTATTTTAATACTTTGCTTAGTATCATAAT

ACGCATGCATTAATGTATTAACCAGTGCTTCGCGAATTGCTACTTTTAAATCTCTTGCATAATTTTGTCTAGTCA
AACCATCATTTAGGCTAAATGATTCCTCAATGTTATCAGTTAATTTTATCAATACTTTTTCAAAAAAACTATACA
CGTTTAAATCTTCATTACCTAAATCTCCACTTGATATTCTATCTTTCCAATCAGTATCTAGGTAATTTGTTTTCTTA
AAATAATCTAATTGAAATCCTGGGAATCTATCACTAATCGCATTATACTTACCAAAGAATAATAAACATGCTGTA
GTCATTTTATACTGTTTGTCTTTCGAACTTCTATCTTTTCTATATGCTCCTAAATCTATCATTAAATCCAGTTGGCT
CATATTCGCATATCTCTCATCATTAGTATTTTTTAATAGAAGTTTCCTATAATTTTCGATAGATTCGTGATTTAAAT
CAGACATGTCATAGTTATCTAATAATTCTGTATCTATATCGTCTTGAGAATTTACAATCATGAATTTATACTCTTCT
TCAGTCGCAATTCTATCAGCATCATCAACTCTTTTATATACTAAATCTTTTTTATTATCTACATATATCGGTTTCTT
CGAATAAGGCGCTTCTGGTACATGAATTTCAATGCACGCTTTATTTTCAATATTAACAATTTTAACATCTTTATT
TGATAAAATATTTTGACTAACTTTACTTCTTCCATATAGTGCATTCCAAAAATCTTCAATCATTTTCTCTGGATCT
TCAACACCAACTGAAACAAAGACTTTTTTGTCCTCGATCTTTTTTTCTTCAATTCCTAAATATATAGTACCACCT
TCAGTATTTGCAAAAGCACTATACGTAGACCAAATATCATTAGATAATCCTTTTCGTGATTTCTTATACTCTATAT
ATTTACTTTCTCTATGCAACGCAATTCCTCCAAACCATAAATATTAAAATACTCCATTAATTTACTCAAACCGTCT
ACCATAGTATTTCACGCGATTTTCGCGCGATTACTACGATGATTTCAAACGATTCCTCTGATTATTTCACGCGAT
TTTCGCGCGATTACTACGATGATTTCAAACGATTCCTCTGATTATTTCACGCGATTTTCGCGCGATTACTACGA
TGATTTCAAACGATTCCTCTGATTATTTCACGTGATTTTCGCGCGATTACTACGATGATTTCAAACGATTCCTCT
GATTATTTCACGTGATTTTCGCGCGATTACTACGATGATTTCAAACGATTCCTCTGATTATTTCACGTGATTTTC
GCGCGATTACTACGATGATTTCAAACGATTCCTCTGATTATTTCACGTGATTTTCGCGCGATTACTACGATGAT
TTCAAACGATTCCTCTGATTATTTCACGTGATTTTCGCGCGATTACTACGATGATTTCAAACGATTCCTCTGATT
ATTTCACGTGATTTTCGCGCGATTACTACGATGATTTCAAACGATTCCTCTGATTATTTCACGCGATTTCCACA
ACATTTTTCGTATTTCAGAACGATTAAGACTACATTATCCTGGCTTAATATTAATTAATTTTAATTATATCTATAAA
TATTTTCAGTGAAAAACTATTTCTTTAAGGCATCTAATGCTTTAAAAAACAACGATACATTGTACTCAATATTGT
TATGCTTTGTTTTTTCTCTAGGAAAGCAGTAGGCTTTATAATAATCAATTTTAACTTTCAACATATTTTGTTCAA
TAGATAAAGTTGCATTTAATAACGATTCTTCTAATCCATTTTTAAATCTTTTAATCTGATCTTTTACGTATCTATAC
TTTTTAATAGATTCTTCTATCAGTGTAATTCTTTTGTAATTATCTTTTTCTCCTTCACATGTATCTACAATGTCTCT
AATAATATATAATCTAACAACAGGTAATCGTATATCTATAAGTAAAATAATGACATATGATTGAATAAATTCGTTT
AATTTTTTCCCAAACTCATAATCTGTTTTCTTTTGTCCTTTAAATGCCTTCAAATACAATTCTATCAAATAAAATA
GTTCATTTAAAAAAGCCCAAGCATATTCCTGTTCTAATGCAACATAACTAATCAATTGATTATTAAGATTATAAA
ACTGAATCTCATTATATAAATATTTCCAATGATTCTCCAATTTTGTATACGATATTTTTTCTCCTAACCGTACATAT
TCATTTTCTTTTTTTGACTGATTATAGAGTGTGTCTATCTTTGTAACTAATCTTTTTTGCGTTTTGTTTATTTCTG
CCAATGCTTTTGAAGTAATATTTGAATCATAACTCATATGTGCATCTACTTTCTTTATCGCGAGTAGTGATTTCA
ATTGTTCTTCATTTATAAATGGATTACGTTTAAAATTATTATTTTGACTATTGATATGCGTCATGATATTTAAATTA
CTGTAAATCTTATTGATACTTCTAAACTCAGTATCATCTAATAAATAATCGAAATTTGAGTTTAAATGTTTTTTTA
TAAATGAAACTCTATCCATCTTTTGATGTTCATTTAAATCGCTTAAACTTCTTAATAACAACAAATCTTCATAAA
CCTCAACTGTTACATCAAATTTCAAATAATCAACTAAGTTTCCATACTTATCAATAATTTCTTCTAGCGTAGGTAT
CAATATATACCCCCCCTCCCCATTTTTTGTTCCGCGTCTAATCATAATGCTTCATTTATGCTTATTATAGCAATCAA
TTGAAGATAGTTTAACCTATATGATATTGATTGACTCCTTAAAAATTATTGGAGGAATTTTAAAATGAATAAGA
AATCTAAGCAGCAAGAAAAGCTTTATAATTTTATAATTGCTAAATCTTTTCAACAACCAGTTGGAAGTACGTTC
ACTTATGGTGAATTAAGAAAGAAATATAATGTGGTTTGTAGCACGAATGATCAACGTGAAGTAGGACGTCGT
TTTGCTTACTGGATTAAGTACACGCCAGGATTACCATTTAAAATTGTAGGAACAAAAAATGGCAGTTTATTAT
ATCAGAAAATAGGGATAAACCCATGCAATAATAGCACGCCTTCGAAAGGAGGTGATTGCTAATGAGTTACAC
ATTGTTTGAAATTGGCCCATTTGCTTTAGATTCCTTCGGTTGGAAAGAAACGATACCACCTAAAAAAGATGGT
GACAGTGAAAAAGTGGTTAGAATGGCTAGCCCTATTATTGTTAATGCAAGATTTTCTGATCCAATTACTGGTG
TTGAAAAGTTGATTATTACTAACAACAATGGTAAAAAAGACATTTTTGAGTCTGATATCTTAACAACACGAAA

CTTACCTTCTTTAATTAAATATGGCTACAGTATTAACGAGAAGTATATTAGATCATTGAGTTATGCCTTGCAATT
GATGCGAGATAGATTACCACTTTCTGAATTATATGAGGGTGTAGGTATACTAGAGACACCTTTTGGCTACTTG
ATATCGCTAGATAAAGTTCTCAAATCTATTCAATTTAATCAGTCCTCACCTTCATACCCTATTGTAGATAGTGCTT
ATGACTTAACTCCTAAAGGTACATTTGATAATTGGTTTAACATGTATATTGACGAGGTTAAAGGTCATCTTCTT
TTAGAATTGGCAGTCATATTTGGGATTTCTGCCTTAGTGACAAGTTTTCTGAAGCATAAACATGAAATCGAAT
TTGCAGGAATTCTCTTTAGTTTTACAGGTCAATCCTCAACCGGTAAGTCAACCGCAGCTGCTTTAGCTGTATC
AGTCGCTGGTAACCCTACCAAAGGTAATGAAACTCTTTTTAGAAGTTGGAATGCCACACGAAACGCACTTGA
AGGTTATTTGAGTAACAATTATGGCATTCCTATAGTATTTGATGAGCTTTCATCTACGACACTTAGAGATACCA
CGGGTTTGTTATATTCCATCGCTGAAGGTCAAGGTCGTCAACGTTCAAACGTTCATGGAGAAGTTAAAACGC
CTAAGAATTGGGGTACTTCTGTCATTTCAACGTCAGAATACAGTATTTTTAATGATTCAGCTCAAAACGATGG
ATTACGTGTAAGAACCATCGAGATTAATGAACAATTCACTACAAATGCGACCAATGCTGACAATATTAAAAAA
GCTGTAGCACTAAACTATGGTCATGTTTTACCGTTAGTAGCTAAATATCTTATAAATCGTGAAGATGAAGTTATT
CAATGGTTTTACAAGGAAGTAGATTGGTTTGAAGCTAAATTGAAAGATGAAACAAACAATACAGGTATTCGT
ATGTTTAAGCGTTATGCAGTCATTACAACATCTGCGAAAATATTAGGTCGTGTTTTATCTACAGATATTGATATC
GCTAATATAAGAGACTACTTTATTGATTATCATACTCATACAGTCTCTGAACGCTCATTAGCTGATAAAGCTATT
GATGTCATCATTCAATTTGTGGCACAAAACAGAGGTAAGTTTTCAGATGAAGGTGCTTTGAAAAATATGTTT
GAGAACTATGGATTAATCTCATTAAAAGATGACCATATCGAAGTAAAAATAATTGCGAACGTTTTTAAACAGA
TGCTAAACAATCATCAATTTCAAGATGTTAATAATGTTGTCAACGCCTTAAGAGATAAAGGTTTCATATTAGCT
GACCGTGGACGCCAAACAACTAAAAGAAGTGTCAAAGATAATAGCGGTAAAAAACAATCACTTGTATTTTAC
CATCTAAAATTAGATGTAGAGTTTGCATCTATCCTCGGATTAACTAAAGATAAGTCGTTACTTCAAAATTGGAC
ACCGTCAAATGATAACAAAGCTGCGAAAGAACTTTTTAAATCCGCTAATGAAGGGATAGGCCCTTCAGGAGT
TCACGAAGATTTCTAATTGTCTTTATCATACAACTGTGAAGCATCAAACTGAATAATCGACGTATTGAAGATAG
AACAAATAACGGAGTGAAAAACATGGATGAACTTATCGAGATATTAGCCGATTTGGTAATTGAAGAAATCAA
TCAAACAAATGACTAATATGGACGCCCAACATCTCATTGATGTTGGGCTTATATATCAATTCATCCATAAATATAT
ATTATCACTGTGAAAGGAGGAGCCGATATGAAACAAGTCATAGGCTATTTACGTCAAAGTACGATGAAACAA
CAATCTCTTGCAGCACAGAAACAAGCTATCGAAGCAATAGCCGAAAAACATCATATTCAACATATCAACTTTT
ATAGCGACAAACAATCAGGTCGCAAAGATAATCGTAGTGGGTATCGACAAATAACACAATTAATTCAACAAG
GACAGTGTGACATATTATGCTGTTATCGTCTTAATAGGTTGCATCGTAATCTGAAAAATGCATTAAAACTCATC
AAATTATGTCAAACGTACCATGTTCATATCTTAAGCGTACATGATGGTTACTTTGATATGGATCAAGCTTTCGAC
CGACTCAAGCTTAATATCTTCATCAGTTTAGCCGAACTTGAATCGGATAACATTGGAGAACAAGTCAGAAATG
GGCTTCAAGAAAAAGCAAAGCAAGGTCGATTGATTACAACACATGCACCTTTTGGTTACGAATATCACAACG
GAACATTCATCATCAATCAAAATGAGTCACCAACGGTAAAGGCTGTATTCAATTATTACATTAAAGGTCATGGT
TATAAGAAAATTGCACAGTTATTAGAAGAAGATAACACGTATATCAATCGACAACCCTATCAAGTTCGTAACAT
TATCATCAATCCTAATTATTGTGGTCGTGTCAACAATCAATATGGCCAATTCGACAATATGTTTCCTTCTATTGTT
TCCACAAGTATATATGAGCAAGCGCAGAGACTTCGATTGCAAAAACAAACCAAACAGACACCTTCGGATAAT
CAACTCAAACAAAAAATCAAATGCCCATGTTGTAATGCAACACTTACAAATATGACCATTAGAAAAAAGAATC
ATACATTACGTTACTACGTCTGTCCTAAAAACATGAATGCTTCACGCTTTGTCTGTGATTTTAAAGGCATCAAT
GCACAAACACTTGAAGATAAAGTATTAGAAGTGTGCCGAGACTTTTATCAAAATCAACGCATCTACACAAAA
ATTAAAAGTGCGATTGACAAACGCATCAAAAGACAAAGAAACATAGAAAAACATCACACATTGACTCAAGA
ACAACTGATAGAAAAGTTGGCACAAGGCATCATTGATGCAGAAACGTTCAGAGAGCAAACACAATCATTAC
GTCAACAACCGCAACGCACTACATCTATCAATGGGCATCAAATACAACACATCATTCAAAATATTATTCAAAAA
CGTTTCACGTTAAACATATTGTACCCCTATATTGAAACCATTCACATTACGAAAGATAAAAATCTTATAGGAATC
TATTTCAAAAATGAACCACTCAATATTGTCAATCAAACCATGCAATCATCGATTGCATAAATGAAGAAAGGAT
GTAACATAATGCAACAACTTAAAACAAAACGTGTCGGTATCTATGTACGTGTATCAACAGAAATGCAAAGCAC

AGAAGGTTATAGTATCGACGGACAAATCAATCAAATCAAAGAATACTGTGACTTCCATCATTTTGAAGTTAAA
GATATATACGCTGACCGTGGTATTTCAGGTAAATCTATGAACAGACCTGAGCTCCAACGTATGTTGAAAGATG
CAAAAGAAGGCAATATCGACTGTGTTATGGTATACAAAACAAACCGATTAGCTCGTAATACATCTGATCTTCTC
AAAATTGTCGAAGATTTACACAAACAAAATGTCGAATTTTTCAGTTTGTCAGAGCGTATGGAAGTCAATACTT
CTTCTGGTAAACTCATGTTACAGATACTTGCGAGTTTCTCAGAATTCGAACGTAATAACATTGTCGAGAACGT
ATTTATGGGTCAAACGAGACGTGCCCAAGAAGGCTATTATCAAGGCAATTTACCACTAGGTTATGACAAAAT
ACCAGATAGTAAACACGAGCTAATGATTAACCAACATGAAGCTAATATTGTAAAATATATATTCGAGTGCTATG
CCAAAGGACATGGCTATCGTAAAATTGCCAATGCATTGAATCACAAAGGATATGTCACTAAAAAGGGGAAAC
CTTTCAGTATTAGTTCAATCACATACATCTTAGCTAACCCTTTCTATATCGGCAAAATTCAATTTGCGAAATACA
AAGATTGGAGTGAAAAACGTCGTAAAGGGCTGAATGATAAACCAGTGATAGCTGAAGGTAAGCATTCCCCC
ATTATTAATCAAGATTTATGGGATAAAGTACAAATGCGTAAGAAACAAGTCAGTCAAAAACCCCAAGTCCATG
GCAAAGGAACGAATCTGCTTACAGGCATTATTCACTGTCCCCAATGTGGCGCACCTATGGCAGCAAGCAATA
CCACGAATACTCTTAAAGACGGGACTAAGAAACGTATTCGTTACTATTCATGTAGTAATTTTCGGAACAAGGG
TTCCAAAGTATGTTCGGCAAACAGTGTAAGAGCTGATGTGATTGAAGATTATGTGATGAAGCAAATACTTGA
AATAGTCAAAAGTGATAAAGTCATTCAACGCGTAGTAACACACGTCAATCAAGAAAATCAAGTTGATGGCGC
TGCACTTCATCACGATATTGCTTATAAGCAACAACAATATGATGAAGTACAAATCAAAATAAATAACTTGATTA
AAACCATCGAGGATAATCCGGACTTAACATCAGTAATCAGACCAAGTATTCAAAAATATGAAAAGCAACTCA
ATGACATTACGAATCAAATCAACCAACTCAAAAATCAACAAAATGAAGATAAGACTTTATTTGATGCCAAAGA
AATCAGTAAACTATTACAACACATCTTTCATGATATTAAGCACATAGAAAAATCTCGACTCAAAGCATTGTATCT
ATCAGTGATTGATCGCATTGATATTAAAAAAGATGGTAATCATAAAAAACAATTCTATGTCACACTCAAACTTA
ATAACGAAATCATTAAACAACTTTTCAATAATAAACAACTCGACGAAGTGCATCTCAGCACTTCGTCTTTATTT
TTGCCCCAAACACTATATCTTACTATTTAGCTAATAGAATCATTGTTATAACAATAATTTTCACTATATTCTTTCGC
TTATTTTATCTAACTATTTTCAAGAATTGCTCTTTATCAATTTCAATAACACTTATCTAATGAAACAGTTATTGTC
GTAATTTTTTCATAACTATACAATGCTTATATTGAAATTGCTAAAGAACATCCCACAATGATTTTTTATCGGGAA
ATGATTGGGATTTGCAGATTCGAAGAAACAGAAGGATTGTGCAAGCACAATCCCAAAATCCCGTTACTTTTT
ATAAATAAATAATATATATAAGGAAGCAGAGACACAACCCTATCATCAATACTTATAAATAATGAGTTGATTGTT
TGTCAAAATAGGCGGAATTTCAGGATTTCCTTGGCATACCAACGTTTGTAGCGGGTTTTTATATGGAATTTTC
TCTTATTCCCACCGGGATTCGTAATAAATGACAAATCAAATATATATTATCTCAATGATGTATATGAACTACATCA
CTATTACACTATCTAGGAGGCAATAATATGAAAACCATCACTCAAGAACTCAAACAATATATAACCCACCTATTC
CAACTATCTAATAACGAAGCTTGGGAATGTGAAGCGCTGGAGGAAGCAGCAGAAAATATTCTACCGGAACG
TTTTATTAATGATTCTCCACTGGTACATCTCACACTCGAGACTTATACCTACTATAATGATGAACTACATGAGCT
AAGTATCTATCCATTTCTAATGTATGCCAATAACCAACTCATCAGTGTCGGATACTTAGACCATTTTGACATGGA
CTTTCTGTACCTTACAGATACTCAAAATATCATTATTGATGAACGTCATTTACTAAAACAAGGAGGTCAGGACC
ATGAATAATTGGATCATAGTTGCTCAATTATCCGTCACAGTTATCAACGAAATCATCGACATCATGAAAGAAAA
GCAGAAGGGAGGGAAATAGCATGAAAATCAATCGATACATCACAAGAGGAATTAATGAAAGTATTCCACTA
GACCTTCAAATCTTACTTTGGCACATGGTAGAAAAAAAAGATAACCAGCCTCATACAGATTACCTACACATTT
TTAAATTACAAGAAGATGAGAATATACTCTCAATCACACATGAACAAGAACAACCCACATACAAATTGGAATA
TCACTATACAAACTATGAAAAAAATCAAAATGCATTACCTAAGAAAGTCTACGTCATTCGTGAAGATGACGTG
GACGCTTTTTATTATGTGATGCTTTTACCAGAAGAATACTAAAGGAAGTGACGATGATGAATACAATCAAAAG
TACGATACACACAGAAGCCATATTTAGCGATGATGAACAGCACCGCTATTTACTCAAGAAAACATGGGATGA
AAAGAAAGCTGCATGCACAGTGATAACGATGTATCCTCATTTAGACGGTGTATTATCACTCGATCTCACAACT
GTTCTTATCCTCAACCAATTAGCCAATTCAGAACGATACGGTGCTGTATATCTTGTAAATCTATTCTCTAATATTA
GAACACCCGAAAACCTCAAACATATCAAAAATCCATACGATGAGCACACTGATATTCATTTGATGAAAGCGAT
TAGTGAAAGTGACACAGTGATTCTTGCTTATGGTGCCTATGCGAAGCGACCAGTTGTTATCGACCGTGTCGA

ACAAGTGATGGAAATGTTAAAACCTCATAAAAAGAAAGTAAAAAAGCTCATCAATCCAGTAACAAATGAAAT
TATGCATCCACTCAACCCTAAGGCACGTCAAAAATGGATTTTGAAATCATAGCAAATACATGGAGGTCAATAT
GGAAACAAAACAAATCAACATCGTTTTACTGTAAATGATATTGTTGAAACAATTCCTGACAATGAATTCGACG
AATTCAGACATCACCGTGGTGCAACTTCGTACCATCCTAAAATGATGTTAAAAGTGATTCTATATGCCTACACA
CAATCTGTATTCTCAGGTCGTAAAATAGAAAAAATGCTTAATGATAGCATCCGAATGATGTGGCTATCACAAA
ATCAAAAACCTTCTTATAAAACAATTAATCAATTTAGAGTAAATCCAAAAGTAGATGCTTTATTAGAATCTTTAT
TTATTCAATTTTACAGTCAGTGTGTAAAACAAAATCTTATAGATGATAAAGCTATTTTTATTGATGGTACAAAAA
TTGAAGCAAATGCCAATCGATATACATTTGTATGGAAAAAGAGTATTCAAAACCATGAATCAAAGATGAATGA
GGATTCTAAAGCCCTCTACCATGAATTGGTAACCAATAAAATCATACCGGAAATTAAAGAAGATCATGATAAT
GAATTAACAAAAGAAGAAATAGATTTGATTGGTAGTCACTTAGATAAAGAAATCGAAGATTTAAACCAACAT
ATCAACAATGAAAAATGTACTAAAACAAGAAAACAAATACGTCTCAAAAGAACTAAAATCAAAAAATACAAA
AAGCAAATCAATGATTATTTTGAGCGAAAGTATCGATACGAATTTCAAAAATCTATTTTAAAGGATAGAAATA
GTTATTCTAAGACAGATTATGATGCGACATTTATGAGAATGAAAGAAGATCACATGAAAAATGGACAACTTAA
GCCAGGGTATAATTTACAAATAGCAACAAATTCCCAATTTGTTTTATCTTATAATGTGTATCAAAATCCAACGG
ATACTAGAACGATGATTCCATTTTTAAATTCAATTCAAGAGACCTACGGTCATTTACCTGAATATATTGTAGCTG
ATGCAGGTTATGGTAGTGAATCAAATTATAAGGCAATTATAGATGACTTTAATCGAACGCCACTCATAACATAT
GGAATGTTTATAAAAGATAAAACTAAAAAATATAAAAGTGACATCTTTAATACTCAAAATTGGAACTATGACG
AAATTAATGACGAATTCATTTGTCCGAATAATAAACGGCTAGGTTTTAAAAGATATGCCTATCGTCATGATAAG
TATGGTTATAAGCGAGACTTCAAATTATATGAATGTGATGATTGTTCAGAATGTCCTCTGAAAAATCAATGTAT
GAACTTCAATTCAAAAACAAACAAAAAAATAATGAAGAATTATAACTGGGAATATTTTAAATCCCAAATTAAC
AAAAAGCTTTCAGAACCAGAAACAAAAAATATCTACAGTCAAAGAAAAATTGATGTGGAACCTGTTTTTGG
ATTTATGAAGGCTATTTTGGGTTTCACTCGGATGTCTGTCCAAGGACTCAATAAAGTCAAAAGAGAACTTGG
TTTTGTATTAATGGCACTTAATATAAGAAAAGTAGTAGCTCAACGAGCTGAAAATAATCAAAAAATTTATAAAA
AAGACAATTTCTATATTATTTCAATAGAAATTGTCTTTTTTCACTTATCCAAGAACTTTATGTCCCGGACTCATT
ATGATTTATTTTTTTATTGCTGACTTGCCATTAATTCTGCTTGCTCTACAACTTGTTCCACAGCCATTTTTTGAA
GATCTGGTGGATAGCCATATTTCTTTAATAGGCGTCTAACTTGAACTCTCATTTTAGCTTTAGCACTGTCTCGTT
TAGACCAATCAACACTCATGTTATCCTTAACTGTTTTAGTTAACTCATGTGCAATGGCTCTTAACTCTTTATCGC
CCAAAGCTTCTTTAGCTGTTTCATGTGAAGCTCGATATACTACATACAATATACGCAACAATACGCTTGTTTCG
ATTAGGTTTAGGCATTGAATCATAAAGTGCAATATACTTAACACGTTCTTTAATATTTGAATTAAAACCTAGTAA
ATATTGTGCTGCCACATTATTTATGTGCTGAGATTTTAAAATAGAGCATTTTAATATCGATTCACCATAACGTATA
TGTTCATGGCGATTCAAAATTTTTAAAACGTTTCTATCACATACTTTTTCACAGTCATTGTCCATCATTGTTTTA
CTTATATATAGTGCAGGATTAAACCAGAATATCATTTTAAAAACAACATAAAGCTGGTTGAATATTAAGTCATG
ACTTTTCACATGTGATAGTTCATGTAGAATAATATATTCAATTTCTTTGTCATTCATGGTTTCGACTACGACAGT
TGGTAGTACAATTTGGGATTTCACTAAACCAAATACCATCGGATTATCAATGTTTGAACTATAACTAATTGTTAT
ATGCTTTTTGTAGAACTGCATCTTACTTTGACATACTTTAAGTCGTTCATTAAGATATGACGATTCCAATGACG
AACTTTTAATAACATCAATTTGTCGGAATGCCTTAATCATATAAAATAAGCACAACAAACTACCAAATACCCATA
TCAAAAGAATCATATACGTTATATTTGAGGTCTCAAACTGATTAACATTAATTGCTAAGTCTTTCGTAACAGAT
GATTGTTGACCATCTAACATATGACTAACCGAAGAAGTCGTGTCAGATACATTTCGATTCATCATATCTTTTGA
AAATGTAAAATTCGATATTTTGTAAAATGGTATTAATGGAATTAACGTGGAGACGAGCACTAATAACCAAATCT
TATGTGACATAATATTTTGAGTATATTTTATATAGAGCATTCTCACTAAAAAAATTACACATATCGTGAGCAATG
AACTGATTATACTTAACATTAAAAAAGATGATAACACCTTCTACACCTCCATATCACAAAAATTATAACATTATT
TTGACATAAATACTACATTTGTAATATACTACAAATGTAGTCTTATATAAGGAGTATATTGATGAAAAAGATAAA
AATTGTTCCACTTATTTTAATAGTTGTAGTTGTCGGGTTTGGTATATATTTTTATGCTTCAAAAGATAAAGAAAT
TAATAATACTATTGATGCAATTGAAGATAAAAATTTCAAACAAGTTTATAAAGATAGCAGTTATATTTCTAAAAG

CGATAATGGTGAAGTAGAAATGACTGAACGTCCGATAAAAATATATAATAGTTTAGGCGTTAAAGATATAAAC
ATTCAGGATCGTAAAATAAAAAAAGTATCTAAAAATAAAAAACGAGTAGATGCTCAATATAAAATTAAAACAA
ACTACGGTAACATTGATCGCAACGTTCAATTTAATTTTGTTAAAGAAGATGGTATGTGGAAGTTAGATTGGGA
TCATAGCGTCATTATTCCAGGAATGCAGAAAGACCAAAGCATACATATTGAAAATTTAAAATCAGAACGTGGT
AAAATTTTAGACCGAAACAATGTGGAATTGGCCAATACAGGAACAGCATATGAGATAGGCATCGTTCCAAAG
AATGTATCTAAAAAAGATTATAAAGCAATCGCTAAAGAACTAAGTATTTCTGAAGACTATATCAAACAACAAAT
GGATCAAAATTGGGTACAAGATGATACCTTCGTTCCACTTAAAACCGTTAAAAAAATGGATGAATATTTAAGT
GATTTCGCAAAAAAATTTCATCTTACAACTAATGAAACAGAAAGTCGTAACTATCCTCTAGGAAAAGCGACTT
CACATCTATTAGGTTATGTTGGTCCCATTAACTCTGAAGAATTAAAACAAAAAGAATATAAAGGCTATAAAGAT
GATGCAGTTATTGGTAAAAAGGGACTCGAAAAACTTTACGATAAAAAGCTCCAACATGAAGATGGCTATCGT
GTCACAATCGTTGACGATAATAGCAATACAATCGCACATACATTAATAGAGAAAAAGAAAAAAGATGGCAAA
GATATTCAACTAACTATTGATGCTAAAGTTCAAAAGAGTATTTATAACAACATGAAAAATGATTATGGCTCAGG
TACTGCTATCCACCCTCAAACAGGTGAATTATTAGCACTTGTAAGCACACCTTCATATGACGTCTATCCATTTAT
GTATGGCATGAGTAACGAAGAATATAATAAATTAACCGAAGATAAAAAAGAACCTCTGCTCAACAAGTTCCA
GATTACAACTTCACCAGGTTCAACTCAAAAAATATTAACAGCAATGATTGGGTTAAATAACAAAACATTAGAC
GATAAAACAAGTTATAAAATCGATGGTAAAGGTTGGCAAAAAGATAAATCTTGGGGTGGTTACAACGTTACA
AGATATGAAGTGGTAAATGGTAATATCGACTTAAAACAAGCAATAGAATCATCAGATAACATTTTCTTTGCTAG
AGTAGCACTCGAATTAGGCAGTAAGAAATTTGAAAAAGGCATGAAAAAACTAGGTGTTGGTGAAGATATAC
CAAGTGATTATCCATTTTATAATGCTCAAATTTCAAACAAAAATTTAGATAATGAAATATTATTAGCTGATTCAG
GTTACGGACAAGGTGAAATACTGATTAACCCAGTACAGATCCTTTCAATCTATAGCGCATTAGAAAATAATGG
CAATATTAACGCACCTCACTTATTAAAAGACACGAAAAACAAAGTTTGGAAGAAAAATATTATTTCCAAAGA
AAATATCAATCTATTAACTGATGGTATGCAACAAGTCGTAAATAAAACACATAAAGAAGATATTTATAGATCTTA
TGCAAACTTAATTGGCAAATCCGGTACTGCAGAACTCAAAATGAAACAAGGAGAAACTGGCAGACAAATTG
GGTGGTTTATATCATATGATAAAGATAATCCAAACATGATGATGGCTATTAATGTTAAAGATGTACAAGATAAA
GGAATGGCTAGCTACAATGCCAAAATCTCAGGTAAAGTGTATGATGAGCTATATGAGAACGGTAATAAAAAA
TACGATATAGATGAATAACAAAACAGTGAAGCAATCCGTAACGATGGTTGCTTCACTGTTTTATTATGAATTAT
TAATAAGTGCTGTTACTTCTCCCTTAAATACAATTTCTTCATTTTCATTGTATGTTGAAAGTGACACTGTAACGA
GTCCATTTTCTTTTTTTATGGATTTCTTATTTGTAATTTCAGCGATAACGTACAATGTATTACCTGGGTATACAG
GTTTAATAAATTTAACGTTATTCATTTGTGTTCCTGCTACAACTTCTTCTCCGTATTTACCTTCTTCTACCCATAAT
TTAAATGATATTGAAAGTGTATGCATGCCAGATGCAATGATACCTTTAAATCTACTTTGTTCTGCTTTTTCTTTAT
CTATATGCATATATTGAGGATCAAAAGTTGTTGCAAATTGGATAATTTCTTCTTCTGTAATATGAAGGCTTTTTG
TTTTGAATGTTTCTCCTACTATAAAATCATCGTATTTCATATATGTCTCTCTTTCTTATTCAAATTAATTTTTTAGTA
TGTAACATGTTAAAGGTAAGTCTACCGTCACTGAAACGTAAGACTCACCTCTAACTTTCTATTGAGACAAATG
CACCATTTTATCTGCATTGTCTGTAAAGATACCATCAACTCCCCAATTAGCAAGTTGGTTTGCACGTGCTGGTT
TGTTTACAGTCCATACGTTCAATTCATAACCCGCTTCTTTTACCATTTTTACTTTTGCTTTAGTAAGTTTGGCAT
CTTCAGTGTTTACTATTTTAGCATTACAGTAATCTAAAAGTGTTCTCCAGTCTTCACGAAACGAAGTTGTATGG
AATATAACTGCTCTGTTATATTGTGGCATGATTTCTTCTGCAAGTTTAACAAGCACAACATTAAAGCTTGAAAT
GAGCACTTCTTGATTCTGATTTAAGTTTGTTAATTGTTCTTCCACTTGCTTAACCATACTTTTAGAAAGTGCTA
GTCCATTCGGTCCAGTAATACCTTTTAATTCTACATTTAAATTCATATTATATTCATTTGCTATTTTTACTACATCA
TCGAAAGTTGGCAAATGTTCATCTTTGAATTTTTCACCAAACCAAGATCCTGCAGAAGCATCTTTAATTTCATC
ATAATTCAATTCAGTTATTTCCCCGGACATATTTGTAGTCCGTTCTAAATAATCATCATGAATGATAATCAGTTGT
TCATCTTTTGTAATTGCAACATCTAACTCCAACCAGTTTATACCTTCTACTTCTGAAGCAGCTTTAAATGATGCA
ATTGTATTTTCCGGAGCTTTACTAGGTAATCCTCTATGTCCATATACAGTTAGCATATTACCTCTCCTTGCATTTT
TATTTTTTTAATTAACGTAACTGTATTATCACATTAATCGCACTTTTATTTCCATTAAAAAGAGATGAATATCATA

AATAAAGAAGTCGATAGATTCGTATTGATTATGGAGTTAATCTACGTCTCATCTCATTTTTAAAAAATCATTTAT
GTCCCAAGCTCCATTTTGTAATCAAGTCTAGTTTTTCTGTACCCCTTATCTGCAATTTTACTTAGGATTGCTTTT
AACTTACCCCTTATCAGCAATTTTACTGAGAACTGCTTTTAACGCACCTCTTATCTGCAATTTTGCCTAGAACT
GCTTTTAACGTACCTCTTATCTGCAATTTTACTGAGAACTGCTTTTAACTTACCCCTTATCAGCAATTTTGCATG
GAATTGCTTTTAACGTACCTCTTATCTGCAATTTTACTTAGAACTGCTTTTAACAAACCTCTTATCTGCAATTTT
ACTTAGAACTGCTTTTAACGTACCTCTTATCTGTAATTTTACTGAGAACTGCTTTTAACAAACCTCTTATCTGCA
ATTTTACTTAGAATTGCTTTTACTATTCCTCTTATTAGTATAATCTCAGTAAGAATGCGTATAAAAATGAAAATTA
CAACCGATTTTGTAAGTGCTGACGCCTGAGGGAATAGTATGTGCGAGAGACTAATGGCTCGAGCCATACCCC
TAGGCAAGCATGCACGTACAAAATCGTAAGATAAAAAAATAAGCATATCACTGTAAACTTTAAAAAATCAGTT
TAGTGATATGCTTATTTATTTCGAGTTAGGATTTATGTCCCAAGCTCATCAAGCACAATCGGCCACTAGTTTATT
TCTCTATCTTATATGTTCTGATATGGTCTTCTATACTGTATAAGTATACTTTTGAATATGGATCTTGTGTCAATTCA
CGTTCGAAATCAAATTCTTGATTATCAAATCTGTTAAAGAATGTTTCGTATTCTTCGACTGATAATTGCTCTCTA
GATTCTAGCATATTTAAGTGTTTCTCTTTATCTAATGCTTTGTCATATCCTTTAACGATTGAACCACTAAAGATTT
CTCCTACTGCTCCTGAACCATAACTAAATAGACATACTTTCTCTTCTGGTTGGAATGTGTGGTTCTGTAATAAC
GAAATTAAACTTAAGTATAATGATCCTGTATAAATGTTACCAACATCTCTATTCCATAATACGGTTCTGTTGCAA
AGTTGAATCTATAGTATAATTTTAACAAAAAGGAGTCTTCTGTATGAACTATTTCAGATATAAACAATTTAACAA
GGATGTTATCACTGTAGCCGTTGGCTACTATCTAAGATATGCATTGAGTTATCGTGATATATCTGAAATATTAAG
GGAACGTGGTGTAAACGTTCATCATTCAACGGTCTACCGTTGGGTTCAAGAATATGCCCCAATTTTATATCAA
ATTTGGAAGAAAAAGCATAAAAAGCTAATTACAAATGGCGTATTGATGAGACGTACATCAAAATAAAAGGAA
AATGGAGCTATTTATATCGTGCCATTGATGCAGAGGGATATACATTAGATATTTGGTTGCGTAAGCAACGAGAT
AATCATTCAGCATATGCGTTTATCAAACGTCTCATTAAACAATTTGGTAAACCTCAAAAGGTAATTACAGATCA
GGCACCTTCAACGAAGGTAGCAATGGCTAAAGTAATTAAAGCTTTTAAACTTAAACCTGACTGTTATTGTACA
TCGAAATATCTGAATAACCTCATTGAGCAAGATCACTGTCATATTAAAATAAGAAAGACAAGGTATCAAAATAT
CAATACAGCAAAGAATACTTTAAAAGGTATTGAATGTATTTACGCTCTATATAAAAAGAACCGCAGGTCTCTTC
AGATCTACGGATTTTCGCCATGCCACGAAATTAGCATCATGCTAGCAAGTTAAGCGAACACTGACATGATAAA
TTAGTGGTTAGCTATATTTTTTTACTTTGCAACAGAACCTATAAAAAACATGGGCTTAAAGGGTTAATTTATAT
AGTATTACTGCAAACATTGATTTGGGTCAAAAATTAGGGGTATTAAAAAAATGAATGAATCATAATTTTATCAA
GCTGATTGGAGAGGTTAAAATGCATTATATAAAATTTATTGAGTCAAAAGATAATACAAAACTTTATATGAAAG
TGAATGATATTCAAGATGCAAAAGCGAATATCATTATAGCTCATGGTGTGGCAGAACATTTAGATCGTTATGAT
GAGATAACAGCATATTTAAATGAAGCGGGTTTTAGTGTTATTAGATATGATCAAAGAGGGCATGGTCGTTCTG
AAGGCAAGCGTGCCTTTTATAGCAATAGTAATGAAATTGTCGAAGATTTAGATGCGATAATAAATTATGTGAA
GTCAAACTTTGAAGGTAAAGTTTACTTAATCGGTCATAGTATGGGTGGTTATACAGTCACTTTATATGGAACG
AAACATCCAAATACAGTGAATGGTATTATAACTTCTGGAGCATTAACACGTTATAATAATAAACTATTTGGCAAT
CCTGATAGAAACATATCACCTGATACTTATATAGAAAACAATTTAAGTGAGGGGGTATGTTCTGATTTAGAGG
TAATGGAAAAATATAAACTTGATGATTTGAATGCGAAACAAATCTCTATGGGGCTCGTCTTTTCAATAATGGAT
GGTGTTAGGTATTTGAAAGACAATGCTCAACAATTTACAGATAATATTTTGATATTGCATGGCAAGGAAGATG
GGCTAGTAAGCTATGTAGATTCTTTACAGCTTTATCAAGAAATAGGATCAGCACATAAATCATTACACATCTAT
GATCGTTTGGAGCATGAAATATTTAATGAAAGTTCTTATAATAGAACTATTTTTAACGAAGTTATTGAATGGCT
TGAAACGGAATTAACTTATAACTAAAACAGTATAGTTCCGTGTATTTGATTATAAGAAATTATGAGGATATTAA
ACATACTAAGATTAGCTATGAAGAAATCTATGACGATAGATTTTTTCATAGCTATTTTTTATAGTTATAGAGAGG
AGTAGACTGTCCAGACTCTTGGATTTTAAATCCGTAAAAAAAACAAGTCAGCTTTACTCTCACCTTTTGAAAT
TCGTTTGTAGTATGTTGGGTTCTTGAAACCGTGTATAGGAAAATGAAATGAGAAAGGTTAAGTAAAGTTTTT
AGCTTCTCAACTATTCAAAGGAGGTTTTTTTATCGATTACTTAGGTGTTGATATTAGTAAAAGGAGTAGTGTA
GTTGCACATTATAAAAATGGAAAATTCCAAAAAGAGTTTTTCATCCAAAATAATAAAAATGGCTACAATTATTT

ACTCAAGTATTTGAATGACTTAGACCACCCACAACTCATTTTTGAATCTACAGGTATCTATTCAAGAGGTATGG
AACGATTTTGTTGTGTAAATCAAATTAACTATATTCAAATGAATCCGTTAGAAGCCAAATTTAAAACGAGCGC
TCTAAGATCATGGAAAACTGATCAGGCAGATGCTCATAAGCTTGCTTGTTTAGGACCGACGCTCAAACAAAC
AGGCAGCTTACCTATACATGAGTTAATATTCTTTGAATTAAGAGAACGTGCCCGTTTTCATCTAGAAATCGAG
AATGAACAAAATCGACTTAAATTTCAGATTCTTGAATTACTCCATCAAACATTCCCTGGTTTAGAAAGATTATT
TAGTAGTCGATATTCAATCATTGCACTCAACATCGCAGAAATTTTTACTCATCCAGACGTGGTTCTTGATATCG
ACAAGGATGTACTTATTACACATATATTCAATTCTACAGATAAGGGAATGTCAATGGATAAAGCTACAAAATAT
GCACTTCAATTAAGAGTGATTGCTCAAGAAAGCTATCCTAATGTCGATAGACATTCCTTTCTAGTCGAAAAAT
TACGCTTACTTATTCAACAATTAAAACAATCTATTCATCATCTCAAACAATTAGATGATGCCATGATTCAATTAG
CACAACAACTCGATTATTTTGAAAATATTCATTCGATACCTGGTATTGGTAAGCTAAGCACAGCTATGATTATT
GGGGAGATTGGTGATATTAAGCGATTTAAATCAAATAAACAACTCAACGCTTTTGTAGGCATTGATATCAAAC
GATATCAATCAGGTCATACACACTGTAGAGATACCATCAACAAGCGTGGTAATAAAAAAGCGAGAAAACTTT
TATTTTGGGTGATTATGAATATAATAAGAGGGCAGCATCATTATGACAATCATGTCGTCGATTATTACTACAAAC
TAAGAAAGCAGCCTAATGAGAAACCTCATAAGACTGCCATCATTGCTTGTATAAATCGATTATTAAAAACGAT
TCATTATCTGGTAATGAATCATAAATTGTACGATTATCAAATGTCACCACATTAGCCAAACGTACAATTAAATAT
ATTTTAATACCTTATTCAAAAAATTAAAATGAACGGTTTAGTTAAGTAATGCTTATTTTAATTATAAGTACTTGA
CTAATCGTAAGAAAGAGCCTAGGACATAAATCAATGTCTCGCGACCACAGCTTAATTTTTGGTGTTCATTCAT
GACTTTAAAAAATCCTTATTGCATAAATGTACATAGTGTAGTACTATTCAAAACGTAATTATTACGATTTGAATT
AAGCGAGGAGAATGAAATGACTAAGACTTATGACGTTTGGTGGCAAAAAGGTCAAGAATCAGATGATGATA
TGGCACGAGACCATCAAGAAGCTTGGGAGAGAACAATAAAAATGCTTGATACATCTGACATCGAAGGGAAA
ACGATTTTAGATGTGGGATGTAATCAAGGCGGATTTTTACGACAGTTATACGATACAACACCGTTTAAAAAAG
GTGTTGGCATAGATTTAGCACGTTTATCTTTGGAAAAGGCAGAGACATTAAAAGGACAACGTCCACTTACAT
ACTATTTAACAGATAAACCGCAAGAAACGAAGCACGTGTTTGATACGGCAGTAAGTACGTCTGTCTTGTACTT
AATAGAAGATATTCCGCAACATGCAAAAGATTTAAAAGAGGTATTGAAACCAGGCGGTGTTTATTACGCTTC
ATTCGCGGATTTAACTAATAACCCAAGTCGTCAGTTTATGGATGACACGATTAATCAATATGGTGCAACACCTT
CTCAGAATCACTCTCTAAAACATATCGTTGATAGCTTTGTGGATGCAGGATTTGAAGTTGCAGTAATGAAAGA
GCATGTACCTGACGTGATTGATTTAACACATTATAGCGATTTTTATTTATCACCGAATGATTATTTACAAACACT
ATATGAAGAATCGTTTTTAATAAAAGCAAGTGTGAAAGAAGGTACTGAGAAATGAGGAAATGTGTATTAATG
ACGGTAGCAGCAAGTGCTACGCTCTTATTGGCAGGTTGTGGCAATGGTCAAAAAGAAGATAAAGATGTTAC
GGTATCGCTACCTACTGAAGCAAAGGCGGATAAACTTGACGCGCAAGGCTATGATGCAGCGATGCCCGTTTA
TAGTGCAGTGTATGATGCATTAGTTAAATATGATAAAGATAAGGGTATTAAAGCAGGTTTAGCAGATAAATGG
AGCGTTGATGAATCAGGGAAAGTTTATGAATTCCATTTGAAAAAGAATGTTAAATTCTCAGATGGTTCAGCAT
TAGATGCTAAGGACGTGAAATTCTCGATTGATCGTGCGAAAGCGATGAACAAAGATTCGACTGTAGAAACGT
TAAAAAAATTAGATAAGGTCGTTGTTAAAAATGAGCACGTGGTCCAAATTAGATTGAAATCTCCTTCAAATCA
AGTGTTAAATGAATTAACACAAGTGAGACCGTTGCGTATTATGAGTCCACATTCAGTAGAAGATGGTAAAGT
AAACGGTAAATTTGAAAAAGCGATTGGAACAGGTGCATTTGTTGTTGATAAAACTGGTAAAGAAAAAACGA
CAATGAAGCCAAATAAATATTTTGACAACGGTCACCCAGTCAATTATCATCTTGCATTCCAAACGATTGAAGA
TGGGGACTCAAGAAATTCTGCAGTACAAAGTGGTTCTGTAGATATTTCTGGTGGTGCTTTAGGTATGCTCTCA
GACGAACAAATCAAACAAGATAAGAAAAATAAGAACTTAACGATTGAAGATAGACCTAGCACAGTAAGTCA
CTTTATGGCATTTAACCCTAAAAATGATGTATTAAATCAACGCACAATTCGTGAAGCGATAAGTAAGAGCATC
GATGCGAAAGACATTGCGGGCAAATCTGTAAATGGTCTGTTCCAGAAGAACGTACAATTTGTGACTAAAAAT
AATCAACAGCCACACGATTATGATATGAAAGCGGCTGAAAGGTTACTTAAATCAGAAGGATATCATAAAAAC
GATGACGGCATCTTTGAAAAGAATGGCAAACCTTTATCATTTAACTTAGTCATTCAAACTGCAGAGTTCCCAA
ATTGGAAAGATAAAGCTGAAAAAGTGCAACGTCAGCTTAAACAAGCCGGTATTAAGTTAAATGTGAAAACG

TTAGATTCACAATCATACTATGATACATTATGGACGAAAAAAGACTATGATTTGATTTTCTATAGAACGTATTCA
GATGCATTAATGCCTTACAACTTTATAAGTTCAGTGTTTAAAAATAATGATGGTCAACCAGGGGTGTTAGCTG
ATGATGAAACATTAACGAAACAGCTAGACGATTTCCCATCAACCGTATCAAAAGAAGACCAACAGTGTTCAT
TTGATGACATATTTAAACACTTTAATCAACAATACTATGGTGTGCCAATTGCTTATCCAAATGAGACGTTTGTA
GTGAGTGATAAAGTAAAACAATTCAAATTCTCTGGACTTACGGATGCACCAATTGATTATAAAGCGTTGAAA
GTTAATGAATAGCAATGCTCAAACGTACAATTAAATTCATACTTTATTTAATCGTAAGTTCGTTTATTATCTTCAT
TTTAGTTGAGAAGACATCTGGTAATCCAGCGATTCTGTATCTACAACGTCATGGTTATACGTCGATTACGCAAG
ACAATATTGAAGCGGCACAACATCAACTTGGCTTAGGACAACATGTGTTACTAAGATATATCGATTGGGTTGG
ACATGCACTCACGGGCAACTTAGGATACGGCTTTAGTACGAACGAAGCAGTTACCGCTATGATAATGGAAGC
CATCGTGCCGACGCTTGTGCTAATCATTGTCTCTAGTTGTATCATGTTGCCATTTGGCTATATTGTTGGTTACTT
CGTTGGGACGCGTCCGCATACACGTTACGCTAATGGAATTCGTGGATTCGCCCAAGTGATGACCTCAATGCC
AGAATACTGGTTAGCTATTTTATTCATTTATTATTTAGGCGTACGTTGGCAATTGTTACCATTTGTAGGTAGTGA
TTCATGGCAACACTTTGTGCTGCCAATCTTCACAATTGTTGTTATAGAAGGGTGTCATATCTTATTGATGACAG
CACATCTGATTACACAAACGTTAGATCAAGATGCGTATCAACTGGCGCAGTTAAGACATTTTTCGTTAAAAGC
GCGTATCATCGTACAAATTAAAGAGATATTTGCACCACTAATGACGATTTCAATTAACAGTATCATTCATTTAAT
TGGAAAAGCCGTAATACTAGAAGTCATCTTCAGCATGTCTGGTATAGGTAAATTGTTGATTAATGCTATTAACC
AACGAGATTATCCACTGATTCAGGGCATTGTCATCTTTATCATTGTCTTTATTATGCTAATGAATTATTTAGGCG
ATGTGATTATTTTGAAGAATGAACCTAGACTTCGACGACGTCATACCCAGCAGTCAGGCAATGAGAAAAGAG
GTACGATGTGATGAAAAAATATCAAACGTACATCGCAATAGGTTCACTATTGAGTTTGATGGTTGTATTAATTA
CGTATGGTTTAATGCAAGACACGCAACATTTGAACCCACTTGAGTCACCTAATGGACAACATTGGTTGGGTA
CCGATCAATTAGGCAGAGACTTCTTAGTAAGACTGATTGTCGGTAGTCTTGTCACATTGAGTTTAACAGGCAT
AGTGATTCTATTAAGCGTTTGTATGGGACTTATCTTTGGCTTAATTGCAGGCATAGAAAGACGATGGTTAGAT
CAAATCATCATGTTTGTTGCCGATATGTTGCTGGCTATTCCGTCATTTATTATCGCATTAGTCATCTTAAGTTTAG
TAAGTAACTCCATGATAGGTTTGATACTTGCTTTAACGATTGGATGGATAGGACGTTATTTACGTTACTTCAGA
AATTTAACGCGAGATATTCAAAAACGTCCATTTGTTCAATATGCACGATTGAGTGGGAACTCAACATTCAAAA
CGACAGTAACACATGTGATTCCACATTTATTAAGTAGTATATTCGCTTTGGTAACGGCTGACTTTGGCAAAATG
ATGCTCAGCATATCTGGACTTGCTTTTCTAGGACTAGGTATTAAACCGCCGACGCCTGAGTTAGGAACAATTC
TTTTTGATGGGAAAAGTTATTTCAACGGCGCACCGTGGCTCTTCTTCTTCCCTGGTGTATTGTTAGGAGGTTT
CGCCTTATTATGTCAAATTATCAACAAAAAAATAACGCAGTAAATACGGTAGTCAACGTCAATCAATTATCGAT
TTTAGATCAAGAGAAAGTATTGTTAAAGGATGTTGATTTGACAGTAACTAAAGGTGCATTTCATTGCATTATA
GGTGAAAGTGGCAGTGGGAAATCACTGTTAACAAGAACAATACTTGGAATGAAACAATCACAATTATGTTAT
CAAGGAGATATTGACATCGATTTAACTCAAACAGATGCAGTGTTTCAAGATGTTCAAAGTAATATGTTTCAAA
ATATAACATTAGCTAAGCATTTCCAATACATTTATGAAGCCAATCGCACACATCTCACTAAACAGCGTATTAAG
GAAGATGTCTTACAGATGATGCAATTACTTGGTTTAAGACAAGGGGAACAATTGCTTGAGCGTTATCCCTTC
GAACTTAGTGGAGGTATGGCACAACGTGTCGCCTTTATAATGTCATTAATTAGACGTCCGAACTACTTATTTTT
AGATGAACCAACGAGTGCACTTGATCAAGAAAATATTAAAAAGTTTATGCATTACCTTCTTAGGGCACAGGA
GCGCTACCAAATGACCATTGTTTTTATCACACATGATATTAACTTAGTGAAAGATTGTGCCACACATATTAGTAT
TATGCAGCAAGGTAAATTGATAGAAAATGGTGAGGCCGCGTCGATCTTAACTAAGCCGACACATAATTACAC
GAAAAAATTAATTACGATTGCACATCGGAGACAACCTTATGCTTAAAATAGAGAGATTAACCAAATATATAGA
CACGCAACTGATATTTAAAGAGATATCATGTACAATTAACGACCAGCACTTACTCATAAGTGGGGAGAGTGGT
TGTGGTAAATCCACATTAGCCAAGATTATCGCTGGCTTAGATACGGATTATCAGGGCGAATTATATCTTAATGG
GCGCTTACGTGAATCTTATACGTCTAAAGAGTGGATGAAGCACATCCAATATGTACCTCAATATCAACGTGATA
CTTTAAATCAGCGTAAAACGGTATTAGCTACATTATTAGAACCACTTAAGAATTATAAGGTAAATAAACAGCGT
TATACATCAAGCATTGAAGCAGTGCTTGATCAGTGTAATTTACCACACGATATACTTAATCATAAAGTTTCGAC

ATTAAGTGGTGGCCAATTTCAACGCGTCTGGATAGCTAAAGCTTTAATATTAGAACCAGAGATTCTCATATTG
GATGAAGCTACAACCAACTTAGATGTCATTAATGAAGAAGCTATACTTCAAATGTTGATTTCCTTAAAGATGA
CACAATTAATCATTATTTCACATGATACATACGTCTTAAGCCAATTTGAAGGAATTCAGTTACAGCTAAATAAAT
TGAATAATTAAGATCACAAATCTTAATATGGTGAATATTTAATGGTACCTAAAAAATAAAATTTAAACTACAATG
TCTAAATCCATATGTTGTTTCATTAGAGGATTTAAAAATGATTATAACACTAAAAGATTTCAAATTATTATATTTA
ATATAAATTTACATATGATAAACGAATAACAATTCCAATATAAATTATTTTTTTGATTATTTTTATTTATAACTATAT
ATTTTATATGAAAAATATAAGTTATAATAATAAGTTTAATATTGCCTCGTGGTTCTGAGCTTGAACTTATCTCTAA
ATCATTTTGAGCTACTTATCTATCAATTCATATATTCTATAACAATATTTGTGACATCACGTGCTATTTCATGAAGT
GATTTTACGATATCACCTTCTTTAGAAAAAATATATTTCAGTAGGCACCGACGTATACAGAATCATTTGAGTATT
AAAAATAAAAACTAGAAAAAGAAACCGCATCATTAACTGATACGCAGAATCATATTATAAATAAAACTAAAAA
TGAGGTTGTATATAACTCACTCTGAAATTGATTGAATATATAGTATCTTTAATAAAATGCAGCTATTGTGGCGTA
GAATTTGAGAATCAAAAAATGATTAATATAGTTTGAAGAGACTGAGCATAAATACTAGAAAAATGGCCAGTA
AATGAGTTTACTATAAACTCATTTACTGGCTTCTCTAATAATTATCAAGACAATTTGCGTTTCTAGGCATACTTT
GAAATGCGCTATTTTCTTTAAGAATATTAATATATGACTTGTGTTGGTAATGATAACCTTGGTTAGAGTGTATTG
TCATGTGATAGTTAAGATTAGATCTTTTAATTAAAACATTGTTTTAAATATCGATGACAAGGTCTAATGTAGGAC
GTGTAGATATAAGGTTCGGAATTATATAAATCCATAAAAGGAGATAAATCGAAAACCTTTATACCCTGGGTGG
GTATATGTTATAGTATAAGTAGCTTTACTATAACATTTTCATTAGGAGGGGTTAATTTGAATAATAATGGTGAAG
AGCATAATCATCAAAATCACATGAATCATTCCAATCAAATGCATCATGATAACCATGCCTCACATGATCATCATA
GTGGCCATGCACATCATCATGGAAATTTTAAAGTTAAGTTTTTTGTTTCATTAATTTTTGCAATACCTATCATTC
TTTTATCGCCACTGATGGGTGTTAACTTACCTTTTCAATTCACATTTCCAGGTTCTGAATGGGTAGTGTTAATAT
TAAGTACAATTTTATTCTTTTATGGTGGTAAACCGTTCTTGTCTGGTGGTAAAGATGAAATTGCTACAAAAAA
ACCAGGCATGATGACCTTAGTTGCCCTAGGTATTTCAGTAGCTTATATTTATAGCTTGTATGCTTTTTATATGAA
TAACTTTAGTAGTGCAACTGGTCATACAATGGACTTTTTTTGGGAATTAGCAACCTTAATTTTAATTATGCTATT
AGGACATTGGATAGAAATGAATGCTGTCGGAAATGCTGGAGATGCTTTAAAGAAAATGGCAGAACTGTTAC
CTAATAGTGCTATTAAAGTTATGGATAATGGCCAACGCGAAGAAGTTAAAATATCAGACATCATGACTGATGA
TATCGTCGAAGTAAAAGCCGGAGAAAGCATTCCAACAGATGGTATTATCGTTCAAGGACAAACATCTATAGAT
GAATCCCTAGTCACTGGAGAATCTAAAAAAGTACAAAAAAATCAAAATGACAACGTCATCGGGGGTTCTATT
AATGGGTCTGGAACAATACAAGTCAAGGTTACAGCTGTGGGAGAAGATGGATATCTTTCTCAAGTTATGGGA
CTTGTTAATCAAGCACAAAATGATAAATCTAGTGCTGAATTGTTATCTGATAAAGTAGCGGGTTATTTATTCTAC
TTTGCTGTAAGTGTTGGCGTGATTTCTTTTATTGTCTGGATGCTCATTCAAAATGATGTTGATTTTGCATTAGA
ACGTCTTGTAACTGTGTTAGTCATTGCTTGTCCACATGCTTTAGGCTTGGCAATACCTTTAGTCACTGCACGTT
CTACTTCAATTGGTGCACATAATGGTTTAATTATTAAAAATAGAGAGTCTGTAGAAATAGCTCAACATATCGAT
TATGTAATGATGGACAAAACTGGTACTTTAACTGAGGGTAACTTTTCTGTGAATCATTATGAGAGCTTTAAAA
ATGATTTGAGTAATGATACAATATTAAGCCTTTTCGCCTCATTAGAAAGTCAATCTAATCACCCATTAGCTATAA
GTATTGTTGATTTTGCGAAAAGTAAAAATGTTTCATTTACTAACCCACAAGACGTTAATAATATTCCAGGTGTC
GGATTAGAAGGTCTAATTGATAATAAAACATATAAAATAACAAATGTCTCTTATCTTGATAAACATAAACTTAAT
TATGACGATGACTTGTTTACTAAATTAGCTCAACAAGGTAATTCAATCAGTTATTTAATTGAGGATCAACAAGT
CATTGGCATGATTGCTCAAGGAGATCAAATTAAAGAAAGCTCAAAACAAATGGTAGCTGATTTACTATCAAG
AAATATTACACCAGTCATGCTTACAGGTGACAATAATGAAGTGGCACACGCTGTCGCAAAAGAATTAGGTATT
AGTGATGTCCACGCACAACTCATGCCAGAAGATAAGGAAAGCATTATAAAAGATTATCAAAGTGACGGTAAT
AAAGTCATGATGGTCGGAGACGGTATCAACGATGCGCCGAGTCTTATAAGAGCGGATATTGGTATAGCAATT
GGTGCAGGTACAGATGTTGCAGTGGATTCAGGTGATATCATACTTGTTAAAAGTAATCCATCAGATATCATTCA
TTTCTTGACCCTTTCAAATAATACTATGAGAAAAATGGTGCAAAACTTATGGTGGGGTGCAGGTTATAATATT
GTTGCTGTACCTTTAGCAGCTGGTATTTTAGCATTTATTGGCTTGATTTTATCACCTGCAATAGGTGCTATTTTA

ATGTCTTTAAGTACAATTATCGTTGCAATTAATGCCTTTACATTAAAATTAAAATAAAAGATAGGAGTTTTATTA
TGATTAAAAAATTATTTTTTATGATATTAGGATCATTACTAATATTATCAGCTTGCTCCAATAATGATGAAAAAGA
TAAAGACACTAATGACCAAAAAAGTGAGAGCCATATGAAGCATAATGATGAAAGTAAAGTTCCAGAAGATAT
GAAATCGACTAATGAGGGTGAATTTAAAGTGGGAGATAAAGTAACGATTACAGCAGGGCATATGCCAGGTA
TGAAAGGTGCAGAAGCTACTGTAAAAGGTGCGTATAAAACATATGCCTATGTTGTAAGTTATAAACCCACAAA
TGGAAATGAAAAAGTAAACAATCATAAATGGGTCGTAAACGAAGAGATTAAAGATGCACCTAAAGATGGATT
TAGTAAGGGCGATACTGTTAAATTAGAAGCAAGTCATATGTCTGGTATGAAAGGTGCTACAGCCAATATAGAT
AACGTGAAAAAGACTACTGTTTACGTAGTTGATTACAAATCCAAAGATAATGGTAAAATCATTAAAAATCATA
AATGGATGACAGGAAATGAGCTGAAAGCACGATAAAAATCTAGTTCTAAATTGAGAAATAAATAGATATAAA
AATATCCTCCTTAATCAATAATTTAAATAACTTATTATTGTTAAGGAGGATATTTTTTAGTGTGTAAATTAAAAAG
AATTTTAGAAGAATAACATTTATCAAAAAACTGTTCATTACCTTATTAATTGAAATTATATAATTAAAAACCGCA
TCATTAACCGATACGCAGAGGCGTATCATAAGT
>Staphylococcus aureus strain AR_0469
GAAGCTTATCATAAGTAATGAGGTTCATGATTTTTGACATAGTTAGCCTCCGCAGTCTTTCATTTCAAGTAAAT
AATAGCGAAATATTCTTTATACTGAATACTTATAGTGAAGCAAAGTTCTAGCTTTGAGAAAATTCTTTCTGCAA
CTAAATATAGTAAATTACGGTAAAATATAAATAAGTACATATTGAAGAAAATGAGACATAATATATTTTATAATAG
GAGGGAATTTCAAATGATAGACAACTTTATGCAGGTCCTTAAATTAATTAAAGAGAAACGTACCAATAATGTA
GTTAAAAAATCTGATTGGGATAAAGGTGATCTATATAAAACTTTAGTCCATGATAAGTTACCCAAGCAGTTAA
AAGTGCATATAAAAGAAGATAAATATTCAGTTGTAGGGAAGGTTGCTACTGGGAACTATAGTAAAGTTCCTT
GGATTTCAATATATGATGAGAATATAACAAAAGAAACAAAGGATGGATATTATTTGGTATATCTTTTTCATCCG
GAAGGAGAAGGCATATACTTATCTTTGAATCAAGGATGGTCAAAGATAAGTGATATGTTTCCGCGGGATAAA
AATGCTGCAAAACAAAGAGCATTAACTTTATCTTCCGAACTCAATAAATATATTACATCAAATGAATTTAATACT
GGAAGATTTTATTACGCAGAAAATAAAGATTCATCTTATGATTTAAAAAATGATTATCCATCAGGATATTCTCAT
GGATCAATAAGATTCAAATATTATGATTTGAATGAAGGATTCACAGAAGAAGATATGCTAGAGGATTTAAAGA
AATTTTTAGAACTATTTAATGAATTAGCTTCAAAAGTTACAAAAACATCCTATGATAGCTTGGTCAATAGCATA
GACGAAATACAGGAAGACAGCGAAATTGAAGAAATTAGAACAGCACAAAAAGATAAGACACTCAAGGAAG
TGGAAGCACCTAAAGGAATAATTCCAAAATATAAAAAAGGTGTATCAAAGACTACTAAAAATGATTCAGAAA
TTGAAAAATCAAATAAAGAGAATAAATTAACCGGTAAAGTTGGAGAAAAATTAGCGCTAAATTACTTTAATG
AGCTAATTGATAATAAAATAGACGAAGATAAGAAAGAACAGTTTAGGAATATTTTAAATGATAATCCAGGCTC
TCAACACGGTCATGGCTATGATTTAGTAGCTTTTGATCCAACAAATACAGATAAAGCTGTAGAAAAATTTATT
GAAATTAAAACATCTACATCTTCTAGTATTGAGGAACCATTTTTTATGTCGCTAAATGAAATGTTTGCTATGAA
AGAATATAAGCAGAAATATTTAATATTAAGAATATTTAATGTTTCCGGTAAAGAACCACAATTTTATTTTATAGA
TCCATATGCAAATTATTCTGAATTTAAAGATGTAGATGATCTCATTGACAAAGTATTTAATGTAGAAGCTATTCA
GTATAAAGTTTTTGGCGAAAAATGATTACTTGAACAAGAGCTAAAATAAAATTGTGATCTAATAAAAATAGAA
ACTGTAATTTAAATAAAACTTTCTAAATAAGCTAACTGATAAAAAATCAGTTTGTCCACAGTCTGAAACAAGAT
TCCTATATTCTTTAGGAATCTTGTTTTTTCTATTTTTATGGTGATAAAGAGCAGATAAGATAATGTGTAATAATCA
CAAAAAAGTTAAATATTTTAAGGCTTGTTTAATTATTAATGATTTTATATATAAAGAGCAGTATAATAAAGTTGT
TAATATATTATGAATAATATTCAAGTAATTTTATTGTTTTTTAATTTGTCGATATTTAAGTTGAGTTAAATTTAAAG
GGTGTAATTTGTTTTACAATGATGAAGATAATTAGTCTATCAAAATAAAGGGGTTGGGACTGTTATGAGTGAT
AATTTGTCATTATTCATTGACTATATCAATGATAATATAATCTATGGTAGTGAAATCAAACGGGAGAAATTAGAG
AATTTATTTAATCAATTTGCTATAAAAAAATGTTGAAAAGAACATTGTCTATGATGAACTGAAATCTTTAGATATT
ACAATCATTGAGTCACAGGATTCATATAAAAATAAATTGAAGAGATTATTTTCGGTTCTGTTGCAAAGTAAAA
AAATATAGCTAACCACTAATTTATCATGTCAGTGTTCGCTTAACTTGCTAGCATGATGCTAATTTCGTGGCATGG
CGAAAATCCGTAGATCTGAAGAGACCTGCGGTTCTTTTTATATAGAGCGTAAATACATTCAATACCTTTTAAAG

TATTCTTTGCTGTATTGATACTTTGATACCTTGTCTTTCTTACTTTAATATGACGGTGATCTTGCTCAATGAGGTT
ATTCAGATATTTCGATGTACAATGACAGTCAGGTTTAAGTTTAAAAGCTTTAATTACTTTAGCCATTGCTACCT
TCGTTGAAGGTGCCTGATCTGTAATTACCTTTTGAGGTTTACCAAATTGTTTAATGAGACGTTTGATAAACGC
ATATGCTGAATGATTATCTCGTTGCTTACGCAACCAAATATCTAATGTATGTCCCTCTGCATCAATGGCACGATA
TAAATAGCTCCATTTTCCTTTTATTTTGATGTACGTCTCATCAATACGCCATTTGTAATAAGCTTTTTTATGCTTT
TTCTTCCAAATTTGATACAAAATTGGGGCATATTCTTGAACCCAACGGTAGACCGTTGAATGATGAACGTTTA
CACCACGTTCCCTTAATATTTCAGATATATCACGATAACTCAATGTATATCTTAGATAGTAGCCAACGGCTACAG
TGATAACATCCTTGTTAAATTGTTTATATCTGAAATAGTTCATACAGAAGACTCCTTTTTGTTAAAATTATACTAT
AAATTCAACTTTGCAACAGAACCATCTAATCTTCAACAAACTGGCCCGTTTGTTGAACTACTCTTTAATAAAAT
AATTTTTCCGTTCCCAATTCCACATTGCAATAATAGAAAATCCATCTTCATCGGCTTTTTCGTCATCATCTGTAT
GAATCAAATCGCCTTCTTCTGTGTCATCAAGGTTTAATTTTTTATGTATTTCTTTTAACAAACCACCATAGGAG
ATTAACCTTTTACGGTGTAAACCTTCCTCCAAATCAGACAAACGTTACAAATTCTTTTCTTCATCATCGGTCATA
AAATCCGTATCCTTTACAGGATATTTTGCAGTTTCGTCAATTGCCGATTGTATATCCGATTTATATTTATTTTTCG
GTCGAATCATTTGAACTTTTACATTTGGATCATAGTCTAATTTCATTGCCTTTTTCCAAAATTGAATCCATTGTT
TTTGATTCACGTAGTTTTCTGTATTCTTAAAATAAGTTGGTTCCACACATACCAATACATGCATGTGCTGATTAT
AAGAATTATCTTTATTATTTATTGTCACTTCCGTTGCACGCATAAAACCAACAAGATTTTTATTAATTTTTTTATA
TTGCATCATTCGGCGAAATCCTTGAGCCATATCTGACAAACTCTTATTTAATTCTTCGCCATCATAAACATTTTT
AACTGTTAATGTGAGAAACAACCAACGAACTGTTGGCTTTTGTTTAATAACTTCAGCAACAACCTTTTGTGAC
TGAATGCCATGTTTCATTGCTCTCCTCCAGTTGCACATTGGACAAAGCCTGGATTTACAAAACCACACTCGAT
ACAACTTTCTTTCGCCTGTTTCACGATTTTGTTTATACTCTAATATTTCAGCACAATCTTTTACTCTTTCAGCCTT
TTTAAATTCAAGAATATGCAGAAGTTCAAAGTAATCAACATTAGCGATTTTCTTTTCTCTCCATGGTCTCACTT
TTCCACTTTTTGTCTTGTCCACTAAAACCCTTGATTTTTCATCTGAATAAATGCTACTATTAGGACACATAATATT
AAAAGAAACCCCCATCTATTTAGTTATTTGTTTAGTCACTTATAACTTTAACAGATGGGGTTTTTCTGTGCAAC
CAATTTTAAGGGTTTTCAATACTTTAAAACACATACATACCAACACTTCAACGCACCTTTCAGCAACTAAAATA
AAAATGACGTTATTTCTATATGTATCAAGATAAGAAAGAACAAGTTCAAAACCATCAAAAAAAGACACCTTTT
CAGGTGCTTTTTTTATTTTATAAACTCATTCCCTGATCTCGACTTCGTTCTTTTTTTACCTCTCGGTTATGAGTTA
GTTCAAATTCGTTCTTTTTAGGTTCTAAATCGTGTTTTTCTTGGAATTGTGCTGTTTTATCCTTTACCTTGTCTA
CAAACCCCTTAAAAACGTTTTTAAAGGCTTTTAAGCCGTCTGTACGTTCCTTAAGGAATTCCTTAGTGCTTTC
ATAGATTAAACTCACATCACGCTTTAAATCGCTTATTTTAGACTTTAAAGACTTGTTTTCTTCAAGCAACTCATT
ATAATCATTTACATTTTCATTAAATCGCTCTACAAGACCACTATATTTTTCTTTAACTTGCCCATGTTCTTTACTTA
ATTTTTTATATTCTCTCGCCATATCAGTACTCATGAGATTTCTAACATGCTGTTTTAACCTATCGTTATCTCTCGC
AGCAGTCACTAAGTTTTTATAATCACGCTCCGATATAACAACATTTTTGGTTGGTTTCTTTTCTGTTTTCATTAT
TTCTTTTCCCAAACCAAACATAGACTTTTCACCCGTTGGCACTTCAACACTTTTCATGTGTCGTTTCGCTGGTA
CTTCTAAATCTGATTTAACTTTATCGCTATAAGCAGTCCATTCATCTTTTTTAACTGCTAAATTTTTTTCTAGAAA
ATCAATCTCTTTTTCCAAAGTTTGTTTTTTAAATTTAGCTGTCTCAATATGTTTACGGTCAGAGCCACGTTCACC
ACGCTTCAACTCAAAACCCTGTTTTTTCATATGCTCGGGGAATTTATCTTGTAGCCATAACAGTTCTTGACGAT
TAAACACATTTTTTCCTTGCAGTTTTCCATCACGCATAGGCACAACACCTAAATGCATGTGAGGGGTTTGCTC
ATCATTATGAACTGTTGCATAAGCAATATTTTGCTTGCCATATCGTTCGGAAAATAATTTATAACTTTCCTCAAA
AAATCGTTTTTGTTCTCCTGGATCCAGTTGCTCAAAAAAATCTCGGTCAGATGTTACTAGCAACTCATTTACAA
GAACAGCATCTTTCCTCGTTTTTCTTGTACCTGTTTTTTGTGATTCAATAATTTCTTTGACACGTTCGTTGTAAT
CAATATTTTTATCATTTTTCAAATCATAATTTTCACGTGTTCGCTCATGGTCAATATCATCATTCGTTCTACTTTTT
CGCTCTCTTTGATTATGAAATTGCATGCCTTTTAGTCCAGCTGATTTCACTTTTTGCATTCTACAAACTGCATAA
CTCATATGTAAATCGCTCCTTTTTAGGTGGCACAAATGTGAGGCATTTTCGCTCTTTCCGGCAACCACTTCCA
AGTAAAGTATAACACACTATACTTTATATTCATAAAGTGTGTGCTCTGCGAGGCTGTCGGCAGTGCCGACCAA

AACCATAAAACCTTTAAGACCTTTCTTTTTTTTACGAGAAAAAAGAAACAAAAAAACCTGCCCTCTGCCACC
TCAGCAAAGGGGGGTTTTGCTCTCGTGCTCGTTTAAAAATCAGCAAGGGACAGGTAGTATTTTTTGAGAAG
ATCACTCAAAAAATCTCCACCTTTAAACCCTTGCCAATTTTTATTTTGTCCGTTTTGTCTAGCTTACCGAAAGC
CAGACTCAGCAAGAATAAAATTTTTATTGTCTTTCGGTTTTCTAGTGTAACGGACAAAACCACTCAAAATAAA
AAAGATACAAGAGAGGTCTCTCGTATCTTTTATTCAGCAATCGCGCCCGATTGCTGAACAGATTAATAATAGA
TTTTAGCTTTTTATTTGTTGAAAAAAGCTAATCAAATTGTTGTCGGGATCAATTACTGCAAAGTCTCGTTCATC
CCACCACTGATCTTTTAATGATGTATTGGGGTGCAAAATGCCCAAAGGCTTAATATGTTGATATAATTCATCAA
TTCCCTCTACTTCAATGCGGCAACTAGCAGTACCAGCAATAAACGACTCCGCACCTGTACAAACCGGTGAATC
ATTACTACGAGAGCGCCAGCCTTCATCACTTGCCTCCCATAGATGAATCCGAACCTCATTACACATTAGAACT
GCGAATCCATCTTCATGGTGAACCAAAGTGAAACCTAGTTTATCGCAATAAAAACCTATACTCTTTTTAATATC
CCCGACTGGCAATGCCGGGATAGACTGTAACATTCTCACGCATAAAATCCCCTTTCATTTTCTAATGTAAATCT
ATTACCTTATTATTAATTCAATTCGCTCATAATTAATCCTTTTTCTTATTACGCAAAATGGCCCGATTTAAGCACA
CCCTTTATTCCGTTAATGCGCCATGACAGCCATGATAATTACTAATACTAGGAGAAGTTAATAAATACGTAACC
AACATGATTAACAATTATTAGAGGTCATCGTTCAAAATGGTATGCGTTTTGACACATCCACTATATATCCGTGTC
GTTCTGTCCACTCCTGAATCCCATTCCAGAAATTCTCTAGCGATTCCAGAAGTTTCTCAGAGTCGGAAAGTTG
ACCAGACATTACGAACTGGCACAGATGGTCATAACCTGAAGGAAGATCTGATTGCTTAACTGCTTCAGTTAA
GACCGAAGCGCTCGTCGTATAACAGATGCGATGATGCAGACCAATCAACATGGCACCTGCCATTGCTACCTG
TACAGTCAAGGATGGTAGAAATGTTGTCGGTCCTTGCACACGAATATTACGCCATTTGCCTGCATATTCAAAC
AGCTCTTCTACGATAAGGGCACAAATCGCATCGTGGAACGTTTGGGCTTCTACCGATTTAGCAGTTTGATACA
CTTTCTCTAAGTATCCACCTGAATCATAAATCGGCAAAATAGAGAAAAATTGACCATGTGTAAGAGGCCAATC
TGATTCCACCTGAGATGCATAATCTAGTAGAATCTCTTCGCTATCAAAATTCACTTCCACCTTCCACTCACCGG
TTGTCCATTCATGGCTGAACTCTGCTTCCTCTGTTGACATGACACACATCATCTCAATATCCGAATAGGGCCCA
TCAGTCTGACGACCAAGAGAGCCATAAACACCAATAGCCTTAACATCATCCCCATATTTATCCAATATTCGTTC
CTTAATTTCATGAACAATCTTCATTCTTTCTTCTCTAGTCATTATTATTGGTCCATTCACTATTCTCATTCCCTTTT
CAGATAATTTTAGATTTGCTTTTCTAAATAAGAATATTTGGAGAGCACCGTTCTTATTCAGCTATTAATAACTCG
TCTTCCTAAGCATCCTTCAATCCTTTTAATAACAATTATAGCATCTAATCGGTTCTGTTGCAAAGTAAAAAAATA
TAGCTAACCACTAATTTATCATGTCAGTGTTCGCTTAACTTGCTAGCATGATGCTAATTTCGTGGCATGGCGAA
AATCCGTAGATCTGAAGAGACCTGCGGTTCTTTTTATATAGAGCGTAAATACATTCAATACCTTTTAAAGTATT
CTTTGCTGTATTGATACTTTGATACCTTGTCTTTCTTACTTTAATATGACGGTGATCTTGCTCAATGAGGTTATTC
AGATATTTCGATGTACAATGACAGTCAGGTTTAAGTTTAAAAGCTTTAATTACTTTAGCCATTGCTACCTTCGT
TGAAGGTGCCTGATCTGTAATTACCTTTTGAGGTTTACCAAATTGTTTAATGAGACGTTTGATAAACGCATATG
CTGAATGATTATCTCGTTGCTTACGCAACCAAATATCTAATGTATGTCCCTCTGCATCAATGGCACGATATAAAT
AGCTCCATTTTCCTTTTATTTTGATGTACGTCTCATCAATACGCCATTTGTAATAAGCTTTTTTATGCTTTTTCTT
CCAAATTTGATACAAAATTGGGGCATATTCTTGAACCCAACGGTAGACCGTTGAATGATGAACGTTTACACCA
CGTTCCCTTAATATTTCAGATATATCACGATAACTCAATGTATATCTTAGATAGTAGCCAACGGCTACAGTGATA
ACATCCTTGTTAAATTGTTTATATCTGAAATAGTTCATACAGAAGACTCCTTTTTGTTAAAATTATACTATAAATT
CAACTTTGCAACAGAACCGAAAAACTAGACTTGATTACAAAATGGAGCTTGGGACATAAATGATTTTTTAAA
AATGAGATGAGACGTAGATTAACTCCATAATCAATACGAATCTATCGACTTCTTTATTTATGATATTCATCTCTTT
TTAATGGAAATAAAAGTGCGATTAATGTGATAATACAGTTACGTTAATTAAAAAAATAAAAATGCAAGGAGAG
GTAATATGCTAACTGTATATGGACATAGAGGATTACCTAGTAAAGCTCCGGAAAATACAATTGCATCATTTAAA
GCTGCTTCAGAAGTAGAAGGTATAAACTGGTTGGAGTTAGATGTTGCAATTACAAAAGATGAACAACTGATT
ATCATTCATGATGATTATTTAGAACGGACTACAAATATGTCCGGGGAAATAACTGAATTGAATTATGATGAAAT
TAAAGATGCTTCTGCAGGATCTTGGTTTGGTGAAAAATTCAAAGATGAACATTTGCCAACTTTCGATGATGTA
GTAAAAATAGCAAATGAATATAATATGAATTTAAATGTAGAATTAAAAGGTATTACTGGACCGAATGGACTAG

CACTTTCTAAAAGTATGGTTAAGCAAGTGGAAGAACAATTAACAAACTTAAATCAGAATCAAGAAGTGCTCA
TTTCAAGCTTTAATGTTGTGCTTGTTAAACTTGCAGAAGAAATCATGCCACAATATAACAGAGCAGTTATATTC
CATACAACTTCGTTTCGTGAAGACTGGAGAACACTTTTAGATTACTGTAATGCTAAAATAGTAAACACTGAAG
ATGCCAAACTTACTAAAGCAAAAGTAAAAATGGTAAAAGAAGCGGGTTATGAATTGAACGTATGGACTGTAA
ACAAACCAGCACGTGCAAACCAACTTGCTAATTGGGGAGTTGATGGTATCTTTACAGACAATGCAGATAAAA
TGGTGCATTTGTCTCAATAGAAAGTTAGAGGTGAGTCTTACGTTTCAGTGACGGTAGACTTACCTTTAACATG
TTACATACTAAAAAATTAATTTGAATAAGAAAGAGAGACATATATGAAATACGATGATTTTATAGTAGGAGAAA
CATTCAAAACAAAAAGCCTTCATATTACAGAAGAAGAAATTATCCAATTTGCAACAACTTTTGATCCTCAATAT
ATGCATATAGATAAAGAAAAAGCAGAACAAAGTAGATTTAAAGGTATCATTGCATCTGGCATGCATACACTTT
CAATATCATTTAAATTATGGGTAGAAGAAGGTAAATACGGAGAAGAAGTTGTAGCAGGAACACAAATGAATA
ACGTTAAATTTATTAAACCTGTATACCCAGGTAATACATTGTACGTTATCGCTGAAATTACAAATAAGAAATCCA
TAAAAAAAGAAAATGGACTCGTTACAGTGTCACTTTCAACATACAATGAAAATGAAGAAATTGTATTTAAGG
GAGAAGTAACAGCACTTATTAATAATTCATAATAAAACAGTGAAGCAACCATCGTTACGGATTGCTTCACTGT
TTTGTTATTCATCTATATCGTATTTTTTATTACCGTTCTCATATAGCTCATCATACACTTTACCTGAGATTTTGGCA
TTGTAGCTAGCCATTCCTTTATCTTGTACATCTTTAACATTAATAGCCATCATCATGTTTGGATTATCTTTATCATA
TGATATAAACCACCCAATTTGTCTGCCAGTTTCTCCTTGTTTCATTTTGAGTTCTGCAGTACCGGATTTGCCAA
TTAAGTTTGCATAAGATCTATAAATATCTTCTTTATGTGTTTTATTTACGACTTGTTGCATACCATCAGTTAATAG
ATTGATATTTTCTTTGGAAATAATATTTTTCTTCCAAACTTTGTTTTTCGTGTCTTTTAATAAGTGAGGTGCGTT
AATATTGCCATTATTTTCTAATGCGCTATAGATTGAAAGGATCTGTACTGGGTTAATCAGTATTTCACCTTGTCC
GTAACCTGAATCAGCTAATAATATTTCATTATCTAAATTTTTGTTTGAAATTTGAGCATTATAAAATGGATAATCA
CTTGGTATATCTTCACCAACACCTAGTTTTTTCATGCCTTTTTCAAATTTCTTACTGCCTAATTCGAGTGCTACT
CTAGCAAAGAAAATGTTATCTGATGATTCTATTGCTTGTTTTAAGTCGATATTACCATTTACCACTTCATATCTTG
TAACGTTGTAACCACCCCAAGATTTATCTTTTTGCCAACCTTTACCATCGATTTTATAACTTGTTTTATCGTCTA
ATGTTTTGTTATTTAACCCAATCATTGCTGTTAATATTTTTTGAGTTGAACCTGGTGAAGTTGTAATCTGGAAC
TTGTTGAGCAGAGGTTCTTTTTTATCTTCGGTTAATTTATTATATTCTTCGTTACTCATGCCATACATAAATGGAT
AGACGTCATATGAAGGTGTGCTTACAAGTGCTAATAATTCACCTGTTTGAGGGTGGATAGCAGTACCTGAGC
CATAATCATTTTTCATGTTGTTATAAATACTCTTTTGAACTTTAGCATCAATAGTTAGTTGAATATCTTTGCCATC
TTTTTTCTTTTTCTCTATTAATGTATGTGCGATTGTATTGCTATTATCGTCAACGATTGTGACACGATAGCCATCT
TCATGTTGGAGCTTTTTATCGTAAAGTTTTTCGAGTCCCTTTTTACCAATAACTGCATCATCTTTATAGCCTTTA
TATTCTTTTTGTTTTAATTCTTCAGAGTTAATGGGACCAACATAACCTAATAGATGTGAAGTCGCTTTTCCTAG
AGGATAGTTACGACTTTCTGTTTCATTAGTTGTAAGATGAAATTTTTTTGCGAAATCACTTAAATATTCATCCAT
TTTTTTAACGGTTTTAAGTGGAACGAAGGTATCATCTTGTACCCAATTTTGATCCATTTGTTGTTTGATATAGTC
TTCAGAAATACTTAGTTCTTTAGCGATTGCTTTATAATCTTTTTTAGATACATTCTTTGGAACGATGCCTATCTC
ATATGCTGTTCCTGTATTGGCCAATTCCACATTGTTTCGGTCTAAAATTTTACCACGTTCTGATTTTAAATTTTC
AATATGTATGCTTTGGTCTTTCTGCATTCCTGGAATAATGACGCTATGATCCCAATCTAACTTCCACATACCATC
TTCTTTAACAAAATTAAATTGAACGTTGCGATCAATGTTACCGTAGTTTGTTTTAATTTTATATTGAGCATCTAC
TCGTTTTTTATTTTTAGATACTTTTTTTATTTTACGATCCTGAATGTTTATATCTTTAACGCCTAAACTATTATATAT
TTTTATCGGACGTTCAGTCATTTCTACTTCACCATTATCGCTTTTAGAAATATAACTGCTATCTTTATAAACTTGT
TTGAAATTTTTATCTTCAATTGCATCAATAGTATTATTAATTTCTTTATCTTTTGAAGCATAAAAATATATACCAAA
CCCGACAACTACAACTATTAAAATAAGTGGAACAATTTTTATCTTTTTCATCAATATCCTCCTTATATAAGACTA
CATTTGTAGTATATTACAAATGTAGTATTTATGTCAAAATAATGTTATAATTTTTGTGATATGGAGGTGTAGAAG
GTGTTATCATCTTTTTTAATGTTAAGTATAATCAGTTCATTGCTCACGATATGTGTAATTTTTTTAGTGAGAATGC
TCTATATAAAATATACTCAAAATATTATGTCACATAAGATTTGGTTATTAGTGCTCGTCTCCACGTTAATTCCATT
AATACCATTTTACAAAATATCGAATTTTACATTTTCAAAAGATATGATGAATCGAAATGTATCTGACACGACTTC

TTCGGTTAGTCATATGTTAGATGGTCAACAATCATCTGTTACGAAAGACTTAGCAATTAATGTTAATCAGTTTG
AGACCTCAAATATAACGTATATGATTCTTTTGATATGGGTATTTGGTAGTTTGTTGTGCTTATTTTATATGATTAA
GGCATTCCGACAAATTGATGTTATTAAAAGTTCGTCATTGGAATCGTCATATCTTAATGAACGACTTAAAGTAT
GTCAAAGTAAGATGCAGTTCTACAAAAAGCATATAACAATTAGTTATAGTTCAAACATTGATAATCCGATGGTA
TTTGGTTTAGTGAAATCCCAAATTGTACTACCAACTGTCGTAGTCGAAACCATGAATGACAAAGAAATTGAAT
ATATTATTCTACATGAACTATCACATGTGAAAAGTCATGACTTAATATTCAACCAGCTTTATGTTGTTTTTAAAAT
GATATTCTGGTTTAATCCTGCACTATATATAAGTAAAACAATGATGGACAATGACTGTGAAAAAGTATGTGATA
GAAACGTTTTAAAAATTTTGAATCGCCATGAACATATACGTTATGGTGAATCGATATTAAAATGCTCTATTTTAA
AATCTCAGCACATAAATAATGTGGCAGCACAATATTTACTAGGTTTTAATTCAAATATTAAAGAACGTGTTAAG
TATATTGCACTTTATGATTCAATGCCTAAACCTAATCGAAACAAGCGTATTGTTGCGTATATTGTATGTAGTATAT
CGCTTTTAATACAAGCACCGTTACTATCTGCACATGTTCAACAAGACAAATATGAAACAAATGTATCATATAAA
AAATTAAATCAACTAGCTCCGTATTTCAAAGGATTTGATGGAAGTTTTGTGCTTTATAATGAACGGGAGCAAG
CTTATTCTATTTATAATGAACCAGAAAGTAAACAACGATATTCACCTAATTCTACTTACAAAATTTATTTAGCGT
TAATGGCATTCGACCAAAATTTACTCTCATTAAATCATACTGAACAACAATGGGATAAACATCAATATCCATTTA
AAGAATGGAACCAAGATCAAAATTTAAATTCTTCAATGAAATATTCAGTAAATTGGTATTACGAAAATTTAAA
CAAACATTTAAGACAAGATGAGGTTAAATCTTATTTAGATCTAATTGAATATGGTAATGAAGAAATATCAGGG
AATGAAAATTATTGGAATGAATCTTCATTAAAAATTTCTGCAATAGAACAGGTTAATTTGTTGAAAAATATGAA
ACAACATAACATGCATTTTGATAATAAGGCTATTGAAAAAGTTGAAAATAGTATGACTTTGAAACAAAAAGAT
ACTTATAAATATGTAGGTAAAACTGGAACAGGAATCGTGAATCACAAAGAAGCAAATGGATGGTTCGTAGGT
TATGTTGAAACGAAAGATAATACGTATTATTTTGCTACACATTTAAAAGGCGAAGACAATGCGAATGGCGAAA
AAGCACAACAAATTTCTGAGCGTATTTTAAAAGAAATGGAATTAATATAATGGATAATAAAACGTATGAAATAT
CATCTGCAGAATGGGAAGTTATGAATATCATTTGGATGAAAAAATATGCAAGTGCGAATAATATAATAGAAGA
AATACAAATGCAAAAGGACTGGAGTCCAAAAACCATTCGTACACTTATAACGAGATTGTATAAAAAGGGATT
TATAGATCGTAAAAAAGACAATAAAATTTTTCAATATTACTCTCTTGTAGAAGAAAGTGATATAAAATATAAAA
CATCTAAAAACTTTATCAATAAAGTATACAAAGGCGGTTTCAATTCACTTGTCTTAAACTTTGTAGAAAAAGA
AGATCTATCACAAGATGAAATAGAAGAATTGAGAAATATATTGAATAAAAAATAAAATTGTTGTGTTTACAAC
AATACATAGAAAACAGAGGAAACAATCAAGTCGTTGAATATTTCCTCTGTTTTTTAGTTGAAAAAATTAACCG
AAAGCCTGAATGCAAGTCTTGATTAAATCAATAATGCTTGTAATAACACCAGTGAAATCCATATGCATACCCTC
TTTCTATTTAAGATACATTAAGTATAATATCAAACAAATAAAAAATGTTAAAAATTCCCTAATTGGCTATTTAGAT
TGCATAAATGTCAAAAATTTGAAAAACATACAACGACTTTGCATAAAAAATCGTCATATTGGAAATACGTAATT
TATTGAAATAATAAAAAAAATAAAAGAACGAAGATGATAACCTAAGTGAGGTTTTAAGTTGTTCTAAGGTTTA
ATTTAATTTATGTTAAAATAGTTGGTATAAAAATACATGATAAACTATAAACTAAATTCAAAATAACTTATGGGG
TAGGCAATTATGGAAAATAAAATATAAATGATAATGAAAAAAGAGTGCTAAGGGAAATTTATAACCATCATAA
TATTTCGCGTACTCAAATATCTAAAAATCTTGAGATTAATAAGGCAACGATTTCTAGTATTTTGAATAAGTTAAA
GTATAAATCTCTTGTTAATGAGGTTGGTGAGGGTGATAGCACGAAGAGTGGTGGTAGAAAACCTATTCTTCT
GAAGGTTAATCATCTTTATGGTTATTTTATTTCTTTGGATTTAACTTATAGTTCTGTTGAAGTGATGTACAATTAT
TTTGATGGTAATGTCATTAAGCATGAATCTTATGATTTACCTGATGAAAAGGTTAGTAGTATATTAAGCATAATA
AAAAAACATATTGATATTCAGGAGAAACTTGATACTTATAACGGACTATTAGGTGTGTCTGTTTCTATACATGG
AGTTGTGGATAATGAGCAGCATGTGACATATTTACCATTCCATGAAACTGAAGGAATTTCAATTGCTAAGAAA
ATAAAAGAAATTACTAATGTTCCAGTCGTAGTTGAAAATGAAGCGAATCTTTCAGCGTTATATGAACGTAATTT
TAATCATAATTTATCCTACAATAATCTTATTGCTTTAAGTATACATAAAGGTATTGGTGCTGGGCTTATTATTAATA
ATCAATTGTATCGTGGTGCAAATGGGGAAGCGGGTGAAATTGGAAAAACACTTGTCTCAAAAGTTAGCGAT
AATGTGGAGATCTTTCATAAGATTGAAGATATTTTTTCACAAGAAGCTTTACTGCATAATTTAAGTAATCAACT
AAATGAGAAGATGACGCTTAGCAAATTAATTCAATTTTATAATGAAAAAAATCCAGTCGTAGTTGAAGAAATG

GAACAATTTATAAATAAAATTGCTGTTTTAATACATAATTTAAACACCCAGTTTAATCCGAATGCAATTTACATT
AACTGTCCATTGTTCAATGAAATGCCTGAAATATTAGAAGCAATTAAGAACCAGTTCAAACAATATTCACGTA
ACGAAATTCAAATAAAGTTAACATCTAATGTCAAATTTGCAACTTTGCTAGGTGGTACATTAGCAATTATCCAA
AAAGTACTACAGATTAATGATATTTACTTAGATATAAAAGCATAAAAAACTAATTCAAATGAATAATCAAAGTT
CGTAATTGTCTTTATAAAAAAATCCCTCAATCCGAATTGAATTTTCGGATTGAGGGATTTTTATAGTTCTATTGC
AGAAGAAAACTATTTTAAAAATGCTGGTAAATGTTGATAGCCACCTCTAACGTTAACAATATTCGTAAATCCTT
TATATTCTAATATTCCTACCGCTATTGAACTTCTAACACCTGATTGACAATGTACATAAATTAGGTCATTTTTATC
GAAAGGTATATCTTCATTTAAAAGTTTACCGTGAGGAATATGAATTGCTTGTTTTAAATGACCTTTACGCCATT
CATCATCATTACGAACATCTAATACATTATGTTCTTCACCAGTCATTTCAGAACTATGAATAGATGATGTGACGA
TATTTGTTTGTGGCAAACGGTAACTTTTTACATTTTCAAAACCAATTAATTGTAAAGCATGAATAGCTTGTTGA
ACGGTAGATTTATCGCCAATTAATTCAATATCTTAGTCATAATCTAAATACCAACCAATTTGATTTATAAAAGTTT
TATTAAAAGGAATATTGATAGTTCCATGCATATGACCACCATGGAATGCTTCTTTACTTCGAAGATCAAAAGCA
GTTTGTGTATTGCTTGAACTAGGGTAAACATTATATGGTTGGTACATTTGCATACCAAATTGATTTATTTTTTTC
ATTTGTGAAAAATGGTGTGGTGGAGCTGGCTGATTGAGTGTTAAAGTTTCGATAAATGAAGTTTCATCTTTA
ACATTAAAAGCCCAGTTGTTTATTTTCTCATAACCCAAAGTAGTTGTAGGTAATGAACCTAGCGCTTTACCACA
AGGACTCCCTGCACCATGACCTGGCCAAATTTGAATATAGTCTGGTAATGTTGCAGCAAATTGTATGGACTGA
TACATTTGTTTTGCTCCGATTTTTGTAGAACCTTCAACATTTACAGCTTTTTCTAATAGATCTGGTCTACCTACA
TCACCAACAAAGATGAAGTCACCGCTAAATAATCCCATTGGTATACTGGAACCCCCACCTTCGTCAGTAAGTA
AAAAACTAATACTCTCAGGGGGATGGCCTGGAGTGTGTAAGACTTCTAATTTAATCTTTCCTAAATAGATAATA
TCTTGATGCTTAACGAAATGTGTTTGTTTAGGCATATTTTTATAATTAAATTCATCTTTACCTTCATCAGATACGT
ATATACTTGCATTCAATTTATTTGCCACATCTCTAATACCTGAAGCAAAATCAGCATGAATATGTGTTTCTGCAG
CTTTAGTAATTGTGAATCCTTCTTTATCTGCAACTTTTAAATATTTTGTTAAATCTCGTATAGGGTCAATAATCAT
TGCTTCTCCTGTACGTTGACATCCAATTAAATAAGATGCTTGTGAAAAATTGTCTTCATAAAATTGTTTGAAAA
ACAAAAAAAACTCCTTTTAAAAATAGATTTTATTGATTAGATAAATAAGTTATGATTTGCTTGCTCAGTATGTCC
AATATAAGTACCTACGCCACCATAATCGACTTCATCTCTTAATTCTTCTTTTGAAATTCCCATAACATCCATACTC
ATGGTACAAGCAATTAACTTTATATCTTGATCGATTGCTTGATCGATAAGTGAGTATAAAGAATCAACATTTTTC
TTGTTCATTACATAACGCATCATAATATTACCTAGTCCAAACATATTCATTTTTGATAATGGCATATGTATTGGAT
CCTTAGGTAACATAAGGTCAAACATTTTGGAAATACCTTTCTTTTTAACGCGAGTTGATTGCGCTTTTTTTAAT
GCGTTGAGGCCCCAAAAAGTAAAGAAAATAGTTACATCTTTACCTGCTGCTTTAGCGCCATTTGCGATGATCA
TTGCTGCTACTGCCTTATCTAACTCACCGCTAAATAAAACAATTGTTGTACCTGTAGCAGTGTCATTGATTTCA
AATTCTTTTGGCTTTTCTTTTTGAATAATTGCATTAATTACATTTGCTTCTTCAGTAAGATTTACAAGGGTATTC
CCTGTTTGTTTCGCCCAACTTTTAATATCACTATTGAAACCAGGATCTGTAACTGTTACCTCGATTTGCTCACCC
GTTGAAATATTGTTAATTTCTTTACTGATATTAACAATAGGTCCAGAGCACTGAAGACCTCTAAAATCAAATTG
TTTACGATTCTCTTTGATTTCAATATCTTTTTCTATTAAAGGAGCACTATTGAAGTTCTTTGCTTCATAATCTTTA
TATCCACCCTTTAAATTCACGACATCATAACCTTGTTTGGCTAAATAATCGCAAGCTTTAGTGCTTCGGTTACC
GCTTTTACAATGTATATAATACGTTTTGTTGCTATTCTTATTGAATGATTTAATCTCTTCTACTGGGTGTAAAGTT
GAACCGTTAATGTGTCCTAATTCATATTCTTCTTTTGTTCTAACATCAATCAATTGACCCATTTTTGCCAATTTTT
CTAATTCTTCTTTGTTTAATGAATTAATGTGTACTTCTTTGTATTGTTCCATACTTACCTCCTATAAATACCTATGA
GGGTATAATAAAACGGATAGAATCATTTGCCAAATACCTATATGGGTATTTGACAATTTTGTTTTAATTTATTAT
TATTAACTTAATCAATTTATGTGGAGGAAATGAATATGACTTATGATAAAAAAATGATTAATCGTATAAATAGAA
TACAAGGTCAATTAAATGGTGTCGTAAAAATGATGGAAGAAGAAAAAGATTGCAAAGATATAATTACGCAAC
TTAGTGCATCTAAAGGTTCTATACAACGTTTAATGGGGATTATAATTAGTGAAAATTTAATAGAATGCGTTAAA
ACAGCAGAAGAAAATAATGAAAGTTCTCAAGAATTAATTAATGAAGCAGTTAATTTATTAGTTAAAAGTAAAT
AATGGATATAGCAAATATGACTATTATGTTGCTAATTGGCGTACTGGGTGGATTTATATCTGGATTAATAGGTAT

TGGGGGCGCAATTATTATTTACCCAGCTATTCTTATATTGCCACCATTAATAGGTATACCTGCGTATAGTGCATAT
ATTGCTTCGGGACTTACCTCTAGTCAAGTATTTTTCAGTACACTTAGTGGATCATTAAATGCAAGAAAACAAC
CAGCTTTCTCTCCTAAACTTGTTATATATATGGGAGGGGGTATGTTGATTGGAAGCATGTTAGGGGCAATTTT
AGCTAGTTTGTTTAATGCTACTTTTGTAAATACGGTATATGTAATAATCGCCATACTTGCTTTAATATTGATGTTT
ATTAAAGTTAAACCTACTACACAAGAGACGAAATCTAAACCTTTGCTATTTATTATAGTTGGATTTGGAATTGG
TGTAATTTCGGGAATTGTGGGTGCAGGTGGAGCATTTATCATCATTCCTGTATTATTAGCATTATTTAAATTACC
AATGAATACGGTAGTGAACAATAGCATAGCAATTGCTTTTATATCTTCAGTAGGGGCATTTTTTATAAAATTAA
TGCAAGGATATATACCAGTAGAAAGTGCAATTTTTTTGATAATTGAATGATGATCATTTTCTGAAAATATTATGT
GGTCATATAATATAATGCCCATCATTTCACTAATCTCTTTTATTCTCTGAGTTATTTTGATATCTCCTGGAGAAGG
TGTTACATCTTATCAAGAGTAAATTACAAAAGAATCATTTAAATCAATACTTTCACTTTGAATACATGTATTTGA
AGTGGAAGGTACTTATTTCAAAAATAGTAAAACCTGTATCTTAAATTACTTAATAGTAACATAAGATACAGGCT
GATTTTTTATTCATTGTTATTTATACTAAAGCACCCGATAGCTCTGAAAACAATCACAAATTCAACTTTTCAAAG
CCACAGCTTTAAGTTATTTTGTCCAGACAACCCCCATTGCCCAACCCATTTTATGGAATTGGCATCCAGGCAA
CAACTTTTCATATAAATCGTAATAATTTTGTTCAGATAGGTACTTATCTGAAGCTAAATGCTCAAGCCATGATTT
AGATGTGTTGTGATTATAAATTCTAATCGCATTTTTTATTCCAAATTTGAAACAATGCGGTAAAAACTCTTGGA
TTGCCCCTAATTTATAAACATAGGTAGGCGGTGTCTCGACTTCAGATACATTATCAAGAATAACTATTCTTCCTT
CCTCATTCAACAGTTCCTTCATTTGCTGTATTACGCTGGCTATATCATCCAAATGATGAAAGGTTGTTCGGCTT
ACAATAAAATCAAACTTCTCATTAAAATTAAGTTGTTCTGCATTCATATTCAGATAGACCGTATTTGTTAGTTGA
CGTTTAGATTTGGCAAGATCGAGCATTTGATTAGAAATATCAATCCCTACCACTTCATCATAATAACTTGCTAAT
TTCTCCACTAACAAACCCGAGCCACATCCGATATCTAATGCTCTGCCTTTCTTTGGAGACATATTAGACACAAA
GAATGAATAATCATTCAAAAGCTCATTCACGAAATCGTAATCTTCTGCAACCTTATCAAACTGTGATTCTATTGT
ATTCAAAAAGATCCCCCATTCCTACTTTATCGACATTCTTTCATTACTTACCACTTTAGATGTTTTTTCGTTGGG
GATAAAACTTCCCTTTAGACAATTTTATCCAAAGACAATACAACAGTGCAACTTTATTAAAGTCACTGTCCTTT
ATCGCAGCCTTTACTTTTTAGTAAAGACAGTGGCTTCTCTTATCAAGTTTCAAAACATATTATTTTGAAGAAAA
CGTCCATCTGAAGTGTCAAGTGCAAAATTACATATAAAGGTTTATTCTAAAATGAAAAGATGATACAATCATAT
TCAGTTACATAAGGAGGTTTCAATTATGTGCACCAGTATCGCAGTAGTAGAAATTACTTTATCTCATTCATAATG
AAAAAAATGGAAAGGAGAAGGTTATAATGAACCAGAAAAACCCTAAAGACACGCAAAATTTTATTACTTCTA
AAAAGCATGTAAAAGAAATATTGAATCACACGAATATCAGTAAACAAGACAACGTAATAGAAATCGGATCAG
GAAAAGGACATTTTACCAAAGAGCTAGTCAAAATGAGTCGATCAGTTACTGCTATAGAAATTGATGGAGGCT
TATGTCAAGTGACTAAAGAAGCGGTAAACCCCTCTGAGAATATAAAAGTGATTCAAACGGATATTCTAAAATT
TTCCTTCCCAAAACATATAAACTATAAGATATATGGTAATATTCCTTATAACATCAGTACGGATATTGTCAAAAG
AATTACCTTTGAAAGTCAGGCTAAATATAGCTATCTTATCGTTGAGAAGGGATTTGCGAAAAGATTGCAAAAT
CTGCAACGAGCTTTGGGTTTACTATTAATGGTGGAGATGGATATAAAAATGCTCAAAAAAGTACCACCACTAT
ATTTTCATCCTAAGCCAAGTGTAGACTCTGTATTGATTGTTCTTGAACGACATCAACCATTGATTTCAAAGAAG
GACTACAAAAAGTATCGATCTTTTGTTTATAAGTGGGTAAACCGTGAATATCGTGTTCTTTTCACTAAAAACCA
ATTCCGACAGGCTTTGAAGCATGCAAATGTCACTAATATTAATAAACTATCGAAGGAACAATTTCTTTCTATTT
TCAATAGTTACAAATTGTTTCACTAAATTAAAGTAATAAAGCGTTCTCTAATTTCACAAGAGGACGCTTTATTC
TTCCCAAAAATTGTTCAATATTTATCAATAAATCAGTAGTTTTAAAAGTAAGCACCTGTTATTGCAATAAAATTA
GCCTAATTGAGAGAAGTTTCTATAGAATTTTTCATATACTTAACGAGTGCTTTCACCTTTGAATATAGTCCTTCC
CACTTATCATCACACTCTCCCCGATAGCCTTTTCTAGCTATATCCAGTAAAGTTACATGCTCTTTAGGTAAAAGA
GGTATAGCCCATTCTGCAGCGACATCTTTCGAGGTAATTTCACCAGTAGTCACTGTTTGCCACATTCGAGCTA
GGGTTAAAATTACATTACGCTCATCACCTTTTATCCCCTCAATTAGTTCTGGCAAAGAATCCTTAATTGCTCTTC
GAATATCTGTCAAAGGTACGGAGACAAGTATACTTGAAGAATCAGGACCAAATAGAGAAATACTATTCTTTCT
TGCTTGTGCTAAAACAATAGCCAAATCAGGATCATAGCTTGGTTCCTGAATTTGTCCATTCTCAAATTCACCCC

TGAGCCACTCACCGTATATAAATTCTCTTTTTGGAGGATATTGCCAAGGGACAACTTCACTCCTATTTATAACC
GTAACTTCAAGTGGTCTAACAGAATCCGTATTTCCAATCTTTCCTGATATAGTCATTAGTCTTTCTGTTAGTTTT
TTTCGAGTTAATTGAGGTAAACTATGATTCACGACGACTAGAACATCTACATCGCTGTTAATGCGTAAACCAC
CATTTACTGCTGAACCAAATAGATATACTCCAACTATTGAACTTCCAAATAAATCTTTTACGATTTTTAATGTTT
GAATCGCTTGATTTGGTATTTTTCCGTTAATCAAATTGCTCATGATTTCACCTCGTTGATTATGTTCATATAAAG
TTTATATTGATACTCAATTTACTTACCCTAGATTGGACATATACTTAAATTACTGTTCAATAAAGCTGACCGTTAG
CGTTTAAGTACATCCTTTCACAATTTGTCTACAGATTAATAATTATTCTTTATTATACAGATCTCCATATAATTTTT
GAATTTGGTTCTGTAATTTTTTATTTTCTTTTTCTAATTCCATTACTCTTCTTTTTAAGGTTTTAATAAGGATTTC
CTCCGAACGAGAACTTTTCTTGGGTTTTGAGACTACATTTGCTGTTATTTGACGCTCACGAAGGGATTCGATT
CTTTGCCTAATATCGTGTTCCTTATAAAGCCATGATTTAGAAACATTAGCTTCCTTTGCTATTGAATTAAAATTA
ATAACTTTACCTTCAATCGAAAATTTAGAAATCGCTTTGTCTACTTTTTCCCTTGTCTTTTTTGATTTCTGCTTC
GCCAAACGTACAATTTCTGTTGTATTTCTAACTTGTTTATCCATTGATAATTACCCCGTCAAACTTCCAATGATT
TGTTCTAAACGCTCTTTAACACGGCTATTAGTCTCTACTTGTCTTTGCCATTGTTTATCCTTAGCTATGGCTAATA
ACTCTTCTGTACGCTCTAACTGTTCTTCGTGCTGTGGTAAGAATTGCTTACTGGTACAGAAGTGAGTGCAATC
TAAGCATGCATTCGCATGTGGACAACCACCTGCTACTACTGGCAATCTACAATAACCATTTGGAAGCACTTGT
GCATTTATATTTTTCTTGAACCATTGAAGCTCTACATCATCGACTTCATTATCTTCATCTAGATCAAGCACATCTC
CATTATTGGTAACCAGTTTTTCCTGAAATTTAGTAAATTCATTTTTTAGAGTTTCATCAAAGATATGAGCGTATC
TGCTTGTCATTTCTGGGCTTTCATGCCCCAAAAATTTCTGCACAATATGCTGGGGCATCCCGTTGTTAATCATT
CTTGTTCCTACTGTATGGCGAAAGGCATGGGCATGGAATCTATAAATCTCACCTGATTTATCCACTATATTTTGC
TCATAAGCTAATTTATTTAACTCACCTCTAAATGTTTCTTGTTTTAATGGCGATCCATCTTTTCTTGGAAAGAGG
TATTCACTATCTGGAAATTCCTCTGAAACTTTATCTTCCCGAACTTTAATAAGTAAAGCTACCTCTTTAGATATT
GGAACTATATGCTCCTTTTTCATTTTCCATTGATAATACTTTAAAAAGAAATCTCCATCTTTGTCCTCTAATAGAC
AGCCTTTTTTCAAGGTGCACAATTCACTTATCCTCATTCCACATTCTTGAACAATCATAGTCATCGTAGCTATAT
ATTCGGGTAATTTATCAAGATGACTGTTCAATTGCTCTAGGACGAATTCATCTATAAAGCGTGGTTTTGCTCTT
GGTATTTTCGGATAGTCCTCAGAATAAATTAATATTTTGGAAGGAACATCATCCCATTCTAGCCTAAGAAGGG
TACTAAATAGTCCTTCCAATATAGAGATCCTCCCAGTTATTGTACTAGGTTTTATTCCCATCATGTTTAGTTCACT
TAAATATGCTTCAATTTCCACTCTCGTTAATTGGTGTACTCTCTGAACTTGTTTAAATTTCATGTCCAGAAAATT
AAAGAACTCTTTAAGTCTTTGGGCAATATCACTTACATAGGAAAAGCTATCCACGTTCAATCTCAACTTACAAT
ATCTTTTTACAAGTTGTTTAAAATATGTATTCCGAAACCCTTTAAAGTTAATTGTATATTCATATTGTGTTGGGTT
AACCTTATCATCTGGCAAAGGTAAGTTACGTCTATCCCAAACGTCTTTATCCCACTCCTCTCCATCAAAATAAA
AGTTCTCATAAAACTCCATAAATTGTTTTAGATTAGTAACATAGTAGGAATTAGCTTTTACAGGTGTTTTTTCTT
GATTAGCAGTAATCTTATAATTAGTAGTGGTAATTCTAACACCCCGTTTTGTCAAATAAGTTCTATACTCCGTCA
TTGCTTTTTCAATAGGAACTTCAGTAATTGAAGTAATGCTAGGATACTTTAAATCTAAGAAATCTAACATTTTAT
TAATTACTGTTCCTTTTCTAATCCAGACAGTTTTTGCATTCCATATTCCATTGTTTAAATGGTAAAAATAAAAAT
ATTTCAATTCTGTTCTTAACCACAGATTTTTAACACGTTCAAAACGAACCCAACGATTCCTTAAAGCAGGATT
CTTACTTAATTCTATGGCAGAAGGATGTGGACATTTTCTTATATCCCAACTATTATTAGCCCAAAACCCCTGCAT
TTCTTCATTCATTACAGCTATTTTTTTGCTAATCTCACTCTGACTAATAATTTTCCTTTTACTAGAAGCATTCATT
TCTTATGCTCCTTTCTCTCGAGGTATTTATTAAACTCATTTTTCATATCCTGATCTGAAAGATGAACATAGGTATT
TAACGTTGTCTGAACATGTGCGTGACCTAATCTCTTTTGAACGAACGCAACATCCCATCCTTCCCTAATTAGCT
GCGTTGCGTGAGTGTGGCGAAGCATATGTGATGTAAATTCTATTCCAGTCCTTTTAACTATTCTTCTAACTAGA
TCAAGAACACTTTGGTACTTTAGTGGTTTCCCAAAATAGCCTTCTTTTAAGGAAATAAAAACATAATCATGCTC
CAATTCCTCACTATACTCATATATCAAGTAATCTGTATAAAGTGACATAAGTTCTTTACTCACATGTATTGTTCTT
TCCTTCCTTAATTTAATATAAGCTTCATTAACATTAACATCTCTAGGTGTTAAATGGATTTGATTGTCCCAAGTG
ACAATATCTTCAAGCCTAAGCGATAACACTTCACCGATTCTTAAACCACCCTCATACATAAGCATTAAAATTAAT

TTATCTCTTTTCGTATGACAAGCATCAATAATTTGCTTAACTTCCTTTGATCTCAATGTTCTTATCTGTTTCTTTT
TAACCCTTAACTTTAAGACATTCTTTTGGTATCTACCCTTATTAACATGATGTAAAAATCCTTTGAAATTTCTTCC
CTTTGCTTGTTTAAATACATCAATTGATTTAAATTCTCCTAATCTACTTAAATAATCAAGAAAACTCATAACTACA
TTTAAAATTGTATTCACTGTCGTTTCTTCTCTTATGGCTTTTTTTGACTGAAGATCAATTACATTTGATGCTGAA
GGATATCTCAACCAACCTACGAAGTCTGCTAACAACTCAAAGTTAATATCATTAAGAATAACACCTCTCTGTTC
CATGAACTCGTACAGCAACTTTAAATGATAGCAGTATGCCTTAATGGTATTAGGAGACTTACCAGTATTATCTA
AGTATTTAATAAATTTCATTACTGGTTCTATTAGCTGGTATTCTTTATCTAGTAATAAATACAATGGATACGGCTT
ATTCTCCACTTCTATCCTTTGAACCTTCACATGTTCCCACCTCTTTAAATACTTTAACTACTACAAATTATAAACC
TTGTTAATTTTTATTTAAAGTAAAATATCCCTTTAATATCTCCTCTTAAACTACTTTAACTACTATTATTTATTATAC
TATGGTTAATACATCTCCACTTGGATGATTATGACCGAGCATTATACTATTTGCGTTACTGAGTATCGCTGTTTT
GAATATTTCTCTAGGGTGAATCACCGTTTGGTTAATAGATCCAATCGATAGTGTTTGAATATGTGTAGGTTCAT
TTTTACTGTTCATACATATGAGAATGAGATGCTCTCGGTCACTGTTTCCAATGAATGAACGCATGATTTCTGCC
GCATCCTCAGGGTTTGAAATACGATTTTTTAGATAACTTAATGTATCTGTTTTTATCATTTGTAGTGAAACAATA
TTGATTTCCTTCATCGTTTACCTCCATATATAGGTTATGCTTTCAAAGTCCATTTTTGACGTGCTTTAGGGTTGA
GTGGGTGCATGATTTCATTTGTGGCTGGATTAATGAGCTTTTTGACTTTCTTTTTATGAGGCTTCAACATTTCC
ATTACTTGTTCGACACGTTCTACAACAACTGGCCGCTTCGCATAAGCACCATAAGCTAGAATCACTGTGTCAC
TTTCGCTAATTGCTTTCATCAAGTGGATGTCTGTGTGTTTATCATAAGGTTCTTTAATATGTTTAAGGTTCTCTG
GTGTTTTAATATTAGAGAATAGATTTACAAGATATACAGCACCGTATCGTTCTGAATTCGCTAATTGATTGAGG
ATAAGAACAGTTGTGAGATCGAGTGATAATACACCATCTAAATGAGGGTACATCGTTATCACTGTACAAGCGG
GTTTCTTTTCATCCCATGTTTTCTTGAGTAAATAGCGGTGTTTTTCATCCTTGCTAAATATAGCTTCTGTGTATAT
CGTATTTTTGATTGTATTCATATATATAATCACTTCCTTTAGTATTCTTCTGGTAAAAGCATCACATAATAAAAAG
CGTCTACGCCATCTTCACGAATGACGTAGACTTTCTTAGGTAATGCATTTTGATTTTTTACATAGTTTGTATAGT
GATATTCCAATTTGTATGCAGGTTGTTCTTGTTCATGTGTGATTGAGAGTATATTCTCATCTTCTTGCAGTTTAA
AAATGTGTAGGTAATCTGTATGAGGTTGATTATCTCTTTCTTTTACCATATTCCAAAGTAAGATTTGAAGGTCTA
GAGATAGGTATTCACTAATACCTCTTGTGATGTATCGATTGATTTTCATGTTATTTTACCTCGTCTTGAATTTCTT
TCATAATGATAATCGCTTGGCTAATAATCGTAACAGATATTTGTGCCACTTTGATCCAGTTATTCATGGTGAGTC
CCTCCTTCTCTTAGTAAATGACGTTCATCGATAATCGTATTTTTAGTATCTGTGAGATATAAAAAGTCCATGTCA
AAATGATTCAGATAACCAACGCTGATGAGTTGGTTATTAGCGTACATAAGAAATGGATAGATACTTAGGTCAT
GTAGTTCATTATTGTAGTAGGTATAAGTTTCAAGTGTAAGATGTGCAAGTGGGGAATCATTAATAAAACGTTC
CGGTAGAATATTTTCTGCTGCTTCTTCTAACGCTTCGCATTCCCATGTTTCATTGTTAGATAGTTGGAATAGAC
GAGTTATATATTGTTTGAGTTCTTGAGTGGTCGTTTTCATATCATTGCCTCCTAGATAGTGTTATAGTGATGTAG
TTTATGTACATCATTGGGATAATATATATTTGATTTGTCATTTATTACGCATCCCGGTGAGAATGAGAGAAAATT
CCATATGAAAAACCGCTTCAAACCTTGGTATGACAAGGAAATCCCGAAATTCCGCCTATTTTGACGAACAATC
AACTCATTCTTTATAACTATTGATGTTAGGGTGGGGCTCTGCTTTCTTATATATTATTTATTTATAAAGAATAACG
GGATTTTGGGATTGTGCTTGCACAATCCTTCTGCTTCTTCGAATCTGCAAATCCCAATCATTTCCCGATAAAAA
ATCATTGTGGGATGTTCTTTAGCAATTTCAATATAAGCATCGTGTAGTTATGAAAAAAATTACGACAATGACTG
TTTCATTAGATAAGTGTTATTGAAATTGATAAAGAGAATTTTAAAAATGGTTAGATAAAATAAATGAAAGAATA
TAATGAAAATTATTGTTATAACAATGATTCTATTAGCTAAATAGTAAGATATAGTGTTTGGGGCAAAAACAAAG
ACGAAGTGCTGAGATGCACTTCGTCGAGTTGTTTATTATTGAAAAGTTGTTTTATGATTTCGTTATTAAGTTTG
AGTGTGACATAGAATTGTTTTTTATGATTACCATCTTTTTTAATATCAATGCGATCAATCACTGATAGATACAAT
GCTTTGAGTCGAGATTTTTCTATGTGCTTAATATCATGAAAGATGTGTTGTAATAGTTTACTGATTTCTTTGGC
ATCAAATAAAGGCTTATCTTCATTTTGTTGATTTTTGAGTTGGTTGATTTGATTCGTAATGTCATTGAGTTGCTT
TTCATATTTTTGAATACTTGGTCTGATTACTGATGTTAAGTCCGGATTATCCTCGATGGTTTTAATCAAGTTATTT
AGTTTGATTTGTACTTCATCATATTGTTGTTGCTTATAAGCAATATCGTGATGAAGTGCAGCGCCATCAACTTG

ATTTTCTTGATTGACGTGTGTTACTACGCGTTGAATGACTTTATCACTTTTGACTATTTCAAGTATTTGCTTCAT
CACATAATCTTCAATCACATCAGCTCTTACACTGTTTGCCGAACATACTTTGGAACCCTTGTTCCGAAAATTAC
TACATGAATAGTAACGAATACGTTTCTTAGTCCCGTCTTTAAGTGTATTCGTGGTATTGCTTGCTGCCATAGGT
GCGCCACATTGGGGACAGTGAATAATGCCTGTAAGCAGATTCGTTCCTTTGCCATGGACTTGGGGTTTTTGA
CTGACTTGTTTTTTACGCATTTGTACTTTATCCCATAAATCTTGATTAATAATGGGGGAATGCTTACCTTCAGCT
ATCACTGGTTTATCATTAAGCCCTTTACGACGTTTTTCACTCCAATCTTTGTATTTCGCAAATTGAATTTTGCCG
ATATAGAATGGGTTAGCTAATATATATGTGATAGAACTAATACTAAAAGGTTTACCCTTTTTAGTGACATAGCCT
TTGTGATTTAATGCATTGGCTATTTTACGATAGCCATGGCCTTTGGCATAGGACTCGAATATATATTTCACAATA
TTAGCTTCATGTTGATTAATCATCAGTTCATGTTTACTATTAGGTATTTTGTCATAGCCCAGCGGCAAATTGCCT
TGATAATAGCCTTCTTGGGCACGTCTCGTTTGACCCATAAATACATTCTCGACAATGTTATTACGTTCGAATTCT
GAGAAACTCGCAAGTATTTGTAACATGAGCTTACCCGATGAAGTATTGACTTCCATACGCTCTGATAAACTGA
AAAATTCGACATTTTGTTTGTGTAAATCTTCGACAATTTTGAGAAGATCAGATGTATTACGAGCTAATCGGTTT
GTTTTGTAGACCATAACACAGTCGATATAGCCTTCTTTCGCATCCTTCAATATACGTTGGAGCTCAGGTCGATT
CATAGATTTACCTGAAATACCACGGTCAGCGTATATATCTTTAACTTCAAAATGATGGAAGTCACAGTATTCTT
TGATTTGATTGATTTGTCCGTCGATACTATAACCTTCTGTGCTTTGCATTTCTGTTGATACACGTACATAGATAC
CGACACGTTTTGTTTTAAGTTGTTGCATTATGTTTCATCCTTTCTTCGTTTATGCAATCGATGATTGCATGGTTT
GATTGACGATATTGAGTGGTTCATTTTTGAAATAGATTCCTATAAGATTTTTATCTTTCGTAATGTGAATGGTTT
CAATATAGGGGTACAATATGTTTAACGTGAAACGTTTTTGAATAATATTTTGAATGGTGTGTTGTATTTGATGT
CCATTGATAGATGTAGTGCGTTGCGGTTGTTGACGTAATGATTGCGTTTGTTCTCTGAACGTTTCTGCATCGAT
GATGCCTTGTGCCAACTTTTCTATCAGTTGTTCTTGAGTCAATGTGTGATGTTTTTCTATGTTTCTTTGTCTTTT
GATGCGTTTGTCAATCGCACCTTTAATTTTTGTGTAGATGCGTTGATTTTGATAAAAGTCTTTGCACACTTCTA
ATACTTTATCTTCAAGTGTTTGTGCATTGATGCCTTTAAAATCACAGACAAAGCGAGAAGCATTCATGTTTTTA
GGACAGACGTAGTAACGTAATGTATGATTCTTTTTTCTAACGGTCATATTCGTAAGTGTTGTATTACAACATGG
GCATTTGATTTTTTGTTTTAGTTGGTTATCCGAAGATGTCTGTTTGATTTGTTTTTGCGATCGAAGTCTCTGCG
CTTGCTCATATATACTTGTGGAAACAATAGAAGGAAACATATTGTCGAATTGGCCATATTGATTGTTGACACGA
CCACAATAATTAGGATTGATGATAATGTTACGAACTTGATAGGGTTGTCGATTGATATACGTGTTATCTTCTTCT
AATAACTGTGCAATTTTCTTATAACCATGACCTTTAATGTAATAATTGAATACAGCCTTTACCGTTGGTGACTCA
TTTTGATTGATGATGAATGCTCCGTTGTGATATTCGTAACCAAAGGGCGCATGGGTTGTAATCAATCGACCTT
GCTTTGCTTTTTCTTGAAGCCCATTTCTGACTTGTTCTCCAATGTTATCTGATTCAAGTTCGGCCAAGCTGATA
AAAATATTAAGCTTGAATCGGTCGAAAGCTTGATCCATATCAAAGTAACCATCGTGTACGCTTAAGATATGAA
CATGGTATGTTTGACATAATTTGATGAGTTTTAATGCATTTTTCAGATTACGATGTAATCTATTAAGACGATAAC
AACATAATATGTCACATTGCCCTTGTTGAATTAATTGCGTCATTTGTCGATACCCACTACGATTATCTTTGCGTC
CTGATTGTTTGTCGCTATAAAAGTTGATATGTTGAATATGATGTTTTTCGGCTATTGCTTCGATAGCCTGTTTCT
GTGCTGCAAGAGATTGTTGTTTCATCGTACTTTGACGTAAATAGCCTATGACTTGTTTCATATCGGCTCCTCCT
TTCACAGTAATAATATATATTTATGGATGAATTGATATATAAGCCCAACATCAATAAGATGTTGGGCGTTCATATT
AGTCATTCATTTGATTGATTTCTTCAATTACCAAATCGGCTAATATCTCGATAAGTTCATCCATGTTTTTCACTCC
GTTATTTGTTCTATCTTCAATACGTCGATTATTCAGTTTGATGCTTCACGGTTGTATGATAAAGACAATCAGAAA
TCTTCGTGAACTCCTGAAGGGCCTATCCCCTCATTAGCGGATTTAAAAAGTTCTTTCGCAGCTTTGTTATCATT
TGCCGGTGTCCAATTTTGAATTAACGACTTATCTTTAGTTAATCCCAGGATAGATGCAAACTCTACATCTAATTT
TAGATGGTAAAATACAAGTGATTGTTTTTTACCGCTATTATCTTTGACACTTCTTTTAGTTGTTTGGCGTCCAC
GGTCAGCTAATATGAAACCTTTATCTCTTAAGGCGTTGACAACATTATTAACATCTTGAAATTGATGATTGTTT
AGCATCTGTTTAAAAACGTTTGCAATCATTTTTACTTCGATATGGTCATCTTTTAATGAGATTAATCCATAGTTC
TCAAACATATTTTTCAAAGCACCTTCATCTGAAAACTTACCTCTGTTTTGTGCTACAAATTGAATGATGACATC
GATAGCTTTATCAGCTAATGAGCGTTCAGAGATTGTATGACCATGATAATCAATAAAGTAGTCTCTTATTTTAG

CGATATCAATATCTGTAGCTAAAACACGACCTAATATTTTTGCAGATGTTGTAATGACTGCATAACGCTTAAAC
ATACGATTGCCTGTGTTGCTTTTATCATCTTTCAATTTAGCTTCAAACCAATCTACTTCCTTGTAAAACCATTGA
ATAACTTCATCTTCACGATTTATAAGATATTTAGCTACTAACGGTAAAACATGACCATAGTTTAGTGCTACAGCT
TTTTTAATATTGTCAGCATTGGTCGCATTTGTAGTGAATTGTTCATTAATCTCGATGGTTCTTACACGTAATCCA
TCGTTTTGAGCTGAATCATTAAAAATACTGTGTTCTGACGTTGAAATGACAGAAGTACCCCAATTCTTAGGCG
TTTTAACTTCTCCATGAACGTTTGAACGTTGACGACCTTGACCTTCAGTGATGGAGTACAATAACCCCGTTGT
ATCTCTAAAAGTTGCTGATGAGAGTTCATCAAATACAATAGGTATACCAAAATTGTTACTCAAGTAACCTTCAA
GTGCATTACGTGTGGCATTCCAATTTCTAAAGAGAGTTTCATTACCTTTGGTAGGGTTACCAGCGACTGATAC
AGCTAAAGAAGCTGCAGTTGACTTACCGGTTGAGGATTGACCTGTAAAACTAAAAATGATTCCGGCAAATTC
GGTTTCATGTTTGTGCTTCAGGAAACTCGTCACTAAGGCAGAAATACCAAATACGACTGCTAATTCTAAAAG
AAGAGAACCTTTAACCTCTTTTAGATACATGTTAAACCAATTATCAAATGTTCCCCTAGGTGCTAAGTCATAAG
TATTCTCACAAATGGCGTCAGATGGAGATTTATTATCAAATTCCGTAGTAGTATAGATTTCATTTAACGATACAA
TAGGACCAAACGGTGTTTCCAGTATACCTACCCCTTCATATAAGTAGGAAATGGGTAATTGGTTGCGCATTTG
TTGCAACGCATAACCTAAATCTTTTGTATATTTTTCATTAATACTAAATCCATATTTCATTAAAGAGGGCAGTTTT
TGTGTTGTTAAAATATCACTAGATTCAACAATTACTTTTTGATCCTCGTCTGTAATAATTACTTTTTCAGTGTTAG
TTTTAGGGTCAATAAACTTATTTTCGATAACGATAGGACCTGCGATTTCAACTTCAGTAGGCATTCCTCCTTTT
TCTTTGGGAGGCTTGTCTTTATACCAACCTTTTTTTGATTTGTATCGTGGTGAAGGATTAAATGAAGGGTTAG
TTTGAGTCATTAGCGAACACCTCCTTTCGAAGGGTTGCTGTTATAGTGTGGATTAGGACCTGTTTTAAGATAA
ACTAAGTGACCGTGAGTATCCTTACCGATAATAATAAATGGAACACGTGGCGCATGTTTTACAAAATATGCGA
ACCAACGTCCAACATTTTGTTGTACAGCTTTTGAACATTGTACATTTGCACGACTGTTCAAGTCATGAAATGT
AAATTCTTCTCCTTCAGGTAAATTGAATGAAATACCTAATATTTTCTTTTTTAACTGCTCGAATTTATCTGATTG
ATTGAAATACATTTAACTTCCTCCTACTAATGAACTAAGATAGGAAAATTAAATATGCGCACAATTAACTTTCTT
AAGTTCATTTTAGCCAAGAAGTTATCATTTGAAAGCTTGTAAAAAATATGTTAGAAATTCCGTACTAAATTAAGA
TGGATTTTTGTTGACAAAATAAAAAACGCTGATTTAACAGCGCTTTAAATAAAAATTAATCTGAAGTTATATAA
AAGTAGTCAGAAGGAGTGGTGTTATTATCATTATAATAATCTAGTGCTTTATTGATTTCTTTTATTAATTTATCAT
GATGTTCTTTTGTTATTTTCTTTGGATATTTGAGTTCTTTTTCTTCTAAGTCTTTTGGAGACACTAGGTCACTAT
TTAAAATTAATGATAACAGCATATTTTTAGGTGCCGAAGCTCTGGTGTCTCGATGAGTAATATATTTTGCTAAAT
TACTGTTTATACTTACTTTTTCATTTTCTAAAAAGTTTGTACTATTTTTAACATGAGCATCAGCAATTTTGGTACT
TATTTCAGTGTTTAAATCGTCTATTTCATCTAAATAATATTCAAGAATCTGAAGTTTATCTTTAGAAGTGATACCT
TTTTCAGTGAAAATCTTAATAATGATAGATTTGATTCCAATTTCTATATTACTGTCTACTAGAAAATAGAGATAAT
AATTAGTCAGAGCATGTTCATAATCTCTTTGATCAGTAATTTCATGATTGATGAAATAATATAAGTTCTCTTTGA
TACGATTAGAAGAATCTATATCTTTGTCATTGATACCGTTTTTAAAAAAGTGTAGATAAACTGAGTTGTTTGAG
AGTATATCATATAATACAAATAAACTTTTTGGTTTAAATACATATGATTTTCCTGATTTAAAGTTTGCTATGTTTAT
TCCTAGAGCGTTGCAGAATTTATTAAAATTAGTTGTAAAATTATTTTTTTCTTGAATAAACATGTCGTTGTCATT
TAATTCTGAACTATGATTTTTAATCATATTGATATTTTCTTCAATCTTGTCACTACTTAAATTTTCCATGCAAAGTA
TACTATATCTTACTATATCACTTATATTTATGGAAAAATCTTTAGGAGAACGATTTAAAGTATCCTTTTTATTATCT
TGATCTACAGCCCTACATGAATCATCAAAAACTTCTTTGACATTTATTTTATCAATGTTCAAAATTATGTCTACTA
AAGCATTTTGAAAATCATCGCTATATATGTCTTTATTATTCATAGTGTCACTACCTTTATTTTTAATAACACTATAT
ATTATATCTTATAAACCTAATACCATGAAGAATAAAAAGAATGATTCAAATTTTATATTTAATCCATAGTTGGCTG
CTATGGTTAGTGTACTAGTCTAGTTTTACTAGTATATAAAACAGTACTCAATTTTTATTTTTTCAAAATTATAAAA
AAGAACACCTATCATCGATAGGCACTGAACCCCTAAAACGGGAACTTAATAAAAACACCATGTTCTAGGCTAT
TAATTCTCTGTATTTTACAGGGGATAAGTAGCCTAGTTTTTGTTGAATTTGATTATTATTATAGTTTTTAATGTAC
TTTTCGACAATATCTATTACAATATGATTAGAGCTATTAAGCTGATTATTGATGTAAAGAGTTTCAGACTTTAGC
GAGGAATGGAAACTTTCTATCGGGGCGTTATCGGCAGGTGTTCCCTTTCGGGACATACTTCTGATAATGCCTT

TTTCTTCGCATAATTGATAATAAGCATAAGATGTATAAACGCTGCCTTGATCACTATGTAATATATACCCCTCAG
GTATATCGATTTGATTTAATGTATCATTAACTAAACGTTGGTCTTGTTTATCATCTATTTTATACGCCACAATTTCT
CCGTTATAAATATCCATTATCGAAGATAAATACAACATAGAATGATCAAATGGTAAATAAGTAATATCGGTTGTT
AATACTTCTATGGGACAATTCGCTTTAAATTATCTTTGTAATAAATTGTCTGTTTTATAATACGGTTTACCTATCC
TTGTCGTCTTTTTAGGTCTAACTCGGCAGTTCAAATGATGCTTCTGCATCATTCTCTGTACTCTCTTATGATTAA
TTGGTGATGTATAACATTGATTAATCAGTGCTGTAATCTTACGATAACCGTAGGTATAATGGTTAGCTTCACATA
ATTCAATAACTTTTTGTGTTACAGTATCATTTTTATAGGTTTTGTTTTTCCATCGGTAATATGTTGATTTAGGTAT
ATTTAATACTTCTAGTATCAATTTGATTGAATAGTTTCCTTTTAATTGATCCACTAAATCTATGACTACTGTTGGT
ACCACTTCCTTTCCAATGCCTTGTACTTTTTTAAAATATCCAATTCTATATCTTTTCTCTTATTTTCTAATTTTAAT
TGTTCTACTTCTGACAGCTCTTCTAATCCTTTACCGTAGGTATATTGTTTACCAACGTGTTGTGAAAATCTATAA
CTTTCCCCATTTCGATACCATCGCCACCAAGTTTCCACTTGTGTTCTATTTTTAATATTTAATTCTTTCATAATTTC
TTTTGTTGAAAATCCTGCTGCTTTCATTTCAACTGCTTTATACTTTGTTTCTACTGAATAAGAAACTCTTTTCAT
AGAAAAAACACCTCCGTATGATTCATTTTAATATGAATTCAACGAAAGTGTTTTTATATAATTCCCACTAATTGG
GGTCAGTCTACTATATGATACGGTTTTTTAATTTAAAGTATCAATAATAATTGGATATAGAGGGAAAGAAGCTA
TAATGATATTTGCTTACTAAGTGGATAAAGATATTAGAAAAACGAGAAGCAGGACAGTTATTAAATCGGTAGA
TATTAATCATAAGTATTTAACTGAATTTTTACCTTTTGTCGAATATATAACTAAAGTTGAAGGTAGTAAAATATTT
ATCTAGTCGATATTAAAACAATTTATCGAAAACGATGGTTTTCCATATCCTCTTTAGTAGCAATTTTCTAACAAT
ATATAATTAATTATATACAAATTAAGAATAATTAAGAAGTATATATTAATTAGAGATAGTTTAAAAATTAGATTTC
AATCAATTAATTAAAACAGGTTAGACTATTAATTTATACTAACCTGTTAAAAGTAACAAAATTAAGAATATATTA
AAACTTGTGTAATGAGTAAGCCTCCACCAAATATTAAAACAAACGCGACAACAGGCCATACGAAACGTAACC
AGTGTGAGTAGCGCACGTTTAACATTTGAAGTGTTGCCATTACAAGTCCAGTAGGCGCTAAGAACAACATTG
CATACTGACCGAATTGATATGTTGTAACAATAACAAATCTTGGTATACCTACTGTATCAGCTAATGGCGCAAAG
ATAGGCATAGATAATACTGCTAATCCTGATGATGATGGTACGATAAATCCTAAACAGAAAAAGATAAAGAGCA
GAACAATGATAAATAAAGGCCCACTCATATGTTGCACGATAGATGATGAAAAGTGCAAGATTGTGTCAGAAA
TCATTCCTTTATTCAATACTAAGTTGATTCCACGAGCTAAACCAATGATTAAAGATACACCTACTAAACTTGAA
GCGCCATTAACGAATGCATCTACAGTGCCTTTTTCGCCTAAACCATATTGTCCTGTTCCAGCAATAAACATGAT
GACAATGGTAAAGATTAAGAATGCAGATGCCATGACTGGGAACCACCATCCTTGTGTCATAACACCCCAAAC
CATAATAGGGAATGGTAGGACGAAAAGCGTAAGAATAATCTTTTTACGTAATGTAAACTCAGAAGAACCGTC
ATCATGGAGCACAGACCACTGTTTTTCAAATGCTGCTTTGTCTTCATAAGAATAAGAGGATTTAGGATCTTTT
TTAATTTTTTTACAATACCAGAATAAATAACTAATAACAAATATGGCACCGATGATACAAGCGCCTATTCTCCAA
TAAAGACCATCAGTAAAAGTTGTTCCTGCTGCATTAGAAGCAATGACGACTGAGAATGGGTTGATTGTTGAG
AATGTACTACCCACAGAGCTTGCTAAGAAAATTGCACCGAAAGTAAGACTTTAGAATCTATGGGAAAGATAA
TAAGGTTTAATAGAAATACAAGAAGTGTAAATAGTAAGCATATAATTTATACATTTTAATATAATATAACAAGAA
AGGTAAACACAAAGATAATAAAAATAAAAATTTAATAAAGATAGAGAATTATTAAATTGTTTTTGATATAAAAC
AACTTAATAAAAAATTTAATTTTAGGGGAGGATAATTATAATATAAAGAAACATAGAATGGAATTGCAATTGG
GAAGTATACGATTTTAACTGCGAATCATATGTTCAACGACTTACAAATGCGTAAATCGATAACACATTCAAAAA
TATATAATCTGAAAATTGATTTACTAAGAGAGGATTATTGTAGTTACAGGTGTTAAGTTGCTCCAATATGGAGT
TGTCGTCTTGTTTTATATTAAACAAGTTTTATTCAAATACATGACAATACGTAAAATAACACTAGAATTATCAAT
CACTAAAATTAAAACAAATGTATCTATTCAAACTTTTCTTTAGGGTTTACTTGCTGAATAATTTAAAAATGAAG
TATTAGGTGTACATGCTTTCAGAATTGCTTTTGAAGAATATTAAAAGTATAATCTGAATACGATGACAGAATGA
ATAGGTAAACTATGACAAGAAGTCATCAAGGTTATCAAATAAATATATCCCAGCGGTTGAGACGACTCACTGC
TGGGATTTTATTGTTAGTTATTTATTCTAAGTGTTGCATTTGGTAGCCGATTCTAGGATGTGTGATGATTAATTT
CTTTTCAGTGTTTGGGTCTTGCGTATAATTCAACTTTTTACGTAAAGAGGCCATATGTACACGTAAGGCAGCC
ATTTCTGTATGATTGACATAGCCATAGAGTGATTTTAATAACACTTGATAGGTTAATACTTTGCCGACATGATG

GCATAATATCGTTAGGAGTTGGAATTCATTCGGTGTTAGATGTACGGACTGTTCATTGACAAGGACTGATTTC
GCATCGAAATCAATGGTTAATGGACCATTTGTGAAACGACTTTGAATTGTCTCAGTAGAACGTGACATACGTA
ATGCTACTCTGATACGTGCTCTTAACTCATCGATATTGAAAGGCTTAGTCATATAATCATTGGCACCGCGATCTA
ATACTTCGACAATGGTTTGTTCCTCTGTTCGTGCACTAATCACAATAATAGGTGTGTCCACAAAGTCTCTGAAT
TGCTGAATGAGAGATAAACCATCAATATCTGGTAAGCCTAAATCTAATAAGATAATATCTGGTTGTTCTGTTCTT
AGGCGAAAGTCCGCTTCTTTCCCCGTCTTCGCCGTAACCACTTTATAATAATTCATAGTTAGCGCAACATCGAT
TAAATGTAAAATCGCTTCATCATCTTCAACGACCAATAATGTTGTTTTCATCCATGGCTCCCTCCATTTCAATTG
GATTAACAGTCAAATAAAAATAAAAAATACTGCCTTGTGGTGTATTCGGTTGATATTCTAATTCACTGTTGTGT
TGTTTCAAGATGAGTTGTACTAAATAGAGCCCTAATCCCAAACTATCTTTTTTATTGTCTTTAAAGTTGTCTCCT
GAATAATAGGGATTAAAAATCAATTGACGTTCTTCTTCTGGAATACCTTTTCCGCAATCTATCATTTCGAATTTT
ATTTTGTTTGTTTCATGTTGAACGTGCAGTTTTATTTCAGAATGTGATTCTGCATGCTTTAAAGCATTATCGATA
AGGTTGAATAGCACTTGCAGTATTAATTTACTGTCGATATTAATGAGTGAAGCGTCATCCTCATTTTCAATAAT
GACATGATTTGCTTGGTGTCTGCGTATGAGGCCTTCTTCAAATTCTTCTAGAAGTTCTTCTACTAAATAAGGG
GTGCGTTGTATTTGAATGTCAGAGCTTTCTAACTTAGTCAAAGATAAAATATTTGTGACTAAGGTATGCAGATA
TTGTGCTTCGCCATAAGAAGCAGTTAAGAGTTCTGCTTGTTGTTGATCGTTTAAATGCTCGTTATGGTATTTCA
GCATATCTAAGTTGCCCATAATGGAAGTCAGTGGTGTTCTAATATCATGTGAAATTGAATGCAAGAAGTTTGA
ACGTGTGGCTTCTCGTTCAGCTTTCAATATGGATTGTCTGGTTTGTTTTAATAGATCCACATTCTCTATTGCCA
GGGTAATATCGTTCAACATAGAGTCTAATATTGAATTGTCATAGGTGTCGATGTAAGTTTCGTCGGTGAAGCG
GATAGAAATCACACCTTTGACTGGATTTGTGCCAATGGGAATACACAAAAATTTACTGCCAGGAAAGGTATC
GGTTAATTTACCGGCACGGCTTTCATTTTCAATGACCCAGCTCAATGTCTCAGCATCATGTGTCTTATCTGAGC
TTGAAATGCTTCTGTTTCCAAATGAGTTGGAAGCAGCGACTTTCTTACTTTGAATTAAGAATACCGTGACATC
CTGATTGAGTAGCTGATGAATCTGATCACCAGCAATATTTAATAAGCGTTCAATTGAATAAGATTCTTTAATGG
ACTGGTTAAATTGCAGCATAATATTGGTCCGATATAACTGCCGTTCTGTTAAGGAATGCTGATGTTTTAAATTC
TTTAAGATGGCACTCGTAAAGATACTAGCAAAAATACTGGTCGCAAACGTAATCGGATATTCAAAGCGATACA
TTTCTAAAGTGAATCTTGGCACCGTAAAGAAATAATTAAAAACAAACACATTTAAGATAGACGCAAAGAATC
CGATTAAATAGGATTGGGTCCAAATAGAAAGCACGATAATACCGATAAAGAACATCAGCAAGATAATGGCAC
TGGATTCGCTTTTATCTAAGTTGTAAACCCATATACCGAGCAAGACACAGATCGTTTGAATTACAAGCATCTTC
ATAATCTCTACAGTTAAACGAGAGGATTTTTGAGCTTGCTTAGGTGTATTTGTAGGTTTGTCTGAATGAATATA
ATGAATCGGTACAATTTCTAACTTGAAATGGTGTGGTACGTGATTAATTTGTTCAATGAGTGACTGTTTAAAG
TAATCTTTCCAACGTGGCTGTTCTGACTGTCCAAGGACTAGCTTCGTCACAAAAGCGAGATCACACCAATCG
GTTAACGCTTTCGCAATATCTTGTGCATACAACACTTTGATTTCTGCACCTAACGCTTTGGCGAGCATTAGATT
TTTATGGACATAATGATCTTGTTTTCTGCTTTCTGACGGTGTCTCAAAGACATCAATATACACAGCTGTGAATT
TAGCATGTTCTTTATAGGCAGCACGTCTTGCTTCTCGTATGACCCGTTCGTTATAAATACTGCCGCTGATAGCT
ACCGCAATATGCGGTGTAATGTCGGTATGTTTAGTTTTATATTGTTGTCCTTTTTGACTCATAATATCTGCGACA
GTTCTGAGTGTAAGTTCACGCAGCTCTGTCAGATTTTCATACGTAAAGAAATTAGAAAAGGCTGTTTCTAAG
CGTTCTTTTTTATATACTTTTCCTGCTTTAAGGCGCTGAATCAACATATTTGGTGAAATATCCACAACTTCAAAG
GCATCTGCTGACGTAATGAATTGGTCGGGCACACGTTCTGTAACTTGAATACCTGTCATTAACGCAATTTGTC
CGCTTAGACTCTCGATATGTTGGATGTTGAGTGTTGTCCAGACATCGATACCATGCGATAGAATTTCTTCTATA
TCCTTATAACGTTTTAAATGGCGCTCTTTTGAAATGTTTGTATGTGCTAGTTCATCAATTAAGACCACATCTGG
ATTAGCTTCTATGATTTTAGAGACATCTATATAGTGAAAGGTGTGGCTGCCAAATTTACGGCTGGAGGTCGAA
ATTTCAGGCAATTGTTGAACCAGTGCATTGGTTTCAGGGCGTTGATGGGGTTCGATATAACCAATTTTAATAT
CTGCACCTTCTTGATACTGATCAATACCATTTGATAACATTTCATACGTTTTACCTACCCCTGGGCTATAGCCTA
AATAAATGGTAAGTTTCCCTCTTTTTTTATATGTACTTTCCATGAGGCACCCCCTCTTAATCACATCATAATTAAT
ATACAACATATTATCCATTTATCTTTCTGTTTTGTTAATCATCTTTATAGTTTCTTTATAACTTTCCTTAAATGTTAA

TACTTCGATTAGATAACTCTTGTTAAGATTTTGATGAAAGTAAGGAGGGAGCGCAATGATTACACTATTAGCT
GTCGTTGTCATCGCATTAATTTTATTTTTATTTTACGCATTAATTTGGAGTGAAAAATTTTAACAGAGAAAGAG
GGAAGCATCATGAGTATTGTGTTGTTTTTGATTGTATTTATCTTGCTCTCACTCATTGTGAGCCGATATTTATAT
TCAGTTGCTTTAAATGTGCCATCTAAAATAGATGTTGTTTTTAATCCGATTGAGAAATTGATTTATCAACTGATT
GGCACGAAATTAGAACACATGTCTGGGAAGACGTATATCAAACATTTTTTGTTGTTTAACGGATTGATGGGC
GGATTGTCCTTTGTATTATTGCTTATTCAACAATGGCTGTTTTTGAATCCTAACCATAATTTAAATCAATCTGTAT
CGTTAGCCTTTAATACTATGGCATCTTTTTTGACCAATACTAACTTACAGCATTATGCAGGTGAAACAGATTTA
AGTTATTTAACACAAATGTGTGTCATCACTTTCTTAATGTTCACGTCAGCAGCGTCAGGTTACGCCGTATGTAT
TGCGATGTTAAGACGTTTGACTGGAATGACAGATGTGATTGGTAATTTCTATCAAGATATTACGCGTTTTATTG
TACGGGTGCTCATACCTTTCGCATTGATCATCAGTTTGTTTTTAATCAGTCAGGGCACACCGCAAACGCTTAA
AGGTAATTTGGTGATTGAGACATTATCAGGTGTGAAACAAACGATTGCATATGGACCGATGGCGTCTTTAGA
ATCTATTAAACATTTAGGGACAAATGGTGGTGGTTTCTTAGGTGCGAACTCTTCTACACCTTTTGAAAATCCG
ACATACTGGTCTAATTACGCTGAAGCTTTAAGTATGATGTTGATTCCAGGTTCATTAGTCTTTCTATTCGGTAG
AATGTTGAAAACTAAACTACAGATTCATCCGCATGCGATTATGATTTTCGTTGCGATGTTTGTAATGTTCATCG
GCTTTTTAGTGACATGTCTCTATTTTGAATTTGCGGGGAATCCAGTGTTGCATCACTTAGGTATTGCCGGTGG
CAATATGGAAGGCAAAGAAACACGTTTCGGTATTGGCTTATCCGCTTTATTTACAACCATTACGACCGCTTTT
ACTACAGGAACAGTTAACAATATGCACGATAGTCTTACACCGCTAGGCGGCATGGTTCCAATGGTATTAATGA
TGTTGAATGCAGTTTTTGGCGGTGAAGGTGTTGGGCTGATGAACATGTTGATTTATGTCATGTTAACGGTCTT
TATCTGTAGTTTGATGATTGGGAAAACACCAAGTTATTTAGGAATGAAGATTGAAGGTAAAGAGATGAAACT
CATTGCGCTTTCTTTCTTAGTACATCCTTTACTTATTTTGGTTTTTTCAGCACTAGCTTTTATTGTGCCAGGGGC
ATCAGATGCGTTAACTAATCCGCAATTCCACGGTGTATCACAAGTGTTGTATGAGTTTACATCATCTTCAGCGA
ATAATGGCTCTGGTTTTGAAGGATTAGGAGACAATACGGTATTTTGGAACATTTCAACAGGCATTGTGATGTT
GCTTGCACGATATATTCCAATCGTTTTACAAATTTTGATTGTATCTAGTTTGGTAAATAAAAAGACCTATCAGCA
ACATACTCAAGATGTACCGATTAATAATTTATTTTTCAGCAGTGTATTGATTATCTTTATTATTTTGTTGAGCGGC
TTAACGTTCTTACCTGACTTAATGCTTGGACCAATAGGCGAACAGCTTTTGCTGCACGCATAGATAAAGGAG
GATTAGAAAATGGCTGAAACTACTAAAATATTTGAATCACATTTGGTCAAACAGGCTCTAAAAGACAGTGTAT
TGAAGCTCTATCCTGTTTATATGATTAAAAATCCGATTATGTTTGTTGTAGAAGTGGGCATGCTGCTTGCCTTA
GGATTAACCATTTATCCGGATTTATTTCACCAAGAAAGTGTATCACGGCTATATGTGTTCAGTATCTTTATCATA
TTATTACTGACACTTGTCTTTGCGAACTTCTCTGAAGCATTAGCTGAAGGTCGCGGTAAAGCACAAGCCAAC
GCTTTACGCCAAACACAAACTGAAATGAAGGCACGTCGTATTAAACAAGACGGCAGTTATGAAATGATTGAC
GCTAGTGACCTGAAAAAAGGACATATCGTACGTGTCGCGACAGGTGAACAAATCCCAAATGACGGTAAAGT
TATTAAGGGCCTCGCAACAGTGGATGAATCTGCGATTACAGGTGAATCTGCACCTGTAATCAAAGAAAGCGG
TGGAGATTTCGATAATGTAATTGGAGGAACTTCTGTAGCTTCAGACTGGTTAGAAGTTGAGATTACTTCAGA
ACCAGGTCATTCATTTTTAGATAAAATGATTGGTTTGGTTGAAGGGGCTACAAGAAAGAAAACACCTAATGA
AATTGCGTTATTTACTTTATTGATGACATTAACGATTATCTTCTTGGTCGTTATTTTAACGATGTATCCATTGGCG
AAATTCCTGAATTTCAATTTATCCATTGCGATGCTGATTGCTTTGGCTGTGTGTTTAATTCCAACAACCATTGG
GGGATTATTATCGGCTATAGGGATTGCAGGGATGGATCGTGTGACACAGTTTAATATCTTGGCTAAAAGCGG
ACGTTCTGTAGAGACTTGTGGTGATGTGAATGTCTTGATTTTAGATAAAACAGGTACCATTACCTACGGCAAC
CGTATGGCAGATGCGTTTATTCCGGTGAAATCATCAAGCTTTGAACGTTTAGTTAAAGCGGCCTATGAAAGTT
CTATCGCAGATGACACACCAGAGGGACGTAGTATTGTGAAATTAGCTTATAAACAACATATCGACTTACCGCA
AGAGGTCGGAGAATATATTCCGTTTACTGCTGAAACACGTATGAGCGGTGTGAAATTTACGACACGTGAAGT
ATATAAAGGTGCACCGAATAGTATGGTTAAGCGTGTGAAAGAAGCAGGGGGACATATTCCAGTTGATTTAGA
CGCTCTTGTCAAAGGGGTGTCTAAAAAAGGTGGCACACCGCTGGTTGTGCTTGAAGATAATGAGATTTTAG
GTGTTATTTATTTGAAAGATGTCATTAAAGATGGACTCGTAGAACGTTTCCGTGAATTACGTGAGATGGGGAT

TGAAACGGTGATGTGTACAGGAGATAACGAATTGACAGCTGCGACAATAGCGAAAGAAGCGGGTGTGGAT
CGCTTTGTGGCAGAGTGTAAACCTGAAGATAAAATCAATGTGATTAGAGAAGAACAAGCGAAAGGTCATAT
TGTTGCGATGACGGGTGACGGTACGAATGACGCGCCAGCTTTAGCAGAAGCTAATGTAGGTTTGGCAATGA
ACTCAGGAACCATGAGTGCCAAAGAAGCGGCGAATTTAATTGATTTAGATTCTAATCCAACCAAACTGATGG
AAGTCGTTCTAATTGGGAAACAATTATTAATGACACGTGGCTCACTCACTACATTTAGTATTGCGAATGACATT
GCGAAATACTTTGCGATTTTACCAGCCATGTTTATGGCGGCTATGCCTGCGATGAATCATTTGAATATTATGCA
TCTGCATTCACCTGAATCAGCAGTATTATCTGCGTTAATCTTTAATGCGTTGATTATTGTATTATTGATTCCGATT
GCGATGAAAGGCGTGAAATTTAAAGGTGCCTCAACGCAAACCATATTGATGAAAAATATGTTAGTTTACGGC
TTAGGCGGTATGATCGTGCCATTTATCGGCATTAAGCTCATTGATCTCATCATCCAACTCTTTGTCTAAAAGGA
GGACAAAACAATGCAGACAATAAGAAAAAGTTTAGGACTAGTACTGATTATGTTTGTTTTATGCGGATTTATC
TTCCCGCTGACTGTCACAGCGCTTGGACAAGTATTATTTCCAGAACAAGCAAACGGCAGTTTAGTGAAACAA
GATGGCAAAGTAATTGGTTCAAAGCTCATTGGACAACAATGGACAGAACCTAAATATTTCCATGGACGTATC
AGTGCAGTCAATTACAATATGAATGCGAATGAAGTGAAAGAAAGTGGCGGACCTGCTTCAGGCGGCTCAAA
CTACGGCAATTCAAATCCTGAATTGAAAAAAAGAGTTCAAGAGACTATTAAACAAGAAGGAAAAAAAATTT
CAAGTGATGCGGTGACCGCTTCTGGCTCTGGTTTAGACCCAGATATTACGGTTGACAATGCGAAACAACAAG
TAAAACGCATTGCGAAAGAAAGAAACATAGATGCTTCAAAAATTAATCACCTTATTGATGAAAACAAACAAG
CATCACCAATGGCAGATGATTATGTTAATGTCTTAAAATTGAATATCACTTTAGATAAACTCTAAATAAACAGG
GAGTGAGGTGAGACATCCATGTGGTTCATTAGCATTATTATTTTAATAGCATTCCTAATTATATTAATGATTGAC
GATTTAATTAAAACATGACAACTTCACGCAAAACAATATGAATTGGATGTAATAGAGAAGGGAGTACCAAGA
AATACTCAGTTTATTGAGGATAATTATTAGGGGAGATTAGAGAAGAATTCTCTATTCACGAACATTACTTTAAC
AGTTGTAATCATTGGTTTCATCATATCGATTAATAGTACAGTCATCTTGAGATTTTCAACACACTATTATAGCTA
GCTAAGATACTTTTAAATTATGACTTATTTTTCATCATTTACGATACATCATTCTCGATAACTTACTATTTGTTATG
GTATTTATAGATTTAAAGAAAGTGAAGGAGATTAAGCAATGTAACTTGGCTTAATCTCTTTTTGATTATATTTAT
TAAGGCAAAGCTTCATTTCCCTTAAATCAAGTATATCTTCTGAATAAGCAAATGAAATCGTGACGCCAACTAC
AGTGATAACTTCGCCTAAAACACAATGAGTGTGGTGTTTTCACTTTTCATAAACTCCTCTAAGTTTGTGTTTTT
AAACTGTGATGGATTTGAATGTTTTTGAATTACGTTGTCGCGTCCTTGGGATTGCAATCCAAGAACGTCAGC
ATAATCTAAATCAATTTTGATTTGGTAGAAGACAAGAGAAGTACTTTTGCCTGTATCATCTTTCGCATTTCTTTT
TGTTGTCTTTCGATCCCGATCGGAAACTAATTTTCCTGCATTATCCAAAGCATCAATAACATTTTTCGTGTCTTC
GAATTGACTGTCTGTTAACATCTTATCGAATACATTTCGGATAATTTTTACTTCGATGTAGTCATCTTTAAGTGA
GAGGATACCAAAGTTTTGAATCATATTAGCAAGACGTGTTTGTTGTGAAAACTTGCCCAGGTTTTCCGCAAT
AAACTGTACGATTGTATCCATCGCTTTATCTGCAAGAGAACGTTCTGACACCGTTTCATTATGATAGCTTAATA
AATAGTTCTTTATGCTGCTAACGTTTAACTGTAGGTCTAGTGATTGATTTAAAATATGGGCTGTTGTTGTTAGA
GTTGCATAATGTTTAATCATTCGAATACCAGTGTTACTGGATTCAGTTGATAGTAATTGATAAAAAACACTGTC
TTCTTTTTGAAAAAGGGAAGTAACATCATTAGTGTTGTCTAATAGGAATTGAGCAATTATAGGTAAAACATGA
CCATTATTTTGACTAACCACTTTTTTTATAGTATCTGAGTTTTGAGCACTTGATGTGAATTTTTCAGACACCTCA
ATAGTACGAACACGCAAACCATCATTTTTAGCTGGATTTTTGAAAATACTATGTTCAGAAGTTGAAATCACTG
ATGTATCCTAGTTTTTAGGTGTTTTGACATCACCATTTATATTTGCTCTTTGACGTCCTTGTCCCTCTGCAAATG
AATATAGAAGACCTGTTGTATCATGAAATGTTGCCGCTGATAATTCATCTAATACTATGGGTACACCATAATTTC
CGCTAAGATATCCTTCTAGTGCATTTCTTGTTGCATTCCAACTCCGGAATAGGTTTTCAGTACCTTTTGTTGGG
TTGCCAGCTACCGATGCGGCTAACATAGCGGCTGTCGATTTTCCTGTTGATGATTGGCCAGTGAAAGAGAAA
ATAGTTCCTGAAAACTCGACGTTATTATGATACTTAAGGAAAGCAGTGACTAATGAAGATACGCCAAACAACA
CATCTAGTTCTAAAGATAAGTTCCCATGGACTTCTTCTTTATACATTTGTAACCATTCTGATAAATTGCCTTTTG
GTATTAAATCGTACTTGTTATCACATATAATAGAAGTGCTATTAGTAATCTCATTAGAAAAATAAGGCTGGTCTA
ATGAAATTAATGGTCCAAGTAATGTATGAAGCATTCCTACACCTGAGTATATTGTTGATAAAGGTGTGGTGCTT

CTCATTTGTTGCAGTACGAACCCTAAATCTCTAATGTGCTTTTCATTAATGGTAAATCCATATTCAATTAAGGAG
GGTAATTGCAAAGCAGTAAGGATTGAAGCCTTTTCGACAAGCTCTATATTATTCAAATCCGAAAGAAATACTT
TTTCTTTACGAGTCGCGGGGTCTATAAAACGATCTGTGAAAGCAATCGGTCCAGATAACATTATTTCAAACTC
CGTACCCCCATCTTTTTTAGGAGGGATGGTTTTGTACCATCCCGAAGGTTTGAGCTTGAATTCGTCAGTTTGA
TATAGAATTTTTTCCATTAACAGTGACCCCCTTGGTTGTAAGAGGAAATTATAATGATATGTCGGTACTTAAGT
GGGTTAGTTCCTATAATAATGAAAGGGATGCTTGGTTTGTACTTTATCCACCAAGCAAAGCGTCTTCCTGCTT
CACGTTGATCGTTGATGTTGCACTGTTCTCTGAATAACTGTAATTATAAGGTTTAGTAATGGCAATTTTTTATTT
AAACAAATTTTTACGAATGGATTGTCTGTTTATTAATTCAGCAACGTTTATTGAAGAAAAATAAGTGTCTTCTG
ATAATTGCAAAGGGTTTAATTCAATGTAAACTGAATTAAGGGTAGGGTAGGTATGCCTGTTTTTTGGTTCTTT
TAAGTTCAATCGATTAATAAAATTGTTGAACCTTTGTTCTAAGTCATACAAAAACATCAGTAATTCATTAGGAG
AATGATTTATTATGCAATCCATATTTTCGTTTATGCTTGAAGTTATACATCTAAAAACAGTATTGGTTTCTTCGTA
TAAAAATAACTTATTAATAGAGGTAACATTGATTAAGACTGAGTATACGTATAATAAGAGTGACTCTGACATCT
CATTTAGATAAATAAGACTGTGATCATTCTTATTTACGATTATTATATGTGATTGAATCAATTCAATAATGTCTAG
AAGAAGACTTGAAAAGTCGCTGTTAATCTTGGTCTTTGAAAAATCATCGTGACCATTTGTCTTATTACTTAGAT
GTAGTTGTTCGTATTTTATAGTTTGGCGGTTGAGGAATTAAGCATTTGATTATTATAAACTATCTCAAAAATTAA
ATTACCTCCTTTTTTCTTCCTCGATGAAGGTTTATCTAATCTTCCAATATACAGGTTTTTGAAGTAAATTATAGAT
ATGATAATCATTAATTCAGTAAATGTATACTTATTAAGGGGTGGAGCTTTAGCTAACTTATCGATAGTACTGCCG
ATGCGTGCTTTGAAACTATTCAAATTGTCGTGACTGTCTATATCTAAATCTTCAAATTTACTAGCAAGGGTTTG
CAAAGTAGAAATTACAACTTCTTCTTTTGTATAATATTTATTGAAAATATCATCTTCTTTATCATCGCTTTGGAGT
TTTAAAGTGCCTGGTTCTAAGATGTCAAATTTTTTGTTTCGATTGGTATTTTTTTAAAATGTGCCTTGTATTTTT
GGGGTTGTTCCAAATGTGTATTTGCTAGACTACCTGTAATTGATATTGCTTTATTTAGCATTTGGTGTGTATTAA
CTGTATCTGATTCTTCTGGAAATTTAAATCTAAAAGTAAAATCTCTACTACCGAATGTACTTTTTACGGCTACAG
TTTTGTACTTATGCTCAGGCAATGTAATTAAACCATCTTGATGCAGGAGGAAAAGTGTGTCATCATTTAATTTC
TCAATTGTATTAAATAAGTAATTATGCATAGCCTCTTTATACATGGCGAATTGATAATTTAGATTATCCCATTCTAT
TAGTTGATTTAAAATACAATATAGACCTTCATTCAATCGGTTTACGAATATCAATACAGCTTCTTCTCGTCCAAC
AAGGCTTATTAACTCCTTCAAGTCATCCTTTAATTTTAATTTTTCTCCTTCAAAATTGTATTGAGTGAAGCCTAT
AGCCTTTTGAAAAGTAACTTCCATGACAGAGTTGGTTTCAGAATATTCTAACGATTCATTTATTGCAAAAATC
AAATTTTTTATAAATATGGAATTACTCATGGTTTGTAATTGCATATACTACCCAACTTTCTTATTTACTTTAAAAC
AAATATAAGCTAAAGTAGTTATTATCATAAAGGGAACGTACGTTCTTATTTAGCTGATTTCTATGAAGAGAGGT
TATGGATATTGAATGAAGAAATCTTACGCGAAGTAGCAGATATTTTTATAGGTGATGACAGAGATAGCATTTAT
GATTATAAAACTGGGAACGAATTAGTGAGGTTTTTTAATCATTACTTTAATAAAGGGGACATATATCAGGCTCC
GTTTCCATCTAGATGGCTATATGTTGTGAAACATTTGCAAACTCTGATTCAGGAGAGAAAGATCAATCAATTT
TTCACATTAATTTTAAGCAATCACTATATTAAGTATGAACTGAAAATTGACGAGGTTGAAGCAGCAAAGCAGG
CTGCTAAAGCACTTAAGTTGTTCAACAAAAGATTAAATCATTATGGGTACTACATAACAGGAACTAACAATGC
TAGATATTTTATGGATAAGGATGAAGATACGGAGTCTATTGGTTATGGAGGGTATGCGAATATTTATTTACAGA
AGTCTACAGGTCTTGCTGTAAAAAAATTGAAAGAGGAGTATCTTACTGATTCTTCAATTAAAAGTAGGTTTAA
GAGAGAATTTGATCTCACTAAATCTTTTGATACAAATCCATTGTTCATTAATGTGTTTGAATTTAATGAATCAGA
TTATTCATACACGATGGAGTTAGCTGATGAAACTTTGAAAGATTACATTGAAAGCAAGACAATTAGTGAGCTA
GAAAAAGTAAAGATAATAATGAAAATTTTAAAAGCGATGAGTCAAGCACATAGTGAAAATAAAATACACAGA
GATATCAGTTCTAAAAATGTATTAATGTTTAGAGGAAAAGTCAAAATATCAGACTTAGGATTAGGTAAAAACC
TTGATGAAATTCATTCGCATCAAACCTTTGATACAAACGGTGTAGGACAATATAAATACTGTGCACCGGAACA
GATGTATAGTTTAAAACAAGCAGATAAACAATCTGATGTTTTTAGTTTAGGAAGATTGATAAATTTTATTATGA
CTGGAAATGTAGTTAACAACCATCACCTATTTAGAGGTGTATCTGATAAGGCTACGAACAGTAGTAAAGAATA
CAGATTTGAAGATGCAAATGAAATGTTGAAAATGCTGCAGAGAATTTTAGAGTATCACAGTAGTGCAAAGCA

CGTCGAAAAATGTCAAGAAAAGCTGAAAAGAGGAGTGTTTGATGATGAAAGCGAAGAATTTATTATGACAC
GAAGTGATGAACAATTATGTCAAATGGTTCTAAGTTCTAATAATAATGAGCAAGCGTGTTTAATTCGTTATATG
CAAAAAAACGAATCTTCAGCATGTGATTTAATAGAGAGTATTAATAGAAAGTATCAAGAGTTTTGTGGAAGG
TTTGAAGACTACGATCCTTTTGCTAAATTAGCATATATGATTTTATGTAATAACTTCAGTTATAGAGTGAATGAA
ACAGCAGCTAGAGTGCTAAATTATGTTGCTTGGTCTGTAAATAGATTTTCGGCACAAGACTTAATTAAAGGTT
TAATTAATAGAGGAGTGGAGCCTTTGATTGAAGAAAAATTAAAGGACAATTAAAAAAACCTCATCATTAACT
GATACGCAGAGGCGTATCATAAGT
>Staphylococcus aureus strain AR_0473
ATGAAAATCACCATTTTAGCTGTAGGGAAACTAAAAGAGAAATATTGGAAGCAAGCCATAGCAGAATATGAA
AAACGTTTAGGCCCATACACCAAGATAGACATCATAGAAGTTCCAGACGAAAAAGCACCAGAAAATATGAG
CGACAAAGAAATTGAGCAAGTAAAAGAAAAAGAAGGCCAACGAATACTAGCCAAAATCAAACCACAATCA
ACAGTCATTACATTAGAAATACAAGGAAAGATGCTATCTTCCGAAGGATTGGCACAAGAATTGAACCAACGC
ATGACCCAAGGGCAAAGCGACTTTGTATTCGTCATTGGTGGATCAAACGGCCTGCACAAGGACGTCTTACA
ACGCAGTAACTACGCACTATCATTCAGCAAAATGACATTTCCACATCAAATGATGCGGGTTGTGTTAATTGAG
CAAGTGTATAGAGCATTTAAGATTATGCGAGGAGAGGCTTATCATAAATAAAACTAAAAATTAGATTGTGTATA
ATTTAAAAATTTAATGAGATGTGGAGGAATTACATATATGAAATATTGGAGTATACCTTGCAATATCATACGATG
TTTATAGAGTGTTTAATAAACCATTTTTCAACTATTGATGATCTAGAATATATAATAACTGTACAAATTATATTGAT
TATGGAACTACAATTCAATTAAGAAATTGATGATGAAATTTTAAATTTAAACTAATGGAATCAAGAAAGAATG
AAAGGAAATATACAATGCCCACGATTAATAAAAGGAAGTTTATTAGATTTTGTGTTAGAAACAGTAGATGTTT
TTGTAAATGGAAGTAAAAATGATTGCGAATTAGTAAATTTTAATTATAAAGAAGGAAAAGTAGTTTCACGTTA
TAGTACAAAATTTGAAAGCAATCCTAAATTAAGAAAAAAGCAATTGATATCGACGGTAAAAGTTGTAAGGTAT
GTTATTTTTGATTTTGAGAAAATTTTATGGGGCATAGGGAAAGTTTTATTGAAGTTCATCATATTAAGGCAATG
TTTACTATAAGAAAGTTGAAATACACTCAGAAACAGACTGGATTCCCGTTTTTTTCAAATTGGCACAAAATGA
TACATAGGCCCACCTATATATTGAAGATTTAAGTTAAACGATAAATAATATTTTTCTCTTTTGTATAGAAATTTTT
TTGGGAATGATATTAATTAGTATAAAAATGAATATGTCTACAAAGAAGGAGAGATTTTATTGAAACACATTAAT
CAATTTTCTGAATCGACACTAGCACAACTAGAAAAAGATCAACAACACGTATTTTATGTATACTGTTTGATGG
ACCCAAGAAATGATGAATGCTTTTATGTTGGTAAAGGTAAGGGGAATAGAATATTTAAACATAAACAAGATG
CGCAGAAAAAAGTATTGTACGAAGATATATTTATAGAAGAAAATAAAGATAACTTGAAATTTAATAGAATTAA
CGAAATTTGTAGCAATGATTTGAATGTTTTAGGATATATAATCAGTTATGGATTAACCGAATCTGAAGCATTTT
CTGCAGAAAATGTTCTTATAAACTTTCTTAACCTAACAAATAAAACTACATTAACAAATATGATTAACGGTCAT
GGATCTAAGGCATACTTAGTTGAAGATTTAGAAAATGAATTTGGCTACGATTCAATTAATCTTGAAAACATAA
ATACGAATGAATTAATTTTAGCAGTGAAAATCAGAGATGCATTTCTATTAGATAAGGATGAAAGTAAAGAGTA
TCCTATTAATGAAAGTAAACGTGATAGAAATAACCTTAAATCGCGTACATTAGGTAGTTGGATAATAGGAAAA
GATAAAATACATAAAATAAAATATATTATTGGTATCAATACAGGTGCTAATAATGCAGTTGTCTCAGCTTATGAA
GTATCATTTGAACAAGCGGAGAGTATCGAAACAAATAATGGTAGAATGAGATATGCGTTTATTGGGCTTTCA
GAAAGAGATGCTACTCTAAAAAAAATTGAATTTATACAAAAAAGCACTACCAGATTTAAGATTTGGTAGTGGTA
GCGCTACAGCATATATAAATAATGGGACAATGAAAGTTGATTAAATGAATAAAAACATCCAATTCCTTAAATTA
GCCTTTGAAAATTTTATACTCGTATATATAAGTGAAAAACTTAAAAGAATTAGATGTTAATTAAAGGAGAGAAT
GCTATGCCATCAGATGATATTGTAAAAAAACTATACTCAAAAGAGGATATTCGATTAGAATTAGGACTTACTCC
GCATAAGTTTAATAAAAAAAATGGAAACTATTGCTAAGCTTTTTAAAATTGATATGAAAATTTTTCACAATTACA
AAGGCCAAGATAAAAATAATCAGTATACATTTAATGGTGTCGCAAAAGAATTAATCAAAGTGTTGCTAAAAAG
TGTTGATTATTACCCAGTGGATATCAATTCCAAAAAAATTTAAACAAAATGGAAAATCTAAAAAAGAAATGATT
GAAAATATAGATAACTCTAGTTACATGAAATATATTTATCAGTTAATGAAATCTATCAATGAAATTCAATACAAA
AGGTTAATTGCTGATATACATATGAAAGATGTGTACCAGAATACAAAAGCATGGTTAAATAATGGTGAATCAAT

TAATAAAAAAGAACAAGAGCTATACCAATATATGACAATTTTACCATTACATAAAAGAGTTGAACTGCAAAAT
GAGGTATTAAAATCTATAGACGAAACAATTTTTCAATTTCTCGCAAAAGAACATAGAAATAATCAAATTGAAG
AAAATAATGAATTAGAAGCATATACAAAAGCAATAAAAGAAGGTAGAAATCCTAAAAATGATTATGAATTAAA
TCATCTCCTATATAAAAAAAATCAATTACCCTTAGATAGTCTAATAGAAGATATATGGGACTATGAGGAAACAG
AGTATACTGAGCTTGATTGGTTAATTGCAGATATGCTAAAACGTTCACAAAGAGCTGATAGTAATTTTATCGA
AAGTTTAGAGAAGAAAAACAAATTGAGAAAAAATATTCATAAAGATTCCATTAAAAGTATATCTAAGTTGATT
GATCATGAATTAGTTAAAAATAATAGAAAGTGGGACGTAGCAGAATTTTATAAAAATCAACAATTTTGGAATA
ATAGTATATTAAGACGTAGCTCTCAAAATTTTGGAATTTTATATTACAAAAAGAATAGGTTACATACAATGAATC
AAAGTAATATTATTAAGAAAATAAAAATTATCGAAGCTAATATAGAGAGTGCAAATAAACAGCCGAGCTATTT
AGATTTCTTTTTACAAGCTAAGTTTGATTTAGAGAGGTTAGAAAGTGAATTAATGAAATATAGCATTAATCCG
AAATATTTTAATAAGATAAATAGTGATTACGTAAGATATGCTATTGAAGATGTACATAATGCAATTATTAAAATT
GACAGATATATAAGTAACGATAAAGTGAAGTTAAACTATTCAAATGTCCAAATGGTAGTGAGAAACGAAATAA
CAGAACAAGGAAGCTACTTTGTAACCCAGGCATTGAATACACTGTCAAAGGTAGAAGAGTCTGGCTACAGC
TTTGATAATTTCATGGTAGGACCTTTCGTAGAAGACTTTAAAAAAGCAATTAAAAATCGTAGATTTGGTTCTA
AAAAAGATTAAGATTGTTTTTTAGAGTTAAACATAAGAATAAATTTAAATAAAAAAATAAACCCAACAAACCT
TGTTATATCAATGTTTGTTGGGTTTATTTTTTTGGTGATTTTTTAATAACTCAATTACATCTTAGGATAATATCGC
TAAATTATACATTGTCGAGGCCAAGACAAATAAAAAATTAAAGCAAAAGTTAGCGCGCAACTTTCTGCTATAA
AGGAGCAAATTTATATGGAGACAAGAGACAAACTGATGTCATTGACTCAAAGTGACAAAACACAGCAATGG
CTAATGGACAAGTCATCTAACCAAGATGACATTCAACAATTGCAGCAACAATTCAGTCAGCAGCTTGATCAA
CAATATAATGCACTTTTAGCTGATGAAAAAGCTAAGTTAGACCAATACGTGGAAGTACATCAAGGATTGGAAT
CATTAAAGGAAGAGATTGAATCAGAACCTATTACGCTTAATATCGATAAATTACCCGATATCAAAGCAACAATG
CTTGAAAGAGCCAAGAATGATGAACATTCTGATAAAATCGAAAAGCTATTTGATAGGTTAGAACAGGCATTA
AATGGTACGAATCGATTATATACGCAATTATCGTTGATTGGTACACGAACACATCGAATTACAACTAAAAATTT
TAATCTTCAAGGCTTACCTAAAGCAGTCCAACATACGATTTTACCTTCAAAATTTAAGAAGGTGTATACAGTC
GATTTTAAATCGTTTGAACCATCAGTTGCAGCGTACATGACTCAAGATTCAAAACTGATTGACTTGTTAAATC
AGAAAGACGGACTGTATGACGCATTGCTAAGTGAATTAGGCTTATCAGATGAGCTACGTGTATTTGTTAAAC
GTGCATTTATTGGTTCGTTTCTATTTGGAGGTAACTTCAAAAATCCTAAATTCAAGCTGAATCAATATGTAAGT
GAAGTACAATGGTTGGATGCGGTCAGCCAATTTACAAAAGTCATTGAACTTAAGAAGCACGTTGAAAAGAG
TAATTCCATGCCTATGCCTTATAGTATTGAGCATGATATGAGCGCATTTCAAGGTAGCAGTATTATGGCAATCTA
CGTACAAACTGTAGCGAGTTATATTTTCAAGCACATTCTGCTAAAAGTGTACAAAGCACAGTGCGATCAAAA
AACGTTCAAGATTATAGTACCTATACACGATGCGATTATGATTGAATGTGAAGATGAAGAAATTGCACAAAAT
GTGGGTCAGTTAATGAAAGATACGGCTAACCAGTTGTTCAATGGTGAATTTGCACATGTGACAGTGGAAGA
AATAGGAGGCGTAGACCATGAATAATGATAGAGGACAAAGCCTACACATCCCAAGTAGTACACCAATCAAAG
AAAATAATATATATGTAGCTACGTTACATTCTGTGATCCAAACAGATTTCTCAGGTGAAATAAAGCACCAATTC
ACGTATGAAATTGAAGTGAACAATCAGATTGTATATGCGAATCGTAATATTCCAACAAAACCGAGCGCTAATC
AGTTGTCAATTCATGATTGGCTGAAACGTCATAGCAACTATAGCGCAAGTCATGAAAACTATGAGCCTTATATT
GATCAGAAACATTTAATTCTATTAGGTCAATATAACGGTAACTATTATGTACAAGATGTAGCATCGTTAGATGC
GTTTGGAGGCGTATTATCATGAATCATATATTACAAATGTTATCTAAGCTATTAAGTGTGGCCAAGGAGGCAAT
CGACCGTCAAGGTCTGATTGCTATCCTAACTATTCCTGTTAATAATAACGATGAAATAGAAGAAACGGCTCAA
GGTGAAACCGTGTATAACGAACTTATCGATCAGTTACGACTTAATATCCCAAAAGATACGGATTATCAACCTAA
CATCTATAGTTATTTTGGTATTAAGAAGAATCCTAATGACACCGTACTCATGGAAATGATGATAAAGGTTTTTC
ATATCAAACGCTTTAATTCAGAACTGTTTATTTTCAAAGTTAACGGGTGGCAAAAGATAAATGGAGATGAATT
ACAAGGGTTGATATCTAAAATGATACAAGTATTGCTTGTAGATTATAAGCCTTCACTAAGCACTCTAAAAAATG
TCGTAGATGGATTGCAAAAATCAACAGATGTAGAAGAACTTGTTGAGAATGAGCGCTATATTGGTTGTGGTG

AAAATATGTTCGATCTTAATACGTTTCAAGTCGTTAAAAATTCAATCGATATCTTTCCAAAAACACGATTGAAT
TTATCATTAAGTACAAATGATGTAATTACTGATAAGATACCGCCTTATTTTAAGCAATATATGTTACAACTTGCG
AATTATGACGATGATTTACAATACTTTCTTTTCCAACATACAGCAGTATTACTTACAGCTGATACTAAATACCGT
AGGGGTCTCATATTATATGGTGGAGCTAAGAATGGTAAATCTGTATATATTGAACTAGTTAAATCATTTTTCTAT
AGTAAAGATATTGTGTCTAAGCCACTTAATGAGCTTGAAGGTCGTTTTGACAAAGAAAGTTTAATTGACAAA
AGTCTAATGGCAAGTCATGAAATTGGGCAATCTAGGATTCAAGAAAAGATCGTAAATGACTTCAAAAAGTTA
TTATCTGTAGAATCAATGCATGTTGATCGTAAAGGAAAAACTCAAGTGGAAGTCATTTTGGATTTGAAACTTA
TTTTTAGTACAAATGCGATACTTAATTTTCCTCCTGAACATGCGAAAGCTTTGGAGCGTCGAATTAATATTATT
CCATGTGAGTATTATGTTGAAAAAGCGGACACTTCATTAATTGATAAGCTCCAGAGTGAGAAGAAAGAAATC
TTTCTTTACTTGATGTATGTGTATCAACAGATTGTAAAAGCAGATATCGAGTATCTTGAAAATAGCCGTGTCAC
TGAAATTACTCACGATTGGTTAAATTTTGGATATGAATTTGTTTCTAGCAGGTCCGTAAGTAATGCAAATCAGA
AAGCATGTATTAATTTACTCAGAAAACTTATAGAAATCAAATCAGGATCACGAATCAAAGTATCCGAGTTAAAT
AAAGTTATTAATGAAGAAATAAAGGTAAGTTCTCAGGTTATTAAACAGTTAATTCAAGCAAACTTTGATACTC
AAACCAAACTATACAATGGCTACGATTATTGGATTGATTTAGGTTGGAAAGAAGCCAATAAAAAAGAGATTC
ATGATATTTCGGAAAAAGATAATATTATTTCATTAGATAAAAATGAAAATATAACAGACGATGAGGCATTAGAT
GAAGAGAATTTGGACTTTGATTGGGAGGACTTTGACGATGAATAATGAACAAATTGAAGCATTTGTAGAAG
TGCTTGTACCTATCATAGAAGAACGTATCAATAAAGGTAATTAAGGCTAATTACGTACTACAGGCAGTTGCCT
GTAGTACTCATATGATTAAGTGGTAAAAGTGATAAAAATGAAACGAAATTATAAATATATATTATCTATATGTTGT
TACAAGACCGATAGTCTGTAGCAATAATCTAATAAAAGGAGCGGTATGATATGAAGGGTAAAATTGCACTTTA
TTCACGCGTTAGTACGTCAGAGCAGTCGGAGCATGGGTACTCAATCCATGAGCAGGAACAAGTACTCATCAA
AGAAGTTGTGAAAAAATTTCCCAGGTTATGACTATGAGACATATACTGACTCAGGCATTTCAGGTAAAAATATT
GAAGGTCGTCCGGCAATGAAACGTCTATTACAAGATGTTAAGGATAATAAAATCGAAATGGTATTAAGTTGG
AAATTGAATCGTATTTCTCGCTCAATGAGAGACGTGTTTAATATTATTCATGAATTCAAAGAACATGACGTAGG
GTATAAATCGATTTCTGAGAATATTGATACATCCAATGCTTCTGGAGAAGTACTCGTTACAATGTTTGGACTAA
TAGGATCTATAGAACGCCAGACTTTGATTTCGAATGTGAAACTTTCTATGAATGCTAAGGCAAGGAGCGGAG
AGGCAATCACCGGTCGTGTTTTAGGCTACAAATTATCACTTAATCCACTTACACAGAAAAATGATTTGGTTATC
GATGAAAATGAAGCTAATATTGTACGTGAAATTTTCGATTTATATTTGAATCATAATAAAGGCCTCAAAGCCAT
TACAACCGTACTTAATCAAAAGGGGTATCGTACTATTAATCAAAAGCCATTTTCAGTGTATGGTGTTAAATACA
TTTTGAATAATCCAGTCTATAAAGGCTATGTCAGATTCAATAACCATCAAAACTGGGCTGTACAGCGAAGAAG
TGGCAAAAGTGATAAAAATGATGTGATATTGGTCAAAGGTAAACATGAAGCCATTATAAGTGAAGAGGTATT
TGATAAAGTTCATGAAAAATTAGCTTCTAAAAGTTTTAAACCGGGCAGACCTATTGGTGGAGATTTCTACTTA
CGTGGCCTTATTAAATGCCCAGAATGCGGAAATAATATGGTATGTCGACGGACGTATTATAAAACGAAAAAGT
CCAAAGAACGGACAATCAAGCGTTATTACATTTGTTCCTTATTCAATCGTTCAGGGAGTTCTGCATGTCACAG
TAATTCCATCAATGCTGAAGTCGTCGAGCGTGTAATTAATGTTCATTTGAATCGTATTCTGTCTCAACCAGATAT
TATCAAGCAGATTGCGTCAAATGTGATAGAAGAACTGAAACAAAAGCATAGTAACCAAACAGAAATTAAATA
TGACATTGATAGTTTAGAAAAACAAAAAGCTAAGCTTAAAACACAACAAGAACGATTGTTAGAATTGTTCTT
AGATGATCAGATGGATAGCGAAATGTTAAAAGCTAAACAAAGTCAAATGAATCAACAGTTAGAAGTATTAGA
TCAACAAATTAAAGAAGCGCAACAAGCAAATCAATCACAGGATGAAATACCTAATTTTGATAAATTAAAAGG
ACGACTCATTTTGATGATAACACGATTCAGCGTGTACTTAAGAAAGGCTACACCCGAAGCTAAAAATCAACTT
ATGAAAATGTTAATTGATTCAATTGAAATTACGACAGATAAACAAGTAAAACTTGTAAGGTATAAAATTGACG
AAAGTCTTATCCCTCAATCTTTGAAAAAAGATTGGGGGTCTTTTTTTATGCCCAAATTCCAATTTGAAATATAT
GGTCAAAATGATTATTTCATCGACCAAATTACCACTTTTACCACTTAGTTATTAGTGACAAAAGTGAGCGAAA
TGAAATAAAAATCAAATATATATTATCAAAATGATGTATCACATGCATACATCAATCAAATACATTAGGAGGTCA
TAACCATGACACTAGAACAACAACTCAAGCACTATATAACCAACTTATTCAATCTGCCAAGGGACGAAGTGTG

GCACTGCGAATCTATCGAGGAAATCGCTGATGATATCTTACCCAATCAATATGTAAGACTTGGCCCACTCAGTA
ATAAAACACTTCAGACTAATACCTACTACTCTGACACACTTCATGAAAGTAATATCTATCCTTTCATTCTCTACTA
TCAGAAACAACTCATAGCCATCGGTTATATCGACGAAAATCACGATATGGATTTCTTATACCTACACAACACTA
TCATGCCTCTTTTGGATCAACGATACTTACTAACAGGAGGACAATAAAATGCATAAATACATCAAAATTACACA
ATTAGTCATTACAATACTAAGTGAAATCATCATTTGGATGAAAGAGTCAGAACGAAAGGAAGTCTCTTATGAA
TAGATATATCACCCGGGGTATCGCCAACAACTTACCTAATATCTTACAACACCAATTATGGCAACTCGTATCTGA
GCGAGAACAAGAACAAACCAAAGATAATACTTCAGTAGATTATTTTCATATATTCCAGTTCAATAAGCATCGC
AATCAATTATATATCAAACACAAACAAGAACGACCTGAATATGCGAAAATCCATAAAGCTAATTATTCAAAAGC
AATCAATATCAATAAGGTCTACATTATTCGAGAAGATGATGTAGACCTTTCTTATTATGTCATGTTATTACCTGA
AGAATACTAGAGGAGTGAAAATTATATGGAAACAATCAAAAGTACATTAAAAACAGAAGCCATATTCAGTGA
TGACAAACAACATCGCTATCTACTTAAGAAAACATGGAATAGTGAAAAACAATCAATCACAATCATTACAATG
TATCCGCATTATGATGGCATTCTCAATATTGACCTAACGACCCAACTCATCATGAACAAAGTTTCAGAAATGGA
TGCATTTGGTTCAATCAATTTTGTGAATCTATACTCTAATATTACAACCCCTATCAATCTCAAACATTTAGAAAAT
GCGTATGATAAGCATACAGATATTCAAATTATGAAGGCAGTGAAAGAGTCAGATGAAGTGATATTAGCTTGG
GGCGCTTACGCTAAAAAGCCCGGTGTTGAAGCACGTGTTAATGAAGTATTAGAGATGTTGAAACCACATAAA
AAGAAAGTAAAACGACTCATGAATCCAGAAACCAATGAAATCATGCATCCCCTTAATTCGAAAGCACGTCAA
AAATGGATATTAAAAGTATAGCAACTAAAATGTTTATTCACTATCCATGAAAATAATTGAATTTCATATATATCTT
TTAATCTACAATATGATTAAAAGATATATTATTTGGTTCTGTTGCAAAGTAAAAAAATATAGCTAACCACTAATT
TATCATGTCAGTGTTCGCTTAACTTGCTAGCATGATGCTAATTTCATGGCATGGCGAAAATCCGCAGATCTGAA
GAGACCTGCGGTTCTTTTTATATAGAGCGTAAATACATTCAATACCTTTTAAAGTATTCTTTGCTGTATTGATAC
TTTGATACCTTGTCTTTCTTACTTTAATATGACGGTGATCTTGCTCAATGAGGTTATTCAGATATTTCGATGTAC
AATGACAGTCAGGTTTAAGTTTAAAAGCTTTAATTACTTTAGCCATTGCTACCTTCGTTGAAGGTGCCTGATC
TGTAATTACCTTTTGAGGTTTACCAAATTGTTTAATGAGACGTTTGATAAACGCATATGCTGAATGATTATCTC
GTTGCTTACGCAACCAAATATCTAATGTATGTCCCTCTGCATCAATGGCACGATATAAATAGCTCCATTTTCCTT
TTATTTTGATGTACGTCTCATCAATACGCCATTTGTAATAAGCTTTTTTATGCTTTTTCTTCCAAATTTGATACAA
AATTGGGGCATATTCTTGAACCCAACGGTAGACCGTTGAATGATGAACGTTTACACCACGTTCCCTTAATATT
TCAGATATATCACGATAACTCAATGTATATCTTAGATAGTAGCCAACGGCTACAGTGATAACATCCTTGTTAAAT
TGTTTATATCTGAAATAGTTCATACAGAAGACTCCTTTTTGTTAAAATTATACTATAAATTCAACTTTGCAACAG
AACCGTATTATGGAATAGAGATGTTGGTAACATTTATACAGGATCATTATACTTAAGTTTAATTTCGTTATTACA
GAACCACACATTCCAACCAGAAGAGAAAGTATGTCTATTTAGTTATGGTTCAGGAGCAGTAGGAGAAATCTT
TAGTGGTTCAATCGTTAAAGGATATGACAAAGCATTAGATAAAGAGAAACACTTAAATATGCTAGAATCTAGA
GAGCAATTATCAGTCGAAGAATACGAAACATTCTTTAACAGATTTGATAATCAAGAATTTGATTTCGAACGTG
AATTGACACAAGATCCATATTCAAAAGTATACTTATACAGTATAGAAGACCATATCAGAACATATAAGATAGAG
AAATAAACTAGTGGCCGATTGTGCTTGATGAGCTTGGGACATAAATCCTAACTCGAAATAAATAAGCATATCA
CTAAACTGATTTTTTAAAGTTTACAGTGATATGCTTATTTTTTTATCTTACGATTTTGTACGTGCATGCTTGCCTA
GGGGTATGGCTCGAGCCATTAGTCTCTCGCACATACTATTCCCTCAGGCGTCAGCACTTACAAAATCGGTTGT
AATTTTCATTTTTATACGCATTCTTACTGAGATTATACTAATAAGAGGAATAGTAAAAGCAATTCTAAGTAAAAT
TGCAGATAAGAGGTTTGTTAAAAGCAGTTCTAAGTAAAATTGCAGATAAGAGGTACGTTAAAAGCAATTCCA
TGCAAAATTGCTGATAAGGGGTAAGTTAAAAGCAGTTCTCAGTAAAATTGCAGATAAGAGGTACGTTAAAA
GCAGTTCTAGGCAAAATTGCAGATAAGAGGTGCGTTAAAAGCAGTTCTCAGTAAAATTGCTGATAAGGGGT
AAGTTAAAAGCAATCCTAAGTAAAATTGCAGATAAGAGGTAAGTTAAAAGCAATCCTAAGTAAAATTGCAGA
TAAGGGGTACAGAAAAACTAGACTTGATTACAAAATGGAGCTTGGGACATAAATGATTTTTTAAAAATGAGA
TGAGACGTAGATTAACTCCATAATCAATACGAATCTATCGACTTCTTTATTTATGATATTCATCTCTTTTTAATGG
AAATAAAAGTGCGATTAATGTGATAATACAGTTACGTTAATTAAAAAAATAAAAATGCAAGGAGAGGTAATAT

GCTAACTGTATATGGACATAGAGGATTACCTAGTAAAGCTCCGGAAAATACAATTGCATCATTTAAAGCTGCT
TCAGAAGTAGAAGGTATAAACTGGTTGGAGTTAGATGTTGCAATTACAAAAGATGAACAACTGATTATCATTC
ATGATGATTATTTAGAACGGACTACAAATATGTCCGGGGAAATAACTGAATTGAATTATGATGAAATTAAAGA
TGCTTCTGCAGGATCTTGGTTTGGTGAAAAATTCAAAGATGAACATTTGCCAACTTTCGATGATGTAGTAAAA
ATAGCAAATGAATATAATATGAATTTAAATGTAGAATTAAAAGGTATTACTGGACCGAATGGACTAGCACTTTC
TAAAAGTATGGTTAAGCAAGTGGAAGAACAATTAACAAACTTAAATCAGAATCAAGAAGTGCTCATTTCAAG
CTTTAATGTTGTGCTTGTTAAACTTGCAGAAGAAATCATGCCACAATATAACAGAGCAGTTATATTCCATACAA
CTTCGTTTCGTGAAGACTGGAGAACACTTTTAGATTACTGTAATGCTAAAATAGTAAACACTGAAGATGCCAA
ACTTACTAAAGCAAAAGTAAAAATGGTAAAAGAAGCGGGTTATGAATTGAACGTATGGACTGTAAACAAAC
CAGCACGTGCAAACCAACTTGCTAATTGGGGAGTTGATGGTATCTTTACAGACAATGCAGATAAAATGGTGC
ATTTGTCTCAATAGAAAGTTAGAGGTGAGTCTTACGTTTCAGTGACGGTAGACTTACCTTTAACATGTTACAT
ACTAAAAAATTAATTTGAATAAGAAAGAGAGACATATATGAAATACGATGATTTTATAGTAGGAGAAACATTC
AAAACAAAAAGCCTTCATATTACAGAAGAAGAAATTATCCAATTTGCAACAACTTTTGATCCTCAATATATGCA
TATAGATAAAGAAAAAGCAGAACAAAGTAGATTTAAAGGTATCATTGCATCTGGCATGCATACACTTTCAATA
TCATTTAAATTATGGGTAGAAGAAGGTAAATACGGAGAAGAAGTTGTAGCAGGAACACAAATGAATAACGT
TAAATTTATTAAACCTGTATACCCAGGTAATACATTGTACGTTATCGCTGAAATTACAAATAAGAAATCCATAAA
AAAAGAAAATGGACTCGTTACAGTGTCACTTTCAACATACAATGAAAATGAAGAAATTGTATTTAAGGGAGA
AGTAACAGCACTTATTAATAATTCATAATAAAACAGTGAAGCAACCATCGTTACGGATTGCTTCACTGTTTTGT
TATTCATCTATATCGTATTTTTTATTACCGTTCTCATATAGCTCATCATACACTTTACCTGAGATTTTGGCATTGTA
GCTAGCCATTCCTTTATCTTGTACATCTTTAACATTAATAGCCATCATCATGTTTGGATTATCTTTATCATATGATA
TAAACCACCCAATTTGTCTGCCAGTTTCTCCTTGTTTCATTTTGAGTTCTGCAGTACCGGATTTGCCAATTAAG
TTTGCATAAGATCTATAAATATCTTCTTTATGTGTTTTATTTACGACTTGTTGCATACCATCAGTTAATAGATTGA
TATTTTCTTTGGAAATAATATTTTTCTTCCAAACTTTGTTTTTCGTGTCTTTTAATAAGTGAGGTGCGTTAATATT
GCCATTATTTTCTAATGCGCTATAGATTGAAAGGATCTGTACTGGGTTAATCAGTATTTCACCTTGTCCGTAACC
TGAATCAGCTAATAATATTTCATTATCTAAATTTTTGTTTGAAATTTGAGCATTATAAAATGGATAATCACTTGGT
ATATCTTCACCAACACCTAGTTTTTTCATGCCTTTTTCAAATTTCTTACTGCCTAATTCGAGTGCTACTCTAGCA
AAGAAAATGTTATCTGATGATTCTATTGCTTGTTTTAAGTCGATATTACCATTTACCACTTCATATCTTGTAACGT
TGTAACCACCCCAAGATTTATCTTTTTGCCAACCTTTACCATCGATTTTATAACTTGTTTTATCGTCTAATGTTTT
GTTATTTAACCCAATCATTGCTGTTAATATTTTTTGAGTTGAACCTGGTGAAGTTGTAATCTGGAACTTGTTGA
GCAGAGGTTCTTTTTTATCTTCGGTTAATTTATTATATTCTTCGTTACTCATGCCATACATAAATGGATAGACGTC
ATATGAAGGTGTGCTTACAAGTGCTAATAATTCACCTGTTTGAGGGTGGATAGCAGTACCTGAGCCATAATCA
TTTTTCATGTTGTTATAAATACTCTTTTGAACTTTAGCATCAATAGTTAGTTGAATATCTTTGCCATCTTTTTTCT
TTTTCTCTATTAATGTATGTGCGATTGTATTGCTATTATCGTCAACGATTGTGACACGATAGCCATCTTCATGTTG
GAGCTTTTTATCGTAAAGTTTTTCGAGTCCCTTTTTACCAATAACTGCATCATCTTTATAGCCTTTATATTCTTTT
TGTTTTAATTCTTCAGAGTTAATGGGACCAACATAACCTAATAGATGTGAAGTCGCTTTTCCTAGAGGATAGT
TACGACTTTCTGTTTCATTAGTTGTAAGATGAAATTTTTTTGCGAAATCTCTTAAATATTCATCCATTTTTTTAAC
GGTTTTAAGTGGAACGAAGGTATCATCTTGTACCCAATTTTGATCCATTTGTTGTTTGATATAGTCTTCAGAAA
TACTTAGTTCTTTAGCGATTGCTTTATAATCTTTTTTAGATACATTCTTTGGAACGATGCCTATCTCATATGCTGT
TCCTGTATTGGCCAATTCCACATTGTTTCGGTCTAAAATTTTACCACGTTCTGATTTTAAATTTTCAATATGTATG
CTTTGGTCTTTCTGCATTCCTGGAATAATGACGCTATGATCCCAATCTAACTTCCACATACCATCTTCTTTAACA
AAATTAAATTGAACGTTGCGATCAATGTTACCGTAGTTTGTTTTAATTTTATATTGAGCATCTACTCGTTTTTTA
TTTTTAGATACTTTTTTTATTTTACGATCCTGAATGTTTATATCTTTAACGCCTAAACTATTATATATTTTTATCGG
ACGTTCAGTCATTTCTACTTCACCATTATCGCTTTTAGAAATATAACTGCTATCTTTATAAACTTGTTTGAAATTT
TTATCTTCAATTGCATCAATAGTATTATTAATTTCTTTATCTTTTGAAGCATAAAAATATATACCAAACCCGACAA

CTACAACTATTAAAATAAGTGGAACAATTTTTATCTTTTTCATCAATATCCTCCTTATATAAGACTACATTTGTAA
TATATTACAAATGTAGTATTTATGTCAAAATAATGTTATAATTTTTGTGATATGGAGGTGTAGAAGGTGTTATCAT
CTTTTTTAATGTTAAGTATAATCAGTTCATTGCTCACGATATGTGTAATTTTTTTAGTGAGAATGCTCTATATAAA
ATATACGGTTCTGTTGCAAAGTTGAATTTATAGTATAATTATAACCAAAAGGAGTCTTCTGTATGAACTATTTCA
GATATAAACAATTTAACAAGGATGTTATCACTGTAGCCGTTGGCTACTATCTAAGATATGCATTGAGTTATCGT
GATATGTCTGAAATATTAAGGGAACGTGGTGTAAACGTTCATCATTTAACGGTCTAGCGTTGAGTTCAAGAAT
ATGCCCCGATTTTATATCAAATTTGGAAGAAAAAGCATAAAAAAGCTTATTACAAATGGCGTATTGATGAGAC
GTACATCAAAATAAAAGGAAAATGGAACTATTTATATCGTGCCATTGATACAGAGGGACATACATTAGATATTT
GGTTGCGTAAGCAACGAGATAATCATTCAGCATATGTATTTATCAAACGTCTCATTAAACAATTTGGTAAACCT
CAAAAGGTAATTACAGATTAGGCACCTTCAACGAAGGTCGCAATGGCTAAAGTCATTAAAGCTTTTAAACTT
AAACCTGACTGTCATTGTACATCGAAATATCTGAATAACCTCATTGAGCAAGATCACCGTCATATTAAAGTAAG
AAAGACAAGATATCAAAGTATCAATACGGCAAAGAATACTTTAAAAGGTATTGAATGTATTTACGGTCTATATA
AAAAGAACCGCAGGTCTCTTCAGATCTGCGGATTTTCGCCATGCCATGAAATTAGCATCATGCTAACAAGTTA
ACACAAAGTATTATTTTAAAATTGAGATTAGACATTTATTTTTCAACTTTGCAGCAGAACCTAATTTTTTCTCTT
TGTAAATAAATGCAAAGAGATACCATAAGGATCTCTTACATAACCATAACCTTCAGTATAGAATTCTGGACTAA
ATGTTTTCAATACCTCACTGCCTTTTTCTATTAACTGGTCATATACATGTTTAGTTTCTTCTACTTGGTCAAAAGT
GAGACAAAGAGATATATTATTACCTTGTGTTATGGGCAAACCTTCAGTGTCATCTGCGATCATAATTTTTATATC
TCCAAATTGAAGTACACATTGATCAATTTTATTTAAATCATTTTCGTCAATATTAAGTTTCTTATCTATAGGTCTA
TCTTTAATACGTTGAATATACAGTGTTTTAGCGCCAAACAGCTCTTCATACAACTTTTTTAAACCCTCTGCATTT
TGAGTGATTAAAAAATATGGACTTACTTGAAATTTCATGTTTTTTCCTCCTTAGATTTGTTATAAATAGAGTATA
TACTTAATTAGTGTCATCTATTGACACTAAAAGGAGAATAATAATGAAAAAATCTGTTAGATTATATAATATGAT
TGAATATTGTAATGAAAATAGGAACTTCAAATTAAATGATTTAATGTCAGAATTTAATATTTCTCGTAGTACCGC
TTTAAGGGATATAAAAGAAATTGAAGCATTAGGAGTACCTTTATATAGTAATCCAGGGAAAAATGGTGGTTAT
ACGATCATAGGTAATCGAGACCAAACGAAAATAGCAATCTCAGATGAAGAGTTGAAAGCTTTAGTATTTACA
CTTTCGAGTATTTCAAATGTGAGTAATCTACCTTTTCAAACAGAATATCAAGAAATATTAAAAAAATTATATAAT
AACTCGAATAAAAAAGAGTTAATAAACCAATATAATGATCTATTTCAATATTTTAATGAAGATAAGTATCAGTTC
AAAAGTTATAAGTTATTTAATGAAATCATTAGATTGATAATTGAGAATAAGTCTTTTGAAACCTGTTATTCACA
AAACTATATTAAAGAACAATATAAAGGTATTGGCATTATGTATAAAAATCATCAATGGTATTTTGTTGTAGCTAA
TATAGAATCAAAGTTAGTGAATCTATTAAATATTTCGAAAATAAAAGAACTATATGAAATGGGAGAGACTCAA
GAGTGTAATGATATAACTATGCAGAATTTTCAACAGTTCATGGTTAAAAATGAAACAGCTATTGATATTCTTATT
AGAAGCAATGTTATGGGATTGAATATCTTGAAAGGCTACCTGTGGAGTGACTATATGATTGAAAATATTGACG
AAGAGACATATTTATTTAAATCAAAAGTGAACGCGAAAGATATAGATTTTATAGCTAAGTTAATTGTCACAGG
TGGTGTCAATGTAAAAGTAGAGACCCCTAATAGTTTGAAAAATGCTGTTAAAGTTGAATTAACTAAAATAATA
AACATGTATTAATAGTAATTTAATCATAATTGTAATAACAAAAAGATGACAACATTAAACAATTTAAATTTATAC
AAAAAAGCATTACCAAATTTAAAATTGGGTAGTGGTAGTGCTACAGCATACATAAATAGTTATAATTAAATAAA
GATGGGTCAATGAAGGTGAATTAAGTGAATAAAAATATTACAGCTCTAAAATCTACTGAAAATACTACATATAT
GCAGTATACGGTTCATATCGATGATCAAATTATAAATAATATAAAAGAGTGTACCAAAAAGTTTAAAATTTTGC
CTATGGAAGATAAAATTCCATTATCACCGTTGTTACAACCAGAATATGCAGGAGAGGTACAAGATTTTATTAG
TACATATGAGCAGTTTATGATTAATTTTGGTAAAGTAATATTGGATAGTCAAGGCATAAAAATACAGTTTGAAA
GTGAATCATTAAGTAGTATTCAACGAGGCATTCAAGAACATTGTTACTTAAATGAGCGAACAAATGACATTGA
TGTGACTAAAGAATGGTATTTATGCAAATTTTCAAATCAAATATTAGAAGAGGATAAAAATCAATTGTATAATG
CTTTAAAGCAACTCATGAATGATTCTAAAAATAAAAAGCAAGCTTTTCAAGAGGTGTTTAAGGTTCATATTGA
TATTTATCTCTACAATAAAGAAAAATCTGAATATAAGTATCAAGTATCTAGTTATTTCAATCTTGTAAAAGAAAA
TCCTAAAATAACTTATAAAAAAAAGACATTTACAAGAGAAACAAGGTGTCAAGGGTACAACTTTCACGAACAA

ATTAAAATTATTGAACAAAATGTATGGTGTAGATGTTGCAAAATATCAGCCATTTTATAATTCTAATAATCCTGA
ATATGAAAGAGGTCAATTTGGTGAAAGATACATTTCTCAAAGATCTAACTATGAATTTAATAGACTTCAATATC
AAATTATAGATATGTTATCGAAAATACTTGATAAGCATCCATTACCAAAGTCAGATAATAATTATAAACATATTCC
AACTATTGAAAAAGCAATATTAAGTGGGGATTCTCATAGTTTTTATGAGTATTTTGAAGATATAATGAAAGAA
ATTATAAGTATGGAAAATTCTTCTTTAAAGGAAAAATTATTGACTGATTTTACATATCAATCTCAATGTAGATGG
TACTCTGAAAGTGAAAAATTAAAATTGCAACTTGAAAGTTTTATGCATAAAGTTTTGGAGAGCAATTATTATG
AGGGTAACAAATTATATAGAATGTTGTCTCATGCAATTGAAGAAACAATCAATGAGGCTGATGAAGATAAGG
TTCATTTCAATTATTTTAAAGATTATTTTTTGACAGACGGTGGGGTGAAGAATTGGGAGCAGATTAGCGAGA
AAATTACTGAATTCAATGGTAAAGTTATTAACGATATCCAAAATGAATATAATAAGATTCAATTTAATAACGCAA
ATCGAAATCAGAAACTAAACTTTAATTATTTATACCATTGCTTTGAGTTTAGTAATAACTTAGTAAAAGCAAGG
GTAAATGGAAATAGAGGTTACATTTTATATTTTGTAGATAAATATAAAATAAAAATGCCCTTTTTGGATCAATTA
TTAATCAATTATTTGAATCAAGAAATAGGTCAGGAAAGTATTAATTATAATATGCAAACATTGTTTGAAAAAGA
AAGATATGATAGAAGTAGTACCATTGAAAAATTAGTAGCAACAAGCAAATTTAAGTATGAAAAAGATGATTCA
GATTTATTCAAACAACTTTTCAATGATGTTGAAAATTCAATAGACAGATTAGGTATTTACTTACTAAATAATGGT
ATAAATTCGAATGATGAAAATGCAAGATATTATAGATCGTTTTTAAAGGAACTTAGTAGAATAAAAAGTAAATT
AACGCCATTTTCTCTTGAAATAAGTAAGTCTAGCGGAAGAGAGCAACATTATCCTGATGATGCTATTGATGAT
AAAGATGAGAGAAGAAAAAAATAAAGAAGAAACATATCATGCTTTTGATGATAAAAGCGATATTGACTCCAAA
TTAAAGAATAAAATAAATGTTTCTATCGATAATTTATTGTCAGTTAAAATTAAGAGCAATTTGTATGATATTAGT
CCAAATACAAAAAAACAATAACTATAATGAGCCAACTCAAGAATGAGTTGGCTTTTTAATTGTAATTTTACGC
TTCCATTACAGGAATCAATGTTATGGAAGCCATTAAGCAATTAATTTGAGTCATTTATAGTATAGGTACAACGT
TGTGATGTTCACATGATAATCAGCCCGGGAGATTATTGTGTAATAACTTATAGAAGGAGCAAATCACCATGTAT
CAAGCAAATATACGTGATTTAATTACAAAATTGCCTCAAAGCAATAAAACAGAACACTTTTTAATGAACAAAT
TTTCAAATCAAGATAAAGTTCAGCAGCTACAAAGACAAATTAGCCAACAGCTAGATCAACAATATAATGAGCT
TTTGGCTAATGAAAAAGCTAAGCTAGACCAATACGTGGAAGTACACCATAATTTAGAACCATTAAAGAAAGA
GATTGAATCAGAATCTATTAACCTTGATACCGATAAATTACCTGATATCAAAGCGACAATGCTTGAAAAGGCTA
AGAACGATGAACATTTTGATAAAATCGAACAGCTATTTGATAGATTAGATCAGTCATTAAATGGTACGAATCG
ATTATATACGCAATTATCGTTGATTGGCACACGAACACATCGGATCACAACGAAAAGATTTAATGTTCAAGGC
TTGCCTAAATTAGTCCAACAAATGATTTTACCTTCGCAATTTAAAAAGGTTTATACAATAGATTTTAAATCATTC
GAACCATCAGTTGCTGCGTATATGACACAAGACGAACAACTGATTGACTACTTGAATCATGAAGAAGGGTTA
TACGATGCATTACTGAGAGACTTATCTTTGTCAAAAGAGAAGCGCGTGAGTGTGAAACGTGCATTTATAGGG
TCATTTCTTTTTGGCGGTCGTTATAGTAGCTCTAAATTCAAAATCAATCAAGAGGTTAGTGAAATTAACTGGCT
ACAAGTAATGAGCAAATTCAAGAAGGTCATTGAATTTAAGGAGCAAGTCGAAAAATATAAAACAATGCCTAC
GCCTTACGGCATTGAACATGATATGAGCGCATTTCAAGGTAGTAGTATTATGGCAATTTATGTACAAACGGTA
GCAAGCTATATTTTCAAGCACATTTTGTTGGAAGTGTACAAAGCACAGTGCGAAAAAAAAACGTTCAAGATT
ATAGTGCCGATACACGATGCGATTATGATTGAATGTAATGATAAGGGGATTGCACAAAATGTAGCACAGCTCA
TGAAAGATACAGCTAATCAACTGTTTAATGGTGAATTTGCACATGTGACAGTGGAAGCTTTAGGAGGTATAG
ACAATGAATAATGATAGAGGAAAAAGTCTTCAAATTCCCCAAAGTACATTGTTAAAAGAAGGATCTATATACG
TTGCTACGTTACATTCTGTGTACGAGAAGAACTTCTCAGGTGATATTAAACATCAGTTTACGTATGAAGTAGA
ACTTAACCAAGAAACGCACTATGTAAATCGCAATATTACCGTAAAATCTATGAGTCACCAATTATCAATTGCTG
ATTGGATTAAACGTCACAGTAACTATAACGTAAACCACATTAATTATGATCCATACATTGATCGAAAACATTTG
GTCCTTGTAGGGCAATATAACGGAAATTATTATATTCAAGATGTAGCACCATTAGATGAATTTGGAGGGGTATT
GTAATGAATCATATTTTAGAAATGTTAATAAAATTATTAAAAGTGGGTATGGAGGCAATCGACCGTAAAGGTC
TGATTGCCATCCTAACAAGTAGTATTGGAAATGATGAAATGGATGATTCTGAACAAGCTGTAATGGTGTATAA
CGAGCTTATCGATAAGCTACAGCTTAACATTCCTAAAGATGTCGACTATAGACCTAACATATACAGTTATTTTG

GTATTCAAAAAAAGCCAAATGACACAATATTAGTGGAAATGATGATATGTATTTTTCATATCAAGCGTTTTGAT
TCAGAGTTGTTTGTTTTCAAAGATAAAGGTTGGCAAAAGGTAAGTGAAGATGAATTGCAAGGGTTGATATCT
AAAATGATACAAGTGTTGCTAGTTGATTATAAACCTTCACTAAGTACTTTGAAAAACGTAGTAGATGGCATAC
AGAAATCAACGGACATAGAAAAACTTGTTGAGCATAGACAGTACATTGGTTGTGGACGAAATATGTTCAATC
TAAAGACCTTTAAAGTGGTTGATAATGACCTTGAAATATTCCCTAAAACACGCTTAGATTTGGAATTAGATATA
AATGACACGATTACGGACAAGATACCCCCGAATTTCAAACAATATATGTTAGAGTTGGCGAATTATGACCATG
ATTTACAATATTTCCTTTTCCAACATATGGCAGTGTTATTGACGGCAGATACTAAACTACGTCGTGGACTTTTT
TTGTATGGAACTGCAAAAAAATGGGAAATCGGTCTATATTAAATTAGTTAAGTCATTCTTTTATAGTAATGATATC
GTATCTAAAACACTTAATGAACTTGGCGGGCGTTTCGATAAGGAAAGTCTAATTGGTAAACGAATTATGGCA
AGTGATGAAGTGGGGAAAGCTAATATTGATGAAGCAACTGTGAATGATTTCAAAAAATTACTATCTGTTGAA
CCAATTCATGCTGACCGTAAAGGAAGAACGCAAGTAGAAGTTACTTTAGATTTAAAACTCATTTTTAATACGA
ATGCTGTACTCAATTTTCCATCATCACATGCAAAAGCATTAGAGCGTAGAATTGCTGTTATTCCATGTGAATATT
ATGTTGAAAAAGCTGACCCTGACTTAATTGAAAAGTTACAGGATGAAAAGAAAGAAATCTTTCTTTACTTGA
TGTATGTGTATAAGCAAATTGTAAAAAATGATATCGAGTACCTCCAAAATGATCGTGTTACTGAAATTTCTCAT
GATTGGTTAAATTTTGGATATGAATTTGTTTCTAGTAAATCAGCAAATATTGCACATCAGAAAGCGTGTATTAA
TTTACTCAGAAAACTTATAGAAATCAAACCAGGGTCACGTATCAAAGTGTCTAGGCTAAATGAGGTTATTAGA
GATGAAATTAAAGTAAGCTCTCAAGTTATTAATGATTTGGTTCAAGCTAACTTTAATGTACAAAGTAGACTAA
ATAATGGTTATAAGTATTGGGTCGATTTAGGATGGAAAGAAACTGATAAAAAAGATGACATGATTTCATTCGA
TAAAAATGAGAATGTAACAGATGATGAATTCTTATACGAAGATGATTTGAACTTAGGTTGGGAGGACTTTGA
CGATGAATAATGAACAAATTGAAGCATTTGTAGAAGTGCTTGTGCCTATCATAGAAGAACGTATCAATAAAGG
TAAGTAATCTAATTACGTACTACAGGCAGTTGCCTGTAGTACTCATATGATTAAGTGGTAAAAGTGATAAAAAT
GAAACGAAATTATAAATATATATTATCTATATGTTGTTACAAGACCGATGGTCTGTAGCAATAATCTAATAAAAG
GAGCGGTATGATATGAAGGGTAAAATTGCACTTTATTCACGTGTTAGTACGTCTGAGCAGTCAGAACATGGT
TATTCTGAAAAGGAGCAGGAACAACTACTCATCAAAGAAGTTATGAAAAATTTCCCAGGTTATGACTATGAG
ACATATACTGACTCAGGCATTTCAGGTAAAAATATTGAAGGTCGTCCGGCAATGAAACGTCTATTACAAGATG
TTAAGGATAATAAAATCGAAATGGTATTAAGTTGGAAATTGAATCGTATTTCTCGCTCAATGAGAGACGTGTT
TAATATTATTCATGAATTCAAAGAACATGACGTAGGGTATAAATCGATTTCTGAGAATATTGATACATCCAATGC
TTCTGGAGAAGTACTCGTTACAATGTTTGGACTAATAGGATCTATAGAACGCCAGACTTTGATTTCCAATGTG
AAACTTTCTATGAATGCTAAGGCAAGGAGCGGAGAGGCAATCACCGGTCGTGTTTTAGGCTACAAATTATCA
CTTAATCCATTGACACAGAAAAATGATTTAGTTATTGATGAAAATGAAGCTCATATTGTACGGGAAATCTTTGA
TTTATATTTGAATCACAATAAAGGATTTAAAGCAATCACGACAATTCTAAATCAAAAAGGATATCGTACCATTA
ATCAAAAACCATTTTCAGTGTTTGGCGTGAAATACATTTTGAATAATCCAGTCTATAAAGGCTATGTCAGATTC
AATAATCATCAAAATTGGGCTGTTCAGCGAAGAAGTGGTAAAAGTGATAAAAATGATGTGATATTGGTCAAA
GGTAAGCATGAAGCCATTATAAGTGAAGATGTATTTGATCAAGTTCATGAGAAACTAGCTTCTAAAAGTTTTA
AACCGGGTCGACCTATTGGTGGAGATTTCTACTTACGTGGCCTTATTAAATGCCCAGAATGCGGAAATAATAT
GGTATGTCGACGGACGTATTATAAAACGAAAAAGTCCAAAGAACGCACAATCAAACGCTATTACATTTGTTC
ATTATTCAATCGCTCAGGAAGTTCTGCCTGTCATAGTAATGCGATTAATGCTGAAGTCGTCGAACGCGTAATC
AATGTTCATTTGAATCGTATTCTTTCACAACCTAATGTTATTAAGCAGATTGCGTCAAGTGTGATAGAAGAACT
GAAACAAAAGCATAGTAAACAAACAGAAATAAAATATGATATTGATAGTCTAGAAAAACAAAAAGCAAAAGT
TAAAACACAACAAGAACGATTATTGGAATTGTTCTTAGATGATGAAATGGATAGCGAAATGTTAAAAGCTAA
ACAAAGTGAAATGAATCAACAGTTAGAAGTATTAGACCAACAAATTAAAGAAGCAAAACAAGCAAATCAAT
CACAGGATGATATACCCAATTTTGATAAGTTAAAAGCACGACTCATTTTGATGATAACACGATTCAGTGTGTAC
TTAAGAAAGGCTACACCCGAAGCTAAAAATCAACTTATGAAAATGTTAATTGATTCAATTGAAATTACGACAG
ATAAACAAGTAAAACTTGTAAGGTATAAAATTGATGAAAGTCTTATCCCTCAATCTTTGAAAAAAGATTGGGG

GTCTTTTTTTATACCTAAATTTAACTTTGTGATAAATGTCACAAAGAAAAATAGGATTGAAAATTTATCACTTTT
ACCACTTTTTTAGAGTGACAAAAGTGGAGGAGTTTTGAAATATTTTATAAATATATATTTTATTTATGGAGTAC
ACATTATTAATTAAGGAGGTCATTATAATGACGCTAAGCAAACAACTTAAAACGTATATCACTGAACGATTTAA
ATTAAATTATCAAGAAACTTGGGCTTGTGAAACCATAGATGCGGTGGCTGAAGATGTATTACCTGAAAAATAT
ATTAAAAATAGTCCACTTGAACATAAAATTTTAAATACTTTTACCTATTACAATGATGAATTACATGAAATCAGC
ATTTACCCTTTTTTATGTTATCTAGATAAGGAATTAGTAGCAATAGGTTATTTAGATAATTTTGATTTAGACTTTA
TATTTTTAAATGACACTCATCAAATTATTATTGATGAACGCTACTTGTTACAAAAAGGGGGCGAGTAATTATGA
ACTGGATCAAGGTTGCTCAGCTATCTGTTACAGTCATTAACGAAGTGATTGAGATCATGAAAGAAAAGCAGA
ATGGAGGAAAATAGTATGAACATCAATCGATACATCACGAGAGGCATTAGTGAACAACTATCTCTAGACCTTC
AAATCTTACTTTGGCACATGGTAGAAGAAAAAGATAACCAGCCTCATACCGATTACCTACACATTTTCAAACT
ACAAGAAGATGATAATATGTTGTCAATTACACATGAACAAGAACAGCCCGCATACAAGTTAGAATATCACTAT
ATAAACTATGAAAAAAATCAAAATGCATTACCTAAGAAAGTCTACGTCATTCGTGAAGATGATGTAGACGTTT
TTTATTATGTGATGCTTTTACCAGAAGAATACTAAAAGGAGTGACGTTGATGAATACAATCAAAAGTACGATA
CACACAGAAGCTATATTTAGCGATGATGAACAACACCGCTACTTACTCAAAAAGACTTGGGATGAAAAGAAA
CCTGTTTGTACAGTGATAACGATGTATCCTCATTTAGATGGCGTATTATCACTCGATCTTACTACTGTTCTTATCC
TCAACCAATTAGCGAATTCTGAACAATATGGCGCTGTATATCTAGTGAATCTATTCTCTAATATTAAAACACCAG
AGAATCTTAAACATATTAAAGAACCTTATGATAAACACACAGACATCCATTTGATGAAAGCAATTAGTGAGAG
TGATACAGTAATTCTAGCTTATGGCGCCTATGCAAAGCGGCCTGTTGTCGTCGAACGTGTTGAGCAAGTGAT
GGAAATGTTAAAACCTCATAAAAAGAAAGTCAAAAAACTCATAAACCCAGCAACAAATGACATTATGCATCC
ACTTAACCCTAAAGCACGTCAAAAATGGATTTTGAAATAAAAGGAGGATTATCTATGAACCATGAAACTACAC
ACTCAGACTGGCGAACGGTTGCTAATTGTTTAGCATCACAAAATTATATATCGATCGTAAAAGGATTAGTACAT
CATTTCACAGCGATTGAAGATGAAGAAATACTAGATAAAATCTATGAAGATTTTATGAATAATGGCTCTATTAC
AACGGTACTTAACAATGATTTACAAGATATTATTAATTATTACCTATCAAAATAAAAATATATTACTTTTAATAAA
ATTAGAGCATCCTTCACTTTGCGGTGAGAGATGCTCTTTTAGTTTATTTTTTTATTGATTACTCGCCATAAGTTC
TGCTTGCTCTACAGCCATTTTTATAAATCTGGTGGATAGCTATATTTTTTAAGTAATCGTCTAACAGCTACGCGC
ATTTTAGCTTTTGCGCTATCACGTTTAGACCAATCACCCCCATATTTTCTTTCACTTTTTTAGTTAGCTCATGAG
CAATCGCACGTAGTTCTTCATCTTCCATTCCTTTTTTTCGCTGTTTCATGTGAAACTAAAGCATCGTAAAAATG
CGATTTCATCTGAATTTAAACCTAATTCATTTCCTCGTTGTTGTTCTTGTTTAATATCTTTGGCGAGTTGAATGA
GTTCTTCAATCACTTTTGATGTTTCAATGGAACGGCTATTATATTTATTAATCGAATTTCTTAACATTTCAGAGA
AACGTTTAGATACCGTCGCATTCGTTTTCATTAATGATTTGACTTGTCCCTTGAGTAATCGATTTAATAATTCTA
CCGCCACATTTTTCTGTTTCAATCCTTCTACATCTTTTAAAAAGTCATCAGATAGGATTGATAAATCGGGTTGT
TCAAGACCTAAAGTTTAATAAATGTCAAGTCAATGAAATCTTCAGTTAATAGTGTATATTTGATTATTATTAGTT
TAAATATATTAAAATTAAATACTTATTATATATGGTTCTTTACTATTTTAGAAGTATATTTTTTATTTTCCTGAACAG
TAGTTATTAAATTTATCTCTTATTTTAAATTAGAAAGGAAGAAAAAGTTAAATTTATATTAAGTTTTATATATGAT
TAATTATTAACGTATTAAATGTAGTATACTCTTAATATATAGAAAATTATCATAATATTTGTCAAAAAAAGTGACA
TTTCATATAGTAGCATGGTGTTAGTTGTCACAGTTTTTGACAGCCAAAATAACACTTGAACCTTTGTGGTTATG
CTATTTATTAAAGTATCTGATCGATAACTACCCCGAATAACAGGGGACGAGAATACTTCTCTCGCCATTTCTGC
TAATTGTTCTACTTTGGATCGATAACTACCCCGAATAACAGGGGACGAGAATTCTATAAGTTCATTAATTCCGA
TACCTAGATTATCTGATCGATAACTACCCCGAATAACAGGGGACGAGAATTTTTTTCCACCCTTTCAGATCATC
TATGATCTTGGATCGATAACTACCCCGAATAACAGGGGACGAGAATAATTTTCTAATTCTATAAGTTCATTAATT
CCGATGATCGATAACTACCCCGAATAACAGGGGACGAGAATTATACTATTTACATAATTTTTTATGTGTCTGTCT
ACGATCGATAACTACCCCGAATAACAGGGGACGAGAATTAATAGTGTTGTTCTCTATTAAAAGATACAATCCT
GTGATCGATAACTACCCCGAATAACAGGGGACGAGAATATAAACCCGTTCAATTCGTTATCTTTAAATTCTTG
GATCGATAACTACCCCGAATAACAGGGGACGAGAATACAACTTCGTCATCTTTCATCATTTCTCTTACATCAGA

TCGATAACTACCCCGAATAACAGGGGACGAGAATATATTTCTTCCATGAATAACACCCTCCTTTTTTCTAGATC
GATAACTACCCCGAATAACAGGGGACGAGAATAAGTTAACGGCATTACCTAATAAAAATATTTTAGGGATCG
ATAACTACCCCGAATAACAGGGGACGAGAACTCATCTTTCATGTCACTGATTAATTCATTTGTAGATCGATAAC
TACCCCGAATAACAGGGGACGAGAACGGTAATAGTTGCTCAATAGGTAATAAAACGTCGGTGATCGATAACT
ATCCCGAATAACAGGGGACAGAGTGTAAATTTAATTACACTCTAAAATTTGTAAATTTTTAATGGAATACGCAT
TGATTAATTTTTAGGGGATGAAAAATGAAAGATGTTATTTATGTAGAAAATCATTACTTTGTTACCGTGAAAG
AAAATAGTATTAAATTTAGAAATGTAATAGATAAAAGTGAGAAATTTTATTTGTTTGAAGAAATAGAAGCGAT
TATTTTTGATCATTATAAAAGCTATTTTTCTCATAAATTAGTAATTAAATGTATAGAAAATGATATCGCTATTATTT
TTTGTGATAAAAAGCACTCTCCATTAACGCAACTTATTTCTTCTTATGGTATGACTCATCGTCTTCAAAGGATTC
AAAGTCAGTTTCAATTATCTGGGAGAACTAGAGATAGAATTTGGAAAAAGATTGTTGTAAATAAAATTATTAA
TCAATCAAAATGTTTAGAAAACAATTTACATAATGAGAATGTGAAGTTATTAGTAAACTTAGCAAAAGATGTT
AGTTCTGGAGATAAAAGTAATAAAGAAGCACAGGCTGCAAGAATTTATTTTAAAGATTTATACGGTAAACAA
TTTAAACGTGGACGGTACAATGATATTATTAACTCAGGGTTGAATTATGGGTATTCGATACTTAGATCTTTTATA
AAAAAAGAACTAGCTTTACATGGATTCGAAATGAGTTTAGGCATTAATCATCGTTCGAAAGAAAATCCATTTA
ATTTAGCAGATGATATTATTGAAGTTTTTCGTCCTTTTGTAGATAATATCGTGTACGAGATAGTTTTTAAGAAAA
ATATTAATACATTTGATGTAAATGAAAAGAAATTATTGTTAAATGTTTTGTATGAAAAGTGCATTATAGATAAAA
AAGTGGTGAGGTTACTTGATAGTGTGAAGATAGTTATTCAATCACTTATTAGATGCTATGAAGAAAACACCCC
TACTTATTTATTACTACCTAAAATGATTGAGGTGGGGAACTAATGTATTTATTAGTTAGTTTTGACTTACCTAGA
GATACTAAATTTGAACGTAGAGTCGCAAGTAAGTATCGTACTCGATTATTAGAACTCGGTTTTAGTATGAAGC
AGTTTAGTTTATACGAGAGGTATGTCAGCGATGTTCAAAAAAAAGATAAGATTTTAGAAATTCTGCAACAAG
AAATTCCTGATACTGGAAGTATTACACTATATGTCTTACCTGATGAGGTAAATAATAGTCAGATTACCATATTAG
GAAAAGAGGTTAAGGTTGTTGTGCGAAAAGAACCTAAGCTAATTTTTCTATAAAGTGGAGGTTACAATGGAT
AAAAAAACGACATTAATGTATGGTTCTTTACTACATGATATAGGTAAAATTATCTATCGGAGTAATGATCATGCA
TTTGCAAGAGGAACGCATTCAAAGTTAGGATACAATTTTTTATCTCAATTTTCAGAATTTAAAGATAACGAAG
TGCTCGATAGTATTGCTTATCATCATTATAAAGAACTTGCAAAAGCTAATTTAGCTAATGATAATACAGCTTATAT
TACCTATATTGCCGATAATATTGCGAGTGGTATTGATAGACGAGATGTTATAGAAGAGGGCGACGAAGAATAC
GAAAAACAAGCATTTAATTTTGATAAGTATACACCTCTATATAGTGTGTTTAATATTGTGAATTCTGAAAAATTG
AAACAAATAAGTGGAAAGTTTAAATTTTCTAATGAAAGTAATATTGAGTATCCTAAAACTGAGAACATTCAAT
ATTCAAGTGGAAATTATACAACACTAATGAAAGATATGAGTTATGATTTAGAGCACAAATTAAGTATTAAAGA
AGATACATTTCCTTCATTATTACAATGGACAGAAAGTCTATGGCAATATGTGCCAAGTTCGACAAATAAAAATC
AATTAATTGATATTTCTCTTTATGATCATAGTCGTATTACATGTGCTATTGCTAGTTGTATATTTGATTATTTAAAT
GAAAATAATATACATAATTACAAAGATGAATTGTTTACAAAGTATGAAAATACCAAAGAATTTTATCAAAAAGA
AGCTTTTTTACTACTTAGTATGGATATGAGTGGTATTCAAGATTTTATTTACAACATAAGTGGTTCTAAAGCATT
AAAAAGTTTAAGATCTCGTAGTTTTTACTTAGAAATCATGCTTGAGGTAATCGTTGATCAATTACTAGAAAAAT
TAGAATTAACACGAGCAAATCTTTTATACACTGGTGGAGGCCATGCATATTTATTAGTCTCTAATACAGATAAA
GGGAAAGAAAAAATAAATCAATTTAATACTGAATTAAAAAATTGGTTTATGTCAGAATTCACTACAGATCTCT
CATTATCAATTGCTTTTGAAAAATGTAGTGGTAATGACTTAATGAATACTAGTGGTAATTATAGAAATATTTGGC
GTAATGTCAGCAGTAAGCTTTCTGATATTAAAGCACATAAGTATTCTTCAGAAGATATATTAAAATTAAATCATT
TTCATTCATATGGGGATCGAGAATGTAAAGAATGTTTAAGAAGTGACATAGATATTAATGATGATGGATTATGC
AGTATATGTGAAGGAATCATTAATATATCAAATGACTTAAGAGATAAATCATTCTTTGTACTGTCAGAAACTGG
AAAATTAAAAATGCCATTCGATAAATTTATATCGGTTATCGATTATGAAGAAGCAGAAAGGTTAGCACAAAAT
AATAATCACATTCGTATTTACAGTAAAAATAAACCTTATATTGGTGTAGGAATATCAACGAATTTATGGATGTGT
GACTACGACTATGCTAGTCAAAATAAAGATATGAGAGAAAAAGGTATTGGAAGTTATGTAGAAAGAGAAGA
AGGCATTAAGCGTTTAGGCGTTGTACGTGCCGACATTGATAATCTTGGCGCTACATTTATATCAGGAATTCCA

GAAAAATATAATTCAATTTCAAGAACAGCTACATTGTCTCGTCAATTATCATTATTTTTAAATATGAATTAAATC
ATTTATTAGAAAATTATCAAATTACAGCTATATATTCAGGTGGCGACGATTTATTTTTGATTGGCGCATGGGAT
GACATTATAGAAGTAAGCGTTTATATAAATGAAAAGTTTAAAGATTTTACTCTTGGTAAATTAACAATGTCTGC
TGGTGTTGGGATGTTCAGTGGTAAGTATCCAATTTCTAAAATGGCTTTTGAGACAGGTCTACTTGAAGAAGC
AGCTAAAACTGATGAAAAAAAATCAGATAGCACTTTGGGTGCAAGAAAAAGTATATAACTGGGATGAGTTTAA
AAAGTATATCTTAGAAGAAAAACTTCTCGTTTTACAACATGGGTTTTCACAAACAGATGAACACGGAAAAGC
CTTTATTTATAAAATGTTAGCTTTACTGCGAAATAATGAAACTATTAATATTGCTCGCTTAGCCTATTTATTAGCA
AGAAGTAAAATGACAGAGGAATTTACATCTAAAATTTTTAATTGGGCTCAAAACGACAAAGATAAAAATCAA
TTAATTACAGCTTTAGAGTATTATGTTTATCAAATAAGGGAGGTTGATTGAGTATGATATTAGCTAAAACAAAA
AGTGGGAAAAAAATAGATTTAACTTTTGCACATGAAGTAGTAAAAAGTAATGTAAAGACAGTTAAAGATAAA
AGAGGTAAAGAAAATCAAGTTTTATTTAACGGTCTTACAACAAGCAAGTTAAGAAATTTGATGGAGCAAGT
GAACCGACTTTATACTATTGCATTTAATTCGACTGAAGATCAATTGAATGAAGAATTTATCGATGAATTAGAGT
ATTTAAAAATTAAATTTTATTATGAAGCAGGTCGAGAAAAAAGTGTTGATGAATTCTTGAAAAAAACATTGAT
GTTCCCAATTATTGATAGAGTGATACAAAAAGAATCAAAAAAATTTTTCTTAGATTACTGTAAATACTTTGAAG
CTCTAGTTGCATACGCTAAATATTATCAAAAGGAGGATTAATATGTATTCAAAAATTAAAATTTCAGGAACAAT
TGAAGTAGTTACTGGTTTACACATCGGCGGGGGCGGTGAATCTAGTATGATTGGCGCAATTGATTCCCCTGT
AGTTAGAGATTTACAAACTAAATTACCTATCATACCTGGCAGTTCAATCAAAGGAAAAATGAGAAGTTTATTA
GCAAAGCATTTTGGCTTGAAAATGAAACAAGAGAATCATAACCAAGACGATGAAAGTGTTTTAAGATTATTC
GGTTCAAGTGAAAAAGGAAATATTCAAAGAGCTCGTCTACAAATTTCTGATGCATTCTTTTCTGAAAAGACG
AAAGAGCATTTTGCACAAAATGATATTGCTTATACAGAAACGAAATTTGAGAATACAATTAATCGTTTAACTG
CAGTTGCAAATCCAAGACAAATTGAGAGAGTAACAAGAGGATCTGAGTTTGACTTTGTATTTATTTACAATG
TCGATGAAGAATCACAAGTTGAGAATGATTTTGAGAATATTGAAAAAGCAATTCACTTATTAGAGAATGACTA
CCTTGGTGGCGGCGGTACCAGAGGTAACGGACGTATCCAATTTAATGATATAACTATCGAGACGGTTGTTGG
AGAATACGACAGTACAAATCTTAAAATTAAGTAGGTGAAAGACATTGACAACAAAAGTATTTAAACTTTCTTT
TAAGACTCCTGTTCATTTTGGGAAAAAAAGGTTGTCAGATGGGGAAATGACAATAACTTCTGATACTTTGTT
TAGTGCGTTATTTATTGAGGCGCTTCAATTGGGTATAGAAACTGATTGGTTATTAAACGATTTAATCATTAGTG
ATACATTTCCTTATGAAAATGAAATTTATTATCTTCCTAAACCTTTGATAAAAATTGAATCTAAAGAAGAAGGC
AACCATAAAGCATTTAAAAAGTTAAAATATGTTCCGGTTCATCACTATAATCAATATTTAAATGGTGAGTTAAG
CGCTGAAGATGCGACAGATTTAAATGATATTTTTAGTATTGGGCATTTTTCTCTACAAACAAAGGTTTCATTAT
CAGCACAAGAAATTGATTCAAGTGCTGACAGTGAACCTTATTCAGTGGGAACATTTACTTTTGAACCTGAAG
CTGGTTTATATTTTATTGCAAAAGGATCAGAAGAAACCCTTGACCAATTAAAAGATATTATGACTTCATTACAG
TACTCAGGTTTAGGTGGTAAACGTAATGCAGGATATGGACAATTTGAATATGAAATAATAAATAATCAACAATT
ATTTAAGTTATTGAATCAAAATGGAGAACATTCTATTCTTTTATCAACGGCAATGGCTAAAGAAGATGAGATA
AAGAGTGCTTTAAAAGAGGCAAGATATATTTTGAATAAACGTTCTGGTTTCATACAATCAACAAATTATTCTG
AAATGCTAGTTAAAAAAAGTGATTTCTATAGCTTTTCTTCTGGTTCAGTTTTTAAAAATATCTTTAACGGTGAC
GTTTTTAATGTTGGACATAATGGTAAACACCCAGTCTATCGTTATGCGAAGCCTTTATGGTTGGAGGTATAAG
TATGACAATAAAAAATTACGAAGTTGTTATTAAAACTTTAGGTCCAGTTCATATTGGTAGTGGTCAAGTTATGA
AGAAACAAGATTACATTTATGATTTCTACAATTCTAAAGTTTATATGATTAATGGAAATAAACTGGTTAAGTTTT
TAAAAAGAAAAAATATACTCGATACATATCAAAACTTTTTGAGATACCCACCAAAAAATCCAAGAGAAAATGG
ACTAAAAGACTATTTAGACGCTCAAAATGTTAAGCAAAGTGAATGGAAAGCATTTGTGAGTTATTCTGAAAA
AGTTAATCAAGGTAAGAAATATGGAAATATACGGCCTAAACCGCTAAATGATTTACATTTAATGGTAAGAGAT
GGACAAAATAAGGTGTATCTTCCGGGAAGTTCAATCAAGGGAGCTATTAAAACAGCACTTGTTTCAAAATAT
AATAATGAAAAAAATACAGATGTATATAGCAAAATTAAAGTCAGCGATTCAGAGCCTATTGATGAAAGACATT
TAGCAATTTATCAAAAAATAGATATTAATAAAAGCGAAAAACCAATGCCTTTATATAGAGAATGTGTAGATGTA

GATACTGAAATAAAATTTAAATTAACCATAGAAGATGAAATTTATTCTATTAATGAAATTGAACAAAGCATCCA
AGATTTTTACAAAAACTATTATGACAAATGGTTGGTCGGTTTCAAAGAAACAAAAGGTGGAAGACGATTTGC
ATTAGAAGGCGGTATGCCCGATGTTCTAAACCAAAATATTTTATTCTTAGGAGCTGGCGCAGGATTTGTTAGT
AAAACGACACATTATCAATTAAAAAGTCGAGAACAAGCAAAACGAGATTCTTTTGATATTTTAACTAAAAAAT
TTCGTAGAACTTATGGGAAAATGAAAGAAATGCCTTCTAACGTACCAGTTGCTCTAAAGGGTACAACTAATC
AAAGTCTTCATGTTTCATATCAACAAGGTATGTGTAAGATTAGTTTTCAAGAGTTAAATAATGAGGTGCTATAA
TGAAAGTACTATTTAGTCCAATAGGTAACTCAGATCCATGGAGTAATGATAGAGATGGTGCAATGCTTCATATT
GTGCGTCATTATAAACCTGATGTAGTCGTTTTATTTTTTACTGAAAGCATTTGGAATGGTAATAGAAATATACCT
GGACGTAAAAACTTCGATTGGGAAAACATTGTTTCAAAAGTATCATCGAGAACAAAAGTGGATATAAAAGT
GGATAGTATTAAATATGAAAATGATTTTGATAGCTATAAAGATATATTTCACTTTTATATAAATGAGATTAGAACT
AAATATTCTGATGCAGAAATTTTATTAAATGTTACAAGCGGAACACCACAAATGGAGTCAACTTTGTGTTTAG
AATATATTTCTAATCCTCATAATATGAAATGTATACAAGTTTCAACACCTGCTCCTATTGAAGGACCTAAACGTT
CATTTGCGAAACTTGAAACTGTGACTGAAGATTTAAATAAAGTTAATGCTAATGAAAAAATGGCTTCTAATAG
AAGTAAATCAATCAATATTATTAGTTTTAGAGAAGTGATGGTGCGCTCTCAAATAAAAAGTTTAGTGAATAATT
ATGATTATGAAGGTGCACTGAATTTAGTAAGTGATCAAAAGTCTTTTCGTAATGGTAAACTATTAAGGAAAAG
ATTATTGGAATTAACAAATCAGATTAAAACGCATGAAGTTTTTCCAGAAATTAATGATAAGTACAGAAGTGTT
GCTTTAAAAAAATCATTATTCCATTATTTATTATTAAATATGCGATATAATCGTCTTGATGTAGCTGAAACGTTAA
TAAGAGTGAAATCTATTGCTGAGTTTATACTTAAAACATATATTGTGGGTCATTGGCCTACTCTAATAATTGAAA
AAGATGATAAACCTTATTTAAATGCTGAAGATAATTTATCTTTTATTTATAAATATAAATTACTATTAGAAAAAAG
AAGACAGAATTTGGATGTTTCAAGGATTCTAGGTCTACCTGCGTTTATAGATATACTTACAGTATTAGAACCTA
ACTCTAAACTATTAAAAGAAGTTAATGCTGTAAACGATATAAATGGTTTAAGAAATTCTATAGCTCACAATTTG
GAAACATTAGATTTGGATAAAAATAAAAATTATAAGAAAATAATGTTATCTGTTGAAGCAATAAAGAATATGTT
GCATATCTCATTTCCCGAGATAGAGGAAAAAGACTATAATTATTTCGAAAGAAAAAACAAGGAATTTAGAGA
ATTATTATGATAAATAAAATTACAGTAGAATTAGACTTACCAGATAGTATTCGTTTTCAATATTTAGGAAGTATTT
TACATGGTGTGTTAATGGACTATCTTCCTAATGATATTGCTGATCAGCTACATCATGAATTTGCTTATAGTCCATT
GAAGCAAAGAATATATCATAAAAACAAAAAAGTAATTTGGGAAATTGTTTGTATGTCAGATCGATTATTTAAT
GAGATAGCCGAGCTATTCACTTCTAAAAACAGGCTGTTTTTAAAATATTACCAAGTTTATATTGAGATTTATTC
TTTTAATATTGAGAAAGTAAACGTTCAAAATATTATGAATCAACTCTTAGAAACAGAGGAATTGAATCGATATG
TAAGAATTAATATACAGACACCTATGTCTTTTAAATATCAGAGTAATTACATGATTTTTCCTGAGGTTAAACGTT
TCTTTAGAAGTATTATGATACAATTTGATGCTTTTTTCGAAGAATATAAAATGTACGATAAAGAAACATTAGATT
TTTTAGAAAAAAATATTAATATTGTTGACTATAAATTGAAAAGTACACGATTTAACTTAGAAAAAGTTAAAATC
CCTTCATTTACGGGAGAAATAGTGTTTAAAATTAAAGGACCCTTACCTTTTCTACAACTAACTCATTTTTTATT
GAAGTTTGGCGAATTTTCTGGATCAGGTATGAAAACAAGCTTAGGCATGGGGAAATATAGTATAATATAACTA
GAGTATAGCTGAAATTTAGTTGTCAAAAAATGTGACATTCTGCACTTATAGTACAAGTGATTTGTCACTATTTT
TGACAGTAAATTTTGCCTTTGAACTATTGATTTGATAGGCTTTAAAGAGCTATCTAAATTCGATAACTACCCCG
TAGAAGAGGGGACGAGAACTCTTCTAAGACGCGATATGATTCTAATTGGTCTTCATTCGATAACTACCCCCGT
AGAAGAGGGGACGAGAACTGATATACTCCTTTACCATGTATTAATTCTGGACCACTATTCGATAACTACCCCG
TAGAAGAGGGGACGAGAACTCATATTCGATCGTGTATATCAAAACTTTATGCATTCGATAACTACCCCGTAGA
AGAGGGGACGAGGATCTCATCTTGATGATCCCAATTGGCTTGGTTAAAAAGAAATCGATAATCCTCGTAGAA
TAGAGAAAAAGTATTAAAAATATATTTGATTTGATAAAATATTCTGCAAAAAAGAGGAACAAAAAATCAAGAT
TTAAACTGTAATTCATTATTATTGAAATTAATCGACATTCAATTTCTAAATAAACAGAAACAAAAATAACTTTCA
TATAAATAATTTCTTTGCGATTAAGTTGGAATTAGAAATACTAAATACAAATAACAGCGAATGAAATGCATATTA
TTGAGGAATTGGATGTGTAGTGCAATTTCGAGAGTTACTACTTAAGAAAATAAAAAGAAGAAAACAAACAA
TAAGAATTAAATCTGAAGCAAGATATAAAAGCAAATATTTTTGATACAACCACATTAGTAAATACTTTATTAATT

AGTATACGACTGAAAGCGAAGTAACAGCTAACAATGCTCCAGATATAGACATTAGTGTTACGAAAGAATAATA
TTACCATCTAGAAAATGTAATTTTTTAAAAATCTGAAGATAAAGTAGATTACTTTGTGAGTTTTCATGTTAAGA
ATTCTGAACTTGGAGACGTAGTATAAATATAGCTTAGTAGAAAATGCCACCGGAGCTGTAGCTGAGATTGACT
ATCAAGAATTAACTAAATTAGGTTATTACCCATCAACAAAAATCCAAGATTTAACACTGTCTAATAATAGTGCG
AATAATGAAGTTTAAAAGTAAACAACTGGTAATAGCGAATCAAACTAAAGTAATCAATTACAGAATTCAAGTA
CTACTCAAGCCTCAAGCAAATATTGAAACTCAAACACAAGTTAAGAATAATACACTGCCTGAAACAGATGAA
CAGTCTAATGCCGATTGAGTCCAATAATAGCCTCTGTATTATTAGCCACACGATTATTATCAATATTTAAATAAA
GTTAATATTAAATAATAAAAATCCTTCAAATTATTTGTTGAAAAATTAATAACTTTGAAGGTTTTTAGCAAAATC
ACTGATAGGGAGAGGCGTATCATAAGT
>Staphylococcus aureus strain BA01611
TTATATTGAATTGATAACATGTTGCATTGTTATTCGTTCTAACCGTGTACTTGGGACAAAATGTTCAGTTATCTC
TTTTTCATTTATGAATTCAGGGTACAAGTGATAAAATTTTAGTAGCTTCTGTTCCGTTTCAGTTAATACATATTG
TTGCATATGGTAGTATCTGTCGATATAATTAACGACAATAGGTTTTTGATGATCATCGACTTCTACTGAATCAAT
TGTGTAATTTAATAAGTTGACGTTTGTTTTCATTTTTATGCCTCCGATTAATTTAATGAGCTACAACGCATAGCG
CGTTGTAGCGGTATAATATTCAAATTTATAGGTCTAAATCATCATTTGCTTCTTTTTCTTTCGCTTGTTTCATAAA
GTTATTAAGCAAATCTGGATTTGTATTACCATTAAATTGCGGTGGTGTAAAAGTTCCAGTATTAGAGCTTAAGC
CGAAAATAGGGGCAAGCTCTTTATCCAATTTAAGATGGTAGAATACAATCGTTTTGGACTTACCATGGGAATC
TTTGATACTGCGTTTGGTTGTCTTTCTGTTAGGATCAGATTGAATATGTCCTTTATCACGCAGCGCATCGATAA
CGTTATTCACATCTTGATATTGGTTTTCTTCCAACATACGTTTAAAGACATCTTTAAGGATTTTGACTTGAATAC
AATTATCCTTTAATTCAATAAGTCCATAATTTTCTACCATGGTTGATAATCTATTATCATCAGAAAATTTCCCTCTA
TTCTGTGCGACAAATTGCGTAATCGTTTCGAGCGCTTTATCAGCAAGAGACCGCTCACTTACTGAATCTGAAT
GGTAAGTGAGTAAGTAATTTCTAATCGCATCCAAATCTATGGGCGTCGCAATAACACGTTCGAATATACGAGC
AGATGTCGTAATCGCTGCATAGCGTTTGAACATACGAATACCTGTATGACTCGTTTCATTTTTTAATTGTTTTTT
GAACCAATCATGTTCCGCGTGAAACCATTTAATTACTTCACCTTCACGATTTAAAAGATATTCAGCTACTAATG
GCATTACATGCCCATAATTGACGGATGTAGCCTTTTTAATCGCATCTGCATTGTCAGCACTTGTTGTAAAGGTT
TCAGATATTTCAATCGTACGGACGTTCAAACCATCATTACGTGCACTATCATTTAAAATACTGTGTTCTGCGGT
ACTTATCACTGTAGTGCCCCAATTTTTGAGTTCTTTGACATTACCATCAATATTAGAGCGTTGTCGGCCTTGTC
CCTCTGCCAAACTGTACAACAATCCAGTAGTGTCTTTGAAGGTTGCAGCTGACAATTCATCTAATACGATAGG
CACACCAAAGTTACTACTTAAATATCCTTCAAGCGCGTTTCTAGTACCATTCCATGATCTAAACAGGGTATTAC
TACCTTTAGTAGGGTTGCCGGCTATTGAAACAGCTAAAGCAGCTGCAGTCGATTTACCTGTGCTTGAGTTTCC
CATAAATGAGAATATGGTTCCCGCAAACTCGACCTCATGTCTTGTTTTTAGGAACGCAGTAACTAATGAAGAA
GCAGCAAATACTACTGCAAGTTCTAGTAGTAAGTTCCCTTTAACTTGTTTTAAGTACATCTTCCACCAGCCTTT
GAAAGTACCTTTTGGTTGTAAATCATAGTGTGTTTCACAAATGATTTCATTAGCTTGAGATTGCTCTATTTCTTT
TGAAAAGTATGGTTTATCCAATGAAATGACCATACCTTCATCATCAGACTGTAATACACCTACGCCGGTATATA
ATTTAGATAATGGAAGTGACTGGCGCATCGATTGTAACGCATAGCTTAATGATTTAATGTACCGTTCATTGATG
TTGAAGCCGTACTTAATCAATCCAGGTAGCTTGAAAGATGTTAAGATATCTGATGCTTCAATACGTTCAATATT
TTTTCCATCTGTAATAATCAACTTCTCAACCCCTGTAGACGGGTCGAGAAATTTATTTTCAATAATGATGGGAC
TAGACAATTGAATAATTTTTTCTACATCATTGTTACTTTTAGGTGGAATCACTTCATAAAAGCCATTTGAATTTA
AAAAATATGGATATTGCTTGAATATGTTGTTAGTCATAATAAATCTCTCCTTTGCTATATAAGCATTAAATTTGTG
TTGCTAAATACATATTAAAGGAGAATTAAACGCAAATAAGTAGGTCGTCCATATACAAAAAAATTGGACGACC
TACTTAGGTAATGCTATTCAAATTTTAAATTGTCCAAGGTTTCACTTAGAGCTTTTGCGATAGACCAACTAGAT
AAATCATTGACTTGTTTACTGACGGTTTCGTCTTTAATTTGAGTAGATTTTATTTCTTCAAATAACTGGAAAGC
GTTCGTTAATTCGGTAAAGTTTATTTTTGAAAGTGGCTCAATATCTTTATTAAATGAATATTTATCTAATAAAAG
ATGAATTCGATACAAGTCATTTAAGAAACTCTCTACTTGATTTGGACTTATACCATCATCAACATAGGATTGGC

GATTAGTATAAAATAGGTTAGAAAATTTAGACAATGATAAATAAAAGTCTTTATAAATATTGAGAAACTTATCG
TTTGATATTAATGGATCGGGGCTATTTATTATATTCTCTATGCGCGATTCAAAATTTTGTAAATCAAAAGTGCTG
TTTTTATATTGATTTATAGTATTAATAATTCTTTGATAGTTAGAAAATAAGTCGTTTTCAATACTGAGAAAGTATC
CATTAACGTTGAATGGGTATTTTACAATTTCTTTTTTTCTTAATTTTTCACGGATTTTCGATTCATCATTTTGTAT
ATCATCAGTCACAATTTTATGATTGATTCTTTTATTCAAACTTGGAAGGTAGAGTTTGTTTAATATATCAATAAT
GAAAAAATCTAATAATTTGTTATCAGTACGTTCACTATACTCATCTATAGTTGGCTCATTTGATATATATCTAGCT
ACTTCATGATTTACATTTGAATACTTGGAATCATCAGGTTCTACTTGGTTGTATATAATAAAATTTTCGGGATTG
TTTTCTTTATTATATTGATGAGCTTTATATAATTGCTTGAAATTGAAGTCATTATCAAATGTAATATTATCTTCCAT
TAATGTTTCAAACAGTAACTCGTCAATTCTATGTGCTAATTTTCTATATAATTTTGAACTCTTATCTAAATCTAGA
GAATTTGTATAATTAAAAAATGAAGACAATGCATCATTACACTTTGACTGAGCACTAAGCCATTCTAAAGTAA
AAGAATAGTTTTCAGATGCATAAATTTGCGCTTTTAATGCATCGTTTTTAATATTATCAATTGAATCGCTGATAT
TAGTAATATAGCTAATAAATTGACTATTATTTTTCTTTAATGCCTCCTCTCTTAAATCTATGTCATCAAATTTTTTA
GCAGATGTAGGAACAGGAAAGTCCTCAATAGTGTTTAGCAGAATTTTAAATAACTCTAATTCAAATAAGTTAA
AACTGTAACCGCTATTGGTTGCTGATGGATTTGATTTGTACCTATTTATATCTAATTTATAAACGCGTTCGAGCT
TATCCATACTATTCTTAAACATTTTTTCACTCAAGTTACATTCCTTCATTAAATCTTTTCCAGTAAAATATTTGTAA
TTTTCAAAATTCAATTTAATTCTCCTTTTTATCGTTATCATGCATGGATAATATGTACTTTACCAATATTATAAAGT
ACAATTAGAATTAACGATAATAGGAACATATGTTCTTTGGAGAGGGAGATTATATGAAACTTATTTATTTTGAT
GAAAGTACTGCAAGCGACTACTTGAAAATTATTCAAAATGGCAAAGAATCAAAACAAACAGTTGAAAAAAA
GAAAAATCAAAATCAAACAAGTTTAGGAGGTAAATTTGGAATAGGACCTATGTTTTCTAAATTGTTTGAAAG
TCTTTTTTCTGTAGAGGGACATATGGGAGGAAATTTAAATCGTACTTCCGAGAAATATATTGAAAAAACTTTA
ACCAATGCAATTTTAAGTGATTTTAAAAATTTAGCAGAAAATGAAGAAATAGGTATCACAAAATTTTCTGAGT
ATAAATTGAGTTATGTTGAGAATTCAATTGCACATTTTCAAACTATTTCACCATATTTAAGTATGGTAGAGGGA
GATATAGTGTTAGATGAAGAAGTGTCAATTAATATTAATAAAATGCATGAGACTTTAAAATTAGGTAAGGGTTA
TTATGAGATTCTTGCTAAGAAGAACGATAATGAATGCATCGTTAGATTTAATAATAAAGCTTTTGTAAATAATTA
TAACTTAACTGATTTGTCTCAAATGCAACTCACTTTATACGGTGTAAAAGTTGGACTAATTGATAAAAATCAAC
TTAATTTTCAAAATGCGATAAATTCACTTGGTTTTAAAAATGAGGTAGTAACTTCTATTTCAGAAGAACCAGT
GAATTCTTCTTTTAATAATGAACAATCGATATTAGAAATGTACGATATTATATTGGCAGGGGTCGAGGTTGAAA
ATGAGTTATAAAATTAAGATAGTTATGGGACCAAAGTCATATTTAAATGAATTTCTTAATAAACCATATATAACT
TTGGAAGATTACGTAATAAAAATAGACTCACTTACTAGAAATTTGGTTGAAAAATTTACAGATAATGATTATGA
AGAAATCTTAAAAGATAAAATTATTCTTGCTAAAACTGAATCATATTCTGGAATTACTTCAGCTGCATTAAGCA
ATTTTATACAAATCGTTAACAAATCTATTGAATTTGGAAACGGAATTATTCTACAGAATCCTCCAAAATCTGTTA
TTAATCAGATTAAAAGTCATTTTGATGAAAATCAATATGCTTTTTGTGAATATAAGTATCCAAAACTAGATTTAG
AGGTATTGTTAAATATTAAAGAGAGGTCAACTGAACAATTATTTGGTCAAGTTAATGCAATAAAGAGAATTTT
AGCTTCACTATACAAACTTACTAAAGAATCTAGAAAACCAATTGTTTTGATGTTATACGGTCCATCAGGAGTC
GGTAAGACTGAAATGAGCAAAATTATTAGTGAATGTATAGGCGGAGAATTATTTAGAAAACAGATGTCAATG
AATAAAACCAATTATATGTTTGACTACATATTTGGTAATAATCACGGTGAGCCTAGTTTAGCTAGAGATTTATTA
GAGCGGGAGAGCAATATTGTATTATTTGATGAATTTGATAAGGGAGTTAATGAAATAAATAGTGCTTTTTACC
AATTGTTTGATGAAGGAATTTTTGAGGATTCTCAGTATGAAGTCAAAATGGGGAATTCAATAATTATATGTAC
GTCTAATTTTAAGGGAGAACTTCAAATAAGAAGAGAGCTAGGTGATCCAATTTATTACAGGTTTGATGATTTT
ATTGAATTTTCTGAGTTAAATAAAGAAGCTAAAAAAAGTATATTAACAAAAATTTTAAGTGATGAATTTATTAA
GTTATCGGATAATGAAAAGTCTTTATTACCTAGTGAAGAAAGTTTATTGGAACAATATGTGATTAAGATAAATC
AATTTACTAATTATAGACATATGCAGAAATTAGTTGAAAATGACATCAATTTAAGGTTAATAAGTACTTTGATG
GGTTTTTAGTAACTTGCAAAGATATTTTATTAGGGTGAATGTACAATAAGGTGTTGAATATATAAAGATATGGA
TAGACAAAAGAAATTAATGGAAAAAGAAAGTTCGACTTTTTATATACTCACATGCTACATTTATTGTAATAATA

CAACATGGTAGGAGTGTGAAACATGGAAATGAATCGCTTTGATATTTACAGAGAGTTATGGACAAAGTCTCG
TACCGCATTTGCTGAGGAACATAATATCGAGTTTAATGATTTGAAGAAAATCATTGAAATCCATAAGATTCCCT
TACCTAATTCTAAGTATTTGTATGATTACAGAAGAGGTATTATTGGTCAGCTAGAACCTATAGAAGGTGAAGAT
TATTCAATTAAACTTGATGAGAAACTGAGCGAAAAAACTAAAATAACAAATAAATGGTCATACTTTGATGATG
ATAAAAAAGAAGAAATTTACAATGTATATACCGAATTAAAGATACCAGATAAGGTTAAACGTCATCATAAAATC
ATCACTAAACATAAAAACTACCTTACATCTGAAAGAAGAAGACAAAGGGAAGCGGAAATGAAGCCTTGGG
GTTATTATCGCTCAGAGCCACAGAAGGTTTTGAATGCCTCTCATATCTCAAAAGAAGCATTAACAGACTTTTA
CAGACTTTGCGATACTTTATTTAAAGCTATAGATTGTTTAAATTATCAGATTACTGTTGATGAAGAATATATTTG
TATTCATATACCTCGTAGAGACAAACAAGTGAAGAAAAGTGGCTTGATGTACCTAAAGTTGAATGTATTTCAT
ATAAAATTAAGGTTTAAAGAAAAGAAGAAAAGAGTCAAAAGAGATGATGATCGTTATTCTTCTTATGATTATC
TCAATACTGGCTATTTCAAGGCTGAACTCATTGGAGTCTATCCTTGGAGTAATAACGTCTATGGTATTAATCGA
GACTATCCTCGTACAGATGACCATAACACATTGATTAAGAAAATTTTTGAGAGAATATTTGAATTACCACAGTT
ATTGAATGATGCAGAGATTGAATATATTGAAAAGCAGAAAGAAGAGGAACGCAAACGAAAAGAAGCAGAA
CAATTAAGAAAAAGTAGGGAATCTGAATTTAATAATATCAAAACACTAATCAGTGATTACAAGTTACATAAAG
AAATAAAAGCGTTAAGCGAGTATATTAATGAGTATAAGCTAAAGAATGCAACAATTGAAGAGACGATGTGGT
ATAAAGAGATACTTACATGGTTGAAAGACTCCAGTAAAGTTGAAAAAGTATTAGGAGATAGAAACCATGAAC
AAATAGTAAAATATTTATTAAATGAAATGAAAGAAAGCGATATTGATTTCCATGATCCATCGAGTTATAGATGG
TGGTGAGACAGTAATCATTGATTCACAGTTAAGTAAGATACATGGAACATAATACTTTTATCAGTTAATGGAAT
TGGTGCTTCATATAAATAACTAAAAAGAATGTTTATAGGTGTGATGGGCTTATATGTCTTAATATAATAAATGCT
TCTCTTTTATAAGTATTTCTATAAAGTATGCCACTGCAAGTGTTTGTGCATCTTACTCACCATGTTTTCGGACTG
ATAGTGAACCCAGGCGAACATTATTTGCTAGTCAATAAGAAAGTTATGATGTTTATCGACGTGCAGTTTACATT
AATACCTATTTGAAAATGCTGAATAGAATTATTAGATAAGAGAAATCTTCTAAATATCAAAGCAGTCATTAGTT
GGCTGCGGATAAAGATTTGTAAGTAAGGAGGGGGTATGTAATGATAAAGAAATTAGACAGTTTAGATTCTGA
GTCAATAGAAAAGATATCAAATATATGGCTTAATTTTAATTTAGATGCGCATGACTTTATAAAACATGATTATTG
GATTGACAACATAGATAAAGTGAAAAAATGCTACCAGATTCAACAATTTACGTGTATTATGCTGATAATCAAAT
CATTGGATTTGCTGGCTTATATGAGCAATATATAGCAGGCATTTTTATTACAGATAATTATCGTAATAAAGGAAG
AGGAAGGTTAATACTAGAAAGATTAAAAACCGATTATAGTGTACTTAATTTACATGTATATGAGAAAAATGAA
GGAGCTACCCGTTTTTATACCAAACACCATTTTGAAATAAGATCTAAGGAGTTTGAAGAAGCTACTCAAGAA
TATGAGTATCTTATGGAATGGAGTAGTAATTGAAATTACAAAAACAACAAATAATAAACTCTATCAAGCTCCAC
AACTTGATAGAACTTTTTGTATATATTATAAGCTTTTATGATACTAGTTGATATTAGATGTAAAGTATTTATAGTA
GTAATATATCGTTTTATCTAGCTGTACGAAAAACCGCATCAATAACTGATAAGCAGAAGCATACCATAAATAAA
ACTAAAAAATAAGTTGCGTATAATGTATTCATAATAATTATTTAACATTTTAATTAAAAAAATATTATAGTTGGAA
AGGTTGGAATCTATATGAAAAATATATTCGTATCATACGCTTGGGATAAAGAAAATGATGAAAAAGTTATTAAA
TTAGCTGAGATGCTAGAAGAGTATAAAGAATTGAATGTTAAATTTGATAAATGGGACATAAATAAAGGTCAA
GAAATACCTTTATTTATGGAAAAAGGGATACAACAATCAGATTTCGTATTAGTTATTTGTAGTAAGCGATATAA
AGAAAATACTGATAAACGAATAGGGGGGAGCGGTTATGAAGCTAGATTAATGGCTAATGAAATATTAAAAAA
CACAGATAAAGATAGATTTCTTCCTGTTCTATTAAATGAAAATGATAAAGATCATATACCTAATTTTTTAACAGG
GAAACTTTGGACTTCACTTTATTATCAAGAAGGTTCAGAAGAATATAATATAGAAATTAATGATTTATTAACAA
CTATCGTGGGTCGAAGCAAAAGAAATATAAAAAAAAGCAAAAGTATTTATGATCAGTTTGAAAATGTGAGTA
CTAAATCAGAAAATATTAGTGAGATAAAAATTTTAGGAATTAACCAGGAAGAAGTAACCGTACCTAAAATGG
ATGGTACTAGAGGATCCGGATTGTATAGTATTCCATTTTTATTAAATCAATGCCCTTCTAGAGAATGGTGTGAG
ATTTTTATAAATAAATGGGATCATCCTCGTCATTATTCTACAATGCATAGACCAGGTATAGCTAAAGTTGTTGAA
GATAAAATTATATTAGAGGGTACTACGATTCAAGAAGTTAAAAAAATATCATAGAGATACTTTAATAATGTGTGT
TAATGATACAAATGAAGAATATAAAATATTAAGAAATAAAGAAATAAAACAACAACAAAATGAATTAAAGAAA

GTAAAAGACTTTAAAAATTCATTAAATAAAAATATAAATGATATTAAATTTTAAATGAATAAAATAGAAACTTTT
AATAAAAAATCACAATAAATTGATAGATATGTAGTGAAAGGAGTATTCATATGAAAGACAGAGTAATTGATAA
AGTTTATTCTAAAGAAGATATTCGATTAGAACTTAAGTTAACAACACATGCTTTTAATAAAAGAATGGATACAA
TAACTAAATTATTTAAAATTAATATGAAAAAATTTCACGATTTTAAAGGAGAATCGAAGAATAATCAATATACTT
TTAACGGAGTCGCCAAGGAACTATTAGTAGTCCTTTTAAAAAGTGTAGAGTATTATCCAATAGATATTAATTCT
AAAAAATTTAAGACACAGCCCAAGAAAGATATTATTGAAGAAATAAGTAGCACCGATTATACGTTATACATAA
GTAAGTTAATAGAAGCAATTAACGAAATAAAATATAAGAGGTTAACAGCTCATATTAAAATGAGTGATGTATTT
CAAAAAACTAAAGCATGGTTTGATATTGGAAATACATTTAGAAAAAAAGAGCAAGAAATTTATCAATTCATG
GAAACATTACCTTTAAATAAAAGAGTAAATCTACAAAATGAAATACTAAAATCAATAGATGAAGCAATTTTCA
AATTCATAATGAAAGAAGAAAAAAAACAACAAATAGAGAAACAGAATGAAGTTGAAAATTATAAAAAAGCA
ATTTTATCTGGTGAAAATCCTAAAAATGATTACGAACTTAATTATCTTATCTATAAAAAATCTTTATTGCCAGTTA
ATACTGTAATTAAAGATGAATGGGATTTTGAAAGAACAAAACATGAGGAGTTAGATTGGTTTATAGCTGATTT
ACTAAATCACTCACAACGTGCTAGTAGATTACATGCAGAAAAAGTAGAAAATAATAATCAATTAAAAACTACT
ATACACAAAAATGCTGGAGTTAATTTAGGAAAACTTTTGGATCATCAATTAATTAAAAATGATAAGACTTGGG
ATATTATAGAATTTTATTTAAAAGAAAAGGCTTGGAAAAATAGTAAATTAATGGAAAGGAGTAACATTCCATC
AGTATTATATTATTATAAAAATAATAAGTTCTTAAAAAAACAACTAAGTGTTGTCCAAAAAATAAAAACTATCGA
TGCACATTTAGAAAATGCTAATAAATTACCTAAATATATACCTCTCCTATTACAAGTCAAATTTGATTTAGAAAA
AATACAAAGTGAGTTAATTAAATATAGTACTAATCTTAATCATGTTGGTCAATTAGATAAAAATTATAATCGTTAT
ATAATTGAAGATATACATACTGCTATTCAAATTATTGATAAGTATTTAAGTAAAGAAGTTGTGAGAAAAAAATA
CCCTAAGCTCCAAATGGTAATAAGAAATGATATAAAAGATTATGGAAATATGTTCATAGATAAAACAAATAAAA
TGACACGTAATTCTCTTGTTAATAATGAAGAATATGAAGGCTTATTAATTACTCCACTTGTCGAAAAAATTTAAA
GATTCATTAAAAAAACGGAGATTAAATACAAAATAATCGCAGTGTATAAAATACAAATAAGTCAAAATTTAAA
ATGAATTTCAACCTCAATAAAGCTTGATATAGAGCTATTATTGAGGTTTTATTTTTTTTTAGATTTTTCAGCGGC
TCGATTACAGAAGCTAATTTTAGTAGTAAATTATAGTCATCGTCATTAAGATATTAAGACGAAATTTATTACTGG
AGTTGAAACTCAAAATGGATGCAAAAGAAAAACTAGCTTTACAACCTAAAGGTAGCAAAGCAGAAAATTGG
ATAATAAAAAAAGAATCTAATCAACATGAAGTACAACAATTAAAAAATAAAATGATGAATAAATTAAAGAAAA
GATATGAAAGTATACCTAATGAAGAAAGAATTAAGTTTGATGAATATAGTGAACTTCATGAGAGTTTATTACCA
TTAAAACAAGATATAGAATCCATTGGATTTAAATTAAATACTCAAATGCTGTCTAAAGTAAAAACTCAATTTTT
AAATAAAGCTAAAAATAATAGTCACCGGGAAAAATTAGAAAAATACTTTGATGCACTAGAATCAGCCATTGAT
GGTAACCAAAAGTTGGATACTAAAATTTCTTTAATTGGGACTCGCACTCATCGTATAACGACTAAAAATTTTA
ATGTTCAAGGATTACCTAAAGAAGTGAAACAACTTATATTACCAACGCAATTTAATAAAGTATTTATGATTGAT
TTCAAGGCGTTTGAGCCATCTGTTGTAGCTTATTTGGCGGATGATGATCATTTGAAAGAGTATCTCAATGGCA
GTCAGGGCTTATATGACACATTACTCAATAACTTATCTCTTCCAGAAACTCATCGTAAGCTTGTGAAGCGTGCA
TTCATAGGGTCATTCTTATTTGGTGGAAAATTCAAAAGCGATACATTTAAATTGAATCAATATATATCAGAAGA
TGAATGGAATAAAGCAATGAGCAAGTTTAGCAATGTAATCAAGCTAAAAGAACAAATTGATAATAAGAAGAC
AATGAAGATGCCTTATGGTATCACTCACGATATGAAGCATTGCCATGGAAGCAGTATTATGGCGCTATATGTTC
AAACAGCTTCTAGTTATATCTTTAAGAATATCTTATGGGAAGTATATCAACATCAGTGTGATCAAGGGGATTTC
AGAATTATGCTCCCAATTCACGATGCCATTATGATTGAGTGTAATACAGATGAAGTTGCAGAGCGGGTTAGAC
AATTAATGGAAACAAGTGCCAATCACTTGTTTGGAGATAACTTTGCTCATGCCACAATTGAATAAATAGGAG
GAAATCATGATGATAAATAATAGAGGACAAAATTTATCAATACCATCATCATCAGCGCTAGTTAAAGATAATATT
TACGTTGCAAAACTACATTCTGTTGGACTATCAGATTTTGAAGATAATAAGCATGTATTTACTTATGAAGTAGT
TCTACATGGCCAAATATATAAACTTCGAAGAAGTATTGCTTTAAACCCTAGTGATAATCAAATTTCTATTTTTGA
GTGGCTAGAACGCCATAGTAATTATAGTCCTAGTCACACTAAATATGGTTCCTATATAGATCATGAACATTTAAT
ACTTGTTGATGAATACGAAGGTCGTTATTTTGTACGTGATGTAGCACCATTAGAAAATATGATGGAGGATAAA

GTAAATGAATAATACGATGAAATTAGCAAAACAATTATTAAGTGAAAATAAGGAGGCAATCGACCGTAATGG
TCTGATTGCACTTCTTCAATCGCAAAATACAAGCGATAATGAGGAAGATTTACCCCAAGCTGAAGCAATATAT
AACTTATTGATTAAAGAATTAAACTTTGAAATAAACGCTGATATTAATTATCGTTCAAATATTTACGAATATTATG
GTATAAAAAATAAACCTAAAGATACTGATCTTGTTCAAATGGCTACACAAATTTTGAATGTTAAACGTTTTGAG
TCAAATTTATTTGTGTTTTCAAAAGAAGGATGGCAAAAGTTAGATGATAGTGAATTAAGAAGTTTAGTTGGG
AAAACTATTCAGGTTATATTAATTGATTATACACCTACCCAAAGCGAATTGAAGAGTGTCATAGATGGCATGAA
AGAATCCGCAGATATAGATGAGTTGGTAGAAGATGAACGATATATAGGTTGTCATCGCTATTTATTCGATTTAG
AACAGTTCCAAGTCATTAATAACTCAATCGATGTCTTTCCGAAAACACGATTAGATGTAGAACTAGATAAAAA
TGATAGTATTACTTCATTTATACCGCCTCATTTTGATAAATATATGTCAGAATTAGCTAATTTTGATGATGATTTA
AGATATTTCCTTATGCAACATACAGCTGTATTACTCACATCAAATCGTAAATTGCGACGAGGATTAATTTTTTAT
GGTACAGCAAATAATGGTAAGTCCGTATATATCAAGTTAATGAGAGCATTTTTCTATCGTCAAGATGTTGTCTC
TAAGACGTTAAACGAACTAGGTGGAAGATTTGATAAAGAAAGCTTAATTGGTAAGCGATTGATGGCAAGTG
ATGAAATTGGAAAAGCAAGAATTGACGAAAAAACCGTTAATGATTTTAAAAAATTATTATCAGTAGAACCCAT
ACATGTTGATCGTAAAGGACGCAGACAAGTAGAAGTCACATTAGATTTAAAACTTTTGTTTAATACGAATGCC
GTTCTGAATTTCCCTCCAGAACATGCGAAAGCTTTGGAACGAAGAATTGCTGTTATACCTTGTGATTATTACG
TAGAAAAAGCTGATATTGATTTAAATGACAAATTAAAAAGTGAGAAGAAAGATATCTTTTTATATCTAATGTAT
ATATATAAACAAATGATGATCGATGATATCAATAGAATTGAAAATGAAAAAGTAACGGAACTGACTCATGATT
GGCTTAACTTCGGTTACACATTCATGGATAAGTCTAATACATCAAGAGATAAACAAAAAGAATGTATTAAATTA
CTTAGAAATACGATAGTTAAAAAAGAAGGTAGCCGACTTAAAGTATCTGATTTAAATAATAAAATTGAGAAAA
TGAGTCGAAGTAGTTTAAGTAGACAAAACATTAATCAACTGGTGAAATTAAATTTTGATGCTCATACGGTACT
TAATGATGGATATAAACATTGGGTTGACTTAGATTGGAAAACAATACAGGAAGAAAACAATATTATTCCGAAT
GATAAAGAAATCAGTGAGTTGATTTATGAAGATGATCAAGATGATTGGATTCCAGAGGAGGATGATGAGCTA
TGAACCAAGAACAACTTGATGCTTTTGTAGAAATTTTATTACCTATGATAGAAGAAAGATTGAAGTCATAAAA
TGAGTGAGGACACTTTGAATGATAGGTTCATCTTCGAACCTATCTCTTTCTCAAAGTGGTAAAAGTGATAAAA
ATGTAATGAAAAAGAAAATAAATTTATATTATAACGTTGAGGTTGAGCAAGACCTTGAAATAAAAAGTAAGG
AATGATGTTATATGAAGCGCAAAGTGGCTATTTATACAAGAGTAAGTACCAAAGAACAATTGAAACAAGGCT
ATTCTATTCAACAACAAGCAGACTTACTGATTAAAAAAGCAAAGCAAATATTTCCGGATGATGAATATGAAAT
TTATAGTGATGAAGGTATCTCTGGTAAGAATATCGAAGCAAGACCTGGAATGAAAAGATTATTAGAAGATATA
GAAAATCGACAAATTAAGGTTGTGATGAGTTGGAAACTTAATCGTCTTTCACGTTCTAATCGAGATATACAAA
ATATTATTTACGAATTTAGAAGAGGTGGCGCCTATTATATCTCTATTTCAGAAAACATTGATACATCAACACAAA
ATGGCGAAATGATGATAGGTGCATTTGGATTAGTTGCACAGATAGAACGAGAAACCATTGTTAGTAACGTAA
AGATGGGAATGAATGCGAAAGCGAAACAAGGTGAAGCTATTACAGGTAGGGTGCTAGGTTATGTTTTAAAA
CCTAATCCTCTAACAGGTAAAAATGAACTTGTCATTGACGAATTTGAAGCCAATATAGTCAGACAAATTTTTG
ACCTGTATTTAAATCAAAATAAAGGATTAAAAGCAATTGCGAATTATTTAAATAAACAGGGCTATCATACGATA
AATAAAAAGCCATTTAGCGTATATGGAGTGAAATATATTCTGAATAATCCCGTGTACAAAGGCTATGTTCGATT
TAATAATTATCAAAATTGGGCAGTGGAACGACGAAGTGGCAAAAATGATAAAAGTAAAGTGATATTAGTTCA
AGGTAAGCACAAAGCGATTATTGATGAAGAGATTTTCGATAAAACACATGAGAAGTTAGCTGCTAAAAGTTT
TAAACCTGGTCGACCAATTGGTGGCGATTTCTGGTTGAGAGGGTTAATCAAATGTCCTGAGTGTGGAAATAA
TATGGTGTGTAGACGAACATATTACAATACAAAGAAATCAAATGAGAGAACGATTAAACGTTATTACATTTGT
TCATTATTCAATCGTGCAGGTAGTGCAGCTTGTCATAGTAATGCGATTAAGGCAGAAGTAGTCGAACGTGTG
GTATATTTTCATTTAAGACGCATCCTATCTCAACCTCATGTGGTCAAAACGATCGCAACACAGGTTATCGATGC
TATGGAAAAGAAAAAAGCAAACCAAACGCCAATGAGTATTGATACAAATTCATTAGAGAATCAAAAGAAAA
AGTTACAAAAAAGAAAAGAACGGTTGATTGATTTATATTTAGATGAAGAAATAGATAAAGAAACGATGCAAA
ACAAACAAGATAAACTTAATGTGCAACTGGAAAAAATAGATGAACAGTTAAAGCAGGCTGAGGTTATGAAT

AAAGCATCTCAAGATATAGCCATACCTAATTATAAGAAAATAAAAGGTCAATTATATGTGATGCTATACCGATTT
ACAGGCTACATGAAAAAAGCAAATCCCGAAGCAAAGAATAAATTAATGCATATGTTAATCGATTCTATAGAAC
TTACGACCGACAAACAAGTTAAACTGATTAAATATAAAATAGATGAATCTCTTCTCCCACAATCCATTAAAAAG
GATTGTGGGAATTTTTTTATGCCTAAATTTAATTTTGTGATAAATGTCACAAAGAAAAATAGGATTGAAAATTT
ATCACTTTTACCACTTTTTTAGAGTGATAAAAGTGGAGAAACTTTGAAATATTTTATAAATATATATTTTATTTAT
GGAGTACACATTATTAATTAAGGAGGTCATTATAATGACGCTAAGCAAACAACTTAAAATGTATATTACTGAAC
GATTTCAATTAAATCATCAAGAAACGTGGGCTTGTGAAACTGTAGACGCGGTGGCTGAAGATGTATTACCTG
AAAAATATATTAAAAATAGTCCACTCGAACATAAAATATTGAATACATTTACTTATTACAATGATGAATTACATG
AAATCAGTATTTATCCTTTTTTATGTTATCTAGATAAGGAATTAGTAGCAATAGGTTATTTAGATAATTTCGATTT
AGACTTTATATTTTTAAATGACACTCATCAAATTATTATTGATGAACGCTACTTATTACAAAAAGGAGACAAGT
AATATGAACTGGATCAAGGTTGCTCAACTATCTGTCACAGTTATCAATGAAGTGATTGACATCATGAAAGAAA
AGCAAAACGGAGGGAAATAGTATGAAAATCAGTCGATATATTACAAGTGGTGTTAGTGAACAACTATCTCTA
GACTTTCAAATATTACTCTGGCATATGGTAGAAGAAAGAGAAAATCCACCTCATACAGATTACCTACACATTTT
TAAACTACAAGAAGATGATAATTTATTATCCGTTACACATGAACAAGAACAGCCTCCATACAAATTGGAATATC
ACTATATAAACTATGTAAAAAATCAAAATGCATTACCTAAGAAAGTCTACGTCATCCGAGAAGATGACGTAGA
CACTTTTTATTATGTGATGCTTTTGCCCGAAGAATACTAAAGGAAGTGACGATATATGAATACAATCAAAAGTA
CGATACACACAGAAGCGATTTTTAGCGATGATGAACAACACCGCTACTTACTCAAAAAGATTTGGGATGAAA
AGAAACCCGCATGTACAGTGATAACGATGTATCCTCATTTAGATGGTGTATTATCACTCGATCTCACAACTGTT
CTAATTCTTAACCAATTAGCGAATTCTGAACAATATGGTGCTGTATATCTAGTGAATCTATTCTCTAATATCAAAA
CACCTGAAAATCTTAAACATATCAAAGAACCTTACGATAAACACACAGACATACATTTAATGAAAGCAATTAG
TGAAAGTGACACAGTGATTTTAGCCTATGGTGCTTATGCAAAGCGACCCGTTGTCGTCGAACGTGTTGAGCA
AGTGATGGAAATGTTAAAACCTCATAAAAAGAAAGTCAAAAAACTCATAAACCCAGCAACGAATGACATTAT
GCATCCGCTTAATCCTAAAGCACGTCAAAAATGGACATTGAAATAAAGGAGGATTATCTATGAACCATAACAC
TACACAATCAGATTGGCGAATAGTTGCTAATTGTTTAGCATCACAAAATTATATATCTATCGTAAAAGGACTAG
TACATCATTTCGCAGCAATTGAAGATGAAGAAATTCTTGATAAAATCTATGATGATTTTATGAATGATGACTCT
ATAACAACGGTGCTTAACAATGATTTACAGATGATTATTAACCAATACCTATCAAAATGAATATCATACTACTTA
CAATAAAACTAGAGCATCCTTCACTTAACGGTGAAGGATGCTATCTATTTATTTTTTTGTTTATCTTTAATCCGT
TTATTAAAACTGATTAATTTATTATCATTAGTTGAAGTTATTACTCAATTAGAATGATACAATAAAATAAAATTTC
ATAAGTAGAGGTTAATTGATGAAAAGATATGTATATGAAAATAACATTAATTTAATAAAGTCTTTATATGCTAGT
GATTTTTGGACGACTTTGAAAGAAGAAGCGAAGTATTATAAGCGTAATAATAAATTAAAAAAAGATAATTCTT
TAAGTAAACTTAAATCTCTAATTAATGTTATATATATAGATCCTGATGCTGTAGATAAAGCACTTGTAGCCGAAA
TGCAAGATTTCTATAATGAAATGCAAGAAACTCAATATATAAATAAGCCCTATTATCTTAGTATTAATAATCATAA
ATGTAGTCTTGATGCTATCATTGGTTGGAAGACACTCTTTCAATATCATAAAGGAGAAGAAATATGGCTAAAG
GATTTAGCACTTATTAGAGGCAGTAGAATGGGACACTTAGCTTTTCCAGTTCAAAAAAATTCTATTAATCAAC
TTAGAGGTAACTTACTAAAAGATAGAATTGATTACACACTTTTTGATATCAAATCCTTTTATAATCATGAAACTA
ATTTAAGATTACAGAAAGCTTATGAACAAAAAAGCACTCGAGATTGGCTATTGTCCTTTGGATCTTTCAATCG
CTTTATAGACCAAATGAAATTAAACTATTTTGTTTATTCAAATTCAAAAGATCTATCCTCGTATGATGTGATTGA
TCTTTCTAAACCATATAGAAATTCTTTAGACCATTGTTTAGAAGCAATCCCTCAAAAAATAAAAATTAAGGAAA
TTTATATTACTATTATCTAGGTACTAATTTTATCTTTTATTTTAAATTAAAAAGAAAAAAAGTTAAATTTTTATTAA
GTTTTACATGTGACTAATTATTAGCCTGTTAAATGTAGTATACTATTAATATAAGAAAAGTCATCGTAATATTTGT
CAAAAAAAGTGACATTCTATATAGTAGTGTGTCGTTAGTTGTCATTCGATAACCACCCCGAAGAACAAGGGA
CGAGAACAAAGATCATGGTTCTGTTGCAAAGTAAAAAAATATAGCTAACCACTAATTTATCATGTCAGTGTTT
GCTTAACTTGCTAGCATGATGCTAATTTCGTGGCATGGGGAAAATCCGTAGATCTGAAGAGACCTACGGTTC
TTTTTATATAGAGCGTAAATACATTCAATACCTTTTAAAGTATTCTTTGCCGTATTGATACTTTGATATCTTGTCT

TTCTTACTTTAATATGACGGTGATCTTGCTCAATGAGGTTATTCAGATATTTCGATGTACAATGACAGTCAGGT
TTAAGTTTAAAAGCTTTAATTATTTTAGCCATTGCTACCTACGTTGAAGGTGCCCGATCTGTAACTACCTTTTG
AGGTTTACCAAATTGTTTAATGAGACGTTTGATAAACGCATATGCTGAATGATTATCTCGTTGCTTACGCAACC
AAATATCTAATGTATGTCCCTCTGCATCAATGGCACGATATAAATAGCTCCATTTTCCTTTTATTTTGATGTACGT
CTCATCAATACGCCATTTGTAATAAGCTTTTTTATGCTTTTTCTTCCAAATTTGATACAAAATTGGGGCATATTC
TTGAACCCAACGGTAGACCGTTGAATGATGAACGTTTACACCACGTTCCCTTAATATTTCAGATATATCACGAT
AACTCAATGTATATCTTAGATAGTAGCCAACGGCTACAGTGATAACATCCTTGTTAAATTGTTTATATCTGAAAT
AGTTCATACAGAAGACTCCTTTTTGTTAAAATTATACTATAAATTCAACTTTGCAACAGAACCGTATTATGGAA
TAGAGATGTTGGTAACATTTATACAGGATCATTATACTTAAGTTTAATTTCGTTATTACAGAACCACACATTCCA
ACCAGAAGAGAAAGTATGTCTATTTAGTTATGGTTCAGGAGCAGTAGGAGAAATCTTTAGTGGTTCAATCGT
TAAAGGATATGACAAAGCATTAGATAAAGAGAAACACTTAAATATGCTAGAATCTAGAGAGCAATTATCAGTC
GAAGAATACGAAACATTCTTTAACAGATTTGATAATCAAGAATTTGATTTCGAACGTGAATTGACACAAGATC
CATATTCAAAAGTATACTTATACAGTATAGAAGACCATATCAGAACATATAAGATAGAGAAATAAACTAGTGGC
CGATTGTGCTTGATGAGCTTGGGACATAAATCCTAACTCGAAATAAATAAGCATATCACTAAACTGATTTTTTT
AAAGTTTACAGTGATATGCTTATTTTTTTATCTTACGATTTTGTACGTGCATGCTTGCCTAGGGGTATGGCTCG
AGCCATTAGTCTCTCGCACATACTATTCCCTCAGGCGTCAGCACTTACAAAATCGGTTGTAATTTTCATTTTTAT
ACGCATTCTTACTGAGATTATACTAATAAGAGGAATAGTAAAAGCAATTCTAAGTAAAATTGCAGATAAGAGG
TTTGTTAAAAGCAGTTCTAAGTAAAATTGCAGATAAGAGGTTTGTTAAAAGCAGTTCTCAGTAAAATTACAG
ATAAGAGGTACGTTAAAAGCAGTTCTAAGTAAAATTGCAGATAAGAGGTTTGTTAAAAGCAGTTCTAAGTAA
AATTGCAGATAAGAGGTACGTTAAAAGCAATTCCATGCAAAATTGCTGATAAGGGGTAAGTTAAAAGCAGTT
CTCAGTAAAATTGCAGATAAGAGGTACGTTAAAAGCAGTTCTAGGCAAAATTGCAGATAAGAGGTGCGTTA
AAAGCAGTTCTCAGTAAAATTGCTGATAAGGGGTAAGTTAAAAGCAATCCTAAGTAAAATTGCAGATAAGAG
GTAAGTTAAAAGCAATCCTAAGTAAAATTGCAGATAAGAGGTAAGTTAAAAGCAATCCTAAGTAAAATTGCA
GATAAGGGGTACAGAAAAACTAGACTTGATTACAAAATGGAGCTTGGGACATAAATGATTTTTTAAAAATGA
GATGAGACGTAGATTAACTCCATAATCAATACGAATCTATCGACTTCTTTATTTATGATATTCATCTCTTTTTAAT
GGAAATAAAAGTGCGATTAATGTGATAATACAGTTACGTTAATTAAAAAAATAAAAATGCAAGGAGAGGTAA
TATGCTAACTGTATATGGACATAGAGGATTACCTAGTAAAGCTCCGGAAAATACAATTGCATCATTTAAAGCTG
CTTCAGAAGTAGAAGGTATAAACTGGTTGGAGTTAGATGTTGCAATTACAAAAGATGAACAACTGATTATCA
TTCATGATGATTATTTAGAACGGACTACAAATATGTCCGGGGAAATAACTGAATTGAATTATGATGAAATTAAA
GATGCTTCTGCAGGATCTTGGTTTGGTGAAAAATTCAAAGATGAACATTTGCCAACTTTCGATGATGTAGTAA
AAATAGCAAATGAATATAATATGAATTTAAATGTAGAATTAAAAGGTATTACTGGACCGAATGGACTAGCACTT
TCTAAAAGTATGGTTAAGCAAGTGGAAGAACAATTAACAAACTTAAATCAGAATCAAGAAGTGCTCATTTCA
AGCTTTAATGTTGTGCTTGTTAAACTTGCAGAAGAAATCATGCCACAATATAACAGAGCAGTTATATTCCATAC
AACTTCGTTTCGTGAAGACTGGAGAACACTTTTAGATTACTGTAATGCTAAAATAGTAAACACTGAAGATGCC
AAACTTACTAAAGCAAAAGTAAAAATGGTAAAAGAAGCGGGTTATGAATTGAACGTATGGACTGTAAACAA
ACCAGCACGTGCAAACCAACTTGCTAATTGGGGAGTTGATGGTATCTTTACAGACAATGCAGATAAAATGGT
GCATTTGTCTCAATAGAAAGTTAGAGGTGAGTCTTACGTTTCAGTGACGGTAGACTTACCTTTAACATGTTAC
ATACTAAAAAATTAATTTGAATAAGAAAGAGAGACATATATGAAATACGATGATTTTATAGTAGGAGAAACATT
CAAAACAAAAAGCCTTCATATTACAGAAGAAGAAATTATCCAATTTGCAACAACTTTTGATCCTCAATATATGC
ATATAGATAAAGAAAAAGCAGAACAAAGTAGATTTAAAGGTATCATTGCATCTGGCATGCATACACTTTCAAT
ATCATTTAAATTATGGGTAGAAGAAGGTAAATACGGAGAAGAAGTTGTAGCAGGAACACAAATGAATAACG
TTAAATTTATTAAACCTGTATACCCAGGTAATACATTGTACGTTATCGCTGAAATTACAAATAAGAAATCCATAA
AAAAAGAAAATGGACTCGTTACAGTGTCACTTTCAACATACAATGAAAATGAAGAAATTGTATTTAAGGGAG
AAGTAACAGCACTTATTAATAATTCATAATAAAACAGTGAAGCAACCATCGTTACGGATTGCTTCACTGTTTTG

TTATTCATCTATATCGTATTTTTTATTACCGTTCTCATATAGCTCATCATACACTTTACCTGAGATTTTGGCATTGT
AGCTAGCCATTCCTTTATCTTGTACATCTTTAACATTAATAGCCATCATCATGTTTGGATTATCTTTATCATATGAT
ATAAACCACCCAATTTGTCTGCCAGTTTCTCCTTGTTTCATTTTGAGTTCTGCAGTACCGGATTTGCCAATTAA
GTTTGCATAAGATCTATAAATATCTTCTTTATGTGTTTTATTTACGACTTGTTGCATACCATCAGTTAATAGATTG
ATATTTTCTTTGGAAATAATATTTTTCTTCCAAACTTTGTTTTTCGTGTCTTTTAATAAGTGAGGTGCGTTAATAT
TGCCATTATTTTCTAATGCGCTATAGATTGAAAGGATCTGTACTGGGTTAATCAGTATTTCACCTTGTCCGTAAC
CTGAATCAGCTAATAATATTTCATTATCTAAATTTTTGTTTGAAATTTGAGCATTATAAAATGGATAATCACTTG
GTATATCTTCACCAACACCTAGTTTTTTCATGCCTTTTTCAAATTTCTTACTGCCTAATTCGAGTGCTACTCTAG
CAAAGAAAATGTTATCTGATGATTCTATTGCTTGTTTTAAGTCGATATTACCATTTACCACTTCATATCTTGTAAC
GTTGTAACCACCCCAAGATTTATCTTTTTGCCAACCTTTACCATCGATTTTATAACTTGTTTTATCGTCTAATGTT
TTGTTATTTAACCCAATCATTGCTGTTAATATTTTTTGAGTTGAACCTGGTGAAGTTGTAATCTGGAACTTGTT
GAGCAGAGGTTCTTTTTTATCTTCGGTTAATTTATTATATTCTTCGTTACTCATGCCATACATAAATGGATAGAC
GTCATATGAAGGTGTGCTTACAAGTGCTAATAATTCACCTGTTTGAGGGTGGATAGCAGTACCTGAGCCATAA
TCATTTTTCATGTTGTTATAAATACTCTTTTGAACTTTAGCATCAATAGTTAGTTGAATATCTTTGCCATCTTTTT
TCTTTTTCTCTATTAATGTATGTGCGATTGTATTGCTATTATCGTCAACGATTGTGACACGATAGCCATCTTCATG
TTGGAGCTTTTTATCGTAAAGTTTTTCGAGTCCCTTTTTACCAATAACTGCATCATCTTTATAGCCTTTATATTCT
TTTTGTTTTAATTCTTCAGAGTTAATGGGACCAACATAACCTAATAGATGTGAAGTCGCTTTTTCTAGAGGATA
GTTACGACTTTCTGTTTCATTAGTTGTAAGATGAAATTTTTTTGCGAAATCTCTTAAATATTCATCCATTTTTTTA
ACGGTTTTAAGTGGAACGAAGGTATCATCTTGTACCCAATTTTGATCCATTTGTTGTTTGATATAGTCTTCAGA
AATACTTAGTTCTTTAGCGATTGCTTTATAATCTTTTTTAGATACATTCTTTGGAACGATGCCTATCTCATATGCT
GTTCCTGTATTGGCCAATTCCACATTGTTTCGGTCTAAAATTTTACCACGTTCTGATTTTAAATTTTCAATATGT
ATGCTTTGGTCTTTCTGCATTCCTGGAATAATGACGCTATGATCCCAATCTAACTTCCACATACCATCTTCTTTA
ACAAAATTAAATTGAACGTTGCGATCAATGTTACCGTAGTTTGTTTTAATTTTATATTGAGCATCTACTCGTTTT
TTATTTTTAGATACTTTTTTTATTTTACGATCCTGAATGTTTATATCTTTAACGCCTAAACTATTATATATTTTTATC
GGACGTTCAGTCATTTCTACTTCACCATTATCGCTTTTAGAAATATAACTGCTATCTTTATAAACTTGTTTGAAA
TTTTTATCTTCAATTGCATCAATAGTATTATTAATTTCTTTATCTTTTGAAGCATAAAAATATATACCAAACCCGA
CAACTACAACTATTAAAATAAGTGGAACAATTTTTATCTTTTTCATCAATATCCTCCTTATATAAGACTACATTTG
TAGTATATTACAAATGTAGTATTTATGTCAAAATAATGTTATAATTTTTGTGATATGGAGGTGTAGAAGGTGTTA
TCATCTTTTTTAATGTTAAGTATAATCAGTTCATTGCTCACGATATGTGTAATTTTTTTAGTGAGAATGCTCTATA
TAAAATATACGGTTCTGTTGCAAAGTTGAATTTATAGTATAATTATAACCAAAAGGAGTCTTCTGTATGAACTAT
TTCAGATATAAACAATTTAACAAGGATGTTATCACTGTAGCCGTTGGCTACTATCTAAGATATGCATTGAGTTAT
CGTGATATATCTGAAATATTAAGGGAACGTGGTGTAAACGTTCATCATTCAACGGTCTACCGTTGGGTTCAAG
AATATGCACCAATTTTATATCAAATTTGGAAGAAAAAGCATAAAAAAGCTTATTACAAATGGCGTATTGATGA
GACGTACATCAAAATAAAAGGAAAATGGAGCTATTTATATCGTGCCATTGATGCAGAGGGACATACATTAGAT
ATTTGGTTGCGTAAGCAACGAGATAATCATTCAGCATATGCGTTTATCAAACGTCTCATTAAACAATTTGGTAA
ACCTCAAAAGGTAATTACAGATCAGGCACCTTCAACGAAGGTAGCAATGGCTAAAGTAATTAAAGCTTTTAA
ACTTAAACCTGACTGTCATTGTACATCGAAATATCTGAATAACCTCATTGAGCAAGATCACCGTCATATTAAAG
TAAGAAAGACAAGGTATCAAAGTATCAATACAGCAAAGAATACTTTAAAAGGTATTGAATGTATTTACGCTCT
ATATAAAAAGAACCGCAGGTCTCTTCAGATCTACGGATTTTCGCCATGCCACGAAATTAGCATCATGCTAGCA
AGTTAAGCGAACACTGACATGATAAATTAGTGGTTAGCTATATTTTTTTACTTTGCAACAGAACCTCTTTTATC
ATATTAGCACTATTTGATGTTGAATATTCAGGTCCTATCCATGCTGTTTTTGGTCGAACTGTTCGAATGTCATCA
CCCTCACCTAATCGCATTTTTATATAAGGCATATATTGTTTTTTAGAATTCATTTTAAAATCTAATTCTGAATCTTT
TTTAGAAAGAAAAGTATCCTTACCCCAACTCCATCTTCCATCTGTACCATTAGAATGAATTGGTAATATTTCAAT
ATATTCATCAGATAATTTATCTAAACTTGAATGATTAGTTTTTGGATTATAATAAATAGGATAATATAAGTTAGGT

CGATCGCTTCTTAATGAATTTTTTCCTGTCTTTTTCAAAGCAACATGTTTATATCTACCAATTGTATCTTTAAATT
TAAATTCCTTTATATAATCCTCTGAAACAGGTAATCCATTAATAATACATTGAGATGACTGTTTAGCATAAACTT
GCATAAATTCACTTGATGTTGCAAAAAATTTTGAATCAGAACGTCCTTTCATATTATTTACAACCGTTATAGTAC
CTAAATCATTCTCTTCCCCAAACACTTCATCCATCAATAACTTTAAGTTCGCTTGTTCGTTATCATCAATCGATAC
AAAAATCACACCTGCATCTGTCATTAAATCTCTTGCTACTTTGAGTCGTTTTTCCATAAATGATAACCATTTACT
ATGTCTAAAACTATCTTCTTTATCTACGATTTTGTCATTATAAACAAACTCATTCCCTGTATTATAAGGCGGATCG
ATATAAATGACATCTACTTTTTTACCATCCTCACGCATTGCAATGAGTGATGCTAAATTATCACCCTCCATTAAA
ATGTTGTTCGTGTGACCATAGCCAGAATCAATACGTAATTCTTTTTCTTCACGTAATACCGGACGGTTATATTTC
ATACGTTCTTCAATATGTTCAGGGTGATCTTCCCAAATAAGTCCGTACTTAGGTTGTTTGATTGCATCTAACAC
ATCTTTAAAATCCTCTGGATTTAATCCTTTTTCTTTAAGTCTCTCTATTAATAGTTCGTTAACATTATCTTTCATTT
TTTCAGTCCTCTTTTCAATACGTTTTCTTTTCTACATCTCTATTATAATATAAATTACGAAAATAATAAGATTTACA
AGGTTAAAGCCTATATTAATGGATCATATGATAATTACAAATATCATTACGTATCACCGATCCGTTGTAGAGAAG
TCAATGAAGATGACACATATAATGCTATGCAAGAATTAGAACGCATGGTAGATGAAAATGCTCACTCAATGAG
CGTGGCTCAATATAACGAGTATAAAGAAAACCGTAAAGTATTAGAGAAAGAATTTAACTCAACAACACCGTT
ATCATTTGTTGAATGTAGATTTCTTACTCAAGTTCCGCATGGTTATGAACCTACACTTTACATCGAACGTCATAT
GCTTGAACCGTTAGATACAGAAGAATCTTTAAAAGTGATGCTAAATGAATTCAGACGTATGGCTGATACTAGA
CCACGTCATAATTATAATAGTCATTTAAATACAATTGTAGAAGATGCTTTAACTGAGATTGTAGAGGGTATATAA
ATAAATGAGGCTATATCAATTGATATAGTCTTTATTTTTCTGTAATATTTAAATATTATCTATTGTGAGAGGTGGT
GACTTTAATGAATAAAAATGGATTATTCAACACCATTATTAATAATTATAATCAAATTTCAGATGAAAGCTATATC
CAAAAATCAGACACAGAAGTGAAACCTAATGGGAACACTGAGAAAAATATTATGAATGATCTTGGTTCTGTT
GCAAAGTTGAATTTATAGTATAATTATAACCAAAAGGAGTCTTCTGTATGAACTATTTCAGATATAAACAATTTA
ACAAGGATGTTATCACTGTAGCCGTTGGCTACTATCTAAGATATGCATTGAGTTATCGTGATATATCTGAAATAT
TAAGGGAACGTGGTGTAAACGTTCATCATTCAACGGTCTACCGTTGGGTTCAAGAATATGCACCAATTTTATA
TCAAATTTGGAAGAAAAAGCATAAAAAAGCTTATTACAAATGGCGTATTGATGAGACGTACATCAAAATAAA
AGGAAAATGGAGCTATTTATATCGTGCCATTGATACAGAGGGACATACATTAGATATTTGGTTGCGTAAGCAA
CGAGATAATCATTCAGCATATGCGTTTATCAAACGTCTCATTAAACAATTTGGTAAACCTCAAAAGGTAGTTAC
AGATCAGGCACCTTCAACGAAGGTAGCAATGGCTAAAATAATTAAAGCTTTTAAACTTAAACCTGACTGTCAT
TGTACATCGAAATATCTGAATAACCTCATTGAGCAAGATCACCGTCATATTAAAGTAAGAAAGACAAGATATCA
AAGTATCAATACGGCAAAGAATACTTTAAAAGGTATTGAATGTATTTACGCTCTATATAAAAAGAACCGTAGG
TCTCTTCAGATCTACGGATTTTCCCCATGCCACGAAATTAGCATCATGCTAGCAAGTTAAGCAAACACTGACA
TGATAAATTAGTGGTTAGCTATATTTTTTTACTTTGCAACAGAACCGGAATAAAGATATTTTTGAAACAGCAAA
ACCTTTACAACATATTGTTGATGTTATTAGAGTGGCTACACCAAACAAAAACGCCAAAGTTTTAGACTTCTTT
GCCGGCTCAGGGACGACGGGACATGCAGTGCTCGAATTAAACAAAGAAGACGGAGGACATCGACAATTTA
TATTATGTACGAATAACGAGAATAATATTTGTCGGGATGTCACATACGAACGTTTAAGACGAGTGATTAATGGT
TACACGACACCAAAAGGTAAAGAAATTGAAAGATTACCAGCAAACTTAATGTATTTAACAGTTGGTGAAACA
CCTAAAGAAAATAATGATGTGATGTTTGATATGGATGAAAATGAGCCATTAAGACCACATATTATTAATAATTTA
CGATTAAAATATCATCTTCACCACCCAGATGAGTTAGTAGAAAATTTATACCGACTCAGAAATGGAAACACAG
TGCATTACGTATATTTCAATGATTATATTGAAGATGGCGTTGAAGAAGCGATTTTAGAATCACTAGATCGTGAT
AAAACAAACATCATGCATACAGTAGAAGGACTGGTTCATGATGAAACATTTAGTATGATGGATGACGTAAAA
GATATAGCAGGATTATTAAACGAGTAGGAGTGAAAATTTGTGTTAAATAATATTAGATATCAAGATAAAGCTGT
AACAGAGTTATTAAAACGAGTCGATAACTGTTTAGATGAGAAAGAACGTGAGTTTTTATTCAAAGCACCAAC
TGGTGCAGGTAAAACAATTACAATGGGTAAATTTTTCGATGCGTATTTTAAAAAACATGAAGATGAATCAATT
GGATTTATCTGGTTATCACCGGGTAAGGGGAATCTTGCTGGACAATCAAGAGATAGCATGGCTAAAACATTT
ACTAATTTCAACAGTATATCACTTCAAGATATGCTGTTGAACCGTGAGATAAGAAATAAAGATGTTGTCTTTAT

TAACTGGGAAGCGGTCAATAAAAAAGGAAATGTATCCATGCGTGAAGGTGAAAAGCTCAATGTAAAAGGA
GCTCTTGAGAATTCGAATCTTGATAAATTAATTATTGTGGTGGATGAATCACACGAAGCAAGAAATGCAACAA
AAGCGATGGAAGTTTTAGAAACGTTTAATGGAGATATTGTTATTGATGTGACAGCGACACCTCGTTCAAACT
CAAGTTACAAAGATAAATTCAATGTAGTAAAAATTGAAATCGATGATGTAATTGAAGAGGGGTTTATTAAAAA
ACAAGTGGTTTTAAATGAAGGACTTTCATCACTCGATACAATGGATGTATTAAAAACAGCCATTGATAAGCGA
GATCAAATTGAACAAGCCTACAAGCAATATGAAGATCATGTGAAAACACCGCTTGTATTAATACAAATAGAAA
ATGATAAAAAAGTGGATGCAGGAAATGGTGTTAAAAAACCACGAGCAGAAGTAATTAAAGAGTATTTAGAT
CGACTTGGTTTAAAAGATGAAGAGATTGCTGTTTGGGTGAGTAATAAAAAACAATGTCAAAATTTAGAAGG
TATTAAACATTCAAATGTTAAAGTGTTAATTTTTAAATCAGCAATCGCAACGGGGTATGATATCGATAGAGCTC
ATATTCTTGTGAAATTAAGAGATGCAAAAAGTGAAGTGTTTAATGCACAAGTGTTAGGACGTGTTCTTAGAA
CACAGTTCAAACAATTTTATGATGATGATTTAGTTGATTCAGCATATGTATATACTGAATTTTCAACATATGATTA
TGAATTAGATATGGACGATGACTTAAAAGAACGTCTTAAAAAACCACGTTCTGAAGCGTATTTGAAAGCTGA
GGTAAAAAAAGATTTACCATCATTTAGTATTCCAATGGAGAAAAAACTGTCACAAAAAGACCATAAAGTAGA
TACTCGTTTACTTAGAAAACGTATAGTAAAAGAGCTATCATCAGATTTTATCGCATCATTGGATTATGATCGTTC
TGTAATTTCAGAACATGTAAAATCAGGCACATTATCAATTAATGATATGGAATCAAATGATTATTTTGAAGATTT
AACAGAACGACAAGTTGTAATGACAGATTTACAAGTACATAGATTGGCACGTAAGTCATTACGACAACTATCA
AAATATATAGACATTGGACACATGGTATTAGGTATTTTACAAAAAGACGACCGTCTCAAAAAAGTGTCTGATC
CAAGTTGGTTCTATATCCAGCATAAAGATACTATTAATAATAAATTACTAGACGCTTTAGCTGCATACTATGAAA
TCAATTTTAAAAAGGCAACTGTTGATAAAATGTACCAACCTCTTCACTCGGTTTATTATAGAGGTGTTCAAAAT
AAAACGAGTGATAATTATGCGTATACATTAGAACCTGATTTATCAACGGCTTCAACCAAGTCGTCATCTGAAG
AAACATTCGCAGCATTTTTAAACCAACATCCAAACGTTTTATATTGGTATAAAAATGATAATAATGGAGATCAT
TTTAGTATTGCGTATGAATCAGATGATTACAGTGCAGCATTATATTATCCTGATTTCATAATTATTACGCGTGATA
TGAAATTAATTATTGTAGATGTGAAAACTCCTATTAAAGGTAGCAAGTCACAAGATATTAGAGATGTGCGATC
TAAATATAATAATGGGAAAAAGTATGAATATAGAGAGCAAGAAGCAATCGAAAATGCAGGGTTTAGTGATAT
AGAGTTTTCAATGATAAAACTAAATGGGGATACACCTTATATTTGTGTAACAGATGAATATTCAGATAAATTCA
CAGATGATGATATTTGGAAAATATTCAATCCATAAAAAAAGAGATCTAAAAGATCTCTTTTTTTGTGTTTAAAT
TTCGTGATAGATAGTATCGACAATAGTTCTAATGGATAACTCATCGACACTACCCAGTACTCGGACGACTTTGG
CATTGCTAATTAAAAATTGAACATCATGCTCTTCAATAAAACAATCAGACACTTCAAAATAAACAGTTTCACCT
GTTTTATTAAAATCATCTTCTATTTTGTTTAACACACTTTTCATTAATTTTTGTTCTCTACTTTCAACAATCACTCT
AACCACTCCTAATTATCAATTTGGTATAACCTAGAAACCGAAGTGTCTGAAAGAAACGAAGGTTTAATTTAAG
GTTTTAATGTAATGAATGTATCGCTCATTTACTCTGGGGTAAACATTTCAGCTTTAATGAGGAATAAAGAATCA
TCCGGCTCATTCTTTAACACATCTAGTTTATTTAAATATTCTTTTGCCATAAGTCTTTCATCCTCTTCGTCTTTTA
AAGGAGGTTCATTATGAATAAGGGTGTTTAAAATATCACATACATCATTGAGTCGATCCTTTGTAACAGGTAA
CAATGTATGTTGTATGATGAATTGTTTATTAAATGATCGTTGGAAGGCTTCTATGATGAATGGATTGTCATAAA
AAGCAATGAGTCTATCTTCTTTATCCATGTCAAAGAATGGTTCATTAATACCCTTACCTATATTTTGTTTAAGAG
TGTTTATAAGACGATAATCAGTAGTAGTTGGACTTGAGATAAAATCTCGTTTTAATGAATTAGGTGTCAATGTA
GAGTAATATGATATTCTATCTTGGTGTTTTTGTTTTTCACTATTTATATTACGTTTTGCTTTTATAAATAAATATTT
ATCAAGAGAACCGTTATTATATTGGTATTCTTGAATTTTAAAATTAGTAAGTCTTGTAAGAAAATCATTATTTTC
TTCTGCAATAAGTAAATCTTTTAATAAAGAAATAGATCCGCTTTTAATTATACAGTCAATTGTGTATAAAACCTT
ATTTAATGAACCCTTATGAATATAATCTTCAATATTGATATTGTTTGGATGATGTGTGTAAGTATATAAGTTTAAA
TCCATAAAAAAACTCCTCCTAATAATTGATAAATAATTTGTCATTTTATAAATACATCTTTTTTTGGTAAAGATTA
TAATTCACTTCGATCACATATGACGAACCATGTATCATCTGGTACATGTGTTAATGATTCTAATTTATATAAAAAT
TGATTTGCTTCTTTTTGTTTACTACGATCACTTATTAAACTTAGGTATTTTGGATCATTAATGTAAGTGTTTAATA
AATAGTACACTTCATTACTTCGATCTTTTGTAGCAGGGTATATTGTATGGTCGGTTGTGAAATGTACATTAAAC

GAATCTTCAAATGCATCTGTTACAAATGGATTATCATCGAAAACTGCAAGGATGTGTTCAAAATTTAAATTAAA
AAGAGATTGCGTCATATATTAACCAATTGAATCTCTAAGAGACACGAGTAATGGATCATTGGATTCGATAACC
GCCCCGAAGAACAGGGGACGAGAATACTTCTGCCATATCATTAATGATTTCATCACATTGATTCGATAACTAC
CTTGTAAAAGAGGGGGGACGAGGACCTCATATTGATGATCCCGATTGGCTTGGTTAAAAAGAAATCGATAAT
CCTCGTAGAATAGAGAAAAAGTATTAAAAATATATTTGGTTTGATAAAATATTCTGCAAAAAAAGAGGAACAA
AAAATCAAGATTTAAACTGTAATTCATTATTTTAGAAATTAATCGACATTCAATTTCTAAATAAACAGAAACAA
AAATAACTTTCATACAAATAATTTCTTTGCGATTAAGTTGAAATTAGAAATACTAAATACAAATAACAGCGTAT
GGAATGTATATTATTGAGGAATTGGATGTGTAGTGCAATTTCGAGAGTTACTACTTAAGAAAATAAAAAGAAG
AAAACAAACAATAAGAATTAAATCTGAAGCAAGTTATAAAAGCAAATATTTTTGCTATTATATTAGTAAATACT
TTATTAATTAGTATACGACTGAAAGCGAAGTAACAGCTAATAATGTTCCAGATATAGACATTAGTGTTGTGAAA
GAACAATATTACCATCTAGAAAATGTAATTTTTTAAAAACCTGAAGATAAAGTAGACTATTCTGTGAGTTTTCA
TGTTAAGAATTCTGAACTTGGGGATGTAGTATAAATATGGTTTAGTAGAAAATAACGCCGGAGCTGTAGCTGA
GATTGACTATCAAGAATTAACTAAAGTAGGTTATTACCCATCAACAAAAATCCAAGATTTAACGCTATCTAATA
ATAGTGCGAATAATGAAGTTTAAAAGTGAAAAACTGATAATAGCGAATCAAACTAAAGTAATCAATTACAGAA
TTCAAGTACTACTCAAGCCTCAAACAAATATTGAAACTCAAACACAAGTTAAGAATAATACACTGCCAGAAAC
AGATGAACAGTCTAATTCCGATTTAGTTCAATAATAGCCTCTGTATTATTAGCCGCACGATTATTATCAATAATTA
AATAAAGCTAATATTAAATAATAAAAATCCTTCAAATTATTTGTTGAAAAAATAATAACTTTGAAGGTTTTTTAG
CAAAATCACTGATAAGCAGAGGCGTATCATAAGT
>Staphylococcus aureus strain BDH17
ATGAAAATCACCATTTTAGCTGTAGGGAAACTAAAAGAGAAATATTGGAAGCAAGCCATAGCAGAATATGAA
AAACGTTTAGGCCCATACACCAAGATAGACATCATAGAAGTTCCAGACGAAAAAGCACCAGAAAATATGAG
CGACAAAGAAATTGAGCAAGTAAAAGAAAAAGAAGGCCAACGAATACTAGCCAAAATCAAACCACAATCC
ACAGTCATTACATTAGAAATACAAGGAAAGATGCTATCTTCCGAAGGATTGGCCCAAGAGTTGAACCAACGC
ATGACCCAAGGGCAAAGCGACTTTGTATTCGTCATTGGCGGATCAAACGGCCTGCACAAGGATGTCTTACA
ACGCAGTAACTACGCACTATCATTCAGCAAAATGACATTTCCACACCAAATGATGCGGGTTGTGTTAATTGAG
CAAGTGTATAGAGCATTTAAGATTATGCGTGGAGAGGCTTATCATAAATAAAACTAAAAATTAGATTGTGTATA
ATTTAAAAATTTAATGAGATGTGGAGGAATTACATATATGAAATATTGGAGTATACCTTGCAATATCATACGATG
TTTATAGAGTGTTTAATAAACCATTTTTCAACTATTGATGATCTAGAATATATAATAACTGTACAAATTATATTGAT
TATGGAACTACAATTCAATTAAGAAATTGATGATGAAATTTTAAATTTAAACTAATGGAATCAAGAAAGAATG
AAAGGAAATATACAATGCCCACGATTAATAAAAGGAAGTTTATTAGATTTTGTGTTAGAAACAGTAGATGTTT
TTGTAAATGGAAGTAAAAATGATTGCGAATTAGTAAATTTTAATTATAAAGAAGGAAAAGTAGTTTCACGTTA
TAGTACAAAATTTGAAAGCAATCCTAAATTAAGAAAAAAGCAATTGATATCGACGGTAAAAGTTGTAAGGTAT
GTTATTTTTGATTTTGAGAAAATTTTATGGGGCATAGGGAAAGTTTTATTGAAGTTCATCATATTAAGGCAATG
TTTACTATAAGAAAGTTGAAATACACTCAGAAACAGACTGGATTCCCGTTTTTTTCAAATTGGCACAAAATGA
TACATAGGCCCACCTATATATTGAAGATTTAAGTTAAACGATAAATAATATTTTTCTCTTTTGTATAGAAATTTTT
TTGGGAATGATATTAATTAGTATAAAAATGAATATGTCTACAAAGAAGGAGAGATTTTATTGAAACACATTAAT
CAATTTTCTGAATCGACACTAGCACAACTAGAAAAAGATCAACAACACGTATTTTATGTATACTGTTTGATGG
ACCCAAGAAATGATGAATGCTTTTATGTTGGTAAAGGTAAGGGGAATAGAATATTTAAACATAAACAAGATG
CGCAGAAAAAAGTATTGTACGAAGATATATTTATAGAAGAAAATAAAGATAACTTGAAATTTAATAGAATTAA
CGAAATTTGTAGCAATGATTTGAATGTTTTAGGATATATAATCAGTTATGGATTAACCGAATCTGAAGCATTTT
CTGCAGAAAATGTTCTTATAAACTTTCTTAACCTAACAAATAAAACTACATTAACAAATATGATTAACGGTCAT
GGATCTAAGGCATACTTAGTTGAAGATTTAGAAAATGAATTTGGCTACGATTCAATTAATCTTGAAAACATAA
ATACGAATGAATTAATTTTAGCAGTGAAAATCAGAGATGCATTTCTATTAGATAAGGATGAAAGTAAAGAGTA
TCCTATTAATGAAAGTAAACGTGATAGAAATAACCTTAAATCGCGTACATTAGGTAGTTGGATAATAGGAAAA

GATAAAATACATAAAATAAAATATATTATTGGTATCAATACAGGTGCTAATAATGCAGTTGTCTCAGCTTATGAA
GTATCATTTGAACAAGCGGAGAGTATCGAAACAAATAATGGTAGAATGAGATATGCGTTTATTGGGCTTTCA
GAAAGAGATGCTACTCTAAAAAAATTGAATTTATACAAAAAAGCACTACCAGATTTAAGATTTGGTAGTGGTA
GCGCTACAGCATATATAAATAATGGGACAATGAAAGTTGATTAAATGAATAAAAACATCCAATTCCTTAAATTA
GCCTTTGAAAATTTTATACTCGTATATATAAGTGAAAAACTTAAAAGAATTAGATGTTAATTAAAGGAGAGAAT
GCTATGCCATCAGATGATATTGTAAAAAAACTATACTCAAAAGAGGATATTCGATTAGAATTAGGACTTACTCC
GCATAAGTTTAATAAAAAAATGGAAACTATTGCTAAGCTTTTTAAAATTGATATGAAAATTTTTCACAATTACA
AAGGCCAAGATAAAAATAATCAGTATACATTTAATGGTGTCGCAAAAGAATTAATCAAAGTGTTGCTAAAAAG
TGTTGATTATTACCCAGTGGATATCAATTCCAAAAAATTTAAACAAAATGGAAAATCTAAAAAAGAAATGATT
GAAAATATAGATAACTCTAGTTACATGAAATATATTTATCAGTTAATGAAATCTATCAATGAAATTCAATACAAA
AGGTTAATTGCTGATATACATATGAAAGATGTGTACCAGAATACAAAAGCATGGTTAAATAATGGTGAATCAAT
TAATAAAAAAGAACAAGAGCTATACCAATATATGACAATTTTACCATTACATAAAAGAGTTGAACTGCAAAAT
GAGGTATTAAAATCTATAGACGAAACAATTTTTCAATTTCTCGCAAAAGAACATAGAAATAATCAAATTGAAG
AAAATAATGAATTAGAAGCATATACAAAAGCAATAAAAGAAGGTAGAAATCCTAAAAATGATTATGAATTAAA
TCATCTCCTATATAAAAAAAATCAATTACCCTTAGATAGTCTAATAGAAGATATATGGGACTATGAGGAAACAG
AGTATACTGAGCTTGATTGGTTAATTGCAGATATGCTAAAACGTTCACAAAGAGCTGATAGTAATTTTATCGA
AAGTTTAGAGAAGAAAAACAAATTGAGAAAAAATATTCATAAAGATTCCATTAAAAGTATATCTAAGTTGATT
GATCATGAATTAGTTAAAAATAATAGAAAGTGGGACGTAGCAGAATTTTATAAAAATCAACAATTTTGGAATA
ATAGTACATTAAGACGTAGCTCTCAAAATTTTGGAATTTTATATTACAAAAAGAATAGGTTACATACAATGAAT
CAAAGTAATATTATTAAGAAAATAAAAATTATCGAAGCTAATATAGAGAGTGCAAATAAACAGCCGAGCTATT
TAGATTTCTTTTTACAAGCTAAGTTTGATTTAGAGAGGTTAGAAAGTGAATTAATGAAATATAGCATTAATCCG
AAATATTTTAATAAGATAAATAGTGATTACGTAAGATATGCTATTGAAGATGTACATAATGCAATTATTAAAATT
GACAGATATATAAGTAACGATAAAGTGAAGTTAAACTATTCAAATGTCCAAATGGTAGTGAGAAACGAAATAA
CAGAACAAGGAAGCTACTTTGTAACCCAGGCATTGAATACACTGTCAAAGGTAGAAGAGTCTGGCTACAGC
TTTGATAATTTCATGGTAGGACCTTTCGTAGAAGACTTTAAAAAAGCAATTAAAAATCGTAGATTTGGTTCTA
AAAAAGATTAAGATTGTTTTTTAGAGTTAAACATAAGAATAAATTTAAATAAAAAAATAAACCCAACAAACCT
TGTTATATCAATGTTTGTTGGGTTTATTTTTTTGGTGATTTTTTAATAACTCAATTACATCTTAGGATAATATCGC
TAAATTATACATTGTCGAGGCCAAGACAAATAAAAAATTAAAGCAAAAGTTAGCGCGCAACTTTCTGCTATAA
AGGAGCAAATTTATATGGAGACAAGAGACAAACTGATGTCATTGACTCAAAGTGACAAAACACAGCAATGG
CTAATGGACAAGTCATCTAACCAAGATGACATTCAACAATTGCAGCAACAATTCAGTCAGCAGCTTGATCAA
CAATATAATGCACTTTTAGCTGATGAAAAAGCTAAGTTAGACCAATACGTGGAAGTACATCAAGGATTGGAAT
CATTAAAGGAAGAGATTGAATCAGAACCTATTACGCTTAATATCGATAAATTACCCGATATCAAAGCAACAATG
CTTGAAAGAGCCAAGAATGATGAACATTCTGATAAAATCGAAAAGCTATTTGATAGGTTAGAACAGGCATTA
AATGGTACGAATCGATTATATACGCAATTATCGTTGATTGGTACACGAACACATCGAATTACAACTAAAAATTT
TAATCTTCAAGGCTTACCTAAAGCAGTCCAACATACGATTTTACCTTCAAAATTTAAGAAGGTGTATACAGTC
GATTTTAAATCGTTTGAACCATCAGTTGCAGCGTACATGACTCAAGATTCAAAACTGATTGACTTGTTAAATC
AGAAAGACGGACTGTATGACGCATTGCTAAGTGAATTAGGCTTATCAGATGAGCTACGTGTATTTGTTAAAC
GTGCATTTATTGGTTCGTTTCTATTTGGAGGTAACTTCAAAAATCCTAAATTCAAGCTGAATCAATATGTAAGT
GAAGTACAATGGTTGGATGCGGTCAGCCAATTTACAAAAGTCATTGAACTTAAGAAGCACGTTGAAAAGAG
TAATTCCATGCCTATGCCTTATAGTATTGAGCATGATATGAGCGCATTTCAAGGTAGCAGTATTATGGCAATCTA
CGTACAAACTGTAGCGAGTTATATTTTCAAGCACATTCTGCTAAAAGTGTACAAAGCACAGTGCGATCAAAA
AACGTTCAAGATTATAGTACCTATACACGATGCGATTATGATTGAATGTGAAGATGAAGAAATTGCACAAAAT
GTGGGTCAGTTAATGAAAGATACGGCTAACCAGTTGTTCAATGGTGAATTTGCACATGTGACAGTGGAAGA
AATAGGAGGCGTAGACCATGAATAATGATAGAGGACAAAGCCTACACATCCCAAGTAGTACACCAATCAAAG

AAAATAATATATATGTAGCTACGTTACATTCTGTGATCCAAACAGATTTCTCAGGTGAAATAAAGCACCAATTC
ACGTATGAAATTGAAGTGAACAATCAGATTGTATATGCGAATCGTAATATTCCAACAAAACCGAGCGCTAATC
AGTTGTCAATTCATGATTGGCTGAAACGTCATAGCAACTATAGCGCAAGTCATGAAAACTATGAGCCTTATATT
GATCAGAAACATTTAATTCTATTAGGTCAATATAACGGTAACTATTATGTACAAGATGTAGCATCGTTAGATGC
GTTTGGAGGCGTATTATCATGAATCATATATTACAAATGTTATCTAAGCTATTAAGTGTGGCCAAGGAGGCAAT
CGACCGTCAAGGTCTGATTGCTATCCTAACTATTCCTGTTAATAATAACGATGAAATAGAAGAAACGGCTCAA
GGTGAAACCGTGTATAACGAACTTATCGATCAGTTACGACTTAATATCCCAAAAGATACGGATTATCAACCTAA
CATCTATAGTTATTTTGGTATTAAGAAGAATCCTAATGACACCGTACTCATGGAAATGATGATAAAGGTTTTTC
ATATCAAACGCTTTAATTCAGAACTGTTTATTTTCAAAGTTAACGGGTGGCAAAAGATAAATGGAGATGAATT
ACAAGGGTTGATATCTAAAATGATACAAGTATTGCTTGTAGATTATAAGCCTTCACTAAGCACTCTAAAAAATG
TCGTAGATGGATTGCAAAAATCAACAGATGTAGAAGAACTTGTTGAGAATGAGCGCTATATTGGTTGTGGTG
AAAATATGTTCGATCTTAATACGTTTCAAGTCGTTAAAAATTCAATCGATATCTTTCCAAAAACACGATTGAAT
TTATCATTAAGTACAAATGATGTAATTACTGATAAGATACCGCCTTATTTTAAGCAATATATGTTACAACTTGCG
AATTATGACGATGATTTACAATACTTTCTTTTCCAACATACAGCAGTATTACTTACAGCTGATACTAAATACCGT
AGGGGTCTCATATTATATGGTGGAGCTAAGAATGGTAAATCTGTATATATTGAACTAGTTAAATCATTTTTCTAT
AGTAAAGATATTGTGTCTAAGCCACTTAATGAGCTTGAAGGTCGTTTTGACAAAGAAAGTTTAATTGACAAA
AGTCTAATGGCAAGTCATGAAATTGGGCAATCTAGGATTCAAGAAAAGATCGTAAATGACTTCAAAAAGTTA
TTATCTGTAGAATCAATGCATGTTGATCGTAAAGGAAAAACTCAAGTGGAAGTCATTTTGGATTTGAAACTTA
TTTTTAGTACAAATGCGATACTTAATTTTCCTCCTGAACATGCGAAAGCTTTGGAGCGTCGAATTAATATTATT
CCATGTGAGTATTATGTTGAAAAAGCGGACACTTCATTAATTGATAAGCTCCAGAGTGAGAAGAAAGAAATC
TTTCTTTACTTGATGTATGTGTATCAACAGATTGTAAAAGCAGATATCGAGTATCTTGAAAATAGCCGTGTCAC
TGAAATTACTCACGATTGGTTAAATTTTGGATATGAATTTGTTTCTAGCAGGTCCGTAAGTAATGCAAATCAGA
AAGCATGTATTAATTTACTCAGAAAACTTATAGAAATCAAATCAGGATCACGAATCAAAGTATCCGAGTTAAAT
AAAGTTATTAATGAAGAAATAAAGGTAAGTTCTCAGGTTATTAAACAGTTAATTCAAGCAAACTTTGATACTC
AAACCAAACTATACAATGGCTACGATTATTGGATTGATTTAGGTTGGAAAGAAGCCAATAAAAAAGAGATTC
ATGATATTTCGGAAAAAGATAATATTATTTCATTAGATAAAAATGAAAATATAACAGACGATGAGGCATTAGAT
GAAGAGAATTTGGACTTTGATTGGGAGGACTTTGACGATGAATAATGAACAAATTGAAGCATTTGTAGAAG
TGCTTGTACCTATCATAGAAGAACGTATCAATAAAGGTAATTAAGGCTAATTACGTACTACAGGCAGTTGCCT
GTAGTACTCATATGATTAAGTGGTAAAAGTGATAAAAATGAAACGAAATTATAAATATATATTATCTATATGTTGT
TACAAGACCGATAGTCTGTAGCAATAATCTAATAAAAGGAGCGGTATGATATGAAGGGTAAAATTGCACTTTA
TTCACGCGTTAGTACGTCAGAGCAGTCGGAGCATGGGTACTCAATCCATGAGCAGGAACAAGTACTCATCAA
AGAAGTTGTGAAAAATTTCCCAGGTTATGACTATGAGACATATACTGACTCAGGCATTTCAGGTAAAAATATT
GAAGGTCGTCCGGCAATGAAACGTCTATTACAAGATGTTAAGGATAATAAAATCGAAATGGTGTTAAGTTGG
AAATTGAATCGTATCTCACGATCAATGAGAGACGTGTTTAATATTATTCATGAATTCAAAGAACATGATGTAGG
GTATAAATCGATTTCTGAGAATATTGATACATCCAATGCTTCTGGAGAAGTACTCGTTACAATGTTTGGGTTAA
TAGGATCTATAGAACGCCAGACTTTGATTTCGAATGTGAAACTTTCTATGAATGCTAAGGCAAGGAGCGGAG
AGGCAATCACCGGTCGTGTTTTAGGCTACAAATTATCACTTAATCCACTTACACAGAAAAATGATTTGGTTATC
GATGAAAATGAAGCTAATATTGTACGTGAAATTTTCGATTTATATTTGAATCATAATAAAGGCCTCAAAGCCAT
TACAACCGTACTTAATCAAAAGGGGTATCGTACTATTAATCAAAAGCCATTTTCAGTGTATGGTGTTAAATACA
TTTTGAATAATCCAGTCTATAAAGGCTATGTCAGATTCAATAACCATCAAAACTGGGCTGTACAGCGAAGAAG
TGGCAAAAGTGATAAAAATGATGTGATATTGGTCAAAGGTAAACATGAAGCCATTATAAGTGAAGAGGTATT
TGATAAAGTTCATGAAAAATTAGCTTCTAAAAGTTTTAAACCGGGCAGACCTATTGGTGGAGATTTCTACTTA
CGTGGCCTTATTAAATGCCCAGAATGCGGAAATAATATGGTATGTCGACGGACGTATTATAAAACGAAAAAGT
CCAAAGAACGGACAATCAAGCGTTATTACATTTGTTCCTTATTCAATCGTTCAGGGAGTTCTGCATGTCACAG

TAATTCCATCAATGCTGAAGTCGTCGAGCGTGTAATTAATGTTCATTTGAATCGTATTCTGTCTCAACCAGATAT
TATCAAGCAGATTGCGTCAAATGTGATAGAAGAACTGAAACAAAAGCATAGTAACCAAACAGAAATTAAATA
TGACATTGATAGTTTAGAAAAACAAAAAGCTAAGCTTAAAACACAACAAGAACGATTGTTAGAATTGTTCTT
AGATGATCAGATGGATAGCGAAATGTTAAAAGCTAAACAAAGTCAAATGAATCAACAGTTAGAAGTATTAGA
TCAACAAATTAAAGAAGCGCAACAAGCAAATCAATCACAGGATGAAATACCTAATTTTGATAAATTAAAAGG
ACGACTCATTTTGATGATAACACGATTCAGCGTGTACTTAAGAAAGGCTACACCCGAAGCTAAAAATCAACTT
ATGAAAATGTTAATTGATTCAATTGAAATTACGACAGATAAACAAGTAAAACTTGTAAGGTATAAAATTGACG
AAAGTCTTATCCCTCAATCTTTGAAAAAAGATTGGGGGTCTTTTTTTATGCCCAAATTCCAATTTGAAATATAT
GGTCAAAATGATTATTTCATCGACCAAATTACCACTTTTACCACTTAGTTATTAGTGACAAAAGTGAGCGAAA
TGAAATAAAAATCAAATATATATTATCAAAATGATGTATCACATGCATACATCAATCAAATACATTAGGAGGTCA
TAACCATGACACTAGAACAACAACTCAAGCACTATATAACCAACTTATTCAATCTGCCAAGGGACGAAGTGTG
GCACTGCGAATCTATCGAGGAAATCGCTGATGATATCTTACCCAATCAATATGTAAGACTTGGCCCACTCAGTA
ATAAAACACTTCAGACTAATACCTACTACTCTGACACACTTCATGAAAGTAATATCTATCCTTTCATTCTCTACTA
TCAGAAACAACTCATAGCCATCGGTTATATCGACGAAAATCACGATATGGATTTCTTATACCTACACAACACTA
TCATGCCTCTTTTGGATCAACGATACTTACTAACAGGAGGACAATAAAATGCATAAATACATCAAAATTACACA
ATTAGTCATTACAATACTAAGTGAAATCATCATTTGGATGAAAGAGTCAGAACGAAAGGAAGTCTCTTATGAA
TAGATATATCACCCGGGGTATCGCCAACAACTTACCTAATATCTTACAACACCAATTATGGCAACTCGTATCTGA
GCGAGAACAAGAACAAACCAAAGATAATACTTCAGTAGATTATTTTCATATATTCCAGTTCAATAAGCATCGC
AATCAATTATATATCAAACACAAACAAGAACGACCTGAATATGCGAAAATCCATAAAGCTAATTATTCAAAAGC
AATCAATATCAATAAGGTCTACATTATTCGAGAAGATGATGTAGACCTTTCTTATTATGTCATGTTATTACCTGA
AGAATACTAGAGGAGTGAAAATTATATGGAAACAATCAAAAGTACATTAAAAACAGAAGCCATATTCAGTGA
TGACAAACAACATCGCTATCTACTTAAGAAAACATGGAATAGTGAAAAACAATCAATCACAATCATTACAATG
TATCCGCATTATGATGGCATTCTCAATATTGACCTAACGACCCAACTCATCATGAACAAAGTTTCAGAAATGGA
TGCATTTGGTTCAATCAATTTTGTGAATCTATACTCTAATATTACAACCCCTATCAATCTCAAACATTTAGAAAAT
GCGTATGATAAGCATACAGATATTCAAATTATGAAGGCAGTGAAAGAGTCAGATGAAGTGATATTAGCTTGG
GGCGCTTACGCTAAAAAGCCCGGTGTTGAAGCACGTGTTAATGAAGTATTAGAGATGTTGAAACCACATAAA
AAGAAAGTAAAACGACTCATGAATCCAGAAACCAATGAAATCATGCATCCCCTTAATTCGAAAGCACGTCAA
AAATGGATATTAAAAGTATAGCAACTAAAATGTTTATTCACTATCCATGAAAATAATTGAATTTCATATATATCTT
TTAATCTACAATATGATTAAAAGATATATTATTTGGTTCTGTTGCAAAGTAAAAAAATATAGCTAACCACTAATT
TATCATGTCAGTGTTCGCTTAACTTGCTAGCATGATGCTAATTTCATGGCATGGCGAAAATCCGCAGATCTGAA
GAGACCTGCGGTTCTTTTTATATAGAGCGTAAATACATTCAATACCTTTTAAAGTATTCTTTGCTGTATTGATAC
TTTGATACCTTGTCTTTCTTACTTTAATATGACGGTGATCTTGCTCAATGAGGTTATTCAGATATTTCGATGTAC
AATGACAGTCAGGTTTAAGTTTAAAAGCTTTAATTACTTTAGCCATTGCTACCTTCGTTGAAGGTGCCTGATC
TGTAATTACCTTTTGAGGTTTACCAAATTGTTTAATGAGACGTTTGATAAACGCATATGCTGAATGATTATCTC
GTTGCTTACGCAACCAAATATCTAATGTATGTCCCTCTGCATCAATGGCACGATATAAATAGCTCCATTTTCCTT
TTATTTTGATGTACGTCTCATCAATACGCCATTTGTAATAAGCTTTTTTATGCTTTTTCTTCCAAATTTGATACAA
AATTGGGGCATATTCTTGAACCCAACGGTAGACCGTTGAATGATGAACGTTTACACCACGTTCCCTTAATATT
TCAGATATATCACGATAACTCAATGTATATCTTAGATAGTAGCCAACGGCTACAGTGATAACATCCTTGTTAAAT
TGTTTATATCTGAAATAGTTCATACAGAAGACTCCTTTTTGTTAAAATTATACTATAAATTCAACTTTGCAACAG
AACCGTATTATGGAATAGAGATGTTGGTAACATTTATACAGGATCATTATACTTAAGTTTAATTTCGTTATTACA
GAACCACACATTCCAACCAGAAGAGAAAGTATGTCTATTTAGTTATGGTTCAGGAGCAGTAGGAGAAATCTT
TAGTGGTTCAATCGTTAAAGGATATGACAAAGCATTAGATAAAGAGAAACACTTAAATATGCTAGAATCTAGA
GAGCAATTATCAGTCGAAGAATACGAAACATTCTTTAACAGATTTGATAATCAAGAATTTGATTTCGAACGTG
AATTGACACAAGATCCATATTCAAAAGTATACTTATACAGTATAGAAGACCATATCAGAACATATAAGATAGAG

AAATAAACTAGTGGCCGATTGTGCTTGATGAGCTTGGGACATAAATCCTAACTCGAAATAAATAAGCATATCA
CTAAACTGATTTTTTAAAGTTTACAGTGATATGCTTATTTTTTTATCTTACGATTTTGTACGTGCATGCTTGCCTA
GGGGTATGGCTCGAGCCATTAGTCTCTCGCACATACTATTCCCTCAGGCGTCAGCACTTACAAAATCGGTTGT
AATTTTCATTTTTATACGCATTCTTACTGAGATTATACTAATAAGAGGAATAGTAAAAGCAATTCTAAGTAAAAT
TGCAGATAAGAGGTTTGTTAAAAGCAGTTCTAAGTAAAATTGCAGATAAGAGGTTTGTTAAAAGCAGTTCTC
AGTAAAATTACAGATAAGAGGTACGTTAAAAGCAGTTCTAAGTAAAATTGCAGATAAGAGGTTTGTTAAAAG
CAGTTCTAAGTAAAATTGCAGATAAGAGGTACGTTAAAAGCAGTTCTAGGCAAAATTGCAGATAAGAGGTG
CGTTAAAAGCAGTTCTCAGTAAAATTGCTGATAAGGGGTAAGTTAAAAGCAATCCTAAGTAAAATTGCAGAT
AAGAGGTAAGTTAAAAGCAATCCTAAGTAAAATTGCAGATAAGGGGTACAGAAAAACTAGACTTGATTACA
AAATGGAGCTTGGGACATAAATGATTTTTTAAAAATGAGATGAGACGTAGATTAACTCCATAATCAATACGAA
TCTATCGACTTCTTTATTTATGATATTCATCTCTTTTTAATGGAAATAAAAGTGCGATTAATGTGATAATACAGTT
ACGTTAATTAAAAAAATAAAAATGCAAGGAGAGGTAATATGCTAACTGTATATGGACATAGAGGATTACCTAG
TAAAGCTCCGGAAAATACAATTGCATCATTTAAAGCTGCTTCAGAAGTAGAAGGTATAAACTGGTTGGAGTT
AGATGTTGCAATTACAAAAGATGAACAACTGATTATCATTCATGATGATTATTTAGAACGGACTACAAATATGT
CCGGGGAAATAACTGAATTGAATTATGATGAAATTAAAGATGCTTCTGCAGGATCTTGGTTTGGTGAAAAAT
TCAAAGATGAACATTTGCCAACTTTCGATGATGTAGTAAAAATAGCAAATGAATATAATATGAATTTAAATGTA
GAATTAAAAGGTATTACTGGACCGAATGGACTAGCACTTTCTAAAAGTATGGTTAAGCAAGTGGAAGAACA
ATTAACAAACTTAAATCAGAATCAAGAAGTGCTCATTTCAAGCTTTAATGTTGTGCTTGTTAAACTTGCAGAA
GAAATCATGCCACAATATAACAGAGCAGTTATATTCCATACAACTTCGTTTCGTGAAGACTGGAGAACACTTT
TAGATTACTGTAATGCTAAAATAGTAAACACTGAAGATGCCAAACTTACTAAAGCAAAAGTAAAAATGGTAAA
AGAAGCGGGTTATGAATTGAACGTATGGACTGTAAACAAACCAGCACGTGCAAACCAACTTGCTAATTGGG
GAGTTGATGGTATCTTTACAGACAATGCAGATAAAATGGTGCATTTGTCTCAATAGAAAGTTAGAGGTGAGT
CTTACGTTTCAGTGACGGTAGACTTACCTTTAACATGTTACATACTAAAAAATTAATTTGAATAAGAAAGAGA
GACATATATGAAATACGATGATTTTATAGTAGGAGAAACATTCAAAACAAAAAGCCTTCATATTACAGAAGAA
GAAATTATCCAATTTGCAACAACTTTTGATCCTCAATATATGCATATAGATAAAGAAAAAGCAGAACAAAGTA
GATTTAAAGGTATCATTGCATCTGGCATGCATACACTTTCAATATCATTTAAATTATGGGTAGAAGAAGGTAAA
TACGGAGAAGAAGTTGTAGCAGGAACACAAATGAATAACGTTAAATTTATTAAACCTGTATACCCAGGTAATA
CATTGTACGTTATCGCTGAAATTACAAATAAGAAATCCATAAAAAAAGAAAATGGACTCGTTACAGTGTCACT
TTCAACATACAATGAAAATGAAGAAATTGTATTTAAGGGAGAAGTAACAGCACTTATTAATAATTCATAATAAA
ACAGTGAAGCAACCATCGTTACGGATTGCTTCACTGTTTTGTTATTCATCTATATCGTATTTTTTATTACCGTTC
TCATATAGCTCATCATACACTTTACCTGAGATTTTGGCATTGTAGCTAGCCATTCCTTTATCTTGTACATCTTTAA
CATTAATAGCCATCATCATGTTTGGATTATCTTTATCATATGATATAAACCACCCAATTTGTCTGCCAGTTTCTCC
TTGTTTCATTTTGAGTTCTGCAGTACCGGATTTGCCAATTAAGTTTGCATAAGATCTATAAATATCTTCTTTATG
TGTTTTATTTACGACTTGTTGCATACCATCAGTTAATAGATTGATATTTTCTTTGGAAATAATATTTTTCTTCCAA
ACTTTGTTTTTCGTGTCTTTTAATAAGTGAGGTGCGTTAATATTGCCATTATTTTCTAATGCGCTATAGATTGAA
AGGATCTGTACTGGGTTAATCAGTATTTCACCTTGTCCGTAACCTGAATCAGCTAATAATATTTCATTATCTAAA
TTTTTGTTTGAAATTTGAGCATTATAAAATGGATAATCACTTGGTATATCTTCACCAACACCTAGTTTTTTCATG
CCTTTTTCAAATTTCTTACTGCCTAATTCGAGTGCTACTCTAGCAAAGAAAATGTTATCTGATGATTCTATTGCT
TGTTTTAAGTCGATATTACCATTTACCACTTCATATCTTGTAACGTTGTAACCACCCCAAGATTTATCTTTTTGCC
AACCTTTACCATCGATTTTATAACTTGTTTTATCGTCTAATGTTTTGTTATTTAACCCAATCATTGCTGTTAATATT
TTTTGAGTTGAACCTGGTGAAGTTGTAATCTGGAACTTGTTGAGCAGAGGTTCTTTTTTATCTTCGGTTAATT
TATTATATTCTTCGTTACTCATGCCATACATAAATGGATAGACGTCATATGAAGGTGTGCTTACAAGTGCTAATA
ATTCACCTGTTTGAGGGTGGATAGCAGTACCTGAGCCATAATCATTTTTCATGTTGTTATAAATACTCTTTTGA
ACTTTAGCATCAATAGTTAGTTGAATATCTTTGCCATCTTTTTTCTTTTTCTCTATTAATGTATGTGCGATTGTATT

GCTATTATCGTCAACGATTGTGACACGATAGCCATCTTCATGTTGGAGCTTTTTATCGTAAAGTTTTTCGAGTC
CCTTTTTACCAATAACTGCATCATCTTTATAGCCTTTATATTCTTTTTGTTTTAATTCTTCAGAGTTAATGGGACC
AACATAACCTAATAGATGTGAAGTCGCTTTTCCTAGAGGATAGTTACGACTTTCTGTTTCATTAGTTGTAAGAT
GAAATTTTTTTGCGAAATCTCTTAAATATTCATCCATTTTTTTAACGGTTTTAAGTGGAACGAAGGTATCATCT
TGTACCCAATTTTGATCCATTTGTTGTTTGATATAGTCTTCAGAAATACTTAGTTCTTTAGCGATTGCTTTATAAT
CTTTTTTAGATACATTCTTTGGAACGATGCCTATCTCATATGCTGTTCCTGTATTGGCCAATTCCACATTGTTTC
GGTCTAAAATTTTACCACGTTCTGATTTTAAATTTTCAATATGTATGCTTTGGTCTTTCTGCATTCCTGGAATAA
TGACGCTATGATCCCAATCTAACTTCCACATACCATCTTCTTTAACAAAATTAAATTGAACGTTGCGATCAATGT
TACCGTAGTTTGTTTTAATTTTATATTGAGCATCTACTCGTTTTTTATTTTTAGATACTTTTTTTATTTTACGATCC
TGAATGTTTATATCTTTAACGCCTAAACTATTATATATTTTTATCGGACGTTCAGTCATTTCTACTTCACCATTATC
GCTTTTAGAAATATAACTGCTATCTTTATAAACTTGTTTGAAATTTTTATCTTCAATTGCATCAATAGTATTATTA
ATTTCTTTATCTTTTGAAGCATAAAAATATATACCAAACCCGACAACTACAACTATTAAAATAAGTGGAACAAT
TTTTATCTTTTTCATCAATATCCTCCTTATATAAGACTACATTTGTAATATATTACAAATGTAGTATTTATGTCAAA
ATAATGTTATAATTTTTGTGATATGGAGGTGTAGAAGGTGTTATCATCTTTTTTAATGTTAAGTATAATCAGTTC
ATTGCTCACGATATGTGTAATTTTTTTAGTGAGAATGCTCTATATAAAATATACGGTTCTGTTGCAAAGTTGAAT
TTATAGTATAATTATAACCAAAAGGAGTCTTCTGTATGAACTATTTCAGATATAAACAATTTAACAAGGATGTTA
TCACTGTAGCCGTTGGCTACTATCTAAGATATGCATTGAGTTATCGTGATATGTCTGAAATATTAAGGGAACGT
GGTGTAAACGTTCATCATTTAACGGTCTAGCGTTGAGTTCAAGAATATGCCCCGATTTTATATCAAATTTGGAA
GAAAAAGCATAAAAAAGCTTATTACAAATGGCGTATTGATGAGACGTACATCAAAATAAAAGGAAAATGGAA
CTATTTATATCGTGCCATTGATACAGAGGGACATACATTAGATATTTGGTTGCGTAAGCAACGAGATAATCATT
CAGCATATGTATTTATCAAACGTCTCATTAAACAATTTGGTAAACCTCAAAAGGTAATTACAGATTAGGCACCT
TCAACGAAGGTCGCAATGGCTAAAGTCATTAAAGCTTTTAAACTTAAACCTGACTGTCATTGTACATCGAAAT
ATCTGAATAACCTCATTGAGCAAGATCACCGTCATATTAAAGTAAGAAAGACAAGATATCAAAGTATCAATAC
GGCAAAGAATACTTTAAAAGGTATTGAATGTATTTACGGTCTATATAAAAAGAACCGCAGGTCTCTTCAGATC
TGCGGATTTTCGCCATGCCATGAAATTAGCATCATGCTAACAAGTTAACACAAAGTATTATTTTAAAATTGAGA
TTAGACATTTATTTTTCAACTTTGCAGCAGAACCTAATTTTTTCTCTTTGTAAATAAATGCAAAGAGATACCATA
AGGATCTCTTACATAACCATAACCTTCAGTATAGAATTCTGGACTAAATGTTTTCAATACCTCACTGCCTTTTTC
TATTAACTGGTCATATACATGTTTAGTTTCTTCTACTTGGTCAAAAGTGAGACAAAGAGATATATTATTACCTTG
TGTTATGGGCAAACCTTCAGTGTCATCTGCGATCATAATTTTTATATCTCCAAATTGAAGTACACATTGATCAAT
TTTATTTAAATCATTTTCGTCAATATTAAGTTTCTTATCTATAGGTCTATCTTTAATACGTTGAATATACAGTGTTT
TAGCGCCAAACAGCTCTTCATACAACTTTTTTAAACCCTCTGCATTTTGAGTGATTAAAAAATATGGACTTACT
TGAAATTTCATGTTTTTTCCTCCTTAGATTTGTTATAAATAGAGTATATACTTAATTAGTGTCATCTATTGACACT
AAAAGGAGAATAATAATGAAAAAATCTGTTAGATTATATAATATGATTGAATATTGTAATGAAAATAGGAACTT
CAAATTAAATGATTTAATGTCAGAATTTAATATTTCTCGTAGTACCGCTTTAAGGGATATAAAAGAAATTGAAG
CATTAGGAGTACCTTTATATAGTAATCCAGGGAAAAATGGTGGTTATACGATCATAGGTAATCGAGACCAAAC
GAAAATAGCAATCTCAGATGAAGAGTTGAAAGCTTTAGTATTTACACTTTCGAGTATTTCAAATGTGAGTAAT
CTACCTTTTCAAACAGAATATCAAGAAATATTAAAAAAATTATATAATAACTCGAATAAAAAAGAGTTAATAAA
CCAATATAATGATCTATTTCAATATTTTAATGAAGATAAGTATCAGTTCAAAAGTTATAAGTTATTTAATGAAATC
ATTAGATTGATAATTGAGAATAAGTCTTTTGAAACCTGTTATTCACAAAACTATATTAAAGAACAATATAAAGG
TATTGGCATTATGTATAAAAATCATCAATGGTATTTTGTTGTAGCTAATATAGAATCAAAGTTAGTGAATCTATTA
AATATTTCGAAAATAAAAGAACTATATGAAATGGGAGAGACTCAAGAGTGTAATGATATAACTATGCAGAATT
TTCAACAGTTCATGGTTAAAAATGAAACAGCTATTGATATTCTTATTAGAAGCAATGTTATGGGATTGAATATC
TTGAAAGGCTACCTGTGGAGTGACTATATGATTGAAAATATTGACGAAGAGACATATTTATTTAAATCAAAAG
TGAACGCGAAAGATATAGATTTTATAGCTAAGTTAATTGTCACAGGTGGTGTCAATGTAAAAGTAGAGACCCC

TAATAGTTTGAAAAATGCTGTTAAAGTTGAATTAACTAAAATAATAAACATGTATTAATAGTAATTTAATCATAA
TTGTAATAACAAAAAGATGACAACATTAAACAATTTAAATTTATACAAAAAAGCATTACCAAATTTAAAATTGG
GTAGTGGTAGTGCTACAGCATACATAAATAGTTATAATTAAATAAAGATGGGTCAATGAAGGTGAATTAAGTG
AATAAAAATATTACAGCTCTAAAATCTACTGAAAATACTACATATATGCAGTATACGGTTCATATCGATGATCAA
ATTATAAATAATATAAAAGAGTGTACCAAAAAGTTTAAAATTTTGCCTATGGAAGATAAAATTCCATTATCACC
GTTGTTACAACCAGAATATGCAGGAGAGGTACAAGATTTTATTAGTACATATGAGCAGTTTATGATTAATTTTG
GTAAAGTAATATTGGATAGTCAAGGCATAAAAATACAGTTTGAAAGTGAATCATTAAGTAGTATTCAACGAGG
CATTCAAGAACATTGTTACTTAAATGAGCGAACAAATGACATTGATGTGACTAAAGAATGGTATTTATGCAAA
TTTTCAAATCAAATATTAGAAGAGGATAAAAATCAATTGTATAATGCTTTAAAGCAACTCATGAATGATTCTAA
AAATAAAAAGCAAGCTTTTCAAGAGGTGTTTAAGGTTCATATTGATATTTATCTCTACAATAAAGAAAAATCT
GAATATAAGTATCAAGTATCTAGTTATTTCAATCTTGTAAAAGAAAATCCTAAAATAACTTATAAAAAAAGACAT
TTACAAGAGAAACAAGGTGTCAAGGGTACAACTTTCACGAACAAATTAAAATTATTGAACAAAATGTATGGT
GTAGATGTTGCAAAATATCAGCCATTTTATAATTCTAATAATCCTGAATATGAAAGAGGTCAATTTGGTGAAAG
ATACATTTCTCAAAGATCTAACTATGAATTTAATAGACTTCAATATCAAATTATAGATATGTTATCGAAAATACTT
GATAAGCATCCATTACCAAAGTCAGATAATAATTATAAACATATTCCAACTATTGAAAAAGCAATATTAAGTGG
GGATTCTCATAGTTTTTATGAGTATTTTGAAGATATAATGAAAGAAATTATAAGTATGGAAAATTCTTCTTTAAA
GGAAAAAATTATTGACTGATTTTACATATCAATCTCAATGTAGATGGTACTCTGAAAGTGAAAAATTAAAATTGC
AACTTGAAAGTTTTATGCATAAAGTTTTGGAGAGCAATTATTATGAGGGTAACAAATTATATAGAATGTTGTCT
CATGCAATTGAAGAAACAATCAATGAGGCTGATGAAGATAAGGTTCATTTCAATTATTTTAAAGATTATTTTTT
GACAGACGGTGGGGTGAAGAATTGGGAGCAGATTAGCGAGAAAATTACTGAATTCAATGGTAAAGTTATTA
ACGATATCCAAAATGAATATAATAAGATTCAATTTAATAACGCAAATCGAAATCAGAAACTAAACTTTAATTATT
TATACCATTGCTTTGAGTTTAGTAATAACTTAGTAAAAGCAAGGGTAAATGGAAATAGAGGTTACATTTTATAT
TTTGTAGATAAATATAAAATAAAAATGCCCTTTTTGGATCAATTATTAATCAATTATTTGAATCAAGAAATAGGT
CAGGAAAGTATTAATTATAATATGCAAACATTGTTTGAAAAAGAAAGATATGATAGAAGTAGTACCATTGAAA
AATTAGTAGCAACAAGCAAATTTAAGTATGAAAAAGATGATTCAGATTTATTCAAACAACTTTTCAATGATGT
TGAAAATTCAATAGACAGATTAGGTATTTACTTACTAAATAATGGTATAAATTCGAATGATGAAAATGCAAGAT
ATTATAGATCGTTTTTAAAGGAACTTAGTAGAATAAAAAGTAAATTAACGCCATTTTCTCTTGAAATAAGTAAG
TCTAGCGGAAGAGAGCAACATTATCCTGATGATGCTATTGATGATAAAGATGAGAGAAGAAAAAATAAAGAA
GAAACATATCATGCTTTTGATGATAAAAGCGATATTGACTCCAAATTAAAGAATAAAATAAATGTTTCTATCGA
TAATTTATTGTCAGTTAAAATTAAGAGCAATTTGTATGATATTAGTCCAAATACAAAAAAACAATAACTATAATG
AGCCAACTCAAGAATGAGTTGGCTTTTTAATTGTAATTTTACGCTTCCATTACAGGAATCAATGTTATGGAAG
CCATTAAGCAATTAATTTGAGTCATTTATAGTATAGGTACAACGTTGTGATGTTCACATGATAATCAGCCCGGG
AGATTATTGTGTAATAACTTATAGAAGGAGCAAATCACCATGTATCAAGCAAATATACGTGATTTAATTACAAA
ATTGCCTCAAAGCAATAAAACAGAACACTTTTTAATGAACAAATTTTCAAATCAAGATAAAGTTCAGCAGCTA
CAAAGACAAATTAGCCAACAGCTAGATCAACAATATAATGAGCTTTTGGCTAATGAAAAAGCTAAGCTAGAC
CAATACGTGGAAGTACACCATAATTTAGAACCATTAAAGAAAGAGATTGAATCAGAATCTATTAACCTTGATA
CCGATAAATTACCTGATATCAAAGCGACAATGCTTGAAAAGGCTAAGAACGATGAACATTTTGATAAAATCGA
ACAGCTATTTGATAGATTAGATCAGTCATTAAATGGTACGAATCGATTATATACGCAATTATCGTTGATTGGCAC
ACGAACACATCGGATCACAACGAAAAGATTTAATGTTCAAGGCTTGCCTAAATTAGTCCAACAAATGATTTTA
CCTTCGCAATTTAAAAAGGTTTATACAATAGATTTTAAATCATTCGAACCATCAGTTGCTGCGTATATGACACA
AGACGAACAACTGATTGACTACTTGAATCATGAAGAAGGGTTATACGATGCATTACTGAGAGACTTATCTTTG
TCAAAAGAGAAGCGCGTGAGTGTGAAACGTGCATTTATAGGGTCATTTCTTTTTGGCGGTCGTTATAGTAGC
TCTAAATTCAAAATCAATCAAGAGGTTAGTGAAATTAACTGGCTACAAGTAATGAGCAAATTCAAGAAGGTC
ATTGAATTTAAGGAGCAAGTCGAAAAATATAAAACAATGCCTACGCCTTACGGCATTGAACATGATATGAGC

GCATTTCAAGGTAGTAGTATTATGGCAATTTATGTACAAACGGTAGCAAGCTATATTTTCAAGCACATTTTGTT
GGAAGTGTACAAAGCACAGTGCGAAAAAAAAACGTTCAAGATTATAGTGCCGATACACGATGCGATTATGAT
TGAATGTAATGATAAGGGGATTGCACAAAATGTAGCACAGCTCATGAAAGATACAGCTAATCAACTGTTTAAT
GGTGAATTTGCACATGTGACAGTGGAAGCTTTAGGAGGTATAGACAATGAATAATGATAGAGGAAAAAGTC
TTCAAATTCCCCAAAGTACATTGTTAAAAGAAGGATCTATATACGTTGCTACGTTACATTCTGTGTACGAGAAG
AACTTCTCAGGTGATATTAAACATCAGTTTACGTATGAAGTAGAACTTAACCAAGAAACGCACTATGTAAATC
GCAATATTACCGTAAAATCTATGAGTCACCAATTATCAATTGCTGATTGGATTAAACGTCACAGTAACTATAAC
GTAAACCACATTAATTATGATCCATACATTGATCGAAAACATTTGGTCCTTGTAGGGCAATATAACGGAAATTA
TTATATTCAAGATGTAGCACCATTAGATGAATTTGGAGGGGTATTGTAATGAATCATATTTTAGAAATGTTAATA
AAATTATTAAAAGTGGGTATGGAGGCAATCGACCGTAAAGGTCTGATTGCCATCCTAACAAGTAGTATTGGA
AATGATGAAATGGATGATTCTGAACAAGCTGTAATGGTGTATAACGAGCTTATCGATAAGCTACAGCTTAACA
TTCCTAAAGATGTCGACTATAGACCTAACATATACAGTTATTTTGGTATTCAAAAAAAGCCAAATGACACAATA
TTAGTGGAAATGATGATATGTATTTTTCATATCAAGCGTTTTGATTCAGAGTTGTTTGTTTTCAAAGATAAAGG
TTGGCAAAAGGTAAGTGAAGATGAATTGCAAGGGTTGATATCTAAAATGATACAAGTGTTGCTAGTTGATTAT
AAACCTTCACTAAGTACTTTGAAAAACGTAGTAGATGGCATACAGAAATCAACGGACATAGAAAAACTTGTT
GAGCATAGACAGTACATTGGTTGTGGACGAAATATGTTCAATCTAAAGACCTTTAAAGTGGTTGATAATGACC
TTGAAATATTCCCTAAAACACGCTTAGATTTGGAATTAGATATAAATGACACGATTACGGACAAGATACCCCC
GAATTTCAAACAATATATGTTAGAGTTGGCGAATTATGACCATGATTTACAATATTTCCTTTTCCAACATATGGC
AGTGTTATTGACGGCAGATACTAAACTACGTCGTGGACTTTTTTTGTATGGAACTGCAAAAAATGGGAAATC
GGTCTATATTAAATTAGTTAAGTCATTCTTTTATAGTAATGATATCGTATCTAAAACACTTAATGAACTTGGCGG
GCGTTTCGATAAGGAAAGTCTAATTGGTAAACGAATTATGGCAAGTGATGAAGTGGGGAAAGCTAATATTGA
TGAAGCAACTGTGAATGATTTCAAAAAATTACTATCTGTTGAACCAATTCATGCTGACCGTAAAGGAAGAAC
GCAAGTAGAAGTTACTTTAGATTTAAAACTCATTTTTAATACGAATGCTGTACTCAATTTTCCATCATCACATGC
AAAAGCATTAGAGCGTAGAATTGCTGTTATTCCATGTGAATATTATGTTGAAAAAGCTGACCCTGACTTAATT
GAAAAGTTACAGGATGAAAAGAAAGAAATCTTTCTTTACTTGATGTATGTGTATAAGCAAATTGTAAAAAATG
ATATCGAGTACCTCCAAAATGATCGTGTTACTGAAATTTCTCATGATTGGTTAAATTTTGGATATGAATTTGTTT
CTAGTAAATCAGCAAATATTGCACATCAGAAAGCGTGTATTAATTTACTCAGAAAACTTATAGAAATCAAACC
AGGGTCACGTATCAAAGTGTCTAGGCTAAATGAGGTTATTAGAGATGAAATTAAAGTAAGCTCTCAAGTTATT
AATGATTTGGTTCAAGCTAACTTTAATGTACAAAGTAGACTAAATAATGGTTATAAGTATTGGGTCGATTTAGG
ATGGAAAGAAACTGATAAAAAAGATGACATGATTTCATTCGATAAAAATGAGAATGTAACAGATGATGAATT
CTTATACGAAGATGATTTGAACTTAGGTTGGGAGGACTTTGACGATGAATAATGAACAAATTGAAGCATTTG
TAGAAGTGCTTGTGCCTATCATAGAAGAACGTATCAATAAAGGTAAGTAATCTAATTACGTACTACAGGCAGT
TGCCTGTAGTACTCATATGATTAAGTGGTAAAAGTGATAAAAATGAAACGAAATTATAAATATATATTATCTATAT
GTTGTTACAAGACCGATGGTCTGTAGCAATAATCTAATAAAAGGAGCGGTATGATATGAAGGGTAAAATTGC
ACTTTATTCACGCGTTAGTACGTCAGAGCAATCGGAGCATGGGTACTCAATCCATGAGCAAGAACAAGTACT
CATCAAAGAGGTTGTGAAAAATTTCCCAGGTTATGACTATGAGACATATACTGACTCAGGCATTTCAGGTAAA
AATATTGAAGGTCGTCCGGCAATGAAACGTCTATTACAAGATGTTAAGGATAATAAAATCGAAATGGTATTAA
GTTGGAAATTGAATCGTATTTCTCGCTCAATGAGAGACGTGTTTAATATTATTCATGAATTCAAAGAACATGAC
GTAGGGTATAAATCAATTTCTGAGAATATTGACACATCCAATGCTTCTGGAGAAGTACTCGTTACAATGTTTG
GGTTAATAGGATCGGTTCTGTTGCAAAGTAAAAAATATAGCTAACCACTAATTTATCATGTCAGTGTTCGCTTA
ACTTGCTAGCATGATGCTAATTTCGTGGCATGGCGAAAATCCGTAGATCTGAAGAGACCTGCGGTTCTTTTTA
TATAGAGCGTAAATACATTCAATACCTTTTAAAGTATTCTTTGCTGTATTGATACTTTGATACCTTGTCTTTCTTA
CTTTAATATGACGGTGATCTTGCTCAATGAGGTTATTCAGATATTTCGATGTACAATGACAGTCAGGTTTAAGT
TTAAAAGCTTTAATTACTTTAGCCATTGCTACCTTCGTTGAAGGTGCCTGATCTGTAATTACCTTTTGAGGTTT

ACCAAATTGTTTAATGAGACGTTTGATAAACGCATATGCTGAATGATTATCTCGTTGCTTACGCAACCAAATAT
CTAATGTATGTCCCTCTGCATCAATGGCACGATATAAATAGCTCCATTTTCCTTTTATTTTGATGTACGTCTCATC
AATACGCCATTTGTAATAAGCTTTTTTATGCTTTTTCTTCCAAATTCGATATAAAATTGGGGCATATTCTTGAAC
CCAACGGTAGACCGTTGAATGATGAACGTTTACACCACGTTCCCTTAATATTTCAGATATATCACGATAACTCA
ATGCATATCTTAGATAGTAGCCAACGGCTACAGTGATAACATCCTTGTTAAATTGTTTATATCTGAAATAGTTCA
TACAGAAGGCTCCTTTTTGTTAAAATTATACTATAAATTCAACTTTGCAACAGAACCACAAAAATGATATATTT
AAACTATTCTAATTTCGGAGGATTTTTTTATGAAGTGTCTATTTAAAAATTTGGGGAATTTATATGAGGTGAAA
GAATAATTTACCCCTATAAACTTTAGTCACCTCAAGTAAAGAGGTAAAATTGTTTAGTTTATATAAAAAATTTA
AAGGTTTGTTTTATAGCGTTTTATTTTGGCTTTGTATTCTTTCATTTTTTAGTGTATTAAATGAAATGGTTTTAA
ATGTTTCTTTACCTGATATTGCAAATCATTTTAATACTACTCCTGGAATTACAAACTGGGTAAACACTGCATATA
TGTTAACTTTTTCGATAGGAACAGCAGTATATGGAAAATTATCTGATTATATAAATATAAAAAAATTGTTAATTA
TTGGTATTAGTTTGAGCTGTCTTGGTTCATTGATTGCTTTTATTGGTCACAATCACTTTTTTATTTTGATTTTTG
GTAGGTTAGTACAAGGAGTAGGATCTGCTGCATTCCCTTCACTGATTATGGTGGTTGTAGCTAGAAATATTAC
AAGAAAAAAACAAGGCAAAGCCTTTGGTTTTATAGGATCAATTGTAGCTTTAGGTGAAGGGTTAGGTCCTT
CAATAGGGGGAATAATAGCACATTATATTCATTGGTCTTACCTACTTATACTTCCTATGATTACAATAGTAACTAT
ACCTTTTCTTATTAAAGTAATGGTACCTGGTAAATCAACAAAAAATACATTAGATATCGTAGGTATTGTTTTAAT
GTCTATAAGTATTATATGTTTTATGTTATTTACGACAAATTATAATTGGACTTTTTTAATACTCTTCACAATCTTTT
TTGTGATTTTTATTAAACATATTTCAAGAGTTTCTAACCCTTTTATTAATCCTAAACTAGGGAAAAACATTCCGT
TTATGCTTGGTTTGTTTTCTGGTGGGCTAATATTTTCTATAGTAGCTGGTTTTATATCAATGGTGCCTTATATGAT
GAAAACTATTTATCATGTAAATGTAGCGACAATAGGTAATAGTGTTATTTTTCCTGGAACCATGAGTGTTATTG
TTTTTGGTTATTTTGGTGGTTTTTTAGTGGATAGAAAAGGATCATTATTTGTTTTTATTTTAGGATCATTGTCTA
TCTCTATAAGTTTTTTAACTATTGCATTTTTTGTTGAGTTTAGTATGTGGTTGACTACTTTTATGTTTATATTTGTT
ATGGGCGGATTATCTTTTACTAAAACAGTTATATCAAAAATAGTATCAAGTAGTCTTTCTGAAGAAGAAGTTG
CTTCTGGAATGAGTTTGCTAAATTTCACAAGTTTTTTATCAGAGGGAACAGGTATAGCAATTGTAGGAGGTT
TATTGTCACTACAATTGATTAATCGTAAACTAGTTCTGGAATTTATAAATTATTCTTCTGGAGTGTATAGTAATAT
TCTTGTAGCCATGGCTATCCTTATTATTTTATGTTGTCTTTTGACGATTATTGTATTTAAACGTTCTGAAAAGCA
GTTTGAATAGTTATATTATATTTTGGTTTAGAACTATGAGTGGCTAGCATTTTGCCACTCATTTTTTGCGTTAGC
AAAAACAGGTTTAAGCCTCGCAGAGCACACGTATTAACGACTTATTAAAAATAAGTCTAGTGTATTAGACTTA
AACTATTAAATACACATGAAACCTTTGTGCTTAGGAGTGATTTTTATATGTCTTATTCCATTGTTAGAGTTTCAA
AAGTTAAATCTGGAACAAATACAACGGGCATACAAAAACATGTTCAAAGAGAAAATAATAATTATGAAAATG
AAGATATAGACCATAGTAAAACTTACTTAAATTATGATTTGGTAAATGCTAATAAACAGAATTTTAATAACTTGA
TTGATGAAAAAAATCGAACAGAATTATACAGGCAAAAGAAAAATTAGAACAGACGCGATTAAACACATTGATG
GTTTAATTACATCAGACAATGATTTCTTTGATAATCAAACGCCAGAAGATACAAAGCAGTTTTTTGAATATGCT
AAAGAGTTTTTAGAACAAGAATACGGTAAAGATAATTTATTATATGCAACAGTTCACATGGACGAAAAAACA
CCACATATGCATTATGGCGTTGTTCCAATAACTGATGATGGTCGTTTAAGTGCTAAAGAAGTTGTAGGTAATAA
AAAAGCTTTAACAGCGTTTCAAGATAGATTTAATGAGCATGTTAAACAACGAGGATATGATTTAGAACGTGG
GCAATCAAGACAAGTAACAAATGCTAAACATGAGCAAATAAGTCAGTATAAACAAAAAACAGAATATCATAA
GCAAGAATATGAACGTGAGAGCCAAAAAACAGACCATATAAAGCAAAAGAACGATAAATTAATGCAAGAGT
ACCAAAAATCGTTAAATACGCTTAAAAAGCCTATAAATGTTCCGTATGAGCAAGAAACTGAAAAAGTAGGTG
GTTTATTTAGCAAAGAAATACAAGAAACTGGAAATGTTGTAATAAGCCAAAAAGATTTCAATGAATTTCAGA
AACAGATAAAAGCTGCTCAAGATATTTCGGAAGATTACGAGTATATAAAGTCTGGTAGAGCCTTAGATGATAA
AGATAAGGAAATACGAGAGAAAGATGATTTATTAAATAAAGCAGTTGAGCGTATTGAAAACGCAGACGATA
ATTTTAACCAACTTTACGAAAATGCAAAGCCACTTAAAGAGAATATAGAAATAGCGTTAAAGCTTTTAAAAAT
CTTACTAAAAGAGTTAGAACGAGTTTTAGGAAGAAATACCTTTGCGGAAAGAGTTAATAAGTTAACAGAAG

ATGAACCAAAACTAAATGGTTTAGCAGGAAACTTAGATAAAAAAATGAATCCAGAATTATATTCAGAACAGG
AACAGCAACAAGAACAACAAAAGAATCAAAAACGAGATAGAGGTATGCACTTATAGAACATGCATTTATGCC
GAGAAAACTTATTGGTTGGAATGGGCTATGTGTTAGCTAACTTGTTAGCGAGTTGGTTGGACTTGAATTGGG
ATTAATCCCAAGAAAGTACCAACTCAACAACACATAAAGCCCTGTAGGTTCCGACCAATAAGGAAATTGGAA
TAAAGCAATAAAAGGAGTTGAAGAAATGAAATTCAGAGAAGCCTTTGAGAATTTTATAACAAGTAAGTATGT
ACTTGGTGTTTTAGTAGTTTTAACTGTTTACCAGATAATACAAATGCTTAAATAAAAAAAGACTTGATCTGATT
AGACCAAGTCTTTTGATAGTGTTATATTAATAACAAAATAAAAAGGAGTCGCTCACGCCCTGACCAAAGTTTG
TGAACGACATCATTCAAAGAAAAAAACACTGAGTTGTTTTTATATATCTTGTATATTTAGATATTAAACGATATT
TAAATATACATCAAGATATATATATTTGGGTGAGCGATTCCTTAAACGAAATTGAGATTAAGGAGTCGATTTTTTA
TGTATAAAAACAATCATGCAAATCATTCAAATCATTTGGAAAATCACGATTTAGACAATTTTTCTAAAACCGGC
TACTCTAATAGCCGGTAATAGCCGGTTGGACGCACATACTGTGTGCATATCTGATCCAAAATTAAGTTTTGATG
CAATGACGATCGGTGGAAATCTCAACCGAGACAACGCTCAAGCCCTTTCTAAATTTATGAGTGTAGAGCCCC
AAATAAGACTTTGGGATATTCTTCAAACAAAGTTTAAAGCTAAAGCACTTCAAGAAAAAGTTTATATTGAATA
TGACAAAGTGAAAGCAGATAGTTGGGATAGACGTAATATGCGTATTGAATTTAATCCAAACAAACTTACACG
AGATGAAATGATTTGGTTAAAACAAAATATAATAAGCTACATGGAAGATGACGGTTTTACAAGATTAGATTTA
GCCTTTGATTTTGAAGATGATTTGAGTGACTACTATGCAATGTCTGATAAAGCAGTTAAGAAAACTATTTTTTA
TGGTCGTAATGGTAAGCCAGAAACAAAATATTTTGGCGTGAGAGATAGTAATAGATTTATTAGAATTTATAATA
AAAAGCAAGAACGTAAAGATAATGCAGATGCTGAAGTTATGTCTGAACATTTATGGCGTGTAGAAATCGAAC
TTAAAAGAGATATGGTGGATTACTGGAATGATTGCTTTAGTGATTTACATATCTTGCAACCAGATTGGAAAAC
TATCCAACGCACTGCGGATAGAGCAATAGTTTTTATGTTATTGAGTGATGAAGAAGAATGGGGAAAGCTTCA
CAGAAATTCTAGAACAAAATATAAGAATTTGATAAAAGAAATTTCGCCAGTCGATTTAACGGACTTAATGAAA
TCGACTTTAAAAGCGAACGAAAAACAATTGCAAAAACAAATCGATTTTTGGCAACATGAATTTAAATTTTGG
AAATAGTGTACATATTAATATTACTGAACAAAAATGGTTCTGTTGCAAAGTAAAAAATATAGCTAACCACTAAT
TTATCATGTCAGTGTTCGCTTAACTTGCTAGCATGATGCTAATTTCGTGGCATGGCGAAAATCCGTAGATCTGA
AGAGACCTGCGGTTCTTTTTATATAGAGCGTAAATACATTCAATACCTTTTAAAGTATTCTTTGCTGTATTGATA
CTTTGATACCTTGTCTTTCTTACTTTAATATGACGGTGATCTTGCTCAATGAGGTTATTCAGATATTTCGATGTA
CAATGACAGTCAGGTTTAAGTTTAAAAGCTTTAATTACTTTAGCCATTGCTACCTTCGTTGAAGGTGCCTGAT
CTGTAATTACCTTTTGAGGTTTACCAAATTGTTTAATGAGACGTTTGATAAACGCATATGCTGAATGATTATCTC
GTTGCTTACGCAACCAAATATCTAATGTATGTCCCTCTGCATCAATGGCACGATATAAATAGCTCCATTTTCCTT
TTATTTTGATGTACGTCTCATCAATACGCCATTTGTAATAAGCTTTTTTATGCTTTTTCTTCCAAATTCGATATAA
AATTGGGGCATATTCTTGAACCCAACGGTAGACCGTTGAATGATGAACGTTTACACCACGTTCCCTTAATATT
TCAGATATATCACGATAACTCAATGCATATCTTAGATAGTAGCCAACGGCTACAGTGATAACATCCTTGTTAAAT
TGTTTATATCTGAAATAGTTCATACAGAAGGCTCCTTTTTGTTAAAATTATACTATAAATTCAACTTTGCAACAG
AACCGATAATAATATATTTAAAAAATTATCAAAAGATAAAGCTACTAAAATACAAGAATTGTTAGAAAAAGATA
GAAATTCTAGAATTTCTAATGTAAGAGAAGCAAAGTACAATATACAAGAAAATATTGATATATATGAAAAATAT
ATTAATGAAATGATTTAAAAATAGAATTATTAGAGTCGATTATAGAGAAGATAAAGTATTAAGCCATTATAACG
ATTTGTTACTTTAGTAATATCATTGATAAGGGGAGGCTTATCATAAAT
>Staphylococcus aureus strain BK20781,SCCmec
AAACGACATGAAAATCACCATTTTAGCTGTAGGGAAACTAAAAGAGAAATATTGGAAGCAAGCCATAGCAG
AATATGAAAAACGTTTAGGCCCATACACCAAGATAGACATCATAGAAGTTCCAGACGAAAAAGCACCAGAA
AATATGAGTGACAAAGAAATTGAGCAAGTAAAAGAAAAAGAAGGCCAACGAATACTAGCCAAAATCAAACC
ACAATCCACAGTCATTACATTAGAAATACAAGGAAAGATGCTATCTTCCGAAGGATTGGCCCAAGAATTGAA
CCAACGCATGACCCAAGGGCAAAGCGACTTTGTTTTCGTCATTGGCGGATCAAACGGCCTGCACAAGGACG
TCTTACAACGCAGTAACTACGCACTATCATTCAGCAAAATGACATTCCCACATCAAATGATGCGGGTTGTGTT

AATTGAACAAGTGTACAGAGCATTTAAGATTATGCGAGGAGAAGCTTATCATAAGTAATGAGGTTCATGATTT
TTGACATAGTTAGCCTCCGCAGTCTTTCATTTCAAGTAAATAATAGCGAAATATTCTTTATACTGAATACTTATA
GTGAAGCAAAGTTCTAGCTTTGAGAAAATTCTTTCTGCAACTAAATATAGTAAATTACGGTAAAATATAAATAA
GTACATATTGAAGAAAATGAGACATAATATATTTTATAATAGGAGGGAATTTCAAATGATAGACAACTTTATGC
AGGTCCTTAAATTAATTAAAGAGAAACGTACCAATAATGTAGTTAAAAAATCTGATTGGGATAAAGGTGATCT
ATATAAAACTTTAGTCCATGATAAGTTACCCAAGCAGTTAAAAGTGCATATAAAAGAAGATAAATATTCAGTTG
TAGGGAAGGTTGCTACTGGGAACTATAGTAAAGTTCCTTGGATTTCAATATATGATGAGAATATAACAAAAGA
AACAAAGGATGGATATTATTTGGTATATCTTTTTCATCCGGAAGGAGAAGGCATATACTTATCTTTGAATCAAG
GATGGTCAAAGATAAGTGATATGTTTCCGCGGGATAAAAATGCTGCAAAACAAAGAGCATTAACTTTATCTTC
CGAACTCAATAAATATATTACATCAAATGAATTTAATACTGGAAGATTTTATTACGCAGAAAATAAAGATTCATC
TTATGATTTAAAAAATGATTATCCATCAGGATATTCTCATGGATCAATAAGATTCAAATATTATGATTTGAATGA
AGGATTCACAGAAGAAGATATGCTAGAGGATTTAAAGAAATTTTTAGAACTATTTAATGAATTAGCTTCAAAA
GTTACAAAAACATCCTATGATAGCTTGGTCAATAGCATAGACGAAATACAGGAAGACAGCGAAATTGAAGAA
ATTAGAACAGCACAAAAAGATAAGACACTCAAGGAAGTGGAAGCACCTAAAGGAATAATTCCAAAATATAA
AAAAGGTGTATCAAAGACTACTAAAAATGATTCAGAAATTGAAAAATCAAATAAAGAGAATAAATTAACCGG
TAAAGTTGGAGAAAAATTAGCGCTAAATTACTTTAATGAGCTAATTGATAATAAAATAGACGAAGATAAGAAA
GAACAGTTTAGGAATATTTTAAATGATAATCCAGGCTCTCAACACGGTCATGGCTATGATTTAGTAGCTTTTGA
TCCAACAAATACAGATAAAGCTGTAGAAAAATTTATTGAAATTAAAACATCTACATCTTCTAGTATTGAGGAAC
CATTTTTTATGTCGCTAAATGAAATGTTTGCTATGAAAGAATATAAGCAGAAATATTTAATATTAAGAATATTTA
ATGTTTCCGGTAAAGAACCACAATTTTATTTTATAGATCCATATGCAAATTATTCTGAATTTAAAGATGTAGATG
ATCTCATTGACAAAGTATTTAATGTAGAAGCTATTCAGTATAAAGTTTTTGGCGAAAAATGATTACTTGAACAA
GAGCTAAAATAAAATTGTGATCTAATAAAAATAGAAACTGTAATTTAAATAAAACTTTCTAAATAAGCTAACTG
ATAAAAAATCAGTTTGTCCACAGTCTGAAACAAGATTCCTATATTCTTTAGGAATCTTGTTTTTTCTATTTTTAT
GGTGATAAAGAGCAGATAAGATAATGTGTAATAATCACAAAAAAGTTAAATATTTTAAGGCTTGTTTAATTATT
AATGATTTTATATATAAAGAGCAGTATAATAAAGTTGTTAATATATTATGAATAATATTCAAGTAATTTTATTGTTT
TTTAATTTGTCGATATTTAAGTTGAGTTAAATTTAAAGGGTGTAATTTGTTTTACAATGATGAAGATAATTAGTC
TATCAAAATAAAGGGGTTGGGACTGTTATGAGTGATAATTTGTCATTATTCATTGACTATATCAATGATAATATA
ATCTATGGTAGTGAAATCAAACGGGAGAAATTAGAGAATTTATTTAATCAATTTGCTATAAAAAATGTTGAAA
AGAACATTGTCTATGATGAACTGAAATCTTTAGATATTACAATCATTGAGTCACAGGATTCATATAAAAATAAAT
TGAAGAGATTATTTTCGGTTCTGTTGCAAAGTAAAAAAATATAGCTAACCACTAATTTATCATGTCAGTGTTCG
CTTAACTTGCTAGCATGATGCTAATTTCGTGGCATGGCGAAAATCCGTAGATCTGAAGAGACCTGCGGTTCTT
TTTATATAGAGCGTAAATACATTCAATACCTTTTAAAGTATTCTTTGCTGTATTGATACTTTGATACCTTGTCTTT
CTTACTTTAATATGACGGTGATCTTGCTCAATGAGGTTATTCAGATATTTCGATGTACAATGACAGTCAGGTTT
AAGTTTAAAAGCTTTAATTACTTTAGCCATTGCTACCTTCGTTGAAGGTGCCTGATCTGTAATTACCTTTTGAG
GTTTACCAAATTGTTTAATGAGACGTTTGATAAACGCATATGCTGAATGATTATCTCGTTGCTTACGCAACCAA
ATATCTAATGTATGTCCCTCTGCATCAATGGCACGATATAAATAGCTCCATTTTCCTTTTATTTTGATGTACGTCT
CATCAATACGCCATTTGTAATAAGCTTTTTTATGCTTTTTCTTCCAAATTTGATACAAAATTGGGGCATATTCTT
GAACCCAACGGTAGACCGTTGAATGATGAACGTTTACACCACGTTCCCTTAATATTTCAGATATATCACGATAA
CTCAATGTATATCTTAGATAGTAGCCAACGGCTACAGTGATAACATCCTTGTTAAATTGTTTATATCTGAAATAG
TTCATACAGAAGACTCCTTTTTGTTAAAATTATACTATAAATTCAACTTTGCAACAGAACCGTATTATGGAATAG
AGATGTTGGTAACATTTATACAGGATCATTATACTTAAGTTTAATTTCGTTATTACAGAACCACACATTCCAACC
AGAAGAGAAAGTATGTCTATTTAGTTATGGTTCAGGAGCAGTAGGAGAAATCTTTAGTGGTTCAATCGTTAA
AGGATATGACAAAGCATTAGATAAAGAGAAACACTTAAATATGCTAGAATCTAGAGAGCAATTATCAGTCGA
AGAATACGAAACATTCTTTAACAGATTTGATAATCAAGAATTTGATTTCGAACGTGAATTGACACAAGATCCA

TATTCAAAAGTATACTTATACAGTATAGAAGACCATATCAGAACATATAAGATAGAGAAATAAACTAGTGGCCG
ATTGTGCTTGATGAGCTTGGGACATAAATCCTAACTCGAAATAAATAAGCATATCACTAAACTGATTTTTTAAA
GTTTACAGTGATATGCTTATTTTTTTATCTTACGATTTTGTACGTGCATGCTTGCCTAGGGGTATGGCTCGAGC
CATTAGTCTCTCGCACATACTATTCCCTCAGGCGTCAGCACTTACAAAATCGGTTGTAATTTTCATTTTTATACG
CATTCTTACTGAGATTATACTAATAAGAGGAATAGTAAAAGCAATTCTAAGTAAAATTGCAGATAAGAGGTTT
GTTAAAAGCAGTTCTAAGTAAAATTGCAGATAAGAGGTTTGTTAAAAGCAGTTCTAAGTAAAATTGCAGATA
AGAGGTTTGTTAAAAGCAGTTCTCAGTAAAATTACAGATAAGAGGTACGTTAAAAGCAGTTCTAAGTAAAAT
TGCAGATAAGAGGTACGTTAAAAGCAGTTCTAGGCAAAATTGCAGATAAGAGGTGCGTTAAAAGCAGTTCT
CAGTAAAATTGCTGATAAGGGGTAAGTTAAAAGCAATCCTAAGTAAAATTGCAGATAAGAGGTAAGTTAAAA
GCAATCCTAAGTAAAATTGCAGATAAGGGGTACAGAAAAACTAGACTTGATTACAAAATGGAGCTTGGGAC
ATAAATGATTTTTTAAAAATGAGATGAGACGTAGATTAACTCCATAATCAATACGAATCTATCGACTTCTTTATT
TATGATATTCATCTCTTTTTAATGGAAATAAAAGTGCGATTAATGTGATAATACAGTTACGTTAATTAAAAAAAT
AAAAATGCAAGGAGAGGTAATATGCTAACTGTATATGGACATAGAGGATTACCTAGTAAAGCTCCGGAAAAT
ACAATTGCATCATTTAAAGCTGCTTCAGAAGTAGAAGGTATAAACTGGTTGGAGTTAGATGTTGCAATTACAA
AAGATGAACAACTGATTATCATTCATGATGATTATTTAGAACGGACTACAAATATGTCCGGGGAAATAACTGA
ATTGAATTATGATGAAATTAAAGATGCTTCTGCAGGATCTTGGTTTGGTGAAAAATTCAAAGATGAACATTTG
CCAACTTTCGATGATGTAGTAAAAATAGCAAATGAATATAATATGAATTTAAATGTAGAATTAAAAGGTATTAC
TGGACCGAATGGACTAGCACTTTCTAAAAGTATGGTTAAGCAAGTGGAAGAACAATTAACAAACTTAAATCA
GAATCAAGAAGTGCTCATTTCAAGCTTTAATGTTGTGCTTGTTAAACTTGCAGAAGAAATCATGCCACAATAT
AACAGAGCAGTTATATTCCATACAACTTCGTTTCGTGAAGACTGGAGAACACTTTTAGATTACTGTAATGCTA
AAATAGTAAACACTGAAGATGCCAAACTTACTAAAGCAAAAGTAAAAATGGTAAAAGAAGCGGGTTATGAA
TTGAACGTATGGACTGTAAACAAACCAGCACGTGCAAACCAACTTGCTAATTGGGGAGTTGATGGTATCTTT
ACAGACAATGCAGATAAAATGGTGCATTTGTCTCAATAGAAAGTTAGAGGTGAGTCTTACGTTTCAGTGACG
GTAGACTTACCTTTAACATGTTACATACTAAAAAATTAATTTGAATAAGAAAGAGAGACATATATGAAATACGA
TGATTTTATAGTAGGAGAAACATTCAAAACAAAAAGCCTTCATATTACAGAAGAAGAAATTATCCAATTTGCA
ACAACTTTTGATCCTCAATATATGCATATAGATAAAGAAAAAGCAGAACAAAGTAGATTTAAAGGTATCATTG
CATCTGGCATGCATACACTTTCAATATCATTTAAATTATGGGTAGAAGAAGGTAAATACGGAGAAGAAGTTGT
AGCAGGAACACAAATGAATAACGTTAAATTTATTAAACCTGTATACCCAGGTAATACATTGTACGTTATCGCTG
AAATTACAAATAAGAAATCCATAAAAAAAGAAAATGGACTCGTTACAGTGTCACTTTCAACATACAATGAAA
ATGAAGAAATTGTATTTAAGGGAGAAGTAACAGCACTTATTAATAATTCATAATAAAACAGTGAAGCAACCAT
CGTTACGGATTGCTTCACTGTTTTGTTATTCATCTATATCGTATTTTTTATTACCGTTCTCATATAGCTCATCATAC
ACTTTACCTGAGATTTTGGCATTGTAGCTAGCCATTCCTTTATCTTGTACATCTTTAACATTAATAGCCATCATCA
TGTTTGGATTATCTTTATCATATGATATAAACCACCCAATTTGTCTGCCAGTTTCTCCTTGTTTCATTTTGAGTTC
TGCAGTACCGGATTTGCCAATTAAGTTTGCATAAGATCTATAAATATCTTCTTTATGTGTTTTATTTACGACTTG
TTGCATACCATCAGTTAATAGATTGATATTTTCTTTGGAAATAATATTTTTCTTCCAAACTTTGTTTTTCGTGTCT
TTTAATAAGTGAGGTGCGTTAATATTGCCATTATTTTCTAATGCGCTATAGATTGAAAGGATCTGTACTGGGTT
AATCAGTATTTCACCTTGTCCGTAACCTGAATCAGCTAATAATATTTCATTATCTAAATTTTTGTTTGAAATTTGA
GCATTATAAAATGGATAATCACTTGGTATATCTTCACCAACACCTAGTTTTTTCATGCCTTTTTCAAATTTCTTAC
TGCCTAATTCGAGTGCTACTCTAGCAAAGAAAATGTTATCTGATGATTCTATTGCTTGTTTTAAGTCGATATTAC
CATTTACCACTTCATATCTTGTAACGTTGTAACCACCCCAAGATTTATCTTTTTGCCAACCTTTACCATCGATTT
TATAACTTGTTTTATCGTCTAATGTTTTGTTATTTAACCCAATCATTGCTGTTAATATTTTTTGAGTTGAACCTGG
TGAAGTTGTAATCTGGAACTTGTTGAGCAGAGGTTCTTTTTTATCTTCGGTTAATTTATTATATATTCTTCGTTACT
CATGCCATACATAAATGGATAGACGTCATATGAAGGTGTGCTTACAAGTGCTAATAATTCACCTGTTTGAGGG
TGGATAGCAGTACCTGAGCCATAATCATTTTTCATGTTGTTATAAATACTCTTTTGAACTTTAGCATCAATAGTT

AGTTGAATATCTTTGCCATCTTTTTTCTTTTTCTCTATTAATGTATGTGCGATTGTATTGCTATTATCGTCAACGAT
TGTGACACGATAGCCATCTTCATGTTGGAGCTTTTTATCGTAAAGTTTTTCGAGTCCCTTTTTACCAATAACTG
CATCATCTTTATAGCCTTTATATTCTTTTTGTTTTAATTCTTCAGAGTTAATGGGACCAACATAACCTAATAGATG
TGAAGTCGCTTTTCCTAGAGGATAGTTACGACTTTCTGTTTCATTAGTTGTAAGATGAAATTTTTTTGCGAAAT
CACTTAAATATTCATCCATTTTTTTAACGGTTTTAAGTGGAACGAAGGTATCATCTTGTACCCAATTTTGATCCA
TTTGTTGTTTGATATAGTCTTCAGAAATACTTAGTTCTTTAGCGATTGCTTTATAATCTTTTTTAGATACATTCTT
TGGAACGATGCCTATCTCATATGCTGTTCCTGTATTGGCCAATTCCACATTGTTTCGGTCTAAAATTTTACCACG
TTCTGATTTTAAATTTTCAATATGTATGCTTTGGTCTTTCTGCATTCCTGGAATAATGACGCTATGATCCCAATCT
AACTTCCACATACCATCTTCTTTAACAAAATTAAATTGAACGTTGCGATCAATGTTACCGTAGTTTGTTTTAATT
TTATATTGAGCATCTACTCGTTTTTTATTTTTAGATACTTTTTTTATTTTACGATCCTGAATGTTTATATCTTTAAC
GCCTAAACTATTATATATTTTTATCGGACGTTCAGTCATTTCTACTTCACCATTATCGCTTTTAGAAATATAACTG
CTATCTTTATAAACTTGTTTGAAATTTTTATCTTCAATTGCATCAATAGTATTATTAATTTCTTTATCTTTTGAAGC
ATAAAAATATATACCAAACCCGACAACTACAACTATTAAAATAAGTGGAACAATTTTTATCTTTTTCATCAATAT
CCTCCTTATATAAGACTACATTTGTAGTATATTACAAATGTAGTATTTATGTCAAAATAATGTTATAATTTTTGTGA
TATGGAGGTGTAGAAGGTGTTATCATCTTTTTTAATGTTAAGTATAATCAGTTCATTGCTCACGATATGTGTAAT
TTTTTTAGTGAGAATGCTCTATATAAAATATACTCAAAATATTATGTCACATAAGATTTGGTTATTAGTGCTCGTC
TCCACGTTAATTCCATTAATACCATTTTACAAAATATCGAATTTTACATTTTCAAAAGATATGATGAATCGAAAT
GTATCTGACACGACTTCTTCGGTTAGTCATATGTTAGATGGTCAACAATCATCTGTTACGAAAGACTTAGCAAT
TAATGTTAATCAGTTTGAGACCTCAAATATAACGTATATGATTCTTTTGATATGGGTATTTGGTAGTTTGTTGTG
CTTATTTTATATGATTAAGGCATTCCGACAAATTGATGTTATTAAAAGTTCGTCATTGGAATCGTCATATCTTAAT
GAACGACTTAAAGTATGTCAAAGTAAGATGCAGTTCTACAAAAAGCATATAACAATTAGTTATAGTTCAAACA
TTGATAATCCGATGGTATTTGGTTTAGTGAAATCCCAAATTGTACTACCAACTGTCGTAGTCGAAACCATGAAT
GACAAAGAAATTGAATATATTATTCTACATGAACTATCACATGTGAAAAGTCATGACTTAATATTCAACCAGCT
TTATGTTGTTTTTAAAATGATATTCTGGTTTAATCCTGCACTATATATAAGTAAAACAATGATGGACAATGACTG
TGAAAAAGTATGTGATAGAAACGTTTTAAAAATTTTGAATCGCCATGAACATATACGTTATGGTGAATCGATAT
TAAAATGCTCTATTTTAAAATCTCAGCACATAAATAATGTGGCAGCACAATATTTACTAGGTTTTAATTCAAATA
TTAAAGAACGTGTTAAGTATATTGCACTTTATGATTCAATGCCTAAACCTAATCGAAACAAGCGTATTGTTGCG
TATATTGTATGTAGTATATCGCTTTTAATACAAGCACCGTTACTATCTGCACATGTTCAACAAGACAAATATGAA
ACAAATGTATCATATAAAAAATTAAATCAACTAGCTCCGTATTTCAAAGGATTTGATGGAAGTTTTGTGCTTTA
TAATGAACGGGAGCAAGCTTATTCTATTTATAATGAACCAGAAAGTAAACAACGATATTCACCTAATTCTACTT
ACAAAATTTATTTAGCGTTAATGGCATTCGACCAAAATTTACTCTCATTAAATCATACTGAACAACAATGGGAT
AAACATCAATATCCATTTAAAGAATGGAACCAAGATCAAAATTTAAATTCTTCAATGAAATATTCAGTAAATTG
GTATTACGAAAATTTAAACAAACATTTAAGACAAGATGAGGTTAAATCTTATTTAGATCTAATTGAATATGGTA
ATGAAGAAATATCAGGGAATGAAAATTATTGGAATGAATCTTCATTAAAAATTTCTGCAATAGAACAGGTTAA
TTTGTTGAAAAATATGAAACAACATAACATGCATTTTGATAATAAGGCTATTGAAAAAGTTGAAAATAGTATG
ACTTTGAAACAAAAAGATACTTATAAATATGTAGGTAAAACTGGAACAGGAATCGTGAATCACAAAGAAGCA
AATGGATGGTTCGTAGGTTATGTTGAAACGAAAGATAATACGTATTATTTTGCTACACATTTAAAAGGCGAAG
ACAATGCGAATGGCGAAAAAGCACAACAAATTTCTGAGCGTATTTTAAAAGAAATGGAGTTAATATAATGGA
TAATAAAACGTATGAAATATCATCTGCAGAATGGGAAGTTATGAATATCATTTGGATGAAAAAATATGCAAGTG
CGAATAATATATATAGAAGAAATACAAATGCAAAAGGACTGGAGTCCAAAAACCATTCGTACACTTATAACGA
GATTGTATAAAAAGGGATTTATAGATCGTAAAAAAGACAATAAAATTTTTCAATATTACTCTCTTGTAGAAGAA
AGTGATATAAAATATAAAACATCTAAAAACTTTATCAATAAAGTATACAAAGGCGGTTTCAATTCACTTGTCTT
AAACTTTGTAGAAAAAGAAGATCTATCACAAGATGAAATAGAAGAATTGAGAAATATATTGAATAAAAAATA
AAATTGTTGTGTTTACAACAATACATAGAAAACAGAGGAAACAATCAAGTCGTTGAATATTTCCTCTGTTTTT

TAGTTGAAAAAATTAACCGAAAGCCTGAATGCAAGTCTTGATTAAATCAATAATGCTTGTAATAACACCAGTG
AAATCCATATGCATACCCTCTTTCTATTTAAGATACATTAAGTATAATATCAAACAAATAAAAAATGTTAAAAATT
CCCTAATTGGCTATTTAGATTGCATAAATGTCAAAAATTTGAAAAACATACAACGACTTTGCATAAAAAAATCGT
CATATTGGAAATACGTAATTTATTGAAATAATAAAAAAAATAAAAGAACGAAGATGATAACCTAAGTGAGGTT
TTAAGTTGTTCTAAGGTTTAATTTAATTTATGTTAAAATAGTTGGTATAAAAATACATGATAAACTATAAACTAA
ATTCAAAATAACTTATGGGGTAGGCAATTATGGAAAATAAAATATAAATGATAATGAAAAAAGAGTGCTAAGG
GAAATTTATAACCATCATAATATTTCGCGTACTCAAATATCTAAAAATCTTGAGATTAATAAGGCAACGATTTCT
AGTATTTTGAATAAGTTAAAGTATAAATCTCTTGTTAATGAGGTTGGTGAGGGTGATAGCACGAAGAGTGGT
GGTAGAAAACCTATTCTTCTGAAGGTTAATCATCTTTATGGTTATTTTATTTCTTTGGATTTAACTTATAGTTCTG
TTGAAGTGATGTACAATTATTTTGATGGTAATGTCATTAAGCATGAATCTTATGATTTACCTGATGAAAAGGTT
AGTAGTATATTAAGCATAATAAAAAAACATATTGATATTCAGGAGAAACTTGATACTTATAACGGACTATTAGG
TGTGTCTGTTTCTATACATGGAGTTGTGGATAATGAGCAGCATGTGACATATTTACCATTCCATGAAACTGAAG
GAATTTCAATTGCTAAGAAAATAAAAGAAATTACTAATGTTCCAGTCGTAGTTGAAAATGAAGCGAATCTTTC
AGCGTTATATGAACGTAATTTTAATCATAATTTATCCTACAATAATCTTATTGCTTTAAGTATACATAAAGGTATT
GGTGCTGGGCTTATTATTAATAATCAATTGTATCGTGGTGCAAATGGGGAAGCGGGTGAAATTGGAAAAACA
CTTGTCTCAAAAGTTAGCGATAATGTGGAGATCTTTCATAAGATTGAAGATATTTTTTCACAAGAAGCTTTACT
GCATAATTTAAGTAATCAACTAAATGAGAAGATGACGCTTAGCAAATTAATTCAATTTTATAATGAAAAAAATC
CAGTCGTAGTTGAAGAAATGGAACAATTTATAAATAAAATTGCTGTTTTAATACATAATTTAAACACCCAGTTT
AATCCGAATGCAATTTACATTAACTGTCCATTGTTCAATGAAATGCCTGAAATATTAGAAGCAATTAAGAACCA
GTTCAAACAATATTCACGTAACGAAATTCAAATAAAGTTAACATCTAATGTCAAATTTGCAACTTTGCTAGGTG
GTACATTAGCAATTATCCAAAAAGTACTACAGATTAATGATATTTACTTAGATATAAAAGCATAAAAAACTAATT
CAAATGAATAATCAAAGTTCGTAATTGTCTTTATAAAAAAATCCCTCAATCCGAATTGAATTTTCGGATTGAGG
GATTTTTATAGTTCTATTGCAGAAGAAAACTATTTTAAAAATGCTGGTAAATGTTGATAGCCACCTCTAACGTT
AACAATATTCGTAAATCCTTTATATTCTAATATTCCTACCGCTATTGAACTTCTAACACCTGATTGACAATGTACA
TAAATTAGGTCATTTTTATCGAAAGGTATATCTTCATTTAAAAGTTTACCGTGAGGAATATGAATTGCTTGTTTT
AAATGACCTTTACGCCATTCATCATCATTACGAACATCTAATACATTATGTTCTTCACCAGTCATTTCAGAACTAT
GAATAGATGATGTGACGATATTTGTTTGTGGCAAACGGTAACTTTTTACATTTTCAAAACCAATTAATTGTAAA
GCATGAATAGCTTGTTGAACGGTAGATTTATCGCCAATTAATTCAATATCTTAGTCATAATCTAAATACCAACCA
ATTTGATTTATAAAAGTTTTATTAAAAGGAATATTGATAGTTCCATGCATATGACCACCATGGAATGCTTCTTTA
CTTCGAAGATCAAAAGCAGTTTGTGTATTGCTTGAACTAGGGTAAACATTATATGGTTGGTACATTTGCATAC
CAAATTGATTTATTTTTTTCATTTGTGAAAAATGGTGTGGTGGAGCTGGCTGATTGAGTGTTAAAGTTTCGAT
AAATGAAGTTTCATCTTTAACATTAAAAGCCCAGTTGTTTATTTTCTCATAACCCAAAGTAGTTGTAGGTAATG
AACCTAGCGCTTTACCACAAGGACTCCCTGCACCATGACCTGGCCAAATTTGAATATAGTCTGGTAATGTTGC
AGCAAATTGTATGGACTGATACATTTGTTTTGCTCCGATTTTTGTAGAACCTTCAACATTTACAGCTTTTTCTA
ATAGATCTGGTCTACCTACATCACCAACAAAGATGAAGTCACCGCTAAATAATCCCATTGGTATACTGGAACCC
CCACCTTCGTCAGTAAGTAAAAAACTAATACTCTCAGGGGGATGGCCTGGAGTGTGTAAGACTTCTAATTTA
ATCTTTCCTAAATAGATAATATCTTGATGCTTAACGAAATGTGTTTGTTTAGGCATATTTTTATAATTAAATTCAT
CTTTACCTTCATCAGATACGTGTATACTTGCATTCAATTTATTTGCCACATCTCTAATACCTGAAGCAAAATCAG
CATGAATATGTGTTTCTGCAGCTTTAGTAATTGTGAATCCTTCTTTATCTGCAACTTTTAAATATTTTGTTAAATC
TCGTATAGGGTCAATAATCATTGCTTCTCCTGTACGTTGACATCCAATTAAATAAGATGCTTGTGAAAAATTGT
CTTCATAAAATTGTTTGAAAAACAAAAAAAACTCCTTTTTAAAATAGATTTTATTGATTAGATAAATAAGTTAT
GATTTGCTTGCTCAGTATGTCCAATATAAGTACCTACGCCACCATAATCGACTTCATCTCTTAATTCTTCTTTTG
AAATTCCCATAACATCCATACTCATGGTACAAGCAATTAACTTTATATCTTGATCGATTGCTTGATCGATAAGTG
AGTATAAAGAATCAACATTTTTCTTGTTCATTACATAACGCATCATAATATTACCTAGTCCAAACATATTCATTTT

TGATAATGGCATATGTATTGGATCCTTAGGTAACATAAGGTCAAACATTTTGGAAATACCTTTCTTTTTAACGC
GAGTTGATTGCGCTTTTTTTAATGCGTTGAGGCCCCAAAAAGTAAAGAAAATAGTTACATCTTTACCTGCTGC
TTTAGCGCCATTTGCGATGATCATTGCTGCTACTGCCTTATCTAACTCACCGCTAAATAAAACAATTGTTGTACC
TGTAGCAGTGTCATTGATTTCAAATTCTTTTGGCTTTTCTTTTTGAATAATTGCATTAATTACATTTGCTTCTTCA
GTAAGATTTACAAGGGTATTCCCTGTTTGTTTCGCCCAACTTTTAATATCACTATTGAAACCAGGATCTGTAAC
TGTTACCTCGATTTGCTCACCCGTTGAAATATTGTTAATTTCTTTACTGATATTAACAATAGGTCCAGGGCACT
GAAGACCTCTAAAATCAAATTGTTTACGATTCTCTTTGATTTCAATATCTTTTTCTATTAAAGGAGCACTATTGA
AGTTCTTTGCTTCATAATCTTTATATCCACCCTTTAAATTCACGACATCATAACCTTGTTGGGCTAAATAATCGC
AAGCTTTAGTGCTTCGGTTACCGCTTTTACAATGTATATAATACGTTTTGTTGCTATTCTTATTGAATGATTTAAT
CTCTTCTACTGGGTGTAAAGTTGAACCGTTAATGTGTCCTAATTCATATTCTTCTTTTGTTCTAACATCAATCAA
TTGACCCATTTTTGCCAATTTTTCTAATTCTTCTTTGTTTAATGAATTAATGTGTACTTCTTTGTATTGTTCCATA
CTTACCTCCTATAAATACCTATGAGGGTATAATAAAACGGATAGAATCATTTGCCAAATACCTATAGGGGTATTT
GACAATTTTGTTTTAATTTATTATTATTAACTTAATCAATTTATGTGGAGGAAATGAATATGACTTATGATAAAA
AAATGATTAATCGTATAAATAGAATACAAGGTCAATTAAATGGTGTCGTAAAAATGATGGAAGAAGAAAAAG
ATTGCAAAGATATAATTACGCAACTTAGTGCATCTAAAGGTTCTATACAACGTTTAATGGGGATTATAATTAGT
GAAAATTTAATAGAATGCGTTAAAACAGCAGAAGAAAATAATGAAAGTTCTCAAGAATTAATTAATGAAGCA
GTTAATTTATTAGTTAAAAGTAAATAATGGATATAGCAAATATGACTATTATGTTGCTAATTGGCGTACTGGGTG
GATTTATATCTGGATTAATAGGTATTGGGGGCGCAATTATTATTTACCCAGCTATTCTTATATTGCCACCATTAAT
AGGTATACCTGCGTATAGTGCATATATTGCTTCGGGACTTACCTCTAGTCAAGTATTTTTCAGTACACTTAGTG
GATCATTAAATGCAAGAAAACAACCAGCTTTCTCTCCTAAACTTGTTATATATATGGGAGGGGGTATGTTGATT
GGAAGCATGTTAGGGGCAATTTTAGCTAGTTTGTTTAATGCTACTTTTGTAAATACGGTATATGTAATAATCGC
CATACTTGCTTTAATATTGATGTTTATTAAAGTTAAACCTACTACACAAGAGACGAAATCTAAACCTTTGCTATT
TATTATAGTTGGATTTGGAATTGGTGTAATTTCGGGAATTGTGGGTGCAGGTGGAGCATTTATCATCATTCCT
GTATTATTAGCATTATTTAAATTACCAATGAATACGGTAGTGAACAATAGCATAGCAATTGCTTTTATATCTTCA
GTAGGGGCATTTTTTATAAAATTAATGCAAGGATATATACCAGTAGAAAGTGCAATTTTTTTGATAATTGAATG
ATGATCATTTTCTGAAAATATTATGTGGTCATATAATATAATGCCCATCATTTCACTAATCTCTTTTATTCTCTGAG
TTATTTTGATATCTCCTGGAGAAGGTGTGATGTCTTATCAAGAGTAAATTACAAAAGAATCATTTAAATCAATA
CTTTCACTTTGAATACATGTATTTGAAGTGGAAGGTACTTATTTCAAAAATAGTAAAACCTGTATCTTAAATTAC
TTAATAGTAACATAAGATACAGGCTGATTTTTTATTCATTGTTATTTATACTAAAGCACCCGATAGCTCTGAAAA
CAATCACAAATTCAACTTTTCAAAGCCACAGCTTTAAGTTATTTTGTCCAGACAACCCCCATTGCCCAACCCA
TTTTATGGAATTGGCATCCAGGCAACAACTTTTCATATAAATCGTAATAATTTTGTTCAGATAGGTACTTATCTG
AAGCTAAATGCTCAAGCCATGATTTAGATGTGTTGTGATTATAAATTCTAATCGCATTTTTTATTCCAAATTTGA
AACAATGCGGTAAAAACTCTTGGATTGCCCCTAATTTATAAACATAGGTAGGCGGTGTCTCGACTTCAGATAC
ATTATCAAGAATAACTATTCTTCCTTCCTCATTCAACAGTTCCTTCATTTGCTGTATTACGCTGGCTATATCATCC
AAATGATGAAAGGTTGTTCGGCTTACAATAAAATCAAACTTCTCATTAAAATTAAGTTGTTCTGCATTCATATT
CAGATAGACCGTATTTGTTAGTTGACGTTTAGATTTGGCAAGATCGAGCATTTGATTAGAAATATCAATCCCTA
CCACTTCATCATAATAACTTGCTAATTTCTCCACTAACAAACCCGAGCCACATCCGATATCTAATGCTCTGCCTT
TCTTTGGAGACATATTAGACACAAAGAATGAATAATCATTCAAAAGCTCATTCACGAAATCGTAATCTTCTGCA
ACCTTATCAAACTGTGATTCTATTGTATTCAAAAAGATCCCCCATTCCTACTTTATCGACATTCTTTCATTACTTA
CCACTTTAGATGTTTTTTCGTTGGGGATAAAACTTCCCTTTAGACAATTTTATCCAAAGACAATACAACAGTG
CAACTTTATTAAAGTCACTGTCCTTTATCGCAGCCTTTACTTTTTAGTAAAGACAGTGGCTTCTCTTATCAAGT
TTCAAAACATATTATTTTGAAGAAAACGTCCATCTGAAGTGTCAAGTGCAAAATTACATATAAAGGTTTATTCT
AAAATGAAAAGATGATACAATCATATTCAGTTACATAAGGAGGTTTCAATTATGTGCACCAGTATCGCAGTAG
TAGAAATTACTTTATCTCATTCATAATGAAAAAAATGGAAAGGAGATAAAAGTATGGGTACTTTTTCTATATTT

GTTATTAATAAAGTTCGTTATCAACCAAATCAAAATTAATTGGTTATAATGAACGCTTAATGTCAGTTCATTATA
ACCAGTAAGGAGAAGGTTATAATGAACCAGTAAGGAGAAGGTTATAATGAACCAGAAAAACCCTAAAGACA
CGCAAAATTTTATTACTTCTAAAAAGCATGTAAAAGAAATATTGAATCACACGAATATCAGTAAACAAGACAA
CGTAATAGAAATCGGATCAGGAAAAGGACATTTTACCAAAGAGCTAGTCAAAATGAGTCGATCAGTTACTGC
TATAGAAATTGATGGAGGCTTATGTCAAGTGACTAAAGAAGCGGTAAACCCCTCTGAGAATATAAAAGTGAT
TCAAACGGATATTCTAAAATTTTCCTTCCCAAAACATATAAACTATAAGATATATGGTAATATTCCTTATAACATC
AGTACGGATATTGTCAAAAGAATTACCTTTGAAAGTCAGGCTAAATATAGCTATCTTATCGTTGAGAAGGGAT
TTGCGAAAAGATTGCAAAATCTGCAACGAGCTTTGGGTTTACTATTAATGGTGGAGATGGATATAAAAATGC
TCAAAAAAGTACCACCACTATATTTTCATCCTAAGCCAAGTGTAGACTCTGTATTGATTGTTCTTGAACGACAT
CAACCATTGATTTCAAAGAAGGACTACAAAAAGTATCGATCTTTTGTTTATAAGTGGGTAAACCGTGAATATC
GTGTTCTTTTCACTAAAAACCAATTCCGACAGGCTTTGAAGCATGCAAATGTCACTAATATTAATAAACTATCG
AAGGAACAATTTCTTTCTATTTTCAATAGTTACAAATTGTTTCACTAAATTAAAGTAATAAAGCGTTCTCTAATT
TCACAAGAGGACGCTTTATTCTTCCCAAAAATTGTTCAATATTTATCAATAAATCAGTAGTTTTAAAAGTAAGC
ACCTGTTATTGCAATAAAATTAGCCTAATTGAGAGAAGTTTCTATAGAATTTTTCATATACTTAACGAGTGCTTT
CACCTTTGAATATAGTCCTTCCCACTTATCATCACACTCTCCCCGATAGCCTTTTCTAGCTATATCCAGTAAAGT
TACATGCTCTTTAGGTAAAAGAGGTATAGCCCATTCTGCAGCGACATCTTTCGAGGTAATTTCACCAGTAGTC
ACTGTTTGCCACATTCGAGCTAGGGTTAAAATTACATTACGCTCATCACCTTTTATCCCCTCAATTAGTTCTGG
CAAAGAATCCTTAATTGCTCTTCGAATATCTGTCAAAGGTACGGAGACAAGTATACTTGAAGAATCAGGACC
AAATAGAGAAATACTATTCTTTCTTGCTTGTGCTAAAACAATAGCCAAATCAGGATCATAGCTTGGTTCCTGA
ATTTGTCCATTCTCAAATTCACCCCTGAGCCACTCACCGTATATAAATTCTCTTTTTGGAGGATATTGCCAAGG
GACAACTTCACTCCTATTTATAACCGTAACTTCAAGTGGTCTAACAGAATCCGTATTTCCAATCTTTCCTGATAT
AGTCATTAGTCTTTCTGTTAGTTTTTTTCGAGTTAATTGAGGTAAACTATGATTCACGACGACTAGAACATCTA
CATCGCTGTTAATGCGTAAACCACCATTTACTGCTGAACCAAATAGATATACTCCAACTATTGAACTTCCAAAT
AAATCTTTTACGATTTTTAATGTTTGAATCGCTTGATTTGGTATTTTTCCGTTAATCAAATTGCTCATGATTTCA
CCTCGTTGATTATGTTCATATAAAGTTTATATTGATACTCAATTTACTTACCCTAGATTGGACATATACTTAAATTA
CTGTTCAATAAAGCTGACCGTTAGCGTTTAAGTACATCCTTTCACAATTTGTCTACAGATTAATAATTATTCTTT
ATTATACAGATCTCCATATAATTTTTGAATTTGGTTCTGTAATTTTTTATTTTCTTTTTCTAATTCCATTACTCTTCT
TTTTAAGGTTTTAATAAGGATTTCCTCCGAACGAGAACTTTTCTTGGGTTTTGAGACTACATTTGCTGTTATTT
GACGCTCACGAAGGGATTCGATTCTTTGCCTAATATCGTGTTCCTTATAAAGCCATGATTTAGAAACATTAGCT
TCCTTTGCTATTGAATTAAAATTAATAACTTTACCTTCAATCGAAAATTTAGAAATCGCTTTGTCTACTTTTTCC
CTTGTCTTTTTTGATTTCTGCTTCGCCAAACGTACAATTTCTGTTGTATTTCTAACTTGTTTATCCATTGATAATT
ACCCCGTCAAACTTCCAATGATTTGTTCTAAACGCTCTTTAACACGGCTATTAGTCTCTACTTGTCTTTGCCATT
GTTTATCCTTAGCTATGGCTAATAACTCTTCTGTACGCTCTAACTGTTCTTCGTGCTGTGGTAAGAATTGCTTAC
TGGTACAGAAGTGAGTGCAATCTAAGCATGCATTCGCATGTGGACAACCACCTGCTACTACTGGCAATCTAC
AATAACCATTTGGAAGCACTTGTGCATTTATATTTTTCTTGAACCATTGAAGCTCTACATCATCGACTTCATTAT
CTTCATCTAGATCAAGCACATCTCCATTATTGGTAACCAGTTTTTCCTGAAATTTAGTAAATTCATTTTTTAGAG
TTTCATCAAAGATATGAGCGTATCTGCTTGTCATTTCTGGGCTTTCATGCCCCAAAAATTTCTGCACAATATGC
TGGGGCATCCCGTTGTTAATCATTCTTGTTCCTACTGTATGGCGAAAGGCATGGGCATGGAATCTATAAATCTC
ACCTGATTTATCCACTATATTTTGCTCATAAGCTAATTTATTTAACTCACCTCTAAATGTTTCTTGTTTTAATGGC
GATCCATCTTTTCTTGGAAAGAGGTATTCACTATCTGGAAATTCCTCTGAAACTTTATCTTCCCGAACTTTAAT
AAGTAAAGCTACCTCTTTAGATATTGGAACTATATGCTCCTTTTTCATTTTCCATTGATAATACTTTAAAAAGAA
ATCTCCATCTTTGTCCTCTAATAGACAGCCTTTTTTCAAGGTGCACAATTCACTTATCCTCATTCCACATTCTTG
AACAATCATAGTCATCGTAGCTATATATTCGGGTAATTTATCAAGATGACTGTTCAATTGCTCTAGGACGAATTC
ATCTATAAAGCGTGGTTTTGCTCTTGGTATTTTCGGATAGTCCTCAGAATAAATTAATATTTTGGAAGGAACAT

CATCCCATTCTAGCCTAAGAAGGGTACTAAATAGTCCTTCCAATATAGAGATCCTCCCAGTTATTGTACTAGGT
TTTATTCCCATCATGTTTAGTTCACTTAAATATGCTTCAATTTCCACTCTCGTTAATTGGTGTACTCTCTGAACTT
GTTTAAATTTCATGTCCAGAAAATTAAAGAACTCTTTAAGTCTTTGGGCAATATCACTTACATAGGAAAAGCT
ATCCACGTTCAATCTCAACTTACAATATCTTTTTACAAGTTGTTTAAAATATGTATTCCGAAACCCTTTAAAGTT
AATTGTATATTCATATTGTGTTGGGTTAACCTTATCATCTGGCAAAGGTAAGTTACGTCTATCCCAAACGTCTTT
ATCCCACTCCTCTCCATCAAAATAAAAGTTCTCATAAAACTCCATAAATTGTTTTAGATTAGTAACATAGTAGGA
ATTAGCTTTTACAGGTGTTTTTTCTTGATTAGCAGTAATCTTATAATTAGTAGTGGTAATTCTAACACCCCGTTT
TGTCAAATAAGTTCTATACTCCGTCATTGCTTTTTCAATAGGAACTTCAGTAATTGAAGTAATGCTAGGATACT
TTAAATCTAAGAAATCTAACATTTTATTAATTACTGTTCCTTTTCTAATCCAGACAGTTTTTGCATTCCATATTCC
ATTGTTTAAATGGTAAAAATAAAAATATTTCAATTCTGTTCTTAACCACAGATTTTTAACACGTTCAAAACGAA
CCCAACGATTCCTTAAAGCAGGATTCTTACTTAATTCTATGGCAGAAGGATGTGGACATTTTCTTATATCCCAA
CTATTATTAGCCCAAAACCCCTGCATTTCTTCATTCATTACAGCTATTTTTTTGCTAATCTCACTCTGACTAATAA
TTTTCCTTTTACTAGAAGCATTCATTTCTTATGCTCCTTTCTCTCGAGGTATTTATTAAACTCATTTTTCATATCCT
GATCTGAAAGATGAACATAGGTATTTAACGTTGTCTGAACATGTGCGTGACCTAATCTCTTTTGAACGAACGC
AACATCCCATCCTTCCCTAATTAGCTGCGTTGCGTGAGTGTGGCGAAGCATATGTGATGTAAATTCTATTCCAG
TCCTTTTAACTATTCTTCTAACTAGATCAAGAACACTTTGGTACTTTAGTGGTTTCCCAAAATAGCCTTCTTTTA
AGGAAATAAAAACATAATCATGCTCCAATTCCTCACTATACTCATATATCAAGTAATCTGTATAAAGTGACATAA
GTTCTTTACTCACATGTATTGTTCTTTCCTTCCTTAATTTAATATAAGCTTCATTAACATTAACATCTCTAGGTGTT
AAATGGATTTGATTGTCCCAAGTGACAATATCTTCAAGCCTAAGCGATAACACTTCACCGATTCTTAAACCAC
CCTCATACATAAGCATTAAAATTAATTTATCTCTTTTCGTATGACAAGCATCAATAATTTGCTTAACTTCCTTTGA
TCTCAATGTTCTTATCTGTTTCTTTTTAACCCTTAACTTTAAGACATTCTTTTGGTATCTACCCTTATTAACATGAT
GTAAAAATCCTTTGAAATTTCTTCCCTTTGCTTGTTTAAATACATCAATTGATTTAAATTCTCCTAATCTACTTAA
ATAATCAAGAAAACTCATAACTACATTTAAAATTGTATTCACTGTCGTTTCTTCTCTTATGGCTTTTTTTGACTG
AAGATCAATTACATTTGATGCTGAAGGATATCTCAACCAACCTACGAAGTCTGCTAACAACTCAAAGTTAATAT
CATTAAGAATAACACCTCTCTGTTCCATGAACTCGTACAGCAACTTTAAATGATAGCAGTATGCCTTAATGGTA
TTAGGAGACTTACCAGTATTATCTAAGTATTTAATAAATTTCATTACTGGTTCTATTAGCTGGTATTCTTTATCTA
GTAATAAATACAATGGATACGGCTTATTCTCCACTTCTATCCTTTGAACCTTCACATGTTCCCACCTCTTTAAAT
ACTTTAACTACTACAAATTATAAACCTTGTTAATTTTTATTTAAAGTAAAATATCCCTTTAATATCTCCTCTTAAAC
TACTTTAACTACTATTATTTATTATACTATGGTTAATACATCTCCACTTGGATGATTATGACCGAGCATTATACTAT
TTGCGTTACTGAGTATCGCTGTTTTGAATATTTCTCTAGGGTGAATCACCGTTTGGTTAATAGATCCAATCGAT
AGTGTTTGAATATGTGTAGGTTCATTTTTACTGTTCATACATATGAGAATGAGATGCTCTCGGTCACTGTTTCC
AATGAATGAACGCATGATTTCTGCCGCATCCTCAGGGTTTGAAATACGATTTTTTAGATAACTTAATGTATCTG
TTTTTATCATTTGTAGTGAAACAATATTGATTTCCTTCATCGTTTACCTCCATATATAGGTTATGCTTTCAAAGTC
CATTTTTGACGTGCTTTAGGGTTGAGTGGGTGCATGATTTCATTTGTGGCTGGATTAATGAGCTTTTTGACTT
TCTTTTTATGAGGCTTCAACATTTCCATTACTTGTTCGACACGTTCTACAACAACTGGCCGCTTCGCATAAGCA
CCATAAGCTAGAATCACTGTGTCACTTTCGCTAATTGCTTTCATCAAGTGGATGTCTGTGTGTTTATCATAAGG
TTCTTTAATATGTTTAAGGTTCTCTGGTGTTTTAATATTAGAGAATAGATTTACAAGATATACAGCACCGTATTG
TTCAGAATTAGCTAATTGGTTGAGAATGAGAACAGTGGTAAGATCGAGTGATAATACGCCATCTAAATGTGG
ATACATCGTTATCACTGTACAAGAGGGTTTCTTTTCATCCCATATTTTCTTGAGTAAGTAGCGGTGTTGTTCATC
GTCGCTAAATATAGCTTCTGTGTGTATCGTACTTTTGATTGTATTCATATATCGTTACCCCTTTTAATATTCTTCTG
GCAAAAGCATCACATAATAAAAAGCGTCTACGTCATCTTCACGAATGACGTAGACTTTCTTAGGTAATGCATT
TTGATTTTTTACATAGTTTGTATAGTGATATTCCAATTTGTATGCAGGTTGTTCTTGTTCATGTGTGATTGAGAG
TATATTCTCATCTTCTTGTAGTCTAAAAATGTGTAGATAATCTGTATGAGATTGATTATCTCTTTCTTTTACCATAT
TCCAAAGTAGGATTTGAAGGTCTAGAGATAGTTGTTCACTTATGCCTCTTGTGATGTATCGATTGATTTTCATG

TTATTTTACCTCGTCTTGAATTTCTTTCATAATGATAATTGCTTGGCTAATAATCGTAACAGATATTTGTGCCACT
TTGATCCAATTATTCATGGTTATTCTCTCCTTGTTTTAGTAAATGACGTTCATCGATAATCGTATTTTTAGTATCT
GTGAGGTATAGAAAGTCCATATCAAAATGATCCAAATAACCAATGCTGATGAGTTGGTCATTGGCGTACATAA
GAAATGGATAGATACTTAGCTCATGTAGCTCATCATTGTAGTAGGTATAAGTGTTGAGTGTGAGATGCGCAAG
TAGAGAATTATTAATAAAACGTTCCGGTAGAATATTTTCTGCTGCTTCCTCAAGTGCTTCACATTCCCATGTTTC
GTTGTTAGATAGTTGGAAGAGACGAGTTATATATTGTTTGAGTTCTTGAGTGGTTGTTTTCATATCATTGCCTC
CTAGATAGTGTGATAGTGATGTAGTTGATATACATCATTGGGATAATATATATTTGATTTATTATTTATTACGAATC
CCGGTGGGAATAAGAGAAAATTCCATATAAAAACCCGTGATAAATGTTGGTGTAACAAGGAAATCCCGGAAT
CCCACTCATTTTGACGAACAATCAACTCATTATTTATAAGTATTGATGATAGGGTTGTGTCTCTGCTTCCTTATA
TATATTATTTATTTATAAAAAATAACGGGATTTTGGGATTGTGCTTGCACAATCCTTCTGCTTCTTCGAATCTGC
AAATCCCATTCCTTTCCCGGTAAAAAATCATTGTGGGATGTTCTTTAGCAATTTCAATATAAGCATTGTGTAGT
CATGAAAAAAATGACGGCAATGACTGTTTCATTAGATAAGTGTTATTGAAATTGATAAAGAGAATTCTAAAAA
TGGTTAGATAAAATAAGTGAAAGAATAGCAGTGTAGTTATTGTTTATTCAATAGTTATATATAAAGTTTGTGGC
AAAAATAAAGACGAAGTGCTAGGGAGCACTTCGTCGAGTGGATGGTTATTAAATAGTTGTTTGATTATATCAT
TATTTAACTTGAGCGTAACATAAAATTGCTTCTTATGATGCTCATCTTTTCTTATGTCAATGCGATCGATGATTG
TTAGATATAGTGATTTCAACTGAGATTTCTCTAATTTGTCTATATCTTTGAATATTGCTTGTAGTACATTCGCAAT
CATATCAGCATCATAATGTGGAGCTTCTGCTTGTTTATCTTGTTCTAGTTGATAGATTTGATTATTTATTTGATTC
AATTCATCTTGGTAGCTAAGTATTGTTGGTTTTAATACGCTATCTAAGTCAGGTGAGTCTTCAATAGTTTTCGTT
AATGTATGCATTTTTGCTTTTATTTCTTCACATTGTGACTGTTTATAGGCAATATCATGGTTCAAAGAAGAGAC
GTCTATTTGACTTTTTTCATTTACCTTTTCAACCAATTGCTTCAATACCTTTTTGCTTTTGATAATTTCCAATATC
TGATCCATAACATATTTTTCTAGTACATCTGCTCTAACGCTATTGGCAGAACAAACTTTTGAACCTTTATTTCTA
AAATTGCTACATGAGTAATATCTGATTCTTTTCTTAGTGCCATCTTTTAATGTATTAGTTGTATTACTTGCCGCCA
TTGCTGCACCACATTTCGGACATTTTACAATCCCAGTCAGTAGGTTTGTTCCTTTGCCGTGAACTTGTGGTTT
CTTGCGACTCTCTTGACGTTTAAACTGTACTTTATCCCATAGGTTTCTATCAATAATAGGCGCATGTTTACCATC
AGCGATAATCGGTTCCTCATTGAGTCCTTTTCGTCTTTTATCGCTCCAGTGTCTATACTTCGCAAACTGTATCTT
TCCAATGTAAAAAGGGTTTGAGATGATGTAGGTAATGGACGAAATACTAAAAGGTTTCCCTTTCTTAGTCACA
TAACCTTTATGATTCAATGCGTTCGCAATCTTACGATAACCATGACCTTTAGCGTATGAATCAAAAATATATTTA
ACAATATTCGCTTCATGTTGATTGATCATGAGCTCTTTTTTACTGTCAGGTACTTTATCGTAGCCTAGAGGTAA
ATTACCTTGATAATAACCTTCAATAGCACGTTGTCTTTGGCCATTGTAGACATTCTCTACAATCGTATTACGTTC
AAATTCTGCGAAGCTGGCTAAAATTTGGAGCATCAATTTACCTGTTGAACTGGCAATTTCTATTTTTTCAGTTA
GACTAAAAAATTCGACATTAATCTTATACAATTCCTCGACAATATTTAACAAATCTGAGGTATTTCTAGCTAAAC
GATTTGTTTTGTAGACCATAATACAATCTAATTTACCTTCGTTGGCATCTTTTAACATACGTTGTAATTCTGGAC
GTTGCATTGTTTTACCTGATATACCACGATCGGTGTATTCATTGACGACTTCATAGCCTTGAAATTGACAATACT
CTGTAAGTTGATTCAATTGACCTTGAATACTGTAACCATCCGTTTGCATTTCAGTCGATACACGTGCATATAATC
CAATACGTTTCTTTTTGAGTTGTTTCATATTACTTCATTCCTTTCAGGAGTTATTAAATGTGATTGTTCAACGAT
ATTGAGTGGACTGTTTTTAAAGTAGATTCCTTGTAATTGTTTAGTTTGAGTTATTTTGATGCAATCGATAAAAG
GTGCTATATCCTGTAAAGTTATTTTATTTTTTATGACATATTTTAATTTGTCATCGATTTGGTAGGTTGAATAAGT
AGAGTAATTCTCTTCACTTTTACAACTAGCAGATAAACGTTTGAACGTTTTTACATCAATACGATTTTGAGCTA
GCTTTTCGATAAGTTGTTCTTGTGTTAGATGGGTTTCTTTATGCTTCATTTGTTGCTGTTTTAGGACTTTGAGT
ATTGTGTTATTCAATCGTTTATGAAACGATTGTTCTTCAAAATACTTTTTACAAGTGTCGAGCACTTCACTTTCA
AGTTCTGGTGCATTGATACTTTTAAATGGGCATGTACGATAAGCGTCATTCATATTTTTAGGACAAACATAGTA
ACGTAGAGAATAGTTCTCTTTTTTTATCGTTAAGTTTGTTAATGTTGATTGACAGTAAGGACATTTGATTCGTC
GCTTTAGCTTATTTCTAGAATTGGATCGATTGAGTTGTTTATGAATACGACGCTCTTGTGCTTCTTCAAATGTAT
CAATATCAATAATAGGTGGAACGATATCATTAAACGTGCCATATTTATTGATGACACGACCGCAATAGTTAGGG

TTCAAGAGAATATTTCTAACTTGATAGGGCTTACGAGGAATAAGGTTAGGATTACTATCCAAATGTTGGGAAA
TCTTTTTGTAGCCTAGACCTTGTAAGTACCAGCGATAAACCGATTTAACTGTATACGCTTCTTCTTCTTGTACA
ACAAAACAACCTTTTCTATAACGATAGCCAAACGGAGCATGAGTTGTGATTAGCTTACCTTGTTTGGCTTTTT
CTCTAATCCCATTTTTTGTTTGTTCGCTGATATTGTTTGATTCCATTTCCGCTAGACTCATAAGTATGTTCAAAC
GAAAGCAATCAAACTCTTTAGACAAATCAAAATATCCATCGTTAACACTGATGATTGTGATGTGATGCTTTTTA
CAAATTTCAAAGAATTGTATGGCATTTTTCAAATTACGATGTAGTCGGTTCAAGCGATAGCAACACAATACTT
TACATTTTCCAGACGTAATTATTTCTACCATTTTTTGATAACCTGAACGTTTTGTATGTCGACCTGTTTTCTTATC
ATCATAAAACGCCACATTAGACCATCCATATTGCTTAGCGGTATCCATAATGAGCGATTTTTGAGTAGCTAAGC
TTTGTTGTTTGAGTGTACTTTGACGTACATAAGCAATCGCTTCTTCCATGTTATACACCTCCAAAAAGATAATAT
ATATTTGTGAGTAAATTAGAATGAAAGGTCCAACGTGCTTTTAACACGTTGGACCGTCGTGATTAGTTATCGT
TCTGTAGCTCTTCAACGACTAAATCAGCTAGTAATTCAATCAATTCATCCATTTTATCTACTCCTGTACGATTTCA
TCTTTAAGTTATTAAAAATCAATTAAATCATCGTTCTGTTTTTTCCATGATTCAAGTATCTTTTTGTTATCCAAGT
TAATTTCAGCTTTAATAGGTTCAGCATCTTTAGTTAGACCAAAAATAGACGCATATTTTGAATCTAGCTTTAAAT
GGTAAAAGACAAGTGACTGTTTCTTGCCATAGTCATCTTTAACTGCTCTTTTCGTAGTTATTCGATCACGGTCA
GATTCGATAAATCCTTTATCCCTTAGTGCATTCACAACATTGTTGACATCTTGGAAATGATGCTCTATCAACATA
TTCTTAAATACAGATGCAATGATTTTAACTTCGATATAATCATCTTTTAAGGCAATTAGTCCATAGTTCTCAATC
ATATTCTTTAATGCTGTATCATCAGAAAACTTACCACGGTTTTGTGCTACAAATTGCGTAATGACTTCAATCGCT
TTATCAGCCAGTGATCGTTCAGAGACTGTATGAGCATGATAATCAATAAAGTAGTCTCTTATTTTAGCGATATC
AATATCTGTAGATAAAACATGACTAAGAATCTTGGCCGAAGTAGTGATAGCCGCATAGCGCTTAAACATACGA
ATACCAGTATTATTTGTTTCATCCTTCAATTTATCTTTAAACCAATGATGTAATTCAAAAACGGATCAAAAAACA
AAAGGCCCTAGAAACTAGGAGCACACTTACACAAGAACAATTGATAGATAAATTAGCTAAAGGAGCCATCG
ATGCAGAAACGTTCAGAAAACAATCTCAATCGTTACTTCAACAATCAAAACCAACACTATCAATAAATGAGCA
ACAAATTCAAAGGTCTTTTGAAAATGTAATTCAACAACACTTCACGTTAAGCATGTTATACCGATATATTGATG
AAATTCATATTTCTAAAAACAAAAGCCTTGTGGGAATCTATTTCAAAAATGAACCGCTAAACATTGTAAACCA
AGCTACGCAATCATCGATTGCTTAATTAATGAAAGGATGAAAAATATGAAAGAAATGAAAAGAAAATCTGTA
GGATGTTATGTTAGAGTTTCAACAATTTCTCAAGACATTGATAAATTTAGTATTAATGGTCAAATTACACAAAT
AAAGGAATATTGCCAACAGGGAAATTATGAATTGCTATACTGATAATTAAAAGTACCCCCGATCGAACGAAAA
TATCTTTTTGATGACTATTAGTGATAATTGTAATTTTATCCATTAATAGATGAATGGATTGGAATAATACTAAGCC
TATTGCTATTCCACCGAGTAAAATTACCCACCCGCCTAATTCAATACTTTCCGGAATCATTTCAAAAAAATAATAA
GCCAATTATAAGGCCTGCACATAATGAATAAATAAAGCCATGACCACTTTGAAAACCTTCCATTATCCAAGCG
AGTCCACCGCCAACACCAATTCCAAGTGCGGAAGCTAACGCCCGATCATCCATACTGCACTCATCCCACTATT
CTTAAAAAGGCTTTGTTTATTTTACTCTAAAAAATCAAAACAAAATATATGAATTCATATTAAATATTATTTGACT
CTTTATTCAGATGGTTATATCATATATATGAACATATATTAATACATGTAGATAAATAGTGATTATTATATATAAGGT
TTTGAATTGAAACCTAAAGTGAGGGAAGGATAATGGAAGAAAAAAAGGAATTAGAGGAAGTAAATAATAA
GGACTTAGATGATGAAACATTATTTGTCGTATCGCAAACATTTAAAGCGTTAGGTGATCCTACGAGAATCCGG
ATTCTCCATTTGCTCTTTTATAAGGAGTATTCGGTAAACGGTATTGCTGAAACGCTACATCTTAGACAATCAAC
AGTTTCCCATCAATTGCGGTTCTTGAAAAATTTACGGTTAGTAAAATTCCGAAGGGAAGGCACAACATTGTT
TTATTCCCATGATGATGAACATACTATGAATATGCTAAAACAGGCGATCGATCACGCCTGTCATCACTAGTATTC
CGTAAACCAGTAATGAGTGTTAATAATAATTCACGTCATTAATATTGTTATATGATCATATAAAAATATTATAGGC
ATATGAAAAGGGGGTTTTGCAATGGATATGGAAAACAAAAAAACAGAATGGAAAGCGTTGTATGATATCTC
GAAGGAAAGTGAGATGGGTGTAGCAGAGAGGGTTAGTGAATACGGTTGATAAATTCATAGGATGTATTATT
GGATACAAAAAGTTTGGATGTGGTCAATTAACGTCCTGGTTTAGTAAAGCTTACTGCGAATATATTGGAACCC
GATATATCATATTTATTTAGAAAGGTGATAGAAATGAAAAATATTCAAGAGCAACAAGCACACGAAAGTCATA
GCCACGATCATAGTCATGATCATGATCACGGAAAAATGCCAATTATTTCATATTTTATTGGCTTAGTGTTGGCT

ATAATTGGGCTTTTTTTAAGTGATGCAAATTTATTAATACAAAACATCTTATTTTCAATTGCCACAATCACAGCC
GGCTACCATGTAATTATTCTCGAAGGAATTGGAGAGACAGTTGAAAATACTAAATTAAAGGGAAAATTCACT
CCTAATTCTCATATTCTAATGGGATTAGCTGCAATCGGGGCTTCTCTGATAGGGAGTTTTTGGGAAGGAACCC
TTTTGATACTTATTTTTTCCGGCGCTCATTTTCTTGAAGATTACGCTGAAGGAAAAAGTAAAAGAGAAATTAC
TAAGCTACTCGAAATGAACCCAACGACAGCTAAATTAATCCTACCTGATGGAAACACAAAAATTGTTGATGTC
AGTGAATTAAAAGTTGGAGATCAACTCCAAGTGCTGAACGGTGATCAAGTTCCAATTGATGGGATTATTTTAT
CCGGTACTACCTCAATTGATGAATCTTCTATTAATGGAGAAAGTATACCGAAAGAGAAGTCTAAGGGTGACG
AAGTTTTTGGAAGTACGATTAATAGAACAGGTACTTTTACTATGGAAGTCACTAAGGAAAACAAGGATACTG
TATTCTCTAAAATTTTACAATTAGTTAGTCAAAACCAAGATAATCAAACAAAAGCTGCCAGTATCATTCAAAAA
TTCGAGCCTAAATATGTTAATATAGTTTTAATCGCAATACCATTAGTAATGTTACTTGCTCCTTTTCTATTTGATT
GGACATGGTCGCAAAGTGTATACAGGGGATTAGTGCTTTTAGTCGCAGCTTCACCGTGTGCTTTGGCAGCA
GCTACTGTATCTGTAACATTGTCTACAACATCTAACCTAGCTAAAAAAGGCGTGCTTTCAAAAGGAAGTACTT
ACCTATCACAATTAGCGGATATAGATGCAATTGCCTTCGATAAAACAGGAACCCTTACGAACGGAGAACCTAA
AGTAACAAATTACTATTTCACTCATTCTGTGAACGAAGAAAATATTATTGATATTATAGTCGCCCTTGAAAAGG
AATCCAATCACCCACTCGCTAATGCTATTTTAGAAAAATTTGAAGTTAAAAATAAAATAGACATCGAAGTTACT
AATCAAATTGGAAAAGGTCTGACAGGAGATTATAATGGAAAAAATTATCGTATTGGTAAGCCTACTTCTTTTG
AAAGTGCTTCTGAAGAGTATACCCAGTTCAATCATGATTGGGCATCAGAAGGAAAGACGGTTGTATACGTAG
CAGAAAATGAAGAAGTTATTGGGATTATAGCTCTAATGGATATTCCGAATGAGCATGCTAAAGAAACAATTAA
TTACTTTAAGAAACTTGGTATCCACACGACTTTAATTACTGGTGATTCGGAAATGACGGGAAAAGCTGTAGG
CGAACAATTGGGAATAGACGAAGTTATCGCTAATGTAATGCCTGAAGATAAATCCAGAATTATAGAAGAACA
AAAAGAAAAAATTTGGAGTTACTGCCATGGTTGGAGATGGTGTGAACGATGCACCGGCCCTTGTTAATGCTG
ATGTTGGTATAGCTATGGGGGGCGGTACTGATGTGGCAGTAGAAGTATCTGATTTGGTTTTAATGCAGAACA
ATTTATCTAAATTAGTACAGTCTCATAAAATTTCCTCAAATATGGGTCGTGTTATTAGGCAAAATATTATTTTTTC
AATGGCAGTTGTTGCCTTTTTAGTTGTCGTTAGTTTGTTAGGATTAACTGATATTACAATCAGTGTAATTGTTC
ATGAAGGAAGTACTTTAGTTGTTATACTAAATGGACTTCGATTATTAAGATCTAAATAATGAACGAATCGATTG
ACATGAATGAACTTTGAAGTGTGGATTCTACAATGTTCCCATAACATTGGACACTAAAAAACAGAGCAATCTA
ATAAAGATGTTATGAGTAAAAACAATGCCTTGCATACCGTTTTTATCATACGGGCGGTCAAATAAGCAATTAA
AGAGCATGGGAAGCATAAAATCATAAACAGTGACTTAAGGCAGCCAGTTTACATTCAAAAGTTAAATTGACT
GTATTAAAAGTTTCGAAACGATTAATATCCGTATGGGGGCAAGCGTCGAGCCAAAGATAATGCCAGGACAGA
GCGTCTTTTTCCGTTCTTTTAAGTGGGAAAGGTTTTACCTTCTCTATGCCAAGACAGTCCCAGAGCTAAAAG
GGCCAGTTGCTCTTTTTGGGCGTTTGTGAACGGTAATCGCAGGAAGGGGATTTTCCCAACCGCACCTGGTT
TTCCATCACAATAAAGTTGGCGGCATTTGTTCTGATATTCTTGGGAATACTTACACTATGAATGGCCGTATTAA
GTGATACAGGAGCTGCTATTATTGTAATACTGAATGCTCTCCGCCTTTTGAGGGTAAAAGAATAAAAGTAAGG
ATAACTAGGTAAAGCTGTTCAATCAAAAATTGAACAGCTTATTTTTCATCAAAATCAAAAACGTTTATTATAAT
ACCTACACTTGTTCTGTTAAATGTACCGATTTTTTAACCTTATTGTATCAGTAATATCTTGAAACGAAGTAAGCG
ACTAAAATTTCTCTTTTATCATACGTTTATAAAAATACACTTTTAAGAACGGTTTGAAAATTTTTGAAATAGATC
AAAATAATCTTCTGGTTAAAAAACCTGTAGATATGATTTCTCTCTTAAATTTTTGTTTGATTAGATTAGACCCTA
ATAAGACGCATTCAATATGTCTACACGTGAATTTAGTCTTTGAAAATGTAAGGACCATTATTATTATAAAAACCC
CAGTATAAAACGATACGCTGAGGCGTATCATAAGTAAAACTAAAAAATTCTGTATGAGGAGATAATAATTTGG
AGGGTGTTAAATGGTGGACATTAAATCCACGTTCATTCAATATATAAGATATATCACGATAATTGCGCATATAAC
TTAAGTAGTAGCTAACAGTTGAAATTAGGCCCTATCAAATTGGTTTATATCTAAAATGATTAATATAGAATGCTT
CTTTTTGTCCTTATTAAATTATAAAAGTAACTTTGCAATAGAAACAGTTATTTCATAATCAACAGTCATTGACGT
AGCTAAGTAATGATAAATAATCATAAATAAAATTACAGATATTGACAAAAAATAGTAAATATACCAATGAAGTTT
CAAAAGAACAATTCCAAGAAATTGAGAATGTAAATAATAAGGTCAAAGAATTTTATTAAGATTTGAAAGAGT

ATCAATCAAGAAAGATGTAGTTTTTTAATAAACTATTTGGAAAATAATTATCATAATTTAAAAACTGACAATTTG
CGAGACTCATAAAATGTAATAATGGAAATAGATGTAAAATATAATTAAGGGGTGTAATATGAAGATTAATATTT
ATAAATCTATTTATAATTTTCAGGAAACAAATACAAATTTTTTAGAGAATCTAGAATCTTTAAATGATGACAATT
ATGAACTGCTTAATGATAAAGAACTTGTTAGTGATTCAAATGAATTAAAATTAATTAGTAAAGTTTATATACGTA
AAAAAGACAAAAAACTATTAGATTGGCAATTATTAATAAAGAATGTATACCTAGATACTGAAGAAGATGACAA
TTTATTTTCAGAATCCGGTCATCATTTTGATGCAATATTATTTCTCAAAGAAGATACAACATTACAAAATAATGT
ATATATTATACCTTTTGGACAAGCATATCATGATATAAATAATTTGATTGATTATGACTTCGGAATTGATTTTGCA
GAAAGAGCAATCAAAAATGAAGACATAGTTAATAAAAATGTTAATTTTTTTCAACAAAACAGGCTTAAAGAG
ATTGTTAATTATAGAAGGAATAGTGTAGATTACGTTAGACCTTCAGAATCTTATATATCAGTCCAAGGACATCC
ACAGA
>Staphylococcus aureus strain BLR-DV
CAAGCAGTTTAGAACAACTTGATTTATTATTATTAGAAATACCTAAATCTAGAAAAATTCATTCAGATGGTATTC
ATTTTCAAGGCTTTAGGTACAGTAATACAAACCTAACTGCTTATGTAGGTGAGTATGTATTAATTCGTTACAAT
CCCAATGATATGGCTGAAATACGCGTTTTTTATAGAGATGAATTTCTTTGTACAGCAATATCCCCTGATTTAGCT
GATTATTCAATTGATATAAAAGAGATACAACACGCACGTAGTCAGAGAAGAAAACACTTAAAACAAAACATT
GCTAGTCCAAGCACCACTGATTTAATTAAGGAAGAAAAAAGTTATGGTTACTCACCTCAAGAAACGACCAAA
AACGTCAAAAAAATTAAAGAGGTATCGTAATGACTAAAAATCAGAATTTCATTGAAACGAAAGAGTATAAACG
ATTTGCTGAATTTTGCGATGCTTGTATCAAATATCAATATATTGGGATCTGCTATGGTCAACCTGGTGTCGGAA
AAACTTTATCTTCTAGGTATTACACAAATTGGAATACTATTGAAAAGCAAGTTAATCATAGAGGGTGGGAAGA
TCTAGCTAGTAAAACGACTGATGATATATTATCGGTGAATAAGATATTTTATACTGCACCGGCTGAAAAACAAA
CAAGATTAAGTAATGATCTATATAGTATTAGTGCAAGTATTGATTTGGGTCAAAAATTACACATCGTAAATAAG
TATGGTCATGATCATAGTAAACATTATAGTGATATGTTTAAATATATTGATTTAATTATAGTAGATGAGATTGATC
GCCTTAAAGTGCAACATTTAGAGCAATTAAGAGCTATTTATGATGAACATAATTTAGCAATGATATTTATTGGT
ATGCCAGGTATAGAGAAAAAACTATCTCGTTATCCTCAACTATATTCGCGTATAGGTTTTGCACATGAATTTGA
TAATCTAAGTAAAGACGAGACGCATCATATATTAGAATATAAGTGGCAAGATTTAGGATTTGATCTAAAACTCG
AAGATTTTACTGATTATGAGGCAATAACGACGATTATTAAAATTACAAAAGGGAATTTTAGGCTGATTCATCG
TTTATTTGCGCAGATAGATAGAATTATGGATATAAATGGCTTAGATAAAATCAGTACAGAAGTTGTAGAAACA
GCTAGAGATAGTTTAGTCATAGGTATTCGTTAATAGAAAAAGCAATGTACTTTAATATAGGACATTGCTTTTCT
CATTTACTAGGTAAAATCATTCTCATATATCAAGTAAAGTAACAAACATAGTGAATGGCTTACAAAAATCAACA
GATGTAGAAGAGCTTATTGAGGATAAGCAGTATATTGGTTGCGGTCCTAGCATGTTTAATCTAAATACATTTGA
AGTTGTTAAAAACTGTATAGATATATTCCCTAAAACACGCTTGAATATAGAAATAGATAGAGGTGATGTTATAA
ATGAAAATATACCTGACCACTTCAGTAAATACATGCTAGAGCTTGCTAACTTTGATACCGATTTAAAGCACTTT
CTGATACAGCATACAGCAATACTGCTTACTGCTAATACCAAACTACGAAGAGGTTTGATTCTCTATGGCGCTG
CTAATAATGGAAAATCAGTATATATAAAGTTGTTGAAATCATTCTTTCATCAAAATGACGTTATATCAAAAACGC
TAAACGAACTTGGTGGACGTTTTGATAAAGAAAGCTTAATTGGGAAACGATTAATGGCAAGTGATGAAATT
GGTGAGGCAAGAATCAATGAGAAGGTAGTAAATGACTTTAAGAAGTTACTTTCAGTTGAACCTATCCATGTT
GATAGAAAGGGACAGACACAAGTAGAAGTCACATTAGATTTAAAACTGATTTTTAATACTAATGCTGTACTAA
ACTTTCCATCTGAGCACGCAAAAGCATTAGAGCGTAGAATTGCTGTTATTCCATGTGAATATTATGTTGAAAA
AGCTGACCCCGACTTAATTGAAAAGTTACAGGATGAAAAGAAAGAAATCTTTCTTTACTTGATGTATGTGTAT
AAGCAAATTGTAAAAAATGATATCGAGTACCTCCAAAATGATCGTGTTACTGAAATTTCTCATGATTGGTTAAA
TTTTGGATATGAATTTGTTTCTAGTAAATCAGCAAATACTGCACATCAGAAAGCGAGTATTAATTTACTCAGAA
AACTTATAGAAATCAAACCAGGATCACGAATCAAAGTATCCGAGTTAAATAAAGTTATTAATGAAGAAATAAA
GGTGAGTTCTCAGGTTATTAAAAAGTTAATTCAAGCAAACTTTGATACTCAAACCAAATTATACAATGGCTAC
GATTATTGGATTGATTTAGGTTGGAAAGAAGCCGATAAAAAAGAGATTCATGATATTTCAGAAAAAGATAATA

TTATTTCATTTGATAAAAATGAAAATATAACAGACGATGAGGCATTAGATGAAGAAAATTTGGACTTTGATTG
GGAGGACTTTGACGATGAATAATGAACAAATCGAAGCATTTGTAGAAGTGCTTGTGCCTATCATAGAAGAAC
GTATCAATAAAGGTAAGTAATCTAATTACGTACTACAGGCAGTTGCCTGTAGTACTCATATGATTAAGTGGTAA
AAGTGATAAAAATGAAACGAAATTATAAATATATATTATCTATATGTTGTTATAAGACCGATAGTCTGTAGCAAT
AATCTAATAAAAGGAGAACGGTATGATATGAAGGGTAAAATTGCACTTTATTCACGTGTTAGTACGTCTGAGC
AGTCAGAACATGGTTATTCTGAAAAAGAGCAGGAACAAGTACTCATCAAAGAAGTTGTGAAAAATTTCCCA
GGTTATGACTATGAGACATATACTGACTCAGGCATTTCAGGTAAAAATATTGAAGGTCGTCCGGCAATGAAAC
GTCTATTACAAGATGTTAAGAATAATAAAATCGAAATGGTATTAAGTTGGAAATTGAATCGTATTTCTCGCTCA
ATGAGAGACGTGTTTAATATTATTCATGAATTCAAAGAGCATGGCGTAGGTTATAAATCGATTTCTGAGAATAT
TGACACATCCAATGCTTCTGGAGAAGTACTCGTTACAATGTTTGGGTTAATAGGATCTATAGAACGCCAGACT
TTGATTTCCAATGTGAAACTTTCTATGAATGCTAAGGCAAGGAGCGGAGAGGCAATCACCGGTCGTGTTTTA
GGCTACAAATTATCACTTAATCCATTGACACAGAAAAATGATTTAGTTATTGATGAAAATGAAGCTCATATTGT
ACGGGAAATCTTTGATTTATATTTGAATCACAATAAAGGACTTAAAGCAATCACGACAATTCTAAATCAAAAA
GGATATCGCACCATTAATCAAAAACCATTTTCAGTGTTTGGCGTGAAATATATTTTGAATAATCCAGTCTATAA
AGGCTATGTCAGATTCAATAACCATCAAAATTGGGCTGTTCAGCGAAGAAGTGGTAAAAGTGATAAAAATGA
TGTGATATTGGTCAAAGGTAAACATGAAGCCATTATAAGTGAAGATGTATTTGATCAAGTTCATGAGAAACTA
GCTTCTAAAAGTTTTAAACCGGGTCGACCTATTGGTGGAGATTTCTACTTACGTGGCCTTATTAAATGCCCAG
AATGCGGAAATAATATGGTATGTCGACGGACGTATTATAAAACGAAAAAGTCCAAAGAACGGACAATCAAAC
GCTATTACATTTGTTCATTATTCAACCGCTCAGGAAGTTCTGCCTGTCATAGTAATGCGATTAATGCTGAAGTC
GTCGAACGCGTAATCAATGTTCATTTGAATCGTATTCTTTCACAACCTAATGTTATTAAGCAGATTGCGTCAAG
TGTGATAGAAGAACTGAAACAAAAGCATAGTAAACAAACAGAAATAAAATATGATATTGATAGTCTAGAAAA
ACGAAAAGCAAAAGTTAAAACACAACAAGAACGATTATTGGAATTGTTCTTAGATGATGAAATGGATAGCG
AAATGTTAAAAGCTAAACAAAGTGAAATGAATCAACAGTTAGAAGTATTAGACCAACAAATTAAAGAAGCA
AAACAAGCAAATCAATCACAGGATGATATACCCAATTTTGATAAGTTAAAAGCACGACTCATTTTGATGATAA
CACGATTCAGCGTATACTTAAGAAAGGCTACACCCGAAGCTAAAAATCAACTTATGAAAATGTTAATTGATTC
AATTGAAATTACGACAGATAAACAAGTAAAACTTGTAAGGTATAAAATTGATGAAAGTCTTATCCCTCAATCTT
TGAAAAAAGATTGGGGGTCTTTTTTTATGCCTAAATTTAACTTTGTGATAAATGTCGCAAATGAAAATAGGAT
TGAAAATTTATCACTTTTACCACTTTTCTAGAGTGACAAAAGTGGAGGAGTTTTGAAATATTTTATAAATATAT
ATTTTATTTATGGAGTGCACATTATTAATTAAGGAGGTCATTATAATGACGCTAAGCAAACAACTTAAAACGTA
TATTACTGAACGATTTAAATTAAATTATCAAGAAACTTGGGCTTGTGAAACCGTAGATGCGGTGGCTGAAGAT
GTATTACCTGAAAAATATATTAAAAATAGTCCACTTGAACATAAAATTTTAAATACTTTTACCTATTACAATGATG
AATTACATGAAATCAGCATTTACCCTTTTTTATGTTATCTAGATAAGGAATTAGTAGCAATAGGTTATTTAGATA
ATTTTGATTTAGACTTTATATTTTTAAATGACACTCATCAAATTATTATTGATGAACGCTACTTGTTACAAAAAG
GAGGGCAGTAATTATGAATTGGATCAAGGTCGCTCAACTATCTGTCACAGTTATCAATGAAGTCATTGAGATC
ATGAAAGAAAAACAGAATGGAGGAAAATAGTATGAACATCAATCGATACATCACAAGAGGCATTAGCGAAC
AACTATCTCTAGACCTTCAAATCTTACTTTGGAACATGGTAAAAGAACGGGACAATCAACCTCATACAGATTA
CCTACACATTTTTAAACTGCAAGAAGATGAGAATATACTCTCAATCACACATGAACAAGAACAACCTGCATAC
AAATTGGAATATCACTATACAAACCATGTAAAAAATCAAAATGCATTACCTAAGAAAGTCTACGTCATCCGAG
AAGATGATGTAGACGTTTTTTATTATGTCATGCTTTTACCTGAAGAATATTAAAAGGAGTGAAGATGATGAATA
CAATCAAAAGTACGATACACACAGAAGCGATTTTTAGCGATGATGAACAACACCGCTACTTACTCAAGAAAA
TTTGGGATGAAAAGAAACCTGTTTGTACGGTGATAACCATGTATCCTCATTTAGATGGCGTATTATCACTCGAT
CTTACGACTGTTCTTATCCTCAATCAATTAGCGAACTCTGAACAATATGGCGCTGTATATCTTGTAAATCTATTC
TCTAATATTAAAACCCCAGAGAATCTTAAACATATTAAAGAACCTTATGATAAACACAGCGACATTCATTTAAT
GAAAGCGATAAGTGAAAGTGACGCAGTGATTTTAGCCTATGGTGCTTATGCGAAGCGTCCCGTTGTTGTCGA

ACGCGTTGAGCAAGTGATGGAAATGTTAAAACCTCATAAAAAGAAAGTAAAAAAGCTCATAAATCCAGCAA
CGAATGAAATTATGCATCCGCTTAATCCTAAAGCGCGTCAAAAATGGACATTGAAATAAAGGAGGATTATCTA
TGAACCATGAAACTAAACAATCAGATTGGCGAACGGTTGCTAATTGTTTAGAATCGCAAAATTATATATCGATT
GTAAAAGGATTAGTACATCATTTCACAGCGATTGAAGATGAAGAAATACTAGATAAAATCTATGAAGATTTTAT
GAATGATGATTCTATTACAATGGTACTTAACAATGATTTACAGACAATCATTAATAATTATCTATTAAAATAAAG
ACTATACTAATTACAATAAAAACAGAGCATCCTTCACTTTGCGGTGAGGGATGCTCTTTTATTTTATTTTTTTAT
TGATTACTCAATACGAGAGTAATATAAAAATTATTTAAGGTTAAATGTATTACTAGTAAATATTTATAATTTTAAT
TTTTCATCACTTAATTGTTAGTTTAAAAATTAAATATATCTCAAAATTCTGTTATAAATAATAATATAATGTAACTA
GTAATAAATATTTATTTATGAATAGGAGTAACTACTAATGAATTTATCATCTATAATTCAAAAAATAGGTAATACA
TATTTAAAAGAGAAAAAATCAGGTAATTTTACGGAAGCACCCGTTGGAAAATTAGTAAGAGATGATTTAGTT
CAAGAATTAAAAAAAATTGAAGAGTTAAAAGGTTTTAAAATTAAAGGAAGTATCGGTAATGGACAATTTGCT
TCTATACCTTGGGTTGCTATGATGAATAAAGAAATAACAAGCTCAACTACAAAAGGTATAGATATTGTTTTCTT
ATTTAGTGGTGATGGAAACAAAGTATATTTAACTCTGAATCAAGGCTCTACATTTTTTAAAAACAAAAATTTAA
AGGAAAAGGAGATATTAAAAATCTCAAGATTAATCTATGAACTAATAGATTCTCCTGAAACTGAACCTATTAGT
ATTGATTTAAAATCCACTACTCCATTAGGCAAGAGCTATGAAAAAACTACAATCAGTGGTTTTGAATACGACA
TTAACGATATGCCATCTTCAATAGCGATAAAAAAAGATATCATTAAACTTTTGAATGACTATAAACAAATAGTTA
CTAAATATAAAGAAAACGGTAATGATATTGAACAATTCTATCGATATGTATTAAATTCAAAATATAATAAATACCA
ACTATTTAAAAATTTATTTGAAAACTTTGTAAAGCAAAGCCAAAATAATATCATTAGTGATAAAAAAAGTAATG
TAATACCAGAAGAAGGTCTTGAGAGTGTAGAAATTAAAAAAATCAAGGACTATAATTCTATATGCATAGATAA
CAATGATTTTCATGTTCATCTGTTTAATAGTGGACAATATGGAAGAAAAGATGGCAATGGAAGTGGAAAAAT
ACCATATATATGCTATCAATCTTCTCATAAAAAATGGATAACTATAAGAGCAACTTTTGAAAATTATAAAATGAT
TAATGTAAGTATTACAATATGGGATGAGTTAAAACAAGTAGACCAAAAAACAGGACGTAATTACAACCTTAAA
GATATGAATTTATTTTCTAGCAATTCGCCTAATGATTATTTCAAACAATTTTATGATGATTATATAAGTTATAAAA
ATGAGAGTGATGTAATGGGAGAAAAAGACTTTGTAAAAGATTTAAATAAAAAGTTAATGAAATCAAAAAATA
TTATTTTAAGAGGATCTCCAGGAACAGGAAAGAGTTATTTAAGTAAAGCAATAGCTTCGGAACTAATTGGCA
CAGCAATAGAAGAATTAGAAGACTCAGATCAATTTGAATTTGTTCAATTTCATCCTAGCTACGATTACACTGAT
TTTGTTGAAGGGATTCGACCTACCGTTTCTCAAACTGGGGAAATGGGTTTCGAATTAAGATCTGGCATATTTA
AAGAATTTTGTAGTAAGGCCGCACGTTCACTTAATAATTCTGAAAATAAGAAATTTGTATTTGTTATAGATGAA
ATAAATAGAGGAGAAATTTCAAAAATATTTGGAGAATTATTTTTCTCAATAGATCCTAGTTATAGAGGAAAAA
AAGGATCAGTTAAAACGCAATATACTAATATGGAGAAATCTAAAGACAAATTTTATATTCCAGATAATGTTTAT
ATTATTGGAACGATGAACGATATTGACCGTTCAATAGATACTTTTGATTTTGCTATGAGACGTAGATTTAGATT
TATTAAAATTGAAGCAAACGAAAATACGCAAATGTTGGCAACTTTAGGTGATAAAAAAGATGAAGCAATTGA
TCGAATGATAAGACTCAATGAAGCTATTTCAAGTGTAGAAGAACTAAACAGTAATTATCATATAGGAGCTGCT
TACTTCTTAAAATTAAGAGCCATGACTTTTGAAGAGCTTTGGAAAGATTATTTACAGCCATTATTAGAAGATTA
TATTAGAGGTATGTTTAACGAAGAAAACATTATGAATAGATTTGAATCTGCATATTATGGTAATTCTTCAGAATT
AGGTGAAGATAATGAAGAAACTTGAAATAGAGGATAATACTTCTTATTCTTCTTTAATATTTAATGATTTACCG
AATATTGTTGATTGTATACTAGATAAGACGCTATTAGAATTAGAACACACAGGCGTTTTTATATTTCCTGATAAT
TTAAAAAATGAGAATAGTATTTCAGAAGAGCAAATAATTCTTCAGAGTAAATACGAAACATACTATACCAAAA
ATATTATGGGCTTTTTGGGATACGGCGATGAAAGCTTAATTATTAAATCTCGTTTTTCAGATAAAGAAGACAA
AAAAGATTATTTTTTTCAATATCTATTAGAAAATGTTTTGAAAATACCACATGTGATCGATTTAAGTACGCTTTT
AGATAAAGAAGATAATACTATTAATTTACTTATCTTTCTATTTCCATATCATCTGAAAAAAGCTATGCAGAAAGG
ACTCTTTAAACAATATACTAGGAATAAATATAATGATGATAATTTAAGAGGTACGATTGATATACCAAGACATAT
CAAACTGAATACACCCTTTGTAGGAAATATTGCATATAATCAAAGAGAATTTTCATATGATAACGATATTACTCA
ATTAATTAGACATACAATAGAGTTTATTCAAATGAAGAAAATAGGCATAAATTTATTAGGTAGCGTTAAACATG

AATCTGAAGTAATACGTGAAGCGACAAATAACTACAATCGTTTGGATAAAAATAAAATTATTATGAGAAACAA
AAAGAGACCACTTCGTCATGCGTTTTATAAAGAATACAGAGAATTACAAAAGCTTTGTTTAATGATTTTACAA
CATAAAAAACATTATGCTGGTGATGGAATCAAAAAGATCTATGGTTTTTTAGTTGATGGAGCCTGGTTATGGG
AAGAATATATTGCTACATTAATAACTGAAGATTATTATCATCCTCAAAATAAAGAAAAAATACGGTGGGCAAAAT
TTATTCACTAAAGAAGATGGTAGAAAATCAGGTAAAATTTATCCTGATTTCATTAGTCGAAATGAGCGTTTTA
GAATTATTGGTGATGCTAAATATAAACCAATTACAAATATTGGTAATCAAGATTATTTTCAAGTTTTAGCGTATA
TGTTTAGGTTTAATTCTAAGACGGGATACTATTTCTACCCAGATCGTAAACATATACAAAAACAAGTATTGTTT
TTAAATGAAGGTCTATCTTATGAAAAAAATGAGCGAAAACGTGGAAATGTTAACTTAATTAAATTAGGACTTA
ATATCCCCAAAAATAATGAAAGTTATGAAGATTTTACAAAAAAAAATGAAACAAGAAGAACAAATATTTCTGA
GTGAATTAAACTAATATAAATAAACTAGAGCATCCTTCACTTTGCAGTGAGGGATGCTCTTTTAGTTTATTTTT
TTATTGATTACTTGCCATAAGTTCTGCTTGCTCCACAACCTGCTCTACAGCCATTTTTTGTAAATCTGGTGGATA
GCCATATTTTTTAAGCAATCGTCTCACAGCTACGCGCATTTTAGCTTTTGCGCTATCACGTTTAGACCAATCAA
CACCCATGTTTTCTTTCACTGTTTTAGTTAGCTCATGAGCAATCGCACGTAGTTCTTTATCTCCCATGGCTTCTT
TTGCTGTTTCATGTGAAGCTAAAGCATCGTAAAACGCAATCTCATCTGAATTCAGGCCTAATTCTTTTCCTCGT
TGTTGTTCTTGTTTAATATCTTTAGCGAGTTGAATGAGTTCTTCAATCACTTTTGATGTTTCAATGGAACGGCT
ATTATATTTATTAATCGAGTTTCCTAACATTTCAGAGAAACGCTTAGATACTGTCGCGTTCGTTTTCATTAAAGA
TTTAACTTGCCCTTTGAGTAATCGATTTAATAATTCTACCGCAACATTTTTTTGTTTCAATCCTTCGACGTCTTT
TAAGAAATCATCTGATAGGATTGATAAATCGGGTTGTTCAAGACCTAGCGTTTGGTAAACATCAATGACATCT
TCAGTCACAATAGATTGTGACACAAGTTGATTAATCTCTGCTTCAACTTCTGCAGGCGTTTTACGTGTTTTCCC
TTCTTTTGGTGGTTGTAATAATTTAACAAGACCTGCTTTAACTGCTTTAAAGAAAGCAATTTCATCATTGAGTT
CTTGGGCTGTTGGTTCAGTCGCACAAAGAGCAAAGGCTTTACCTAACTCTGTGACCGTTTTAATAAAACGTT
GACGTTCATCTTCACCTAAACCAATCACATAATCCATCGTATTCGATATTGTATAATAACGTTCCGATTTTTTATT
TGAATTAAATTTAGAATAATCAAGATTATATAACATATCTTGAATTACATCATATTTCAATAACATCAATTCAACC
GCTTTATCTGTATCTATCGCTGTTTGAGCTTGATCAGATTCTGTATATTCTTTAAGGGCCTCTTTTAAGCTTTCA
GCGATACCCACATAGTCGACAATTAATCCGCCTGGTTTATCTTTAAACACTCGATTGACACGAGCAATCGCTT
GCATTAAATTATGACCTTTCATCGGTTTATCGATATACATGGTATGCATGGAAGGTACATCAAATCCTGTGAGC
CACATATCTCGAACAATCACGAGTTGTAATTCATCATTCACATCTTTCATACGTTTTTCTAATAAATTACGGCGT
TTTTTAGGACCAATATGTCTTTGGAAAGAAGCTGGGTCACTAGAGGAGCCCGTCATTACCACTTTAATGACC
CCTTTATCATCATCATCTGAATGCCATTCTGGTTTTAGACGAATGATTTCATCATATAAATCAACAGCAATTCGA
CGACTCATCGTTACAATCATTCCTTTGCCTTTCATCGCTTGTTGACGTGTTTCAAAGTGTTGGATGATATCTTT
GGCTAGGGCTTCGATACGAGGTTTTGCACCTGCTAAAGATTCAATACGAGACCATTTTGATTTTAAACGCTGT
TTTACATCCTCTTCTTGATCTTCAGTAATGTCATTATACGCTTCATCTAAATCTAAACTTTGAGGTAGATTTAATG
GAATTACACGACTTTCATAGTAAATTTTAACCGTACTTCCATCAGCTACGGCTTGTGTCATATCATAAACATCGA
TATAGTTTCCGAAAACCATTTGCGTATTTTTATCCGTTGAAGCTACAGGTGTGCCTGTAAATCCTACGAATGTT
GCATTCGGTAAAGCATCTCTTAAATATTTGGCATAACCATATTTAATGCCTTCACCTTTATCATCGTATTTTGCGT
TAAAGCCATATTGTGTACGATGTGCTTCATCTGCCATAACAATCACATTTTTACGTTCAGTTAGAGCAGCCATG
GTCGTTTCATTTTGTTCAGGTTCAAATTTTTGCATTGTTGTAAATACAATACCACCCGACTCAACAGATAATAA
CGATTTTAATTCTTTACGTGTTTCAGCTTGTTTTGGTGTTTGTCTTAATAATCCTTTACCAGAGCGACCTTTCG
ATTTAACAAACGTACTGTACAGTTGGTTATCTAAATCATTACGATCTGTTACGACAACTAAGGTAGGGTTATTA
AGCATTTGAATTAATTTTCCAGAGAAAAAGACCATGGTTAAACTTTTACCAGACCCTTGAGTATGCCAAATAA
CGCCGCCTTTACCATCACCCGGCCCAGATGAAGCTAATAAAGCTCTATCAACAGCTTTATTAACAGCATAGTAT
TGATGATACGCTGCTAGAATTTTACTGATATGTCCTTTGCCATCATCTTGAAATACGACAAAATAGCGAATTAA
ATCAAGTAAAGTTTCTGGATTCAACATGCCATGTATGAGTATGTCTAAGCTAGATAAATTTGATGATGATTCAG
TTTCTCCATCTTTAGAACGCCAAGTCATAAAACGATCATAATTTGCGGTTAGGGAGCCCCCCTTTGTATTAATA

CCATCACTTGTAATAAGTACTTCATTAAATGTAAATAATTGTGGAATACGCATCTTATACGTTTCTAATTGATGAT
AACCGTCTTCGACGCCTACGGTTTCATTGGTTGAATTTTTAAGTTCGATCACAACGATAGGCAAGCCATTGAT
AAAGAGTACAATATCGGGACGTTTTGTATAGTCTCCATTAACGACCGTGAATTGATTGACTGCTAAAAAATCA
TTGTTTTGTGGATGTTCAAAATCAACGATTTTAACAATTTCTACTACCGATTGCCCTTCGTCATCATAATCTTCA
ACTTCAATACCATTGATTAAATTTTCATGAAAGGTAAGGTTATTTTCTAAAAGGTTGGGTGACTTTTCTAAAGT
TAGTTCATGTATCGCTTTTTCAATAAAACGAGGATGGATGTCTAAGTTGATTTTTCTTAATGCTTTCTCTAAAC
GTTCATGAAGGACAACGTCTTTATCACTTTTACGTTCTGGCGTTAGACCTGTCAAACTAATCTCATTACCTTTT
TTATAGTCATAGCCTAGTGATTGTAACCATTCTAATGCGACTTGTTCTAAATCATCTTCAGTAAATTGAAAGTTC
ATCTGTATTCACCTCAACATCATCAGGTATTTCTATTTCACCAGACATAAGTTTAGGAAGGAGTGTGTCTCTTA
AAATCGCTAACTTTCTATTTTGCTTTAACAACGAGATGGATAACTCAAAATTGCTTTTAATCTTACTATTAAATT
GATTTAAGTAATTTAAAGGAGGTTTAATCACTTTAATTTTATCAAGAACACCTTCCCATACCGCATAAGGCATA
GTTGAACCTTTAGAATTGTTTGAAGCATATTCAATAAATTCATCTTTATTAATTAAATTAATTAAATAATAAGTAT
ACCTTTCATCTTTTGGTCTTAAAGTGAAAACAGTGTTCCTAGTTACACCCTCAAAAGGCGCCAAACATACTCT
ATGAAAATAAACTCTCATATTGCCTAATAAAATGTCATTTCTTTTAAATTTAATCAAACTACTTTTCGCTTCTTTT
CCATCTTTATAATTATAAAATGTTAATTTTCTTTTAGGTATTAAATCAATAGGTACGTAAGGAAAATTACTACTTA
CTGATTTGGCATTAATTCTATCTTTAACATTATCTATAAGCATTTCTAATTTCACTACTTGCCATTCTTTCGGTATT
TCTCCTAATTCACTACCAACCATTTCTCCACCACTAGATTTATAAGGATTACCATTTTCATCTGGGAATTCAAAA
TCAACAAACCAACGTTTGAATAATGTTTGTGAAAGTTCTTCTAGGTTTGCTATGATTTTATTATTGAGTTCTATT
TTATTATCTATATTTCTCAGTAAAGTGCCTATAAACTTTTGAAATTTCTCAATTGGGATTTTTATTTCTAAATTTTC
TAAAGTACTTTTATTAATTGAATTGAATATAGTACCAGTAGATTTTGAATGAATATCTTGCAATTTATATATTAAT
AAATAATGTAGAAATTTATTATCTTTATTTTTAGATCTAATGGCACTTAAACCACGGCCTATACATAATTCAAATG
GAGCTATATTTATTTCTCCAACAGGAGCTCTTACGGAAAATAAAATATCATTTTTTTTTGCTGTTTTTGTGATTT
TAGTTGTATAAGTATCAATAGTTGGATACAAATTATTAAATGTCCTGTTTCCTTGTAAAAAAGCTGTGCCATTAA
AATCACTATAATACTCTGATTTAGGGGACTGACCCATTATAATGGTGGCAATATCTTTTAATAAATATTTATTAAA
TTCCATAACCTAACCCCTCCAATGATTTACGGATTTGGTCTTCAAGTTCTTTTGATTTTGCAAATTGTTCACTTA
ATTCAGAAGTAATACGTTCCATTTTTTGTTCAAAAGGTTCTTCATCTTCTTCAACGTCAGCTAAACCAACGTAT
CGTCCAGGTGTTAAAATGTATTCATTATTCTTAACTTCTTCAAGGTTAGCTACTTTACAAAAACCAGCTATATCT
TCATAAGATTTATCGTTTGTGCCTCTCCAAGCATGATACGTTTGTGCTACTTTTTGAATATCTTCATCAGAAAAT
TCTTTCAATGTTCTAGATACCATATGACCAATTTCACGAGCATCAATAAATAAAATTTCATTTTTACGTTCTTTTT
TACCATTTTGACCTTTATTATTGCTAATGAACCAAAGGCATACTGGAATTTGTGTAGAATAAAAGAGTTGACC
AGGTAAAGTAACAATACATTCCACTAAATCTTGTTCAATAAGATTTTTTCGAACTTCTAATTCATCTTTTCCACT
TGTAGACATTGATCCATTAGCTAATACAAATCCTGCTGTCCCGTTAGGTGCTAATTTTGAAATCATATGTTCAAT
CCATGCATAGTTGGCATTACCTTTTGGGGGAATACCAAATTGCCAACGGTAATCATCAAGTAATCGTTCTTGA
CCCCAATCACTTGCGTTAAAAGGAGGATTGGCTAATATGTAATCTGCTTTTAATCCTTTATGTAAATCGTTATG
GAATGTATCGGCATTACGTTCACCTAAATCATTATCAATTCCACGAATAGCTAAATTCATTTTAGCTAATTTCCA
AGTGGTAGGATTAGACTCTTGTCCGTAAATTGCAATATCGTCTAATCGACCTTGATGTTTTTTAACAAAACGTT
CACTTTGTACAAACATTCCTCCTGAACCACAACATGGATCGTAGATACGACCTTTATAAGGTTCAATCATTTCA
ACTAATAATTTTACAATTGATGACGGCGTATAGAATTCTCCAGCATTTTTACCTTCGGCACTCGCAAACTTGGC
AATAAAGTACTCATATACTCTGCCTAATACATCTTGTTTACGACTTTCAATATCACCAACTTTAAATGTAAATAAA
TCAATAATATCGCCTAATTTTTCTTTGTCTAACGCAGGACGTGCGTATTCTTTAGGTAACACACCTTTTAATGAT
TCGTTTTCATTTTCAATCGCAATCATGGCTTTATCAATCAATTGTCCAATTTCAGGTTTCTTAGCATTATCATTGA
TATATTGCCATCTTGCTTCTTTTGGTACCCAGAAAATGTTTTCTGCTAAATATTCATCTTGATCCTCTTCGTCTGC
ATAAGGATCTTGCTTCAATTCTTCATACTTTTCTTCAAAAGAATCTGATACATATTTTAAAAAGATTAAATCTAA
TGCTACATTTTTATATTCAGCAGCATCCATACTTCCCCTCAATTTATCGGCAGCTTGCCATAACTTTTCTTCAAAT

CCGATTGTCGCCATTTATATTACACCTCTATATTATCCTTTATACAAATATAGCAAATATTAAGTCTTTATGAGACT
TTTCTTGTGATTTTCTTTGTGAAAAATATTGTTTGTTATATTATTTAAGTAATATTAAGTAGCTAATTATAAAAGT
ATAGATTATTATGTAGTTAAACTGAGTATGAATGGGGGAGCAAATTTGTTATTTACTGAAGAACAATTAAAATT
ATATTCTAAACCATTGTCAGAATCTGAAAAAGAAAAGTGTGAAAATGCAATAAGAATTATCCAAGAATCTCTG
GAATCATTAGGGTATGAAATAAAAAAAGGTCTACATAGAAACAATGAAGATACGCTCTCATATCAAATTAAAA
TGACTAATCCATCGAAAGATTATGAACTAAGTATATTTGTGAAAGGTTCGTATGCAACAAATACCAATGTAAGA
CAAAATAGTGACGTTGATATTGCAGTGGTAAAAGAAAGTGAGTTTTTTGATAAATATAGAGAAGGTAAAACT
AGAGAAAATTATAAATTTATTTCTAGTAATAAGCCTCCGTATCATTTTAAAGATGAAGTGGAAGAAGCTTTGAT
TGAAAGATTTGGAAGAAGTGAGGTAAGAAGAGGTAATAAAGCAATTAGAATCAATGGCAATACTTACCGTA
AAGAAACAGATTGTGTACCTTGTTTTAGATATAGAGATTATAGTAATGATTATATGGATGATCCAAATAATTTCA
TTGGAGGAATCACAATTTATTCAGATAAAGGTGAACGAATTATAAATTATCCGGAACAGCATATAAATAATAGT
ATTATAAAAAATAACAATACAAATTATAAATATAAAAAGATGGTTAGAATAATAAAGGAAATAAGATATCAATTA
ATAGATAGTAAAAATAGAAACGCGGAACAAACTTCTTCATTTGGAGTTGAAGGTTTGTTTTGGAACATACCG
GATTACAAATATAGCAATGATGAAATGTTAGGTGATACATTTAATACATTAATTGCATTTTTAATAGATAATATAG
ATAAATTAAGTGAATTTAAAGAACCTAATGACATTCTATATTTATGCGATTCTCAAGAAAAAAATAATGTTTATA
AAAATTTTATTCTAGATGTAAAGAATTATTTTGAATATAGTGGTGAGAAAAAATATGAATGGCAAAATAAATCA
TTTGATTAAAATAACAGGATGGTTTACAGGAATCTTTTTCTTAGTACTAATTATAGTATCATGTACGGTATGGCA
AATAGGATGGATAGATATCATAAGTTATACTGTAACAATATCAACATTTATTACTATAGGGTATGAAAGGCTTAT
GTGGAAGTGGAAATTGTTTCGTATATTTAATAAGCAAGCTGATATATCAGGAGATTATGAGGCAACACTCAGA
CATTTTCATGGAGAAGGTGGAATTAAAAAGGTAAGTGTGGAAATAAAACAAACTTTTTTAACAGTAAATATT
GAATTAAGAAGCGATGAAATTACTAGTAATAGTATTACAGCAGATATTATTGAAGAACATGGTAAAAATATATT
GTACTATACTTATATAACAAATCCAAAAGCTGAATTCGAAAAGAAAAATCCTATTAATCGAGGAACAACAAGA
TTAATAGTTGGAGAAAATGAATTGACTGGAAAGTATTGGACTGGAGCCAAAACCACAGGTGATCTAAAACT
CAAAAAACTAAAATAAAGTATTGATACCAAAATAAATTCAATTTTATTTTGGTATTTTTAGCAATATCACTAATA
AGGAGAAGCGTACCACAAATGATGCGGTTTTTTATCCAGTTTTTTGTTTAATGAACAAGGTAAATTACGAGAT
AATATTTGAAGAAAACAATAAAGTAGAGATGGATTTCCATATCCTCTTTAGTAGCGGTTTTTATCTGTAAGGTT
TATTAATAATTAAATAAATAGGCGGGATAGTTATATATAGCTTATTAATGAAAGAATATGATTATTAATTTAGTATT
ATATTTTAATATTAAAAAGAAGATATGAAATAATTATTCATACCTTCCACCTTACAATAATTAGTTTTCAATCGAA
TATTAAGATTATTAGTAGTCTTAAAAGTTAAGACTTCCTTATATTAATGACCTAATTTATTATTTGCCTCATGAAT
TATCTTTTTATTTCTTTGATATGTCCCAAACCACATCGTGATATACACTACAATAAATATTATGATGAAACTAATA
ATATTCTCAAAGTTCAGATGGAACCAACCTGCTAGAATAGCGAGTGGGAAGAATAGGATTATCATCAATATAA
AGTGAACTACAGTCTGTTTTGTTATACTCCAATCGGTATCTGTAAATATCAAATTACCATAAGTAAACAAAATTC
CAATCAATGCCCATAGTGCTACACATATTAGCATAATAACCGCTTCATTAAAGTTTTCATAATAAATTTTACCCAT
AAAAGAATCTGGATATAGTGGTACATATTTATCCCTTGAAAAAAATAAGTGAAGTAATGACAGAAATCATAAG
ACCAGTGAACGCACCTTTTTGAACAGCGTGGAATAATTTTTTCATAGTGAGATGGACCATTCCATTTGTTTCT
AACTTCAAGTGATCAATGTAATTTAGATTGATAATTTCTGATTTTGAAATACGCACGAATATTGAACCGACAAG
CTCTTCAATTTGGTAAAGTCGCTGATAAAGTTTTAAAGCTTTATTATTCATTGTTATCGCATACCTGTTTATCTTC
TACTATGAACTGTGCAATTTGTTCTAGATCAATTGGGTAAACATGATGGTTCTGTTGCAAAGTAAAAAAATATA
GCTAACCACTAATTTATCATGTCAGTGTTCGCTTAACTTGCTAGCATGATGCTAATTTCGTGGCATGGCGAAAA
TCCGTAGATCTGATGAGACCTGCGGTTCTTTTTATATAGAGCGTAAATACATTCAATACCTTTTAAAGTATTCTT
TGCTGTATTGATACTTTGATACCTTGTCTTTCTTACTTTAATATGACGGTGATCTTGCTCAATGAGGTTATTCAG
ATATTTCGATGTACAATGACAGTCAGGTTTAAGTTTAAAAGCTTTAATTACTTTAGCCATTGCTACCTTCGTTG
AAGGTGCCTGATCTGTAATTACCTTTTGAGGTTTACCAAATTGTTTAATGAGACGTTTGATAAACGCATATGCT
GAATGATTATCTCGTTGCTTACGCAACCAAATATCTAATGTATGTCCCTCTGCATCAATGGCACGATATAAATAG

CTCCATTTTCCTTTTATTTTGATGTACGTCTCATCAATACGCCATTTGTAATAAGCTTTTTATGCTTTTTCTTCC
AAATTTGATACAAAATTGGGGCATATTCTTGAACCCAACGGTAGACCGTTGAATGATGAACGTTTACACCAC
GTTCCCTTAATATTTCAGATATATCACGATAACTCAATGTATATCTTAGATAGTAGCCAACGGCTACAGTGATAA
CATCCTTGTTAAATTGTTTATATCTGAAATAGTTCATACAGAAGACTCCTTTTTGTTAAAATTATACTATAAATTC
AACTTTGCAACAGAACCACAAAAATGATATATTTAAACTATTCTAATTTAGGAGGATTTTTTTATGAAGTGTCT
ATTTAAAAAATTTGGGGAATTTATATGAGGTGAAAGAATAATTTACCCCTATAAACTTTAGTCACCTCAAGTAAA
GAGGTAAAATTGTTTAGTTTATATAAAAAAATTTAAAGGTTTGTTTTATAGCGTTTTATTTTGGCTTTGTATTCTT
TCATTTTTTAGTGTATTAAATGAAATGGTTTTAAATGTTTCTTTACCTGATATTGCAAATCATTTTAATACTACTC
CTGGAATTACAAACTGGGTAAACACTGCATATATGTTAACTTTTTCGATAGGAACAGCAGTATATGGAAAATT
ATCTGATTATATAAATATAAAAAAATTGTTAATTATTGGTATTAGTTTGAGCTGTCTTGGTTCATTGATTGCTTTT
ATTGGTCACAATCACTTTTTTATTTTGATTTTTGGTAGGTTAGTACAAGGAGTAGGATCTGCTGCATTCCCTTC
ACTGATTATGGTGGTTGTAGCTAGAAATATTACAAGAAAAAAACAAGGCAAAGCCTTTGGTTTTATAGGATC
AATTGTAGCTTTAGGTGAAGGGTTAGGTCCTTCAATAGGGGGAATAATAGCACATTATATTCATTGGTCTTAC
CTACTTATACTTCCTATGATTACAATAGTAACTATACCTTTTCTTATTAAAGTAATGGTACCTGGTAAATCAACAA
AAAATACATTAGATATCGTAGGTATTGTTTTAATGTCTATAAGTATTATATGTTTTATGTTATTTACGACAAATTAT
AATTGGACTTTTTTAATACTCTTCACAATCTTTTTTGTGATTTTTATTAAACATATTTCAAGAGTTTCTAACCCTT
TTATTAATCCTAAACTAGGGAAAAACATTCCGTTTATGCTTGGTTTGTTTTCTGGTGGGCTAATATTTTCTATAG
TAGCTGGTTTTATATCAATGGTGCCTTATATGATGAAAACTATTTATCATGTAAATGTAGCGACAATAGGTAATA
GTGTTATTTTTCCTGGAACCATGAGTGTTATTGTTTTTGGTTATTTTGGTGGTTTTTTAGTGGATAGAAAAGG
ATCATTATTTGTTTTTATTTTAGGATCATTGTCTATCTCTATAAGTTTTTTAACTATTGCATTTTTTGTTGAGTTTA
GTATGTGGTTGACTACTTTTATGTTTATATTTGTTATGGGCGGATTATCTTTTACTAAAACAGTTATATCAAAAAT
AGTATCAAGTAGTCTTTCTGAAGAAGAAGTTGCTTCTGGAATGAGTTTGCTAAATTTCACAAGTTTTTTATCA
GAGGGAACAGGTATAGCAATTGTAGGAGGTTTATTGTCACTACAATTGATTAATCGTAAACTAGTTCTGGAAT
TTATAAATTATTCTTCTGGAGTGTATAGTAATATTCTTGTAGCCATGGCTATCCTTATTATTTTATGTTGTCTTTTG
ACGATTATTGTATTTAAACGTTCTGAAAAGCAGTTTGAATAGTTATATTATATTTTGGTTTAGAACTATGAGTGG
CTAGCATTTTGCCACTCATTTTTTGCGTTAGCAAAAACAGGTTTAAGCCTCGCAGAGCACACGTATTAACGAC
TTATTAAAAATAAGTCTAGTGTGTTTAGACTTAAACTATTAAATACACATGAAACCTTTGTGCTTAGGAGTGAT
TTTTATATGTCTTATTCCATTGTTAGAGTTTCAAAAGTTAAATCTGGAACAAATACAACGGGCATACAAAAACA
TGTTCAAAGAGAAAATAATAATTATGAAAATGAAGATATAGACCATAGTAAAACTTACTTAAATTATGATTTGG
TAAATGCTAATAAACAGAATTTTAATAACTTGATTGATGAAAAAATCGAACAGAATTATACAGGCAAAAGAAA
AATTAGAACAGACGCGATTAAACACATTGATGGTTTAATTACATCAGACAATGATTTCTTTGATAATCAAACGC
CAGAAGATACAAAGCAGTTTTTTGAATATGCTAAAGAGTTTTTAGAACAAGAATACGGTAAAGATAATTTATT
ATATGCAACAGTTCACATGGACGAAAAAACACCACATATGCATTATGGCGTTGTTCCAATAACTGATGATGGT
CGTTTAAGTGCTAAAGAAGTTGTAGGTAATAAAAAAGCTTTAACAGCGTTTCAAGATAGATTTAATGAGCAT
GTTAAACAACGAGGATATGATTTAGAACGTGGGCAATCAAGACAAGTAACAAATGCTAAACATGAGCAAATA
AGTCAGTATAAACAAAAAACAGAATATCATAAGCAAGAATATGAACGTGAGAGCCAAAAAACAGACCATATA
AAGCAAAAGAACGATAAATTAATGCAAGAGTACCAAAAATCGTTAAATACGCTTAAAAAGCCTATAAATGTTC
CGTATGAGCAAGAAACTGAAAAAGTAGGTGGTTTATTTAGCAAAGAAATACAAGAAACTGGAAATGTTGTA
ATAAGCCAAAAAGATTTCAATGAATTTCAGAAACAGATAAAAGCTGCTCAAGATATTTCGGAAGATTACGAG
TATATAAAGTCTGGTAGAGCCTTAGATGATAAAGATAAGGAAATACGAGAGAAAGATGATTTATTAAATAAAG
CAGTTGAGCGTATTGAAAACGCAGACGATAATTTTAACCAACTTTACGAAAATGCAAAGCCACTTAAAGAGA
ATATAGAAATAGCGTTAAAGCTTTTAAAAATCTTACTAAAAGAGTTAGAACGAGTTTTAGGAAGAAATACCTT
TGCGGAAAGAGTTAATAAGTTAACAGAAGATGAACCAAAACTAAATGGTTTAGCAGGAAACTTAGATAAAA
AAATGAATCCAGAATTATATTCAGAACAGGAACAGCAACAAGAACAACAAAAGAATCAAAAACGAGATAGA

GGTATGCACTTATAGAACATGCATTTATGCCGAGAAAACTTATTGGTTGGAATGGGCTATGTGTTAGCTAACT
TGTTAGCGAGCTGGTTGGACTTGAATTGGGATTAATCCCAAGAAAGTACCAACTCAACAACACATAAAGCCC
TGTAGGTTCCGACCAATAAGGAAATTGGAATAAAGCAATAAAAGGAGTTGAAGAAATGAAATTCAGAGAAG
CCTTTGAGAATTTTATAACAAGTAAGTATGTACTTGGTGTTTTAGTAGTTTTAACTGTTTACCAGATAATACAA
ATGCTTAAATAAAAAAAGACTTGATCTGATTAGACCAAGTCTTTTGATAGTGTTATATTAATAACAAAATAAAA
AGGAGTCGCTCACGCCCTGACCAAAGTTTGTGAACGACATCATTCAAAGAAAAAAACACTGAGTTGTTTTT
ATAATCTTGTATATTTAGATATTAAACGATATTTAAATATACATCAAGATATATATTTGGGTGAGCGATTCCTTAA
ACGAAATTGAGATTAAGGAGTCGATTTTTTATGTATAAAAACAATCATGCAAATCATTCAAATCATTTGGAAA
ATCACGATTTAGACAATTTTTCTAAAACCGGCTACTCTAATAGCCGGTAATAGCCGGTTGGACGCACATACTG
TGTGCATATCTGATCCAAAATTAAGTTTTGATGCAATGACGATCGTTGGAAATCTCAACCGAGACAACGCTCA
AGCCCTTTCTAAATTTATGAGTGTAGAGCCCCAAATAAGACTTTGGGATATTCTTCAAACAAAGTTTAAAGCT
AAAGCACTTCAAGAAAAAGTTTATATTGAATATGACAAAGTGAAAGCAGATAGTTGGGATAGACGTAATATG
CGTATTGAATTTAATCCAAACAAACTTACACGAGATGAAATGATTTGGTTAAAACAAAATATAATAAGCTACAT
GGAAGATGACGGTTTTACAAGATTAGATTTAGCCTTTGATTTTGAAGATGATTTGAGTGACTACTATGCAATG
TCTGATAAAGCAGTTAAGAAAACTATTTTTTATGGTCGTAATGGTAAGCCAGAAACAAAATATTTTGGCGTGA
GAGATAGTAATAGATTTATTAGAATTTATAATAAAAAGCAAGAACGTAAAGATAATGCAGATGCTGAAGTTAT
GTCTGAACATTTATGGCGTGTAGAAATCGAACTTAAAAGAGATATGGTGGATTACTGGAATGATTGCTTTAGT
GATTTACATATCTTGCAACCAGATTGGAAAACTATCCAACGCACTGCGGATAGAGCAATAGTTTTTATGTTATT
GAGTGATGAAGAAGAATGGGGAAAGCTTCACAGAAATTCTAGAACAAAATATAAGAATTTGATAAAAGAAA
TTTCGCCAGTCGATTTAACGGACTTAATGAAATCGACTTTAAAAGCGAACGAAAAACAATTGCAAAAACAAA
TCGATTTTTGGCAACATGAATTTAAATTTTGGAAATAGTGTACATATTAATATTACTGAACAAAAATGGTTCTG
TTGCAAAGTAAAAAAATATAGCTAACCACTAATTTATCATGTCAGTGTTCGCTTAACTTGCTAGCATGATGCTA
ATTTCGTGGCATGGCGAAAATCCGTAGATCTGAAGAGACCTGCGGTTCTTTTTATATAGAGCGTAAATACATT
CAATACCTTTTAAAGTATTCTTTGCTGTATTGATACTTTGATACCTTGTCTTTCTTACTTTAATATGACGGTGATC
TTGCTCAATGAGGTTATTCAGATATTTCGATGTACAATGACAGTCAGGTTTAAGTTTAAAAGCTTTAATTACTT
TAGCCATTGCTACCTTCGTTGAAGGTGCCTGATCTGTAATTACCTTTTGAGGTTTACCAAATTGTTTAATGAGA
CGTTTGATAAACGCATATGCTGAATGATTATCTCGTTGCTTACGCAACCAAATATCTAATGTATGTCCCTCTGCA
TCAATGGCACGATATAAATAGCTCCATTTTCCTTTTATTTTGATGTACGTCTCATCAATACGCCATTTGTAATAAG
CTTTTTTATGCTTTTTCTTCCAAATTTGATACAAAATTGGGGCATATTCTTGAACCCAACGGTAGACCGTTGAA
TGATGAACGTTTACACCACGTTCCCTTAATATTTCAGATATATCACGATAACTCAATGTATATCTTAGATAGTAGC
CAACGGCTACAGTGATAACATCCTTGTTAAATTGTTTATATCTGAAATAGTTCATACAGAAGACTCCTTTTTGT
TAAAATTATACTATAAATTCAACTTTGCAACAGAACCGTATTATGGAATAGAGATGTTGGTAACATTTATACAG
GATCATTATACTTAAGTTTAATTTCGTTATTACAGAACCACACATTCCAACCAGAAGAGAAAGTATGTCTATTT
AGTTATGGTTCAGGAGCAGTAGGAGAAATCTTTAGTGGTTCAATCGTTAAAGGATATGACAAAGCATTAGAT
AAAGAGAAACACTTAAATATGCTAGAATCTAGAGAGCAATTATCAGTCGAAGAATACGAAACATTCTTTAACA
GATTTGATAATCAAGAATTTGATTTCGAACGTGAATTGACACAAGATCCATATTCAAAAGTATACTTATACAGT
ATAGAAGACCATATCAGAACATATAAGATAGAGAAATAAACTAGTGGCCGATTGTGCTTGATGAGCTTGGGA
CATAAATCCTAACTCGAAATAAATAAGCATATCACTAAACTGATTTTTTAAAGTTTACAGTGATATGCTTATTTT
TTTATCTTACGATTTTGTACGTGCATGCTTGCCTAGGGGTATGGCTCGAGCCATTAGTCTCTCGCACATACTATT
CCCTCAGGCGTCAGCACTTACAAAATCGGTTGTAATTTTCATTTTTATACGCATTCTTACTGAGATTATACTAAT
AAGAGGAATAGTAAAAGCAATTCTAAGTAAAATTGCAGATAAGAGGTTTGTTAAAAGCAGTTCTAAGTAAAA
TTGCAGATAAGAGGTTTGTTAAAAGCAGTTCTCAGTAAAATTACAGATAAGAGGTACGTTAAAAGCAGTTCT
AAGTAAAATTGCAGATAAGAGGTTTGTTAAAAGCAGTTCTAAGTAAAATTGCAGATAAGAGGTACGTTAAAA
GCAATTCCATGCAAAATTGCTGATAAGGGGTAAGTTAAAAGCAGTTCTCAGTAAAATTGCAGATAAGAGGTA

CGTTAAAAGCAGTTCTAGGCAAAATTGCAGATAAGAGGTTTGTTAAAAGCAGTTCTCAGTAAAATTGCTGAT
AAGGGGTAAGTTAAAAGCAATCCTAAGTAAAATTGCAGATAAGAGGTAAGTTAAAAGCAATCCTAAGTAAA
ATTGCAGATAAGGGGTACAGAAAAACTAGACTTGATTACAAAATGGAGCTTGGGACATAAATGATTTTTTAA
AAATGAGATGAGACGTAGATTAACTCCATAATCAATACGAATCTATCGACTTCTTTATTTATGATATTCATCTCTT
TTTAATGGAAATAAAAGTGCGATTAATGTGATAATACAGTTACGTTAATTAAAAAAATAAAAATGCAAGGAGA
GGTAATATGCTAACTGTATATGGACATAGAGGATTACCTAGTAAAGCTCCGGAAAATACAATTGCATCATTTAA
AGCTGCTTCAGAAGTAGAAGGTATAAACTGGTTGGAGTTAGATGTTGCAATTACAAAAGATGAACAACTGAT
TATCATTCATGATGATTATTTAGAACGGACTACAAATATGTCCGGGGAAATAACTGAATTGAATTATGATGAAA
TTAAAGATGCTTCTGCAGGATCTTGGTTTGGTGAAAAATTCAAAGATGAACATTTGCCAACTTTCGATGATGT
AGTAAAAATAGCAAATGAATATAATATGAATTTAAATGTAGAATTAAAAGGTATTACTGGACCGAATGGACTA
GCACTTTCTAAAAGTATGGTTAAGCAAGTGGAAGAACAATTAACAAACTTAAATCAGAATCAAGAAGTGCTC
ATTTCAAGCTTTAATGTTGTGCTTGTTAAACTTGCAGAAGAAATCATGCCACAATATAACAGAGCAGTTATATT
CCATACAACTTCGTTTCGTGAAGACTGGAGAACACTTTTAGATTACTGTAATGCTAAAATAGTAAACACTGAA
GATGCCAAACTTACTAAAGCAAAAGTAAAAATGGTAAAAGAAGCGGGTTATGAATTGAACGTATGGACTGT
AAACAAACCAGCACGTGCAAACCAACTTGCTAATTGGGGAGTTGATGGTATCTTTACAGACAATGCAGATAA
AATGGTGCATTTGTCTCAATAGAAAGTTAGAGGTGAGTCTTACGTTTCAGTGACGGTAGACTTACCTTTAACA
TGTTACATACTAAAAAATTAATTTGAATAAGAAAGAGAGACATATATGAAATACGATGATTTTATAGTAGGAGA
AACATTCAAAACAAAAAGCCTTCATATTACAGAAGAAGAAATTATCCAATTTGCAACAACTTTTGATCCTCAA
TATATGCATATAGATAAAGAAAAAGCAGAACAAAGTAGATTTAAAGGTATCATTGCATCTGGCATGCATACAC
TTTCAATATCATTTAAATTATGGGTAGAAGAAGGTAAATACGGAGAAGAAGTTGTAGCAGGAACACAAATGA
ATAACGTTAAATTTATTAAACCTGTATACCCAGGTAATACATTGTACGTTATCGCTGAAATTACAAATAAGAAAT
CCATAAAAAAAGAAAATGGACTCGTTACAGTGTCACTTTCAACATACAATGAAAATGAAGAAATTGTATTTAA
GGGAGAAGTAACAGCACTTATTAATAATTCATAATAAAACAGTGAAGCAACCATCGTTACGGATTGCTTCACT
GTTTTGTTATTCATCTATATCGTATTTTTTATTACCGTTCTCATATAGCTCATCATACACTTTACCTGAGATTTTGG
CATTGTAGCTAGCCATTCCTTTATCTTGTACATCTTTAACATTAATAGCCATCATCATGTTTGGATTATCTTTATCA
TATGATATAAACCACCCAATTTGTCTGCCAGTTTCTCCTTGTTTCATTTTGAGTTCTGCAGTACCGGATTTGCC
AATTAAGTTTGCATAAGATCTATAAATATCTTCTTTATGTGTTTTATTTACGACTTGTTGCATACCATCAGTTAAT
AGATTGATATTTTCTTTGGAAATAATATTTTTCTTCCAAACTTTGTTTTTCGTGTCTTTTAATAAGTGAGGTGCG
TTAATATTGCCATTATTTTCTAATGCGCTATAGATTGAAAGGATCTGTACTGGGTTAATCAGTATTTCACCTTGT
CCGTAACCTGAATCAGCTAATAATATTTCATTATCTAAATTTTTGTTTGAAATTTGAGCATTATAAAATGGATAAT
CACTTGGTATATCTTCACCAACACCTAGTTTTTTCATGCCTTTTTCAAATTTCTTACTGCCTAATTCGAGTGCTA
CTCTAGCAAAGAAAATGTTATCTGATGATTCTATTGCTTGTTTTAAGTCGATATTACCATTTACCACTTCATATCT
TGTAACGTTGTAACCACCCCAAGATTTATCTTTTTGCCAACCTTTACCATCGATTTTATAACTTGTTTTATCGTC
TAATGTTTTGTTATTTAACCCAATCATTGCTGTTAATATTTTTTGAGTTGAACCTGGTGAAGTTGTAATCTGGA
ACTTGTTGAGCAGAGGTTCTTTTTTATCTTCGGTTAATTTATTATATTCTTCGTTACTCATGCCATACATAAATGG
ATAGACGTCATATGAAGGTGTGCTTACAAGTGCTAATAATTCACCTGTTTGAGGGTGGATAGCAGTACCTGAG
CCATAATCATTTTTCATGTTGTTATAAATACTCTTTTGAACTTTAGCATCAATAGTTAGTTGAATATCTTTGCCAT
CTTTTTTCTTTTTCTCTATTAATGTATGTGCGATTGTATTGCTATTATCGTCAACGATTGTGACACGATAGCCATC
TTCATGTTGGAGCTTTTTATCGTAAAGTTTTTCGAGTCCCTTTTTACCAATAACTGCATCATCTTTATAGCCTTT
ATATTCTTTTTGTTTTAATTCTTCAGAGTTAATGGGACCAACATAACCTAATAGATGTGAAGTCGCTTTTCCTA
GAGGATAGTTACGACTTTCTGTTTCATTAATTGTAAGATGAAATTTTTTTGCGAAATCACTTAAATATTCATCCA
TTTTTTTAACGGTTTTAAGTGGAACGAAGGTATCATCTTGTACCCACTTTTGATCCATTTGTTGTTTGATATAGT
CTTCAGAAATACTTAGTTCTTTAGCGATTGCTTTATAATCTTTTTTAGATACATTCTTTGGAACGATGCCTATCT
CATATGCTGTTCCTGTATTGGCCAATTCCACATTGTTTCGGTCTAAAATTTTACCACGTTCTGATTTTAAATTTT

CAATATGTATGCTTTGGTCTTTCTGCATTCCTGGAATAATGACGCTATGATCCCAATCTAACTTCCACATACCAT
CTTCTTTAACAAAATTAAATTGAACGTTGCGATCAATGTTACCGTAGTTTGTTTTAATTTTATATTGAGCATCTA
CTCGTTTTTTATTTTTAGATACTTTTTTTATTTTACGATCCTGAATGTTTATATCTTTAACGCCTAAACTATTATAT
ATTTTTATCGGACGTTCAGTCATTTCTACTTCACCATTATCGCTTTTAGAAATATAACTGCTATCTTTATAAACTT
GTTTGAAATTTTTATCTTCAATTGCATCAATAGTATTATTAATTTCTTTATCTTTTGAAGCATAAAAATATATACCA
AACCCGACAACTACAACTATTAAAATAAGTGGAACAATTTTTATCTTTTTCATCAATATCCTCCTTATATAAGAC
TACATTTGTAGTATATTACAAATGTAGTATTTATGTCAAAATAATGTTATAATTTTTGTGATATGGAGGTGTAGAA
GGTGTTATCATCTTTTTTAATGTTAAGTATAATCAGTTCATTGCTCACGATATGTGTAATTTTTTTAGTGAGAAT
GCTCTATATAAAATATACTCAAAATATTATGTCACATAAGATTTGGTTATTAGTGCTCGTCTCCACGTTAATTCCA
TTAATACCATTTTACAAAATATCGAATTTTACATTTTCAAAAGATATGATGAATCGAAATGTATCTGACACGACT
TCTTCGGTTAGTCATATGTTAGATGGTCAACAATCATCTGTTACGAAAGACTTAGCAATTAATGTTAATCAGTT
TGAGACCTCAAATATAACGTATATGATTCTTTTGATATGGGTATTTGGTAGTTTGTTGTGCTTATTTTATATGATT
AAGGCATTCCGACAAATTGATGTTATTAAAAGTTCGTCATTGGAATCGTCATATCTTAATGAACGACTTAAAGT
ATGTCAAAGTAAGATGCAGTTCTACAAAAAGCATATAACAATTAGTTATAGTTCAAACATAGATAATCCGATGG
TATTTGGTTTAGTGAAATCCCAAATTGTACTACCAACTGTCGTAGTCGAAACCATGAATGACAAAGAAATTGA
ATATATTATTCTACATGAACTATCACATGTGAAAAGTCATGACTTAATATTCAACCAGCTTTATGTTGTTTTTAAA
ATGATATTCTGGTTTAATCCTGCACTATATATAAGTAAAACAATGATGGACAATGACTGTGAAAAAGTATGTGA
TAGAAACGTTTTAAAAATTTTGAATCGCCATGAACATATACGTTATGGTGAATCGATATTAAAATGCTCTATTTT
AAAATCTCAGCACATAAATAATGTGGCAGCACAATATTTACTAGGTTTTAATTCAAATATTAAAGAACGTGTTA
AGTATATTGCACTTTATGATTCAATGCCTAAACCTAATCGAAACAAGCGTATTGTTGCGTATATTGTATGTAGTA
TATCGCTTTTAATACAAGCACCGTTACTATCTGCACATGTTCAACAAGACAAATATGAAACAAATGTATCATATA
AAAAATTAAATCAACTAGCTCCGTATTTCAAAGGATTTGATGGAAGTTTTGTGCTTTATAATGAACGGGAGCA
AGCTTATTCTATTTATAATGAACCAGAAAGTAAACAACGATATTCACCTAATTCTACTTACAAAATTTATTTAGC
GTTAATGGCATTCGACCAAAATTTACTCTCATTAAATCATACTGAACAACAATGGGATAAACATCAATATCCATT
TAAAGAATGGAACCAAGATCAAAATTTAAATTCTTCAATGAAATATTCAGTAAATTGGTATTACGAAAATTTAA
ACAAACATTTAAGACAAGATGAGGTTAAATCTTATTTAGATCTAATTGAATATGGTAATGAAGAAATATCAGG
GAATGAAAATTATTGGAATGAATCTTCATTAAAAATTTCTGCAATAGAACAGGTTAATTTGTTGAAAAAATATGA
AACAACATAACATGCATTTTGATAATAAGGCTATTGAAAAAGTTGAAAATAGTATGACTTTGAAACAAAAAGA
TACTTATAAATATGTAGGTAAAACTGGAACAGGAATCGTGAATCACAAAGAAGCAAATGGATGGTTCGTAGG
TTATGTTGAAACGAAAGATAATACGTATTATTTTGCTACACATTTAAAAGGCGAAGACAATGCGAATGGCGAA
AAAGCACAACAAATTTCTGAGCGTATTTTAAAAGAAATGGAGTTAATATAATGGATAATAAAACGTATGAAAT
ATCATCTGCAGAATGGGAAGTTATGAATATCATTTGGATGAAAAAATATGCAAGTGCGAATAATATAATAGAA
GAAATACAAATGCAAAAGGACTGGAGTCCAAAAACCATTCGTACACTTATAACGAGATTGTATAAAAAGGGA
TTTATAGATCGTAAAAAAGACAATAAAATTTTTTAATATTACTCTCTTGTAGAAGAAAGTGATATAAAATATAAA
ACATCTAAAAACTTTATCAATAAAGTATACAAAGGCGGTTTCAATTCACTTGTCTTAAACTTTGTAGAAAAAG
AAGATCTATCACAAGATGAAATAGAAGAATTGAGAAATATATTGAATAAAAAATAAAATTGTTGTGTTTACAA
CAATACATAGAAAACAGAGGAAACAATCAAGTCGTTGAATATTTCCTCTGTTTTTTAGTTGAAAAAATTAACC
GAAAGCCTGAATGCAAGTCTTGATTAAATCAATAATGCTTGTAATAACACCAGTGAAATCCATATGCATACCCT
CTTTCTATTTAAGATACATTAAGTATAATATCAAACAAATAAAAAATGTTAAAAATTCCCTAATTGGCTATTTAG
ATTGCATAAATGTCAAAAATTTGAAAAACATACAACGACTTTGCATAAAAAATCGTCATATTGGAAATACGTA
ATTTATTGAAATAATAAAAAAAAATAAAAGAACGAAGATGATAACCTAAGTGAGGTTTTAAGTTGTTCTAAGGT
TTAATTTAATTTATGTTAAAATAGTTGGTATAAAAATACATGATAAACTATAAACTAAATTCAAAATAACTTATGG
GGTAGGCAATTATGGAAAATATTTTAAATATAAATGATAATGAAAAAAGAGTGCTAAGGGAAATTTATAACCA
TCATAATATTTCGCGTACTCAAATATCTAAAAATCTTGAGATTAATAAGGCAACGATTTCTAGTATTTTGAATAA

GTTAAAGTATAAATCTCTTGTTAATGAGGTTGGTGAGGGTGATAGCACGAAGAGTGGTGGTAGAAAACCTAT
TCTTCTGAAGGTTAATCATCTTTATGGTTATTTTATTTCTTTGGATTTAACTTATAGTTCTGTTGAAGTGATGTAC
AATTATTTTGATGGTAATGTCATTAAGCATGAATCTTATGATTTACCTGATGAAAAGGTTAGTAGTATATTAAGC
ATAATAAAAAAACATATTGATATTCAGGAGAAACTTGATACTTATAACGGACTATTAGGTGTGTCTGTTTCTATA
CATGGAGTTGTGGATAATGAGCAGCATGTGACATATTTACCATTCCATGAAACTGAAGGAATTTCAATTGCTA
AGAAAATAAAAGAAATTACTAATGTTCCAGTCGTAGTTGAAAATGAAGCGAATCTTTCAGCGTTATATGAACG
TAATTTTAATCATAATTTATCCTACAATAATCTTATTGCTTTAAGTATACATAAAGGTATTGGTGCTGGGCTTATT
ATTAATAATCAATTGTATCGTGGTGCAAATGGGGAAGCGGGTGAAATTGGAAAAACACTTGTCTCAAAAGTT
AGCGATAATGTGGAGATCTTTCATAAGATTGAAGATATTTTTTCACAAGAAGCTTTACTGCATAATTTAAGTAA
TCAACTAAATGAGAAGATGACGCTTAGCAAATTAATTCAATTTTATAATGAAAAAAATCCAGTCGTAGTTGAA
GAAATGGAACAATTTATAAATAAAATTGCTGTTTTAATACATAATTTAAACACCCAGTTTAATCCGAATGCAAT
TTACATTAACTGTCCATTGTTCAATGAAATGCCTGAAATATTAGAAGCAATTAAGAACCAGTTCAAACAATATT
CACGTAACGAAATTCAAATAAAGTTAACATCTAATGTCAAATTTGCAACTTTGCTAGGTGGTACATTAGCAATT
ATCCAAAAAGTACTACAGATTAATGATATTTACTTAGATATAAAAGCATAAAAAACTAATTCAAATGAATAATCA
AAGTTCGTAATTGTCTTTATAAAAAAATCCCTCAATCCGAATTGAATTTTCGGATTGAGGGATTTTTATAGTTC
TATTGCAGAAGAAAACTATTTTAAAAATGCTGGTAAATGTTGATAGCCACCTCTAACGTTAACAATATTCGTAA
ATCCTTTATATTCTAATATTCCTACCGCTATTGAACTTCTAACACCTGATTGACAATGTACATAAATTAGGTCATT
TTTATCGAAAGGTATATCTTCATTTAAAAGTTTACCGTGAGGAATATGAATTGCTTGTTTTAAATGACCTTTAC
GCCATTCATCATCATTACGAACATCTAATACATTATGTTCTTCACCAGTCATTTCAGAACTATGAATAGATGATG
TGACGATATTTGTTTGTGGCAAACGGTAACTTTTTACATTTTCAAAACCAATTAATTGTAAAGCATGAATAGCT
TGTTGAACGGTAGATTTATCGCCAATTAATTCAATATCTTAGTCATAATCTAAATACCAACCAATTTGATTTATAA
AAGTTTTATTAAAAGGAATATTGATAGTTCCATGCATATGACCACCATGGAATGCTTCTTTACTTCGAAGATCA
AAAGCAGTTTGTGTATTGCTTGAACTAGGGTAAACATTATATGGTTGGTACATTTGCATACCAAATTGATTTAT
TTTTTTCATTTGTGAAAAATGGTGTGGTGGAGCTGGCTGATTGAGTGTTAAAGTTTCGATAAATGAAGTTTC
ATCTTTAACATTAAAAGCCCAGTTGTTTATTTTCTCATAACCCAAAGTAGTTGTAGGTAATGAACCTAGCGCTT
TACCACAAGGACTCCCTGCACCATGACCTGGCCAAATTTGAATATAGTCTGGTAATGTTGCAGCAAATTGTAT
GGACTGATACATTTGTTTTGCTCCGATTTTTGTAGAACCTTCAACATTTACAGCTTTTTCTAATAGATCTGGTCT
ACCTACATCACCAACAAAGATGAAGTCACCGCTAAATAATCCCATTGGTATACTGGAACCCCCACCTTCGTCA
GTAAGTAAAAAACTAATACTCTCAGGGGGATGGCCTGGAGTGTGTAAGACTTCTAATTTAATCTTTCCTAAAT
AGATAATATCTTGATGCTTAACGAAATGTGTTTGTTTAGGCATATTTTTATAATTAAATTCATCTTTACCTTCATC
AGATACGTATATACTTGCATTCAATTTATTTGCCACATCTCTAATACCTGAAGCAAAATCAGCATGAATATGTGT
TTCTGCAGCTTTAGTAATTGTGAATCCTTCTTTATCTGCAACTTTTAAATATTTTGTTAAATCTCGTATAGGGTC
AATAATCATTGCTTCTCCTGTACGTTGACATCCAATTAAATAAGATGCTTGTGAAAAAATTGTCTTCATAAAATTG
TTTGAAAAACAAAAAAAACTCCTTTTTAAAATAGATTTTATTGATTAGATAAATAAGTTATGATTTGCTTGCTC
AGTATGTCCAATATAAGTACCTACGCCACCATAATCGACTTCATCTCTTAATTCTTCTTTTGAAATTCCCATAACA
TCCATACTCATGGTACAAGCAATTAACTTTATATCTTGATCGATTGCTTGATCGATAAGTGAGTATAAAGAATCA
ACATTTTTCTTGTTCATTACATAACGCATCATAATATTACCTAGTCCAAACATATTCATTTTTGATAATGGCATAT
GTATTGGATCTTTAGGTAACATAAGGTCAAACATTTTGGAAATACCTTTCTTTTTAACGCGAGTTGATTGCGCT
TTTTTTAATGCGTTGAGGCCCCAAAAAGTAAAGAAAATAGTTACATCTTTACCTGCTGCTTTAGCGCCATTTG
CGATGATCATTGCTGCTACTGCCTTATCTAACTCACCGCTAAATAAAACAATTGTTGAACCTGTAGCAGTGTCA
TTGATTTCAAATTCTTTTGGCTTTTCTTTTTGAATAATTGCATTAATTACATTTGCTTCTTCAGTAAGATTTACAA
GGGTATTCCCTGTTTGTTTCGCCCAACTTTTAATATCACTATTGAAACCAGGATCTGTAACTGTTACCTCGATTT
GCTCACCCGTTGAAATATTGTTAATTTCTTTACTGATATTAACAATAGGTCCAGGGCACTGAAGACCTCTAAAA
TCAAATTGTTTACGATTCTCTTTGATTTCAATATCTTTTTCTATTAAAGGAGCACTATTGAAGTTCTTTGCTTCAT

AATCTTTATATCCACCCTTTAAATTCACGACATCATAACCTTGTTTGGCTAAATAATCGCAAGCTTTAGTGCTTC
GGTTACCGCTTTTACAATGTATATAATACGTTTTGTTGCTATTCTTATTGAATGATTTAATCTCTTCTACTGGGTG
TAAAGTTGAACCGTTAATGTGTCCTAATTCATATTCTTCTTTTGTTCTAACATCAATCAATTGACCCATTTTTGC
CAATTTTTCTAATTCTTCTTTGTTTAATGAATTAATGTGTACTTCTTTGTATTGTTCCATACTTACCTCCTATAAAT
ACCTATGAGGGTATAATAAAACGGATAGAATCATTTGCCAAATACCTATAGGGGTATTTGACAATTTTGTTTTA
ATTTATTATTATTAACTTAATCAATTTATGTGGAGGAAATGAATATGACTTATGATAAAAAAATGATTAATCGTAT
AAATAGAATACAAGGTCAATTAAATGGTGTCGTAAAAATGATGGAAGAAGAAAAAGATTGCAAAGATATAAT
TACGCAACTTAGTGCATCTAAAGGTTCTATACAACGTTTAATGGGGATTATAATTAGTGAAAATTTAATAGAAT
GCGTTAAAACAGCAGAAGAAAATAATGAAAGTTCTCAAGAATTAATTAATGAAGCAGTTAATTTATTAGTTAA
AAGTAAATAATGGATATAGCAAATATGACTATTATGTTGCTAATTGGCGTACTGGGTGGATTTATATCTGGATTA
ATAGGTATTGGGGGCGCAATTATTATTTACCCAGCTATTCTTATATTGCCACCATTAATAGGTATACCTGCGTATA
GTGCATATATTGCTTCGGGACTTACCTCTAGTCAAGTATTTTTCAGTACACTTAGTGGATCATTAAATGCAAGA
AAACAACCAGCTTTCTCTCCTAAACTTGTTATATATATGGGAGGGGGTATGTTGATTGGAAGCATGTTAGGGG
CAATTTTAGCTAGTTTGTTTAATGCTACTTTTGTAAATACGGTATATGTAATAATCGCCATACTTGCTTTAATATT
GATGTTTATTAAAGTTAAACCTACTACACAAGAGACGAAATCTAAACCTTTGCTATTTATTATAGTTGGATTTG
GAATTGGTGTAATTTCGGGAATTGTGGGTGCAGGTGGAGCATTTATCATCATTCCTGTATTATTAGCATTATTT
AAATTACCAATGAATACGGTAGTGAACAATAGCATAGCAATTGCTTTTATATCTTCAGTAGGGGCATTTTTTAT
AAAATTAATGCAAGGATATATACCAGTAGAAAGTGCAATTTTTTTGATAATTGAATGATGATCATTTTCTGAAA
ATATTATGTGGTCATATAATATAATGCCCATCATTTCACTAATCTCTTTTATTCTCTGAGTTATTTTGATATCTCCTG
GAGAAGGTGTCGGATCTTATCAAGAGTAAATTATGAAAAAAATGGTTTAAATCAATGTTCTTACTTTAAGTAC
ATGTGTTTAACGTAAATGATAATTAGTTAAATATAAAAAGGTTATCCCAATCAATAGGATAACCTCTTTATACTT
TACTTTATGGTTTGGATTCGCCACTTCAAAGAAGAAATGAAATCAGAGCATTGAATGTTCCATTTTCAAACAA
TATATAAATACCTAGCCCAATGAATACAATTGGTACAATCCAACGTTCATATTTCTCAATTGTTTCCGATATAAAA
TCGAAGGAAGCTAGACGGTAACTGACATAGCACAAAACTCCAACCATAATTAGAAAGACAATAGTGACAATA
AAGATTTCAGACATACTTAAGGTCGTGAAGTACGGTATATAAATGGAAAAGTCATCCGCACTGGAAGCCAAT
ACGATGAAAATCATCGTCAAAAATAACTGATTAAATTTTCCAGAGGAGAATAAAGATAAAATGCTACTTTCAT
CTTCATCCTCTTCTCCTTTAATCCATATTTTCACGCCTAGGTAAAGTGGTAAAAGTCCAAGTAGTCCGATAACC
CATTGCTGAGGAATTAAATTTACAACCCCCTGTGCAACTAAAAGACTTGCTCCTATCACAATTGCAGTCCCTAT
ATATTGTCCTATCCAAATATGTTTTACCTGACCTTTTTTTACTTGCGAAAACAAAAGAATTAATATGACGAGATA
ATCAATTCCTGTTGCTACATATACCGCAGTAGCCGTCAGTATCGTCGCGATCATTTTATCATCTCCAAATATTTTA
GGGATAGGACTTTTCTTCAAAATGAAAAGTCCTTCCGTAAATTGCACACATATAGTACTTATTTATCCTTCACT
CTCATCAGTCGCAAACTATTTAATGCTACCAAAATAGTGGCTCCCATATCGGAAAGAATCGCAATCCAAAGGG
TTAGCCAGCCTGGAATAACCAATAGTAAGGCAATTATCTTAATTCCGATGGCAAACGTGATGTTCGCTTTGAT
GATATTTAGCGTTTTCCTGCTAAGTCTTACTGCAAATGGAAGCTTACTTAAATCATCTCCCATTAATGCAATATT
AGCTGTCTCGATGGCAGTATCTGTTCCAGCACCGCCCATTGCAATGCCAACAGTGGATGCAGCAAGTGCAG
GAGCATCATTGACGCCATCGCCAATCATAGCTACATTACCATGCTCGGCTTTCATTTTTTTAATATAGTCCAACT
TATCCTGTGGCAACAATTCGGACTGAATATCAGAAACGCCTACATGAGCACCGATTGCTTCTGCGGTACCTTG
ATTATCACCTGTCAGCATAATTGTTTGCTTGATTCCTAACTGATGAAGTTTTAGAATCACATTTTTACTTGTTTC
GCGGACCTCATCTGCTACAGCAATCACGCCGAGGATTGTTTGGTCCGTTCCAATAATCATGGCCGTTTTCCCT
TGGTTTTGTAAAACTTTCACTTTATTTTCAAACTCAAGGCTAAAATCGGAAACATTTAATTCTTTAAAAAGCCT
TGGACTGCCAATGTAATAGGTTGTTCCATCTATATTCCCTTGAATGCCCCGACCTGTAATAGAAGTGAAGTCCT
TCACTCTAACATCGGAATAAGTAATATTATCTTGCTCTGCTTTCTTCATTATTGCTGAAGCAAGTGGATGTTGTG
ATCGATATTCTAAAGCTGTAATAATGGAAAACAGCTCTTTTTCTTCCACTTGATCATTTAACACTTTAAAATCTG
TTACCACTGGTACACCTTTTGTCAGTGTTCCTGTTTTATCAAATGCGATTGCCTTAATGGCTCCTAATTCCTCTA

GATAGACACCGCCTTTAATCAACACACCTTTTTTAGCTGCATTTCCAATTGCCGAGACAATCGAGATTGGAGT
AGTAATAACTAATGCACACGGACATCCAACTACAAGTACCGCTAATCCTTGATAAACCCAAGTATCCCAACTTC
CACCAAAGAATAAAGGTGGAACGACTGCAACGAGCGCCGCAATAACCATAATGATCGGCGTATAATATTTCG
CAAATTTATCTACGAATGCTTGCGCTGGAGCGCGCTCCCCTTGTGCTTCCTCAACCAGATGAATAATCTTGGA
GATAGTTGTATCCTCTACGTATTTGGTGATTTTTACTTCAAGTAGTCCCTCTTCGTTAAGCGTGCCTGCAAATAC
TTCATCATCTACCGTTTTGGCAACAGGGACAGATTCTCCTGTTATAGCAGCCTGGTTGACAGCCGACACACC
ATTTATAATGATCCCATCCATGGCAATTTTCTCCCCTGGTTTGACGATCATAATATCACCCACGGCAATATCGTC
CACATGGATCATTATTTCCTGACCATTCCGCCTAACAAGTGCTTCTTTTGGGGCAATATCCATCAATGAACGAA
TGGACTGTCTTGCTCTATCCATAGAAAAACGTTCAAGTGCTTCACTGATTGCAAAGAGAATGACAACAATGG
ATGCCTCTGCCCATTCACCAATGATGGCAGCTCCAATAACTGCAACGGTCATCAGGGTTTTCATGTCGAAATC
AAAGCGTATCAAATTTTGAAAACCAACTTTAAATAGTGAATATCCGCCAATTACAATCGAACTTACAAATAACA
TGGACGTTACAAGGTTATCTTCTCCATTTACAAAGTGAGAAAGGTAACCAAAAGCAATCAGTAATGTGGCAA
ACAGCAATGTGCTGTGTTTTTTATAAAACGGTATTTTCTCTTCTTTAGGAGCCTTAGTGTCTTCTTTGACCGCT
TGTATCGATGGATTCGCCAGTTTTTCAGGAATTACCTTAAGATTCTCGAAAGCACCTGCTTTTTCAAGCTCTTC
AACCGATGCGTTTCCATATACATCAATTTTAGAAGCGCCAAAGTTCACTTTTGCATCCTGAACTCCAGCTAGTT
GTTTTACATTTTTTTCAAACTTCCCAGCACAGTTTGCGCAAGAAAATCCCTCCACACGGTAAACATTTTTATCT
TCTGTTACCACTGGTTCGACCCTTCTTCTAGCCTTCTCTGGTGCCACTTTAAGATTCTCGAAAGCACCAGCCT
TTTCCAGATCTTCAACGGTTGCACTGCCAAAGACATCAATTTTGGAAGCTCCGAAATTGACTTTAGCATCATG
CACCCCTGATAGTTCTTTTACATTTTTTTCAAACTTCCCAGCACAATTCGCACACGAGAAACCCTCCACACGG
TAAACCTGTTTATCTTCTGTTAATGTTTTTGTTGAACTATCCAATACTAGCAACCTCCCTTTGATGCAAGAAAG
CTTTTTCTACAAGCTGTTTAACATGCTCATCATCTAGTGAATAATAGACTAATTTTCCTTCTTTACGGTATTTTGC
TATACCTAAATTTTTCAATAATCTTAAATGATGGGATGCCGTAGCCGTTGAAGATTCAATGATATTAGCTACATC
ACAAACACATAACTCTCCCTCTAAAGACAAAACATAAGCAATTTTAACTCTTGTATCATCTGATAGAGCCTTAA
AAACTTTCGCTACATCCATAGGATTCTGTTTAGCAAGGTCTTTTTTAGCCCTGTTTACCTTATCTTCATGAATAT
AGGTAACTTCACACATATCTTTTGTCATAATTACCCTCCTTATTCAAATGACTGTTTGTTTGATGATTATAATATA
TCCCACTATAAACAAATAGTCAAATGATTGTTTGAATGATATATAATTAATATTAAAAAGGATTGGTTTCTAATG
TTAGAAACCAATCCTTTCGGAGATTTTAACCAAATTTTAAAGTATCTTAAACATAACTGCCCCGTTAGTTTAAG
TGCATCCTTTCACAATCTGTCTACAGATTAATAATAAAAACTACTCTTTATTATACAGATCTCCATATAATTTTTG
AATTTGGTTCTGTAATTTTTTATTTTCTTTTTCTAATTCCATAACTCTTCTTTTTAAGGTTTTTATAAGGATTTCCT
CCGAACGAGAACTTTTCTTGGGTTTTGAGACTACATTTGATGTTATTTGACGCTCACGAAGGGATTCGATTCT
TTGCCTAATATCGTGTTCCTTATAAAGCCATGATTTAGAAACATTAGCTTCCTTTGCTATTGAATTAAAATTAATA
GCTTTACCTTCAATCGAAAATTTAGAAATCGCTTTGTCTACTTTTTCTCTTGTCTTTTGTGATTTCTGCTTCGCC
AAACGTACAATTTCTGTTGTATTTCTAACTTGTTTATTCATTGATAATTACCCCGTTAAACTTCCAATGATTTGTT
CTAAACGCTCTTTAACACGGCTATTAGTCTCTATTTGTCTTTGCCATTGTTTATCCTTTGCTATGGTTAATAACTC
TTCTGTACGCTCTAACTGTTCTTCGTGCTGTGGTAAGAATTGCTTACTGGTACAGAAGTGAGTGCAATCTAAG
CATGCATTCGCATGTGGACAACCACCTGCTATTACTGGCAATCTACAATAACCATTTGGAAGCACTTGTGCATT
TATATTTTTCTTGAACCATTGAAGCTCTACATCATCGACTTCACTATCATCATCTAGATTGAGCACATCTCCATTA
TTGGTAACCAGTTTTTCTTGAAATTTAGTAAATTCATTTTTTAGAGTTTCATCAAAGATATGAGCGTATCTGCTT
GTCATTTCTGGGCTTTCATGTCCCAAAAATTTCTGCACAATATGCTGGGGCACCCCGTTGTTAATCATTCTTGT
TCCTACTGAATGGCGAAAGGCATGGGCATGGAATCTATAAATCTCGCCTAATTTATCCACTATATTTTGCTCATA
AGCTAATTTATTTAATTCGCCTCTAAATGTTTCTTGTTTTAATGGCGATCCATCTTTTCTTGGAAAGAGGTATTC
ACTATCTGGAAATTCCTCTGAAACTTTATCTTCCCGAACTTTAATAAGTAAAACTACCTCTTTAGATATTGGAAC
TATATGCTCCTTTTTCATTTTCCATTGATAATACTTTAAAAAGTAATCTCCATCTTTGTCCTCTAATAAACAGCCT
TTTTTCAAGGTGCACAATTCACTTATCCTCATTCCACATTCTTGAACAATCATAGTCATCGTAGCTATATATTCG

GGTAATTTATCAAGATGACTGTTCAATTGCTCTAGAACGAATTCGTCTATAAAGCGAGGTTTTGCTCTTGGTAT
TTTCGGATAGTCCTCGGGATAAATTAATAATTTGGAAGGAACATCATCCCATTCTAGCCTATGAAGGGTACTAA
ATAGTCCTTCCAATATAGAGATCCTCCCAGTTATTGTACTAGGTTTTATCCCCATCATGTTTAGTTCACTTAAATA
TGCTTCAATTTCCACTCTCGTTAATTGGTGTACTCTCTGAACGTGTTTAAACTTTATGTCCAGAAAATTAAAGA
ACTCTTTAAGTTTTTGGGCAATATCACTTACATAGGAAAAGCTATCCATGTTCAATCTTAACTTACAATATCTTT
TTACAAGCTGTTTAAAATATGTATTCCGAAACCCTTTAAAGTTAATTGTATATTCATATTGTGTTGGGTTAACCT
TATCATCTGGCAAAGGTAAGTTTCGTCTATCCCAAACGTCTTTATCCCACTCCTCTCCATCAAAATAAAAATCCT
CATAAAACTCAATAAATTGTTTAAGATTAGTAACATAGTAAGAATTAGCTTTTACAGGTGTTTTTTCTTGATTA
GCAGTAATCTTATAATTAGTGGTGGTAATTCTAACACCCTGTTTTGTCAAATATGTTCTATACTCCGTCATTGCT
TTATCAATAGGAACTTCAGTAATTGAAGTAATGCTGGGATACTTTAAATCTAAAAAATCCAACATTTTATTAATT
ACTGTTCCTTTTCTAATCCAGACAGTTTTTGCATTCCATATTCCATTGTTTAAATGGTAAAAATAAAAATATTTC
AATTCTGTTCTTAACCACAGATTTTTAACACGTTCAAAACGAACCCAACGATTTCTTAAAGCAGGATTCTTAC
TTAATTCTATGGCAGAAGGATGTGGACATTTTCTTATATCCCAACTATTATTAGCCCAAAACCCTTGCATTTCTT
CATTCATTACAGCTATTTTTTTGCTGATCTCACTCTGACTGATAATTTTCCTTTTACTAGAAGCATTCATTTAGCT
GGTATTCTTTATCTAGTAATAAATACAATGGATACGGCTTATTCTCCACTTCTATCCTTTGAACCCTCACACGCG
CCCCCCTCTTTAAACACTTTAAGTACTACAAATTATAAACCTTGTTAATTTTTATTTAAAGTAAAATCTCCCTTTA
ACATCTCCTCCTGAACTACTTTAACTACTATTATTTATTATACTATGGTTAATATGTCTCCACTTGGGTGGTTATG
GCCAATAATCATACCCGTTGCGTTACTTAATATGGCTGTTTTAAATATTTCTCGAGGATGTATAACCGCAAAATT
AATGGTTCCTATAGAGACGGTTTCAATATGTGTAGGTTCATTTTTGCTATTCAAGCAAATTAAAATGAAGTGTT
CTCGGTCAGCATTTCCTATGAAGTCACGCATAATATCAGCAGCGTCTTTTGGTTCTTCAATACGTCTTTTTAAA
TACCAAAGTGTATCTTCTTTAACCATTTGGACTTTGACAACATCTATTCTTTTTCTACTCATTATGGATAAGCTCT
ATACTTTCTATTAAGTCAGGGTGAACAATGGATGTGATAGAATCATCATTCATATAAATGGAATATAGCTCATCT
AATTCTGAATTATTTTTTTTGTTTGATGAAAAGGCGATAATGCCTTTAATAACATCGATGTGTGGTTGATGCAT
TAAATATTGGTATACTTCTTTCCATGTTTGATTCATTCTATTTAGTCTCCTTTAATGGTTTAATTGTCCATGCCTTT
CTAGCGTATGGATTTAATGGATGCATGATTTCATTTGTTTGAGGATTTGTTAATATACTGATTTTCTTACTGTGA
GGTTTTAACATGTCTAATACTTCATTCACTCGATTTTCCACAAGAGGTTTTTTTCCGTAGGAACCCCAAGCCA
ATAAAACACTATCGGCTTCTTTGACAGCTTTCATAATGTGAATATCCGTGTGCTTGTCATGGCTGTTTTCGATAT
GTTTTAAATTAATGGGGGTATCAATATTTGAAAAAAGATTTATAAAATTAACGCCACCATATTCATCTTTTTCAG
CTACTTTATTGACAATAAGTTGTGTTGTAAGGTCAACGTTGATAACACCATCATAATGAGGATACATCGTTATA
ATTGTAAGTACTTCTTTATCAATATCCCAAGTTTTCTTGAGAAGATAACGATGCATTTTATCCTGACTAAATATC
GCTTCAGTTTCTAATACACTTTTGATTGATTTCATTTTATCTAACTCCTTTATTTAATATTCTTCAGGTAACAACA
TGACGTAATAAGAAAGGTCTACATCATCTTCTCGGATAATGTAGACCTTATTAATATTGATAGCTTTTGAATAAT
TTGCCTTATGAATTTTTACGTACTCTGGACGTTCTTGCTTATGTTTGATATATAATTGACTATTATGCATATTGAA
CTGGAATATATGAAAGTAATCAATCTCTTCTAGTTCCTTGAATTGTTCGTTTTCGCGTTGCGCTACAAGTTTCC
ATAACTGTTGCTGCAACATTGCTGGCAAGTAATTGGCAATACTACGTGTAATATAACGTTTCATGCTCATTTCT
CCTTTTCTATATCTTTCATTATTGTAATAACTTCTGTAATGACTGTGACGCTTATCTGAGCTATGTGTATCCATTTA
TTCATTATTATAATTCTCCTTTTCAAGTAAGTGTCTTTGATCTAGTAGTGGCATAACTGTGTTATGAAGATATAA
GAAGTCCATATCGTTTGTTTCATCAATATAACCAATCGCAATAAGTTGTTTTTGATAGTAGAGGATAAAGGGGT
ATATATGCCTTTCATGAAGTGTGTCAGAGTAGTAAGTATACGTATGAAGGATTTTGTTGGTAAGCGGACCAAG
TCTGATATATTGATCAGGAAGGATATTATCTGCCACTTCCTCGATAGATTCACATTCCCATTTTTCGTCCTTTGG
CAGATTGAATAAGTTAGTTATATACTCTTTGAGTTGTTTTTCTAGTGTCATGGTTATGACCTCCTATAATGTTTTT
GATGTATACATGTAATACACCATATAGATAATATATGCTTCAAAAAAATTTTGTTTCGTTTGTGAATCCCATTGTT
ATTAAGAAAATATCCCAATAGAAAATTATCACAATTGTTGATATGACAAGGAAATCCCATTATCCCGCTCATTTC
TCAGGAAATATAGCGATATATTTATATATCATTTGGATTTGGTGGTGTCAGTACGACTTACTTTTTCTTTTTATAT

TATTTATTTATAAATAATAATGGGATTTCGGGATTACGCTTGCGTAATCCTTATCCTACATGTATTTATCAAATCCC
ATTGCTATCCCGTTCATATTTTATTTTGGGATGTTCTTTCGTCATACAATATAAGCTTCACATTGAGTTGTGACTT
GTTAGTATGTTGAAAAGATTCGTTAGATGGGTGTTATTGAATGACGAAAGGACAGTACATAGATATTAGATTT
GAAGAAAGAGCGTTTGAGGCAAAAATAAAGACGAAGTGCTGAGGAGCACTTCGTCGAGAGGGGTATTATT
GAAAAGTTGTTTAATAATTTCATTATTGAGTTTTAGTGTAACGTAGAACTGTTTTTTATGATTACCGTCTTTACG
AATATCAATACGGTCAATGACTGTAAGATATAATGCTTTGAGTTGTGCTTTATCCATTGATTCTATATTTTGAAAT
ATTCGTTGTAATAGGGCAGCGATTTGTTTCGTATCATAAGATAGTTTCTCTTGATTTTGTTGCTGTTTGAGTTG
ATTCATTTGATTTGTAATGTCATTGAGTTGTGTTTCATATTGATGAATAGTTGCTTTCAATGCAGATGTTAGGTC
CGGATTATCTTCAATGGTTTTAACTAAATTATGGAGTTTCCCGCTGACTTCATCGTATTGTTGTTGTTTATAAGC
GATATCGTGGTTCAATGCACCAATATCGACTTTATTTTCTTGATTGACACGTTCTAAGACTTGGTTAATGACTT
TATCACTTTTGACAATTTCGAGTATTTGATCCATGACGTATTTCTCAATCACATCAGCTCTAACGCTATTCGCAG
AACATACTTTTGAGCCTTTGTTTCGGAAGTTACTGCAAGAATAATAACGTATTCGCTTCTTGGTACCATCTTTC
AATGTGTTCGTTGTGTTACTAGCTGCCATTGGTGCACCACATTGTGGACAATGAACGATACCTGTTAATAGAT
TAGTTCCTTTACCGTGGACTTGAGGTTTTTGACTGACTTGTTTTTTACGTAATTGGACTTTATCCCATAAGTCT
TGAATAATAATAGGGGAATGCTTACCTTCAGCTATTATTGGTTTATCATTCAGCCCTTTACGACGCTTTTCATTC
CAATCTTTGTACTTTGCGAATTGAATTTTACCAACATAGAATGGATTAGATAAGATATAGGTCACTGAACCAAT
ACTGAAAGGCTTTCCTTTTTTAGTCACGTATCCTTTGTGATTGAGTGCATTCGCAATTTTACGATATCCGTGGC
CTTTAGCATATGACTCAAATATATATTTGACAATATTCGCTTCATGTTGGTTTATCATGAGTTCATGCTTGCTATC
CGGTATTTTGTCATAGCCCAGCGGCAAATTGCCTTGATAATAGCCTTCTTGAGCGCGTCGGGTTTGACCCATG
AATACATTTTCGACAATATTATTTCTTTCAAATTCTGAAAAACTCGCTAGAATTTGTAGCATCAATTTACCACTG
CTTGTATTGACTTCCATACGCTCAGATAAGCTGAAGAATTCGACATTTTGACGATGAAGGTCTTCAACAATTT
TGAGTAAGTCAGAAGTGTTACGTGCTAGTCGGTTTGTTTTGTAGACCATAACAGAATCAATCTGACCTTCGTT
CGCATCTTTTAACAAACGTTGTAGTTCTGGTCGGTTCATAGATTTTCCAGAGATACCTCTATCCGCGTATACAT
CTACAACAACAAAGTTATTGAAATCACAATATTCTCGAATTTGATTGATTTGTCCATCGATACTATAGCCTTCAG
TACTTTGGATTTCCGTTGATACACGAACATAGATACCGACACGTTTTTGTTTGAGTTGTTGCATGATTTTTTCA
TCCTTTCTGATTAAGCAATCGATGATTGCGAGGTTTGGTTCACAATGTTCAATGGTTCATTTTTGAAATAGATC
CCAACAAGGGCTTTATTTTTTGTAATGCGAATTTCATCAATATAGGGATGCAGCATGTTTAACGTGAAACTTTT
CTGTATAACCTTTTGTAGTGATGTTTGTAACTGATTATCACTTATGGAGGATATGGTTTTGTGCTTTTGATTCAT
CAAATGAGTCTGTTTTCTGAATGATTCAGCATCAATCATACCTTTGGCAAGTTTATCTATCAGTTGTTCTTGAG
TTAGCGTACTTTTAGCTTCTATCACTCTTTGTTTTTTGAGGCGTTGATGAATTGCATTATTAATTTTTGAATAGA
GCTGTTGGTTTTGAAAGAAGTTCTGACATGTAGCTAAGACTTGAACTTCTAATTTTTGTGCATTTATTCCTTTG
AATGAACAGACAAAGCGAGATTCATTCATATTTTTAGGACAAATATAATATCGCAATGTATGTTTTTTTCTTATT
GTCATATTTGTCAGTGTTGAGTCACAACAAGGACATTTGATCTTTTGTTTCAGCTGATTCTCTGAAGGTATACA
GTGAAGTTGCTTCTTATTACGGATTGCTTGAGCATGTTCATATTTCGTTGCCGAAACAATAGGTGGTACCATAT
TGTTATATTGACCATATTGATTGATGACACGACCACAATAATTTGGGTTCATAATTATATTTCGTACCTGATAAG
GCTTGCGGGTAATAAGTTTATTATCGTCTTCTAAATATTGTGCAATCTTCTTGTAGCCATATCCTTGAAGATAAT
AATTGAATACAGCTTTGACGGTAGGTGATTCATCATTATTAATGATGAAAGTACCATTTTGATAGTGATAACCG
AAAGGCGCATGGGTCGTTATGAGTTTACCTTGTTTTGCCTTTTCTCTAAGTCCATTTTTGACTTGTTCTCCAAT
ATTATCGGATTCAAGTTCAGCCAGACTCATGAATATATTGAGTTTTAGGCGATCAAACGCTTTATCCATATCAA
AATAGCCATCATGAACACTTAGAATATGAACATGATATTTTTGACAGAGTTTCATGAGTTTTAATGCATTTTTC
AAGTTGCGATGAAGTCGATTCAAGCGATAACAACATAATACGTCACATTGTCTTTGTTGGATGCGTTCGGTGA
CTTGTTGATAGCCTGTTCGATTATCTGTTCTGCCTGATTGCTTATCACTAATGTATTGGATATTTTGAATATTGTG
CTTTGGAGCTAATAATTCTATTGCTTGCTTTTGAGCTGGGAGTGATTGTTGTTTCGTTGTACTTTGGCGCAAA
TAACCAATGGCTCGTTTCATAGTATTTCCTCCTTTCAAAGAGATAATATATATTTATGAACGAAATTATAGATAA

GCCCAACACCAGTCGGGTGTTGGGCGGTATTATTAGTCATCAGCGTGATTGATTTCTTCAATAACCAGATCGG
CTAATAGTATGATTAATTCGTCCATGTATATCACCTCATTATTCATAGTATTAACAACAATTCTTTAGTTGCATCTC
AAAAGTAGTAACGAATTCGATATTCTTCTTTGAGCAAATTTTTTTAAATGAATGTAATCTTCATATTCTCCATAT
GCAACAATGGACGTAGGTTTGATTACTTTCTCCAAAATGTCATTTATTAACTTGTACTCCTCAGTGTAAGTGTT
ACTTATTTGACGACCTTCTTGTTTACAGCATTCCTTCACAGTATCTAGGGGTGTGCTTTCTAAGTATTCAAATCT
TTCATCTTCAATTTCGTGAGCTAGTGCTACTGTATTCATAGTACAATTCCTCCATTGATTTAATATTTGTGCTACA
ACACCTTTAAATAGGTGTTGTAGCAAATTTATTTAATAACATATTTTAAGCTTTAAAAGTCAATATCACCGTTTT
TATCCCATTCTCTTGCTTTTGAAATCATGTCTCGTAGTATTGGATGTTGGTGTTCAGAGATAAATGATAAGGGT
ATATTGAATTTACTTTTTTCAGATAGACCAAAAATAGGAGCGTATGATTTATCAAGCTTAAGATGATAAAATAC
GATGGTTTTTGGTTTGCCTTCTTTGTCTTTGACACTACGTTTTGTTGTTTTACGATTGGCGTCTGACTGGATGT
AATCTTTATCCCTCAGTGCATCAATGACATTATTTGTATCTTGGAAATTATGTTCTTTTAACATATTTTTAAAAAC
CGAGGCAATCATTTTCACCTGGATATGATCATCTTCAAGTGAAATCATTCCATAATTTTCAATAAGCGTTGATA
ACCTAGTGTCGTCAGAAAACTTACCACGATTTTGTGCTACAAATTGGACAATGACTTCAATTGCTTTATCAGC
TAGGCTTCGCTCACTAACAGAATCTAAATGATAGTTAATAAGATAGTCCCTTACAGCATTCAAATCAACAGGT
GTGGCAATGACACGTTCCAATATACGTGCTGATGTTGTAATGGTTGCATAGCGTTTGAACATCCGAATTCCTG
TATTGCTGGTTTCCTTTTCTAGTTGAGTTTTGAACCTTAGGTGCTCATTTTGGAACCATTTAATGACTTCTTGT
TCACGATTGAGTAGATATTCAGCTACTAATGGCATGATATGACCATAATTAGCTGATGTTGCTTTTTTAATAGTA
TCTGCGTTGTCAGCGTTAGTTGTAAAAGCTTCAGATATTTCAATGGTACGTACATTCAAACCATCATTACGTGC
ACTATTATTGAAAATACTGTGTTCGGCAGTACTAATTACTGAGGTTCCCCAGTTTTTTAGTGACTTGACTTCTC
CGTGTATATTAGAACGTTGTCTTCCTTGACCTTCGGCAATTGAATAGAGCAAACCAGTTGTATCTTTAAAGGT
TGCAGATGACAGTTCGTCCAATACAATAGGTACACCATAGTTGTTACTTAGATATCCTTCAAGCGCGTTTCTAG
TGCCATTCCATGATCTAAACAGCGTATTACTACCTTTAGTAGGGTTGCCGGTTATTGAGACTCCTAAAGCCGCT
GCAGTTGATTTACCCGTGCTTGAATTTCCCGTAAATGAGAAAATCGTACCTGCGAATTCAACCTCATGTTTTG
TTTTTAAAAACGCAGTTACTAATGAAGAAATACCAAACACTACAGCAAGTTCGAGTAACAAGTTTCCTTTTAC
TTCATCAAGATACATTTGCCACCAGTCTTCAAAAGTGCCTTTAGGTTGTAGATCGTACTGTGTTTCACAAATG
ATTTCATCCGCCTGCGACTGTGTAATTTCTTTTGAAAGATATGGTTCATCTAATGAAATGACGACACCTTTATC
AGTATTTAACACACCGACACCCGTATACAACGATGACAGAGGCAGTGATTGGCGCATTAATTGTAGTGCATTG
CTTAATGATTTAATGTACTTCTCATTGATGCTGAAGCCATACATGATTAGTGAAGGTAACTTTTGCGATGTTAA
AATATCTGATGTTTCAATACGTTCAATATTTTGTCCGTTGGAAATAACTAGTTTCTCAACCCCAGATTTGGGGT
CAAGAAACTTGTTTTTAATTAAAATTGGACTAGATAAGCGTATGGACTTTTGCTGATCATTTTTACCTTTCGGT
GGTATAACTTCATACCATGAATCTTCAGTAAGCCAGTATGGATATTCACTAAAGATGATGTTAGTCATTAGTTGT
TACCACCTTTCCAGTTATTTTGATGATTCATCGGATTTGGTGCAGTTTTAAGGTAGAGCAAAGATGAACCTTT
TTTTCCAACTACAATGAATGGAAGGTTAGGTGTATATTTTACAAAATGTGCAAACCAACGACCAACATCTTGT
TGAATTTCAGTCGAGCACAAAACATTAGCCCTCACATTCAAATCGTGATACATAAACATGGTGCCTACAGGTA
AATTAAATGCGATGCTTGTAACAGCACGTTTTAGTGTATAAAATTCTTTTTCTTTCATCATAAGTTCCTCCAAAT
AAGTCTTATTAGTCAATCAATACTGTAGATGCTTAACACCTCAATGGTTGATTGCTATAATAAGCATAAATGAA
GCATTTTAATTAGACGCGGAACAAAAAATAGGGAGGGGGGTATATATGATACCTACGATAGAAGAAATTATT
GACAAGTATGGCAGTTTAGTAGATTGTCTAAAGTTTGATATATCAGGTGAGGTTTATGAAGACTTATTGTTATT
AAGAAGTTTAAGCGATTTAAATGAACACCAAAAGATGCATAGAGTTTCATTTATACAAAAACACTTTAACCCT
AATTTTGATTATTTGTTAGATGACTCAGAATTTAGAAGTATGAATAAAATTTATAGAAATTTAAATACTATGACG
TATATCAATAGTCAAAACAATGAATTTAAACTTAAACCATTTATAAATGAAGAACAATTAAAATCACTACTTGC
GATAAAGAAAGTAGATGAACATTTGAGTTATGACCCATCTATTACCTCAAAAGCATTAGCAGATATAAACAAG
ACGCAAAAAAGATTGGTTACAAAGCCAAATATTCTCTATAATCGATCCAAAAAAGAAAATGAGTATGTACGGT
TAGGAGGAAAAATATCATATACAAAGATGGAGAAGCATTGGGAATATTTACATAATGAGATTCAGTATTATAAT

CTTACTAATCAATTAATTAGTTATGTTGCTTTAGAAAAGGAATATGCATGGGCTTTTTAAATGAATTATTTTAT
TTAATAGAATTATATTTGAAGGCATTTAAAGGACAAAAGAAAACTGGCCATGAATTTGAAGAAGGATTGAAC
GCATTTATTCAATCATATGTCATTATTTTACTTATAGATATAAGATTACCGACTGTTAGGTTATATATTATTAGGCG
TATTGTGGATGAATGCGAAAAAGAATCAGATAATCAAAAAAGAATTAAACTGATAGAGGAATCCATTAAAAA
GTACAGATATGTAAAAGAACAGATTGAAAGATTTGGAAATGCGTTGAAAGAAGAATTATCAATCGCGACTTT
GTCTATTGAACAGAATATGTTAGAAGCTAAAATTGATTATTATAAGAATTACTACTATCCTAGAAAAAAAACAA
AGCATAACAATATTGAGTACAACGTATCAATGTTTTTAAAGCTGTAGATTCTTTAAAGAAATAGTATTCGACG
GACAATATTTATAGATAATAATTAATTAGAGAGGTTTTAAGTTGTGTTTAGATTAAAAAAATGGTATTTAAAAC
AAAAAGGCTATTTTTGCCGTGTCGGTGAAAAGTGGGACAATAGTAATTTATCCCATGAAAATATACATAGTTA
TGTAGATAGAGTACTTGAATATGAGACTGAAACACGTAGAAACGGCCAACCAACTGAAGAATATATATTAGCT
AGTAGTGGGGAAAAAATAAATGAAGATTTAAGAAGTGGTACTAATGATCACAAAGAAATTCTAGAGGTATTT
GATAAATATAAAGTAAAAGAGGATATAACTACTTATAGAACGGTTAGTCCTCGGATATACAAATTAATGGAGA
AAAATGCTAAAAACTTAAATGGAGTAGATTTGTTTGAACTTGGAATTTTACACACTTCATTAATAAAAGGGTA
CGAATCAAGAGAAGAAGGTTACAAATTACGGGTAAAAGTAAAAAAAGGAACACCAGCTTTCTATGTAGGTA
ATTTAACAGGCGAAGAAAGCCATTATTATGAGGTGATTGTAGTTAATAATTTAAAATTAAAAATCATATCTATT
GAAGATTACTATATAAATTGTGAAGTAGTATAATTGGAATCAATTCTGTTAGTTGTTCATAATAAGCCAATTATT
TTTTTAATGAACTTATTACTTAAAAACCGCATCATCAACTGATAAGCAGAAGCGTATCATAAGTAGCGGAGGA
GTTTTTTACCTTGTGACTTATCATAAAGTACGATGTTTATGTAAGTGATTATCATTATTTAAGCAGGTTTTTCAA
ATTAAATAATAACAAGAATAAAATGCACTTAGCGACATTGAAATTTATTAATCTAGTAAACTAATAGATTTATAG
AAAATTTTATTTGCAAGGGGATAATTTTGAAAAGTAGTATTTTCTATCTTTCCATAATACATTGTAATTACAACG
GAGGGGATATTGTGATGAAGTGTATAGATAAAACGTGGGT
>Staphylococcus aureus strain BSN07S
ATGAAAATCACCATTTTAGCTGTAGGGAAACTAAAAGAGAAATATTGGAAGCAAGCCATAGCAGAATATGAA
AAACGTTTAGGCCCATACACCAAGATAGACATCATAGAAGTTCCAGACGAAAAAGCACCAGAAAATATGAGT
GACAAAGAAATTGAGCAAGTAAAAGAAAAAGAAGGCCAACGAATACTAGCCAAAATCAAACCACAATCCA
CAGTCATTACATTAGAAATACAAGGAAAGATGCTATCTTCCGAAGGATTGGCCCAAGAATTGAACCAACGCA
TGACCCAAGGGCAAAGCGACTTTGTTTTCGTCATTGGCGGATCAAACGGCCTGCACAAGGACGTCTTACAA
CGCAGTAACTACGCACTATCATTCAGCAAAATGACATTCCCACATCAAATGATGCGGGTTGTGTTAATTGAAC
AAGTGTACAGAGCATTTAAGATTATGCGAGGAGAAGCATATCATAAATGATGCGGTTTTTTCAGCCGCTTCAT
AAAGGGATTTTGAATGTATCAGAACATATGAGGTTTATGTGAATTGCTGTTATGTTTTTAAGAAGCTTATCATA
AGTAATGAGGTTCATGATTTTTGACATAGTTAGCCTCCGCAGTCTTTCATTTCAAGTAAATAATAGCGAAATAT
TCTTTATACTGAATACTTATAGTGAAGCAAAGTTCTAGCTTTGAGAAAATTCTTTCTGCAACTAAATATAGTAA
ATTACGGTAAAATATAAATAAGTACATATTGAAGAAAATGAGACATAATATATTTTATAATAGGAGGGAATTTC
AAATGATAGACAACTTTATGCAGGTCCTTAAATTAATTAAAGAGAAACGTACCAATAATGTAGTTAAAAAATC
TGATTGGGATAAAGGTGATCTATATAAAACTTTAGTCCATGATAAGTTACCCAAGCAGTTAAAAGTGCATATAA
AAGAAGATAAATATTCAGTTGTAGGGAAGGTTGCTACTGGGAACTATAGTAAAGTTCCTTGGATTTCAATATA
TGATGAGAATATAACAAAAGAAACAAAGGATGGATATTATTTGGTATATCTTTTTCATCCGGAAGGAGAAGG
CATATACTTATCTTTGAATCAAGGATGGTCAAAGATAAGTGATATGTTTCCGCGGGATAAAAATGCTGCAAAA
CAAAGAGCATTAACTTTATCTTCCGAACTCAATAAATATATTACATCAAATGAATTTAATACTGGAAGATTTTAT
TACGCAGAAAATAAAGATTCATCTTATGATTTAAAAAATGATTATCCATCAGGATATTCTCATGGATCAATAAGA
TTCAAATATTATGATTTGAATGAAGGATTCACAGAAGAAGATATGCTAGAGGATTTAAAGAAATTTTTAGAAC
TATTTAATGAATTAGCTTCAAAAGTTACAAAAACATCCTATGATAGCTTGGTCAATAGCATAGACGAAATACAG
GAAGACAGCGAAATTGAAGAAATTAGAACAGCACAAAAAGATAAGACACTCAAGGAAGTGGAAGCACCTA
AAGGAATAATTCCAAAATATAAAAAAGGTGTATCAAAGACTACTAAAAATGATTCAGAAATTGAAAAATCAA

ATAAAGAGAATAAATTAACCGGTAAAGTTGGAGAAAAATTAGCGCTAAATTACTTTAATGAGCTAATTGATAA
TAAAATAGACGAAGATAAGAAAGAACAGTTTAGGAATATTTTAAATGATAATCCAGGCTCTCAACACGGTCAT
GGCTATGATTTAGTAGCTTTTGATCCAACAAATACAGATAAAGCTGTAGAAAAATTTATTGAAATTAAAACATC
TACATCTTCTAGTATTGAGGAACCATTTTTTATGTCGCTAAATGAAATGTTTGCTATGAAAGAATATAAGCAGA
AATATTTAATATTAAGAATATTTAATGTTTCCGGTAAAGAACCACAATTTTATTTTATAGATCCATATGCAAATTA
TTCTGAATTTAAAGATGTAGATGATCTCATTGACAAAGTATTTAATGTAGAAGCTATTCAGTATAAAGTTTTTG
GCGAAAAAATGATTACTTGAACAAGAGCTAAAATAAAATTGTGATCTAATAAAAATAGAAGGTTCTGTTGCAA
AGTAAAAAAATATAGCTAACCACTAATTTATCATGTCAGTGTTCGCTTAACTTGCTAGCATGATGCTAATTTCGT
GGCATGGCGAAAATCCGTAGATCTGAAGAGACCTGCGGTTCTTTTTATATAGAGCGTAAATACATTCAATACC
TTTTAAAGTATTCTTTGCTGTATTGATAYTTTGATACCTTGTCTTTCTTAYTTTAATATGACRGTGATCTTGCTCA
ATGAGGTTATTCAGATATTTCGATGTACAATRACAGTCAGGTTTAAGTTTAAAAGCTTTAATTACTTTAGCCAT
TGCTACCTTCGTTGAAGGTGCCTGATCTGTAATTACCTTTTGAGGTTTACCAAATTGTTTAATGAGACGTTTG
ATAAACGCATATGCTGAATGATTATCTCGTTGCTTACGCAACCAAATATCTAATGTATGTCCCTCTGCATCAATG
GCACGATATAAATAGCTCCATTTTCCTTTTATTTTGATGTACGTCTCATCAATACGCCATTTGTAATAAGCTTTTT
TATGCTTTTTCTTCCAAATTTGATACAAAATTGGGGCATATTCTTGAACCCAACGGTAGACCGTTGAATGATG
AACGTTTACACCACGTTCCCTTAATATTTCAGATATATCACGATAACTCAATGTATATCTTAGATAGTAGCCAAC
GGCTACAGTGATAACATCCTTGTTAAATTGTTTATATCTGAAATAGTTCATACAGAAGACTCCTTTTTGTTAAA
ATTATACTATAAATTCAACTTTGCAACAGAACCGTATTATGGAATAGAGATGTTGGTAACATTTATACAGGATC
ATTATACTTAAGTTTAATTTCGTTATTACAGAACCACACATTCCAACCAGAAGAGAAAGTATGTCTATTTAGTT
ATGGTTCAGGAGCAGTAGGAGAAATCTTTAGTGGTTCAATCGTTAAAGGATATGACAAAGCATTAGATAAAG
AGAAACACTTAAATATGCTAGAATCTAGAGAGCAATTATCAGTCGAAGAATACGAAACATTCTTTAACAGATT
TGATAATCAAGAATTTGATTTCGAACGTGAATTGACACAAGATCCATATTCAAAAGTATACTTATACAGTATAG
AAGACCATATCAGAACATATAAGATAGAGAAATAAACTAGTGGCCGATTGTGCTTGATGAGCTTGGGACATA
AATCCTAACTCGAAATAAATAAGCATATCACTAAACTGATTTTTTAAAGTTTACAGTGATATGCTTATTTTTTTAT
CTTACGATTTTGTACGTGCATGCTTGCCTAGGGGTATGGCTCGAGCCATTAGTCTCTCGCACATACTATTCCCT
CAGGCGTCAGCACTTACAAAATCGGTTGTAATTTTCATTTTTATACGCATTCTTACTGAGATTATACTAATAAGA
GGAATAGTAAAAGCAATTCTAAGTAAAATTGCAGATAAGAGGTTTGTTAAAAGCAGTTCTCAGTAAAATTAC
AGATAAGAGGTACGTTAAAAGCAGTTCTAAGTAAAATTGCAGATAAGAGGTTTGTTAAAAGCAGTTCTAAGT
AAAATTGCAGATAAGAGGTACGTTAAAAGCAATTCCATGCAAAATTGCAGATAAGGGGTAAGTTAAAAGCA
GTTCTCAGTAAAATTGCAGATAAGAGGTACGTTAAAAGCAGTTCTAGGCAAAATTGCAGATAAGAGGTGCG
TTAAAAGCAGTTCTCAGTAAAATTGCTGATAAGGGGTAAGTTAAAAGCAATCCTAAGTAAAATTGCAGATAA
GGGGTACAGAAAAACTAGACTTGATTACAAAATGGAGCTTGGGACATAAATGATTTTTTAAAAATGAGATGA
GACGTAGATTAACTCCATAATCAATACGAATCTATCGACTTCTTTATTTATGATATTCATCTCTTTTTAATGGAAA
TAAAAGTGCGATTAATGTGATAATACAGTTACGTTAATTAAAAAAATAAAAATGCAAGGAGAGGTAATATGCT
AACTGTATATGGACATAGAGGATTACCTAGTAAAGCTCCGGAAAATACAATTGCATCATTTAAAGCTGCTTCA
GAAGTAGAAGGTATAAACTGGTTGGAGTTAGATGTTGCAATTACAAAAGATGAACAACTGATTATCATTCATG
ATGATTATTTAGAACGGACTACAAATATGTCCGGGGAAATAACTGAATTGAATTATGATGAAATTAAAGATGC
TTCTGCAGGATCTTGGTTTGGTGAAAAATTCAAAGATGAACATTTGCCAACTTTCGATGATGTAGTAAAAATA
GCAAATGAATATAATATGAATTTAAATGTAGAATTAAAAGGTATTACTGGACCGAATGGACTAGCACTTTCTAA
AAGTATGGTTAAGCAAGTGGAAGAACAATTAACAAACTTAAATCAGAATCAAGAAGTGCTCATTTCAAGCTT
TAATGTTGTGCTTGTTAAACTTGCAGAAGAAATCATGCCACAATATAACAGAGCAGTTATATTCCATACAACTT
CGTTTCGTGAAGACTGGAGAACACTTTTAGATTACTGTAATGCTAAAATAGTAAACACTGAAGATGCCAAAC
TTACTAAAGCAAAAGTAAAAATGGTAAAAGAAGCGGGTTATGAATTGAACGTATGGACTGTAAACAAACCA
GCACGTGCAAACCAACTTGCTAATTGGGGAGTTGATGGTATCTTTACAGACAATGCAGATAAAATGGTGCAT

TTGTCTCAATAGAAAGTTAGAGGTGAGTCTTACGTTTCAGTGACGGTAGACTTACCTTTAACATGTTACATAC
TAAAAAATTAATTTGAATAAGAAAGAGAGACATATATGAAATACGATGATTTTATAGTAGGAGAAACATTCAA
AACAAAAAGCCTTCATATTACAGAAGAAGAAATTATCCAATTTGCAACAACTTTTGATCCTCAATATATGCATA
TAGATAAAGAAAAAGCAGAACAAAGTAGATTTAAAGGTATCATTGCATCTGGCATGCATACACTTTCAATATC
ATTTAAATTATGGGTAGAAGAAGGTAAATACGGAGAAGAAGTTGTAGCAGGAACACAAATGAATAACGTTA
AATTTATTAAACCTGTATACCCAGGTAATACATTGTACGTTATCGCTGAAATTACAAATAAGAAATCCATAAAAA
AAGAAAATGGACTCGTTACAGTGTCACTTTCAACATACAATGAAAATGAAGAAATTGTATTTAAGGGAGAAG
TAACAGCACTTATTAATAATTCATAATAAAACAGTGAAGCAACCATCGTTACGGATTGCTTCACTGTTTTGTTA
TTCATCTATATCGTATTTTTTATTACCGTTCTCATATAGCTCATCATACACTTTACCTGAGATTTTGGCATTGTAGC
TAGCCATTCCTTTATCTTGTACATCTTTAACATTAATAGCCATCATCATGTTTGGATTATCTTTATCATATGATATA
AACCACCCAATTTGTCTGCCAGTTTCTCCTTGTTTCATTTTGAGTTCTGCAGTACCGGATTTGCCAATTAAGTT
TGCATAAGATCTATAAATATCTTCTTTATGTGTTTTATTTACGACTTGTTGCATACCATCAGTTAATAGATTGATAT
TTTCTTTGGAAATAATATTTTTCTTCCAAACTTTGTTTTTCGTGTCTTTTAATAAGTGAGGTGCGTTAATATTGC
CATTATTTTCTAATGCGCTATAGATTGAAAGGATCTGTACTGGGTTAATCAGTATTTCACCTTGTCCGTAACCTG
AATCAGCTAATAATATTTCATTATCTAAATTTTTGTTTGAAATTTGAGCATTATAAAATGGATAATCACTTGGTAT
ATCTTCACCAACACCTAGTTTTTTCATGCCTTTTTCAAATTTCTTACTGCCTAATTCGAGTGCTACTCTAGCAAA
GAAAATGTTATCTGATGATTCTATTGCTTGTTTTAAGTCGATATTACCATTTACCACTTCATATCTTGTAACGTTG
TAACCACCCCAAGATTTATCTTTTTGCCAACCTTTACCATCGATTTTATAACTTGTTTTATCGTCTAATGTTTTGT
TATTTAACCCAATCATTGCTGTTAATATTTTTTGAGTTGAACCTGGTGAAGTTGTAATCTGGAACTTGTTGAGC
AGAGGTTCTTTTTTATCTTCGGTTAATTTATTATATTCTTCGTTACTCATGCCATACATAAATGGATAGACGTCAT
ATGAAGGTGTGCTTACAAGTGCTAATAATTCACCTGTTTGAGGGTGGATAGCAGTACCTGAGCCATAATCATT
TTTCATGTTGTTATAAATACTCTTTTGAACTTTAGCATCAATAGTTAGTTGAATATCTTTGCCATCTTTTTTCTTT
TTCTCTATTAATGTATGTGCGATTGTATTGCTATTATCGTCAACGATTGTGACACGATAGCCATCTTCATGTTGG
AGCTTTTTATCGTAAAGTTTTTCGAGTCCCTTTTTACCAATAACTGCATCATCTTTATAGCCTTTATATTCTTTTT
GTTTTAATTCTTCAGAGTTAATGGGACCAACATAACCTAATAGATGTGAAGTCGCTTTTCCTAGAGGATAGTT
ACGACTTTCTGTTTCATTAGTTGTAAGATGAAATTTTTTTGCGAAATCACTTAAATATTCATCCATTTTTTTAAC
GGTTTTAAGTGGAACGAAGGTATCATCTTGTACCCAATTTTGATCCATTTGTTGTTTGATATAGTCTTCAGAAA
TACTTAGTTCTTTAGCGATTGCTTTATAATCTTTTTTAGATACATTCTTTGGAACGATGCCTATCTCATATGCTGT
TCCTGTATTGGCCAATTCCACATTGTTTCGGTCTAAAATTTTACCACGTTCTGATTTTAAATTTTCAATATGTATG
CTTTGGTCTTTCTGCATTCCTGGAATAATGACGCTATGATCCCAATCTAACTTCCACATACCATCTTCTTTAACA
AAATTAAATTGAACGTTGCGATCAATGTTACCGTAGTTTGTTTTAATTTTATATTGAGCATCTACTCGTTTTTTA
TTTTTAGATACTTTTTTTATTTTACGATCCTGAATGTTTATATCTTTAACGCCTAAACTATTATATATTTTTATCGG
ACGTTCAGTCATTTCTACTTCACCATTATCGCTTTTAGAAATATAACTGCTATCTTTATAAACTTGTTTGAAATTT
TTATCTTCAATTGCATCAATAGTATTATTAATTTCTTTATCTTTTGAAGCATAAAAATATATACCAAACCCGACAA
CTACAACTATTAAAATAAGTGGAACAATTTTTATCTTTTTCATCAATATACTCCTTATATAAGACTACATTTGTAG
TATATTACAAATGTAGTATTTATGTCAAAATAATGTTATAATTTTTGTGATATGGAGGTGTAGAAGGTGTTATCAT
CTTTTTTAATGTTAAGTATAATCAGTTCATTGCTCACGATATGTGTAATTTTTTTAGTGAGAATGCTCTATATAAA
ATATACTCAAAATATTATGTCACATAAGATTTGGTTATTAGTGCTCGTCTCCACGTTAATTCCATTAATACCATTT
TACAAAATATCGAATTTTACATTTTCAAAAGATATGATGAATCGAAATGTATCTGACACGACTTCTTCGGTTAG
TCATATGTTAGATGGTCAACAATCATCTGTTACGAAAGACTTAGCAATTAATGTTAATCAGTTTGAGACCTCAA
ATATAACGTATATGATTCTTTTGATATGGGTATTTGGTAGTTTGTTGTGCTTATTTTATATGATTAAGGCATTCCG
ACAAATTGATGTTATTAAAAGTTCGTCATTGGAATCGTCATATCTTAATGAACGACTTAAAGTATGTCAAAGTA
AGATGCAGTTCTACAAAAAGCATATAACAATTAGTTATAGTTCAAACATTGATAATCCGATGGTATTTGGTTTA
GTGAAATCCCAAATTGTACTACCAACTGTCGTAGTCGAAACCATGAATGACAAAGAAATTGAATATATTATTCT

ACATGAACTATCACATGTGAAAAGTCATGACTTAATATTCAACCAGCTTTATGTTGTTTTTAAAATGATATTCTG
GTTTAATCCTGCACTATATATAAGTAAAACAATGATGGACAATGACTGTGAAAAAGTATGTGATAGAAACGTT
TTAAAAATTTTGAATCGCCATGAACATATACGTTATGGTGAATCGATATTAAAATGCTCTATTTTAAAATCTCAG
CACATAAATAATGTGGCAGCACAATATTTACTAGGTTTTAATTCAAATATTAAAGAACGTGTTAAGTATATTGCA
CTTTATGATTCAATGCCTAAACCTAATCGAAACAAGCGTATTGTTGCGTATATTGTATGTAGTATATCGAGCTTC
ACATGAAACAGCTAAAGAAGCTTTGGGCGATAAAGAGTTAAGAGCCATTGCACATGAGTTAACTAAAACAG
TTAAGGATAACATGAGTGTTGATTGGTCTAAACGAGACAGTGCTAAAGCTAAAATGAGAGTTCAAGTTAGAC
GCCTATTAAAGAAATATGGCTATCCACCAGATCTTCAAAAAATGGCTGTGGAACAAGTTGTAGAGCAAGCAG
AATTAATGGCAAGTCAGCAATAAAAAAATAAATCATAATGAGTCCGGGACATAAAGTTCTTGGATAAGTGAA
AAAAGACAATTTCTATTGAAATAATATAGAAATTGTCTTTTTTATAAATTTTTTGATTATTTTCAGCTCGTTGAG
CTACTACTTTTCTTATATTAAGTGCCATTAATACAAAACCAAGTTCTCTTTTGACTTTATTGAGTCCTCGGACAG
ACATCCGAGTGAAACCCAAAATAGCCTTCATAAATCCAAAAACAGGTTCCACATCAATTTTTCTTTGACTGTA
GATATTTTTTGTTTCTGGTTCTGAAAGCTTTTTGTTAATTTGGGATTTAAAATATTCCCAGTTATAATTCTTCATT
ATTTTTTTGTTTGTTTTTGAATTGAAGTTCATACATTGATTTTTCAGAGGACATTCTGAACAATCATCACATTCA
TATAATTTGAAGTCTCGCTTATAACCATACTTATCATGACGATAGGCATATCTTTTAAAACCTAGCCGTTTATTAT
TCGGACAAATGAATTCGTCATTAATTTCGTCATAGTTCCAATTTTGAGTATTAAAGATGTCACTTTTATATTTTT
TAGTTTTATCTTTTATAAACATTCCATATGTTATGAGTGGCGTTCGATTAAAGTCATCTATAATTGCCTTATAATTT
GATTCACTACCATAACCTGCATCAGCTACAATATATTCAGGTAAATGACCGTAGGTCTCTTGAATTGAATTTAA
AAATGGAATCATCGTTCTAGTATCCGTTGGATTTTGATACACATTATAAGATAAAACAAATTGGGAATTTGTTG
CTATTTGTAAATTATACCCTGGCTTAAGTTGTCCATTTTTCATGTGATCTTCTTTCATTCTCATAAATGTCGCATC
ATAATCTGTCTTAGAATAACTATTTCTATCCTTTAAAATAGATTTTTGAAATTCGTATCGATACTTTCGCTCAAAA
TAATCATTGATTTGCTTTTTGTATTTTTTGATTTAGTTCTTTTGAGACGTATTTGTTTTCTTGTTTTAGTACATT
TTTCATTGTTGATATGTTGGTTTAAATCTTCGATTTCTTTATCTAAGTGACTACCAATCAAATCTATTTCTTCTTT
TGTTAATTCATTATCATGATCTTCTTTAATTTCCGGTATGATTTTATTGGTTACCAATTCATGGTAGAGGGCTTTA
GAATCCTCATTCATCTTTGATTCATGGTTTTGAATACTCTTTTTCCATACAAATGTATATCGATTGGCATTTGCTT
CAATTTTTGTACCATCAATAAAAAATAGCTTTATCATCTATAAGATTTTGTTTTACACACTGACTGTAAAATTGAA
TAAATAAAGATTCTAATAAAGCATCTACTTTTGGATTTACTCTAAATTGATTAATTGTTTTATAAGAAGGTTTTT
GATTTTGTGATAGCCACATCATTCGGATGCTATCATTAAGCATTTTTTCTATTTTACGACCTGAGAATACAGATT
GTGTGTAGGCATATAGAATCACTTTTAACATCATTTTAGGATGGTACGAAGTTGCACCACGGTGATGTCTGAA
TTCGTCGAATTCATTGTCAGGAATTGTTTCAACAATATCATTTACAGTAAAACGATGTTGATTTGTTTTGTTTCC
ATATTGACCTCCATGTATTTGCTATGATTTCAAAATCCATTTTTGACGTGCCTTAGGGTTGAGTGGATGCATAAT
TTCATTTGTTACTGGATTGATGAGCTTTTTTACTTTCTTTTTATGAGGTTTTAACATTTCCATCACTTGTTCGAC
ACGGTCGATAACAACTGGTCGCTTCGCATAGGCACCATAAGCAAGAATCACTGTGTCACTTTCACTAATCGCT
TTCATCAAATGAATATCAGTGTGCTCATCGTATGGATTTTTGATATGTTTGAGGTTTTCGGGTGTTCTAATATTA
GAGAATAGATTTACAAGATATACAGCACCGTATCGTTCTGAATTGGCTAATTGGTTGAGGATAAGAACAGTTG
TGAGATCGAGTGATAATACACCGTCTAAATGAGGATACATCGTTATCACTGTGCATGCAGCTTTCTTTTCATCC
CATGTTTTCTTGAGTAAATAGCGGTGCTGTTCATCATCGCTAAATATGGCTTCTGTGTGTATCGTACTTTTGATT
GTATTCATCATCGTCACTTCCTTTAGTATTCTTCTGGTAAAAGCATCACATAATAAAAAGCGTCCACGTCATCTT
CACGAATGACGTAGACTTTCTTAGGTAATGCATTTTGATTTTTTTCATAGTTTGTATAGTGATATTCCAATTTGT
ATGTGGGTTGTTCTTGTTCATGTGTGATTGAGAGTATATTCTCATCTTCTTGTAATTTAAAAATGTGTAGGTAAT
CTGTATGAGGCTGGTTATCTTTTTTTTCTACCATGTGCCAAAGTAAGATTTGAAGGTCTAGTGGAATACTTTCA
TTAATTCCTCTTGTGATGTATCGATTGATTTTCATGCTATTTCCCTCCCTTCTGCTTTTCTTTCATGATGTCGATG
ATTTCGTTGATAACTGTGACGGATAATTGAGCAACTATGATCCAATTATTCATGGTCCTGACCTCCTTGTTTTA
GTAAATGACGTTCATCAATAATGATATTTTGAGTATCTGTAAGGTACAGAAAGTCCATGTCAAAATGGTCTAAG

TATCCGACACTGATGAGTTGGTTATTGGCATACATTAGAAATGGATAGATACTTAGCTCATGTAGTTCATCATTA
TAGTAGGTATAAGTCTCGAGTGTGAGATGTACCAGTGGAGAATCATTAATAAAACGTTCCGGTAGAATATTTT
CTGCTGCTTCCTCCAGCGCTTCACATTCCCAAGCTTCGTTATTAGATAGTTGGAATAGGTGGGTTATATATTGT
TTGAGTTCTTGAGTGATGGTTTTCATATTATTGCCTCCTAGATAGTGTAATAGTGATGTAGTTCATATACATCATT
GAGATAATATATATATTTGATTTGTCATTTATTACGAATCCCGGTGGGAATAAGAGAAAATTCCATATAAAAACCC
GCTACAAACGTTGGTATGCCAAGGAAATCCTGAAATTCCGCCTATTTTGACAAACAATCAACTCATTATTTATA
AGTATTGATGATAGGGTTGTGTCTCTGCTTCCTTATATATATTATTTATTTATAAAAAGTAACGGGATTTTGGGA
TTGTGCTTGCACAATCCTTCTGTTTCTTCGAATCTGCAAATCCCAATCATTTCCCGATAAAAAAATCATTGTGGG
ATGTTCTTTAGCAATTTCAATATAAGCATTGTATAGTTATGAAAAAATTACGACAATAACTGTTTCATTAGATAA
GTGTTATTGAAATTGATAAAGAGCAATTCTTGAAAATAGTTAGATAAAATAAGCGAAAGAATATAGTGAAAAT
TATTGTTATAACAATGATTCTATTAGCTAAATAGTAAGATATAGTGTTTGGGGCAAAAATAAAGACGAAGTGCT
GAGATGCACTTCGTCGAGTTGTTTATTATTGAAAAGTTGTTTAATGATTTCGTTATTAAGTTTGAGTGTGACAT
AGAATTGTTTTTTATGATTACCATCTTTTTTAATATCAATGCGATCAATCACTGATAGATACAATGCTTTGAGTC
GAGATTTTTCTATGTGCTTAATATCATGAAAGATGTGTTGTAATAGTTTACTGATTTCTTTGGCATCAAATAAAG
TCTTATCTTCATTTTGTTGATTTTTGAGTTGGTTGATTTGATTCGTAATGTCATTGAGTTGCTTTTCATATTTTTG
AATACTTGGTCTGATTACTGATGTTAAGTCCGGATTATCCTCGATGGTTTTAATCAAGTTATTTATTTTGATTTG
TACTTCATCATATTGTTGTTGCTTATAAGCAATATCGTGATGAAGTGCAGCGCCATCAACTTGATTTTCTTGATT
GACGTGTGTTACTACGCGTTGAATGACTTTATCACTTTTGACTATTTCAAGTATTTGCTTCATCACATAATCTTC
AATCACATCAGCTCTTACACTGTTTGCCGAACATACTTTGGAACCCCTTGTTCCGAAAATTACTACATGAATAGT
AACGAATACGTTTCTTAGTCCCGTCTTTAAGAGTATTCGTGGTATTGCTTGCTGCCATAGGTGCGCCACATTG
GGGACAGTGAATAATGCCTGTAAGCAGATTCGTTCCTTTGCCATGGACTTGGGGTTTTTGACTGACTTGTTT
CTTACGCATTTGTACTTTATCCCATAAATCTTGATTAATAATGGGGGAATGCTTACCTTCAGCTATCACTGGTTT
ATCATTCAGCCCTTTACGACGTTTTTCACTCCAATCTTTGTATTTCGCAAATTGAATTTTGCCGATATAGAAAG
GGTTAGCTAAGATGTATGTGATTGAACTAATACTGAAAGGTTTCCCCTTTTTAGTGACATATCCTTTGTGATTC
AATGCATTGGCAATTTTACGATAGCCATGTCCTTTGGCATAGCACTCGAATATATATTTTACAATATTAGCTTCA
TGTTGGTTAATCATTAGCTCGTGTTTACTATCTGGTATTTTGTCATAACCTAGTGGTAAATTGCCTTGATAATAG
CCTTCTTGGGCACGTCTCGTTTGACCCATAAATACGTTCTCGACAATGTTATTACGTTCGAATTCTGAGAAACT
CGCAAGTATCTGTAACATGAGTTTACCAGAAGAAGTATTGACTTCCATACGCTCTGACAAACTGAAAAATTCG
ACATTTTGTTTGTGTAAATCTTCGACAATTTTGAGAAGATCAGATGTATTACGAGCTAATCGGTTTGTTTTGTA
TACCATAACACAGTCGATATTGCCTTCTTTTGCATCTTTCAACATACGTTGGAGCTCAGGTCTGTTCATAGATT
TACCTGAAATACCACGGTCAGCGTATATATCTTTAACTTCAAAATGATGGAAGTCACAGTATTCTTTGATTTGA
TTGATTTGTCCGTCGATACTATAACCTTCTGTGCTTTGCATTTCTGTTGATACACGTACATAGATACCGACACGT
TTTGTTTTAAGTTGTTGCATTATGTTACATCCTTTCTTCATTTATGCAATCGATGATTGCATGGTTTGATTGACA
ATATTGAGTGGTTCATTTTTGAAATAGATTCCTATAAGATTTTTATCTTTCGTAATGTGAATGGTTTCAATATAG
GGGTACAATATGTTTAACGTGAAACGTTTTTGAATAATATTTTGAATGATGTGTTGTATTTGATGCCCATTGATA
GATGTAGTGCGTTGCGGTTGTTGACGTAATGATTGTGTTTGCTCTCTGAACGTTTCTGCATCAATGATGCCTT
GTGCCAACTTTTCTATCAGTTGTTCTTGAGTCAATGTGTGATGTTTTTCTATGTTTCTTTGTCTTTTGATGCGTT
TGTCAATCGCACTTTTAATTTTTGTGTAGATGCGTTGATTTTGATAAAAGTCTCGGCACACTTCTAATACTTTAT
CTTCAAGTGTTTGTGCATTGATGCCTTTAAAATCACAGACAAAGCGTGAAGCATTCATGTTTTTAGGACAGA
CGTAGTAACGTAATGTATGATTCTTTTTTCTAATGGTCATATTTGTAAGTGTTGCATTACAACATGGGCATTTGA
TTTTTTGTTTGAGTTGATTATCCGAAGGTGTCTGTTTGGTTTGTTTTTGCAATCGAAGTCTCTGCGCTTGCTCA
TATATACTTGTGGAAACAATAGAAGGAAACATATTGTCGAATTGGCCATATTGATTGTTGACACGACCACAAT
AATTAGGATTGATGATAATGTTACGAACTTGATAGGGTTGTCGATTGATATACGTGTTATCTTCTTCTAATAACT
GTGCAATTTTCTTATAACCATGACCTTTAATGTAATAATTGAATACAGCCTTTACCGTTGGTGACTCATTTTGAT

TGATGATGAATGTTCCGTTGTGATATTCGTAACCAAAAGGTGCATGTGTTGTAATCAATCGACCTTGCTTTGCT
TTTTCTTGAAGCCCATTTCTGACTTGTTCTCCAATGTTATCCGATTCAAGTTCGGCTAAACTGATGAAGATATT
AAGCTTGAGTCGGTCGAAAGCTTGATCCATATCAAAGTAACCATCATGTACGCTTAAGATATGAACATGGTAC
GTTTGACATAATTTGATGAGTTTTAATGCATTTTTCAGATTACGATGCAACCTATTAAGACGATAACAGCATAA
TATGTCACACTGTCCTTGTTGAATTAATTGTGTTATTTGTCGATACCCACTACGATTATCTTTGCGACCTGATTG
TTTGTCGCTATAAAAGTTGATATGTTGAATATGATGTTTTTCGGCTATTGCTTCGATAGCTTGTTTCTGTGCTGC
AAGAGATTGTTGTTTCATCGTACTTTGACGTAAATAGCCTATGACTTGTTTCATATCGGCTCCTCCTTTCACAG
TGATAATATATATTTATGGATGAATTGATATATAAGCCCAACATCAATGAGATGTTGGGCGTCCATATTAGTCATT
TGTTTGATTGATTTCTTCAATTACCAAATCGGCTAATATCTCGATAAGTTCATCCATGTTTTTCACTCCGTTATTT
GTTCTATCTTCAATACGTCGATTATTCAGTTTGATGCTTCACAGTTGTATGATAAAGACAATTAGAAATCTTCGT
GAACTCCTGAAGGGCCTATCCCTTCATTAGCGGATTTAAAAAGTTCTTTCGCAGCTTTGTTATCATTTGACGG
TGTCCAATTTTGAAGTAACGACTTATCTTTAGTTAATCCGAGGATAGATGCAAACTCTACATCTAATTTTAGAT
GGTAAAATACAAGTGATTGTTTTTTACCGCTATTATCTTTGACACTTCTTTTAGTTGTTTGGCGTCCACGGTCA
GCTAATATGAAACCTTTATCTCTTAAGGCGTTGACAACATTATTAACATCTTGAAATTGATGATTGTTTAGCATC
TGTTTAAAAACGTTCGCAATTATTTTTACTTCGATATGGTCATCTTTTAATGAGATTAATCCATAGTTCTCAAAC
ATATTTTTCAAAGCACCTTCATCTGAAAACTTACCTCTGTTTTGTGCCACAAATTGAATGATGACATCAATAGC
TTTATCAGCTAATGAGCGTTCAGAGACTGTATGAGTATGATAATCAATAAAGTAGTCTCTTATATTAGCGATATC
AATATCTGTAGATAAAACACGACCTAATATTTTCGCAGATGTTGTAATGACTGCATAACGCTTAAACATACGAA
TACCTGTATTGTTTGTTTCATCTTTCAATTTAGCTTCAAACCAATCTACTTCCTTGTAAAACCATTGAATAACTTC
ATCTTCACGATTTATAAGATATTTAGCTACTAACGGTAAAACATGACCATAGTTTAGTGCTACAGCTTTTTTAAT
ATTGTCAGCATTGGTCGCATTTGTAGTGAATTGTTCATTAATCTCGATGGTTCTTACACGTAATCCATCGTTTTG
AGCTGAATCATTAAAAATACTGTATTCTGACGTTGAAATGACAGAAGTACCCCAATTCTTAGGCGTTTTAACT
TCTCCATGAACGTTTGAACGTTGACGACCTTGACCTTCAGCGATGGAATATAACAAACCCGTGGTATCTCTAA
GTGTCGTAGATGAAAGCTCATCAAATACTATAGGAATGCCATAATTGTTACTCAAATAACCTTCAAGTGCGTTT
CGTGTGGCATTCCAACTTCTAAAAAGAGTTTCATTACCTTTGGTAGGGTTACCAGCGACTGATACAGCTAAA
GCAGCTGCGGTTGACTTACCGGTTGAGGATTGACCTGTAAAACTAAAGAGAATTCCTGCAAATTCGATTTCA
TGTTTATGCTTCAGAAAACTTGTCACTAAGGCAGAAATCCCAAATATGACTGCCAATTCTAAAAGAAGATGAC
CTTTAACCTCGTCAATATACATGTTAAACCAATTATCAAATGTACCTTTAGGAGTTAAGTCATAAGCACTATCTA
CAATAGGGTATGAAGGTGAGGACTGATTAAATTGAATAGATTTGAGAACTTTATCTAGCGATATCAAGTAGCC
AAAAGGTGTCTCTAGTATACCTACACCCTCATATAATTCAGAAAGTGGTAATCTATCTCGCATCAATTGCAAGG
CATAACTCAATGATCTAATATACTTCTCGTTAATACTGTAGCCATATTTAATTAAAGAAGGTAAGTTTCGTGTTG
TTAAGATATCAGACTCAAAAATGTCTTTTTTACCATTGTTGTTAGTAATAATCAACTTTTCAACACCAGTAATTG
GATCAGAAAATCTTGCATTAACAATAATAGGGCTAGCCATTCTAACCACTTTTTCACTGTCACCATCTTTTTTA
GGTGGTATCGTTTCTTTCCAACCGAAGGAATCTAAAGCAAATGGGCCAATTTCAAACAATGTGTAACTCATTA
GCAATCACCTCCTTTCGAAGGCGTGCTATTATTGCATGGGTTTATCCCTATTTTCTGATATAATAAACTGCCATT
TTTTGTTCCTACAATTTTAAATGGTAATCCTGGCGTGTACTTAATCCAGTAAGCAAAACGACGTCCTACTTCAC
GTTGATCATTCGTGCTACAAACCACATTATATTTCTTTCTTAATTCACCATAAGTGAACGTACTTCCAACTGGTT
GTTGAAAAGATTTAGCAATTATAAAATTATAAAGCTTTTCTTGCTGCTTAGATTTCTTATTCATTTTAAAATTCC
TCCAATAATTTTTAAGGAGTCAATCAATATCATATAGGTTAAACTATCTTCAATTGATTGCTATAATAAGCATAAA
TGAAGCATTATGATTAGACGCGGAACAAAAAATGGGGAGGGGGGGTATATATTGATACCTACGCTAGAAGA
AATTATTGATAAGTATGGAAACTTAGTTGATTATTTGAAATTTGATGTAACAGTTGAGGTTTATGAAGATTTGT
TGTTATTAAGAAGTTTAAGCGATTTAAATGAACATCAAAAGATGGATAGAGTTTCATTTATAAAAAAACATTTA
AACTCAAATTTCGATTATTTATTAGATGATACTGAGTTTAGAAGTATCAATAAGATTTACAGTAATTTAAATATC
ATGACGCATATCAATAGTCAAAATAATAATTTTAAACGTAATCCATTTATAAATGAAGAACAATTGAAATCACTA

CTCGCGATAAAGAAAGTAGATGCACATATGAGTTATGATTCAAATATTACTTCAAAAGCATTGGCAGAAATAA
ACAAAACGCAAAAAAGATTAGTTACAAAGATAGACACACTCTATAATCAGTCAAAAAAAGAAAATGAATATG
TACGGTTAGGAGAAAAAATATCGTATACAAAATTGGAGAATCATTGGAAATATTTATATAATGAGATTCAGTTT
TATAATCTTAATAATCAATTGATTAGTTATGTTGCATTAGAACAGGAATATGCTTGGGCTTTTTTAAATGAACTA
TTTTATTTGATAGAATTGTATTTGAAGGCATTTAAAGGACAAAAGAAAACAGATTATGAGTTTGGGAAAAAA
TTAAACGAATTTATTCAATCATATGTCATTATTTTACTTATAGATATACGATTACCTGTTGTTAGATTATATATTATT
AGAGACATTGTAGATACATGTGAAGGAGAAAAAGATAATTACAAAAGAATTACACTGATAGAAGAATCTATT
AAAAAGTATAGATACGTAAAAGATCAGATTAAAAGATTTAAAAATGGATTAGAAGAATCGTTATTAAATGCAA
CTTTATCTATTGAACAAAATATGTTGAAAGTTAAAATTGATTATTATAAAGCCTACTGCTTTCCTAGAGAAAAA
ACAAAGCATAACAATATTGAGTACAATGTATCGTTGTTTTTAAAGCATTAGATGCCTTAAAGAAATAGTTTTT
CACTGAAAATATTTATAGATATAATTAAAATTAATTAATATTAAGCCAGGATAATGTAGTCTTAATCGTTCTGAAA
TACGAAAAATGTTGTGGAAATCGCGTGAAATAATCAGAGGAATCGTTTGAAATCATCGTAGTAATCGCGCGA
AAATCACGTGAAATAATCAGAGGAATCGTTTGAAATCATCGTAGTAATCGCGCGAAAATCACGTGAAATAATC
AGAGGAATCGTTTGAAATCATCGTAGTAATCGCGCGAAAATCACGTGAAATAATCAGAGGAATCGTTTGAAA
TCATCGTAGTAATCGCGCGAAAATCGCGTGAAATAATCAGAGGAATCGTTTGAAATCATCGTAGTAATCGCGC
GAAAATCGCGTGAAATAATCAGAGGAATCGTTTGAAATCATCGTAGTAATCGCGCGAAAATCGCGTGAAATA
CTATGGTAGACGGTTTGAGTAAATTAATGGAGTATTTTAATATTTATGGTTTGGAGGAATTGCGTTGCATAGA
GAAAGTAAATATATAGAGTATAAGAAATCACGAAAAGGATTATCTAATGATATTTGGTCTACGTATAGTGCTTT
TGCAAATACTGAAGGTGGTACTATATATTTAGGAATTGAAGAAAAAAAGATCGAGGACAAAAAAGTCTTTGT
TTCAGTTGGTGTTGAAGATCCAGAGAAAATGATTGAAGATTTTTGGAATGCACTATATGGAAGAAGTAAAGT
TAGTCAAAATATTTTATCAAATAAAGATGTTAAAATTGTTAATATTGAAAATAAAGCGTGCATTGAAATTCATGT
ACCAGAAGCGCCTTATTCGAAGAAACCGATATATGTAGATAATAAAAAAGATTTAGTATATAAAAGAGTTGAT
GATGCTGATAGAATTGCGACTGAAGAAGAGTATAAATTCATGATTGTAAATTCTCAAGACGATATAGATACAG
AATTATTAGATAACTATGACATGTCTGATTTAAATCACGAATCTATCGAAAATTATAGGAAACTTCTATTAAAAA
ATACTAATGATGAGAGATATGCGAATATGAGCCAACTGGATTTAATGATAGATTTAGGAGCATATAGAAAAGA
TAGAAGTTCGAAAGACAAACAGTATAAAATGACTACAGCATGTTTATTATTCTTTGGTAAGTATAATGCGATTA
GTGATAGATTCCCAGGATTTCAATTAGATTATTTTAAGAAAACAAATTACCTAGATACTGATTGGAAAGATAG
AATATCAAGTGGAGATTTAGGTAATGAAGATTTAAACGTGTATAGTTTTTTTGAAAAAGTATTGATAAAATTAA
CTGATAACATTGAGGAATCATTTAGCCTAAATGATGGTTTGACTAGACAAAATTATGCAAGAGATTTAAAAGT
AGCAATTCGCGAAGCACTGGTTAATACATTAATGCATGCGTATTATGATACTAAGCAAAGTATTAAAATAGTTA
ATTGTGAAGATTTTATAGAGTTTTATAATCCGGGTAATATGAGAATAAATAAAGAAGATTTTATTCATGGAGGG
CATTCAAAGGACAGAAATAGTATATTATCGACGCTTTTCAGAAGAGTAGGATATTCAGAAAAAGCTGGATCT
GGAGGACCAAGGATATTCGATGTAGTTAATAGACATAAGCTTAAAACGCCTGAAATAGAATTAACGGACATG
GACACTAATGTAGTACTTTGGAAACAAGATTTAATGAAGGAGTTTGAAAAATATCCTGAGTTAGACAAAAAA
GTAATAAAGTATATTATTGACTATGGATCAATAAGTAAGGGTGAAGCCTTAAAAATGGAAAATATGACAGAAT
ATCAGTTTAGAAATATTTTAAAAAAACTAAAAGATGATAACTTGATAAAAAAAGAAGGTGAAGGTCCGGCTA
CTAAATATGTGTTAATAGAATCAAAAGAAGCTGATATATTGCGAACTAAAAAAGTAATTAAAAGTTTAGAGTC
TTTCTTTAGGAATAAATAAAAAAACAAGAGATAGGTGCGAAGTGTTTGGATTATACAAATGCTTCGCATCTTA
TATTTATTAATAAATATCATAGAAAACCGTATCATTAACCGATACGCAGAGATGCGGTTTTTTAGACACTTCATA
AAGGGATTTTGAACGTATCAGAACATATGAGGTTTATAGGAATTGCTGTTATGTTTTTTGATCACATCAATAAA
CAAAAAAGGTATGTACTATGTAAAATATTTATTAAATGATATAAAGCGAGGGTATATAAATGATTTTTAAATAGA
TATTTATCCAATAATATAAAAAGGAACTATAAGCTATATCTAAAAGCTATATCTAACTACTTATAGTCCTTCTTCAT
TAGTATAAATATAATTATTAAGAAAGGTATATATCTTTGTAACCTTCGTTTACATTAATAATGTTGTGATAACCTTT
GTGTTCTAAAATACCAATAGCTATAGAACTTCTAATGCCAGACTGACAGTCTACATAAATAACATCGTTTCTATT

GAAAGGTAAATCTGTTTCTAAAAGTTTGCCGTGTGGCACATGAACCGCTTGGGATAAGTGGCCATTATTCCA
TTCATTATCATTACGTACATCTAAGACATGTGCTTCATTACCAGTTATGTCTTTACTATGAACAGATTGTGTTTG
AATTTGAGCTTGTGGTAACTGATATCCAGACACATTATCATATCCAATAAGTTGTAAAGTATGTTATGTGTTGCT
TTTGAAACAAGGTGATAGTCTCCAATCAAGTTAATTTCTTGATTATAGTTTAGATACCAGCCAATTTGATTGAT
GAAATTTTTATCATATGGAATATTGATTGTACCTTCAATATGTCCACCATGATAAGCCTCCTTACTGCGGAGATC
AAAAGTTAATCTGTTTGTACTTGTAGCTGGATAAACCGTATAAGGTTGATATAAATTCATACCGAATTGATTAAT
TTTTTTCATTTGTGCAAAATGATGTGGTGGTGCAGGTTGGTCAGAAATGAGTTTATCGATAAAGGTAGCTTCA
TTATTTTCAGAAAAAGCCCAGTTCGTTTGTTTTTCATAGCCAAGCGTAGATGTTGGAATAGCACCTAAAGATT
TACCACAAGGACTACCAGCGCCATGACCAGGCCAAATTTGAATGTAGTCTGGCAAGTCTTTAATACTTTCAAT
AGATTTAAACATTTGTTTTGCGCCTATTTCAGATAATCCTTCTACTTTAACAGCTTTTTCTAGTAAATCAGGTCT
ACCGATATCTCCTACAAAAATAAAATCACCACTGAATAGTCCCATTGGAACTTGTGCTCCAGCACCTTCGTCA
GTAAGTAAAAAACTTATACTTTCTGGCGTGTGACCAGGTGTATGAAGCACTTTTAATTTTATATTTCCTACATA
AATATCATCATTATGTTGAACAAAATGAGTGTGGTTAGGCATATTTTTATAACCTAACATGTCATCACTTTCGCC
CGATACATAAATACTAGCATTTAACTTTATAGCAACATCTCTAATTCCTGAAACAAAATTTGCATGTATATGTGTT
TCAGCTGCATGAGTAATGGTTAAACTCTCTTCATCGGCAACTCGAATATATGAAGATAAGTCACAAATAGGAT
CAATGATCATGGCTTCTCCAGTTTTTTGACAACCGATTAAATAAGATGCTTGAGATAAATGTTTATCATAAATT
GATGATAGTCAATTTGTTATCAAGGGTGATAATTATATTAACTATGGTAATTACATGGAATTAAAGTTATTAAAC
AAACTTGATGATGACCAATTAGATTTAGAATTTTTACAAGATAATGGGTTAAAAGGTAATGTTCAAGGTCCTA
TGACTTTAAAGGAACCTTTAAAAAGTTATATACAAAAGTCTTGAAAACGAATATTTTAAATTTAGATAATGAGT
TAGAAAACACTGAATATGTCGAAGGTAAACCATATGTACAGTACGGTATTAAATATGAAAGAAATCAGGCATT
AAGAAATGAAGCTATTAAAATTCATGGAACTACATGTAAAGTATGTGGATTTGATTTTAAAGCTAAGTATGGC
GATTTAGGTGAGGGTTTTATTGAAATTCATCATTTAAAACCAATGTTTTCAATAAAAAGAGAAATAAAAGTAA
ATCCACAAAAAGATTTAGTCCCACTATGTTCTAATTGCCATAAAATGATTCATAGAAATACTAAAAAACCTTTA
ACGATTAAAGAATTAACCAAAATAGTTAATTATAATAGCAAATAATTTAATATTTTATAAACTATCATTCAACCCT
CTTAATTTATTAGGAGGTTTTTTGTATTTATGCTTTCAAATGTGTGATATACTTTGTTTGTTGAAATATAGAGTAT
CTATAGATAGGGTGATTGAGTATGAAATTCACTGAAGTGGAAGTTATCGAACATCTTGTAAAGGCATATAAAG
AAGCAGGAAAGCCTACTTATCCTCATGAAAATTTATATCGAGGACGTAATCATAGTATTTCAGGTATTGGAGA
AGACTTGCTGGGTGCTTATTTGATTAGTAGATTGGAAGGTGTCCAAATATTTATTGATCAGCCTTTATCTATGA
TTGATAAATCTTTAAGTACAAGATATCCGGATTTATTAATTTGTGAAGATAATGAAATTAAAAATATACTAGAAG
TTAAAATGGACTTAGGATATCAAAGAAAAGATTTTATAGATTATTGCCGAAAGAAAGAAGAATGGATTTCAA
ATATCGTAGGAAAACAGTGTGTATTGTCTAGAAAGAGAGAAGACAAAATTCCTATGAATATAGCTGATGATAT
TAAATTTCATGTTGTGATTTACAGTGAAAACAATGGACCGAAGCGGTTTGATGAAGAAATCATGCCTATCGTT
AATGAAACATGTCCACATATTGAAGTATATGTCCTAACAAGCGGTCAACACCCTAATTTAGTAAATGTTAATCT
TGAAGGTATTAATATTAATAAAGATGAATTTGAAATATTAGTAAATGCGTTATAAAAAAATAGAGCATCCTCCA
CGTTATGGAGGTGCTCTGTTTTTTATTGAAAAGTATCAAGTTAATTAATTTAATATGCTTAATAAGTTCTACCTT
GACCTTTTTCTCTAGCTTCTGTTCGATCTCTTATGTACTCAGTACATACTGGATTTTCTGTTAATTCATCAACTGT
ATTAGTTTAATCAGAAGACGTGTCTACTTTGTAAGCTTCTAAGAACTTATTATATGATATAGCGTTTGAGTTTTG
TTGTTCTTCTATAAATTTCTGTAGTTATTTTTCAAAAACCGCATCATTAACTGATAAGCAGAAGCGTATCACAA
ATAAAACTAAAAAATAGATTGTGTATAATATAAAAGGAAGGGATTTATATTAAAATTTTGAATTCAAAAATTATT
GAAAGGGAAGTTACCTTAGAAATTGAATCTATGGCCACTAACTGCTTTTTTATATCACCACTTATTAAAAAATG
TAAGAATATTCCTCAGTGCTAAGTGAAGGAGGTTCTAGTTTTATTTAATATTCATATTGATAGGTAATGGTTAAT
AATTGCCTGTAAATTATTGTTAAATATCGTTGTTATAGTATCATCATTCATAAAATTTTTATAGATTTTATCAAGTA
TTTCCTCATCTTCAATAGCTGTAAAGCGATGTACTAGTCTTTTTATGAGCTATATATAATTTTGCGAAGCTAAAC
TGTTTGTAATTATTCACCAATCAGGTTATGTAGTGTCACGGTTCATAGGTAATTCTCTTTTTAAAATCATCTAATA

TTTTATATAATTCATATAAGTGTAGTATTAACGTTTTCTACTTTATCTATAAAATATTTAGAATTACATTAGAATTTT
AAATTATACGGCTGGAAATTCAATGCTGTAAAAATAGACAGATTTTAAAAAAGTAGTAATAAAAAGTCTAAGT
TTTATCTTTGTAATAAACTTCGACTATATTTAAAAATAAATATGAATGTCTTTTAATTTCTATAATATCAAAGCTAT
TATATGATTTGAAATTTTGTAACTTTTTCTCTAATTTGGAAGTAGATAATGACTTAAAGGTTTTAACTTTTACCC
AACGACTTGGCATTTTATCACTCCTATGTAATGTATTACTTCAATATACCCTTAAATTTTTATAATTAATTATATTG
TAATATTATTTTATTTTATAAAAATAGTTTAATAAAATGTTTAATTTTTATAAAAAATAATTAAATATTATAGACATT
ACGATAGTTTAATTAGAAAATATATTTAAGAATTATGAATTTAGTTCTTATTAATGTTAAAGATCATCAATTATTT
TGTCACATTATCTTGAGAGGATTACCTTATGAGGTAGCCTTTTATTTGTCACCTAATATTGCTCTAAGGCTAATA
ATACATATATAAAATAAATAATTATCAGAATATATTTAGAAATTGATATTTAATAATTAAATAAAAGTATGCTAAAT
ATTTATAATTTACAAGTGAGCTGATAGAAAGATGAGTATAAGCGAATATAAAAATAGATACTTAGAGTCACTAT
ATAACACCTTTAAAACTGAGTTAGAAAACGAACGCACTCGCTATTTTATATTAGATACCAACTATACAAATAGT
GATGATACTGAAAAGGAGTTTACTTGGGATGTAAAACAGAATAATAAGATCAGAGAAGGCGATTTGTTCATT
TTTAGACGGCCAACTAATTTATCTCAAATTGCTCAACAATTTTATTTCTTTGGAGCTGGGAAAATAGAAAAAA
TTGAGCGAAAAAATAATGTAGCAACCGCTTACATTTCAAAACCCTTACTTTTTGTGGATCGTGTTTTAAAAGA
TAATATTGAAAATTTAAAATGGGAGTTCAAAGAAAGAATTAAAGAAGAGTGGGAGCAGTTTTTCTTTAAAA
ATAAAATCAGTAATATTACAAAAAATGATTTTTTAAAACTACTAAGTTTGAGTAGAAATGTAGTGGAAATAGA
AAGTAATTATATTAATAATGATATTTCGCTAACTTTTAAACAAATGGCTGATATGGCAACTCGAATAAAGAAAA
ATATTTACTACGTAGGGAATCATGAAGCATTTGTTCAAGCTAAGGGAAGTGCTCATTTTGAGTTTTCAAAAAG
GATTAAACAAAATTATAGTTATAGATGTGCAATTACCGGGATTAAAACTAAAGACTTTTTAGTCGTTACACATA
TTATTCCTTGGCATGAAAATGAGTTTATTAGACTAGATCCTTCAAATGGTATTTGTTTGTCTTTATTTTTAGCAA
AAGCATTTAAGAAAGGCTTTATCACTTTTTCTAACAGTTATAGAGTTGTTTTATCTAAAGAAGCAGAGAAAGA
TGCTGCTTTATATGAAGAATTAAAGATTTATGAAAATCAAAAAATCGAACTACCAGATTGTCAGAAACCTAATT
TGAAATATTTAGATTGGCATAGAGAACATATTTTTAAAAATTAATTTTGGGAGAGGTTGTATGACAGATATTAG
TCAATTAGTAGAGGTGCCATACATCTTTCATTAGTAGATTGAAAAAGAGCACTTGAAACAGATAATTTGTATTA
TTTAGCCTATGGATTAACAACTTTAGATAATTGTTTTAAATATTACCAATTAATTACACAAAATGAACATTTTTG
CTATTCTCAACAAGAAGAAAGAGAAAGTTATCAGCAAAAATTTCAAAGTATGATTAATGGTTTAAAAATTAAA
ATTGGCATACAAAGTTCAAATGATGATGCGCAATAAGCTACAAAGTTTGATTTTTTAATTGAACGTTATATGTT
TTTAAAAGAAGAAAGAGCTTCGAATTAATATCTTAATGGAGGAAGATTAGTAAATGGCTAGAACGTATAATGT
GATTAAATATGTCATTAAGAAAAATAAGGATGATCTTAAAATACATGGATTAAGATTATATGAATTTAATATAAA
AGATGAAAAAGCTATAAATATTAGCCAACCCATTTTTGTGAACAGAGCAACATTGATTGAGAAAATACAAAAT
GGTGAAGCATTTACGCACGCCTTTAGAGTAAATCACAATACTTATTATGCAGGCCAATTAATTTCTTTATCATT
GAATAAAAATGGTTATATTAATAACAACTTTGAGGCCTCACGAGATATTATTAGAAACATTGAAGTAAGTATTT
TAGACTATGAATAAAGGTAATACTGATAGAATACATTACAATTAAAAAAGGGAGTATAGGACTCCCTTAATAA
GTTGATATATAAGTGACTTATTTTTTTAATAGAATTTTAATTCCTCTGTTAATGTTCAAGATATATAATATTAAAGC
AATTAAAAAGAATATAATAGTAATAATTAAAAGAATGCTAAATATTAATAATTGATGATCCAAAGGTTTATCAAA
GACTTCTTTAGAAAAAATAAATGCCAATCTACCTATAAAAAAACTAACCCAAGTCATAATATGATTAATGAATG
CTTTTTTGCCATGTTCTGATGTTGGTTCATCAGCAAGTATCCATACAAATAAAGGTAAAATGACAGGTGCGAA
AAAGATACTTAAATAACTCAATGCTGATAAAACGTAACTATTGTCATTTTTTATGCATATTACTATTTGTATTTTCC
ACTTTTTTCACCTTCTTTACTTTTCTTTACTATTATAAAAACATTAATAGCGAGCTATTACAACAGTTATAAGAG
AAGCTTACGATATTGTAAGTATGTGTTGTTATAATCAATATAAAATTAAGAAAGACCTGAGAATTTATATTATCA
TCCACAATCGAAGTGAGTAGCGAAATTTATTGGAAAAGGTACTTATATTGAAGATATTGTTAATGGTTCATTTT
TAAAAAGCCAAGACGATTTGAAGTTAAAAGCTTCTGACTTCACTGTCGAAGATAACTATAACTACGGTGTACT
TATATTTCCAGAGAATCTTTCTCTTACAGATAATAATAAGAAATTGGAATCTTTAATAAAAACTATACTATTTACA
GGTAAACGATATCAATATACTTATTTTAAAATTTGATCTTAATAGATAAGGATATAATGATTTAAACAATTTAAG

AGATATTATTTTCACAAAAAAAGAATGCTTTTTGTTAGAGGCACTGAACCCTTAAAATGGGAACTTAATAAAA
ACACTATGTTCTAGGCTGCTAATTCTCTGTATTTTACAGGGGATAAGTAGCCTAGTTTTTGTTGAATTCGATTAT
TATTATAGTTTTTAATGTATTTTCGACAATATCTATTACAATATGATTAGAGTTATTAAGCTGATTATTGATGTAAA
AAGTTTCAGACTTTAGCGAGGAATGGAAACTTTCTATCGGGGCGTTATCGGCAGGTGTTCCCTTTCGGGAC
ATACTTCTGATAATGCCTTTTTCTTCGCATAATTGATAATAAGCATAAGATGTATAAACGCTGCCTTGATCACTAT
GTAATATACACCCCTCAGGTATATCGATTTGATTTAATGTATCATTAACTAAACGTTGGTCTTGTTTATCATCTAT
TTTATACGCCACAATTTCTCCGTTATAAATGTCCATTATCGAAGATAAATACAACATAGAATGACCAAATGGTAA
ATAAGTAATATCGGTTGTTAATACTTCCATGGGACAACTCGCTTTAAATTGTCTTTGTAATAAATTGTCCGTTTT
ATAATACGGTTTACCTATTCTTGTCGTCTTTTTAGGTCTAACTCGGCAGTTCAAATGATGCTTCTGCATCACTCT
CTGTACTCTCTTATGATTAATTGGTGATGTATAACATTGATTAATCAATGCTGTAATCTTACGATAACCGTAGGTA
TATTGTTTACCAACATGTTGTGAAAATCTATAACTTTCCCCATTTCGATACCATCGCCACCAATTTTTCACTTGT
GTTCTATTTCTAATATTTAATTCTTTCATAATTTCTTTTGTTGGAAATCCTGCTGCTTTCATTTCAACTGCTTTATA
CTTTGTTTCTACTGAATAAGAAGCTCTTTTCATAGAAAAAAAACACCTCCGTATGATTCATTTTAATATGAATTC
AACGAAAGTGTTTTTATATAATTCCCACTATTGGGGTCAGTCTAAAATTAAAATTGATAACTTTTTTAATAAAAT
AGATACACTAATTTTAAAACAAGGTAAAAAAATAGAATTATTAAAACAACGCAAACAAGGCTTACTACAAAA
AATGTTTGTGTAATTCTGATAACGTCCTATTATGTTAATTGAAAATAGACAAAAATAAATTGAGAGCATCCCTT
ACCGCAAAGTGAAGGATGCTCTATTTTTGTTGAAATTAATATAAGCTTCATTCTGATAGATAGTCATTAATAATT
GTTTGCAAATCGTTATTAAGTACCGTTGTTACAGCGTCATCATTCATAAAATCCTCGTAGATTTTATCAAGAATT
TATTCCTCTTACAGACATTCGCGAGAAGTCCGTTTTAATTTTATTAGAAGTAATTCAGGTTTGAACCTACCTAA
ATGAATATATGAGTTATCTTTTATACTACAAAATATATTCAGATTTCAATAATGACATAAAATAGGCATCTTTATAT
TTACCTTTAGTGTAGAATTGCTCTTTAAGTAATCCTTCTGTTTTAAATCCTTGTGACTCGTATATATGAATAGCTT
TTTTGTTATCTGCATCAACATATAGATAGATCTTGTGCATATTTAAAATATTGAATGCATAAATTATCGCTTTTTC
GAATGCGAATTTTGCATAACCTTTACCACTGAACTCAGGTTTAATAATTATTTGTATTTCACAATTACGATGGAT
GTAATTAATTTCTACTAATTCAACAATACCTACGACTTGATTTTCATCTTCAACAATAAAACGTCTTTCCGATTC
ATCTAATAAATGCTTATCAAATAAATGTTGAAGTTCCGTTAAGGATTCATAAGGCTCTTCAAACCAATAAGACA
TAATAGAATATTCATTATTTAATTCATGAACAAAAAGTAAATCACTATACTCTAATGCTCTTAGTTTCATAATCCC
ACTCCCAAAATTTTCTTATATATTTGCATTATAAATATAAATAACGAATAAGTCATTATTCACTATGAATAAATCTA
TTTTAACAATTCACTACATACTAATTCTCATTTTCTTATTATTCTCTAATATCTCTGATTTATTACTCAGTGAAAGA
TGCCCTATTTTTATCAGTATATAGTTTTTATTTTGATAGATATTGATTAATAATCGTCTGTAAATCATTGTTAAGTA
AGGTTGTGATAGCGTCGTTATTCATAAAATCTTCATAAATTTTATCAAGTATTTCTTTAATATATTTTTTAATCTTC
ACACCTTAAATTCACTTGAAAACCAAATCACTTCTAAATTATGATTTTACATAATAAATTTTTAAAAATTGGTAT
TATATAAAATAATCTCAATAATCAGAATTTTTTGTAAATAATAATATTAAAGTGTAAAATAACAGATAAGAATAG
ACTTAATGTATATAAATTAAAGGAGTGTTGGAAGTGAAAACTAGAGCAGCAGTTTTACATGAAATGGGGGCT
AAAAAGCCCTATAAAGAATCTAAACCTTTAAAAATTGAATACTTAGAACTTGATAATCCTAGCGAACACGAAG
TATTAATTAAAATTCATGCAGCTGGATTATGTCACTCTGATTTGTCAGTAATTAATGGTAACAGACCTAGACCTT
TACCTATGGCACTTGGTCATGAAGCTTCCGGTGAAGTAATTAAAGTTGGAAAAGCTGTTACAAGAGTTAGCG
AAGGAGATCATGTGGTATGCACATTTATTCCCAGTTGTGGAAAATGTATCCCATGTAAAGAAGGACGTCCTGC
ATTATGTGAAAACGGAGCAATATCTAATGAAAAAGGCGAAATGTTAGAGGGAGGGATGCGTTTATCTAATGA
TGAAGGAAAAGTATATCATCACTTGGGGATATCAGGTTTTGCTGAATATTCTGTTGTCTCTGAAAACTCTATAG
TTAAAATTGATAAAAAAAATACCTTTTGAACGTGCAGCTGCATTTGGTTGTGCTATTATCACAGGTATCGGTGCT
GTGGTGAATACAGCCCAAATTCGTTCTGGTAGTAATGTAGCGGTTGTTGGTTTAGGAGGTATTGGATTGAAT
GCTATTATTGGAGCTAAACTAGCGGGAGCCAATGAAATTATTGCTTTAGATATTAACGAAGATAAATTTGAATT
AGCAAAGCAATTTGGGGCTACAGCAACATTTAATTCAAGCGATAAAGATATCGATGAGCAAATTAAAGAATA
TATTCCTGGCGGAGTAGAATATGCTTTTGAAACAGCGGGTGTAGTGCCAGCTATGAAAGTTGCTTATCAGATT

ACTAAACGAGGGGGGACAACTGTAACAACAGGACTGCCTAATCCTAAAGATAATTTTTCTTTCCCTCAAGTT
ACTTTAGCGGCTGAAGAACGTACCATTAAAGGATCATATGTAGGAAGTTGTGTACCTGACAGAGATATACCA
AGGTTCGTTAATCTATACAACCAAGGACGTTTAAATATTGATTCACTCATCAGTGAGGTTATCACTTTAGATGA
AATTAATGAGGGATTTGATCGTTTGTCTAATGGTGAAGTAGGTAGAATTATAATGAAGATGCATTGAATATAAT
AGAATTCAAGTCGTTCCTCCTCTTGATTTCTATGAAAAGAATAACAATTTGAGTAGGAAGATTACTGACAAAT
ATTACATTTGCATTGTGACTGCCAATCTAATTGTATTTTTACATTTCATATCTATATATTAGAAGATAAATGTTTTA
AAACTAATTACTAATAGTTTTTTTAGATAAAAAAAGATATAGGAGCATCCTTCACCGCAAAAGTGAAGGATGC
TCTAGTTTTATTAGAATATAGTTTTCATTTTGATAGATAATGATTAATAATTGTTTGTAAATCATTGTTAAGTACC
GTTGTAATAGAGTCATCATTTATAAACTCTTCATAGATTTTATCAAGTATTTCCTCGTCATCGATGGCTGTGAAA
TGATGCACCAGACCTTTTACTATTGAAATGTAATTTTGTGATGCTAAACAACTAGCAACCGTTCGCCAATTGG
ATTGTATAGTGTTGTCATGGTTCATAGATAATACTCCTTTACTTTAGTGTCCATTTTTGACGTGCTTTAGGGTTG
AGTGGATGCATAACTTCGTTCGTTACTGGATTTATGAGCTTTTTTACTTTCTTTTTATGAGGTTTTAACATTTCC
ATCACTTGTTCGACACGGTCGATAACAACTGGTCGCTTCGCATAGGCACCATAAGCAAGAATGACTGTGTCA
CTTTCACTAATTGCTTTCATTAAGTGTATATCTGTGTGCTCATCATAAGGCTCTTTGATATGTTTAAGGTTCTCTG
GTGTTTTAATATTAGAGAAAAGATTAACGAGATAAACAGCGCCATATTGCTCAGAATTAGCTAATTGATTGAG
GATGAGAACCGTAGTAAGATCGAGTGATAATACACCATCTAAATGAAGATACATCGTTATCACAGTACAAGCC
GGTTTCTTGTCATCCCATATCTTTTTGAGTAAGTAGCGATGTTGTTCATCATCGCTAAATATCGCTTCTGTGTGT
ATCGTACTTTTGATTGTATTCATATATCGTCACTTCCTTTAGTATTCTTCTGGCAAAAGCATCACATAATAAAAA
GCGTCTACATCATCTTCTCGGATGACGTAGACTTTCTTAGGTAATGCATTTTGATTTTTTATATAGTTTGTATAG
TGATATTCCAATTTGTATGCGGGTTGTTCTTGTTCATGTGTAATTGACAACATATTATCATCTTCTTGTAGTTTGA
AAATGTGTAGGTAATCTGTATTAAGTTGATTGTCTCGATCTTTTACCATGTTCCAAAGTAAGATTTGAAGGTCT
AGAGATAGTTGTTCACTAATGCCTCTTGTGATGTATCGATTGATTTTCATACTATTTTCCTCCATTTTGCTTTTCT
TTCATGATGTCAATCACTTCGTTAATGACTGTAACAGATATTTGTGCCACTTTGATCCAATTATTCATGGTTATT
CCCTCCTTCTTTTAGTAAATGACGTTCATCGATAATCGTATTTTTAGTATCTGTGAGGTATAGAAAGTCCATATC
AAAATGATCCAGATAACCAATACTGATGAGTTGGTTATTAGAATACATTAGAAATGGATAGATACTTAGTTCAT
GTAGCTCATTATTATAGTAGGTATAAGTTTCAAGTATAAGATGTGCAAGTGGGGAATCATTAATAAAACGTTTG
GGTAGAATATTTTCTGCCGCTTCCTCCAGTGTTTCACATTCCCATGTTTCATTGTTAGATAATTGGAATAAGCG
AGTCATATATTGTTTGAGTTCTTGAGTAGTTGTTTTCATATCATTGCCTCCCTAGATAGTGTGATAGTGATGTAC
TTCATATACATCATTGAGATAATATATATTTGATTTGTCATTTATTACTGATCCCGTTGACAATATGAGAAAATTC
CATATAAAAAAACCGCTACAAACCTTGGTATGACAAGGAAATCCCGAAATTCCGCCTATTTTGACGAACAATCA
ACTCATTATTTATAAGTATTGATGATAGGGTGGGGTCTCTGCTTCCTTATATATATTATTTATTTATAAAGAATAAC
GGGATTTTGGGATTGTGCTTGCACAATCCTTCTGCTTCTTCTAATCTGCAAATCCCATTCCTTTCCCGATAAAA
AATCATCGTGGGATGTTCTTTAGCAATTTCAATATAAGCCTCGTGTAGTTATGAAAAAATTACGACAATGACTG
TTTCATTAGATAAGTGTTATTGAAATTGATAAAGAGAATTCTCAAAAATTTTTAGAAAAAGAAAGAGGAAGT
ATGTAGATATAAAATAGATTTATTAAATAGTAAGGTATAATGTTTGCGGTAAAAATAAAGACGAAGTGCTGGA
ATACACTTCGTCGATAATAAGGTAGTTGAAGTTTCGATTATTTTAAATTGATCATTTTAAGCCTACAAATCCCTC
TAATGTTTCACCAGATTTGTACACTTTTGTCGCCTTCTTTTTCCTAAATAACGTAAATGCCAAGGTTCATATTGA
TATCCTGTGATGCTTTCTTTGTTTTTAGGATATCTTATAATAAATCCATAATTATGGGCATTCTTAGCTATCCAGC
GTCCTTCCTCAGTTTTACCAAAACTAGCATATAGATTTTCACAACAAGTTGGTAAACAATATACCTACGGTAAA
GGATTAGAAGAGCTGTCAGAAGTAGAACAATTAAAATTAGAAAATAAGAGAAAAGATATAGAATTGGATATT
TTAAAAAAGTACAAGGTATTGGAAAGGAAGTGGTACCAACAGTAGTCATAGATTTAGTGGATCAATTAAAAG
TAAAATATTCAATCAAATTGCTACTAAAAGTATTAAACATACCTAAATCAACATATTACCGATGGAAAAACAAA
ACCCATAAAAATGATACCGTAACACAAAAAGTTATTGAATTATGTAAAGCTAACCACTATACCTACGGTTATCG
TAAGATTACAGCATTGATTAATCAATGTTATACATCACCAATTAATCATAAGAGAGTACAGAGAATGATGCAGA

AGCATCATTTGAACTGCCGAGTTAGACCTAAAAAGATGACAAAAATAGGTAAACCGTATTATAAAACGGACA
ATTTATTACAAAGACAATTTAAAGCGAGTTGTCCAATGGAAGTATTAACAACCGATATTACTTATTTACCATTT
GGTCATTCTATGTTGTATTTATCTTCGATAATGGATATTTATAACGGAGAAATTGTGGCGTATAAAATAGATGAT
AAACAAGACCAAAGTTTAGTTAATGATACATTAAATCAAATCGATATACCTGAGGGTTGTATATTACATAGTGA
TCAAGGCAGCGTTTATACATCTTATGCTTATTATCAATTGTGCGAAGAAAAAGGCATTATCAGAAGTATGTCCC
GAAAGGGAACACCTGCCGATAACGCCCCGATAGAAAGTTTCCATTCCTCGCTAAAGTCTGAAACTTTTTACA
TCAATAATGAGCTTAATCACTCTAATCACATTGTAATAGATATTGTCGAAAAGTACATTAAAAACAATAATAATA
ATAATAATAATCGAATTCAACAAAAACTAGGCTACTTATCCCCTGTAAAATACAGAGAATTAATAGCCTAGAAC
ATGGTGTTTTTATTAAGTTCCCGTTTTAAGGGTTCAGTGCCCTAGGATTATAGGCTCTTTTGTTTATAAAGGTA
ATTGAACTAAAGTATTATAATTTCAATTCTTAATTAATGTTCCTATTTTACCGTCTAAAGCGTCCCCTAATCCTGC
TAGAGATGTAATCAAAACACTGCCTTTAGTATTTTTTTCAAGAAATTGAAGTGCAGCTTCAACTTTTGGAAGC
ATACTTCCTTTAGCAAATTGACCATCAGAGATATGTTTTTTCATTTCATCCACAGACACTTCATCGAGACCTCTT
TGGTTTTCTTTTCCATAGTTAATGTAAACATGGTCCACAGCAGTTAATATGATTAATTGATCAGATTGTAAATGT
GCTGCTAATAAAGCACTCGTTTTATCTTTATCAATAACTGCATCAACACCTGTATAAACTTCATTTTCCTTAATTA
CTGGAATTCCACCACCGCCGGCAGCGATAACTAGTGTTCCATGAGTGATTAATGTTTCTATACTATCTAACTCA
ACTATACTTATAGGTTGTGGGGAAGGGACAACGCGACGATAGCCGCGTCCAGAATCTTCTACAAAAGTATAA
CCTTTTTCTTTCGTAAATTTATCAGCCTGCTCTTTTGTATAAAATAATCCAATTGGTTTAGTAGGGTTATTGAAA
GCAGAATCATCGCTTGCAACTTGAACTTGTGTTACTAGCGTAACAACTTGTTTATCTATGCCCATTGAATGAA
GTTCGTTTTGTAAACTTTCTTGCATCTGATAGCCAATATAAGCTTGACTCATAGCACCACATTCAGGGAAAGG
AAAAGGAGGACCTTGTTTGTGTTCCGCCGCATAATTTAAACCTAAGTTAATACTTCCAACTTGTGGACCATTA
CCATGACTAATTACAATTTCGTATCCTTTATCGATTAAACTTACTAGAGATTTAGATGTACTTTTTAATAAATCTA
ATTGTTCTTTAGGCGATTGTCCTAAAGCGTTACCACCCAAAGCTACGACGATTTTAGACATATTTATATCCTCCT
TTCATTTACTCTCCTAATGTTGCTACCATGACTGCTTTTATTGTATGTGCTCTATTTTCTGCTTCTTGGAAAACAA
CTGATTGTTCACTTTCAAATACTTCATTTGTTACTTCCATTTCAGTTAGACCATATTTTTCTTGAATTTGTTTACC
GATTATTGTTTCAGTATCATGGAATGATGGTAAGCAATGTTCAAAAAATTGTATGTGGATTACCTGTTTTTTTCAT
TAATTCTTTAGTTACACGGTATGGTTCTAATAATTTGATACGTTTTTCCCAAACTTCATCAGGTTCACCCATAGA
TACCCAAACATCTGTGTAAATTACATCAGATCCTTTGACACCTTCATCAATATCATCAGTTATAAGGATTTCACC
ACCGTTTTTGTCAGCTATATCATTACACCGATTTAATAATTCATCAGTTGGATTTAGTTCTTTAGGACATACAAG
ATGGAAAGTCATGCCCATGATTGCTGCTCCTTGCATTAAGGCATTTGCAACGTTATTACGTCCATCTCCAACAT
ATGTGAAGTTAATTTCATTATATGGTTTTTTCAATACTTCTTTAGCTGTTAAAAAAATCAGCAAGTACTTGTGTAG
GATGATCTTCATCTGTTAAACCATTCCATACGGGAACGCCAGAATATTTTGCCAAATCCTCAACTACTCTTTGT
GAGAATCCACGATATTCTATGCCATCATACATTCCACCTAAAACACGAGCAGTATCTTTGGTAGACTCTTTTTT
ACCCATTTGAGAACCTGTTGGCCCAAGGTATGTTACATGTGCACCTTGATCATAAGCCGCTGTTTCAAATGCA
CATCGAGTGCGTGTTGAATCTTTTTCAAAAAGTAGAGCGATATTTTTACCTTTCATTTTTTGTTGTTCTATTCCT
GCATATTTTGCGCGTTTAAGATCTTCAGATAAATTAAGTAAAAATTCCATTTCTTTTTGTGTGAAGTCTAACAA
AGTTAAAAAGTTTCTATTTCTTAAATTTTTCATTTTGAATATCTCCTTTTCAATTAATATTTTATTCATGTTATTTT
TTTATAATCCAATTTCTTTTATCTTGATATATAATATTTTCTATTTTTAATTCTCTGATTATTTCACCAGTAGTTTTT
CTGTTAAGTCCAGATAAATTACTTATTAATTGAACAGTCATAATTTGTTTTAATTCATAGAATTCTCCATTATCAT
CCCCAATTGAAAGGCATAAAATTTGTAGTATTCTTTCAATTCTTTCTTTAGCTGAATTAGCTCTTAACGCCATAA
TATATTCTACTTGAAATTGAATAGTCTCATTAAGTTTCTTGAAGAGACTTTCAAATATTTCATTATGCTTTCTACA
TAAATACTCAAGTAAATCTTTCGGTAAAGTTAATATTTTACAATCTGTCAAAGCTGTACATATTTCATATGGTGC
AGGGGTTTCATTAAATATGAAGTTCATTGGAAATATATTTTCGTCTTTACTTAATCTTAAATAATTGTCACCAGT
AATATTAGAAGATTCATGTAAAATACAACCATTTACTAAAAAGTATACAAATTTTATTTGATCAGTTGAATGATA
TATGACTTGTCCTTTTTTATATTGATAAAGTGTTAAATCCTCTTTATAAGGTCTAACAATACTAACAGGAATATTT

AAGTATGATGCTAATTGTTTAAGATTATTATCAAATTCATATTCTGAATTTTTAATATAAATATTTTCTTCATACAT
AACTATAAACCCCTTAAAAATTACTTAGTTTAAATCATAACTTAAAATACAGAAATATTACCTGTAATCAAACGA
ACTATTCCTATTATTGCAAGTACAATGATGAATATGAATAACGTATAATCCAATTTTGTCAAATGTTTGTTATTAT
CCCTTTGTACGTAGCTGTATACGAGTAATCCAGGTATATATAACAACATCGTTAATAGTAAATAATCTAATCCAG
CTGCATAAACCAACCAAATTGTGTAAATAGATGCAATAATTCCTATTATCCATTGTTTTAAATTAGCTTTAGATT
TATTTTGAATAGTATATTTAACCTGGTAAAAAGCACTGAGTGTATATGGAATTAAGATTGCACTTGATGCAAGT
GAAAACGCAAACTGATAGGCACTATCTGTAAACAACATACTAATTAAAAATAACTGAACTAATATATTAGTAAT
AATTAAAGCGTTGACCGGAGCTTTATTCTTATTTTCTTTAGCAAACCATTTCGGGAAAAGTCCATCTTTAGCTA
CAATGAATGGTAATTCACCAGCTAGTAATGTCCATCCTAACCAAGCTCCTAAAACAGAGATAATTAAGCCTATA
TTAACTAACACTGAACCCCAATGACCTACAATATGTTCTAATACTTGTGCCATTGATGGATTAGCAAGTTTTGA
AATTTGGTTCTGCTGAATGACACCTTGGGCTAGTACAGTCATTAAGAAATAAATGACTAGCACAGAAATCAA
ACCAATAACGGTAGCAGTTCCTACATCCTTTTTAGACTTTGCACGTCCAGAAAAGACAACGGCTCCTTCAATC
CCTGTGAATACCCATACAGTTACTAACATAGTACTTTTTACTTGTGCCATTGTATCTCCCCAACTAAAAACGCCA
ACACTTCCACTAGTCATACCATAAAAACCGGATTTAAAAGTACTGAAGTTGAATACAACTATCATGCATATAAT
AACTAGAAATATAGGTATTAATTTAGCTACTGTAACAATACTATTTATAAACGCTGCAGTTTCTACACCTCTAAG
TATTAAAAAATGTACACCCCATAATAAAATTGATGCTATGATAATACTTGGAAGTGTGTTACCTCCTTTAAATAT
AGGGAAAAAGTTACCCACAGCTGACATTAATAGGGTTGCATAAGCCACATTACCTAGAAATGCTGCAAACCA
ATATCCCCAAGCACTTGAAAAACCAATAAAATCTCCAAACCCTGTTTGAGCATAACTATAAATTCCTCCATCAA
GATCTGGTCGCTCATTTGTTAAATTTTGAAATACGAAAGCAAGAGAAATCATACCAATAGCAGTTATTATCCA
ACCGATAATTATTGCAAGTCCACCAGCTTGGCCACCCATATCTGAGATGATATTGAATGCACCACCGCCTATCA
TAGAGCCTATGACTAAACCAATTAAGGAAGTTTTACCTAATTTATTTTCATCCATATTAATCTCCCCTAATAAAG
GTGGTAAAAATCTTGTTATTTGATTTAATGAATGAATTACTTGTGACTTTTACCACCTTTCAAATTTAAATATCT
TCTCTAAATAACGGCTGACTCATACATCTTGGGCCTCCGCGTCCACGTACAAGTTCACTACCAGTAATTTCAAT
CACTTTAATTCCTTTGTCGCGTAAAAGTTGGTTTGATACATAGTTGCGATCGTATGTCACCACAACCCCTGGTC
GAATACATAATGTGTTTGAGCCATCATTCCATTGTTCACGTGCACCATCAATAACGTCGCCATTACCTGTTGGA
ATAAAGTCCACTTTTTCTACTTCTAAAACTTCAGCAAGTGTTTCACGTAACTTGCTAGAACGAGTAATTTTTAT
ATCGTCCTTACCATCATTTTGTTCTATGGTAAATATATTCATATTATTTTCTTCTTTAAATATTGCTGCATGTACTGT
AAACTTATCGTAGTCAATCATAGTTAGTACTGTATCTAGGTGCATAAATGTACGTGTATTAGGTATTTCAATAGC
TACGATTTTTTTAAAACTTGTGTTTGCATCTTTGAAAATATTACGTGCTAACTTTTCTATTGCTTGAGCTGATGT
ACGTTCTGATATACCAATAGCTAAAACATCTTTCGATAATACTAATTCATCTCCACCTTCAATATTAAATGGTGA
GTTACGATCTAACCATACTGGTACATCTTTATCTTTAAATCTTGGATGATGTTTCAGTATATATGTCATAAAAATA
GATTCTCTACGTCGTGCTCTCCAATACATTCTGTTAATTGTCATTCCTCTACCAATTGAAGCTTGGGGATCTCTT
GTAAAATAAAGGTTGGGCATTGGATCTAAGTAAAATGGATATCTATCATCCATATATTCTACTAAATGGGTTGTT
TCAAGTTGAATTTCTTCTTTACGTACGCCAGCCATGATTTTATTTACAAGTTCTTGGTCAGATAACTTTGAAAA
GAATTCTTTAATTTCAGTTTCATGACCTAATATTGTCTTTTTAGATTCTGTTAATATGTCGTTTATGAAGTTCTCG
CGTACTTCTGGCTCAGTAATAGATTCTGCTGCAAGTTTTTCTAAATAAACTACTTCGATTCCTTCATCTCTCAAA
GTTTGAGCAAATTTGTCATGCTCTTCTTGTGCAACTTTTAAGTAGGGAATATCATCGAATAATAAACCACTTAA
ATGATCAGGTACTAAATTTTCTAATTCTTTTCCTGGTCTTTTTAACAACACAGTTTTCAATTTGCCTATTTCACT
ATTTACTTGAATGGGTCCTTGTACCATTTCAAATTCCTCCTTTGCTTTATTACATTCACATTATAAAACGCTTTCA
AAAAACATCGTGTGATTTAATTCACAATTGTTTAGGGAGGGTTTTCACATATATAGATTTATTCAATGCAAAAT
TTTGTATGAATATGTATTTTGTACTATATAATAGTGAATAATTTGAAAAATCTCATTGTTAAAACCTTATCTTATTC
AAGCAAAAAGATTTTTTACTCATTTTTTTGTATTAGTAATCATTCCTATTAATATAATCATTTAATTTTTAAGGGA
ATAATTGATAACAAACAAATTCTGCTATTTCATTTGAAGAAGTCAAAATCATCAAAGTATCGTTACCTCCAATA
ATTCCTAATATTTCTTTCATTTGTAATTGATCTATGTAATAACTTATACTTTGAGCAAAGCCAGGAGATGTTTTTA

TTAAGACATAGTTATTTAGCGTTATAAATTCAATAATCTCATCACTAAATATTTCTAATTGTTTTTTTGCACTTAAT
TGATTTGTTTGATTTATTTTCTTGTAAATATACTTTTTATTTTCAACAGGGATTTTGTAAATTTCTAATTCTTGTA
AGTCACGAGAAATAGTTGTCAAGCTATAGTAAACTCCAAAATGTCTTGCCATGTAATCCACTATTTGTTGTTTT
TTATTAAACTGATTCTGTTGTATAACAGTTAAGATAAGATTTAAACGTTTTTCTTTTTTCATTTTTATTACCCCCT
TTATTATTTTTGATTATGAAATAGATTTTAAATAACTGTATAAAAATCACTTATAAATCTCAATTTACAAATAATTT
TGAAATAGTGGATGCTTTAATTATAAGTAGATTATTTTATGCCTAAAAGGAGAGATTAATTAGAATAGATTATA
GTCATCATAATTATAAAATCAAAATAATTTGCGTAATAATAGCGAAATGTGTAAATTGTAAATGTCAGGTAATAA
CCACAATATCTCATAAAAGAAGTTTGGAAAAAGGCATTGAAATAAAAAGTAGTTTATAAGTTGAAGATATTTA
GGTATGTATTAGAGCGCTTTATAAATAAATAGGTTTATACAAAGAATTACAAAAATAATATAAAAAATAGAATCT
AATACATGATAAGGAATATATTTGAATAATTTGAATATGAGAATAAGCATACATATTTAGGGTATATATAAGTAAT
AGACATCTAAATAATAAGTAATAGGAGGCTGGTGTAGATGTTCAAAAATATATTATTACCCTATGATTTCGAAA
ATGATTTTAGTGCTATCCCTGACTATTTAGAAAAAGTCACCGATGAAGATTCAGTTGTTGTAATTTATCACGTT
GTAACAGAAAATGATCTTGCAATTAGTGTCAAGTATTATAATAAGCATAAAGAAGATATTATTAGAGAAAAAG
AGAAAAAACTCACTCCATTTTTACGTGAATTAGAAAAAAGAGATATTCAATATAAAATAGATGTAGATTTTGG
GCATATTAAAGATACAATCTTAGAAAAAATTACTTCTGGAGATATAAATAATGGTGAATTTGATTTAGTAATTAT
GAGTAATCATAGAGTCGATTTGAATATTAAACATGTTTTAGGAGATGTTACACATAAGATTGCTAAAAGAAGT
TCTGTCCCAGTACTAATTGTTAAATAAACATAAGAAGTAAGAAATTTATTAATTCAAAAAGCCTAAATACTTTC
TCACAAATCGAGAGAGTATTTAGGCTTTTTTATTTTTTTAATAAACGTAATGAATTTAATATAACTAATATTGCT
GCACCTGTATCACTTAGAACAGCTAACCAAAGTGTTAGTAATCCAGGGAATACTAACACAAAGGCAATTAATT
TAATAATTATAGCAAAATATAGGTTCTGTTGCAAAGTTGAATCTATAGTATAATTTTAACAAAAAGGAGTCTTCT
GTATGAACTATTTCAGATATAAACAATTTAACAAGGATGTTATCACTGTAGCCGTTGGCTACTATCTAAGATATG
CATTGAGTTATCGTGATATATCTGAAATATTAAGGGAACGTGGTGTAAACGTTCATCATTCAACGGTCTACCGT
TGGGTTCAAGAATATGCCCCAATTTTATATCAAATTTGGAAGAAAAAGCATAAAAAGCTAATTACAAATGGCG
TATTGATGAGACGTACATCAAAATAAAAGGAAAATGGAGCTATTTATATCGTGCCATTGATGCAGAGGGATAT
ACATTAGATATTTGGTTGCGTAAGCAACGAGATAATCATTCAGCATATGCGTTTATCAAACGTCTCATTAAACA
ATTTGGTAAACCTCAAAAGGTAATTACAGATCAGGCACCTTCAACGAAGGTAGCAATGGCTAAAGTAATTAA
AGCTTTTAAACTTAAACCTGACTGTYATTGTACATCGAAATATCTGAATAACCTCATTGAGCAAGATCACYGTC
ATATTAAARTAAGAAAGACAAGGTATCAAARTATCAATACAGCAAAGAATACTTTAAAAGGTATTGAATGTAT
TTACGCTCTATATAAAAAGAACCGCAGGTCTCTTCAGATCTACGGATTTTCGCCATGCCACGAAATTAGCATC
ATGCTAGCAAGTTAAGCGAACACTGACATGATAAATTAGTGGTTAGCTATATTTTTTTACTTTGCAACAGAAC
CTATAAAAAACATGGGCTTAAAGGGTTAATTTATATAGTATTACTGCAAACATTGATTTGGGTCAAAAATTAGG
GGTATTAAAAAAATGAATGAATCATAATTTTATCAAGCTGATTGGAGAGGTTAAAATGCATTATATAAAATTTA
TTGAGTCAAAAGATAATACAAAACTTTATATGAAAGTGAATGATATTCAAGATGCAAAAGCGAATATCATTATA
GCTCATGGTGTGGCAGAACATTTAGATCGTTATGATGAGATAACAGCATATTTAAATGAAGCGGGTTTTAGTG
TTATTAGATATGATCAAAGAGGGCATGGTCGTTCTGAAGGCAAGCGTGCCTTTTATAGCAATAGTAATGAAAT
TGTCGAAGATTTAGATGCGATAATAAATTATGTGAAGTCAAACTTTGAAGGTAAAGTTTACTTAATCGGTCATA
GTATGGGTGGTTATACAGTCACTTTATATGGAACGAAACATCCAAATACAGTGAATGGTATTATAACTTCTGGA
GCATTAACACGTTATAATAATAAACTATTTGGCAATCCTGATAGAAACATATCACCTGATACTTATATAGAAAAC
AATTTAAGTGAGGGGGTATGTTCTGATTTAGAGGTAATGGAAAAATATAAACTTGATGATTTGAATGCGAAA
CAAATCTCTATGGGGCTCGTCTTTTCAATAATGGATGGTGTTAGGTATTTGAAAGACAATGCTCAACAATTTAC
AGATAATATTTTGATATTGCATGGCAAGGAAGATGGGCTAGTAAGCTATGTAGATTCTTTACAGCTTTATCAAG
AAATAGGATCAGCACATAAATCATTACACATCTATGATCGTTTGGAGCATGAAATATTTAATGAAAGTTCTTATA
ATAGAACTATTTTTAACGAAGTTATTGAATGGCTTGAAACGGAATTAACTTATAACTAAAACAGTATAGTTCCG
TGTATTTGATTATAAGAAATTATGAGGATATTAAACATACTAAGATTAGCTATGAAGAAATCTATGACGATAGAT

TTTTTCATAGCTATTTTTTATAGTTATAGAGAGGAGTAGACTGTCCAGACTCTTGGATTTTAAATCCGTAAAAA
AAACAAGTCAGCTTTACTCTCACCTTTTGAAATTCGTTTGTAGTATGTTGGGTTCTTGAAACCGTGTATAGGA
AAATGAAATGAGAAAGGTTAAGTAAAGTTTTTAGCTTCTCAACTATTCAAAGGAGGTTTTTTTATCGATTACT
TAGGTGTTGATATTAGTAAAAGGAGTAGTGTAGTTGCACATTATAAAAATGGAAAATTCCAAAAAGAGTTTTT
CATCCAAAATAATAAAAATGGCTACAATTATTTACTCAAGTATTTGAATGACTTAGACCACCCACAACTCATTT
TTGAATCTACAGGTATCTATTCAAGAGGTATGGAACGATTTTGTTGTGTAAATCAAATTAACTATATTCAAATG
AATCCGTTAGAAGCCAAATTTAAAACGAGCGCTCTAAGATCATGGAAAACTGATCAGGCAGATGCTCATAAG
CTTGCTTGTTTAGGACCGACGCTCAAACAAACAGGCAGCTTACCTATACATGAGTTAATATTCTTTGAATTAA
GAGAACGTGCCCGTTTTCATCTAGAAATCGAGAATGAACAAAATCGACTTAAATTTCAGATTCTTGAATTACT
CCATCAAACATTCCCTGGTTTAGAAAGATTATTTAGTAGTCGATATTCAATCATTGCACTCAACATCGCAGAAA
TTTTTACTCATCCAGACGTGGTTCTTGATATCGACAAGGATGTACTTATTACACATATATTCAATTCTACAGATA
AGGGAATGTCAATGGATAAAGCTACAAAATATGCACTTCAATTAAGAGTGATTGCTCAAGAAAGCTATCCTAA
TGTCGATAGACATTCCTTTCTAGTCGAAAAATTACGCTTACTTATTCAACAATTAAAACAATCTATTCATCATCT
CAAACAATTAGATGATGCCATGATTCAATTAGCACAACAACTCGATTATTTTGAAAATATTCATTCGATACCTG
GTATTGGTAAGCTAAGCACAGCTATGATTATTGGGGAGATTGGTGATATTAAGCGATTTAAATCAAATAAACA
ACTCAACGCTTTTGTAGGCATTGATATCAAACGATATCAATCAGGTCATACACACTGTAGAGATACCATCAACA
AGCGTGGTAATAAAAAAGCGAGAAAACTTTTATTTTGGGTGATTATGAATATAATAAGAGGGCAGCATCATTA
TGACAATCATGTCGTCGATTATTACTACAAACTAAGAAAGCAGCCTAATGAGAAACCTCATAAGACTGCCATC
ATTGCTTGTATAAATCGATTATTAAAAACGATTCATTATCTGGTAATGAATCATAAATTGTACGATTATCAAATGT
CACCACATTAGCCAAACGTACAATTAAATATATTTTAATACCTTATTCAAAAAATTAAAATGAACGGTTTAGTTA
AGTAATGCTTATTTTAATTATAAGTACTTGACTAATCGTAAGAAAGAGCCTAGGACATAAATCAATGTCTCGCG
ACCACAGCTTAATTTTTGGTGTTCATTCATGACTTTAAAAAATCCTTATTGCATAAATGTACATAGTGTAGTACT
ATTCAAAACGTAATTATTACGATTTGAATTAAGCGAGGAGAATGAAATGACTAAGACTTATGACGTTTGGTGG
CAAAAAGGTCAAGAATCAGATGATGATATGGCACGAGACCATCAAGAAGCTTGGGAGAGAACAATAAAAAT
GCTTGATACATCTGACATCGAAGGGAAAACGATTTTAGATGTGGGATGTAATCAAGGCGGATTTTTACGACA
GTTATACGATACAACACCGTTTAAAAAAGGTGTTGGCATAGATTTAGCACGTTTATCTTTGGAAAAGGCAGA
GACATTAAAAGGACAACGTCCACTTACATACTATTTAACAGATAAACCGCAAGAAACGAAGCACGTGTTTGA
TACGGCAGTAAGTACGTCTGTCTTGTACTTAATAGAAGATATTCCGCAACATGCAAAAGATTTAAAAGAGGTA
TTGAAACCAGGCGGTGTTTATTACGCTTCATTCGCGGATTTAACTAATAACCCAAGTCGTCAGTTTATGGATG
ACACGATTAATCAATATGGTGCAACACCTTCTCAGAATCACTCTCTAAAACATATCGTTGATAGCTTTGTGGAT
GCAGGATTTGAAGTTGCAGTAATGAAAGAGCATGTACCTGACGTGATTGATTTAACACATTATAGCGATTTTT
ATTTATCACCGAATGATTATTTACAAACACTATATGAAGAATCGTTTTTAATAAAAGCAAGTGTGAAAGAAGG
TACTGAGAAATGAGGAAATGTGTATTAATGACGGTAGCAGCAAGTGCTACGCTCTTATTGGCAGGTTGTGGC
AATGGTCAAAAAGAAGATAAAGATGTTACGGTATCGCTACCTACTGAAGCAAAGGCGGATAAACTTGACGC
GCAAGGCTATGATGCAGCGATGCCCGTTTATAGTGCAGTGTATGATGCATTAGTTAAATATGATAAAGATAAG
GGTATTAAAGCAGGTTTAGCAGATAAATGGAGCGTTGATGAATCAGGGAAAGTTTATGAATTCCATTTGAAA
AAGAATGTTAAATTCTCAGATGGTTCAGCATTAGATGCTAAGGACGTGAAATTCTCGATTGATCGTGCGAAA
GCGATGAACAAAGATTCGACTGTAGAAACGTTAAAAAAATTAGATAAGGTCGTTGTTAAAAATGAGCACGT
GGTCCAAATTAGATTGAAATCTCCTTCAAATCAAGTGTTAAATGAATTAACACAAGTGAGACCGTTGCGTATT
ATGAGTCCACATTCAGTAGAAGATGGTAAAGTAAACGGTAAATTTGAAAAAGCGATTGGAACAGGTGCATT
TGTTGTTGATAAAACTGGTAAAGAAAAAACGACAATGAAGCCAAATAAATATTTTGACAACGGTCACCCAGT
CAATTATCATCTTGCATTCCAAACGATTGAAGATGGGGACTCAAGAAATTCTGCAGTACAAAGTGGTTCTGTA
GATATTTCTGGTGGTGCTTTAGGTATGCTCTCAGACGAACAAATCAAACAAGATAAGAAAAATAAGAACTTA
ACGATTGAAGATAGACCTAGCACAGTAAGTCACTTTATGGCATTTAACCCTAAAAATGATGTATTAAATCAAC

GCACAATTCGTGAAGCGATAAGTAAGAGCATCGATGCGAAAGACATTGCGGGCAAATCTGTAAATGGTCTG
TTCCAGAAGAACGTACAATTTGTGACTAAAAATAATCAACAGCCACACGATTATGATATGAAAGCGGCTGAA
AGGTTACTTAAATCAGAAGGATATCATAAAAACGATGACGGCATCTTTGAAAAGAATGGCAAACCTTTATCAT
TTAACTTAGTCATTCAAACTGCAGAGTTCCCAAATTGGAAAGATAAAGCTGAAAAAGTGCAACGTCAGCTTA
AACAAGCCGGTATTAAGTTAAATGTGAAAACGTTAGATTCACAATCATACTATGATACATTATGGACGAAAAA
AGACTATGATTTGATTTTCTATAGAACGTATTCAGATGCATTAATGCCTTACAACTTTATAAGTTCAGTGTTTAA
AAATAATGATGGTCAACCAGGGGTGTTAGCTGATGATGAAACATTAACGAAACAGCTAGACGATTTCCCATC
AACCGTATCAAAAGAAGACCAACAGTGTTCATTTGATGACATATTTAAACACTTTAATCAACAATACTATGGTG
TGCCAATTGCTTATCCAAATGAGACGTTTGTAGTGAGTGATAAAGTAAAACAATTCAAATTCTCTGGACTTAC
GGATGCACCAATTGATTATAAAGCGTTGAAAGTTAATGAATAGCAATGCTCAAACGTACAATTAAATTCATACT
TTATTTAATCGTAAGTTCGTTTATTATCTTCATTTTAGTTGAGAAGACATCTGGTAATCCAGCGATTCTGTATCTA
CAACGTCATGGTTATACGTCGATTACGCAAGACAATATTGAAGCGGCACAACATCAACTTGGCTTAGGACAA
CATGTGTTACTAAGATATATCGATTGGGTTGGACATGCACTCACGGGCAACTTAGGATACGGCTTTAGTACGA
ACGAAGCAGTTACCGCTATGATAATGGAAGCCATCGTGCCGACGCTTGTGCTAATCATTGTCTCTAGTTGTAT
CATGTTGCCATTTGGCTATATTGTTGGTTACTTCGTTGGGACGCGTCCGCATACACGTTACGCTAATGGAATTC
GTGGATTCGCCCAAGTGATGACCTCAATGCCAGAATACTGGTTAGCTATTTTATTCATTTATTATTTAGGCGTA
CGTTGGCAATTGTTACCATTTGTAGGTAGTGATTCATGGCAACACTTTGTGCTGCCAATCTTCACAATTGTTGT
TATAGAAGGGTGTCATATCTTATTGATGACAGCACATCTGATTACACAAACGTTAGATCAAGATGCGTATCAAC
TGGCGCAGTTAAGACATTTTTCGTTAAAAGCGCGTATCATCGTACAAATTAAAGAGATATTTGCACCACTAAT
GACGATTTCAATTAACAGTATCATTCATTTAATTGGAAAAGCCGTAATACTAGAAGTCATCTTCAGCATGTCTG
GTATAGGTAAATTGTTGATTAATGCTATTAACCAACGAGATTATCCACTGATTCAGGGCATTGTCATCTTTATCA
TTGTCTTTATTATGCTAATGAATTATTTAGGCGATGTGATTATTTTGAAGAATGAACCTAGACTTCGACGACGT
CATACCCAGCAGTCAGGCAATGAGAAAAGAGGTACGATGTGATGAAAAAATATCAAACGTACATCGCAATAG
GTTCACTATTGAGTTTGATGGTTGTATTAATTACGTATGGTTTAATGCAAGACACGCAACATTTGAACCCACTT
GAGTCACCTAATGGACAACATTGGTTGGGTACCGATCAATTAGGCAGAGACTTCTTAGTAAGACTGATTGTC
GGTAGTCTTGTCACATTGAGTTTAACAGGCATAGTGATTCTATTAAGCGTTTGTATGGGACTTATCTTTGGCTT
AATTGCAGGCATAGAAAGACGATGGTTAGATCAAATCATCATGTTTGTTGCCGATATGTTGCTGGCTATTCCG
TCATTTATTATCGCATTAGTCATCTTAAGTTTAGTAAGTAACTCCATGATAGGTTTGATACTTGCTTTAACGATT
GGATGGATAGGACGTTATTTACGTTACTTCAGAAATTTAACGCGAGATATTCAAAAACGTCCATTTGTTCAAT
ATGCACGATTGAGTGGGAACTCAACATTCAAAACGACAGTAACACATGTGATTCCACATTTATTAAGTAGTAT
ATTCGCTTTGGTAACGGCTGACTTTGGCAAAATGATGCTCAGCATATCTGGACTTGCTTTTCTAGGACTAGGT
ATTAAACCGCCGACGCCTGAGTTAGGAACAATTCTTTTTGATGGGAAAAGTTATTTCAACGGCGCACCGTGG
CTCTTCTTCTTCCCTGGTGTATTGTTAGGAGGTTTCGCCTTATTATGTCAAATTATCAACAAAAAAATAACGCA
GTAAATACGGTAGTCAACGTCAATCAATTATCGATTTTAGATCAAGAGAAAGTATTGTTAAAGGATGTTGATT
TGACAGTAACTAAAGGTGCATTTCATTGCATTATAGGTGAAAGTGGCAGTGGGAAATCACTGTTAACAAGAA
CAATACTTGGAATGAAACAATCACAATTATGTTATCAAGGAGATATTGACATCGATTTAACTCAAACAGATGC
AGTGTTTCAAGATGTTCAAAGTAATATGTTTCAAAATATAACATTAGCTAAGCATTTCCAATACATTTATGAAGC
CAATCGCACACATCTCACTAAACAGCGTATTAAGGAAGATGTCTTACAGATGATGCAATTACTTGGTTTAAGA
CAAGGGGAACAATTGCTTGAGCGTTATCCCTTCGAACTTAGTGGAGGTATGGCACAACGTGTCGCCTTTATA
ATGTCATTAATTAGACGTCCGAACTACTTATTTTTAGATGAACCAACGAGTGCACTTGATCAAGAAAATATTAA
AAAGTTTATGCATTACCTTCTTAGGGCACAGGAGCGCTACCAAATGACCATTGTTTTTATCACACATGATATTA
ACTTAGTGAAAGATTGTGCCACACATATTAGTATTATGCAGCAAGGTAAATTGATAGAAAATGGTGAGGCCG
CGTCGATCTTAACTAAGCCGACACATAATTACACGAAAAAATTAATTACGATTGCACATCGGAGACAACCTTA
TGCTTAAAATAGAGAGATTAACCAAATATATAGACACGCAACTGATATTTAAAGAGATATCATGTACAATTAAC

GACCAGCACTTACTCATAAGTGGGGAGAGTGGTTGTGGTAAATCCACATTAGCCAAGATTATCGCTGGCTTA
GATACGGATTATCAGGGCGAATTATATCTTAATGGGCGCTTACGTGAATCTTATACGTCTAAAGAGTGGATGA
AGCACATCCAATATGTACCTCAATATCAACGTGATACTTTAAATCAGCGTAAAACGGTATTAGCTACATTATTAG
AACCACTTAAGAATTATAAGGTAAATAAACAGCGTTATACATCAAGCATTGAAGCAGTGCTTGATCAGTGTAA
TTTACCACACGATATACTTAATCATAAAGTTTCGACATTAAGTGGTGGCCAATTTCAACGCGTCTGGATAGCTA
AAGCTTTAATATTAGAACCAGAGATTCTCATATTGGATGAAGCTACAACCAACTTAGATGTCATTAATGAAGA
AGCTATACTTCAAATGTTGATTTCCTTAAAGATGACACAATTAATCATTATTTCACATGATACATACGTCTTAAG
CCAATTTGAAGGAATTCAGTTACAGCTAAATAAATTGAATAATTAAGATCACAAATCTTAATATGGTGAATATT
TAATGGTACCTAAAAAATAAAATTTAAACTACAATGTCTAAATCCATATGTTGTTTCATTAGAGGATTTAAAAAT
GATTATAACACTAAAAGATTTCAAATTATTATATTTAATATAAATTTACATATGATAAACGAATAACAATTCCAAT
ATAAATTATTTTTTGATTATTTTTATTTATAACTATATATTTTATATGAAAAATATAAGTTATAATAATAAGTTTAATA
TTGCCTCGTGGTTCTGAGCTTGAACTTATCTCTAAATCATTTTGAGCTACTTATCTATCAATTCATATATTCTATA
ACAATATTTGTGACATCACGTGCTATTTCATGAAGTGATTTTACGATATCACCTTCTTTAGAAAAAATATATTTC
AGTAGGCACCGACGTATACAGAATCATTTGAGTATTAAAAATAAAAACTAGAAAAAGAAACCGCATCATTAA
CTGATACGCAGAATCATATTATAAATAAAACTAAAAATGAGGTTGTATATAACTCACTCTGAAATTGATTGAATA
TATAGTATCTTTAATAAAATGCAGCTATTGTGGCGTAGAATTTGAGAATCAAAAAATGATTAATATAGTTTGAA
GAGACTGAGCATAAATACTAGAAAAATGGCCAGTAAATGAGTTTACTATAAACTCATTTACTGGCTTCTCTAAT
AATTATCAAGACAATTTGCGTTTCTAGGCATACTTTGAAATGCGCTATTTTCTTTAAGAATATTAATATATGACT
TGTGTTGGTAATGATAACCTTGGTTAGAGTGTATTGTCATGTGATAGTTAAGATTAGATCTTTTAATTAAAACAT
TGTTTTAAATATCGATGACAAGGTCTAATGTAGGACGTGTAGATATAAGGTTCGGAATTATATAAATCCATAAA
AGGAGATAAATCGAAAACCTTTATACCCTGGGTGGGTATATGTTATAGTATAAGTAGCTTTACTATAACATTTTC
ATTAGGAGGGGTTAATTTGAATAATAATGGTGAAGAGCATAATCATCAAAATCACATGAATCATTCCAATCAA
ATGCATCATGATAACCATGCCTCACATGATCATCATAGTGGCCATGCACATCATCATGGAAATTTTAAAGTTAA
GTTTTTTGTTTCATTAATTTTTGCAATACCTATCATTCTTTTATCGCCACTGATGGGTGTTAACTTACCTTTTCAA
TTCACATTTCCAGGTTCTGAATGGGTAGTGTTAATATTAAGTACAATTTTATTCTTTTATGGTGGTAAACCGTTC
TTGTCTGGTGGTAAAGATGAAATTGCTACAAAAAAACCAGGCATGATGACCTTAGTTGCCCTAGGTATTTCA
GTAGCTTATATTTATAGCTTGTATGCTTTTTATATGAATAACTTTAGTAGTGCAACTGGTCATACAATGGACTTTT
TTTGGGAATTAGCAACCTTAATTTTAATTATGCTATTAGGACATTGGATAGAAATGAATGCTGTCGGAAATGCT
GGAGATGCTTTAAAGAAAATGGCAGAACTGTTACCTAATAGTGCTATTAAAGTTATGGATAATGGCCAACGC
GAAGAAGTTAAAATATCAGACATCATGACTGATGATATCGTCGAAGTAAAAGCCGGAGAAAGCATTCCAACA
GATGGTATTATCGTTCAAGGACAAACATCTATAGATGAATCCCTAGTCACTGGAGAATCTAAAAAAGTACAAA
AAAATCAAAATGACAACGTCATCGGGGGTTCTATTAATGGGTCTGGAACAATACAAGTCAAGGTTACAGCTG
TGGGAGAAGATGGATATCTTTCTCAAGTTATGGGACTTGTTAATCAAGCACAAAATGATAAATCTAGTGCTGA
ATTGTTATCTGATAAAGTAGCGGGTTATTTATTCTACTTTGCTGTAAGTGTTGGCGTGATTTCTTTTATTGTCTG
GATGCTCATTCAAAATGATGTTGATTTTGCATTAGAACGTCTTGTAACTGTGTTAGTCATTGCTTGTCCACATG
CTTTAGGCTTGGCAATACCTTTAGTCACTGCACGTTCTACTTCAATTGGTGCACATAATGGTTTAATTATTAAA
AATAGAGAGTCTGTAGAAATAGCTCAACATATCGATTATGTAATGATGGACAAAACTGGTACTTTAACTGAGG
GTAACTTTTCTGTGAATCATTATGAGAGCTTTAAAAATGATTTGAGTAATGATACAATATTAAGCCTTTTCGCC
TCATTAGAAAGTCAATCTAATCACCCATTAGCTATAAGTATTGTTGATTTTGCGAAAAGTAAAAATGTTTCATTT
ACTAACCCACAAGACGTTAATAATATTCCAGGTGTCGGATTAGAAGGTCTAATTGATAATAAAACATATAAAAT
AACAAATGTCTCTTATCTTGATAAACATAAACTTAATTATGACGATGACTTGTTTACTAAATTAGCTCAACAAG
GTAATTCAATCAGTTATTTAATTGAGGATCAACAAGTCATTGGCATGATTGCTCAAGGAGATCAAATTAAAGA
AAGCTCAAAACAAATGGTAGCTGATTTACTATCAAGAAATATTACACCAGTCATGCTTACAGGTGACAATAAT
GAAGTGGCACACGCTGTCGCAAAAGAATTAGGTATTAGTGATGTCCACGCACAACTCATGCCAGAAGATAA

GGAAAGCATTATAAAAGATTATCAAAGTGACGGTAATAAAGTCATGATGGTCGGAGACGGTATCAACGATGC
GCCGAGTCTTATAAGAGCGGATATTGGTATAGCAATTGGTGCAGGTACAGATGTTGCAGTGGATTCAGGTGA
TATCATACTTGTTAAAAGTAATCCATCAGATATCATTCATTTCTTGACCCTTTCAAATAATACTATGAGAAAAATG
GTGCAAAACTTATGGTGGGGTGCAGGTTATAATATTGTTGCTGTACCTTTAGCAGCTGGTATTTTAGCATTTAT
TGGCTTGATTTTATCACCTGCAATAGGTGCTATTTTAATGTCTTTAAGTACAATTATCGTTGCAATTAATGCCTT
TACATTAAAATTAAAATAAAAGATAGGAGTTTTATTATGATTAAAAAATTATTTTTTATGATATTAGGATCATTAC
TAATATTATCAGCTTGCTCCAATAATGATGAAAAAGATAAAGACACTAATGACCAAAAAAGTGAGAGCCATAT
GAAGCATAATGATGAAAGTAAAGTTCCAGAAGATATGAAATCGACTAATGAGGGTGAATTTAAAGTGGGAG
ATAAAGTAACGATTACAGCAGGGCATATGCCAGGTATGAAAGGTGCAGAAGCTACTGTAAAAGGTGCGTATA
AAACATATGCCTATGTTGTAAGTTATAAACCCACAAATGGAAATGAAAAAGTAAACAATCATAAATGGGTCGT
AAACGAAGAGATTAAAGATGCACCTAAAGATGGATTTAGTAAGGGCGATACTGTTAAATTAGAAGCAAGTC
ATATGTCTGGTATGAAAGGTGCTACAGCCAATATAGATAACGTGAAAAAGACTACTGTTTACGTAGTTGATTA
CAAATCCAAAGATAATGGTAAAATCATTAAAAATCATAAATGGATGACAGGAAATGAGCTGAAAGCACGATA
AAAATCTAGTTCTAAATTGAGAAATAAATAGATATAAAAATATCCTCCTTAATCAATAATTTAAATAACTTATTAT
TGTTAAGGAGGATATTTTTTAGTGTGTAAATTAAAAAGAATTTTAGAAGAATAACATTTATCAAAAAACTGTT
CATTACCTTATTAATTGAAATTATATAATTAAAAACCGCATCATTAACCGATACGCAGAGGCGTATCATAAGT
>Staphylococcus aureus strain BSN14R1
ATGAAAATCACCATTTTAGCTGTAGGGAAACTAAAAGAGAAATATTGGAAGCAAGCCATAGCAGAATATGAA
AAACGTTTAGGCCCATACACCAAGATAGACATCATAGAAGTTCCAGACGAAAAAGCACCAGAAAATATGAGT
GACAAAGAAATTGAGCAAGTAAAAGAAAAAGAAGGCCAACGAATACTAGCCAAAATCAAACCACAATCCA
CAGTCATTACATTAGAAATACAAGGAAAGATGCTATCTTCCGAAGGATTGGCCCAAGAATTGAACCAACGCA
TGACCCAAGGGCAAAGCGACTTTGTTTTCGTCATTGGCGGATCAAACGGCCTGCACAAGGACGTCTTACAA
CGCAGTAACTACGCACTATCATTCAGCAAAATGACATTCCCACATCAAATGATGCGGGTTGTGTTAATTGAAC
AAGTGTACAGAGCATTTAAGATTATGCGAGGAGAAGCATATCATAAGTGATGCGGTTTTTATTAATTAGTTGC
TAAAAAATGAAGTATGCAATATTAATTATTATTAAATTTTGATATATTTAAAGAAAGATTAAGTTTAGGGTGAAT
GAATGGCTTATCAAAGTGAATATGCATTAGAAAATGAAGTACTTCAACAACTTGAGGAATTGAACTATGAAA
GAGTAAATATACATAATATTAAATTAGAAATTAATGAATATCTCAAAGAACTAGGAGTGTTGAAAAATGAATAA
GCAGACAAATACTCCAGAACTAAGATTTCCAGAGTTTGATGAGGAATGGAAAAAAAGGAAATTAGGTGAA
GTAGTAAATTATAAAAATGGTGGTTCATTTGAAAGTTTAGTGAAAAACCATGGTGTATATAAACTCATAACTCT
TAAATCTGTTAATACAGAAGGAAAGTTGTGTAATTCTGGAAAATATATCGATGATAAATGTGTTGAAACATTGT
GTAATGATACTTTAGTAATGATACTGAGCGAGCAAGCACCAGGACTAGTTGGAATGACTGCAATTATACCTAA
TAATAATGAGTATGTACTAAATCAACGAGTAGCAGCACTAGTGCCTAAACAATTTATAGATAGTCAATTTCTATC
TAAGTTAATTAATAGAAACCAGAAATATTTCAGTGTGAGATCTGCTGGAACAAAAGTGAAAAATATTTCTAAA
GGACATGTAGAAAACTTTAATTTTTTATCTCCTAATTACACTGAACAACAAAAAATAGGTAATTTCTTCAGCAA
ACTCGACCGCCAGATTGAGTTAGAAGAAGAGAAACTTGAACTCTTAGAGCAACAAAAGCGTGGATATATTC
AGAAGATTTTTTCTCAAGATTTAAGATTTAAAGATGAAAATGGAAACAGTTATCCTGATTGGTCTATTAAAAA
GATTGAAGATATTTCTAAAGTTAATAAAGGGTTTACTCCAAATACAAAAAATGATAAATACTGGGATGAATTA
AATGAAAATTGGTTATCTATAGCAGGTATGACACAGAAATATTTGTATAAAGGAAATAAAGGAATTACTGAAA
AAGGTGCATCAAAGCATGTAAAAGTAGATAAAGATACTCTAATAATGAGTTTTAAATTGACTTTAGGTAAGTT
AGCTATAGTAAAAGAGCCTATCTATACAAATGAAGCTATATGCCATTTCGTATGGAAAGAAAGTAATGTTAATA
CTGAGTATATGTACTACTATTTAAATTCTATAAATATAAGTACTTTTGGTGCACAGGCAGTTAAAGGAGTAACAT
TAAATAACGATGCAATTAATAGTATTATAGTAAAGTTACCAGTGATACAAGAACAAAATAAAATAGCATACTTT
TTCAATAAATTAGATAAATTAATTGAAAAACAATCTTCTAAAGTAGAATTATTAAAACAACGCAAACAAGGATT
TTTACAGAAAATGTTTGTTTAATTCTTATAAAGTTCTATTATGTAAAATATTAAATAGAGATAACATTATGAAAG

CGAGCCCAAGACATAAAGTTTTTGAATAAATAAAAAAGATAATTTCTATCAAATTAATATAGAAATTGTCTTTT
TTATAAATTTTTTGATTATTTTTAGCTGATTGAGCTGTTACTTTTCTTATAATAAGTGCTATTAGCACAAATCCTA
GTTCTCTTTTGGCTTTGTTTATTCCTCTTACGGACATTCGAGTGAAACCCATTTTAATTTTATTAGAAGTAATTT
AGGTTTGAACCCACCTAAATAAATATATGAGTTATTTTTTTATGCTACAAAATATATTCAGATTTCAATAATGAC
ATAAAATAGGCATCTTTATATTTACCTTTAGTGTAGAATTGCTCTTTGAGTAATCCTTCTGTTTTAAATCCTTGTG
ACTCGTATATATGCACAGCTTTTTTGTTATCTGTATCAACATATAGATAAATTTTGTGCATGTTTAATATATCGAAT
GCATAATTTATCGCTTTTTCGAATGCGAATTTTGCATAACCTTTACCACTGAACTCAGGTTTAATAATTATTTGT
ATTTCACAATTACGATGGATGTAATTAATTTCTACTAATTCAACAATACCTACGACTTGATTTTCATCTTCAACA
ATAAAACGTCTCTCTGATTCATCTAATAAATGCTTATCAAATAAATATTGAAGTTCCGTTAAGGATTCATATGGT
TCTTCAAACCAATAAGACATAATAGAATATTCATTATTTAATTCATGAACAAAAAGTAAATCACTATACTCTAAT
GCTCTTAGTTTCATAATTCCACTCCCAAAATTTTCTCATATATTTGCATTATAAATATAAATAACGAATAAGTCAT
CATTCACTGTGAATACTCTATTTTAACAATTCACCACATACTAATTCTCATTTTCTTGTTATTCTCGATTTATTACT
CTTACTATGAAACCTATAAAATTCTCACATTTGTTTGTATTAAGAATAAATACGTCGATAGTAACAATAAAAAAA
TAAATAATAAAGCATCCCTCACCGTAAAAGTGAAGGATGCTCTAGTTTTATTGAAATATACATTTCATTTTGTTA
AATAATTATTAATAATATTTTGAAAATCATTATTACGTGAAATCTTCATAGATTTTATCAAGTATTTCTTTGCCTTC
AATTGCTGTGAAGTGATGTACCAATCTATTTTTACAATCATATGTAATTTTGTGACGCTAGGTAATTAGTAATTG
TTCGTCAGTCTGATTGTATAGTATCAAGTTTCATAGATAATACTCTTTGATTTTAATGTCCACTTTGACGTGCTT
TAAGATTGAGTATATACATAATGTCATTGTGGAATGTTAAAAATCCTACAAATGTTTATTCATCTGCAGGATTTT
TAAATCTCCAAGAATAAAAATCATCATAGGACAACTGGATTATTGTTTGGATAAATAACGTAAACAATAATTAG
GTACTATTATTTATTTTTGTTTATTCTTTTTCCTAACAAAATAAAGAAAAGAATAAACGCAATTGTTAAAAATAT
GTGTCCTAAACCAGCAATACCAGCAATAGCAGGACTTACACTTAGATCTTTAATGGTAGAAATACCGTTCACG
AATTGCATTGCCACAGTAACAAGCACACCTAAATGGTATATATAAAAGAAACTGTTAAACAGCTTTGTATGAG
TTGTTAATTTGAATTGGCCCTCGATAATCATGAAAATTAAGAACATAATTGTACCTAGTACTAATAAATGTGTAT
GTGTAACATTTAATTGAGAAAAACCGCTAAAATCTTCCGCTTTTGTCATTTCTCTATAAAATAGACCACTTAATA
ACCCTAATAGTGTATAGAGCGCTGAACTATACATTAATCTTTTCATTTTAATTCCCCCTATTTTTAATTACGAGAT
AAGTATAGCGGTAGTTTATGAACTGAGTATGAACTTACAACAAAAAAATTAATGAAGTACTTTACAATAAACT
CAATTTATTAGATGGTGGAGGGACGAAAAAGGATTTTAGAAAAATAAATTAATATATTTTTATTTTGATAAGTA
ATAATTAATAATATCTTGGAAATCATTGTTAAGTATTGTTGTAATACAATCGTCATTCATAAAATCTTCATAGATT
TTATCAAGAATTTCTTCATCTTCGATAGATGTGAAATGATTAGCTAACCCTTTTATAATTTAAGTGTAATTTGTG
AATCTAAACAACTAGCAACTGTTCGCCAATCTGATTGTGTAGTGTCATGGTTCATAGATAATCCTCCCTTTATTT
TAATGTCCATTTTTGACGTGCTTTAGGGTTAAGTGGATGCATAACTTCGTTCGTTACTGGATTAATGAGTTTTT
TGACTTTCTTTTTATGAGGTTTTAACATTTCCATCACTTGCTCAACACGTTCGACGACAACAGGCCGCTTCGC
ATAAGCTCCATAGGCTAGAATCACTGTGTCACTTTCACTAATCGCTTTCATTAAGTGAATGTCTGTGTGTTTGT
CATAAGGCTCTTTAATATGTTTAAGGTTTTCAGGTGTTTTAATATTAGAGAATAGATTTACTAGATATACAGCAC
CGTATTGTTCAGAATTAGCTAATTGGTTGAGAATGAGAACAGTGGTAAGATCGAGTGATAATACGCCATCTAA
ATGTGGATACATCGTTATCACTGTACAAGAGGGTTTCTTTTCATCCCATATTTTCTTGAGTAAGTAGCGGTGTT
GTTCATCGTCGCTAAATATAGCTTCTGTGTGTATCGTACTTTTGATTGTATTCATATATCGTTACCCCTTTTAATAT
TCTTCTGGCAAAAGCATCACATAATAAAAAGCGTCTACGTCATCTTCACGAATGACGTAGACTTTCTTAGGTA
ATGCATTTTGATTTTTTACATAGTTTGTATAGTGATATTCCAATTTGTATGCAGGTTGTTCTTGTTCATGTGTGAT
TGAGAGTATATTCTCATCTTCTTGTAGTCTAAAAATGTGTAGATAATCTGTATGAGATTGATTATCTCTTTCTTTT
ACCATATTCCAAAGTAGGATTTGAAGGTCTAGAGATAGTTGTTCACTTATGCCTCTTGTGATGTATCGATTGAT
TTTCATGTTATTTTACCTCGTCTTGAATTTCTTTCATAATGATAATTGCTTGGCTAATAATCGTAACAGATATTTG
TGCCACTTTGATCCAATTATTCATGGTTATTCTCTCCTTGTTTTAGTAAATGACGTTCATCGATAATCGTATTTTT
AGTATCTGTGAGGTATAGAAAGTCCATATCAAAATGATCCAAATAACCAATGCTGATGAGTTGGTCATTGGCG

TACATAAGAAATGGATAGATACTTAGCTCATGTAGCTCATCATTGTAGTAGGTATAAGTGTTGAGTGTGAGATG
CGCAAGTAGAGAATTATTAATAAAACGTTCCGGTAGAATATTTTCTGCTGCTTCCTCAAGTGCTTCACATTCCC
ATGTTTCGTTGTTAGATAGTTGGAAGAGACGAGTTATATATTGTTTGAGTTCTTGAGTGGTTGTTTTCATATCA
TTGCCTCCTAGATAGTGTGATAGTGATGTAGTTGATATACATCATTGGGATAATATATATTTGATTTATTATTTATT
ACGAATCCCGGTGGGAATAAGAGAAAATTCCATATAAAAACCCGTGATAAATGTTGGTGTAACAAGGAAATC
CCGGAATCCCACTCATTTTGACGAACAATCAACTCATTATTTATAAGTATTGATGATAGGGTTGTGTCTCTGCT
TCCTTATATATATTATTTATTTATAAAAAATAACGGGATTTTGGGATTGTGCTTGCACAATCCTTCTGCTTCTTCG
AATCTGCAAATCCCATTCCTTTCCCGGTAAAAAATCATTGTGGGATGTTCTTTAGCAATTTCAATATAAGCATT
GTGTAGTCATGAAAAAAATGACGGCAATGACTGTTTCATTAGATAAGTGTTATTGAAATTGATAAAGAGAATT
CTAAAAATGGTTAGATAAAATAAGTGAAAGAATAGCAGTGTAGTTATTGTTTATTCAATAGTTATATATAAAGT
TTGTGGCAAAAATAAAGACGAAGTGCTAGGGAGCACTTCGTCGAGTGGATGGTTATTAAATAGTTGTTTGAT
TATATCATTATTTAACTTGAGCGTAACATAAAATTGCTTCTTATGATGCTCATCTTTTCTTATGTCAATGCGATCG
ATGATTGTTAGATATAGTGATTTCAACTGAGATTTCTCTAATTTGTCTATATCTTTGAATATTGCTTGTAGTACAT
TCGCAATCATATCAGCATCATAATGTGGAGCTTCTGCTTGTTTATCTTGTTCTAGTTGATAGATTTGATTATTTAT
TTGATTCAATTCATCTTGGTAGCTAAGTATTGTTGGTTTTAATACGCTATCTAAGTCAGGTGAGTCTTCAATAGT
TTTCGTTAATGTATGCATTTTTGCTTTTATTTCTTCACATTGTGACTGTTTATAGGCAATATCATGGTTCAAAGA
AGAGACGTCTATTTGACTTTTTTCATTTACCTTTTCAACCAATTGCTTCAATACCTTTTTGCTTTTGTTAATTTC
CAATATCTGATCCATAACATATTTTTCTAGTACATCTGCTCTAACGCTATTGGCAGAACAAACTTTTGAACCTTT
ATTTCTAAAATTGCTACATGAGTAATATCTGATTCTTTTCTTAGTGCCATCTTTTAATGTATTAGTTGTATTACTT
GCCGCCATTGCTGCACCACATTTCGGACATTTTACAATCCCAGTCAGTAGGTTTGTTCCTTTGCCGTGAACTT
GTGGTTTCTTGCGACTCTCTTGACGTTTAAACTGTACTTTATCCCATAGGTTTCTATCAATAATAGGCGCATGTT
TACCATCAGCGATAATCGGTTCCTCATTGAGTCCTTTTCGTCTTTTATCGCTCCAGTGTCTATACTTCGCAAACT
GTATCTTTCCAATGTAAAAAGGGTTTGAGATGATGTAGGTAATGGACGAAATACTAAAAGGTTTCCCTTTCTT
AGTCACATAACCTTTATGATTCAATGCGTTCGCAATCTTACGATAACCATGACCTTTAGCGTATGAATCAAAAA
TATATTTAACAATATTCGCTTCATGTTGATTGATCATGAGCTCTTTTTTACTGTCAGGTACTTTATCGTAGCCTAG
AGGTAAATTACCTTGATAATAACCTTCAATAGCACGTTGTCTTTGGCCATTGTAGACATTCTCTACAATCGTATT
ACGTTCAAATTCTGCGAAGCTGGCTAAAATTTGGAGCATCAATTTACCTGTTGAACTGGCAATTTCTATTTTTT
CAGTTAGACTAAAAAATTCGACATTAATCTTATACAATTCCTCGACAATATTTAACAAATCTGAGGTATTTCTAG
CTAAACGATTTGTTTTGTAGACCATAATACAATCTAATTTACCTTCGTTGGCATCTTTTAACATACGTTGTAATT
CTGGACGTTGCATTGTTTTACCTGATATACCACGATCGGTGTATTCATTGACGACTTCATAGCCTTGAAATTGA
CAATACTCTGTAAGTTGATTCAATTGACCTTGAATACTGTAACCATCCGTTTGCATTTCAGTCGATACACGTGC
ATATAATCCAATACGTTTCTTTTTGAGTTGTTTCATATTACTTCATTCCTTTCAGGAGTTATTAAATGTGATTGTT
CAACGATATTGAGTGGACTGTTTTTAAAGTAGATTCCTTGTAATTGTTTAGTTTGAGTTATTTTGATGCAATCG
ATAAAAGGTGCTATATCCTGTAAAGTTATTTTATTTTTTATGACATATTTTAATTTGTCATCGATTTGGTAGGTTG
AATAAGTAGAGTAATTCTCTTCACTTTTACAACTAGCAGATAAACGTTTGAACGTTTTTACATCAATACGATTT
TGAGCTAGCTTTTCGATAAGTTGTTCTTGTGTTAGATGGGTTTCTTTATGCTTCATTTGTTGCTGTTTTAGGAC
TTTGAGTATTGTGTTATTCAATCGTTTATGAAACGATTGTTCTTCAAAATACTTTTTACAAGTGTCGAGCACTT
CACTTTCAAGTTCTGGTGCATTGATACTTTTAAATGGGCATGTACGATAAGCGTCATTCATATTTTTAGGACAA
ACATAGTAACGTAGAGAATAGTTCTCTTTTTTTATCGTTAAGTTTGTTAATGTTGATTGACAGTAAGGACATTT
GATTCGTCGCTTTAGCTTATTTCTAGAATTGGATCGATTGAGTTGTTTATGAATACGACGCTCTTGTGCTTCTT
CAAATGTATCAATATCAATAATAGGTGGAACGATATCATTAAACGTGCCATATTTATTGATGACACGACCGCAAT
AGTTAGGGTTCAAGAGAATATTTCTAACTTGATAGGGCTTACGAGGAATAAGGTTAGGATTACTATCCAAATG
TTGGGAAATCTTTTTGTAGCCTAGACCTTGTAAGTACCAGCGATAAACCGATTTAACTGTATACGCTTCTTCTT
CTTGTACAACAAAACAACCTTTTCTATAACGATAGCCAAACGGAGCATGAGTTGTGATTAGCTTACCTTGTTT

GGCTTTTTCTCTAATCCCATTTTTTGTTTGTTCGCTGATATTGTTTGATTCCATTTCCGCTAGACTCATAAGTATG
TTCAAACGAAAGCAATCAAACTCTTTAGACAAATCAAAATATCCATCGTTAACACTGATGATTGTGATGTGAT
GCTTTTTACAAATTTCAAAGAATTGTATGGCATTTTTCAAATTACGATGTAGTCGGTTCAAGCGATAGCAACA
CAATACTTTACATTTTCCAGACGTAATTATTTCTACCATTTTTTGATAACCTGAACGTTTTGTATGTCGACCTGT
TTTCTTATCATCATAAAACGCCACATTAGACCATCCATATTGCTTAGCGGTATCCATAATGAGCGATTTTTGAGT
AGCTAAGCTTTGTTGTTTGAGTGTACTTTGACGTACATAAGCAATCGCTTCTTCCATGTTATACACCTCCAAAA
AGATAATATATATTTGTGAGTAAATTAGAATGAAAGGTCCAACGTGCTTTTAACACGTTGGACCGTCGTGATT
AGTTATCGTTCTGTAGCTCTTCAACGACTAAATCAGCTAGTAATTCAATCAATTCATCCATTTTATCTACTCCTGT
ACGATTTCATCTTTAAGTTATTAAAAATCAATTAAATCATCGTTCTGTTTTTTCCATGATTCAAGTATCTTTTTGT
TATCCAAGTTAATTTCAGCTTTAATAGGTTCAGCATCTTTAGTTAGACCAAAAATAGACGCATATTTTGAATCTA
GCTTTAAATGGTAAAAGACAAGTGACTGTTTCTTGCCATAGTCATCTTTAACTGCTCTTTTCGTAGTTATTCGA
TCACGGTCAGATTCGATAAATCCTTTATCCCTTAGTGCATTCACAACATTGTTGACATCTTGGAAATGATGCTC
TATCAACATATTCTTAAATACAGATGCAATGATTTTAACTTCGATATAATCATCTTTTAAGGCAATTAGTCCATAG
TTCTCAATCATATTCTTTAATGCTGTATCATCAGAAAACTTACCACGGTTTTGTGCTACAAATTGCGTAATGACT
TCAATCGCTTTATCAGCCAGTGATCGTTCAGAGACTGTATGAGCATGATAATCAATAAAGTAGTCTCTTATTTT
AGCGATATCAATATCTGTAGATAAAACATGACTAAGAATCTTGGCCGAAGTAGTGATAGCCGCATAGCGCTTA
AACATACGAATACCAGTATTATTTGTTTCATCCTTCAATTTATCTTTAAACCAATGATGTAATTCAAAAACGGAT
CAAAAAACAAAAGGCCCTAGAAACTAGGAGCACACTTACACAAGAACAATTGATAGATAAATTAGCTAAAG
GAGCCATCGATGCAGAAACGTTCAGAAAACAATCTCAATCGTTACTTCAACAATCAAAACCAACACTATCAAT
AAATGAGCAACAAATTCAAAGGTCTTTTGAAAATGTAATTCAACAACACTTCACGTTAAGCATGTTATACCGA
TATATTGATGAAATTCATATTTCTAAAAACAAAAGCCTTGTGGGAATCTATTTCAAAAATGAACCGCTAAACAT
TGTAAACCAAGCTACGCAATCATCGATTGCTTAATTAATGAAAGGATGAAAAATATGAAAGAAATGAAAAGA
AAATCTGTAGGATGTTATGTTAGAGTTTCAACAATTTCTCAAGACATTGATAAATTTAGTATTAATGGTCAAAT
TACACAAATAAAGGAATATTGCCAACAGGGAAATTATGAATTGCTATACTGATAATTAAAAGTACCCCCGATC
GAACGAAAATATCTTTTTGATGACTATTAGTGATAATTGTAATTTTATCCATTAATAGATGAATGGATTGGAATA
ATACTAAGCCTATTGCTATTCCACCGAGTAAAATTACCCACCCGCCTAATTCAATACTTTCCGGAATCATTTCAA
AAAATAATAAGCCAATTATAAGGCCTGCACATAATGAATAAATAAAGCCATGACCACTTTGAAAACCTTCCATT
ATCCAAGCGAGTCCACCGCCAACACCAATTCCAAGTGCGGAAGCTAACGCCCGATCATCCATACTGCACTCA
TCCCACTATTCTTAAAAAGGCTTTGTTTATTTTACTCTAAAAAATCAAAACAAAATATATGAATTCATATTAAATA
TTATTTGACTCTTTATTCAGATGGTTATATCATATATATGAACATATATTAATACATGTAGATAAATAGTGATTATTA
TATATAAGGTTTTGAATTGAAACCTAAAGTGAGGGAAGGATAATGGAAGAAAAAAAGGAATTAGAGGAAGT
AAATAATAAGGACTTAGATGATGAAACATTATTTGTCGTATCGCAAACATTTAAAGCGTTAGGTGATCCTACG
AGAATCCGGATTCTCCATTTGCTCTTTTATAAGGAGTATTCGGTAAACGGTATTGCTGAAACGCTACATCTTAG
ACAATCAACAGTTTCCCATCAATTGCGGTTCTTGAAAAATTTACGGTTAGTAAAATTCCGAAGGGAAGGCAC
AACATTGTTTTATTCCCATGATGATGAACATACTATGAATATGCTAAAACAGGCGATCGATCACGCCTGTCATC
ACTAGTATTCCGTAAACCAGTAATGAGTGTTAATAATAATTCACGTCATTAATATTGTTATATGATCATATAAAAA
TATTATAGGCATATGAAAAGGGGGTTTTGCAATGGATATGGAAAACAAAAAAACAGAATGGAAAGCGTTGT
ATGATATCTCGAAGGAAAGTGAGATGGGTGTAGCAGAGAGGGTTAGTGAATACGGTTGATAAATTCATAGG
ATGTATTATTGGATACAAAAAGTTTGGATGTGGTCAATTAACGTCCTGGTTTAGTAAAGCTTACTGCGAATATA
TTGGAACCCGATATATCATATTTATTTAGAAAGGTGATAGAAATGAAAAATATTCAAGAGCAACAAGCACACG
AAAGTCATAGCCACGATCATAGTCATGATCATGATCACGGAAAAATGCCAATTATTTCATATTTTATTGGCTTA
GTGTTGGCTATAATTGGGCTTTTTTAAGTGATGCAAATTTATTAATACAAAACATCTTATTTTCAATTGCCACAA
TCACAGCCGGCTACCATGTAATTATTCTCGAAGGAATTGGAGAGACAGTTGAAAATACTAAATTAAAGGGAA
AATTCACTCCTAATTCTCATATTCTAATGGGATTAGCTGCAATCGGGGCTTCTCTGATAGGGAGTTTTTGGGA

AGGAACCCTTTTGATACTTATTTTTTCCGGCGCTCATTTTCTTGAAGATTACGCTGAAGGAAAAAGTAAAAG
AGAAATTACTAAGCTACTCGAAATGAACCCAACGACAGCTAAATTAATCCTACCTGATGGAAACACAAAAATT
GTTGATGTCAGTGAATTAAAAGTTGGAGATCAACTCCAAGTGCTGAACGGTGATCAAGTTCCAATTGATGGG
ATTATTTTATCCGGTACTACCTCAATTGATGAATCTTCTATTAATGGAGAAAGTATACCGAAAGAGAAGTCTAA
GGGTGACGAAGTTTTTGGAAGTACGATTAATGGAACAGGTACTTTTACTATGGAAGTCACTAAGGAAAACA
AGGATACTGTATTCTCTAAAATTTTACAATTAGTTAGTCAAAACCAAGATAATCAAACAAAAGCTGCCAGTATC
ATTCAAAAATTCGAGCCTAAATATGTTAATATAGTTTTAATCGCAATACCATTAGTAATGTTACTTGCTCCTTTTC
TATTTGATTGGACATGGTCGCAAAGTGTATACAGGGGATTAGTGCTTTTAGTCGCAGCTTCACCGTGTGCTTT
GGCAGCAGCTACTGTATCTGTAACATTGTCTACAACATCTAACCTAGCTAAAAAAGGCGTGCTTTCAAAAGG
AAGTACTTACCTATCACAATTAGCGGATATAGATGCAATTGCCTTCGATAAAACAGGAACCCTTACGAACGGA
GAACCTAAAGTAACAAATTACTATTTCACTCATTCTGTGAACGAAGAAAATATTATTGATATTATAGTCGCCCTT
GAAAAGGAATCCAATCACCCACTCGCTAATGCTATTTTAGAAAAATTTGAAGTTAAAAATAAAATAGACATCG
AAGTTACTAATCAAATTGGAAAAGGTCTGACAGGAGATTATAATGGAAAAAATTATCGTATTGGTAAGCCTAC
TTCTTTTGAAAGTGCTTCTGAAGAGTATACCCAGTTCAATCATGATTGGGCATCAGAAGGAAAGACGGTTGT
ATACGTAGCAGAAAATGAAGAAGTTATTGGGATTATAGCTCTAATGGATATTCCGAATGAGCATGCTAAAGAA
ACAATTAATTACTTTAAGAAACTTGGTATCCACACGACTTTAATTACTGGTGATTCGGAAATGACGGGAAAAG
CTGTAGGCGAACAATTGGGAATAGACGAAGTTATCGCTAATGTAATGCCTGAAGATAAATCCAGAATTATAGA
AGAACAAAAAGAAAAATTTGGAGTTACTGCCATGGTTGGAGATGGTGTGAACGATGCACCGGCCCTTGTTA
ATGCTGATGTTGGTATAGCTATGGGGGGCGGTACTGATGTGGCAGTAGAAGTATCTGATTTGGTTTTAATGCA
GAACAATTTATCTAAATTAGTACAGTCTCATAAAATTTCCTCAAATATGGGTCGTGTTATTAGGCAAAATATTAT
TTTTTCAATGGCAGTTGTTGCCTTTTTAGTTGTCGTTAGTTTGTTAGGATTAACTGATATTACAATCAGTGTAAT
TGTTCATGAAGGAAGTACTTTAGTTGTTATACTAAATGGACTTCGATTATTAAGATCTAAATAATGAACGAATC
GATTGACATGAATGAACTTTGAAGTGTGGATTCTACAATGTTCCCATAACATTGGACACTAAAAAACAGAGC
AATCTAATAAAGATGTTATGAGTAAAAACAATGCCTTGCATACCGTTTTTATCATACGGGCGGTCAAATAAGCA
ATTAAAGAGCATGGGAAGCATAAAATCATAAACAGTGACTTAAGGCAGCCAGTTTACATTCAAAAGTTAAAT
TGACTGTATTAAAAGTTTCGAAACGATTAATATCCGTATGGGGGCAAGCGTCGAGCCAAAGATAATGCCAGG
ACAGAGCGTCTTTTTCCGTTCTTTTAAGTGGGAAAGGTTTTACCTTCTCTATGCCAAGACAGTCCCAGAGCT
AAAAGGGCCAGTTGCTCTTTTTGGGCGTTTGTGAACGGTAATCGCAGGAAGGGGATTTTCCCAACCGCACC
TGGTTTTCCATCACAATAAAGTTGGCGGCATTTGTTCTGATATTCTTGGGAATACTTACACTATGAATGGCCGT
ATTAAGTGATACAGGAGCTGCTATTATTGTAATACTGAATGCTCTCCGCCTTTTGAGGGTAAAAGAATAAAAG
TAAGGATAACTAGGTAAAGCTGTTCAATCAAAAATTGAACAGCTTATTTTTCATCAAAATCAAAAACGTTTATT
ATAATACCTACACTTGTTCTGTTAAATGTACCGATTTTTTAACCTTATTGTATCAGTAATATCTTGAAACGAAGTA
AGCGACTAAAATTTCTCTTTTATCATACGTTTATAAAAATACACTTTTAAGAACGGTTTGAAAATTTTTGAAAT
AGATCAAAATAATCTTCTGGTTAAAAAACCTGTAGATATGATTTCTCTCTTAAATTTTTGTTTGATTAGATTAGA
CCCTAATAAGACGCATTCAATATGTCTACACGTGAATTTAGTCTTTGAAAATGTAAGGACCATTATTATTATAAA
AACCCCAGTATAAAACGATACGCTGAAGCTTATCATAAGTAATGAGGTTCATGATTTTTGACATAGTTAGCCTC
CGCAGTCTTTCATTTCAAGTAAATAATAGCGAAATATTCTTTATACTGAATACTTATAGTGAAGCAAAGTTCTA
GCTTTGAGAAAATTCTTTCTGCAACTAAATATAGTAAATTACGGTAAAATATAAATAAGTACATATTGAAGAAA
ATGAGACATAATATATTTTATAATAGGAGGGAATTTCAAATGATAGACAACTTTATGCAGGTCCTTAAATTAATT
AAAGAGAAACGTACCAATAATGTAGTTAAAAAATTTGATTGGGATAAAGGTGATCTATATAAAACTTTAGTCC
ATGATAAGTTACCCAAGCAGTTAAAAGTGCATATAAAAGAAGATAAATATTCAGTTGTAGGGAAGGTTGCTA
CTGGGAACTATAGTAAAGTTCCTTGGATTTCAATATATGATGAGAATATAACAAAAGAAACAAAGGATGGATA
TTATTTGGTATATCTTTTTCATCCGGAAGGAGAAGGCATATACTTATCTTTGAATCAAGGATGGTCAAAGATAA
GTGATATGTTTCCGCGGGATAAAAATGCTGCAAAACAAAGAGCATTAACTTTATCTTCCGAACTCAATAAATA

TATTACATCAAATGAATTTAATACTGGAAGATTTTATTACGCAGAAAATAAAGATTCATCTTATGATTTAAAAAA
TGATTATCCATCAGGATATTCTCATGGATCAATAAGATTCAAATATTATGATTTGAATGAAGGATTCACAGAAG
AAGATATGCTAGAGGATTTAAAGAAATTTTTAGAACTATTTAATGAATTAGCTTCAAAAGTTACAAAAACATC
CTATGATAGCTTGGTCAATAGCATAGACGAAATACAGGAAGACAGCGAAATTGAAGAAATTAGAACAGCAC
AAAAAGATAAGACACTCAAGGAAGTGGAAGCACCTAAAGGAATAATTCCAAAATATAAAAAAGGTGTATCA
AAGACTACTAAAAATGATTCAGAAATTGAAAAATCAAATAAAGAGAATAAATTAACCGGTAAAGTTGGAGAA
AAATTAGCGCTAAATTACTTTAATGAGCTAATTGATAATAAAATAGACGAAGATAAGAAAGAACAGTTTAGGA
ATATTTTAAATGATAATCCAGGCTCTCAACACGGTCATGGCTATGATTTAGTAGCTTTTGATCCAACAAATACA
GATAAAGCTGTAGAAAAATTTATTGAAATTAAAACATCTACATCTTCTAGTATTGAGGAACCATTTTTTATGTC
GCTAAATGAAATGTTTGCTATGAAAGAATATAAGCAGAAATATTTAATATTAAGAATATTTAATGTTTCCGGTAA
AGAACCACAATTTTATTTTATAGATCCATATGCAAATTATTCTGAATTTAAAGATGTAGATGATCTCATTGACAA
AGTATTTAATGTAGAAGCTATTCAGTATAAAGTTTTTGGCGAAAAATGATTACTTGAACAAGAGCTAAAATAA
AATTGTGATCTAATAAAAATAGAAACTGTAATTTAAATAAAACTTTCTAAATAAGCTAACTGATAAAAAATCAG
TTTGTCCACAGTCTGAAACAAGATTCCTATATTCTTTAGGAATCTTGTTTTTTCTATTTTTATGGTGATAAAGAG
CAGATAAGATAATGTGTAATAATCACAAAAAAGTTAAATATTTTAAGGCTTGTTTAATTATTAATGATTTTATATA
TAAAGAGCAGTATAATAAAGTTGTTAATATATTATGAATAATATTCAAGTAATTTTATTGTTTTTTAATTTGTCGA
TATTTAAGTTGAGTTAAATTTAAAGGGTGTAATTTGTTTTACAATGATGAAGATAATTAGTCTATCAAAATAAA
GGGGTTGGGACTGTTATGAGTGATAATTTGTCATTATTCATTGACTATATCAATGATAATATAATCTATGGTAGT
GAAATCAAACGGGAGAAATTAGAGAATTTATTTAATCAATTTGCTATAAAAAAATGTTGAAAAGAACATTGTCT
ATGATGAACTGAAATCTTTAGATATTACAATCATTGAGTCACAGGATTCATATAAAAATAAATTGAAGAGATTA
TTTTCGGTTCTGTTGCAAAGTAAAAAAATATAGCTAACCACTAATTTATCATGTCAGTGTTCGCTTAACTTGCT
AGCATGATGCTAATTTCGTGGCATGGCGAAAATCCGTAGATCTGAAGAGACCTGCGGTTCTTTTTATATAGAG
CGTAAATACATTCAATACCTTTTAAAGTATTCTTTGCTGTATTGATACTTTGATACCTTGTCTTTCTTACTTTAATA
TGACGGTGATCTTGCTCAATGAGGTTATTCAGATATTTCGATGTACAATGACAGTCAGGTTTAAGTTTAAAAG
CTTTAATTACTTTAGCCATTGCTACCTTCGTTGAAGGTGCCTGATCTGTAATTACCTTTTGAGGTTTACCAAATT
GTTTAATGAGACGTTTGATAAACGCATATGCTGAATGATTATCTCGTTGCTTACGCAACCAAATATCTAATGTAT
GTCCCTCTGCATCAATGGCACGATATAAATAGCTCCATTTTCCTTTTATTTTGATGTACGTCTCATCAATACGCC
ATTTGTAATAAGCTTTTTTATGCTTTTTCTTCCAAATTTGATACAAAATTGGGGCATATTCTTGAACCCAACGG
TAGACCGTTGAATGATGAACGTTTACACCACGTTCCCTTAATATTTCAGATATATCACGATAACTCAATGTATAT
CTTAGATAGTAGCCAACGGCTACAGTGATAACATCCTTGTTAAATTGTTTATATCTGAAATAGTTCATACAGAA
GACTCCTTTTTGTTAAAATTATACTATAAATTCAACTTTGCAACAGAACCGTATTATGGAATAGAGATGTTGGT
AACATTTATACAGGATCATTATACTTAAGTTTAATTTCGTTATTACAGAACCACACATTCCAACCAGAAGAGAA
AGTATGTCTATTTAGTTATGGTTCAGGAGCAGTAGGAGAAATCTTTAGTGGTTCAATCGTTAAAGGATATGAC
AAAGCATTAGATAAAGAGAAACACTTAAATATGCTAGAATCTAGAGAGCAATTATCAGTCGAAGAATACGAA
ACATTCTTTAACAGATTTGATAATCAAGAATTTGATTTCGAACGTGAATTGACACAAGATCCATATTCAAAAGT
ATACTTATACAGTATAGAAGACCATATCAGAACATATAAGATAGAGAAATAAACTAGTGGCCGATTGTGCTTGA
TGAGCTTGGGACATAAATCCTAACTCGAAATAAATAAGCATATCACTAAACTGATTTTTTAAAGTTTACAGTGA
TATGCTTATTTTTTTATCTTACGATTTTGTACGTGCATGCTTGCCTAGGGGTATGGCTCGAGCCATTAGTCTCTC
GCACATACTATTCCCTCAGGCGTCAGCACTTACAAAATCGGTTGTAATTTTCATTTTTATACGCATTCTTACTGA
GATTATACTAATAAGAGGAATAGTAAAAGCAATTCTAAGTAAAATTGCAGATAAGAGGTTTGTTAAAAGCAGT
TCTCAGTAAAATTGCAGATAAGAGGTTTGTTAAAAGCAGTTCTCAGTAAAATTACAGATAAGAGGTACGTTA
AAAGCAGTTCTAAGTAAAATTGCAGATAAGAGGTTTGTTAAAAGCAGTTCTAAGTAAAATTGCAGATAAGAG
GTACGTTAAAAGCAATTCCATGCAAAATTGCTGATAAGGGGTAAGTTAAAAGCAGTTCTCAGTAAAATTGCA
GATAAGAGGTACGTTAAAAGCAGTTCTAGGCAAAATTGCAGATAAGAGGTGCGTTAAAAGCAGTTCTCAGT

AAAATTGCTGATAAGGGGTAAGTTAAAAGCAATCCTAAGTAAAATTGCAGATAAGGGGTACAGAAAAACTA
GACTTGATTACAAAATGGAGCTTGGGACATAAATGATTTTTTAAAAATGAGATGAGACGTAGATTAACTCCAT
AATCAATACGAATCTATCGACTTCTTTATTTATGATATTCATCTCTTTTTAATGGAAATAAAAGTGCGATTAATGT
GATAATACAGTTACGTTAATTAAAAAAATAAAAATGCAAGGAGAGGTAATATGCTAACTGTATATGGACATAG
AGGATTACCTAGTAAAGCTCCGGAAAATACAATTGCATCATTTAAAGCTGCTTCAGAAGTAGAAGGTATAAA
CTGGTTGGAGTTAGATGTTGCAATTACAAAAGATGAACAACTGATTATCATTCATGATGATTATTTAGAACGG
ACTACAAATATGTCCGGGGAAATAACTGAATTGAATTATGATGAAATTAAAGATGCTTCTGCAGGATCTTGGT
TTGGTGAAAAAATTCAAAGATGAACATTTGCCAACTTTCGATGATGTAGTAAAAATAGCAAATGAATATAATAT
GAATTTAAATGTAGAATTAAAAGGTATTACTGGACCGAATGGACTAGCACTTTCTAAAAGTATGGTTAAGCAA
GTGGAAGAACAATTAACAAACTTAAATCAGAATCAAGAAGTGCTCATTTCAAGCTTTAATGTTGTGCTTGTTA
AACTTGCAGAAGAAATCATGCCACAATATAACAGAGCAGTTATATTCCATACAACTTCGTTTCGTGAAGACTG
GAGAACACTTTTAGATTACTGTAATGCTAAAATAGTAAACACTGAAGATGCCAAACTTACTAAAGCAAAAGTA
AAAATGGTAAAAGAAGCGGGTTATGAATTGAACGTATGGACTGTAAACAAACCAGCACGTGCAAACCAACT
TGCTAATTGGGGAGTTGATGGTATCTTTACAGACAATGCAGATAAAATGGTGCATTTGTCTCAATAGAAAGTT
AGAGGTGAGTCTTACGTTTCAGTGACGGTAGACTTACCTTTAACATGTTACATACTAAAAAATTAATTTGAATA
AGAAAGAGAGACATATATGAAATACGATGATTTTATAGTAGGAGAAACATTCAAAACAAAAAGCCTTCATATT
ACAGAAGAAGAAATTATCCAATTTGCAACAACTTTTGATCCTCAATATATGCATATAGATAAAGAAAAAGCAG
AACAAAGTAGATTTAAAGGTATCATTGCATCTGGCATGCATACACTTTCAATATCATTTAAATTATGGGTAGAA
GAAGGTAAATACGGAGAAGAAGTTGTAGCAGGAACACAAATGAATAACGTTAAATTTATTAAACCTGTATAC
CCAGGTAATACATTGTACGTTATCGCTGAAATTACAAATAAGAAATCCATAAAAAAAGAAAATGGACTCGTTA
CAGTGTCACTTTCAACATACAATGAAAATGAAGAAATTGTATTTAAGGGAGAAGTAACAGCACTTATTAATAA
TTCATAATAAAACAGTGAAGCAACCATCGTTACGGATTGCTTCACTGTTTTGTTATTCATCTATATCGTATTTTT
TATTACCGTTCTCATATAGCTCATCATACACTTTACCTGAGATTTTGGCATTGTAGCTAGCCATTCCTTTATCTTG
TACATCTTTAACATTAATAGCCATCATCATGTTTGGATTATCTTTATCATATGATATAAACCACCCAATTTGTCTGC
CAGTTTCTCCTTGTTTCATTTTGAGTTCTGCAGTACCGGATTTGCCAATTAAGTTTGCATAAGATCTATAAATAT
CTTCTTTATGTGTTTTATTTACGACTTGTTGCATACCATCAGTTAATAGATTGATATTTTCTTTGGAAATAATATT
TTTCTTCCAAACTTTGTTTTTCGTGTCTTTTAATAAGTGAGGTGCGTTAATATTGCCATTATTTTCTAATGCGCT
ATAGATTGAAAGGATCTGTACTGGGTTAATCAGTATTTCACCTTGTCCGTAACCTGAATCAGCTAATAATATTTC
ATTATCTAAATTTTTGTTTGAAATTTGAGCATTATAAAATGGATAATCACTTGGTATATCTTCACCAACACCTAG
TTTTTTCATGCCTTTTTCAAATTTCTTACTGCCTAATTCGAGTGCTACTCTAGCAAAGAAAATGTTATCTGATGA
TTCTATTGCTTGTTTTAAGTCGATATTACCATTTACCACTTCATATCTTGTAACGTTGTAACCACCCCAAGATTTA
TCTTTTTGCCAACCTTTACCATCGATTTTATAACTTGTTTTATCGTCTAATGTTTTGTTATTTAACCCAATCATTG
CTGTTAATATTTTTTGAGTTGAACCTGGTGAAGTTGTAATCTGGAACTTGTTGAGCAGAGGTTCTTTTTTATCT
TCGGTTAATTTATTATATTCTTCGTTACTCATGCCATACATAAATGGATAGACGTCATATGAAGGTGTGCTTACA
AGTGCTAATAATTCACCTGTTTGAGGGTGGATAGCAGTACCTGAGCCATAATCATTTTTCATGTTGTTATAAAT
ACTCTTTTGAACTTTAGCATCAATAGTTAGTTGAATATCTTTGCCATCTTTTTTCTTTTTCTCTATTAATGTATGT
GCGATTGTATTGCTATTATCGTCAACGATTGTGACACGATAGCCATCTTCATGTTGGAGCTTTTTATCGTAAAG
TTTTTCGAGTCCCTTTTTACCAATAACTGCATCATCTTTATAGCCTTTATATTCTTTTTGTTTTAATTCTTCAGAG
TTAATGGGACCAACATAACCTAATAGATGTGAAGTCGCTTTTCCTAGAGGATAGTTACGACTTTCTGTTTCATT
AGTTGTAAGATGAAATTTTTTTGCGAAATCACTTAAATATTCATCCATTTTTTTAACGGTTTTAAGTGGAACGA
AGGTATCATCTTGTACCCAATTTTGATCCATTTGTTGTTTGATATAGTCTTCAGAAATACTTAGTTCTTTAGCGA
TTGCTTTATAATCTTTTTTAGATACATTCTTTGGAACGATGCCTATCTCATATGCTGTTCCTGTATTGGCCAATTC
CACATTGTTTCGGTCTAAAATTTTACCACGTTCTGATTTTAAATTTTCAATATGTATGCTTTGGTCTTTCTGCATT
CCTGGAATAATGACGCTATGATCCCAATCTAACTTCCACATACCATCTTCTTTAACAAAATTAAATTGAACGTTG

CGATCAATGTTACCGTAGTTTGTTTTAATTTTATATTGAGCATCTACTCGTTTTTTATTTTTAGATACTTTTTTTAT
TTTACGATCCTGAATGTTTATATCTTTAACGCCTAAACTATTATATATTTTTATCGGACGTTCAGTCATTTCTACTT
CACCATTATCGCTTTTAGAAATATAACTGCTATCTTTATAAACTTGTTTGAAATTTTTATCTTCAATTGCATCAAT
AGTATTATTAATTTCTTTATCTTTTGAAGCATAAAAATATATACCAAACCCGACAACTACAACTATTAAAATAAG
TGGAACAATTTTTATCTTTTTCATCAATATACTCCTTATATAAGACTACATTTGTAGTATATTACAAATGTAGTATT
TATGTCAAAATAATGTTATAATTTTTGTGATATGGAGGTGTAGAAGGTGTTATCATCTTTTTTAATGTTAAGTAT
AATCAGTTCATTGCTCACGATATGTGTAATTTTTTTAGTGAGAATGCTCTATATAAAATATACTCAAAATATTATG
TCACATAAGATTTGGTTATTAGTGCTCGTCTCCACGTTAATTCCATTAATACCATTTTACAAAATATCGAATTTTA
CATTTTCAAAAGATATGATGAATCGAAATGTATCTGACACGACTTCTTCGGTTAGTCATATGTTAGATGGTCAA
CAATCATCTGTTACGAAAGACTTAGCAATTAATGTTAATCAGTTTGAGACCTCAAATATAACGTATATGATTCTT
TTGATATGGGTATTTGGTAGTTTGTTGTGCTTATTTTATATGATTAAGGCATTCCGACAAATTGATGTTATTAAA
AGTTCGTCATTGGAATCGTCATATCTTAATGAACGACTTAAAGTATGTCAAAGTAAGATGCAGTTCTACAAAA
AGCATATAACAATTAGTTATAGTTCAAACATTGATAATCCGATGGTATTTGGTTTAGTGAAATCCCAAATTGTAC
TACCAACTGTCGTAGTCGAAACCATGAATGACAAAGAAATTGAATATATTATTCTACATGAACTATCACATGTG
AAAAGTCATGACTTAATATTCAACCAGCTTTATGTTGTTTTTAAAATGATATTCTGGTTTAATCCTGCACTATATA
TAAGTAAAACAATGATGGACAATGACTGTGAAAAAGTATGTGATAGAAACGTTTTAAAAATTTTGAATCGCC
ATGAACATATACGTTATGGTGAATCGATATTAAAATGCTCTATTTTAAAATCTCAGCACATAAATAATGTGGCAG
CACAATATTTACTAGGTTTTAATTCAAATATTAAAGAACGTGTTAAGTATATTGCACTTTATGATTCAATGCCTA
AACCTAATCGAAACAAGCGTATTGTTGCGTATATTGTATGTAGTATATCGAGCTTCACATGAAACAGCTAAAGA
AGCTTTGGGCGATAAAGAGTTAAGAGCCATTGCACATGAGTTAACTAAAACAGTTAAGGATAACATGAGTGT
TGATTGGTCTAAACGAGACAGTGCTAAAGCTAAATGAGAGTTCAAGTTAGACGCCTATTAAAGAAATATGGC
TATCCACCAGATCTTCAAAAAATGGCTGTGGAACAAGTTGTAGAGCAAGCAGAATTAATGGCAAGTCAGCA
ATAAAAAAATAAATCATAATGAGTCCGGGACATAAAGTTCTTGGATAAGTGAAAAAAGACAATTTCTATTGAA
ATAATATAGAAATTGTCTTTTTTATAAATTTTTTGATTATTTTCAGCTCGTTGAGCTACTACTTTTCTTATATTAAG
TGCCATTAATACAAAACCAAGTTCTCTTTTGACTTTATTGAGTCCTCGGACAGACATCCGAGTGAAACCCAAA
ATAGCCTTCATAAATCCAAAAACAGGTTCCACATCAATTTTTCTTTGACTGTAGATATTTTTTGTTTCTGGTTCT
GAAAGCTTTTTGTTAATTTGGGATTTAAAATATTCCCAGTTATAATTCTTCATTATTTTTTTGTTTGTTTTTGAAT
TGAAGTTCATACATTGATTTTTCAGAGGACATTCTGAACAATCATCACATTCATATAATTTGAAGTCTCGCTTAT
AACCATACTTATCATGACGATAGGCATATCTTTTAAAACCTAGCCGTTTATTATTCGGACAAATGAATTCGTCAT
TAATTTCGTCATAGTTCCAATTTTGAGTATTAAAGATGTCACTTTTATATTTTTTAGTTTTATCTTTTATAAACATT
CCATATGTTATGAGTGGCGTTCGATTAAAGTCATCTATAATTGCCTTATAATTTGATTCACTACCATAACCTGCAT
CAGCTACAATATATTCAGGTAAATGACCGTAGGTCTCTTGAATTGAATTTAAAAATGGAATCATCGTTCTAGTA
TCCGTTGGATTTTGATACACATTATAAGATAAAACAAATTGGGAATTTGTTGCTATTTGTAAATTATACCCTGGC
TTAAGTTGTCCATTTTTCATGTGATCTTCTTTCATTCTCATAAATGTCGCATCATAATCTGTCTTAGAATAACTAT
TTCTATCCTTTAAAATAGATTTTTGAAATTCGTATCGATACTTTCGCTCAAAATAATCATTGATTTGCTTTTTGTA
TTTTTTGATTTTAGTTCTTTTGAGACGTATTTGTTTTCTTGTTTTAGTACATTTTTCATTGTTGATATGTTGGTTT
AAATCTTCGATTTCTTTATCTAAGTGACTACCAATCAAATCTATTTCTTCTTTTGTTAAGTCATTATCATGATCTT
CTTTAATTTCCGGTATGATTTTATTGGTTACCAATTCATGGTAGAGGGCTTTAGAATCCTCATTCATCTTTGATT
CATGGTTTTGAATACTCTTTTTCCATACAAATGTATATCGATTGGCATTTGCTTCAATTTTTGTACCATCAATAAA
AATAGCTTTATCATCTATAAGATTTTGTTTTACACACTGACTGTAAAATTGAATAAATAAAGATTCTAATAAAGC
ATCTACTTTTGGATTTACTCTAAATTGATTAATTGTTTTATAAGAAGGTTTTTGATTTTGTGATAGCCACATCAT
TCGGATGCTATCATTAAGCATTTTTTCTATTTTACGACCTGAGAATACAGATTGTGTGTAGGCATATAGAATCAC
TTTTAACATCATTTTAGGATGGTACGAAGTTGCACCACGGTGATGTCTGAATTCGTCGAATTCATTGTCAGGA
ATTGTTTCAACAATATCATTTACAGTAAAACGATGTTGATTTGTTTTGTTTCCATATTGACCTCCATGTATTTGCT

ATGATTTCAAAATCCATTTTTGACGTGCCTTAGGGTTGAGTGGATGCATAATTTCATTGTTACTGGATTGATG
AGCTTTTTTACTTTCTTTTTATGAGGTTTTAACATTTCCATCACTTGTTCGACACGGTCGATAACAACTGGTCG
CTTCGCATAGGCACCATAAGCAAGAATCACTGTGTCACTTTCACTAATCGCTTTCATCAAATGAATATCAGTGT
GCTCATCGTATGGATTTTTGATATGTTTGAGGTTTTCGGGTGTTCTAATATTAGAGAATAGATTTACAAGATATA
CAGCACCGTATCGTTCTGAATTGGCTAATTGGTTGAGGATAAGAACAGTTGTGAGATCGAGTGATAATACAC
CGTCTAAATGAGGATACATCGTTATCACTGTGCATGCAGCTTTCTTTTCATCCCATGTTTTCTTGAGTAAATAGC
GGTGCTGTTCATCATCGCTAAATATGGCTTCTGTGTGTATCGTACTTTTGATTGTATTCATCATCGTCACTTCCTT
TAGTATTCTTCTGGTAAAAGCATCACATAATAAAAAGCGTCCACGTCATCTTCACGAATGACGTAGACTTTCTT
AGGTAATGCATTTTGATTTTTTTCATAGTTTGTATAGTGATATTCCAATTTGTATGTGGGTTGTTCTTGTTCATGT
GTGATTGAGAGTATATTCTCATCTTCTTGTAATTTAAAAATGTGTAGGTAATCTGTATGAGGCTGGTTATCTTTT
TTTTCTACCATGTGCCAAAGTAAGATTTGAAGGTCTAGTGGAATACTTTCATTAATTCCTCTTGTGATGTATCG
ATTGATTTTCATGCTATTTCCCTCCCTTCTGCTTTTCTTTCATGATGTCGATGATTTCGTTGATAACTGTGACGG
ATAATTGAGCAACTATGATCCAATTATTCATGGTCCTGACCTCCTTGTTTTAGTAAATGACGTTCATCAATAATG
ATATTTTGAGTATCTGTAAGGTACAGAAAGTCCATGTCAAAATGGTCTAAGTATCCGACACTGATGAGTTGGT
TATTGGCATACATTAGAAATGGATAGATACTTAGCTCATGTAGTTCATCATTATAGTAGGTATAAGTCTCGAGTG
TGAGATGTACCAGTGGAGAATCATTAATAAAACGTTCCGGTAGAATATTTTCTGCTGCTTCCTCCAGCGCTTC
ACATTCCCAAGCTTCGTTATTAGATAGTTGGAATAGGTGGGTTATATATTGTTTGAGTTCTTGAGTGATGGTTT
TCATATTATTGCCTCCTAGATAGTGTAATAGTGATGTAGTTCATATACATCATTGAGATAATATATATTTGATTTGT
CATTTATTACGAATCCCGGTGGGAATAAGAGAAAATTCCATATAAAAACCCGCTACAAACGTTGGTATGCCAA
GGAAATCCTGAAATTCCGCCTATTTTGACAAACAATCAACTCATTATTTATAAGTATTGATGATAGGGTTGTGT
CTCTGCTTCCTTATATATATTATTTATTTATAAAAAGTAACGGGATTTTGGGATTGTGCTTGCACAATCCTTCTGT
TTCTTCGAATCTGCAAATCCCAATCATTTCCCGATAAAAAATCATTGTGGGATGTTCTTTAGCAATTTCAATATA
AGCATTGTATAGTTATGAAAAAATTACGACAATAACTGTTTCATTAGATAAGTGTTATTGAAATTGATAAAGAG
CAATTCTTGAAAATAGTTAGATAAAATAAGCGAAAGAATATAGTGAAAATTATTGTTATAACAATGATTCTATTA
GCTAAATAGTAAGATATAGTGTTTGGGGCAAAAATAAAGACGAAGTGCTGAGATGCACTTCGTCGAGTTGTT
TATTATTGAAAAGTTGTTTAATGATTTCGTTATTAAGTTTGAGTGTGACATAGAATTGTTTTTTATGATTACCAT
CTTTTTTAATATCAATGCGATCAATCACTGATAGATACAATGCTTTGAGTCGAGATTTTTCTATGTGCTTAATATC
ATGAAAGATGTGTTGTAATAGTTTACTGATTTCTTTGGCATCAAATAAAGTCTTATCTTCATTTTGTTGATTTTT
GAGTTGGTTGATTTGATTCGTAATGTCATTGAGTTGCTTTTCATATTTTTGAATACTTGGTCTGATTACTGATGT
TAAGTCCGGATTATCCTCGATGGTTTTAATCAAGTTATTTATTTTGATTTGTACTTCATCATATTGTTGTTGCTTA
TAAGCAATATCGTGATGAAGTGCAGCGCCATCAACTTGATTTTCTTGATTGACGTGTGTTACTACGCGTTGAA
TGACTTTATCACTTTTGACTATTTCAAGTATTTGCTTCATCACATAATCTTCAATCACATCAGCTCTTACACTGTT
TGCCGAACATACTTTGGAACCCTTGTTCCGAAAATTACTACATGAATAGTAACGAATACGTTTCTTAGTCCCGT
CTTTAAGAGTATTCGTGGTATTGCTTGCTGCCATAGGTGCGCCACATTGGGGACAGTGAATAATGCCTGTAAG
CAGATTCGTTCCTTTGCCATGGACTTGGGGTTTTTGACTGACTTGTTTCTTACGCATTTGTACTTTATCCCATA
AATCTTGATTAATAATGGGGGAATGCTTACCTTCAGCTATCACTGGTTTATCATTCAGCCCTTTACGACGTTTT
TCACTCCAATCTTTGTATTTCGCAAATTGAATTTTGCCGATATAGAAAGGGTTAGCTAAGATGTATGTGATTGA
ACTAATACTGAAAGGTTTCCCCTTTTTAGTGACATATCCTTTGTGATTCAATGCATTGGCAATTTTACGATAGC
CATGTCCTTTGGCATAGCACTCGAATATATATTTTACAATATTAGCTTCATGTTGGTTAATCATTAGCTCGTGTTT
ACTATCTGGTATTTTGTCATAACCTAGTGGTAAATTGCCTTGATAATAGCCTTCTTGGGCACGTCTCGTTTGAC
CCATAAATACGTTCTCGACAATGTTATTACGTTCGAATTCTGAGAAACTCGCAAGTATCTGTAACATGAGTTTA
CCAGAAGAAGTATTGACTTCCATACGCTCTGACAAACTGAAAAATTCGACATTTTGTTTGTGTAAATCTTCGA
CAATTTTGAGAAGATCAGATGTATTACGAGCTAATCGGTTTGTTTTGTATACCATAACACAGTCGATATTGCCT
TCTTTTGCATCTTTCAACATACGTTGGAGCTCAGGTCTGTTCATAGATTTACCTGAAATACCACGGTCAGCGTA

TATATCTTTAACTTCAAAATGATGGAAGTCACAGTATTCTTTGATTTGATTGATTTGTCCGTCGATACTATAACC
TTCTGTGCTTTGCATTTCTGTTGATACACGTACATAGATACCGACACGTTTTGTTTTAAGTTGTTGCATTATGTT
ACATCCTTTCTTCATTTATGCAATCGATGATTGCATGGTTTGATTGACGATATTGAGTGGTTCATTTTTGAAATA
GATTCCTATAAGATTTTTATCTTTCGTAATGTGAATGGTTTCAATATAGGGGTACAATATGTTTAACGTGAAACG
TTTTTGAATAATATTTTGAATGGTGTGTTGTATTTGATGTCCATTGATAGATGTAGTGCGTTGAGGTTGTTGAC
GTAATGATTGCGTTTGTTCTCTGAACGTTTCTGCATCGATGATGCCTTGTGCCAACTTTTCTATCAGTTGTTCT
TGAGTCAATGTGTGATGTTTTTCTATGTTTCTTTGTCTTTTGATGCGTTTGTCAATCGCACCTTTAATTTTTGTG
TAGATGCGTTGATTTTGATAAAAGTCTCGGCACACTTCTAATACTTTATCTTCAAATGTTTGTGCATTGATGCCT
TTGAAATCACATACAAAGCGTGAAGCATTCATGTTTTTAGGACAGACGTAGTAACGTAATATATGATTCTTTTT
TCTAACGGTCATATTTGTAAGTGTTGCATTACAACATGGGCAATTGATTTTTTGTTTGAGTTGATTATCCGAAG
ATGTCTGTTTGGTTTGTTTTTGCAATCGAAGTCTCTGCGCTTGCTCATATATACTTGTGGAAACAATAGAAGG
AAACATATTGTCGAATTGGCCATATTGATTGTTGACACGACCACAATAATTAGGATTGATGATAATGTTACGAA
CTTGATAGGGTTGTCGATTGATATACGTGTTATCTTCTTCTAATAACTGTGCAATTTTCTTATAACCATGACCTTT
AATGTAATAATTGAATACAGCCTTTACCGTTGGTGACTCATTTTGATTGATGATGAATGTTCCGTTGTGATATTC
GTAACCAAAAGGTGCATGTGTTGTAATCAATCGACCTTGCTTTGCTTTTTCTTGAAGCCCATTTCTGACTTGTT
CTCCAATGTTATCCGATTCAAGTTCGGCTAAACTGATGAAGATATTAAGCTTGAGTCGGTCGAAAGCTTGATC
CATATCAAAGTAACCATCATGTACGCTTAAGATATGAACATGGTACGTTTGACATAATTTGATGAGTTTTAATG
CATTTTTCAGATTACGATGCAACCTATTAAGACGATAACAGCATAATATGTCACACTGTCCTTGTTGAATTAATT
GTGTTATTTGTCGATACCCACTACGATTATCTTTGCGACCTGATTGTTTGTCGCTATAAAAGTTGATATGTTGAA
TATGATGTTTTTCGGCTATTGCTTCGATAGCTTGTTTCTGTGCTGCAAGAGATTGTTGTTTCATCGTACTTTGA
CGTAAATAGCCTATGACTTGTTTCATATCGGCTCCTCCTTTCACAGTGATAATATATATTTATGGATGAATTGATA
TATAAGCCCAACATCAATGAGATGTTGGGCGTCCATATTAGTCATTTGTTTGATTGATTTCTTCAATTACCAAAT
CGGCTAATATCTCGATAAGTTCATCCATGTTTTTCACTCCGTTATTTGTTCTATCTTCAATACGTCGATTATTCAG
TTTGATGCTTCACAGTTGTATGATAAAGACAATTAGAAATCTTCGTGAACTCCTGAAGGGCCTATCCCTTCATT
AGCGGATTTAAAAAGTTCTTTCGCAGCTTTGTTATCATTTGACGGTGTCCAATTTTGAAGTAACGACTTATCTT
TAGTTAATCCGAGGATAGATGCAAACTCTACATCTAATTTTAGATGGTAAAATACAAGTGATTGTTTTTTACCG
CTATTATCTTTGACACTTCTTTTAGTTGTTTGGCGTCCACGGTCAGCTAATATGAAACCTTTATCTCTTAAGGC
GTTGACAACATTATTAACATCTTGAAATTGATGATTGTTTAGCATCTGTTTAAAAACGTTCGCAATTATTTTTAC
TTCGATATGGTCATCTTTTAATGAGATTAATCCATAGTTCTCAAACATATTTTTCAAAGCACCTTCATCTGAAAA
CTTACCTCTGTTTTGTGCCACAAATTGAATGATGACATCAATAGCTTTATCAGCTAATGAGCGTTCAGAGACT
GTATGAGTATGATAATCAATAAAGTAGTCTCTTATATTAGCGATATCAATATCTGTAGATAAAACACGACCTAATA
TTTTCGCAGATGTTGTAATGACTGCATAACGCTTAAACATACGAATACCTGTATTGTTTGTTTCATCTTTCAATT
TAGCTTCAAACCAATCTACTTCCTTGTAAAACCATTGAATAACTTCATCTTCACGATTTATAAGATATTTAGCTA
CTAACGGTAAAACATGACCATAGTTTAGTGCTACAGCTTTTTTAATATTGTCAGCATTGGTCGCATTTGTAGTG
AATTGTTCATTAATCTCGATGGTTCTTACACGTAATCCATCGTTTTGAGCTGAATCATTAAAAATACTGTATTCT
GACGTTGAAATGACAGAAGTACCCCAATTCTTAGGCGTTTTAACTTCTCCATGAACGTTTGAACGTTGACGA
CCTTGACCTTCAGCGATGGAATATAACAAACCCGTGGTATCTCTAAGTGTCGTAGATGAAAGCTCATCAAATA
CTATAGGAATGCCATAATTGTTACTCAAATAACCTTCAAGTGCGTTTCGTGTGGCATTCCAACTTCTAAAAAGA
GTTTCATTACCTTTGGTAGGGTTACCAGCGACTGATACAGCTAAAGCAGCTGCGGTTGACTTACCGGTTGAG
GATTGACCTGTAAAACTAAAGAGAATTCCTGCAAATTCGATTTCATGTTTATGCTTCAGAAAACTTGTCACTA
AGGCAGAAATCCCAAATATGACTGCCAATTCTAAAAGAAGATGACCTTTAACCTCGTCAATATACATGTTAAA
CCAATTATCAAATGTTCCCCTAGGTGCTAAGTCATAAGTATTCTCACAAATGGCGTCAGATGGAGATTTATTAT
CAAATTCCGTAGTAGTATAGATTTCATTTAACGATACAATAGGACCAAACGGTGTTTCCAGTATACCTACCCCT
TCATATAAGTAGGAAATGGGTAATTGGTTGCGCATTTGTTGCAACGCATAACCTAAATCTTTTGTATATTTTTC

ATTAATACTAAATCCATATTTCATTAAAGAGGGCAGTTTTTGTGTTGTTAAAATATCACTAGATTCAACAATTAC
TTTTTGATCCTCGTCTGTAATAATTACTTTTTCAGTGTTAGTTTTAGGGTCAATAAACTTATTTTCGATAACGAT
AGGACCTGCGATTTCAACTTCAGTAGGCATTCCTCCTTTTTCTTTGGGAGGCTTGTCTTTATACCAACCTTTTT
TTGATTTGTATCGTGGTGAAGGATTAAATGAAGGGTTAGTTTGAGTCATTAGCGAACACCTCCTTTCGAAGG
GTTGCTGTTATGGTGTGGATTAGGACCTGTTTTAAGATAAACTAAATGACCGTGAGTATCCTTACCGATAATAA
TAAATGGAACACGTGGCGCATGTTTTACAAAATATGCGAACCAACGTCCAACATTTTGTTGTACTTCTTTAGA
ACACGTTACATTTGCACGACTGTTCAAGTCATGAAATGTAAATTCTTCTCCTTCAGGTAAATTGAATGCAATAC
CTAAAACCTTGCGTTTTAACAATAAAAAGTGGAGACTCTTTTTATTCATTTGGATCATCCTTTGTACATTAGTC
ATCATTTAAATGATGCACTTGTATCTTATATCTTTACTGAGATATAAAGTTCTAAATTTGAGAGGAAAATATTAG
GACATTGGTATTACACCTTTTGATAAGTAAAAAAACAACGATTTTTTTAGACTGACCCCAATTAGTGGGAATT
ATATAAAAACACTTTCGTTGAATTCATATTAAAATGAATCATACGGGGGGTGTTTTTTTCTATGAAAAGAGTTT
CTTATTCAGTAGAAACAAAGTATAAAGCAGTTGAAATGAAAGCAGCAGGATTTTCAACAAAAGAAATTATGA
AAGAATTAAATATTAGAAATAGAACACAAGTGAAAACTTGGTGGCGATGGTATCGAAATGGGGAAAGTTATA
GATTTTCACAACACGTTGGTAAACAATATACCTACGGTAAAGGATTAGAAGAGCTGTTAGAAGTAGAACAAT
TAAAATTAGAAAATAAGAGAAAAGATATAGAATTGGATATTTTAAAAAAGTACAAGGCATTGGAGAGGAAGT
GGTACCAACAGTAGTCGTAGATTTAGTGGATCAATTAAAAGTAAAATATTCAATCAAATTGATACTAGAAGTAT
TAAACATACCTAAATCAACATATTACCGATGGAAAAACAAAACCCATAAAAATGATACCGTAACACAAAAAGT
TATTGAATTATGTAAAGCTAACCACTATACCTACGGTTATCGTAAGATTACAGCATTGATTAATCAATGTTATAC
ATCACCAATTAATCATAAGAGAGTACAGAGAATGATGCAGAAGCATCATTTGAACTGCCGAGTTACCTAAAA
AGACGACAAGAATAGGTAAACCGTATTATAAAACGGACAATTTATTACAAAGACAATTTAAAGCGAGTTGTC
CAATGGAAGTATTAACAACCGATATTACTTATTTACCATTTGGTCATTCTATGTTGTATTTATCTTCGATAATGGA
TATTTATAACGGAGAAATTGTGGCGTATAAAATAGATGATAAACAAGACCAAAGTTTAGTTAATGATACATTAA
ATCAAATCGATATACCTGAGGGTTGTATATTACATAGTGATCAAGGCAGCGTTTATACATCTTATGCTTATTATC
AATTGTACGAAGAAAAAGGCATTATCAGAAGTATGTCCCGAAAGGGAACACCCGCCGATAACGCCCCGATA
GAAAGTTTCCATTCCTCGCTAAAGTCTGAAACTTTTTACATCAATAATGAGCTTAATCGCTCTAATCATATTGTA
ATAGATATTGTCGAAAAGTACATTAAAAACTATAATAATAATCGAATTCAACAAAAACTAGGCTACTTATCCCCT
GTGAAATACAGAGAATTAATAGCCTAGAACATGGTGTTTTTATTAAGTTCCCGTTTTAAGGGTTCAGTGCCTT
TAATCGTTGGTTTTTTTTATTGAATTAATAATATAAATTTGGTCCATCGTTAATATCTTCTAAACGTGTCTTGTAA
AACTTACTGATTATTTGAATCATTAGTTTATCGAGAAAATCATTAGGATTATTATTAAAGTTGTCTCTTTTATAAA
AGTTGCTAATATTATATTTTTTTAGATCGGTGAGTAAAGACCTTTCTACTTTTTCTGTCATTTTTATATCATTCATA
ATAATATTATGAATTAAATCTTTTAATCTAGTATTTCTTACATAACCATCAATAAGACTTTTAAAACGGGTTAATG
ATCGTTCGTGTTTGTGTGCTAATTTATTCAAATCATTTTTTAAATTAGAAATACTATATAAAGCGACTAATACATC
GTATTGTGTCGCACTTTGAATGATTTTACTTGTTACTAAAACTAATTCATCCATTAAATCGGAAATGGCAGTGA
ATAATGCAAAGTGGAACGTATCTTCAATAGTTGTTAAATAATATAATTCATTGTCGCTAATTAGATCTTGTTTTT
TCATAAATTCATTTATAGTGTTTTGAGACTCAATAAGTCCATCTATTGTTAAATTAACTTTACGTGACTTTTCTGT
CAGAATACTACGGATGAATTTAAAGTATGTATCATTTTGATCATATAAGTTGAGAGAAATGCCGATAACAGCTT
GCCTAAATTGGTCTAACTCATCAGATTGATTTAAGCTATACTTGTTATGAATCGAAGGTTTGATACTATTAGCA
ATTTCAAAGTATTGAGAATTACTTGTTCGTTCTTTGATTAAACCTTCCGTCACTAATTGTTTAAAGTAGTTAGA
CTTATTTAAGTTTGAGATATTTTTCTTAACATAAGAGTCTTGTTTTTCTTTATAAATGAGATATTTCGTTAAAAA
ATCACTTATTAATACATCGTCAAAATTTTCTTTAGATAAATCTTGATGAGTTTCTAATATTGTATCTTTCAGATTTA
TAAATGATTCCAAAGAAGATACATCGTCAATAGCTTTTTCATAATCTATTCTATAGTTAGCTTTTTTTAATTCAAT
ATTTTCTCGAATCTGTTTTATTGTATTGTTAGCTATCTTTTTAGCAAAATCTGTTGTTTTATTGATTGTGGTTAAG
AACTCGGTGAAATATGTATCTAGAGTTGTTGATTCTAACACATCTAGAGCATCTACACCTTGGTCAATGATTTC
ATTTGAAAGTTCATCTATGTCCACCATACTTTGACTATCAATAGAATACGTCAGATTATCTTTAGTAAAGCCACT

AGGAATTTTATCGATAAATATACCCTCTTCACTACATTTCACAGTGTGTTGTTCAATATTTTGATTAGAGGTATT
TACAAAGTAGCTTATATATATAGTTTCATTAGGTTGTGCTTCTCCCACTACATAATTTGTTCTCATTTCTTCAGGT
AAAATATGTTGTGCATATTCTTTTAGTATAAATTCGGAATCAATTTTGTGTATAAAATAACTGAAGAATTGAAAC
CACAGCTCTATTTTCCACTTATTTTTAATCATGATAATCACTCTCTTTCAATATTTGAATTAAGTATATCAAATCAA
TATTAATTGATATGATAGTTCTTTTACTAATTATTTTTATAAGATAAAATAGATAAAAAAGGAACAAATGTTCTCT
TTGGGTATATCATATGATTAGAGGAGATCGATTCATTTAATGAATTATTTTATTTAATAGAATTATATTTGAAGGC
ATTTAAAGGACAAAAGAAAACAGGCTATGAATTTGAAGAAAGATTGAACACATTTATTCAATCATATGTCATT
ATTTTACTTATAGATATAAGGTTACCGACTGTTAGGTTATATATTATTAGGCATATTGTGGATACATGCGAAAAA
GAAGTGGACAATCAAAAAAGAATTATACTAATAGAGAAATCTATTAAAAAGTACAGATATGTAAAAGAACAG
ATTAAAAGATTTGGAAATGCGTTGAAAGAAGAATTATCTAACGCGACTTTGTCTATTGAATAGAATATGTTAG
AAAATATCATTGATTATTATAAGGATTACTTTTATCCTAGAGAAAAAATAAAGCATAATAATGTTGAGTGCAATG
TATCATTGTTTTTTAAAGCTGTAGATGCTTTTAAGAAATAGTTTTTCACTGACAATATTTATGGATATAATTAAAT
TTAAGCAATGTTAATTTATTCCAGTCTTAGTAGTTTTTGAATTTCACAAACACAATCTATGAAGTGAGTTAATAT
TAAACGATTTTAATAGGAGAGGGTTAGTATGGCTAGTTATTTATTTTTTCATCCTAAACCAGCGTGTGATACTT
ATGGGGATATGAATATTTATCACGACAAATTTGGAAATAATGAGGACCCATACGTATGGAGTGAACGGTTTTT
GCATAGCTTTTGTAAAATAACGGATTATGCATATAGTAAATCTACTCAAAAGGACATTATTTTTTGGATATCAAT
AAATAAAGAAGGCAATAATTTAAAATATTTATGTGATTTAGTATTTAAAATAGAAAAGTGGGATTTTTGGTATA
AAACTTTTAGTGAACAAAAAGATGCCATAGCTACAAATAAAGAATTAACGATAAATGACGCAGTAGTAGAAG
GCGATGAAGAAGCATATGAATATCATTATTCTTGGATCAACAGAGGGGAACACAAGTGGGAACCAACTTATC
GAAGGCGAAGACTCACATTAAAAGCAGATCCCGTCTTGAGTTTTCAGCCTCAAAATCGACAAGGTAATTTAC
TAGATGTCACGGAATTACTAAAAACAATTGTGAATTTTAATGTTGAAAAATCACCTGCAAAAAGTGGGACTTC
TTATAAAGCTTTCGAACTTGAAGAAGAGCAGGCAAGCAAACTATATGAGGAAATAAAACGATTATCTTTTATT
CGATTAAAGGGAAGGGACTTAAAAAACTTAAGAAGAAACTTTTCTTGATATATCTTAATAGTTGTTGTTATTA
AAACCATAAAGAAAGCTACCATTTTTAAGATACAAGCATTGATATGTACAATCTGAATGGAGTGTAAAAAGTA
AGTGATGTTGTGATGGACATAAAGTTGATACTCTATTTATACGAAAAATATTGATAAAGATTAATTATGATAAAC
ATGTAGTAATAGAAAATGAATTCTATAGTTCAGGACTTAGCAACTAATATTATGGTAATGGGAAGAATAAACAA
ATTAATTTTTATAAAAAATATATAAATTAAAACTTAGAACAGTTAGAAACAGAATTATTTGATTATGTAAATTGG
TACAACAATTTTAGACCACATTCTTCGTTACAGTATTTAACGCCAATGGCGTATAAAGATATACACATGAAAAG
TGTCTAAAAAAACTGTTGACATTCCACTCTATATTTGATTGTAGTTGTTTATAGATAGTAAGATCATTCATTATACA
TGGATTTTTGTCCGATTTCTTGACACAATTCCAGACAAACTAATAAACTAATAAAATAAAAAAATAAAGGCGC
GAAAACAGGAATTATTAAATAAAATATTAATTTAATAAGGGGAACAAATGAAAAATATAGATATCGCAATTTAT
GATATAGATAAAGTTATTTGTAAAAGTATTGAGAATAACTCATCAGATTTAGGTTATTTATCACAAAGTATCTTA
TCACATTTAAGAAACTACGTAGAACATATTGGTATGAAATATTATTTCGAAAGTGTAAATGAAGATATGACTAA
TAGTTCTTCAAATATCTATAATGAAATACAAATGGGAATAGGTTATTTTAGGAAGCATTACAAATTGAATTGGC
TTAAAAAATTTTCATGAATTACTTCAGCAATCTGTTTCTCATTATACATTTGATGAAGATAGATCCGAACGGTTAT
TTATAAAGTATTATGACTTAATGTTAGAATTAAAAGAAAAGTTAAAGAAAGATTTTGAAATGGATTTATTGCAA
AATTTATATAAAGTACCGCTGAATATTGATAGTGATTTAAAAGGATATTATCATACAATATCTCAATTAATAGGTA
ATAAATACACTGATAATGAATATGAAGTAGATAATTCAAGGTATTATATTGAAAAAACAAAACCATTTGTTATTG
AAAATAAAGTATATTATGAAATTACATTTAGAAATGCAAATGATAAAACTAGTAAATACGAAAGGCTGATTGGT
TATTCAAAACATAGAATAAACACAAATTATGCTGTAAAATTCGCATTAGAAATGCAAACAATAAGTTATTTAGG
GATTAAAGCTAACGTAATAATAATAAATGATTACGAAATATCTATAAGACCTTGTGAATTTAATAATTTTGCTAA
AATATTAGATTACGATTTAAAATTACAATCAAATCATAATGAATATAAAAAATTAATGAAATTGTTAAAAGAATT
TAATTTACATTTACTTGATATTGTTTTGTTAGATGACGATGAATTTAATGAATTGGAGTGTTCAGTGAATAGCG
AATCAGCAGTTATTAATATTTTTAATGTGTTGAGACTAGCAAGAAGATATATTTTAACAAATAAATCAGGCGCA

AATGTTCTTAGGTATTTATTATTTGTTTTTAACAATAGGATAATTCGTTTACAGTTACCTTATAATAGGAATAAAT
GTTATAAATTATCTAACCTTATATTGGATTATAAATGTGTTCCATTTGATCAAATGCCATTTACAGCCTCTTTAAA
AGGCCATAATCCTAATATCTATACTTTGTTACAATGTATAGAATACAAAGGGAGAGAATATGAATTATTAGTCAG
GAAGATTCAAAAGAATACTTTGAAGAATAAGAAAGTTTATACAAGCAAAGAAGAAATTGAGCAGTATGGTG
TTGTAAACGAACTAATCGATAAATATAATAATAATTTGTATTATAAGCATCGACCGAATAGGGAAATACATTCAT
TTGGTGATAAATATTATTTATATGAAAATGAGCAGAGTATTATGAGTATAATAAAGTCAATTAAACTATTGTCAA
ATGAAAGCGTAGAGGGCTATTCAAATTCAGTAGAGTTTTGGCTAAATAATGAATATACATCGTTGGATTGTAA
AGAAAAGAAGGAAATACTCTTAAGAATGTTTTCAAATAGCAAAATATCGATGGTATACGGAGCTGCTGGAAC
AGGGAAATCAACTTTAATAAACCATATTTGCAATTTTTTCTATGATAAAGATGTTATCGTCATAGCAAATACTAA
TACTGCAGTAGACAATATTAAAAGAAAAATCAAATTATCTAACATTAAAACGTCTACTATTTCTAAATTTTTATA
TAATGATAAAGAAAAGTATGACTTGTTAATAATCGATGAGGCAGGTACAGTTAGTAATAAGGACATGAATCGA
ATTCTTGAAAACAAGCAATTTGAATTATTATTAATTGTCGGTGATAATTATCAAATCGAATCGATAGATTTTGGA
AATTGGTTCGAAATTGCCAAAGATGTTTTGTCAAAAAATATAATCAACGAACTAACTGATATGTATCGAACTAA
AAATGATGATTTACTTTACTTTTGGAAATCTGTTAGAGAAAAAAAGAGTAATTTAAATGAAATTATTAATATGA
ATAAATATTCTACAAGATTAGATGAAAGTATATTTAATGAATTCAATAAAGATGAAATTATTCTTTGTTTAAATTA
CGATGGTATATATGGTATCAATAACATTAATAGATTATTACAAGCAAATAATAAAAATGATTCTGTAATTTGGGG
TGTGAAAGAATATAAAGTTGGTGATCCTATTCTATTTAATGAAACTAATAAGTACTCACCAATACTTTTTAATAA
TTTAAAAGGGTCAATAATTGAAATACATGTTTTTGAAGAATATATATTGTTTGATTTAGAAATAAATAAAGTTAT
AAATGAACTTGATATTATTTCGTTAGAAATTGATTTAATAAGTTCTTCTGAAAATAGTTCGGTGATTAGGATTA
GAGTAGAAAAAAGTGATGGGTTGAATGATGATGATAATGATTCATCTGATAGTATAGTTCCTTTTCAAGTGAG
TTATGCAATTTCAATACATAAAGCTCAAGGGTTAGAGTTTAATTCAGTAAAAATTGTTATATCTGATGATTTAG
ACGAACAAATCACTAACAATATTTTTTATACTGCAATTACACGAGCTAGAGAAAATTTAAAGATTTATTGGTCA
CCACGAACTGAAAAGAAAATTATTGATAATATAATTTCTAAAAGAAACTTGAAGGATCTTTCTATATTAAAATC
TCGAATAAAAAAACAAAATACTTAGAAATTTTCATACGACGCTTATATAGAACCATAAAAATCGTTTGACTAGT
ACCAGTGATTTTATTTAATTTTTCTCAACGACTCTACTTCTAAAAATATTTTAAATGACTTTGATACAGCACGAA
TTAAAATCGCATAAGCGATGACTTTTCCACTTAATTTCTTATGCCATTCTACGGAAGTCATTTGAGAACAAAGT
ATTAATGATTTTTCACGGCTTCTTATTTCGAATACCTCCATGAGATACTATTGTTCTTGTTCAGTTGTATACGGC
AATAAAAAATCATCATTTACTAAAATATCCGTTCTGCTAATCACTTTTAATAGTTTATCTATGCTATTATAATCAGC
TTGTTGTAGCTTACTTAATAAATCTGTCAATCTATAGAATAATACACTATACCGATTATCAATCGCATGATTTACT
AAAGTAAAAGCGATGTAACTTTTACCATTTCCGGTGACACCTTTTTAAACCGCCATTTACATAAATACTATCTT
TAAATATAACTTGATTATTTGAAAGTATGTGAAAGTAAATTAAGGATTGGTACGTTTTGGTTAATTAAATATCTA
CCTAAATATTCCTCTAATTGACTTGTTGCATGTTCATTCAGCAAAACAGGAATAATCCATGCTGTATATAAATAA
TCATCTAGAAATTCTCTACATGTAGAATATTGTTTTTTTAATTCATCGTCTTTTATATACAATGAAGATAATATTTT
TCCGGTACCTTTATCTTCATCCATCTTCTTAATAAGTCACCTTCTTGATTTTTAACTAATTTATATTCATTACCCTT
AAATTCATATTTGAAATGTGATTTTCCAACTACGCAAACCATGCCATCTTTTCTTTGAGTAACATAGATGTAATT
TATTATTGGGTTATTTCCATTAGCTCTATTATTATTTGTTAATGTGTTACTTATATTAGACAATAAATCCTCAAAAT
ATGTAATAATATATTCATCTAAGGGTTTATTGTTATTACTAATATTACAATTTTGTTTGATAGCATAAAAAGCTTCT
AGGTTTAATTCAAATGGTTCCGCGCTAACGATTTTGCTATCTTTGTTGTCTATAATAAATTCGATTAGTAATTTC
TTAATCATTTCGTGATGTTTTCTAGGCTTATTGTAATGACAGGTTTTATAAAGCTGTTTATTAATTCTATTAAACA
TTTCTATTTGCAAGTCAGCAAGAATAGTTCTACAGTCTTCATAGATATCATGTACTGGGTATGGTTTATTAGGA
AACATGCTATATATTTCTTTATATGTTGTATTGAACACGTTAATTATACCTCCTACTATCTATTAATACTATTGTAAT
TATCAGATATAACTAAGTAAATGAATCGTCTACACTTAATTGGACAAATTCTATGAGAATAGATATTGTTAATTT
AAGAAAGAAATTACGAATGAACGCATTCGCAAAAATTGCATTTAAATAATGAAATATAGAGTAACTTGAGAA
AGTTGTTTCATCTAAAATATATAAACAGTAGAGGGGAGAACTTATGCACTGGAAAGAAAATAGAATAAAATC

AGCCAAAAATGACACGAATCCGATGGTAATCAAAGAATTAAAAGGTAGTTATGTGGTTTTTGGAGATGTTCA
GTTTCTTCCAGGTTATTGTGTATTACTTCCTAAAAGAGAGGTAAGATTATTGAATGATCTTACTTTAGAAGAAC
GACAAGATTACTTATTAGATATGAGCTTTGTTGGTGATGCTATGATGAAAGCATTGAAACCTACAAGAGTAAA
TTATGAAATACTAGGTAATAAAAATCACTTTCTTCATGCACATTTATTCCAAAGATATGAATGGGAAGATGAAT
CTGTTAGGTATATGCCAGTGTGGTGTATGATGCTTCTAATTGGTCTAATGAAGAAACGTCTTACGATTCTGATA
AACATGATGAAATTAGAAATAAGATAAAGAACGAACTTGAACAGTTGTATAACATATAAAACCAAAGTCGATA
TCATCATTTTGATATCGACTTTAATTATAAAAAACCGCACTCTTAACCGATACGCAGAGGCGTATCATAAGT
>Staphylococcus aureus strain BSN14S3
ATGAAAATCACCATTTTAGCTGTAGGGAAACTAAAAGAGAAATATTGGAAGCAAGCCATAGCAGAATATGAA
AAACGTTTAGGCCCATACACCAAGATAGACATCATAGAAGTTCCAGACGAAAAAGCACCAGAAAATATGAGT
GACAAAGAAATTGAGCAAGTAAAAGAAAAAGAAGGCCAACGAATACTAGCCAAAATCAAACCACAATCCA
CAGTCATTACATTAGAAATACAAGGAAAGATGCTATCTTCCGAAGGATTGGCCCAAGAATTGAACCAACGCA
TGACCCAAGGGCAAAGCGACTTTGTTTTCGTCATTGGCGGATCAAACGGCCTGCACAAGGACGTCTTACAA
CGCAGTAACTACGCACTATCATTCAGCAAAATGACATTCCCACATCAAATGATGCGGGTTGTGTTAATTGAAC
AAGTGTACAGAGCATTTAAGATTATGCGAGGAGAAGCATATCATAAGTGATGCGGTTTTTATTAATTAGTTGC
TAAAAAATGAAGTATGCAATATTAATTATTATTAAATTTTGATATATTTAAAGAAAGATTAAGTTTAGGGTGAAT
GAATGGCTTATCAAAGTGAATATGCATTAGAAAATGAAGTACTTCAACAACTTGAGGAATTGAACTATGAAA
GAGTAAATATACATAATATTAAATTAGAAATTAATGAATATCTCAAAGAACTAGGAGTGTTGAAAAATGAATAA
GCAGACAAATACTCCAGAACTAAGATTTCCAGAGTTTGATGAGGAATGGAAAAAAAGGAAATTAGGTGAA
GTAGTAAATTATAAAAATGGTGGTTCATTTGAAAGTTTAGTGAAAAACCATGGTGTATATAAACTCATAACTCT
TAAATCTGTTAATACAGAAGGAAAGTTGTGTAATTCTGGAAAATATATCGATGATAAATGTGTTGAAACATTGT
GTAATGATACTTTAGTAATGATACTGAGCGAGCAAGCACCAGGACTAGTTGGAATGACTGCAATTATACCTAA
TAATAATGAGTATGTACTAAATCAACGAGTAGCAGCACTAGTGCCTAAACAATTTATAGATAGTCAATTTCTATC
TAAGTTAATTAATAGAAACCAGAAATATTTCAGTGTGAGATCTGCTGGAACAAAAGTGAAAAATATTTCTAAA
GGACATGTAGAAAACTTTAATTTTTTATCTCCTAATTACACTGAACAACAAAAAATAGGTAATTTCTTCAGCAA
ACTCGACCGCCAGATTGAGTTAGAAGAAGAGAAACTTGAACTCTTAGAGCAACAAAAGCGTGGATATATTC
AGAAGATTTTTTCTCAAGATTTAAGATTTAAAGATGAAAATGGAAACAGTTATCCTGATTGGTCTATTAAAAA
GATTGAAGATATTTCTAAAGTTAATAAAGGGTTTACTCCAAATACAAAAAATGATAAATACTGGGATGAATTA
AATGAAAATTGGTTATCTATAGCAGGTATGACACAGAAATATTTGTATAAAGGAAATAAAGGAATTACTGAAA
AAGGTGCATCAAAGCATGTAAAAGTAGATAAAGATACTCTAATAATGAGTTTTAAATTGACTTTAGGTAAGTT
AGCTATAGTAAAAGAGCCTATCTATACAAATGAAGCTATATGCCATTTCGTATGGAAAGAAAGTAATGTTAATA
CTGAGTATATGTACTACTATTTAAATTCTATAAATATAAGTACTTTTGGTGCACAGGCAGTTAAAGGAGTAACAT
TAAATAACGATGCAATTAATAGTATTATAGTAAAGTTACCAGTGATACAAGAACAAAATAAAATAGCATACTTT
TTCAATAAATTAGATAAATTAATTGAAAAACAATCTTCTAAAGTAGAATTATTAAAACAACGCAAACAAGGATT
TTTACAGAAAATGTTTGTTTAATTCTTATAAAGTTCTATTATGTAAAATATTAAATAGAGATAACATTATGAAAG
CGAGCCCAAGACATAAAGTTTTTGAATAAATAAAAAAGATAATTTCTATCAAATTAATATAGAAATTGTCTTTT
TTATAAATTTTTTGATTATTTTTAGCTGATTGAGCTGTTACTTTTCTTATAATAAGTGCTATTAGCACAAATCCTA
GTTCTCTTTTGGCTTTGTTTATTCCTCTTACGGACATTCGAGTGAAACCCATTTTAATTTTATTAGAAGTAATTT
AGGTTTGAACCCACCTAAATAAATATATGAGTTATTTTTTTATGCTACAAAATATATTCAGATTTCAATAATGAC
ATAAAATAGGCATCTTTATATTTACCTTTAGTGTAGAATTGCTCTTTGAGTAATCCTTCTGTTTTAAATCCTTGTG
ACTCGTATATATGCACAGCTTTTTTGTTATCTGTATCAACATATAGATAAATTTTGTGCATGTTTAATATATCGAAT
GCATAATTTATCGCTTTTTCGAATGCGAATTTTGCATAACCTTTACCACTGAACTCAGGTTTAATAATTATTTGT
ATTTCACAATTACGATGGATGTAATTAATTTCTACTAATTCAACAATACCTACGACTTGATTTTCATCTTCAACA
ATAAAACGTCTCTCTGATTCATCTAATAAATGCTTATCAAATAAATATTGAAGTTCCGTTAAGGATTCATATGGT

TCTTCAAACCAATAAGACATAATAGAATATTCATTATTTAATTCATGAACAAAAAGTAAATCACTATACTCTAAT
GCTCTTAGTTTCATAATTCCACTCCCAAAATTTTCTCATATATTTGCATTATAAATATAAATAACGAATAAGTCAT
CATTCACTGTGAATACTCTATTTTAACAATTCACCACATACTAATTCTCATTTTCTTGTTATTCTCGATTTATTACT
CTTACTATGAAACCTATAAAATTCTCACATTTGTTTGTATTAAGAATAAATACGTCGATAGTAACAATAAAAAAA
TAAATAATAAAGCATCCCTCACCGTAAAAGTGAAGGATGCTCTAGTTTTATTGAAATATACATTTCATTTTGTTA
AATAATTATTAATAATATTTTGAAAATCATTATTACGTGAAATCTTCATAGATTTTATCAAGTATTTCTTTGCCTTC
AATTGCTGTGAAGTGATGTACCAATCTATTTTTACAATCATATGTAATTTTGTGACGCTAGGTAATTAGTAATTG
TTCGTCAGTCTGATTGTATAGTATCAAGTTTCATAGATAATACTCTTTGATTTTAATGTCCACTTTGACGTGCTT
TAAGATTGAGTATATACATAATGTCATTGTGGAATGTTAAAAATCCTACAAATGTTTATTCATCTGCAGGATTTT
TAAATCTCCAAGAATAAAAATCATCATAGGACAACTGGATTATTGTTTGGATAAATAACGTAAACAATAATTAG
GTACTATTATTTATTTTTGTTTATTCTTTTTCCTAACAAAATAAAGAAAAGAATAAACGCAATTGTTAAAAATAT
GTGTCCTAAACCAGCAATACCAGCAATAGCAGGACTTACACTTAGATCTTTAATGGTAGAAATACCGTTCACG
AATTGCATTGCCACAGTAACAAGCACACCTAAATGGTATATATAAAAGAAACTGTTAAACAGCTTTGTATGAG
TTGTTAATTTGAATTGGCCCTCGATAATCATGAAAATTAAGAACATAATTGTACCTAGTACTAATAAATGTGTAT
GTGTAACATTTAATTGAGAAAAACCGCTAAAATCTTCCGCTTTTGTCATTTCTCTATAAAATAGACCACTTAATA
ACCCTAATAGTGTATAGAGCGCTGAACTATACATTAATCTTTTCATTTTAATTCCCCCTATTTTTAATTACGAGAT
AAGTATAGCGGTAGTTTATGAACTGAGTATGAACTTACAACAAAAAAATTAATGAAGTACTTTACAATAAACT
CAATTTATTAGATGGTGGAGGGACGAAAAAGGATTTTAGAAAAATAAATTAATATATTTTTATTTTGATAAGTA
ATAATTAATAATATCTTGGAAATCATTGTTAAGTATTGTTGTAATACAATCGTCATTCATAAAATCTTCATAGATT
TTATCAAGAATTTCTTCATCTTCGATAGATGTGAAATGATTAGCTAACCCTTTTATAATTTAAGTGTAATTTGTG
AATCTAAACAACTAGCAACTGTTCGCCAATCTGATTGTGTAGTGTCATGGTTCATAGATAATCCTCCCTTTATTT
TAATGTCCATTTTTGACGTGCTTTAGGGTTAAGTGGATGCATAACTTCGTTCGTTACTGGATTAATGAGTTTTT
TGACTTTCTTTTTATGAGGTTTTAACATTTCCATCACTTGCTCAACACGTTCGACGACAACAGGCCGCTTCGC
ATAAGCTCCATAGGCTAGAATCACTGTGTCACTTTCACTAATCGCTTTCATTAAGTGAATGTCTGTGTGTTTGT
CATAAGGCTCTTTAATATGTTTAAGGTTTTCAGGTGTTTTAATATTAGAGAATAGATTTACTAGATATACAGCAC
CGTATTGTTCAGAATTAGCTAATTGGTTGAGAATGAGAACAGTGGTAAGATCGAGTGATAATACGCCATCTAA
ATGTGGATACATCGTTATCACTGTACAAGAGGGTTTCTTTTCATCCCATATTTTCTTGAGTAAGTAGCGGTGTT
GTTCATCGTCGCTAAATATAGCTTCTGTGTGTATCGTACTTTTGATTGTATTCATATATCGTTACCCCTTTTAATAT
TCTTCTGGCAAAAGCATCACATAATAAAAAGCGTCTACGTCATCTTCACGAATGACGTAGACTTTCTTAGGTA
ATGCATTTTGATTTTTTACATAGTTTGTATAGTGATATTCCAATTTGTATGCAGGTTGTTCTTGTTCATGTGTGAT
TGAGAGTATATTCTCATCTTCTTGTAGTCTAAAAATGTGTAGATAATCTGTATGAGATTGATTATCTCTTTCTTTT
ACCATATTCCAAAGTAGGATTTGAAGGTCTAGAGATAGTTGTTCACTTATGCCTCTTGTGATGTATCGATTGAT
TTTCATGTTATTTTACCTCGTCTTGAATTTCTTTCATAATGATAATTGCTTGGCTAATAATCGTAACAGATATTTG
TGCCACTTTGATCCAATTATTCATGGTTATTCTCTCCTTGTTTTAGTAAATGACGTTCATCGATAATCGTATTTTT
AGTATCTGTGAGGTATAGAAAGTCCATATCAAAATGATCCAAATAACCAATGCTGATGAGTTGGTCATTGGCG
TACATAAGAAATGGATAGATACTTAGCTCATGTAGCTCATCATTGTAGTAGGTATAAGTGTTGAGTGTGAGATG
CGCAAGTAGAGAATTATTAATAAAACGTTCCGGTAGAATATTTTCTGCTGCTTCCTCAAGTGCTTCACATTCCC
ATGTTTCGTTGTTAGATAGTTGGAAGAGACGAGTTATATATTGTTTGAGTTCTTGAGTGGTTGTTTTCATATCA
TTGCCTCCTAGATAGTGTGATAGTGATGTAGTTGATATACATCATTGGGATAATATATATTTGATTTATTATTTATT
ACGAATCCCGGTGGGAATAAGAGAAAATTCCATATAAAAACCCGTGATAAATGTTGGTGTAACAAGGAAATC
CCGGAATCCCACTCATTTTGACGAACAATCAACTCATTATTTATAAGTATTGATGATAGGGTTGTGTCTCTGCT
TCCTTATATATATTATTTATTTATAAAAAATAACGGGATTTTGGGATTGTGCTTGCACAATCCTTCTGCTTCTTCG
AATCTGCAAATCCCATTCCTTTCCCGGTAAAAAATCATTGTGGGATGTTCTTTAGCAATTTCAATATAAGCATT
GTGTAGTCATGAAAAAAATGACGGCAATGACTGTTTCATTAGATAAGTGTTATTGAAATTGATAAAGAGAATT

CTAAAAATGGTTAGATAAAATAAGTGAAAGAATAGCAGTGTAGTTATTGTTTATTCAATAGTTATATATAAAGT
TTGTGGCAAAAATAAAGACGAAGTGCTAGGGAGCACTTCGTCGAGTGGATGGTTATTAAATAGTTGTTTGAT
TATATCATTATTTAACTTGAGCGTAACATAAAATTGCTTCTTATGATGCTCATCTTTTCTTATGTCAATGCGATCG
ATGATTGTTAGATATAGTGATTTCAACTGAGATTTCTCTAATTTGTCTATATCTTTGAATATTGCTTGTAGTACAT
TCGCAATCATATCAGCATCATAATGTGGAGCTTCTGCTTGTTTATCTTGTTCTAGTTGATAGATTTGATTATTTAT
TTGATTCAATTCATCTTGGTAGCTAAGTATTGTTGGTTTTAATACGCTATCTAAGTCAGGTGAGTCTTCAATAGT
TTTCGTTAATGTATGCATTTTTGCTTTTATTTCTTCACATTGTGACTGTTTATAGGCAATATCATGGTTCAAAGA
AGAGACGTCTATTTGACTTTTTTCATTTACCTTTTCAACCAATTGCTTCAATACCTTTTTGCTTTTGTTAATTTC
CAATATCTGATCCATAACATATTTTTCTAGTACATCTGCTCTAACGCTATTGGCAGAACAAACTTTTGAACCTTT
ATTTCTAAAATTGCTACATGAGTAATATCTGATTCTTTTCTTAGTGCCATCTTTTAATGTATTAGTTGTATTACTT
GCCGCCATTGCTGCACCACATTTCGGACATTTTACAATCCCAGTCAGTAGGTTTGTTCCTTTGCCGTGAACTT
GTGGTTTCTTGCGACTCTCTTGACGTTTAAACTGTACTTTATCCCATAGGTTTCTATCAATAATAGGCGCATGTT
TACCATCAGCGATAATCGGTTCCTCATTGAGTCCTTTTCGTCTTTTATCGCTCCAGTGTCTATACTTCGCAAACT
GTATCTTTCCAATGTAAAAAGGGTTTGAGATGATGTAGGTAATGGACGAAATACTAAAAGGTTTCCCTTTCTT
AGTCACATAACCTTTATGATTCAATGCGTTCGCAATCTTACGATAACCATGACCTTTAGCGTATGAATCAAAAA
TATATTTAACAATATTCGCTTCATGTTGATTGATCATGAGCTCTTTTTTACTGTCAGGTACTTTATCGTAGCCTAG
AGGTAAATTACCTTGATAATAACCTTCAATAGCACGTTGTCTTTGGCCATTGTAGACATTCTCTACAATCGTATT
ACGTTCAAATTCTGCGAAGCTGGCTAAAATTTGGAGCATCAATTTACCTGTTGAACTGGCAATTTCTATTTTTT
CAGTTAGACTAAAAAATTCGACATTAATCTTATACAATTCCTCGACAATATTTAACAAATCTGAGGTATTTCTAG
CTAAACGATTTGTTTTGTAGACCATAATACAATCTAATTTACCTTCGTTGGCATCTTTTAACATACGTTGTAATT
CTGGACGTTGCATTGTTTTACCTGATATACCACGATCGGTGTATTCATTGACGACTTCATAGCCTTGAAATTGA
CAATACTCTGTAAGTTGATTCAATTGACCTTGAATACTGTAACCATCCGTTTGCATTTCAGTCGATACACGTGC
ATATAATCCAATACGTTTCTTTTTGAGTTGTTTCATATTACTTCATTCCTTTCAGGAGTTATTAAATGTGATTGTT
CAACGATATTGAGTGGACTGTTTTTAAAGTAGATTCCTTGTAATTGTTTAGTTTGAGTTATTTTGATGCAATCG
ATAAAAGGTGCTATATCCTGTAAAGTTATTTTATTTTTTATGACATATTTTAATTTGTCATCGATTTGGTAGGTTG
AATAAGTAGAGTAATTCTCTTCACTTTTACAACTAGCAGATAAACGTTTGAACGTTTTTACATCAATACGATTT
TGAGCTAGCTTTTCGATAAGTTGTTCTTGTGTTAGATGGGTTTCTTTATGCTTCATTTGTTGCTGTTTTAGGAC
TTTGAGTATTGTGTTATTCAATCGTTTATGAAACGATTGTTCTTCAAAATACTTTTTACAAGTGTCGAGCACTT
CACTTTCAAGTTCTGGTGCATTGATACTTTTAAATGGGCATGTACGATAAGCGTCATTCATATTTTTAGGACAA
ACATAGTAACGTAGAGAATAGTTCTCTTTTTTTATCGTTAAGTTTGTTAATGTTGATTGACAGTAAGGACATTT
GATTCGTCGCTTTAGCTTATTTCTAGAATTGGATCGATTGAGTTGTTTATGAATACGACGCTCTTGTGCTTCTT
CAAATGTATCAATATCAATAATAGGTGGAACGATATCATTAAACGTGCCATATTTATTGATGACACGACCGCAAT
AGTTAGGGTTCAAGAGAATATTTCTAACTTGATAGGGCTTACGAGGAATAAGGTTAGGATTACTATCCAAATG
TTGGGAAATCTTTTTGTAGCCTAGACCTTGTAAGTACCAGCGATAAACCGATTTAACTGTATACGCTTCTTCTT
CTTGTACAACAAAACAACCTTTTCTATAACGATAGCCAAACGGAGCATGAGTTGTGATTAGCTTACCTTGTTT
GGCTTTTTCTCTAATCCCATTTTTTGTTTGTTCGCTGATATTGTTTGATTCCATTTCCGCTAGACTCATAAGTATG
TTCAAACGAAAGCAATCAAACTCTTTAGACAAATCAAAATATCCATCGTTAACACTGATGATTGTGATGTGAT
GCTTTTTACAAATTTCAAAGAATTGTATGGCATTTTTCAAATTACGATGTAGTCGGTTCAAGCGATAGCAACA
CAATACTTTACATTTTCCAGACGTAATTATTTCTACCATTTTTTGATAACCTGAACGTTTTGTATGTCGACCTGT
TTTCTTATCATCATAAAACGCCACATTAGACCATCCATATTGCTTAGCGGTATCCATAATGAGCGATTTTTGAGT
AGCTAAGCTTTGTTGTTTGAGTGTACTTTGACGTACATAAGCAATCGCTTCTTCCATGTTATACACCTCCAAAA
AGATAATATATATTTGTGAGTAAATTAGAATGAAAGGTCCAACGTGCTTTTAACACGTTGGACCGTCGTGATT
AGTTATCGTTCTGTAGCTCTTCAACGACTAAATCAGCTAGTAATTCAATCAATTCATCCATTTTATCTACTCCTGT
ACGATTTCATCTTTAAGTTATTAAAAATCAATTAAATCATCGTTCTGTTTTTTCCATGATTCAAGTATCTTTTTGT

TATCCAAGTTAATTTCAGCTTTAATAGGTTCAGCATCTTTAGTTAGACCAAAAATAGACGCATATTTTGAATCTA
GCTTTAAATGGTAAAAGACAAGTGACTGTTTCTTGCCATAGTCATCTTTAACTGCTCTTTTCGTAGTTATTCGA
TCACGGTCAGATTCGATAAATCCTTTATCCCTTAGTGCATTCACAACATTGTTGACATCTTGGAAATGATGCTC
TATCAACATATTCTTAAATACAGATGCAATGATTTTAACTTCGATATAATCATCTTTTAAGGCAATTAGTCCATAG
TTCTCAATCATATTCTTTAATGCTGTATCATCAGAAAACTTACCACGGTTTTGTGCTACAAATTGCGTAATGACT
TCAATCGCTTTATCAGCCAGTGATCGTTCAGAGACTGTATGAGCATGATAATCAATAAAGTAGTCTCTTATTTT
AGCGATATCAATATCTGTAGATAAAACATGACTAAGAATCTTGGCCGAAGTAGTGATAGCCGCATAGCGCTTA
AACATACGAATACCAGTATTATTTGTTTCATCCTTCAATTTATCTTTAAACCAATGATGTAATTCAAAAACGGAT
CAAAAAACAAAAGGCCCTAGAAACTAGGAGCACACTTACACAAGAACAATTGATAGATAAATTAGCTAAAG
GAGCCATCGATGCAGAAACGTTCAGAAAACAATCTCAATCGTTACTTCAACAATCAAAACCAACACTATCAAT
AAATGAGCAACAAATTCAAAGGTCTTTTGAAAATGTAATTCAACAACACTTCACGTTAAGCATGTTATACCGA
TATATTGATGAAATTCATATTTCTAAAAACAAAAGCCTTGTGGGAATCTATTTCAAAAATGAACCGCTAAACAT
TGTAAACCAAGCTACGCAATCATCGATTGCTTAATTAATGAAAGGATGAAAAATATGAAAGAAATGAAAAGA
AAATCTGTAGGATGTTATGTTAGAGTTTCAACAATTTCTCAAGACATTGATAAATTTAGTATTAATGGTCAAAT
TACACAAATAAAGGAATATTGCCAACAGGGAAATTATGAATTGCTATACTGATAATTAAAAGTACCCCCGATC
GAACGAAAATATCTTTTTGATGACTATTAGTGATAATTGTAATTTTATCCATTAATAGATGAATGGATTGGAATA
ATACTAAGCCTATTGCTATTCCACCGAGTAAAATTACCCACCCGCCTAATTCAATACTTTCCGGAATCATTTCAA
AAAATAATAAGCCAATTATAAGGCCTGCACATAATGAATAAATAAAGCCATGACCACTTTGAAAACCTTCCATT
ATCCAAGCGAGTCCACCGCCAACACCAATTCCAAGTGCGGAAGCTAACGCCCGATCATCCATACTGCACTCA
TCCCACTATTCTTAAAAAGGCTTTGTTTATTTTACTCTAAAAAATCAAAACAAAATATATGAATTCATATTAAATA
TTATTTGACTCTTTATTCAGATGGTTATATCATATATATGAACATATATTAATACATGTAGATAAATAGTGATTATTA
TATATAAGGTTTTGAATTGAAACCTAAAGTGAGGGAAGGATAATGGAAGAAAAAAAGGAATTAGAGGAAGT
AAATAATAAGGACTTAGATGATGAAACATTATTTGTCGTATCGCAAACATTTAAAGCGTTAGGTGATCCTACG
AGAATCCGGATTCTCCATTTGCTCTTTTATAAGGAGTATTCGGTAAACGGTATTGCTGAAACGCTACATCTTAG
ACAATCAACAGTTTCCCATCAATTGCGGTTCTTGAAAAATTTACGGTTAGTAAAATTCCGAAGGGAAGGCAC
AACATTGTTTTATTCCCATGATGATGAACATACTATGAATATGCTAAAACAGGCGATCGATCACGCCTGTCATC
ACTAGTATTCCGTAAACCAGTAATGAGTGTTAATAATAATTCACGTCATTAATATTGTTATATGATCATATAAAAA
TATTATAGGCATATGAAAAGGGGGTTTTGCAATGGATATGGAAAACAAAAAAACAGAATGGAAAGCGTTGT
ATGATATCTCGAAGGAAAGTGAGATGGGTGTAGCAGAGAGGGTTAGTGAATACGGTTGATAAATTCATAGG
ATGTATTATTGGATACAAAAAGTTTGGATGTGGTCAATTAACGTCCTGGTTTAGTAAAGCTTACTGCGAATATA
TTGGAACCCGATATATCATATTTATTTAGAAAGGTGATAGAAATGAAAAATATTCAAGAGCAACAAGCACACG
AAAGTCATAGCCACGATCATAGTCATGATCATGATCACGGAAAAATGCCAATTATTTCATATTTTATTGGCTTA
GTGTTGGCTATAATTGGGCTTTTTTAAGTGATGCAAATTTATTAATACAAAACATCTTATTTTCAATTGCCACAA
TCACAGCCGGCTACCATGTAATTATTCTCGAAGGAATTGGAGAGACAGTTGAAAATACTAAATTAAAGGGAA
AATTCACTCCTAATTCTCATATTCTAATGGGATTAGCTGCAATCGGGGCTTCTCTGATAGGGAGTTTTTGGGA
AGGAACCCTTTTGATACTTATTTTTTCCGGCGCTCATTTTCTTGAAGATTACGCTGAAGGAAAAAGTAAAAG
AGAAATTACTAAGCTACTCGAAATGAACCCAACGACAGCTAAATTAATCCTACCTGATGGAAACACAAAAATT
GTTGATGTCAGTGAATTAAAAGTTGGAGATCAACTCCAAGTGCTGAACGGTGATCAAGTTCCAATTGATGGG
ATTATTTTATCCGGTACTACCTCAATTGATGAATCTTCTATTAATGGAGAAAGTATACCGAAAGAGAAGTCTAA
GGGTGACGAAGTTTTTGGAAGTACGATTAATGGAACAGGTACTTTTACTATGGAAGTCACTAAGGAAAACA
AGGATACTGTATTCTCTAAAATTTTACAATTAGTTAGTCAAAACCAAGATAATCAAACAAAAGCTGCCAGTATC
ATTCAAAAATTCGAGCCTAAATATGTTAATATAGTTTTAATCGCAATACCATTAGTAATGTTACTTGCTCCTTTTC
TATTTGATTGGACATGGTCGCAAAGTGTATACAGGGGATTAGTGCTTTTAGTCGCAGCTTCACCGTGTGCTTT
GGCAGCAGCTACTGTATCTGTAACATTGTCTACAACATCTAACCTAGCTAAAAAAGGCGTGCTTTCAAAAGG

AAGTACTTACCTATCACAATTAGCGGATATAGATGCAATTGCCTTCGATAAAACAGGAACCCTTACGAACGGA
GAACCTAAAGTAACAAATTACTATTTCACTCATTCTGTGAACGAAGAAAATATTATTGATATTATAGTCGCCCTT
GAAAAGGAATCCAATCACCCACTCGCTAATGCTATTTTAGAAAAATTTGAAGTTAAAAATAAAATAGACATCG
AAGTTACTAATCAAATTGGAAAAGGTCTGACAGGAGATTATAATGGAAAAAATTATCGTATTGGTAAGCCTAC
TTCTTTTGAAAGTGCTTCTGAAGAGTATACCCAGTTCAATCATGATTGGGCATCAGAAGGAAAGACGGTTGT
ATACGTAGCAGAAAATGAAGAAGTTATTGGGATTATAGCTCTAATGGATATTCCGAATGAGCATGCTAAAGAA
ACAATTAATTACTTTAAGAAACTTGGTATCCACACGACTTTAATTACTGGTGATTCGGAAATGACGGGAAAAG
CTGTAGGCGAACAATTGGGAATAGACGAAGTTATCGCTAATGTAATGCCTGAAGATAAATCCAGAATTATAGA
AGAACAAAAAGAAAAATTTGGAGTTACTGCCATGGTTGGAGATGGTGTGAACGATGCACCGGCCCTTGTTA
ATGCTGATGTTGGTATAGCTATGGGGGGCGGTACTGATGTGGCAGTAGAAGTATCTGATTTGGTTTTAATGCA
GAACAATTTATCTAAATTAGTACAGTCTCATAAAATTTCCTCAAATATGGGTCGTGTTATTAGGCAAAATATTAT
TTTTTCAATGGCAGTTGTTGCCTTTTTAGTTGTCGTTAGTTTGTTAGGATTAACTGATATTACAATCAGTGTAAT
TGTTCATGAAGGAAGTACTTTAGTTGTTATACTAAATGGACTTCGATTATTAAGATCTAAATAATGAACGAATC
GATTGACATGAATGAACTTTGAAGTGTGGATTCTACAATGTTCCCATAACATTGGACACTAAAAAACAGAGC
AATCTAATAAAGATGTTATGAGTAAAAACAATGCCTTGCATACCGTTTTTATCATACGGGCGGTCAAATAAGCA
ATTAAAGAGCATGGGAAGCATAAAATCATAAACAGTGACTTAAGGCAGCCAGTTTACATTCAAAAGTTAAAT
TGACTGTATTAAAAGTTTCGAAACGATTAATATCCGTATGGGGGCAAGCGTCGAGCCAAAGATAATGCCAGG
ACAGAGCGTCTTTTTCCGTTCTTTTAAGTGGGAAAGGTTTTACCTTCTCTATGCCAAGACAGTCCCAGAGCT
AAAAGGGCCAGTTGCTCTTTTTGGGCGTTTGTGAACGGTAATCGCAGGAAGGGGATTTTCCCAACCGCACC
TGGTTTTCCATCACAATAAAGTTGGCGGCATTTGTTCTGATATTCTTGGGAATACTTACACTATGAATGGCCGT
ATTAAGTGATACAGGAGCTGCTATTATTGTAATACTGAATGCTCTCCGCCTTTTGAGGGTAAAAGAATAAAAG
TAAGGATAACTAGGTAAAGCTGTTCAATCAAAAATTGAACAGCTTATTTTTCATCAAAATCAAAAACGTTTATT
ATAATACCTACACTTGTTCTGTTAAATGTACCGATTTTTTAACCTTATTGTATCAGTAATATCTTGAAACGAAGTA
AGCGACTAAAATTTCTCTTTTATCATACGTTTATAAAAATACACTTTTAAGAACGGTTTGAAAATTTTTGAAAT
AGATCAAAATAATCTTCTGGTTAAAAAACCTGTAGATATGATTTCTCTCTTAAATTTTTGTTTGATTAGATTAGA
CCCTAATAAGACGCATTCAATATGTCTACACGTGAATTTAGTCTTTGAAAATGTAAGGACCATTATTATTATAAA
AACCCCAGTATAAAACGATACGCTGAAGCTTATCATAAGTAATGAGGTTCATGATTTTTGACATAGTTAGCCTC
CGCAGTCTTTCATTTCAAGTAAATAATAGCGAAATATTCTTTATACTGAATACTTATAGTGAAGCAAAGTTCTA
GCTTTGAGAAAATTCTTTCTGCAACTAAATATAGTAAATTACGGTAAAATATAAATAAGTACATATTGAAGAAA
ATGAGACATAATATATTTTATAATAGGAGGGAATTTCAAATGATAGACAACTTTATGCAGGTCCTTAAATTAATT
AAAGAGAAACGTACCAATAATGTAGTTAAAAAATTTGATTGGGATAAAGGTGATCTATATAAAACTTTAGTCC
ATGATAAGTTACCCAAGCAGTTAAAAGTGCATATAAAAGAAGATAAATATTCAGTTGTAGGGAAGGTTGCTA
CTGGGAACTATAGTAAAGTTCCTTGGATTTCAATATATGATGAGAATATAACAAAAGAAACAAAGGATGGATA
TTATTTGGTATATCTTTTTCATCCGGAAGGAGAAGGCATATACTTATCTTTGAATCAAGGATGGTCAAAGATAA
GTGATATGTTTCCGCGGGATAAAAATGCTGCAAAACAAAGAGCATTAACTTTATCTTCCGAACTCAATAAATA
TATTACATCAAATGAATTTAATACTGGAAGATTTTATTACGCAGAAAATAAAGATTCATCTTATGATTTAAAAAA
TGATTATCCATCAGGATATTCTCATGGATCAATAAGATTCAAATATTATGATTTGAATGAAGGATTCACAGAAG
AAGATATGCTAGAGGATTTAAAGAAATTTTTAGAACTATTTAATGAATTAGCTTCAAAAGTTACAAAAACATC
CTATGATAGCTTGGTCAATAGCATAGACGAAATACAGGAAGACAGCGAAATTGAAGAAATTAGAACAGCAC
AAAAAGATAAGACACTCAAGGAAGTGGAAGCACCTAAAGGAATAATTCCAAAATATAAAAAAGGTGTATCA
AAGACTACTAAAAATGATTCAGAAATTGAAAAATCAAATAAAGAGAATAAATTAACCGGTAAAGTTGGAGAA
AAATTAGCGCTAAATTACTTTAATGAGCTAATTGATAATAAAATAGACGAAGATAAGAAAGAACAGTTTAGGA
ATATTTTAAATGATAATCCAGGCTCTCAACACGGTCATGGCTATGATTTAGTAGCTTTTGATCCAACAAATACA
GATAAAGCTGTAGAAAAATTTATTGAAATTAAAACATCTACATCTTCTAGTATTGAGGAACCATTTTTTATGTC

GCTAAATGAAATGTTTGCTATGAAAGAATATAAGCAGAAATATTTAATATTAAGAATATTTAATGTTTCCGGTAA
AGAACCACAATTTTATTTTATAGATCCATATGCAAATTATTCTGAATTTAAAGATGTAGATGATCTCATTGACAA
AGTATTTAATGTAGAAGCTATTCAGTATAAAGTTTTTGGCGAAAAATGATTACTTGAACAAGAGCTAAAATAA
AATTGTGATCTAATAAAAATAGAAACTGTAATTTAAATAAAACTTTCTAAATAAGCTAACTGATAAAAAATCAG
TTTGTCCACAGTCTGAAACAAGATTCCTATATTCTTTAGGAATCTTGTTTTTTCTATTTTTATGGTGATAAAGAG
CAGATAAGATAATGTGTAATAATCACAAAAAAGTTAAATATTTTAAGGCTTGTTTAATTATTAATGATTTTATATA
TAAAGAGCAGTATAATAAAGTTGTTAATATATTATGAATAATATTCAAGTAATTTTATTGTTTTTTAATTTGTCGA
TATTTAAGTTGAGTTAAATTTAAAGGGTGTAATTTGTTTTACAATGATGAAGATAATTAGTCTATCAAAATAAA
GGGGTTGGGACTGTTATGAGTGATAATTTGTCATTATTCATTGACTATATCAATGATAATATAATCTATGGTAGT
GAAATCAAACGGGAGAAATTAGAGAATTTATTTAATCAATTTGCTATAAAAAATGTTGAAAAGAACATTGTCT
ATGATGAACTGAAATCTTTAGATATTACAATCATTGAGTCACAGGATTCATATAAAAATAAATTGAAGAGATTA
TTTTCGGTTCTGTTGCAAAGTAAAAAAATATAGCTAACCACTAATTTATCATGTCAGTGTTCGCTTAACTTGCT
AGCATGATGCTAATTTCGTGGCATGGCGAAAATCCGTAGATCTGAAGAGACCTGCGGTTCTTTTTATATAGAG
CGTAAATACATTCAATACCTTTTAAAGTATTCTTTGCTGTATTGATACTTTGATACCTTGTCTTTCTTACTTTAATA
TGACGGTGATCTTGCTCAATGAGGTTATTCAGATATTTCGATGTACAATGACAGTCAGGTTTAAGTTTAAAAG
CTTTAATTACTTTAGCCATTGCTACCTTCGTTGAAGGTGCCTGATCTGTAATTACCTTTTGAGGTTTACCAAATT
GTTTAATGAGACGTTTGATAAACGCATATGCTGAATGATTATCTCGTTGCTTACGCAACCAAATATCTAATGTAT
GTCCCTCTGCATCAATGGCACGATATAAATAGCTCCATTTTCCTTTTATTTTGATGTACGTCTCATCAATACGCC
ATTTGTAATAAGCTTTTTTATGCTTTTTCTTCCAAATTTGATACAAAATTGGGGCATATTCTTGAACCCAACGG
TAGACCGTTGAATGATGAACGTTTACACCACGTTCCCTTAATATTTCAGATATATCACGATAACTCAATGTATAT
CTTAGATAGTAGCCAACGGCTACAGTGATAACATCCTTGTTAAATTGTTTATATCTGAAATAGTTCATACAGAA
GACTCCTTTTTGTTAAAATTATACTATAAATTCAACTTTGCAACAGAACCGTATTATGGAATAGAGATGTTGGT
AACATTTATACAGGATCATTATACTTAAGTTTAATTTCGTTATTACAGAACCACACATTCCAACCAGAAGAGAA
AGTATGTCTATTTAGTTATGGTTCAGGAGCAGTAGGAGAAATCTTTAGTGGTTCAATCGTTAAAGGATATGAC
AAAGCATTAGATAAAGAGAAACACTTAAATATGCTAGAATCTAGAGAGCAATTATCAGTCGAAGAATACGAA
ACATTCTTTAACAGATTTGATAATCAAGAATTTGATTTCGAACGTGAATTGACACAAGATCCATATTCAAAAGT
ATACTTATACAGTATAGAAGACCATATCAGAACATATAAGATAGAGAAATAAACTAGTGGCCGATTGTGCTTGA
TGAGCTTGGGACATAAATCCTAACTCGAAATAAATAAGCATATCACTAAACTGATTTTTTAAAGTTTACAGTGA
TATGCTTATTTTTTTATCTTACGATTTTGTACGTGCATGCTTGCCTAGGGGTATGGCTCGAGCCATTAGTCTCTC
GCACATACTATTCCCTCAGGCGTCAGCACTTACAAAATCGGTTGTAATTTTCATTTTTATACGCATTCTTACTGA
GATTATACTAATAAGAGGAATAGTAAAAGCAATTCTAAGTAAAATTGCAGATAAGAGGTTTGTTAAAAGCAGT
TCTCAGTAAAATTGCAGATAAGAGGTTTGTTAAAAGCAGTTCTCAGTAAAATTACAGATAAGAGGTACGTTA
AAAGCAGTTCTAAGTAAAATTGCAGATAAGAGGTTTGTTAAAAGCAGTTCTAAGTAAAATTGCAGATAAGAG
GTACGTTAAAAGCAATTCCATGCAAAATTGCTGATAAGGGGTAAGTTAAAAGCAGTTCTCAGTAAAATTGCA
GATAAGAGGTACGTTAAAAGCAGTTCTAGGCAAAATTGCAGATAAGAGGTGCGTTAAAAGCAGTTCTCAGT
AAAATTGCTGATAAGGGGTAAGTTAAAAGCAATCCTAAGTAAAATTGCAGATAAGGGGTACAGAAAAACTA
GACTTGATTACAAAATGGAGCTTGGGACATAAATGATTTTTTAAAAATGAGATGAGACGTAGATTAACTCCAT
AATCAATACGAATCTATCGACTTCTTTATTTATGATATTCATCTCTTTTTAATGGAAATAAAAGTGCGATTAATGT
GATAATACAGTTACGTTAATTAAAAAAAATAAAAATGCAAGGAGAGGTAATATGCTAACTGTATATGGACATAG
AGGATTACCTAGTAAAGCTCCGGAAAATACAATTGCATCATTTAAAGCTGCTTCAGAAGTAGAAGGTATAAA
CTGGTTGGAGTTAGATGTTGCAATTACAAAAGATGAACAACTGATTATCATTCATGATGATTATTTAGAACGG
ACTACAAATATGTCCGGGGAAATAACTGAATTGAATTATGATGAAATTAAAGATGCTTCTGCAGGATCTTGGT
TTGGTGAAAAATTCAAAGATGAACATTTGCCAACTTTCGATGATGTAGTAAAAATAGCAAATGAATATAATAT
GAATTTAAATGTAGAATTAAAAGGTATTACTGGACCGAATGGACTAGCACTTTCTAAAAGTATGGTTAAGCAA

GTGGAAGAACAATTAACAAACTTAAATCAGAATCAAGAAGTGCTCATTTCAAGCTTTAATGTTGTGCTTGTTA
AACTTGCAGAAGAAATCATGCCACAATATAACAGAGCAGTTATATTCCATACAACTTCGTTTCGTGAAGACTG
GAGAACACTTTTAGATTACTGTAATGCTAAAATAGTAAACACTGAAGATGCCAAACTTACTAAAGCAAAAGTA
AAAATGGTAAAAGAAGCGGGTTATGAATTGAACGTATGGACTGTAAACAAACCAGCACGTGCAAACCAACT
TGCTAATTGGGGAGTTGATGGTATCTTTACAGACAATGCAGATAAAATGGTGCATTTGTCTCAATAGAAAGTT
AGAGGTGAGTCTTACGTTTCAGTGACGGTAGACTTACCTTTAACATGTTACATACTAAAAAATTAATTTGAATA
AGAAAGAGAGACATATATGAAATACGATGATTTTATAGTAGGAGAAACATTCAAAACAAAAAGCCTTCATATT
ACAGAAGAAGAAATTATCCAATTTGCAACAACTTTTGATCCTCAATATATGCATATAGATAAAGAAAAAGCAG
AACAAAGTAGATTTAAAGGTATCATTGCATCTGGCATGCATACACTTTCAATATCATTTAAATTATGGGTAGAA
GAAGGTAAATACGGAGAAGAAGTTGTAGCAGGAACACAAATGAATAACGTTAAATTTATTAAACCTGTATAC
CCAGGTAATACATTGTACGTTATCGCTGAAATTACAAATAAGAAATCCATAAAAAAAGAAAATGGACTCGTTA
CAGTGTCACTTTCAACATACAATGAAAATGAAGAAATTGTATTTAAGGGAGAAGTAACAGCACTTATTAATAA
TTCATAATAAAACAGTGAAGCAACCATCGTTACGGATTGCTTCACTGTTTTGTTATTCATCTATATCGTATTTTT
TATTACCGTTCTCATATAGCTCATCATACACTTTACCTGAGATTTTGGCATTGTAGCTAGCCATTCCTTTATCTTG
TACATCTTTAACATTAATAGCCATCATCATGTTTGGATTATCTTTATCATATGATATAAACCACCCAATTTGTCTGC
CAGTTTCTCCTTGTTTCATTTTGAGTTCTGCAGTACCGGATTTGCCAATTAAGTTTGCATAAGATCTATAAATAT
CTTCTTTATGTGTTTTATTTACGACTTGTTGCATACCATCAGTTAATAGATTGATATTTTCTTTGGAAATAATATT
TTTCTTCCAAACTTTGTTTTTCGTGTCTTTTAATAAGTGAGGTGCGTTAATATTGCCATTATTTTCTAATGCGCT
ATAGATTGAAAGGATCTGTACTGGGTTAATCAGTATTTCACCTTGTCCGTAACCTGAATCAGCTAATAATATTTC
ATTATCTAAATTTTTGTTTGAAATTTGAGCATTATAAAATGGATAATCACTTGGTATATCTTCACCAACACCTAG
TTTTTTCATGCCTTTTTCAAATTTCTTACTGCCTAATTCGAGTGCTACTCTAGCAAAGAAAATGTTATCTGATGA
TTCTATTGCTTGTTTTAAGTCGATATTACCATTTACCACTTCATATCTTGTAACGTTGTAACCACCCCAAGATTTA
TCTTTTTGCCAACCTTTACCATCGATTTTATAACTTGTTTTATCGTCTAATGTTTTGTTATTTAACCCAATCATTG
CTGTTAATATTTTTTGAGTTGAACCTGGTGAAGTTGTAATCTGGAACTTGTTGAGCAGAGGTTCTTTTTTATCT
TCGGTTAATTTATTATATTCTTCGTTACTCATGCCATACATAAATGGATAGACGTCATATGAAGGTGTGCTTACA
AGTGCTAATAATTCACCTGTTTGAGGGTGGATAGCAGTACCTGAGCCATAATCATTTTTCATGTTGTTATAAAT
ACTCTTTTGAACTTTAGCATCAATAGTTAGTTGAATATCTTTGCCATCTTTTTTCTTTTTCTCTATTAATGTATGT
GCGATTGTATTGCTATTATCGTCAACGATTGTGACACGATAGCCATCTTCATGTTGGAGCTTTTTATCGTAAAG
TTTTTCGAGTCCCTTTTTACCAATAACTGCATCATCTTTATAGCCTTTATATTCTTTTTGTTTTAATTCTTCAGAG
TTAATGGGACCAACATAACCTAATAGATGTGAAGTCGCTTTTCCTAGAGGATAGTTACGACTTTCTGTTTCATT
AGTTGTAAGATGAAATTTTTTTGCGAAATCACTTAAATATTCATCCATTTTTTTAACGGTTTTAAGTGGAACGA
AGGTATCATCTTGTACCCAATTTTGATCCATTTGTTGTTTGATATAGTCTTCAGAAATACTTAGTTCTTTAGCGA
TTGCTTTATAATCTTTTTTAGATACATTCTTTGGAACGATGCCTATCTCATATGCTGTTCCTGTATTGGCCAATTC
CACATTGTTTCGGTCTAAAATTTTACCACGTTCTGATTTTAAATTTTCAATATGTATGCTTTGGTCTTTCTGCATT
CCTGGAATAATGACGCTATGATCCCAATCTAACTTCCACATACCATCTTCTTTAACAAAATTAAATTGAACGTTG
CGATCAATGTTACCGTAGTTTGTTTTAATTTTATATTGAGCATCTACTCGTTTTTTATTTTTAGATACTTTTTTTAT
TTTACGATCCTGAATGTTTATATCTTTAACGCCTAAACTATTATATATTTTTATCGGACGTTCAGTCATTTCTACTT
CACCATTATCGCTTTTAGAAATATAACTGCTATCTTTATAAACTTGTTTGAAATTTTTATCTTCAATTGCATCAAT
AGTATTATTAATTTCTTTATCTTTTGAAGCATAAAAATATATACCAAACCCGACAACTACAACTATTAAAATAAG
TGGAACAATTTTTATCTTTTTCATCAATATACTCCTTATATAAGACTACATTTGTAGTATATTACAAATGTAGTATT
TATGTCAAAATAATGTTATAATTTTTGTGATATGGAGGTGTAGAAGGTGTTATCATCTTTTTTAATGTTAAGTAT
AATCAGTTCATTGCTCACGATATGTGTAATTTTTTTAGTGAGAATGCTCTATATAAAATATACTCAAAATATTATG
TCACATAAGATTTGGTTATTAGTGCTCGTCTCCACGTTAATTCCATTAATACCATTTTACAAAATATCGAATTTTA
CATTTTCAAAAGATATGATGAATCGAAATGTATCTGACACGACTTCTTCGGTTAGTCATATGTTAGATGGTCAA

CAATCATCTGTTACGAAAGACTTAGCAATTAATGTTAATCAGTTTGAGACCTCAAATATAACGTATATGATTCTT
TTGATATGGGTATTTGGTAGTTTGTTGTGCTTATTTTATATGATTAAGGCATTCCGACAAATTGATGTTATTAAA
AGTTCGTCATTGGAATCGTCATATCTTAATGAACGACTTAAAGTATGTCAAAGTAAGATGCAGTTCTACAAAA
AGCATATAACAATTAGTTATAGTTCAAACATTGATAATCCGATGGTATTTGGTTTAGTGAAATCCCAAATTGTAC
TACCAACTGTCGTAGTCGAAACCATGAATGACAAAGAAATTGAATATATTATTCTACATGAACTATCACATGTG
AAAAGTCATGACTTAATATTCAACCAGCTTTATGTTGTTTTTAAAATGATATTCTGGTTTAATCCTGCACTATATA
TAAGTAAAACAATGATGGACAATGACTGTGAAAAAGTATGTGATAGAAACGTTTTAAAAATTTTGAATCGCC
ATGAACATATACGTTATGGTGAATCGATATTAAAATGCTCTATTTTAAAATCTCAGCACATAAATAATGTGGCAG
CACAATATTTACTAGGTTTTAATTCAAATATTAAAGAACGTGTTAAGTATATTGCACTTTATGATTCAATGCCTA
AACCTAATCGAAACAAGCGTATTGTTGCGTATATTGTATGTAGTATATCGAGCTTCACATGAAACAGCTAAAGA
AGCTTTGGGCGATAAAGAGTTAAGAGCCATTGCACATGAGTTAACTAAAACAGTTAAGGATAACATGAGTGT
TGATTGGTCTAAACGAGACAGTGCTAAAGCTAAATGAGAGTTCAAGTTAGACGCCTATTAAAGAAATATGGC
TATCCACCAGATCTTCAAAAAATGGCTGTGGAACAAGTTGTAGAGCAAGCAGAATTAATGGCAAGTCAGCA
ATAAAAAAATAAATCATAATGAGTCCGGGACATAAAGTTCTTGGATAAGTGAAAAAAGACAATTTCTATTGAA
ATAATATAGAAATTGTCTTTTTTATAAATTTTTTGATTATTTTCAGCTCGTTGAGCTACTACTTTTCTTATATTAAG
TGCCATTAATACAAAACCAAGTTCTCTTTTGACTTTATTGAGTCCTCGGACAGACATCCGAGTGAAACCCAAA
ATAGCCTTCATAAATCCAAAAACAGGTTCCACATCAATTTTTCTTTGACTGTAGATATTTTTTGTTTCTGGTTCT
GAAAGCTTTTTGTTAATTTGGGATTTAAAATATTCCCAGTTATAATTCTTCATTATTTTTTTGTTTGTTTTTGAAT
TGAAGTTCATACATTGATTTTTCAGAGGACATTCTGAACAATCATCACATTCATATAATTTGAAGTCTCGCTTAT
AACCATACTTATCATGACGATAGGCATATCTTTTAAAACCTAGCCGTTTATTATTCGGACAAATGAATTCGTCAT
TAATTTCGTCATAGTTCCAATTTTGAGTATTAAAGATGTCACTTTTATATTTTTTAGTTTTATCTTTTATAAACATT
CCATATGTTATGAGTGGCGTTCGATTAAAGTCATCTATAATTGCCTTATAATTTGATTCACTACCATAACCTGCAT
CAGCTACAATATATTCAGGTAAATGACCGTAGGTCTCTTGAATTGAATTTAAAAATGGAATCATCGTTCTAGTA
TCCGTTGGATTTTGATACACATTATAAGATAAAACAAATTGGGAATTTGTTGCTATTTGTAAATTATACCCTGGC
TTAAGTTGTCCATTTTTCATGTGATCTTCTTTCATTCTCATAAATGTCGCATCATAATCTGTCTTAGAATAACTAT
TTCTATCCTTTAAAATAGATTTTTGAAATTCGTATCGATACTTTCGCTCAAAATAATCATTGATTTGCTTTTTGTA
TTTTTTGATTTTAGTTCTTTTGAGACGTATTTGTTTTCTTGTTTTAGTACATTTTTCATTGTTGATATGTTGGTTT
AAATCTTCGATTTCTTTATCTAAGTGACTACCAATCAAATCTATTTCTTCTTTTGTTAAGTCATTATCATGATCTT
CTTTAATTTCCGGTATGATTTTATTGGTTACCAATTCATGGTAGAGGGCTTTAGAATCCTCATTCATCTTTGATT
CATGGTTTTGAATACTCTTTTTCCATACAAATGTATATCGATTGGCATTTGCTTCAATTTTTGTACCATCAATAAA
AATAGCTTTATCATCTATAAGATTTTGTTTTACACACTGACTGTAAAATTGAATAAATAAAGATTCTAATAAAGC
ATCTACTTTTGGATTTACTCTAAATTGATTAATTGTTTTATAAGAAGGTTTTTGATTTTGTGATAGCCACATCAT
TCGGATGCTATCATTAAGCATTTTTTCTATTTTACGACCTGAGAATACAGATTGTGTGTAGGCATATAGAATCAC
TTTTAACATCATTTTAGGATGGTACGAAGTTGCACCACGGTGATGTCTGAATTCGTCGAATTCATTGTCAGGA
ATTGTTTCAACAATATCATTTACAGTAAAACGATGTTGATTTGTTTTGTTTCCATATTGACCTCCATGTATTTGCT
ATGATTTCAAAATCCATTTTTGACGTGCCTTAGGGTTGAGTGGATGCATAATTTCATTTGTTACTGGATTGATG
AGCTTTTTTACTTTCTTTTTATGAGGTTTTAACATTTCCATCACTTGTTCGACACGGTCGATAACAACTGGTCG
CTTCGCATAGGCACCATAAGCAAGAATCACTGTGTCACTTTCACTAATCGCTTTCATCAAATGAATATCAGTGT
GCTCATCGTATGGATTTTTGATATGTTTGAGGTTTTCGGGTGTTCTAATATTAGAGAATAGATTTACAAGATATA
CAGCACCGTATCGTTCTGAATTGGCTAATTGGTTGAGGATAAGAACAGTTGTGAGATCGAGTGATAATACAC
CGTCTAAATGAGGATACATCGTTATCACTGTGCATGCAGCTTTCTTTTCATCCCATGTTTTCTTGAGTAAATAGC
GGTGCTGTTCATCATCGCTAAATATGGCTTCTGTGTGTATCGTACTTTTGATTGTATTCATCATCGTCACTTCCTT
TAGTATTCTTCTGGTAAAAGCATCACATAATAAAAAGCGTCCACGTCATCTTCACGAATGACGTAGACTTTCTT
AGGTAATGCATTTTGATTTTTTTCATAGTTTGTATAGTGATATTCCAATTTGTATGTGGGTTGTTCTTGTTCATGT

GTGATTGAGAGTATATTCTCATCTTCTTGTAATTTAAAAATGTGTAGGTAATCTGTATGAGGCTGGTTATCTTTT
TTTTCTACCATGTGCCAAAGTAAGATTTGAAGGTCTAGTGGAATACTTTCATTAATTCCTCTTGTGATGTATCG
ATTGATTTTCATGCTATTTCCCTCCCTTCTGCTTTTCTTTCATGATGTCGATGATTTCGTTGATAACTGTGACGG
ATAATTGAGCAACTATGATCCAATTATTCATGGTCCTGACCTCCTTGTTTTAGTAAATGACGTTCATCAATAATG
ATATTTTGAGTATCTGTAAGGTACAGAAAGTCCATGTCAAAATGGTCTAAGTATCCGACACTGATGAGTTGGT
TATTGGCATACATTAGAAATGGATAGATACTTAGCTCATGTAGTTCATCATTATAGTAGGTATAAGTCTCGAGTG
TGAGATGTACCAGTGGAGAATCATTAATAAAACGTTCCGGTAGAATATTTTCTGCTGCTTCCTCCAGCGCTTC
ACATTCCCAAGCTTCGTTATTAGATAGTTGGAATAGGTGGGTTATATATTGTTTGAGTTCTTGAGTGATGGTTT
TCATATTATTGCCTCCTAGATAGTGTAATAGTGATGTAGTTCATATACATCATTGAGATAATATATATTTGATTTGT
CATTTATTACGAATCCCGGTGGGAATAAGAGAAAATTCCATATAAAAACCCGCTACAAACGTTGGTATGCCAA
GGAAATCCTGAAATTCCGCCTATTTTGACAAACAATCAACTCATTATTTATAAGTATTGATGATAGGGTTGTGT
CTCTGCTTCCTTATATATATTATTTATTTATAAAAAGTAACGGGATTTTGGGATTGTGCTTGCACAATCCTTCTGT
TTCTTCGAATCTGCAAATCCCAATCATTTCCCGATAAAAAATCATTGTGGGATGTTCTTTAGCAATTTCAATATA
AGCATTGTATAGTTATGAAAAAATTACGACAATAACTGTTTCATTAGATAAGTGTTATTGAAATTGATAAAGAG
CAATTCTTGAAAATAGTTAGATAAAATAAGCGAAAGAATATAGTGAAAATTATTGTTATAACAATGATTCTATTA
GCTAAATAGTAAGATATAGTGTTTGGGGCAAAAATAAAGACGAAGTGCTGAGATGCACTTCGTCGAGTTGTT
TATTATTGAAAAGTTGTTTAATGATTTCGTTATTAAGTTTGAGTGTGACATAGAATTGTTTTTTATGATTACCAT
CTTTTTTAATATCAATGCGATCAATCACTGATAGATACAATGCTTTGAGTCGAGATTTTTCTATGTGCTTAATATC
ATGAAAGATGTGTTGTAATAGTTTACTGATTTCTTTGGCATCAAATAAAGTCTTATCTTCATTTTGTTGATTTTT
GAGTTGGTTGATTTGATTCGTAATGTCATTGAGTTGCTTTTCATATTTTTGAATACTTGGTCTGATTACTGATGT
TAAGTCCGGATTATCCTCGATGGTTTTAATCAAGTTATTTATTTTGATTTGTACTTCATCATATTGTTGTTGCTTA
TAAGCAATATCGTGATGAAGTGCAGCGCCATCAACTTGATTTTCTTGATTGACGTGTGTTACTACGCGTTGAA
TGACTTTATCACTTTTGACTATTTCAAGTATTTGCTTCATCACATAATCTTCAATCACATCAGCTCTTACACTGTT
TGCCGAACATACTTTGGAACCCTTGTTCCGAAAATTACTACATGAATAGTAACGAATACGTTTCTTAGTCCCGT
CTTTAAGAGTATTCGTGGTATTGCTTGCTGCCATAGGTGCGCCACATTGGGGACAGTGAATAATGCCTGTAAG
CAGATTCGTTCCTTTGCCATGGACTTGGGGTTTTTGACTGACTTGTTTCTTACGCATTTGTACTTTATCCCATA
AATCTTGATTAATAATGGGGGAATGCTTACCTTCAGCTATCACTGGTTTATCATTCAGCCCTTTACGACGTTTT
TCACTCCAATCTTTGTATTTCGCAAATTGAATTTTGCCGATATAGAAAGGGTTAGCTAAGATGTATGTGATTGA
ACTAATACTGAAAGGTTTCCCCTTTTTAGTGACATATCCTTTGTGATTCAATGCATTGGCAATTTTACGATAGC
CATGTCCTTTGGCATAGCACTCGAATATATATTTTACAATATTAGCTTCATGTTGGTTAATCATTAGCTCGTGTTT
ACTATCTGGTATTTTGTCATAACCTAGTGGTAAATTGCCTTGATAATAGCCTTCTTGGGCACGTCTCGTTTGAC
CCATAAATACGTTCTCGACAATGTTATTACGTTCGAATTCTGAGAAACTCGCAAGTATCTGTAACATGAGTTTA
CCAGAAGAAGTATTGACTTCCATACGCTCTGACAAACTGAAAAATTCGACATTTTGTTTGTGTAAATCTTCGA
CAATTTTGAGAAGATCAGATGTATTACGAGCTAATCGGTTTGTTTTGTATACCATAACACAGTCGATATTGCCT
TCTTTTGCATCTTTCAACATACGTTGGAGCTCAGGTCTGTTCATAGATTTACCTGAAATACCACGGTCAGCGTA
TATATCTTTAACTTCAAAATGATGGAAGTCACAGTATTCTTTGATTTGATTGATTTGTCCGTCGATACTATAACC
TTCTGTGCTTTGCATTTCTGTTGATACACGTACATAGATACCGACACGTTTTGTTTTAAGTTGTTGCATTATGTT
ACATCCTTTCTTCATTTATGCAATCGATGATTGCATGGTTTGATTGACGATATTGAGTGGTTCATTTTTGAAATA
GATTCCTATAAGATTTTTATCTTTCGTAATGTGAATGGTTTCAATATAGGGGTACAATATGTTTAACGTGAAACG
TTTTTGAATAATATTTTGAATGGTGTGTTGTATTTGATGTCCATTGATAGATGTAGTGCGTTGAGGTTGTTGAC
GTAATGATTGCGTTTGTTCTCTGAACGTTTCTGCATCGATGATGCCTTGTGCCAACTTTTCTATCAGTTGTTCT
TGAGTCAATGTGTGATGTTTTTCTATGTTTCTTTGTCTTTTGATGCGTTTGTCAATCGCACCTTTAATTTTTGTG
TAGATGCGTTGATTTTGATAAAAGTCTCGGCACACTTCTAATACTTTATCTTCAAATGTTTGTGCATTGATGCCT
TTGAAATCACATACAAAGCGTGAAGCATTCATGTTTTTAGGACAGACGTAGTAACGTAATATATGATTCTTTTT

TCTAACGGTCATATTTGTAAGTGTTGCATTACAACATGGGCAATTGATTTTTTGTTTGAGTTGATTATCCGAAG
ATGTCTGTTTGGTTTGTTTTTGCAATCGAAGTCTCTGCGCTTGCTCATATATACTTGTGGAAACAATAGAAGG
AAACATATTGTCGAATTGGCCATATTGATTGTTGACACGACCACAATAATTAGGATTGATGATAATGTTACGAA
CTTGATAGGGTTGTCGATTGATATACGTGTTATCTTCTTCTAATAACTGTGCAATTTTCTTATAACCATGACCTTT
AATGTAATAATTGAATACAGCCTTTACCGTTGGTGACTCATTTTGATTGATGATGAATGTTCCGTTGTGATATTC
GTAACCAAAAGGTGCATGTGTTGTAATCAATCGACCTTGCTTTGCTTTTTCTTGAAGCCCATTTCTGACTTGTT
CTCCAATGTTATCCGATTCAAGTTCGGCTAAACTGATGAAGATATTAAGCTTGAGTCGGTCGAAAGCTTGATC
CATATCAAAGTAACCATCATGTACGCTTAAGATATGAACATGGTACGTTTGACATAATTTGATGAGTTTTAATG
CATTTTTCAGATTACGATGCAACCTATTAAGACGATAACAGCATAATATGTCACACTGTCCTTGTTGAATTAATT
GTGTTATTTGTCGATACCCACTACGATTATCTTTGCGACCTGATTGTTTGTCGCTATAAAAGTTGATATGTTGAA
TATGATGTTTTTCGGCTATTGCTTCGATAGCTTGTTTCTGTGCTGCAAGAGATTGTTGTTTCATCGTACTTTGA
CGTAAATAGCCTATGACTTGTTTCATATCGGCTCCTCCTTTCACAGTGATAATATATATTTATGGATGAATTGATA
TATAAGCCCAACATCAATGAGATGTTGGGCGTCCATATTAGTCATTTGTTTGATTGATTTCTTCAATTACCAAAT
CGGCTAATATCTCGATAAGTTCATCCATGTTTTTCACTCCGTTATTTGTTCTATCTTCAATACGTCGATTATTCAG
TTTGATGCTTCACAGTTGTATGATAAAGACAATTAGAAATCTTCGTGAACTCCTGAAGGGCCTATCCCTTCATT
AGCGGATTTAAAAAGTTCTTTCGCAGCTTTGTTATCATTTGACGGTGTCCAATTTTGAAGTAACGACTTATCTT
TAGTTAATCCGAGGATAGATGCAAACTCTACATCTAATTTTAGATGGTAAAATACAAGTGATTGTTTTTTACCG
CTATTATCTTTGACACTTCTTTTAGTTGTTTGGCGTCCACGGTCAGCTAATATGAAACCTTTATCTCTTAAGGC
GTTGACAACATTATTAACATCTTGAAATTGATGATTGTTTAGCATCTGTTTAAAAACGTTCGCAATTATTTTTAC
TTCGATATGGTCATCTTTTAATGAGATTAATCCATAGTTCTCAAACATATTTTTCAAAGCACCTTCATCTGAAAA
CTTACCTCTGTTTTGTGCCACAAATTGAATGATGACATCAATAGCTTTATCAGCTAATGAGCGTTCAGAGACT
GTATGAGTATGATAATCAATAAAGTAGTCTCTTATATTAGCGATATCAATATCTGTAGATAAAACACGACCTAATA
TTTTCGCAGATGTTGTAATGACTGCATAACGCTTAAACATACGAATACCTGTATTGTTTGTTTCATCTTTCAATT
TAGCTTCAAACCAATCTACTTCCTTGTAAAACCATTGAATAACTTCATCTTCACGATTTATAAGATATTTAGCTA
CTAACGGTAAAACATGACCATAGTTTAGTGCTACAGCTTTTTTAATATTGTCAGCATTGGTCGCATTTGTAGTG
AATTGTTCATTAATCTCGATGGTTCTTACACGTAATCCATCGTTTTGAGCTGAATCATTAAAAATACTGTATTCT
GACGTTGAAATGACAGAAGTACCCCAATTCTTAGGCGTTTTAACTTCTCCATGAACGTTTGAACGTTGACGA
CCTTGACCTTCAGCGATGGAATATAACAAACCCGTGGTATCTCTAAGTGTCGTAGATGAAAGCTCATCAAATA
CTATAGGAATGCCATAATTGTTACTCAAATAACCTTCAAGTGCGTTTCGTGTGGCATTCCAACTTCTAAAAAGA
GTTTCATTACCTTTGGTAGGGTTACCAGCGACTGATACAGCTAAAGCAGCTGCGGTTGACTTACCGGTTGAG
GATTGACCTGTAAAACTAAAGAGAATTCCTGCAAATTCGATTTCATGTTTATGCTTCAGAAAACTTGTCACTA
AGGCAGAAATCCCAAATATGACTGCCAATTCTAAAAGAAGATGACCTTTAACCTCGTCAATATACATGTTAAA
CCAATTATCAAATGTTCCCCTAGGTGCTAAGTCATAAGTATTCTCACAAATGGCGTCAGATGGAGATTTATTAT
CAAATTCCGTAGTAGTATAGATTTCATTTAACGATACAATAGGACCAAACGGTGTTTCCAGTATACCTACCCCT
TCATATAAGTAGGAAATGGGTAATTGGTTGCGCATTTGTTGCAACGCATAACCTAAATCTTTTGTATATTTTTC
ATTAATACTAAATCCATATTTCATTAAAGAGGGCAGTTTTTGTGTTGTTAAAATATCACTAGATTCAACAATTAC
TTTTTGATCCTCGTCTGTAATAATTACTTTTTCAGTGTTAGTTTTAGGGTCAATAAACTTATTTTCGATAACGAT
AGGACCTGCGATTTCAACTTCAGTAGGCATTCCTCCTTTTTCTTTGGGAGGCTTGTCTTTATACCAACCTTTTT
TTGATTTGTATCGTGGTGAAGGATTAAATGAAGGGTTAGTTTGAGTCATTAGCGAACACCTCCTTTCGAAGG
GTTGCTGTTATGGTGTGGATTAGGACCTGTTTTAAGATAAACTAAATGACCGTGAGTATCCTTACCGATAATAA
TAAATGGAACACGTGGCGCATGTTTTACAAAATATGCGAACCAACGTCCAACATTTTGTTGTACTTCTTTAGA
ACACGTTACATTTGCACGACTGTTCAAGTCATGAAATGTAAATTCTTCTCCTTCAGGTAAATTGAATGCAATAC
CTAAAACCTTGCGTTTTAACAATAAAAAGTGGAGACTCTTTTTATTCATTTGGATCATCCTTTGTACATTAGTC
ATCATTTAAATGATGCACTTGTATCTTATATCTTTACTGAGATATAAAGTTCTAAATTTGAGAGGAAAATATTAG

GACATTGGTATTACACCTTTTGATAAGTAAAAAAACAACGATTTTTTAGACTGACCCCAATTAGTGGGAATT
ATATAAAAACACTTTCGTTGAATTCATATTAAAATGAATCATACGGGGGGTGTTTTTTTCTATGAAAAGAGTTT
CTTATTCAGTAGAAACAAAGTATAAAGCAGTTGAAATGAAAGCAGCAGGATTTTCAACAAAAGAAATTATGA
AAGAATTAAATATTAGAAATAGAACACAAGTGAAAACTTGGTGGCGATGGTATCGAAATGGGGAAAGTTATA
GATTTTCACAACACGTTGGTAAACAATATACCTACGGTAAAGGATTAGAAGAGCTGTTAGAAGTAGAACAAT
TAAAATTAGAAAATAAGAGAAAAGATATAGAATTGGATATTTTAAAAAAGTACAAGGCATTGGAGAGGAAGT
GGTACCAACAGTAGTCGTAGATTTAGTGGATCAATTAAAAGTAAAATATTCAATCAAATTGATACTAGAAGTAT
TAAACATACCTAAATCAACATATTACCGATGGAAAAACAAAACCCATAAAAATGATACCGTAACACAAAAAGT
TATTGAATTATGTAAAGCTAACCACTATACCTACGGTTATCGTAAGATTACAGCATTGATTAATCAATGTTATAC
ATCACCAATTAATCATAAGAGAGTACAGAGAATGATGCAGAAGCATCATTTGAACTGCCGAGTTACCTAAAA
AGACGACAAGAATAGGTAAACCGTATTATAAAACGGACAATTTATTACAAAGACAATTTAAAGCGAGTTGTC
CAATGGAAGTATTAACAACCGATATTACTTATTTACCATTTGGTCATTCTATGTTGTATTTATCTTCGATAATGGA
TATTTATAACGGAGAAATTGTGGCGTATAAAATAGATGATAAACAAGACCAAAGTTTAGTTAATGATACATTAA
ATCAAATCGATATACCTGAGGGTTGTATATTACATAGTGATCAAGGCAGCGTTTATACATCTTATGCTTATTATC
AATTGTACGAAGAAAAAGGCATTATCAGAAGTATGTCCCGAAAGGGAACACCCGCCGATAACGCCCCGATA
GAAAGTTTCCATTCCTCGCTAAAGTCTGAAACTTTTTACATCAATAATGAGCTTAATCGCTCTAATCATATTGTA
ATAGATATTGTCGAAAAGTACATTAAAAACTATAATAATAATCGAATTCAACAAAAACTAGGCTACTTATCCCCT
GTGAAATACAGAGAATTAATAGCCTAGAACATGGTGTTTTTATTAAGTTCCCGTTTTAAGGGTTCAGTGCCTT
TAATCGTTGGTTTTTTTTATTGAATTAATAATATAAATTTGGTCCATCGTTAATATCTTCTAAACGTGTCTTGTAA
AACTTACTGATTATTTGAATCATTAGTTTATCGAGAAAATCATTAGGATTATTATTAAAGTTGTCTCTTTTATAAA
AGTTGCTAATATTATATTTTTTTAGATCGGTGAGTAAAGACCTTTCTACTTTTTCTGTCATTTTTATATCATTCATA
ATAATATTATGAATTAAATCTTTTAATCTAGTATTTCTTACATAACCATCAATAAGACTTTTAAAACGGGTTAATG
ATCGTTCGTGTTTGTGTGCTAATTTATTCAAATCATTTTTTAAATTAGAAATACTATATAAAGCGACTAATACATC
GTATTGTGTCGCACTTTGAATGATTTTACTTGTTACTAAAACTAATTCATCCATTAAATCGGAAATGGCAGTGA
ATAATGCAAAGTGGAACGTATCTTCAATAGTTGTTAAATAATATAATTCATTGTCGCTAATTAGATCTTGTTTTT
TCATAAATTCATTTATAGTGTTTTGAGACTCAATAAGTCCATCTATTGTTAAATTAACTTTACGTGACTTTTCTGT
CAGAATACTACGGATGAATTTAAAGTATGTATCATTTTGATCATATAAGTTGAGAGAAATGCCGATAACAGCTT
GCCTAAATTGGTCTAACTCATCAGATTGATTTAAGCTATACTTGTTATGAATCGAAGGTTTGATACTATTAGCA
ATTTCAAAGTATTGAGAATTACTTGTTCGTTCTTTGATTAAACCTTCCGTCACTAATTGTTTAAAGTAGTTAGA
CTTATTTAAGTTTGAGATATTTTTCTTAACATAAGAGTCTTGTTTTTCTTTATAAATGAGATATTTCGTTAAAAA
ATCACTTATTAATACATCGTCAAAATTTTCTTTAGATAAATCTTGATGAGTTTCTAATATTGTATCTTTCAGATTTA
TAAATGATTCCAAAGAAGATACATCGTCAATAGCTTTTTCATAATCTATTCTATAGTTAGCTTTTTTTAATTCAAT
ATTTTCTCGAATCTGTTTTATTGTATTGTTAGCTATCTTTTTAGCAAAATCTGTTGTTTTATTGATTGTGGTTAAG
AACTCGGTGAAATATGTATCTAGAGTTGTTGATTCTAACACATCTAGAGCATCTACACCTTGGTCAATGATTTC
ATTTGAAAGTTCATCTATGTCCACCATACTTTGACTATCAATAGAATACGTCAGATTATCTTTAGTAAAGCCACT
AGGAATTTTATCGATAAATATACCCTCTTCACTACATTTCACAGTGTGTTGTTCAATATTTTGATTAGAGGTATT
TACAAAGTAGCTTATATATATAGTTTCATTAGGTTGTGCTTCTCCCACTACATAATTTGTTCTCATTTCTTCAGGT
AAAATATGTTGTGCATATTCTTTTAGTATAAATTCGGAATCAATTTTGTGTATAAAATAACTGAAGAATTGAAAC
CACAGCTCTATTTTCCACTTATTTTTAATCATGATAATCACTCTCTTTCAATATTTGAATTAAGTATATCAAATCAA
TATTAATTGATATGATAGTTCTTTTACTAATTATTTTTATAAGATAAAATAGATAAAAAAGGAACAAATGTTCTCT
TTGGGTATATCATATGATTAGAGGAGATCGATTCATTTAATGAATTATTTTATTTAATAGAATTATATTTGAAGGC
ATTTAAAGGACAAAAGAAAACAGGCTATGAATTTGAAGAAAGATTGAACACATTTATTCAATCATATGTCATT
ATTTTACTTATAGATATAAGGTTACCGACTGTTAGGTTATATATTATTAGGCATATTGTGGATACATGCGAAAAA
GAAGTGGACAATCAAAAAAGAATTATACTAATAGAGAAATCTATTAAAAAGTACAGATATGTAAAAGAACAG

ATTAAAAGATTTGGAAATGCGTTGAAAGAAGAATTATCTAACGCGACTTTGTCTATTGAATAGAATATGTTAG
AAAATATCATTGATTATTATAAGGATTACTTTTATCCTAGAGAAAAAATAAAGCATAATAATGTTGAGTGCAATG
TATCATTGTTTTTTAAAGCTGTAGATGCTTTTAAGAAATAGTTTTTCACTGACAATATTTATGGATATAATTAAAT
TTAAGCAATGTTAATTTATTCCAGTCTTAGTAGTTTTTGAATTTCACAAACACAATCTATGAAGTGAGTTAATAT
TAAACGATTTTAATAGGAGAGGGTTAGTATGGCTAGTTATTTATTTTTTCATCCTAAACCAGCGTGTGATACTT
ATGGGGATATGAATATTTATCACGACAAATTTGGAAATAATGAGGACCCATACGTATGGAGTGAACGGTTTTT
GCATAGCTTTTGTAAAATAACGGATTATGCATATAGTAAATCTACTCAAAAGGACATTATTTTTTGGATATCAAT
AAATAAAGAAGGCAATAATTTAAAATATTTATGTGATTTAGTATTTAAAATAGAAAAGTGGGATTTTTGGTATA
AAACTTTTAGTGAACAAAAAGATGCCATAGCTACAAATAAAGAATTAACGATAAATGACGCAGTAGTAGAAG
GCGATGAAGAAGCATATGAATATCATTATTCTTGGATCAACAGAGGGGAACACAAGTGGGAACCAACTTATC
GAAGGCGAAGACTCACATTAAAAGCAGATCCCGTCTTGAGTTTTCAGCCTCAAAATCGACAAGGTAATTTAC
TAGATGTCACGGAATTACTAAAAACAATTGTGAATTTTAATGTTGAAAAATCACCTGCAAAAAGTGGGACTTC
TTATAAAGCTTTCGAACTTGAAGAAGAGCAGGCAAGCAAACTATATGAGGAAATAAAACGATTATCTTTTATT
CGATTAAAGGGAAGGGACTTAAAAAACTTAAGAAGAAACTTTTCTTGATATATCTTAATAGTTGTTGTTATTA
AAACCATAAAGAAAGCTACCATTTTTAAGATACAAGCATTGATATGTACAATCTGAATGGAGTGTAAAAAGTA
AGTGATGTTGTGATGGACATAAAGTTGATACTCTATTTATACGAAAAATATTGATAAAGATTAATTATGATAAAC
ATGTAGTAATAGAAAATGAATTCTATAGTTCAGGACTTAGCAACTAATATTATGGTAATGGGAAGAATAAACAA
ATTAATTTTTATAAAAAATATATAAATTAAAACTTAGAACAGTTAGAAACAGAATTATTTGATTATGTAAATTGG
TACAACAATTTTAGACCACATTCTTCGTTACAGTATTTAACGCCAATGGCGTATAAAGATATACACATGAAAAG
TGTCTAAAAAACTGTTGACATTCCACTCTATATTTGATTGTAGTTGTTTATAGATAGTAAGATCATTCATTATACA
TGGATTTTTGTCCGATTTCTTGACACAATTCCAGACAAACTAATAAACTAATAAAATAAAAAAATAAAGGCGC
GAAAACAGGAATTATTAAATAAAATATTAATTTAATAAGGGGAACAAATGAAAAATATAGATATCGCAATTTAT
GATATAGATAAAGTTATTTGTAAAAGTATTGAGAATAACTCATCAGATTTAGGTTATTTATCACAAAGTATCTTA
TCACATTTAAGAAACTACGTAGAACATATTGGTATGAAATATTATTTCGAAAGTGTAAATGAAGATATGACTAA
TAGTTCTTCAAATATCTATAATGAAATACAAATGGGAATAGGTTATTTTAGGAAGCATTACAAATTGAATTGGC
TTAAAAATTTTCATGAATTACTTCAGCAATCTGTTTCTCATTATACATTTGATGAAGATAGATCCGAACGGTTAT
TTATAAAGTATTATGACTTAATGTTAGAATTAAAAGAAAAGTTAAAGAAAGATTTTGAAATGGATTTATTGCAA
AATTTATATAAAGTACCGCTGAATATTGATAGTGATTTAAAAGGATATTATCATACAATATCTCAATTAATAGGTA
ATAAATACACTGATAATGAATATGAAGTAGATAATTCAAGGTATTATATTGAAAAAACAAAACCATTTGTTATTG
AAAATAAAGTATATTATGAAATTACATTTAGAAATGCAAATGATAAAACTAGTAAATACGAAAGGCTGATTGGT
TATTCAAAACATAGAATAAACACAAATTATGCTGTAAAATTCGCATTAGAAATGCAAACAATAAGTTATTTAGG
GATTAAAGCTAACGTAATAATAATAAATGATTACGAAATATCTATAAGACCTTGTGAATTTAATAATTTTGCTAA
AATATTAGATTACGATTTAAAATTACAATCAAATCATAATGAATATAAAAAATTAATGAAATTGTTAAAAGAATT
TAATTTACATTTACTTGATATTGTTTTGTTAGATGACGATGAATTTAATGAATTGGAGTGTTCAGTGAATAGCG
AATCAGCAGTTATTAATATTTTTAATGTGTTGAGACTAGCAAGAAGATATATTTTAACAAATAAATCAGGCGCA
AATGTTCTTAGGTATTTATTATTTGTTTTTAACAATAGGATAATTCGTTTACAGTTACCTTATAATAGGAATAAAT
GTTATAAATTATCTAACCTTATATTGGATTATAAATGTGTTCCATTTGATCAAATGCCATTTACAGCCTCTTTAAA
AGGCCATAATCCTAATATCTATACTTTGTTACAATGTATAGAATACAAAGGGAGAGAATATGAATTATTAGTCAG
GAAGATTCAAAAGAATACTTTGAAGAATAAGAAAGTTTATACAAGCAAAGAAGAAATTGAGCAGTATGGTG
TTGTAAACGAACTAATCGATAAATATAATAATAATTTGTATTATAAGCATCGACCGAATAGGGAAATACATTCAT
TTGGTGATAAATATTATTTATATGAAAATGAGCAGAGTATTATGAGTATAATAAAGTCAATTAAACTATTGTCAA
ATGAAAGCGTAGAGGGCTATTCAAATTCAGTAGAGTTTTGGCTAAATAATGAATATACATCGTTGGATTGTAA
AGAAAAGAAGGAAATACTCTTAAGAATGTTTTCAAATAGCAAAATATCGATGGTATACGGAGCTGCTGGAAC
AGGGAAATCAACTTTAATAAACCATATTTGCAATTTTTTCTATGATAAAGATGTTATCGTCATAGCAAATACTAA

TACTGCAGTAGACAATATTAAAAGAAAAATCAAATTATCTAACATTAAAACGTCTACTATTTCTAAATTTTTATA
TAATGATAAAGAAAAGTATGACTTGTTAATAATCGATGAGGCAGGTACAGTTAGTAATAAGGACATGAATCGA
ATTCTTGAAAACAAGCAATTTGAATTATTATTAATTGTCGGTGATAATTATCAAATCGAATCGATAGATTTTGGA
AATTGGTTCGAAATTGCCAAAGATGTTTTGTCAAAAAATATAATCAACGAACTAACTGATATGTATCGAACTAA
AAATGATGATTTACTTTACTTTTGGAAATCTGTTAGAGAAAAAAAGAGTAATTTAAATGAAATTATTAATATGA
ATAAATATTCTACAAGATTAGATGAAAGTATATTTAATGAATTCAATAAAGATGAAATTATTCTTTGTTTAAATTA
CGATGGTATATATGGTATCAATAACATTAATAGATTATTACAAGCAAATAATAAAAATGATTCTGTAATTTGGGG
TGTGAAAGAATATAAAGTTGGTGATCCTATTCTATTTAATGAAACTAATAAGTACTCACCAATACTTTTTAATAA
TTTAAAAGGGTCAATAATTGAAATACATGTTTTTGAAGAATATATATTGTTTGATTTAGAAATAAATAAAGTTAT
AAATGAACTTGATATTATTTCGTTAGAAATTGATTTAATAAGTTCTTCTGAAAATAGTTCGGTGATTAGGATTA
GAGTAGAAAAAAGTGATGGGTTGAATGATGATGATAATGATTCATCTGATAGTATAGTTCCTTTTCAAGTGAG
TTATGCAATTTCAATACATAAAGCTCAAGGGTTAGAGTTTAATTCAGTAAAAATTGTTATATCTGATGATTTAG
ACGAACAAATCACTAACAATATTTTTTATACTGCAATTACACGAGCTAGAGAAAATTTAAAGATTTATTGGTCA
CCACGAACTGAAAAGAAAATTATTGATAATATAATTTCTAAAAGAAACTTGAAGGATCTTTCTATATTAAAATC
TCGAATAAAAAAACAAAATACTTAGAAATTTTCATACGACGCTTATATAGAACCATAAAAATCGTTTGACTAGT
ACCAGTGATTTTATTTAATTTTTCTCAACGACTCTACTTCTAAAAATATTTAAATGACTTTGATACAGCACGAA
TTAAAATCGCATAAGCGATGACTTTTCCACTTAATTTCTTATGCCATTCTACGGAAGTCATTTGAGAACAAAGT
ATTAATGATTTTTCACGGCTTCTTATTTCGAATACCTCCATGAGATACTATTGTTCTTGTTCAGTTGTATACGGC
AATAAAAAATCATCATTTACTAAAATATCCGTTCTGCTAATCACTTTTAATAGTTTATCTATGCTATTATAATCAGC
TTGTTGTAGCTTACTTAATAAATCTGTCAATCTATAGAATAATACACTATACCGATTATCAATCGCATGATTTACT
AAAGTAAAAGCGATGTAACTTTTACCATTTCCGGTGACACCTTTTTAAACCGCCATTTACATAAATACTATCTT
TAAATATAACTTGATTATTTGAAAGTATGTGAAAGTAAATTAAGGATTGGTACGTTTTGGTTAATTAAATATCTA
CCTAAATATTCCTCTAATTGACTTGTTGCATGTTCATTCAGCAAAACAGGAATAATCCATGCTGTATATAAATAA
TCATCTAGAAATTCTCTACATGTAGAATATTGTTTTTTTAATTCATCGTCTTTTATATACAATGAAGATAATATTTT
TCCGGTACCTTTATCTTCATCCATCTTCTTAATAAGTCACCTTCTTGATTTTTAACTAATTTATATTCATTACCCTT
AAATTCATATTTGAAATGTGATTTTCCAACTACGCAAACCATGCCATCTTTTCTTTGAGTAACATAGATGTAATT
TATTATTGGGTTATTTCCATTAGCTCTATTATTATTTGTTAATGTGTTACTTATATTAGACAATAAATCCTCAAAAT
ATGTAATAATATATTCATCTAAGGGTTTATTGTTATTACTAATATTACAATTTTGTTTGATAGCATAAAAAGCTTCT
AGGTTTAATTCAAATGGTTCCGCGCTAACGATTTTGCTATCTTTGTTGTCTATAATAAATTCGATTAGTAATTTC
TTAATCATTTCGTGATGTTTTCTAGGCTTATTGTAATGACAGGTTTTATAAAGCTGTTTATTAATTCTATTAAACA
TTTCTATTTGCAAGTCAGCAAGAATAGTTCTACAGTCTTCATAGATATCATGTACTGGGTATGGTTTATTAGGA
AACATGCTATATATTTCTTTATATGTTGTATTGAACACGTTAATTATACCTCCTACTATCTATTAATACTATTGTAAT
TATCAGATATAACTAAGTAAATGAATCGTCTACACTTAATTGGACAAATTCTATGAGAATAGATATTGTTAATTT
AAGAAAGAAATTACGAATGAACGCATTCGCAAAAATTGCATTTAAATAATGAAATATAGAGTAACTTGAGAA
AGTTGTTTCATCTAAAATATATAAACAGTAGAGGGGAGAACTTATGCACTGGAAAGAAAATAGAATAAAATC
AGCCAAAAATGACACGAATCCGATGGTAATCAAAGAATTAAAAGGTAGTTATGTGGTTTTTGGAGATGTTCA
GTTTCTTCCAGGTTATTGTGTATTACTTCCTAAAAGAGAGGTAAGATTATTGAATGATCTTACTTTAGAAGAAC
GACAAGATTACTTATTAGATATGAGCTTTGTTGGTGATGCTATGATGAAAGCATTGAAACCTACAAGAGTAAA
TTATGAAATACTAGGTAATAAAAATCACTTTCTTCATGCACATTTATTCCAAAGATATGAATGGGAAGATGAAT
CTGTTAGGTATATGCCAGTGTGGTGTATGATGCTTCTAATTGGTCTAATGAAGAAACGTCTTACGATTCTGATA
AACATGATGAAATTAGAAATAAGATAAAGAACGAACTTGAACAGTTGTATAACATATAAAACCAAAGTCGATA
TCATCATTTTGATATCGACTTTAATTATAAAAAACCGCACTCTTAACCGATACGCAGAGGCGTATCATAAGT
>Staphylococcus aureus strain BSN42
ATGAAAATCACCATTTTAGCTGTAGGGAAACTAAAAGAGAAATATTGGAAGCAAGCCATAGCAGAATATGAA

AAACGTTTAGGCCCATACACCAAGATAGACATCATAGAAGTTCCAGACGAAAAAGCACCAGAAAATATGAGT
GACAAAGAAATTGAGCAAGTAAAAGAAAAAGAAGGCCAACGAATACTAGCCAAAATCAAACCACAATCCA
CAGTCATTACATTAGAAATACAAGGAAAGATGCTATCTTCCGAAGGATTGGCCCAAGAATTGAACCAACGCA
TGACCCAAGGGCAAAGCGACTTTGTTTTCGTCATTGGCGGATCAAACGGCCTGCACAAGGACGTCTTACAA
CGCAGTAACTACGCACTATCATTCAGCAAAATGACATTCCCACATCAAATGATGCGGGTTGTGTTAATTGAAC
AAGTGTACAGAGCATTTAAGATTATGCGAGGAGAAGCATATCATAAATGATGCGGTTTTTTCAGCCGCTTCAT
AAAGGGATTTTGAATGTATCAGAACATATGAGGTTTATGTGAATTGCTGTTATGTTTTTAAGAAGCTTATCATA
AGTAATGAGGTTCATGATTTTTGACATAGTTAGCCTCCGCAGTCTTTCATTTCAAGTAAATAATAGCGAAATAT
TCTTTATACTGAATACTTATAGTGAAGCAAAGTTCTAGCTTTGAGAAAATTCTTTCTGCAACTAAATATAGTAA
ATTACGGTAAAATATAAATAAGTACATATTGAAGAAAATGAGACATAATATATTTTATAATAGGAGGGAATTTC
AAATGATAGACAACTTTATGCAGGTCCTTAAATTAATTAAAGAGAAACGTACCAATAATGTAGTTAAAAAATC
TGATTGGGATAAAGGTGATCTATATAAAACTTTAGTCCATGATAAGTTACCCAAGCAGTTAAAAGTGCATATAA
AAGAAGATAAATATTCAGTTGTAGGGAAGGTTGCTACTGGGAACTATAGTAAAGTTCCTTGGATTTCAATATA
TGATGAGAATATAACAAAAGAAACAAAGGATGGATATTATTTGGTATATCTTTTTCATCCGGAAGGAGAAGG
CATATACTTATCTTTGAATCAAGGATGGTCAAAGATAAGTGATATGTTTCCGCGGGATAAAAATGCTGCAAAA
CAAAGAGCATTAACTTTATCTTCCGAACTCAATAAATATATTACATCAAATGAATTTAATACTGGAAGATTTTAT
TACGCAGAAAATAAAGATTCATCTTATGATTTAAAAAATGATTATCCATCAGGATATTCTCATGGATCAATAAGA
TTCAAATATTATGATTTGAATGAAGGATTCACAGAAGAAGATATGCTAGAGGATTTAAAGAAATTTTTAGAAC
TATTTAATGAATTAGCTTCAAAAGTTACAAAAACATCCTATGATAGCTTGGTCAATAGCATAGACGAAATACAG
GAAGACAGCGAAATTGAAGAAATTAGAACAGCACAAAAAGATAAGACACTCAAGGAAGTGGAAGCACCTA
AAGGAATAATTCCAAAATATAAAAAAGGTGTATCAAAGACTACTAAAAATGATTCAGAAATTGAAAAATCAA
ATAAAGAGAATAAATTAACCGGTAAAGTTGGAGAAAAATTAGCGCTAAATTACTTTAATGAGCTAATTGATAA
TAAAATAGACGAAGATAAGAAAGAACAGTTTAGGAATATTTTAAATGATAATCCAGGCTCTCAACACGGTCAT
GGCTATGATTTAGTAGCTTTTGATCCAACAAATACAGATAAAGCTGTAGAAAAATTTATTGAAATTAAAACATC
TACATCTTCTAGTATTGAGGAACCATTTTTTATGTCGCTAAATGAAATGTTTGCTATGAAAGAATATAAGCAGA
AATATTTAATATTAAGAATATTTAATGTTTCCGGTAAAGAACCACAATTTTATTTTATAGATCCATATGCAAATTA
TTCTGAATTTAAAGATGTAGATGATCTCATTGACAAAGTATTTAATGTAGAAGCTATTCAGTATAAAGTTTTTG
GCGAAAAAATGATTACTTGAACAAGAGCTAAAATAAAATTGTGATCTAATAAAAATAGAAGGTTCTGTTGCAA
AGTAAAAAAATATAGCTAACCACTAATTTATCATGTCAGTGTTCGCTTAACTTGCTAGCATGATGCTAATTTCGT
GGCATGGCGAAAATCCGTAGATCTGAAGAGACCTGCGGTTCTTTTTATATAGAGCGTAAATACATTCAATACC
TTTTAAAGTATTCTTTGCTGTATTGATACTTTGATACCTTGTCTTTCTTACTTTAATATGACGGTGATCTTGCTCA
ATGAGGTTATTCAGATATTTCGATGTACAATGACAGTCAGGTTTAAGTTTAAAAGCTTTAATTACTTTAGCCAT
TGCTACCTTCGTTGAAGGTGCCTGATCTGTAATTACCTTTTGAGGTTTACCAAATTGTTTAATGAGACGTTTG
ATAAACGCATATGCTGAATGATTATCTCGTTGCTTACGCAACCAAATATCTAATGTATGTCCCTCTGCATCAATG
GCACGATATAAATAGCTCCATTTTCCTTTTATTTTGATGTACGTCTCATCAATACGCCATTTGTAATAAGCTTTTT
TATGCTTTTTCTTCCAAATTTGATACAAAATTGGGGCATATTCTTGAACCCAACGGTAGACCGTTGAATGATG
AACGTTTACACCACGTTCCCTTAATATTTCAGATATATCACGATAACTCAATGTATATCTTAGATAGTAGCCAAC
GGCTACAGTGATAACATCCTTGTTAAATTGTTTATATCTGAAATAGTTCATACAGAAGACTCCTTTTTGTTAAA
ATTATACTATAAATTCAACTTTGCAACAGAACCGTATTATGGAATAGAGATGTTGGTAACATTTATACAGGATC
ATTATACTTAAGTTTAATTTCGTTATTACAGAACCACACATTCCAACCAGAAGAGAAAGTATGTCTATTTAGTT
ATGGTTCAGGAGCAGTAGGAGAAATCTTTAGTGGTTCAATCGTTAAAGGATATGACAAAGCATTAGATAAAG
AGAAACACTTAAATATGCTAGAATCTAGAGAGCAATTATCAGTCGAAGAATACGAAACATTCTTTAACAGATT
TGATAATCAAGAATTTGATTTCGAACGTGAATTGACACAAGATCCATATTCAAAAGTATACTTATACAGTATAG
AAGACCATATCAGAACATATAAGATAGAGAAATAAACTAGTGGCCGATTGTGCTTGATGAGCTTGGGACATA

AATCCTAACTCGAAATAAATAAGCATATCACTAAACTGATTTTTTAAAGTTTACAGTGATATGCTTATTTTTTTAT
CTTACGATTTTGTACGTGCATGCTTGCCTAGGGGTATGGCTCGAGCCATTAGTCTCTCGCACATACTATTCCCT
CAGGCGTCAGCACTTACAAAATCGGTTGTAATTTTCATTTTTATACGCATTCTTACTGAGATTATACTAATAAGA
GGAATAGTAAAAGCAATTCTAAGTAAAATTGCAGATAAGAGGTTTGTTAAAAGCAGTTCTCAGTAAAATTAC
AGATAAGAGGTACGTTAAAAGCAGTTCTAAGTAAAATTGCAGATAAGAGGTTTGTTAAAAGCAGTTCTAAGT
AAAATTGCAGATAAGAGGTACGTTAAAAGCAATTCCATGCAAAATTGCTGATAAGGGGTAAGTTAAAAGCA
GTTCTCAGTAAAATTGCAGATAAGAGGTACGTTAAAAGCAGTTCTAGGCAAAATTGCAGATAAGAGGTGCG
TTAAAAGCAGTTCTCAGTAAAATTGCTGATAAGGGGTAAGTTAAAAGCAATCCTAAGTAAAATTGCAGATAA
GGGGTACAGAAAAACTAGACTTGATTACAAAATGGAGCTTGGGACATAAATGATTTTTTAAAAATGAGATGA
GACGTAGATTAACTCCATAATCAATACGAATCTATCGACTTCTTTATTTATGATATTCATCTCTTTTTAATGGAAA
TAAAAGTGCGATTAATGTGATAATACAGTTACGTTAATTAAAAAAATAAAAATGCAAGGAGAGGTAATATGCT
AACTGTATATGGACATAGAGGATTACCTAGTAAAGCTCCGGAAAATACAATTGCATCATTTAAAGCTGCTTCA
GAAGTAGAAGGTATAAACTGGTTGGAGTTAGATGTTGCAATTACAAAAGATGAACAACTGATTATCATTCATG
ATGATTATTTAGAACGGACTACAAATATGTCCGGGGAAATAACTGAATTGAATTATGATGAAATTAAAGATGC
TTCTGCAGGATCTTGGTTTGGTGAAAAATTCAAAGATGAACATTTGCCAACTTTCGATGATGTAGTAAAAATA
GCAAATGAATATAATATGAATTTAAATGTAGAATTAAAAGGTATTACTGGACCGAATGGACTAGCACTTTCTAA
AAGTATGGTTAAGCAAGTGGAAGAACAATTAACAAACTTAAATCAGAATCAAGAAGTGCTCATTTCAAGCTT
TAATGTTGTGCTTGTTAAACTTGCAGAAGAAATCATGCCACAATATAACAGAGCAGTTATATTCCATACAACTT
CGTTTCGTGAAGACTGGAGAACACTTTTAGATTACTGTAATGCTAAAATAGTAAACACTGAAGATGCCAAAC
TTACTAAAGCAAAAGTAAAAATGGTAAAAGAAGCGGGTTATGAATTGAACGTATGGACTGTAAACAAACCA
GCACGTGCAAACCAACTTGCTAATTGGGGAGTTGATGGTATCTTTACAGACAATGCAGATAAAATGGTGCAT
TTGTCTCAATAGAAAGTTAGAGGTGAGTCTTACGTTTCAGTGACGGTAGACTTACCTTTAACATGTTACATAC
TAAAAAATTAATTTGAATAAGAAAGAGAGACATATATGAAATACGATGATTTTATAGTAGGAGAAACATTCAA
AACAAAAAGCCTTCATATTACAGAAGAAGAAATTATCCAATTTGCAACAACTTTTGATCCTCAATATATGCATA
TAGATAAAGAAAAAGCAGAACAAAGTAGATTTAAAGGTATCATTGCATCTGGCATGCATACACTTTCAATATC
ATTTAAATTATGGGTAGAAGAAGGTAAATACGGAGAAGAAGTTGTAGCAGGAACACAAATGAATAACGTTA
AATTTATTAAACCTGTATACCCAGGTAATACATTGTACGTTATCGCTGAAATTACAAATAAGAAATCCATAAAAA
AAGAAAATGGACTCGTTACAGTGTCACTTTCAACATACAATGAAAATGAAGAAATTGTATTTAAGGGAGAAG
TAACAGCACTTATTAATAATTCATAATAAAACAGTGAAGCAACCATCGTTACGGATTGCTTCACTGTTTTGTTA
TTCATCTATATCGTATTTTTTATTACCGTTCTCATATAGCTCATCATACACTTTACCTGAGATTTTGGCATTGTAGC
TAGCCATTCCTTTATCTTGTACATCTTTAACATTAATAGCCATCATCATGTTTGGATTATCTTTATCATATGATATA
AACCACCCAATTTGTCTGCCAGTTTCTCCTTGTTTCATTTTGAGTTCTGCAGTACCGGATTTGCCAATTAAGTT
TGCATAAGATCTATAAATATCTTCTTTATGTGTTTTATTTACGACTTGTTGCATACCATCAGTTAATAGATTGATAT
TTTCTTTGGAAATAATATTTTTCTTCCAAACTTTGTTTTTCGTGTCTTTTAATAAGTGAGGTGCGTTAATATTGC
CATTATTTTCTAATGCGCTATAGATTGAAAGGATCTGTACTGGGTTAATCAGTATTTCACCTTGTCCGTAACCTG
AATCAGCTAATAATATTTCATTATCTAAATTTTTGTTTGAAATTTGAGCATTATAAAATGGATAATCACTTGGTAT
ATCTTCACCAACACCTAGTTTTTTCATGCCTTTTTCAAATTTCTTACTGCCTAATTCGAGTGCTACTCTAGCAAA
GAAAATGTTATCTGATGATTCTATTGCTTGTTTTAAGTCGATATTACCATTTACCACTTCATATCTTGTAACGTTG
TAACCACCCCAAGATTTATCTTTTTGCCAACCTTTACCATCGATTTTATAACTTGTTTTATCGTCTAATGTTTTGT
TATTTAACCCAATCATTGCTGTTAATATTTTTTGAGTTGAACCTGGTGAAGTTGTAATCTGGAACTTGTTGAGC
AGAGGTTCTTTTTTATCTTCGGTTAATTTATTATATTCTTCGTTACTCATGCCATACATAAATGGATAGACGTCAT
ATGAAGGTGTGCTTACAAGTGCTAATAATTCACCTGTTTGAGGGTGGATAGCAGTACCTGAGCCATAATCATT
TTTCATGTTGTTATAAATACTCTTTTGAACTTTAGCATCAATAGTTAGTTGAATATCTTTGCCATCTTTTTTCTTT
TTCTCTATTAATGTATGTGCGATTGTATTGCTATTATCGTCAACGATTGTGACACGATAGCCATCTTCATGTTGG

AGCTTTTTATCGTAAAGTTTTTCGAGTCCCTTTTTACCAATAACTGCATCATCTTTATAGCCTTTATATTCTTTTT
GTTTTAATTCTTCAGAGTTAATGGGACCAACATAACCTAATAGATGTGAAGTCGCTTTTCCTAGAGGATAGTT
ACGACTTTCTGTTTCATTAGTTGTAAGATGAAATTTTTTTGCGAAATCACTTAAATATTCATCCATTTTTTTAAC
GGTTTTAAGTGGAACGAAGGTATCATCTTGTACCCAATTTTGATCCATTTGTTGTTTGATATAGTCTTCAGAAA
TACTTAGTTCTTTAGCGATTGCTTTATAATCTTTTTTAGATACATTCTTTGGAACGATGCCTATCTCATATGCTGT
TCCTGTATTGGCCAATTCCACATTGTTTCGGTCTAAAATTTTACCACGTTCTGATTTTAAATTTTCAATATGTATG
CTTTGGTCTTTCTGCATTCCTGGAATAATGACGCTATGATCCCAATCTAACTTCCACATACCATCTTCTTTAACA
AAATTAAATTGAACGTTGCGATCAATGTTACCGTAGTTTGTTTTAATTTTATATTGAGCATCTACTCGTTTTTTA
TTTTTAGATACTTTTTTTATTTTACGATCCTGAATGTTTATATCTTTAACGCCTAAACTATTATATATTTTTATCGG
ACGTTCAGTCATTTCTACTTCACCATTATCGCTTTTAGAAATATAACTGCTATCTTTATAAACTTGTTTGAAATTT
TTATCTTCAATTGCATCAATAGTATTATTAATTTCTTTATCTTTTGAAGCATAAAAATATATACCAAACCCGACAA
CTACAACTATTAAAATAAGTGGAACAATTTTTATCTTTTTCATCAATATACTCCTTATATAAGACTACATTTGTAG
TATATTACAAATGTAGTATTTATGTCAAAATAATGTTATAATTTTTGTGATATGGAGGTGTAGAAGGTGTTATCAT
CTTTTTTAATGTTAAGTATAATCAGTTCATTGCTCACGATATGTGTAATTTTTTTAGTGAGAATGCTCTATATAAA
ATATACTCAAAATATTATGTCACATAAGATTTGGTTATTAGTGCTCGTCTCCACGTTAATTCCATTAATACCATTT
TACAAAATATCGAATTTTACATTTTCAAAAGATATGATGAATCGAAATGTATCTGACACGACTTCTTCGGTTAG
TCATATGTTAGATGGTCAACAATCATCTGTTACGAAAGACTTAGCAATTAATGTTAATCAGTTTGAGACCTCAA
ATATAACGTATATGATTCTTTTGATATGGGTATTTGGTAGTTTGTTGTGCTTATTTTATATGATTAAGGCATTCCG
ACAAATTGATGTTATTAAAAGTTCGTCATTGGAATCGTCATATCTTAATGAACGACTTAAAGTATGTCAAAGTA
AGATGCAGTTCTACAAAAAGCATATAACAATTAGTTATAGTTCAAACATTGATAATCCGATGGTATTTGGTTTA
GTGAAATCCCAAATTGTACTACCAACTGTCGTAGTCGAAACCATGAATGACAAAGAAATTGAATATATTATTCT
ACATGAACTATCACATGTGAAAAGTCATGACTTAATATTCAACCAGCTTTATGTTGTTTTTAAAATGATATTCTG
GTTTAATCCTGCACTATATATAAGTAAAACAATGATGGACAATGACTGTGAAAAAGTATGTGATAGAAACGTT
TTAAAAATTTTGAATCGCCATGAACATATACGTTATGGTGAATCGATATTAAAATGCTCTATTTTAAAATCTCAG
CACATAAATAATGTGGCAGCACAATATTTACTAGGTTTTAATTCAAATATTAAAGAACGTGTTAAGTATATTGCA
CTTTATGATTCAATGCCTAAACCTAATCGAAACAAGCGTATTGTTGCGTATATTGTATGTAGTATATCGAGCTTC
ACATGAAACAGCTAAAGAAGCTTTGGGCGATAAAGAGTTAAGAGCCATTGCACATGAGTTAACTAAAACAG
TTAAGGATAACATGAGTGTTGATTGGTCTAAACGAGACAGTGCTAAAGCTAAAATGAGAGTTCAAGTTAGAC
GCCTATTAAAGAAATATGGCTATCCACCAGATCTTCAAAAAATGGCTGTGGAACAAGTTGTAGAGCAAGCAG
AATTAATGGCAAGTCAGCAATAAAAAAATAAATCATAATGAGTCCGGGACATAAAGTTCTTGGATAAGTGAA
AAAAGACAATTTCTATTGAAATAATATAGAAATTGTCTTTTTTATAAATTTTTTGATTATTTTCAGCTCGTTGAG
CTACTACTTTTCTTATATTAAGTGCCATTAATACAAAACCAAGTTCTCTTTTGACTTTATTGAGTCCTCGGACAG
ACATCCGAGTGAAACCCAAAATAGCCTTCATAAATCCAAAAACAGGTTCCACATCAATTTTTCTTTGACTGTA
GATATTTTTTGTTTCTGGTTCTGAAAGCTTTTTGTTAATTTGGGATTTAAAATATTCCCAGTTATAATTCTTCATT
ATTTTTTTGTTTGTTTTTGAATTGAAGTTCATACATTGATTTTTCAGAGGACATTCTGAACAATCATCACATTCA
TATAATTTGAAGTCTCGCTTATAACCATACTTATCATGACGATAGGCATATCTTTTAAAACCTAGCCGTTTATTAT
TCGGACAAATGAATTCGTCATTAATTTCGTCATAGTTCCAATTTTGAGTATTAAAGATGTCACTTTTATATTTTT
TAGTTTTATCTTTTATAAACATTCCATATGTTATGAGTGGCGTTCGATTAAAGTCATCTATAATTGCCTTATAATTT
GATTCACTACCATAACCTGCATCAGCTACAATATATTCAGGTAAATGACCGTAGGTCTCTTGAATTGAATTTAA
AAATGGAATCATCGTTCTAGTATCCGTTGGATTTTGATACACATTATAAGATAAAACAAATTGGGAATTTGTTG
CTATTTGTAAATTATACCCTGGCTTAAGTTGTCCATTTTTCATGTGATCTTCTTTCATTCTCATAAATGTCGCATC
ATAATCTGTCTTAGAATAACTATTTCTATCCTTTAAAATAGATTTTTGAAATTCGTATCGATACTTTCGCTCAAAA
TAATCATTGATTTGCTTTTTGTATTTTTTGATTTAGTTCTTTTGAGACGTATTTGTTTTCTTGTTTTAGTACATT
TTTCATTGTTGATATGTTGGTTTAAATCTTCGATTTCTTTATCTAAGTGACTACCAATCAAATCTATTTCTTCTTT

TGTTAATTCATTATCATGATCTTCTTTAATTTCCGGTATGATTTTATTGGTTACCAATTCATGGTAGAGGGCTTTA
GAATCCTCATTCATCTTTGATTCATGGTTTTGAATACTCTTTTTCCATACAAATGTATATCGATTGGCATTTGCTT
CAATTTTTGTACCATCAATAAAAATAGCTTTATCATCTATAAGATTTTGTTTTACACACTGACTGTAAAATTGAA
TAAATAAAGATTCTAATAAAGCATCTACTTTTGGATTTACTCTAAATTGATTAATTGTTTTATAAGAAGGTTTTT
GATTTTGTGATAGCCACATCATTCGGATGCTATCATTAAGCATTTTTTCTATTTTACGACCTGAGAATACAGATT
GTGTGTAGGCATATAGAATCACTTTTAACATCATTTTAGGATGGTACGAAGTTGCACCACGGTGATGTCTGAA
TTCGTCGAATTCATTGTCAGGAATTGTTTCAACAATATCATTTACAGTAAAACGATGTTGATTTGTTTTGTTTCC
ATATTGACCTCCATGTATTTGCTATGATTTCAAAATCCATTTTTGACGTGCCTTAGGGTTGAGTGGATGCATAAT
TTCATTTGTTACTGGATTGATGAGCTTTTTTACTTTCTTTTTATGAGGTTTTAACATTTCCATCACTTGTTCGAC
ACGGTCGATAACAACTGGTCGCTTCGCATAGGCACCATAAGCAAGAATCACTGTGTCACTTTCACTAATCGCT
TTCATCAAATGAATATCAGTGTGCTCATCGTATGGATTTTTGATATGTTTGAGGTTTTCGGGTGTTCTAATATTA
GAGAATAGATTTACAAGATATACAGCACCGTATCGTTCTGAATTGGCTAATTGGTTGAGGATAAGAACAGTTG
TGAGATCGAGTGATAATACACCGTCTAAATGAGGATACATCGTTATCACTGTGCATGCAGCTTTCTTTTCATCC
CATGTTTTCTTGAGTAAATAGCGGTGCTGTTCATCATCGCTAAATATGGCTTCTGTGTGTATCGTACTTTTGATT
GTATTCATCATCGTCACTTCCTTTAGTATTCTTCTGGTAAAAGCATCACATAATAAAAAGCGTCCACGTCATCTT
CACGAATGACGTAGACTTTCTTAGGTAATGCATTTTGATTTTTTTCATAGTTTGTATAGTGATATTCCAATTTGT
ATGTGGGTTGTTCTTGTTCATGTGTGATTGAGAGTATATTCTCATCTTCTTGTAATTTAAAAATGTGTAGGTAAT
CTGTATGAGGCTGGTTATCTTTTTTTTCTACCATGTGCCAAAGTAAGATTTGAAGGTCTAGTGGAATACTTTCA
TTAATTCCTCTTGTGATGTATCGATTGATTTTCATGCTATTTCCCTCCCTTCTGCTTTTCTTTCATGATGTCGATG
ATTTCGTTGATAACTGTGACGGATAATTGAGCAACTATGATCCAATTATTCATGGTCCTGACCTCCTTGTTTTA
GTAAATGACGTTCATCAATAATGATATTTTGAGTATCTGTAAGGTACAGAAAGTCCATGTCAAAATGGTCTAAG
TATCCGACACTGATGAGTTGGTTATTGGCATACATTAGAAATGGATAGATACTTAGCTCATGTAGTTCATCATTA
TAGTAGGTATAAGTCTCGAGTGTGAGATGTACCAGTGGAGAATCATTAATAAAACGTTCCGGTAGAATATTTT
CTGCTGCTTCCTCCAGCGCTTCACATTCCCAAGCTTCGTTATTAGATAGTTGGAATAGGTGGGTTATATATTGT
TTGAGTTCTTGAGTGATGGTTTTCATATTATTGCCTCCTAGATAGTGTAATAGTGATGTAGTTCATATACATCATT
GAGATAATATATATTTGATTTGTCATTTATTACGAATCCCGGTGGGAATAAGAGAAAATTCCATATAAAAACCC
GCTACAAACGTTGGTATGCCAAGGAAATCCTGAAATTCCGCCTATTTTGACAAACAATCAACTCATTATTTATA
AGTATTGATGATAGGGTTGTGTCTCTGCTTCCTTATATATATTATTTATTTATAAAAAGTAACGGGATTTTGGGA
TTGTGCTTGCACAATCCTTCTGTTTCTTCGAATCTGCAAATCCCAATCATTTCCCGATAAAAAATCATTGTGGG
ATGTTCTTTAGCAATTTCAATATAAGCATTGTATAGTTATGAAAAAATTACGACAATAACTGTTTCATTAGATAA
GTGTTATTGAAATTGATAAAGAGCAATTCTTGAAAATAGTTAGATAAAATAAGCGAAAGAATATAGTGAAAAT
TATTGTTATAACAATGATTCTATTAGCTAAATAGTAAGATATAGTGTTTGGGGCAAAAATAAAGACGAAGTGCT
GAGATGCACTTCGTCGAGTTGTTTATTATTGAAAAGTTGTTTAATGATTTCGTTATTAAGTTTGAGTGTGACAT
AGAATTGTTTTTTATGATTACCATCTTTTTTAATATCAATGCGATCAATCACTGATAGATACAATGCTTTGAGTC
GAGATTTTTCTATGTGCTTAATATCATGAAAGATGTGTTGTAATAGTTTACTGATTTCTTTGGCATCAAATAAAG
TCTTATCTTCATTTTGTTGATTTTTGAGTTGGTTGATTTGATTCGTAATGTCATTGAGTTGCTTTTCATATTTTTG
AATACTTGGTCTGATTACTGATGTTAAGTCCGGATTATCCTCGATGGTTTTAATCAAGTTATTTATTTTGATTTG
TACTTCATCATATTGTTGTTGCTTATAAGCAATATCGTGATGAAGTGCAGCGCCATCAACTTGATTTTCTTGATT
GACGTGTGTTACTACGCGTTGAATGACTTTATCACTTTTGACTATTTCAAGTATTTGCTTCATCACATAATCTTC
AATCACATCAGCTCTTACACTGTTTGCCGAACATACTTTGGAACCCTTGTTCCGAAAATTACTACATGAATAGT
AACGAATACGTTTCTTAGTCCCGTCTTTAAGAGTATTCGTGGTATTGCTTGCTGCCATAGGTGCGCCACATTG
GGGACAGTGAATAATGCCTGTAAGCAGATTCGTTCCTTTGCCATGGACTTGGGGTTTTTGACTGACTTGTTT
CTTACGCATTTGTACTTTATCCCATAAATCTTGATTAATAATGGGGGAATGCTTACCTTCAGCTATCACTGGTTT
ATCATTCAGCCCTTTACGACGTTTTTCACTCCAATCTTTGTATTTCGCAAATTGAATTTTGCCGATATAGAAAG

GGTTAGCTAAGATGTATGTGATTGAACTAATACTGAAAGGTTTCCCCTTTTTAGTGACATATCCTTTGTGATTC
AATGCATTGGCAATTTTACGATAGCCATGTCCTTTGGCATAGCACTCGAATATATATTTTACAATATTAGCTTCA
TGTTGGTTAATCATTAGCTCGTGTTTACTATCTGGTATTTTGTCATAACCTAGTGGTAAATTGCCTTGATAATAG
CCTTCTTGGGCACGTCTCGTTTGACCCATAAATACGTTCTCGACAATGTTATTACGTTCGAATTCTGAGAAACT
CGCAAGTATCTGTAACATGAGTTTACCAGAAGAAGTATTGACTTCCATACGCTCTGACAAACTGAAAAATTCG
ACATTTTGTTTGTGTAAATCTTCGACAATTTTGAGAAGATCAGATGTATTACGAGCTAATCGGTTTGTTTTGTA
TACCATAACACAGTCGATATTGCCTTCTTTTGCATCTTTCAACATACGTTGGAGCTCAGGTCTGTTCATAGATT
TACCTGAAATACCACGGTCAGCGTATATATCTTTAACTTCAAAATGATGGAAGTCACAGTATTCTTTGATTTGA
TTGATTTGTCCGTCGATACTATAACCTTCTGTGCTTTGCATTTCTGTTGATACACGTACATAGATACCGACACGT
TTTGTTTTAAGTTGTTGCATTATGTTACATCCTTTCTTCATTTATGCAATCGATGATTGCATGGTTTGATTGACA
ATATTGAGTGGTTCATTTTTGAAATAGATTCCTATAAGATTTTTATCTTTCGTAATGTGAATGGTTTCAATATAG
GGGTACAATATGTTTAACGTGAAACGTTTTTGAATAATATTTTGAATGATGTGTTGTATTTGATGCCCATTGATA
GATGTAGTGCGTTGCGGTTGTTGACGTAATGATTGTGTTTGCTCTCTGAACGTTTCTGCATCAATGATGCCTT
GTGCCAACTTTTCTATCAGTTGTTCTTGAGTCAATGTGTGATGTTTTTCTATGTTTCTTTGTCTTTTGATGCGTT
TGTCAATCGCACTTTTAATTTTTGTGTAGATGCGTTGATTTTGATAAAAGTCTCGGCACACTTCTAATACTTTAT
CTTCAAGTGTTTGTGCATTGATGCCTTTAAAATCACAGACAAAGCGTGAAGCATTCATGTTTTTAGGACAGA
CGTAGTAACGTAATGTATGATTCTTTTTTCTAATGGTCATATTTGTAAGTGTTGCATTACAACATGGGCATTTGA
TTTTTTGTTTGAGTTGATTATCCGAAGGTGTCTGTTTGGTTTGTTTTTGCAATCGAAGTCTCTGCGCTTGCTCA
TATATACTTGTGGAAACAATAGAAGGAAACATATTGTCGAATTGGCCATATTGATTGTTGACACGACCACAAT
AATTAGGATTGATGATAATGTTACGAACTTGATAGGGTTGTCGATTGATATACGTGTTATCTTCTTCTAATAACT
GTGCAATTTTCTTATAACCATGACCTTTAATGTAATAATTGAATACAGCCTTTACCGTTGGTGACTCATTTTGAT
TGATGATGAATGTTCCGTTGTGATATTCGTAACCAAAAGGTGCATGTGTTGTAATCAATCGACCTTGCTTTGCT
TTTTCTTGAAGCCCATTTCTGACTTGTTCTCCAATGTTATCCGATTCAAGTTCGGCTAAACTGATGAAGATATT
AAGCTTGAGTCGGTCGAAAGCTTGATCCATATCAAAGTAACCATCATGTACGCTTAAGATATGAACATGGTAC
GTTTGACATAATTTGATGAGTTTTAATGCATTTTTCAGATTACGATGCAACCTATTAAGACGATAACAGCATAA
TATGTCACACTGTCCTTGTTGAATTAATTGTGTTATTTGTCGATACCCACTACGATTATCTTTGCGACCTGATTG
TTTGTCGCTATAAAAGTTGATATGTTGAATATGATGTTTTTCGGCTATTGCTTCGATAGCTTGTTTCTGTGCTGC
AAGAGATTGTTGTTTCATCGTACTTTGACGTAAATAGCCTATGACTTGTTTCATATCGGCTCCTCCTTTCACAG
TGATAATATATATTTATGGATGAATTGATATATAAGCCCAACATCAATGAGATGTTGGGCGTCCATATTAGTCATT
TGTTTGATTGATTTCTTCAATTACCAAATCGGCTAATATCTCGATAAGTTCATCCATGTTTTTCACTCCGTTATTT
GTTCTATCTTCAATACGTCGATTATTCAGTTTGATGCTTCACAGTTGTATGATAAAGACAATTAGAAATCTTCGT
GAACTCCTGAAGGGCCTATCCCTTCATTAGCGGATTTAAAAAGTTCTTTCGCAGCTTTGTTATCATTTGACGG
TGTCCAATTTTGAAGTAACGACTTATCTTTAGTTAATCCGAGGATAGATGCAAACTCTACATCTAATTTTAGAT
GGTAAAATACAAGTGATTGTTTTTTACCGCTATTATCTTTGACACTTCTTTTAGTTGTTTGGCGTCCACGGTCA
GCTAATATGAAACCTTTATCTCTTAAGGCGTTGACAACATTATTAACATCTTGAAATTGATGATTGTTTAGCATC
TGTTTAAAAACGTTCGCAATTATTTTTACTTCGATATGGTCATCTTTTAATGAGATTAATCCATAGTTCTCAAAC
ATATTTTTCAAAGCACCTTCATCTGAAAACTTACCTCTGTTTTGTGCCACAAATTGAATGATGACATCAATAGC
TTTATCAGCTAATGAGCGTTCAGAGACTGTATGAGTATGATAATCAATAAAGTAGTCTCTTATATTAGCGATATC
AATATCTGTAGATAAAACACGACCTAATATTTTCGCAGATGTTGTAATGACTGCATAACGCTTAAACATACGAA
TACCTGTATTGTTTGTTTCATCTTTCAATTTAGCTTCAAACCAATCTACTTCCTTGTAAAACCATTGAATAACTTC
ATCTTCACGATTTATAAGATATTTAGCTACTAACGGTAAAACATGACCATAGTTTAGTGCTACAGCTTTTTTAAT
ATTGTCAGCATTGGTCGCATTTGTAGTGAATTGTTCATTAATCTCGATGGTTCTTACACGTAATCCATCGTTTTG
AGCTGAATCATTAAAAATACTGTATTCTGACGTTGAAATGACAGAAGTACCCCAATTCTTAGGCGTTTTAACT
TCTCCATGAACGTTTGAACGTTGACGACCTTGACCTTCAGCGATGGAATATAACAAACCCGTGGTATCTCTAA

GTGTCGTAGATGAAAGCTCATCAAATACTATAGGAATGCCATAATTGTTACTCAAATAACCTTCAAGTGCGTTT
CGTGTGGCATTCCAACTTCTAAAAAGAGTTTCATTACCTTTGGTAGGGTTACCAGCGACTGATACAGCTAAA
GCAGCTGCGGTTGACTTACCGGTTGAGGATTGACCTGTAAAACTAAAGAGAATTCCTGCAAATTCGATTTCA
TGTTTATGCTTCAGAAAACTTGTCACTAAGGCAGAAATCCCAAATATGACTGCCAATTCTAAAAGAAGATGAC
CTTTAACCTCGTCAATATACATGTTAAACCAATTATCAAATGTACCTTTAGGAGTTAAGTCATAAGCACTATCTA
CAATAGGGTATGAAGGTGAGGACTGATTAAATTGAATAGATTTGAGAACTTTATCTAGCGATATCAAGTAGCC
AAAAGGTGTCTCTAGTATACCTACACCCTCATATAATTCAGAAAGTGGTAATCTATCTCGCATCAATTGCAAGG
CATAACTCAATGATCTAATATACTTCTCGTTAATACTGTAGCCATATTTAATTAAAGAAGGTAAGTTTCGTGTTG
TTAAGATATCAGACTCAAAAATGTCTTTTTTACCATTGTTGTTAGTAATAATCAACTTTTCAACACCAGTAATTG
GATCAGAAAATCTTGCATTAACAATAATAGGGCTAGCCATTCTAACCACTTTTTCACTGTCACCATCTTTTTTA
GGTGGTATCGTTTCTTTCCAACCGAAGGAATCTAAAGCAAATGGGCCAATTTCAAACAATGTGTAACTCATTA
GCAATCACCTCCTTTCGAAGGCGTGCTATTATTGCATGGGTTTATCCCTATTTTCTGATATAATAAACTGCCATT
TTTTGTTCCTACAATTTTAAATGGTAATCCTGGCGTGTACTTAATCCAGTAAGCAAAACGACGTCCTACTTCAC
GTTGATCATTCGTGCTACAAACCACATTATATTTCTTTCTTAATTCACCATAAGTGAACGTACTTCCAACTGGTT
GTTGAAAAGATTTAGCAATTATAAAATTATAAAGCTTTTCTTGCTGCTTAGATTTCTTATTCATTTTAAAATTCC
TCCAATAATTTTTAAGGAGTCAATCAATATCATATAGGTTAAACTATCTTCAATTGATTGCTATAATAAGCATAAA
TGAAGCATTATGATTAGACGCGGAACAAAAAAATGGGGAGGGGGGGTATATATTGATACCTACGCTAGAAGA
AATTATTGATAAGTATGGAAACTTAGTTGATTATTTGAAATTTGATGTAACAGTTGAGGTTTATGAAGATTTGT
TGTTATTAAGAAGTTTAAGCGATTTAAATGAACATCAAAAGATGGATAGAGTTTCATTTATAAAAAAACATTTA
AACTCAAATTTCGATTATTTATTAGATGATACTGAGTTTAGAAGTATCAATAAGATTTACAGTAATTTAAATATC
ATGACGCATATCAATAGTCAAAATAATAATTTTAAACGTAATCCATTTATAAATGAAGAACAATTGAAATCACTA
CTCGCGATAAAGAAAGTAGATGCACATATGAGTTATGATTCAAATATTACTTCAAAAGCATTGGCAGAAATAA
ACAAAACGCAAAAAAGATTAGTTACAAAGATAGACACACTCTATAATCAGTCAAAAAAAGAAAATGAATATG
TACGGTTAGGAGAAAAAATATCGTATACAAAATTGGAGAATCATTGGAAATATTTATATAATGAGATTCAGTTT
TATAATCTTAATAATCAATTGATTAGTTATGTTGCATTAGAACAGGAATATGCTTGGGCTTTTTTAAATGAACTA
TTTTATTTGATAGAATTGTATTTGAAGGCATTTAAAGGACAAAAGAAAACAGATTATGAGTTTGGGAAAAAA
TTAAACGAATTTATTCAATCATATGTCATTATTTTACTTATAGATATACGATTACCTGTTGTTAGATTATATATTATT
AGAGACATTGTAGATACATGTGAAGGAGAAAAAGATAATTACAAAAGAATTACACTGATAGAAGAATCTATT
AAAAAGTATAGATACGTAAAAGATCAGATTAAAAGATTTAAAAATGGATTAGAAGAATCGTTATTAAATGCAA
CTTTATCTATTGAACAAAATATGTTGAAAGTTAAAATTGATTATTATAAAGCCTACTGCTTTCCTAGAGAAAAA
ACAAAGCATAACAATATTGAGTACAATGTATCGTTGTTTTTTAAAGCATTAGATGCCTTAAAGAAATAGTTTTT
CACTGAAAATATTTATAGATATAATTAAAATTAATTAATATTAAGCCAGGATAATGTAGTCTTAATCGTTCTGAAA
TACGAAAAATGTTGTGGAAATCGCGTGAAATAATCAGAGGAATCGTTTGAAATCATCGTAGTAATCGCGCGA
AAATCACGTGAAATAATCAGAGGAATCGTTTGAAATCATCGTAGTAATCGCGCGAAAATCACGTGAAATAATC
AGAGGAATCGTTTGAAATCATCGTAGTAATCGCGCGAAAATCACGTGAAATAATCAGAGGAATCGTTTGAAA
TCATCGTAGTAATCGCGCGAAAATCACGTGAAATAATCAGAGGAATCGTTTGAAATCATCGTAGTAATCGCGC
GAAAATCGCGTGAAATAATCAGAGGAATCGTTTGAAATCATCGTAGTAATCGCGCGAAAATCGCGTGAAATA
ATCAGAGGAATCGTTTGAAATCATCGTAGTAATCGCGCGAAAATCGCGTGAAATACTATGGTAGACGGTTTG
AGTAAATTAATGGAGTATTTTAATATTTATGGTTTGGAGGAATTGCGTTGCATAGAGAAAGTAAATATATAGAG
TATAAGAAATCACGAAAAGGATTATCTAATGATATTTGGTCTACGTATAGTGCTTTTGCAAATACTGAAGGTGG
TACTATATATTTAGGAATTGAAGAAAAAAAGATCGAGGACAAAAAAGTCTTTGTTTCAGTTGGTGTTGAAGA
TCCAGAGAAAATGATTGAAGATTTTTGGAATGCACTATATGGAAGAAGTAAAGTTAGTCAAAATATTTTATCA
AATAAAGATGTTAAAATTGTTAATATTGAAAATAAAGCGTGCATTGAAATTCATGTACCAGAAGCGCCTTATTC
GAAGAAACCGATATATGTAGATAATAAAAAAGATTTAGTATATAAAAGAGTTGATGATGCTGATAGAATTGCG

ACTGAAGAAGAGTATAAATTCATGATTGTAAATTCTCAAGACGATATAGATACAGAATTATTAGATAACTATGA
CATGTCTGATTTAAATCACGAATCTATCGAAAATTATAGGAAACTTCTATTAAAAAATACTAATGATGAGAGAT
ATGCGAATATGAGCCAACTGGATTTAATGATAGATTTAGGAGCATATAGAAAAGATAGAAGTTCGAAAGACA
AACAGTATAAAATGACTACAGCATGTTTATTATTCTTTGGTAAGTATAATGCGATTAGTGATAGATTCCCAGGAT
TTCAATTAGATTATTTTAAGAAAACAAATTACCTAGATACTGATTGGAAAGATAGAATATCAAGTGGAGATTTA
GGTAATGAAGATTTAAACGTGTATAGTTTTTTTGAAAAAGTATTGATAAAATTAACTGATAACATTGAGGAATC
ATTTAGCCTAAATGATGGTTTGACTAGACAAAATTATGCAAGAGATTTAAAAGTAGCAATTCGCGAAGCACT
GGTTAATACATTAATGCATGCGTATTATGATACTAAGCAAAGTATTAAAATAGTTAATTGTGAAGATTTTATAGA
GTTTTATAATCCGGGTAATATGAGAATAAATAAAGAAGATTTTATTCATGGAGGGCATTCAAAGGACAGAAAT
AGTATATTATCGACGCTTTTCAGAAGAGTAGGATATTCAGAAAAAGCTGGATCTGGAGGACCAAGGATATTC
GATGTAGTTAATAGACATAAGCTTAAAACGCCTGAAATAGAATTAACGGACATGGACACTAATGTAGTACTTT
GGAAACAAGATTTAATGAAGGAGTTTGAAAAATATCCTGAGTTAGACAAAAAAGTAATAAAGTATATTATTG
ACTATGGATCAATAAGTAAGGGTGAAGCCTTAAAAATGGAAAATATGACAGAATATCAGTTTAGAAATATTTT
AAAAAAACTAAAAGATGATAACTTGATAAAAAAAGAAGGTGAAGGTCCGGCTACTAAATATGTGTTAATAGA
ATCAAAAGAAGCTGATATATTGCGAACTAAAAAAGTAATTAAAAGTTTAGAGTCTTTCTTTAGGAATAAATAA
AAAAACAAGAGATAGGTGCGAAGTGTTTGGATTATACAAATGCTTCGCATCTTATATTTATTAATAAATATCATA
GAAAACCGTATCATTAACCGATACGCAGAGATGCGGTTTTTTAGACACTTCATAAAGGGATTTTGAACGTATC
AGAACATATGAGGTTTATAGGAATTGCTGTTATGTTTTTTGATCACATCAATAAACAAAAAAGGTATGTACTAT
GTAAAATATTTATTAAATGATATAAAGCGAGGGTATATAAATGATTTTTAAATAGATATTTATCCAATAATATAAA
AAGGAACTATAAGCTATATCTAAAAGCTATATCTAACTACTTATAGTCCTTCTTCATTAGTATAAATATAATTATTA
AGAAAGGTATATATCTTTGTAACCTTCGTTTACATTAATAATGTTGTGATAACCTTTGTGTTCTAAAATACCAAT
AGCTATAGAACTTCTAATGCCAGACTGACAGTCTACATAAATAACATCGTTTCTATTGAAAGGTAAATCTGTTT
CTAAAAGTTTGCCGTGTGGCACATGAACCGCTTGGGATAAGTGGCCATTATTCCATTCATTATCATTACGTACA
TCTAAGACATGTGCTTCATTACCAGTTATGTCTTTACTATGAACAGATTGTGTTTGAATTTGAGCTTGTGGTAA
CTGATATCCAGACACATTATCATATCCAATAAGTTGTAAAGTATGTTATGTGTTGCTTTTGAAACAAGGTGATA
GTCTCCAATCAAGTTAATTTCTTGATTATAGTTTAGATACCAGCCAATTTGATTGATGAAATTTTTATCATATGG
AATATTGATTGTACCTTCAATATGTCCACCATGATAAGCCTCCTTACTGCGGAGATCAAAAGTTAATCTGTTTGT
ACTTGTAGCTGGATAAACCGTATAAGGTTGATATAAATTCATACCGAATTGATTAATTTTTTTCATTTGTGCAAA
ATGATGTGGTGGTGCAGGTTGGTCAGAAATGAGTTTATCGATAAAGGTAGCTTCATTATTTTCAGAAAAAGC
CCAGTTCGTTTGTTTTTCATAGCCAAGCGTAGATGTTGGAATAGCACCTAAAGATTTACCACAAGGACTACCA
GCGCCATGACCAGGCCAAATTTGAATGTAGTCTGGCAAGTCTTTAATACTTTCAATAGATTTAAACATTTGTTT
TGCGCCTATTTCAGATAATCCTTCTACTTTAACAGCTTTTTCTAGTAAATCAGGTCTACCGATATCTCCTACAAA
AATAAAATCACCACTGAATAGTCCCATTGGAACTTGTGCTCCAGCACCTTCGTCAGTAAGTAAAAAACTTATA
CTTTCTGGCGTGTGACCAGGTGTATGAAGCACTTTTAATTTTATATTTCCTACATAAATATCATCATTATGTTGA
ACAAAATGAGTGTGGTTAGGCATATTTTTATAACCTAACATGTCATCACTTTCGCCCGATACATAAATACTAGC
ATTTAACTTTATAGCAACATCTCTAATTCCTGAAACAAAATTTGCATGTATATGTGTTTCAGCTGCATGAGTAAT
GGTTAAACTCTCTTCATCGGCAACTCGAATATATGAAGATAAGTCACAAATAGGATCAATGATCATGGCTTCTC
CAGTTTTTTGACAACCGATTAAATAAGATGCTTGAGATAAATGTTTATCATAAATTGATGATAGTCAATTTGTTA
TCAAGGGTGATAATTATATTAACTATGGTAATTACATGGAATTAAAGTTATTAAACAAACTTGATGATGACCAAT
TAGATTTAGAATTTTTACAAGATAATGGGTTAAAAGGTAATGTTCAAGGTCCTATGACTTTAAAGGAACCTTT
AAAAAGTTATATACAAAAGTCTTGAAAACGAATATTTTAAATTTAGATAATGAGTTAGAAAACACTGAATATGT
CGAAGGTAAACCATATGTACAGTACGGTATTAAATATGAAAGAAATCAGGCATTAAGAAATGAAGCTATTAAA
ATTCATGGAACTACATGTAAAGTATGTGGATTTGATTTTAAAGCTAAGTATGGCGATTTAGGTGAGGGTTTTAT
TGAAATTCATCATTTAAAACCAATGTTTTCAATAAAAAGAGAAATAAAAGTAAATCCACAAAAAGATTTAGTC

CCACTATGTTCTAATTGCCATAAAATGATTCATAGAAATACTAAAAAACCTTTAACGATTAAAGAATTAACCAA
AATAGTTAATTATAATAGCAAATAATTTAATATTTTATAAACTATCATTCAACCCTCTTAATTTATTAGGAGGTTTT
TTGTATTTATGCTTTCAAATGTGTGATATACTTTGTTTGTTGAAATATAGAGTATCTATAGATAGGGTGATTGAG
TATGAAATTCACTGAAGTGGAAGTTATCGAACATCTTGTAAAGGCATATAAAGAAGCAGGAAAGCCTACTTAT
CCTCATGAAAATTTATATCGAGGACGTAATCATAGTATTTCAGGTATTGGAGAAGACTTGCTGGGTGCTTATTT
GATTAGTAGATTGGAAGGTGTCCAAATATTTATTGATCAGCCTTTATCTATGATTGATAAATCTTTAAGTACAAG
ATATCCGGATTTATTAATTTGTGAAGATAATGAAATTAAAAATATACTAGAAGTTAAAATGGACTTAGGATATCA
AAGAAAAGATTTTATAGATTATTGCCGAAAGAAAGAAGAATGGATTTCAAATATCGTAGGAAAACAGTGTGT
ATTGTCTAGAAAGAGAGAAGACAAAATTCCTATGAATATAGCTGATGATATTAAATTTCATGTTGTGATTTACA
GTGAAAACAATGGACCGAAGCGGTTTGATGAAGAAATCATGCCTATCGTTAATGAAACATGTCCACATATTG
AAGTATATGTCCTAACAAGCGGTCAACACCCTAATTTAGTAAATGTTAATCTTGAAGGTATTAATATTAATAAAG
ATGAATTTGAAATATTAGTAAATGCGTTATAAAAAAATAGAGCATCCTCCACGTTATGGAGGTGCTCTGTTTTT
TATTGAAAAGTATCAAGTTAATTAATTTAATATGCTTAATAAGTTCTACCTTGACCTTTTTCTCTAGCTTCTGTTC
GATCTCTTATGTACTCAGTACATACTGGATTTTCTGTTAATTCATCAACTGTATTAGTTTAATCAGAAGACGTGT
CTACTTTGTAAGCTTCTAAGAACTTATTATATGATATAGCGTTTGAGTTTTGTTGTTCTTCTATAAATTTCTGTAG
TTATTTTTCAAAAACCGCATCATTAACTGATAAGCAGAAGCGTATCACAAATAAAACTAAAAAATAGATTGTG
TATAATATAAAAGGAAGGGATTTATATTAAAATTTTGAATTCAAAAATTATTGAAAGGGAAGCTACCTTAGAA
ATTGAATCTATGGCCACTAACTGCTTTTTTATATCACCACTTATTAAAAAATGTAAGAATATTCCTCAGTGCTAA
GTGAAGGAGGTTCTAGTTTTATTTAATATTCATATTGATAGGTAATGGTTAATAATTGCCTGTAAATTATTGTTA
AATATCGTTGTTATAGTATCATCATTCATAAAATTTTTATAGATTTTATCAAGTATTTCCTCATCTTCAATAGCTGT
AAAGCGATGTACTAGTCTTTTTATGAGCTATATATAATTTTGCGAAGCTAAACTGTTTGTAATTATTCACCAATC
AGGTTATGTAGTGTCACGGTTCATAGGTAATTCTCTTTTTAAAATCATCTAATATTTTATATAATTCATATAAGTG
TAGTATTAACGTTTTCTACTTTATCTATAAAATATTTAGAATTACATTAGAATTTTAAATTATACGGCTGGAAATT
CAATGCTGTAAAAATAGACAGATTTTAAAAAAGTAGTAATAAAAAGTCTAAGTTTTATCTTTGTAATAAACTTC
GACTATATTTAAAAATAAATATGAATGTCTTTTAATTTCTATAATATCAAAGCTATTATATGATTTGAAATTTTGTA
ACTTTTTCTCTAATTTGGAAGTAGATAATGACTTAAAGGTTTTAACTTTTACCCAACGACTTGGCATTTTATCA
CTCCTATGTAATGTATTACTTCAATATACCCTTAAATTTTTATAATTAATTATATTGTAATATTATTTTATTTTATAAA
AATAGTTTAATAAAATGTTTAATTTTTATAAAAAATAATTAAATATTATAGACATTACGATAGTTTAATTAGAAAA
TATATTTAAGAATTATGAATTTAGTTCTTATTAATGTTAAAGATCATCAATTATTTTGTCACATTATCTTGAGAGG
ATTACCTTATGAGGTAGCCTTTTATTTGTCACCTAATATTGCTCTAAGGCTAATAATACATATATAAAATAAATAA
TTATCAGAATATATTTAGAAATTGATATTTAATAATTAAATAAAAGTATGCTAAATATTTATAATTTACAAGTGAG
CTGATAGAAAGATGAGTATAAGCGAATATAAAAATAGATACTTAGAGTCACTATATAACACCTTTAAAACTGAG
TTAGAAAACGAACGCACTCGCTATTTTATATTAGATACCAACTATACAAATAGTGATGATACTGAAAAGGAGTT
TACTTGGGATGTAAAACAGAATAATAAGATCAGAGAAGGCGATTTGTTCATTTTTAGACGGCCAACTAATTTA
TCTCAAATTGCTCAACAATTTTATTTCTTTGGAGCTGGGAAAATAGAAAAAATTGAGCGAAAAAATAATGTA
GCAACCGCTTACATTTCAAAACCCTTACTTTTTGTGGATCGTGTTTTAAAAGATAATATTGAAAATTTAAAATG
GGAGTTCAAAGAAAGAATTAAAGAAGAGTGGGAGCAGTTTTTCTTTAAAAATAAAATCAGTAATATTACAAA
AAATGATTTTTTAAAACTACTAAGTTTGAGTAGAAATGTAGTGGAAATAGAAAGTAATTATATTAATAATGATA
TTTCGCTAACTTTTAAACAAATGGCTGATATGGCAACTCGAATAAAGAAAAATATTTACTACGTAGGGAATCA
TGAAGCATTTGTTCAAGCTAAGGGAAGTGCTCATTTTGAGTTTTCAAAAAGGATTAAACAAAATTATAGTTAT
AGATGTGCAATTACCGGGATTAAAACTAAAGACTTTTTAGTCGTTACACATATTATTCCTTGGCATGAAAATGA
GTTTATTAGACTAGATCCTTCAAATGGTATTTGTTTGTCTTTATTTTTAGCAAAAGCATTTAAGAAAGGCTTTAT
CACTTTTTCTAACAGTTATAGAGTTGTTTTATCTAAAGAAGCAGAGAAAGATGCTGCTTTATATGAAGAATTA
AAGATTTATGAAAATCAAAAAATCGAACTACCAGATTGTCAGAAACCTAATTTGAAATATTTAGATTGGCATA

GAGAACATATTTTTAAAAATTAATTTTGGGAGAGGTTGTATGACAGATATTAGTCAATTAGTAGAGGTGCCAT
ACATCTTTCATTAGTAGATTGAAAAAGAGCACTTGAAACAGATAATTTGTATTATTTAGCCTATGGATTAACAA
CTTTAGATAATTGTTTTAAATATTACCAATTAATTACACAAAATGAACATTTTTGCTATTCTCAACAAGAAGAAA
GAGAAAGTTATCAGCAAAAATTTCAAAGTATGATTAATGGTTTAAAAATTAAAATTGGCATACAAAGTTCAAA
TGATGATGCGCAATAAGCTACAAAGTTTGATTTTTTAATTGAACGTTATATGTTTTTAAAAGAAGAAAGAGCT
TCGAATTAATATCTTAATGGAGGAAGATTAGTAAATGGCTAGAACGTATAATGTGATTAAATATGTCATTAAGA
AAAATAAGGATGATCTTAAAATACATGGATTAAGATTATATGAATTTAATATAAAAGATGAAAAAGCTATAAAT
ATTAGCCAACCCATTTTTGTGAACAGAGCAACATTGATTGAGAAAATACAAAATGGTGAAGCATTTACGCAC
GCCTTTAGAGTAAATCACAATACTTATTATGCAGGCCAATTAATTTCTTTATCATTGAATAAAAATGGTTATATTA
ATAACAACTTTGAGGCCTCACGAGATATTATTAGAAACATTGAAGTAAGTATTTTAGACTATGAATAAAGGTA
ATACTGATAGAATACATTACAATTAAAAAAGGGAGTATAGGACTCCCTTAATAAGTTGATATATAAGTGACTTA
TTTTTTTAATAGAATTTTAATTCCTCTGTTAATGTTCAAGATATATAATATTAAAGCAATTAAAAAGAATATAATA
GTAATAATTAAAAGAATGCTAAATATTAATAATTGATGATCCAAAGGTTTATCAAAGACTTCTTTAGAAAAAAT
AAATGCCAATCTACCTATAAAAAAACTAACCCAAGTCATAATATGATTAATGAATGCTTTTTTGCCATGTTCTGA
TGTTGGTTCATCAGCAAGTATCCATACAAATAAAGGTAAAATGACAGGTGCGAAAAAGATACTTAAATAACTC
AATGCTGATAAAACGTAACTATTGTCATTTTTATGCATATTACTATTTGTATTTTCCACTTTTTTCACCTTCTTTAC
TTTTCTTTACTATTATAAAAACATTAATAGCGAGCTATTACAACAGTTATAAGAGAAGCTTACGATATTGTAAGT
ATGTGTTGTTATAATCAATATAAAATTAAGAAAGACCTGAGAATTTATATTATCATCCACAATCGAAGTGAGTA
GCGAAATTTATTGGAAAAGGTACTTATATTGAAGATATTGTTAATGGTTCATTTTTAAAAAGCCAAGACGATTT
GAAGTTAAAAGCTTCTGACTTCACTGTCGAAGATAACTATAACTACGGTGTACTTATATTTCCAGAGAATCTTT
CTCTTACAGATAATAATAAGAAATTGGAATCTTTAATAAAAACTATACTATTTACAGGTAAACGATATCAATATA
CTTATTTTAAAATTTGATCTTAATAGATAAGGATATAATGATTTAAACAATTTAAGAGATATTATTTTCACAAAA
AAAGAATGCTTTTTGTTAGAGGCACTGAACCCTTAAAATGGGAACTTAATAAAAACACTATGTTCTAGGCTG
CTAATTCTCTGTATTTTACAGGGGATAAGTAGCCTAGTTTTTGTTGAATTCGATTATTATTATAGTTTTTAATGTA
TTTTCGACAATATCTATTACAATATGATTAGAGTTATTAAGCTGATTATTGATGTAAAAAGTTTCAGACTTTAGC
GAGGAATGGAAACTTTCTATCGGGGCGTTATCGGCAGGTGTTCCCTTTCGGGACATACTTCTGATAATGCCTT
TTTCTTCGCATAATTGATAATAAGCATAAGATGTATAAACGCTGCCTTGATCACTATGTAATATACACCCCTCAG
GTATATCGATTTGATTTAATGTATCATTAACTAAACGTTGGTCTTGTTTATCATCTATTTTATACGCCACAATTTCT
CCGTTATAAATGTCCATTATCGAAGATAAATACAACATAGAATGACCAAATGGTAAATAAGTAATATCGGTTGT
TAATACTTCCATGGGACAACTCGCTTTAAATTGTCTTTGTAATAAATTGTCCGTTTTATAATACGGTTTACCTATT
CTTGTCGTCTTTTTAGGTCTAACTCGGCAGTTCAAATGATGCTTCTGCATCACTCTCTGTACTCTCTTATGATTA
ATTGGTGATGTATAACATTGATTAATCAATGCTGTAATCTTACGATAACCGTAGGTATATTGTTTACCAACATGT
TGTGAAAATCTATAACTTTCCCCATTTCGATACCATCGCCACCAATTTTTCACTTGTGTTCTATTTCTAATATTTA
ATTCTTTCATAATTTCTTTTGTTGGAAATCCTGCTGCTTTCATTTCAACTGCTTTATACTTTGTTTCTACTGAATA
AGAAGCTCTTTTCATAGAAAAAAAACACCTCCGTATGATTCATTTTAATATGAATTCAACGAAAGTGTTTTTAT
ATAATTCCCACTATTGGGGTCAGTCTAAAATTAAAATTGATAACTTTTTTAATAAAATAGATACACTAATTTTAA
AACAAGGTAAAAAAATAGAATTATTAAAACAACGCAAACAAGGCTTACTACAAAAAATGTTTGTGTAATTCT
GATAACGTCCTATTATGTTAATTGAAAATAGACAAAAATAAATTGAGAGCATCCCTTACCGCAAAGTGAAGGA
TGCTCTATTTTTGTTGAAATTAATATAAGCTTCATTCTGATAGATAGTCATTAATAATTGTTTGCAAATCGTTATT
AAGTACCGTTGTTACAGCGTCATCATTCATAAAATCCTCGTAGATTTTATCAAGAATTTATTCCTCTTACAGACA
TTCGCGAGAAGTCCGTTTTAATTTTATTAGAAGTAATTCAGGTTTGAACCTACCTAAATGAATATATGAGTTAT
CTTTTATACTACAAAATATATTCAGATTTCAATAATGACATAAAATAGGCATCTTTATATTTACCTTTAGTGTAGA
ATTGCTCTTTAAGTAATCCTTCTGTTTTAAATCCTTGTGACTCGTATATATGAATAGCTTTTTTGTTATCTGCATC
AACATATAGATAGATCTTGTGCATATTTAAAATATTGAATGCATAAATTATCGCTTTTTCGAATGCGAATTTTGC

ATAACCTTTACCACTGAACTCAGGTTTAATAATTATTTGTATTTCACAATTACGATGGATGTAATTAATTTCTACT
AATTCAACAATACCTACGACTTGATTTTCATCTTCAACAATAAAACGTCTTTCCGATTCATCTAATAAATGCTTA
TCAAATAAATGTTGAAGTTCCGTTAAGGATTCATAAGGCTCTTCAAACCAATAAGACATAATAGAATATTCATT
ATTTAATTCATGAACAAAAAGTAAATCACTATACTCTAATGCTCTTAGTTTCATAATCCCACTCCCAAAATTTTC
TTATATATTTGCATTATAAATATAAATAACGAATAAGTCATTATTCACTATGAATAAATCTATTTTAACAATTCACT
ACATACTAATTCTCATTTTCTTATTATTCTCTAATATCTCTGATTTATTACTCAGTGAAAGATGCCCTATTTTTATC
AGTATATAGTTTTTATTTTGATAGATATTGATTAATAATCGTCTGTAAATCATTGTTAAGTAAGGTTGTGATAGCG
TCGTTATTCATAAAATCTTCATAAATTTTATCAAGTATTTCTTTAATATATTTTTTAATCTTCACACCTTAAATTCA
CTTGAAAACCAAATCACTTCTAAATTATGATTTTACATAATAAATTTTTAAAAATTGGTATTATATAAAATAATCT
CAATAATCAGAATTTTTTGTAAATAATAATATTAAAGTGTAAAATAACAGATAAGAATAGACTTAATGTATATAA
ATTAAAGGAGTGTTGGAAGTGAAAACTAGAGCAGCAGTTTTACATGAAATGGGGGCTAAAAAGCCCTATAA
AGAATCTAAACCTTTAAAAATTGAATACTTAGAACTTGATAATCCTAGCGAACACGAAGTATTAATTAAAATTC
ATGCAGCTGGATTATGTCACTCTGATTTGTCAGTAATTAATGGTAACAGACCTAGACCTTTACCTATGGCACTT
GGTCATGAAGCTTCCGGTGAAGTAATTAAAGTTGGAAAAGCTGTTACAAGAGTTAGCGAAGGAGATCATGT
GGTATGCACATTTATTCCCAGTTGTGGAAAATGTATCCCATGTAAAGAAGGACGTCCTGCATTATGTGAAAAC
GGAGCAATATCTAATGAAAAAGGCGAAATGTTAGAGGGAGGGATGCGTTTATCTAATGATGAAGGAAAAGT
ATATCATCACTTGGGGATATCAGGTTTTGCTGAATATTCTGTTGTCTCTGAAAACTCTATAGTTAAAATTGATAA
AAAAATACCTTTTGAACGTGCAGCTGCATTTGGTTGTGCTATTATCACAGGTATCGGTGCTGTGGTGAATACA
GCCCAAATTCGTTCTGGTAGTAATGTAGCGGTTGTTGGTTTAGGAGGTATTGGATTGAATGCTATTATTGGAG
CTAAACTAGCGGGAGCCAATGAAATTATTGCTTTAGATATTAACGAAGATAAATTTGAATTAGCAAAGCAATT
TGGGGCTACAGCAACATTTAATTCAAGCGATAAAGATATCGATGAGCAAATTAAAGAATATATTCCTGGCGGA
GTAGAATATGCTTTTGAAACAGCGGGTGTAGTGCCAGCTATGAAAGTTGCTTATCAGATTACTAAACGAGGG
GGGACAACTGTAACAACAGGACTGCCTAATCCTAAAGATAATTTTTCTTTCCCTCAAGTTACTTTAGCGGCTG
AAGAACGTACCATTAAAGGATCATATGTAGGAAGTTGTGTACCTGACAGAGATATACCAAGGTTCGTTAATCT
ATACAACCAAGGACGTTTAAATATTGATTCACTCATCAGTGAGGTTATCACTTTAGATGAAATTAATGAGGGA
TTTGATCGTTTGTCTAATGGTGAAGTAGGTAGAATTATAATGAAGATGCATTGAATATAATAGAATTCAAGTCG
TTCCTCCTCTTGATTTCTATGAAAAGAATAACAATTTGAGTAGGAAGATTACTGACAAATATTACATTTGCATT
GTGACTGCCAATCTAATTGTATTTTTACATTTCATATCTATATATTAGAAGATAAATGTTTTAAAACTAATTACTA
ATAGTTTTTTTAGATAAAAAAAGATATAGGAGCATCCTTCACCGCAAAAGTGAAGGATGCTCTAGTTTTATTA
GAATATAGTTTTCATTTTGATAGATAATGATTAATAATTGTTTGTAAATCATTGTTAAGTACCGTTGTAATAGAGT
CATCATTTATAAACTCTTCATAGATTTTATCAAGTATTTCCTCGTCATCGATGGCTGTGAAATGATGCACCAGAC
CTTTTACTATTGAAATGTAATTTTGTGATGCTAAACAACTAGCAACCGTTCGCCAATTGGATTGTATAGTGTTG
TCATGGTTCATAGATAATACTCCTTTACTTTAGTGTCCATTTTTGACGTGCTTTAGGGTTGAGTGGATGCATAA
CTTCGTTCGTTACTGGATTTATGAGCTTTTTTACTTTCTTTTTATGAGGTTTTAACATTTCCATCACTTGTTCGA
CACGGTCGATAACAACTGGTCGCTTCGCATAGGCACCATAAGCAAGAATGACTGTGTCACTTTCACTAATTGC
TTTCATTAAGTGTATATCTGTGTGCTCATCATAAGGCTCTTTGATATGTTTAAGGTTCTCTGGTGTTTTAATATTA
GAGAAAAGATTAACGAGATAAACAGCGCCATATTGCTCAGAATTAGCTAATTGATTGAGGATGAGAACCGTA
GTAAGATCGAGTGATAATACACCATCTAAATGAAGATACATCGTTATCACAGTACAAGCCGGTTTCTTGTCATC
CCATATCTTTTTGAGTAAGTAGCGATGTTGTTCATCATCGCTAAATATCGCTTCTGTGTGTATCGTACTTTTGAT
TGTATTCATATATCGTCACTTCCTTTAGTATTCTTCTGGCAAAAGCATCACATAATAAAAAGCGTCTACATCATCT
TCTCGGATGACGTAGACTTTCTTAGGTAATGCATTTTGATTTTTTATATAGTTTGTATAGTGATATTCCAATTTGT
ATGCGGGTTGTTCTTGTTCATGTGTAATTGACAACATATTATCATCTTCTTGTAGTTTGAAAATGTGTAGGTAAT
CTGTATTAAGTTGATTGTCTCGATCTTTTACCATGTTCCAAAGTAAGATTTGAAGGTCTAGAGATAGTTGTTCA
CTAATGCCTCTTGTGATGTATCGATTGATTTTCATACTATTTTCCTCCATTTTGCTTTTCTTTCATGATGTCAATC

ACTTCGTTAATGACTGTAACAGATATTTGTGCCACTTTGATCCAATTATTCATGGTTATTCCCTCCTTCTTTTAGT
AAATGACGTTCATCGATAATCGTATTTTTAGTATCTGTGAGGTATAGAAAGTCCATATCAAAATGATCCAGATA
ACCAATACTGATGAGTTGGTTATTAGAATACATTAGAAATGGATAGATACTTAGTTCATGTAGCTCATTATTATA
GTAGGTATAAGTTTCAAGTATAAGATGTGCAAGTGGGGAATCATTAATAAAACGTTTGGGTAGAATATTTTCT
GCCGCTTCCTCCAGTGTTTCACATTCCCATGTTTCATTGTTAGATAATTGGAATAAGCGAGTCATATATTGTTTG
AGTTCTTGAGTAGTTGTTTTCATATCATTGCCTCCCTAGATAGTGTGATAGTGATGTACTTCATATACATCATTG
AGATAATATATATTTGATTTGTCATTTATTACTGATCCCGTTGACAATATGAGAAAATTCCATATAAAAAACCGCT
ACAAACCTTGGTATGACAAGGAAATCCCGAAATTCCGCCTATTTTGACGAACAATCAACTCATTATTTATAAGT
ATTGATGATAGGGTGGGGTCTCTGCTTCCTTATATATATTATTTATTTATAAAGAATAACGGGATTTTGGGATTG
TGCTTGCACAATCCTTCTGCTTCTTCTAATCTGCAAATCCCATTCCTTTCCCGATAAAAAATCATCGTGGGATGT
TCTTTAGCAATTTCAATATAAGCCTCGTGTAGTTATGAAAAAATTACGACAATGACTGTTTCATTAGATAAGTG
TTATTGAAATTGATAAAGAGAATTCTCAAAAATTTTTAGAAAAAGAAAGAGGAAGTATGTAGATATAAAATAG
ATTTATTAAATAGTAAGGTATAATGTTTGCGGTAAAAATAAAGACGAAGTGCTGGAATACACTTCGTCGATAAT
AAGGTAGTTGAAGTTTCGATTATTTTAAATTGATCATTTTAAGCCTACAAATCCCTCTAATGTTTCACCAGATTT
GTACACTTTTGTCGCCTTCTTTTTCCTAAATAACGTAAATGCCAAGGTTCATATTGATATCCTGTGATGCTTTCT
TTGTTTTTAGGATATCTTATAATAAATCCATAATTATGGGCATTCTTAGCTATCCAGCGTCCTTCCTCAGTTTTAC
CAAAACTAGCATATAGATTTTCACAACAAGTTGGTAAACAATATACCTACGGTAAAGGATTAGAAGAGCTGTC
AGAAGTAGAACAATTAAAATTAGAAAATAAGAGAAAAGATATAGAATTGGATATTTTAAAAAAGTACAAGGT
ATTGGAAAGGAAGTGGTACCAACAGTAGTCATAGATTTAGTGGATCAATTAAAAGTAAAATATTCAATCAAAT
TGCTACTAAAAGTATTAAACATACCTAAATCAACATATTACCGATGGAAAAACAAAACCCATAAAAATGATACC
GTAACACAAAAAGTTATTGAATTATGTAAAGCTAACCACTATACCTACGGTTATCGTAAGATTACAGCATTGAT
TAATCAATGTTATACATCACCAATTAATCATAAGAGAGTACAGAGAATGATGCAGAAGCATCATTTGAACTGCC
GAGTTAGACCTAAAAAGATGACAAAAATAGGTAAACCGTATTATAAAACGGACAATTTATTACAAAGACAATT
TAAAGCGAGTTGTCCAATGGAAGTATTAACAACCGATATTACTTATTTACCATTTGGTCATTCTATGTTGTATTT
ATCTTCGATAATGGATATTTATAACGGAGAAATTGTGGCGTATAAAATAGATGATAAACAAGACCAAAGTTTA
GTTAATGATACATTAAATCAAATCGATATACCTGAGGGTTGTATATTACATAGTGATCAAGGCAGCGTTTATACA
TCTTATGCTTATTATCAATTGTGCGAAGAAAAAGGCATTATCAGAAGTATGTCCCGAAAGGGAACACCTGCCG
ATAACGCCCCGATAGAAAGTTTCCATTCCTCGCTAAAGTCTGAAACTTTTTACATCAATAATGAGCTTAATCAC
TCTAATCACATTGTAATAGATATTGTCGAAAAGTACATTAAAAACAATAATAATAATAATAATAATCGAATTCAA
CAAAAACTAGGCTACTTATCCCCTGTAAAATACAGAGAATTAATAGCCTAGAACATGGTGTTTTTATTAAGTTC
CCGTTTTAAGGGTTCAGTGCCCTAGGATTATAGGCTCTTTTGTTTATAAAGGTAATTGAACTAAAGTATTATAA
TTTCAATTCTTAATTAATGTTCCTATTTTACCGTCTAAAGCGTCCCCTAATCCTGCTAGAGATGTAATCAAAACA
CTGCCTTTAGTATTTTTTTCAAGAAATTGAAGTGCAGCTTCAACTTTTGGAAGCATACTTCCTTTAGCAAATT
GACCATCAGAGATATGTTTTTTCATTTCATCCACAGACACTTCATCGAGACCTCTTTGGTTTTCTTTTCCATAG
TTAATGTAAACATGGTCCACAGCAGTTAATATGATTAATTGATCAGATTGTAAATGTGCTGCTAATAAAGCACT
CGTTTTATCTTTATCAATAACTGCATCAACACCTGTATAAACTTCATTTTCCTTAATTACTGGAATTCCACCACC
GCCGGCAGCGATAACTAGTGTTCCATGAGTGATTAATGTTTCTATACTATCTAACTCAACTATACTTATAGGTTG
TGGGGAAGGGACAACGCGACGATAGCCGCGTCCAGAATCTTCTACAAAAGTATAACCTTTTTCTTTCGTAAA
TTTATCAGCCTGCTCTTTTGTATAAAATAATCCAATTGGTTTAGTAGGGTTATTGAAAGCAGAATCATCGCTTG
CAACTTGAACTTGTGTTACTAGCGTAACAACTTGTTTATCTATGCCCATTGAATGAAGTTCGTTTTGTAAACTT
TCTTGCATCTGATAGCCAATATAAGCTTGACTCATAGCACCACATTCAGGGAAAGGAAAAGGAGGACCTTGT
TTGTGTTCCGCCGCATAATTTAAACCTAAGTTAATACTTCCAACTTGTGGACCATTACCATGACTAATTACAATT
TCGTATCCTTTATCGATTAAACTTACTAGAGATTTAGATGTACTTTTTAATAAATCTAATTGTTCTTTAGGCGATT
GTCCTAAAGCGTTACCACCCAAAGCTACGACGATTTTAGACATATTTATATCCTCCTTTCATTTACTCTCCTAAT

GTTGCTACCATGACTGCTTTTATTGTATGTGCTCTATTTTCTGCTTCTTGGAAAACAACTGATTGTTCACTTTCA
AATACTTCATTTGTTACTTCCATTTCAGTTAGACCATATTTTTCTTGAATTTGTTTACCGATTATTGTTTCAGTAT
CATGGAATGATGGTAAGCAATGTTCAAAAATTGTATGTGGATTACCTGTTTTTTTCATTAATTCTTTAGTTACAC
GGTATGGTTCTAATAATTTGATACGTTTTTCCCAAACTTCATCAGGTTCACCCATAGATACCCAAACATCTGTGT
AAATTACATCAGATCCTTTGACACCTTCATCAATATCATCAGTTATAAGGATTTCACCACCGTTTTTGTCAGCTA
TATCATTACACCGATTTAATAATTCATCAGTTGGATTTAGTTCTTTAGGACATACAAGATGGAAAGTCATGCCC
ATGATTGCTGCTCCTTGCATTAAGGCATTTGCAACGTTATTACGTCCATCTCCAACATATGTGAAGTTAATTTCA
TTATATGGTTTTTTCAATACTTCTTTAGCTGTTAAAAAATCAGCAAGTACTTGTGTAGGATGATCTTCATCTGTT
AAACCATTCCATACGGGAACGCCAGAATATTTTGCCAAATCCTCAACTACTCTTTGTGAGAATCCACGATATTC
TATGCCATCATACATTCCACCTAAAACACGAGCAGTATCTTTGGTAGACTCTTTTTTACCCATTTGAGAACCTG
TTGGCCCAAGGTATGTTACATGTGCACCTTGATCATAAGCCGCTGTTTCAAATGCACATCGAGTGCGTGTTGA
ATCTTTTTCAAAAAGTAGAGCGATATTTTTACCTTTCATTTTTTGTTGTTCTATTCCTGCATATTTTGCGCGTTTA
AGATCTTCAGATAAATTAAGTAAAAATTCCATTTCTTTTTGTGTGAAGTCTAACAAAGTTAAAAAGTTTCTATT
TCTTAAATTTTTCATTTTGAATATCTCCTTTTCAATTAATATTTTATTCATGTTATTTTTTTATAATCCAATTTCTTT
TATCTTGATATATAATATTTTCTATTTTTAATTCTCTGATTATTTCACCAGTAGTTTTTCTGTTAAGTCCAGATAAA
TTACTTATTAATTGAACAGTCATAATTTGTTTTAATTCATAGAATTCTCCATTATCATCCCCAATTGAAAGGCATA
AAATTTGTAGTATTCTTTCAATTCTTTCTTTAGCTGAATTAGCTCTTAACGCCATAATATATTCTACTTGAAATTG
AATAGTCTCATTAAGTTTCTTGAAGAGACTTTCAAATATTTCATTATGCTTTCTACATAAATACTCAAGTAAATC
TTTCGGTAAAGTTAATATTTTACAATCTGTCAAAGCTGTACATATTTCATATGGTGCAGGGGTTTCATTAAATAT
GAAGTTCATTGGAAATATATTTTCGTCTTTACTTAATCTTAAATAATTGTCACCAGTAATATTAGAAGATTCATG
TAAAATACAACCATTTACTAAAAAGTATACAAATTTTATTTGATCAGTTGAATGATATATGACTTGTCCTTTTTTA
TATTGATAAAGTGTTAAATCCTCTTTATAAGGTCTAACAATACTAACAGGAATATTTAAGTATGATGCTAATTGT
TTAAGATTATTATCAAATTCATATTCTGAATTTTTAATATAAATATTTTCTTCATACATAACTATAAACCCCTTAAA
AATTACTTAGTTTAAATCATAACTTAAAATACAGAAATATTACCTGTAATCAAACGAACTATTCCTATTATTGCAA
GTACAATGATGAATATGAATAACGTATAATCCAATTTTGTCAAATGTTTGTTATTATCCCTTTGTACGTAGCTGTA
TACGAGTAATCCAGGTATATATAACAACATCGTTAATAGTAAATAATCTAATCCAGCTGCATAAACCAACCAAAT
TGTGTAAATAGATGCAATAATTCCTATTATCCATTGTTTTAAATTAGCTTTAGATTTATTTTGAATAGTATATTTAA
CCTGGTAAAAAGCACTGAGTGTATATGGAATTAAGATTGCACTTGATGCAAGTGAAAACGCAAACTGATAGG
CACTATCTGTAAACAACATACTAATTAAAAATAACTGAACTAATATATTAGTAATAATTAAAGCGTTGACCGGA
GCTTTATTCTTATTTTCTTTAGCAAACCATTTCGGGAAAAGTCCATCTTTAGCTACAATGAATGGTAATTCACC
AGCTAGTAATGTCCATCCTAACCAAGCTCCTAAAACAGAGATAATTAAGCCTATATTAACTAACACTGAACCCC
AATGACCTACAATATGTTCTAATACTTGTGCCATTGATGGATTAGCAAGTTTTGAAATTTGGTTCTGCTGAATG
ACACCTTGGGCTAGTACAGTCATTAAGAAATAAATGACTAGCACAGAAATCAAACCAATAACGGTAGCAGTT
CCTACATCCTTTTTAGACTTTGCACGTCCAGAAAAGACAACGGCTCCTTCAATCCCTGTGAATACCCATACAG
TTACTAACATAGTACTTTTTACTTGTGCCATTGTATCTCCCCAACTAAAAACGCCAACACTTCCACTAGTCATAC
CATAAAAACCGGATTTAAAAGTACTGAAGTTGAATACAACTATCATGCATATAATAACTAGAAATATAGGTATT
AATTTAGCTACTGTAACAATACTATTTATAAACGCTGCAGTTTCTACACCTCTAAGTATTAAAAAATGTACACCC
CATAATAAAATTGATGCTATGATAATACTTGGAAGTGTGTTACCTCCTTTAAATATAGGGAAAAAGTTACCCAC
AGCTGACATTAATAGGGGTGCATAAGCCACATTACCTAGAAATGCTGCAAACCAATATCCCCAAGCACTTGAA
AAACCAATAAAATCTCCAAACCCTGTTTGAGCATAACTATAAATTCCTCCATCAAGATCTGGTCGCTCATTTGT
TAAATTTTGAAATACGAAAGCAAGAGAAATCATACCAATAGCAGTTATTATCCAACCGATAATTATTGCAAGTC
CACCAGCTTGGCCACCCATATCTGAGATGATATTGAATGCACCACCGCCTATCATAGAGCCTATGACTAAACCA
ATTAAGGAAGTTTTACCTAATTTATTTTCATCCATATTAATCTCCCCTAATAAAGGTGGTAAAAATCTTGTTATTT
GATTTAATGAATGAATTACTTGTGACTTTTACCACCTTTCAAATTTAAATATCTTCTCTAAATAACGGCTGACTC

ATACATCTTGGGCCTCCGCGTCCACGTACAAGTTCACTACCAGTAATTTCAATCACTTTAATTCCTTTGTCGCG
TAAAAGTTGGTTTGATACATAGTTGCGATCGCATGTCACCACAACCCCTGGTCGAATACATAATGTGTTTGAG
CCATCATTCCATTGTTCACGTGCACCATCAATAACGTCGCCATTACCTGTTGGAATAAAGTCCACTTTTTCTACT
TCTAAAACTTCAGCAAGTGTTTCACGTAACTTGCTAGAACGAGTAATTTTTATATCGTCCTTACCATCATTTTGT
TCTATGGTAAATATATTCATATTATTTTCTTCTTTAAATATTGCTGCATGTACTGTAAACTTATCGTAGTCAATCAT
AGTTAGTACTGTATCTAGGTGCATAAATGTACGTGTATTAGGTATTTCAATAGCTACGATTTTTTTAAAACTTGT
GTTTGCATCTTTGAAAATATTACGTGCTAACTTTTCTATTGCTTGAGCTGATGTACGTTCTGATATACCAATAGC
TAAAACATCTTTCGATAATACTAATTCATCTCCACCTTCAATATTAAATGGTGAGTTACGATCTAACCATACTGG
TACATCTTTATCTTTAAATCTTGGATGATGTTTCAGTATATATGTCATAAAAAATAGATTCTCTACGTCGTGCTCTC
CAATACATTCTGTTAATTGTCATTCCTCTACCAATTGAAGCTTGGGGATCTCTTGTAAAATAAAGGTTGGGCAT
TGGATCTAAGTAAAATGGATATCTATCATCCATATATTCTACTAAATGGGTTGTTTCAAGTTGAATTTCTTCTTTA
CGTACGCCAGCCATGATTTTATTTACAAGTTCTTGGTCAGATAACTTTGAAAAGAATTCTTTAATTTCAGTTTC
ATGACCTAATATTGTCTTTTTAGATTCTGTTAATATGTCGTTTATGAAGTTCTCGCGTACTTCTGGCTCAGTAATA
GATTCTGCTGCAAGTTTTTCTAAATAAACTACTTCGATTCCTTCATCTCTCAAAGTTTGAGCAAATTTGTCATG
CTCTTCTTGTGCAACTTTTAAGTAGGGAATATCATCGAATAATAAACCACTTAAATGATCAGGTACTAAATTTT
CTAATTCTTTTCCTGGTCTTTTTAACAACACAGTTTTCAATTTGCCTATTTCACTATTTACTTGAATGGGTCCTT
GTACCATTTCAAATTCCTCCTTTGCTTTATTACATTCACATTATAAAACGCTTTCAAAAAACATCGTGTGATTTA
ATTCACAATTGTTTAGGGAGGGTTTTCACATATATAGATTTATTCAATGCAAAATTTTGTATGAATATGTATTTT
GTACTATATAATAGTGAATAATTTGAAAAATCTCATTGTTAAAACCTTATCTTATTCAAGCAAAAAGATTTTTTA
CTCATTTTTTTGTATTAGTAATCATTCCTATTAATATAATCATTTAATTTTTAAGGGAATAATTGATAACAAACAA
ATTCTGCTATTTCATTTGAAGAAGTCAAAATCATCAAAGTATCGTTACCTCCAATAATTCCTAATATTTCTTTCAT
TTGTAATTGATCTATGTAATAACTTATACTTTGAGCAAAGCCAGGAGATGTTTTTATTAAGACATAGTTATTTAG
CGTTATAAATTCAATAATCTCATCACTAAATATTTCTAATTGTTTTTTTGCACTTAATTGATTTGTTTGATTTATTT
TCTTGTAAATATACTTTTTATTTTCAACAGGGATTTTGTAAATTTCTAATTCTTGTAAGTCACGAGAAATAGTTG
TCAAGCTATAGTAAACTCCAAAATGTCTTGCCATGTAATCCACTATTTGTTGTTTTTTATTAAACTGATTCTGTT
GTATAACAGTTAAGATAAGATTTAAACGTTTTTCTTTTTTCATTTTTATTACCCCCTTTATTATTTTTGATTATGA
AATAGATTTTAAATAACTGTATAAAAATCACTTATAAATCTCAATTTACAAATAATTTTGAAATAGTGGATGCTT
TAATTATAAGTAGATTATTTTATGCCTAAAAGGAGAGATTAATTAGAATAGATTATAGTCATCATAATTATAAAAT
CAAAATAATTTGCGTAATAATAGCGAAATGTGTAAATTGTAAATGTCAGGTAATAACCACAATATCTCATAAAA
GAAGTTTGGAAAAAGGCATTGAAATAAAAAGTAGTTTATAAGTTGAAGATATTTAGGTATGTATTAGAGCGC
TTTATAAATAAATAGGTTTATACAAAGAATTACAAAAATAATATAAAAAATAGAATCTAATACATGATAAGGAAT
ATATTTGAATAATTTGAATATGAGAATAAGCATACATATTTAGGGTATATATAAGTAATAGACATCTAAATAATAA
GTAATAGGAGGCTGGTGTAGATGTTCAAAAATATATTATTACCCTATGATTTCGAAAATGATTTTAGTGCTATCC
CTGACTATTTAGAAAAAGTCACCGATGAAGATTCAGTTGTTGTAATTTATCACGTTGTAACAGAAAATGATCT
TGCAATTAGTGTCAAGTATTATAATAAGCATAAAGAAGATATTATTAGAGAAAAAGAGAAAAAACTCACTCCA
TTTTTACGTGAATTAGAAAAAAGAGATATTCAATATAAAATAGATGTAGATTTTGGGCATATTAAAGATACAAT
CTTAGAAAAAAATTACTTCTGGAGATATAAATAATGGTGAATTTGATTTAGTAATTATGAGTAATCATAGAGTCG
ATTTGAATATTAAACATGTTTTAGGAGATGTTACACATAAGATTGCTAAAAGAAGTTCTGTCCCAGTACTAATT
GTTAAATAAACATAAGAAGTAAGAAATTTATTAATTCAAAAAGCCTAAATACTTTCTCACAAATCGAGAGAGT
ATTTAGGCTTTTTTATTTTTTTAATAAACGTAATGAATTTAATATAACTAATATTGCTGCACCTGTATCACTTAGA
ACAGCTAACCAAAGTGTTAGTAATCCAGGGAATACTAACACAAAGGCAATTAATTTAATAATTATAGCAAAAT
ATAGGTTCTGTTGCAAAGTTGAATCTATAGTATAATTTTAACAAAAAGGAGTCTTCTGTATGAACTATTTCAGA
TATAAACAATTTAACAAGGATGTTATCACTGTAGCCGTTGGCTACTATCTAAGATATGCATTGAGTTATCGTGAT
ATATCTGAAATATTAAGGGAACGTGGTGTAAACGTTCATCATTCAACGGTCTACCGTTGGGTTCAAGAATATG

CCCCAATTTTATATCAAATTTGGAAGAAAAAGCATAAAAAGCTAATTACAAATGGCGTATTGATGAGACGTAC
ATCAAAATAAAAGGAAAATGGAGCTATTTATATCGTGCCATTGATGCAGAGGGATATACATTAGATATTTGGTT
GCGTAAGCAACGAGATAATCATTCAGCATATGCGTTTATCAAACGTCTCATTAAACAATTTGGTAAACCTCAA
AAGGTAATTACAGATCAGGCACCTTCAACGAAGGTAGCAATGGCTAAAGTAATTAAAGCTTTTAAACTTAAA
CCTGACTGTTATTGTACATCGAAATATCTGAATAACCTCATTGAGCAAGATCACTGTCATATTAAAATAAGAAA
GACAAGGTATCAAAATATCAATACAGCAAAGAATACTTTAAAAGGTATTGAATGTATTTACGCTCTATATAAAA
AGAACCGCAGGTCTCTTCAGATCTACGGATTTTCGCCATGCCACGAAATTAGCATCATGCTAGCAAGTTAAGC
GAACACTGACATGATAAATTAGTGGTTAGCTATATTTTTTTACTTTGCAACAGAACCTATAAAAAACATGGGCT
TAAAGGGTTAATTTATATAGTATTACTGCAAACATTGATTTGGGTCAAAAATTAGGGGTATTAAAAAAATGAAT
GAATCATAATTTTATCAAGCTGATTGGAGAGGTTAAAATGCATTATATAAAATTTATTGAGTCAAAAGATAATA
CAAAACTTTATATGAAAGTGAATGATATTCAAGATGCAAAAGCGAATATCATTATAGCTCATGGTGTGGCAGA
ACATTTAGATCGTTATGATGAGATAACAGCATATTTAAATGAAGCGGGTTTTAGTGTTATTAGATATGATCAAA
GAGGGCATGGTCGTTCTGAAGGCAAGCGTGCCTTTTATAGCAATAGTAATGAAATTGTCGAAGATTTAGATG
CGATAATAAATTATGTGAAGTCAAACTTTGAAGGTAAAGTTTACTTAATCGGTCATAGTATGGGTGGTTATACA
GTCACTTTATATGGAACGAAACATCCAAATACAGTGAATGGTATTATAACTTCTGGAGCATTAACACGTTATAA
TAATAAACTATTTGGCAATCCTGATAGAAACATATCACCTGATACTTATATAGAAAACAATTTAAGTGAGGGGG
TATGTTCTGATTTAGAGGTAATGGAAAAATATAAACTTGATGATTTGAATGCGAAACAAATCTCTATGGGGCT
CGTCTTTTCAATAATGGATGGTGTTAGGTATTTGAAAGACAATGCTCAACAATTTACAGATAATATTTTGATATT
GCATGGCAAGGAAGATGGGCTAGTAAGCTATGTAGATTCTTTACAGCTTTATCAAGAAATAGGATCAGCACA
TAAATCATTACACATCTATGATCGTTTGGAGCATGAAATATTTAATGAAAGTTCTTATAATAGAACTATTTTTAA
CGAAGTTATTGAATGGCTTGAAACGGAATTAACTTATAACTAAAACAGTATAGTTCCGTGTATTTGATTATAAG
AAATTATGAGGATATTAAACATACTAAGATTAGCTATGAAGAAATCTATGACGATAGATTTTTTCATAGCTATTT
TTTATAGTTATAGAGAGGAGTAGACTGTCCAGACTCTTGGATTTTAAATCCGTAAAAAAAAACAAGTCAGCTTT
ACTCTCACCTTTTGAAATTCGTTTGTAGTATGTTGGGTTCTTGAAACCGTGTATAGGAAAATGAAATGAGAAA
GGTTAAGTAAAGTTTTTAGCTTCTCAACTATTCAAAGGAGGTTTTTTTATCGATTACTTAGGTGTTGATATTAG
TAAAAGGAGTAGTGTAGTTGCACATTATAAAAATGGAAAATTCCAAAAAGAGTTTTTCATCCAAAATAATAAA
AATGGCTACAATTATTTACTCAAGTATTTGAATGACTTAGACCACCCACAACTCATTTTTGAATCTACAGGTAT
CTATTCAAGAGGTATGGAACGATTTTGTTGTGTAAATCAAATTAACTATATTCAAATGAATCCGTTAGAAGCCA
AATTTAAAACGAGCGCTCTAAGATCATGGAAAACTGATCAGGCAGATGCTCATAAGCTTGCTTGTTTAGGAC
CGACGCTCAAACAAACAGGCAGCTTACCTATACATGAGTTAATATTCTTTGAATTAAGAGAACGTGCCCGTTT
TCATCTAGAAATCGAGAATGAACAAAATCGACTTAAATTTCAGATTCTTGAATTACTCCATCAAACATTCCCTG
GTTTAGAAAGATTATTTAGTAGTCGATATTCAATCATTGCACTCAACATCGCAGAAATTTTTACTCATCCAGAC
GTGGTTCTTGATATCGACAAGGATGTACTTATTACACATATATTCAATTCTACAGATAAGGGAATGTCAATGGA
TAAAGCTACAAAATATGCACTTCAATTAAGAGTGATTGCTCAAGAAAGCTATCCTAATGTCGATAGACATTCCT
TTCTAGTCGAAAAATTACGCTTACTTATTCAACAATTAAAACAATCTATTCATCATCTCAAACAATTAGATGATG
CCATGATTCAATTAGCACAACAACTCGATTATTTTGAAAATATTCATTCGATACCTGGTATTGGTAAGCTAAGC
ACAGCTATGATTATTGGGGAGATTGGTGATATTAAGCGATTTAAATCAAATAAACAACTCAACGCTTTTGTAG
GCATTGATATCAAACGATATCAATCAGGTCATACACACTGTAGAGATACCATCAACAAGCGTGGTAATAAAAA
AGCGAGAAAACTTTTATTTTGGGTGATTATGAATATAATAAGAGGGCAGCATCATTATGACAATCATGTCGTC
GATTATTACTACAAACTAAGAAAGCAGCCTAATGAGAAACCTCATAAGACTGCCATCATTGCTTGTATAAATCG
ATTATTAAAAACGATTCATTATCTGGTAATGAATCATAAATTGTACGATTATCAAATGTCACCACATTAGCCAAA
CGTACAATTAAATATATTTTAATACCTTATTCAAAAAATTAAAATGAACGGTTTAGTTAAGTAATGCTTATTTTAA
TTATAAGTACTTGACTAATCGTAAGAAAGAGCCTAGGACATAAATCAATGTCTCGCGACCACAGCTTAATTTT
TGGTGTTCATTCATGACTTTAAAAAATCCTTATTGCATAAATGTACATAGTGTAGTACTATTCAAAACGTAATTA

TTACGATTTGAATTAAGCGAGGAGAATGAAATGACTAAGACTTATGACGTTTGGTGGCAAAAAGGTCAAGA
ATCAGATGATGATATGGCACGAGACCATCAAGAAGCTTGGGAGAGAACAATAAAAATGCTTGATACATCTGA
CATCGAAGGGAAAACGATTTTAGATGTGGGATGTAATCAAGGCGGATTTTTACGACAGTTATACGATACAAC
ACCGTTTAAAAAAGGTGTTGGCATAGATTTAGCACGTTTATCTTTGGAAAAGGCAGAGACATTAAAAGGAC
AACGTCCACTTACATACTATTTAACAGATAAACCGCAAGAAACGAAGCACGTGTTTGATACGGCAGTAAGTA
CGTCTGTCTTGTACTTAATAGAAGATATTCCGCAACATGCAAAAGATTTAAAAGAGGTATTGAAACCAGGCG
GTGTTTATTACGCTTCATTCGCGGATTTAACTAATAACCCAAGTCGTCAGTTTATGGATGACACGATTAATCAA
TATGGTGCAACACCTTCTCAGAATCACTCTCTAAAACATATCGTTGATAGCTTTGTGGATGCAGGATTTGAAG
TTGCAGTAATGAAAGAGCATGTACCTGACGTGATTGATTTAACACATTATAGCGATTTTTATTTATCACCGAAT
GATTATTTACAAACACTATATGAAGAATCGTTTTTAATAAAAGCAAGTGTGAAAGAAGGTACTGAGAAATGA
GGAAATGTGTATTAATGACGGTAGCAGCAAGTGCTACGCTCTTATTGGCAGGTTGTGGCAATGGTCAAAAAG
AAGATAAAGATGTTACGGTATCGCTACCTACTGAAGCAAAGGCGGATAAACTTGACGCGCAAGGCTATGATG
CAGCGATGCCCGTTTATAGTGCAGTGTATGATGCATTAGTTAAATATGATAAAGATAAGGGTATTAAAGCAGG
TTTAGCAGATAAATGGAGCGTTGATGAATCAGGGAAAGTTTATGAATTCCATTTGAAAAAGAATGTTAAATTC
TCAGATGGTTCAGCATTAGATGCTAAGGACGTGAAATTCTCGATTGATCGTGCGAAAGCGATGAACAAAGAT
TCGACTGTAGAAACGTTAAAAAAATTAGATAAGGTCGTTGTTAAAAATGAGCACGTGGTCCAAATTAGATTG
AAATCTCCTTCAAATCAAGTGTTAAATGAATTAACACAAGTGAGACCGTTGCGTATTATGAGTCCACATTCAG
TAGAAGATGGTAAAGTAAACGGTAAATTTGAAAAAGCGATTGGAACAGGTGCATTTGTTGTTGATAAAACT
GGTAAAGAAAAAACGACAATGAAGCCAAATAAATATTTTGACAACGGTCACCCAGTCAATTATCATCTTGCAT
TCCAAACGATTGAAGATGGGGACTCAAGAAATTCTGCAGTACAAAGTGGTTCTGTAGATATTTCTGGTGGTG
CTTTAGGTATGCTCTCAGACGAACAAATCAAACAAGATAAGAAAAATAAGAACTTAACGATTGAAGATAGAC
CTAGCACAGTAAGTCACTTTATGGCATTTAACCCTAAAAATGATGTATTAAATCAACGCACAATTCGTGAAGC
GATAAGTAAGAGCATCGATGCGAAAGACATTGCGGGCAAATCTGTAAATGGTCTGTTCCAGAAGAACGTAC
AATTTGTGACTAAAAATAATCAACAGCCACACGATTATGATATGAAAGCGGCTGAAAGGTTACTTAAATCAGA
AGGATATCATAAAAACGATGACGGCATCTTTGAAAAGAATGGCAAACCTTTATCATTTAACTTAGTCATTCAA
ACTGCAGAGTTCCCAAATTGGAAAGATAAAGCTGAAAAAGTGCAACGTCAGCTTAAACAAGCCGGTATTAA
GTTAAATGTGAAAACGTTAGATTCACAATCATACTATGATACATTATGGACGAAAAAAGACTATGATTTGATTT
TCTATAGAACGTATTCAGATGCATTAATGCCTTACAACTTTATAAGTTCAGTGTTTAAAAATAATGATGGTCAAC
CAGGGGTGTTAGCTGATGATGAAACATTAACGAAACAGCTAGACGATTTCCCATCAACCGTATCAAAAGAAG
ACCAACAGTGTTCATTTGATGACATATTTAAACACTTTAATCAACAATACTATGGTGTGCCAATTGCTTATCCAA
ATGAGACGTTTGTAGTGAGTGATAAAGTAAAACAATTCAAATTCTCTGGACTTACGGATGCACCAATTGATTA
TAAAGCGTTGAAAGTTAATGAATAGCAATGCTCAAACGTACAATTAAATTCATACTTTATTTAATCGTAAGTTC
GTTTATTATCTTCATTTTAGTTGAGAAGACATCTGGTAATCCAGCGATTCTGTATCTACAACGTCATGGTTATAC
GTCGATTACGCAAGACAATATTGAAGCGGCACAACATCAACTTGGCTTAGGACAACATGTGTTACTAAGATAT
ATCGATTGGGTTGGACATGCACTCACGGGCAACTTAGGATACGGCTTTAGTACGAACGAAGCAGTTACCGCT
ATGATAATGGAAGCCATCGTGCCGACGCTTGTGCTAATCATTGTCTCTAGTTGTATCATGTTGCCATTTGGCTA
TATTGTTGGTTACTTCGTTGGGACGCGTCCGCATACACGTTACGCTAATGGAATTCGTGGATTCGCCCAAGTG
ATGACCTCAATGCCAGAATACTGGTTAGCTATTTTATTCATTTATTATTTAGGCGTACGTTGGCAATTGTTACCA
TTTGTAGGTAGTGATTCATGGCAACACTTTGTGCTGCCAATCTTCACAATTGTTGTTATAGAAGGGTGTCATAT
CTTATTGATGACAGCACATCTGATTACACAAACGTTAGATCAAGATGCGTATCAACTGGCGCAGTTAAGACAT
TTTTCGTTAAAAGCGCGTATCATCGTACAAATTAAAGAGATATTTGCACCACTAATGACGATTTCAATTAACAG
TATCATTCATTTAATTGGAAAAGCCGTAATACTAGAAGTCATCTTCAGCATGTCTGGTATAGGTAAATTGTTGA
TTAATGCTATTAACCAACGAGATTATCCACTGATTCAGGGCATTGTCATCTTTATCATTGTCTTTATTATGCTAAT
GAATTATTTAGGCGATGTGATTATTTTGAAGAATGAACCTAGACTTCGACGACGTCATACCCAGCAGTCAGGC

AATGAGAAAAGAGGTACGATGTGATGAAAAAATATCAAACGTACATCGCAATAGGTTCACTATTGAGTTTGA
TGGTTGTATTAATTACGTATGGTTTAATGCAAGACACGCAACATTTGAACCCACTTGAGTCACCTAATGGACA
ACATTGGTTGGGTACCGATCAATTAGGCAGAGACTTCTTAGTAAGACTGATTGTCGGTAGTCTTGTCACATTG
AGTTTAACAGGCATAGTGATTCTATTAAGCGTTTGTATGGGACTTATCTTTGGCTTAATTGCAGGCATAGAAA
GACGATGGTTAGATCAAATCATCATGTTTGTTGCCGATATGTTGCTGGCTATTCCGTCATTTATTATCGCATTAG
TCATCTTAAGTTTAGTAAGTAACTCCATGATAGGTTTGATACTTGCTTTAACGATTGGATGGATAGGACGTTAT
TTACGTTACTTCAGAAATTTAACGCGAGATATTCAAAAACGTCCATTTGTTCAATATGCACGATTGAGTGGGA
ACTCAACATTCAAAACGACAGTAACACATGTGATTCCACATTTATTAAGTAGTATATTCGCTTTGGTAACGGCT
GACTTTGGCAAAATGATGCTCAGCATATCTGGACTTGCTTTTCTAGGACTAGGTATTAAACCGCCGACGCCTG
AGTTAGGAACAATTCTTTTTGATGGGAAAAGTTATTTCAACGGCGCACCGTGGCTCTTCTTCTTCCCTGGTGT
ATTGTTAGGAGGTTTCGCCTTATTATGTCAAATTATCAACAAAAAAATAACGCAGTAAATACGGTAGTCAACG
TCAATCAATTATCGATTTTAGATCAAGAGAAAGTATTGTTAAAGGATGTTGATTTGACAGTAACTAAAGGTGC
ATTTCATTGCATTATAGGTGAAAGTGGCAGTGGGAAATCACTGTTAACAAGAACAATACTTGGAATGAAACA
ATCACAATTATGTTATCAAGGAGATATTGACATCGATTTAACTCAAACAGATGCAGTGTTTCAAGATGTTCAAA
GTAATATGTTTCAAAATATAACATTAGCTAAGCATTTCCAATACATTTATGAAGCCAATCGCACACATCTCACTA
AACAGCGTATTAAGGAAGATGTCTTACAGATGATGCAATTACTTGGTTTAAGACAAGGGGAACAATTGCTTG
AGCGTTATCCCTTCGAACTTAGTGGAGGTATGGCACAACGTGTCGCCTTTATAATGTCATTAATTAGACGTCC
GAACTACTTATTTTTAGATGAACCAACGAGTGCACTTGATCAAGAAAATATTAAAAAGTTTATGCATTACCTTC
TTAGGGCACAGGAGCGCTACCAAATGACCATTGTTTTTATCACACATGATATTAACTTAGTGAAAGATTGTGC
CACACATATTAGTATTATGCAGCAAGGTAAATTGATAGAAAATGGTGAGGCCGCGTCGATCTTAACTAAGCCG
ACACATAATTACACGAAAAAATTAATTACGATTGCACATCGGAGACAACCTTATGCTTAAAATAGAGAGATTA
ACCAAATATATAGACACGCAACTGATATTTAAAGAGATATCATGTACAATTAACGACCAGCACTTACTCATAAG
TGGGGAGAGTGGTTGTGGTAAATCCACATTAGCCAAGATTATCGCTGGCTTAGATACGGATTATCAGGGCGA
ATTATATCTTAATGGGCGCTTACGTGAATCTTATACGTCTAAAGAGTGGATGAAGCACATCCAATATGTACCTC
AATATCAACGTGATACTTTAAATCAGCGTAAAACGGTATTAGCTACATTATTAGAACCACTTAAGAATTATAAG
GTAAATAAACAGCGTTATACATCAAGCATTGAAGCAGTGCTTGATCAGTGTAATTTACCACACGATATACTTAA
TCATAAAGTTTCGACATTAAGTGGTGGCCAATTTCAACGCGTCTGGATAGCTAAAGCTTTAATATTAGAACCA
GAGATTCTCATATTGGATGAAGCTACAACCAACTTAGATGTCATTAATGAAGAAGCTATACTTCAAATGTTGAT
TTCCTTAAAGATGACACAATTAATCATTATTTCACATGATACATACGTCTTAAGCCAATTTGAAGGAATTCAGTT
ACAGCTAAATAAATTGAATAATTAAGATCACAAATCTTAATATGGTGAATATTTAATGGTACCTAAAAAATAAA
ATTTAAACTACAATGTCTAAATCCATATGTTGTTTCATTAGAGGATTTAAAAATGATTATAACACTAAAAGATTT
CAAATTATTATATTTAATATAAATTTACATATGATAAACGAATAACAATTCCAATATAAATTATTTTTTGATTATTTT
TATTTATAACTATATATTTTATATGAAAAATATAAGTTATAATAATAAGTTTAATATTGCCTCGTGGTTCTGAGCTT
GAACTTATCTCTAAATCATTTTGAGCTACTTATCTATCAATTCATATATTCTATAACAATATTTGTGACATCACGTG
CTATTTCATGAAGTGATTTTACGATATCACCTTCTTTAGAAAAAATATATTTCAGTAGGCACCGACGTATACAG
AATCATTTGAGTATTAAAAATAAAAACTAGAAAAAGAAACCGCATCATTAACTGATACGCAGAATCATATTATA
AATAAAACTAAAAATGAGGTTGTATATAACTCACTCTGAAATTGATTGAATATATAGTATCTTTAATAAAATGCA
GCTATTGTGGCGTAGAATTTGAGAATCAAAAAATGATTAATATAGTTTGAAGAGACTGAGCATAAATACTAGA
AAAATGGCCAGTAAATGAGTTTACTATAAACTCATTTACTGGCTTCTCTAATAATTATCAAGACAATTTGCGTTT
CTAGGCATACTTTGAAATGCGCTATTTTCTTTAAGAATATTAATATATGACTTGTGTTGGTAATGATAACCTTGG
TTAGAGTGTATTGTCATGTGATAGTTAAGATTAGATCTTTTAATTAAAACATTGTTTTAAATATCGATGACAAGG
TCTAATGTAGGACGTGTAGATATAAGGTTCGGAATTATATAAATCCATAAAAGGAGATAAATCGAAAACCTTTA
TACCCTGGGTGGGTATATGTTATAGTATAAGTAGCTTTACTATAACATTTTCATTAGGAGGGGTTAATTTGAATA
ATAATGGTGAAGAGCATAATCATCAAAATCACATGAATCATTCCAATCAAATGCATCATGATAACCATGCCTCA

CATGATCATCATAGTGGCCATGCACATCATCATGGAAATTTTAAAGTTAAGTTTTTTGTTTCATTAATTTTTGCA
ATACCTATCATTCTTTTATCGCCACTGATGGGTGTTAACTTACCTTTTCAATTCACATTTCCAGGTTCTGAATGG
GTAGTGTTAATATTAAGTACAATTTTATTCTTTTATGGTGGTAAACCGTTCTTGTCTGGTGGTAAAGATGAAAT
TGCTACAAAAAAACCAGGCATGATGACCTTAGTTGCCCTAGGTATTTCAGTAGCTTATATTTATAGCTTGTATG
CTTTTTATATGAATAACTTTAGTAGTGCAACTGGTCATACAATGGACTTTTTTTGGGAATTAGCAACCTTAATT
TTAATTATGCTATTAGGACATTGGATAGAAATGAATGCTGTCGGAAATGCTGGAGATGCTTTAAAGAAAATGG
CAGAACTGTTACCTAATAGTGCTATTAAAGTTATGGATAATGGCCAACGCGAAGAAGTTAAAATATCAGACAT
CATGACTGATGATATCGTCGAAGTAAAAGCCGGAGAAAGCATTCCAACAGATGGTATTATCGTTCAAGGACA
AACATCTATAGATGAATCCCTAGTCACTGGAGAATCTAAAAAAGTACAAAAAAATCAAAATGACAACGTCATC
GGGGGTTCTATTAATGGGTCTGGAACAATACAAGTCAAGGTTACAGCTGTGGGAGAAGATGGATATCTTTCT
CAAGTTATGGGACTTGTTAATCAAGCACAAAATGATAAATCTAGTGCTGAATTGTTATCTGATAAAGTAGCGG
GTTATTTATTCTACTTTGCTGTAAGTGTTGGCGTGATTTCTTTTATTGTCTGGATGCTCATTCAAAATGATGTTG
ATTTTGCATTAGAACGTCTTGTAACTGTGTTAGTCATTGCTTGTCCACATGCTTTAGGCTTGGCAATACCTTTA
GTCACTGCACGTTCTACTTCAATTGGTGCACATAATGGTTTAATTATTAAAAATAGAGAGTCTGTAGAAATAGC
TCAACATATCGATTATGTAATGATGGACAAAACTGGTACTTTAACTGAGGGTAACTTTTCTGTGAATCATTATG
AGAGCTTTAAAAATGATTTGAGTAATGATACAATATTAAGCCTTTTCGCCTCATTAGAAAGTCAATCTAATCAC
CCATTAGCTATAAGTATTGTTGATTTTGCGAAAAGTAAAAATGTTTCATTTACTAACCCACAAGACGTTAATAA
TATTCCAGGTGTCGGATTAGAAGGTCTAATTGATAATAAAACATATAAAATAACAAATGTCTCTTATCTTGATAA
ACATAAACTTAATTATGACGATGACTTGTTTACTAAATTAGCTCAACAAGGTAATTCAATCAGTTATTTAATTGA
GGATCAACAAGTCATTGGCATGATTGCTCAAGGAGATCAAATTAAAGAAAGCTCAAAACAAATGGTAGCTG
ATTTACTATCAAGAAATATTACACCAGTCATGCTTACAGGTGACAATAATGAAGTGGCACACGCTGTCGCAAA
AGAATTAGGTATTAGTGATGTCCACGCACAACTCATGCCAGAAGATAAGGAAAGCATTATAAAAGATTATCAA
AGTGACGGTAATAAAGTCATGATGGTCGGAGACGGTATCAACGATGCGCCGAGTCTTATAAGAGCGGATATT
GGTATAGCAATTGGTGCAGGTACAGATGTTGCAGTGGATTCAGGTGATATCATACTTGTTAAAAGTAATCCAT
CAGATATCATTCATTTCTTGACCCTTTCAAATAATACTATGAGAAAAATGGTGCAAAACTTATGGTGGGGTGC
AGGTTATAATATTGTTGCTGTACCTTTAGCAGCTGGTATTTTAGCATTTATTGGCTTGATTTTATCACCTGCAAT
AGGTGCTATTTTAATGTCTTTAAGTACAATTATCGTTGCAATTAATGCCTTTACATTAAAATTAAAATAAAAGAT
AGGAGTTTTATTATGATTAAAAAATTATTTTTTATGATATTAGGATCATTACTAATATTATCAGCTTGCTCCAATA
ATGATGAAAAAGATAAAGACACTAATGACCAAAAAAGTGAGAGCCATATGAAGCATAATGATGAAAGTAAA
GTTCCAGAAGATATGAAATCGACTAATGAGGGTGAATTTAAAGTGGGAGATAAAGTAACGATTACAGCAGG
GCATATGCCAGGTATGAAAGGTGCAGAAGCTACTGTAAAAGGTGCGTATAAAACATATGCCTATGTTGTAAGT
TATAAACCCACAAATGGAAATGAAAAAGTAAACAATCATAAATGGGTCGTAAACGAAGAGATTAAAGATGCA
CCTAAAGATGGATTTAGTAAGGGCGATACTGTTAAATTAGAAGCAAGTCATATGTCTGGTATGAAAGGTGCTA
CAGCCAATATAGATAACGTGAAAAAGACTACTGTTTACGTAGTTGATTACAAATCCAAAGATAATGGTAAAAT
CATTAAAAATCATAAATGGATGACAGGAAATGAGCTGAAAGCACGATAAAAATCTAGTTCTAAATTGAGAAA
TAAATAGATATAAAAATATCCTCCTTAATCAATAATTTAAATAACTTATTATTGTTAAGGAGGATATTTTTTAGTG
TGTAAATTAAAAAGAATTTTAGAAGAATAACATTTATCAAAAAACTGTTCATTACCTTATTAATTGAAATTATAT
AATTAAAAACCGCATCATTAACCGATACGCAGAGGCGTATCATAAGT
>Staphylococcus aureus strain BSN9R
ATGAAAATCACCATTTTAGCTGTAGGGAAACTAAAAGAGAAATATTGGAAGCAAGCCATAGCAGAATATGAA
AAACGTTTAGGCCCATACACCAAGATAGACATCATAGAAGTTCCAGACGAAAAAGCACCAGAAAATATGAGT
GACAAAGAAATTGAGCAAGTAAAAGAAAAAGAAGGCCAACGAATACTAGCCAAAATCAAACCACAATCCA
CAGTCATTACATTAGAAATACAAGGAAAGATGCTATCTTCCGAAGGATTGGCCCAAGAATTGAACCAACGCA
TGACCCAAGGGCAAAGCGACTTTGTTTTCGTCATTGGCGGATCAAACGGCCTGCACAAGGACGTCTTACAA

CGCAGTAACTACGCACTATCATTCAGCAAAATGACATTCCCACATCAAATGATGCGGGTTGTGTTAATTGAAC
AAGTGTACAGAGCATTTAAGATTATGCGAGGAGAAGCATATCATAAATGATGCGGTTTTTTCAGCCGCTTCAT
AAAGGGATTTTGAATGTATCAGAACATATGAGGTTTATGTGAATTGCTGTTATGTTTTTAAGAAGCTTATCATA
AGTAATGAGGTTCATGATTTTTGACATAGTTAGCCTCCGCAGTCTTTCATTTCAAGTAAATAATAGCGAAATAT
TCTTTATACTGAATACTTATAGTGAAGCAAAGTTCTAGCTTTGAGAAAATTCTTTCTGCAACTAAATATAGTAA
ATTACGGTAAAATATAAATAAGTACATATTGAAGAAAATGAGACATAATATATTTTATAATAGGAGGGAATTTC
AAATGATAGACAACTTTATGCAGGTCCTTAAATTAATTAAAGAGAAACGTACCAATAATGTAGTTAAAAAATC
TGATTGGGATAAAGGTGATCTATATAAAACTTTAGTCCATGATAAGTTACCCAAGCAGTTAAAAGTGCATATAA
AAGAAGATAAATATTCAGTTGTAGGGAAGGTTGCTACTGGGAACTATAGTAAAGTTCCTTGGATTTCAATATA
TGATGAGAATATAACAAAAGAAACAAAGGATGGATATTATTTGGTATATCTTTTTCATCCGGAAGGAGAAGG
CATATACTTATCTTTGAATCAAGGATGGTCAAAGATAAGTGATATGTTTCCGCGGGATAAAAATGCTGCAAAA
CAAAGAGCATTAACTTTATCTTCCGAACTCAATAAATATATTACATCAAATGAATTTAATACTGGAAGATTTTAT
TACGCAGAAAATAAAGATTCATCTTATGATTTAAAAAATGATTATCCATCAGGATATTCTCATGGATCAATAAGA
TTCAAATATTATGATTTGAATGAAGGATTCACAGAAGAAGATATGCTAGAGGATTTAAAGAAATTTTTAGAAC
TATTTAATGAATTAGCTTCAAAAGTTACAAAAACATCCTATGATAGCTTGGTCAATAGCATAGACGAAATACAG
GAAGACAGCGAAATTGAAGAAATTAGAACAGCACAAAAAGATAAGACACTCAAGGAAGTGGAAGCACCTA
AAGGAATAATTCCAAAATATAAAAAAGGTGTATCAAAGACTACTAAAAATGATTCAGAAATTGAAAAATCAA
ATAAAGAGAATAAATTAACCGGTAAAGTTGGAGAAAAATTAGCGCTAAATTACTTTAATGAGCTAATTGATAA
TAAAATAGACGAAGATAAGAAAGAACAGTTTAGGAATATTTTAAATGATAATCCAGGCTCTCAACACGGTCAT
GGCTATGATTTAGTAGCTTTTGATCCAACAAATACAGATAAAGCTGTAGAAAAATTTATTGAAATTAAAACATC
TACATCTTCTAGTATTGAGGAACCATTTTTTATGTCGCTAAATGAAATGTTTGCTATGAAAGAATATAAGCAGA
AATATTTAATATTAAGAATATTTAATGTTTCCGGTAAAGAACCACAATTTTATTTTATAGATCCATATGCAAATTA
TTCTGAATTTAAAGATGTAGATGATCTCATTGACAAAGTATTTAATGTAGAAGCTATTCAGTATAAAGTTTTTG
GCGAAAAATGATTACTTGAACAAGAGCTAAAATAAAATTGTGATCTAATAAAAATAGAAGGTTCTGTTGCAA
AGTAAAAAAATATAGCTAACCACTAATTTATCATGTCAGTGTTCGCTTAACTTGCTAGCATGATGCTAATTTCGT
GGCATGGCGAAAATCCGTAGATCTGAAGAGACCTGCGGTTCTTTTTATATAGAGCGTAAATACATTCAATACC
TTTTAAAGTATTCTTTGCTGTATTGATACTTTGATACCTTGTCTTTCTTACTTTAATATGACGGTGATCTTGCTCA
ATGAGGTTATTCAGATATTTCGATGTACAATGACAGTCAGGTTTAAGTTTAAAAGCTTTAATTACTTTAGCCAT
TGCTACCTTCGTTGAAGGTGCCTGATCTGTAATTACCTTTTGAGGTTTACCAAATTGTTTAATGAGACGTTTG
ATAAACGCATATGCTGAATGATTATCTCGTTGCTTACGCAACCAAATATCTAATGTATGTCCCTCTGCATCAATG
GCACGATATAAATAGCTCCATTTTCCTTTTATTTTGATGTACGTCTCATCAATACGCCATTTGTAATAAGCTTTTT
TATGCTTTTTCTTCCAAATTTGATACAAAATTGGGGCATATTCTTGAACCCAACGGTAGACCGTTGAATGATG
AACGTTTACACCACGTTCCCTTAATATTTCAGATATATCACGATAACTCAATGTATATCTTAGATAGTAGCCAAC
GGCTACAGTGATAACATCCTTGTTAAATTGTTTATATCTGAAATAGTTCATACAGAAGACTCCTTTTTGTTAAA
ATTATACTATAAATTCAACTTTGCAACAGAACCGTATTATGGAATAGAGATGTTGGTAACATTTATACAGGATC
ATTATACTTAAGTTTAATTTCGTTATTACAGAACCACACATTCCAACCAGAAGAGAAAGTATGTCTATTTAGTT
ATGGTTCAGGAGCAGTAGGAGAAATCTTTAGTGGTTCAATCGTTAAAGGATATGACAAAGCATTAGATAAAG
AGAAACACTTAAATATGCTAGAATCTAGAGAGCAATTATCAGTCGAAGAATACGAAACATTCTTTAACAGATT
TGATAATCAAGAATTTGATTTCGAACGTGAATTGACACAAGATCCATATTCAAAAGTATACTTATACAGTATAG
AAGACCATATCAGAACATATAAGATAGAGAAATAAACTAGTGGCCGATTGTGCTTGATGAGCTTGGGACATA
AATCCTAACTCGAAATAAATAAGCATATCACTAAACTGATTTTTTAAAGTTTACAGTGATATGCTTATTTTTTTAT
CTTACGATTTTGTACGTGCATGCTTGCCTAGGGGTATGGCTCGAGCCATTAGTCTCTCGCACATACTATTCCCT
CAGGCGTCAGCACTTACAAAATCGGTTGTAATTTTCATTTTTATACGCATTCTTACTGAGATTATACTAATAAGA
GGAATAGTAAAAGCAATTCTAAGTAAAATTGCAGATAAGAGGTTTGTTAAAAGCAGTTCTCAGTAAAATTAC

AGATAAGAGGTACGTTAAAAGCAGTTCTAAGTAAAATTGCAGATAAGAGGTTTGTTAAAAGCAGTTCTAAGT
AAAATTGCAGATAAGAGGTACGTTAAAAGCAATTCCATGCAAAATTGCTGATAAGGGGTAAGTTAAAAGCA
GTTCTCAGTAAAATTGCAGATAAGAGGTACGTTAAAAGCAGTTCTAGGCAAAATTGCAGATAAGAGGTGCG
TTAAAAGCAGTTCTCAGTAAAATTGCTGATAAGGGGTAAGTTAAAAGCAATCCTAAGTAAAATTGCAGATAA
GGGGTACAGAAAAACTAGACTTGATTACAAAATGGAGCTTGGGACATAAATGATTTTTTAAAAATGAGATGA
GACGTAGATTAACTCCATAATCAATACGAATCTATCGACTTCTTTATTTATGATATTCATCTCTTTTTAATGGAAA
TAAAAGTGCGATTAATGTGATAATACAGTTACGTTAATTAAAAAAATAAAAATGCAAGGAGAGGTAATATGCT
AACTGTATATGGACATAGAGGATTACCTAGTAAAGCTCCGGAAAATACAATTGCATCATTTAAAGCTGCTTCA
GAAGTAGAAGGTATAAACTGGTTGGAGTTAGATGTTGCAATTACAAAAGATGAACAACTGATTATCATTCATG
ATGATTATTTAGAACGGACTACAAATATGTCCGGGGAAATAACTGAATTGAATTATGATGAAATTAAAGATGC
TTCTGCAGGATCTTGGTTTGGTGAAAAATTCAAAGATGAACATTTGCCAACTTTCGATGATGTAGTAAAAATA
GCAAATGAATATAATATGAATTTAAATGTAGAATTAAAAGGTATTACTGGACCGAATGGACTAGCACTTTCTAA
AAGTATGGTTAAGCAAGTGGAAGAACAATTAACAAACTTAAATCAGAATCAAGAAGTGCTCATTTCAAGCTT
TAATGTTGTGCTTGTTAAACTTGCAGAAGAAATCATGCCACAATATAACAGAGCAGTTATATTCCATACAACTT
CGTTTCGTGAAGACTGGAGAACACTTTTAGATTACTGTAATGCTAAAATAGTAAACACTGAAGATGCCAAAC
TTACTAAAGCAAAAGTAAAAATGGTAAAAGAAGCGGGTTATGAATTGAACGTATGGACTGTAAACAAACCA
GCACGTGCAAACCAACTTGCTAATTGGGGAGTTGATGGTATCTTTACAGACAATGCAGATAAAATGGTGCAT
TTGTCTCAATAGAAAGTTAGAGGTGAGTCTTACGTTTCAGTGACGGTAGACTTACCTTTAACATGTTACATAC
TAAAAAATTAATTTGAATAAGAAAGAGAGACATATATGAAATACGATGATTTTATAGTAGGAGAAACATTCAA
AACAAAAAGCCTTCATATTACAGAAGAAGAAATTATCCAATTTGCAACAACTTTTGATCCTCAATATATGCATA
TAGATAAAGAAAAAGCAGAACAAAGTAGATTTAAAGGTATCATTGCATCTGGCATGCATACACTTTCAATATC
ATTTAAATTATGGGTAGAAGAAGGTAAATACGGAGAAGAAGTTGTAGCAGGAACACAAATGAATAACGTTA
AATTTATTAAACCTGTATACCCAGGTAATACATTGTACGTTATCGCTGAAATTACAAATAAGAAATCCATAAAAA
AAGAAAATGGACTCGTTACAGTGTCACTTTCAACATACAATGAAAATGAAGAAATTGTATTTAAGGGAGAAG
TAACAGCACTTATTAATAATTCATAATAAAACAGTGAAGCAACCATCGTTACGGATTGCTTCACTGTTTTGTTA
TTCATCTATATCGTATTTTTTATTACCGTTCTCATATAGCTCATCATACACTTTACCTGAGATTTTGGCATTGTAGC
TAGCCATTCCTTTATCTTGTACATCTTTAACATTAATAGCCATCATCATGTTTGGATTATCTTTATCATATGATATA
AACCACCCAATTTGTCTGCCAGTTTCTCCTTGTTTCATTTTGAGTTCTGCAGTACCGGATTTGCCAATTAAGTT
TGCATAAGATCTATAAATATCTTCTTTATGTGTTTTATTTACGACTTGTTGCATACCATCAGTTAATAGATTGATAT
TTTCTTTGGAAATAATATTTTTCTTCCAAACTTTGTTTTTCGTGTCTTTTAATAAGTGAGGTGCGTTAATATTGC
CATTATTTTCTAATGCGCTATAGATTGAAAGGATCTGTACTGGGTTAATCAGTATTTCACCTTGTCCGTAACCTG
AATCAGCTAATAATATTTCATTATCTAAATTTTTGTTTGAAATTTGAGCATTATAAAATGGATAATCACTTGGTAT
ATCTTCACCAACACCTAGTTTTTTCATGCCTTTTTCAAATTTCTTACTGCCTAATTCGAGTGCTACTCTAGCAAA
GAAAATGTTATCTGATGATTCTATTGCTTGTTTTAAGTCGATATTACCATTTACCACTTCATATCTTGTAACGTTG
TAACCACCCCAAGATTTATCTTTTTGCCAACCTTTACCATCGATTTTATAACTTGTTTTATCGTCTAATGTTTTGT
TATTTAACCCAATCATTGCTGTTAATATTTTTTGAGTTGAACCTGGTGAAGTTGTAATCTGGAACTTGTTGAGC
AGAGGTTCTTTTTTATCTTCGGTTAATTTATTATATTCTTCGTTACTCATGCCATACATAAATGGATAGACGTCAT
ATGAAGGTGTGCTTACAAGTGCTAATAATTCACCTGTTTGAGGGTGGATAGCAGTACCTGAGCCATAATCATT
TTTCATGTTGTTATAAATACTCTTTTGAACTTTAGCATCAATAGTTAGTTGAATATCTTTGCCATCTTTTTTCTTT
TTCTCTATTAATGTATGTGCGATTGTATTGCTATTATCGTCAACGATTGTGACACGATAGCCATCTTCATGTTGG
AGCTTTTTATCGTAAAGTTTTTCGAGTCCCTTTTTACCAATAACTGCATCATCTTTATAGCCTTTATATTCTTTTT
GTTTTAATTCTTCAGAGTTAATGGGACCAACATAACCTAATAGATGTGAAGTCGCTTTTCCTAGAGGATAGTT
ACGACTTTCTGTTTCATTAGTTGTAAGATGAAATTTTTTTGCGAAATCACTTAAATATTCATCCATTTTTTTAAC
GGTTTTAAGTGGAACGAAGGTATCATCTTGTACCCAATTTTGATCCATTTGTTGTTTGATATAGTCTTCAGAAA

TACTTAGTTCTTTAGCGATTGCTTTATAATCTTTTTTAGATACATTCTTTGGAACGATGCCTATCTCATATGCTGT
TCCTGTATTGGCCAATTCCACATTGTTTCGGTCTAAAATTTTACCACGTTCTGATTTTAAATTTTCAATATGTATG
CTTTGGTCTTTCTGCATTCCTGGAATAATGACGCTATGATCCCAATCTAACTTCCACATACCATCTTCTTTAACA
AAATTAAATTGAACGTTGCGATCAATGTTACCGTAGTTTGTTTTAATTTTATATTGAGCATCTACTCGTTTTTTA
TTTTTAGATACTTTTTTTATTTTACGATCCTGAATGTTTATATCTTTAACGCCTAAACTATTATATATTTTTATCGG
ACGTTCAGTCATTTCTACTTCACCATTATCGCTTTTAGAAATATAACTGCTATCTTTATAAACTTGTTTGAAATTT
TTATCTTCAATTGCATCAATAGTATTATTAATTTCTTTATCTTTTGAAGCATAAAAATATATACCAAACCCGACAA
CTACAACTATTAAAATAAGTGGAACAATTTTTATCTTTTTCATCAATATACTCCTTATATAAGACTACATTTGTAG
TATATTACAAATGTAGTATTTATGTCAAAATAATGTTATAATTTTTGTGATATGGAGGTGTAGAAGGTGTTATCAT
CTTTTTTAATGTTAAGTATAATCAGTTCATTGCTCACGATATGTGTAATTTTTTTAGTGAGAATGCTCTATATAAA
ATATACTCAAAATATTATGTCACATAAGATTTGGTTATTAGTGCTCGTCTCCACGTTAATTCCATTAATACCATTT
TACAAAATATCGAATTTTACATTTTCAAAAGATATGATGAATCGAAATGTATCTGACACGACTTCTTCGGTTAG
TCATATGTTAGATGGTCAACAATCATCTGTTACGAAAGACTTAGCAATTAATGTTAATCAGTTTGAGACCTCAA
ATATAACGTATATGATTCTTTTGATATGGGTATTTGGTAGTTTGTTGTGCTTATTTTATATGATTAAGGCATTCCG
ACAAATTGATGTTATTAAAAGTTCGTCATTGGAATCGTCATATCTTAATGAACGACTTAAAGTATGTCAAAGTA
AGATGCAGTTCTACAAAAAGCATATAACAATTAGTTATAGTTCAAACATTGATAATCCGATGGTATTTGGTTTA
GTGAAATCCCAAATTGTACTACCAACTGTCGTAGTCGAAACCATGAATGACAAAGAAATTGAATATATTATTCT
ACATGAACTATCACATGTGAAAAGTCATGACTTAATATTCAACCAGCTTTATGTTGTTTTTAAAATGATATTCTG
GTTTAATCCTGCACTATATATAAGTAAAACAATGATGGACAATGACTGTGAAAAAGTATGTGATAGAAACGTT
TTAAAAATTTTGAATCGCCATGAACATATACGTTATGGTGAATCGATATTAAAATGCTCTATTTTAAAATCTCAG
CACATAAATAATGTGGCAGCACAATATTTACTAGGTTTTAATTCAAATATTAAAGAACGTGTTAAGTATATTGCA
CTTTATGATTCAATGCCTAAACCTAATCGAAACAAGCGTATTGTTGCGTATATTGTATGTAGTATATCGAGCTTC
ACATGAAACAGCTAAAGAAGCTTTGGGCGATAAAGAGTTAAGAGCCATTGCACATGAGTTAACTAAAACAG
TTAAGGATAACATGAGTGTTGATTGGTCTAAACGAGACAGTGCTAAAGCTAAAATGAGAGTTCAAGTTAGAC
GCCTATTAAAGAAATATGGCTATCCACCAGATCTTCAAAAAATGGCTGTGGAACAAGTTGTAGAGCAAGCAG
AATTAATGGCAAGTCAGCAATAAAAAAATAAATCATAATGAGTCCGGGACATAAAGTTCTTGGATAAGTGAA
AAAAGACAATTTCTATTGAAATAATATAGAAATTGTCTTTTTTATAAATTTTTTGATTATTTTCAGCTCGTTGAG
CTACTACTTTTCTTATATTAAGTGCCATTAATACAAAACCAAGTTCTCTTTTGACTTTATTGAGTCCTCGGACAG
ACATCCGAGTGAAACCCAAAATAGCCTTCATAAATCCAAAAACAGGTTCCACATCAATTTTTCTTTGACTGTA
GATATTTTTTGTTTCTGGTTCTGAAAGCTTTTTGTTAATTTGGGATTTAAAATATTCCCAGTTATAATTCTTCATT
ATTTTTTTGTTTGTTTTTGAATTGAAGTTCATACATTGATTTTTCAGAGGACATTCTGAACAATCATCACATTCA
TATAATTTGAAGTCTCGCTTATAACCATACTTATCATGACGATAGGCATATCTTTTAAAACCTAGCCGTTTATTAT
TCGGACAAATGAATTCGTCATTAATTTCGTCATAGTTCCAATTTTGAGTATTAAAGATGTCACTTTTATATTTTT
TAGTTTTATCTTTTATAAACATTCCATATGTTATGAGTGGCGTTCGATTAAAGTCATCTATAATTGCCTTATAATTT
GATTCACTACCATAACCTGCATCAGCTACAATATATTCAGGTAAATGACCGTAGGTCTCTTGAATTGAATTTAA
AAATGGAATCATCGTTCTAGTATCCGTTGGATTTTGATACACATTATAAGATAAAACAAATTGGGAATTTGTTG
CTATTTGTAAATTATACCCTGGCTTAAGTTGTCCATTTTTCATGTGATCTTCTTTCATTCTCATAAATGTCGCATC
ATAATCTGTCTTAGAATAACTATTTCTATCCTTTAAAATAGATTTTTGAAATTCGTATCGATACTTTCGCTCAAAA
TAATCATTGATTTGCTTTTTGTATTTTTTGATTTAGTTCTTTTGAGACGTATTTGTTTTCTTGTTTTAGTACATT
TTTCATTGTTGATATGTTGGTTTAAATCTTCGATTTCTTTATCTAAGTGACTACCAATCAAATCTATTTCTTCTTT
TGTTAATTCATTATCATGATCTTCTTTAATTTCCGGTATGATTTTATTGGTTACCAATTCATGGTAGAGGGCTTTA
GAATCCTCATTCATCTTTGATTCATGGTTTTGAATACTCTTTTTCCATACAAATGTATATCGATTGGCATTTGCTT
CAATTTTTGTACCATCAATAAAAATAGCTTTATCATCTATAAGATTTTGTTTTACACACTGACTGTAAAATTGAA
TAAATAAAGATTCTAATAAAGCATCTACTTTTGGATTTACTCTAAATTGATTAATTGTTTTATAAGAAGGTTTTT

GATTTTGTGATAGCCACATCATTCGGATGCTATCATTAAGCATTTTTTCTATTTTACGACCTGAGAATACAGATT
GTGTGTAGGCATATAGAATCACTTTTAACATCATTTTAGGATGGTACGAAGTTGCACCACGGTGATGTCTGAA
TTCGTCGAATTCATTGTCAGGAATTGTTTCAACAATATCATTTACAGTAAAACGATGTTGATTTGTTTTGTTTCC
ATATTGACCTCCATGTATTTGCTATGATTTCAAAATCCATTTTTGACGTGCCTTAGGGTTGAGTGGATGCATAAT
TTCATTTGTTACTGGATTGATGAGCTTTTTTACTTTCTTTTTATGAGGTTTTAACATTTCCATCACTTGTTCGAC
ACGGTCGATAACAACTGGTCGCTTCGCATAGGCACCATAAGCAAGAATCACTGTGTCACTTTCACTAATCGCT
TTCATCAAATGAATATCAGTGTGCTCATCGTATGGATTTTTGATATGTTTGAGGTTTTCGGGTGTTCTAATATTA
GAGAATAGATTTACAAGATATACAGCACCGTATCGTTCTGAATTGGCTAATTGGTTGAGGATAAGAACAGTTG
TGAGATCGAGTGATAATACACCGTCTAAATGAGGATACATCGTTATCACTGTGCATGCAGCTTTCTTTTCATCC
CATGTTTTCTTGAGTAAATAGCGGTGCTGTTCATCATCGCTAAATATGGCTTCTGTGTGTATCGTACTTTTGATT
GTATTCATCATCGTCACTTCCTTTAGTATTCTTCTGGTAAAAGCATCACATAATAAAAAGCGTCCACGTCATCTT
CACGAATGACGTAGACTTTCTTAGGTAATGCATTTTGATTTTTTTCATAGTTTGTATAGTGATATTCCAATTTGT
ATGTGGGTTGTTCTTGTTCATGTGTGATTGAGAGTATATTCTCATCTTCTTGTAATTTAAAAATGTGTAGGTAAT
CTGTATGAGGCTGGTTATCTTTTTTTTCTACCATGTGCCAAAGTAAGATTTGAAGGTCTAGTGGAATACTTTCA
TTAATTCCTCTTGTGATGTATCGATTGATTTTCATGCTATTTCCCTCCCTTCTGCTTTTCTTTCATGATGTCGATG
ATTTCGTTGATAACTGTGACGGATAATTGAGCAACTATGATCCAATTATTCATGGTCCTGACCTCCTTGTTTTA
GTAAATGACGTTCATCAATAATGATATTTTGAGTATCTGTAAGGTACAGAAAGTCCATGTCAAAATGGTCTAAG
TATCCGACACTGATGAGTTGGTTATTGGCATACATTAGAAATGGATAGATACTTAGCTCATGTAGTTCATCATTA
TAGTAGGTATAAGTCTCGAGTGTGAGATGTACCAGTGGAGAATCATTAATAAAACGTTCCGGTAGAATATTTT
CTGCTGCTTCCTCCAGCGCTTCACATTCCCAAGCTTCGTTATTAGATAGTTGGAATAGGTGGGTTATATATTGT
TTGAGTTCTTGAGTGATGGTTTTCATATTATTGCCTCCTAGATAGTGTAATAGTGATGTAGTTCATATACATCATT
GAGATAATATATATTTGATTTGTCATTTATTACGAATCCCGGTGGGAATAAGAGAAAATTCCATATAAAAACCC
GCTACAAACGTTGGTATGCCAAGGAAATCCTGAAATTCCGCCTATTTTGACAAACAATCAACTCATTATTTATA
AGTATTGATGATAGGGTTGTGTCTCTGCTTCCTTATATATATTATTTATTTATAAAAAGTAACGGGATTTTGGGA
TTGTGCTTGCACAATCCTTCTGTTTCTTCGAATCTGCAAATCCCAATCATTTCCCGATAAAAAATCATTGTGGG
ATGTTCTTTAGCAATTTCAATATAAGCATTGTATAGTTATGAAAAAATTACGACAATAACTGTTTCATTAGATAA
GTGTTATTGAAATTGATAAAGAGCAATTCTTGAAAATAGTTAGATAAAATAAGCGAAAGAATATAGTGAAAAT
TATTGTTATAACAATGATTCTATTAGCTAAATAGTAAGATATAGTGTTTGGGGCAAAAATAAAGACGAAGTGCT
GAGATGCACTTCGTCGAGTTGTTTATTATTGAAAAGTTGTTTAATGATTTCGTTATTAAGTTTGAGTGTGACAT
AGAATTGTTTTTTATGATTACCATCTTTTTTAATATCAATGCGATCAATCACTGATAGATACAATGCTTTGAGTC
GAGATTTTTCTATGTGCTTAATATCATGAAAGATGTGTTGTAATAGTTTACTGATTTCTTTGGCATCAAATAAAG
TCTTATCTTCATTTTGTTGATTTTTGAGTTGGTTGATTTGATTCGTAATGTCATTGAGTTGCTTTTCATATTTTTG
AATACTTGGTCTGATTACTGATGTTAAGTCCGGATTATCCTCGATGGTTTTAATCAAGTTATTTATTTTGATTTG
TACTTCATCATATTGTTGTTGCTTATAAGCAATATCGTGATGAAGTGCAGCGCCATCAACTTGATTTTCTTGATT
GACGTGTGTTACTACGCGTTGAATGACTTTATCACTTTTGACTATTTCAAGTATTTGCTTCATCACATAATCTTC
AATCACATCAGCTCTTACACTGTTTGCCGAACATACTTTGGAACCCTTGTTCCGAAAATTACTACATGAATAGT
AACGAATACGTTTCTTAGTCCCGTCTTTAAGAGTATTCGTGGTATTGCTTGCTGCCATAGGTGCGCCACATTG
GGGACAGTGAATAATGCCTGTAAGCAGATTCGTTCCTTTGCCATGGACTTGGGGTTTTTGACTGACTTGTTT
CTTACGCATTTGTACTTTATCCCATAAATCTTGATTAATAATGGGGGAATGCTTACCTTCAGCTATCACTGGTTT
ATCATTCAGCCCTTTACGACGTTTTTCACTCCAATCTTTGTATTTCGCAAATTGAATTTTGCCGATATAGAAAG
GGTTAGCTAAGATGTATGTGATTGAACTAATACTGAAAGGTTTCCCCTTTTTAGTGACATATCCTTTGTGATTC
AATGCATTGGCAATTTTACGATAGCCATGTCCTTTGGCATAGCACTCGAATATATATTTTACAATATTAGCTTCA
TGTTGGTTAATCATTAGCTCGTGTTTACTATCTGGTATTTTGTCATAACCTAGTGGTAAATTGCCTTGATAATAG
CCTTCTTGGGCACGTCTCGTTTGACCCATAAATACGTTCTCGACAATGTTATTACGTTCGAATTCTGAGAAACT

CGCAAGTATCTGTAACATGAGTTTACCAGAAGAAGTATTGACTTCCATACGCTCTGACAAACTGAAAAATTCG
ACATTTTGTTTGTGTAAATCTTCGACAATTTTGAGAAGATCAGATGTATTACGAGCTAATCGGTTTGTTTTGTA
TACCATAACACAGTCGATATTGCCTTCTTTTGCATCTTTCAACATACGTTGGAGCTCAGGTCTGTTCATAGATT
TACCTGAAATACCACGGTCAGCGTATATATCTTTAACTTCAAAATGATGGAAGTCACAGTATTCTTTGATTTGA
TTGATTTGTCCGTCGATACTATAACCTTCTGTGCTTTGCATTTCTGTTGATACACGTACATAGATACCGACACGT
TTTGTTTTAAGTTGTTGCATTATGTTACATCCTTTCTTCATTTATGCAATCGATGATTGCATGGTTTGATTGACA
ATATTGAGTGGTTCATTTTTGAAATAGATTCCTATAAGATTTTTATCTTTCGTAATGTGAATGGTTTCAATATAG
GGGTACAATATGTTTAACGTGAAACGTTTTTGAATAATATTTTGAATGATGTGTTGTATTTGATGCCCATTGATA
GATGTAGTGCGTTGCGGTTGTTGACGTAATGATTGTGTTTGCTCTCTGAACGTTTCTGCATCAATGATGCCTT
GTGCCAACTTTTCTATCAGTTGTTCTTGAGTCAATGTGTGATGTTTTTCTATGTTTCTTTGTCTTTTGATGCGTT
TGTCAATCGCACTTTTAATTTTTGTGTAGATGCGTTGATTTTGATAAAAGTCTCGGCACACTTCTAATACTTTAT
CTTCAAGTGTTTGTGCATTGATGCCTTTAAAATCACAGACAAAGCGTGAAGCATTCATGTTTTTAGGACAGA
CGTAGTAACGTAATGTATGATTCTTTTTTCTAATGGTCATATTTGTAAGTGTTGCATTACAACATGGGCATTTGA
TTTTTTGTTTGAGTTGATTATCCGAAGGTGTCTGTTTGGTTTGTTTTTGCAATCGAAGTCTCTGCGCTTGCTCA
TATATACTTGTGGAAACAATAGAAGGAAACATATTGTCGAATTGGCCATATTGATTGTTGACACGACCACAAT
AATTAGGATTGATGATAATGTTACGAACTTGATAGGGTTGTCGATTGATATACGTGTTATCTTCTTCTAATAACT
GTGCAATTTTCTTATAACCATGACCTTTAATGTAATAATTGAATACAGCCTTTACCGTTGGTGACTCATTTTGAT
TGATGATGAATGTTCCGTTGTGATATTCGTAACCAAAAGGTGCATGTGTTGTAATCAATCGACCTTGCTTTGCT
TTTTCTTGAAGCCCATTTCTGACTTGTTCTCCAATGTTATCCGATTCAAGTTCGGCTAAACTGATGAAGATATT
AAGCTTGAGTCGGTCGAAAGCTTGATCCATATCAAAGTAACCATCATGTACGCTTAAGATATGAACATGGTAC
GTTTGACATAATTTGATGAGTTTTAATGCATTTTTCAGATTACGATGCAACCTATTAAGACGATAACAGCATAA
TATGTCACACTGTCCTTGTTGAATTAATTGTGTTATTTGTCGATACCCACTACGATTATCTTTGCGACCTGATTG
TTTGTCGCTATAAAAGTTGATATGTTGAATATGATGTTTTTCGGCTATTGCTTCGATAGCTTGTTTCTGTGCTGC
AAGAGATTGTTGTTTCATCGTACTTTGACGTAAATAGCCTATGACTTGTTTCATATCGGCTCCTCCTTTCACAG
TGATAATATATATTTATGGATGAATTGATATATAAGCCCAACATCAATGAGATGTTGGGCGTCCATATTAGTCATT
TGTTTGATTGATTTCTTCAATTACCAAATCGGCTAATATCTCGATAAGTTCATCCATGTTTTTCACTCCGTTATTT
GTTCTATCTTCAATACGTCGATTATTCAGTTTGATGCTTCACAGTTGTATGATAAAGACAATTAGAAATCTTCGT
GAACTCCTGAAGGGCCTATCCCTTCATTAGCGGATTTAAAAAGTTCTTTCGCAGCTTTGTTATCATTTGACGG
TGTCCAATTTTGAAGTAACGACTTATCTTTAGTTAATCCGAGGATAGATGCAAACTCTACATCTAATTTTAGAT
GGTAAAATACAAGTGATTGTTTTTTACCGCTATTATCTTTGACACTTCTTTTAGTTGTTTGGCGTCCACGGTCA
GCTAATATGAAACCTTTATCTCTTAAGGCGTTGACAACATTATTAACATCTTGAAATTGATGATTGTTTAGCATC
TGTTTAAAAACGTTCGCAATTATTTTTACTTCGATATGGTCATCTTTTAATGAGATTAATCCATAGTTCTCAAAC
ATATTTTTCAAAGCACCTTCATCTGAAAACTTACCTCTGTTTTGTGCCACAAATTGAATGATGACATCAATAGC
TTTATCAGCTAATGAGCGTTCAGAGACTGTATGAGTATGATAATCAATAAAGTAGTCTCTTATATTAGCGATATC
AATATCTGTAGATAAAACACGACCTAATATTTTCGCAGATGTTGTAATGACTGCATAACGCTTAAACATACGAA
TACCTGTATTGTTTGTTTCATCTTTCAATTTAGCTTCAAACCAATCTACTTCCTTGTAAAACCATTGAATAACTTC
ATCTTCACGATTTATAAGATATTTAGCTACTAACGGTAAAACATGACCATAGTTTAGTGCTACAGCTTTTTTAAT
ATTGTCAGCATTGGTCGCATTTGTAGTGAATTGTTCATTAATCTCGATGGTTCTTACACGTAATCCATCGTTTTG
AGCTGAATCATTAAAAATACTGTATTCTGACGTTGAAATGACAGAAGTACCCCAATTCTTAGGCGTTTTAACT
TCTCCATGAACGTTTGAACGTTGACGACCTTGACCTTCAGCGATGGAATATAACAAACCCGTGGTATCTCTAA
GTGTCGTAGATGAAAGCTCATCAAATACTATAGGAATGCCATAATTGTTACTCAAATAACCTTCAAGTGCGTTT
CGTGTGGCATTCCAACTTCTAAAAAGAGTTTCATTACCTTTGGTAGGGTTACCAGCGACTGATACAGCTAAA
GCAGCTGCGGTTGACTTACCGGTTGAGGATTGACCTGTAAAACTAAAGAGAATTCCTGCAAATTCGATTTCA
TGTTTATGCTTCAGAAAACTTGTCACTAAGGCAGAAATCCCAAATATGACTGCCAATTCTAAAAGAAGATGAC

CTTTAACCTCGTCAATATACATGTTAAACCAATTATCAAATGTACCTTTAGGAGTTAAGTCATAAGCACTATCTA
CAATAGGGTATGAAGGTGAGGACTGATTAAATTGAATAGATTTGAGAACTTTATCTAGCGATATCAAGTAGCC
AAAAGGTGTCTCTAGTATACCTACACCCTCATATAATTCAGAAAGTGGTAATCTATCTCGCATCAATTGCAAGG
CATAACTCAATGATCTAATATACTTCTCGTTAATACTGTAGCCATATTTAATTAAAGAAGGTAAGTTTCGTGTTG
TTAAGATATCAGACTCAAAAATGTCTTTTTTACCATTGTTGTTAGTAATAATCAACTTTTCAACACCAGTAATTG
GATCAGAAAATCTTGCATTAACAATAATAGGGCTAGCCATTCTAACCACTTTTTCACTGTCACCATCTTTTTTA
GGTGGTATCGTTTCTTTCCAACCGAAGGAATCTAAAGCAAATGGGCCAATTTCAAACAATGTGTAACTCATTA
GCAATCACCTCCTTTCGAAGGCGTGCTATTATTGCATGGGTTTATCCCTATTTTCTGATATAATAAACTGCCATT
TTTTGTTCCTACAATTTTAAATGGTAATCCTGGCGTGTACTTAATCCAGTAAGCAAAACGACGTCCTACTTCAC
GTTGATCATTCGTGCTACAAACCACATTATATTTCTTTCTTAATTCACCATAAGTGAACGTACTTCCAACTGGTT
GTTGAAAAGATTTAGCAATTATAAAATTATAAAGCTTTTCTTGCTGCTTAGATTTCTTATTCATTTTAAAATTCC
TCCAATAATTTTTAAGGAGTCAATCAATATCATATAGGTTAAACTATCTTCAATTGATTGCTATAATAAGCATAAA
TGAAGCATTATGATTAGACGCGGAACAAAAAATGGGGAGGGGGGGTATATATTGATACCTACGCTAGAAGA
AATTATTGATAAGTATGGAAACTTAGTTGATTATTTGAAATTTGATGTAACAGTTGAGGTTTATGAAGATTTGT
TGTTATTAAGAAGTTTAAGCGATTTAAATGAACATCAAAAGATGGATAGAGTTTCATTTATAAAAAAACATTTA
AACTCAAATTTCGATTATTTATTAGATGATACTGAGTTTAGAAGTATCAATAAGATTTACAGTAATTTAAATATC
ATGACGCATATCAATAGTCAAAATAATAATTTTAAACGTAATCCATTTATAAATGAAGAACAATTGAAATCACTA
CTCGCGATAAAGAAAGTAGATGCACATATGAGTTATGATTCAAATATTACTTCAAAAGCATTGGCAGAAATAA
ACAAAACGCAAAAAAGATTAGTTACAAAGATAGACACACTCTATAATCAGTCAAAAAAAGAAAATGAATATG
TACGGTTAGGAGAAAAAATATCGTATACAAAATTGGAGAATCATTGGAAATATTTATATAATGAGATTCAGTTT
TATAATCTTAATAATCAATTGATTAGTTATGTTGCATTAGAACAGGAATATGCTTGGGCTTTTTTAAATGAACTA
TTTTATTTGATAGAATTGTATTTGAAGGCATTTAAAGGACAAAAGAAAACAGATTATGAGTTTGGGAAAAAA
TTAAACGAATTTATTCAATCATATGTCATTATTTTACTTATAGATATACGATTACCTGTTGTTAGATTATATATTATT
AGAGACATTGTAGATACATGTGAAGGAGAAAAAGATAATTACAAAAGAATTACACTGATAGAAGAATCTATT
AAAAAGTATAGATACGTAAAAGATCAGATTAAAAGATTTAAAAATGGATTAGAAGAATCGTTATTAAATGCAA
CTTTATCTATTGAACAAAATATGTTGAAAGTTAAAATTGATTATTATAAAGCCTACTGCTTTCCTAGAGAAAAA
ACAAAGCATAACAATATTGAGTACAATGTATCGTTGTTTTTTAAAGCATTAGATGCCTTAAAGAAATAGTTTTT
CACTGAAAATATTTATAGATATAATTAAAATTAATTAATATTAAGCCAGGATAATGTAGTCTTAATCGTTCTGAAA
TACGAAAAATGTTGTGGAAATCGCGTGAAATAATCAGAGGAATCGTTTGAAATCATCGTAGTAATCGCGCGA
AAATCACGTGAAATAATCAGAGGAATCGTTTGAAATCATCGTAGTAATCGCGCGAAAATCACGTGAAATAATC
AGAGGAATCGTTTGAAATCATCGTAGTAATCGCGCGAAAATCACGTGAAATAATCAGAGGAATCGTTTGAAA
TCATCGTAGTAATCGCGCGAAAATCACGTGAAATAATCAGAGGAATCGTTTGAAATCATCGTAGTAATCGCGC
GAAAATCACGTGAAATAATCAGAGGAATCGTTTGAAATCATCGTAGTAATCGCGCGAAAATCACGTGAAATA
ATCAGAGGAATCGTTTGAAATCATCGTAGTAATCGCGCGAAAATCGCGTGAAATAATCAGAGGAATCGTTTG
AAATCATCGTAGTAATCGCGCGAAAATCGCGTGAAATAATCAGAGGAATCGTTTGAAATCATCGTAGTAATCG
CGCGAAAATCGCGTGAAATACTATGGTAGACGGTTTGAGTAAATTAATGGAGTATTTTAATATTTATGGTTTG
GAGGAATTGCGTTGCATAGAGAAAGTAAATATATAGAGTATAAGAAATCACGAAAAGGATTATCTAATGATAT
TTGGTCTACGTATAGTGCTTTTGCAAATACTGAAGGTGGTACTATATATTTAGGAATTGAAGAAAAAAAGATC
GAGGACAAAAAAGTCTTTGTTTCAGTTGGTGTTGAAGATCCAGAGAAAATGATTGAAGATTTTTGGAATGC
ACTATATGGAAGAAGTAAAGTTAGTCAAAATATTTTATCAAATAAAGATGTTAAAATTGTTAATATTGAAAATA
AAGCGTGCATTGAAATTCATGTACCAGAAGCGCCTTATTCGAAGAAACCGATATATGTAGATAATAAAAAAGA
TTTAGTATATAAAAGAGTTGATGATGCTGATAGAATTGCGACTGAAGAAGAGTATAAATTCATGATTGTAAATT
CTCAAGACGATATAGATACAGAATTATTAGATAACTATGACATGTCTGATTTAAATCACGAATCTATCGAAAATT
ATAGGAAACTTCTATTAAAAAATACTAATGATGAGAGATATGCGAATATGAGCCAACTGGATTTAATGATAGAT

TTAGGAGCATATAGAAAAGATAGAAGTTCGAAAGACAAACAGTATAAAATGACTACAGCATGTTTATTATTCT
TTGGTAAGTATAATGCGATTAGTGATAGATTCCCAGGATTTCAATTAGATTATTTTAAGAAAACAAATTACCTA
GATACTGATTGGAAAGATAGAATATCAAGTGGAGATTTAGGTAATGAAGATTTAAACGTGTATAGTTTTTTTG
AAAAAGTATTGATAAAATTAACTGATAACATTGAGGAATCATTTAGCCTAAATGATGGTTTGACTAGACAAAA
TTATGCAAGAGATTTAAAAGTAGCAATTCGCGAAGCACTGGTTAATACATTAATGCATGCGTATTATGATACTA
AGCAAAGTATTAAAATAGTTAATTGTGAAGATTTTATAGAGTTTTATAATCCGGGTAATATGAGAATAAATAAA
GAAGATTTTATTCATGGAGGGCATTCAAAGGACAGAAATAGTATATTATCGACGCTTTTCAGAAGAGTAGGA
TATTCAGAAAAAGCTGGATCTGGAGGACCAAGGATATTCGATGTAGTTAATAGACATAAGCTTAAAACGCCT
GAAATAGAATTAACGGACATGGACACTAATGTAGTACTTTGGAAACAAGATTTAATGAAGGAGTTTGAAAAA
TATCCTGAGTTAGACAAAAAAGTAATAAAGTATATTATTGACTATGGATCAATAAGTAAGGGTGAAGCCTTAA
AAATGGAAAATATGACAGAATATCAGTTTAGAAATATTTTAAAAAAACTAAAAGATGATAACTTGATAAAAAA
AGAAGGTGAAGGTCCGGCTACTAAATATGTGTTAATAGAATCAAAAGAAGCTGATATATTGCGAACTAAAAA
AGTAATTAAAAGTTTAGAGTCTTTCTTTAGGAATAAATAAAAAAACAAGAGATAGGTGCGAAGTGTTTGGAT
TATACAAATGCTTCGCATCTTATATTTATTAATAAATATCATAGAAAACCGTATCATTAACCGATACGCAGAGATG
CGGTTTTTTAGACACTTCATAAAGGGATTTTGAACGTATCAGAACATATGAGGTTTATAGGAATTGCTGTTAT
GTTTTTTGATCACATCAATAAACAAAAAAGGTATGTACTATGTAAAATATTTATTAAATGATATAAAGCGAGGG
TATATAAATGATTTTTAAATAGATATTTATCCAATAATATAAAAAGGAACTATAAGCTATATCTAAAAGCTATATCT
AACTACTTATAGTCCTTCTTCATTAGTATAAATATAATTATTAAGAAAGGTATATATCTTTGTAACCTTCGTTTACA
TTAATAATGTTGTGATAACCTTTGTGTTCTAAAATACCAATAGCTATAGAACTTCTAATGCCAGACTGACAGTCT
ACATAAATAACATCGTTTCTATTGAAAGGTAAATCTGTTTCTAAAAGTTTGCCGTGTGGCACATGAACCGCTT
GGGATAAGTGGCCATTATTCCATTCATTATCATTACGTACATCTAAGACATGTGCTTCATTACCAGTTATGTCTT
TACTATGAACAGATTGTGTTTGAATTTGAGCTTGTGGTAACTGATATCCAGACACATTATCATATCCAATAAGTT
GTAAAGTATGTTATGTGTTGCTTTTGAAACAAGGTGATAGTCTCCAATCAAGTTAATTTCTTGATTATAGTTTA
GATACCAGCCAATTTGATTGATGAAATTTTTATCATATGGAATATTGATTGTACCTTCAATATGTCCACCATGATA
AGCCTCCTTACTGCGGAGATCAAAAGTTAATCTGTTTGTACTTGTAGCTGGATAAACCGTATAAGGTTGATATA
AATTCATACCGAATTGATTAATTTTTTTCATTTGTGCAAAATGATGTGGTGGTGCAGGTTGGTCAGAAATGAG
TTTATCGATAAAGGTAGCTTCATTATTTTCAGAAAAAGCCCAGTTCGTTTGTTTTTCATAGCCAAGCGTAGATG
TTGGAATAGCACCTAAAGATTTACCACAAGGACTACCAGCGCCATGACCAGGCCAAATTTGAATGTAGTCTG
GCAAGTCTTTAATACTTTCAATAGATTTAAACATTTGTTTTGCGCCTATTTCAGATAATCCTTCTACTTTAACAG
CTTTTTCTAGTAAATCAGGTCTACCGATATCTCCTACAAAAATAAAATCACCACTGAATAGTCCCATTGGAACT
TGTGCTCCAGCACCTTCGTCAGTAAGTAAAAAACTTATACTTTCTGGCGTGTGACCAGGTGTATGAAGCACTT
TTAATTTTATATTTCCTACATAAATATCATCATTATGTTGAACAAAATGAGTGTGGTTAGGCATATTTTTATAACC
TAACATGTCATCACTTTCGCCCGATACATAAATACTAGCATTTAACTTTATAGCAACATCTCTAATTCCTGAAAC
AAAATTTGCATGTATATGTGTTTCAGCTGCATGAGTAATGGTTAAACTCTCTTCATCGGCAACTCGAATATATG
AAGATAAGTCACAAATAGGATCAATGATCATGGCTTCTCCAGTTTTTTGACAACCGATTAAATAAGATGCTTG
AGATAAATGTTTATCATAAATTGATGATAGTCAATTTGTTATCAAGGGTGATAATTATATTAACTATGGTAATTAC
ATGGAATTAAAGTTATTAAACAAACTTGATGATGACCAATTAGATTTAGAATTTTTACAAGATAATGGGTTAAA
AGGTAATGTTCAAGGTCCTATGACTTTAAAGGAACCTTTAAAAAGTTATATACAAAAGTCTTGAAAACGAATA
TTTTAAATTTAGATAATGAGTTAGAAAACACTGAATATGTCGAAGGTAAACCATATGTACAGTACGGTATTAAA
TATGAAAGAAATCAGGCATTAAGAAATGAAGCTATTAAAATTCATGGAACTACATGTAAAGTATGTGGATTTG
ATTTTAAAGCTAAGTATGGCGATTTAGGTGAGGGTTTTATTGAAATTCATCATTTAAAACCAATGTTTTCAATA
AAAAGAGAAATAAAAGTAAATCCACAAAAAGATTTAGTCCCACTATGTTCTAATTGCCATAAAATGATTCATA
GAAATACTAAAAAACCTTTAACGATTAAAGAATTAACCAAAATAGTTAATTATAATAGCAAATAATTTAATATTT
TATAAACTATCATTCAACCCTCTTAATTTATTAGGAGGTTTTTTGTATTTATGCTTTCAAATGTGTGATATACTTT

GTTTGTTGAAATATAGAGTATCTATAGATAGGGTGATTGAGTATGAAATTCACTGAAGTGGAAGTTATCGAAC
ATCTTGTAAAGGCATATAAAGAAGCAGGAAAGCCTACTTATCCTCATGAAAATTTATATCGAGGACGTAATCAT
AGTATTTCAGGTATTGGAGAAGACTTGCTGGGTGCTTATTTGATTAGTAGATTGGAAGGTGTCCAAATATTTA
TTGATCAGCCTTTATCTATGATTGATAAATCTTTAAGTACAAGATATCCGGATTTATTAATTTGTGAAGATAATG
AAATTAAAAATATACTAGAAGTTAAAATGGACTTAGGATATCAAAGAAAAGATTTTATAGATTATTGCCGAAA
GAAAGAAGAATGGATTTCAAATATCGTAGGAAAACAGTGTGTATTGTCTAGAAAGAGAGAAGACAAAATTC
CTATGAATATAGCTGATGATATTAAATTTCATGTTGTGATTTACAGTGAAAACAATGGACCGAAGCGGTTTGAT
GAAGAAATCATGCCTATCGTTAATGAAACATGTCCACATATTGAAGTATATGTCCTAACAAGCGGTCAACACCC
TAATTTAGTAAATGTTAATCTTGAAGGTATTAATATTAATAAAGATGAATTTGAAATATTAGTAAATGCGTTATAA
AAAAATAGAGCATCCTCCACGTTATGGAGGTGCTCTGTTTTTTATTGAAAAGTATCAAGTTAATTAATTTAATA
TGCTTAATAAGTTCTACCTTGACCTTTTTCTCTAGCTTCTGTTCGATCTCTTATGTACTCAGTACATACTGGATTT
TCTGTTAATTCATCAACTGTATTAGTTTAATCAGAAGACGTGTCTACTTTGTAAGCTTCTAAGAACTTATTATAT
GATATAGCGTTTGAGTTTTGTTGTTCTTCTATAAATTTCTGTAGTTATTTTTCAAAAACCGCATCATTAACTGAT
AAGCAGAAGCGTATCACAAATAAAACTAAAAAATAGATTGTGTATAATATAAAAGGAAGGGATTTATATTAAA
ATTTTGAATTCAAAAATTATTGAAAGGGAAGCTACCTTAGAAATTGAATCTATGGCCACTAACTGCTTTTTTAT
ATCACCACTTATTAAAAAATGTAAGAATATTCCTCAGTGCTAAGTGAAGGAGGTTCTAGTTTTATTTAATATTC
ATATTGATAGGTAATGGTTAATAATTGCCTGTAAATTATTGTTAAATATCGTTGTTATAGTATCATCATTCATAAA
ATTTTTATAGATTTTATCAAGTATTTCCTCATCTTCAATAGCTGTAAAGCGATGTACTAGTCTTTTTATGAGCTAT
ATATAATTTTGCGAAGCTAAACTGTTTGTAATTATTCACCAATCAGGTTATGTAGTGTCACGGTTCATAGGTAAT
TCTCTTTTTAAAATCATCTAATATTTTATATAATTCATATAAGTGTAGTATTAACGTTTTCTACTTTATCTATAAAAT
ATTTAGAATTACATTAGAATTTTAAATTATACGGCTGGAAATTCAATGCTGTAAAAATAGACAGATTTTAAAAA
AGTAGTAATAAAAAGTCTAAGTTTTATCTTTGTAATAAACTTCGACTATATTTAAAAATAAATATGAATGTCTTT
TAATTTCTATAATATCAAAGCTATTATATGATTTGAAATTTTGTAACTTTTTCTCTAATTTGGAAGTAGATAATGA
CTTAAAGGTTTTAACTTTTACCCAACGACTTGGCATTTTATCACTCCTATGTAATGTATTACTTCAATATACCCTT
AAATTTTTATAATTAATTATATTGTAATATTATTTTATTTTATAAAAATAGTTTAATAAAATGTTTAATTTTTATAAA
AAATAATTAAATATTATAGACATTACGATAGTTTAATTAGAAAATATATTTAAGAATTATGAATTTAGTTCTTATTA
ATGTTAAAGATCATCAATTATTTTGTCACATTATCTTGAGAGGATTACCTTATGAGGTAGCCTTTTATTTGTCAC
CTAATATTGCTCTAAGGCTAATAATACATATATAAAATAAATAATTATCAGAATATATTTAGAAATTGATATTTAAT
AATTAAATAAAAGTATGCTAAATATTTATAATTTACAAGTGAGCTGATAGAAAGATGAGTATAAGCGAATATAA
AAATAGATACTTAGAGTCACTATATAACACCTTTAAAACTGAGTTAGAAAACGAACGCACTCGCTATTTTATAT
TAGATACCAACTATACAAATAGTGATGATACTGAAAAGGAGTTTACTTGGGATGTAAAACAGAATAATAAGAT
CAGAGAAGGCGATTTGTTCATTTTTAGACGGCCAACTAATTTATCTCAAATTGCTCAACAATTTTATTTCTTTG
GAGCTGGGAAAATAGAAAAAAATTGAGCGAAAAAATAATGTAGCAACCGCTTACATTTCAAAACCCTTACTTT
TTGTGGATCGTGTTTTAAAAGATAATATTGAAAATTTAAAATGGGAGTTCAAAGAAAGAATTAAAGAAGAGT
GGGAGCAGTTTTTCTTTAAAAATAAAATCAGTAATATTACAAAAAATGATTTTTTAAAACTACTAAGTTTGAGT
AGAAATGTAGTGGAAATAGAAAGTAATTATATTAATAATGATATTTCGCTAACTTTTAAACAAATGGCTGATAT
GGCAACTCGAATAAAGAAAAATATTTACTACGTAGGGAATCATGAAGCATTTGTTCAAGCTAAGGGAAGTGC
TCATTTTGAGTTTTCAAAAAGGATTAAACAAAATTATAGTTATAGATGTGCAATTACCGGGATTAAAACTAAA
GACTTTTTAGTCGTTACACATATTATTCCTTGGCATGAAAATGAGTTTATTAGACTAGATCCTTCAAATGGTATT
TGTTTGTCTTTATTTTTAGCAAAAGCATTTAAGAAAGGCTTTATCACTTTTTCTAACAGTTATAGAGTTGTTTTA
TCTAAAGAAGCAGAGAAAGATGCTGCTTTATATGAAGAATTAAAGATTTATGAAAATCAAAAAATCGAACTA
CCAGATTGTCAGAAACCTAATTTGAAATATTTAGATTGGCATAGAGAACATATTTTTAAAAATTAATTTTGGGA
GAGGTTGTATGACAGATATTAGTCAATTAGTAGAGGTGCCATACATCTTTCATTAGTAGATTGAAAAAGAGCA
CTTGAAACAGATAATTTGTATTATTTAGCCTATGGATTAACAACTTTAGATAATTGTTTTAAATATTACCAATTAA

TTACACAAAATGAACATTTTTGCTATTCTCAACAAGAAGAAAGAGAAAGTTATCAGCAAAAATTTCAAAGTAT
GATTAATGGTTTAAAAATTAAAATTGGCATACAAAGTTCAAATGATGATGCGCAATAAGCTACAAAGTATGAT
TTTTTAATTGAACGTTATATGTTTTTAAAAGAAGAAAGAGCTTCGAATTAATATCTTAATGGAGGAAGATTAGT
AAATGGCTAGAACGTATAATGTGATTAAATATGTCATTAAGAAAAATAAGGATGATCTTAAAATACATGGATTA
AGATTATATGAATTTAATATAAAAGATGAAAAAGCTATAAATATTAGCCAACCCATTTTTGTGAACAGAGCAAC
ATTGATTGAGAAAATACAAAATGGTGAAGCATTTACGCACGCCTTTAGAGTAAATCACAATACTTATTATGCA
GGCCAATTAATTTCTTTATCATTGAATAAAAATGGTTATATTAATAACAACTTTGAGGCCTCACGAGATATTATT
AGAAACATTGAAGTAAGTATTTTAGACTATGAATAAAGGTAATACTGATAGAATACATTACAATTAAAAAAGG
GAGTATAGGACTCCCTTAATAAGTTGATATATAAGTGACTTATTTTTTTAATAGAATTTTAATTCCTCTGTTAATG
TTCAAGATATATAATATTAAAGCAATTAAAAAGAATATAATAGTAATAATTAAAAGAATGCTAAATATTAATAATT
GATGATCCAAAGGTTTATCAAAGACTTCTTTAGAAAAAATAAATGCCAATCTACCTATAAAAAAACTAACCCA
AGTCATAATATGATTAATGAATGCTTTTTTGCCATGTTCTGATGTTGGTTCATCAGCAAGTATCCATACAAATAA
AGGTAAAATGACAGGTGCGAAAAAGATACTTAAATAACTCAATGCTGATAAAACGTAACTATTGTCATTTTTA
TGCATATTACTATTTGTATTTTCCACTTTTTTCACCTTCTTTACTTTTCTTTACTATTATAAAAACATTAATAGCGA
GCTATTACAACAGTTATAAGAGAAGCTTACGATATTGTAAGTATGTGTTGTTATAATCAATATAAAATTAAGAAA
GACCTGAGAATTTATATTATCATCCACAATCGAAGTGAGTAGCGAAATTTATTGGAAAAGGTACTTATATTGAA
GATATTGTTAATGGTTCATTTTTAAAAAGCCAAGACGATTTGAAGTTAAAAGCTTCTGACTTCACTGTCGAAG
ATAACTATAACTACGGTGTACTTATATTTCCAGAGAATCTTTCTCTTACAGATAATAATAAGAAATTGGAATCTT
TAATAAAAACTATACTATTTACAGGTAAACGATATCAATATACTTATTTTAAAATTTGATCTTAATAGATAAGGAT
ATAATGATTTAAACAATTTAAGAGATATTATTTTCACAAAAAAAGAATGCTTTTTGTTAGAGGCACTGAACCCT
TAAAATGGGAACTTAATAAAAACACTATGTTCTAGGCTGCTAATTCTCTGTATTTTACAGGGGATAAGTAGCCT
AGTTTTTGTTGAATTCGATTATTATTATAGTTTTTAATGTATTTTCGACAATATCTATTACAATATGATTAGAGTTA
TTAAGCTGATTATTGATGTAAAAAGTTTCAGACTTTAGCGAGGAATGGAAACTTTCTATCGGGGCGTTATCGG
CAGGTGTTCCCTTTCGGGACATACTTCTGATAATGCCTTTTTCTTCGCATAATTGATAATAAGCATAAGATGTAT
AAACGCTGCCTTGATCACTATGTAATATACACCCCTCAGGTATATCGATTTGATTTAATGTATCATTAACTAAAC
GTTGGTCTTGTTTATCATCTATTTTATACGCCACAATTTCTCCGTTATAAATGTCCATTATCGAAGATAAATACAA
CATAGAATGACCAAATGGTAAATAAGTAATATCGGTTGTTAATACTTCCATGGGACAACTCGCTTTAAATTGTC
TTTGTAATAAATTGTCCGTTTTATAATACGGTTTACCTATTCTTGTCGTCTTTTTAGGTCTAACTCGGCAGTTCA
AATGATGCTTCTGCATCACTCTCTGTACTCTCTTATGATTAATTGGTGATGTATAACATTGATTAATCAATGCTGT
AATCTTACGATAACCGTAGGTATATTGTTTACCAACATGTTGTGAAAATCTATAACTTTCCCCATTTCGATACCA
TCGCCACCAATTTTTCACTTGTGTTCTATTTCTAATATTTAATTCTTTCATAATTTCTTTTGTTGGAAATCCTGCT
GCTTTCATTTCAACTGCTTTATACTTTGTTTCTACTGAATAAGAAGCTCTTTTCATAGAAAAAAAACACCTCCG
TATGATTCATTTTAATATGAATTCAACGAAAGTGTTTTTATATAATTCCCACTATTGGGGTCAGTCTAAAATTAA
AATTGATAACTTTTTTAATAAAATAGATACACTAATTTTAAAACAAGGTAAAAAAATAGAATTATTAAAACAAC
GCAAACAAGGCTTACTACAAAAAATGTTTGTGTAATTCTGATAACGTCCTATTATGTTAATTGAAAATAGACAA
AAATAAATTGAGAGCATCCCTTACCGCAAAGTGAAGGATGCTCTATTTTTGTTGAAATTAATATAAGCTTCATT
CTGATAGATAGTCATTAATAATTGTTTGCAAATCGTTATTAAGTACCGTTGTTACAGCGTCATCATTCATAAAAT
CCTCGTAGATTTTATCAAGAATTTATTCCTCTTACAGACATTCGCGAGAAGTCCGTTTTAATTTTATTAGAAGTA
ATTCAGGTTTGAACCTACCTAAATGAATATATGAGTTATCTTTTATACTACAAAATATATTCAGATTTCAATAATG
ACATAAAATAGGCATCTTTATATTTACCTTTAGTGTAGAATTGCTCTTTAAGTAATCCTTCTGTTTTAAATCCTTG
TGACTCGTATATATGAATAGCTTTTTTGTTATCTGCATCAACATATAGATAGATCTTGTGCATATTTAAAATATTG
AATGCATAAATTATCGCTTTTTCGAATGCGAATTTTGCATAACCTTTACCACTGAACTCAGGTTTAATAATTATT
TGTATTTCACAATTACGATGGATGTAATTAATTTCTACTAATTCAACAATACCTACGACTTGATTTTCATCTTCAA
CAATAAAACGTCTTTCCGATTCATCTAATAAATGCTTATCAAATAAATGTTGAAGTTCCGTTAAGGATTCATAA

GGCTCTTCAAACCAATAAGACATAATAGAATATTCATTATTTAATTCATGAACAAAAAGTAAATCACTATACTCT
AATGCTCTTAGTTTCATAATCCCACTCCCAAAATTTTCTTATATATTTGCATTATAAATATAAATAACGAATAAGT
CATTATTCACTATGAATAAATCTATTTTAACAATTCACTACATACTAATTCTCATTTTCTTATTATTCTCTAATATCT
CTGATTTATTACTCAGTGAAAGATGCCCTATTTTTATCAGTATATAGTTTTTATTTTGATAGATATTGATTAATAAT
CGTCTGTAAATCATTGTTAAGTAAGGTTGTGATAGCGTCGTTATTCATAAAATCTTCATAAATTTTATCAAGTAT
TTCTTTAATATATTTTTTAATCTTCACACCTTAAATTCACTTGAAAACCAAATCACTTCTAAATTATGATTTTACAT
AATAAATTTTTAAAAATTGGTATTATATAAAATAATCTCAATAATCAGAATTTTTTGTAAATAATAATATTAAAGT
GTAAAATAACAGATAAGAATAGACTTAATGTATATAAATTAAAGGAGTGTTGGAAGTGAAAACTAGAGCAGC
AGTTTTACATGAAATGGGGGCTAAAAAGCCCTATAAAGAATCTAAACCTTTAAAAATTGAATACTTAGAACTT
GATAATCCTAGCGAACACGAAGTATTAATTAAAATTCATGCAGCTGGATTATGTCACTCTGATTTGTCAGTAAT
TAATGGTAACAGACCTAGACCTTTACCTATGGCACTTGGTCATGAAGCTTCCGGTGAAGTAATTAAAGTTGGA
AAAGCTGTTACAAGAGTTAGCGAAGGAGATCATGTGGTATGCACATTTATTCCCAGTTGTGGAAAATGTATCC
CATGTAAAGAAGGACGTCCTGCATTATGTGAAAACGGAGCAATATCTAATGAAAAAGGCGAAATGTTAGAG
GGAGGGATGCGTTTATCTAATGATGAAGGAAAAGTATATCATCACTTGGGGATATCAGGTTTTGCTGAATATT
CTGTTGTCTCTGAAAACTCTATAGTTAAAATTGATAAAAAAAATACCTTTTGAACGTGCAGCTGCATTTGGTTGT
GCTATTATCACAGGTATCGGTGCTGTGGTGAATACAGCCCAAATTCGTTCTGGTAGTAATGTAGCGGTTGTTG
GTTTAGGAGGTATTGGATTGAATGCTATTATTGGAGCTAAACTAGCGGGAGCCAATGAAATTATTGCTTTAGA
TATTAACGAAGATAAATTTGAATTAGCAAAGCAATTTGGGGCTACAGCAACATTTAATTCAAGCGATAAAGAT
ATCGATGAGCAAATTAAAGAATATATTCCTGGCGGAGTAGAATATGCTTTTGAAACAGCGGGTGTAGTGCCA
GCTATGAAAGTTGCTTATCAGATTACTAAACGAGGGGGGACAACTGTAACAACAGGACTGCCTAATCCTAAA
GATAATTTTTCTTTCCCTCAAGTTACTTTAGCGGCTGAAGAACGTACCATTAAAGGATCATATGTAGGAAGTT
GTGTACCTGACAGAGATATACCAAGGTTCGTTAATCTATACAACCAAGGACGTTTAAATATTGATTCACTCATC
AGTGAGGTTATCACTTTAGATGAAATTAATGAGGGATTTGATCGTTTGTCTAATGGTGAAGTAGGTAGAATTA
TAATGAAGATGCATTGAATATAATAGAATTCAAGTCGTTCCTCCTCTTGATTTCTATGAAAAGAATAACAATTT
GAGTAGGAAGATTACTGACAAATATTACATTTGCATTGTGACTGCCAATCTAATTGTATTTTTACATTTCATATC
TATATATTAGAAGATAAATGTTTTAAAACTAATTACTAATAGTTTTTTAGATAAAAAAAGATATAGGAGCATCC
TTCACCGCAAAAGTGAAGGATGCTCTAGTTTTATTAGAATATAGTTTTCATTTTGATAGATAATGATTAATAATT
GTTTGTAAATCATTGTTAAGTACCGTTGTAATAGAGTCATCATTTATAAACTCTTCATAGATTTTATCAAGTATTT
CCTCGTCATCGATGGCTGTGAAATGATGCACCAGACCTTTTACTATTGAAATGTAATTTTGTGATGCTAAACAA
CTAGCAACCGTTCGCCAATTGGATTGTATAGTGTTGTCATGGTTCATAGATAATACTCCTTTACTTTAGTGTCCA
TTTTTGACGTGCTTTAGGGTTGAGTGGATGCATAACTTCGTTCGTTACTGGATTTATGAGCTTTTTTACTTTCT
TTTTATGAGGTTTTAACATTTCCATCACTTGTTCGACACGGTCGATAACAACTGGTCGCTTCGCATAGGCACC
ATAAGCAAGAATGACTGTGTCACTTTCACTAATTGCTTTCATTAAGTGTATATCTGTGTGCTCATCATAAGGCTC
TTTGATATGTTTAAGGTTCTCTGGTGTTTTAATATTAGAGAAAAGATTAACGAGATAAACAGCGCCATATTGCT
CAGAATTAGCTAATTGATTGAGGATGAGAACCGTAGTAAGATCGAGTGATAATACACCATCTAAATGAAGATA
CATCGTTATCACAGTACAAGCCGGTTTCTTGTCATCCCATATCTTTTTGAGTAAGTAGCGATGTTGTTCATCATC
GCTAAATATCGCTTCTGTGTGTATCGTACTTTTGATTGTATTCATATATCGTCACTTCCTTTAGTATTCTTCTGGC
AAAAGCATCACATAATAAAAAGCGTCTACATCATCTTCTCGGATGACGTAGACTTTCTTAGGTAATGCATTTTG
ATTTTTTATATAGTTTGTATAGTGATATTCCAATTTGTATGCGGGTTGTTCTTGTTCATGTGTAATTGACAACATA
TTATCATCTTCTTGTAGTTTGAAAATGTGTAGGTAATCTGTATTAAGTTGATTGTCTCGATCTTTTACCATGTTCC
AAAGTAAGATTTGAAGGTCTAGAGATAGTTGTTCACTAATGCCTCTTGTGATGTATCGATTGATTTTCATACTA
TTTTCCTCCATTTTGCTTTTCTTTCATGATGTCAATCACTTCGTTAATGACTGTAACAGATATTTGTGCCACTTT
GATCCAATTATTCATGGTTATTCCCTCCTTCTTTTAGTAAATGACGTTCATCGATAATCGTATTTTTAGTATCTGT
GAGGTATAGAAAGTCCATATCAAAATGATCCAGATAACCAATACTGATGAGTTGGTTATTAGAATACATTAGAA

ATGGATAGATACTTAGTTCATGTAGCTCATTATTATAGTAGGTATAAGTTTCAAGTATAAGATGTGCAAGTGGG
GAATCATTAATAAAACGTTTGGGTAGAATATTTTCTGCCGCTTCCTCCAGTGTTTCACATTCCCATGTTTCATTG
TTAGATAATTGGAATAAGCGAGTCATATATTGTTTGAGTTCTTGAGTAGTTGTTTTCATATCATTGCCTCCCTAG
ATAGTGTGATAGTGATGTACTTCATATACATCATTGAGATAATATATATTTGATTTGTCATTTATTACTGATCCCGT
TGACAATATGAGAAAATTCCATATAAAAAACCGCTACAAACCTTGGTATGACAAGGAAATCCCGAAATTCCGC
CTATTTTGACGAACAATCAACTCATTATTTATAAGTATTGATGATAGGGTGGGGTCTCTGCTTCCTTATATATATT
ATTTATTTATAAAGAATAACGGGATTTTGGGATTGTGCTTGCACAATCCTTCTGCTTCTTCTAATCTGCAAATCC
CATTCCTTTCCCGATAAAAAATCATCGTGGGATGTTCTTTAGCAATTTCAATATAAGCCTCGTGTAGTTATGAA
AAAATTACGACAATGACTGTTTCATTAGATAAGTGTTATTGAAATTGATAAAGAGAATTCTCAAAAATTTTTAG
AAAAAGAAAGAGGAAGTATGTAGATATAAAATAGATTTATTAAATAGTAAGGTATAATGTTTGCGGTAAAAAT
AAAGACGAAGTGCTGGAATACACTTCGTCGATAATAAGGTAGTTGAAGTTTCGATTATTTTAAATTGATCATT
TTAAGCCTACAAATCCCTCTAATGTTTCACCAGATTTGTACACTTTTGTCGCCTTCTTTTTCCTAAATAACGTAA
ATGCCAAGGTTCATATTGATATCCTGTGATGCTTTCTTTGTTTTTAGGATATCTTATAATAAATCCATAATTATGG
GCATTCTTAGCTATCCAGCGTCCTTCCTCAGTTTTACCAAAACTAGCATATAGATTTTCACAACAAGTTGGTAA
ACAATATACCTACGGTAAAGGATTAGAAGAGCTGTCAGAAGTAGAACAATTAAAATTAGAAAATAAGAGAAA
AGATATAGAATTGGATATTTTAAAAAAGTACAAGGTATTGGAAAGGAAGTGGTACCAACAGTAGTCATAGAT
TTAGTGGATCAATTAAAAGTAAAATATTCAATCAAATTGCTACTAAAAGTATTAAACATACCTAAATCAACATAT
TACCGATGGAAAAACAAAACCCATAAAAATGATACCGTAACACAAAAAGTTATTGAATTATGTAAAGCTAACC
ACTATACCTACGGTTATCGTAAGATTACAGCATTGATTAATCAATGTTATACATCACCAATTAATCATAAGAGAG
TACAGAGAATGATGCAGAAGCATCATTTGAACTGCCGAGTTAGACCTAAAAAGATGACAAAAATAGGTAAA
CCGTATTATAAAACGGACAATTTATTACAAAGACAATTTAAAGCGAGTTGTCCAATGGAAGTATTAACAACCG
ATATTACTTATTTACCATTTGGTCATTCTATGTTGTATTTATCTTCGATAATGGATATTTATAACGGAGAAATTGT
GGCGTATAAAATAGATGATAAACAAGACCAAAGTTTAGTTAATGATACATTAAATCAAATCGATATACCTGAGG
GTTGTATATTACATAGTGATCAAGGCAGCGTTTATACATCTTATGCTTATTATCAATTGTGCGAAGAAAAAGGC
ATTATCAGAAGTATGTCCCGAAAGGGAACACCTGCCGATAACGCCCCGATAGAAAGTTTCCATTCCTCGCTAA
AGTCTGAAACTTTTTACATCAATAATGAGCTTAATCACTCTAATCACATTGTAATAGATATTGTCGAAAAGTACA
TTAAAAACAATAATAATAATAATAATAATCGAATTCAACAAAAACTAGGCTACTTATCCCCTGTAAAATACAGA
GAATTAATAGCCTAGAACATGGTGTTTTTATTAAGTTCCCGTTTTAAGGGTTCAGTGCCCTAGGATTATAGGCT
CTTTTGTTTATAAAGGTAATTGAACTAAAGTATTATAATTTCAATTCTTAATTAATGTTCCTATTTTACCGTCTAA
AGCGTCCCCTAATCCTGCTAGAGATGTAATCAAAACACTGCCTTTAGTATTTTTTTCAAGAAATTGAAGTGCA
GCTTCAACTTTTGGAAGCATACTTCCTTTAGCAAATTGACCATCAGAGATATGTTTTTCATTTCATCCACAGA
CACTTCATCGAGACCTCTTTGGTTTTCTTTTCCATAGTTAATGTAAACATGGTCCACAGCAGTTAATATGATTA
ATTGATCAGATTGTAAATGTGCTGCTAATAAAGCACTCGTTTTATCTTTATCAATAACTGCATCAACACCTGTAT
AAACTTCATTTTCCTTAATTACTGGAATTCCACCACCGCCGGCAGCGATAACTAGTGTTCCATGAGTGATTAAT
GTTTCTATACTATCTAACTCAACTATACTTATAGGTTGTGGGGAAGGGACAACGCGACGATAGCCGCGTCCAG
AATCTTCTACAAAAGTATAACCTTTTTCTTTCGTAAATTTATCAGCCTGCTCTTTTGTATAAAATAATCCAATTG
GTTTAGTAGGGTTATTGAAAGCAGAATCATCGCTTGCAACTTGAACTTGTGTTACTAGCGTAACAACTTGTTT
ATCTATGCCCATTGAATGAAGTTCGTTTTGTAAACTTTCTTGCATCTGATAGCCAATATAAGCTTGACTCATAGC
ACCACATTCAGGGAAAGGAAAAGGAGGACCTTGTTTGTGTTCCGCCGCATAATTTAAACCTAAGTTAATACT
TCCAACTTGTGGACCATTACCATGACTAATTACAATTTCGTATCCTTTATCGATTAAACTTACTAGAGATTTAGA
TGTACTTTTTAATAAATCTAATTGTTCTTTAGGCGATTGTCCTAAAGCGTTACCACCCAAAGCTACGACGATTT
TAGACATATTTATATCCTCCTTTCATTTACTCTCCTAATGTTGCTACCATGACTGCTTTTATTGTATGTGCTCTATT
TTCTGCTTCTTGGAAAACAACTGATTGTTCACTTTCAAATACTTCATTTGTTACTTCCATTTCAGTTAGACCATA
TTTTTCTTGAATTTGTTTACCGATTATTGTTTCAGTATCATGGAATGATGGTAAGCAATGTTCAAAAATTGTATG

TGGATTACCTGTTTTTTTCATTAATTCTTTAGTTACACGGTATGGTTCTAATAATTTGATACGTTTTTCCCAAACT
TCATCAGGTTCACCCATAGATACCCAAACATCTGTGTAAATTACATCAGATCCTTTGACACCTTCATCAATATCA
TCAGTTATAAGGATTTCACCACCGTTTTTGTCAGCTATATCATTACACCGATTTAATAATTCATCAGTTGGATTT
AGTTCTTTAGGACATACAAGATGGAAAGTCATGCCCATGATTGCTGCTCCTTGCATTAAGGCATTTGCAACGT
TATTACGTCCATCTCCAACATATGTGAAGTTAATTTCATTATATGGTTTTTTCAATACTTCTTTAGCTGTTAAAAA
ATCAGCAAGTACTTGTGTAGGATGATCTTCATCTGTTAAACCATTCCATACGGGAACGCCAGAATATTTTGCC
AAATCCTCAACTACTCTTTGTGAGAATCCACGATATTCTATGCCATCATACATTCCACCTAAAACACGAGCAGT
ATCTTTGGTAGACTCTTTTTTACCCATTTGAGAACCTGTTGGCCCAAGGTATGTTACATGTGCACCTTGATCAT
AAGCCGCTGTTTCAAATGCACATCGAGTGCGTGTTGAATCTTTTTCAAAAAGTAGAGCGATATTTTTACCTTT
CATTTTTTGTTGTTCTATTCCTGCATATTTTGCGCGTTTAAGATCTTCAGATAAATTAAGTAAAAATTCCATTTCT
TTTTGTGTGAAGTCTAACAAAGTTAAAAAGTTTCTATTTCTTAAATTTTTCATTTTGAATATCTCCTTTTCAATT
AATATTTTATTCATGTTATTTTTTTATAATCCAATTTCTTTTATCTTGATATATAATATTTTCTATTTTTAATTCTCTG
ATTATTTCACCAGTAGTTTTTCTGTTAAGTCCAGATAAATTACTTATTAATTGAACAGTCATAATTTGTTTTAATT
CATAGAATTCTCCATTATCATCCCCAATTGAAAGGCATAAAATTTGTAGTATTCTTTCAATTCTTTCTTTAGCTG
AATTAGCTCTTAACGCCATAATATATTCTACTTGAAATTGAATAGTCTCATTAAGTTTCTTGAAGAGACTTTCAA
ATATTTCATTATGCTTTCTACATAAATACTCAAGTAAATCTTTCGGTAAAGTTAATATTTTACAATCTGTCAAAGC
TGTACATATTTCATATGGTGCAGGGGTTTCATTAAATATGAAGTTCATTGGAAATATATTTTCGTCTTTACTTAAT
CTTAAATAATTGTCACCAGTAATATTAGAAGATTCATGTAAAATACAACCATTTACTAAAAAGTATACAAATTTT
ATTTGATCAGTTGAATGATATATGACTTGTCCTTTTTTATATTGATAAAGTGTTAAATCCTCTTTATAAGGTCTAA
CAATACTAACAGGAATATTTAAGTATGATGCTAATTGTTTAAGATTATTATCAAATTCATATTCTGAATTTTTAAT
ATAAATATTTTCTTCATACATAACTATAAACCCCTTAAAAATTACTTAGTTTAAATCATAACTTAAAATACAGAAA
TATTACCTGTAATCAAACGAACTATTCCTATTATTGCAAGTACAATGATGAATATGAATAACGTATAATCCAATTT
TGTCAAATGTTTGTTATTATCCCTTTGTACGTAGCTGTATACGAGTAATCCAGGTATATATAACAACATCGTTAAT
AGTAAATAATCTAATCCAGCTGCATAAACCAACCAAATTGTGTAAATAGATGCAATAATTCCTATTATCCATTGT
TTTAAATTAGCTTTAGATTTATTTTGAATAGTATATTTAACCTGGTAAAAAGCACTGAGTGTATATGGAATTAAG
ATTGCACTTGATGCAAGTGAAAACGCAAACTGATAGGCACTATCTGTAAACAACATACTAATTAAAAATAACT
GAACTAATATATTAGTAATAATTAAAGCGTTGACCGGAGCTTTATTCTTATTTTCTTTAGCAAACCATTTCGGG
AAAAGTCCATCTTTAGCTACAATGAATGGTAATTCACCAGCTAGTAATGTCCATCCTAACCAAGCTCCTAAAAC
AGAGATAATTAAGCCTATATTAACTAACACTGAACCCCAATGACCTACAATATGTTCTAATACTTGTGCCATTGA
TGGATTAGCAAGTTTTGAAATTTGGTTCTGCTGAATGACACCTTGGGCTAGTACAGTCATTAAGAAATAAATG
ACTAGCACAGAAATCAAACCAATAACGGTAGCAGTTCCTACATCCTTTTTAGACTTTGCACGTCCAGAAAAG
ACAACGGCTCCTTCAATCCCTGTGAATACCCATACAGTTACTAACATAGTACTTTTTACTTGTGCCATTGTATCT
CCCCAACTAAAAACGCCAACACTTCCACTAGTCATACCATAAAAACCGGATTTAAAAGTACTGAAGTTGAATA
CAACTATCATGCATATAATAACTAGAAATATAGGTATTAATTTAGCTACTGTAACAATACTATTTATAAACGCTGC
AGTTTCTACACCTCTAAGTATTAAAAAATGTACACCCCATAATAAAATTGATGCTATGATAATACTTGGAAGTGT
GTTACCTCCTTTAAATATAGGGAAAAAGTTACCCACAGCTGACATTAATAGGGTTGCATAAGCCACATTACCT
AGAAATGCTGCAAACCAATATCCCCAAGCACTTGAAAAACCAATAAAATCTCCAAACCCTGTTTGAGCATAA
CTATAAATTCCTCCATCAAGATCTGGTCGCTCATTTGTTAAATTTTGAAATACGAAAGCAAGAGAAATCATACC
AATAGCAGTTATTATCCAACCGATAATTATTGCAAGTCCACCAGCTTGGCCACCCATATCTGAGATGATATTGA
ATGCACCACCGCCTATCATAGAGCCTATGACTAAACCAATTAAGGAAGTTTTACCTAATTTATTTTCATCCATAT
TAATCTCCCCTAATAAAGGTGGTAAAAATCTTGTTATTTGATTTAATGAATGAATTACTTGTGACTTTTACCACC
TTTCAAATTTAAATATCTTCTCTAAATAACGGCTGACTCATACATCTTGGGCCTCCGCGTCCACGTACAAGTTC
ACTACCAGTAATTTCAATCACTTTAATTCCTTTGTCGCGTAAAAGTTGGTTTGATACATAGTTGCGATCGTATGT
CACCACAACCCCTGGTCGAATACATAATGTGTTTGAGCCATCATTCCATTGTTCACGTGCACCATCAATAACGT

CGCCATTACCTGTTGGAATAAAGTCCACTTTTTCTACTTCTAAAACTTCAGCAAGTGTTTCACGTAACTTGCTA
GAACGAGTAATTTTTATATCGTCCTTACCATCATTTTGTTCTATGGTAAATATATTCATATTATTTTCTTCTTTAAA
TATTGCTGCATGTACTGTAAACTTATCGTAGTCAATCATAGTTAGTACTGTATCTAGGTGCATAAATGTACGTGT
ATTAGGTATTTCAATAGCTACGATTTTTTTAAAACTTGTGTTTGCATCTTTGAAAATATTACGTGCTAACTTTTC
TATTGCTTGAGCTGATGTACGTTCTGATATACCAATAGCTAAAACATCTTTCGATAATACTAATTCATCTCCACCT
TCAATATTAAATGGTGAGTTACGATCTAACCATACTGGTACATCTTTATCTTTAAATCTTGGATGATGTTTCAGT
ATATATGTCATAAAAATAGATTCTCTACGTCGTGCTCTCCAATACATTCTGTTAATTGTCATTCCTCTACCAATTG
AAGCTTGGGGATCTCTTGTAAAATAAAGGTTGGGCATTGGATCTAAGTAAAATGGATATCTATCATCCATATAT
TCTACTAAATGGGTTGTTTCAAGTTGAATTTCTTCTTTACGTACGCCAGCCATGATTTTATTTACAAGTTCTTG
GTCAGATAACTTTGAAAAGAATTCTTTAATTTCAGTTTCATGACCTAATATTGTCTTTTTAGATTCTGTTAATAT
GTCGTTTATGAAGTTCTCGCGTACTTCTGGCTCAGTAATAGATTCTGCTGCAAGTTTTTCTAAATAAACTACTT
CGATTCCTTCATCTCTCAAAGTTTGAGCAAATTTGTCATGCTCTTCTTGTGCAACTTTTAAGTAGGGAATATCA
TCGAATAATAAACCACTTAAATGATCAGGTACTAAATTTTCTAATTCTTTTCCTGGTCTTTTTAACAACACAGTT
TTCAATTTGCCTATTTCACTATTTACTTGAATGGGTCCTTGTACCATTTCAAATTCCTCCTTTGCTTTATTACATT
CACATTATAAAACGCTTTCAAAAAACATCGTGTGATTTAATTCACAATTGTTTAGGGAGGGTTTTCACATATAT
AGATTTATTCAATGCAAAATTTTGTATGAATATGTATTTTGTACTATATAATAGTGAATAATTTGAAAAATCTCAT
TGTTAAAACCTTATCTTATTCAAGCAAAAAGATTTTTTACTCATTTTTTTGTATTAGTAATCATTCCTATTAATATA
ATCATTTAATTTTTAAGGGAATAATTGATAACAAACAAATTCTGCTATTTCATTTGAAGAAGTCAAAATCATCA
AAGTATCGTTACCTCCAATAATTCCTAATATTTCTTTCATTTGTAATTGATCTATGTAATAACTTATACTTTGAGCA
AAGCCAGGAGATGTTTTTATTAAGACATAGTTATTTAGCGTTATAAATTCAATAATCTCATCACTAAATATTTCT
AATTGTTTTTTTGCACTTAATTGATTTGTTTGATTTATTTTCTTGTAAATATACTTTTTATTTTCAACAGGGATTT
TGTAAATTTCTAATTCTTGTAAGTCACGAGAAATAGTTGTCAAGCTATAGTAAACTCCAAAATGTCTTGCCATG
TAATCCACTATTTGTTGTTTTTTATTAAACTGATTCTGTTGTATAACAGTTAAGATAAGATTTAAACGTTTTTCTT
TTTTCATTTTTATTACCCCCTTTATTATTTTTGATTATGAAATAGATTTTAAATAACTGTATAAAAATCACTTATAA
ATCTCAATTTACAAATAATTTTGAAATAGTGGATGCTTTAATTATAAGTAGATTATTTTATGCCTAAAAGGAGAG
ATTAATTAGAATAGATTATAGTCATCATAATTATAAAATCAAAATAATTTGCGTAATAATAGCGAAATGTGTAAAT
TGTAAATGTCAGGTAATAACCACAATATCTCATAAAAGAAGTTTGGAAAAAGGCATTGAAATAAAAAGTAGT
TTATAAGTTGAAGATATTTAGGTATGTATTAGAGCGCTTTATAAATAAATAGGTTTATACAAAGAATTACAAAA
ATAATATAAAAAATAGAATCTAATACATGATAAGGAATATATTTGAATAATTTGAATATGAGAATAAGCATACATA
TTTAGGGTATATATAAGTAATAGACATCTAAATAATAAGTAATAGGAGGCTGGTGTAGATGTTCAAAAATATATT
ATTACCCTATGATTTCGAAAATGATTTTAGTGCTATCCCTGACTATTTAGAAAAAGTCACCGATGAAGATTCAG
TTGTTGTAATTTATCACGTTGTAACAGAAAATGATCTTGCAATTAGTGTCAAGTATTATAATAAGCATAAAGAA
GATATTATTAGAGAAAAAGAGAAAAAACTCACTCCATTTTTACGTGAATTAGAAAAAAGAGATATTCAATATA
AAATAGATGTAGATTTTGGGCATATTAAAGATACAATCTTAGAAAAAATTACTTCTGGAGATATAAATAATGGT
GAATTTGATTTAGTAATTATGAGTAATCATAGAGTCGATTTGAATATTAAACATGTTTTAGGAGATGTTACACAT
AAGATTGCTAAAAGAAGTTCTGTCCCAGTACTAATTGTTAAATAAACATAAGAAGTAAGAAATTTATTAATTCA
AAAAGCCTAAATACTTTCTCACAAATCGAGAGAGTATTTAGGCTTTTTTATTTTTTTAATAAACGTAATGAATT
TAATATAACTAATATTGCTGCACCTGTATCACTTAGAACAGCTAACCAAAGTGTTAGTAATCCAGGGAATACTA
ACACAAAGGCAATTAATTTAATAATTATAGCAAAATATAGGTTCTGTTGCAAAGTTGAATCTATAGTATAATTTT
AACAAAAAGGAGTCTTCTGTATGAACTATTTCAGATATAAACAATTTAACAAGGATGTTATCACTGTAGCCGT
TGGCTACTATCTAAGATATGCATTGAGTTATCGTGATATATCTGAAATATTAAGGGAACGTGGTGTAAACGTTC
ATCATTCAACGGTCTACCGTTGGGTTCAAGAATATGCCCCAATTTTATATCAAATTTGGAAGAAAAAGCATAA
AAAGCTAATTACAAATGGCGTATTGATGAGACGTACATCAAAATAAAAGGAAAATGGAGCTATTTATATCGTG
CCATTGATGCAGAGGGATATACATTAGATATTTGGTTGCGTAAGCAACGAGATAATCATTCAGCATATGCGTTT

ATCAAACGTCTCATTAAACAATTTGGTAAACCTCAAAAGGTAATTACAGATCAGGCACCTTCAACGAAGGTA
GCAATGGCTAAAGTAATTAAAGCTTTTAAACTTAAACCTGACTGTTATTGTACATCGAAATATCTGAATAACCT
CATTGAGCAAGATCACTGTCATATTAAAATAAGAAAGACAAGGTATCAAAATATCAATACAGCAAAGAATACT
TTAAAAGGTATTGAATGTATTTACGCTCTATATAAAAAGAACCGCAGGTCTCTTCAGATCTACGGATTTTCGCC
ATGCCACGAAATTAGCATCATGCTAGCAAGTTAAGCGAACACTGACATGATAAATTAGTGGTTAGCTATATTTT
TTTACTTTGCAACAGAACCTATAAAAAACATGGGCTTAAAGGGTTAATTTATATAGTATTACTGCAAACATTGA
TTTGGGTCAAAAATTAGGGGTATTAAAAAAATGAATGAATCATAATTTTATCAAGCTGATTGGAGAGGTTAAA
ATGCATTATATAAAATTTATTGAGTCAAAAGATAATACAAAACTTTATATGAAAGTGAATGATATTCAAGATGCA
AAAGCGAATATCATTATAGCTCATGGTGTGGCAGAACATTTAGATCGTTATGATGAGATAACAGCATATTTAAA
TGAAGCGGGTTTTAGTGTTATTAGATATGATCAAAGAGGGCATGGTCGTTCTGAAGGCAAGCGTGCCTTTTA
TAGCAATAGTAATGAAATTGTCGAAGATTTAGATGCGATAATAAATTATGTGAAGTCAAACTTTGAAGGTAAA
GTTTACTTAATCGGTCATAGTATGGGTGGTTATACAGTCACTTTATATGGAACGAAACATCCAAATACAGTGAA
TGGTATTATAACTTCTGGAGCATTAACACGTTATAATAATAAACTATTTGGCAATCCTGATAGAAACATATCACC
TGATACTTATATAGAAAACAATTTAAGTGAGGGGGTATGTTCTGATTTAGAGGTAATGGAAAAATATAAACTT
GATGATTTGAATGCGAAACAAATCTCTATGGGGCTCGTCTTTTCAATAATGGATGGTGTTAGGTATTTGAAAG
ACAATGCTCAACAATTTACAGATAATATTTTGATATTGCATGGCAAGGAAGATGGGCTAGTAAGCTATGTAGA
TTCTTTACAGCTTTATCAAGAAATAGGATCAGCACATAAATCATTACACATCTATGATCGTTTGGAGCATGAAA
TATTTAATGAAAGTTCTTATAATAGAACTATTTTTAACGAAGTTATTGAATGGCTTGAAACGGAATTAACTTATA
ACTAAAACAGTATAGTTCCGTGTATTTGATTATAAGAAATTATGAGGATATTAAACATACTAAGATTAGCTATGA
AGAAATCTATGACGATAGATTTTTTCATAGCTATTTTTTATAGTTATAGAGAGGAGTAGACTGTCCAGACTCTT
GGATTTTAAATCCGTAAAAAAAACAAGTCAGCTTTACTCTCACCTTTTGAAATTCGTTTGTAGTATGTTGGGT
TCTTGAAACCGTGTATAGGAAAATGAAATGAGAAAGGTTAAGTAAAGTTTTTAGCTTCTCAACTATTCAAAG
GAGGTTTTTTTATCGATTACTTAGGTGTTGATATTAGTAAAAGGAGTAGTGTAGTTGCACATTATAAAAATGG
AAAATTCCAAAAAGAGTTTTTCATCCAAAATAATAAAAATGGCTACAATTATTTACTCAAGTATTTGAATGACT
TAGACCACCCACAACTCATTTTTGAATCTACAGGTATCTATTCAAGAGGTATGGAACGATTTTGTTGTGTAAAT
CAAATTAACTATATTCAAATGAATCCGTTAGAAGCCAAATTTAAAACGAGCGCTCTAAGATCATGGAAAACTG
ATCAGGCAGATGCTCATAAGCTTGCTTGTTTAGGACCGACGCTCAAACAAACAGGCAGCTTACCTATACATG
AGTTAATATTCTTTGAATTAAGAGAACGTGCCCGTTTTCATCTAGAAATCGAGAATGAACAAAATCGACTTAA
ATTTCAGATTCTTGAATTACTCCATCAAACATTCCCTGGTTTAGAAAGATTATTTAGTAGTCGATATTCAATCAT
TGCACTCAACATCGCAGAAATTTTTACTCATCCAGACGTGGTTCTTGATATCGACAAGGATGTACTTATTACAC
ATATATTCAATTCTACAGATAAGGGAATGTCAATGGATAAAGCTACAAAATATGCACTTCAATTAAGAGTGATT
GCTCAAGAAAGCTATCCTAATGTCGATAGACATTCCTTTCTAGTCGAAAAATTACGCTTACTTATTCAACAATT
AAAACAATCTATTCATCATCTCAAACAATTAGATGATGCCATGATTCAATTAGCACAACAACTCGATTATTTTGA
AAATATTCATTCGATACCTGGTATTGGTAAGCTAAGCACAGCTATGATTATTGGGGAGATTGGTGATATTAAGC
GATTTAAATCAAATAAACAACTCAACGCTTTTGTAGGCATTGATATCAAACGATATCAATCAGGTCATACACAC
TGTAGAGATACCATCAACAAGCGTGGTAATAAAAAAGCGAGAAAACTTTTATTTTGGGTGATTATGAATATAA
TAAGAGGGCAGCATCATTATGACAATCATGTCGTCGATTATTACTACAAACTAAGAAAGCAGCCTAATGAGAA
ACCTCATAAGACTGCCATCATTGCTTGTATAAATCGATTATTAAAAACGATTCATTATCTGGTAATGAATCATAA
ATTGTACGATTATCAAATGTCACCACATTAGCCAAACGTACAATTAAATATATTTTAATACCTTATTCAAAAAATT
AAAATGAACGGTTTAGTTAAGTAATGCTTATTTTAATTATAAGTACTTGACTAATCGTAAGAAAGAGCCTAGG
ACATAAATCAATGTCTCGCGACCACAGCTTAATTTTTGGTGTTCATTCATGACTTTAAAAAATCCTTATTGCATA
AATGTACATAGTGTAGTACTATTCAAAACGTAATTATTACGATTTGAATTAAGCGAGGAGAATGAAATGACTAA
GACTTATGACGTTTGGTGGCAAAAAGGTCAAGAATCAGATGATGATATGGCACGAGACCATCAAGAAGCTT
GGGAGAGAACAATAAAAATGCTTGATACATCTGACATCGAAGGGAAAACGATTTTAGATGTGGGATGTAATC

AAGGCGGATTTTTACGACAGTTATACGATACAACACCGTTTAAAAAAGGTGTTGGCATAGATTTAGCACGTT
TATCTTTGGAAAAGGCAGAGACATTAAAAGGACAACGTCCACTTACATACTATTTAACAGATAAACCGCAAG
AAACGAAGCACGTGTTTGATACGGCAGTAAGTACGTCTGTCTTGTACTTAATAGAAGATATTCCGCAACATGC
AAAAGATTTAAAAGAGGTATTGAAACCAGGCGGTGTTTATTACGCTTCATTCGCGGATTTAACTAATAACCCA
AGTCGTCAGTTTATGGATGACACGATTAATCAATATGGTGCAACACCTTCTCAGAATCACTCTCTAAAACATAT
CGTTGATAGCTTTGTGGATGCAGGATTTGAAGTTGCAGTAATGAAAGAGCATGTACCTGACGTGATTGATTT
AACACATTATAGCGATTTTTATTTATCACCGAATGATTATTTACAAACACTATATGAAGAATCGTTTTTAATAAA
AGCAAGTGTGAAAGAAGGTACTGAGAAATGAGGAAATGTGTATTAATGACGGTAGCAGCAAGTGCTACGCT
CTTATTGGCAGGTTGTGGCAATGGTCAAAAAGAAGATAAAGATGTTACGGTATCGCTACCTACTGAAGCAAA
GGCGGATAAACTTGACGCGCAAGGCTATGATGCAGCGATGCCCGTTTATAGTGCAGTGTATGATGCATTAGT
TAAATATGATAAAGATAAGGGTATTAAAGCAGGTTTAGCAGATAAATGGAGCGTTGATGAATCAGGGAAAGT
TTATGAATTCCATTTGAAAAAGAATGTTAAATTCTCAGATGGTTCAGCATTAGATGCTAAGGACGTGAAATTC
TCGATTGATCGTGCGAAAGCGATGAACAAAGATTCGACTGTAGAAACGTTAAAAAAATTAGATAAGGTCGTT
GTTAAAAATGAGCACGTGGTCCAAATTAGATTGAAATCTCCTTCAAATCAAGTGTTAAATGAATTAACACAAG
TGAGACCGTTGCGTATTATGAGTCCACATTCAGTAGAAGATGGTAAAGTAAACGGTAAATTTGAAAAAGCGA
TTGGAACAGGTGCATTTGTTGTTGATAAAACTGGTAAAGAAAAAACGACAATGAAGCCAAATAAATATTTTG
ACAACGGTCACCCAGTCAATTATCATCTTGCATTCCAAACGATTGAAGATGGGGACTCAAGAAATTCTGCAG
TACAAAGTGGTTCTGTAGATATTTCTGGTGGTGCTTTAGGTATGCTCTCAGACGAACAAATCAAACAAGATAA
GAAAAATAAGAACTTAACGATTGAAGATAGACCTAGCACAGTAAGTCACTTTATGGCATTTAACCCTAAAAAT
GATGTATTAAATCAACGCACAATTCGTGAAGCGATAAGTAAGAGCATCGATGCGAAAGACATTGCGGGCAA
ATCTGTAAATGGTCTGTTCCAGAAGAACGTACAATTTGTGACTAAAAATAATCAACAGCCACACGATTATGAT
ATGAAAGCGGCTGAAAGGTTACTTAAATCAGAAGGATATCATAAAAACGATGACGGCATCTTTGAAAAGAAT
GGCAAACCTTTATCATTTAACTTAGTCATTCAAACTGCAGAGTTCCCAAATTGGAAAGATAAAGCTGAAAAA
GTGCAACGTCAGCTTAAACAAGCCGGTATTAAGTTAAATGTGAAAACGTTAGATTCACAATCATACTATGATA
CATTATGGACGAAAAAAGACTATGATTTGATTTTCTATAGAACGTATTCAGATGCATTAATGCCTTACAACTTT
ATAAGTTCAGTGTTTAAAAATAATGATGGTCAACCAGGGGTGTTAGCTGATGATGAAACATTAACGAAACAG
CTAGACGATTTCCCATCAACCGTATCAAAAGAAGACCAACAGTGTTCATTTGATGACATATTTAAACACTTTA
ATCAACAATACTATGGTGTGCCAATTGCTTATCCAAATGAGACGTTTGTAGTGAGTGATAAAGTAAAACAATT
CAAATTCTCTGGACTTACGGATGCACCAATTGATTATAAAGCGTTGAAAGTTAATGAATAGCAATGCTCAAAC
GTACAATTAAATTCATACTTTATTTAATCGTAAGTTCGTTTATTATCTTCATTTTAGTTGAGAAGACATCTGGTAA
TCCAGCGATTCTGTATCTACAACGTCATGGTTATACGTCGATTACGCAAGACAATATTGAAGCGGCACAACAT
CAACTTGGCTTAGGACAACATGTGTTACTAAGATATATCGATTGGGTTGGACATGCACTCACGGGCAACTTA
GGATACGGCTTTAGTACGAACGAAGCAGTTACCGCTATGATAATGGAAGCCATCGTGCCGACGCTTGTGCTA
ATCATTGTCTCTAGTTGTATCATGTTGCCATTTGGCTATATTGTTGGTTACTTCGTTGGGACGCGTCCGCATACA
CGTTACGCTAATGGAATTCGTGGATTCGCCCAAGTGATGACCTCAATGCCAGAATACTGGTTAGCTATTTTATT
CATTTATTATTTAGGCGTACGTTGGCAATTGTTACCATTTGTAGGTAGTGATTCATGGCAACACTTTGTGCTGC
CAATCTTCACAATTGTTGTTATAGAAGGGTGTCATATCTTATTGATGACAGCACATCTGATTACACAAACGTTA
GATCAAGATGCGTATCAACTGGCGCAGTTAAGACATTTTTCGTTAAAAGCGCGTATCATCGTACAAATTAAAG
AGATATTTGCACCACTAATGACGATTTCAATTAACAGTATCATTCATTTAATTGGAAAAGCCGTAATACTAGAA
GTCATCTTCAGCATGTCTGGTATAGGTAAATTGTTGATTAATGCTATTAACCAACGAGATTATCCACTGATTCA
GGGCATTGTCATCTTTATCATTGTCTTTATTATGCTAATGAATTATTTAGGCGATGTGATTATTTTGAAGAATGA
ACCTAGACTTCGACGACGTCATACCCAGCAGTCAGGCAATGAGAAAAGAGGTACGATGTGATGAAAAAATA
TCAAACGTACATCGCAATAGGTTCACTATTGAGTTTGATGGTTGTATTAATTACGTATGGTTTAATGCAAGACA
CGCAACATTTGAACCCACTTGAGTCACCTAATGGACAACATTGGTTGGGTACCGATCAATTAGGCAGAGACT

TCTTAGTAAGACTGATTGTCGGTAGTCTTGTCACATTGAGTTTAACAGGCATAGTGATTCTATTAAGCGTTTGT
ATGGGACTTATCTTTGGCTTAATTGCAGGCATAGAAAGACGATGGTTAGATCAAATCATCATGTTTGTTGCCG
ATATGTTGCTGGCTATTCCGTCATTTATTATCGCATTAGTCATCTTAAGTTTAGTAAGTAACTCCATGATAGGTTT
GATACTTGCTTTAACGATTGGATGGATAGGACGTTATTTACGTTACTTCAGAAATTTAACGCGAGATATTCAAA
AACGTCCATTTGTTCAATATGCACGATTGAGTGGGAACTCAACATTCAAAACGACAGTAACACATGTGATTCC
ACATTTATTAAGTAGTATATTCGCTTTGGTAACGGCTGACTTTGGCAAAATGATGCTCAGCATATCTGGACTTG
CTTTTCTAGGACTAGGTATTAAACCGCCGACGCCTGAGTTAGGAACAATTCTTTTTGATGGGAAAAGTTATTT
CAACGGCGCACCGTGGCTCTTCTTCTTCCCTGGTGTATTGTTAGGAGGTTTCGCCTTATTATGTCAAATTATCA
ACAAAAAAATAACGCAGTAAATACGGTAGTCAACGTCAATCAATTATCGATTTTAGATCAAGAGAAAGTATTG
TTAAAGGATGTTGATTTGACAGTAACTAAAGGTGCATTTCATTGCATTATAGGTGAAAGTGGCAGTGGGAAA
TCACTGTTAACAAGAACAATACTTGGAATGAAACAATCACAATTATGTTATCAAGGAGATATTGACATCGATTT
AACTCAAACAGATGCAGTGTTTCAAGATGTTCAAAGTAATATGTTTCAAAATATAACATTAGCTAAGCATTTCC
AATACATTTATGAAGCCAATCGCACACATCTCACTAAACAGCGTATTAAGGAAGATGTCTTACAGATGATGCA
ATTACTTGGTTTAAGACAAGGGGAACAATTGCTTGAGCGTTATCCCTTCGAACTTAGTGGAGGTATGGCACA
ACGTGTCGCCTTTATAATGTCATTAATTAGACGTCCGAACTACTTATTTTTAGATGAACCAACGAGTGCACTTG
ATCAAGAAAATATTAAAAAGTTTATGCATTACCTTCTTAGGGCACAGGAGCGCTACCAAATGACCATTGTTTT
TATCACACATGATATTAACTTAGTGAAAGATTGTGCCACACATATTAGTATTATGCAGCAAGGTAAATTGATAG
AAAATGGTGAGGCCGCGTCGATCTTAACTAAGCCGACACATAATTACACGAAAAAATTAATTACGATTGCAC
ATCGGAGACAACCTTATGCTTAAAATAGAGAGATTAACCAAATATATAGACACGCAACTGATATTTAAAGAGA
TATCATGTACAATTAACGACCAGCACTTACTCATAAGTGGGGAGAGTGGTTGTGGTAAATCCACATTAGCCAA
GATTATCGCTGGCTTAGATACGGATTATCAGGGCGAATTATATCTTAATGGGCGCTTACGTGAATCTTATACGT
CTAAAGAGTGGATGAAGCACATCCAATATGTACCTCAATATCAACGTGATACTTTAAATCAGCGTAAAACGGT
ATTAGCTACATTATTAGAACCACTTAAGAATTATAAGGTAAATAAACAGCGTTATACATCAAGCATTGAAGCAG
TGCTTGATCAGTGTAATTTACCACACGATATACTTAATCATAAAGTTTCGACATTAAGTGGTGGCCAATTTCAA
CGCGTCTGGATAGCTAAAGCTTTAATATTAGAACCAGAGATTCTCATATTGGATGAAGCTACAACCAACTTAG
ATGTCATTAATGAAGAAGCTATACTTCAAATGTTGATTTCCTTAAAGATGACACAATTAATCATTATTTCACATG
ATACATACGTCTTAAGCCAATTTGAAGGAATTCAGTTACAGCTAAATAAATTGAATAATTAAGATCACAAATCT
TAATATGGTGAATATTTAATGGTACCTAAAAAATAAAATTTAAACTACAATGTCTAAATCCATATGTTGTTTCATT
AGAGGATTTAAAAATGATTATAACACTAAAAGATTTCAAATTATTATATTTAATATAAATTTACATATGATAAACG
AATAACAATTCCAATATAAATTATTTTTTGATTATTTTTATTTATAACTATATATTTTATATGAAAAATATAAGTTAT
AATAATAAGTTTAATATTGCCTCGTGGTTCTGAGCTTGAACTTATCTCTAAATCATTTTGAGCTACTTATCTATCA
ATTCATATATTCTATAACAATATTTGTGACATCACGTGCTATTTCATGAAGTGATTTTACGATATCACCTTCTTTA
GAAAAAATATATTTCAGTAGGCACCGACGTATACAGAATCATTTGAGTATTAAAAATAAAAACTAGAAAAAGA
AACCGCATCATTAACTGATACGCAGAATCATATTATAAATAAAACTAAAAATGAGGTTGTATATAACTCACTCTG
AAATTGATTGAATATATAGTATCTTTAATAAAATGCAGCTATTGTGGCGTAGAATTTGAGAATCAAAAAATGAT
TAATATAGTTTGAAGAGACTGAGCATAAATACTAGAAAAATGGCCAGTAAATGAGTTTACTATAAACTCATTTA
CTGGCTTCTCTAATAATTATCAAGACAATTTGCGTTTCTAGGCATACTTTGAAATGCGCTATTTTCTTTAAGAAT
ATTAATATATGACTTGTGTTGGTAATGATAACCTTGGTTAGAGTGTATTGTCATGTGATAGTTAAGATTAGATCT
TTTAATTAAAACATTGTTTTAAATATCGATGACAAGGTCTAATGTAGGACGTGTAGATATAAGGTTCGGAATTA
TATAAATCCATAAAAGGAGATAAATCGAAAACCTTTATACCCTGGGTGGGTATATGTTATAGTATAAGTAGCTT
TACTATAACATTTTCATTAGGAGGGGTTAATTTGAATAATAATGGTGAAGAGCATAATCATCAAAATCACATGA
ATCATTCCAATCAAATGCATCATGATAACCATGCCTCACATGATCATCATAGTGGCCATGCACATCATCATGGAA
ATTTTAAAGTTAAGTTTTTTGTTTCATTAATTTTTGCAATACCTATCATTCTTTTATCGCCACTGATGGGTGTTAA
CTTACCTTTTCAATTCACATTTCCAGGTTCTGAATGGGTAGTGTTAATATTAAGTACAATTTTATTCTTTTATGG

TGGTAAACCGTTCTTGTCTGGTGGTAAAGATGAAATTGCTACAAAAAAACCAGGCATGATGACCTTAGTTGC
CCTAGGTATTTCAGTAGCTTATATTTATAGCTTGTATGCTTTTTATATGAATAACTTTAGTAGTGCAACTGGTCAT
ACAATGGACTTTTTTTGGGAATTAGCAACCTTAATTTTAATTATGCTATTAGGACATTGGATAGAAATGAATGC
TGTCGGAAATGCTGGAGATGCTTTAAAGAAAATGGCAGAACTGTTACCTAATAGTGCTATTAAAGTTATGGAT
AATGGCCAACGCGAAGAAGTTAAAATATCAGACATCATGACTGATGATATCGTCGAAGTAAAAGCCGGAGA
AAGCATTCCAACAGATGGTATTATCGTTCAAGGACAAACATCTATAGATGAATCCCTAGTCACTGGAGAATCT
AAAAAAGTACAAAAAAATCAAAATGACAACGTCATCGGGGGTTCTATTAATGGGTCTGGAACAATACAAGTC
AAGGTTACAGCTGTGGGAGAAGATGGATATCTTTCTCAAGTTATGGGACTTGTTAATCAAGCACAAAATGAT
AAATCTAGTGCTGAATTGTTATCTGATAAAGTAGCGGGTTATTTATTCTACTTTGCTGTAAGTGTTGGCGTGAT
TTCTTTTATTGTCTGGATGCTCATTCAAAATGATGTTGATTTTGCATTAGAACGTCTTGTAACTGTGTTAGTCAT
TGCTTGTCCACATGCTTTAGGCTTGGCAATACCTTTAGTCACTGCACGTTCTACTTCAATTGGTGCACATAATG
GTTTAATTATTAAAAATAGAGAGTCTGTAGAAATAGCTCAACATATCGATTATGTAATGATGGACAAAACTGGT
ACTTTAACTGAGGGTAACTTTTCTGTGAATCATTATGAGAGCTTTAAAAATGATTTGAGTAATGATACAATATT
AAGCCTTTTCGCCTCATTAGAAAGTCAATCTAATCACCCATTAGCTATAAGTATTGTTGATTTTGCGAAAAGTA
AAAATGTTTCATTTACTAACCCACAAGACGTTAATAATATTCCAGGTGTCGGATTAGAAGGTCTAATTGATAAT
AAAACATATAAAATAACAAATGTCTCTTATCTTGATAAACATAAACTTAATTATGACGATGACTTGTTTACTAAA
TTAGCTCAACAAGGTAATTCAATCAGTTATTTAATTGAGGATCAACAAGTCATTGGCATGATTGCTCAAGGAG
ATCAAATTAAAGAAAGCTCAAAACAAATGGTAGCTGATTTACTATCAAGAAATATTACACCAGTCATGCTTAC
AGGTGACAATAATGAAGTGGCACACGCTGTCGCAAAAGAATTAGGTATTAGTGATGTCCACGCACAACTCAT
GCCAGAAGATAAGGAAAGCATTATAAAAGATTATCAAAGTGACGGTAATAAAGTCATGATGGTCGGAGACG
GTATCAACGATGCGCCGAGTCTTATAAGAGCGGATATTGGTATAGCAATTGGTGCAGGTACAGATGTTGCAG
TGGATTCAGGTGATATCATACTTGTTAAAAGTAATCCATCAGATATCATTCATTTCTTGACCCTTTCAAATAATA
CTATGAGAAAAAATGGTGCAAAACTTATGGTGGGGTGCAGGTTATAATATTGTTGCTGTACCTTTAGCAGCTG
GTATTTTAGCATTTATTGGCTTGATTTTATCACCTGCAATAGGTGCTATTTTAATGTCTTTAAGTACAATTATCGT
TGCAATTAATGCCTTTACATTAAAATTAAAATAAAAGATAGGAGTTTTATTATGATTAAAAAAATTATTTTTTATG
ATATTAGGATCATTACTAATATTATCAGCTTGCTCCAATAATGATGAAAAAGATAAAGACACTAATGACCAAAA
AAGTGAGAGCCATATGAAGCATAATGATGAAAGTAAAGTTCCAGAAGATATGAAATCGACTAATGAGGGTGA
ATTTAAAGTGGGAGATAAAGTAACGATTACAGCAGGGCATATGCCAGGTATGAAAGGTGCAGAAGCTACTG
TAAAAGGTGCGTATAAAACATATGCCTATGTTGTAAGTTATAAACCCACAAATGGAAATGAAAAAGTAAACAA
TCATAAATGGGTCGTAAACGAAGAGATTAAAGATGCACCTAAAGATGGATTTAGTAAGGGCGATACTGTTAA
ATTAGAAGCAAGTCATATGTCTGGTATGAAAGGTGCTACAGCCAATATAGATAACGTGAAAAAGACTACTGTT
TACGTAGTTGATTACAAATCCAAAGATAATGGTAAAATCATTAAAAATCATAAATGGATGACAGGAAATGAGC
TGAAAGCACGATAAAAATCTAGTTCTAAATTGAGAAATAAATAGATATAAAAATATCCTCCTTAATCAATAATTT
AAATAACTTATTATTGTTAAGGAGGATATTTTTTAGTGTGTAAATTAAAAAGAATTTTAGAAGAATAACATTTAT
CAAAAAACTGTTCATTACCTTATTAATTGAAATTATATAATTAAAAACCGCATCATTAACCGATACGCAGAGGC
GTATCATAAGT
>Staphylococcus aureus strain C308
ATGAAAATCACCATTTTAGCTGTAGGGAAACTAAAAGAGAAATATTGGAAGCAAGCCATAGCAGAATATGAA
AAACGTTTAGGCCCATACACCAAGATAGACATCATAGAAGTTCCAGACGAAAAAGCACCAGAAAATATGAG
CGACAAAGAAATTGAGCAAGTAAAAGAAAAAGAAGGCCAACGAATACTAGCCAAAATCAAACCACAATCA
ACAGTCATTACATTAGAAATACAAGGAAAGATGCTATCTTCCGAAGGATTGGCCCAAGAATTGAACCAACGC
ATGACCCAAGGGCAAAGCGACTTTGTATTCGTCATTGGCGGATCAAACGGCCTGCACAAGGACGTCTTACA
ACGTAGTAACTACGCACTATCATTCAGCAAAATGACATTCCCACATCAAATGATGCGGGTTGTGTTAATTGAG
CAAGTGTATAGAGCATTTAAGATTATGCGTGGAGAAGCATATCATAAATGATGCGGTTTTTTCAGCCGCTTCA

TAAAGGGATTTTGAATGTATCAGAACATATGAGGTTTATGTGAATTGCTGTTATGTTTTTAAGAAGCTTATCAT
AAGTAATGAGGTTCATGATTTTTGACATAGTTAGCCTCCGCAGTCTTTCATTTCAAGTAAATAATAGCGAAATA
TTCTTTATACTGAATACTTATAGTGAAGCAAAGTTCTAGCTTTGAGAAAATTCTTTCTGCAACTAAATATAGTA
AATTACGGTAAAATATAAATAAGTACATATTGAAGAAAATGAGACATAATATATTTTATAATAGGAGGGAATTT
CAAATGATAGACAACTTTATGCAGGTCCTTAAATTAATTAAAGAGAAACGTACCAATAATGTAGTTAAAAAAT
CTGATTGGGATAAAGGTGATCTATATAAAACTTTAGTCCATGATAAGTTACCCAAGCAGTTAAAAGTGCATATA
AAAGAAGATAAATATTTAGTTGTAGGGAAGGTTGCTACTGGGAACTATAGTAAAGTTCCTTGGATTTCAATAT
ATGATGAGAATATAACAAAAGAAACAAAGGATGGATATTATTTGGTATATCTTTTTCATCCGGAAGGAGAAGG
CATATACTTATCTTTGAATCAAGGATGGTCAAAGATAAGTGATATGTTTCCGCGGGATAAAAATGCTGCAAAA
CAAAGAGCATTAACTTTATCTTCCGAACTCAATAAATATATTACATCAAATGAATTTAATACTGGAAGATTTTAT
TACGCAGAAAATAAAGATTCATCTTATGATTTAAAAAATGATTATCCATCAGGATATTCTCATGGATCAATAAGA
TTCAAATATTATGATTTGAATGAAGGATTCACAGAAGAAGATATGCTAGAGGATTTAAAGAAATTTTTAGAAC
TATTTAATGAATTAGCTTCAAAAGTTACAAAAACATCCTATGATAGCTTGGTCAATAGCATAGACGAAATACAG
GAAGACAGCGAAATTGAAGAAATTAGAACAGCACAAAAAGATAAGACACTCAAGGAAGTGGAAGCACCTA
AAGGAATAATTCCAAAATATAAAAAAGGTGTATCAAAGACTACTAAAAATGATTCAGAAATTGAAAAATCAA
ATAAAGAGAATAAATTAACCGGTAAAGTTGGAGAAAAATTAGCGCTAAATTACTTTAATGAGCTAATTGATAA
TAAAATAGACGAAGATAAGAAAGAACAGTTTAGGAATATTTTAAATGATAATCCAGGCTCTCAACACGGTCAT
GGCTATGATTTAGTAGCTTTTGATCCAACAAATACAGATAAAGCTGTAGAAAAATTTATTGAAATTAAAACATC
TACATCTTCTAGTATTGAGGAACCATTTTTTATGTCGCTAAATGAAATGTTTGCTATGAAAGAATATAAGCAGA
AATATTTAATATTAAGAATATTTAATGTTTCCGGTAAAGAACCACAATTTTATTTTATAGATCCATATGCAAATTA
TTCTGAATTTAAAGATGTAGATGATCTCATTGACAAAGTATTTAATGTAGAAGCTATTCAGTATAAAGTTTTTG
GCGAAAAAATGATTACTTGAACAAGAGCTAAAATAAAATTGTGATCTAATAAAAATAGAAACTGTAATTTAAAT
AAAACTTTCTAAATAAGCTAACTGATAAAAAATCAGTTTGTCCACAGTCTGAAACAAGATTCCTATATTCTTTA
GGAATCTTGTTTTTTCTATTTTTATGGTGATAAAGAGCAGATAAGATAATGTGTAATAATCACAAAAAAGTTAA
ATATTTTAAGGCTTGTTTAATTATTAATGATTTTATATATAAAGAGCAGTATAATAAAGTTGTTAATATATTATGAA
TAATATTCAAGTAATTTTATTGTTTTTTAATTTGTCGATATTTAAGTTGAGTTAAATTTAAAGGGTGTAATTTGTT
TTACAATGATGAAGATAATTAGTCTATCAAAATAAAGGGGTTGGGACTGTTATGAGTGATAATTTGTCATTATT
CATTGACTATATCAATGATAATATAATCTATGGTAGTGAAATCAAACGGGAGAAATTAGAGAATTTATTTAATCA
ATTTGCTATAAAAAAATGTTGAAAAGAACATTGTCTATGATGAACTGAAATCTTTAGATATTACAATCATTGAGT
CACAGGATTCATATAAAAATAAATTGAAGAGATTATTTTCGGTTCTGTTGCAAAGTAAAAAAATATAGCTAACC
ACTAATTTATCATGTCAGTGTTCGCTTAACTTGCTAGCATGATGCTAATTTCGTGGCATGGCGAAAATCCGTAG
ATCTGAAGAGACCTGCGGTTCTTTTTATATAGAGCGTAAATACATTCAATACCTTTTAAAGTATTCTTTGCTGTA
TTGATACTTTGATACCTTGTCTTTCTTACTTTAATATGACGGTGATCTTGCTCAATGAGGTTATTCAGATATTTC
GATGTACAATGACAGTCAGGTTTAAGTTTAAAAGCTTTAATTACTTTAGCCATTGCTACCTTCGTTGAAGGTG
CCTGATCTGTAATTACCTTTTGAGGTTTACCAAATTGTTTAATGAGACGTTTGATAAACGCATATGCTGAATGA
TTATCTCGTTGCTTACGCAACCAAATATCTAATGTATGTCCCTCTGCATCAATGGCACGATATAAATAGCTCCAT
TTTCCTTTTATTTTGATGTACGTCTCATCAATACGCCATTTGTAATAAGCTTTTTTATGCTTTTTCTTCCAAATTT
GATACAAAATTGGGGCATATTCTTGAACCCAACGGTAGACCGTTGAATGATGAACGTTTACACCACGTTCCCT
TAATATTTCAGATATATCACGATAACTCAATGTATATCTTAGATAGTAGCCAACGGCTACAGTGATAACATCCTT
GTTAAATTGTTTATATCTGAAATAGTTCATACAGAAGACTCCTTTTTGTTAAAATTATACTATAAATTCAACTTTG
CAACAGAACCGTATTATGGAATAGAGATGTTGGTAACATTTATACAGGATCATTATACTTAAGTTTAATTTCGTT
ATTACAGAACCACACATTCCAACCAGAAGAGAAAGTATGTCTATTTAGTTATGGTTCAGGAGCAGTAGGAGA
AATCTTTAGTGGTTCAATCGTTAAAGGATATGACAAAGCATTAGATAAAGAGAAACACTTAAATATGCTAGAA
TCTAGAGAGCAATTATCAGTCGAAGAATACGAAACATTCTTTAACAGATTTGATAATCAAGAATTTGATTTCG

AACGTGAATTGACACAAGATCCATATTCAAAAGTATACTTATACAGTATAGAAGACCATATCAGAACATATAAG
ATAGAGAAATAAACTAGTGGCCGATTGTGCTTGATGAGCTTGGGACATAAATCCTAACTCGAAATAAATAAGC
ATATCACTAAACTGATTTTTTAAAGTTTACAGTGATATGCTTATTTTTTTATCTTACGATTTTGTACGTGCATGCT
TGCCTAGGGGTATGGCTCGAGCCATTAGTCTCTCGCACATACTATTCCCTCAGGCGTCAGCACTTACAAAATC
GGTTGTAATTTTCATTTTTATACGCATTCTTACTGAGATTATACTAATAAGAGGAATAGTAAAAGCAATTCTAAG
TAAAATTGCAGATAAGAGGTTTGTTAAAAGCAGTTCTAAGTAAAATTGCAGATAAGAGGTTTGTTAAAAGCA
GTTCTCAGTAAAATTACAGATAAGAGGTACGTTAAAAGCAGTTCTAAGTAAAATTGCAGATAAGAGGTTTGT
TAAAAGCAGTTCTAAGTAAAATTGCAGATAAGAGGTACGTTAAAAGCAATTCCATGCAAAATTGCTGATAAG
GGGTAAGTTAAAAGCAGTTCTCAGTAAAATTGCAGATAAGAGGTACGTTAAAAGCAGTTCTAGGCAAAATT
GCAGATAAGAGGTGCGTTAAAAGCAGTTCTCAGTAAAATTGCTGATAAGGGGTAAGTTAAAAGCAATCCTA
AGTAAAATTGCAGATAAGGGGTACAGAAAAACTAGACTTGATTACAAAATGGAGCTTGGGACATAAATGATT
TTTTAAAAATGAGATGAGACGTAGATTAACTCCATAATCAATACGAATCTATCGACTTCTTTATTTATGATATTC
ATCTCTTTTTAATGGAAATAAAAGTGCGATTAATGTGATAATACAGTTACGTTAATTAAAAAAATAAAAATGCA
AGGAGAGGTAATATGCTAACTGTATATGGACATAGAGGATTACCTAGTAAAGCTCCGGAAAATACAATTGCAT
CATTTAAAGCTGCTTCAGAAGTAGAAGGTATAAACTGGTTGGAGTTAGATGTTGCAATTACAAAAGATGAAC
AACTGATTATCATTCATGATGATTATTTAGAACGGACTACAAATATGTCCGGGGAAATAACTGAATTGAATTAT
GATGAAATTAAAGATGCTTCTGCAGGATCTTGGTTTGGTGAAAAATTCAAAGATGAACATTTGCCAACTTTC
GATGATGTAGTAAAAATAGCAAATGAATATAATATGAATTTAAATGTAGAATTAAAAGGTATTACTGGACCGAA
TGGACTAGCACTTTCTAAAAGTATGGTTAAGCAAGTGGAAGAACAATTAACAAACTTAAATCAGAATCAAGA
AGTGCTCATTTCAAGCTTTAATGTTGTGCTTGTTAAACTTGCAGAAGAAATCATGCCACAATATAACAGAGCA
GTTATATTCCATACAACTTCGTTTCGTGAAGACTGGAGAACACTTTTAGATTACTGTAATGCTAAAATAGTAAA
CACTGAAGATGCCAAACTTACTAAAGCAAAAGTAAAAATGGTAAAAGAAGCGGGTTATGAATTGAACGTAT
GGACTGTAAACAAACCAGCACGTGCAAACCAACTTGCTAATTGGGGAGTTGATGGTATCTTTACAGACAATG
CAGATAAAATGGTGCATTTGTCTCAATAGAAAGTTAGAGGTGAGTCTTACGTTTCAGTGACGGTAGACTTAC
CTTTAACATGTTACATACTAAAAAATTAATTTGAATAAGAAAGAGAGACATATATGAAATACGATGATTTTATA
GTAGGAGAAACATTCAAAACAAAAAGCCTTCATATTACAGAAGAAGAAATTATCCAATTTGCAACAACTTTT
GATCCTCAATATATGCATATAGATAAAGAAAAAGCAGAACAAAGTAGATTTAAAGGTATCATTGCATCTGGCA
TGCATACACTTTCAATATCATTTAAATTATGGGTAGAAGAAGGTAAATACGGAGAAGAAGTTGTAGCAGGAA
CACAAATGAATAACGTTAAATTTATTAAACCTGTATACCCAGGTAATACATTGTACGTTATCGCTGAAATTACAA
ATAAGAAATCCATAAAAAAAGAAAATGGACTCGTTACAGTGTCACTTTCAACATACAATGAAAATGAAGAAA
TTGTATTTAAGGGAGAAGTAACAGCACTTATTAATAATTCATAATAAAACAGTGAAGCAACCATCGTTACGGA
TTGCTTCACTGTTTTGTTATTCATCTATATCGTATTTTTTATTACCGTTCTCATATAGCTCATCATACACTTTACCT
GAGATTTTGGCATTGTAGCTAGCCATTCCTTTATCTTGTACATCTTTAACATTAATAGCCATCATCATGTTTGGA
TTATCTTTATCATATGATATAAACCACCCAATTTGTCTGCCAGTTTCTCCTTGTTTCATTTTGAGTTCTGCAGTAC
CGGATTTGCCAATTAAGTTTGCATAAGATCTATAAATATCTTCTTTATGTGTTTTATTTACGACTTGTTGCATACC
ATCAGTTAATAGATTGATATTTTCTTTGGAAATAATATTTTTCTTCCAAACTTTGTTTTTCGTGTCTTTTAATAAG
TGAGGTGCGTTAATATTGCCATTATTTTCTAATGCGCTATAGATTGAAAGGATCTGTACTGGGTTAATCAGTATT
TCACCTTGTCCGTAACCTGAATCAGCTAATAATATTTCATTATCTAAATTTTTGTTTGAAATTTGAGCATTATAAA
ATGGATAATCACTTGGTATATCTTCACCAACACCTAGTTTTTTCATGCCTTTTTCAAATTTCTTACTGCCTAATTC
GAGTGCTACTCTAGCAAAGAAAATGTTATCTGATGATTCTATTGCTTGTTTTAAGTCGATATTACCATTTACCAC
TTCATATCTTGTAACGTTGTAACCACCCCAAGATTTATCTTTTTGCCAACCTTTACCATCGATTTTATAACTTGTT
TTATCGTCTAATGTTTTGTTATTTAACCCAATCATTGCTGTTAATATTTTTTGAGTTGAACCTGGTGAAGTTGTA
ATCTGGAACTTGTTGAGCAGAGGTTCTTTTTTATCTTCGGTTAATTTATTATATTCTTCGTTACTCATGCCATAC
ATAAATGGATAGACGTCATATGAAGGTGTGCTTACAAGTGCTAATAATTCACCTGTTTGAGGGTGGATAGCAG

TACCTGAGCCATAATCATTTTTCATGTTGTTATAAATACTCTTTTGAACTTTAGCATCAATAGTTAGTTGAATATC
TTTGCCATCTTTTTTCTTTTTCTCTATTAATGTATGTGCGATTGTATTGCTATTATCGTCAACGATTGTGACACGA
TAGCCATCTTCATGTTGGAGCTTTTTATCGTAAAGTTTTTCGAGTCCCTTTTTACCAATAACTGCATCATCTTTA
TAGCCTTTATATTCTTTTTGTTTTAATTCTTCAGAGTTAATGGGACCAACATAACCTAATAGATGTGAAGTCGCT
TTTTCTAGAGGATAGTTACGACTTTCTGTTTCATTAGTTGTAAGATGAAATTTTTTTGCGAAATCACTTAAATAT
TCATCCATTTTTTTAACGGTTTTAAGTGGAACGAAGGTATCATCTTGTACCCAATTTTGATCCATTTGTTGTTTG
ATATAGTCTTCAGAAATACTTAGTTCTTTAGCGATTGCTTTATAATCTTTTTTAGATACATTCTTTGGAACGATG
CCTATCTCATATGCTGTTCCTGTATTGGCCAATTCCACATTGTTTCGGTCTAAAATTTTACCACGTTCTGATTTTA
AATTTTCAATATGTATGCTTTGGTCTTTCTGCATTCCTGGAATAATGACGCTATGATCCCAATCTAACTTCCACA
TACCATCTTCTTTAACAAAATTAAATTGAACGTTGCGATCAATGTTACCGTAGTTTGTTTTAATTTTATATTGAG
CATCTACTCGTTTTTTATTTTTAGATACTTTTTTTATTTTACGATCCTGAATGTTTATATCTTTAACGCCTAAACTA
TTATATATTTTTATCGGACGTTCAGTCATTTCTACTTCACCATTATCGCTTTTAGAAATATAACTGCTATCTTTATA
AACTTGTTTGAAATTTTTATCTTCAATTGCATCAATAGTATTATTAATTTCTTTATCTTTTGAAGCATAAAAATAT
ATACCAAACCCGACAACTACAACTATTAAAATAAGTGGAACAATTTTTATCTTTTTCATCAATATCCTCCTTATAT
AAGACTACATTTGTAGTATATTACAAATGTAGTATTTATGTCAAAATAATGTTATAATTTTTGTGATATGGAGGT
GTAGAAGGTGTTATCATCTTTTTTAATGTTAAGTATAATCAGTTCATTGCTCACGATATGTGTAATTTTTTTAGT
GAGAATGCTCTATATAAAATATACTCAAAATATTATGTCACATAAGATTTGGTTATTAGTGCTCGTCTCCACGTT
AATTCCATTAATACCATTTTACAAAATATCGAATTTTACATTTTCAAAAGATATGATGAATCGAAATGTATCTGA
CACGACTTCTTCGGTTAGTCATATGTTAGATGGTCAACAATCATCTGTTACGAAAGACTTAGCAATTAATGTTA
ATCAGTTTGAGACCTCAAATATAACGTATATGATTCTTTTGATATGGGTATTTGGTAGTTTGTTGTGCTTATTTT
ATATGATTAAGGCATTCCGACAAATTGATGTTATTAAAAGTTCGTCATTGGAATCGTCATATCTTAATGAACGA
CTTAAAGTATGTCAAAGTAAGATGCAGTTCTACAAAAAGCATATAACAATTAGTTATAGTTCAAACATTGATAA
TCCGATGGTATTTGGTTTAGTGAAATCCCAAATTGTACTACCAACTGTCGTAGTCGAAACCATGAATGACAAA
GAAATTGAATATATTATTCTACATGAACTATCACATGTGAAAAGTCATGACTTAATATTCAACCAGCTTTATGTT
GTTTTTAAAATGATATTCTGGTTTAATCCTGCACTATATATAAGTAAAACAATGATGGACAATGACTGTGAAAA
AGTATGTGATAGAAACGTTTTAAAAATTTTGAATCGCCATGAACATATACGTTATGGTGAATCGATATTAAAAT
GCTCTATTTTAAAATCTCAGCACATAAATAATGTGGCAGCACAATATTTACTAGGTTTTAATTCAAATATTAAAG
AACGTGTTAAGTATATTGCACTTTATGATTCAATGCCTAAACCTAATCGAAACAAGCGTATTGTTGCGTATATTG
TATGTAGTATATCGAGCTTCACATGAAACAGCTAAAGAAGCTTTGGGCGATAAAGAGTTAAGAGCCATTGCA
CATGAGTTAACTAAAACAGTTAAGGATAACATGAGTGTTGATTGGTCTAAACGAGACAGTGCTAAAGCTAAA
ATGAGAGTTCAAGTTAGACGCCTATTAAAGAAATATGGCTATCCACCAGATCTTCAAAAAATGGCTGTGGAA
CAAGTTGTAGAGCAAGCAGAATTAATGGCAAGTCAGCAATAAAAAAATAAATCATAATGAGTCCGGGACATA
AAGTTCTTGGATAAGTGAAAAAAGACAATTTCTATTGAAATAATATAGAAATTGTCTTTTTTATAAATTTTTTG
ATTATTTTCAGCTCGTTGAGCTACTACTTTTCTTATATTAAGTGCCATTAATACAAAACCAAGTTCTCTTTTGAC
TTTATTGAGTCCTCGGACAGACATCCGAGTGAAACCCAAAATAGCCTTCATAAATCCAAAAACAGGTTCCAC
ATCAATTTTTCTTTGACTGTAGATATTTTTTGTTTCTGGTTCTGAAAGCTTTTTGTTAATTTGGGATTTAAAATA
TTCCCAGTTATAATTCTTCATTATTTTTTTGTTTGTTTTTGAATTGAAGTTCATACATTGATTTTTCAGAGGACAT
TCTGAACAATCATCACATTCATATAATTTGAAGTCTCGCTTATAACCATACTTATCATGACGATAGGCATATCTTT
TAAAACCTAGCCGTTTATTATTCGGACAAATGAATTCGTCATTAATTTCGTCATAGTTCCAATTTTGAGTATTAA
AGATGTCACTTTTATATTTTTTAGTTTTATCTTTTATAAACATTCCATATGTTATGAGTGGCGTTCGATTAAAGTC
ATCTATAATTGCCTTATAATTTGATTCACTACCATAACCTGCATCAGCTACAATATATTCAGGTAAATGACCGTAG
GTCTCTTGAATTGAATTTAAAAATGGAATCATCGTTCTAGTATCCGTTGGATTTTGATACACATTATAAGATAAA
ACAAATTGGGAATTTGTTGCTATTTGTAAATTATACCCTGGCTTAAGTTGTCCATTTTTCATGTGATCTTCTTTC
ATTCTCATAAATGTCGCATCATAATCTGTCTTAGAATAACTATTTCTATCCTTTAAAATAGATTTTTGAAATTCGT

ATCGATACTTTCGCTCAAAATAATCATTGATTTGCTTTTTGTATTTTTTGATTTTAGTTCTTTTGAGACGTATTTG
TTTTCTTGTTTTAGTACATTTTTCATTGTTGATATGTTGGTTTAAATCTTCGATTTCTTTATCTAAGTGACTACCA
ATCAAATCTATTTCTTCTTTTGTTAATTCATTATCATGATCTTCTTTAATTTCCGGTATGATTTTATTGGTTACCAA
TTCATGGTAGAGGGCTTTAGAATCCTCATTCATCTTTGATTCATGGTTTTGAATACTCTTTTTCCATACAAATGT
ATATCGATTGGCATTTGCTTCAATTTTTGTACCATCAATAAAAATAGCTTTATCATCTATAAGATTTTGTTTTACA
CACTGACTGTAAAATTGAATAAATAAAGATTCTAATAAAGCATCTACTTTTGGATTTACTCTAAATTGATTAATT
GTTTTATAAGAAGGTTTTTGATTTTGTGATAGCCACATCATTCGGATGCTATCATTAAGCATTTTTTCTATTTTA
CGACCTGAGAATACAGATTGTGTGTAGGCATATAGAATCACTTTTAACATCATTTTAGGATGGTACGAAGTTG
CACCACGGTGATGTCTGAATTCGTCGAATTCATTGTCAGGAATTGTTTCAACAATATCATTTACAGTAAAACG
ATGTTGATTTGTTTTGTTTCCATATTGACCTCCATGTATTTGCTATGATTTCAAAATCCATTTTTGACGTGCCTTA
GGGTTGAGTGGATGCATAATTTCATTTGTTACTGGATTGATGAGCTTTTTTACTTTCTTTTTATGAGGTTTTAA
CATTTCCATCACTTGTTCGACACGGTCGATAACAACTGGTCGCTTCGCATAGGCACCATAAGCAAGAATCACT
GTGTCACTTTCACTAATCGCTTTCATCAAATGAATATCTGTGTGCTCATCGTATGGCTCTTTGATATGTTTAAGA
TTCTCTGGAGTTTTAATATTAGAGAATAGATTTACAAGATATACAGCACCGTATCGTTCTGAATTGGCTAATTG
GTTGAGGATAAGAACAGTTGTGAGATCGAGTGATAATACACCGTCTAAATGAGGATACATCGTTATCACTGTG
CATGCAGCTTTCTTTTCATCCCATGTTTTCTTGAGTAAATAGCGGTGCTGTTCATCATCGCTAAATATGGCTTCT
GTGTGTATCGTACTTTTGATTGTATTCATCATCGTCACTTCCTTTAGTATTCTTCTGGTAAAAGCATCACATAATA
AAAAGCGTCCACGTCATCTTCACGAATGACGTAGACTTTCTTAGGTAATGCATTTTGATTTTTTTCATAGTTTG
TATAGTGATATTCCAATTTGTATGTGGGTTGTTCTTGTTCATGTGTGATTGAGAGTATATTCTCATCTTCTTGTAA
TTTAAAAATGTGTAGGTAATCTGTATGAGGCTGGTTATCTTTTTTTTCTACCATGTGCCAAAGTAAGATTTGAA
GGTCTAGTGGAATACTTTCATTAATTCCTCTTGTGATGTATCGATTGATTTTCATGCTATTTCCCTCCCTTCTGCT
TTTCTTTCATGATGTCGATGATTTCGTTGATAACTGTGACGGATAATTGAGCAACTATGATCCAATTATTCATGG
TCCTGACCTCCTTGTTTTAGTAAATGACGTTCATCAATAATGATATTTTGAGTATCTGTAAGGTACAGAAAGTC
CATGTCAAAATGGTCTAAGTATCCGACACTGATGAGTTGGTTATTGGCATACATTAGAAATGGATAGATACTTA
GCTCATGTAGTTCATCATTATAGTAGGTATAAGTCTCGAGTGTGAGATGTACCAGTGGAGAATCATTAATAAAA
CGTTCCGGTAGAATATTTTCTGCTGCTTCCTCCAGCGCTTCACATTCCCAAGCTTCGTTATTAGATAGTTGGAA
TAGGTGGGTTATATATTGTTTGAGTTCTTGAGTGATGGTTTTCATATTATTGCCTCCTAGATAGTGTAATAGTGA
TGTAGTTCATATACATCATTGAGATAATATATATTTGATTTGTCATTTATTACGAATCCCGGTGGGAATAAGAGA
AAATTCCATATAAAAACCCGCTACAAACGTTGGTATGCCAAGGAAATCCTGAAATTCCGCCTATTTTGACAAA
CAATCAACTCATTATTTATAAGTATTGATGATAGGGTTGTGTCTCTGCTTCCTTATATATATTATTTATTTATAAAA
AGTAACGGGATTTTGGGATTGTGCTTGCACAATCCTTCTGTTTCTTCGAATCTGCAAATCCCAATCATTTCCCG
ATAAAAAATCATTGTGGGATGTTCTTTAGCAATTTCAATATAAGCATTGTATAGTTATGAAAAAATTACGACAA
TAACTGTTTCATTAGATAAGTGTTATTGAAATTGATAAAGAGCAATTCTTGAAAATAGTTAGATAAAATAAGCG
AAAGAATATAGTGAAAATTATTGTTATAACAATGATTCTATTAGCTAAATAGTAAGATATAGTGTTTGGGGCAA
AAATAAAGACGAAGTGCTGAGATGCACTTCGTCGAGTTGTTTATTATTGAAAAGTTGTTTAATGATTTCGTTA
TTAAGTTTGAGTGTGACATAGAATTGTTTTTTATGATTACCATCTTTTTTAATATCAATGCGATCAATCACTGAT
AGATACAATGCTTTGAGTCGAGATTTTTCTATGTGTTTAATATCATGAAAGATGTGTTGTAATAGTTTACTGATT
TCTTTGGCATCAAATAAAGTCTTATCTTCATTTTGTTGATTTTTGAGTTGGTTGATTTGATTCGTAATGTCATTG
AGTTGCTTTTCATATTTTTGAATACTTGGTCTGATTACTGATGTTAAGTCCGGATTATCCTCGATGGTTTTAATC
AAGTTATTTATTTTGATTTGTACTTCATCATATTGTTGTTGCTTATAAGCAATATCGTGATGAAGTGCAGCGCCA
TCAACTTGATTTTCTTGATTGACGTGTGTTACTACGCGTTGAATGACTTTATCACTTTTGACTATTTCAAGTATT
TGCTTCATCACATAATCTTCAATCACATCAGCTCTTACACTGTTTGCCGAACATACTTTGGAACCCTTGTTCCG
AAAATTACTACATGAATAGTAACGAATACGTTTCTTAGTCCCGTCTTTAAGAGTATTCGTGGTATTGCTTGCTG
CCATAGGTGCGCCACATTGGGGACAGTGAATAATGCCTGTAAGCAGATTCGTTCCTTTGCCATGGACTTGGG

GTTTTTGACTGACTTGTTTCTTACGCATTTGTACTTTATCCCATAAATCTTGATTAATAATGGGGGAATGCTTAC
CTTCAGCTATCACTGGTTTATCATTCAGCCCTTTACGACGTTTTTCACTCCAATCTTTGTATTTCGCAAATTGAA
TTTTGCCGATATAGAAAGGGTTAGCTAAGATGTATGTGATTGAACTAATACTGAAAGGTTTCCCCTTTTTAGT
GACATATCCTTTGTGATTCAATGCATTGGCAATTTTACGATAGCCATGTCCTTTGGCATAGCACTCGAATATATA
TTTTACAATATTAGCTTCATGTTGGTTAATCATTAGCTCGTGTTTACTATCTGGTATTTTGTCATAACCTAGTGGT
AAATTGCCTTGATAATAGCCTTCTTGGGCACGTCTCGTTTGACCCATAAATACGTTCTCGACAATGTTATTACG
TTCGAATTCTGAGAAACTCGCAAGTATCTGTAACATGAGTTTACCAGAAGAAGTATTGACTTCCATACGCTCT
GACAAACTGAAAAAATTCGACATTTTGTTTGTGTAAATCTTCGACAATTTTGAGAAGATCAGATGTATTACGAG
CTAATCGGTTTGTTTTGTATACCATAACACAGTCGATATTGCCTTCTTTTGCATCTTTCAACATACGTTGGAGCT
CAGGTCTGTTCATAGATTTACCTGAAATACCACGGTCAGCGTATATATCTTTAACTTCAAAATGATGGAAGTCA
CAGTATTCTTTGATTTGATTGATTTGTCCGTCGATACTATAACCTTCTGTGCTTTGCATTTCTGTTGATACACGT
ACATAGATACCGACACGTTTTGTTTTAAGTTGTTGCATTATGTTACATCCTTTCTTCATTTATGCAATCGATGAT
TGCATGGTTTGATTGACAATATTGAGTGGTTCATTTTTGAAATAGATTCCTATAAGATTTTTATCTTTCGTAATG
TGAATGGTTTCAATATAGGGGTACAATATGTTTAACGTGAAACGTTTTTGAATAATATTTTGAATGATGTGTTG
TATTTGATGCCCATTGATAGATGTAGTGCGTTGCGGTTGTTGACGTAATGATTGTGTTTGCTCTCTGAACGTTT
CTGCATCAATGATGCCTTGTGCCAACTTTTCTATCAGTTGTTCTTGAGTCAATGTGTGATGTTTTTCTATGTTTC
TTTGTCTTTTGATGCGTTTGTCAATCGCACTTTTAATTTTTGTGTAGATGCGTTGATTTTGATAAAAGTCTCGG
CACACTTCTAATACTTTATCTTCAAGTGTTTGTGCATTGATGCCTTTAAAATCACAGACAAAGCGTGAAGCATT
CATGTTTTTAGGACAGACGTAGTAACGTAATGTATGATTCTTTTTTCTAATGGTCATATTTGTAAGTGTTGCATT
ACAACATGGGCATTTGATTTTTTGTTTGAGTTGATTATCCGAAGATGTCTGTTTGGTTTGTTTTTGCGATCGA
AGTCTCTGCGCTTGCTCATATATACTTGTGGAAACAATAGAAGGAAACATATTGTCGAATTGGCCATATTGATT
GTTGACACGACCACAATAATTAGGATTGATGATAATGTTACGAACTTGATAGGGTTGTCGATTGATATACGTGT
TATCTTCTTCTAATAACTGTGCAATTTTCTTATAACCATGGCCTTTAATGTAATAATTGAATACAGCCTTTACCGT
TGGTGACTCATTTTGATTGATGATGAATGTTCCGTGGTGATAATCGTAACCAAAAGGTGCATGTGTTGTAATC
AATCGACCTTGCTTTGCTTTTTCTTGAAGCCCATTTCTGACTTGTTCTCCAATGTTATCCGATTCAAGTTCGGC
TAAACTGATGAAGATATTAAGCTTGAGTCGGTCGAAAGCTTGATCCATATCAAAGTAACCATCATGTACGCTT
AAGATATGAACATGGTACGTTTGACATAATTTGATGAGTTTTAATGCATTTTTCAGATTACGATGCAACCTATT
AAGACGATAACAGCATAATATGTCACACTGTCCTTGTTGAATTAATTGTGTTATTTGTCGATACCCACTACGATT
ATCTTTGCGACCTGATTGTTTGTCGCTATAAAAGTTGATATGTTGAATATGATGTTTTTCGGCTATTGCTTCGAT
AGCTTGTTTCTGTGCTGCAAGAGATTGTTGTTTCATCGTACTTTGACGTAAATAGCCTATGACTTGTTTCATAT
CGGCTCCTCCTTTCACAGTGATAATATATATTTATGGATGAATTGATATATAAGCCCAACATCAATGAGATGTTG
GGCGTCCATATTAGTCATTTGTTTGATTGATTTCTTCAATTACCAAATCGGCTAATATCTCGATAAGTTCATCCAT
GTTTTTCACTCCGTTATTTGTTCTATCTTCAATACGTCGATTATTCAGTTTGATGCTTCACAGTTGTATGATAAA
GACAATTAGAAATCTTCGTGAACTCCTGAAGGGCCTATCCCTTCATTAGCGGATTTAAAAAGTTCTTTCGCAG
CTTTGTTATCATTTGACGGTGTCCAATTTTGAAGTAACGACTTATCTTTAGTTAATCCGAGGATAGATGCAAAC
TCTACATCTAATTTTAGATGGTAAAATACAAGTGATTGTTTTTTACCGCTATTATCTTTGACACTTCTTTTAGTTG
TTTGGCGTCCACGGTCAGCTAATATGAAACCTTTATCTCTTAAGGCGTTGACAACATTATTAACATCTTGAAAT
TGATGATTGTTTAGCATCTGTTTAAAAACGTTCGCAATTATTTTTACTTCGATATGGTCATCTTTTAATGAGATT
AATCCATAGTTCTCAAACATATTTTTCAAAGCACCTTCATCTGAAAACTTACCTCTGTTTTGTGCTACAAATTG
AATGATGACATCGATAGCTTTATCAGCTAATGAGCGTTCAGAGATTGTATGACCATGATAATCAATAAAGTAGT
CTCTTATTTTAGCGATATCAATATCTGTAGCTAAAACACGACCTAATATTTTTGCAGATGTTGTAATGACTGCAT
AACGCTTAAACATACGATTGCCTGTGTTGCTTTTATCATCTTTCAATTTAGCTTCAAACCAATCTACTTCCTTGT
AAAACCATTGAATAACTTCATCTTCACGATTTATAAGATATTTAGCTACTAACGGTAAAACATGACCATAGTTTA
GTGCTACAGCTTTTTTAATATTGTCAGCATTGGTCGCATTTGTAGTGAATTGTTCATTAATCTCGATGGTTCTTA

CACGTAATCCATCGTTTTGAGCTGAATCATTAAAAATACTGTGTTCTGACGTTGAAATGACAGAAGTACCCCA
ATTCTTAGGCGTTTTAACTTCTCCATGAACGTTTGAACGTTGACGACCTTGACCTTCAGTGATGGAGTACAAT
AACCCCGTTGTATCTCTAAAAGTTGCTGATGAGAGTTCATCAAATACAATAGGTATACCAAAATTGTTACTCAA
GTAACCTTCAAGTGCATTACGTGTGGCATTCCAATTTCTAAAGAGAGTTTCATTACCTTTGGTAGGGTTACCA
GCGACTGATACAGCTAAAGAAGCTGCAGTTGACTTACCGGTTGAGGATTGACCTGTAAAACTAAAAATGATT
CCGGCAAATTCGGTTTCATGTTTGTGCTTCAGGAAACTCGTCACTAAGGCAGAAATACCAAATACGACTGCT
AATTCTAAAAGAAGAGAACCTTTAACCTCTTTTAGATACATGTTAAACCAATTATCAAATGTACCTTTAGGAGT
TAAGTCATAAGCATTATCACAAATGGCGTCAGATGGAGATTTATTATCAAATTCTTTAGTAGTATAGATTTCACT
TAACGATACAATAGGACCAAACGGTGTTTCCAGTATACCTACCCCTTCATATAAGTAGGAAATGGGTAATTGAT
TGCGCATTTGTTGCAACGCATAACCTAAATCTTTTGTATATTTTTCATTAATACTAAATCCATACTTCATTAAAGA
TGGCAGTTTTTGTGTTGTTAAAATATCACTAGATTCAACAATTTCTTTTTGATCCTCGTCTGTAATAATTACTTT
TTCAGTGTTAGTTTTAGGGTCAATAAACTTATTTTCGATAACGATAGGACCTGCGATTTCAACTTCAGTAGGC
ATTCCTCCTTTTTCTTTGGGAGGCTTGTCTTTATACCAACCTTTTTTTGATTTGTATCGTGGTGAAGGATTAAA
TGAAGGGTTAGTTTGAGTCATTAGCGAACACCTCCTTTCGAAGGGTTGCTGTTATTATATGGATTTGGCCCTG
TTTTAAGATAAACCAAGTGTCCATGAGTATTTTTACCAATAATAACAAATGGAATACGTGGCGCATGTTTTACA
AAGTACGCGAACCAACGTCCGACATTTTGTTGTACAGCTTTTGAGCATTGTACATTTGCACGACTGTTCAGAT
CATGAAATGTAAACGTTTGCCCCTCAGGTAAATCGAACACAATTCCTAAAATTCTTCTTTTCAACTCTTTAAAA
TCATCTTTTTGATTGTAAGTCATATGAGTTCCTCCCAAATATTATTAATTAAGTCTATCTTCTGGTTTTTTTCAGA
AGATTACATTGCTTATAATAGAGATGTACTGTCTTTATTGCTCGAATAGTATTGAGCAAAAGACGAACATATAA
TTGCAAGACTTTTCGCTTGACAAATTAGAAAAGAGGTAACCAAAGTGTATGATTTTTTAGAATATTTACATCG
TTATTGTGTAAAATCGATTAATAATTGGGAGGCTGTTGCAGATATATCATGCAAAGTTGTACGTGAAAAAGTT
AGTAAAGGTTATTATGTTAAACAAGATAGCGTTGTAATTGATGATAATGATATACCTTCAATTATTAAAATAAGT
GGAAATTATGTAGCAGCAGATATATTAGTTACTAGATATTTAGATGGAATGGGTGAATTTATAAAGGTATCAGA
ATTAGATCAGTCGAGCTATTACGAATGGTTTATGTTTGTTGAAGAAACTAGAAGAATATTTAGAGGTACAGAA
ATAGTTTATCGATTTTTAAAATATCTATTTACGCATACCTATGAACATAAAAACGAAGAACAAAAAATAGAAAA
ATATATGAAGAATATTATCATAGATAATCATTTTAGAGGGGGGCATAGTAATGATATAGTTCGTTTAGATATGAA
AGATGATAATGTGTTATTTTATAATTTTGATATATACGATACAAATATTCTTTATGAAAAAATAGACGAGGAAAA
ATTACTTAGAATTTTAGATGAAATAAGTAATACTGACAATTACAAAGCGCTATTTAACATTGTAAGTGAGAACC
AAAAGAAATTTTGGAAATCAAATATTAAGCCAATAGTAAGTGAATGTTTAGAAGATATAAATACGATACCATTT
GATTTATATGTTTATGTAAATAAGTATATAAGTAATAAAATATTTAACTGTGATAATTATTTAATTACTAAAAATGA
GGTTGTGGATTTTTATTTTAATCATGGTAGTGACTTTAATAATTTCAAGATGGGACAGAAATGGACAAAGTCT
AAATTTAAGCAGGAAAATAAACTTAAAATACACGAATATATTATTGCAGTAACTTATGTGAATTTATTAAATCAT
TCATATATGAAATATATCAATGCTGAAAATTACTATGACCCTAGCAAAAAAATAACAAAAGGAATGAAAAATAA
TTATCATAAATTTACAGATTCATTCAGTGATTTTGTAGAAATGGTGGACGATATAACATGGAGAATCCGTGATT
CATTTGATTATATTGGAGGATTAAAAAACAAAGAATTTAACGAAGATGAGAATGAATATATCGAACAATTAAT
TCAATTTGTTATATTATCTAAATTCGGCTATATGAATGTAGAAAATCACTTTGATTATATTGAGAACTTAAATACG
CTTAATAATGAATTAACGACTATATTAAAAAGTGAGTTCGCGCACATTTCAAATGATATGTATAATAATTTATTC
AGTAATTTGTTTGATGTTGTAAATACATGGAAAAAGGATTATTCAAATAACTTTTATAATAATACTCAGAATACT
ATAACTACAGAAAAGTTAGACATAATATCTCGATTTATAAAACAATATGATAAACCAGTTGACCGAGACAAAC
ATGGTAAACGAGTTAAAGCAGAAGTGTTTGAAGGTATAAGTGATTTTCATAGGCGTGTAAAAGAAGGATTT
GATAATAATGATGTGCTATTACAATTAAGATTAGAAATGAGCGTACATGAAAATGATAAATTTACTAGTTAAGT
AATGGGGATTTTTTGTAAAATCTAATTCAAAAGCAATATAAATATTTAAATATAAAAAAGGGGTAAGTTATGGA
CTTAAAAAAATTTGAAGAACTATATAAAGATTATAATGATGATGATGTAATAGAACAATTTGCCATATATAAAAT
AAAAGATGAAAAGCAAGTAATGGAAATTACTGATAAAAAGCTAACCAGTGAAGATAAACAAGTTTTTCAGA

GAATAGCTAATTTAAGTTATCAATTTAATGTAGTAAAGGAAATGTATGATTACTATCTTTATGATTTTCAAGTAAT
TACTGATTATATCAAAGATAAAAGAAACCTAAATAATAAGTTTAAATCTGTTGACTTTGTCAACCTATATTTTTT
ACATTTCTTATCTACTGCAAGGACTTTTTATCAATATACTGAAAATTTATTTAAGTCTATAGGTAAGAAAGAATT
TGAGAAATGGAAAAATAATATATCAAATATTTATGATAATAATTTTTCTTATAGATTATGTTATCAAATGAGGAAT
TTTACACAGCATCATGGTTTGGCAATTACGAATATTAAAGAGAAATTAAATTTAGATTCTAATGGTAAAGAAAT
AAATGACTATCAATTGTTCATTGATCCATCTATATTACTAAATACTAACTATAATTGGAAGAATTCAATTAAGAG
CGATTTAGAAAATCATAATGGATTAATTGATTTTTATCAATTAATAATAGATTATGACAAGTGTATAAAAGATATA
CTTTTGTATTCATATATAGGGTTAATAGATAAGTATAGCACTGAATTAAACCAATTAATGACAAAAGCTATGGCA
ATAGAAGGAGAGGCAGGTATACCTTTTTTAAGAAAAGCTAAAAAGAAAGAACTTAGAGAATATTATAAAAAT
ATTGAATTTACGGAATTACAAGGAAGTAAAGCAATAAATGATATATTTAATATGATGTGTGATTTATATACTCAA
CTCAATACATCTAAAATTTGTTTCAATAGATGAATATATTTAAACTGAGTAAAAGATGAATAGATAAATGGACC
AATTAGCAAAATAACAGATTACAAGTCTAATAATATTAAAATATTGTGAAGATATTTATGTTTTTTTTATAGAATT
ACTTTAGAGTTAATCCAATATACAGGGTATTTAACTATAGAAGAGATTACTATGTTTGTTTTTTATCAGAGATCC
TTCTGAAATTTCTTTGAAAATAAAGCAAACTGAAAATTTTAGAAAACTTAAAAGGGTTTAAAGCGAAGATTT
AGTTAATACTTTTAAAAAAGTCCAAAAATAGGTAATACAACTTTAAATAAAGTTCCAAGTGCTGTATATTTTAT
GTAATTTTGTGTAGGTTTAGATATTAAAAAAGTATAGTTAAAATAGGGAATTCATCAAAAAAAGTAAGTGCAT
TATTTAATAAAGAAGTGAAAATAGAATGGGTAAAAGAAACACTGATTCAATATAAAAGTATTGAACCATGTAA
TTTTAATAATGATTTAAATTTTTGGATTGAATATATTGAATATGTTGACAAAAAGCTTCCTCCTTTCGATACAAA
TACTGTTAATGACGAAAATTTTGATGTGTTTTATGAGTTGTTTGATCAATCAAAATCAATAAAAGATTTAAATAT
AATTTCCTCTGCTTCTAATCATATTAGGTTATATAAAAATAAAATTAATAATAAAATAGTTGTTAAAGAGATATTT
TGTGTACCTATTGTAAATGAAAGGATATACAACACAAAGAAAAGCATACTTAAAGAATCAAACACTGATATTG
TGTCGCAAAAAATCCTATAATTGCTAGATTTACTAGAAGAAAGTACAAAGAAGAATTTAAATAATTCATTAGAT
GAAGTATAAAAAAAGCTTTTTATTAGTGATTAATAATTTTTATAATTTAAATTGCTGCAAAAATGTGAAAGATG
CTGCACCTTTTAAAAGTAATAAAAAATAAATATTTAAACTTCTAGAGGAGTTAGGAAAAGTGCAATTTTTACA
ATTAATGCATATCCTACAAAATTCATTGAGAACAATTTTTCGAAAAAGAAGAACTATATGAAATGATTATAAGA
AAAATTACATTATTATGGTATTAATGAATTATTATTTATTGACCTAGATAGTCATATATATTGGATAATAGAAGGA
AATACGTATGGTTATAATAATATAACAAATGGGTTAAATTTAATTAACGAATAATAGCTAAAATCTGATAAGTAA
ATGGTATAATATTTGTAAATATAAATTGTTTGGAGGTATCATATGTCTTTATTTGATAGACTAAGTGTTCTAAAAA
ATAAATCAGAAATAAAACTTATAGAAAGTAAGCAAGTAGTTGAAGATAAAGTATTTGAAATACGACATGGTAA
ATTAGATGATATACCAATGACTAAAGAAAGAAAGCGTTCAGAAAAAACGAGGAAAAAACTTGAAAAACATG
AATCAAAGGTAAAAGAAAAAGAAAAAAGAAAGCAACAAAGAGAAGAAGAAATAGAAAAAAGGAGAATA
GCAAAAGAAGAGCAAGAAAAGCGTGATGTTTGGATTAAAGAGAATGTATTTAAAATTATAGTAAATGAGGA
AGGTAAACGAATAATTTTTTGTCCTAAATGTGGTAGTGAGAATAATGTAACTGCTACTACTTGGAACGTAGAT
GAAGCTGAAAATAGTGAGGATATTGGAACATATTTTAAGAGAACTTGTAATTTATGCGGACATTCTCAAAAAT
TTAATAAAGATAACCCTAAAAAAATTTATGAATAGGAATAAAGAATTGATACATTTTCTGGATAAGTGAAAAA
GACAAATTCTATTGAAATAATATAGAATTTGTCTTTTTTTTATAATTTTTAGTTGTTTAAATGTTAGAATTTATAAA
ACCTCATTATCAACCGATACGCAGAGGCGTATCATAAGT
>Staphylococcus aureus strain CC1153-MRSA
ATGAAAATCACCATTTTAGCTGTAGGGAAACTAAAAGAGAAATATTGGAAGCAAGCCATAGCAGAATATGAA
AAACGTTTAGGCCCATACACCAAGATAGACATCATAGAAGTTCCAGACGAAAAAGCACCAGAAAATATGAG
CGACAAAGAAATTGAGCAAGTAAAAGAAAAAGAAGGCCAACGAATACTAGCCAAAATCAAACCACAATCA
ACAGTCATTACATTAGAAATACAAGGAAAGATGCTATCTTCCGAAGGATTGGCCCAAGAATTGAACCAACGC
ATGACCCAAGGGCAAAGCGACTTTGTATTCATCATTGGCGGATCAAACGGCCTGCACAAGGACGTCCTACA
ACGCAGTAACTACGCACTATCATTCAGCAAAATGACATTTCCACATCAAATGATGCGGGTTGTGTTAATTGAG

CAAGTGTATAGAGCGTTTAAGATTATGCGAGGAGAAGCATATCATAAATGATGCGGTTTTTTCAGCCGCTTCA
TAAAGGGATTTTGAATGTATCAGAACATATGAGGTTTATGTGAATTGCTGTTATGTTTTTAAGAAGCTTATCAT
AAGTAATGAGGTTCATGATTTTTGACATAGTTAGCCTCCGCAGTCTTTCATTTCAAGTAAATAATAGCGAAATA
TTCTTTATACTGAATACTTATAGTGAAGCAAAGTTCTAGCTTTGAGAAAATTCTTTCTGCAACTAAATATAGTA
AATTACGGTAAAATATAAATAAGTACATATTGAAGAAAATGAGACATAATATATTTTATAATAGGAGGGAATTT
CAAATGATAGACAACTTTATGCAGGTCCTTAAATTAATTAAAGAGAAACGTACCAATAATGTAGTTAAAAAAT
CTGATTGGGATAAAGGTGATCTATATAAAACTTTAGTCCATGATAAGTTACCCAAGCAGTTAAAAGTGCATATA
AAAGAAGATAAATATTCAGTTGTAGGGAAGGTTGCTACTGGGAACTATAGTAAAGTTCCTTGGATTTCAATAT
ATGATGAGAATATAACAAAAGAAACAAAGGATGGATATTATTTGGTATATCTTTTTCATCCGGAAGGAGAAGG
CATATACTTATCTTTGAATCAAGGATGGTCAAAGATAAGTATATGTTTCCGCGGGATAAAAATGCTGCAAAAC
AAAGAGCATTAACTTTATCTTCCGAACTCAATAAATATATTACATCAAATGAATTTAATACTGGAAGATTTTATT
ACGCAGAAAATAAAGATTCATCTTATGATTTAAAAAATGATTATCCATCAGGATATTCTCATGGATCAATAAGAT
TCAAATATTATGATTTGAATGAAGGATTCACAGAAGAAGATATGCTAGAGGATTTAAAGAAATTTTTAGAACT
ATTTAATGAATTAGCTTCAAAAGTTACAAAAACATCCTATGATAGCTTGGTCAATAGCATAGACGAAATACAG
GAAGACAGCGAAATTGAAGAAATTAGAACAGCACAAAAAGATAAGACACTCAAGGAAGTGGAAGCACCTA
AAGGAATAATTCCAAAATATAAAAAAGGTGTATCAAAGACTACTAAAAATGATTCAGAAATTGAAAAATCAA
ATAAAGAGAATAAATTAACCGGTAAAGTTGGAGAAAAATTAGCGCTAAATTACTTTAATGAGCTAATTGATAA
TAAAATAGACGAAGATAAGAAAGAACAGTTTAGGAATATTTTAAATGATAATCCAGGCTCTCAACACGGTCAT
GGCTATGATTTAGTAGCTTTTGATCCAACAAATACAGATAAAGCTGTAGAAAAATTTATTGAAATTAAAACATC
TACATCTTCTAGTATTGAGGAACCATTTTTTATGTCGCTAAATGAAATGTTTGCTATGAAAGAATATAAGCAGA
AATATTTAATATTAAGAATATTTAATGTTTCCGGTAAAGAACCACAATTTTATTTTATAGATCCATATGCAAATTA
TTCTGAATTTAAAGATGTAGATGATCTCATTGACAAAGTATTTAATGTAGAAGCTATTCAGTATAAAGTTTTTG
GCGAAAAAATGATTACTTGAACAAGAGCTAAAATAAAATTGTGATCTAATAAAAATAGAAACTGTAATTTAAAT
AAAACTTTCTAAATAAGCTAACTGATAAAAAATCAGTTTGTCCACAGTCTGAAACAAGATTCCTATATTCTTTA
GGAATCTTGTTTTTTCTATTTTTATGGTGATAAAGAGCAGATAAGATAATGTGTAATAATCACAAAAAAGTTAA
ATATTTTAAGGCTTGTTTAATTATTAATGATTTTATATATAAAGAGCAGTATAATAAAGTTGTTAATATATTATGAA
TAATATTCAAGTAATTTTATTGTTTTTTAATTTGTCGATATTTAAGTTGAGTTAAATTTAAAGGGTGTAATTTGTT
TTACAATGATGAAGATAATTAGTCTATCAAAATAAAGGGGTTGGGACTGTTATGAGTGATAATTTGTCATTATT
CATTGACTATATCAATGATAATATAATCTATGGTAGTGAAATCAAACGGGAGAAATTAGAGAATTTATTTAATCA
ATTTGCTATAAAAAAATGTTGAAAAGAACATTGTCTATGATGAACTGAAATCTTTAGATATTACAATCATTGAGT
CACAGGATTCATATAAAAATAAATTGAAGAGATTATTTTCGGTTCTGTTGCAAAGTAAAAAAATATAGCTAACC
ACTAATTTATCATGTCAGTGTTCGCTTAACTTGCTAGCATGATGCTAATTTCGTGGCATGGCGAAAATCCGTAG
ATCTGAAGAGACCTGCGGTTCTTTTTATATAGAGCGTAAATACATTCAATACCTTTTAAAGTATTCTTTGCTGTA
TTGATACTTTGATACCTTGTCTTTCTTACTTTAATATGACGGTGATCTTGCTCAATGAGGTTATTCAGATATTTC
GATGTACAATGACAGTCAGGTTTAAGTTTAAAAGCTTTAATTACTTTAGCCATTGCTACCTTCGTTGAAGGTG
CCTGATCTGTAATTACCTTTTGAGGTTTACCAAATTGTTTAATGAGACGTTTGATAAACGCATATGCTGAATGA
TTATCTCGTTGCTTACGCAACCAAATATCTAATGTATGTCCCTCTGCATCAATGGCACGATATAAATAGCTCCAT
TTTCCTTTTATTTTGATGTACGTCTCATCAATACGCCATTTGTAATAAGCTTTTTTATGCTTTTTCTTCCAAATTT
GATACAAAATTGGGGCATATTCTTGAACCCAACGGTAGACCGTTGAATGATGAACGTTTACACCACGTTCCCT
TAATATTTCAGATATATCACGATAACTCAATGTATATCTTAGATAGTAGCCAACGGCTACAGTGATAACATCCTT
GTTAAATTGTTTATATCTGAAATAGTTCATACAGAAGACTCCTTTTTGTTAAAATTATACTATAAATTCAACTTTG
CAACAGAACCGTATTATGGAATAGAGATGTTGGTAACATTTATACAGGATCATTATACTTAAGTTTAATTTCGTT
ATTACAGAACCACACATTCCAACCAGAAGAGAAAGTATGTCTATTTAGTTATGGTTCAGGAGCAGTAGGAGA
AATCTTTAGTGGTTCAATCGTTAAAGGATATGACAAAGCATTAGATAAAGAGAAACACTTAAATATGCTAGAA

TCTAGAGAGCAATTATCAGTCGAAGAATACGAAACATTCTTTAACAGATTTGATAATCAAGAATTTGATTTCG
AACGTGAATTGACACAAGATCCATATTCAAAAGTATACTTATACAGTATAGAAGACCATATCAGAACATATAAG
ATAGAGAAATAAACTAGTGGCCGATTGTGCTTGATGAGCTTGGGACATAAATCCTAACTCGAAATAAATAAGC
ATATCACTAAACTGATTTTTTAAAGTTTACAGTGATATGCTTATTTTTTTATCTTACGATTTTGTACGTGCATGCT
TGCCTAGGGGTATGGCTCGAGCCATTAGTCTCTCGCACATACTATTCCCTCAGGCGTCAGCACTTACAAAATC
GGTTGTAATTTTCATTTTTATACGCATTCTTACTGAGATTATACTAATAAGAGGAATAGTAAAAGCAATTCTAAG
TAAAATTGCAGATAAGAGGTTTGTTAAAAGCAGTTCTAAGTAAAATTGCAGATAAGAGGTTTGTTAAAAGCA
GTTCTCAGTAAAATTACAGATAAGAGGTACGTTAAAAGCAGTTCTAAGTAAAATTGCAGATAAGAGGTTTGT
TAAAAGCAGTTCTAAGTAAAATTGCAGATAAGAGGTACGTTAAAAGCAATTCCATGCAAAATTGCTGATAAG
GGGTAAGTTAAAAGCAGTTCTCAGTAAAATTGCAGATAAGAGGTACGTTAAAAGCAGTTCTAGGCAAAATT
GCAGATAAGAGGTGCGTTAAAAGCAGTTCTCAGTAAAATTGCTGATAAGGGGTAAGTTAAAAGCAATCCTA
AGTAAAATTGCAGATAAGGGGTACAGAAAAACTAGACTTGATTACAAAATGGAGCTTGGGACATAAATGATT
TTTTAAAAATGAGATGAGACGTAGATTAACTCCATAATCAATACGAATCTATCGACTTCTTTATTTATGATATTC
ATCTCTTTTTAATGGAAATAAAAGTGCGATTAATGTGATAATACAGTTACGTTAATTAAAAAAATAAAAATGCA
AGGAGAGGTAATATGCTAACTGTATATGGACATAGAGGATTACCTAGTAAAGCTCCGGAAAATACAATTGCAT
CATTTAAAGCTGCTTCAGAAGTAGAAGGTATAAACTGGTTGGAGTTAGATGTTGCAATTACAAAAGATGAAC
AACTGATTATCATTCATGATGATTATTTAGAACGGACTACAAATATGTCCGGGGAAATAACTGAATTGAATTAT
GATGAAATTAAAGATGCTTCTGCAGGATCTTGGTTTGGTGAAAAATTCAAAGATGAACATTTGCCAACTTTC
GATGATGTAGTAAAAATAGCAAATGAATATAATATGAATTTAAATGTAGAATTAAAAGGTATTACTGGACCGAA
TGGACTAGCACTTTCTAAAAGTATGGTTAAGCAAGTGGAAGAACAATTAACAAACTTAAATCAGAATCAAGA
AGTGCTCATTTCAAGCTTTAATGTTGTGCTTGTTAAACTTGCAGAAGAAATCATGCCACAATATAACAGAGCA
GTTATATTCCATACAACTTCGTTTCGTGAAGACTGGAGAACACTTTTAGATTACTGTAATGCTAAAATAGTAAA
CACTGAAGATGCCAAACTTACTAAAGCAAAAGTAAAAATGGTAAAAGAAGCGGGTTATGAATTGAACGTAT
GGACTGTAAACAAACCAGCACGTGCAAACCAACTTGCTAATTGGGGAGTTGATGGTATCTTTACAGACAATG
CAGATAAAATGGTGCATTTGTCTCAATAGAAAGTTAGAGGTGAGTCTTACGTTTCAGTGACGGTAGACTTAC
CTTTAACATGTTACATACTAAAAAATTAATTTGAATAAGAAAGAGAGACATATATGAAATACGATGATTTTATA
GTAGGAGAAACATTCAAAACAAAAAGCCTTCATATTACAGAAGAAGAAATTATCCAATTTGCAACAACTTTT
GATCCTCAATATATGCATATAGATAAAGAAAAAGCAGAACAAAGTAGATTTAAAGGTATCATTGCATCTGGCA
TGCATACACTTTCAATATCATTTAAATTATGGGTAGAAGAAGGTAAATACGGAGAAGAAGTTGTAGCAGGAA
CACAAATGAATAACGTTAAATTTATTAAACCTGTATACCCAGGTAATACATTGTACGTTATCGCTGAAATTACAA
ATAAGAAATCCATAAAAAAAGAAAATGGACTCGTTACAGTGTCACTTTCAACATACAATGAAAATGAAGAAA
TTGTATTTAAGGGAGAAGTAACAGCACTTATTAATAATTCATAATAAAACAGTGAAGCAACCATCGTTACGGA
TTGCTTCACTGTTTTGTTATTCATCTATATCGTATTTTTTATTACCGTTCTCATATAGCTCATCATACACTTTACCT
GAGATTTTGGCATTGTAGCTAGCCATTCCTTTATCTTGTACATCTTTAACATTAATAGCCATCATCATGTTTGGA
TTATCTTTATCATATGATATAAACCACCCAATTTGTCTGCCAGTTTCTCCTTGTTTCATTTTGAGTTCTGCAGTAC
CGGATTTGCCAATTAAGTTTGCATAAGATCTATAAATATCTTCTTTATGTGTTTTATTTACGACTTGTTGCATACC
ATCAGTTAATAGATTGATATTTTCTTTGGAAATAATATTTTTCTTCCAAACTTTGTTTTTCGTGTCTTTTAATAAG
TGAGGTGCGTTAATATTGCCATTATTTTCTAATGCGCTATAGATTGAAAGGATCTGTACTGGGTTAATCAGTATT
TCACCTTGTCCGTAACCTGAATCAGCTAATAATATTTCATTATCTAAATTTTTGTTTGAAATTTGAGCATTATAAA
ATGGATAATCACTTGGTATATCTTCACCAACACCTAGTTTTTTCATGCCTTTTTCAAATTTCTTACTGCCTAATTC
GAGTGCTACTCTAGCAAAGAAAATGTTATCTGATGATTCTATTGCTTGTTTTAAGTCGATATTACCATTTACCAC
TTCATATCTTGTAACGTTGTAACCACCCCAAGATTTATCTTTTTGCCAACCTTTACCATCGATTTTATAACTTGTT
TTATCGTCTAATGTTTTGTTATTTAACCCAATCATTGCTGTTAATATTTTTTGAGTTGAACCTGGTGAAGTTGTA
ATCTGGAACTTGTTGAGCAGAGGTTCTTTTTTATCTTCGGTTAATTTATTATATTCTTCGTTACTCATGCCATAC

ATAAATGGATAGACGTCATATGAAGGTGTGCTTACAAGTGCTAATAATTCACCTGTTTGAGGGTGGATAGCAG
TACCTGAGCCATAATCATTTTTCATGTTGTTATAAATACTCTTTTGAACTTTAGCATCAATAGTTAGTTGAATATC
TTTGCCATCTTTTTTCTTTTTCTCTATTAATGTATGTGCGATTGTATTGCTATTATCGTCAACGATTGTGACACGA
TAGCCATCTTCATGTTGGAGCTTTTTATCGTAAAGTTTTTCGAGTCCCTTTTTACCAATAACTGCATCATCTTTA
TAGCCTTTATATTCTTTTTGTTTTAATTCTTCAGAGTTAATGGGACCAACATAACCTAATAGATGTGAAGTCGCT
TTTCCTAGAGGATAGTTACGACTTTCTGTTTCATTAGTTGTAAGATGAAATTTTTTTGCGAAATCACTTAAATAT
TCATCCATTTTTTTAACGGTTTTAAGTGGAACGAAGGTATCATCTTGTACCCAATTTTGATCCATTTGTTGTTTG
ATATAGTCTTCAGAAATACTTAGTTCTTTAGCGATTGCTTTATAATCTTTTTTAGATACATTCTTTGGAACGATG
CCTATCTCATATGCTGTTCCTGTATTGGCCAATTCCACATTGTTTCGGTCTAAAATTTTACCACGTTCTGATTTTA
AATTTTCAATATGTATGCTTTGGTCTTTCTGCATTCCTGGAATAATGACGCTATGATCCCAATCTAACTTCCACA
TACCATCTTCTTTAACAAAATTAAATTGAACGTTGCGATCAATGTTACCGTAGTTTGTTTTAATTTTATATTGAG
CATCTACTCGTTTTTTATTTTTAGATACTTTTTTTATTTTACGATCCTGAATGTTTATATCTTTAACGCCTAAACTA
TTATATATTTTTATCGGACGTTCAGTCATTTCTACTTCACCATTATCGCTTTTAGAAATATAACTGCTATCTTTATA
AACTTGTTTGAAATTTTTATCTTCAATTGCATCAATAGTATTATTAATTTCTTTATCTTTTGAAGCATAAAAATAT
ATACCAAACCCGACAACTACAACTATTAAAATAAGTGGAACAATTTTTATCTTTTTCATCAATATACTCCTTATAT
AAGACTACATTTGTAGTATATTACAAATGTAGTATTTATGTCAAAATAATGTTATAATTTTTGTGATATGGAGGT
GTAGAAGGTGTTATCATCTTTTTTAATGTTAAGTATAATCAGTTCATTGCTCACGATATGTGTAATTTTTTTAGT
GAGAATGCTCTATATAAAATATACTCAAAATATTATGTCACATAAGATTTGGTTATTAGTGCTCGTCTCCACGTT
AATTCCATTAATACCATTTTACAAAATATCGAATTTTACATTTTCAAAAGATATGATGAATCGAAATGTATCTGA
CACGACTTCTTCGGTTAGTCATATGTTAGATGGTCAACAATCATCTGTTACGAAAGACTTAGCAATTAATGTTA
ATCAGTTTGAGACCTCAAATATAACGTATATGATTCTTTTGATATGGGTATTTGGTAGTTTGTTGTGCTTATTTT
ATATGATTAAGGCATTCCGACAAATTGATGTTATTAAAAGTTCGTCATTGGAATCGTCATATCTTAATGAACGA
CTTAAAGTATGTCAAAGTAAGATGCAGTTCTACAAAAAGCATATAACAATTAGTTATAGTTCAAACATTGATAA
TCCGATGGTATTTGGTTTAGTGAAATCCCAAATTGTACTACCAACTGTCGTAGTCGAAACCATGAATGACAAA
GAAATTGAATATATTATTCTACATGAACTATCACATGTGAAAAGTCATGACTTAATATTCAACCAGCTTTATGTT
GTTTTTAAAATGATATTCTGGTTTAATCCTGCACTATATATAAGTAAAACAATGATGGACAATGACTGTGAAAA
AGTATGTGATAGAAACGTTTTAAAAATTTTGAATCGCCATGAACATATACGTTATGGTGAATCGATATTAAAAT
GCTCTATTTTAAAATCTCAGCACATAAATAATGTGGCAGCACAATATTTACTAGGTTTTAATTCAAATATTAAAG
AACGTGTTAAGTATATTGCACTTTATGATTCAATGCCTAAACCTAATCGAAACAAGCGTATTGTTGCGTATATTG
TATGTAGTATATCGAGCTTCACATGAAACAGCTAAAGAAGCTTTGGGCGATAAAGAGTTAAGAGCCATTGCA
CATGAGTTAACTAAAACAGTTAAGGATAACATGAGTGTTGATTGGTCTAAACGAGACAGTGCTAAAGCTAAA
ATGAGAGTTCAAGTTAGACGCCTATTAAAGAAATATGGCTATCCACCAGATCTTCAAAAAATGGCTGTGGAA
CAAGTTGTAGAGCAAGCAGAATTAATGGCAAGTCAGCAATAAAAAAATAAATCATAATGAGTCCGGGACATA
AAGTTCTTGGATAAGTGAAAAAAGACAATTTCTATTGAAATAATATAGAAATTGTCTTTTTTATAAATTTTTTG
ATTATTTTCAGCTCGTTGAGCTACTACTTTTCTTATATTAAGTGCCATTAATACAAAACCAAGTTCTCTTTTGAC
TTTATTGAGTCCTCGGACAGACATCCGAGTGAAACCCAAAATAGCCTTCATAAATCCAAAAACAGGTTCCAC
ATCAATTTTTCTTTGACTGTAGATATTTTTTGTTTCTGGTTCTGAAAGCTTTTTGTTAATTTGGGATTTAAAATA
TTCCCAGTTATAATTCTTCATTATTTTTTTGTTTGTTTTTGAATTGAAGTTCATACATTGATTTTTCAGAGGACAT
TCTGAACAATCATCACATTCATATAATTTGAAGTCTCGCTTATAACCATACTTATCATGACGATAGGCATATCTTT
TAAAACCTAGCCGTTTATTATTCGGACAAATGAATTCGTCATTAATTTCGTCATAGTTCCAATTTTGAGTATTAA
AGATGTCACTTTTATATTTTTTAGTTTTATCTTTTATAAACATTCCATATGTTATGAGTGGCGTTCGATTAAAGTC
ATCTATAATTGCCTTATAATTTGATTCACTACCATAACCTGCATCAGCTACAATATATTCAGGTAAATGACCGTAG
GTCTCTTGAATTGAATTTAAAAATGGAATCATCGTTCTAGTATCCGTTGGATTTTGATACACATTATAAGATAAA
ACAAATTGGGAATTTGTTGCTATTTGTAAATTATACCCTGGCTTAAGTTGTCCATTTTTCATGTGATCTTCTTTC

ATTCTCATAAATGTCGCATCATAATCTGTCTTAGAATAACTATTTCTATCCTTTAAAATAGATTTTTGAAATTCGT
ATCGATACTTTCGCTCAAAATAATCATTGATTTGCTTTTTGTATTTTTTGATTTTAGTTCTTTTGAGACGTATTTG
TTTTCTTGTTTTAGTACATTTTTCATTGTTGATATGTTGGTTTAAATCTTCGATTTCTTTATCTAAGTGACTACCA
ATCAAATCTATTTCTTCTTTTGTTAATTCATTATCATGATCTTCTTTAATTTCCGGTATGATTTTATTGGTTACCAA
TTCATGGTAGAGGGCTTTAGAATCCTCATTCATCTTTGATTCATGGTTTTGAATACTCTTTTTCCATACAAATGT
ATATCGATTGGCATTTGCTTCAATTTTTGTACCATCAATAAAAATAGCTTTATCATCTATAAGATTTTGTTTTACA
CACTGACTGTAAAATTGAATAAATAAAGATTCTAATAAAGCATCTACTTTTGGATTTACTCTAAATTGATTAATT
GTTTTATAAGAAGGTTTTTGATTTTGTGATAGCCACATCATTCGGATGCTATCATTAAGCATTTTTTCTATTTTA
CGACCTGAGAATACAGATTGTGTGTAGGCATATAGAATCACTTTTAACATCATTTTAGGATGGTACGAAGTTG
CACCACGGTGATGTCTGAATTCGTCGAATTCATTGTCAGGAATTGTTTCAACAATATCATTTACAGTAAAACG
ATGTTGATTTGTTTTGTTTCCATATTGACCTCCATGTATTTGCTATGATTTCAAAATCCATTTTTGACGTGCCTTA
GGGTTGAGTGGATGCATAATTTCATTTGTTACTGGATTGATGAGCTTTTTTACTTTCTTTTTATGAGGTTTTAA
CATTTCCATCACTTGTTCGACACGGTCGATAACAACTGGTCGCTTCGCATAGGCACCATAAGCAAGAATCACT
GTGTCACTTTCACTAATCGCTTTCATCAAATGAATATCAGTGTGCTCATCGTATGGATTTTTGATATGTTTGAGG
TTTTCGGGTGTTCTAATATTAGAGAATAGATTTACAAGATATACAGCACCGTATCGTTCTGAATTGGCTAATTG
GTTGAGGATAAGAACAGTTGTGAGATCGAGTGATAATACACCGTCTAAATGAGGATACATCGTTATCACTGTG
CATGCAGCTTTCTTTTCATCCCATGTTTTCTTGAGTAAATAGCGGTGCTGTTCATCATCGCTAAATATAGCTTCT
GTGTGTATCGTACTTTTGATTGTATTCATATATCGTCACTTCCTTTAGTATTCTTCTGGTAAAAGCATCACATAAT
AAAAAGCGTCTACATCATCTTCTCGGATGACGTAGACTTTCTTAGGTAATGCATTTTGATTTTTTACATAGTTT
GTATAGTGATATTCCAATTTGTATGCGGGTTGTTCTTGTTCATGTGTGATTGAGAGTATATTCTCATCTTCTTGC
AGTCTAAAAATGTGTAGGTAATCTGTATGAGGTTGATTGTCCCGTTCTTTTACCATGTTCCAAAGTAAGATTTG
AAGGTCTAGAGATAGTTGTTCACTAATGCCTCTTGTGATGTATCGATTGATTTTCATGTTATTTTACCTCATCTT
GAATTTCTTTCATGATGATAATCGCTTGGCTAATAATCGTAACAGATATTTGTGCCACTTTAATCCAATTATTCAT
GATGATTCCCTCCTTGTTTTAGTAAATGACGTTCATCGATAAGCGTGTTTTTAGTGTCTGTGAGGTAGAGAAA
GTCCATATCAAAATGATCCAGGTAACCAATACTGATGAGTTGGTTATTAGCATATATTAGAAATGGATAGATAC
TTAGCTCATGTAGTTCATCGTTATAGTAGGTATAAGTTTCAAGTGTAAGATGCGCAAGTGGAGTATGGTCTAC
AAAGCGAGTAGGAAGTATATTTTCTGCTACTTCTTCTAACGCTTCACATTCCCATGTTTCATTGTTAGATAGTT
GGAATAGACGAGTTATATATTGTTTGAGTTCTTGAGTGGTTGTTTTCATATCATTGCCTCCTAGATAGTGTTAG
CGATGTATTTCATATACATCACTGAGATAATATATATTTGATTTATCATTTATTACGAATCCCGGTGGGAATAAGA
GAAAATCCCATATGAAAAACTGCTACAAACATTGATATGACAAGAAAATCCCGTTATCCCGCTCATTTGTTGG
GAAATATAGCTATATATTTATATATCATTTGGATTTGGTGGTGTTATTACGACTTACTTTTTCTTTTTATATTATTTA
TTTATAAATAATAACGGGATTTTGGGATTACGCTTGCGTAATCCTTCTCCTACATGAAGTTAGCCAATCCCGTT
ACTATCCCACTTATATTTTGTTTTGGGATGTTCTTTCGTCATATCAATATAAGCTTCACATTGAGCCATGATTTGT
TAGCGTGTTGAATATATTTGTTAGATGAGTGTTATTGAATGACGAAAGGACAGTACATAGATATTATATTTGAA
AGTAGAGTGTTTGAGGCAAAAACAAAGACGAAGTGCTGAGGAGCACTTCGTCTAGATTGTTATTATTAAAA
AGTTGTTTAATAATTTCATTATTGAGCTTGAGAGTGACATAGAATTGTTTTTTATGATTCTCGTCTTTACGAATA
TCAATACGGTCAATAACCGTTAGGTACAAAGCTTTGAGCTGTGATTTATCCATGAATTCTATATTTTGAAATATT
CGTTGTAATAGGGCAGCGATTTGTTTCGTATCATAAGATGGTTTCTCTTGATTTTGTTGGTGCTTGAGCTGATT
CATTTGATTTGTAATGTCATTGAGTTGTGTTTCATATTGATGAATGGTTGGTTTGAGTGCAGATGTTAAGTCTG
GATTGTCTTCGATGGTTTGAACTAGATTTTTAAGTTTAGTGTTAATTTCATCAAATTGTTGTTGTTTATAAGCG
ATATCATGATTGAGCGCAGCTACATCGACTTGATTTTCTTGATTAACACGTTCGACAACTTGTTTGATAACTTT
ATCACTTTTGACTATTTCGAGTATTTGATCCATAACATACTTTTCGATAACATCAGCTCTAACACTATTGGCTGA
ACATACCTTTGATCCTTTGTTACGAAAGTTACTACATGAATAATACCGAATGCGTTTTTTAGTACCGTCTTTGA
GTGTGTTGGTTGTATTTGAAGCTGCATATGCTGCACCACATTTTTCGCAGACAATTATTCCAGTTAACAGATTT

GTCCCTTTACCATGTACCTGTGGCTTTTTGCTTACTTGCTTCTTACGTGCTTGCACTTTATCCCATAAATCTTGA
CTAATAATAGGCGTGTGTTTACCTTCAGCGATTACTGGCTTATCGTTTAATCCTTTACGTCGTTTATCATTCCAA
TCTTTGTATTTTGCGAATTGAATTTTACCAATATAGAATGGGTTTGAGAGAATATAAGTAACAGCTGAAATACT
AAATGGATTACCCTTCTTAGTGACATAACCTTTATGATTGAGTGCATTGGCAATTTTACGATAGCCATGACCTT
TAGCGTAAGATTCAAAGATATACTTCACAATATTGGCTTCATGTTGATTAATCATTAATTCTTTTTTATTATCAGG
TATGTTATTATATCCTAATGGAAGATTGCCTTGATAATAGCCCTCTAAAGCTCTTTGACGTTGTCCAGTGTAAAT
GTTCTCTAAAATTGTATTTCTTTCAAATTCGGAAAAACTTGCAAGTATCTGGAGCATTAACTTGCCTGTTGAAT
TTTTGACTTCCATGCGTTCAGACAAGCTAAAAAATTCAACATTTTGGCGATGAAGTTCTTCGACGATTGTAAG
TAAATCGGAAGTATTACGTGCCAAACGATTTGTTTTATAAACCATAACACAGTCTAATTTTCCATTTTTAGCATC
ATTTAACATGCGCTGTAATTCAGGACGGTTCATAGATTTTCCTGATATACCCCGATCTGCATATATATCAACGAG
TTCATAGCCATTAAATTGGCAATATTGCTCTATTTGTGTAATTTGTCCCTCGATGCTATAACCCTCTACTTGTCTC
TCTGTGGATACGCGAATGTAGCCTCCTACAAGCTTTTTCTTCATTTTATCCATTATAATCCATCCTTTCTTTAATT
ATACAATTGATAATTCCATAGTCTGCTTTACAATATTCAGTGGTTCATTTTTGAAATAGATGCCAGCGAGGCTT
TTATTTTTAGAAATATTAATTTCATCAATATAGGGGTACAACATGTTTAGCGTGAAACGTTGTTGAATGATGTTT
TGGAAAGCTTTTCGAATTTGATACGTACTGATTGATGATATAGGTTTTGATTGCTGACGTAATGATTGCGTTTG
TTCTCTGAACGTTTCTGCATCAATTTTGCCTTGGGCTAATTTTTCTATCAGCTGCTCGTGATTGAGTGTAGTTT
TAGTTTCTATATCTCTTTGTCTTTTGAGTCGTTGTTGAATAGTATGGTTTATTTTTGAATAGAGCTGTTGATTTT
GAAAGAAGTCCTGACAAGTCGCTAAAACACTTGTTTCTAATTCTTGTGCGTTGATCCCTTTGAATTCACAGAC
AAAACGAGATGCATTCATATTTTGAGGACAAACATAATAACGTAATGTATGGTGCTTTTTTCTAATGGTCATAT
TAGTTAGTGTTGAATCACAATAAGGACATTTGATTTTTTGTTTGAGTTGGTTTTCTGACGGCTTACGTTTTACT
GGTTTCTGAGTTCGGGTAACTTGAGCTTCTTCGTATATCGTTGTACTGACAATAGCTGGGAACATGTTTTCAT
ATTGTCCGTATTGATTGATAACACGGCCACAGTAATTAGGGTTAAGGATAATATTACGCACTTGATAGGGCTTA
CGATTAATGAATTTATCATCAGCTTCTAAGTATTGCGCAATTTTTTTATAACCATAACCTTGAAGGTAATAATTG
AACACAGCTTTTACTGTTGGTGCTTTTACTGTGTCTATCGTGAAAGTACCATTATGATAGTGATACCCAAAGG
GTGCATGTGTTGTAATCATTTTACCTTGTTTCGCTTTTTCTTTGATTCCATTTTTGACTTGTTCGCCTATATTATC
AGATTCTAGTTCGGCCAAGCTGATGAAAATATTGAGTTTGAGCCGATCGAATGCTTTATCCATATCAAAATAG
CCATCATGAACGCTTAAGATATGGACATGGTATTTTTGACACAATTTCATGAGTTTTAATGCATTTTTAAGATT
GCGATGAAGTCGGTTTAATCTGTAACAACATAATACATCACATTGTCCTTGTTGAATCAGTTCAGTAATTTGTT
GGTAACCGTTCCGCTTATCAGTGCGTCCTGATTGCTTATCGCTATAAAAGGTAATGTATTGAATATTATGTTTTT
TGGCTAATGCCTCGATGGTTTGTTTTTGTGCTGCTAAGGATTGTTGCTTTGTAGTGCTCTGTCGTAAGTAACCT
ATTGCTTGTTTCATCGTATTTCCTCCTTCCAAAGTGATAATATATATTTATGAACGAATTTATAGATGAGCCCAAC
ACCTGCTGGTGTTGGGCGTTATTATTAGTCATCAGCATGATTAATTTCTTCAACAACTAAATCAGCTAGTAATT
CAATCAATTCATCCATTTTATTCACTCCTGTACGATTTCATCTTTAAGTTATTAAAAATCAATTAAATCATCATTCT
GTTTTTTCCATGATTCAAGTATCTTTTTGTTATCCAAGTTAATTTCAGCTTTAATAGGTTCAGCATCTTTAGTTA
GACCAAAAATAGACGCATATTCTGAATCTAGCTTTAAATGGTAAAAGACAAGTGACTGTTTTTTGCCATTGCC
ATCTTTGACTGTTCTTTTTGTTGTAATTCGATCATGGTCAGATTCGATAAATCCTTTATCCCTTAGTGCATTAAC
AACATTATTAACATCTTGGAAGTGATGCTCTAACAACATATTTTTAAATACAGACGCAATGATTTTGACTTCGA
TATGATTATCTTTTAAGGCAATTAGTCCATAGTTCTCGAACATATTCTTTAACGCTGTATCATCAGAAAATTTAC
CACGGTTTTGTGCTACAAATTGAGTAATGACTTCGATCGCTTTATCTGCCAGTGATCGTTCAGAGACTGTATG
AGCATGATAATCAATAAAGTAGTCTCTGATTTTAGCGATATCAATATCTGTAGCTAAAACACGACCTAATATTTT
CGCAGATGTTGTAATGACTGCATAACGCTTAAACATACGAATACCTGTATTGTTTGTTTCACTGTTCAATTTAG
CTTCAAACCAATCTACTTCCTTGTAAAACCATTGAATAACTTCATCTTCACGATTTATAAGGTATTGAGCTACTA
ACGGTAAAACATGACCATGGTTTAGTGCCACAGCTTTTTTGATATTGTCAGCATTGGTCGCATTTGTAGTAAA
TTGTTCATTAATCTCGATGGTTCTTACACGTAATCCATCGTTTTGAGCAGAATCAGTAAAGATACTGTATTCAG

AGGTTGAAATCACAGAAGTGCCCCAATTCTTAGGCGTTTTAACTTCTCCGTGTACATTGGAACGTTGACGCC
CTTGACCTTCAGCGATAGAGTACAATAACCCCGTTGTATCTTTAAAAGTTGCTGATGAGAGTTCATCAAATAC
AATAGGTATACCAAAATTGTTACTCAAGTAACTTTCAAGTGCATTACGTGTGGCATTCCAACCTCGAAAAAGT
GTTTGATTACCTTTAGTTGGATTTCCAGCGACGGATACTGCTAAAGCTGCTGCTGTTGATTTACCAGTTGATG
ACTGGCCTGTAAAACTAAAGATAATTCCTGCAAATTCGATTTCACGTTTGTACTTCAGAAAGCTTGTCACTAA
GGCAGAAATTCCAAATACGACCGCTAACTCTAGAAGAAGATGACCTTTGACTTCGTTAATATACATATTTAAC
CAATCTTTAAATGTACCTTTAGGCTCTAATGGATAAGCACTATCGACAATAGGATCTAAAGATGATAATTGATC
ATATTGTTTAGATGTATAAATGGTATCTATCATTACAATATAACCGTGGGGTGTTTCTATAATGCCCGAGCCATC
ATATAAATCAGAAGTGGGCAGTTCATCGCGCATTAATTGTAGTGCATAGCTCAAATCTTTTATATAATTTTCATT
GATACTGTGACCATATTTAATCAAGGAAGGTAACTTTCGAGCTGTTAAAATATCAGAGTCAAATGTATGTTCTT
TATCTTTACCGTTAGAAATAATTATCTTTTCTGTATTTTCTGAGGCATGCCAAAATTTAGCTTCAACAGTGATAC
AACTAGACATAGTCACCACTTTTTTCTCATCTCCATCTTTTTTAGGTGGGATAGTTTTATGCCAACCCATTGTAT
CTAAATGGTATAGGCCTAGTCTAAAAATGTCATGATGACTCATTAGCGAACACCTCCTTTCGAAGGGGTGCTG
TTATTGAGTGGATTAGGACCTGTTTTTTTATACAATAAACTACCATTTCTCTTACCAATTATAATAAAAGGAATG
CTAGGTGTATGTTTTACGAAGTATGCGAACCAACGTCCTACCTCGCGTTGAACATCTCTAGAACAGTTAATCC
CTGACTTTTTAGTTAAATCTCCAATCGTAAAATCAGTTCCTTCCTGTAAATTCCATGCAATAGCTAAATCTACAT
TTTTTAAATTTATAAATTCATGACTTTTATAAGTCATAAAAATCACTCCTTACTTAATTGCTAGTAAAGTGACTAA
TTACTTAATCAACTTTACACTTGAATTATTGCAAGGATCAGAATTTTAAGTCATAGAGAGTTTTAATAACACTT
TTTATCGATTTAATAAATTAATGTATTAACTCAGTAAAGTATAAAAGGAGAGATAAAATGAATAATTGGAAAAA
TGAGTTGTTCGAAAGATTAAGAGATATTATTGAACTTGAGATTGCAATACATCTAATGATCAGGAAACATGAA
CGTAATTCGATAGATAAATATAAGAACATTAAGGAGTTTTACCATAATCTAATACAGAAGGATATAGTTATTTAT
GATTATGAGACATTTCATCGATTCTATAAAACAAGCAATATTCACAATTACTTACGGGTAAAACTAAAGCCAAA
AAAAGCTTGGGAAGAAGAAAAAGTCATTCATCCAGAATCTTTTAAAAGTATCTTAGCAATACGTTTAGGTGA
AACAAAATTGATGGCTGGGACTAAATACATTGACCCCAATATTATTAAAAGCTTACGCCAAACTTATAAACAA
CTAAATTCTAATATTGAACAAATTTTAGATAAAATAAGTAAGAATACATTTGACATAAAATTGGAGCCAAAGTT
GATTGCTTATAAAAATTTATATGAATTATATAGTTATTTTATAGATGAAATAGATAACTATAGCAATGACTTTGAA
CAATTACGAGCACTTCTATATTTAGAATTAGATTACTCATTTGTACTTTATAATGAGTTACTAAGGATGATACATT
TAATCAAAAAGTATAAACTAGCTACTAGTAGCAAGATAATTGAAAAAGAAATAGGCGAAATTAGTATGAAGTT
AGCAAATATAGAATTTCCGTGGCTAAGATTAGTAGTTCTAAGAGCTTTTATTGATAATATAAAAAAATATCATAA
TATAAATTCATTGATTAACGATATTAGTCATATTACTAATTATATATTATTCGAAATCGAACAGTGGGTTAAACGA
GCAGAAATTGAAGAAGGAATTAGAGATTTAATGAATGTGAAATATGGTAGGATCCCTTATCTAGATGCAATTA
TAGAAAACAGATATAAAAGAAATAGTATTAAAGAATATTTTATCTTACTCAAACATGAATTTGAAATTAGATGG
TTAAGAGGTCGAGACTTTAATGATCAAGATAGGAATAAACTTCATAGAGCTATTAAAGATATATCTCTTAATCT
TATTGGATACAATAACTAACTTAAACACATTATCTACAAGTAAAAACTTGTGAAAAGAACATGTGTTCTTAATT
GGTGTATAATTAGTTTAAATTAATCTTGGGGTGATGTAATGAATGAGAGTAATCAAATTATATTCGATCATATAA
CAAAGGCTATTGAAAATATGAAAAATAAAGGCTGGGAAGCAAGGGATGGGCAAGAAACTTTAATGTATGAT
GTATTTGATGCTTATGATTCAAGAGAAAATTTAATAGTAGAAGCGCAAGTAGGTATAGGAAAATCATTTGGAT
ATTTAATTCCTGGTATATTGATTTCAAAAAATACTAAAAAGCCTTTAATTGTAACGACTTCCTCAATTCAATTAA
CTGAGCAACTAGTCAATGATATACAGAAAGTTGAAGAAATTTTACATATATCTGTTGATTGTATAGTAGGTAAA
GGTGTAACTAACTATCCTTGTTTTAAAGAAATTCACCGTAAAAATTTAAGTAAATTAAATTTAATGGAAGCTTT
AGATATAGCAAATAAAGGTCTAACAAAACAAACTGTACGTGAGACTAATTTAAAATGGAATCAAATATCTACC
AATAATTGTATTATGAGTAAATGTCATTATAAAAATGAATGCGCTTATTTTAAAATGAGAAATAAGTTAAAAGA
GGGAAATAGATATATTAGACTCAATGAATATAAGCCGAAAGTCATAATCGTTAATCAAGATATGTTAATGATGA
ATTTTAAAAAGTTAACCTTTGGAAAAGAGAGTTTAATCTATGATGATCCTTGTATGCTAATCGTAGATGAAGTT

CACAACTTAGAAGAGAAACAACGTGCTAACATGACTAAAACTATTAACTCTAAAACTGTGATAAATAAAATAA
AAGAAGGCGCAAACCGAGTTGGTAGTAGATCTCGGTATTTAAAAAATATAAAAATGATTGAAGATTGGTTTA
ATCTTCAAAAGGATAGTGCCAAAAGTGAGATATACAAAAATGGTAATTGTTTATCTACTGGACGAGTTGATAT
AAAACCTGTTAGTCATCATCAAATGGCCAAATTAATTAGTATGACAAAGGAAATAGTCGAAGAATTCGATGTT
AATAATATTGTTGTATTTAAACAATCTTCGTTAGTTAGTAATGAACAATTAGAAATAGCTAATAATCTGTTAACA
TTATTTAAAAATCTTCAAAATAACAGCGACAAATATATATTTTGGACTGAAATAACTAAGCAAGACCAAATTGA
TATTTCATTTTGTCCCAATAATATTGCTGAAACACTTAGAAAAACAGTTTTTAGTACAAGTTACCCAGTTGTAT
GTCTTTCTGCAACAATTACTAATAAAACTAATAATGAAAATAGCTATGAGTACATTAAAGAAATAATTGGTTTT
AAAGGCTATGAGGAAGATATTAAGTATAATGATTTTCCGTACAAGGAAAGCAGGCTCTACATACCTCCAAACT
TACCAAAATTTGATAAACGTGATGTTAAATATTATGAAGAAATAGGTAAACATATTTTTGAATTGGCAAGCCAA
AATAAGGGAGGCACTTTGATATTGTTTACTGCTAAAGATGACATTGACGGTGTATATAATGATTTATCAAAAA
GGAAATTTAATAAAACTATCTATGTAGATGATGGTAGTAAAAGTCAAAATGAAATTATTGAATCATTTAAAAAA
ACTAAAGGTGTAATATTGGGTACGGGAGTATTTTGGGAAGGAATAGACTTAAAAAATGAATTATTAACATTAT
TAGTTATTGTCAGATTACCTTTTCCTACGATTGATCCAATTACAAAATATAAGATTACTAAATTAAATGATAGTAA
TGAAGCAGTTATAGTTCCTGAAATGATAATTAAACTGAAACAAGGTGTAGGAAGATTAGTAAGAACAAAGCA
GGATAAAGGTTTATTAGTGTTATTAGATTCAAGAATGAATAAACCCATTTATAAGCATAAAGAAGCAGTATTAG
ATGCATTACCAATTAAAAATATGATTCATAAAGATGAAGTCAAAGATTTCCTTAATAACATTTAAAGTAAGTAG
TAATAGTTATCCAATATTTTCTAGTTGATTCTCTTATAATATGTCCAATTAAGTGTAGACGATTCAAGATGATATT
GAGGAAAGGATGAGAATGTGTCTTTCAGGTAGCTCATTTGATGATAACCTTAAACATAATATTTACTTTCATCC
TGCTGAAAAAAGAGGTTACAGTTTCAAGCCGATAGAATACATTGGGCTATATAAAGACAAAGCAATAAAAGC
TATCGGAAAAGTAGACAAAGTAATAGTGACGGAAATAAATGAGAGCTCATTATCCTTGAAGACAGTTTATCCT
GTAGGTACTGAGTTATCGATTGATGAGTATGAAATAGTTAAAAATAAAATAATGGTGCTTGGGAAAGAAAAG
TGGACTAATCTATTGAACGAACCCCATTATTATTACTTAATTGAAGATTTTATAGAGACAGACTACAAGAAAAC
ATCTAAAGGTGGATTGATGGGAGTTAAGTATTTTAATGTGAATGAAATTTTGAATAGGGATTGCTTAACCACC
GAACAAATCGCTAAAGAATTATGTAATAAAGATTGGGAATGATAAATAGTGAATGAAGCTATAATGCAATAAT
GTATGTATAACATAATCAAGTAATGAATTTAATGATAAGAGAGTATGGAGTAAAGAATTGAATTAGTTTGCATG
GTCTATGTATCTTAAAGGTGTTGAAGGAGATACACCGAAGTATGCAGTACCAATTCTTGAAACAGACTTTAGT
GATTTACCATCTACACATACGACAGTCGGATCACTTGATCGGTTTCGAGATAAGACAATGAGCATTAACAGAA
GCGGGAGTAACTGTTGAATTCCATTTATATCCAGTTAGATACTAGAGTTTGAAGTGATAAATCTTGATTCGGAT
GCTGAAATAAGTAAAAGAGCAGTTACTGAATATAATATAAGTATAAATATGCCACTAGATATTTTAAAGGGTAT
TTATTCACTTTTATAAAATCATCTGCTAAGATAGTATGGAAGATTTTTATAGGAGCTGATTATAATGCGACATATA
AATATGTCATACATCATACGCTATAGTACTCTAACTCAAAGATAAGAAGTACTTAGTTATGAATAGCATTCATTT
CACAAAGTACTTCAACAAAAATGGAGGAATATGAAATGAATAAAATAGAAGTGTATAAGTTTGTTAAAGTAA
AGCAGTTAGTATATCAATTGATTAAGTTATATCGTACAAACGATATGAATTCCCATAAAACACAAAAAGATTTT
TTACTAAATGAAATTAATGATATCTTTAAAGAAAAAGATATTGATATCTCGGACTTTATTACATCGATTGACGAT
GTAAAATTAACTAAGAAAAAAGCAGAACATCTTTTAAATGAATTAAAAGTGTACATCCAAGATTTTGAAATAC
CTTCATCAAGTCAACTGGAGAAAATTTTTCGTAAAGTAAAAAAATTAAAGAGACCAGATATAAATTTAATTGA
TACAAAAGAAATTTCATATTTAGGATGGAATGATAATTCTTCTAACCGAAAATATATCGTTTATAAAAATTTAGA
TGATAAATTCGAAGGTATATATGGCGAAATTTCACCAAATAAAGTAAAAGGATTCTGTAAAATTTGTAATCAG
GAATCTGATACATCACTCTTTCTCAATAAAACTAAACATAATAAGAGTAGTGGAACATATACTAAAAAAGGAG
ATTACATTTGTTATGACAGTTTTAAATGTAATCAGAACCTAGATGATATAAATAATCTTTACGAATTTATTGTTAA
AATAAAATAGATCCAAACAGCCCTGATCTTTAGAACTAATGATCAGGGTTGTTATATTTTGTACCTCACTCTTA
ATCGATACGCTGAAAGTCCCTATAAATAGAACGTACGTTCCGTTTGTAGTATAATGACAAAAATATTAAATTAG
AGGATTAAAAATGGGATTATAATTTTTTAATGTACCAAACTGAAAAAGGCTAAATAAAGATAAATATAAGATTA

GAAAAAACTGTATGGATTAGGCAATATTTATATATAATGAAATGAATTAACAGAAAATTGAATTATCCGGAAAA
ACCGTACTGTTCGATTGGAAGGGAATAGAGAAGGTGAAAAGAATTAACTTCAATTATCATCTTATTTCTGAAA
AAAGATATGAAAAACGGACGCAAGACACTTTTGGACTGACCCCAATGAGTGGGAATGATATAAAAACACTT
TCGTTGAATTCATATTAAAATGAATCATGCGAAGGTGTTTTTTCTATGAAAAGAGTTTCTTATTCAGTAGAAAC
AAAGTATAAAACTGTTGAAATGAAAGCAGCAGGATTTTCAACAAAAGAAATTATGGAAGAATTAAATATTAG
AAATAGAACACAAGTGGAAACTTGGTGGCGATGGTATCGAAATGGGGAAAGTTATAGATTTTCACAACACG
TTGGTAAACAATATACCTACGGTAAAGGATTAGAAGAGCTGTCAAAAGTAGAACAATTAAAATTAGAAAATA
AGAGAAAAGATATAGAATTGGATATTTTAAAAAAGTACAAGGCATTGGAAAGGAAGTGGTACCAACAGTAG
TCATAGATTTAGTAGATCAATTAAAAGGAAACTATTCAATCAAATTGATACTAGAAGTATTAAACATACCTAAAT
CAACATATTACCGATGGAAAAACAAAACCTATAAAAATGATACTGTAACACAAAAAGTTATTGAATTATGTGA
AGCTAACCACTATACCTACGGTTATCGTAAGATTACAGCACTGATTAATCAATGTTATACATCATCAATTAATCAT
AAGAGCGTACAGAGAATGATGCAGAAGCATCATTTGAACCGCCGAGTTAGACCTAAAAAGACGACAAGGA
TAGGTAAACCGTATTATAAAACAGACAATTTATTACAAAGACAATTTAAAGCGAGTTGTCCCATGGAAGTATT
AACAACCGATATTACTTATTTACCATTTGGTCATTCTATGTTGTATTTATCTTCGATAATGGATATTTATAACGGA
GAAATTGTGGCGTATAAAATAGATAATAAACAAGACCAACGTTTAGTTAATGATACATTAAATCAAATCGATAT
ACCTGAGGGGTGTATATTACATAGTGATCAAGGCAGCGTTTATACATCTTATGCTTATTATCAATTATGCGAAG
AAAAAAGCATTATCAGAAGTATGTCCCGAAAGGGAACACCTGCCGATAACGCCCCGATAGAAAGTTTCCATT
CCTTGCTAAAGTCTGAAACTTTTTACATCAATAATCAGCTTAATAGCTCTAATCATATTGTAATAGATATTGTCG
AAAAATACATTAAAAACTATAATAATAATCGAATTCAACAAAAGCTAGGCTACTTATCCCCTGTAAAATACAGA
GAATTAGCAGCCTAGAACATAGTGTTTTTATTAAGTTCCCATTTTAAGGGTTCAGTGCCCATAATGTCATGGCT
TTTTATATTGAAAAAACATGCGATAATTTGTTCCCAAATGTCCTTATAGTTGTACTAATATCTGTAATAGAACAC
TAGAAACTTGGTTTAACCTTTCCTTTTCGTAAATAAATGTAAAGATATACCATAAGGATCTCTTACATAACCATA
ACCTTCAGTATAGAACTCTGGGCTGAATGCTTTTAATACTTCACTGCCTTTTTCTATTAACTGGTCATACACAC
GCTTAGTTTCTTCTACATGATCAAAAGTAATACAAAGCGATATATTATTACCTTGGGTTATAGGTAAACCTTCG
GTATCATCCGCAATCATAATTTTTATATCTCCAAATTGAAGTACACATTGATCAATTTTATTTAAATCTTTTTTATC
GATATTAAGTTGCTTATCTGTCGGTCTATCCTTGATACGTTGGATATACAGTGTTTTAGCGCCAAACAGCTCTTC
ATACAACCTTTTCAAACCATCTGCATTTTCTATAATTAAAAAATAGGGACTTACTTGAAAATTCATAGTTTTTCC
TCCTTAAATTTGTTATAAATAAGTATATACTGTATTAGTGTCACCTATTGATACTAAAAGGGGAATAATAATGAA
AAAATCTGTTAGATTATATCATATGATTGAATACTGTAATGAAAAAAGGAATTTCAAGTTAAATGATTTAATGTC
AGAATTTAATATTTCTCGTAGTACTGCTTTAAGGGATATAAAGGAAATTGAAGCATTAGGAGTGCCTTTATATA
GTAGTACAGGGAAAAATGGCGGTTATATGACCATAGGTAATCGAAACCAAACGAAAATAGCAATCACAGAC
GAAGAATTGAAAGCTTTAGTATTTACACTTTCAAGTATCTCCAATGTTAGTAAGTTACCTTTTCAAACAGAATA
TCAAGAAATATTAAAAAAATTATATAATAATTCGAATAAAAAAGAATTGATAAACCAGTATAATGAGATATTTCA
ATATTTTAATGAAGATACATATCAGTTCAAAAGTTATAAGATATTTAATGAAATCATTAGATTAACAATTGGTAG
TAAGTCATTTAAAATCTGTTATTCACAAAACTACTTGAAAGAACAATATAAAGGTATTGGTATCTTATATAAAAA
TCAACAATGGTACTTCGTTGTTGTTAATATAGCATCAAAATTAGTAAAGTTGTTAAACATTTCTAAAATAAAAG
AAGTATATGAAATGGGAGAAACTAAAGAATGTAATGAAATAACTATGCAGAATTTTCAACAGTTTATGGTTGA
AAACGAAAATACGATTGATATACAAATTAGAAGTAATATTATTGGATTAAATATCTTGAAAGGATACTTATGGA
GTGACTATATGATTGAAAATATTAACGAAGAGACATATTTGTTTAAATCAAAAGTGAACATGCAAGATATAAAT
TTTATAGCTAAATTAATAGTCACATGTGGTGCCAATGTCAAAGTAGAATTTCCTATTAGTTTGAAAAAAGCTAT
TAAAACTGAACTAACCAAAATAATAAACTTATACTAATGGTAATTTAATTATGATTGCAATAACAAATAAAAGAT
AATCTTAAACATATTAAATTGATCCGTAGTATAAAAACCGCATCATTAACCGATACGCAGAGGCGTATCATAAA
T
>Staphylococcus aureus strain CHU15-080

ATGAAAATCACCATTTTAGCTGTAGGGAAACTAAAAGAGAAATATTGGAAGCAAGCCATAGCAGAATATGAA
AAACGTTTAGGCCCATACACCAAGATAGACATCATAGAAGTTCCAGACGAAAAAGCACCAGAAAATATGAG
CGACAAAGAAATTGAGCAAGTAAAAGAAAAAGAAGGCCAACGAATACTAGCCAAAATTAAACCACAATCCA
CAGTCATTACATTAGAAATACAAGGAAAGATGCTATCTTCCGAAGGATTGGCCCAAGAATTGAACCAACGCA
TGACCCACTTTGTATTCGTCATTGGCGGATCAAACGGCCTGCACAAGGACGTCTTACAACGCAGTAACTACG
CACTATCATTCAGCAAAATGACATTCCCACATCAAATGATGCGGGTTGTGTTAATTGAGCAAGTGTATAGCCC
CAGTATAAAACGATACGCTGAAGCATATCATAAATGATGCGGTTTTTTCAGCCGCTTCATAAAGGGATTTTGA
ATGTATCAGAACATATGAGGTTTATGTGAATTGCTGTTATGTTTTTAAGAAGCTTATCATAAGTAATGAGGTTC
ATGATTTTTGACATAGTTAGCCTCCGCAGTCTTTCATTTCAAGTAAATAATAGCGAAATATTCTTTATACTGAAT
ACTTATAGTGAAGCAAAGTTCTAGCTTTGAGAAAATTCTTTCTGCAACTAAATATAGTAAATTACGGTAAAATA
TAAATAAGTACATATTGAAGAAAATGAGACATAATATATTTTATAATAGGAGGGAATTTCAAATGATAGACAAC
TTTATGCAGGTCCTTAAATTAATTAAAGAGAAACGTACCAATAATGTAGTTAAAAAATCTGATTGGGATAAAG
GTGATCTATATAAAACTTTAGTCCATGATAAGTTACCCAAGCAGTTAAAAGTGCATATAAAAGAAGATAAATAT
TCAGTTGTAGGGAAGGTTGCTACTGGGAACTATAGTAAAGTTCCTTGGATTTCAATATATGATGAGAATATAA
CAAAAGAAACAAAGGATGGATATTATTTGGTATATCTTTTTCATCCGGAAGGAGAAGGCATATACTTATCTTT
GAATCAAGGATGGTCAAAGATAAGTGATATGTTTCCGCGGGATAAAAATGCTGCAAAACAAAGAGCATTAAC
TTTATCTTCCGAACTCAATAAATATATTACATCAAATGAATTTAATACTGGAAGATTTTATTACGCAGAAAATAA
AGATTCATCTTATGATTTAAAAAATGATTATCCATCAGGATATTCTCATGGATCAATAAGATTCAAATATTATGAT
TTGAATGAAGGATTCACAGAAGAAGATATGCTAGAGGATTTAAAGAAATTTTTAGAACTATTTAATGAATTAG
CTTCAAAAGTTACAAAAACATCCTATGATAGCTTGGTCAATAGCATAGACGAAATACAGGAAGACAGCGAAA
TTGAAGAAATTAGAACAGCACAAAAAGATAAGACACTCAAGGAAGTGGAAGCACCTAAAGGAATAATTCCA
AAATATAAAAAAGGTGTATCAAAGACTACTAAAAATGATTCAGAAATTGAAAAATCAAATAAAGAGAATAAAT
TAACCGGTAAAGTTGGAGAAAAATTAGCGCTAAATTACTTTAATGAGCTAATTGATAATAAAATAGACGAAGA
TAAGAAAGAACAGTTTAGGAATATTTTAAATGATAATCCAGGCTCTCAACACGGTCATGGCTATGATTTAGTA
GCTTTTGATCCAACAAATACAGATAAAGCTGTAGAAAAATTTATTGAAATTAAAACATCTACATCTTCTAGTAT
TGAGGAACCATTTTTTATGTCGCTAAATGAAATGTTTGCTATGAAAGAATATAAGCAGAAATATTTAATATTAA
GAATATTTAATGTTTCCGGTAAAGAACCACAATTTTATTTTATAGATCCATATGCAAATTATTCTGAATTTAAAG
ATGTAGATGATCTCATTGACAAAGTATTTAATGTAGAAGCTATTCAGTATAAAGTTTTTGGCGAAAAATGATTA
CTTGAACAAGAGCTAAAATAAAATTGTGATCTAATAAAAATAGAAACTGTAATTTAAATAAAACTTTCTAAATA
AGCTAACTGATAAAAAATCAGTTTGTCCACAGTCTGAAACAAGATTCCTATATTCTTTAGGAATCTTGTTTTTT
CTATTTTTATGGTGATAAAGAGCAGATAAGATAATGTGTAATAATCACAAAAAAGTTAAATATTTTAAGGCTTG
TTTAATTATTAATGATTTTATATATAAAGAGCAGTATAATAAAGTTGTTAATATATTATGAATAATATTCAAGTAAT
TTTATTGTTTTTTAATTTGTCGATATTTAAGTTGAGTTAAATTTAAAGGGTGTAATTTGTTTTACAATGATGAAG
ATAATTAGTCTATCAAAATAAAGGGGTTGGGACTGTTATGAGTGATAATTTGTCATTATTCATTGACTATATCAA
TGATAATATAATCTATGGTAGTGAAATCAAACGGGAGAAATTAGAGAATTTATTTAATCAATTTGCTATAAAAA
ATGTTGAAAAGAACATTGTCTATGATGAACTGAAATCTTTAGATATTACAATCATTGAGTCACAGGATTCATAT
AAAAATAAATTGAAGAGATTATTTTCGGTTCTGTTGCAAAGTAAAAAAATATAGCTAACCACTAATTTATCATG
TCAGTGTTCGCTTAACTTGCTAGCATGATGCTAATTTCGTGGCATGGCGAAAATCCGTAGATCTGAAGAGACC
TGCGGTTCTTTTTATATAGAGCGTAAATACATTCAATACCTTTTAAAGTATTCTTTGCTGTATTGATACTTTGATA
CCTTGTCTTTCTTACTTTAATATGACGGTGATCTTGCTCAATGAGGTTATTCAGATATTTCGATGTACAATGACA
GTCAGGTTTAAGTTTAAAAGCTTTAATTACTTTAGCCATTGCTACCTTCGTTGAAGGTGCCTGATCTGTAATTA
CCTTTTGAGGTTTACCAAATTGTTTAATGAGACGTTTGATAAACGCATATGCTGAATGATTATCTCGTTGCTTA
CGCAACCAAATATCTAATGTATGTCCCTCTGCATCAATGGCACGATATAAATAGCTCCATTTTCCTTTTATTTTG
ATGTACGTCTCATCAATACGCCATTTGTAATAAGCTTTTTTATGCTTTTTCTTCCAAATTTGATACAAAATTGGG

GCATATTCTTGAACCCAACGGTAGACCGTTGAATGATGAACGTTTACACCACGTTCCCTTAATATTTCAGATAT
ATCACGATAACTCAATGTATATCTTAGATAGTAGCCAACGGCTACAGTGATAACATCCTTGTTAAATTGTTTATA
TCTGAAATAGTTCATACAGAAGACTCCTTTTTGTTAAAATTATACTATAAATTCAACTTTGCAACAGAACCATCT
AATCTTCAACAAACTGGCCCGTTTGTTGAACTACTCTTTAATAAAATAATTTTTCCGTTCCCAATTCCACATTGC
AATAATAGAAAATCCATCTTCATCGGCTTTTTCGTCATCATCTGTATGAATCAAATCGCCTTCTTCTGTGTCATC
AAGGTTTAATTTTTTATGTATTTCTTTTAACAAACCACCATAGGAGATTAACCTTTTACGGTGTAAACCTTCCTC
CAAATCAGACAAACGTTACAAATTCTTTTCTTCATCATCGGTCATAAAATCCGTATCCTTTACAGGATATTTTGC
AGTTTCGTCAATTGCCGATTGTATATCCGATTTATATTTATTTTTCGGTCGAATCATTTGAACTTTTACATTTGGA
TCATAGTCTAATTTCATTGCCTTTTTCCAAAATTGAATCCATTGTTTTTGATTCACGTAGTTTTCTGTATTCTTAA
AATAAGTTGGTTCCACACATACCAATACATGCATGTGCTGATTATAAGAATTATCTTTATTATTTATTGTCACTTC
CGTTGCACGCATAAAACCAACAAGATTTTTATTAATTTTTTTATATTGCATCATTCGGCGAAATCCTTGAGCCA
TATCTGACAAACTCTTATTTAATTCTTCGCCATCATAAACATTTTTAACTGTTAATGTGAGAAACAACCAACGA
ACTGTTGGCTTTTGTTTAATAACTTCAGCAACAACCTTTTGTGACTGAATGCCATGTTTCATTGCTCTCCTCCA
GTTGCACATTGGACAAAGCCTGGATTTACAAAACCACACTCGATACAACTTTCTTTCGCCTGTTTCACGATTT
TGTTTATACTCTAATATTTCAGCACAATCTTTTACTCTTTCAGCCTTTTTAAATTCAAGAATATGCAGAAGTTCA
AAGTAATCAACATTAGCGATTTTCTTTTCTCTCCATGGTCTCACTTTTCCACTTTTTGTCTTGTCCACTAAAACC
CTTGATTTTTCATCTGAATAAATGCTACTATTAGGACACATAATATTAAAAGAAACCCCCATCTATTTAGTTATTT
GTTTAGTCACTTATAACTTTAACAGATGGGGTTTTTCTGTGCAACCAATTTTAAGGGTTTTCAATACTTTAAAA
CACATACATACCAACACTTCAACGCACCTTTCAGCAACTAAAATAAAAATGACGTTATTTCTATATGTATCAAG
ATAAGAAAGAACAAGTTCAAAACCATCAAAAAAAGACACCTTTTCAGGTGCTTTTTTTATTTTATAAACTCAT
TCCCTGATCTCGACTTCGTTCTTTTTTTACCTCTCGGTTATGAGTTAGTTCAAATTCGTTCTTTTTAGGTTCTAA
ATCGTGTTTTTCTTGGAATTGTGCTGTTTTATCCTTTACCTTGTCTACAAACCCCTTAAAAACGTTTTTAAAGG
CTTTTAAGCCGTCTGTACGTTCCTTAAGGAATTCCTTAGTGCTTTCATAGATTAAACTCACATCACGCTTTAAA
TCGCTTATTTTAGACTTTAAAGACTTGTTTTCTTCAAGCAACTCATTATAATCATTTACATTTTCATTAAATCGCT
CTACAAGACCACTATATTTTTCTTTAACTTGCCCATGTTCTTTACTTAATTTTTTATATTCTCTCGCCATATCAGTA
CTCATGAGATTTCTAACATGCTGTTTTAACCTATCGTTATCTCTCGCAGCAGTCACTAAGTTTTTATAATCACGC
TCCGATATAACAACATTTTTGGTTGGTTTCTTTTCTGTTTTCATTATTTCTTTTCCCAAACCAAACATAGACTTT
TCACCCGTTGGCACTTCAACACTTTTCATGTGTCGTTTCGCTGGTACTTCTAAATCTGATTTAACTTTATCGCTA
TAAGCAGTCCATTCATCTTTTTTAACTGCTAAATTTTTTTCTAGAAAATCAATCTCTTTTTCCAAAGTTTGTTTT
TTAAATTTAGCTGTCTCAATATGTTTACGGTCAGAGCCACGTTCACCACGCTTCAACTCAAAACCCTGTTTTTT
CATATGCTCGGGGAATTTATCTTGTAGCCATAACAGTTCTTGACGATTAAACACATTTTTTCCTTGCAGTTTTC
CATCACGCATAGGCACAACACCTAAATGCATGTGAGGGGTTTGCTCATCATTATGAACTGTTGCATAAGCAAT
ATTTTGCTTGCCATATCGTTCGGAAAATAATTTATAACTTTCCTCAAAAAATCGTTTTTGTTCTCCTGGATCCAG
TTGCTCAAAAAAATCTCGGTCAGATGTTACTAGCAACTCATTTACAAGAACAGCATCTTTCCTCGTTTTTCTTG
TACCTGTTTTTTGTGATTCAATAATTTCTTTGACACGTTCGTTGTAATCAATATTTTTATCATTTTTCAAATCATA
ATTTTCACGTGTTCGCTCATGGTCAATATCATCATTCGTTCTACTTTTTCGCTCTCTTTGATTATGAAATTGCATG
CCTTTTAGTCCAGCTGATTTCACTTTTTGCATTCTACAAACTGCATAACTCATATGTAAATCGCTCCTTTTTAGG
TGGCACAAATGTGAGGCATTTTCGCTCTTTCCGGCAACCACTTCCAAGTAAAGTATAAACACACTATACTTTA
TATTCATAAAGTGTGTGCTCTGCGAGGCTGTCGGCAGTGCCGACCAAAACCATAAAACCTTTAAGACCTTTC
TTTTTTTTACGAGAAAAAAGAAACAAAAAAAACCTGCCCTCTGCCACCTCAGCAAAGGGGGGTTTTGCTCTC
GTGCTCGTTTAAAAATCAGCAAGGGACAGGTAGTATTTTTTGAGAAGATCACTCAAAAAATCTCCACCTTTA
AACCCTTGCCAATTTTTATTTTGTCCGTTTTGTCTAGCTTACCGAAAGCCAGACTCAGCAAGAATAAAATTTTT
ATTGTCTTTCGGTTTTCTAGTGTAACGGACAAAACCACTCAAAATAAAAAAGATACAAGAGAGGTCTCTCGT
ATCTTTTATTCAGCAATCGCGCCCGATTGCTGAACAGATTAATAATAGATTTTAGCTTTTTATTTGTTGAAAAA

AGCTAATCAAATTGTTGTCGGGATCAATTACTGCAAAGTCTCGTTCATCCCACCACTGATCTTTTAATGATGTA
TTGGGGTGCAAAATGCCCAAAGGCTTAATATGTTGATATAATTCATCAATTCCCTCTACTTCAATGCGGCAACT
AGCAGTACCAGCAATAAACGACTCCGCACCTGTACAAACCGGTGAATCATTACTACGAGAGCGCCAGCCTTC
ATCACTTGCCTCCCATAGATGAATCCGAACCTCATTACACATTAGAACTGCGAATCCATCTTCATGGTGAACCA
AAGTGAAACCTAGTTTATCGCAATAAAAACCTATACTCTTTTTAATATCCCCGACTGGCAATGCCGGGATAGAC
TGTAACATTCTCACGCATAAAATCCCCTTTCATTTTCTAATGTAAATCTATTACCTTATTATTAATTCAATTCGCTC
ATAATTAATCCTTTTTCTTATTACGCAAAATGGCCCGATTTAAGCACACCCTTTATTCCGTTAATGCGCCATGAC
AGCCATGATAATTACTAATACTAGGAGAAGTTAATAAATACGTAACCAACATGATTAACAATTATTAGAGGTCA
TCGTTCAAAATGGTATGCGTTTTGACACATCCACTATATATCCGTGTCGTTCTGTCCACTCCTGAATCCCATTCC
AGAAATTCTCTAGCGATTCCAGAAGTTTCTCAGAGTCGGAAAGTTGACCAGACATTACGAACTGGCACAGA
TGGTCATAACCTGAAGGAAGATCTGATTGCTTAACTGCTTCAGTTAAGACCGAAGCGCTCGTCGTATAACAG
ATGCGATGATGCAGACCAATCAACATGGCACCTGCCATTGCTACCTGTACAGTCAAGGATGGTAGAAATGTT
GTCGGTCCTTGCACACGAATATTACGCCATTTGCCTGCATATTCAAACAGCTCTTCTACGATAAGGGCACAAA
TCGCATCGTGGAACGTTTGGGCTTCTACCGATTTAGCAGTTTGATACACTTTCTCTAAGTATCCACCTGAATCA
TAAATCGGCAAAATAGAGAAAAATTGACCATGTGTAAGAGGCCAATCTGATTCCACCTGAGATGCATAATCTA
GTAGAATCTCTTCGCTATCAAAATTCACTTCCACCTTCCACTCACCGGTTGTCCATTCATGGCTGAACTCTGCT
TCCTCTGTTGACATGACACACATCATCTCAATATCCGAATAGGGCCCATCAGTCTGACGACCAAGAGAGCCAT
AAACACCAATAGCCTTAACATCATCCCCATATTTATCCAATATTCGTTCCTTAATTTCATGAACAATCTTCATTCT
TTCTTCTCTAGTCATTATTATTGGTCCATTCACTATTCTCATTCCCTTTTCAGATAATTTTAGATTTGCTTTTCTAA
ATAAGAATATTTGGAGAGCACCGTTCTTATTCAGCTATTAATAACTCGTCTTCCTAAGCATCCTTCAATCCTTTT
AATAACAATTATAGCATCTAATCGGTTCTGTTGCAAAGTAAAAAAATATAGCTAACCACTAATTTATCATGTCAG
TGTTCGCTTAACTTGCTAGCATGATGCTAATTTCGTGGCATGGCGAAAATCCGTAGATCTGAAGAGACCTGCG
GTTCTTTTTATATAGAGCGTAAATACATTCAATACCTTTTAAAGTATTCTTTGCTGTATTGATACTTTGATACCTT
GTCTTTCTTACTTTAATATGACGGTGATCTTGCTCAATGAGGTTATTCAGATATTTCGATGTACAATGACAGTCA
GGTTTAAGTTTAAAAGCTTTAATTACTTTAGCCATTGCTACCTTCGTTGAAGGTGCCTGATCTGTAATTACCTT
TTGAGGTTTACCAAATTGTTTAATGAGACGTTTGATAAACGCATATGCTGAATGATTATCTCGTTGCTTACGCA
ACCAAATATCTAATGTATGTCCCTCTGCATCAATGGCACGATATAAATAGCTCCATTTTCCTTTTATTTTGATGTA
CGTCTCATCAATACGCCATTTGTAATAAGCTTTTTTATGCTTTTTCTTCCAAATTTGATACAAAATTGGGGCATA
TTCTTGAACCCAACGGTAGACCGTTGAATGATGAACGTTTACACCACGTTCCCTTAATATTTCAGATATATCAC
GATAACTCAATGTATATCTTAGATAGTAGCCAACGGCTACAGTGATAACATCCTTGTTAAATTGTTTATATCTGA
AATAGTTCATACAGAAGACTCCTTTTTGTTAAAATTATACTATAAATTCAACTTTGCAACAGAACCGAAAAACT
AGACTTGATTACAAAATGGAGCTTGGGACATAAATGATTTTTTAAAAATGAGATGAGACGTAGATTAACTCC
ATAATCAATACGAATCTATCGACTTCTTTATTTATGATATTCATCTCTTTTTAATGGAAATAAAAGTGCGATTAAT
GTGATAATACAGTTACGTTAATTAAAAAAATAAAAATGCAAGGAGAGGTAATATGCTAACTGTATATGGACATA
GAGGATTACCTAGTAAAGCTCCGGAAAATACAATTGCATCATTTAAAGCTGCTTCAGAAGTAGAAGGTATAA
ACTGGTTGGAGTTAGATGTTGCAATTACAAAAGATGAACAACTGATTATCATTCATGATGATTATTTAGAACG
GACTACAAATATGTCCGGGGAAATAACTGAATTGAATTATGATGAAATTAAAGATGCTTCTGCAGGATCTTGG
TTTGGTGAAAAATTCAAAGATGAACATTTGCCAACTTTCGATGATGTAGTAAAAATAGCAAATGAATATAATAT
GAATTTAAATGTAGAATTAAAAGGTATTACTGGACCGAATGGACTAGCACTTTCTAAAAGTATGGTTAAGCAA
GTGGAAGAACAATTAACAAACTTAAATCAGAATCAAGAAGTGCTCATTTCAAGCTTTAATGTTGTGCTTGTTA
AACTTGCAGAAGAAATCATGCCACAATATAACAGAGCAGTTATATTCCATACAACTTCGTTTCGTGAAGACTG
GAGAACACTTTTAGATTACTGTAATGCTAAAATAGTAAACACTGAAGATGCCAAACTTACTAAAGCAAAAGTA
AAAATGGTAAAAGAAGCGGGTTATGAATTGAACGTATGGACTGTAAACAAACCAGCACGTGCAAACCAACT
TGCTAATTGGGGAGTTGATGGTATCTTTACAGACAATGCAGATAAAATGGTGCATTTGTCTCAATAGAAAGTT

AGAGGTGAGTCTTACGTTTCAGTGACGGTAGACTTACCTTTAACATGTTACATACTAAAAAATTAATTTGAATA
AGAAAGAGAGACATATATGAAATACGATGATTTTATAGTAGGAGAAACATTCAAAACAAAAAGCCTTCATATT
ACAGAAGAAGAAATTATCCAATTTGCAACAACTTTTGATCCTCAATATATGCATATAGATAAAGAAAAAGCAG
AACAAAGTAGATTTAAAGGTATCATTGCATCTGGCATGCATACACTTTCAATATCATTTAAATTATGGGTAGAA
GAAGGTAAATACGGAGAAGAAGTTGTAGCAGGAACACAAATGAATAACGTTAAATTTATTAAACCTGTATAC
CCAGGTAATACATTGTACGTTATCGCTGAAATTACAAATAAGAAATCCATAAAAAAAGAAAATGGACTCGTTA
CAGTGTCACTTTCAACATACAATGAAAATGAAGAAATTGTATTTAAGGGAGAAGTAACAGCACTTATTAATAA
TTCATAATAAAACAGTGAAGCAACCATCGTTACGGATTGCTTCACTGTTTTGTTATTCATCTATATCGTATTTTT
TATTACCGTTCTCATATAGCTCATCATACACTTTACCTGAGATTTTGGCATTGTAGCTAGCCATTCCTTTATCTTG
TACATCTTTAACATTAATAGCCATCATCATGTTTGGATTATCTTTATCATATGATATAAACCACCCAATTTGTCTGC
CAGTTTCTCCTTGTTTCATTTTGAGTTCTGCAGTACCGGATTTGCCAATTAAGTTTGCATAAGATCTATAAATAT
CTTCTTTATGTGTTTTATTTACGACTTGTTGCATACCATCAGTTAATAGATTGATATTTTCTTTGGAAATAATATT
TTTCTTCCAAACTTTGTTTTTCGTGTCTTTTAATAAGTGAGGTGCGTTAATATTGCCATTATTTTCTAATGCGCT
ATAGATTGAAAGGATCTGTACTGGGTTAATCAGTATTTCACCTTGTCCGTAACCTGAATCAGCTAATAATATTTC
ATTATCTAAATTTTTGTTTGAAATTTGAGCATTATAAAATGGATAATCACTTGGTATATCTTCACCAACACCTAG
TTTTTTCATGCCTTTTTCAAATTTCTTACTGCCTAATTCGAGTGCTACTCTAGCAAAGAAAATGTTATCTGATGA
TTCTATTGCTTGTTTTAAGTCGATATTACCATTTACCACTTCATATCTTGTAACGTTGTAACCACCCCAAGATTTA
TCTTTTTGCCAACCTTTACCATCGATTTTATAACTTGTTTTATCGTCTAATGTTTTGTTATTTAACCCAATCATTG
CTGTTAATATTTTTTGAGTTGAACCTGGTGAAGTTGTAATCTGGAACTTGTTGAGCAGAGGTTCTTTTTTATCT
TCGGTTAATTTATTATATTCTTCGTTACTCATGCCATACATAAATGGATAGACGTCATATGAAGGTGTGCTTACA
AGTGCTAATAATTCACCTGTTTGAGGGTGGATAGCAGTACCTGAGCCATAATCATTTTTCATGTTGTTATAAAT
ACTCTTTTGAACTTTAGCATCAATAGTTAGTTGAATATCTTTGCCATCTTTTTTCTTTTTCTCTATTAATGTATGT
GCGATTGTATTGCTATTATCGTCAACGATTGTGACACGATAGCCATCTTCATGTTGGAGCTTTTTATCGTAAAG
TTTTTCGAGTCCCTTTTTACCAATAACTGCATCATCTTTATAGCCTTTATATTCTTTTTGTTTTAATTCTTCAGAG
TTAATGGGACCAACATAACCTAATAGATGTGAAGTCGCTTTTCCTAGAGGATAGTTACGACTTTCTGTTTCATT
AGTTGTAAGATGAAATTTTTTTGCGAAATCACTTAAATATTCATCCATTTTTTTAACGGTTTTAAGTGGAACGA
AGGTATCATCTTGTACCCAATTTTGATCCATTTGTTGTTTGATATAGTCTTCAGAAATACTTAGTTCTTTAGCGA
TTGCTTTATAATCTTTTTTAGATACATTCTTTGGAACGATGCCTATCTCATATGCTGTTCCTGTATTGGCCAATTC
CACATTGTTTCGGTCTAAAATTTTACCACGTTCTGATTTTAAATTTTCAATATGTATGCTTTGGTCTTTCTGCATT
CCTGGAATAATGACGCTATGATCCCAATCTAACTTCCACATACCATCTTCTTTAACAAAATTAAATTGAACGTTG
CGATCAATGTTACCGTAGTTTGTTTTAATTTTATATTGAGCATCTACTCGTTTTTTATTTTTAGATACTTTTTTTAT
TTTACGATCCTGAATGTTTATATCTTTAACGCCTAAACTATTATATATTTTTATCGGACGTTCAGTCATTTCTACTT
CACCATTATCGCTTTTAGAAATATAACTGCTATCTTTATAAACTTGTTTGAAATTTTTATCTTCAATTGCATCAAT
AGTATTATTAATTTCTTTATCTTTTGAAGCATAAAAATATATACCAAACCCGACAACTACAACTATTAAAATAAG
TGGAACAATTTTTATCTTTTTCATCAATATCCTCCTTATATAAGACTACATTTGTAGTATATTACAAATGTAGTATT
TATGTCAAAATAATGTTATAATTTTTGTGATATGGAGGTGTAGAAGGTGTTATCATCTTTTTTAATGTTAAGTAT
AATCAGTTCATTGCTCACGATATGTGTAATTTTTTTAGTGAGAATGCTCTATATAAAATATACTCAAAATATTATG
TCACATAAGATTTGGTTATTAGTGCTCGTCTCCACGTTAATTCCATTAATACCATTTTACAAAATATCGAATTTTA
CATTTTCAAAAGATATGATGAATCGAAATGTATCTGACACGACTTCTTCGGTTAGTCATATGTTAGATGGTCAA
CAATCATCTGTTACGAAAGACTTAGCAATTAATGTTAATCAGTTTGAGACCTCAAATATAACGTATATGATTCTT
TTGATATGGGTATTTGGTAGTTTGTTGTGCTTATTTTATATGATTAAGGCATTCCGACAAATTGATGTTATTAAA
AGTTCGTCATTGGAATCGTCATATCTTAATGAACGACTTAAAGTATGTCAAAGTAAGATGCAGTTCTACAAAA
AGCATATAACAATTAGTTATAGTTCAAACATTGATAATCCGATGGTATTTGGTTTAGTGAAATCCCAAATTGTAC
TACCAACTGTCGTAGTCGAAACCATGAATGACAAAGAAATTGAATATATTATTCTACATGAACTATCACATGTG

AAAAGTCATGACTTAATATTCAACCAGCTTTATGTTGTTTTTAAAATGATATTCTGGTTTAATCCTGCACTATATA
TAAGTAAAACAATGATGGACAATGACTGTGAAAAAGTATGTGATAGAAACGTTTTAAAAATTTTGAATCGCC
ATGAACATATACGTTATGGTGAATCGATATTAAAATGCTCTATTTTAAAATCTCAGCACATAAATAATGTGGCAG
CACAATATTTACTAGGTTTTAATTCAAATATTAAAGAACGTGTTAAGTATATTGCACTTTATGATTCAATGCCTA
AACCTAATCGAAACAAGCGTATTGTTGCGTATATTGTATGTAGTATATCGCTTTTAATACAAGCACCGTTACTAT
CTGCACATGTTCAACAAGACAAATATGAAACAAATGTATCATATAAAAAATTAAATCAACTAGCTCCGTATTTC
AAAGGATTTGATGGAAGTTTTGTGCTTTATAATGAACGGGAGCAAGCTTATTCTATTTATAATGAACCAGAAA
GTAAACAACGATATTCACCTAATTCTACTTACAAAATTTATTTAGCGTTAATGGCATTCGACCAAAATTTACTCT
CATTAAATCATACTGAACAACAATGGGATAAACATCAATATCCATTTAAAGAATGGAACCAAGATCAAAATTTA
AATTCTTCAATGAAATATTCAGTAAATTGGTATTACGAAAATTTAAACAAACATTTAAGACAAGATGAGGTTA
AATCTTATTTAGATCTAATTGAATATGGTAATGAAGAAATATCAGGGAATGAAAATTATTGGAATGAATCTTCA
TTAAAAATTTCTGCAATAGAACAGGTTAATTTGTTGAAAAATATGAAACAACATAACATGCATTTTGATAATAA
GGCTATTGAAAAAGTTGAAAATAGTATGACTTTGAAACAAAAAGATACTTATAAATATGTAGGTAAAACTGGA
ACAGGAATCGTGAATCACAAAGAAGCAAATGGATGGTTCGTAGGTTATGTTGAAACGAAAGATAATACGTAT
TATTTTGCTACACATTTAAAAGGCGAAGACAATGCGAATGGCGAAAAAGCACAACAAATTTCTGAGCGTATT
TTAAAAGAAATGGAATTAATATAATGGATAATAAAACGTATGAAATATCATCTGCAGAATGGGAAGTTATGAAT
ATCATTTGGATGAAAAAATATGCAAGTGCGAATAATATAATAGAAGAAATACAAATGCAAAAGGACTGGAGT
CCAAAAACCATTCGTACACTTATAACGAGATTGTATAAAAAGGGATTTATAGATCGTAAAAAAGACAATAAAA
TTTTTCAATATTACTCTCTTGTAGAAGAAAGTGATATAAAATATAAAACATCTAAAAACTTTATCAATAAAGTAT
ACAAAGGCGGTTTCAATTCACTTGTCTTAAACTTTGTAGAAAAAGAAGATCTATCACAAGATGAAATAGAAG
AATTGAGAAATATATTGAATAAAAAATAAAATTGTTGTGTTTACAACAATACATAGAAAACAGAGGAAACAAT
CAAGTCGTTGAATATTTCCTCTGTTTTTTAGTTGAAAAAATTAACCGAAAGCCTGAATGCAAGTCTTGATTAA
ATCAATAATGCTTGTAATAACACCAGTGAAATCCATATGCATACCCTCTTTCTATTTAAGATACATTAAGTATAAT
ATCAAACAAATAAAAAATGTTAAAAATTCCCTAATTGGCTATTTAGATTGCATAAATGTCAAAAATTTGAAAAA
CATACAACGACTTTGCATAAAAAATCGTCATATTGGAAATACGTAATTTATTGAAATAATAAAAAAAATAAAAG
AACGAAGATGATAACCTAAGTGAGGTTTTAAGTTGTTCTAAGGTTTAATTTAATTTATGTTAAAATAGTTGGTA
TAAAAATACATGATAAACTATAAACTAAATTCAAAATAACTTATGGGGTAGGCAATTATGGAAAATAAAATATA
AATGATAATGAAAAAAGAGTGCTAAGGGAAATTTATAACCATCATAATATTTCGCGTACTCAAATATCTAAAAA
TCTTGAGATTAATAAGGCAACGATTTCTAGTATTTTGAATAAGTTAAAGTATAAATCTCTTGTTAATGAGGTTG
GTGAGGGTGATAGCACGAAGAGTGGTGGTAGAAAACCTATTCTTCTGAAGGTTAATCATCTTTATGGTTATTT
TATTTCTTTGGATTTAACTTATAGTTCTGTTGAAGTGATGTACAATTATTTTGATGGTAATGTCATTAAGCATGA
ATCTTATGATTTACCTGATGAAAAGGTTAGTAGTATATTAAGCATAATAAAAAAACATATTGATATTCAGGAGA
AACTTGATACTTATAACGGACTATTAGGTGTGTCTGTTTCTATACATGGAGTTGTGGATAATGAGCAGCATGTG
ACATATTTACCATTCCATGAAACTGAAGGAATTTCAATTGCTAAGAAAATAAAAGAAATTACTAATGTTCCAGT
CGTAGTTGAAAATGAAGCGAATCTTTCAGCGTTATATGAACGTAATTTTAATCATAATTTATCCTACAATAATCT
TATTGCTTTAAGTATACATAAAGGTATTGGTGCTGGGCTTATTATTAATAATCAATTGTATCGTGGTGCAAATGG
GGAAGCGGGTGAAATTGGAAAAACACTTGTCTCAAAAGTTAGCGATAATGTGGAGATCTTTCATAAGATTG
AAGATATTTTTTCACAAGAAGCTTTACTGCATAATTTAAGTAATCAACTAAATGAGAAGATGACGCTTAGCAA
ATTAATTCAATTTTATAATGAAAAAAATCCAGTCGTAGTTGAAGAAATGGAACAATTTATAAATAAAATTGCTG
TTTTAATACATAATTTAAACACCCAGTTTAATCCGAATGCAATTTACATTAACTGTCCATTGTTCAATGAAATGC
CTGAAATATTAGAAGCAATTAAGAACCAGTTCAAACAATATTCACGTAACGAAATTCAAATAAAGTTAACATC
TAATGTCAAATTTGCAACTTTGCTAGGTGGTACATTAGCAATTATCCAAAAAGTACTACAGATTAATGATATTTA
CTTAGATATAAAAGCATAAAAAACTAATTCAAATGAATAATCAAAGTTCGTAATTGTCTTTATAAAAAAATCCC
TCAATCCGAATTGAATTTTCGGATTGAGGGATTTTTATAGTTCTATTGCAGAAGAAAACTATTTTAAAAATGCT

GGTAAATGTTGATAGCCACCTCTAACGTTAACAATATTCGTAAATCCTTTATATTCTAATATTCCTACCGCTATTG
AACTTCTAACACCTGATTGACAATGTACATAAATTAGGTCATTTTTATCGAAAGGTATATCTTCATTTAAAAGTT
TACCGTGAGGAATATGAATTGCTTGTTTTAAATGACCTTTACGCCATTCATCATCATTACGAACATCTAATACAT
TATGTTCTTCACCAGTCATTTCAGAACTATGAATAGATGATGTGACGATATTTGTTTGTGGCAAACGGTAACTT
TTTACATTTTCAAAACCAATTAATTGTAAAGCATGAATAGCTTGTTGAACGGTAGATTTATCGCCAATTAATTC
AATATCTTAGTCATAATCTAAATACCAACCAATTTGATTTATAAAAGTTTTATTAAAAGGAATATTGATAGTTCCA
TGCATATGACCACCATGGAATGCTTCTTTACTTCGAAGATCAAAAGCAGTTTGTGTATTGCTTGAACTAGGGT
AAACATTATATGGTTGGTACATTTGCATACCAAATTGATTTATTTTTTTCATTTGTGAAAAATGGTGTGGTGGA
GCTGGCTGATTGAGTGTTAAAGTTTCGATAAATGAAGTTTCATCTTTAACATTAAAAGCCCAGTTGTTTATTTT
CTCATAACCCAAAGTAGTTGTAGGTAATGAACCTAGCGCTTTACCACAAGGACTCCCTGCACCATGACCTGG
CCAAATTTGAATATAGTCTGGTAATGTTGCAGCAAATTGTATGGACTGATACATTTGTTTTGCTCCGATTTTTG
TAGAACCTTCAACATTTACAGCTTTTTCTAATAGATCTGGTCTACCTACATCACCAACAAAGATGAAGTCACCG
CTAAATAATCCCATTGGTATACTGGAACCCCCACCTTCGTCAGTAAGTAAAAAACTAATACTCTCAGGGGGAT
GGCCTGGAGTGTGTAAGACTTCTAATTTAATCTTTCCTAAATAGATAATATCTTGATGCTTAACGAAATGTGTT
TGTTTAGGCATATTTTTATAATTAAATTCATCTTTACCTTCATCAGATACGTATATACTTGCATTCAATTTATTTGC
CACATCTCTAATACCTGAAGCAAAATCAGCATGAATATGTGTTTCTGCAGCTTTAGTAATTGTGAATCCTTCTT
TATCTGCAACTTTTAAATATTTTGTTAAATCTCGTATAGGGTCAATAATCATTGCTTCTCCTGTACGTTGACATCC
AATTAAATAAGATGCTTGTGAAAAATTGTCTTCATAAAATTGTTTGAAAAACAAAAAAAACTCCTTTTTAAAA
TAGATTTTATTGATTAGATAAATAAGTTATGATTTGCTTGCTCAGTATGTCCAATATAAGTACCTACGCCACCATA
ATCGACTTCATCTCTTAATTCTTCTTTTGAAATTCCCATAACATCCATACTCATGGTACAAGCAATTAACTTTATA
TCTTGATCGATTGCTTGATCGATAAGTGAGTATAAAGAATCAACATTTTTCTTGTTCATTACATAACGCATCATA
ATATTACCTAGTCCAAACATATTCATTTTTGATAATGGCATATGTATTGGATCCTTAGGTAACATAAGGTCAAAC
ATTTTGGAAATACCTTTCTTTTTAACGCGAGTTGATTGCGCTTTTTTTAATGCGTTGAGGCCCCAAAAAGTAA
AGAAAATAGTTACATCTTTACCTGCTGCTTTAGCGCCATTTGCGATGATCATTGCTGCTACTGCCTTATCTAACT
CACCGCTAAATAAAACAATTGTTGTACCTGTAGCAGTGTCATTGATTTCAAATTCTTTTGGCTTTTCTTTTTGA
ATAATTGCATTAATTACATTTGCTTCTTCAGTAAGATTTACAAGGGTATTCCCTGTTTGTTTCGCCCAACTTTTA
ATATCACTATTGAAACCAGGATCTGTAACTGTTACCTCGATTTGCTCACCCGTTGAAATATTGTTAATTTCTTTA
CTGATATTAACAATAGGTCCAGGGCACTGAAGACCTCTAAAATCAAATTGTTTACGATTCTCTTTGATTTCAAT
ATCTTTTTCTATTAAAGGAGCACTATTGAAGTTCTTTGCTTCATAATCTTTATATCCACCCTTTAAATTCACGAC
ATCATAACCTTGTTTGGCTAAATAATCGCAAGCTTTAGTGCTTCGGTTACCGCTTTTACAATGTATATAATACGT
TTTGTTGCTATTCTTATTGAATGATTTAATCTCTTCTACTGGGTGTAAAGTTGAACCGTTAATGTGTCCTAATTC
ATATTCTTCTTTTGTTCTAACATCAATCAATTGACCCATTTTTGCCAATTTTTCTAATTCTTCTTTGTTTAATGAAT
TAATGTGTACTTCTTTGTATTGTTCCATACTTACCTCCTATAAATACCTATGAGGGTATAATAAAACGGATAGAAT
CATTTGCCAAATACCTATATGGGTATTTGACAATTTTGTTTTAATTTATTATTATTAACTTAATCAATTTATGTGGA
GGAAATGAATATGACTTATGATAAAAAAATGATTAATCGTATAAATAGAATACAAGGTCAATTAAATGGTGTCG
TAAAAATGATGGAAGAAGAAAAAGATTGCAAAGATATAATTACGCAACTTAGTGCATCTAAAGGTTCTATACA
ACGTTTAATGGGGATTATAATTAGTGAAAATTTAATAGAATGCGTTAAAACAGCAGAAGAAAATAATGAAAG
TTCTCAAGAATTAATTAATGAAGCAGTTAATTTATTAGTTAAAAGTAAATAATGGATATAGCAAATATGACTATT
ATGTTGCTAATTGGCGTACTGGGTGGATTTATATCTGGATTAATAGGTATTGGGGGCGCAATTATTATTTACCC
AGCTATTCTTATATTGCCACCATTAATAGGTATACCTGCGTATAGTGCATATATTGCTTCGGGACTTACCTCTAGT
CAAGTATTTTTCAGTACACTTAGTGGATCATTAAATGCAAGAAAACAACCAGCTTTCTCTCCTAAACTTGTTAT
ATATATGGGAGGGGGTATGTTGATTGGAAGCATGTTAGGGGCAATTTTAGCTAGTTTGTTTAATGCTACTTTT
GTAAATACGGTATATGTAATAATCGCCATACTTGCTTTAATATTGATGTTTATTAAAGTTAAACCTACTACACAAG
AGACGAAATCTAAACCTTTGCTATTTATTATAGTTGGATTTGGAATTGGTGTAATTTCGGGAATTGTGGGTGC

AGGTGGAGCATTTATCATCATTCCTGTATTATTAGCATTATTTAAATTACCAATGAATACGGTAGTGAACAATAG
CATAGCAATTGCTTTTATATCTTCAGTAGGGGCATTTTTTATAAAATTAATGCAAGGATATATACCAGTAGAAA
GTGCAATTTTTTTGATAATTGAATGATGATCATTTTCTGAAAATATTATGTGGTCATATAATATAATGCCCATCAT
TTCACTAATCTCTTTTATTCTCTGAGTTATTTTGATATCTCCTGGAGAAGGTGTTACATCTTATCAAGAGTAAAT
TACAAAAGAATCATTTAAATCAATACTTTCACTTTGAATACATGTATTTGAAGTGGAAGGTACTTATTTCAAAA
ATAGTAAAACCTGTATCTTAAATTACTTAATAGTAACATAAGATACAGGCTGATTTTTTATTCATTGTTATTTATA
CTAAAGCACCCGATAGCTCTGAAAACAATCACAAATTCAACTTTTCAAAGCCACAGCTTTAAGTTATTTTGTC
CAGACAACCCCCATTGCCCAACCCATTTTATGGAATTGGCATCCAGGCAACAACTTTTCATATAAATCGTAATA
ATTTTGTTCAGATAGGTACTTATCTGAAGCTAAATGCTCAAGCCATGATTTAGATGTGTTGTGATTATAAATTCT
AATCGCATTTTTTATTCCAAATTTGAAACAATGCGGTAAAAACTCTTGGATTGCCCCTAATTTATAAACATAGG
TAGGCGGTGTCTCGACTTCAGATACATTATCAAGAATAACTATTCTTCCTTCCTCATTCAACAGTTCCTTCATTT
GCTGTATTACGCTGGCTATATCATCCAAWTAATGAGAAGGTTGTTCGGCTTACAATAAAATCAAACTTCTCATT
AAAATTAAGTTGTTCTGCATTCATATTCAGATAGACCGTATTTGTTAGTTGACGTTTAGATTTGGCAAGATCGA
GCATTTGATTAGAAATATCAATCCCTACCACTTCATCATAATAACTTGCTAATTTCTCCACTAACAAACCCGAGC
CACATCCGATATCTAATGCTCTGCCTTTCTTTGGAGACATATTAGACACAAAGAATGAATAATCATTCAAAAGC
TCATTCACGAAATCGTAATCTTCTGCAACCTTATCAAACTGTGATTCTATTGTATTCAAAAAGATCCCCCATTCC
TACTTTATCGACATTCTTTCATTACTTACCACTTTAGATGTTTTTTCGTTGGGGATAAAACTTCCCTTTAGACAA
TTTTATCCAAAGACAATACAACAGTGCAACTTTATTAAAGTCACTCTCCTTTATCGCAGCCTTTACTTTTTAGTA
AAGACAGTGGCTTCTCTTATCAAGTTTCAAAACATATTATTTTGAAGAAAACGTCCATCTGAAGTGTCAAGTG
CAAAATTACATATAAAGGTTTATTCTAAAATGAAAAGATGATACAATCATATTCAGTTACATAAGGAGGTTTCA
ATTATGTGCACCAGTATCGCAGTAGTAGAAATTACTTTATCTCATTCATAATGAAAAAAATGGAAAGGAGATA
AAAGTATGGGTACATTAATAAAGTTCGTTATCAACCAAATCAAAATTAATTGGTTATAATAGTAAGGAGAAGG
TTATAATGAACCAGAAAAACCCTAAAGACACGCAAAATTTTATTACTTCTAAAAAGCATGTAAAAGAAATATT
GAATCACACGAATATCAGTAAACAAGACAACGTAATAGAAATCGGATCAGGAAAAGGACATTTTACCAAAG
AGCTAGTCAAAATGAGTCGATCAGTTACTGCTATAGAAATTGATGGAGGCTTATGTCAAGTGACTAAAGAAG
CGGTAAACCCCTCTGAGAATATAAAAGTGATTCAAACGGATATTCTAAAATTTTCCTTCCCAAAACATATAAAC
TATAAGATATATGGTAATATTCCTTATAACATCAGTACGGATATTGTCAAAAGAATTACCTTTGAAAGTCAGGCT
AAATATAGCTATCTTATCGTTGAGAAGGGATTTGCGAAAAGATTGCAAAATCTGCAACGAGCTTTGGGTTTA
CTATTAATGGTGGAGATGGATATAAAAATGCTCAAAAAAGTACCACCACTATATTTTCATCCTAAGCCAAGTGT
AGACTCTGTATTGATTGTTCTTGAACGACATCAACCATTGATTTCAAAGAAGGACTACAAAAAGTATCGATCT
TTTGTTTATAAGTGGGTAAACCGTGAATATCGTGTTCTTTTCACTAAAAACCAATTCCGACAGGCTTTGAAGC
ATGCAAATGTCACTAATATTAATAAACTATCGAAGGAACAATTTCTTTCTATTTTCAATAGTTACAAATTGTTTC
ACTAAATTAAAGTAATAAAGCGTTCTCTAATTTCACAAGAGGACGCTTTATTCTTCCCAAAAATTGTTCAATAT
TTATCAATAAATCAGTAGTTTTAAAAGTAAGCACCTGTTATTGCAATAAAATTAGCCTAATTGAGAGAAGTTTC
TATAGAATTTTTCATATACTTAACGAGTGCTTTCACCTTTGAATATAGTCCTTCCCACTTATCATCACACTCTCCC
CGATAGCCTTTTCTAGCTATATCCAGTAAAGTTACATGCTCTTTAGGTAAAAGAGGTATAGCCCATTCTGCAGC
GACATCTTTCGAGGTAATTTCACCAGTAGTCACTGTTTGCCACATTCGAGCTAGGGTTAAAATTACATTACGC
TCATCACCTTTTATCCCCTCAATTAGTTCTGGCAAAGAATCCTTAATTGCTCTTCGAATATCTGTCAAAGGTACG
GAGACAAGTATACTTGAAGAATCAGGACCAAATAGAGAAATACTATTCTTTCTTGCTTGTGCTAAAACAATAG
CCAAATCAGGATCATAGCTTGGTTCCTGAATTTGTCCATTCTCAAATTCACCCCTGAGCCACTCACCGTATATA
AATTCTCTTTTTGGAGGATATTGCCAAGGGACAACTTCACTCCTATTTATAACCGTAACTTCAAGTGGTCTAAC
AGAATCCGTATTTCCAATCTTTCCTGATATAGNGTCATTAGTCTTTCTGTTAGTTTTTTTCGAGTTAATTGAGGT
AAACTATGATTCACGACGACTAGAACATCTACATCGCTGTTAATGCGTAAACCACCATTTACTGCTGAACCAA
ATAGATATACTCCAACTATTGAACTTCCAAATAAATCTTTTACGATTTTTAATGTTTGAATCGCTTGATTTGGTAT

TTTTCCGTTAATCAAATTGCTCATGATTTCACCTCGTTGATTATGTTCATATAAAGTTTATATTGATACTCAATTTA
CTTACCCTAGATTGGACATATACTTAAATTACTGTTCAATAAAGCTGACCGTTAGCGTTTAAGTACATCCTTTCA
CAATTTGTCTACAGATTAATAATTATTCTTTATTATACAGATCTCCATATAATTTTTGAATTTGGTTCTGTAATTTT
TTATTTTCTTTTTCTAATTCCATTACTCTTCTTTTTAAGGTTTTAATAAGGATTTCCTCCGAACGAGAACTTTTCT
TGGGTTTTGAGACTACATTTGCTGTTATTTGACGCTCACGAAGGGATTCGATTCTTTGCCTAATATCGTGTTCC
TTATAAAGCCATGATTTAGAAACATTAGCTTCCTTTGCTATTGAATTAAAATTAATAACTTTACCTTCAATCGAA
AATTTAGAAATCGCTTTGTCTACTTTTTCCCTTGTCTTTTTTGATTTCTGCTTCGCCAAACGTACAATTTCTGTT
GTATTTCTAACTTGTTTATCCATTGATAATTACCCCGTCAAACTTCCAATGATTTGTTCTAAACGCTCTTTAACA
CGGCTATTAGTCTCTACTTGTCTTTGCCATTGTTTATCCTTAGCTATGGCTAATAACTCTTCTGTACGCTCTAACT
GTTCTTCGTGCTGTGGTAAGAATTGCTTACTGGTACAGAAGTGAGTGCAATCTAAGCATGCATTCGCATGTG
GACAACCACCTGCTACTACTGGCAATCTACAATAACCATTTGGAAGCACTTGTGCATTTATATTTTTCTTGAAC
CATTGAAGCTCTACATCATCGACTTCATTATCTTCATCTAGATCAAGCACATCTCCATTATTGGTAACCAGTTTT
TCCTGAAATTTAGTAAATTCATTTTTTAGAGTTTCATCAAAGATATGAGCGTATCTGCTTGTCATTTCTGGGCTT
TCATGCCCCAAAAATTTCTGCACAATATGCTGGGGCATCCCGTTGTTAATCATTCTTGTTCCTACTGTATGGCG
AAAGGCATGGGCATGGAATCTATAAATCTCACCTGATTTATCCACTATATTTTGCTCATAAGCTAATTTATTTAA
CTCACCTCTAAATGTTTCTTGTTTTAATGGCGATCCATCTTTTCTTGGAAAGAGGTATTCACTATCTGGAAATT
CCTCTGAAACTTTATCTTCCCGAACTTTAATAAGTAAAGCTACCTCTTTAGATATTGGAACTATATGCTCCTTTT
TCATTTTCCATTGATAATACTTTAAAAAGAAATCTCCATCTTTGTCCTCTAATAGACAGCCTTTTTTCAAGGTGC
ACAATTCACTTATCCTCATTCCACATTTCTAATAGACAGCCTTTTTTCAAGGTGCACAATTCACTTATCCTCATT
CCACATTCTTGAACAATCATAGTCATCGTAGCTATATATTCGGGTAATTTATCAAGATGACTGTTCAATTGCTCT
AGGACGAATTCATCTATAAAGCGTGGTTTTGCTCTTGGTATTTTCGGATAGTCCTCAGAATAAATTAATATTTT
GGAAGGAACATCATCCCATTCTAGCCTAAGAAGGGTACTAAATAGTCCTTCCAATATAGAGATCCTCCCAGTT
ATTGTACTAGGTTTTATTCCCATCATGTTTAGTTCACTTAAATATGCTTCAATTTCCACTCTCCGTTAATTGGTGT
ACTCTCTGAACTTGTTTAAATTTCATGTCCAGAAAATTAAAGAACTCTTTAAGTCTTTGGGCAATATCACTTAC
ATAGGAAAAGCTATCCACGTTCAATCTCAACTTACAATATCTTTTTACAAGTTGTTTAAAATATGTATTCCGAAA
CCCTTTAAAGTTAATTGTATATTCATATTGTGTTGGGTTAACCTTATCATCTGGCAAAGGTAAGTTACGTCTATC
CCAAACGTCTTTATCCCACTCCTCTCCATCAAAATAAAAGTTCTCATAAAACTCCATAAATTGTTTTAGATTAGT
AACATAGTAGGAATTAGCTTTTACAGGTGTTTTTTCTTGATTAGCAGTAATCTTATAATTAGTAGTGGTAATTCT
AACACCCCGTTTTGTCAAATAAGGTTCTATACTCCGTCATTGCTTTTTCAATAGGAACTTCAGTAATTGAAGTA
ATGCTAGGATACTTTAAATCTAAGAAATCTAACATTTTATTAATTACTGTTCCTTTTCTAATCCAGACAGTTTTT
GCATTCCATATTCCATTGTTTAAATGGTAAAAATAAAAATATTTCAATTCTGTTCTTAACCACAGATTTTTAACA
CGTTCAAAACGAACCCAACGATTCCTTAAAGCAGGATTCTTACTTAATTCTATGGCAGAAGGATGTGGACATT
TTCTTATATCCCAACTATTATTAGCCCAAAACCCCTGCATTTCTTCATTCATTACAGCTATTTTTTTGCTAATCTC
ACTCTGACTAATAATTTTCCTTTTACTAGAAGCATTCATTTCTTATGCTCCTTTCTCTCGAGGTATTTATTAAACT
CATTTTTCATATCCTGATCTGAAAGATGAACATAGGTATTTAACGTTGTCTGAACATGTGCGTGACCTAATCTC
TTTTGAACGAACGCAACATCCCATCCTTCCCTAATTAGCTGCGTTGCGTGAGTGTGGCGAAGCATATGTGATG
TAAATTCTATTCCAGTCCTTTTAACTATTCTTCTAACTAGATCAAGAACACTTTGGTACTTTAGTGGTTTCCCAA
AATAGCCTTCTTTTAAGGAAATAAAAACATAATCATGCTCCAATTCCTCACTATACTCATATATCAAGTAATCTGT
ATAAAGTGACATAAGTTCTTTACTCACATGTATTGTTCTTTCCTTCCTTAATTTAATATAAGCTTCATTAACATTA
ACATCTCTAGGTGTTAAATGGATTTGATTGTCCCAAGTGACAATATCTTCAAGCCTAAGCGATAACACTTCACC
GATTCTTAAACCACCCTCATACATAAGCATTAAAATTAATTTATCTCTTTTCGTATGACAAGCATCAATAATTTGC
TTAACTTCCTTTGATCTCAATGTTCTTATCTGTTTCTTTTTAACCCTTAACTTTAAGACATTCTTTTGGTATCTAC
CCTTATTAACATGATGTAAAAATCCTTTGAAATTTCTTCCCTTTGCTTGTTTAAATACATCAATTGATTTAAATTC
TCCTAATCTACTTAAATAATCAAGAAAACTCATAACTACATTTAAAATTGTATTCACTGTCGTTTCTTCTCTTATG

GCTTTTTTTGACTGAAGATCAATTACATTTGATGCTGAAGGATATCTCAACCAACCTACGAAGTCTGCTAACA
ACTCAAAGTTAATATCATTAAGAATAACACCTCTCTGTTCCATGAACTCGTACAGCAACTTTAAATGATAGCAG
TATGCCTTAATGGTATTAGGAGGCTTACCAGTATTATCTAAGTATTTAATAAATTTCATTACTGGTTCTATTAGCT
GGTATTCTTTATCTAGTAATAAATACAATGGATACGGCTTATTCTCCACTTCTATCCTTTGAACCTTCACATGTTC
CCACCTCTTTAAATACTTTAACTACTACAAATTATAAACCTTGTTAATTTTTATTTAAAGTAAAATATCCCTTTAA
TATCTCCTCTTAAACTACTTTAACTACTATTATTTATTATACTATGGTYMWTWMATATCTCCTCTTAAACTACTT
TAACTACTATTATTTATTATACTATGGTTAATACATCTCCACTTGGATGATTATGACCGAGCATTATACTATTTGCG
TTACTGAGTATCGCTGTTTTGAATATTTCTCTAGGGTGAATCACCGTTTGGTTAATAGATCCAATCGATAGTGT
TTGAATATGTGTAGGTTCATTTTTACTGTTCATACATATGAGAATGAGATGCTCTCGGTCACTGTTTCCAATGA
ATGAACGCATGATTTCTGCCGCATCCTCAGGGTTTGAAATACGATTTTTTAGATAACTTAATGTATCTGTTTTTA
TCATTTGTAGTGAAACAATATTGATTTCCTTCATCGTTTACCTCCATATATAGGTTATGCTTTCAAAGTCCATTTT
TGACGTGCTTTAGGGTTGAGTGGGTGCATGATTTCATTTGTGGCTGGATTAATGAGCTTTTTGACTTTCTTTT
TATGAGGCTTCAACATTTCCATTACTTGTTCGACACGTTCTACAACAACTGGCCGCTTCGCATAAGCACCATA
AGCTAGAATCACTGTGTCACTTTCACTAATCGCTTTCATCAAGTGGATGTCTGTGTGTTTATCATAAGGTTCTT
TAATATGTTTAAGGTTCTCTGGTGTTTTAATATTAGAGAATAGATTTACAAGATATACAGCACCGTATCGTTCTG
AATTCGCTAATTGATTGAGGATAAGAACAGTTGTGAGATCGAGTGATAATACACCATCTAAATGTGGATACAT
CGTTATCACTGTACAAGAGGGTTTCTTTTCATCCCATATTTTCTTGAGTAAGTAGCGGTGTTGTTCATCGTCGC
TAAATATAGCTTCTGTGTGTATCGTACTTTTGATTGTATTCATATATCGTTACCCCTTTTAATATTCTTCTGGCAAA
AGCATCACATAATAAAAAGCGTCTACGYCATCTTCACGAATGACGTAGACTTTCTTAGGTAATGCATTTTGATT
TTTTACATAGTTTGTAACGAATGACGTAGACTTTCTTAGGTAATGCATTTTGATTTTTTACATAGTTTGTATAGT
GATATTCCAATTTGTATGCAGGTTGTTCTTGTTCATGTGTGATTGAGAGTATATTCTCATCTTCTTGCAGTTTAA
AAATGTGTAGGTAATCTGTATGAGGTTGATTATCTCTTTCTTTTACCATATTCCAAAGTAAGATTTGAAGGTCTA
GAGATAGGTATTCACTAATACCTCTTGTGATGTATCGATTGATTTTCATGTTATTTTACCTCGTCTTGAATTTCTT
TCATAATGATAATTGCTTGGCTAATAATCGTAACAGATATTTGTGCCACTTTGATCCAGTTATTCATGGTGAGTC
CCTCCTTCTCTTAGTAAATGACGTTCATCGATAATCGTATTTTTAGTATCTGTGAGATATAAAAAGTCCATGTCA
AAATGATTCAGATAACCAACGCTGATGAGTTGGTTATTAGCGTACATAAGAAATGGATAGATACTTAGGTCAT
GTAGTTCATTATTGTAGTAGGTATAAGTTTCAAGTGTAAGATGTGCAAGTGGGGAATCATTAATAAAACGTTC
CGGTAGAATATTTTCTGCTGCTTCTTCTAACGCTTCGCATTCCCATGTTTCATTGTTAGATAGTTGGAATAGAC
GAGTTATATATTGTTTGAGTTCTTGAGTGGTCGTTTTCATATCATTGCCTCCTAGATAGTGTTATAGTGATGTAG
TTTATGTACATCATTGGGATAATATATATTTGATTTGTCATTTATTACGCATCCCGGTGAGAATGAGAGAAAATT
CCATATGAAAAACCGCTTCAAACCTTGGTATGACAAGGAAATCCCGAAATTCCGCCTATTTTGACGAACAATC
AACTCATTCTTTATAACTATTGATGTTAGGGTGGGGCTCTGCTTTCTTATATATATTATTTATTTATAAAGAATAA
CGGGATTTTGGGATTGTGCTTGCACAATCCTTCTGCTTCTTCGAATCTGCAAATCCCAATCMTTTCCCGGTAA
AAAATCATTGTGGGATGTTCTTTAGCAATTTCAATATAAGCATCGTGTAGTCATGAAAAAAATGACGGCAATG
ACTGTTTCATTAGATAAGTGTTATTGAAATTGATAAAGAGAATTTTAAAAATGGTTAGATAAAATAAATGAAA
GAATATAATGAAAATTATTGTTATAACAATGATTCTATTAGCTAAATAGTAAGATATAGTGTTTGGGGCAAAAA
CAAAGACGAAGTGCTGAGATGCACTTCGTCGAGTTGTTTATTATTGAAAAGTTGTTTTATGATTTCGTTATTA
AGTTTGAGTGTGACATAGAATTGTTTTTTATGATTACCATCTTTTTTAATATCAATGCGATCAATCACTGATAGA
TACAATGCTTTGAGTCGAGATTTTTCTATGTGCTTAATATCATGAAAGATGTGTTGTAATAGTTTACTGATTTCT
TTGGCATCAAATAAAGGCTTATCTTCATTTTGTTGATTTTTGAGTTGGTTGATTTGATTCGTAATGTCATTGAG
TTGCTTTTCATATTTTTGAATACTTGGTCTGATTACTGATGTTAAGTCCGGATTATCCTCGATGGTTTTAATCAA
GTTATTTAGTTTGATTTGTACTTCATCATATTGTTGTTGCTTATAAGCAATATCGTGATGAAGTGCAGCGCCATC
AACTTGATTTTCTTGATTGACGTGTGTTACTACGCGTTGAATGACTTTATCACTTTTGACTATTTCAAGTATTTG
CTTCATCACATAATCTTCAATCACATCAGCTCTTACACTGTTTGCCGAACATACTTTGGAACCCTTGTTCCGAA

AATTACTACATGAATAGTAACGAATACGTTTCTTAGTCCCGTCTTTAAGTGTATTCGTGGTATTGCTTGCTGCCA
TAGGTGCGCCACATTGGGGACAGTGAATAATGCCTGTAAGCAGATTCGTTCCTTTGCCATGGACTTGGGGTT
TTTGACTGACTTGTTTTTTACGCATTTGTACTTTATCCCATAAATCTTGATTAATAATGGGGGAATGCTTACCTT
CAGCTATCACTGGTTTATCATTAAGCCCTTTACGACGTTTTTCACTCCAATCTTTGTATTTCGCAAATTGAATTT
TGCCGATATAGAATGGGTTAGCTAATATATATGTGATAGAACTAATACTAAAAGGTTTACCCTTTTTAGTGACAT
AGCCTTTGTGATTTAATGCATTGGCTATTTTACGATAGCCATGGCCTTTGGCATAGGACTCGAATATATATTTCA
CAATATTAGCTTCATGTTGATTAATCATCAGTTCATGTTTACTATTAGGTATTTTGTCATAGCCCAGCGGCAAAT
TGCCTTGATAATAGCCTTCTTGGGCACGTCTCGTTTGACCCATAAATACATTCTCGACAATGTTATTACGTTCG
AATTCTGAGAAACTCGCAAGTATTTGTAACATGAGCTTACCCGATGAAGTATTGACTTCCATACGCTCTGATAA
ACTGAAAAATTCGACATTTTGTTTGTGTAAATCTTCGACAATTTTGAGAAGATCAGATGTATTACGAGCTAATC
GGTTTGTTTTGTAGACCATAACACAGTCGATATAGCCTTCTTTCGCATCCTTCAATATACGTTGGAGCTCAGGT
CGATTCATAGATTTACCTGAAATACCACGGTCAGCGTATATATCTTTAACTTCAAAATGATGGAAGTCACAGTA
TTCTTTGATTTGATTGATTTGTCCGTCGATACTATAACCTTCTGTGCTTTGCATTTCTGTTGATACACGTACATA
GATACCGACACGTTTTGTTTTAAGTTGTTGCATTATGTTTCATCCTTTCTTCGTTTATGCAATCGATGATTGCAT
GGTTTGATTGACGATATTGAGTGGTTCATTTTTGAAATAGATTCCTATAAGATTTTTATCTTTCGTAATGTGAAT
GGTTTCAATATAGGGGTACAATATGTTTAACGTGAAACGTTTTTGAATAATATTTTGAATGGTGTGTTGTATTT
GATGTCCATTGATAGATGTAGTGCGTTGCGGTTGTTGACGTAATGATTGCGTTTGTTCTCTGAACGTTTCTGC
ATCGATGATGCCTTGTGCCAACTTTTCTATCAGTTGTTCTTGAGTCAATGTGTGATGTTTTTCTATGTTTCTTTG
TCTTTTGATGCGTTTGTCAATCGCACCTTTAATTTTTGTGTAGATGCGTTGATTTTGATAAAAGTCTTTGCACA
CTTCTAATACTTTATCTTCAAGTGTTTGTGCATTGATGCCTTTAAAATCACAGACAAAGCGAGAAGCATTCATG
TTTTTAGGACAGACGTAGTAACGTAATGTATGATTCTTTTTTCTAACGGTCATATTCGTAAGTGTTGTATTACAA
CATGGGCATTTGATTTTTTGTTTTAGTTGGTTATCCGAAGATGTCTGTTTGATTTGTTTTTGCGATCGAAGTCT
CTGCGCTTGCTCATATATACTTGTGGAAACAATAGAAGGAAACATATTGTCGAATTGGCCATATTGATTGTTGA
CACGACCACAATAATTAGGATTGATGATAATGTTACGAACTTGATAGGGTTGTCGATTGATATACGTGTTATCT
TCTTCTAATAACTGTGCAATTTTCTTATAACCATGACCTTTAATGTAATAATTGAATACAGCCTTTACCGTTGGT
GACTCATTTTGATTGATGATGAATGCTCCGTTGTGATATTCGTAACCAAAGGGCGCATGGGTTGTAATCAATC
GACCTTGCTTTGCTTTTTCTTGAAGCCCATTTCTGACTTGTTCTCCAATGTTATCTGATTCAAGTTCGGCCAAG
CTGATAAAAATATTAAGCTTGAATCGGTCGAAAGCTTGATCCATATCAAAGTAACCATCGTGTACGCTTAAGAT
ATGAACATGGTATGTTTGACATAATTTGATGAGTTTTAATGCATTTTTCAGATTACGATGTAATCTATTAAGACG
ATAACAACATAATATGTCACATTGCCCTTGTTGAATTAATTGCGTCATTTGTCGATACCCACTACGATTATCTTT
GCGTCCTGATTGTTTGTCGCTATAAAAGTTGATATGTTGAATATGATGTTTTTCGGCTATTGCTTCGATAGCCT
GTTTCTGTGCTGCAAGAGATTGTTGTTTCATCGTACTTTGACGTAAATAGCCTATGACTTGTTTCATATCGGCT
CCTCCTTTCACAGTAATAATATATATTTATGGATGAATTGATATATAAGCCCAACATCAATAAGATGTTGGGCGT
TCATATTAGTCATTCATTTGATTGATTTCTTCAATTACCAAATCGGCTAATATCTCGATAAGTTCATCCATGTTTT
TCACTCCGTTATTTGTTCTATCTTCAATACGTCGATTATTCAGTTTGATGCTTCACGGTTGTATGATAAAGACAA
TCAGAAATCTTCGTGAACTCCTGAAGGGCCTATCCCCTCATTAGCGGATTTAAAAAGTTCTTTCGCAGCTTTG
TTATCATTTGCCGGTGTCCAATTTTGAATTAACGACTTATCTTTAGTTAATCCCAGGATAGATGCAAACTCTACA
TCTAATTTTAGATGGTAAAATACAAGTGATTGTTTTTTACCGCTATTATCTTTGACACTTCTTTTAGTTGTTTGG
CGTCCACGGTCAGCTAATATGAAACCTTTATCTCTTAAGGCGTTGACAACATTATTAACATCTTGAAATTGATG
ATTGTTTAGCATCTGTTTAAAAACGTTTGCAATCATTTTTACTTCGATATGGTCATCTTTTAATGAGATTAATCC
ATAGTTCTCAAACATATTTTTCAAAGCACCTTCATCTGAAAACTTACCTCTGTTTTGTGCTACAAATTGAATGAT
GACATCGATAGCTTTATCAGCTAATGAGCGTTCAGAGATTGTATGACCATGATAATCAATAAAGTAGTCTCTTA
TTTTAGCGATATCAATATCTGTAGCTAAAACACGACCTAATATTTTTGCAGATGTTGTAATGACTGCATAACGCT
TAAACATACGATTGCCTGTGTTGCTTTTATCATCTTTCAATTTAGCTTCAAACCAATCTACTTCCTTGTAAAACC

ATTGAATAACTTCATCTTCACGATTTATAAGATATTTAGCTACTAACGGTAAAACATGACCATAGTTTAGTGCTA
CAGCTTTTTTAATATTGTCAGCATTGGTCGCATTTGTAGTGAATTGTTCATTAATCTCGATGGTTCTTACACGTA
ATCCATCGTTTTGAGCTGAATCATTAAAAATACTGTGTTCTGACGTTGAAATGACAGAAGTACCCCAATTCTTA
GGCGTTTTAACTTCTCCATGAACGTTTGAACGTTGACGACCTTGACCTTCAGTGATGGAGTACAATAACCCC
GTTGTATCTCTAAAAGTTGCTGATGAGAGTTCATCAAATACAATAGGTATACCAAAATTGTTACTCAAGTAACC
TTCAAGTGCATTACGTGTGGCATTCCAATTTCTAAAGAGAGTTTCATTACCTTTGGTAGGGTTACCAGCGACT
GATACAGCTAAAGAAGCTGCAGTTGACTTACCGGTTGAGGATTGACCTGTAAAACTAAAAATGATTCCGGCA
AATTCGGTTTCATGTTTGTGCTTCAGGAAACTCGTCACTAAGGCAGAAATACCAAATACGACTGCTAATTCTA
AAAGAAGAGAACCTTTAACCTCTTTTAGATACATGTTAAACCAATTATCAAATGTTCCCCTAGGTGCTAAGTC
ATAAGTATTCTCACAAATGGCGTCAGATGGAGATTTATTATCAAATTCCGTAGTAGTATAGATTTCATTTAACGA
TACAATAGGACCAAACGGTGTTTCCAGTATACCTACCCCTTCATATAAGTAGGAAATGGGTAATTGGTTGCGC
ATTTGTTGCAACGCATAACCTAAATCTTTTGTATATTTTTCATTAATACTAAATCCATATTTCATTAAAGAGGGCA
GTTTTTGTGTTGTTAAAATATCACTAGATTCAACAATTACTTTTTGATCCTCGTCTGTAATAATTACTTTTTCAGT
GTTAGTTTTAGGGTCAATAAACTTATTTTCGATAACGATAGGACCTGCGATTTCAACTTCAGTAGGCATTCCTC
CTTTTTCTTTGGGAGGCTTGTCTTTATACCAACCTTTTTTTGATTTGTATCGTGGTGAAGGATTAAATGAAGG
GTTAGTTTGAGTCATTAGCGAACACCTCCTTTCGAAGGGTTGCTGTTATAGTGTGGATTAGGACCTGTTTTAA
GATAAACTAAGTGACCGTGAGTATCCTTACCGATAATAATAAATGGAACACGTGGCGCATGTTTTACAAAATA
TGCGAACCAACGTCCAACATTTTGTTGTACAGCTTTTGAACATTGTACATTTGCACGACTGTTCAAGTCATGA
AATGTAAATTCTTCTCCTTCAGGTAAATTGAATGAAATACCTAATATTTTCTTTTTTAACTGCTCGAATTTATCTG
ATTGATTGAAATACATTTAACTTCCTCCTACTAATGAACTAAGATAGGAAAATTAAATATGCGCACAATTAACTT
TCTTAAGTTCATTTTAGCCAAGAAGTTATCATTTGAAAGCTTGTAAAAATATGTTAGAAATTCCGTACTAAATT
AAGATGGATTTTTGTTGACAAAATAAAAAACGCTGATTTAACAGCGCTTTAAATAAAAATTAATCTGAAGTTA
TATAAAAGTAGTCAGAAGGAGTGGTGTTATTATCATTATAATAATCTAGTGCTTTATTGATTTCTTTTATTAATTT
ATCATGATGTTCTTTTGTTATTTTCTTTGGATATTTGAGTTCTTTTTCTTCTAAGTCTTTTGGAGACACTAGGTC
ACTATTTAAAATTAATGATAACAGCATATTTTTAGGTGCCGAAGCTCTGGTGTCTCGATGAGTAATATATTTTGC
TAAATTACTGTTTATACTTACTTTTTCATTTTCTAAAAAGTTTGTACTATTTTTAACATGAGCATCAGCAATTTTG
GTACTTATTTCAGTGTTTAAATCGTCTATTTCATCTAAATAATATTCAAGAATCTGAAGTTTATCTTTAGAAGTG
ATACCTTTTTCAGTGAAAATCTTAATAATGATAGATTTGATTCCAATTTCTATATTACTGTCTACTAGAAAATAGA
GATAATAATTAGTCAGAGCATGTTCATAATCTCTTTGATCAGTAATTTCATGATTGATGAAATAATATAAGTTCTC
TTTGATACGATTAGAAGAATCTATATCTTTGTCATTGATACCGTTTTTAAAAAAGTGTAGATAAACTGAGTTGT
TTGAGAGTATATCATATAATACAAATAAACTTTTTGGTTTAAATACATATGATTTTCCTGATTTAAAGTTTGCTAT
GTTTATTCCTAGAGCGTTGCAGAATTTATTAAAATTAGTTGTAAAATTATTTTTTTCTTGAATAAACATGTCGTT
GTCATTTAATTCTGAACTATGATTTTTAATCATATTGATATTTTCTTCAATCTTGTCACTACTTAAATTTTCCATGC
AAAGTATACTATATCTTACTATATCACTTATATTTATGGAAAAATCTTTAGGAGAACGATTTAAAGTATCCTTTTT
ATTATCTTGATCTACAGCCCTACATGAATCATCAAAAACTTCTTTGACATTTATTTTATCAATGTTCAAAATTATG
TCTACTAAAGCATTTTGAAAATCATCGCTATATATGTCTTTATTATTCATAGTGTCACTACCTTTATTTTTAATAAC
ACTATATATTATATCTTATAAACCTAATACCATGAAGAATAAAAAGAATGATTCAAATTTTATATTTAATCCATAGT
TGGCTGCTATGGTTAGTGTACTAGTCTAGTTTTACTAGTATATAAAACAGTACTCAATTTTTATTTTTTCAAAAT
TATAAAAAAGAACACCTATCATCGATAGGCACTGAACCCCTAAAACGGGAACTTAATAAAAACACCATGTTCT
AGGCTATTAATTCTCTGTATTTTACAGGGGATAAGTAGCCTAGTTTTTGTTGAATTTGATTATTATTATAGTTTTT
AATGTACTTTTCGACAATATCTATTACAATATGATTAGAGCTATTAAGCTGATTATTGATGTAAAGAGTTTCAGA
CTTTAGCGAGGAATGGAAACTTTCTATCGGGGCGTTATCGGCAGGTGTTCCCTTTCGGGACATACTTCTGAT
AATGCCTTTTTCTTCGCATAATTGATAATAAGCATAAGATGTATAAACGCTGCCTTGATCACTATGTAATATATAC
CCCTCAGGTATATCGATTTGATTTAATGTATCATTAACTAAACGTTGGTCTTGTTTATCATCTATTTTATACGCCA

CAATTTCTCCGTTATAAATATCCATTATCGAAGATAAATACAACATAGAATGATCAAATGGTAAATAAGTAATAT
CGGTTGTTAATACTTCTATGGGACAATTCGCTTTAAATTATCTTTGTAATAAATTGTCTGTTTTATAATACGGTTT
ACCTATCCTTGTCGTCTTTTTAGGTCTAACTCGGCAGTTCAAATGATGCTTCTGCATCATTCTCTGTACTCTCTT
ATGATTAATTGGTGATGTATAACATTGATTAATCAGTGCTGTAATCTTACGATAACCGTAGGTATAATGGTTAGC
TTCACATAATTCAATAACTTTTTGTGTTACAGTATCATTTTTATAGGTTTTGTTTTTCCATCGGTAATATGTTGAT
TTAGGTATATTTAATACTTCTAGTATCAATTTGATTGAATAGTTTCCTTTTAATTGATCCACTAAATCTATGACTAC
TGTTGGTACCACTTCCTTTCCAATGCCTTGTACTTTTTTAAAATATCCAATTCTATATCTTTTCTCTTATTTTCTAA
TTTTAATTGTTCTACTTCTGACAGCTCTTCTAATCCTTTACCGTAGGTATATTGTTTACCAACGTGTTGTGAAAA
TCTATAACTTTCCCCATTTCGATACCATCGCCACCAAGTTTCCACTTGTGTTCTATTTTTAATATTTAATTCTTTC
ATAATTTCTTTTGTTGAAAATCCTGCTGCTTTCATTTCAACTGCTTTATACTTTGTTTCTACTGAATAAGAAACT
CTTTTCATAGAAAAAACACCTCCGTATGATTCATTTTAATATGAATTCAACGAAAGTGTTTTTATATAATTCCCA
CTAATTGGGGTCAGTCTACTATATGATACGGTTTTTTAATTTAAAGTATCAATAATAATTGGATATAGAGGGAA
AGAAGCTATAATGATATTTGCTTACTAAGTGGATAAAGATATTAGAAAAACGAGAAGCAGGACAGTTATTAAA
TCGGTAGATATTAATCATAAGTATTTAACTGAATTTTTACCTTTTGTCGAATATATAACTAAAGTTGAAGGTAGT
AAAATATTTATCTAGTCGATATTAAAACAATTTATCGAAAACGATGGTTTTCCATATCCTCTTTAGTAGCAATTT
TCTAACAATATATAATTAATTATATACAAATTAAGAATAATTAAGAAGTATATATTAATTAGAGATAGTTTAAAAAT
TAGATTTCAATCAATTAATTAAAACAGGTTAGACTATTAATTTATACTAACCTGTTAAAAGTAACAAAATTAAGA
ATATATTAAAACTTGTGTAATGAGTAAGCCTCCACCAAATATTAAAACAAACGCGACAACAGGCCATACGAAA
CGTAACCAGTGTGAGTAGCGCACGTTTAACATTTGAAGTGTTGCCATTACAAGTCCAGTAGGCGCTAAGAAC
AACATTGCATACTGACCGAATTGATATGTTGTAACAATAACAAATCTTGGTATACCTACTGTATCAGCTAATGGC
GCAAAGATAGGCATAGATAATACTGCTAATCCTGATGATGATGGTACGATAAATCCTAAACAGAAAAAGATAA
AGAGCAGAACAATGATAAATAAAGGCCCACTCATATGTTGCACGATAGATGATGAAAAGTGCAAGATTGTGT
CAGAAATCATTCCTTTATTCAATACTAAGTTGATTCCACGAGCTAAACCAATGATTAAAGATACACCTACTAAA
CTTGAAGCGCCATTAACGAATGCATCTACAGTGCCTTTTTCGCCTAAACCATATTGTCCTGTTCCAGCAATAAA
CATGATGACAATGGTAAAGATTAAGAATGCAGATGCCATGACTGGGAACCACCATCCTTGTGTCATAACACCC
CAAACCATAATAGGGAATGGTAGGACGAAAAGCGTAAGAATAATCTTTTTACGTAATGTAAACTCAGAAGAA
CCGTCATCATGGAGCACAGACCACTGTTTTTCAAATGCTGCTTTGTCTTCATAAGAATAAGAGGATTTAGGAT
CTTTTTTAATTTTTTTACAATACCAGAATAAATAACTAATAACAAATATGGCACCGATGATACAAGCGCCTATTC
TCCAATAAAGACCATCAGTAAAAGTTGTTCCTGCTGCATTAGAAGCAATGACGACTGAGAATGGGTTGATTG
TTGAGAATGTACTACCCACAGAGCTTGCTAAGAAAATTGCACCGAAAGTAAGACTTTAGAATCTATGGGAAA
GATAATAAGGTTTAATAGAAATACAAGAAGTGTAAATAGTAAGCATATAATTTATACATTTTAATATAATATAACA
AGAAAGGTAAACACAAAGATAATAAAAATAAAAATTTAATAAAGATAGAGAATTATTAAATTGTTTTTGATATA
AAACAACTTAATAAAAAAATTTAATTTTAGGGGAGGATAATTATAATATAAAGAAACATAGAATGGAATTGCAA
TTGGGAAGTATACGATTTTAACTGCGAATCATATGTTCAACGACTTACAAATGCGTAAATCGATAACACATTCA
AAAATATATAATCTGAAAATTGATTTACTAAGAGAGGATTATTGTAGTTACAGGTGTTAAGTTGCTCCAATATG
GAGTTGTCGTCTTGTTTTATATTAAACAAGTTTTATTCAAATACATGACAATACGTAAAATAACACTAGAATTAT
CAATCACTAAAATTAAAACAAATGTATCTATTCAAACTTTTCTTTAGGGTTTACTTGCTGAATAATTTAAAAATG
AAGTATTAGGTGTACATGCTTTCAGAATTGCTTTTGAAGAATATTAAAAGTATAATCTGAATACGATGACAGAA
TGAATAGGTAAACTATGACAAGAAGTCATCAAGGTTATCAAATAAATATATCCCAGCGGTTGAGACGACTCAC
TGCTGGGATTTTATTGTTAGTTATTTATTCTAAGTGTTGCATTTGGTAGCCGATTCTAGGATGTGTGATGATTAA
TTTCTTTTCAGTGTTTGGGTCTTGCGTATAATTCAACTTTTTACGTAAAGAGGCCATATGTACACGTAAGGCA
GCCATTTCTGTATGATTGACATAGCCATAGAGTGATTTTAATAACACTTGATAGGTTAATACTTTGCCGACATG
ATGGCATAATATCGTTAGGAGTTGGAATTCATTCGGTGTTAGATGTACGGACTGTTCATTGACAAGGACTGAT
TTCGCATCGAAATCAATGGTTAATGGACCATTTGTGAAACGACTTTGAATTGTCTCAGTAGAACGTGACATAC

GTAATGCTACTCTGATACGTGCTCTTAACTCATCGATATTGAAAGGCTTAGTCATATAATCATTGGCACCGCGAT
CTAATACTTCGACAATGGTTTGTTCCTCTGTTCGTGCACTAATCACAATAATAGGTGTGTCCACAAAGTCTCTG
AATTGCTGAATGAGAGATAAACCATCAATATCTGGTAAGCCTAAATCTAATAAGATAATATCTGGTTGTTCTGT
TCTTAGGCGAAAGTCCGCTTCTTTCCCCGTCTTCGCCGTAACCACTTTATAATAATTCATAGTTAGCGCAACAT
CGATTAAATGTAAAATCGCTTCATCATCTTCAACGACCAATAATGTTGTTTTCATCCATGGCTCCCTCCATTTCA
ATTGGATTAACAGTCAAATAAAAATAAAAAATACTGCCTTGTGGTGTATTCGGTTGATATTCTAATTCACTGTT
GTGTTGTTTCAAGATGAGTTGTACTAAATAGAGCCCTAATCCCAAACTATCTTTTTTATTGTCTTTAAAGTTGTC
TCCTGAATAATAGGGATTAAAAATCAATTGACGTTCTTCTTCTGGAATACCTTTTCCGCAATCTATCATTTCGAA
TTTTATTTTGTTTGTTTCATGTTGAACGTGCAGTTTTATTTCAGAATGTGATTCTGCATGCTTTAAAGCATTATC
GATAAGGTTGAATAGCACTTGCAGTATTAATTTACTGTCGATATTAATGAGTGAAGCGTCATCCTCATTTTCAA
TAATGACATGATTTGCTTGGTGTCTGCGTATGAGGCCTTCTTCAAATTCTTCTAGAAGTTCTTCTACTAAATAA
GGGGTGCGTTGTATTTGAATGTCAGAGCTTTCTAACTTAGTCAAAGATAAAATATTTGTGACTAAGGTATGCA
GATATTGTGCTTCGCCATAAGAAGCAGTTAAGAGTTCTGCTTGTTGTTGATCGTTTAAATGCTCGTTATGGTAT
TTCAGCATATCTAAGTTGCCCATAATGGAAGTCAGTGGTGTTCTAATATCATGTGAAATTGAATGCAAGAAGT
TTGAACGTGTGGCTTCTCGTTCAGCTTTCAATATGGATTGTCTGGTTTGTTTTAATAGATCCACATTCTCTATTG
CCAGGGTAATATCGTTCAACATAGAGTCTAATATTGAATTGTCATAGGTGTCGATGTAAGTTTCGTCGGTGAA
GCGGATAGAAATCACACCTTTGACTGGATTTGTGCCAATGGGAATACACAAAAATTTACTGCCAGGAAAGGT
ATCGGTTAATTTACCGGCACGGCTTTCATTTTCAATGACCCAGCTCAATGTCTCAGCATCATGTGTCTTATCTG
AGCTTGAAATGCTTCTGTTTCCAAATGAGTTGGAAGCAGCGACTTTCTTACTTTGAATTAAGAATACCGTGAC
ATCCTGATTGAGTAGCTGATGAATCTGATCACCAGCAATATTTAATAAGCGTTCAATTGAATAAGATTCTTTAAT
GGACTGGTTAAATTGCAGCATAATATTGGTCCGATATAACTGCCGTTCTGTTAAGGAATGCTGATGTTTTAAAT
TCTTTAAGATGGCACTCGTAAAGATACTAGCAAAAATACTGGTCGCAAACGTAATCGGATATTCAAAGCGATA
CATTTCTAAAGTGAATCTTGGCACCGTAAAGAAATAATTAAAAACAAACACATTTAAGATAGACGCAAAGAA
TCCGATTAAATAGGATTGGGTCCAAATAGAAAGCACGATAATACCGATAAAGAACATCAGCAAGATAATGGC
ACTGGATTCGCTTTTATCTAAGTTGTAAACCCATATACCGAGCAAGACACAGATCGTTTGAATTACAAGCATCT
TCATAATCTCTACAGTTAAACGAGAGGATTTTTGAGCTTGCTTAGGTGTATTTGTAGGTTTGTCTGAATGAATA
TAATGAATCGGTACAATTTCTAACTTGAAATGGTGTGGTACGTGATTAATTTGTTCAATGAGTGACTGTTTAAA
GTAATCTTTCCAACGTGGCTGTTCTGACTGTCCAAGGACTAGCTTCGTCACAAAAGCGAGATCACACCAATC
GGTTAACGCTTTCGCAATATCTTGTGCATACAACACTTTGATTTCTGCACCTAACGCTTTGGCGAGCATTAGAT
TTTTATGGACATAATGATCTTGTTTTCTGCTTTCTGACGGTGTCTCAAAGACATCAATATACACAGCTGTGAAT
TTAGCATGTTCTTTATAGGCAGCACGTCTTGCTTCTCGTATGACCCGTTCGTTATAAATACTGCCGCTGATAGC
TACCGCAATATGCGGTGTAATGTCGGTATGTTTAGTTTTATATTGTTGTCCTTTTTGACTCATAATATCTGCGAC
AGTTCTGAGTGTAAGTTCACGCAGCTCTGTCAGATTTTCATACGTAAAGAAATTAGAAAAGGCTGTTTCTAA
GCGTTCTTTTTTATATACTTTTCCTGCTTTAAGGCGCTGAATCAACATATTTGGTGAAATATCCACAACTTCAAA
GGCATCTGCTGACGTAATGAATTGGTCGGGCACACGTTCTGTAACTTGAATACCTGTCATTAACGCAATTTGT
CCGCTTAGACTCTCGATATGTTGGATGTTGAGTGTTGTCCAGACATCGATACCATGCGATAGAATTTCTTCTAT
ATCCTTATAACGTTTTAAATGGCGCTCTTTTGAAATGTTTGTATGTGCTAGTTCATCAATTAAGACCACATCTGG
ATTAGCTTCTATGATTTTAGAGACATCTATATAGTGAAAGGTGTGGCTGCCAAATTTACGGCTGGAGGTCGAA
ATTTCAGGCAATTGTTGAACCAGTGCATTGGTTTCAGGGCGTTGATGGGGTTCGATATAACCAATTTTAATAT
CTGCACCTTCTTGATACTGATCAATACCATTTGATAACATTTCATACGTTTTACCTACCCCTGGGCTATAGCCTA
AATAAATGGTAAGTTTCCCTCTTTTTTTATATGTACTTTCCATGAGGCACCCCCTCTTAATCACATCATAATTAAT
ATACAACATATTATCCATTTATCTTTCTGTTTTGTTAATCATCTTTATAGTTTCTTTATAACTTTCCTTAAATGTTAA
TACTTCGATTAGATAACTCTTGTTAAGATTTTGATGAAAGTAAGGAGGGAGCGCAATGATTACACTATTAGCT
GTCGTTGTCATCGCATTAATTTTATTTTTATTTTACGCATTAATTTGGAGTGAAAAATTTTAACAGAGAAAGAG

GGAAGCATCATGAGTATTGTGTTGTTTTTGATTGTATTTATCTTGCTCTCACTCATTGTGAGCCGATATTTATAT
TCAGTTGCTTTAAATGTGCCATCTAAAATAGATGTTGTTTTTAATCCGATTGAGAAATTGATTTATCAACTGATT
GGCACGAAATTAGAACACATGTCTGGGAAGACGTATATCAAACATTTTTTGTTGTTTAACGGATTGATGGGC
GGATTGTCCTTTGTATTATTGCTTATTCAACAATGGCTGTTTTTGAATCCTAACCATAATTTAAATCAATCTGTAT
CGTTAGCCTTTAATACTATGGCATCTTTTTTGACCAATACTAACTTACAGCATTATGCAGGTGAAACAGATTTA
AGTTATTTAACACAAATGTGTGTCATCACTTTCTTAATGTTCACGTCAGCAGCGTCAGGTTACGCCGTATGTAT
TGCGATGTTAAGACGTTTGACTGGAATGACAGATGTGATTGGTAATTTCTATCAAGATATTACGCGTTTTATTG
TACGGGTGCTCATACCTTTCGCATTGATCATCAGTTTGTTTTTAATCAGTCAGGGCACACCGCAAACGCTTAA
AGGTAATTTGGTGATTGAGACATTATCAGGTGTGAAACAAACGATTGCATATGGACCGATGGCGTCTTTAGA
ATCTATTAAACATTTAGGGACAAATGGTGGTGGTTTCTTAGGTGCGAACTCTTCTACACCTTTTGAAAATCCG
ACATACTGGTCTAATTACGCTGAAGCTTTAAGTATGATGTTGATTCCAGGTTCATTAGTCTTTCTATTCGGTAG
AATGTTGAAAACTAAACTACAGATTCATCCGCATGCGATTATGATTTTCGTTGCGATGTTTGTAATGTTCATCG
GCTTTTTAGTGACATGTCTCTATTTTGAATTTGCGGGGAATCCAGTGTTGCATCACTTAGGTATTGCCGGTGG
CAATATGGAAGGCAAAGAAACACGTTTCGGTATTGGCTTATCCGCTTTATTTACAACCATTACGACCGCTTTT
ACTACAGGAACAGTTAACAATATGCACGATAGTCTTACACCGCTAGGCGGCATGGTTCCAATGGTATTAATGA
TGTTGAATGCAGTTTTTGGCGGTGAAGGTGTTGGGCTGATGAACATGTTGATTTATGTCATGTTAACGGTCTT
TATCTGTAGTTTGATGATTGGGAAAACACCAAGTTATTTAGGAATGAAGATTGAAGGTAAAGAGATGAAACT
CATTGCGCTTTCTTTCTTAGTACATCCTTTACTTATTTTGGTTTTTTCAGCACTAGCTTTTATTGTGCCAGGGGC
ATCAGATGCGTTAACTAATCCGCAATTCCACGGTGTATCACAAGTGTTGTATGAGTTTACATCATCTTCAGCGA
ATAATGGCTCTGGTTTTGAAGGATTAGGAGACAATACGGTATTTTGGAACATTTCAACAGGCATTGTGATGTT
GCTTGCACGATATATTCCAATCGTTTTACAAATTTTGATTGTATCTAGTTTGGTAAATAAAAAGACCTATCAGCA
ACATACTCAAGATGTACCGATTAATAATTTATTTTTCAGCAGTGTATTGATTATCTTTATTATTTTGTTGAGCGGC
TTAACGTTCTTACCTGACTTAATGCTTGGACCAATAGGCGAACAGCTTTTGCTGCACGCATAGATAAAGGAG
GATTAGAAAATGGCTGAAACTACTAAAATATTTGAATCACATTTGGTCAAACAGGCTCTAAAAGACAGTGTAT
TGAAGCTCTATCCTGTTTATATGATTAAAAATCCGATTATGTTTGTTGTAGAAGTGGGCATGCTGCTTGCCTTA
GGATTAACCATTTATCCGGATTTATTTCACCAAGAAAGTGTATCACGGCTATATGTGTTCAGTATCTTTATCATA
TTATTACTGACACTTGTCTTTGCGAACTTCTCTGAAGCATTAGCTGAAGGTCGCGGTAAAGCACAAGCCAAC
GCTTTACGCCAAACACAAACTGAAATGAAGGCACGTCGTATTAAACAAGACGGCAGTTATGAAATGATTGAC
GCTAGTGACCTGAAAAAAGGACATATCGTACGTGTCGCGACAGGTGAACAAATCCCAAATGACGGTAAAGT
TATTAAGGGCCTCGCAACAGTGGATGAATCTGCGATTACAGGTGAATCTGCACCTGTAATCAAAGAAAGCGG
TGGAGATTTCGATAATGTAATTGGAGGAACTTCTGTAGCTTCAGACTGGTTAGAAGTTGAGATTACTTCAGA
ACCAGGTCATTCATTTTTAGATAAAATGATTGGTTTGGTTGAAGGGGCTACAAGAAAGAAAACACCTAATGA
AATTGCGTTATTTACTTTATTGATGACATTAACGATTATCTTCTTGGTCGTTATTTTAACGATGTATCCATTGGCG
AAATTCCTGAATTTCAATTTATCCATTGCGATGCTGATTGCTTTGGCTGTGTGTTTAATTCCAACAACCATTGG
GGGATTATTATCGGCTATAGGGATTGCAGGGATGGATCGTGTGACACAGTTTAATATCTTGGCTAAAAGCGG
ACGTTCTGTAGAGACTTGTGGTGATGTGAATGTCTTGATTTTAGATAAAACAGGTACCATTACCTACGGCAAC
CGTATGGCAGATGCGTTTATTCCGGTGAAATCATCAAGCTTTGAACGTTTAGTTAAAGCGGCCTATGAAAGTT
CTATCGCAGATGACACACCAGAGGGACGTAGTATTGTGAAATTAGCTTATAAACAACATATCGACTTACCGCA
AGAGGTCGGAGAATATATTCCGTTTACTGCTGAAACACGTATGAGCGGTGTGAAATTTACGACACGTGAAGT
ATATAAAGGTGCACCGAATAGTATGGTTAAGCGTGTGAAAGAAGCAGGGGGACATATTCCAGTTGATTTAGA
CGCTCTTGTCAAAGGGGTGTCTAAAAAAGGTGGCACACCGCTGGTTGTGCTTGAAGATAATGAGATTTTAG
GTGTTATTTATTTGAAAGATGTCATTAAAGATGGACTCGTAGAACGTTTCCGTGAATTACGTGAGATGGGGAT
TGAAACGGTGATGTGTACAGGAGATAACGAATTGACAGCTGCGACAATAGCGAAAGAAGCGGGTGTGGAT
CGCTTTGTGGCAGAGTGTAAACCTGAAGATAAAATCAATGTGATTAGAGAAGAACAAGCGAAAGGTCATAT

TGTTGCGATGACGGGTGACGGTACGAATGACGCGCCAGCTTTAGCAGAAGCTAATGTAGGTTTGGCAATGA
ACTCAGGAACCATGAGTGCCAAAGAAGCGGCGAATTTAATTGATTTAGATTCTAATCCAACCAAACTGATGG
AAGTCGTTCTAATTGGGAAACAATTATTAATGACACGTGGCTCACTCACTACATTTAGTATTGCGAATGACATT
GCGAAATACTTTGCGATTTTACCAGCCATGTTTATGGCGGCTATGCCTGCGATGAATCATTTGAATATTATGCA
TCTGCATTCACCTGAATCAGCAGTATTATCTGCGTTAATCTTTAATGCGTTGATTATTGTATTATTGATTCCGATT
GCGATGAAAGGCGTGAAATTTAAAGGTGCCTCAACGCAAACCATATTGATGAAAAATATGTTAGTTTACGGC
TTAGGCGGTATGATCGTGCCATTTATCGGCATTAAGCTCATTGATCTCATCATCCAACTCTTTGTCTAAAAGGA
GGACAAAACAATGCAGACAATAAGAAAAAGTTTAGGACTAGTACTGATTATGTTTGTTTTATGCGGATTTATC
TTCCCGCTGACTGTCACAGCGCTTGGACAAGTATTATTTCCAGAACAAGCAAACGGCAGTTTAGTGAAACAA
GATGGCAAAGTAATTGGTTCAAAGCTCATTGGACAACAATGGACAGAACCTAAATATTTCCATGGACGTATC
AGTGCAGTCAATTACAATATGAATGCGAATGAAGTGAAAGAAAGTGGCGGACCTGCTTCAGGCGGCTCAAA
CTACGGCAATTCAAATCCTGAATTGAAAAAAAGAGTTCAAGAGACTATTAAACAAGAAGGAAAAAAAATTT
CAAGTGATGCGGTGACCGCTTCTGGCTCTGGTTTAGACCCAGATATTACGGTTGACAATGCGAAACAACAAG
TAAAACGCATTGCGAAAGAAAGAAACATAGATGCTTCAAAAATTAATCACCTTATTGATGAAAACAAACAAG
CATCACCAATGGCAGATGATTATGTTAATGTCTTAAAATTGAATATCACTTTAGATAAACTCTAAATAAACAGG
GAGTGAGGTGAGACATCCATGTGGTTCATTAGCATTATTATTTTAATAGCATTCCTAATTATATTAATGATTGAC
GATTTAATTAAAACATGACAACTTCACGCAAAACAATATGAATTGGATGTAATAGAGAAGGGAGTACCAAGA
AATACTCAGTTTATTGAGGATAATTATTAGGGGAGATTAGAGAAGAATTCTCTATTCACGAACATTACTTTAAC
AGTTGTAATCATTGGTTTCATCATATCGATTAATAGTACAGTCATCTTGAGATTTTCAACACACTATTATAGCTA
GCTAAGATACTTTTAAATTATGACTTATTTTTCATCATTTACGATACATCATTCTCGATAACTTACTATTTGTTATG
GTATTTATAGATTTAAAGAAAGTGAAGGAGATTAAGCAATGTAACTTGGCTTAATCTCTTTTTGATTATATTTAT
TAAGGCAAAGCTTCATTTCCCTTAAATCAAGTATATCTTCTGAATAAGCAAATGAAATCGTGACGCCAACTAC
AGTGATAACTTCGCCTAAAACACAATGAGTGTGGTGTTTTCACTTTTCATAAACTCCTCTAAGTTTGTGTTTTT
AAACTGTGATGGATTTGAATGTTTTTGAATTACGTTGTCGCGTCCTTGGGATTGCAATCCAAGAACGTCAGC
ATAATCTAAATCAATTTTGATTTGGTAGAAGACAAGAGAAGTACTTTTGCCTGTATCATCTTTCGCATTTCTTTT
TGTTGTCTTTCGATCCCGATCGGAAACTAATTTTCCTGCATTATCCAAAGCATCAATAACATTTTTCGTGTCTTC
GAATTGACTGTCTGTTAACATCTTATCGAATACATTTCGGATAATTTTTACTTCGATGTAGTCATCTTTAAGTGA
GAGGATACCAAAGTTTTGAATCATATTAGCAAGACGTGTTTGTTGTGAAAACTTGCCCAGGTTTTCCGCAAT
AAACTGTACGATTGTATCCATCGCTTTATCTGCAAGAGAACGTTCTGACACCGTTTCATTATGATAGCTTAATA
AATAGTTCTTTATGCTGCTAACGTTTAACTGTAGGTCTAGTGATTGATTTAAAATATGGGCTGTTGTTGTTAGA
GTTGCATAATGTTTAATCATTCGAATACCAGTGTTACTGGATTCAGTTGATAGTAATTGATAAAAAACACTGTC
TTCTTTTTGAAAAAGGGAAGTAACATCATTAGTGTTGTCTAATAGGAATTGAGCAATTATAGGTAAAACATGA
CCATTATTTTGACTAACCACTTTTTTTATAGTATCTGAGTTTTGAGCACTTGATGTGAATTTTTCAGACACCTCA
ATAGTACGAACACGCAAACCATCATTTTTAGCTGGATTTTTGAAAATACTATGTTCAGAAGTTGAAATCACTG
ATGTATCCTAGTTTTTAGGTGTTTTGACATCACCATTTATATTTGCTCTTTGACGTCCTTGTCCCTCTGCAAATG
AATATAGAAGACCTGTTGTATCATGAAATGTTGCCGCTGATAATTCATCTAATACTATGGGTACACCATAATTTC
CGCTAAGATATCCTTCTAGTGCATTTCTTGTTGCATTCCAACTCCGGAATAGGTTTTCAGTACCTTTTGTTGGG
TTGCCAGCTACCGATGCGGCTAACATAGCGGCTGTCGATTTTCCTGTTGATGATTGGCCAGTGAAAGAGAAA
ATAGTTCCTGAAAACTCGACGTTATTATGATACTTAAGGAAAGCAGTGACTAATGAAGATACGCCAAACAACA
CATCTAGTTCTAAAGATAAGTTCCCATGGACTTCTTCTTTATACATTTGTAACCATTCTGATAAATTGCCTTTTG
GTATTAAATCGTACTTGTTATCACATATAATAGAAGTGCTATTAGTAATCTCATTAGAAAAATAAGGCTGGTCTA
ATGAAATTAATGGTCCAAGTAATGTATGAAGCATTCCTACACCTGAGTATATTGTTGATAAAGGTGTGGTGCTT
CTCATTTGTTGCAGTACGAACCCTAAATCTCTAATGTGCTTTTCATTAATGGTAAATCCATATTCAATTAAGGAG
GGTAATTGCAAAGCAGTAAGGATTGAAGCCTTTTCGACAAGCTCTATATTATTCAAATCCGAAAGAAATACTT

TTTCTTTACGAGTCGCGGGGTCTATAAAACGATCTGTGAAAGCAATCGGTCCAGATAACATTATTTCAAACTC
CGTACCCCCATCTTTTTTAGGAGGGATGGTTTTGTACCATCCCGAAGGTTTGAGCTTGAATTCGTCAGTTTGA
TATAGAATTTTTTCCATTAACAGTGACCCCCTTGGTTGTAAGAGGAAATTATAATGATATGTCGGTACTTAAGT
GGGTTAGTTCCTATAATAATGAAAGGGATGCTTGGTTTGTACTTTATCCACCAAGCAAAGCGTCTTCCTGCTT
CACGTTGATCGTTGATGTTGCACTGTTCTCTGAATAACTGTAATTATAAGGTTTAGTAATGGCAATTTTTTATTT
AAACAAATTTTTACGAATGGATTGTCTGTTTATTAATTCAGCAACGTTTATTGAAGAAAAATAAGTGTCTTCTG
ATAATTGCAAAGGGTTTAATTCAATGTAAACTGAATTAAGGGTAGGGTAGGTATGCCTGTTTTTTGGTTCTTT
TAAGTTCAATCGATTAATAAAATTGTTGAACCTTTGTTCTAAGTCATACAAAAACATCAGTAATTCATTAGGAG
AATGATTTATTATGCAATCCATATTTTCGTTTATGCTTGAAGTTATACATCTAAAAACAGTATTGGTTTCTTCGTA
TAAAAATAACTTATTAATAGAGGTAACATTGATTAAGACTGAGTATACGTATAATAAGAGTGACTCTGACATCT
CATTTAGATAAATAAGACTGTGATCATTCTTATTTACGATTATTATATGTGATTGAATCAATTCAATAATGTCTAG
AAGAAGACTTGAAAAGTCGCTGTTAATCTTGGTCTTTGAAAAATCATCGTGACCATTTGTCTTATTACTTAGAT
GTAGTTGTTCGTATTTTATAGTTTGGCGGTTGAGGAATTAAGCATTTGATTATTATAAACTATCTCAAAAATTAA
ATTACCTCCTTTTTTCTTCCTCGATGAAGGTTTATCTAATCTTCCAATATACAGGTTTTTGAAGTAAATTATAGAT
ATGATAATCATTAATTCAGTAAATGTATACTTATTAAGGGGTGGAGCTTTAGCTAACTTATCGATAGTACTGCCG
ATGCGTGCTTTGAAACTATTCAAATTGTCGTGACTGTCTATATCTAAATCTTCAAATTTACTAGCAAGGGTTTG
CAAAGTAGAAATTACAACTTCTTCTTTTGTATAATATTTATTGAAAATATCATCTTCTTTATCATCGCTTTGGAGT
TTTAAAGTGCCTGGTTCTAAGATGTCAAATTTTTTGTTTCGATTGGTATTTTTTTAAAATGTGCCTTGTATTTTT
GGGGTTGTTCCAAATGTGTATTTGCTAGACTACCTGTAATTGATATTGCTTTATTTAGCATTTGGTGTGTATTAA
CTGTATCTGATTCTTCTGGAAATTTAAATCTAAAAGTAAAATCTCTACTACCGAATGTACTTTTTACGGCTACAG
TTTTGTACTTATGCTCAGGCAATGTAATTAAACCATCTTGATGCAGGAGGAAAAGTGTGTCATCATTTAATTTC
TCAATTGTATTAAATAAGTAATTATGCATAGCCTCTTTATACATGGCGAATTGATAATTTAGATTATCCCATTCTAT
TAGTTGATTTAAAATACAATATAGACCTTCATTCAATCGGTTTACGAATATCAATACAGCTTCTTCTCGTCCAAC
AAGGCTTATTAACTCCTTCAAGTCATCCTTTAATTTTAATTTTTCTCCTTCAAAATTGTATTGAGTGAAGCCTAT
AGCCTTTTGAAAAGTAACTTCCATGACAGAGTTGGTTTCAGAATATTCTAACGATTCATTTATTGCAAAAATC
AAATTTTTTATAAATATGGAATTACTCATGGTTTGTAATTGCATATACTACCCAACTTTCTTATTTACTTTAAAAC
AAATATAAGCTAAAGTAGTTATTATCATAAAGGGAACGTACGTTCTTATTTAGCTGATTTCTATGAAGAGAGGT
TATGGATATTGAATGAAGAAATCTTACGCGAAGTAGCAGATATTTTTATAGGTGATGACAGAGATAGCATTTAT
GATTATAAAACTGGGAACGAATTAGTGAGGTTTTTTAATCATTACTTTAATAAAGGGGACATATATCAGGCTCC
GTTTCCATCTAGATGGCTATATGTTGTGAAACATTTGCAAACTCTGATTCAGGAGAGAAAGATCAATCAATTT
TTCACATTAATTTTAAGCAATCACTATATTAAGTATGAACTGAAAATTGACGAGGTTGAAGCAGCAAAGCAGG
CTGCTAAAGCACTTAAGTTGTTCAACAAAAGATTAAATCATTATGGGTACTACATAACAGGAACTAACAATGC
TAGATATTTTATGGATAAGGATGAAGATGCGGAGTCTATTGGTTATGGAGGGTATGCGAATATTTATTTACAGA
AGTCTACAGGTCTTGCTGTAAAAAAATTGAAAGAGGAGTATCTTACTGATTCTTCAATTAAAAGTAGGTTTAA
GAGAGAATTTGATCTCACTAAATCTTTTGATACAAATCCATTGTTCATTAATGTGTTTGAATTTAATGAATCAGA
TTATTCATACACGATGGAGTTAGCTGATGAAACTTTGAAAGATTACATTGAAAGCAAGACAATTAGTGAGCTA
GAAAAAGTAAAGATAATAATGAAAATTTTAAAAGCGATGAGTCAAGCACATAGTGAAAATAAAATACACAGA
GATATCAGTTCTAAAAATGTATTAATGTTTAGAGGAAAAGTCAAAATATCAGACTTAGGATTAGGTAAAAACC
TTGATGAAATTCATTCGCATCAAACCTTTGATACAAACGGTGTAGGACAATATAAATACTGTGCACCGGAACA
GATGTATAGTTTAAAACAAGCAGATAAACAATCTGATGTTTTTAGTTTAGGAAGATTGATAAATTTTATTATGA
CTGGAAATGTAGTTAACAACCATCACCTATTTAGAGGTGTATCTGATAAGGCTACGAACAGTAGTAAAGAATA
CAGATTTGAAGATGCAAATGAAATGTTGAAAATGCTGCAGAGAATTTTAGAGTATCACAGTAGTGCAAAGCA
CGTCGAAAAATGTCAAGAAAAGCTGAAAAGAGGAGTGTTTGATGATGAAAGCGAAGAATTTATTATGACAC
GAAGTGATGAACAATTATGTCAAATGGTTCTAAGTTCTAATAATAATGAGCAAGCGTGTTTAATTCGTTATATG

CAAAAAAACGAATCTTCAGCATGTGATTTAATAGAGAGTATTAATAGAAAGTATCAAGAGTTTTGTGGAAGG
TTTGAAGACTACGATCCTTTTGCTAAATTAGCATATATGATTTTATGTAATAACTTCAGTTATAGAGTGAATGAA
ACAGCAGCTAGAGTGCTAAATTATGTTGCTTGGTCTGTAAATAGATTTTCGGCACAAGACTTAATTAAAGGTT
TAATTAATAGAGGAGTGGAGCCTTTGATTGAAGAAAAATTAAAGGACAATTAAAAAAACCTCATCATTAACT
GATACGCAGAGGCGTATCATAAGT
>Staphylococcus aureus strain CMRSA-3
ATGAAAATCACCATTTTAGCTGTAGGGAAACTAAAAGAGAAATATTGGAAGCAAGCCATAGCAGAATATGAA
AAACGTTTAGGCCCATACACCAAGATAGACATCATAGAAGTTCCAGACGAAAAAGCACCAGAAAATATGAG
CGACAAAGAAATTGAGCAAGTAAAAGAAAAAGAAGGCCAACGAATACTAGCCAAAATCAAACCACAATCA
ACAGTCATTACATTAGAAATACAAGGAAAGATGCTATCTTCCGAAGGATTGGCCCAAGAATTGAACCAACGC
ATGACCCAAGGGCAAAGCGACTTTGTATTCGTCATTGGCGGATCAAACGGCCTGCACAAGGACGTCTTACA
ACGCAGTAACTACGCACTATCATTCAGCAAAATGACATTCCCACATCAAATGATGCGGGTTGTGTTAATTGAA
CAAGTGTACAGAGCATTTAAGATTATGCGTGGAGAAGCGTATCATAAATAAAACTAAAAATTAGGTTGTGTAT
AATTTAAAAATTTAATGAGATGTGGAGGAATTACATATATGAAATATTGGATTATACCTTGCAATATCATACGAT
GTTTATAGAGTGTTTAATAAACCATTTTTCAACTATTGATGATCTAGAATATATAATAACTGTACAAATTATATTG
ATTATGGAACTACAATTAAATTAAGAAATTGATGATGAAATTTTAAATTTAAACTAATGGAATCAAGAAAGAAT
GAAAGGAAATATACAATGCCTACGATTAATAAAAGGAAGTTTATTAGATTTTGTGTTAGAAACAGTGGATGTT
TTTGTAAATGGAAGTAAAAATGATTGTGAATTAGTAAATTTTAATTATAAAGAAGGAAAAGTAGTTTCACGTT
ATAGTACAAAATTTGAAAGCAATCCTAAATTAAGAAAAGAGCAATTGATATCGACGGTAAAAGTTGTAAGGT
ATGTTATTTTTGATTTTGAGAAAATTTTATGGGGCATAGGGAAAGTTTTATTGAAGTTCATCATATTAAGACAA
TGTTTACTATAAGAAAGTTGAAATAAACTCAGAAACAGACTTGATTCCCGTTTATTCAAATTGGCGCAAAATG
ATACATATAAACACCTATATATTGAAGATTTAAGTCAAACGGTAAATAATATTTTTCACTTTTAAACAGAAACTT
TTTTGGAAATTATATTAATTAGTATAAAAATGAATATGTTCACAAAGAAGGAGAGATTTTATTGAAACACATTA
ATCATTTTTCTGAATCGACACTAGCATACTTGGAAAAAGACCATGAACATGCATTTTATGTATATTGTTTAATAG
ACCCAAGAAATGATGAATGCTTTTATGTTGGTAAAGGTAAGGGGAATAGAATATTTAAACATAAACAAGATG
CGCAGAGACAATTATTGTACGAAGATATATTCAGAGAAGAAAATAAAGATAACTTGAAATTTAATAGAATTAA
CGAAATTTGTAGCAATGATTTGAATGTTTTAGGATATATAATCAGTTATGGATTAACCGAATCTGAAGCATTTT
CTGCAGAAAATGTTCTTATAAACTTTCTTAACCTAACAAATAAAACTACATTAACAAATATGATTAACGGTCAT
GGATCTAAGGCATACTTAGTTGAAGATTTAGAAAATGAATTTGGCTACGATTCAATTAATCTTGAAAACATAA
ATACGAATGAATTAATTTTAGCAGTGAAAATCAGAGATGCATTTCTATTAGATAAGGATGAAAGTAAAGAGTA
TCCTATTAATGAAAGTAAACGTGATAGAAATAACCTTAAATCGCGTACATTAGGTAGTTGGATAATAGGAAAA
GATAAAATACATAAAATAAAATATATTATTGGTATCAATACAGGTGCTAATAATGCAGTTGTCTCAGCTTATGAA
GTATCATTTGAACAAGCGGAGAGTATCGAAACAAATAATGGTAGAATGAGATATGCGTTTATTGGGCTTTCA
GAAAGAGATGCTACTCTAAAAAAAATTGAATTTATACAAAAAAGCACTACCAGATTTAAGATTTGGTAGTGGTA
GCGCTACAGCATATATAAATAATGGGACAATGAAAGTTGATTAAATGAATAAAAACATCCAATTCCTTAAATTA
GCCTTTGAAAATTTTATACTCGTATATATAAGTGAAAAACTTAAAAGAATTAGATGTTAATTAAAGGAGAGAAT
GCTATGCCATCAGATGATATTGTAAAAAAACTATACTCAAAAGAGGATATTCGATTAGAATTAGGACTTACTCC
GCATAAGTTTAATAAAAAAATGGAAACTATTGCTAAGCTTTTTAAAATTGATATGAAAATTTTTCACAGTTACA
AAGGCCAAGATAAAAATAATCAGTATACATTTAATGGTGTCGCAAAAGAATTAATCAAAGTGTTGCTAAAAAG
TGTTGATTATTACCCAGTGGATATCAATTCCAAAAAATTTAAACAAAATGGAAAATCTAAAAAAGAAATGATT
GAAAATATAGATAACTCTAGTTACATGAAATATATTTATCAGTTAATGAAATCTATCAATGAAATTCAATACAAA
AGGTTAATTGCTGATATACATATGAAAGATGTGTACCAGAATACAAAAGCATGGTTAAATAATGGTGAATCAAT
TAATAAAAAAGAACAAGAGCTATACCAATATATGACAATTTTACCATTACATAAAAGAGTTGAACTGCAAAAT
GAGGTATTAAAATCTATAGACGAAACAATTTTTCAATTTCTCGCAAAAGAACATAGAAATAATCAAATTGAAG

AAAATAATGAATTAGAAGCATATACAAAAGCAATAAAAGAAGGTAGAAATCCTAAAAATGATTATGAATTAAA
TCATCTCCTATATAAAAAAAATCAATTACCCTTAGATAGTCTAATAGAAGATATATGGGACTATGAGGAAACAG
AGTATACTGAGCTTGATTGGTTAATTGCAGATATGCTAAAACGTTCACAAAGAGCTGATAGTAATTTTATCGA
AAGTTTAGAGAAGAAAAACAAATTGAGAAAAAAATATTCATAAAGATTCCATTAAAAGTATATCTAAGTTGATT
GATCATGAATTAGTTAAAAATAATAGAAAGTGGGACGTAGCAGAATTTTATAAAAATCAACAATTTTGGAATA
ATAGTACATTAAGACGTAGCTCTCAAAATTTTGGAATTTTATATTACAAAAAGAATAGGTTACATACAATGAAT
CAAAGTAATATTATTAAGAAAATAAAAATTATCGAAGCTAATATAGAGAGTGCAAATAAACAGCCGAGCTATT
TAGATTTCTTTTTACAAGCTAAGTTTGATTTAGAGAGGTTAGAAAGTGAATTAATGAAATATAGCATTAATCCG
AAATATTTTAATAAGATAAATAGTGATTACGTAAGATATGCTATTGAAGATGTACATAATGCAATTATTAAAATT
GACAGATATATAAGTAACGATAAAGTGAAGTTAAACTATTCAAATGTCCAAATGGTAGTGAGAAACGAAATAA
CAGAACAAGGAAGCTACTTTGTAACCCAGGCATTGAATACACTGTCAAAGGTAGAAGAGTCTGGCTACAGC
TTTGATAATTTCATGGTAGGACCTTTCGTAGAAGACTTTAAAAAGCAATTAAAAATCGTAGATTTGGTTCTAA
AAAAGATTAAGATTGTTTTTTAGAGTTAAACATAAGAATAAATTTAAATAAAAAAATAAACCCAACAAACCTC
GTTATATCAATGTTTGTTGGGTTTATTTTTTTTGGTGATTTTTTAATAACTCAATTACATCTTAGGATAATATCGC
TAAATTATACATTGTCGAGGCCAAGACAAATAAAAAATTAAAGCAAAAGTTAGCGCGCAACTTTCTGCTATAA
AGGAGCAAATTTATATGGAGACAAGAGACAAACTGATGTCATTGACTCAAAGTGACAAAACACAGCAATGG
CTAATGGACAAGTCATCTAACCAAGATGACATTCAACAATTGCAGCAACAATTCAGTCAGCAGCTTGATCAA
CAATATAATGCACTTTTAGCTGATGAAAAAGCTAAGTTAGACCAATACGTGGAAGTACATCAAGGATTGGAAT
CATTAAAGGAAGAGATTGAATCAGAACCTATTACGCTTAATATCGATAAATTACCCGATATCAAAGCAACAATG
CTTGAAAGAGCCAAGAATGATGAACATTCTGATAAAATCGAAAAGCTATTTGATAGGTTAGAACAGGCATTA
AATGGTACGAATCGATTATATACGCAATTATCGTTGATTGGTACACGAACACATCGAATTACAACTAAAAATTT
TAATCTTCAAGGCTTACCTAAAGCAGTCCAACATACGATTTTACCTTCAAAATTTAAGAAGGTGTATACAGTC
GATTTTAAATCGTTTGAACCATCAGTTGCAGCGTACATGACTCAAGATTCAAAACTGATTGACTTGTTAAATC
AGAAAGACGGACTGTATGACGCATTGCTAAGTGAATTAGGCTTATCAGATGAGCTACGTGTATTTGTTAAAC
GTGCATTTATTGGTTCGTTTCTATTTGGAGGTAACTTCAAAAATCCTAAATTCAAGCTGAATCAATATGTAAGT
GAAGTACAATGGTTGGATGCGGTCAGCCAATTTACAAAAGTCATTGAACTTAAGAAGCACGTTGAAAAGAG
TAATTCCATGCCTATGCCTTATAGTATTGAGCATGATATGAGCGCATTTCAAGGTAGCAGTATTATGGCAATCTA
CGTACAAACTGTAGCGAGTTATATTTTCAAGCACATTCTGCTAAAAGTGTACAAAGCACAGTGCGATCAAAA
AACGTTCAAGATTATAGTACCTATACACGATGCGATTATGATTGAATGTGAAGATGAAGAAATTGCACAAAAT
GTGGGTCAGTTAATGAAAGATACGGCTAACCAGTTGTTCAATGGTGAATTTGCACATGTGACAGTGGAAGA
AATAGGAGGCGTAGACCATGAATAATGATAGAGGACAAAGCCTACACATCCCAAGTAGTACACCAATCAAAG
AAAATAATATATATGTAGCTACGTTACATTCTGTGATCCAAACAGATTTCTCAGGTGAAATAAAGCACCAATTC
ACGTATGAAATTGAAGTGAACAATCAGATTGTATATGCGAATCGTAATATTCTAACAAAACCGAGCGCTAATC
AGTTGTCAATTCATGATTGGCTGAAACGTCATAGCAACTATAGCGTGGGTCATGATACGTATGAACCTTATATT
GACCGTAAGCATTTAATCCATATTGGTCAATATAGCGGTAACTATTATGTACAAGATGTAGCATCGTTAGATGC
GTTTGGAGGCGTATTATCATGAATCATATATTACAAATGTTATCTAAGCTATTAAGTGTGGCCAAGGAGGCAAT
CGACCGTCAAGGTCTGATTGCTATCCTAACTATTCCTGTTAATAATAACGATGAAATAGAAGAAACGGCTCAA
GGTGAAACCGTGTATAACGAACTTATCGATCAGTTACGACTTAATATCCCAAAAGATACGGATTATCAACCTAA
CATCTATAGTTATTTTGGTATTAAGAAGAATCCTAATGACACCGTACTCATGGAAATGATGATAAAGGTTTTTC
ATATCAAACGCTTTAATTCAGAACTGTTTATTTTCAAAGTTAACGGGTGGCAAAAGATAAATGGAGATGAATT
ACAAGGGTTGATATCTAAAATGATACAAGTATTGCTTGTAGATTATAAGCCTTCACTAAGCACTCTAAAAAATG
TCGTAGATGGATTGCAAAAATCAACAGATGTAGAAGAACTTGTTGAGAATGAGCGCTATATTGGTTGTGGTG
AAAATATGTTCGATCTTAATACGTTTCAAGTCGTTAAAAATTCAATCGATATCTTTCCAAAAACACGATTGAAT
TTATCATTAAGTACAAATGATGTAATTACTGATAAGATACCGCCTTATTTTAAGCAATATATGTTACAACTTGCG

AATTATGACGATGATTTACAATACTTTCTTTTCCAACATACAGCAGTATTACTTACAGCTGATACTAAATACCGT
AGGGGTCTCATATTATATGGTGGAGCTAAGAATGGTAAATCTGTATATATTGAACTAGTTAAATCATTTTTCTAT
AGTAAAGATATTGTGTCTAAGCCACTTAATGAGCTTGAAGGTCGTTTTGACAAAGAAAGTTTAATTGACAAA
AGTCTAATGGCAAGTCATGAAATTGGGCAATCTAGGATTCAAGAAAAGATCGTAAATGACTTCAAAAAGTTA
TTATCTGTAGAATCAATGCATGTTGATCGTAAAGGAAAAACTCAAGTGGAAGTCATTTTGGATTTGAAACTTA
TTTTTAGTACAAATGCGATACTTAATTTTCCTCCTGAACATGCGAAAGCTTTGGAGCGTCGAATTAATATTATT
CCATGTGAGTATTATGTTGAAAAAGCGGACACTTCATTAATTGATAAGCTCCAGAGTGAGAAGAAAGAAATC
TTTCTTTACTTGATGTATGTGTATCAACAGATTGTAAAAGCAGATATCGAGTATCTTGAAAATAGCCGTGTCAC
TGAAATTACTCACGATTGGTTAAATTTTGGATATGAATTTGTTTCTAGCAGGTCCGTAAGTAATGCAAATCAGA
AAGCATGTATTAATTTACTCAGAAAACTTATAGAAATCAAATCAGGATCACGAATCAAAGTATCCGAGTTAAAT
AAAGTTATTAATGAAGAAATAAAGGTGAGTTCTCAGGTTATTAATCAGTTAATTCAAGCAAACTTTGATACTC
AAACCAAACTATACAATGGCTACGATTATTGGATTGATTTAGGTTGGAAAGAAGCCAATAAAAAAGAGATTC
ATGATATTTCGGAAAAAGATAATATTATTTCATTAGATAAAAATGAAAATATAACAGACGATGAGGCATTAGAT
GAAGAGAATTTGGACTTTGATTGGGAGGACTTTGACGATGAATAATGAACAAATTGAAGCATTTGTAGAAG
TGCTTGTGCCTATCATAGAAGAACGTATCAATAAAGGTAAGTAATCTAATTACGTACTACAGGCAACTGCCTGT
AGTACTCATATAATTAAGTGGTAAAAGTGATAAAAATGAAACGAAATTATAAATATATATTATCTATATGTTGTTA
CAAGACCGATAGTCTGTAGCAATAATCTAATAAAAGGAGCGGTATGAAATGAAGGGTAAAATTGCACTTTATT
CACGTGTTAGTACGTCTGAGCAGTCAGAACATGGTTATTCTGTAAAAGAGCAGGAACAAGTACTCATCAAAG
AAGTTGTGAAAAATTTCCCAGGTTATGACTATGAGACATATACTGACTCAGGCATTTCAGGTAAAAATATTGA
AGGTCGTCCGGCAATGAAACGTCTATTACAAGATGTTAAGGATAATAAAATCGAAATGGTGTTAAGTTGGAA
ATTGAATCGTATCTCACGATCAATGAGAGACGTGTTTAATATTATTCATGAATTCAAAGAACATGATGTAGGGT
ATAAATCGATTTCTGAGAATATTGATACATCCAATGCTTCTGGAGAAGTACTCGTTACAATGTTTGGGTTAATA
GGATCTATAGAACGCCAGACTTTGATTTCGAATGTGAAACTTTCTATGAATGCTAAGGCAAGGAGCGGAGA
GGCAATCACCGGTCGTGTTTTAGGCTACAAATTATCACTTAATCCACTTACACAGAAAAATGATTTAGTTATTA
ATGAAAATGAAGCTCATATTGTACGGGAAATCTTTGATTTATATTTGAATCACAATAAAGGACTTAAAGCAATC
ACGACAATTCTAAATCAAAAAGGATATCGCACCATTAATCAAAAACCATTTTCAGTGTTTGGCGTGAAATATAT
TTTGAATAATCCAGTCTATAAAGGCTATGTCAGATTCAATAACCATCAAAATTGGGCTGTTCAGCGAAGAAGT
GGTAAAAGTGATAAAAATGATGTGATATTGGTCAAAGGTAAGCATGAAGCCATTATAAGTGAAGATGTATTTG
ATCAAGTTCATGAGAAACTAGCTTCTAAAAGTTTTAAACCGGGTCGACCTATTGGTGGAGATTTCTACTTACG
TGGCCTTATTAAATGCCCAGAATGCGGAAATAATATGGTATGTCGACGGACGTATTATAAAACGAAAAAGTCC
AAAGAACGAACAATCAAGCGTTATTACATTTGTTCCTTATTCAATCGTTCAGGGAGTTCTGCATGTCACAGTA
ATTCCATCAATGCTGAAGTCGTCGAACGCGTAATCAATGTTCATTTGAATCGTATTCTTTCACAACCTAATGTTA
TTAAGCAGATTGCGTCAAGTGTGATAGAAGAACTGAAACAAAAGCATAGTAAACAAACAGAAATAAAATAT
GATATTGATAGTCTAGACAAACAAAAAGCAAAAGTTAAAACACAACAAGAACGATTATTGGAATTGTTTTTA
GATGATGAAATGGATAGCGAAATGTTAAAAGCTAAACAAAGTAAAATGAATCAACAGTTAGAAGTATTAGAC
CAACAAATTAAAGAAGCAAAACAAGCAAATCAATCACAGGATGATATACCCAATTTTGATAAGTTAAAAGCA
CGACTCATTTTGATGATAACACGATTCAGCGTGTACTTAAGAAAGGCTACACCCGAAGCTAAAAATCAACTTA
TGAAAATGTTAATTGATTCAATTGAAATTACGACAGATAAACAAGTAAAACTTGTAAGGTATAAAATTGATGA
AAGTCTTATCCCTCAATCTTTGAAAAAAGATTGGGGGTCTTTTTGTAAAAAGAAAACCATACGCTATGCGTAT
GGTTCAGAAAAGGTTCTACCATTGTCACAAAAAATGCATCTCTACGTGCTAGAATAAATATTGGTCAGCCAAC
CAAAATAATCAACACGAGGAGATGCTATTTAATGTCATCTGACACAAACAGTTTAGCACATACAAAATGGAAT
TGTAAGTATCATATTGTCTTTGCACCTAAATACAGAAGACAAGTGATATATGGAAAAATAAAAAAAGATATAG
GGATTATATTGCGTCAATTATGTGAAAGAAAAGGTGTAGAGATAATTGAAGCAGAGGCATGTAAAGATCATAT
CCATATGTTAGTTAGTATACCACCCAAACTTGGGGTATCATCATTTGTTGGCTATTTAAAAGGAAAAAGTAGTT

TAATGATATTTGATAGACATGCTAACTTAAAGTATAGATATGGAAATAGAAAGTTTTGGTGTAAAGGATTTTAT
GTGGATACAGTAGGTAGAAATAAAAAAGTGATTGAAAATTATATTCGTAATCAATTACAAGAGGATATCGTTG
CAGACCAAATCTCAATGGAAGAATATTTAGATCCTTTCACTGGAGAGAAAAATAAAAAAAGAAAGAAAAAA
GAGTAACCCTTTAGGGTTGCTGGAATAGTAGTGCAGTTGGCTGACTTGTCAGTGCCCTTTTAGGGCTGGCCA
GTAAGGAAGGCTTATAGCCGCAGAACAAACCACCCGTTCACACGGGTGGTTTTGATTTTATGCCTAAATTTA
GCTTTGTGATAAATGTCACAAATGAAAATAGGATTGAAAATTTATCACTTTTACCACTTTTTTAGAGTGACAA
AAGTGGAGGGATTTTGAAATATTTTATAAATATATATTTTATTTATGGAGTACACATTATTAATTAAGGAGGTCA
TTATAATGACGCTAAGCAAACAACTTAAAACGTATATTACTGAACGATTTAAATTAAATTATCAAGAAACTTGG
GCTCGTGAAACCGTAGATGCGGTGGCTGAAGATGTATTACCTGAAAAATATATTAAAAATAGTCCACTTGAAA
ATAAAATTTTAAATACTTTTACCTATTACAATGATGAATTACATGAAATCAGCATTTACCCTTTTTTATGTTATCTA
GATAAAGAATTAGTAGCAATAGGTTATTTAGATAATTTTGATTTAGACTTTATATTTTTAAATGACACTCATCAA
GTCATTATTGATGAACGCTACTTGTTACAAAAAGGGGGTGAGTAATTATGAATTGGATTAAGGTCGCTCAACT
ATCTGTCACAGTTATCAATGAAGTCATTGAGATTATGAAAGAAAAACAAAATGGAGGGGAATAGTATGCAAA
TCAATCGATACATTACACGAGGCATTAGTGAACACCTATCTCTAGACCTTCAAATCTTACTTTGGAATATGGTA
AAAGAAAGAGATAATCAACCTGATACAGATTACCTACACATTTTTAGACTGCAAGAAGATGAGAATATACTCT
CAATCACACATGAACAAGAACAACCTGCATACAAATTGGAATATCACTATACAAACTATGAAAAAAATCAAAA
CGCATTACCTAAGAAAGTCTACGTCATTCGAGAAGATGATGTAGACCTTTCTTATTATGTCATGTTACTACCAG
AAGAATACTAGAGGAGTGAAAATTACGTGAAAACAATCGAAAGTACATTAGAAACCGAAGCTATATTCAGTG
ATGACCAACAACATCGCTATCTACTTAAGAAAACATGGGACAGTGAAAAACAAAAGATCACAATCATCACAA
TGTATCCAAATTATACGGGCCTTCTTCGTATAGATTTAACTACCCAATTGATAATGAACAAAGTTTCAGAAATG
GATGCATTTGGTTCCATCAATTTTGTGAATCTATACTCTAATATTACAACTCCTATCAATCTCAAACATTTAGAAA
ACGCCTATGATAATCATACAGATATTCAAATTATGAAAGCCGTGAAAGAGTCAGATGAAGTGATACTAGCTTG
GGGTGCTTACGCTAAAAAGCCCGTTGTTGAATCACGCGTCAATGAAGTATTAGAGATGTTGAAACCACATAA
GAAAAAAGTGAAACGACTCATGAACCCCGCAACCAATGAAATCATGCATCCCCTTAATCCGAAAGCGAGAC
AGAAATGGACTTTGAAAGCATAACCTATATATGGAGGTAAACGATGAAGGAAATCAATATTGTTTCACTACAA
ATGATAAAAACAGACACATTAAGTTATCTAAAAAATCGTATTTCAAACCCTGAGGATGCGGCAGAAATCTTGC
GTTCATTCATTGGAAACAGTGACCGAGAACATCTCATTCTCATATGTATGAATAGTAAAAATGAACCTACACAC
ATTCAAATACTATCGATTGGATCTATTAACCAAACAGTGATTCATCCCAGAGAAATATTCAAAACAGCAATACT
CAGTAACGCCAACAGTATTATGCTCGGTCATAATCATCCAAGTGGGGATATATTAACCATAGTATAATAAATAAT
AGTAGTTAAAGTAGTTTAAGAGGAGATATTAAAGGGATATTTTACTTTAAATAAAAATTAACAAGGTTTATAAT
TTGTAGTAGTTAAAGTATTTAAAGAGGTGGGAACATGTGAAGGTTCAAAGGATAGAAGTGGAGAATAAGCC
GTATCCATTGTATTTATTACTAGATAAAGAATACCAGCTAATAGAACCAGTAATGAAATTTATTAAATACTTAGA
TAATACTGGTAAGTCTCCTAATACCATTAAGGCATACTGCTATCATTTAAAGTTGCTGTACGAGTTCATGGAAC
AGAGAGGTGTTATTCTTAATGATATTAACTTTGAGTTGTTAGCAGACTTCGTAGGTTGGTTGAGATATCCTTCA
GCATCAAATGTAATTGATCTTCAGTCAAAAAAAGCCATAAGAGAAGAAACGACAGTGAATACAATTTTAAAT
GTAGTTATGAGTTTTCTTGATTATTTAAGTAGATTAGGAGAATTTAAATCAATTGATGTATTTAAACAAGCAAA
GGGAAGAAATTTCAAAGGATTTTTACATCATGTTAATAAGGGTAGATACCAAAAGAATGTCTTAAAGTTAAG
GGTTAAAAAGAAACAGATAAGAACATTGAGATCAAAGGAAGTTAAGCAAATTATTGATGCTTGTCATACGAA
AAGAGATAAATTAATTTTAATGCTTATGTATGAGGGTGGTTTAAGAATCGGTGAAGTGTTATCGCTTAGGCTT
GAAGATATTGTCACTTGGGACAATCAAATCCATTTAACACCTAGAGATGTTAATGTTAATGAAGCTTATATTAA
ATTAAGGAAGGAAAGAACAATACATGTGAGTAAAGAACTTATGTCACTTTATACAGATTACTTGATATATGAG
TATAGTGAGGAATTGGAGCATGATTATGTTTTTATTTCCTTAAAAGAAGGCTATTTTGGGAAACCACTAAAGT
ACCAAAGTGTTCTTGATCTAGTTAGAAGAATAGTTAAAAGGACTGGAATAGAATTTACATCACATATGCTTCG
CCACACTCACGCAACGCAGCTAATTAGGGAAGGATGGGATGTTGCGTTCGTTCAAAAGAGATTAGGTCACG

CACATGTTCAGACAACGTTAAATACCTATGTTCATCTTTCAGATCAGGATATGAAAAATGAGTTTAATAAATAC
CTCGAGAGAAAGGAGCATAAGAAATGAATGCTTCTAGTAAAAGGAAAATTATTAGTCAGAGTGAGATTAGC
AAAAAAATAGCTGTAATGAATGAAGAAATGCAGGGGTTTTGGGCTAATAATAGTTGGGATATAAGAAAATGT
CCACATCCTTCTGCCATAGAATTAAGTAAGAATCCTGCTTTAAGGAATCGTTGGGTTCGTTTTGAACGTGTTA
AAAATCTGTGGTTAAGAACAGAATTGAAATATTTTTATTTTTACCATTTAAACAATGGAATATGGAATGCAAAA
ACTGTCTGGATTAGAAAAGGAACAGTAATTAATAAAATGTTAGATTTCTTAGATTTAAAGTATCCTAGCATTAC
TTCAATTACTGAAGTTCCTATTGAAAAAGCAATGACGGAGTATAGAACTTATTTGACAAAACGGGGTGTTAG
AATTACCACTACTAATTATAAGATTACTGCTAATCAAGAAAAAACACCTGTAAAAGCTAATTCCTACTATGTTAC
TAATCTAAAACAATTTATGGAGTTTTATGAGAACTTTTATTTTGATGGAGAGGAGTGGGATAAAGACGTTTG
GGATAGACGTAACTTACCTTTGCCAGATGATAAGGTTAACCCAACACAATATGAATATACAATTAACTTTAAAG
GGTTTCGGAATACATATTTTAAACAACTTGTAAAAAGATATTGTAAGTTGAGATTGAACGTGGATAGCTTTTC
CTATGTAAGTGATATTGCCCAAAGACTTAAAGAGTTCTTTAATTTTCTGGACATGAAATTTAAACAAGTTCAG
AGAGTACACCAATTAACGAGAGTGGAAATTGAAGCATATTTAAGTGAACTAAACATGATGGGAATAAAACCT
AGTACAATAACTGGGAGGATCTCTATATTGGAAGGACTATTTAGTACCCTTCTTAGGCTAGAATGGGATGATG
TTCCTTCCAAAATATTAATTTATTCTGAGGACTATCCGAAAATACCAAGAGCAAAACCACGCTTTATAGATGAA
TTCGTCCTAGAGCAATTGAACAGTCATCTTGATAAATTACCCGAATATATAGCTACGATGACTATGATTGTTCAA
GAATGTGGAATGAGGATAAGTGAATTGTGCACCTTGAAAAAAGGCTGTCTATTAGAGGACAAAGATGGAGA
TTTCTTTTTAAAGTATTATCAATGGAAAATGAAAAAGGAGCATATAGTTCCAATATCTAAAGAGGTAGCTTTAC
TTATTAAAGTTCGGGAAGATAAAGTTTCAGAGGAATTTCCAGATAGTGAATACCTCTTTCCAAGAAAAGATG
GATCGCCATTAAAACAAGAAACATTTAGAGGTGAGTTAAATAAATTAGCTTATGAGCAAAATATAGTGGATAA
ATCAGGTGAGATTTATAGATTCCATGCCCATGCCTTTCGCCATACAGTAGGAACAAGAATGATTAACAACGGG
ATGCCCCAGCATATTGTGCAGAAATTTTTGGGGCATGAAAGCCCAGAAATGACAAGCAGATACGCTCATATC
TTTGATGAAACTCTAAAAAAATGAATTTACTAAATTTTAGGAAAAACTGGTTACCAATAATGGAGATGTGCTTG
ATCTAGATGAAGATAATGAAGTCGATGATGTAGAGCTTCAATGGTTCAAGAAAAATATAAATGCACAAGTGCT
TCCAAATGGTTATTGTAGATTGCCAGTAATAGCAGGTGGTTGTCCACATGCGAATGCATGCTTAGATTGCACT
CACTTCTGTACCAGTAAGCAATTCTTACCACAGCACGAAGAACAGTTAGAGCGTACAGAAGAGTTATTAACC
ATAGCAAAGGATAAACAATGGCAAAGACAAATAGAGACTAATAGCCGTGTTAAAGAGCGTTTAGAACAAAT
CATTGGAAGTTTAACGGGGTAATTATCAATGAATAAACAAGTTAGAAATACAACAGAAATTGTACGTTTGGC
GAAGCAGAAATCACAAAAGACAAGAGAAAAAGTAGACAAAGCGATTTCTAAATTTTCGATTGAAGGTAAA
GCTATTAATTTTAATTCAATAGCAAAGGAAGCTAATGTTTCTAAATCATGGCTTTATAAGGAACACGATATTAG
GCAAAGAATCGAATCCCTTCGTGAGCGTCAAATAACATCAAATGTAGTCTCAAAACCCAAGAAAAGTTCTCG
TTCGGAGGAAATCCTTATAAAAACCTTAAAAAGAAGAGTTATGGAATTAGAAAAAGAAAATAAAAAATTACA
GAACCAAATTCAAAAATTATATGGAGATCTGTATAATAAAGAGTAGTTTTTATTATTAATCTGTAGACAGATTGT
GAAAGGATGCACTTAAACTAACGGGGCAGTTATGTTTAAGATACTTTAAAATTTGGTTAAAATCTCCGAAAG
GATTGGTTTCTAACATTAGAAACCAATCCTTTTTAATATTAATTATATATCATTCAAACAATCATTTGAATAAGGA
GGGTAATTATGACAAAAGATATGTGTGAAGTTACCTATATTCATGAAGATAAGGTAAACAGGGCTAAAAAAG
ACCTTGCTAAACAGAATCCTATGGATGTAGCGAAAGTTTTTAAGGCTCTATCAGATGATACAAGAGTTAAAAT
TGCTTATGTTTTGTCTTTAGAGGGAGAGTTATGTGTTTGTGATGTAGCTAATATCATTGAATCTTCAACGGCTA
CGGCATCCCATCATTTAAGATTATTGAAAAATTTAGGTATAGCAAAATACCGTAAAGAAGGAAAATTAGTCTAT
TATTCACTAGATGATGAGCATGTTAAACAGCTTGTAGAAAAAGCTTTCTTGCATCAAAGGGAGGTTGCTAGT
ATTGGATAGTTCAACAAAAACATTAACAGAAGATAAACAGGTTTACCGTGTGGAGGGTTTCTCGTGTGCGAA
TTGTGCTGGGAAGTTTGAAAAAAATGTAAAAGAACTATCAGGGGTGCATGATGCTAAAGTCAATTTCGGAG
CTTCCAAAATTGATGTCTTTGGCAGTGCAACCGTTGAAGATCTGGAAAAGGCTGGTGCTTTCGAGAATCTTA
AAGTGGCACCAGAGAAGGCTAGAAGAAGGGTCGAACCAGTGGTAACAGAAGATAAAAAATGTTTACCGTGT

GGAGGGATTTTCTTGCGCAAACTGTGCTGGGAAGTTTGAAAAAAATGTAAAACAACTAGCTGGAGTTCAG
GATGCAAAAGTGAACTTTGGCGCTTCTAAAATTGATGTATATGGAAACGCATCGGTTGAAGAGCTTGAAAAA
GCAGGTGCTTTCGAGAATCTTAAGGTAATTCCTGAAAAACTGGCGAATCCATCGATACAAGCGGTCAAAGA
AGACACTAAGGCTCCTAAAGAAGAGAAAATACCGTTTTATAAAAAACACAGCACATTGCTGTTTGCCACATT
ACTGATTGCTTTTGGTTACCTTTCTCACTTTGTAAATGGAGAAGATAACCTTGTAACGTCCATGTTATTTGTAA
GTTCGATTGTAATTGGCGGATATTCACTATTTAAAGTTGGTTTTCAAAATTTGATACGCTTTGATTTCGACATG
AAAACCCTGATGACCGTTGCAGTTATTGGAGCTGCCATCATTGGTGAATGGGCAGAGGCATCCATTGTTGTC
ATTCTCTTTGCAATCAGTGAAGCACTTGAACGTTTTTCTATGGATAGAGCAAGACAGTCCATTCGTTCATTGA
TGGATATTGCCCCAAAAGAAGCACTTGTTAGGCGGAATGGTCAGGAAATAATGATCCATGTGGACGATATTG
CCGTGGGTGATATTATGATCGTCAAACCAGGGGAGAAAATTGCCATGGATGGGATCATTATAAATGGTGTGT
CGGCTGTCAACCAGGCTGCTATAACAGGAGAATCTGTCCCTGTTGCCAAAACGGTAGATGATGAAGTATTTG
CAGGTACGCTTAACGAAGAGGGACTACTTGAAGTAAAAATCACCAAATACGTAGAGGATACAACTATCTCCA
AGATTATTCATCTGGTTGAGGAAGCACAAGGGGAGCGCGCTCCAGCGCAAGCATTCGTAGATAAATTTGCG
AAATATTATACGCCGATCATTATGGTTATTGCGGCGCTCGTTGCAGTCGTTCCACCTTTATTCTTTGGTGGAAG
TTGGGATACTTGGGTTTATCAAGGATTAGCGGTACTTGTAGTTGGATGTCCGTGTGCATTAGTTATTACTACTC
CAATCTCGATTGTCTCGGCAATTGGAAATGCAGCTAAAAAAGGTGTGTTGATTAAAGGCGGTGTCTATCTAG
AGGAATTAGGAGCCATTAAGGCAATCGCATTTGATAAAACAGGAACACTGACAAAAGGTGTACCAGTGGTA
ACAGATTTTAAAGTGTTAAATGATCAAGTGGAAGAAAAAGAGCTGTTTTCCATTATTACAGCTTTAGAATATC
GATCACAACATCCACTTGCTTCAGCAATAATGAAGAAAGCAGAGCAAGATAATATTACTTATTCCGATGTTAG
AGTGAAGGACTTCACTTCTATTACAGGTCGGGGCATTCAAGGGAATATAGATGGAACAACCTATTACATTGG
CAGTCCAAGGCTTTTTAAAGAATTAAATGTTTCCGATTTTAGCCTTGAGTTTGAAAATAAAGTGAAAGTTTTA
CAAAACCAAGGGAAAACGGCCATGATTATTGGAACGGACCAAACAATCCTCGGCGTGATTGCTGTAGCAGA
TGAGGTCCGCGAAACAAGTAAAAATGTGATTCTAAAACTTCATCAGTTAGGAATCAAGCAAACAATTATGCT
GACAGGTGATAATCAAGGTACCGCAGAAGCAATCGGTGCTCATGTAGGCGTTTCTGATATTCAGTCCGAATT
GTTGCCACAGGATAAGTTGGACTATATTAAAAAAATGAAAGCCGAGCATGGTAATGTAGCTATGATTGGCGA
TGGCGTCAATGATGCTCCTGCACTTGCTGCATCCACTGTTGGCATTGCAATGGGCGGTGCTGGAACAGATAC
TGCCATCGAGACAGCTGATATTGCATTAATGGGAGATGATTTAAGTAAGCTTCCATTTGCAGTAAGACTTAGC
AGGAAAACGCTAAATATCATCAAAGCGAACATCACGTTTGCCATCGGAATTAAGATAATTGCCTTACTATTGG
TTATTCCAGGCTGGCTAACCCTTTGGATTGCGATTCTTTCCGATATGGGAGCCACTATTTTGGTAGCATTAAAT
AGTTTGCGACTGATGAGAGTGAAGGATAAATAAGTACTATATGTGTGCAATTTACGGAAGGACTTTTCATTTT
GAAGAAAAGTCCTATCCCTAAAATATTTGGAGATGATAAAATGATCGCGACGATACTGACGGCTACTGCGGT
ATATGTAGCAACAGGAATTGATTATCTCGTCATATTAATTCTTTTGTTTTCGCAAGTAAAAAAAGGTCAGGTAA
AACATATTTGGATAGGACAATATATAGGGACTGCAATTGTGATAGGAGCAAGTCTTTTAGTTGCACAGGGGG
TTGTAAATTTAATTCCTCAGCAATGGGTTATCGGACTACTTGGACTTTTACCACTTTACCTAGGCGTGAAAATA
TGGATTAAAGGAGAAGAGGATGAAGATGAAAGTAGCATTTTATCTTTATTCTCCTCTGGAAAATTTAATCAGT
TATTTTTGACGATGATTTTCATCGTATTGGCTTCCAGTGCGGATGACTTTTCCATTTATATACCGTACTTCACGA
CCTTAAGTATGTCTGAAATCTTTATTGTCACTATTGTCTTTCTAATTATGGTTGGAGTTTTGTGCTATGTCAGTTA
CCGTCTAGCTTCCTTCGATTTTATATCGGAAACAATTGAGAAATATGAACGTTGGATTGTACCAATTGTATTCA
TTGGGCTAGGTATTTATATATTGTTTGAAAATGGAACATTCAATGCTCTGATTTCATTTCTTCTTTGAAGTGGC
GAATCCAAACCATAAAGTAAAGTATAAAGAGGTTATCCTATTGATTGGGATAACCTTTTTATATTTAACTAATTA
TCATTTACGTTAAACACATGTACTTAAAGTAAGAACATTGATTTAAACCATTTTTTTCATAATTTACTCTTGACA
AGATCCGACACCTTCTCCAGGAGATATCAAAATAACTCAGAGAATAAAAGAGATTAGTGAAATGATGGGCAT
TATATTATATGACCACATAATATTTTCAGAAAATGATCATCATTCAATTATCAAAAAAATTGCACTTTCTACTGGT
ATATATCCTTGCATTAATTTTATAAAAAATGCCCCTACTGAAGATATAAAAGCAATTGCTATGCTATTGTTCACTA

CCGTATTCATTGGTAATTTAAATAATGCTAATAATACAGGAATGATGATAAATGCTCCACCTGCACCCACAATTC
CCGAAATTACACCAATTCCAAATCCAACTATAATAAATAGCAAAGGTTTAGATTTCGTCTCTTGTGTAGTAGGT
TTAACTTTAATAAACATCAATATTAAAGCAAGTATGGCGATTATTACATATACCGTATTTACAAAAGTAGCATTA
AACAAACTAGCTAAAATTGCCCCTAACATGCTTCCAATCAACATACCCCCTCCCATATATATAACAAGTTTAGG
AGAGAAAGCTGGTTGTTTTCTTGCATTTAATGATCCACTAAGTGTACTGAAAAATACTTGACTAGAGGTAAGT
CCCGAAGCAATATATGCACTATACGCAGGTATACCTATTAATGGTGGCAATATAAGAATAGCTGGGTAAATAAT
AATTGCGCCCCCAATACCTATTAATCCAGATATAAATCCACCCAGTACGCCAATTAGCAACATAATAGTCATATT
TGCTATATCCATTATTTACTTTTAACTAATAAATTAACTGCTTCATTAATTAATTCTTGAGAACTTTCATTATTTTC
TTCTGCTGTTTTAACGCATTCTATTAAATTTTCACTAATTATAATCCCCATTAAACGTTGTATAGAACCTTTAGAT
GCACTAAGTTGCGTAATTATATCTTTGCAATCTTTTTCTTCTTCCATCATTTTTACGACACCATTTAATTGACCTT
GTATTCTATTTATACGATTAATCATTTTTTTATCATAAGTCATATTCATTTCCTCCACATAAATTGATTAAGTTAATA
ATAATAAATTAAAACAAAATTGTCAAATACCCCTATAGGTATTTGGCAAATGATTCTATCCGTTTTATTATACCCT
CATAGGTATTTATAGGAGGTAAGTATGGAACAATACAAAGAAGTACACATTAATTCATTAAACAAAGAAGAAT
TAGAAAAATTGGCAAAAATGGGTCAATTGATTGATGTTAGAACAAAAGAAGAATATGAATTAGGACACATTA
ACGGTTCAACTTTACACCCAGTAGAAGAGATTAAATCATTCAATAAGAATAGCAACAAAACGTATTATATACAT
TGTAAAAGCGGTAACCGAAGCACTAAAGCTTGCGATTATTTAGCCAAACAAGGTTATGATGTCGTGAATTTA
AAGGGTGGATATAAAGATTATGAAGCAAAGAACTTCAATAGTGCTCCTTTAATAGAAAAAGATATTGAAATCA
AAGAGAATCGTAAACAATTTGATTTTAGAGGTCTTCAGTGCCCTGGACCTATTGTTAATATCAGTAAAGAAAT
TAACAATATTTCAACGGGTGAGCAAATCGAGGTAACAGTTACAGATCCTGGTTTCAATAGTGATATTAAAAGT
TGGGCGAAACAAACAGGGAATACCCTTGTAAATCTTACTGAAGAAGCAAATGTAATTAATGCAATTATTCAA
AAAGAAAAGCCAAAAGAATTTGAAATCAATGACACTGCTACAGGTACAACAATTGTTTTATTTAGCGGTGAG
TTAGATAAGGCAGTAGCAGCAATGATCATCGCAAATGGCGCTAAAGCAGCAGGTAAAGATGTAACTATTTTC
TTTACTTTTTGGGGCCTCAACGCATTAAAAAAAGCGCAATCAACTCGCGTTAAAAAGAAAGGTATTTCCAAA
ATGTTTGACCTTATGTTACCTAAGGATCCAATACATATGCCATTATCAAAAATGAATATGTTTGGACTAGGTAAT
ATTATGATGCGTTATGTAATGAACAAGAAAAATGTTGATTCTTTATACTCACTTATCGATCAAGCAATCGATCAA
GATATAAAGTTAATTGCTTGTACCATGAGTATGGATGTTATGGGAATTTCAAAAGAAGAATTAAGAGATGAAG
TCGATTATGGTGGCGTAGGTACTTATATTGGACATACTGAGCAAGCAAATCATAACTTATTTATCTAATCAATAA
AATCTATTTTAAAAAGGAGTTTTTTTGTTTTTCAAACAATTTTATGAAGACAATTTTTCACAAGCATCTTATTTA
ATTGGATGTCAACGTACAGGAGAAGCAATGATTATTGACCCTATACGAGATTTAACAAAATATTTAAAAGTTG
CAGATAAAGAAGGATTCACAATTACTAAAGCTGCAGAAACACATATTCATGCTGATTTTGCTTCAGGTATTAG
AGATGTGGCAAATAAATTGAATGCAAGTATATACGTATCTGATGAAGGTAAAGATGAATTTAATTATAAAAATA
TGCCTAAACAAACACATTTCGTTAAGCATCAAGATATTATCTATTTAGGAAAGATTAAATTAGAAGTCTTACAC
ACTCCAGGCCATCCCCCTGAGAGTATTAGTTTTTTACTTACTGACGAAGGTGGGGGTTCCAGTATACCAATGG
GATTATTTAGCGGTGACTTCATCTTTGTTGGTGATGTAGGTAGACCAGATCTATTAGAAAAAGCTGTAAATGT
TGAAGGTTCTACAAAAATCGGAGCAAAACAAATGTATCAGTCCATACAATTTGCTGCAACATTACCAGACTAT
ATTCAAATTTGGCCAGGTCATGGTGCAGGGAGTCCTTGTGGTAAAGCGCTAGGTTCATTACCTACAACTACT
TTGGGTTATGAGAAAATAAACAACTGGGCTTTTAATGTTAAAGATGAAACTTCATTTATCGAAACTTTAACAC
TCAATCAGCCAGCTCCACCACACCATTTTTCACAAATGAAAAAAATAAATCAATTTGGTATGCAAATGTACCA
ACCATATAATGTTTACCCTAGTTCAAGCAATACACAAACTGCTTTTGATCTTCGAAGTAAAGAAGCATTCCATG
GTGGTCATATGCATGGAACTATCAATATTCCTTTTAATAAAACTTTTATAAATCAAATTGGTTGGTATTTAGATTA
TGACTAAGATATTGAATTAATTGGCGATAAATCTACCGTTCAACAAGCTATTCATGCTTTACAATTAATTGGTTT
TGAAAATGTAAAAAGTTACCGTTTGCCACAAACAAATATCGTCACATCATCTATTCATAGTTCTGAAATGACTG
GTGAAGAACATAATGTATTAGATGTTCGTAATGATGATGAATGGCGTAAAGGTCATTTAAAACAAGCAATTCA
TATTCCTCACGGTAAACTTTTAAATGAAGATATACCTTTCGATAAAAATGACCTAATTTATGTACATTGTCAATC

AGGTGTTAGAAGTTCAATAGCGGTAGGAATATTAGAATATAAAGGATTTACGAATATTGTTAACGTTAGAGGT
GGCTATCAACATTTACCAGCATTTTTAAAATAGTTTTCTTCTGCAATAGAACTATAAAAATCCCTCAATCCGAA
AATTCAATTCGGATTGAGGGATTTTTTTATAAAGACAATTACGAACTTTGATTATTCATTTGAATTAGTTTTTTA
TGCTTTTATATCTAAGTAAATATCATTAATCTGTAGTACTTTTTGGATAATTGCTAATGTACCACCTAGCAAAGTT
GCAAATTTGACATTAGATGTTAACTTTATTTGAATTTCGTTACGTGAATATTGTTTGAACTGGTTCTTAATTGCT
TCTAATATTTCAGGCATTTCATTGAACAATGGACAGTTAATGTAAATTGCATTCGGATTAAACTGGGTGTTTAA
ATTATGTATTAAAACAGCAATTTTATTTATAAATTGTTCCATTTCTTCAACTACGACTGGATTTTTTTCATTATAA
AATTGAATTAATTTGCTAAGCGTCATCTTCTCATTTAGTTGATTACTTAAATTATGCAGTAAAGCTTCTTGTGAA
AAAATATCTTCAATCTTATGAAAGATCTCCACATTATCGCTAACTTTTGAGACAAGTGTTTTTCCAATTTCACCC
GCTTCCCCATTTGCACCACGATACAATTGATTATTAATAATAAGCCCAGCACCAATACCTTTATGTATACTTAAA
GCAATAAGATTATTGTAGGATAAATTATGATTAAAATTACGTTCATATAACGCTGAAAGATTCGCTTCATTTTCA
ACTACGACTGGAACATTAGTAATTTCTTTTATTTTCTTAGCAATTGAAATTCCTTCAGTTTCATGGAATGGTAA
ATATGTCACATGCTGCTCATTATCCACAACTCCATGTATAGAAACAGACACACCTAATAGTCCGTTATAAGTATC
AAGTTTCTCCTGAATATCAATATGTTTTTTTATTATGCTTAATATACTACTAACCTTTTCATCAGGTAAATCATAA
GATTCATGCTTAATGACATTACCATCAAAATAATTGTACATCACTTCAACAGAACTATAAGTTAAATCCAAAGA
AATAAAATAACCATAAAGATGATTAACCTTCAGAAGAATAGGTTTTCTACCACCACTCTTCGTGCTATCACCCT
CACCAACCTCATTAACAAGAGATTTATACTTTAACTTATTCAAAATACTAGAAATCGTTGCCTTATTAATCTCAA
GATTTTTAGATATTTGAGTACGCGAAATATTATGATGGTTATAAATTTCCCTTAGCACTCTTTTTTCATTATCATT
TATATTTAAAATATTTTCCATAATTGCCTACCCCATAAGTTATTTTGAATTTAGTTTATAGTTTATCATGTATTTTTA
TACCAACTATTTTAACATAAATTAAATTAAACCTTAGAACAACTTAAAACCTCACTTAGGTTATCATCTTCGTTC
TTTTATTTTTTTTATTATTTCAATAAATTACGTATTTCCAATATGACGATTTTTTATGCAAAGTCGTTGTATGTTTT
TCAAATTTTTGACATTTATGCAATCTAAATAGCCAATTAGGGAATTTTTAACATTTTTTATTTGTTTGATATTATA
CTTAATGTATCTTAAATAGAAAGAGGGTATGCATATGGATTTCACTGGTGTTATTACAAGCATTATTGATTTAAT
CAAGACTTGCATTCAGGCTTTCGGTTAATTTTTTCAACTAAAAAACAGAGGAAATATTCAACGACTTGATTGT
TTCCTCTGTTTTCTATGTATTGTTGTAAACACAACAATTTTATTTTTTATTCAATATATTTCTCAATTCTTCTATTTC
ATCTTGTGATAGATCTTCTTTTTCTACAAAGTTTAAGACAAGTGAATTGAAACCGCCTTTGTATACTTTATTGAT
AAAGTTTTTAGATGTTTTATATTTTATATCACTTTCTTCTACAAGAGAGTAATATTAAAAAATTTTATTGTCTTTT
TTACGATCTATAAATCCCTTTTTATACAATCTCGTTATAAGTGTACGAATGGTTTTTGGACTCCAGTCCTTTTGC
ATTTGTATTTCTTCTATTATATTATTCGCACTTGCATATTTTTTCATCCAAATGATATTCATAACTTCCCATTCTGCA
GATGATATTTCATACGTTTTATTATCCATTATATTAACTCCATTTCTTTTAAAATACGCTCAGAAATTTGTTGTGCT
TTTTCGCCATTCGCATTGTCTTCGCCTTTTAAATGTGTAGCAAAATAATACGTATTATCTTTCGTTTCAACATAA
CCTACGAACCATCCATTTGCTTCTTTGTGATTCACGATTCCTGTTCCAGTTTTACCTACATATTTATAAGTATCTT
TTTGTTTCAAAGTCATACTATTTTCAACTTTTTCAATAGCCTTATTATCAAAATGCATGTTATGTTGTTTCATATT
TTTCAACAAATTAACCTGTTCTATTGCAGAAATTTTTAATGAAGATTCATTCCAATAATTTTCATTCCCTGATATT
TCTTCATTACCATATTCAATTAGATCTAAATAAGATTTAACCTCATCTTGTCTTAAATGTTTGTTTAAATTTTCGT
AATACCAATTTACTGAATATTTCATTGAAGAATTTAAATTTTGATCTTGGTTCCATTCTTTAAATGGATATTGAT
GTTTATCCCATTGTTGTTCAGTATGATTTAATGAGAGTAAATTTTGGTCGAATGCCATTAACGCTAAATAAATTT
TGTAAGTAGAATTAGGTGAATATCGTTGTTTACTTTCTGGTTCATTATAAATAGAATAAGCTTGCTCCCGTTCAT
TATAAAGCACAAAACTTCCATCAAATCCTTTGAAATACGGAGCTAGTTGATTTAATTTTTTATATGATACATTTG
TTTCATATTTGTCTTGTTGAACATGTGCAGATAGTAACGGTGCTTGTATTAAAAGCGATATACTACATACAATAT
ACGCAACAATACGCTTGTTTCGATTAGGTTTAGGCATTGAATCATAAAGTGCAATATACTTAACACGTTCTTTA
ATATTTGAATTAAAACCTAGTAAATATTGTGCTGCCACATTATTTATGTGCTGAGATTTTAAAATAGAGCATTTT
AATATCGATTCACCATAACGTATATGTTCATGGCGATTCAAAATTTTTAAAACGTTTCTATCACATACTTTTTCAC
AGTCATTGTCCATCATTGTTTTACTTATATATAGTGCAGGATTAAACCAGAATATCATTTTAAAAACAACATAAA

GCTGGTTGAATATTAAGTCATGACTTTTCACATGTGATAGTTCATGTAGAATAATATATTCAATTTCTTTGTCATT
CATGGTTTCGACTACGACAGTTGGTAGTACAATTTGGGATTTCACTAAACCAAATACCATCGGATTATCAATGT
TTGAACTATAACTAATTGTTATATGCTTTTTGTAGAACTGCATCTTACTTTGACATACTTTAAGTCGTTCATTAA
GATATGACGATTCCAATGACGAACTTTTAATAACATCAATTTGTCGGAATGCCTTAATCATATAAAATAAGCAC
AACAAACTACCAAATACCCATATCAAAAGAATCATATACGTTATATTTGAGGTCTCAAACTGATTAACATTAATT
GCTAAGTCTTTCGTAACAGATGATTGTTGACCATCTAACATATGACTAACCGAAGAAGTCGTGTCAGATACAT
TTCGATTCATCATATCTTTTGAAAATGTAAAATTCGATATTTTGTAAAATGGTATTAATGGAATTAACGTGGAGA
CGAGCACTAATAACCAAATCTTATGTGACATAATATTTTGAGTATATTTTATATAGAGCATTCTCACTAAAAAAA
TTACACATATCGTGAGCAATGAACTGATTATACTTAACATTAAAAAAGATGATAACACCTTCTACACCTCCATAT
CACAAAAATTATAACATTATTTTGACATAAATACTACATTTGTAATATACTACAAATGTAGTCTTATATAAGGAGG
ATATTGATGAAAAAGATAAAAATTGTTCCACTTATTTTAATAGTTGTAGTTGTCGGGTTTGGTATATATTTTTAT
GCTTCAAAAGATAAAGAAATTAATAATACTATTGATGCAATTGAAGATAAAAATTTCAAACAAGTTTATAAAG
ATAGCAGTTATATTTCTAAAAGCGATAATGGTGAAGTAGAAATGACTGAACGTCCGATAAAAATATATAATAGT
TTAGGCGTTAAAGATATAAACATTCAGGATCGTAAAATAAAAAAAGTATCTAAAAATAAAAAACGAGTAGATG
CTCAATATAAAATTAAAACAAACTACGGTAACATTGATCGCAACGTTCAATTTAATTTTGTTAAAGAAGATGGT
ATGTGGAAGTTAGATTGGGATCATAGCGTCATTATTCCAGGAATGCAGAAAGACCAAAGCATACATATTGAA
AAATTAAAATCAGAACGTGGTAAAATTTTAGACCGAAACAATGTGGAATTGGCCAATACAGGAACAGCATAT
GAGATAGGCATCGTTCCAAAGAATGTATCTAAAAAAGATTATAAAGCAATCGCTAAAGAACTAAGTATTTCTG
AAGACTATATCAAACAACAAATGGATCAAAAGTGGGTACAAGATGATACCTTCGTTCCACTTAAAACCGTTAA
AAAAATGGATGAATATTTAAGTGATTTCGCAAAAAAATTTCATCTTACAACTAATGAAACAGAAAGTCGTAAC
TATCCTCTAGAAAAAGCGACTTCACATCTATTAGGTTATGTTGGTCCCATTAACTCTGAAGAATTAAAACAAAA
AGAATATAAAGGCTATAAAGATGATGCAGTTATTGGTAAAAAGGGACTCGAAAAACTTTACGATAAAAAGCT
CCAACATGAAGATGGCTATCGTGTCACAATCGTTGACGATAATAGCAATACAATCGCACATACATTAATAGAG
AAAAAGAAAAAAGATGGCAAAGATATTCAACTAACTATTGATGCTAAAGTTCAAAAGAGTATTTATAACAAC
ATGAAAAATGATTATGGCTCAGGTACTGCTATCCACCCTCAAACAGGTGAATTATTAGCACTTGTAAGCACAC
CTTCATATGACGTCTATCCATTTATGTATGGCATGAGTAACGAAGAATATAATAAATTAACCGAAGATAAAAAA
GAACCTCTGCTCAACAAGTTCCAGATTACAACTTCACCAGGTTCAACTCAAAAAATATTAACAGCAATGATTG
GGTTAAATAACAAAACATTAGACGATAAAACAAGTTATAAAATCGATGGTAAAGGTTGGCAAAAAGATAAAT
CTTGGGGTGGTTACAACGTTACAAGATATGAAGTGGTAAATGGTAATATCGACTTAAAACAAGCAATAGAAT
CATCAGATAACATTTTCTTTGCTAGAGTAGCACTCGAATTAGGCAGTAAGAAATTTGAAAAAGGCATGAAAA
AACTAGGTGTTGGTGAAGATATACCAAGTGATTATCCATTTTATAATGCTCAAATTTCAAACAAAAATTTAGAT
AATGAAATATTATTAGCTGATTCAGGTTACGGACAAGGTGAAATACTGATTAACCCAGTACAGATCCTTTCAAT
CTATAGCGCATTAGAAAATAATGGCAATATTAACGCACCTCACTTATTAAAAGACACGAAAAACAAAGTTTGG
AAGAAAAATATTATTTCCAAAGAAAATATCAATCTATTAACTGATGGTATGCAACAAGTCGTAAATAAAACACA
TAAAGAAGATATTTATAGATCTTATGCAAACTTAATTGGCAAATCCGGTACTGCAGAACTCAAAATGAAACAA
GGAGAAACTGGCAGACAAATTGGGTGGTTTATATCATATGATAAAGATAATCCAAACATGATGATGGCTATTA
ATGTTAAAGATGTACAAGATAAAGGAATGGCTAGCTACAATGCCAAAATCTCAGGTAAAGTGTATGATGAGC
TATATGAGAACGGTAATAAAAAATACGATATAGATGAATAACAAAACAGTGAAGCAATCCGTAACGATGGTTG
CTTCACTGTTTTATTATGAATTATTAATAAGTGCTGTTACTTCTCCCTTAAATACAATTTCTTCATTTTCATTGTAT
GTTGAAAGTGACACTGTAACGAGTCCATTTTCTTTTTTTATGGATTTCTTATTTGTAATTTCAGCGATAACGTA
CAATGTATTACCTGGGTATACAGGTTTAATAAATTTAACGTTATTCATTTGTGTTCCTGCTACAACTTCTTCTCC
GTATTTACCTTCTTCTACCCATAATTTAAATGATATTGAAAGTGTATGCATGCCAGATGCAATGATACCTTTAAA
TCTACTTTGTTCTGCTTTTTCTTTATCTATATGCATATATTGAGGATCAAAAGTTGTTGCAAATTGGATAATTTCT
TCTTCTGTAATATGAAGGCTTTTTGTTTTGAATGTTTCTCCTACTATAAAATCATCGTATTTCATATATGTCTCTCT

TTCTTATTCAAATTAATTTTTTAGTATGTAACATGTTAAAGGTAAGTCTACCGTCACTGAAACGTAAGACTCAC
CTCTAACTTTCTATTGAGACAAATGCACCATTTTATCTGCATTGTCTGTAAAGATACCATCAACTCCCCAATTAG
CAAGTTGGTTTGCACGTGCTGGTTTGTTTACAGTCCATACGTTCAATTCATAACCCGCTTCTTTTACCATTTTT
ACTTTTGCTTTAGTAAGTTTGGCATCTTCAGTGTTTACTATTTTAGCATTACAGTAATCTAAAAGTGTTCTCCAG
TCTTCACGAAACGAAGTTGTATGGAATATAACTGCTCTGTTATATTGTGGCATGATTTCTTCTGCAAGTTTAAC
AAGCACAACATTAAAGCTTGAAATGAGCACTTCTTGATTCTGATTTAAGTTTGTTAATTGTTCTTCCACTTGCT
TAACCATACTTTTAGAAAGTGCTAGTCCATTCGGTCCAGTAATACCTTTTAATTCTACATTTAAATTCATATTATA
TTCATTTGCTATTTTTACTACATCATCGAAAGTTGGCAAATGTTCATCTTTGAATTTTTCACCAAACCAAGATCC
TGCAGAAGCATCTTTAATTTCATCATAATTCAATTCAGTTATTTCCCCGGACATATTTGTAGTCCGTTCTAAATA
ATCATCATGAATGATAATCAGTTGTTCATCTTTTGTAATTGCAACATCTAACTCCAACCAGTTTATACCTTCTACT
TCTGAAGCAGCTTTAAATGATGCAATTGTATTTTCCGGAGCTTTACTAGGTAATCCTCTATGTCCATATACAGTT
AGCATATTACCTCTCCTTGCATTTTTATTTTTTTAATTAACGTAACTGTATTATCACATTAATCGCACTTTTATTTC
CATTAAAAAGAGATGAATATCATAAATAAAGAAGTCGATAGATTCGTATTGATTATGGAGTTAATCTACGTCTC
ATCTCATTTTTAAAAAATCATTTATGTCCCAAGCTCCATTTTGTAATCAAGTCTAGTTTTTCTGTACCCCTTATCT
GCAATTTTACTTAGGATTGCTTTTAACTTACCTCTTATCTGCAATTTTACTTAGGATTGCTTTTAACTTACCCCTT
ATCAGCAATTTTACTGAGAACTGCTTTTAACGCACCTCTTATCTGCAATTTTGCCTAGAACTGCTTTTAACGTA
CCTCTTATCTGCAATTTTACTGAGAACTGCTTTTAACTTACCCCTTATCAGCAATTTTGCATGGAATTGCTTTTA
ACGTACCTCTTATCTGCAATTTTACTTAGAATTGCTTTTACTATTCCTCTTATTAGTATAATCTCAGTAAGAATGC
GTATAAAAATGAAAATTACAACCGATTTTGTAAGTGCTGACGCCTGAGGGAATAGTATGTGCGAGAGACTAA
TGGCTCGAGCCATACCCCTAGGCAAGCATGCACGTACAAAATCGTAAGATAAAAAAATAAGCATATCACTGTA
AACTTTAAAAAATCAGTTTAGTGATATGCTTATTTATTTCGAGTTAGGATTTATGTCCCAAGCTCATCAAGCAC
AATCGGCCACTAGTTTATTTCTCTATCTTATATGTTCTGATATGGTCTTCTATACTGTATAAGTATACTTTTGAATA
TGGATCTTGTGTCAATTCACGTTCGAAATCAAATTCTTGATTATCAAATCTGTTAAAGAATGTTTCGTATTCTTC
GACTGATAATTGCTCTCTAGATTCTAGCATATTTAAGTGTTTCTCTTTATCTAATGCTTTGTCATATCCTTTAACG
ATTGAACCACTAAAGATTTCTCCTACTGCTCCTGAACCATAACTAAATAGACATACTTTCTCTTCTGGTTGGAA
TGTGTGGTTCTGTAATAACGAAATTAAACTTAAGTATAATGATCCTGTATAAATGTTACCAACATCTCTATTCCAT
AATACGGTTCTGTTGCAAAGTTGAATTTATAGTATAATTTTAACAAAAAGGAGTCTTCTGTATGAACTATTTCA
GATATAAACAATTTAACAAGGATGTTATCACTGTAGCCGTTGGCTACTATCTAAGATATACATTGAGTTATCGTG
ATATATCTGAAATATTAAGGGAACGTGGTGTAAACGTTCATCATTCAACGGTCTACCGTTGGGTTCAAGAATAT
GCCCCAATTTTGTATCAAATTTGGAAGAAAAAGCATAAAAAAGCTTATTACAAATGGCGTATTGATGAGACGT
ACATCAAAATAAAAGGAAAATGGAGCTATTTATATCGTGCCATTGATGCAGAGGGACATACATTAGATATTTG
GTTGCGTAAGCAACGAGATAATCATTCAGCATATGCGTTTATCAAACGTCTCATTAAACAATTTGGTAAACCTC
AAAAGGTAATTACAGATCAGGCACCTTCAACGAAGGTAGCAATGGCTAAAGTAATTAAAGCTTTTAAACTTA
AACCTGACTGTCATTGTACATCGAAATATCTGAATAACCTCATTGAGCAAGATCACCGTCATATTAAAGTAAGA
AAGACAAGGTATCAAAGTATCAATACAGCAAAGAATACTTTAAAAGGTATTGAATGTATTTACGCTCTATATAA
AAAGAACCGCAGGTCTCTTCAGATCTACGGATTTTCGCCATGCCACGAAATTAGCATCATGCTAGCAAGTTAA
GCGAACACTGACATGATAAATTAGTGGTTAGCTATATTTTTTTACTTTGCAACAGAACCATTTTTGTTCAGTAA
TATTAATATGTACACTATTTCCAAAATTTAAATTCATGTTGCCAAAAATCGATTTGTTTTTGCAATTGTTTTTCGT
TCGCTTTTAAAGTCGATTTCATTAAGTCCGTTAAATCGACTGGCGAAATTTCTTTTATCAAATTCTTATATTTTG
TTCTAGAATTTCTGTGAAGCTTTCCCCATTCTTCTTCATCACTCAATAACATAAAAACTATTGCTCTATCCGCAG
TGCGTTGGATAGTTTTCCAATCTGGTTGCAAGATATGTAAATCACTAAAGCAATCATTCCAGTAATCCACCATA
TCTCTTTTAAGTTCGATTTCTACACGCCATAAATGTTCAGACATAACTTCAGCATCTGCATTATCTTTACGTTCT
TGCTTTTTATTATAAATTCTAATAAATCTATTACTATCTCTCACGCCAAAATATTTTGTTTCTGGCTTACCATTACG
ACCATAAAAAAATAGTTTTCTTAACTGCTTTATCAGACATTGCATAGTAGTCACTCAAATCATCTTCAAAATCAA

AGGCTAAATCTAATCTTGTAAAACCGTCATCTTCCATGTAGCTTATTATATTTTGTTTTAACCAAATCATTTCATC
TCGTGTAAGTTTGTTTGGATTAAATTCAATACGCATATTACGTCTATCCCAACTATCTGCTTTCACTTTGTCATAT
TCAATATAAACTTTTTCTTGAAGTGCTTTAGCTTTAAACTTTGTTTGAAGAATATCCCAAAGTCTTATTTGGGG
CTCTACACTCATAAATTTAGAAAGGGCTTGAGCGTTGTCTCGGTTGAGATTTCCAACGATCGTCATTGCATCA
AAACTTAATTTTGGATCAGATATGCACACAGTATGTGCGTCCAACCGGCTATTACCGGCTATTAGAGTAGCCG
GTTTTAGAAAAATTGTCTAAATCGTGATTTTCCAAATGATTTGAATGATTTGCATGATTGTTTTTATACATAAAA
AATCGACTCCTTAATCTCAATTTCGTTTAAGGAATCGCTCACCCAAATATATATCTTGATGTATATTTAAATATCG
TTTAATATCTAAATATACAAGATTATAAAAACAACTCAGTGTTTTTTTCTTTGAATGATGTCGTTCACAAACTTT
GGTCAGGGCGTGAGCGACTCCTTTTTATTTTGTTATTAATATAACACTATCAAAAGACTTGGTCTAATCAGATC
AAGTCTTTTTTTATTTAAGCATTTGTATTATCTGGTAAACAGTTAAAACTACTAAAACACCAAGTACATACTTAC
TTGTTATAAAATTCTCAAAGGCTTCTCTGAATTTCATTTCTTCAACTCCTTTCGTTGCTTTATTCCAATTTCCTTA
TTGGTCGGAACCTACAGGGCTTTATGTGTTGTTGAGTTGGTACTTTCTTGGGATTAATCCCAATTCAAGTCCA
ACCAACTCGCTAACAAGTTAGCTAACACATAGCCCATTCCAACCAATAAGTTTTCTCGGCATAAATGCATGTTC
TATAAGTGCATACCTCTATCTCGTTTTTGATTCTTTTGTTGTTCTTGTTGCTGTTCCTGTTCTGAATATAATTCTG
GATTCATTTTTTTATCTAAGTTTCCTGCTAAACCATTTAGTTTTGGTTCATCTTCTGTTAACTTATTAACTCTTTC
CGCAAAGGTATTTCTTCCTAAAACTCGTTCTAACTCTTTTAGTAAGATTTTTAAAAGCTTTAACGCTATTTCTAT
ATTCTCTTTAAGTGGCTTTGCATTTTCGTAAAGTTGGTTAAAATTATCGTCTGCGTTTTCAATACGCTCAACTG
CTTTATTTAATAAATCATCTTTCTCTCGTATTTCCTTATCTTTATCATCTAAGGCTCTACCAGACTTTATATACTCG
TAATCTTCCGAAATATCTTGAGCAGCTTTTATCTGTTTCTGAAATTCATTGAAATCTTTTTGGCTTATTACAACA
TTTCCAGTTTCTTGTATTTCTTTGCTAAATAAACCACCTACTTTTTCAGTTTCTTGCTCATACGGAACATTTATA
GGCTTTTTAAGCGTATTTAACGATTTTTGGTACTCTTGCATTAATTTATCGTTCTTTTGCTTTATATGGTCTGTTT
TTTGGCTCTCACGTTCATATTCTTGCTTATGATATTCTGTTTTTTGTTTATACTGACTTATTTGCTCATGTTTAGC
ATTTGTTACTTGTCTTGATTGCCCACGTTCTAAATCATATCCTCGTTGTTTAACATGCTCATTAAATCTATCTTGA
AACGCTGTTAAAGCTTTTTTATTACCTACAACTTCTTTAGCACTTAAACGACCATCATCAGTTATTGGAACAAC
GCCATAATGCATATGTGGTGTTTTTTCGTCCATGTGAACTGTTGCATATAATAAATTATCTTTACCGTATTCTTGT
TCTAAAAACTCTTTAGCATATTCAAAAAACTGCTTTGTATCTTCTGGCGTTTGATTATCAAAGAAATCATTGTCT
GATGTAATTAAACCATCAATGTGTTTAATCGCGTCTGTTCTAATTTTTCTTTTGCCTGTATAATTCTGTTCGATTT
TTTCATCAATCAAGTTATTAAAATTCTGTTTATTAGCATTTACCAAATCATAATTTAAGTAAGTTTTACTATGGTC
TATATCTTCATTTTCATAATTATTATTTTCTCTTTGAACATGTTTTTGTATGCCCGTTGTATTTGTTCCAGATTTAA
CTTTTGAAACTCTAACAATGGAATAAGACATATAAAAATCACTCCTAAGCACAAAGGTTTCATGTGTATTTAAT
AGTTTAAGTCTAATAACACACTAGACTTATTTTTAATAAGTCGTTAATACGTGTGCTCTGCGAGGCTTAAACCT
GTTTTTGCTAACGCAAAAAAATGAGTGGCAAAATGCTAGCCACTCATAGTTCTAAACCAAAATATAATATAACTA
TTCAAACTGCTTTTCAGAACGTTTAAATACAATAATCGTCAAAAGACAACATAAAATAATAAGGATAGCCATG
GCTACAAGAATATTACTATACACTCCAGAAGAATAATTTATAAATTCCAGAACTAGTTTACGATTAATCAATTGT
AGTGACAATAAACCTCCTACAATTGCTATACCTGTTCCCTCTGATAAAAAACTTGTGAAATTTAGCAAACTCAT
TCCAGAAGCAACTTCTTCTTCAGAAAGACTACTTGATACTATTTTTGATATAACTGTTTTAGTAAAAGATAATC
CGCCCATAACAAATATAAACATAAAAGTAGTCAACCACATACTAAACTCAACAAAAAATGCAATAGTTAAAAA
ACTTATAGAGATAGACAATGATCCTAAAATAAAAACAAATAATGATCCTTTTCTATCCACTAAAAAACCACCAA
AATAACCAAAAACAATAACACTCATGGTTCCAGGAAAAATAACACTATTACCTATTGTCGCTACATTTACATGA
TAAATAGTTTTCATCATATAAGGCACCATTGATATAAAACCAGCTACTATAGAAAATATTAGCCCACCAGAAAA
CAAACCAAGCATAAACGGAATGTTTTTCCCTAGTTTAGGATTAATAAAAGGGTTAGAAACTCTTGAAATATGT
TTAATAAAAATCACAAAAAAGATTGTGAAGAGTATTAAAAAAGTCCAATTATAATTTGTCGTAAATAACATAAA
ACATATAATACTTATAGACATTAAAACAATACCTACGATATCTAATGTATTTTTTGTTGATTTACCAGGTACCATT
ACTTTAATAAGAAAAGGTATAGTTACTATTGTAATCATAGGAAGTATAAGTAGGTAAGACCAATGAATATAATG

TGCTATTATTCCCCCTATTGAAGGACCTAACCCTTCACCTAAAGCTACAATTGATCCTATAAAACCAAAGGCTT
TGCCTTGTTTTTTTCTTGTAATATTTCTAGCTACAACCACCATAATCAGTGAAGGGAATGCAGCAGATCCTACT
CCTTGTACTAACCTACCAAAAATCAAAATAAAAAAGTGATTGTGACCAATAAAAGCAATCAATGAACCAAGA
CAGCTCAAACTAATACCAATAATTAACAATTTTTTTATATTTATATAATCAGATAATTTTCCATATACTGCTGTTCC
TATCGAAAAAGTTAACATATATGCAGTGTTTACCCAGTTTGTAATTCCAGGAGTAGTATTAAAATGATTTGCAA
TATCAGGTAAAGAAACATTTAAAACCATTTCATTTAATACACTAAAAAATGAAAGAATACAAAGCCAAAATAA
AACGCTATAAAACAAACCTTTAAATTTTTTATATAAACTAAACAATTTTACCTCTTTACTTGAGGTGACTAAAG
TTTATAGGGGTAAATTATTCTTTCACCTCATATAAATTCCCCAAATTTTTAAATAGACACTTCATAAAAAAATCC
TCCTAAATTAGAATAGTTTAAATATATCATTTTTGTGGTTCTGTTGCAAAGTTGAATTTATAGTATAATTTTAACA
AAAAGGAGTCTTCTGTATGAACTATTTCAGATATAAACAATTTAACAAGGATGTTATCACTGTAGCCGTTGGC
TACTATCTAAGATATACATTGAGTTATCGTGATATATCTGAAATATTAAGGGAACGTGGTGTAAACGTTCATCAT
TCAACGGTCTACCGTTGGGTTCAAGAATATGCCCCAATTTTGTATCAAATTTGGAAGAAAAAGCATAAAAAA
GCTTATTACAAATGGCGTATTGATGAGACGTACATCAAAATAAAAGGAAAATGGAGCTATTTATATCGTGCCA
TTGATGCAGAGGGACATACATTAGATATTTGGTTGCGTAAGCAACGAGATAATCATTCAGCATATGCGTTTATC
AAACGTCTCATTAAACAATTTGGTAAACCTCAAAAGGTAATTACAGATCAGGCACCTTCAACGAAGGTAGCA
ATGGCTAAAGTAATTAAAGCTTTTAAACTTAAACCTGACTGTCATTGTACATCGAAATATCTGAATAACCTCAT
TGAGCAAGATCACCGTCATATTAAAGTAAGAAAGACAAGGTATCAAAGTATCAATACAGCAAAGAATACTTTA
AAAGGTATTGAATGTATTTACGCTCTATATAAAAAGAACCGCAGGTCTCATCAGATCTACGGATTTTCGCCATG
CCACGAAATTAGCATCATGCTAGCAAGTTAAGCGAACACTGACATGATAAATTAGTGGTTAGCTATATTTTTTT
ACTTTGCAACAGAACCATCATGTTTACCCAATTGATCTAGAACAAATTGCACAGTTCATAGTAGAAGATAAAC
AGGTATGCGATAACAATGAATAATAAAGCTTTAAAACTTTATCAGCGACTTTACCAAATTGAAGAGCTTGTCG
GTTCAATATTCGTGCGTATTTCAAAATCAGAAATTATCAATCTAAATTACATTGATCACTTGAAGTTAGAAACA
AATGGAATGGTCCATCTCACTATGAAAAAATTATTCCACGCTGTTCAAAAAGGTGCGTTCACTGGTCTTATGA
TTGCTGTCATTACTTCACTTATTTTTTTCAAGGGATAAATATGTACCACTATATCCAGATTCTTTTATGGGTAAA
ATTTATTATGAAAACTTTAATGAAGCGGTTATTATGCTAATATGTGTAGCACTATGGGCATTGATTGGAATTTTG
TTTACTTATGGTAATTTGATATTTACAGATACCGATTGGAGTATAACAAAACAGACTGTAGTTCACTTTATATTG
ATGATAATCCTATTCTTCCCACTCGCTATTCTAGCAGGTTGGTTCCATCTGAACTTTGAGAATATTATTAGTTTC
ATCATAATATTTATTGTAGTGTATATCACGATGTGGTTTGGGACATATCAAAGAAATAAAAAGATAATTCATGAG
GCAAATAATAAATTAGGTCATTAATATAAGGAAGTCTTAACTTTTAAGACTACTAATAATCTTAATATTCGATTG
AAAACTAATTATTGTAAGGTGGAAGGTATGAATAATTATTTCATATCTTCTTTTTAATATTAAAATATAATACTAA
ATTAATAATCATATTCTTTCATTAATAAGCTATATATAACTATCCCGCCTATTTATTTAATTATTAATAAACCTTACA
GATAAAAACCGCTACTAAAGAGGATATGGAAATCCATCTCTACTTTATTGTTTTCTTCAAATATTATCTCGTAAT
TTACCTTGTTCATTAAACAAAAAACTGGATAAAAAACCGCATCATTTGTGGTACGCTTCACCCTATCAGTGATA
TGGCTAAAAAGATATGCAATAAGGTTCTGTTGCAAAGTTGAATTTATAGTATAATTTTAACAAAAAGGAGTCT
TCTGTATGAACTATTTCAGATATAAACAATTTAACAAGGATGTTATCACTGTAGCCGTTGGCTACTATCTAAGAT
ATGCATTGAGTTATCGTGATATATCTGAAATATTAAGGGAACGTGGTGTAAACGTTCATCATTCAACGGTCTAC
CGTTGGGTTCAAGAATATGCCCCAATTTTGTATCAAATTTGGAAGAAAAAGCATAAAAAAGCTTATTACAAAT
GGCGTATTGATGAGACGTACATCAAAATAAAAGGAAAATGGAGCTATTTATATCGTGCCATTGATGCAGAGG
GACATACATTAGATATTTGGTTGCGTAAGCAACGAGATAATCATTCAGCATATGCGTTTATCAAACGTCTCATT
AAACAATTTGGTAAACCTCAAAAGGTAATTACAGATCAGGCACCTTCAACGAAGGTAGCAATGGCTAAAGTA
ATTAAAGCTTTTAAACTTAAACCTGACTGTCATTGTACATCGAAATATCTGAATAACCTCATTGAGCAAGATCA
CCGTCATATTAAAGTAAGAAAGACAAGGTATCAAAGTATCAATACAGCAAAGAATACTTTAAAAGGTATTGAA
TGTATTTACACTCTATATAAAAAGAACCGCAGGTCTCTTCAGATCTACGGATTTTCGCCATGCCACGAAATTAG
CATCATGCTAGCAAGTTAAGCGAACACTGACATGATAAATTAGTGGTTAGCTATATTTTTTTACTTTGCAACAG

AACCCCTAAAATTCATTTATTGGGCATACAATGCTTTTAGGCAAATGTTTAGGAGGTATGGCAATGGATAGTC
ATTTAAATCACATTTTGTCCTATTGGCAGGGACCACCGTTGGAAGGGGCAAAAACCATAATTAGTAAGTATG
GATATCCCCAGGAAGCAACAATGAGTCGATTAATTTGGTACAATAGCGGGCCATGGAAACGCACGATAGTAT
ATAAGGATCCAGTCCCTCATAACTTCCCTACCCCTCACCTTGATTTCCTTAAGCAGACCATCGATTATAAAGTG
CCCGTACACTTATATGATGCGATTGCCGCTTTTGATGGAAGTGTCTACTTAGATCGAACTACAGGTGAGGCAT
CAGCAAAGTGTCATGAGGAAGCGATGAATTTTTTATCTTTAAACTTGTTAAATGACATTGTAACTGGCAAACG
TGATGTTCAAGGAGCAAAAGCTTTCTACGCCCAAACTGCCGAACAGTTTACCAAATATCATATAACATCTCCT
TATACAGAAGGTTTTCTTTTTCCAATGCAATATAATACGGCAGACCTTGGCGTTACATATTTTAAATAAGGTGC
CATTTTGGCACCTTATTTACTCCTATATTCAAGAATCCACTTTCTCGCTTTATTAACTCCCCGATATAGGCACAA
GCAATTGATCTTTTTATTTATTTGGTTTCCCATAAAATTGGTTTCACTAGATAGAGCTTCGGTTACAGGACATC
ATCGTAAGGCCAAGATTGACGCAGGCGTAATTGCCTAAGATTAAATGTTACAACAGGACCCATTAGTAGGTC
CTGACTCCTCATAACGCGCTCCCAAATGGCGACCCAGTTCAAAGGCATCTTCAACGGGCAAAACGCTCGACT
CAGGGTGTTGGTTAAGCCAGTCTTGGGCTGCACTCGGTGAACTGAAAAAGTGAACGTCGTTACAGAAGGC
CGACCGAACCGAGGCCATTTCATCTGGTGTAACAATCGAGACAACGGCTGTTGAAGGCTCGACGCTTACAA
CGCGGTCCGGTTCCACCGTCAACCGGACGGACTTACCGGTGCCGTGACAAGGCGAAGCGATGTGGACCGT
CCGGCCGATGAGTGCTGGGAACATAAGTGTGTCAAGGGCGCACCATGCATATAGTTGCTTCCCATCAACCTC
GAAGCGATGGGGGGTAGGGAACAGTGTGAGGCCATAACCGACGACACGGCCCTGCTCATCAAGTTCCACA
CTAGGTAGAGTCTGTAGGACTTGCTTAACTTCCTCGACGGGCTTCCCGGTCTCCGCCGCGATGTCCTCAACG
GGGACTGGATCGCCCTCCGCCAGCATTTTTAGCAACGGACGGAATAACCACTCCATAGAGACGGCTTCCCCT
TGATCAAAAGTTTGATCAAGTTGGGCTGAGAATTCTGAAATATTTTTCATAAGCTTATTCCTCCTTATATTTTCT
CCACTTCATTGTTTTAAGTGCCTCCTGCAACATAGCATTAGAATCAAAAGGAACCATTAGCCTGCACAACAAG
ATAATTTCGATACGTCTTTATCAAACGTCAAGGCTGCAAGCTTTAATCCTTCAGCCATCGTTAAATAAGGCGCA
AAGCTATCCGTAAGGTCTTCAATGGTCAATCCAAATTGAACCGCTAACGTTGCCGCATAAATCACATCTCCAG
CATTTTCACTCACAATGTGCGCTCCGATCAATTTCTGGGTTTGGGCGTTGACTACAAGTTTATAGACCCCTGT
TGTTTCGTGATTGACTAAGGCCCGCGGTACAGCGTCCAACGGAAGGACCGATGTTTTGACATCGTAACCTTT
TTCTTTTGCCTGTTGTTCAGTCAAGCCGACTGTGGCGATCGATGGATTGGTGAAGGTTACGCCGGGAACAA
AGCGAAGATCGATTTTGCGTTTCGCTAGACCCAACGCATTATTTGCCACAATCCCGCCTTCATAAGCTGCAAC
ATAAACGAATTGCGGACCGAGGGTCACATCGCCCGCGGCATATATTCGGTTATTCGACGTTTGCAAATATTCA
TTGGTCAGCACTTCGCCTTTTTTCCCTGTTTTCACACCTGCTGATTCAAGGTTTAAAGTCTCTGTGTTCGGCTT
TCTTCCTGTTGCCACGAGGACTTGATCGGCTTCGATGACTTGTTCTTGACCGTTCACTTCAATATAAATGCTTG
TCGACTTACCGTTTTGCTCAACCTTTTGATAAGTGACCCCAGTGATCAGGTTAAGTCCTTGCTCAGTTAAGGA
TTCATCGATGGCTTCGGAAATTTCAGGATCGTAGGTTTTAAACAGACGCTCGCTTCTTTGCATGAGAGTCACT
TCTGTTCCGAGGTTGTGAAACATTTGACCTAATTCCGCTGCGATATAGCCAGAACCGATCACTGCCAATCGTT
GTGGAACCTCTTTTAATTCGAGTGCGGATGTACTTGTTAAATAATCAACCTCATTCATTCCCGGGATTTCCGGA
ACAGCCGGAGAAGCCCCCGTTGCGATTAAAAAGCTTTTAGACGTGATGTTTTGTCCATTCACTTGTATCGTCT
TATCGTCGATAAACGAGGCCTCGCCACGAATGAGATCAAATCCATATTCTTCGATCAAGTCTATATATTTTTCTT
GACGCATTTGACTGACTAATCCATCTTTTTGTTCGGTCAATTGGGCAAGGTCCGCAGCACCGGTACTCGTTT
GAAGTCCGGTAAACGGATTGTTTTGGGCGAGACCGTTTATTTCACCGGCACGAAGCATGGTTTTTGACGGT
ACACAACCGATGTTAACGCAGGTCCCCCCGACGGTTCCCCGTTCTACCATGGCCACTTTCGCCCCGTTTTCAT
TGGCCTTGATAGCTGCAGAAAACGCCGCACCGCCGGAACCAATAATCAGAAGATCGTAATCGCCATCCCGAT
TGAAATCTACACTGTTTTCAGAGGGCTGGCTTTCTTCCTCTCCGGGTTGATAGCCGGCTGCCGAAATATTTTG
CTTAGCCTTTTCGATCTGATCATCGCTGAGTTCAAAAATGGCCTCACCGCGTCGGAAATCGGCCGAAACATCT
TTAGCTCCGGCTTGTTCCAATGCTTCGGTTACATGTTCTTCACAGCCTGTGCATGTCATGCCTTGAATGGGTAT
TTTATATGAATTTTGAGTCATTTGAATCCCTCTTTCTATTTAAAGTTTGATAGACAGTTCTGTACATTTATCTCAT

ATTTATCTTGTCTCATGTTTCCGATCCACGCTCTCTGGAGGCGTACAGCAATCCGTTTTATTTTTTGTTTTTCCA
TTGCCTTCAGGAGAATTGTATTTGTTCTTCAACAACTTGCTGAGGATCAGAGCAATCATAACAATGGCCAATG
CTGCAAAAATGCCGGTCAACCACCAATTTCCTGTCGCACCAGCAAAGAGGGCACCAATACCTGTGCTCCCTA
ATGCAACAAGGAGAATCGGTCCTGCGCAACAAAGTAAAGGAACCAATAAAGGAACCAATAATATTCCGAAA
AAGGCCCAAACACCCCAGCGGTTACCTTTCGAGCGATTCTCATCCATCATTCTCACCACTCTTTCTAAAAAAA
ATTTTATTCTTTGGTTAAGGTACATACATGGCCTATAGCCTAACTTGAAATTTGTTCGTTTAGCTTTTTTACATC
TTTCATCTATTTTTTGTTACACCTGATCGGTTGGATTTTAAAATGTACAAATGCCACCAGAACCGAAATACCAG
TAATAAGCCATGTACAAAACGGAGAGGAAAATCACAACAGCTGCTATTTTTTGGATAGAACCCATATAGTTGT
GCAGAAACTTCTGTACCAATTGTCGTGAAATCAATGAGACCATCGTGATCGCTGTCACCACAATTCCCATACC
AAGGGAGTAGATGATGAACTTGATGATCACGGCCGTTACGCTATTGTCATTCAGCGATGCAGAGACCACCAA
CATAAAGGCTGGCAAGGTACAACCAAGTGATGTCACGGCATAGGCTATTCCGTAAAAATAGATAGACCATTT
CCCTGGCTTGACTTGAAAAGATCCTATTTTAATCGGCAAATGCTTCCCGAATAGCATGCCCAACCCCAATAAA
GCAATGAGTATACCCATAACCAATGAAAGAATCGGAAAAATCCCTGTTAGTGCGCTTCCCAATCCTCCTATCA
ATAAACCAGCCAATACAAAAATCGTTAAAAACCCCGTGGTCATCGCTCCACCAAGCCCTAATCCTTTAAAAAT
TGCATAGCGGAACGAATGATCCTTTGTTTCGCCTCCAATTAAATAGGAGATATAAGAAGGAAGCAAGGCGAT
ACCACAAGGATTAAAGGCCGCAACCATTCCAGCGGTCAGAATGAAGAGAAACGAAAAACTCATAATTCCAC
TCCTATTTGAGTCAACGCTTCTTTTAAATCATCAAAGGACGGTCGAACAGAACGATAGAATACCTCATTTTCA
GAATTGACCAGTACGATCTCTTCCAATTGTTTGACGCCATAGGTATCCGCAATTTCTTTACCATTCTTTAAAAC
ATGGGGCCAATCCCCGCCATAATCCTGTTTAAATTTCGCAAGGCTTTCTTTTGTATCTGTGTTAGGGTCTAAAC
TAACTGTGATCAATTGAACATCGTTCGGGTTTAGTTGATGCATTTCCTTAAAGATTTCCTCATTGTATATACAAG
ATGGACACCAGGTTGCCATAAAATAAATGAGTGTGGGTTTCTCATTAGGTAGGCTGACTGGATTTCCTTGAAT
GTCTTGAACCTTTGATTCATTCGCGGTATCAGATTCTGCTCCACAAGCTGTTAAACCGATTAACAACACCGTC
ATTATAGCGGTGAAGGAAATGCGTTTTTTCATTCAAATCTTCCCCCATTTATTTATCAGGCCCTCCCATTAACGT
TTCAATAATAGGACAGGTATACATCGCCTTTTCATCTGGACACTTTTCTTTTAATTCCTCTAATAACCGTTGGAT
TCGTAACAAACCCTGCACTTTCCGCTCGATTTCTTTGGTTTTTTGAACGGTAAAGGCGTACATATCTTTACATC
TCTCCCCATCTTGATCAACCACACCAAACAACAGGTGGATTTCCTTTAGCGAGAAATCCAATTCCTTCATTCG
TTTAATAAACCGTACCCGATCTGCTGTTTCCTCTGAATATATTCGATACCCTGATTCGTTTCTGGGAGGCCCGG
CTATCAATCCTTTCCGCTCGTAATACCGAACGGTTTCTTTATTCACATCACACGCTTTAGCCAATTCACTGATTT
TCATCCCCATATTGTCACCTCAATAAAAAGTATAAACCCTGTACCATAGTACACGGTCAAGTCATACATTATTTT
TTCTTTTATAGAATAATCTTATAAAATAGGTCAGATAAAAAACCTTATTTATCAAGTGTTCACATAAGCTTTTTTA
GAAGTTTAAGAATAAAGATATCTGCTCTTCACGCCAAGAAGCCACATTCTTCCGTTGAAAAATGCGGCTGTC
AAAAAAGTTATTTTCTTTTTGATTCTAAGATCATATAACAAGATTAGACGATAAAGTGGTTTATAAACATCTCA
CAAAACGCAAACTTACAACATGTGTGCAATGTCCGAAGCTGCCGTTGGATAGGCAAATATCATTTGTTTCAAT
TCTTTGGTTGAAATCCCAAAACGAATGGCTGTTGCAAAATGATTAATCAGTTCATCGGCTTCATTACTAATCAA
ATGAGCACCAACAATTTGATCATGATCTTCGTCAATCAGCACTTTAAACGCAGCAAAGTCCTCATTTGTCCGT
TTATACGTAAACCAGTCGGAGATGTTTTTCTGCTTTACTTTAATATTCCGGCCAGAGTTTTTGGCTTCCTCCTC
GCTCATACCTACCGATGCCATTTTAGGTACGGTAAATACAGCAGATGGAATCACGGGATATTCAATTTTTTTGC
TGTTCCCTTTCAATAAATTAGATGCTACGACATGAGAATCTGCACTGGCTACAGGTGTGAGGGGCAAGCCAT
CCGTTGCTGCAGCATCTCCAGCTGCATAGACATTCGGGTTACTTACACTTTGCAAATACTCATTAACATGGAC
ACCATGTTTTTTCCTTTCTATATTCCCTTTTTCAAGATTCATATCTAAGGCAGGGCCCCGTCCAGCACCATGAAT
AACAATGTCTGCTTCAAACCGCGTTATATCCTCTTTTTTTCGAGCGTACACATGAAACTTTCCTTGTTCTTTTTC
AATGGATTCAACGGAATGTTGAAGATGTACCTGTATGCCAATCTCTTTCGACTTTTCTAAAAGGATATCTACAA
GATCTATATCAAAATTCTCCAAAGGTCTTTGACCTCGATGGATAATATGAACTTCTGATCCAGCACGTGCCGCG
ATATGAGCAAATTCAAACGAAATATACCCACCACCAACAAAGACGATTCTTTGTGGTAATTCATCCAACTCCA

AAAACTCATCACTATAAGTAAGATGTTCTTCTCCTTTTATGGGTAAAGGGGTAGGTTTCGCACCACTAGCGAT
CAAGAAATGACTCCCCTCGAGCACTTCTTTATTCACTTCAAGTTTGTCCTCACTCACGAAAGATGCATTGCCA
TGATACGTATCGATTCCCTGTTTATTCAATGCTTCCTCTTTTTTTTCCGGGACATCATCTGTAAAGGTTCTTTTA
AAATTCATAAGATCTTTCCAGTTGATGGAGACTTCAGACGGTACTCCATTCTTAACCATCCGTTTGTTCCAATC
AATAAGCTCGGCAGCACCATGTAGAACCTTTTTTGGGTCACATCCACGTAGTGCACACGTGCCTCCAAACGG
CCTGTCATCTACCATAGCTACATTCCAGCCGGCTTTATTACATTTTGCTGCTGTGATTGACCCCGCAGAACCTG
TACCAATCACAATAAGATCGTATTTCTTTGTCATCATTATCCTCCTCACAGAAATCACTCTTTAATAAGTTTTCCC
TGTTTTTATCTAATGAAACTCTGTACCTCATCCACAGTTTGAAAAAATCCATCATCTTAACTAAACGGTGGATT
CACTGTGTTGTTATCCCGGACAACATTGTTAAATATATGGCAAGGGGTTCTTTTAAATCTTCTACAGTTAAGCA
AATTAACGGCTAATGTCGCCTCGTTGTATCGCAAATGGTTCTGTTGCAAAGTTGAATTTATAGTATAATTTTAA
CAAAAAGGAGTCTTCTGTATGAACTATTTCAGATATAAACAATTTAACAAGGATGTTATCACTGTAGCCGTTG
GCTACTATCTAAGATATGCATTGAGTTATCGTGATATATCTGAAATATTAAGGGAACGTGGTGTAAACGTTCAT
CATTCAACGGTCTACCGTTGGGTTCAAGAATATGCCCCAATTTTGTATCAAATTTGGAAGAAAAAGCATAAAA
AAGCTTATTACAAATGGCGTATTGATGAGACGTACATCAAAATAAAAGGAAAATGGAGCTATTTATATCGTGC
CATTGATGCAGAGGGACATACATTAGATATTTGGTTGCGTAAGCAACGAGATAATCATTCAGCATATGCGTTT
ATCAAACGTCTCATTAAACAATTTGGTAAACCTCAAAAGGTAATTACAGATCAGGCACCTTCAACGAAGGTA
GCAATGGCTAAAGTAATTAAAGCTTTTAAACTTAAACCTGACTGTCATTGTACATCGAAATATCTGAATAACCT
CATTGAGCAAGATCACCGTCATATTAAAGTAAGAAAGACAAGGTATCAAAGTATCAATACAGCAAAGAATAC
TTTAAAAGGTATTGAATGTATTTACGCTCTATATAAAAAGAACCGCAGGTCTCTTCAGATCTACGGATTTTCGC
CATGCCACGAAATTAGCATCATGCTAGCAAGTTAAGCGAACACTGACATGATAAATTAGTGGTTAGCTATATTT
TTTTACTTTGCAACAGAACCCTCAAGGGTCTGGTAAAAGTTTAACCATGGTCTTTTTCTCTGGAAAATTAATT
CAAATGCTTAATAATCCTACCTTAGTTGTCGTAACAGATCGTAATGATTTAGATAACCAACTATACAGTACGTTT
GTTAAATCGAAAGGACGGTCTGGTAAAGGTTTATTAAGACAAACACCAAAACAAGCTGAAACACGTAAAGA
ATTAAAATCGTTATTATCTGTTGAGTCGGGCGGTATTGTATTTACAACGATGCAAAAATTTGAACCTGAACAA
AACGAAACGACCATGTCTGCTCTAACAGAACGTAAAAATGTGATTGTTATGGCAGATGAAGCGCATCGTACA
CAATATGGTTTTAATGCAAAATACGATGATAAAGGGGAAGGCATTAAATATGGTTATGCGAAGTATTTAAGAG
ATGCTTTACCGAATGCGACGTTTGTAGGATTTACGGGCACGCCCGTAGCTTCAACGGATAAAAATACGCAAA
TGGTCTTTGGGAACTATATCGATGTTTATGATATGACACAAGCCGTAGCTGATGGAAGTACGGTGAAAATTTA
CTATGAAAGTCGCATAATTCCATTAAATTTACCTCAAAATTTAGACTTAGACGAAGCATATAATGACATTACAG
AGGATCAAGAAGAGGATGTAAAACAGCGTTTAAAATCAAAATGGTCACGAATTGAAGCCTTAGCAGGCGCA
AAACCTCGTGTAGAAGCTTTAGCCAAAGACATCATCCAACATTTTGAAACACGTCAGCAAGCGATGAAAGG
TAAAGGAATGATCGTAACGATGAGTCGTCGAATTGCTGTTGATTTATACGATGAAATCATTCGTCTAAAACCA
GAATGGCATTCAGATGATGATGATAAAGGGGTCATTAAAGTGGTGATGACTGGCTCATCTAGTGACCCAACT
TCTTTCCAAAGACATATTGGTCCTAAAAAACGTCGTAATTTATTAGAAAAACGTATGAAAGATATGAATGATG
AATTACAACTCGTGATTGTTCGAGATATGTGGCTCACAGGATTTGATGTACCTTCCATGCATACCATGTATATCG
ATAAACCGATGAAAGGTCATAATTTAATGCAAGCGATTGCTCGTGTCAATCGAGTGTTTAAAGATAAACCGG
GTGGATTGATTGTCGATTATGTGGGTATCGCTGAAAGTTTAAAAGAGGCACTTAAAGAATATACAGAATCTGA
TCAAGCACAAACGGCGATAGATACAGATAAAGCGGTTGAATTGATGTTATTGAAATATGATGTGATTCAAGAT
ATGTTATATAATCTTGACTATTCTAAGTTTAATTCAGAGAAAAAGTCTGAACGTTATTATGCGATTTCAGATACG
ATGGATTATGTGATTGGTTTAGGCGAAGATGAACGCCAGCGTTTTATTAAAACGGTCACAGAGTTAGGGAAA
GCTTTTGCGCTTTGTGCGACTGAACCTACAGCCCAAGAACTCAATGATGAAATTGCCTTCTTTAAAGCAGTT
AAAGCAGGACTTGTTAAATTGTTACAACCGCCAAAAGAAGGAAAAACACGTAAAACACCTGCAGAAGTTG
AAGCAGAGATTAATCAACTTGTGTCACAATCTGTTGTGACAGAAGATGTGATTGATGTTTACCAAACACTAG
GTCTTGAACAACCCGATTTATCAATCCTATCAGATGACTTCCTAAAAGATGTCGAAGGATTGAAACAAAAAAA

TGTTGCGGTAGAATTATTAAATCGATTACTCAAAGGGCAAGTAAAATCATTAATGAAAACGAACGCGACAGT
ATCTAAGCGTTTCTCTGAAATGTTAGGAAATTCGATTAATAAATATAATAGCCGTTCTATTGAAACATCAAAAG
TGATTGAAGAACTCATTCAACTCGCTAAAGATATTAAACAAGAACAACAACGAGGAAATGAATTAGGCCTGA
ATTCAGATGAGATTGCGTTTTACGATGCTTTAGCTTCACATGAAACAGCAAAAGAAGCCATGGGAGATAAAG
AACTACGTGCGATTGCTCATGAGCTAACTAAAACAGTGAAAGAAAACATGGGTGTTGATTGGTCTAAACGCG
ATAGCGCAAAAGCTAAAATGCGCGTAGCTGTTAGACGATTGCTTAAAAAATATGGCTATCCACCAGATTTACA
AAAAATGGCTGTAGAGCAGGTTGTAGAGCAAGCAGAACTTATGGCGAGTAATCAGTAAAAAAATAAACTAA
AAGAGCATCCTTCACTTTGCAGTGAGGGATGCTCTTATATTAAAGATGATAATTCATATATATACTTCATTCATT
TATTTTATGACTAAAATCTTTTCTGAAAGCATCGTATGGACCTTTATAAAGAGTACTTTCTTTAGCATGTTGGAC
ATGACGCTCTTTATCTTCATCGCTTCTATCATCAATATAATCAATAAATTCTCCTGTTCGCTTATCAATAATAACAG
CAGCTTGCATAGGATAACCTTCTGCATCTTGAGAAGTAAATTTGATAGCGTACACATTATTATCAGTTCTAGTA
GTATCTATACGATAGTCATTTCCTGTTCGTGTAGATGGAAACTCATCTTTTACCTTTTGAATAGCTTCATTTTCT
GTTATTATTTGCGGATTTTGTTGTGAACTTGAATTATTATTAGCTGGTTGATTTTGTTCTGTTTGATTTATAGTAT
TTTCTTGAGATGATTGTTTAGAAGTATCTGATTCATTCGAACTTGTATTGTTTGAATCAGTATGAGTGTTTTCTT
TCTGTTTATTTTTCTCACTATTTTCCTCATCCCGCTCAATATTAATTGTACAAGCGGATAGTAAAAAAGAAGTTA
AAAGTACTAAACTAATTATATATTTTTTCATAAATTAAATGTCACTCCATTATTTTAAATTATACAATACATTTTAA
ATAAAATTAAATATATATAATGGTAAATAGCAAAAAAATTAAATAGAGAGCATCCCTCACTATAAAAGTGAAGG
GATGCTCTGGTTTTACTATTATATACATTTTATTTTGATAAGTAGCGATTGATAATATCTTGAAAATCATTATTAA
GTACCGTTGTTATAGAGTCATCATTCATAAAATCGTCATAGGTTTTATCAAGTATTTCCTCATCATCGATGTTTGT
GAAATGATGAACTAAACCTTTCACAATTGAAATATAGTTTTGTGACGCTAAGCACTTAGCAACTGTTTGCCAA
TCTGATTGAATTGAATCTTGGTTCATAGATAATTCTCCTTTAGTTTAATGTCCATTTTTGACGTGCTTTAGGGTT
GAGTGGATGCATAATTTTATTGGTTGCTGGTTTAATGAGCCAGTTAACTTTTTGTTTATGCGGTTTCAAAATTT
TGGTGATTAATAAGTAGGGTGAAATCAGAATATAGATTAATTCAAATTCATCCATTTCTGAATTTTGTTCATAAT
TAATTGTGTCGTTAAGCTTAGAGTAAGGATCTCTTCGTAATGCATTTACATAATAATAACTGTGGTAAATTATTT
TTCATGGTTCCATCTTGTTTTAAGTAAGCGTTGTGGTTATTGGTGATGAATGAATATCATTTATTTTCATAATAC
GCTTTGAATGTTACATATAATTTTTCACCCGACTAGTATTCATGGCAAAGTAACTTGATATGATAAGAGAGCTC
TACATCACTCGTAAAGGTGATGTAGAGCTTGTTAATCGCTTTCACTATAATTCAATTTTAGTTTCATGATTCATA
GATGTTTTAGGTCCATTCCATATTTGAACTGTTCCATCATCTTTAACTACGATGGTGTTAGCTCCAGCACCTGAT
TTATTATTTGCATTAATTGTCCATTCTGACCCATTGTATTCCGGTTCTTGGAATTTTAATAAATCAGCATCCCCTA
CTTCATTTACAGCAGCAATAGCGTAATCAAACATATTATCTCTAGTCACTTGTTGTTCATCATTTGTATTTTCTTC
TGTTGTAGCTTGATCTTGACTATTCTTTTGACTTTGATTCGATTGATTTTCATTATTAGATGTTTCACTAGTAGTT
TGAGAATTACTTTCTTCGTTTTCTTTTTGCTTAGCCTGTAATTTATTTTTTCTAATTCTACTTCTTGATATTTGTTG
TAACAAAAAAAACAACAGGTGCTAATATGCCAAAAATTATAATTATCGCAATTGTAGCTAGTGCTTTTTTATTT
TATTGTTTCTCCAGGTTCAGGAACTTCGCCACGAGCAGCTTTTTTGTACATTTCTTGTTGTTTTTTATATGATTC
ATCATAGTCATATGTATTATCTACATTATTTTTATTCTTAGTCCACTCTTCAACCATTCTATCTGAAGATGGCTGTA
GTTTACCTTCATCAACTAATTTTTGAGCTTCTGGTGTTACTTCACTAGTGGAGATAAATCCATCTCCGTTTGTAT
CAAATCTAAGCACACCATTAGGATCATTTTTATCCGTACTTTTTTCTTGAACTCCTAGGTTATTGCCATATTGTG
CTTTTTCATTTGGTGCTGTAATTGTTTGATTATTAATCTGGTCTTGATTTTCATTTTCTGTAGTTTTGTTTTTATC
GATTTTCTTATTCTTCTTATCCTCTAGTTTAGCTTCTTCTTTTTTTAGTTCTAAATCTTTGTAACTCCCATATGCAA
ATACAACAGCAACTCCCAATATTGCAACTAATAAAATAACACCTAAGCTTGCAAAAATTTTCTTCACATTGCTA
ACCCCATTCTATATTTATTTTAATTTTATATTAATCTATTTTGCTACGCTTTTCTATATTTATTCTAAAATAATTGAA
AAAGAACACATTTTGAAATCAATGTGTCTTTGAAAATAGTTACTTTCTATTATTTTTTAATTTCTATCGGTTCTC
CTAATTCATTTTCTATAATTTGTGAAATTTCTTTTCCTAATGGTCCTTTGATTACAAGTGTACGTCTTGCAACTG
AGGTAAGTATTCCAAATACTGCTCCAATGGCTAATATACTTCCACCTGAAGTCAAAGCAATTAGCCATCCTAAT

GCAATTTTGCCCATTTGTTTAAAAGGTTTTTTGTTAAATATGACTGTTAATCCAATAATATTAGGCGCTTTTGTT
TCTCCCATAATAATTAAATAATTACTTTTATGACTCTTAAAATGATAAGTTGTTATCTGTGCATTATCTTTAAATAC
TTTTAATAGTTCGCGCTCATGGCTATTCGATTTTTCAAGGATTTCATCTTCTTTAAGGACTCCGGTTTCCTTACT
GAATGTATATTTTTTATTTAAAGCAAGTGTTTTCAGTTCATTTTCAAAATCATTTAAAGATTTCGTTTCATAATAT
GTAAATTCCATTTATTTCACTCCTTACTATTTTTCCCACTTGTTCGTTTCGTGATTATAAGTGAATCCTCTCTCTT
GTAATAAAGATTTAGCTGCAATATAAGTATCATAATCCCCATCATCTTTCTTTTGTATCTCATATATTTCCATCAAA
TCTTGATTAGAACTATCACTTAAAGCATCTCTCATTAAACCTCTATCGCCTGTTGTTTCCCAAATCGCACCGATA
GCTGCGCCAACAGTTGCAACTGAATAATATGCTGCTGCACCGACACCTTTTCCAATACCTTTGCCTATTGTTTT
TATGATGTTACTCATAATTCCCATTCCTTTCATTATTATTAATGTTATTTATTAAATATTGTATGTTATTTCTACTTTT
GAACCTGTTGTTTTAAATAGGTTGTTTGTAATAAAAGTAATTCATCAATCACTTGTTCCTTCAAATCAGGGGGT
GAAATGATACGTATATTGGATCGATACATAAAACAGAGTTGAATCGCTTCGATCCTTGTCATCTTAAAAGTGAC
GATAAGATAATGCGCATCATATTTTTCTATCACATGAATTTGATAATATCGATGCATTTTTGACCACACATCTTTA
GAAATTTCAAACGTGATATAGGTTTGTACCTTTGCTTTTTCATGTGTGTCCACAGATTTTTCAGAGAAACTCA
GATTCATTTGATTAATATCATCGATATAAATTTCATCGTTCATTCGATATACCAAGTGAAAAGCATGCATTTGATA
GTACATCGATAAAGGTAATAATATTTTTTGATTGTATTGAAGATATTTATTTTCGTTCATTGCTTGAACAACTAAT
GATGATGTCGAAATACCTGTTTGATTCTTATCAATTTCAAAGCGCTCAAGATATTTTAATAATGTAGTTCGATGA
TTTGAATGATGTTTTATGATTAATAATTTAAGATATTGATAAAAGTAATGACTTAATTTAGGTGTCGTCAGGTAT
AAAGCAGTCAAAATATTATACATAAAATGCACATTTTTACTCTTATCTTGTTCGAGCTTATAACTTTCAGATTTAT
AATCGTACGTGATTTCTTTAGGTGTACTTATCCATGATTTACTTTCATATAAGAAGTTTCTTATATCGTCGATATC
TCTTTGTATCGTCCGAGGACTTGTATCGAGTTCTAATGTGAGTGATTGCTTGTTGACGGATTTATTTTGTAGTA
AACGATTGTATATCGTAAGTATTCGAAATGCACGATCCAATATGTCACCTCCTTTAAGTTATATATTACAAAATA
GGTTTGTCACAATAAGTCAATCCTACGTATATTTAATAAAAGTTGTAAAAAATATTTTTCAGTTGAAAATGTTG
TTATAAAGGGATTTTATAAATATTGATAAGTGAAGAAATAACGGTAGTGACAAAATACGGCTATACCTCTACTT
CTAAATCATGTTGTCTAATGGAATAGGTGTTACTATCTGAAAAAATAATATGATCATAAAATAAAATGCCCATCA
TTTCACTGATGAGCATTAACCGTTCGGTTACATTGATATCTTCAGGAGAGGGGGTTAAACTTTATCAAGAGTA
AATTACAAAAGAATCATTTAAATCAATACTTTCACTTTGAATACATGTATTTGAAGTGGAAGGTACTTATTTCA
AAAATAGTAAAACCTGTATCTTAAATTACTTAATAGTAACATAAGATACAGGCTGATTTTTTATTCATTGTTATTT
ATACTAAAGCACCCGATAGCTCTGAAAACAATCACAAATTCAACTTTTCAAAGCCACAGCTTTAAGTTATTTT
GTCCAGACAACCCCCATTGCCCAACCCATTTTATGGAATTGGCATCCAGGCAACAACTTTTCATATAAATCGTA
ATAATTTTGTTCAGATAGGTACTTATCTGAAGCTAAATGCTCAAGCCATGATTTAGATGTGTTGTGATTATAAAT
TCTAATCGCATTTTTTATTCCAAATTTGAAACAATGCGGTAAAAACTCTTGGATTGCCCCTAATTTATAAACATA
GGTAGGCGGTGTCTCGACTTCAGATACATTATCAAGAATAACTATTCTTCCTTCCTCATTCAACAGTTCCTTCA
TTTGCTGTATTACGCTGGCTATATCATCCAAATGATGAAAGGTTGTTCGGCTTACAATAAAATCAAACTTCTCA
TTAAAATTAAGTTGTTCTGCATTCATATTCAGATAGACCGTATTTGTTAGTTGACGTTTAGATTTGGCAAGATC
GAGCATTTGATTAGAAATATCAATCCCTACCACTTCATCATAATAACTTGCTAATTTCTCCACTAACAAACCCGA
GCCACATCCGATATCTAATGCTCTGCCTTTCTTTGGAGACATATTAGACACAAAGAATGAATAATCATTCAAAA
GCTCATTCACGAAATCGTAATCTTCTGCAACCTTATCAAACTGTGATTCTATTGTATTCAAAAAGATCCCCCATT
CCTACTTTATCGACATTCTTTCATTACTTACCACTTTAGATGTTTTTTCGTTGGGGATAAAACTTCCCTTTAGAC
AATTTTATCCAAAGACAATACAACAGTGCAACTTTATTAAAGTCACTGTCCTTTATCGCAGCCTTTACTTTTTA
GTAAAGACAGTGGCTTCTCTTATCAAGTTTCAAAACATATTATTTTGAAGAAAACGTCCATCTGAAGTGTCAA
GTGCAAAATTACATATAAAGGTTTATTCTAAAATGAAAAGATGATACAATCATATTCAGTTACATAAGGAGGTT
TCAATTATGTGCACCAGTATCGCAGTAGTAGAAATTACTTTATCTCATTCATAATGAAAAAAATGGAAAGGAG
ATAAAAGTATGGGTACTTTTTCTATATTTGTTATTAATAAAGTTCGTTATCAACCAAATCAAAATTAATTGGTTAT
AATGAACGCTTAATGTCAGTTCATTATAACCAGTAAGGAGAAGGTTATAATGAACCAGAAAAACCCTAAAGA

CACGCAAAATTTTATTACTTCTAAAAAGCATGTAAAAGAAATATTGAATCACACGAATATCAGTAAACAAGAC
AACGTAATAGAAATCGGATCAGGAAAAGGACATTTTACCAAAGAGCTAGTCAAAATGAGTCGATCAGTTACT
GCTATAGAAATTGAAGGAGAAGGTTATAATGAACCAGAAAAACCCTAAAGACACGCAAAATTTTATTACTTC
TAAAAAGCATGTAAAAGAAATATTGAATCACACGAATATCAGTAAACAAGACAACGTAATAGAAATCGGATC
AGGAAAAGGACATTTTACCAAAGAGCTAGTCAAAATGAGTCGATCAGTTACTGCTATAGAAATTGATGGAG
GCTTATGTCAAGTGACTAAAGAAGCGGTAAACCCCTCTGAGAATATAAAAGTGATTCAAACGGATATTCTAAA
ATTTTCCTTCCCAAAACATATAAACTATAAGATATATGGTAATATTCCTTATAACATCAGTACGGATATTGTCAAA
AGAATTACCTTTGAAAGTCAGGCTAAATATAGCTATCTTATCGTTGAGAAGGGATTTGCGAAAAGATTGCAA
AATCTGCAACGAGCTTTGGGTTTACTATTAATGGTGGAGATGGATATAAAAATGCTCAAAAAAGTACCACCAC
TATATTTTCATCCTAAGCCAAGTGTAGACTCTGTATTGATTGTTCTTGAACGACATCAACCATTGATTTCAAAG
AAGGACTACAAAAAGTATCGATCTTTTGTTTATAAGTGGGTAAACCGTGAATATCGTGTTCTTTTCACTAAAA
ACCAATTCCGACAGGCTTTGAAGCATGCAAATGTCACTAATATTAATAAACTATCGAAGGAACAATTTCTTTCT
ATTTTCAATAGTTACAAATTGTTTCACTAAATTAAAGTAATAAAGCGTTCTCTAATTTCACAAGAGGACGCTTT
ATTCTTCCCAAAAATTGTTCAATATTTATCAATAAATCAGTAGTTTTAAAAGTAAGCACCTGTTATTGCAATAAA
ATTAGCCTAATTGAGAGAAGTTTCTATAGAATTTTTCATATACTTAACGAGTGCTTTCACCTTTGAATATAGTCC
TTCCCACTTATCATCACACTCTCCCCGATAGCCTTTTCTAGCTATATCCAGTAAAGTTACATGCTCTTTAGGTAA
AAGAGGTATAGCCCATTCTGCAGCGACATCTTTCGAGGTAATTTCACCAGTAGTCACTGTTTGCCACATTCGA
GCTAGGGTTAAAATTACATTACGCTCATCACCTTTTATCCCCTCAATTAGTTCTGGCAAAGAATCCTTAATTGC
TCTTCGAATATCTGTCAAAGGTACGGAGACAAGTATACTTGAAGAATCAGGACCAAATAGAGAAATACTATTC
TTTCTTGCTTGTGCTAAAACAATAGCCAAATCAGGATCATAGCTTGGTTCCTGAATTTGTCCATTCTCAAATTC
ACCCCTGAGCCACTCACCGTATATAAATTCTCTTTTTGGAGGATATTACCAAGGGACAACTTCACTCCTATTTA
TAACCGTAACTTCAAGTGGTCTAACAGAATCCGTATTTCCAATCTTTCCTGATATAGTCATTAGTCTTTCTGTTA
GTTTTTTTCGAGTTAATTGAGGTAAACTATGATTCACGACGACTAGAACATCTACATCGCTGTTAATGCGTAAA
CCACCATTTACTGCTGAACCAAATAGATATACTCCAACTATTGAACTTCCAAATAAATCTTTTACGATTTTTAAT
GTTTGAATCGCTTGATTTGGTATTTTTCCGTTAATCAAATTGCTCATGATTTCACCTCGTTGATTATGTTCATATA
AAGTTTATATTGATACTCAATTTACTTACCCTAGATTGGACATATACTTAAATTACTGTTCAATAAAGCTGACCG
TTAGCGTTTAAGTACATCCTTTCACAATTTGTCTACAGATTAATAATTATTCTTTATTATACAGATCTCCATATAAT
TTTTGAATTTGGTTCTGTAATTTTTTATTTTCTTTTTCTAATTCCATTACTCTTCTTTTTAAGGTTTTAATAAGGA
TTTCCTCCGAACGAGAACTTTTCTTGGGTTTTGAGACTACATTTGCTGTTATTTGACGCTCACGAAGGGATTC
GATTCTTTGCCTAATATCGTGTTCCTTATAAAGCCATGATTTAGAAACATTAGCTTCCTTTGCTATTGAATTAAA
ATTAATAACTTTACCTTCAATCGAAAATTTAGAAATCGCTTTGTCTACTTTTTCCCTTGTCTTTTTTGATTTCTGC
TTCGCCAAACGTACAATTTCTGTTGTATTTCTAACTTGTTTATCCATTGATAATTACCCCGTCAAACTTCCAATG
ATTTGTTCTAAACGCTCTTTAACACGGCTATTAGTCTCTACTTGTCTTTGCCATTGTTTATCCTTAGCTATGGCT
AATAACTCTTCTGTACGCTCTAACTGTTCTTCGTGCTGTGGTAAGAATTGCTTACTGGTACAGAAGTGAGTGC
AATCTAAGCATGCATTCGCATGTGGACAACCACCTGCTACTACTGGCAATCTACAATAACCATTTGGAAGCAC
TTGTGCATTTATATTTTTCTTGAACCATTGAAGCTCTACATCATCGACTTCACTATCATCATCTAGATTGAGCAC
ATCTCCATTATTGGTAACCAGTTTTTCTTGAAATTTAGTAAATTCATTTTTTAGAGTTTCATCAAAGATATGAGC
GTATCTGCTTGTCATTTCTGGGCTTTCATGTCCCAAAAATTTCTGCACAATATGCTGGGGCACCCCGTTGTTAA
TCATTCTTGTTCCTACTGAATGGCGAAAGGCATGGGCATGGAATCTATAAATCTCGCCTAATTTATCCACTATAT
TTTGCTCATAAGCTAATTTATTTAATTCGCCTCTAAATGTTTCTTGTTTTAATGGCGATCCATCTTTTCTTGGAA
AGAGGTATTCACTATCTGGAAATTCCTCTGAAACTTTATCTTCCCGAACTTTAATAAGTAAAACTACCTCTTTA
GATATTGGAACTATATGCTCCTTTTTCATTTTCCATTGATAATACTTTAAAAAGTAATCTCCATCTTTGTCCTCTA
ATAAACAGCCTTTTTTCAAGGTGCACAATTCACTTATCCTCATTCCACATTCTTGAACAATCATAGTCATCGTA
GCTATATATTCGGGTAATTTATCAAGATGACTGTTCAATTGCTCTAGAACGAATTCGTCTATAAAGCGAGGTTT

TGCTCTTGGTATTTTCGGATAGTCCTCGGGATAAATTAATAATTTGGAAGGAACATCATCCCATTCTAGCCTAT
GAAGGGTACTAAATAGTCCTTCCAATATAGAGATCCTCCCAGTTATTGTACTAGGTTTTATCCCCATCATGTTTA
GTTCACTTAAATATGCTTCAATTTCCACTCTCGTTAATTGGTGTACTCTCTGAACGTGTTTAAACTTTATGTCCA
GAAAATTAAAGAACTCTTTAAGTTTTTGGGCAATATCACTTACATAGGAAAAGCTATCCATGTTCAATCTTAAC
TTACAATATCTTTTTACAAGCTGTTTAAAATATGTATTCCGAAACCCTTTAAAGTTAATTGTATATTCATATTGTG
TTGGGTTAACCTTATCATCTGGCAAAGGTAAGTTTCGTCTATCCCAAACGTCTTTATCCCACTCCTCTCCATCA
AAATAAAAGTTCTCATAAAACTCCATAAATTGTTTTAGATTAGTAACATAGTAGGAATTAGCTTTTACAGGTGT
TTTTTCTTGATTAGCAGTAATCTTATAATTAGTAGTGGTAATTCTAACACCCCGTTTTGTCAAATAAGTTCTATAC
TCCGTCATTGCTTTATCAATAGGAACTTCAGTAATTGAAGTAATGCTGGGATACTTTAAATCTAAAAAATCCAA
CATTTTATTAATTACTGTTCCTTTTCTAATCCAGACAGTTTTTGCATTCCATATTCCATTGTTTAAATGGTAAAAA
TAAAAATATTTCAATTCTGTTCTTAACCACAGATTTTTAACACGTTCAAAACGAACCCAACGATTTCTTAAAGC
AGGATTCTTACTTAATTCTATGGCAGAAGGATGTGGACATTTTCTTATATCCCAACTATTATTAGCCCAAAACC
CTTGCATTTCTTCATTCATTACAGCTATTTTTTTGCTGATCTCACTCTGACTGATAATTTTCCTTTTACTAGAAGC
ATTCATTTAGCTGGTATTCTTTATCTAGTAATAAATACAATGGATACGGCTTATTCTCCACTTCTATCCTTTGAAC
CCTCACACGCGCCCCCTCTTTAAACACTTTAAGTACTACAAATTATAAACCTTGTTAATTTTTATTTAAAGTAAA
ATCTCCCTTTAACATCTCCTCCTGAACTACTTTAACTACTATTATTTATTATACTATGGTTAATATGTCTCCACTTG
GGTGGTTATGGCCAATAATCATACCCGTTGCGTTACTTAATATGGCTGTTTTAAATATTTCTCGAGGATGTATAA
CCGCAAAATTAATGGTTCCTATAGAGACGGTTTCAATATGTGTAGGTTCATTTTTGCTATTCAAGCAAATTAAA
ATGAAGTGTTCTCGGTCAGCATTTCCTATGAAGTCACGCATAATATCAGCAGCGTCTTTTGGTTCTTCAATACG
TCTTTTTAAATACCAAAGTGTATCTTCTTTAACCATTTGGACTTTGACAACATCTATTCTTTTTCTACTCATTATG
GATAAGCTCTATACTTTCTATTAAGTCAGGGTGAACAATGGATGTGATAGAATCATCATTCATATAAATGGAAT
ATAGCTCATCTAATTCTGAATTATTTTTTTTGTTTGATGAAAAGGCGATAATGCCTTTAATAACATCGATGTGTG
GTTGATGCATTAAATATTGGTATACTTCTTTCCATGTTTGATTCATTCTATTTAGTCTCCTTTAATGGTTTAATTGT
CCATGCCTTTCTAGCGTATGGATTTAATGGATGCATGATTTCATTTGTTTGAGGATTTGTTAATATACTGATTTT
CTTACTGTGAGGTTTTAACATGTCTAATACTTCATTCACTCGATTTTCCACAAGAGGTTTTTTTCCGTAGGAAC
CCCAAGCCAATAAAACACTATCGGCTTCTTTGACAGCTTTCATAATGTGAATATCCGTGTGCTTGTCATGGCTG
TTTTCGATATGTTTTAAATTAATGGGGGTATCAATATTTGAAAAAAGATTTATAAAATTAACGCCACCATATTCA
TCTTTTTCAGCTACTTTATTGACAATAAGTTGTGTTGTAAGGTCAACGTTGATAACACCATCATAATGAGGATA
CATCGTTATAATTGTAAGTACTTCTTTATCAATATCCCAAGTTTTCTTGAGAAGATAACGATGCATTTTATCCTG
ACTAAATATCGCTTCAGTTTCTAATACACTTTTGATTGATTTCATTTTATCTAACTCCTTTATTTAATATTCTTCAG
GTAACAACATGACGTAATAAGAAAGGTCTACATCATCTTCTCGGATAATGTAGACCTTATTAATATTGATAGCTT
TTGAATAATTTGCCTTATGAATTTTTACGTACTCTGGACGTTCTTGCTTATGTTTGATATATAATTGACTATTATG
CATATTGAACTGGAATATATGAAAGTAATCAATCTCTTCTAGTTCCTTGAATTGTTCGTTTTCGCGTTGCGCTAC
AAGTTTCCATAACTGTTGCTGCAACATTGCTGGCAAGTAATTGGCAATACTACGTGTAATATAACGTTTCATGC
TCATTTCTCCTTTTCTATATCTTTCATTATTGTAATAACTTCTGTAATGACTGTGACGCTTATCTGAGCTATGTGTA
TCCATTTATTCATTATTATAATTCTCCTTTTCAAGTAAGTGTCTTTGATCTAGTAGTGGCATAACTGTGTTATGAA
GATATAAGAAGTCCATATCGTTTGTTTCATCAATATAACCAATCGCAATAAGTTGTTTTTGATAGTAGAGGATA
AAGGGGTATATATGCCTTTCATGAAGTGTGTCAGAGTAGTAAGTATACGTATGAAGGATTTTGTTGGTAAGCG
GACCAAGTCTGATATATTGATCAGGAAGGATATTATCTGCCACTTCCTCGATAGATTCACATTCCCATTTTTCGT
CCTTTGGCAGATTGAATAAGTTAGTTATATACTCTTTGAGTTGTTTTTCTAGTGTCATGGTTATGACCTCCTATA
ATGTTTTTGATGTATACATGTAATACACCATATAGATAATATATGCTTCAAAAAAATTTTGTTTCGTTTGTGAATC
CCATTGTTATTAAGAAAATATCCCAATAGAAAATTATCACAATTGTTGATATGACAAGGAAATCCCATTATCCCG
CTCATTTCTCAGGAAATATAGCGATATATTTATATATCATTTGGATTTGGTGGTGTCAGTACGACTTACTTTTTCT
TTTTATATTATTTATTTATAAATAATAATGGGATTTCGGGATTACGCTTGCGTAATCCTTATCCTACATGTATTTAT

CAAATCCCATTGCTATCCCGTTCATATTTTATTTTGGGATGTTCTTTCGTCATACAATATAAGCTTCACATTGAGT
TGTGACTTGTTAGTATGTTGAAAAGATTCGTTAGATGGGTGTTATTGAATGACGAAAGGACAGTACATAGATA
TTAGATTTGAAGAAAGAGCGTTTGAGGCAAAAATAAAGACGAAGTGCTGAGGAGCACTTCGTCGAGAGGG
GTATTATTGAAAAGTTGTTTAATAATTTCATTATTGAGTTTTAGTGTAACGTAGAACTGTTTTTTATGATTACCG
TCTTTACGAATATCAATACGGTCAATGACTGTAAGATATAATGCTTTGAGTTGTGCTTTATCCATTGATTCTATAT
TTTGAAATATTCGTTGTAATAGGGCAGCGATTTGTTTCGTATCATAAGATAGTTTCTCTTGATTTTGTTGCTGTT
TGAGTTGATTCATTTGATTTGTAATGTCATTGAGTTGTGTTTCATATTGATGAATAGTTGCTTTCAATGCAGATG
TTAGGTCCGGATTATCTTCAATGGTTTTAACTAAATTATGGAGTTTCCCGCTGACTTCATCGTATTGTTGTTGTT
TATAAGCGATATCGTGGTTCAATGCACCAATATCGACTTTATTTTCTTGATTGACACGTTCTAAGACTTGGTTA
ATGACTTTATCACTTTTGACAATTTCGAGTATTTGATCCATGACGTATTTTTCAATCACATCAGCTCTAACGCTA
TTCGCAGAACATACTTTTGAGCCTTTGTTTCGGAAGTTACTGCAAGAATAATAACGTATTCGCTTCTTGGTAC
CATCTTTCAATGTGTTCGTTGTGTTACTAGCTGCCATTGGTGCACCACATTGTGGACAATGAACGATACCTGTT
AATAGATTAGTTCCTTTACCGTGGACTTGAGGTTTTTGACTGACTTGTTTTTTACGTAATTGGACTTTATCCCA
TAAGTCTTGAATAATAATAGGGGAATGCTTACCTTCAGCTATTATTGGTTTATCATTCAGCCCTTTACGACGCTT
TTCATTCCAATCTTTGTACTTTGCGAATTGAATTTTACCAACATAGAATGGATTAGATAAGATATAGGTCACTG
AACCAATACTGAAAGGCTTTCCTTTTTTAGTCACGTATCCTTTGTGATTGAGTGCATTCGCAATTTTACGATAT
CCGTGGCCTTTAGCATATGACTCAAATATATATTTGACAATATTCGCTTCATGTTGGTTTATCATGAGTTCATGC
TTGCTATCCGGTATTTTGTCATAGCCCAGCGGCAAATTGCCTTGATAATAGCCTTCTTGAGCGCGTCGGGTTT
GACCCATGAATACATTTTCGACAATATTATTTCTTTCAAATTCTGAAAAACTCGCTAGAATTTGTAGCATCAATT
TACCACTGCTTGTATTGACTTCCATACGCTCAGATAAGCTGAAGAATTCGACATTTTGACGATGAAGGTCTTC
AACAATTTTGAGTAAGTCAGAAGTGTTACGTGCTAGTCGGTTTGTTTTGTAGACCATAACAGAATCAATCTGA
CCTTCGTTCGCATCTTTTAACAAACGTTGTAGTTCTGGTCGGTTCATAGATTTTCCAGAGATACCTCTATCCGC
GTATACATCTACAACAACAAAGTTATTGAAATCACAATATTCTCGAATTTGATTGATTTGTCCATCGATACTATA
GCCTTCAGTACTTTGGATTTCCGTTGATACACGAACATAGATACCGACACGTTTTTGTTTGAGTTGTTGCATG
ATTTTTTCATCCTTTCTGATTAAGCAATCGATGATTGCGAGGTTTGGTTCACAATGTTCAATGGTTCATTTTTG
AAATAGATCCCAACAAGGGCTTTATTTTTTGTAATGCGAATTTCATCAATATAGGGATGCAGCATGTTTAACGT
GAAACTTTTCTGTATAACCTTTTGTAGTGATGTTTGTAACTGATTATCACTTATGGAGGATATGGTTTTGTGCTT
TTGATTCATCAAATGAGTCTGTTTTCTGAATGATTCAGCATCAATCATACCTTTGGCAAGTTTATCTATCAGTTG
TTCTTGAGTTAGCGTACTTTTAGCTTCTATCACTCTTTGTTTTTTGAGGCGTTGATGAATTGCATTATTAATTTT
TGAATAGAGCTGTTGGTTTTGAAAGAAGTTCTGACATGTAGCTAAGACTTGAACTTCTAATTTTTGTGCATTT
ATTCCTTTGAATGAACAGACAAAGCGAGATTCATTCATATTTTTAGGACAAATATAATATCGCAATGTATGTTTT
TTTCTTATTGTCATATTTGTCAGTGTTGAGTCACAACAAGGACATTTGATCTTTTGTTTCAGCTGATTCTCTGA
AGGTATACAGTGAAGTTGCTTCTTATTACGGATTGCTTGAGCATGTTCATATTTCGTTGCCGAAACAATAGGT
GGTACCATATTGTTATATTGACCATATTGATTGATGACACGACCACAATAATTTGGGTTCATAATTATATTTCGTA
CCTGATAAGGCTTGCGGGTAATAAGTTTATTATCGTCTTCTAAATATTGTGCAATCTTCTTGTAGCCATATCCTT
GAAGATAATAATTGAATACAGCTTTGACGGTAGGTGATTCATCATTATTAATGATGAAAGTACCATTTTGATAG
TGATAACCGAAAGGCGCATGGGTCGTTATGAGTTTACCTTGTTTTGCCTTTTCTCTAAGTCCATTTTTGACTTG
TTCTCCAATATTATCGGATTCAAGTTCAGCCAGACTCATGAATATATTGAGTTTTAGGCGATCAAACGCTTTAT
CCATATCAAAATAGCCATCATGAACACTTAGAATATGAACATGATATTTTTGACAGAGTTTCATGAGTTTTAAT
GCATTTTTCAAGTTGCGATGAAGTCGATTCAAGCGATAACAACATAATACGTCACATTGTCTTTGTTGGATGC
GTTCGGTGACTTGTTGATAGCCTGTTCGATTATCTGTTCTGCCTGATTGCTTATCACTAATGTATTGGATATTTT
GAATATTGTGCTTTGGAGCTAATAATTCTATTGCTTGCTTTTGAGCTGGGAGTGATTGTTGTTTCGTTGTACTT
TGGCGCAAATAACCAATGGCTCGTTTCATAGTATTTCCTCCTTTCAAAGAGATAATATATATTTATGAACGAAAT
TATAGATAAGCCCAACACCAGTCGGGTGTTGGGCGGTATTATTAGTCATCAGCGTGATTGATTTCTTCAATAA

CCAGATCGGCTAATAGTATGATTAATTCGTCCATGTATATCACCTCATTATTCATAGTATTAACAACAATTCTTTA
GTTGCATCTCAAAAGTAGTAACGAATTCGATATTCTTCTTTGAGCAAATTTTTTTAAATGAATGTAATCTTCATA
TTCTCCATATGCAACAATGGACGTAGGTTTGATTACTTTCTCCAAAATGTCATTTATTAACTTGTACTCCTCAGT
GTAAGTGTTACTTATTTGACGACCTTCTTGTTTACAGCATTCCTTCACAGTATCTAGGGGTGTGCTTTCTAAGT
ATTCAAATCTTTCATCTTCAATTTCGTGAGCTAGTGCTACTGTATTCATAGTACAATTCCTCCATTGATTTAATAT
TTGTGCTACAACACCTTTAAATAGGTGTTGTAGCAAATTTATTTAATAACATATTTTAAGCTTTAAAAGTCAATA
TCACCGTTTTTATCCCATTCTCTTGCTTTTGAAATCATGTCTCGTAGTATTGGATGTTGGTGTTCAGAGATAAAT
GATAAGGGTATATTGAATTTACTTTTTTCAGATAGACCAAAAATAGGAGCGTATGATTTATCAAGCTTAAGATG
ATAAAATACGATGGTTTTTGGTTTGCCTTCTTTGTCTTTGACACTACGTTTTGTTGTTTTACGATTGGCGTCTG
ACTGGATGTAATCTTTATCCCTCAGTGCATCAATGACATTATTTGTATCTTGGAAATTATGTTCTTTTAACATATT
TTTAAAAACCGAGGCAATCATTTTCACCTGGATATGATCATCTTCAAGTGAAATCATTCCATAATTTTCAATAA
GCGTTGATAACCTAGTGTCGTCAGAAAACTTACCACGATTTTGTGCTACAAATTGGACAATGACTTCAATTGC
TTTATCAGCTAGGCTTCGCTCACTAACAGAATCTAAATGATAGTTAATAAGATAGTCCCTTACAGCATTCAAAT
CAACAGGTGTGGCAATGACACGTTCCAATATACGTGCTGATGTTGTAATGGTTGCATATCGTTTGAACATCCG
AATTCCTGTATTGCTGGTTTCCTTTTCTAGTTGAGTTTTGAACCTTAGGTGCTCATTTTGGAACCATTTAATGA
CTTCTTGTTCACGATTGAGTAGATATTCAGCTACTAATGGCATGATATGACCATAATTAGCTGATGTTGCTTTTT
TAATAGTATCTGCGTTGTCAGCGTTAGTTGTAAAAGCTTCAGATATTTCAATGGTACGTACATTCAAACCATCA
TTACGTGCACTATTATTGAAAATACTGTGTTCGGCAGTACTAATTACTGAGGTTCCCCAGTTTTTTAGTGACTT
GACTTCTCCGTGTATATTAGAACGTTGTCTTCCTTGACCTTCGGCAATTGAATAGAGCAAACCAGTTGTATCTT
TAAAGGTTGCAGATGACAGTTCGTCCAATACAATAGGTACACCATAGTTGTTACTTAGATATCCTTCAAGCGC
GTTTCTAGTGCCATTCCATGATCTAAACAGCGTATTACTACCTTTAGTAGGGTTGCCGGTTATTGAGACTCCTA
AAGCCGCTGCAGTTGATTTACCCGTGCTTGAATTTCCCGTAAATGAGAAAATCGTACCTGCGAATTCAACCTC
ATGTTTTGTTTTTAAAAACGCAGTTACTAATGAAGAAATACCAAACACTACAGCAAGTTCGAGTAACAAGTTT
CCTTTTACTTCATCAAGATACATTTGCCACCAGTCTTCAAAAGTGCCTTTAGGTTGTAGATCGTACTGTGTTTC
ACAAATGATTTCATCCGCCTGCGACTGTGTAATTTCTTTTGAAAGATATGGTTCATCTAATGAAATGACGACAC
CTTTATCAGTATTTAACACACCGACACCCGTATACAACGATGACAGAGGCAGTGATTGGCGCATTAATTGTAG
TGCATTGCTTAATGATTTAATGTACTTCTCATTGATGCTGAAGCCATACATGATTAGTGAAGGTAACTTTTGCG
ATGTTAAAATATCTGATGTTTCAATACGTTCAATATTTTGTCCGTTGGAAATAACTAGTTTCTCAACCCCAGATT
TGGGGTCAAGAAACTTGTTTTTAATTAAAATTGGACTAGATAAGCGTATGGACTTTTGCTGATCATTTTTACCT
TTCGGTGGTATAACTTCATACCATGAATCTTCAGTAAGCCAGTATGGATATTCACTAAAGATGATGTTAGTCATT
AGTTGTTACCACCTTTCCAGTTATTTTGATGATTCATCGGATTTGGTGCAGTTTTAAGGTAGAGCAAAGATGA
ACCTTTTTTTCCAACTACAATGAATGGAAGGTTAGGTGTATATTTTACAAAATGTGCAAACCAACGACCAACA
TCTTGTTGAATTTCAGTCGAGCACAAAACATTAGCCCTCACATTCAAATCGTGATACATAAACATGGTGCCTAC
AGGTAAATTAAATGCGATGCTTGTAACAGCACGTTTTAGTGTATAAAATTCTTTTTCTTTCATCATAAGTTCCTC
CAAATAAGTCTTATTAGTCAATCAATACTGTAGATGCTTAACACCTCAATGGTTGATTGCTATAATAAGCATAAA
TGAAGCATTTTAATTAGACGCGGAACAAAAAATAGGGAGGGGGGTATATATGATACCTACGATAGAAGAAAT
TATTGACAAGTATGGCAGTTTAGTAGATTGTCTAAAGTTTGATATATCAGGTGAGGTTTATGAAGACTTATTGT
TATTAAGAAGTTTAAGCGATTTAAATGAACACCAAAAGATGCATAGAGTTTCATTTATACAAAAACACTTTAA
CCCTAATTTTGATTATTTGTTAGATGACTCAGAATTTAGAAGTATGAATAAAATTTATAGAAATTTAAATACTAT
GACGTATATCAATAGTCAAAACAATGAATTTAAACTTAAACCATTTATAAATGAAGAACAATTAAAATCACTAC
TTGCGATAAAGAAAGTAGATGAACATTTGAGTTATGACCCATCTATTACCTCAAAAGCATTAGCAGATATAAA
CAAGACGCAAAAAAGATTGGTTACAAAGCCAAATATTCTCTATAATCGATCCAAAAAAGAAAATGAGTATGT
ACGGTTAGGAGGAAAAATATCATATACAAAGATGGAGAAGCATTGGGAATATTTACATAATGAGATTCAGTAT
TATAATCTTACTAATCAATTAATTAGTTATGTTGCTTTAGAAAAGGAATATGCATGGGCTTTTTTAAATGAATTAT

TTTATTTAATAGAATTATATTTGAAGGCATTTAAAGGACAAAAGAAAACTGGCCATGAATTTGAAGAAGGATT
GAACGCATTTATTCAATCATATGTCATTATTTTACTTATAGATATAAGATTACCGACTGTTAGGTTATATATTATTA
GGCGTATTGTGGATGAATGCGAAAAAGAATCAGATAATCAAAAAAGAATTAAACTGATAGAGGAATCCATTA
AAAAGTACAGATATGTAAAAGAACAGATTGAAAGATTTGGAAATGCGTTGAAAGAAGAATTATCAATCGCG
ACTTTGTCTATTGAACAGAATATGTTAGAAGCTAAAATTGATTATTATAAGAATTACTACTATCCTAGAAAAAA
AACAAAGCATAACAATATTGAGTACAACGTATCAATGTTTTTTAAAGCTGTAGATTCTTTAAAGAAATAGTATT
CGACGGACAATATTTATAGATAATAATTAATTAGAGAGGTTTTAAGTTGTGTTTAGATTAAAAAAATGGTATTT
AAAACAAAAAGGCTATTTTTGCCGTGTCGGTGAAAAGTGGGACAATAGTAATTTATCCCATGAAAATATACAT
AGTTATGTAGATAGAGTACTTGAATATGAGACTGAAACACGTAGAAACGGCCAACCAACTGAAGAATATATAT
TAGCTAGTAGTGGGGAAAAAATAAATGAAGATTTAAGAAGTGGTACTAATGATCACAAAGAAATTCTAGAGG
TATTTGATAAATATAAAGTAAAAGAGGATATAACTACTTATAGAACGGTTAGTCCTCGGATATACAAATTAATG
GAGAAAAATGCTAAAAACTTAAATGGAGTAGATTTGTTTGAACTTGGAATTTTACACACTTCATTAATAAAAG
GGTACGAATCAAGAGAAGAAGGTTACAAATTACGGGTAAAAGTAAAAAAAGGAACACCAGCTTTCTATGTA
GGTAATTTAACAGGCGAAGAAAGCCATTATTATGAGGTGATTGTAGTTAATAATTTAAAATTAAAAATCATATC
TATTGAAGATTACTATATAAATTGTGAAGTAGTATAATTGGAATCAATTCTGTTAGTTGTTCATAATAAGCCAAT
TATTTTTTTAATGAACTTATTACTTAAAAACCGCATCATCAACTGATAAGCAGAAGCGTATCATAAGT
>Staphylococcus aureus strain CUBIST-2
ATGAAAATCACCATTTTAGCTGTAGGGAAACTAAAAGAGAAATATTGGAAGCAAGCCATAGCAGAATATGAA
AAACGTTTAGGCCCATACACCAAGATAGACATCATAGAAGTTCCAGACGAAAAAGCACCAGAAAATATGAG
CGACAAAGAAATTGAGCAAGTAAAAGAAAAAGAAGGCCAACGAATACTAGCCAAAATTAAACCACAATCCA
CAGTCATTACATTAGAAATACAAGGAAAGATGCTATCTTCCGAAGGATTGGCCCAAGAATTGAACCAACGCA
TGACCCAAGGGCAAAGCGACTTTGTATTCGTCATTGGCGGATCAAACGGCCTGCACAAGGACGTCTTACAA
CGCAGTAACTACGCACTATCATTCAGCAAAATGACATTCCCACATCAAATGATGCGGGTTGTGTTAATTGAGC
AAGTGTATAGAGCATTTAAGATTATGCGTGGAGAAGCATATCATAAATGATGCGGTTTTTTCAGCCGCTTCAT
AAAGGGATTTTGAATGTATCAGAACATATGAGGTTTATGTGAATTGCTGTTATGTTTTTAAGAAGCTTATCATA
AGTAATGAGGTTCATGATTTTTGACATAGTTAGCCTCCGCAGTCTTTCATTTCAAGTAAATAATAGCGAAATAT
TCTTTATACTGAATACTTATAGTGAAGCAAAGTTCTAGCTTTGAGAAAATTCTTTCTGCAACTAAATATAGTAA
ATTACGGTAAAATATAAATAAGTACATATTGAAGAAAATGAGACATAATATATTTTATAATAGGAGGGAATTTC
AAATGATAGACAACTTTATGCAGGTCCTTAAATTAATTAAAGAGAAACGTACCAATAATGTAGTTAAAAAATC
TGATTGGGATAAAGGTGATCTATATAAAACTTTAGTCCATGATAAGTTACCCAAGCAGTTAAAAGTGCATATAA
AAGAAGATAAATATTCAGTTGTAGGGAAGGTTGCTACTGGGAACTATAGTAAAGTTCCTTGGATTTCAATATA
TGATGAGAATATAACAAAAGAAACAAAGGATGGATATTATTTGGTATATCTTTTTCATCCGGAAGGAGAAGG
CATATACTTATCTTTGAATCAAGGATGGTCAAAGATAAGTGATATGTTTCCGCGGGATAAAAATGCTGCAAAA
CAAAGAGCATTAACTTTATCTTCCGAACTCAATAAATATATTACATCAAATGAATTTAATACTGGAAGATTTTAT
TACGCAGAAAATAAAGATTCATCTTATGATTTAAAAAATGATTATCCATCAGGATATTCTCATGGATCAATAAGA
TTCAAATATTATGATTTGAATGAAGGATTCACAGAAGAAGATATGCTAGAGGATTTAAAGAAATTTTTAGAAC
TATTTAATGAATTAGCTTCAAAAGTTACAAAAACATCCTATGATAGCTTGGTCAATAGCATAGACGAAATACAG
GAAGACAGCGAAATTGAAGAAATTAGAACAGCACAAAAAGATAAGACACTCAAGGAAGTGGAAGCACCTA
AAGGAATAATTCCAAAATATAAAAAAGGTGTATCAAAGACTACTAAAAATGATTCAGAAATTGAAAAATCAA
ATAAAGAGAATAAATTAACCGGTAAAGTTGGAGAAAAATTAGCGCTAAATTACTTTAATGAGCTAATTGATAA
TAAAATAGACGAAGATAAGAAAGAACAGTTTAGGAATATTTTAAATGATAATCCAGGCTCTCAACACGGTCAT
GGCTATGATTTAGTAGCTTTTGATCCAACAAATACAGATAAAGCTGTAGAAAAATTTATTGAAATTAAAACATC
TACATCTTCTAGTATTGAGGAACCATTTTTTATGTCGCTAAATGAAATGTTTGCTATGAAAGAATATAAGCAGA
AATATTTAATATTAAGAATATTTAATGTTTCCGGTAAAGAACCACAATTTTATTTTATAGATCCATATGCAAATTA

TTCTGAATTTAAAGATGTAGATGATCTCATTGACAAAGTATTTAATGTAGAAGCTATTCAGTATAAAGTTTTTG
GCGAAAAAATGATTACTTGAACAAGAGCTAAAATAAAATTGTGATCTAATAAAAATAGAAACTGTAATTTAAAT
AAAACTTTCTAAATAAGCTAACTGATAAAAAATCAGTTTGTCCACAGTCTGAAACAAGATTCCTATATTCTTTA
GGAATCTTGTTTTTTCTATTTTTATGGTGATAAAGAGCAGATAAGATAATGTGTAATAATCACAAAAAAGTTAA
ATATTTTAAGGCTTGTTTAATTATTAATGATTTTATATATAAAGAGCAGTATAATAAAGTTGTTAATATATTATGAA
TAATATTCAAGTAATTTTATTGTTTTTTAATTTGTCGATATTTAAGTTGAGTTAAATTTAAAGGGTGTAATTTGTT
TTACAATGATGAAGATAATTAGTCTATCAAAATAAAGGGGTTGGGACTGTTATGAGTGATAATTTGTCATTATT
CATTGACTATATCAATGATAATATAATCTATGGTAGTGAAATCAAACGGGAGAAATTAGAGAATTTATTTAATCA
ATTTGCTATAAAAAAATGTTGAAAAGAACATTGTCTATGATGAACTGAAATCTTTAGATATTACAATCATTGAGT
CACAGGATTCATATAAAAATAAATTGAAGAGATTATTTTCGGTTCTGTTGCAAAGTAAAAAAATATAGCTAACC
ACTAATTTATCATGTCAGTGTTCGCTTAACTTGCTAGCATGATGCTAATTTCGTGGCATGGCGAAAATCCGTAG
ATCTGAAGAGACCTGCGGTTCTTTTTATATAGAGCGTAAATACATTCAATACCTTTTAAAGTATTCTTTGCTGTA
TTGATACTTTGATACCTTGTCTTTCTTACTTTAATATGACGGTGATCTTGCTCAATGAGGTTATTCAGATATTTC
GATGTACAATGACAGTCAGGTTTAAGTTTAAAAGCTTTAATTACTTTAGCCATTGCTACCTTCGTTGAAGGTG
CCTGATCTGTAATTACCTTTTGAGGTTTACCAAATTGTTTAATGAGACGTTTGATAAACGCATATGCTGAATGA
TTATCTCGTTGCTTACGCAACCAAATATCTAATGTATGTCCCTCTGCATCAATGGCACGATATAAATAGCTCCAT
TTTCCTTTTATTTTGATGTACGTCTCATCAATACGCCATTTGTAATAAGCTTTTTTATGCTTTTTCTTCCAAATTT
GATACAAAATTGGGGCATATTCTTGAACCCAACGGTAGACCGTTGAATGATGAACGTTTACACCACGTTCCCT
TAATATTTCAGATATATCACGATAACTCAATGTATATCTTAGATAGTAGCCAACGGCTACAGTGATAACATCCTT
GTTAAATTGTTTATATCTGAAATAGTTCATACAGAAGACTCCTTTTTGTTAAAATTATACTATAAATTCAACTTTG
CAACAGAACCATCTAATCTTCAACAAACTGGCCCGTTTGTTGAACTACTCTTTAATAAAATAATTTTTCCGTTC
CCAATTCCACATTGCAATAATAGAAAATCCATCTTCATCGGCTTTTTCGTCATCATCTGTATGAATCAAATCGCC
TTCTTCTGTGTCATCAAGGTTTAATTTTTTATGTATTTCTTTTAACAAACCACCATAGGAGATTAACCTTTTACG
GTGTAAACCTTCCTCCAAATCAGACAAACGTTACAAATTCTTTTCTTCATCATCGGTCATAAAATCCGTATCCTT
TACAGGATATTTTGCAGTTTCGTCAATTGCCGATTGTATATCCGATTTATATTTATTTTTCGGTCGAATCATTTGA
ACTTTTACATTTGGATCATAGTCTAATTTCATTGCCTTTTTCCAAAATTGAATCCATTGTTTTTGATTCACGTAG
TTTTCTGTATTCTTAAAATAAGTTGGTTCCACACATACCAATACATGCATGTGCTGATTATAAGAATTATCTTTAT
TATTTATTGTCACTTCCGTTGCACGCATAAAACCAACAAGATTTTTATTAATTTTTTTATATTGCATCATTCGGC
GAAATCCTTGAGCCATATCTGACAAACTCTTATTTAATTCTTCGCCATCATAAACATTTTTAACTGTTAATGTGA
GAAACAACCAACGAACTGTTGGCTTTTGTTTAATAACTTCAGCAACAACCTTTTGTGACTGAATGCCATGTTT
CATTGCTCTCCTCCAGTTGCACATTGGACAAAGCCTGGATTTACAAAACCACACTCGATACAACTTTCTTTCG
CCTGTTTCACGATTTTGTTTATACTCTAATATTTCAGCACAATCTTTTACTCTTTCAGCCTTTTTAAATTCAAGAA
TATGCAGAAGTTCAAAGTAATCAACATTAGCGATTTTCTTTTCTCTCCATGGTCTCACTTTTCCACTTTTTGTCT
TGTCCACTAAAACCCTTGATTTTTCATCTGAATAAATGCTACTATTAGGACACATAATATTAAAAGAAACCCCC
ATCTATTTAGTTATTTGTTTAGTCACTTATAACTTTAACAGATGGGGTTTTTCTGTGCAACCAATTTTAAGGGTT
TTCAATACTTTAAAACACATACATACCAACACTTCAACGCACCTTTCAGCAACTAAAATAAAAATGACGTTATT
TCTATATGTATCAAGATAAGAAAGAACAAGTTCAAAACCATCAAAAAAAGACACCTTTTCAGGTGCTTTTTTT
ATTTTATAAACTCATTCCCTGATCTCGACTTCGTTCTTTTTTTACCTCTCGGTTATGAGTTAGTTCAAATTCGTTC
TTTTTAGGTTCTAAATCGTGTTTTTCTTGGAATTGTGCTGTTTTATCCTTTACCTTGTCTACAAACCCCTTAAAA
ACGTTTTTAAAGGCTTTTAAGCCGTCTGTACGTTCCTTAAGGAATTCCTTAGTGCTTTCATAGATTAAACTCAC
ATCACGCTTTAAATCGCTTATTTTAGACTTTAAAGACTTGTTTTCTTCAAGCAACTCATTATAATCATTTACATTT
TCATTAAATCGCTCTACAAGACCACTATATTTTTCTTTAACTTGCCCATGTTCTTTACTTAATTTTTTATATTCTCT
CGCCATATCAGTACTCATGAGATTTCTAACATGCTGTTTTAACCTATCGTTATCTCTCGCAGCAGTCACTAAGTT
TTTATAATCACGCTCCGATATAACAACATTTTTGGTTGGTTTCTTTTCTGTTTTCATTATTTCTTTTCCCAAACCA

AACATAGACTTTTCACCCGTTGGCACTTCAACACTTTTCATGTGTCGTTTCGCTGGTACTTCTAAATCTGATTT
AACTTTATCGCTATAAGCAGTCCATTCATCTTTTTTAACTGCTAAATTTTTTTCTAGAAAATCAATCTCTTTTTCC
AAAGTTTGTTTTTTAAATTTAGCTGTCTCAATATGTTTACGGTCAGAGCCACGTTCACCACGCTTCAACTCAA
AACCCTGTTTTTTCATATGCTCGGGGAATTTATCTTGTAGCCATAACAGTTCTTGACGATTAAACACATTTTTTC
CTTGCAGTTTTCCATCACGCATAGGCACAACACCTAAATGCATGTGAGGGGTTTGCTCATCATTATGAACTGT
TGCATAAGCAATATTTTGCTTGCCATATCGTTCGGAAAATAATTTATAACTTTCCTCAAAAAATCGTTTTTGTTC
TCCTGGATCCAGTTGCTCAAAAAAATCTCGGTCAGATGTTACTAGCAACTCATTTACAAGAACAGCATCTTTC
CTCGTTTTTCTTGTACCTGTTTTTTGTGATTCAATAATTTCTTTGACACGTTCGTTGTAATCAATATTTTTATCAT
TTTTCAAATCATAATTTTCACGTGTTCGCTCATGGTCAATATCATCATTCGTTCTACTTTTTCGCTCTCTTTGATT
ATGAAATTGCATGCCTTTTAGTCCAGCTGATTTCACTTTTTGCATTCTACAAACTGCATAACTCATATGTAAATC
GCTCCTTTTTAGGTGGCACAAATGTGAGGCATTTTCGCTCTTTCCGGCAACCACTTCCAAGTAAAGTATAACA
CACTATACTTTATATTCATAAAGTGTGTGCTCTGCGAGGCTGTCGGCAGTGCCGACCAAAACCATAAAACCTT
TAAGACCTTTCTTTTTTTTACGAGAAAAAAGAAACAAAAAAACCTGCCCTCTGCCACCTCAGCAAAGGGGG
GTTTTGCTCTCGTGCTCGTTTAAAAATCAGCAAGGGACAGGTAGTATTTTTTGAGAAGATCACTCAAAAAAT
CTCCACCTTTAAACCCTTGCCAATTTTTATTTTGTCCGTTTTGTCTAGCTTACCGAAAGCCAGACTCAGCAAG
AATAAAATTTTTATTGTCTTTCGGTTTTCTAGTGTAACGGACAAAACCACTCAAAATAAAAAAGATACAAGAG
AGGTCTCTCGTATCTTTTATTCAGCAATCGCGCCCGATTGCTGAACAGATTAATAATAGATTTTAGCTTTTTATT
TGTTGAAAAAAGCTAATCAAATTGTTGTCGGGATCAATTACTGCAAAGTCTCGTTCATCCCACCACTGATCTT
TTAATGATGTATTGGGGTGCAAAATGCCCAAAGGCTTAATATGTTGATATAATTCATCAATTCCCTCTACTTCAA
TGCGGCAACTAGCAGTACCAGCAATAAACGACTCCGCACCTGTACAAACCGGTGAATCATTACTACGAGAGC
GCCAGCCTTCATCACTTGCCTCCCATAGATGAATCCGAACCTCATTACACATTAGAACTGCGAATCCATCTTCA
TGGTGAACCAAAGTGAAACCTAGTTTATCGCAATAAAAACCTATACTCTTTTTAATATCCCCGACTGGCAATGC
CGGGATAGACTGTAACATTCTCACGCATAAAATCCCCTTTCATTTTCTAATGTAAATCTATTACCTTATTATTAAT
TCAATTCGCTCATAATTAATCCTTTTTCTTATTACGCAAAATGGCCCGATTTAAGCACACCCTTTATTCCGTTAA
TGCGCCATGACAGCCATGATAATTACTAATACTAGGAGAAGTTAATAAATACGTAACCAACATGATTAACAATT
ATTAGAGGTCATCGTTCAAAATGGTATGCGTTTTGACACATCCACTATATATCCGTGTCGTTCTGTCCACTCCTG
AATCCCATTCCAGAAATTCTCTAGCGATTCCAGAAGTTTCTCAGAGTCGGAAAGTTGACCAGACATTACGAA
CTGGCACAGATGGTCATAACCTGAAGGAAGATCTGATTGCTTAACTGCTTCAGTTAAGACCGAAGCGCTCGT
CGTATAACAGATGCGATGATGCAGACCAATCAACATGGCACCTGCCATTGCTACCTGTACAGTCAAGGATGG
TAGAAATGTTGTCGGTCCTTGCACACGAATATTACGCCATTTGCCTGCATATTCAAACAGCTCTTCTACGATAA
GGGCACAAATCGCATCGTGGAACGTTTGGGCTTCTACCGATTTAGCAGTTTGATACACTTTCTCTAAGTATCC
ACCTGAATCATAAATCGGCAAAATAGAGAAAAATTGACCATGTGTAAGAGGCCAATCTGATTCCACCTGAGA
TGCATAATCTAGTAGAATCTCTTCGCTATCAAAATTCACTTCCACCTTCCACTCACCGGTTGTCCATTCATGGCT
GAACTCTGCTTCCTCTGTTGACATGACACACATCATCTCAATATCCGAATAGGGCCCATCAGTCTGACGACCA
AGAGAGCCATAAACACCAATAGCCTTAACATCATCCCCATATTTATCCAATATTCGTTCCTTAATTTCATGAACA
ATCTTCATTCTTTCTTCTCTAGTCATTATTATTGGTCCATTCACTATTCTCATTCCCTTTTCAGATAATTTTAGATT
TGCTTTTCTAAATAAGAATATTTGGAGAGCACCGTTCTTATTCAGCTATTAATAACTCGTCTTCCTAAGCATCCT
TCAATCCTTTTAATAACAATTATAGCATCTAATCGGTTCTGTTGCAAAGTAAAAAAATATAGCTAACCACTAATT
TATCATGTCAGTGTTCGCTTAACTTGCTAGCATGATGCTAATTTCGTGGCATGGCGAAAATCCGTAGATCTGAA
GAGACCTGCGGTTCTTTTTATATAGAGCGTAAATACATTCAATACCTTTTAAAGTATTCTTTGCTGTATTGATAC
TTTGATACCTTGTCTTTCTTACTTTAATATGACGGTGATCTTGCTCAATGAGGTTATTCAGATATTTCGATGTAC
AATGACAGTCAGGTTTAAGTTTAAAAGCTTTAATTACTTTAGCCATTGCTACCTTCGTTGAAGGTGCCTGATC
TGTAATTACCTTTTGAGGTTTACCAAATTGTTTAATGAGACGTTTGATAAACGCATATGCTGAATGATTATCTC
GTTGCTTACGCAACCAAATATCTAATGTATGTCCCTCTGCATCAATGGCACGATATAAATAGCTCCATTTTCCTT

TTATTTTGATGTACGTCTCATCAATACGCCATTTGTAATAAGCTTTTTTATGCTTTTTCTTCCAAATTTGATACAA
AATTGGGGCATATTCTTGAACCCAACGGTAGACCGTTGAATGATGAACGTTTACACCACGTTCCCTTAATATT
TCAGATATATCACGATAACTCAATGTATATCTTAGATAGTAGCCAACGGCTACAGTGATAACATCCTTGTTAAAT
TGTTTATATCTGAAATAGTTCATACAGAAGACTCCTTTTTGTTAAAATTATACTATAAATTCAACTTTGCAACAG
AACCGAAAAACTAGACTTGATTACAAAATGGAGCTTGGGACATAAATGATTTTTTAAAAATGAGATGAGACG
TAGATTAACTCCATAATCAATACGAATCTATCGACTTCTTTATTTATGATATTCATCTCTTTTTAATGGAAATAAA
AGTGCGATTAATGTGATAATACAGTTACGTTAATTAAAAAAATAAAAATGCAAGGAGAGGTAATATGCTAACT
GTATATGGACATAGAGGATTACCTAGTAAAGCTCCGGAAAATACAATTGCATCATTTAAAGCTGCTTCAGAAG
TAGAAGGTATAAACTGGTTGGAGTTAGATGTTGCAATTACAAAAGATGAACAACTGATTATCATTCATGATGA
TTATTTAGAACGGACTACAAATATGTCCGGGGAAATAACTGAATTGAATTATGATGAAATTAAAGATGCTTCT
GCAGGATCTTGGTTTGGTGAAAAATTCAAAGATGAACATTTGCCAACTTTCGATGATGTAGTAAAAATAGCA
AATGAATATAATATGAATTTAAATGTAGAATTAAAAGGTATTACTGGACCGAATGGACTAGCACTTTCTAAAAG
TATGGTTAAGCAAGTGGAAGAACAATTAACAAACTTAAATCAGAATCAAGAAGTGCTCATTTCAAGCTTTAA
TGTTGTGCTTGTTAAACTTGCAGAAGAAATCATGCCACAATATAACAGAGCAGTTATATTCCATACAACTTCGT
TTCGTGAAGACTGGAGAACACTTTTAGATTACTGTAATGCTAAAATAGTAAACACTGAAGATGCCAAACTTAC
TAAAGCAAAAGTAAAAATGGTAAAAGAAGCGGGTTATGAATTGAACGTATGGACTGTAAACAAACCAGCAC
GTGCAAACCAACTTGCTAATTGGGGAGTTGATGGTATCTTTACAGACAATGCAGATAAAATGGTGCATTTGTC
TCAATAGAAAGTTAGAGGTGAGTCTTACGTTTCAGTGACGGTAGACTTACCTTTAACATGTTACATACTAAAA
AATTAATTTGAATAAGAAAGAGAGACATATATGAAATACGATGATTTTATAGTAGGAGAAACATTCAAAACAA
AAAGCCTTCATATTACAGAAGAAGAAATTATCCAATTTGCAACAACTTTTGATCCTCAATATATGCATATAGATA
AAGAAAAAGCAGAACAAAGTAGATTTAAAGGTATCATTGCATCTGGCATGCATACACTTTCAATATCATTTAA
ATTATGGGTAGAAGAAGGTAAATACGGAGAAGAAGTTGTAGCAGGAACACAAATGAATAACGTTAAATTTAT
TAAACCTGTATACCCAGGTAATACATTGTACGTTATCGCTGAAATTACAAATAAGAAATCCATAAAAAAAGAAA
ATGGACTCGTTACAGTGTCACTTTCAACATACAATGAAAATGAAGAAATTGTATTTAAGGGAGAAGTAACAG
CACTTATTAATAATTCATAATAAAACAGTGAAGCAACCATCGTTACGGATTGCTTCACTGTTTTGTTATTCATCT
ATATCGTATTTTTTATTACCGTTCTCATATAGCTCATCATACACTTTACCTGAGATTTTGGCATTGTAGCTAGCCA
TTCCTTTATCTTGTACATCTTTAACATTAATAGCCATCATCATGTTTGGATTATCTTTATCATATGATATAAACCAC
CCAATTTGTCTGCCAGTTTCTCCTTGTTTCATTTTGAGTTCTGCAGTACCGGATTTGCCAATTAAGTTTGCATA
AGATCTATAAATATCTTCTTTATGTGTTTTATTTACGACTTGTTGCATACCATCAGTTAATAGATTGATATTTTCTT
TGGAAATAATATTTTTCTTCCAAACTTTGTTTTTCGTGTCTTTTAATAAGTGAGGTGCGTTAATATTGCCATTAT
TTTCTAATGCGCTATAGATTGAAAGGATCTGTACTGGGTTAATCAGTATTTCACCTTGTCCGTAACCTGAATCA
GCTAATAATATTTCATTATCTAAATTTTTGTTTGAAATTTGAGCATTATAAAATGGATAATCACTTGGTATATCTT
CACCAACACCTAGTTTTTTCATGCCTTTTTCAAATTTCTTACTGCCTAATTCGAGTGCTACTCTAGCAAAGAAA
ATGTTATCTGATGATTCTATTGCTTGTTTTAAGTCGATATTACCATTTACCACTTCATATCTTGTAACGTTGTAAC
CACCCCAAGATTTATCTTTTTGCCAACCTTTACCATCGATTTTATAACTTGTTTTATCGTCTAATGTTTTGTTATT
TAACCCAATCATTGCTGTTAATATTTTTTGAGTTGAACCTGGTGAAGTTGTAATCTGGAACTTGTTGAGCAGA
GGTTCTTTTTTATCTTCGGTTAATTTATTATATTCTTCGTTACTCATGCCATACATAAATGGATAGACGTCATATG
AAGGTGTGCTTACAAGTGCTAATAATTCACCTGTTTGAGGGTGGATAGCAGTACCTGAGCCATAATCATTTTT
CATGTTGTTATAAATACTCTTTTGAACTTTAGCATCAATAGTTAGTTGAATATCTTTGCCATCTTTTTTCTTTTTC
TCTATTAATGTATGTGCGATTGTATTGCTATTATCGTCAACGATTGTGACACGATAGCCATCTTCATGTTGGAGC
TTTTTATCGTAAAGTTTTTCGAGTCCCTTTTTACCAATAACTGCATCATCTTTATAGCCTTTATATTCTTTTTGTTT
TAATTCTTCAGAGTTAATGGGACCAACATAACCTAATAGATGTGAAGTCGCTTTTCCTAGAGGATAGTTACGA
CTTTCTGTTTCATTAGTTGTAAGATGAAATTTTTTTGCGAAATCACTTAAATATTCATCCATTTTTTTAACGGTT
TTAAGTGGAACGAAGGTATCATCTTGTACCCAATTTTGATCCATTTGTTGTTTGATATAGTCTTCAGAAATACT

TAGTTCTTTAGCGATTGCTTTATAATCTTTTTTAGATACATTCTTTGGAACGATGCCTATCTCATATGCTGTTCCT
GTATTGGCCAATTCCACATTGTTTCGGTCTAAAATTTTACCACGTTCTGATTTTAAATTTTCAATATGTATGCTT
TGGTCTTTCTGCATTCCTGGAATAATGACGCTATGATCCCAATCTAACTTCCACATACCATCTTCTTTAACAAAA
TTAAATTGAACGTTGCGATCAATGTTACCGTAGTTTGTTTTAATTTTATATTGAGCATCTACTCGTTTTTTATTTT
TAGATACTTTTTTTATTTTACGATCCTGAATGTTTATATCTTTAACGCCTAAACTATTATATATTTTTATCGGACGT
TCAGTCATTTCTACTTCACCATTATCGCTTTTAGAAATATAACTGCTATCTTTATAAACTTGTTTGAAATTTTTAT
CTTCAATTGCATCAATAGTATTATTAATTTCTTTATCTTTTGAAGCATAAAAATATATACCAAACCCGACAACTAC
AACTATTAAAATAAGTGGAACAATTTTTATCTTTTTCATCAATATCCTCCTTATATAAGACTACATTTGTAGTATAT
TACAAATGTAGTATTTATGTCAAAATAATGTTATAATTTTTGTGATATGGAGGTGTAGAAGGTGTTATCATCTTT
TTTAATGTTAAGTATAATCAGTTCATTGCTCACGATATGTGTAATTTTTTTAGTGAGAATGCTCTATATAAAATAT
ACTCAAAATATTATGTCACATAAGATTTGGTTATTAGTGCTCGTCTCCACGTTAATTCCATTAATACCATTTTACA
AAATATCGAATTTTACATTTTCAAAAGATATGATGAATCGAAATGTATCTGACACGACTTCTTCGGTTAGTCAT
ATGTTAGATGGTCAACAATCATCTGTTACGAAAGACTTAGCAATTAATGTTAATCAGTTTGAGACCTCAAATAT
AACGTATATGATTCTTTTGATATGGGTATTTGGTAGTTTGTTGTGCTTATTTTATATGATTAAGGCATTCCGACA
AATTGATGTTATTAAAAGTTCGTCATTGGAATCGTCATATCTTAATGAACGACTTAAAGTATGTCAAAGTAAGA
TGCAGTTCTACAAAAAGCATATAACAATTAGTTATAGTTCAAACATTGATAATCCGATGGTATTTGGTTTAGTG
AAATCCCAAATTGTACTACCAACTGTCGTAGTCGAAACCATGAATGACAAAGAAATTGAATATATTATTCTACA
TGAACTATCACATGTGAAAAGTCATGACTTAATATTCAACCAGCTTTATGTTGTTTTTAAAATGATATTCTGGTT
TAATCCTGCACTATATATAAGTAAAACAATGATGGACAATGACTGTGAAAAAGTATGTGATAGAAACGTTTTAA
AAATTTTGAATCGCCATGAACATATACGTTATGGTGAATCGATATTAAAATGCTCTATTTTAAAATCTCAGCACA
TAAATAATGTGGCAGCACAATATTTACTAGGTTTTAATTCAAATATTAAAGAACGTGTTAAGTATATTGCACTTT
ATGATTCAATGCCTAAACCTAATCGAAACAAGCGTATTGTTGCGTATATTGTATGTAGTATATCGCTTTTAATAC
AAGCACCGTTACTATCTGCACATGTTCAACAAGACAAATATGAAACAAATGTATCATATAAAAAATTAAATCAA
CTAGCTCCGTATTTCAAAGGATTTGATGGAAGTTTTGTGCTTTATAATGAACGGGAGCAAGCTTATTCTATTTA
TAATGAACCAGAAAGTAAACAACGATATTCACCTAATTCTACTTACAAAATTTATTTAGCGTTAATGGCATTCG
ACCAAAATTTACTCTCATTAAATCATACTGAACAACAATGGGATAAACATCAATATCCATTTAAAGAATGGAAC
CAAGATCAAAATTTAAATTCTTCAATGAAATATTCAGTAAATTGGTATTACGAAAATTTAAACAAACATTTAAG
ACAAGATGAGGTTAAATCTTATTTAGATCTAATTGAATATGGTAATGAAGAAATATCAGGGAATGAAAATTATT
GGAATGAATCTTCATTAAAAATTTCTGCAATAGAACAGGTTAATTTGTTGAAAAATATGAAACAACATAACAT
GCATTTTGATAATAAGGCTATTGAAAAAGTTGAAAATAGTATGACTTTGAAACAAAAAGATACTTATAAATATG
TAGGTAAAACTGGAACAGGAATCGTGAATCACAAAGAAGCAAATGGATGGTTCGTAGGTTATGTTGAAACG
AAAGATAATACGTATTATTTTGCTACACATTTAAAAGGCGAAGACAATGCGAATGGCGAAAAAGCACAACAA
ATTTCTGAGCGTATTTTAAAAGAAATGGAATTAATATAATGGATAATAAAACGTATGAAATATCATCTGCAGAA
TGGGAAGTTATGAATATCATTTGGATGAAAAAATATGCAAGTGCGAATAATATAATAGAAGAAATACAAATGC
AAAAGGACTGGAGTCCAAAAACCATTCGTACACTTATAACGAGATTGTATAAAAAGGGATTTATAGATCGTAA
AAAAGACAATAAAATTTTTCAATATTACTCTCTTGTAGAAGAAAGTGATATAAAATATAAAACATCTAAAAACT
TTATCAATAAAGTATACAAAGGCGGTTTCAATTCACTTGTCTTAAACTTTGTAGAAAAAGAAGATCTATCACA
AGATGAAATAGAAGAATTGAGAAATATATTGAATAAAAAATAAAATTGTTGTGTTTACAACAATACATAGAAA
ACAGAGGAAACAATCAAGTCGTTGAATATTTCCTCTGTTTTTTAGTTGAAAAAATTAACCGAAAGCCTGAAT
GCAAGTCTTGATTAAATCAATAATGCTTGTAATAACACCAGTGAAATCCATATGCATACCCTCTTTCTATTTAAG
ATACATTAAGTATAATATCAAACAAATAAAAAATGTTAAAAATTCCCTAATTGGCTATTTAGATTGCATAAATGT
CAAAAATTTGAAAAACATACAACGACTTTGCATAAAAAATCGTCATATTGGAAATACGTAATTTATTGAAATAA
TAAAAAAAATAAAAGAACGAAGATGATAACCTAAGTGAGGTTTTAAGTTGTTCTAAGGTTTAATTTAATTTAT
GTTAAAATAGTTGGTATAAAAATACATGATAAACTATAAACTAAATTCAAAATAACTTATGGGGTAGGCAATTA

TGGAAAATAAAATATAAATGATAATGAAAAAAGAGTGCTAAGGGAAATTTATAACCATCATAATATTTCGCGTA
CTCAAATATCTAAAAATCTTGAGATTAATAAGGCAACGATTTCTAGTATTTTGAATAAGTTAAAGTATAAATCTC
TTGTTAATGAGGTTGGTGAGGGTGATAGCACGAAGAGTGGTGGTAGAAAACCTATTCTTCTGAAGGTTAATC
ATCTTTATGGTTATTTTATTTCTTTGGATTTAACTTATAGTTCTGTTGAAGTGATGTACAATTATTTTGATGGTAA
TGTCATTAAGCATGAATCTTATGATTTACCTGATGAAAAGGTTAGTAGTATATTAAGCATAATAAAAAAACATAT
TGATATTCAGGAGAAACTTGATACTTATAACGGACTATTAGGTGTGTCTGTTTCTATACATGGAGTTGTGGATA
ATGAGCAGCATGTGACATATTTACCATTCCATGAAACTGAAGGAATTTCAATTGCTAAGAAAATAAAAGAAAT
TACTAATGTTCCAGTCGTAGTTGAAAATGAAGCGAATCTTTCAGCGTTATATGAACGTAATTTTAATCATAATTT
ATCCTACAATAATCTTATTGCTTTAAGTATACATAAAGGTATTGGTGCTGGGCTTATTATTAATAATCAATTGTAT
CGTGGTGCAAATGGGGAAGCGGGTGAAATTGGAAAAACACTTGTCTCAAAAGTTAGCGATAATGTGGAGA
TCTTTCATAAGATTGAAGATATTTTTTCACAAGAAGCTTTACTGCATAATTTAAGTAATCAACTAAATGAGAAG
ATGACGCTTAGCAAATTAATTCAATTTTATAATGAAAAAAATCCAGTCGTAGTTGAAGAAATGGAACAATTTA
TAAATAAAATTGCTGTTTTAATACATAATTTAAACACCCAGTTTAATCCGAATGCAATTTACATTAACTGTCCAT
TGTTCAATGAAATGCCTGAAATATTAGAAGCAATTAAGAACCAGTTCAAACAATATTCACGTAACGAAATTCA
AATAAAGTTAACATCTAATGTCAAATTTGCAACTTTGCTAGGTGGTACATTAGCAATTATCCAAAAAGTACTAC
AGATTAATGATATTTACTTAGATATAAAAGCATAAAAAACTAATTCAAATGAATAATCAAAGTTCGTAATTGTCT
TTATAAAAAAATCCCTCAATCCGAATTGAATTTTCGGATTGAGGGATTTTTATAGTTCTATTGCAGAAGAAAAC
TATTTTAAAAATGCTGGTAAATGTTGATAGCCACCTCTAACGTTAACAATATTCGTAAATCCTTTATATTCTAATA
TTCCTACCGCTATTGAACTTCTAACACCTGATTGACAATGTACATAAATTAGGTCATTTTTATCGAAAGGTATAT
CTTCATTTAAAAGTTTACCGTGAGGAATATGAATTGCTTGTTTTAAATGACCTTTACGCCATTCATCATCATTAC
GAACATCTAATACATTATGTTCTTCACCAGTCATTTCAGAACTATGAATAGATGATGTGACGATATTTGTTTGTG
GCAAACGGTAACTTTTTACATTTTCAAAACCAATTAATTGTAAAGCATGAATAGCTTGTTGAACGGTAGATTT
ATCGCCAATTAATTCAATATCTTAGTCATAATCTAAATACCAACCAATTTGATTTATAAAAGTTTTATTAAAAGG
AATATTGATAGTTCCATGCATATGACCACCATGGAATGCTTCTTTACTTCGAAGATCAAAAGCAGTTTGTGTAT
TGCTTGAACTAGGGTAAACATTATATGGTTGGTACATTTGCATACCAAATTGATTTATTTTTTTCATTTGTGAAA
AATGGTGTGGTGGAGCTGGCTGATTGAGTGTTAAAGTTTCGATAAATGAAGTTTCATCTTTAACATTAAAAG
CCCAGTTGTTTATTTTCTCATAACCCAAAGTAGTTGTAGGTAATGAACCTAGCGCTTTACCACAAGGACTCCC
TGCACCATGACCTGGCCAAATTTGAATATAGTCTGGTAATGTTGCAGCAAATTGTATGGACTGATACATTTGTT
TTGCTCCGATTTTTGTAGAACCTTCAACATTTACAGCTTTTTCTAATAGATCTGGTCTACCTACATCACCAACAA
AGATGAAGTCACCGCTAAATAATCCCATTGGTATACTGGAACCCCCACCTTCGTCAGTAAGTAAAAAACTAAT
ACTCTCAGGGGGATGGCCTGGAGTGTGTAAGACTTCTAATTTAATCTTTCCTAAATAGATAATATCTTGATGCT
TAACGAAATGTGTTTGTTTAGGCATATTTTTATAATTAAATTCATCTTTACCTTCATCAGATACGTATATACTTGC
ATTCAATTTATTTGCCACATCTCTAATACCTGAAGCAAAATCAGCATGAATATGTGTTTCTGCAGCTTTAGTAAT
TGTGAATCCTTCTTTATCTGCAACTTTTAAATATTTTGTTAAATCTCGTATAGGGTCAATAATCATTGCTTCTCCT
GTACGTTGACATCCAATTAAATAAGATGCTTGTGAAAAATTGTCTTCATAAAATTGTTTGAAAAACAAAAAAA
ACTCCTTTTAAAAATAGATTTTATTGATTAGATAAATAAGTTATGATTTGCTTGCTCAGTATGTCCAATATAAGTA
CCTACGCCACCATAATCGACTTCATCTCTTAATTCTTCTTTTGAAATTCCCATAACATCCATACTCATGGTACAA
GCAATTAACTTTATATCTTGATCGATTGCTTGATCGATAAGTGAGTATAAAGAATCAACATTTTTCTTGTTCATT
ACATAACGCATCATAATATTACCTAGTCCAAACATATTCATTTTTGATAATGGCATATGTATTGGATCCTTAGGTA
ACATAAGGTCAAACATTTTGGAAATACCTTTCTTTTTAACGCGAGTTGATTGCGCTTTTTTTAATGCGTTGAG
GCCCCAAAAAGTAAAGAAAATAGTTACATCTTTACCTGCTGCTTTAGCGCCATTTGCGATGATCATTGCTGCT
ACTGCCTTATCTAACTCACCGCTAAATAAAACAATTGTTGTACCTGTAGCAGTGTCATTGATTTCAAATTCTTTT
GGCTTTTCTTTTTGAATAATTGCATTAATTACATTTGCTTCTTCAGTAAGATTTACAAGGGTATTCCCTGTTTGT
TTCGCCCAACTTTTAATATCACTATTGAAACCAGGATCTGTAACTGTTACCTCGATTTGCTCACCCGTTGAAAT

ATTGTTAATTTCTTTACTGATATTAACAATAGGTCCAGGGCACTGAAGACCTCTAAAATCAAATTGTTTACGAT
TCTCTTTGATTTCAATATCTTTTTCTATTAAAGGAGCACTATTGAAGTTCTTTGCTTCATAATCTTTATATCCACC
CTTTAAATTCACGACATCATAACCTTGTTTGGCTAAATAATCGCAAGCTTTAGTGCTTCGGTTACCGCTTTTAC
AATGTATATAATACGTTTTGTTGCTATTCTTATTGAATGATTTAATCTCTTCTACTGGGTGTAAAGTTGAACCGTT
AATGTGTCCTAATTCATATTCTTCTTTTGTTCTAACATCAATCAATTGACCCATTTTTGCCAATTTTTCTAATTCTT
CTTTGTTTAATGAATTAATGTGTACTTCTTTGTATTGTTCCATACTTACCTCCTATAAATACCTATGAGGGTATAA
TAAAACGGATAGAATCATTTGCCAAATACCTATATGGGTATTTGACAATTTTGTTTTAATTTATTATTATTAACTT
AATCAATTTATGTGGAGGAAATGAATATGACTTATGATAAAAAAATGATTAATCGTATAAATAGAATACAAGGT
CAATTAAATGGTGTCGTAAAAATGATGGAAGAAGAAAAAGATTGCAAAGATATAATTACGCAACTTAGTGCA
TCTAAAGGTTCTATACAACGTTTAATGGGGATTATAATTAGTGAAAATTTAATAGAATGCGTTAAAACAGCAG
AAGAAAATAATGAAAGTTCTCAAGAATTAATTAATGAAGCAGTTAATTTATTAGTTAAAAGTAAATAATGGATA
TAGCAAATATGACTATTATGTTGCTAATTGGCGTACTGGGTGGATTTATATCTGGATTAATAGGTATTGGGGGC
GCAATTATTATTTACCCAGCTATTCTTATATTGCCACCATTAATAGGTATACCTGCGTATAGTGCATATATTGCTTC
GGGACTTACCTCTAGTCAAGTATTTTTCAGTACACTTAGTGGATCATTAAATGCAAGAAAACAACCAGCTTTC
TCTCCTAAACTTGTTATATATATGGGAGGGGGTATGTTGATTGGAAGCATGTTAGGGGCAATTTTAGCTAGTT
TGTTTAATGCTACTTTTGTAAATACGGTATATGTAATAATCGCCATACTTGCTTTAATATTGATGTTTATTAAAGT
TAAACCTACTACACAAGAGACGAAATCTAAACCTTTGCTATTTATTATAGTTGGATTTGGAATTGGTGTAATTT
CGGGAATTGTGGGTGCAGGTGGAGCATTTATCATCATTCCTGTATTATTAGCATTATTTAAATTACCAATGAAT
ACGGTAGTGAACAATAGCATAGCAATTGCTTTTATATCTTCAGTAGGGGCATTTTTTATAAAATTAATGCAAGG
ATATATACCAGTAGAAAGTGCAATTTTTTTGATAATTGAATGATGATCATTTTCTGAAAATATTATGTGGTCATAT
AATATAATGCCCATCATTTCACTAATCTCTTTTATTCTCTGAGTTATTTTGATATCTCCTGGAGAAGGTGTTACAT
CTTATCAAGAGTAAATTACAAAAGAATCATTTAAATCAATACTTTCACTTTGAATACATGTATTTGAAGTGGAA
GGTACTTATTTCAAAAATAGTAAAACCTGTATCTTAAATTACTTAATAGTAACATAAGATACAGGCTGATTTTTT
ATTCATTGTTATTTATACTAAAGCACCCGATAGCTCTGAAAACAATCACAAATTCAACTTTTCAAAGCCACAGC
TTTAAGTTATTTTGTCCAGACAACCCCCATTGCCCAACCCATTTTATGGAATTGGCATCCAGGCAACAACTTTT
CATATAAATCGTAATAATTTTGTTCAGATAGGTACTTATCTGAAGCTAAATGCTCAAGCCATGATTTAGATGTGT
TGTGATTATAAATTCTAATCGCATTTTTTATTCCAAATTTGAAACAATGCGGTAAAAACTCTTGGATTGCCCCTA
ATTTATAAACATAGGTAGGCGGTGTCTCGACTTCAGATACATTATCAAGAATAACTATTCTTCCTTCCTCATTCA
ACAGTTCCTTCATTTGCTGTATTACGCTGGCTATATCATCCAAATGATGAAAGGTTGTTCGGCTTACAATAAAA
TCAAACTTCTCATTAAAATTAAGTTGTTCTGCATTCATATTCAGATAGACCGTATTTGTTAGTTGACGTTTAGAT
TTGGCAAGATCGAGCATTTGATTAGAAATATCAATCCCTACCACTTCATCATAATAACTTGCTAATTTCTCCACT
AACAAACCCGAGCCACATCCGATATCTAATGCTCTGCCTTTCTTTGGAGACATATTAGACACAAAGAATGAAT
AATCATTCAAAAGCTCATTCACGAAATCGTAATCTTCTGCAACCTTATCAAACTGTGATTCTATTGTATTCAAAA
AGATCCCCCATTCCTACTTTATCGACATTCTTTCATTACTTACCACTTTAGATGTTTTTTCGTTGGGGATAAAAC
TTCCCTTTAGACAATTTTATCCAAAGACAATACAACAGTGCAACTTTATTAAAGTCACTGTCCTTTATCGCAGC
CTTTACTTTTTAGTAAAGACAGTGGCTTCTCTTATCAAGTTTCAAAACATATTATTTTGAAGAAAACGTCCATC
TGAAGTGTCAAGTGCAAAATTACATATAAAGGTTTATTCTAAAATGAAAAGATGATACAATCATATTCAGTTAC
ATAAGGAGGTTTCAATTATGTGCACCAGTATCGCAGTAGTAGAAATTACTTTATCTCATTCATAATGAAAAAAA
TGGAAAGGAGAAGGTTATAATGAACCAGAAAAACCCTAAAGACACGCAAAATTTTATTACTTCTAAAAAGC
ATGTAAAAGAAATATTGAATCACACGAATATCAGTAAACAAGACAACGTAATAGAAATCGGATCAGGAAAAG
GACATTTTACCAAAGAGCTAGTCAAAATGAGTCGATCAGTTACTGCTATAGAAATTGATGGAGGCTTATGTCA
AGTGACTAAAGAAGCGGTAAACCCCTCTGAGAATATAAAAGTGATTCAAACGGATATTCTAAAATTTTCCTTC
CCAAAACATATAAACTATAAGATATATGGTAATATTCCTTATAACATCAGTACGGATATTGTCAAAAGAATTACC
TTTGAAAGTCAGGCTAAATATAGCTATCTTATCGTTGAGAAGGGATTTGCGAAAAGATTGCAAAATCTGCAA

CGAGCTTTGGGTTTACTATTAATGGTGGAGATGGATATAAAAATGCTCAAAAAAGTACCACCACTATATTTTC
ATCCTAAGCCAAGTGTAGACTCTGTATTGATTGTTCTTGAACGACATCAACCATTGATTTCAAAGAAGGACTA
CAAAAAGTATCGATCTTTTGTTTATAAGTGGGTAAACCGTGAATATCGTGTTCTTTTCACTAAAAACCAATTCC
GACAGGCTTTGAAGCATGCAAATGTCACTAATATTAATAAACTATCGAAGGAACAATTTCTTTCTATTTTCAAT
AGTTACAAATTGTTTCACTAAATTAAAGTAATAAAGCGTTCTCTAATTTCACAAGAGGACGCTTTATTCTTCCC
AAAAATTGTTCAATATTTATCAATAAATCAGTAGTTTTAAAAGTAAGCACCTGTTATTGCAATAAAATTAGCCTA
ATTGAGAGAAGTTTCTATAGAATTTTTCATATACTTAACGAGTGCTTTCACCTTTGAATATAGTCCTTCCCACTT
ATCATCACACTCTCCCCGATAGCCTTTTCTAGCTATATCCAGTAAAGTTACATGCTCTTTAGGTAAAAGAGGTA
TAGCCCATTCTGCAGCGACATCTTTCGAGGTAATTTCACCAGTAGTCACTGTTTGCCACATTCGAGCTAGGGT
TAAAATTACATTACGCTCATCACCTTTTATCCCCTCAATTAGTTCTGGCAAAGAATCCTTAATTGCTCTTCGAAT
ATCTGTCAAAGGTACGGAGACAAGTATACTTGAAGAATCAGGACCAAATAGAGAAATACTATTCTTTCTTGCT
TGTGCTAAAACAATAGCCAAATCAGGATCATAGCTTGGTTCCTGAATTTGTCCATTCTCAAATTCACCCCTGA
GCCACTCACCGTATATAAATTCTCTTTTTGGAGGATATTGCCAAGGGACAACTTCACTCCTATTTATAACCGTA
ACTTCAAGTGGTCTAACAGAATCCGTATTTCCAATCTTTCCTGATATAGTCATTAGTCTTTCTGTTAGTTTTTTT
CGAGTTAATTGAGGTAAACTATGATTCACGACGACTAGAACATCTACATCGCTGTTAATGCGTAAACCACCAT
TTACTGCTGAACCAAATAGATATACTCCAACTATTGAACTTCCAAATAAATCTTTTACGATTTTTAATGTTTGAA
TCGCTTGATTTGGTATTTTTCCGTTAATCAAATTGCTCATGATTTCACCTCGTTGATTATGTTCATATAAAGTTTA
TATTGATACTCAATTTACTTACCCTAGATTGGACATATACTTAAATTACTGTTCAATAAAGCTGACCGTTAGCGT
TTAAGTACATCCTTTCACAATTTGTCTACAGATTAATAATTATTCTTTATTATACAGATCTCCATATAATTTTTGAA
TTTGGTTCTGTAATTTTTTATTTTCTTTTTCTAATTCCATTACTCTTCTTTTTAAGGTTTTAATAAGGATTTCCTCC
GAACGAGAACTTTTCTTGGGTTTTGAGACTACATTTGCTGTTATTTGACGCTCACGAAGGGATTCGATTCTTT
GCCTAATATCGTGTTCCTTATAAAGCCATGATTTAGAAACATTAGCTTCCTTTGCTATTGAATTAAAATTAATAA
CTTTACCTTCAATCGAAAATTTAGAAATCGCTTTGTCTACTTTTTCCCTTGTCTTTTTTGATTTCTGCTTCGCCA
AACGTACAATTTCTGTTGTATTTCTAACTTGTTTATCCATTGATAATTACCCCGTCAAACTTCCAATGATTTGTTC
TAAACGCTCTTTAACACGGCTATTAGTCTCTACTTGTCTTTGCCATTGTTTATCCTTAGCTATGGCTAATAACTCT
TCTGTACGCTCTAACTGTTCTTCGTGCTGTGGTAAGAATTGCTTACTGGTACAGAAGTGAGTGCAATCTAAGC
ATGCATTCGCATGTGGACAACCACCTGCTACTACTGGCAATCTACAATAACCATTTGGAAGCACTTGTGCATT
TATATTTTTCTTGAACCATTGAAGCTCTACATCATCGACTTCATTATCTTCATCTAGATCAAGCACATCTCCATTA
TTGGTAACCAGTTTTTCCTGAAATTTAGTAAATTCATTTTTTAGAGTTTCATCAAAGATATGAGCGTATCTGCT
TGTCATTTCTGGGCTTTCATGCCCCAAAAATTTCTGCACAATATGCTGGGGCATCCCGTTGTTAATCATTCTTG
TTCCTACTGTATGGCGAAAGGCATGGGCATGGAATCTATAAATCTCACCTGATTTATCCACTATATTTTGCTCAT
AAGCTAATTTATTTAACTCACCTCTAAATGTTTCTTGTTTTAATGGCGATCCATCTTTTCTTGGAAAGAGGTATT
CACTATCTGGAAATTCCTCTGAAACTTTATCTTCCCGAACTTTAATAAGTAAAGCTACCTCTTTAGATATTGGA
ACTATATGCTCCTTTTTCATTTTCCATTGATAATACTTTAAAAAGAAATCTCCATCTTTGTCCTCTAATAGACAGC
CTTTTTTCAAGGTGCACAATTCACTTATCCTCATTCCACATTCTTGAACAATCATAGTCATCGTAGCTATATATTC
GGGTAATTTATCAAGATGACTGTTCAATTGCTCTAGGACGAATTCATCTATAAAGCGTGGTTTTGCTCTTGGTA
TTTTCGGATAGTCCTCAGAATAAATTAATATTTTGGAAGGAACATCATCCCATTCTAGCCTAAGAAGGGTACTA
AATAGTCCTTCCAATATAGAGATCCTCCCAGTTATTGTACTAGGTTTTATTCCCATCATGTTTAGTTCACTTAAAT
ATGCTTCAATTTCCACTCTCGTTAATTGGTGTACTCTCTGAACTTGTTTAAATTTCATGTCCAGAAAATTAAAG
AACTCTTTAAGTCTTTGGGCAATATCACTTACATAGGAAAAGCTATCCACGTTCAATCTCAACTTACAATATCTT
TTTACAAGTTGTTTAAAATATGTATTCCGAAACCCTTTAAAGTTAATTGTATATTCATATTGTGTTGGGTTAACC
TTATCATCTGGCAAAGGTAAGTTACGTCTATCCCAAACGTCTTTATCCCACTCCTCTCCATCAAAATAAAAGTT
CTCATAAAACTCCATAAATTGTTTTAGATTAGTAACATAGTAGGAATTAGCTTTTACAGGTGTTTTTTCTTGATT
AGCAGTAATCTTATAATTAGTAGTGGTAATTCTAACACCCCGTTTTGTCAAATAAGTTCTATACTCCGTCATTGC

TTTTTCAATAGGAACTTCAGTAATTGAAGTAATGCTAGGATACTTTAAATCTAAGAAATCTAACATTTTATTAAT
TACTGTTCCTTTTCTAATCCAGACAGTTTTTGCATTCCATATTCCATTGTTTAAATGGTAAAAATAAAAATATTTC
AATTCTGTTCTTAACCACAGATTTTTAACACGTTCAAAACGAACCCAACGATTCCTTAAAGCAGGATTCTTAC
TTAATTCTATGGCAGAAGGATGTGGACATTTTCTTATATCCCAACTATTATTAGCCCAAAACCCCTGCATTTCTT
CATTCATTACAGCTATTTTTTTGCTAATCTCACTCTGACTAATAATTTTCCTTTTACTAGAAGCATTCATTTCTTAT
GCTCCTTTCTCTCGAGGTATTTATTAAACTCATTTTTCATATCCTGATCTGAAAGATGAACATAGGTATTTAACG
TTGTCTGAACATGTGCGTGACCTAATCTCTTTTGAACGAACGCAACATCCCATCCTTCCCTAATTAGCTGCGTT
GCGTGAGTGTGGCGAAGCATATGTGATGTAAATTCTATTCCAGTCCTTTTAACTATTCTTCTAACTAGATCAAG
AACACTTTGGTACTTTAGTGGTTTCCCAAAATAGCCTTCTTTTAAGGAAATAAAAACATAATCATGCTCCAATT
CCTCACTATACTCATATATCAAGTAATCTGTATAAAGTGACATAAGTTCTTTACTCACATGTATTGTTCTTTCCTT
CCTTAATTTAATATAAGCTTCATTAACATTAACATCTCTAGGTGTTAAATGGATTTGATTGTCCCAAGTGACAAT
ATCTTCAAGCCTAAGCGATAACACTTCACCGATTCTTAAACCACCCTCATACATAAGCATTAAAATTAATTTATC
TCTTTTCGTATGACAAGCATCAATAATTTGCTTAACTTCCTTTGATCTCAATGTTCTTATCTGTTTCTTTTTAACC
CTTAACTTTAAGACATTCTTTTGGTATCTACCCTTATTAACATGATGTAAAAATCCTTTGAAATTTCTTCCCTTTG
CTTGTTTAAATACATCAATTGATTTAAATTCTCCTAATCTACTTAAATAATCAAGAAAACTCATAACTACATTTAA
AATTGTATTCACTGTCGTTTCTTCTCTTATGGCTTTTTTTGACTGAAGATCAATTACATTTGATGCTGAAGGATA
TCTCAACCAACCTACGAAGTCTGCTAACAACTCAAAGTTAATATCATTAAGAATAACACCTCTCTGTTCCATGA
ACTCGTACAGCAACTTTAAATGATAGCAGTATGCCTTAATGGTATTAGGAGACTTACCAGTATTATCTAAGTAT
TTAATAAATTTCATTACTGGTTCTATTAGCTGGTATTCTTTATCTAGTAATAAATACAATGGATACGGCTTATTCT
CCACTTCTATCCTTTGAACCTTCACATGTTCCCACCTCTTTAAATACTTTAACTACTACAAATTATAAACCTTGTT
AATTTTTATTTAAAGTAAAATATCCCTTTAATATCTCCTCTTAAACTACTTTAACTACTATTATTTATTATACTATGG
TTAATACATCTCCACTTGGATGATTATGACCGAGCATTATACTATTTGCGTTACTGAGTATCGCTGTTTTGAATA
TTTCTCTAGGGTGAATCACCGTTTGGTTAATAGATCCAATCGATAGTGTTTGAATATGTGTAGGTTCATTTTTA
CTGTTCATACATATGAGAATGAGATGCTCTCGGTCACTGTTTCCAATGAATGAACGCATGATTTCTGCCGCATC
CTCAGGGTTTGAAATACGATTTTTTAGATAACTTAATGTATCTGTTTTTATCATTTGTAGTGAAACAATATTGAT
TTCCTTCATCGTTTACCTCCATATATAGGTTATGCTTTCAAAGTCCATTTTTGACGTGCTTTAGGGTTGAGTGG
GTGCATGATTTCATTTGTGGCTGGATTAATGAGCTTTTTGACTTTCTTTTTATGAGGCTTCAACATTTCCATTAC
TTGTTCGACACGTTCTACAACAACTGGCCGCTTCGCATAAGCACCATAAGCTAGAATCACTGTGTCACTTTCG
CTAATTGCTTTCATCAAGTGGATGTCTGTGTGTTTATCATAAGGTTCTTTAATATGTTTAAGGTTCTCTGGTGTT
TTAATATTAGAGAATAGATTTACAAGATATACAGCACCGTATCGTTCTGAATTCGCTAATTGATTGAGGATAAG
AACAGTTGTGAGATCGAGTGATAATACACCATCTAAATGAGGGTACATCGTTATCACTGTACAAGCGGGTTTC
TTTTCATCCCATGTTTTCTTGAGTAAATAGCGGTGTTTTTCATCCTTGCTAAATATAGCTTCTGTGTATATCGTAT
TTTTGATTGTATTCATATATATAATCACTTCCTTTAGTATTCTTCTGGTAAAAGCATCACATAATAAAAAGCGTCT
ACGCCATCTTCACGAATGACGTAGACTTTCTTAGGTAATGCATTTTGATTTTTTACATAGTTTGTATAGTGATAT
TCCAATTTGTATGCAGGTTGTTCTTGTTCATGTGTGATTGAGAGTATATTCTCATCTTCTTGCAGTTTAAAAATG
TGTAGGTAATCTGTATGAGGTTGATTATCTCTTTCTTTTACCATATTCCAAAGTAAGATTTGAAGGTCTAGAGA
TAGGTATTCACTAATACCTCTTGTGATGTATCGATTGATTTTCATGTTATTTTACCTCGTCTTGAATTTCTTTCATA
ATGATAATCGCTTGGCTAATAATCGTAACAGATATTTGTGCCACTTTGATCCAGTTATTCATGGTGAGTCCCTCC
TTCTCTTAGTAAATGACGTTCATCGATAATCGTATTTTTAGTATCTGTGAGATATAAAAAGTCCATGTCAAAATG
ATTCAGATAACCAACGCTGATGAGTTGGTTATTAGCGTACATAAGAAATGGATAGATACTTAGGTCATGTAGT
TCATTATTGTAGTAGGTATAAGTTTCAAGTGTAAGATGTGCAAGTGGGGAATCATTAATAAAACGTTCCGGTA
GAATATTTTCTGCTGCTTCTTCTAACGCTTCGCATTCCCATGTTTCATTGTTAGATAGTTGGAATAGACGAGTT
ATATATTGTTTGAGTTCTTGAGTGGTCGTTTTCATATCATTGCCTCCTAGATAGTGTTATAGTGATGTAGTTTATG
TACATCATTGGGATAATATATATTTGATTTGTCATTTATTACGCATCCCGGTGAGAATGAGAGAAAATTCCATAT

GAAAAACCGCTTCAAACCTTGGTATGACAAGGAAATCCCGAAATTCCGCCTATTTTGACGAACAATCAACTC
ATTCTTTATAACTATTGATGTTAGGGTGGGGCTCTGCTTTCTTATATATTATTTATTTATAAAGAATAACGGGATT
TTGGGATTGTGCTTGCACAATCCTTCTGCTTCTTCGAATCTGCAAATCCCAATCATTTCCCGATAAAAAATCAT
TGTGGGATGTTCTTTAGCAATTTCAATATAAGCATCGTGTAGTTATGAAAAAAATTACGACAATGACTGTTTCA
TTAGATAAGTGTTATTGAAATTGATAAAGAGAATTTTAAAAATGGTTAGATAAAATAAATGAAAGAATATAATG
AAAATTATTGTTATAACAATGATTCTATTAGCTAAATAGTAAGATATAGTGTTTGGGGCAAAAACAAAGACGA
AGTGCTGAGATGCACTTCGTCGAGTTGTTTATTATTGAAAAGTTGTTTTATGATTTCGTTATTAAGTTTGAGTG
TGACATAGAATTGTTTTTTATGATTACCATCTTTTTTAATATCAATGCGATCAATCACTGATAGATACAATGCTTT
GAGTCGAGATTTTTCTATGTGCTTAATATCATGAAAGATGTGTTGTAATAGTTTACTGATTTCTTTGGCATCAA
ATAAAGGCTTATCTTCATTTTGTTGATTTTTGAGTTGGTTGATTTGATTCGTAATGTCATTGAGTTGCTTTTCAT
ATTTTTGAATACTTGGTCTGATTACTGATGTTAAGTCCGGATTATCCTCGATGGTTTTAATCAAGTTATTTAGTT
TGATTTGTACTTCATCATATTGTTGTTGCTTATAAGCAATATCGTGATGAAGTGCAGCGCCATCAACTTGATTTT
CTTGATTGACGTGTGTTACTACGCGTTGAATGACTTTATCACTTTTGACTATTTCAAGTATTTGCTTCATCACAT
AATCTTCAATCACATCAGCTCTTACACTGTTTGCCGAACATACTTTGGAACCCTTGTTCCGAAAATTACTACAT
GAATAGTAACGAATACGTTTCTTAGTCCCGTCTTTAAGTGTATTCGTGGTATTGCTTGCTGCCATAGGTGCGCC
ACATTGGGGACAGTGAATAATGCCTGTAAGCAGATTCGTTCCTTTGCCATGGACTTGGGGTTTTTGACTGAC
TTGTTTTTTACGCATTTGTACTTTATCCCATAAATCTTGATTAATAATGGGGGAATGCTTACCTTCAGCTATCAC
TGGTTTATCATTAAGCCCTTTACGACGTTTTTCACTCCAATCTTTGTATTTCGCAAATTGAATTTTGCCGATATA
GAATGGGTTAGCTAATATATATGTGATAGAACTAATACTAAAAGGTTTACCCTTTTTAGTGACATAGCCTTTGT
GATTTAATGCATTGGCTATTTTACGATAGCCATGGCCTTTGGCATAGGACTCGAATATATATTTCACAATATTAG
CTTCATGTTGATTAATCATCAGTTCATGTTTACTATTAGGTATTTTGTCATAGCCCAGCGGCAAATTGCCTTGAT
AATAGCCTTCTTGGGCACGTCTCGTTTGACCCATAAATACATTCTCGACAATGTTATTACGTTCGAATTCTGAG
AAACTCGCAAGTATTTGTAACATGAGCTTACCCGATGAAGTATTGACTTCCATACGCTCTGATAAACTGAAAA
ATTCGACATTTTGTTTGTGTAAATCTTCGACAATTTTGAGAAGATCAGATGTATTACGAGCTAATCGGTTTGTT
TTGTAGACCATAACACAGTCGATATAGCCTTCTTTCGCATCCTTCAATATACGTTGGAGCTCAGGTCGATTCAT
AGATTTACCTGAAATACCACGGTCAGCGTATATATCTTTAACTTCAAAATGATGGAAGTCACAGTATTCTTTGA
TTTGATTGATTTGTCCGTCGATACTATAACCTTCTGTGCTTTGCATTTCTGTTGATACACGTACATAGATACCGA
CACGTTTTGTTTTAAGTTGTTGCATTATGTTTCATCCTTTCTTCGTTTATGCAATCGATGATTGCATGGTTTGAT
TGACGATATTGAGTGGTTCATTTTTGAAATAGATTCCTATAAGATTTTTATCTTTCGTAATGTGAATGGTTTCAA
TATAGGGGTACAATATGTTTAACGTGAAACGTTTTTGAATAATATTTTGAATGGTGTGTTGTATTTGATGTCCAT
TGATAGATGTAGTGCGTTGCGGTTGTTGACGTAATGATTGCGTTTGTTCTCTGAACGTTTCTGCATCGATGAT
GCCTTGTGCCAACTTTTCTATCAGTTGTTCTTGAGTCAATGTGTGATGTTTTTCTATGTTTCTTTGTCTTTTGAT
GCGTTTGTCAATCGCACCTTTAATTTTTGTGTAGATGCGTTGATTTTGATAAAAGTCTTTGCACACTTCTAATA
CTTTATCTTCAAGTGTTTGTGCATTGATGCCTTTAAAATCACAGACAAAGCGAGAAGCATTCATGTTTTTAGG
ACAGACGTAGTAACGTAATGTATGATTCTTTTTTCTAACGGTCATATTCGTAAGTGTTGTATTACAACATGGGC
ATTTGATTTTTTGTTTTAGTTGGTTATCCGAAGATGTCTGTTTGATTTGTTTTTGCGATCGAAGTCTCTGCGCT
TGCTCATATATACTTGTGGAAACAATAGAAGGAAACATATTGTCGAATTGGCCATATTGATTGTTGACACGACC
ACAATAATTAGGATTGATGATAATGTTACGAACTTGATAGGGTTGTCGATTGATATACGTGTTATCTTCTTCTAA
TAACTGTGCAATTTTCTTATAACCATGACCTTTAATGTAATAATTGAATACAGCCTTTACCGTTGGTGACTCATT
TTGATTGATGATGAATGCTCCGTTGTGATATTCGTAACCAAAGGGCGCATGGGTTGTAATCAATCGACCTTGC
TTTGCTTTTTCTTGAAGCCCATTTCTGACTTGTTCTCCAATGTTATCTGATTCAAGTTCGGCCAAGCTGATAAA
AATATTAAGCTTGAATCGGTCGAAAGCTTGATCCATATCAAAGTAACCATCGTGTACGCTTAAGATATGAACAT
GGTATGTTTGACATAATTTGATGAGTTTTAATGCATTTTTCAGATTACGATGTAATCTATTAAGACGATAACAAC
ATAATATGTCACATTGCCCTTGTTGAATTAATTGCGTCATTTGTCGATACCCACTACGATTATCTTTGCGTCCTG

ATTGTTTGTCGCTATAAAAGTTGATATGTTGAATATGATGTTTTTCGGCTATTGCTTCGATAGCCTGTTTCTGTG
CTGCAAGAGATTGTTGTTTCATCGTACTTTGACGTAAATAGCCTATGACTTGTTTCATATCGGCTCCTCCTTTC
ACAGTAATAATATATATTTATGGATGAATTGATATATAAGCCCAACATCAATAAGATGTTGGGCGTTCATATTAG
TCATTCATTTGATTGATTTCTTCAATTACCAAATCGGCTAATATCTCGATAAGTTCATCCATGTTTTTCACTCCGT
TATTTGTTCTATCTTCAATACGTCGATTATTCAGTTTGATGCTTCACGGTTGTATGATAAAGACAATCAGAAATC
TTCGTGAACTCCTGAAGGGCCTATCCCCTCATTAGCGGATTTAAAAAGTTCTTTCGCAGCTTTGTTATCATTTG
CCGGTGTCCAATTTTGAATTAACGACTTATCTTTAGTTAATCCCAGGATAGATGCAAACTCTACATCTAATTTTA
GATGGTAAAATACAAGTGATTGTTTTTTACCGCTATTATCTTTGACACTTCTTTTAGTTGTTTGGCGTCCACGG
TCAGCTAATATGAAACCTTTATCTCTTAAGGCGTTGACAACATTATTAACATCTTGAAATTGATGATTGTTTAGC
ATCTGTTTAAAAACGTTTGCAATCATTTTTACTTCGATATGGTCATCTTTTAATGAGATTAATCCATAGTTCTCA
AACATATTTTTCAAAGCACCTTCATCTGAAAACTTACCTCTGTTTTGTGCTACAAATTGAATGATGACATCGAT
AGCTTTATCAGCTAATGAGCGTTCAGAGATTGTATGACCATGATAATCAATAAAGTAGTCTCTTATTTTAGCGA
TATCAATATCTGTAGCTAAAACACGACCTAATATTTTTGCAGATGTTGTAATGACTGCATAACGCTTAAACATAC
GATTGCCTGTGTTGCTTTTATCATCTTTCAATTTAGCTTCAAACCAATCTACTTCCTTGTAAAACCATTGAATAA
CTTCATCTTCACGATTTATAAGATATTTAGCTACTAACGGTAAAACATGACCATAGTTTAGTGCTACAGCTTTTT
TAATATTGTCAGCATTGGTCGCATTTGTAGTGAATTGTTCATTAATCTCGATGGTTCTTACACGTAATCCATCGT
TTTGAGCTGAATCATTAAAAATACTGTGTTCTGACGTTGAAATGACAGAAGTACCCCAATTCTTAGGCGTTTT
AACTTCTCCATGAACGTTTGAACGTTGACGACCTTGACCTTCAGTGATGGAGTACAATAACCCCGTTGTATCT
CTAAAAGTTGCTGATGAGAGTTCATCAAATACAATAGGTATACCAAAATTGTTACTCAAGTAACCTTCAAGTG
CATTACGTGTGGCATTCCAATTTCTAAAGAGAGTTTCATTACCTTTGGTAGGGTTACCAGCGACTGATACAGC
TAAAGAAGCTGCAGTTGACTTACCGGTTGAGGATTGACCTGTAAAACTAAAAATGATTCCGGCAAATTCGGT
TTCATGTTTGTGCTTCAGGAAACTCGTCACTAAGGCAGAAATACCAAATACGACTGCTAATTCTAAAAGAAG
AGAACCTTTAACCTCTTTTAGATACATGTTAAACCAATTATCAAATGTTCCCCTAGGTGCTAAGTCATAAGTATT
CTCACAAATGGCGTCAGATGGAGATTTATTATCAAATTCCGTAGTAGTATAGATTTCATTTAACGATACAATAG
GACCAAACGGTGTTTCCAGTATACCTACCCCTTCATATAAGTAGGAAATGGGTAATTGGTTGCGCATTTGTTG
CAACGCATAACCTAAATCTTTTGTATATTTTTCATTAATACTAAATCCATATTTCATTAAAGAGGGCAGTTTTTGT
GTTGTTAAAATATCACTAGATTCAACAATTACTTTTTGATCCTCGTCTGTAATAATTACTTTTTCAGTGTTAGTTT
TAGGGTCAATAAACTTATTTTCGATAACGATAGGACCTGCGATTTCAACTTCAGTAGGCATTCCTCCTTTTTCT
TTGGGAGGCTTGTCTTTATACCAACCTTTTTTTGATTTGTATCGTGGTGAAGGATTAAATGAAGGGTTAGTTT
GAGTCATTAGCGAACACCTCCTTTCGAAGGGTTGCTGTTATAGTGTGGATTAGGACCTGTTTTAAGATAAACT
AAGTGACCGTGAGTATCCTTACCGATAATAATAAATGGAACACGTGGCGCATGTTTTACAAAATATGCGAACC
AACGTCCAACATTTTGTTGTACAGCTTTTGAACATTGTACATTTGCACGACTGTTCAAGTCATGAAATGTAAAT
TCTTCTCCTTCAGGTAAATTGAATGAAATACCTAATATTTTCTTTTTTAACTGCTCGAATTTATCTGATTGATTGA
AATACATTTAACTTCCTCCTACTAATGAACTAAGATAGGAAAATTAAATATGCGCACAATTAACTTTCTTAAGTT
CATTTTAGCCAAGAAGTTATCATTTGAAAGCTTGTAAAAATATGTTAGAAATTCCGTACTAAATTAAGATGGAT
TTTTGTTGACAAAATAAAAAACGCTGATTTAACAGCGCTTTAAATAAAAATTAATCTGAAGTTATATAAAAGTA
GTCAGAAGGAGTGGTGTTATTATCATTATAATAATCTAGTGCTTTATTGATTTCTTTTATTAATTTATCATGATGT
TCTTTTGTTATTTTCTTTGGATATTTGAGTTCTTTTTCTTCTAAGTCTTTTGGAGACACTAGGTCACTATTTAAA
ATTAATGATAACAGCATATTTTTAGGTGCCGAAGCTCTGGTGTCTCGATGAGTAATATATTTTGCTAAATTACTG
TTTATACTTACTTTTTCATTTTCTAAAAAGTTTGTACTATTTTTAACATGAGCATCAGCAATTTTGGTACTTATTT
CAGTGTTTAAATCGTCTATTTCATCTAAATAATATTCAAGAATCTGAAGTTTATCTTTAGAAGTGATACCTTTTT
CAGTGAAAATCTTAATAATGATAGATTTGATTCCAATTTCTATATTACTGTCTACTAGAAAATAGAGATAATAATT
AGTCAGAGCATGTTCATAATCTCTTTGATCAGTAATTTCATGATTGATGAAATAATATAAGTTCTCTTTGATACG
ATTAGAAGAATCTATATCTTTGTCATTGATACCGTTTTTAAAAAAGTGTAGATAAACTGAGTTGTTTGAGAGTA

TATCATATAATACAAATAAACTTTTTGGTTTAAATACATATGATTTTCCTGATTTAAAGTTTGCTATGTTTATTCCT
AGAGCGTTGCAGAATTTATTAAAATTAGTTGTAAAATTATTTTTTTCTTGAATAAACATGTCGTTGTCATTTAAT
TCTGAACTATGATTTTTAATCATATTGATATTTTCTTCAATCTTGTCACTACTTAAATTTTCCATGCAAAGTATACT
ATATCTTACTATATCACTTATATTTATGGAAAAATCTTTAGGAGAACGATTTAAAGTATCCTTTTTATTATCTTGAT
CTACAGCCCTACATGAATCATCAAAAACTTCTTTGACATTTATTTTATCAATGTTCAAAATTATGTCTACTAAAG
CATTTTGAAAATCATCGCTATATATGTCTTTATTATTCATAGTGTCACTACCTTTATTTTTAATAACACTATATATTA
TATCTTATAAACCTAATACCATGAAGAATAAAAAGAATGATTCAAATTTTATATTTAATCCATAGTTGGCTGCTAT
GGTTAGTGTACTAGTCTAGTTTTACTAGTATATAAAACAGTACTCAATTTTTATTTTTTCAAAATTATAAAAAAG
AACACCTATCATCGATAGGCACTGAACCCCTAAAACGGGAACTTAATAAAAACACCATGTTCTAGGCTATTAA
TTCTCTGTATTTTACAGGGGATAAGTAGCCTAGTTTTTGTTGAATTTGATTATTATTATAGTTTTTAATGTACTTT
TCGACAATATCTATTACAATATGATTAGAGCTATTAAGCTGATTATTGATGTAAAGAGTTTCAGACTTTAGCGA
GGAATGGAAACTTTCTATCGGGGCGTTATCGGCAGGTGTTCCCTTTCGGGACATACTTCTGATAATGCCTTTT
TCTTCGCATAATTGATAATAAGCATAAGATGTATAAACGCTGCCTTGATCACTATGTAATATATACCCCTCAGGT
ATATCGATTTGATTTAATGTATCATTAACTAAACGTTGGTCTTGTTTATCATCTATTTTATACGCCACAATTTCTCC
GTTATAAATATCCATTATCGAAGATAAATACAACATAGAATGATCAAATGGTAAATAAGTAATATCGGTTGTTAA
TACTTCTATGGGACAATTCGCTTTAAATTATCTTTGTAATAAATTGTCTGTTTTATAATACGGTTTACCTATCCTT
GTCGTCTTTTTAGGTCTAACTCGGCAGTTCAAATGATGCTTCTGCATCATTCTCTGTACTCTCTTATGATTAATT
GGTGATGTATAACATTGATTAATCAGTGCTGTAATCTTACGATAACCGTAGGTATAATGGTTAGCTTCACATAAT
TCAATAACTTTTTGTGTTACAGTATCATTTTTATAGGTTTTGTTTTTCCATCGGTAATATGTTGATTTAGGTATAT
TTAATACTTCTAGTATCAATTTGATTGAATAGTTTCCTTTTAATTGATCCACTAAATCTATGACTACTGTTGGTAC
CACTTCCTTTCCAATGCCTTGTACTTTTTTAAAATATCCAATTCTATATCTTTTCTCTTATTTTCTAATTTTAATTGT
TCTACTTCTGACAGCTCTTCTAATCCTTTACCGTAGGTATATTGTTTACCAACGTGTTGTGAAAATCTATAACTT
TCCCCATTTCGATACCATCGCCACCAAGTTTCCACTTGTGTTCTATTTTTAATATTTAATTCTTTCATAATTTCTTT
TGTTGAAAATCCTGCTGCTTTCATTTCAACTGCTTTATACTTTGTTTCTACTGAATAAGAAACTCTTTTCATAGA
AAAAACACCTCCGTATGATTCATTTTAATATGAATTCAACGAAAGTGTTTTTATATAATTCCCACTAATTGGGGT
CAGTCTACTATATGATACGGTTTTTTAATTTAAAGTATCAATAATAATTGGATATAGAGGGAAAGAAGCTATAAT
GATATTTGCTTACTAAGTGGATAAAGATATTAGAAAAACGAGAAGCAGGACAGTTATTAAATCGGTAGATATT
AATCATAAGTATTTAACTGAATTTTTACCTTTTGTCGAATATATAACTAAAGTTGAAGGTAGTAAAATATTTATCT
AGTCGATATTAAAACAATTTATCGAAAACGATGGTTTTCCATATCCTCTTTAGTAGCAATTTTCTAACAATATAT
AATTAATTATATACAAATTAAGAATAATTAAGAAGTATATATTAATTAGAGATAGTTTAAAAATTAGATTTCAATC
AATTAATTAAAACAGGTTAGACTATTAATTTATACTAACCTGTTAAAAGTAACAAAATTAAGAATATATTAAAAC
TTGTGTAATGAGTAAGCCTCCACCAAATATTAAAACAAACGCGACAACAGGCCATACGAAACGTAACCAGTG
TGAGTAGCGCACGTTTAACATTTGAAGTGTTGCCATTACAAGTCCAGTAGGCGCTAAGAACAACATTGCATA
CTGACCGAATTGATATGTTGTAACAATAACAAATCTTGGTATACCTACTGTATCAGCTAATGGCGCAAAGATAG
GCATAGATAATACTGCTAATCCTGATGATGATGGTACGATAAATCCTAAACAGAAAAAGATAAAGAGCAGAAC
AATGATAAATAAAGGCCCACTCATATGTTGCACGATAGATGATGAAAAGTGCAAGATTGTGTCAGAAATCATT
CCTTTATTCAATACTAAGTTGATTCCACGAGCTAAACCAATGATTAAAGATACACCTACTAAACTTGAAGCGCC
ATTAACGAATGCATCTACAGTGCCTTTTTCGCCTAAACCATATTGTCCTGTTCCAGCAATAAACATGATGACAA
TGGTAAAGATTAAGAATGCAGATGCCATGACTGGGAACCACCATCCTTGTGTCATAACACCCCAAACCATAAT
AGGGAATGGTAGGACGAAAAGCGTAAGAATAATCTTTTTACGTAATGTAAACTCAGAAGAACCGTCATCATG
GAGCACAGACCACTGTTTTTCAAATGCTGCTTTGTCTTCATAAGAATAAGAGGATTTAGGATCTTTTTTAATTT
TTTTACAATACCAGAATAAATAACTAATAACAAATATGGCACCGATGATACAAGCGCCTATTCTCCAATAAAGA
CCATCAGTAAAAGTTGTTCCTGCTGCATTAGAAGCAATGACGACTGAGAATGGGTTGATTGTTGAGAATGTA
CTACCCACAGAGCTTGCTAAGAAAATTGCACCGAAAGTAAGACTTTAGAATCTATGGGAAAGATAATAAGGT

TTAATAGAAATACAAGAAGTGTAAATAGTAAGCATATAATTTATACATTTTAATATAATATAACAAGAAAGGTAA
ACACAAAGATAATAAAAATAAAAATTTAATAAAGATAGAGAATTATTAAATTGTTTTTGATATAAAACAACTTA
ATAAAAAATTTAATTTTAGGGGAGGATAATTATAATATAAAGAAACATAGAATGGAATTGCAATTGGGAAGTA
TACGATTTTAACTGCGAATCATATGTTCAACGACTTACAAATGCGTAAATCGATAACACATTCAAAAATATATAA
TCTGAAAATTGATTTACTAAGAGAGGATTATTGTAGTTACAGGTGTTAAGTTGCTCCAATATGGAGTTGTCGT
CTTGTTTTATATTAAACAAGTTTTATTCAAATACATGACAATACGTAAAATAACACTAGAATTATCAATCACTAA
AATTAAAACAAATGTATCTATTCAAACTTTTCTTTAGGGTTTACTTGCTGAATAATTTAAAAATGAAGTATTAG
GTGTACATGCTTTCAGAATTGCTTTTGAAGAATATTAAAAGTATAATCTGAATACGATGACAGAATGAATAGGT
AAACTATGACAAGAAGTCATCAAGGTTATCAAATAAATATATCCCAGCGGTTGAGACGACTCACTGCTGGGAT
TTTATTGTTAGTTATTTATTCTAAGTGTTGCATTTGGTAGCCGATTCTAGGATGTGTGATGATTAATTTCTTTTCA
GTGTTTGGGTCTTGCGTATAATTCAACTTTTTACGTAAAGAGGCCATATGTACACGTAAGGCAGCCATTTCTG
TATGATTGACATAGCCATAGAGTGATTTTAATAACACTTGATAGGTTAATACTTTGCCGACATGATGGCATAATA
TCGTTAGGAGTTGGAATTCATTCGGTGTTAGATGTACGGACTGTTCATTGACAAGGACTGATTTCGCATCGA
AATCAATGGTTAATGGACCATTTGTGAAACGACTTTGAATTGTCTCAGTAGAACGTGACATACGTAATGCTAC
TCTGATACGTGCTCTTAACTCATCGATATTGAAAGGCTTAGTCATATAATCATTGGCACCGCGATCTAATACTTC
GACAATGGTTTGTTCCTCTGTTCGTGCACTAATCACAATAATAGGTGTGTCCACAAAGTCTCTGAATTGCTGA
ATGAGAGATAAACCATCAATATCTGGTAAGCCTAAATCTAATAAGATAATATCTGGTTGTTCTGTTCTTAGGCG
AAAGTCCGCTTCTTTCCCCGTCTTCGCCGTAACCACTTTATAATAATTCATAGTTAGCGCAACATCGATTAAAT
GTAAAATCGCTTCATCATCTTCAACGACCAATAATGTTGTTTTCATCCATGGCTCCCTCCATTTCAATTGGATTA
ACAGTCAAATAAAAATAAAAAATACTGCCTTGTGGTGTATTCGGTTGATATTCTAATTCACTGTTGTGTTGTTT
CAAGATGAGTTGTACTAAATAGAGCCCTAATCCCAAACTATCTTTTTTATTGTCTTTAAAGTTGTCTCCTGAATA
ATAGGGATTAAAAATCAATTGACGTTCTTCTTCTGGAATACCTTTTCCGCAATCTATCATTTCGAATTTTATTTT
GTTTGTTTCATGTTGAACGTGCAGTTTTATTTCAGAATGTGATTCTGCATGCTTTAAAGCATTATCGATAAGGT
TGAATAGCACTTGCAGTATTAATTTACTGTCGATATTAATGAGTGAAGCGTCATCCTCATTTTCAATAATGACAT
GATTTGCTTGGTGTCTGCGTATGAGGCCTTCTTCAAATTCTTCTAGAAGTTCTTCTACTAAATAAGGGGTGCG
TTGTATTTGAATGTCAGAGCTTTCTAACTTAGTCAAAGATAAAATATTTGTGACTAAGGTATGCAGATATTGTG
CTTCGCCATAAGAAGCAGTTAAGAGTTCTGCTTGTTGTTGATCGTTTAAATGCTCGTTATGGTATTTCAGCATA
TCTAAGTTGCCCATAATGGAAGTCAGTGGTGTTCTAATATCATGTGAAATTGAATGCAAGAAGTTTGAACGTG
TGGCTTCTCGTTCAGCTTTCAATATGGATTGTCTGGTTTGTTTTAATAGATCCACATTCTCTATTGCCAGGGTA
ATATCGTTCAACATAGAGTCTAATATTGAATTGTCATAGGTGTCGATGTAAGTTTCGTCGGTGAAGCGGATAG
AAATCACACCTTTGACTGGATTTGTGCCAATGGGAATACACAAAAATTTACTGCCAGGAAAGGTATCGGTTA
ATTTACCGGCACGGCTTTCATTTTCAATGACCCAGCTCAATGTCTCAGCATCATGTGTCTTATCTGAGCTTGAA
ATGCTTCTGTTTCCAAATGAGTTGGAAGCAGCGACTTTCTTACTTTGAATTAAGAATACCGTGACATCCTGAT
TGAGTAGCTGATGAATCTGATCACCAGCAATATTTAATAAGCGTTCAATTGAATAAGATTCTTTAATGGACTGG
TTAAATTGCAGCATAATATTGGTCCGATATAACTGCCGTTCTGTTAAGGAATGCTGATGTTTTAAATTCTTTAAG
ATGGCACTCGTAAAGATACTAGCAAAAATACTGGTCGCAAACGTAATCGGATATTCAAAGCGATACATTTCTA
AAGTGAATCTTGGCACCGTAAAGAAATAATTAAAAACAAACACATTTAAGATAGACGCAAAGAATCCGATTA
AATAGGATTGGGTCCAAATAGAAAGCACGATAATACCGATAAAGAACATCAGCAAGATAATGGCACTGGATT
CGCTTTTATCTAAGTTGTAAACCCATATACCGAGCAAGACACAGATCGTTTGAATTACAAGCATCTTCATAATC
TCTACAGTTAAACGAGAGGATTTTTGAGCTTGCTTAGGTGTATTTGTAGGTTTGTCTGAATGAATATAATGAAT
CGGTACAATTTCTAACTTGAAATGGTGTGGTACGTGATTAATTTGTTCAATGAGTGACTGTTTAAAGTAATCTT
TCCAACGTGGCTGTTCTGACTGTCCAAGGACTAGCTTCGTCACAAAAGCGAGATCACACCAATCGGTTAACG
CTTTCGCAATATCTTGTGCATACAACACTTTGATTTCTGCACCTAACGCTTTGGCGAGCATTAGATTTTTATGG
ACATAATGATCTTGTTTTCTGCTTTCTGACGGTGTCTCAAAGACATCAATATACACAGCTGTGAATTTAGCATG

TTCTTTATAGGCAGCACGTCTTGCTTCTCGTATGACCCGTTCGTTATAAATACTGCCGCTGATAGCTACCGCAA
TATGCGGTGTAATGTCGGTATGTTTAGTTTTATATTGTTGTCCTTTTTGACTCATAATATCTGCGACAGTTCTGA
GTGTAAGTTCACGCAGCTCTGTCAGATTTTCATACGTAAAGAAATTAGAAAAGGCTGTTTCTAAGCGTTCTTT
TTTATATACTTTTCCTGCTTTAAGGCGCTGAATCAACATATTTGGTGAAATATCCACAACTTCAAAGGCATCTG
CTGACGTAATGAATTGGTCGGGCACACGTTCTGTAACTTGAATACCTGTCATTAACGCAATTTGTCCGCTTAG
ACTCTCGATATGTTGGATGTTGAGTGTTGTCCAGACATCGATACCATGCGATAGAATTTCTTCTATATCCTTATA
ACGTTTTAAATGGCGCTCTTTTGAAATGTTTGTATGTGCTAGTTCATCAATTAAGACCACATCTGGATTAGCTT
CTATGATTTTAGAGACATCTATATAGTGAAAGGTGTGGCTGCCAAATTTACGGCTGGAGGTCGAAATTTCAG
GCAATTGTTGAACCAGTGCATTGGTTTCAGGGCGTTGATGGGGTTCGATATAACCAATTTTAATATCTGCACC
TTCTTGATACTGATCAATACCATTTGATAACATTTCATACGTTTTACCTACCCCTGGGCTATAGCCTAAATAAATG
GTAAGTTTCCCTCTTTTTTTATATGTACTTTCCATGAGGCACCCCCTCTTAATCACATCATAATTAATATACAACA
TATTATCCATTTATCTTTCTGTTTTGTTAATCATCTTTATAGTTTCTTTATAACTTTCCTTAAATGTTAATACTTCGA
TTAGATAACTCTTGTTAAGATTTTGATGAAAGTAAGGAGGGAGCGCAATGATTACACTATTAGCTGTCGTTGT
CATCGCATTAATTTTATTTTTATTTTACGCATTAATTTGGAGTGAAAAATTTTAACAGAGAAAGAGGGAAGCA
TCATGAGTATTGTGTTGTTTTTGATTGTATTTATCTTGCTCTCACTCATTGTGAGCCGATATTTATATTCAGTTGC
TTTAAATGTGCCATCTAAAATAGATGTTGTTTTTAATCCGATTGAGAAATTGATTTATCAACTGATTGGCACGA
AATTAGAACACATGTCTGGGAAGACGTATATCAAACATTTTTTGTTGTTTAACGGATTGATGGGCGGATTGTC
CTTTGTATTATTGCTTATTCAACAATGGCTGTTTTTGAATCCTAACCATAATTTAAATCAATCTGTATCGTTAGCC
TTTAATACTATGGCATCTTTTTTGACCAATACTAACTTACAGCATTATGCAGGTGAAACAGATTTAAGTTATTTA
ACACAAATGTGTGTCATCACTTTCTTAATGTTCACGTCAGCAGCGTCAGGTTACGCCGTATGTATTGCGATGTT
AAGACGTTTGACTGGAATGACAGATGTGATTGGTAATTTCTATCAAGATATTACGCGTTTTATTGTACGGGTG
CTCATACCTTTCGCATTGATCATCAGTTTGTTTTTAATCAGTCAGGGCACACCGCAAACGCTTAAAGGTAATTT
GGTGATTGAGACATTATCAGGTGTGAAACAAACGATTGCATATGGACCGATGGCGTCTTTAGAATCTATTAAA
CATTTAGGGACAAATGGTGGTGGTTTCTTAGGTGCGAACTCTTCTACACCTTTTGAAAATCCGACATACTGGT
CTAATTACGCTGAAGCTTTAAGTATGATGTTGATTCCAGGTTCATTAGTCTTTCTATTCGGTAGAATGTTGAAA
ACTAAACTACAGATTCATCCGCATGCGATTATGATTTTCGTTGCGATGTTTGTAATGTTCATCGGCTTTTTAGTG
ACATGTCTCTATTTTGAATTTGCGGGGAATCCAGTGTTGCATCACTTAGGTATTGCCGGTGGCAATATGGAAG
GCAAAGAAACACGTTTCGGTATTGGCTTATCCGCTTTATTTACAACCATTACGACCGCTTTTACTACAGGAAC
AGTTAACAATATGCACGATAGTCTTACACCGCTAGGCGGCATGGTTCCAATGGTATTAATGATGTTGAATGCA
GTTTTTGGCGGTGAAGGTGTTGGGCTGATGAACATGTTGATTTATGTCATGTTAACGGTCTTTATCTGTAGTT
TGATGATTGGGAAAACACCAAGTTATTTAGGAATGAAGATTGAAGGTAAAGAGATGAAACTCATTGCGCTTT
CTTTCTTAGTACATCCTTTACTTATTTTGGTTTTTTCAGCACTAGCTTTTATTGTGCCAGGGGCATCAGATGCG
TTAACTAATCCGCAATTCCACGGTGTATCACAAGTGTTGTATGAGTTTACATCATCTTCAGCGAATAATGGCTC
TGGTTTTGAAGGATTAGGAGACAATACGGTATTTTGGAACATTTCAACAGGCATTGTGATGTTGCTTGCACG
ATATATTCCAATCGTTTTACAAATTTTGATTGTATCTAGTTTGGTAAATAAAAAGACCTATCAGCAACATACTCA
AGATGTACCGATTAATAATTTATTTTTCAGCAGTGTATTGATTATCTTTATTATTTTGTTGAGCGGCTTAACGTTC
TTACCTGACTTAATGCTTGGACCAATAGGCGAACAGCTTTTGCTGCACGCATAGATAAAGGAGGATTAGAAA
ATGGCTGAAACTACTAAAATATTTGAATCACATTTGGTCAAACAGGCTCTAAAAGACAGTGTATTGAAGCTCT
ATCCTGTTTATATGATTAAAAATCCGATTATGTTTGTTGTAGAAGTGGGCATGCTGCTTGCCTTAGGATTAACC
ATTTATCCGGATTTATTTCACCAAGAAAGTGTATCACGGCTATATGTGTTCAGTATCTTTATCATATTATTACTGA
CACTTGTCTTTGCGAACTTCTCTGAAGCATTAGCTGAAGGTCGCGGTAAAGCACAAGCCAACGCTTTACGCC
AAACACAAACTGAAATGAAGGCACGTCGTATTAAACAAGACGGCAGTTATGAAATGATTGACGCTAGTGAC
CTGAAAAAAGGACATATCGTACGTGTCGCGACAGGTGAACAAATCCCAAATGACGGTAAAGTTATTAAGGG
CCTCGCAACAGTGGATGAATCTGCGATTACAGGTGAATCTGCACCTGTAATCAAAGAAAGCGGTGGAGATTT

CGATAATGTAATTGGAGGAACTTCTGTAGCTTCAGACTGGTTAGAAGTTGAGATTACTTCAGAACCAGGTCA
TTCATTTTTAGATAAAATGATTGGTTTGGTTGAAGGGGCTACAAGAAAGAAAACACCTAATGAAATTGCGTT
ATTTACTTTATTGATGACATTAACGATTATCTTCTTGGTCGTTATTTTAACGATGTATCCATTGGCGAAATTCCTG
AATTTCAATTTATCCATTGCGATGCTGATTGCTTTGGCTGTGTGTTTAATTCCAACAACCATTGGGGGATTATT
ATCGGCTATAGGGATTGCAGGGATGGATCGTGTGACACAGTTTAATATCTTGGCTAAAAGCGGACGTTCTGT
AGAGACTTGTGGTGATGTGAATGTCTTGATTTTAGATAAAACAGGTACCATTACCTACGGCAACCGTATGGCA
GATGCGTTTATTCCGGTGAAATCATCAAGCTTTGAACGTTTAGTTAAAGCGGCCTATGAAAGTTCTATCGCAG
ATGACACACCAGAGGGACGTAGTATTGTGAAATTAGCTTATAAACAACATATCGACTTACCGCAAGAGGTCG
GAGAATATATTCCGTTTACTGCTGAAACACGTATGAGCGGTGTGAAATTTACGACACGTGAAGTATATAAAGG
TGCACCGAATAGTATGGTTAAGCGTGTGAAAGAAGCAGGGGGACATATTCCAGTTGATTTAGACGCTCTTGT
CAAAGGGGTGTCTAAAAAAGGTGGCACACCGCTGGTTGTGCTTGAAGATAATGAGATTTTAGGTGTTATTTA
TTTGAAAGATGTCATTAAAGATGGACTCGTAGAACGTTTCCGTGAATTACGTGAGATGGGGATTGAAACGGT
GATGTGTACAGGAGATAACGAATTGACAGCTGCGACAATAGCGAAAGAAGCGGGTGTGGATCGCTTTGTG
GCAGAGTGTAAACCTGAAGATAAAATCAATGTGATTAGAGAAGAACAAGCGAAAGGTCATATTGTTGCGAT
GACGGGTGACGGTACGAATGACGCGCCAGCTTTAGCAGAAGCTAATGTAGGTTTGGCAATGAACTCAGGA
ACCATGAGTGCCAAAGAAGCGGCGAATTTAATTGATTTAGATTCTAATCCAACCAAACTGATGGAAGTCGTT
CTAATTGGGAAACAATTATTAATGACACGTGGCTCACTCACTACATTTAGTATTGCGAATGACATTGCGAAATA
CTTTGCGATTTTACCAGCCATGTTTATGGCGGCTATGCCTGCGATGAATCATTTGAATATTATGCATCTGCATTC
ACCTGAATCAGCAGTATTATCTGCGTTAATCTTTAATGCGTTGATTATTGTATTATTGATTCCGATTGCGATGAA
AGGCGTGAAATTTAAAGGTGCCTCAACGCAAACCATATTGATGAAAAATATGTTAGTTTACGGCTTAGGCGG
TATGATCGTGCCATTTATCGGCATTAAGCTCATTGATCTCATCATCCAACTCTTTGTCTAAAAGGAGGACAAAA
CAATGCAGACAATAAGAAAAAGTTTAGGACTAGTACTGATTATGTTTGTTTTATGCGGATTTATCTTCCCGCTG
ACTGTCACAGCGCTTGGACAAGTATTATTTCCAGAACAAGCAAACGGCAGTTTAGTGAAACAAGATGGCAA
AGTAATTGGTTCAAAGCTCATTGGACAACAATGGACAGAACCTAAATATTTCCATGGACGTATCAGTGCAGTC
AATTACAATATGAATGCGAATGAAGTGAAAGAAAGTGGCGGACCTGCTTCAGGCGGCTCAAACTACGGCAA
TTCAAATCCTGAATTGAAAAAAAGAGTTCAAGAGACTATTAAACAAGAAGGAAAAAAAATTTCAAGTGATG
CGGTGACCGCTTCTGGCTCTGGTTTAGACCCAGATATTACGGTTGACAATGCGAAACAACAAGTAAAACGCA
TTGCGAAAGAAAGAAACATAGATGCTTCAAAAATTAATCACCTTATTGATGAAAACAAACAAGCATCACCAA
TGGCAGATGATTATGTTAATGTCTTAAAATTGAATATCACTTTAGATAAACTCTAAATAAACAGGGAGTGAGG
TGAGACATCCATGTGGTTCATTAGCATTATTATTTTAATAGCATTCCTAATTATATTAATGATTGACGATTTAATT
AAAACATGACAACTTCACGCAAAACAATATGAATTGGATGTAATAGAGAAGGGAGTACCAAGAAATACTCAG
TTTATTGAGGATAATTATTAGGGGAGATTAGAGAAGAATTCTCTATTCACGAACATTACTTTAACAGTTGTAAT
CATTGGTTTCATCATATCGATTAATAGTACAGTCATCTTGAGATTTTCAACACACTATTATAGCTAGCTAAGATA
CTTTTAAATTATGACTTATTTTTCATCATTTACGATACATCATTCTCGATAACTTACTATTTGTTATGGTATTTATA
GATTTAAAGAAAGTGAAGGAGATTAAGCAATGTAACTTGGCTTAATCTCTTTTTGATTATATTTATTAAGGCAA
AGCTTCATTTCCCTTAAATCAAGTATATCTTCTGAATAAGCAAATGAAATCGTGACGCCAACTACAGTGATAAC
TTCGCCTAAAACACAATGAGTGTGGTGTTTTCACTTTTCATAAACTCCTCTAAGTTTGTGTTTTTAAACTGTGA
TGGATTTGAATGTTTTTGAATTACGTTGTCGCGTCCTTGGGATTGCAATCCAAGAACGTCAGCATAATCTAAA
TCAATTTTGATTTGGTAGAAGACAAGAGAAGTACTTTTGCCTGTATCATCTTTCGCATTTCTTTTTGTTGTCTT
TCGATCCCGATCGGAAACTAATTTTCCTGCATTATCCAAAGCATCAATAACATTTTTCGTGTCTTCGAATTGAC
TGTCTGTTAACATCTTATCGAATACATTTCGGATAATTTTTACTTCGATGTAGTCATCTTTAAGTGAGAGGATAC
CAAAGTTTTGAATCATATTAGCAAGACGTGTTTGTTGTGAAAACTTGCCCAGGTTTTCCGCAATAAACTGTAC
GATTGTATCCATCGCTTTATCTGCAAGAGAACGTTCTGACACCGTTTCATTATGATAGCTTAATAAATAGTTCTT
TATGCTGCTAACGTTTAACTGTAGGTCTAGTGATTGATTTAAAATATGGGCTGTTGTTGTTAGAGTTGCATAAT

GTTTAATCATTCGAATACCAGTGTTACTGGATTCAGTTGATAGTAATTGATAAAAAACACTGTCTTCTTTTTGA
AAAAGGGAAGTAACATCATTAGTGTTGTCTAATAGGAATTGAGCAATTATAGGTAAAACATGACCATTATTTT
GACTAACCACTTTTTTTATAGTATCTGAGTTTTGAGCACTTGATGTGAATTTTTCAGACACCTCAATAGTACGA
ACACGCAAACCATCATTTTTAGCTGGATTTTTGAAAATACTATGTTCAGAAGTTGAAATCACTGATGTATCCTA
GTTTTTAGGTGTTTTGACATCACCATTTATATTTGCTCTTTGACGTCCTTGTCCCTCTGCAAATGAATATAGAAG
ACCTGTTGTATCATGAAATGTTGCCGCTGATAATTCATCTAATACTATGGGTACACCATAATTTCCGCTAAGATA
TCCTTCTAGTGCATTTCTTGTTGCATTCCAACTCCGGAATAGGTTTTCAGTACCTTTTGTTGGGTTGCCAGCTA
CCGATGCGGCTAACATAGCGGCTGTCGATTTTCCTGTTGATGATTGGCCAGTGAAAGAGAAAATAGTTCCTG
AAAACTCGACGTTATTATGATACTTAAGGAAAGCAGTGACTAATGAAGATACGCCAAACAACACATCTAGTTC
TAAAGATAAGTTCCCATGGACTTCTTCTTTATACATTTGTAACCATTCTGATAAATTGCCTTTTGGTATTAAATC
GTACTTGTTATCACATATAATAGAAGTGCTATTAGTAATCTCATTAGAAAAATAAGGCTGGTCTAATGAAATTAA
TGGTCCAAGTAATGTATGAAGCATTCCTACACCTGAGTATATTGTTGATAAAGGTGTGGTGCTTCTCATTTGTT
GCAGTACGAACCCTAAATCTCTAATGTGCTTTTCATTAATGGTAAATCCATATTCAATTAAGGAGGGTAATTGC
AAAGCAGTAAGGATTGAAGCCTTTTCGACAAGCTCTATATTATTCAAATCCGAAAGAAATACTTTTTCTTTAC
GAGTCGCGGGGTCTATAAAACGATCTGTGAAAGCAATCGGTCCAGATAACATTATTTCAAACTCCGTACCCCC
ATCTTTTTTAGGAGGGATGGTTTTGTACCATCCCGAAGGTTTGAGCTTGAATTCGTCAGTTTGATATAGAATT
TTTTCCATTAACAGTGACCCCCTTGGTTGTAAGAGGAAATTATAATGATATGTCGGTACTTAAGTGGGTTAGTT
CCTATAATAATGAAAGGGATGCTTGGTTTGTACTTTATCCACCAAGCAAAGCGTCTTCCTGCTTCACGTTGATC
GTTGATGTTGCACTGTTCTCTGAATAACTGTAATTATAAGGTTTAGTAATGGCAATTTTTTATTTAAACAAATTT
TTACGAATGGATTGTCTGTTTATTAATTCAGCAACGTTTATTGAAGAAAAATAAGTGTCTTCTGATAATTGCAA
AGGGTTTAATTCAATGTAAACTGAATTAAGGGTAGGGTAGGTATGCCTGTTTTTTGGTTCTTTTAAGTTCAAT
CGATTAATAAAATTGTTGAACCTTTGTTCTAAGTCATACAAAAACATCAGTAATTCATTAGGAGAATGATTTAT
TATGCAATCCATATTTTCGTTTATGCTTGAAGTTATACATCTAAAAACAGTATTGGTTTCTTCGTATAAAAATAAC
TTATTAATAGAGGTAACATTGATTAAGACTGAGTATACGTATAATAAGAGTGACTCTGACATCTCATTTAGATAA
ATAAGACTGTGATCATTCTTATTTACGATTATTATATGTGATTGAATCAATTCAATAATGTCTAGAAGAAGACTT
GAAAAGTCGCTGTTAATCTTGGTCTTTGAAAAATCATCGTGACCATTTGTCTTATTACTTAGATGTAGTTGTTC
GTATTTTATAGTTTGGCGGTTGAGGAATTAAGCATTTGATTATTATAAACTATCTCAAAAATTAAATTACCTCCT
TTTTTCTTCCTCGATGAAGGTTTATCTAATCTTCCAATATACAGGTTTTTGAAGTAAATTATAGATATGATAATCA
TTAATTCAGTAAATGTATACTTATTAAGGGGTGGAGCTTTAGCTAACTTATCGATAGTACTGCCGATGCGTGCT
TTGAAACTATTCAAATTGTCGTGACTGTCTATATCTAAATCTTCAAATTTACTAGCAAGGGTTTGCAAAGTAGA
AATTACAACTTCTTCTTTTGTATAATATTTATTGAAAATATCATCTTCTTTATCATCGCTTTGGAGTTTTAAAGTG
CCTGGTTCTAAGATGTCAAATTTTTTGTTTCGATTGGTATTTTTTTAAAATGTGCCTTGTATTTTTGGGGTTGTT
CCAAATGTGTATTTGCTAGACTACCTGTAATTGATATTGCTTTATTTAGCATTTGGTGTGTATTAACTGTATCTGA
TTCTTCTGGAAATTTAAATCTAAAAGTAAAATCTCTACTACCGAATGTACTTTTTACGGCTACAGTTTTGTACTT
ATGCTCAGGCAATGTAATTAAACCATCTTGATGCAGGAGGAAAAGTGTGTCATCATTTAATTTCTCAATTGTAT
TAAATAAGTAATTATGCATAGCCTCTTTATACATGGCGAATTGATAATTTAGATTATCCCATTCTATTAGTTGATT
TAAAATACAATATAGACCTTCATTCAATCGGTTTACGAATATCAATACAGCTTCTTCTCGTCCAACAAGGCTTAT
TAACTCCTTCAAGTCATCCTTTAATTTTAATTTTTCTCCTTCAAAATTGTATTGAGTGAAGCCTATAGCCTTTTG
AAAAGTAACTTCCATGACAGAGTTGGTTTCAGAATATTCTAACGATTCATTTATTGCAAAAATCAAATTTTTTA
TAAATATGGAATTACTCATGGTTTGTAATTGCATATACTACCCAACTTTCTTATTTACTTTAAAACAAATATAAGC
TAAAGTAGTTATTATCATAAAGGGAACGTACGTTCTTATTTAGCTGATTTCTATGAAGAGAGGTTATGGATATT
GAATGAAGAAATCTTACGCGAAGTAGCAGATATTTTTATAGGTGATGACAGAGATAGCATTTATGATTATAAA
ACTGGGAACGAATTAGTGAGGTTTTTTAATCATTACTTTAATAAAGGGGACATATATCAGGCTCCGTTTCCATC
TAGATGGCTATATGTTGTGAAACATTTGCAAACTCTGATTCAGGAGAGAAAGATCAATCAATTTTTCACATTA

ATTTTAAGCAATCACTATATTAAGTATGAACTGAAAATTGACGAGGTTGAAGCAGCAAAGCAGGCTGCTAAA
GCACTTAAGTTGTTCAACAAAAGATTAAATCATTATGGGTACTACATAACAGGAACTAACAATGCTAGATATTT
TATGGATAAGGATGAAGATACGGAGTCTATTGGTTATGGAGGGTATGCGAATATTTATTTACAGAAGTCTACA
GGTCTTGCTGTAAAAAAATTGAAAGAGGAGTATCTTACTGATTCTTCAATTAAAAGTAGGTTTAAGAGAGAA
TTTGATCTCACTAAATCTTTTGATACAAATCCATTGTTCATTAATGTGTTTGAATTTAATGAATCAGATTATTCAT
ACACGATGGAGTTAGCTGATGAAACTTTGAAAGATTACATTGAAAGCAAGACAATTAGTGAGCTAGAAAAA
GTAAAGATAATAATGAAAATTTTAAAAGCGATGAGTCAAGCACATAGTGAAAATAAAATACACAGAGATATCA
GTTCTAAAAATGTATTAATGTTTAGAGGAAAAGTCAAAATATCAGACTTAGGATTAGGTAAAAACCTTGATGA
AATTCATTCGCATCAAACCTTTGATACAAACGGTGTAGGACAATATAAATACTGTGCACCGGAACAGATGTAT
AGTTTAAAACAAGCAGATAAACAATCTGATGTTTTTAGTTTAGGAAGATTGATAAATTTTATTATGACTGGAA
ATGTAGTTAACAACCATCACCTATTTAGAGGTGTATCTGATAAGGCTACGAACAGTAGTAAAGAATACAGATT
TGAAGATGCAAATGAAATGTTGAAAATGCTGCAGAGAATTTTAGAGTATCACAGTAGTGCAAAGCACGTCG
AAAAATGTCAAGAAAAGCTGAAAAGAGGAGTGTTTGATGATGAAAGCGAAGAATTTATTATGACACGAAGT
GATGAACAATTATGTCAAATGGTTCTAAGTTCTAATAATAATGAGCAAGCGTGTTTAATTCGTTATATGCAAAA
AAACGAATCTTCAGCATGTGATTTAATAGAGAGTATTAATAGAAAGTATCAAGAGTTTTGTGGAAGGTTTGA
AGACTACGATCCTTTTGCTAAATTAGCATATATGATTTTATGTAATAACTTCAGTTATAGAGTGAATGAAACAGC
AGCTAGAGTGCTAAATTATGTTGCTTGGTCTGTAAATAGATTTTCGGCACAAGACTTAATTAAAGGTTTAATTA
ATAGAGGAGTGGAGCCTTTGATTGAAGAAAAATTAAAGGACAATTAAAAAAACCTCATCATTAACTGATACG
CAGAGGCGTATCATAAGT
>Staphylococcus aureus strain ER00951.3
ATGAAAATCACCATTTTAGCTGTAGGGAAACTAAAAGAGAAATATTGGAAGCAAGCCATAGCAGAATATGAA
AAACGTTTAGGCCCATACACCAAGATAGACATCATAGAAGTTCCAGACGAAAAAGCACCAGAAAATATGAG
CGACAAAGAAATTGAGCAAGTAAAAGAAAAAGAAGGCCAACGAATACTAGCCAAAATTAAACCACAATCCA
CAGTCATTACATTAGAAATACAAGGAAAGATGCTATCTTCCGAAGGATTGGCCCAAGAATTGAACCAACGCA
TGACCCAAGGGCAAAGCGACTTTGTATTCGTCATTGGCGGATCAAACGGCCTGCACAAGGACGTCTTACAA
CGCAGTAACTACGCACTATCATTCAGCAAAATGACATTCCCACATCAAATGATGCGGGTTGTGTTAATTGAGC
AAGTGTATAGAGCATTTAAGATTATGCGTGGAGAAGCATATCATAAGTGATGCGGTTTTTATTAATTAGTTGCT
AAAAAATGAAGTATGCAATATTAATTATTATTAAATTTTGATATATTTAAAGAAAGATTAAGTTTAGGGTGAATG
AATGGCTTATCAAAGTGAATATGCATTAGAAAATGAAGTACTTCAACAACTTGAGGAATTGAACTATGAAAG
AGTAAATATACATAATATTAAATTAGAAATTAATGAATATCTCAAAGAACTAGGAGTGTTGAAAAATGAATAAG
CAGACAAATACTCCAGAACTAAGATTTCCAGAGTTTGATGAGGAATGGAAAAAAAGGAAATTAGGTGAAGT
AGTAAATTATAAAAATGGTGGTTCATTTGAAAGTTTAGTGAAAAACCATGGTGTATATAAACTCATAACTCTTA
AATCTGTTAATACAGAAGGAAAGTTGTGTAATTCTGGAAAATATATCGATGATAAATGTGTTGAAACATTGTGT
AATGATACTTTAGTAATGATACTGAGCGAGCAAGCACCAGGACTAGTTGGAATGACTGCAATTATACCTAATA
ATAATGAGTATGTACTAAATCAACGAGTAGCAGCACTAGTGCCTAAACAATTTATAGATAGTCAATTTCTATCTA
AGTTAATTAATAGAAACCAGAAATATTTCAGTGTGAGATCTGCTGGAACAAAAGTGAAAAATATTTCTAAAG
GACATGTAGAAAACTTTAATTTTTTATCTCCTAATTACACTGAACAACAAAAAATAGGTAATTTCTTCAGCAAA
CTCGACCGCCAGATTGAGTTAGAAGAAGAGAAACTTGAACTCTTAGAGCAACAAAAGCGTGGATATATTCA
GAAGATTTTTTCTCAAGATTTAAGATTTAAAGATGAAAATGGAAACAGTTATCCTGATTGGTCTATTAAAAAG
ATTGAAGATATTTCTAAAGTTAATAAAGGGTTTACTCCAAATACAAAAAATGATAAATACTGGGATGAATTAAA
TGAAAATTGGTTATCTATAGCAGGTATGACACAGAAATATTTGTATAAAGGAAATAAAGGAATTACTGAAAAA
GGTGCATCAAAGCATGTAAAAGTAGATAAAGATACTCTAATAATGAGTTTTAAATTGACTTTAGGTAAGTTAG
CTATAGTAAAAGAGCCTATCTATACAAATGAAGCTATATGCCATTTCGTATGGAAAGAAAGTAATGTTAATACT
GAGTATATGTACTACTATTTAAATTCTATAAATATAAGTACTTTTGGTGCACAGGCAGTTAAAGGAGTAACATTA

AATAACGATGCAATTAATAGTATTATAGTAAAGTTACCAGTGATACAAGAACAAAATAAAATAGCATACTTTTT
CAATAAATTAGATAAATTAATTGAAAAACAATCTTCTAAAGTAGAATTATTAAAACAACGCAAACAAGGATTT
TTACAGAAAATGTTTGTTTAATTCTTATAAAGTTCTATTATGTAAAATATTAAATAGAGATAACATTATGAAAGC
GAGCCCAAGACATAAAGTTTTTGAATAAATAAAAAAGATAATTTCTATCAAATTAATATAGAAATTGTCTTTTT
TATAAATTTTTTGATTATTTTTAGCTGATTGAGCTGTTACTTTTCTTATAATAAGTGCTATTAGCACAAATCCTAG
TTCTCTTTTGGCTTTGTTTATTCCTCTTACGGACATTCGAGTGAAACCCATTTTAATTTTATTAGAAGTAATTTA
GGTTTGAACCCACCTAAATAAATATATGAGTTATTTTTTTATGCTACAAAATATATTCAGATTTCAATAATGACAT
AAAATAGGCATCTTTATATTTACCTTTAGTGTAGAATTGCTCTTTGAGTAATCCTTCTGTTTTAAATCCTTGTGA
CTCGTATATATGCACAGCTTTTTTGTTATCTGTATCAACATATAGATAAATTTTGTGCATGTTTAATATATCGAATG
CATAATTTATCGCTTTTTCGAATGCGAATTTTGCATAACCTTTACCACTGAACTCAGGTTTAATAATTATTTGTAT
TTCACAATTACGATGGATGTAATTAATTTCTACTAATTCAACAATACCTACGACTTGATTTTCATCTTCAACAATA
AAACGTCTCTCTGATTCATCTAATAAATGCTTATCAAATAAATATTGAAGTTCCGTTAAGGATTCATATGGTTCT
TCAAACCAATAAGACATAATAGAATATTCATTATTTAATTCATGAACAAAAAGTAAATCACTATACTCTAATGCT
CTTAGTTTCATAATTCCACTCCCAAAATTTTCTCATATATTTGCATTATAAATATAAATAACGAATAAGTCATCATT
CACTGTGAATACTCTATTTTAACAATTCACCACATACTAATTCTCATTTTCTTGTTATTCTCGATTTATTACTCTTA
CTATGAAACCTATAAAATTCTCACATTTGTTTGTATTAAGAATAAATACGTCGATAGTAACAATAAAAAAATAAA
TAATAAAGCATCCCTCACCGTAAAAGTGAAGGATGCTCTAGTTTTATTGAAATATACATTTCATTTTGTTAAATA
ATTATTAATAATATTTTGAAAATCATTATTACGTGAAATCTTCATAGATTTTATCAAGTATTTCTTTGCCTTCAATT
GCTGTGAAGTGATGTACCAATCTATTTTTACAATCATATGTAATTTTGTGACGCTAGGTAATTAGTAATTGTTCG
TCAGTCTGATTGTATAGTATCAAGTTTCATAGATAATACTCTTTGATTTTAATGTCCACTTTGACGTGCTTTAAG
ATTGAGTATATACATAATGTCATTGTGGAATGTTAAAAATCCTACAAATGTTTATTCATCTGCAGGATTTTTAAA
TCTCCAAGAATAAAAATCATCATAGGACAACTGGATTATTGTTTGGATAAATAACGTAAACAATAATTAGGTAC
TATTATTTATTTTTGTTTATTCTTTTTCCTAACAAAATAAAGAAAAGAATAAACGCAATTGTTAAAAATATGTGT
CCTAAACCAGCAATACCAGCAATAGCAGGACTTACACTTAGATCTTTAATGGTAGAAATACCGTTCACGAATT
GCATTGCCACAGTAACAAGCACACCTAAATGGTATATATAAAAGAAACTGTTAAACAGCTTTGTATGAGTTGT
TAATTTGAATTGGCCCTCGATAATCATGAAAATTAAGAACATAATTGTACCTAGTACTAATAAATGTGTATGTGT
AACATTTAATTGAGAAAAACCGCTAAAATCTTCCGCTTTTGTCATTTCTCTATAAAATAGACCACTTAATAACC
CTAATAGTGTATAGAGCGCTGAACTATACATTAATCTTTTCATTTTAATTCCCCCTATTTTTAATTACGAGATAAG
TATAGCGGTAGTTTATGAACTGAGTATGAACTTACAACAAAAAAATTAATGAAGTACTTTACAATAAACTCAAT
TTATTAGATGGTGGAGGGACGAAAAAGGATTTTAGAAAAATAAATTAATATATTTTTATTTTGATAAGTAATAA
TTAATAATATCTTGGAAATCATTGTTAAGTATTGTTGTAATACAATCGTCATTCATAAAATCTTCATAGATTTTATC
AAGAATTTCTTCATCTTCGATAGATGTGAAATGATTAGCTAACCCTTTTATAATTTAAGTGTAATTTGTGAATCT
AAACAACTAGCAACTGTTCGCCAATCTGATTGTGTAGTGTCATGGTTCATAGATAATCCTCCCTTTATTTTAATG
TCCATTTTTGACGTGCTTTAGGGTTAAGTGGATGCATAACTTCGTTCGTTACTGGATTAATGAGTTTTTTGACT
TTCTTTTTATGAGGTTTTAACATTTCCATCACTTGCTCAACACGTTCGACGACAACAGGCCGCTTCGCATAAG
CTCCATAGGCTAGAATCACTGTGTCACTTTCACTAATCGCTTTCATTAAGTGAATGTCTGTGTGTTTGTCATAA
GGCTCTTTAATATGTTTAAGGTTTTCAGGTGTTTTAATATTAGAGAATAGATTTACTAGATATACAGCACCGTAT
TGTTCAGAATTAGCTAATTGGTTGAGAATGAGAACAGTGGTAAGATCGAGTGATAATACGCCATCTAAATGTG
GATACATCGTTATCACTGTACAAGAGGGTTTCTTTTCATCCCATATTTTCTTGAGTAAGTAGCGGTGTTGTTCA
TCGTCGCTAAATATAGCTTCTGTGTGTATCGTACTTTTGATTGTATTCATATATCGTTACCCCTTTTAATATTCTTC
TGGCAAAAGCATCACATAATAAAAAGCGTCTACGTCATCTTCACGAATGACGTAGACTTTCTTAGGTAATGCA
TTTTGATTTTTTACATAGTTTGTATAGTGATATTCCAATTTGTATGCAGGTTGTTCTTGTTCATGTGTGATTGAG
AGTATATTCTCATCTTCTTGTAGTCTAAAAATGTGTAGATAATCTGTATGAGATTGATTATCTCTTTCTTTTACCA
TATTCCAAAGTAGGATTTGAAGGTCTAGAGATAGTTGTTCACTTATGCCTCTTGTGATGTATCGATTGATTTTC

ATGTTATTTTACCTCGTCTTGAATTTCTTTCATAATGATAATTGCTTGGCTAATAATCGTAACAGATATTTGTGCC
ACTTTGATCCAATTATTCATGGTTATTCTCTCCTTGTTTTAGTAAATGACGTTCATCGATAATCGTATTTTTAGTA
TCTGTGAGGTATAGAAAGTCCATATCAAAATGATCCAAATAACCAATGCTGATGAGTTGGTCATTGGCGTACA
TAAGAAATGGATAGATACTTAGCTCATGTAGCTCATCATTGTAGTAGGTATAAGTGTTGAGTGTGAGATGCGC
AAGTAGAGAATTATTAATAAAACGTTCCGGTAGAATATTTTCTGCTGCTTCCTCAAGTGCTTCACATTCCCATG
TTTCGTTGTTAGATAGTTGGAAGAGACGAGTTATATATTGTTTGAGTTCTTGAGTGGTTGTTTTCATATCATTG
CCTCCTAGATAGTGTGATAGTGATGTAGTTGATATACATCATTGGGATAATATATATTTGATTTATTATTTATTACG
AATCCCGGTGGGAATAAGAGAAAATTCCATATAAAAACCCGTGATAAATGTTGGTGTAACAAGGAAATCCCG
GAATCCCACTCATTTTGACGAACAATCAACTCATTATTTATAAGTATTGATGATAGGGTTGTGTCTCTGCTTCCT
TATATATATTATTTATTTATAAAAAATAACGGGATTTTGGGATTGTGCTTGCACAATCCTTCTGCTTCTTCGAATC
TGCAAATCCCATTCCTTTCCCGGTAAAAAATCATTGTGGGATGTTCTTTAGCAATTTCAATATAAGCATTGTGT
AGTCATGAAAAAAATGACGGCAATGACTGTTTCATTAGATAAGTGTTATTGAAATTGATAAAGAGAATTCTAA
AAATGGTTAGATAAAATAAGTGAAAGAATAGCAGTGTAGTTATTGTTTATTCAATAGTTATATATAAAGTTTGT
GGCAAAAATAAAGACGAAGTGCTAGGGAGCACTTCGTCGAGTGGATGGTTATTAAATAGTTGTTTGATTATA
TCATTATTTAACTTGAGCGTAACATAAAATTGCTTCTTATGATGCTCATCTTTTCTTATGTCAATGCGATCGATG
ATTGTTAGATATAGTGATTTCAACTGAGATTTCTCTAATTTGTCTATATCTTTGAATATTGCTTGTAGTACATTCG
CAATCATATCAGCATCATAATGTGGAGCTTCTGCTTGTTTATCTTGTTCTAGTTGATAGATTTGATTATTTATTTG
ATTCAATTCATCTTGGTAGCTAAGTATTGTTGGTTTTAATACGCTATCTAAGTCAGGTGAGTCTTCAATAGTTTT
CGTTAATGTATGCATTTTTGCTTTTATTTCTTCACATTGTGACTGTTTATAGGCAATATCATGGTTCAAAGAAGA
GACGTCTATTTGACTTTTTTCATTTACCTTTTCAACCAATTGCTTCAATACCTTTTTGCTTTTGATAATTTCCAAT
ATCTGATCCATAACATATTTTTCTAGTACATCTGCTCTAACGCTATTGGCAGAACAAACTTTTGAACCTTTATTT
CTAAAATTGCTACATGAGTAATATCTGATTCTTTTCTTAGTGCCATCTTTTAATGTATTAGTTGTATTACTTGCCG
CCATTGCTGCACCACATTTCGGACATTTTACAATCCCAGTCAGTAGGTTTGTTCCTTTGCCGTGAACTTGTGG
TTTCTTGCGACTCTCTTGACGTTTAAACTGTACTTTATCCCATAGGTTTCTATCAATAATAGGCGCATGTTTACC
ATCAGCGATAATCGGTTCCTCATTGAGTCCTTTTCGTCTTTTATCGCTCCAGTGTCTATACTTCGCAAACTGTAT
CTTTCCAATGTAAAAAGGGTTTGAGATGATGTAGGTAATGGACGAAATACTAAAAGGTTTCCCTTTCTTAGTC
ACATAACCTTTATGATTCAATGCGTTCGCAATCTTACGATAACCATGACCTTTAGCGTATGAATCAAAAATATAT
TTAACAATATTCGCTTCATGTTGATTGATCATGAGCTCTTTTTTACTGTCAGGTACTTTATCGTAGCCTAGAGGT
AAATTACCTTGATAATAACCTTCAATAGCACGTTGTCTTTGGCCATTGTAGACATTCTCTACAATCGTATTACGT
TCAAATTCTGCGAAGCTGGCTAAAATTTGGAGCATCAATTTACCTGTTGAACTGGCAATTTCTATTTTTTCAGT
TAGACTAAAAAATTCGACATTAATCTTATACAATTCCTCGACAATATTTAACAAATCTGAGGTATTTCTAGCTAA
ACGATTTGTTTTGTAGACCATAATACAATCTAATTTACCTTCGTTGGCATCTTTTAACATACGTTGTAATTCTGG
ACGTTGCATTGTTTTACCTGATATACCACGATCGGTGTATTCATTGACGACTTCATAGCCTTGAAATTGACAAT
ACTCTGTAAGTTGATTCAATTGACCTTGAATACTGTAACCATCCGTTTGCATTTCAGTCGATACACGTGCATATA
ATCCAATACGTTTCTTTTTGAGTTGTTTCATATTACTTCATTCCTTTCAGGAGTTATTAAATGTGATTGTTCAAC
GATATTGAGTGGACTGTTTTTAAAGTAGATTCCTTGTAATTGTTTAGTTTGAGTTATTTTGATGCAATCGATAA
AAGGTGCTATATCCTGTAAAGTTATTTTATTTTTTATGACATATTTTAATTTGTCATCGATTTGGTAGGTTGAATA
AGTAGAGTAATTCTCTTCACTTTTACAACTAGCAGATAAACGTTTGAACGTTTTTACATCAATACGATTTTGAG
CTAGCTTTTCGATAAGTTGTTCTTGTGTTAGATGGGTTTCTTTATGCTTCATTTGTTGCTGTTTTAGGACTTTG
AGTATTGTGTTATTCAATCGTTTATGAAACGATTGTTCTTCAAAATACTTTTTACAAGTGTCGAGCACTTCACT
TTCAAGTTCTGGTGCATTGATACTTTTAAATGGGCATGTACGATAAGCGTCATTCATATTTTTAGGACAAACAT
AGTAACGTAGAGAATAGTTCTCTTTTTTTATCGTTAAGTTTGTTAATGTTGATTGACAGTAAGGACATTTGATT
CGTCGCTTTAGCTTATTTCTAGAATTGGATCGATTGAGTTGTTTATGAATACGACGCTCTTGTGCTTCTTCAAA
TGTATCAATATCAATAATAGGTGGAACGATATCATTAAACGTGCCATATTTATTGATGACACGACCGCAATAGTT

AGGGTTCAAGAGAATATTTCTAACTTGATAGGGCTTACGAGGAATAAGGTTAGGATTACTATCCAAATGTTG
GGAAATCTTTTTGTAGCCTAGACCTTGTAAGTACCAGCGATAAACCGATTTAACTGTATACGCTTCTTCTTCTT
GTACAACAAAACAACCTTTTCTATAACGATAGCCAAACGGAGCATGAGTTGTGATTAGCTTACCTTGTTTGGC
TTTTTCTCTAATCCCATTTTTTGTTTGTTCGCTGATATTGTTTGATTCCATTTCCGCTAGACTCATAAGTATGTTC
AAACGAAAGCAATCAAACTCTTTAGACAAATCAAAATATCCATCGTTAACACTGATGATTGTGATGTGATGCT
TTTTACAAATTTCAAAGAATTGTATGGCATTTTTCAAATTACGATGTAGTCGGTTCAAGCGATAGCAACACAAT
ACTTTACATTTTCCAGACGTAATTATTTCTACCATTTTTTGATAACCTGAACGTTTTGTATGTCGACCTGTTTTC
TTATCATCATAAAACGCCACATTAGACCATCCATATTGCTTAGCGGTATCCATAATGAGCGATTTTTGAGTAGCT
AAGCTTTGTTGTTTGAGTGTACTTTGACGTACATAAGCAATCGCTTCTTCCATGTTATACACCTCCAAAAAGAT
AATATATATTTGTGAGTAAATTAGAATGAAAGGTCCAACGTGCTTTTAACACGTTGGACCGTCGTGATTAGTT
ATCGTTCTGTAGCTCTTCAACGACTAAATCAGCTAGTAATTCAATCAATTCATCCATTTTATCTACTCCTGTACG
ATTTCATCTTTAAGTTATTAAAAATCAATTAAATCATCGTTCTGTTTTTTCCATGATTCAAGTATCTTTTTGTTATC
CAAGTTAATTTCAGCTTTAATAGGTTCAGCATCTTTAGTTAGACCAAAAATAGACGCATATTTTGAATCTAGCT
TTAAATGGTAAAAGACAAGTGACTGTTTCTTGCCATAGTCATCTTTAACTGCTCTTTTCGTAGTTATTCGATCA
CGGTCAGATTCGATAAATCCTTTATCCCTTAGTGCATTCACAACATTGTTGACATCTTGGAAATGATGCTCTATC
AACATATTCTTAAATACAGATGCAATGATTTTAACTTCGATATAATCATCTTTTAAGGCAATTAGTCCATAGTTCT
CAATCATATTCTTTAATGCTGTATCATCAGAAAACTTACCACGGTTTTGTGCTACAAATTGCGTAATGACTTCA
ATCGCTTTATCAGCCAGTGATCGTTCAGAGACTGTATGAGCATGATAATCAATAAAGTAGTCTCTTATTTTAGC
GATATCAATATCTGTAGATAAAACATGACTAAGAATCTTGGCCGAAGTAGTGATAGCCGCATAGCGCTTAAAC
ATACGAATACCAGTATTATTTGTTTCATCCTTCAATTTATCTTTAAACCAATGATGTAATTCAAAAACGGATCAA
AAAACAAAAGGCCCTAGAAACTAGGAGCACACTTACACAAGAACAATTGATAGATAAATTAGCTAAAGGAG
CCATCGATGCAGAAACGTTCAGAAAACAATCTCAATCGTTACTTCAACAATCAAAACCAACACTATCAATAAA
TGAGCAACAAATTCAAAGGTCTTTTGAAAATGTAATTCAACAACACTTCACGTTAAGCATGTTATACCGATATA
TTGATGAAATTCATATTTCTAAAAACAAAAGCCTTGTGGGAATCTATTTCAAAAATGAACCGCTAAACATTGTA
AACCAAGCTACGCAATCATCGATTGCTTAATTAATGAAAGGATGAAAAATATGAAAGAAATGAAAAGAAAAT
CTGTAGGATGTTATGTTAGAGTTTCAACAATTTCTCAAGACATTGATAAATTTAGTATTAATGGTCAAATTACA
CAAATAAAGGAATATTGCCAACAGGGAAATTATGAATTGCTATACTGATAATTAAAAGTACCCCCGATCGAAC
GAAAATATCTTTTTGATGACTATTAGTGATAATTGTAATTTTATCCATTAATAGATGAATGGATTGGAATAATACT
AAGCCTATTGCTATTCCACCGAGTAAAATTACCCACCCGCCTAATTCAATACTTTCCGGAATCATTTCAAAAAA
TAATAAGCCAATTATAAGGCCTGCACATAATGAATAAATAAAGCCATGACCACTTTGAAAACCTTCCATTATCC
AAGCGAGTCCACCGCCAACACCAATTCCAAGTGCGGAAGCTAACGCCCGATCATCCATACTGCACTCATCCC
ACTATTCTTAAAAAGGCTTTGTTTATTTTACTCTAAAAAATCAAAACAAAATATATGAATTCATATTAAATATTAT
TTGACTCTTTATTCAGATGGTTATATCATATATATGAACATATATTAATACATGTAGATAAATAGTGATTATTATATA
TAAGGTTTTGAATTGAAACCTAAAGTGAGGGAAGGATAATGGAAGAAAAAAAGGAATTAGAGGAAGTAAA
TAATAAGGACTTAGATGATGAAACATTATTTGTCGTATCGCAAACATTTAAAGCGTTAGGTGATCCTACGAGA
ATCCGGATTCTCCATTTGCTCTTTTATAAGGAGTATTCGGTAAACGGTATTGCTGAAACGCTACATCTTAGACA
ATCAACAGTTTCCCATCAATTGCGGTTCTTGAAAAATTTACGGTTAGTAAAATTCCGAAGGGAAGGCACAAC
ATTGTTTTATTCCCATGATGATGAACATACTATGAATATGCTAAAACAGGCGATCGATCACGCCTGTCATCACTA
GTATTCCGTAAACCAGTAATGAGTGTTAATAATAATTCACGTCATTAATATTGTTATATGATCATATAAAAATATT
ATAGGCATATGAAAAGGGGGTTTTGCAATGGATATGGAAAACAAAAAAACAGAATGGAAAGCGTTGTATGA
TATCTCGAAGGAAAGTGAGATGGGTGTAGCAGAGAGGGTTAGTGAATACGGTTGATAAATTCATAGGATGTA
TTATTGGATACAAAAAGTTTGGATGTGGTCAATTAACGTCCTGGTTTAGTAAAGCTTACTGCGAATATATTGG
AACCCGATATATCATATTTATTTAGAAAGGTGATAGAAATGAAAAATATTCAAGAGCAACAAGCACACGAAAG
TCATAGCCACGATCATAGTCATGATCATGATCACGGAAAAATGCCAATTATTTCATATTTTATTGGCTTAGTGTT

GGCTATAATTGGGCTTTTTTTAAGTGATGCAAATTTATTAATACAAAACATCTTATTTTCAATTGCCACAATCAC
AGCCGGCTACCATGTAATTATTCTCGAAGGAATTGGAGAGACAGTTGAAAATACTAAATTAAAGGGAAAATT
CACTCCTAATTCTCATATTCTAATGGGATTAGCTGCAATCGGGGCTTCTCTGATAGGGAGTTTTTGGGAAGGA
ACCCTTTTGATACTTATTTTTTCCGGCGCTCATTTTCTTGAAGATTACGCTGAAGGAAAAAGTAAAAGAGAAA
TTACTAAGCTACTCGAAATGAACCCAACGACAGCTAAATTAATCCTACCTGATGGAAACACAAAAATTGTTGA
TGTCAGTGAATTAAAAGTTGGAGATCAACTCCAAGTGCTGAACGGTGATCAAGTTCCAATTGATGGGATTAT
TTTATCCGGTACTACCTCAATTGATGAATCTTCTATTAATGGAGAAAGTATACCGAAAGAGAAGTCTAAGGGT
GACGAAGTTTTTGGAAGTACGATTAATGGAACAGGTACTTTTACTATGGAAGTCACTAAGGAAAACAAGGA
TACTGTATTCTCTAAAATTTTACAATTAGTTAGTCAAAACCAAGATAATCAAACAAAAGCTGCCAGTATCATTC
AAAAATTCGAGCCTAAATATGTTAATATAGTTTTAATCGCAATACCATTAGTAATGTTACTTGCTCCTTTTCTATT
TGATTGGACATGGTCGCAAAGTGTATACAGGGGATTAGTGCTTTTAGTCGCAGCTTCACCGTGTGCTTTGGC
AGCAGCTACTGTATCTGTAACATTGTCTACAACATCTAACCTAGCTAAAAAAGGCGTGCTTTCAAAAGGAAGT
ACTTACCTATCACAATTAGCGGATATAGATGCAATTGCCTTCGATAAAACAGGAACCCTTACGAACGGAGAAC
CTAAAGTAACAAATTACTATTTCACTCATTCTGTGAACGAAGAAAATATTATTGATATTATAGTCGCCCTTGAAA
AGGAATCCAATCACCCACTCGCTAATGCTATTTTAGAAAAATTTGAAGTTAAAAATAAAATAGACATCGAAGT
TACTAATCAAATTGGAAAAGGTCTGACAGGAGATTATAATGGAAAAAATTATCGTATTGGTAAGCCTACTTCT
TTTGAAAGTGCTTCTGAAGAGTATACCCAGTTCAATCATGATTGGGCATCAGAAGGAAAGACGGTTGTATAC
GTAGCAGAAAATGAAGAAGTTATTGGGATTATAGCTCTAATGGATATTCCGAATGAGCATGCTAAAGAAACA
ATTAATTACTTTAAGAAACTTGGTATCCACACGACTTTAATTACTGGTGATTCGGAAATGACGGGAAAAGCTG
TAGGCGAACAATTGGGAATAGACGAAGTTATCGCTAATGTAATGCCTGAAGATAAATCCAGAATTATAGAAG
AACAAAAAGAAAAATTTGGAGTTACTGCCATGGTTGGAGATGGTGTGAACGATGCACCGGCCCTTGTTAAT
GCTGATGTTGGTATAGCTATGGGGGGCGGTACTGATGTGGCAGTAGAAGTATCTGATTTGGTTTTAATGCAG
AACAATTTATCTAAATTAGTACAGTCTCATAAAATTTCCTCAAATATGGGTCGTGTTATTAGGCAAAATATTATT
TTTTCAATGGCAGTTGTTGCCTTTTTAGTTGTCGTTAGTTTGTTAGGATTAACTGATATTACAATCAGTGTAATT
GTTCATGAAGGAAGTACTTTAGTTGTTATACTAAATGGACTTCGATTATTAAGATCTAAATAATGAACGAATCG
ATTGACATGAATGAACTTTGAAGTGTGGATTCTACAATGTTCCCATAACATTGGACACTAAAAAACAGAGCA
ATCTAATAAAGATGTTATGAGTAAAAACAATGCCTTGCATACCGTTTTTATCATACGGGCGGTCAAATAAGCAA
TTAAAGAGCATGGGAAGCATAAAATCATAAACAGTGACTTAAGGCAGCCAGTTTACATTCAAAAGTTAAATT
GACTGTATTAAAAGTTTCGAAACGATTAATATCCGTATGGGGGCAAGCGTCGAGCCAAAGATAATGCCAGGA
CAGAGCGTCTTTTTCCGTTCTTTTAAGTGGGAAAGGTTTTACCTTCTCTATGCCAAGACAGTCCCAGAGCTA
AAAGGGCCAGTTGCTCTTTTTGGGCGTTTGTGAACGGTAATCGCAGGAAGGGGATTTTCCCAACCGCACCT
GGTTTTCCATCACAATAAAGTTGGCGGCATTTGTTCTGATATTCTTGGGAATACTTACACTATGAATGGCCGTA
TTAAGTGATACAGGAGCTGCTATTATTGTAATACTGAATGCTCTCCGCCTTTTGAGGGTAAAAGAATAAAAGT
AAGGATAACTAGGTAAAGCTGTTCAATCAAAAATTGAACAGCTTATTTTTCATCAAAATCAAAAACGTTTATT
ATAATACCTACACTTGTTCTGTTAAATGTACCGATTTTTTAACCTTATTGTATCAGTAATATCTTGAAACGAAGTA
AGCGACTAAAATTTCTCTTTTATCATACGTTTATAAAAATACACTTTTAAGAACGGTTTGAAAATTTTTGAAAT
AGATCAAAATAATCTTCTGGTTAAAAAACCTGTAGATATGATTTCTCTCTTAAATTTTTGTTTGATTAGATTAGA
CCCTAATAAGACGCATTCAATATGTCTACACGTGAATTTAGTCTTTGAAAATGTAAGGACCATTATTATTATAAA
AACCCCAGTATAAAACGATACGCTGAAGCGTACCACAAATAAAACTAAAAAATATGAGAAAATTATTAAATTA
GCTCAAATCTTTGAAGAATAAAAAGTGAATATTAAGTTTGATAATTTAGGTACAAGTAAAGATTAAGAATTTC
CATTATTTAATACATGGTGTGTAAATCGACTTCTTTTTGTATTAGATGTTTGCAGTAAGCGATGTAAAGAAGAT
GCTAATAAATATGTGAGGAATGATTACGATACTAGATAAGCGGCTAATGAAATTTTTTAAAGTACATATATAGA
CATATTTTTCATTTAGTAAAATTTTGAATTTCACTTTGCTAAGACTAGTGTCTAGAAATTTATAATGATTTATTAA
CACCTATTTGAAACTTAAGTATAATAAATGATTCGGATTTTATTTTTAATAAAGACAAACTTGAACGTAGCAAA

GTAGTTTTTATGATAAATAATAAGTTTTAATAATGTGACGCTTTTATATAAGCACATTATTATGAACAATGTGAAT
TGAGCATCTACAATTACATTAATAAATATATAAATGATGATTTAAATTCACATATATTTATAATACACATACTATATG
AAAGTTTTGATTATCCGAATAAATGCTAAAATTAATAAAATAATTAAAGGAATCATACTTATTATACGTATACGTT
TAGCTACTGAACTACTGGATTCATTTGGAGATTCTAGTAGTTCTTTTTCAATCTCTAAATCTAAATCAGTTTTGT
AATAACCATTAATTCCTAATCTTTCATCTAGCTCTGTACTTTTTTCATCATTTTTATCTTTGTTGATATGTTCCATT
TTCTCGCCTCTTTTTAATCAAGTAGAAAACCTTACAAATATATGCTATCAATTAACTATAGAAATTACAATGAAG
AAAATATTTAGATTATTAAACTTGTTAAGTTTCTATGAATTTTAAATTAATAAAAATATATAAATAATACATTTTAA
TATAACGTAGTATTGCGAAATAATTGTACTTCTGCTAATTTTATAATGTAGGGTTTAGAATTCTATATAAATTTAG
AAATGGAGTGTTTTTGTTGAAAAAGAAATTGGACTTTTTACCTAATAAGTTGAATAAGTATTCAATTAGAAGA
TTTACAGTAGGTACGGCATCTATATTAGTAGGATCTACTCTATTATTTGGTATTGGGAATGAGGCACATGCAGC
AGAAGAGCATCAAAAATCAAACACTACAGAAAATGTAGCTGACAGTAATGCAAGCGAAGCACCAACAAAA
GAAGAAGCGCCAAGCAATGAAGCAACAAGCGAAGCACCAACAAAAGAAGAAGTGCCAAGCAATGAAGCA
ACAAGCGAAGCACCAACAAAAGAAGAAGCGCCAAGCAATGAAGCAACAAGTGAGGCACCAACAAAAGAA
GAAGCGCCAAGCAATGAAGCAACAAGTGAGGCATCAACAAAAGAAGAAGCGCCAGCAGCTGAAGAAACA
GACAAAGCAACAGAAGAAGCACCAAAAACTGAAGAAACAGACAAAGCAACAAAAGAAGAAGCGCCAGC
AGTTGAAGAAACAAGCAAAGCAGCAACAGAAGAAGCGCCAAAAGCTGAAGAAACAGACAAAGCAACAG
AAGAAGCACCAAAAACTGAAGAAACAAGCAAAGCAGCAACAGAAAAAGCACCAAAAGCTGAAGAAACA
AACAAAGTAGAAACAGAAGAAGCGCCAGCAGCTGAAGAAACAAACAAAGCAGCAACAGAAGAAACACCA
GCAGTTGAAGACACAAATGCTAAGAGCAATTCAAATGCTCAACCATCAGAAACTGAGAGAACTCAAGTTGT
AGATACAGTTGCTAAAGATTTATATAAAAAATCTGAAGTTACAGAAGCAGAAAAAGCTGAAATTGAAAAAGT
ATTACCAAAAGATATTTCAAACTTATCTAATGAAGAAATTAAAAAAATAGCTTTAAGTGAAGTACTTAAAGAA
ACAGCTAACAAAGAAAACGCACAACCAAGAGCAACATTCCGTTCAGTAAGCAGCAATGCTAGAACAACAAA
TGTTAACTATTCAGCAACAGCATTAAGAGCAGCTGCACAAGACACAGTTACTAAAAAAGGAACTGGTAACTT
TACTGCGCATGGAGATATAATCCATAAAACTTATAAAGAAGAATTCCCTAATGAAGGTAAGCTAACTGCATTCA
ATACAAACTTCAATCCTGATACAGGAACTAAAGGCGCATTAGAATATAATGATAAAATAGATTTTAATAAAGAC
TTTACAATTACTGTTCCAGTAGCAAACAACAACCAAGGCAATACAACAGGAGCAGATGGCTGGGGCTTCAT
GTTTACTCAAGGGAATGGCCAAGACTTCTTAAACCAAGGTGGTATTTTAAGAGACAAAGGTATGGCAAATG
CATCTGGTTTTAAAATTGATACGGCATATAATAATGTTAATGGTAAAGTCGATAAACTCGATGCAGATAAAACA
AACAATCTAAGTCAAATTGGCGCAGCAAAAGTTGGTTACGGTACATTTGTTAAAAATGGTGCAGATGGTGTG
ACTAACCAAGTTGGTCAAAATGCCCTAAATACAAAAGATAAACCTGTAAATAAAATAATTTATGCAGATAATAC
AACTAATCATCTTGATGGTCAATTCCATGGCCAAAGATTAAATGATGTAGTATTAAATTATGATGCAGCAACAA
GTACAATAACTGCTACATATGCAGGAAAAACATGGAAAGCTACTACAGATGATTTAGGAATTGATAAATCACA
AAAATATAATTTCTTAATTACTTCAAGTCATATGCAAAATAGATATTCTAATGGAATTATGAGAACAAATCTTGA
AGGTGTAACAATTACAACGCCTCAAGCTGATTTAATTGATGATGTGGAAGTAACGAAACAACCAATTCCTCAT
AAAACTATTCGTGAGTTTGATCCAACTCTAGAACCAGGCTCACCTGATGTTATTGTACAAAAAGGTGAAGAT
GGAGAGAAAACAACAACTACACCAACTAAAGTTGACCCTGATACAGGAGATGTAGTTGAACGTGGTGAACC
AACAACAGAAGTTACAAAAAATCCAGTTGACGAGATTGTACACTTTGCACCTGAAGAAGTACCACAAGGTC
ATAAAGATGAGTTCGATCCAAACTTACCAATTGACGGTACAGAAGAAGTACCAGGTAAACCAGGCATTAAGA
ACCCAGAAACAGGTAAAGTGGTAACACCTCCGGTTGACGATGTCACAAAACATGGTCCAAAAGCAGGCGA
ACCAGAGGTTACTAAAGAAGAAATACCATTCGAGAAAAAACGTGAGTTCAATCCAGACTTAAAACCAGGTG
AAGAGAAAGTAACGCAAGAAGGACAAACTGGAGAGAAAACAACAACAACGCCAACAACAATTAATCCATT
AACGGGAGAAAAAGTAGGCGAAGGTGAACCAACAACAGAAGTAACAAAAGAACCAGTAGATGAAATCAC
ACAATTCGGTGGAGAAGAAGTACCACAAGGTCATAAAGATGAGTTCGATCCAAACTTACCAATTGACGGTAC
AGAAGAAGTACCAGGTAAACCAGGCATTAAGAACCCAGAAACAGGTAAAGTGGTAACACCTCCGGTTGAC

GATGTCACAAAACATGGTCCAAAAGCAGGCGAACCAGAGGTTACTAAAGAAGAAATACCATTCGAGAAAA
AACGTGAGTTCAATCCAGACTTAAAACCAGGTGAAGAGAAAGTAACGCAAGAAGGACAAACTGGAGAGA
AAACAACAACAACGCCAACAACAATTAATCCATTAACGGGAGAAAAAGTAGGCGAAGGTGAACCAACAAC
AGAAGTAACAAAAGAACCAGTAGATGAAATCACACAATTCGGTGGAGAAGAAGTACCACAAGGTCATAAA
GATGAGTTCGATCCAAACTTACCAATTGACGGTACAGAAGAAGTACCAGGTAAACCAGGCATTAAGAACCC
AGAAACAGGTAAAGTGGTAACACCTCCGGTTGACGATGTCACAAAACATGGTCCAAAAGCAGGCGAACCA
GAGGTTACTAAAGAAGAAATACCATTCGAGAAAAAACGTGAGTTCAATCCAGACTTAAAACCAGGTGAAGA
GAAAGTAACGCAAGAAGGACAAACTGGAGAGAAAACAACAACAACGCCAACAACAATTAATCCATTAACG
GGAGAAAAAGTAGGCGAAGGTGAACCAACAACAGAAGTAACAAAAGAACCAGTAGATGAAATCACACAAT
TCGGTGGAGAAGAAGTACCACAAGGTCATAAAGATGAGTTCGATCCAAACTTACCAATTGACGGTACAGAA
GAAGTACCAGGTAAACCAGGCATTAAGAACCCAGAAACAGGTAAAGTGGTAACACCTCCGGTTGACGATGT
CACAAAACATGGTCCAAAAGCAGGCGAACCAGAGGTTACTAAAGAAGAAATACCATTCGAGAAAAAACGT
GAGTTCAATCCAGACTTAAAACCAGGTGAAGAGAAAGTAACGCAAGAAGGACAAACTGGAGAGAAAACA
ACAACAACGCCAACAACAATTAATCCATTAACGGGAGAAAAAGTAGGCGAAGGTGAACCAACAACAGAAG
TAACAAAAGAACCAGTAGATGAAATCACACAATTCGGTGGAGAAGAAGTACCACAAGGTCATAAAGATGAG
TTCGATCCAAACTTACCAATTGACGGTACAGAAGAAGTACCAGGTAAACCAGGCATTAAGAACCCAGAAAC
AGGTAAAGTGGTAACACCTCCGGTTGACGATGTCACAAAACATGGTCCAAAAGCAGGCGAACCAGAGGTT
ACTAAAGAAGAAATACCATTCGAGAAAAAACGTGAGTTCAATCCAGACTTAAAACCAGGTGAAGAGAAAGT
AACGCAAGAAGGACAAACTGGAGAGAAAACAACAACAACGCCAACAACAATTAATCCATTAACGGGAGAA
AAAGTAGGCGAAGGTGAACCAACAACAGAAGTAACAAAAGAACCAGTAGATGAAATCACACAATTCGGTG
GAGAAGAAGTACCACAAGGTCATAAAGATGAGTTCGATCCAAACTTACCAATTGACGGTACAGAAGAAGTA
CCAGGTAAACCAGGCATTAAGAACCCAGAAACAGGTAAAGTGGTAACACCTCCGGTTGACGATGTCACAAA
ACATGGTCCAAAAGCAGGCGAACCAGAGGTTACTAAAGAAGAAATTCCATATGAAACTAAACGCGTATTAG
ATCCAACAATGGAACCAGGTAGTCCTGATAAAGTAGCTCAAAAAGGTGAAAATGGTGAAAAAACAACAACA
ACACCAACTACAATTAATCCATTAACGGGAGAAAAAGTAGGCGAAGGCGAACCAACAACGGAAGTAACGA
AAGAACCAATAGACGAAATTGTTAACTATGCACCTGAAATTATTCCTCATGGTACACGTGAAGAAATTGATCC
AAACTTACCAGAAGGTGAAACTAAAGTTATCCCAGGTAAAGATGGCTTGAAAGATCCTGAAACTGGAGAAA
TCATTGAAGAACCACAAGATGAAGTAATCATCCATGGTGCTAAAGATGATTCAGATTCAGATGCGGACAGCG
ACTCAGACGCAGATAGCGATTCAGATGCGGACAGCGACTCAGATTCAGACAGCGACTCAGACGCAGATAGC
GACTCAGACGCAGATAGCGACTCTGATGCGGACAGCGACTCAGACGCAGATAGCGATTCTGATGCGGACAG
CGATTCAGACGCAGATAGCGATTCTGATGCGGACAGCGATTCAGACGCAGATAGCGATTCAGATTCAGACA
GCGACTCAGACGCAGATAGCGATTCAGACGCAGATAGCGACTCTGATGCGGACAGCGACTCAGATTCAGAC
AGCGATTCAGACGCAGATAGCGATTCAGATTCAGACAGCGACTCAGACGCAGATAGCGATTCAGATGCGGA
CAGCGATTCAGACGCAGATAGCGATTCTGATGCGGACAGCGATTCAGACGCAGATAGCGATTCAGATTCAG
ATAGCGATTCTGATGCGGACAGCGATTCAGACGCAGATAGCGATTCTGATGCGGACAGCGATTCAGACGCA
GATAGCGATTCAGATTCAGATAGCGACTCTGATGCGGACAGCGACTCAGACGCAGATAGCGACTCAGACGC
AGATAGCGACTCTGATGCGGACAGCGACTCAGATTCAGACAGCGATTCAGACGCAGATAGCGATTCAGATG
CGGACAGCGATTCAGACGCAGATAGCGATTCTGATGCGGACAGCGATTCAGACGCAGATAGCGATTCAGAT
TCAGACAGCGACTCAGACGCAGATAGCGATTCAGACGCAGATAGCGACTCTGATGCGGACAGCGACTCAG
ACGCAGATAGAGATCATAATGACAAAACAGATAAACCAAATAATAAAGAGTTACCAGATACTGGTAATGATGC
TCAAAATAATGGCACATTATTTGGTTCACTATTCGCTGCGCTTGGAGGATTATTCTTAGTTGGCAGACGTCGTA
AAAACAAAAATAATGAAGAAAAATAATATTTAAACTTCATAATTTTGGGTAAAATTTAAAACCAGGCCTTACA
TGGCCTGGTTTTATTTTAAATACTATGATATAAATTAATGGAAATAGAACAAATATATAAAAATAAGGAGGAAA
CAATGGAATTTGAACATAAATTAGAGAAATTAATATCTGAAGTAAATAATAAGACTGAAATTAATAATTATGTT

TTTTTAAGTTTAGGTAAGTCAAGTGTTAAAGCACAGGTTAAGTTATTAAAAAAAACTAATTATCTCAAACAAG
ATATTTCAAAATTGGCACTTAAATTTAAAAAGAAATCTGGGGAATTTCCTGAGTGGATTAAATTAGATATTGTA
ACCTCGACTGAAAAAATATTATTTAAAGAATTAAAAAAAACACTGATCAATACAAGAAGAAATTATGTAGATT
TTGGTATAGCATTTGATAGTCAATGGAATTTTGCAGTATTACCTGAAGAAATTAACGCGAATGCTTTTGTTCGA
CCAGATAATACTACTAAAGAGTTATTTCTTTCGGAAAAAAACATCAATAATTATTTACGCAAATATACTACAAAT
AAAAAGGCATTTTCTAGTGAGTTCTATAATGAAAAAGAAGTTATTAAATTCTATACACAAGGTTTCTTTATAGG
TGATGAAGAAGTACATGAACTATATAGCGAAGGCTATAAAAAAGGTTTAAGAAAAGTAAATGATTTAAATAAT
GAAATTGATCAATTAATTGAAAGTAGTACTAATTTCTTACAAAATATGTTGTTAGATACTGGTAAATATATTTAT
GGATATTTTCCTCATTTTGATAATGAAATTGGATTCTACAATGTTTTAAGACACTCTTCTTCTACTTATGCCTTAA
TTGAGGGTCTGTCCTATTTAGGTAAAAGCTTACAACCTGTTGAAAAAGCAATTGATTATATCATTTTAAATCAA
CTGTTTGAGATTGGTGATAAAGCCTATATCTTTGATGATACAGAGGAAGCAAATGAAATCAAATTAGGACAA
AATGCTTCATTTATATTCGCAGTTTGTGAATATTTAAAGCATGAAGATAATCCTAAATTTCTTGAGTCAGCTCAG
AAAGTGGCTAAAGGTATTCTTTCAATGATAGATGAAGATACATATGAGACAACTCACTTATTGAATTATCCTGA
TTTAAGCGTAAAAGAGAAGTTTAGAATTATTTATTACGATGGTGAAGCAGCGCTTGCTTTATTGAGATTGTAT
CAAAAGGATGAAAATGAACTATGGCTAAAAACTGTAGAAAATTTGATGGACCGTTTCATTGAGAAGAAATAT
TGGCAATATCATGATCATTGGTTAGGATATTGTACGAATGAATTAGTTCAAATTAATCCACAAGACAAATATTTT
GAATTTGGAATCAAAAATGTGAATAATTACTTAGATTATATTAAAAATCGTGAAACAACATTTCCAACATTCTT
AGAAATGTTAATGGCCACATATAGATTAGTTCAAAAAGCGAAAGATACGGGTCGCGAGGAATTGGTAAACA
ATTTAATAGATGAACAATATTTGATAGATGTAATTAATATTAGAGCAGATTATCAAAGAGTTGGCTTCTTTTATC
CTGAAATTGCTATGTATTTTAAAAACCCATCAAGAATACTAGGAAGTTTCTTTATTAAACACCACGGTTATCGT
GTTCGAATTGATGATATCGAACATTATTTATCTGGATATGTACAATATCAGCTTGCATTTAATAGGTAAAATAAG
TATTTGAAATTTAAATTAATGACTTACAAAAAATGGTTGATTTACATAAAAACGAAATCAACCATTTTTAATTT
GCTCTTTAATAATTATTTTTCATATAACTAAAATTCTAATTTCATCAAGAATAACTGAAAAAACTAAACAACATG
TGAGCTATTCATAACTTGAATTAATTGTAGAAAATCTATCTTCCCATAACTTCATAACAATTTTACTTGAAAAAG
CTTTAGAGTGCTTGATACATTCTTGAGAAAAAGTATCTCTTAAAGTCTTATCTAATAATAGGTTTCGCACTCGA
TTATATAATTCATCTTTATCATTTTTGTTAATTAAATAACCATTTTCATTATTTAATATTAATTCGGAAGGACCATA
CTTCGAATTATAGCCAACTGGAGGTATGCCTTCAGTTATAGTTTCTAATAAACCTAAATTAAATCCTTCCATATT
ACTAGTAATTAGAGACATATAAGCATCTTGAATCTCAGCACTTAAATTTCTTCTAAACCCTCGTAAAAACACAT
TGTTTTCTAAATTATATTCAGTGATTAGTTGCTTGTATTTTTCTTCTTCTTTACCAAACCCATACAAATGTAGTTG
GATGTTAGGAAATTCTTTAATTAATTTAGAAACTAATTCAATTTGATGATTCAACTGTTTTTCAGGAGAATAAC
GTGCAACGGAAATAATTTTATTATTATTTATACTATGATTATTTCTTTTTAAATTAGTAAAATGTTCATCAATATAT
CCTACTGGTATAGTATGAACAGGGATTTCATTATTTATTCGAGCTGATATATCCAATTGTTGCTGTTTAGTCGAT
ACAATTATCCCAGAGTATCTATTTAGATTGTTAAAAACATGTTTATAAGTATTTTTGATGTCCGATTCATATACCA
TGTCAATATTCTTGACATGAGTACTATGCAAAACAGCTAAAACAGGTATTGTTTCACTAGTATTATTGAATATTG
GTGCAGTATTTATATTTTTATCACTCAGGAAAATATCTCCATTTTTATACAATTTTTCAATTGCAAATGCTAGTAA
TTCCTGATCATTATTAAAATACTTAAGTTGAGAATCGGTGTTATAAATAATAAGTTGTGATTGTGAATTTTCAAG
TTCTGGATTAAAAAATTTTTGGAACTTTATAGTACCTTCAGGAGTGTAATAATGTTCACAAACTACTTTTTGAT
TTGTTGTTAAAGTTCTTGAACAACTTAAAAAGCCTCTTGAATCATATAGATCTCGACGTATTTTACGTTGATTT
ACATCAAAATGATTTATGTAATCCAAATGTTGGTAATTACTATCATTAAAATGGACATACATTCTATAGTTGCTTT
TTTCATATATCTTTATATCATTGGTATTTTCAATAAATTTTAGCGTATAATTACACTCTTTAGTCCAATGAGTGATC
CAGTCCTTATGGATGCTTTTTATATTTATACTGTCTTGTAGCACATCATATAAGCTTAATATATCGTCATCTTCAAT
TTGAAATAGACTTGCATTAAAATGTAAACGAGGTGACCACGATGCAAAAATCAATTTACTAGTTATCGAGTGA
TTTTTAAATAAGTTGTGTCTATTAATCATCGCTTTTTCAATTCCGGTTAATTTATTTCCTAACCTATTCCCTACTG
AATATATCATTTTAATTCAACCTCATTTTTTGTTATTTATTATTTAGTTTATTTTTAATTTCATTTAAGTTTGGGTAA

GAGGTAAATATAATAAATTTATCTTTATCTTTTATAATACCGCTATTATATTTCCTGCCAATAATTATTTCGTTAGTA
AATTTAAATAGGGTATTTTTATATGCTTTTAAAATTTTAAATAAGTCGTCAACAGTGAAGTTTTGTCGTTCTCTC
ATAGTATGACGTCCAGTTAAATTTATTGCTACAGAATTTGAAAGACTTAAATGTTCTATTTTTGAATTGATATAT
TCATCGCGCTTTGGACCGTTTTCAAATAAATACTTAGATAATTCATAAGAAGGTGTATACATAGGTGAATTAAG
CATGGCTTTCATCAATTCGTAAGTTGTCGTTTCTTTTCCTTCATAATAAAGTTTGTTATTTGTTGCCCAGTTAGC
AATAAAAATAGTATCTTCTGATACATTATCATCTATAGCTTTTAAATAATTAAAGATAGGGCCTAAGCCTTCAATT
GTACCTGAATGTTCTCTGTTATGAGATTCAATGACATTATAATTTTCATCAATAATCATATAACTGCGACCGTTC
AAGGATTGACCATCAAATAAAGAAGTGTTCGAATTATTAATATTATATTTATTTAATTTCTCAGGTAATTCTTTT
GCTAATTTTGGAAATTCTGTACCACCTGCTGATGATTTTAATAGAGTTAATGAATCTGGTTTATTTAGGTACAAT
AAATCTTTTTCTAATAGATGTCTATTAGAATACCATGTTATATTTTTGCTTTTTACTCCTATAACGACACTCTTTAA
AGCATCATCCATACATAGAATATAATCTGCATTACTATTTTCTAGAACATCAACTAATTGCAAATGCAATGATTTA
GAGTGCTTACCTAAGTCACTAATTTGCCCAATCGCAATAATTTTATTACCTTTAAAAAATTTTGTTTGAGTATTA
AATGCTTTTATAGCATTAATCATTGCTGGTAATGAAGCGTTGTGTGTATCATCTATTAAGTTAACTTTATAGTTA
GGTGTTTCAACTTCTTTTAGGTTTAAAACTTTTTCAAATGGTTTAAATGTACTTAAGTTCTCTAAGGCTCTTTC
TAGTGGTATATCTAAATGCGATAATGTAGCAAATGTGGCTAGAGAGTTTTCAACCATACCGTCACTTATTGAAT
TAATTCTATAAGTATATTTTTGTCCGTTAAAATCAATTGTTATAACTGTATAGCCTTTACTATATTGAATAGATTTA
GGACAAATTGTTGCGCTAGAGTCATGTGTACTATAAGTAATAACATTACTTGTATTTTGTTTGGCGCGTTCGAT
TAATATATCTGAATGTAATGTATCTTTATTAATTATTGCTACTCCCTCAGGAGTTAAACCATCAAAAATACTTGCT
TTCACTTCTACAATATTTAGAATATCTTTAAATGTAGACATATGAGCAGCACCTATACCAGTAACTATAGCAATAT
TTGGTTTTATTAAATATGATGAATTACCAACTGCATTTAATGCGTTTAATGATACTTCTAAAACAGCAAAATTTG
GTTGTCTAATCATTTTGCATAATAATAAATATACTGCGGATCTTATGTTATTATTAAATCTATTTTGAAGAGGATA
ATAATCTTTAAGACCAGCACTTATCAGCATTCTAGTTGAACTTTTCCCATTGATCCTGTTATAGCAACAACAGG
GTGTTTATACTGTAGTCTCATTTCTTCAGCTAATTTAGTTAGTGTGTTTGCAGAATCATTGATAACTAATTGAGG
AGTTTTAAACTTCATGTCAGGAACATAGGTTTCAGTAATAATTAATCCTAAATCTTCCTTATCCTTCTTTATATAT
TCATTACCTTCTTTTGCGTTTGCATCTCTACCAAAACGTCTCCATGTATGCTTATTGGGCGATATGTAGGCTGTG
CTTTTATTATGAATAAAGGGGAACCAATTCTCAAAATTATTTATAACATTGTCCTCTTTACCTTCAGCATCAAGG
AGATTACCTCCTAAAATATTATGAAGTTCTTTAATTGTTAATGACATTTTGTACTCCTTTATAAAATGTAAAATTG
TGTTTAATTTCTAAATATATATATAGTATAGCATGTTGATATACTGAAATGATTTGAAAAAATTTTTGAAACTTAAT
GACTTATAAATTCATATTAGTTTAAAATTCGATACTATTCCATAATGATGGAATGTGATGTATATGATTAATCAAT
ATACATCACATTTCATTTAATGCAACTATTGCACAAGTTAATAGTATATTTAAAAATAGGTTAGTGAGATTGATT
AATTATTGATTATGTTGGTATCAATGAAAGCTTGGAAGAAGGGCTTAAAAATATAAAAACTTGGATAAATCAC
AATACAATCATAGACACAGATAAGTTCGTCAATCTGTTAATGAATTATGATGTGATAGAGTTAAGTGAGGATAA
ATGTTAACGTTTTATTAAAACAGTCACAGAATTAGATAGAGCATTTGCAATTTGTGCTACTGAACTTATAATCT
AAGAGCTCAATAGTGAAATTGTCTTTTTTAAAGTTGTTAAAGCATAAATTTGTTGAATTATTACAGAAAAAGA
TCGAAATTTAGTTACTGCTAATGGAACGGCAGCATTAGAGTTTGCCGAACTCATTTTAAAAGGACTTGAATTT
GATAGTAAAGAAAATATTGAAAGGCAAATCTATATGTATCAAAGAGGCTTCTATGAGTATTGTAATAAGTATGG
CAATCCTTATCAATAATTAAAAACCGCATCATTATCTGATACGCAGAGGCTTATCATAAATAAAACTAAAAATTA
GATTGTGTATAATTTAAAAATTTAATGAGATGTGGAGGAATTACATATATGAAATATTGGAGTATACCTTGCAAT
ATCATACGATGTTTATAGAGTGTTTAATAAACCATTTTTCAACTATTGATGATCTAGAATATATAATAACTGTACA
AATTATATTGATTATGGAACTACAATTCAATTAAGAAATTGATGATGAAATTTTAAATTTAAACTAATGGAATCA
AGAAAGAATGAAAGGAAATATACAATGCCCACGATTAATAAAAGGAAGTTTATTAGATTTTGTGTTAGAAAC
AGTAGATGTTTTTGTAAATGGAAGTAAAAATGATTGCGAATTAGTAAATTTTAATTATAAAGAAGGAAAAGTA
GTTTCACGTTATAGTACAAAATTTGAAAGCAATCCTAAATTAAGAAAAAAGCAATTGATATCGACGGTAAAAG
TTGTAAGGTATGTTATTTTTGATTTTGAGAAAATTTTATGGGGCATAGGGAAAGTTTTATTGAAGTTCATCATA

TTAAGGCAATGTTTACTATAAGAAAGTTGAAATACACTCAGAAACAGACTGGATTCCCGTTTTTTTCAAATTG
GCACAAAATGATACATAGGCCCACCTATATATTGAAGATTTAAGTTAAACGATAAATAATATTTTTCTCTTTTGT
ATAGAAATTTTTTTGGGAATGATATTAATTAGTATAAAAATGAATATGTCTACAAAGAAGGAGAGATTTTATTG
AAACACATTAATCAATTTTCTGAATCGACACTAGCACAACTAGAAAAAGATCAACAACACGTATTTTATGTATA
CTGTTTGATGGACCCAAGAAATGATGAATGCTTTTATGTTGGTAAAGGTAAGGGGAATAGAATATTTAAACAT
AAACAAGATGCGCAGAAAAAAGTATTGTACGAAGATATATTTATAGAAGAAAATAAAGATAACTTGAAATTTA
ATAGAATTAACGAAATTTGTAGCAATGATTTGAATGTTTTAGGATATATAATCAGTTATGGATTAACCGAATCTG
AAGCATTTTCTGCAGAAAATGTTCTTATAAACTTTCTTAACCTAACAAATAAAACTACATTAACAAATATGATTA
ACGGTCATGGATCTAAGGCATACTTAGTTGAAGATTTAGAAAATGAATTTGGCTACGATTCAATTAATCTTGA
AAACATAAATACGAATGAATTAATTTTAGCAGTGAAAATCAGAGATGCATTTCTATTAGATAAGGATGAAAGT
AAAGAGTATCCTATTAATGAAAGTAAACGTGATAGAAATAACCTTAAATCGCGTACATTAGGTAGTTGGATAAT
AGGAAAAGATAAAATACATAAAATAAAATATATTATTGGTATCAATACAGGTGCTAATAATGCAGTTGTCTCAG
CTTATGAAGTATCATTTGAACAAGCGGAGAGTATCGAAACAAATAATGGTAGAATGAGATATGCGTTTATTGG
GCTTTCAGAAAGAGATGCTACTCTAAAAAAATTGAATTTATACAAAAAAGCACTACCAGATTTAAGATTTGGT
AGTGGTAGCGCTACAGCATATATAAATAATGGGACAATGAAAGTTGATTAAATGAATAAAAACATCCAATTCC
TTAAATTAGCCTTTGAAAATTTTATACTCGTATATATAAGTGAAAAACTTAAAAGAATTAGATGTTAATTAAAG
GAGAGAATGCTATGCCATCAGATGATATTGTAAAAAAACTATACTCAAAAGAGGATATTCGATTAGAATTAGG
ACTTACTCCGCATAAGTTTAATAAAAAAAATGGAAACTATTGCTAAGCTTTTTAAAATTGATATGAAAATTTTTC
ACAATTACAAAGGCCAAGATAAAAATAATCAGTATACATTTAATGGTGTCGCAAAAGAATTAATCAAAGTGTT
GCTAAAAAGTGTTGATTATTACCCAGTGGATATCAATTCCAAAAAATTTAAACAAAATGGAAAATCTAAAAAA
GAAATGATTGAAAATATAGATAACTCTAGTTACATGAAATATATTTATCAGTTAATGAAATCTATCAATGAAATT
CAATACAAAAGGTTAATTGCTGATATACATATGAAAGATGTGTACCAGAATACAAAAGCATGGTTAAATAATG
GTGAATCAATTAATAAAAAAGAACAAGAGCTATACCAATATATGACAATTTTACCATTACATAAAAGAGTTGA
ACTGCAAAATGAGGTATTAAAATCTATAGACGAAACAATTTTTCAATTTCTCGCAAAAGAACATAGAAATAAT
CAAATTGAAGAAAATAATGAATTAGAAGCATATACAAAAGCAATAAAAGAAGGTAGAAATCCTAAAAATGAT
TATGAATTAAATCATCTCCTATATAAAAAAAATCAATTACCCTTAGATAGTCTAATAGAAGATATATGGGACTATG
AGGAAACAGAGTATACTGAGCTTGATTGGTTAATTGCAGATATGCTAAAACGTTCACAAAGAGCTGATAGTA
ATTTTATCGAAAGTTTAGAGAAGAAAAACAAATTGAGAAAAAATATTCATAAAGATTCCATTAAAAGTATATC
TAAGTTGATTGATCATGAATTAGTTAAAAATAATAGAAAGTGGGACGTAGCAGAATTTTATAAAAATCAACAA
TTTTGGAATAATAGTACATTAAGACGTAGCTCTCAAAATTTTGGAATTTTATATTACAAAAAGAATAGGTTACA
TACAATGAATCAAAGTAATATTATTAAGAAAATAAAAATTATCGAAGCTAATATAGAGAGTGCAAATAAACAGC
CGAGCTATTTAGATTTCTTTTTACAAGCTAAGTTTGATTTAGAGAGGTTAGAAAGTGAATTAATGAAATATAG
CATTAATCCGAAATATTTTAATAAGATAAATAGTGATTACGTAAGATATGCTATTGAAGATGTACATAATGCAAT
TATTAAAATTGACAGATATATAAGTAACGATAAAGTGAAGTTAAACTATTCAAATGTCCAAATGGTAGTGAGA
AACGAAATAACAGAACAAGGAAGCTACTTTGTAACCCAGGCATTGAATACACTGTCAAAGGTAGAAGAGTC
TGGCTACAGCTTTGATAATTTCATGGTAGGACCTTTCGTAGAAGACTTTAAAAAAGCAATTAAAAATCGTAGA
TTTGGTTCTAAAAAAGATTAAGATTGTTTTTTAGAGTTAAACATAAGAATAAATTTAAATAAAAAAATAAACCC
AACAAACCTTGTTATATCAATGTTTGTTGGGTTTATTTTTTTGGTGATTTTTTAATAACTCAATTACATCTTAGG
ATAATATCGCTAAATTATACATTGTCGAGGCCAAGACAAATAAAAAATTAAAGCAAAAGTTAGCGCGCAACTT
TCTGCTATAAAGGAGCAAATTTATATGGAGACAAGAGACAAACTGATGTCATTGACTCAAAGTGACAAAACA
CAGCAATGGCTAATGGACAAGTCATCTAACCAAGATGACATTCAACAATTGCAGCAACAATTCAGTCAGCAG
CTTGATCAACAATATAATGCACTTTTAGCTGATGAAAAAGCTAAGTTAGACCAATACGTGGAAGTACATCAAG
GATTGGAATCATTAAAGGAAGAGATTGAATCAGAACCTATTACGCTTAATATCGATAAATTACCCGATATCAAA
GCAACAATGCTTGAAAGAGCCAAGAATGATGAACATTCTGATAAAATCGAAAAGCTATTTGATAGGTTAGAA

CAGGCATTAAATGGTACGAATCGATTATATACGCAATTATCGTTGATTGGTACACGAACACATCGAATTACAAC
TAAAAATTTTAATCTTCAAGGCTTACCTAAAGCAGTCCAACATACGATTTTACCTTCAAAATTTAAGAAGGTGT
ATACAGTCGATTTTAAATCGTTTGAACCATCAGTTGCAGCGTACATGACTCAAGATTCAAAACTGATTGACTT
GTTAAATCAGAAAGACGGACTGTATGACGCATTGCTAAGTGAATTAGGCTTATCAGATGAGCTACGTGTATTT
GTTAAACGTGCATTTATTGGTTCGTTTCTATTTGGAGGTAACTTCAAAAATCCTAAATTCAAGCTGAATCAATA
TGTAAGTGAAGTACAATGGTTGGATGCGGTCAGCCAATTTACAAAAGTCATTGAACTTAAGAAGCACGTTGA
AAAGAGTAATTCCATGCCTATGCCTTATAGTATTGAGCATGATATGAGCGCATTTCAAGGTAGCAGTATTATGG
CAATCTACGTACAAACTGTAGCGAGTTATATTTTCAAGCACATTCTGCTAAAAGTGTACAAAGCACAGTGCGA
TCAAAAAACGTTCAAGATTATAGTACCTATACACGATGCGATTATGATTGAATGTGAAGATGAAGAAATTGCA
CAAAATGTGGGTCAGTTAATGAAAGATACGGCTAACCAGTTGTTCAATGGTGAATTTGCACATGTGACAGTG
GAAGAAATAGGAGGCGTAGACCATGAATAATGATAGAGGACAAAGCCTACACATCCCAAGTAGTACACCAA
TCAAAGAAAATAATATATATGTAGCTACGTTACATTCTGTGATCCAAACAGATTTCTCAGGTGAAATAAAGCAC
CAATTCACGTATGAAATTGAAGTGAACAATCAGATTGTATATGCGAATCGTAATATTCCAACAAAACCGAGCG
CTAATCAGTTGTCAATTCATGATTGGCTGAAACGTCATAGCAACTATAGCGCAAGTCATGAAAACTATGAGCC
TTATATTGATCAGAAACATTTAATTCTATTAGGTCAATATAACGGTAACTATTATGTACAAGATGTAGCATCGTTA
GATGCGTTTGGAGGCGTATTATCATGAATCATATATTACAAATGTTATCTAAGCTATTAAGTGTGGCCAAGGAG
GCAATCGACCGTCAAGGTCTGATTGCTATCCTAACTATTCCTGTTAATAATAACGATGAAATAGAAGAAACGG
CTCAAGGTGAAACCGTGTATAACGAACTTATCGATCAGTTACGACTTAATATCCCAAAAGATACGGATTATCA
ACCTAACATCTATAGTTATTTTGGTATTAAGAAGAATCCTAATGACACCGTACTCATGGAAATGATGATAAAGG
TTTTTCATATCAAACGCTTTAATTCAGAACTGTTTATTTTCAAAGTTAACGGGTGGCAAAAGATAAATGGAGA
TGAATTACAAGGGTTGATATCTAAAATGATACAAGTATTGCTTGTAGATTATAAGCCTTCACTAAGCACTCTAA
AAAATGTCGTAGATGGATTGCAAAAATCAACAGATGTAGAAGAACTTGTTGAGAATGAGCGCTATATTGGTT
GTGGTGAAAATATGTTCGATCTTAATACGTTTCAAGTCGTTAAAAATTCAATCGATATCTTTCCAAAAACACGA
TTGAATTTATCATTAAGTACAAATGATGTAATTACTGATAAGATACCGCCTTATTTTAAGCAATATATGTTACAAC
TTGCGAATTATGACGATGATTTACAATACTTTCTTTTCCAACATACAGCAGTATTACTTACAGCTGATACTAAAT
ACCGTAGGGGTCTCATATTATATGGTGGAGCTAAGAATGGTAAATCTGTATATATTGAACTAGTTAAATCATTTT
TCTATAGTAAAGATATTGTGTCTAAGCCACTTAATGAGCTTGAAGGTCGTTTTGACAAAGAAAGTTTAATTGA
CAAAAGTCTAATGGCAAGTCATGAAATTGGGCAATCTAGGATTCAAGAAAAGATCGTAAATGACTTCAAAAA
GTTATTATCTGTAGAATCAATGCATGTTGATCGTAAAGGAAAAACTCAAGTGGAAGTCATTTTGGATTTGAAA
CTTATTTTTAGTACAAATGCGATACTTAATTTTCCTCCTGAACATGCGAAAGCTTTGGAGCGTCGAATTAATAT
TATTCCATGTGAGTATTATGTTGAAAAAGCGGACACTTCATTAATTGATAAGCTCCAGAGTGAGAAGAAAGA
AATCTTTCTTTACTTGATGTATGTGTATCAACAGATTGTAAAAGCAGATATCGAGTATCTTGAAAATAGCCGTG
TCACTGAAATTACTCACGATTGGTTAAATTTTGGATATGAATTTGTTTCTAGCAGGTCCGTAAGTAATGCAAAT
CAGAAAGCATGTATTAATTTACTCAGAAAACTTATAGAAATCAAATCAGGATCACGAATCAAAGTATCCGAGT
TAAATAAAGTTATTAATGAAGAAATAAAGGTAAGTTCTCAGGTTATTAAACAGTTAATTCAAGCAAACTTTGA
TACTCAAACCAAACTATACAATGGCTACGATTATTGGATTGATTTAGGTTGGAAAGAAGCCAATAAAAAAGA
GATTCATGATATTTCGGAAAAAGATAATATTATTTCATTAGATAAAAATGAAAATATAACAGACGATGAGGCAT
TAGATGAAGAGAATTTGGACTTTGATTGGGAGGACTTTGACGATGAATAATGAACAAATTGAAGCATTTGTA
GAAGTGCTTGTACCTATCATAGAAGAACGTATCAATAAAGGTAATTAAGGCTAATTACGTACTACAGGCAGTT
GCCTGTAGTACTCATATGATTAAGTGGTAAAAGTGATAAAAATGAAACGAAATTATAAATATATATTATCTATAT
GTTGTTACAAGACCGATAGTCTGTAGCAATAATCTAATAAAAGGAGCGGTATGATATGAAGGGTAAAATTGCA
CTTTATTCACGCGTTAGTACGTCAGAGCAGTCGGAGCATGGGTACTCAATCCATGAGCAGGAACAAGTACTC
ATCAAAGAAGTTGTGAAAAATTTCCCAGGTTATGACTATGAGACATATACTGACTCAGGCATTTCAGGTAAAA
ATATTGAAGGTCGTCCGGCAATGAAACGTCTATTACAAGATGTTAAGGATAATAAAATCGAAATGGTGTTAAG

TTGGAAATTGAATCGTATCTCACGATCAATGAGAGACGTGTTTAATATTATTCATGAATTCAAAGAACATGATG
TAGGGTATAAATCGATTTCTGAGAATATTGATACATCCAATGCTTCTGGAGAAGTACTCGTTACAATGTTTGGG
TTAATAGGATCTATAGAACGCCAGACTTTGATTTCGAATGTGAAACTTTCTATGAATGCTAAGGCAAGGAGCG
GAGAGGCAATCACCGGTCGTGTTTTAGGCTACAAATTATCACTTAATCCACTTACACAGAAAAATGATTTGGT
TATCGATGAAAATGAAGCTAATATTGTACGTGAAATTTTCGATTTATATTTGAATCATAATAAAGGCCTCAAAG
CCATTACAACCGTACTTAATCAAAAGGGGTATCGTACTATTAATCAAAAGCCATTTTCAGTGTATGGTGTTAAA
TACATTTTGAATAATCCAGTCTATAAAGGCTATGTCAGATTCAATAACCATCAAAACTGGGCTGTACAGCGAA
GAAGTGGCAAAAGTGATAAAAATGATGTGATATTGGTCAAAGGTAAACATGAAGCCATTATAAGTGAAGAG
GTATTTGATAAAGTTCATGAAAAATTAGCTTCTAAAAGTTTTAAACCGGGCAGACCTATTGGTGGAGATTTCT
ACTTACGTGGCCTTATTAAATGCCCAGAATGCGGAAATAATATGGTATGTCGACGGACGTATTATAAAACGAA
AAAGTCCAAAGAACGGACAATCAAGCGTTATTACATTTGTTCCTTATTCAATCGTTCAGGGAGTTCTGCATGT
CACAGTAATTCCATCAATGCTGAAGTCGTCGAGCGTGTAATTAATGTTCATTTGAATCGTATTCTGTCTCAACC
AGATATTATCAAGCAGATTGCGTCAAATGTGATAGAAGAACTGAAACAAAAGCATAGTAACCAAACAGAAAT
TAAATATGACATTGATAGTTTAGAAAAACAAAAAGCTAAGCTTAAAACACAACAAGAACGATTGTTAGAATT
GTTCTTAGATGATCAGATGGATAGCGAAATGTTAAAAGCTAAACAAAGTCAAATGAATCAACAGTTAGAAGT
ATTAGATCAACAAATTAAAGAAGCGCAACAAGCAAATCAATCACAGGATGAAATACCTAATTTTGATAAATTA
AAAGGACGACTCATTTTGATGATAACACGATTCAGCGTGTACTTAAGAAAGGCTACACCCGAAGCTAAAAAT
CAACTTATGAAAATGTTAATTGATTCAATTGAAATTACGACAGATAAACAAGTAAAACTTGTAAGGTATAAAAT
TGACGAAAGTCTTATCCCTCAATCTTTGAAAAAAGATTGGGGGTCTTTTTTTATGCCCAAATTCCAATTTGAA
ATATATGGTCAAAATGATTATTTCATCGACCAAATTACCACTTTTACCACTTAGTTATTAGTGACAAAAGTGAG
CGAAATGAAATAAAAATCAAATATATATTATCAAAATGATGTATCACATGCATACATCAATCAAATACATTAGGA
GGTCATAACCATGACACTAGAACAACAACTCAAGCACTATATAACCAACTTATTCAATCTGCCAAGGGACGAA
GTGTGGCACTGCGAATCTATCGAGGAAATCGCTGATGATATCTTACCCAATCAATATGTAAGACTTGGCCCAC
TCAGTAATAAAACACTTCAGACTAATACCTACTACTCTGACACACTTCATGAAAGTAATATCTATCCTTTCATTC
TCTACTATCAGAAACAACTCATAGCCATCGGTTATATCGACGAAAATCACGATATGGATTTCTTATACCTACACA
ACACTATCATGCCTCTTTTGGATCAACGATACTTACTAACAGGAGGACAATAAAATGCATAAATACATCAAAAT
TACACAATTAGTCATTACAATACTAAGTGAAATCATCATTTGGATGAAAGAGTCAGAACGAAAGGAAGTCTCT
TATGAATAGATATATCACCCGGGGTATCGCCAACAACTTACCTAATATCTTACAACACCAATTATGGCAACTCGT
ATCTGAGCGAGAACAAGAACAAACCAAAGATAATACTTCAGTAGATTATTTTCATATATTCCAGTTCAATAAGC
ATCGCAATCAATTATATATCAAACACAAACAAGAACGACCTGAATATGCGAAAATCCATAAAGCTAATTATTCA
AAAGCAATCAATATCAATAAGGTCTACATTATTCGAGAAGATGATGTAGACCTTTCTTATTATGTCATGTTATTA
CCTGAAGAATACTAGAGGAGTGAAAATTATATGGAAACAATCAAAAGTACATTAAAAACAGAAGCCATATTC
AGTGATGACAAACAACATCGCTATCTACTTAAGAAAACATGGAATAGTGAAAAACAATCAATCACAATCATTA
CAATGTATCCGCATTATGATGGCATTCTCAATATTGACCTAACGACCCAACTCATCATGAACAAAGTTTCAGAA
ATGGATGCATTTGGTTCAATCAATTTTGTGAATCTATACTCTAATATTACAACCCCTATCAATCTCAAACATTTAG
AAAATGCGTATGATAAGCATACAGATATTCAAATTATGAAGGCAGTGAAAGAGTCAGATGAAGTGATATTAGC
TTGGGGCGCTTACGCTAAAAAGCCCGGTGTTGAAGCACGTGTTAATGAAGTATTAGAGATGTTGAAACCAC
ATAAAAAGAAAGTAAAACGACTCATGAATCCAGAAACCAATGAAATCATGCATCCCCTTAATTCGAAAGCAC
GTCAAAAATGGATATTAAAAGTATAGCAACTAAAATGTTTATTCACTATCCATGAAAATAATTGAATTTCATATA
TATCTTTTAATCTACAATATGATTAAAAGATATATTATTTGGTTCTGTTGCAAAGTAAAAAAATATAGCTAACCAC
TAATTTATCATGTCAGTGTTCGCTTAACTTGCTAGCATGATGCTAATTTCATGGCATGGCGAAAATCCGCAGAT
CTGAAGAGACCTGCGGTTCTTTTTATATAGAGCGTAAATACATTCAATACCTTTTAAAGTATTCTTTGCTGTATT
GATACTTTGATACCTTGTCTTTCTTACTTTAATATGACGGTGATCTTGCTCAATGAGGTTATTCAGATATTTCGA
TGTACAATGACAGTCAGGTTTAAGTTTAAAAGCTTTAATTACTTTAGCCATTGCTACCTTCGTTGAAGGTGCC

TGATCTGTAATTACCTTTTGAGGTTTACCAAATTGTTTAATGAGACGTTTGATAAACGCATATGCTGAATGATT
ATCTCGTTGCTTACGCAACCAAATATCTAATGTATGTCCCTCTGCATCAATGGCACGATATAAATAGCTCCATTT
TCCTTTTATTTTGATGTACGTCTCATCAATACGCCATTTGTAATAAGCTTTTTTATGCTTTTTCTTCCAAATTTGA
TACAAAATTGGGGCATATTCTTGAACCCAACGGTAGACCGTTGAATGATGAACGTTTACACCACGTTCCCTTA
ATATTTCAGATATATCACGATAACTCAATGTATATCTTAGATAGTAGCCAACGGCTACAGTGATAACATCCTTGT
TAAATTGTTTATATCTGAAATAGTTCATACAGAAGACTCCTTTTTGTTAAAATTATACTATAAATTCAACTTTGCA
ACAGAACCGTATTATGGAATAGAGATGTTGGTAACATTTATACAGGATCATTATACTTAAGTTTAATTTCGTTAT
TACAGAACCACACATTCCAACCAGAAGAGAAAGTATGTCTATTTAGTTATGGTTCAGGAGCAGTAGGAGAAA
TCTTTAGTGGTTCAATCGTTAAAGGATATGACAAAGCATTAGATAAAGAGAAACACTTAAATATGCTAGAATC
TAGAGAGCAATTATCAGTCGAAGAATACGAAACATTCTTTAACAGATTTGATAATCAAGAATTTGATTTCGAA
CGTGAATTGACACAAGATCCATATTCAAAAGTATACTTATACAGTATAGAAGACCATATCAGAACATATAAGAT
AGAGAAATAAACTAGTGGCCGATTGTGCTTGATGAGCTTGGGACATAAATCCTAACTCGAAATAAATAAGCA
TATCACTAAACTGATTTTTTAAAGTTTACAGTGATATGCTTATTTTTTTATCTTACGATTTTGTACGTGCATGCTT
GCCTAGGGGTATGGCTCGAGCCATTAGTCTCTCGCACATACTATTCCCTCAGGCGTCAGCACTTACAAAATCG
GTTGTAATTTTCATTTTTATACGCATTCTTACTGAGATTATACTAATAAGAGGAATAGTAAAAGCAATTCTAAGT
AAAATTGCAGATAAGAGGTTTGTTAAAAGCAGTTCTAAGTAAAATTGCAGATAAGAGGTTTGTTAAAAGCA
GTTCTCAGTAAAATTACAGATAAGAGGTACGTTAAAAGCAGTTCTAAGTAAAATTGCAGATAAGAGGTTTGT
TAAAAGCAGTTCTAAGTAAAATTGCAGATAAGAGGTACGTTAAAAGCAATTCCATGCAAAATTGCTGATAAG
GGGTAAGTTAAAAGCAGTTCTCAGTAAAATTGCAGATAAGAGGTACGTTAAAAGCAGTTCTAGGCAAAATT
GCAGATAAGAGGTGCGTTAAAAGCAGTTCTCAGTAAAATTGCTGATAAGGGGTAAGTTAAAAGCAATCCTA
AGTAAAATTGCAGATAAGAGGTAAGTTAAAAGCAATCCTAAGTAAAATTGCAGATAAGGGGTACAGAAAAA
CTAGACTTGATTACAAAATGGAGCTTGGGACATAAATGATTTTTTAAAAATGAGATGAGACGTAGATTAACTC
CATAATCAATACGAATCTATCGACTTCTTTATTTATGATATTCATCTCTTTTTAATGGAAATAAAAGTGCGATTAA
TGTGATAATACAGTTACGTTAATTAAAAAAATAAAAATGCAAGGAGAGGTAATATGCTAACTGTATATGGACAT
AGAGGATTACCTAGTAAAGCTCCGGAAAATACAATTGCATCATTTAAAGCTGCTTCAGAAGTAGAAGGTATA
AACTGGTTGGAGTTAGATGTTGCAATTACAAAAGATGAACAACTGATTATCATTCATGATGATTATTTAGAAC
GGACTACAAATATGTCCGGGGAAATAACTGAATTGAATTATGATGAAATTAAAGATGCTTCTGCAGGATCTTG
GTTTGGTGAAAAATTCAAAGATGAACATTTGCCAACTTTCGATGATGTAGTAAAAATAGCAAATGAATATAAT
ATGAATTTAAATGTAGAATTAAAAGGTATTACTGGACCGAATGGACTAGCACTTTCTAAAAGTATGGTTAAGC
AAGTGGAAGAACAATTAACAAACTTAAATCAGAATCAAGAAGTGCTCATTTCAAGCTTTAATGTTGTGCTTG
TTAAACTTGCAGAAGAAATCATGCCACAATATAACAGAGCAGTTATATTCCATACAACTTCGTTTCGTGAAGA
CTGGAGAACACTTTTAGATTACTGTAATGCTAAAATAGTAAACACTGAAGATGCCAAACTTACTAAAGCAAAA
GTAAAAATGGTAAAAGAAGCGGGTTATGAATTGAACGTATGGACTGTAAACAAACCAGCACGTGCAAACCA
ACTTGCTAATTGGGGAGTTGATGGTATCTTTACAGACAATGCAGATAAAATGGTGCATTTGTCTCAATAGAAA
GTTAGAGGTGAGTCTTACGTTTCAGTGACGGTAGACTTACCTTTAACATGTTACATACTAAAAAATTAATTTG
AATAAGAAAGAGAGACATATATGAAATACGATGATTTTATAGTAGGAGAAACATTCAAAACAAAAAGCCTTC
ATATTACAGAAGAAGAAATTATCCAATTTGCAACAACTTTTGATCCTCAATATATGCATATAGATAAAGAAAAA
GCAGAACAAAGTAGATTTAAAGGTATCATTGCATCTGGCATGCATACACTTTCAATATCATTTAAATTATGGGT
AGAAGAAGGTAAATACGGAGAAGAAGTTGTAGCAGGAACACAAATGAATAACGTTAAATTTATTAAACCTG
TATACCCAGGTAATACATTGTACGTTATCGCTGAAATTACAAATAAGAAATCCATAAAAAAAGAAAATGGACT
CGTTACAGTGTCACTTTCAACATACAATGAAAATGAAGAAATTGTATTTAAGGGAGAAGTAACAGCACTTATT
AATAATTCATAATAAAACAGTGAAGCAACCATCGTTACGGATTGCTTCACTGTTTTGTTATTCATCTATATCGTA
TTTTTTATTACCGTTCTCATATAGCTCATCATACACTTTACCTGAGATTTTGGCATTGTAGCTAGCCATTCCTTTA
TCTTGTACATCTTTAACATTAATAGCCATCATCATGTTTGGATTATCTTTATCATATGATATAAACCACCCAATTTG

TCTGCCAGTTTCTCCTTGTTTCATTTTGAGTTCTGCAGTACCGGATTTGCCAATTAAGTTTGCATAAGATCTATA
AATATCTTCTTTATGTGTTTTATTTACGACTTGTTGCATACCATCAGTTAATAGATTGATATTTTCTTTGGAAATA
ATATTTTTCTTCCAAACTTTGTTTTTCGTGTCTTTTAATAAGTGAGGTGCGTTAATATTGCCATTATTTTCTAATG
CGCTATAGATTGAAAGGATCTGTACTGGGTTAATCAGTATTTCACCTTGTCCGTAACCTGAATCAGCTAATAAT
ATTTCATTATCTAAATTTTTGTTTGAAATTTGAGCATTATAAAATGGATAATCACTTGGTATATCTTCACCAACAC
CTAGTTTTTTCATGCCTTTTTCAAATTTCTTACTGCCTAATTCGAGTGCTACTCTAGCAAAGAAAATGTTATCTG
ATGATTCTATTGCTTGTTTTAAGTCGATATTACCATTTACCACTTCATATCTTGTAACGTTGTAACCACCCCAAG
ATTTATCTTTTTGCCAACCTTTACCATCGATTTTATAACTTGTTTTATCGTCTAATGTTTTGTTATTTAACCCAATC
ATTGCTGTTAATATTTTTTGAGTTGAACCTGGTGAAGTTGTAATCTGGAACTTGTTGAGCAGAGGTTCTTTTT
TATCTTCGGTTAATTTATTATATTCTTCGTTACTCATGCCATACATAAATGGATAGACGTCATATGAAGGTGTGCT
TACAAGTGCTAATAATTCACCTGTTTGAGGGTGGATAGCAGTACCTGAGCCATAATCATTTTTCATGTTGTTAT
AAATACTCTTTTGAACTTTAGCATCAATAGTTAGTTGAATATCTTTGCCATCTTTTTTCTTTTTCTCTATTAATGT
ATGTGCGATTGTATTGCTATTATCGTCAACGATTGTGACACGATAGCCATCTTCATGTTGGAGCTTTTTATCGTA
AAGTTTTTCGAGTCCCTTTTTACCAATAACTGCATCATCTTTATAGCCTTTATATTCTTTTTGTTTTAATTCTTCA
GAGTTAATGGGACCAACATAACCTAATAGATGTGAAGTCGCTTTTCCTAGAGGATAGTTACGACTTTCTGTTT
CATTAGTTGTAAGATGAAATTTTTTTGCGAAATCACTTAAATATTCATCCATTTTTTTAACGGTTTTAAGTGGA
ACGAAGGTATCATCTTGTACCCAATTTTGATCCATTTGTTGTTTGATATAGTCTTCAGAAATACTTAGTTCTTTA
GCGATTGCTTTATAATCTTTTTTAGATACATTCTTTGGAACGATGCCTATCTCATATGCTGTTCCTGTATTGGCC
AATTCCACATTGTTTCGGTCTAAAATTTTACCACGTTCTGATTTTAAATTTTCAATATGTATGCTTTGGTCTTTCT
GCATTCCTGGAATAATGACGCTATGATCCCAATCTAACTTCCACATACCATCTTCTTTAACAAAATTAAATTGAA
CGTTGCGATCAATGTTACCGTAGTTTGTTTTAATTTTATATTGAGCATCTACTCGTTTTTTATTTTTAGATACTTT
TTTTATTTTACGATCCTGAATGTTTATATCTTTAACGCCTAAACTATTATATATATTTTTATCGGACGTTCAGTCATTT
CTACTTCACCATTATCGCTTTTAGAAATATAACTGCTATCTTTATAAACTTGTTTGAAATTTTTATCTTCAATTGC
ATCAATAGTATTATTAATTTCTTTATCTTTTGAAGCATAAAAATATATACCAAACCCGACAACTACAACTATTAAA
ATAAGTGGAACAATTTTTATCTTTTTCATCAATATCCTCCTTATATAAGACTACATTTGTAGTATATTACAAATGTA
GTATTTATGTCAAAATAATGTTATAATTTTTGTGATATGGAGGTGTAGAAGGTGTTATCATCTTTTTTAATGTTA
AGTATAATCAGTTCATTGCTCACGATATGTGTAATTTTTTTAGTGAGAATGCTCTATATAAAATATACTCAAAATA
TTATGTCACATAAGATTTGGTTATTAGTGCTCGTCTCCACGTTAATTCCATTAATACCATTTTACAAAATATCGAA
TTTTACATTTTCAAAAGATATGATGAATCGAAATGTATCTGACACGACTTCTTCGGTTAGTCATATGTTAGATG
GTCAACAATCATCTGTTACGAAAGACTTAGCAATTAATGTTAATCAGTTTGAGACCTCAAATATAACGTATATG
ATTCTTTTGATATGGGTATTTGGTAGTTTGTTGTGCTTATTTTATATGATTAAGGCATTCCGACAAATTGATGTT
ATTAAAAGTTCGTCATTGGAATCGTCATATCTTAATGAACGACTTAAAGTATGTCAAAGTAAGATGCAGTTCTA
CAAAAAGCATATAACAATTAGTTATAGTTCAAACATTGATAATCCGATGGTATTTGGTTTAGTGAAATCCCAAA
TTGTACTACCAACTGTCGTAGTCGAAACCATGAATGACAAAGAAATTGAATATATTATTCTACATGAACTATCA
CATGTGAAAAGTCATGACTTAATATTCAACCAGCTTTATGTTGTTTTTAAAATGATATTCTGGTTTAATCCTGCA
CTATATATAAGTAAAACAATGATGGACAATGACTGTGAAAAAGTATGTGATAGAAACGTTTTAAAAAATTTTGA
ATCGCCATGAACATATACGTTATGGTGAATCGATATTAAAATGCTCTATTTTAAAATCTCAGCACATAAATAATG
TGGCAGCACAATATTTACTAGGTTTTAATTCAAATATTAAAGAACGTGTTAAGTATATTGCACTTTATGATTCAA
TGCCTAAACCTAATCGAAACAAGCGTATTGTTGCGTATATTGTATGTAGTATATCGCTTTTAATACAAGCACCGT
TACTATCTGCACATGTTCAACAAGACAAATATGAAACAAATGTATCATATAAAAAATTAAATCAACTAGCTCCG
TATTTCAAAGGATTTGATGGAAGTTTTGTGCTTTATAATGAACGGGAGCAAGCTTATTCTATTTATAATGAACC
AGAAAGTAAACAACGATATTCACCTAATTCTACTTACAAAATTTATTTAGCGTTAATGGCATTCGACCAAAATT
TACTCTCATTAAATCATACTGAACAACAATGGGATAAACATCAATATCCATTTAAAGAATGGAACCAAGATCAA
AATTTAAATTCTTCAATGAAATATTCAGTAAATTGGTATTACGAAAATTTAAACAAACATTTAAGACAAGATGA

GGTTAAATCTTATTTAGATCTAATTGAATATGGTAATGAAGAAATATCAGGGAATGAAAATTATTGGAATGAAT
CTTCATTAAAAATTTCTGCAATAGAACAGGTTAATTTGTTGAAAAATATGAAACAACATAACATGCATTTTGAT
AATAAGGCTATTGAAAAAGTTGAAAATAGTATGACTTTGAAACAAAAAGATACTTATAAATATGTAGGTAAAA
CTGGAACAGGAATCGTGAATCACAAAGAAGCAAATGGATGGTTCGTAGGTTATGTTGAAACGAAAGATAAT
ACGTATTATTTTGCTACACATTTAAAAGGCGAAGACAATGCGAATGGCGAAAAAGCACAACAAATTTCTGAG
CGTATTTTAAAAGAAATGGAATTAATATAATGGATAATAAAACGTATGAAATATCATCTGCAGAATGGGAAGTT
ATGAATATCATTTGGATGAAAAAATATGCAAGTGCGAATAATATAATAGAAGAAATACAAATGCAAAAGGACT
GGAGTCCAAAAACCATTCGTACACTTATAACGAGATTGTATAAAAAGGGATTTATAGATCGTAAAAAAGACA
ATAAAATTTTTCAATATTACTCTCTTGTAGAAGAAAGTGATATAAAATATAAAACATCTAAAAACTTTATCAATA
AAGTATACAAAGGCGGTTTCAATTCACTTGTCTTAAACTTTGTAGAAAAAGAAGATCTATCACAAGATGAAAT
AGAAGAATTGAGAAATATATTGAATAAAAAATAAAATTGTTGTGTTTACAACAATACATAGAAAACAGAGGA
AACAATCAAGTCGTTGAATATTTCCTCTGTTTTTTAGTTGAAAAAATTAACCGAAAGCCTGAATGCAAGTCTT
GATTAAATCAATAATGCTTGTAATAACACCAGTGAAATCCATATGCATACCCTCTTTCTATTTAAGATACATTAA
GTATAATATCAAACAAATAAAAAATGTTAAAAATTCCCTAATTGGCTATTTAGATTGCATAAATGTCAAAAATTT
GAAAAACATACAACGACTTTGCATAAAAAATCGTCATATTGGAAATACGTAATTTATTGAAATAATAAAAAAA
ATAAAAGAACGAAGATGATAACCTAAGTGAGGTTTTAAGTTGTTCTAAGGTTTAATTTAATTTATGTTAAAATA
GTTGGTATAAAAATACATGATAAACTATAAACTAAATTCAAAATAACTTATGGGGTAGGCAATTATGGAAAATA
AAATATAAATGATAATGAAAAAAGAGTGCTAAGGGAAATTTATAACCATCATAATATTTCGCGTACTCAAATAT
CTAAAAATCTTGAGATTAATAAGGCAACGATTTCTAGTATTTTGAATAAGTTAAAGTATAAATCTCTTGTTAATG
AGGTTGGTGAGGGTGATAGCACGAAGAGTGGTGGTAGAAAACCTATTCTTCTGAAGGTTAATCATCTTTATG
GTTATTTTATTTCTTTGGATTTAACTTATAGTTCTGTTGAAGTGATGTACAATTATTTTGATGGTAATGTCATTAA
GCATGAATCTTATGATTTACCTGATGAAAAGGTTAGTAGTATATTAAGCATAATAAAAAAACATATTGATATTCA
GGAGAAACTTGATACTTATAACGGACTATTAGGTGTGTCTGTTTCTATACATGGAGTTGTGGATAATGAGCAG
CATGTGACATATTTACCATTCCATGAAACTGAAGGAATTTCAATTGCTAAGAAAATAAAAGAAATTACTAATGT
TCCAGTCGTAGTTGAAAATGAAGCGAATCTTTCAGCGTTATATGAACGTAATTTTAATCATAATTTATCCTACAA
TAATCTTATTGCTTTAAGTATACATAAAGGTATTGGTGCTGGGCTTATTATTAATAATCAATTGTATCGTGGTGC
AAATGGGGAAGCGGGTGAAATTGGAAAAACACTTGTCTCAAAAGTTAGCGATAATGTGGAGATCTTTCATA
AGATTGAAGATATTTTTTCACAAGAAGCTTTACTGCATAATTTAAGTAATCAACTAAATGAGAAGATGACGCT
TAGCAAATTAATTCAATTTTATAATGAAAAAAATCCAGTCGTAGTTGAAGAAATGGAACAATTTATAAATAAAA
TTGCTGTTTTAATACATAATTTAAACACCCAGTTTAATCCGAATGCAATTTACATTAACTGTCCATTGTTCAATG
AAATGCCTGAAATATTAGAAGCAATTAAGAACCAGTTCAAACAATATTCACGTAACGAAATTCAAATAAAGTT
AACATCTAATGTCAAATTTGCAACTTTGCTAGGTGGTACATTAGCAATTATCCAAAAAGTACTACAGATTAATG
ATATTTACTTAGATATAAAAGCATAAAAAACTAATTCAAATGAATAATCAAAGTTCGTAATTGTCTTTATAAAAA
AATCCCTCAATCCGAATTGAATTTTCGGATTGAGGGATTTTTATAGTTCTATTGCAGAAGAAAACTATTTTAAA
AATGCTGGTAAATGTTGATAGCCACCTCTAACGTTAACAATATTCGTAAATCCTTTATATTCTAATATTCCTACCG
CTATTGAACTTCTAACACCTGATTGACAATGTACATAAATTAGGTCATTTTTATCGAAAGGTATATCTTCATTTA
AAAGTTTACCGTGAGGAATATGAATTGCTTGTTTTAAATGACCTTTACGCCATTCATCATCATTACGAACATCT
AATACATTATGTTCTTCACCAGTCATTTCAGAACTATGAATAGATGATGTGACGATATTTGTTTGTGGCAAACG
GTAACTTTTTACATTTTCAAAACCAATTAATTGTAAAGCATGAATAGCTTGTTGAACGGTAGATTTATCGCCAA
TTAATTCAATATCTTAGTCATAATCTAAATACCAACCAATTTGATTTATAAAAGTTTTATTAAAAGGAATATTGAT
AGTTCCATGCATATGACCACCATGGAATGCTTCTTTACTTCGAAGATCAAAAGCAGTTTGTGTATTGCTTGAA
CTAGGGTAAACATTATATGGTTGGTACATTTGCATACCAAATTGATTTATTTTTTTCATTTGTGAAAAATGGTGT
GGTGGAGCTGGCTGATTGAGTGTTAAAGTTTCGATAAATGAAGTTTCATCTTTAACATTAAAAGCCCAGTTG
TTTATTTTCTCATAACCCAAAGTAGTTGTAGGTAATGAACCTAGCGCTTTACCACAAGGACTCCCTGCACCAT

GACCTGGCCAAATTTGAATATAGTCTGGTAATGTTGCAGCAAATTGTATGGACTGATACATTTGTTTTGCTCCG
ATTTTTGTAGAACCTTCAACATTTACAGCTTTTTCTAATAGATCTGGTCTACCTACATCACCAACAAAGATGAA
GTCACCGCTAAATAATCCCATTGGTATACTGGAACCCCCACCTTCGTCAGTAAGTAAAAAACTAATACTCTCAG
GGGGATGGCCTGGAGTGTGTAAGACTTCTAATTTAATCTTTCCTAAATAGATAATATCTTGATGCTTAACGAAA
TGTGTTTGTTTAGGCATATTTTTATAATTAAATTCATCTTTACCTTCATCAGATACGTATATACTTGCATTCAATTT
ATTTGCCACATCTCTAATACCTGAAGCAAAATCAGCATGAATATGTGTTTCTGCAGCTTTAGTAATTGTGAATC
CTTCTTTATCTGCAACTTTTAAATATTTTGTTAAATCTCGTATAGGGTCAATAATCATTGCTTCTCCTGTACGTTG
ACATCCAATTAAATAAGATGCTTGTGAAAAATTGTCTTCATAAAATTGTTTGAAAAACAAAAAAAACTCCTTT
TTAAAATAGATTTTATTGATTAGATAAATAAGTTATGATTTGCTTGCTCAGTATGTCCAATATAAGTACCTACGCC
ACCATAATCGACTTCATCTCTTAATTCTTCTTTTGAAATTCCCATAACATCCATACTCATGGTACAAGCAATTAAC
TTTATATCTTGATCGATTGCTTGATCGATAAGTGAGTATAAAGAATCAACATTTTTCTTGTTCATTACATAACGC
ATCATAATATTACCTAGTCCAAACATATTCATTTTTGATAATGGCATATGTATTGGATCCTTAGGTAACATAAGGT
CAAACATTTTGGAAATACCTTTCTTTTTAACGCGAGTTGATTGCGCTTTTTTTAATGCGTTGAGGCCCCAAAA
AGTAAAGAAAATAGTTACATCTTTACCTGCTGCTTTAGCGCCATTTGCGATGATCATTGCTGCTACTGCCTTAT
CTAACTCACCGCTAAATAAAACAATTGTTGTACCTGTAGCAGTGTCATTGATTTCAAATTCTTTTGGCTTTTCT
TTTTGAATAATTGCATTAATTACATTTGCTTCTTCAGTAAGATTTACAAGGGTATTCCCTGTTTGTTTCGCCCAA
CTTTTAATATCACTATTGAAACCAGGATCTGTAACTGTTACCTCGATTTGCTCACCCGTTGAAATATTGTTAATT
TCTTTACTGATATTAACAATAGGTCCAGGGCACTGAAGACCTCTAAAATCAAATTGTTTACGATTCTCTTTGAT
TTCAATATCTTTTTCTATTAAAGGAGCACTATTGAAGTTCTTTGCTTCATAATCTTTATATCCACCCTTTAAATTC
ACGACATCATAACCTTGTTTGGCTAAATAATCGCAAGCTTTAGTGCTTCGGTTACCGCTTTTACAATGTATATA
ATACGTTTTGTTGCTATTCTTATTGAATGATTTAATCTCTTCTACTGGGTGTAAAGTTGAACCGTTAATGTGTCC
TAATTCATATTCTTCTTTTGTTCTAACATCAATCAATTGACCCATTTTTGCCAATTTTTCTAATTCTTCTTTGTTTA
ATGAATTAATGTGTACTTCTTTGTATTGTTCCATACTTACCTCCTATAAATACCTATGAGGGTATAATAAAACGGA
TAGAATCATTTGCCAAATACCTATATGGGTATTTGACAATTTTGTTTTAATTTATTATTATTAACTTAATCAATTTA
TGTGGAGGAAATGAATATGACTTATGATAAAAAAATGATTAATCGTATAAATAGAATACAAGGTCAATTAAATG
GTGTCGTAAAAATGATGGAAGAAGAAAAAGATTGCAAAGATATAATTACGCAACTTAGTGCATCTAAAGGTT
CTATACAACGTTTAATGGGGATTATAATTAGTGAAAATTTAATAGAATGCGTTAAAACAGCAGAAGAAAATAA
TGAAAGTTCTCAAGAATTAATTAATGAAGCAGTTAATTTATTAGTTAAAAGTAAATAATGGATATAGCAAATAT
GACTATTATGTTGCTAATTGGCGTACTGGGTGGATTTATATCTGGATTAATAGGTATTGGGGGCGCAATTATTA
TTTACCCAGCTATTCTTATATTGCCACCATTAATAGGTATACCTGCGTATAGTGCATATATTGCTTCGGGACTTAC
CTCTAGTCAAGTATTTTTCAGTACACTTAGTGGATCATTAAATGCAAGAAAACAACCAGCTTTCTCTCCTAAAC
TTGTTATATATATGGGAGGGGGTATGTTGATTGGAAGCATGTTAGGGGCAATTTTAGCTAGTTTGTTTAATGC
TACTTTTGTAAATACGGTATATGTAATAATCGCCATACTTGCTTTAATATTGATGTTTATTAAAGTTAAACCTACT
ACACAAGAGACGAAATCTAAACCTTTGCTATTTATTATAGTTGGATTTGGAATTGGTGTAATTTCGGGAATTG
TGGGTGCAGGTGGAGCATTTATCATCATTCCTGTATTATTAGCATTATTTAAATTACCAATGAATACGGTAGTG
AACAATAGCATAGCAATTGCTTTTATATCTTCAGTAGGGGCATTTTTTATAAAATTAATGCAAGGATATATACCA
GTAGAAAGTGCAATTTTTTTGATAATTGAATGATGATCATTTTCTGAAAATATTATGTGGTCATATAATATAATG
CCCATCATTTCACTAATCTCTTTTATTCTCTGAGTTATTTTGATATCTCCTGGAGAAGGTGTTACATCTTATCAAG
AGTAAATTACAAAAGAATCATTTAAATCAATACTTTCACTTTGAATACATGTATTTGAAGTGGAAGGTACTTAT
TTCAAAAATAGTAAAACCTGTATCTTAAATTACTTAATAGTAACATAAGATACAGGCTGATTTTTTATTCATTGT
TATTTATACTAAAGCACCCGATAGCTCTGAAAACAATCACAAATTCAACTTTTCAAAGCCACAGCTTTAAGTTA
TTTTGTCCAGACAACCCCCATTGCCCAACCCATTTTATGGAATTGGCATCCAGGCAACAACTTTTCATATAAAT
CGTAATAATTTTGTTCAGATAGGTACTTATCTGAAGCTAAATGCTCAAGCCATGATTTAGATGTGTTGTGATTAT
AAATTCTAATCGCATTTTTTATTCCAAATTTGAAACAATGCGGTAAAAACTCTTGGATTGCCCCTAATTTATAAA

CATAGGTAGGCGGTGTCTCGACTTCAGATACATTATCAAGAATAACTATTCTTCCTTCCTCATTCAACAGTTCC
TTCATTTGCTGTATTACGCTGGCTATATCATCCAAATGATGAAAGGTTGTTCGGCTTACAATAAAATCAAACTT
CTCATTAAAATTAAGTTGTTCTGCATTCATATTCAGATAGACCGTATTTGTTAGTTGACGTTTAGATTTGGCAA
GATCGAGCATTTGATTAGAAATATCAATCCCTACCACTTCATCATAATAACTTGCTAATTTCTCCACTAACAAAC
CCGAGCCACATCCGATATCTAATGCTCTGCCTTTCTTTGGAGACATATTAGACACAAAGAATGAATAATCATTC
AAAAGCTCATTCACGAAATCGTAATCTTCTGCAACCTTATCAAACTGTGATTCTATTGTATTCAAAAAGATCCC
CCATTCCTACTTTATCGACATTCTTTCATTACTTACCACTTTAGATGTTTTTTCGTTGGGGATAAAACTTCCCTT
TAGACAATTTTATCCAAAGACAATACAACAGTGCAACTTTATTAAAGTCACTGTCCTTTATCGCAGCCTTTACT
TTTTAGTAAAGACAGTGGCTTCTCTTATCAAGTTTCAAAACATATTATTTTGAAGAAAACGTCCATCTGAAGT
GTCAAGTGCAAAATTACATATAAAGGTTTATTCTAAAATGAAAAGATGATACAATCATATTCAGTTACATAAGG
AGGTTTCAATTATGTGCACCAGTATCGCAGTAGTAGAAATTACTTTATCTCATTCATAATGAAAAAAATGGAAA
GGAGATAAAAGTATGGGTACTTTTTCTATATTTGTTATTAATAAAGTAATCAAAATTAATTGGTTATAATGAACG
CTTAATGTCAGTTCATTATAACCAGTAAGGAGAAGGTTATAATGAACCAGAAAAACCCTAAAGACACGCAAA
ATTTTATTACTTCTAAAAAGCATGTAAAAGAAATATTGAATCACACGAATATCAGTAAACAAGACAACGTAATA
GAAATCGGATCAGGAAAAGGACATTTTACCAAAGAGCTAGTCAAAATGAGTCGATCAGTTACTGCTATAGAA
ATTGATGGAGGCTTATGTCAAGTGACTAAAGAAGCGGTAAACCCCTCTGAGAATATAAAAGTGATTCAAACG
GATATTCTAAAATTTTCCTTCCCAAAACATATAAACTATAAGATATATGGTAATATTCCTTATAACATCAGTACGG
ATATTGTCAAAAGAATTACCTTTGAAAGTCAGGCTAAATATAGCTATCTTATCGTTGAGAAGGGATTTGCGAA
AAGATTGCAAAATCTGCAACGAGCTTTGGGTTTACTATTAATGGTGGAGATGGATATAAAAATGCTCAAAAA
AGTACCACCACTATATTTTCATCCTAAGCCAAGTGTAGACTCTGTATTGATTGTTCTTGAACGACATCAACCAT
TGATTTCAAAGAAGGACTACAAAAAGTATCGATCTTTTGTTTATAAGTGGGTAAACCGTGAATATCGTGTTCT
TTTCACTAAAAACCAATTCCGACAGGCTTTGAAGCATGCAAATGTCACTAATATTAATAAACTATCGAAGGAA
CAATTTCTTTCTATTTTCAATAGTTACAAATTGTTTCACTAAATTAAAGTAATAAAGCGTTCTCTAATTTCACAA
GAGGACGCTTTATTCTTCCCAAAAATTGTTCAATATTTATCAATAAATCAGTAGTTTTAAAAGTAAGCACCTGT
TATTGCAATAAAATTAGCCTAATTGAGAGAAGTTTCTATAGAATTTTTCATATACTTAACGAGTGCTTTCACCTT
TGAATATAGTCCTTCCCACTTATCATCACACTCTCCCCGATAGCCTTTTCTAGCTATATCCAGTAAAGTTACATGC
TCTTTAGGTAAAAGAGGTATAGCCCATTCTGCAGCGACATCTTTCGAGGTAATTTCACCAGTAGTCACTGTTT
GCCACATTCGAGCTAGGGTTAAAATTACATTACGCTCATCACCTTTTATCCCCTCAATTAGTTCTGGCAAAGAA
TCCTTAATTGCTCTTCGAATATCTGTCAAAGGTACGGAGACAAGTATACTTGAAGAATCAGGACCAAATAGAG
AAATACTATTCTTTCTTGCTTGTGCTAAAACAATAGCCAAATCAGGATCATAGCTTGGTTCCTGAATTTGTCCA
TTCTCAAATTCACCCCTGAGCCACTCACCGTATATAAATTCTCTTTTTGGAGGATATTGCCAAGGGACAACTTC
ACTCCTATTTATAACCGTAACTTCAAGTGGTCTAACAGAATCCGTATTTCCAATCTTTCCTGATATAGTCATTAG
TCTTTCTGTTAGTTTTTTTCGAGTTAATTGAGGTAAACTATGATTCACGACGACTAGAACATCTACATCGCTGT
TAATGCGTAAACCACCATTTACTGCTGAACCAAATAGATATACTCCAACTATTGAACTTCCAAATAAATCTTTTA
CGATTTTTAATGTTTGAATCGCTTGATTTGGTATTTTTCCGTTAATCAAATTGCTCATGATTTCACCTCGTTGAT
TATGTTCATATAAAGTTTATATTGATACTCAATTTACTTACCCTAGATTGGACATATACTTAAATTACTGTTCAATA
AAGCTGACCGTTAGCGTTTAAGTACATCCTTTCACAATTTGTCTACAGATTAATAATTATTCTTTATTATACAGA
TCTCCATATAATTTTTGAATTTGGTTCTGTAATTTTTTATTTTCTTTTTCTAATTCCATTACTCTTCTTTTTAAGGT
TTTAATAAGGATTTCCTCCGAACGAGAACTTTTCTTGGGTTTTGAGACTACATTTGCTGTTATTTGACGCTCA
CGAAGGGATTCGATTCTTTGCCTAATATCGTGTTCCTTATAAAGCCATGATTTAGAAACATTAGCTTCCTTTGC
TATTGAATTAAAATTAATAACTTTACCTTCAATCGAAAATTTAGAAATCGCTTTGTCTACTTTTTCCCTTGTCTTT
TTTGATTTCTGCTTCGCCAAACGTACAATTTCTGTTGTATTTCTAACTTGTTTATCCATTGATAATTACCCCGTCA
AACTTCCAATGATTTGTTCTAAACGCTCTTTAACACGGCTATTAGTCTCTACTTGTCTTTGCCATTGTTTATCCT
TAGCTATGGCTAATAACTCTTCTGTACGCTCTAACTGTTCTTCGTGCTGTGGTAAGAATTGCTTACTGGTACAG

AAGTGAGTGCAATCTAAGCATGCATTCGCATGTGGACAACCACCTGCTACTACTGGCAATCTACAATAACCAT
TTGGAAGCACTTGTGCATTTATATTTTTCTTGAACCATTGAAGCTCTACATCATCGACTTCATTATCTTCATCTA
GATCAAGCACATCTCCATTATTGGTAACCAGTTTTTCCTGAAATTTAGTAAATTCATTTTTTAGAGTTTCATCAA
AGATATGAGCGTATCTGCTTGTCATTTCTGGGCTTTCATGCCCCAAAAATTTCTGCACAATATGCTGGGGCATC
CCGTTGTTAATCATTCTTGTTCCTACTGTATGGCGAAAGGCATGGGCATGGAATCTATAAATCTCACCTGATTT
ATCCACTATATTTTGCTCATAAGCTAATTTATTTAACTCACCTCTAAATGTTTCTTGTTTTAATGGCGATCCATCT
TTTCTTGGAAAGAGGTATTCACTATCTGGAAATTCCTCTGAAACTTTATCTTCCCGAACTTTAATAAGTAAAGC
TACCTCTTTAGATATTGGAACTATATGCTCCTTTTTCATTTTCCATTGATAATACTTTAAAAAGAAATCTCCATCT
TTGTCCTCTAATAGACAGCCTTTTTTCAAGGTGCACAATTCACTTATCCTCATTCCACATTCTTGAACAATCATA
GTCATCGTAGCTATATATTCGGATAATTTATCAAGATGACTGTTCAATTGCTCTAGGACGAATTCATCTATAAAG
CGTGGTTTTGCTCTTGGTATTTTCGGATAGTCCTCAGAATAAATTAATATTTTGGAAGGAACATCATCCCATTC
TAGCCTAAGAAGGGTACTAAATAGTCCTTCCAATATAGAGATCCTCCCAGTTATTGTACTAGGTTTTATTCCCAT
CATGTTTAGTTCACTTAAATATGCTTCAATTTCCACTCTCGTTAATTGGTGTACTCTCTGAACTTGTTTAAATTTC
ATGTCCAGAAAATTAAAGAACTCTTTAAGTCTTTGGGCAATATCACTTACATAGGAAAAGCTATCCACGTTCA
ATCTCAACTTACAATATCTTTTTACAAGTTGTTTAAAATATGTATTCCGAAACCCTTTAAAGTTAATTGTATATTC
ATATTGTGTTGGGTTAACCTTATCATCTGGCAAAGGTAAGTTACGTCTATCCCAAACGTCTTTATCCCACTCCTC
TCCATCAAAATAAAAGTTCTCATAAAACTCCATAAATTGTTTTAGATTAGTAACATAGTAGGAATTAGCTTTTAC
AGGTGTTTTTTCTTGATTAGCAGTAATCTTATAATTAGTAGTGGTAATTCTAACACCCCGTTTTGTCAAATAAGT
TCTATACTCCGTCATTGCTTTTTCAATAGGAACTTCAGTAATTGAAGTAATGCTAGGATACTTTAAATCTAAGA
AATCTAACATTTTATTAATTACTGTTCCTTTTCTAATCCAGACAGTTTTTGCATTCCATATTCCATTGTTTAAATG
GTAAAAATAAAAATATTTCAATTCTGTTCTTAACCACAGATTTTTAACACGTTCAAAACGAACCCAACGATTCC
TTAAAGCAGGATTCTTACTTAATTCTATGGCAGAAGGATGTGGACATTTTCTTATATCCCAACTATTATTAGCCC
AAAACCCCTGCATTTCTTCATTCATTACAGCTATTTTTTTGCTAATCTCACTCTGACTAATAATTTTCCTTTTACT
AGAAGCATTCATTTCTTATGCTCCTTTCTCTCGAGGTATTTATTAAACTCATTTTTCATATCCTGATCTGAAAGAT
GAACATAGGTATTTAACGTTGTCTGAACATGTGCGTGACCTAATCTCTTTTGAACGAACGCAACATCCCATCC
TTCCCTAATTAGCTGCGTTGCGTGAGTGTGGCGAAGCATATGTGATGTAAATTCTATTCCAGTCCTTTTAACTA
TTCTTCTAACTAGATCAAGAACACTTTGGTACTTTAGTGGTTTCCCAAAATAGCCTTCTTTTAAGGAAATAAAA
ACATAATCATGCTCCAATTCCTCACTATACTCATATATCAAGTAATCTGTATAAAGTGACATAAGTTCTTTACTCA
CATGTATTGTTCTTTCCTTCCTTAATTTAATATAAGCTTCATTAACATTAACATCTCTAGGTGTTAAATGGATTTG
ATTGTCCCAAGTGACAATATCTTCAAGCCTAAGCGATAACACTTCACCGATTCTTAAACCACCCTCATACATAA
GCATTAAAATTAATTTATCTCTTTTCGTATGACAAGCATCAATAATTTGCTTAACTTCCTTTGATCTCAATGTTCT
TATCTGTTTCTTTTTAACCCTTAACTTTAAGACATTCTTTTGGTATCTACCCTTATTAACATGATGTAAAAATCCT
TTGAAATTTCTTCCCTTTGCTTGTTTAAATACATCAATTGATTTAAATTCTCCTAATCTACTTAAATAATCAAGAA
AACTCATAACTACATTTAAAATTGTATTCACTGTCGTTTCTTCTCTTATGGCTTTTTTTGACTGAAGATCAATTA
CATTTGATGCTGAAGGATATCTCAACCAACCTACGAAGTCTGCTAACAACTCAAAGTTAATATCATTAAGAATA
ACACCTCTCTGTTCCATGAACTCGTACAGCAACTTTAAATGATAGCAGTATGCCTTAATGGTATTAGGAGACTT
ACCAGTATTATCTAAGTATTTAATAAATTTCATTACTGGTTCTATTAGCTGGTATTCTTTATCTAGTAATAAATACA
ATGGATACGGCTTATTCTCCACTTCTATCCTTTGAACCTTCACATGTTCCCACCTCTTTAAATACTTTAACTACTA
CAAATTATAAACCTTGTTAATTTTTATTTAAAGTAAAATATCCCTTTAATATCTCCTCTTAAACTACTTTAACTACT
ATTATTTATTATACTATGGTTAATACATCTCCACTTGGATGATTATGACCGAGCATTATACTATTTGCGTTACTGA
GTATCGCTGTTTTGAATATTTCTCTAGGGTGAATCACCGTTTGGTTAATAGATCCAATCGATAGTGTTTGAATAT
GTGTAGGTTCATTTTTACTGTTCATACATATGAGAATGAGATGCTCTCGGTCACTGTTTCCAATGAATGAACGC
ATGATTTCTGCCGCATCCTCAGGGTTTGAAATACGATTTTTTAGATAACTTAATGTATCTGTTTTTATCATTTGT
AGTGAAACAATATTGATTTCCTTCATCGTTTACCTCCATATATAGGTTATGCTTTCAAAGTCCATTTTTGACGTG

CTTTAGGGTTGAGTGGGTGCATGATTTCATTTGTGGCTGGATTAATGAGCTTTTTGACTTTCTTTTTATGAGG
CTTCAACATTTCCATTACTTGTTCGACACGTTCTACAACAACTGGCCGCTTCGCATAAGCACCATAAGCTAGA
ATCACTGTGTCACTTTCGCTAATTGCTTTCATCAAGTGGATGTCTGTGTGTTTATCATAAGGTTCTTTAATATGT
TTAAGGTTCTCTGGTGTTTTAATATTAGAGAATAGATTTACAAGATATACAGCACCGTATCGTTCTGAATTCGC
TAATTGATTGAGGATAAGAACAGTTGTGAGATCGAGTGATAATACACCATCTAAATGAGGGTACATCGTTATC
ACTGTACAAGCGGGTTTCTTTTCATCCCATGTTTTCTTGAGTAAATAGCGGTGTTTTTCATCCTTGCTAAATATA
GCTTCTGTGTATATCGTATTTTTGATTGTATTCATATATATAATCACTTCCTTTAGTATTCTTCTGGTAAAAGCATC
ACATAATAAAAAGCGTCTACGCCATCTTCACGAATGACGTAGACTTTCTTAGGTAATGCATTTTGATTTTTTAC
ATAGTTTGTATAGTGATATTCCAATTTGTATGCAGGTTGTTCTTGTTCATGTGTGATTGAGAGTATATTCTCATCT
TCTTGCAGTTTAAAAATGTGTAGGTAATCTGTATGAGGTTGATTATCTCTTTCTTTTACCATATTCCAAAGTAAG
ATTTGAAGGTCTAGAGATAGGTATTCACTAATACCTCTTGTGATGTATCGATTGATTTTCATGTTATTTTACCTC
GTCTTGAATTTCTTTCATAATGATAATCGCTTGGCTAATAATCGTAACAGATATTTGTGCCACTTTGATCCAGTT
ATTCATGGTGAGTCCCTCCTTCTCTTAGTAAATGACGTTCATCGATAATCGTATTTTTAGTATCTGTGAGATATA
AAAAGTCCATGTCAAAATGATTCAGATAACCAACGCTGATGAGTTGGTTATTAGCGTACATAAGAAATGGATA
GATACTTAGGTCATGTAGTTCATTATTGTAGTAGGTATAAGTTTCAAGTGTAAGATGTGCAAGTGGGGAATCA
TTAATAAAACGTTCCGGTAGAATATTTTCTGCTGCTTCTTCTAACGCTTCGCATTCCCATGTTTCATTGTTAGAT
AGTTGGAATAGACGAGTTATATATTGTTTGAGTTCTTGAGTGGTCGTTTTCATATCATTGCCTCCTAGATAGTG
TTATAGTGATGTAGTTTATGTACATCATTGGGATAATATATATTTGATTTGTCATTTATTACGCATCCCGGTGAGA
ATGAGAGAAAATTCCATATGAAAAACCGCTTCAAACCTTGGTATGACAAGGAAATCCCGAAATTCCGCCTATT
TTGACGAACAATCAACTCATTCTTTATAACTATTGATGTTAGGGTGGGGCTCTGCTTTCTTATATATTATTTATTT
ATAAAGAATAACGGGATTTTGGGATTGTGCTTGCACAATCCTTCTGCTTCTTCGAATCTGCAAATCCCAATCAT
TTCCCGATAAAAAATCATTGTGGGATGTTCTTTAGCAATTTCAATATAAGCATCGTGTAGTTATGAAAAAAATT
ACGACAATGACTGTTTCATTAGATAAGTGTTATTGAAATTGATAAAGAGAATTTTAAAAATGGTTAGATAAAA
TAAATGAAAGAATATAATGAAAATTATTGTTATAACAATGATTCTATTAGCTAAATAGTAAGATATAGTGTTTGG
GGCAAAAACAAAGACGAAGTGCTGAGATGCACTTCGTCGAGTTGTTTATTATTGAAAAGTTGTTTTATGATT
TCGTTATTAAGTTTGAGTGTGACATAGAATTGTTTTTTATGATTACCATCTTTTTTAATATCAATGCGATCAATCA
CTGATAGATACAATGCTTTGAGTCGAGATTTTTCTATGTGCTTAATATCATGAAAGATGTGTTGTAATAGTTTAC
TGATTTCTTTGGCATCAAATAAAGGCTTATCTTCATTTTGTTGATTTTTGAGTTGGTTGATTTGATTCGTAATGT
CATTGAGTTGCTTTTCATATTTTTGAATACTTGGTCTGATTACTGATGTTAAGTCCGGATTATCCTCGATGGTTT
TAATCAAGTTATTTAGTTTGATTTGTACTTCATCATATTGTTGTTGCTTATAAGCAATATCGTGATGAAGTGCAG
CGCCATCAACTTGATTTTCTTGATTGACGTGTGTTACTACGCGTTGAATGACTTTATCACTTTTGACTATTTCAA
GTATTTGCTTCATCACATAATCTTCAATCACATCAGCTCTTACACTGTTTGCCGAACATACTTTGGAACCCTTGT
TCCGAAAATTACTACATGAATAGTAACGAATACGTTTCTTAGTCCCGTCTTTAAGTGTATTCGTGGTATTGCTT
GCTGCCATAGGTGCGCCACATTGGGGACAGTGAATAATGCCTGTAAGCAGATTCGTTCCTTTGCCATGGACT
TGGGGTTTTTGACTGACTTGTTTTTTACGCATTTGTACTTTATCCCATAAATCTTGATTAATAATGGGGGAATG
CTTACCTTCAGCTATCACTGGTTTATCATTAAGCCCTTTACGACGTTTTTCACTCCAATCTTTGTATTTCGCAAA
TTGAATTTTGCCGATATAGAATGGGTTAGCTAATATATATGTGATAGAACTAATACTAAAAGGTTTACCCTTTTT
AGTGACATAGCCTTTGTGATTTAATGCATTGGCTATTTTACGATAGCCATGGCCTTTGGCATAGGACTCGAATA
TATATTTCACAATATTAGCTTCATGTTGATTAATCATCAGTTCATGTTTACTATTAGGTATTTTGTCATAGCCCAG
CGGCAAATTGCCTTGATAATAGCCTTCTTGGGCACGTCTCGTTTGACCCATAAATACATTCTCGACAATGTTAT
TACGTTCGAATTCTGAGAAACTCGCAAGTATTTGTAACATGAGCTTACCCGATGAAGTATTGACTTCCATACG
CTCTGATAAACTGAAAAATTCGACATTTTGTTTGTGTAAATCTTCGACAATTTTGAGAAGATCAGATGTATTAC
GAGCTAATCGGTTTGTTTTGTAGACCATAACACAGTCGATATAGCCTTCTTTCGCATCCTTCAATATACGTTGG
AGCTCAGGTCGATTCATAGATTTACCTGAAATACCACGGTCAGCGTATATATCTTTAACTTCAAAATGATGGAA

GTCACAGTATTCTTTGATTTGATTGATTTGTCCGTCGATACTATAACCTTCTGTGCTTTGCATTTCTGTTGATAC
ACGTACATAGATACCGACACGTTTTGTTTTAAGTTGTTGCATTATGTTTCATCCTTTCTTCGTTTATGCAATCGA
TGATTGCATGGTTTGATTGACGATATTGAGTGGTTCATTTTTGAAATAGATTCCTATAAGATTTTTATCTTTCGT
AATGTGAATGGTTTCAATATAGGGGTACAATATGTTTAACGTGAAACGTTTTTGAATAATATTTTGAATGGTGT
GTTGTATTTGATGTCCATTGATAGATGTAGTGCGTTGCGGTTGTTGACGTAATGATTGCGTTTGTTCTCTGAAC
GTTTCTGCATCGATGATGCCTTGTGCCAACTTTTCTATCAGTTGTTCTTGAGTCAATGTGTGATGTTTTTCTATG
TTTCTTTGTCTTTTGATGCGTTTGTCAATCGCACCTTTAATTTTTGTGTAGATGCGTTGATTTTGATAAAAGTCT
TTGCACACTTCTAATACTTTATCTTCAAGTGTTTGTGCATTGATGCCTTTAAAATCACAGACAAAGCGAGAAG
CATTCATGTTTTTAGGACAGACGTAGTAACGTAATGTATGATTCTTTTTTCTAACGGTCATATTCGTAAGTGTTG
TATTACAACATGGGCATTTGATTTTTTGTTTTAGTTGGTTATCCGAAGATGTCTGTTTGATTTGTTTTTGCGATC
GAAGTCTCTGCGCTTGCTCATATATACTTGTGGAAACAATAGAAGGAAACATATTGTCGAATTGGCCATATTG
ATTGTTGACACGACCACAATAATTAGGATTGATGATAATGTTACGAACTTGATAGGGTTGTCGATTGATATACG
TGTTATCTTCTTCTAATAACTGTGCAATTTTCTTATAACCATGACCTTTAATGTAATAATTGAATACAGCCTTTAC
CGTTGGTGACTCATTTTGATTGATGATGAATGCTCCGTTGTGATATTCGTAACCAAAGGGCGCATGGGTTGTA
ATCAATCGACCTTGCTTTGCTTTTTCTTGAAGCCCATTTCTGACTTGTTCTCCAATGTTATCTGATTCAAGTTCG
GCCAAGCTGATAAAAATATTAAGCTTGAATCGGTCGAAAGCTTGATCCATATCAAAGTAACCATCGTGTACGC
TTAAGATATGAACATGGTATGTTTGACATAATTTGATGAGTTTTAATGCATTTTTCAGATTACGATGTAATCTAT
TAAGACGATAACAACATAATATGTCACATTGCCCTTGTTGAATTAATTGCGTCATTTGTCGATACCCACTACGAT
TATCTTTGCGTCCTGATTGTTTGTCGCTATAAAAGTTGATATGTTGAATATGATGTTTTTCGGCTATTGCTTCGA
TAGCCTGTTTCTGTGCTGCAAGAGATTGTTGTTTCATCGTACTTTGACGTAAATAGCCTATGACTTGTTTCATA
TCGGCTCCTCCTTTCACAGTAATAATATATATTTATGGATGAATTGATATATAAGCCCAACATCAATAAGATGTTG
GGCGTTCATATTAGTCATTCATTTGATTGATTTCTTCAATTACCAAATCGGCTAATATCTCGATAAGTTCATCCAT
GTTTTTCACTCCGTTATTTGTTCTATCTTCAATACGTCGATTATTCAGTTTGATGCTTCACGGTTGTATGATAAA
GACAATCAGAAATCTTCGTGAACTCCTGAAGGGCCTATCCCCTCATTAGCGGATTTAAAAAGTTCTTTCGCAG
CTTTGTTATCATTTGCCGGTGTCCAATTTTGAATTAACGACTTATCTTTAGTTAATCCCAGGATAGATGCAAACT
CTACATCTAATTTTAGATGGTAAAATACAAGTGATTGTTTTTTACCGCTATTATCTTTGACACTTCTTTTAGTTGT
TTGGCGTCCACGGTCAGCTAATATGAAACCTTTATCTCTTAAGGCGTTGACAACATTATTAACATCTTGAAATT
GATGATTGTTTAGCATCTGTTTAAAAACGTTTGCAATCATTTTTACTTCGATATGGTCATCTTTTAATGAGATTA
ATCCATAGTTCTCAAACATATTTTTCAAAGCACCTTCATCTGAAAACTTACCTCTGTTTTGTGCTACAAATTGA
ATGATGACATCGATAGCTTTATCAGCTAATGAGCGTTCAGAGATTGTATGACCATGATAATCAATAAAGTAGTC
TCTTATTTTAGCGATATCAATATCTGTAGCTAAAACACGACCTAATATTTTTGCAGATGTTGTAATGACTGCATA
ACGCTTAAACATACGATTGCCTGTGTTGCTTTTATCATCTTTCAATTTAGCTTCAAACCAATCTACTTCCTTGTA
AAACCATTGAATAACTTCATCTTCACGATTTATAAGATATTTAGCTACTAACGGTAAAACATGACCATAGTTTAG
TGCTACAGCTTTTTTAATATTGTCAGCATTGGTCGCATTTGTAGTGAATTGTTCATTAATCTCGATGGTTCTTAC
ACGTAATCCATCGTTTTGAGCTGAATCATTAAAAATACTGTGTTCTGACGTTGAAATGACAGAAGTACCCCAA
TTCTTAGGCGTTTTAACTTCTCCATGAACGTTTGAACGTTGACGACCTTGACCTTCAGTGATGGAGTACAATA
ACCCCGTTGTATCTCTAAAAGTTGCTGATGAGAGTTCATCAAATACAATAGGTATACCAAAATTGTTACTCAAG
TAACCTTCAAGTGCATTACGTGTGGCATTCCAATTTCTAAAGAGAGTTTCATTACCTTTGGTAGGGTTACCAG
CGACTGATACAGCTAAAGAAGCTGCAGTTGACTTACCGGTTGAGGATTGACCTGTAAAACTAAAAATGATTC
CGGCAAATTCGGTTTCATGTTTGTGCTTCAGGAAACTCGTCACTAAGGCAGAAATACCAAATACGACTGCTA
ATTCTAAAAGAAGAGAACCTTTAACCTCTTTTAGATACATGTTAAACCAATTATCAAATGTTCCCCTAGGTGCT
AAGTCATAAGTATTCTCACAAATGGCGTCAGATGGAGATTTATTATCAAATTCCGTAGTAGTATAGATTTCATTT
AACGATACAATAGGACCAAACGGTGTTTCCAGTATACCTACCCCTTCATATAAGTAGGAAATGGGTAATTGGT
TGCGCATTTGTTGCAACGCATAACCTAAATCTTTTGTATATTTTTCATTAATACTAAATCCATATTTCATTAAAGA

GGGCAGTTTTTGTGTTGTTAAAATATCACTAGATTCAACAATTACTTTTTGATCCTCGTCTGTAATAATTACTTT
TTCAGTGTTAGTTTTAGGGTCAATAAACTTATTTTCGATAACGATAGGACCTGCGATTTCAACTTCAGTAGGC
ATTCCTCCTTTTTCTTTGGGAGGCTTGTCTTTATACCAACCTTTTTTTGATTTGTATCGTGGTGAAGGATTAAA
TGAAGGGTTAGTTTGAGTCATTAGCGAACACCTCCTTTCGAAGGGTTGCTGTTATAGTGTGGATTAGGACCT
GTTTTAAGATAAACTAAGTGACCGTGAGTATCCTTACCGATAATAATAAATGGAACACGTGGCGCATGTTTTA
CAAAATATGCGAACCAACGTCCAACATTTTGTTGTACAGCTTTTGAACATTGTACATTTGCACGACTGTTCAA
GTCATGAAATGTAAATTCTTCTCCTTCAGGTAAATTGAATGAAATACCTAATATTTTCTTTTTTAACTGCTCGAA
TTTATCTGATTGATTGAAATACATTTAACTTCCTCCTACTAATGAACTAAGATAGGAAAATTAAATATGCGCACA
ATTAACTTTCTTAAGTTCATTTTAGCCAAGAAGTTATCATTTGAAAGCTTGTAAAAATATGTTAGAAATTCCGT
ACTAAATTAAGATGGATTTTTGTTGACAAAATAAAAAACGCTGATTTAACAGCGCTTTAAATAAAAATTAATCT
GAAGTTATATAAAAGTAGTCAGAAGGAGTGGTGTTATTATCATTATAATAATCTAGTGCTTTATTGATTTCTTTT
ATTAATTTATCATGATGTTCTTTTGTTATTTTCTTTGGATATTTGAGTTCTTTTTCTTCTAAGTCTTTTGGAGACA
CTAGGTCACTATTTAAAATTAATGATAACAGCATATTTTTAGGTGCCGAAGCTCTGGTGTCTCGATGAGTAATA
TATTTTGCTAAATTACTGTTTATACTTACTTTTTCATTTTCTAAAAAGTTTGTACTATTTTTAACATGAGCATCAG
CAATTTTGGTACTTATTTCAGTGTTTAAATCGTCTATTTCATCTAAATAATATTCAAGAATCTGAAGTTTATCTTT
AGAAGTGATACCTTTTTCAGTGAAAATCTTAATAATGATAGATTTGATTCCAATTTCTATATTACTGTCTACTAG
AAAATAGAGATAATAATTAGTCAGAGCATGTTCATAATCTCTTTGATCAGTAATTTCATGATTGATGAAATAATA
TAAGTTCTCTTTGATACGATTAGAAGAATCTATATCTTTGTCATTGATACCGTTTTTAAAAAAGTGTAGATAAAC
TGAGTTGTTTGAGAGTATATCATATAATACAAATAAACTTTTTGGTTTAAATACATATGATTTTCCTGATTTAAA
GTTTGCTATGTTTATTCCTAGAGCGTTGCAGAATTTATTAAAATTAGTTGTAAAATTATTTTTTTCTTGAATAAA
CATGTCGTTGTCATTTAATTCTGAACTATGATTTTTAATCATATTGATATTTTCTTCAATCTTGTCACTACTTAAAT
TTTCCATGCAAAGTATACTATATCTTACTATATCACTTATATTTATGGAAAAATCTTTAGGAGAACGATTTAAAGT
ATCCTTTTTATTATCTTGATCTACAGCCCTACATGAATCATCAAAAACTTCTTTGACATTTATTTTATCAATGTTC
AAAATTATGTCTACTAAAGCATTTTGAAAATCATCGCTATATATGTCTTTATTATTCATAGTGTCACTACCTTTATT
TTTAATAACACTATATATTATATCTTATAAACCTAATACCATGAAGAATAAAAAGAATGATTCAAATTTTATATTTA
ATCCATAGTTGGCTGCTATGGTTAGTGTACTAGTCTAGTTTTACTAGTATATAAAACAGTACTCAATTTTTATTTT
TTCAAAATTATAAAAAAGAACACCTATCATCGATAGGCACTGAACCCCTAAAACGGGAACTTAATAAAAACAC
CATGTTCTAGGCTATTAATTCTCTGTATTTTACAGGGGATAAGTAGCCTAGTTTTTGTTGAATTTGATTATTATT
ATAGTTTTTAATGTACTTTTCGACAATATCTATTACAATATGATTAGAGCTATTAAGCTGATTATTGATGTAAAGA
GTTTCAGACTTTAGCGAGGAATGGAAACTTTCTATCGGGGCGTTATCGGCAGGTGTTCCCTTTCGGGACATA
CTTCTGATAATGCCTTTTTCTTCGCATAATTGATAATAAGCATAAGATGTATAAACGCTGCCTTGATCACTATGT
AATATATACCCCTCAGGTATATCGATTTGATTTAATGTATCATTAACTAAACGTTGGTCTTGTTTATCATCTATTTT
ATACGCCACAATTTCTCCGTTATAAATATCCATTATCGAAGATAAATACAACATAGAATGATCAAATGGTAAATA
AGTAATATCGGTTGTTAATACTTCTATGGGACAATTCGCTTTAAATTATCTTTGTAATAAATTGTCTGTTTTATAA
TACGGTTTACCTATCCTTGTCGTCTTTTTAGGTCTAACTCGGCAGTTCAAATGATGCTTCTGCATCATTCTCTGT
ACTCTCTTATGATTAATTGGTGATGTATAACATTGATTAATCAGTGCTGTAATCTTACGATAACCGTAGGTATAAT
GGTTAGCTTCACATAATTCAATAACTTTTTGTGTTACAGTATCATTTTTATAGGTTTTGTTTTTCCATCGGTAATA
TGTTGATTTAGGTATATTTAATACTTCTAGTATCAATTTGATTGAATAGTTTCCTTTTAATTGATCCACTAAATCTA
TGACTACTGTTGGTACCACTTCCTTTCCAATGCCTTGTACTTTTTTAAAATATCCAATTCTATATCTTTTCTCTTAT
TTTCTAATTTTAATTGTTCTACTTCTGACAGCTCTTCTAATCCTTTACCGTAGGTATATTGTTTACCAACGTGTTG
TGAAAATCTATAACTTTCCCCATTTCGATACCATCGCCACCAAGTTTCCACTTGTGTTCTATTTTTAATATTTAAT
TCTTTCATAATTTCTTTTGTTGAAAATCCTGCTGCTTTCATTTCAACTGCTTTATACTTTGTTTCTACTGAATAAG
AAACTCTTTTCATAGAAAAAACACCTCCGTATGATTCATTTTAATATGAATTCAACGAAAGTGTTTTTATATAAT
TCCCACTAATTGGGGTCAGTCTACTATATGATACGGTTTTTTAATTTAAAGTATCAATAATAATTGGATATAGAG

GGAAAGAAGCTATAATGATATTTGCTTACTAAGTGGATAAAGATATTAGAAAAACGAGAAGCAGGACAGTTA
TTAAATCGGTAGATATTAATCATAAGTATTTAACTGAATTTTTACCTTTTGTCGAATATATAACTAAAGTTGAAG
GTAGTAAAATATTTATCTAGTCGATATTAAAACAATTTATCGAAAACGATGGTTTTCCATATCCTCTTTAGTAGC
AATTTTCTAACAATATATAATTAATTATATACAAATTAAGAATAATTAAGAAGTATATATTAATTAGAGATAGTTTA
AAAATTAGATTTCAATCAATTAATTAAAACAGGTTAGACTATTAATTTATACTAACCTGTTAAAAGTAACAAAAT
TAAGAATATATTAAAACTTGTGTAATGAGTAAGCCTCCACCAAATATTAAAACAAACGCGACAACAGGCCATA
CGAAACGTAACCAGTGTGAGTAGCGCACGTTTAACATTTGAAGTGTTGCCATTACAAGTCCAGTAGGCGCTA
AGAACAACATTGCATACTGACCGAATTGATATGTTGTAACAATAACAAATCTTGGTATACCTACTGTATCAGCT
AATGGCGCAAAGATAGGCATAGATAATACTGCTAATCCTGATGATGATGGTACGATAAATCCTAAACAGAAAA
AGATAAAGAGCAGAACAATGATAAATAAAGGCCCACTCATATGTTGCACGATAGATGATGAAAAGTGCAAGA
TTGTGTCAGAAATCATTCCTTTATTCAATACTAAGTTGATTCCACGAGCTAAACCAATGATTAAAGATACACCT
ACTAAACTTGAAGCGCCATTAACGAATGCATCTACAGTGCCTTTTTCGCCTAAACCATATTGTCCTGTTCCAGC
AATAAACATGATGACAATGGTAAAGATTAAGAATGCAGATGCCATGACTGGGAACCACCATCCTTGTGTCATA
ACACCCCAAACCATAATAGGGAATGGTAGGACGAAAAGCGTAAGAATAATCTTTTTACGTAATGTAAACTCA
GAAGAACCGTCATCATGGAGCACAGACCACTGTTTTTCAAATGCTGCTTTGTCTTCATAAGAATAAGAGGAT
TTAGGATCTTTTTTAATTTTTTTACAATACCAGAATAAATAACTAATAACAAATATGGCACCGATGATACAAGCG
CCTATTCTCCAATAAAGACCATCAGTAAAAGTTGTTCCTGCTGCATTAGAAGCAATGACGACTGAGAATGGG
TTGATTGTTGAGAATGTACTACCCACAGAGCTTGCTAAGAAAATTGCACCGAAAGTAAGACTTTAGAATCTAT
GGGAAAGATAATAAGGTTTAATAGAAATACAAGAAGTGTAAATAGTAAGCATATAATTTATACATTTTAATATA
ATATAACAAGAAAGGTAAACACAAAGATAATAAAAATAAAAATTTAATAAAGATAGAGAATTATTAAATTGTTT
TTGATATAAAACAACTTAATAAAAAATTTAATTTTAGGGGAGGATAATTATAATATAAAGAAACATAGAATGGA
ATTGCAATTGGGAAGTATACGATTTTAACTGCGAATCATATGTTCAACGACTTACAAATGCGTAAATCGATAAC
ACATTCAAAAATATATAATCTGAAAATTGATTTACTAAGAGAGGATTATTGTAGTTACAGGTGTTAAGTTGCTC
CAATATGGAGTTGTCGTCTTGTTTTATATTAAACAAGTTTTATTCAAATACATGACAATACGTAAAATAACACTA
GAATTATCAATCACTAAAATTAAAACAAATGTATCTATTCAAACTTTTCTTTAGGGTTTACTTGCTGAATAATTT
AAAAATGAAGTATTAGGTGTACATGCTTTCAGAATTGCTTTTGAAGAATATTAAAAGTATAATCTGAATACGAT
GACAGAATGAATAGGTAAACTATGACAAGAAGTCATCAAGGTTATCAAATAAATATATCCCAGCGGTTGAGA
CGACTCACTGCTGGGATTTTATTGTTAGTTATTTATTCTAAGTGTTGCATTTGGTAGCCGATTCTAGGATGTGT
GATGATTAATTTCTTTTCAGTGTTTGGGTCTTGCGTATAATTCAACTTTTTACGTAAAGAGGCCATATGTACAC
GTAAGGCAGCCATTTCTGTATGATTGACATAGCCATAGAGTGATTTTAATAACACTTGATAGGTTAATACTTTG
CCGACATGATGGCATAATATCGTTAGGAGTTGGAATTCATTCGGTGTTAGATGTACGGACTGTTCATTGACAA
GGACTGATTTCGCATCGAAATCAATGGTTAATGGACCATTTGTGAAACGACTTTGAATTGTCTCAGTAGAAC
GTGACATACGTAATGCTACTCTGATACGTGCTCTTAACTCATCGATATTGAAAGGCTTAGTCATATAATCATTGG
CACCGCGATCTAATACTTCGACAATGGTTTGTTCCTCTGTTCGTGCACTAATCACAATAATAGGTGTGTCCACA
AAGTCTCTGAATTGCTGAATGAGAGATAAACCATCAATATCTGGTAAGCCTAAATCTAATAAGATAATATCTGG
TTGTTCTGTTCTTAGGCGAAAGTCCGCTTCTTTCCCCGTCTTCGCCGTAACCACTTTATAATAATTCATAGTTAG
CGCAACATCGATTAAATGTAAAATCGCTTCATCATCTTCAACGACCAATAATGTTGTTTTCATCCATGGCTCCCT
CCATTTCAATTGGATTAACAGTCAAATAAAAATAAAAAATACTGCCTTGTGGTGTATTCGGTTGATATTCTAATT
CACTGTTGTGTTGTTTCAAGATGAGTTGTACTAAATAGAGCCCTAATCCCAAACTATCTTTTTTATTGTCTTTAA
AGTTGTCTCCTGAATAATAGGGATTAAAAATCAATTGACGTTCTTCTTCTGGAATACCTTTTCCGCAATCTATC
ATTTCGAATTTTATTTTGTTTGTTTCATGTTGAACGTGCAGTTTTATTTCAGAATGTGATTCTGCATGCTTTAAA
GCATTATCGATAAGGTTGAATAGCACTTGCAGTATTAATTTACTGTCGATATTAATGAGTGAAGCGTCATCCTC
ATTTTCAATAATGACATGATTTGCTTGGTGTCTGCGTATGAGGCCTTCTTCAAATTCTTCTAGAAGTTCTTCTAC
TAAATAAGGGGTGCGTTGTATTTGAATGTCAGAGCTTTCTAACTTAGTCAAAGATAAAATATTTGTGACTAAG

GTATGCAGATATTGTGCTTCGCCATAAGAAGCAGTTAAGAGTTCTGCTTGTTGTTGATCGTTTAAATGCTCGT
TATGGTATTTCAGCATATCTAAGTTGCCCATAATGGAAGTCAGTGGTGTTCTAATATCATGTGAAATTGAATGC
AAGAAGTTTGAACGTGTGGCTTCTCGTTCAGCTTTCAATATGGATTGTCTGGTTTGTTTTAATAGATCCACATT
CTCTATTGCCAGGGTAATATCGTTCAACATAGAGTCTAATATTGAATTGTCATAGGTGTCGATGTAAGTTTCGT
CGGTGAAGCGGATAGAAATCACACCTTTGACTGGATTTGTGCCAATGGGAATACACAAAAATTTACTGCCAG
GAAAGGTATCGGTTAATTTACCGGCACGGCTTTCATTTTCAATGACCCAGCTCAATGTCTCAGCATCATGTGT
CTTATCTGAGCTTGAAATGCTTCTGTTTCCAAATGAGTTGGAAGCAGCGACTTTCTTACTTTGAATTAAGAAT
ACCGTGACATCCTGATTGAGTAGCTGATGAATCTGATCACCAGCAATATTTAATAAGCGTTCAATTGAATAAG
ATTCTTTAATGGACTGGTTAAATTGCAGCATAATATTGGTCCGATATAACTGCCGTTCTGTTAAGGAATGCTGA
TGTTTTAAATTCTTTAAGATGGCACTCGTAAAGATACTAGCAAAAATACTGGTCGCAAACGTAATCGGATATTC
AAAGCGATACATTTCTAAAGTGAATCTTGGCACCGTAAAGAAATAATTAAAAACAAACACATTTAAGATAGAC
GCAAAGAATCCGATTAAATAGGATTGGGTCCAAATAGAAAGCACGATAATACCGATAAAGAACATCAGCAAG
ATAATGGCACTGGATTCGCTTTTATCTAAGTTGTAAACCCATATACCGAGCAAGACACAGATCGTTTGAATTAC
AAGCATCTTCATAATCTCTACAGTTAAACGAGAGGATTTTTGAGCTTGCTTAGGTGTATTTGTAGGTTTGTCT
GAATGAATATAATGAATCGGTACAATTTCTAACTTGAAATGGTGTGGTACGTGATTAATTTGTTCAATGAGTGA
CTGTTTAAAGTAATCTTTCCAACGTGGCTGTTCTGACTGTCCAAGGACTAGCTTCGTCACAAAAGCGAGATC
ACACCAATCGGTTAACGCTTTCGCAATATCTTGTGCATACAACACTTTGATTTCTGCACCTAACGCTTTGGCG
AGCATTAGATTTTTATGGACATAATGATCTTGTTTTCTGCTTTCTGACGGTGTCTCAAAGACATCAATATACAC
AGCTGTGAATTTAGCATGTTCTTTATAGGCAGCACGTCTTGCTTCTCGTATGACCCGTTCGTTATAAATACTGC
CGCTGATAGCTACCGCAATATGCGGTGTAATGTCGGTATGTTTAGTTTTATATTGTTGTCCTTTTTGACTCATAA
TATCTGCGACAGTTCTGAGTGTAAGTTCACGCAGCTCTGTCAGATTTTCATACGTAAAGAAATTAGAAAAGG
CTGTTTCTAAGCGTTCTTTTTTATATACTTTTCCTGCTTTAAGGCGCTGAATCAACATATTTGGTGAAATATCCA
CAACTTCAAAGGCATCTGCTGACGTAATGAATTGGTCGGGCACACGTTCTGTAACTTGAATACCTGTCATTAA
CGCAATTTGTCCGCTTAGACTCTCGATATGTTGGATGTTGAGTGTTGTCCAGACATCGATACCATGCGATAGA
ATTTCTTCTATATCCTTATAACGTTTTAAATGGCGCTCTTTTGAAATGTTTGTATGTGCTAGTTCATCAATTAAGA
CCACATCTGGATTAGCTTCTATGATTTTAGAGACATCTATATAGTGAAAGGTGTGGCTGCCAAATTTACGGCTG
GAGGTCGAAATTTCAGGCAATTGTTGAACCAGTGCATTGGTTTCAGGGCGTTGATGGGGTTCGATATAACCA
ATTTTAATATCTGCACCTTCTTGATACTGATCAATACCATTTGATAACATTTCATACGTTTTACCTACCCCTGGGC
TATAGCCTAAATAAATGGTAAGTTTCCCTCTTTTTTTATATGTACTTTCCATGAGGCACCCCCTCTTAATCACATC
ATAATTAATATACAACATATTATCCATTTATCTTTCTGTTTTGTTAATCATCTTTATAGTTTCTTTATAACTTTCCTT
AAATGTTAATACTTCGATTAGATAACTCTTGTTAAGATTTTGATGAAAGTAAGGAGGGAGCGCAATGATTACA
CTATTAGCTGTCGTTGTCATCGCATTAATTTTATTTTTATTTTACGCATTAATTTGGAGTGAAAAATTTTAACAG
AGAAAGAGGGAAGCATCATGAGTATTGTGTTGTTTTTGATTGTATTTATCTTGCTCTCACTCATTGTGAGCCG
ATATTTATATTCAGTTGCTTTAAATGTGCCATCTAAAATAGATGTTGTTTTTAATCCGATTGAGAAATTGATTTAT
CAACTGATTGGCACGAAATTAGAACACATGTCTGGGAAGACGTATATCAAACATTTTTTGTTGTTTAACGGAT
TGATGGGCGGATTGTCCTTTGTATTATTGCTTATTCAACAATGGCTGTTTTTGAATCCTAACCATAATTTAAATC
AATCTGTATCGTTAGCCTTTAATACTATGGCATCTTTTTTGACCAATACTAACTTACAGCATTATGCAGGTGAAA
CAGATTTAAGTTATTTAACACAAATGTGTGTCATCACTTTCTTAATGTTCACGTCAGCAGCGTCAGGTTACGCC
GTATGTATTGCGATGTTAAGACGTTTGACTGGAATGACAGATGTGATTGGTAATTTCTATCAAGATATTACGCG
TTTTATTGTACGGGTGCTCATACCTTTCGCATTGATCATCAGTTTGTTTTTAATCAGTCAGGGCACACCGCAAA
CGCTTAAAGGTAATTTGGTGATTGAGACATTATCAGGTGTGAAACAAACGATTGCATATGGACCGATGGCGT
CTTTAGAATCTATTAAACATTTAGGGACAAATGGTGGTGGTTTCTTAGGTGCGAACTCTTCTACACCTTTTGA
AAATCCGACATACTGGTCTAATTACGCTGAAGCTTTAAGTATGATGTTGATTCCAGGTTCATTAGTCTTTCTATT
CGGTAGAATGTTGAAAACTAAACTACAGATTCATCCGCATGCGATTATGATTTTCGTTGCGATGTTTGTAATGT

TCATCGGCTTTTTAGTGACATGTCTCTATTTTGAATTTGCGGGGAATCCAGTGTTGCATCACTTAGGTATTGCC
GGTGGCAATATGGAAGGCAAAGAAACACGTTTCGGTATTGGCTTATCCGCTTTATTTACAACCATTACGACC
GCTTTTACTACAGGAACAGTTAACAATATGCACGATAGTCTTACACCGCTAGGCGGCATGGTTCCAATGGTAT
TAATGATGTTGAATGCAGTTTTTGGCGGTGAAGGTGTTGGGCTGATGAACATGTTGATTTATGTCATGTTAAC
GGTCTTTATCTGTAGTTTGATGATTGGGAAAACACCAAGTTATTTAGGAATGAAGATTGAAGGTAAAGAGAT
GAAACTCATTGCGCTTTCTTTCTTAGTACATCCTTTACTTATTTTGGTTTTTTCAGCACTAGCTTTTATTGTGCC
AGGGGCATCAGATGCGTTAACTAATCCGCAATTCCACGGTGTATCACAAGTGTTGTATGAGTTTACATCATCT
TCAGCGAATAATGGCTCTGGTTTTGAAGGATTAGGAGACAATACGGTATTTTGGAACATTTCAACAGGCATT
GTGATGTTGCTTGCACGATATATTCCAATCGTTTTACAAATTTTGATTGTATCTAGTTTGGTAAATAAAAAGACC
TATCAGCAACATACTCAAGATGTACCGATTAATAATTTATTTTTCAGCAGTGTATTGATTATCTTTATTATTTTGT
TGAGCGGCTTAACGTTCTTACCTGACTTAATGCTTGGACCAATAGGCGAACAGCTTTTGCTGCACGCATAGA
TAAAGGAGGATTAGAAAATGGCTGAAACTACTAAAATATTTGAATCACATTTGGTCAAACAGGCTCTAAAAG
ACAGTGTATTGAAGCTCTATCCTGTTTATATGATTAAAAATCCGATTATGTTTGTTGTAGAAGTGGGCATGCTG
CTTGCCTTAGGATTAACCATTTATCCGGATTTATTTCACCAAGAAAGTGTATCACGGCTATATGTGTTCAGTATC
TTTATCATATTATTACTGACACTTGTCTTTGCGAACTTCTCTGAAGCATTAGCTGAAGGTCGCGGTAAAGCACA
AGCCAACGCTTTACGCCAAACACAAACTGAAATGAAGGCACGTCGTATTAAACAAGACGGCAGTTATGAAA
TGATTGACGCTAGTGACCTGAAAAAAGGACATATCGTACGTGTCGCGACAGGTGAACAAATCCCAAATGAC
GGTAAAGTTATTAAGGGCCTCGCAACAGTGGATGAATCTGCGATTACAGGTGAATCTGCACCTGTAATCAAA
GAAAGCGGTGGAGATTTCGATAATGTAATTGGAGGAACTTCTGTAGCTTCAGACTGGTTAGAAGTTGAGAT
TACTTCAGAACCAGGTCATTCATTTTTAGATAAAATGATTGGTTTGGTTGAAGGGGCTACAAGAAAGAAAAC
ACCTAATGAAATTGCGTTATTTACTTTATTGATGACATTAACGATTATCTTCTTGGTCGTTATTTTAACGATGTAT
CCATTGGCGAAATTCCTGAATTTCAATTTATCCATTGCGATGCTGATTGCTTTGGCTGTGTGTTTAATTCCAAC
AACCATTGGGGGATTATTATCGGCTATAGGGATTGCAGGGATGGATCGTGTGACACAGTTTAATATCTTGGCT
AAAAGCGGACGTTCTGTAGAGACTTGTGGTGATGTGAATGTCTTGATTTTAGATAAAACAGGTACCATTACC
TACGGCAACCGTATGGCAGATGCGTTTATTCCGGTGAAATCATCAAGCTTTGAACGTTTAGTTAAAGCGGCC
TATGAAAGTTCTATCGCAGATGACACACCAGAGGGACGTAGTATTGTGAAATTAGCTTATAAACAACATATCG
ACTTACCGCAAGAGGTCGGAGAATATATTCCGTTTACTGCTGAAACACGTATGAGCGGTGTGAAATTTACGA
CACGTGAAGTATATAAAGGTGCACCGAATAGTATGGTTAAGCGTGTGAAAGAAGCAGGGGGACATATTCCA
GTTGATTTAGACGCTCTTGTCAAAGGGGTGTCTAAAAAAGGTGGCACACCGCTGGTTGTGCTTGAAGATAA
TGAGATTTTAGGTGTTATTTATTTGAAAGATGTCATTAAAGATGGACTCGTAGAACGTTTCCGTGAATTACGT
GAGATGGGGATTGAAACGGTGATGTGTACAGGAGATAACGAATTGACAGCTGCGACAATAGCGAAAGAAG
CGGGTGTGGATCGCTTTGTGGCAGAGTGTAAACCTGAAGATAAAATCAATGTGATTAGAGAAGAACAAGCG
AAAGGTCATATTGTTGCGATGACGGGTGACGGTACGAATGACGCGCCAGCTTTAGCAGAAGCTAATGTAGG
TTTGGCAATGAACTCAGGAACCATGAGTGCCAAAGAAGCGGCGAATTTAATTGATTTAGATTCTAATCCAAC
CAAACTGATGGAAGTCGTTCTAATTGGGAAACAATTATTAATGACACGTGGCTCACTCACTACATTTAGTATT
GCGAATGACATTGCGAAATACTTTGCGATTTTACCAGCCATGTTTATGGCGGCTATGCCTGCGATGAATCATT
TGAATATTATGCATCTGCATTCACCTGAATCAGCAGTATTATCTGCGTTAATCTTTAATGCGTTGATTATTGTATT
ATTGATTCCGATTGCGATGAAAGGCGTGAAATTTAAAGGTGCCTCAACGCAAACCATATTGATGAAAAATAT
GTTAGTTTACGGCTTAGGCGGTATGATCGTGCCATTTATCGGCATTAAGCTCATTGATCTCATCATCCAACTCT
TTGTCTAAAAGGAGGACAAAACAATGCAGACAATAAGAAAAAGTTTAGGACTAGTACTGATTATGTTTGTTT
TATGCGGATTTATCTTCCCGCTGACTGTCACAGCGCTTGGACAAGTATTATTTCCAGAACAAGCAAACGGCA
GTTTAGTGAAACAAGATGGCAAAGTAATTGGTTCAAAGCTCATTGGACAACAATGGACAGAACCTAAATATT
TCCATGGACGTATCAGTGCAGTCAATTACAATATGAATGCGAATGAAGTGAAAGAAAGTGGCGGACCTGCTT
CAGGCGGCTCAAACTACGGCAATTCAAATCCTGAATTGAAAAAAAGAGTTCAAGAGACTATTAAACAAGAA

GGAAAAAAAATTTCAAGTGATGCGGTGACCGCTTCTGGCTCTGGTTTAGACCCAGATATTACGGTTGACAAT
GCGAAACAACAAGTAAAACGCATTGCGAAAGAAAGAAACATAGATGCTTCAAAAATTAATCACCTTATTGAT
GAAAACAAACAAGCATCACCAATGGCAGATGATTATGTTAATGTCTTAAAATTGAATATCACTTTAGATAAACT
CTAAATAAACAGGGAGTGAGGTGAGACATCCATGTGGTTCATTAGCATTATTATTTTAATAGCATTCCTAATTA
TATTAATGATTGACGATTTAATTAAAACATGACAACTTCACGCAAAACAATATGAATTGGATGTAATAGAGAA
GGGAGTACCAAGAAATACTCAGTTTATTGAGGATAATTATTAGGGGAGATTAGAGAAGAATTCTCTATTCAC
GAACATTACTTTAACAGTTGTAATCATTGGTTTCATCATATCGATTAATAGTACAGTCATCTTGAGATTTTCAAC
ACACTATTATAGCTAGCTAAGATACTTTTAAATTATGACTTATTTTTCATCATTTACGATACATCATTCTCGATAAC
TTACTATTTGTTATGGTATTTATAGATTTAAAGAAAGTGAAGGAGATTAAGCAATGTAACTTGGCTTAATCTCT
TTTTGATTATATTTATTAAGGCAAAGCTTCATTTCCCTTAAATCAAGTATATCTTCTGAATAAGCAAATGAAATC
GTGACGCCAACTACAGTGATAACTTCGCCTAAAACACAATGAGTGTGGTGTTTTCACTTTTCATAAACTCCTC
TAAGTTTGTGTTTTTAAACTGTGATGGATTTGAATGTTTTTGAATTACGTTGTCGCGTCCTTGGGATTGCAATC
CAAGAACGTCAGCATAATCTAAATCAATTTTGATTTGGTAGAAGACAAGAGAAGTACTTTTGCCTGTATCATC
TTTCGCATTTCTTTTTGTTGTCTTTCGATCCCGATCGGAAACTAATTTTCCTGCATTATCCAAAGCATCAATAAC
ATTTTTCGTGTCTTCGAATTGACTGTCTGTTAACATCTTATCGAATACATTTCGGATAATTTTTACTTCGATGTA
GTCATCTTTAAGTGAGAGGATACCAAAGTTTTGAATCATATTAGCAAGACGTGTTTGTTGTGAAAACTTGCCC
AGGTTTTCCGCAATAAACTGTACGATTGTATCCATCGCTTTATCTGCAAGAGAACGTTCTGACACCGTTTCATT
ATGATAGCTTAATAAATAGTTCTTTATGCTGCTAACGTTTAACTGTAGGTCTAGTGATTGATTTAAAATATGGGC
TGTTGTTGTTAGAGTTGCATAATGTTTAATCATTCGAATACCAGTGTTACTGGATTCAGTTGATAGTAATTGATA
AAAAACACTGTCTTCTTTTTGAAAAAGGGAAGTAACATCATTAGTGTTGTCTAATAGGAATTGAGCAATTATA
GGTAAAACATGACCATTATTTTGACTAACCACTTTTTTTATAGTATCTGAGTTTTGAGCACTTGATGTGAATTT
TTCAGACACCTCAATAGTACGAACACGCAAACCATCATTTTTAGCTGGATTTTTGAAAATACTATGTTCAGAA
GTTGAAATCACTGATGTATCCTAGTTTTTAGGTGTTTTGACATCACCATTTATATTTGCTCTTTGACGTCCTTGT
CCCTCTGCAAATGAATATAGAAGACCTGTTGTATCATGAAATGTTGCCGCTGATAATTCATCTAATACTATGGG
TACACCATAATTTCCGCTAAGATATCCTTCTAGTGCATTTCTTGTTGCATTCCAACTCCGGAATAGGTTTTCAGT
ACCTTTTGTTGGGTTGCCAGCTACCGATGCGGCTAACATAGCGGCTGTCGATTTTCCTGTTGATGATTGGCCA
GTGAAAGAGAAAATAGTTCCTGAAAACTCGACGTTATTATGATACTTAAGGAAAGCAGTGACTAATGAAGAT
ACGCCAAACAACACATCTAGTTCTAAAGATAAGTTCCCATGGACTTCTTCTTTATACATTTGTAACCATTCTGAT
AAATTGCCTTTTGGTATTAAATCGTACTTGTTATCACATATAATAGAAGTGCTATTAGTAATCTCATTAGAAAAA
TAAGGCTGGTCTAATGAAATTAATGGTCCAAGTAATGTATGAAGCATTCCTACACCTGAGTATATTGTTGATAA
AGGTGTGGTGCTTCTCATTTGTTGCAGTACGAACCCTAAATCTCTAATGTGCTTTTCATTAATGGTAAATCCAT
ATTCAATTAAGGAGGGTAATTGCAAAGCAGTAAGGATTGAAGCCTTTTCGACAAGCTCTATATTATTCAAATC
CGAAAGAAATACTTTTTCTTTACGAGTCGCGGGGTCTATAAAACGATCTGTGAAAGCAATCGGTCCAGATAA
CATTATTTCAAACTCCGTACCCCCATCTTTTTTAGGAGGGATGGTTTTGTACCATCCCGAAGGTTTGAGCTTG
AATTCGTCAGTTTGATATAGAATTTTTTCCATTAACAGTGACCCCCTTGGTTGTAAGAGGAAATTATAATGATA
TGTCGGTACTTAAGTGGGTTAGTTCCTATAATAATGAAAGGGATGCTTGGTTTGTACTTTATCCACCAAGCAA
AGCGTCTTCCTGCTTCACGTTGATCGTTGATGTTGCACTGTTCTCTGAATAACTGTAATTATAAGGTTTAGTAA
TGGCAATTTTTTATTTAAACAAATTTTTACGAATGGATTGTCTGTTTATTAATTCAGCAACGTTTATTGAAGAA
AAATAAGTGTCTTCTGATAATTGCAAAGGGTTTAATTCAATGTAAACTGAATTAAGGGTAGGGTAGGTATGCC
TGTTTTTTGGTTCTTTTAAGTTCAATCGATTAATAAAATTGTTGAACCTTTGTTCTAAGTCATACAAAAACATCA
GTAATTCATTAGGAGAATGATTTATTATGCAATCCATATTTTCGTTTATGCTTGAAGTTATACATCTAAAAACAG
TATTGGTTTCTTCGTATAAAAATAACTTATTAATAGAGGTAACATTGATTAAGACTGAGTATACGTATAATAAGA
GTGACTCTGACATCTCATTTAGATAAATAAGACTGTGATCATTCTTATTTACGATTATTATATGTGATTGAATCAA
TTCAATAATGTCTAGAAGAAGACTTGAAAAGTCGCTGTTAATCTTGGTCTTTGAAAAATCATCGTGACCATTT

GTCTTATTACTTAGATGTAGTTGTTCGTATTTTATAGTTTGGCGGTTGAGGAATTAAGCATTTGATTATTATAAA
CTATCTCAAAAATTAAATTACCTCCTTTTTTCTTCCTCGATGAAGGTTTATCTAATCTTCCAATATACAGGTTTTT
GAAGTAAATTATAGATATGATAATCATTAATTCAGTAAATGTATACTTATTAAGGGGTGGAGCTTTAGCTAACTT
ATCGATAGTACTGCCGATGCGTGCTTTGAAACTATTCAAATTGTCGTGACTGTCTATATCTAAATCTTCAAATTT
ACTAGCAAGGGTTTGCAAAGTAGAAATTACAACTTCTTCTTTTGTATAATATTTATTGAAAATATCATCTTCTTT
ATCATCGCTTTGGAGTTTTAAAGTGCCTGGTTCTAAGATGTCAAATTTTTTGTTTCGATTGGTATTTTTTTAAA
ATGTGCCTTGTATTTTTGGGGTTGTTCCAAATGTGTATTTGCTAGACTACCTGTAATTGATATTGCTTTATTTAG
CATTTGGTGTGTATTAACTGTATCTGATTCTTCTGGAAATTTAAATCTAAAAGTAAAATCTCTACTACCGAATGT
ACTTTTTACGGCTACAGTTTTGTACTTATGCTCAGGCAATGTAATTAAACCATCTTGATGCAGGAGGAAAAGT
GTGTCATCATTTAATTTCTCAATTGTATTAAATAAGTAATTATGCATAGCCTCTTTATACATGGCGAATTGATAAT
TTAGATTATCCCATTCTATTAGTTGATTTAAAATACAATATAGACCTTCATTCAATCGGTTTACGAATATCAATAC
AGCTTCTTCTCGTCCAACAAGGCTTATTAACTCCTTCAAGTCATCCTTTAATTTTAATTTTTCTCCTTCAAAATT
GTATTGAGTGAAGCCTATAGCCTTTTGAAAAGTAACTTCCATGACAGAGTTGGTTTCAGAATATTCTAACGAT
TCATTTATTGCAAAAATCAAATTTTTTATAAATATGGAATTACTCATGGTTTGTAATTGCATATACTACCCAACTT
TCTTATTTACTTTAAAACAAATATAAGCTAAAGTAGTTATTATCATAAAGGGAACGTACGTTCTTATTTAGCTGA
TTTCTATGAAGAGAGGTTATGGATATTGAATGAAGAAATCTTACGCGAAGTAGCAGATATTTTTATAGGTGAT
GACAGAGATAGCATTTATGATTATAAAACTGGGAACGAATTAGTGAGGTTTTTTAATCATTACTTTAATAAAG
GGGACATATATCAGGCTCCGTTTCCATCTAGATGGCTATATGTTGTGAAACATTTGCAAACTCTGATTCAGGA
GAGAAAGATCAATCAATTTTTCACATTAATTTTAAGCAATCACTATATTAAGTATGAACTGAAAATTGACGAGG
TTGAAGCAGCAAAGCAGGCTGCTAAAGCACTTAAGTTGTTCAACAAAAGATTAAATCATTATGGGTACTACA
TAACAGGAACTAACAATGCTAGATATTTTATGGATAAGGATGAAGATACGGAGTCTATTGGTTATGGAGGGTA
TGCGAATATTTATTTACAGAAGTCTACAGGTCTTGCTGTAAAAAAATTGAAAGAGGAGTATCTTACTGATTCT
TCAATTAAAAGTAGGTTTAAGAGAGAATTTGATCTCACTAAATCTTTTGATACAAATCCATTGTTCATTAATGT
GTTTGAATTTAATGAATCAGATTATTCATACACGATGGAGTTAGCTGATGAAACTTTGAAAGATTACATTGAA
AGCAAGACAATTAGTGAGCTAGAAAAAGTAAAGATAATAATGAAAATTTTAAAAGCGATGAGTCAAGCACAT
AGTGAAAATAAAATACACAGAGATATCAGTTCTAAAAATGTATTAATGTTTAGAGGAAAAGTCAAAATATCAG
ACTTAGGATTAGGTAAAAACCTTGATGAAATTCATTCGCATCAAACCTTTGATACAAACGGTGTAGGACAATA
TAAATACTGTGCACCGGAACAGATGTATAGTTTAAAACAAGCAGATAAACAATCTGATGTTTTTAGTTTAGGA
AGATTGATAAATTTTATTATGACTGGAAATGTAGTTAACAACCATCACCTATTTAGAGGTGTATCTGATAAGGC
TACGAACAGTAGTAAAGAATACAGATTTGAAGATGCAAATGAAATGTTGAAAATGCTGCAGAGAATTTTAGA
GTATCACAGTAGTGCAAAGCACGTCGAAAAATGTCAAGAAAAGCTGAAAAGAGGAGTGTTTGATGATGAA
AGCGAAGAATTTATTATGACACGAAGTGATGAACAATTATGTCAAATGGTTCTAAGTTCTAATAATAATGAGC
AAGCGTGTTTAATTCGTTATATGCAAAAAAACGAATCTTCAGCATGTGATTTAATAGAGAGTATTAATAGAAA
GTATCAAGAGTTTTGTGGAAGGTTTGAAGACTACGATCCTTTTGCTAAATTAGCATATATGATTTTATGTAATA
ACTTCAGTTATAGAGTGAATGAAACAGCAGCTAGAGTGCTAAATTATGTTGCTTGGTCTGTAAATAGATTTTC
GGCACAAGACTTAATTAAAGGTTTAATTAATAGAGGAGTGGAGCCTTTGATTGAAGAAAAATTAAAGGACA
ATTAAAAAAACCTCATCATTAACTGATACGCAGAGGCGTATCATAAGT
>Staphylococcus aureus strain ER01109.3
ATGAAAATCACCATTTTAGCTGTAGGGAAACTAAAAGAGAAATATTGGAAGCAAGCCATAGCAGAATATGAA
AAACGTTTAGGCCCATACACCAAGATAGACATCATAGAAGTTCCAGACGAAAAAGCACCAGAAAATATGAGT
GACAAAGAAATTGAGCAAGTAAAAGAAAAAGAAGGCCAACGAATACTAGCCAAAATCAAACCACAATCCA
CAGTCATTACATTAGAAATACAAGGAAAGATGCTATCTTCCGAAGGATTGGCCCAAGAATTGAACCAACGCA
TGACCCAAGGGCAAAGCGACTTTGTTTTCGTCATTGGCGGATCAAACGGCCTGCACAAGGACGTCTTACAA
CGCAGTAACTACGCACTATCATTCAGCAAAATGACATTCCCACATCAAATGATGCGGGTTGTGTTAATTGAAC

AAGTGTACAGAGCATTTAAGATTATGCGAGGAGAAGCTTATCATAAGTAATGAGGTTCATGATTTTTGACATA
GTTAGCCTCCGCAGTCTTTCATTTCAAGTAAATAATAGCGAAATATTCTTTATACTGAATACTTATAGTGAAGCA
AAGTTCTAGCTTTGAGAAAATTCTTTCTGCAACTAAATATAGTAAATTACGGTAAAATATAAATAAGTACATATT
GAAGAAAATGAGACATAATATATTTTATAATAGGAGGGAATTTCAAATGATAGACAACTTTATGCAGGTCCTT
AAATTAATTAAAGAGAAACGTACCAATAATGTAGTTAAAAAATCTGATTGGGATAAAGGTGATCTATATAAAA
CTTTAGTCCATGATAAGTTACCCAAGCAGTTAAAAGTGCATATAAAAGAAGATAAATATTCAGTTGTAGGGAA
GGTTGCTACTGGGAACTATAGTAAAGTTCCTTGGATTTCAATATATGATGAGAATATAACAAAAGAAACAAAG
GATGGATATTATTTGGTATATCTTTTTCATCCGGAAGGAGAAGGCATATACTTATCTTTGAATCAAGGATGGTC
AAAGATAAGTGATATGTTTCCGCGGGATAAAAATGCTGCAAAACAAAGAGCATTAACTTTATCTTCCGAACTC
AATAAATATATTACATCAAATGAATTTAATACTGGAAGATTTTATTACGCAGAAAATAAAGATTCATCTTATGAT
TTAAAAAATGATTATCCATCAGGATATTCTCATGGATCAATAAGATTCAAATATTATGATTTGAATGAAGGATTC
ACAGAAGAAGATATGCTAGAGGATTTAAAGAAATTTTTAGAACTATTTAATGAATTAGCTTCAAAAGTTACAA
AAACATCCTATGATAGCTTGGTCAATAGCATAGACGAAATACAGGAAGACAGCGAAATTGAAGAAATTAGAA
CAGCACAAAAAGATAAGACACTCAAGGAAGTGGAAGCACCTAAAGGAATAATTCCAAAATATAAAAAAGGT
GTATCAAAGACTACTAAAAATGATTCAGAAATTGAAAAATCAAATAAAGAGAATAAATTAACCGGTAAAGTT
GGAGAAAAATTAGCGCTAAATTACTTTAATGAGCTAATTGATAATAAAATAGACGAAGATAAGAAAGAACAG
TTTAGGAATATTTTAAATGATAATCCAGGCTCTCAACACGGTCATGGCTATGATTTAGTAGCTTTTGATCCAAC
AAATACAGATAAAGCTGTAGAAAAATTTATTGAAATTAAAACATCTACATCTTCTAGTATTGAGGAACCATTTT
TTATGTCGCTAAATGAAATGTTTGCTATGAAAGAATATAAGCAGAAATATTTAATATTAAGAATATTTAATGTTT
CCGGTAAAGAACCACAATTTTATTTTATAGATCCATATGCAAATTATTCTGAATTTAAAGATGTAGATGATCTCA
TTGACAAAGTATTTAATGTAGAAGCTATTCAGTATAAAGTTTTTGGCGAAAAATGATTACTTGAACAAGAGCT
AAAATAAAATTGTGATCTAATAAAAATAGAAACTGTAATTTAAATAAAACTTTCTAAATAAGCTAACTGATAAA
AAATCAGTTTGTCCACAGTCTGAAACAAGATTCCTATATTCTTTAGGAATCTTGTTTTTTCTATTTTTATGGTGA
TAAAGAGCAGATAAGATAATGTGTAATAATCACAAAAAAGTTAAATATTTTAAGGCTTGTTTAATTATTAATGA
TTTTATATATAAAGAGCAGTATAATAAAGTTGTTAATATATTATGAATAATATTCAAGTAATTTTATTGTTTTTTAA
TTTGTCGATATTTAAGTTGAGTTAAATTTAAAGGGTGTAATTTGTTTTACAATGATGAAGATAATTAGTCTATCA
AAATAAAGGGGTTGGGACTGTTATGAGTGATAATTTGTCATTATTCATTGACTATATCAATGATAATATAATCTA
TGGTAGTGAAATCAAACGGGAGAAATTAGAGAATTTATTTAATCAATTTGCTATAAAAAAATGTTGAAAAGAA
CATTGTCTATGATGAACTGAAATCTTTAGATATTACAATCATTGAGTCACAGGATTCATATAAAAATAAATTGAA
GAGATTATTTTCGGTTCTGTTGCAAAGTAAAAAAATATAGCTAACCACTAATTTATCATGTCAGTGTTCGCTTA
ACTTGCTAGCATGATGCTAATTTCGTGGCATGGCGAAAATCCGTAGATCTGAAGAGACCTGCGGTTCTTTTTA
TATAGAGCGTAAATACATTCAATACCTTTTAAAGTATTCTTTGCTGTATTGATACTTTGATACCTTGTCTTTCTTA
CTTTAATATGACGGTGATCTTGCTCAATGAGGTTATTCAGATATTTCGATGTACAATGACAGTCAGGTTTAAGT
TTAAAAGCTTTAATTACTTTAGCCATTGCTACCTTCGTTGAAGGTGCCTGATCTGTAATTACCTTTTGAGGTTT
ACCAAATTGTTTAATGAGACGTTTGATAAACGCATATGCTGAATGATTATCTCGTTGCTTACGCAACCAAATAT
CTAATGTATGTCCCTCTGCATCAATGGCACGATATAAATAGCTCCATTTTCCTTTTATTTTGATGTACGTCTCATC
AATACGCCATTTGTAATAAGCTTTTTTATGCTTTTTCTTCCAAATTTGATACAAAATTGGGGCATATTCTTGAAC
CCAACGGTAGACCGTTGAATGATGAACGTTTACACCACGTTCCCTTAATATTTCAGATATATCACGATAACTCA
ATGTATATCTTAGATAGTAGCCAACGGCTACAGTGATAACATCCTTGTTAAATTGTTTATATCTGAAATAGTTCA
TACAGAAGACTCCTTTTTGTTAAAATTATACTATAAATTCAACTTTGCAACAGAACCGTATTATGGAATAGAGA
TGTTGGTAACATTTATACAGGATCATTATACTTAAGTTTAATTTCGTTATTACAGAACCACACATTCCAACCAGA
AGAGAAAGTATGTCTATTTAGTTATGGTTCAGGAGCAGTAGGAGAAATCTTTAGTGGTTCAATCGTTAAAGG
ATATGACAAAGCATTAGATAAAGAGAAACACTTAAATATGCTAGAATCTAGAGAGCAATTATCAGTCGAAGAA
TACGAAACATTCTTTAACAGATTTGATAATCAAGAATTTGATTTCGAACGTGAATTGACACAAGATCCATATTC

AAAAGTATACTTATACAGTATAGAAGACCATATCAGAACATATAAGATAGAGAAATAAACTAGTGGCCGATTG
TGCTTGATGAGCTTGGGACATAAATCCTAACTCGAAATAAATAAGCATATCACTAAACTGATTTTTTAAAGTTT
ACAGTGATATGCTTATTTTTTTATCTTACGATTTTGTACGTGCATGCTTGCCTAGGGGTATGGCTCGAGCCATT
AGTCTCTCGCACATACTATTCCCTCAGGCGTCAGCACTTACAAAATCGGTTGTAATTTTCATTTTTATACGCATT
CTTACTGAGATTATACTAATAAGAGGAATAGTAAAAGCAATTCTAAGTAAAATTGCAGATAAGAGGTTTGTTA
AAAGCAGTTCTCAGTAAAATTGCAGATAAGAGGTACGTTAAAAGCAATTCCATGCAAAATTGCTGATAAGGG
GTAAGTTAAAAGCAGTTCTCAGTAAAATTGCAGATAAGAGGTACGTTAAAAGCAGTTCTAGGCAAAATTGCA
GATAAGAGGTGCGTTAAAAGCAGTTCTCAGTAAAATTGCTGATAAGGGGTAAGTTAAAAGCAATCCTAAGTA
AAATTGCAGATAAGGGGTACAGAAAAACTAGACTTGATTACAAAATGGAGCTTGGGACATAAATGATTTTTT
AAAAATGAGATGAGACGTAGATTAACTCCATAATCAATACGAATCTATCGACTTCTTTATTTATGATATTCATCT
CTTTTTAATGGAAATAAAAGTGCGATTAATGTGATAATACAGTTACGTTAATTAAAAAAATAAAAATGCAAGG
AGAGGTAATATGCTAACTGTATATGGACATAGAGGATTACCTAGTAAAGCTCCGGAAAATACAATTGCATCATT
TAAAGCTGCTTCAGAAGTAGAAGGTATAAACTGGTTGGAGTTAGATGTTGCAATTACAAAAGATGAACAACT
GATTATCATTCATGATGATTATTTAGAACGGACTACAAATATGTCCGGGGAAATAACTGAATTGAATTATGATG
AAATTAAAGATGCTTCTGCAGGATCTTGGTTTGGTGAAAAATTCAAAGATGAACATTTGCCAACTTTCGATG
ATGTAGTAAAAATAGCAAATGAATATAATATGAATTTAAATGTAGAATTAAAAGGTATTACTGGACCGAATGGA
CTAGCACTTTCTAAAAGTATGGTTAAGCAAGTGGAAGAACAATTAACAAACTTAAATCAGAATCAAGAAGTG
CTCATTTCAAGCTTTAATGTTGTGCTTGTTAAACTTGCAGAAGAAATCATGCCACAATATAACAGAGCAGTTAT
ATTCCATACAACTTCGTTTCGTGAAGACTGGAGAACACTTTTAGATTACTGTAATGCTAAAATAGTAAACACT
GAAGATGCCAAACTTACTAAAGCAAAAGTAAAAATGGTAAAAGAAGCGGGTTATGAATTGAACGTATGGAC
TGTAAACAAACCAGCACGTGCAAACCAACTTGCTAATTGGGGAGTTGATGGTATCTTTACAGACAATGCAGA
TAAAATGGTGCATTTGTCTCAATAGAAAGTTAGAGGTGAGTCTTACGTTTCAGTGACGGTAGACTTACCTTTA
ACATGTTACATACTAAAAAATTAATTTGAATAAGAAAGAGAGACATATATGAAATACGATGATTTTATAGTAGG
AGAAACATTCAAAACAAAAAGCCTTCATATTACAGAAGAAGAAATTATCCAATTTGCAACAACTTTTGATCCT
CAATATATGCATATAGATAAAGAAAAAGCAGAACAAAGTAGATTTAAAGGTATCATTGCATCTGGCATGCATA
CACTTTCAATATCATTTAAATTATGGGTAGAAGAAGGTAAATACGGAGAAGAAGTTGTAGCAGGAACACAAA
TGAATAACGTTAAATTTATTAAACCTGTATACCCAGGTAATACATTGTACGTTATCGCTGAAATTACAAATAAGA
AATCCATAAAAAAAGAAAATGGACTCGTTACAGTGTCACTTTCAACATACAATGAAAATGAAGAAATTGTATT
TAAGGGAGAAGTAACAGCACTTATTAATAATTCATAATAAAACAGTGAAGCAACCATCGTTACGGATTGCTTC
ACTGTTTTGTTATTCATCTATATCGTATTTTTTATTACCGTTCTCATATAGCTCATCATACACTTTACCTGAGATTT
TGGCATTGTAGCTAGCCATTCCTTTATCTTGTACATCTTTAACATTAATAGCCATCATCATGTTTGGATTATCTTT
ATCATATGATATAAACCACCCAATTTGTCTGCCAGTTTCTCCTTGTTTCATTTTGAGTTCTGCAGTACCGGATTT
GCCAATTAAGTTTGCATAAGATCTATAAATATCTTCTTTATGTGTTTTATTTACGACTTGTTGCATACCATCAGTT
AATAGATTGATATTTTCTTTGGAAATAATATTTTTCTTCCAAACTTTGTTTTTCGTGTCTTTTAATAAGTGAGGT
GCGTTAATATTGCCATTATTTTCTAATGCGCTATAGATTGAAAGGATCTGTACTGGGTTAATCAGTATTTCACCT
TGTCCGTAACCTGAATCAGCTAATAATATTTCATTATCTAAATTTTTGTTTGAAATTTGAGCATTATAAAATGGA
TAATCACTTGGTATATCTTCACCAACACCTAGTTTTTTCATGCCTTTTTCAAATTTCTTACTGCCTAATTCGAGT
GCTACTCTAGCAAAGAAAATGTTATCTGATGATTCTATTGCTTGTTTTAAGTCGATATTACCATTTACCACTTCA
TATCTTGTAACGTTGTAACCACCCCAAGATTTATCTTTTTGCCAACCTTTACCATCGATTTTATAACTTGTTTTAT
CGTCTAATGTTTTGTTATTTAACCCAATCATTGCTGTTAATATTTTTTGAGTTGAACCTGGTGAAGTTGTAATCT
GGAACTTGTTGAGCAGAGGTTCTTTTTTATCTTCGGTTAATTTATTATATTCTTCGTTACTCATGCCATACATAA
ATGGATAGACGTCATATGAAGGTGTGCTTACAAGTGCTAATAATTCACCTGTTTGAGGGTGGATAGCAGTACC
TGAGCCATAATCATTTTTCATGTTGTTATAAATACTCTTTTGAACTTTAGCATCAATAGTTAGTTGAATATCTTTG
CCATCTTTTTTCTTTTTCTCTATTAATGTATGTGCGATTGTATTGCTATTATCGTCAACGATTGTGACACGATAGC

CATCTTCATGTTGGAGCTTTTTATCGTAAAGTTTTTCGAGTCCCTTTTTACCAATAACTGCATCATCTTTATAGC
CTTTATATTCTTTTTGTTTTAATTCTTCAGAGTTAATGGGACCAACATAACCTAATAGATGTGAAGTCGCTTTTC
CTAGAGGATAGTTACGACTTTCTGTTTCATTAGTTGTAAGATGAAATTTTTTTGCGAAATCACTTAAATATTCAT
CCATTTTTTTAACGGTTTTAAGTGGAACGAAGGTATCATCTTGTACCCAATTTTGATCCATTTGTTGTTTGATAT
AGTCTTCAGAAATACTTAGTTCTTTAGCGATTGCTTTATAATCTTTTTTAGATACATTCTTTGGAACGATGCCTA
TCTCATATGCTGTTCCTGTATTGGCCAATTCCACATTGTTTCGGTCTAAAATTTTACCACGTTCTGATTTTAAAT
TTTCAATATGTATGCTTTGGTCTTTCTGCATTCCTGGAATAATGACGCTATGATCCCAATCTAACTTCCACATAC
CATCTTCTTTAACAAAATTAAATTGAACGTTGCGATCAATGTTACCGTAGTTTGTTTTAATTTTATATTGAGCAT
CTACTCGTTTTTTATTTTTAGATACTTTTTTTATTTTACGATCCTGAATGTTTATATCTTTAACGCCTAAACTATTA
TATATTTTTATCGGACGTTCAGTCATTTCTACTTCACCATTATCGCTTTTAGAAATATAACTGCTATCTTTATAAAC
TTGTTTGAAATTTTTATCTTCAATTGCATCAATAGTATTATTAATTTCTTTATCTTTTGAAGCATAAAAATATATAC
CAAACCCGACAACTACAACTATTAAAATAAGTGGAACAATTTTTATCTTTTTCATCAATATACTCCTTATATAAG
ACTACATTTGTAGTATATTACAAATGTAGTATTTATGTCAAAATAATGTTATAATTTTTGTGATATGGAGGTGTAG
AAGGTGTTATCATCTTTTTTAATGTTAAGTATAATCAGTTCATTGCTCACGATATGTGTAATTTTTTTAGTGAGA
ATGCTCTATATAAAATATACTCAAAATATTATGTCACATAAGATTTGGTTATTAGTGCTCGTCTCCACGTTAATTC
CATTAATACCATTTTACAAAATATCGAATTTTACATTTTCAAAAGATATGATGAATCGAAATGTATCTGACACGA
CTTCTTCGGTTAGTCATATGTTAGATGGTCAACAATCATCTGTTACGAAAGACTTAGCAATTAATGTTAATCAG
TTTGAGACCTCAAATATAACGTATATGATTCTTTTGATATGGGTATTTGGTAGTTTGTTGTGCTTATTTTATATGA
TTAAGGCATTCCGACAAATTGATGTTATTAAAAGTTCGTCATTGGAATCGTCATATCTTAATGAACGACTTAAA
GTATGTCAAAGTAAGATGCAGTTCTACAAAAAGCATATAACAATTAGTTATAGTTCAAACATTGATAATCCGAT
GGTATTTGGTTTAGTGAAATCCCAAATTGTACTACCAACTGTCGTAGTCGAAACCATGAATGACAAAGAAATT
GAATATATTATTCTACATGAACTATCACATGTGAAAAGTCATGACTTAATATTCAACCAGCTTTATGTTGTTTTTA
AAATGATATTCTGGTTTAATCCTGCACTATATATAAGTAAAACAATGATGGACAATGACTGTGAAAAAGTATGT
GATAGAAACGTTTTAAAAATTTTGAATCGCCATGAACATATACGTTATGGTGAATCGATATTAAAATGCTCTATT
TTAAAATCTCAGCACATAAATAATGTGGCAGCACAATATTTACTAGGTTTTAATTCAAATATTAAAGAACGTGT
TAAGTATATTGCACTTTATGATTCAATGCCTAAACCTAATCGAAACAAGCGTATTGTTGCGTATATTGTATGTAG
TATATCGAGCTTCACATGAAACAGCTAAAGAAGCTTTGGGCGATAAAGAGTTAAGAGCCATTGCACATGAGT
TAACTAAAACAGTTAAGGATAACATGAGTGTTGATTGGTCTAAACGAGACAGTGCTAAAGCTAAAATGAGAG
TTCAAGTTAGACGCCTATTAAAGAAATATGGCTATCCACCAGATCTTCAAAAAATGGCTGTGGAACAAGTTGT
AGAGCAAGCAGAATTAATGGCAAGTCAGCAATAAAAAAATAAATCATAATGAGTCCGGGACATAAAGTTCTT
GGATAAGTGAAAAAAGACAATTTCTATTGAAATAATATAGAAATTGTCTTTTTTATAAATTTTTTGATTATTTTC
AGCTCGTTGAGCTACTACTTTTCTTATATTAAGTGCCATTAATACAAAACCAAGTTCTCTTTTGACTTTATTGAG
TCCTCGGACAGACATCCGAGTGAAACCCAAAATAGCCTTCATAAATCCAAAAACAGGTTCCACATCAATTTTT
CTTTGACTGTAGATATTTTTTGTTTCTGGTTCTGAAAGCTTTTTGTTAATTTGGGATTTAAAATATTCCCAGTTA
TAATTCTTCATTATTTTTTTGTTTGTTTTTGAATTGAAGTTCATACATTGATTTTTCAGAGGACATTCTGAACAA
TCATCACATTCATATAATTTGAAGTCTCGCTTATAACCATACTTATCATGACGATAGGCATATCTTTTAAAACCTA
GCCGTTTATTATTCGGACAAATGAATTCGTCATTAATTTCGTCATAGTTCCAATTTTGAGTATTAAAGATGTCAC
TTTTATATTTTTTAGTTTTATCTTTTATAAACATTCCATATGTTATGAGTGGCGTTCGATTAAAGTCATCTATAATT
GCCTTATAATTTGATTCACTACCATAACCTGCATCAGCTACAATATATTCAGGTAAATGACCGTAGGTCTCTTGA
ATTGAATTTAAAAATGGAATCATCGTTCTAGTATCCGTTGGATTTTGATACACATTATAAGATAAAACAAATTG
GGAATTTGTTGCTATTTGTAAATTATACCCTGGCTTAAGTTGTCCATTTTTCATGTGATCTTCTTTCATTCTCATA
AATGTCGCATCATAATCTGTCTTAGAATAACTATTTCTATCCTTTAAAATAGATTTTTGAAATTCGTATCGATACT
TTCGCTCAAAATAATCATTGATTTGCTTTTTGTATTTTTTGATTTTAGTTCTTTTGAGACGTATTTGTTTTCTTGT
TTTAGTACATTTTTCATTGTTGATATGTTGGTTTAAATCTTCGATTTCTTTATCTAAGTGACTACCAATCAAATCT

ATTTCTTCTTTTGTTAAGTCATTATCATGATCTTCTTTAATTTCCGGTATGATTTTATTGGTTACCAATTCATGGTA
GAGGGCTTTAGAATCCTCATTCATCTTTGATTCATGGTTTTGAATACTCTTTTTCCATACAAATGTATATCGATT
GGCATTTGCTTCAATTTTTGTACCATCAATAAAAATAGCTTTATCATCTATAAGATTTTGTTTTACACACTGACT
GTAAAATTGAATAAATAAAGATTCTAATAAAGCATCTACTTTTGGATTTACTCTAAATTGATTAATTGTTTTATA
AGAAGGTTTTTGATTTTGTGATAGCCACATCATTCGGATGCTATCATTAAGCATTTTTTCTATTTTACGACCTGA
GAATACAGATTGTGTGTAGGCATATAGAATCACTTTTAACATCATTTTAGGATGGTACGAAGTTGCACCACGG
TGATGTCTGAATTCGTCGAATTCATTGTCAGGAATTGTTTCAACAATATCATTTACAGTAAAACGATGTTGATT
TGTTTTGTTTCCATATTGACCTCCATGTATTTGCTATGATTTCAAAATCCATTTTTGACGTGCCTTAGGGTTGAG
TGGATGCATAATTTCATTTGTTACTGGATTGATGAGCTTTTTTACTTTCTTTTTATGAGGTTTTAACATTTCCAT
CACTTGTTCGACACGGTCGATAACAACTGGTCGCTTCGCATAGGCACCATAAGCAAGAATCACTGTGTCACT
TTCACTAATCGCTTTCATCAAATGAATATCAGTGTGCTCATCGTATGGATTTTTGATATGTTTGAGGTTTTCGGG
TGTTCTAATATTAGAGAATAGATTTACAAGATATACAGCACCGTATCGTTCTGAATTGGCTAATTGGTTGAGGA
TAAGAACAGTTGTGAGATCGAGTGATAATACACCGTCTAAATGAGGATACATCGTTATCACTGTGCATGCAGC
TTTCTTTTCATCCCATGTTTTCTTGAGTAAATAGCGGTGCTGTTCATCATCGCTAAATATGGCTTCTGTGTGTAT
CGTACTTTTGATTGTATTCATCATCGTCACTTCCTTTAGTATTCTTCTGGTAAAAGCATCACATAATAAAAAGCG
TCCACGTCATCTTCACGAATGACGTAGACTTTCTTAGGTAATGCATTTTGATTTTTTTCATAGTTTGTATAGTGA
TATTCCAATTTGTATGTGGGTTGTTCTTGTTCATGTGTGATTGAGAGTATATTCTCATCTTCTTGTAATTTAAAA
ATGTGTAGGTAATCTGTATGAGGCTGGTTATCTTTTTTTTCTACCATGTGCCAAAGTAAGATTTGAAGGTCTAG
TGGAATACTTTCATTAATTCCTCTTGTGATGTATCGATTGATTTTCATGCTATTTCCCTCCCTTCTGCTTTTCTTT
CATGATGTCGATGATTTCGTTGATAACTGTGACGGATAATTGAGCAACTATGATCCAATTATTCATGGTCCTGA
CCTCCTTGTTTTAGTAAATGACGTTCATCAATAATGATATTTTGAGTATCTGTAAGGTACAGAAAGTCCATGTC
AAAATGGTCTAAGTATCCGACACTGATGAGTTGGTTATTGGCATACATTAGAAATGGATAGATACTTAGCTCAT
GTAGTTCATCATTATAGTAGGTATAAGTCTCGAGTGTGAGATGTACCAGTGGAGAATCATTAATAAAACGTTCC
GGTAGAATATTTTCTGCTGCTTCCTCCAGCGCTTCACATTCCCAAGCTTCGTTATTAGATAGTTGGAATAGGTG
GGTTATATATTGTTTGAGTTCTTGAGTGATGGTTTTCATATTATTGCCTCCTAGATAGTGTAATAGTGATGTAGT
TCATATACATCATTGAGATAATATATATTTGATTTGTCATTTATTACGAATCCCGGTGGGAATAAGAGAAAATTC
CATATAAAAACCCGCTACAAACGTTGGTATGCCAAGGAAATCCTGAAATTCCGCCTATTTTGACAAACAATCA
ACTCATTATTTATAAGTATTGATGATAGGGTTGTGTCTCTGCTTCCTTATATATATTATTTATTTATAAAAAGTAAC
GGGATTTTGGGATTGTGCTTGCACAATCCTTCTGTTTCTTCGAATCTGCAAATCCCAATCATTTCCCGATAAAA
AATCATTGTGGGATGTTCTTTAGCAATTTCAATATAAGCATTGTATAGTTATGAAAAAATTACGACAATAACTGT
TTCATTAGATAAGTGTTATTGAAATTGATAAAGAGCAATTCTTGAAAATAGTTAGATAAAATAAGCGAAAGAA
TATAGTGAAAATTATTGTTATAACAATGATTCTATTAGCTAAATAGTAAGATATAGTGTTTGGGGCAAAAATAAA
GACGAAGTGCTGAGATGCACTTCGTCGAGTTGTTTATTATTGAAAAGTTGTTTAATGATTTCGTTATTAAGTTT
GAGTGTGACATAGAATTGTTTTTTATGATTACCATCTTTTTTAATATCAATGCGATCAATCACTGATAGATACAA
TGCTTTGAGTCGAGATTTTTCTATGTGCTTAATATCATGAAAGATGTGTTGTAATAGTTTACTGATTTCTTTGGC
ATCAAATAAAGTCTTATCTTCATTTTGTTGATTTTTGAGTTGGTTGATTTGATTCGTAATGTCATTGAGTTGCTT
TTCATATTTTTGAATACTTGGTCTGATTACTGATGTTAAGTCCGGATTATCCTCGATGGTTTTAATCAAGTTATTT
ATTTTGATTTGTACTTCATCATATTGTTGTTGCTTATAAGCAATATCGTGATGAAGTGCAGCGCCATCAACTTGA
TTTTCTTGATTGACGTGTGTTACTACGCGTTGAATGACTTTATCACTTTTGACTATTTCAAGTATTTGCTTCATC
ACATAATCTTCAATCACATCAGCTCTTACACTGTTTGCCGAACATACTTTGGAACCCTTGTTCCGAAAATTACT
ACATGAATAGTAACGAATACGTTTCTTAGTCCCGTCTTTAAGAGTATTCGTGGTATTGCTTGCTGCCATAGGTG
CGCCACATTGGGGACAGTGAATAATGCCTGTAAGCAGATTCGTTCCTTTGCCATGGACTTGGGGTTTTTGAC
TGACTTGTTTCTTACGCATTTGTACTTTATCCCATAAATCTTGATTAATAATGGGGGAATGCTTACCTTCAGCTA
TCACTGGTTTATCATTCAGCCCTTTACGACGTTTTTCACTCCAATCTTTGTATTTCGCAAATTGAATTTTGCCGA

TATAGAAAGGGTTAGCTAAGATGTATGTGATTGAACTAATACTGAAAGGTTTCCCCTTTTTAGTGACATATCCT
TTGTGATTCAATGCATTGGCAATTTTACGATAGCCATGTCCTTTGGCATAGCACTCGAATATATATTTTACAATA
TTAGCTTCATGTTGGTTAATCATTAGCTCGTGTTTACTATCTGGTATTTTGTCATAACCTAGTGGTAAATTGCCT
TGATAATAGCCTTCTTGGGCACGTCTCGTTTGACCCATAAATACGTTCTCGACAATGTTATTACGTTCGAATTC
TGAGAAACTCGCAAGTATCTGTAACATGAGTTTACCAGAAGAAGTATTGACTTCCATACGCTCTGACAAACTG
AAAAATTCGACATTTTGTTTGTGTAAATCTTCGACAATTTTGAGAAGATCAGATGTATTACGAGCTAATCGGT
TTGTTTTGTATACCATAACACAGTCGATATTGCCTTCTTTTGCATCTTTCAACATACGTTGGAGCTCAGGTCTGT
TCATAGATTTACCTGAAATACCACGGTCAGCGTATATATCTTTAACTTCAAAATGATGGAAGTCACAGTATTCTT
TGATTTGATTGATTTGTCCGTCGATACTATAACCTTCTGTGCTTTGCATTTCTGTTGATACACGTACATAGATAC
CGACACGTTTTGTTTTAAGTTGTTGCATTATGTTACATCCTTTCTTCATTTATGCAATCGATGATTGCATGGTTT
GATTGACGATATTGAGTGGTTCATTTTTGAAATAGATTCCTATAAGATTTTTATCTTTCGTAATGTGAATGGTTT
CAATATAGGGGTACAATATGTTTAACGTGAAACGTTTTTGAATAATATTTTGAATGGTGTGTTGTATTTGATGT
CCATTGATAGATGTAGTGCGTTGAGGTTGTTGACGTAATGATTGCGTTTGTTCTCTGAACGTTTCTGCATCGA
TGATGCCTTGTGCCAACTTTTCTATCAGTTGTTCTTGAGTCAATGTGTGATGTTTTTCTATGTTTCTTTGTCTTT
TGATGCGTTTGTCAATCGCACCTTTAATTTTTGTGTAGATGCGTTGATTTTGATAAAAGTCTCGGCACACTTCT
AATACTTTATCTTCAAATGTTTGTGCATTGATGCCTTTGAAATCACATACAAAGCGTGAAGCATTCATGTTTTT
AGGACAGACGTAGTAACGTAATATATGATTCTTTTTTCTAACGGTCATATTTGTAAGTGTTGCATTACAACATG
GGCAATTGATTTTTTGTTTGAGTTGATTATCCGAAGATGTCTGTTTGGTTTGTTTTTGCAATCGAAGTCTCTGC
GCTTGCTCATATATACTTGTGGAAACAATAGAAGGAAACATATTGTCGAATTGGCCATATTGATTGTTGACAC
GACCACAATAATTAGGATTGATGATAATGTTACGAACTTGATAGGGTTGTCGATTGATATACGTGTTATCTTCT
TCTAATAACTGTGCAATTTTCTTATAACCATGACCTTTAATGTAATAATTGAATACAGCCTTTACCGTTGGTGAC
TCATTTTGATTGATGATGAATGTTCCGTTGTGATATTCGTAACCAAAAGGTGCATGTGTTGTAATCAATCGACC
TTGCTTTGCTTTTTCTTGAAGCCCATTTCTGACTTGTTCTCCAATGTTATCCGATTCAAGTTCGGCTAAACTGA
TGAAGATATTAAGCTTGAGTCGGTCGAAAGCTTGATCCATATCAAAGTAACCATCATGTACGCTTAAGATATG
AACATGGTACGTTTGACATAATTTGATGAGTTTTAATGCATTTTTCAGATTACGATGCAACCTATTAAGACGAT
AACAGCATAATATGTCACACTGTCCTTGTTGAATTAATTGTGTTATTTGTCGATACCCACTACGATTATCTTTGC
GACCTGATTGTTTGTCGCTATAAAAGTTGATATGTTGAATATGATGTTTTTCGGCTATTGCTTCGATAGCTTGTT
TCTGTGCTGCAAGAGATTGTTGTTTCATCGTACTTTGACGTAAATAGCCTATGACTTGTTTCATATCGGCTCCT
CCTTTCACAGTGATAATATATATTTATGGATGAATTGATATATAAGCCCAACATCAATGAGATGTTGGGCGTCCA
TATTAGTCATTTGTTTGATTGATTTCTTCAATTACCAAATCGGCTAATATCTCGATAAGTTCATCCATGTTTTTCA
CTCCGTTATTTGTTCTATCTTCAATACGTCGATTATTCAGTTTGATGCTTCACAGTTGTATGATAAAGACAATTA
GAAATCTTCGTGAACTCCTGAAGGGCCTATCCCTTCATTAGCGGATTTAAAAAGTTCTTTCGCAGCTTTGTTA
TCATTTGACGGTGTCCAATTTTGAAGTAACGACTTATCTTTAGTTAATCCGAGGATAGATGCAAACTCTACATC
TAATTTTAGATGGTAAAATACAAGTGATTGTTTTTTACCGCTATTATCTTTGACACTTCTTTTAGTTGTTTGGCG
TCCACGGTCAGCTAATATGAAACCTTTATCTCTTAAGGCGTTGACAACATTATTAACATCTTGAAATTGATGAT
TGTTTAGCATCTGTTTAAAAACGTTCGCAATTATTTTTACTTCGATATGGTCATCTTTTAATGAGATTAATCCATA
GTTCTCAAACATATTTTTCAAAGCACCTTCATCTGAAAACTTACCTCTGTTTTGTGCCACAAATTGAATGATGA
CATCAATAGCTTTATCAGCTAATGAGCGTTCAGAGACTGTATGAGTATGATAATCAATAAAGTAGTCTCTTATAT
TAGCGATATCAATATCTGTAGATAAAACACGACCTAATATTTTCGCAGATGTTGTAATGACTGCATAACGCTTAA
ACATACGAATACCTGTATTGTTTGTTTCATCTTTCAATTTAGCTTCAAACCAATCTACTTCCTTGTAAAACCATT
GAATAACTTCATCTTCACGATTTATAAGATATTTAGCTACTAACGGTAAAACATGACCATAGTTTAGTGCTACA
GCTTTTTTAATATTGTCAGCATTGGTCGCATTTGTAGTGAATTGTTCATTAATCTCGATGGTTCTTACACGTAAT
CCATCGTTTTGAGCTGAATCATTAAAAATACTGTATTCTGACGTTGAAATGACAGAAGTACCCCAATTCTTAG
GCGTTTTAACTTCTCCATGAACGTTTGAACGTTGACGACCTTGACCTTCAGCGATGGAATATAACAAACCCGT

GGTATCTCTAAGTGTCGTAGATGAAAGCTCATCAAATACTATAGGAATGCCATAATTGTTACTCAAATAACCTT
CAAGTGCGTTTCGTGTGGCATTCCAACTTCTAAAAAGAGTTTCATTACCTTTGGTAGGGTTACCAGCGACTG
ATACAGCTAAAGCAGCTGCGGTTGACTTACCGGTTGAGGATTGACCTGTAAAACTAAAGAGAATTCCTGCAA
ATTCGATTTCATGTTTATGCTTCAGAAAACTTGTCACTAAGGCAGAAATCCCAAATATGACTGCCAATTCTAAA
AGAAGATGACCTTTAACCTCGTCAATATACATGTTAAACCAATTATCAAATGTTCCCCTAGGTGCTAAGTCATA
AGTATTCTCACAAATGGCGTCAGATGGAGATTTATTATCAAATTCCGTAGTAGTATAGATTTCATTTAACGATAC
AATAGGACCAAACGGTGTTTCCAGTATACCTACCCCTTCATATAAGTAGGAAATGGGTAATTGGTTGCGCATT
TGTTGCAACGCATAACCTAAATCTTTTGTATATTTTTCATTAATACTAAATCCATATTTCATTAAAGAGGGCAGT
TTTTGTGTTGTTAAAATATCACTAGATTCAACAATTACTTTTTGATCCTCGTCTGTAATAATTACTTTTTCAGTGT
TAGTTTTAGGGTCAATAAACTTATTTTCGATAACGATAGGACCTGCGATTTCAACTTCAGTAGGCATTCCTCCT
TTTTCTTTGGGAGGCTTGTCTTTATACCAACCTTTTTTTGATTTGTATCGTGGTGAAGGATTAAATGAAGGGT
TAGTTTGAGTCATTAGCGAACACCTCCTTTCGAAGGGTTGCTGTTATGGTGTGGATTAGGACCTGTTTTAAG
ATAAACTAAATGACCGTGAGTATCCTTACCGATAATAATAAATGGAACACGTGGCGCATGTTTTACAAAATATG
CGAACCAACGTCCAACATTTTGTTGTACTTCTTTAGAACACGTTACATTTGCACGACTGTTCAAGTCATGAAA
TGTAAATTCTTCTCCTTCAGGTAAATTGAATGCAATACCTAAAACCTTGCGTTTTAACAATAAAAAGTGGAGA
CTCTTTTTATTCATTTGGATCATCCTTTGTACATTAGTCATCATTTAAATGATGCACTTGTATCTTATATCTTTACT
GAGATATAAAGTTCTAAATTTGAGAGGAAAATATTAGGACATTGGTATTACACCTTTTGATAAGTAAAAAAAC
AACGATTTTTTTAGACTGACCCCAATTAGTGGGAATTATATAAAAACACTTTCGTTGAATTCATATTAAAATGA
ATCATACGGGGGGTGTTTTTTTCTATGAAAAGAGTTTCTTATTCAGTAGAAACAAAGTATAAAGCAGTTGAA
ATGAAAGCAGCAGGATTTTCAACAAAAGAAATTATGAAAGAATTAAATATTAGAAATAGAACACAAGTGAAA
ACTTGGTGGCGATGGTATCGAAATGGGGAAAGTTATAGATTTTCACAACACGTTGGTAAACAATATACCTACG
GTAAAGGATTAGAAGAGCTGTTAGAAGTAGAACAATTAAAATTAGAAAATAAGAGAAAAGATATAGAATTG
GATATTTTAAAAAAGTACAAGGCATTGGAGAGGAAGTGGTACCAACAGTAGTCGTAGATTTAGTGGATCAAT
TAAAAGTAAAATATTCAATCAAATTGATACTAGAAGTATTAAACATACCTAAATCAACATATTACCGATGGAAA
AACAAAACCCATAAAAATGATACCGTAACACAAAAAGTTATTGAATTATGTAAAGCTAACCACTATACCTACG
GTTATCGTAAGATTACAGCATTGATTAATCAATGTTATACATCACCAATTAATCATAAGAGAGTACAGAGAATG
ATGCAGAAGCATCATTTGAACTGCCGAGTTACCTAAAAAGACGACAAGAATAGGTAAACCGTATTATAAAAC
GGACAATTTATTACAAAGACAATTTAAAGCGAGTTGTCCAATGGAAGTATTAACAACCGATATTACTTATTTAC
CATTTGGTCATTCTATGTTGTATTTATCTTCGATAATGGATATTTATAACGGAGAAATTGTGGCGTATAAAATAG
ATGATAAACAAGACCAAAGTTTAGTTAATGATACATTAAATCAAATCGATATACCTGAGGGTTGTATATTACATA
GTGATCAAGGCAGCGTTTATACATCTTATGCTTATTATCAATTGTACGAAGAAAAAGGCATTATCAGAAGTATG
TCCCGAAAGGGAACACCCGCCGATAACGCCCCGATAGAAAGTTTCCATTCCTCGCTAAAGTCTGAAACTTTT
TACATCAATAATGAGCTTAATCGCTCTAATCATATTGTAATAGATATTGTCGAAAAGTACATTAAAAACTATAATA
ATAATCGAATTCAACAAAAACTAGGCTACTTATCCCCTGTGAAATACAGAGAATTAATAGCCTAGAACATGGT
GTTTTTATTAAGTTCCCGTTTTAAGGGTTCAGTGCCTTTAATCGTTGGTTTTTTTTATTGAATTAATAATATAAA
TTTGGTCCATCGTTAATATCTTCTAAACGTGTCTTGTAAAACTTACTGATTATTTGAATCATTAGTTTATCGAGA
AAATCATTAGGATTATTATTAAAGTTGTCTCTTTTATAAAAGTTGCTAATATTATATTTTTTTAGATCGGTGAGTA
AAGACCTTTCTACTTTTTCTGTCATTTTTATATCATTCATAATAATATTATGAATTAAATCTTTTAATCTAGTATTTC
TTACATAACCATCAATAAGACTTTTAAAACGGGTTAATGATCGTTCGTGTTTGTGTGCTAATTTATTCAAATCAT
TTTTTAAATTAGAAATACTATATAAAGCGACTAATACATCGTATTGTGTCGCACTTTGAATGATTTTACTTGTTA
CTAAAACTAATTCATCCATTAAATCGGAAATGGCAGTGAATAATGCAAAGTGGAACGTATCTTCAATAGTTGT
TAAATAATATAATTCATTGTCGCTAATTAGATCTTGTTTTTTCATAAATTCATTTATAGTGTTTTGAGACTCAATA
AGTCCATCTATTGTTAAATTAACTTTACGTGACTTTTCTGTCAGAATACTACGGATGAATTTAAAGTATGTATCA
TTTTGATCATATAAGTTGAGAGAAATGCCGATAACAGCTTGCCTAAATTGGTCTAACTCATCAGATTGATTTAA

GCTATACTTGTTATGAATCGAAGGTTTGATACTATTAGCAATTTCAAAGTATTGAGAATTACTTGTTCGTTCTTT
GATTAAACCTTCCGTCACTAATTGTTTAAAGTAGTTAGACTTATTTAAGTTTGAGATATTTTTCTTAACATAAGA
GTCTTGTTTTTCTTTATAAATGAGATATTTCGTTAAAAAATCACTTATTAATACATCGTCAAAATTTTCTTTAGAT
AAATCTTGATGAGTTTCTAATATTGTATCTTTCAGATTTATAAATGATTCCAAAGAAGATACATCGTCAATAGCT
TTTTCATAATCTATTCTATAGTTAGCTTTTTTTAATTCAATATTTTCTCGAATCTGTTTTATTGTATTGTTAGCTATC
TTTTTAGCAAAATCTGTTGTTTTATTGATTGTGGTTAAGAACTCGGTGAAATATGTATCTAGAGTTGTTGATTC
TAACACATCTAGAGCATCTACACCTTGGTCAATGATTTCATTTGAAAGTTCATCTATGTCCACCATACTTTGACT
ATCAATAGAATACGTCAGATTATCTTTAGTAAAGCCACTAGGAATTTTATCGATAAATATACCCTCTTCACTACA
TTTCACAGTGTGTTGTTCAATATTTTGATTAGAGGTATTTACAAAGTAGCTTATATATATAGTTTCATTAGGTTG
TGCTTCTCCCACTACATAATTTGTTCTCATTTCTTCAGGTAAAATATGTTGTGCATATTCTTTTAGTATAAATTCG
GAATCAATTTTGTGTATAAAATAACTGAAGAATTGAAACCACAGCTCTATTTTCCACTTATTTTTAATCATGATA
ATCACTCTCTTTCAATATTTGAATTAAGTATATCAAATCAATATTAATTGATATGATAGTTCTTTTACTAATTATTTT
TATAAGATAAAATAGATAAAAAAGGAACAAATGTTCTCTTTGGGTATATCATATGATTAGAGGAGATCGATTCA
TTTAATGAATTATTTTATTTAATAGAATTATATTTGAAGGCATTTAAAGGACAAAAGAAAACAGGCTATGAATT
TGAAGAAAGATTGAACACATTTATTCAATCATATGTCATTATTTTACTTATAGATATAAGGTTACCGACTGTTAG
GTTATATATTATTAGGCATATTGTGGATACATGCGAAAAAGAAGTGGACAATCAAAAAAGAATTATACTAATAG
AGAAATCTATTAAAAAGTACAGATATGTAAAAGAACAGATTAAAAGATTTGGAAATGCGTTGAAAGAAGAAT
TATCTAACGCGACTTTGTCTATTGAATAGAATATGTTAGAAAATATCATTGATTATTATAAGGATTACTTTTATCC
TAGAGAAAAAAATAAAGCATAATAATGTTGAGTGCAATGTATCATTGTTTTTTAAAGCTGTAGATGCTTTTAAGA
AATAGTTTTTCACTGACAATATTTATGGATATAATTAAATTTAAGCAATGTTAATTTATTCCAGTCTTAGTAGTTT
TTGAATTTCACAAACACAATCTATGAAGTGAGTTAATATTAAACGATTTTAATAGGAGAGGGTTAGTATGGCT
AGTTATTTATTTTTTCATCCTAAACCAGCGTGTGATACTTATGGGGATATGAATATTTATCACGACAAATTTGGA
AATAATGAGGACCCATACGTATGGAGTGAACGGTTTTTGCATAGCTTTTGTAAAATAACGGATTATGCATATAG
TAAATCTACTCAAAAGGACATTATTTTTTGGATATCAATAAATAAAGAAGGCAATAATTTAAAATATTTATGTGA
TTTAGTATTTAAAATAGAAAAGTGGGATTTTTGGTATAAAACTTTTAGTGAACAAAAAGATGCCATAGCTACA
AATAAAGAATTAACGATAAATGACGCAGTAGTAGAAGGCGATGAAGAAGCATATGAATATCATTATTCTTGGA
TCAACAGAGGGGAACACAAGTGGGAACCAACTTATCGAAGGCGAAGACTCACATTAAAAGCAGATCCCGT
CTTGAGTTTTCAGCCTCAAAATCGACAAGGTAATTTACTAGATGTCACGGAATTACTAAAAACAATTGTGAAT
TTTAATGTTGAAAAATCACCTGCAAAAAGTGGGACTTCTTATAAAGCTTTCGAACTTGAAGAAGAGCAGGC
AAGCAAACTATATGAGGAAATAAAACGATTATCTTTTATTCGATTAAAGGGAAGGGACTTAAAAAACTTAAG
AAGAAACTTTTCTTGATATATCTTAATAGTTGTTGTTATTAAAACCATAAAGAAAGCTACCATTTTTAAGATACA
AGCATTGATATGTACAATCTGAATGGAGTGTAAAAAGTAAGTGATGTTGTGATGGACATAAAGTTGATACTCT
ATTTATACGAAAAATATTGATAAAGATTAATTATGATAAACATGTAGTAATAGAAAATGAATTCTATAGTTCAGG
ACTTAGCAACTAATATTATGGTAATGGGAAGAATAAACAAATTAATTTTTATAAAAAATATATAAATTAAAACTT
AGAACAGTTAGAAACAGAATTATTTGATTATGTAAATTGGTACAACAATTTTAGACCACATTCTTCGTTACAGT
ATTTAACGCCAATGGCGTATAAAGATATACACATGAAAAGTGTCTAAAAAACTGTTGACATTCCACTCTATATT
TGATTGTAGTTGTTTATAGATAGTAAGATCATTCATTATACATGGATTTTTGTCCGATTTCTTGACACAATTCCA
GACAAACTAATAAACTAATAAAATAAAAAAATAAAGGCGCGAAAACAGGAATTATTAAATAAAATATTAATTT
AATAAGGGGAACAAATGAAAAATATAGATATCGCAATTTATGATATAGATAAAGTTATTTGTAAAAGTATTGAG
AATAACTCATCAGATTTAGGTTATTTATCACAAAGTATCTTATCACATTTAAGAAACTACGTAGAACATATTGGT
ATGAAATATTATTTCGAAAGTGTAAATGAAGATATGACTAATAGTTCTTCAAATATCTATAATGAAATACAAATG
GGAATAGGTTATTTTAGGAAGCATTACAAATTGAATTGGCTTAAAAATTTTCATGAATTACTTCAGCAATCTGT
TTCTCATTATACATTTGATGAAGATAGATCCGAACGGTTATTTATAAAGTATTATGACTTAATGTTAGAATTAAA
AGAAAAGTTAAAGAAAGATTTTGAAATGGATTTATTGCAAAATTTATATAAAGTACCGCTGAATATTGATAGT

GATTTAAAAGGATATTATCATACAATATCTCAATTAATAGGTAATAAATACACTGATAATGAATATGAAGTAGATA
ATTCAAGGTATTATATTGAAAAAACAAAACCATTTGTTATTGAAAATAAAGTATATTATGAAATTACATTTAGAA
ATGCAAATGATAAAACTAGTAAATACGAAAGGCTGATTGGTTATTCAAAACATAGAATAAACACAAATTATGC
TGTAAAATTCGCATTAGAAATGCAAACAATAAGTTATTTAGGGATTAAAGCTAACGTAATAATAATAAATGATT
ACGAAATATCTATAAGACCTTGTGAATTTAATAATTTTGCTAAAATATTAGATTACGATTTAAAATTACAATCAA
ATCATAATGAATATAAAAAATTAATGAAATTGTTAAAAGAATTTAATTTACATTTACTTGATATTGTTTTGTTAGA
TGACGATGAATTTAATGAATTGGAGTGTTCAGTGAATAGCGAATCAGCAGTTATTAATATTTTTAATGTGTTGA
GACTAGCAAGAAGATATATTTTAACAAATAAATCAGGCGCAAATGTTCTTAGGTATTTATTATTTGTTTTTAAC
AATAGGATAATTCGTTTACAGTTACCTTATAATAGGAATAAATGTTATAAATTATCTAACCTTATATTGGATTATA
AATGTGTTCCATTTGATCAAATGCCATTTACAGCCTCTTTAAAAGGCCATAATCCTAATATCTATACTTTGTTAC
AATGTATAGAATACAAAGGGAGAGAATATGAATTATTAGTCAGGAAGATTCAAAAGAATACTTTGAAGAATA
AGAAAGTTTATACAAGCAAAGAAGAAATTGAGCAGTATGGTGTTGTAAACGAACTAATCGATAAATATAATAA
TAATTTGTATTATAAGCATCGACCGAATAGGGAAATACATTCATTTGGTGATAAATATTATTTATATGAAAATGA
GCAGAGTATTATGAGTATAATAAAGTCAATTAAACTATTGTCAAATGAAAGCGTAGAGGGCTATTCAAATTCA
GTAGAGTTTTGGCTAAATAATGAATATACATCGTTGGATTGTAAAGAAAAGAAGGAAATACTCTTAAGAATGT
TTTCAAATAGCAAAATATCGATGGTATACGGAGCTGCTGGAACAGGGAAATCAACTTTAATAAACCATATTTG
CAATTTTTTCTATGATAAAGATGTTATCGTCATAGCAAATACTAATACTGCAGTAGACAATATTAAAAGAAAAAT
CAAATTATCTAACATTAAAACGTCTACTATTTCTAAATTTTTATATAATGATAAAGAAAAGTATGACTTGTTAATA
ATCGATGAGGCAGGTACAGTTAGTAATAAGGACATGAATCGAATTCTTGAAAACAAGCAATTTGAATTATTAT
TAATTGTCGGTGATAATTATCAAATCGAATCGATAGATTTTGGAAATTGGTTCGAAATTGCCAAAGATGTTTTG
TCAAAAAATATAATCAACGAACTAACTGATATGTATCGAACTAAAAATGATGATTTACTTTACTTTTGGAAATCT
GTTAGAGAAAAAAAGAGTAATTTAAATGAAATTATTAATATGAATAAATATTCTACAAGATTAGATGAAAGTAT
ATTTAATGAATTCAATAAAGATGAAATTATTCTTTGTTTAAATTACGATGGTATATATGGTATCAATAACATTAAT
AGATTATTACAAGCAAATAATAAAAATGATTCTGTAATTTGGGGTGTGAAAGAATATAAAGTTGGTGATCCTAT
TCTATTTAATGAAACTAATAAGTACTCACCAATACTTTTTAATAATTTAAAAGGGTCAATAATTGAAATACATGT
TTTTGAAGAATATATATTGTTTGATTTAGAAATAAATAAAGTTATAAATGAACTTGATATTATTTCGTTAGAAATT
GATTTAATAAGTTCTTCTGAAAATAGTTCGGTGATTAGGATTAGAGTAGAAAAAAGTGATGGGTTGAATGAT
GATGATAATGATTCATCTGATAGTATAGTTCCTTTTCAAGTGAGTTATGCAATTTCAATACATAAAGCTCAAGG
GTTAGAGTTTAATTCAGTAAAAATTGTTATATCTGATGATTTAGACGAACAAATCACTAACAATATTTTTTATAC
TGCAATTACACGAGCTAGAGAAAATTTAAAGATTTATTGGTCACCACGAACTGAAAAGAAAATTATTGATAAT
ATAATTTCTAAAAGAAACTTGAAGGATCTTTCTATATTAAAATCTCGAATAAAAAAACAAAATACTTAGAAATT
TTCATACGACGCTTATATAGAACCATAAAAATCGTTTGACTAGTACCAGTGATTTTATTTAATTTTTCTCAACGA
CTCTACTTCTAAAAATATTTTAAATGACTTTGATACAGCACGAATTAAAATCGCATAAGCGATGACTTTTCCAC
TTAATTTCTTATGCCATTCTACGGAAGTCATTTGAGAACAAAGTATTAATGATTTTTCACGGCTTCTTATTTCGA
ATACCTCCATGAGATACTATTGTTCTTGTTCAGTTGTATACGGCAATAAAAAATCATCATTTACTAAAATATCCG
TTCTGCTAATCACTTTTAATAGTTTATCTATGCTATTATAATCAGCTTGTTGTAGCTTACTTAATAAATCTGTCAAT
CTATAGAATAATACACTATACCGATTATCAATCGCATGATTTACTAAAGTAAAAGCGATGTAACTTTTACCATTT
CCGGTGACACCTTTTTAAACCGCCATTTACATAAATACTATCTTTAAATATAACTTGATTATTTGAAAGTATGTG
AAAGTAAATTAAGGATTGGTACGTTTTGGTTAATTAAATATCTACCTAAATATTCCTCTAATTGACTTGTTGCAT
GTTCATTCAGCAAAACAGGAATAATCCATGCTGTATATAAATAATCATCTAGAAATTCTCTACATGTAGAATATT
GTTTTTTTAATTCATCGTCTTTTATATACAATGAAGATAATATTTTTCCGGTACCTTTATCTTCATCCATCTTCTTA
ATAAGTCACCTTCTTGATTTTTAACTAATTTATATTCATTACCCTTAAATTCATATTTGAAATGTGATTTTCCAACT
ACGCAAACCATGCCATCTTTTCTTTGAGTAACATAGATGTAATTTATTATTGGGTTATTTCCATTAGCTCTATTAT
TATTTGTTAATGTGTTACTTATATTAGACAATAAATCCTCAAAATATGTAATAATATATTCATCTAAGGGTTTATTG

TTATTACTAATATTACAATTTTGTTTGATAGCATAAAAAGCTTCTAGGTTTAATTCAAATGGTTCCGCGCTAACG
ATTTTGCTATCTTTGTTGTCTATAATAAATTCGATTAGTAATTTCTTAATCATTTCGTGATGTTTTCTAGGCTTATT
GTAATGACAGGTTTTATAAAGCTGTTTATTAATTCTATTAAACATTTCTATTTGCAAGTCAGCAAGAATAGTTCT
ACAGTCTTCATAGATATCATGTACTGGGTATGGTTTATTAGGAAACATGCTATATATTTCTTTATATGTTGTATTG
AACACGTTAATTATACCTCCTACTATCTATTAATACTATTGTAATTATCAGATATAACTAAGTAAATGAATCGTCTA
CACTTAATTGGACAAATTCTATGAGAATAGATATTGTTAATTTAAGAAAGAAATTACGAATGAACGCATTCGC
AAAAATTGCATTTAAATAATGAAATATAGAGTAACTTGAGAAAGTTGTTTCATCTAAAATATATAAACAGTAGA
GGGGAGAACTTATGCACTGGAAAGAAAATAGAATAAAATCAGCCAAAAATGACACGAATCCGATGGTAATC
AAAGAATTAAAAGGTAGTTATGTGGTTTTTGGAGATGTTCAGTTTCTTCCAGGTTATTGTGTATTACTTCCTAA
AAGAGAGGTAAGATTATTGAATGATCTTACTTTAGAAGAACGACAAGATTACTTATTAGATATGAGCTTTGTT
GGTGATGCTATGATGAAAGCATTGAAACCTACAAGAGTAAATTATGAAATACTAGGTAATAAAAATCACTTTC
TTCATGCACATTTATTCCAAAGATATGAATGGGAAGATGAATCTGTTAGGTATATGCCAGTGTGGTGTATGATG
CTTCTAATTGGTCTAATGAAGAAACGTCTTACGATTCTGATAAACATGATGAAATTAGAAATAAGATAAAGAA
CGAACTTGAACAGTTGTATAACATATAAAACCAAAGTCGATATCATCATTTTGATATCGACTTTAATTATAAAAA
ACCGCACTCTTAACCGATACGCAGAGGCGTATCATAAGT
>Staphylococcus aureus strain ER01560.3
ATGAAAATCACCATTTTAGCTGTAGGGAAACTAAAAGAGAAATATTGGAAGCAAGCCATAGCAGAATATGAA
AAACGTTTAGGCCCATACACCAAGATAGACATCATAGAAGTTCCAGACGAAAAAGCACCAGAAAATATGAGT
GACAAAGAAATTGAGCAAGTAAAAGAAAAAGAAGGCCAACGAATACTAGCCAAAATCAAACCACAATCCA
CAGTCATTACATTAGAAATACAAGGAAAGATGCTATCTTCCGAAGGATTGGCCCAAGAATTGAACCAACGCA
TGACCCAAGGGCAAAGCGACTTTGTTTTCGTCATTGGCGGATCAAACGGCCTGCACAAGGACGTCTTACAA
CGCAGTAACTACGCACTATCATTCAGCAAAATGACATTCCCACATCAAATGATGCGGGTTGTGTTAATTGAAC
AAGTGTACAGAGCATTTAAGATTATGCGAGGAGAAGCATATCATAAATGATGCGGTTTTTTCAGCCGCTTCAT
AAAGGGATTTTGAATGTATCAGAACATATGAGGTTTATGTGAATTGCTGTTATGTTTTTAAGAAGCTTATCATA
AGTAATGAGGTTCATGATTTTTGACATAGTTAGCCTCCGCAGTCTTTCATTTCAAGTAAATAATAGCGAAATAT
TCTTTATACTGAATACTTATAGTGAAGCAAAGTTCTAGCTTTGAGAAAATTCTTTCTGCAACTAAATATAGTAA
ATTACGGTAAAATATAAATAAGTACATATTGAAGAAAATGAGACATAATATATTTTATAATAGGAGGGAATTTC
AAATGATAGACAACTTTATGCAGGTCCTTAAATTAATTAAAGAGAAACGTACCAATAATGTAGTTAAAAAATC
TGATTGGGATAAAGGTGATCTATATAAAACTTTAGTCCATGATAAGTTACCCAAGCAGTTAAAAGTGCATATAA
AAGAAGATAAATATTCAGTTGTAGGGAAGGTTGCTACTGGGAACTATAGTAAAGTTCCTTGGATTTCAATATA
TGATGAGAATATAACAAAAGAAACAAAGGATGGATATTATTTGGTATATCTTTTTCATCCGGAAGGAGAAGG
CATATACTTATCTTTGAATCAAGGATGGTCAAAGATAAGTGATATGTTTCCGCGGGATAAAAATGCTGCAAAA
CAAAGAGCATTAACTTTATCTTCCGAACTCAATAAATATATTACATCAAATGAATTTAATACTGGAAGATTTTAT
TACGCAGAAAATAAAGATTCATCTTATGATTTAAAAAATGATTATCCATCAGGATATTCTCATGGATCAATAAGA
TTCAAATATTATGATTTGAATGAAGGATTCACAGAAGAAGATATGCTAGAGGATTTAAAGAAATTTTTAGAAC
TATTTAATGAATTAGCTTCAAAAGTTACAAAAACATCCTATGATAGCTTGGTCAATAGCATAGACGAAATACAG
GAAGACAGCGAAATTGAAGAAATTAGAACAGCACAAAAAGATAAGACACTCAAGGAAGTGGAAGCACCTA
AAGGAATAATTCCAAAATATAAAAAAGGTGTATCAAAGACTACTAAAAATGATTCAGAAATTGAAAAATCAA
ATAAAGAGAATAAATTAACCGGTAAAGTTGGAGAAAAATTAGCGCTAAATTACTTTAATGAGCTAATTGATAA
TAAAATAGACGAAGATAAGAAAGAACAGTTTAGGAATATTTTAAATGATAATCCAGGCTCTCAACACGGTCAT
GGCTATGATTTAGTAGCTTTTGATCCAACAAATACAGATAAAGCTGTAGAAAAATTTATTGAAATTAAAACATC
TACATCTTCTAGTATTGAGGAACCATTTTTTATGTCGCTAAATGAAATGTTTGCTATGAAAGAATATAAGCAGA
AATATTTAATATTAAGAATATTTAATGTTTCCGGTAAAGAACCACAATTTTATTTTATAGATCCATATGCAAATTA
TTCTGAATTTAAAGATGTAGATGATCTCATTGACAAAGTATTTAATGTAGAAGCTATTCAGTATAAAGTTTTTG

GCGAAAAATGATTACTTGAACAAGAGCTAAAATAAAATTGTGATCTAATAAAAATAGAAGGTTCTGTTGCAA
AGTAAAAAAATATAGCTAACCACTAATTTATCATGTCAGTGTTCGCTTAACTTGCTAGCATGATGCTAATTTCGT
GGCATGGCGAAAATCCGTAGATCTGAAGAGACCTGCGGTTCTTTTTATATAGAGCGTAAATACATTCAATACC
TTTTAAAGTATTCTTTGCTGTATTGATACTTTGATACCTTGTCTTTCTTACTTTAATATGACGGTGATCTTGCTCA
ATGAGGTTATTCAGATATTTCGATGTACAATGACAGTCAGGTTTAAGTTTAAAAGCTTTAATTACTTTAGCCAT
TGCTACCTTCGTTGAAGGTGCCTGATCTGTAATTACCTTTTGAGGTTTACCAAATTGTTTAATGAGACGTTTG
ATAAACGCATATGCTGAATGATTATCTCGTTGCTTACGCAACCAAATATCTAATGTATGTCCCTCTGCATCAATG
GCACGATATAAATAGCTCCATTTTCCTTTTATTTTGATGTACGTCTCATCAATACGCCATTTGTAATAAGCTTTTT
TATGCTTTTTCTTCCAAATTTGATACAAAATTGGGGCATATTCTTGAACCCAACGGTAGACCGTTGAATGATG
AACGTTTACACCACGTTCCCTTAATATTTCAGATATATCACGATAACTCAATGTATATCTTAGATAGTAGCCAAC
GGCTACAGTGATAACATCCTTGTTAAATTGTTTATATCTGAAATAGTTCATACAGAAGACTCCTTTTTGTTAAA
ATTATACTATAAATTCAACTTTGCAACAGAACCGTATTATGGAATAGAGATGTTGGTAACATTTATACAGGATC
ATTATACTTAAGTTTAATTTCGTTATTACAGAACCACACATTCCAACCAGAAGAGAAAGTATGTCTATTTAGTT
ATGGTTCAGGAGCAGTAGGAGAAATCTTTAGTGGTTCAATCGTTAAAGGATATGACAAAGCATTAGATAAAG
AGAAACACTTAAATATGCTAGAATCTAGAGAGCAATTATCAGTCGAAGAATACGAAACATTCTTTAACAGATT
TGATAATCAAGAATTTGATTTCGAACGTGAATTGACACAAGATCCATATTCAAAAGTATACTTATACAGTATAG
AAGACCATATCAGAACATATAAGATAGAGAAATAAACTAGTGGCCGATTGTGCTTGATGAGCTTGGGACATA
AATCCTAACTCGAAATAAATAAGCATATCACTAAACTGATTTTTTAAAGTTTACAGTGATATGCTTATTTTTTTAT
CTTACGATTTTGTACGTGCATGCTTGCCTAGGGGTATGGCTCGAGCCATTAGTCTCTCGCACATACTATTCCCT
CAGGCGTCAGCACTTACAAAATCGGTTGTAATTTTCATTTTTATACGCATTCTTACTGAGATTATACTAATAAGA
GGAATAGTAAAAGCAATTCTAAGTAAAATTGCAGATAAGAGGTTTGTTAAAAGCAGTTCTCAGTAAAATTAC
AGATAAGAGGTACGTTAAAAGCAGTTCTAAGTAAAATTGCAGATAAGAGGTTTGTTAAAAGCAGTTCTAAGT
AAAATTGCAGATAAGAGGTACGTTAAAAGCAATTCCATGCAAAATTGCTGATAAGGGGTAAGTTAAAAGCA
GTTCTCAGTAAAATTGCAGATAAGAGGTACGTTAAAAGCAGTTCTAGGCAAAATTGCAGATAAGAGGTGCG
TTAAAAGCAGTTCTCAGTAAAATTGCTGATAAGGGGTAAGTTAAAAGCAATCCTAAGTAAAATTGCAGATAA
GGGGTACAGAAAAACTAGACTTGATTACAAAATGGAGCTTGGGACATAAATGATTTTTTAAAAATGAGATGA
GACGTAGATTAACTCCATAATCAATACGAATCTATCGACTTCTTTATTTATGATATTCATCTCTTTTTAATGGAAA
TAAAAGTGCGATTAATGTGATAATACAGTTACGTTAATTAAAAAAATAAAAATGCAAGGAGAGGTAATATGCT
AACTGTATATGGACATAGAGGATTACCTAGTAAAGCTCCGGAAAATACAATTGCATCATTTAAAGCTGCTTCA
GAAGTAGAAGGTATAAACTGGTTGGAGTTAGATGTTGCAATTACAAAAGATGAACAACTGATTATCATTCATG
ATGATTATTTAGAACGGACTACAAATATGTCCGGGGAAATAACTGAATTGAATTATGATGAAATTAAAGATGC
TTCTGCAGGATCTTGGTTTGGTGAAAAATTCAAAGATGAACATTTGCCAACTTTCGATGATGTAGTAAAAATA
GCAAATGAATATAATATGAATTTAAATGTAGAATTAAAAGGTATTACTGGACCGAATGGACTAGCACTTTCTAA
AAGTATGGTTAAGCAAGTGGAAGAACAATTAACAAACTTAAATCAGAATCAAGAAGTGCTCATTTCAAGCTT
TAATGTTGTGCTTGTTAAACTTGCAGAAGAAATCATGCCACAATATAACAGAGCAGTTATATTCCATACAACTT
CGTTTCGTGAAGACTGGAGAACACTTTTAGATTACTGTAATGCTAAAATAGTAAACACTGAAGATGCCAAAC
TTACTAAAGCAAAAGTAAAAATGGTAAAAGAAGCGGGTTATGAATTGAACGTATGGACTGTAAACAAACCA
GCACGTGCAAACCAACTTGCTAATTGGGGAGTTGATGGTATCTTTACAGACAATGCAGATAAAATGGTGCAT
TTGTCTCAATAGAAAGTTAGAGGTGAGTCTTACGTTTCAGTGACGGTAGACTTACCTTTAACATGTTACATAC
TAAAAAATTAATTTGAATAAGAAAGAGAGACATATATGAAATACGATGATTTTATAGTAGGAGAAACATTCAA
AACAAAAAGCCTTCATATTACAGAAGAAGAAATTATCCAATTTGCAACAACTTTTGATCCTCAATATATGCATA
TAGATAAAGAAAAAGCAGAACAAAGTAGATTTAAAGGTATCATTGCATCTGGCATGCATACACTTTCAATATC
ATTTAAATTATGGGTAGAAGAAGGTAAATACGGAGAAGAAGTTGTAGCAGGAACACAAATGAATAACGTTA
AATTTATTAAACCTGTATACCCAGGTAATACATTGTACGTTATCGCTGAAATTACAAATAAGAAATCCATAAAAA

AAGAAAATGGACTCGTTACAGTGTCACTTTCAACATACAATGAAAATGAAGAAATTGTATTTAAGGGAGAAG
TAACAGCACTTATTAATAATTCATAATAAAACAGTGAAGCAACCATCGTTACGGATTGCTTCACTGTTTTGTTA
TTCATCTATATCGTATTTTTTATTACCGTTCTCATATAGCTCATCATACACTTTACCTGAGATTTTGGCATTGTAGC
TAGCCATTCCTTTATCTTGTACATCTTTAACATTAATAGCCATCATCATGTTTGGATTATCTTTATCATATGATATA
AACCACCCAATTTGTCTGCCAGTTTCTCCTTGTTTCATTTTGAGTTCTGCAGTACCGGATTTGCCAATTAAGTT
TGCATAAGATCTATAAATATCTTCTTTATGTGTTTTATTTACGACTTGTTGCATACCATCAGTTAATAGATTGATAT
TTTCTTTGGAAATAATATTTTTCTTCCAAACTTTGTTTTTCGTGTCTTTTAATAAGTGAGGTGCGTTAATATTGC
CATTATTTTCTAATGCGCTATAGATTGAAAGGATCTGTACTGGGTTAATCAGTATTTCACCTTGTCCGTAACCTG
AATCAGCTAATAATATTTCATTATCTAAATTTTTGTTTGAAATTTGAGCATTATAAAATGGATAATCACTTGGTAT
ATCTTCACCAACACCTAGTTTTTTCATGCCTTTTTCAAATTTCTTACTGCCTAATTCGAGTGCTACTCTAGCAAA
GAAAATGTTATCTGATGATTCTATTGCTTGTTTTAAGTCGATATTACCATTTACCACTTCATATCTTGTAACGTTG
TAACCACCCCAAGATTTATCTTTTTGCCAACCTTTACCATCGATTTTATAACTTGTTTTATCGTCTAATGTTTTGT
TATTTAACCCAATCATTGCTGTTAATATTTTTTGAGTTGAACCTGGTGAAGTTGTAATCTGGAACTTGTTGAGC
AGAGGTTCTTTTTTATCTTCGGTTAATTTATTATATTCTTCGTTACTCATGCCATACATAAATGGATAGACGTCAT
ATGAAGGTGTGCTTACAAGTGCTAATAATTCACCTGTTTGAGGGTGGATAGCAGTACCTGAGCCATAATCATT
TTTCATGTTGTTATAAATACTCTTTTGAACTTTAGCATCAATAGTTAGTTGAATATCTTTGCCATCTTTTTTCTTT
TTCTCTATTAATGTATGTGCGATTGTATTGCTATTATCGTCAACGATTGTGACACGATAGCCATCTTCATGTTGG
AGCTTTTTATCGTAAAGTTTTTCGAGTCCCTTTTTACCAATAACTGCATCATCTTTATAGCCTTTATATTCTTTTT
GTTTTAATTCTTCAGAGTTAATGGGACCAACATAACCTAATAGATGTGAAGTCGCTTTTCCTAGAGGATAGTT
ACGACTTTCTGTTTCATTAGTTGTAAGATGAAATTTTTTTGCGAAATCACTTAAATATTCATCCATTTTTTTAAC
GGTTTTAAGTGGAACGAAGGTATCATCTTGTACCCAATTTTGATCCATTTGTTGTTTGATATAGTCTTCAGAAA
TACTTAGTTCTTTAGCGATTGCTTTATAATCTTTTTTAGATACATTCTTTGGAACGATGCCTATCTCATATGCTGT
TCCTGTATTGGCCAATTCCACATTGTTTCGGTCTAAAATTTTACCACGTTCTGATTTTAAATTTTCAATATGTATG
CTTTGGTCTTTCTGCATTCCTGGAATAATGACGCTATGATCCCAATCTAACTTCCACATACCATCTTCTTTAACA
AAATTAAATTGAACGTTGCGATCAATGTTACCGTAGTTTGTTTTAATTTTATATTGAGCATCTACTCGTTTTTTA
TTTTTAGATACTTTTTTTATTTTACGATCCTGAATGTTTATATCTTTAACGCCTAAACTATTATATATTTTTATCGG
ACGTTCAGTCATTTCTACTTCACCATTATCGCTTTTAGAAATATAACTGCTATCTTTATAAACTTGTTTGAAATTT
TTATCTTCAATTGCATCAATAGTATTATTAATTTCTTTATCTTTTGAAGCATAAAAATATATACCAAACCCGACAA
CTACAACTATTAAAATAAGTGGAACAATTTTTATCTTTTTCATCAATATACTCCTTATATAAGACTACATTTGTAG
TATATTACAAATGTAGTATTTATGTCAAAATAATGTTATAATTTTTGTGATATGGAGGTGTAGAAGGTGTTATCAT
CTTTTTTAATGTTAAGTATAATCAGTTCATTGCTCACGATATGTGTAATTTTTTTAGTGAGAATGCTCTATATAAA
ATATACTCAAAATATTATGTCACATAAGATTTGGTTATTAGTGCTCGTCTCCACGTTAATTCCATTAATACCATTT
TACAAAATATCGAATTTTACATTTTCAAAAGATATGATGAATCGAAATGTATCTGACACGACTTCTTCGGTTAG
TCATATGTTAGATGGTCAACAATCATCTGTTACGAAAGACTTAGCAATTAATGTTAATCAGTTTGAGACCTCAA
ATATAACGTATATGATTCTTTTGATATGGGTATTTGGTAGTTTGTTGTGCTTATTTTATATGATTAAGGCATTCCG
ACAAATTGATGTTATTAAAAGTTCGTCATTGGAATCGTCATATCTTAATGAACGACTTAAAGTATGTCAAAGTA
AGATGCAGTTCTACAAAAAGCATATAACAATTAGTTATAGTTCAAACATTGATAATCCGATGGTATTTGGTTTA
GTGAAATCCCAAATTGTACTACCAACTGTCGTAGTCGAAACCATGAATGACAAAGAAATTGAATATATTATTCT
ACATGAACTATCACATGTGAAAAGTCATGACTTAATATTCAACCAGCTTTATGTTGTTTTTAAAATGATATTCTG
GTTTAATCCTGCACTATATATAAGTAAAACAATGATGGACAATGACTGTGAAAAAGTATGTGATAGAAACGTT
TTAAAAATTTTGAATCGCCATGAACATATACGTTATGGTGAATCGATATTAAAATGCTCTATTTTAAAATCTCAG
CACATAAATAATGTGGCAGCACAATATTTACTAGGTTTTAATTCAAATATTAAAGAACGTGTTAAGTATATTGCA
CTTTATGATTCAATGCCTAAACCTAATCGAAACAAGCGTATTGTTGCGTATATTGTATGTAGTATATCGAGCTTC
ACATGAAACAGCTAAAGAAGCTTTGGGCGATAAAGAGTTAAGAGCCATTGCACATGAGTTAACTAAAACAG

TTAAGGATAACATGAGTGTTGATTGGTCTAAACGAGACAGTGCTAAAGCTAAAATGAGAGTTCAAGTTAGAC
GCCTATTAAAGAAATATGGCTATCCACCAGATCTTCAAAAAATGGCTGTGGAACAAGTTGTAGAGCAAGCAG
AATTAATGGCAAGTCAGCAATAAAAAAATAAATCATAATGAGTCCGGGACATAAAGTTCTTGGATAAGTGAA
AAAAGACAATTTCTATTGAAATAATATAGAAATTGTCTTTTTTATAAATTTTTTGATTATTTTCAGCTCGTTGAG
CTACTACTTTTCTTATATTAAGTGCCATTAATACAAAACCAAGTTCTCTTTTGACTTTATTGAGTCCTCGGACAG
ACATCCGAGTGAAACCCAAAATAGCCTTCATAAATCCAAAAACAGGTTCCACATCAATTTTTCTTTGACTGTA
GATATTTTTTGTTTCTGGTTCTGAAAGCTTTTTGTTAATTTGGGATTTAAAATATTCCCAGTTATAATTCTTCATT
ATTTTTTTGTTTGTTTTTGAATTGAAGTTCATACATTGATTTTTCAGAGGACATTCTGAACAATCATCACATTCA
TATAATTTGAAGTCTCGCTTATAACCATACTTATCATGACGATAGGCATATCTTTTAAAACCTAGCCGTTTATTAT
TCGGACAAATGAATTCGTCATTAATTTCGTCATAGTTCCAATTTTGAGTATTAAAGATGTCACTTTTATATTTTT
TAGTTTTATCTTTTATAAACATTCCATATGTTATGAGTGGCGTTCGATTAAAGTCATCTATAATTGCCTTATAATTT
GATTCACTACCATAACCTGCATCAGCTACAATATATTCAGGTAAATGACCGTAGGTCTCTTGAATTGAATTTAA
AAATGGAATCATCGTTCTAGTATCCGTTGGATTTTGATACACATTATAAGATAAAACAAATTGGGAATTTGTTG
CTATTTGTAAATTATACCCTGGCTTAAGTTGTCCATTTTTCATGTGATCTTCTTTCATTCTCATAAATGTCGCATC
ATAATCTGTCTTAGAATAACTATTTCTATCCTTTAAAATAGATTTTTGAAATTCGTATCGATACTTTCGCTCAAAA
TAATCATTGATTTGCTTTTTGTATTTTTTGATTTAGTTCTTTTGAGACGTATTTGTTTTCTTGTTTTAGTACATT
TTTCATTGTTGATATGTTGGTTTAAATCTTCGATTTCTTTATCTAAGTGACTACCAATCAAATCTATTTCTTCTTT
TGTTAATTCATTATCATGATCTTCTTTAATTTCCGGTATGATTTTATTGGTTACCAATTCATGGTAGAGGGCTTTA
GAATCCTCATTCATCTTTGATTCATGGTTTTGAATACTCTTTTTCCATACAAATGTATATCGATTGGCATTTGCTT
CAATTTTTGTACCATCAATAAAAATAGCTTTATCATCTATAAGATTTTGTTTTACACACTGACTGTAAAATTGAA
TAAATAAAGATTCTAATAAAGCATCTACTTTTGGATTTACTCTAAATTGATTAATTGTTTTATAAGAAGGTTTTT
GATTTTGTGATAGCCACATCATTCGGATGCTATCATTAAGCATTTTTTCTATTTTACGACCTGAGAATACAGATT
GTGTGTAGGCATATAGAATCACTTTTAACATCATTTTAGGATGGTACGAAGTTGCACCACGGTGATGTCTGAA
TTCGTCGAATTCATTGTCAGGAATTGTTTCAACAATATCATTTACAGTAAAACGATGTTGATTTGTTTTGTTTCC
ATATTGACCTCCATGTATTTGCTATGATTTCAAAATCCATTTTTGACGTGCCTTAGGGTTGAGTGGATGCATAAT
TTCATTTGTTACTGGATTGATGAGCTTTTTTACTTTCTTTTTATGAGGTTTTAACATTTCCATCACTTGTTCGAC
ACGGTCGATAACAACTGGTCGCTTCGCATAGGCACCATAAGCAAGAATCACTGTGTCACTTTCACTAATCGCT
TTCATCAAATGAATATCAGTGTGCTCATCGTATGGATTTTTGATATGTTTGAGGTTTTCGGGTGTTCTAATATTA
GAGAATAGATTTACAAGATATACAGCACCGTATCGTTCTGAATTGGCTAATTGGTTGAGGATAAGAACAGTTG
TGAGATCGAGTGATAATACACCGTCTAAATGAGGATACATCGTTATCACTGTGCATGCAGCTTTCTTTTCATCC
CATGTTTTCTTGAGTAAATAGCGGTGCTGTTCATCATCGCTAAATATGGCTTCTGTGTGTATCGTACTTTTGATT
GTATTCATCATCGTCACTTCCTTTAGTATTCTTCTGGTAAAAGCATCACATAATAAAAAGCGTCCACGTCATCTT
CACGAATGACGTAGACTTTCTTAGGTAATGCATTTTGATTTTTTTCATAGTTTGTATAGTGATATTCCAATTTGT
ATGTGGGTTGTTCTTGTTCATGTGTGATTGAGAGTATATTCTCATCTTCTTGTAATTTAAAAATGTGTAGGTAAT
CTGTATGAGGCTGGTTATCTTTTTTTTCTACCATGTGCCAAAGTAAGATTTGAAGGTCTAGTGGAATACTTTCA
TTAATTCCTCTTGTGATGTATCGATTGATTTTCATGCTATTTCCCTCCCTTCTGCTTTTCTTTCATGATGTCGATG
ATTTCGTTGATAACTGTGACGGATAATTGAGCAACTATGATCCAATTATTCATGGTCCTGACCTCCTTGTTTTA
GTAAATGACGTTCATCAATAATGATATTTTGAGTATCTGTAAGGTACAGAAAGTCCATGTCAAAATGGTCTAAG
TATCCGACACTGATGAGTTGGTTATTGGCATACATTAGAAATGGATAGATACTTAGCTCATGTAGTTCATCATTA
TAGTAGGTATAAGTCTCGAGTGTGAGATGTACCAGTGGAGAATCATTAATAAAACGTTCCGGTAGAATATTTT
CTGCTGCTTCCTCCAGCGCTTCACATTCCCAAGCTTCGTTATTAGATAGTTGGAATAGGTGGGTTATATATTGT
TTGAGTTCTTGAGTGATGGTTTTCATATTATTGCCTCCTAGATAGTGTAATAGTGATGTAGTTCATATACATCATT
GAGATAATATATATTTGATTTGTCATTTATTACGAATCCCGGTGGGAATAAGAGAAAATTCCATATAAAAACCC
GCTACAAACGTTGGTATGCCAAGGAAATCCTGAAATTCCGCCTATTTTGACAAACAATCAACTCATTATTTATA

AGTATTGATGATAGGGTTGTGTCTCTGCTTCCTTATATATATTATTTATTTATAAAAAGTAACGGGATTTTGGGA
TTGTGCTTGCACAATCCTTCTGTTTCTTCGAATCTGCAAATCCCAATCATTTCCCGATAAAAAATCATTGTGGG
ATGTTCTTTAGCAATTTCAATATAAGCATTGTATAGTTATGAAAAAATTACGACAATAACTGTTTCATTAGATAA
GTGTTATTGAAATTGATAAAGAGCAATTCTTGAAAATAGTTAGATAAAATAAGCGAAAGAATATAGTGAAAAT
TATTGTTATAACAATGATTCTATTAGCTAAATAGTAAGATATAGTGTTTGGGGCAAAAATAAAGACGAAGTGCT
GAGATGCACTTCGTCGAGTTGTTTATTATTGAAAAGTTGTTTAATGATTTCGTTATTAAGTTTGAGTGTGACAT
AGAATTGTTTTTTATGATTACCATCTTTTTTAATATCAATGCGATCAATCACTGATAGATACAATGCTTTGAGTC
GAGATTTTTCTATGTGCTTAATATCATGAAAGATGTGTTGTAATAGTTTACTGATTTCTTTGGCATCAAATAAAG
TCTTATCTTCATTTTGTTGATTTTTGAGTTGGTTGATTTGATTCGTAATGTCATTGAGTTGCTTTTCATATTTTTG
AATACTTGGTCTGATTACTGATGTTAAGTCCGGATTATCCTCGATGGTTTTAATCAAGTTATTTATTTTGATTTG
TACTTCATCATATTGTTGTTGCTTATAAGCAATATCGTGATGAAGTGCAGCGCCATCAACTTGATTTTCTTGATT
GACGTGTGTTACTACGCGTTGAATGACTTTATCACTTTTGACTATTTCAAGTATTTGCTTCATCACATAATCTTC
AATCACATCAGCTCTTACACTGTTTGCCGAACATACTTTGGAACCCTTGTTCCGAAAATTACTACATGAATAGT
AACGAATACGTTTCTTAGTCCCGTCTTTAAGAGTATTCGTGGTATTGCTTGCTGCCATAGGTGCGCCACATTG
GGGACAGTGAATAATGCCTGTAAGCAGATTCGTTCCTTTGCCATGGACTTGGGGTTTTTGACTGACTTGTTT
CTTACGCATTTGTACTTTATCCCATAAATCTTGATTAATAATGGGGGAATGCTTACCTTCAGCTATCACTGGTTT
ATCATTCAGCCCTTTACGACGTTTTTCACTCCAATCTTTGTATTTCGCAAATTGAATTTTGCCGATATAGAAAG
GGTTAGCTAAGATGTATGTGATTGAACTAATACTGAAAGGTTTCCCCTTTTTAGTGACATATCCTTTGTGATTC
AATGCATTGGCAATTTTACGATAGCCATGTCCTTTGGCATAGCACTCGAATATATATTTTACAATATTAGCTTCA
TGTTGGTTAATCATTAGCTCGTGTTTACTATCTGGTATTTTGTCATAACCTAGTGGTAAATTGCCTTGATAATAG
CCTTCTTGGGCACGTCTCGTTTGACCCATAAATACGTTCTCGACAATGTTATTACGTTCGAATTCTGAGAAACT
CGCAAGTATCTGTAACATGAGTTTACCAGAAGAAGTATTGACTTCCATACGCTCTGACAAACTGAAAAATTCG
ACATTTTGTTTGTGTAAATCTTCGACAATTTTGAGAAGATCAGATGTATTACGAGCTAATCGGTTTGTTTTGTA
TACCATAACACAGTCGATATTGCCTTCTTTTGCATCTTTCAACATACGTTGGAGCTCAGGTCTGTTCATAGATT
TACCTGAAATACCACGGTCAGCGTATATATCTTTAACTTCAAAATGATGGAAGTCACAGTATTCTTTGATTTGA
TTGATTTGTCCGTCGATACTATAACCTTCTGTGCTTTGCATTTCTGTTGATACACGTACATAGATACCGACACGT
TTTGTTTTAAGTTGTTGCATTATGTTACATCCTTTCTTCATTTATGCAATCGATGATTGCATGGTTTGATTGACA
ATATTGAGTGGTTCATTTTTGAAATAGATTCCTATAAGATTTTTATCTTTCGTAATGTGAATGGTTTCAATATAG
GGGTACAATATGTTTAACGTGAAACGTTTTTGAATAATATTTTGAATGATGTGTTGTATTTGATGCCCATTGATA
GATGTAGTGCGTTGCGGTTGTTGACGTAATGATTGTGTTTGCTCTCTGAACGTTTCTGCATCAATGATGCCTT
GTGCCAACTTTTCTATCAGTTGTTCTTGAGTCAATGTGTGATGTTTTTCTATGTTTCTTTGTCTTTTGATGCGTT
TGTCAATCGCACTTTTAATTTTTGTGTAGATGCGTTGATTTTGATAAAAGTCTCGGCACACTTCTAATACTTTAT
CTTCAAGTGTTTGTGCATTGATGCCTTTAAAATCACAGACAAAGCGTGAAGCATTCATGTTTTTAGGACAGA
CGTAGTAACGTAATGTATGATTCTTTTTTCTAATGGTCATATTTGTAAGTGTTGCATTACAACATGGGCATTTGA
TTTTTTGTTTGAGTTGATTATCCGAAGGTGTCTGTTTGGTTTGTTTTTGCAATCGAAGTCTCTGCGCTTGCTCA
TATATACTTGTGGAAACAATAGAAGGAAACATATTGTCGAATTGGCCATATTGATTGTTGACACGACCACAAT
AATTAGGATTGATGATAATGTTACGAACTTGATAGGGTTGTCGATTGATATACGTGTTATCTTCTTCTAATAACT
GTGCAATTTTCTTATAACCATGACCTTTAATGTAATAATTGAATACAGCCTTTACCGTTGGTGACTCATTTTGAT
TGATGATGAATGTTCCGTTGTGATATTCGTAACCAAAAGGTGCATGTGTTGTAATCAATCGACCTTGCTTTGCT
TTTTCTTGAAGCCCATTTCTGACTTGTTCTCCAATGTTATCCGATTCAAGTTCGGCTAAACTGATGAAGATATT
AAGCTTGAGTCGGTCGAAAGCTTGATCCATATCAAAGTAACCATCATGTACGCTTAAGATATGAACATGGTAC
GTTTGACATAATTTGATGAGTTTTAATGCATTTTTCAGATTACGATGCAACCTATTAAGACGATAACAGCATAA
TATGTCACACTGTCCTTGTTGAATTAATTGTGTTATTTGTCGATACCCACTACGATTATCTTTGCGACCTGATTG
TTTGTCGCTATAAAAGTTGATATGTTGAATATGATGTTTTTCGGCTATTGCTTCGATAGCTTGTTTCTGTGCTGC

AAGAGATTGTTGTTTCATCGTACTTTGACGTAAATAGCCTATGACTTGTTTCATATCGGCTCCTCCTTTCACAG
TGATAATATATATTTATGGATGAATTGATATATAAGCCCAACATCAATGAGATGTTGGGCGTCCATATTAGTCATT
TGTTTGATTGATTTCTTCAATTACCAAATCGGCTAATATCTCGATAAGTTCATCCATGTTTTTCACTCCGTTATTT
GTTCTATCTTCAATACGTCGATTATTCAGTTTGATGCTTCACAGTTGTATGATAAAGACAATTAGAAATCTTCGT
GAACTCCTGAAGGGCCTATCCCTTCATTAGCGGATTTAAAAAGTTCTTTCGCAGCTTTGTTATCATTTGACGG
TGTCCAATTTTGAAGTAACGACTTATCTTTAGTTAATCCGAGGATAGATGCAAACTCTACATCTAATTTTAGAT
GGTAAAATACAAGTGATTGTTTTTTACCGCTATTATCTTTGACACTTCTTTTAGTTGTTTGGCGTCCACGGTCA
GCTAATATGAAACCTTTATCTCTTAAGGCGTTGACAACATTATTAACATCTTGAAATTGATGATTGTTTAGCATC
TGTTTAAAAACGTTCGCAATTATTTTTACTTCGATATGGTCATCTTTTAATGAGATTAATCCATAGTTCTCAAAC
ATATTTTTCAAAGCACCTTCATCTGAAAACTTACCTCTGTTTTGTGCCACAAATTGAATGATGACATCAATAGC
TTTATCAGCTAATGAGCGTTCAGAGACTGTATGAGTATGATAATCAATAAAGTAGTCTCTTATATTAGCGATATC
AATATCTGTAGATAAAACACGACCTAATATTTTCGCAGATGTTGTAATGACTGCATAACGCTTAAACATACGAA
TACCTGTATTGTTTGTTTCATCTTTCAATTTAGCTTCAAACCAATCTACTTCCTTGTAAAACCATTGAATAACTTC
ATCTTCACGATTTATAAGATATTTAGCTACTAACGGTAAAACATGACCATAGTTTAGTGCTACAGCTTTTTTAAT
ATTGTCAGCATTGGTCGCATTTGTAGTGAATTGTTCATTAATCTCGATGGTTCTTACACGTAATCCATCGTTTTG
AGCTGAATCATTAAAAATACTGTATTCTGACGTTGAAATGACAGAAGTACCCCAATTCTTAGGCGTTTTAACT
TCTCCATGAACGTTTGAACGTTGACGACCTTGACCTTCAGCGATGGAATATAACAAACCCGTGGTATCTCTAA
GTGTCGTAGATGAAAGCTCATCAAATACTATAGGAATGCCATAATTGTTACTCAAATAACCTTCAAGTGCGTTT
CGTGTGGCATTCCAACTTCTAAAAAGAGTTTCATTACCTTTGGTAGGGTTACCAGCGACTGATACAGCTAAA
GCAGCTGCGGTTGACTTACCGGTTGAGGATTGACCTGTAAAACTAAAGAGAATTCCTGCAAATTCGATTTCA
TGTTTATGCTTCAGAAAACTTGTCACTAAGGCAGAAATCCCAAATATGACTGCCAATTCTAAAAGAAGATGAC
CTTTAACCTCGTCAATATACATGTTAAACCAATTATCAAATGTACCTTTAGGAGTTAAGTCATAAGCACTATCTA
CAATAGGGTATGAAGGTGAGGACTGATTAAATTGAATAGATTTGAGAACTTTATCTAGCGATATCAAGTAGCC
AAAAGGTGTCTCTAGTATACCTACACCCTCATATAATTCAGAAAGTGGTAATCTATCTCGCATCAATTGCAAGG
CATAACTCAATGATCTAATATACTTCTCGTTAATACTGTAGCCATATTTAATTAAAGAAGGTAAGTTTCGTGTTG
TTAAGATATCAGACTCAAAAATGTCTTTTTTACCATTGTTGTTAGTAATAATCAACTTTTCAACACCAGTAATTG
GATCAGAAAATCTTGCATTAACAATAATAGGGCTAGCCATTCTAACCACTTTTTCACTGTCACCATCTTTTTTA
GGTGGTATCGTTTCTTTCCAACCGAAGGAATCTAAAGCAAATGGGCCAATTTCAAACAATGTGTAACTCATTA
GCAATCACCTCCTTTCGAAGGCGTGCTATTATTGCATGGGTTTATCCCTATTTTCTGATATAATAAACTGCCATT
TTTTGTTCCTACAATTTTAAATGGTAATCCTGGCGTGTACTTAATCCAGTAAGCAAAACGACGTCCTACTTCAC
GTTGATCATTCGTGCTACAAACCACATTATATTTCTTTCTTAATTCACCATAAGTGAACGTACTTCCAACTGGTT
GTTGAAAAGATTTAGCAATTATAAAATTATAAAGCTTTTCTTGCTGCTTAGATTTCTTATTCATTTTAAAATTCC
TCCAATAATTTTTAAGGAGTCAATCAATATCATATAGGTTAAACTATCTTCAATTGATTGCTATAATAAGCATAAA
TGAAGCATTATGATTAGACGCGGAACAAAAAATGGGGAGGGGGGGTATATATTGATACCTACGCTAGAAGA
AATTATTGATAAGTATGGAAACTTAGTTGATTATTTGAAATTTGATGTAACAGTTGAGGTTTATGAAGATTTGT
TGTTATTAAGAAGTTTAAGCGATTTAAATGAACATCAAAAGATGGATAGAGTTTCATTTATAAAAAAACATTTA
AACTCAAATTTCGATTATTTATTAGATGATACTGAGTTTAGAAGTATCAATAAGATTTACAGTAATTTAAATATC
ATGACGCATATCAATAGTCAAAATAATAATTTTAAACGTAATCCATTTATAAATGAAGAACAATTGAAATCACTA
CTCGCGATAAAGAAAGTAGATGCACATATGAGTTATGATTCAAATATTACTTCAAAAGCATTGGCAGAAATAA
ACAAAACGCAAAAAAGATTAGTTACAAAGATAGACACACTCTATAATCAGTCAAAAAAAGAAAATGAATATG
TACGGTTAGGAGAAAAAATATCGTATACAAAATTGGAGAATCATTGGAAATATTTATATAATGAGATTCAGTTT
TATAATCTTAATAATCAATTGATTAGTTATGTTGCATTAGAACAGGAATATGCTTGGGCTTTTTTAAATGAACTA
TTTTATTTGATAGAATTGTATTTGAAGGCATTTAAAGGACAAAAGAAAACAGATTATGAGTTTGGGAAAAAA
TTAAACGAATTTATTCAATCATATGTCATTATTTTACTTATAGATATACGATTACCTGTTGTTAGATTATATATTATT

AGAGACATTGTAGATACATGTGAAGGAGAAAAAGATAATTACAAAAGAATTACACTGATAGAAGAATCTATT
AAAAAGTATAGATACGTAAAAGATCAGATTAAAAGATTTAAAAATGGATTAGAAGAATCGTTATTAAATGCAA
CTTTATCTATTGAACAAAATATGTTGAAAGTTAAAATTGATTATTATAAAGCCTACTGCTTTCCTAGAGAAAAA
ACAAAGCATAACAATATTGAGTACAATGTATCGTTGTTTTTTAAAGCATTAGATGCCTTAAAGAAATAGTTTTT
CACTGAAAATATTTATAGATATAATTAAAATTAATTAATATTAAGCCAGGATAATGTAGTCTTAATCGTTCTGAAA
TACGAAAAATGTTGTGGAAATCGCGTGAAATAATCAGAGGAATCGTTTGAAATCATCGTAGTAATCGCGCGA
AAATCACGTGAAATAATCAGAGGAATCGTTTGAAATCATCGTAGTAATCGCGCGAAAATCACGTGAAATAATC
AGAGGAATCGTTTGAAATCATCGTAGTAATCGCGCGAAAATCACGTGAAATAATCAGAGGAATCGTTTGAAA
TCATCGTAGTAATCGCGCGAAAATCACGTGAAATAATCAGAGGAATCGTTTGAAATCATCGTAGTAATCGCGC
GAAAATCACGTGAAATAATCAGAGGAATCGTTTGAAATCATCGTAGTAATCGCGCGAAAATCGCGTGAAATA
ATCAGAGGAATCGTTTGAAATCATCGTAGTAATCGCGCGAAAATCGCGTGAAATACTATGGTAGACGGTTTG
AGTAAATTAATGGAGTATTTTAATATTTATGGTTTGGAGGAATTGCGTTGCATAGAGAAAGTAAATATATAGAG
TATAAGAAATCACGAAAAGGATTATCTAATGATATTTGGTCTACGTATAGTGCTTTTGCAAATACTGAAGGTGG
TACTATATATTTAGGAATTGAAGAAAAAAAGATCGAGGACAAAAAAGTCTTTGTTTCAGTTGGTGTTGAAGA
TCCAGAGAAAATGATTGAAGATTTTTGGAATGCACTATATGGAAGAAGTAAAGTTAGTCAAAATATTTTATCA
AATAAAGATGTTAAAATTGTTAATATTGAAAATAAAGCGTGCATTGAAATTCATGTACCAGAAGCGCCTTATTC
GAAGAAACCGATATATGTAGATAATAAAAAAGATTTAGTATATAAAAGAGTTGATGATGCTGATAGAATTGCG
ACTGAAGAAGAGTATAAATTCATGATTGTAAATTCTCAAGACGATATAGATACAGAATTATTAGATAACTATGA
CATGTCTGATTTAAATCACGAATCTATCGAAAATTATAGGAAACTTCTATTAAAAAATACTAATGATGAGAGAT
ATGCGAATATGAGCCAACTGGATTTAATGATAGATTTAGGAGCATATAGAAAAGATAGAAGTTCGAAAGACA
AACAGTATAAAATGACTACAGCATGTTTATTATTCTTTGGTAAGTATAATGCGATTAGTGATAGATTCCCAGGAT
TTCAATTAGATTATTTTAAGAAAACAAATTACCTAGATACTGATTGGAAAGATAGAATATCAAGTGGAGATTTA
GGTAATGAAGATTTAAACGTGTATAGTTTTTTTGAAAAAGTATTGATAAAATTAACTGATAACATTGAGGAATC
ATTTAGCCTAAATGATGGTTTGACTAGACAAAATTATGCAAGAGATTTAAAAGTAGCAATTCGCGAAGCACT
GGTTAATACATTAATGCATGCGTATTATGATACTAAGCAAAGTATTAAAATAGTTAATTGTGAAGATTTTATAGA
GTTTTATAATCCGGGTAATATGAGAATAAATAAAGAAGATTTTATTCATGGAGGGCATTCAAAGGACAGAAAT
AGTATATTATCGACGCTTTTCAGAAGAGTAGGATATTCAGAAAAAGCTGGATCTGGAGGACCAAGGATATTC
GATGTAGTTAATAGACATAAGCTTAAAACGCCTGAAATAGAATTAACGGACATGGACACTAATGTAGTACTTT
GGAAACAAGATTTAATGAAGGAGTTTGAAAAATATCCTGAGTTAGACAAAAAAGTAATAAAGTATATTATTG
ACTATGGATCAATAAGTAAGGGTGAAGCCTTAAAAATGGAAAATATGACAGAATATCAGTTTAGAAATATTTT
AAAAAAACTAAAAGATGATAACTTGATAAAAAAAGAAGGTGAAGGTCCGGCTACTAAATATGTGTTAATAGA
ATCAAAAGAAGCTGATATATTGCGAACTAAAAAAGTAATTAAAAGTTTAGAGTCTTTCTTTAGGAATAAATAA
AAAAACAAGAGATAGGTGCGAAGTGTTTGGATTATACAAATGCTTCGCATCTTATATTTATTAATAAATATCATA
GAAAACCGTATCATTAACCGATACGCAGAGATGCGGTTTTTTAGACACTTCATAAAGGGATTTTGAACGTATC
AGAACATATGAGGTTTATAGGAATTGCTGTTATGTTTTTTGATCACATCAATAAACAAAAAAGGTATGTACTAT
GTAAAATATTTATTAAATGATATAAAGCGAGGGTATATAAATGATTTTTAAATAGATATTTATCCAATAATATAAA
AAGGAACTATAAGCTATATCTAAAAGCTATATCTAACTACTTATAGTCCTTCTTCATTAGTATAAATATAATTATTA
AGAAAGGTATATATCTTTGTAACCTTCGTTTACATTAATAATGTTGTGATAACCTTTGTGTTCTAAAATACCAAT
AGCTATAGAACTTCTAATGCCAGACTGACAGTCTACATAAATAACATCGTTTCTATTGAAAGGTAAATCTGTTT
CTAAAAGTTTGCCGTGTGGCACATGAACCGCTTGGGATAAGTGGCCATTATTCCATTCATTATCATTACGTACA
TCTAAGACATGTGCTTCATTACCAGTTATGTCTTTACTATGAACAGATTGTGTTTGAATTTGAGCTTGTGGTAA
CTGATATCCAGACACATTATCATATCCAATAAGTTGTAAAGTATGTTATGTGTTGCTTTTGAAACAAGGTGATA
GTCTCCAATCAAGTTAATTTCTTGATTATAGTTTAGATACCAGCCAATTTGATTGATGAAATTTTTATCATATGG
AATATTGATTGTACCTTCAATATGTCCACCATGATAAGCCTCCTTACTGCGGAGATCAAAAGTTAATCTGTTTGT

ACTTGTAGCTGGATAAACCGTATAAGGTTGATATAAATTCATACCGAATTGATTAATTTTTTTCATTTGTGCAAA
ATGATGTGGTGGTGCAGGTTGGTCAGAAATGAGTTTATCGATAAAGGTAGCTTCATTATTTTCAGAAAAAGC
CCAGTTCGTTTGTTTTTCATAGCCAAGCGTAGATGTTGGAATAGCACCTAAAGATTTACCACAAGGACTACCA
GCGCCATGACCAGGCCAAATTTGAATGTAGTCTGGCAAGTCTTTAATACTTTCAATAGATTTAAACATTTGTTT
TGCGCCTATTTCAGATAATCCTTCTACTTTAACAGCTTTTTCTAGTAAATCAGGTCTACCGATATCTCCTACAAA
AATAAAATCACCACTGAATAGTCCCATTGGAACTTGTGCTCCAGCACCTTCGTCAGTAAGTAAAAAACTTATA
CTTTCTGGCGTGTGACCAGGTGTATGAAGCACTTTTAATTTTATATTTCCTACATAAATATCATCATTATGTTGA
ACAAAATGAGTGTGGTTAGGCATATTTTTATAACCTAACATGTCATCACTTTCGCCCGATACATAAATACTAGC
ATTTAACTTTATAGCAACATCTCTAATTCCTGAAACAAAATTTGCATGTATATGTGTTTCAGCTGCATGAGTAAT
GGTTAAACTCTCTTCATCGGCAACTCGAATATATGAAGATAAGTCACAAATAGGATCAATGATCATGGCTTCTC
CAGTTTTTTGACAACCGATTAAATAAGATGCTTGAGATAAATGTTTATCATAAATTGATGATAGTCAATTTGTTA
TCAAGGGTGATAATTATATTAACTATGGTAATTACATGGAATTAAAGTTATTAAACAAACTTGATGATGACCAAT
TAGATTTAGAATTTTTACAAGATAATGGGTTAAAAGGTAATGTTCAAGGTCCTATGACTTTAAAGGAACCTTT
AAAAAGTTATATACAAAAGTCTTGAAAACGAATATTTTAAATTTAGATAATGAGTTAGAAAACACTGAATATGT
CGAAGGTAAACCATATGTACAGTACGGTATTAAATATGAAAGAAATCAGGCATTAAGAAATGAAGCTATTAAA
ATTCATGGAACTACATGTAAAGTATGTGGATTTGATTTTAAAGCTAAGTATGGCGATTTAGGTGAGGGTTTTAT
TGAAATTCATCATTTAAAACCAATGTTTTCAATAAAAAGAGAAATAAAAGTAAATCCACAAAAAGATTTAGTC
CCACTATGTTCTAATTGCCATAAAATGATTCATAGAAATACTAAAAAACCTTTAACGATTAAAGAATTAACCAA
AATAGTTAATTATAATAGCAAATAATTTAATATTTTATAAACTATCATTCAACCCTCTTAATTTATTAGGAGGTTTT
TTGTATTTATGCTTTCAAATGTGTGATATACTTTGTTTGTTGAAATATAGAGTATCTATAGATAGGGTGATTGAG
TATGAAATTCACTGAAGTGGAAGTTATCGAACATCTTGTAAAGGCATATAAAGAAGCAGGAAAGCCTACTTAT
CCTCATGAAAATTTATATCGAGGACGTAATCATAGTATTTCAGGTATTGGAGAAGACTTGCTGGGTGCTTATTT
GATTAGTAGATTGGAAGGTGTCCAAATATTTATTGATCAGCCTTTATCTATGATTGATAAATCTTTAAGTACAAG
ATATCCGGATTTATTAATTTGTGAAGATAATGAAATTAAAAATATACTAGAAGTTAAAATGGACTTAGGATATCA
AAGAAAAGATTTTATAGATTATTGCCGAAAGAAAGAAGAATGGATTTCAAATATCGTAGGAAAACAGTGTGT
ATTGTCTAGAAAGAGAGAAGACAAAATTCCTATGAATATAGCTGATGATATTAAATTTCATGTTGTGATTTACA
GTGAAAACAATGGACCGAAGCGGTTTGATGAAGAAATCATGCCTATCGTTAATGAAACATGTCCACATATTG
AAGTATATGTCCTAACAAGCGGTCAACACCCTAATTTAGTAAATGTTAATCTTGAAGGTATTAATATTAATAAAG
ATGAATTTGAAATATTAGTAAATGCGTTATAAAAAAAATAGAGCATCCTCCACGTTATGGAGGTGCTCTGTTTTT
TATTGAAAAGTATCAAGTTAATTAATTTAATATGCTTAATAAGTTCTACCTTGACCTTTTTCTCTAGCTTCTGTTC
GATCTCTTATGTACTCAGTACATACTGGATTTTCTGTTAATTCATCAACTGTATTAGTTTAATCAGAAGACGTGT
CTACTTTGTAAGCTTCTAAGAACTTATTATATGATATAGCGTTTGAGTTTTGTTGTTCTTCTATAAATTTCTGTAG
TTATTTTTCAAAAACCGCATCATTAACTGATAAGCAGAAGCGTATCACAAATAAAACTAAAAAATAGATTGTG
TATAATATAAAAGGAAGGGATTTATATTAAAATTTTGAATTCAAAAATTATTGAAAGGGAAGCTACCTTAGAA
ATTGAATCTATGGCCACTAACTGCTTTTTTATATCACCACTTATTAAAAAATGTAAGAATATTCCTCAGTGCTAA
GTGAAGGAGGTTCTAGTTTTATTTAATATTCATATTGATAGGTAATGGTTAATAATTGCCTGTAAATTATTGTTA
AATATCGTTGTTATAGTATCATCATTCATAAAATTTTTATAGATTTTATCAAGTATTTCCTCATCTTCAATAGCTGT
AAAGCGATGTACTAGTCTTTTTATGAGCTATATATAATTTTGCGAAGCTAAACTGTTTGTAATTATTCACCAATC
AGGTTATGTAGTGTCACGGTTCATAGGTAATTCTCTTTTTAAAATCATCTAATATTTTATATAATTCATATAAGTG
TAGTATTAACGTTTTCTACTTTATCTATAAAATATTTAGAATTACATTAGAATTTTAAATTATACGGCTGGAAATT
CAATGCTGTAAAAATAGACAGATTTTAAAAAAGTAGTAATAAAAAGTCTAAGTTTTATCTTTGTAATAAACTTC
GACTATATTTAAAAATAAATATGAATGTCTTTTAATTTCTATAATATCAAAGCTATTATATGATTTGAAATTTTGTA
ACTTTTTCTCTAATTTGGAAGTAGATAATGACTTAAAGGTTTTAACTTTTACCCAACGACTTGGCATTTTATCA
CTCCTATGTAATGTATTACTTCAATATACCCTTAAATTTTTATAATTAATTATATTGTAATATTATTTTATTTTATAAA

AATAGTTTAATAAAATGTTTAATTTTTATAAAAAATAATTAAATATTATAGACATTACGATAGTTTAATTAGAAAA
TATATTTAAGAATTATGAATTTAGTTCTTATTAATGTTAAAGATCATCAATTATTTTGTCACATTATCTTGAGAGG
ATTACCTTATGAGGTAGCCTTTTATTTGTCACCTAATATTGCTCTAAGGCTAATAATACATATATAAAATAAATAA
TTATCAGAATATATTTAGAAATTGATATTTAATAATTAAATAAAAGTATGCTAAATATTTATAATTTACAAGTGAG
CTGATAGAAAGATGAGTATAAGCGAATATAAAAATAGATACTTAGAGTCACTATATAACACCTTTAAAACTGAG
TTAGAAAACGAACGCACTCGCTATTTTATATTAGATACCAACTATACAAATAGTGATGATACTGAAAAGGAGTT
TACTTGGGATGTAAAACAGAATAATAAGATCAGAGAAGGCGATTTGTTCATTTTTAGACGGCCAACTAATTTA
TCTCAAATTGCTCAACAATTTTATTTCTTTGGAGCTGGGAAAATAGAAAAAATTGAGCGAAAAAATAATGTA
GCAACCGCTTACATTTCAAAACCCTTACTTTTTGTGGATCGTGTTTTAAAAGATAATATTGAAAATTTAAAATG
GGAGTTCAAAGAAAGAATTAAAGAAGAGTGGGAGCAGTTTTTCTTTAAAAATAAAATCAGTAATATTACAAA
AAATGATTTTTTAAAACTACTAAGTTTGAGTAGAAATGTAGTGGAAATAGAAAGTAATTATATTAATAATGATA
TTTCGCTAACTTTTAAACAAATGGCTGATATGGCAACTCGAATAAAGAAAAATATTTACTACGTAGGGAATCA
TGAAGCATTTGTTCAAGCTAAGGGAAGTGCTCATTTTGAGTTTTCAAAAAGGATTAAACAAAATTATAGTTAT
AGATGTGCAATTACCGGGATTAAAACTAAAGACTTTTTAGTCGTTACACATATTATTCCTTGGCATGAAAATGA
GTTTATTAGACTAGATCCTTCAAATGGTATTTGTTTGTCTTTATTTTTAGCAAAAGCATTTAAGAAAGGCTTTAT
CACTTTTTCTAACAGTTATAGAGTTGTTTTATCTAAAGAAGCAGAGAAAGATGCTGCTTTATATGAAGAATTA
AAGATTTATGAAAATCAAAAAATCGAACTACCAGATTGTCAGAAACCTAATTTGAAATATTTAGATTGGCATA
GAGAACATATTTTTAAAAATTAATTTTGGGAGAGGTTGTATGACAGATATTAGTCAATTAGTAGAGGTGCCAT
ACATCTTTCATTAGTAGATTGAAAAAGAGCACTTGAAACAGATAATTTGTATTATTTAGCCTATGGATTAACAA
CTTTAGATAATTGTTTTAAATATTACCAATTAATTACACAAAATGAACATTTTTGCTATTCTCAACAAGAAGAAA
GAGAAAGTTATCAGCAAAAATTTCAAAGTATGATTAATGGTTTAAAAATTAAAATTGGCATACAAAGTTCAAA
TGATGATGCGCAATAAGCTACAAAGTTTGATTTTTTAATTGAACGTTATATGTTTTTAAAAGAAGAAAGAGCT
TCGAATTAATATCTTAATGGAGGAAGATTAGTAAATGGCTAGAACGTATAATGTGATTAAATATGTCATTAAGA
AAAATAAGGATGATCTTAAAATACATGGATTAAGATTATATGAATTTAATATAAAAGATGAAAAAGCTATAAAT
ATTAGCCAACCCATTTTTGTGAACAGAGCAACATTGATTGAGAAAATACAAAATGGTGAAGCATTTACGCAC
GCCTTTAGAGTAAATCACAATACTTATTATGCAGGCCAATTAATTTCTTTATCATTGAATAAAAATGGTTATATTA
ATAACAACTTTGAGGCCTCACGAGATATTATTAGAAACATTGAAGTAAGTATTTTAGACTATGAATAAAGGTA
ATACTGATAGAATACATTACAATTAAAAAAGGGAGTATAGGACTCCCTTAATAAGTTGATATATAAGTGACTTA
TTTTTTTAATAGAATTTTAATTCCTCTGTTAATGTTCAAGATATATAATATTAAAGCAATTAAAAAGAATATAATA
GTAATAATTAAAAGAATGCTAAATATTAATAATTGATGATCCAAAGGTTTATCAAAGACTTCTTTAGAAAAAAT
AAATGCCAATCTACCTATAAAAAAACTAACCCAAGTCATAATATGATTAATGAATGCTTTTTTGCCATGTTCTGA
TGTTGGTTCATCAGCAAGTATCCATACAAATAAAGGTAAAATGACAGGTGCGAAAAAGATACTTAAATAACTC
AATGCTGATAAAACGTAACTATTGTCATTTTTATGCATATTACTATTTGTATTTTCCACTTTTTTCACCTTCTTTAC
TTTTCTTTACTATTATAAAAACATTAATAGCGAGCTATTACAACAGTTATAAGAGAAGCTTACGATATTGTAAGT
ATGTGTTGTTATAATCAATATAAAATTAAGAAAGACCTGAGAATTTATATTATCATCCACAATCGAAGTGAGTA
GCGAAATTTATTGGAAAAGGTACTTATATTGAAGATATTGTTAATGGTTCATTTTTAAAAAGCCAAGACGATTT
GAAGTTAAAAGCTTCTGACTTCACTGTCGAAGATAACTATAACTACGGTGTACTTATATTTCCAGAGAATCTTT
CTCTTACAGATAATAATAAGAAATTGGAATCTTTAATAAAAACTATACTATTTACAGGTAAACGATATCAATATA
CTTATTTTAAAATTTGATCTTAATAGATAAGGATATAATGATTTAAACAATTTAAGAGATATTATTTTCACAAAA
AAAGAATGCTTTTTGTTAGAGGCACTGAACCCTTAAAATGGGAACTTAATAAAAACACTATGTTCTAGGCTG
CTAATTCTCTGTATTTTACAGGGGATAAGTAGCCTAGTTTTTGTTGAATTCGATTATTATTATAGTTTTTAATGTA
TTTTCGACAATATCTATTACAATATGATTAGAGTTATTAAGCTGATTATTGATGTAAAAAGTTTCAGACTTTAGC
GAGGAATGGAAACTTTCTATCGGGGTGTTATCGGCAGGTGTTCCCTTTCGGGACATACTTCTGATAATGCCTT
TTTCTTCGCATAATTGATAATAAGCATAAGATGTATAAACGCTGCCTTGATCACTATGTAATATACACCCCTCAG

GTATATCGATTTGATTTAATGTATCATTAACTAAACGTTGGTCTTGTTTATCATCTATTTTATACGCCACAATTTCT
CCGTTATAAATGTCCATTATCGAAGATAAATACAACATAGAATGACCAAATGGTAAATAAGTAATATCGGTTGT
TAATACTTCCATGGGACAACTCGCTTTAAATTGTCTTTGTAATAAATTGTCCGTTTTATAATACGGTTTACCTATT
CTTGTCGTCTTTTTAGGTCTAACTCGGCAGTTCAAATGATGCTTCTGCATCACTCTCTGTACTCTCTTATGATTA
ATTGGTGATGTATAACATTGATTAATCAATGCTGTAATCTTACGATAACCGTAGGTATATTGTTTACCAACATGT
TGTGAAAATCTATAACTTTCCCCATTTCGATACCATCGCCACCAATTTTTCACTTGTGTTCTATTTCTAATATTTA
ATTCTTTCATAATTTCTTTTGTTGGAAATCCTGCTGCTTTCATTTCAACTGCTTTATACTTTGTTTCTACTGAATA
AGAAGCTCTTTTCATAGAAAAAAACACCTCCGTATGATTCATTTTAATATGAATTCAACGAAAGTGTTTTTATA
TAATTCCCACTATTGGGGTCAGTCTAAAATTAAAATTGATAACTTTTTTAATAAAATAGATACACTAATTTTAAA
ACAAGGTAAAAAAATAGAATTATTAAAACAACGCAAACAAGGCTTACTACAAAAAATGTTTGTGTAATTCTG
ATAACGTCCTATTATGTTAATTGAAAATAGACAAAAATAAATTGAGAGCATCCCTTACCGCAAAGTGAAGGAT
GCTCTATTTTTGTTGAAATTAATATAAGCTTCATTCTGATAGATAGTCATTAATAATTGTTTGCAAATCGTTATTA
AGTACCGTTGTTACAGCGTCATCATTCATAAAATCCTCGTAGATTTTATCAAGAATTTATTCCTCTTACAGACAT
TCGCGAGAAGTCCGTTTTAATTTTATTAGAAGTAATTCAGGTTTGAACCTACCTAAATGAATATATGAGTTATC
TTTTATACTACAAAATATATTCAGATTTCAATAATGACATAAAATAGGCATCTTTATATTTACCTTTAGTGTAGAA
TTGCTCTTTAAGTAATCCTTCTGTTTTAAATCCTTGTGACTCGTATATATGAATAGCTTTTTTGTTATCTGCATCA
ACATATAGATAGATCTTGTGCATATTTAAAATATTGAATGCATAAATTATCGCTTTTTCGAATGCGAATTTTGCAT
AACCTTTACCACTGAACTCAGGTTTAATAATTATTTGTATTTCACAATTACGATGGATGTAATTAATTTCTACTA
ATTCAACAATACCTACGACTTGATTTTCATCTTCAACAATAAAACGTCTTTCCGATTCATCTAATAAATGCTTATC
AAATAAATGTTGAAGTTCCGTTAAGGATTCATAAGGCTCTTCAAACCAATAAGACATAATAGAATATTCATTAT
TTAATTCATGAACAAAAAGTAAATCACTATACTCTAATGCTCTTAGTTTCATAATCCCACTCCCAAAATTTTCTT
ATATATTTGCATTATAAATATAAATAACGAATAAGTCATTATTCACTATGAATAAATCTATTTTAACAATTCACTAC
ATACTAATTCTCATTTTCTTATTATTCTCTAATATCTCTGATTTATTACTCAGTGAAAGATGCCCTATTTTTATCAG
TATATAGTTTTTATTTTGATAGATATTGATTAATAATCGTCTGTAAATCATTGTTAAGTAAGGTTGTGATAGCGTC
GTTATTCATAAAATCTTCATAAATTTTATCAAGTATTTCTTTAATATATTTTTTAATCTTCACACCTTAAATTCACT
TGAAAACCAAATCACTTCTAAATTATGATTTTACATAATAAATTTTTAAAAATTGGTATTATATAAAATAATCTCA
ATAATCAGAATTTTTTGTAAATAATAATATTAAAGTGTAAAATAACAGATAAGAATAGACTTAATGTATATAAATT
AAAGGAGTGTTGGAAGTGAAAACTAGAGCAGCAGTTTTACATGAAATGGGGGCTAAAAAGCCCTATAAAG
AATCTAAACCTTTAAAAATTGAATACTTAGAACTTGATAATCCTAGCGAACACGAAGTATTAATTAAAATTCAT
GCAGCTGGATTATGTCACTCTGATTTGTCAGTAATTAATGGTAACAGACCTAGACCTTTACCTATGGCACTTGG
TCATGAAGCTTCCGGTGAAGTAATTAAAGTTGGAAAAGCTGTTACAAGAGTTAGCGAAGGAGATCATGTGG
TATGCACATTTATTCCCAGTTGTGGAAAATGTATCCCATGTAAAGAAGGACGTCCTGCATTATGTGAAAACGG
AGCAATATCTAATGAAAAAGGCGAAATGTTAGAGGGAGGGATGCGTTTATCTAATGATGAAGGAAAAGTATA
TCATCACTTGGGGATATCAGGTTTTGCTGAATATTCTGTTGTCTCTGAAAACTCTATAGTTAAAATTGATAAAA
AAATACCTTTTGAACGTGCAGCTGCATTTGGTTGTGCTATTATCACAGGTATCGGTGCTGTGGTGAATACAGC
CCAAATTCGTTCTGGTAGTAATGTAGCGGTTGTTGGTTTAGGAGGTATTGGATTGAATGCTATTATTGGAGCT
AAACTAGCGGGAGCCAATGAAATTATTGCTTTAGATATTAACGAAGATAAATTTGAATTAGCAAAGCAATTTG
GGGCTACAGCAACATTTAATTCAAGCGATAAAGATATCGATGAGCAAATTAAAGAATATATTCCTGGCGGAGT
AGAATATGCTTTTGAAACAGCGGGTGTAGTGCCAGCTATGAAAGTTGCTTATCAGATTACTAAACGAGGGGG
GACAACTGTAACAACAGGACTGCCTAATCCTAAAGATAATTTTTCTTTCCCTCAAGTTACTTTAGCGGCTGAA
GAACGTACCATTAAAGGATCATATGTAGGAAGTTGTGTACCTGACAGAGATATACCAAGGTTCGTTAATCTAT
ACAACCAAGGACGTTTAAATATTGATTCACTCATCAGTGAGGTTATCACTTTAGATGAAATTAATGAGGGATT
TGATCGTTTGTCTAATGGTGAAGTAGGTAGAATTATAATGAAGATGCATTGAATATAATAGAATTCAAGTCGTT
CCTCCTCTTGATTTCTATGAAAAGAATAACAATTTGAGTAGGAAGATTACTGACAAATATTACATTTGCATTGT

GACTGCCAATCTAATTGTATTTTTACATTTCATATCTATATATTAGAAGATAAATGTTTTAAAACTAATTACTAATA
GTTTTTTTAGATAAAAAAAGATATAGGAGCATCCTTCACCGCAAAAGTGAAGGATGCTCTAGTTTTATTAGAA
TATAGTTTTCATTTTGATAGATAATGATTAATAATTGTTTGTAAATCATTGTTAAGTACCGTTGTAATAGAGTCAT
CATTTATAAACTCTTCATAGATTTTATCAAGTATTTCCTCGTCATCGATGGCTGTGAAATGATGCACCAGACCTT
TTACTATTGAAATGTAATTTTGTGATGCTAAACAACTAGCAACCGTTCGCCAATTGGATTGTATAGTGTTGTCA
TGGTTCATAGATAATACTCCTTTACTTTAGTGTCCATTTTTGACGTGCTTTAGGGTTGAGTGGATGCATAACTT
CGTTCGTTACTGGATTTATGAGCTTTTTTACTTTCTTTTTATGAGGTTTTAACATTTCCATCACTTGTTCGACAC
GGTCGATAACAACTGGTCGCTTCGCATAGGCACCATAAGCAAGAATGACTGTGTCACTTTCACTAATTGCTTT
CATTAAGTGTATATCTGTGTGCTCATCATAAGGCTCTTTGATATGTTTAAGGTTCTCTGGTGTTTTAATATTAGA
GAAAAGATTAACGAGATAAACAGCGCCATATTGCTCAGAATTAGCTAATTGATTGAGGATGAGAACCGTAGT
AAGATCGAGTGATAATACACCATCTAAATGAAGATACATCGTTATCACAGTACAAGCCGGTTTCTTGTCATCCC
ATATCTTTTTGAGTAAGTAGCGATGTTGTTCATCATCGCTAAATATCGCTTCTGTGTGTATCGTACTTTTGATTG
TATTCATATATCGTCACTTCCTTTAGTATTCTTCTGGCAAAAGCATCACATAATAAAAAGCGTCTACATCATCTTC
TCGGATGACGTAGACTTTCTTAGGTAATGCATTTTGATTTTTTATATAGTTTGTATAGTGATATTCCAATTTGTAT
GCGGGTTGTTCTTGTTCATGTGTAATTGACAACATATTATCATCTTCTTGTAGTTTGAAAATGTGTAGGTAATCT
GTATTAAGTTGATTGTCTCGATCTTTTACCATGTTCCAAAGTAAGATTTGAAGGTCTAGAGATAGTTGTTCACT
AATGCCTCTTGTGATGTATCGATTGATTTTCATACTATTTTCCTCCATTTTGCTTTTCTTTCATGATGTCAATCAC
TTCGTTAATGACTGTAACAGATATTTGTGCCACTTTGATCCAATTATTCATGGTTATTCCCTCCTTCTTTTAGTAA
ATGACGTTCATCGATAATCGTATTTTTAGTATCTGTGAGGTATAGAAAGTCCATATCAAAATGATCCAGATAACC
AATACTGATGAGTTGGTTATTAGAATACATTAGAAATGGATAGATACTTAGTTCATGTAGCTCATTATTATAGTA
GGTATAAGTTTCAAGTATAAGATGTGCAAGTGGGGAATCATTAATAAAACGTTTGGGTAGAATATTTTCTGCC
GCTTCCTCCAGTGTTTCACATTCCCATGTTTCATTGTTAGATAATTGGAATAAGCGAGTCATATATTGTTTGAGT
TCTTGAGTAGTTGTTTTCATATCATTGCCTCCCTAGATAGTGTGATAGTGATGTACTTCATATACATCATTGAGAT
AATATATATTTGATTTGTCATTTATTACTGATCCCGTTGACAATATGAGAAAATTCCATATAAAAAACCGCTACA
AACCTTGGTATGACAAGGAAATCCCGAAATTCCGCCTATTTTGACGAACAATCAACTCATTATTTATAAGTATT
GATGATAGGGTGGGGTCTCTGCTTCCTTATATATATTATTTATTTATAAAGAATAACGGGATTTTGGGATTGTGC
TTGCACAATCCTTCTGCTTCTTCTAATCTGCAAATCCCATTCCTTTCCCGATAAAAAATCATCGTGGGATGTTCT
TTAGCAATTTCAATATAAGCCTCGTGTAGTTATGAAAAAATTACGACAATGACTGTTTCATTAGATAAGTGTTA
TTGAAATTGATAAAGAGAATTCTCAAAAATTTTTAGAAAAAGAAAGAGGAAGTATGTAGATATAAAATAGAT
TTATTAAATAGTAAGGTATAATGTTTGCGGTAAAAATAAAGACGAAGTGCTGGAATACACTTCGTCGATAATA
AGGTAGTTGAAGTTTCGATTATTTTAAATTGATCATTTTAAGCCTACAAATCCCTCTAATGTTTCACCAGATTTG
TACACTTTTGTCGCCTTCTTTTTCCTAAATAACGTAAATGCCAAGGTTCATATTGATATCCTGTGATGCTTTCTT
TGTTTTTAGGATATCTTATAATAAATCCATAATTATGGGCATTCTTAGCTATCCAGCGTCCTTCCTCAGTTTTACC
AAAACTAGCATATAGATTTTCACAACAAGTTGGTAAACAATATACCTACGGTAAAGGATTAGAAGAGCTGTCA
GAAGTAGAACAATTAAAATTAGAAAATAAGAGAAAAGATATAGAATTGGATATTTTAAAAAAGTACAAGGTA
TTGGAAAGGAAGTGGTACCAACAGTAGTCATAGATTTAGTGGATCAATTAAAAGTAAAATATTCAATCAAATT
GCTACTAAAAGTATTAAACATACCTAAATCAACATATTACCGATGGAAAAACAAAACCCATAAAAATGATACCG
TAACACAAAAAGTTATTGAATTATGTAAAGCTAACCACTATACCTACGGTTATCGTAAGATTACAGCATTGATT
AATCAATGTTATACATCACCAATTAATCATAAGAGAGTACAGAGAATGATGCAGAAGCATCATTTGAACTGCC
GAGTTAGACCTAAAAAGATGACAAAAATAGGTAAACCGTATTATAAAACGGACAATTTATTACAAAGACAATT
TAAAGCGAGTTGTCCAATGGAAGTATTAACAACCGATATTACTTATTTACCATTTGGTCATTCTATGTTGTATTT
ATCTTCGATAATGGATATTTATAACGGAGAAATTGTGGCGTATAAAATAGATGATAAACAAGACCAAAGTTTA
GTTAATGATACATTAAATCAAATCGATATACCTGAGGGTTGTATATTACATAGTGATCAAGGCAGCGTTTATACA
TCTTATGCTTATTATCAATTGTGCGAAGAAAAAGGCATTATCAGAAGTATGTCCCGAAAGGGAACACCTGCCG

ATAACGCCCCGATAGAAAGTTTCCATTCCTCGCTAAAGTCTGAAACTTTTTACATCAATAATGAGCTTAATCAC
TCTAATCACATTGTAATAGATATTGTCGAAAAGTACATTAAAAACAATAATAATAATAATCGAATTCAACAAAAA
CTAGGCTACTTATCCCCTGTAAAATACAGAGAATTAATAGCCTAGAACATGGTGTTTTTATTAAGTTCCCGTTT
TAAGGGTTCAGTGCCCTAGGATTATAGGCTCTTTTGTTTATAAAGGTAATTGAACTAAAGTATTATAATTTCAA
TTCTTAATTAATGTTCCTATTTTACCGTCTAAAGCGTCCCCTAATCCTGCTAGAGATGTAATCAAAACACTGCCT
TTAGTATTTTTTTCAAGAAATTGAAGTGCAGCTTCAACTTTTGGAAGCATACTTCCTTTAGCAAATTGACCATC
AGAGATATGTTTTTTCATTTCATCCACAGACACTTCATCGAGACCTCTTTGGTTTTCTTTTCCATAGTTAATGTA
AACATGGTCCACAGCAGTTAATATGATTAATTGATCAGATTGTAAATGTGCTGCTAATAAAGCACTCGTTTTAT
CTTTATCAATAACTGCATCAACACCTGTATAAACTTCATTTTCCTTAATTACTGGAATTCCACCACCGCCGGCA
GCGATAACTAGTGTTCCATGAGTGATTAATGTTTCTATACTATCTAACTCAACTATACTTATAGGTTGTGGGGAA
GGGACAACGCGACGATAGCCGCGTCCAGAATCTTCTACAAAAGTATAACCTTTTTCTTTCGTAAATTTATCAG
CCTGCTCTTTTGTATAAAATAATCCAATTGGTTTAGTAGGGTTATTGAAAGCAGAATCATCGCTTGCAACTTGA
ACTTGTGTTACTAGCGTAACAACTTGTTTATCTATGCCCATTGAATGAAGTTCGTTTTGTAAACTTTCTTGCATC
TGATAGCCAATATAAGCTTGACTCATAGCACCACATTCAGGGAAAGGAAAAGGAGGACCTTGTTTGTGTTCC
GCCGCATAATTTAAACCTAAGTTAATACTTCCAACTTGTGGACCATTACCATGACTAATTACAATTTCGTATCCT
TTATCGATTAAACTTACTAGAGATTTAGATGTACTTTTTAATAAATCTAATTGTTCTTTAGGCGATTGTCCTAAA
GCGTTACCACCCAAAGCTACGACGATTTTAGACATATTTATATCCTCCTTTCATTTACTCTCCTAATGTTGCTAC
CATGACTGCTTTTATTGTATGTGCTCTATTTTCTGCTTCTTGGAAAACAACTGATTGTTCACTTTCAAATACTTC
ATTTGTTACTTCCATTTCAGTTAGACCATATTTTTCTTGAATTTGTTTACCGATTATTGTTTCAGTATCATGGAAT
GATGGTAAGCAATGTTCAAAAATTGTATGTGGATTACCTGTTTTTTTCATTAATTCTTTAGTTACACGGTATGG
TTCTAATAATTTGATACGTTTTTCCCAAACTTCATCAGGTTCACCCATAGATACCCAAACATCTGTGTAAATTAC
ATCAGATCCTTTGACACCTTCATCAATATCATCAGTTATAAGGATTTCACCACCGTTTTTGTCAGCTATATCATTA
CACCGATTTAATAATTCATCAGTTGGATTTAGTTCTTTAGGACATACAAGATGGAAAGTCATGCCCATGATTGC
TGCTCCTTGCATTAAGGCATTTGCAACGTTATTACGTCCATCTCCAACATATGTGAAGTTAATTTCATTATATGG
TTTTTTCAATACTTCTTTAGCTGTTAAAAAATCAGCAAGTACTTGTGTAGGATGATCTTCATCTGTTAAACCATT
CCATACGGGAACGCCAGAATATTTTGCCAAATCCTCAACTACTCTTTGTGAGAATCCACGATATTCTATGCCAT
CATACATTCCACCTAAAACACGAGCAGTATCTTTGGTAGACTCTTTTTTACCCATTTGAGAACCTGTTGGCCC
AAGGTATGTTACATGTGCACCTTGATCATAAGCCGCTGTTTCAAATGCACATCGAGTGCGTGTTGAATCTTTT
TCAAAAAGTAGAGCGATATTTTTACCTTTCATTTTTTGTTGTTCTATTCCTGCATATTTTGCGCGTTTAAGATCT
TCAGATAAATTAAGTAAAAATTCCATTTCTTTTTGTGTGAAGTCTAACAAAGTTAAAAAGTTTCTATTTCTTAA
ATTTTTCATTTTGAATATCTCCTTTTCAATTAATATTTTATTCATGTTATTTTTTTATAATCCAATTTCTTTTATCTTG
ATATATAATATTTTCTATTTTTAATTCTCTGATTATTTCACCAGTAGTTTTTCTGTTAAGTCCAGATAAATTACTTA
TTAATTGAACAGTCATAATTTGTTTTAATTCATAGAATTCTCCATTATCATCCCCAATTGAAAGGCATAAAATTT
GTAGTATTCTTTCAATTCTTTCTTTAGCTGAATTAGCTCTTAACGCCATAATATATTCTACTTGAAATTGAATAGT
CTCATTAAGTTTCTTGAAGAGACTTTCAAATATTTCATTATGCTTTCTACATAAATACTCAAGTAAATCTTTCGG
TAAAGTTAATATTTTACAATCTGTCAAAGCTGTACATATTTCATATGGTGCAGGGGTTTCATTAAATATGAAGTT
CATTGGAAATATATTTTCGTCTTTACTTAATCTTAAATAATTGTCACCAGTAATATTAGAAGATTCATGTAAAATA
CAACCATTTACTAAAAAGTATACAAATTTTATTTGATCAGTTGAATGATATATGACTTGTCCTTTTTTATATTGAT
AAAGTGTTAAATCCTCTTTATAAGGTCTAACAATACTAACAGGAATATTTAAGTATGATGCTAATTGTTTAAGAT
TATTATCAAATTCATATTCTGAATTTTTAATATAAATATTTTCTTCATACATAACTATAAACCCCTTAAAAATTACTT
AGTTTAAATCATAACTTAAAATACAGAAATATTACCTGTAATCAAACGAACTATTCCTATTATTGCAAGTACAAT
GATGAATATGAATAACGTATAATCCAATTTTGTCAAATGTTTGTTATTATCCCTTTGTACGTAGCTGTATACGAG
TAATCCAGGTATATATAACAACATCGTTAATAGTAAATAATCTAATCCAGCTGCATAAACCAACCAAATTGTGTA
AATAGATGCAATAATTCCTATTATCCATTGTTTTAAATTAGCTTTAGATTTATTTTGAATAGTATATTTAACCTGGT

AAAAAGCACTGAGTGTATATGGAATTAAGATTGCACTTGATGCAAGTGAAAACGCAAACTGATAGGCACTAT
CTGTAAACAACATACTAATTAAAAATAACTGAACTAATATATTAGTAATAATTAAAGCGTTGACCGGAGCTTTAT
TCTTATTTTCTTTAGCAAACCATTTCGGGAAAAGTCCATCTTTAGCTACAATGAATGGTAATTCACCAGCTAGT
AATGTCCATCCTAACCAAGCTCCTAAAACAGAGATAATTAAGCCTATATTAACTAACACTGAACCCCAATGACC
TACAATATGTTCTAATACTTGTGCCATTGATGGATTAGCAAGTTTTGAAATTTGGTTCTGCTGAATGACACCTT
GGGCTAGTACAGTCATTAAGAAATAAATGACTAGCACAGAAATCAAACCAATAACGGTAGCAGTTCCTACAT
CCTTTTTAGACTTTGCACGTCCAGAAAAGACAACGGCTCCTTCAATCCCTGTGAATACCCATACAGTTACTAA
CATAGTACTTTTTACTTGTGCCATTGTATCTCCCCAACTAAAAACGCCAACACTTCCACTAGTCATACCATAAAA
ACCGGATTTAAAAGTACTGAAGTTGAATACAACTATCATGCATATAATAACTAGAAATATAGGTATTAATTTAGC
TACTGTAACAATACTATTTATAAACGCTGCAGTTTCTACACCTCTAAGTATTAAAAAATGTACACCCCATAATAA
AATTGATGCTATGATAATACTTGGAAGTGTGTTACCTCCTTTAAATATAGGGAAAAAGTTACCCACAGCTGAC
ATTAATAGGGTTGCATAAGCCACATTACCTAGAAATGCTGCAAACCAATATCCCCAAGCACTTGAAAAACCAA
TAAAATCTCCAAACCCTGTTTGAGCATAACTATAAATTCCTCCATCAAGATCTGGTCGCTCATTTGTTAAATTTT
GAAATACGAAAGCAAGAGAAATCATACCAATAGCAGTTATTATCCAACCGATAATTATTGCAAGTCCACCAGC
TTGGCCACCCATATCTGAGATGATATTGAATGCACCACCGCCTATCATAGAGCCTATGACTAAACCAATTAAGG
AAGTTTTACCTAATTTATTTTCATCCATATTAATCTCCCCTAATAAAGGTGGTAAAAATCTTGTTATTTGATTTAA
TGAATGAATTACTTGTGACTTTTACCACCTTTCAAATTTAAATATCTTCTCTAAATAACGGCTGACTCATACATC
TTGGGCCTCCGCGTCCACGTACAAGTTCACTACCAGTAATTTCAATCACTTTAATTCCTTTGTCGCGTAAAAGT
TGGTTTGATACATAGTTGCGATCGTATGTCACCACAACCCCTGGTCGAATACATAATGTGTTTGAGCCATCATT
CCATTGTTCACGTGCACCATCAATAACGTCGCCATTACCTGTTGGAATAAAGTCCACTTTTTCTACTTCTAAAA
CTTCAGCAAGTGTTTCACGTAACTTGCTAGAACGAGTAATTTTTATATCGTCCTTACCATCATTTTGTTCTATGG
TAAATATATTCATATTATTTTCTTCTTTAAATATTGCTGCATGTACTGTAAACTTATCGTAGTCAATCATAGTTAGT
ACTGTATCTAGGTGCATAAATGTACGTGTATTAGGTATTTCAATAGCTACGATTTTTTTAAAACTTGTGTTTGCA
TCTTTGAAAATATTACGTGCTAACTTTTCTATTGCTTGAGCTGATGTACGTTCTGATATACCAATAGCTAAAACA
TCTTTCGATAATACTAATTCATCTCCACCTTCAATATTAAATGGTGAGTTACGATCTAACCATACTGGTACATCTT
TATCTTTAAATCTTGGATGATGTTTCAGTATATATGTCATAAAAATAGATTCTCTACGTCGTGCTCTCCAATACAT
TCTGTTAATTGTCATTCCTCTACCAATTGAAGCTTGGGGATCTCTTGTAAAATAAAGGTTGGGCATTGGATCTA
AGTAAAATGGATATCTATCATCCATATATTCTACTAAATGGGTTGTTTCAAGTTGAATTTCTTCTTTACGTACGC
CAGCCATGATTTTATTTACAAGTTCTTGGTCAGATAACTTTGAAAAGAATTCTTTAATTTCAGTTTCATGACCT
AATATTGTCTTTTTAGATTCTGTTAATATGTCGTTTATGAAGTTCTCGCGTACTTCTGGCTCAGTAATAGATTCT
GCTGCAAGTTTTTCTAAATAAACTACTTCGATTCCTTCATCTCTCAAAGTTTGAGCAAATTTGTCATGCTCTTC
TTGTGCAACTTTTAAGTAGGGAATATCATCGAATAATAAACCACTTAAATGATCAGGTACTAAATTTTCTAATTC
TTTTCCTGGTCTTTTTAACAACACAGTTTTCAATTTGCCTATTTCACTATTTACTTGAATGGGTCCTTGTACCAT
TTCAAATTCCTCCTTTGCTTTATTACATTCACATTATAAAACGCTTTCAAAAACATCGTGTGATTTAATTCACAA
TTGTTTAGGGAGGGTTTTCACATATATAGATTTATTCAATGCAAAATTTTGTATGAATATGTATTTTGTACTATAT
AATAGTGAATAATTTGAAAAATCTCATTGTTAAAACCTTATCTTATTCAAGCAAAAAGATTTTTTACTCATTTTT
TTGTATTAGTAATCATTCCTATTAATATAATCATTTAATTTTTAAGGGAATAATTGATAACAAACAAATTCTGCTA
TTTCATTTGAAGAAGTCAAAATCATCAAAGTATCGTTACCTCCAATAATTCCTAATATTTCTTTCATTTGTAATTG
ATCTATGTAATAACTTATACTTTGAGCAAAGCCAGGAGATGTTTTTATTAAGACATAGTTATTTAGCGTTATAAA
TTCAATAATCTCATCACTAAATATTTCTAATTGTTTTTTTGCACTTAATTGATTTGTTTGATTTATTTTCTTGTAAA
TATACTTTTTATTTTCAACAGGGATTTTGTAAATTTCTAATTCTTGTAAGTCACGAGAAATAGTTGTCAAGCTAT
AGTAAACTCCAAAATGTCTTGCCATGTAATCCACTATTTGTTGTTTTTTATTAAACTGATTCTGTTGTATAACAG
TTAAGATAAGATTTAAACGTTTTTCTTTTTTCATTTTTATTACCCCCTTTATTATTTTTGATTATGAAATAGATTTT
AAATAACTGTATAAAAATCACTTATAAATCTCAATTTACAAATAATTTTGAAATAGTGGATGCTTTAATTATAAG

TAGATTATTTTATGCCTAAAAGGAGAGATTAATTAGAATAGATTATAGTCATCATAATTATAAAATCAAAATAATT
TGCGTAATAATAGCGAAATGTGTAAATTGTAAATGTCAGGTAATAACCACAATATCTCATAAAAGAAGTTTGG
AAAAAGGCATTGAAATAAAAAGTAGTTTATAAGTTGAAGATATTTAGGTATGTATTAGAGCGCTTTATAAATAA
ATAGGTTTATACAAAGAATTACAAAAATAATATAAAAAATAGAATCTAATACATGATAAGGAATATATTTGAATA
ATTTGAATATGAGAATAAGCATACATATTTAGGGTATATATAAGTAATAGACATCTAAATAATAAGTAATAGGAG
GCTGGTGTAGATGTTCAAAAATATATTATTACCCTATGATTTCGAAAATGATTTTAGTGCTATCCCTGACTATTT
AGAAAAAGTCACCGATGAAGATTCAGTTGTTGTAATTTATCACGTTGTAACAGAAAATGATCTTGCAATTAGT
GTCAAGTATTATAATAAGCATAAAGAAGATATTATTAGAGAAAAAGAGAAAAAACTCACTCCATTTTTACGTG
AATTAGAAAAAAGAGATATTCAATATAAAATAGATGTAGATTTTGGGCATATTAAAGATACAATCTTAGAAAAA
ATTACTTCTGGAGATATAAATAATGGTGAATTTGATTTAGTAATTATGAGTAATCATAGAGTCGATTTGAATATT
AAACATGTTTTAGGAGATGTTACACATAAGATTGCTAAAAGAAGTTCTGTCCCAGTACTAATTGTTAAATAAA
CATAAGAAGTAAGAAATTTATTAATTCAAAAAGCCTAAATACTTTCTCACAAATCGAGAGAGTATTTAGGCTT
TTTTATTTTTTTAATAAACGTAATGAATTTAATATAACTAATATTGCTGCACCTGTATCACTTAGAACAGCTAACC
AAAGTGTTAGTAATCCAGGGAATACTAACACAAAGGCAATTAATTTAATAATTATAGCAAAATATAGGTTCTGT
TGCAAAGTTGAATCTATAGTATAATTTTAACAAAAAGGAGTCTTCTGTATGAACTATTTCAGATATAAACAATTT
AACAAGGATGTTATCACTGTAGCCGTTGGCTACTATCTAAGATATGCATTGAGTTATCGTGATATATCTGAAATA
TTAAGGGAACGTGGTGTAAACGTTCATCATTCAACGGTCTACCGTTGGGTTCAAGAATATGCCCCAATTTTAT
ATCAAATTTGGAAGAAAAAGCATAAAAAGCTAATTACAAATGGCGTATTGATGAGACGTACATCAAAATAAA
AGGAAAATGGAGCTATTTATATCGTGCCATTGATGCAGAGGGATATACATTAGATATTTGGTTGCGTAAGCAA
CGAGATAATCATTCAGCATATGCGTTTATCAAACGTCTCATTAAACAATTTGGTAAACCTCAAAAGGTAATTAC
AGATCAGGCACCTTCAACGAAGGTAGCAATGGCTAAAGTAATTAAAGCTTTTAAACTTAAACCTGACTGTTAT
TGTACATCGAAATATCTGAATAACCTCATTGAGCAAGATCACTGTCATATTAAAATAAGAAAGACAAGGTATCA
AAATATCAATACAGCAAAGAATACTTTAAAAGGTATTGAATGTATTTACGCTCTATATAAAAAGAACCGCAGGT
CTCTTCAGATCTACGGATTTTCGCCATGCCACGAAATTAGCATCATGCTAGCAAGTTAAGCGAACACTGACAT
GATAAATTAGTGGTTAGCTATATTTTTTTACTTTGCAACAGAACCTATAAAAAACATGGGCTTAAAGGGTTAAT
TTATATAGTATTACTGCAAACATTGATTTGGGTCAAAAATTAGGGGTATTAAAAAAATGAATGAATCATAATTT
TATCAAGCTGATTGGAGAGGTTAAAATGCATTATATAAAATTTATTGAGTCAAAAGATAATACAAAACTTTATA
TGAAAGTGAATGATATTCAAGATGCAAAAGCGAATATCATTATAGCTCATGGTGTGGCAGAACATTTAGATCG
TTATGATGAGATAACAGCATATTTAAATGAAGCGGGTTTTAGTGTTATTAGATATGATCAAAGAGGGCATGGT
CGTTCTGAAGGCAAGCGTGCCTTTTATAGCAATAGTAATGAAATTGTCGAAGATTTAGATGCGATAATAAATT
ATGTGAAGTCAAACTTTGAAGGTAAAGTTTACTTAATCGGTCATAGTATGGGTGGTTATACAGTCACTTTATAT
GGAACGAAACATCCAAATACAGTGAATGGTATTATAACTTCTGGAGCATTAACACGTTATAATAATAAACTATT
TGGCAATCCTGATAGAAACATATCACCTGATACTTATATAGAAAACAATTTAAGTGAGGGGGTATGTTCTGATT
TAGAGGTAATGGAAAAATATAAACTTGATGATTTGAATGCGAAACAAATCTCTATGGGGCTCGTCTTTTCAAT
AATGGATGGTGTTAGGTATTTGAAAGACAATGCTCAACAATTTACAGATAATATTTTGATATTGCATGGCAAG
GAAGATGGGCTAGTAAGCTATGTAGATTCTTTACAGCTTTATCAAGAAATAGGATCAGCACATAAATCATTAC
ACATCTATGATCGTTTGGAGCATGAAATATTTAATGAAAGTTCTTATAATAGAACTATTTTTAACGAAGTTATTG
AATGGCTTGAAACGGAATTAACTTATAACTAAAACAGTATAGTTCCGTGTATTTGATTATAAGAAATTATGAGG
ATATTAAACATACTAAGATTAGCTATGAAGAAATCTATGACGATAGATTTTTTCATAGCTATTTTTTATAGTTATA
GAGAGGAGTAGACTGTCCAGACTCTTGGATTTTAAATCCGTAAAAAAAACAAGTCAGCTTTACTCTCACCTT
TTGAAATTCGTTTGTAGTATGTTGGGTTCTTGAAACCGTGTATAGGAAAATGAAATGAGAAAGGTTAAGTAA
AGTTTTTAGCTTCTCAACTATTCAAAGGAGGTTTTTTTATCGATTACTTAGGTGTTGATATTAGTAAAAGGAGT
AGTGTAGTTGCACATTATAAAAATGGAAAATTCCAAAAAGAGTTTTTCATCCAAAATAATAAAAATGGCTACA
ATTATTTACTCAAGTATTTGAATGACTTAGACCACCCACAACTCATTTTTGAATCTACAGGTATCTATTCAAGAG

GTATGGAACGATTTTGTTGTGTAAATCAAATTAACTATATTCAAATGAATCCGTTAGAAGCCAAATTTAAAACG
AGCGCTCTAAGATCATGGAAAACTGATCAGGCAGATGCTCATAAGCTTGCTTGTTTAGGACCGACGCTCAAA
CAAACAGGCAGCTTACCTATACATGAGTTAATATTCTTTGAATTAAGAGAACGTGCCCGTTTTCATCTAGAAAT
CGAGAATGAACAAAATCGACTTAAATTTCAGATTCTTGAATTACTCCATCAAACATTCCCTGGTTTAGAAAGA
TTATTTAGTAGTCGATATTCAATCATTGCACTCAACATCGCAGAAATTTTTACTCATCCAGACGTGGTTCTTGAT
ATCGACAAGGATGTACTTATTACACATATATTCAATTCTACAGATAAGGGAATGTCAATGGATAAAGCTACAAA
ATATGCACTTCAATTAAGAGTGATTGCTCAAGAAAGCTATCCTAATGTCGATAGACATTCCTTTCTAGTCGAAA
AATTACGCTTACTTATTCAACAATTAAAACAATCTATTCATCATCTCAAACAATTAGATGATGCCATGATTCAAT
TAGCACAACAACTCGATTATTTTGAAAATATTCATTCGATACCTGGTATTGGTAAGCTAAGCACAGCTATGATT
ATTGGGGAGATTGGTGATATTAAGCGATTTAAATCAAATAAACAACTCAACGCTTTTGTAGGCATTGATATCA
AACGATATCAATCAGGTCATACACACTGTAGAGATACCATCAACAAGCGTGGTAATAAAAAAGCGAGAAAAC
TTTTATTTTGGGTGATTATGAATATAATAAGAGGGCAGCATCATTATGACAATCATGTCGTCGATTATTACTACA
AACTAAGAAAGCAGCCTAATGAGAAACCTCATAAGACTGCCATCATTGCTTGTATAAATCGATTATTAAAAAC
GATTCATTATCTGGTAATGAATCATAAATTGTACGATTATCAAATGTCACCACATTAGCCAAACGTACAATTAAA
TATATTTTAATACCTTATTCAAAAAATTAAAATGAACGGTTTAGTTAAGTAATGCTTATTTTAATTATAAGTACTT
GACTAATCGTAAGAAAGAGCCTAGGACATAAATCAATGTCTCGCGACCACAGCTTAATTTTTGGTGTTCATTC
ATGACTTTAAAAAATCCTTATTGCATAAATGTACATAGTGTAGTACTATTCAAAACGTAATTATTACGATTTGAA
TTAAGCGAGGAGAATGAAATGACTAAGACTTATGACGTTTGGTGGCAAAAAGGTCAAGAATCAGATGATGA
TATGGCACGAGACCATCAAGAAGCTTGGGAGAGAACAATAAAAATGCTTGATACATCTGACATCGAAGGGA
AAACGATTTTAGATGTGGGATGTAATCAAGGCGGATTTTTACGACAGTTATACGATACAACACCGTTTAAAAA
AGGTGTTGGCATAGATTTAGCACGTTTATCTTTGGAAAAGGCAGAGACATTAAAAGGACAACGTCCACTTAC
ATACTATTTAACAGATAAACCGCAAGAAACGAAGCACGTGTTTGATACGGCAGTAAGTACGTCTGTCTTGTAC
TTAATAGAAGATATTCCGCAACATGCAAAAGATTTAAAAGAGGTATTGAAACCAGGCGGTGTTTATTACGCTT
CATTCGCGGATTTAACTAATAACCCAAGTCGTCAGTTTATGGATGACACGATTAATCAATATGGTGCAACACCT
TCTCAGAATCACTCTCTAAAACATATCGTTGATAGCTTTGTGGATGCAGGATTTGAAGTTGCAGTAATGAAAG
AGCATGTACCTGACGTGATTGATTTAACACATTATAGCGATTTTTATTTATCACCGAATGATTATTTACAAACAC
TATATGAAGAATCGTTTTTAATAAAAGCAAGTGTGAAAGAAGGTACTGAGAAATGAGGAAATGTGTATTAAT
GACGGTAGCAGCAAGTGCTACGCTCTTATTGGCAGGTTGTGGCAATGGTCAAAAAGAAGATAAAGATGTTA
CGGTATCGCTACCTACTGAAGCAAAGGCGGATAAACTTGACGCGCAAGGCTATGATGCAGCGATGCCCGTTT
ATAGTGCAGTGTATGATGCATTAGTTAAATATGATAAAGATAAGGGTATTAAAGCAGGTTTAGCAGATAAATG
GAGCGTTGATGAATCAGGGAAAGTTTATGAATTCCATTTGAAAAAGAATGTTAAATTCTCAGATGGTTCAGC
ATTAGATGCTAAGGACGTGAAATTCTCGATTGATCGTGCGAAAGCGATGAACAAAGATTCGACTGTAGAAAC
GTTAAAAAAATTAGATAAGGTCGTTGTTAAAAATGAGCACGTGGTCCAAATTAGATTGAAATCTCCTTCAAAT
CAAGTGTTAAATGAATTAACACAAGTGAGACCGTTGCGTATTATGAGTCCACATTCAGTAGAAGATGGTAAA
GTAAACGGTAAATTTGAAAAAGCGATTGGAACAGGTGCATTTGTTGTTGATAAAACTGGTAAAGAAAAAAC
GACAATGAAGCCAAATAAATATTTTGACAACGGTCACCCAGTCAATTATCATCTTGCATTCCAAACGATTGAA
GATGGGGACTCAAGAAATTCTGCAGTACAAAGTGGTTCTGTAGATATTTCTGGTGGTGCTTTAGGTATGCTCT
CAGACGAACAAATCAAACAAGATAAGAAAAATAAGAACTTAACGATTGAAGATAGACCTAGCACAGTAAGT
CACTTTATGGCATTTAACCCTAAAAATGATGTATTAAATCAACGCACAATTCGTGAAGCGATAAGTAAGAGCA
TCGATGCGAAAGACATTGCGGGCAAATCTGTAAATGGTCTGTTCCAGAAGAACGTACAATTTGTGACTAAAA
ATAATCAACAGCCACACGATTATGATATGAAAGCGGCTGAAAGGTTACTTAAATCAGAAGGATATCATAAAAA
CGATGACGGCATCTTTGAAAAGAATGGCAAACCTTTATCATTTAACTTAGTCATTCAAACTGCAGAGTTCCCA
AATTGGAAAGATAAAGCTGAAAAAGTGCAACGTCAGCTTAAACAAGCCGGTATTAAGTTAAATGTGAAAAC
GTTAGATTCACAATCATACTATGATACATTATGGACGAAAAAAGACTATGATTTGATTTTCTATAGAACGTATTC

AGATGCATTAATGCCTTACAACTTTATAAGTTCAGTGTTTAAAAATAATGATGGTCAACCAGGGGTGTTAGCT
GATGATGAAACATTAACGAAACAGCTAGACGATTTCCCATCAACCGTATCAAAAGAAGACCAACAGTGTTCA
TTTGATGACATATTTAAACACTTTAATCAACAATACTATGGTGTGCCAATTGCTTATCCAAATGAGACGTTTGTA
GTGAGTGATAAAGTAAAACAATTCAAATTCTCTGGACTTACGGATGCACCAATTGATTATAAAGCGTTGAAA
GTTAATGAATAGCAATGCTCAAACGTACAATTAAATTCATACTTTATTTAATCGTAAGTTCGTTTATTATCTTCAT
TTTAGTTGAGAAGACATCTGGTAATCCAGCGATTCTGTATCTACAACGTCATGGTTATACGTCGATTACGCAAG
ACAATATTGAAGCGGCACAACATCAACTTGGCTTAGGACAACATGTGTTACTAAGATATATCGATTGGGTTGG
ACATGCACTCACGGGCAACTTAGGATACGGCTTTAGTACGAACGAAGCAGTTACCGCTATGATAATGGAAGC
CATCGTGCCGACGCTTGTGCTAATCATTGTCTCTAGTTGTATCATGTTGCCATTTGGCTATATTGTTGGTTACTT
CGTTGGGACGCGTCCGCATACACGTTACGCTAATGGAATTCGTGGATTCGCCCAAGTGATGACCTCAATGCC
AGAATACTGGTTAGCTATTTTATTCATTTATTATTTAGGCGTACGTTGGCAATTGTTACCATTTGTAGGTAGTGA
TTCATGGCAACACTTTGTGCTGCCAATCTTCACAATTGTTGTTATAGAAGGGTGTCATATCTTATTGATGACAG
CACATCTGATTACACAAACGTTAGATCAAGATGCGTATCAACTGGCGCAGTTAAGACATTTTTCGTTAAAAGC
GCGTATCATCGTACAAATTAAAGAGATATTTGCACCACTAATGACGATTTCAATTAACAGTATCATTCATTTAAT
TGGAAAAGCCGTAATACTAGAAGTCATCTTCAGCATGTCTGGTATAGGTAAATTGTTGATTAATGCTATTAACC
AACGAGATTATCCACTGATTCAGGGCATTGTCATCTTTATCATTGTCTTTATTATGCTAATGAATTATTTAGGCG
ATGTGATTATTTTGAAGAATGAACCTAGACTTCGACGACGTCATACCCAGCAGTCAGGCAATGAGAAAAGAG
GTACGATGTGATGAAAAAAATATCAAACGTACATCGCAATAGGTTCACTATTGAGTTTGATGGTTGTATTAATTA
CGTATGGTTTAATGCAAGACACGCAACATTTGAACCCACTTGAGTCACCTAATGGACAACATTGGTTGGGTA
CCGATCAATTAGGCAGAGACTTCTTAGTAAGACTGATTGTCGGTAGTCTTGTCACATTGAGTTTAACAGGCAT
AGTGATTCTATTAAGCGTTTGTATGGGACTTATCTTTGGCTTAATTGCAGGCATAGAAAGACGATGGTTAGAT
CAAATCATCATGTTTGTTGCCGATATGTTGCTGGCTATTCCGTCATTTATTATCGCATTAGTCATCTTAAGTTTAG
TAAGTAACTCCATGATAGGTTTGATACTTGCTTTAACGATTGGATGGATAGGACGTTATTTACGTTACTTCAGA
AATTTAACGCGAGATATTCAAAAACGTCCATTTGTTCAATATGCACGATTGAGTGGGAACTCAACATTCAAAA
CGACAGTAACACATGTGATTCCACATTTATTAAGTAGTATATTCGCTTTGGTAACGGCTGACTTTGGCAAAATG
ATGCTCAGCATATCTGGACTTGCTTTTCTAGGACTAGGTATTAAACCGCCGACGCCTGAGTTAGGAACAATTC
TTTTTGATGGGAAAAGTTATTTCAACGGCGCACCGTGGCTCTTCTTCTTCCCTGGTGTATTGTTAGGAGGTTT
CGCCTTATTATGTCAAATTATCAACAAAAAAATAACGCAGTAAATACGGTAGTCAACGTCAATCAATTATCGAT
TTTAGATCAAGAGAAAGTATTGTTAAAGGATGTTGATTTGACAGTAACTAAAGGTGCATTTCATTGCATTATA
GGTGAAAGTGGCAGTGGGAAATCACTGTTAACAAGAACAATACTTGGAATGAAACAATCACAATTATGTTAT
CAAGGAGATATTGACATCGATTTAACTCAAACAGATGCAGTGTTTCAAGATGTTCAAAGTAATATGTTTCAAA
ATATAACATTAGCTAAGCATTTCCAATACATTTATGAAGCCAATCGCACACATCTCACTAAACAGCGTATTAAG
GAAGATGTCTTACAGATGATGCAATTACTTGGTTTAAGACAAGGGGAACAATTGCTTGAGCGTTATCCCTTC
GAACTTAGTGGAGGTATGGCACAACGTGTCGCCTTTATAATGTCATTAATTAGACGTCCGAACTACTTATTTTT
AGATGAACCAACGAGTGCACTTGATCAAGAAAATATTAAAAAGTTTATGCATTACCTTCTTAGGGCACAGGA
GCGCTACCAAATGACCATTGTTTTTATCACACATGATATTAACTTAGTGAAAGATTGTGCCACACATATTAGTAT
TATGCAGCAAGGTAAATTGATAGAAAATGGTGAGGCCGCGTCGATCTTAACTAAGCCGACACATAATTACAC
GAAAAAATTAATTACGATTGCACATCGGAGACAACCTTATGCTTAAAATAGAGAGATTAACCAAATATATAGA
CACGCAACTGATATTTAAAGAGATATCATGTACAATTAACGACCAGCACTTACTCATAAGTGGGGAGAGTGGT
TGTGGTAAATCCACATTAGCCAAGATTATCGCTGGCTTAGATACGGATTATCAGGGCGAATTATATCTTAATGG
GCGCTTACGTGAATCTTATACGTCTAAAGAGTGGATGAAGCACATCCAATATGTACCTCAATATCAACGTGATA
CTTTAAATCAGCGTAAAACGGTATTAGCTACATTATTAGAACCACTTAAGAATTATAAGGTAAATAAACAGCGT
TATACATCAAGCATTGAAGCAGTGCTTGATCAGTGTAATTTACCACACGATATACTTAATCATAAAGTTTCGAC
ATTAAGTGGTGGCCAATTTCAACGCGTCTGGATAGCTAAAGCTTTAATATTAGAACCAGAGATTCTCATATTG

GATGAAGCTACAACCAACTTAGATGTCATTAATGAAGAAGCTATACTTCAAATGTTGATTTCCTTAAAGATGA
CACAATTAATCATTATTTCACATGATACATACGTCTTAAGCCAATTTGAAGGAATTCAGTTACAGCTAAATAAAT
TGAATAATTAAGATCACAAATCTTAATATGGTGAATATTTAATGGTACCTAAAAAATAAAATTTAAACTACAATG
TCTAAATCCATATGTTGTTTCATTAGAGGATTTAAAAATGATTATAACACTAAAAGATTTCAAATTATTATATTTA
ATATAAATTTACATATGATAAACGAATAACAATTCCAATATAAATTATTTTTTGATTATTTTTATTTATAACTATATA
TTTTATATGAAAAATATAAGTTATAATAATAAGTTTAATATTGCCTCGTGGTTCTGAGCTTGAACTTATCTCTAAA
TCATTTTGAGCTACTTATCTATCAATTCATATATTCTATAACAATATTTGTGACATCACGTGCTATTTCATGAAGT
GATTTTACGATATCACCTTCTTTAGAAAAAATATATTTCAGTAGGCACCGACGTATACAGAATCATTTGAGTATT
AAAAATAAAAACTAGAAAAAGAAACCGCATCATTAACTGATACGCAGAATCATATTATAAATAAAACTAAAAA
TGAGGTTGTATATAACTCACTCTGAAATTGATTGAATATATAGTATCTTTAATAAAATGCAGCTATTGTGGCGTA
GAATTTGAGAATCAAAAAATGATTAATATAGTTTGAAGAGACTGAGCATAAATACTAGAAAAATGGCCAGTA
AATGAGTTTACTATAAACTCATTTACTGGCTTCTCTAATAATTATCAAGACAATTTGCGTTTCTAGGCATACTTT
GAAATGCGCTATTTTCTTTAAGAATATTAATATATGACTTGTGTTGGTAATGATAACCTTGGTTAGAGTGTATTG
TCATGTGATAGTTAAGATTAGATCTTTTAATTAAAACATTGTTTTAAATATCGATGACAAGGTCTAATGTAGGAC
GTGTAGATATAAGGTTCGGAATTATATAAATCCATAAAAGGAGATAAATCGAAAACCTTTATACCCTGGGTGG
GTATATGTTATAGTATAAGTAGCTTTACTATAACATTTTCATTAGGAGGGGTTAATTTGAATAATAATGGTGAAG
AGCATAATCATCAAAATCACATGAATCATTCCAATCAAATGCATCATGATAACCATGCCTCACATGATCATCATA
GTGGCCATGCACATCATCATGGAAATTTTAAAGTTAAGTTTTTTGTTTCATTAATTTTTGCAATACCTATCATTC
TTTTATCGCCACTGATGGGTGTTAACTTACCTTTTCAATTCACATTTCCAGGTTCTGAATGGGTAGTGTTAATAT
TAAGTACAATTTTATTCTTTTATGGTGGTAAACCGTTCTTGTCTGGTGGTAAAGATGAAATTGCTACAAAAAA
ACCAGGCATGATGACCTTAGTTGCCCTAGGTATTTCAGTAGCTTATATTTATAGCTTGTATGCTTTTTATATGAA
TAACTTTAGTAGTGCAACTGGTCATACAATGGACTTTTTTTGGGAATTAGCAACCTTAATTTTAATTATGCTATT
AGGACATTGGATAGAAATGAATGCTGTCGGAAATGCTGGAGATGCTTTAAAGAAAATGGCAGAACTGTTAC
CTAATAGTGCTATTAAAGTTATGGATAATGGCCAACGCGAAGAAGTTAAAATATCAGACATCATGACTGATGA
TATCGTCGAAGTAAAAGCCGGAGAAAGCATTCCAACAGATGGTATTATCGTTCAAGGACAAACATCTATAGAT
GAATCCCTAGTCACTGGAGAATCTAAAAAAGTACAAAAAAATCAAAATGACAACGTCATCGGGGGTTCTATT
AATGGGTCTGGAACAATACAAGTCAAGGTTACAGCTGTGGGAGAAGATGGATATCTTTCTCAAGTTATGGGA
CTTGTTAATCAAGCACAAAATGATAAATCTAGTGCTGAATTGTTATCTGATAAAGTAGCGGGTTATTTATTCTAC
TTTGCTGTAAGTGTTGGCGTGATTTCTTTTATTGTCTGGATGCTCATTCAAAATGATGTTGATTTTGCATTAGA
ACGTCTTGTAACTGTGTTAGTCATTGCTTGTCCACATGCTTTAGGCTTGGCAATACCTTTAGTCACTGCACGTT
CTACTTCAATTGGTGCACATAATGGTTTAATTATTAAAAATAGAGAGTCTGTAGAAATAGCTCAACATATCGAT
TATGTAATGATGGACAAAACTGGTACTTTAACTGAGGGTAACTTTTCTGTGAATCATTATGAGAGCTTTAAAA
ATGATTTGAGTAATGATACAATATTAAGCCTTTTCGCCTCATTAGAAAGTCAATCTAATCACCCATTAGCTATAA
GTATTGTTGATTTTGCGAAAAGTAAAAATGTTTCATTTACTAACCCACAAGACGTTAATAATATTCCAGGTGTC
GGATTAGAAGGTCTAATTGATAATAAAACATATAAAATAACAAATGTCTCTTATCTTGATAAACATAAACTTAAT
TATGACGATGACTTGTTTACTAAATTAGCTCAACAAGGTAATTCAATCAGTTATTTAATTGAGGATCAACAAGT
CATTGGCATGATTGCTCAAGGAGATCAAATTAAAGAAAGCTCAAAACAAATGGTAGCTGATTTACTATCAAG
AAATATTACACCAGTCATGCTTACAGGTGACAATAATGAAGTGGCACACGCTGTCGCAAAAGAATTAGGTATT
AGTGATGTCCACGCACAACTCATGCCAGAAGATAAGGAAAGCATTATAAAAGATTATCAAAGTGACGGTAAT
AAAGTCATGATGGTCGGAGACGGTATCAACGATGCGCCGAGTCTTATAAGAGCGGATATTGGTATAGCAATT
GGTGCAGGTACAGATGTTGCAGTGGATTCAGGTGATATCATACTTGTTAAAAGTAATCCATCAGATATCATTCA
TTTCTTGACCCTTTCAAATAATACTATGAGAAAAATGGTGCAAAACTTATGGTGGGGTGCAGGTTATAATATT
GTTGCTGTACCTTTAGCAGCTGGTATTTTAGCATTTATTGGCTTGATTTTATCACCTGCAATAGGTGCTATTTTA
ATGTCTTTAAGTACAATTATCGTTGCAATTAATGCCTTTACATTAAAATTAAAATAAAAGATAGGAGTTTTATTA

TGATTAAAAAATTATTTTTTATGATATTAGGATCATTACTAATATTATCAGCTTGCTCCAATAATGATGAAAAAGA
TAAAGACACTAATGACCAAAAAAGTGAGAGCCATATGAAGCATAATGATGAAAGTAAAGTTCCAGAAGATAT
GAAATCGACTAATGAGGGTGAATTTAAAGTGGGAGATAAAGTAACGATTACAGCAGGGCATATGCCAGGTA
TGAAAGGTGCAGAAGCTACTGTAAAAGGTGCGTATAAAACATATGCCTATGTTGTAAGTTATAAACCCACAAA
TGGAAATGAAAAAGTAAACAATCATAAATGGGTCGTAAACGAAGAGATTAAAGATGCACCTAAAGATGGATT
TAGTAAGGGCGATACTGTTAAATTAGAAGCAAGTCATATGTCTGGTATGAAAGGTGCTACAGCCAATATAGAT
AACGTGAAAAAGACTACTGTTTACGTAGTTGATTACAAATCCAAAGATAATGGTAAAATCATTAAAAATCATA
AATGGATGACAGGAAATGAGCTGAAAGCACGATAAAAATCTAGTTCTAAATTGAGAAATAAATAGATATAAA
AATATCCTCCTTAATCAATAATTTAAATAACTTATTATTGTTAAGGAGGATATTTTTTAGTGTGTAAATTAAAAAG
AATTTTAGAAGAATAACATTTATCAAAAAACTGTTCATTACCTTATTAATTGAAATTATATAATTAAAAACCGCA
TCATTAACCGATACGCAGAGGCGTATCATAAGT
>Staphylococcus aureus strain ER06690.3
ATGAAAATCACCATTTTAGCTGTAGGGAAACTAAAAGAGAAATATTGGAAGCAAGCCATAGCAGAATATGAA
AAACGTTTAGGCCCATACACCAAGATAGACATCATAGAAGTTACAGACGAAAAAGCACCAGAAAATATGAG
CGACAAAGAAATCGAGCAAGTAAAAGAAAAAGAAGGCCAACGAATACTAGCCAAAATCAAACCACAATCC
ACAGTCATTACATTAGAAATACAAGGAAAGATGCTATCTTCCGAAGGATTGGCCCAAGAATTGAACCAACGC
ATGACCCAAGGGCAAAGCGACTTTGTATTCGTCATTGGCGGATCAAACGGCCTGCACAAGGACGTCTTACA
ACGTAGTAACTACGCACTATCATTCAGCAAAATGACATTTCCACATCAAATGATGCGGGTTGTGTTAATTGAA
CAAGTGTACAGAGCATTTAAGATTATGCGTGGAGAAGCATATCATAAATGATGCGGTTTTTTCAGCCGCTTCA
TAAAGGGATTTTGAATGTATCAGAACATATGAGGTTTATGTGAATTGCTGTTATGTTTTTAAGAAGCTTATCAT
AAGTAATGAGGTTCATGATTTTTGACATAGTTAGCCTCCGCAGTCTTTCATTTCAAGTAAATAATAGCGAAATA
TTCTTTATACTGAATACTTATAGTGAAGCAAAGTTCTAGCTTTGAGAAAATTCTTTCTGCAACTAAATATAGTA
AATTACGGTAAAATATAAATAAGTACATATTGAAGAAAATGAGACATAATATATTTTATAATAGGAGGGAATTT
CAAATGATAGACAACTTTATGCAGGTCCTTAAATTAATTAAAGAGAAACGTACCAATAATGTAGTTAAAAAAT
CTGATTGGGATAAAGGTGATCTATATAAAACTTTAGTCCATGATAAGTTACCCAAGCAGTTAAAAGTGCATATA
AAAGAAGATAAATATTCAGTTGTAGGGAAGGTTGCTACTGGGAACTATAGTAAAGTTCCTTGGATTTCAATAT
ATGATGAGAATATAACAAAAGAAACAAAGGATGGATATTATTTGGTATATCTTTTTCATCCGGAAGGAGAAGG
CATATACTTATCTTTGAATCAAGGATGGTCAAAGATAAGTGATATGTTTCCGCGGGATAAAAATGCTGCAAAA
CAAAGAGCATTAACTTTATCTTCCGAACTCAATAAATATATTACATCAAATGAATTTAATACTGGAAGATTTTAT
TACGCAGAAAATAAAGATTCATCTTATGATTTAAAAAATGATTATCCATCAGGATATTCTCATGGATCAATAAGA
TTCAAATATTATGATTTGAATGAAGGATTCACAGAAGAAGATATGCTAGAGGATTTAAAGAAATTTTTAGAAC
TATTTAATGAATTAGCTTCAAAAGTTACAAAAACATCCTATGATAGCTTGGTCAATAGCATAGACGAAATACAG
GAAGACAGCGAAATTGAAGAAATTAGAACAGCACAAAAAGATAAGACACTCAAGGAAGTGGAAGCACCTA
AAGGAATAATTCCAAAATATAAAAAAGGTGTATCAAAGACTACTAAAAATGATTCAGAAATTGAAAAATCAA
ATAAAGAGAATAAATTAACCGGTAAAGTTGGAGAAAAATTAGCGCTAAATTACTTTAATGAGCTAATTGATAA
TAAAATAGACGAAGATAAGAAAGAACAGTTTAGGAATATTTTAAATGATAATCCAGGCTCTCAACACGGTCAT
GGCTATGATTTAGTAGCTTTTGATCCAACAAATACAGATAAAGCTGTAGAAAAATTTATTGAAATTAAAACATC
TACATCTTCTAGTATTGAGGAACCATTTTTTATGTCGCTAAATGAAATGTTTGCTATGAAAGAATATAAGCAGA
AATATTTAATATTAAGAATATTTAATGTTTCCGGTAAAGAACCACAATTTTATTTTATAGATCCATATGCAAATTA
TTCTGAATTTAAAGATGTAGATGATCTCATTGACAAAGTATTTAATGTAGAAGCTATTCAGTATAAAGTTTTTG
GCGAAAAATGATTACTTGAACAAGAGCTAAAATAAAATTGTGATCTAATAAAAATAGAAACTGTAATTTAAAT
AAAACTTTCTAAATAAGCTAACTGATAAAAAATCAGTTTGTCCACAGTCTGAAACAAGATTCCTATATTCTTTA
GGAATCTTGTTTTTTCTATTTTTATGGTGATAAAGAGCAGATAAGATAATGTGTAATAATCACAAAAAAGTTAA
ATATTTTAAGGCTTGTTTAATTATTAATGATTTTATATATAAAGAGCAGTATAATAAAGTTGTTAATATATTATGAA

TAATATTCAAGTAATTTTATTGTTTTTTAATTTGTCGATATTTAAGTTGAGTTAAATTTAAAGGGTGTAATTTGTT
TTACAATGATGAAGATAATTAGTCTATCAAAATAAAGGGGTTGGGACTGTTATGAGTGATAATTTGTCATTATA
CATTGACTATATCAATGATAATATAATCTATGGTAGTGAAATCAAACGGGAGAAATTAGAGAATTTATTTAATCA
ATTTGCTATAAAAAATGTTGAAAAGAACATTGTCTATGATGAACTGAAATCTTTAGATATTACAATCATTGAGT
CACAGGATTCATATAAAAATAAATTGAAGAGATTATTTTCGGTTCTGTTGCAAAGTAAAAAAATATAGCTAACC
ACTAATTTATCATGTCAGTGTTCGCTTAACTTGCTAGCATGATGCTAATTTCGTGGCATGGCGAAAATCCGTAG
ATCTGAAGAGACCTGCGGTTCTTTTTATATAGAGCGTAAATACATTCAATACCTTTTAAAGTATTCTTTGCTGTA
TTGATACTTTGATACCTTGTCTTTCTTACTTTAATATGACGGTGATCTTGCTCAATGAGGTTATTCAGATATTTC
GATGTACAATGACAGTCAGGTTTAAGTTTAAAAGCTTTAATTACTTTAGCCATTGCTACCTTCGTTGAAGGTG
CCTGATCTGTAATTACCTTTTGAGGTTTACCAAATTGTTTAATGAGACGTTTGATAAACGCATATGCTGAATGA
TTATCTCGTTGCTTACGCAACCAAATATCTAATGTATGTCCCTCTGCATCAATGGCACGATATAAATAGCTCCAT
TTTCCTTTTATTTTGATGTACGTCTCATCAATACGCCATTTGTAATAAGCTTTTTTATGCTTTTTCTTCCAAATTT
GATACAAAATTGGGGCATATTCTTGAACCCAACGGTAGACCGTTGAATGATGAACGTTTACACCACGTTCCCT
TAATATTTCAGATATATCACGATAACTCAATGTATATCTTAGATAGTAGCCAACGGCTACAGTGATAACATCCTT
GTTAAATTGTTTATATCTGAAATAGTTCATACAGAAGACTCCTTTTTGTTAAAATTATACTATAAATTCAACTTTG
CAACAGAACCGTATTATGGAATAGAGATGTTGGTAACATTTATACAGGATCATTATACTTAAGTTTAATTTCGTT
ATTACAGAACCACACATTCCAACCAGAAGAGAAAGTATGTCTATTTAGTTATGGTTCAGGAGCAGTAGGAGA
AATCTTTAGTGGTTCAATCGTTAAAGGATATGACAAAGCATTAGATAAAGAGAAACACTTAAATATGCTAGAA
TCTAGAGAGCAATTATCAGTCGAAGAATACGAAACATTCTTTAACAGATTTGATAATCAAGAATTTGATTTCG
AACGTGAATTGACACAAGATCCATATTCAAAAGTATACTTATACAGTATAGAAGACCATATCAGAACATATAAG
ATAGAGAAATAAACTAGTGGCCGATTGTGCTTGATGAGCTTGGGACATAAATCCTAACTCGAAATAAATAAGC
ATATCACTAAACTGATTTTTTAAAGTTTACAGTGATATGCTTATTTTTTTATCTTACGATTTTGTACGTGCATGCT
TGCCTAGGGGTATGGCTCGAGCCATTAGTCTCTCGCACATACTATTCCCTCAGGCGTCAGCACTTACAAAATC
GGTTGTAATTTTCATTTTTATACGCATTCTTACTGAGATTATACTAATAAGAGGAATAGTAAAAGCAATTCTAAG
TAAAATTGCAGATAAGAGGTTTGTTAAAAGCAGTTCTAAGTAAAATTGCAGATAAGAGGTTTGTTAAAAGCA
GTTCTCAGTAAAATTACAGATAAGAGGTACGTTAAAAGCAGTTCTAAGTAAAATTGCAGATAAGAGGTTTGT
TAAAAGCAGTTCTCAGTAAAATTGCAGATAAGAGGTACGTTAAAAGCAGTTCTAGGCAAAATTGCAGATAAG
AGGTGCGTTAAAAGCAGTTCTCAGTAAAATTGCTGATAAGGGGTAAGTTAAAAGCAATCCTAAGTAAAATTG
CAGATAAGGGGTACAGAAAAACTAGACTTGATTACAAAATGGAGCTTGGGACATAAATGATTTTTTAAAAAT
GAGATGAGACGTAGATTAACTCCATAATCAATACGAATCTATCGACTTCTTTATTTATGATATTCATCTCTTTTTA
ATGGAAATAAAAGTGCGATTAATGTGATAATACAGTTACGTTAATTAAAAAAATAAAAATGCAAGGAGAGGT
AATATGCTAACTGTATATGGACATAGAGGATTACCTAGTAAAGCTCCGGAAAATACAATTGCATCATTTAAAGC
TGCTTCAGAAGTAGAAGGTATAAACTGGTTGGAGTTAGATGTTGCAATTACAAAAGATGAACAACTGATTAT
CATTCATGATGATTATTTAGAACGGACTACAAATATGTCCGGGGAAATAACTGAATTGAATTATGATGAAATTA
AAGATGCTTCTGCAGGATCTTGGTTTGGTGAAAAATTCAAAGATGAACATTTGCCAACTTTCGATGATGTAG
TAAAAATAGCAAATGAATATAATATGAATTTAAATGTAGAATTAAAAGGTATTACTGGACCGAATGGACTAGCA
CTTTCTAAAAGTATGGTTAAGCAAGTGGAAGAACAATTAACAAACTTAAATCAGAATCAAGAAGTGCTCATT
TCAAGCTTTAATGTTGTGCTTGTTAAACTTGCAGAAGAAATCATGCCACAATATAACAGAGCAGTTATATTCCA
TACAACTTCGTTTCGTGAAGACTGGAGAACACTTTTAGATTACTGTAATGCTAAAATAGTAAACACTGAAGAT
GCCAAACTTACTAAAGCAAAAGTAAAAATGGTAAAAGAAGCGGGTTATGAATTGAACGTATGGACTGTAAA
CAAACCAGCACGTGCAAACCAACTTGCTAATTGGGGAGTTGATGGTATCTTTACAGACAATGCAGATAAAAT
GGTGCATTTGTCTCAATAGAAAGTTAGAGGTGAGTCTTACGTTTCAGTGACGGTAGACTTACCTTTAACATGT
TACATACTAAAAAATTAATTTGAATAAGAAAGAGAGACATATATGAAATACGATGATTTTATAGTAGGAGAAA
CATTCAAAACAAAAAGCCTTCATATTACAGAAGAAGAAATTATCCAATTTGCAACAACTTTTGATCCTCAATAT

ATGCATATAGATAAAGAAAAAGCAGAACAAAGTAGATTTAAAGGTATCATTGCATCTGGCATGCATACACTTT
CAATATCATTTAAATTATGGGTAGAAGAAGGTAAATACGGAGAAGAAGTTGTAGCAGGAACACAAATGAATA
ACGTTAAATTTATTAAACCTGTATACCCAGGTAATACATTGTACGTTATCGCTGAAATTACAAATAAGAAATCCA
TAAAAAAAGAAAATGGACTCGTTACAGTGTCACTTTCAACATACAATGAAAATGAAGAAATTGTATTTAAGG
GAGAAGTAACAGCACTTATTAATAATTCATAATAAAACAGTGAAGCAACCATCGTTACGGATTGCTTCACTGT
TTTGTTATTCATCTATATCGTATTTTTTATTACCGTTCTCATATAGCTCATCATACACTTTACCTGAGATTTTGGCA
TTGTAGCTAGCCATTCCTTTATCTTGTACATCTTTAACATTAATAGCCATCATCATGTTTGGATTATCTTTATCATA
TGATATAAACCACCCAATTTGTCTGCCAGTTTCTCCTTGTTTCATTTTGAGTTCTGCAGTACCGGATTTGCCAA
TTAAGTTTGCATAAGATCTATAAATATCTTCTTTATGTGTTTTATTTACGACTTGTTGCATACCATCAGTTAATAG
ATTGATATTTTCTTTGGAAATAATATTTTTCTTCCAAACTTTGTTTTTCGTGTCTTTTAATAAGTGAGGTGCGTT
AATATTGCCATTATTTTCTAATGCGCTATAGATTGAAAGGATCTGTACTGGGTTAATCAGTATTTCACCTTGTCC
GTAACCTGAATCAGCTAATAATATTTCATTATCTAAATTTTTGTTTGAAATTTGAGCATTATAAAATGGATAATCA
CTTGGTATATCTTCACCAACACCTAGTTTTTTCATGCCTTTTTCAAATTTCTTACTGCCTAATTCGAGTGCTACT
CTAGCAAAGAAAATGTTATCTGATGATTCTATTGCTTGTTTTAAGTCGATATTACCATTTACCACTTCATATCTTG
TAACGTTGTAACCACCCCAAGATTTATCTTTTTGCCAACCTTTACCATCGATTTTATAACTTGTTTTATCGTCTA
ATGTTTTGTTATTTAACCCAATCATTGCTGTTAATATTTTTTGAGTTGAACCTGGTGAAGTTGTAATCTGGAAC
TTGTTGAGCAGAGGTTCTTTTTTATCTTCGGTTAATTTATTATATTCTTCGTTACTCATGCCATACATAAATGGAT
AGACGTCATATGAAGGTGTGCTTACAAGTGCTAATAATTCACCTGTTTGAGGGTGGATAGCAGTACCTGAGC
CATAATCATTTTTCATGTTGTTATAAATACTCTTTTGAACTTTAGCATCAATAGTTAGTTGAATATCTTTGCCATC
TTTTTTCTTTTTCTCTATTAATGTATGTGCGATTGTATTGCTATTATCGTCAACGATTGTGACACGATAGCCATCT
TCATGTTGGAGCTTTTTATCGTAAAGTTTTTCGAGTCCCTTTTTACCAATAACTGCATCATCTTTATAGCCTTTA
TATTCTTTTTGTTTTAATTCTTCAGAGTTAATGGGACCAACATAACCTAATAGATGTGAAGTCGCTTTTTCTAG
AGGATAGTTACGACTTTCTGTTTCATTAGTTGTAAGATGAAATTTTTTTGCGAAATCACTTAAATATTCATCCAT
TTTTTTAACGGTTTTAAGTGGAACGAAGGTATCATCTTGTACCCAATTTTGATCCATTTGTTGTTTGATATAGTC
TTCAGAAATACTTAGTTCTTTAGCGATTGCTTTATAATCTTTTTTAGATACATTCTTTGGAACGATGCCTATCTC
ATATGCTGTTCCTGTATTGGCCAATTCCACATTGTTTCGGTCTAAAATTTTACCACGTTCTGATTTTAAATTTTC
AATATGTATGCTTTGGTCTTTCTGCATTCCTGGAATAATGACGCTATGATCCCAATCTAACTTCCACATACCATC
TTCTTTAACAAAATTAAATTGAACGTTGCGATCAATGTTACCGTAGTTTGTTTTAATTTTATATTGAGCATCTAC
TCGTTTTTTATTTTTAGATACTTTTTTTATTTTACGATCCTGAATGTTTATATCTTTAACGCCTAAACTATTATATAT
TTTTATCGGACGTTCAGTCATTTCTACTTCACCATTATCGCTTTTAGAAATATAACTGCTATCTTTATAAACTTGT
TTGAAATTTTTATCTTCAATTGCATCAATAGTATTATTAATTTCTTTATCTTTTGAAGCATAAAAATATATACCAAA
CCCGACAACTACAACTATTAAAATAAGTGGAACAATTTTTATCTTTTTCATCAATATCCTCCTTATATAAGACTA
CATTTGTAGTATATTACAAATGTAGTATTTATGTCAAAATAATGTTATAATTTTTGTGATATGGAGGTGTAGAAG
GTGTTATCATCTTTTTTAATGTTAAGTATAATCAGTTCATTGCTCACGATATGTGTAATTTTTTTAGTGAGAATGC
TCTATATAAAATATACTCAAAATATTATGTCACATAAGATTTGGTTATTAGTGCTCGTCTCCACGTTAATTCCATT
AATACCATTTTACAAAATATCGAATTTTACATTTTCAAAAGATATGATGAATCGAAATGTATCTGACACGACTTC
TTCGGTTAGTCATATGTTAGATGGTCAACAATCATCTGTTACGAAAGACTTAGCAATTAATGTTAATCAGTTTG
AGACCTCAAATATAACGTATATGATTCTTTTGATATGGGTATTTGGTAGTTTGTTGTGCTTATTTTATATGATTAA
GGCATTCCGACAAATTGATGTTATTAAAAGTTCGTCATTGGAATCGTCATATCTTAATGAACGACTTAAAGTAT
GTCAAAGTAAGATGCAGTTCTACAAAAAGCATATAACAATTAGTTATAGTTCAAACATTGATAATCCGATGGTA
TTTGGTTTAGTGAAATCCCAAATTGTACTACCAACTGTCGTAGTCGAAACCATGAATGACAAAGAAATTGAAT
ATATTATTCTACATGAACTATCACATGTGAAAAGTCATGACTTAATATTCAACCAGCTTTATGTTGTTTTTAAAAT
GATATTCTGGTTTAATCCTGCACTATATATAAGTAAAACAATGATGGACAATGACTGTGAAAAAGTATGTGATA
GAAACGTTTTAAAAATTTTGAATCGCCATGAACATATACGTTATGGTGAATCGATATTAAAATGCTCTATTTTAA

AATCTCAGCACATAAATAATGTGGCAGCACAATATTTACTAGGTTTTAATTCAAATATTAAAGAACGTGTTAAG
TATATTGCACTTTATGATTCAATGCCTAAACCTAATCGAAACAAGCGTATTGTTGCGTATATTGTATGTAGTATAT
CGAGCTTCACATGAAACAGCTAAAGAAGCTTTGGGCGATAAAGAGTTAAGAGCCATTGCACATGAGTTAAC
TAAAACAGTTAAGGATAACATGAGTGTTGATTGGTCTAAACGAGACAGTGCTAAAGCTAAAATGAGAGTTCA
AGTTAGACGCCTATTAAAGAAATATGGCTATCCACCAGATCTTCAAAAAATGGCTGTGGAACAAGTTGTAGA
GCAAGCAGAATTAATGGCAAGTCAGCAATAAAAAAATAAATCATAATGAGTCCGGGACATAAAGTTCTTGGA
TAAGTGAAAAAAGACAATTTCTATTGAAATAATATAGAAATTGTCTTTTTTATAAATTTTTTGATTATTTTCAGC
TCGTTGAGCTACTACTTTTCTTATATTAAGTGCCATTAATACAAAACCAAGTTCTCTTTTGACTTTATTGAGTCC
TCGGACAGACATCCGAGTGAAACCCAAAATAGCCTTCATAAATCCAAAAACAGGTTCCACATCAATTTTTCTT
TGACTGTAGATATTTTTTGTTTCTGGTTCTGAAAGCTTTTTGTTAATTTGGGATTTAAAATATTCCCAGTTATAA
TTCTTCATTATTTTTTTGTTTGTTTTTGAATTGAAGTTCATACATTGATTTTTCAGAGGACATTCTGAACAATCA
TCACATTCATATAATTTGAAGTCTCGCTTATAACCATACTTATCATGACGATAGGCATATCTTTTAAAACCTAGCC
GTTTATTATTCGGACAAATGAATTCGTCATTAATTTCGTCATAGTTCCAATTTTGAGTATTAAAGATGTCACTTT
TATATTTTTTAGTTTTATCTTTTATAAACATTCCATATGTTATGAGTGGCGTTCGATTAAAGTCATCTATAATTGCC
TTATAATTTGATTCACTACCATAACCTGCATCAGCTACAATATATTCAGGTAAATGACCGTAGGTCTCTTGAATT
GAATTTAAAAATGGAATCATCGTTCTAGTATCCGTTGGATTTTGATACACATTATAAGATAAAACAAATTGGGA
ATTTGTTGCTATTTGTAAATTATACCCTGGCTTAAGTTGTCCATTTTTCATGTGATCTTCTTTCATTCTCATAAAT
GTCGCATCATAATCTGTCTTAGAATAACTATTTCTATCCTTTAAAATAGATTTTTGAAATTCGTATCGATACTTTC
GCTCAAAATAATCATTGATTTGCTTTTTGTATTTTTTGATTTTAGTTCTTTTGAGACGTATTTGTTTTCTTGTTTT
AGTACATTTTTCATTGTTGATATGTTGGTTTAAATCTTCGATTTCTTTATCTAAGTGACTACCAATCAAATCTATT
TCTTCTTTTGTTAATTCATTATCATGATCTTCTTTAATTTCCGGTATGATTTTATTGGTTACCAATTCATGGTAGA
GGGCTTTAGAATCCTCATTCATCTTTGATTCATGGTTTTGAATACTCTTTTTCCATACAAATGTATATCGATTGG
CATTTGCTTCAATTTTTGTACCATCAATAAAAATAGCTTTATCATCTATAAGATTTTGTTTTACACACTGACTGTA
AAATTGAATAAATAAAGATTCTAATAAAGCATCTACTTTTGGATTTACTCTAAATTGATTAATTGTTTTATAAGA
AGGTTTTTGATTTTGTGATAGCCACATCATTCGGATGCTATCATTAAGCATTTTTTCTATTTTACGACCTGAGAA
TACAGATTGTGTGTAGGCATATAGAATCACTTTTAACATCATTTTAGGATGGTACGAAGTTGCACCACGGTGA
TGTCTGAATTCGTCGAATTCATTGTCAGGAATTGTTTCAACAATATCATTTACAGTAAAACGATGTTGATTTGT
TTTGTTTCCATATTGACCTCCATGTATTTGCTATGATTTCAAAATCCATTTTTGACGTGCCTTAGGGTTGAGTGG
ATGCATAATTTCATTTGTTACTGGATTGATGAGCTTTTTTACTTTCTTTTTATGAGGTTTTAACATTTCCATCACT
TGTTCGACACGGTCGATAACAACTGGTCGCTTCGCATAGGCACCATAAGCAAGAATCACTGTGTCACTTTCA
CTAATCGCTTTCATCAAATGAATATCAGTGTGCTCATCGTATGGATTTTTGATATGTTTGAGGTTTTCGGGTGTT
CTAATATTTGAAAATAGATTTACAAGATATACAGCACCATATTGTTCAGTATTAGCTAATTGATTGAGGATAAGA
ACAGTTGTGAGATCGAGTGATAATACACCATCTAAATGAGGGTACATCGTTATCACTGTACAAGCGGGTTTCT
TTTCATCCCATGTTTTCTTGAGTAAATAGCGGTGTTGTTCATCATCGCTAAATATGGCTTCTGTGTTTATCGTAC
TTTTGATTGTATTCATCATCGTCACTCCTTTTAGTATTCTTCTGGTAAAAGCATCACATAATAAAAAACGTCTAC
ATCATCTTCTCGAATGACGTAGACTTTCTTAGGTAATGCATTTTGATTTTTTACATAGTTTGTATAGTGATATTCC
AATTTGTATGCGGGTTGTTCTTGTTCATGTGTGATTGAGAGTATATTTTCATCTTCTTGTAGTTTAAAAATGTGT
AGGTAATCTGTATTAAGTTGATTGTCTCGATCTTTTACCATATTCCAAAGTAGGATTTGAAGGTCTAGAGATAG
TTGTTTACTAATGCCTTTTGTGATGTATCGATTGATTTTCATACTATTTTCCTCCATTTTGCTTTTCTTTCATGATG
TCAATTACTTCGTTAATAACTGTAACAGATAGTTGAGCGATCTTGATCCAGTTCATATTATTCTCCTCCTTTTTGT
AATAGATGACGTTCATCGATAATTGTATTTTTAGTATCTGTGAGGTATAGAAAGTCCATATCAAAATGATCCAG
ATAACCAATACTGATGAGTTGGTTATTGGCGTACATAAGAAATGGATAGATACTTAGCTCATGTAGTTCATCGT
TATAGTAGGTATAAGTTTCAAGTGTAAGATGCGCAAGTGGGGTATGGTCTACAAAGCGAGTAGGAAGTATAT
TTTCTGCTGCTTCTTCTAACGCTTCACATTCCCATGTTTCATTGTTAGATAGTTGGAATAGACGAGTTATATATT

GTTTGAGTTCTTGAGTGGTTGTTTTCATATCATTGCCTCCTAGATAGTGTTAGCGATGTATTTCATATACATCAC
TGAGATAATATATATTTGATTTATCATTTATTACGAATCCCGGTGGGAATAAGAGAAAATCCCATATGAAAAACT
GCTACAAACATTGATATGACAAGAAAATCCCGTTATCCCGCTCATTTGTTGGGAAATATAGGTATATATTTATAT
ATTATTTGGATTTGGTGGTGTTATTACGACTTACTTTTTCTTTTTATATTATTTATTTATAAATAATAACGGGATTT
TGGGATTACGCTTGCGTAATCCTTCTCCTACATGAAGTTAGCCAATCCCGTTACTATCCCACTTATATTTTGTTT
TGGGATGTTCTTTCGTCATATCAATATAAGCTTCACATTGAGCCATGATTTGTTAGCGTGTTGAATATATTTGTT
AGATGAGTGTTATTGAATGACGAAAGGACAGTACATAGATATTATATTTGAAAGTAGAGTGTTTGAGGCAAA
AACAAAGACGAAGTGCTGAGGAGCACTTCGTCTAGATTGTTATTATTAAAAAGTTGTTTAATAATTTCATTATT
GAGCTTGAGAGTGACATAGAATTGTTTTTTATGATTCTCGTCTTTACGAATATCAATACGGTCAATAACCGTTA
GGTATAATGCTTTGAGCTGTGATTTATCCATGGATTCTATATTTTGAAATGTTCGTTGTAATAGGGCAGCGATT
TGTTTCGTATCATAAGATGGTTTCTCTTGATTTTGTTGCTGCTTGAGTTGATTCATTTGGTTTGTAATATCATTC
AGTTTTGTTTCATATTGATGAATGGTTGGTTTGAGTGCAGATGTTAAGTCTGGATTGTCTTCGATGGTTTGAA
CGAGATTTTTAAGTTTGATGTTTACTTCATCAAATTGTTGTTGTTTATAAGCAATATCATGATTGAGTGCAGCTA
CATCGACTTGATTATCTTGATTAACACGTTCGACAACTTGTTTGAGAACTTTATCGCTTTTGACAATTTCAAGT
ATTTGATCCATAACGTATTTCTCAATGACATCGGCTCTAACGCTATTCGCAGAACATACTTTTGAGCCTTTGTTT
CGGAAGTTACTACAAGAATAATAACGTATTCGCTTCTTAGTACCATCTTTCAATGTGTTCGTTGTGTTACTAGC
TGCCATTGGTGCACCACATTGTGGGCAGTGAATAATACCTGTTAATAGATTGGTTCCTTTCCCATGAACTTGA
GGTTTTTGACTGACCTGTTTTTTGCGTGATTGGACTTTATCCCATAATTCTTGACTAATAATAGGAGGGTGTTT
ACCATCAGCTATAATAGGTTTATCATTTAGTCCTTTACGACGCTTTTCATTCCAATCTTTGTACTTCGCAAATTG
AATTTTACCAATGTAGAATGGATTGACTAAGATATAGGTTATTGAATTGATACTAAATGGATTGCCTTTTTTAGT
CACATAGCCTTTGTGATTGAGTGCATTGGCTATTTTACGATAACCATGACCTTTGGCATAAGATTCAAAGATAT
ATTTAACGATGTTAGCTTCATGTTGATTAATCATTAATTCTTTTTTATTATCAGGTATGTTATTATATCCTAATGGA
AGATTGCCTTGATAATAGCCCTTTAAAGCTCTTTGATGTTGCCCGTTGTAAATATTCTCTAAAATTGTATTTCTT
TCAAATTCGGAAAAACTTGCAAGCATCTGGAGCATTAACTTGCCTGTTGAATTTTTGACTTCCATACGTTCAG
ACAAGCTAAAAAATTCAACATTTTGACGATGAAGTTCTTCGACGATTGTAAGTAAATCGGAAGTATTACGTGC
CAAACGATTTGTTTTATAAACCATAACACAGTCTAATTTTCCATTTTTAGCATCATTTAACATGCGCTGTAATTC
AGGACGGTTCATAGATTTTCCTGATATACCCCGATCTGCATATATATCAACGAGTTCATAGCCGTTGAATTGGC
AATATTGCTCTATTTGTGTAATTTGTCCCTCGATGCTATAACCCTCTACTTGTCTCTCTGTGGACACGCGAATGT
AGCCTCCTATAAGCTTTTTCTTCATTTTATCCATTATAATCCATCCTTTCTTTAATTATACAATTGATAATTTCATA
GTCTGCTTTACAATATTCAGTGGTTCGTTTTTGAAATAGATGCCAGCGAGGCTTTTATTTTTAGAAATATTAAT
TTCATCAATATAGGGGTACAACATGTTTAGCGTGAAACGTTGTTGAATGATGTTTTGGAAAGCTTTTCGAATT
TGATACGCACTGATTGATGATATAGGTTTTGATTGTTGATGTAATGAGTGCGTTTGTTCTTTGAACGTTTCTGC
ATCAATTTTGCCTTGGGCTAATTTTTCTATCAGCTGCTCGTGATTGAGTGTAGTTTTAATTTCTATATCTCTTTGT
CTTTTGAGTCGTTGTTGAATAGTATGGTTTATTTTTGAATAGAGCTGTTGATTTTGAAAGAAGTCCTGACAAG
TCGCTAAAACACTTGTTTCTAATTCTTGTGCGTTGATTCCTTTAAATTCACAAACAAAGCGAGCGTTATTCATA
TTTTGCGGACAGACATAGTAACGCAATGAATGATCTGGTTTTCGGACAGTCATGTTTGTGAGTGTCGAATGA
CAATAGGGACATTTGATTTTTTGTTTGAGTTGATTTTCTGACGGCTTACATTTTACTTGTTTCTGAGTTCGGGT
AACTTGAGCTTCTTCGTATATCGTTGTACTGACAATAGCTGGGAACATGTTTTCATATTGTCCGTATTGATTGAT
AACACGGCCACAGTAATTAGGATTGAGGATAATATTACGCACTTGATAAGGCTTGCGATTAATGAAGTTATCA
TCAGCTTCTAAGTATTGCGCAATTTTTTTATAACCATAACCTTGAAGGTAATAATTGAACACAGCTTTTACTGT
TGGTGACTTTACTGTGTCTATCGTGAAAGTACCATTATGATAGTGATACCCAAAGGGCGCATGTGTTGTAATTA
GTTTACCTTGTCTCGCTTTTTCTTTGATTCCATTTTTGACTTGTTCGCCTATATTATCAGATTCTAGTTCGGCCA
AGCTGATGAAAATATTGAGTTTGAGCCGATCGAATGCTTTATCCATGTCGAAGTAGCCATCATGAACGCTTAA
GATATGAACATGATACGTTTGACATAATTTCATGAGCTTTAATGCATTTTTTAGATTACGATGAAGTCGATTTAG

ACGATAGCAACATAATACGTCACATTGTCCTTGTTGAATCAGTTCAGTAATTTGTTGATAACCATTCCGCTTATC
AGTACGTCCTGATTGTTTATCGCTATAAAAGGTAATGTGTTGAATATTATGTTTTTCGGCTAATGCCTTGATGGT
TTGTTTTTGTGCTGCTAAGGATTGTTGCTTTGTAGTGCTCTGTCGTAAGTAACCTATTGCTTGTTTCATAGTATT
TCCTCCTTCCAAAGTGATAATATATATTTATGAACGAATTTATAGATGAGCCCAACACCTGCTGGTGTTGGGCG
TTATTATTAGTCATCAGCATGATTAATTTCTTCAACAACTAAATCAGCTAGTAATTCAATCAATTCATCCATTTTA
TCTAATCCTGTACGATTGCATCTTTAAGTTATTAAAAATCAATTAAATCATCATTCTGTTTTTTCCATGATTCAAG
CATCTTTTTGTTATCCAAGTTAATTTTAGCTTTAATAGGTTCAGCATCTTTAGTTAGACCAAAAATAGACGCATA
TTCTGAATCTAACTTTAAATGGTAAAAAACAAGTGACTGTTTCTTGCCATAGTCATCTTTTACTGTTCTTTTTGT
CGTGATTCGATCACGGTCAGATTCGATAAATCCTTTATCTCTTAGTGCATTCACGACATTGTTGACATCCTGAA
AGTGTTGTTCTTTCAGCATATCTTTAAATACAGTCGCAATGATTTTGACCTCGATATAATCATCTTTTAAGGCAA
TTAGTCCATAATTCTCAATCATATTCTTTAACGCTGTGTCATCAGAAAACTTACCACGGTTTTGTGCTACAAATT
GTGTAATGACTTCAATCGCTTTATCTGCCAGTGATCGTTCAGAGACTGTATGAGTATGATAATCAATAAAGTAG
TCTCTTATTTTAGCGATATCAATATTTGTAGCTAAAACACGACCTAATATTTGCGCAGATGTTGTAATGACTGCA
TAACGCTTAAACATACGAATACCTGTATTGTTTGCTTCACTCTTCAATTTAGCTTTAAACCAATGATGTTCATCT
TTAAACCATTGAATAACTTCCTCTTCACGATTTATAAGGTATTGAGCTACTAACGGTAAAACATGACCATAGTT
TAGCGCCACAGCTTTTTTAATATTGTCAGCATTGGTCGCATTTGTAGTAAATTGCTCATTAATTTCAATGGTTCT
TACACGTAACCCATCGTTTTGAGCTGAATCAGTAAAAATACTGTATTCTGACGTTGAAATGACAGAAGTTCCC
CAATTCTTAGGTGTTTTAACTTCTCCATGAACGTTTGAACGTTGACGCCCTTGACCTTCAGCGATGGAGTACA
ATAACCCTGTTGTATCTTTAAAAGTTGCTGAGGAGAGCTCGTCAAATACCATAGGTATGCCATAATTGTTACTC
AAGTAACCTTCAAGAGCGTTACGTGTGCCATTCCAACTTCGAAAAAGCGTTTTATTACCTTTGGTTGGATTTC
CAGCGACGGATACCGCTAAAGCTGCCGCTGTTGATTTACCGGTTGATGACTGGCCTGTAAAACTAAAGATGA
TACCAGCAAATTCAACCTCATGTTTATGCTTCAGAAAGGCTGTCACTAAGGCAGAAATTCCAAATATGACCGC
TAACTCTAGAAGAAGATGACCTTTGACTTCGTTCATATACATATTTAACCAATCTTTAAATGTCCCTTTAGGCTC
TAATAGATAAGCACTATCGACAATAGGCTCTAAAGATGCTAACTGATCATATTGTTTAGATGTGTAAACGGTAT
CTACCATTACAATGTAACCGTGGGGTGTTTCTATGATGCCCGAGCCATCATATAAATCAGAAGTGGGCAGTTC
ATCGCGCATTAACTGTAGTGCATAGCTCAAATCTCTTATATAACTTTCATTGATACTGTGACCATATTTAATCAA
GGAAGGTAAGTTTCGAGTTGTTAAAATATCAGATTCAAATGTATGTTCTTTATCCTTACCGTTAGAAATAATTAT
TTTTTCTGTATTTTCTGAGGCATGCCAAAATTTAGCTTCAACAACGATACAACTAGATATAGGCACTACTTTTT
CCTTATCTCCATCTTTTTTAGGTGGAAAAGTTTTACGCCACGCCTTTGCATCTAATTGGTATGGACCTGCTTCA
AAAATGATGTAATGACTCATTAGCGAACACCTCCTTTCGAAGGGTTGCTATCTTGGAGTGGATTAGGTCCGA
CTTTCATATATACTAAATGACCATTGGTATTTTTACCGATAATAATAAATGGAACACTTGGCGCATTTTTAACAA
AATATGCGAACCAACGTCCAACATTTTGTTGTACAGCTTTTGAGCATTGTACATTTGCACGACGGTTCAAGTC
ATGGAAAGTAAATACTTGTCCCTCCGGTAAAACGAATCCAATTCCTAAAATTCTTCTTTTCAACAGTTTAAAAT
CATCTTCTTGCTCGTAAGTCATTTGGATCATCCTTTGTATATTAGTCATCATTTAAATGATGCCTTTGTATCTTATA
TCTACACTGAGATATAAAGTTCTAAATTTGAGAGGAAAATATTAGGACATTGGTATTACACCTTTTGTTAATTA
AAAAACCCAACGATAAAAAAATCGTTGGGTTTAAAAAGGTATGTTAATAATATAATTTAGATGCACTATTCAAT
TTTTTTAAGCGTAATTTATAAAAATCATTAATTACCTGAATCATTAATTTATCAAGAATACCATTGTAATCGTTAT
TAAAAGTCTCCCTACTATATATTTCACTAATATTATAATCATTAAGGTTTGAAATTAATGAATCTTCTATTTTCATT
GTTAGTTCTGGGTGCTGTTCAATTGTATTGCGAACTAATATTTTTAATTGAGTATTTCTTACATAACCATTAATA
AGACTTTCGAAACGAATTAAATTTTCTTCGTGTTTATCTATTATTGTATTCAACTCATTTTTTAAACTATTCAAAC
TGTGTAAGGCAATTAATATATCGTATTGAGATGCTTCTTCGATAATACGACTTGAAACATTGACTACTTTATCAA
TTAAATCAGTTATAGCAGTGAATAATGTAAAATGAAATGTATCCTCAATTGTTGTTAAATAAAATAACTCATTAT
TACTAATTAACTTATTTTTCTTCATAAATTTATCTATATTATTTTTAGATTTAATAATATCGTCTATTGTTAAATTAAC
TTTACGTGAACTTTTTTTCATAATACTACGGATAAATTTGAAGTAAGTATCATTATGATCGTATAGATTAAGAGA

AATACCAATTACAGCCTGTCTAAATTGATCTATTTCATCAGATTGATTTAAGCTATTATTATTGTGAGATGAAGG
TTTGATACTATTAGCTATTTCAAAGTATTGAGAGTTACTCTTTCTTTCTCTAATTAAACCTTCGATTACTAACTTT
TTAAAATACTGAGATTTATTTATACTTGAGATATCTTTTTTTACATACGAATCTTGTTTTTCTTTATAAATGAGGT
ATCTTGTTAAAAAGTCGCTTATTATAATATCATCAACATTTTTTAACGATAAATCTCGGTGTTGTTTTAATATCGT
ATGCTTTAAATCCTTAAAGCTTTCTAAAGAAGAGACTTGATCTGCATTTTTCATATAACTTACTTGCTGTTTAAT
TTGCTCTAACTCAATATTTTTTCGAATTTCTTTCATAGCTTTGTTAGCTATTTTTTTAGCGAATTCTGTTGTATGG
TTGATTGTTTCTAAGTAATCATTAAAATATGTTTCTAGGTCATTTAAATCTAATATATTCAAAATGGCTGTACCTT
GATCAATAAACTCATTTAATAAGTCTTTAATATCAATTTTACTTTGACTATCAATAAAATAATACATTTTATTTTTT
ATAAAATGTTTAGGGATTTCATCTATAAAAAAACCAGTTTTGTCACATTCCACAGTATGTTGCTCCACAGTTTG
TTCATCCTGATTTATAAAATAACTTACATATATAGTTTCGCTAGGTTGGGCTTTTCCACATATAAAATTTGGTCG
GTATTCTTTAGGTAAATAATCCTCTAAGTATTCTTCTAATATAAATTCTGAATCAATTTTATGTATAAAATAACTAA
AATTCCGAAACCACATTTCTATTTTCCATTTATTTTTAATCATAGTATTACTCACTTTCAATAATTAAATGTATAGA
CATAATGCTAGTTTTATTTTTAAGATATTTCAATAATGTTTACTAATTTTTCCCTATAAGATAAGATATACAATATA
AAAGGGAACAAACGTTCCTATTGGAGGGGTCCTATGAAAAGGTCAGTCGAGTCTTTCAGTGAGTTACATCA
AGTTTTAAAGAATTATAGAGATAATAGCTTATGGTTATTTAGAGGTCATAGTAATTTGAATTGGAGTTTAGTTC
CGAAAGCTGGTCGAGCACCTTATGATAAATATGATGATGAAGAAACATTTTATGCTTGGAAACGTCAAGCAA
TGATATTTTTAAAAGAGACTTATAGTGATTGGGATATGCTAACTATAGCGCAACATCACGGATTAGCTACTAGA
CTATTGGATTGGAGTTTTAATCCTTTAGTTGCTGCCTTTTTTGCGGTGCAAACATATGAGGATTGTGATGCTGT
TATTTATGCCTATTTAAAAAAATCGGATATGAATATGAAAGGGGCTACGCCTTTTAAGAGTAAATGGATAAAA
AAAGTAAAGCCTAACGGTCTAGCTTCGAGAGTGGTTAGACAAAGTGGAATTTTTACTATTCATGAGCCTCCT
ACAAATTCTATAGAAGCTAATCTTTCAGAGAAAGAAGAACTACACAAAATCATCATAAAGAAACATTATCGCA
AAAAATTAAGAGAAGAGTTATCTTATTATGGTATCAACGAATTATCATTATTTCCTGACTTAGATGGTTTATCTA
GCCATGTTAATTGGATAATGGAGATAAATCCATACGCTTCAAATAATATAACGCAAGAATTAAATTCAATTAAT
GAATAAGATTATAAAAAGCGAGGTATTATATGTCAGTATTAGAAACTAAATTAAAAAGTCAAATGTCAAAATCT
GCAAAAATAGCTAGAAATATGAATAAGTTACCAGATGAAATTGATAGATTAAGAAAAAGGATAGAAAGAATA
AATAAAAAAAGAAAACCTACTTCCAGTAATATTAGAGATTTGGAAAAATCTAATAAGCAATTAGTTACAAAGC
AGCAAAAACTTGCAGATTTACAAGTTGAGTATACTAAGATTGAGAAAAAAATTAATGAAACAAAAATTAATC
TTCAAAAAGAGCAGTCTAGGAATCAAAAGAAATTATCCAGTATGTTAGATAAAAATACAAAAGGAAATGAAG
AGATTATGGAAAAATTATTAACTAATTCAGATCAAATAAATGAGATATCAAATCAGATAAAAAAAGCAGTGAA
TCAAAAAGAGATAATTGAATATGATGTCTTTCTATCCCATTCTAGTTTAGATAAAGAAGATTATGTATCTAAGAT
AAGTGAAAAACTAATAGAAAAAGGACTAAAAGTCTTTGAAGATGTTAAAGTTTTTGAAATCGGAAAAAGTC
AAACAGAAACTATGAATATGGGTATACTAAACTCTCGGTTTGTTGTAGTATTTTTATCCCCTAATTTTATTGAAA
GTGGTTGGAGCAGGTACGAATTTTTATCCTTTCTAAACAGAGAAATAAATGAAGAGCATGTTATTATATTACC
TATTTGGCATAAAGTGAGTGTTGAAGATGTACGTGCATACAACCCATATTTAGTAGATAAGTATGCATTGAATA
CAAGTGATTTTAGTATAGAAGAAATAGTAGAAAAAATTTATCAAGTAATCGTTAATTCTAAGAATTAGTAATAG
TATTGGTTATCTACTTTAAAAGCAAAGAGGGAATTAAATGAAAAAAGCAGTGTTTTTATTACTAAATGAGTAT
GCAGAATGGTAAGCAAGCTACTGTTCCTCTATTTTAAACCAAAGCAGTGAATGGAAAATAGAAACAACTTCA
ATTTATGAATATTTTACCTCGATTGGTGGATTTACAATTAAAGTAGTAATAGTTATCCAATATTTTCTAGTTGATT
CTCTTATAATATGTCCAATTAAGTGTAGATGATTCAAGATGATATTGAGGGAAGGATGAGAATGTGTCTTTCAG
GTAGCTCATTTGATGATAACCTTAAACATAATATTTACTTTCATCCTGCTGAAAAAGAGGTTACAGTTTCAAGC
CGATAGAATACATTGGGTTATATAAAGACAAAGCAATAAAAGCTATCGGAAAAGTAGACAAAGTAATAGTGA
CGGAAATAAATGAGAGCTCATTATCCTTGAAGACAGTTTATCCTGTAGGTACTGAGTTATCGATTGATGAGTAT
GAAATAGTTAAAAATAAAATAATGGTGCTTGGGAAAGAAAAGTGGACTAATCTATTGAACGAACCCCATTATT
ATTACTTAATTGAAGATTTTATAGAGACAGACTACAAGAAAACATCTAAAGGTGGATTGATGGGAGTTAAGT

ATTTTAATGTGAATGAAATTTTGAATAGGGATTGCTTAACCACCGAACAAATCGCTAAAGAATTATGTAATAAA
GATTGGGAATGATAAATAGTGAATGAAGCTATAATGCAATAATGTATGTATAACATAATCAAGTAATGAATTTA
ATGATAAGAGAGTATGGAGTAAAGAATTGAATTAGTTTGCATGGTCTATGTATCTTAAAGGTGTTGAAGGAG
ATACACCGAAGTATGCAGTACCAATTCTTGAAACAGACTTTAGTGATTTACCATCTACACATACGACAGTCGG
ATCACTTGATCGGTTTCGAGATAAGACAATGAGCATTAACAGAAGCGGGAGTAACTGTTGAATTCCATTTATA
TCCAGTTAGATACTAGAGTTTGAAGTGATAAATCTTGATTCGGATGCTGAAATAAGTAAAAGAGCAGTTACTG
AATATAATATAAGTATAAATATGCCACTAGATATTTTAAGGAGTATTTATTCACTTTTATAAAATCATCTGCTAAG
ATAGTATGGAAGATTTTTATAGGAGCTGATTATAATGCGACATATAAATATGTCATACATCATACGCTATAGTACT
CTAACTCAAAGATAAGAAGTACTTAGTTATGAATAGCATTCATTTCACAAAGTACTTCAACAAAAATGGAGGA
ATATGAAATGAATAAAATAGAAGTGTATAAGTTTGTTAAAGTAAAGCAGTTAGTATATCAATTGATTAAGTTAT
ATCGTACAAACGATATGAATTCCCATAAAACACAAAAAGATTTTTTACTAAATGAAATTAATGATATCTTTAAA
GAAAAAGATATTGATATCTCGGACTTTATTACATCGATTGACGATGTAAAATTAACTAAGAAAAAAGCAGAAC
ATCTTTTAAATGAATTAAAAGTGTACATCCAAGATTTTGAAATACCTTCATCAAGTCAACTGGAGAAAATTTTT
CGTAAAGTAAAAAAATTAAAGAGACCAGATATAAATTTAATTGATACAAAAGAAATTTCATATTTAGGATGGA
ATGATAATTCTTCTAACCGAAAATATATCGTTTATAAAAATTTAGATGATAAATTCGAAGGTATATATGGCGAAA
TTTCACCAAATAAAGTAAAAGGATTCTGTAAAATTTGTAATCAGGAATCTGATACATCACTCTTTCTCAATAAA
ACTAAACATAATAAGAGTAGTGGAACATATACTAAAAAAGGAGATTACATTTGTTATGACAGTTTTAAATGTA
ATCAGAACCTAGATGATATAAATAATCTTTACGAATTTATTGTTAAAATAAAATAGATCCAAACAGCCCTGATCT
TTAGAACTAATGATCAGGGTTGTTATATTTTGTACCTCACTCTTAATCGATACGCTGAAAGTCCCTATAAATAGA
ACGTACGTTCCGTTTGTAGTATAATGACAAAAATATTAAATTAGAGGATTAAAAATGGGATTATAATTTTTTAAT
GTACCAAACTGAAAAAGGCTAAATAAAGATAAATATAAGATTAGAAAAAACTGTATGGATTAGGCAATATTTA
TATATAATGAAATGAATTAACAGAAAATCGAATTATCCGGAAAAACCGTACTGTTCGATTGGAAGGGAATAGA
GAAGGTGAAAAGAATTAACTTCAATTATCATCTTATTTCTGAAAAAAGATATGAAAAACGGACGCAAGACAC
TTTAAATATTAGTAACAACGTAAAGTGAATAAAATCTATATTAAGGCATCGTGATGAATTTAATATAATTGATAA
AAATGATTAATTATCCATTTTATTTATAATATATTATTTTTAGCAATTGTATGTATATTTTTCTTGATTTACTCTAATA
GTACAGAAACTATAAACAACAATCTTCTAAAATACGCTGAAAGTTATCATAAGTGATGGTAAAAAAATGAGTA
AGTAGATGAAGAGTGAAAATCAGATTAATTAATAATAATGTATCAAATTTAAATAAAGGGGGTCTTTAAGTAT
GAATTTAAGAGGTCATGAAAATAGACTTAAATTTCTTGAGAAATATGATGTGACACCTATATCACATTTAAAAT
TATTAGAAGGTCAAAAGAAAGACGGTGAAGGCGGTAGACTGACAGATAGCTATTATTGTTTTTCATACAGTT
TAAAAGATAATTCTAAAAAAGTTTTAGGTACGTTTAATTGTGGTTATCATATTGCTGAAGATTTACTAAAATTAT
CAAATCAAGACAAATTACCTTTATTTAACCCGTTTAAAGTAATTAATAAAGGTAATCAATTGCAAGGCGTAAC
GAATAAAGGTAATTTAGATATTAATAGGCAAAGAAAACAGTATAATGAAGTGGCTTTACAGCTTTCAAACGCT
ATTAATTTAATCATAATTTGTTATGAGGGTAATATTAAAGAACCACTTTCAACGATAAAATACGAAACCGATAA
ATATTATTATAGTGAACCATATGATAGTAAAATTAAAGCAGTAAATACTATTATAGGTAATTTGTTTGATAAGAA
ATTAGTTGAGAAGATATCTGAAATTAATGTAAATCAGGAAATTAGAAAATTTGATTTTTCATTACTCACTAATA
AAATTAAAAAATATGAGATACAAAATAATTTTGAATAAGTGGAGGTAAGTCAATATTAAATATAGAAGCTAGTA
TTAAGGATATTCAAGATATTATAAATTATTTTATTAAATGTGACGTTAATAATAAGTATTATGAAAGTAAGCAAA
GAAGCAGTCTCGAAAAGTTAGCTTTAAAATTGGATAAAGTAAGTAATCAGTTAAACAATGAAAACATTGAAG
GTATGAAAAAGATAATTGAAAAAGTAGCAGAAGCAAATAGAGTTATCGCTAAATCTTCTTTAAGAAAAAATT
GAGTGTTTTAGTAAGATGATTGAAGTTGATAATAAATCTACTAATCAAAAGAAATTTTAAATATATATTGAGTTA
AAACTTGAAAAACTTATATCTAAATGAAGAAACGGGAAACCATTCTGAATCCTCACCCTTTCCCGTTCCAAAA
TCATTGATAATGAAAAACATCGCTTATACGGGGTATTGCATATTTTGCCTTAAGTTCAAATACTATACATATAATA
GAATCACAACTAAAAGCACGTCATAACTGTACTGTTCATTCTGTACAGTAGGTATTAAACTATTAAATAGGTAT
AAGATTAAACGAAAGCCAAGATGCATAATGTCATGGCTTTTTATATTGAAAAAACATGCGATAATTTGTTCCC

AAATGTCCTTATAGTTGTACCAATATCTGTAATAGAACACTAGAAACTTGGTTTAACCTTTCCTTTTCGTAAATA
AATGTAAAGATATACCATAAGGATCTCTTACATAACCATAACCTTCAGTATAGAACTCTGGGCTGAATGCTTTT
AATACTTCACTGCCTTTTTCTATTAACTGGTCATACACACGCTTAGTTTCTTCTACATGGTCAAAAGTAATACAA
AGCGATATATTATTACCTTGGGTTATAGGTAAACCTTCGGTATCATCCGCAATCATAATTTTTATATCTCCAAATT
GAAGTACACATTGATCAATTTTATTTAAATCTTTTTTATCGATATTAAGTTGCTTATCTGTCGGTCTATCCTTGAT
ACGTTGGATATACAGTGTTTTAGCGCCAAACAGCTCTTCATACAACCTTTTCAAACCATCTGCATTTTCTATAAT
TAAAAAATAGGGACTTACTTGAAAATTCATAGTTTTTCCTCCTTAAATTTGTTATAAATAAGTATATACTGTATTA
GTGTCACCTATTGATACTAAAAGGGGAATAATAATGAAAAAATCTGTTAGATTATATCATATGATTGAATACTGT
AATGAAAAAAGGAATTTCAAGTTAAATGATTTAATGTCAGAATTTAATATTTCTCGTAGTACTGCTTTAAGGG
ATATAAAGGAAATTGAAGCATTAGGAGTGCCTTTATATAGTAGTACAGGGAAAAATGGCGGTTATATGACCAT
AGGTAATCGAAACCAAACGAAAATAGCAATCACAGACGAAGAATTGAAAGCTTTAGTATTTACACTTTCAAG
TATCTCCAATGTTAGTAAGTTACCTTTTCAAACAGAATATCAAGAAATATTAAAAAAGTTATATAATAATTCGAA
TAAAAAAGAATTGATAAACCAGTATAATGAGATATTTCAATATTTTAATGAAGATACATATCAGTTCAAAAGTT
ATAAGATATTTAATGAAATCATTAGATTAACAATTGGTAGTAAGTCATTTAAAATCTGTTATTCACAAAACTATT
TGAAAGAACAATATAAAGGTATTGGTATCTTATATAAAAATCAACAATGGTACTTCGTTGTTGTTAATATAGCAT
CAAAATTAGTAAAGTTGTTAAACATTTCTAAAATAAAAGAAGTATATGAAATGGGAGAAACTAAAGAATGTA
ATGAAATAACTATGCAGAATTTTCAACAGTTTATGGTTGAAAACGAAAATACGATTGATATACAAATTAGAAG
TAATATTATTGGATTAAATATCTTGAAAGGATACTTGTGGAGTGACTATATGATTGAAAATATTAACGAAGAGA
CATATTTGTTTAAATCAAAAGTGAACATGCAAGATATAAATTTTATAGCTAAATTAATAGTCACATGTGGTGCCA
ATGTCAAAGTAGAATTTCCTATTAGTTTGAAAAAAGCTATTAAAACTGAACTAACCAAAATAATAAACTTATAC
TAATGGTAATTTAATTATGATTGCAATAACAAAGAAAAGATAATCTTAAACATATTAAATTGATCCGTAGTTTAA
AAACCGCATCACTAACCGAGACGCAGAGGCGTATCATAAGT
>Staphylococcus aureus strain ER09654.3
GAGGCTTATCATAAATAAAACTAAAAATTAGATTGTGTATAATTTAAAAATTTAATGAGATGTGGAGGAATTAC
ATATATGAAATATTGGAGTATACCTTGCAATATCATACGATGTTTATAGAGTGTTTAATAAACCATTTTTCAACTA
TTGATGATCTAGAATATATAATAACTGTACAAATTATATTGATTATGGAACTACAATTCAATTAAGAAATTGATGA
TGAAATTTTAAATTTAAACTAATGGAATCAAGAAAGAATGAAAGGAAATATACAATGCCCACGATTAATAAAA
GGAAGTTTATTAGATTTTGTGTTAGAAACAGTAGATGTTTTTGTAAATGGAAGTAAAAATGATTGCGAATTAG
TAAATTTTAATTATAAAGAAGGAAAAGTAGTTTCACGTTATAGTACAAAATTTGAAAGCAATCCTAAATTAAG
AAAAAAGCAATTGATATCGACGGTAAAAGTTGTAAGGTATGTTATTTTTGATTTTGAGAAAATTTTATGGGGC
ATAGGGAAAGTTTTATTGAAGTTCATCATATTAAGGCAATGTTTACTATAAGAAAGTTGAAATACACTCAGAA
ACAGACTGGATTCCCGTTTTTTTCAAATTGGCACAAAATGATACATAGGCCCACCTATATATTGAAGATTTAAG
TTAAACGATAAATAATATTTTTCTCTTTTGTATAGAAATTTTTTTGGGAATGATATTAATTAGTATAAAAATGAAT
ATGTCTACAAAGAAGGAGAGATTTTATTGAAACACATTAATCAATTTTCTGAATCGACACTAGCACAACTAGA
AAAAGATCAACAACACGTATTTTATGTATACTGTTTGATGGACCCAAGAAATGATGAATGCTTTTATGTTGGTA
AAGGTAAGGGGAATAGAATATTTAAACATAAACAAGATGCGCAGAAAAAAGTATTGTACGAAGATATATTTAT
AGAAGAAAATAAAGATAACTTGAAATTTAATAGAATTAACGAAATTTGTAGCAATGATTTGAATGTTTTAGGA
TATATAATCAGTTATGGATTAACCGAATCTGAAGCATTTTCTGCAGAAAATGTTCTTATAAACTTTCTTAACCTA
ACAAATAAAACTACATTAACAAATATGATTAACGGTCATGGATCTAAGGCATACTTAGTTGAAGATTTAGAAA
ATGAATTTGGCTACGATTCAATTAATCTTGAAAACATAAATACGAATGAATTAATTTTAGCAGTGAAAATCAGA
GATGCATTTCTATTAGATAAGGATGAAAGTAAAGAGTATCCTATTAATGAAAGTAAACGTGATAGAAATAACC
TTAAATCGCGTACATTAGGTAGTTGGATAATAGGAAAAGATAAAATACATAAAATAAAATATATTATTGGTATCA
ATACAGGTGCTAATAATGCAGTTGTCTCAGCTTATGAAGTATCATTTGAACAAGCGGAGAGTATCGAAACAA
ATAATGGTAGAATGAGATATGCGTTTATTGGGCTTTCAGAAAGAGATGCTACTCTAAAAAAATTGAATTTATAC

AAAAAAGCACTACCAGATTTAAGATTTGGTAGTGGTAGCGCTACAGCATATATAAATAATGGGACAATGAAA
GTTGATTAAATGAATAAAAACATCCAATTCCTTAAATTAGCCTTTGAAAATTTTATACTCGTATATATAAGTGAA
AAACTTAAAAGAATTAGATGTTAATTAAAGGAGAGAATGCTATGCCATCAGATGATATTGTAAAAAAACTATA
CTCAAAAGAGGATATTCGATTAGAATTAGGACTTACTCCGCATAAGTTTAATAAAAAAATGGAAACTATTGCT
AAGCTTTTTAAAATTGATATGAAAATTTTTCACAATTACAAAGGCCAAGATAAAAATAATCAGTATACATTTAA
TGGTGTCGCAAAAGAATTAATCAAAGTGTTGCTAAAAAGTGTTGATTATTACCCAGTGGATATCAATTCCAAA
AAATTTAAACAAAATGGAAAATCTAAAAAAGAAATGATTGAAAATATAGATAACTCTAGTTACATGAAATATAT
TTATCAGTTAATGAAATCTATCAATGAAATTCAATACAAAAGGTTAATTGCTGATATACATATGAAAGATGTGTA
CCAGAATACAAAAGCATGGTTAAATAATGGTGAATCAATTAATAAAAAAGAACAAGAGCTATACCAATATATG
ACAATTTTACCATTACATAAAAGAGTTGAACTGCAAAATGAGGTATTAAAATCTATAGACGAAACAATTTTTC
AATTTCTCGCAAAAGAACATAGAAATAATCAAATTGAAGAAAATAATGAATTAGAAGCATATACAAAAGCAAT
AAAAGAAGGTAGAAATCCTAAAAATGATTATGAATTAAATCATCTCCTATATAAAAAAAATCAATTACCCTTAG
ATAGTCTAATAGAAGATATATGGGACTATGAGGAAACAGAGTATACTGAGCTTGATTGGTTAATTGCAGATAT
GCTAAAACGTTCACAAAGAGCTGATAGTAATTTTATCGAAAGTTTAGAGAAGAAAAACAAATTGAGAAAAA
ATATTCATAAAGATTCCATTAAAAGTATATCTAAGTTGATTGATCATGAATTAGTTAAAAATAATAGAAAGTGG
GACGTAGCAGAATTTTATAAAAATCAACAATTTTGGAATAATAGTACATTAAGACGTAGCTCTCAAAATTTTG
GAATTTTATATTACAAAAAGAATAGGTTACATACAATGAATCAAAGTAATATTATTAAGAAAATAAAAATTATCG
AAGCTAATATAGAGAGTGCAAATAAACAGCCGAGCTATTTAGATTTCTTTTTACAAGCTAAGTTTGATTTAGA
GAGGTTAGAAAGTGAATTAATGAAATATAGCATTAATCCGAAATATTTTAATAAGATAAATAGTGATTACGTAA
GATATGCTATTGAAGATGTACATAATGCAATTATTAAAATTGACAGATATATAAGTAACGATAAAGTGAAGTTA
AACTATTCAAATGTCCAAATGGTAGTGAGAAACGAAATAACAGAACAAGGAAGCTACTTTGTAACCCAGGC
ATTGAATACACTGTCAAAGGTAGAAGAGTCTGGCTACAGCTTTGATAATTTCATGGTAGGACCTTTCGTAGAA
GACTTTAAAAAAGCAATTAAAAATCGTAGATTTGGTTCTAAAAAAGATTAAGATTGTTTTTTAGAGTTAAACA
TAAGAATAAATTTAAATAAAAAAATAAACCCAACAAACCTTGTTATATCAATGTTTGTTGGGTTTATTTTTTTG
GTGATTTTTTAATAACTCAATTACATCTTAGGATAATATCGCTAAATTATACATTGTCGAGGCCAAGACAAATAA
AAAATTAAAGCAAAAGTTAGCGCGCAACTTTCTGCTATAAAGGAGCAAATTTATATGGAGACAAGAGACAA
ACTGATGTCATTGACTCAAAGTGACAAAACACAGCAATGGCTAATGGACAAGTCATCTAACCAAGATGACAT
TCAACAATTGCAGCAACAATTCAGTCAGCAGCTTGATCAACAATATAATGCACTTTTAGCTGATGAAAAAGCT
AAGTTAGACCAATACGTGGAAGTACATCAAGGATTGGAATCATTAAAGGAAGAGATTGAATCAGAACCTATT
ACGCTTAATATCGATAAATTACCCGATATCAAAGCAACAATGCTTGAAAGAGCCAAGAATGATGAACATTCTG
ATAAAATCGAAAAGCTATTTGATAGGTTAGAACAGGCATTAAATGGTACGAATCGATTATATACGCAATTATCG
TTGATTGGTACACGAACACATCGAATTACAACTAAAAAATTTTAATCTTCAAGGCTTACCTAAAGCAGTCCAAC
ATACGATTTTACCTTCAAAATTTAAGAAGGTGTATACAGTCGATTTTAAATCGTTTGAACCATCAGTTGCAGCG
TACATGACTCAAGATTCAAAACTGATTGACTTGTTAAATCAGAAAGACGGACTGTATGACGCATTGCTAAGT
GAATTAGGCTTATCAGATGAGCTACGTGTATTTGTTAAACGTGCATTTATTGGTTCGTTTCTATTTGGAGGTAA
CTTCAAAAAATCCTAAATTCAAGCTGAATCAATATGTAAGTGAAGTACAATGGTTGGATGCGGTCAGCCAATTT
ACAAAAGTCATTGAACTTAAGAAGCACGTTGAAAAGAGTAATTCCATGCCTATGCCTTATAGTATTGAGCATG
ATATGAGCGCATTTCAAGGTAGCAGTATTATGGCAATCTACGTACAAACTGTAGCGAGTTATATTTTCAAGCAC
ATTCTGCTAAAAGTGTACAAAGCACAGTGCGATCAAAAAACGTTCAAGATTATAGTACCTATACACGATGCGA
TTATGATTGAATGTGAAGATGAAGAAATTGCACAAAATGTGGGTCAGTTAATGAAAGATACGGCTAACCAGT
TGTTCAATGGTGAATTTGCACATGTGACAGTGGAAGAAATAGGAGGCGTAGACCATGAATAATGATAGAGG
ACAAAGCCTACACATCCCAAGTAGTACACCAATCAAAGAAAATAATATATATGTAGCTACGTTACATTCTGTGA
TCCAAACAGATTTCTCAGGTGAAATAAAGCACCAATTCACGTATGAAATTGAAGTGAACAATCAGATTGTATA
TGCGAATCGTAATATTCCAACAAAACCGAGCGCTAATCAGTTGTCAATTCATGATTGGCTGAAACGTCATAGC

AACTATAGCGCAAGTCATGAAAACTATGAGCCTTATATTGATCAGAAACATTTAATTCTATTAGGTCAATATAAC
GGTAACTATTATGTACAAGATGTAGCATCGTTAGATGCGTTTGGAGGCGTATTATCATGAATCATATATTACAAA
TGTTATCTAAGCTATTAAGTGTGGCCAAGGAGGCAATCGACCGTCAAGGTCTGATTGCTATCCTAACTATTCCT
GTTAATAATAACGATGAAATAGAAGAAACGGCTCAAGGTGAAACCGTGTATAACGAACTTATCGATCAGTTA
CGACTTAATATCCCAAAAGATACGGATTATCAACCTAACATCTATAGTTATTTTGGTATTAAGAAGAATCCTAAT
GACACCGTACTCATGGAAATGATGATAAAGGTTTTTCATATCAAACGCTTTAATTCAGAACTGTTTATTTTCAA
AGTTAACGGGTGGCAAAAGATAAATGGAGATGAATTACAAGGGTTGATATCTAAAATGATACAAGTATTGCT
TGTAGATTATAAGCCTTCACTAAGCACTCTAAAAAATGTCGTAGATGGATTGCAAAAATCAACAGATGTAGAA
GAACTTGTTGAGAATGAGCGCTATATTGGTTGTGGTGAAAATATGTTCGATCTTAATACGTTTCAAGTCGTTA
AAAATTCAATCGATATCTTTCCAAAAACACGATTGAATTTATCATTAAGTACAAATGATGTAATTACTGATAAGA
TACCGCCTTATTTTAAGCAATATATGTTACAACTTGCGAATTATGACGATGATTTACAATACTTTCTTTTCCAAC
ATACAGCAGTATTACTTACAGCTGATACTAAATACCGTAGGGGTCTCATATTATATGGTGGAGCTAAGAATGGT
AAATCTGTATATATTGAACTAGTTAAATCATTTTTCTATAGTAAAGATATTGTGTCTAAGCCACTTAATGAGCTT
GAAGGTCGTTTTGACAAAGAAAGTTTAATTGACAAAAGTCTAATGGCAAGTCATGAAATTGGGCAATCTAG
GATTCAAGAAAAGATCGTAAATGACTTCAAAAAGTTATTATCTGTAGAATCAATGCATGTTGATCGTAAAGGA
AAAACTCAAGTGGAAGTCATTTTGGATTTGAAACTTATTTTTAGTACAAATGCGATACTTAATTTTCCTCCTGA
ACATGCGAAAGCTTTGGAGCGTCGAATTAATATTATTCCATGTGAGTATTATGTTGAAAAAGCGGACACTTCA
TTAATTGATAAGCTCCAGAGTGAGAAGAAAGAAATCTTTCTTTACTTGATGTATGTGTATCAACAGATTGTAA
AAGCAGATATCGAGTATCTTGAAAATAGCCGTGTCACTGAAATTACTCACGATTGGTTAAATTTTGGATATGA
ATTTGTTTCTAGCAGGTCCGTAAGTAATGCAAATCAGAAAGCATGTATTAATTTACTCAGAAAACTTATAGAA
ATCAAATCAGGATCACGAATCAAAGTATCCGAGTTAAATAAAGTTATTAATGAAGAAATAAAGGTAAGTTCTC
AGGTTATTAAACAGTTAATTCAAGCAAACTTTGATACTCAAACCAAACTATACAATGGCTACGATTATTGGATT
GATTTAGGTTGGAAAGAAGCCAATAAAAAAGAGATTCATGATATTTCGGAAAAAGATAATATTATTTCATTAG
ATAAAAATGAAAATATAACAGACGATGAGGCATTAGATGAAGAGAATTTGGACTTTGATTGGGAGGACTTTG
ACGATGAATAATGAACAAATTGAAGCATTTGTAGAAGTGCTTGTACCTATCATAGAAGAACGTATCAATAAAG
GTAATTAAGGCTAATTACGTACTACAGGCAGTTGCCTGTAGTACTCATATGATTAAGTGGTAAAAGTGATAAA
AATGAAACGAAATTATAAATATATATTATCTATATGTTGTTACAAGACCGATAGTCTGTAGCAATAATCTAATAAA
AGGAGCGGTATGATATGAAGGGTAAAATTGCACTTTATTCACGCGTTAGTACGTCAGAGCAGTCGGAGCATG
GGTACTCAATCCATGAGCAGGAACAAGTACTCATCAAAGAAGTTGTGAAAAATTTCCCAGGTTATGACTATG
AGACATATACTGACTCAGGCATTTCAGGTAAAAATATTGAAGGTCGTCCGGCAATGAAACGTCTATTACAAGA
TGTTAAGGATAATAAAATCGAAATGGTGTTAAGTTGGAAATTGAATCGTATCTCACGATCAATGAGAGACGTG
TTTAATATTATTCATGAATTCAAAGAACATGATGTAGGGTATAAATCGATTTCTGAGAATATTGATACATCCAAT
GCTTCTGGAGAAGTACTCGTTACAATGTTTGGGTTAATAGGATCTATAGAACGCCAGACTTTGATTTCGAATG
TGAAACTTTCTATGAATGCTAAGGCAAGGAGCGGAGAGGCAATCACCGGTCGTGTTTTAGGCTACAAATTAT
CACTTAATCCACTTACACAGAAAAATGATTTGGTTATCGATGAAAATGAAGCTAATATTGTACGTGAAATTTTC
GATTTATATTTGAATCATAATAAAGGCCTCAAAGCCATTACAACCGTACTTAATCAAAAGGGGTATCGTACTAT
TAATCAAAAGCCATTTTCAGTGTATGGTGTTAAATACATTTTGAATAATCCAGTCTATAAAGGCTATGTCAGATT
CAATAACCATCAAAACTGGGCTGTACAGCGAAGAAGTGGCAAAAGTGATAAAAATGATGTGATATTGGTCAA
AGGTAAACATGAAGCCATTATAAGTGAAGAGGTATTTGATAAAGTTCATGAAAAATTAGCTTCTAAAAGTTTT
AAACCGGGCAGACCTATTGGTGGAGATTTCTACTTACGTGGCCTTATTAAATGCCCAGAATGCGGAAATAATA
TGGTATGTCGACGGACGTATTATAAAACGAAAAAGTCCAAAGAACGGACAATCAAGCGTTATTACATTTGTT
CCTTATTCAATCGTTCAGGGAGTTCTGCATGTCACAGTAATTCCATCAATGCTGAAGTCGTCGAGCGTGTAAT
TAATGTTCATTTGAATCGTATTCTGTCTCAACCAGATATTATCAAGCAGATTGCGTCAAATGTGATAGAAGAAC
TGAAACAAAAGCATAGTAACCAAACAGAAATTAAATATGACATTGATAGTTTAGAAAAACAAAAAGCTAAGC

TTAAAACACAACAAGAACGATTGTTAGAATTGTTCTTAGATGATCAGATGGATAGCGAAATGTTAAAAGCTA
AACAAAGTCAAATGAATCAACAGTTAGAAGTATTAGATCAACAAATTAAAGAAGCGCAACAAGCAAATCAAT
CACAGGATGAAATACCTAATTTTGATAAATTAAAAGGACGACTCATTTTGATGATAACACGATTCAGCGTGTA
CTTAAGAAAGGCTACACCCGAAGCTAAAAATCAACTTATGAAAATGTTAATTGATTCAATTGAAATTACGACA
GATAAACAAGTAAAACTTGTAAGGTATAAAATTGACGAAAGTCTTATCCCTCAATCTTTGAAAAAAGATTGG
GGGTCTTTTTTTATGCCCAAATTCCAATTTGAAATATATGGTCAAAATGATTATTTCATCGACCAAATTACCACT
TTTACCACTTAGTTATTAGTGACAAAAGTGAGCGAAATGAAATAAAAATCAAATATATATTATCAAAATGATGT
ATCACATGCATACATCAATCAAATACATTAGGAGGTCATAACCATGACACTAGAACAACAACTCAAGCACTATA
TAACCAACTTATTCAATCTGCCAAGGGACGAAGTGTGGCACTGCGAATCTATCGAGGAAATCGCTGATGATAT
CTTACCCAATCAATATGTAAGACTTGGCCCACTCAGTAATAAAACACTTCAGACTAATACCTACTACTCTGACA
CACTTCATGAAAGTAATATCTATCCTTTCATTCTCTACTATCAGAAACAACTCATAGCCATCGGTTATATCGACG
AAAATCACGATATGGATTTCTTATACCTACACAACACTATCATGCCTCTTTTGGATCAACGATACTTACTAACAG
GAGGACAATAAAATGCATAAATACATCAAAATTACACAATTAGTCATTACAATACTAAGTGAAATCATCATTTG
GATGAAAGAGTCAGAACGAAAGGAAGTCTCTTATGAATAGATATATCACCCGGGGTATCGCCAACAACTTAC
CTAATATCTTACAACACCAATTATGGCAACTCGTATCTGAGCGAGAACAAGAACAAACCAAAGATAATACTTC
AGTAGATTATTTTCATATATTCCAGTTCAATAAGCATCGCAATCAATTATATATCAAACACAAACAAGAACGACC
TGAATATGCGAAAATCCATAAAGCTAATTATTCAAAAGCAATCAATATCAATAAGGTCTACATTATTCGAGAAG
ATGATGTAGACCTTTCTTATTATGTCATGTTATTACCTGAAGAATACTAGAGGAGTGAAAATTATATGGAAACA
ATCAAAAGTACATTAAAAACAGAAGCCATATTCAGTGATGACAAACAACATCGCTATCTACTTAAGAAAACAT
GGAATAGTGAAAAACAATCAATCACAATCATTACAATGTATCCGCATTATGATGGCATTCTCAATATTGACCTA
ACGACCCAACTCATCATGAACAAAGTTTCAGAAATGGATGCATTTGGTTCAATCAATTTTGTGAATCTATACTC
TAATATTACAACCCCTATCAATCTCAAACATTTAGAAAATGCGTATGATAAGCATACAGATATTCAAATTATGAA
GGCAGTGAAAGAGTCAGATGAAGTGATATTAGCTTGGGGCGCTTACGCTAAAAAGCCCGGTGTTGAAGCA
CGTGTTAATGAAGTATTAGAGATGTTGAAACCACATAAAAAGAAAGTAAAACGACTCATGAATCCAGAAACC
AATGAAATCATGCATCCCCTTAATTCGAAAGCACGTCAAAAATGGATATTAAAAGTATAGCAACTAAAATGTTT
ATTCACTATCCATGAAAATAATTGAATTTCATATATATCTTTTAATCTACAATATGATTAAAAGATATATTATTTGG
TTCTGTTGCAAAGTAAAAAAATATAGCTAACCACTAATTTATCATGTCAGTGTTCGCTTAACTTGCTAGCATGA
TGCTAATTTCATGGCATGGCGAAAATCCGCAGATCTGAAGAGACCTGCGGTTCTTTTTATATAGAGCGTAAAT
ACATTCAATACCTTTTAAAGTATTCTTTGCTGTATTGATACTTTGATACCTTGTCTTTCTTACTTTAATATGACGG
TGATCTTGCTCAATGAGGTTATTCAGATATTTCGATGTACAATGACAGTCAGGTTTAAGTTTAAAAGCTTTAAT
TACTTTAGCCATTGCTACCTTCGTTGAAGGTGCCTGATCTGTAATTACCTTTTGAGGTTTACCAAATTGTTTAA
TGAGACGTTTGATAAACGCATATGCTGAATGATTATCTCGTTGCTTACGCAACCAAATATCTAATGTATGTCCCT
CTGCATCAATGGCACGATATAAATAGCTCCATTTTCCTTTTATTTTGATGTACGTCTCATCAATACGCCATTTGTA
ATAAGCTTTTTTATGCTTTTTCTTCCAAATTTGATACAAAATTGGGGCATATTCTTGAACCCAACGGTAGACCG
TTGAATGATGAACGTTTACACCACGTTCCCTTAATATTTCAGATATATCACGATAACTCAATGTATATCTTAGATA
GTAGCCAACGGCTACAGTGATAACATCCTTGTTAAATTGTTTATATCTGAAATAGTTCATACAGAAGACTCCTT
TTTGTTAAAATTATACTATAAATTCAACTTTGCAACAGAACCGTATTATGGAATAGAGATGTTGGTAACATTTAT
ACAGGATCATTATACTTAAGTTTAATTTCGTTATTACAGAACCACACATTCCAACCAGAAGAGAAAGTATGTCT
ATTTAGTTATGGTTCAGGAGCAGTAGGAGAAATCTTTAGTGGTTCAATCGTTAAAGGATATGACAAAGCATTA
GATAAAGAGAAACACTTAAATATGCTAGAATCTAGAGAGCAATTATCAGTCGAAGAATACGAAACATTCTTTA
ACAGATTTGATAATCAAGAATTTGATTTCGAACGTGAATTGACACAAGATCCATATTCAAAAGTATACTTATAC
AGTATAGAAGACCATATCAGAACATATAAGATAGAGAAATAAACTAGTGGCCGATTGTGCTTGATGAGCTTGG
GACATAAATCCTAACTCGAAATAAATAAGCATATCACTAAACTGATTTTTTAAAGTTTACAGTGATATGCTTATT
TTTTTATCTTACGATTTTGTACGTGCATGCTTGCCTAGGGGTATGGCTCGAGCCATTAGTCTCTCGCACATACT

ATTCCCTCAGGCGTCAGCACTTACAAAATCGGTTGTAATTTTCATTTTTATACGCATTCTTACTGAGATTATACT
AATAAGAGGAATAGTAAAAGCAATTCTAAGTAAAATTGCAGATAAGAGGTTTGTTAAAAGCAGTTCTAAGTA
AAATTGCAGATAAGAGGTTTGTTAAAAGCAGTTCTCAGTAAAATTACAGATAAGAGGTACGTTAAAAGCAGT
TCTAAGTAAAATTGCAGATAAGAGGTTTGTTAAAAGCAGTTCTAAGTAAAATTGCAGATAAGAGGTACGTTA
AAAGCAATTCCATGCAAAATTGCTGATAAGGGGTAAGTTAAAAGCAGTTCTCAGTAAAATTGCAGATAAGAG
GTACGTTAAAAGCAGTTCTAGGCAAAATTGCAGATAAGAGGTGCGTTAAAAGCAGTTCTCAGTAAAATTGCT
GATAAGGGGTAAGTTAAAAGCAATCCTAAGTAAAATTGCAGATAAGGGGTACAGAAAAACTAGACTTGATT
ACAAAATGGAGCTTGGGACATAAATGATTTTTTAAAAATGAGATGAGACGTAGATTAACTCCATAATCAATAC
GAATCTATCGACTTCTTTATTTATGATATTCATCTCTTTTTAATGGAAATAAAAGTGCGATTAATGTGATAATACA
GTTACGTTAATTAAAAAAATAAAAATGCAAGGAGAGGTAATATGCTAACTGTATATGGACATAGAGGATTACC
TAGTAAAGCTCCGGAAAATACAATTGCATCATTTAAAGCTGCTTCAGAAGTAGAAGGTATAAACTGGTTGGA
GTTAGATGTTGCAATTACAAAAGATGAACAACTGATTATCATTCATGATGATTATTTAGAACGGACTACAAATA
TGTCCGGGGAAATAACTGAATTGAATTATGATGAAATTAAAGATGCTTCTGCAGGATCTTGGTTTGGTGAAA
AATTCAAAGATGAACATTTGCCAACTTTCGATGATGTAGTAAAAATAGCAAATGAATATAATATGAATTTAAAT
GTAGAATTAAAAGGTATTACTGGACCGAATGGACTAGCACTTTCTAAAAGTATGGTTAAGCAAGTGGAAGAA
CAATTAACAAACTTAAATCAGAATCAAGAAGTGCTCATTTCAAGCTTTAATGTTGTGCTTGTTAAACTTGCAG
AAGAAATCATGCCACAATATAACAGAGCAGTTATATTCCATACAACTTCGTTTCGTGAAGACTGGAGAACACT
TTTAGATTACTGTAATGCTAAAATAGTAAACACTGAAGATGCCAAACTTACTAAAGCAAAAGTAAAAATGGTA
AAAGAAGCGGGTTATGAATTGAACGTATGGACTGTAAACAAACCAGCACGTGCAAACCAACTTGCTAATTG
GGGAGTTGATGGTATCTTTACAGACAATGCAGATAAAATGGTGCATTTGTCTCAATAGAAAGTTAGAGGTGA
GTCTTACGTTTCAGTGACGGTAGACTTACCTTTAACATGTTACATACTAAAAAATTAATTTGAATAAGAAAGA
GAGACATATATGAAATACGATGATTTTATAGTAGGAGAAACATTCAAAACAAAAAGCCTTCATATTACAGAAG
AAGAAATTATCCAATTTGCAACAACTTTTGATCCTCAATATATGCATATAGATAAAGAAAAAGCAGAACAAAG
TAGATTTAAAGGTATCATTGCATCTGGCATGCATACACTTTCAATATCATTTAAATTATGGGTAGAAGAAGGTA
AATACGGAGAAGAAGTTGTAGCAGGAACACAAATGAATAACGTTAAATTTATTAAACCTGTATACCCAGGTA
ATACATTGTACGTTATCGCTGAAATTACAAATAAGAAATCCATAAAAAAAGAAAATGGACTCGTTACAGTGTC
ACTTTCAACATACAATGAAAATGAAGAAATTGTATTTAAGGGAGAAGTAACAGCACTTATTAATAATTCATAAT
AAAACAGTGAAGCAACCATCGTTACGGATTGCTTCACTGTTTTGTTATTCATCTATATCGTATTTTTTATTACCG
TTCTCATATAGCTCATCATACACTTTACCTGAGATTTTGGCATTGTAGCTAGCCATTCCTTTATCTTGTACATCTT
TAACATTAATAGCCATCATCATGTTTGGATTATCTTTATCATATGATATAAACCACCCAATTTGTCTGCCAGTTTC
TCCTTGTTTCATTTTGAGTTCTGCAGTACCGGATTTGCCAATTAAGTTTGCATAAGATCTATAAATATCTTCTTT
ATGTGTTTTATTTACGACTTGTTGCATACCATCAGTTAATAGATTGATATTTTCTTTGGAAATAATATTTTTCTTC
CAAACTTTGTTTTTCGTGTCTTTTAATAAGTGAGGTGCGTTAATATTGCCATTATTTTCTAATGCGCTATAGATT
GAAAGGATCTGTACTGGGTTAATCAGTATTTCACCTTGTCCGTAACCTGAATCAGCTAATAATATTTCATTATCT
AAATTTTTGTTTGAAATTTGAGCATTATAAAATGGATAATCACTTGGTATATCTTCACCAACACCTAGTTTTTTC
ATGCCTTTTTCAAATTTCTTACTGCCTAATTCGAGTGCTACTCTAGCAAAGAAAATGTTATCTGATGATTCTATT
GCTTGTTTTAAGTCGATATTACCATTTACCACTTCATATCTTGTAACGTTGTAACCACCCCAAGATTTATCTTTTT
GCCAACCTTTACCATCGATTTTATAACTTGTTTTATCGTCTAATGTTTTGTTATTTAACCCAATCATTGCTGTTAA
TATTTTTTGAGTTGAACCTGGTGAAGTTGTAATCTGGAACTTGTTGAGCAGAGGTTCTTTTTTATCTTCGGTT
AATTTATTATATTCTTCGTTACTCATGCCATACATAAATGGATAGACGTCATATGAAGGTGTGCTTACAAGTGCT
AATAATTCACCTGTTTGAGGGTGGATAGCAGTACCTGAGCCATAATCATTTTTCATGTTGTTATAAATACTCTTT
TGAACTTTAGCATCAATAGTTAGTTGAATATCTTTGCCATCTTTTTTCTTTTTCTCTATTAATGTATGTGCGATTG
TATTGCTATTATCGTCAACGATTGTGACACGATAGCCATCTTCATGTTGGAGCTTTTTATCGTAAAGTTTTTCGA
GTCCCTTTTTACCAATAACTGCATCATCTTTATAGCCTTTATATTCTTTTTGTTTTAATTCTTCAGAGTTAATGGG

ACCAACATAACCTAATAGATGTGAAGTCGCTTTTCCTAGAGGATAGTTACGACTTTCTGTTTCATTAGTTGTAA
GATGAAATTTTTTTGCGAAATCACTTAAATATTCATCCATTTTTTTAACGGTTTTAAGTGGAACGAAGGTATCA
TCTTGTACCCAATTTTGATCCATTTGTTGTTTGATATAGTCTTCAGAAATACTTAGTTCTTTAGCGATTGCTTTAT
AATCTTTTTTAGATACATTCTTTGGAACGATGCCTATCTCATATGCTGTTCCTGTATTGGCCAATTCCACATTGT
TTCGGTCTAAAATTTTACCACGTTCTGATTTTAAATTTTCAATATGTATGCTTTGGTCTTTCTGCATTCCTGGAA
TAATGACGCTATGATCCCAATCTAACTTCCACATACCATCTTCTTTAACAAAATTAAATTGAACGTTGCGATCAA
TGTTACCGTAGTTTGTTTTAATTTTATATTGAGCATCTACTCGTTTTTTATTTTTAGATACTTTTTTTATTTTACGA
TCCTGAATGTTTATATCTTTAACGCCTAAACTATTATATATTTTTATCGGACGTTCAGTCATTTCTACTTCACCATT
ATCGCTTTTAGAAATATAACTGCTATCTTTATAAACTTGTTTGAAATTTTTATCTTCAATTGCATCAATAGTATTAT
TAATTTCTTTATCTTTTGAAGCATAAAAATATATACCAAACCCGACAACTACAACTATTAAAATAAGTGGAACA
ATTTTTATCTTTTTCATCAATATCCTCCTTATATAAGACTACATTTGTAGTATATTACAAATGTAGTATTTATGTCA
AAATAATGTTATAATTTTTGTGATATGGAGGTGTAGAAGGTGTTATCATCTTTTTTAATGTTAAGTATAATCAGT
TCATTGCTCACGATATGTGTAATTTTTTTAGTGAGAATGCTCTATATAAAATATACTCAAAATATTATGTCACATA
AGATTTGGTTATTAGTGCTCGTCTCCACGTTAATTCCATTAATACCATTTTACAAAATATCGAATTTTACATTTTC
AAAAGATATGATGAATCGAAATGTATCTGACACGACTTCTTCGGTTAGTCATATGTTAGATGGTCAACAATCAT
CTGTTACGAAAGACTTAGCAATTAATGTTAATCAGTTTGAGACCTCAAATATAACGTATATGATTCTTTTGATAT
GGGTATTTGGTAGTTTGTTGTGCTTATTTTATATGATTAAGGCATTCCGACAAATTGATGTTATTAAAAGTTCGT
CATTGGAATCGTCATATCTTAATGAACGACTTAAAGTATGTCAAAGTAAGATGCAGTTCTACAAAAAGCATATA
ACAATTAGTTATAGTTCAAACATTGATAATCCGATGGTATTTGGTTTAGTGAAATCCCAAATTGTACTACCAAC
TGTCGTAGTCGAAACCATGAATGACAAAGAAATTGAATATATTATTCTACATGAACTATCACATGTGAAAAGTC
ATGACTTAATATTCAACCAGCTTTATGTTGTTTTTAAAATGATATTCTGGTTTAATCCTGCACTATATATAAGTAA
AACAATGATGGACAATGACTGTGAAAAAGTATGTGATAGAAACGTTTTAAAAATTTTGAATCGCCATGAACA
TATACGTTATGGTGAATCGATATTAAAATGCTCTATTTTAAAATCTCAGCACATAAATAATGTGGCAGCACAATA
TTTACTAGGTTTTAATTCAAATATTAAAGAACGTGTTAAGTATATTGCACTTTATGATTCAATGCCTAAACCTAA
TCGAAACAAGCGTATTGTTGCGTATATTGTATGTAGTATATCGCTTTTAATACAAGCACCGTTACTATCTGCACA
TGTTCAACAAGACAAATATGAAACAAATGTATCATATAAAAAATTAAATCAACTAGCTCCGTATTTCAAAGGAT
TTGATGGAAGTTTTGTGCTTTATAATGAACGGGAGCAAGCTTATTCTATTTATAATGAACCAGAAAGTAAACA
ACGATATTCACCTAATTCTACTTACAAAATTTATTTAGCGTTAATGGCATTCGACCAAAATTTACTCTCATTAAAT
CATACTGAACAACAATGGGATAAACATCAATATCCATTTAAAGAATGGAACCAAGATCAAAATTTAAATTCTTC
AATGAAATATTCAGTAAATTGGTATTACGAAAATTTAAACAAACATTTAAGACAAGATGAGGTTAAATCTTATT
TAGATCTAATTGAATATGGTAATGAAGAAATATCAGGGAATGAAAATTATTGGAATGAATCTTCATTAAAAATT
TCTGCAATAGAACAGGTTAATTTGTTGAAAAATATGAAACAACATAACATGCATTTTGATAATAAGGCTATTGA
AAAAGTTGAAAATAGTATGACTTTGAAACAAAAAGATACTTATAAATATGTAGGTAAAACTGGAACAGGAAT
CGTGAATCACAAAGAAGCAAATGGATGGTTCGTAGGTTATGTTGAAACGAAAGATAATACGTATTATTTTGCT
ACACATTTAAAAGGCGAAGACAATGCGAATGGCGAAAAAGCACAACAAATTTCTGAGCGTATTTTAAAAGA
AATGGAATTAATATAATGGATAATAAAACGTATGAAATATCATCTGCAGAATGGGAAGTTATGAATATCATTTG
GATGAAAAAAATATGCAAGTGCGAATAATATAATAGAAGAAATACAAATGCAAAAGGACTGGAGTCCAAAAAC
CATTCGTACACTTATAACGAGATTGTATAAAAAGGGATTTATAGATCGTAAAAAAGACAATAAAATTTTTCAAT
ATTACTCTCTTGTAGAAGAAAGTGATATAAAATATAAAACATCTAAAAACTTTATCAATAAAGTATACAAAGGC
GGTTTCAATTCACTTGTCTTAAACTTTGTAGAAAAAGAAGATCTATCACAAGATGAAATAGAAGAATTGAGA
AATATATTGAATAAAAAAATAAAATTGTTGTGTTTACAACAATACATAGAAAACAGAGGAAACAATCAAGTCGT
TGAATATTTCCTCTGTTTTTTAGTTGAAAAAATTAACCGAAAGCCTGAATGCAAGTCTTGATTAAATCAATAAT
GCTTGTAATAACACCAGTGAAATCCATATGCATACCCTCTTTCTATTTAAGATACATTAAGTATAATATCAAACA
AATAAAAAAATGTTAAAAATTCCCTAATTGGCTATTTAGATTGCATAAATGTCAAAAATTTGAAAAACATACAAC

GACTTTGCATAAAAAAATCGTCATATTGGAAATACGTAATTTATTGAAATAATAAAAAAAATAAAAGAACGAAG
ATGATAACCTAAGTGAGGTTTTAAGTTGTTCTAAGGTTTAATTTAATTTATGTTAAAATAGTTGGTATAAAAATA
CATGATAAACTATAAACTAAATTCAAAATAACTTATGGGGTAGGCAATTATGGAAAATAAAATATAAATGATAA
TGAAAAAAGAGTGCTAAGGGAAATTTATAACCATCATAATATTTCGCGTACTCAAATATCTAAAAATCTTGAGA
TTAATAAGGCAACGATTTCTAGTATTTTGAATAAGTTAAAGTATAAATCTCTTGTTAATGAGGTTGGTGAGGGT
GATAGCACGAAGAGTGGTGGTAGAAAACCTATTCTTCTGAAGGTTAATCATCTTTATGGTTATTTTATTTCTTT
GGATTTAACTTATAGTTCTGTTGAAGTGATGTACAATTATTTTGATGGTAATGTCATTAAGCATGAATCTTATGA
TTTACCTGATGAAAAGGTTAGTAGTATATTAAGCATAATAAAAAAACATATTGATATTCAGGAGAAACTTGATA
CTTATAACGGACTATTAGGTGTGTCTGTTTCTATACATGGAGTTGTGGATAATGAGCAGCATGTGACATATTTA
CCATTCCATGAAACTGAAGGAATTTCAATTGCTAAGAAAATAAAAGAAATTACTAATGTTCCAGTCGTAGTTG
AAAATGAAGCGAATCTTTCAGCGTTATATGAACGTAATTTTAATCATAATTTATCCTACAATAATCTTATTGCTTT
AAGTATACATAAAGGTATTGGTGCTGGGCTTATTATTAATAATCAATTGTATCGTGGTGCAAATGGGGAAGCG
GGTGAAATTGGAAAAACACTTGTCTCAAAAGTTAGCGATAATGTGGAGATCTTTCATAAGATTGAAGATATTT
TTTCACAAGAAGCTTTACTGCATAATTTAAGTAATCAACTAAATGAGAAGATGACGCTTAGCAAATTAATTCA
ATTTTATAATGAAAAAAATCCAGTCGTAGTTGAAGAAATGGAACAATTTATAAATAAAATTGCTGTTTTAATAC
ATAATTTAAACACCCAGTTTAATCCGAATGCAATTTACATTAACTGTCCATTGTTCAATGAAATGCCTGAAATAT
TAGAAGCAATTAAGAACCAGTTCAAACAATATTCACGTAACGAAATTCAAATAAAGTTAACATCTAATGTCAA
ATTTGCAACTTTGCTAGGTGGTACATTAGCAATTATCCAAAAAGTACTACAGATTAATGATATTTACTTAGATAT
AAAAGCATAAAAAACTAATTCAAATGAATAATCAAAGTTCGTAATTGTCTTTATAAAAAAATCCCTCAATCCGA
ATTGAATTTTCGGATTGAGGGATTTTTATAGTTCTATTGCAGAAGAAAACTATTTTAAAAATGCTGGTAAATGT
TGATAGCCACCTCTAACGTTAACAATATTCGTAAATCCTTTATATTCTAATATTCCTACCGCTATTGAACTTCTAA
CACCTGATTGACAATGTACATAAATTAGGTCATTTTTATCGAAAGGTATATCTTCATTTAAAAGTTTACCGTGA
GGAATATGAATTGCTTGTTTTAAATGACCTTTACGCCATTCATCATCATTACGAACATCTAATACATTATGTTCTT
CACCAGTCATTTCAGAACTATGAATAGATGATGTGACGATATTTGTTTGTGGCAAACGGTAACTTTTTACATTT
TCAAAACCAATTAATTGTAAAGCATGAATAGCTTGTTGAACGGTAGATTTATCGCCAATTAATTCAATATCTTA
GTCATAATCTAAATACCAACCAATTTGATTTATAAAAGTTTTATTAAAAGGAATATTGATAGTTCCATGCATATG
ACCACCATGGAATGCTTCTTTACTTCGAAGATCAAAAGCAGTTTGTGTATTGCTTGAACTAGGGTAAACATTA
TATGGTTGGTACATTTGCATACCAAATTGATTTATTTTTTTCATTTGTGAAAAATGGTGTGGTGGAGCTGGCTG
ATTGAGTGTTAAAGTTTCGATAAATGAAGTTTCATCTTTAACATTAAAAGCCCAGTTGTTTATTTTCTCATAAC
CCAAAGTAGTTGTAGGTAATGAACCTAGCGCTTTACCACAAGGACTCCCTGCACCATGACCTGGCCAAATTT
GAATATAGTCTGGTAATGTTGCAGCAAATTGTATGGACTGATACATTTGTTTTGCTCCGATTTTTGTAGAACCT
TCAACATTTACAGCTTTTTCTAATAGATCTGGTCTACCTACATCACCAACAAAGATGAAGTCACCGCTAAATAA
TCCCATTGGTATACTGGAACCCCCACCTTCGTCAGTAAGTAAAAAACTAATACTCTCAGGGGGATGGCCTGG
AGTGTGTAAGACTTCTAATTTAATCTTTCCTAAATAGATAATATCTTGATGCTTAACGAAATGTGTTTGTTTAGG
CATATTTTTATAATTAAATTCATCTTTACCTTCATCAGATACGTATATACTTGCATTCAATTTATTTGCCACATCTCT
AATACCTGAAGCAAAATCAGCATGAATATGTGTTTCTGCAGCTTTAGTAATTGTGAATCCTTCTTTATCTGCAA
CTTTTAAATATTTTGTTAAATCTCGTATAGGGTCAATAATCATTGCTTCTCCTGTACGTTGACATCCAATTAAATA
AGATGCTTGTGAAAAATTGTCTTCATAAAATTGTTTGAAAAACAAAAAAAACTCCTTTTTAAAATAGATTTTAT
TGATTAGATAAATAAGTTATGATTTGCTTGCTCAGTATGTCCAATATAAGTACCTACGCCACCATAATCGACTTC
ATCTCTTAATTCTTCTTTTGAAATTCCCATAACATCCATACTCATGGTACAAGCAATTAACTTTATATCTTGATCG
ATTGCTTGATCGATAAGTGAGTATAAAGAATCAACATTTTTCTTGTTCATTACATAACGCATCATAATATTACCT
AGTCCAAACATATTCATTTTTGATAATGGCATATGTATTGGATCCTTAGGTAACATAAGGTCAAACATTTTGGA
AATACCTTTCTTTTTAACGCGAGTTGATTGCGCTTTTTTTAATGCGTTGAGGCCCCAAAAAGTAAAGAAAATA
GTTACATCTTTACCTGCTGCTTTAGCGCCATTTGCGATGATCATTGCTGCTACTGCCTTATCTAACTCACCGCTA

AATAAAACAATTGTTGTACCTGTAGCAGTGTCATTGATTTCAAATTCTTTTGGCTTTTCTTTTTGAATAATTGCA
TTAATTACATTTGCTTCTTCAGTAAGATTTACAAGGGTATTCCCTGTTTGTTTCGCCCAACTTTTAATATCACTA
TTGAAACCAGGATCTGTAACTGTTACCTCGATTTGCTCACCCGTTGAAATATTGTTAATTTCTTTACTGATATTA
ACAATAGGTCCAGGGCACTGAAGACCTCTAAAATCAAATTGTTTACGATTCTCTTTGATTTCAATATCTTTTTC
TATTAAAGGAGCACTATTGAAGTTCTTTGCTTCATAATCTTTATATCCACCCTTTAAATTCACGACATCATAACC
TTGTTTGGCTAAATAATCGCAAGCTTTAGTGCTTCGGTTACCGCTTTTACAATGTATATAATACGTTTTGTTGCT
ATTCTTATTGAATGATTTAATCTCTTCTACTGGGTGTAAAGTTGAACCGTTAATGTGTCCTAATTCATATTCTTCT
TTTGTTCTAACATCAATCAATTGACCCATTTTTGCCAATTTTTCTAATTCTTCTTTGTTTAATGAATTAATGTGTA
CTTCTTTGTATTGTTCCATACTTACCTCCTATAAATACCTATGAGGGTATAATAAAACGGATAGAATCATTTGCC
AAATACCTATATGGGTATTTGACAATTTTGTTTTAATTTATTATTATTAACTTAATCAATTTATGTGGAGGAAATG
AATATGACTTATGATAAAAAAATGATTAATCGTATAAATAGAATACAAGGTCAATTAAATGGTGTCGTAAAAAT
GATGGAAGAAGAAAAAGATTGCAAAGATATAATTACGCAACTTAGTGCATCTAAAGGTTCTATACAACGTTTA
ATGGGGATTATAATTAGTGAAAATTTAATAGAATGCGTTAAAACAGCAGAAGAAAATAATGAAAGTTCTCAA
GAATTAATTAATGAAGCAGTTAATTTATTAGTTAAAAGTAAATAATGGATATAGCAAATATGACTATTATGTTGC
TAATTGGCGTACTGGGTGGATTTATATCTGGATTAATAGGTATTGGGGGCGCAATTATTATTTACCCAGCTATT
CTTATATTGCCACCATTAATAGGTATACCTGCGTATAGTGCATATATTGCTTCGGGACTTACCTCTAGTCAAGTAT
TTTTCAGTACACTTAGTGGATCATTAAATGCAAGAAAACAACCAGCTTTCTCTCCTAAACTTGTTATATATATG
GGAGGGGGTATGTTGATTGGAAGCATGTTAGGGGCAATTTTAGCTAGTTTGTTTAATGCTACTTTTGTAAATA
CGGTATATGTAATAATCGCCATACTTGCTTTAATATTGATGTTTATTAAAGTTAAACCTACTACACAAGAGACGA
AATCTAAACCTTTGCTATTTATTATAGTTGGATTTGGAATTGGTGTAATTTCGGGAATTGTGGGTGCAGGTGG
AGCATTTATCATCATTCCTGTATTATTAGCATTATTTAAATTACCAATGAATACGGTAGTGAACAATAGCATAGC
AATTGCTTTTATATCTTCAGTAGGGGCATTTTTTATAAAATTAATGCAAGGATATATACCAGTAGAAAGTGCAA
TTTTTTTGATAATTGAATGATGATCATTTTCTGAAAATATTATGTGGTCATATAATATAATGCCCATCATTTCACTA
ATCTCTTTTATTCTCTGAGTTATTTTGATATCTCCTGGAGAAGGTGTTACATCTTATCAAGAGTAAATTACAAAA
GAATCATTTAAATCAATACTTTCACTTTGAATACATGTATTTGAAGTGGAAGGTACTTATTTCAAAAATAGTAA
AACCTGTATCTTAAATTACTTAATAGTAACATAAGATACAGGCTGATTTTTTATTCATTGTTATTTATACTAAAGC
ACCCGATAGCTCTGAAAACAATCACAAATTCAACTTTTCAAAGCCACAGCTTTAAGTTATTTTGTCCAGACAA
CCCCCATTGCCCAACCCATTTTATGGAATTGGCATCCAGGCAACAACTTTTCATATAAATCGTAATAATTTTGTT
CAGATAGGTACTTATCTGAAGCTAAATGCTCAAGCCATGATTTAGATGTGTTGTGATTATAAATTCTAATCGCAT
TTTTTATTCCAAATTTGAAACAATGCGGTAAAAACTCTTGGATTGCCCCTAATTTATAAACATAGGTAGGCGGT
GTCTCGACTTCAGATACATTATCAAGAATAACTATTCTTCCTTCCTCATTCAACAGTTCCTTCATTTGCTGTATTA
CGCTGGCTATATCATCCAAATGATGAAAGGTTGTTCGGCTTACAATAAAATCAAACTTCTCATTAAAATTAAGT
TGTTCTGCATTCATATTCAGATAGACCGTATTTGTTAGTTGACGTTTAGATTTGGCAAGATCGAGCATTTGATT
AGAAATATCAATCCCTACCACTTCATCATAATAACTTGCTAATTTCTCCACTAACAAACCCGAGCCACATCCGAT
ATCTAATGCTCTGCCTTTCTTTGGAGACATATTAGACACAAAGAATGAATAATCATTCAAAAGCTCATTCACGA
AATCGTAATCTTCTGCAACCTTATCAAACTGTGATTCTATTGTATTCAAAAAGATCCCCCATTCCTACTTTATCG
ACATTCTTTCATTACTTACCACTTTAGATGTTTTTTCGTTGGGGATAAAACTTCCCTTTAGACAATTTTATCCAA
AGACAATACAACAGTGCAACTTTATTAAAGTCACTGTCCTTTATCGCAGCCTTTACTTTTTAGTAAAGACAGT
GGCTTCTCTTATCAAGTTTCAAAACATATTATTTTGAAGAAAACGTCCATCTGAAGTGTCAAGTGCAAAATTA
CATATAAAGGTTTATTCTAAAATGAAAAGATGATACAATCATATTCAGTTACATAAGGAGGTTTCAATTATGTG
CACCAGTATCGCAGTAGTAGAAATTACTTTATCTCATTCATAATGAAAAAAATGGAAAGGAGATAAAAGTATG
GGTACTTTTTCTATATTTGTTATTAATAAAGTAATCAAAATTAATTGGTTATAATGAACGCTTAATGTCAGTTCAT
TATAACCAGTAAGGAGAAGGTTATAATGAACCAGAAAAACCCTAAAGACACGCAAAATTTTATTACTTCTAAA
AAGCATGTAAAAGAAATATTGAATCACACGAATATCAGTAAACAAGACAACGTAATAGAAATCGGATCAGGA

AAAGGACATTTTACCAAAGAGCTAGTCAAAATGAGTCGATCAGTTACTGCTATAGAAATTGATGGAGGCTTA
TGTCAAGTGACTAAAGAAGCGGTAAACCCCTCTGAGAATATAAAAGTGATTCAAACGGATATTCTAAAATTTT
CCTTCCCAAAACATATAAACTATAAGATATATGGTAATATTCCTTATAACATCAGTACGGATATTGTCAAAAGAA
TTACCTTTGAAAGTCAGGCTAAATATAGCTATCTTATCGTTGAGAAGGGATTTGCGAAAAGATTGCAAAATCT
GCAACGAGCTTTGGGTTTACTATTAATGGTGGAGATGGATATAAAAATGCTCAAAAAAGTACCACCACTATAT
TTTCATCCTAAGCCAAGTGTAGACTCTGTATTGATTGTTCTTGAACGACATCAACCATTGATTTCAAAGAAGG
ACTACAAAAAGTATCGATCTTTTGTTTATAAGTGGGTAAACCGTGAATATCGTGTTCTTTTCACTAAAAACCAA
TTCCGACAGGCTTTGAAGCATGCAAATGTCACTAATATTAATAAACTATCGAAGGAACAATTTCTTTCTATTTT
CAATAGTTACAAATTGTTTCACTAAATTAAAGTAATAAAGCGTTCTCTAATTTCACAAGAGGACGCTTTATTCT
TCCCAAAAATTGTTCAATATTTATCAATAAATCAGTAGTTTTAAAAGTAAGCACCTGTTATTGCAATAAAATTAG
CCTAATTGAGAGAAGTTTCTATAGAATTTTTCATATACTTAACGAGTGCTTTCACCTTTGAATATAGTCCTTCCC
ACTTATCATCACACTCTCCCCGATAGCCTTTTCTAGCTATATCCAGTAAAGTTACATGCTCTTTAGGTAAAAGAG
GTATAGCCCATTCTGCAGCGACATCTTTCGAGGTAATTTCACCAGTAGTCACTGTTTGCCACATTCGAGCTAG
GGTTAAAATTACATTACGCTCATCACCTTTTATCCCCTCAATTAGTTCTGGCAAAGAATCCTTAATTGCTCTTCG
AATATCTGTCAAAGGTACGGAGACAAGTATACTTGAAGAATCAGGACCAAATAGAGAAATACTATTCTTTCTT
GCTTGTGCTAAAACAATAGCCAAATCAGGATCATAGCTTGGTTCCTGAATTTGTCCATTCTCAAATTCACCCCT
GAGCCACTCACCGTATATAAATTCTCTTTTTGGAGGATATTGCCAAGGGACAACTTCACTCCTATTTATAACCG
TAACTTCAAGTGGTCTAACAGAATCCGTATTTCCAATCTTTCCTGATATAGTCATTAGTCTTTCTGTTAGTTTTT
TTCGAGTTAATTGAGGTAAACTATGATTCACGACGACTAGAACATCTACATCGCTGTTAATGCGTAAACCACC
ATTTACTGCTGAACCAAATAGATATACTCCAACTATTGAACTTCCAAATAAATCTTTTACGATTTTTAATGTTTG
AATCGCTTGATTTGGTATTTTTCCGTTAATCAAATTGCTCATGATTTCACCTCGTTGATTATGTTCATATAAAGTT
TATATTGATACTCAATTTACTTACCCTAGATTGGACATATACTTAAATTACTGTTCAATAAAGCTGACCGTTAGC
GTTTAAGTACATCCTTTCACAATTTGTCTACAGATTAATAATTATTCTTTATTATACAGATCTCCATATAATTTTTG
AATTTGGTTCTGTAATTTTTTATTTTCTTTTTCTAATTCCATTACTCTTCTTTTTAAGGTTTTAATAAGGATTTCCT
CCGAACGAGAACTTTTCTTGGGTTTTGAGACTACATTTGCTGTTATTTGACGCTCACGAAGGGATTCGATTCT
TTGCCTAATATCGTGTTCCTTATAAAGCCATGATTTAGAAACATTAGCTTCCTTTGCTATTGAATTAAAATTAATA
ACTTTACCTTCAATCGAAAATTTAGAAATCGCTTTGTCTACTTTTTCCCTTGTCTTTTTTGATTTCTGCTTCGCC
AAACGTACAATTTCTGTTGTATTTCTAACTTGTTTATCCATTGATAATTACCCCGTCAAACTTCCAATGATTTGTT
CTAAACGCTCTTTAACACGGCTATTAGTCTCTACTTGTCTTTGCCATTGTTTATCCTTAGCTATGGCTAATAACT
CTTCTGTACGCTCTAACTGTTCTTCGTGCTGTGGTAAGAATTGCTTACTGGTACAGAAGTGAGTGCAATCTAA
GCATGCATTCGCATGTGGACAACCACCTGCTACTACTGGCAATCTACAATAACCATTTGGAAGCACTTGTGCA
TTTATATTTTTCTTGAACCATTGAAGCTCTACATCATCGACTTCATTATCTTCATCTAGATCAAGCACATCTCCAT
TATTGGTAACCAGTTTTTCCTGAAATTTAGTAAATTCATTTTTTAGAGTTTCATCAAAGATATGAGCGTATCTGC
TTGTCATTTCTGGGCTTTCATGCCCCAAAAATTTCTGCACAATATGCTGGGGCATCCCGTTGTTAATCATTCTT
GTTCCTACTGTATGGCGAAAGGCATGGGCATGGAATCTATAAATCTCACCTGATTTATCCACTATATTTTGCTC
ATAAGCTAATTTATTTAACTCACCTCTAAATGTTTCTTGTTTTAATGGCGATCCATCTTTTCTTGGAAAGAGGTA
TTCACTATCTGGAAATTCCTCTGAAACTTTATCTTCCCGAACTTTAATAAGTAAAGCTACCTCTTTAGATATTGG
AACTATATGCTCCTTTTTCATTTTCCATTGATAATACTTTAAAAAGAAATCTCCATCTTTGTCCTCTAATAGACAG
CCTTTTTTCAAGGTGCACAATTCACTTATCCTCATTCCACATTCTTGAACAATCATAGTCATCGTAGCTATATATT
CGGGTAATTTATCAAGATGACTGTTCAATTGCTCTAGGACGAATTCATCTATAAAGCGTGGTTTTGCTCTTGGT
ATTTTCGGATAGTCCTCAGAATAAATTAATATTTTGGAAGGAACATCATCCCATTCTAGCCTAAGAAGGGTACT
AAATAGTCCTTCCAATATAGAGATCCTCCCAGTTATTGTACTAGGTTTTATTCCCATCATGTTTAGTTCACTTAA
ATATGCTTCAATTTCCACTCTCGTTAATTGGTGTACTCTCTGAACTTGTTTAAATTTCATGTCCAGAAAATTAAA
GAACTCTTTAAGTCTTTGGGCAATATCACTTACATAGGAAAAGCTATCCACGTTCAATCTCAACTTACAATATC

TTTTTACAAGTTGTTTAAAATATGTATTCCGAAACCCTTTAAAGTTAATTGTATATTCATATTGTGTTGGGTTAA
CCTTATCATCTGGCAAAGGTAAGTTACGTCTATCCCAAACGTCTTTATCCCACTCCTCTCCATCAAAATAAAAG
TTCTCATAAAACTCCATAAATTGTTTTAGATTAGTAACATAGTAGGAATTAGCTTTTACAGGTGTTTTTTCTTGA
TTAGCAGTAATCTTATAATTAGTAGTGGTAATTCTAACACCCCGTTTTGTCAAATAAGTTCTATACTCCGTCATT
GCTTTTTCAATAGGAACTTCAGTAATTGAAGTAATGCTAGGATACTTTAAATCTAAGAAATCTAACATTTTATTA
ATTACTGTTCCTTTTCTAATCCAGACAGTTTTTGCATTCCATATTCCATTGTTTAAATGGTAAAAATAAAAATATT
TCAATTCTGTTCTTAACCACAGATTTTTAACACGTTCAAAACGAACCCAACGATTCCTTAAAGCAGGATTCTT
ACTTAATTCTATGGCAGAAGGATGTGGACATTTTCTTATATCCCAACTATTATTAGCCCAAAACCCCTGCATTTC
TTCATTCATTACAGCTATTTTTTTGCTAATCTCACTCTGACTAATAATTTTCCTTTTACTAGAAGCATTCATTTCT
TATGCTCCTTTCTCTCGAGGTATTTATTAAACTCATTTTTCATATCCTGATCTGAAAGATGAACATAGGTATTTAA
CGTTGTCTGAACATGTGCGTGACCTAATCTCTTTTGAACGAACGCAACATCCCATCCTTCCCTAATTAGCTGCG
TTGCGTGAGTGTGGCGAAGCATATGTGATGTAAATTCTATTCCAGTCCTTTTAACTATTCTTCTAACTAGATCA
AGAACACTTTGGTACTTTAGTGGTTTCCCAAAATAGCCTTCTTTTAAGGAAATAAAAACATAATCATGCTCCA
ATTCCTCACTATACTCATATATCAAGTAATCTGTATAAAGTGACATAAGTTCTTTACTCACATGTATTGTTCTTTC
CTTCCTTAATTTAATATAAGCTTCATTAACATTAACATCTCTAGGTGTTAAATGGATTTGATTGTCCCAAGTGAC
AATATCTTCAAGCCTAAGCGATAACACTTCACCGATTCTTAAACCACCCTCATACATAAGCATTAAAATTAATTT
ATCTCTTTTCGTATGACAAGCATCAATAATTTGCTTAACTTCCTTTGATCTCAATGTTCTTATCTGTTTCTTTTTA
ACCCTTAACTTTAAGACATTCTTTTGGTATCTACCCTTATTAACATGATGTAAAAATCCTTTGAAATTTCTTCCCT
TTGCTTGTTTAAATACATCAATTGATTTAAATTCTCCTAATCTACTTAAATAATCAAGAAAACTCATAACTACATT
TAAAATTGTATTCACTGTCGTTTCTTCTCTTATGGCTTTTTTTGACTGAAGATCAATTACATTTGATGCTGAAGG
ATATCTCAACCAACCTACGAAGTCTGCTAACAACTCAAAGTTAATATCATTAAGAATAACACCTCTCTGTTCCAT
GAACTCGTACAGCAACTTTAAATGATAGCAGTATGCCTTAATGGTATTAGGAGACTTACCAGTATTATCTAAGT
ATTTAATAAATTTCATTACTGGTTCTATTAGCTGGTATTCTTTATCTAGTAATAAATACAATGGATACGGCTTATT
CTCCACTTCTATCCTTTGAACCTTCACATGTTCCCACCTCTTTAAATACTTTAACTACTACAAATTATAAACCTTG
TTAATTTTTATTTAAAGTAAAATATCCCTTTAATATCTCCTCTTAAACTACTTTAACTACTATTATTTATTATACTAT
GGTTAATACATCTCCACTTGGATGATTATGACCGAGCATTATACTATTTGCGTTACTGAGTATCGCTGTTTTGAA
TATTTCTCTAGGGTGAATCACCGTTTGGTTAATAGATCCAATCGATAGTGTTTGAATATGTGTAGGTTCATTTTT
ACTGTTCATACATATGAGAATGAGATGCTCTCGGTCACTGTTTCCAATGAATGAACGCATGATTTCTGCCGCAT
CCTCAGGGTTTGAAATACGATTTTTTAGATAACTTAATGTATCTGTTTTTATCATTTGTAGTGAAACAATATTGA
TTTCCTTCATCGTTTACCTCCATATATAGGTTATGCTTTCAAAGTCCATTTTTGACGTGCTTTAGGGTTGAGTGG
GTGCATGATTTCATTTGTGGCTGGATTAATGAGCTTTTTGACTTTCTTTTTATGAGGCTTCAACATTTCCATTAC
TTGTTCGACACGTTCTACAACAACTGGCCGCTTCGCATAAGCACCATAAGCTAGAATCACTGTGTCACTTTCG
CTAATTGCTTTCATCAAGTGGATGTCTGTGTGTTTATCATAAGGTTCTTTAATATGTTTAAGGTTCTCTGGTGTT
TTAATATTAGAGAATAGATTTACAAGATATACAGCACCGTATCGTTCTGAATTCGCTAATTGATTGAGGATAAG
AACAGTTGTGAGATCGAGTGATAATACACCATCTAAATGAGGGTACATCGTTATCACTGTACAAGCGGGTTTC
TTTTCATCCCATGTTTTCTTGAGTAAATAGCGGTGTTTTTCATCCTTGCTAAATATAGCTTCTGTGTATATCGTAT
TTTTGATTGTATTCATATATATAATCACTTCCTTTAGTATTCTTCTGGTAAAAGCATCACATAATAAAAAGCGTCT
ACGCCATCTTCACGAATGACGTAGACTTTCTTAGGTAATGCATTTTGATTTTTTACATAGTTTGTATAGTGATAT
TCCAATTTGTATGCAGGTTGTTCTTGTTCATGTGTGATTGAGAGTATATTCTCATCTTCTTGCAGTTTAAAAATG
TGTAGGTAATCTGTATGAGGTTGATTATCTCTTTCTTTTACCATATTCCAAAGTAAGATTTGAAGGTCTAGAGA
TAGGTATTCACTAATACCTCTTGTGATGTATCGATTGATTTTCATGTTATTTTACCTCGTCTTGAATTTCTTTCATA
ATGATAATCGCTTGGCTAATAATCGTAACAGATATTTGTGCCACTTTGATCCAGTTATTCATGGTGAGTCCCTCC
TTCTCTTAGTAAATGACGTTCATCGATAATCGTATTTTTAGTATCTGTGAGATATAAAAAGTCCATGTCAAAATG
ATTCAGATAACCAACGCTGATGAGTTGGTTATTAGCGTACATAAGAAATGGATAGATACTTAGGTCATGTAGT

TCATTATTGTAGTAGGTATAAGTTTCAAGTGTAAGATGTGCAAGTGGGGAATCATTAATAAAACGTTCCGGTA
GAATATTTTCTGCTGCTTCTTCTAACGCTTCGCATTCCCATGTTTCATTGTTAGATAGTTGGAATAGACGAGTT
ATATATTGTTTGAGTTCTTGAGTGGTCGTTTTCATATCATTGCCTCCTAGATAGTGTTATAGTGATGTAGTTTATG
TACATCATTGGGATAATATATATTTGATTTGTCATTTATTACGCATCCCGGTGAGAATGAGAGAAAATTCCATAT
GAAAAACCGCTTCAAACCTTGGTATGACAAGGAAATCCCGAAATTCCGCCTATTTTGACGAACAATCAACTC
ATTCTTTATAACTATTGATGTTAGGGTGGGGCTCTGCTTTCTTATATATTATTTATTTATAAAGAATAACGGGATT
TTGGGATTGTGCTTGCACAATCCTTCTGCTTCTTCGAATCTGCAAATCCCAATCATTTCCCGATAAAAAATCAT
TGTGGGATGTTCTTTAGCAATTTCAATATAAGCATCGTGTAGTTATGAAAAAAATTACGACAATGACTGTTTCA
TTAGATAAGTGTTATTGAAATTGATAAAGAGAATTTTAAAAATGGTTAGATAAAATAAATGAAAGAATATAATG
AAAATTATTGTTATAACAATGATTCTATTAGCTAAATAGTAAGATATAGTGTTTGGGGCAAAAACAAAGACGA
AGTGCTGAGATGCACTTCGTCGAGTTGTTTATTATTGAAAAGTTGTTTTATGATTTCGTTATTAAGTTTGAGTG
TGACATAGAATTGTTTTTTATGATTACCATCTTTTTTAATATCAATGCGATCAATCACTGATAGATACAATGCTTT
GAGTCGAGATTTTTCTATGTGCTTAATATCATGAAAGATGTGTTGTAATAGTTTACTGATTTCTTTGGCATCAA
ATAAAGGCTTATCTTCATTTTGTTGATTTTTGAGTTGGTTGATTTGATTCGTAATGTCATTGAGTTGCTTTTCAT
ATTTTTGAATACTTGGTCTGATTACTGATGTTAAGTCCGGATTATCCTCGATGGTTTTAATCAAGTTATTTAGTT
TGATTTGTACTTCATCATATTGTTGTTGCTTATAAGCAATATCGTGATGAAGTGCAGCGCCATCAACTTGATTTT
CTTGATTGACGTGTGTTACTACGCGTTGAATGACTTTATCACTTTTGACTATTTCAAGTATTTGCTTCATCACAT
AATCTTCAATCACATCAGCTCTTACACTGTTTGCCGAACATACTTTGGAACCCTTGTTCCGAAAATTACTACAT
GAATAGTAACGAATACGTTTCTTAGTCCCGTCTTTAAGTGTATTCGTGGTATTGCTTGCTGCCATAGGTGCGCC
ACATTGGGGACAGTGAATAATGCCTGTAAGCAGATTCGTTCCTTTGCCATGGACTTGGGGTTTTTGACTGAC
TTGTTTTTTACGCATTTGTACTTTATCCCATAAATCTTGATTAATAATGGGGGAATGCTTACCTTCAGCTATCAC
TGGTTTATCATTAAGCCCTTTACGACGTTTTTCACTCCAATCTTTGTATTTCGCAAATTGAATTTTGCCGATATA
GAATGGGTTAGCTAATATATATGTGATAGAACTAATACTAAAAGGTTTACCCTTTTTAGTGACATAGCCTTTGT
GATTTAATGCATTGGCTATTTTACGATAGCCATGGCCTTTGGCATAGGACTCGAATATATATTTCACAATATTAG
CTTCATGTTGATTAATCATCAGTTCATGTTTACTATTAGGTATTTTGTCATAGCCCAGCGGCAAATTGCCTTGAT
AATAGCCTTCTTGGGCACGTCTCGTTTGACCCATAAATACATTCTCGACAATGTTATTACGTTCGAATTCTGAG
AAACTCGCAAGTATTTGTAACATGAGCTTACCCGATGAAGTATTGACTTCCATACGCTCTGATAAACTGAAAA
ATTCGACATTTTGTTTGTGTAAATCTTCGACAATTTTGAGAAGATCAGATGTATTACGAGCTAATCGGTTTGTT
TTGTAGACCATAACACAGTCGATATAGCCTTCTTTCGCATCCTTCAATATACGTTGGAGCTCAGGTCGATTCAT
AGATTTACCTGAAATACCACGGTCAGCGTATATATCTTTAACTTCAAAATGATGGAAGTCACAGTATTCTTTGA
TTTGATTGATTTGTCCGTCGATACTATAACCTTCTGTGCTTTGCATTTCTGTTGATACACGTACATAGATACCGA
CACGTTTTGTTTTAAGTTGTTGCATTATGTTTCATCCTTTCTTCGTTTATGCAATCGATGATTGCATGGTTTGAT
TGACGATATTGAGTGGTTCATTTTTGAAATAGATTCCTATAAGATTTTTATCTTTCGTAATGTGAATGGTTTCAA
TATAGGGGTACAATATGTTTAACGTGAAACGTTTTTGAATAATATTTTGAATGGTGTGTTGTATTTGATGTCCAT
TGATAGATGTAGTGCGTTGCGGTTGTTGACGTAATGATTGCGTTTGTTCTCTGAACGTTTCTGCATCGATGAT
GCCTTGTGCCAACTTTTCTATCAGTTGTTCTTGAGTCAATGTGTGATGTTTTTCTATGTTTCTTTGTCTTTTGAT
GCGTTTGTCAATCGCACCTTTAATTTTTGTGTAGATGCGTTGATTTTGATAAAAGTCTTTGCACACTTCTAATA
CTTTATCTTCAAGTGTTTGTGCATTGATGCCTTTAAAATCACAGACAAAGCGAGAAGCATTCATGTTTTTAGG
ACAGACGTAGTAACGTAATGTATGATTCTTTTTTCTAACGGTCATATTCGTAAGTGTTGTATTACAACATGGGC
ATTTGATTTTTTGTTTTAGTTGGTTATCCGAAGATGTCTGTTTGATTTGTTTTTGCGATCGAAGTCTCTGCGCT
TGCTCATATATACTTGTGGAAACAATAGAAGGAAACATATTGTCGAATTGGCCATATTGATTGTTGACACGACC
ACAATAATTAGGATTGATGATAATGTTACGAACTTGATAGGGTTGTCGATTGATATACGTGTTATCTTCTTCTAA
TAACTGTGCAATTTTCTTATAACCATGACCTTTAATGTAATAATTGAATACAGCCTTTACCGTTGGTGACTCATT
TTGATTGATGATGAATGCTCCGTTGTGATATTCGTAACCAAAGGGCGCATGGGTTGTAATCAATCGACCTTGC

TTTGCTTTTTCTTGAAGCCCATTTCTGACTTGTTCTCCAATGTTATCTGATTCAAGTTCGGCCAAGCTGATAAA
AATATTAAGCTTGAATCGGTCGAAAGCTTGATCCATATCAAAGTAACCATCGTGTACGCTTAAGATATGAACAT
GGTATGTTTGACATAATTTGATGAGTTTTAATGCATTTTTCAGATTACGATGTAATCTATTAAGACGATAACAAC
ATAATATGTCACATTGCCCTTGTTGAATTAATTGCGTCATTTGTCGATACCCACTACGATTATCTTTGCGTCCTG
ATTGTTTGTCGCTATAAAAGTTGATATGTTGAATATGATGTTTTTCGGCTATTGCTTCGATAGCCTGTTTCTGTG
CTGCAAGAGATTGTTGTTTCATCGTACTTTGACGTAAATAGCCTATGACTTGTTTCATATCGGCTCCTCCTTTC
ACAGTAATAATATATATTTATGGATGAATTGATATATAAGCCCAACATCAATAAGATGTTGGGCGTTCATATTAG
TCATTCATTTGATTGATTTCTTCAATTACCAAATCGGCTAATATCTCGATAAGTTCATCCATGTTTTTCACTCCGT
TATTTGTTCTATCTTCAATACGTCGATTATTCAGTTTGATGCTTCACGGTTGTATGATAAAGACAATCAGAAATC
TTCGTGAACTCCTGAAGGGCCTATCCCCTCATTAGCGGATTTAAAAAGTTCTTTCGCAGCTTTGTTATCATTTG
CCGGTGTCCAATTTTGAATTAACGACTTATCTTTAGTTAATCCCAGGATAGATGCAAACTCTACATCTAATTTTA
GATGGTAAAATACAAGTGATTGTTTTTTACCGCTATTATCTTTGACACTTCTTTTAGTTGTTTGGCGTCCACGG
TCAGCTAATATGAAACCTTTATCTCTTAAGGCGTTGACAACATTATTAACATCTTGAAATTGATGATTGTTTAGC
ATCTGTTTAAAAACGTTTGCAATCATTTTTACTTCGATATGGTCATCTTTTAATGAGATTAATCCATAGTTCTCA
AACATATTTTTCAAAGCACCTTCATCTGAAAACTTACCTCTGTTTTGTGCTACAAATTGAATGATGACATCGAT
AGCTTTATCAGCTAATGAGCGTTCAGAGATTGTATGACCATGATAATCAATAAAGTAGTCTCTTATTTTAGCGA
TATCAATATCTGTAGCTAAAACACGACCTAATATTTTTGCAGATGTTGTAATGACTGCATAACGCTTAAACATAC
GATTGCCTGTGTTGCTTTTATCATCTTTCAATTTAGCTTCAAACCAATCTACTTCCTTGTAAAACCATTGAATAA
CTTCATCTTCACGATTTATAAGATATTTAGCTACTAACGGTAAAACATGACCATAGTTTAGTGCTACAGCTTTTT
TAATATTGTCAGCATTGGTCGCATTTGTAGTGAATTGTTCATTAATCTCGATGGTTCTTACACGTAATCCATCGT
TTTGAGCTGAATCATTAAAAATACTGTGTTCTGACGTTGAAATGACAGAAGTACCCCAATTCTTAGGCGTTTT
AACTTCTCCATGAACGTTTGAACGTTGACGACCTTGACCTTCAGTGATGGAGTACAATAACCCCGTTGTATCT
CTAAAAGTTGCTGATGAGAGTTCATCAAATACAATAGGTATACCAAAATTGTTACTCAAGTAACCTTCAAGTG
CATTACGTGTGGCATTCCAATTTCTAAAGAGAGTTTCATTACCTTTGGTAGGGTTACCAGCGACTGATACAGC
TAAAGAAGCTGCAGTTGACTTACCGGTTGAGGATTGACCTGTAAAACTAAAAATGATTCCGGCAAATTCGGT
TTCATGTTTGTGCTTCAGGAAACTCGTCACTAAGGCAGAAATACCAAATACGACTGCTAATTCTAAAAGAAG
AGAACCTTTAACCTCTTTTAGATACATGTTAAACCAATTATCAAATGTTCCCCTAGGTGCTAAGTCATAAGTATT
CTCACAAATGGCGTCAGATGGAGATTTATTATCAAATTCCGTAGTAGTATAGATTTCATTTAACGATACAATAG
GACCAAACGGTGTTTCCAGTATACCTACCCCTTCATATAAGTAGGAAATGGGTAATTGGTTGCGCATTTGTTG
CAACGCATAACCTAAATCTTTTGTATATTTTTCATTAATACTAAATCCATATTTCATTAAAGAGGGCAGTTTTTGT
GTTGTTAAAATATCACTAGATTCAACAATTACTTTTTGATCCTCGTCTGTAATAATTACTTTTTCAGTGTTAGTTT
TAGGGTCAATAAACTTATTTTCGATAACGATAGGACCTGCGATTTCAACTTCAGTAGGCATTCCTCCTTTTTCT
TTGGGAGGCTTGTCTTTATACCAACCTTTTTTTGATTTGTATCGTGGTGAAGGATTAAATGAAGGGTTAGTTT
GAGTCATTAGCGAACACCTCCTTTCGAAGGGTTGCTGTTATAGTGTGGATTAGGACCTGTTTTAAGATAAACT
AAGTGACCGTGAGTATCCTTACCGATAATAATAAATGGAACACGTGGCGCATGTTTTACAAAATATGCGAACC
AACGTCCAACATTTTGTTGTACAGCTTTTGAACATTGTACATTTGCACGACTGTTCAAGTCATGAAATGTAAAT
TCTTCTCCTTCAGGTAAATTGAATGAAATACCTAATATTTTCTTTTTTAACTGCTCGAATTTATCTGATTGATTGA
AATACATTTAACTTCCTCCTACTAATGAACTAAGATAGGAAAATTAAATATGCGCACAATTAACTTTCTTAAGTT
CATTTTAGCCAAGAAGTTATCATTTGAAAGCTTGTAAAAATATGTTAGAAATTCCGTACTAAATTAAGATGGAT
TTTTGTTGACAAAATAAAAAACGCTGATTTAACAGCGCTTTAAATAAAAATTAATCTGAAGTTATATAAAAGTA
GTCAGAAGGAGTGGTGTTATTATCATTATAATAATCTAGTGCTTTATTGATTTCTTTTATTAATTTATCATGATGT
TCTTTTGTTATTTTCTTTGGATATTTGAGTTCTTTTTCTTCTAAGTCTTTTGGAGACACTAGGTCACTATTTAAA
ATTAATGATAACAGCATATTTTTAGGTGCCGAAGCTCTGGTGTCTCGATGAGTAATATATTTTGCTAAATTACTG
TTTATACTTACTTTTTCATTTTCTAAAAAGTTTGTACTATTTTTAACATGAGCATCAGCAATTTTGGTACTTATTT

CAGTGTTTAAATCGTCTATTTCATCTAAATAATATTCAAGAATCTGAAGTTTATCTTTAGAAGTGATACCTTTTT
CAGTGAAAATCTTAATAATGATAGATTTGATTCCAATTTCTATATTACTGTCTACTAGAAAATAGAGATAATAATT
AGTCAGAGCATGTTCATAATCTCTTTGATCAGTAATTTCATGATTGATGAAATAATATAAGTTCTCTTTGATACG
ATTAGAAGAATCTATATCTTTGTCATTGATACCGTTTTTAAAAAAGTGTAGATAAACTGAGTTGTTTGAGAGTA
TATCATATAATACAAATAAACTTTTTGGTTTAAATACATATGATTTTCCTGATTTAAAGTTTGCTATGTTTATTCCT
AGAGCGTTGCAGAATTTATTAAAATTAGTTGTAAAATTATTTTTTTCTTGAATAAACATGTCGTTGTCATTTAAT
TCTGAACTATGATTTTTAATCATATTGATATTTTCTTCAATCTTGTCACTACTTAAATTTTCCATGCAAAGTATACT
ATATCTTACTATATCACTTATATTTATGGAAAAATCTTTAGGAGAACGATTTAAAGTATCCTTTTTATTATCTTGAT
CTACAGCCCTACATGAATCATCAAAAACTTCTTTGACATTTATTTTATCAATGTTCAAAATTATGTCTACTAAAG
CATTTTGAAAATCATCGCTATATATGTCTTTATTATTCATAGTGTCACTACCTTTATTTTTAATAACACTATATATTA
TATCTTATAAACCTAATACCATGAAGAATAAAAAGAATGATTCAAATTTTATATTTAATCCATAGTTGGCTGCTAT
GGTTAGTGTACTAGTCTAGTTTTACTAGTATATAAAACAGTACTCAATTTTTATTTTTTCAAAATTATAAAAAAG
AACACCTATCATCGATAGGCACTGAACCCCTAAAACGGGAACTTAATAAAAACACCATGTTCTAGGCTATTAA
TTCTCTGTATTTTACAGGGGATAAGTAGCCTAGTTTTTGTTGAATTTGATTATTATTATAGTTTTTAATGTACTTT
TCGACAATATCTATTACAATATGATTAGAGCTATTAAGCTGATTATTGATGTAAAGAGTTTCAGACTTTAGCGA
GGAATGGAAACTTTCTATCGGGGCGTTATCGGCAGGTGTTCCCTTTCGGGACATACTTCTGATAATGCCTTTT
TCTTCGCATAATTGATAATAAGCATAAGATGTATAAACGCTGCCTTGATCACTATGTAATATATACCCTCAGGTAT
ATCGATTTGATTTAATGTATCATTAACTAAACGTTGGTCTTGTTTATCATCTATTTTATACGCCACAATTTCTCCG
TTATAAATATCCATTATCGAAGATAAATACAACATAGAATGATCAAATGGTAAATAAGTAATATCGGTTGTTAAT
ACTTCTATGGGACAATTCGCTTTAAATTATCTTTGTAATAAATTGTCTGTTTTATAATACGGTTTACCTATCCTTG
TCGTCTTTTTAGGTCTAACTCGGCAGTTCAAATGATGCTTCTGCATCATTCTCTGTACTCTCTTATGATTAATTG
GTGATGTATAACATTGATTAATCAGTGCTGTAATCTTACGATAACCGTAGGTATAATGGTTAGCTTCACATAATT
CAATAACTTTTTGTGTTACAGTATCATTTTTATAGGTTTTGTTTTTCCATCGGTAATATGTTGATTTAGGTATATT
TAATACTTCTAGTATCAATTTGATTGAATAGTTTCCTTTTAATTGATCCACTAAATCTATGACTACTGTTGGTACC
ACTTCCTTTCCAATGCCTTGTACTTTTTTAAAATATCCAATTCTATATCTTTTCTCTTATTTTCTAATTTTAATTGTT
CTACTTCTGACAGCTCTTCTAATCCTTTACCGTAGGTATATTGTTTACCAACGTGTTGTGAAAATCTATAACTTT
CCCCATTTCGATACCATCGCCACCAAGTTTCCACTTGTGTTCTATTTTTAATATTTAATTCTTTCATAATTTCTTTT
GTTGAAAATCCTGCTGCTTTCATTTCAACTGCTTTATACTTTGTTTCTACTGAATAAGAAACTCTTTTCATAGAA
AAAACACCTCCGTATGATTCATTTTAATATGAATTCAACGAAAGTGTTTTTATATAATTCCCACTAATTGGGGTC
AGTCTACTATATGATACGGTTTTTTAATTTAAAGTATCAATAATAATTGGATATAGAGGGAAAGAAGCTATAATG
ATATTTGCTTACTAAGTGGATAAAGATATTAGAAAAACGAGAAGCAGGACAGTTATTAAATCGGTAGATATTA
ATCATAAGTATTTAACTGAATTTTTACCTTTTGTCGAATATATAACTAAAGTTGAAGGTAGTAAAATATTTATCTA
GTCGATATTAAAACAATTTATCGAAAACGATGGTTTTCCATATCCTCTTTAGTAGCAATTTTCTAACAATATATA
ATTAATTATATACAAATTAAGAATAATTAAGAAGTATATATTAATTAGAGATAGTTTAAAAATTAGATTTCAATCA
ATTAATTAAAACAGGTTAGACTATTAATTTATACTAACCTGTTAAAAGTAACAAAATTAAGAATATATTAAAACT
TGTGTAATGAGTAAGCCTCCACCAAATATTAAAACAAACGCGACAACAGGCCATACGAAACGTAACCAGTGT
GAGTAGCGCACGTTTAACATTTGAAGTGTTGCCATTACAAGTCCAGTAGGCGCTAAGAACAACATTGCATAC
TGACCGAATTGATATGTTGTAACAATAACAAATCTTGGTATACCTACTGTATCAGCTAATGGCGCAAAGATAGG
CATAGATAATACTGCTAATCCTGATGATGATGGTACGATAAATCCTAAACAGAAAAAGATAAAGAGCAGAACA
ATGATAAATAAAGGCCCACTCATATGTTGCACGATAGATGATGAAAAGTGCAAGATTGTGTCAGAAATCATTC
CTTTATTCAATACTAAGTTGATTCCACGAGCTAAACCAATGATTAAAGATACACCTACTAAACTTGAAGCGCCA
TTAACGAATGCATCTACAGTGCCTTTTTCGCCTAAACCATATTGTCCTGTTCCAGCAATAAACATGATGACAAT
GGTAAAGATTAAGAATGCAGATGCCATGACTGGGAACCACCATCCTTGTGTCATAACACCCCAAACCATAATA
GGGAATGGTAGGACGAAAAGCGTAAGAATAATCTTTTTACGTAATGTAAACTCAGAAGAACCGTCATCATGG

AGCACAGACCACTGTTTTTCAAATGCTGCTTTGTCTTCATAAGAATAAGAGGATTTAGGATCTTTTTTAATTTT
TTTACAATACCAGAATAAATAACTAATAACAAATATGGCACCGATGATACAAGCGCCTATTCTCCAATAAAGAC
CATCAGTAAAAGTTGTTCCTGCTGCATTAGAAGCAATGACGACTGAGAATGGGTTGATTGTTGAGAATGTAC
TACCCACAGAGCTTGCTAAGAAAATTGCACCGAAAGTAAGACTTTAGAATCTATGGGAAAGATAATAAGGTT
TAATAGAAATACAAGAAGTGTAAATAGTAAGCATATAATTTATACATTTTAATATAATATAACAAGAAAGGTAAA
CACAAAGATAATAAAAATAAAAATTTAATAAAGATAGAGAATTATTAAATTGTTTTTGATATAAAACAACTTAA
TAAAAAATTTAATTTTAGGGGAGGATAATTATAATATAAAGAAACATAGAATGGAATTGCAATTGGGAAGTAT
ACGATTTTAACTGCGAATCATATGTTCAACGACTTACAAATGCGTAAATCGATAACACATTCAAAAATATATAAT
CTGAAAATTGATTTACTAAGAGAGGATTATTGTAGTTACAGGTGTTAAGTTGCTCCAATATGGAGTTGTCGTC
TTGTTTTATATTAAACAAGTTTTATTCAAATACATGACAATACGTAAAATAACACTAGAATTATCAATCACTAAA
ATTAAAACAAATGTATCTATTCAAACTTTTCTTTAGGGTTTACTTGCTGAATAATTTAAAAATGAAGTATTAGGT
GTACATGCTTTCAGAATTGCTTTTGAAGAATATTAAAAGTATAATCTGAATACGATGACAGAATGAATAGGTAA
ACTATGACAAGAAGTCATCAAGGTTATCAAATAAATATATCCCAGCGGTTGAGACGACTCACTGCTGGGATTT
TATTGTTAGTTATTTATTCTAAGTGTTGCATTTGGTAGCCGATTCTAGGATGTGTGATGATTAATTTCTTTTCAG
TGTTTGGGTCTTGCGTATAATTCAACTTTTTACGTAAAGAGGCCATATGTACACGTAAGGCAGCCATTTCTGTA
TGATTGACATAGCCATAGAGTGATTTTAATAACACTTGATAGGTTAATACTTTGCCGACATGATGGCATAATATC
GTTAGGAGTTGGAATTCATTCGGTGTTAGATGTACGGACTGTTCATTGACAAGGACTGATTTCGCATCGAAA
TCAATGGTTAATGGACCATTTGTGAAACGACTTTGAATTGTCTCAGTAGAACGTGACATACGTAATGCTACTC
TGATACGTGCTCTTAACTCATCGATATTGAAAGGCTTAGTCATATAATCATTGGCACCGCGATCTAATACTTCGA
CAATGGTTTGTTCCTCTGTTCGTGCACTAATCACAATAATAGGTGTGTCCACAAAGTCTCTGAATTGCTGAATG
AGAGATAAACCATCAATATCTGGTAAGCCTAAATCTAATAAGATAATATCTGGTTGTTCTGTTCTTAGGCGAAA
GTCCGCTTCTTTCCCCGTCTTCGCCGTAACCACTTTATAATAATTCATAGTTAGCGCAACATCGATTAAATGTAA
AATCGCTTCATCATCTTCAACGACCAATAATGTTGTTTTCATCCATGGCTCCCTCCATTTCAATTGGATTAACAG
TCAAATAAAAATAAAAAATACTGCCTTGTGGTGTATTCGGTTGATATTCTAATTCACTGTTGTGTTGTTTCAAG
ATGAGTTGTACTAAATAGAGCCCTAATCCCAAACTATCTTTTTTATTGTCTTTAAAGTTGTCTCCTGAATAATAG
GGATTAAAAATCAATTGACGTTCTTCTTCTGGAATACCTTTTCCGCAATCTATCATTTCGAATTTTATTTTGTTT
GTTTCATGTTGAACGTGCAGTTTTATTTCAGAATGTGATTCTGCATGCTTTAAAGCATTATCGATAAGGTTGAA
TAGCACTTGCAGTATTAATTTACTGTCGATATTAATGAGTGAAGCGTCATCCTCATTTTCAATAATGACATGATT
TGCTTGGTGTCTGCGTATGAGGCCTTCTTCAAATTCTTCTAGAAGTTCTTCTACTAAATAAGGGGTGCGTTGT
ATTTGAATGTCAGAGCTTTCTAACTTAGTCAAAGATAAAATATTTGTGACTAAGGTATGCAGATATTGTGCTTC
GCCATAAGAAGCAGTTAAGAGTTCTGCTTGTTGTTGATCGTTTAAATGCTCGTTATGGTATTTCAGCATATCTA
AGTTGCCCATAATGGAAGTCAGTGGTGTTCTAATATCATGTGAAATTGAATGCAAGAAGTTTGAACGTGTGG
CTTCTCGTTCAGCTTTCAATATGGATTGTCTGGTTTGTTTTAATAGATCCACATTCTCTATTGCCAGGGTAATAT
CGTTCAACATAGAGTCTAATATTGAATTGTCATAGGTGTCGATGTAAGTTTCGTCGGTGAAGCGGATAGAAAT
CACACCTTTGACTGGATTTGTGCCAATGGGAATACACAAAAATTTACTGCCAGGAAAGGTATCGGTTAATTTA
CCGGCACGGCTTTCATTTTCAATGACCCAGCTCAATGTCTCAGCATCATGTGTCTTATCTGAGCTTGAAATGCT
TCTGTTTCCAAATGAGTTGGAAGCAGCGACTTTCTTACTTTGAATTAAGAATACCGTGACATCCTGATTGAGT
AGCTGATGAATCTGATCACCAGCAATATTTAATAAGCGTTCAATTGAATAAGATTCTTTAATGGACTGGTTAAA
TTGCAGCATAATATTGGTCCGATATAACTGCCGTTCTGTTAAGGAATGCTGATGTTTTAAATTCTTTAAGATGG
CACTCGTAAAGATACTAGCAAAAATACTGGTCGCAAACGTAATCGGATATTCAAAGCGATACATTTCTAAAGT
GAATCTTGGCACCGTAAAGAAATAATTAAAAACAAACACATTTAAGATAGACGCAAAGAATCCGATTAAATA
GGATTGGGTCCAAATAGAAAGCACGATAATACCGATAAAGAACATCAGCAAGATAATGGCACTGGATTCGCT
TTTATCTAAGTTGTAAACCCATATACCGAGCAAGACACAGATCGTTTGAATTACAAGCATCTTCATAATCTCTA
CAGTTAAACGAGAGGATTTTTGAGCTTGCTTAGGTGTATTTGTAGGTTTGTCTGAATGAATATAATGAATCGG

TACAATTTCTAACTTGAAATGGTGTGGTACGTGATTAATTTGTTCAATGAGTGACTGTTTAAAGTAATCTTTCC
AACGTGGCTGTTCTGACTGTCCAAGGACTAGCTTCGTCACAAAAGCGAGATCACACCAATCGGTTAACGCTT
TCGCAATATCTTGTGCATACAACACTTTGATTTCTGCACCTAACGCTTTGGCGAGCATTAGATTTTTATGGACA
TAATGATCTTGTTTTCTGCTTTCTGACGGTGTCTCAAAGACATCAATATACACAGCTGTGAATTTAGCATGTTC
TTTATAGGCAGCACGTCTTGCTTCTCGTATGACCCGTTCGTTATAAATACTGCCGCTGATAGCTACCGCAATAT
GCGGTGTAATGTCGGTATGTTTAGTTTTATATTGTTGTCCTTTTTGACTCATAATATCTGCGACAGTTCTGAGTG
TAAGTTCACGCAGCTCTGTCAGATTTTCATACGTAAAGAAATTAGAAAAGGCTGTTTCTAAGCGTTCTTTTTT
ATATACTTTTCCTGCTTTAAGGCGCTGAATCAACATATTTGGTGAAATATCCACAACTTCAAAGGCATCTGCTG
ACGTAATGAATTGGTCGGGCACACGTTCTGTAACTTGAATACCTGTCATTAACGCAATTTGTCCGCTTAGACT
CTCGATATGTTGGATGTTGAGTGTTGTCCAGACATCGATACCATGCGATAGAATTTCTTCTATATCCTTATAACG
TTTTAAATGGCGCTCTTTTGAAATGTTTGTATGTGCTAGTTCATCAATTAAGACCACATCTGGATTAGCTTCTAT
GATTTTAGAGACATCTATATAGTGAAAGGTGTGGCTGCCAAATTTACGGCTGGAGGTCGAAATTTCAGGCAA
TTGTTGAACCAGTGCATTGGTTTCAGGGCGTTGATGGGGTTCGATATAACCAATTTTAATATCTGCACCTTCTT
GATACTGATCAATACCATTTGATAACATTTCATACGTTTTACCTACCCCTGGGCTATAGCCTAAATAAATGGTAA
GTTTCCCTCTTTTTTTATATGTACTTTCCATGAGGCACCCCCTCTTAATCACATCATAATTAATATACAACATATTA
TCCATTTATCTTTCTGTTTTGTTAATCATCTTTATAGTTTCTTTATAACTTTCCTTAAATGTTAATACTTCGATTAG
ATAACTCTTGTTAAGATTTTGATGAAAGTAAGGAGGGAGCGCAATGATTACACTATTAGCTGTCGTTGTCATC
GCATTAATTTTATTTTTATTTTACGCATTAATTTGGAGTGAAAAATTTTAACAGAGAAAGAGGGAAGCATCAT
GAGTATTGTGTTGTTTTTGATTGTATTTATCTTGCTCTCACTCATTGTGAGCCGATATTTATATTCAGTTGCTTTA
AATGTGCCATCTAAAATAGATGTTGTTTTTAATCCGATTGAGAAATTGATTTATCAACTGATTGGCACGAAATT
AGAACACATGTCTGGGAAGACGTATATCAAACATTTTTTGTTGTTTAACGGATTGATGGGCGGATTGTCCTTT
GTATTATTGCTTATTCAACAATGGCTGTTTTTGAATCCTAACCATAATTTAAATCAATCTGTATCGTTAGCCTTTA
ATACTATGGCATCTTTTTTGACCAATACTAACTTACAGCATTATGCAGGTGAAACAGATTTAAGTTATTTAACA
CAAATGTGTGTCATCACTTTCTTAATGTTCACGTCAGCAGCGTCAGGTTACGCCGTATGTATTGCGATGTTAAG
ACGTTTGACTGGAATGACAGATGTGATTGGTAATTTCTATCAAGATATTACGCGTTTTATTGTACGGGTGCTCA
TACCTTTCGCATTGATCATCAGTTTGTTTTTAATCAGTCAGGGCACACCGCAAACGCTTAAAGGTAATTTGGT
GATTGAGACATTATCAGGTGTGAAACAAACGATTGCATATGGACCGATGGCGTCTTTAGAATCTATTAAACAT
TTAGGGACAAATGGTGGTGGTTTCTTAGGTGCGAACTCTTCTACACCTTTTGAAAATCCGACATACTGGTCTA
ATTACGCTGAAGCTTTAAGTATGATGTTGATTCCAGGTTCATTAGTCTTTCTATTCGGTAGAATGTTGAAAACT
AAACTACAGATTCATCCGCATGCGATTATGATTTTCGTTGCGATGTTTGTAATGTTCATCGGCTTTTTAGTGAC
ATGTCTCTATTTTGAATTTGCGGGGAATCCAGTGTTGCATCACTTAGGTATTGCCGGTGGCAATATGGAAGGC
AAAGAAACACGTTTCGGTATTGGCTTATCCGCTTTATTTACAACCATTACGACCGCTTTTACTACAGGAACAG
TTAACAATATGCACGATAGTCTTACACCGCTAGGCGGCATGGTTCCAATGGTATTAATGATGTTGAATGCAGTT
TTTGGCGGTGAAGGTGTTGGGCTGATGAACATGTTGATTTATGTCATGTTAACGGTCTTTATCTGTAGTTTGA
TGATTGGGAAAACACCAAGTTATTTAGGAATGAAGATTGAAGGTAAAGAGATGAAACTCATTGCGCTTTCTT
TCTTAGTACATCCTTTACTTATTTTGGTTTTTTCAGCACTAGCTTTTATTGTGCCAGGGGCATCAGATGCGTTA
ACTAATCCGCAATTCCACGGTGTATCACAAGTGTTGTATGAGTTTACATCATCTTCAGCGAATAATGGCTCTGG
TTTTGAAGGATTAGGAGACAATACGGTATTTTGGAACATTTCAACAGGCATTGTGATGTTGCTTGCACGATAT
ATTCCAATCGTTTTACAAATTTTGATTGTATCTAGTTTGGTAAATAAAAAGACCTATCAGCAACATACTCAAGA
TGTACCGATTAATAATTTATTTTTCAGCAGTGTATTGATTATCTTTATTATTTTGTTGAGCGGCTTAACGTTCTTA
CCTGACTTAATGCTTGGACCAATAGGCGAACAGCTTTTGCTGCACGCATAGATAAAGGAGGATTAGAAAATG
GCTGAAACTACTAAAATATTTGAATCACATTTGGTCAAACAGGCTCTAAAAGACAGTGTATTGAAGCTCTATC
CTGTTTATATGATTAAAAATCCGATTATGTTTGTTGTAGAAGTGGGCATGCTGCTTGCCTTAGGATTAACCATT
TATCCGGATTTATTTCACCAAGAAAGTGTATCACGGCTATATGTGTTCAGTATCTTTATCATATTATTACTGACAC

TTGTCTTTGCGAACTTCTCTGAAGCATTAGCTGAAGGTCGCGGTAAAGCACAAGCCAACGCTTTACGCCAAA
CACAAACTGAAATGAAGGCACGTCGTATTAAACAAGACGGCAGTTATGAAATGATTGACGCTAGTGACCTG
AAAAAAGGACATATCGTACGTGTCGCGACAGGTGAACAAATCCCAAATGACGGTAAAGTTATTAAGGGCCT
CGCAACAGTGGATGAATCTGCGATTACAGGTGAATCTGCACCTGTAATCAAAGAAAGCGGTGGAGATTTCG
ATAATGTAATTGGAGGAACTTCTGTAGCTTCAGACTGGTTAGAAGTTGAGATTACTTCAGAACCAGGTCATTC
ATTTTTAGATAAAATGATTGGTTTGGTTGAAGGGGCTACAAGAAAGAAAACACCTAATGAAATTGCGTTATT
TACTTTATTGATGACATTAACGATTATCTTCTTGGTCGTTATTTTAACGATGTATCCATTGGCGAAATTCCTGAA
TTTCAATTTATCCATTGCGATGCTGATTGCTTTGGCTGTGTGTTTAATTCCAACAACCATTGGGGATTATTATCG
GCTATAGGGATTGCAGGGATGGATCGTGTGACACAGTTTAATATCTTGGCTAAAAGCGGACGTTCTGTAGAG
ACTTGTGGTGATGTGAATGTCTTGATTTTAGATAAAACAGGTACCATTACCTACGGCAACCGTATGGCAGATG
CGTTTATTCCGGTGAAATCATCAAGCTTTGAACGTTTAGTTAAAGCGGCCTATGAAAGTTCTATCGCAGATGA
CACACCAGAGGGACGTAGTATTGTGAAATTAGCTTATAAACAACATATCGACTTACCGCAAGAGGTCGGAGA
ATATATTCCGTTTACTGCTGAAACACGTATGAGCGGTGTGAAATTTACGACACGTGAAGTATATAAAGGTGCA
CCGAATAGTATGGTTAAGCGTGTGAAAGAAGCAGGGGGACATATTCCAGTTGATTTAGACGCTCTTGTCAAA
GGGGTGTCTAAAAAAGGTGGCACACCGCTGGTTGTGCTTGAAGATAATGAGATTTTAGGTGTTATTTATTTG
AAAGATGTCATTAAAGATGGACTCGTAGAACGTTTCCGTGAATTACGTGAGATGGGGATTGAAACGGTGAT
GTGTACAGGAGATAACGAATTGACAGCTGCGACAATAGCGAAAGAAGCGGGTGTGGATCGCTTTGTGGCA
GAGTGTAAACCTGAAGATAAAATCAATGTGATTAGAGAAGAACAAGCGAAAGGTCATATTGTTGCGATGAC
GGGTGACGGTACGAATGACGCGCCAGCTTTAGCAGAAGCTAATGTAGGTTTGGCAATGAACTCAGGAACCA
TGAGTGCCAAAGAAGCGGCGAATTTAATTGATTTAGATTCTAATCCAACCAAACTGATGGAAGTCGTTCTAAT
TGGGAAACAATTATTAATGACACGTGGCTCACTCACTACATTTAGTATTGCGAATGACATTGCGAAATACTTTG
CGATTTTACCAGCCATGTTTATGGCGGCTATGCCTGCGATGAATCATTTGAATATTATGCATCTGCATTCACCTG
AATCAGCAGTATTATCTGCGTTAATCTTTAATGCGTTGATTATTGTATTATTGATTCCGATTGCGATGAAAGGCG
TGAAATTTAAAGGTGCCTCAACGCAAACCATATTGATGAAAAATATGTTAGTTTACGGCTTAGGCGGTATGAT
CGTGCCATTTATCGGCATTAAGCTCATTGATCTCATCATCCAACTCTTTGTCTAAAAGGAGGACAAAACAATG
CAGACAATAAGAAAAAGTTTAGGACTAGTACTGATTATGTTTGTTTTATGCGGATTTATCTTCCCGCTGACTGT
CACAGCGCTTGGACAAGTATTATTTCCAGAACAAGCAAACGGCAGTTTAGTGAAACAAGATGGCAAAGTAA
TTGGTTCAAAGCTCATTGGACAACAATGGACAGAACCTAAATATTTCCATGGACGTATCAGTGCAGTCAATTA
CAATATGAATGCGAATGAAGTGAAAGAAAGTGGCGGACCTGCTTCAGGCGGCTCAAACTACGGCAATTCAA
ATCCTGAATTGAAAAAAAGAGTTCAAGAGACTATTAAACAAGAAGGAAAAAAAATTTCAAGTGATGCGGTG
ACCGCTTCTGGCTCTGGTTTAGACCCAGATATTACGGTTGACAATGCGAAACAACAAGTAAAACGCATTGCG
AAAGAAAGAAACATAGATGCTTCAAAAATTAATCACCTTATTGATGAAAACAAACAAGCATCACCAATGGCA
GATGATTATGTTAATGTCTTAAAATTGAATATCACTTTAGATAAACTCTAAATAAACAGGGAGTGAGGTGAGA
CATCCATGTGGTTCATTAGCATTATTATTTTAATAGCATTCCTAATTATATTAATGATTGACGATTTAATTAAAACA
TGACAACTTCACGCAAAACAATATGAATTGGATGTAATAGAGAAGGGAGTACCAAGAAATACTCAGTTTATT
GAGGATAATTATTAGGGGAGATTAGAGAAGAATTCTCTATTCACGAACATTACTTTAACAGTTGTAATCATTG
GTTTCATCATATCGATTAATAGTACAGTCATCTTGAGATTTTCAACACACTATTATAGCTAGCTAAGATACTTTTA
AATTATGACTTATTTTTCATCATTTACGATACATCATTCTCGATAACTTACTATTTGTTATGGTATTTATAGATTTA
AAGAAAGTGAAGGAGATTAAGCAATGTAACTTGGCTTAATCTCTTTTTGATTATATTTATTAAGGCAAAGCTT
CATTTCCCTTAAATCAAGTATATCTTCTGAATAAGCAAATGAAATCGTGACGCCAACTACAGTGATAACTTCGC
CTAAAACACAATGAGTGTGGTGTTTTCACTTTTCATAAACTCCTCTAAGTTTGTGTTTTTAAACTGTGATGGAT
TTGAATGTTTTTGAATTACGTTGTCGCGTCCTTGGGATTGCAATCCAAGAACGTCAGCATAATCTAAATCAATT
TTGATTTGGTAGAAGACAAGAGAAGTACTTTTGCCTGTATCATCTTTCGCATTTCTTTTTGTTGTCTTTCGATC
CCGATCGGAAACTAATTTTCCTGCATTATCCAAAGCATCAATAACATTTTTCGTGTCTTCGAATTGACTGTCTG

TTAACATCTTATCGAATACATTTCGGATAATTTTTACTTCGATGTAGTCATCTTTAAGTGAGAGGATACCAAAGT
TTTGAATCATATTAGCAAGACGTGTTTGTTGTGAAAACTTGCCCAGGTTTTCCGCAATAAACTGTACGATTGT
ATCCATCGCTTTATCTGCAAGAGAACGTTCTGACACCGTTTCATTATGATAGCTTAATAAATAGTTCTTTATGCT
GCTAACGTTTAACTGTAGGTCTAGTGATTGATTTAAAATATGGGCTGTTGTTGTTAGAGTTGCATAATGTTTAA
TCATTCGAATACCAGTGTTACTGGATTCAGTTGATAGTAATTGATAAAAAACACTGTCTTCTTTTTGAAAAAG
GGAAGTAACATCATTAGTGTTGTCTAATAGGAATTGAGCAATTATAGGTAAAACATGACCATTATTTTGACTAA
CCACTTTTTTTATAGTATCTGAGTTTTGAGCACTTGATGTGAATTTTTCAGACACCTCAATAGTACGAACACGC
AAACCATCATTTTTAGCTGGATTTTTGAAAATACTATGTTCAGAAGTTGAAATCACTGATGTATCCTAGTTTTT
AGGTGTTTTGACATCACCATTTATATTTGCTCTTTGACGTCCTTGTCCCTCTGCAAATGAATATAGAAGACCTG
TTGTATCATGAAATGTTGCCGCTGATAATTCATCTAATACTATGGGTACACCATAATTTCCGCTAAGATATCCTTC
TAGTGCATTTCTTGTTGCATTCCAACTCCGGAATAGGTTTTCAGTACCTTTTGTTGGGTTGCCAGCTACCGAT
GCGGCTAACATAGCGGCTGTCGATTTTCCTGTTGATGATTGGCCAGTGAAAGAGAAAATAGTTCCTGAAAAC
TCGACGTTATTATGATACTTAAGGAAAGCAGTGACTAATGAAGATACGCCAAACAACACATCTAGTTCTAAAG
ATAAGTTCCCATGGACTTCTTCTTTATACATTTGTAACCATTCTGATAAATTGCCTTTTGGTATTAAATCGTACTT
GTTATCACATATAATAGAAGTGCTATTAGTAATCTCATTAGAAAAATAAGGCTGGTCTAATGAAATTAATGGTCC
AAGTAATGTATGAAGCATTCCTACACCTGAGTATATTGTTGATAAAGGTGTGGTGCTTCTCATTTGTTGCAGTA
CGAACCCTAAATCTCTAATGTGCTTTTCATTAATGGTAAATCCATATTCAATTAAGGAGGGTAATTGCAAAGCA
GTAAGGATTGAAGCCTTTTCGACAAGCTCTATATTATTCAAATCCGAAAGAAATACTTTTTCTTTACGAGTCGC
GGGGTCTATAAAACGATCTGTGAAAGCAATCGGTCCAGATAACATTATTTCAAACTCCGTACCCCCATCTTTTT
TAGGAGGGATGGTTTTGTACCATCCCGAAGGTTTGAGCTTGAATTCGTCAGTTTGATATAGAATTTTTTCCAT
TAACAGTGACCCCCTTGGTTGTAAGAGGAAATTATAATGATATGTCGGTACTTAAGTGGGTTAGTTCCTATAAT
AATGAAAGGGATGCTTGGTTTGTACTTTATCCACCAAGCAAAGCGTCTTCCTGCTTCACGTTGATCGTTGATG
TTGCACTGTTCTCTGAATAACTGTAATTATAAGGTTTAGTAATGGCAATTTTTTATTTAAACAAATTTTTACGAA
TGGATTGTCTGTTTATTAATTCAGCAACGTTTATTGAAGAAAAATAAGTGTCTTCTGATAATTGCAAAGGGTTT
AATTCAATGTAAACTGAATTAAGGGTAGGGTAGGTATGCCTGTTTTTTGGTTCTTTTAAGTTCAATCGATTAAT
AAAATTGTTGAACCTTTGTTCTAAGTCATACAAAAACATCAGTAATTCATTAGGAGAATGATTTATTATGCAAT
CCATATTTTCGTTTATGCTTGAAGTTATACATCTAAAAACAGTATTGGTTTCTTCGTATAAAAATAACTTATTAAT
AGAGGTAACATTGATTAAGACTGAGTATACGTATAATAAGAGTGACTCTGACATCTCATTTAGATAAATAAGAC
TGTGATCATTCTTATTTACGATTATTATATGTGATTGAATCAATTCAATAATGTCTAGAAGAAGACTTGAAAAGT
CGCTGTTAATCTTGGTCTTTGAAAAATCATCGTGACCATTTGTCTTATTACTTAGATGTAGTTGTTCGTATTTTA
TAGTTTGGCGGTTGAGGAATTAAGCATTTGATTATTATAAACTATCTCAAAAATTAAATTACCTCCTTTTTTCTT
CCTCGATGAAGGTTTATCTAATCTTCCAATATACAGGTTTTTGAAGTAAATTATAGATATGATAATCATTAATTCA
GTAAATGTATACTTATTAAGGGGTGGAGCTTTAGCTAACTTATCGATAGTACTGCCGATGCGTGCTTTGAAAC
TATTCAAATTGTCGTGACTGTCTATATCTAAATCTTCAAATTTACTAGCAAGGGTTTGCAAAGTAGAAATTACA
ACTTCTTCTTTTGTATAATATTTATTGAAAATATCATCTTCTTTATCATCGCTTTGGAGTTTTAAAGTGCCTGGTT
CTAAGATGTCAAATTTTTTGTTTCGATTGGTATTTTTTTAAAATGTGCCTTGTATTTTTGGGGTTGTTCCAAATG
TGTATTTGCTAGACTACCTGTAATTGATATTGCTTTATTTAGCATTTGGTGTGTATTAACTGTATCTGATTCTTCT
GGAAATTTAAATCTAAAAGTAAAATCTCTACTACCGAATGTACTTTTTACGGCTACAGTTTTGTACTTATGCTC
AGGCAATGTAATTAAACCATCTTGATGCAGGAGGAAAAGTGTGTCATCATTTAATTTCTCAATTGTATTAAATA
AGTAATTATGCATAGCCTCTTTATACATGGCGAATTGATAATTTAGATTATCCCATTCTATTAGTTGATTTAAAAT
ACAATATAGACCTTCATTCAATCGGTTTACGAATATCAATACAGCTTCTTCTCGTCCAACAAGGCTTATTAACTC
CTTCAAGTCATCCTTTAATTTTAATTTTTCTCCTTCAAAATTGTATTGAGTGAAGCCTATAGCCTTTTGAAAAGT
AACTTCCATGACAGAGTTGGTTTCAGAATATTCTAACGATTCATTTATTGCAAAAATCAAATTTTTTATAAATAT
GGAATTACTCATGGTTTGTAATTGCATATACTACCCAACTTTCTTATTTACTTTAAAACAAATATAAGCTAAAGT

AGTTATTATCATAAAGGGAACGTACGTTCTTATTTAGCTGATTTCTATGAAGAGAGGTTATGGATATTGAATGA
AGAAATCTTACGCGAAGTAGCAGATATTTTTATAGGTGATGACAGAGATAGCATTTATGATTATAAAACTGGG
AACGAATTAGTGAGGTTTTTTAATCATTACTTTAATAAAGGGGACATATATCAGGCTCCGTTTCCATCTAGATG
GCTATATGTTGTGAAACATTTGCAAACTCTGATTCAGGAGAGAAAGATCAATCAATTTTTCACATTAATTTTAA
GCAATCACTATATTAAGTATGAACTGAAAATTGACGAGGTTGAAGCAGCAAAGCAGGCTGCTAAAGCACTTA
AGTTGTTCAACAAAAGATTAAATCATTATGGGTACTACATAACAGGAACTAACAATGCTAGATATTTTATGGAT
AAGGATGAAGATACGGAGTCTATTGGTTATGGAGGGTATGCGAATATTTATTTACAGAAGACTACAGGTCTT
GCTGTAAAAAAATTGAAAGAGGAGTATCTTACTGATTCTTCAATTAAAAGTAGGTTTAAGAGAGAATTTGAT
CTCACTAAATCTTTTGATACAAATCCATTGTTCATTAATGTGTTTGAATTTAATGAATCAGATTATTCATACACGA
TGGAGTTAGCTGATGAAACTTTGAAAGATTACATTGAAAGCAAGACAATTAGTGAGCTAGAAAAAGTAAAG
ATAATAATGAAAATTTTAAAAGCGATGAGTCAAGCACATAGTGAAAATAAAATACACAGAGATATCAGTTCTA
AAAATGTATTAATGTTTAGAGGAAAAGTCAAAATATCAGACTTAGGATTAGGTAAAAACCTTGATGAAATTCA
TTCGCATCAAACCTTTGATACAAACGGTGTAGGACAATATAAATACTGTGCACCGGAACAGATGTATAGTTTA
AAACAAGCAGATAAACAATCTGATGTTTTTAGTTTAGGAAGATTGATAAATTTTATTATGACTGGAAATGTAG
TTAACAACCATCACCTATTTAGAGGTGTATCTGATAAGGCTACGAACAGTAGTAAAGAATACAGATTTGAAGA
TGCAAATGAAATGTTGAAAATGCTGCAGAGAATTTTAGAGTATCACAGTAGTGCAAAGCACGTCGAAAAAT
GTCAAGAAAAGCTGAAAAGAGGAGTGTTTGATGATGAAAGCGAAGAATTTATTATGACACGAAGTGATGAA
CAATTATGTCAAATGGTTCTAAGTTCTAATAATAATGAGCAAGCGTGTTTAATTCGTTATATGCAAAAAAACGA
ATCTTCAGCATGTGATTTAATAGAGAGTATTAATAGAAAGTATCAAGAGTTTTGTGGAAGGTTTGAAGACTAC
GATCCTTTTGCTAAATTAGCATATATGATTTTATGTAATAACTTCAGTTATAGAGTGAATGAAACAGCAGCTAG
AGTGCTAAATTATGTTGCTTGGTCTGTAAATAGATTTTCGGCACAAGACTTAATTAAAGGTTTAATTAATAGAG
GAGTGGAGCCTTTGATTGAAGAAAAATTAAAGGACAATTAAAAAAACCTCATCATTAACTGATACGCAGAG
GCGTATCATAAGT
>Staphylococcus aureus strain ER11501.3
GAGGCTTATCATAAATAAAACTAAAAATTAGATTGTGTATAATTTAAAAATTTAATGAGATGTGGAGGAATTAC
ATATATGAAATATTGGAGTATACCTTGCAATATCATACGATGTTTATAGAGTGTTTAATAAACCATTTTTCAACTA
TTGATGATCTAGAATATATAATAACTGTACAAATTATATTGATTATGGAACTACAATTCAATTAAGAAATTGATGA
TGAAATTTTAAATTTAAACTAATGGAATCAAGAAAGAATGAAAGGAAATATACAATGCCCACGATTAATAAAA
GGAAGTTTATTAGATTTTGTGTTAGAAACAGTAGATGTTTTTGTAAATGGAAGTAAAAATGATTGCGAATTAG
TAAATTTTAATTATAAAGAAGGAAAAGTAGTTTCACGTTATAGTACAAAATTTGAAAGCAATCCTAAATTAAG
AAAAAAGCAATTGATATCGACGGTAAAAGTTGTAAGGTATGTTATTTTTGATTTTGAGAAAATTTTATGGGGC
ATAGGGAAAGTTTTATTGAAGTTCATCATATTAAGGCAATGTTTACTATAAGAAAGTTGAAATACACTCAGAA
ACAGACTGGATTCCCGTTTTTTTCAAATTGGCACAAAATGATACATAGGCCCACCTATATATTGAAGATTTAAG
TTAAACGATAAATAATATTTTTCTCTTTTGTATAGAAATTTTTTTGGGAATGATATTAATTAGTATAAAAATGAAT
ATGTCTACAAAGAAGGAGAGATTTTATTGAAACACATTAATCAATTTTCTGAATCGACACTAGCACAACTAGA
AAAAGATCAACAACACGTATTTTATGTATACTGTTTGATGGACCCAAGAAATGATGAATGCTTTTATGTTGGTA
AAGGTAAGGGGAATAGAATATTTAAACATAAACAAGATGCGCAGAAAAAAGTATTGTACGAAGATATATTTAT
AGAAGAAAATAAAGATAACTTGAAATTTAATAGAATTAACGAAATTTGTAGCAATGATTTGAATGTTTTAGGA
TATATAATCAGTTATGGATTAACCGAATCTGAAGCATTTTCTGCAGAAAATGTTCTTATAAACTTTCTTAACCTA
ACAAATAAAACTACATTAACAAATATGATTAACGGTCATGGATCTAAGGCATACTTAGTTGAAGATTTAGAAA
ATGAATTTGGCTACGATTCAATTAATCTTGAAAACATAAATACGAATGAATTAATTTTAGCAGTGAAAATCAGA
GATGCATTTCTATTAGATAAGGATGAAAGTAAAGAGTATCCTATTAATGAAAGTAAACGTGATAGAAATAACC
TTAAATCGCGTACATTAGGTAGTTGGATAATAGGAAAAGATAAAATACATAAAATAAAATATATTATTGGTATCA
ATACAGGTGCTAATAATGCAGTTGTCTCAGCTTATGAAGTATCATTTGAACAAGCGGAGAGTATCGAAACAA

ATAATGGTAGAATGAGATATGCGTTTATTGGGCTTTCAGAAAGAGATGCTACTCTAAAAAAATTGAATTTATAC
AAAAAAGCACTACCAGATTTAAGATTTGGTAGTGGTAGCGCTACAGCATATATAAATAATGGGACAATGAAA
GTTGATTAAATGAATAAAAACATCCAATTCCTTAAATTAGCCTTTGAAAATTTTATACTCGTATATATAAGTGAA
AAACTTAAAAGAATTAGATGTTAATTAAAGGAGAGAATGCTATGCCATCAGATGATATTGTAAAAAAACTATA
CTCAAAAGAGGATATTCGATTAGAATTAGGACTTACTCCGCATAAGTTTAATAAAAAAATGGAAACTATTGCT
AAGCTTTTTAAAATTGATATGAAAATTTTTCACAATTACAAAGGCCAAGATAAAAATAATCAGTATACATTTAA
TGGTGTCGCAAAAGAATTAATCAAAGTGTTGCTAAAAAGTGTTGATTATTACCCAGTGGATATCAATTCCAAA
AAATTTAAACAAAATGGAAAATCTAAAAAAGAAATGATTGAAAATATAGATAACTCTAGTTACATGAAATATAT
TTATCAGTTAATGAAATCTATCAATGAAATTCAATACAAAAGGTTAATTGCTGATATACATATGAAAGATGTGTA
CCAGAATACAAAAGCATGGTTAAATAATGGTGAATCAATTAATAAAAAAGAACAAGAGCTATACCAATATATG
ACAATTTTACCATTACATAAAAGAGTTGAACTGCAAAATGAGGTATTAAAATCTATAGACGAAACAATTTTTC
AATTTCTCGCAAAAGAACATAGAAATAATCAAATTGAAGAAAATAATGAATTAGAAGCATATACAAAAGCAAT
AAAAGAAGGTAGAAATCCTAAAAATGATTATGAATTAAATCATCTCCTATATAAAAAAAATCAATTACCCTTAG
ATAGTCTAATAGAAGATATATGGGACTATGAGGAAACAGAGTATACTGAGCTTGATTGGTTAATTGCAGATAT
GCTAAAACGTTCACAAAGAGCTGATAGTAATTTTATCGAAAGTTTAGAGAAGAAAAACAAATTGAGAAAAA
ATATTCATAAAGATTCCATTAAAAGTATATCTAAGTTGATTGATCATGAATTAGTTAAAAATAATAGAAAGTGG
GACGTAGCAGAATTTTATAAAAATCAACAATTTTGGAATAATAGTACATTAAGACGTAGCTCTCAAAATTTTG
GAATTTTATATTACAAAAAGAATAGGTTACATACAATGAATCAAAGTAATATTATTAAGAAAATAAAAATTATCG
AAGCTAATATAGAGAGTGCAAATAAACAGCCGAGCTATTTAGATTTCTTTTTACAAGCTAAGTTTGATTTAGA
GAGGTTAGAAAGTGAATTAATGAAATATAGCATTAATCCGAAATATTTTAATAAGATAAATAGTGATTACGTAA
GATATGCTATTGAAGATGTACATAATGCAATTATTAAAATTGACAGATATATAAGTAACGATAAAGTGAAGTTA
AACTATTCAAATGTCCAAATGGTAGTGAGAAACGAAATAACAGAACAAGGAAGCTACTTTGTAACCCAGGC
ATTGAATACACTGTCAAAGGTAGAAGAGTCTGGCTACAGCTTTGATAATTTCATGGTAGGACCTTTCGTAGAA
GACTTTAAAAAAGCAATTAAAAATCGTAGATTTGGTTCTAAAAAAGATTAAGATTGTTTTTTAGAGTTAAACA
TAAGAATAAATTTAAATAAAAAAATAAACCCAACAAACCTTGTTATATCAATGTTTGTTGGGTTTATTTTTTTG
GTGATTTTTTAATAACTCAATTACATCTTAGGATAATATCGCTAAATTATACATTGTCGAGGCCAAGACAAATAA
AAAATTAAAGCAAAAGTTAGCGCGCAACTTTCTGCTATAAAGGAGCAAATTTATATGGAGACAAGAGACAA
ACTGATGTCATTGACTCAAAGTGACAAAACACAGCAATGGCTAATGGACAAGTCATCTAACCAAGATGACAT
TCAACAATTGCAGCAACAATTCAGTCAGCAGCTTGATCAACAATATAATGCACTTTTAGCTGATGAAAAAGCT
AAGTTAGACCAATACGTGGAAGTACATCAAGGATTGGAATCATTAAAGGAAGAGATTGAATCAGAACCTATT
ACGCTTAATATCGATAAATTACCCGATATCAAAGCAACAATGCTTGAAAGAGCCAAGAATGATGAACATTCTG
ATAAAATCGAAAAGCTATTTGATAGGTTAGAACAGGCATTAAATGGTACGAATCGATTATATACGCAATTATCG
TTGATTGGTACACGAACACATCGAATTACAACTAAAAATTTTAATCTTCAAGGCTTACCTAAAGCAGTCCAAC
ATACGATTTTACCTTCAAAATTTAAGAAGGTGTATACAGTCGATTTTAAATCGTTTGAACCATCAGTTGCAGCG
TACATGACTCAAGATTCAAAACTGATTGACTTGTTAAATCAGAAAGACGGACTGTATGACGCATTGCTAAGT
GAATTAGGCTTATCAGATGAGCTACGTGTATTTGTTAAACGTGCATTTATTGGTTCGTTTCTATTTGGAGGTAA
CTTCAAAAAATCCTAAATTCAAGCTGAATCAATATGTAAGTGAAGTACAATGGTTGGATGCGGTCAGCCAATTT
ACAAAAGTCATTGAACTTAAGAAGCACGTTGAAAAGAGTAATTCCATGCCTATGCCTTATAGTATTGAGCATG
ATATGAGCGCATTTCAAGGTAGCAGTATTATGGCAATCTACGTACAAACTGTAGCGAGTTATATTTTCAAGCAC
ATTCTGCTAAAAGTGTACAAAGCACAGTGCGATCAAAAAACGTTCAAGATTATAGTACCTATACACGATGCGA
TTATGATTGAATGTGAAGATGAAGAAATTGCACAAAATGTGGGTCAGTTAATGAAAGATACGGCTAACCAGT
TGTTCAATGGTGAATTTGCACATGTGACAGTGGAAGAAATAGGAGGCGTAGACCATGAATAATGATAGAGG
ACAAAGCCTACACATCCCAAGTAGTACACCAATCAAAGAAAATAATATATATGTAGCTACGTTACATTCTGTGA
TCCAAACAGATTTCTCAGGTGAAATAAAGCACCAATTCACGTATGAAATTGAAGTGAACAATCAGATTGTATA

TGCGAATCGTAATATTCCAACAAAACCGAGCGCTAATCAGTTGTCAATTCATGATTGGCTGAAACGTCATAGC
AACTATAGCGCAAGTCATGAAAACTATGAGCCTTATATTGATCAGAAACATTTAATTCTATTAGGTCAATATAAC
GGTAACTATTATGTACAAGATGTAGCATCGTTAGATGCGTTTGGAGGCGTATTATCATGAATCATATATTACAAA
TGTTATCTAAGCTATTAAGTGTGGCCAAGGAGGCAATCGACCGTCAAGGTCTGATTGCTATCCTAACTATTCCT
GTTAATAATAACGATGAAATAGAAGAAACGGCTCAAGGTGAAACCGTGTATAACGAACTTATCGATCAGTTA
CGACTTAATATCCCAAAAGATACGGATTATCAACCTAACATCTATAGTTATTTTGGTATTAAGAAGAATCCTAAT
GACACCGTACTCATGGAAATGATGATAAAGGTTTTTCATATCAAACGCTTTAATTCAGAACTGTTTATTTTCAA
AGTTAACGGGTGGCAAAAGATAAATGGAGATGAATTACAAGGGTTGATATCTAAAATGATACAAGTATTGCT
TGTAGATTATAAGCCTTCACTAAGCACTCTAAAAAATGTCGTAGATGGATTGCAAAAATCAACAGATGTAGAA
GAACTTGTTGAGAATGAGCGCTATATTGGTTGTGGTGAAAATATGTTCGATCTTAATACGTTTCAAGTCGTTA
AAAATTCAATCGATATCTTTCCAAAAACACGATTGAATTTATCATTAAGTACAAATGATGTAATTACTGATAAGA
TACCGCCTTATTTTAAGCAATATATGTTACAACTTGCGAATTATGACGATGATTTACAATACTTTCTTTTCCAAC
ATACAGCAGTATTACTTACAGCTGATACTAAATACCGTAGGGGTCTCATATTATATGGTGGAGCTAAGAATGGT
AAATCTGTATATATTGAACTAGTTAAATCATTTTTCTATAGTAAAGATATTGTGTCTAAGCCACTTAATGAGCTT
GAAGGTCGTTTTGACAAAGAAAGTTTAATTGACAAAAGTCTAATGGCAAGTCATGAAATTGGGCAATCTAG
GATTCAAGAAAAGATCGTAAATGACTTCAAAAAGTTATTATCTGTAGAATCAATGCATGTTGATCGTAAAGGA
AAAACTCAAGTGGAAGTCATTTTGGATTTGAAACTTATTTTTAGTACAAATGCGATACTTAATTTTCCTCCTGA
ACATGCGAAAGCTTTGGAGCGTCGAATTAATATTATTCCATGTGAGTATTATGTTGAAAAAGCGGACACTTCA
TTAATTGATAAGCTCCAGAGTGAGAAGAAAGAAATCTTTCTTTACTTGATGTATGTGTATCAACAGATTGTAA
AAGCAGATATCGAGTATCTTGAAAATAGCCGTGTCACTGAAATTACTCACGATTGGTTAAATTTTGGATATGA
ATTTGTTTCTAGCAGGTCCGTAAGTAATGCAAATCAGAAAGCATGTATTAATTTACTCAGAAAACTTATAGAA
ATCAAATCAGGATCACGAATCAAAGTATCCGAGTTAAATAAAGTTATTAATGAAGAAATAAAGGTAAGTTCTC
AGGTTATTAAACAGTTAATTCAAGCAAACTTTGATACTCAAACCAAACTATACAATGGCTACGATTATTGGATT
GATTTAGGTTGGAAAGAAGCCAATAAAAAAGAGATTCATGATATTTCGGAAAAAGATAATATTATTTCATTAG
ATAAAAATGAAAATATAACAGACGATGAGGCATTAGATGAAGAGAATTTGGACTTTGATTGGGAGGACTTTG
ACGATGAATAATGAACAAATTGAAGCATTTGTAGAAGTGCTTGTACCTATCATAGAAGAACGTATCAATAAAG
GTAATTAAGGCTAATTACGTACTACAGGCAGTTGCCTGTAGTACTCATATGATTAAGTGGTAAAAGTGATAAA
AATGAAACGAAATTATAAATATATATTATCTATATGTTGTTACAAGACCGATAGTCTGTAGCAATAATCTAATAAA
AGGAGCGGTATGATATGAAGGGTAAAATTGCACTTTATTCACGCGTTAGTACGTCAGAGCAGTCGGAGCATG
GGTACTCAATCCATGAGCAGGAACAAGTACTCATCAAAGAAGTTGTGAAAAATTTCCCAGGTTATGACTATG
AGACATATACTGACTCAGGCATTTCAGGTAAAAATATTGAAGGTCGTCCGGCAATGAAACGTCTATTACAAGA
TGTTAAGGATAATAAAATCGAAATGGTGTTAAGTTGGAAATTGAATCGTATCTCACGATCAATGAGAGACGTG
TTTAATATTATTCATGAATTCAAAGAACATGATGTAGGGTATAAATCGATTTCTGAGAATATTGATACATCCAAT
GCTTCTGGAGAAGTACTCGTTACAATGTTTGGGTTAATAGGATCTATAGAACGCCAGACTTTGATTTCGAATG
TGAAACTTTCTATGAATGCTAAGGCAAGGAGCGGAGAGGCAATCACCGGTCGTGTTTTAGGCTACAAATTAT
CACTTAATCCACTTACACAGAAAAATGATTTGGTTATCGATGAAAATGAAGCTAATATTGTACGTGAAATTTTC
GATTTATATTTGAATCATAATAAAGGCCTCAAAGCCATTACAACCGTACTTAATCAAAAGGGGTATCGTACTAT
TAATCAAAAGCCATTTTCAGTGTATGGTGTTAAATACATTTTGAATAATCCAGTCTATAAAGGCTATGTCAGATT
CAATAACCATCAAAACTGGGCTGTACAGCGAAGAAGTGGCAAAAGTGATAAAAATGATGTGATATTGGTCAA
AGGTAAACATGAAGCCATTATAAGTGAAGAGGTATTTGATAAAGTTCATGAAAAATTAGCTTCTAAAAGTTTT
AAACCGGGCAGACCTATTGGTGGAGATTTCTACTTACGTGGCCTTATTAAATGCCCAGAATGCGGAAATAATA
TGGTATGTCGACGGACGTATTATAAAACGAAAAAGTCCAAAGAACGGACAATCAAGCGTTATTACATTTGTT
CCTTATTCAATCGTTCAGGGAGTTCTGCATGTCACAGTAATTCCATCAATGCTGAAGTCGTCGAGCGTGTAAT
TAATGTTCATTTGAATCGTATTCTGTCTCAACCAGATATTATCAAGCAGATTGCGTCAAATGTGATAGAAGAAC

TGAAACAAAAGCATAGTAACCAAACAGAAATTAAATATGACATTGATAGTTTAGAAAAACAAAAAGCTAAGC
TTAAAACACAACAAGAACGATTGTTAGAATTGTTCTTAGATGATCAGATGGATAGCGAAATGTTAAAAGCTA
AACAAAGTCAAATGAATCAACAGTTAGAAGTATTAGATCAACAAATTAAAGAAGCGCAACAAGCAAATCAAT
CACAGGATGAAATACCTAATTTTGATAAATTAAAAGGACGACTCATTTTGATGATAACACGATTCAGCGTGTA
CTTAAGAAAGGCTACACCCGAAGCTAAAAATCAACTTATGAAAATGTTAATTGATTCAATTGAAATTACGACA
GATAAACAAGTAAAACTTGTAAGGTATAAAATTGACGAAAGTCTTATCCCTCAATCTTTGAAAAAAGATTGG
GGGTCTTTTTTTATGCCCAAATTCCAATTTGAAATATATGGTCAAAATGATTATTTCATCGACCAAATTACCACT
TTTACCACTTAGTTATTAGTGACAAAAGTGAGCGAAATGAAATAAAAATCAAATATATATTATCAAAATGATGT
ATCACATGCATACATCAATCAAATACATTAGGAGGTCATAACCATGACACTAGAACAACAACTCAAGCACTATA
TAACCAACTTATTCAATCTGCCAAGGGACGAAGTGTGGCACTGCGAATCTATCGAGGAAATCGCTGATGATAT
CTTACCCAATCAATATGTAAGACTTGGCCCACTCAGTAATAAAACACTTCAGACTAATACCTACTACTCTGACA
CACTTCATGAAAGTAATATCTATCCTTTCATTCTCTACTATCAGAAACAACTCATAGCCATCGGTTATATCGACG
AAAATCACGATATGGATTTCTTATACCTACACAACACTATCATGCCTCTTTTGGATCAACGATACTTACTAACAG
GAGGACAATAAAATGCATAAATACATCAAAATTACACAATTAGTCATTACAATACTAAGTGAAATCATCATTTG
GATGAAAGAGTCAGAACGAAAGGAAGTCTCTTATGAATAGATATATCACCCGGGGTATCGCCAACAACTTAC
CTAATATCTTACAACACCAATTATGGCAACTCGTATCTGAGCGAGAACAAGAACAAACCAAAGATAATACTTC
AGTAGATTATTTTCATATATTCCAGTTCAATAAGCATCGCAATCAATTATATATCAAACACAAACAAGAACGACC
TGAATATGCGAAAATCCATAAAGCTAATTATTCAAAAGCAATCAATATCAATAAGGTCTACATTATTCGAGAAG
ATGATGTAGACCTTTCTTATTATGTCATGTTATTACCTGAAGAATACTAGAGGAGTGAAAATTATATGGAAACA
ATCAAAAGTACATTAAAAACAGAAGCCATATTCAGTGATGACAAACAACATCGCTATCTACTTAAGAAAACAT
GGAATAGTGAAAAACAATCAATCACAATCATTACAATGTATCCGCATTATGATGGCATTCTCAATATTGACCTA
ACGACCCAACTCATCATGAACAAAGTTTCAGAAATGGATGCATTTGGTTCAATCAATTTTGTGAATCTATACTC
TAATATTACAACCCCTATCAATCTCAAACATTTAGAAAATGCGTATGATAAGCATACAGATATTCAAATTATGAA
GGCAGTGAAAGAGTCAGATGAAGTGATATTAGCTTGGGGCGCTTACGCTAAAAAGCCCGGTGTTGAAGCA
CGTGTTAATGAAGTATTAGAGATGTTGAAACCACATAAAAAGAAAGTAAAACGACTCATGAATCCAGAAACC
AATGAAATCATGCATCCCCTTAATTCGAAAGCACGTCAAAAATGGATATTAAAAGTATAGCAACTAAAATGTTT
ATTCACTATCCATGAAAATAATTGAATTTCATATATATCTTTTAATCTACAATATGATTAAAAGATATATTATTTGG
TTCTGTTGCAAAGTAAAAAAATATAGCTAACCACTAATTTATCATGTCAGTGTTCGCTTAACTTGCTAGCATGA
TGCTAATTTCATGGCATGGCGAAAATCCGCAGATCTGAAGAGACCTGCGGTTCTTTTTATATAGAGCGTAAAT
ACATTCAATACCTTTTAAAGTATTCTTTGCTGTATTGATACTTTGATACCTTGTCTTTCTTACTTTAATATGACGG
TGATCTTGCTCAATGAGGTTATTCAGATATTTCGATGTACAATGACAGTCAGGTTTAAGTTTAAAAGCTTTAAT
TACTTTAGCCATTGCTACCTTCGTTGAAGGTGCCTGATCTGTAATTACCTTTTGAGGTTTACCAAATTGTTTAA
TGAGACGTTTGATAAACGCATATGCTGAATGATTATCTCGTTGCTTACGCAACCAAATATCTAATGTATGTCCCT
CTGCATCAATGGCACGATATAAATAGCTCCATTTTCCTTTTATTTTGATGTACGTCTCATCAATACGCCATTTGTA
ATAAGCTTTTTTATGCTTTTTCTTCCAAATTTGATACAAAATTGGGGCATATTCTTGAACCCAACGGTAGACCG
TTGAATGATGAACGTTTACACCACGTTCCCTTAATATTTCAGATATATCACGATAACTCAATGTATATCTTAGATA
GTAGCCAACGGCTACAGTGATAACATCCTTGTTAAATTGTTTATATCTGAAATAGTTCATACAGAAGACTCCTT
TTTGTTAAAATTATACTATAAATTCAACTTTGCAACAGAACCGTATTATGGAATAGAGATGTTGGTAACATTTAT
ACAGGATCATTATACTTAAGTTTAATTTCGTTATTACAGAACCACACATTCCAACCAGAAGAGAAAGTATGTCT
ATTTAGTTATGGTTCAGGAGCAGTAGGAGAAATCTTTAGTGGTTCAATCGTTAAAGGATATGACAAAGCATTA
GATAAAGAGAAACACTTAAATATGCTAGAATCTAGAGAGCAATTATCAGTCGAAGAATACGAAACATTCTTTA
ACAGATTTGATAATCAAGAATTTGATTTCGAACGTGAATTGACACAAGATCCATATTCAAAAGTATACTTATAC
AGTATAGAAGACCATATCAGAACATATAAGATAGAGAAATAAACTAGTGGCCGATTGTGCTTGATGAGCTTGG
GACATAAATCCTAACTCGAAATAAATAAGCATATCACTAAACTGATTTTTTAAAGTTTACAGTGATATGCTTATT

TTTTTATCTTACGATTTTGTACGTGCATGCTTGCCTAGGGGTATGGCTCGAGCCATTAGTCTCTCGCACATACT
ATTCCCTCAGGCGTCAGCACTTACAAAATCGGTTGTAATTTTCATTTTTATACGCATTCTTACTGAGATTATACT
AATAAGAGGAATAGTAAAAGCAATTCTAAGTAAAATTGCAGATAAGAGGTTTGTTAAAAGCAGTTCTAAGTA
AAATTGCAGATAAGAGGTTTGTTAAAAGCAGTTCTCAGTAAAATTACAGATAAGAGGTACGTTAAAAGCAGT
TCTAAGTAAAATTGCAGATAAGAGGTTTGTTAAAAGCAGTTCTAAGTAAAATTGCAGATAAGAGGTACGTTA
AAAGCAGTTCTAAGTAAAATTGCAGATAAGAGGTTTGTTAAAAGCAGTTCTCAGTAAAATTACAGATAAGAG
GTACGTTAAAAGCAGTTCTAAGTAAAATTGCAGATAAGAGGTTTGTTAAAAGCAGTTCTAAGTAAAATTGCA
GATAAGAGGTACGTTAAAAGCAATTCCATGCAAAATTGCTGATAAGGGGTAAGTTAAAAGCAGTTCTCAGTA
AAATTGCAGATAAGAGGTACGTTAAAAGCAGTTCTAGGCAAAATTGCAGATAAGAGGTGCGTTAAAAGCAG
TTCTCAGTAAAATTGCTGATAAGGGGTAAGTTAAAAGCAATCCTAAGTAAAATTGCAGATAAGGGGTACAGA
AAAACTAGACTTGATTACAAAATGGAGCTTGGGACATAAATGATTTTTTAAAAATGAGATGAGACGTAGATT
AACTCCATAATCAATACGAATCTATCGACTTCTTTATTTATGATATTCATCTCTTTTTAATGGAAATAAAAGTGCG
ATTAATGTGATAATACAGTTACGTTAATTAAAAAAATAAAAATGCAAGGAGAGGTAATATGCTAACTGTATATG
GACATAGAGGATTACCTAGTAAAGCTCCGGAAAATACAATTGCATCATTTAAAGCTGCTTCAGAAGTAGAAG
GTATAAACTGGTTGGAGTTAGATGTTGCAATTACAAAAGATGAACAACTGATTATCATTCATGATGATTATTTA
GAACGGACTACAAATATGTCCGGGGAAATAACTGAATTGAATTATGATGAAATTAAAGATGCTTCTGCAGGA
TCTTGGTTTGGTGAAAAATTCAAAGATGAACATTTGCCAACTTTCGATGATGTAGTAAAAATAGCAAATGAAT
ATAATATGAATTTAAATGTAGAATTAAAAGGTATTACTGGACCGAATGGACTAGCACTTTCTAAAAGTATGGTT
AAGCAAGTGGAAGAACAATTAACAAACTTAAATCAGAATCAAGAAGTGCTCATTTCAAGCTTTAATGTTGTG
CTTGTTAAACTTGCAGAAGAAATCATGCCACAATATAACAGAGCAGTTATATTCCATACAACTTCGTTTCGTGA
AGACTGGAGAACACTTTTAGATTACTGTAATGCTAAAATAGTAAACACTGAAGATGCCAAACTTACTAAAGC
AAAAGTAAAAATGGTAAAAGAAGCGGGTTATGAATTGAACGTATGGACTGTAAACAAACCAGCACGTGCAA
ACCAACTTGCTAATTGGGGAGTTGATGGTATCTTTACAGACAATGCAGATAAAATGGTGCATTTGTCTCAATA
GAAAGTTAGAGGTGAGTCTTACGTTTCAGTGACGGTAGACTTACCTTTAACATGTTACATACTAAAAAATTAA
TTTGAATAAGAAAGAGAGACATATATGAAATACGATGATTTTATAGTAGGAGAAACATTCAAAACAAAAAGC
CTTCATATTACAGAAGAAGAAATTATCCAATTTGCAACAACTTTTGATCCTCAATATATGCATATAGATAAAGAA
AAAGCAGAACAAAGTAGATTTAAAGGTATCATTGCATCTGGCATGCATACACTTTCAATATCATTTAAATTATG
GGTAGAAGAAGGTAAATACGGAGAAGAAGTTGTAGCAGGAACACAAATGAATAACGTTAAATTTATTAAAC
CTGTATACCCAGGTAATACATTGTACGTTATCGCTGAAATTACAAATAAGAAATCCATAAAAAAAGAAAATGG
ACTCGTTACAGTGTCACTTTCAACATACAATGAAAATGAAGAAATTGTATTTAAGGGAGAAGTAACAGCACT
TATTAATAATTCATAATAAAACAGTGAAGCAACCATCGTTACGGATTGCTTCACTGTTTTGTTATTCATCTATATC
GTATTTTTTATTACCGTTCTCATATAGCTCATCATACACTTTACCTGAGATTTTGGCATTGTAGCTAGCCATTCCT
TTATCTTGTACATCTTTAACATTAATAGCCATCATCATGTTTGGATTATCTTTATCATATGATATAAACCACCCAAT
TTGTCTGCCAGTTTCTCCTTGTTTCATTTTGAGTTCTGCAGTACCGGATTTGCCAATTAAGTTTGCATAAGATC
TATAAATATCTTCTTTATGTGTTTTATTTACGACTTGTTGCATACCATCAGTTAATAGATTGATATTTTCTTTGGA
AATAATATTTTTCTTCCAAACTTTGTTTTTCGTGTCTTTTAATAAGTGAGGTGCGTTAATATTGCCATTATTTTCT
AATGCGCTATAGATTGAAAGGATCTGTACTGGGTTAATCAGTATTTCACCTTGTCCGTAACCTGAATCAGCTAA
TAATATTTCATTATCTAAATTTTTGTTTGAAATTTGAGCATTATAAAATGGATAATCACTTGGTATATCTTCACCA
ACACCTAGTTTTTTCATGCCTTTTTCAAATTTCTTACTGCCTAATTCGAGTGCTACTCTAGCAAAGAAAATGTT
ATCTGATGATTCTATTGCTTGTTTTAAGTCGATATTACCATTTACCACTTCATATCTTGTAACGTTGTAACCACCC
CAAGATTTATCTTTTTGCCAACCTTTACCATCGATTTTATAACTTGTTTTATCGTCTAATGTTTTGTTATTTAACC
CAATCATTGCTGTTAATATTTTTTGAGTTGAACCTGGTGAAGTTGTAATCTGGAACTTGTTGAGCAGAGGTTC
TTTTTTATCTTCGGTTAATTTATTATATTCTTCGTTACTCATGCCATACATAAATGGATAGACGTCATATGAAGGT
GTGCTTACAAGTGCTAATAATTCACCTGTTTGAGGGTGGATAGCAGTACCTGAGCCATAATCATTTTTCATGTT

GTTATAAATACTCTTTTGAACTTTAGCATCAATAGTTAGTTGAATATCTTTGCCATCTTTTTTCTTTTTCTCTATTA
ATGTATGTGCGATTGTATTGCTATTATCGTCAACGATTGTGACACGATAGCCATCTTCATGTTGGAGCTTTTTAT
CGTAAAGTTTTTCGAGTCCCTTTTTACCAATAACTGCATCATCTTTATAGCCTTTATATTCTTTTTGTTTTAATTC
TTCAGAGTTAATGGGACCAACATAACCTAATAGATGTGAAGTCGCTTTTCCTAGAGGATAGTTACGACTTTCT
GTTTCATTAGTTGTAAGATGAAATTTTTTTGCGAAATCACTTAAATATTCATCCATTTTTTTAACGGTTTTAAGT
GGAACGAAGGTATCATCTTGTACCCAATTTTGATCCATTTGTTGTTTGATATAGTCTTCAGAAATACTTAGTTCT
TTAGCGATTGCTTTATAATCTTTTTTAGATACATTCTTTGGAACGATGCCTATCTCATATGCTGTTCCTGTATTGG
CCAATTCCACATTGTTTCGGTCTAAAATTTTACCACGTTCTGATTTTAAATTTTCAATATGTATGCTTTGGTCTTT
CTGCATTCCTGGAATAATGACGCTATGATCCCAATCTAACTTCCACATACCATCTTCTTTAACAAAATTAAATTG
AACGTTGCGATCAATGTTACCGTAGTTTGTTTTAATTTTATATTGAGCATCTACTCGTTTTTTATTTTTAGATACT
TTTTTTATTTTACGATCCTGAATGTTTATATCTTTAACGCCTAAACTATTATATATTTTTATCGGACGTTCAGTCAT
TTCTACTTCACCATTATCGCTTTTAGAAATATAACTGCTATCTTTATAAACTTGTTTGAAATTTTTATCTTCAATT
GCATCAATAGTATTATTAATTTCTTTATCTTTTGAAGCATAAAAATATATACCAAACCCGACAACTACAACTATTA
AAATAAGTGGAACAATTTTTATCTTTTTCATCAATATCCTCCTTATATAAGACTACATTTGTAGTATATTACAAAT
GTAGTATTTATGTCAAAATAATGTTATAATTTTTGTGATATGGAGGTGTAGAAGGTGTTATCATCTTTTTTAATG
TTAAGTATAATCAGTTCATTGCTCACGATATGTGTAATTTTTTTAGTGAGAATGCTCTATATAAAATATACTCAAA
ATATTATGTCACATAAGATTTGGTTATTAGTGCTCGTCTCCACGTTAATTCCATTAATACCATTTTACAAAATATC
GAATTTTACATTTTCAAAAGATATGATGAATCGAAATGTATCTGACACGACTTCTTCGGTTAGTCATATGTTAG
ATGGTCAACAATCATCTGTTACGAAAGACTTAGCAATTAATGTTAATCAGTTTGAGACCTCAAATATAACGTAT
ATGATTCTTTTGATATGGGTATTTGGTAGTTTGTTGTGCTTATTTTATATGATTAAGGCATTCCGACAAATTGAT
GTTATTAAAAGTTCGTCATTGGAATCGTCATATCTTAATGAACGACTTAAAGTATGTCAAAGTAAGATGCAGTT
CTACAAAAAGCATATAACAATTAGTTATAGTTCAAACATTGATAATCCGATGGTATTTGGTTTAGTGAAATCCC
AAATTGTACTACCAACTGTCGTAGTCGAAACCATGAATGACAAAGAAATTGAATATATTATTCTACATGAACTA
TCACATGTGAAAAGTCATGACTTAATATTCAACCAGCTTTATGTTGTTTTTAAAATGATATTCTGGTTTAATCCT
GCACTATATATAAGTAAAACAATGATGGACAATGACTGTGAAAAAGTATGTGATAGAAACGTTTTAAAAATTT
TGAATCGCCATGAACATATACGTTATGGTGAATCGATATTAAAATGCTCTATTTTAAAATCTCAGCACATAAATA
ATGTGGCAGCACAATATTTACTAGGTTTTAATTCAAATATTAAAGAACGTGTTAAGTATATTGCACTTTATGATT
CAATGCCTAAACCTAATCGAAACAAGCGTATTGTTGCGTATATTGTATGTAGTATATCGCTTTTAATACAAGCAC
CGTTACTATCTGCACATGTTCAACAAGACAAATATGAAACAAATGTATCATATAAAAAATTAAATCAACTAGCT
CCGTATTTCAAAGGATTTGATGGAAGTTTTGTGCTTTATAATGAACGGGAGCAAGCTTATTCTATTTATAATGA
ACCAGAAAGTAAACAACGATATTCACCTAATTCTACTTACAAAATTTATTTAGCGTTAATGGCATTCGACCAAA
ATTTACTCTCATTAAATCATACTGAACAACAATGGGATAAACATCAATATCCATTTAAAGAATGGAACCAAGAT
CAAAATTTAAATTCTTCAATGAAATATTCAGTAAATTGGTATTACGAAAATTTAAACAAACATTTAAGACAAGA
TGAGGTTAAATCTTATTTAGATCTAATTGAATATGGTAATGAAGAAATATCAGGGAATGAAAATTATTGGAATG
AATCTTCATTAAAAATTTCTGCAATAGAACAGGTTAATTTGTTGAAAAATATGAAACAACATAACATGCATTTT
GATAATAAGGCTATTGAAAAAGTTGAAAATAGTATGACTTTGAAACAAAAAGATACTTATAAATATGTAGGTA
AAACTGGAACAGGAATCGTGAATCACAAAGAAGCAAATGGATGGTTCGTAGGTTATGTTGAAACGAAAGAT
AATACGTATTATTTTGCTACACATTTAAAAGGCGAAGACAATGCGAATGGCGAAAAAGCACAACAAATTTCT
GAGCGTATTTTAAAAGAAATGGAATTAATATAATGGATAATAAAACGTATGAAATATCATCTGCAGAATGGGA
AGTTATGAATATCATTTGGATGAAAAAATATGCAAGTGCGAATAATATAATAGAAGAAATACAAATGCAAAAG
GACTGGAGTCCAAAAACCATTCGTACACTTATAACGAGATTGTATAAAAAGGGATTTATAGATCGTAAAAAAG
ACAATAAAATTTTTCAATATTACTCTCTTGTAGAAGAAAGTGATATAAAATATAAAACATCTAAAAACTTTATCA
ATAAAGTATACAAAGGCGGTTTCAATTCACTTGTCTTAAACTTTGTAGAAAAAGAAGATCTATCACAAGATGA
AATAGAAGAATTGAGAAATATATTGAATAAAAAATAAAATTGTTGTGTTTACAACAATACATAGAAAACAGAG

GAAACAATCAAGTCGTTGAATATTTCCTCTGTTTTTTAGTTGAAAAAATTAACCGAAAGCCTGAATGCAAGTC
TTGATTAAATCAATAATGCTTGTAATAACACCAGTGAAATCCATATGCATACCCTCTTTCTATTTAAGATACATTA
AGTATAATATCAAACAAATAAAAAAATGTTAAAAATTCCCTAATTGGCTATTTAGATTGCATAAATGTCAAAAATT
TGAAAAACATACAACGACTTTGCATAAAAAAATCGTCATATTGGAAATACGTAATTTATTGAAATAATAAAAAAA
ATAAAAGAACGAAGATGATAACCTAAGTGAGGTTTTAAGTTGTTCTAAGGTTTAATTTAATTTATGTTAAAATA
GTTGGTATAAAAATACATGATAAACTATAAACTAAATTCAAAATAACTTATGGGGTAGGCAATTATGGAAAATA
AAATATAAATGATAATGAAAAAAGAGTGCTAAGGGAAATTTATAACCATCATAATATTTCGCGTACTCAAATAT
CTAAAAATCTTGAGATTAATAAGGCAACGATTTCTAGTATTTTGAATAAGTTAAAGTATAAATCTCTTGTTAATG
AGGTTGGTGAGGGTGATAGCACGAAGAGTGGTGGTAGAAAACCTATTCTTCTGAAGGTTAATCATCTTTATG
GTTATTTTATTTCTTTGGATTTAACTTATAGTTCTGTTGAAGTGATGTACAATTATTTTGATGGTAATGTCATTAA
GCATGAATCTTATGATTTACCTGATGAAAAGGTTAGTAGTATATTAAGCATAATAAAAAAACATATTGATATTCA
GGAGAAACTTGATACTTATAACGGACTATTAGGTGTGTCTGTTTCTATACATGGAGTTGTGGATAATGAGCAG
CATGTGACATATTTACCATTCCATGAAACTGAAGGAATTTCAATTGCTAAGAAAATAAAAGAAATTACTAATGT
TCCAGTCGTAGTTGAAAATGAAGCGAATCTTTCAGCGTTATATGAACGTAATTTTAATCATAATTTATCCTACAA
TAATCTTATTGCTTTAAGTATACATAAAGGTATTGGTGCTGGGCTTATTATTAATAATCAATTGTATCGTGGTGC
AAATGGGGAAGCGGGTGAAATTGGAAAAACACTTGTCTCAAAAGTTAGCGATAATGTGGAGATCTTTCATA
AGATTGAAGATATTTTTTCACAAGAAGCTTTACTGCATAATTTAAGTAATCAACTAAATGAGAAGATGACGCT
TAGCAAATTAATTCAATTTTATAATGAAAAAAATCCAGTCGTAGTTGAAGAAATGGAACAATTTATAAATAAAA
TTGCTGTTTTAATACATAATTTAAACACCCAGTTTAATCCGAATGCAATTTACATTAACTGTCCATTGTTCAATG
AAATGCCTGAAATATTAGAAGCAATTAAGAACCAGTTCAAACAATATTCACGTAACGAAATTCAAATAAAGTT
AACATCTAATGTCAAATTTGCAACTTTGCTAGGTGGTACATTAGCAATTATCCAAAAAGTACTACAGATTAATG
ATATTTACTTAGATATAAAAGCATAAAAAACTAATTCAAATGAATAATCAAAGTTCGTAATTGTCTTTATAAAAA
AATCCCTCAATCCGAATTGAATTTTCGGATTGAGGGATTTTTATAGTTCTATTGCAGAAGAAAACTATTTTAAA
AATGCTGGTAAATGTTGATAGCCACCTCTAACGTTAACAATATTCGTAAATCCTTTATATTCTAATATTCCTACCG
CTATTGAACTTCTAACACCTGATTGACAATGTACATAAATTAGGTCATTTTTATCGAAAGGTATATCTTCATTTA
AAAGTTTACCGTGAGGAATATGAATTGCTTGTTTTAAATGACCTTTACGCCATTCATCATCATTACGAACATCT
AATACATTATGTTCTTCACCAGTCATTTCAGAACTATGAATAGATGATGTGACGATATTTGTTTGTGGCAAACG
GTAACTTTTTACATTTTCAAAACCAATTAATTGTAAAGCATGAATAGCTTGTTGAACGGTAGATTTATCGCCAA
TTAATTCAATATCTTAGTCATAATCTAAATACCAACCAATTTGATTTATAAAAGTTTTATTAAAAGGAATATTGAT
AGTTCCATGCATATGACCACCATGGAATGCTTCTTTACTTCGAAGATCAAAAGCAGTTTGTGTATTGCTTGAA
CTAGGGTAAACATTATATGGTTGGTACATTTGCATACCAAATTGATTTATTTTTTTCATTTGTGAAAAATGGTGT
GGTGGAGCTGGCTGATTGAGTGTTAAAGTTTCGATAAATGAAGTTTCATCTTTAACATTAAAAGCCCAGTTG
TTTATTTTCTCATAACCCAAAGTAGTTGTAGGTAATGAACCTAGCGCTTTACCACAAGGACTCCCTGCACCAT
GACCTGGCCAAATTTGAATATAGTCTGGTAATGTTGCAGCAAATTGTATGGACTGATACATTTGTTTTGCTCCG
ATTTTTGTAGAACCTTCAACATTTACAGCTTTTTCTAATAGATCTGGTCTACCTACATCACCAACAAAGATGAA
GTCACCGCTAAATAATCCCATTGGTATACTGGAACCCCCACCTTCGTCAGTAAGTAAAAAACTAATACTCTCAG
GGGGATGGCCTGGAGTGTGTAAGACTTCTAATTTAATCTTTCCTAAATAGATAATATCTTGATGCTTAACGAAA
TGTGTTTGTTTAGGCATATTTTTATAATTAAATTCATCTTTACCTTCATCAGATACGTATATACTTGCATTCAATTT
ATTTGCCACATCTCTAATACCTGAAGCAAAATCAGCATGAATATGTGTTTCTGCAGCTTTAGTAATTGTGAATC
CTTCTTTATCTGCAACTTTTAAATATTTTGTTAAATCTCGTATAGGGTCAATAATCATTGCTTCTCCTGTACGTTG
ACATCCAATTAAATAAGATGCTTGTGAAAAATTGTCTTCATAAAATTGTTTGAAAAACAAAAAAAACTCCTTT
TTAAAATAGATTTTATTGATTAGATAAATAAGTTATGATTTGCTTGCTCAGTATGTCCAATATAAGTACCTACGCC
ACCATAATCGACTTCATCTCTTAATTCTTCTTTTGAAATTCCCATAACATCCATACTCATGGTACAAGCAATTAAC
TTTATATCTTGATCGATTGCTTGATCGATAAGTGAGTATAAAGAATCAACATTTTTCTTGTTCATTACATAACGC

ATCATAATATTACCTAGTCCAAACATATTCATTTTTGATAATGGCATATGTATTGGATCCTTAGGTAACATAAGGT
CAAACATTTTGGAAATACCTTTCTTTTTAACGCGAGTTGATTGCGCTTTTTTTAATGCGTTGAGGCCCCAAAA
AGTAAAGAAAATAGTTACATCTTTACCTGCTGCTTTAGCGCCATTTGCGATGATCATTGCTGCTACTGCCTTAT
CTAACTCACCGCTAAATAAAACAATTGTTGTACCTGTAGCAGTGTCATTGATTTCAAATTCTTTTGGCTTTTCT
TTTTGAATAATTGCATTAATTACATTTGCTTCTTCAGTAAGATTTACAAGGGTATTCCCTGTTTGTTTCGCCCAA
CTTTTAATATCACTATTGAAACCAGGATCTGTAACTGTTACCTCGATTTGCTCACCCGTTGAAATATTGTTAATT
TCTTTACTGATATTAACAATAGGTCCAGGGCACTGAAGACCTCTAAAATCAAATTGTTTACGATTCTCTTTGAT
TTCAATATCTTTTTCTATTAAAGGAGCACTATTGAAGTTCTTTGCTTCATAATCTTTATATCCACCCTTTAAATTC
ACGACATCATAACCTTGTTTGGCTAAATAATCGCAAGCTTTAGTGCTTCGGTTACCGCTTTTACAATGTATATA
ATACGTTTTGTTGCTATTCTTATTGAATGATTTAATCTCTTCTACTGGGTGTAAAGTTGAACCGTTAATGTGTCC
TAATTCATATTCTTCTTTTGTTCTAACATCAATCAATTGACCCATTTTTGCCAATTTTTCTAATTCTTCTTTGTTTA
ATGAATTAATGTGTACTTCTTTGTATTGTTCCATACTTACCTCCTATAAATACCTATGAGGGTATAATAAAACGGA
TAGAATCATTTGCCAAATACCTATATGGGTATTTGACAATTTTGTTTTAATTTATTATTATTAACTTAATCAATTTA
TGTGGAGGAAATGAATATGACTTATGATAAAAAAATGATTAATCGTATAAATAGAATACAAGGTCAATTAAATG
GTGTCGTAAAAATGATGGAAGAAGAAAAAGATTGCAAAGATATAATTACGCAACTTAGTGCATCTAAAGGTT
CTATACAACGTTTAATGGGGATTATAATTAGTGAAAATTTAATAGAATGCGTTAAAACAGCAGAAGAAAATAA
TGAAAGTTCTCAAGAATTAATTAATGAAGCAGTTAATTTATTAGTTAAAAGTAAATAATGGATATAGCAAATAT
GACTATTATGTTGCTAATTGGCGTACTGGGTGGATTTATATCTGGATTAATAGGTATTGGGGGCGCAATTATTA
TTTACCCAGCTATTCTTATATTGCCACCATTAATAGGTATACCTGCGTATAGTGCATATATTGCTTCGGGACTTAC
CTCTAGTCAAGTATTTTTCAGTACACTTAGTGGATCATTAAATGCAAGAAAACAACCAGCTTTCTCTCCTAAAC
TTGTTATATATATGGGAGGGGGTATGTTGATTGGAAGCATGTTAGGGGCAATTTTAGCTAGTTTGTTTAATGC
TACTTTTGTAAATACGGTATATGTAATAATCGCCATACTTGCTTTAATATTGATGTTTATTAAAGTTAAACCTACT
ACACAAGAGACGAAATCTAAACCTTTGCTATTTATTATAGTTGGATTTGGAATTGGTGTAATTTCGGGAATTG
TGGGTGCAGGTGGAGCATTTATCATCATTCCTGTATTATTAGCATTATTTAAATTACCAATGAATACGGTAGTG
AACAATAGCATAGCAATTGCTTTTATATCTTCAGTAGGGGCATTTTTTATAAAATTAATGCAAGGATATATACCA
GTAGAAAGTGCAATTTTTTTGATAATTGAATGATGATCATTTTCTGAAAATATTATGTGGTCATATAATATAATG
CCCATCATTTCACTAATCTCTTTTATTCTCTGAGTTATTTTGATATCTCCTGGAGAAGGTGTTACATCTTATCAAG
AGTAAATTACAAAAGAATCATTTAAATCAATACTTTCACTTTGAATACATGTATTTGAAGTGGAAGGTACTTAT
TTCAAAAATAGTAAAACCTGTATCTTAAATTACTTAATAGTAACATAAGATACAGGCTGATTTTTTATTCATTGT
TATTTATACTAAAGCACCCGATAGCTCTGAAAACAATCACAAATTCAACTTTTCAAAGCCACAGCTTTAAGTTA
TTTTGTCCAGACAACCCCCATTGCCCAACCCATTTTATGGAATTGGCATCCAGGCAACAACTTTTCATATAAAT
CGTAATAATTTTGTTCAGATAGGTACTTATCTGAAGCTAAATGCTCAAGCCATGATTTAGATGTGTTGTGATTAT
AAATTCTAATCGCATTTTTTATTCCAAATTTGAAACAATGCGGTAAAAACTCTTGGATTGCCCCTAATTTATAAA
CATAGGTAGGCGGTGTCTCGACTTCAGATACATTATCAAGAATAACTATTCTTCCTTCCTCATTCAACAGTTCC
TTCATTTGCTGTATTACGCTGGCTATATCATCCAAATGATGAAAGGTTGTTCGGCTTACAATAAAATCAAACTT
CTCATTAAAATTAAGTTGTTCTGCATTCATATTCAGATAGACCGTATTTGTTAGTTGACGTTTAGATTTGGCAA
GATCGAGCATTTGATTAGAAATATCAATCCCTACCACTTCATCATAATAACTTGCTAATTTCTCCACTAACAAAC
CCGAGCCACATCCGATATCTAATGCTCTGCCTTTCTTTGGAGACATATTAGACACAAAGAATGAATAATCATTC
AAAAGCTCATTCACGAAATCGTAATCTTCTGCAACCTTATCAAACTGTGATTCTATTGTATTCAAAAAGATCCC
CCATTCCTACTTTATCGACATTCTTTCATTACTTACCACTTTAGATGTTTTTTCGTTGGGGATAAAACTTCCCTT
TAGACAATTTTATCCAAAGACAATACAACAGTGCAACTTTATTAAAGTCACTGTCCTTTATCGCAGCCTTTACT
TTTTAGTAAAGACAGTGGCTTCTCTTATCAAGTTTCAAAACATATTATTTTGAAGAAAACGTCCATCTGAAGT
GTCAAGTGCAAAATTACATATAAAGGTTTATTCTAAAATGAAAAGATGATACAATCATATTCAGTTACATAAGG
AGGTTTCAATTATGTGCACCAGTATCGCAGTAGTAGAAATTACTTTATCTCATTCATAATGAAAAAAATGGAAA

GGAGATAAAAGTATGGGTACTTTTTCTATATTTGTTATTAATAAAGTAATCAAAATTAATTGGTTATAATGAACG
CTTAATGTCAGTTCATTATAACCAGTAAGGAGAAGGTTATAATGAACCAGAAAAACCCTAAAGACACGCAAA
ATTTTATTACTTCTAAAAAGCATGTAAAAGAAATATTGAATCACACGAATATCAGTAAACAAGACAACGTAATA
GAAATCGGATCAGGAAAAGGACATTTTACCAAAGAGCTAGTCAAAATGAGTCGATCAGTTACTGCTATAGAA
ATTGATGGAGGCTTATGTCAAGTGACTAAAGAAGCGGTAAACCCCTCTGAGAATATAAAAGTGATTCAAACG
GATATTCTAAAATTTTCCTTCCCAAAACATATAAACTATAAGATATATGGTAATATTCCTTATAACATCAGTACGG
ATATTGTCAAAAGAATTACCTTTGAAAGTCAGGCTAAATATAGCTATCTTATCGTTGAGAAGGGATTTGCGAA
AAGATTGCAAAATCTGCAACGAGCTTTGGGTTTACTATTAATGGTGGAGATGGATATAAAAATGCTCAAAAA
AGTACCACCACTATATTTTCATCCTAAGCCAAGTGTAGACTCTGTATTGATTGTTCTTGAACGACATCAACCAT
TGATTTCAAAGAAGGACTACAAAAAGTATCGATCTTTTGTTTATAAGTGGGTAAACCGTGAATATCGTGTTCT
TTTCACTAAAAACCAATTCCGACAGGCTTTGAAGCATGCAAATGTCACTAATATTAATAAACTATCGAAGGAA
CAATTTCTTTCTATTTTCAATAGTTACAAATTGTTTCACTAAATTAAAGTAATAAAGCGTTCTCTAATTTCACAA
GAGGACGCTTTATTCTTCCCAAAAATTGTTCAATATTTATCAATAAATCAGTAGTTTTAAAAGTAAGCACCTGT
TATTGCAATAAAATTAGCCTAATTGAGAGAAGTTTCTATAGAATTTTTCATATACTTAACGAGTGCTTTCACCTT
TGAATATAGTCCTTCCCACTTATCATCACACTCTCCCCGATAGCCTTTTCTAGCTATATCCAGTAAAGTTACATGC
TCTTTAGGTAAAAGAGGTATAGCCCATTCTGCAGCGACATCTTTCGAGGTAATTTCACCAGTAGTCACTGTTT
GCCACATTCGAGCTAGGGTTAAAATTACATTACGCTCATCACCTTTTATCCCCTCAATTAGTTCTGGCAAAGAA
TCCTTAATTGCTCTTCGAATATCTGTCAAAGGTACGGAGACAAGTATACTTGAAGAATCAGGACCAAATAGAG
AAATACTATTCTTTCTTGCTTGTGCTAAAACAATAGCCAAATCAGGATCATAGCTTGGTTCCTGAATTTGTCCA
TTCTCAAATTCACCCCTGAGCCACTCACCGTATATAAATTCTCTTTTTGGAGGATATTGCCAAGGGACAACTTC
ACTCCTATTTATAACCGTAACTTCAAGTGGTCTAACAGAATCCGTATTTCCAATCTTTCCTGATATAGTCATTAG
TCTTTCTGTTAGTTTTTTTCGAGTTAATTGAGGTAAACTATGATTCACGACGACTAGAACATCTACATCGCTGT
TAATGCGTAAACCACCATTTACTGCTGAACCAAATAGATATACTCCAACTATTGAACTTCCAAATAAATCTTTTA
CGATTTTTAATGTTTGAATCGCTTGATTTGGTATTTTTCCGTTAATCAAATTGCTCATGATTTCACCTCGTTGAT
TATGTTCATATAAAGTTTATATTGATACTCAATTTACTTACCCTAGATTGGACATATACTTAAATTACTGTTCAATA
AAGCTGACCGTTAGCGTTTAAGTACATCCTTTCACAATTTGTCTACAGATTAATAATTATTCTTTATTATACAGA
TCTCCATATAATTTTTGAATTTGGTTCTGTAATTTTTTATTTTCTTTTTCTAATTCCATTACTCTTCTTTTTAAGGT
TTTAATAAGGATTTCCTCCGAACGAGAACTTTTCTTGGGTTTTGAGACTACATTTGCTGTTATTTGACGCTCA
CGAAGGGATTCGATTCTTTGCCTAATATCGTGTTCCTTATAAAGCCATGATTTAGAAACATTAGCTTCCTTTGC
TATTGAATTAAAATTAATAACTTTACCTTCAATCGAAAATTTAGAAATCGCTTTGTCTACTTTTTCCCTTGTCTTT
TTTGATTTCTGCTTCGCCAAACGTACAATTTCTGTTGTATTTCTAACTTGTTTATCCATTGATAATTACCCCGTCA
AACTTCCAATGATTTGTTCTAAACGCTCTTTAACACGGCTATTAGTCTCTACTTGTCTTTGCCATTGTTTATCCT
TAGCTATGGCTAATAACTCTTCTGTACGCTCTAACTGTTCTTCGTGCTGTGGTAAGAATTGCTTACTGGTACAG
AAGTGAGTGCAATCTAAGCATGCATTCGCATGTGGACAACCACCTGCTACTACTGGCAATCTACAATAACCAT
TTGGAAGCACTTGTGCATTTATATTTTTCTTGAACCATTGAAGCTCTACATCATCGACTTCATTATCTTCATCTA
GATCAAGCACATCTCCATTATTGGTAACCAGTTTTTCCTGAAATTTAGTAAATTCATTTTTTAGAGTTTCATCAA
AGATATGAGCGTATCTGCTTGTCATTTCTGGGCTTTCATGCCCCAAAAATTTCTGCACAATATGCTGGGGCATC
CCGTTGTTAATCATTCTTGTTCCTACTGTATGGCGAAAGGCATGGGCATGGAATCTATAAATCTCACCTGATTT
ATCCACTATATTTTGCTCATAAGCTAATTTATTTAACTCACCTCTAAATGTTTCTTGTTTTAATGGCGATCCATCT
TTTCTTGGAAAGAGGTATTCACTATCTGGAAATTCCTCTGAAACTTTATCTTCCCGAACTTTAATAAGTAAAGC
TACCTCTTTAGATATTGGAACTATATGCTCCTTTTTCATTTTCCATTGATAATACTTTAAAAAGAAATCTCCATCT
TTGTCCTCTAATAGACAGCCTTTTTTCAAGGTGCACAATTCACTTATCCTCATTCCACATTCTTGAACAATCATA
GTCATCGTAGCTATATATTCGGGTAATTTATCAAGATGACTGTTCAATTGCTCTAGGACGAATTCATCTATAAAG
CGTGGTTTTGCTCTTGGTATTTTCGGATAGTCCTCAGAATAAATTAATATTTTGGAAGGAACATCATCCCATTC

TAGCCTAAGAAGGGTACTAAATAGTCCTTCCAATATAGAGATCCTCCCAGTTATTGTACTAGGTTTTATTCCCAT
CATGTTTAGTTCACTTAAATATGCTTCAATTTCCACTCTCGTTAATTGGTGTACTCTCTGAACTTGTTTAAATTTC
ATGTCCAGAAAATTAAAGAACTCTTTAAGTCTTTGGGCAATATCACTTACATAGGAAAAGCTATCCACGTTCA
ATCTCAACTTACAATATCTTTTTACAAGTTGTTTAAAATATGTATTCCGAAACCCTTTAAAGTTAATTGTATATTC
ATATTGTGTTGGGTTAACCTTATCATCTGGCAAAGGTAAGTTACGTCTATCCCAAACGTCTTTATCCCACTCCTC
TCCATCAAAATAAAAGTTCTCATAAAACTCCATAAATTGTTTTAGATTAGTAACATAGTAGGAATTAGCTTTTAC
AGGTGTTTTTTCTTGATTAGCAGTAATCTTATAATTAGTAGTGGTAATTCTAACACCCCGTTTTGTCAAATAAGT
TCTATACTCCGTCATTGCTTTTTCAATAGGAACTTCAGTAATTGAAGTAATGCTAGGATACTTTAAATCTAAGA
AATCTAACATTTTATTAATTACTGTTCCTTTTCTAATCCAGACAGTTTTTGCATTCCATATTCCATTGTTTAAATG
GTAAAAATAAAAATATTTCAATTCTGTTCTTAACCACAGATTTTTAACACGTTCAAAACGAACCCAACGATTCC
TTAAAGCAGGATTCTTACTTAATTCTATGGCAGAAGGATGTGGACATTTTCTTATATCCCAACTATTATTAGCCC
AAAACCCCTGCATTTCTTCATTCATTACAGCTATTTTTTTGCTAATCTCACTCTGACTAATAATTTTCCTTTTACT
AGAAGCATTCATTTCTTATGCTCCTTTCTCTCGAGGTATTTATTAAACTCATTTTTCATATCCTGATCTGAAAGAT
GAACATAGGTATTTAACGTTGTCTGAACATGTGCGTGACCTAATCTCTTTTGAACGAACGCAACATCCCATCC
TTCCCTAATTAGCTGCGTTGCGTGAGTGTGGCGAAGCATATGTGATGTAAATTCTATTCCAGTCCTTTTAACTA
TTCTTCTAACTAGATCAAGAACACTTTGGTACTTTAGTGGTTTCCCAAAATAGCCTTCTTTTAAGGAAATAAAA
ACATAATCATGCTCCAATTCCTCACTATACTCATATATCAAGTAATCTGTATAAAGTGACATAAGTTCTTTACTCA
CATGTATTGTTCTTTCCTTCCTTAATTTAATATAAGCTTCATTAACATTAACATCTCTAGGTGTTAAATGGATTTG
ATTGTCCCAAGTGACAATATCTTCAAGCCTAAGCGATAACACTTCACCGATTCTTAAACCACCCTCATACATAA
GCATTAAAATTAATTTATCTCTTTTCGTATGACAAGCATCAATAATTTGCTTAACTTCCTTTGATCTCAATGTTCT
TATCTGTTTCTTTTTAACCCTTAACTTTAAGACATTCTTTTGGTATCTACCCTTATTAACATGATGTAAAAATCCT
TTGAAATTTCTTCCCTTTGCTTGTTTAAATACATCAATTGATTTAAATTCTCCTAATCTACTTAAATAATCAAGAA
AACTCATAACTACATTTAAAATTGTATTCACTGTCGTTTCTTCTCTTATGGCTTTTTTTGACTGAAGATCAATTA
CATTTGATGCTGAAGGATATCTCAACCAACCTACGAAGTCTGCTAACAACTCAAAGTTAATATCATTAAGAATA
ACACCTCTCTGTTCCATGAACTCGTACAGCAACTTTAAATGATAGCAGTATGCCTTAATGGTATTAGGAGACTT
ACCAGTATTATCTAAGTATTTAATAAATTTCATTACTGGTTCTATTAGCTGGTATTCTTTATCTAGTAATAAATACA
ATGGATACGGCTTATTCTCCACTTCTATCCTTTGAACCTTCACATGTTCCCACCTCTTTAAATACTTTAACTACTA
CAAATTATAAACCTTGTTAATTTTTATTTAAAGTAAAATATCCCTTTAATATCTCCTCTTAAACTACTTTAACTACT
ATTATTTATTATACTATGGTTAATACATCTCCACTTGGATGATTATGACCGAGCATTATACTATTTGCGTTACTGA
GTATCGCTGTTTTGAATATTTCTCTAGGGTGAATCACCGTTTGGTTAATAGATCCAATCGATAGTGTTTGAATAT
GTGTAGGTTCATTTTTACTGTTCATACATATGAGAATGAGATGCTCTCGGTCACTGTTTCCAATGAATGAACGC
ATGATTTCTGCCGCATCCTCAGGGTTTGAAATACGATTTTTTAGATAACTTAATGTATCTGTTTTTATCATTTGT
AGTGAAACAATATTGATTTCCTTCATCGTTTACCTCCATATATAGGTTATGCTTTCAAAGTCCATTTTTGACGTG
CTTTAGGGTTGAGTGGGTGCATGATTTCATTTGTGGCTGGATTAATGAGCTTTTTGACTTTCTTTTTATGAGG
CTTCAACATTTCCATTACTTGTTCGACACGTTCTACAACAACTGGCCGCTTCGCATAAGCACCATAAGCTAGA
ATCACTGTGTCACTTTCGCTAATTGCTTTCATCAAGTGGATGTCTGTGTGTTTATCATAAGGTTCTTTAATATGT
TTAAGGTTCTCTGGTGTTTTAATATTAGAGAATAGATTTACAAGATATACAGCACCGTATCGTTCTGAATTCGC
TAATTGATTGAGGATAAGAACAGTTGTGAGATCGAGTGATAATACACCATCTAAATGAGGGTACATCGTTATC
ACTGTACAAGCGGGTTTCTTTTCATCCCATGTTTTCTTGAGTAAATAGCGGTGTTTTTCATCCTTGCTAAATATA
GCTTCTGTGTATATCGTATTTTTGATTGTATTCATATATATAATCACTTCCTTTAGTATTCTTCTGGTAAAAGCATC
ACATAATAAAAAGCGTCTACGCCATCTTCACGAATGACGTAGACTTTCTTAGGTAATGCATTTTGATTTTTTAC
ATAGTTTGTATAGTGATATTCCAATTTGTATGCAGGTTGTTCTTGTTCATGTGTGATTGAGAGTATATTCTCATCT
TCTTGCAGTTTAAAAATGTGTAGGTAATCTGTATGAGGTTGATTATCTCTTTCTTTTACCATATTCCAAAGTAAG
ATTTGAAGGTCTAGAGATAGGTATTCACTAATACCTCTTGTGATGTATCGATTGATTTTCATGTTATTTTACCTC

GTCTTGAATTTCTTTCATAATGATAATCGCTTGGCTAATAATCGTAACAGATATTTGTGCCACTTTGATCCAGTT
ATTCATGGTGAGTCCCTCCTTCTCTTAGTAAATGACGTTCATCGATAATCGTATTTTTAGTATCTGTGAGATATA
AAAAGTCCATGTCAAAATGATTCAGATAACCAACGCTGATGAGTTGGTTATTAGCGTACATAAGAAATGGATA
GATACTTAGGTCATGTAGTTCATTATTGTAGTAGGTATAAGTTTCAAGTGTAAGATGTGCAAGTGGGGAATCA
TTAATAAAACGTTCCGGTAGAATATTTTCTGCTGCTTCTTCTAACGCTTCGCATTCCCATGTTTCATTGTTAGAT
AGTTGGAATAGACGAGTTATATATTGTTTGAGTTCTTGAGTGGTCGTTTTCATATCATTGCCTCCTAGATAGTG
TTATAGTGATGTAGTTTATGTACATCATTGGGATAATATATATTTGATTTGTCATTTATTACGCATCCCGGTGAGA
ATGAGAGAAAATTCCATATGAAAAACCGCTTCAAACCTTGGTATGACAAGGAAATCCCGAAATTCCGCCTATT
TTGACGAACAATCAACTCATTCTTTATAACTATTGATGTTAGGGTGGGGCTCTGCTTTCTTATATATTATTTATTT
ATAAAGAATAACGGGATTTTGGGATTGTGCTTGCACAATCCTTCTGCTTCTTCGAATCTGCAAATCCCAATCAT
TTCCCGATAAAAAATCATTGTGGGATGTTCTTTAGCAATTTCAATATAAGCATCGTGTAGTTATGAAAAAAATT
ACGACAATGACTGTTTCATTAGATAAGTGTTATTGAAATTGATAAAGAGAATTTTAAAAATGGTTAGATAAAA
TAAATGAAAGAATATAATGAAAATTATTGTTATAACAATGATTCTATTAGCTAAATAGTAAGATATAGTGTTTGG
GGCAAAAACAAAGACGAAGTGCTGAGATGCACTTCGTCGAGTTGTTTATTATTGAAAAGTTGTTTTATGATT
TCGTTATTAAGTTTGAGTGTGACATAGAATTGTTTTTTATGATTACCATCTTTTTTAATATCAATGCGATCAATCA
CTGATAGATACAATGCTTTGAGTCGAGATTTTTCTATGTGCTTAATATCATGAAAGATGTGTTGTAATAGTTTAC
TGATTTCTTTGGCATCAAATAAAGGCTTATCTTCATTTTGTTGATTTTTGAGTTGGTTGATTTGATTCGTAATGT
CATTGAGTTGCTTTTCATATTTTTGAATACTTGGTCTGATTACTGATGTTAAGTCCGGATTATCCTCGATGGTTT
TAATCAAGTTATTTAGTTTGATTTGTACTTCATCATATTGTTGTTGCTTATAAGCAATATCGTGATGAAGTGCAG
CGCCATCAACTTGATTTTCTTGATTGACGTGTGTTACTACGCGTTGAATGACTTTATCACTTTTGACTATTTCAA
GTATTTGCTTCATCACATAATCTTCAATCACATCAGCTCTTACACTGTTTGCCGAACATACTTTGGAACCCTTGT
TCCGAAAATTACTACATGAATAGTAACGAATACGTTTCTTAGTCCCGTCTTTAAGTGTATTCGTGGTATTGCTT
GCTGCCATAGGTGCGCCACATTGGGGACAGTGAATAATGCCTGTAAGCAGATTCGTTCCTTTGCCATGGACT
TGGGGTTTTTGACTGACTTGTTTTTTACGCATTTGTACTTTATCCCATAAATCTTGATTAATAATGGGGGAATG
CTTACCTTCAGCTATCACTGGTTTATCATTAAGCCCTTTACGACGTTTTTCACTCCAATCTTTGTATTTCGCAAA
TTGAATTTTGCCGATATAGAATGGGTTAGCTAATATATATGTGATAGAACTAATACTAAAAGGTTTACCCTTTTT
AGTGACATAGCCTTTGTGATTTAATGCATTGGCTATTTTACGATAGCCATGGCCTTTGGCATAGGACTCGAATA
TATATTTCACAATATTAGCTTCATGTTGATTAATCATCAGTTCATGTTTACTATTAGGTATTTTGTCATAGCCCAG
CGGCAAATTGCCTTGATAATAGCCTTCTTGGGCACGTCTCGTTTGACCCATAAATACATTCTCGACAATGTTAT
TACGTTCGAATTCTGAGAAACTCGCAAGTATTTGTAACATGAGCTTACCCGATGAAGTATTGACTTCCATACG
CTCTGATAAACTGAAAAATTCGACATTTTGTTTGTGTAAATCTTCGACAATTTTGAGAAGATCAGATGTATTAC
GAGCTAATCGGTTTGTTTTGTAGACCATAACACAGTCGATATAGCCTTCTTTCGCATCCTTCAATATACGTTGG
AGCTCAGGTCGATTCATAGATTTACCTGAAATACCACGGTCAGCGTATATATCTTTAACTTCAAAATGATGGAA
GTCACAGTATTCTTTGATTTGATTGATTTGTCCGTCGATACTATAACCTTCTGTGCTTTGCATTTCTGTTGATAC
ACGTACATAGATACCGACACGTTTTGTTTTAAGTTGTTGCATTATGTTTCATCCTTTCTTCGTTTATGCAATCGA
TGATTGCATGGTTTGATTGACGATATTGAGTGGTTCATTTTTGAAATAGATTCCTATAAGATTTTTATCTTTCGT
AATGTGAATGGTTTCAATATAGGGGTACAATATGTTTAACGTGAAACGTTTTTGAATAATATTTTGAATGGTGT
GTTGTATTTGATGTCCATTGATAGATGTAGTGCGTTGCGGTTGTTGACGTAATGATTGCGTTTGTTCTCTGAAC
GTTTCTGCATCGATGATGCCTTGTGCCAACTTTTCTATCAGTTGTTCTTGAGTCAATGTGTGATGTTTTTCTATG
TTTCTTTGTCTTTTGATGCGTTTGTCAATCGCACCTTTAATTTTTGTGTAGATGCGTTGATTTTGATAAAAGTCT
TTGCACACTTCTAATACTTTATCTTCAAGTGTTTGTGCATTGATGCCTTTAAAATCACAGACAAAGCGAGAAG
CATTCATGTTTTTAGGACAGACGTAGTAACGTAATGTATGATTCTTTTTTCTAACGGTCATATTCGTAAGTGTTG
TATTACAACATGGGCATTTGATTTTTTGTTTTAGTTGGTTATCCGAAGATGTCTGTTTGATTTGTTTTTGCGATC
GAAGTCTCTGCGCTTGCTCATATATACTTGTGGAAACAATAGAAGGAAACATATTGTCGAATTGGCCATATTG

ATTGTTGACACGACCACAATAATTAGGATTGATGATAATGTTACGAACTTGATAGGGTTGTCGATTGATATACG
TGTTATCTTCTTCTAATAACTGTGCAATTTTCTTATAACCATGACCTTTAATGTAATAATTGAATACAGCCTTTAC
CGTTGGTGACTCATTTTGATTGATGATGAATGCTCCGTTGTGATATTCGTAACCAAAGGGCGCATGGGTTGTA
ATCAATCGACCTTGCTTTGCTTTTTCTTGAAGCCCATTTCTGACTTGTTCTCCAATGTTATCTGATTCAAGTTCG
GCCAAGCTGATAAAAATATTAAGCTTGAATCGGTCGAAAGCTTGATCCATATCAAAGTAACCATCGTGTACGC
TTAAGATATGAACATGGTATGTTTGACATAATTTGATGAGTTTTAATGCATTTTTCAGATTACGATGTAATCTAT
TAAGACGATAACAACATAATATGTCACATTGCCCTTGTTGAATTAATTGCGTCATTTGTCGATACCCACTACGAT
TATCTTTGCGTCCTGATTGTTTGTCGCTATAAAAGTTGATATGTTGAATATGATGTTTTTCGGCTATTGCTTCGA
TAGCCTGTTTCTGTGCTGCAAGAGATTGTTGTTTCATCGTACTTTGACGTAAATAGCCTATGACTTGTTTCATA
TCGGCTCCTCCTTTCACAGTAATAATATATATTTATGGATGAATTGATATATAAGCCCAACATCAATAAGATGTTG
GGCGTTCATATTAGTCATTCATTTGATTGATTTCTTCAATTACCAAATCGGCTAATATCTCGATAAGTTCATCCAT
GTTTTTCACTCCGTTATTTGTTCTATCTTCAATACGTCGATTATTCAGTTTGATGCTTCACGGTTGTATGATAAA
GACAATCAGAAATCTTCGTGAACTCCTGAAGGGCCTATCCCCTCATTAGCGGATTTAAAAAGTTCTTTCGCAG
CTTTGTTATCATTTGCCGGTGTCCAATTTTGAATTAACGACTTATCTTTAGTTAATCCCAGGATAGATGCAAACT
CTACATCTAATTTTAGATGGTAAAATACAAGTGATTGTTTTTTACCGCTATTATCTTTGACACTTCTTTTAGTTGT
TTGGCGTCCACGGTCAGCTAATATGAAACCTTTATCTCTTAAGGCGTTGACAACATTATTAACATCTTGAAATT
[truncated: 5,661,757 more chars]
