# Supplementary material for: In Silico Exploration of Staphylococcal Cassette Chromosome mec (SCCmec) Evolution Based on Phylogenetic Relationship of ccrAB/C
Source: Microorganisms. 2025 Jan 13;13(1):153. doi: 10.3390/microorganisms13010153 (PMC11767417; doi:10.3390/microorganisms13010153)
Supplement: Supplementary file 1 [file Supplementary Data S4.pdf › microorganisms-3393230-supplementary/Supplementary Data S4.pdf]

```
LOCUS       Staphylococcus          41022 bp    DNA     linear   UNK
DEFINITION  Staphylococcus Staphylococcus aureus PM1
ACCESSION   Staphylococcus
KEYWORDS    .
SOURCE      Staphylococcus Staphylococcus aureus
  ORGANISM  Staphylococcus Staphylococcus aureus
            Bacteria; Bacillati; Bacillota; Bacilli; Bacillales;
            Staphylococcaceae; Staphylococcus; Staphylococcus aureus.
FEATURES             Location/Qualifiers
     source          1..41022
                     /db_xref="taxon:703339"
                     /genome_id="703339.249"
                     /mol_type="genomic DNA"
                     /organism="Staphylococcus Staphylococcus aureus"
                     /genome_md5=""
                     /project="hwwang_703339"
     CDS             1..369
                     /EC_number="2.3.3.10"
                     /transl_table=11
                     /translation="VLWNRDVGNIYTGSLYLSLISLLQNHTFQPEEKVCLFSYGSGAV

GEIFSGSIVKGYDKALDKEKHLNMLESREQLSVEEYETFFNRFDNQEFDFERELTQDP
                     YSKVYLYSIEDHIRTYKIEK"
                     /db_xref="SEED:fig|703339.249.peg.1"
                     /product="Hydroxymethylglutaryl-CoA synthase N-domain
                     homolog (EC 2.3.3.10)"
     CDS             1326..2069
                     /EC_number="3.1.4.46"
                     /transl_table=11
                     /product="Glycerophosphoryl diester phosphodiesterase (EC
                     3.1.4.46)"
                     /db_xref="SEED:fig|703339.249.peg.2"
                     /db_xref="GO:0008889"
                     /translation="MLTVYGHRGLPSKAPENTIASFKAASEVEGINWLELDVAITKDE

QLIIIHDDYLERTTNMSGEITELNYDEIKDASAGSWFGEKFKDEHLPTFDDVVKIANE

YNMNLNVELKGITGPNGLALSKSMVKQVEEQLTNLNQNQEVLISSFNVVLVKLAEEIM

PQYNRAVIFHTTSFREDWRTLLDYCNAKIVNTEDAKLTKAKVKMVKEAGYELNVWTVN
                     KPARANQLANWGVDGIFTDNADKMVHLSQ"
     CDS             2166..2594
                     /translation="MKYDDFIVGETFKTKSLHITEEEIIQFATTFDPQYMHIDKEKAE
```

QSRFKGIIASGMHTLSISFKLWVEEGKYGEEVVAGTQMNNVKFIKPVYPGNTLYVIAE
ITNKKSIKKENGLVTVSLSTYNENEEIVFKGEVTALINNS"
/db_xref="SEED:fig|703339.249.peg.3"
/product="MaoC domain protein"
/transl_table=11
CDS complement(2640..4646)
/transl_table=11
/db_xref="SEED:fig|703339.249.peg.4"
/db_xref="GO:0008658"
/db_xref="GO:0046677"
/translation="MKKIKIVPLILIVVVVGFGIYFYASKDKEINNTIDAIEDKNFKQ

VYKDSSYISKSDNGEVEMTERPIKIYNSLGVKDINIQDRKIKKVSKNKKRVDAQYKIK

TNYGNIDRNVQFNFVKEDGMWKLDWDHSVIIPGMQKDQSIHIENLKSERGKILDRNNV

ELANTGTAYEIGIVPKNVSKKDYKAIAKELSISEDYIKQQMDQNWVQDDTFVPLKTVK

KMDEYLRDFAKKFHLTTNETESRNYPLGKATSHLLGYVGPINSEELKQKEYKGYKDDA

VIGKKGLEKLYDKKLQHEDGYRVTIVDDNSNTIAHTLIEKKKKDGKDIQLTIDAKVQK

SIYNNMKNDYGSGTAIHPQTGELLALVSTPSYDVYPFMYGMSNEEYNKLTEDKKEPLL

NKFQITTSPGSTQKILTAMIGLNNKTLDDKTSYKIDGKGWQKDKSWGGYNVTRYEVVN

GNIDLKQAIESSDNIFFARVALELGSKKFEKGMKKLGVGEDIPSDYPFYNAQISNKNL

DNEILLADSGYGQGEILINPVQILSIYSALENNGNINAPHLLKDTKNKVWKKNIISKE

NINLLTDGMQQVVNKTHKEDIYRSYANLIGKSGTAELKMKQGETGRQIGWFISYDKDN
PNMMMAINVKDVQDKGMASYNAKISGKVYDELYENGNKKYDIDE"
/product="Penicillin-binding protein PBP2a, methicillin
resistance determinant MecA, transpeptidase"
CDS 4894..5037
/transl_table=11
/db_xref="SEED:fig|703339.249.peg.5"

/translation="MNYFRYKQFNKDVITVAVGYYLRYALSYRDMSEILRERGVNVHH
LTV"
/product="Mobile element protein"
CDS 5317..5568
/product="Mobile element protein"
/translation="MAKVIKAFKLKPDCHCTSKYLNNLIEQDHRHIKVRKTRYQSINT

AKNTLKGIECIYGLYKKNRRSLQICGFSPCHEISIMLTS"
/db_xref="SEED:fig|703339.249.peg.6"
/transl_table=11
CDS complement(5628..6056)
/transl_table=11
/product="PhnB protein; putative DNA binding
3-demethylubiquinone-9 3-methyltransferase domain
protein"
/db_xref="SEED:fig|703339.249.peg.7"
/translation="MKFQVSPYFLITQNAEGLKKLYEELFGAKTLYIQRIKDRPIDKK

LNIDENDLNKIDQCVLQFGDIKIMIADDTEGLPITQGNNISLCLTFDQVEETKHVYDQ
LIEKGSEVLKTFSPEFYTEGYGYVRDPYGISLHLFTKRKN"
CDS 6137..7066
/transl_table=11
/db_xref="SEED:fig|703339.249.peg.8"
/translation="MKKSVRLYNMIEYCNENRNFKLNDLMSEFNISRSTALRDIKEIE

ALGVPLYSNPGKNGGYTIIGNRDQTKIAISDEELKALVFTLSSISNVSNLPFQTEYQE
ILKKLYNNSNKKELINQYNDLFQYFNEDKYQFKSYKLFNEIIRLIIENKSFETCYSQN

YIKEQYKGIGIMYKNHQWYFVVANIESKLVNLLNISKIKELYEMGETQECNDITMQNF

QQFMVKNETAIDILIRSNVMGLNILKGYLWSDYMIENIDEETYLFKSKVNAKDIDFIA
KLIVTGGVNVKVETPNSLKNAVKVELTKIINMY"
/product="Transcriptional regulator, YafY family"
CDS 7228..7710
/product="Similarity"
/db_xref="SEED:fig|703339.249.peg.9"
/translation="MNKNITALKSTENTTYMQYTVHIDDQIINNIKECTKKFKILPME
DKIPLSPLLQPEYAGEVQDFISTYEQFMINFGKVILDSQGIKIQFESESLSSIQRGIQ

EHCYLNERTNDIDVTKEWYLCKFSNQILEEDKNQLYNALKQLMNDSKNKKQAFWTDPN
"
/transl_table=11
CDS 7778..8092
/transl_table=11
/product="Mobile element protein"
/db_xref="SEED:fig|703339.249.peg.10"

/translation="MKRVSYSVETKYKAVEMKIAGFSTKEIMKELNIKNRTQVETWWR

WYRNEESYRFSQHVGKQYTYGKGLEELSEIERLKLENKRKDIELDILKKYKALERKWY
QQ"

CDS 8077..8919
/translation="MVPAVVIDLVDQLKVKYSIKLILEVLNIPKSTYYRWKNKTYKND

TVTQKVIELCEANHYTYGYRKITALINQCYTSPINHKRVQRIMQKHHLNCRVRPKKTT

RIGKPYYKTDNLLQRQFKASCPMEVLTTDITYLPFGHSMLYLSSIMDIYNGEIVAYKI

DDKQDQSLVNDTLNQIDIPEGCILHSDQGSVYTSYAYYQLCEEKGIIRSMSRKGTPAD

NAPIESFHSSLKSETFYINNQLNSSNHIVIDIVEKYIKNYNNNRIQQKLGYLSPVKYR
ELAA"
/db_xref="SEED:fig|703339.249.peg.11"
/product="Mobile element protein"
/transl_table=11
CDS 8926..10491
/transl_table=11
/db_xref="SEED:fig|703339.249.peg.12"
/translation="MFLLSSRLRGSVPFQEVFKVHIDIYLYNKEKSEYKYQVSSYFNL

VKENPKITYKKRHLQEKQGVKGTTFTNKLKLLNKMYGVDVAKYQPFYNSNNPEYERGQ

FGERYISQRSNYEFNRLQYQIIDMLSKILDKHPLPKSDNNYKHIPTIEKAILSGDSHS

FYEYFEDIMKEIISMENSSLKEKLLTDFTYQSQCRWYSESEKLKLQLESFMHKVLESN

YYEGNKLYRMLSHAIEETINEADEDKVHFNYFKDYFLTDGGVKNWEQISEKITEFNGK

VINDIQNEYNKIQFNNANRNQKLNFNYLYHCFEFSNNLVKARVNGNRGYILYFVDKYK

IKMPFLDQLLINYLNQEIGQESINYNMQTLFEKERYDRSSTIEKLVATSKFKYEKDDS
DLFKQLFNDVENSIDRLGIYLLNNGINSNDENARYYRSFLKELSRIKSKLTPFSLEIS

KSSGREQHYPDDAIDDKDERRKNKEETYHAFDDKSDIDSKLKNKINVSIDNLLSVKIK
SNLYDISPNTKKQ"
/product="Similarity"
CDS 10686..11795
/product="hypothetical protein"

/translation="MYQANIRDLITKLPQSNKTEHFLMNKFSNQDKVQQLQRQISQQL

DQQYNELLANEKAKLDQYVEVHHNLEPLKKEIESESINLDTDKLPDIKATMLEKAKND

EHFDKIEQLFDRLDQSLNGTNRLYTQLSLIGTRTHRITTKRFNVQGLPKLVQQMILPS

QFKKVYTIDFKSFEPSVAAYMTQDEQLIDYLNHEEGLYDALLRDLSLSKEKRVSVKRA

FIGSFLFGGRYSSSKFKINQEVSEINWLQVMSKFKKVIEFKEQVEKYKTMPTPYGIEH

DMSAFQGSSIMAIYVQTVASYIFKHILLEVYKAQCEKKTFKIIVPIHDAIMIECNDKG
IAQNVAQLMKDTANQLFNGEFAHVTVEALGGIDNE"
/db_xref="SEED:fig|703339.249.peg.13"
/transl_table=11
CDS 11788..12156
/translation="MNNDRGKSLQIPQSTLLKEGSIYVATLHSVYEKNFSGDIKHQFT

YEVELNQETHYVNRNITVKSMSHQLSIADWIKRHSNYNVNHINYDPYIDRKHLVLVGQ
YNGNYYIQDVAPLDEFGGVL"
/db_xref="SEED:fig|703339.249.peg.14"
/product="hypothetical protein"
/transl_table=11
CDS 12156..13772
/db_xref="SEED:fig|703339.249.peg.15"
/translation="MNHILEMLIKLLKVGMEAIDRKGLIAILTSSIGNDEMDDSEQAV

MVYNELIDKLQLNIPKDVDYRPNIYSYFGIQKKPNDTILVEMMICIFHIKRFDSELFV

FKDKGWQKVSEDELQGLISKMIQVLLVDYKPSLSTLKNVVDGIQKSTDIEKLVEHRQY

IGCGRNMFNLKTFKVVDNDLEIFPKTRLDLELDINDTITDKIPPNFKQYMLELANYDH

DLQYFLFQHMAVLLTADTKLRRGLFLYGTAKNGKSVYIKLVKSFFYSNDIVSKTLNEL

GGRFDKESLIGKRIMASDEVGKANIDEATVNDFKKLLSVEPIHADRKGRTQVEVTLDL

KLIFNTNAVLNFPSSHAKALERRIAVIPCEYYVEKADPDLIEKLQDEKKEIFLYLMYV

YKQIVKNDIEYLQNDRVTEISHDWLNFGYEFVSSKSANIAHQKACINLLRKLIEIKPG

SRIKVSRLNEVIRDEIKVSSQVINDLVQANFNVQSRLNNGYKYWVDLGWKETDKKDDM
ISFDKNENVTDDEFLYEDDLNLGWEDFDDE"
/product="putative primase"
/transl_table=11
CDS 13997..15676
/product="Cassette chromosome recombinase B"
/db_xref="SEED:fig|703339.249.peg.16"
/translation="MKGKIALYSRVSTSEQSEHGYSEKEQEQLLIKEVMKNFPGYDYE

TYTDSGISGKNIEGRPAMKRLLQDVKDNKIEMVLSWKLNRISRSMRDVFNIIHEFKEH

DVGYKSISENIDTSNASGEVLVTMFGLIGSIERQTLISNVKLSMNAKARSGEAITGRV

LGYKLSLNPLTQKNDLVIDENEAHIVREIFDLYLNHNKGFKAITTILNQKGYRTINQK

PFSVFGVKYILNNPVYKGYVRFNNHQNWAVQRRSGKSDKNDVILVKGKHEAIISEDVF

DQVHEKLASKSFKPGRPIGGDFYLRGLIKCPECGNNMVCRRTYYKTKKSKERTIKRYY

ICSLFNRSGSSACHSNAINAEVVERVINVHLNRILSQPNVIKQIASSVIEELKQKHSK

QTEIKYDIDSLEKQKAKVKTQQERLLELFLDDEMDSEMLKAKQSEMNQQLEVLDQQIK

EAKQANQSQDDIPNFDKLKARLILMITRFSVYLRKATPEAKNQLMKMLIDSIEITTDK
QVKLVRYKIDESLIPQSLKKDWGSFFIPKFNFVINVTKKNRIENLSLLPLF"
/transl_table=11
CDS 15765..16103
/translation="MTLSKQLKTYITERFKLNYQETWACETIDAVAEDVLPEKYIKNS
PLEHKILNTFTYYNDELHEISIYPFLCYLDKELVAIGYLDNFDLDFIFLNDTHQVIID
ERYLLQKGGE"
/db_xref="SEED:fig|703339.249.peg.17"
/product="hypothetical protein"
/transl_table=11
CDS 16106..16195
/translation="MNWIKVAQLSVTVINEVIEIMKEKQNGGE"
/db_xref="SEED:fig|703339.249.peg.18"
/product="hypothetical protein"
/transl_table=11
CDS 16197..16508
/transl_table=11
/product="hypothetical protein"
/translation="MKINRYITRGVSEHLSLDLQILLWNMVKDRDNQLHTDYLHIFKL

QEDDNILSITHEQEQPAYKLEYHYTNYVKNQNALPKKVYVIREDDVDAFYYVMLLPEE
Y"
/db_xref="SEED:fig|703339.249.peg.19"
CDS 16520..17026
/transl_table=11
/db_xref="SEED:fig|703339.249.peg.20"

/translation="MMNTIKNTIHTEAIFSDDEQHRYLLKKTWDEKKPACTVITMYPH

LDGVLSLDLTTVLILNQLANSEQYGAVYLVNLFSNIKTPENLKHIKEPYDKHTDIHLM

KAISESDTVILAYGAYAKRPVVVERVEQVMEMLKPHKKKVKKLINPATNEIMHPLNPK
ARQKWTLK"
/product="hypothetical protein"
CDS 17040..17261
/transl_table=11
/db_xref="SEED:fig|703339.249.peg.21"
/translation="MNHETKQSDWRTVASCLASQNYISIVKGLVHHFTAIEDEEILDK
IYDDFMNDDSITTVLNNDLQTIINHYLSK"
/product="hypothetical protein"
CDS complement(17336..20455)
/db_xref="SEED:fig|703339.249.peg.22"

/translation="MNFQFNEDDLEQVALEWLQSLGYDYKKGNEISMTSLTPERKSDK
DVVLHERLEKALRKINSDIHPRFIEKALHELTLEKSPNLLENNLTFHENLINGIEIED
YDDEGQSIVEIVKIIDFEYPQNNDFLAVNQLTIVNGDYKKRPDIVLFINGLPIVVIEL

KNSTNETVGVEDGYHQLETYKMRIPQLFTFNEVLVTSDGINTKAGSLTANYDRFMTWR

SKDGQTESSPGLASLDVLIHGMLNPETLLDLIRYFVLFQDDGKGHISKILAAYHQYYA

VNKAVDRALLASSEQGDGKGGVIWHTQGSGKSLTMVFFSGKLIQMLNNPTLVVVTDRN

DLDNQLYSTFVKSKGRSGKGLLRQTPKQAETRKELKSLLSVESGGIVFTTMQKFEPEQ

NETTMAALTERKNVIVMADEAHRTQYGFNAKYDDKGEGIKYGYAKYLRDALPNATFVG

FTGTPVASTDKNTQMVFGNYIDVYDMTQAVADGSTVKIYYESRVIPLNLPQSLDLDEA

YNDITEDQEEDVKQRLKSKWSRIEALAGAKPRVEALAKDIIQHFETRQQAMKGKGMIV

TMSRRIAVDLYDEIIRLKPEWHSDDDDKGVIKVVMTGSSSDPTSFQRHIGPKKRRNLL

EKRMKDVDDELQLVIVRDMWLTGFDVPSMHTMYIDKPMKGHNLMQAIARVNRVFKDKP

GGLIVDYVGIAESLKEALKEYTESDQAQTAIDTDKAVELMLLKYDVIQDMLYNLDYSK

FNSDKKSERYYTISNTMDYVIGLGEDERQRFIKTVTELGKAFALCATEPTAQELNDEI

AFFKAVKAGLVKLLQPPKEGKTRKTPAEVEAEINQLVSQSVVTEDVIDVYQTLGLEQP

DLSILSDDFLKDVEGLKQKNVAVELLNRLLKGQVKSLMKTNATVSKRFSEMLGNSINK

YNSRSIETSKVIEELIQLAKDIKQEQQRGNELGLNSDEIAFYDALASHETAKEAMGDK

ELRAIAHELTKTVKENMGVDWSKRDSAKAKMRVAVRRLLKKYGYPPDLQKMAVEQVVE
QAELMANNQ"
/product="Type I restriction-modification system,
restriction subunit R (EC 3.1.21.3)"
/transl_table=11
/EC_number="3.1.21.3"
CDS complement(20439..21740)
/transl_table=11
/product="Type I restriction-modification system,
specificity subunit S"
/db_xref="SEED:fig|703339.249.peg.23"
/translation="MEYNTIFFGDVAKIKNGYAFKSKEFQEKGIPVIKIKNIISPIVD

TKDSQKVSIKTYEKTKGFSLKKNDILISLTGSGVNQMSSAVGKVGRIEFDYPALQNQR
VGKFELKYSNSADLDFLFYYFLQPKITEYLVRNSTGSANQANINSKLIETVKIPNFSL

IKQKSISKFLNQITRKIETNQKMIANLKELSQTLFKHWFVDFEFPDEDGNPYKSSGGE

MIDSELGKIPSNWKIYKLKDIASHKKETFNPKKSEEVTVKHFSLPAYDNEEQAIEEEV

NKIKSNKWIINNNCVLFSKMNPDTKRIWLPVIDNKKLNVASSEFVVMESPNNKINSFI

YNICLNSQFIDYLKANTTGSTNSRQRVKPTIAVNYKLAIEDSIVKKYSEIITPYMEEM
KILRSEIGKLTQLRDTLLPKLMSGELEISDDIEVNSDELSI"
CDS complement(21730..23244)
/EC_number="2.1.1.72"
/transl_table=11

/translation="MATIGFEEKLWQAADKLRGSMDAAEYKNVALGLIFLKYVSDSFE

EKYEELKQDPYADEEDQDEYLAENIFWVPKEARWQYINDNAKKPEIGQIIDKAMIAIE

NENESLKGVLPKEYARPALDKEKLGDIIDLFTFKVGDTESRKQDVLGRVYEYFIAKFA

SAEGKNAGEFYTPSSIVKLLVEMIEPYKGRIYDPCCGSGGMFVQSERFVEKHQGRLDD

IAIYGQESNPTTWKLAKMNLAIRGIDNDLGERNADTFHNDLHKGLKADYILANPPFNA

SDWGQERLLDDYRWQFGIPPKGNANYAWIEHMISKLAPNGTAGFVLANGSMSTSGKDE

LEIRKNLIEQDLVECIVTLPGQLFYSTQIPVCLWFISNNKGQNGKKERENEILFIDAR

EIGHMVSRTLKEFSDEDIQKVAQTYHAWRGTNDKPYEDIAGFCKVANLEEVKNNEYIL
TPGRYVGLADVEEDEEPFEQKMERITSELSEQFAKSKELEDQIRKSLEGLGYGV"

/db_xref="SEED:fig|703339.249.peg.24"
/product="Type I restriction-modification system, DNA-methyltransferase subunit M (EC 2.1.1.72)"

CDS 23440..23586
/transl_table=11
/db_xref="SEED:fig|703339.249.peg.25"
/translation="MDGVTNVKIHILTFVKIVVIVDIVAIVNTRLDKLGIFNSINKKF
KCSQ"
/product="hypothetical protein"

CDS 23686..25749
/product="FIG131328: Predicted ATP-dependent endonuclease of the OLD family"
/db_xref="SEED:fig|703339.249.peg.26"
/translation="MYISSVKIKNFRNFDNIDIDFHEGVNVLIGHNNSGKSNLLRALS

LIFDGSVRKQLSVEDFNNSLTKESLKKEAPKIVISVHITQSENERLMSDELITVSNWL

TKLEEPYEARIQYEFFLPKDEEKNYIDLVKNIDEKEEIWNLIRSQFIRLYVNKIWVGN

PEHQIPIDNDSLNKFDFQFLDAIRDVERDMFSGKNTLLKSVIDFFIDYDIKSDETITE

EEQKEKLEERRKEFSDNSSDLIETIHKRLDSGNQKILSYTNGIGASYDKSTPDFKGNL

TESEIYTVLQLIIKHETGMTLPITHNGLGYNNLIFMALLLSKMQADSDGNFLGSNAKV
FPILAIEEPEAHLHPTMQNEFIKFLKNNIREKKVKQIFITTHSTHISSSTNIDDIICL

YTDDSKTNVSYPGKVFDKNNQSKKYVQRFLDATKSNMLFAEKVIFVEGIAEQLLLHIF

ADYLNKPLEKNHVAVINVGGRYFNHFLSLFDSNNKYAINRKVSCITDLDPMRKEKNEN

GQKNNFKACYPFEYEMDLDKFDYSTNNSLDKYLNNEHSNIRVFVQPNKYGKTFEYQLM

FDNPSLKLLLTDSISNSQELAELMDHYEENESLQKLIDTLSPSNENQRIIKSLKDTND

SWNEDNKKKALIASRYLNSIGKGENALELASVLKDNLELKGQSDYEDFVVPKYIEGAI
RWVCE"
/transl_table=11

CDS 25751..27436
/transl_table=11
/db_xref="SEED:fig|703339.249.peg.27"
/translation="MIINAQTKLNIEQDFKIQAGPGAGKTEFLVNHIKNVIQTSEKLE

RMKKIACITYTNTAAQTVLQRLGKSVSNRVDISTIHSFLYRNVVKPYCSFLPKEYGIC

VQKLKGHSDPIVINKYVNAWLKTEYFTKLKPPSNRNQLLKMPVLKKALQNWLLSMKLS

YENNDVQFICDNTKAQAIDKKSKKRMGINKSNLNILEKQLLNYKKIYWEKGIIDHDDV
LFFSYVLIRDYPFILTILCAKYPYFFIDEFQDTSQLQDFIIDKIRQKGCIVGVIGDKA
QAIYSFQGARVSLFEKFKVDLINSHTILENHRSSYQIVTFLNSIREDITQTVYSGIEN

DKVTILIGNRIRSYTKVVNICNKESLVTLSRDNITSNAMRIKLEGNNFDKKLLEKFQE
IDSNSKRRNTIISLIKAIELAKNTKYKDALKIVESIYKDENNPKKIALNSLINMINKY

DKYGNGTLMNFYNTLCKTLDIKLSGFRKGQVKDFYENTLYKNMVICTNIVEDTSNHIT
IHKAKGAEFENVFVIGNKDTLSLLLKPDLMNDEEHRIFYVAMSRAKKKLFFTI"
/product="ATP-dependent DNA helicase UvrD/PcrA,
clostridial paralog 2"
CDS 27764..27892
/transl_table=11
/translation="MIIFNVIMLVSSIIQMILGKGRRITPAKPTKDAKLHLDNQSS"
/db_xref="SEED:fig|703339.249.peg.28"
/product="Multidrug resistance protein"
CDS complement(27960..29384)
/transl_table=11
/translation="MEMEFNENNIDLETIIRDEVNKYLSRDIGDLPATQQAPLELREK

YEKMEVPNKGRDIYEVLKDLNNEVLNYLYRPNHPRSFSFIPGPASRLSWLGDILTTAN

NIHASNFANATLPINIERNLINYLVGKIGYEIKPAGGVFVSGGSMANLTAIVAARDAQ

VEMEDIKKATVYLTSQTHHSVGKALHVAGFLKNNIRRIEYNDDFTMNTDSLKAAIDKD

IEEGYKPAMVIATAGTTNTGSVDDFTTLGDTCDHYNLWLHVDGAYGLSHILSDKAQHL

FKGIERSDSASWDAHKLLFQTYSCAMVIVKEKNHLLQSFGEDAEYLDDIASDDDVIDP
EMLGIELTRPARALKLWITLQVIGEDEIIERIKYGEGLAEYAEEYVSGLDNWRIISHA

NLSIVNFRYENSDLTEAQNNQLNSMAAQKIADSGYAIAYTTVLNNQRVIRLCTTNPLT
THDDIRETINRLDQYIGDYDMKDA"
/db_xref="SEED:fig|703339.249.peg.29"
/product="Decarboxylase, pyridoxal-dependent"
CDS complement(29467..29679)
/EC_number="1.1.1.95"
/db_xref="SEED:fig|703339.249.peg.30"
/db_xref="GO:0004617"
/translation="MRSSSPVLRGSYRIPKDKHQYKLKITDDNLQVDYPRQFLKGAHT
PSVKAQHVIHWGDTFWVSSLWLAWFY"
/product="D-3-phosphoglycerate dehydrogenase (EC

1.1.1.95)"
/transl_table=11
CDS 30329..30496
/product="hypothetical protein"
/translation="MKFKSEKLIIANQTKVINYRIQVLLKPQTNIETQTQVKNNTLSE
TDEQSNADLVQ"
/db_xref="SEED:fig|703339.249.peg.31"
/transl_table=11
CDS 30702..31988
/transl_table=11
/translation="MGIYHLNKDKDVLTDLKSNEKQEQVATFINKHLSANNLTIFIGS

GCSTGAVPLMSTTMKNILEENESVLNYVKKFLNSKGIKEFIKYVEEQEQEKIQEKERK

ALHTIMDQLEAENFKNLEEYSGWLDMQDSEYKEEILNFLDCYYLNYSNIEELLNWIQN

GLHYDNNNGDLKDVFTTLKSEFIKTIPKVGDKEYSTETYEIYKDFYRYVFDKRTEQKS

KVSIFTTNYDLFNEYALENNNIIYSTGIQNTILKKFDINQFKYRVVDDTNRYKEKWQP

VSKEANLYKIHGSINWKSNEEGELQQIDFNDEDDQVVIYPTMLKHKETAQAPYSELFR

EFSNCLQIKDTTLIIIGYGFPDEHINNIIAQNLKNQDFNLIIFGDVKEENVKNFYDNF
KNFNLHLIGGNSSKAEQKAHYFQFIVENFLKNQRRR"
/db_xref="SEED:fig|703339.249.peg.32"
/product="hypothetical protein"
CDS 31994..33268
/translation="MHSIGKVTSVTFEKLIFEVSDFEKLNYNLLGQIYIAKGVIDYVT

IKNEYSEKFIYQVVKVEDKEIPLSSEEHSKFKYHGRFECVPVGMIKHGKIEFNLKKYP

FLQDKVYLTSQEEMEMVFSHFHNGNDITIGLIDDQYPAYFNTAKLLTNHTAIIGNTGS

GKSTTVRQIISKINNLNTQNLHFHIFDVHDEYKDINGVKIVDVINDFKINIKNLEMQD

WINLIKPSELVQLPILQMGLKYANAIENKIIEEEWLKCYIALSLYRNQQTDAVTKRTK

ILSILDGTNIDTEKYDSKYGNMDSNTEKKFIESLKNVVDNGGNIFTLSEVIEKAKYNV

SSFNKLLEGLNYVFLLEESKGNNQARSYSATLETRIKNVQTRFSNLFGNNDTELEDKS
IVYSVSELDDDLLLFFTTFILKKEFEKNKKRN"
/db_xref="SEED:fig|703339.249.peg.33"
/product="hypothetical protein"
/transl_table=11

CDS 33280..33681
/db_xref="SEED:fig|703339.249.peg.34"
/translation="MNVFIFEEAHRYISKFKESSQFNEVEAFKKIAREGRKFGCFLML

SSQRPSELSSTVLSQCNNYIVHRVKNNVDLEYLLNSIPYINKFQLNRFSYLPTGTAYI
VGELFPIPVEIEIFEEFSKNSTITPEIVYRS"
/product="Bipolar DNA helicase HerA"
/transl_table=11
CDS 33842..33979
/transl_table=11
/product="hypothetical protein"
/translation="MAKLIHLLSILYRLSSDKKFTVKQISDTCHNGGKSLYSAITNLK
K"
/db_xref="SEED:fig|703339.249.peg.35"
CDS 34242..34853
/transl_table=11
/product="hypothetical protein"
/db_xref="SEED:fig|703339.249.peg.36"
/translation="MKTPIYYTIVNKGNNIENTIFQTPPNTVSDFDHLDVMRYLPSIV
DISEICLLAEAKKIPEFKKSLDFILNKFETYTFNIVNDLIEITLTKDALLNYQKYLLH
QSIIISKSLALKNSNNGLCTQELKSQLYGYEKLHYEPAFHILDNDLIPNINEEQSELI
PLEHVVLLSDGISYLMDYFDKNPEATFTLYIVKDYTSYYEVEL"
CDS complement(35192..35599)
/transl_table=11
/db_xref="SEED:fig|703339.249.peg.37"
/translation="MTLLEYSNIGNTPFEKLLGHNKNLLKYWNNLSDFLSSDGAISKE

LKEELRRLLAQQNGCQYCKAKGSPTNKFLNEKSLLCVGFVEVYLKSKQNIPESSISLL
KETMTDEEIIELITFVLFTTAQQHLGAILRLSE"
/product="hypothetical protein"
CDS 35700..36113
/transl_table=11
/product="Leucine-responsive regulatory protein,
regulator for leucine (or lrp) regulon and high-affinity
branched-chain amino acid transport system"
/translation="MNDLINEKIINVLLNNSRITINELSKQVNLSAPAVRERVNKLED
QGIIKGYTINIDYKELGYDVEILIELTIKNNRYKDFKEFISKQDNVEFCYRVSGEACF
VFKVRLENMDEVETFVDTIQPYGQTKCQFIFSSVV"
/db_xref="SEED:fig|703339.249.peg.38"
CDS 36175..36648
/transl_table=11
/translation="MEYKSYNKMSNNILSILLSEKNFDNKMVVSTGSYILSELPCIVA
YKNNDIVGLLTYKVYDEYIEIISLDSFVENKGIGSHLLNYAEIIASDMSKRSIRVITT
NENIKALYFYQKNKYRITDVIFDAVTEARKIKPSIPAIDKNGIEIRDEIVLRKCL"

/db_xref="SEED:fig|703339.249.peg.39"
/product="Acetyltransferase, GNAT family"
CDS complement(36756..37742)
/transl_table=11
/product="tRNA-dihydrouridine synthase 2"
/translation="MKENFWSELPRPFFILAPMEDVTDIVFRHVVSEAARPDVFFTEF

TNTESFCHPEGIHSVRGRLTFSEDEQPMVAHIWGDKPEQFRETSIQLAKMGFKGIDLN

MGCPVANVAKKGKGSGLILRPDVAAEIIQATKAGGLPVSVKTRLGYYEIDEWKDWLKH

VFEQDIANLSIHLRTRKEMSKVDAHWELIEAIKNLRDEIAPNTLLTINGDIPDRKTGL

ELAEKYGIDGVMIGRGIFHNPFAFEKEPREHTSKELLDLLRLHLSLFNKYEKDEIRQF
KSLRRFFKIYVRGIRGASELRHQLMNTQSIAEARALLDEFEAQMDEDVKIEL"
/db_xref="SEED:fig|703339.249.peg.40"
CDS 38050..38253
/product="hypothetical protein"
/translation="MEDLKESLKSLGWWDLFFAIPMFLLFAYLPNYNFITIFLNIVII
IFFSIGLILTTHIIIDKIKSNTK"
/db_xref="SEED:fig|703339.249.peg.41"
/transl_table=11
CDS 38302..38598
/transl_table=11
/db_xref="SEED:fig|703339.249.peg.42"

/translation="MATEKDVNDLFLNHVNSNAVKTRKMMGEYIVYYDGVVIGGLYDN
RLLVKATKSARHQFQDNTLVSPYPGAKEMILIPDITEVTNLTDLFELIKNDLQK"
/product="hypothetical protein"
CDS complement(38660..39880)
/translation="MTDLLKIKNFRLFFLAEIISAFGVGISTVGANWYLIDKTNDSQL
LGIMLALNVLSGFLASPIIGGLADKYNRRNIILITYLLQVILYLLIVIALLMIGFETY

LVIGFAIVNGIGWTTYMATSRSLVKQILKPDQYTDANSLLEISLQTGMFIAGGLSGIL

YEINGFTLIIAMTIMMFLISIFMLFRLHVDKPTHSEEESTNSLLQEYLLGWKFLKDNM
MIFIFGVISIIPMVFTMIFNISLPGYVYNVLKLSSVQFGFSDMLYGIGGLCAGLISAI
LSKKISTKALIFLLYFILVINSALFIWINSAFYLFIGSFILGYSISSIRIYMNTAIMN
TVSDKYVGRSFTIWTSISLLLQSFIAPFLGRWINEINDKFGFYIILILSLLIFVTLLL
VNKTDKIKYAHKEE"
/db_xref="SEED:fig|703339.249.peg.43"
/product="FIG01108443: hypothetical protein"
/transl_table=11
CDS complement(39873..40424)

/transl_table=11
/db_xref="SEED:fig|703339.249.peg.44"
/translation="MITCLFGSSRKDGNSAIAVENLLKNLNVNFVNLYQSNIEKVTDN

RHSKDANFHNDDYEQILIKVLESDIIIFSTPLYWYSMSASLKLFIDRWTESLRDTQID

NFKEIMSQKKYLILIIGGDNPHTKAQPLVNQFKLIFEFMNITNYRFLIGEGNKPFDIL
NDSQFIEELANINLALKKGDIYD"
/product="hypothetical protein"
CDS 40495..41022
/transl_table=11
/db_xref="SEED:fig|703339.249.peg.45"
/translation="MQNSIKVNPKGGTTLNLNILKSFLVTSETKNLTKASELLNYSQS

TVSTHIEKLEKQLDVKLFYRKKYGMELTEEGLAYVKYAKVILDSNSEYEREIKGLYNK

KVNISINMQESQYLYRYYNKISEWLAEHPYVNLKFKSAHSNFYIKEEIANFKSDISLI
TDEKIINSNLTAIPIT"
/product="LysR-family regulatory protein"
ORIGIN
1 gtattatgga atagagatgt tggtaacatt tatacaggat cattatactt aagtttaatt
61 tcgttattac agaaccacac attccaacca gaagagaaag tatgtctatt tagttatggt
121 tcaggagcag taggagaaat ctttagtggt tcaatcgtta aaggatatga caaagcatta
181 gataaagaga aacacttaaa tatgctagaa tctagagagc aattatcagt cgaagaatac
241 gaaacattct ttaacagatt tgataatcaa gaatttgatt tcgaacgtga attgacacaa
301 gatccatatt caaaagtata cttatacagt atagaagacc atatcagaac atataagata
361 gagaaataaa ctagtggccg attgtgcttg atgagcttgg gacataaatc ctaactcgaa
421 ataaataagc atatcactaa actgattttt taaagtttac agtgatatgc ttattttttt
481 atcttacgat tttgtacgtg catgcttgcc taggggtatg gctcgagcca ttagtctctc
541 gcacatacta ttccctcagg cgtcagcact tacaaaatcg gttgtaattt tcatttttat
601 acgcattctt actgagatta tactaataag aggaatagta aaagcaattc taagtaaaat
661 tgcagataag aggtttgtta aaagcagttc taagtaaaat tgcagataag aggtttgtta
721 aaagcagttc taagtaaaat tgcagataag aggtttgtta aaagcagttc tcagtaaaat
781 tacagataag aggtttgtta aaagcagttc taagtaaaat tgcagataag aggtttgtta
841 aaagcagttc taagtaaaat tgcagataag aggtacgtta aaagcaattc catgcaaaat
901 tgctgataag gggtaagtta aaagcagttc tcagtaaaat tgcagataag aggtacgtta
961 aaagcagttc taggcaaaat tgcagataag aggtgcgtta aaagcagttc taagtaaaat
1021 tgctgataag gggtaagtta aaagcaatcc taagtaaaat tgcagataag aggtaagtta
1081 aaagcaatcc taagtaaaat tgcagataag gggtacagaa aaactagact tgattacaaa
1141 atggagcttg ggacataaat gatttttaa aaatgagatg agacgtagat taactccata
1201 atcaatacga atctatcgac ttctttattt atgatattca tctcttttta atggaaataa
1261 aagtgcgatt aatgtgataa tacagttacg ttaattaaaa aaataaaaat gcaaggagag
1321 gtaatatgct aactgtatat ggacatagag gattacctag taaagctccg gaaaatacaa
1381 ttgcatcatt taaagctgct tcagaagtag aaggtataaa ctggttggag ttagatgttg

1441 caattacaaa agatgaacaa ctgattatca ttcatgatga ttatttagaa cggactacaa
1501 atatgtccgg ggaaataact gaattgaatt atgatgaaat taaagatgct tctgcaggat
1561 cttggtttgg tgaaaaattc aaagatgaac atttgccaac tttcgatgat gtagtaaaaa
1621 tagcaaatga atataatatg aatttaaatg tagaattaaa aggtattact ggaccgaatg
1681 gactagcact ttctaaaagt atggttaagc aagtggaaga acaattaaca aacttaaatc
1741 agaatcaaga agtgctcatt tcaagcttta atgttgtgct tgttaaactt gcagaagaaa
1801 tcatgccaca atataacaga gcagttatat tccatacaac ttcgtttcgt gaagactgga
1861 gaacactttt agattactgt aatgctaaaa tagtaaacac tgaagatgcc aaacttacta
1921 aagcaaaagt aaaaatggta aaagaagcgg gttatgaatt gaacgtatgg actgtaaaca
1981 aaccagcacg tgcaaaccaa cttgctaatt ggggagttga tggtatcttt acagacaatg
2041 cagataaaat ggtgcatttg tctcaataga aagttagagg tgagtcttac gtttcagtga
2101 cggtagactt acctttaaca tgttacatac taaaaaatta atttgaataa gaaagagaga
2161 catatatgaa atacgatgat tttatagtag gagaaacatt caaaacaaaa agccttcata
2221 ttacagaaga agaaattatc caatttgcaa caacttttga tcctcaatat atgcatatag
2281 ataaagaaaa agcagaacaa agtagattta aaggtatcat tgcatctggc atgcatacac
2341 tttcaatatc atttaaatta tgggtagaag aaggtaaata cggagaagaa gttgtagcag
2401 gaacacaaat gaataacgtt aaatttatta aacctgtata cccaggtaat acattgtacg
2461 ttatcgctga aattacaaat aagaaatcca taaaaaaaga aaatggactc gttacagtgt
2521 cactttcaac atacaatgaa aatgaagaaa ttgtatttaa gggagaagta acagcactta
2581 ttaataattc ataataaaac agtgaagcaa ccatcgttac ggattgcttc actgttttgt
2641 tattcatcta tatcgtattt tttattaccg ttctcatata gctcatcata cactttacct
2701 gagattttgg cattgtagct agccattcct ttatcttgta catctttaac attaatagcc
2761 atcatcatgt ttggattatc tttatcatat gatataaacc acccaatttg tctgccagtt
2821 tctccttgtt tcattttgag ttctgcagta ccggatttgc caattaagtt tgcataagat
2881 ctataaatat cttctttatg tgttttattt acgacttgtt gcataccatc agttaataga
2941 ttgatatttt ctttggaaat aatatttttc ttccaaactt tgtttttcgt gtcttttaat
3001 aagtgaggtg cgttaatatt gccattattt tctaatgcgc tatagattga aaggatctgt
3061 actgggttaa tcagtatttc accttgtccg taacctgaat cagctaataa tatttcatta
3121 tctaaatttt tgtttgaaat ttgagcatta taaaatggat aatcacttgg tatatcttca
3181 ccaacaccta gtttttcat gcctttttca aatttcttac tgcctaattc gagtgctact
3241 ctagcaaaga aaatgttatc tgatgattct attgcttgtt ttaagtcgat attaccattt
3301 accacttcat atcttgtaac gttgtaacca ccccaagatt tatctttttg ccaaccttta
3361 ccatcgattt tataacttgt tttatcgtct aatgttttgt tatttaaccc aatcattgct
3421 gttaatattt tttgagttga acctggtgaa gttgtaatct ggaacttgtt gagcagaggt
3481 tcttttttat cttcggttaa tttattatat tcttcgttac tcatgccata cataaatgga
3541 tagacgtcat atgaaggtgt gcttacaagt gctaataatt cacctgtttg agggtggata
3601 gcagtacctg agccataatc atttttcatg ttgttataaa tactcttttg aactttagca
3661 tcaatagtta gttgaatatc tttgccatct tttttctttt tctctattaa tgtatgtgcg
3721 attgtattgc tattatcgtc aacgattgtg acacgatagc catcttcatg ttggagcttt
3781 ttatcgtaaa gtttttcgag tccctttta ccaataactg catcatcttt atagccttta
3841 tattcttttt gttttaattc ttcagagtta atgggaccaa cataacctaa tagatgtgaa
3901 gtcgcttttc ctagaggata gttacgactt tctgtttcat tagttgtaag atgaaatttt
3961 tttgcgaaat ctcttaaata ttcatccatt tttttaacgg ttttaagtgg aacgaaggta
4021 tcatcttgta cccaattttg atccatttgt tgtttgatat agtcttcaga aatacttagt

4081 tctttagcga ttgctttata atctttttta gatacattct ttggaacgat gcctatctca
4141 tatgctgttc ctgtattggc caattccaca ttgtttcggt ctaaaatttt accacgttct
4201 gattttaaat tttcaatatg tatgctttgg tctttctgca ttcctggaat aatgacgcta
4261 tgatcccaat ctaacttcca cataccatct tctttaacaa aattaaattg aacgttgcga
4321 tcaatgttac cgtagtttgt tttaatttta tattgagcat ctactcgttt tttattttta
4381 gatacttttt ttattttacg atcctgaatg tttatatctt taacgcctaa actattatat
4441 atttttatcg gacgttcagt catttctact tcaccattat cgcttttaga aatataactg
4501 ctatctttat aaacttgttt gaaattttta tcttcaattg catcaatagt attattaatt
4561 tctttatctt ttgaagcata aaaatatata ccaaacccga caactacaac tattaaaata
4621 agtggaacaa tttttatctt tttcatcaat atcctcctta tataagacta catttgtaat
4681 atactacaaa tgtagtattt atgtcaaaat aatgttataa tttttgtgat atggaggtgt
4741 agaaggtgtt atcatctttt ttaatgttaa gtataatcag ttcattgctc acgatatgtg
4801 taattttttt agtgagaatg ctctatataa aatatacggt tctgttgcaa agttgaattt
4861 atagtataat tataaccaaa aggagtcttc tgtatgaact atttcagata taaacaattt
4921 aacaaggatg ttatcactgt agccgttggc tactatctaa gatatgcatt gagttatcgt
4981 gatatgtctg aaatattaag ggaacgtggt gtaaacgttc atcatttaac ggtctagcgt
5041 tgagttcaag aatatgcccc gattttatat caaatttgga agaaaaagca taaaaaagct
5101 tattacaaat ggcgtattga tgagacgtac atcaaaataa aaggaaaatg gaactattta
5161 tatcgtgcca ttgatacaga gggacataca ttagatattt ggttgcgtaa gcaacgagat
5221 aatcattcag catatgtatt tatcaaacgt ctcattaaac aatttggtaa acctcaaaag
5281 gtaattacag attaggcacc ttcaacgaag gtcgcaatgg ctaaagtcat taaagctttt
5341 aaacttaaac ctgactgtca ttgtacatcg aaatatctga ataacctcat tgagcaagat
5401 caccgtcata ttaaagtaag aaagacaaga tatcaaagta tcaatacggc aaagaatact
5461 ttaaaaggta ttgaatgtat ttacggtcta tataaaaaga accgcaggtc tcttcagatc
5521 tgcggatttt cgccatgcca tgaaattagc atcatgctaa caagttaaca caaagtatta
5581 ttttaaaatt gagattagac atttattttt caactttgca gcagaaccta attttttctc
5641 tttgtaaata aatgcaaaga gataccataa ggatctctta cataaccata accttcagta
5701 tagaattctg gactaaatgt tttcaatacc tcactgcctt tttctattaa ctggtcatat
5761 acatgtttag tttcttctac ttggtcaaaa gtgagacaaa gagatatatt attaccttgt
5821 gttatgggca aaccttcagt gtcatctgcg atcataattt ttatatctcc aaattgaagt
5881 acacattgat caattttatt taaatcattt tcgtcaatat taagtttctt atctataggt
5941 ctatctttaa tacgttgaat atacagtgtt ttagcgccaa acagctcttc atacaacttt
6001 tttaaaccct ctgcattttg agtgattaaa aaatatggac ttacttgaaa tttcatgttt
6061 tttcctcctt agatttgtta taaatagagt atatacttaa ttagtgtcat ctattgacac
6121 taaaaggaga ataataatga aaaaatctgt tagattatat aatatgattg aatattgtaa
6181 tgaaaatagg aacttcaaat taaatgattt aatgtcagaa tttaatattt ctcgtagtac
6241 cgctttaagg gatataaaag aaattgaagc attaggagta cctttatata gtaatccagg
6301 gaaaaatggt ggttatacga tcataggtaa tcgagaccaa acgaaaatag caatctcaga
6361 tgaagagttg aaagctttag tatttacact ttcgagtatt tcaaatgtga gtaatctacc
6421 ttttcaaaca gaatatcaag aaatattaaa aaaattatat aataactcga ataaaaaaga
6481 gttaataaac caatataatg atctatttca atattttaat gaagataagt atcagttcaa
6541 aagttataag ttatttaatg aaatcattag attgataatt gagaataagt cttttgaaac
6601 ctgttattca caaaactata ttaaagaaca atataaaggt attggcatta tgtataaaaa
6661 tcatcaatgg tattttgttg tagctaatat agaatcaaag ttagtgaatc tattaaatat

6721 ttcgaaaata aaagaactat atgaaatggg agagactcaa gagtgtaatg atataactat
6781 gcagaatttt caacagttca tggttaaaaa tgaaacagct attgatattc ttattagaag
6841 caatgttatg ggattgaata tcttgaaagg ctacctgtgg agtgactata tgattgaaaa
6901 tattgacgaa gagacatatt tatttaaatc aaaagtgaac gcgaaagata tagattttat
6961 agctaagtta attgtcacag gtggtgtcaa tgtaaaagta gagaccccta atagtttgaa
7021 aaatgctgtt aaagttgaat taactaaaat aataaacatg tattaatagt aatttaatca
7081 taattgtaat aacaaaaaga tgacaacatt aaacaattta aatttataca aaaaagcatt
7141 accaaattta aaattgggta gtggtagtgc tacagcatac ataaatagtt ataattaaat
7201 aaagatgggt caatgaaggt gaattaagtg aataaaaata ttacagctct aaaatctact
7261 gaaaatacta catatatgca gtatacggtt catatcgatg atcaaattat aaataatata
7321 aaagagtgta ccaaaaagtt taaaattttg cctatggaag ataaaattcc attatcaccg
7381 ttgttacaac cagaatatgc aggagaggta caagatttta ttagtacata tgagcagttt
7441 atgattaatt ttggtaaagt aatattggat agtcaaggca taaaaataca gtttgaaagt
7501 gaatcattaa gtagtattca acgaggcatt caagaacatt gttacttaaa tgagcgaaca
7561 aatgacattg atgtgactaa agaatggtat ttatgcaaat tttcaaatca aatattagaa
7621 gaggataaaa atcaattgta taatgcttta aagcaactca tgaatgattc taaaaataaa
7681 aagcaagctt tttggactga ccccaattag tgggaattat ataaaaacac tttcgttgaa
7741 ttcataataa aatgaatcat gcggaggtgt tttttctatg aaaagagttt cttattcagt
7801 agaaacaaag tataaagcag ttgaaatgaa aatagctggt ttttcgacaa aagaaattat
7861 gaaagaatta aatattaaaa atagaacaca agtggaaact tggtggcgat ggtatcgaaa
7921 tgaggaaagt tatagatttt cacaacacgt tgggaaacaa tatacctacg gtaaaggatt
7981 agaagaattg tcagaaatag aacgattaaa attagaaaat aagagaaaag atatagaatt
8041 ggatatttta aaaaagtaca aggcattgga aaggaagtgg taccagcagt agtcatagat
8101 ttagtggatc aattaaaagt aaaatattca atcaaattga tactagaagt attaaacata
8161 cctaaatcaa catattaccg atggaaaaac aaaacctata aaaatgatac tgtaacacaa
8221 aaagttattg aattatgtga agctaaccac tatacctacg gttatcgtaa gattacagca
8281 ctgattaatc aatgttatac atcaccaatt aatcataaga gagtacagag aattatgcag
8341 aagcatcatt tgaactgccg agttagacct aaaaagacga caaggatagg taaaccgtat
8401 tataaaactg acaatttatt acaaagacaa tttaaagcga gttgtcccat ggaagtatta
8461 acaaccgata ttacttattt accatttggt cattctatgt tgtatttatc ttcgataatg
8521 gatatttata acggagaaat tgtggcgtat aaaatagatg ataaacaaga ccaaagttta
8581 gttaatgata cattaaatca aatcgatata cccgaggggt gtatattaca tagtgatcaa
8641 ggcagcgttt atacatctta tgcttattat caattatgcg aagaaaaagg cattatcaga
8701 agtatgtccc gaaagggaac acctgctgat aacgccccga tagaaagttt tcattcctcg
8761 ctaaagtctg aaacttttta catcaataat cagcttaata gctctaatca tattgtaata
8821 gatattgtcg aaaaatacat taaaaactat aataataatc gaattcaaca aaaactaggc
8881 tacttatctc ctgtaaaata cagagaatta gcagcctaga acatagtgtt tttattaagt
8941 tcccgtttaa gggggtcagt accttttcaa gaggtgttta aggttcatat tgatatttat
9001 ctctacaata aagaaaaatc tgaatataag tatcaagtat ctagttattt caatcttgta
9061 aaagaaaatc ctaaaataac ttataaaaaa agacatttac aagagaaaca aggtgtcaag
9121 ggtacaactt tcacgaacaa attaaaatta ttgaacaaaa tgtatggtgt agatgttgca
9181 aaatatcagc cattttataa ttctaataat cctgaatatg aaagaggtca atttggtgaa
9241 agatacattt ctcaaagatc taactatgaa tttaatagac ttcaatatca aattatagat
9301 atgttatcga aaatacttga taagcatcca ttaccaaagt cagataataa ttataaacat

9361 attccaacta ttgaaaaagc aatattaagt ggggattctc atagttttta tgagtatttt
9421 gaagatataa tgaaagaaat tataagtatg gaaaattctt ctttaaagga aaaattattg
9481 actgatttta catatcaatc tcaatgtaga tggtactctg aaagtgaaaa attaaaattg
9541 caacttgaaa gttttatgca taaagttttg gagagcaatt attatgaggg taacaaatta
9601 tatagaatgt tgtctcatgc aattgaagaa acaatcaatg aggctgatga agataaggtt
9661 catttcaatt attttaaaga ttattttttg acagacggtg gggtgaagaa ttgggagcag
9721 attagcgaga aaattactga attcaatggt aaagttatta acgatatcca aaatgaatat
9781 aataagattc aatttaataa cgcaaatcga aatcagaaac taaactttaa ttatttatac
9841 cattgctttg agtttagtaa taacttagta aaagcaaggg taaatggaaa tagaggttac
9901 attttatatt ttgtagataa atataaaata aaaatgccct ttttggatca attattaatc
9961 aattatttga atcaagaaat aggtcaggaa agtattaatt ataatatgca aacattgttt
10021 gaaaaagaaa gatatgatag aagtagtacc attgaaaaat tagtagcaac aagcaaattt
10081 aagtatgaaa aagatgattc agatttattc aaacaacttt tcaatgatgt tgaaaattca
10141 atagacagat taggtattta cttactaaat aatggtataa attcgaatga tgaaaatgca
10201 agatattata gatcgttttt aaaggaactt agtagaataa aaagtaaatt aacgccattt
10261 tctcttgaaa taagtaagtc tagcggaaga gagcaacatt atcctgatga tgctattgat
10321 gataaagatg agagaagaaa aaataaagaa gaaacatatc atgcttttga tgataaaagc
10381 gatattgact ccaaattaaa gaataaaata aatgtttcta tcgataattt attgtcagtt
10441 aaaattaaga gcaatttgta tgatattagt ccaaatacaa aaaaacaata actataatga
10501 gccaactcaa gaatgagttg gctttttaat tgtaatttta cgcttccatt acaggaatca
10561 atgttatgga agccattaag caattaattt gagtcattta tagtataggt acaacgttgt
10621 gatgttcaca tgataatcag cccgggagat tattgtgtaa taacttatag aaggagcaaa
10681 tcaccatgta tcaagcaaat atacgtgatt taattacaaa attgcctcaa agcaataaaa
10741 cagaacactt tttaatgaac aaattttcaa atcaagataa agttcagcag ctacaaagac
10801 aaattagcca acagctagat caacaatata atgagctttt ggctaatgaa aaagctaagc
10861 tagaccaata cgtggaagta caccataatt tagaaccatt aaagaaagag attgaatcag
10921 aatctattaa ccttgatacc gataaattac ctgatatcaa agcgacaatg cttgaaaagg
10981 ctaagaacga tgaacatttt gataaaatcg aacagctatt tgatagatta gatcagtcat
11041 taaatggtac gaatcgatta tatacgcaat tatcgttgat tggcacacga acacatcgga
11101 tcacaacgaa aagatttaat gttcaaggct tgcctaaatt agtccaacaa atgattttac
11161 cttcgcaatt taaaaaggtt tatacaatag attttaaatc attcgaacca tcagttgctg
11221 cgtatatgac acaagacgaa caactgattg actacttgaa tcatgaagaa gggttatacg
11281 atgcattact gagagactta tctttgtcaa aagagaagcg cgtgagtgtg aaacgtgcat
11341 ttatagggtc atttcttttt ggcggtcgtt atagtagctc taaattcaaa atcaatcaag
11401 aggttagtga aattaactgg ctacaagtaa tgagcaaatt caagaaggtc attgaattta
11461 aggagcaagt cgaaaaatat aaaacaatgc ctacgcctta cggcattgaa catgatatga
11521 gcgcatttca aggtagtagt attatggcaa tttatgtaca aacggtagca agctatattt
11581 tcaagcacat tttgttggaa gtgtacaaag cacagtgcga aaaaaaaacg ttcaagatta
11641 tagtgccgat acacgatgcg attatgattg aatgtaatga taaggggatt gcacaaaatg
11701 tagcacagct catgaaagat acagctaatc aactgtttaa tggtgaattt gcacatgtga
11761 cagtggaagc tttaggaggt atagacaatg aataatgata gaggaaaaag tcttcaaatt
11821 ccccaaagta cattgttaaa agaaggatct atatacgttg ctacgttaca ttctgtgtac
11881 gagaagaact tctcaggtga tattaaacat cagtttacgt atgaagtaga acttaaccaa
11941 gaaacgcact atgtaaatcg caatattacc gtaaaatcta tgagtcacca attatcaatt

12001 gctgattgga ttaaacgtca cagtaactat aacgtaaacc acattaatta tgatccatac
12061 attgatcgaa aacatttggt ccttgtaggg caatataacg gaaattatta tattcaagat
12121 gtagcaccat tagatgaatt tggaggggta ttgtaatgaa tcatatttta gaaatgttaa
12181 taaaattatt aaaagtgggt atggaggcaa tcgaccgtaa aggtctgatt gccatcctaa
12241 caagtagtat tggaaatgat gaaatggatg attctgaaca agctgtaatg gtgtataacg
12301 agcttatcga taagctacag cttaacattc ctaaagatgt cgactataga cctaacatat
12361 acagttattt tggtattcaa aaaaagccaa atgacacaat attagtggaa atgatgatat
12421 gtatttttca tatcaagcgt tttgattcag agttgtttgt tttcaaagat aaaggttggc
12481 aaaaggtaag tgaagatgaa ttgcaagggt tgatatctaa aatgatacaa gtgttgctag
12541 ttgattataa accttcacta agtactttga aaaacgtagt agatggcata cagaaatcaa
12601 cggacataga aaaacttgtt gagcatagac agtacattgg ttgtggacga aatatgttca
12661 atctaaagac ctttaaagtg gttgataatg accttgaaat attccctaaa acacgcttag
12721 atttggaatt agatataaat gacacgatta cggacaagat acccccgaat ttcaaacaat
12781 atatgttaga gttggcgaat tatgaccatg atttacaata tttccttttc caacatatgg
12841 cagtgttatt gacggcagat actaaactac gtcgtggact ttttttgtat ggaactgcaa
12901 aaaatgggaa atcggtctat attaaattag ttaagtcatt cttttatagt aatgatatcg
12961 tatctaaaac acttaatgaa cttggcgggc gtttcgataa ggaaagtcta attggtaaac
13021 gaattatggc aagtgatgaa gtggggaaag ctaatattga tgaagcaact gtgaatgatt
13081 tcaaaaaatt actatctgtt gaaccaattc atgctgaccg taaaggaaga acgcaagtag
13141 aagttacttt agatttaaaa ctcattttta atacgaatgc tgtactcaat tttccatcat
13201 cacatgcaaa agcattagag cgtagaattg ctgttattcc atgtgaatat tatgttgaaa
13261 aagctgaccc tgacttaatt gaaaagttac aggatgaaaa gaaagaaatc tttctttact
13321 tgatgtatgt gtataagcaa attgtaaaaa atgatatcga gtacctccaa aatgatcgtg
13381 ttactgaaat ttctcatgat tggttaaatt ttggatatga atttgtttct agtaaatcag
13441 caaatattgc acatcagaaa gcgtgtatta atttactcag aaaacttata gaaatcaaac
13501 cagggtcacg tatcaaagtg tctaggctaa atgaggttat tagagatgaa attaaagtaa
13561 gctctcaagt tattaatgat ttggttcaag ctaactttaa tgtacaaagt agactaaata
13621 atggttataa gtattgggtc gatttaggat ggaaagaaac tgataaaaaa gatgacatga
13681 tttcattcga taaaaatgag aatgtaacag atgatgaatt cttatacgaa gatgatttga
13741 acttaggttg ggaggacttt gacgatgaat aatgaacaaa ttgaagcatt tgtagaagtg
13801 cttgtgccta tcatagaaga acgtatcaat aaaggtaagt aatctaatta cgtactacag
13861 gcagttgcct gtagtactca tatgattaag tggtaaaagt gataaaaatg aaacgaaatt
13921 ataaatatat attatctata tgttgttaca agaccgatgg tctgtagcaa taatctaata
13981 aaaggagcgg tatgatatga agggtaaaat tgcactttat tcacgtgtta gtacgtctga
14041 gcagtcagaa catggttatt ctgaaaagga gcaggaacaa ctactcatca aagaagttat
14101 gaaaaatttc ccaggttatg actatgagac atatactgac tcaggcattt caggtaaaaa
14161 tattgaaggt cgtccggcaa tgaaacgtct attacaagat gttaaggata ataaaatcga
14221 aatggtatta agttggaaat tgaatcgtat ttctcgctca atgagagacg tgtttaatat
14281 tattcatgaa ttcaaagaac atgacgtagg gtataaatcg atttctgaga atattgatac
14341 atccaatgct tctggagaag tactcgttac aatgtttgga ctaataggat ctatagaacg
14401 ccagactttg atttccaatg tgaaactttc tatgaatgct aaggcaagga gcggagaggc
14461 aatcaccggt cgtgttttag gctacaaatt atcacttaat ccattgacac agaaaaatga
14521 tttagttatt gatgaaaatg aagctcatat tgtacgggaa atctttgatt tatatttgaa
14581 tcacaataaa ggatttaaag caatcacgac aattctaaat caaaaaggat atcgtaccat

14641 taatcaaaaa ccattttcag tgtttggcgt gaaatacatt ttgaataatc cagtctataa
14701 aggctatgtc agattcaata atcatcaaaa ttgggctgtt cagcgaagaa gtggtaaaag
14761 tgataaaaat gatgtgatat tggtcaaagg taagcatgaa gccattataa gtgaagatgt
14821 atttgatcaa gttcatgaga aactagcttc taaaagtttt aaaccgggtc gacctattgg
14881 tggagatttc tacttacgtg gccttattaa atgcccagaa tgcggaaata atatggtatg
14941 tcgacggacg tattataaaa cgaaaaagtc caaagaacgc acaatcaaac gctattacat
15001 ttgttcatta ttcaatcgct caggaagttc tgcctgtcat agtaatgcga ttaatgctga
15061 agtcgtcgaa cgcgtaatca atgttcattt gaatcgtatt ctttcacaac ctaatgttat
15121 taagcagatt gcgtcaagtg tgatagaaga actgaaacaa aagcatagta aacaaacaga
15181 aataaaatat gatattgata gtctagaaaa acaaaaagca aaagttaaaa cacaacaaga
15241 acgattattg gaattgttct tagatgatga aatggatagc gaaatgttaa aagctaaaca
15301 aagtgaaatg aatcaacagt tagaagtatt agaccaacaa attaaagaag caaaacaagc
15361 aaatcaatca caggatgata tacccaattt tgataagtta aaagcacgac tcattttgat
15421 gataacacga ttcagtgtgt acttaagaaa ggctacaccc gaagctaaaa atcaacttat
15481 gaaaatgtta attgattcaa ttgaaattac gacagataaa caagtaaaac ttgtaaggta
15541 taaaattgat gaaagtctta tccctcaatc tttgaaaaaa gattgggggt cttttttat
15601 acctaaattt aactttgtga taaatgtcac aaagaaaaat aggattgaaa atttatcact
15661 tttaccactt ttttagagtg acaaaagtgg aggagttttg aaatatttta taaatatata
15721 ttttatttat ggagtacaca ttattaatta aggaggtcat tataatgacg ctaagcaaac
15781 aacttaaaac gtatatcact gaacgattta aattaaatta tcaagaaact tgggcttgtg
15841 aaaccataga tgcggtggct gaagatgtat tacctgaaaa atatattaaa aatagtccac
15901 ttgaacataa aattttaaat acttttacct attacaatga tgaattacat gaaatcagca
15961 tttacccttt tttatgttat ctagataagg aattagtagc aataggttat ttagataatt
16021 ttgatttaga ctttatattt ttaaatgaca ctcatcaagt cattattgat gaacgctact
16081 tgttacaaaa agggggcgag taattatgaa ttggatcaag gtcgctcaac tatctgtcac
16141 agttatcaat gaagtcattg agattatgaa agaaaaacaa aatggagggg aatagtatga
16201 aaatcaatcg atacattaca agaggtgtta gtgaacattt atctctagac cttcaaatct
16261 tactttggaa catggtaaaa gatcgagaca atcaacttca tacagattac ctacacattt
16321 ttaaactaca agaagatgat aatatactct caatcacaca tgaacaagaa caacccgcat
16381 acaaattgga atatcactat acaaactatg taaaaaatca aaatgcatta cctaagaaag
16441 tctacgtcat ccgagaagat gatgtagacg ctttttatta tgtgatgctt ttaccagaag
16501 aatactaaaa ggagtgacaa tgatgaatac aatcaaaaat acaatacaca cagaagcgat
16561 atttagcgat gatgaacaac accgctactt actcaaaaag acttgggatg aaaagaaacc
16621 tgcatgtacg gtaatcacca tgtaccctca tctagatggc gtattatcac tcgatcttac
16681 aactgttctt atcctcaatc aattagcgaa ttctgaacaa tatggcgctg tatatcttgt
16741 aaatctattt tctaatatta aaaccccaga gaaccttaaa catattaaag aaccttatga
16801 taaacacaca gacattcatt taatgaaagc gataagtgaa agtgacacag tgattttagc
16861 ctatggtgct tatgcgaagc gacccgttgt tgtcgaacgc gttgagcaag tgatggaaat
16921 gttaaaacct cataaaaaga aagtaaaaaa gctcataaat ccggcaacga atgaaattat
16981 gcatccgctt aatcctaaag cgcgtcaaaa atggacattg aaataaagga ggattatcta
17041 tgaaccatga aactaaacaa tcagattggc gaacggttgc tagttgttta gcatcacaaa
17101 attatatatc gattgtaaaa ggtttagtac atcattttac agcgatcgaa gatgaggaaa
17161 ttcttgataa aatctatgat gattttatga atgatgactc tataacaacg gtacttaaca
17221 atgatttaca gacaatcatt aaccattatc tatcaaaata aagactatac taattacaat

17281 aaaaacagag catccttcac ttttatggtg agggatgctc ttttaattta tttttttatt
17341 gattattcgc cataagttct gcttgctcta caacctgctc tacagccatt ttttgtaaat
17401 ctggtggata gccatatttt ttaagcaatc gtctaacagc tacgcgcatt ttagcttttg
17461 cgctatcacg tttagaccaa tcaacaccca tgttttcttt cactgtttta gttagctcat
17521 gagcaatcgc acgtagttct ttatctccca tggcttcttt tgctgtttca tgtgaagcta
17581 aagcatcgta aaacgcaatc tcatctgaat tcaggcctaa ttcatttcct cgttgttgtt
17641 cttgtttaat atctttagcg agttgaataa gttcttcaat cactttagat gtttcaatgg
17701 aacgactatt atatttatta atcgaatttc ctaacatctc agagaaacgc ttagataccg
17761 ttgcattcgt tttcattaat gatttaactt gtcctttgag taatcgattt aataattcta
17821 ccgccacatt tttctgtttc aatccttcga catcttttaa aaagtcatca gataagattg
17881 ataaatcggg ttgttcaaga cctagcgttt gataaacatc aatgacatct tcagtcacaa
17941 cagattgtga cacaagttga ttaatctctg cttcaacttc tgcaggtgtt ttacgtgttt
18001 ttccttcttt tggcggttgt aacaatttaa caagtcctgc tttaactgct ttaaagaagg
18061 caatctcatc gttgagttct tgggctgtag gttcagtcgc acaaagagca aaggctttcc
18121 ctaattctgt gactgtttta ataaaacgtt gacgttcatc ttcacctaaa ccaattacat
18181 aatccatcgt attcgatatt gtataataac gttcagattt tttatctgaa ttaaatttag
18241 agtaatcaag attatataac atatcttgaa ttacatcata cttcaataac atcaattcaa
18301 ccgctttatc cgtatctatt gccgtttgag cttgatcaga ttctgtatat tctttaagtg
18361 cctcttttaa actttcggca atccccacat aatcgacaat taaaccaccc ggcttatctt
18421 taaacactcg attaactcga gcaattgctt gcatcaaatt atgccctttc atcggtttat
18481 caatatacat cgtatgcata gaaggaacat caaaccctgt cagccacata tctcgaacaa
18541 tcacgagttg taactcatca tccacatctt tcatacgttt ttctaataaa ttacggcgtt
18601 ttttaggacc aatgtgtctt tggaaagaag ttgggtcact agatgagcct gtcattacca
18661 ctttaatgac tcctttatca tcatcatctg aatgccattc tggttttaga cgaatgattt
18721 catcatataa atcaacagca attcgacgac tcatcgttac aatcattcct ttgcctttca
18781 tcgcttgttg acgtgtttca aagtgttgga tgatatcttt ggctagggct tctacacgag
18841 gttttgcgcc tgctaaagct tcaatacggg accattttga ttttaaacgc tgttttacat
18901 cctcttcttg atcttcagta atgtcattat acgcttcatc taaatctaaa ctttgaggta
18961 gatttaatgg aattacacga ctttcatagt aaattttaac ggtgcttcca tcagcgacgg
19021 cttgtgtcat atcataaaca tcgatatagt ttccgaaaac catttgcgta tttttatccg
19081 ttgaagctac gggtgtgcct gtaaatccta cgaatgttgc attcggtaaa gcatctctta
19141 aatatttggc ataaccatat ttaatgcctt cacctttatc atcgtatttt gcgttaaagc
19201 catattgtgt acgatgagct tcgtctgcca tgacaatcac attttttcgt tctgttaggg
19261 cagccatggt cgtttcgttt tgttcaggtt caaatttttg catcgttgta aatacaatac
19321 cacccgactc aacagataat aatgatttta attctttacg tgtttcagct tgttttggtg
19381 tttgtcttaa taatccttta ccagaacgtc cttttgattt aacaaacgta ctgtacagtt
19441 ggttatctaa atcattacga tccgttacga cgactaaggt agggttattc agcatttgaa
19501 ttaattttcc agagaaaaag accatggtta aacttttacc ggacccttgg gtatgccaaa
19561 taacgccgcc tttaccatca ccttgctcag atgaagctaa taaagctctg tcaacggctt
19621 tattaacggc ataatattga tgatacgctg ctagaatttt actgatatgt cctttgccat
19681 catcttgaaa taaaacaaaa tatcgaatta aatcaagtaa tgtttctgga tttaacatgc
19741 catgaatgag tacgtctaag ctagctaaac ctggtgatga ttcagtttga ccatctttac
19801 tgcgccaagt cataaaccga tcataatttg cggttagaga gccagccttt gtattaatac
19861 catcacttgt aacaagcact tcgttaaacg taaataattg tggaatacgc atcttatacg

19921 tttctaattg atgataaccg tcttcgacgc ccacggtttc atttgttgaa tttttaagtt
19981 caatcactac gataggcaag ccattgataa agagtacaat atcgggacgt tttttatagt
20041 ctccattaac gatagttaat tgattgactg ctaaaaaatc attgttttgt ggatattcaa
20101 aatcaataat tttaacaatt tctacaatcg attgtccttc atcatcatag tcttcgattt
20161 caataccatt aatcaaattt tcatgaaagg taaggttatt ttctaaaaga ttgggcgact
20221 tctctaaagt tagttcatgt agcgcttttt caataaaacg aggatggata tctgagttga
20281 tttttcttaa tgctttctct aaacgttcat gaaggacaac atctttatca cttttacgtt
20341 ctggtgttag acttgtcata ctaatctcat taccttttt atagtcatag cctagtgatt
20401 gtagccattc taatgcgact tgttctaaat catcttcatt aaattgaaag ttcatcacta
20461 ttcacctcaa tatcatcaga tatttctagt tctccagaca ttagtttagg aagaagagta
20521 tctcgtaatt gagttaattt accaatttct gatcttaata ttttcatttc ttccatatat
20581 ggagtgatta tttcagaata tttttttaca atactatctt caattgctaa tttataatta
20641 acagctattg ttggttttac tctttgtcta ctatttgttg aaccggtagt attagctttt
20701 aaataatcta taaattgaga attaagacat atattatata tgaatgaatt aattttatta
20761 ttaggagatt ccattacaac aaattctgat gaagcaacat ttaatttttt attatctatt
20821 acaggtaacc aaatcctttt agtatcagga ttcattttg aaaataatac gcaattgtta
20881 ttaataatcc atttattact ttttatttta ttgacttctt cctctatagc ttgttcttca
20941 ttatcataag caggtaaact aaaatgttta actgttactt cttcactctt tttaggatta
21001 aaagtttcct ttttatgact tgctatatct tttaatttat agattttcca attacttggt
21061 atcttaccta actcactatc aatcatttct cctccactag atttataagg attaccatct
21121 tcatctggaa attcaaaatc tacaaaccaa tgcttaaata gtgtttgtga aagttcttta
21181 aggtttgcta tcattttttg gttagtttct attttcttg tgatttgatt taaaaattta
21241 gaaatgcttt tttgttttat taaagaaaaa ttaggaattt tgacagtttc aataagttta
21301 gaatttatat tagcttgatt agcactccct gtagaatttc taactaaata ctcagttatt
21361 ttaggttgta gaaaataata aaataaaaaa tctaagtctg cactattact atattttaat
21421 tcgaattttc ccacacgttg attttgtaat gctggataat caaattcaat tcttccaact
21481 tttcctactg cagaagacat ttgattcacg cctgatccag ttaatgatat taatatatca
21541 tttttcttta atgaaaaacc tttagttttt tcatatgttt taatagagac tttttgagag
21601 tcttttgtat caacaatggg tgaaattata ttcttaattt taataaccgg tatacctttt
21661 tcttgaaatt cttttgattt aaaagcatat ccatttttaa ttttagctac atcaccgaaa
21721 aaaattgtat tatactccat atcctaaccc ctccaatgat tttcggattt ggtcttcaag
21781 ttcttttgat tttgcgaatt gttcacttaa ttcagaagta atacgttcca tttttttgttc
21841 aaaaggttct tcatcttctt caacgtcagc taaaccaaca tatcgtccag gtgttaaaat
21901 gtattcatta ttcttaactt cttctaaatt agctacttta caaaaaccag ctatatcttc
21961 ataaggttta tcgtttgtac ctctccacgc atgatacgtc tgtgctactt tttgaatatc
22021 ttcatctgaa aattctttta atgttctaga taccatatgg ccgatttcac gagcatcgat
22081 aaataaaatt tcattttcac gctctttttt accattttga cctttattat tactaatgaa
22141 ccaaagacat acaggtattt gggttgagta aaagagttgt ccaggtaaag taacaataca
22201 ttcaactaaa tcttgttcaa ttagattttt acgaatttct aattcatctt ttccgcttgt
22261 agacattgat ccgttggcta atacgaatcc tgctgtacca ttaggtgcta attttgaaat
22321 catatgttca atccatgcat agttagcatt accttttggt ggaataccaa attgccaacg
22381 gtaatcatca agtaatcgtt cttgacccca atcacttgcg ttaaaaggag gattggctaa
22441 tatgtaatct gcttttaatc ctttatgtaa atcattatgg aatgtatcag catttcgttc
22501 acctaagtca ttatcaatac cacgaatcgc taagttcatt tttgctaatt tccaagtcgt

22561 agggttagat tcttgtccgt aaatcgcaat atcgtctaat cgaccttgat gtttctcaac
22621 gaagcgttca ctttgtacaa acatcccacc tgaaccgcaa cacggatcgt aaatacggcc
22681 tttataaggc tcaatcatct caactaataa ctttacaatt gatgacgggg tatagaattc
22741 tccagcattt ttaccttcgg cacttgcgaa tttagcaata aagtactcat acacacgacc
22801 taacacatct tgtttacgac tctctgtgtc gccaacttta aatgtaaata aatcaataat
22861 atcgcctaat ttttctttat ctaatgcagg tcttgcatat tctttaggta atacaccttt
22921 gagtgattca ttttcatttt caattgcaat cattgcttta tcaataattt gtccaatttc
22981 tggtttttc gcattatcat tgatatattg ccatcttgct tcttttggca cccagaaaat
23041 gttttctgct aagtattcat cttggtcctc ttcatctgca tatggatcct gcttcaattc
23101 ttcatacttt tcttcaaaag aatctgatac atattttaaa aagattaaac ctagtgctac
23161 atttttatac tcagcagcat ccatacttcc tctcaattta tcggcagctt gccataattt
23221 ttcttcaaaa cctatagtgg ccattaataa cacctcttaa aattaatgtt aaatcaacta
23281 tagcaaaaaa tagaagatga ttgtgtagtg ttttattaat atttaattta tctagactat
23341 caaaaaaata tagattatta ttatcttaaa ttaataaaca ggagtgaatt aaaatgaata
23401 aaaaatataa attaaaacga tacgataaaa gatttagagg tggatggtgt tacaaatgtg
23461 aagattcata ttcttacctt tgtgaagatt gtggttattg tagatattgt tgccattgtt
23521 aatacaagac tagataaatt gggtatcttt aactcaatta ataaaaaatt taaatgtagt
23581 caatagcttg ctaactttat ttgatgtaat ataattttgg cttttagcat tcagttaaaa
23641 agaagtaaat aattgacaga ttttttagag aactggagaa aaaagatgta catttctagt
23701 gttaaaatta agaattttag aaattttgat aatatcgata ttgatttcca tgagggagtt
23761 aatgtattaa tcggacataa taattcgggg aaatcaaatt tattacgtgc tttatcttta
23821 atttttgacg gttcagttag aaagcaactg tcagtggaag attttaataa ctctttaaca
23881 aaagaatctt taaaaaaaga ggctccaaaa atagtaattt cagtacatat aacacaatct
23941 gaaaacgaaa gactgatgtc tgatgagtta attacggtaa gtaattggct gactaaatta
24001 gaagaacctt atgaagctag aatacaatat gaattctttt taccaaaaga tgaggaaaaa
24061 aattacatag atttagtaaa aaatatagat gaaaaagaag aaatatggaa tttaattaga
24121 agccagttta ttaggttgta tgtcaataaa atctgggtgg gtaatccaga acatcagatt
24181 cctatagata atgatagttt aaataaattt gatttccaat ttttagacgc aataagagat
24241 gtagagagag atatgtttag tggtaaaaat acattattaa aaagtgtaat agattttttt
24301 atagattacg atataaaatc cgatgaaaca atcacagaag aagaacagaa agaaaaatta
24361 gaagaaagaa gaaaagaatt ttctgataat tctagtgatt tgattgaaac tattcataaa
24421 agattagata gtgggaatca aaaaattttg tcttatacaa atggcatagg agcatcttat
24481 gataaatcaa caccggattt taaaggtaat cttacagaaa gtgaaattta tactgtattg
24541 caattgataa ttaaacatga aactggaatg actttaccta taactcataa cgggttgggt
24601 tacaataatt taattttcat ggccttgcta ttgtctaaaa tgcaagctga ttctgatgga
24661 aattttctag gtagtaatgc aaaagtattt ccgatactag ctattgaaga accagaggct
24721 catctacatc ctactatgca aaatgaattt attaaatttt taaaaaataa cattagagaa
24781 aaaaaggtaa agcagatttt cataactact cattcaacac atatttcttc ttctacaaat
24841 attgatgata ttatttgtct ttacacagat gatagtaaaa ctaatgttag ttatcctgga
24901 aaagtttttg ataagaataa tcaaagtaag aagtacgtac aacggttttt agatgccact
24961 aagtcaaata tgctatttgc agaaaaggta atatttgtgg aaggtatagc agaacaatta
25021 ctattacata tatttgcaga ctatttaaat aaaccgttag aaaagaatca tgttgcagta
25081 ataaatgtag gcgggagata ttttaatcat tttttatctt tgtttgatag taataataaa
25141 tatgcaatta atcgtaaggt ttcttgtata acagatttag atccaatgcg aaaagaaaaa

25201 aatgaaaatg gacaaaaaaa taattttaaa gcatgctatc cttttgaata tgaaatggat
25261 ttagataaat ttgattattc aactaataat tctctagata aatatctaaa taatgagcac
25321 agtaatatac gagtatttgt tcaacctaat aaatatggaa aaacatttga gtaccaactt
25381 atgtttgaca atccttcatt aaaactgtta cttacagact cgatttctaa tagtcaagag
25441 ctagcagaat taatggatca ctatgaagaa aatgaatctt tacaaaaatt aattgataca
25501 ttatcaccat ctaatgaaaa tcagagaata ataaaatctt taaaagacac aaatgacagt
25561 tggaatgaag ataataaaaa gaaagcatta atagcatcaa gatacctaaa ttccatagga
25621 aaaggagaaa atgctctaga gctagcttca gtattaaaag ataatttaga gttaaaaggc
25681 cagagtgact atgaagattt tgtagtacca aaatatatag aaggtgcaat tcgatgggtg
25741 tgtgaataat atgattatta atgctcaaac gaaattaaat atagaacaag attttaaaat
25801 acaggctggt cctggtgctg gaaaaacaga atttttggtg aatcatatca aaaatgtaat
25861 acaaacttct gaaaagctcg aacgtatgaa gaaaatagcg tgtattactt atacgaatac
25921 ggctgctcag acagttttac aaagattagg aaaaagtgtt tcaaatagag tagatatatc
25981 tacaatacat agctttttgt atagaaacgt agttaaacca tattgttctt ttctacccaa
26041 agaatacgga atttgtgtgc aaaaattaaa aggacatagt gatccaattg tgattaacaa
26101 atatgtaaat gcatggttaa aaactgaata ttttacgaaa ttaaaaccgc ctagtaatag
26161 aaatcaacta ctaaaaatgc cagtactaaa gaaagctttg caaaactggc tactatctat
26221 gaaactttct tacgaaaata atgatgtgca atttatttgt gataatacca aagctcaagc
26281 aattgataaa aaaagtaaaa aaagaatggg aataaataaa tcaaacttaa atatactaga
26341 aaagcaatta ttaaattata aaaaaatata ttgggagaaa ggaattatcg atcatgatga
26401 tgttttgttt ttttcatatg tattaattag agattaccca ttcattctca ctattttatg
26461 tgctaaatat ccctactttt ttatagatga atttcaagat actagtcaat tacaagattt
26521 tattatagat aaaataagac agaaaggctg catagtgggg gttattggcg ataaggcaca
26581 ggcaatttac agctttcaag gtgcacgggt ttccttattt gagaagttta aagttgattt
26641 aataaattct cacactatct tagaaaatca tagaagttca tatcaaatag ttactttttt
26701 aaatagtatc agagaagata ttactcaaac agtatatagt ggtattgaaa atgataaagt
26761 tacaattttg attggaaatc ggattcgcag ttatactaag gtagttaata tttgtaacaa
26821 ggagtcactt gttaccttgt caagagataa tattacttct aatgcaatga gaataaagtt
26881 ggaaggaaat aattttgata aaaagttatt ggaaaaattt caagaaattg atagtaatag
26941 taaaagaagg aatactatta tttcgcttat aaaagctatt gaattagcta agaatactaa
27001 atataaagat gccttaaaaa tagtcgaatc gatttataaa gatgaaaata atcctaaaaa
27061 aatagctctt aattctctaa taaatatgat taataaatat gataaatacg gtaatggtac
27121 attaatgaat ttctacaata ctttatgtaa aactttagat attaaacttt cagggtttag
27181 aaaggggcaa gtaaaagatt tttacgaaaa cactttatat aaaaaatatgg tgatatgtac
27241 aaatatcgtt gaagacacaa gtaatcatat tactatccac aaagcgaaag gcgcagaatt
27301 cgaaaatgta tttgttatag gtaataaaga cactttaagt ctacttttga aaccagattt
27361 aatgaacgat gaagagcatc gtatattcta tgtagctatg agtcgagcaa agaagaaact
27421 tttttttaca atttgattat cttgataata atgaacaaaa gaaaattaag gaacgttatg
27481 atgtaagtat tatacaataa cctaatctaa gaatatttaa atccaatata tttcttgatt
27541 ataattcaat aatagataaa atctataaac gattaaaaag aaaatatatt tgctaagact
27601 tataaaaatg aagagataat agattttcct aaattacagt taaaagaagt aaatagattg
27661 aacactttga aagattctaa tccaaaattt gaaccactaa aattgaaaga tgaagaagat
27721 gcaagatgta gccatcaacg ctgatcacaa agtatttttc ttaatgatta tttttaatgt
27781 tatcatgctc gtttcttcta ttattcaaat gattttagga aaaggcagac gtatcacacc

27841 agcaaaacct actaaagatg ctaagttaca cttagataat caatcctcat aaagagaaaa
27901 aagaacccct aaaaagttca actttttggg ggttacttac tattaagaag tatttttgat
27961 taagcatctt tcatatcgta atcaccaata tactgatcaa ggcgatttat tgtttctctg
28021 atatcatcat gtgtcgttaa tgggttcgtt gtacataaac gaatgacacg ttggttattc
28081 aatactgtgg tataagcaat ggcataacca gaatctgcaa ttttttgcgc agccatgcta
28141 ttgagttgat tattttgtgc ttcagttaaa tcactgtttt catatctgaa gttcacaatg
28201 gaaagattag cgtgtgagat aatacgccag ttatcgagac ctgacacgta ttcttctgca
28261 tattctgcta aaccttcgcc gtatttaatg cgctcgataa tttcatcttc accaatgact
28321 tgcagagtaa tccataattt taaagcacgt gcagggcgtg taagttcaat gcctaacatt
28381 tctggatcaa tcacatcatc atctgaagca atatcatcca agtactcagc atcttcgcca
28441 aagctttgca gcagatgatt cttttctttc acgattacca tagcgcaact atatgtttgg
28501 aacagtaatt tgtgagcatc ccaactggca ctatctgaac gttcaatgcc tttaaataag
28561 tgttgtgctt tatcggataa gatatgtgat agtccataag caccgtctac atggagccat
28621 agattgtagt gatcacatgt atctccaagt gtggtgaaat catcgacaga acctgtatta
28681 gtcgtgcctg cagtcgcaat caccatagca ggtttgtaac cttcttcaat atctttatca
28741 attgcggctt ttaagctatc tgtattcatt gtaaagtcat cgttgtactc aattctgcgg
28801 atattatttt taaggaagcc tgcaacatgc aaggctttgc ctacagaatg atgcgtttga
28861 gatgtaagat aaacggttgc ttttttaata tcttccattt ctacctgtgc atctctagcc
28921 gcaacgatag ctgttaaatt ggccattgaa ccgccagata caaagacacc accggccggt
28981 ttgatctcat aaccgatttt tcctaccaag tagttaatta aattacgctc aatattaatt
29041 ggcagcgtcg cattggcaaa attagaggca tggatattat ttgctgttgt taaaatgtct
29101 ccaagccatg atagacgtga tgctggacct ggtataaatg agaatgaacg tggatggttc
29161 ggacgatata aataattcag tacttcatta tttaaatctt tgagtacttc atagatatct
29221 cgcccctttat tcggcacttc catcttttca tatttctctc tcaattcgag cggtgcctgt
29281 tgtgtcgcag gtaagtctcc aatatctcta gataaatatt tattaacttc atctctgata
29341 atcgtctcta agtcgatatt gttctcattg aattccattt ccatactctc tccttcaatt
29401 atatttttaa taaaagacag acgtattaat tacgacgttt tactacaata taaatgatag
29461 cagatattaa taaaaccacg ctaaccataa tgacgaaacc cagaaggtgt cgccccaatg
29521 aataacgtgc tgtgctttaa cactcggtgt atgtgcgcct ttcagaaatt gacgagggta
29581 atcgacttgc agattatcat ctgtgatttt caatttgtat tgatgcttgt cttttggaat
29641 tcgataacta ccccgtagaa caggggacga ggacctcatc ttgatgatcc caattggctt
29701 ggttaaaaag aaatcgataa tcctcgtaga atagagaaaa agtattaaaa atatatttga
29761 tttgataaaa tattttgcaa aaaagagaaa caaaaataac tttcatataa ataatttctt
29821 tgcgattaag ttggaattag aaatactaaa tacaaataac agcgaatgga atgcatatta
29881 ttgaggaatt ggatgtgtag tgcaatttcg agagttacta cttaacttaa gaaaataaaa
29941 agaagaaaac aaacaataag aattaaatct gaagcaagat ataaaagcaa atatttttgc
30001 tacaattata ttagtaaata ctttattaat tagcatacga ctgaaagcga agtaacagct
30061 aataatgttc cagatataga cattagtgtt atgaaagaac aatattacca tctagaaaat
30121 gtaattttta aaaacctgaa gataaagtag actattctgt gagttttcat gttaagaatt
30181 ctgaacttgg ggatgtagta taaatatggt ttagtagaaa atgacgctgg agctgtagct
30241 gagattgact atcaagaatt aactaaagta gattattacc catcaacaaa aatccaagat
30301 ttaacactat ctaataatag tgcgaataat gaagtttaaa agtgaaaaac tgataatagc
30361 gaatcaaact aaagtaatca attacagaat tcaagtacta ctcaagcctc aaacaaatat
30421 tgaaactcaa acacaagtta agaataatac actgtcagaa acagatgaac agtctaatgc

30481 cgatttagtc cagtaatagc ctctgtatta ttagccgcac gattattatc aataattaaa
30541 taaagttaat attaaataat aaaaatcctt caaattattt gttgaaaaaa taataacttt
30601 gaaggttttt tagcaaaatc actgataggg agaagcgtat cataaatgat ggggttttaa
30661 gtacgattta ataaactaaa ataagatagg ggaattaaga aatgggaatt tatcatttaa
30721 ataaagataa agatgtattg acagatttaa agtcaaatga aaaacaagag caggttgcta
30781 catttataaa taaacatctt tcagcaaata atttaacgat atttatcggt tcaggatgct
30841 cgacaggtgc tgttccttta atgtctacta caatgaaaaa catattagaa gaaaatgaga
30901 gtgtcttaaa ttatgtaaaa aaatttctga attctaaagg gattaaagaa tttataaagt
30961 atgtagaaga acaagaacaa gaaaaaatac aagagaaaga aagaaaagcg ttacatacaa
31021 taatggatca attagaggct gaaaatttta aaaatttaga agaatatagt ggttggttag
31081 atatgcaaga tagtgaatat aaagaagaaa tattgaattt tttagattgt tattatctaa
31141 attatagtaa tattgaggaa ttactaaatt ggatacaaaa tggacttcat tatgacaata
31201 ataatggaga tttgaaagac gtgtttacta ctttaaaaag tgagtttata aaaactattc
31261 ctaaagtggg tgataaagaa tacagtacag aaacttatga aatatataag gacttttatc
31321 gttatgtatt tgataaaaga actgaacaaa aatctaaagt atcaattttc actacaaatt
31381 atgatttgtt taatgaatat gcacttgaga ataataatat tatctatagt acggggattc
31441 aaaatacaat acttaaaaaa tttgacatta atcaatttaa atacagagtt gtagatgata
31501 caaacaggta taaagaaaaa tggcaaccag tatctaaaga ggcaaattta tataaaatac
31561 atggttcgat aaattggaag tccaatgaag aaggggaatt gcaacaaatc gattttaatg
31621 atgaagatga tcaggtggtc atttatccaa ccatgttgaa acacaaggaa acagcacaag
31681 caccgtattc agaattgttc agagaattct ctaattgttt acagataaaa gatactactt
31741 taattattat agggtatgga ttcccagatg aacatattaa taatataata gctcagaatt
31801 taaagaatca agattttaat ttaataatat ttggtgatgt taaagaagaa aacgtgaaga
31861 atttctatga taattttaag aattttaatt tacatcttat cggaggtaat tcaagtaaag
31921 cagagcaaaa agcacattat ttccagttta tcgtagaaaa ttttctgaaa aatcaaagga
31981 gaagataaaa taaatgcata gtattggaaa agtcacaagt gttacatttg aaaagttaat
32041 attcgaagtt agtgattttg aaaaactaaa ttataatttg ttaggtcaaa tttacatagc
32101 taaaggtgta atagactatg taacaataaa aaatgaatat agtgaaaaat ttatatatca
32161 agttgtaaaa gtagaagata aagaaattcc attatcttct gaagagcact ctaaatttaa
32221 atatcatggt aggtttgagt gtgtaccagt aggaatgatt aaacatggaa aaattgagtt
32281 taatttaaag aaatatcctt ttttacaaga taaagtttat ttaacaagtc aagaagaaat
32341 ggaaatggta ttttcacatt ttcataatgg aaatgatata actattggac tcattgatga
32401 ccaatatcca gcttatttta atactgctaa attattaacg aatcatacag ctataattgg
32461 aaatactgga tctggcaaat ctacaacagt acgtcagatt atttcaaaaa tcaacaatct
32521 aaacacccaa aatttacact ttcacatatt cgatgtccat gatgaatata aagatataaa
32581 tggagtgaaa atagtagatg taataaatga ctttaaaatt aatattaaaa atttagagat
32641 gcaagactgg ataaatttga ttaaaccatc ggaactagtt caattaccta ttttacaaat
32701 gggattaaaa tatgccaatg ctattgaaaa taaaattata gaggaagaat ggttgaagtg
32761 ttatatagcg ctatcattat atagaaatca gcaaacagat gctgttacaa agcgaactaa
32821 gattttaagc atattagatg gaacaaatat tgacacagaa aaatatgatt ctaaatatgg
32881 aaatatggac agcaatactg aaaaaaaatt tatcgagagt ttgaaaaatg tagtagataa
32941 tggtggtaat atatttactt taagcgaagt aattgaaaaa gcaaaatata atgtatcgag
33001 ttttaataaa ttattagaag gattaaacta tgtattttta cttgaagaaa gtaaaggaaa
33061 taatcaagca agatcatatt cagcaacact tgaaacgcgt ataaaaaatg ttcaaacacg

33121 attttcaaat ttatttggaa ataatgatac tgaactggaa gataaatcaa ttgtttattc
33181 agtttcagaa ttagatgatg atttgttatt atttttcaca acatttattt taaaaaaaga
33241 atttgaaaaa aacaaaaaaa gaaattagaa gatcgttcag tgaacgtttt tatatttgaa
33301 gaagctcata gatatatatc gaaatttaaa gaaagtagcc aatttaatga agtagaagca
33361 ttcaaaaaga tagctagaga aggaaggaaa ttcggttgtt ttttaatgtt atctagtcaa
33421 aggccaagtg agctgtcttc tactgtctta tcacaatgca ataattatat agtccatagg
33481 gtaaaaaata atgtagattt agaatattta ctaaattcca ttccctacat aaataaattt
33541 caattaaatc gtttttctta tttacctact ggaacagctt atatagtagg agaattattt
33601 ccaatccctg tagaaataga aatatttgaa gagttttcga aaaacagcac gattacacct
33661 gaaatagtat atagatcttg aataataact acgctcattt tggatgatta tgtgttatac
33721 gaaattaata aacataacta cgcgttgata acaaaaataa atgactttaa aattactact
33781 tgacatattt tataaagact agaggttaaa atttttttac tctcttaatg agcaggaatt
33841 tatggccaaa ttaatacatt tactttcaat tttatataga cttagttcag ataagaaatt
33901 tactgtgaaa caaatttcag acacctgtca taatggtggg aaatctttat atagtgctat
33961 tacaaatttg aaaaaataat cacatttgga gcaagtaatt gatatagaaa agcatgccgc
34021 tagtcttgaa atagcaaatg aagagttgga taaaatctaa agtaagttat aataaaaaag
34081 caattaatat aaatgtatca ttaattatat aatacagaaa acgttgtttt ttgacattga
34141 aatatttgta tatactttga tgagaaatag ttgtgtgaaa gttacgaaat agaaatatat
34201 cccaaaaagc gtatgagaac taagaaagga agtctcgaat aatgaaaaca cctatttatt
34261 atacgattgt aaataaaggc aataatattg aaaatacaat atttcagaca ccaccaaata
34321 cagttagtga ctttgaccat ttggatgtta tgcgttactt accatcaatt gttgatatta
34381 gtgagatttg tttattagcc gaagcaaaaa aaataccaga gtttaaaaaa tcgctagact
34441 tcattcttaa taaatttgaa acatatacat ttaatattgt taatgattta attgaaataa
34501 cactaacgaa agacgcatta ttaaattatc aaaagtactt gttacatcaa tcaatcataa
34561 taagtaaatc attagcttta aaaaatagta acaatggttt atgtacacaa gaattaaaat
34621 cccaattata tggatatgaa aagcttcatt atgaaccagc ctttcatata ttagataacg
34681 atttaattcc taatattaat gaagagcaaa gtgaattgat accccttgag catgttgttt
34741 tattatctga tgggattagt tatttaatgg attattttga caaaaatcct gaagcaactt
34801 tcacattata cattgtgaaa gattacacaa gttattacga ggtagaatta taaaagcatt
34861 tttattaatt aaaatattta ggcgagctaa atgcatagat tgtggcaagt attgtattaa
34921 ccatattggt caacattaaa ttgggaatgt actaaaccta acacacaaag ttatgtgaat
34981 aaaaaagatg atcttacttg atttacattt cctgagttaa ataatgaaat atagagcaac
35041 ttttcaagag gcattaaagt atcttgaaaa gttgcttatt aaataataat tgctcaaata
35101 aaaaaacata taaatgctct taccaaaaaa atattcgtaa ctaaaatatt tcaaagtaat
35161 taaaaagtgt gattacccaa aaatccattt tttattcaga tagccttaat attgctccta
35221 aatgttgctg tgcagtagta aataatacga atgtaattaa ttcaatgatt tcttcatctg
35281 tcatcgtttc ttttaataaa cttatacttg attcgggtat gttttgttta gattttaaat
35341 aaacttcaac aaagccaaca caaagtagag atttttcgtt tagaaattta tttgtaggac
35401 taccttttgc tttgcaatat tgacatccgt tttgctgggc cagtaacctt cttaactcct
35461 cttttaattc tttacttatt gccccatcgc tacttaaaaa atcggataaa ttattccagt
35521 attttaataa gtttttatta tgacctaata gcttttcaaa tggtgtgttt ccaatattac
35581 tgtattctaa taatgtcata tcctcatctc ctatcttaat gatatacatg ataaaattgt
35641 atggaaataa gattttgaaa gtttatatca tttaatactt tgaaaagaga ggattttcta
35701 tgaatgactt aataaatgaa aaaataatca atgttttgtt aaataatagt agaattacta

35761 ttaatgaatt aagtaaacaa gtgaacttat ctgctccagc tgtaagagaa agggtaaata
35821 aacttgaaga tcaaggtatt ataaaaggtt atacaattaa catagattat aaggaattag
35881 gttacgatgt agaaatatta attgagttaa ctataaagaa taatagatac aaagatttta
35941 aagaatttat ttcaaaacaa gataatgttg agttttgtta tcgtgtatca ggagaggcat
36001 gttttgtatt taaggttcgg ttggaaaata tggatgaagt ggaaacattt gttgatacca
36061 tacaaccata tgggcagaca aaatgtcagt ttatcttttc atcagttgtc taaactacat
36121 ggaaaaagtt taataagcat aggaaagatt tttaacagag agaagtgact tactttggaa
36181 tataaatctt acaacaaaat gagtaataat attttatcta tcttgttgag tgaaaaaaat
36241 ttcgataata aaatggtagt ctcaacaggt agttacattt tgtctgagtt gccatgtatt
36301 gttgcctata aaaataatga tatcgtgggg ctactaacgt ataaagttta tgatgagtat
36361 attgaaatca tttctttaga tagttttgta gagaacaagg gtattggtag tcatttactt
36421 aattatgctg aaataattgc ctcagatatg agtaaaagaa gtatccgtgt tattactaca
36481 aatgaaaata ttaaggcact ctatttctat caaaaaaata aatatagaat cacagacgtt
36541 atttttgatg ctgtaacaga agctaggaaa ataaagccgt cgattcctgc aattgataaa
36601 aatggcattg aaattagaga tgaaattgtt ttgaggaaat gtttgtaatg tataaatata
36661 ctcagcttta aatcacccta gttcatacct aaaagacttt cacacaaaca aaaaaggagg
36721 aacattaaat tcctcctcaa actttattac tcatactata attcaatttt aacgtcttcg
36781 tccatttggg cttcaaattc atctagtagt gctcgtgctt ctgcaattga ttgtgtgttc
36841 atcaattgat gtcgaagttc actagcgcct cttatgccac gcacatagat tttaaagaat
36901 ctacgcaagc tcttgaattg tcgtatttca tctttttcat atttgttaaa caatgataaa
36961 tgcaatctca acagatctaa tagttctttg cttgtgtgtt cgcgtggttc tttttcaaaa
37021 gcgaatggat tgtggaaaat gcctctacca atcatgacgc catcaatgcc atatttttct
37081 gcaagttcaa gtcctgtttt tctatcggga atatcaccgt taattgttaa caatgtgttt
37141 ggtgcaattt cgtcacgtaa atttttaata gcttcgatta attcccaatg tgcatctact
37201 ttactcattt ctttacgtgt acgaagatga atagataaat ttgcaatgtc ttgttcgaag
37261 acatgcttca accaatcttt ccattcatcg atttcatagt agccaaggcg tgttttaaca
37321 cttactggaa gcccgcctgc tttagttgct tgaataattt cggcagcgac gtcaggtctt
37381 aagattaagc cggaaccctt accctttta gcaacatttg ctacaggaca tcccatattt
37441 aagtcaatgc ctttaaagcc cattttagct aattgaatac tcgtttcacg gaactgttct
37501 ggcttatctc cccatatgtg agcgaccatc ggctgttcat cttcactaaa agttaagcgt
37561 ccgcgcacac tatgtatgcc ttcagggtga caaaagctct cggtatttgt aaattcagtg
37621 aaaaacacat ccggtctagc tgcttcactt acaacgtgtc gaaagacgat atctgtaacg
37681 tcttccattg gcgccaaaat aaaaaatgga cgtggtaatt cactccaaaa attttccttc
37741 ataatatatt tataccctct ttataattag tatctcgatt ttttatgcat gatgatatta
37801 ccacaaaaga cgaacttata caaaaggaat tttagtagat acaaccattc gaaagggaag
37861 tctacgagta gtctaaaatg aatgttgtgt aagttgatta atatacaaat caaggattat
37921 cgcgttaaat tgttcattat taatgataca ctgcttatta ttatgattca gaattttctt
37981 tagctacata tacagtaatg tgatttatac gttatcttat aacaaagaca aatttataaa
38041 ggtgatatta tggaagattt aaaggaatct ttaaaaagtt taggttggtg ggatttattt
38101 tttgcgatac ctatgtttct gctattcgca taccttccaa actataattt tataacgata
38161 tttcttaaca ttgttatcat tattttcttt tccataggtt tgattttaac tacgcatata
38221 attatagata aaattaagag caacacgaaa tgaatcatta atacgaatgt gattaaacat
38281 aaaactgaag gagcgattac aatggcgact gagaaagatg taaatgattt atttttaaat
38341 catgtgaatt caaatgccgt taaaactaga aagatgatgg gagaatatat tgtttattat

38401 gatggtgtgg ttataggtgg tttgtatgat aatagactat tggtcaaggc gactaaaagt
38461 gcacgtcatc aatttcaaga taatacatta gtatcgccgt atcctggtgc taaagaaatg
38521 atattaattc cagacattac cgaagtaaca aatctcactg atttatttga gctcataaaa
38581 aatgatttgc aaaagtgaag ttcggatgga gtaaactaag cacatcgata aatggtcgct
38641 tgttaaagaa gagtgacggt cactcttctt tatgtgcata ttttattttg tctgttttgt
38701 taacgagcag cagtgtaaca aatatgagta aggataaaat gagtataata tagaaaccga
38761 atttatcatt aatttcatta atccatcttc ctaaaaatgg agcaatgaaa ctttgcagta
38821 acaatgaaat tgacgtccat atcgtaaatg agcgaccgac atatttatct gaaacagtgt
38881 tcattatagc tgtattcata taaattctga ttgatgaaat tgagtagcct agtataaatg
38941 atcctatgaa taagtaaaat gctgagttta tccaaataaa tagtgctgaa tttatgacta
39001 atatgaaata taacaaaaat atcaatgctt tagttgagat ttttttcgaa agaatagctg
39061 aaattaaacc tgcacataat cctccaatgc catataacat atctgaaaaa ccaaattgta
39121 cagacgaaag ttttaaaaca ttatatacat atcctggtaa tgatatgtta aagatcattg
39181 taaacaccat tggtatgatt gaaataactc caaaaataaa tatcatcatg ttgtctttta
39241 aaaatttcca tcctaataaa tattcttgca ataagctatt tgttgattct tcctctgaat
39301 gagttggttt atctacatgc aatctaaata acataaaaat gctgattaaa aacatcatta
39361 tagtcatcgc tataattaga gtgaatccat ttatttcata taatattcct gataatccac
39421 ctgcaataaa catacctgtt tgcaaactaa tctctaatag agaatttgca tctgtatatt
39481 gatctggttt taaaatctgt ttaactaaac ttctagatgt tgccatatac gtagtccaac
39541 ctatcccatt aacaatcgca aatccaatca ctaaataggt ctcaaagcct atcattaaga
39601 gtgctatgac aatgagtaaa tataatatta cctgaagaag ataggtgatt aaaataatat
39661 ttcgtctatt atatttatct gctagtccac ctattatagg agaagctaag aaaccggata
39721 atacatttaa agctaacata atacctaata gttgggagtc gttagttttg tcgattaaat
39781 accaattagc cccaactgta ctaattccaa ctccgaatgc tgaaattatt tctgctaaaa
39841 agaacaatct gaagttttta attttcaaca aatcagtcat aaatatcacc ttttttcaat
39901 gcaagattta tatttgcgag ctcttctata aattgagagt cgtttaaaat atcgaatggt
39961 ttattgcctt cgccgatcaa aaatctatag ttagtaatat tcataaactc aaatattaat
40021 ttgaattggt tgactaaagg ttgtgctttg gtatgcggat tgtcgccacc tatgattaaa
40081 atcaaatact ttttttgtga catgatttct ttgaagttat ctatttgcgt atctcgcaat
40141 gactcggtcc atctatcaat gaagagtttt aaagacgcac tcatggagta ccaatagagt
40201 ggtgttgaaa aaataataat atctgattct aaaactttga ttaaaatttg ttcgtaatca
40261 tcattatgaa agttggcatc tttgctatga cgattatctg taaccttttc aatgttactt
40321 tgatataaat tcacaaagtt gacatttaaa ttcttcaata gattctctac tgcgatagct
40381 gaattgccat cttttctact acttccaaat aaacaagtaa tcatagtcat aactcctttc
40441 gatttacctt tataataata tttaatatat tattattaaa tcagaattct tagaatgcag
40501 aattcgataa aagtgaatcc taaaggaggt actactttga acttaaacat tttaaaatca
40561 tttttagtaa ctagtgaaac aaagaacctt actaaagcat cagaattact taactattca
40621 cagtcaactg tatctacaca tattgaaaaa ttagaaaagc aattagatgt taaattattt
40681 tatagaaaaa aatatggtat ggaactaacg gaagaaggct tagcatacgt taaatacgct
40741 aaagtgattt tagatagtaa tagcgaatac gagagagaaa taaaaggact ttacaataag
40801 aaggtaaata taagtattaa catgcaagaa agtcagtatt tgtatcgcta ctataataag
40861 attagtgaat ggttagctga acacccatat gtaaacttaa agtttaaatc cgcacattct
40921 aatttctata ttaaagaaga aattgctaat tttaaatcgg atattagcct tatcacagac
40981 gaaaagatta ttaatagtaa cttaactgct attcctataa ct

//
LOCUS Staphylococcus 39003 bp DNA linear UNK
DEFINITION Staphylococcus Staphylococcus aureus strain 110900
ACCESSION Staphylococcus
KEYWORDS .
SOURCE Staphylococcus Staphylococcus aureus
ORGANISM Staphylococcus Staphylococcus aureus
Bacteria; Bacillati; Bacillota; Bacilli; Bacillales;
Staphylococcaceae; Staphylococcus; Staphylococcus aureus.
FEATURES Location/Qualifiers
source 1..39003
/genome_md5=""
/organism="Staphylococcus Staphylococcus aureus"
/mol_type="genomic DNA"
/project="hwwang_703339"
/genome_id="703339.257"
/db_xref="taxon:703339"
CDS 122..601
/product="23S rRNA
(pseudouridine(1915)-N(3))-methyltransferase (EC
2.1.1.177)"
/EC_number="2.1.1.177"
/translation="MKITILAVGKLKEKYWKQAIAEYEKRLGPYTKIDIIEVPDEKAP

ENMSDKEIEQVKEKEGQRILAKIKPQSTVITLEIQGKMLSSEGLAQELNQRMTQGQSD

FVFVIGGSNGLHKDVLQRSNYALSFSKMTFPHQMMRVVLIEQVYRAFKIMRGEAYHK"
/transl_table=11
/db_xref="SEED:fig|703339.257.peg.1"
CDS 655..2406
/transl_table=11
/db_xref="SEED:fig|703339.257.peg.2"
/translation="MNSKIIEREATLEIESMATNTLKINPDINSNDTKMSFDGELEVY

DSENLSKKNFVGKIQVQVKGKEVAKRGGKVIHRSNVKMNDLKAYQREGGVYYFVVYLI

VENKKVVEKQVYGKQLHQLDLQFLLQKKQKSVTIKMYEIENEKILYNNCVKYINEKRL

QNQVGQVKVKNIEKALSYIATPENIVMDHRGLPLNDFYGYIKINSSELDVTIPDGVLS
MEKVKRVNKKQIIKEGKLLFEGMVGIETSKESISITIDDIFKIQTFESDNKSTYTMLP
FKKLNIAEQSFNVINELSKGGEFFLDQIQLVIQPFEINIIEIKETINKLNIKLSEYSN

LLSFDVSLKSTEFDKQMNEIKGLLELLEYKNFKDFKMHNNGYYKMKFCGKFILLFKDN

TSLYNVYSNDFVDRFEAVTKERVVQMPIVYTLTRDMIVDVLNFDINVIKECIESDKIA

IQSDIKWEKLNNFALELIAAYDETQRTDLLELAEYVLNNLLNFDNDKIFMNLINKAQI
IKRRRNDVDEKLLIKLFKLRDKLIDIDKEIATLYINVVSGSKQEAKIRYETLNAADLE
IFNAYPIFNLYKKLIES"
/product="hypothetical protein"
CDS 2411..3736
/product="hypothetical protein"
/translation="MTKNTFRERIQKTYTKKDLEKALGLKNKAQLQKRIDKLVDIYKV

DMKKFQKNSGESERAAYSFNGIAFDILCVLLNNMTDDMLPKAITDEDVKLRKNAIEHI

DIDQYAHFIKSIKNDIDKIEFKPLKLHIHAQKAYQDVKKVFDSGNKINEKLQLVSEVM

NQLPIDKQVELSDKIAFAIHETIYSTFAEIKYNERIAEHNQTSENSSQYDFRFNYFVN

QLTDNVITEDINEEKAFIYDNDEQLDWLLVNILNKVQRAADGWGMRNAKPHHRTQKFR

DIEHYFLRDVSKMIDKELNKKENLNKQSKKYDFDNKIKVINDNIEFFNEDEQIRKHLL

ELRKRLDFHSAAVLNQKNHSHIGNEMFQYFMDDMQQVVTLIDRYINKTEHYNDMKNGY
INEYKSFYNAYNNEIEVTKTYENNIPMAMLMKQFKNQLLKRRNNRKSSN"
/transl_table=11
/db_xref="SEED:fig|703339.257.peg.3"
CDS 3947..5047
/transl_table=11
/db_xref="SEED:fig|703339.257.peg.4"

/translation="METRDKLMSLTQSDKTQQWLMDKSSNQDDIQQLQQQFSQQLDQK

YNALLADNQAKLDQYVEVHQGLEALKEEIESESIKLNIDKLPDIKATMLERAKNDEHS

DKIEKLFDRLEQALNGTNRLYTQLSLIGTRTHRITTKNFNLQGLPKAVQHTILPSKFK

KVYTVDFKSFEPSVAAYMTQDSKLIDYLNHKEGLYDALLEDLSLSKKEKKFVKRTFIG

SFLFGGNFDSPKFKLKHYVSEEQWLDTVSQFTKVNELKKQIEEHKTMPMPYGIEHDMS

AFQGSSIMAIYVQTVASYIFKHILLKVYKAQCDQKTFKIIVPIHDAIMIECEDEEIAQ
NVGQLMKDTANQLFNGEFAHVTVEEIGGVDHE"
/product="hypothetical protein"
CDS 5040..5411
/product="hypothetical protein"
/translation="MNNDRGQSLHIPSSTPIKENNIYVATLHSVIQTDFSGEIKHQFT

YEIEVNNQIVYANRNIPTKPSANQLSIHDWLKRHSNYSASHENYEPYIDQKHLILLGQ
YNGNYYVQDVASLDAFGGVLS"
/db_xref="SEED:fig|703339.257.peg.5"
/transl_table=11
CDS 5408..5536
/product="putative primase"
/translation="MNHILQMLSKLLSVAKEAIDRQGLIAILTIPVNNNDEIEETA"
/transl_table=11
/db_xref="SEED:fig|703339.257.peg.6"
CDS 5546..7051
/product="putative primase"
/transl_table=11
/db_xref="SEED:fig|703339.257.peg.7"

/translation="MYNELIDQLRLNIPKDTDYQPNIYSYFGIKKNPNDTVLMEMMIK

VFHIKRFNSELFIFKVNGWQKINGDELQGLISKMIQVLLVDYKPSLSTLKNVVDGLQK

STDVEELVENERYIGCGENMFDLNTFQVVKNSIDIFPKTRLNLSLSTNDVITDKIPPY

FKQYMLQLANYDDDLQYFLFQHTAVLLTADTKYRRGLILYGGAKNGKSVYIELVKSFF

YSKDIVSKPLNELEGRFDKESLIDKSLMASHEIGQSRIQEKIVNDFKKLLSVESMHVD
RKGKTQVEVILDLKLIFSTNAILNFPPEHAKALERRINIIPCEYYVEKADTSLIDKLQ

SEKKEIFLYLMYVYQQIVKADIEYLENSRVTEITHDWLNFGYEFVSSRSVSNANQKAC
INLLRKLIEIKSGSRIKVSELNKVINEEIKVSSQVIKQLIQANFDTQTKLYNGYDYWI
DLGWKEANKKEIHDISEKDNIISLDKNENITDDEALDEENLDFDWEDFDDE"
CDS 7277..8953
/translation="MKGKIALYSRVSTSEQSEHGYSIHEQEQVLIKEVVKNFPGYDYE

TYTDSGISGKNIEGRPAMKRLLQDVKDNKIEMVLSWKLNRISRSMRDVFNIIHEFKEH

DVGYKSISENIDTSNASGEVLVTMFGLIGSIERQTLISNVKLSMNAKARSGEAITGRV

LGYKLSLNPLTQKNDLVIDENEANIVREIFDLYLNHNKGLKAITTVLNQKGYRTINQK

PFSVYGVKYILNNPVYKGYVRFNNHQNWAVQRRSGKSDKNDVILVKGKHEAIISEEVF

DKVHEKLASKSFKPGRPIGGDFYLRGLIKCPECGNNMVCRRTYYKTKKSKERTIKRYY

ICSLFNRSGSSACHSNSINAEVVERVINVHLNRILSQPDIIKQIASNVIEELKQKHSN

QTEIKYDIDSLEKQKAKLKTQQERLLELFLDDQMDSEMLKAKQSQMNQQLEVLDQQIK

EAQQANQSQDEIPNFDKLKGRLILMITRFSVYLRKATPEAKNQLMKMLIDSIEITTDK
QVKLVRYKIDESLIPQSLKKDWGSFFMPKFQFEIYGQNDYFIDQITTFTT"
/transl_table=11
/db_xref="SEED:fig|703339.257.peg.8"
/product="Cassette chromosome recombinase B"
CDS 9059..9397
/translation="MTLEQQLKHYITNLFNLPRDEVWHCESIEEIADDILPNQYVRLG

PLSNKTLQTNTYYSDTLHESNIYPFILYYQKQLIAIGYIDENHDMDFLYLHNTIMPLL
DQRYLLTGGQ"
/db_xref="SEED:fig|703339.257.peg.9"
/transl_table=11
/product="FIG01108299: hypothetical protein"
CDS 9493..9804

/translation="MNRYITRGIANNLPNILQHQLWQLVSEREQEQTKDNTSVDYFHI

FQFNKHRNQLYIKHKQERPEYAKIHKANYSKAININKVYIIREDDVDLSYYVMLLPEE
Y"
/transl_table=11
/db_xref="SEED:fig|703339.257.peg.10"
/product="FIG01107894: hypothetical protein"
CDS 9820..10326
/translation="METIKSTLKTEAIFSDDKQHRYLLKKTWNSEKQSITIITMYPHY

DGILNIDLTTQLIMNKVSEMDAFGSINFVNLYSNITTPINLKHLENAYDKHTDIQIMK

AVKESDEVILAWGAYAKKPGVEARVNEVLEMLKPHKKKVKRLMNPETNEIMHPLNSKA
RQKWILKV"
/transl_table=11
/db_xref="SEED:fig|703339.257.peg.11"
/product="hypothetical protein"
CDS complement(10475..11149)
/product="Transposase for insertion sequence-like element
IS431mec"
/translation="MNYFRYKQFNKDVITVAVGYYLRYTLSYRDISEILRERGVNVHH

STVYRWVQEYAPILYQIWKKKHKKAYYKWRIDETYIKIKGKWSYLYRAIDAEGHTLDI

WLRKQRDNHSAYAFIKRLIKQFGKPQKVITDQAPSTKVAMAKVIKAFKLKPDCHCTSK
YLNNLIEQDHRHIKVRKTRYQSINTAKNTLKGIECIYALYKKNRRSLQICGFSPCHEI
SIMLAS"

/db_xref="SEED:fig|703339.257.peg.12"
/transl_table=11
CDS 11407..11574
/product="Hydroxymethylglutaryl-CoA synthase N-domain homolog (EC 2.3.3.10)"
/translation="MLESREQLSVEEYETFFNRFDNQEFDFERELTQDPYSKVYLYSI
EDHIRTYKIEK"
/EC_number="2.3.3.10"
/transl_table=11
/db_xref="SEED:fig|703339.257.peg.13"
CDS 12491..13234
/product="Glycerophosphoryl diester phosphodiesterase (EC 3.1.4.46)"
/translation="MLTVYGHRGLPSKAPENTIASFKAASEVEGINWLELDVAITKDE

QLIIIHDDYLERTTNMSGEITELNYDEIKDASAGSWFGEKFKDEHLPTFDDVVKIANE

YNMNLNVELKGITGPNGLALSKSMVKQVEEQLTNLNQNQEVLISSFNVVLVKLAEEIM

PQYNRAVIFHTTSFREDWRTLLDYCNAKIVNTEDAKLTKAKVKMVKEAGYELNVWTVN
KPARANQLANWGVDGIFTDNADKMVHLSQ"
/EC_number="3.1.4.46"
/transl_table=11
/db_xref="SEED:fig|703339.257.peg.14"
/db_xref="GO:0008889"
CDS 13331..13759
/translation="MKYDDFIVGETFKTKSLHITEEEIIQFATTFDPQYMHIDKEKAE

QSRFKGIIASGMHTLSISFKLWVEEGKYGEEVVAGTQMNNVKFIKPVYPGNTLYVIAE
ITNKKSIKKENGLVTVSLSTYNENEEIVFKGEVTALINNS"
/transl_table=11
/db_xref="SEED:fig|703339.257.peg.15"
/product="MaoC domain protein"
CDS complement(13805..15811)
/product="Penicillin-binding protein PBP2a, methicillin resistance determinant MecA, transpeptidase"
/translation="MKKIKIVPLILIVVVVGFGIYFYASKDKEINNTIDAIEDKNFKQ

VYKDSSYISKSDNGEVEMTERPIKIYNSLGVKDINIQDRKIKKVSKNKKRVDAQYKIK

TNYGNIDRNVQFNFVKEDGMWKLDWDHSVIIPGMQKDQSIHIENLKSERGKILDRNNV

ELANTGTAYEIGIVPKNVSKKDYKAIAKELSISEDYIKQQMDQNWVQDDTFVPLKTVK

KMDEYLRDFAKKFHLTTNETESRNYPLGKATSHLLGYVGPINSEELKQKEYKGYKDDA

VIGKKGLEKLYDKKLQHEDGYRVTIVDDNSNTIAHTLIEKKKKDGKDIQLTIDAKVQK

SIYNNMKNDYGSGTAIHPQTGELLALVSTPSYDVYPFMYGMSNEEYNKLTEDKKEPLL

NKFQITTSPGSTQKILTAMIGLNNKTLDDKTSYKIDGKGWQKDKSWGGYNVTRYEVVN

GNIDLKQAIESSDNIFFARVALELGSKKFEKGMKKLGVGEDIPSDYPFYNAQISNKNL

DNEILLADSGYGQGEILINPVQILSIYSALENNGNINAPHLLKDTKNKVWKKNIISKE

NINLLTDGMQQVVNKTHKEDIYRSYANLIGKSGTAELKMKQGETGRQIGWFISYDKDN
PNMMMAINVKDVQDKGMASYNAKISGKVYDELYENGNKKYDIDE"
/transl_table=11
/db_xref="SEED:fig|703339.257.peg.16"
/db_xref="GO:0008658"
/db_xref="GO:0046677"
CDS 16059..16202
/product="Mobile element protein"
/db_xref="SEED:fig|703339.257.peg.17"
/transl_table=11

/translation="MNYFRYKQFNKDVITVAVGYYLRYALSYRDMSEILRERGVNVHH
LTV"
CDS 16482..16733
/translation="MAKVIKAFKLKPDCHCTSKYLNNLIEQDHRHIKVRKTRYQSINT
AKNTLKGIECIYGLYKKNRRSLQICGFSPCHEISIMLTS"
/transl_table=11
/db_xref="SEED:fig|703339.257.peg.18"
/product="Mobile element protein"
CDS complement(16793..17221)
/db_xref="SEED:fig|703339.257.peg.19"
/transl_table=11
/translation="MKFQVSPYFLITQNAEGLKKLYEELFGAKTLYIQRIKDRPIDKK

LNIDENDLNKIDQCVLQFGDIKIMIADDTEGLPITQGNNISLCLTFDQVEETKHVYDQ
LIEKGSEVLKTFSPEFYTEGYGYVRDPYGISLHLFTKRKN"
/product="PhnB protein; putative DNA binding
3-demethylubiquinone-9 3-methyltransferase domain
protein"
CDS 17302..18231
/product="Transcriptional regulator, YafY family"
/db_xref="SEED:fig|703339.257.peg.20"

/transl_table=11
/translation="MKKSVRLYNMIEYCNENRNFKLNDLMSEFNISRSTALRDIKEIE

ALGVPLYSNPGKNGGYTIIGNRDQTKIAISDEELKALVFTLSSISNVSNLPFQTEYQE
ILKKLYNNSNKKELINQYNDLFQYFNEDKYQFKSYKLFNEIIRLIIENKSFETCYSQN

YIKEQYKGIGIMYKNHQWYFVVANIESKLVNLLNISKIKELYEMGETQECNDITMQNF

QQFMVKNETAIDILIRSNVMGLNILKGYLWSDYMIENIDEETYLFKSKVNAKDIDFIA
KLIVTGGVNVKVETPNSLKNAVKVELTKIINMY"
CDS 18393..20381
/db_xref="SEED:fig|703339.257.peg.21"
/transl_table=11
/translation="MNKNITALKSTENTTYMQYTVHIDDQIINNIKECTKKFKILPME
DKIPLSPLLQPEYAGEVQDFISTYEQFMINFGKVILDSQGIKIQFESESLSSIQRGIQ

EHCYLNERTNDIDVTKEWYLCKFSNQILEEDKNQLYNALKQLMNDSKNKKQAFQEVFK

VHIDIYLYNKEKSEYKYQVSSYFNLVKENPKITYKKRHLQEKQGVKGTTFTNKLKLLN

KMYGVDVAKYQPFYNSNNPEYERGQFGERYISQRSNYEFNRLQYQIIDMLSKILDKHP
LPKSDNNYKHIPTIEKAILSGDSHSFYEYFEDIMKEIISMENSSLKEKLLTDFTYQSQ

CRWYSESEKLKLQLESFMHKVLESNYYEGNKLYRMLSHAIEETINEADEDKVHFNYFK

DYFLTDGGVKNWEQISEKITEFNGKVINDIQNEYNKIQFNNANRNQKLNFNYLYHCFE

FSNNLVKARVNGNRGYILYFVDKYKIKMPFLDQLLINYLNQEIGQESINYNMQTLFEK

ERYDRSSTIEKLVATSKFKYEKDDSDLFKQLFNDVENSIDRLGIYLLNNGINSNDENA

RYYRSFLKELSRIKSKLTPFSLEISKSSGREQHYPDDAIDDKDERRKNKEETYHAFDD
KSDIDSKLKNKINVSIDNLLSVKIKSNLYDISPNTKKQ"
/product="Similarity"
CDS 20576..21685
/transl_table=11
/db_xref="SEED:fig|703339.257.peg.22"

/translation="MYQANIRDLITKLPQSNKTEHFLMNKFSNQDKVQQLQRQISQQL

DQQYNELLANEKAKLDQYVEVHHNLEPLKKEIESESINLDTDKLPDIKATMLEKAKND

EHFDKIEQLFDRLDQSLNGTNRLYTQLSLIGTRTHRITTKRFNVQGLPKLVQQMILPS

QFKKVYTIDFKSFEPSVAAYMTQDEQLIDYLNHEEGLYDALLRDLSLSKEKRVSVKRA

FIGSFLFGGRYSSSKFKINQEVSEINWLQVMSKFKKVIEFKEQVEKYKTMPTPYGIEH

DMSAFQGSSIMAIYVQTVASYIFKHILLEVYKAQCEKKTFKIIVPIHDAIMIECNDKG
IAQNVAQLMKDTANQLFNGEFAHVTVEALGGIDNE"
/product="hypothetical protein"
CDS 21678..22046
/product="hypothetical protein"
/translation="MNNDRGKSLQIPQSTLLKEGSIYVATLHSVYEKNFSGDIKHQFT

YEVELNQETHYVNRNITVKSMSHQLSIADWIKRHSNYNVNHINYDPYIDRKHLVLVGQ
YNGNYYIQDVAPLDEFGGVM"
/transl_table=11
/db_xref="SEED:fig|703339.257.peg.23"
CDS 22046..23662
/product="putative primase"
/translation="MNHILEMLIKLLKVGMEAIDRKGLIAILTSSIGNDEMDDSEQAV

MVYNELIDKLQLNIPKDVDYRPNIYSYFGIQKKPNDTILVEMMICIFHIKRFDSELFV

FKDKGWQKVSEDELQGLISKMIQVLLVDYKPSLSTLKNVVDGIQKSTDIEKLVEHRQY

IGCGRNMFNLKTFKVVDNDLEIFPKTRLDLELDINDTITDKIPPNFKQYMLELANYDH

DLQYFLFQHMAVLLTADTKLRRGLFLYGTAKNGKSVYIKLVKSFFYSNDIVSKTLNEL

GGRFDKESLIGKRIMASDEVGKANIDEATVNDFKKLLSVEPIHADRKGRTQVEVTLDL

KLIFNTNAVLNFPSSHAKALERRIAVIPCEYYVEKADPDLIEKLQDEKKEIFLYLMYV

YKQIVKNDIEYLQNDRVTEISHDWLNFGYEFVSSKSANIAHQKACINLLRKLIEIKPG

SRIKVSRLNEVIRDEIKVSSQVINDLVQANFNVQSRLNNGYKYWVDLGWKETDKKDDM
ISFDKNENVTDDEFLYEDDLNLGWEDFDDE"
/db_xref="SEED:fig|703339.257.peg.24"
/transl_table=11
CDS 23887..25566
/product="Cassette chromosome recombinase B"
/db_xref="SEED:fig|703339.257.peg.25"
/transl_table=11
/translation="MKGKIALYSRVSTSEQSEHGYSEKEQEQLLIKEVMKNFPGYDYE

TYTDSGISGKNIEGRPAMKRLLQDVKDNKIEMVLSWKLNRISRSMRDVFNIIHEFKEH

DVGYKSISENIDTSNASGEVLVTMFGLIGSIERQTLISNVKLSMNAKARSGEAITGRV

LGYKLSLNPLTQKNDLVIDENEAHIVREIFDLYLNHNKGFKAITTILNQKGYRTINQK

PFSVFGVKYILNNPVYKGYVRFNNHQNWAVQRRSGKSDKNDVILVKGKHEAIISEDVF

DQVHEKLASKSFKPGRPIGGDFYLRGLIKCPECGNNMVCRRTYYKTKKSKERTIKRYY

ICSLFNRSGSSACHSNAINAEVVERVINVHLNRILSQPNVIKQIASSVIEELKQKHSK

QTEIKYDIDSLEKQKAKVKTQQERLLELFLDDEMDSEMLKAKQSEMNQQLEVLDQQIK

EAKQANQSQDDIPNFDKLKARLILMITRFSVYLRKATPEAKNQLMKMLIDSIEITTDK
QVKLVRYKIDESLIPQSLKKDWGSFFIPKFNFVINVTKKNRIENLSLLPLF"
CDS 25655..25993
/db_xref="SEED:fig|703339.257.peg.26"
/transl_table=11
/translation="MTLSKQLKTYITERFKLNYQETWACETIDAVAEDVLPEKYIKNS
PLEHKILNTFTYYNDELHEISIYPFLCYLDKELVAIGYLDNFDLDFIFLNDTHQIIID
ERYLLQKGGE"
/product="hypothetical protein"
CDS 25996..26085
/product="FIG01108299: hypothetical protein"
/transl_table=11
/db_xref="SEED:fig|703339.257.peg.27"
/translation="MNWIKVAQLSVTVINEVIEIMKEKQNGGK"
CDS 26087..26398
/product="hypothetical protein"
/transl_table=11
/db_xref="SEED:fig|703339.257.peg.28"
/translation="MNINRYITRGISEQLSLDLQILLWHMVEEKDNQPHTDYLHIFKL

QEDDNMLSITHEQEQPAYKLEYHYINYEKNQNALPKKVYVIREDDVDVFYYVMLLPEE
Y"
CDS 26413..26916
/product="hypothetical protein"
/db_xref="SEED:fig|703339.257.peg.29"
/transl_table=11
/translation="MNTIKSTIHTEAIFSDDEQHRYLLKKTWDEKKPVCTVITMYPHL

DGVLSLDLTTVLILNQLANSEQYGAVYLVNLFSNIKTPENLKHIKEPYDKHTDIHLMK

AISESDTVILAYGAYAKRPVVVERVEQVMEMLKPHKKKVKKLINPATNDIMHPLNPKA

RQKWILK"
CDS 26931..27152
/product="hypothetical protein"
/db_xref="SEED:fig|703339.257.peg.30"
/transl_table=11
/translation="MNHETTHSDWRTVANCLASQNYISIVKGLVHHFTAIEDEEILDK
IYEDFMNNGSITTVLNNDLQDIINYYLSK"
CDS complement(27316..27447)
/product="hypothetical protein"
/transl_table=11
/db_xref="SEED:fig|703339.257.peg.31"

/translation="MKQRKKGMEDEELRAIAHELTKKVKENMGVIGLNVIAQKLKCA"
CDS complement(27444..27704)
/product="Type I restriction-modification system,
restriction subunit R (EC 3.1.21.3)"
/EC_number="3.1.21.3"
/translation="MAVELLNRLLKGQVKSLMKTNATVSKRFSEMLRNSINKYNSRSI
ETSKVIEELIQLAKDIKQEQQRGNELGLNSDEIAFLRCFSFT"
/db_xref="SEED:fig|703339.257.peg.32"
/transl_table=11
CDS 29131..30036
/transl_table=11
/db_xref="SEED:fig|703339.257.peg.33"
/translation="MKDVIYVENHYFVTVKENSIKFRNVIDKSEKFYLFEEIEAIIFD
HYKSYFSHKLVIKCIENDIAIIFCDKKHSPLTQLISSYGMTHRLQRIQSQFQLSGRTR

DRIWKKIVVNKIINQSKCLENNLHNENVKLLVNLAKDVSSGDKSNKEAQAARIYFKDL

YGKQFKRGRYNDIINSGLNYGYSILRSFIKKELALHGFEMSLGINHRSKENPFNLADD
IIEVFRPFVDNIVYEIVFKKNINTFDVNEKKLLLNVLYEKCIIDKKVVRLLDSVKIVI
QSLIRCYEENTPTYLLLPKMIEVGN"
/product="CRISPR-associated protein Cas1"
CDS 30036..30341
/product="CRISPR-associated protein Cas2"
/translation="MYLLVSFDLPRDTKFERRVASKYRTRLLELGFSMKQFSLYERYV
SDVQKKDKILEILQQEIPDTGSITLYVLPDEVNNSQITILGKEVKVVVRKEPKLIFL"
/db_xref="SEED:fig|703339.257.peg.34"
/transl_table=11
CDS 30355..32628
/db_xref="SEED:fig|703339.257.peg.35"
/transl_table=11
/translation="MDKKTTLMYGSLLHDIGKIIYRSNDHAFARGTHSKLGYNFLSQF

SEFKDNEVLDSIAYHHYKELAKANLANDNTAYITYIADNIASGIDRRDVIEEGDEEYE

KQAFNFDKYTPLYSVFNIVNSEKLKQISGKFKFSNESNIEYPKTENIQYSSGNYTTLM

KDMSYDLEHKLSIKEDTFPSLLQWTESLWQYVPSSTNKNQLIDISLYDHSRITCAIAS

CIFDYLNENNIHNYKDELFTKYENTKEFYQKEAFLLLSMDMSGIQDFIYNISGSKALK

SLRSRSFYLEIMLEVIVDQLLEKLELTRANLLYTGGGHAYLLVSNTDKGKEKINQFNT

ELKNWFMSEFTTDLSLSIAFEKCSGNDLMNTSGNYRNIWRNVSSKLSDIKAHKYSSED
ILKLNHFHSYGDRECKECLRSDIDINDDGLCSICEGIINISNDLRDKSFFVLSETGKL

KMPFDKFISVIDYEEAERLAQNNNHIRIYSKNKPYIGVGISTNLWMCDYDYASQNKDM
REKGIGSYVEREEGIKRLGVVRADIDNLGATFISGIPEKYNSISRTATLSRQLSLFFK

YELNHLLENYQITAIYSGGDDLFLIGAWDDIIEVSVYINEKFKDFTLGKLTMSAGVGM

FSGKYPISKMAFETGLLEEAAKTDEKNQIALWVQEKVYNWDEFKKYILEEKLLVLQHG

FSQTDEHGKAFIYKMLALLRNNETINIARLAYLLARSKMTEEFTSKIFNWAQNDKDKN
QLITALEYYVYQIREVD"
/product="CRISPR-associated protein, Csm1 family"
CDS 32631..33056
/transl_table=11
/db_xref="SEED:fig|703339.257.peg.36"
/translation="MILAKTKSGKKIDLTFAHEVVKSNVKTVKDKRGKENQVLFNGLT

TSKLRNLMEQVNRLYTIAFNSTEDQLNEEFIDELEYLKIKFYYEAGREKSVDEFLKKT
LMFPIIDRVIQKESKKFFLDYCKYFEALVAYAKYYQKED"
/product="CRISPR-associated protein, Csm2 family"
CDS 33058..33702
/product="CRISPR-associated RAMP Csm3"
/db_xref="SEED:fig|703339.257.peg.37"
/transl_table=11
/translation="MYSKIKISGTIEVVTGLHIGGGGESSMIGAIDSPVVRDLQTKLP

IIPGSSIKGKMRSLLAKHFGLKMKQENHNQDDESVLRLFGSSEKGNIQRARLQISDAF

FSEKTKEHFAQNDIAYTETKFENTINRLTAVANPRQIERVTRGSEFDFVFIYNVDEES
QVENDFENIEKAIHLLENDYLGGGGTRGNGRIQFNDITIETVVGEYDSTNLKIK"
CDS 33713..34621
/db_xref="SEED:fig|703339.257.peg.38"
/transl_table=11

/translation="MTTKVFKLSFKTPVHFGKKRLSDGEMTITSDTLFSALFIEALQL

GIETDWLLNDLIISDTFPYENEIYYLPKPLIKIESKEEGNHKAFKKLKYVPVHHYNQY

LNGELSAEDATDLNDIFSIGHFSLQTKVSLSAQEIDSSADSEPYSVGTFTFEPEAGLY

FIAKGSEETLDQLKDIMTSLQYSGLGGKRNAGYGQFEYEIINNQQLFKLLNQNGEHSI

LLSTAMAKEDEIKSALKEARYILNKRSGFIQSTNYSEMLVKKSDFYSFSSGSVFKNIF
NGDVFNVGHNGKHPVYRYAKPLWLEV"
/product="CRISPR-associated RAMP protein, Csm4 family"
CDS 34624..35646
/product="CRISPR-associated protein, Csm5 family"

/translation="MTIKNYEVVIKTLGPVHIGSGQVMKKQDYIYDFYNSKVYMINGN

KLVKFLKRKNILDTYQNFLRYPPKNPRENGLKDYLDAQNVKQSEWKAFVSYSEKVNQG

KKYGNIRPKPLNDLHLMVRDGQNKVYLPGSSIKGAIKTALVSKYNNEKNTDVYSKIKV
SDSEPIDERHLAIYQKIDINKSEKPMPLYRECVDVDTEIKFKLTIEDEIYSINEIEQS

IQDFYKNYYDKWLVGFKETKGGRRFALEGGMPDVLNQNILFLGAGAGFVSKTTHYQLK

SREQAKRDSFDILTKKFRRTYGKMKEMPSNVPVALKGTTNQSLHVSYQQGMCKISFQE
LNNEVL"
/transl_table=11
/db_xref="SEED:fig|703339.257.peg.39"
CDS 35646..36914
/product="CRISPR-associated protein Csm6"

/translation="MKVLFSPIGNSDPWSNDRDGAMLHIVRHYKPDVVVLFFTESIWN

GNRNIPGRKNFDWENIVSKVSSRTKVDIKVDSIKYENDFDSYKDIFHFYINEIRTKYS

DAEILLNVTSGTPQMESTLCLEYISNPHNMKCIQVSTPAPIEGPKRSFAKLETVTEDL

NKVNANEKMASNRSKSINIISFREVMVRSQIKSLVNNYDYEGALNLVSDQKSFRNGKL

LRKRLLELTNQIKTHEVFPEINDKYRSVALKKSLFHYLLLNMRYNRLDVAETLIRVKS
IAEFILKTYIVGHWPTLIIEKDDKPYLNAEDNLSFIYKYKLLLEKRRQNLDVSRILGL

PAFIDILTVLEPNSKLLKEVNAVNDINGLRNSIAHNLETLDLDKNKNYKKIMLSVEAI
KNMLHISFPEIEEKDYNYFERKNKEFRELL"
/transl_table=11

/db_xref="SEED:fig|703339.257.peg.40"
CDS 36911..37645
/product="CRISPR-associated endoribonuclease Cas6"
/transl_table=11
/db_xref="SEED:fig|703339.257.peg.41"
/translation="MINKITVELDLPDSIRFQYLGSILHGVLMDYLPNDIADQLHHEF
AYSPLKQRIYHKNKKVIWEIVCMSDRLFNEIAELFTSKNRLFLKYYQVYIEIYSFNIE

KVNVQNIMNQLLETEELNRYVRINIQTPMSFKYQSNYMIFPEVKRFFRSIMIQFDAFF
EEYKMYDKETLDFLEKNINIVDYKLKSTRFNLEKVKIPSFTGEIVFKIKGPLPFLQLT
HFLLKFGEFSGSGMKTSLGMGKYSII"
CDS 38686..38844
/transl_table=11
/db_xref="SEED:fig|703339.257.peg.42"
/translation="MKFKSKQLVIANQTKVINYRIQVLLKPQANIETQTQVKNNTLPE
TDEQSNAD"
/product="hypothetical protein"
ORIGIN
1 ctaaaaatcg ggcataaatg tcaggaaaat atcaaaaact gcaaaaaata ttggtataat
61 aagagggaac agtgtgaaca agttaataac ttgtggataa caatttggag gaccaaacga
121 catgaaaatc accattttag ctgtagggaa actaaaagag aaatattgga agcaagccat
181 agcagaatat gaaaaacgtt taggcccata caccaagata gacatcatag aagttccaga
241 cgaaaaagca ccagaaaata tgagcgacaa agaaattgag caagtaaaag aaaaagaagg
301 ccaacgaata ctagccaaaa ttaaaccaca atccacagtc attacattag aaatacaagg
361 aaagatgcta tcttccgaag gattggccca agaattgaac caacgcatga cccaagggca
421 aagcgacttt gtattcgtca ttggcggatc aaacggcctg cacaaggacg tcttacaacg
481 cagtaactac gcactatcat tcagcaaaat gacattccca catcaaatga tgcgggttgt
541 gttaattgag caagtgtata gagcatttaa gattatgcgt ggagaagcgt atcacaaata
601 aaactaaaaa atagattgtg cataatataa aaggagcgga tttatattaa aactttgaat
661 tcaaaaatta ttgaaaggga agctacctta gaaattgaat ctatggcaac taatacattg
721 aaaataaacc cggatattaa ttcaaacgat acaaaaatgt ctttcgatgg agaattggaa
781 gtgtatgatt ctgaaaattt gagtaaaaaa aatttcgttg gaaaaataca agttcaagtt
841 aaaggaaagg aagtagctaa aagaggaggt aaggttattc atcgaagtaa tgtcaaaatg
901 aatgatttaa aggcatacca acgagaaggt ggtgtgtatt actttgtcgt gtatttaatc
961 gttgagaata aaaaagttgt tgagaagcag gtttatggta aacaattaca tcaattagat
1021 ttacaatttt tactgcaaaa aaagcagaaa agcgtcacta taaaaatgta tgaaattgaa
1081 aatgaaaaaa ttttatataa taattgcgta aaatatataa atgaaaagag attacaaaac
1141 caagtaggtc aagttaaagt aaagaacata gaaaaagctt tatcatatat agctacacct
1201 gaaaatattg tgatggatca tagaggttta ccattaaatg atttttatgg ctatataaaa
1261 attaattcat ctgaattaga tgtaactata ccagatggag tattaagtat ggaaaaagta
1321 aaaagagtga acaaaaagca gataataaaa gaaggcaagt tattatttga aggtatggta
1381 ggtatagaaa cttcaaagga atccatctct ataactatag atgatatttt caaaattcaa
1441 acatttgaaa gtgataacaa aagtacatat acaatgttac catttaaaaa attaaatata
1501 gccgaacaat cttttaatgt tataaacgaa ttgtcgaaag gcggcgaatt ctttttggat

1561 caaatccaat tagtaatcca accttttgaa attaatatta ttgaaataaa agaaacaata
1621 aataagttga atattaaatt atcagagtat agtaacttgc tttcgtttga tgtgagtcta
1681 aaatctacag aatttgataa gcagatgaat gagataaaag gtttattaga attattggaa
1741 tataaaaatt ttaaagattt taaaatgcat aataatggat actataaaat gaagttttgt
1801 ggaaaattta tattattatt taaagacaat acatcattgt ataatgtcta ctctaatgac
1861 tttgtagata gatttgaggc tgttacaaaa gaaagagttg tacagatgcc aattgtttac
1921 acattaacaa gagatatgat tgtagatgta ctgaattttg atataaatgt tattaaagaa
1981 tgtattgaat cagataaaat tgctattcaa tctgacatta aatgggagaa attaaataac
2041 tttgcattag aattaatcgc agcctatgat gaaactcaaa gaaccgattt attagaatta
2101 gctgaatatg tattaaataa tttgctgaat tttgataatg ataaaatatt tatgaactta
2161 atcaacaaag ctcaaattat aaagagaaga agaaatgatg tagatgaaaa attgttaatt
2221 aagttattca aattgagaga taaattaata gacattgata aagaaatagc aacattgtat
2281 atcaacgtag tatcagggag taagcaagag gcaaaaaataa gatatgaaac cctcaatgct
2341 gcagatttgg aaatatttaa tgcttacccg atttttaatt tatataaaaa attaatagaa
2401 agttgagatt atgacgaaaa atacttttag agaaagaatt caaaaaacat atacaaaaaa
2461 agatcttgag aaagctttag gtcttaaaaa caaagctcaa ttgcagaaac gaattgataa
2521 attagttgat atttataagg ttgatatgaa gaaatttcaa aaaaattctg gcgaaagtga
2581 aagagctgct tatagtttca atgggattgc ttttgatata ctatgtgtct tattaaataa
2641 tatgacagat gatatgctcc ctaaagcaat aacagatgaa gacgtaaagt taagaaaaaa
2701 tgcgatagag catatcgata ttgaccaata tgcacatttt attaaaagca ttaaaaatga
2761 tatagataaa attgaattta agccattaaa gttacatata catgcacaaa aagcatatca
2821 agatgtaaaa aaagtatttg attcaggtaa taaaataaat gaaaagttgc aattagtttc
2881 agaagtaatg aatcaattac ctattgataa acaagttgaa ttaagtgata aaatcgcatt
2941 tgctatacat gaaactattt attctacatt cgcagagatc aagtataatg aaaggattgc
3001 tgagcataat caaacaagtg aaaactcaag tcagtatgat tttagattta actatttcgt
3061 aaatcaatta actgataatg taataacaga agatataaat gaagaaaaag cctttatata
3121 tgataatgat gaacaattag attggttatt ggttaacata ctaaataaag ttcaacgggc
3181 tgctgatggt tgggggatga gaaacgctaa gcctcatcat agaacacaaa agtttagaga
3241 tattgaacat tatttcctta gagacgtatc gaaaatgatt gataaagagc ttaacaaaaa
3301 ggaaaactta aataaacaat ctaaaaaata tgacttcgat aataagataa aggtcattaa
3361 tgacaatatt gagtttttta atgaggatga acaaattaga aagcatttat tagagttacg
3421 taaacgatta gactttcatt ctgcagcagt attaaatcag aaaaatcatt cacacatagg
3481 caatgaaatg ttccaatatt ttatggatga tatgcaacaa gttgtaacat tgatagaccg
3541 atacattaat aagactgaac attataatga tatgaaaaat ggctatatta atgaatataa
3601 atcattttat aatgcatata ataatgaaat tgaagtgact aagacgtacg aaaataacat
3661 cccaatggca atgttaatga aacaatttaa gaatcaactg ttaaaaagaa ggaataaccg
3721 aaaaagctct aattgaagtt aagtagaaaa ttaataaata attggtaagc tcaatccctc
3781 atataaaggg attgagctta tttatttggt ggaatttttt ttgaaatttt ttgccacttc
3841 atcaatgacc tctatttagt attaggattg tcttatcaaa acgttttgaa caaattttaa
3901 gttgaaagtg agcgcgccac tttcaaccat aaaggagaag atttacatgg agacaagaga
3961 caaactgatg tcattgactc aaagtgacaa aacacagcaa tggctaatgg acaagtcatc
4021 taaccaagat gacattcaac aattgcagca acaattcagc cagcagttag atcaaaaata
4081 taatgcactt ttagctgaca atcaagctaa gttagaccaa tatgtggaag tacatcaagg
4141 attggaagca ttaaaggaag agattgaatc agaatccatt aaacttaata tagataaatt

4201 acccgatatc aaagcaacaa tgcttgaaag agccaagaat gatgaacatt ctgataaaat
4261 cgaaaagcta tttgataggt tagaacaggc attaaatggt acgaatcgat tatatacgca
4321 attatcgttg attggtacac gaacacatcg aattacaact aaaaatttta atcttcaagg
4381 cttacctaaa gcagtccaac atacgatttt accttcaaaa tttaagaagg tgtatacagt
4441 cgattttaaa tcgtttgaac catcagttgc agcgtacatg actcaagatt ctaaactgat
4501 tgactatttg aatcataaag aaggtttata tgacgcatta ttagaagatt tatcactgtc
4561 caaaaaggag aaaaagttcg ttaaacgtac atttattggt tcgtttttat ttggcggcaa
4621 cttcgatagc cccaaattca agttgaaaca ttatgtaagt gaagagcaat ggttggatac
4681 ggtcagccaa tttacaaaag tcaatgaact taagaaacaa atcgaagagc ataaaaccat
4741 gccgatgccc tacggcattg aacatgatat gagcgcattt caaggtagca gtattatggc
4801 aatctacgta caaactgtag cgagttatat tttcaagcac attctgctaa aagtgtacaa
4861 agcacagtgc gatcaaaaaa cgttcaagat tatagtacct atacacgatg cgattatgat
4921 tgaatgtgaa gatgaagaaa ttgcacaaaa tgtgggtcag ttaatgaaag atacggctaa
4981 ccagttgttc aatggtgaat ttgcacatgt gacagtggaa gaaataggag gcgtagacca
5041 tgaataatga tagaggacaa agcctacaca tcccaagtag tacaccaatc aaagaaaata
5101 atatatatgt agctacgtta cattctgtga tccaaacaga tttctcaggt gaaataaagc
5161 accaattcac gtatgaaatt gaagtgaaca atcagattgt atatgcgaat cgtaatattc
5221 caacaaaacc gagcgctaat cagttgtcaa ttcatgattg gctgaaacgt catagcaact
5281 atagcgcaag tcatgaaaac tatgagcctt atattgatca gaaacattta attctattag
5341 gtcaatataa cggtaactat tatgtacaag atgtagcatc gttagatgcg tttggaggcg
5401 tattatcatg aatcatatat tacaaatgtt atctaagcta ttaagtgtgg ccaaggaggc
5461 aatcgaccgt caaggtctga ttgctatcct aactattcct gttaataata acgatgaaat
5521 agaagaaacg gcttaaggtg aaaccgtgta taacgaactt atcgatcagt tacgacttaa
5581 tatcccaaaa gatacggatt atcaacctaa catctatagt tattttggta ttaagaagaa
5641 tcctaatgac accgtactca tggaaatgat gataaaggtt tttcatatca aacgctttaa
5701 ttcagaactg tttattttca aagttaacgg gtggcaaaag ataaatggag atgaattaca
5761 agggttgata tctaaaatga tacaagtatt gcttgtagat tataagcctt cactaagcac
5821 tctaaaaaat gtcgtagatg gattgcaaaa atcaacagat gtagaagaac ttgttgagaa
5881 tgagcgctat attggttgtg gtgaaaatat gttcgatctt aatacgtttc aagtcgttaa
5941 aaattcaatc gatatctttc caaaaacacg attgaattta tcattaagta caaatgatgt
6001 aattactgat aagataccgc cttattttaa gcaatatatg ttacaacttg cgaattatga
6061 cgatgattta caatactttc ttttccaaca tacagcagta ttacttacag ctgatactaa
6121 ataccgtagg ggtctcatat tatatggtgg agctaagaat ggtaaatctg tatatattga
6181 actagttaaa tcatttttct atagtaaaga tattgtgtct aagccactta atgagcttga
6241 aggtcgtttt gacaaagaaa gtttaattga caaaagtcta atggcaagtc atgaaattgg
6301 gcaatctagg attcaagaaa agatcgtaaa tgacttcaaa aagttattat ctgtagaatc
6361 aatgcatgtt gatcgtaaag gaaaaactca agtggaagtc attttggatt tgaaacttat
6421 ttttagtaca aatgcgatac ttaattttcc tcctgaacat gcgaaagctt tggagcgtcg
6481 aattaatatt attccatgtg agtattatgt tgaaaaagcg gacacttcat taattgataa
6541 gctccagagt gagaagaaag aaatctttct ttacttgatg tatgtgtatc aacagattgt
6601 aaaagcagat atcgagtatc ttgaaaatag ccgtgtcact gaaattactc acgattggtt
6661 aaattttgga tatgaatttg tttctagcag gtccgtaagt aatgcaaatc agaaagcatg
6721 tattaattta ctcagaaaac ttatagaaat caaatcagga tcacgaatca aagtatccga
6781 gttaaataaa gttattaatg aagaaataaa ggtaagttct caggttatta aacagttaat

6841 tcaagcaaac tttgatactc aaaccaaact atacaatggc tacgattatt ggattgattt
6901 aggttggaaa gaagccaata aaaaagagat tcatgatatt tcggaaaaag ataatattat
6961 ttcattagat aaaaatgaaa atataacaga cgatgaggca ttagatgaag agaatttgga
7021 ctttgattgg gaggactttg acgatgaata atgaacaaat tgaagcattt gtagaagtgc
7081 ttgtacctat catagaagaa cgtatcaata aaggtaatta aggctaatta cgtactacag
7141 gcagttgcct gtagtactca tatgattaag tggtaaaagt gataaaaatg aaacgaaatt
7201 ataaatatat attatctata tgttgttaca agaccgatag tctgtagcaa taatctaata
7261 aaaggagcgg tatgatatga agggtaaaat tgcactttat tcacgcgtta gtacgtcaga
7321 gcagtcggag catgggtact caatccatga gcaggaacaa gtactcatca aagaagttgt
7381 gaaaaatttc ccaggttatg actatgagac atatactgac tcaggcattt caggtaaaaa
7441 tattgaaggt cgtccggcaa tgaaacgtct attacaagat gttaaggata ataaaatcga
7501 aatggtgtta agttggaaat tgaatcgtat ctcacgatca atgagagacg tgtttaatat
7561 tattcatgaa ttcaaagaac atgatgtagg gtataaatcg atttctgaga atattgatac
7621 atccaatgct tctggagaag tactcgttac aatgtttggg ttaataggat ctatagaacg
7681 ccagactttg atttcgaatg tgaaactttc tatgaatgct aaggcaagga gcggagaggc
7741 aatcaccggt cgtgttttag gctacaaatt atcacttaat ccacttacac agaaaaatga
7801 tttggttatc gatgaaaatg aagctaatat tgtacgtgaa attttcgatt tatatttgaa
7861 tcataataaa ggcctcaaag ccattacaac cgtacttaat caaaaggggt atcgtactat
7921 taatcaaaag ccattttcag tgtatggtgt taaatacatt ttgaataatc cagtctataa
7981 aggctatgtc agattcaata accatcaaaa ctgggctgta cagcgaagaa gtggcaaaag
8041 tgataaaaat gatgtgatat tggtcaaagg taaacatgaa gccattataa gtgaagaggt
8101 atttgataaa gttcatgaaa aattagcttc taaaagtttt aaaccgggca gacctattgg
8161 tggagatttc tacttacgtg gccttattaa atgcccagaa tgcggaaata atatggtatg
8221 tcgacggacg tattataaaa cgaaaaagtc caaagaacgg acaatcaagc gttattacat
8281 ttgttcctta ttcaatcgtt cagggagttc tgcatgtcac agtaattcca tcaatgctga
8341 agtcgtcgag cgtgtaatta atgttcattt gaatcgtatt ctgtctcaac cagatattat
8401 caagcagatt gcgtcaaatg tgatagaaga actgaaacaa aagcatagta accaaacaga
8461 aattaaatat gacattgata gtttagaaaa acaaaaagct aagcttaaaa cacaacaaga
8521 acgattgtta gaattgttct tagatgatca gatggatagc gaaatgttaa aagctaaaca
8581 aagtcaaatg aatcaacagt tagaagtatt agatcaacaa attaaagaag cgcaacaagc
8641 aaatcaatca caggatgaaa tacctaattt tgataaatta aaaggacgac tcattttgat
8701 gataacacga ttcagcgtgt acttaagaaa ggctacaccc gaagctaaaa atcaacttat
8761 gaaaatgtta attgattcaa ttgaaattac gacagataaa caagtaaaac ttgtaaggta
8821 taaaattgac gaaagtctta tccctcaatc tttgaaaaaa gattgggggt cttttttat
8881 gcccaaattc caatttgaaa tatatggtca aaatgattat ttcatcgacc aaattaccac
8941 ttttaccact tagttattag tgacaaaagt gagcgaaatg aaataaaaat caaatatata
9001 ttatcaaaat gatgtatcac atgcatacat caatcaaata cattaggagg tcataaccat
9061 gacactagaa caacaactca agcactatat aaccaactta ttcaatctgc caagggacga
9121 agtgtggcac tgcgaatcta tcgaggaaat cgctgatgat atcttaccca atcaatatgt
9181 aagacttggc ccactcagta ataaaacact tcagactaat acctactact ctgacacact
9241 tcatgaaagt aatatctatc ctttcattct ctactatcag aaacaactca tagccatcgg
9301 ttatatcgac gaaaatcacg atatggattt cttataccta cacaacacta tcatgcctct
9361 tttggatcaa cgatacttac taacaggagg acaataaaat gcataaatac atcaaaatta
9421 cacaattagt cattacaata ctaagtgaaa tcatcatttg gatgaaagag tcagaacgaa

9481 aggaagtctc ttatgaatag atatatcacc cggggtatcg ccaacaactt acctaatatc
9541 ttacaacacc aattatggca actcgtatct gagcgagaac aagaacaaac caaagataat
9601 acttcagtag attattttca tatattccag ttcaataagc atcgcaatca attatatatc
9661 aaacacaaac aagaacgacc tgaatatgcg aaaatccata aagctaatta ttcaaaagca
9721 atcaatatca ataaggtcta cattattcga gaagatgatg tagacctttc ttattatgtc
9781 atgttattac ctgaagaata ctagaggaat gaaaattata tggaaacaat caaaagtaca
9841 ttaaaaacag aagccatatt cagtgatgac aaacaacatc gctatctact taagaaaaca
9901 tggaatagtg aaaaacaatc aatcacaatc attacaatgt atccgcatta tgatggcatt
9961 ctcaatattg acctaacgac ccaactcatc atgaacaaag tttcagaaat ggatgcattt
10021 ggttcaatca attttgtgaa tctatactct aatattacaa cccctatcaa tctcaaacat
10081 ttagaaaatg cgtatgataa gcatacagat attcaaatta tgaaggcagt gaaagagtca
10141 gatgaagtga tattagcttg gggcgcttac gctaaaaagc ccggtgttga agcacgtgtt
10201 aatgaagtat tagagatgtt gaaaccacat aaaaagaaag taaaacgact catgaatcca
10261 gaaaccaatg aaatcatgca tccccttaat tcgaaagcac gtcaaaaatg gatattaaaa
10321 gtatagcaac taaaatgttt attcactatc catgaaaata attgaatttc atatatatct
10381 tttaatctac aatatgatta aaagatatat tatttggttc tgttgcaaag taaaaaaata
10441 tagctaacca ctaatttatc atgtcagtgt tcgcttaact tgctagcatg atgctaattt
10501 catggcatgg cgaaaatccg cagatctgaa gagacctgcg gttcttttta tatagagcgt
10561 aaatacattc aatacctttt aaagtattct ttgctgtatt gatactttga taccttgtct
10621 ttcttacttt aatatgacgg tgatcttgct caatgaggtt attcagatat ttcgatgtac
10681 aatgacagtc aggtttaagt ttaaaagctt taattacttt agccattgct accttcgttg
10741 aaggtgcctg atctgtaatt accttttgag gtttaccaaa ttgtttaatg agacgtttga
10801 taaacgcata tgctgaatga ttatctcgtt gcttacgcaa ccaaatatct aatgtatgtc
10861 cctctgcatc aatggcacga tataaatagc tccattttcc ttttattttg atgtacgtct
10921 catcaatacg ccatttgtaa taagcttttt tatgcttttt cttccaaatt tgatacaaaa
10981 ttggggcata ttcttgaacc caacggtaga ccgttgaatg atgaacgttt acaccacgtt
11041 cccttaatat ttcagatata tcacgataac tcaatgtata tcttagatag tagccaacgg
11101 ctacagtgat aacatccttg ttaaattgtt tatatctgaa atagttcata cagaagactc
11161 ctttttgtta aaattatact ataaattcaa ctttgcaaca gaaccgtatt atggaataga
11221 gatgttggta acatttatac aggatcatta tacttaagtt taatttcgtt attacagaac
11281 cacacattcc aaccagaaga gaaagtatgt ctatttagtt atggttcagg agcagtagga
11341 gaaatcttta gtggttcaat cgttaaagga tatgacaaag cattagataa agagaaacac
11401 ttaaatatgc tagaatctag agagcaatta tcagtcgaag aatacgaaac attctttaac
11461 agatttgata atcaagaatt tgatttcgaa cgtgaattga cacaagatcc atattcaaaa
11521 gtatacttat acagtataga agaccatatc agaacatata agatagagaa ataaactagt
11581 ggccgattgt gcttgatgag cttgggacat aaatcctaac tcgaaataaa taagcatatc
11641 actaaactga ttttttaaag tttacagtga tatgcttatt tttttatctt acgattttgt
11701 acgtgcatgc ttgcctaggg gtatggctcg agccattagt ctctcgcaca tactattccc
11761 tcaggcgtca gcacttacaa aatcggttgt aattttcatt tttatacgca ttcttactga
11821 gattatacta ataagaggaa tagtaaaagc aattctaagt aaaattgcag ataagaggtt
11881 tgttaaaagc agttctaagt aaaattgcag ataagaggtt tgttaaaagc agttctcagt
11941 aaaattacag ataagaggta cgttaaaagc agttctaagt aaaattgcag ataagaggtt
12001 tgttaaaagc agttctaagt aaaattgcag ataagaggta cgttaaaagc aattccatgc
12061 aaaattgctg ataaggggta agttaaaagc agttctcagt aaaattgcag ataagaggta

12121 cgttaaaagc agttctaggc aaaattgcag ataagaggtg cgttaaaagc agttctcagt
12181 aaaattgctg ataaggggta agttaaaagc aatcctaagt aaaattgcag ataagaggta
12241 agttaaaagc aatcctaagt aaaattgcag ataaggggta cagaaaaact agacttgatt
12301 acaaaatgga gcttgggaca taaatgattt tttaaaaatg agatgagacg tagattaact
12361 ccataatcaa tacgaatcta tcgacttctt tatttatgat attcatctct ttttaatgga
12421 aataaaagtg cgattaatgt gataatacag ttacgttaat taaaaaaata aaaatgcaag
12481 gagaggtaat atgctaactg tatatggaca tagaggatta cctagtaaag ctccggaaaa
12541 tacaattgca tcatttaaag ctgcttcaga agtagaaggt ataaactggt tggagttaga
12601 tgttgcaatt acaaaagatg aacaactgat tatcattcat gatgattatt tagaacggac
12661 tacaaatatg tccggggaaa taactgaatt gaattatgat gaaattaaag atgcttctgc
12721 aggatcttgg tttggtgaaa aattcaaaga tgaacatttg ccaactttcg atgatgtagt
12781 aaaaatagca aatgaatata atatgaattt aaatgtagaa ttaaaaggta ttactggacc
12841 gaatggacta gcactttcta aaagtatggt taagcaagtg gaagaacaat taacaaactt
12901 aaatcagaat caagaagtgc tcatttcaag ctttaatgtt gtgcttgtta aacttgcaga
12961 agaaatcatg ccacaatata acagagcagt tatattccat acaacttcgt ttcgtgaaga
13021 ctggagaaca cttttagatt actgtaatgc taaaatagta aacactgaag atgccaaact
13081 tactaaagca aaagtaaaaa tggtaaaaga agcgggttat gaattgaacg tatggactgt
13141 aaacaaacca gcacgtgcaa accaacttgc taattgggga gttgatggta tctttacaga
13201 caatgcagat aaaatggtgc atttgtctca atagaaagtt agaggtgagt cttacgtttc
13261 agtgacggta gacttacctt taacatgtta catactaaaa aattaatttg aataagaaag
13321 agagacatat atgaaatacg atgattttat agtaggagaa acattcaaaa caaaaagcct
13381 tcatattaca gaagaagaaa ttatccaatt tgcaacaact tttgatcctc aatatatgca
13441 tatagataaa gaaaaagcag aacaaagtag atttaaaggt atcattgcat ctggcatgca
13501 tacactttca atatcattta aattatgggt agaagaaggt aaatacggag aagaagttgt
13561 agcaggaaca caaatgaata acgttaaatt tattaaacct gtataccag gtaatacatt
13621 gtacgttatc gctgaaatta caaataagaa atccataaaa aaagaaaatg gactcgttac
13681 agtgtcactt tcaacataca atgaaaatga agaaattgta tttaagggag aagtaacagc
13741 acttattaat aattcataat aaaacagtga agcaaccatc gttacggatt gcttcactgt
13801 tttgttattc atctatatcg tattttttat taccgttctc atatagctca tcatacactt
13861 tacctgagat tttggcattg tagctagcca ttcctttatc ttgtacatct ttaacattaa
13921 tagccatcat catgtttgga ttatctttat catatgatat aaaccaccca atttgtctgc
13981 cagtttctcc ttgtttcatt ttgagttctg cagtaccgga tttgccaatt aagtttgcat
14041 aagatctata aatatcttct ttatgtgttt tatttacgac ttgttgcata ccatcagtta
14101 atagattgat attttctttg gaaataatat ttttcttcca aactttgttt ttcgtgtctt
14161 ttaataagtg aggtgcgtta atattgccat tattttctaa tgcgctatag attgaaagga
14221 tctgtactgg gttaatcagt atttcacctt gtccgtaacc tgaatcagct aataatattt
14281 cattatctaa atttttgttt gaaatttgag cattataaaa tggataatca cttggtatat
14341 cttcaccaac acctagtttt ttcatgcctt tttcaaattt cttactgcct aattcgagtg
14401 ctactctagc aaagaaaatg ttatctgatg attctattgc ttgttttaag tcgatattac
14461 catttaccac ttcatatctt gtaacgttgt aaccacccca agatttatct ttttgccaac
14521 ctttaccatc gattttataa cttgttttat cgtctaatgt tttgttattt aacccaatca
14581 ttgctgttaa tattttttga gttgaacctg gtgaagttgt aatctggaac ttgttgagca
14641 gaggttcttt tttatcttcg gttaatttat tatattcttc gttactcatg ccatacataa
14701 atggatagac gtcatatgaa ggtgtgctta caagtgctaa taattcacct gtttgagggt

14761 ggatagcagt acctgagcca taatcatttt tcatgttgtt ataaatactc ttttgaactt
14821 tagcatcaat agttagttga atatctttgc catctttttt ctttttctct attaatgtat
14881 gtgcgattgt attgctatta tcgtcaacga ttgtgacacg atagccatct tcatgttgga
14941 gctttttatc gtaaagtttt tcgagtccct ttttaccaat aactgcatca tctttatagc
15001 ctttatattc tttttgtttt aattcttcag agttaatggg accaacataa cctaatagat
15061 gtgaagtcgc ttttcctaga ggatagttac gactttctgt ttcattagtt gtaagatgaa
15121 atttttttgc gaaatctctt aaatattcat ccattttttt aacggtttta agtggaacga
15181 aggtatcatc ttgtacccaa ttttgatcca tttgttgttt gatatagtct tcagaaatac
15241 ttagttcttt agcgattgct ttataatctt ttttagatac attctttgga acgatgccta
15301 tctcatatgc tgttcctgta ttggccaatt ccacattgtt tcggtctaaa attttaccac
15361 gttctgattt taaattttca atatgtatgc tttggtcttt ctgcattcct ggaataatga
15421 cgctatgatc ccaatctaac ttccacatac catcttcttt aacaaaatta aattgaacgt
15481 tgcgatcaat gttaccgtag tttgttttaa ttttatattg agcatctact cgttttttat
15541 ttttagatac ttttttttatt ttacgatcct gaatgtttat atctttaacg cctaaactat
15601 tatatatttt tatcggacgt tcagtcattt ctacttcacc attatcgctt ttagaaatat
15661 aactgctatc tttataaact tgtttgaaat ttttatcttc aattgcatca atagtattat
15721 taatttcttt atcttttgaa gcataaaaat atataccaaa cccgacaact acaactatta
15781 aaataagtgg aacaattttt atctttttca tcaatatcct ccttatataa gactacattt
15841 gtaatatatt acaaatgtag tatttatgtc aaaataatgt tataattttt gtgatatgga
15901 ggtgtagaag gtgttatcat cttttttaat gttaagtata atcagttcat tgctcacgat
15961 atgtgtaatt tttttagtga gaatgctcta tataaaatat acggttctgt tgcaaagttg
16021 aatttatagt ataattataa ccaaaaggag tcttctgtat gaactatttc agatataaac
16081 aatttaacaa ggatgttatc actgtagccg ttggctacta tctaagatat gcattgagtt
16141 atcgtgatat gtctgaaata ttaagggaac gtggtgtaaa cgttcatcat ttaacggtct
16201 agcgttgagt tcaagaatat gccccgattt tatatcaaat ttggaagaaa aagcataaaa
16261 aagcttatta caaatggcgt attgatgaga cgtacatcaa aataaaagga aaatggaact
16321 atttatatcg tgccattgat acagagggac atacattaga tatttggttg cgtaagcaac
16381 gagataatca ttcagcatat gtatttatca aacgtctcat taaacaattt ggtaaacctc
16441 aaaaggtaat tacagattag gcaccttcaa cgaaggtcgc aatggctaaa gtcattaaag
16501 cttttaaact taaacctgac tgtcattgta catcgaaata tctgaataac ctcattgagc
16561 aagatcaccg tcatattaaa gtaagaaaga caagatatca aagtatcaat acggcaaaga
16621 atactttaaa aggtattgaa tgtatttacg gtctatataa aaagaaccgc aggtctcttc
16681 agatctgcgg attttcgcca tgccatgaaa ttagcatcat gctaacaagt taacacaaag
16741 tattatttta aaattgagat tagacattta tttttcaact ttgcagcaga acctaatttt
16801 ttctctttgt aaataaatgc aaagagatac cataaggatc tcttacataa ccataacctt
16861 cagtatagaa ttctggacta aatgttttca atacctcact gcctttttct attaactggt
16921 catatacatg tttagtttct tctacttggt caaaagtgag acaaagagat atattattac
16981 cttgtgttat gggcaaacct tcagtgtcat ctgcgatcat aatttttata tctccaaatt
17041 gaagtacaca ttgatcaatt ttatttaaat cattttcgtc aatattaagt ttcttatcta
17101 taggtctatc tttaatacgt tgaatataca gtgttttagc gccaaacagc tcttcataca
17161 acttttttaa accctctgca ttttgagtga ttaaaaaata tggacttact tgaaatttca
17221 tgtttttttcc tccttagatt tgttataaat agagtatata cttaattagt gtcatctatt
17281 gacactaaaa ggagaataat aatgaaaaaa tctgttagat tatataatat gattgaatat
17341 tgtaatgaaa ataggaactt caaattaaat gatttaatgt cagaatttaa tatttctcgt

17401 agtaccgctt taagggatat aaaagaaatt gaagcattag gagtaccttt atatagtaat
17461 ccagggaaaa atggtggtta tacgatcata ggtaatcgag accaaacgaa aatagcaatc
17521 tcagatgaag agttgaaagc tttagtattt acactttcga gtatttcaaa tgtgagtaat
17581 ctaccttttc aaacagaata tcaagaaata ttaaaaaaat tatataataa ctcgaataaa
17641 aaagagttaa taaaccaata taatgatcta tttcaatatt ttaatgaaga taagtatcag
17701 ttcaaaagtt ataagttatt taatgaaatc attagattga taattgagaa taagtctttt
17761 gaaacctgtt attcacaaaa ctatattaaa gaacaatata aaggtattgg cattatgtat
17821 aaaaatcatc aatggtattt tgttgtagct aatatagaat caaagttagt gaatctatta
17881 aatatttcga aaataaaaga actatatgaa atgggagaga ctcaagagtg taatgatata
17941 actatgcaga attttcaaca gttcatggtt aaaaatgaaa cagctattga tattcttatt
18001 agaagcaatg ttatgggatt gaatatcttg aaaggctacc tgtggagtga ctatatgatt
18061 gaaaatattg acgaagagac atatttattt aaatcaaaag tgaacgcgaa agatatagat
18121 tttatagcta agttaattgt cacaggtggt gtcaatgtaa aagtagagac ccctaatagt
18181 ttgaaaaatg ctgttaaagt tgaattaact aaaataataa acatgtatta atagtaattt
18241 aatcataatt gtaataacaa aaagatgaca acattaaaca atttaaattt atacaaaaaa
18301 gcattaccaa atttaaaatt gggtagtggt agtgctacag catacataaa tagttataat
18361 taaataaaga tgggtcaatg aaggtgaatt aagtgaataa aaatattaca gctctaaaat
18421 ctactgaaaa tactacatat atgcagtata cggttcatat cgatgatcaa attataaata
18481 atataaaaga gtgtaccaaa aagtttaaaa ttttgcctat ggaagataaa attccattat
18541 caccgttgtt acaaccagaa tatgcaggag aggtacaaga ttttattagt acatatgagc
18601 agtttatgat taattttggt aaagtaatat tggatagtca aggcataaaa atacagtttg
18661 aaagtgaatc attaagtagt attcaacgag gcattcaaga acattgttac ttaaatgagc
18721 gaacaaatga cattgatgtg actaaagaat ggtatttatg caaattttca aatcaaatat
18781 tagaagagga taaaaatcaa ttgtataatg ctttaaagca actcatgaat gattctaaaa
18841 ataaaaagca agcttttcaa gaggtgttta aggttcatat tgatatttat ctctacaata
18901 aagaaaaatc tgaatataag tatcaagtat ctagttattt caatcttgta aaagaaaatc
18961 ctaaaataac ttataaaaaa agacatttac aagagaaaca aggtgtcaag ggtacaactt
19021 tcacgaacaa attaaaatta ttgaacaaaa tgtatggtgt agatgttgca aaatatcagc
19081 cattttataa ttctaataat cctgaatatg aaagaggtca atttggtgaa agatacattt
19141 ctcaaagatc taactatgaa tttaatagac ttcaatatca aattatagat atgttatcga
19201 aaatacttga taagcatcca ttaccaaagt cagataataa ttataaacat attccaacta
19261 ttgaaaaagc aatattaagt ggggattctc atagttttta tgagtatttt gaagatataa
19321 tgaaagaaat tataagtatg gaaaattctt ctttaaagga aaaattattg actgatttta
19381 catatcaatc tcaatgtaga tggtactctg aaagtgaaaa attaaaattg caacttgaaa
19441 gttttatgca taaagttttg gagagcaatt attatgaggg taacaaatta tatagaatgt
19501 tgtctcatgc aattgaagaa acaatcaatg aggctgatga agataaggtt catttcaatt
19561 attttaaaga ttatttttttg acagacggtg gggtgaagaa ttgggagcag attagcgaga
19621 aaattactga attcaatggt aaagttatta acgatatcca aaatgaatat aataagattc
19681 aatttaataa cgcaaatcga aatcagaaac taaactttaa ttatttatac cattgctttg
19741 agtttagtaa taacttagta aaagcaaggg taaatggaaa tagaggttac attttatatt
19801 ttgtagataa atataaaata aaaatgccct ttttggatca attattaatc aattatttga
19861 atcaagaaat aggtcaggaa agtattaatt ataatatgca aacattgttt gaaaaagaaa
19921 gatatgatag aagtagtacc attgaaaaat tagtagcaac aagcaaattt aagtatgaaa
19981 aagatgattc agatttattc aaacaacttt tcaatgatgt tgaaaattca atagacagat

20041 taggtattta cttactaaat aatggtataa attcgaatga tgaaaatgca agatattata
20101 gatcgttttt aaaggaactt agtagaataa aaagtaaatt aacgccattt tctcttgaaa
20161 taagtaagtc tagcggaaga gagcaacatt atcctgatga tgctattgat gataaagatg
20221 agagaagaaa aaataaagaa gaaacatatc atgcttttga tgataaaagc gatattgact
20281 ccaaattaaa gaataaaata aatgtttcta tcgataattt attgtcagtt aaaattaaga
20341 gcaatttgta tgatattagt ccaaatacaa aaaaacaata actataatga gccaactcaa
20401 gaatgagttg gctttttaat tgtaatttta cgcttccatt acaggaatca atgttatgga
20461 agccattaag caattaattt gagtcattta tagtataggt acaacgttgt gatgttcaca
20521 tgataatcag cccgggagat tattgtgtaa taacttatag aaggagcaaa tcaccatgta
20581 tcaagcaaat atacgtgatt taattacaaa attgcctcaa agcaataaaa cagaacactt
20641 tttaatgaac aaattttcaa atcaagataa agttcagcag ctacaaagac aaattagcca
20701 acagctagat caacaatata atgagctttt ggctaatgaa aaagctaagc tagaccaata
20761 cgtggaagta caccataatt tagaaccatt aaagaaagag attgaatcag aatctattaa
20821 ccttgatacc gataaattac ctgatatcaa agcgacaatg cttgaaaagg ctaagaacga
20881 tgaacatttt gataaaatcg aacagctatt tgatagatta gatcagtcat taaatggtac
20941 gaatcgatta tatacgcaat tatcgttgat tggcacacga acacatcgga tcacaacgaa
21001 aagatttaat gttcaaggct tgcctaaatt agtccaacaa atgattttac cttcgcaatt
21061 taaaaaggtt tatacaatag attttaaatc attcgaacca tcagttgctg cgtatatgac
21121 acaagacgaa caactgattg actacttgaa tcatgaagaa gggttatacg atgcattact
21181 gagagactta tctttgtcaa aagagaagcg cgtgagtgtg aaacgtgcat ttatagggtc
21241 atttcttttt ggcggtcgtt atagtagctc taaattcaaa atcaatcaag aggttagtga
21301 aattaactgg ctacaagtaa tgagcaaatt caagaaggtc attgaattta aggagcaagt
21361 cgaaaaatat aaaacaatgc ctacgcctta cggcattgaa catgatatga gcgcatttca
21421 aggtagtagt attatggcaa tttatgtaca aacggtagca agctatattt tcaagcacat
21481 tttgttggaa gtgtacaaag cacagtgcga aaaaaaaacg ttcaagatta tagtgccgat
21541 acacgatgcg attatgattg aatgtaatga taaggggatt gcacaaaatg tagcacagct
21601 catgaaagat acagctaatc aactgtttaa tggtgaattt gcacatgtga cagtggaagc
21661 tttaggaggt atagacaatg aataatgata gaggaaaaag tcttcaaatt ccccaaagta
21721 cattgttaaa agaaggatct atatacgttg ctacgttaca ttctgtgtac gagaagaact
21781 tctcaggtga tattaaacat cagtttacgt atgaagtaga acttaaccaa gaaacgcact
21841 atgtaaatcg caatattacc gtaaaatcta tgagtcacca attatcaatt gctgattgga
21901 ttaaacgtca cagtaactat aacgtaaacc acattaatta tgatccatac attgatcgaa
21961 aacatttggt ccttgtaggg caatataacg gaaattatta tattcaagat gtagcaccat
22021 tagatgaatt tggaggggta atgtaatgaa tcatatttta gaaatgttaa taaaattatt
22081 aaaagtgggt atggaggcaa tcgaccgtaa aggtctgatt gccatcctaa caagtagtat
22141 tggaaatgat gaaatggatg attctgaaca agctgtaatg gtgtataacg agcttatcga
22201 taagctacag cttaacattc ctaaagatgt cgactataga cctaacatat acagttattt
22261 tggtattcaa aaaaagccaa atgacacaat attagtggaa atgatgatat gtatttttca
22321 tatcaagcgt tttgattcag agttgtttgt tttcaaagat aaaggttggc aaaaggtaag
22381 tgaagatgaa ttgcaagggt tgatatctaa aatgatacaa gtgttgctag ttgattataa
22441 accttcacta agtactttga aaaacgtagt agatggcata cagaaatcaa cggacataga
22501 aaaacttgtt gagcatagac agtacattgg ttgtggacga aatatgttca atctaaagac
22561 ctttaaagtg gttgataatg accttgaaat attccctaaa acacgcttag atttggaatt
22621 agatataaat gacacgatta cggacaagat acccccgaat ttcaaacaat atatgttaga

22681 gttggcgaat tatgaccatg atttacaata tttccttttc caacatatgg cagtgttatt
22741 gacggcagat actaaactac gtcgtggact ttttttgtat ggaactgcaa aaaatgggaa
22801 atcggtctat attaaattag ttaagtcatt cttttatagt aatgatatcg tatctaaaac
22861 acttaatgaa cttggcgggc gtttcgataa ggaaagtcta attggtaaac gaattatggc
22921 aagtgatgaa gtggggaaag ctaatattga tgaagcaact gtgaatgatt tcaaaaaatt
22981 actatctgtt gaaccaattc atgctgaccg taaaggaaga acgcaagtag aagttacttt
23041 agatttaaaa ctcattttta atacgaatgc tgtactcaat tttccatcat cacatgcaaa
23101 agcattagag cgtagaattg ctgttattcc atgtgaatat tatgttgaaa aagctgaccc
23161 tgacttaatt gaaaagttac aggatgaaaa gaaagaaatc tttctttact tgatgtatgt
23221 gtataagcaa attgtaaaaa atgatatcga gtacctccaa aatgatcgtg ttactgaaat
23281 ttctcatgat tggttaaatt ttggatatga atttgtttct agtaaatcag caaatattgc
23341 acatcagaaa gcgtgtatta atttactcag aaaacttata gaaatcaaac cagggtcacg
23401 tatcaaagtg tctaggctaa atgaggttat tagagatgaa attaaagtaa gctctcaagt
23461 tattaatgat ttggttcaag ctaactttaa tgtacaaagt agactaaata atggttataa
23521 gtattgggtc gatttaggat ggaaagaaac tgataaaaaa gatgacatga tttcattcga
23581 taaaaatgag aatgtaacag atgatgaatt cttatacgaa gatgatttga acttaggttg
23641 ggaggacttt gacgatgaat aatgaacaaa ttgaagcatt tgtagaagtg cttgtgccta
23701 tcatagaaga acgtatcaat aaaggtaagt aatctaatta cgtactacag gcagttgcct
23761 gtagtactca tatgattaag tggtaaaagt gataaaaatg aaacgaaatt ataaatatat
23821 attatctata tgttgttaca agaccgatgg tctgtagcaa taatctaata aaaggagcgg
23881 tatgatatga agggtaaaat tgcactttat tcacgtgtta gtacgtctga gcagtcagaa
23941 catggttatt ctgaaaagga gcaggaacaa ctactcatca aagaagttat gaaaaatttc
24001 ccaggttatg actatgagac atatactgac tcaggcattt caggtaaaaa tattgaaggt
24061 cgtccggcaa tgaaacgtct attacaagat gttaaggata ataaaatcga aatggtatta
24121 agttggaaat tgaatcgtat ttctcgctca atgagagacg tgtttaatat tattcatgaa
24181 ttcaaagaac atgacgtagg gtataaatcg atttctgaga atattgatac atccaatgct
24241 tctggagaag tactcgttac aatgtttgga ctaataggat ctatagaacg ccagactttg
24301 atttccaatg tgaaactttc tatgaatgct aaggcaagga gcggagaggc aatcaccggt
24361 cgtgttttag gctacaaatt atcacttaat ccattgacac agaaaaatga tttagttatt
24421 gatgaaaatg aagctcatat tgtacgggaa atctttgatt tatatttgaa tcacaataaa
24481 ggatttaaag caatcacgac aattctaaat caaaaaggat atcgtaccat taatcaaaaa
24541 ccattttcag tgtttggcgt gaaatacatt ttgaataatc cagtctataa aggctatgtc
24601 agattcaata atcatcaaaa ttgggctgtt cagcgaagaa gtggtaaaag tgataaaaat
24661 gatgtgatat tggtcaaagg taagcatgaa gccattataa gtgaagatgt atttgatcaa
24721 gttcatgaga aactagcttc taaaagtttt aaaccgggtc gacctattgg tggagatttc
24781 tacttacgtg gccttattaa atgcccagaa tgcggaaata atatggtatg tcgacggacg
24841 tattataaaa cgaaaaagtc caaagaacgc acaatcaaac gctattacat ttgttcatta
24901 ttcaatcgct caggaagttc tgcctgtcat agtaatgcga ttaatgctga agtcgtcgaa
24961 cgcgtaatca atgttcattt gaatcgtatt ctttcacaac ctaatgttat taagcagatt
25021 gcgtcaagtg tgatagaaga actgaaacaa aagcatagta aacaaacaga aataaaatat
25081 gatattgata gtctagaaaa acaaaaagca aaagttaaaa cacaacaaga acgattattg
25141 gaattgttct tagatgatga aatggatagc gaaatgttaa aagctaaaca aagtgaaatg
25201 aatcaacagt tagaagtatt agaccaacaa attaaagaag caaaacaagc aaatcaatca
25261 caggatgata tacccaattt tgataagtta aaagcacgac tcattttgat gataacacga

25321 ttcagtgtgt acttaagaaa ggctacaccc gaagctaaaa atcaacttat gaaaatgtta
25381 attgattcaa ttgaaattac gacagataaa caagtaaaac ttgtaaggta taaaattgat
25441 gaaagtctta tccctcaatc tttgaaaaaa gattgggggt cttttttat acctaaattt
25501 aactttgtga taaatgtcac aaagaaaaat aggattgaaa atttatcact tttaccactt
25561 ttttagagtg acaaaagtgg aggagttttg aaatatttta taaatatata ttttatttat
25621 ggagtacaca ttattaatta aggaggtcat tataatgacg ctaagcaaac aacttaaaac
25681 gtatatcact gaacgattta aattaaatta tcaagaaact tgggcttgtg aaaccataga
25741 tgcggtggct gaagatgtat tacctgaaaa atatattaaa aatagtccac ttgaacataa
25801 aattttaaat acttttacct attacaatga tgaattacat gaaatcagca tttacccttt
25861 tttatgttat ctagataagg aattagtagc aataggttat ttagataatt ttgatttaga
25921 ctttatattt ttaaatgaca ctcatcaaat tattattgat gaacgctact tgttacaaaa
25981 agggggcgag taattatgaa ctggatcaag gttgctcagc tatctgttac agtcattaac
26041 gaagtgattg agatcatgaa agaaaagcag aatggaggaa aatagtatga acatcaatcg
26101 atacatcacg agaggcatta gtgaacaact atctctagac cttcaaatct tactttggca
26161 catggtagaa gaaaaagata accagcctca taccgattac ctacacattt tcaaactaca
26221 agaagatgat aatatgttgt caattacaca tgaacaagaa cagcccgcat acaagttaga
26281 atatcactat ataaactatg aaaaaaatca aaatgcatta cctaagaaag tctacgtcat
26341 tcgtgaagat gatgtagacg ttttttatta tgtgatgctt ttaccagaag aatactaaaa
26401 ggagtgacgt tgatgaatac aatcaaaagt acgatacaca cagaagctat atttagcgat
26461 gatgaacaac accgctactt actcaaaaag acttgggatg aaaagaaacc tgtttgtaca
26521 gtgataacga tgtatcctca tttagatggc gtattatcac tcgatcttac tactgttctt
26581 atcctcaacc aattagcgaa ttctgaacaa tatggcgctg tatatctagt gaatctattc
26641 tctaatatta aaacaccaga gaatcttaaa catattaaag aaccttatga taaacacaca
26701 gacatccatt tgatgaaagc aattagtgag agtgatacag taattctagc ttatggcgcc
26761 tatgcaaagc ggcctgttgt cgtcgaacgt gttgagcaag tgatggaaat gttaaaacct
26821 cataaaaaga aagtcaaaaa actcataaac ccagcaacaa atgacattat gcatccactt
26881 aaccctaaag cacgtcaaaa atggattttg aaataaaagg aggattatct atgaaccatg
26941 aaactacaca ctcagactgg cgaacggttg ctaattgttt agcatcacaa aattatatat
27001 cgatcgtaaa aggattagta catcatttca cagcgattga agatgaagaa atactagata
27061 aaatctatga agattttatg aataatggct ctattacaac ggtacttaac aatgatttac
27121 aagatattat taattattac ctatcaaaat aaaaatatat tacttttaat aaaattagag
27181 catccttcac tttgcggtga gagatgctct tttagtttat ttttttattg attactcgcc
27241 ataagttctg cttgctctac agccattttt ataaatctgg tggatagcta tattttttaa
27301 gtaatcgtct aacagctacg cgcattttag cttttgcgct atcacgttta gaccaatcac
27361 ccccatattt tctttcactt ttttagttag ctcatgagca atcgcacgta gttcttcatc
27421 ttccattcct ttttttcgct gtttcatgtg aaactaaagc atcgtaaaaa tgcgatttca
27481 tctgaattta aacctaattc atttcctcgt tgttgttctt gtttaatatc tttggcgagt
27541 tgaatgagtt cttcaatcac ttttgatgtt tcaatggaac ggctattata tttattaatc
27601 gaatttctta acatttcaga gaaacgttta gataccgtcg cattcgtttt cattaatgat
27661 ttgacttgtc ccttgagtaa tcgatttaat aattctaccg ccacattttt ctgtttcaat
27721 ccttctacat cttttaaaaa gtcatcagat aggattgata aatcgggttg ttcaagacct
27781 aaagtttaat aaatgtcaag tcaatgaaat cttcagttaa tagtgtatat ttgattatta
27841 ttagtttaaa tatattaaaa ttaaatactt attatatatg gttctttact attttagaag
27901 tatatttttt attttcctga acagtagtta ttaaatttat ctcttatttt aaattagaaa

27961 ggaagaaaaa gttaaattta tattaagttt tatatatgat taattattaa cgtattaaat
28021 gtagtatact cttaatatat agaaaattat cataatattt gtcaaaaaaa gtgacatttc
28081 atatagtagc atggtgttag ttgtcacagt ttttgacagc caaaataaca cttgaacctt
28141 tgtggttatg ctatttatta aagtatctga tcgataacta ccccgaataa caggggacga
28201 gaattctata agttcattaa ttccgatacc tagattatct gatcgataac taccccgaat
28261 aacaggggac gagaattttt ttccaccctt tcagatcatc tatgatcttg gatcgataac
28321 taccccgaat aacaggggac gagaataatt ttctaattct ataagttcat taattccgat
28381 gatcgataac taccccgaat aacaggggac gagaattata ctatttacat aattttttat
28441 gtgtctgtct acgatcgata actaccccga ataacagggg acgagaatta atagtgttgt
28501 tctctattaa aagatacaat cctgtgatcg ataactaccc cgaataacag gggacgagaa
28561 ttagaatgtt attatctaag tggtcgatgt attccgatcg ataactaccc cgaataacag
28621 gggacgagaa ttagaccatt tacctcatta tatttatagt ctttattaga tcgataacta
28681 ccccgaataa caggggacga gaatttttct ttaactgttt ttactgccca tttaatagtg
28741 atcgataact accccgaata acaggggacg agaatataaa cccgttcaat tcgttatctt
28801 taaattcttg gatcgataac taccccgaat aacaggggac gagaataagt taacggcatt
28861 acctaataaa aatattttag ggatcgataa ctaccccgaa taacagggga cgagaactca
28921 tctttcatgt cactgattaa ttcatttgta gatcgataac taccccgaat aacaggggac
28981 gagaacggta atagttgctc aataggtaat aaaacgtcgg tgatcgataa ctatcccgaa
29041 taacagggga cagagtgtaa atttaattac actctaaaat ttgtaaattt ttaatggaat
29101 acgcattgat taatttttag gggatgaaaa atgaaagatg ttatttatgt agaaaatcat
29161 tactttgtta ccgtgaaaga aaatagtatt aaatttagaa atgtaataga taaaagtgag
29221 aaattttatt tgtttgaaga aatagaagcg attatttttg atcattataa aagctatttt
29281 tctcataaat tagtaattaa atgtatagaa aatgatatcg ctattatttt ttgtgataaa
29341 aagcactctc cattaacgca acttatttct tcttatggta tgactcatcg tcttcaaagg
29401 attcaaagtc agtttcaatt atctgggaga actagagata gaatttggaa aaagattgtt
29461 gtaaataaaa ttattaatca atcaaaatgt ttagaaaaca atttacataa tgagaatgtg
29521 aagttattag taaacttagc aaaagatgtt agttctggag ataaaagtaa taaagaagca
29581 caggctgcaa gaatttattt taaagattta tacggtaaac aatttaaacg tggacggtac
29641 aatgatatta ttaactcagg gttgaattat gggtattcga tacttagatc ttttataaaa
29701 aaagaactag ctttacatgg attcgaaatg agtttaggca ttaatcatcg ttcgaaagaa
29761 aatccattta atttagcaga tgatattatt gaagtttttc gtccttttgt agataatatc
29821 gtgtacgaga tagtttttaa gaaaaatatt aatacatttg atgtaaatga aaagaaatta
29881 ttgttaaatg ttttgtatga aaagtgcatt atagataaaa aagtggtgag gttacttgat
29941 agtgtgaaga tagttattca atcacttatt agatgctatg aagaaaacac ccctacttat
30001 ttattactac ctaaaatgat tgaggtgggg aactaatgta tttattagtt agttttgact
30061 tacctagaga tactaaattt gaacgtagag tcgcaagtaa gtatcgtact cgattattag
30121 aactcggttt tagtatgaag cagtttagtt tatacgagag gtatgtcagc gatgttcaaa
30181 aaaaagataa gattttagaa attctgcaac aagaaattcc tgatactgga agtattacac
30241 tatatgtctt acctgatgag gtaaataata gtcagattac catattagga aaagaggtta
30301 aggttgttgt gcgaaaagaa cctaagctaa tttttctata aagtggaggt tacaatggat
30361 aaaaaaacga cattaatgta tggttcttta ctacatgata taggtaaaat tatctatcgg
30421 agtaatgatc atgcatttgc aagaggaacg cattcaaagt taggatacaa ttttttatct
30481 caattttcag aatttaaaga taacgaagtg ctcgatagta ttgcttatca tcattataaa
30541 gaacttgcaa aagctaattt agctaatgat aatacagctt atattaccta tattgccgat

30601 aatattgcga gtggtattga tagacgagat gttatagaag agggcgacga agaatacgaa
30661 aaacaagcat ttaattttga taagtataca cctctatata gtgtgtttaa tattgtgaat
30721 tctgaaaaat tgaaacaaat aagtggaaag tttaaatttt ctaatgaaag taatattgag
30781 tatcctaaaa ctgagaacat tcaatattca agtggaaatt atacaacact aatgaaagat
30841 atgagttatg atttagagca caaattaagt attaaagaag atacatttcc ttcattatta
30901 caatggacag aaagtctatg gcaatatgtg ccaagttcga caaataaaaa tcaattaatt
30961 gatatttctc tttatgatca tagtcgtatt acatgtgcta ttgctagttg tatatttgat
31021 tatttaaatg aaaataatat acataattac aaagatgaat tgtttacaaa gtatgaaaat
31081 accaaagaat tttatcaaaa agaagctttt ttactactta gtatggatat gagtggtatt
31141 caagatttta tttacaacat aagtggttct aaagcattaa aaagtttaag atctcgtagt
31201 ttttacttag aaatcatgct tgaggtaatc gttgatcaat tactagaaaa attagaatta
31261 acacgagcaa atcttttata cactggtgga ggccatgcat atttattagt ctctaataca
31321 gataaaggga aagaaaaaat aaatcaattt aatactgaat taaaaaattg gtttatgtca
31381 gaattcacta cagatctctc attatcaatt gcttttgaaa aatgtagtgg taatgactta
31441 atgaatacta gtggtaatta tagaaatatt tggcgtaatg tcagcagtaa gctttctgat
31501 attaaagcac ataagtattc ttcagaagat atattaaaat taaatcattt tcattcatat
31561 ggggatcgag aatgtaaaga atgtttaaga agtgacatag atattaatga tgatggatta
31621 tgcagtatat gtgaaggaat cattaatata tcaaatgact taagagataa atcattcttt
31681 gtactgtcag aaactggaaa attaaaaatg ccattcgata aatttatatc ggttatcgat
31741 tatgaagaag cagaaaggtt agcacaaaat aataatcaca ttcgtattta cagtaaaaat
31801 aaaccttata ttggtgtagg aatatcaacg aatttatgga tgtgtgacta cgactatgct
31861 agtcaaaata aagatatgag agaaaaaggt attggaagtt atgtagaaag agaagaaggc
31921 attaagcgtt taggcgttgt acgtgccgac attgataatc ttggcgctac atttatatca
31981 ggaattccag aaaaatataa ttcaatttca agaacagcta cattgtctcg tcaattatca
32041 ttatttttta aatatgaatt aaatcattta ttagaaaatt atcaaattac agctatatat
32101 tcaggtggcg acgatttatt tttgattggc gcatgggatg acattataga agtaagcgtt
32161 tatataaatg aaaagtttaa agattttact cttggtaaat taacaatgtc tgctggtgtt
32221 gggatgttca gtggtaagta tccaatttct aaaatggctt ttgagacagg tctacttgaa
32281 gaagcagcta aaactgatga aaaaaatcag atagcacttt gggtgcaaga aaaagtatat
32341 aactgggatg agtttaaaaa gtatatctta gaagaaaaac ttctcgtttt acaacatggg
32401 ttttcacaaa cagatgaaca cggaaaagcc tttatttata aaatgttagc tttactgcga
32461 aataatgaaa ctattaatat tgctcgctta gcctatttat tagcaagaag taaaatgaca
32521 gaggaattta catctaaaat ttttaattgg gctcaaaacg acaaagataa aaatcaatta
32581 attacagctt tagagtatta tgtttatcaa ataagggagg ttgattgagt atgatattag
32641 ctaaaacaaa aagtgggaaa aaaatagatt taacttttgc acatgaagta gtaaaaagta
32701 atgtaaagac agttaaagat aaaagaggta aagaaaatca agttttattt aacggtctta
32761 caacaagcaa gttaagaaat ttgatggagc aagtgaaccg actttatact attgcattta
32821 attcgactga agatcaattg aatgaagaat ttatcgatga attagagtat ttaaaaatta
32881 aattttatta tgaagcaggt cgagaaaaaa gtgttgatga attcttgaaa aaaacattga
32941 tgttcccaat tattgataga gtgatacaaa aagaatcaaa aaaatttttc ttagattact
33001 gtaaatactt tgaagctcta gttgcatacg ctaaatatta tcaaaaggag gattaatatg
33061 tattcaaaaa ttaaaatttc aggaacaatt gaagtagtta ctggtttaca catcggcggg
33121 ggcggtgaat ctagtatgat tggcgcaatt gattcccctg tagttagaga tttacaaact
33181 aaattaccta tcatacctgg cagttcaatc aaaggaaaaa tgagaagttt attagcaaag

33241 cattttggct tgaaaatgaa acaagagaat cataaccaag acgatgaaag tgttttaaga
33301 ttattcggtt caagtgaaaa aggaaatatt caaagagctc gtctacaaat ttctgatgca
33361 ttcttttctg aaaagacgaa agagcatttt gcacaaaatg atattgctta tacagaaacg
33421 aaatttgaga atacaattaa tcgtttaact gcagttgcaa atccaagaca aattgagaga
33481 gtaacaagag gatctgagtt tgactttgta tttatttaca atgtcgatga agaatcacaa
33541 gttgagaatg attttgagaa tattgaaaaa gcaattcact tattagagaa tgactacctt
33601 ggtggcggcg gtaccagagg taacggacgt atccaattta atgatataac tatcgagacg
33661 gttgttggag aatacgacag tacaaatctt aaaattaagt aggtgaaaga cattgacaac
33721 aaaagtattt aaactttctt ttaagactcc tgttcatttt gggaaaaaaa ggttgtcaga
33781 tggggaaatg acaataactt ctgatacttt gtttagtgcg ttatttattg aggcgcttca
33841 attgggtata gaaactgatt ggttattaaa cgatttaatc attagtgata catttcctta
33901 tgaaaatgaa atttattatc ttcctaaacc tttgataaaa attgaatcta aagaagaagg
33961 caaccataaa gcatttaaaa agttaaaata tgttccggtt catcactata atcaatattt
34021 aaatggtgag ttaagcgctg aagatgcgac agatttaaat gatattttta gtattgggca
34081 tttttctcta caaacaaagg tttcattatc agcacaagaa attgattcaa gtgctgacag
34141 tgaaccttat tcagtgggaa catttacttt tgaacctgaa gctggtttat attttattgc
34201 aaaaggatca gaagaaaccc ttgaccaatt aaaagatatt atgacttcat tacagtactc
34261 aggtttaggt ggtaaacgta atgcaggata tggacaattt gaatatgaaa taataaataa
34321 tcaacaatta tttaagttat tgaatcaaaa tggagaacat tctattcttt tatcaacggc
34381 aatggctaaa gaagatgaga taaagagtgc tttaaaagag gcaagatata ttttgaataa
34441 acgttctggt ttcatacaat caacaaatta ttctgaaatg ctagttaaaa aaagtgattt
34501 ctatagcttt tcttctggtt cagtttttaa aaatatcttt aacggtgacg tttttaatgt
34561 tggacataat ggtaaacacc cagtctatcg ttatgcgaag cctttatggt tggaggtata
34621 agtatgacaa taaaaaatta cgaagttgtt attaaaactt taggtccagt tcatattggt
34681 agtggtcaag ttatgaagaa acaagattac atttatgatt tctacaattc taaagtttat
34741 atgattaatg gaaataaact ggttaagttt ttaaaaagaa aaaatatact cgatacatat
34801 caaaactttt tgagataccc accaaaaaat ccaagagaaa atggactaaa agactattta
34861 gacgctcaaa atgttaagca aagtgaatgg aaagcatttg tgagttattc tgaaaaagtt
34921 aatcaaggta agaaatatgg aaatatacgg cctaaaccgc taaatgattt acatttaatg
34981 gtaagagatg gacaaaataa ggtgtatctt ccgggaagtt caatcaaggg agctattaaa
35041 acagcacttg tttcaaaata taataatgaa aaaaatacag atgtatatag caaaattaaa
35101 gtcagcgatt cagagcctat tgatgaaaga catttagcaa tttatcaaaa aatagatatt
35161 aataaaagcg aaaaaccaat gcctttatat agagaatgtg tagatgtaga tactgaaata
35221 aaatttaaat taaccataga agatgaaatt tattctatta atgaaattga acaaagcatc
35281 caagattttt acaaaaaact a ttatgacaaa tggttggtcg gtttcaaaga aacaaaaggt
35341 ggaagacgat ttgcattaga aggcggtatg cccgatgttc taaaccaaaa tattttattc
35401 ttaggagctg gcgcaggatt tgttagtaaa acgacacatt atcaattaaa aagtcgagaa
35461 caagcaaaac gagattcttt tgatatttta actaaaaaat ttcgtagaac ttatgggaaa
35521 atgaaagaaa tgccttctaa cgtaccagtt gctctaaagg gtacaactaa tcaaagtctt
35581 catgtttcat atcaacaagg tatgtgtaag attagttttc aagagttaaa taatgaggtg
35641 ctataatgaa agtactattt agtccaatag gtaactcaga tccatggagt aatgatagag
35701 atggtgcaat gcttcatatt gtgcgtcatt ataaacctga tgtagtcgtt ttatttttta
35761 ctgaaagcat ttggaatggt aatagaaata tacctggacg taaaaacttc gattgggaaa
35821 acattgtttc aaaagtatca tcgagaacaa aagtggatat aaaagtggat agtattaaat

35881 atgaaaatga ttttgatagc tataaagata tatttcactt ttatataaat gagattagaa
35941 ctaaatattc tgatgcagaa attttattaa atgttacaag cggaacacca caaatggagt
36001 caactttgtg tttagaatat atttctaatc ctcataatat gaaatgtata caagtttcaa
36061 cacctgctcc tattgaagga cctaaacgtt catttgcgaa acttgaaact gtgactgaag
36121 atttaaataa agttaatgct aatgaaaaaa tggcttctaa tagaagtaaa tcaatcaata
36181 ttattagttt tagagaagtg atggtgcgct ctcaaataaa aagtttagtg aataattatg
36241 attatgaagg tgcactgaat ttagtaagtg atcaaaagtc ttttcgtaat ggtaaactat
36301 taaggaaaag attattggaa ttaacaaatc agattaaaac gcatgaagtt tttccagaaa
36361 ttaatgataa gtacagaagt gttgctttaa aaaaatcatt attccattat ttattattaa
36421 atatgcgata taatcgtctt gatgtagctg aaacgttaat aagagtgaaa tctattgctg
36481 agtttatact taaaacatat attgtgggtc attggcctac tctaataatt gaaaaagatg
36541 ataaacctta tttaaatgct gaagataatt tatcttttat ttataaatat aaattactat
36601 tagaaaaaag aagacagaat ttggatgttt caaggattct aggtctacct gcgtttatag
36661 atatacttac agtattagaa cctaactcta aactattaaa agaagttaat gctgtaaacg
36721 atataaatgg tttaagaaat tctatagctc acaatttgga aacattagat ttggataaaa
36781 ataaaaatta taagaaaata atgttatctg ttgaagcaat aaagaatatg ttgcatatct
36841 catttcccga gatagaggaa aaagactata attatttcga aagaaaaaac aaggaattta
36901 gagaattatt atgataaata aaattacagt agaattagac ttaccagata gtattcgttt
36961 tcaatattta ggaagtattt tacatggtgt gttaatggac tatcttccta atgatattgc
37021 tgatcagcta catcatgaat ttgcttatag tccattgaag caaagaatat atcataaaaa
37081 caaaaaagta atttgggaaa ttgtttgtat gtcagatcga ttatttaatg agatagccga
37141 gctattcact tctaaaaaca ggctgttttt aaaatattac caagtttata ttgagattta
37201 ttcttttaat attgagaaag taaacgttca aaatattatg aatcaactct tagaaacaga
37261 ggaattgaat cgatatgtaa gaattaatat acagacacct atgtctttta aatatcagag
37321 taattacatg atttttcctg aggttaaacg tttctttaga agtattatga tacaatttga
37381 tgctttttc gaagaatata aaatgtacga taaagaaaca ttagattttt tggaaaaaaa
37441 tattaatatt gttgactata aattgaaaag tacacgattt aacttagaaa aagttaaaat
37501 cccttcattt acgggagaaa tagtgtttaa aattaaagga cccttacctt ttctacaact
37561 aactcatttt ttattgaagt ttggcgaatt ttctggatca ggtatgaaaa caagcttagg
37621 catggggaaa tatagtataa tataactaga gtatagctga aatttagttg tcaaaaaatg
37681 tgacattctg cacttatagt acaagtgatt tgtcactatt tttgacagta aattttgcct
37741 ttgaactatt gatttgatag gctttaaaga gctatctaaa ttcgataact accccgtaga
37801 agaggggacg agaactcttc taagacgcga tatgattcta attggtcttc attcgataac
37861 tacccccgta gaagagggga cgagaactga tatactcctt taccatgtat taattctgga
37921 ccactattcg ataactaccc cgtagaagag gggacgagga tctcatcttg atgatcccaa
37981 ttggcttggt taaaaagaaa tcgataatcc tcgtagaata gagaaaaagt attaaaaata
38041 tatttgattt gataaaatat tctgcaaaaa agaggaacaa aaaatcaaga tttaaactgt
38101 aattcattat tattgaaatt aatcgacatt caatttctaa ataaacagaa acaaaaataa
38161 ctttcatata aataatttct ttgcgattaa gttggaatta gaaatactaa atacaaataa
38221 cagcgaatga aatgcatatt attgaggaat tggatgtgta gtgcaatttc gagagttact
38281 acttaagaaa ataaaaagaa gaaaacaaac aataagaatt aaatctgaag caagatataa
38341 aagcaaatat ttttgataca accacattag taaatacttt attaattagt atacgactga
38401 aagcgaagta acagctaaca atgctccaga tatagacatt agtgttacga aagaataata
38461 ttaccatcta gaaaatgtaa ttttttaaaa atctgaagat aaagtagatt actttgtgag

38521 ttttcatgtt aagaattctg aacttggaga cgtagtataa atatagctta gtagaaaatg
38581 ccaccggagc tgtagctgag attgactatc aagaattaac taaattaggt tattacccat
38641 caacaaaaat ccaagattta acactgtcta ataatagtgc gaataatgaa gtttaaaagt
38701 aaacaactgg taatagcgaa tcaaactaaa gtaatcaatt acagaattca agtactactc
38761 aagcctcaag caaatattga aactcaaaca caagttaaga ataatacact gcctgaaaca
38821 gatgaacagt ctaatgccga ttgagtccaa taatagcctc tgtattatta gccacacgat
38881 tattatcaat atttaaataa agttaatatt aaataataaa aatccttcaa attatttgtt
38941 gaaaaattaa taactttgaa ggtttttagc aaaatcactg atagggagaa gcatatcata
39001 aat

//

LOCUS CP030670 82501 bp DNA linear BCT 10-OCT-2019
DEFINITION Staphylococcus aureus strain ER00951.3 chromosome, complete genome.
ACCESSION CP030670 REGION: 33176..115676
VERSION CP030670.1 GI:1760398406
DBLINK BioProject: PRJNA470993
BioSample: SAMN09483934
KEYWORDS .
SOURCE Staphylococcus aureus
ORGANISM Staphylococcus aureus
Bacteria; Bacillati; Bacillota; Bacilli; Bacillales;
Staphylococcaceae; Staphylococcus.
REFERENCE 1 (bases 1 to 82501)
AUTHORS Sullivan,M.J., Altman,D.R., Chacko,K., Cliferri,B., Webster,E.,
Deikus,G., Lewis,M., Khan,Z., Beckford,C., Rendo,A., Samaroo,F.,
Sebra,R., Karam-Howlin,R., Southwick,K., Adams,E., Ying,L.,
Kornblum,J., Factor,S., Danesh Yazdi,M., Dingle,T., Hamula,C.,
Bashir,A., Schadt,E., Kasarskis,A., Patel,G., Wallach,F., Gibbs,K.
and van Bakel,H.
TITLE Whole genome sequencing to identify and define MRSA outbreaks
JOURNAL Unpublished
REFERENCE 2 (bases 1 to 82501)
AUTHORS Sullivan,M.J., Altman,D.R., Chacko,K., Cliferri,B., Webster,E.,
Deikus,G., Lewis,M., Khan,Z., Beckford,C., Rendo,A., Samaroo,F.,
Sebra,R., Karam-Howlin,R., Southwick,K., Adams,E., Ying,L.,
Kornblum,J., Factor,S., Danesh Yazdi,M., Dingle,T., Hamula,C.,
Bashir,A., Schadt,E., Kasarskis,A., Patel,G., Wallach,F., Gibbs,K.
and van Bakel,H.
TITLE Direct Submission
JOURNAL Submitted (28-JUN-2018) Genetics and Genomic Sciences, Icahn School
of Medicine, One Gustave L. Levy Place Box 1498, New York, NY
10029, USA
COMMENT Annotation was added by the NCBI Prokaryotic Genome Annotation
Pipeline (released 2013). Information about the Pipeline can be
found here: https://www.ncbi.nlm.nih.gov/genome/annotation_prok/

```
##Genome-Assembly-Data-START##
Assembly Method          :: HGAP v. 2.2.0
Genome Representation    :: Full
Expected Final Version :: Yes
Genome Coverage          :: 293.624506523x
Sequencing Technology    :: PacBio
##Genome-Assembly-Data-END##

##Genome-Annotation-Data-START##
Annotation Provider                 :: NCBI
Annotation Date                     :: 06/29/2018 15:27:13
Annotation Pipeline                 :: NCBI Prokaryotic Genome
                                       Annotation Pipeline
Annotation Method                   :: Best-placed reference protein
                                       set; GeneMarkS+
Annotation Software revision        :: 4.5
Features Annotated                  :: Gene; CDS; rRNA; tRNA; ncRNA;
                                       repeat_region
Genes (total)                       :: 3,103
CDS (total)                         :: 3,020
Genes (coding)                      :: 2,921
CDS (coding)                        :: 2,921
Genes (RNA)                         :: 83
rRNAs                               :: 7, 6, 6 (5S, 16S, 23S)
complete rRNAs                      :: 7, 6, 6 (5S, 16S, 23S)
tRNAs                               :: 60
ncRNAs                              :: 4
Pseudo Genes (total)                :: 99
Pseudo Genes (ambiguous residues) :: 0 of 99
Pseudo Genes (frameshifted)         :: 50 of 99
Pseudo Genes (incomplete)           :: 38 of 99
Pseudo Genes (internal stop)        :: 26 of 99
Pseudo Genes (multiple problems)    :: 15 of 99
##Genome-Annotation-Data-END##
FEATURES             Location/Qualifiers
     source          1..82501
                     /organism="Staphylococcus aureus"
                     /mol_type="genomic DNA"
                     /strain="ER00951.3"
                     /isolation_source="blood"
                     /host="Homo sapiens"
                     /db_xref="taxon:1280"
                     /geo_loc_name="USA:New York City"
```

/lat_lon="40.7903114 N 73.9528738 W"
/collection_date="2015"
/collected_by="Mount Sinai Hospital"
gene 1..480
/locus_tag="DQU53_00305"
CDS 1..480
/locus_tag="DQU53_00305"
/inference="COORDINATES: similar to AA
sequence:RefSeq:WP_020363758.1"
/note="Derived by automated computational analysis using
gene prediction method: Protein Homology."
/codon_start=1
/transl_table=11
/product="23S rRNA
(pseudouridine(1915)-N(3))-methyltransferase RlmH"
/protein_id="QFM29048.1"
/db_xref="GI:1760398432"
/translation="MKITILAVGKLKEKYWKQAIAEYEKRLGPYTKIDIIEVPDEKAP

ENMSDKEIEQVKEKEGQRILAKIKPQSTVITLEIQGKMLSSEGLAQELNQRMTQGQSD

FVFVIGGSNGLHKDVLQRSNYALSFSKMTFPHQMMRVVLIEQVYRAFKIMRGEAYHK"
gene 581..724
/locus_tag="DQU53_00310"
CDS 581..724
/locus_tag="DQU53_00310"
/inference="COORDINATES: similar to AA
sequence:RefSeq:NP_763598.1"
/note="Derived by automated computational analysis using
gene prediction method: Protein Homology."
/codon_start=1
/transl_table=11
/product="restriction endonuclease subunit R"
/protein_id="QFM29049.1"
/db_xref="GI:1760398433"
/translation="MAYQSEYALENEVLQQLEELNYERVNIHNIKLEINEYLKELGVL
KNE"
gene 717..1919
/locus_tag="DQU53_00315"
CDS 717..1919
/locus_tag="DQU53_00315"
/inference="COORDINATES: similar to AA
sequence:RefSeq:NP_763599.1"
/note="Derived by automated computational analysis using

gene prediction method: Protein Homology."
/codon_start=1
/transl_table=11
/product="restriction endonuclease subunit S"
/protein_id="QFM29050.1"
/db_xref="GI:1760398434"

/translation="MNKQTNTPELRFPEFDEEWKKRKLGEVVNYKNGGSFESLVKNHG

VYKLITLKSVNTEGKLCNSGKYIDDKCVETLCNDTLVMILSEQAPGLVGMTAIIPNNN

EYVLNQRVAALVPKQFIDSQFLSKLINRNQKYFSVRSAGTKVKNISKGHVENFNFLSP

NYTEQQKIGNFFSKLDRQIELEEEKLELLEQQKRGYIQKIFSQDLRFKDENGNSYPDW

SIKKIEDISKVNKGFTPNTKNDKYWDELNENWLSIAGMTQKYLYKGNKGITEKGASKH

VKVDKDTLIMSFKLTLGKLAIVKEPIYTNEAICHFVWKESNVNTEYMYYYLNSINIST

FGAQAVKGVTLNNDAINSIIVKLPVIQEQNKIAYFFNKLDKLIEKQSSKVELLKQRKQ
GFLQKMFV"
gene complement(1964..2174)
/locus_tag="DQU53_00320"
/pseudo
CDS complement(1964..2174)
/locus_tag="DQU53_00320"
/inference="COORDINATES: similar to AA
sequence:RefSeq:WP_015365352.1"
/note="frameshifted; incomplete; partial on complete
genome; missing start; Derived by automated computational
analysis using gene prediction method: Protein Homology."
/pseudo
/codon_start=1
/transl_table=11
/product="IS5/IS1182 family transposase"
gene complement(2242..2739)
/locus_tag="DQU53_00325"
CDS complement(2242..2739)
/locus_tag="DQU53_00325"
/inference="COORDINATES: similar to AA
sequence:RefSeq:NP_763601.1"
/note="Derived by automated computational analysis using
gene prediction method: Protein Homology."
/codon_start=1

/transl_table=11
/product="GNAT family N-acetyltransferase"
/protein_id="QFM29051.1"
/db_xref="GI:1760398435"
/translation="MKLRALEYSDLLFVHELNNEYSIMSYWFEEPYESLTELQYLFDK
HLLDESERRFIVEDENQVVGIVELVEINYIHRNCEIQIIIKPEFSGKGYAKFAFEKAI

NYAFDILNMHKIYLYVDTDNKKAVHIYESQGFKTEGLLKEQFYTKGKYKDAYFMSLLK
SEYIL"
gene complement(3018..3146)
/locus_tag="DQU53_00330"
CDS complement(3018..3146)
/locus_tag="DQU53_00330"
/inference="COORDINATES: similar to AA
sequence:RefSeq:NP_763602.1"
/note="Derived by automated computational analysis using
gene prediction method: Protein Homology."
/codon_start=1
/transl_table=11
/product="hypothetical protein"
/protein_id="QFM29052.1"
/db_xref="GI:1760398436"
/translation="MIVKIDWYITSQQLKAKKYLIKSMKISRNNDFQNIINNYLTK"
gene complement(3411..3821)
/locus_tag="DQU53_00335"
CDS complement(3411..3821)
/locus_tag="DQU53_00335"
/inference="COORDINATES: similar to AA
sequence:RefSeq:NP_763603.1"
/note="Derived by automated computational analysis using
gene prediction method: Protein Homology."
/codon_start=1
/transl_table=11
/product="DUF2871 domain-containing protein"
/protein_id="QFM29053.1"
/db_xref="GI:1760398437"
/translation="MKRLMYSSALYTLLGLLSGLFYREMTKAEDFSGFSQLNVTHTHL
LVLGTIMFLIFMIIEGQFKLTTHTKLFNSFFYIYHLGVLVTVAMQFVNGISTIKDLSV
SPAIAGIAGLGHIFLTIAFILFFILLGKRINKNK"
gene complement(4127..4234)
/locus_tag="DQU53_00340"
CDS complement(4127..4234)
/locus_tag="DQU53_00340"
/inference="COORDINATES: similar to AA

sequence:RefSeq:NP_763604.1"
/note="Derived by automated computational analysis using
gene prediction method: Protein Homology."
/codon_start=1
/transl_table=11
/product="hypothetical protein"
/protein_id="QFM29054.1"
/db_xref="GI:1760398438"
/translation="MDIKIKGGLSMNHDTTQSDWRTVASCLDSQITLKL"
gene complement(4219..4722)
/locus_tag="DQU53_00345"
CDS complement(4219..4722)
/locus_tag="DQU53_00345"
/inference="COORDINATES: similar to AA
sequence:RefSeq:WP_012086592.1"
/note="Derived by automated computational analysis using
gene prediction method: Protein Homology."
/codon_start=1
/transl_table=11
/product="DUF1643 domain-containing protein"
/protein_id="QFM29055.1"
/db_xref="GI:1760398439"
/translation="MNTIKSTIHTEAIFSDDEQHRYLLKKIWDEKKPSCTVITMYPHL

DGVLSLDLTTVLILNQLANSEQYGAVYLVNLFSNIKTPENLKHIKEPYDKHTDIHLMK

AISESDTVILAYGAYAKRPVVVERVEQVMEMLKPHKKKVKKLINPVTNEVMHPLNPKA
RQKWTLK"
gene complement(4738..5049)
/locus_tag="DQU53_00350"
CDS complement(4738..5049)
/locus_tag="DQU53_00350"
/inference="COORDINATES: similar to AA
sequence:RefSeq:NP_763608.1"
/note="Derived by automated computational analysis using
gene prediction method: Protein Homology."
/codon_start=1
/transl_table=11
/product="hypothetical protein"
/protein_id="QFM29056.1"
/db_xref="GI:1760398440"
/translation="MKINRYITRGISEQLSLDLQILLWNMVKERDNQSHTDYLHIFRL

QEDENILSITHEQEQPAYKLEYHYTNYVKNQNALPKKVYVIREDDVDAFYYVMLLPEE

Y"
gene complement(5136..5486)
/locus_tag="DQU53_00355"
CDS complement(5136..5486)
/locus_tag="DQU53_00355"
/inference="COORDINATES: similar to AA
sequence:RefSeq:NP_763610.1"
/note="Derived by automated computational analysis using
gene prediction method: Protein Homology."
/codon_start=1
/transl_table=11
/product="hypothetical protein"
/protein_id="QFM29057.1"
/db_xref="GI:1760398441"
/translation="MKTTTQELKQYITRLFQLSNNETWECEALEEAAENILPERFINN
SLLAHLTLNTYTYYNDELHELSIYPFLMYANDQLISIGYLDHFDMDFLYLTDTKNTII
DERHLLKQGENNHE"
gene complement(5987..7615)
/locus_tag="DQU53_00360"
CDS complement(5987..7615)
/locus_tag="DQU53_00360"
/inference="COORDINATES: similar to AA
sequence:RefSeq:NP_763611.1"
/note="Derived by automated computational analysis using
gene prediction method: Protein Homology."
/codon_start=1
/transl_table=11
/product="recombinase family protein"
/protein_id="QFM29058.1"
/db_xref="GI:1760398442"

/translation="MKQLKKKRIGLYARVSTEMQTDGYSIQGQLNQLTEYCQFQGYEV

VNEYTDRGISGKTMQRPELQRMLKDANEGKLDCIMVYKTNRLARNTSDLLNIVEELYK

INVEFFSLTEKIEIASSTGKLMLQILASFAEFERNTIVENVYNGQRQRAIEGYYQGNL

PLGYDKVPDSKKELMINQHEANIVKYIFDSYAKGHGYRKIANALNHKGYVTKKGKPFS

ISSITYIISNPFYIGKIQFAKYRHWSDKRRKGLNEEPIIADGKHAPIIDRNLWDKVQF

KRQESRKKPQVHGKGTNLLTGIVKCPKCGAAMAASNTTNTLKDGTKKRIRYYSCSNFR

NKGSKVCSANSVRADVLEKYVMDQILEIIKSKKVLKQLVEKVNEKSQIDVSSLNHDIA

YKQSQCEEIKAKMHTLTKTIEDSPDLDSVLKPTILSYQDELNQINNQIYQLEQDKQAE

APHYDADMIANVLQAIFKDIDKLEKSQLKSLYLTIIDRIDIRKDEHHKKQFYVTLKLN
NDIIKQLFNNHPLDEVLPSTSSLFLPQTLYITIE"
gene complement(7612..8973)
/locus_tag="DQU53_00365"
CDS complement(7612..8973)
/locus_tag="DQU53_00365"
/inference="COORDINATES: similar to AA
sequence:RefSeq:NP_763612.1"
/note="Derived by automated computational analysis using
gene prediction method: Protein Homology."
/codon_start=1
/transl_table=11
/product="recombinase family protein"
/protein_id="QFM29059.1"
/db_xref="GI:1760398443"

/translation="MEEAIAYVRQSTLKQQSLATQKSLIMDTAKQYGWSNVAFYDDKK

TGRHTKRSGYQKMVEIITSGKCKVLCCYRLNRLHRNLKNAIQFFEICKKHHITIISVN

DGYFDLSKEFDCFRLNILMSLAEMESNNISEQTKNGIREKAKQGKLITTHAPFGYRYR

KGCFVVQEEEAYTVKSVYRWYLQGLGYKKISQHLDSNPNLIPRKPYQVRNILLNPNYC

GRVINKYGTFNDIVPPIIDIDTFEEAQERRIHKQLNRSNSRNKLKRRIKCPYCQSTLT

NLTIKKENYSLRYYVCPKNMNDAYRTCPFKSINAPELESEVLDTCKKYFEEQSFHKRL

NNTILKVLKQQQMKHKETHLTQEQLIEKLAQNRIDVKTFKRLSASCKSEENYSTYSTY
QIDDKLKYVIKNKITLQDIAPFIDCIKITQTKQLQGIYFKNSPLNIVEQSHLITPERN
EVI"
gene complement(9160..9741)
/locus_tag="DQU53_00370"
CDS complement(9160..9741)
/locus_tag="DQU53_00370"
/inference="COORDINATES: similar to AA
sequence:RefSeq:NP_763613.1"
/note="Derived by automated computational analysis using
gene prediction method: Protein Homology."
/codon_start=1
/transl_table=11

/product="hypothetical protein"
/protein_id="QFM31773.1"
/db_xref="GI:1760401157"
/translation="MFKRYAAITTSAKILSHVLSTDIDIAKIRDYFIDYHAHTVSERS

LADKAIEVITQFVAQNRGKFSDDTALKNMIENYGLIALKDDYIEVKIIASVFKNMLIE

HHFQDVNNVVNALRDKGFIESDRDRITTKRAVKDDYGKKQSLVFYHLKLDSKYASIFG
LTKDAEPIKAEINLDNKKILESWKKQNDDLIDF"
gene 9798..10136
/locus_tag="DQU53_00375"
/pseudo
CDS 9798..10136
/locus_tag="DQU53_00375"
/inference="COORDINATES: similar to AA
sequence:RefSeq:WP_000806246.1"
/note="incomplete; partial on complete genome; missing
start; Derived by automated computational analysis using
gene prediction method: Protein Homology."
/pseudo
/codon_start=1
/transl_table=11
/product="recombinase family protein"
gene complement(10248..10607)
/locus_tag="DQU53_00380"
CDS complement(10248..10607)
/locus_tag="DQU53_00380"
/inference="COORDINATES: similar to AA
sequence:RefSeq:NP_763615.1"
/note="Derived by automated computational analysis using
gene prediction method: Protein Homology."
/codon_start=1
/transl_table=11
/product="hypothetical protein"
/protein_id="QFM29060.1"
/db_xref="GI:1760398444"
/translation="MDDRALASALGIGVGGGLAWIMEGFQSGHGFIYSLCAGLIIGLL
FFEMIPESIELGGWVILLGGIAIGLVLFQSIHLLMDKITIITNSHQKDIFVRSGVLLI
ISIAIHNFPVGNIPLFV"
gene 10814..11140
/locus_tag="DQU53_00385"
CDS 10814..11140
/locus_tag="DQU53_00385"
/inference="COORDINATES: similar to AA

sequence:RefSeq:WP_000391495.1"
/note="Derived by automated computational analysis using
gene prediction method: Protein Homology."
/codon_start=1
/transl_table=11
/product="ArsR family transcriptional regulator"
/protein_id="QFM29061.1"
/db_xref="GI:1760398445"
/translation="MEEKKELEEVNNKDLDDETLFVVSQTFKALGDPTRIRILHLLFY

KEYSVNGIAETLHLRQSTVSHQLRFLKNLRLVKFRREGTTLFYSHDDEHTMNMLKQAI
DHACHH"
gene 11243..11341
/locus_tag="DQU53_00390"
CDS 11243..11341
/locus_tag="DQU53_00390"
/inference="COORDINATES: similar to AA
sequence:RefSeq:NP_763616.1"
/note="Derived by automated computational analysis using
gene prediction method: Protein Homology."
/codon_start=1
/transl_table=11
/product="hypothetical protein"
/protein_id="QFM29062.1"
/db_xref="GI:1760398446"
/translation="MDMENKKTEWKALYDISKESEMGVAERVSEYG"
gene 11461..13395
/locus_tag="DQU53_00395"
CDS 11461..13395
/locus_tag="DQU53_00395"
/inference="COORDINATES: similar to AA
sequence:RefSeq:NP_763617.1"
/note="Derived by automated computational analysis using
gene prediction method: Protein Homology."
/codon_start=1
/transl_table=11
/product="heavy metal translocating P-type ATPase"
/protein_id="QFM29063.1"
/db_xref="GI:1760398447"
/translation="MIEMKNIQEQQAHESHSHDHSHDHDHGKMPIISYFIGLVLAIIG
LFLSDANLLIQNILFSIATITAGYHVIILEGIGETVENTKLKGKFTPNSHILMGLAAI
GASLIGSFWEGTLLILIFSGAHFLEDYAEGKSKREITKLLEMNPTTAKLILPDGNTKI

VDVSELKVGDQLQVLNGDQVPIDGIILSGTTSIDESSINGESIPKEKSKGDEVFGSTI

NGTGTFTMEVTKENKDTVFSKILQLVSQNQDNQTKAASIIQKFEPKYVNIVLIAIPLV

MLLAPFLFDWTWSQSVYRGLVLLVAASPCALAAATVSVTLSTTSNLAKKGVLSKGSTY

LSQLADIDAIAFDKTGTLTNGEPKVTNYYFTHSVNEENIIDIIVALEKESNHPLANAI

LEKFEVKNKIDIEVTNQIGKGLTGDYNGKNYRIGKPTSFESASEEYTQFNHDWASEGK

TVVYVAENEEVIGIIALMDIPNEHAKETINYFKKLGIHTTLITGDSEMTGKAVGEQLG

IDEVIANVMPEDKSRIIEEQKEKFGVTAMVGDGVNDAPALVNADVGIAMGGGTDVAVE

VSDLVLMQNNLSKLVQSHKISSNMGRVIRQNIIFSMAVVAFLVVVSLLGLTDITISVI
VHEGSTLVVILNGLRLLRSK"
gene complement(14951..15184)
/locus_tag="DQU53_00400"
CDS complement(14951..15184)
/locus_tag="DQU53_00400"
/inference="COORDINATES: similar to AA
sequence:RefSeq:WP_002454331.1"
/note="Derived by automated computational analysis using
gene prediction method: Protein Homology."
/codon_start=1
/transl_table=11
/product="hypothetical protein"
/protein_id="QFM29064.1"
/db_xref="GI:1760398448"
/translation="MEHINKDKNDEKSTELDERLGINGYYKTDLDLEIEKELLESPNE
SSSSVAKRIRIISMIPLIILLILAFIRIIKTFI"
gene 15430..16128
/locus_tag="DQU53_00405"
/pseudo
CDS 15430..16128
/locus_tag="DQU53_00405"
/inference="COORDINATES: similar to AA
sequence:RefSeq:WP_089205030.1"
/note="incomplete; partial on complete genome; missing
stop; Derived by automated computational analysis using
gene prediction method: Protein Homology."
/pseudo
/codon_start=1
/transl_table=11
/product="YSIRK signal domain/LPXTG anchor domain surface

protein"
gene 16243..19929
/locus_tag="DQU53_00410"
/pseudo
CDS 16243..19929
/locus_tag="DQU53_00410"
/inference="COORDINATES: similar to AA sequence:RefSeq:WP_001208316.1"
/note="incomplete; partial on complete genome; missing stop; Derived by automated computational analysis using gene prediction method: Protein Homology."
/pseudo
/codon_start=1
/transl_table=11
/product="Accumulation-associated protein"
gene 19933..20910
/locus_tag="DQU53_00415"
/pseudo
CDS 19933..20910
/locus_tag="DQU53_00415"
/inference="COORDINATES: similar to AA sequence:RefSeq:NP_765950.1"
/note="incomplete; partial on complete genome; missing start; Derived by automated computational analysis using gene prediction method: Protein Homology."
/pseudo
/codon_start=1
/transl_table=11
/product="YSIRK signal domain/LPXTG anchor domain surface protein"
gene 21034..22650
/locus_tag="DQU53_00420"
CDS 21034..22650
/locus_tag="DQU53_00420"
/inference="COORDINATES: similar to AA sequence:RefSeq:WP_002448877.1"
/note="Derived by automated computational analysis using gene prediction method: Protein Homology."
/codon_start=1
/transl_table=11
/product="poly(glycerol-phosphate) alpha-glucosyltransferase"
/protein_id="QFM29065.1"
/db_xref="GI:1760398449"

/translation="MEFEHKLEKLISEVNNKTEINNYVFLSLGKSSVKAQVKLLKKTN
YLKQDISKLALKFKKKSGEFPEWIKLDIVTSTEKILFKELKKTLINTRRNYVDFGIAF

DSQWNFAVLPEEINANAFVRPDNTTKELFLSEKNINNYLRKYTTNKKAFSSEFYNEKE

VIKFYTQGFFIGDEEVHELYSEGYKKGLRKVNDLNNEIDQLIESSTNFLQNMLLDTGK
YIYGYFPHFDNEIGFYNVLRHSSSTYALIEGLSYLGKSLQPVEKAIDYIILNQLFEIG

DKAYIFDDTEEANEIKLGQNASFIFAVCEYLKHEDNPKFLESAQKVAKGILSMIDEDT

YETTHLLNYPDLSVKEKFRIIYYDGEAALALLRLYQKDENELWLKTVENLMDRFIEKK

YWQYHDHWLGYCTNELVQINPQDKYFEFGIKNVNNYLDYIKNRETTFPTFLEMLMATY

RLVQKAKDTGREELVNNLIDEQYLIDVINIRADYQRVGFFYPEIAMYFKNPSRILGSF
FIKHHGYRVRIDDIEHYLSGYVQYQLAFNR"

gene complement(22810..24321)
/locus_tag="DQU53_00425"

CDS complement(22810..24321)
/locus_tag="DQU53_00425"
/inference="COORDINATES: similar to AA
sequence:RefSeq:WP_000640645.1"
/note="Derived by automated computational analysis using
gene prediction method: Protein Homology."
/codon_start=1
/transl_table=11
/product="glycosyltransferase"
/protein_id="QFM29066.1"
/db_xref="GI:1760398450"
/translation="MIYSVGNRLGNKLTGIEKAMINRHNLFKNHSITSKLIFASWSPR
LHFNASLFQIEDDDILSLYDVLQDSINIKSIHKDWITHWTKECNYTLKFIENTNDIKI

YEKSNYRMYVHFNDSNYQHLDYINHFDVNQRKIRRDLYDSRGFLSCSRTLTTNQKVVC

EHYYTPEGTIKFQKFFNPELENSQSQLIIYNTDSQLKYFNNDQELLAFAIEKLYKNGD

IFLSDKNINTAPIFNNTSETIPVLAVLHSTHVKNIDMVYESDIKNTYKHVFNNLNRYS

GIIVSTKQQQLDISARINNEIPVHTIPVGYIDEHFTNLKRNNHSINNNKIISVARYSP

EKQLNHQIELVSKLIKEFPNIQLHLYGFGKEEEKYKQLITEYNLENNVFLRGFRRNLS

AEIQDAYMSLITSNMEGFNLGLLETITEGIPPVGYNSKYGPSELILNNENGYLINKND
KDELYNRVRNLLLDKTLRDTFSQECIKHSKAFSSKIVMKLWEDRFSTINSSYE"

gene complement(24344..26002)
/locus_tag="DQU53_00430"
CDS complement(24344..26002)
/locus_tag="DQU53_00430"
/inference="COORDINATES: similar to AA
sequence:RefSeq:WP_001621813.1"
/note="Derived by automated computational analysis using
gene prediction method: Protein Homology."
/codon_start=1
/transl_table=11
/product="glutamate ligase"
/protein_id="QFM29067.1"
/db_xref="GI:1760398451"
/translation="MLISAGLKDYYPLQNRFNNNIRSAVYLLLCKMIRQPNFAVLEVS
LNALNAVGNSSYLIKPNIAIVTGIGAAHMSTFKDILNIVEVKASIFDGLTPEGVAIIN
KDTLHSDILIERAKQNTSNVITYSTHDSSATICPKSIQYSKGYTVITIDFNGQKYTYR
INSISDGMVENSLATFATLSHLDIPLERALENLSTFKPFEKVLNLKEVETPNYKVNLI

DDTHNASLPAMINAIKAFNTQTKFFKGNKIIAIGQISDLGKHSKSLHLQLVDVLENSN

ADYILCMDDALKSVVIGVKSKNITWYSNRHLLEKDLLYLNKPDSLTLLKSSAGGTEFP

KLAKELPEKLNKYNINNSNTSLFDGQSLNGRSYMIIDENYNVIESHNREHSGTIEGLG

PIFNYLKAIDDNVSEDTIFIANWATNNKLYYEGKETTTYELMKAMLNSPMYTPSYELS

KYLFENGPKRDEYINSKIEHLSLSNSVAINLTGRHTMRERQNFTVDDLFKILKAYKNT
LFKFTNEIIIGRKYNSGIIKDKDKFIIFTSYPNLNEIKNKLNNK"
gene complement(25996..26394)
/locus_tag="DQU53_00435"
CDS complement(25996..26394)
/locus_tag="DQU53_00435"
/inference="COORDINATES: similar to AA
sequence:RefSeq:WP_001621810.1"
/note="Derived by automated computational analysis using
gene prediction method: Protein Homology."
/codon_start=1
/transl_table=11
/product="hypothetical protein"
/protein_id="QFM29068.1"
/db_xref="GI:1760398452"
/translation="MSLTIKELHNILGGNLLDAEGKEDNVINNFENWFPFIHNKSTAY

ISPNKHTWRRFGRDANAKEGNEYIKKDKEDLGLIITETYVPDMKFKTPQLVINDSANT

LTKLAEEMRLQYKHPVVAITGSMGKVQLEC"
gene 27400..27705
/locus_tag="DQU53_00440"
CDS 27400..27705
/locus_tag="DQU53_00440"
/inference="COORDINATES: similar to AA
sequence:RefSeq:WP_000677988.1"
/note="Derived by automated computational analysis using
gene prediction method: Protein Homology."
/codon_start=1
/transl_table=11
/product="hypothetical protein"
/protein_id="QFM29069.1"
/db_xref="GI:1760398453"
/translation="MKGNIQCPRLIKGSLLDFVLETVDVFVNGSKNDCELVNFNYKEG
KVVSRYSTKFESNPKLRKKQLISTVKVVRYVIFDFEKILWGIGKVLLKFIILRQCLL"
gene 27904..28767
/locus_tag="DQU53_00445"
CDS 27904..28767
/locus_tag="DQU53_00445"
/inference="COORDINATES: similar to AA
sequence:RefSeq:WP_020220483.1"
/note="Derived by automated computational analysis using
gene prediction method: Protein Homology."
/codon_start=1
/transl_table=11
/product="hypothetical protein"
/protein_id="QFM29070.1"
/db_xref="GI:1760398454"

/translation="MKHINQFSESTLAQLEKDQQHVFYVYCLMDPRNDECFYVGKGKG
NRIFKHKQDAQKKVLYEDIFIEENKDNLKFNRINEICSNDLNVLGYIISYGLTESEAF

SAENVLINFLNLTNKTTLTNMINGHGSKAYLVEDLENEFGYDSINLENINTNELILAV

KIRDAFLLDKDESKEYPINESKRDRNNLKSRTLGSWIIGKDKIHKIKYIIGINTGANN

AVVSAYEVSFEQAESIETNNGRMRYAFIGLSERDATLKKLNLYKKALPDLRFGSGSAT
AYINNGTMKVD"
gene 28875..30350
/locus_tag="DQU53_00450"
CDS 28875..30350
/locus_tag="DQU53_00450"
/inference="COORDINATES: similar to AA

sequence:RefSeq:WP_001138664.1"
/note="Derived by automated computational analysis using
gene prediction method: Protein Homology."
/codon_start=1
/transl_table=11
/product="hypothetical protein"
/protein_id="QFM29071.1"
/db_xref="GI:1760398455"
/translation="MPSDDIVKKLYSKEDIRLELGLTPHKFNKKMETIAKLFKIDMKI

FHNYKGQDKNNQYTFNGVAKELIKVLLKSVDYYPVDINSKKFKQNGKSKKEMIENIDN

SSYMKYIYQLMKSINEIQYKRLIADIHMKDVYQNTKAWLNNGESINKKEQELYQYMTI

LPLHKRVELQNEVLKSIDETIFQFLAKEHRNNQIEENNELEAYTKAIKEGRNPKNDYE

LNHLLYKKNQLPLDSLIEDIWDYEETEYTELDWLIADMLKRSQRADSNFIESLEKKNK

LRKNIHKDSIKSISKLIDHELVKNNRKWDVAEFYKNQQFWNNSTLRRSSQNFGILYYK

KNRLHTMNQSNIIKKIKIIEANIESANKQPSYLDFFLQAKFDLERLESELMKYSINPK

YFNKINSDYVRYAIEDVHNAIIKIDRYISNDKVKLNYSNVQMVVRNEITEQGSYFVTQ
ALNTLSKVEESGYSFDNFMVGPFVEDFKKAIKNRRFGSKKD"
gene 30576..31676
/locus_tag="DQU53_00455"
CDS 30576..31676
/locus_tag="DQU53_00455"
/inference="COORDINATES: similar to AA
sequence:RefSeq:WP_002511801.1"
/note="Derived by automated computational analysis using
gene prediction method: Protein Homology."
/codon_start=1
/transl_table=11
/product="hypothetical protein"
/protein_id="QFM29072.1"
/db_xref="GI:1760398456"

/translation="METRDKLMSLTQSDKTQQWLMDKSSNQDDIQQLQQQFSQQLDQQ

YNALLADEKAKLDQYVEVHQGLESLKEEIESEPITLNIDKLPDIKATMLERAKNDEHS

DKIEKLFDRLEQALNGTNRLYTQLSLIGTRTHRITTKNFNLQGLPKAVQHTILPSKFK

KVYTVDFKSFEPSVAAYMTQDSKLIDLLNQKDGLYDALLSELGLSDELRVFVKRAFIG

SFLFGGNFKNPKFKLNQYVSEVQWLDAVSQFTKVIELKKHVEKSNSMPMPYSIEHDMS

AFQGSSIMAIYVQTVASYIFKHILLKVYKAQCDQKTFKIIVPIHDAIMIECEDEEIAQ
NVGQLMKDTANQLFNGEFAHVTVEEIGGVDHE"
gene 31669..32040
/locus_tag="DQU53_00460"
CDS 31669..32040
/locus_tag="DQU53_00460"
/inference="COORDINATES: similar to AA
sequence:RefSeq:WP_001058156.1"
/note="Derived by automated computational analysis using
gene prediction method: Protein Homology."
/codon_start=1
/transl_table=11
/product="hypothetical protein"
/protein_id="QFM29073.1"
/db_xref="GI:1760398457"
/translation="MNNDRGQSLHIPSSTPIKENNIYVATLHSVIQTDFSGEIKHQFT

YEIEVNNQIVYANRNIPTKPSANQLSIHDWLKRHSNYSASHENYEPYIDQKHLILLGQ
YNGNYYVQDVASLDAFGGVLS"
gene 32037..33680
/locus_tag="DQU53_00465"
CDS 32037..33680
/locus_tag="DQU53_00465"
/inference="COORDINATES: similar to AA
sequence:RefSeq:WP_011302047.1"
/note="Derived by automated computational analysis using
gene prediction method: Protein Homology."
/codon_start=1
/transl_table=11
/product="DNA primase"
/protein_id="QFM29074.1"
/db_xref="GI:1760398458"
/translation="MNHILQMLSKLLSVAKEAIDRQGLIAILTIPVNNNDEIEETAQG

ETVVYNELIDQLRLNIPKDTDYQPNIYSYFGIKKNPNDTVLMEMMIKVFHIKRFNSELF

IFKVNGWQKINGDELQGLISKMIQVLLVDYKPSLSTLKNVVDGLQKSTDVEELVENER

YIGCGENMFDLNTFQVVKNSIDIFPKTRLNLSLSTNDVITDKIPPYFKQYMLQLANYD

DDLQYFLFQHTAVLLTADTKYRRGLILYGGAKNGKSVYIELVKSFFYSKDIVSKPLNE

LEGRFDKESLIDKSLMASHEIGQSRIQEKIVNDFKKLLSVESMHVDRKGKTQVEVILD
LKLIFSTNAILNFPPEHAKALERRINIIPCEYYVEKADTSLIDKLQSEKKEIFLYLMY

VYQQIVKADIEYLENSRVTEITHDWLNFGYEFVSSRSVSNANQKACINLLRKLIEIKS

GSRIKVSELNKVINEEIKVSSQVIKQLIQANFDTQTKLYNGYDYWIDLGWKEANKKEI
HDISEKDNIISLDKNENITDDEALDEENLDFDWEDFDDE"
gene 33906..35582
/locus_tag="DQU53_00470"
CDS 33906..35582
/locus_tag="DQU53_00470"
/inference="COORDINATES: similar to AA
sequence:RefSeq:WP_000676594.1"
/note="Derived by automated computational analysis using
gene prediction method: Protein Homology."
/codon_start=1
/transl_table=11
/product="recombinase family protein"
/protein_id="QFM29075.1"
/db_xref="GI:1760398459"
/translation="MKGKIALYSRVSTSEQSEHGYSIHEQEQVLIKEVVKNFPGYDYE

TYTDSGISGKNIEGRPAMKRLLQDVKDNKIEMVLSWKLNRISRSMRDVFNIIHEFKEH

DVGYKSISENIDTSNASGEVLVTMFGLIGSIERQTLISNVKLSMNAKARSGEAITGRV

LGYKLSLNPLTQKNDLVIDENEANIVREIFDLYLNHNKGLKAITTVLNQKGYRTINQK

PFSVYGVKYILNNPVYKGYVRFNNHQNWAVQRRSGKSDKNDVILVKGKHEAIISEEVF

DKVHEKLASKSFKPGRPIGGDFYLRGLIKCPECGNNMVCRRTYYKTKKSKERTIKRYY

ICSLFNRSGSSACHSNSINAEVVERVINVHLNRILSQPDIIKQIASNVIEELKQKHSN

QTEIKYDIDSLEKQKAKLKTQQERLLELFLDDQMDSEMLKAKQSQMNQQLEVLDQQIK

EAQQANQSQDEIPNFDKLKGRLILMITRFSVYLRKATPEAKNQLMKMLIDSIEITTDK
QVKLVRYKIDESLIPQSLKKDWGSFFMPKFQFEIYGQNDYFIDQITTFTT"
gene 35688..36026
/locus_tag="DQU53_00475"
CDS 35688..36026
/locus_tag="DQU53_00475"

/inference="COORDINATES: similar to AA
sequence:RefSeq:WP_000171559.1"
/note="Derived by automated computational analysis using
gene prediction method: Protein Homology."
/codon_start=1
/transl_table=11
/product="hypothetical protein"
/protein_id="QFM29076.1"
/db_xref="GI:1760398460"
/translation="MTLEQQLKHYITNLFNLPRDEVWHCESIEEIADDILPNQYVRLG

PLSNKTLQTNTYYSDTLHESNIYPFILYYQKQLIAIGYIDENHDMDFLYLHNTIMPLL
DQRYLLTGGQ"
gene 36122..36433
/locus_tag="DQU53_00480"
CDS 36122..36433
/locus_tag="DQU53_00480"
/inference="COORDINATES: similar to AA
sequence:RefSeq:WP_002511765.1"
/note="Derived by automated computational analysis using
gene prediction method: Protein Homology."
/codon_start=1
/transl_table=11
/product="hypothetical protein"
/protein_id="QFM29077.1"
/db_xref="GI:1760398461"

/translation="MNRYITRGIANNLPNILQHQLWQLVSEREQEQTKDNTSVDYFHI

FQFNKHRNQLYIKHKQERPEYAKIHKANYSKAININKVYIIREDDVDLSYYVMLLPEE
Y"
gene 36449..36955
/locus_tag="DQU53_00485"
CDS 36449..36955
/locus_tag="DQU53_00485"
/inference="COORDINATES: similar to AA
sequence:RefSeq:WP_000447786.1"
/note="Derived by automated computational analysis using
gene prediction method: Protein Homology."
/codon_start=1
/transl_table=11
/product="DUF1643 domain-containing protein"
/protein_id="QFM29078.1"
/db_xref="GI:1760398462"

/translation="METIKSTLKTEAIFSDDKQHRYLLKKTWNSEKQSITIITMYPHY

DGILNIDLTTQLIMNKVSEMDAFGSINFVNLYSNITTPINLKHLENAYDKHTDIQIMK

AVKESDEVILAWGAYAKKPGVEARVNEVLEMLKPHKKKVKRLMNPETNEIMHPLNSKA
RQKWILKV"
gene complement(37104..37778)
/locus_tag="DQU53_00490"
CDS complement(37104..37778)
/locus_tag="DQU53_00490"
/inference="COORDINATES: similar to AA
sequence:RefSeq:WP_001106057.1"
/note="Derived by automated computational analysis using
gene prediction method: Protein Homology."
/codon_start=1
/transl_table=11
/product="IS6-like element IS257 family transposase"
/protein_id="QFM29079.1"
/db_xref="GI:1760398463"
/translation="MNYFRYKQFNKDVITVAVGYYLRYTLSYRDISEILRERGVNVHH

STVYRWVQEYAPILYQIWKKKHKKAYYKWRIDETYIKIKGKWSYLYRAIDAEGHTLDI

WLRKQRDNHSAYAFIKRLIKQFGKPQKVITDQAPSTKVAMAKVIKAFKLKPDCHCTSK
YLNNLIEQDHRHIKVRKTRYQSINTAKNTLKGIECIYALYKKNRRSLQICGFSPCHEI
SIMLAS"
gene 37814..38203
/locus_tag="DQU53_00495"
CDS 37814..38203
/locus_tag="DQU53_00495"
/inference="COORDINATES: similar to AA
sequence:RefSeq:WP_001797647.1"
/note="Derived by automated computational analysis using
gene prediction method: Protein Homology."
/codon_start=1
/transl_table=11
/product="nucleoid-structuring protein H-NS"
/protein_id="QFM29080.1"
/db_xref="GI:1760398464"
/translation="MQLCNRTVLWNRDVGNIYTGSLYLSLISLLQNHTFQPEEKVCLF

SYGSGAVGEIFSGSIVKGYDKALDKEKHLNMLESREQLSVEEYETFFNRFDNQEFDFE
RELTQDPYSKVYLYSIEDHIRTYKIEK"
gene 38301..38531

/locus_tag="DQU53_00500"
CDS 38301..38531
/locus_tag="DQU53_00500"
/inference="COORDINATES: similar to AA
sequence:RefSeq:WP_001797648.1"
/note="Derived by automated computational analysis using
gene prediction method: Protein Homology."
/codon_start=1
/transl_table=11
/product="hypothetical protein"
/protein_id="QFM29081.1"
/db_xref="GI:1760398465"
/translation="MLIFLSYDFVRACLPRGMARAISLSHILFPQASALTKSVVIFIF
IRILTEIILIRGIVKAILSKIADKRFVKSSSK"
gene 39120..39863
/locus_tag="DQU53_00505"
CDS 39120..39863
/locus_tag="DQU53_00505"
/inference="COORDINATES: similar to AA
sequence:RefSeq:WP_001830227.1"
/note="Derived by automated computational analysis using
gene prediction method: Protein Homology."
/codon_start=1
/transl_table=11
/product="glycerophosphoryl diester phosphodiesterase"
/protein_id="QFM29082.1"
/db_xref="GI:1760398466"
/translation="MLTVYGHRGLPSKAPENTIASFKAASEVEGINWLELDVAITKDE

QLIIIHDDYLERTTNMSGEITELNYDEIKDASAGSWFGEKFKDEHLPTFDDVVKIANE

YNMNLNVELKGITGPNGLALSKSMVKQVEEQLTNLNQNQEVLISSFNVVLVKLAEEIM

PQYNRAVIFHTTSFREDWRTLLDYCNAKIVNTEDAKLTKAKVKMVKEAGYELNVWTVN
KPARANQLANWGVDGIFTDNADKMVHLSQ"
gene 39960..40388
/locus_tag="DQU53_00510"
CDS 39960..40388
/locus_tag="DQU53_00510"
/inference="COORDINATES: similar to AA
sequence:RefSeq:WP_000872606.1"
/note="Derived by automated computational analysis using
gene prediction method: Protein Homology."
/codon_start=1

/transl_table=11
/product="hypothetical protein"
/protein_id="QFM29083.1"
/db_xref="GI:1760398467"
/translation="MKYDDFIVGETFKTKSLHITEEEIIQFATTFDPQYMHIDKEKAE

QSRFKGIIASGMHTLSISFKLWVEEGKYGEEVVAGTQMNNVKFIKPVYPGNTLYVIAE
ITNKKSIKKENGLVTVSLSTYNENEEIVFKGEVTALINNS"
gene complement(40434..42443)
/locus_tag="DQU53_00515"
CDS complement(40434..42443)
/locus_tag="DQU53_00515"
/inference="COORDINATES: similar to AA
sequence:RefSeq:WP_001801873.1"
/note="Derived by automated computational analysis using
gene prediction method: Protein Homology."
/codon_start=1
/transl_table=11
/product="PBP2a family beta-lactam-resistant peptidoglycan
transpeptidase MecA"
/protein_id="QFM29084.1"
/db_xref="GI:1760398468"
/translation="MMKKIKIVPLILIVVVVGFGIYFYASKDKEINNTIDAIEDKNFK

QVYKDSSYISKSDNGEVEMTERPIKIYNSLGVKDINIQDRKIKKVSKNKKRVDAQYKI

KTNYGNIDRNVQFNFVKEDGMWKLDWDHSVIIPGMQKDQSIHIENLKSERGKILDRNN

VELANTGTAYEIGIVPKNVSKKDYKAIAKELSISEDYIKQQMDQNWVQDDTFVPLKTV

KKMDEYLSDFAKKFHLTTNETESRNYPLGKATSHLLGYVGPINSEELKQKEYKGYKDD

AVIGKKGLEKLYDKKLQHEDGYRVTIVDDNSNTIAHTLIEKKKKDGKDIQLTIDAKVQ

KSIYNNMKNDYGSGTAIHPQTGELLALVSTPSYDVYPFMYGMSNEEYNKLTEDKKEPL

LNKFQITTSPGSTQKILTAMIGLNNKTLDDKTSYKIDGKGWQKDKSWGGYNVTRYEVV

NGNIDLKQAIESSDNIFFARVALELGSKKFEKGMKKLGVGEDIPSDYPFYNAQISNKN

LDNEILLADSGYGQGEILINPVQILSIYSALENNGNINAPHLLKDTKNKVWKKNIISK

ENINLLTDGMQQVVNKTHKEDIYRSYANLIGKSGTAELKMKQGETGRQIGWFISYDKD
NPNMMMAINVKDVQDKGMASYNAKISGKVYDELYENGNKKYDIDE"

gene 42540..44297
/locus_tag="DQU53_00520"
CDS 42540..44297
/locus_tag="DQU53_00520"
/inference="COORDINATES: similar to AA
sequence:RefSeq:WP_000952924.1"
/note="Derived by automated computational analysis using
gene prediction method: Protein Homology."
/codon_start=1
/transl_table=11
/product="beta-lactam sensor/signal transducer MecR1"
/protein_id="QFM29085.1"
/db_xref="GI:1760398469"
/translation="MLSSFLMLSIISSLLTICVIFLVRMLYIKYTQNIMSHKIWLLVL

VSTLIPLIPFYKISNFTFSKDMMNRNVSDTTSSVSHMLDGQQSSVTKDLAINVNQFET

SNITYMILLIWVFGSLLCLFYMIKAFRQIDVIKSSSLESSYLNERLKVCQSKMQFYKK

HITISYSSNIDNPMVFGLVKSQIVLPTVVVETMNDKEIEYIILHELSHVKSHDLIFNQ

LYVVFKMIFWFNPALYISKTMMDNDCEKVCDRNVLKILNRHEHIRYGESILKCSILKS

QHINNVAAQYLLGFNSNIKERVKYIALYDSMPKPNRNKRIVAYIVCSISLLIQAPLLS

AHVQQDKYETNVSYKKLNQLAPYFKGFDGSFVLYNEREQAYSIYNEPESKQRYSPNST

YKIYLALMAFDQNLLSLNHTEQQWDKHQYPFKEWNQDQNLNSSMKYSVNWYYENLNKH

LRQDEVKSYLDLIEYGNEEISGNENYWNESSLKISAIEQVNLLKNMKQHNMHFDNKAI

EKVENSMTLKQKDTYKYVGKTGTGIVNHKEANGWFVGYVETKDNTYYFATHLKGEDNA
NGEKAQQISERILKEMELI"
gene 44297..44668
/gene="mecI"
/locus_tag="DQU53_00525"
CDS 44297..44668
/gene="mecI"
/locus_tag="DQU53_00525"
/inference="COORDINATES: similar to AA
sequence:RefSeq:WP_000369216.1"
/note="Derived by automated computational analysis using
gene prediction method: Protein Homology."
/codon_start=1

/transl_table=11
/product="mecA-type methicillin resistance repressor MecI"
/protein_id="QFM29086.1"
/db_xref="GI:1760398470"

/translation="MDNKTYEISSAEWEVMNIIWMKKYASANNIIEEIQMQKDWSPKT
IRTLITRLYKKGFIDRKKDNKIFQYYSLVEESDIKYKTSKNFINKVYKGGFNSLVLNF
VEKEDLSQDEIEELRNILNKK"
gene complement(44753..44821)
/gene="psm-mec"
/locus_tag="DQU53_00530"
CDS complement(44753..44821)
/gene="psm-mec"
/locus_tag="DQU53_00530"
/inference="COORDINATES: similar to AA
sequence:RefSeq:WP_014532405.1"
/note="Derived by automated computational analysis using
gene prediction method: Protein Homology."
/codon_start=1
/transl_table=11
/product="phenol-soluble modulin PSM-mec"
/protein_id="QFM31774.1"
/db_xref="GI:1760401158"
/translation="MDFTGVITSIIDLIKTCIQAFG"
gene 45155..46285
/locus_tag="DQU53_00535"
CDS 45155..46285
/locus_tag="DQU53_00535"
/inference="COORDINATES: similar to AA
sequence:RefSeq:WP_010922808.1"
/note="Derived by automated computational analysis using
gene prediction method: Protein Homology."
/codon_start=1
/transl_table=11
/product="ROK family transcriptional regulator"
/protein_id="QFM29087.1"
/db_xref="GI:1760398471"
/translation="MNDNEKRVLREIYNHHNISRTQISKNLEINKATISSILNKLKYK

SLVNEVGEGDSTKSGGRKPILLKVNHLYGYFISLDLTYSSVEVMYNYFDGNVIKHESY

DLPDEKVSSILSIIKKHIDIQEKLDTYNGLLGVSVSIHGVVDNEQHVTYLPFHETEGI

SIAKKIKEITNVPVVVENEANLSALYERNFNHNLSYNNLIALSIHKGIGAGLIINNQL

YRGANGEAGEIGKTLVSKVSDNVEIFHKIEDIFSQEALLHNLSNQLNEKMTLSKLIQF

YNEKNPVVVEEMEQFINKIAVLIHNLNTQFNPNAIYINCPLFNEMPEILEAIKNQFKQ
YSRNEIQIKLTSNVKFATLLGGTLAIIQKVLQINDIYLDIKA"
gene complement(46399..46704)
/locus_tag="DQU53_00540"
CDS complement(46399..46704)
/locus_tag="DQU53_00540"
/inference="COORDINATES: similar to AA
sequence:RefSeq:WP_001136661.1"
/note="Derived by automated computational analysis using
gene prediction method: Protein Homology."
/codon_start=1
/transl_table=11
/product="rhodanese-like domain-containing protein"
/protein_id="QFM29088.1"
/db_xref="GI:1760398472"

/translation="MPQTNIVTSSIHSSEMTGEEHNVLDVRNDDEWRKGHLKQAIHIP

HGKLLNEDIPFDKNDLIYVHCQSGVRSSIAVGILEYKGFTNIVNVRGGYQHLPAFLK"
gene complement(46795..47733)
/locus_tag="DQU53_00545"
CDS complement(46795..47733)
/locus_tag="DQU53_00545"
/inference="COORDINATES: similar to AA
sequence:RefSeq:WP_000465480.1"
/note="Derived by automated computational analysis using
gene prediction method: Protein Homology."
/codon_start=1
/transl_table=11
/product="MBL fold metallo-hydrolase"
/protein_id="QFM29089.1"
/db_xref="GI:1760398473"
/translation="MFFKQFYEDNFSQASYLIGCQRTGEAMIIDPIRDLTKYLKVADK

EGFTITKAAETHIHADFASGIRDVANKLNASIYVSDEGKDEFNYKNMPKQTHFVKHQD
IIYLGKIKLEVLHTPGHPPESISFLLTDEGGGSSIPMGLFSGDFIFVGDVGRPDLLEK

AVNVEGSTKIGAKQMYQSIQFAATLPDYIQIWPGHGAGSPCGKALGSLPTTTLGYEKI

NNWAFNVKDETSFIETLTLNQPAPPHHFSQMKKINQFGMQMYQPYNVYPSSSNTQTAF
DLRSKEAFHGGHMHGTINIPFNKTFINQIGWYLDYD"

gene complement(47766..48830)
/locus_tag="DQU53_00550"
CDS complement(47766..48830)
/locus_tag="DQU53_00550"
/inference="COORDINATES: similar to AA
sequence:RefSeq:YP_498641.1"
/note="Derived by automated computational analysis using
gene prediction method: Protein Homology."
/codon_start=1
/transl_table=11
/product="dihydroneopterin aldolase"
/protein_id="QFM29090.1"
/db_xref="GI:1760398474"
/translation="MEQYKEVHINSLNKEELEKLAKMGQLIDVRTKEEYELGHINGST

LHPVEEIKSFNKNSNKTYYIHCKSGNRSTKACDYLAKQGYDVVNLKGGYKDYEAKNFN
SAPLIEKDIEIKENRKQFDFRGLQCPGPIVNISKEINNISTGEQIEVTVTDPGFNSDI

KSWAKQTGNTLVNLTEEANVINAIIQKEKPKEFEINDTATGTTIVLFSGELDKAVAAM

IIANGAKAAGKDVTIFFTFWGLNALKKAQSTRVKKKGISKMFDLMLPKDPIHMPLSKM

NMFGLGNIMMRYVMNKKNVDSLYSLIDQAIDQDIKLIACTMSMDVMGISKEELRDEVD
YGGVGTYIGHTEQANHNLFI"
gene 48966..49226
/locus_tag="DQU53_00555"
CDS 48966..49226
/locus_tag="DQU53_00555"
/inference="COORDINATES: similar to AA
sequence:RefSeq:YP_498640.1"
/note="Derived by automated computational analysis using
gene prediction method: Protein Homology."
/codon_start=1
/transl_table=11
/product="metal-sensitive transcriptional regulator"
/protein_id="QFM29091.1"
/db_xref="GI:1760398475"

/translation="MTYDKKMINRINRIQGQLNGVVKMMEEEKDCKDIITQLSASKGS
IQRLMGIIISENLIECVKTAEENNESSQELINEAVNLLVKSK"
gene 49226..49870
/locus_tag="DQU53_00560"
CDS 49226..49870
/locus_tag="DQU53_00560"

/inference="COORDINATES: similar to AA
sequence:RefSeq:WP_000350222.1"
/note="Derived by automated computational analysis using
gene prediction method: Protein Homology."
/codon_start=1
/transl_table=11
/product="sulfite exporter TauE/SafE family protein"
/protein_id="QFM29092.1"
/db_xref="GI:1760398476"
/translation="MDIANMTIMLLIGVLGGFISGLIGIGGAIIIYPAILILPPLIGI

PAYSAYIASGLTSSQVFFSTLSGSLNARKQPAFSPKLVIYMGGGMLIGSMLGAILASL
FNATFVNTVYVIIAILALILMFIKVKPTTQETKSKPLLFIIVGFGIGVISGIVGAGGA
FIIIPVLLALFKLPMNTVVNNSIAIAFISSVGAFFIKLMQGYIPVESAIFLIIE"
gene complement(49855..49980)
/locus_tag="DQU53_00565"
/pseudo
CDS complement(49855..49980)
/locus_tag="DQU53_00565"
/inference="COORDINATES: similar to AA
sequence:RefSeq:WP_016911187.1"
/note="incomplete; partial on complete genome; missing
start and stop; Derived by automated computational
analysis using gene prediction method: Protein Homology."
/pseudo
/codon_start=1
/transl_table=11
/product="DNA repair protein RadC"
gene complement(50209..50871)
/locus_tag="DQU53_00570"
CDS complement(50209..50871)
/locus_tag="DQU53_00570"
/inference="COORDINATES: similar to AA
sequence:RefSeq:WP_016922249.1"
/note="Derived by automated computational analysis using
gene prediction method: Protein Homology."
/codon_start=1
/transl_table=11
/product="methyltransferase domain-containing protein"
/protein_id="QFM29093.1"
/db_xref="GI:1760398477"

/translation="MNTIESQFDKVAEDYDFVNELLNDYSFFVSNMSPKKGRALDIGC

GSGLLVEKLASYYDEVVGIDISNQMLDLAKSKRQLTNTVYLNMNAEQLNFNEKFDFIV

SRTTFHHLDDIASVIQQMKELLNEEGRIVILDNVSEVETPPTYVYKLGAIQEFLPHCF

KFGIKNAIRIYNHNTSKSWLEHLASDKYLSEQNYYDLYEKLLPGCQFHKMGWAMGVVW

TK"

gene complement(50921..51022)
/locus_tag="DQU53_00575"
/pseudo

CDS complement(50921..51022)
/locus_tag="DQU53_00575"
/inference="COORDINATES: similar to AA
sequence:RefSeq:WP_017466700.1"
/note="incomplete; partial on complete genome; missing
start; Derived by automated computational analysis using
gene prediction method: Protein Homology."
/pseudo
/codon_start=1
/transl_table=11
/product="zeta toxin"

gene 51190..51319
/locus_tag="DQU53_00580"
/pseudo

CDS 51190..51319
/locus_tag="DQU53_00580"
/inference="COORDINATES: similar to AA
sequence:RefSeq:WP_000554414.1"
/note="frameshifted; Derived by automated computational
analysis using gene prediction method: Protein Homology."
/pseudo
/codon_start=1
/transl_table=11
/product="hypothetical protein"

gene 51367..52098
/locus_tag="DQU53_00585"

CDS 51367..52098
/locus_tag="DQU53_00585"
/inference="COORDINATES: similar to AA
sequence:RefSeq:WP_001072197.1"
/note="Derived by automated computational analysis using
gene prediction method: Protein Homology."
/codon_start=1
/transl_table=11
/product="23S rRNA (adenine(2058)-N(6))-methyltransferase

Erm(A)"
/protein_id="QFM29094.1"
/db_xref="GI:1760398478"
/translation="MNQKNPKDTQNFITSKKHVKEILNHTNISKQDNVIEIGSGKGHF
TKELVKMSRSVTAIEIDGGLCQVTKEAVNPSENIKVIQTDILKFSFPKHINYKIYGNI

PYNISTDIVKRITFESQAKYSYLIVEKGFAKRLQNLQRALGLLLMVEMDIKMLKKVPP

LYFHPKPSVDSVLIVLERHQPLISKKDYKKYRSFVYKWVNREYRVLFTKNQFRQALKH
ANVTNINKLSKEQFLSIFNSYKLFH"
gene complement(52224..53006)
/locus_tag="DQU53_00590"
CDS complement(52224..53006)
/locus_tag="DQU53_00590"
/inference="COORDINATES: similar to AA
sequence:RefSeq:WP_000067268.1"
/note="Derived by automated computational analysis using
gene prediction method: Protein Homology."
/codon_start=1
/transl_table=11
/product="aminoglycoside nucleotidyltransferase ANT(9)-Ia"
/protein_id="QFM29095.1"
/db_xref="GI:1760398479"
/translation="MSNLINGKIPNQAIQTLKIVKDLFGSSIVGVYLFGSAVNGGLRI

NSDVDVLVVVNHSLPQLTRKKLTERLMTISGKIGNTDSVRPLEVTVINRSEVVPWQYP

PKREFIYGEWLRGEFENGQIQEPSYDPDLAIVLAQARKNSISLFGPDSSSILVSVPLT

DIRRAIKDSLPELIEGIKGDERNVILTLARMWQTVTTGEITSKDVAAEWAIPLLPKEH
VTLLDIARKGYRGECDDKWEGLYSKVKALVKYMKNSIETSLN"
gene complement(53157..53534)
/locus_tag="DQU53_00595"
CDS complement(53157..53534)
/locus_tag="DQU53_00595"
/inference="COORDINATES: similar to AA
sequence:RefSeq:WP_000361059.1"
/note="Derived by automated computational analysis using
gene prediction method: Protein Homology."
/codon_start=1
/transl_table=11
/product="transposase"
/protein_id="QFM29096.1"
/db_xref="GI:1760398480"

/translation="MDKQVRNTTEIVRLAKQKSKKTREKVDKAISKFSIEGKVINFNS

IAKEANVSKSWLYKEHDIRQRIESLRERQITANVVSKPKKSSRSEEILIKTLKRRVME
LEKENKKLQNQIQKLYGDLYNKE"
gene complement(53541..55433)
/locus_tag="DQU53_00600"
CDS complement(53541..55433)
/locus_tag="DQU53_00600"
/inference="COORDINATES: similar to AA
sequence:RefSeq:WP_001833801.1"
/note="Derived by automated computational analysis using
gene prediction method: Protein Homology."
/codon_start=1
/transl_table=11
/product="transposase"
/protein_id="QFM29097.1"
/db_xref="GI:1760398481"

/translation="MNASSKRKIISQSEISKKIAVMNEEMQGFWANNSWDIRKCPHPS

AIELSKNPALRNRWVRFERVKNLWLRTELKYFYFYHLNNGIWNAKTVWIRKGTVINKM
LDFLDLKYPSITSITEVPIEKAMTEYRTYLTKRGVRITTTNYKITANQEKTPVKANSY

YVTNLKQFMEFYENFYFDGEEWDKDVWDRRNLPLPDDKVNPTQYEYTINFKGFRNTYF

KQLVKRYCKLRLNVDSFSYVSDIAQRLKEFFNFLDMKFKQVQRVHQLTRVEIEAYLSE
LNMMGIKPSTITGRISILEGLFSTLLRLEWDDVPSKILIYSEDYPKIPRAKPRFIDEF

VLEQLNSHLDKLSEYIATMTMIVQECGMRISELCTLKKGCLLEDKDGDFFLKYYQWKM

KKEHIVPISKEVALLIKVREDKVSEEFPDSEYLFPRKDGSPLKQETFRGELNKLAYEQ

NIVDKSGEIYRFHAHAFRHTVGTRMINNGMPQHIVQKFLGHESPEMTSRYAHIFDETL

KNEFTKFQEKLVTNNGDVLDLDEDNEVDDVELQWFKKNINAQVLPNGYCRLPVVAGGC

PHANACLDCTHFCTSKQFLPQHEEQLERTEELLAIAKDKQWQRQVETNSRVKERLEQI
IGSLTG"
gene complement(55430..56515)
/locus_tag="DQU53_00605"
CDS complement(55430..56515)
/locus_tag="DQU53_00605"
/inference="COORDINATES: similar to AA
sequence:RefSeq:WP_002300186.1"

/note="Derived by automated computational analysis using
gene prediction method: Protein Homology."
/codon_start=1
/transl_table=11
/product="transposase"
/protein_id="QFM29098.1"
/db_xref="GI:1760398482"
/translation="MKVQRIEVENKPYPLYLLLDKEYQLIEPVMKFIKYLDNTGKSPN

TIKAYCYHLKLLYEFMEQRGVILNDINFELLADFVGWLRYPSASNVIDLQSKKAIREE

TTVNTILNVVMSFLDYLSRLGEFKSIDVFKQAKGRNFKGFLHHVNKGRYQKNVLKLRV

KKKQIRTLRSKEVKQIIDACHTKRDKLILMLMYEGGLRIGEVLSLRLEDIVTWDNQIH
LTPRDVNVNEAYIKLRKERTIHVSKELMSLYTDYLIYEYSEELEHDYVFISLKEGYFG

KPLKYQSVLDLVRRIVKRTGIEFTSHMLRHTHATQLIREGWDVAFVQKRLGHAHVQTT
LNTYVHLSDQDMKNEFNKYLERKEHKK"
gene complement(56634..56951)
/locus_tag="DQU53_00610"
CDS complement(56634..56951)
/locus_tag="DQU53_00610"
/inference="COORDINATES: similar to AA
sequence:RefSeq:WP_000659046.1"
/note="Derived by automated computational analysis using
gene prediction method: Protein Homology."
/codon_start=1
/transl_table=11
/product="DNA recombinase"
/protein_id="QFM29099.1"
/db_xref="GI:1760398483"
/translation="MKEINIVSLQMIKTDTLSYLKNRISNPEDAAEIMRSFIGNSDRE

HLILICMNSKNEPTHIQTLSIGSINQTVIHPREIFKTAILSNANSIMLGHNHPSGDVL
TIV"
gene complement(56972..57478)
/locus_tag="DQU53_00615"
CDS complement(56972..57478)
/locus_tag="DQU53_00615"
/inference="COORDINATES: similar to AA
sequence:RefSeq:WP_001092170.1"
/note="Derived by automated computational analysis using
gene prediction method: Protein Homology."
/codon_start=1

/transl_table=11
/product="DUF1643 domain-containing protein"
/protein_id="QFM29100.1"
/db_xref="GI:1760398484"
/translation="MNTIKNTIYTEAIFSKDEKHRYLLKKTWDEKKPACTVITMYPHL

DGVLSLDLTTVLILNQLANSERYGAVYLVNLFSNIKTPENLKHIKEPYDKHTDIHLMK

AISESDTVILAYGAYAKRPVVVERVEQVMEMLKPHKKKVKKLINPATNEIMHPLNPKA
RQKWTLKA"
gene complement(57496..57807)
/locus_tag="DQU53_00620"
CDS complement(57496..57807)
/locus_tag="DQU53_00620"
/inference="COORDINATES: similar to AA
sequence:RefSeq:NP_763608.1"
/note="Derived by automated computational analysis using
gene prediction method: Protein Homology."
/codon_start=1
/transl_table=11
/product="hypothetical protein"
/protein_id="QFM29101.1"
/db_xref="GI:1760398485"
/translation="MKINRYITRGISEYLSLDLQILLWNMVKERDNQPHTDYLHIFKL

QEDENILSITHEQEQPAYKLEYHYTNYVKNQNALPKKVYVIREDGVDAFYYVMLLPEE
Y"
gene complement(57894..58244)
/locus_tag="DQU53_00625"
CDS complement(57894..58244)
/locus_tag="DQU53_00625"
/inference="COORDINATES: similar to AA
sequence:RefSeq:NP_763610.1"
/note="Derived by automated computational analysis using
gene prediction method: Protein Homology."
/codon_start=1
/transl_table=11
/product="hypothetical protein"
/protein_id="QFM29102.1"
/db_xref="GI:1760398486"
/translation="MKTTTQELKQYITRLFQLSNNETWECEALEEAAENILPERFIND

SPLAHLTLETYTYYNNELHDLSIYPFLMYANNQLISVGYLNHFDMDFLYLTDTKNTII
DERHLLREGGTHHE"

gene complement(58762..60390)
/locus_tag="DQU53_00630"
CDS complement(58762..60390)
/locus_tag="DQU53_00630"
/inference="COORDINATES: similar to AA
sequence:RefSeq:NP_763591.1"
/note="Derived by automated computational analysis using
gene prediction method: Protein Homology."
/codon_start=1
/transl_table=11
/product="recombinase family protein"
/protein_id="QFM29103.1"
/db_xref="GI:1760398487"

/translation="MQQLKTKRVGIYVRVSTEMQSTEGYSIDGQINQIKEYCDFHHFE

VKDIYADRGISGKSMNRPELQRILKDAKEGYIDCVMVYKTNRLARNTSDLLKIVEDLH

KQNVEFFSLSERMEVNTSSGKLMLQILASFSEFERNNIVENVFMGQTRRAQEGYYQGN

LPLGYDKIPNSKHELMINQHEANIVKYIFESYAKGHGYRKIANALNHKGYVTKKGKPF

SISSITYILANPFYIGKIQFAKYKDWSEKRRKGLNDKPVIAEGKHSPIINQDLWDKVQ

MRKKQVSQKPQVHGKGTNLLTGIIHCPQCGAPMAASNTTNTLKDGTKKRIRYYSCSNF

RNKGSKVCSANSVRADVIEDYVMKQILEIVKSDKVIQRVVTHVNQENQVDGAALHHDI

AYKQQQYDEVQIKLNNLIKTIEDNPDLTSVIRPSIQKYEKQLNDITNQINQLKNQQNE
DKPLFDAKEISKLLQHIFHDIKHIEKSRLKALYLSVIDRIDIKKDGNHKKQFYVTLKL
NNEIIKQLFNNKQLDEVHLSTSSLFLPQTLYLTI"
gene complement(60412..61761)
/locus_tag="DQU53_00635"
CDS complement(60412..61761)
/locus_tag="DQU53_00635"
/inference="COORDINATES: similar to AA
sequence:RefSeq:NP_763592.1"
/note="Derived by automated computational analysis using
gene prediction method: Protein Homology."
/codon_start=1
/transl_table=11
/product="recombinase family protein"
/protein_id="QFM29104.1"
/db_xref="GI:1760398488"

/translation="MKQVIGYLRQSTMKQQSLAAQKQAIEAIAEKHHIQHINFYSDKQ

SGRKDNRSGYRQMTQLIQQGQCDILCCYRLNRLHRNLKNALKLIKLCQTYHVHILSVH

DGYFDMDQAFDRFKLNIFISLAELESDNIGEQVRNGLQEKAKQGRLITTHAPFGYEYH

NGAFIINQNESPTVKAVFNYYIKGHGYKKIAQLLEEDNTYINRQPYQVRNIIINPNYC

GRVNNQYGQFDNMFPSIVSTSIYEQAQRLRSQKQIKQTSSDNQLKQKIKCPCCNTTLT

NMTVRKKNHTLRYYVCPKNMNASRFVCDFKGINAQTLEDKVLEVCKDFYQNQRIYTKI

KGAIDKRIKRQRNIEKHHTLTQEQLIEKLAQGIIDAETFREQTQSLRQQPQRTTSING
HQIQHTIQNIIQKRFTLNILYPYIETIHITKDKNLIGIYFKNEPLNIVNQTMQSSIA"
gene complement(61995..63788)
/locus_tag="DQU53_00640"
CDS complement(61995..63788)
/locus_tag="DQU53_00640"
/inference="COORDINATES: similar to AA
sequence:RefSeq:NP_763593.1"
/note="Derived by automated computational analysis using
gene prediction method: Protein Homology."
/codon_start=1
/transl_table=11
/product="DUF927 domain-containing protein"
/protein_id="QFM29105.1"
/db_xref="GI:1760398489"

/translation="MTQTNPSFNPSPRYKSKKGWYKDKPPKEKGGMPTEVEIAGPIVI
ENKFIDPKTNTEKVIITDEDQKVIVESSDILTTQKLPSLMKYGFSINEKYTKDLGYAL

QQMRNQLPISYLYEGVGILETPFGPIVSLNEIYTTTEFDNKSPSDAICENTYDLAPRG

TFDNWFNMYLKEVKGSLLLELAVVFGISALVTSFLKHKHETEFAGIIFSFTGQSSTGK

STAASLAVSVAGNPTKGNETLFRNWNATRNALEGYLSNNFGIPIVFDELSSATFRDTT

GLLYSITEGQGRQRSNVHGEVKTPKNWGTSVISTSEHSIFNDSAQNDGLRVRTIEINE

QFTTNATNADNIKKAVALNYGHVLPLVAKYLINREDEVIQWFYKEVDWFEAKLKDDKS
NTGNRMFKRYAVITTSAKILGRVLATDIDIAKIRDYFIDYHGHTISERSLADKAIDVI

IQFVAQNRGKFSDEGALKNMFENYGLISLKDDHIEVKMIANVFKQMLNNHQFQDVNNV

VNALRDKGFILADRGRQTTKRSVKDNSGKKQSLVFYHLKLDVEFASILGLTKDKSLIQ
NWTPANDNKAAKELFKSANEGIGPSGVHEDF"
gene complement(63788..64084)
/locus_tag="DQU53_00645"
CDS complement(63788..64084)
/locus_tag="DQU53_00645"
/inference="COORDINATES: similar to AA
sequence:RefSeq:WP_000273824.1"
/note="Derived by automated computational analysis using
gene prediction method: Protein Homology."
/codon_start=1
/transl_table=11
/product="DUF1413 domain-containing protein"
/protein_id="QFM29106.1"
/db_xref="GI:1760398490"
/translation="MYFNQSDKFEQLKKKILGISFNLPEGEEFTFHDLNSRANVQCSK

AVQQNVGRWFAYFVKHAPRVPFIIIGKDTHGHLVYLKTGPNPHYNSNPSKGGVR"
gene complement(64277..65323)
/locus_tag="DQU53_00650"
CDS complement(64277..65323)
/locus_tag="DQU53_00650"
/inference="COORDINATES: similar to AA
sequence:RefSeq:NP_763595.1"
/note="Derived by automated computational analysis using
gene prediction method: Protein Homology."
/codon_start=1
/transl_table=11
/product="hypothetical protein"
/protein_id="QFM29107.1"
/db_xref="GI:1760398491"

/translation="MNNKDIYSDDFQNALVDIILNIDKINVKEVFDDSCRAVDQDNKK

DTLNRSPKDFSINISDIVRYSILCMENLSSDKIEENINMIKNHSSELNDNDMFIQEKN

NFTTNFNKFCNALGINIANFKSGKSYVFKPKSLFVLYDILSNNSVYLHFFKNGINDKD
IDSSNRIKENLYYFINHEITDQRDYEHALTNYYLYFLVDSNIEIGIKSIIIKIFTEKG
ITSKDKLQILEYYLDEIDDLNTEISTKIADAHVKNSTNFLENEKVSINSNLAKYITHR

DTRASAPKNMLLSLILNSDLVSPKDLEEKELKYPKKITKEHHDKLIKEINKALDYYND
NNTTPSDYFYITSD"
gene complement(65574..66715)

/locus_tag="DQU53_00655"
/pseudo
CDS complement(65574..66715)
/locus_tag="DQU53_00655"
/inference="COORDINATES: similar to AA
sequence:RefSeq:WP_086558285.1"
/note="programmed frameshift; internal stop; Derived by
automated computational analysis using gene prediction
method: Protein Homology."
/pseudo
/codon_start=1
/transl_table=11
/product="IS3-like element ISSau4 family transposase"
gene complement(67226..68152)
/locus_tag="DQU53_00660"
/pseudo
CDS complement(67226..68152)
/locus_tag="DQU53_00660"
/inference="COORDINATES: similar to AA
sequence:RefSeq:NP_763782.1"
/note="incomplete; partial on complete genome; missing
start; Derived by automated computational analysis using
gene prediction method: Protein Homology."
/pseudo
/codon_start=1
/transl_table=11
/product="YfcC family protein"
gene complement(68874..69569)
/locus_tag="DQU53_00665"
CDS complement(68874..69569)
/locus_tag="DQU53_00665"
/inference="COORDINATES: similar to AA
sequence:RefSeq:WP_000858967.1"
/note="Derived by automated computational analysis using
gene prediction method: Protein Homology."
/codon_start=1
/transl_table=11
/product="DNA-binding response regulator"
/protein_id="QFM29108.1"
/db_xref="GI:1760398492"
/translation="MKTTLLVVEDDEAILHLIDVALTMNYYKVVTAKTGKEADFRLRT

EQPDIILLDLGLPDIDGLSLIQQFRDFVDTPIIVISARTEEQTIVEVLDRGANDYMTK

PFNIDELRARIRVALRMSRSTETIQSRFTNGPLTIDFDAKSVLVNEQSVHLTPNEFQL

LTILCHHVGKVLTYQVLLKSLYGYVNHTEMAALRVHMASLRKKLNYTQDPNTEKKLII
THPRIGYQMQHLE"
gene complement(69544..72213)
/locus_tag="DQU53_00670"
CDS complement(69544..72213)
/locus_tag="DQU53_00670"
/inference="COORDINATES: similar to AA
sequence:RefSeq:WP_000446429.1"
/note="Derived by automated computational analysis using
gene prediction method: Protein Homology."
/codon_start=1
/transl_table=11
/product="sensor histidine kinase KdpD"
/protein_id="QFM29109.1"
/db_xref="GI:1760398493"
/translation="MESTYKKRGKLTIYLGYSPGVGKTYEMLSNGIDQYQEGADIKIG
YIEPHQRPETNALVQQLPEISTSSRKFGSHTFHYIDVSKIIEANPDVVLIDELAHTNI

SKERHLKRYKDIEEILSHGIDVWTTLNIQHIESLSGQIALMTGIQVTERVPDQFITSA

DAFEVVDISPNMLIQRLKAGKVYKKERLETAFSNFFTYENLTELRELTLRTVADIMSQ

KGQQYKTKHTDITPHIAVAISGSIYNERVIREARRAAYKEHAKFTAVYIDVFETPSES

RKQDHYVHKNLMLAKALGAEIKVLYAQDIAKALTDWCDLAFVTKLVLGQSEQPRWKDY

FKQSLIEQINHVPHHFKLEIVPIHYIHSDKPTNTPKQAQKSSRLTVEIMKMLVIQTIC
VLLGIWVYNLDKSESSAIILLMFFIGIIVLSIWTQSYLIGFFASILNVFVFNYFFTVP
RFTLEMYRFEYPITFATSIFASIFTSAILKNLKHQHSLTERQLYRTNIMLQFNQSIKE

SYSIERLLNIAGDQIHQLLNQDVTVFLIQSKKVAASNSFGNRSISSSDKTHDAETLSW
VIENESRAGKLTDTFPGSKFLCIPIGTNPVKGVISIRFTDETYIDTYDNSILDSMLND

ITLAIENVDLLKQTRQSILKAEREATRSNFLHSISHDIRTPLTSIMGNLDMLKYHNEH
LNDQQQAELLTASYGEAQYLHTLVTNILSLTKLESSDIQIQRTPYLVEELLEEFEEGL

IRRHQANHVIIENEDDASLINIDSKLILQVLFNLIDNALKHAESHSEIKLHVQHETNK

IKFEMIDCGKGIPEEERQLIFNPYYSGDNFKDNKKDSLGLGLYLVQLILKQHNSELEY
QPNTPQGSIFYFYLTVNPIEMEGAMDENNIIGR"
gene 72380..72460
/gene="kdpF"

/locus_tag="DQU53_00675"
CDS 72380..72460
/gene="kdpF"
/locus_tag="DQU53_00675"
/EC_number="7.2.2.6"
/inference="COORDINATES: similar to AA
sequence:RefSeq:WP_000631574.1"
/note="Derived by automated computational analysis using
gene prediction method: Protein Homology."
/codon_start=1
/transl_table=11
/product="K(+)-transporting ATPase subunit F"
/protein_id="QFM29110.1"
/db_xref="GI:1760398494"
/translation="MITLLAVVVIALILFLFYALIWSEKF"
gene 72481..74157
/gene="kdpA"
/locus_tag="DQU53_00680"
CDS 72481..74157
/gene="kdpA"
/locus_tag="DQU53_00680"
/EC_number="7.2.2.6"
/inference="COORDINATES: similar to AA
sequence:RefSeq:WP_002460931.1"
/note="Derived by automated computational analysis using
gene prediction method: Protein Homology."
/codon_start=1
/transl_table=11
/product="potassium-transporting ATPase subunit A"
/protein_id="QFM29111.1"
/db_xref="GI:1760398495"
/translation="MSIVLFLIVFILLSLIVSRYLYSVALNVPSKIDVVFNPIEKLIY

QLIGTKLEHMSGKTYIKHFLLFNGLMGGLSFVLLLIQQWLFLNPNHNLNQSVSLAFNT

MASFLTNTNLQHYAGETDLSYLTQMCVITFLMFTSAASGYAVCIAMLRRLTGMTDVIG
NFYQDITRFIVRVLIPFALIISLFLISQGTPQTLKGNLVIETLSGVKQTIAYGPMASL

ESIKHLGTNGGGFLGANSSTPFENPTYWSNYAEALSMMLIPGSLVFLFGRMLKTKLQI

HPHAIMIFVAMFVMFIGFLVTCLYFEFAGNPVLHHLGIAGGNMEGKETRFGIGLSALF

TTITTAFTTGTVNNMHDSLTPLGGMVPMVLMMLNAVFGGEGVGLMNMLIYVMLTVFIC

SLMIGKTPSYLGMKIEGKEMKLIALSFLVHPLLILVFSALAFIVPGASDALTNPQFHG

VSQVLYEFTSSSANNGSGFEGLGDNTVFWNISTGIVMLLARYIPIVLQILIVSSLVNK
KTYQQHTQDVPINNLFFSSVLIIFIILLSGLTFLPDLMLGPIGEQLLLHA"
gene 74176..76197
/locus_tag="DQU53_00685"
CDS 74176..76197
/locus_tag="DQU53_00685"
/inference="COORDINATES: similar to AA
sequence:RefSeq:WP_000852430.1"
/note="Derived by automated computational analysis using
gene prediction method: Protein Homology."
/codon_start=1
/transl_table=11
/product="potassium-transporting ATPase subunit B"
/protein_id="QFM29112.1"
/db_xref="GI:1760398496"
/translation="MAETTKIFESHLVKQALKDSVLKLYPVYMIKNPIMFVVEVGMLL
ALGLTIYPDLFHQESVSRLYVFSIFIILLLTLVFANFSEALAEGRGKAQANALRQTQT

EMKARRIKQDGSYEMIDASDLKKGHIVRVATGEQIPNDGKVIKGLATVDESAITGESA

PVIKESGGDFDNVIGGTSVASDWLEVEITSEPGHSFLDKMIGLVEGATRKKTPNEIAL
FTLLMTLTIIFLVVILTMYPLAKFLNFNLSIAMLIALAVCLIPTTIGGLLSAIGIAGM

DRVTQFNILAKSGRSVETCGDVNVLILDKTGTITYGNRMADAFIPVKSSSFERLVKAA

YESSIADDTPEGRSIVKLAYKQHIDLPQEVGEYIPFTAETRMSGVKFTTREVYKGAPN

SMVKRVKEAGGHIPVDLDALVKGVSKKGGTPLVVLEDNEILGVIYLKDVIKDGLVERF

RELREMGIETVMCTGDNELTAATIAKEAGVDRFVAECKPEDKINVIREEQAKGHIVAM

TGDGTNDAPALAEANVGLAMNSGTMSAKEAANLIDLDSNPTKLMEVVLIGKQLLMTRG

SLTTFSIANDIAKYFAILPAMFMAAMPAMNHLNIMHLHSPESAVLSALIFNALIIVLL
IPIAMKGVKFKGASTQTILMKNMLVYGLGGMIVPFIGIKLIDLIIQLFV"
gene 76213..76770
/locus_tag="DQU53_00690"
CDS 76213..76770
/locus_tag="DQU53_00690"
/EC_number="7.2.2.6"
/inference="COORDINATES: similar to AA
sequence:RefSeq:WP_001646386.1"

/note="one of the components of the high-affinity
ATP-driven potassium transport (or KDP)system, which
catalyzes the hydrolysis of ATP coupled with the exchange
of hydrogen and potassium ions; the C subunit may be
involved in assembly of the KDP complex; Derived by
automated computational analysis using gene prediction
method: Protein Homology."
/codon_start=1
/transl_table=11
/product="potassium-transporting ATPase subunit C"
/protein_id="QFM29113.1"
/db_xref="GI:1760398497"
/translation="MQTIRKSLGLVLIMFVLCGFIFPLTVTALGQVLFPEQANGSLVK

QDGKVIGSKLIGQQWTEPKYFHGRISAVNYNMNANEVKESGGPASGGSNYGNSNPELK

KRVQETIKQEGKKISSDAVTASGSGLDPDITVDNAKQQVKRIAKERNIDASKINHLID
ENKQASPMADDYVNVLKLNITLDKL"
gene complement(77315..78184)
/locus_tag="DQU53_00695"
CDS complement(77315..78184)
/locus_tag="DQU53_00695"
/inference="COORDINATES: similar to AA
sequence:RefSeq:WP_000626133.1"
/note="Derived by automated computational analysis using
gene prediction method: Protein Homology."
/codon_start=1
/transl_table=11
/product="hypothetical protein"
/protein_id="QFM29114.1"
/db_xref="GI:1760398498"
/translation="MISTSEHSIFKNPAKNDGLRVRTIEVSEKFTSSAQNSDTIKKVV

SQNNGHVLPIIAQFLLDNTNDVTSLFQKEDSVFYQLLSTESSNTGIRMIKHYATLTTT

AHILNQSLDLQLNVSSIKNYLLSYHNETVSERSLADKAMDTIVQFIAENLGKFSQQTR

LANMIQNFGILSLKDDYIEVKIIRNVFDKMLTDSQFEDTKNVIDALDNAGKLVSDRDR

KTTKRNAKDDTGKSTSLVFYQIKIDLDYADVLGLQSQGRDNVIQKHSNPSQFKNTNLE
EFMKSENTTLIVF"
gene complement(78194..79090)
/locus_tag="DQU53_00700"
CDS complement(78194..79090)

/locus_tag="DQU53_00700"
/inference="COORDINATES: similar to AA
sequence:RefSeq:WP_000411461.1"
/note="Derived by automated computational analysis using
gene prediction method: Protein Homology."
/codon_start=1
/transl_table=11
/product="DUF927 domain-containing protein"
/protein_id="QFM29115.1"
/db_xref="GI:1760398499"
/translation="MEKILYQTDEFKLKPSGWYKTIPPKKDGGTEFEIMLSGPIAFTD
RFIDPATRKEKVFLSDLNNIELVEKASILTALQLPSLIEYGFTINEKHIRDLGFVLQQ
MRSTTPLSTIYSGVGMLHTLLGPLISLDQPYFSNEITNSTSIICDNKYDLIPKGNLSE

WLQMYKEEVHGNLSLELDVLFGVSSLVTAFLKYHNNVEFSGTIFSFTGQSSTGKSTAA

MLAASVAGNPTKGTENLFRSWNATRNALEGYLSGNYGVPIVLDELSAATFHDTTGLLY
SFAEGQGRQRANINGDVKTPKN"

gene complement(79832..80119)
/locus_tag="DQU53_00705"

CDS complement(79832..80119)
/locus_tag="DQU53_00705"
/inference="COORDINATES: similar to AA
sequence:RefSeq:WP_001193834.1"
/note="Derived by automated computational analysis using
gene prediction method: Protein Homology."
/codon_start=1
/transl_table=11
/product="hypothetical protein"
/protein_id="QFM29116.1"
/db_xref="GI:1760398500"
/translation="MQTLASKFEDLDIDSHDNLNSFKARIGSTIDKLAKAPPLNKYTF
TELMIIISIIYFKNLYIGRLDKPSSRKKKGGNLIFEIVYNNQMLNSSTAKL"

gene complement(80209..80835)
/locus_tag="DQU53_00710"

CDS complement(80209..80835)
/locus_tag="DQU53_00710"
/inference="COORDINATES: similar to AA
sequence:RefSeq:WP_001176969.1"
/note="Derived by automated computational analysis using
gene prediction method: Protein Homology."
/codon_start=1
/transl_table=11
/product="hypothetical protein"

/protein_id="QFM29117.1"
/db_xref="GI:1760398501"
/translation="MQLQTMSNSIFIKNLIFAINESLEYSETNSVMEVTFQKAIGFTQ

YNFEGEKLKLKDDLKELISLVGREEAVLIFVNRLNEGLYCILNQLIEWDNLNYQFAMY

KEAMHNYLFNTIEKLNDDTLFLLHQDGLITLPEHKYKTVAVKSTFGSRDFTFRFKFPE
ESDTVNTHQMLNKAISITGSLANTHLEQPQKYKAHFKKIPIETKNLTS"
gene 80950..82458
/locus_tag="DQU53_00715"
CDS 80950..82458
/locus_tag="DQU53_00715"
/inference="COORDINATES: similar to AA
sequence:RefSeq:WP_001001347.1"
/note="Derived by automated computational analysis using
gene prediction method: Protein Homology."
/codon_start=1
/transl_table=11
/product="kinase"
/protein_id="QFM29118.1"
/db_xref="GI:1760398502"
/translation="MNEEILREVADIFIGDDRDSIYDYKTGNELVRFFNHYFNKGDIY

QAPFPSRWLYVVKHLQTLIQERKINQFFTLILSNHYIKYELKIDEVEAAKQAAKALKL

FNKRLNHYGYYITGTNNARYFMDKDEDTESIGYGGYANIYLQKSTGLAVKKLKEEYLT
DSSIKSRFKREFDLTKSFDTNPLFINVFEFNESDYSYTMELADETLKDYIESKTISEL

EKVKIIMKILKAMSQAHSENKIHRDISSKNVLMFRGKVKISDLGLGKNLDEIHSHQTF

DTNGVGQYKYCAPEQMYSLKQADKQSDVFSLGRLINFIMTGNVVNNHHLFRGVSDKAT

NSSKEYRFEDANEMLKMLQRILEYHSSAKHVEKCQEKLKRGVFDDESEEFIMTRSDEQ

LCQMVLSSNNNEQACLIRYMQKNESSACDLIESINRKYQEFCGRFEDYDPFAKLAYMI
LCNNFSYRVNETAARVLNYVAWSVNRFSAQDLIKGLINRGVEPLIEEKLKDN"
ORIGIN
1 atgaaaatca ccattttagc tgtagggaaa ctaaaagaga aatattggaa gcaagccata
61 gcagaatatg aaaaacgttt aggcccatac accaagatag acatcataga agttccagac
121 gaaaaagcac cagaaaatat gagcgacaaa gaaattgagc aagtaaaaga aaaagaaggc
181 caacgaatac tagccaaaat taaaccacaa tccacagtca ttacattaga aatacaagga
241 aagatgctat cttccgaagg attggcccaa gaattgaacc aacgcatgac ccaagggcaa
301 agcgactttg tattcgtcat tggcggatca aacggcctgc acaaggacgt cttacaacgc
361 agtaactacg cactatcatt cagcaaaatg acattcccac atcaaatgat gcgggttgtg

421 ttaattgagc aagtgtatag agcatttaag attatgcgtg gagaagcata tcataagtga
481 tgcggttttt attaattagt tgctaaaaaa tgaagtatgc aatattaatt attattaaat
541 tttgatatat ttaaagaaag attaagttta gggtgaatga atggcttatc aaagtgaata
601 tgcattagaa aatgaagtac ttcaacaact tgaggaattg aactatgaaa gagtaaatat
661 acataatatt aaattagaaa ttaatgaata tctcaaagaa ctaggagtgt tgaaaaatga
721 ataagcagac aaatactcca gaactaagat ttccagagtt tgatgaggaa tggaaaaaaa
781 ggaaattagg tgaagtagta aattataaaa atggtggttc atttgaaagt ttagtgaaaa
841 accatggtgt atataaactc ataactctta aatctgttaa tacagaagga aagttgtgta
901 attctggaaa atatatcgat gataaatgtg ttgaaacatt gtgtaatgat actttagtaa
961 tgatactgag cgagcaagca ccaggactag ttggaatgac tgcaattata cctaataata
1021 atgagtatgt actaaatcaa cgagtagcag cactagtgcc taaacaattt atagatagtc
1081 aatttctatc taagttaatt aatagaaacc agaaatattt cagtgtgaga tctgctggaa
1141 caaaagtgaa aaatatttct aaaggacatg tagaaaactt taatttttta tctcctaatt
1201 acactgaaca acaaaaaata ggtaatttct tcagcaaact cgaccgccag attgagttag
1261 aagaagagaa acttgaactc ttagagcaac aaaagcgtgg atatattcag aagatttttt
1321 ctcaagattt aagatttaaa gatgaaaatg gaaacagtta tcctgattgg tctattaaaa
1381 agattgaaga tatttctaaa gttaataaag ggtttactcc aaatacaaaa aatgataaat
1441 actgggatga attaaatgaa aattggttat ctatagcagg tatgacacag aaatatttgt
1501 ataaaggaaa taaaggaatt actgaaaaag gtgcatcaaa gcatgtaaaa gtagataaag
1561 atactctaat aatgagtttt aaattgactt taggtaagtt agctatagta aaagagccta
1621 tctatacaaa tgaagctata tgccatttcg tatggaaaga aagtaatgtt aatactgagt
1681 atatgtacta ctatttaaat tctataaata taagtacttt tggtgcacag gcagttaaag
1741 gagtaacatt aaataacgat gcaattaata gtattatagt aaagttacca gtgatacaag
1801 aacaaaataa aatagcatac tttttcaata aattagataa attaattgaa aaacaatctt
1861 ctaaagtaga attattaaaa caacgcaaac aaggattttt acagaaaatg tttgtttaat
1921 tcttataaag ttctattatg taaaatatta aatagagata acattatgaa agcgagccca
1981 agacataaag tttttgaata aataaaaaag ataatttcta tcaaattaat atagaaattg
2041 tctttttat aaatttttg attattttta gctgattgag ctgttacttt tcttataata
2101 agtgctatta gcacaaatcc tagttctctt ttggctttgt ttattcctct tacggacatt
2161 cgagtgaaac ccattttaat tttattagaa gtaatttagg tttgaaccca cctaaataaa
2221 tatatgagtt atttttttat gctacaaaat atattcagat ttcaataatg acataaaata
2281 ggcatcttta tatttacctt tagtgtagaa ttgctctttg agtaatcctt ctgttttaaa
2341 tccttgtgac tcgtatatat gcacagcttt tttgttatct gtatcaacat atagataaat
2401 tttgtgcatg tttaatatat cgaatgcata atttatcgct ttttcgaatg cgaattttgc
2461 ataaccttta ccactgaact caggtttaat aattatttgt atttcacaat tacgatggat
2521 gtaattaatt tctactaatt caacaatacc tacgacttga ttttcatctt caacaataaa
2581 acgtctctct gattcatcta ataaatgctt atcaaataaa tattgaagtt ccgttaagga
2641 ttcatatggt tcttcaaacc aataagacat aatagaatat tcattattta attcatgaac
2701 aaaaagtaaa tcactatact ctaatgctct tagtttcata attccactcc caaaattttc
2761 tcatatattt gcattataaa tataaataac gaataagtca tcattcactg tgaatactct
2821 attttaacaa ttcaccacat actaattctc attttcttgt tattctcgat ttattactct
2881 tactatgaaa cctataaaat tctcacattt gtttgtatta agaataaata cgtcgatagt
2941 aacaataaaa aaataaataa taaagcatcc ctcaccgtaa aagtgaagga tgctctagtt
3001 ttattgaaat atacatttca ttttgttaaa taattattaa taatattttg aaaatcatta

3061 ttacgtgaaa tcttcataga ttttatcaag tatttctttg ccttcaattg ctgtgaagtg
3121 atgtaccaat ctatttttac aatcatatgt aattttgtga cgctaggtaa ttagtaattg
3181 ttcgtcagtc tgattgtata gtatcaagtt tcatagataa tactctttga ttttaatgtc
3241 cactttgacg tgctttaaga ttgagtatat acataatgtc attgtggaat gttaaaaatc
3301 ctacaaatgt ttattcatct gcaggatttt taaatctcca agaataaaaa tcatcatagg
3361 acaactggat tattgtttgg ataaataacg taaacaataa ttaggtacta ttatttattt
3421 ttgtttattc tttttcctaa caaaataaag aaaagaataa acgcaattgt taaaaatatg
3481 tgtcctaaac cagcaatacc agcaatagca ggacttacac ttagatcttt aatggtagaa
3541 ataccgttca cgaattgcat tgccacagta acaagcacac ctaaatggta tatataaaag
3601 aaactgttaa acagctttgt atgagttgtt aatttgaatt ggccctcgat aatcatgaaa
3661 attaagaaca taattgtacc tagtactaat aaatgtgtat gtgtaacatt taattgagaa
3721 aaaccgctaa aatcttccgc ttttgtcatt tctctataaa atagaccact taataaccct
3781 aatagtgtat agagcgctga actatacatt aatcttttca ttttaattcc ccctattttt
3841 aattacgaga taagtatagc ggtagtttat gaactgagta tgaacttaca acaaaaaaat
3901 taatgaagta ctttacaata aactcaattt attagatggt ggagggacga aaaaggattt
3961 tagaaaaata aattaatata tttttatttt gataagtaat aattaataat atcttggaaa
4021 tcattgttaa gtattgttgt aatacaatcg tcattcataa aatcttcata gattttatca
4081 agaatttctt catcttcgat agatgtgaaa tgattagcta acccttttat aatttaagtg
4141 taatttgtga atctaaacaa ctagcaactg ttcgccaatc tgattgtgta gtgtcatggt
4201 tcatagataa tcctcccttt attttaatgt ccatttttga cgtgctttag ggttaagtgg
4261 atgcataact tcgttcgtta ctggattaat gagtttttttg actttctttt tatgaggttt
4321 taacatttcc atcacttgct caacacgttc gacgacaaca ggccgcttcg cataagctcc
4381 ataggctaga atcactgtgt cactttcact aatcgctttc attaagtgaa tgtctgtgtg
4441 tttgtcataa ggctctttaa tatgtttaag gttttcaggt gttttaatat tagagaatag
4501 atttactaga tatacagcac cgtattgttc agaattagct aattggttga gaatgagaac
4561 agtggtaaga tcgagtgata atacgccatc taaatgtgga tacatcgtta tcactgtaca
4621 agagggtttc ttttcatccc atattttctt gagtaagtag cggtgttgtt catcgtcgct
4681 aaatatagct tctgtgtgta tcgtactttt gattgtattc atatatcgtt accccttttta
4741 atattcttct ggcaaaagca tcacataata aaaagcgtct acgtcatctt cacgaatgac
4801 gtagactttc ttaggtaatg cattttgatt ttttacatag tttgtatagt gatattccaa
4861 tttgtatgca ggttgttctt gttcatgtgt gattgagagt atattctcat cttcttgtag
4921 tctaaaaatg tgtagataat ctgtatgaga ttgattatct ctttctttta ccatattcca
4981 aagtaggatt tgaaggtcta gagatagttg ttcacttatg cctcttgtga tgtatcgatt
5041 gattttcatg ttattttacc tcgtcttgaa tttctttcat aatgataatt gcttggctaa
5101 taatcgtaac agatatttgt gccactttga tccaattatt catggttatt ctctccttgt
5161 tttagtaaat gacgttcatc gataatcgta tttttagtat ctgtgaggta tagaaagtcc
5221 atatcaaaat gatccaaata accaatgctg atgagttggt cattggcgta cataagaaat
5281 ggatagatac ttagctcatg tagctcatca ttgtagtagg tataagtgtt gagtgtgaga
5341 tgcgcaagta gagaattatt aataaaacgt tccggtagaa tattttctgc tgcttcctca
5401 agtgcttcac attcccatgt ttcgttgtta gatagttgga agagacgagt tatatattgt
5461 ttgagttctt gagtggttgt tttcatatca ttgcctccta gatagtgtga tagtgatgta
5521 gttgatatac atcattggga taatatatat ttgatttatt atttattacg aatcccggtg
5581 ggaataagag aaaattccat ataaaaaccc gtgataaatg ttggtgtaac aaggaaatcc
5641 cggaatccca ctcattttga cgaacaatca actcattatt tataagtatt gatgataggg

5701 ttgtgtctct gcttccttat atatattatt tatttataaa aaataacggg attttgggat
5761 tgtgcttgca caatccttct gcttcttcga atctgcaaat cccattcctt tcccggtaaa
5821 aaatcattgt gggatgttct ttagcaattt caatataagc attgtgtagt catgaaaaaa
5881 atgacggcaa tgactgtttc attagataag tgttattgaa attgataaag agaattctaa
5941 aaatggttag ataaaataag tgaaagaata gcagtgtagt tattgtttat tcaatagtta
6001 tatataaagt ttgtggcaaa aataaagacg aagtgctagg gagcacttcg tcgagtggat
6061 ggttattaaa tagttgtttg attatatcat tatttaactt gagcgtaaca taaaattgct
6121 tcttatgatg ctcatctttt cttatgtcaa tgcgatcgat gattgttaga tatagtgatt
6181 tcaactgaga tttctctaat ttgtctatat ctttgaatat tgcttgtagt acattcgcaa
6241 tcatatcagc atcataatgt ggagcttctg cttgtttatc ttgttctagt tgatagattt
6301 gattatttat ttgattcaat tcatcttggt agctaagtat tgttggtttt aatacgctat
6361 ctaagtcagg tgagtcttca atagttttcg ttaatgtatg catttttgct tttatttctt
6421 cacattgtga ctgtttatag gcaatatcat ggttcaaaga agagacgtct atttgacttt
6481 tttcatttac cttttcaacc aattgcttca ataccttttt gcttttgata atttccaata
6541 tctgatccat aacatatttt tctagtacat ctgctctaac gctattggca gaacaaactt
6601 ttgaaccttt atttctaaaa ttgctacatg agtaatatct gattcttttc ttagtgccat
6661 cttttaatgt attagttgta ttacttgccg ccattgctgc accacatttc ggacatttta
6721 caatcccagt cagtaggttt gttcctttgc cgtgaacttg tggtttcttg cgactctctt
6781 gacgtttaaa ctgtacttta tcccataggt ttctatcaat aataggcgca tgtttaccat
6841 cagcgataat cggttcctca ttgagtcctt ttcgtctttt atcgctccag tgtctatact
6901 tcgcaaactg tatctttcca atgtaaaaag ggtttgagat gatgtaggta atggacgaaa
6961 tactaaaagg tttccctttc ttagtcacat aacctttatg attcaatgcg ttcgcaatct
7021 tacgataacc atgaccttta gcgtatgaat caaaaatata tttaacaata ttcgcttcat
7081 gttgattgat catgagctct tttttactgt caggtacttt atcgtagcct agaggtaaat
7141 taccttgata ataaccttca atagcacgtt gtctttggcc attgtagaca ttctctacaa
7201 tcgtattacg ttcaaattct gcgaagctgg ctaaaatttg gagcatcaat ttacctgttg
7261 aactggcaat ttctattttt tcagttagac taaaaaattc gacattaatc ttatacaatt
7321 cctcgacaat atttaacaaa tctgaggtat ttctagctaa acgatttgtt ttgtagacca
7381 taatacaatc taatttacct tcgttggcat cttttaacat acgttgtaat tctggacgtt
7441 gcattgtttt acctgatata ccacgatcgg tgtattcatt gacgacttca tagccttgaa
7501 attgacaata ctctgtaagt tgattcaatt gaccttgaat actgtaacca tccgtttgca
7561 tttcagtcga tacacgtgca tataatccaa tacgtttctt tttgagttgt ttcatattac
7621 ttcattcctt tcaggagtta ttaaatgtga ttgttcaacg atattgagtg gactgttttt
7681 aaagtagatt ccttgtaatt gtttagtttg agttattttg atgcaatcga taaaaggtgc
7741 tatatcctgt aaagttattt tattttttat gacatatttt aatttgtcat cgatttggta
7801 ggttgaataa gtagagtaat tctcttcact tttacaacta gcagataaac gtttgaacgt
7861 ttttacatca atacgatttt gagctagctt ttcgataagt tgttcttgtg ttagatgggt
7921 ttctttatgc ttcatttgtt gctgttttag gactttgagt attgtgttat tcaatcgttt
7981 atgaaacgat tgttcttcaa aatacttttt acaagtgtcg agcacttcac tttcaagttc
8041 tggtgcattg atacttttaa atgggcatgt acgataagcg tcattcatat ttttaggaca
8101 aacatagtaa cgtagagaat agttctcttt ttttatcgtt aagtttgtta atgttgattg
8161 acagtaagga catttgattc gtcgctttag cttatttcta gaattggatc gattgagttg
8221 tttatgaata cgacgctctt gtgcttcttc aaatgtatca atatcaataa taggtggaac
8281 gatatcatta aacgtgccat atttattgat gacacgaccg caatagttag ggttcaagag

8341 aatatttcta acttgatagg gcttacgagg aataaggtta ggattactat ccaaatgttg
8401 ggaaatcttt ttgtagccta gaccttgtaa gtaccagcga taaaccgatt taactgtata
8461 cgcttcttct tcttgtacaa caaaacaacc ttttctataa cgatagccaa acggagcatg
8521 agttgtgatt agcttacctt gtttggcttt ttctctaatc ccatttttg tttgttcgct
8581 gatattgttt gattccattt ccgctagact cataagtatg ttcaaacgaa agcaatcaaa
8641 ctctttagac aaatcaaaat atccatcgtt aacactgatg attgtgatgt gatgcttttt
8701 acaaatttca aagaattgta tggcattttt caaattacga tgtagtcggt tcaagcgata
8761 gcaacacaat actttacatt ttccagacgt aattatttct accatttttt gataacctga
8821 acgttttgta tgtcgacctg ttttcttatc atcataaaac gccacattag accatccata
8881 ttgcttagcg gtatccataa tgagcgattt ttgagtagct aagctttgtt gtttgagtgt
8941 actttgacgt acataagcaa tcgcttcttc catgttatac acctccaaaa agataatata
9001 tatttgtgag taaattagaa tgaaaggtcc aacgtgcttt taacacgttg gaccgtcgtg
9061 attagttatc gttctgtagc tcttcaacga ctaaatcagc tagtaattca atcaattcat
9121 ccattttatc tactcctgta cgatttcatc tttaagttat taaaaatcaa ttaaatcatc
9181 gttctgtttt ttccatgatt caagtatctt tttgttatcc aagttaattt cagctttaat
9241 aggttcagca tctttagtta gaccaaaaat agacgcatat tttgaatcta gctttaaatg
9301 gtaaaagaca agtgactgtt tcttgccata gtcatcttta actgctcttt tcgtagttat
9361 tcgatcacgg tcagattcga taaatccttt atcccttagt gcattcacaa cattgttgac
9421 atcttggaaa tgatgctcta tcaacatatt cttaaataca gatgcaatga ttttaacttc
9481 gatataatca tcttttaagg caattagtcc atagttctca atcatattct ttaatgctgt
9541 atcatcagaa aacttaccac ggttttgtgc tacaaattgc gtaatgactt caatcgcttt
9601 atcagccagt gatcgttcag agactgtatg agcatgataa tcaataaagt agtctcttat
9661 tttagcgata tcaatatctg tagataaaac atgactaaga atcttggccg aagtagtgat
9721 agccgcatag cgcttaaaca tacgaatacc agtattattt gtttcatcct tcaatttatc
9781 tttaaaccaa tgatgtaatt caaaaacgga tcaaaaaaca aaaggcccta gaaactagga
9841 gcacacttac acaagaacaa ttgatagata aattagctaa aggagccatc gatgcagaaa
9901 cgttcagaaa acaatctcaa tcgttacttc aacaatcaaa accaacacta tcaataaatg
9961 agcaacaaat tcaaaggtct tttgaaaatg taattcaaca acacttcacg ttaagcatgt
10021 tataccgata tattgatgaa attcatattt ctaaaaacaa aagccttgtg ggaatctatt
10081 tcaaaaatga accgctaaac attgtaaacc aagctacgca atcatcgatt gcttaattaa
10141 tgaaaggatg aaaaatatga aagaaatgaa aagaaaatct gtaggatgtt atgttagagt
10201 ttcaacaatt tctcaagaca ttgataaatt tagtattaat ggtcaaatta cacaaataaa
10261 ggaatattgc caacagggaa attatgaatt gctatactga taattaaaag tacccccgat
10321 cgaacgaaaa tatctttttg atgactatta gtgataattg taattttatc cattaataga
10381 tgaatggatt ggaataatac taagcctatt gctattccac cgagtaaaat tacccacccg
10441 cctaattcaa tactttccgg aatcatttca aaaaataata agccaattat aaggcctgca
10501 cataatgaat aaataaagcc atgaccactt tgaaaacctt ccattatcca agcgagtcca
10561 ccgccaacac caattccaag tgcggaagct aacgcccgat catccatact gcactcatcc
10621 cactattctt aaaaaggctt tgtttatttt actctaaaaa atcaaaacaa aatatatgaa
10681 ttcatattaa atattatttg actctttatt cagatggtta tatcatatat atgaacatat
10741 attaatacat gtagataaat agtgattatt atatataagg ttttgaattg aaacctaaag
10801 tgagggaagg ataatggaag aaaaaaagga attagaggaa gtaaataata aggacttaga
10861 tgatgaaaca ttatttgtcg tatcgcaaac atttaaagcg ttaggtgatc ctacgagaat
10921 ccggattctc catttgctct tttataagga gtattcggta aacggtattg ctgaaacgct

10981 acatcttaga caatcaacag tttcccatca attgcggttc ttgaaaaatt tacggttagt
11041 aaaattccga agggaaggca caacattgtt ttattcccat gatgatgaac atactatgaa
11101 tatgctaaaa caggcgatcg atcacgcctg tcatcactag tattccgtaa accagtaatg
11161 agtgttaata ataattcacg tcattaatat tgttatatga tcatataaaa atattatagg
11221 catatgaaaa gggggttttg caatggatat ggaaaacaaa aaaacagaat ggaaagcgtt
11281 gtatgatatc tcgaaggaaa gtgagatggg tgtagcagag agggttagtg aatacggttg
11341 ataaattcat aggatgtatt attggataca aaaagtttgg atgtggtcaa ttaacgtcct
11401 ggtttagtaa agcttactgc gaatatattg gaacccgata tatcatattt atttagaaag
11461 gtgatagaaa tgaaaaatat tcaagagcaa caagcacacg aaagtcatag ccacgatcat
11521 agtcatgatc atgatcacgg aaaaatgcca attatttcat attttattgg cttagtgttg
11581 gctataattg ggcttttttt aagtgatgca aatttattaa tacaaaacat cttattttca
11641 attgccacaa tcacagccgg ctaccatgta attattctcg aaggaattgg agagacagtt
11701 gaaaatacta aattaaaggg aaaattcact cctaattctc atattctaat gggattagct
11761 gcaatcgggg cttctctgat agggagtttt tgggaaggaa cccttttgat acttattttt
11821 tccggcgctc attttcttga agattacgct gaaggaaaaa gtaaaagaga aattactaag
11881 ctactcgaaa tgaacccaac gacagctaaa ttaatcctac ctgatggaaa cacaaaaatt
11941 gttgatgtca gtgaattaaa agttggagat caactccaag tgctgaacgg tgatcaagtt
12001 ccaattgatg ggattatttt atccggtact acctcaattg atgaatcttc tattaatgga
12061 gaaagtatac cgaaagagaa gtctaagggt gacgaagttt ttggaagtac gattaatgga
12121 acaggtactt ttactatgga agtcactaag gaaaacaagg atactgtatt ctctaaaatt
12181 ttacaattag ttagtcaaaa ccaagataat caaacaaaag ctgccagtat cattcaaaaa
12241 ttcgagccta aatatgttaa tatagtttta atcgcaatac cattagtaat gttacttgct
12301 ccttttctat ttgattggac atggtcgcaa agtgtataca ggggattagt gcttttagtc
12361 gcagcttcac cgtgtgcttt ggcagcagct actgtatctg taacattgtc tacaacatct
12421 aacctagcta aaaaaggcgt gctttcaaaa ggaagtactt acctatcaca attagcggat
12481 atagatgcaa ttgccttcga taaaacagga acccttacga acggagaacc taaagtaaca
12541 aattactatt tcactcattc tgtgaacgaa gaaaatatta ttgatattat agtcgccctt
12601 gaaaaggaat ccaatcaccc actcgctaat gctattttag aaaaatttga agttaaaaat
12661 aaaatagaca tcgaagttac taatcaaatt ggaaaaggtc tgacaggaga ttataatgga
12721 aaaaattatc gtattggtaa gcctacttct tttgaaagtg cttctgaaga gtatacccag
12781 ttcaatcatg attgggcatc agaaggaaag acggttgtat acgtagcaga aaatgaagaa
12841 gttattggga ttatagctct aatggatatt ccgaatgagc atgctaaaga aacaattaat
12901 tactttaaga aacttggtat ccacacgact ttaattactg gtgattcgga aatgacggga
12961 aaagctgtag gcgaacaatt gggaatagac gaagttatcg ctaatgtaat gcctgaagat
13021 aaatccagaa ttatagaaga acaaaaagaa aaatttggag ttactgccat ggttggagat
13081 ggtgtgaacg atgcaccggc ccttgttaat gctgatgttg gtatagctat ggggggcggt
13141 actgatgtgg cagtagaagt atctgatttg gttttaatgc agaacaattt atctaaatta
13201 gtacagtctc ataaaatttc ctcaaatatg ggtcgtgtta ttaggcaaaa tattattttt
13261 tcaatggcag ttgttgcctt tttagttgtc gttagtttgt taggattaac tgatattaca
13321 atcagtgtaa ttgttcatga aggaagtact ttagttgtta tactaaatgg acttcgatta
13381 ttaagatcta aataatgaac gaatcgattg acatgaatga actttgaagt gtggattcta
13441 caatgttccc ataacattgg acactaaaaa acagagcaat ctaataaaga tgttatgagt
13501 aaaaacaatg ccttgcatac cgtttttatc atacgggcgg tcaaataagc aattaaagag
13561 catgggaagc ataaaatcat aaacagtgac ttaaggcagc cagtttacat tcaaaagtta

13621 aattgactgt attaaaagtt tcgaaacgat taatatccgt atgggggcaa gcgtcgagcc
13681 aaagataatg ccaggacaga gcgtcttttt ccgttctttt aagtgggaaa ggttttacct
13741 tctctatgcc aagacagtcc cagagctaaa agggccagtt gctctttttg ggcgtttgtg
13801 aacggtaatc gcaggaaggg gattttccca accgcacctg gttttccatc acaataaagt
13861 tggcggcatt tgttctgata ttcttgggaa tacttacact atgaatggcc gtattaagtg
13921 atacaggagc tgctattatt gtaatactga atgctctccg ccttttgagg gtaaaagaat
13981 aaaagtaagg ataactaggt aaagctgttc aatcaaaaat tgaacagctt atttttcatc
14041 aaaatcaaaa acgtttatta taatacctac acttgttctg ttaaatgtac cgattttta
14101 accttattgt atcagtaata tcttgaaacg aagtaagcga ctaaaatttc tcttttatca
14161 tacgtttata aaaatacact tttaagaacg gtttgaaaat ttttgaaata gatcaaaata
14221 atcttctggt taaaaaacct gtagatatga tttctctctt aaatttttgt ttgattagat
14281 tagaccctaa taagacgcat tcaatatgtc tacacgtgaa tttagtcttt gaaaatgtaa
14341 ggaccattat tattataaaa accccagtat aaaacgatac gctgaagcgt accacaaata
14401 aaactaaaaa atatgagaaa attattaaat tagctcaaat ctttgaagaa taaaaagtga
14461 atattaagtt tgataattta ggtacaagta aagattaaga atttccatta tttaatacat
14521 ggtgtgtaaa tcgacttctt tttgtattag atgtttgcag taagcgatgt aaagaagatg
14581 ctaataaata tgtgaggaat gattacgata ctagataagc ggctaatgaa atttttaaa
14641 gtacatatat agacatattt ttcatttagt aaaattttga atttcacttt gctaagacta
14701 gtgtctagaa atttataatg atttattaac acctatttga aacttaagta taataaatga
14761 ttcggatttt atttttaata aagacaaact tgaacgtagc aaagtagttt ttatgataaa
14821 taataagttt taataatgtg acgcttttat ataagcacat tattatgaac aatgtgaatt
14881 gagcatctac aattacatta ataaatatat aaatgatgat ttaaattcac atatatttat
14941 aatacacata ctatatgaaa gttttgatta tccgaataaa tgctaaaatt aataaaataa
15001 ttaaaggaat catacttatt atacgtatac gtttagctac tgaactactg gattcatttg
15061 gagattctag tagttctttt tcaatctcta aatctaaatc agttttgtaa taaccattaa
15121 ttcctaatct ttcatctagc tctgtacttt tttcatcatt tttatctttg ttgatatgtt
15181 ccattttctc gcctcttttt aatcaagtag aaaaccttac aaatatatgc tatcaattaa
15241 ctatagaaat tacaatgaag aaaatattta gattattaaa cttgttaagt ttctatgaat
15301 tttaaattaa taaaaatata taaataatac attttaatat aacgtagtat tgcgaaataa
15361 ttgtacttct gctaatttta taatgtaggg tttagaattc tatataaatt tagaaatgga
15421 gtgtttttgt tgaaaaagaa attggacttt ttacctaata agttgaataa gtattcaatt
15481 agaagattta cagtaggtac ggcatctata ttagtaggat ctactctatt atttggtatt
15541 gggaatgagg cacatgcagc agaagagcat caaaaatcaa acactacaga aaatgtagct
15601 gacagtaatg caagcgaagc accaacaaaa gaagaagcgc caagcaatga agcaacaagc
15661 gaagcaccaa caaaagaaga agtgccaagc aatgaagcaa caagcgaagc accaacaaaa
15721 gaagaagcgc caagcaatga agcaacaagt gaggcaccaa caaaagaaga agcgccaagc
15781 aatgaagcaa caagtgaggc atcaacaaaa gaagaagcgc cagcagctga agaaacagac
15841 aaagcaacag aagaagcacc aaaaactgaa gaaacagaca aagcaacaaa agaagaagcg
15901 ccagcagttg aagaaacaag caaagcagca acagaagaag cgccaaaagc tgaagaaaca
15961 gacaaagcaa cagaagaagc accaaaaact gaagaaacaa gcaaagcagc aacagaaaaa
16021 gcaccaaaag ctgaagaaac aaacaaagta gaaacagaag aagcgccagc agctgaagaa
16081 acaaacaaag cagcaacaga agaaacacca gcagttgaag acacaaatgc taagagcaat
16141 tcaaatgctc aaccatcaga aactgagaga actcaagttg tagatacagt tgctaaagat
16201 ttatataaaa aatctgaagt tacagaagca gaaaaagctg aaattgaaaa agtattacca

16261 aaagatattt caaacttatc taatgaagaa attaaaaaaa tagctttaag tgaagtactt
16321 aaagaaacag ctaacaaaga aaacgcacaa ccaagagcaa cattccgttc agtaagcagc
16381 aatgctagaa caacaaatgt taactattca gcaacagcat taagagcagc tgcacaagac
16441 acagttacta aaaaaggaac tggtaacttt actgcgcatg gagatataat ccataaaact
16501 tataaagaag aattccctaa tgaaggtaag ctaactgcat tcaatacaaa cttcaatcct
16561 gatacaggaa ctaaaggcgc attagaatat aatgataaaa tagattttaa taaagacttt
16621 acaattactg ttccagtagc aaacaacaac caaggcaata caacaggagc agatggctgg
16681 ggcttcatgt ttactcaagg gaatggccaa gacttcttaa accaaggtgg tattttaaga
16741 gacaaaggta tggcaaatgc atctggtttt aaaattgata cggcatataa taatgttaat
16801 ggtaaagtcg ataaactcga tgcagataaa acaaacaatc taagtcaaat tggcgcagca
16861 aaagttggtt acggtacatt tgttaaaaat ggtgcagatg gtgtgactaa ccaagttggt
16921 caaaatgccc taaatacaaa agataaacct gtaaataaaa taatttatgc agataataca
16981 actaatcatc ttgatggtca attccatggc caaagattaa atgatgtagt attaaattat
17041 gatgcagcaa caagtacaat aactgctaca tatgcaggaa aaacatggaa agctactaca
17101 gatgatttag gaattgataa atcacaaaaa tataatttct taattacttc aagtcatatg
17161 caaaatagat attctaatgg aattatgaga acaaatcttg aaggtgtaac aattacaacg
17221 cctcaagctg atttaattga tgatgtggaa gtaacgaaac aaccaattcc tcataaaact
17281 attcgtgagt ttgatccaac tctagaacca ggctcacctg atgttattgt acaaaaaggt
17341 gaagatggag agaaaacaac aactacacca actaaagttg accctgatac aggagatgta
17401 gttgaacgtg gtgaaccaac aacagaagtt acaaaaaatc cagttgacga gattgtacac
17461 tttgcacctg aagaagtacc acaaggtcat aaagatgagt tcgatccaaa cttaccaatt
17521 gacggtacag aagaagtacc aggtaaacca ggcattaaga acccagaaac aggtaaagtg
17581 gtaacacctc cggttgacga tgtcacaaaa catggtccaa aagcaggcga accagaggtt
17641 actaaagaag aaataccatt cgagaaaaaa cgtgagttca atccagactt aaaaccaggt
17701 gaagagaaag taacgcaaga aggacaaact ggagagaaaa caacaacaac gccaacaaca
17761 attaatccat taacgggaga aaaagtaggc gaaggtgaac caacaacaga agtaacaaaa
17821 gaaccagtag atgaaatcac acaattcggt ggagaagaag taccacaagg tcataaagat
17881 gagttcgatc caaacttacc aattgacggt acagaagaag taccaggtaa accaggcatt
17941 aagaacccag aaacaggtaa agtggtaaca cctccggttg acgatgtcac aaaacatggt
18001 ccaaaagcag gcgaaccaga ggttactaaa gaagaaatac cattcgagaa aaaacgtgag
18061 ttcaatccag acttaaaacc aggtgaagag aaagtaacgc aagaaggaca aactggagag
18121 aaaacaacaa caacgccaac aacaattaat ccattaacgg gagaaaaagt aggcgaaggt
18181 gaaccaacaa cagaagtaac aaaagaacca gtagatgaaa tcacacaatt cggtggagaa
18241 gaagtaccac aaggtcataa agatgagttc gatccaaact taccaattga cggtacagaa
18301 gaagtaccag gtaaaccagg cattaagaac ccagaaacag gtaaagtggt aacacctccg
18361 gttgacgatg tcacaaaaca tggtccaaaa gcaggcgaac cagaggttac taaagaagaa
18421 ataccattcg agaaaaaacg tgagttcaat ccagacttaa aaccaggtga agagaaagta
18481 acgcaagaag gacaaactgg agagaaaaca acaacaacgc caacaacaat taatccatta
18541 acgggagaaa aagtaggcga aggtgaacca acaacagaag taacaaaaga accagtagat
18601 gaaatcacac aattcggtgg agaagaagta ccacaaggtc ataaagatga gttcgatcca
18661 aacttaccaa ttgacggtac agaagaagta ccaggtaaac caggcattaa gaacccagaa
18721 acaggtaaag tggtaacacc tccggttgac gatgtcacaa aacatggtcc aaaagcaggc
18781 gaaccagagg ttactaaaga agaaatacca ttcgagaaaa aacgtgagtt caatccagac
18841 ttaaaaccag gtgaagagaa agtaacgcaa gaaggacaaa ctggagagaa aacaacaaca

18901 acgccaacaa caattaatcc attaacggga gaaaaagtag gcgaaggtga accaacaaca
18961 gaagtaacaa aagaaccagt agatgaaatc acacaattcg gtggagaaga agtaccacaa
19021 ggtcataaag atgagttcga tccaaactta ccaattgacg gtacagaaga agtaccaggt
19081 aaaccaggca ttaagaaccc agaaacaggt aaagtggtaa cacctccggt tgacgatgtc
19141 acaaaacatg gtccaaaagc aggcgaacca gaggttacta aagaagaaat accattcgag
19201 aaaaaacgtg agttcaatcc agacttaaaa ccaggtgaag agaaagtaac gcaagaagga
19261 caaactggag agaaaacaac aacaacgcca acaacaatta atccattaac gggagaaaaa
19321 gtaggcgaag gtgaaccaac aacagaagta acaaaagaac cagtagatga aatcacacaa
19381 ttcggtggag aagaagtacc acaaggtcat aaagatgagt tcgatccaaa cttaccaatt
19441 gacggtacag aagaagtacc aggtaaacca ggcattaaga acccagaaac aggtaaagtg
19501 gtaacacctc cggttgacga tgtcacaaaa catggtccaa aagcaggcga accagaggtt
19561 actaaagaag aaattccata tgaaactaaa cgcgtattag atccaacaat ggaaccaggt
19621 agtcctgata aagtagctca aaaaggtgaa aatggtgaaa aaacaacaac aacaccaact
19681 acaattaatc cattaacggg agaaaaagta ggcgaaggcg aaccaacaac ggaagtaacg
19741 aaagaaccaa tagacgaaat tgttaactat gcacctgaaa ttattcctca tggtacacgt
19801 gaagaaattg atccaaactt accagaaggt gaaactaaag ttatcccagg taaagatggc
19861 ttgaaagatc ctgaaactgg agaaatcatt gaagaaccac aagatgaagt aatcatccat
19921 ggtgctaaag atgattcaga ttcagatgcg gacagcgact cagacgcaga tagcgattca
19981 gatgcggaca gcgactcaga ttcagacagc gactcagacg cagatagcga ctcagacgca
20041 gatagcgact ctgatgcgga cagcgactca gacgcagata gcgattctga tgcggacagc
20101 gattcagacg cagatagcga ttctgatgcg gacagcgatt cagacgcaga tagcgattca
20161 gattcagaca gcgactcaga cgcagatagc gattcagacg cagatagcga ctctgatgcg
20221 gacagcgact cagattcaga cagcgattca gacgcagata gcgattcaga ttcagacagc
20281 gactcagacg cagatagcga ttcagatgcg gacagcgatt cagacgcaga tagcgattct
20341 gatgcggaca gcgattcaga cgcagatagc gattcagatt cagatagcga ttctgatgcg
20401 gacagcgatt cagacgcaga tagcgattct gatgcggaca gcgattcaga cgcagatagc
20461 gattcagatt cagatagcga ctctgatgcg gacagcgact cagacgcaga tagcgactca
20521 gacgcagata gcgactctga tgcggacagc gactcagatt cagacagcga ttcagacgca
20581 gatagcgatt cagatgcgga cagcgattca gacgcagata gcgattctga tgcggacagc
20641 gattcagacg cagatagcga ttcagattca gacagcgact cagacgcaga tagcgattca
20701 gacgcagata gcgactctga tgcggacagc gactcagacg cagatagaga tcataatgac
20761 aaaacagata aaccaaataa taaagagtta ccagatactg gtaatgatgc tcaaaataat
20821 ggcacattat ttggttcact attcgctgcg cttggaggat tattcttagt tggcagacgt
20881 cgtaaaaaca aaaataatga agaaaaataa tatttaaact tcataatttt gggtaaaatt
20941 taaaaccagg ccttacatgg cctggtttta ttttaaatac tatgatataa attaatggaa
21001 atagaacaaa tatataaaaa taaggaggaa acaatggaat ttgaacataa attagagaaa
21061 ttaatatctg aagtaaataa taagactgaa attaataatt atgttttttt aagtttaggt
21121 aagtcaagtg ttaaagcaca ggttaagtta ttaaaaaaaa ctaattatct caaacaagat
21181 atttcaaaat tggcacttaa atttaaaaag aaatctgggg aatttcctga gtggattaaa
21241 ttagatattg taacctcgac tgaaaaaata ttatttaaag aattaaaaaa aacactgatc
21301 aatacaagaa gaaattatgt agattttggt atagcatttg atagtcaatg gaattttgca
21361 gtattacctg aagaaattaa cgcgaatgct tttgttcgac cagataatac tactaaagag
21421 ttatttcttt cggaaaaaaa catcaataat tatttacgca aatatactac aaataaaaag
21481 gcattttcta gtgagttcta taatgaaaaa gaagttatta aattctatac acaaggtttc

21541 tttataggtg atgaagaagt acatgaacta tatagcgaag gctataaaaa aggtttaaga
21601 aaagtaaatg atttaaataa tgaaattgat caattaattg aaagtagtac taatttctta
21661 caaaatatgt tgttagatac tggtaaatat atttatggat attttcctca ttttgataat
21721 gaaattggat tctacaatgt tttaagacac tcttcttcta cttatgcctt aattgagggt
21781 ctgtcctatt taggtaaaag cttacaacct gttgaaaaag caattgatta tatcatttta
21841 aatcaactgt ttgagattgg tgataaagcc tatatctttg atgatacaga ggaagcaaat
21901 gaaatcaaat taggacaaaa tgcttcattt atattcgcag tttgtgaata tttaaagcat
21961 gaagataatc ctaaatttct tgagtcagct cagaaagtgg ctaaaggtat tctttcaatg
22021 atagatgaag atacatatga gacaactcac ttattgaatt atcctgattt aagcgtaaaa
22081 gagaagttta gaattattta ttacgatggt gaagcagcgc ttgctttatt gagattgtat
22141 caaaaggatg aaaatgaact atggctaaaa actgtagaaa atttgatgga ccgtttcatt
22201 gagaagaaat attggcaata tcatgatcat tggttaggat attgtacgaa tgaattagtt
22261 caaattaatc cacaagacaa atattttgaa tttggaatca aaaatgtgaa taattactta
22321 gattatatta aaaatcgtga aacaacattt ccaacattct tagaaatgtt aatggccaca
22381 tatagattag ttcaaaaagc gaaagatacg ggtcgcgagg aattggtaaa caatttaata
22441 gatgaacaat atttgataga tgtaattaat attagagcag attatcaaag agttggcttc
22501 ttttatcctg aaattgctat gtattttaaa aacccatcaa gaatactagg aagtttcttt
22561 attaaacacc acggttatcg tgttcgaatt gatgatatcg aacattattt atctggatat
22621 gtacaatatc agcttgcatt taataggtaa aataagtatt tgaaatttaa attaatgact
22681 tacaaaaaat ggttgattta cataaaaacg aaatcaacca tttttaattt gctctttaat
22741 aattattttt catataacta aaattctaat ttcatcaaga ataactgaaa aaactaaaca
22801 acatgtgagc tattcataac ttgaattaat tgtagaaaat ctatcttccc ataacttcat
22861 aacaatttta cttgaaaaag ctttagagtg cttgatacat tcttgagaaa aagtatctct
22921 taaagtctta tctaataata ggtttcgcac tcgattatat aattcatctt tatcattttt
22981 gttaattaaa taaccatttt cattatttaa tattaattcg gaaggaccat acttcgaatt
23041 atagccaact ggaggtatgc cttcagttat agtttctaat aaacctaaat taaatccttc
23101 catattacta gtaattagag acatataagc atcttgaatc tcagcactta aatttcttct
23161 aaaccctcgt aaaaacacat tgttttctaa attatattca gtgattagtt gcttgtattt
23221 ttcttcttct ttaccaaacc catacaaatg tagttggatg ttaggaaatt ctttaattaa
23281 tttagaaact aattcaattt gatgattcaa ctgtttttca ggagaataac gtgcaacgga
23341 aataatttta ttattattta tactatgatt atttcttttt aaattagtaa aatgttcatc
23401 aatatatcct actggtatag tatgaacagg gatttcatta tttattcgag ctgatatatc
23461 caattgttgc tgtttagtcg atacaattat cccagagtat ctatttagat tgttaaaaac
23521 atgtttataa gtatttttga tgtccgattc atataccatg tcaatattct tgacatgagt
23581 actatgcaaa acagctaaaa caggtattgt ttcactagta ttattgaata ttggtgcagt
23641 atttatattt ttatcactca ggaaaatatc tccattttta tacaattttt caattgcaaa
23701 tgctagtaat tcctgatcat tattaaaata cttaagttga gaatcggtgt tataaataat
23761 aagttgtgat tgtgaatttt caagttctgg attaaaaaat ttttggaact ttatagtacc
23821 ttcaggagtg taataatgtt cacaaactac tttttgattt gttgttaaag ttcttgaaca
23881 acttaaaaag cctcttgaat catatagatc tcgacgtatt ttacgttgat ttacatcaaa
23941 atgatttatg taatccaaat gttggtaatt actatcatta aaatggacat acattctata
24001 gttgcttttt tcatatatct ttatatcatt ggtattttca ataaatttta gcgtataatt
24061 acactcttta gtccaatgag tgatccagtc cttatggatg ctttttatat ttatactgtc
24121 ttgtagcaca tcatataagc ttaatatatc gtcatcttca atttgaaata gacttgcatt

24181 aaaatgtaaa cgaggtgacc acgatgcaaa aatcaattta ctagttatcg agtgattttt
24241 aaataagttg tgtctattaa tcatcgcttt ttcaattccg gttaatttat ttcctaacct
24301 attccctact gaatatatca ttttaattca acctcatttt ttgttattta ttatttagtt
24361 tatttttaat ttcatttaag tttgggtaag aggtaaatat aataaattta tctttatctt
24421 ttataatacc gctattatat ttcctgccaa taattatttc gttagtaaat ttaaataggg
24481 tatttttata tgcttttaaa attttaaata agtcgtcaac agtgaagttt tgtcgttctc
24541 tcatagtatg acgtccagtt aaatttattg ctacagaatt tgaaagactt aaatgttcta
24601 tttttgaatt gatatattca tcgcgctttg gaccgttttc aaataaatac ttagataatt
24661 cataagaagg tgtatacata ggtgaattaa gcatggcttt catcaattcg taagttgtcg
24721 tttcttttcc ttcataataa agtttgttat ttgttgccca gttagcaata aaaatagtat
24781 cttctgatac attatcatct atagctttta aataattaaa gatagggcct aagccttcaa
24841 ttgtacctga atgttctctg ttatgagatt caatgacatt ataattttca tcaataatca
24901 tataactgcg accgttcaag gattgaccat caaataaaga agtgttcgaa ttattaatat
24961 tatatttatt taatttctca ggtaattctt ttgctaattt tggaaattct gtaccacctg
25021 ctgatgattt taatagagtt aatgaatctg gtttatttag gtacaataaa tctttttcta
25081 atagatgtct attagaatac catgttatat ttttgctttt tactcctata acgacactct
25141 ttaaagcatc atccatacat agaatataat ctgcattact attttctaga acatcaacta
25201 attgcaaatg caatgattta gagtgcttac ctaagtcact aatttgccca atcgcaataa
25261 ttttattacc tttaaaaaat tttgtttgag tattaaatgc ttttatagca ttaatcattg
25321 ctggtaatga agcgttgtgt gtatcatcta ttaagttaac tttatagtta ggtgtttcaa
25381 cttcttttag gtttaaaact ttttcaaatg gtttaaatgt acttaagttc tctaaggctc
25441 tttctagtgg tatatctaaa tgcgataatg tagcaaatgt ggctagagag ttttcaacca
25501 taccgtcact tattgaatta attctataag tatattttttg tccgttaaaa tcaattgtta
25561 taactgtata gcctttacta tattgaatag atttaggaca aattgttgcg ctagagtcat
25621 gtgtactata agtaataaca ttacttgtat tttgtttggc gcgttcgatt aatatatctg
25681 aatgtaatgt atctttatta attattgcta ctccctcagg agttaaacca tcaaaaatac
25741 ttgctttcac ttctacaata tttagaatat ctttaaatgt agacatatga gcagcaccta
25801 taccagtaac tatagcaata tttggtttta ttaaatatga tgaattacca actgcattta
25861 atgcgtttaa tgatacttct aaaacagcaa aatttggttg tctaatcatt ttgcataata
25921 ataaatatac tgcggatctt atgttattat taaatctatt ttgaagagga taataatctt
25981 taagaccagc acttatcagc attctagttg aacttttccc attgatcctg ttatagcaac
26041 aacagggtgt ttatactgta gtctcatttc ttcagctaat ttagttagtg tgtttgcaga
26101 atcattgata actaattgag gagttttaaa cttcatgtca ggaacatagg tttcagtaat
26161 aattaatcct aaatcttcct tatccttctt tatatattca ttaccttctt ttgcgtttgc
26221 atctctacca aaacgtctcc atgtatgctt attgggcgat atgtaggctg tgcttttatt
26281 atgaataaag gggaaccaat tctcaaaatt atttataaca ttgtcctctt taccttcagc
26341 atcaaggaga ttacctccta aaatattatg aagttcttta attgttaatg acattttgta
26401 ctcctttata aaatgtaaaa ttgtgtttaa tttctaaata tatatatagt atagcatgtt
26461 gatatactga aatgatttga aaaatttttg aaacttaatg acttataaat tcatattagt
26521 ttaaaattcg atactattcc ataatgatgg aatgtgatgt atatgattaa tcaatataca
26581 tcacatttca tttaatgcaa ctattgcaca agttaatagt atatttaaaa ataggttagt
26641 gagattgatt aattattgat tatgttggta tcaatgaaag cttggaagaa gggcttaaaa
26701 atataaaaac ttggataaat cacaatacaa tcatagacac agataagttc gtcaatctgt
26761 taatgaatta tgatgtgata gagttaagtg aggataaatg ttaacgtttt attaaaacag

26821 tcacagaatt agatagagca tttgcaattt gtgctactga acttataatc taagagctca
26881 atagtgaaat tgtctttttt aaagttgtta aagcataaat ttgttgaatt attacagaaa
26941 aagatcgaaa tttagttact gctaatggaa cggcagcatt agagtttgcc gaactcattt
27001 taaaaggact tgaatttgat agtaaagaaa atattgaaag gcaaatctat atgtatcaaa
27061 gaggcttcta tgagtattgt aataagtatg gcaatcctta tcaataatta aaaaccgcat
27121 cattatctga tacgcagagg cttatcataa ataaaactaa aaattagatt gtgtataatt
27181 taaaaattta atgagatgtg gaggaattac atatatgaaa tattggagta taccttgcaa
27241 tatcatacga tgtttataga gtgtttaata aaccattttt caactattga tgatctagaa
27301 tatataataa ctgtacaaat tatattgatt atggaactac aattcaatta agaaattgat
27361 gatgaaattt taaatttaaa ctaatggaat caagaaagaa tgaaaggaaa tatacaatgc
27421 ccacgattaa taaaaggaag tttattagat tttgtgttag aaacagtaga tgtttttgta
27481 aatggaagta aaaatgattg cgaattagta aattttaatt ataaagaagg aaaagtagtt
27541 tcacgttata gtacaaaatt tgaaagcaat cctaaattaa gaaaaaagca attgatatcg
27601 acggtaaaag ttgtaaggta tgttattttt gattttgaga aaattttatg gggcataggg
27661 aaagttttat tgaagttcat catattaagg caatgtttac tataagaaag ttgaaataca
27721 ctcagaaaca gactggattc ccgttttttt caaattggca caaaatgata cataggccca
27781 cctatatatt gaagatttaa gttaaacgat aaataatatt tttctctttt gtatagaaat
27841 ttttttggga atgatattaa ttagtataaa aatgaatatg tctacaaaga aggagagatt
27901 ttattgaaac acattaatca attttctgaa tcgacactag cacaactaga aaaagatcaa
27961 caacacgtat tttatgtata ctgtttgatg gacccaagaa atgatgaatg cttttatgtt
28021 ggtaaaggta aggggaatag aatatttaaa cataaacaag atgcgcagaa aaaagtattg
28081 tacgaagata tatttataga agaaaataaa gataacttga aatttaatag aattaacgaa
28141 atttgtagca atgatttgaa tgttttagga tatataatca gttatggatt aaccgaatct
28201 gaagcatttt ctgcagaaaa tgttcttata aactttctta acctaacaaa taaaactaca
28261 ttaacaaata tgattaacgg tcatggatct aaggcatact tagttgaaga tttagaaaat
28321 gaatttggct acgattcaat taatcttgaa aacataaata cgaatgaatt aattttagca
28381 gtgaaaatca gagatgcatt tctattagat aaggatgaaa gtaaagagta tcctattaat
28441 gaaagtaaac gtgatagaaa taaccttaaa tcgcgtacat taggtagttg gataatagga
28501 aaagataaaa tacataaaat aaaatatatt attggtatca atacaggtgc taataatgca
28561 gttgtctcag cttatgaagt atcatttgaa caagcggaga gtatcgaaac aaataatggt
28621 agaatgagat atgcgtttat tgggctttca gaaagagatg ctactctaaa aaaattgaat
28681 ttatacaaaa aagcactacc agatttaaga tttggtagtg gtagcgctac agcatatata
28741 aataatggga caatgaaagt tgattaaatg aataaaaaca tccaattcct taaattagcc
28801 tttgaaaatt ttatactcgt atatataagt gaaaaactta aaagaattag atgttaatta
28861 aaggagagaa tgctatgcca tcagatgata ttgtaaaaaa actatactca aaagaggata
28921 ttcgattaga attaggactt actccgcata agtttaataa aaaaatggaa actattgcta
28981 agctttttaa aattgatatg aaaatttttc acaattacaa aggccaagat aaaaataatc
29041 agtatacatt taatggtgtc gcaaaagaat taatcaaagt gttgctaaaa agtgttgatt
29101 attacccagt ggatatcaat tccaaaaaat ttaaacaaaa tggaaaatct aaaaaagaaa
29161 tgattgaaaa tatagataac tctagttaca tgaaatatat ttatcagtta atgaaatcta
29221 tcaatgaaat tcaatacaaa aggttaattg ctgatataca tatgaaagat gtgtaccaga
29281 atacaaaagc atggttaaat aatggtgaat caattaataa aaaagaacaa gagctatacc
29341 aatatatgac aattttacca ttacataaaa gagttgaact gcaaaatgag gtattaaaat
29401 ctatagacga aacaattttt caatttctcg caaaagaaca tagaaataat caaattgaag

29461 aaaataatga attagaagca tatacaaaag caataaaaga aggtagaaat cctaaaaatg
29521 attatgaatt aaatcatctc ctatataaaa aaaatcaatt acccttagat agtctaatag
29581 aagatatatg ggactatgag gaaacagagt atactgagct tgattggtta attgcagata
29641 tgctaaaacg ttcacaaaga gctgatagta attttatcga aagtttagag aagaaaaaca
29701 aattgagaaa aaatattcat aaagattcca ttaaaagtat atctaagttg attgatcatg
29761 aattagttaa aaataataga aagtgggacg tagcagaatt ttataaaaat caacaatttt
29821 ggaataatag tacattaaga cgtagctctc aaaattttgg aattttatat tacaaaaaga
29881 ataggttaca tacaatgaat caaagtaata ttattaagaa aataaaaatt atcgaagcta
29941 atatagagag tgcaaataaa cagccgagct atttagattt ctttttacaa gctaagtttg
30001 atttagagag gttagaaagt gaattaatga aatatagcat taatccgaaa tattttaata
30061 agataaatag tgattacgta agatatgcta ttgaagatgt acataatgca attattaaaa
30121 ttgacagata tataagtaac gataaagtga agttaaacta ttcaaatgtc caaatggtag
30181 tgagaaacga aataacagaa caaggaagct actttgtaac ccaggcattg aatacactgt
30241 caaaggtaga agagtctggc tacagctttg ataatttcat ggtaggacct ttcgtagaag
30301 actttaaaaa agcaattaaa aatcgtagat ttggttctaa aaaagattaa gattgttttt
30361 tagagttaaa cataagaata aatttaaata aaaaaataaa cccaacaaac cttgttatat
30421 caatgtttgt tgggtttatt tttttggtga ttttttaata actcaattac atcttaggat
30481 aatatcgcta aattatacat tgtcgaggcc aagacaaata aaaaattaaa gcaaaagtta
30541 gcgcgcaact ttctgctata aaggagcaaa tttatatgga gacaagagac aaactgatgt
30601 cattgactca aagtgacaaa acacagcaat ggctaatgga caagtcatct aaccaagatg
30661 acattcaaca attgcagcaa caattcagtc agcagcttga tcaacaatat aatgcacttt
30721 tagctgatga aaaagctaag ttagaccaat acgtggaagt acatcaagga ttggaatcat
30781 taaaggaaga gattgaatca gaacctatta cgcttaatat cgataaatta cccgatatca
30841 aagcaacaat gcttgaaaga gccaagaatg atgaacattc tgataaaatc gaaaagctat
30901 ttgataggtt agaacaggca ttaaatggta cgaatcgatt atatacgcaa ttatcgttga
30961 ttggtacacg aacacatcga attacaacta aaaattttaa tcttcaaggc ttacctaaag
31021 cagtccaaca tacgatttta ccttcaaaat ttaagaaggt gtatacagtc gattttaaat
31081 cgtttgaacc atcagttgca gcgtacatga ctcaagattc aaaactgatt gacttgttaa
31141 atcagaaaga cggactgtat gacgcattgc taagtgaatt aggcttatca gatgagctac
31201 gtgtatttgt taaacgtgca tttattggtt cgtttctatt tggaggtaac ttcaaaaatc
31261 ctaaattcaa gctgaatcaa tatgtaagtg aagtacaatg gttggatgcg gtcagccaat
31321 ttacaaaagt cattgaactt aagaagcacg ttgaaaagag taattccatg cctatgcctt
31381 atagtattga gcatgatatg agcgcatttc aaggtagcag tattatggca atctacgtac
31441 aaactgtagc gagttatatt ttcaagcaca ttctgctaaa agtgtacaaa gcacagtgcg
31501 atcaaaaaac gttcaagatt atagtaccta tacacgatgc gattatgatt gaatgtgaag
31561 atgaagaaat tgcacaaaat gtgggtcagt taatgaaaga tacggctaac cagttgttca
31621 atggtgaatt tgcacatgtg acagtggaag aaataggagg cgtagaccat gaataatgat
31681 agaggacaaa gcctacacat cccaagtagt acaccaatca aagaaaataa tatatatgta
31741 gctacgttac attctgtgat ccaaacagat ttctcaggtg aaataaagca ccaattcacg
31801 tatgaaattg aagtgaacaa tcagattgta tatgcgaatc gtaatattcc aacaaaaccg
31861 agcgctaatc agttgtcaat tcatgattgg ctgaaacgtc atagcaacta tagcgcaagt
31921 catgaaaact atgagcctta tattgatcag aaacatttaa ttctattagg tcaatataac
31981 ggtaactatt atgtacaaga tgtagcatcg ttagatgcgt ttggaggcgt attatcatga
32041 atcatatatt acaaatgtta tctaagctat taagtgtggc caaggaggca atcgaccgtc

32101 aaggtctgat tgctatccta actattcctg ttaataataa cgatgaaata gaagaaacgg
32161 ctcaaggtga aaccgtgtat aacgaactta tcgatcagtt acgacttaat atcccaaaag
32221 atacggatta tcaacctaac atctatagtt attttggtat taagaagaat cctaatgaca
32281 ccgtactcat ggaaatgatg ataaaggttt ttcatatcaa acgctttaat tcagaactgt
32341 ttattttcaa agttaacggg tggcaaaaga taaatggaga tgaattacaa gggttgatat
32401 ctaaaatgat acaagtattg cttgtagatt ataagccttc actaagcact ctaaaaaatg
32461 tcgtagatgg attgcaaaaa tcaacagatg tagaagaact tgttgagaat gagcgctata
32521 ttggttgtgg tgaaaatatg ttcgatctta atacgtttca agtcgttaaa aattcaatcg
32581 atatctttcc aaaaacacga ttgaatttat cattaagtac aaatgatgta attactgata
32641 agataccgcc ttattttaag caatatatgt tacaacttgc gaattatgac gatgatttac
32701 aatactttct tttccaacat acagcagtat tacttacagc tgatactaaa taccgtaggg
32761 gtctcatatt atatggtgga gctaagaatg gtaaatctgt atatattgaa ctagttaaat
32821 catttttcta tagtaaagat attgtgtcta agccacttaa tgagcttgaa ggtcgttttg
32881 acaaagaaag tttaattgac aaaagtctaa tggcaagtca tgaaattggg caatctagga
32941 ttcaagaaaa gatcgtaaat gacttcaaaa agttattatc tgtagaatca atgcatgttg
33001 atcgtaaagg aaaaactcaa gtggaagtca ttttggattt gaaacttatt tttagtacaa
33061 atgcgatact taattttcct cctgaacatg cgaaagcttt ggagcgtcga attaatatta
33121 ttccatgtga gtattatgtt gaaaaagcgg acacttcatt aattgataag ctccagagtg
33181 agaagaaaga aatctttctt tacttgatgt atgtgtatca acagattgta aaagcagata
33241 tcgagtatct tgaaaatagc cgtgtcactg aaattactca cgattggtta aattttggat
33301 atgaatttgt ttctagcagg tccgtaagta atgcaaatca gaaagcatgt attaatttac
33361 tcagaaaact tatagaaatc aaatcaggat cacgaatcaa agtatccgag ttaaataaag
33421 ttattaatga agaaataaag gtaagttctc aggttattaa acagttaatt caagcaaact
33481 ttgatactca aaccaaacta tacaatggct acgattattg gattgattta ggttggaaag
33541 aagccaataa aaaagagatt catgatattt cggaaaaaga taatattatt tcattagata
33601 aaaatgaaaa tataacagac gatgaggcat tagatgaaga gaatttggac tttgattggg
33661 aggactttga cgatgaataa tgaacaaatt gaagcatttg tagaagtgct tgtacctatc
33721 atagaagaac gtatcaataa aggtaattaa ggctaattac gtactacagg cagttgcctg
33781 tagtactcat atgattaagt ggtaaaagtg ataaaaatga aacgaaatta taaatatata
33841 ttatctatat gttgttacaa gaccgatagt ctgtagcaat aatctaataa aaggagcggt
33901 atgatatgaa gggtaaaatt gcactttatt cacgcgttag tacgtcagag cagtcggagc
33961 atgggtactc aatccatgag caggaacaag tactcatcaa agaagttgtg aaaaatttcc
34021 caggttatga ctatgagaca tatactgact caggcatttc aggtaaaaat attgaaggtc
34081 gtccggcaat gaaacgtcta ttacaagatg ttaaggataa taaaatcgaa atggtgttaa
34141 gttggaaatt gaatcgtatc tcacgatcaa tgagagacgt gtttaatatt attcatgaat
34201 tcaaagaaca tgatgtaggg tataaatcga tttctgagaa tattgataca tccaatgctt
34261 ctggagaagt actcgttaca atgtttgggt taataggatc tatagaacgc cagactttga
34321 tttcgaatgt gaaactttct atgaatgcta aggcaaggag cggagaggca atcaccggtc
34381 gtgttttagg ctacaaatta tcacttaatc cacttacaca gaaaaatgat ttggttatcg
34441 atgaaaatga agctaatatt gtacgtgaaa ttttcgattt atatttgaat cataataaag
34501 gcctcaaagc cattacaacc gtacttaatc aaaaggggta tcgtactatt aatcaaaagc
34561 cattttcagt gtatggtgtt aaatacattt tgaataatcc agtctataaa ggctatgtca
34621 gattcaataa ccatcaaaac tgggctgtac agcgaagaag tggcaaaagt gataaaaatg
34681 atgtgatatt ggtcaaaggt aaacatgaag ccattataag tgaagaggta tttgataaag

34741 ttcatgaaaa attagcttct aaaagtttta aaccgggcag acctattggt ggagatttct
34801 acttacgtgg ccttattaaa tgcccagaat gcggaaataa tatggtatgt cgacggacgt
34861 attataaaac gaaaaagtcc aaagaacgga caatcaagcg ttattacatt tgttccttat
34921 tcaatcgttc agggagttct gcatgtcaca gtaattccat caatgctgaa gtcgtcgagc
34981 gtgtaattaa tgttcatttg aatcgtattc tgtctcaacc agatattatc aagcagattg
35041 cgtcaaatgt gatagaagaa ctgaaacaaa agcatagtaa ccaaacagaa attaaatatg
35101 acattgatag tttagaaaaa caaaaagcta agcttaaaac acaacaagaa cgattgttag
35161 aattgttctt agatgatcag atggatagcg aaatgttaaa agctaaacaa agtcaaatga
35221 atcaacagtt agaagtatta gatcaacaaa ttaaagaagc gcaacaagca aatcaatcac
35281 aggatgaaat acctaatttt gataaattaa aaggacgact cattttgatg ataacacgat
35341 tcagcgtgta cttaagaaag gctacacccg aagctaaaaa tcaacttatg aaaatgttaa
35401 ttgattcaat tgaaattacg acagataaac aagtaaaact tgtaaggtat aaaattgacg
35461 aaagtcttat ccctcaatct ttgaaaaaag attgggggtc ttttttatg cccaaattcc
35521 aatttgaaat atatggtcaa aatgattatt tcatcgacca aattaccact tttaccactt
35581 agttattagt gacaaaagtg agcgaaatga aataaaaatc aaatatatat tatcaaaatg
35641 atgtatcaca tgcatacatc aatcaaatac attaggaggt cataaccatg acactagaac
35701 aacaactcaa gcactatata accaacttat tcaatctgcc aagggacgaa gtgtggcact
35761 gcgaatctat cgaggaaatc gctgatgata tcttacccaa tcaatatgta agacttggcc
35821 cactcagtaa taaaacactt cagactaata cctactactc tgacacactt catgaaagta
35881 atatctatcc tttcattctc tactatcaga aacaactcat agccatcggt tatatcgacg
35941 aaaatcacga tatggatttc ttatacctac acaacactat catgcctctt ttggatcaac
36001 gatacttact aacaggagga caataaaatg cataaataca tcaaaattac acaattagtc
36061 attacaatac taagtgaaat catcatttgg atgaaagagt cagaacgaaa ggaagtctct
36121 tatgaataga tatatcaccc ggggtatcgc caacaactta cctaatatct tacaacacca
36181 attatggcaa ctcgtatctg agcgagaaca agaacaaacc aaagataata cttcagtaga
36241 ttattttcat atattccagt tcaataagca tcgcaatcaa ttatatatca aacacaaaca
36301 agaacgacct gaatatgcga aaatccataa agctaattat tcaaaagcaa tcaatatcaa
36361 taaggtctac attattcgag aagatgatgt agacctttct tattatgtca tgttattacc
36421 tgaagaatac tagaggagtg aaaattatat ggaaacaatc aaaagtacat taaaaacaga
36481 agccatattc agtgatgaca aacaacatcg ctatctactt aagaaaacat ggaatagtga
36541 aaaacaatca atcacaatca ttacaatgta tccgcattat gatggcattc tcaatattga
36601 cctaacgacc caactcatca tgaacaaagt ttcagaaatg gatgcatttg gttcaatcaa
36661 ttttgtgaat ctatactcta atattacaac ccctatcaat ctcaaacatt tagaaaatgc
36721 gtatgataag catacagata ttcaaattat gaaggcagtg aaagagtcag atgaagtgat
36781 attagcttgg ggcgcttacg ctaaaaagcc cggtgttgaa gcacgtgtta atgaagtatt
36841 agagatgttg aaaccacata aaaagaaagt aaaacgactc atgaatccag aaaccaatga
36901 aatcatgcat ccccttaatt cgaaagcacg tcaaaaatgg atattaaaag tatagcaact
36961 aaaatgttta ttcactatcc atgaaaataa ttgaatttca tatatatctt ttaatctaca
37021 atatgattaa aagatatatt atttggttct gttgcaaagt aaaaaaatat agctaaccac
37081 taatttatca tgtcagtgtt cgcttaactt gctagcatga tgctaatttc atggcatggc
37141 gaaaatccgc agatctgaag agacctgcgg ttctttttat atagagcgta aatacattca
37201 ataccttttа aagtattctt tgctgtattg atactttgat accttgtctt tcttacttta
37261 atatgacggt gatcttgctc aatgaggtta ttcagatatt tcgatgtaca atgacagtca
37321 ggtttaagtt taaaagcttt aattacttta gccattgcta ccttcgttga aggtgcctga

37381 tctgtaatta ccttttgagg tttaccaaat tgtttaatga gacgtttgat aaacgcatat
37441 gctgaatgat tatctcgttg cttacgcaac caaatatcta atgtatgtcc ctctgcatca
37501 atggcacgat ataaatagct ccattttcct tttattttga tgtacgtctc atcaatacgc
37561 catttgtaat aagctttttt atgctttttc ttccaaattt gatacaaaat tggggcatat
37621 tcttgaaccc aacggtagac cgttgaatga tgaacgttta caccacgttc ccttaatatt
37681 tcagatatat cacgataact caatgtatat cttagatagt agccaacggc tacagtgata
37741 acatccttgt taaattgttt atatctgaaa tagttcatac agaagactcc tttttgttaa
37801 aattatacta taaattcaac tttgcaacag aaccgtatta tggaatagag atgttggtaa
37861 catttataca ggatcattat acttaagttt aatttcgtta ttacagaacc acacattcca
37921 accagaagag aaagtatgtc tatttagtta tggttcagga gcagtaggag aaatctttag
37981 tggttcaatc gttaaaggat atgacaaagc attagataaa gagaaacact taaatatgct
38041 agaatctaga gagcaattat cagtcgaaga atacgaaaca ttctttaaca gatttgataa
38101 tcaagaattt gatttcgaac gtgaattgac acaagatcca tattcaaaag tatacttata
38161 cagtatagaa gaccatatca gaacatataa gatagagaaa taaactagtg gccgattgtg
38221 cttgatgagc ttgggacata aatcctaact cgaaataaat aagcatatca ctaaactgat
38281 tttttaaagt ttacagtgat atgcttattt ttttatctta cgattttgta cgtgcatgct
38341 tgcctagggg tatggctcga gccattagtc tctcgcacat actattccct caggcgtcag
38401 cacttacaaa atcggttgta attttcattt ttatacgcat tcttactgag attatactaa
38461 taagaggaat agtaaaagca attctaagta aaattgcaga taagaggttt gttaaaagca
38521 gttctaagta aaattgcaga taagaggttt gttaaaagca gttctcagta aaattacaga
38581 taagaggtac gttaaaagca gttctaagta aaattgcaga taagaggttt gttaaaagca
38641 gttctaagta aaattgcaga taagaggtac gttaaaagca attccatgca aaattgctga
38701 taaggggtaa gttaaaagca gttctcagta aaattgcaga taagaggtac gttaaaagca
38761 gttctaggca aaattgcaga taagaggtgc gttaaaagca gttctcagta aaattgctga
38821 taaggggtaa gttaaaagca atcctaagta aaattgcaga taagaggtaa gttaaaagca
38881 atcctaagta aaattgcaga taaggggtac agaaaaacta gacttgatta caaaatggag
38941 cttgggacat aaatgatttt ttaaaaatga gatgagacgt agattaactc cataatcaat
39001 acgaatctat cgacttcttt atttatgata ttcatctctt tttaatggaa ataaaagtgc
39061 gattaatgtg ataatacagt tacgttaatt aaaaaaataa aaatgcaagg agaggtaata
39121 tgctaactgt atatggacat agaggattac ctagtaaagc tccggaaaat acaattgcat
39181 catttaaagc tgcttcagaa gtagaaggta taaactggtt ggagttagat gttgcaatta
39241 caaaagatga acaactgatt atcattcatg atgattattt agaacggact acaaatatgt
39301 ccggggaaat aactgaattg aattatgatg aaattaaaga tgcttctgca ggatcttggt
39361 ttggtgaaaa attcaaagat gaacatttgc caactttcga tgatgtagta aaaatagcaa
39421 atgaatataa tatgaattta aatgtagaat taaaaggtat tactggaccg aatggactag
39481 cactttctaa aagtatggtt aagcaagtgg aagaacaatt aacaaactta aatcagaatc
39541 aagaagtgct catttcaagc tttaatgttg tgcttgttaa acttgcagaa gaaatcatgc
39601 cacaatataa cagagcagtt atattccata caacttcgtt tcgtgaagac tggagaacac
39661 ttttagatta ctgtaatgct aaaatagtaa acactgaaga tgccaaactt actaaagcaa
39721 aagtaaaaat ggtaaaagaa gcgggttatg aattgaacgt atggactgta aacaaaccag
39781 cacgtgcaaa ccaacttgct aattggggag ttgatggtat ctttacagac aatgcagata
39841 aaatggtgca tttgtctcaa tagaaagtta gaggtgagtc ttacgtttca gtgacggtag
39901 acttaccttt aacatgttac atactaaaaa attaatttga ataagaaaga gagacatata
39961 tgaaatacga tgattttata gtaggagaaa cattcaaaac aaaaagcctt catattacag

40021 aagaagaaat tatccaattt gcaacaactt ttgatcctca atatatgcat atagataaag
40081 aaaaagcaga acaaagtaga tttaaaggta tcattgcatc tggcatgcat acactttcaa
40141 tatcatttaa attatgggta gaagaaggta aatacggaga agaagttgta gcaggaacac
40201 aaatgaataa cgttaaattt attaaacctg tatacccagg taatacattg tacgttatcg
40261 ctgaaattac aaataagaaa tccataaaaa aagaaaatgg actcgttaca gtgtcacttt
40321 caacatacaa tgaaaatgaa gaaattgtat ttaagggaga agtaacagca cttattaata
40381 attcataata aaacagtgaa gcaaccatcg ttacggattg cttcactgtt ttgttattca
40441 tctatatcgt attttttatt accgttctca tatagctcat catacacttt acctgagatt
40501 ttggcattgt agctagccat tcctttatct tgtacatctt taacattaat agccatcatc
40561 atgtttggat tatctttatc atatgatata aaccacccaa tttgtctgcc agtttctcct
40621 tgtttcattt tgagttctgc agtaccggat ttgccaatta agtttgcata agatctataa
40681 atatcttctt tatgtgtttt atttacgact tgttgcatac catcagttaa tagattgata
40741 ttttctttgg aaataatatt tttcttccaa actttgtttt tcgtgtcttt taataagtga
40801 ggtgcgttaa tattgccatt attttctaat gcgctataga ttgaaaggat ctgtactggg
40861 ttaatcagta tttcaccttg tccgtaacct gaatcagcta ataatatttc attatctaaa
40921 tttttgtttg aaatttgagc attataaaat ggataatcac ttggtatatc ttcaccaaca
40981 cctagttttt tcatgccttt ttcaaatttc ttactgccta attcgagtgc tactctagca
41041 aagaaaatgt tatctgatga ttctattgct tgttttaagt cgatattacc atttaccact
41101 tcatatcttg taacgttgta accaccccaa gatttatctt tttgccaacc tttaccatcg
41161 attttataac ttgttttatc gtctaatgtt ttgttattta acccaatcat tgctgttaat
41221 attttttgag ttgaacctgg tgaagttgta atctggaact tgttgagcag aggttctttt
41281 ttatcttcgg ttaatttatt atattcttcg ttactcatgc catacataaa tggatagacg
41341 tcatatgaag gtgtgcttac aagtgctaat aattcacctg tttgagggtg gatagcagta
41401 cctgagccat aatcattttt catgttgtta taaatactct tttgaacttt agcatcaata
41461 gttagttgaa tatctttgcc atcttttttc tttttctcta ttaatgtatg tgcgattgta
41521 ttgctattat cgtcaacgat tgtgacacga tagccatctt catgttggag ctttttatcg
41581 taaagttttt cgagtccctt tttaccaata actgcatcat ctttatagcc tttatattct
41641 ttttgtttta attcttcaga gttaatggga ccaacataac ctaatagatg tgaagtcgct
41701 tttcctagag gatagttacg actttctgtt tcattagttg taagatgaaa tttttttgcg
41761 aaatcactta aatattcatc catttttttta acggttttaa gtggaacgaa ggtatcatct
41821 tgtacccaat tttgatccat ttgttgtttg atatagtctt cagaaatact tagttcttta
41881 gcgattgctt tataatcttt tttagataca ttctttggaa cgatgcctat ctcatatgct
41941 gttcctgtat tggccaattc cacattgttt cggtctaaaa ttttaccacg ttctgatttt
42001 aaattttcaa tatgtatgct ttggtctttc tgcattcctg gaataatgac gctatgatcc
42061 caatctaact tccacatacc atcttcttta acaaaattaa attgaacgtt gcgatcaatg
42121 ttaccgtagt ttgttttaat tttatattga gcatctactc gtttttttatt tttagatact
42181 ttttttattt tacgatcctg aatgtttata tctttaacgc ctaaactatt atatattttt
42241 atcggacgtt cagtcatttc tacttcacca ttatcgcttt tagaaatata actgctatct
42301 ttataaactt gtttgaaatt tttatcttca attgcatcaa tagtattatt aatttcttta
42361 tcttttgaag cataaaaata tataccaaac ccgacaacta caactattaa aataagtgga
42421 acaattttta tctttttcat caatatcctc cttatataag actacatttg tagtatatta
42481 caaatgtagt atttatgtca aaataatgtt ataatttttg tgatatggag gtgtagaagg
42541 tgttatcatc ttttttaatg ttaagtataa tcagttcatt gctcacgata tgtgtaattt
42601 ttttagtgag aatgctctat ataaaatata ctcaaaatat tatgtcacat aagatttggt

42661 tattagtgct cgtctccacg ttaattccat taataccatt ttacaaaata tcgaatttta
42721 cattttcaaa agatatgatg aatcgaaatg tatctgacac gacttcttcg gttagtcata
42781 tgttagatgg tcaacaatca tctgttacga aagacttagc aattaatgtt aatcagtttg
42841 agacctcaaa tataacgtat atgattcttt tgatatgggt atttggtagt ttgttgtgct
42901 tattttatat gattaaggca ttccgacaaa ttgatgttat taaaagttcg tcattggaat
42961 cgtcatatct taatgaacga cttaaagtat gtcaaagtaa gatgcagttc tacaaaaagc
43021 atataacaat tagttatagt tcaaacattg ataatccgat ggtatttggt ttagtgaaat
43081 cccaaattgt actaccaact gtcgtagtcg aaaccatgaa tgacaaagaa attgaatata
43141 ttattctaca tgaactatca catgtgaaaa gtcatgactt aatattcaac cagctttatg
43201 ttgtttttaa aatgatattc tggtttaatc ctgcactata tataagtaaa acaatgatgg
43261 acaatgactg tgaaaaagta tgtgatagaa acgttttaaa aattttgaat cgccatgaac
43321 atatacgtta tggtgaatcg atattaaaat gctctatttt aaaatctcag cacataaata
43381 atgtggcagc acaatattta ctaggtttta attcaaatat taaagaacgt gttaagtata
43441 ttgcacttta tgattcaatg cctaaaccta atcgaaacaa gcgtattgtt gcgtatattg
43501 tatgtagtat atcgctttta atacaagcac cgttactatc tgcacatgtt caacaagaca
43561 aatatgaaac aaatgtatca tataaaaaat taaatcaact agctccgtat ttcaaaggat
43621 ttgatggaag ttttgtgctt tataatgaac gggagcaagc ttattctatt tataatgaac
43681 cagaaagtaa acaacgatat tcacctaatt ctacttacaa aatttattta gcgttaatgg
43741 cattcgacca aaatttactc tcattaaatc atactgaaca acaatgggat aaacatcaat
43801 atccatttaa agaatggaac caagatcaaa atttaaattc ttcaatgaaa tattcagtaa
43861 attggtatta cgaaaattta aacaaacatt taagacaaga tgaggttaaa tcttatttag
43921 atctaattga atatggtaat gaagaaatat cagggaatga aaattattgg aatgaatctt
43981 cattaaaaat ttctgcaata gaacaggtta atttgttgaa aaatatgaaa caacataaca
44041 tgcattttga taataaggct attgaaaaag ttgaaaatag tatgactttg aaacaaaaag
44101 atacttataa atatgtaggt aaaactggaa caggaatcgt gaatcacaaa gaagcaaatg
44161 gatggttcgt aggttatgtt gaaacgaaag ataatacgta ttattttgct acacatttaa
44221 aaggcgaaga caatgcgaat ggcgaaaaag cacaacaaat ttctgagcgt attttaaaag
44281 aaatggaatt aatataatgg ataataaaac gtatgaaata tcatctgcag aatgggaagt
44341 tatgaatatc atttggatga aaaaatatgc aagtgcgaat aatataatag aagaaataca
44401 aatgcaaaag gactggagtc caaaaaccat tcgtacactt ataacgagat tgtataaaaa
44461 gggatttata gatcgtaaaa aagacaataa aatttttcaa tattactctc ttgtagaaga
44521 aagtgatata aaatataaaa catctaaaaa ctttatcaat aaagtataca aaggcggttt
44581 caattcactt gtcttaaact ttgtagaaaa agaagatcta tcacaagatg aaatagaaga
44641 attgagaaat atattgaata aaaaataaaa ttgttgtgtt tacaacaata catagaaaac
44701 agaggaaaca atcaagtcgt tgaatatttc ctctgttttt tagttgaaaa aattaaccga
44761 aagcctgaat gcaagtcttg attaaatcaa taatgcttgt aataacacca gtgaaatcca
44821 tatgcatacc ctctttctat ttaagataca ttaagtataa tatcaaacaa ataaaaaatg
44881 ttaaaaattc cctaattggc tatttagatt gcataaatgt caaaaatttg aaaaacatac
44941 aacgactttg cataaaaaat cgtcatattg gaaatacgta atttattgaa ataataaaaa
45001 aaataaaaga acgaagatga taacctaagt gaggttttaa gttgttctaa ggtttaattt
45061 aatttatgtt aaaatagttg gtataaaaat acatgataaa ctataaacta aattcaaaat
45121 aacttatggg gtaggcaatt atggaaaata aaatataaat gataatgaaa aaagagtgct
45181 aagggaaatt tataaccatc ataatatttc gcgtactcaa atatctaaaa atcttgagat
45241 taataaggca acgatttcta gtattttgaa taagttaaag tataaatctc ttgttaatga

45301 ggttggtgag ggtgatagca cgaagagtgg tggtagaaaa cctattcttc tgaaggttaa
45361 tcatctttat ggttatttta tttctttgga tttaacttat agttctgttg aagtgatgta
45421 caattatttt gatggtaatg tcattaagca tgaatcttat gatttacctg atgaaaaggt
45481 tagtagtata ttaagcataa taaaaaaaca tattgatatt caggagaaac ttgatactta
45541 taacggacta ttaggtgtgt ctgtttctat acatggagtt gtggataatg agcagcatgt
45601 gacatattta ccattccatg aaactgaagg aatttcaatt gctaagaaaa taaaagaaat
45661 tactaatgtt ccagtcgtag ttgaaaatga agcgaatctt tcagcgttat atgaacgtaa
45721 ttttaatcat aatttatcct acaataatct tattgcttta agtatacata aaggtattgg
45781 tgctgggctt attattaata atcaattgta tcgtggtgca aatggggaag cgggtgaaat
45841 tggaaaaaca cttgtctcaa aagttagcga taatgtggag atctttcata agattgaaga
45901 tattttttca caagaagctt tactgcataa tttaagtaat caactaaatg agaagatgac
45961 gcttagcaaa ttaattcaat tttataatga aaaaaatcca gtcgtagttg aagaaatgga
46021 acaatttata aataaaattg ctgttttaat acataattta aacacccagt ttaatccgaa
46081 tgcaatttac attaactgtc cattgttcaa tgaaatgcct gaaatattag aagcaattaa
46141 gaaccagttc aaacaatatt cacgtaacga aattcaaata aagttaacat ctaatgtcaa
46201 atttgcaact ttgctaggtg gtacattagc aattatccaa aaagtactac agattaatga
46261 tatttactta gatataaaag cataaaaaac taattcaaat gaataatcaa agttcgtaat
46321 tgtctttata aaaaaatccc tcaatccgaa ttgaattttc ggattgaggg atttttatag
46381 ttctattgca gaagaaaact attttaaaaa tgctggtaaa tgttgatagc cacctctaac
46441 gttaacaata ttcgtaaatc ctttatattc taatattcct accgctattg aacttctaac
46501 acctgattga caatgtacat aaattaggtc atttttatcg aaaggtatat cttcatttaa
46561 aagtttaccg tgaggaatat gaattgcttg ttttaaatga cctttacgcc attcatcatc
46621 attacgaaca tctaatacat tatgttcttc accagtcatt tcagaactat gaatagatga
46681 tgtgacgata tttgtttgtg gcaaacggta actttttaca ttttcaaaac caattaattg
46741 taaagcatga atagcttgtt gaacggtaga tttatcgcca attaattcaa tatcttagtc
46801 ataatctaaa taccaaccaa tttgatttat aaaagtttta ttaaaaggaa tattgatagt
46861 tccatgcata tgaccaccat ggaatgcttc tttacttcga agatcaaaag cagtttgtgt
46921 attgcttgaa ctagggtaaa cattatatgg ttggtacatt tgcataccaa attgatttat
46981 ttttttcatt tgtgaaaaat ggtgtggtgg agctggctga ttgagtgtta aagtttcgat
47041 aaatgaagtt tcatctttaa cattaaaagc ccagttgttt attttctcat aacccaaagt
47101 agttgtaggt aatgaaccta gcgctttacc acaaggactc cctgcaccat gacctggcca
47161 aatttgaata tagtctggta atgttgcagc aaattgtatg gactgataca tttgttttgc
47221 tccgattttt gtagaacctt caacatttac agctttttct aatagatctg gtctacctac
47281 atcaccaaca aagatgaagt caccgctaaa taatcccatt ggtatactgg aacccccacc
47341 ttcgtcagta agtaaaaaac taatactctc agggggatgg cctggagtgt gtaagacttc
47401 taatttaatc tttcctaaat agataatatc ttgatgctta acgaaatgtg tttgtttagg
47461 catattttta taattaaatt catctttacc ttcatcagat acgtatatac ttgcattcaa
47521 tttatttgcc acatctctaa tacctgaagc aaaatcagca tgaatatgtg tttctgcagc
47581 tttagtaatt gtgaatcctt ctttatctgc aacttttaaa tattttgtta aatctcgtat
47641 agggtcaata atcattgctt ctcctgtacg ttgacatcca attaaataag atgcttgtga
47701 aaaattgtct tcataaaatt gtttgaaaaa caaaaaaaac tcctttttaa aatagatttt
47761 attgattaga taaataagtt atgatttgct tgctcagtat gtccaatata agtacctacg
47821 ccaccataat cgacttcatc tcttaattct tcttttgaaa ttcccataac atccatactc
47881 atggtacaag caattaactt tatatcttga tcgattgctt gatcgataag tgagtataaa

47941 gaatcaacat ttttcttgtt cattacataa cgcatcataa tattacctag tccaaacata
48001 ttcatttttg ataatggcat atgtattgga tccttaggta acataaggtc aaacattttg
48061 gaaatacctt tctttttaac gcgagttgat tgcgcttttt ttaatgcgtt gaggccccaa
48121 aaagtaaaga aaatagttac atctttacct gctgctttag cgccatttgc gatgatcatt
48181 gctgctactg ccttatctaa ctcaccgcta aataaaacaa ttgttgtacc tgtagcagtg
48241 tcattgattt caaattcttt tggcttttct ttttgaataa ttgcattaat tacatttgct
48301 tcttcagtaa gatttacaag ggtattccct gtttgtttcg cccaactttt aatatcacta
48361 ttgaaaccag gatctgtaac tgttacctcg atttgctcac ccgttgaaat attgttaatt
48421 tctttactga tattaacaat aggtccaggg cactgaagac ctctaaaatc aaattgttta
48481 cgattctctt tgatttcaat atctttttct attaaaggag cactattgaa gttctttgct
48541 tcataatctt tatatccacc ctttaaattc acgacatcat aaccttgttt ggctaaataa
48601 tcgcaagctt tagtgcttcg gttaccgctt ttacaatgta tataatacgt tttgttgcta
48661 ttcttattga atgatttaat ctcttctact gggtgtaaag ttgaaccgtt aatgtgtcct
48721 aattcatatt cttcttttgt tctaacatca atcaattgac ccatttttgc caatttttct
48781 aattcttctt tgtttaatga attaatgtgt acttctttgt attgttccat acttacctcc
48841 tataaatacc tatgagggta taataaaacg gatagaatca tttgccaaat acctatatgg
48901 gtatttgaca attttgtttt aatttattat tattaactta atcaatttat gtggaggaaa
48961 tgaatatgac ttatgataaa aaaatgatta atcgtataaa tagaatacaa ggtcaattaa
49021 atggtgtcgt aaaaaatgatg gaagaagaaa aagattgcaa agatataatt acgcaactta
49081 gtgcatctaa aggttctata caacgtttaa tggggattat aattagtgaa aatttaatag
49141 aatgcgttaa aacagcagaa gaaaataatg aaagttctca agaattaatt aatgaagcag
49201 ttaatttatt agttaaaagt aaataatgga tatagcaaat atgactatta tgttgctaat
49261 tggcgtactg ggtggattta tatctggatt aataggtatt gggggcgcaa ttattattta
49321 cccagctatt cttatattgc caccattaat aggtatacct gcgtatagtg catatattgc
49381 ttcgggactt acctctagtc aagtattttt cagtacactt agtggatcat taaatgcaag
49441 aaaacaacca gctttctctc ctaaacttgt tatatatatg ggaggggggta tgttgattgg
49501 aagcatgtta ggggcaattt tagctagttt gtttaatgct acttttgtaa atacggtata
49561 tgtaataatc gccatacttg ctttaatatt gatgtttatt aaagttaaac ctactacaca
49621 agagacgaaa tctaaacctt tgctatttat tatagttgga tttggaattg gtgtaatttc
49681 gggaattgtg ggtgcaggtg gagcatttat catcattcct gtattattag cattatttaa
49741 attaccaatg aatacggtag tgaacaatag catagcaatt gcttttatat cttcagtagg
49801 ggcatttttt ataaaattaa tgcaaggata tataccagta gaaagtgcaa ttttttgat
49861 aattgaatga tgatcatttt ctgaaaatat tatgtggtca tataatataa tgcccatcat
49921 ttcactaatc tcttttattc tctgagttat tttgatatct cctggagaag gtgttacatc
49981 ttatcaagag taaattacaa aagaatcatt taaatcaata ctttcacttt gaatacatgt
50041 atttgaagtg gaaggtactt atttcaaaaa tagtaaaacc tgtatcttaa attacttaat
50101 agtaacataa gatacaggct gattttttat tcattgttat ttatactaaa gcacccgata
50161 gctctgaaaa caatcacaaa ttcaactttt caaagccaca gctttaagtt attttgtcca
50221 gacaaccccc attgcccaac ccattttatg gaattggcat ccaggcaaca acttttcata
50281 taaatcgtaa taattttgtt cagataggta cttatctgaa gctaaatgct caagccatga
50341 tttagatgtg ttgtgattat aaattctaat cgcatttttt attccaaatt tgaaacaatg
50401 cggtaaaaac tcttggattg cccctaattt ataaacatag gtaggcggtg tctcgacttc
50461 agatacatta tcaagaataa ctattcttcc ttcctcattc aacagttcct tcatttgctg
50521 tattacgctg gctatatcat ccaaatgatg aaaggttgtt cggcttacaa taaaatcaaa

50581 cttctcatta aaattaagtt gttctgcatt catattcaga tagaccgtat ttgttagttg
50641 acgtttagat ttggcaagat cgagcatttg attagaaata tcaatcccta ccacttcatc
50701 ataataactt gctaatttct ccactaacaa acccgagcca catccgatat ctaatgctct
50761 gcctttcttt ggagacatat tagacacaaa gaatgaataa tcattcaaaa gctcattcac
50821 gaaatcgtaa tcttctgcaa ccttatcaaa ctgtgattct attgtattca aaaagatccc
50881 ccattcctac tttatcgaca ttctttcatt acttaccact ttagatgttt tttcgttggg
50941 gataaaactt ccctttagac aattttatcc aaagacaata caacagtgca actttattaa
51001 agtcactgtc ctttatcgca gcctttactt tttagtaaag acagtggctt ctcttatcaa
51061 gtttcaaaac atattatttt gaagaaaacg tccatctgaa gtgtcaagtg caaaattaca
51121 tataaaggtt tattctaaaa tgaaaagatg atacaatcat attcagttac ataaggaggt
51181 ttcaattatg tgcaccagta tcgcagtagt agaaattact ttatctcatt cataatgaaa
51241 aaaatggaaa ggagataaaa gtatgggtac tttttctata tttgttatta ataaagtaat
51301 caaaattaat tggttataat gaacgcttaa tgtcagttca ttataaccag taaggagaag
51361 gttataatga accagaaaaa ccctaaagac acgcaaaatt ttattacttc taaaaagcat
51421 gtaaaagaaa tattgaatca cacgaatatc agtaaacaag acaacgtaat agaaatcgga
51481 tcaggaaaag gacattttac caaagagcta gtcaaaatga gtcgatcagt tactgctata
51541 gaaattgatg gaggcttatg tcaagtgact aaagaagcgg taaacccctc tgagaatata
51601 aaagtgattc aaacggatat tctaaaattt tccttcccaa aacatataaa ctataagata
51661 tatggtaata ttccttataa catcagtacg gatattgtca aaagaattac ctttgaaagt
51721 caggctaaat atagctatct tatcgttgag aagggatttg cgaaaagatt gcaaaatctg
51781 caacgagctt tgggtttact attaatggtg gagatggata taaaaatgct caaaaaagta
51841 ccaccactat attttcatcc taagccaagt gtagactctg tattgattgt tcttgaacga
51901 catcaaccat tgatttcaaa gaaggactac aaaaagtatc gatcttttgt ttataagtgg
51961 gtaaaccgtg aatatcgtgt tcttttcact aaaaaccaat tccgacaggc tttgaagcat
52021 gcaaatgtca ctaatattaa taaactatcg aaggaacaat ttctttctat tttcaatagt
52081 tacaaattgt ttcactaaat taaagtaata aagcgttctc taatttcaca agaggacgct
52141 ttattcttcc caaaaattgt tcaatattta tcaataaatc agtagtttta aaagtaagca
52201 cctgttattg caataaaatt agcctaattg agagaagttt ctatagaatt tttcatatac
52261 ttaacgagtg ctttcacctt tgaatatagt ccttcccact tatcatcaca ctctccccga
52321 tagccttttc tagctatatc cagtaaagtt acatgctctt taggtaaaag aggtatagcc
52381 cattctgcag cgacatcttt cgaggtaatt tcaccagtag tcactgtttg ccacattcga
52441 gctagggtta aaattacatt acgctcatca ccttttatcc cctcaattag ttctggcaaa
52501 gaatccttaa ttgctcttcg aatatctgtc aaaggtacgg agacaagtat acttgaagaa
52561 tcaggaccaa atagagaaat actattcttt cttgcttgtg ctaaaacaat agccaaatca
52621 ggatcatagc ttggttcctg aatttgtcca ttctcaaatt cacccctgag ccactcaccg
52681 tatataaatt ctctttttgg aggatattgc caagggacaa cttcactcct atttataacc
52741 gtaacttcaa gtggtctaac agaatccgta tttccaatct ttcctgatat agtcattagt
52801 ctttctgtta gttttttcg agttaattga ggtaaactat gattcacgac gactagaaca
52861 tctacatcgc tgttaatgcg taaaccacca tttactgctg aaccaaatag atatactcca
52921 actattgaac ttccaaataa atcttttacg atttttaatg tttgaatcgc ttgatttggt
52981 atttttccgt taatcaaatt gctcatgatt tcacctcgtt gattatgttc atataaagtt
53041 tatattgata ctcaatttac ttaccctaga ttggacatat acttaaatta ctgttcaata
53101 aagctgaccg ttagcgttta agtacatcct ttcacaattt gtctacagat taataattat
53161 tctttattat acagatctcc atataatttt tgaatttggt tctgtaattt tttattttct

53221 ttttctaatt ccattactct tctttttaag gttttaataa ggatttcctc cgaacgagaa
53281 cttttcttgg gttttgagac tacatttgct gttatttgac gctcacgaag ggattcgatt
53341 ctttgcctaa tatcgtgttc cttataaagc catgatttag aaacattagc ttcctttgct
53401 attgaattaa aattaataac tttaccttca atcgaaaatt tagaaatcgc tttgtctact
53461 ttttcccttg tcttttttga tttctgcttc gccaaacgta caatttctgt tgtatttcta
53521 acttgtttat ccattgataa ttaccccgtc aaacttccaa tgatttgttc taaacgctct
53581 ttaacacggc tattagtctc tacttgtctt tgccattgtt tatccttagc tatggctaat
53641 aactcttctg tacgctctaa ctgttcttcg tgctgtggta agaattgctt actggtacag
53701 aagtgagtgc aatctaagca tgcattcgca tgtggacaac cacctgctac tactggcaat
53761 ctacaataac catttggaag cacttgtgca tttatatttt tcttgaacca ttgaagctct
53821 acatcatcga cttcattatc ttcatctaga tcaagcacat ctccattatt ggtaaccagt
53881 ttttcctgaa atttagtaaa ttcatttttt agagtttcat caaagatatg agcgtatctg
53941 cttgtcattt ctgggctttc atgccccaaa aatttctgca caatatgctg gggcatcccg
54001 ttgttaatca ttcttgttcc tactgtatgg cgaaaggcat gggcatggaa tctataaatc
54061 tcacctgatt tatccactat attttgctca taagctaatt tatttaactc acctctaaat
54121 gtttcttgtt ttaatggcga tccatctttt cttggaaaga ggtattcact atctggaaat
54181 tcctctgaaa ctttatcttc ccgaacttta ataagtaaag ctacctcttt agatattgga
54241 actatatgct cctttttcat tttccattga taatacttta aaaagaaatc tccatctttg
54301 tcctctaata gacagccttt tttcaaggtg cacaattcac ttatcctcat tccacattct
54361 tgaacaatca tagtcatcgt agctatatat tcggataatt tatcaagatg actgttcaat
54421 tgctctagga cgaattcatc tataaagcgt ggttttgctc ttggtatttt cggatagtcc
54481 tcagaataaa ttaatatttt ggaaggaaca tcatcccatt ctagcctaag aagggtacta
54541 aatagtcctt ccaatataga gatcctccca gttattgtac taggttttat tcccatcatg
54601 tttagttcac ttaaatatgc ttcaatttcc actctcgtta attggtgtac tctctgaact
54661 tgtttaaatt tcatgtccag aaaattaaag aactctttaa gtctttgggc aatatcactt
54721 acataggaaa agctatccac gttcaatctc aacttacaat atctttttac aagttgttta
54781 aaatatgtat tccgaaaccc tttaaagtta attgtatatt catattgtgt tgggttaacc
54841 ttatcatctg gcaaaggtaa gttacgtcta tcccaaacgt ctttatccca ctcctctcca
54901 tcaaaataaa agttctcata aaactccata aattgtttta gattagtaac atagtaggaa
54961 ttagctttta caggtgtttt ttcttgatta gcagtaatct tataattagt agtggtaatt
55021 ctaacacccc gttttgtcaa ataagttcta tactccgtca ttgctttttc aataggaact
55081 tcagtaattg aagtaatgct aggatacttt aaatctaaga aatctaacat tttattaatt
55141 actgttcctt ttctaatcca gacagttttt gcattcccata ttccattgtt taaatggtaa
55201 aaataaaaat atttcaattc tgttcttaac cacagatttt taacacgttc aaaacgaacc
55261 caacgattcc ttaaagcagg attcttactt aattctatgg cagaaggatg tggacatttt
55321 cttatatccc aactattatt agcccaaaac ccctgcattt cttcattcat tacagctatt
55381 tttttgctaa tctcactctg actaataatt ttccttttac tagaagcatt catttcttat
55441 gctcctttct ctcgaggtat ttattaaact catttttcat atcctgatct gaaagatgaa
55501 cataggtatt taacgttgtc tgaacatgtg cgtgacctaa tctcttttga acgaacgcaa
55561 catcccatcc ttccctaatt agctgcgttg cgtgagtgtg gcgaagcata tgtgatgtaa
55621 attctattcc agtcctttta actattcttc taactagatc aagaacactt tggtacttta
55681 gtggtttccc aaaatagcct tcttttaagg aaataaaaac ataatcatgc tccaattcct
55741 cactatactc atatatcaag taatctgtat aaagtgacat aagttcttta ctcacatgta
55801 ttgttctttc cttccttaat ttaatataag cttcattaac attaacatct ctaggtgtta

55861 aatggatttg attgtcccaa gtgacaatat cttcaagcct aagcgataac acttcaccga
55921 ttcttaaacc accctcatac ataagcatta aaattaattt atctcttttc gtatgacaag
55981 catcaataat ttgcttaact tcctttgatc tcaatgttct tatctgtttc tttttaaccc
56041 ttaactttaa gacattcttt tggtatctac ccttattaac atgatgtaaa aatcctttga
56101 aatttcttcc ctttgcttgt ttaaatacat caattgattt aaattctcct aatctactta
56161 aataatcaag aaaactcata actacattta aaattgtatt cactgtcgtt tcttctctta
56221 tggctttttt tgactgaaga tcaattacat ttgatgctga aggatatctc aaccaaccta
56281 cgaagtctgc taacaactca aagttaatat cattaagaat aacacctctc tgttccatga
56341 actcgtacag caactttaaa tgatagcagt atgccttaat ggtattagga gacttaccag
56401 tattatctaa gtatttaata aatttcatta ctggttctat tagctggtat tctttatcta
56461 gtaataaata caatggatac ggcttattct ccacttctat cctttgaacc ttcacatgtt
56521 cccacctctt taaatacttt aactactaca aattataaac cttgttaatt tttatttaaa
56581 gtaaaatatc cctttaatat ctcctcttaa actactttaa ctactattat ttattatact
56641 atggttaata catctccact tggatgatta tgaccgagca ttatactatt tgcgttactg
56701 agtatcgctg ttttgaatat ttctctaggg tgaatcaccg tttggttaat agatccaatc
56761 gatagtgttt gaatatgtgt aggttcattt ttactgttca tacatatgag aatgagatgc
56821 tctcggtcac tgtttccaat gaatgaacgc atgatttctg ccgcatcctc agggtttgaa
56881 atacgatttt ttagataact taatgtatct gtttttatca tttgtagtga aacaatattg
56941 atttccttca tcgtttacct ccatatatag gttatgcttt caaagtccat ttttgacgtg
57001 ctttagggtt gagtgggtgc atgatttcat ttgtggctgg attaatgagc tttttgactt
57061 tctttttatg aggcttcaac atttccatta cttgttcgac acgttctaca acaactggcc
57121 gcttcgcata agcaccataa gctagaatca ctgtgtcact ttcgctaatt gctttcatca
57181 agtggatgtc tgtgtgttta tcataaggtt ctttaatatg tttaaggttc tctggtgttt
57241 taatattaga gaatagattt acaagatata cagcaccgta tcgttctgaa ttcgctaatt
57301 gattgaggat aagaacagtt gtgagatcga gtgataatac accatctaaa tgagggtaca
57361 tcgttatcac tgtacaagcg ggtttctttt catcccatgt tttcttgagt aaatagcggt
57421 gtttttcatc cttgctaaat atagcttctg tgtatatcgt atttttgatt gtattcatat
57481 atataatcac ttcctttagt attcttctgg taaaagcatc acataataaa aagcgtctac
57541 gccatcttca cgaatgacgt agactttctt aggtaatgca ttttgatttt ttacatagtt
57601 tgtatagtga tattccaatt tgtatgcagg ttgttcttgt tcatgtgtga ttgagagtat
57661 attctcatct tcttgcagtt taaaaatgtg taggtaatct gtatgaggtt gattatctct
57721 ttcttttacc atattccaaa gtaagatttg aaggtctaga gataggtatt cactaatacc
57781 tcttgtgatg tatcgattga ttttcatgtt attttacctc gtcttgaatt tctttcataa
57841 tgataatcgc ttggctaata atcgtaacag atatttgtgc cactttgatc cagttattca
57901 tggtgagtcc ctccttctct tagtaaatga cgttcatcga taatcgtatt tttagtatct
57961 gtgagatata aaaagtccat gtcaaaatga ttcagataac caacgctgat gagttggtta
58021 ttagcgtaca taagaaatgg atagatactt aggtcatgta gttcattatt gtagtaggta
58081 taagtttcaa gtgtaagatg tgcaagtggg gaatcattaa taaaacgttc cggtagaata
58141 ttttctgctg cttcttctaa cgcttcgcat tcccatgttt cattgttaga tagttggaat
58201 agacgagtta tatattgttt gagttcttga gtggtcgttt tcatatcatt gcctcctaga
58261 tagtgttata gtgatgtagt ttatgtacat cattgggata atatatattt gatttgtcat
58321 ttattacgca tcccggtgag aatgagagaa aattccatat gaaaaaccgc ttcaaacctt
58381 ggtatgacaa ggaaatcccg aaattccgcc tattttgacg aacaatcaac tcattcttta
58441 taactattga tgttagggtg gggctctgct ttcttatata ttatttattt ataaagaata

58501 acgggatttt gggattgtgc ttgcacaatc cttctgcttc ttcgaatctg caaatcccaa
58561 tcatttcccg ataaaaaatc attgtgggat gttctttagc aatttcaata taagcatcgt
58621 gtagttatga aaaaaattac gacaatgact gtttcattag ataagtgtta ttgaaattga
58681 taaagagaat tttaaaaatg gttagataaa ataaatgaaa gaatataatg aaaattattg
58741 ttataacaat gattctatta gctaaatagt aagatatagt gtttggggca aaaacaaaga
58801 cgaagtgctg agatgcactt cgtcgagttg tttattattg aaaagttgtt ttatgatttc
58861 gttattaagt ttgagtgtga catagaattg ttttttatga ttaccatctt ttttaatatc
58921 aatgcgatca atcactgata gatacaatgc tttgagtcga gatttttcta tgtgcttaat
58981 atcatgaaag atgtgttgta atagtttact gatttctttg gcatcaaata aaggcttatc
59041 ttcattttgt tgatttttga gttggttgat ttgattcgta atgtcattga gttgcttttc
59101 atatttttga atacttggtc tgattactga tgttaagtcc ggattatcct cgatggtttt
59161 aatcaagtta tttagtttga tttgtacttc atcatattgt tgttgcttat aagcaatatc
59221 gtgatgaagt gcagcgccat caacttgatt ttcttgattg acgtgtgtta ctacgcgttg
59281 aatgacttta tcacttttga ctatttcaag tatttgcttc atcacataat cttcaatcac
59341 atcagctctt acactgtttg ccgaacatac tttggaaccc ttgttccgaa aattactaca
59401 tgaatagtaa cgaatacgtt tcttagtccc gtctttaagt gtattcgtgg tattgcttgc
59461 tgccataggt gcgccacatt ggggacagtg aataatgcct gtaagcagat tcgttccttt
59521 gccatggact tggggttttt gactgacttg ttttttacgc atttgtactt tatcccataa
59581 atcttgatta ataatggggg aatgcttacc ttcagctatc actggtttat cattaagccc
59641 tttacgacgt ttttcactcc aatctttgta tttcgcaaat tgaattttgc cgatatagaa
59701 tgggttagct aatatatatg tgatagaact aatactaaaa ggtttaccct ttttagtgac
59761 atagcctttg tgatttaatg cattggctat tttacgatag ccatggcctt tggcatagga
59821 ctcgaatata tatttcacaa tattagcttc atgttgatta atcatcagtt catgtttact
59881 attaggtatt ttgtcatagc ccagcggcaa attgccttga taatagcctt cttgggcacg
59941 tctcgtttga cccataaata cattctcgac aatgttatta cgttcgaatt ctgagaaact
60001 cgcaagtatt tgtaacatga gcttacccga tgaagtattg acttccatac gctctgataa
60061 actgaaaaat tcgacatttt gtttgtgtaa atcttcgaca attttgagaa gatcagatgt
60121 attacgagct aatcggtttg ttttgtagac cataacacag tcgatatagc cttctttcgc
60181 atccttcaat atacgttgga gctcaggtcg attcatagat ttacctgaaa taccacggtc
60241 agcgtatata tctttaactt caaaatgatg gaagtcacag tattctttga tttgattgat
60301 ttgtccgtcg atactataac cttctgtgct ttgcatttct gttgatacac gtacatagat
60361 accgacacgt tttgttttaa gttgttgcat tatgtttcat cctttcttcg tttatgcaat
60421 cgatgattgc atggtttgat tgacgatatt gagtggttca tttttgaaat agattcctat
60481 aagattttta tctttcgtaa tgtgaatggt ttcaatatag gggtacaata tgtttaacgt
60541 gaaacgtttt tgaataatat tttgaatggt gtgttgtatt tgatgtccat tgatagatgt
60601 agtgcgttgc ggttgttgac gtaatgattg cgtttgttct ctgaacgttt ctgcatcgat
60661 gatgccttgt gccaactttt ctatcagttg ttcttgagtc aatgtgtgat gtttttctat
60721 gtttctttgt cttttgatgc gtttgtcaat cgcaccttta atttttgtgt agatgcgttg
60781 attttgataa aagtctttgc acacttctaa tactttatct tcaagtgttt gtgcattgat
60841 gcctttaaaa tcacagacaa agcgagaagc attcatgttt ttaggacaga cgtagtaacg
60901 taatgtatga ttcttttttc taacggtcat attcgtaagt gttgtattac aacatgggca
60961 tttgattttt tgttttagtt ggttatccga agatgtctgt ttgatttgtt tttgcgatcg
61021 aagtctctgc gcttgctcat atatacttgt ggaaacaata gaaggaaaca tattgtcgaa
61081 ttggccatat tgattgttga cacgaccaca ataattagga ttgatgataa tgttacgaac

61141 ttgatagggt tgtcgattga tatacgtgtt atcttcttct aataactgtg caattttctt
61201 ataaccatga cctttaatgt aataattgaa tacagccttt accgttggtg actcattttg
61261 attgatgatg aatgctccgt tgtgatattc gtaaccaaag ggcgcatggg ttgtaatcaa
61321 tcgaccttgc tttgcttttt cttgaagccc atttctgact tgttctccaa tgttatctga
61381 ttcaagttcg gccaagctga taaaaatatt aagcttgaat cggtcgaaag cttgatccat
61441 atcaaagtaa ccatcgtgta cgcttaagat atgaacatgg tatgtttgac ataatttgat
61501 gagttttaat gcatttttca gattacgatg taatctatta agacgataac aacataatat
61561 gtcacattgc ccttgttgaa ttaattgcgt catttgtcga tacccactac gattatcttt
61621 gcgtcctgat tgtttgtcgc tataaaagtt gatatgttga atatgatgtt tttcggctat
61681 tgcttcgata gcctgtttct gtgctgcaag agattgttgt ttcatcgtac tttgacgtaa
61741 atagcctatg acttgtttca tatcggctcc tcctttcaca gtaataatat atatttatgg
61801 atgaattgat atataagccc aacatcaata agatgttggg cgttcatatt agtcattcat
61861 ttgattgatt tcttcaatta ccaaatcggc taatatctcg ataagttcat ccatgttttt
61921 cactccgtta tttgttctat cttcaatacg tcgattattc agtttgatgc ttcacggttg
61981 tatgataaag acaatcagaa atcttcgtga actcctgaag ggcctatccc ctcattagcg
62041 gatttaaaaa gttctttcgc agctttgtta tcatttgccg gtgtccaatt ttgaattaac
62101 gacttatctt tagttaatcc caggatagat gcaaactcta catctaattt tagatggtaa
62161 aatacaagtg attgtttttt accgctatta tctttgacac ttcttttagt tgtttggcgt
62221 ccacggtcag ctaatatgaa acctttatct cttaaggcgt tgacaacatt attaacatct
62281 tgaaattgat gattgtttag catctgttta aaaacgtttg caatcatttt tacttcgata
62341 tggtcatctt ttaatgagat taatccatag ttctcaaaca tatttttcaa agcaccttca
62401 tctgaaaact tacctctgtt ttgtgctaca aattgaatga tgacatcgat agctttatca
62461 gctaatgagc gttcagagat tgtatgacca tgataatcaa taaagtagtc tcttatttta
62521 gcgatatcaa tatctgtagc taaaacacga cctaatattt ttgcagatgt tgtaatgact
62581 gcataacgct taaacatacg attgcctgtg ttgcttttat catctttcaa tttagcttca
62641 aaccaatcta cttccttgta aaaccattga ataacttcat cttcacgatt tataagatat
62701 ttagctacta acggtaaaac atgaccatag tttagtgcta cagctttttt aatattgtca
62761 gcattggtcg catttgtagt gaattgttca ttaatctcga tggttcttac acgtaatcca
62821 tcgttttgag ctgaatcatt aaaaatactg tgttctgacg ttgaaatgac agaagtaccc
62881 caattcttag gcgttttaac ttctccatga acgtttgaac gttgacgacc ttgaccttca
62941 gtgatggagt acaataaccc cgttgtatct ctaaaagttg ctgatgagag ttcatcaaat
63001 acaataggta taccaaaatt gttactcaag taaccttcaa gtgcattacg tgtggcattc
63061 caatttctaa agagagtttc attacctttg gtagggttac cagcgactga tacagctaaa
63121 gaagctgcag ttgacttacc ggttgaggat tgacctgtaa aactaaaaat gattccggca
63181 aattcggttt catgtttgtg cttcaggaaa ctcgtcacta aggcagaaat accaaatacg
63241 actgctaatt ctaaaagaag agaaccttta acctctttta gatacatgtt aaaccaatta
63301 tcaaatgttc ccctaggtgc taagtcataa gtattctcac aaatggcgtc agatggagat
63361 ttattatcaa attccgtagt agtatagatt tcatttaacg atacaatagg accaaacggt
63421 gtttccagta tacctacccc ttcatataag taggaaatgg gtaattggtt gcgcatttgt
63481 tgcaacgcat aacctaaatc ttttgtatat ttttcattaa tactaaatcc atatttcatt
63541 aaagagggca gtttttgtgt tgttaaaata tcactagatt caacaattac tttttgatcc
63601 tcgtctgtaa taattacttt ttcagtgtta gttttagggt caataaactt attttcgata
63661 acgataggac ctgcgatttc aacttcagta ggcattcctc ctttttcttt gggaggcttg
63721 tctttatacc aacctttttt tgatttgtat cgtggtgaag gattaaatga agggttagtt

63781 tgagtcatta gcgaacacct cctttcgaag ggttgctgtt atagtgtgga ttaggacctg
63841 ttttaagata aactaagtga ccgtgagtat ccttaccgat aataataaat ggaacacgtg
63901 gcgcatgttt tacaaaatat gcgaaccaac gtccaacatt ttgttgtaca gcttttgaac
63961 attgtacatt tgcacgactg ttcaagtcat gaaatgtaaa ttcttctcct tcaggtaaat
64021 tgaatgaaat acctaatatt ttctttttta actgctcgaa tttatctgat tgattgaaat
64081 acatttaact tcctcctact aatgaactaa gataggaaaa ttaaatatgc gcacaattaa
64141 ctttcttaag ttcattttag ccaagaagtt atcatttgaa agcttgtaaa aatatgttag
64201 aaattccgta ctaaattaag atggattttt gttgacaaaa taaaaaacgc tgatttaaca
64261 gcgctttaaa taaaaattaa tctgaagtta tataaaagta gtcagaagga gtggtgttat
64321 tatcattata ataatctagt gctttattga tttcttttat taatttatca tgatgttctt
64381 ttgttatttt ctttggatat ttgagttctt tttcttctaa gtcttttgga gacactaggt
64441 cactatttaa aattaatgat aacagcatat ttttaggtgc cgaagctctg gtgtctcgat
64501 gagtaatata ttttgctaaa ttactgttta tacttacttt ttcattttct aaaaagtttg
64561 tactattttt aacatgagca tcagcaattt tggtacttat ttcagtgttt aaatcgtcta
64621 tttcatctaa ataatattca agaatctgaa gtttatcttt agaagtgata cctttttcag
64681 tgaaaatctt aataatgata gatttgattc caatttctat attactgtct actagaaaat
64741 agagataata attagtcaga gcatgttcat aatctctttg atcagtaatt tcatgattga
64801 tgaaataata taagttctct ttgatacgat tagaagaatc tatatctttg tcattgatac
64861 cgtttttaaa aaagtgtaga taaactgagt tgtttgagag tatatcatat aatacaaata
64921 aactttttgg tttaaataca tatgattttc ctgatttaaa gtttgctatg tttattccta
64981 gagcgttgca gaatttatta aaattagttg taaaattatt tttttcttga ataaacatgt
65041 cgttgtcatt taattctgaa ctatgatttt taatcatatt gatattttct tcaatcttgt
65101 cactacttaa attttccatg caaagtatac tatatcttac tatatcactt atatttatgg
65161 aaaaatcttt aggagaacga tttaaagtat cctttttatt atcttgatct acagccctac
65221 atgaatcatc aaaaacttct ttgacattta ttttatcaat gttcaaaatt atgtctacta
65281 aagcattttg aaaatcatcg ctatatatgt ctttattatt catagtgtca ctacctttat
65341 ttttaataac actatatatt atatcttata aacctaatac catgaagaat aaaaagaatg
65401 attcaaattt tatatttaat ccatagttgg ctgctatggt tagtgtacta gtctagtttt
65461 actagtatat aaaacagtac tcaattttta tttttcaaa attataaaaa agaacaccta
65521 tcatcgatag gcactgaacc cctaaaacgg gaacttaata aaaacaccat gttctaggct
65581 attaattctc tgtattttac aggggataag tagcctagtt tttgttgaat ttgattatta
65641 ttatagtttt taatgtactt ttcgacaata tctattacaa tatgattaga gctattaagc
65701 tgattattga tgtaaagagt ttcagacttt agcgaggaat ggaaactttc tatcggggcg
65761 ttatcggcag gtgttccctt tcgggacata cttctgataa tgcctttttc ttcgcataat
65821 tgataataag cataagatgt ataaacgctg ccttgatcac tatgtaatat atacccctca
65881 ggtatatcga tttgatttaa tgtatcatta actaaacgtt ggtcttgttt atcatctatt
65941 ttatacgcca caatttctcc gttataaata tccattatcg aagataaata caacatagaa
66001 tgatcaaatg gtaaataagt aatatcggtt gttaatactt ctatgggaca attcgcttta
66061 aattatcttt gtaataaatt gtctgtttta taatacggtt tacctatcct tgtcgtcttt
66121 ttaggtctaa ctcggcagtt caaatgatgc ttctgcatca ttctctgtac tctcttatga
66181 ttaattggtg atgtataaca ttgattaatc agtgctgtaa tcttacgata accgtaggta
66241 taatggttag cttcacataa ttcaataact ttttgtgtta cagtatcatt tttataggtt
66301 ttgtttttcc atcggtaata tgttgattta ggtatattta atacttctag tatcaatttg
66361 attgaatagt ttccttttaa ttgatccact aaatctatga ctactgttgg taccacttcc

66421 tttccaatgc cttgtacttt tttaaaatat ccaattctat atcttttctc ttattttcta
66481 attttaattg ttctacttct gacagctctt ctaatccttt accgtaggta tattgtttac
66541 caacgtgttg tgaaaatcta taactttccc catttcgata ccatcgccac caagtttcca
66601 cttgtgttct atttttaata tttaattctt tcataatttc ttttgttgaa aatcctgctg
66661 ctttcatttc aactgcttta tactttgttt ctactgaata agaaactctt ttcatagaaa
66721 aaacacctcc gtatgattca ttttaatatg aattcaacga aagtgttttt atataattcc
66781 cactaattgg ggtcagtcta ctatatgata cggtttttta atttaaagta tcaataataa
66841 ttggatatag agggaaagaa gctataatga tatttgctta ctaagtggat aaagatatta
66901 gaaaaacgag aagcaggaca gttattaaat cggtagatat taatcataag tatttaactg
66961 aatttttacc ttttgtcgaa tatataacta aagttgaagg tagtaaaata tttatctagt
67021 cgatattaaa acaatttatc gaaaacgatg gttttccata tcctctttag tagcaatttt
67081 ctaacaatat ataattaatt atatacaaat taagaataat taagaagtat atattaatta
67141 gagatagttt aaaaattaga tttcaatcaa ttaattaaaa caggttagac tattaattta
67201 tactaacctg ttaaaagtaa caaaattaag aatatattaa aacttgtgta atgagtaagc
67261 ctccaccaaa tattaaaaca aacgcgacaa caggccatac gaaacgtaac cagtgtgagt
67321 agcgcacgtt taacatttga agtgttgcca ttacaagtcc agtaggcgct aagaacaaca
67381 ttgcatactg accgaattga tatgttgtaa caataacaaa tcttggtata cctactgtat
67441 cagctaatgg cgcaaagata ggcatagata atactgctaa tcctgatgat gatggtacga
67501 taaatcctaa acagaaaaag ataaagagca gaacaatgat aaataaaggc ccactcatat
67561 gttgcacgat agatgatgaa aagtgcaaga ttgtgtcaga aatcattcct ttattcaata
67621 ctaagttgat tccacgagct aaaccaatga ttaaagatac acctactaaa cttgaagcgc
67681 cattaacgaa tgcatctaca gtgccttttt cgcctaaacc atattgtcct gttccagcaa
67741 taaacatgat gacaatggta aagattaaga atgcagatgc catgactggg aaccaccatc
67801 cttgtgtcat aacaccccaa accataatag ggaatggtag gacgaaaagc gtaagaataa
67861 tctttttacg taatgtaaac tcagaagaac cgtcatcatg gagcacagac cactgttttt
67921 caaatgctgc tttgtcttca taagaataag aggatttagg atctttttta atttttttac
67981 aataccagaa taaataacta ataacaaata tggcaccgat gatacaagcg cctattctcc
68041 aataaagacc atcagtaaaa gttgttcctg ctgcattaga agcaatgacg actgagaatg
68101 ggttgattgt tgagaatgta ctacccacag agcttgctaa gaaaattgca ccgaaagtaa
68161 gactttagaa tctatgggaa agataataag gtttaataga aatacaagaa gtgtaaatag
68221 taagcatata atttatacat tttaatataa tataacaaga aaggtaaaca caaagataat
68281 aaaaataaaa atttaataaa gatagagaat tattaaattg tttttgatat aaaacaactt
68341 aataaaaaat ttaattttag gggaggataa ttataatata aagaaacata gaatggaatt
68401 gcaattggga agtatacgat tttaactgcg aatcatatgt tcaacgactt acaaatgcgt
68461 aaatcgataa cacattcaaa aatatataat ctgaaaattg atttactaag agaggattat
68521 tgtagttaca ggtgttaagt tgctccaata tggagttgtc gtcttgtttt atattaaaca
68581 agttttattc aaatacatga caatacgtaa aataacacta gaattatcaa tcactaaaat
68641 taaaacaaat gtatctattc aaacttttct ttagggttta cttgctgaat aatttaaaaa
68701 tgaagtatta ggtgtacatg ctttcagaat tgcttttgaa gaatattaaa agtataatct
68761 gaatacgatg acagaatgaa taggtaaact atgacaagaa gtcatcaagg ttatcaaata
68821 aatatatccc agcggttgag acgactcact gctgggattt tattgttagt tatttattct
68881 aagtgttgca tttggtagcc gattctagga tgtgtgatga ttaatttctt ttcagtgttt
68941 gggtcttgcg tataattcaa ctttttacgt aaagaggcca tatgtacacg taaggcagcc
69001 atttctgtat gattgacata gccatagagt gattttaata acacttgata ggttaatact

69061 ttgccgacat gatggcataa tatcgttagg agttggaatt cattcggtgt tagatgtacg
69121 gactgttcat tgacaaggac tgatttcgca tcgaaatcaa tggttaatgg accatttgtg
69181 aaacgacttt gaattgtctc agtagaacgt gacatacgta atgctactct gatacgtgct
69241 cttaactcat cgatattgaa aggcttagtc atataatcat tggcaccgcg atctaatact
69301 tcgacaatgg tttgttcctc tgttcgtgca ctaatcacaa taataggtgt gtccacaaag
69361 tctctgaatt gctgaatgag agataaacca tcaatatctg gtaagcctaa atctaataag
69421 ataatatctg gttgttctgt tcttaggcga aagtccgctt ctttccccgt cttcgccgta
69481 accactttat aataattcat agttagcgca acatcgatta aatgtaaaat cgcttcatca
69541 tcttcaacga ccaataatgt tgttttcatc catggctccc tccatttcaa ttggattaac
69601 agtcaaataa aaataaaaaa tactgccttg tggtgtattc ggttgatatt ctaattcact
69661 gttgtgttgt ttcaagatga gttgtactaa atagagccct aatcccaaac tatcttttt
69721 attgtcttta aagttgtctc ctgaataata gggattaaaa atcaattgac gttcttcttc
69781 tggaatacct tttccgcaat ctatcatttc gaattttatt ttgtttgttt catgttgaac
69841 gtgcagtttt atttcagaat gtgattctgc atgctttaaa gcattatcga taaggttgaa
69901 tagcacttgc agtattaatt tactgtcgat attaatgagt gaagcgtcat cctcattttc
69961 aataatgaca tgatttgctt ggtgtctgcg tatgaggcct tcttcaaatt cttctagaag
70021 ttcttctact aaataagggg tgcgttgtat ttgaatgtca gagctttcta acttagtcaa
70081 agataaaata tttgtgacta aggtatgcag atattgtgct tcgccataag aagcagttaa
70141 gagttctgct tgttgttgat cgtttaaatg ctcgttatgg tatttcagca tatctaagtt
70201 gcccataatg gaagtcagtg gtgttctaat atcatgtgaa attgaatgca agaagtttga
70261 acgtgtggct tctcgttcag ctttcaatat ggattgtctg gtttgtttta atagatccac
70321 attctctatt gccagggtaa tatcgttcaa catagagtct aatattgaat tgtcataggt
70381 gtcgatgtaa gtttcgtcgg tgaagcggat agaaatcaca cctttgactg gatttgtgcc
70441 aatgggaata cacaaaaatt tactgccagg aaaggtatcg gttaatttac cggcacggct
70501 ttcattttca atgacccagc tcaatgtctc agcatcatgt gtcttatctg agcttgaaat
70561 gcttctgttt ccaaatgagt tggaagcagc gactttctta ctttgaatta agaataccgt
70621 gacatcctga ttgagtagct gatgaatctg atcaccagca atatttaata agcgttcaat
70681 tgaataagat tctttaatgg actggttaaa ttgcagcata atattggtcc gatataactg
70741 ccgttctgtt aaggaatgct gatgttttaa attctttaag atggcactcg taaagatact
70801 agcaaaaata ctggtcgcaa acgtaatcgg atattcaaag cgatacattt ctaaagtgaa
70861 tcttggcacc gtaaagaaat aattaaaaac aaacacattt aagatagacg caaagaatcc
70921 gattaaatag gattgggtcc aaatagaaag cacgataata ccgataaaga acatcagcaa
70981 gataatggca ctggattcgc ttttatctaa gttgtaaacc catataccga gcaagacaca
71041 gatcgtttga attacaagca tcttcataat ctctacagtt aaacgagagg atttttgagc
71101 ttgcttaggt gtatttgtag gtttgtctga atgaatataa tgaatcggta caatttctaa
71161 cttgaaatgg tgtggtacgt gattaatttg ttcaatgagt gactgtttaa agtaatcttt
71221 ccaacgtggc tgttctgact gtccaaggac tagcttcgtc acaaaagcga gatcacacca
71281 atcggttaac gctttcgcaa tatcttgtgc atacaacact ttgatttctg cacctaacgc
71341 tttggcgagc attagatttt tatggacata atgatcttgt tttctgcttt ctgacggtgt
71401 ctcaaagaca tcaatataca cagctgtgaa tttagcatgt tctttatagg cagcacgtct
71461 tgcttctcgt atgacccgtt cgttataaat actgccgctg atagctaccg caatatgcgg
71521 tgtaatgtcg gtatgtttag ttttatattg ttgtcctttt tgactcataa tatctgcgac
71581 agttctgagt gtaagttcac gcagctctgt cagattttca tacgtaaaga aattagaaaa
71641 ggctgtttct aagcgttctt ttttatatac ttttcctgct ttaaggcgct gaatcaacat

71701 atttggtgaa atatccacaa cttcaaaggc atctgctgac gtaatgaatt ggtcgggcac
71761 acgttctgta acttgaatac ctgtcattaa cgcaatttgt ccgcttagac tctcgatatg
71821 ttggatgttg agtgttgtcc agacatcgat accatgcgat agaatttctt ctatatcctt
71881 ataacgtttt aaatggcgct cttttgaaat gtttgtatgt gctagttcat caattaagac
71941 cacatctgga ttagcttcta tgattttaga gacatctata tagtgaaagg tgtggctgcc
72001 aaatttacgg ctggaggtcg aaatttcagg caattgttga accagtgcat tggtttcagg
72061 gcgttgatgg ggttcgatat aaccaatttt aatatctgca ccttcttgat actgatcaat
72121 accatttgat aacatttcat acgttttacc tacccctggg ctatagccta aataaatggt
72181 aagtttccct cttttttat atgtactttc catgaggcac cccctcttaa tcacatcata
72241 attaatatac aacatattat ccatttatct ttctgttttg ttaatcatct ttatagtttc
72301 tttataactt tccttaaatg ttaatacttc gattagataa ctcttgttaa gattttgatg
72361 aaagtaagga gggagcgcaa tgattacact attagctgtc gttgtcatcg cattaatttt
72421 atttttattt tacgcattaa tttggagtga aaaattttaa cagagaaaga gggaagcatc
72481 atgagtattg tgttgttttt gattgtattt atcttgctct cactcattgt gagccgatat
72541 ttatattcag ttgctttaaa tgtgccatct aaaatagatg ttgtttttaa tccgattgag
72601 aaattgattt atcaactgat tggcacgaaa ttagaacaca tgtctgggaa gacgtatatc
72661 aaacattttt tgttgtttaa cggattgatg ggcggattgt cctttgtatt attgcttatt
72721 caacaatggc tgtttttgaa tcctaaccat aatttaaatc aatctgtatc gttagccttt
72781 aatactatgg catctttttt gaccaatact aacttacagc attatgcagg tgaaacagat
72841 ttaagttatt taacacaaat gtgtgtcatc actttcttaa tgttcacgtc agcagcgtca
72901 ggttacgccg tatgtattgc gatgttaaga cgtttgactg gaatgacaga tgtgattggt
72961 aatttctatc aagatattac gcgttttatt gtacgggtgc tcataccttt cgcattgatc
73021 atcagtttgt ttttaatcag tcagggcaca ccgcaaacgc ttaaaggtaa tttggtgatt
73081 gagacattat caggtgtgaa acaaacgatt gcatatggac cgatggcgtc tttagaatct
73141 attaaacatt tagggacaaa tggtggtggt ttcttaggtg cgaactcttc tacacctttt
73201 gaaaatccga catactggtc taattacgct gaagctttaa gtatgatgtt gattccaggt
73261 tcattagtct ttctattcgg tagaatgttg aaaactaaac tacagattca tccgcatgcg
73321 attatgattt tcgttgcgat gtttgtaatg ttcatcggct ttttagtgac atgtctctat
73381 tttgaatttg cggggaatcc agtgttgcat cacttaggta ttgccggtgg caatatggaa
73441 ggcaaagaaa cacgtttcgg tattggctta tccgctttat ttacaaccat tacgaccgct
73501 tttactacag gaacagttaa caatatgcac gatagtctta caccgctagg cggcatggtt
73561 ccaatggtat taatgatgtt gaatgcagtt tttggcggtg aaggtgttgg gctgatgaac
73621 atgttgattt atgtcatgtt aacggtcttt atctgtagtt tgatgattgg gaaaacacca
73681 agttatttag gaatgaagat tgaaggtaaa gagatgaaac tcattgcgct ttctttctta
73741 gtacatcctt tacttatttt ggtttttca gcactagctt ttattgtgcc aggggcatca
73801 gatgcgttaa ctaatccgca attccacggt gtatcacaag tgttgtatga gtttacatca
73861 tcttcagcga ataatggctc tggttttgaa ggattaggag acaatacggt attttggaac
73921 atttcaacag gcattgtgat gttgcttgca cgatatattc caatcgtttt acaaattttg
73981 attgtatcta gtttggtaaa taaaaagacc tatcagcaac atactcaaga tgtaccgatt
74041 aataatttat ttttcagcag tgtattgatt atctttatta ttttgttgag cggcttaacg
74101 ttcttacctg acttaatgct tggaccaata ggcgaacagc ttttgctgca cgcatagata
74161 aaggaggatt agaaaatggc tgaaactact aaaatatttg aatcacattt ggtcaaacag
74221 gctctaaaag acagtgtatt gaagctctat cctgtttata tgattaaaaa tccgattatg
74281 tttgttgtag aagtgggcat gctgcttgcc ttaggattaa ccatttatcc ggatttattt

74341 caccaagaaa gtgtatcacg gctatatgtg ttcagtatct ttatcatatt attactgaca
74401 cttgtctttg cgaacttctc tgaagcatta gctgaaggtc gcggtaaagc acaagccaac
74461 gctttacgcc aaacacaaac tgaaatgaag gcacgtcgta ttaaacaaga cggcagttat
74521 gaaatgattg acgctagtga cctgaaaaaa ggacatatcg tacgtgtcgc gacaggtgaa
74581 caaatcccaa atgacggtaa agttattaag ggcctcgcaa cagtggatga atctgcgatt
74641 acaggtgaat ctgcacctgt aatcaaagaa agcggtggag atttcgataa tgtaattgga
74701 ggaacttctg tagcttcaga ctggttagaa gttgagatta cttcagaacc aggtcattca
74761 tttttagata aaatgattgg tttggttgaa ggggctacaa gaaagaaaac acctaatgaa
74821 attgcgttat ttactttatt gatgacatta acgattatct tcttggtcgt tattttaacg
74881 atgtatccat tggcgaaatt cctgaatttc aatttatcca ttgcgatgct gattgctttg
74941 gctgtgtgtt taattccaac aaccattggg ggattattat cggctatagg gattgcaggg
75001 atggatcgtg tgacacagtt taatatcttg gctaaaagcg gacgttctgt agagacttgt
75061 ggtgatgtga atgtcttgat tttagataaa acaggtacca ttacctacgg caaccgtatg
75121 gcagatgcgt ttattccggt gaaatcatca agctttgaac gtttagttaa agcggcctat
75181 gaaagttcta tcgcagatga cacaccagag ggacgtagta ttgtgaaatt agcttataaa
75241 caacatatcg acttaccgca agaggtcgga gaatatattc cgtttactgc tgaaacacgt
75301 atgagcggtg tgaaatttac gacacgtgaa gtatataaag gtgcaccgaa tagtatggtt
75361 aagcgtgtga aagaagcagg gggacatatt ccagttgatt tagacgctct tgtcaaaggg
75421 gtgtctaaaa aaggtggcac accgctggtt gtgcttgaag ataatgagat tttaggtgtt
75481 atttatttga aagatgtcat taaagatgga ctcgtagaac gtttccgtga attacgtgag
75541 atggggattg aaacggtgat gtgtacagga gataacgaat tgacagctgc gacaatagcg
75601 aaagaagcgg gtgtggatcg ctttgtggca gagtgtaaac ctgaagataa aatcaatgtg
75661 attagagaag aacaagcgaa aggtcatatt gttgcgatga cgggtgacgg tacgaatgac
75721 gcgccagctt tagcagaagc taatgtaggt ttggcaatga actcaggaac catgagtgcc
75781 aaagaagcgg cgaatttaat tgatttagat tctaatccaa ccaaactgat ggaagtcgtt
75841 ctaattggga aacaattatt aatgacacgt ggctcactca ctacatttag tattgcgaat
75901 gacattgcga aatactttgc gattttacca gccatgttta tggcggctat gcctgcgatg
75961 aatcatttga atattatgca tctgcattca cctgaatcag cagtattatc tgcgttaatc
76021 tttaatgcgt tgattattgt attattgatt ccgattgcga tgaaaggcgt gaaatttaaa
76081 ggtgcctcaa cgcaaaccat attgatgaaa aatatgttag tttacggctt aggcggtatg
76141 atcgtgccat ttatcggcat taagctcatt gatctcatca tccaactctt tgtctaaaag
76201 gaggacaaaa caatgcagac aataagaaaa agtttaggac tagtactgat tatgtttgtt
76261 ttatgcggat ttatcttccc gctgactgtc acagcgcttg gacaagtatt atttccagaa
76321 caagcaaacg gcagtttagt gaaacaagat ggcaaagtaa ttggttcaaa gctcattgga
76381 caacaatgga cagaacctaa atatttccat ggacgtatca gtgcagtcaa ttacaatatg
76441 aatgcgaatg aagtgaaaga aagtggcgga cctgcttcag gcggctcaaa ctacggcaat
76501 tcaaatcctg aattgaaaaa aagagttcaa gagactatta aacaagaagg aaaaaaaatt
76561 tcaagtgatg cggtgaccgc ttctggctct ggtttagacc cagatattac ggttgacaat
76621 gcgaaacaac aagtaaaacg cattgcgaaa gaaagaaaca tagatgcttc aaaaattaat
76681 caccttattg atgaaaacaa acaagcatca ccaatggcag atgattatgt taatgtctta
76741 aaattgaata tcactttaga taaactctaa ataaacaggg agtgaggtga gacatccatg
76801 tggttcatta gcattattat tttaatagca ttcctaatta tattaatgat tgacgattta
76861 attaaaacat gacaacttca cgcaaaacaa tatgaattgg atgtaataga gaagggagta
76921 ccaagaaata ctcagtttat tgaggataat tattagggga gattagagaa gaattctcta

76981 ttcacgaaca ttactttaac agttgtaatc attggtttca tcatatcgat taatagtaca
77041 gtcatcttga gattttcaac acactattat agctagctaa gatactttta aattatgact
77101 tatttttcat catttacgat acatcattct cgataactta ctatttgtta tggtatttat
77161 agatttaaag aaagtgaagg agattaagca atgtaacttg gcttaatctc tttttgatta
77221 tatttattaa ggcaaagctt catttccctt aaatcaagta tatcttctga ataagcaaat
77281 gaaatcgtga cgccaactac agtgataact tcgcctaaaa cacaatgagt gtggtgtttt
77341 cacttttcat aaactcctct aagtttgtgt ttttaaactg tgatggattt gaatgttttt
77401 gaattacgtt gtcgcgtcct tgggattgca atccaagaac gtcagcataa tctaaatcaa
77461 ttttgatttg gtagaagaca agagaagtac ttttgcctgt atcatctttc gcatttcttt
77521 ttgttgtctt tcgatcccga tcggaaacta attttcctgc attatccaaa gcatcaataa
77581 catttttcgt gtcttcgaat tgactgtctg ttaacatctt atcgaataca tttcggataa
77641 tttttacttc gatgtagtca tctttaagtg agaggatacc aaagttttga atcatattag
77701 caagacgtgt ttgttgtgaa aacttgccca ggttttccgc aataaactgt acgattgtat
77761 ccatcgcttt atctgcaaga gaacgttctg acaccgtttc attatgatag cttaataaat
77821 agttctttat gctgctaacg tttaactgta ggtctagtga ttgatttaaa atatgggctg
77881 ttgttgttag agttgcataa tgtttaatca ttcgaatacc agtgttactg gattcagttg
77941 atagtaattg ataaaaaaca ctgtcttctt tttgaaaaag ggaagtaaca tcattagtgt
78001 tgtctaatag gaattgagca attataggta aaacatgacc attattttga ctaaccactt
78061 tttttatagt atctgagttt tgagcacttg atgtgaattt ttcagacacc tcaatagtac
78121 gaacacgcaa accatcattt ttagctggat ttttgaaaat actatgttca gaagttgaaa
78181 tcactgatgt atcctagttt ttaggtgttt tgacatcacc atttatattt gctctttgac
78241 gtccttgtcc ctctgcaaat gaatatagaa gacctgttgt atcatgaaat gttgccgctg
78301 ataattcatc taatactatg ggtacaccat aatttccgct aagatatcct tctagtgcat
78361 ttcttgttgc attccaactc cggaataggt tttcagtacc ttttgttggg ttgccagcta
78421 ccgatgcggc taacatagcg gctgtcgatt ttcctgttga tgattggcca gtgaaagaga
78481 aaatagttcc tgaaaactcg acgttattat gatacttaag gaaagcagtg actaatgaag
78541 atacgccaaa caacacatct agttctaaag ataagttccc atggacttct tctttataca
78601 tttgtaacca ttctgataaa ttgccttttg gtattaaatc gtacttgtta tcacatataa
78661 tagaagtgct attagtaatc tcattagaaa aataaggctg gtctaatgaa attaatggtc
78721 caagtaatgt atgaagcatt cctacacctg agtatattgt tgataaaggt gtggtgcttc
78781 tcatttgttg cagtacgaac cctaaatctc taatgtgctt ttcattaatg gtaaatccat
78841 attcaattaa ggagggtaat tgcaaagcag taaggattga agccttttcg acaagctcta
78901 tattattcaa atccgaaaga aatacttttt ctttacgagt cgcggggtct ataaaacgat
78961 ctgtgaaagc aatcggtcca gataacatta tttcaaactc cgtacccccca tcttttttag
79021 gagggatggt tttgtaccat cccgaaggtt tgagcttgaa ttcgtcagtt tgatatagaa
79081 ttttttccat taacagtgac ccccttggtt gtaagaggaa attataatga tatgtcggta
79141 cttaagtggg ttagttccta taataatgaa agggatgctt ggtttgtact ttatccacca
79201 agcaaagcgt cttcctgctt cacgttgatc gttgatgttg cactgttctc tgaataactg
79261 taattataag gtttagtaat ggcaattttt tatttaaaca aatttttacg aatggattgt
79321 ctgtttatta attcagcaac gtttattgaa gaaaaataag tgtcttctga taattgcaaa
79381 gggtttaatt caatgtaaac tgaattaagg gtagggtagg tatgcctgtt ttttggttct
79441 tttaagttca atcgattaat aaaattgttg aacctttgtt ctaagtcata caaaaacatc
79501 agtaattcat taggagaatg atttattatg caatccatat tttcgtttat gcttgaagtt
79561 atacatctaa aaacagtatt ggtttcttcg tataaaaata acttattaat agaggtaaca

79621 ttgattaaga ctgagtatac gtataataag agtgactctg acatctcatt tagataaata
79681 agactgtgat cattcttatt tacgattatt atatgtgatt gaatcaattc aataatgtct
79741 agaagaagac ttgaaaagtc gctgttaatc ttggtctttg aaaaatcatc gtgaccattt
79801 gtcttattac ttagatgtag ttgttcgtat tttatagttt ggcggttgag gaattaagca
79861 tttgattatt ataaactatc tcaaaaatta aattacctcc tttttcttc ctcgatgaag
79921 gtttatctaa tcttccaata tacaggtttt tgaagtaaat tatagatatg ataatcatta
79981 attcagtaaa tgtatactta ttaaggggtg gagctttagc taacttatcg atagtactgc
80041 cgatgcgtgc tttgaaacta ttcaaattgt cgtgactgtc tatatctaaa tcttcaaatt
80101 tactagcaag ggtttgcaaa gtagaaatta caacttcttc ttttgtataa tatttattga
80161 aaatatcatc ttctttatca tcgctttgga gttttaaagt gcctggttct aagatgtcaa
80221 attttttgtt tcgattggta ttttttaaa atgtgccttg tatttttggg gttgttccaa
80281 atgtgtattt gctagactac ctgtaattga tattgcttta tttagcattt ggtgtgtatt
80341 aactgtatct gattcttctg gaaatttaaa tctaaaagta aaatctctac taccgaatgt
80401 actttttacg gctacagttt tgtacttatg ctcaggcaat gtaattaaac catcttgatg
80461 caggaggaaa agtgtgtcat catttaattt ctcaattgta ttaaataagt aattatgcat
80521 agcctcttta tacatggcga attgataatt tagattatcc cattctatta gttgatttaa
80581 aatacaatat agaccttcat tcaatcggtt tacgaatatc aatacagctt cttctcgtcc
80641 aacaaggctt attaactcct tcaagtcatc ctttaatttt aatttttctc cttcaaaatt
80701 gtattgagtg aagcctatag ccttttgaaa agtaacttcc atgacagagt tggtttcaga
80761 atattctaac gattcattta ttgcaaaaat caaatttttt ataaatatgg aattactcat
80821 ggtttgtaat tgcatatact acccaacttt cttatttact ttaaaacaaa tataagctaa
80881 agtagttatt atcataaagg gaacgtacgt tcttatttag ctgatttcta tgaagagagg
80941 ttatggatat tgaatgaaga aatcttacgc gaagtagcag atatttttat aggtgatgac
81001 agagatagca tttatgatta taaaactggg aacgaattag tgaggttttt taatcattac
81061 tttaataaag gggacatata tcaggctccg tttccatcta gatggctata tgttgtgaaa
81121 catttgcaaa ctctgattca ggagagaaag atcaatcaat ttttcacatt aattttaagc
81181 aatcactata ttaagtatga actgaaaatt gacgaggttg aagcagcaaa gcaggctgct
81241 aaagcactta agttgttcaa caaaagatta aatcattatg ggtactacat aacaggaact
81301 aacaatgcta gatattttat ggataaggat gaagatacgg agtctattgg ttatggaggg
81361 tatgcgaata tttatttaca gaagtctaca ggtcttgctg taaaaaaatt gaaagaggag
81421 tatcttactg attcttcaat taaaagtagg tttaagagag aatttgatct cactaaatct
81481 tttgatacaa atccattgtt cattaatgtg tttgaattta atgaatcaga ttattcatac
81541 acgatggagt tagctgatga aactttgaaa gattacattg aaagcaagac aattagtgag
81601 ctagaaaaag taaagataat aatgaaaatt ttaaaagcga tgagtcaagc acatagtgaa
81661 aataaaatac acagagatat cagttctaaa aatgtattaa tgtttagagg aaaagtcaaa
81721 atatcagact taggattagg taaaaacctt gatgaaattc attcgcatca aacctttgat
81781 acaaacggtg taggacaata taaatactgt gcaccggaac agatgtatag tttaaaacaa
81841 gcagataaac aatctgatgt ttttagttta ggaagattga taaattttat tatgactgga
81901 aatgtagtta acaaccatca cctatttaga ggtgtatctg ataaggctac gaacagtagt
81961 aaagaataca gatttgaaga tgcaaatgaa atgttgaaaa tgctgcagag aattttagag
82021 tatcacagta gtgcaaagca cgtcgaaaaa tgtcaagaaa agctgaaaag aggagtgttt
82081 gatgatgaaa gcgaagaatt tattatgaca cgaagtgatg aacaattatg tcaaatggtt
82141 ctaagttcta ataataatga gcaagcgtgt ttaattcgtt atatgcaaaa aaacgaatct
82201 tcagcatgtg atttaataga gagtattaat agaaagtatc aagagttttg tggaaggttt

82261 gaagactacg atccttttgc taaattagca tatatgattt tatgtaataa cttcagttat
82321 agagtgaatg aaacagcagc tagagtgcta aattatgttg cttggtctgt aaatagattt
82381 tcggcacaag acttaattaa aggtttaatt aatagaggag tggagccttt gattgaagaa
82441 aaattaaagg acaattaaaa aaacctcatc attaactgat acgcagaggc gtatcataag
82501 t
//
LOCUS Staphylococcus 55323 bp DNA linear UNK
DEFINITION Staphylococcus Staphylococcus aureus strain ER09654.3
ACCESSION Staphylococcus
KEYWORDS .
SOURCE Staphylococcus Staphylococcus aureus
ORGANISM Staphylococcus Staphylococcus aureus
Bacteria; Bacillati; Bacillota; Bacilli; Bacillales;
Staphylococcaceae; Staphylococcus; Staphylococcus aureus.
FEATURES Location/Qualifiers
source 1..55323
/mol_type="genomic DNA"
/db_xref="taxon:703339"
/genome_id="703339.280"
/organism="Staphylococcus Staphylococcus aureus"
/genome_md5=""
/project="hwwang_703339"
CDS 264..569
/translation="MKGNIQCPRLIKGSLLDFVLETVDVFVNGSKNDCELVNFNYKEG
KVVSRYSTKFESNPKLRKKQLISTVKVVRYVIFDFEKILWGIGKVLLKFIILRQCLL"
/transl_table=11
/product="hypothetical protein"
/db_xref="SEED:fig|703339.280.peg.1"
CDS 768..1631
/db_xref="SEED:fig|703339.280.peg.2"
/product="hypothetical protein"
/transl_table=11

/translation="MKHINQFSESTLAQLEKDQQHVFYVYCLMDPRNDECFYVGKGKG
NRIFKHKQDAQKKVLYEDIFIEENKDNLKFNRINEICSNDLNVLGYIISYGLTESEAF

SAENVLINFLNLTNKTTLTNMINGHGSKAYLVEDLENEFGYDSINLENINTNELILAV

KIRDAFLLDKDESKEYPINESKRDRNNLKSRTLGSWIIGKDKIHKIKYIIGINTGANN

AVVSAYEVSFEQAESIETNNGRMRYAFIGLSERDATLKKLNLYKKALPDLRFGSGSAT
AYINNGTMKVD"
CDS 1739..3214
/transl_table=11

/translation="MPSDDIVKKLYSKEDIRLELGLTPHKFNKKMETIAKLFKIDMKI

FHNYKGQDKNNQYTFNGVAKELIKVLLKSVDYYPVDINSKKFKQNGKSKKEMIENIDN

SSYMKYIYQLMKSINEIQYKRLIADIHMKDVYQNTKAWLNNGESINKKEQELYQYMTI

LPLHKRVELQNEVLKSIDETIFQFLAKEHRNNQIEENNELEAYTKAIKEGRNPKNDYE

LNHLLYKKNQLPLDSLIEDIWDYEETEYTELDWLIADMLKRSQRADSNFIESLEKKNK

LRKNIHKDSIKSISKLIDHELVKNNRKWDVAEFYKNQQFWNNSTLRRSSQNFGILYYK

KNRLHTMNQSNIIKKIKIIEANIESANKQPSYLDFFLQAKFDLERLESELMKYSINPK

YFNKINSDYVRYAIEDVHNAIIKIDRYISNDKVKLNYSNVQMVVRNEITEQGSYFVTQ
ALNTLSKVEESGYSFDNFMVGPFVEDFKKAIKNRRFGSKKD"
/db_xref="SEED:fig|703339.280.peg.3"
/product="hypothetical protein"
CDS 3440..4540
/transl_table=11

/translation="METRDKLMSLTQSDKTQQWLMDKSSNQDDIQQLQQQFSQQLDQQ

YNALLADEKAKLDQYVEVHQGLESLKEEIESEPITLNIDKLPDIKATMLERAKNDEHS

DKIEKLFDRLEQALNGTNRLYTQLSLIGTRTHRITTKNFNLQGLPKAVQHTILPSKFK

KVYTVDFKSFEPSVAAYMTQDSKLIDLLNQKDGLYDALLSELGLSDELRVFVKRAFIG

SFLFGGNFKNPKFKLNQYVSEVQWLDAVSQFTKVIELKKHVEKSNSMPMPYSIEHDMS

AFQGSSIMAIYVQTVASYIFKHILLKVYKAQCDQKTFKIIVPIHDAIMIECEDEEIAQ
NVGQLMKDTANQLFNGEFAHVTVEEIGGVDHE"
/db_xref="SEED:fig|703339.280.peg.4"
/product="hypothetical protein"
CDS 4533..4904
/db_xref="SEED:fig|703339.280.peg.5"
/product="hypothetical protein"
/transl_table=11
/translation="MNNDRGQSLHIPSSTPIKENNIYVATLHSVIQTDFSGEIKHQFT

YEIEVNNQIVYANRNIPTKPSANQLSIHDWLKRHSNYSASHENYEPYIDQKHLILLGQ
YNGNYYVQDVASLDAFGGVLS"
CDS 4901..6544

/translation="MNHILQMLSKLLSVAKEAIDRQGLIAILTIPVNNNDEIEETAQG

ETVYNELIDQLRLNIPKDTDYQPNIYSYFGIKKNPNDTVLMEMMIKVFHIKRFNSELF

IFKVNGWQKINGDELQGLISKMIQVLLVDYKPSLSTLKNVVDGLQKSTDVEELVENER

YIGCGENMFDLNTFQVVKNSIDIFPKTRLNLSLSTNDVITDKIPPYFKQYMLQLANYD

DDLQYFLFQHTAVLLTADTKYRRGLILYGGAKNGKSVYIELVKSFFYSKDIVSKPLNE

LEGRFDKESLIDKSLMASHEIGQSRIQEKIVNDFKKLLSVESMHVDRKGKTQVEVILD
LKLIFSTNAILNFPPEHAKALERRINIIPCEYYVEKADTSLIDKLQSEKKEIFLYLMY

VYQQIVKADIEYLENSRVTEITHDWLNFGYEFVSSRSVSNANQKACINLLRKLIEIKS

GSRIKVSELNKVINEEIKVSSQVIKQLIQANFDTQTKLYNGYDYWIDLGWKEANKKEI
HDISEKDNIISLDKNENITDDEALDEENLDFDWEDFDDE"
/transl_table=11
/product="putative primase"
/db_xref="SEED:fig|703339.280.peg.6"
CDS 6770..8446
/translation="MKGKIALYSRVSTSEQSEHGYSIHEQEQVLIKEVVKNFPGYDYE

TYTDSGISGKNIEGRPAMKRLLQDVKDNKIEMVLSWKLNRISRSMRDVFNIIHEFKEH

DVGYKSISENIDTSNASGEVLVTMFGLIGSIERQTLISNVKLSMNAKARSGEAITGRV

LGYKLSLNPLTQKNDLVIDENEANIVREIFDLYLNHNKGLKAITTVLNQKGYRTINQK

PFSVYGVKYILNNPVYKGYVRFNNHQNWAVQRRSGKSDKNDVILVKGKHEAIISEEVF

DKVHEKLASKSFKPGRPIGGDFYLRGLIKCPECGNNMVCRRTYYKTKKSKERTIKRYY

ICSLFNRSGSSACHSNSINAEVVERVINVHLNRILSQPDIIKQIASNVIEELKQKHSN

QTEIKYDIDSLEKQKAKLKTQQERLLELFLDDQMDSEMLKAKQSQMNQQLEVLDQQIK

EAQQANQSQDEIPNFDKLKGRLILMITRFSVYLRKATPEAKNQLMKMLIDSIEITTDK
QVKLVRYKIDESLIPQSLKKDWGSFFMPKFQFEIYGQNDYFIDQITTFTT"
/transl_table=11
/product="Cassette chromosome recombinase B"
/db_xref="SEED:fig|703339.280.peg.7"
CDS 8552..8890
/product="FIG01108299: hypothetical protein"

/db_xref="SEED:fig|703339.280.peg.8"
/translation="MTLEQQLKHYITNLFNLPRDEVWHCESIEEIADDILPNQYVRLG

PLSNKTLQTNTYYSDTLHESNIYPFILYYQKQLIAIGYIDENHDMDFLYLHNTIMPLL
DQRYLLTGGQ"
/transl_table=11
CDS 8892..8993
/translation="MHKYIKITQLVITILSEIIIWMKESERKEVSYE"
/transl_table=11
/product="hypothetical protein"
/db_xref="SEED:fig|703339.280.peg.9"
CDS 8986..9297
/product="FIG01107894: hypothetical protein"
/db_xref="SEED:fig|703339.280.peg.10"

/translation="MNRYITRGIANNLPNILQHQLWQLVSEREQEQTKDNTSVDYFHI

FQFNKHRNQLYIKHKQERPEYAKIHKANYSKAININKVYIIREDDVDLSYYVMLLPEE
Y"
/transl_table=11
CDS 9313..9819
/transl_table=11
/translation="METIKSTLKTEAIFSDDKQHRYLLKKTWNSEKQSITIITMYPHY

DGILNIDLTTQLIMNKVSEMDAFGSINFVNLYSNITTPINLKHLENAYDKHTDIQIMK

AVKESDEVILAWGAYAKKPGVEARVNEVLEMLKPHKKKVKRLMNPETNEIMHPLNSKA
RQKWILKV"
/db_xref="SEED:fig|703339.280.peg.11"
/product="hypothetical protein"
CDS complement(9968..10642)
/product="Transposase for insertion sequence-like element
IS431mec"
/db_xref="SEED:fig|703339.280.peg.12"
/translation="MNYFRYKQFNKDVITVAVGYYLRYTLSYRDISEILRERGVNVHH

STVYRWVQEYAPILYQIWKKKHKKAYYKWRIDETYIKIKGKWSYLYRAIDAEGHTLDI

WLRKQRDNHSAYAFIKRLIKQFGKPQKVITDQAPSTKVAMAKVIKAFKLKPDCHCTSK
YLNNLIEQDHRHIKVRKTRYQSINTAKNTLKGIECIYALYKKNRRSLQICGFSPCHEI
SIMLAS"
/transl_table=11
CDS 10900..11067
/transl_table=11

/translation="MLESREQLSVEEYETFFNRFDNQEFDFERELTQDPYSKVYLYSI
EDHIRTYKIEK"
/EC_number="2.3.3.10"
/db_xref="SEED:fig|703339.280.peg.13"
/product="Hydroxymethylglutaryl-CoA synthase N-domain
homolog (EC 2.3.3.10)"
CDS 11165..11395
/translation="MLIFLSYDFVRACLPRGMARAISLSHILFPQASALTKSVVIFIF
IRILTEIILIRGIVKAILSKIADKRFVKSSSK"
/transl_table=11
/product="hypothetical protein"
/db_xref="SEED:fig|703339.280.peg.14"
CDS 11944..12687
/product="Glycerophosphoryl diester phosphodiesterase (EC
3.1.4.46)"
/db_xref="SEED:fig|703339.280.peg.15"
/db_xref="GO:0008889"
/EC_number="3.1.4.46"
/translation="MLTVYGHRGLPSKAPENTIASFKAASEVEGINWLELDVAITKDE

QLIIIHDDYLERTTNMSGEITELNYDEIKDASAGSWFGEKFKDEHLPTFDDVVKIANE

YNMNLNVELKGITGPNGLALSKSMVKQVEEQLTNLNQNQEVLISSFNVVLVKLAEEIM

PQYNRAVIFHTTSFREDWRTLLDYCNAKIVNTEDAKLTKAKVKMVKEAGYELNVWTVN
KPARANQLANWGVDGIFTDNADKMVHLSQ"
/transl_table=11
CDS 12784..13212
/db_xref="SEED:fig|703339.280.peg.16"
/product="MaoC domain protein"
/transl_table=11
/translation="MKYDDFIVGETFKTKSLHITEEEIIQFATTFDPQYMHIDKEKAE

QSRFKGIIASGMHTLSISFKLWVEEGKYGEEVVAGTQMNNVKFIKPVYPGNTLYVIAE
ITNKKSIKKENGLVTVSLSTYNENEEIVFKGEVTALINNS"
CDS complement(13258..15264)
/product="Penicillin-binding protein PBP2a, methicillin
resistance determinant MecA, transpeptidase"
/db_xref="SEED:fig|703339.280.peg.17"
/db_xref="GO:0008658"
/db_xref="GO:0046677"
/translation="MKKIKIVPLILIVVVVGFGIYFYASKDKEINNTIDAIEDKNFKQ

VYKDSSYISKSDNGEVEMTERPIKIYNSLGVKDINIQDRKIKKVSKNKKRVDAQYKIK

TNYGNIDRNVQFNFVKEDGMWKLDWDHSVIIPGMQKDQSIHIENLKSERGKILDRNNV

ELANTGTAYEIGIVPKNVSKKDYKAIAKELSISEDYIKQQMDQNWVQDDTFVPLKTVK

KMDEYLSDFAKKFHLTTNETESRNYPLGKATSHLLGYVGPINSEELKQKEYKGYKDDA

VIGKKGLEKLYDKKLQHEDGYRVTIVDDNSNTIAHTLIEKKKKDGKDIQLTIDAKVQK

SIYNNMKNDYGSGTAIHPQTGELLALVSTPSYDVYPFMYGMSNEEYNKLTEDKKEPLL

NKFQITTSPGSTQKILTAMIGLNNKTLDDKTSYKIDGKGWQKDKSWGGYNVTRYEVVN

GNIDLKQAIESSDNIFFARVALELGSKKFEKGMKKLGVGEDIPSDYPFYNAQISNKNL

DNEILLADSGYGQGEILINPVQILSIYSALENNGNINAPHLLKDTKNKVWKKNIISKE

NINLLTDGMQQVVNKTHKEDIYRSYANLIGKSGTAELKMKQGETGRQIGWFISYDKDN
PNMMMAINVKDVQDKGMASYNAKISGKVYDELYENGNKKYDIDE"
/transl_table=11
CDS 15559..17121

/translation="MMNRNVSDTTSSVSHMLDGQQSSVTKDLAINVNQFETSNITYMI
LLIWVFGSLLCLFYMIKAFRQIDVIKSSSLESSYLNERLKVCQSKMQFYKKHITISYS

SNIDNPMVFGLVKSQIVLPTVVVETMNDKEIEYIILHELSHVKSHDLIFNQLYVVFKM

IFWFNPALYIISKTMMDNDCEKVCDRNVLKILNRHEHIRYGESILKCSILKSQHINNVA

AQYLLGFNSNIKERVKYIALYDSMPKPNRNKRIVAYIVCSISLLIQAPLLSAHVQQDK

YETNVSYKKLNQLAPYFKGFDGSFVLYNEREQAYSIYNEPESKQRYSPNSTYKIYLAL

MAFDQNLLSLNHTEQQWDKHQYPFKEWNQDQNLNSSMKYSVNWYYENLNKHLRQDEVK

SYLDLIEYGNEEISGNENYWNESSLKISAIEQVNLLKNMKQHNMHFDNKAIEKVENSM

TLKQKDTYKYVGKTGTGIVNHKEANGWFVGYVETKDNTYYFATHLKGEDNANGEKAQQ
ISERILKEMELI"
/transl_table=11
/product="Methicillin resistance regulatory
sensor-transducer MecR1"
/db_xref="SEED:fig|703339.280.peg.18"
/db_xref="GO:0008658"

CDS 17121..17492

/translation="MDNKTYEISSAEWEVMNIIWMKKYASANNIIEEIQMQKDWSPKT
IRTLITRLYKKGFIDRKKDNKIFQYYSLVEESDIKYKTSKNFINKVYKGGFNSLVLNF
VEKEDLSQDEIEELRNILNKK"
/transl_table=11
/product="Methicillin resistance repressor MecI"
/db_xref="SEED:fig|703339.280.peg.19"
/db_xref="GO:0003677"
/db_xref="GO:0016481"
/db_xref="GO:0016566"
CDS 17593..17805
/db_xref="SEED:fig|703339.280.peg.20"
/product="hypothetical protein"
/transl_table=11
/translation="MQVLIKSIMLVITPVKSICIPSFYLRYIKYNIKQIKNVKNSLIG
YLDCINVKNLKNIQRLCIKNRHIGNT"
CDS 18000..19109
/translation="MLREIYNHHNISRTQISKNLEINKATISSILNKLKYKSLVNEVG

EGDSTKSGGRKPILLKVNHLYGYFISLDLTYSSVEVMYNYFDGNVIKHESYDLPDEKV
SSILSIIKKHIDIQEKLDTYNGLLGVSVSIHGVVDNEQHVTYLPFHETEGISIAKKIK

EITNVPVVVENEANLSALYERNFNHNLSYNNLIALSIHKGIGAGLIINNQLYRGANGE

AGEIGKTLVSKVSDNVEIFHKIEDIFSQEALLHNLSNQLNEKMTLSKLIQFYNEKNPV

VVEEMEQFINKIAVLIHNLNTQFNPNAIYINCPLFNEMPEILEAIKNQFKQYSRNEIQ
IKLTSNVKFATLLGGTLAIIQKVLQINDIYLDIKA"
/transl_table=11
/product="Putative ROK-family transcriptional regulator"
/db_xref="SEED:fig|703339.280.peg.21"
CDS complement(19223..19528)
/product="Polysulfide binding protein"
/db_xref="SEED:fig|703339.280.peg.22"

/translation="MPQTNIVTSSIHSSEMTGEEHNVLDVRNDDEWRKGHLKQAIHIP

HGKLLNEDIPFDKNDLIYVHCQSGVRSSIAVGILEYKGFTNIVNVRGGYQHLPAFLK"
/transl_table=11
CDS complement(19619..20557)
/db_xref="SEED:fig|703339.280.peg.23"
/product="Zn-dependent hydroxyacylglutathione hydrolase /
Polysulfide binding protein"

/transl_table=11
/translation="MFFKQFYEDNFSQASYLIGCQRTGEAMIIDPIRDLTKYLKVADK

EGFTITKAAETHIHADFASGIRDVANKLNASIYVSDEGKDEFNYKNMPKQTHFVKHQD
IIYLGKIKLEVLHTPGHPPESISFLLTDEGGGSSIPMGLFSGDFIFVGDVGRPDLLEK

AVNVEGSTKIGAKQMYQSIQFAATLPDYIQIWPGHGAGSPCGKALGSLPTTTLGYEKI

NNWAFNVKDETSFIETLTLNQPAPPHHFSQMKKINQFGMQMYQPYNVYPSSSNTQTAF
DLRSKEAFHGGHMHGTINIPFNKTFINQIGWYLDYD"
CDS complement(20590..21654)
/product="Disulfide bond regulator"
/db_xref="SEED:fig|703339.280.peg.24"
/translation="MEQYKEVHINSLNKEELEKLAKMGQLIDVRTKEEYELGHINGST

LHPVEEIKSFNKNSNKTYYIHCKSGNRSTKACDYLAKQGYDVVNLKGGYKDYEAKNFN
SAPLIEKDIEIKENRKQFDFRGLQCPGPIVNISKEINNISTGEQIEVTVTDPGFNSDI

KSWAKQTGNTLVNLTEEANVINAIIQKEKPKEFEINDTATGTTIVLFSGELDKAVAAM

IIANGAKAAGKDVTIFFTFWGLNALKKAQSTRVKKKGISKMFDLMLPKDPIHMPLSKM

NMFGLGNIMMRYVMNKKNVDSLYSLIDQAIDQDIKLIACTMSMDVMGISKEELRDEVD
YGGVGTYIGHTEQANHNLFI"
/transl_table=11
CDS 21790..22050

/translation="MTYDKKMINRINRIQGQLNGVVKMMEEEKDCKDIITQLSASKGS
IQRLMGIIISENLIECVKTAEENNESSQELINEAVNLLVKSK"
/transl_table=11
/product="Metal-sensitive transcriptional repressor"
/db_xref="SEED:fig|703339.280.peg.25"
CDS 22050..22694
/product="FIG003846: hypothetical protein"
/db_xref="SEED:fig|703339.280.peg.26"
/translation="MDIANMTIMLLIGVLGGFISGLIGIGGAIIIYPAILILPPLIGI

PAYSAYIASGLTSSQVFFSTLSGSLNARKQPAFSPKLVIYMGGGMLIGSMLGAILASL
FNATFVNTVYVIIAILALILMFIKVKPTTQETKSKPLLFIIVGFGIGVISGIVGAGGA
FIIIPVLLALFKLPMNTVVNNSIAIAFISSVGAFFIKLMQGYIPVESAIFLIIE"
/transl_table=11
CDS 22795..22914
/db_xref="SEED:fig|703339.280.peg.27"
/product="Mobile element protein"

/transl_table=11
/translation="MLHLIKSKLQKNHLNQYFHFEYMYLKWKVLISKIVKPVS"
CDS complement(23033..23695)

/translation="MNTIESQFDKVAEDYDFVNELLNDYSFFVSNMSPKKGRALDIGC

GSGLLVEKLASYYDEVVGIDISNQMLDLAKSKRQLTNTVYLNMNAEQLNFNEKFDFIV

SRTTFHHLDDIASVIQQMKELLNEEGRIVILDNVSEVETPPTYVYKLGAIQEFLPHCF

KFGIKNAIRIYNHNTSKSWLEHLASDKYLSEQNYYDLYEKLLPGCQFHKMGWAMGVVW
TK"
/transl_table=11
/product="Mobile element protein"
/db_xref="SEED:fig|703339.280.peg.28"
CDS 24191..24922
/EC_number="2.1.1.184"
/translation="MNQKNPKDTQNFITSKKHVKEILNHTNISKQDNVIEIGSGKGHF
TKELVKMSRSVTAIEIDGGLCQVTKEAVNPSENIKVIQTDILKFSFPKHINYKIYGNI

PYNISTDIVKRITFESQAKYSYLIVEKGFAKRLQNLQRALGLLLMVEMDIKMLKKVPP

LYFHPKPSVDSVLIVLERHQPLISKKDYKKYRSFVYKWVNREYRVLFTKNQFRQALKH
ANVTNINKLSKEQFLSIFNSYKLFH"
/transl_table=11
/product="23S rRNA
(adenine(2058)-N(6))-dimethyltransferase (EC 2.1.1.184)
=> Erm(A)"
/db_xref="SEED:fig|703339.280.peg.29"
CDS complement(25048..25830)
/db_xref="SEED:fig|703339.280.peg.30"
/product="Aminoglycoside 9-nucleotidyltransferase (EC
2.7.7.-) => ANT(9)-I"
/transl_table=11
/translation="MSNLINGKIPNQAIQTLKIVKDLFGSSIVGVYLFGSAVNGGLRI

NSDVDVLVVVNHSLPQLTRKKLTERLMTISGKIGNTDSVRPLEVTVINRSEVVPWQYP

PKREFIYGEWLRGEFENGQIQEPSYDPDLAIVLAQARKNSISLFGPDSSSILVSVPLT

DIRRAIKDSLPELIEGIKGDERNVILTLARMWQTVTTGEITSKDVAAEWAIPLLPKEH
VTLLDIARKGYRGECDDKWEGLYSKVKALVKYMKNSIETSLN"
CDS complement(25981..26358)
/transl_table=11

/translation="MDKQVRNTTEIVRLAKQKSKKTREKVDKAISKFSIEGKVINFNS

IAKEANVSKSWLYKEHDIRQRIESLRERQITANVVSKPKKSSRSEEILIKTLKRRVME
LEKENKKLQNQIQKLYGDLYNKE"
/db_xref="SEED:fig|703339.280.peg.31"
/product="Mobile element protein"
CDS complement(26365..28257)
/db_xref="SEED:fig|703339.280.peg.32"
/product="Transposase B from transposon Tn554"
/transl_table=11

/translation="MNASSKRKIISQSEISKKIAVMNEEMQGFWANNSWDIRKCPHPS

AIELSKNPALRNRWVRFERVKNLWLRTELKYFYFYHLNNGIWNAKTVWIRKGTVINKM
LDFLDLKYPSITSITEVPIEKAMTEYRTYLTKRGVRITTTNYKITANQEKTPVKANSY

YVTNLKQFMEFYENFYFDGEEWDKDVWDRRNLPLPDDKVNPTQYEYTINFKGFRNTYF

KQLVKRYCKLRLNVDSFSYVSDIAQRLKEFFNFLDMKFKQVQRVHQLTRVEIEAYLSE
LNMMGIKPSTITGRISILEGLFSTLLRLEWDDVPSKILIYSEDYPKIPRAKPRFIDEF

VLEQLNSHLDKLPEYIATMTMIVQECGMRISELCTLKKGCLLEDKDGDFFLKYYQWKM

KKEHIVPISKEVALLIKVREDKVSEEFPDSEYLFPRKDGSPLKQETFRGELNKLAYEQ

NIVDKSGEIYRFHAHAFRHTVGTRMINNGMPQHIVQKFLGHESPEMTSRYAHIFDETL

KNEFTKFQEKLVTNNGDVLDLDEDNEVDDVELQWFKKNINAQVLPNGYCRLPVVAGGC

PHANACLDCTHFCTSKQFLPQHEEQLERTEELLAIAKDKQWQRQVETNSRVKERLEQI
IGSLTG"
CDS complement(28254..29339)
/transl_table=11
/translation="MKVQRIEVENKPYPLYLLLDKEYQLIEPVMKFIKYLDNTGKSPN

TIKAYCYHLKLLYEFMEQRGVILNDINFELLADFVGWLRYPSASNVIDLQSKKAIREE

TTVNTILNVVMSFLDYLSRLGEFKSIDVFKQAKGRNFKGFLHHVNKGRYQKNVLKLRV

KKKQIRTLRSKEVKQIIDACHTKRDKLILMLMYEGGLRIGEVLSLRLEDIVTWDNQIH
LTPRDVNVNEAYIKLRKERTIHVSKELMSLYTDYLIYEYSEELEHDYVFISLKEGYFG

KPLKYQSVLDLVRRIVKRTGIEFTSHMLRHTHATQLIREGWDVAFVQKRLGHAHVQTT
LNTYVHLSDQDMKNEFNKYLERKEHKK"

/db_xref="SEED:fig|703339.280.peg.33"
/product="Transposase A from transposon Tn554"
CDS complement(29458..29775)
/translation="MKEINIVSLQMIKTDTLSYLKNRISNPEDAAEIMRSFIGNSDRE

HLILICMNSKNEPTHIQTLSIGSINQTVIHPREIFKTAILSNANSIMLGHNHPSGDVL
TIV"
/transl_table=11
/product="DNA repair protein RadC truncated by transposon
Tn554"
/db_xref="SEED:fig|703339.280.peg.34"
CDS complement(29796..30302)
/db_xref="SEED:fig|703339.280.peg.35"
/product="hypothetical protein"
/transl_table=11
/translation="MNTIKNTIYTEAIFSKDEKHRYLLKKTWDEKKPACTVITMYPHL

DGVLSLDLTTVLILNQLANSERYGAVYLVNLFSNIKTPENLKHIKEPYDKHTDIHLMK

AISESDTVILAYGAYAKRPVVVERVEQVMEMLKPHKKKVKKLINPATNEIMHPLNPKA
RQKWTLKA"
CDS complement(30320..30631)
/db_xref="SEED:fig|703339.280.peg.36"
/product="hypothetical protein"
/transl_table=11
/translation="MKINRYITRGISEYLSLDLQILLWNMVKERDNQPHTDYLHIFKL

QEDENILSITHEQEQPAYKLEYHYTNYVKNQNALPKKVYVIREDGVDAFYYVMLLPEE
Y"
CDS complement(30633..30725)
/transl_table=11
/translation="MNNWIKVAQISVTIISQAIIIMKEIQDEVK"
/db_xref="SEED:fig|703339.280.peg.37"
/product="hypothetical protein"
CDS complement(30718..31068)
/product="hypothetical protein"
/db_xref="SEED:fig|703339.280.peg.38"
/translation="MKTTTQELKQYITRLFQLSNNETWECEALEEAAENILPERFIND

SPLAHLTLETYTYYNNELHDLSIYPFLMYANNQLISVGYLNHFDMDFLYLTDTKNTII
DERHLLREGGTHHE"
/transl_table=11
CDS complement(31586..33214)
/product="Site-specific recombinase"

/db_xref="SEED:fig|703339.280.peg.39"

/translation="MQQLKTKRVGIYVRVSTEMQSTEGYSIDGQINQIKEYCDFHHFE

VKDIYADRGISGKSMNRPELQRILKDAKEGYIDCVMVYKTNRLARNTSDLLKIVEDLH

KQNVEFFSLSERMEVNTSSGKLMLQILASFSEFERNNIVENVFMGQTRRAQEGYYQGN

LPLGYDKIPNSKHELMINQHEANIVKYIFESYAKGHGYRKIANALNHKGYVTKKGKPF

SISSITYILANPFYIGKIQFAKYKDWSEKRRKGLNDKPVIAEGKHSPIINQDLWDKVQ

MRKKQVSQKPQVHGKGTNLLTGIIHCPQCGAPMAASNTTNTLKDGTKKRIRYYSCSNF

RNKGSKVCSANSVRADVIEDYVMKQILEIVKSDKVIQRVVTHVNQENQVDGAALHHDI

AYKQQQYDEVQIKLNNLIKTIEDNPDLTSVIRPSIQKYEKQLNDITNQINQLKNQQNE
DKPLFDAKEISKLLQHIFHDIKHIEKSRLKALYLSVIDRIDIKKDGNHKKQFYVTLKL
NNEIIKQLFNNKQLDEVHLSTSSLFLPQTLYLTI"
/transl_table=11
CDS complement(33236..34585)

/translation="MKQVIGYLRQSTMKQQSLAAQKQAIEAIAEKHHIQHINFYSDKQ

SGRKDNRSGYRQMTQLIQQGQCDILCCYRLNRLHRNLKNALKLIKLCQTYHVHILSVH

DGYFDMDQAFDRFKLNIFISLAELESDNIGEQVRNGLQEKAKQGRLITTHAPFGYEYH

NGAFIINQNESPTVKAVFNYYIKGHGYKKIAQLLEEDNTYINRQPYQVRNIIINPNYC

GRVNNQYGQFDNMFPSIVSTSIYEQAQRLRSQKQIKQTSSDNQLKQKIKCPCCNTTLT

NMTVRKKNHTLRYYVCPKNMNASRFVCDFKGINAQTLEDKVLEVCKDFYQNQRIYTKI

KGAIDKRIKRQRNIEKHHTLTQEQLIEKLAQGIIDAETFREQTQSLRQQPQRTTSING
HQIQHTIQNIIQKRFTLNILYPYIETIHITKDKNLIGIYFKNEPLNIVNQTMQSSIA"
/transl_table=11
/product="Cassette chromosome recombinase A"
/db_xref="SEED:fig|703339.280.peg.40"
CDS complement(34819..36612)
/product="hypothetical protein"
/db_xref="SEED:fig|703339.280.peg.41"

/translation="MTQTNPSFNPSPRYKSKKGWYKDKPPKEKGGMPTEVEIAGPIVI

ENKFIDPKTNTEKVIITDEDQKVIVESSDILTTQKLPSLMKYGFSINEKYTKDLGYAL

QQMRNQLPISYLYEGVGILETPFGPIVSLNEIYTTTEFDNKSPSDAICENTYDLAPRG

TFDNWFNMYLKEVKGSLLLELAVVFGISALVTSFLKHKHETEFAGIIFSFTGQSSTGK

STAASLAVSVAGNPTKGNETLFRNWNATRNALEGYLSNNFGIPIVFDELSSATFRDTT

GLLYSITEGQGRQRSNVHGEVKTPKNWGTSVISTSEHSIFNDSAQNDGLRVRTIEINE

QFTTNATNADNIKKAVALNYGHVLPLVAKYLINREDEVIQWFYKEVDWFEAKLKDDKS
NTGNRMFKRYAVITTSAKILGRVLATDIDIAKIRDYFIDYHGHTISERSLADKAIDVI

IQFVAQNRGKFSDEGALKNMFENYGLISLKDDHIEVKMIANVFKQMLNNHQFQDVNNV

VNALRDKGFILADRGRQTTKRSVKDNSGKKQSLVFYHLKLDVEFASILGLTKDKSLIQ
NWTPANDNKAAKELFKSANEGIGPSGVHEDF"
/transl_table=11
CDS complement(36612..36908)
/transl_table=11
/translation="MYFNQSDKFEQLKKKILGISFNLPEGEEFTFHDLNSRANVQCSK

AVQQNVGRWFAYFVKHAPRVPFIIIGKDTHGHLVYLKTGPNPHYNSNPSKGGVR"
/db_xref="SEED:fig|703339.280.peg.42"
/product="hypothetical protein"
CDS complement(37101..38147)
/transl_table=11

/translation="MNNKDIYSDDFQNALVDIILNIDKINVKEVFDDSCRAVDQDNKK

DTLNRSPKDFSINISDIVRYSILCMENLSSDKIEENINMIKNHSSELNDNDMFIQEKN

NFTTNFNKFCNALGINIANFKSGKSYVFKPKSLFVLYDILSNNSVYLHFFKNGINDKD
IDSSNRIKENLYYFINHEITDQRDYEHALTNYYLYFLVDSNIEIGIKSIIIKIFTEKG
ITSKDKLQILEYYLDEIDDLNTEISTKIADAHVKNSTNFLENEKVSINSNLAKYITHR

DTRASAPKNMLLSLILNSDLVSPKDLEEKELKYPKKITKEHHDKLIKEINKALDYYND
NNTTPSDYFYITSD"
/db_xref="SEED:fig|703339.280.peg.43"
/product="hypothetical protein"
CDS complement(38398..38613)
/translation="MSRKGTPADNAPIESFHSSLKSETLYINNQLNSSNHIVIDIVEK
YIKNYNNNQIQQKLGYLSPVKYRELIA"
/transl_table=11

/product="Mobile element protein"
/db_xref="SEED:fig|703339.280.peg.44"
CDS complement(38574..38798)
/db_xref="SEED:fig|703339.280.peg.45"
/product="Mobile element protein"
/transl_table=11
/translation="MDIYNGEIVAYKIDDKQDQRLVNDTLNQIDIPEGIYYIVIKAAF
IHLMLIINYAKKKALSEVCPEREHLPITPR"
CDS complement(38886..39239)
/transl_table=11
/translation="MVPTVVIDLVDQLKGNYSIKLILEVLNIPKSTYYRWKNKTYKND

TVTQKVIELCEANHYTYGYRKITALINQCYTSPINHKRVQRMMQKHHLNCRVRPKKTT
RIGKPYYKTDNLLQR"
/db_xref="SEED:fig|703339.280.peg.46"
/product="Mobile element protein"
CDS complement(39224..39538)
/db_xref="SEED:fig|703339.280.peg.47"
/product="Mobile element protein"
/transl_table=11

/translation="MKRVSYSVETKYKAVEMKAAGFSTKEIMKELNIKNRTQVETWWR

WYRNGESYRFSQHVGKQYTYGKGLEELSEVEQLKLENKRKDIELDILKKYKALERKWY
QQ"
CDS complement(40049..40951)
/db_xref="SEED:fig|703339.280.peg.48"
/product="Arginine/ornithine antiporter ArcD"
/transl_table=11
/translation="MGSTFSTINPFSVVIASNAAGTTFTDGLYWRIGACIIGAIFVIS

YLFWYCKKIKKDPKSSYSYEDKAAFEKQWSVLHDDGSSEFTLRKKIILTLFVLPFPIM

VWGVMTQGWWFPVMASAFLIFTIVIMFIAGTGQYGLGEKGTVDAFVNGASSLVGVSLI
IGLARGINLVLNKGMISDTILHFSSSIVQHMSGPLFIIVLLFIFFCLGFIVPSSSGLA

VLSMPIFAPLADTVGIPRFVIVTTYQFGQYAMLFLAPTGLVMATLQMLNVRYSHWLRF
VWPVVAFVLIFGGGLLITQVLIYS"
CDS complement(41697..42392)
/product="DNA-binding response regulator KdpE"
/db_xref="SEED:fig|703339.280.peg.49"
/db_xref="GO:0000156"
/db_xref="GO:0003677"
/translation="MKTTLLVVEDDEAILHLIDVALTMNYYKVVTAKTGKEADFRLRT

EQPDIILLDLGLPDIDGLSLIQQFRDFVDTPIIVISARTEEQTIVEVLDRGANDYMTK

PFNIDELRARIRVALRMSRSTETIQSRFTNGPLTIDFDAKSVLVNEQSVHLTPNEFQL

LTILCHHVGKVLTYQVLLKSLYGYVNHTEMAALRVHMASLRKKLNYTQDPNTEKKLII
THPRIGYQMQHLE"
/transl_table=11
CDS complement(42367..45087)
/product="Osmosensitive K+ channel histidine kinase KdpD"
/db_xref="SEED:fig|703339.280.peg.50"

/translation="MDNMLYINYDVIKRGCLMESTYKKRGKLTIYLGYSPGVGKTYEM
LSNGIDQYQEGADIKIGYIEPHQRPETNALVQQLPEISTSSRKFGSHTFHYIDVSKII

EANPDVVLIDELAHTNISKERHLKRYKDIEEILSHGIDVWTTLNIQHIESLSGQIALM

TGIQVTERVPDQFITSADAFEVVDISPNMLIQRLKAGKVYKKERLETAFSNFFTYENL

TELRELTLRTVADIMSQKGQQYKTKHTDITPHIAVAISGSIYNERVIREARRAAYKEH

AKFTAVYIDVFETPSESRKQDHYVHKNLMLAKALGAEIKVLYAQDIAKALTDWCDLAF

VTKLVLGQSEQPRWKDYFKQSLIEQINHVPHHFKLEIVPIHYIHSDKPTNTPKQAQKS
SRLTVEIMKMLVIQTICVLLGIWVYNLDKSESSAIILLMFFIGIIVLSIWTQSYLIGF
FASILNVFVFNYFFTVPRFTLEMYRFEYPITFATSIFASIFTSAILKNLKHQHSLTER

QLYRTNIMLQFNQSIKESYSIERLLNIAGDQIHQLLNQDVTVFLIQSKKVAASNSFGN
RSISSSDKTHDAETLSWVIENESRAGKLTDTFPGSKFLCIPIGTNPVKGVISIRFTDE
TYIDTYDNSILDSMLNDITLAIENVDLLKQTRQSILKAEREATRSNFLHSISHDIRTP

LTSIMGNLDMLKYHNEHLNDQQQAELLTASYGEAQYLHTLVTNILSLTKLESSDIQIQ
RTPYLVEELLEEFEEGLIRRHQANHVIIENEDDASLINIDSKLILQVLFNLIDNALKH

AESHSEIKLHVQHETNKIKFEMIDCGKGIPEEERQLIFNPYYSGDNFKDNKKDSLGLG
LYLVQLILKQHNSELEYQPNTPQGSIFYFYLTVNPIEMEGAMDENNIIGR"
/transl_table=11
CDS complement(45088..45231)
/db_xref="SEED:fig|703339.280.peg.51"
/product="hypothetical protein"
/transl_table=11
/translation="MTTTANSVIIALPPYFHQNLNKSYLIEVLTFKESYKETIKMINK
TER"
CDS 45304..46980

/product="Potassium-transporting ATPase A chain (EC
3.6.3.12) (TC 3.A.3.7.1)"
/db_xref="SEED:fig|703339.280.peg.52"
/db_xref="GO:0008556"
/EC_number="3.6.3.12"
/translation="MSIVLFLIVFILLSLIVSRYLYSVALNVPSKIDVVFNPIEKLIY

QLIGTKLEHMSGKTYIKHFLLFNGLMGGLSFVLLLIQQWLFLNPNHNLNQSVSLAFNT

MASFLTNTNLQHYAGETDLSYLTQMCVITFLMFTSAASGYAVCIAMLRRLTGMTDVIG
NFYQDITRFIVRVLIPFALIISLFLISQGTPQTLKGNLVIETLSGVKQTIAYGPMASL

ESIKHLGTNGGGFLGANSSTPFENPTYWSNYAEALSMMLIPGSLVFLFGRMLKTKLQI

HPHAIMIFVAMFVMFIGFLVTCLYFEFAGNPVLHHLGIAGGNMEGKETRFGIGLSALF

TTITTAFTTGTVNNMHDSLTPLGGMVPMVLMMLNAVFGGEGVGLMNMLIYVMLTVFIC

SLMIGKTPSYLGMKIEGKEMKLIALSFLVHPLLILVFSALAFIVPGASDALTNPQFHG

VSQVLYEFTSSSANNGSGFEGLGDNTVFWNISTGIVMLLARYIPIVLQILIVSSLVNK
KTYQQHTQDVPINNLFFSSVLIIFIILLSGLTFLPDLMLGPIGEQLLLHA"
/transl_table=11
CDS 46999..47811
/transl_table=11
/EC_number="3.6.3.12"
/translation="MAETTKIFESHLVKQALKDSVLKLYPVYMIKNPIMFVVEVGMLL
ALGLTIYPDLFHQESVSRLYVFSIFIILLLTLVFANFSEALAEGRGKAQANALRQTQT

EMKARRIKQDGSYEMIDASDLKKGHIVRVATGEQIPNDGKVIKGLATVDESAITGESA

PVIKESGGDFDNVIGGTSVASDWLEVEITSEPGHSFLDKMIGLVEGATRKKTPNEIAL
FTLLMTLTIIFLVVILTMYPLAKFLNFNLSIAMLIALAVCLIPTTIGDYYRL"
/db_xref="SEED:fig|703339.280.peg.53"
/db_xref="GO:0008556"
/product="Potassium-transporting ATPase B chain (EC
3.6.3.12) (TC 3.A.3.7.1)"
CDS 47823..49019
/transl_table=11
/EC_number="3.6.3.12"

/translation="MDRVTQFNILAKSGRSVETCGDVNVLILDKTGTITYGNRMADAF
IPVKSSSFERLVKAAYESSIADDTPEGRSIVKLAYKQHIDLPQEVGEYIPFTAETRMS

GVKFTTREVYKGAPNSMVKRVKEAGGHIPVDLDALVKGVSKKGGTPLVVLEDNEILGV

IYLKDVIKDGLVERFRELREMGIETVMCTGDNELTAATIAKEAGVDRFVAECKPEDKI

NVIREEQAKGHIVAMTGDGTNDAPALAEANVGLAMNSGTMSAKEAANLIDLDSNPTKL

MEVVLIGKQLLMTRGSLTTFSIANDIAKYFAILPAMFMAAMPAMNHLNIMHLHSPESA
VLSALIFNALIIVLLIPIAMKGVKFKGASTQTILMKNMLVYGLGGMIVPFIGIKLIDL
IIQLFV"
/db_xref="SEED:fig|703339.280.peg.54"
/db_xref="GO:0008556"
/product="Potassium-transporting ATPase B chain (EC
3.6.3.12) (TC 3.A.3.7.1)"
CDS 49035..49592
/db_xref="SEED:fig|703339.280.peg.55"
/db_xref="GO:0008556"
/product="Potassium-transporting ATPase C chain (EC
3.6.3.12) (TC 3.A.3.7.1)"
/transl_table=11
/translation="MQTIRKSLGLVLIMFVLCGFIFPLTVTALGQVLFPEQANGSLVK

QDGKVIGSKLIGQQWTEPKYFHGRISAVNYNMNANEVKESGGPASGGSNYGNSNPELK

KRVQETIKQEGKKISSDAVTASGSGLDPDITVDNAKQQVKRIAKERNIDASKINHLID
ENKQASPMADDYVNVLKLNITLDKL"
/EC_number="3.6.3.12"
CDS complement(50137..51006)
/db_xref="SEED:fig|703339.280.peg.56"
/product="hypothetical protein"
/transl_table=11
/translation="MISTSEHSIFKNPAKNDGLRVRTIEVSEKFTSSAQNSDTIKKVV

SQNNGHVLPIIAQFLLDNTNDVTSLFQKEDSVFYQLLSTESSNTGIRMIKHYATLTTT

AHILNQSLDLQLNVSSIKNYLLSYHNETVSERSLADKAMDTIVQFIAENLGKFSQQTR

LANMIQNFGILSLKDDYIEVKIIRNVFDKMLTDSQFEDTKNVIDALDNAGKLVSDRDR

KTTKRNAKDDTGKSTSLVFYQIKIDLDYADVLGLQSQGRDNVIQKHSNPSQFKNTNLE
EFMKSENTTLIVF"
CDS complement(51016..51912)
/translation="MEKILYQTDEFKLKPSGWYKTIPPKKDGGTEFEIMLSGPIAFTD
RFIDPATRKEKVFLSDLNNIELVEKASILTALQLPSLIEYGFTINEKHIRDLGFVLQQ
MRSTTPLSTIYSGVGMLHTLLGPLISLDQPYFSNEITNSTSIICDNKYDLIPKGNLSE

WLQMYKEEVHGNLSLELDVLFGVSSLVTAFLKYHNNVEFSGTIFSFTGQSSTGKSTAA

MLAASVAGNPTKGTENLFRSWNATRNALEGYLSGNYGVPIVLDELSAATFHDTTGLLY
SFAEGQGRQRANINGDVKTPKN"
/transl_table=11
/product="hypothetical protein"
/db_xref="SEED:fig|703339.280.peg.57"
CDS complement(52112..52549)
/product="hypothetical protein"
/db_xref="SEED:fig|703339.280.peg.58"
/translation="MIQSHIIIVNKNDHSLIYLNEMSESLLLYVYSVLINVTSINKLF

LYEETNTVFRCITSSINENMDCIINHSPNELLMFLYDLEQRFNNFINRLNLKEPKNRH
TYPTLNSVYIELNPLQLSEDTYFSSINVAELINRQSIRKNLFK"
/transl_table=11
CDS complement(52654..52941)
/translation="MQTLASKFEDLDIDSHDNLNSFKARIGSTIDKLAKAPPLNKYTF
TELMIIISIIYFKNLYIGRLDKPSSRKKKGGNLIFEIVYNNQMLNSSTAKL"
/transl_table=11
/product="hypothetical protein"
/db_xref="SEED:fig|703339.280.peg.59"
CDS complement(53031..53657)
/product="hypothetical protein"
/db_xref="SEED:fig|703339.280.peg.60"
/translation="MQLQTMSNSIFIKNLIFAINESLEYSETNSVMEVTFQKAIGFTQ

YNFEGEKLKLKDDLKELISLVGREEAVLIFVNRLNEGLYCILNQLIEWDNLNYQFAMY

KEAMHNYLFNTIEKLNDDTLFLLHQDGLITLPEHKYKTVAVKSTFGSRDFTFRFKFPE
ESDTVNTHQMLNKAISITGSLANTHLEQPQKYKAHFKKIPIETKNLTS"
/transl_table=11
CDS 53772..55280
/transl_table=11
/translation="MNEEILREVADIFIGDDRDSIYDYKTGNELVRFFNHYFNKGDIY

QAPFPSRWLYVVKHLQTLIQERKINQFFTLILSNHYIKYELKIDEVEAAKQAAKALKL

FNKRLNHYGYYITGTNNARYFMDKDEDTESIGYGGYANIYLQKTTGLAVKKLKEEYLT
DSSIKSRFKREFDLTKSFDTNPLFINVFEFNESDYSYTMELADETLKDYIESKTISEL

EKVKIIMKILKAMSQAHSENKIHRDISSKNVLMFRGKVKISDLGLGKNLDEIHSHQTF

DTNGVGQYKYCAPEQMYSLKQADKQSDVFSLGRLINFIMTGNVVNNHHLFRGVSDKAT

NSSKEYRFEDANEMLKMLQRILEYHSSAKHVEKCQEKLKRGVFDDESEEFIMTRSDEQ

LCQMVLSSNNNEQACLIRYMQKNESSACDLIESINRKYQEFCGRFEDYDPFAKLAYMI
LCNNFSYRVNETAARVLNYVAWSVNRFSAQDLIKGLINRGVEPLIEEKLKDN"
/db_xref="SEED:fig|703339.280.peg.61"
/product="hypothetical protein"

ORIGIN

1 gaggcttatc ataaataaaa ctaaaaatta gattgtgtat aatttaaaaa tttaatgaga
61 tgtggaggaa ttacatatat gaaatattgg agtatacctt gcaatatcat acgatgttta
121 tagagtgttt aataaaccat ttttcaacta ttgatgatct agaatatata ataactgtac
181 aaattatatt gattatggaa ctacaattca attaagaaat tgatgatgaa attttaaatt
241 taaactaatg gaatcaagaa agaatgaaag gaaatataca atgcccacga ttaataaaag
301 gaagtttatt agattttgtg ttagaaacag tagatgtttt tgtaaatgga agtaaaaatg
361 attgcgaatt agtaaatttt aattataaag aaggaaaagt agtttcacgt tatagtacaa
421 aatttgaaag caatcctaaa ttaagaaaaa agcaattgat atcgacggta aaagttgtaa
481 ggtatgttat ttttgatttt gagaaaattt tatggggcat agggaaagtt ttattgaagt
541 tcatcatatt aaggcaatgt ttactataag aaagttgaaa tacactcaga aacagactgg
601 attcccgttt ttttcaaatt ggcacaaaat gatacatagg cccacctata tattgaagat
661 ttaagttaaa cgataaataa tatttttctc ttttgtatag aaattttttt gggaatgata
721 ttaattagta taaaaatgaa tatgtctaca aagaaggaga gattttattg aaacacatta
781 atcaattttc tgaatcgaca ctagcacaac tagaaaaaga tcaacaacac gtattttatg
841 tatactgttt gatggaccca agaaatgatg aatgctttta tgttggtaaa ggtaagggga
901 atagaatatt taaacataaa caagatgcgc agaaaaaagt attgtacgaa gatatattta
961 tagaagaaaa taaagataac ttgaaattta atagaattaa cgaaatttgt agcaatgatt
1021 tgaatgtttt aggatatata atcagttatg gattaaccga atctgaagca ttttctgcag
1081 aaaatgttct tataaacttt cttaacctaa caaataaaac tacattaaca aatatgatta
1141 acggtcatgg atctaaggca tacttagttg aagatttaga aaatgaattt ggctacgatt
1201 caattaatct tgaaaacata aatacgaatg aattaatttt agcagtgaaa atcagagatg
1261 catttctatt agataaggat gaaagtaaag agtatcctat taatgaaagt aaacgtgata
1321 gaaataacct taaatcgcgt acattaggta gttggataat aggaaaagat aaaatacata
1381 aaataaaata tattattggt atcaatacag gtgctaataa tgcagttgtc tcagcttatg
1441 aagtatcatt tgaacaagcg gagagtatcg aaacaaataa tggtagaatg agatatgcgt
1501 ttattgggct ttcagaaaga gatgctactc taaaaaaatt gaatttatac aaaaaagcac
1561 taccagattt aagatttggt agtggtagcg ctacagcata tataaataat gggacaatga
1621 aagttgatta aatgaataaa aacatccaat tccttaaatt agcctttgaa aattttatac
1681 tcgtatatat aagtgaaaaa cttaaaagaa ttagatgtta attaaaggag agaatgctat
1741 gccatcagat gatattgtaa aaaaactata ctcaaaagag gatattcgat tagaattagg
1801 acttactccg cataagttta ataaaaaaat ggaaactatt gctaagcttt ttaaaattga
1861 tatgaaaatt tttcacaatt acaaaggcca agataaaaat aatcagtata catttaatgg
1921 tgtcgcaaaa gaattaatca aagtgttgct aaaaagtgtt gattattacc cagtggatat
1981 caattccaaa aaatttaaac aaaatggaaa atctaaaaaa gaaatgattg aaaatataga
2041 taactctagt tacatgaaat atatttatca gttaatgaaa tctatcaatg aaattcaata
2101 caaaaggtta attgctgata tacatatgaa agatgtgtac cagaatacaa aagcatggtt

2161 aaataatggt gaatcaatta ataaaaaaga acaagagcta taccaatata tgacaatttt
2221 accattacat aaaagagttg aactgcaaaa tgaggtatta aaatctatag acgaaacaat
2281 ttttcaattt ctcgcaaaag aacatagaaa taatcaaatt gaagaaaata atgaattaga
2341 agcatataca aaagcaataa aagaaggtag aaatcctaaa aatgattatg aattaaatca
2401 tctcctatat aaaaaaaatc aattaccctt agatagtcta atagaagata tatgggacta
2461 tgaggaaaca gagtatactg agcttgattg gttaattgca gatatgctaa aacgttcaca
2521 aagagctgat agtaatttta tcgaaagttt agagaagaaa aacaaattga gaaaaaatat
2581 tcataaagat tccattaaaa gtatatctaa gttgattgat catgaattag ttaaaaataa
2641 tagaaagtgg gacgtagcag aattttataa aaatcaacaa ttttggaata atagtacatt
2701 aagacgtagc tctcaaaatt ttggaatttt atattacaaa aagaataggt tacatacaat
2761 gaatcaaagt aatattatta agaaaataaa aattatcgaa gctaatatag agagtgcaaa
2821 taaacagccg agctatttag atttcttttt acaagctaag tttgatttag agaggttaga
2881 aagtgaatta atgaaatata gcattaatcc gaaatatttt aataagataa atagtgatta
2941 cgtaagatat gctattgaag atgtacataa tgcaattatt aaaattgaca gatatataag
3001 taacgataaa gtgaagttaa actattcaaa tgtccaaatg gtagtgagaa acgaaataac
3061 agaacaagga agctactttg taacccaggc attgaataca ctgtcaaagg tagaagagtc
3121 tggctacagc tttgataatt tcatggtagg acctttcgta gaagacttta aaaaagcaat
3181 taaaaatcgt agatttggtt ctaaaaaaga ttaagattgt tttttagagt taaacataag
3241 aataaattta aataaaaaaa taaacccaac aaaccttgtt atatcaatgt ttgttgggtt
3301 tattttttg gtgatttttt aataactcaa ttacatctta ggataatatc gctaaattat
3361 acattgtcga ggccaagaca aataaaaaat taaagcaaaa gttagcgcgc aactttctgc
3421 tataaaggag caaatttata tggagacaag agacaaactg atgtcattga ctcaaagtga
3481 caaaacacag caatggctaa tggacaagtc atctaaccaa gatgacattc aacaattgca
3541 gcaacaattc agtcagcagc ttgatcaaca atataatgca cttttagctg atgaaaaagc
3601 taagttagac caatacgtgg aagtacatca aggattggaa tcattaaagg aagagattga
3661 atcagaacct attacgctta atatcgataa attacccgat atcaaagcaa caatgcttga
3721 aagagccaag aatgatgaac attctgataa aatcgaaaag ctatttgata ggttagaaca
3781 ggcattaaat ggtacgaatc gattatatac gcaattatcg ttgattggta cacgaacaca
3841 tcgaattaca actaaaaatt ttaatcttca aggcttacct aaagcagtcc aacatacgat
3901 tttaccttca aaatttaaga aggtgtatac agtcgatttt aaatcgtttg aaccatcagt
3961 tgcagcgtac atgactcaag attcaaaact gattgacttg ttaaatcaga aagacggact
4021 gtatgacgca ttgctaagtg aattaggctt atcagatgag ctacgtgtat ttgttaaacg
4081 tgcatttatt ggttcgtttc tatttggagg taacttcaaa aatcctaaat tcaagctgaa
4141 tcaatatgta agtgaagtac aatggttgga tgcggtcagc caatttacaa aagtcattga
4201 acttaagaag cacgttgaaa agagtaattc catgcctatg ccttatagta ttgagcatga
4261 tatgagcgca tttcaaggta gcagtattat ggcaatctac gtacaaactg tagcgagtta
4321 tattttcaag cacattctgc taaaagtgta caaagcacag tgcgatcaaa aaacgttcaa
4381 gattatagta cctatacacg atgcgattat gattgaatgt gaagatgaag aaattgcaca
4441 aaatgtgggt cagttaatga aagatacggc taaccagttg ttcaatggtg aatttgcaca
4501 tgtgacagtg gaagaaatag gaggcgtaga ccatgaataa tgatagagga caaagcctac
4561 acatcccaag tagtacacca atcaaagaaa ataatatata tgtagctacg ttacattctg
4621 tgatccaaac agatttctca ggtgaaataa agcaccaatt cacgtatgaa attgaagtga
4681 acaatcagat tgtatatgcg aatcgtaata ttccaacaaa accgagcgct aatcagttgt
4741 caattcatga ttggctgaaa cgtcatagca actatagcgc aagtcatgaa aactatgagc

4801 cttatattga tcagaaacat ttaattctat taggtcaata taacggtaac tattatgtac
4861 aagatgtagc atcgttagat gcgtttggag gcgtattatc atgaatcata tattacaaat
4921 gttatctaag ctattaagtg tggccaagga ggcaatcgac cgtcaaggtc tgattgctat
4981 cctaactatt cctgttaata ataacgatga aatagaagaa acggctcaag gtgaaaccgt
5041 gtataacgaa cttatcgatc agttacgact taatatccca aaagatacgg attatcaacc
5101 taacatctat agttattttg gtattaagaa gaatcctaat gacaccgtac tcatggaaat
5161 gatgataaag gtttttcata tcaaacgctt taattcagaa ctgtttattt tcaaagttaa
5221 cgggtggcaa aagataaatg gagatgaatt acaagggttg atatctaaaa tgatacaagt
5281 attgcttgta gattataagc cttcactaag cactctaaaa aatgtcgtag atggattgca
5341 aaaatcaaca gatgtagaag aacttgttga gaatgagcgc tatattggtt gtggtgaaaa
5401 tatgttcgat cttaatacgt ttcaagtcgt taaaaattca atcgatatct ttccaaaaac
5461 acgattgaat ttatcattaa gtacaaatga tgtaattact gataagatac cgccttattt
5521 taagcaatat atgttacaac ttgcgaatta tgacgatgat ttacaatact ttcttttcca
5581 acatacagca gtattactta cagctgatac taaataccgt aggggtctca tattatatgg
5641 tggagctaag aatggtaaat ctgtatatat tgaactagtt aaatcatttt tctatagtaa
5701 agatattgtg tctaagccac ttaatgagct tgaaggtcgt tttgacaaag aaagtttaat
5761 tgacaaaagt ctaatggcaa gtcatgaaat tgggcaatct aggattcaag aaaagatcgt
5821 aaatgacttc aaaaagttat tatctgtaga atcaatgcat gttgatcgta aaggaaaaac
5881 tcaagtggaa gtcattttgg atttgaaact tatttttagt acaaatgcga tacttaattt
5941 tcctcctgaa catgcgaaag ctttggagcg tcgaattaat attattccat gtgagtatta
6001 tgttgaaaaa gcggacactt cattaattga taagctccag agtgagaaga aagaaatctt
6061 tctttacttg atgtatgtgt atcaacagat tgtaaaagca gatatcgagt atcttgaaaa
6121 tagccgtgtc actgaaatta ctcacgattg gttaaatttt ggatatgaat ttgtttctag
6181 caggtccgta agtaatgcaa atcagaaagc atgtattaat ttactcagaa aacttataga
6241 aatcaaatca ggatcacgaa tcaaagtatc cgagttaaat aaagttatta atgaagaaat
6301 aaaggtaagt tctcaggtta ttaaacagtt aattcaagca aactttgata ctcaaaccaa
6361 actatacaat ggctacgatt attggattga tttaggttgg aaagaagcca ataaaaaaga
6421 gattcatgat atttcggaaa aagataatat tatttcatta gataaaaatg aaaatataac
6481 agacgatgag gcattagatg aagagaattt ggactttgat tgggaggact ttgacgatga
6541 ataatgaaca aattgaagca tttgtagaag tgcttgtacc tatcatagaa gaacgtatca
6601 ataaaggtaa ttaaggctaa ttacgtacta caggcagttg cctgtagtac tcatatgatt
6661 aagtggtaaa agtgataaaa atgaaacgaa attataaata tatattatct atatgttgtt
6721 acaagaccga tagtctgtag caataatcta ataaaaggag cggtatgata tgaagggtaa
6781 aattgcactt tattcacgcg ttagtacgtc agagcagtcg gagcatgggt actcaatcca
6841 tgagcaggaa caagtactca tcaaagaagt tgtgaaaaat ttcccaggtt atgactatga
6901 gacatatact gactcaggca tttcaggtaa aaatattgaa ggtcgtccgg caatgaaacg
6961 tctattacaa gatgttaagg ataataaaat cgaaatggtg ttaagttgga aattgaatcg
7021 tatctcacga tcaatgagag acgtgtttaa tattattcat gaattcaaag aacatgatgt
7081 agggtataaa tcgatttctg agaatattga tacatccaat gcttctggag aagtactcgt
7141 tacaatgttt gggttaatag gatctataga acgccagact ttgatttcga atgtgaaact
7201 ttctatgaat gctaaggcaa ggagcggaga ggcaatcacc ggtcgtgttt taggctacaa
7261 attatcactt aatccactta cacagaaaaa tgatttggtt atcgatgaaa atgaagctaa
7321 tattgtacgt gaaattttcg atttatattt gaatcataat aaaggcctca aagccattac
7381 aaccgtactt aatcaaaagg ggtatcgtac tattaatcaa aagccatttt cagtgtatgg

7441 tgttaaatac attttgaata atccagtcta taaaggctat gtcagattca ataaccatca
7501 aaactgggct gtacagcgaa gaagtggcaa aagtgataaa aatgatgtga tattggtcaa
7561 aggtaaacat gaagccatta taagtgaaga ggtatttgat aaagttcatg aaaaattagc
7621 ttctaaaagt tttaaaccgg gcagacctat tggtggagat ttctacttac gtggccttat
7681 taaatgccca gaatgcggaa ataatatggt atgtcgacgg acgtattata aaacgaaaaa
7741 gtccaaagaa cggacaatca agcgttatta catttgttcc ttattcaatc gttcagggag
7801 ttctgcatgt cacagtaatt ccatcaatgc tgaagtcgtc gagcgtgtaa ttaatgttca
7861 tttgaatcgt attctgtctc aaccagatat tatcaagcag attgcgtcaa atgtgataga
7921 agaactgaaa caaaagcata gtaaccaaac agaaattaaa tatgacattg atagtttaga
7981 aaaacaaaaa gctaagctta aaacacaaca agaacgattg ttagaattgt tcttagatga
8041 tcagatggat agcgaaatgt taaaagctaa acaaagtcaa atgaatcaac agttagaagt
8101 attagatcaa caaattaaag aagcgcaaca agcaaatcaa tcacaggatg aaatacctaa
8161 ttttgataaa ttaaaaggac gactcatttt gatgataaca cgattcagcg tgtacttaag
8221 aaaggctaca cccgaagcta aaaatcaact tatgaaaatg ttaattgatt caattgaaat
8281 tacgacagat aaacaagtaa aacttgtaag gtataaaatt gacgaaagtc ttatccctca
8341 atctttgaaa aaagattggg ggtctttttt tatgcccaaa ttccaatttg aaatatatgg
8401 tcaaaatgat tatttcatcg accaaattac cacttttacc acttagttat tagtgacaaa
8461 agtgagcgaa atgaaataaa aatcaaatat atattatcaa aatgatgtat cacatgcata
8521 catcaatcaa atacattagg aggtcataac catgacacta gaacaacaac tcaagcacta
8581 tataaccaac ttattcaatc tgccaaggga cgaagtgtgg cactgcgaat ctatcgagga
8641 aatcgctgat gatatcttac ccaatcaata tgtaagactt ggcccactca gtaataaaac
8701 acttcagact aatacctact actctgacac acttcatgaa agtaatatct atcctttcat
8761 tctctactat cagaaacaac tcatagccat cggttatatc gacgaaaatc acgatatgga
8821 tttcttatac ctacacaaca ctatcatgcc tcttttggat caacgatact tactaacagg
8881 aggacaataa aatgcataaa tacatcaaaa ttacacaatt agtcattaca atactaagtg
8941 aaatcatcat ttggatgaaa gagtcagaac gaaaggaagt ctcttatgaa tagatatatc
9001 acccggggta tcgccaacaa cttacctaat atcttacaac accaattatg gcaactcgta
9061 tctgagcgag aacaagaaca aaccaaagat aatacttcag tagattattt tcatatattc
9121 cagttcaata agcatcgcaa tcaattatat atcaaacaca aacaagaacg acctgaatat
9181 gcgaaaatcc ataaagctaa ttattcaaaa gcaatcaata tcaataaggt ctacattatt
9241 cgagaagatg atgtagacct ttcttattat gtcatgttat tacctgaaga atactagagg
9301 agtgaaaatt atatggaaac aatcaaaagt acattaaaaa cagaagccat attcagtgat
9361 gacaaacaac atcgctatct acttaagaaa acatggaata gtgaaaaaca atcaatcaca
9421 atcattacaa tgtatccgca ttatgatggc attctcaata ttgacctaac gacccaactc
9481 atcatgaaca aagtttcaga aatggatgca tttggttcaa tcaattttgt gaatctatac
9541 tctaatatta caacccctat caatctcaaa catttagaaa atgcgtatga taagcataca
9601 gatattcaaa ttatgaaggc agtgaaagag tcagatgaag tgatattagc ttggggcgct
9661 tacgctaaaa agcccggtgt tgaagcacgt gttaatgaag tattagagat gttgaaacca
9721 cataaaaaga aagtaaaacg actcatgaat ccagaaacca atgaaatcat gcatcccctt
9781 aattcgaaag cacgtcaaaa atggatatta aaagtatagc aactaaaatg tttattcact
9841 atccatgaaa ataattgaat ttcatatata tcttttaatc tacaatatga ttaaaagata
9901 tattatttgg ttctgttgca aagtaaaaaa atatagctaa ccactaattt atcatgtcag
9961 tgttcgctta acttgctagc atgatgctaa tttcatggca tggcgaaaat ccgcagatct
10021 gaagagacct gcggttcttt ttatatagag cgtaaataca ttcaatacct tttaaagtat

10081 tctttgctgt attgatactt tgataccttg tctttcttac tttaatatga cggtgatctt
10141 gctcaatgag gttattcaga tatttcgatg tacaatgaca gtcaggttta agtttaaaag
10201 ctttaattac tttagccatt gctaccttcg ttgaaggtgc ctgatctgta attacctttt
10261 gaggtttacc aaattgttta atgagacgtt tgataaacgc atatgctgaa tgattatctc
10321 gttgcttacg caaccaaata tctaatgtat gtccctctgc atcaatggca cgatataaat
10381 agctccattt tccttttatt ttgatgtacg tctcatcaat acgccatttg taataagctt
10441 ttttatgctt tttcttccaa atttgataca aaattggggc atattcttga acccaacggt
10501 agaccgttga atgatgaacg tttacaccac gttcccttaa tatttcagat atatcacgat
10561 aactcaatgt atatcttaga tagtagccaa cggctacagt gataacatcc ttgttaaatt
10621 gtttatatct gaaatagttc atacagaaga ctcctttttg ttaaaattat actataaatt
10681 caactttgca acagaaccgt attatggaat agagatgttg gtaacattta tacaggatca
10741 ttatacttaa gtttaatttc gttattacag aaccacacat tccaaccaga agagaaagta
10801 tgtctattta gttatggttc aggagcagta ggagaaatct ttagtggttc aatcgttaaa
10861 ggatatgaca aagcattaga taaagagaaa cacttaaata tgctagaatc tagagagcaa
10921 ttatcagtcg aagaatacga aacattcttt aacagatttg ataatcaaga atttgatttc
10981 gaacgtgaat tgacacaaga tccatattca aaagtatact tatacagtat agaagaccat
11041 atcagaacat ataagataga gaaataaact agtggccgat tgtgcttgat gagcttggga
11101 cataaatcct aactcgaaat aaataagcat atcactaaac tgatttttta aagtttacag
11161 tgatatgctt atttttttat cttacgattt tgtacgtgca tgcttgccta ggggtatggc
11221 tcgagccatt agtctctcgc acatactatt ccctcaggcg tcagcactta caaaatcggt
11281 tgtaattttc atttttatac gcattcttac tgagattata ctaataagag gaatagtaaa
11341 agcaattcta agtaaaattg cagataagag gtttgttaaa agcagttcta agtaaaattg
11401 cagataagag gtttgttaaa agcagttctc agtaaaatta cagataagag gtacgttaaa
11461 agcagttcta agtaaaattg cagataagag gtttgttaaa agcagttcta agtaaaattg
11521 cagataagag gtacgttaaa agcaattcca tgcaaaattg ctgataaggg gtaagttaaa
11581 agcagttctc agtaaaattg cagataagag gtacgttaaa agcagttcta ggcaaaattg
11641 cagataagag gtgcgttaaa agcagttctc agtaaaattg ctgataaggg gtaagttaaa
11701 agcaatccta agtaaaattg cagataaggg gtacagaaaa actagacttg attacaaaat
11761 ggagcttggg acataaatga ttttttaaaa atgagatgag acgtagatta actccataat
11821 caatacgaat ctatcgactt ctttatttat gatattcatc tctttttaat ggaaataaaa
11881 gtgcgattaa tgtgataata cagttacgtt aattaaaaaa ataaaaatgc aaggagaggt
11941 aatatgctaa ctgtatatgg acatagagga ttacctagta aagctccgga aaatacaatt
12001 gcatcattta aagctgcttc agaagtagaa ggtataaact ggttggagtt agatgttgca
12061 attacaaaag atgaacaact gattatcatt catgatgatt atttagaacg gactacaaat
12121 atgtccgggg aaataactga attgaattat gatgaaatta aagatgcttc tgcaggatct
12181 tggtttggtg aaaaattcaa agatgaacat ttgccaactt tcgatgatgt agtaaaaata
12241 gcaaatgaat ataatatgaa tttaaatgta gaattaaaag gtattactgg accgaatgga
12301 ctagcacttt ctaaaagtat ggttaagcaa gtggaagaac aattaacaaa cttaaatcag
12361 aatcaagaag tgctcatttc aagctttaat gttgtgcttg ttaaacttgc agaagaaatc
12421 atgccacaat ataacagagc agttatattc catacaactt cgtttcgtga agactggaga
12481 acacttttag attactgtaa tgctaaaata gtaaacactg aagatgccaa acttactaaa
12541 gcaaaagtaa aaatggtaaa agaagcgggt tatgaattga acgtatggac tgtaaacaaa
12601 ccagcacgtg caaaccaact tgctaattgg ggagttgatg gtatctttac agacaatgca
12661 gataaaatgg tgcatttgtc tcaatagaaa gttagaggtg agtcttacgt ttcagtgacg

12721 gtagacttac ctttaacatg ttacatacta aaaaattaat ttgaataaga aagagagaca
12781 tatatgaaat acgatgattt tatagtagga gaaacattca aaacaaaaag ccttcatatt
12841 acagaagaag aaattatcca atttgcaaca acttttgatc ctcaatatat gcatatagat
12901 aaagaaaaag cagaacaaag tagatttaaa ggtatcattg catctggcat gcatacactt
12961 tcaatatcat ttaaattatg ggtagaagaa ggtaaatacg gagaagaagt tgtagcagga
13021 acacaaatga ataacgttaa atttattaaa cctgtatacc caggtaatac attgtacgtt
13081 atcgctgaaa ttacaaataa gaaatccata aaaaaagaaa atggactcgt tacagtgtca
13141 ctttcaacat acaatgaaaa tgaagaaatt gtatttaagg gagaagtaac agcacttatt
13201 aataattcat aataaaacag tgaagcaacc atcgttacgg attgcttcac tgttttgtta
13261 ttcatctata tcgtattttt tattaccgtt ctcatatagc tcatcataca ctttacctga
13321 gattttggca ttgtagctag ccattccttt atcttgtaca tctttaacat taatagccat
13381 catcatgttt ggattatctt tatcatatga tataaaccac ccaatttgtc tgccagtttc
13441 tccttgtttc attttgagtt ctgcagtacc ggatttgcca attaagtttg cataagatct
13501 ataaatatct tctttatgtg ttttatttac gacttgttgc ataccatcag ttaatagatt
13561 gatattttct ttggaaataa tatttttctt ccaaactttg tttttcgtgt cttttaataa
13621 gtgaggtgcg ttaatattgc cattattttc taatgcgcta tagattgaaa ggatctgtac
13681 tgggttaatc agtatttcac cttgtccgta acctgaatca gctaataata tttcattatc
13741 taaatttttg tttgaaattt gagcattata aaatggataa tcacttggta tatcttcacc
13801 aacacctagt tttttcatgc ctttttcaaa tttcttactg cctaattcga gtgctactct
13861 agcaaagaaa atgttatctg atgattctat tgcttgtttt aagtcgatat taccatttac
13921 cacttcatat cttgtaacgt tgtaaccacc ccaagattta tctttttgcc aacctttacc
13981 atcgatttta taacttgttt tatcgtctaa tgttttgtta tttaacccaa tcattgctgt
14041 taatattttt tgagttgaac ctggtgaagt tgtaatctgg aacttgttga gcagaggttc
14101 ttttttatct tcggttaatt tattatattc ttcgttactc atgccataca taaatggata
14161 gacgtcatat gaaggtgtgc ttacaagtgc taataattca cctgtttgag ggtggatagc
14221 agtacctgag ccataatcat ttttcatgtt gttataaata ctcttttgaa ctttagcatc
14281 aatagttagt tgaatatctt tgccatcttt tttctttttc tctattaatg tatgtgcgat
14341 tgtattgcta ttatcgtcaa cgattgtgac acgatagcca tcttcatgtt ggagcttttt
14401 atcgtaaagt ttttcgagtc cctttttacc aataactgca tcatctttat agcctttata
14461 ttctttttgt tttaattctt cagagttaat gggaccaaca taacctaata gatgtgaagt
14521 cgcttttcct agaggatagt tacgactttc tgtttcatta gttgtaagat gaaatttttt
14581 tgcgaaatca cttaaatatt catccatttt tttaacggtt ttaagtggaa cgaaggtatc
14641 atcttgtacc caattttgat ccatttgttg tttgatatag tcttcagaaa tacttagttc
14701 tttagcgatt gctttataat cttttttaga tacattcttt ggaacgatgc ctatctcata
14761 tgctgttcct gtattggcca attccacatt gtttcggtct aaaattttac cacgttctga
14821 ttttaaattt tcaatatgta tgctttggtc tttctgcatt cctggaataa tgacgctatg
14881 atcccaatct aacttccaca taccatcttc tttaacaaaa ttaaattgaa cgttgcgatc
14941 aatgttaccg tagtttgttt taattttata ttgagcatct actcgttttt tatttttaga
15001 tacttttttt attttacgat cctgaatgtt tatatcttta acgcctaaac tattatatat
15061 ttttatcgga cgttcagtca tttctacttc accattatcg cttttagaaa tataactgct
15121 atctttataa acttgtttga aatttttatc ttcaattgca tcaatagtat tattaatttc
15181 tttatctttt gaagcataaa aatatatacc aaacccgaca actacaacta ttaaaataag
15241 tggaacaatt tttatctttt tcatcaatat cctccttata taagactaca tttgtagtat
15301 attacaaatg tagtatttat gtcaaaataa tgttataatt tttgtgatat ggaggtgtag

15361 aaggtgttat catctttttt aatgttaagt ataatcagtt cattgctcac gatatgtgta
15421 attttttag tgagaatgct ctatataaaa tatactcaaa atattatgtc acataagatt
15481 tggttattag tgctcgtctc cacgttaatt ccattaatac cattttacaa aatatcgaat
15541 tttacatttt caaaagatat gatgaatcga aatgtatctg acacgacttc ttcggttagt
15601 catatgttag atggtcaaca atcatctgtt acgaaagact tagcaattaa tgttaatcag
15661 tttgagacct caaatataac gtatatgatt cttttgatat gggtatttgg tagtttgttg
15721 tgcttatttt atatgattaa ggcattccga caaattgatg ttattaaaag ttcgtcattg
15781 gaatcgtcat atcttaatga acgacttaaa gtatgtcaaa gtaagatgca gttctacaaa
15841 aagcatataa caattagtta tagttcaaac attgataatc cgatggtatt tggtttagtg
15901 aaatcccaaa ttgtactacc aactgtcgta gtcgaaacca tgaatgacaa agaaattgaa
15961 tatattattc tacatgaact atcacatgtg aaaagtcatg acttaatatt caaccagctt
16021 tatgttgttt ttaaaatgat attctggttt aatcctgcac tatatataag taaaacaatg
16081 atggacaatg actgtgaaaa agtatgtgat agaaacgttt taaaaatttt gaatcgccat
16141 gaacatatac gttatggtga atcgatatta aaatgctcta ttttaaaatc tcagcacata
16201 aataatgtgg cagcacaata tttactaggt tttaattcaa atattaaaga acgtgttaag
16261 tatattgcac tttatgattc aatgcctaaa cctaatcgaa acaagcgtat tgttgcgtat
16321 attgtatgta gtatatcgct tttaatacaa gcaccgttac tatctgcaca tgttcaacaa
16381 gacaaatatg aaacaaatgt atcatataaa aaattaaatc aactagctcc gtatttcaaa
16441 ggatttgatg gaagttttgt gctttataat gaacgggagc aagcttattc tatttataat
16501 gaaccagaaa gtaaacaacg atattcacct aattctactt acaaaattta tttagcgtta
16561 atggcattcg accaaaattt actctcatta aatcatactg aacaacaatg ggataaacat
16621 caatatccat ttaaagaatg gaaccaagat caaaatttaa attcttcaat gaaatattca
16681 gtaaattggt attacgaaaa tttaaacaaa catttaagac aagatgaggt taaatcttat
16741 ttagatctaa ttgaatatgg taatgaagaa atatcaggga atgaaaatta ttggaatgaa
16801 tcttcattaa aaatttctgc aatagaacag gttaatttgt tgaaaaatat gaaacaacat
16861 aacatgcatt ttgataataa ggctattgaa aaagttgaaa atagtatgac tttgaaacaa
16921 aaagatactt ataaatatgt aggtaaaact ggaacaggaa tcgtgaatca caaagaagca
16981 aatggatggt tcgtaggtta tgttgaaacg aaagataata cgtattattt tgctacacat
17041 ttaaaaggcg aagacaatgc gaatggcgaa aaagcacaac aaatttctga gcgtatttta
17101 aaagaaatgg aattaatata atggataata aaacgtatga aatatcatct gcagaatggg
17161 aagttatgaa tatcatttgg atgaaaaaat atgcaagtgc gaataatata atagaagaaa
17221 tacaaatgca aaaggactgg agtccaaaaa ccattcgtac acttataacg agattgtata
17281 aaaagggatt tatagatcgt aaaaaagaca ataaaatttt tcaatattac tctcttgtag
17341 aagaaagtga tataaaatat aaaacatcta aaaactttat caataaagta tacaaaggcg
17401 gtttcaattc acttgtctta aactttgtag aaaaagaaga tctatcacaa gatgaaatag
17461 aagaattgag aaatatattg aataaaaaat aaaattgttg tgtttacaac aatacataga
17521 aaacagagga aacaatcaag tcgttgaata tttcctctgt tttttagttg aaaaaaattaa
17581 ccgaaagcct gaatgcaagt cttgattaaa tcaataatgc ttgtaataac accagtgaaa
17641 tccatatgca taccctcttt ctatttaaga tacattaagt ataatatcaa acaaataaaa
17701 aatgttaaaa attccctaat tggctattta gattgcataa atgtcaaaaa tttgaaaaac
17761 atacaacgac tttgcataaa aaatcgtcat attggaaata cgtaatttat tgaaataata
17821 aaaaaaataa aagaacgaag atgataacct aagtgaggtt ttaagttgtt ctaaggttta
17881 atttaattta tgttaaaata gttggtataa aaatacatga taaactataa actaaattca
17941 aaataactta tggggtaggc aattatggaa aataaaatat aaatgataat gaaaaaagag

18001 tgctaaggga aatttataac catcataata tttcgcgtac tcaaatatct aaaaatcttg
18061 agattaataa ggcaacgatt tctagtattt tgaataagtt aaagtataaa tctcttgtta
18121 atgaggttgg tgagggtgat agcacgaaga gtggtggtag aaaacctatt cttctgaagg
18181 ttaatcatct ttatggttat tttatttctt tggatttaac ttatagttct gttgaagtga
18241 tgtacaatta ttttgatggt aatgtcatta agcatgaatc ttatgattta cctgatgaaa
18301 aggttagtag tatattaagc ataataaaaa aacatattga tattcaggag aaacttgata
18361 cttataacgg actattaggt gtgtctgttt ctatacatgg agttgtggat aatgagcagc
18421 atgtgacata tttaccattc catgaaactg aaggaatttc aattgctaag aaaataaaag
18481 aaattactaa tgttccagtc gtagttgaaa atgaagcgaa tctttcagcg ttatatgaac
18541 gtaattttaa tcataattta tcctacaata atcttattgc tttaagtata cataaaggta
18601 ttggtgctgg gcttattatt aataatcaat tgtatcgtgg tgcaaatggg gaagcgggtg
18661 aaattggaaa aacacttgtc tcaaaagtta gcgataatgt ggagatcttt cataagattg
18721 aagatatttt ttcacaagaa gctttactgc ataatttaag taatcaacta aatgagaaga
18781 tgacgcttag caaattaatt caattttata atgaaaaaaa tccagtcgta gttgaagaaa
18841 tggaacaatt tataaataaa attgctgttt taatacataa tttaaacacc cagtttaatc
18901 cgaatgcaat ttacattaac tgtccattgt tcaatgaaat gcctgaaata ttagaagcaa
18961 ttaagaacca gttcaaacaa tattcacgta acgaaattca aataaagtta acatctaatg
19021 tcaaatttgc aactttgcta ggtggtacat tagcaattat ccaaaaagta ctacagatta
19081 atgatattta cttagatata aaagcataaa aaactaattc aaatgaataa tcaaagttcg
19141 taattgtctt tataaaaaaa tccctcaatc cgaattgaat tttcggattg agggattttt
19201 atagttctat tgcagaagaa aactatttta aaaatgctgg taaatgttga tagccacctc
19261 taacgttaac aatattcgta aatcctttat attctaatat tcctaccgct attgaacttc
19321 taacacctga ttgacaatgt acataaatta ggtcattttt atcgaaaggt atatcttcat
19381 ttaaaagttt accgtgagga atatgaattg cttgttttaa atgaccttta cgccattcat
19441 catcattacg aacatctaat acattatgtt cttcaccagt catttcagaa ctatgaatag
19501 atgatgtgac gatatttgtt tgtggcaaac ggtaactttt tacattttca aaaccaatta
19561 attgtaaagc atgaatagct tgttgaacgg tagatttatc gccaattaat tcaatatctt
19621 agtcataatc taaataccaa ccaatttgat ttataaaagt tttattaaaa ggaatattga
19681 tagttccatg catatgacca ccatggaatg cttctttact tcgaagatca aaagcagttt
19741 gtgtattgct tgaactaggg taaacattat atggttggta catttgcata ccaaattgat
19801 ttattttttt catttgtgaa aaatggtgtg gtggagctgg ctgattgagt gttaaagttt
19861 cgataaatga agtttcatct ttaacattaa aagcccagtt gtttattttc tcataaccca
19921 aagtagttgt aggtaatgaa cctagcgctt taccacaagg actccctgca ccatgacctg
19981 gccaaatttg aatatagtct ggtaatgttg cagcaaattg tatggactga tacatttgtt
20041 ttgctccgat ttttgtagaa ccttcaacat ttacagcttt ttctaataga tctggtctac
20101 ctacatcacc aacaaagatg aagtcaccgc taaataatcc cattggtata ctggaacccc
20161 caccttcgtc agtaagtaaa aaactaatac tctcaggggg atggcctgga gtgtgtaaga
20221 cttctaattt aatctttcct aaatagataa tatcttgatg cttaacgaaa tgtgtttgtt
20281 taggcatatt tttataatta aattcatctt taccttcatc agatacgtat atacttgcat
20341 tcaatttatt tgccacatct ctaatacctg aagcaaaatc agcatgaata tgtgtttctg
20401 cagctttagt aattgtgaat ccttctttat ctgcaacttt taaatatttt gttaaatctc
20461 gtatagggtc aataatcatt gcttctcctg tacgttgaca tccaattaaa taagatgctt
20521 gtgaaaaatt gtcttcataa aattgtttga aaaacaaaaa aaactccttt ttaaaataga
20581 ttttattgat tagataaata agttatgatt tgcttgctca gtatgtccaa tataagtacc

20641 tacgccacca taatcgactt catctcttaa ttcttctttt gaaattccca taacatccat
20701 actcatggta caagcaatta actttatatc ttgatcgatt gcttgatcga taagtgagta
20761 taaagaatca acatttttct tgttcattac ataacgcatc ataatattac ctagtccaaa
20821 catattcatt tttgataatg gcatatgtat tggatcctta ggtaacataa ggtcaaacat
20881 tttggaaata cctttctttt taacgcgagt tgattgcgct ttttttaatg cgttgaggcc
20941 ccaaaaagta aagaaaatag ttacatcttt acctgctgct ttagcgccat ttgcgatgat
21001 cattgctgct actgccttat ctaactcacc gctaaataaa acaattgttg tacctgtagc
21061 agtgtcattg atttcaaatt cttttggctt ttctttttga ataattgcat taattacatt
21121 tgcttcttca gtaagattta caagggtatt ccctgtttgt ttcgcccaac ttttaatatc
21181 actattgaaa ccaggatctg taactgttac ctcgatttgc tcacccgttg aaatattgtt
21241 aatttcttta ctgatattaa caataggtcc agggcactga agacctctaa aatcaaattg
21301 tttacgattc tctttgattt caatatcttt ttctattaaa ggagcactat tgaagttctt
21361 tgcttcataa tctttatatc caccctttaa attcacgaca tcataacctt gtttggctaa
21421 ataatcgcaa gctttagtgc ttcggttacc gcttttacaa tgtatataat acgttttgtt
21481 gctattctta ttgaatgatt taatctcttc tactgggtgt aaagttgaac cgttaatgtg
21541 tcctaattca tattcttctt ttgttctaac atcaatcaat tgacccattt ttgccaattt
21601 ttctaattct tctttgttta atgaattaat gtgtacttct ttgtattgtt ccatacttac
21661 ctcctataaa tacctatgag ggtataataa aacggataga atcatttgcc aaatacctat
21721 atgggtattt gacaattttg ttttaattta ttattattaa cttaatcaat ttatgtggag
21781 gaaatgaata tgacttatga taaaaaaatg attaatcgta taaatagaat acaaggtcaa
21841 ttaaatggtg tcgtaaaaat gatggaagaa gaaaaagatt gcaaagatat aattacgcaa
21901 cttagtgcat ctaaaggttc tatacaacgt ttaatgggga ttataattag tgaaaattta
21961 atagaatgcg ttaaaacagc agaagaaaat aatgaaagtt ctcaagaatt aattaatgaa
22021 gcagttaatt tattagttaa aagtaaataa tggatatagc aaatatgact attatgttgc
22081 taattggcgt actgggtgga tttatatctg gattaatagg tattgggggc gcaattatta
22141 tttacccagc tattcttata ttgccaccat taataggtat acctgcgtat agtgcatata
22201 ttgcttcggg acttacctct agtcaagtat ttttcagtac acttagtgga tcattaaatg
22261 caagaaaaca accagctttc tctcctaaac ttgttatata tatgggaggg ggtatgttga
22321 ttggaagcat gttaggggca attttagcta gtttgtttaa tgctactttt gtaaatacgg
22381 tatatgtaat aatcgccata cttgctttaa tattgatgtt tattaaagtt aaacctacta
22441 cacaagagac gaaatctaaa cctttgctat ttattatagt tggatttgga attggtgtaa
22501 tttcgggaat tgtgggtgca ggtggagcat ttatcatcat tcctgtatta ttagcattat
22561 ttaaattacc aatgaatacg gtagtgaaca atagcatagc aattgctttt atatcttcag
22621 taggggcatt ttttataaaa ttaatgcaag gatatatacc agtagaaagt gcaatttttt
22681 tgataattga atgatgatca ttttctgaaa atattatgtg gtcatataat ataatgccca
22741 tcatttcact aatctctttt attctctgag ttattttgat atctcctgga gaaggtgtta
22801 catcttatca agagtaaatt acaaaagaat catttaaatc aatactttca ctttgaatac
22861 atgtatttga agtggaaggt acttatttca aaaatagtaa aacctgtatc ttaaattact
22921 taatagtaac ataagataca ggctgatttt ttattcattg ttatttatac taaagcaccc
22981 gatagctctg aaaacaatca caaattcaac ttttcaaagc cacagcttta agttattttg
23041 tccagacaac ccccattgcc caacccattt tatggaattg gcatccaggc aacaactttt
23101 catataaatc gtaataattt tgttcagata ggtacttatc tgaagctaaa tgctcaagcc
23161 atgatttaga tgtgttgtga ttataaattc taatcgcatt ttttattcca aatttgaaac
23221 aatgcggtaa aaactcttgg attgccccta atttataaac ataggtaggc ggtgtctcga

23281 cttcagatac attatcaaga ataactattc ttccttcctc attcaacagt tccttcattt
23341 gctgtattac gctggctata tcatccaaat gatgaaaggt tgttcggctt acaataaaat
23401 caaacttctc attaaaatta agttgttctg cattcatatt cagatagacc gtatttgtta
23461 gttgacgttt agatttggca agatcgagca tttgattaga aatatcaatc cctaccactt
23521 catcataata acttgctaat ttctccacta acaaacccga gccacatccg atatctaatg
23581 ctctgccttt ctttggagac atattagaca caaagaatga ataatcattc aaaagctcat
23641 tcacgaaatc gtaatcttct gcaaccttat caaactgtga ttctattgta ttcaaaaaga
23701 tcccccattc ctactttatc gacattcttt cattacttac cactttagat gttttttcgt
23761 tggggataaa acttcccttt agacaatttt atccaaagac aatacaacag tgcaacttta
23821 ttaaagtcac tgtcctttat cgcagccttt actttttagt aaagacagtg gcttctctta
23881 tcaagtttca aaacatatta ttttgaagaa aacgtccatc tgaagtgtca agtgcaaaat
23941 tacatataaa ggtttattct aaaatgaaaa gatgatacaa tcatattcag ttacataagg
24001 aggtttcaat tatgtgcacc agtatcgcag tagtagaaat tactttatct cattcataat
24061 gaaaaaaatg gaaaggagat aaaagtatgg gtactttttc tatatttgtt attaataaag
24121 taatcaaaat taattggtta taatgaacgc ttaatgtcag ttcattataa ccagtaagga
24181 gaaggttata atgaaccaga aaaaccctaa agacacgcaa aattttatta cttctaaaaa
24241 gcatgtaaaa gaaatattga atcacacgaa tatcagtaaa caagacaacg taatagaaat
24301 cggatcagga aaaggacatt ttaccaaaga gctagtcaaa atgagtcgat cagttactgc
24361 tatagaaatt gatggaggct tatgtcaagt gactaaagaa gcggtaaacc cctctgagaa
24421 tataaaagtg attcaaacgg atattctaaa attttccttc ccaaaacata taaactataa
24481 gatatatggt aatattcctt ataacatcag tacggatatt gtcaaaagaa ttacctttga
24541 aagtcaggct aaatatagct atcttatcgt tgagaaggga tttgcgaaaa gattgcaaaa
24601 tctgcaacga gctttgggtt tactattaat ggtggagatg gatataaaaa tgctcaaaaa
24661 agtaccacca ctatattttc atcctaagcc aagtgtagac tctgtattga ttgttcttga
24721 acgacatcaa ccattgattt caaagaagga ctacaaaaag tatcgatctt ttgtttataa
24781 gtgggtaaac cgtgaatatc gtgttctttt cactaaaaac caattccgac aggctttgaa
24841 gcatgcaaat gtcactaata ttaataaact atcgaaggaa caatttcttt ctattttcaa
24901 tagttacaaa ttgtttcact aaattaaagt aataaagcgt tctctaattt cacaagagga
24961 cgctttattc ttcccaaaaa ttgttcaata tttatcaata aatcagtagt tttaaaagta
25021 agcacctgtt attgcaataa aattagccta attgagagaa gtttctatag aatttttcat
25081 atacttaacg agtgctttca cctttgaata tagtccttcc cacttatcat cacactctcc
25141 ccgatagcct tttctagcta tatccagtaa agttacatgc tctttaggta aaagaggtat
25201 agcccattct gcagcgacat ctttcgaggt aatttcacca gtagtcactg tttgccacat
25261 tcgagctagg gttaaaatta cattacgctc atcacctttt atcccctcaa ttagttctgg
25321 caaagaatcc ttaattgctc ttcgaatatc tgtcaaaggt acggagacaa gtatacttga
25381 agaatcagga ccaaatagag aaatactatt ctttcttgct tgtgctaaaa caatagccaa
25441 atcaggatca tagcttggtt cctgaatttg tccattctca aattcacccc tgagccactc
25501 accgtatata aattctcttt ttggaggata ttgccaaggg acaacttcac tcctatttat
25561 aaccgtaact tcaagtggtc taacagaatc cgtatttcca atctttcctg atatagtcat
25621 tagtctttct gttagttttt ttcgagttaa ttgaggtaaa ctatgattca cgacgactag
25681 aacatctaca tcgctgttaa tgcgtaaacc accatttact gctgaaccaa atagatatac
25741 tccaactatt gaacttccaa ataaatcttt tacgattttt aatgtttgaa tcgcttgatt
25801 tggtattttt ccgttaatca aattgctcat gatttcacct cgttgattat gttcatataa
25861 agtttatatt gatactcaat ttacttaccc tagattggac atatacttaa attactgttc

25921 aataaagctg accgttagcg tttaagtaca tcctttcaca atttgtctac agattaataa
25981 ttattcttta ttatacagat ctccatataa tttttgaatt tggttctgta attttttatt
26041 ttctttttct aattccatta ctcttctttt taaggtttta ataaggattt cctccgaacg
26101 agaacttttc ttgggttttg agactacatt tgctgttatt tgacgctcac gaagggattc
26161 gattctttgc ctaatatcgt gttccttata aagccatgat ttagaaacat tagcttcctt
26221 tgctattgaa ttaaaattaa taactttacc ttcaatcgaa aatttagaaa tcgctttgtc
26281 tactttttcc cttgtctttt ttgatttctg cttcgccaaa cgtacaattt ctgttgtatt
26341 tctaacttgt ttatccattg ataattaccc cgtcaaactt ccaatgattt gttctaaacg
26401 ctctttaaca cggctattag tctctacttg tctttgccat tgtttatcct tagctatggc
26461 taataactct tctgtacgct ctaactgttc ttcgtgctgt ggtaagaatt gcttactggt
26521 acagaagtga gtgcaatcta agcatgcatt cgcatgtgga caaccacctg ctactactgg
26581 caatctacaa taaccatttg gaagcacttg tgcatttata tttttcttga accattgaag
26641 ctctacatca tcgacttcat tatcttcatc tagatcaagc acatctccat tattggtaac
26701 cagtttttcc tgaaatttag taaattcatt ttttagagtt tcatcaaaga tatgagcgta
26761 tctgcttgtc atttctgggc tttcatgccc caaaaatttc tgcacaatat gctggggcat
26821 cccgttgtta atcattcttg ttcctactgt atggcgaaag gcatgggcat ggaatctata
26881 aatctcacct gatttatcca ctatattttg ctcataagct aatttattta actcacctct
26941 aaatgtttct tgttttaatg gcgatccatc ttttcttgga aagaggtatt cactatctgg
27001 aaattcctct gaaactttat cttcccgaac tttaataagt aaagctacct ctttagatat
27061 tggaactata tgctcctttt tcattttcca ttgataatac tttaaaaaga aatctccatc
27121 tttgtcctct aatagacagc ctttttttcaa ggtgcacaat tcacttatcc tcattccaca
27181 ttcttgaaca atcatagtca tcgtagctat atattcgggt aatttatcaa gatgactgtt
27241 caattgctct aggacgaatt catctataaa gcgtggtttt gctcttggta ttttcggata
27301 gtcctcagaa taaattaata ttttggaagg aacatcatcc cattctagcc taagaagggt
27361 actaaatagt ccttccaata tagagatcct cccagttatt gtactaggtt ttattcccat
27421 catgtttagt tcacttaaat atgcttcaat ttccactctc gttaattggt gtactctctg
27481 aacttgttta aatttcatgt ccagaaaatt aaagaactct ttaagtcttt gggcaatatc
27541 acttacatag gaaaagctat ccacgttcaa tctcaactta caatatcttt ttacaagttg
27601 tttaaaatat gtattccgaa accctttaaa gttaattgta tattcatatt gtgttgggtt
27661 aaccttatca tctggcaaag gtaagttacg tctatcccaa acgtctttat cccactcctc
27721 tccatcaaaa taaaagttct cataaaactc cataaattgt tttagattag taacatagta
27781 ggaattagct tttacaggtg ttttttcttg attagcagta atcttataat tagtagtggt
27841 aattctaaca ccccgttttg tcaaataagt tctatactcc gtcattgctt tttcaatagg
27901 aacttcagta attgaagtaa tgctaggata ctttaaatct aagaaatcta acattttatt
27961 aattactgtt ccttttctaa tccagacagt ttttgcattc catattccat tgtttaaatg
28021 gtaaaaataa aaatatttca attctgttct taaccacaga tttttaacac gttcaaaacg
28081 aacccaacga ttccttaaag caggattctt acttaattct atggcagaag gatgtggaca
28141 ttttcttata tcccaactat tattagccca aaaccccctgc atttcttcat tcattacagc
28201 tatttttttg ctaatctcac tctgactaat aattttcctt ttactagaag cattcatttc
28261 ttatgctcct ttctctcgag gtatttatta aactcatttt tcatatcctg atctgaaaga
28321 tgaacatagg tatttaacgt tgtctgaaca tgtgcgtgac ctaatctctt ttgaacgaac
28381 gcaacatccc atccttccct aattagctgc gttgcgtgag tgtggcgaag catatgtgat
28441 gtaaattcta ttccagtcct tttaactatt cttctaacta gatcaagaac actttggtac
28501 tttagtggtt tcccaaaata gccttctttt aaggaaataa aaacataatc atgctccaat

28561 tcctcactat actcatatat caagtaatct gtataaagtg acataagttc tttactcaca
28621 tgtattgttc tttccttcct taatttaata taagcttcat taacattaac atctctaggt
28681 gttaaatgga tttgattgtc ccaagtgaca atatcttcaa gcctaagcga taacacttca
28741 ccgattctta aaccaccctc atacataagc attaaaatta atttatctct tttcgtatga
28801 caagcatcaa taatttgctt aacttccttt gatctcaatg ttcttatctg tttcttttta
28861 acccttaact ttaagacatt cttttggtat ctacccttat taacatgatg taaaaatcct
28921 ttgaaatttc ttccctttgc ttgtttaaat acatcaattg atttaaattc tcctaatcta
28981 cttaaataat caagaaaact cataactaca tttaaaattg tattcactgt cgtttcttct
29041 cttatggctt tttttgactg aagatcaatt acatttgatg ctgaaggata tctcaaccaa
29101 cctacgaagt ctgctaacaa ctcaaagtta atatcattaa gaataacacc tctctgttcc
29161 atgaactcgt acagcaactt taaatgatag cagtatgcct taatggtatt aggagactta
29221 ccagtattat ctaagtattt aataaatttc attactggtt ctattagctg gtattcttta
29281 tctagtaata aatacaatgg atacggctta ttctccactt ctatcctttg aaccttcaca
29341 tgttcccacc tctttaaata ctttaactac tacaaattat aaaccttgtt aatttttatt
29401 taaagtaaaa tatcccttta atatctcctc ttaaactact ttaactacta ttatttatta
29461 tactatggtt aatacatctc cacttggatg attatgaccg agcattatac tatttgcgtt
29521 actgagtatc gctgttttga atatttctct agggtgaatc accgtttggt taatagatcc
29581 aatcgatagt gtttgaatat gtgtaggttc atttttactg ttcatacata tgagaatgag
29641 atgctctcgg tcactgtttc caatgaatga acgcatgatt tctgccgcat cctcagggtt
29701 tgaaatacga ttttttagat aacttaatgt atctgttttt atcatttgta gtgaaacaat
29761 attgatttcc ttcatcgttt acctccatat ataggttatg ctttcaaagt ccatttttga
29821 cgtgctttag ggttgagtgg gtgcatgatt tcatttgtgg ctggattaat gagctttttg
29881 actttctttt tatgaggctt caacatttcc attacttgtt cgacacgttc tacaacaact
29941 ggccgcttcg cataagcacc ataagctaga atcactgtgt cactttcgct aattgctttc
30001 atcaagtgga tgtctgtgtg tttatcataa ggttctttaa tatgtttaag gttctctggt
30061 gttttaatat tagagaatag atttacaaga tatacagcac cgtatcgttc tgaattcgct
30121 aattgattga ggataagaac agttgtgaga tcgagtgata atacaccatc taaatgaggg
30181 tacatcgtta tcactgtaca agcgggtttc ttttcatccc atgttttctt gagtaaatag
30241 cggtgttttt catccttgct aaatatagct tctgtgtata tcgtattttt gattgtattc
30301 atatatataa tcacttcctt tagtattctt ctggtaaaag catcacataa taaaaagcgt
30361 ctacgccatc ttcacgaatg acgtagactt tcttaggtaa tgcattttga ttttttacat
30421 agtttgtata gtgatattcc aatttgtatg caggttgttc ttgttcatgt gtgattgaga
30481 gtatattctc atcttcttgc agtttaaaaa tgtgtaggta atctgtatga ggttgattat
30541 ctctttcttt taccatattc caaagtaaga tttgaaggtc tagagatagg tattcactaa
30601 tacctcttgt gatgtatcga ttgattttca tgttatttta cctcgtcttg aatttctttc
30661 ataatgataa tcgcttggct aataatcgta acagatattt gtgccacttt gatccagtta
30721 ttcatggtga gtccctcctt ctcttagtaa atgacgttca tcgataatcg tatttttagt
30781 atctgtgaga tataaaaagt ccatgtcaaa atgattcaga taaccaacgc tgatgagttg
30841 gttattagcg tacataagaa atggatagat acttaggtca tgtagttcat tattgtagta
30901 ggtataagtt tcaagtgtaa gatgtgcaag tggggaatca ttaataaaac gttccggtag
30961 aatattttct gctgcttctt ctaacgcttc gcattcccat gtttcattgt tagatagttg
31021 gaatagacga gttatatatt gtttgagttc ttgagtggtc gttttcatat cattgcctcc
31081 tagatagtgt tatagtgatg tagtttatgt acatcattgg gataatatat atttgatttg
31141 tcatttatta cgcatcccgg tgagaatgag agaaaattcc atatgaaaaa ccgcttcaaa

31201 ccttggtatg acaaggaaat cccgaaattc cgcctatttt gacgaacaat caactcattc
31261 tttataacta ttgatgttag ggtggggctc tgctttctta tatattattt atttataaag
31321 aataacggga ttttgggatt gtgcttgcac aatccttctg cttcttcgaa tctgcaaatc
31381 ccaatcattt cccgataaaa aatcattgtg ggatgttctt tagcaatttc aatataagca
31441 tcgtgtagtt atgaaaaaaa ttacgacaat gactgtttca ttagataagt gttattgaaa
31501 ttgataaaga gaattttaaa aatggttaga taaaataaat gaaagaatat aatgaaaatt
31561 attgttataa caatgattct attagctaaa tagtaagata tagtgtttgg ggcaaaaaca
31621 aagacgaagt gctgagatgc acttcgtcga gttgtttatt attgaaaagt tgttttatga
31681 tttcgttatt aagtttgagt gtgacataga attgtttttt atgattacca tcttttttaa
31741 tatcaatgcg atcaatcact gatagataca atgctttgag tcgagatttt tctatgtgct
31801 taatatcatg aaagatgtgt tgtaatagtt tactgatttc tttggcatca aataaaggct
31861 tatcttcatt ttgttgattt ttgagttggt tgatttgatt cgtaatgtca ttgagttgct
31921 tttcatattt ttgaatactt ggtctgatta ctgatgttaa gtccggatta tcctcgatgg
31981 ttttaatcaa gttatttagt ttgatttgta cttcatcata ttgttgttgc ttataagcaa
32041 tatcgtgatg aagtgcagcg ccatcaactt gattttcttg attgacgtgt gttactacgc
32101 gttgaatgac tttatcactt ttgactattt caagtatttg cttcatcaca taatcttcaa
32161 tcacatcagc tcttacactg tttgccgaac atactttgga acccttgttc cgaaaattac
32221 tacatgaata gtaacgaata cgtttcttag tcccgtcttt aagtgtattc gtggtattgc
32281 ttgctgccat aggtgcgcca cattggggac agtgaataat gcctgtaagc agattcgttc
32341 ctttgccatg gacttggggt ttttgactga cttgtttttt acgcatttgt actttatccc
32401 ataaatcttg attaataatg ggggaatgct taccttcagc tatcactggt ttatcattaa
32461 gccctttacg acgtttttca ctccaatctt tgtatttcgc aaattgaatt ttgccgatat
32521 agaatgggtt agctaatata tatgtgatag aactaatact aaaaggttta cccttttttag
32581 tgacatagcc tttgtgattt aatgcattgg ctattttacg atagccatgg cctttggcat
32641 aggactcgaa tatatatttc acaatattag cttcatgttg attaatcatc agttcatgtt
32701 tactattagg tattttgtca tagcccagcg gcaaattgcc ttgataatag ccttcttggg
32761 cacgtctcgt ttgacccata aatacattct cgacaatgtt attacgttcg aattctgaga
32821 aactcgcaag tatttgtaac atgagcttac ccgatgaagt attgacttcc atacgctctg
32881 ataaactgaa aaattcgaca ttttgtttgt gtaaatcttc gacaattttg agaagatcag
32941 atgtattacg agctaatcgg tttgttttgt agaccataac acagtcgata tagccttctt
33001 tcgcatcctt caatatacgt tggagctcag gtcgattcat agatttacct gaaataccac
33061 ggtcagcgta tatatcttta acttcaaaat gatggaagtc acagtattct ttgatttgat
33121 tgatttgtcc gtcgatacta taaccttctg tgctttgcat ttctgttgat acacgtacat
33181 agataccgac acgttttgtt ttaagttgtt gcattatgtt tcatcctttc ttcgtttatg
33241 caatcgatga ttgcatggtt tgattgacga tattgagtgg ttcatttttg aaatagattc
33301 ctataagatt tttatctttc gtaatgtgaa tggtttcaat ataggggtac aatatgttta
33361 acgtgaaacg tttttgaata atattttgaa tggtgtgttg tatttgatgt ccattgatag
33421 atgtagtgcg ttgcggttgt tgacgtaatg attgcgtttg ttctctgaac gtttctgcat
33481 cgatgatgcc ttgtgccaac ttttctatca gttgttcttg agtcaatgtg tgatgttttt
33541 ctatgtttct ttgtcttttg atgcgtttgt caatcgcacc tttaattttt gtgtagatgc
33601 gttgattttg ataaaagtct ttgcacactt ctaatacttt atcttcaagt gtttgtgcat
33661 tgatgccttt aaaatcacag acaaagcgag aagcattcat gtttttagga cagacgtagt
33721 aacgtaatgt atgattcttt tttctaacgg tcatattcgt aagtgttgta ttacaacatg
33781 ggcatttgat tttttgtttt agttggttat ccgaagatgt ctgtttgatt tgtttttgcg

33841 atcgaagtct ctgcgcttgc tcatatatac ttgtggaaac aatagaagga aacatattgt
33901 cgaattggcc atattgattg ttgacacgac cacaataatt aggattgatg ataatgttac
33961 gaacttgata gggttgtcga ttgatatacg tgttatcttc ttctaataac tgtgcaattt
34021 tcttataacc atgaccttta atgtaataat tgaatacagc ctttaccgtt ggtgactcat
34081 tttgattgat gatgaatgct ccgttgtgat attcgtaacc aaagggcgca tgggttgtaa
34141 tcaatcgacc ttgctttgct ttttcttgaa gcccatttct gacttgttct ccaatgttat
34201 ctgattcaag ttcggccaag ctgataaaaa tattaagctt gaatcggtcg aaagcttgat
34261 ccatatcaaa gtaaccatcg tgtacgctta agatatgaac atggtatgtt tgacataatt
34321 tgatgagttt taatgcattt ttcagattac gatgtaatct attaagacga taacaacata
34381 atatgtcaca ttgcccttgt tgaattaatt gcgtcatttg tcgataccca ctacgattat
34441 ctttgcgtcc tgattgtttg tcgctataaa agttgatatg ttgaatatga tgtttttcgg
34501 ctattgcttc gatagcctgt ttctgtgctg caagagattg ttgtttcatc gtactttgac
34561 gtaaatagcc tatgacttgt ttcatatcgg ctcctccttt cacagtaata atatatattt
34621 atggatgaat tgatatataa gcccaacatc aataagatgt tgggcgttca tattagtcat
34681 tcatttgatt gatttcttca attaccaaat cggctaatat ctcgataagt tcatccatgt
34741 ttttcactcc gttatttgtt ctatcttcaa tacgtcgatt attcagtttg atgcttcacg
34801 gttgtatgat aaagacaatc agaaatcttc gtgaactcct gaagggccta tcccctcatt
34861 agcggattta aaaagttctt tcgcagcttt gttatcattt gccggtgtcc aattttgaat
34921 taacgactta tctttagtta atcccaggat agatgcaaac tctacatcta attttagatg
34981 gtaaaataca agtgattgtt ttttaccgct attatctttg acacttcttt tagttgtttg
35041 gcgtccacgg tcagctaata tgaaaccttt atctcttaag gcgttgacaa cattattaac
35101 atcttgaaat tgatgattgt ttagcatctg tttaaaaacg tttgcaatca tttttacttc
35161 gatatggtca tcttttaatg agattaatcc atagttctca aacatatttt tcaaagcacc
35221 ttcatctgaa aacttacctc tgttttgtgc tacaaattga atgatgacat cgatagcttt
35281 atcagctaat gagcgttcag agattgtatg accatgataa tcaataaagt agtctcttat
35341 tttagcgata tcaatatctg tagctaaaac acgacctaat atttttgcag atgttgtaat
35401 gactgcataa cgcttaaaca tacgattgcc tgtgttgctt ttatcatctt tcaatttagc
35461 ttcaaaccaa tctacttcct tgtaaaacca ttgaataact tcatcttcac gatttataag
35521 atatttagct actaacggta aaacatgacc atagtttagt gctacagctt ttttaatatt
35581 gtcagcattg gtcgcatttg tagtgaattg ttcattaatc tcgatggttc ttacacgtaa
35641 tccatcgttt tgagctgaat cattaaaaat actgtgttct gacgttgaaa tgacagaagt
35701 accccaattc ttaggcgttt taacttctcc atgaacgttt gaacgttgac gaccttgacc
35761 ttcagtgatg gagtacaata accccgttgt atctctaaaa gttgctgatg agagttcatc
35821 aaatacaata ggtataccaa aattgttact caagtaacct tcaagtgcat tacgtgtggc
35881 attccaattt ctaaagagag tttcattacc tttggtaggg ttaccagcga ctgatacagc
35941 taaagaagct gcagttgact taccggttga ggattgacct gtaaaactaa aaatgattcc
36001 ggcaaattcg gtttcatgtt tgtgcttcag gaaactcgtc actaaggcag aaataccaaa
36061 tacgactgct aattctaaaa gaagagaacc tttaacctct tttagataca tgttaaacca
36121 attatcaaat gttcccctag gtgctaagtc ataagtattc tcacaaatgg cgtcagatgg
36181 agatttatta tcaaattccg tagtagtata gatttcattt aacgatacaa taggaccaaa
36241 cggtgtttcc agtataccta ccccttcata taagtaggaa atgggtaatt ggttgcgcat
36301 ttgttgcaac gcataaccta aatcttttgt atatttttca ttaatactaa atccatattt
36361 cattaaagag ggcagttttt gtgttgttaa aatatcacta gattcaacaa ttactttttg
36421 atcctcgtct gtaataatta ctttttcagt gttagtttta gggtcaataa acttattttc

36481 gataacgata ggacctgcga tttcaacttc agtaggcatt cctccttttt ctttgggagg
36541 cttgtcttta taccaacctt tttttgattt gtatcgtggt gaaggattaa atgaagggtt
36601 agtttgagtc attagcgaac acctcctttc gaagggttgc tgttatagtg tggattagga
36661 cctgttttaa gataaactaa gtgaccgtga gtatccttac cgataataat aaatggaaca
36721 cgtggcgcat gttttacaaa atatgcgaac caacgtccaa cattttgttg tacagctttt
36781 gaacattgta catttgcacg actgttcaag tcatgaaatg taaattcttc tccttcaggt
36841 aaattgaatg aaatacctaa tattttcttt tttaactgct cgaatttatc tgattgattg
36901 aaatacattt aacttcctcc tactaatgaa ctaagatagg aaaattaaat atgcgcacaa
36961 ttaactttct taagttcatt ttagccaaga agttatcatt tgaaagcttg taaaaatatg
37021 ttagaaattc cgtactaaat taagatggat ttttgttgac aaaataaaaa acgctgattt
37081 aacagcgctt taaataaaaa ttaatctgaa gttatataaa agtagtcaga aggagtggtg
37141 ttattatcat tataataatc tagtgcttta ttgatttctt ttattaattt atcatgatgt
37201 tcttttgtta ttttctttgg atatttgagt tctttttctt ctaagtcttt tggagacact
37261 aggtcactat ttaaaattaa tgataacagc atatttttag gtgccgaagc tctggtgtct
37321 cgatgagtaa tatattttgc taaattactg tttatactta ctttttcatt ttctaaaaag
37381 tttgtactat ttttaacatg agcatcagca attttggtac ttatttcagt gtttaaatcg
37441 tctatttcat ctaaataata ttcaagaatc tgaagtttat ctttagaagt gataccttttt
37501 tcagtgaaaa tcttaataat gatagatttg attccaattt ctatattact gtctactaga
37561 aaatagagat aataattagt cagagcatgt tcataatctc tttgatcagt aatttcatga
37621 ttgatgaaat aatataagtt ctctttgata cgattagaag aatctatatc tttgtcattg
37681 ataccgtttt taaaaaagtg tagataaact gagttgtttg agagtatatc atataataca
37741 aataaacttt ttggtttaaa tacatatgat tttcctgatt taaagtttgc tatgtttatt
37801 cctagagcgt tgcagaattt attaaaatta gttgtaaaat tatttttttc ttgaataaac
37861 atgtcgttgt catttaattc tgaactatga tttttaatca tattgatatt ttcttcaatc
37921 ttgtcactac ttaaattttc catgcaaagt atactatatc ttactatatc acttatattt
37981 atggaaaaat ctttaggaga acgatttaaa gtatcctttt tattatcttg atctacagcc
38041 ctacatgaat catcaaaaac ttctttgaca tttattttat caatgttcaa aattatgtct
38101 actaaagcat tttgaaaatc atcgctatat atgtctttat tattcatagt gtcactacct
38161 ttatttttaa taacactata tattatatct tataaaccta ataccatgaa gaataaaaag
38221 aatgattcaa attttatatt taatccatag ttggctgcta tggttagtgt actagtctag
38281 ttttactagt atataaaaca gtactcaatt tttatttttt caaaattata aaaaagaaca
38341 cctatcatcg ataggcactg aacccctaaa acgggaactt aataaaaaca ccatgttcta
38401 ggctattaat tctctgtatt ttacagggga taagtagcct agtttttgtt gaatttgatt
38461 attattatag tttttaatgt acttttcgac aatatctatt acaatatgat tagagctatt
38521 aagctgatta ttgatgtaaa gagtttcaga ctttagcgag gaatggaaac tttctatcgg
38581 ggcgttatcg gcaggtgttc cctttcggga catacttctg ataatgcctt tttcttcgca
38641 taattgataa taagcataag atgtataaac gctgccttga tcactatgta atatatacc
38701 tcaggtatat cgatttgatt taatgtatca ttaactaaac gttggtcttg tttatcatct
38761 attttatacg ccacaatttc tccgttataa atatccatta tcgaagataa atacaacata
38821 gaatgatcaa atggtaaata agtaatatcg gttgttaata cttctatggg acaattcgct
38881 ttaaattatc tttgtaataa attgtctgtt ttataatacg gtttacctat ccttgtcgtc
38941 tttttaggtc taactcggca gttcaaatga tgcttctgca tcattctctg tactctctta
39001 tgattaattg gtgatgtata acattgatta atcagtgctg taatcttacg ataaccgtag
39061 gtataatggt tagcttcaca taattcaata actttttgtg ttacagtatc atttttatag

39121 gttttgtttt tccatcggta atatgttgat ttaggtatat ttaatacttc tagtatcaat
39181 ttgattgaat agtttccttt taattgatcc actaaatcta tgactactgt tggtaccact
39241 tcctttccaa tgccttgtac tttttaaaa tatccaattc tatatctttt ctcttatttt
39301 ctaattttaa ttgttctact tctgacagct cttctaatcc tttaccgtag gtatattgtt
39361 taccaacgtg ttgtgaaaat ctataacttt ccccatttcg ataccatcgc caccaagttt
39421 ccacttgtgt tctattttta atatttaatt ctttcataat ttcttttgtt gaaaatcctg
39481 ctgctttcat ttcaactgct ttatactttg tttctactga ataagaaact cttttcatag
39541 aaaaaacacc tccgtatgat tcattttaat atgaattcaa cgaaagtgtt tttatataat
39601 tcccactaat tggggtcagt ctactatatg atacggtttt ttaatttaaa gtatcaataa
39661 taattggata tagagggaaa gaagctataa tgatatttgc ttactaagtg gataaagata
39721 ttagaaaaac gagaagcagg acagttatta aatcggtaga tattaatcat aagtatttaa
39781 ctgaattttt accttttgtc gaatatataa ctaaagttga aggtagtaaa atatttatct
39841 agtcgatatt aaaacaattt atcgaaaacg atggttttcc atatcctctt tagtagcaat
39901 tttctaacaa tatataatta attatataca aattaagaat aattaagaag tatatattaa
39961 ttagagatag tttaaaaatt agatttcaat caattaatta aaacaggtta gactattaat
40021 ttatactaac ctgttaaaag taacaaaatt aagaatatat taaaacttgt gtaatgagta
40081 agcctccacc aaatattaaa acaaacgcga caacaggcca tacgaaacgt aaccagtgtg
40141 agtagcgcac gtttaacatt tgaagtgttg ccattacaag tccagtaggc gctaagaaca
40201 acattgcata ctgaccgaat tgatatgttg taacaataac aaatcttggt atacctactg
40261 tatcagctaa tggcgcaaag ataggcatag ataatactgc taatcctgat gatgatggta
40321 cgataaatcc taaacagaaa aagataaaga gcagaacaat gataaataaa ggcccactca
40381 tatgttgcac gatagatgat gaaaagtgca agattgtgtc agaaatcatt cctttattca
40441 atactaagtt gattccacga gctaaaccaa tgattaaaga tacacctact aaacttgaag
40501 cgccattaac gaatgcatct acagtgcctt tttcgcctaa accatattgt cctgttccag
40561 caataaacat gatgacaatg gtaaagatta agaatgcaga tgccatgact gggaaccacc
40621 atccttgtgt cataacaccc caaaccataa tagggaatgg taggacgaaa agcgtaagaa
40681 taatcttttt acgtaatgta aactcagaag aaccgtcatc atggagcaca gaccactgtt
40741 tttcaaatgc tgctttgtct tcataagaat aagaggattt aggatctttt ttaatttttt
40801 tacaatacca gaataaataa ctaataacaa atatggcacc gatgatacaa gcgcctattc
40861 tccaataaag accatcagta aaagttgttc ctgctgcatt agaagcaatg acgactgaga
40921 atgggttgat tgttgagaat gtactaccca cagagcttgc taagaaaatt gcaccgaaag
40981 taagacttta gaatctatgg gaaagataat aaggtttaat agaaatacaa gaagtgtaaa
41041 tagtaagcat ataatttata cattttaata taatataaca agaaaggtaa acacaaagat
41101 aataaaaata aaaatttaat aaagatagag aattattaaa ttgtttttga tataaaacaa
41161 cttaataaaa aatttaattt taggggagga taattataat ataaagaaac atagaatgga
41221 attgcaattg ggaagtatac gattttaact gcgaatcata tgttcaacga cttacaaatg
41281 cgtaaatcga taacacattc aaaaatatat aatctgaaaa ttgatttact aagagaggat
41341 tattgtagtt acaggtgtta agttgctcca atatggagtt gtcgtcttgt tttatattaa
41401 acaagtttta ttcaaataca tgacaatacg taaaataaca ctagaattat caatcactaa
41461 aattaaaaca aatgtatcta ttcaaacttt tctttagggt ttacttgctg aataatttaa
41521 aaatgaagta ttaggtgtac atgctttcag aattgctttt gaagaatatt aaaagtataa
41581 tctgaatacg atgacagaat gaataggtaa actatgacaa gaagtcatca aggttatcaa
41641 ataaatatat cccagcggtt gagacgactc actgctggga ttttattgtt agttatttat
41701 tctaagtgtt gcatttggta gccgattcta ggatgtgtga tgattaattt cttttcagtg

41761 tttgggtctt gcgtataatt caacttttta cgtaaagagg ccatatgtac acgtaaggca
41821 gccatttctg tatgattgac atagccatag agtgatttta ataacacttg ataggttaat
41881 actttgccga catgatggca taatatcgtt aggagttgga attcattcgg tgttagatgt
41941 acggactgtt cattgacaag gactgatttc gcatcgaaat caatggttaa tggaccattt
42001 gtgaaacgac tttgaattgt ctcagtagaa cgtgacatac gtaatgctac tctgatacgt
42061 gctcttaact catcgatatt gaaaggctta gtcatataat cattggcacc gcgatctaat
42121 acttcgacaa tggtttgttc ctctgttcgt gcactaatca caataatagg tgtgtccaca
42181 aagtctctga attgctgaat gagagataaa ccatcaatat ctggtaagcc taaatctaat
42241 aagataatat ctggttgttc tgttcttagg cgaaagtccg cttctttccc cgtcttcgcc
42301 gtaaccactt tataataatt catagttagc gcaacatcga ttaaatgtaa aatcgcttca
42361 tcatcttcaa cgaccaataa tgttgttttc atccatggct ccctccattt caattggatt
42421 aacagtcaaa taaaaataaa aaatactgcc ttgtggtgta ttcggttgat attctaattc
42481 actgttgtgt tgtttcaaga tgagttgtac taaatagagc cctaatccca aactatcttt
42541 tttattgtct ttaaagttgt ctcctgaata atagggatta aaaatcaatt gacgttcttc
42601 ttctggaata ccttttccgc aatctatcat ttcgaatttt attttgtttg tttcatgttg
42661 aacgtgcagt tttatttcag aatgtgattc tgcatgcttt aaagcattat cgataaggtt
42721 gaatagcact tgcagtatta atttactgtc gatattaatg agtgaagcgt catcctcatt
42781 ttcaataatg acatgatttg cttggtgtct gcgtatgagg ccttcttcaa attcttctag
42841 aagttcttct actaaataag gggtgcgttg tatttgaatg tcagagcttt ctaacttagt
42901 caaagataaa atatttgtga ctaaggtatg cagatattgt gcttcgccat aagaagcagt
42961 taagagttct gcttgttgtt gatcgtttaa atgctcgtta tggtatttca gcatatctaa
43021 gttgcccata atggaagtca gtggtgttct aatatcatgt gaaattgaat gcaagaagtt
43081 tgaacgtgtg gcttctcgtt cagctttcaa tatggattgt ctggtttgtt ttaatagatc
43141 cacattctct attgccaggg taatatcgtt caacatagag tctaatattg aattgtcata
43201 ggtgtcgatg taagtttcgt cggtgaagcg gatagaaatc acacctttga ctggatttgt
43261 gccaatggga atacacaaaa atttactgcc aggaaaggta tcggttaatt taccggcacg
43321 gctttcattt tcaatgaccc agctcaatgt ctcagcatca tgtgtcttat ctgagcttga
43381 aatgcttctg tttccaaatg agttggaagc agcgactttc ttactttgaa ttaagaatac
43441 cgtgacatcc tgattgagta gctgatgaat ctgatcacca gcaatattta ataagcgttc
43501 aattgaataa gattctttaa tggactggtt aaattgcagc ataatattgg tccgatataa
43561 ctgccgttct gttaaggaat gctgatgttt taaattcttt aagatggcac tcgtaaagat
43621 actagcaaaa atactggtcg caaacgtaat cggatattca aagcgataca tttctaaagt
43681 gaatcttggc accgtaaaga aataattaaa aacaaacaca tttaagatag acgcaaagaa
43741 tccgattaaa taggattggg tccaaataga aagcacgata ataccgataa agaacatcag
43801 caagataatg gcactggatt cgcttttatc taagttgtaa acccatatac cgagcaagac
43861 acagatcgtt tgaattacaa gcatcttcat aatctctaca gttaaacgag aggatttttg
43921 agcttgctta ggtgtatttg taggtttgtc tgaatgaata taatgaatcg gtacaatttc
43981 taacttgaaa tggtgtggta cgtgattaat ttgttcaatg agtgactgtt taaagtaatc
44041 tttccaacgt ggctgttctg actgtccaag gactagcttc gtcacaaaag cgagatcaca
44101 ccaatcggtt aacgctttcg caatatcttg tgcatacaac actttgattt ctgcacctaa
44161 cgctttggcg agcattagat ttttatggac ataatgatct tgttttctgc tttctgacgg
44221 tgtctcaaag acatcaatat acacagctgt gaatttagca tgttctttat aggcagcacg
44281 tcttgcttct cgtatgaccc gttcgttata aatactgccg ctgatagcta ccgcaatatg
44341 cggtgtaatg tcggtatgtt tagttttata ttgttgtcct ttttgactca taatatctgc

44401 gacagttctg agtgtaagtt cacgcagctc tgtcagattt tcatacgtaa agaaattaga
44461 aaaggctgtt tctaagcgtt cttttttata tacttttcct gctttaaggc gctgaatcaa
44521 catatttggt gaaatatcca caacttcaaa ggcatctgct gacgtaatga attggtcggg
44581 cacacgttct gtaacttgaa tacctgtcat taacgcaatt tgtccgctta gactctcgat
44641 atgttggatg ttgagtgttg tccagacatc gataccatgc gatagaattt cttctatatc
44701 cttataacgt tttaaatggc gctcttttga aatgtttgta tgtgctagtt catcaattaa
44761 gaccacatct ggattagctt ctatgatttt agagacatct atatagtgaa aggtgtggct
44821 gccaaattta cggctggagg tcgaaatttc aggcaattgt tgaaccagtg cattggtttc
44881 agggcgttga tggggttcga tataaccaat tttaatatct gcaccttctt gatactgatc
44941 aataccattt gataacattt catacgtttt acctacccct gggctatagc ctaaataaat
45001 ggtaagtttc cctctttttt tatatgtact ttccatgagg cacccccctct taatcacatc
45061 ataattaata tacaacatat tatccattta tctttctgtt ttgttaatca tctttatagt
45121 ttctttataa ctttccttaa atgttaatac ttcgattaga taactcttgt taagattttg
45181 atgaaagtaa ggagggagcg caatgattac actattagct gtcgttgtca tcgcattaat
45241 tttattttta ttttacgcat taatttggag tgaaaaattt taacagagaa agagggaagc
45301 atcatgagta ttgtgttgtt tttgattgta tttatcttgc tctcactcat tgtgagccga
45361 tatttatatt cagttgcttt aaatgtgcca tctaaaatag atgttgtttt taatccgatt
45421 gagaaattga tttatcaact gattggcacg aaattagaac acatgtctgg gaagacgtat
45481 atcaaacatt ttttgttgtt taacggattg atgggcggat tgtcctttgt attattgctt
45541 attcaacaat ggctgttttt gaatcctaac cataatttaa atcaatctgt atcgttagcc
45601 tttaatacta tggcatcttt tttgaccaat actaacttac agcattatgc aggtgaaaca
45661 gatttaagtt atttaacaca aatgtgtgtc atcactttct taatgttcac gtcagcagcg
45721 tcaggttacg ccgtatgtat tgcgatgtta agacgtttga ctggaatgac agatgtgatt
45781 ggtaatttct atcaagatat tacgcgtttt attgtacggg tgctcatacc tttcgcattg
45841 atcatcagtt tgttttttaat cagtcagggc acaccgcaaa cgcttaaagg taatttggtg
45901 attgagacat tatcaggtgt gaaacaaacg attgcatatg gaccgatggc gtctttagaa
45961 tctattaaac atttagggac aaatggtggt ggtttcttag gtgcgaactc ttctacacct
46021 tttgaaaatc cgacatactg gtctaattac gctgaagctt taagtatgat gttgattcca
46081 ggttcattag tctttctatt cggtagaatg ttgaaaacta aactacagat tcatccgcat
46141 gcgattatga ttttcgttgc gatgtttgta atgttcatcg gctttttagt gacatgtctc
46201 tattttgaat ttgcggggaa tccagtgttg catcacttag gtattgccgg tggcaatatg
46261 gaaggcaaag aaacacgttt cggtattggc ttatccgctt tatttacaac cattacgacc
46321 gcttttacta caggaacagt taacaatatg cacgatagtc ttacaccgct aggcggcatg
46381 gttccaatgg tattaatgat gttgaatgca gtttttggcg gtgaaggtgt tgggctgatg
46441 aacatgttga tttatgtcat gttaacggtc tttatctgta gtttgatgat tgggaaaaca
46501 ccaagttatt taggaatgaa gattgaaggt aaagagatga aactcattgc gctttctttc
46561 ttagtacatc ctttacttat tttggttttt tcagcactag cttttattgt gccaggggca
46621 tcagatgcgt taactaatcc gcaattccac ggtgtatcac aagtgttgta tgagtttaca
46681 tcatcttcag cgaataatgg ctctggtttt gaaggattag gagacaatac ggtattttgg
46741 aacatttcaa caggcattgt gatgttgctt gcacgatata ttccaatcgt tttacaaatt
46801 ttgattgtat ctagtttggt aaataaaaag acctatcagc aacatactca agatgtaccg
46861 attaataatt tatttttcag cagtgtattg attatcttta ttattttgtt gagcggctta
46921 acgttcttac ctgacttaat gcttggacca ataggcgaac agcttttgct gcacgcatag
46981 ataaaggagg attagaaaat ggctgaaact actaaaatat ttgaatcaca tttggtcaaa

47041 caggctctaa aagacagtgt attgaagctc tatcctgttt atatgattaa aaatccgatt
47101 atgtttgttg tagaagtggg catgctgctt gccttaggat taaccattta tccggattta
47161 tttcaccaag aaagtgtatc acggctatat gtgttcagta tctttatcat attattactg
47221 acacttgtct ttgcgaactt ctctgaagca ttagctgaag gtcgcggtaa agcacaagcc
47281 aacgctttac gccaaacaca aactgaaatg aaggcacgtc gtattaaaca agacggcagt
47341 tatgaaatga ttgacgctag tgacctgaaa aaaggacata tcgtacgtgt cgcgacaggt
47401 gaacaaatcc caaatgacgg taaagttatt aagggcctcg caacagtgga tgaatctgcg
47461 attacaggtg aatctgcacc tgtaatcaaa gaaagcggtg gagatttcga taatgtaatt
47521 ggaggaactt ctgtagcttc agactggtta gaagttgaga ttacttcaga accaggtcat
47581 tcatttttag ataaaatgat tggtttggtt gaaggggcta caagaaagaa aacacctaat
47641 gaaattgcgt tatttacttt attgatgaca ttaacgatta tcttcttggt cgttatttta
47701 acgatgtatc cattggcgaa attcctgaat ttcaatttat ccattgcgat gctgattgct
47761 ttggctgtgt gtttaattcc aacaaccatt ggggattatt atcggctata gggattgcag
47821 ggatggatcg tgtgacacag tttaatatct tggctaaaag cggacgttct gtagagactt
47881 gtggtgatgt gaatgtcttg attttagata aaacaggtac cattacctac ggcaaccgta
47941 tggcagatgc gtttattccg gtgaaatcat caagctttga acgtttagtt aaagcggcct
48001 atgaaagttc tatcgcagat gacacaccag agggacgtag tattgtgaaa ttagcttata
48061 aacaacatat cgacttaccg caagaggtcg gagaatatat tccgtttact gctgaaacac
48121 gtatgagcgg tgtgaaattt acgacacgtg aagtatataa aggtgcaccg aatagtatgg
48181 ttaagcgtgt gaaagaagca gggggacata ttccagttga tttagacgct cttgtcaaag
48241 gggtgtctaa aaaaggtggc acaccgctgg ttgtgcttga agataatgag attttaggtg
48301 ttatttattt gaaagatgtc attaaagatg gactcgtaga acgtttccgt gaattacgtg
48361 agatggggat tgaaacggtg atgtgtacag gagataacga attgacagct gcgacaatag
48421 cgaaagaagc gggtgtggat cgctttgtgg cagagtgtaa acctgaagat aaaatcaatg
48481 tgattagaga agaacaagcg aaaggtcata ttgttgcgat gacgggtgac ggtacgaatg
48541 acgcgccagc tttagcagaa gctaatgtag gtttggcaat gaactcagga accatgagtg
48601 ccaaagaagc ggcgaattta attgatttag attctaatcc aaccaaactg atggaagtcg
48661 ttctaattgg gaaacaatta ttaatgacac gtggctcact cactacattt agtattgcga
48721 atgacattgc gaaatacttt gcgattttac cagccatgtt tatggcggct atgcctgcga
48781 tgaatcattt gaatattatg catctgcatt cacctgaatc agcagtatta tctgcgttaa
48841 tctttaatgc gttgattatt gtattattga ttccgattgc gatgaaaggc gtgaaattta
48901 aaggtgcctc aacgcaaacc atattgatga aaaatatgtt agtttacggc ttaggcggta
48961 tgatcgtgcc atttatcggc attaagctca ttgatctcat catccaactc tttgtctaaa
49021 aggaggacaa aacaatgcag acaataagaa aaagtttagg actagtactg attatgtttg
49081 ttttatgcgg atttatcttc ccgctgactg tcacagcgct tggacaagta ttatttccag
49141 aacaagcaaa cggcagttta gtgaaacaag atggcaaagt aattggttca aagctcattg
49201 gacaacaatg gacagaacct aaatatttcc atggacgtat cagtgcagtc aattacaata
49261 tgaatgcgaa tgaagtgaaa gaaagtggcg gacctgcttc aggcggctca aactacggca
49321 attcaaatcc tgaattgaaa aaaagagttc aagagactat taaacaagaa ggaaaaaaaa
49381 tttcaagtga tgcggtgacc gcttctggct ctggtttaga cccagatatt acggttgaca
49441 atgcgaaaca acaagtaaaa cgcattgcga aagaaagaaa catagatgct tcaaaaatta
49501 atcaccttat tgatgaaaac aaacaagcat caccaatggc agatgattat gttaatgtct
49561 taaaattgaa tatcacttta gataaactct aaataaacag ggagtgaggt gagacatcca
49621 tgtggttcat tagcattatt attttaatag cattcctaat tatattaatg attgacgatt

49681 taattaaaac atgacaactt cacgcaaaac aatatgaatt ggatgtaata gagaagggag
49741 taccaagaaa tactcagttt attgaggata attattaggg gagattagag aagaattctc
49801 tattcacgaa cattacttta acagttgtaa tcattggttt catcatatcg attaatagta
49861 cagtcatctt gagattttca acacactatt atagctagct aagatacttt taaattatga
49921 cttatttttc atcatttacg atacatcatt ctcgataact tactatttgt tatggtattt
49981 atagatttaa agaaagtgaa ggagattaag caatgtaact tggcttaatc tctttttgat
50041 tatatttatt aaggcaaagc ttcatttccc ttaaatcaag tatatcttct gaataagcaa
50101 atgaaatcgt gacgccaact acagtgataa cttcgcctaa aacacaatga gtgtggtgtt
50161 ttcacttttc ataaactcct ctaagtttgt gtttttaaac tgtgatggat ttgaatgttt
50221 ttgaattacg ttgtcgcgtc cttgggattg caatccaaga acgtcagcat aatctaaatc
50281 aattttgatt tggtagaaga caagagaagt acttttgcct gtatcatctt tcgcatttct
50341 ttttgttgtc tttcgatccc gatcggaaac taattttcct gcattatcca aagcatcaat
50401 aacatttttc gtgtcttcga attgactgtc tgttaacatc ttatcgaata catttcggat
50461 aatttttact tcgatgtagt catctttaag tgagaggata ccaaagtttt gaatcatatt
50521 agcaagacgt gtttgttgtg aaaacttgcc caggttttcc gcaataaact gtacgattgt
50581 atccatcgct ttatctgcaa gagaacgttc tgacaccgtt tcattatgat agcttaataa
50641 atagttcttt atgctgctaa cgtttaactg taggtctagt gattgattta aaatatgggc
50701 tgttgttgtt agagttgcat aatgtttaat cattcgaata ccagtgttac tggattcagt
50761 tgatagtaat tgataaaaaa cactgtcttc tttttgaaaa agggaagtaa catcattagt
50821 gttgtctaat aggaattgag caattatagg taaaacatga ccattatttt gactaaccac
50881 ttttttata gtatctgagt tttgagcact tgatgtgaat ttttcagaca cctcaatagt
50941 acgaacacgc aaaccatcat ttttagctgg atttttgaaa atactatgtt cagaagttga
51001 aatcactgat gtatcctagt ttttaggtgt tttgacatca ccatttatat ttgctctttg
51061 acgtccttgt ccctctgcaa atgaatatag aagacctgtt gtatcatgaa atgttgccgc
51121 tgataattca tctaatacta tgggtacacc ataatttccg ctaagatatc cttctagtgc
51181 atttcttgtt gcattccaac tccggaatag gttttcagta ccttttgttg ggttgccagc
51241 taccgatgcg gctaacatag cggctgtcga ttttcctgtt gatgattggc cagtgaaaga
51301 gaaaatagtt cctgaaaact cgacgttatt atgatactta aggaaagcag tgactaatga
51361 agatacgcca aacaacacat ctagttctaa agataagttc ccatggactt cttctttata
51421 catttgtaac cattctgata aattgccttt tggtattaaa tcgtacttgt tatcacatat
51481 aatagaagtg ctattagtaa tctcattaga aaaataaggc tggtctaatg aaattaatgg
51541 tccaagtaat gtatgaagca ttcctacacc tgagtatatt gttgataaag gtgtggtgct
51601 tctcatttgt tgcagtacga accctaaatc tctaatgtgc ttttcattaa tggtaaatcc
51661 atattcaatt aaggagggta attgcaaagc agtaaggatt gaagcctttt cgacaagctc
51721 tatattattc aaatccgaaa gaaatacttt ttctttacga gtcgcggggt ctataaaacg
51781 atctgtgaaa gcaatcggtc cagataacat tatttcaaac tccgtacccc catctttttt
51841 aggagggatg gttttgtacc atcccgaagg tttgagcttg aattcgtcag tttgatatag
51901 aattttttcc attaacagtg acccccttgg ttgtaagagg aaattataat gatatgtcgg
51961 tacttaagtg ggttagttcc tataataatg aaagggatgc ttggtttgta ctttatccac
52021 caagcaaagc gtcttcctgc ttcacgttga tcgttgatgt tgcactgttc tctgaataac
52081 tgtaattata aggtttagta atggcaattt tttatttaaa caaattttta cgaatggatt
52141 gtctgtttat taattcagca acgtttattg aagaaaaata agtgtcttct gataattgca
52201 aagggtttaa ttcaatgtaa actgaattaa gggtagggta ggtatgcctg ttttttggtt
52261 cttttaagtt caatcgatta ataaaattgt tgaacctttg ttctaagtca tacaaaaaca

52321 tcagtaattc attaggagaa tgatttatta tgcaatccat attttcgttt atgcttgaag
52381 ttatacatct aaaaacagta ttggtttctt cgtataaaaa taacttatta atagaggtaa
52441 cattgattaa gactgagtat acgtataata agagtgactc tgacatctca tttagataaa
52501 taagactgtg atcattctta tttacgatta ttatatgtga ttgaatcaat tcaataatgt
52561 ctagaagaag acttgaaaag tcgctgttaa tcttggtctt tgaaaaatca tcgtgaccat
52621 ttgtcttatt acttagatgt agttgttcgt attttatagt ttggcggttg aggaattaag
52681 catttgatta ttataaacta tctcaaaaat taaattacct cctttttct tcctcgatga
52741 aggtttatct aatcttccaa tatacaggtt tttgaagtaa attatagata tgataatcat
52801 taattcagta aatgtatact tattaagggg tggagcttta gctaacttat cgatagtact
52861 gccgatgcgt gctttgaaac tattcaaatt gtcgtgactg tctatatcta aatcttcaaa
52921 tttactagca agggtttgca aagtagaaat tacaacttct tcttttgtat aatatttatt
52981 gaaaatatca tcttctttat catcgctttg gagttttaaa gtgcctggtt ctaagatgtc
53041 aaattttttg tttcgattgg tattttttta aaatgtgcct tgtatttttg gggttgttcc
53101 aaatgtgtat ttgctagact acctgtaatt gatattgctt tatttagcat ttggtgtgta
53161 ttaactgtat ctgattcttc tggaaattta aatctaaaag taaaatctct actaccgaat
53221 gtacttttta cggctacagt tttgtactta tgctcaggca atgtaattaa accatcttga
53281 tgcaggagga aaagtgtgtc atcatttaat ttctcaattg tattaaataa gtaattatgc
53341 atagcctctt tatacatggc gaattgataa tttagattat cccattctat tagttgattt
53401 aaaatacaat atagaccttc attcaatcgg tttacgaata tcaatacagc ttcttctcgt
53461 ccaacaaggc ttattaactc cttcaagtca tcctttaatt ttaatttttc tccttcaaaa
53521 ttgtattgag tgaagcctat agccttttga aaagtaactt ccatgacaga gttggtttca
53581 gaatattcta acgattcatt tattgcaaaa atcaaatttt ttataaatat ggaattactc
53641 atggtttgta attgcatata ctacccaact ttcttattta ctttaaaaca aatataagct
53701 aaagtagtta ttatcataaa gggaacgtac gttcttattt agctgatttc tatgaagaga
53761 ggttatggat attgaatgaa gaaatcttac gcgaagtagc agatattttt ataggtgatg
53821 acagagatag catttatgat tataaaactg ggaacgaatt agtgaggttt tttaatcatt
53881 actttaataa aggggacata tatcaggctc cgtttccatc tagatggcta tatgttgtga
53941 aacatttgca aactctgatt caggagagaa agatcaatca atttttcaca ttaattttaa
54001 gcaatcacta tattaagtat gaactgaaaa ttgacgaggt tgaagcagca aagcaggctg
54061 ctaaagcact taagttgttc aacaaaagat taaatcatta tgggtactac ataacaggaa
54121 ctaacaatgc tagatatttt atggataagg atgaagatac ggagtctatt ggttatggag
54181 ggtatgcgaa tatttattta cagaagacta caggtcttgc tgtaaaaaaa ttgaaagagg
54241 agtatcttac tgattcttca attaaaagta ggtttaagag agaatttgat ctcactaaat
54301 cttttgatac aaatccattg ttcattaatg tgtttgaatt taatgaatca gattattcat
54361 acacgatgga gttagctgat gaaactttga aagattacat tgaaagcaag acaattagtg
54421 agctagaaaa agtaaagata ataatgaaaa ttttaaaagc gatgagtcaa gcacatagtg
54481 aaaataaaat acacagagat atcagttcta aaaatgtatt aatgtttaga ggaaaagtca
54541 aaatatcaga cttaggatta ggtaaaaacc ttgatgaaat tcattcgcat caaacctttg
54601 atacaaacgg tgtaggacaa tataaatact gtgcaccgga acagatgtat agtttaaaac
54661 aagcagataa acaatctgat gtttttagtt taggaagatt gataaatttt attatgactg
54721 gaaatgtagt taacaaccat cacctattta gaggtgtatc tgataaggct acgaacagta
54781 gtaaagaata cagatttgaa gatgcaaatg aaatgttgaa aatgctgcag agaattttag
54841 agtatcacag tagtgcaaag cacgtcgaaa aatgtcaaga aaagctgaaa agaggagtgt
54901 ttgatgatga aagcgaagaa tttattatga cacgaagtga tgaacaatta tgtcaaatgg

54961 ttctaagttc taataataat gagcaagcgt gtttaattcg ttatatgcaa aaaaacgaat
55021 cttcagcatg tgatttaata gagagtatta atagaaagta tcaagagttt tgtggaaggt
55081 ttgaagacta cgatcctttt gctaaattag catatatgat tttatgtaat aacttcagtt
55141 atagagtgaa tgaaacagca gctagagtgc taaattatgt tgcttggtct gtaaatagat
55201 tttcggcaca agacttaatt aaaggtttaa ttaatagagg agtggagcct ttgattgaag
55261 aaaaattaaa ggacaattaa aaaaacctca tcattaactg atacgcagag gcgtatcata
55321 agt
//
LOCUS Staphylococcus 55485 bp DNA linear UNK
DEFINITION Staphylococcus Staphylococcus aureus strain ER11501.3
ACCESSION Staphylococcus
KEYWORDS .
SOURCE Staphylococcus Staphylococcus aureus
ORGANISM Staphylococcus Staphylococcus aureus
Bacteria; Bacillati; Bacillota; Bacilli; Bacillales;
Staphylococcaceae; Staphylococcus; Staphylococcus aureus.
FEATURES Location/Qualifiers
source 1..55485
/mol_type="genomic DNA"
/db_xref="taxon:703339"
/genome_id="703339.281"
/project="hwwang_703339"
/organism="Staphylococcus Staphylococcus aureus"
/genome_md5=""
CDS 264..569
/transl_table=11
/translation="MKGNIQCPRLIKGSLLDFVLETVDVFVNGSKNDCELVNFNYKEG
KVVSRYSTKFESNPKLRKKQLISTVKVVRYVIFDFEKILWGIGKVLLKFIILRQCLL"
/db_xref="SEED:fig|703339.281.peg.1"
/product="hypothetical protein"
CDS 768..1631
/db_xref="SEED:fig|703339.281.peg.2"
/product="hypothetical protein"

/translation="MKHINQFSESTLAQLEKDQQHVFYVYCLMDPRNDECFYVGKGKG
NRIFKHKQDAQKKVLYEDIFIEENKDNLKFNRINEICSNDLNVLGYIISYGLTESEAF

SAENVLINFLNLTNKTTLTNMINGHGSKAYLVEDLENEFGYDSINLENINTNELILAV

KIRDAFLLDKDESKEYPINESKRDRNNLKSRTLGSWIIGKDKIHKIKYIIGINTGANN

AVVSAYEVSFEQAESIETNNGRMRYAFIGLSERDATLKKLNLYKKALPDLRFGSGSAT
AYINNGTMKVD"
/transl_table=11

CDS 1739..3214
/product="hypothetical protein"
/db_xref="SEED:fig|703339.281.peg.3"
/transl_table=11
/translation="MPSDDIVKKLYSKEDIRLELGLTPHKFNKKMETIAKLFKIDMKI

FHNYKGQDKNNQYTFNGVAKELIKVLLKSVDYYPVDINSKKFKQNGKSKKEMIENIDN

SSYMKYIYQLMKSINEIQYKRLIADIHMKDVYQNTKAWLNNGESINKKEQELYQYMTI

LPLHKRVELQNEVLKSIDETIFQFLAKEHRNNQIEENNELEAYTKAIKEGRNPKNDYE

LNHLLYKKNQLPLDSLIEDIWDYEETEYTELDWLIADMLKRSQRADSNFIESLEKKNK

LRKNIHKDSIKSISKLIDHELVKNNRKWDVAEFYKNQQFWNNSTLRRSSQNFGILYYK

KNRLHTMNQSNIIKKIKIIEANIESANKQPSYLDFFLQAKFDLERLESELMKYSINPK

YFNKINSDYVRYAIEDVHNAIIKIDRYISNDKVKLNYSNVQMVVRNEITEQGSYFVTQ
ALNTLSKVEESGYSFDNFMVGPFVEDFKKAIKNRRFGSKKD"
CDS 3440..4540
/transl_table=11

/translation="METRDKLMSLTQSDKTQQWLMDKSSNQDDIQQLQQQFSQQLDQQ

YNALLADEKAKLDQYVEVHQGLESLKEEIESEPITLNIDKLPDIKATMLERAKNDEHS

DKIEKLFDRLEQALNGTNRLYTQLSLIGTRTHRITTKNFNLQGLPKAVQHTILPSKFK

KVYTVDFKSFEPSVAAYMTQDSKLIDLLNQKDGLYDALLSELGLSDELRVFVKRAFIG

SFLFGGNFKNPKFKLNQYVSEVQWLDAVSQFTKVIELKKHVEKSNSMPMPYSIEHDMS

AFQGSSIMAIYVQTVASYIFKHILLKVYKAQCDQKTFKIIVPIHDAIMIECEDEEIAQ
NVGQLMKDTANQLFNGEFAHVTVEEIGGVDHE"
/db_xref="SEED:fig|703339.281.peg.4"
/product="hypothetical protein"
CDS 4533..4904
/db_xref="SEED:fig|703339.281.peg.5"
/product="hypothetical protein"
/translation="MNNDRGQSLHIPSSTPIKENNIYVATLHSVIQTDFSGEIKHQFT

YEIEVNNQIVYANRNIPTKPSANQLSIHDWLKRHSNYSASHENYEPYIDQKHLILLGQ
YNGNYYVQDVASLDAFGGVLS"

/transl_table=11
CDS 4901..6544
/db_xref="SEED:fig|703339.281.peg.6"
/product="putative primase"
/translation="MNHILQMLSKLLSVAKEAIDRQGLIAILTIPVNNNDEIEETAQG

ETVYNELIDQLRLNIPKDTDYQPNIYSYFGIKKNPNDTVLMEMMIKVFHIKRFNSELF

IFKVNGWQKINGDELQGLISKMIQVLLVDYKPSLSTLKNVVDGLQKSTDVEELVENER

YIGCGENMFDLNTFQVVKNSIDIFPKTRLNLSLSTNDVITDKIPPYFKQYMLQLANYD

DDLQYFLFQHTAVLLTADTKYRRGLILYGGAKNGKSVYIELVKSFFYSKDIVSKPLNE

LEGRFDKESLIDKSLMASHEIGQSRIQEKIVNDFKKLLSVESMHVDRKGKTQVEVILD
LKLIFSTNAILNFPPEHAKALERRINIIPCEYYVEKADTSLIDKLQSEKKEIFLYLMY

VYQQIVKADIEYLENSRVTEITHDWLNFGYEFVSSRSVSNANQKACINLLRKLIEIKS

GSRIKVSELNKVINEEIKVSSQVIKQLIQANFDTQTKLYNGYDYWIDLGWKEANKKEI
HDISEKDNIISLDKNENITDDEALDEENLDFDWEDFDDE"
/transl_table=11
CDS 6770..8446
/translation="MKGKIALYSRVSTSEQSEHGYSIHEQEQVLIKEVVKNFPGYDYE

TYTDSGISGKNIEGRPAMKRLLQDVKDNKIEMVLSWKLNRISRSMRDVFNIIHEFKEH

DVGYKSISENIDTSNASGEVLVTMFGLIGSIERQTLISNVKLSMNAKARSGEAITGRV

LGYKLSLNPLTQKNDLVIDENEANIVREIFDLYLNHNKGLKAITTVLNQKGYRTINQK

PFSVYGVKYILNNPVYKGYVRFNNHQNWAVQRRSGKSDKNDVILVKGKHEAIISEEVF

DKVHEKLASKSFKPGRPIGGDFYLRGLIKCPECGNNMVCRRTYYKTKKSKERTIKRYY

ICSLFNRSGSSACHSNSINAEVVERVINVHLNRILSQPDIIKQIASNVIEELKQKHSN

QTEIKYDIDSLEKQKAKLKTQQERLLELFLDDQMDSEMLKAKQSQMNQQLEVLDQQIK

EAQQANQSQDEIPNFDKLKGRLILMITRFSVYLRKATPEAKNQLMKMLIDSIEITTDK
QVKLVRYKIDESLIPQSLKKDWGSFFMPKFQFEIYGQNDYFIDQITTFTT"
/transl_table=11
/db_xref="SEED:fig|703339.281.peg.7"
/product="Cassette chromosome recombinase B"

CDS 8552..8890
/transl_table=11
/translation="MTLEQQLKHYITNLFNLPRDEVWHCESIEEIADDILPNQYVRLG

PLSNKTLQTNTYYSDTLHESNIYPFILYYQKQLIAIGYIDENHDMDFLYLHNTIMPLL
DQRYLLTGGQ"
/db_xref="SEED:fig|703339.281.peg.8"
/product="FIG01108299: hypothetical protein"
CDS 8892..8993
/transl_table=11
/translation="MHKYIKITQLVITILSEIIIWMKESERKEVSYE"
/db_xref="SEED:fig|703339.281.peg.9"
/product="hypothetical protein"
CDS 8986..9297
/product="FIG01107894: hypothetical protein"
/db_xref="SEED:fig|703339.281.peg.10"

/translation="MNRYITRGIANNLPNILQHQLWQLVSEREQEQTKDNTSVDYFHI

FQFNKHRNQLYIKHKQERPEYAKIHKANYSKAININKVYIIREDDVDLSYYVMLLPEE
Y"
/transl_table=11
CDS 9313..9819
/product="hypothetical protein"
/db_xref="SEED:fig|703339.281.peg.11"
/transl_table=11
/translation="METIKSTLKTEAIFSDDKQHRYLLKKTWNSEKQSITIITMYPHY

DGILNIDLTTQLIMNKVSEMDAFGSINFVNLYSNITTPINLKHLENAYDKHTDIQIMK

AVKESDEVILAWGAYAKKPGVEARVNEVLEMLKPHKKKVKRLMNPETNEIMHPLNSKA
RQKWILKV"
CDS complement(9968..10642)
/db_xref="SEED:fig|703339.281.peg.12"
/product="Transposase for insertion sequence-like element
IS431mec"
/translation="MNYFRYKQFNKDVITVAVGYYLRYTLSYRDISEILRERGVNVHH

STVYRWVQEYAPILYQIWKKKHKKAYYKWRIDETYIKIKGKWSYLYRAIDAEGHTLDI

WLRKQRDNHSAYAFIKRLIKQFGKPQKVITDQAPSTKVAMAKVIKAFKLKPDCHCTSK
YLNNLIEQDHRHIKVRKTRYQSINTAKNTLKGIECIYALYKKNRRSLQICGFSPCHEI
SIMLAS"
/transl_table=11

CDS 10900..11067
/translation="MLESREQLSVEEYETFFNRFDNQEFDFERELTQDPYSKVYLYSI
EDHIRTYKIEK"
/transl_table=11
/EC_number="2.3.3.10"
/db_xref="SEED:fig|703339.281.peg.13"
/product="Hydroxymethylglutaryl-CoA synthase N-domain
homolog (EC 2.3.3.10)"
CDS 12104..12847
/db_xref="SEED:fig|703339.281.peg.14"
/db_xref="GO:0008889"
/product="Glycerophosphoryl diester phosphodiesterase (EC
3.1.4.46)"
/transl_table=11
/translation="MLTVYGHRGLPSKAPENTIASFKAASEVEGINWLELDVAITKDE

QLIIIHDDYLERTTNMSGEITELNYDEIKDASAGSWFGEKFKDEHLPTFDDVVKIANE

YNMNLNVELKGITGPNGLALSKSMVKQVEEQLTNLNQNQEVLISSFNVVLVKLAEEIM

PQYNRAVIFHTTSFREDWRTLLDYCNAKIVNTEDAKLTKAKVKMVKEAGYELNVWTVN
KPARANQLANWGVDGIFTDNADKMVHLSQ"
/EC_number="3.1.4.46"
CDS 12944..13372
/translation="MKYDDFIVGETFKTKSLHITEEEIIQFATTFDPQYMHIDKEKAE

QSRFKGIIASGMHTLSISFKLWVEEGKYGEEVVAGTQMNNVKFIKPVYPGNTLYVIAE
ITNKKSIKKENGLVTVSLSTYNENEEIVFKGEVTALINNS"
/transl_table=11
/db_xref="SEED:fig|703339.281.peg.15"
/product="MaoC domain protein"
CDS complement(13418..15424)
/product="Penicillin-binding protein PBP2a, methicillin
resistance determinant MecA, transpeptidase"
/db_xref="SEED:fig|703339.281.peg.16"
/db_xref="GO:0008658"
/db_xref="GO:0046677"
/translation="MKKIKIVPLILIVVVVGFGIYFYASKDKEINNTIDAIEDKNFKQ

VYKDSSYISKSDNGEVEMTERPIKIYNSLGVKDINIQDRKIKKVSKNKKRVDAQYKIK

TNYGNIDRNVQFNFVKEDGMWKLDWDHSVIIPGMQKDQSIHIENLKSERGKILDRNNV

ELANTGTAYEIGIVPKNVSKKDYKAIAKELSISEDYIKQQMDQNWVQDDTFVPLKTVK

KMDEYLSDFAKKFHLTTNETESRNYPLGKATSHLLGYVGPINSEELKQKEYKGYKDDA

VIGKKGLEKLYDKKLQHEDGYRVTIVDDNSNTIAHTLIEKKKKDGKDIQLTIDAKVQK

SIYNNMKNDYGSGTAIHPQTGELLALVSTPSYDVYPFMYGMSNEEYNKLTEDKKEPLL

NKFQITTSPGSTQKILTAMIGLNNKTLDDKTSYKIDGKGWQKDKSWGGYNVTRYEVVN

GNIDLKQAIESSDNIFFARVALELGSKKFEKGMKKLGVGEDIPSDYPFYNAQISNKNL

DNEILLADSGYGQGEILINPVQILSIYSALENNGNINAPHLLKDTKNKVWKKNIISKE

NINLLTDGMQQVVNKTHKEDIYRSYANLIGKSGTAELKMKQGETGRQIGWFISYDKDN
PNMMMAINVKDVQDKGMASYNAKISGKVYDELYENGNKKYDIDE"
/transl_table=11
CDS 15524..17281
/product="Methicillin resistance regulatory
sensor-transducer MecR1"
/db_xref="SEED:fig|703339.281.peg.17"
/db_xref="GO:0008658"
/transl_table=11
/translation="MLSSFLMLSIISSLLTICVIFLVRMLYIKYTQNIMSHKIWLLVL

VSTLIPLIPFYKISNFTFSKDMMNRNVSDTTSSVSHMLDGQQSSVTKDLAINVNQFET

SNITYMILLIWVFGSLLCLFYMIKAFRQIDVIKSSSLESSYLNERLKVCQSKMQFYKK

HITISYSSNIDNPMVFGLVKSQIVLPTVVVETMNDKEIEYIILHELSHVKSHDLIFNQ

LYVVFKMIFWFNPALYISKTMMDNDCEKVCDRNVLKILNRHEHIRYGESILKCSILKS

QHINNVAAQYLLGFNSNIKERVKYIALYDSMPKPNRNKRIVAYIVCSISLLIQAPLLS

AHVQQDKYETNVSYKKLNQLAPYFKGFDGSFVLYNEREQAYSIYNEPESKQRYSPNST

YKIYLALMAFDQNLLSLNHTEQQWDKHQYPFKEWNQDQNLNSSMKYSVNWYYENLNKH

LRQDEVKSYLDLIEYGNEEISGNENYWNESSLKISAIEQVNLLKNMKQHNMHFDNKAI

EKVENSMTLKQKDTYKYVGKTGTGIVNHKEANGWFVGYVETKDNTYYFATHLKGEDNA
NGEKAQQISERILKEMELI"
CDS 17281..17652

/translation="MDNKTYEISSAEWEVMNIIWMKKYASANNIIEEIQMQKDWSPKT
IRTLITRLYKKGFIDRKKDNKIFQYYSLVEESDIKYKTSKNFINKVYKGGFNSLVLNF
VEKEDLSQDEIEELRNILNKK"
/transl_table=11
/db_xref="SEED:fig|703339.281.peg.18"
/db_xref="GO:0003677"
/db_xref="GO:0016481"
/db_xref="GO:0016566"
/product="Methicillin resistance repressor MecI"
CDS 17753..17965
/db_xref="SEED:fig|703339.281.peg.19"
/product="hypothetical protein"
/translation="MQVLIKSIMLVITPVKSICIPSFYLRYIKYNIKQIKNVKNSLIG
YLDCINVKNLKNIQRLCIKNRHIGNT"
/transl_table=11
CDS 18160..19269
/translation="MLREIYNHHNISRTQISKNLEINKATISSILNKLKYKSLVNEVG

EGDSTKSGGRKPILLKVNHLYGYFISLDLTYSSVEVMYNYFDGNVIKHESYDLPDEKV
SSILSIIKKHIDIQEKLDTYNGLLGVSVSIHGVVDNEQHVTYLPFHETEGISIAKKIK

EITNVPVVVENEANLSALYERNFNHNLSYNNLIALSIHKGIGAGLIINNQLYRGANGE

AGEIGKTLVSKVSDNVEIFHKIEDIFSQEALLHNLSNQLNEKMTLSKLIQFYNEKNPV

VVEEMEQFINKIAVLIHNLNTQFNPNAIYINCPLFNEMPEILEAIKNQFKQYSRNEIQ
IKLTSNVKFATLLGGTLAIIQKVLQINDIYLDIKA"
/transl_table=11
/product="Putative ROK-family transcriptional regulator"
/db_xref="SEED:fig|703339.281.peg.20"
CDS complement(19383..19688)

/translation="MPQTNIVTSSIHSSEMTGEEHNVLDVRNDDEWRKGHLKQAIHIP

HGKLLNEDIPFDKNDLIYVHCQSGVRSSIAVGILEYKGFTNIVNVRGGYQHLPAFLK"
/transl_table=11
/product="Polysulfide binding protein"
/db_xref="SEED:fig|703339.281.peg.21"
CDS complement(19779..20717)
/transl_table=11
/translation="MFFKQFYEDNFSQASYLIGCQRTGEAMIIDPIRDLTKYLKVADK

EGFTITKAAETHIHADFASGIRDVANKLNASIYVSDEGKDEFNYKNMPKQTHFVKHQD
IIYLGKIKLEVLHTPGHPPESISFLLTDEGGGSSIPMGLFSGDFIFVGDVGRPDLLEK

AVNVEGSTKIGAKQMYQSIQFAATLPDYIQIWPGHGAGSPCGKALGSLPTTTLGYEKI

NNWAFNVKDETSFIETLTLNQPAPPHHFSQMKKINQFGMQMYQPYNVYPSSSNTQTAF
DLRSKEAFHGGHMHGTINIPFNKTFINQIGWYLDYD"
/product="Zn-dependent hydroxyacylglutathione hydrolase / Polysulfide binding protein"
/db_xref="SEED:fig|703339.281.peg.22"
CDS complement(20750..21814)
/product="Disulfide bond regulator"
/db_xref="SEED:fig|703339.281.peg.23"
/transl_table=11
/translation="MEQYKEVHINSLNKEELEKLAKMGQLIDVRTKEEYELGHINGST

LHPVEEIKSFNKNSNKTYYIHCKSGNRSTKACDYLAKQGYDVVNLKGGYKDYEAKNFN
SAPLIEKDIEIKENRKQFDFRGLQCPGPIVNISKEINNISTGEQIEVTVTDPGFNSDI

KSWAKQTGNTLVNLTEEANVINAIIQKEKPKEFEINDTATGTTIVLFSGELDKAVAAM

IIANGAKAAGKDVTIFFTFWGLNALKKAQSTRVKKKGISKMFDLMLPKDPIHMPLSKM

NMFGLGNIMMRYVMNKKNVDSLYSLIDQAIDQDIKLIACTMSMDVMGISKEELRDEVD
YGGVGTYIGHTEQANHNLFI"
CDS 21950..22210
/product="Metal-sensitive transcriptional repressor"
/db_xref="SEED:fig|703339.281.peg.24"

/translation="MTYDKKMINRINRIQGQLNGVVKMMEEEKDCKDIITQLSASKGS
IQRLMGIIISENLIECVKTAEENNESSQELINEAVNLLVKSK"
/transl_table=11
CDS 22210..22854
/translation="MDIANMTIMLLIGVLGGFISGLIGIGGAIIIYPAILILPPLIGI

PAYSAYIASGLTSSQVFFSTLSGSLNARKQPAFSPKLVIYMGGGMLIGSMLGAILASL
FNATFVNTVYVIIAILALILMFIKVKPTTQETKSKPLLFIIVGFGIGVISGIVGAGGA
FIIIPVLLALFKLPMNTVVNNSIAIAFISSVGAFFIKLMQGYIPVESAIFLIIE"
/transl_table=11
/db_xref="SEED:fig|703339.281.peg.25"
/product="FIG003846: hypothetical protein"
CDS 22955..23074
/translation="MLHLIKSKLQKNHLNQYFHFEYMYLKWKVLISKIVKPVS"
/transl_table=11
/db_xref="SEED:fig|703339.281.peg.26"
/product="Mobile element protein"

CDS complement(23193..23855)
/transl_table=11

/translation="MNTIESQFDKVAEDYDFVNELLNDYSFFVSNMSPKKGRALDIGC

GSGLLVEKLASYYDEVVGIDISNQMLDLAKSKRQLTNTVYLNMNAEQLNFNEKFDFIV

SRTTFHHLDDIASVIQQMKELLNEEGRIVILDNVSEVETPPTYVYKLGAIQEFLPHCF

KFGIKNAIRIYNHNTSKSWLEHLASDKYLSEQNYYDLYEKLLPGCQFHKMGWAMGVVW
TK"
/db_xref="SEED:fig|703339.281.peg.27"
/product="Mobile element protein"
CDS 24351..25082
/product="23S rRNA
(adenine(2058)-N(6))-dimethyltransferase (EC 2.1.1.184)
=> Erm(A)"
/db_xref="SEED:fig|703339.281.peg.28"
/EC_number="2.1.1.184"
/translation="MNQKNPKDTQNFITSKKHVKEILNHTNISKQDNVIEIGSGKGHF
TKELVKMSRSVTAIEIDGGLCQVTKEAVNPSENIKVIQTDILKFSFPKHINYKIYGNI

PYNISTDIVKRITFESQAKYSYLIVEKGFAKRLQNLQRALGLLLMVEMDIKMLKKVPP

LYFHPKPSVDSVLIVLERHQPLISKKDYKKYRSFVYKWVNREYRVLFTKNQFRQALKH
ANVTNINKLSKEQFLSIFNSYKLFH"
/transl_table=11
CDS complement(25208..25990)
/db_xref="SEED:fig|703339.281.peg.29"
/product="Aminoglycoside 9-nucleotidyltransferase (EC
2.7.7.-) => ANT(9)-I"
/transl_table=11
/translation="MSNLINGKIPNQAIQTLKIVKDLFGSSIVGVYLFGSAVNGGLRI

NSDVDVLVVVNHSLPQLTRKKLTERLMTISGKIGNTDSVRPLEVTVINRSEVVPWQYP

PKREFIYGEWLRGEFENGQIQEPSYDPDLAIVLAQARKNSISLFGPDSSSILVSVPLT

DIRRAIKDSLPELIEGIKGDERNVILTLARMWQTVTTGEITSKDVAAEWAIPLLPKEH
VTLLDIARKGYRGECDDKWEGLYSKVKALVKYMKNSIETSLN"
CDS complement(26141..26518)
/db_xref="SEED:fig|703339.281.peg.30"
/product="Mobile element protein"
/translation="MDKQVRNTTEIVRLAKQKSKKTREKVDKAISKFSIEGKVINFNS

IAKEANVSKSWLYKEHDIRQRIESLRERQITANVVSKPKKSSRSEEILIKTLKRRVME
LEKENKKLQNQIQKLYGDLYNKE"
/transl_table=11
CDS complement(26525..28417)
/transl_table=11

/translation="MNASSKRKIISQSEISKKIAVMNEEMQGFWANNSWDIRKCPHPS

AIELSKNPALRNRWVRFERVKNLWLRTELKYFYFYHLNNGIWNAKTVWIRKGTVINKM
LDFLDLKYPSITSITEVPIEKAMTEYRTYLTKRGVRITTTNYKITANQEKTPVKANSY

YVTNLKQFMEFYENFYFDGEEWDKDVWDRRNLPLPDDKVNPTQYEYTINFKGFRNTYF

KQLVKRYCKLRLNVDSFSYVSDIAQRLKEFFNFLDMKFKQVQRVHQLTRVEIEAYLSE
LNMMGIKPSTITGRISILEGLFSTLLRLEWDDVPSKILIYSEDYPKIPRAKPRFIDEF

VLEQLNSHLDKLPEYIATMTMIVQECGMRISELCTLKKGCLLEDKDGDFFLKYYQWKM

KKEHIVPISKEVALLIKVREDKVSEEFPDSEYLFPRKDGSPLKQETFRGELNKLAYEQ

NIVDKSGEIYRFHAHAFRHTVGTRMINNGMPQHIVQKFLGHESPEMTSRYAHIFDETL

KNEFTKFQEKLVTNNGDVLDLDEDNEVDDVELQWFKKNINAQVLPNGYCRLPVVAGGC

PHANACLDCTHFCTSKQFLPQHEEQLERTEELLAIAKDKQWQRQVETNSRVKERLEQI
IGSLTG"
/product="Transposase B from transposon Tn554"
/db_xref="SEED:fig|703339.281.peg.31"
CDS complement(28414..29499)
/transl_table=11
/translation="MKVQRIEVENKPYPLYLLLDKEYQLIEPVMKFIKYLDNTGKSPN

TIKAYCYHLKLLYEFMEQRGVILNDINFELLADFVGWLRYPSASNVIDLQSKKAIREE

TTVNTILNVVMSFLDYLSRLGEFKSIDVFKQAKGRNFKGFLHHVNKGRYQKNVLKLRV

KKKQIRTLRSKEVKQIIDACHTKRDKLILMLMYEGGLRIGEVLSLRLEDIVTWDNQIH
LTPRDVNVNEAYIKLRKERTIHVSKELMSLYTDYLIYEYSEELEHDYVFISLKEGYFG

KPLKYQSVLDLVRRIVKRTGIEFTSHMLRHTHATQLIREGWDVAFVQKRLGHAHVQTT
LNTYVHLSDQDMKNEFNKYLERKEHKK"
/db_xref="SEED:fig|703339.281.peg.32"
/product="Transposase A from transposon Tn554"

CDS complement(29618..29935)
/translation="MKEINIVSLQMIKTDTLSYLKNRISNPEDAAEIMRSFIGNSDRE

HLILICMNSKNEPTHIQTLSIGSINQTVIHPREIFKTAILSNANSIMLGHNHPSGDVL
TIV"
/transl_table=11
/product="DNA repair protein RadC truncated by transposon
Tn554"
/db_xref="SEED:fig|703339.281.peg.33"
CDS complement(29956..30462)
/db_xref="SEED:fig|703339.281.peg.34"
/product="hypothetical protein"
/translation="MNTIKNTIYTEAIFSKDEKHRYLLKKTWDEKKPACTVITMYPHL

DGVLSLDLTTVLILNQLANSERYGAVYLVNLFSNIKTPENLKHIKEPYDKHTDIHLMK

AISESDTVILAYGAYAKRPVVVERVEQVMEMLKPHKKKVKKLINPATNEIMHPLNPKA
RQKWTLKA"
/transl_table=11
CDS complement(30480..30791)
/translation="MKINRYITRGISEYLSLDLQILLWNMVKERDNQPHTDYLHIFKL

QEDENILSITHEQEQPAYKLEYHYTNYVKNQNALPKKVYVIREDGVDAFYYVMLLPEE
Y"
/transl_table=11
/product="hypothetical protein"
/db_xref="SEED:fig|703339.281.peg.35"
CDS complement(30793..30885)
/product="hypothetical protein"
/db_xref="SEED:fig|703339.281.peg.36"
/transl_table=11
/translation="MNNWIKVAQISVTIISQAIIIMKEIQDEVK"
CDS complement(30878..31228)
/transl_table=11
/translation="MKTTTQELKQYITRLFQLSNNETWECEALEEAAENILPERFIND

SPLAHLTLETYTYYNNELHDLSIYPFLMYANNQLISVGYLNHFDMDFLYLTDTKNTII
DERHLLREGGTHHE"
/db_xref="SEED:fig|703339.281.peg.37"
/product="hypothetical protein"
CDS complement(31746..33374)
/product="Site-specific recombinase"
/db_xref="SEED:fig|703339.281.peg.38"
/transl_table=11

/translation="MQQLKTKRVGIYVRVSTEMQSTEGYSIDGQINQIKEYCDFHHFE

VKDIYADRGISGKSMNRPELQRILKDAKEGYIDCVMVYKTNRLARNTSDLLKIVEDLH

KQNVEFFSLSERMEVNTSSGKLMLQILASFSEFERNNIVENVFMGQTRRAQEGYYQGN

LPLGYDKIPNSKHELMINQHEANIVKYIFESYAKGHGYRKIANALNHKGYVTKKGKPF

SISSITYILANPFYIGKIQFAKYKDWSEKRRKGLNDKPVIAEGKHSPIINQDLWDKVQ

MRKKQVSQKPQVHGKGTNLLTGIIHCPQCGAPMAASNTTNTLKDGTKKRIRYYSCSNF

RNKGSKVCSANSVRADVIEDYVMKQILEIVKSDKVIQRVVTHVNQENQVDGAALHHDI

AYKQQQYDEVQIKLNNLIKTIEDNPDLTSVIRPSIQKYEKQLNDITNQINQLKNQQNE
DKPLFDAKEISKLLQHIFHDIKHIEKSRLKALYLSVIDRIDIKKDGNHKKQFYVTLKL
NNEIIKQLFNNKQLDEVHLSTSSLFLPQTLYLTI"
CDS complement(33396..34745)
/db_xref="SEED:fig|703339.281.peg.39"
/product="Cassette chromosome recombinase A"

/translation="MKQVIGYLRQSTMKQQSLAAQKQAIEAIAEKHHIQHINFYSDKQ

SGRKDNRSGYRQMTQLIQQGQCDILCCYRLNRLHRNLKNALKLIKLCQTYHVHILSVH

DGYFDMDQAFDRFKLNIFISLAELESDNIGEQVRNGLQEKAKQGRLITTHAPFGYEYH

NGAFIINQNESPTVKAVFNYYIKGHGYKKIAQLLEEDNTYINRQPYQVRNIIINPNYC

GRVNNQYGQFDNMFPSIVSTSIYEQAQRLRSQKQIKQTSSDNQLKQKIKCPCCNTTLT

NMTVRKKNHTLRYYVCPKNMNASRFVCDFKGINAQTLEDKVLEVCKDFYQNQRIYTKI

KGAIDKRIKRQRNIEKHHTLTQEQLIEKLAQGIIDAETFREQTQSLRQQPQRTTSING
HQIQHTIQNIIQKRFTLNILYPYIETIHITKDKNLIGIYFKNEPLNIVNQTMQSSIA"
/transl_table=11
CDS complement(34979..36772)
/db_xref="SEED:fig|703339.281.peg.40"
/product="hypothetical protein"

/translation="MTQTNPSFNPSPRYKSKKGWYKDKPPKEKGGMPTEVEIAGPIVI
ENKFIDPKTNTEKVIITDEDQKVIVESSDILTTQKLPSLMKYGFSINEKYTKDLGYAL

QQMRNQLPISYLYEGVGILETPFGPIVSLNEIYTTTEFDNKSPSDAICENTYDLAPRG

TFDNWFNMYLKEVKGSLLLELAVVFGISALVTSFLKHKHETEFAGIIFSFTGQSSTGK

STAASLAVSVAGNPTKGNETLFRNWNATRNALEGYLSNNFGIPIVFDELSSATFRDTT

GLLYSITEGQGRQRSNVHGEVKTPKNWGTSVISTSEHSIFNDSAQNDGLRVRTIEINE

QFTTNATNADNIKKAVALNYGHVLPLVAKYLINREDEVIQWFYKEVDWFEAKLKDDKS
NTGNRMFKRYAVITTSAKILGRVLATDIDIAKIRDYFIDYHGHTISERSLADKAIDVI

IQFVAQNRGKFSDEGALKNMFENYGLISLKDDHIEVKMIANVFKQMLNNHQFQDVNNV

VNALRDKGFILADRGRQTTKRSVKDNSGKKQSLVFYHLKLDVEFASILGLTKDKSLIQ
NWTPANDNKAAKELFKSANEGIGPSGVHEDF"
/transl_table=11
CDS complement(36772..37068)
/transl_table=11
/translation="MYFNQSDKFEQLKKKILGISFNLPEGEEFTFHDLNSRANVQCSK

AVQQNVGRWFAYFVKHAPRVPFIIIGKDTHGHLVYLKTGPNPHYNSNPSKGGVR"
/db_xref="SEED:fig|703339.281.peg.41"
/product="hypothetical protein"
CDS complement(37261..38307)
/db_xref="SEED:fig|703339.281.peg.42"
/product="hypothetical protein"

/translation="MNNKDIYSDDFQNALVDIILNIDKINVKEVFDDSCRAVDQDNKK

DTLNRSPKDFSINISDIVRYSILCMENLSSDKIEENINMIKNHSSELNDNDMFIQEKN

NFTTNFNKFCNALGINIANFKSGKSYVFKPKSLFVLYDILSNNSVYLHFFKNGINDKD
IDSSNRIKENLYYFINHEITDQRDYEHALTNYYLYFLVDSNIEIGIKSIIIKIFTEKG
ITSKDKLQILEYYLDEIDDLNTEISTKIADAHVKNSTNFLENEKVSINSNLAKYITHR

DTRASAPKNMLLSLILNSDLVSPKDLEEKELKYPKKITKEHHDKLIKEINKALDYYND
NNTTPSDYFYITSD"
/transl_table=11
CDS complement(38558..38959)
/translation="MDIYNGEIVAYKIDDKQDQRLVNDTLNQIDIPEGYILHSDQGSV
YTSYAYYQLCEEKGIIRSMSRKGTPADNAPIESFHSSLKSETLYINNQLNSSNHIVID
IVEKYIKNYNNNQIQQKLGYLSPVKYRELIA"
/transl_table=11
/product="Mobile element protein"

/db_xref="SEED:fig|703339.281.peg.43"
CDS complement(39047..39400)
/product="Mobile element protein"
/db_xref="SEED:fig|703339.281.peg.44"
/transl_table=11
/translation="MVPTVVIDLVDQLKGNYSIKLILEVLNIPKSTYYRWKNKTYKND

TVTQKVIELCEANHYTYGYRKITALINQCYTSPINHKRVQRMMQKHHLNCRVRPKKTT
RIGKPYYKTDNLLQR"
CDS complement(39385..39699)
/transl_table=11

/translation="MKRVSYSVETKYKAVEMKAAGFSTKEIMKELNIKNRTQVETWWR

WYRNGESYRFSQHVGKQYTYGKGLEELSEVEQLKLENKRKDIELDILKKYKALERKWY
QQ"
/db_xref="SEED:fig|703339.281.peg.45"
/product="Mobile element protein"
CDS complement(40210..41112)
/db_xref="SEED:fig|703339.281.peg.46"
/product="Arginine/ornithine antiporter ArcD"
/transl_table=11
/translation="MGSTFSTINPFSVVIASNAAGTTFTDGLYWRIGACIIGAIFVIS

YLFWYCKKIKKDPKSSYSYEDKAAFEKQWSVLHDDGSSEFTLRKKIILTLFVLPFPIM

VWGVMTQGWWFPVMASAFLIFTIVIMFIAGTGQYGLGEKGTVDAFVNGASSLVGVSLI
IGLARGINLVLNKGMISDTILHFSSSIVQHMSGPLFIIVLLFIFFCLGFIVPSSSGLA

VLSMPIFAPLADTVGIPRFVIVTTYQFGQYAMLFLAPTGLVMATLQMLNVRYSHWLRF
VWPVVAFVLIFGGGLLITQVLIYS"
CDS complement(41858..42553)
/translation="MKTTLLVVEDDEAILHLIDVALTMNYYKVVTAKTGKEADFRLRT

EQPDIILLDLGLPDIDGLSLIQQFRDFVDTPIIVISARTEEQTIVEVLDRGANDYMTK

PFNIDELRARIRVALRMSRSTETIQSRFTNGPLTIDFDAKSVLVNEQSVHLTPNEFQL

LTILCHHVGKVLTYQVLLKSLYGYVNHTEMAALRVHMASLRKKLNYTQDPNTEKKLII
THPRIGYQMQHLE"
/transl_table=11
/db_xref="SEED:fig|703339.281.peg.47"
/db_xref="GO:0000156"
/db_xref="GO:0003677"

/product="DNA-binding response regulator KdpE"
CDS complement(42528..45248)

/translation="MDNMLYINYDVIKRGCLMESTYKKRGKLTIYLGYSPGVGKTYEM
LSNGIDQYQEGADIKIGYIEPHQRPETNALVQQLPEISTSSRKFGSHTFHYIDVSKII

EANPDVVLIDELAHTNISKERHLKRYKDIEEILSHGIDVWTTLNIQHIESLSGQIALM

TGIQVTERVPDQFITSADAFEVVDISPNMLIQRLKAGKVYKKERLETAFSNFFTYENL

TELRELTLRTVADIMSQKGQQYKTKHTDITPHIAVAISGSIYNERVIREARRAAYKEH

AKFTAVYIDVFETPSESRKQDHYVHKNLMLAKALGAEIKVLYAQDIAKALTDWCDLAF

VTKLVLGQSEQPRWKDYFKQSLIEQINHVPHHFKLEIVPIHYIHSDKPTNTPKQAQKS
SRLTVEIMKMLVIQTICVLLGIWVYNLDKSESSAIILLMFFIGIIVLSIWTQSYLIGF
FASILNVFVFNYFFTVPRFTLEMYRFEYPITFATSIFASIFTSAILKNLKHQHSLTER

QLYRTNIMLQFNQSIKESYSIERLLNIAGDQIHQLLNQDVTVFLIQSKKVAASNSFGN
RSISSSDKTHDAETLSWVIENESRAGKLTDTFPGSKFLCIPIGTNPVKGVISIRFTDE
TYIDTYDNSILDSMLNDITLAIENVDLLKQTRQSILKAEREATRSNFLHSISHDIRTP

LTSIMGNLDMLKYHNEHLNDQQQAELLTASYGEAQYLHTLVTNILSLTKLESSDIQIQ
RTPYLVEELLEEFEEGLIRRHQANHVIIENEDDASLINIDSKLILQVLFNLIDNALKH

AESHSEIKLHVQHETNKIKFEMIDCGKGIPEEERQLIFNPYYSGDNFKDNKKDSLGLG
LYLVQLILKQHNSELEYQPNTPQGSIFYFYLTVNPIEMEGAMDENNIIGR"
/transl_table=11
/product="Osmosensitive K+ channel histidine kinase KdpD"
/db_xref="SEED:fig|703339.281.peg.48"
CDS complement(45249..45392)
/translation="MTTTANSVIIALPPYFHQNLNKSYLIEVLTFKESYKETIKMINK
TER"
/transl_table=11
/product="hypothetical protein"
/db_xref="SEED:fig|703339.281.peg.49"
CDS 45465..47141
/EC_number="3.6.3.12"
/translation="MSIVLFLIVFILLSLIVSRYLYSVALNVPSKIDVVFNPIEKLIY

QLIGTKLEHMSGKTYIKHFLLFNGLMGGLSFVLLLIQQWLFLNPNHNLNQSVSLAFNT

MASFLTNTNLQHYAGETDLSYLTQMCVITFLMFTSAASGYAVCIAMLRRLTGMTDVIG
NFYQDITRFIVRVLIPFALIISLFLISQGTPQTLKGNLVIETLSGVKQTIAYGPMASL

ESIKHLGTNGGGFLGANSSTPFENPTYWSNYAEALSMMLIPGSLVFLFGRMLKTKLQI

HPHAIMIFVAMFVMFIGFLVTCLYFEFAGNPVLHHLGIAGGNMEGKETRFGIGLSALF

TTITTAFTTGTVNNMHDSLTPLGGMVPMVLMMLNAVFGGEGVGLMNMLIYVMLTVFIC

SLMIGKTPSYLGMKIEGKEMKLIALSFLVHPLLILVFSALAFIVPGASDALTNPQFHG

VSQVLYEFTSSSANNGSGFEGLGDNTVFWNISTGIVMLLARYIPIVLQILIVSSLVNK
KTYQQHTQDVPINNLFFSSVLIIFIILLSGLTFLPDLMLGPIGEQLLLHA"
/transl_table=11
/product="Potassium-transporting ATPase A chain (EC
3.6.3.12) (TC 3.A.3.7.1)"
/db_xref="SEED:fig|703339.281.peg.50"
/db_xref="GO:0008556"
CDS 47160..49181
/product="Potassium-transporting ATPase B chain (EC
3.6.3.12) (TC 3.A.3.7.1)"
/db_xref="SEED:fig|703339.281.peg.51"
/db_xref="GO:0008556"
/EC_number="3.6.3.12"
/translation="MAETTKIFESHLVKQALKDSVLKLYPVYMIKNPIMFVVEVGMLL
ALGLTIYPDLFHQESVSRLYVFSIFIILLLTLVFANFSEALAEGRGKAQANALRQTQT

EMKARRIKQDGSYEMIDASDLKKGHIVRVATGEQIPNDGKVIKGLATVDESAITGESA

PVIKESGGDFDNVIGGTSVASDWLEVEITSEPGHSFLDKMIGLVEGATRKKTPNEIAL
FTLLMTLTIIFLVVILTMYPLAKFLNFNLSIAMLIALAVCLIPTTIGGLLSAIGIAGM

DRVTQFNILAKSGRSVETCGDVNVLILDKTGTITYGNRMADAFIPVKSSSFERLVKAA

YESSIADDTPEGRSIVKLAYKQHIDLPQEVGEYIPFTAETRMSGVKFTTREVYKGAPN

SMVKRVKEAGGHIPVDLDALVKGVSKKGGTPLVVLEDNEILGVIYLKDVIKDGLVERF

RELREMGIETVMCTGDNELTAATIAKEAGVDRFVAECKPEDKINVIREEQAKGHIVAM

TGDGTNDAPALAEANVGLAMNSGTMSAKEAANLIDLDSNPTKLMEVVLIGKQLLMTRG

SLTTFSIANDIAKYFAILPAMFMAAMPAMNHLNIMHLHSPESAVLSALIFNALIIVLL
IPIAMKGVKFKGASTQTILMKNMLVYGLGGMIVPFIGIKLIDLIIQLFV"
/transl_table=11
CDS 49197..49754

/translation="MQTIRKSLGLVLIMFVLCGFIFPLTVTALGQVLFPEQANGSLVK

QDGKVIGSKLIGQQWTEPKYFHGRISAVNYNMNANEVKESGGPASGGSNYGNSNPELK

KRVQETIKQEGKKISSDAVTASGSGLDPDITVDNAKQQVKRIAKERNIDASKINHLID
ENKQASPMADDYVNVLKLNITLDKL"
/transl_table=11
/EC_number="3.6.3.12"
/db_xref="SEED:fig|703339.281.peg.52"
/db_xref="GO:0008556"
/product="Potassium-transporting ATPase C chain (EC
3.6.3.12) (TC 3.A.3.7.1)"
CDS complement(50299..51168)
/translation="MISTSEHSIFKNPAKNDGLRVRTIEVSEKFTSSAQNSDTIKKVV

SQNNGHVLPIIAQFLLDNTNDVTSLFQKEDSVFYQLLSTESSNTGIRMIKHYATLTTT

AHILNQSLDLQLNVSSIKNYLLSYHNETVSERSLADKAMDTIVQFIAENLGKFSQQTR

LANMIQNFGILSLKDDYIEVKIIRNVFDKMLTDSQFEDTKNVIDALDNAGKLVSDRDR

KTTKRNAKDDTGKSTSLVFYQIKIDLDYADVLGLQSQGRDNVIQKHSNPSQFKNTNLE
EFMKSENTTLIVF"
/transl_table=11
/db_xref="SEED:fig|703339.281.peg.53"
/product="hypothetical protein"
CDS complement(51178..52074)
/transl_table=11
/translation="MEKILYQTDEFKLKPSGWYKTIPPKKDGGTEFEIMLSGPIAFTD
RFIDPATRKEKVFLSDLNNIELVEKASILTALQLPSLIEYGFTINEKHIRDLGFVLQQ
MRSTTPLSTIYSGVGMLHTLLGPLISLDQPYFSNEITNSTSIICDNKYDLIPKGNLSE

WLQMYKEEVHGNLSLELDVLFGVSSLVTAFLKYHNNVEFSGTIFSFTGQSSTGKSTAA

MLAASVAGNPTKGTENLFRSWNATRNALEGYLSGNYGVPIVLDELSAATFHDTTGLLY
SFAEGQGRQRANINGDVKTPKN"
/db_xref="SEED:fig|703339.281.peg.54"
/product="hypothetical protein"
CDS complement(52274..52648)
/product="hypothetical protein"
/db_xref="SEED:fig|703339.281.peg.55"
/transl_table=11
/translation="MSESLLLYVYSVLINVTSINKLFLYEETNTVFRCITSSINENMD

CIINHSPNELLMFLYDLEQRFNNFINRLNLKEPKNRHTYPTLNSVYIELNPLQLSEDT
YFSSINVAELINRQSIRKNLFK"
CDS complement(52816..53103)
/translation="MQTLASKFEDLDIDSHDNLNSFKARIGSTIDKLAKAPPLNKYTF
TELMIIISIIYFKNLYIGRLDKPSSRKKKGGNLIFEIVYNNQMLNSSTAKL"
/transl_table=11
/product="hypothetical protein"
/db_xref="SEED:fig|703339.281.peg.56"
CDS complement(53193..53819)
/translation="MQLQTMSNSIFIKNLIFAINESLEYSETNSVMEVTFQKAIGFTQ

YNFEGEKLKLKDDLKELISLVGREEAVLIFVNRLNEGLYCILNQLIEWDNLNYQFAMY

KEAMHNYLFNTIEKLNDDTLFLLHQDGLITLPEHKYKTVAVKSTFGSRDFTFRFKFPE
ESDTVNTHQMLNKAISITGSLANTHLEQPQKYKAHFKKIPIETKNLTS"
/transl_table=11
/product="hypothetical protein"
/db_xref="SEED:fig|703339.281.peg.57"
CDS 53934..55442
/translation="MNEEILREVADIFIGDDRDSIYDYKTGNELVRFFNHYFNKGDIY

QAPFPSRWLYVVKHLQTLIQERKINQFFTLILSNHYIKYELKIDEVEAAKQAAKALKL

FNKRLNHYGYYITGTNNARYFMDKDEDTESIGYGGYANIYLQKTTGLAVKKLKEEYLT
DSSIKSRFKREFDLTKSFDTNPLFINVFEFNESDYSYTMELADETLKDYIESKTISEL

EKVKIIMKILKAMSQAHSENKIHRDISSKNVLMFRGKVKISDLGLGKNLDEIHSHQTF

DTNGVGQYKYCAPEQMYSLKQADKQSDVFSLGRLINFIMTGNVVNNHHLFRGVSDKAT

NSSKEYRFEDANEMLKMLQRILEYHSSAKHVEKCQEKLKRGVFDDESEEFIMTRSDEQ

LCQMVLSSNNNEQACLIRYMQKNESSACDLIESINRKYQEFCGRFEDYDPFAKLAYMI
LCNNFSYRVNETAARVLNYVAWSVNRFSAQDLIKGLINRGVEPLIEEKLKDN"
/transl_table=11
/product="hypothetical protein"
/db_xref="SEED:fig|703339.281.peg.58"
ORIGIN
1 gaggcttatc ataaataaaa ctaaaaatta gattgtgtat aatttaaaaa tttaatgaga
61 tgtggaggaa ttacatatat gaaatattgg agtatacctt gcaatatcat acgatgttta
121 tagagtgttt aataaaccat ttttcaacta ttgatgatct agaatatata ataactgtac
181 aaattatatt gattatggaa ctacaattca attaagaaat tgatgatgaa attttaaatt
241 taaactaatg gaatcaagaa agaatgaaag gaaatataca atgcccacga ttaataaaag
301 gaagtttatt agattttgtg ttagaaacag tagatgtttt tgtaaatgga agtaaaaatg

361 attgcgaatt agtaaatttt aattataaag aaggaaaagt agtttcacgt tatagtacaa
421 aatttgaaag caatcctaaa ttaagaaaaa agcaattgat atcgacggta aaagttgtaa
481 ggtatgttat ttttgatttt gagaaaattt tatggggcat agggaaagtt ttattgaagt
541 tcatcatatt aaggcaatgt ttactataag aaagttgaaa tacactcaga aacagactgg
601 attcccgttt ttttcaaatt ggcacaaaat gatacatagg cccacctata tattgaagat
661 ttaagttaaa cgataaataa tatttttctc ttttgtatag aaattttttt gggaatgata
721 ttaattagta taaaaatgaa tatgtctaca aagaaggaga gattttattg aaacacatta
781 atcaattttc tgaatcgaca ctagcacaac tagaaaaaga tcaacaacac gtattttatg
841 tatactgttt gatggaccca agaaatgatg aatgctttta tgttggtaaa ggtaagggga
901 atagaatatt taaacataaa caagatgcgc agaaaaaagt attgtacgaa gatatattta
961 tagaagaaaa taaagataac ttgaaattta atagaattaa cgaaatttgt agcaatgatt
1021 tgaatgtttt aggatatata atcagttatg gattaaccga atctgaagca ttttctgcag
1081 aaaatgttct tataaacttt cttaacctaa caaataaaac tacattaaca aatatgatta
1141 acggtcatgg atctaaggca tacttagttg aagatttaga aaatgaattt ggctacgatt
1201 caattaatct tgaaaacata aatacgaatg aattaatttt agcagtgaaa atcagagatg
1261 catttctatt agataaggat gaaagtaaag agtatcctat taatgaaagt aaacgtgata
1321 gaaataacct taaatcgcgt acattaggta gttggataat aggaaaagat aaaatacata
1381 aaataaaata tattattggt atcaatacag gtgctaataa tgcagttgtc tcagcttatg
1441 aagtatcatt tgaacaagcg gagagtatcg aaacaaataa tggtagaatg agatatgcgt
1501 ttattgggct ttcagaaaga gatgctactc taaaaaaatt gaatttatac aaaaaagcac
1561 taccagattt aagatttggt agtggtagcg ctacagcata tataaataat gggacaatga
1621 aagttgatta aatgaataaa aacatccaat tccttaaatt agcctttgaa aattttatac
1681 tcgtatatat aagtgaaaaa cttaaaagaa ttagatgtta attaaaggag agaatgctat
1741 gccatcagat gatattgtaa aaaaactata ctcaaaagag gatattcgat tagaattagg
1801 acttactccg cataagttta ataaaaaaat ggaaactatt gctaagcttt ttaaaattga
1861 tatgaaaatt tttcacaatt acaaaggcca agataaaaat aatcagtata catttaatgg
1921 tgtcgcaaaa gaattaatca aagtgttgct aaaaagtgtt gattattacc cagtggatat
1981 caattccaaa aaatttaaac aaaatggaaa atctaaaaaa gaaatgattg aaaatataga
2041 taactctagt tacatgaaat atatttatca gttaatgaaa tctatcaatg aaattcaata
2101 caaaaggtta attgctgata tacatatgaa agatgtgtac cagaatacaa aagcatggtt
2161 aaataatggt gaatcaatta ataaaaaaga acaagagcta taccaatata tgacaatttt
2221 accattacat aaaagagttg aactgcaaaa tgaggtatta aaatctatag acgaaacaat
2281 ttttcaattt ctcgcaaaag aacatagaaa taatcaaatt gaagaaaata atgaattaga
2341 agcatataca aaagcaataa aagaaggtag aaatcctaaa aatgattatg aattaaatca
2401 tctcctatat aaaaaaaatc aattaccctt agatagtcta atagaagata tatgggacta
2461 tgaggaaaca gagtatactg agcttgattg gttaattgca gatatgctaa aacgttcaca
2521 aagagctgat agtaatttta tcgaaagttt agagaagaaa aacaaattga gaaaaaatat
2581 tcataaagat tccattaaaa gtatatctaa gttgattgat catgaattag ttaaaaataa
2641 tagaaagtgg gacgtagcag aattttataa aaatcaacaa ttttggaata atagtacatt
2701 aagacgtagc tctcaaaatt ttggaatttt atattacaaa aagaataggt tacatacaat
2761 gaatcaaagt aatattatta agaaaataaa aattatcgaa gctaatatag agagtgcaaa
2821 taaacagccg agctatttag atttcttttt acaagctaag tttgatttag agaggttaga
2881 aagtgaatta atgaaatata gcattaatcc gaaatatttt aataagataa atagtgatta
2941 cgtaagatat gctattgaag atgtacataa tgcaattatt aaaattgaca gatatataag

3001 taacgataaa gtgaagttaa actattcaaa tgtccaaatg gtagtgagaa acgaaataac
3061 agaacaagga agctactttg taacccaggc attgaataca ctgtcaaagg tagaagagtc
3121 tggctacagc tttgataatt tcatggtagg acctttcgta gaagacttta aaaaagcaat
3181 taaaaatcgt agatttggtt ctaaaaaaga ttaagattgt tttttagagt taaacataag
3241 aataaattta aataaaaaaa taaacccaac aaaccttgtt atatcaatgt ttgttgggtt
3301 tatttttttg gtgatttttt aataactcaa ttacatctta ggataatatc gctaaattat
3361 acattgtcga ggccaagaca aataaaaaat taaagcaaaa gttagcgcgc aactttctgc
3421 tataaaggag caaatttata tggagacaag agacaaactg atgtcattga ctcaaagtga
3481 caaaacacag caatggctaa tggacaagtc atctaaccaa gatgacattc aacaattgca
3541 gcaacaattc agtcagcagc ttgatcaaca atataatgca cttttagctg atgaaaaagc
3601 taagttagac caatacgtgg aagtacatca aggattggaa tcattaaagg aagagattga
3661 atcagaacct attacgctta atatcgataa attacccgat atcaaagcaa caatgcttga
3721 aagagccaag aatgatgaac attctgataa aatcgaaaag ctatttgata ggttagaaca
3781 ggcattaaat ggtacgaatc gattatatac gcaattatcg ttgattggta cacgaacaca
3841 tcgaattaca actaaaaatt ttaatcttca aggcttacct aaagcagtcc aacatacgat
3901 tttaccttca aaatttaaga aggtgtatac agtcgatttt aaatcgtttg aaccatcagt
3961 tgcagcgtac atgactcaag attcaaaact gattgacttg ttaaatcaga aagacggact
4021 gtatgacgca ttgctaagtg aattaggctt atcagatgag ctacgtgtat ttgttaaacg
4081 tgcatttatt ggttcgtttc tatttggagg taacttcaaa aatcctaaat tcaagctgaa
4141 tcaatatgta agtgaagtac aatggttgga tgcggtcagc caatttacaa aagtcattga
4201 acttaagaag cacgttgaaa agagtaattc catgcctatg ccttatagta ttgagcatga
4261 tatgagcgca tttcaaggta gcagtattat ggcaatctac gtacaaactg tagcgagtta
4321 tattttcaag cacattctgc taaaagtgta caaagcacag tgcgatcaaa aaacgttcaa
4381 gattatagta cctatacacg atgcgattat gattgaatgt gaagatgaag aaattgcaca
4441 aaatgtgggt cagttaatga aagatacggc taaccagttg ttcaatggtg aatttgcaca
4501 tgtgacagtg gaagaaatag gaggcgtaga ccatgaataa tgatagagga caaagcctac
4561 acatcccaag tagtacacca atcaaagaaa ataatatata tgtagctacg ttacattctg
4621 tgatccaaac agatttctca ggtgaaataa agcaccaatt cacgtatgaa attgaagtga
4681 acaatcagat tgtatatgcg aatcgtaata ttccaacaaa accgagcgct aatcagttgt
4741 caattcatga ttggctgaaa cgtcatagca actatagcgc aagtcatgaa aactatgagc
4801 cttatattga tcagaaacat ttaattctat taggtcaata taacggtaac tattatgtac
4861 aagatgtagc atcgttagat gcgtttggag gcgtattatc atgaatcata tattacaaat
4921 gttatctaag ctattaagtg tggccaagga ggcaatcgac cgtcaaggtc tgattgctat
4981 cctaactatt cctgttaata ataacgatga aatagaagaa acggctcaag gtgaaaccgt
5041 gtataacgaa cttatcgatc agttacgact taatatccca aaagatacgg attatcaacc
5101 taacatctat agttattttg gtattaagaa gaatcctaat gacaccgtac tcatggaaat
5161 gatgataaag gtttttcata tcaaacgctt taattcagaa ctgtttattt tcaaagttaa
5221 cgggtggcaa aagataaatg gagatgaatt acaagggttg atatctaaaa tgatacaagt
5281 attgcttgta gattataagc cttcactaag cactctaaaa aatgtcgtag atggattgca
5341 aaaatcaaca gatgtagaag aacttgttga gaatgagcgc tatattggtt gtggtgaaaa
5401 tatgttcgat cttaatacgt ttcaagtcgt taaaaattca atcgatatct ttccaaaaac
5461 acgattgaat ttatcattaa gtacaaatga tgtaattact gataagatac cgccttattt
5521 taagcaatat atgttacaac ttgcgaatta tgacgatgat ttacaatact ttcttttcca
5581 acatacagca gtattactta cagctgatac taaataccgt aggggtctca tattatatgg

5641 tggagctaag aatggtaaat ctgtatatat tgaactagtt aaatcatttt tctatagtaa
5701 agatattgtg tctaagccac ttaatgagct tgaaggtcgt tttgacaaag aaagtttaat
5761 tgacaaaagt ctaatggcaa gtcatgaaat tgggcaatct aggattcaag aaaagatcgt
5821 aaatgacttc aaaaagttat tatctgtaga atcaatgcat gttgatcgta aaggaaaaac
5881 tcaagtggaa gtcattttgg atttgaaact tatttttagt acaaatgcga tacttaattt
5941 tcctcctgaa catgcgaaag ctttggagcg tcgaattaat attattccat gtgagtatta
6001 tgttgaaaaa gcggacactt cattaattga taagctccag agtgagaaga aagaaatctt
6061 tctttacttg atgtatgtgt atcaacagat tgtaaaagca gatatcgagt atcttgaaaa
6121 tagccgtgtc actgaaatta ctcacgattg gttaaatttt ggatatgaat ttgtttctag
6181 caggtccgta agtaatgcaa atcagaaagc atgtattaat ttactcagaa aacttataga
6241 aatcaaatca ggatcacgaa tcaaagtatc cgagttaaat aaagttatta atgaagaaat
6301 aaaggtaagt tctcaggtta ttaaacagtt aattcaagca aactttgata ctcaaaccaa
6361 actatacaat ggctacgatt attggattga tttaggttgg aaagaagcca ataaaaaaga
6421 gattcatgat atttcggaaa aagataatat tatttcatta gataaaaatg aaaatataac
6481 agacgatgag gcattagatg aagagaattt ggactttgat tgggaggact ttgacgatga
6541 ataatgaaca aattgaagca tttgtagaag tgcttgtacc tatcatagaa gaacgtatca
6601 ataaaggtaa ttaaggctaa ttacgtacta caggcagttg cctgtagtac tcatatgatt
6661 aagtggtaaa agtgataaaa atgaaacgaa attataaata tatattatct atatgttgtt
6721 acaagaccga tagtctgtag caataatcta ataaaaggag cggtatgata tgaagggtaa
6781 aattgcactt tattcacgcg ttagtacgtc agagcagtcg gagcatgggt actcaatcca
6841 tgagcaggaa caagtactca tcaaagaagt tgtgaaaaat ttcccaggtt atgactatga
6901 gacatatact gactcaggca tttcaggtaa aaatattgaa ggtcgtccgg caatgaaacg
6961 tctattacaa gatgttaagg ataataaaat cgaaatggtg ttaagttgga aattgaatcg
7021 tatctcacga tcaatgagag acgtgtttaa tattattcat gaattcaaag aacatgatgt
7081 agggtataaa tcgatttctg agaatattga tacatccaat gcttctggag aagtactcgt
7141 tacaatgttt gggttaatag gatctataga acgccagact ttgatttcga atgtgaaact
7201 ttctatgaat gctaaggcaa ggagcggaga ggcaatcacc ggtcgtgttt taggctacaa
7261 attatcactt aatccactta cacagaaaaa tgatttggtt atcgatgaaa atgaagctaa
7321 tattgtacgt gaaattttcg atttatattt gaatcataat aaaggcctca aagccattac
7381 aaccgtactt aatcaaaagg ggtatcgtac tattaatcaa aagccatttt cagtgtatgg
7441 tgttaaatac attttgaata atccagtcta taaaggctat gtcagattca ataaccatca
7501 aaactgggct gtacagcgaa gaagtggcaa aagtgataaa aatgatgtga tattggtcaa
7561 aggtaaacat gaagccatta taagtgaaga ggtatttgat aaagttcatg aaaaaattagc
7621 ttctaaaagt tttaaaccgg gcagacctat tggtggagat ttctacttac gtggccttat
7681 taaatgccca gaatgcggaa ataatatggt atgtcgacgg acgtattata aaacgaaaaa
7741 gtccaaagaa cggacaatca agcgttatta catttgttcc ttattcaatc gttcagggag
7801 ttctgcatgt cacagtaatt ccatcaatgc tgaagtcgtc gagcgtgtaa ttaatgttca
7861 tttgaatcgt attctgtctc aaccagatat tatcaagcag attgcgtcaa atgtgataga
7921 agaactgaaa caaaagcata gtaaccaaac agaaattaaa tatgacattg atagtttaga
7981 aaaacaaaaa gctaagctta aaacacaaca agaacgattg ttagaattgt tcttagatga
8041 tcagatggat agcgaaatgt taaaagctaa acaaagtcaa atgaatcaac agttagaagt
8101 attagatcaa caaattaaag aagcgcaaca agcaaatcaa tcacaggatg aaatacctaa
8161 ttttgataaa ttaaaaggac gactcatttt gatgataaca cgattcagcg tgtacttaag
8221 aaaggctaca cccgaagcta aaaatcaact tatgaaaatg ttaattgatt caattgaaat

8281 tacgacagat aaacaagtaa aacttgtaag gtataaaatt gacgaaagtc ttatccctca
8341 atctttgaaa aaagattggg ggtctttttt tatgcccaaa ttccaatttg aaatatatgg
8401 tcaaaatgat tatttcatcg accaaattac cacttttacc acttagttat tagtgacaaa
8461 agtgagcgaa atgaaataaa aatcaaatat atattatcaa aatgatgtat cacatgcata
8521 catcaatcaa atacattagg aggtcataac catgacacta gaacaacaac tcaagcacta
8581 tataaccaac ttattcaatc tgccaaggga cgaagtgtgg cactgcgaat ctatcgagga
8641 aatcgctgat gatatcttac ccaatcaata tgtaagactt ggcccactca gtaataaaac
8701 acttcagact aatacctact actctgacac acttcatgaa agtaatatct atcctttcat
8761 tctctactat cagaaacaac tcatagccat cggttatatc gacgaaaatc acgatatgga
8821 tttcttatac ctacacaaca ctatcatgcc tcttttggat caacgatact tactaacagg
8881 aggacaataa aatgcataaa tacatcaaaa ttacacaatt agtcattaca atactaagtg
8941 aaatcatcat ttggatgaaa gagtcagaac gaaaggaagt ctcttatgaa tagatatatc
9001 acccggggta tcgccaacaa cttacctaat atcttacaac accaattatg gcaactcgta
9061 tctgagcgag aacaagaaca aaccaaagat aatacttcag tagattattt tcatatattc
9121 cagttcaata agcatcgcaa tcaattatat atcaaacaca aacaagaacg acctgaatat
9181 gcgaaaatcc ataaagctaa ttattcaaaa gcaatcaata tcaataaggt ctacattatt
9241 cgagaagatg atgtagacct ttcttattat gtcatgttat tacctgaaga atactagagg
9301 agtgaaaatt atatggaaac aatcaaaagt acattaaaaa cagaagccat attcagtgat
9361 gacaaacaac atcgctatct acttaagaaa acatggaata gtgaaaaaca atcaatcaca
9421 atcattacaa tgtatccgca ttatgatggc attctcaata ttgacctaac gacccaactc
9481 atcatgaaca aagtttcaga aatggatgca tttggttcaa tcaattttgt gaatctatac
9541 tctaatatta caacccctat caatctcaaa catttagaaa atgcgtatga taagcataca
9601 gatattcaaa ttatgaaggc agtgaaagag tcagatgaag tgatattagc ttggggcgct
9661 tacgctaaaa agcccggtgt tgaagcacgt gttaatgaag tattagagat gttgaaacca
9721 cataaaaaga aagtaaaacg actcatgaat ccagaaacca atgaaatcat gcatcccctt
9781 aattcgaaag cacgtcaaaa atggatatta aaagtatagc aactaaaatg tttattcact
9841 atccatgaaa ataattgaat ttcatatata tcttttaatc tacaatatga ttaaaagata
9901 tattatttgg ttctgttgca aagtaaaaaa atatagctaa ccactaattt atcatgtcag
9961 tgttcgctta acttgctagc atgatgctaa tttcatggca tggcgaaaat ccgcagatct
10021 gaagagacct gcggttcttt ttatatagag cgtaaataca ttcaatacct tttaaagtat
10081 tctttgctgt attgatactt tgataccttg tctttcttac tttaatatga cggtgatctt
10141 gctcaatgag gttattcaga tatttcgatg tacaatgaca gtcaggttta agtttaaaag
10201 ctttaattac tttagccatt gctaccttcg ttgaaggtgc ctgatctgta attacctttt
10261 gaggtttacc aaattgttta atgagacgtt tgataaacgc atatgctgaa tgattatctc
10321 gttgcttacg caaccaaata tctaatgtat gtccctctgc atcaatggca cgatataaat
10381 agctccattt tccttttatt ttgatgtacg tctcatcaat acgccatttg taataagctt
10441 ttttatgctt tttcttccaa atttgataca aaattggggc atattcttga acccaacggt
10501 agaccgttga atgatgaacg tttacaccac gttcccttaa tatttcagat atatcacgat
10561 aactcaatgt atatcttaga tagtagccaa cggctacagt gataacatcc ttgttaaatt
10621 gtttatatct gaaatagttc atacagaaga ctcctttttg ttaaaattat actataaatt
10681 caactttgca acagaaccgt attatggaat agagatgttg gtaacattta tacaggatca
10741 ttatacttaa gtttaatttc gttattacag aaccacacat tccaaccaga agagaaagta
10801 tgtctattta gttatggttc aggagcagta ggagaaatct ttagtggttc aatcgttaaa
10861 ggatatgaca aagcattaga taaagagaaa cacttaaata tgctagaatc tagagagcaa

10921 ttatcagtcg aagaatacga aacattcttt aacagatttg ataatcaaga atttgatttc
10981 gaacgtgaat tgacacaaga tccatattca aaagtatact tatacagtat agaagaccat
11041 atcagaacat ataagataga gaaataaact agtggccgat tgtgcttgat gagcttggga
11101 cataaatcct aactcgaaat aaataagcat atcactaaac tgatttttta aagtttacag
11161 tgatatgctt atttttttat cttacgattt tgtacgtgca tgcttgccta ggggtatggc
11221 tcgagccatt agtctctcgc acatactatt ccctcaggcg tcagcactta caaaatcggt
11281 tgtaattttc atttttatac gcattcttac tgagattata ctaataagag gaatagtaaa
11341 agcaattcta agtaaaattg cagataagag gtttgttaaa agcagttcta agtaaaattg
11401 cagataagag gtttgttaaa agcagttctc agtaaaatta cagataagag gtacgttaaa
11461 agcagttcta agtaaaattg cagataagag gtttgttaaa agcagttcta agtaaaattg
11521 cagataagag gtacgttaaa agcagttcta agtaaaattg cagataagag gtttgttaaa
11581 agcagttctc agtaaaatta cagataagag gtacgttaaa agcagttcta agtaaaattg
11641 cagataagag gtttgttaaa agcagttcta agtaaaattg cagataagag gtacgttaaa
11701 agcaattcca tgcaaaattg ctgataaggg gtaagttaaa agcagttctc agtaaaattg
11761 cagataagag gtacgttaaa agcagttcta ggcaaaattg cagataagag gtgcgttaaa
11821 agcagttctc agtaaaattg ctgataaggg gtaagttaaa agcaatccta agtaaaattg
11881 cagataaggg gtacagaaaa actagacttg attacaaaat ggagcttggg acataaatga
11941 ttttttaaaa atgagatgag acgtagatta actccataat caatacgaat ctatcgactt
12001 ctttatttat gatattcatc tctttttaat ggaaataaaa gtgcgattaa tgtgataata
12061 cagttacgtt aattaaaaaa ataaaaatgc aaggagaggt aatatgctaa ctgtatatgg
12121 acatagagga ttacctagta aagctccgga aaatacaatt gcatcattta aagctgcttc
12181 agaagtagaa ggtataaact ggttggagtt agatgttgca attacaaaag atgaacaact
12241 gattatcatt catgatgatt atttagaacg gactacaaat atgtccgggg aaataactga
12301 attgaattat gatgaaatta aagatgcttc tgcaggatct tggtttggtg aaaaattcaa
12361 agatgaacat ttgccaactt tcgatgatgt agtaaaaata gcaaatgaat ataatatgaa
12421 tttaaatgta gaattaaaag gtattactgg accgaatgga ctagcacttt ctaaaagtat
12481 ggttaagcaa gtggaagaac aattaacaaa cttaaatcag aatcaagaag tgctcatttc
12541 aagctttaat gttgtgcttg ttaaacttgc agaagaaatc atgccacaat ataacagagc
12601 agttatattc catacaactt cgtttcgtga agactggaga acacttttag attactgtaa
12661 tgctaaaata gtaaacactg aagatgccaa acttactaaa gcaaaagtaa aaatggtaaa
12721 agaagcgggt tatgaattga acgtatggac tgtaaacaaa ccagcacgtg caaaccaact
12781 tgctaattgg ggagttgatg gtatctttac agacaatgca gataaaatgg tgcatttgtc
12841 tcaatagaaa gttagaggtg agtcttacgt ttcagtgacg gtagacttac ctttaacatg
12901 ttacatacta aaaaattaat ttgaataaga aagagagaca tatatgaaat acgatgattt
12961 tatagtagga gaaacattca aaacaaaaag ccttcatatt acagaagaag aaattatcca
13021 atttgcaaca acttttgatc ctcaatatat gcatatagat aaagaaaaag cagaacaaag
13081 tagatttaaa ggtatcattg catctggcat gcatacactt tcaatatcat ttaaattatg
13141 ggtagaagaa ggtaaatacg gagaagaagt tgtagcagga acacaaatga ataacgttaa
13201 atttattaaa cctgtatacc caggtaatac attgtacgtt atcgctgaaa ttacaaataa
13261 gaaatccata aaaaaagaaa atggactcgt tacagtgtca ctttcaacat acaatgaaaa
13321 tgaagaaatt gtatttaagg gagaagtaac agcacttatt aataattcat aataaaacag
13381 tgaagcaacc atcgttacgg attgcttcac tgttttgtta ttcatctata tcgtattttt
13441 tattaccgtt ctcatatagc tcatcataca ctttacctga gattttggca ttgtagctag
13501 ccattccttt atcttgtaca tctttaacat taatagccat catcatgttt ggattatctt

13561 tatcatatga tataaaccac ccaatttgtc tgccagtttc tccttgtttc attttgagtt
13621 ctgcagtacc ggatttgcca attaagtttg cataagatct ataaatatct tctttatgtg
13681 ttttatttac gacttgttgc ataccatcag ttaatagatt gatattttct ttggaaataa
13741 tatttttctt ccaaactttg tttttcgtgt cttttaataa gtgaggtgcg ttaatattgc
13801 cattattttc taatgcgcta tagattgaaa ggatctgtac tgggttaatc agtatttcac
13861 cttgtccgta acctgaatca gctaataata tttcattatc taaatttttg tttgaaattt
13921 gagcattata aaatggataa tcacttggta tatcttcacc aacacctagt tttttcatgc
13981 ctttttcaaa tttcttactg cctaattcga gtgctactct agcaaagaaa atgttatctg
14041 atgattctat tgcttgtttt aagtcgatat taccatttac cacttcatat cttgtaacgt
14101 tgtaaccacc ccaagattta tctttttgcc aacctttacc atcgatttta taacttgttt
14161 tatcgtctaa tgttttgtta tttaacccaa tcattgctgt taatattttt tgagttgaac
14221 ctggtgaagt tgtaatctgg aacttgttga gcagaggttc ttttttatct tcggttaatt
14281 tattatattc ttcgttactc atgccataca taaatggata gacgtcatat gaaggtgtgc
14341 ttacaagtgc taataattca cctgtttgag ggtggatagc agtacctgag ccataatcat
14401 ttttcatgtt gttataaata ctcttttgaa ctttagcatc aatagttagt tgaatatctt
14461 tgccatcttt tttctttttc tctattaatg tatgtgcgat tgtattgcta ttatcgtcaa
14521 cgattgtgac acgatagcca tcttcatgtt ggagcttttt atcgtaaagt ttttcgagtc
14581 cctttttacc aataactgca tcatctttat agcctttata ttctttttgt tttaattctt
14641 cagagttaat gggaccaaca taacctaata gatgtgaagt cgcttttcct agaggatagt
14701 tacgactttc tgtttcatta gttgtaagat gaaatttttt tgcgaaatca cttaaatatt
14761 catccatttt tttaacggtt ttaagtggaa cgaaggtatc atcttgtacc caattttgat
14821 ccatttgttg tttgatatag tcttcagaaa tacttagttc tttagcgatt gctttataat
14881 ctttttttaga tacattcttt ggaacgatgc ctatctcata tgctgttcct gtattggcca
14941 attccacatt gtttcggtct aaaattttac cacgttctga ttttaaattt tcaatatgta
15001 tgctttggtc tttctgcatt cctggaataa tgacgctatg atcccaatct aacttccaca
15061 taccatcttc tttaacaaaa ttaaattgaa cgttgcgatc aatgttaccg tagtttgttt
15121 taattttata ttgagcatct actcgttttt tatttttaga tactttttttt attttacgat
15181 cctgaatgtt tatatcttta acgcctaaac tattatatat ttttatcgga cgttcagtca
15241 tttctacttc accattatcg cttttagaaa tataactgct atctttataa acttgtttga
15301 aatttttatc ttcaattgca tcaatagtat tattaatttc tttatctttt gaagcataaa
15361 aatatatacc aaacccgaca actacaacta ttaaaataag tggaacaatt tttatctttt
15421 tcatcaatat cctccttata taagactaca tttgtagtat attacaaatg tagtatttat
15481 gtcaaaataa tgttataatt tttgtgatat ggaggtgtag aaggtgttat catctttttt
15541 aatgttaagt ataatcagtt cattgctcac gatatgtgta atttttttag tgagaatgct
15601 ctatataaaa tatactcaaa atattatgtc acataagatt tggttattag tgctcgtctc
15661 cacgttaatt ccattaatac cattttacaa aatatcgaat tttacatttt caaaagatat
15721 gatgaatcga aatgtatctg acacgacttc ttcggttagt catatgttag atggtcaaca
15781 atcatctgtt acgaaagact tagcaattaa tgttaatcag tttgagacct caaatataac
15841 gtatatgatt cttttgatat gggtatttgg tagtttgttg tgcttatttt atatgattaa
15901 ggcattccga caaattgatg ttattaaaag ttcgtcattg gaatcgtcat atcttaatga
15961 acgacttaaa gtatgtcaaa gtaagatgca gttctacaaa aagcatataa caattagtta
16021 tagttcaaac attgataatc cgatggtatt tggtttagtg aaatcccaaa ttgtactacc
16081 aactgtcgta gtcgaaacca tgaatgacaa agaaattgaa tatattattc tacatgaact
16141 atcacatgtg aaaagtcatg acttaatatt caaccagctt tatgttgttt ttaaaatgat

16201 attctggttt aatcctgcac tatatataag taaaacaatg atggacaatg actgtgaaaa
16261 agtatgtgat agaaacgttt taaaaatttt gaatcgccat gaacatatac gttatggtga
16321 atcgatatta aaatgctcta ttttaaaatc tcagcacata aataatgtgg cagcacaata
16381 tttactaggt tttaattcaa atattaaaga acgtgttaag tatattgcac tttatgattc
16441 aatgcctaaa cctaatcgaa acaagcgtat tgttgcgtat attgtatgta gtatatcgct
16501 tttaatacaa gcaccgttac tatctgcaca tgttcaacaa gacaaatatg aaacaaatgt
16561 atcatataaa aaattaaatc aactagctcc gtatttcaaa ggatttgatg gaagttttgt
16621 gctttataat gaacgggagc aagcttattc tatttataat gaaccagaaa gtaaacaacg
16681 atattcacct aattctactt acaaaattta tttagcgtta atggcattcg accaaaattt
16741 actctcatta aatcatactg aacaacaatg ggataaacat caatatccat ttaaagaatg
16801 gaaccaagat caaaatttaa attcttcaat gaaatattca gtaaattggt attacgaaaa
16861 tttaaacaaa catttaagac aagatgaggt taaatcttat ttagatctaa ttgaatatgg
16921 taatgaagaa atatcaggga atgaaaatta ttggaatgaa tcttcattaa aaatttctgc
16981 aatagaacag gttaatttgt tgaaaaatat gaaacaacat aacatgcatt ttgataataa
17041 ggctattgaa aaagttgaaa atagtatgac tttgaaacaa aaagatactt ataaatatgt
17101 aggtaaaact ggaacaggaa tcgtgaatca caaagaagca aatggatggt tcgtaggtta
17161 tgttgaaacg aaagataata cgtattattt tgctacacat ttaaaaggcg aagacaatgc
17221 gaatggcgaa aaagcacaac aaatttctga gcgtatttta aaagaaatgg aattaatata
17281 atggataata aaacgtatga aatatcatct gcagaatggg aagttatgaa tatcatttgg
17341 atgaaaaaat atgcaagtgc gaataatata atagaagaaa tacaaatgca aaaggactgg
17401 agtccaaaaa ccattcgtac acttataacg agattgtata aaaagggatt tatagatcgt
17461 aaaaaagaca ataaaatttt tcaatattac tctcttgtag aagaaagtga tataaaatat
17521 aaaacatcta aaaactttat caataaagta tacaaaggcg gtttcaattc acttgtctta
17581 aactttgtag aaaaagaaga tctatcacaa gatgaaatag aagaattgag aaatatattg
17641 aataaaaaat aaaattgttg tgtttacaac aatacataga aaacagagga aacaatcaag
17701 tcgttgaata tttcctctgt tttttagttg aaaaaattaa ccgaaagcct gaatgcaagt
17761 cttgattaaa tcaataatgc ttgtaataac accagtgaaa tccatatgca taccctcttt
17821 ctatttaaga tacattaagt ataatatcaa acaaataaaa aatgttaaaa attccctaat
17881 tggctattta gattgcataa atgtcaaaaa tttgaaaaac atacaacgac tttgcataaa
17941 aaatcgtcat attggaaata cgtaatttat tgaaataata aaaaaaataa aagaacgaag
18001 atgataacct aagtgaggtt ttaagttgtt ctaaggttta atttaattta tgttaaaata
18061 gttggtataa aaatacatga taaactataa actaaattca aaataactta tggggtaggc
18121 aattatggaa aataaaatat aaatgataat gaaaaaagag tgctaaggga aatttataac
18181 catcataata tttcgcgtac tcaaatatct aaaaatcttg agattaataa ggcaacgatt
18241 tctagtattt tgaataagtt aaagtataaa tctcttgtta atgaggttgg tgagggtgat
18301 agcacgaaga gtggtggtag aaaacctatt cttctgaagg ttaatcatct ttatggttat
18361 tttatttctt tggatttaac ttatagttct gttgaagtga tgtacaatta ttttgatggt
18421 aatgtcatta agcatgaatc ttatgattta cctgatgaaa aggttagtag tatattaagc
18481 ataataaaaa aacatattga tattcaggag aaacttgata cttataacgg actattaggt
18541 gtgtctgttt ctatacatgg agttgtggat aatgagcagc atgtgacata tttaccattc
18601 catgaaactg aaggaatttc aattgctaag aaaataaaag aaattactaa tgttccagtc
18661 gtagttgaaa atgaagcgaa tctttcagcg ttatatgaac gtaattttaa tcataattta
18721 tcctacaata atcttattgc tttaagtata cataaaggta ttggtgctgg gcttattatt
18781 aataatcaat tgtatcgtgg tgcaaatggg gaagcgggtg aaattggaaa aacacttgtc

18841 tcaaaagtta gcgataatgt ggagatcttt cataagattg aagatatttt ttcacaagaa
18901 gctttactgc ataatttaag taatcaacta aatgagaaga tgacgcttag caaattaatt
18961 caattttata atgaaaaaaa tccagtcgta gttgaagaaa tggaacaatt tataaataaa
19021 attgctgttt taatacataa tttaaacacc cagtttaatc cgaatgcaat ttacattaac
19081 tgtccattgt tcaatgaaat gcctgaaata ttagaagcaa ttaagaacca gttcaaacaa
19141 tattcacgta acgaaattca aataaagtta acatctaatg tcaaatttgc aactttgcta
19201 ggtggtacat tagcaattat ccaaaaagta ctacagatta atgatattta cttagatata
19261 aaagcataaa aaactaattc aaatgaataa tcaaagttcg taattgtctt tataaaaaaa
19321 tccctcaatc cgaattgaat tttcggattg agggattttt atagttctat tgcagaagaa
19381 aactatttta aaaatgctgg taaatgttga tagccacctc taacgttaac aatattcgta
19441 aatcctttat attctaatat tcctaccgct attgaacttc taacacctga ttgacaatgt
19501 acataaatta ggtcattttt atcgaaaggt atatcttcat ttaaaagttt accgtgagga
19561 atatgaattg cttgttttaa atgaccttta cgccattcat catcattacg aacatctaat
19621 acattatgtt cttcaccagt catttcagaa ctatgaatag atgatgtgac gatatttgtt
19681 tgtggcaaac ggtaactttt tacattttca aaaccaatta attgtaaagc atgaatagct
19741 tgttgaacgg tagatttatc gccaattaat tcaatatctt agtcataatc taaataccaa
19801 ccaatttgat ttataaaagt tttattaaaa ggaatattga tagttccatg catatgacca
19861 ccatggaatg cttctttact tcgaagatca aaagcagttt gtgtattgct tgaactaggg
19921 taaacattat atggttggta catttgcata ccaaattgat ttattttttt catttgtgaa
19981 aaatggtgtg gtggagctgg ctgattgagt gttaaagttt cgataaatga agtttcatct
20041 ttaacattaa aagcccagtt gtttattttc tcataaccca aagtagttgt aggtaatgaa
20101 cctagcgctt taccacaagg actccctgca ccatgacctg gccaaatttg aatatagtct
20161 ggtaatgttg cagcaaattg tatggactga tacatttgtt ttgctccgat ttttgtagaa
20221 ccttcaacat ttacagcttt ttctaataga tctggtctac ctacatcacc aacaaagatg
20281 aagtcaccgc taaataatcc cattggtata ctggaacccc caccttcgtc agtaagtaaa
20341 aaactaatac tctcaggggg atggcctgga gtgtgtaaga cttctaattt aatctttcct
20401 aaatagataa tatcttgatg cttaacgaaa tgtgtttgtt taggcatatt tttataatta
20461 aattcatctt taccttcatc agatacgtat atacttgcat tcaatttatt tgccacatct
20521 ctaatacctg aagcaaaatc agcatgaata tgtgtttctg cagctttagt aattgtgaat
20581 ccttctttat ctgcaacttt taaatatttt gttaaatctc gtatagggtc aataatcatt
20641 gcttctcctg tacgttgaca tccaattaaa taagatgctt gtgaaaaatt gtcttcataa
20701 aattgtttga aaaacaaaaa aaactccttt ttaaaataga ttttattgat tagataaata
20761 agttatgatt tgcttgctca gtatgtccaa tataagtacc tacgccacca taatcgactt
20821 catctcttaa ttcttctttt gaaattccca taacatccat actcatggta caagcaatta
20881 actttatatc ttgatcgatt gcttgatcga taagtgagta taaagaatca acatttttct
20941 tgttcattac ataacgcatc ataatattac ctagtccaaa catattcatt tttgataatg
21001 gcatatgtat tggatcctta ggtaacataa ggtcaaacat tttggaaata cctttctttt
21061 taacgcgagt tgattgcgct ttttttaatg cgttgaggcc ccaaaaagta aagaaaatag
21121 ttacatcttt acctgctgct ttagcgccat ttgcgatgat cattgctgct actgccttat
21181 ctaactcacc gctaaataaa acaattgttg tacctgtagc agtgtcattg atttcaaatt
21241 cttttggctt ttcttttga ataattgcat taattacatt tgcttcttca gtaagattta
21301 caagggtatt ccctgtttgt ttcgcccaac ttttaatatc actattgaaa ccaggatctg
21361 taactgttac ctcgatttgc tcacccgttg aaatattgtt aatttcttta ctgatattaa
21421 caataggtcc agggcactga agacctctaa aatcaaattg tttacgattc tctttgattt

21481 caatatcttt ttctattaaa ggagcactat tgaagttctt tgcttcataa tctttatatc
21541 cacccttttaa attcacgaca tcataacctt gtttggctaa ataatcgcaa gctttagtgc
21601 ttcggttacc gcttttacaa tgtatataat acgttttgtt gctattctta ttgaatgatt
21661 taatctcttc tactgggtgt aaagttgaac cgttaatgtg tcctaattca tattcttctt
21721 ttgttctaac atcaatcaat tgacccattt ttgccaattt ttctaattct tctttgttta
21781 atgaattaat gtgtacttct ttgtattgtt ccatacttac ctcctataaa tacctatgag
21841 ggtataataa aacggataga atcatttgcc aaatacctat atgggtattt gacaattttg
21901 ttttaattta ttattattaa cttaatcaat ttatgtggag gaaatgaata tgacttatga
21961 taaaaaaatg attaatcgta taaatagaat acaaggtcaa ttaaatggtg tcgtaaaaat
22021 gatggaagaa gaaaaagatt gcaaagatat aattacgcaa cttagtgcat ctaaaggttc
22081 tatacaacgt ttaatgggga ttataattag tgaaaattta atagaatgcg ttaaaacagc
22141 agaagaaaat aatgaaagtt ctcaagaatt aattaatgaa gcagttaatt tattagttaa
22201 aagtaaataa tggatatagc aaatatgact attatgttgc taattggcgt actgggtgga
22261 tttatatctg gattaatagg tattgggggc gcaattatta tttacccagc tattcttata
22321 ttgccaccat taataggtat acctgcgtat agtgcatata ttgcttcggg acttacctct
22381 agtcaagtat ttttcagtac acttagtgga tcattaaatg caagaaaaca accagctttc
22441 tctcctaaac ttgttatata tatgggaggg ggtatgttga ttggaagcat gttaggggca
22501 attttagcta gtttgtttaa tgctactttt gtaaatacgg tatatgtaat aatcgccata
22561 cttgctttaa tattgatgtt tattaaagtt aaacctacta cacaagagac gaaatctaaa
22621 cctttgctat ttattatagt tggatttgga attggtgtaa tttcgggaat tgtgggtgca
22681 ggtggagcat ttatcatcat tcctgtatta ttagcattat ttaaattacc aatgaatacg
22741 gtagtgaaca atagcatagc aattgctttt atatcttcag taggggcatt ttttataaaa
22801 ttaatgcaag gatatatacc agtagaaagt gcaatttttt tgataattga atgatgatca
22861 ttttctgaaa atattatgtg gtcatataat ataatgccca tcatttcact aatctctttt
22921 attctctgag ttattttgat atctcctgga gaaggtgtta catcttatca agagtaaatt
22981 acaaaagaat catttaaatc aatactttca ctttgaatac atgtatttga agtggaaggt
23041 acttatttca aaaatagtaa aacctgtatc ttaaattact taatagtaac ataagataca
23101 ggctgatttt ttattcattg ttatttatac taaagcaccc gatagctctg aaaacaatca
23161 caaattcaac ttttcaaagc cacagcttta agttattttg tccagacaac ccccattgcc
23221 caacccattt tatggaattg gcatccaggc aacaactttt catataaatc gtaataattt
23281 tgttcagata ggtacttatc tgaagctaaa tgctcaagcc atgatttaga tgtgttgtga
23341 ttataaattc taatcgcatt ttttattcca aatttgaaac aatgcggtaa aaactcttgg
23401 attgccccta atttataaac ataggtaggc ggtgtctcga cttcagatac attatcaaga
23461 ataactattc ttccttcctc attcaacagt tccttcattt gctgtattac gctggctata
23521 tcatccaaat gatgaaaggt tgttcggctt acaataaaat caaacttctc attaaaatta
23581 agttgttctg cattcatatt cagatagacc gtatttgtta gttgacgttt agatttggca
23641 agatcgagca tttgattaga aatatcaatc cctaccactt catcataata acttgctaat
23701 ttctccacta acaaacccga gccacatccg atatctaatg ctctgccttt ctttggagac
23761 atattagaca caaagaatga ataatcattc aaaagctcat tcacgaaatc gtaatcttct
23821 gcaaccttat caaactgtga ttctattgta ttcaaaaaga tcccccattc ctactttatc
23881 gacattcttt cattacttac cactttagat gttttttcgt tggggataaa acttcccttt
23941 agacaatttt atccaaagac aatacaacag tgcaacttta ttaaagtcac tgtcctttat
24001 cgcagccttt actttttagt aaagacagtg gcttctctta tcaagtttca aaacatatta
24061 ttttgaagaa aacgtccatc tgaagtgtca agtgcaaaat tacatataaa ggtttattct

24121 aaaatgaaaa gatgatacaa tcatattcag ttacataagg aggtttcaat tatgtgcacc
24181 agtatcgcag tagtagaaat tactttatct cattcataat gaaaaaaatg gaaaggagat
24241 aaaagtatgg gtactttttc tatatttgtt attaataaag taatcaaaat taattggtta
24301 taatgaacgc ttaatgtcag ttcattataa ccagtaagga gaaggttata atgaaccaga
24361 aaaaccctaa agacacgcaa aattttatta cttctaaaaa gcatgtaaaa gaaatattga
24421 atcacacgaa tatcagtaaa caagacaacg taatagaaat cggatcagga aaaggacatt
24481 ttaccaaaga gctagtcaaa atgagtcgat cagttactgc tatagaaatt gatggaggct
24541 tatgtcaagt gactaaagaa gcggtaaacc cctctgagaa tataaaagtg attcaaacgg
24601 atattctaaa attttccttc ccaaaacata taaactataa gatatatggt aatattcctt
24661 ataacatcag tacggatatt gtcaaaagaa ttacctttga aagtcaggct aaatatagct
24721 atcttatcgt tgagaaggga tttgcgaaaa gattgcaaaa tctgcaacga gctttgggtt
24781 tactattaat ggtggagatg gatataaaaa tgctcaaaaa agtaccacca ctatattttc
24841 atcctaagcc aagtgtagac tctgtattga ttgttcttga acgacatcaa ccattgattt
24901 caaagaagga ctacaaaaag tatcgatctt ttgtttataa gtgggtaaac cgtgaatatc
24961 gtgttctttt cactaaaaac caattccgac aggctttgaa gcatgcaaat gtcactaata
25021 ttaataaact atcgaaggaa caatttcttt ctattttcaa tagttacaaa ttgtttcact
25081 aaattaaagt aataaagcgt tctctaattt cacaagagga cgctttattc ttcccaaaaa
25141 ttgttcaata tttatcaata aatcagtagt tttaaaagta agcacctgtt attgcaataa
25201 aattagccta attgagagaa gtttctatag aatttttcat atacttaacg agtgctttca
25261 cctttgaata tagtccttcc cacttatcat cacactctcc ccgatagcct tttctagcta
25321 tatccagtaa agttacatgc tctttaggta aaagaggtat agcccattct gcagcgacat
25381 ctttcgaggt aatttcacca gtagtcactg tttgccacat tcgagctagg gttaaaatta
25441 cattacgctc atcacctttt atcccctcaa ttagttctgg caaagaatcc ttaattgctc
25501 ttcgaatatc tgtcaaaggt acggagacaa gtatacttga agaatcagga ccaaatagag
25561 aaatactatt ctttcttgct tgtgctaaaa caatagccaa atcaggatca tagcttggtt
25621 cctgaatttg tccattctca aattcacccc tgagccactc accgtatata aattctcttt
25681 ttggaggata ttgccaaggg acaacttcac tcctatttat aaccgtaact tcaagtggtc
25741 taacagaatc cgtatttcca atctttcctg atatagtcat tagtctttct gttagttttt
25801 ttcgagttaa ttgaggtaaa ctatgattca cgacgactag aacatctaca tcgctgttaa
25861 tgcgtaaacc accatttact gctgaaccaa atagatatac tccaactatt gaacttccaa
25921 ataaatcttt tacgattttt aatgtttgaa tcgcttgatt tggtattttt ccgttaatca
25981 aattgctcat gatttcacct cgttgattat gttcatataa agtttatatt gatactcaat
26041 ttacttaccc tagattggac atatacttaa attactgttc aataaagctg accgttagcg
26101 tttaagtaca tcctttcaca atttgtctac agattaataa ttattcttta ttatacagat
26161 ctccatataa tttttgaatt tggttctgta attttttatt ttctttttct aattccatta
26221 ctcttctttt taaggtttta ataaggattt cctccgaacg agaacttttc ttgggttttg
26281 agactacatt tgctgttatt tgacgctcac gaagggattc gattctttgc ctaatatcgt
26341 gttccttata aagccatgat ttagaaacat tagcttcctt tgctattgaa ttaaaattaa
26401 taactttacc ttcaatcgaa aatttagaaa tcgctttgtc tactttttcc cttgtctttt
26461 ttgatttctg cttcgccaaa cgtacaattt ctgttgtatt tctaacttgt ttatccattg
26521 ataattaccc cgtcaaactt ccaatgattt gttctaaacg ctctttaaca cggctattag
26581 tctctacttg tctttgccat tgtttatcct tagctatggc taataactct tctgtacgct
26641 ctaactgttc ttcgtgctgt ggtaagaatt gcttactggt acagaagtga gtgcaatcta
26701 agcatgcatt cgcatgtgga caaccacctg ctactactgg caatctacaa taaccatttg

26761 gaagcacttg tgcatttata tttttcttga accattgaag ctctacatca tcgacttcat
26821 tatcttcatc tagatcaagc acatctccat tattggtaac cagtttttcc tgaaatttag
26881 taaattcatt ttttagagtt tcatcaaaga tatgagcgta tctgcttgtc atttctgggc
26941 tttcatgccc caaaaatttc tgcacaatat gctggggcat cccgttgtta atcattcttg
27001 ttcctactgt atggcgaaag gcatgggcat ggaatctata aatctcacct gatttatcca
27061 ctatattttg ctcataagct aatttattta actcacctct aaatgtttct tgttttaatg
27121 gcgatccatc ttttcttgga aagaggtatt cactatctgg aaattcctct gaaactttat
27181 cttcccgaac tttaataagt aaagctacct ctttagatat tggaactata tgctcctttt
27241 tcattttcca ttgataatac tttaaaaaga aatctccatc tttgtcctct aatagacagc
27301 cttttttcaa ggtgcacaat tcacttatcc tcattccaca ttcttgaaca atcatagtca
27361 tcgtagctat atattcgggt aatttatcaa gatgactgtt caattgctct aggacgaatt
27421 catctataaa gcgtggtttt gctcttggta ttttcggata gtcctcagaa taaattaata
27481 ttttggaagg aacatcatcc cattctagcc taagaagggt actaaatagt ccttccaata
27541 tagagatcct cccagttatt gtactaggtt ttattcccat catgtttagt tcacttaaat
27601 atgcttcaat ttccactctc gttaattggt gtactctctg aacttgttta aatttcatgt
27661 ccagaaaatt aaagaactct ttaagtcttt gggcaatatc acttacatag gaaaagctat
27721 ccacgttcaa tctcaactta caatatcttt ttacaagttg tttaaaatat gtattccgaa
27781 accctttaaa gttaattgta tattcatatt gtgttgggtt aaccttatca tctggcaaag
27841 gtaagttacg tctatcccaa acgtctttat cccactcctc tccatcaaaa taaaagttct
27901 cataaaactc cataaattgt tttagattag taacatagta ggaattagct tttacaggtg
27961 ttttttcttg attagcagta atcttataat tagtagtggt aattctaaca ccccgttttg
28021 tcaaataagt tctatactcc gtcattgctt tttcaatagg aacttcagta attgaagtaa
28081 tgctaggata ctttaaatct aagaaatcta acattttatt aattactgtt ccttttctaa
28141 tccagacagt ttttgcattc catattccat tgtttaaatg gtaaaaataa aaatatttca
28201 attctgttct taaccacaga tttttaacac gttcaaaacg aacccaacga ttccttaaag
28261 caggattctt acttaattct atggcagaag gatgtggaca ttttcttata tcccaactat
28321 tattagccca aaaccccctgc atttcttcat tcattacagc tattttttttg ctaatctcac
28381 tctgactaat aattttcctt ttactagaag cattcatttc ttatgctcct ttctctcgag
28441 gtatttatta aactcatttt tcatatcctg atctgaaaga tgaacatagg tatttaacgt
28501 tgtctgaaca tgtgcgtgac ctaatctctt ttgaacgaac gcaacatccc atccttccct
28561 aattagctgc gttgcgtgag tgtggcgaag catatgtgat gtaaattcta ttccagtcct
28621 tttaactatt cttctaacta gatcaagaac actttggtac tttagtggtt tcccaaaata
28681 gccttctttt aaggaaataa aaacataatc atgctccaat tcctcactat actcatatat
28741 caagtaatct gtataaagtg acataagttc tttactcaca tgtattgttc tttccttcct
28801 taatttaata taagcttcat taacattaac atctctaggt gttaaatgga tttgattgtc
28861 ccaagtgaca atatcttcaa gcctaagcga taacacttca ccgattctta aaccaccctc
28921 atacataagc attaaaatta atttatctct tttcgtatga caagcatcaa taatttgctt
28981 aacttccttt gatctcaatg ttcttatctg tttcttttta acccttaact ttaagacatt
29041 cttttggtat ctacccttat taacatgatg taaaaatcct ttgaaatttc ttccctttgc
29101 ttgtttaaat acatcaattg atttaaattc tcctaatcta cttaaataat caagaaaact
29161 cataactaca tttaaaattg tattcactgt cgtttcttct cttatggctt tttttgactg
29221 aagatcaatt acatttgatg ctgaaggata tctcaaccaa cctacgaagt ctgctaacaa
29281 ctcaaagtta atatcattaa gaataacacc tctctgttcc atgaactcgt acagcaactt
29341 taaatgatag cagtatgcct taatggtatt aggagactta ccagtattat ctaagtattt

29401 aataaatttc attactggtt ctattagctg gtattcttta tctagtaata aatacaatgg
29461 atacggctta ttctccactt ctatcctttg aaccttcaca tgttcccacc tctttaaata
29521 ctttaactac tacaaattat aaaccttgtt aatttttatt taaagtaaaa tatcccttta
29581 atatctcctc ttaaactact ttaactacta ttatttatta tactatggtt aatacatctc
29641 cacttggatg attatgaccg agcattatac tatttgcgtt actgagtatc gctgttttga
29701 atatttctct agggtgaatc accgtttggt taatagatcc aatcgatagt gtttgaatat
29761 gtgtaggttc atttttactg ttcatacata tgagaatgag atgctctcgg tcactgtttc
29821 caatgaatga acgcatgatt tctgccgcat cctcagggtt tgaaatacga ttttttagat
29881 aacttaatgt atctgttttt atcatttgta gtgaaacaat attgatttcc ttcatcgttt
29941 acctccatat ataggttatg ctttcaaagt ccatttttga cgtgctttag ggttgagtgg
30001 gtgcatgatt tcatttgtgg ctggattaat gagctttttg actttctttt tatgaggctt
30061 caacatttcc attacttgtt cgacacgttc tacaacaact ggccgcttcg cataagcacc
30121 ataagctaga atcactgtgt cactttcgct aattgctttc atcaagtgga tgtctgtgtg
30181 tttatcataa ggttctttaa tatgtttaag gttctctggt gttttaatat tagagaatag
30241 atttacaaga tatacagcac cgtatcgttc tgaattcgct aattgattga ggataagaac
30301 agttgtgaga tcgagtgata atacaccatc taaatgaggg tacatcgtta tcactgtaca
30361 agcgggtttc ttttcatccc atgttttctt gagtaaatag cggtgttttt catccttgct
30421 aaatatagct tctgtgtata tcgtattttt gattgtattc atatatataa tcacttcctt
30481 tagtattctt ctggtaaaag catcacataa taaaaagcgt ctacgccatc ttcacgaatg
30541 acgtagactt tcttaggtaa tgcattttga ttttttacat agtttgtata gtgatattcc
30601 aatttgtatg caggttgttc ttgttcatgt gtgattgaga gtatattctc atcttcttgc
30661 agtttaaaaa tgtgtaggta atctgtatga ggttgattat ctctttcttt taccatattc
30721 caaagtaaga tttgaaggtc tagagatagg tattcactaa tacctcttgt gatgtatcga
30781 ttgattttca tgttatttta cctcgtcttg aatttctttc ataatgataa tcgcttggct
30841 aataatcgta acagatattt gtgccacttt gatccagtta ttcatggtga gtccctcctt
30901 ctcttagtaa atgacgttca tcgataatcg tatttttagt atctgtgaga tataaaaagt
30961 ccatgtcaaa atgattcaga taaccaacgc tgatgagttg gttattagcg tacataagaa
31021 atggatagat acttaggtca tgtagttcat tattgtagta ggtataagtt tcaagtgtaa
31081 gatgtgcaag tggggaatca ttaataaaac gttccggtag aatattttct gctgcttctt
31141 ctaacgcttc gcattcccat gtttcattgt tagatagttg gaatagacga gttatatatt
31201 gtttgagttc ttgagtggtc gttttcatat cattgcctcc tagatagtgt tatagtgatg
31261 tagtttatgt acatcattgg gataatatat atttgatttg tcatttatta cgcatcccgg
31321 tgagaatgag agaaaattcc atatgaaaaa ccgcttcaaa ccttggtatg acaaggaaat
31381 cccgaaattc cgcctatttt gacgaacaat caactcattc tttataacta ttgatgttag
31441 ggtggggctc tgctttctta tatattattt atttataaag aataacggga ttttgggatt
31501 gtgcttgcac aatccttctg cttcttcgaa tctgcaaatc ccaatcattt cccgataaaa
31561 aatcattgtg ggatgttctt tagcaatttc aatataagca tcgtgtagtt atgaaaaaaa
31621 ttacgacaat gactgtttca ttagataagt gttattgaaa ttgataaaga gaattttaaa
31681 aatggttaga taaaataaat gaaagaatat aatgaaaatt attgttataa caatgattct
31741 attagctaaa tagtaagata tagtgtttgg ggcaaaaaca aagacgaagt gctgagatgc
31801 acttcgtcga gttgtttatt attgaaaagt tgttttatga tttcgttatt aagtttgagt
31861 gtgacataga attgtttttt atgattacca tcttttttaa tatcaatgcg atcaatcact
31921 gatagataca atgctttgag tcgagatttt tctatgtgct taatatcatg aaagatgtgt
31981 tgtaatagtt tactgatttc tttggcatca aataaaggct tatcttcatt ttgttgattt

32041 ttgagttggt tgatttgatt cgtaatgtca ttgagttgct tttcatattt ttgaatactt
32101 ggtctgatta ctgatgttaa gtccggatta tcctcgatgg ttttaatcaa gttatttagt
32161 ttgatttgta cttcatcata ttgttgttgc ttataagcaa tatcgtgatg aagtgcagcg
32221 ccatcaactt gattttcttg attgacgtgt gttactacgc gttgaatgac tttatcactt
32281 ttgactattt caagtatttg cttcatcaca taatcttcaa tcacatcagc tcttacactg
32341 tttgccgaac atactttgga acccttgttc cgaaaattac tacatgaata gtaacgaata
32401 cgtttcttag tcccgtcttt aagtgtattc gtggtattgc ttgctgccat aggtgcgcca
32461 cattggggac agtgaataat gcctgtaagc agattcgttc ctttgccatg gacttggggt
32521 ttttgactga cttgtttttt acgcatttgt actttatccc ataaatcttg attaataatg
32581 ggggaatgct taccttcagc tatcactggt ttatcattaa gccctttacg acgtttttca
32641 ctccaatctt tgtatttcgc aaattgaatt ttgccgatat agaatgggtt agctaatata
32701 tatgtgatag aactaatact aaaaggttta cccttttttag tgacatagcc tttgtgattt
32761 aatgcattgg ctattttacg atagccatgg cctttggcat aggactcgaa tatatatttc
32821 acaatattag cttcatgttg attaatcatc agttcatgtt tactattagg tattttgtca
32881 tagcccagcg gcaaattgcc ttgataatag ccttcttggg cacgtctcgt ttgacccata
32941 aatacattct cgacaatgtt attacgttcg aattctgaga aactcgcaag tatttgtaac
33001 atgagcttac ccgatgaagt attgacttcc atacgctctg ataaactgaa aaattcgaca
33061 ttttgtttgt gtaaatcttc gacaattttg agaagatcag atgtattacg agctaatcgg
33121 tttgttttgt agaccataac acagtcgata tagccttctt tcgcatcctt caatatacgt
33181 tggagctcag gtcgattcat agatttacct gaaataccac ggtcagcgta tatatcttta
33241 acttcaaaat gatggaagtc acagtattct ttgatttgat tgatttgtcc gtcgatacta
33301 taaccttctg tgctttgcat ttctgttgat acacgtacat agataccgac acgttttgtt
33361 ttaagttgtt gcattatgtt tcatcctttc ttcgtttatg caatcgatga ttgcatggtt
33421 tgattgacga tattgagtgg ttcatttttg aaatagattc ctataagatt tttatctttc
33481 gtaatgtgaa tggtttcaat ataggggtac aatatgttta acgtgaaacg tttttgaata
33541 atattttgaa tggtgtgttg tatttgatgt ccattgatag atgtagtgcg ttgcggttgt
33601 tgacgtaatg attgcgtttg ttctctgaac gtttctgcat cgatgatgcc ttgtgccaac
33661 ttttctatca gttgttcttg agtcaatgtg tgatgttttt ctatgtttct ttgtcttttg
33721 atgcgtttgt caatcgcacc tttaattttt gtgtagatgc gttgattttg ataaaagtct
33781 ttgcacactt ctaatacttt atcttcaagt gtttgtgcat tgatgccttt aaaatcacag
33841 acaaagcgag aagcattcat gtttttagga cagacgtagt aacgtaatgt atgattcttt
33901 tttctaacgg tcatattcgt aagtgttgta ttacaacatg ggcatttgat tttttgtttt
33961 agttggttat ccgaagatgt ctgtttgatt tgtttttgcg atcgaagtct ctgcgcttgc
34021 tcatatatac ttgtggaaac aatagaagga aacatattgt cgaattggcc atattgattg
34081 ttgacacgac cacaataatt aggattgatg ataatgttac gaacttgata gggttgtcga
34141 ttgatatacg tgttatcttc ttctaataac tgtgcaattt tcttataacc atgaccttta
34201 atgtaataat tgaatacagc ctttaccgtt ggtgactcat tttgattgat gatgaatgct
34261 ccgttgtgat attcgtaacc aaagggcgca tgggttgtaa tcaatcgacc ttgctttgct
34321 ttttcttgaa gcccatttct gacttgttct ccaatgttat ctgattcaag ttcggccaag
34381 ctgataaaaa tattaagctt gaatcggtcg aaagcttgat ccatatcaaa gtaaccatcg
34441 tgtacgctta agatatgaac atggtatgtt tgacataatt tgatgagttt taatgcattt
34501 ttcagattac gatgtaatct attaagacga taacaacata atatgtcaca ttgcccttgt
34561 tgaattaatt gcgtcatttg tcgataccca ctacgattat ctttgcgtcc tgattgtttg
34621 tcgctataaa agttgatatg ttgaatatga tgtttttcgg ctattgcttc gatagcctgt

34681 ttctgtgctg caagagattg ttgtttcatc gtactttgac gtaaatagcc tatgacttgt
34741 ttcatatcgg ctcctccttt cacagtaata atatatattt atggatgaat tgatatataa
34801 gcccaacatc aataagatgt tgggcgttca tattagtcat tcatttgatt gatttcttca
34861 attaccaaat cggctaatat ctcgataagt tcatccatgt ttttcactcc gttatttgtt
34921 ctatcttcaa tacgtcgatt attcagtttg atgcttcacg gttgtatgat aaagacaatc
34981 agaaatcttc gtgaactcct gaagggccta tcccctcatt agcggattta aaaagttctt
35041 tcgcagcttt gttatcattt gccggtgtcc aattttgaat taacgactta tctttagtta
35101 atcccaggat agatgcaaac tctacatcta attttagatg gtaaaataca agtgattgtt
35161 ttttaccgct attatctttg acacttcttt tagttgtttg gcgtccacgg tcagctaata
35221 tgaaaccttt atctcttaag gcgttgacaa cattattaac atcttgaaat tgatgattgt
35281 ttagcatctg tttaaaaacg tttgcaatca tttttacttc gatatggtca tcttttaatg
35341 agattaatcc atagttctca aacatatttt tcaaagcacc ttcatctgaa aacttacctc
35401 tgttttgtgc tacaaattga atgatgacat cgatagcttt atcagctaat gagcgttcag
35461 agattgtatg accatgataa tcaataaagt agtctcttat tttagcgata tcaatatctg
35521 tagctaaaac acgacctaat atttttgcag atgttgtaat gactgcataa cgcttaaaca
35581 tacgattgcc tgtgttgctt ttatcatctt tcaatttagc ttcaaaccaa tctacttcct
35641 tgtaaaacca ttgaataact tcatcttcac gatttataag atatttagct actaacggta
35701 aaacatgacc atagtttagt gctacagctt ttttaatatt gtcagcattg gtcgcatttg
35761 tagtgaattg ttcattaatc tcgatggttc ttacacgtaa tccatcgttt tgagctgaat
35821 cattaaaaat actgtgttct gacgttgaaa tgacagaagt accccaattc ttaggcgttt
35881 taacttctcc atgaacgttt gaacgttgac gaccttgacc ttcagtgatg gagtacaata
35941 accccgttgt atctctaaaa gttgctgatg agagttcatc aaatacaata ggtataccaa
36001 aattgttact caagtaacct tcaagtgcat tacgtgtggc attccaattt ctaaagagag
36061 tttcattacc tttggtaggg ttaccagcga ctgatacagc taaagaagct gcagttgact
36121 taccggttga ggattgacct gtaaaactaa aaatgattcc ggcaaattcg gtttcatgtt
36181 tgtgcttcag gaaactcgtc actaaggcag aaataccaaa tacgactgct aattctaaaa
36241 gaagagaacc tttaacctct tttagataca tgttaaacca attatcaaat gttcccctag
36301 gtgctaagtc ataagtattc tcacaaatgg cgtcagatgg agatttatta tcaaattccg
36361 tagtagtata gatttcattt aacgatacaa taggaccaaa cggtgtttcc agtataccta
36421 ccccttcata taagtaggaa atgggtaatt ggttgcgcat ttgttgcaac gcataaccta
36481 aatcttttgt atatttttca ttaatactaa atccatattt cattaaagag ggcagttttt
36541 gtgttgttaa aatatcacta gattcaacaa ttactttttg atcctcgtct gtaataatta
36601 ctttttcagt gttagtttta gggtcaataa acttattttc gataacgata ggacctgcga
36661 tttcaacttc agtaggcatt cctccttttt ctttgggagg cttgtcttta taccaacctt
36721 tttttgattt gtatcgtggt gaaggattaa atgaagggtt agtttgagtc attagcgaac
36781 acctcctttc gaagggttgc tgttatagtg tggattagga cctgttttaa gataaactaa
36841 gtgaccgtga gtatccttac cgataataat aaatggaaca cgtggcgcat gttttacaaa
36901 atatgcgaac caacgtccaa cattttgttg tacagctttt gaacattgta catttgcacg
36961 actgttcaag tcatgaaatg taaattcttc tccttcaggt aaattgaatg aaatacctaa
37021 tattttcttt tttaactgct cgaatttatc tgattgattg aaatacattt aacttcctcc
37081 tactaatgaa ctaagatagg aaaattaaat atgcgcacaa ttaactttct taagttcatt
37141 ttagccaaga agttatcatt tgaaagcttg taaaaatatg ttagaaattc cgtactaaat
37201 taagatggat ttttgttgac aaaataaaaa acgctgattt aacagcgctt taaataaaaa
37261 ttaatctgaa gttatataaa agtagtcaga aggagtggtg ttattatcat tataataatc

37321 tagtgcttta ttgatttctt ttattaattt atcatgatgt tcttttgtta ttttctttgg
37381 atatttgagt tctttttctt ctaagtcttt tggagacact aggtcactat ttaaaattaa
37441 tgataacagc atatttttag gtgccgaagc tctggtgtct cgatgagtaa tatattttgc
37501 taaattactg tttatactta ctttttcatt ttctaaaaag tttgtactat ttttaacatg
37561 agcatcagca attttggtac ttatttcagt gtttaaatcg tctatttcat ctaaataata
37621 ttcaagaatc tgaagtttat ctttagaagt gataccttttt tcagtgaaaa tcttaataat
37681 gatagatttg attccaattt ctatattact gtctactaga aaatagagat aataattagt
37741 cagagcatgt tcataatctc tttgatcagt aatttcatga ttgatgaaat aatataagtt
37801 ctctttgata cgattagaag aatctatatc tttgtcattg ataccgtttt taaaaaagtg
37861 tagataaact gagttgtttg agagtatatc atataataca aataaacttt ttggtttaaa
37921 tacatatgat tttcctgatt taaagtttgc tatgtttatt cctagagcgt tgcagaattt
37981 attaaaatta gttgtaaaat tatttttttc ttgaataaac atgtcgttgt catttaattc
38041 tgaactatga tttttaatca tattgatatt ttcttcaatc ttgtcactac ttaaattttc
38101 catgcaaagt atactatatc ttactatatc acttatattt atggaaaaat ctttaggaga
38161 acgatttaaa gtatcctttt tattatcttg atctacagcc ctacatgaat catcaaaaac
38221 ttctttgaca tttattttat caatgttcaa aattatgtct actaaagcat tttgaaaatc
38281 atcgctatat atgtctttat tattcatagt gtcactacct ttatttttaa taacactata
38341 tattatatct tataaaccta ataccatgaa gaataaaaag aatgattcaa attttatatt
38401 taatccatag ttggctgcta tggttagtgt actagtctag ttttactagt atataaaaca
38461 gtactcaatt tttatttttt caaaattata aaaaagaaca cctatcatcg ataggcactg
38521 aacccctaaa acgggaactt aataaaaaca ccatgttcta ggctattaat tctctgtatt
38581 ttacagggga taagtagcct agtttttgtt gaatttgatt attattatag tttttaatgt
38641 acttttcgac aatatctatt acaatatgat tagagctatt aagctgatta ttgatgtaaa
38701 gagtttcaga ctttagcgag gaatggaaac tttctatcgg ggcgttatcg gcaggtgttc
38761 cctttcggga catacttctg ataatgcctt tttcttcgca taattgataa taagcataag
38821 atgtataaac gctgccttga tcactatgta atatataccc ctcaggtata tcgatttgat
38881 ttaatgtatc attaactaaa cgttggtctt gtttatcatc tattttatac gccacaattt
38941 ctccgttata aatatccatt atcgaagata aatacaacat agaatgatca aatggtaaat
39001 aagtaatatc ggttgttaat acttctatgg gacaattcgc tttaaattat ctttgtaata
39061 aattgtctgt tttataatac ggtttaccta tccttgtcgt ctttttaggt ctaactcggc
39121 agttcaaatg atgcttctgc atcattctct gtactctctt atgattaatt ggtgatgtat
39181 aacattgatt aatcagtgct gtaatcttac gataaccgta ggtataatgg ttagcttcac
39241 ataattcaat aactttttgt gttacagtat catttttata ggttttgttt ttccatcggt
39301 aatatgttga tttaggtata tttaatactt ctagtatcaa tttgattgaa tagtttcctt
39361 ttaattgatc cactaaatct atgactactg ttggtaccac ttcctttcca atgccttgta
39421 cttttttaaa atatccaatt ctatatcttt tctcttattt tctaatttta attgttctac
39481 ttctgacagc tcttctaatc ctttaccgta ggtatattgt ttaccaacgt gttgtgaaaa
39541 tctataactt tccccatttc gataccatcg ccaccaagtt tccacttgtg ttctattttt
39601 aatatttaat tctttcataa tttcttttgt tgaaaatcct gctgctttca tttcaactgc
39661 tttatacttt gtttctactg aataagaaac tcttttcata gaaaaaacac ctccgtatga
39721 ttcattttaa tatgaattca acgaaagtgt ttttatataa ttcccactaa ttggggtcag
39781 tctactatat gatacggttt tttaatttaa agtatcaata ataattggat atagagggaa
39841 agaagctata atgatatttg cttactaagt ggataaagat attagaaaaa cgagaagcag
39901 gacagttatt aaatcggtag atattaatca taagtattta actgaatttt taccttttgt

39961 cgaatatata actaaagttg aaggtagtaa aatatttatc tagtcgatat taaaacaatt
40021 tatcgaaaac gatggttttc catatcctct ttagtagcaa ttttctaaca atatataatt
40081 aattatatac aaattaagaa taattaagaa gtatatatta attagagata gtttaaaaat
40141 tagatttcaa tcaattaatt aaaacaggtt agactattaa tttatactaa cctgttaaaa
40201 gtaacaaaat taagaatata ttaaaacttg tgtaatgagt aagcctccac caaatattaa
40261 aacaaacgcg acaacaggcc atacgaaacg taaccagtgt gagtagcgca cgtttaacat
40321 ttgaagtgtt gccattacaa gtccagtagg cgctaagaac aacattgcat actgaccgaa
40381 ttgatatgtt gtaacaataa caaatcttgg tatacctact gtatcagcta atggcgcaaa
40441 gataggcata gataatactg ctaatcctga tgatgatggt acgataaatc ctaaacagaa
40501 aaagataaag agcagaacaa tgataaataa aggcccactc atatgttgca cgatagatga
40561 tgaaaagtgc aagattgtgt cagaaatcat tcctttattc aatactaagt tgattccacg
40621 agctaaacca atgattaaag atacacctac taaacttgaa gcgccattaa cgaatgcatc
40681 tacagtgcct ttttcgccta aaccatattg tcctgttcca gcaataaaca tgatgacaat
40741 ggtaaagatt aagaatgcag atgccatgac tgggaaccac catccttgtg tcataacacc
40801 ccaaaccata atagggaatg gtaggacgaa aagcgtaaga ataatctttt tacgtaatgt
40861 aaactcagaa gaaccgtcat catggagcac agaccactgt ttttcaaatg ctgctttgtc
40921 ttcataagaa taagaggatt taggatcttt tttaattttt ttacaatacc agaataaata
40981 actaataaca aatatggcac cgatgataca agcgcctatt ctccaataaa gaccatcagt
41041 aaaagttgtt cctgctgcat tagaagcaat gacgactgag aatgggttga ttgttgagaa
41101 tgtactaccc acagagcttg ctaagaaaat tgcaccgaaa gtaagacttt agaatctatg
41161 ggaaagataa taaggtttaa tagaaataca agaagtgtaa atagtaagca tataatttat
41221 acattttaat ataatataac aagaaaggta aacacaaaga taataaaaat aaaaatttaa
41281 taaagataga gaattattaa attgtttttg atataaaaca acttaataaa aaatttaatt
41341 ttaggggagg ataattataa tataaagaaa catagaatgg aattgcaatt gggaagtata
41401 cgattttaac tgcgaatcat atgttcaacg acttacaaat gcgtaaatcg ataacacatt
41461 caaaaatata taatctgaaa attgatttac taagagagga ttattgtagt tacaggtgtt
41521 aagttgctcc aatatggagt tgtcgtcttg ttttatatta aacaagtttt attcaaatac
41581 atgacaatac gtaaaataac actagaatta tcaatcacta aaattaaaac aaatgtatct
41641 attcaaactt ttctttaggg tttacttgct gaataattta aaaatgaagt attaggtgta
41701 catgctttca gaattgcttt tgaagaatat taaaagtata atctgaatac gatgacagaa
41761 tgaataggta aactatgaca agaagtcatc aaggttatca aataaatata tcccagcggt
41821 tgagacgact cactgctggg attttattgt tagttattta ttctaagtgt tgcatttggt
41881 agccgattct aggatgtgtg atgattaatt tcttttcagt gtttgggtct tgcgtataat
41941 tcaacttttt acgtaaagag gccatatgta cacgtaaggc agccatttct gtatgattga
42001 catagccata gagtgatttt aataacactt gataggttaa tactttgccg acatgatggc
42061 ataatatcgt taggagttgg aattcattcg gtgttagatg tacggactgt tcattgacaa
42121 ggactgattt cgcatcgaaa tcaatggtta atggaccatt tgtgaaacga ctttgaattg
42181 tctcagtaga acgtgacata cgtaatgcta ctctgatacg tgctcttaac tcatcgatat
42241 tgaaaggctt agtcatataa tcattggcac cgcgatctaa tacttcgaca atggtttgtt
42301 cctctgttcg tgcactaatc acaataatag gtgtgtccac aaagtctctg aattgctgaa
42361 tgagagataa accatcaata tctggtaagc ctaaatctaa taagataata tctggttgtt
42421 ctgttcttag gcgaaagtcc gcttctttcc ccgtcttcgc cgtaaccact ttataataat
42481 tcatagttag cgcaacatcg attaaatgta aaatcgcttc atcatcttca acgaccaata
42541 atgttgtttt catccatggc tccctccatt tcaattggat taacagtcaa ataaaaataa

42601 aaaatactgc cttgtggtgt attcggttga tattctaatt cactgttgtg ttgtttcaag
42661 atgagttgta ctaaatagag ccctaatccc aaactatctt ttttattgtc tttaaagttg
42721 tctcctgaat aatagggatt aaaaatcaat tgacgttctt cttctggaat accttttccg
42781 caatctatca tttcgaattt tattttgttt gtttcatgtt gaacgtgcag ttttatttca
42841 gaatgtgatt ctgcatgctt taaagcatta tcgataaggt tgaatagcac ttgcagtatt
42901 aatttactgt cgatattaat gagtgaagcg tcatcctcat tttcaataat gacatgattt
42961 gcttggtgtc tgcgtatgag gccttcttca aattcttcta gaagttcttc tactaaataa
43021 ggggtgcgtt gtatttgaat gtcagagctt tctaacttag tcaaagataa aatatttgtg
43081 actaaggtat gcagatattg tgcttcgcca taagaagcag ttaagagttc tgcttgttgt
43141 tgatcgttta aatgctcgtt atggtatttc agcatatcta agttgcccat aatggaagtc
43201 agtggtgttc taatatcatg tgaaattgaa tgcaagaagt ttgaacgtgt ggcttctcgt
43261 tcagctttca atatggattg tctggtttgt tttaatagat ccacattctc tattgccagg
43321 gtaatatcgt tcaacataga gtctaatatt gaattgtcat aggtgtcgat gtaagtttcg
43381 tcggtgaagc ggatagaaat cacacctttg actggatttg tgccaatggg aatacacaaa
43441 aatttactgc caggaaaggt atcggttaat ttaccggcac ggctttcatt ttcaatgacc
43501 cagctcaatg tctcagcatc atgtgtctta tctgagcttg aaatgcttct gtttccaaat
43561 gagttggaag cagcgacttt cttactttga attaagaata ccgtgacatc ctgattgagt
43621 agctgatgaa tctgatcacc agcaatattt aataagcgtt caattgaata agattcttta
43681 atggactggt taaattgcag cataatattg gtccgatata actgccgttc tgttaaggaa
43741 tgctgatgtt ttaaattctt taagatggca ctcgtaaaga tactagcaaa aatactggtc
43801 gcaaacgtaa tcggatattc aaagcgatac atttctaaag tgaatcttgg caccgtaaag
43861 aaataattaa aaacaaacac atttaagata gacgcaaaga atccgattaa ataggattgg
43921 gtccaaatag aaagcacgat aataccgata aagaacatca gcaagataat ggcactggat
43981 tcgcttttat ctaagttgta aacccatata ccgagcaaga cacagatcgt ttgaattaca
44041 agcatcttca taatctctac agttaaacga gaggattttt gagcttgctt aggtgtattt
44101 gtaggtttgt ctgaatgaat ataatgaatc ggtacaattt ctaacttgaa atggtgtggt
44161 acgtgattaa tttgttcaat gagtgactgt ttaaagtaat ctttccaacg tggctgttct
44221 gactgtccaa ggactagctt cgtcacaaaa gcgagatcac accaatcggt taacgctttc
44281 gcaatatctt gtgcatacaa cactttgatt tctgcaccta acgctttggc gagcattaga
44341 tttttatgga cataatgatc ttgttttctg ctttctgacg gtgtctcaaa gacatcaata
44401 tacacagctg tgaatttagc atgttcttta taggcagcac gtcttgcttc tcgtatgacc
44461 cgttcgttat aaatactgcc gctgatagct accgcaatat gcggtgtaat gtcggtatgt
44521 ttagttttat attgttgtcc tttttgactc ataatatctg cgacagttct gagtgtaagt
44581 tcacgcagct ctgtcagatt ttcatacgta aagaaattag aaaaggctgt ttctaagcgt
44641 tcttttttat atacttttcc tgctttaagg cgctgaatca acatatttgg tgaaatatcc
44701 acaacttcaa aggcatctgc tgacgtaatg aattggtcgg gcacacgttc tgtaacttga
44761 atacctgtca ttaacgcaat ttgtccgctt agactctcga tatgttggat gttgagtgtt
44821 gtccagacat cgataccatg cgatagaatt tcttctatat ccttataacg ttttaaatgg
44881 cgctcttttg aaatgtttgt atgtgctagt tcatcaatta agaccacatc tggattagct
44941 tctatgattt tagagacatc tatatagtga aaggtgtggc tgccaaattt acggctggag
45001 gtcgaaattt caggcaattg ttgaaccagt gcattggttt cagggcgttg atggggttcg
45061 atataaccaa ttttaatatc tgcaccttct tgatactgat caataccatt tgataacatt
45121 tcatacgttt tacctacccc tgggctatag cctaaataaa tggtaagttt ccctcttttt
45181 ttatatgtac tttccatgag gcaccccctc ttaatcacat cataattaat atacaacata

45241 ttatccattt atctttctgt tttgttaatc atctttatag tttctttata actttcctta
45301 aatgttaata cttcgattag ataactcttg ttaagatttt gatgaaagta aggagggagc
45361 gcaatgatta cactattagc tgtcgttgtc atcgcattaa ttttattttt attttacgca
45421 ttaatttgga gtgaaaaatt ttaacagaga aagagggaag catcatgagt attgtgttgt
45481 ttttgattgt atttatcttg ctctcactca ttgtgagccg atatttatat tcagttgctt
45541 taaatgtgcc atctaaaata gatgttgttt ttaatccgat tgagaaattg atttatcaac
45601 tgattggcac gaaattagaa cacatgtctg ggaagacgta tatcaaacat tttttgttgt
45661 ttaacggatt gatgggcgga ttgtcctttg tattattgct tattcaacaa tggctgtttt
45721 tgaatcctaa ccataattta aatcaatctg tatcgttagc ctttaatact atggcatctt
45781 ttttgaccaa tactaactta cagcattatg caggtgaaac agatttaagt tatttaacac
45841 aaatgtgtgt catcactttc ttaatgttca cgtcagcagc gtcaggttac gccgtatgta
45901 ttgcgatgtt aagacgtttg actggaatga cagatgtgat tggtaatttc tatcaagata
45961 ttacgcgttt tattgtacgg gtgctcatac ctttcgcatt gatcatcagt ttgtttttaa
46021 tcagtcaggg cacaccgcaa acgcttaaag gtaatttggt gattgagaca ttatcaggtg
46081 tgaaacaaac gattgcatat ggaccgatgg cgtctttaga atctattaaa catttaggga
46141 caaatggtgg tggtttctta ggtgcgaact cttctacacc ttttgaaaat ccgacatact
46201 ggtctaatta cgctgaagct ttaagtatga tgttgattcc aggttcatta gtctttctat
46261 tcggtagaat gttgaaaact aaactacaga ttcatccgca tgcgattatg attttcgttg
46321 cgatgtttgt aatgttcatc ggctttttag tgacatgtct ctattttgaa tttgcgggga
46381 atccagtgtt gcatcactta ggtattgccg gtggcaatat ggaaggcaaa gaaacacgtt
46441 tcggtattgg cttatccgct ttatttacaa ccattacgac cgcttttact acaggaacag
46501 ttaacaatat gcacgatagt cttacaccgc taggcggcat ggttccaatg gtattaatga
46561 tgttgaatgc agtttttggc ggtgaaggtg ttgggctgat gaacatgttg atttatgtca
46621 tgttaacggt ctttatctgt agtttgatga ttgggaaaac accaagttat ttaggaatga
46681 agattgaagg taaagagatg aaactcattg cgctttcttt cttagtacat cctttactta
46741 ttttggtttt ttcagcacta gcttttattg tgccaggggc atcagatgcg ttaactaatc
46801 cgcaattcca cggtgtatca caagtgttgt atgagtttac atcatcttca gcgaataatg
46861 gctctggttt tgaaggatta ggagacaata cggtattttg gaacatttca acaggcattg
46921 tgatgttgct tgcacgatat attccaatcg ttttacaaat tttgattgta tctagtttgg
46981 taaataaaaa gacctatcag caacatactc aagatgtacc gattaataat ttatttttca
47041 gcagtgtatt gattatcttt attattttgt tgagcggctt aacgttctta cctgacttaa
47101 tgcttggacc aataggcgaa cagcttttgc tgcacgcata gataaaggag gattagaaaa
47161 tggctgaaac tactaaaata tttgaatcac atttggtcaa acaggctcta aaagacagtg
47221 tattgaagct ctatcctgtt tatatgatta aaaatccgat tatgtttgtt gtagaagtgg
47281 gcatgctgct tgccttagga ttaaccattt atccggattt atttcaccaa gaaagtgtat
47341 cacggctata tgtgttcagt atctttatca tattattact gacacttgtc tttgcgaact
47401 tctctgaagc attagctgaa ggtcgcggta aagcacaagc caacgcttta cgccaaacac
47461 aaactgaaat gaaggcacgt cgtattaaac aagacggcag ttatgaaatg attgacgcta
47521 gtgacctgaa aaaaggacat atcgtacgtg tcgcgacagg tgaacaaatc ccaaatgacg
47581 gtaaagttat taagggcctc gcaacagtgg atgaatctgc gattacaggt gaatctgcac
47641 ctgtaatcaa agaaagcggt ggagatttcg ataatgtaat tggaggaact tctgtagctt
47701 cagactggtt agaagttgag attacttcag aaccaggtca ttcattttta gataaaatga
47761 ttggtttggt tgaagggggct acaagaaaga aaacacctaa tgaaattgcg ttatttactt
47821 tattgatgac attaacgatt atcttcttgg tcgttatttt aacgatgtat ccattggcga

47881 aattcctgaa tttcaattta tccattgcga tgctgattgc tttggctgtg tgtttaattc
47941 caacaaccat tgggggatta ttatcggcta tagggattgc agggatggat cgtgtgacac
48001 agtttaatat cttggctaaa agcggacgtt ctgtagagac ttgtggtgat gtgaatgtct
48061 tgattttaga taaaacaggt accattacct acggcaaccg tatggcagat gcgtttattc
48121 cggtgaaatc atcaagcttt gaacgtttag ttaaagcggc ctatgaaagt tctatcgcag
48181 atgacacacc agagggacgt agtattgtga aattagctta taaacaacat atcgacttac
48241 cgcaagaggt cggagaatat attccgttta ctgctgaaac acgtatgagc ggtgtgaaat
48301 ttacgacacg tgaagtatat aaaggtgcac cgaatagtat ggttaagcgt gtgaaagaag
48361 caggggggaca tattccagtt gatttagacg ctcttgtcaa aggggtgtct aaaaaaggtg
48421 gcacaccgct ggttgtgctt gaagataatg agattttagg tgttatttat ttgaaagatg
48481 tcattaaaga tggactcgta gaacgtttcc gtgaattacg tgagatgggg attgaaacgg
48541 tgatgtgtac aggagataac gaattgacag ctgcgacaat agcgaaagaa gcgggtgtgg
48601 atcgctttgt ggcagagtgt aaacctgaag ataaaatcaa tgtgattaga gaagaacaag
48661 cgaaaggtca tattgttgcg atgacgggtg acggtacgaa tgacgcgcca gctttagcag
48721 aagctaatgt aggtttggca atgaactcag gaaccatgag tgccaaagaa gcggcgaatt
48781 taattgattt agattctaat ccaaccaaac tgatggaagt cgttctaatt gggaaacaat
48841 tattaatgac acgtggctca ctcactacat ttagtattgc gaatgacatt gcgaaatact
48901 ttgcgatttt accagccatg tttatggcgg ctatgcctgc gatgaatcat ttgaatatta
48961 tgcatctgca ttcacctgaa tcagcagtat tatctgcgtt aatctttaat gcgttgatta
49021 ttgtattatt gattccgatt gcgatgaaag gcgtgaaatt taaaggtgcc tcaacgcaaa
49081 ccatattgat gaaaaatatg ttagtttacg gcttaggcgg tatgatcgtg ccatttatcg
49141 gcattaagct cattgatctc atcatccaac tctttgtcta aaaggaggac aaaacaatgc
49201 agacaataag aaaaagttta ggactagtac tgattatgtt tgttttatgc ggatttatct
49261 tcccgctgac tgtcacagcg cttggacaag tattatttcc agaacaagca aacggcagtt
49321 tagtgaaaca agatggcaaa gtaattggtt caaagctcat tggacaacaa tggacagaac
49381 ctaaatattt ccatggacgt atcagtgcag tcaattacaa tatgaatgcg aatgaagtga
49441 aagaaagtgg cggacctgct tcaggcggct caaactacgg caattcaaat cctgaattga
49501 aaaaaagagt tcaagagact attaaacaag aaggaaaaaa aatttcaagt gatgcggtga
49561 ccgcttctgg ctctggttta gacccagata ttacggttga caatgcgaaa caacaagtaa
49621 aacgcattgc gaaagaaaga aacatagatg cttcaaaaat taatcacctt attgatgaaa
49681 acaaacaagc atcaccaatg gcagatgatt atgttaatgt cttaaaattg aatatcactt
49741 tagataaact ctaaataaac agggagtgag gtgagacatc catgtggttc attagcatta
49801 ttattttaat agcattccta attatattaa tgattgacga tttaattaaa acatgacaac
49861 ttcacgcaaa acaatatgaa ttggatgtaa tagagaaggg agtaccaaga aatactcagt
49921 ttattgagga taattattag gggagattag agaagaattc tctattcacg aacattactt
49981 taacagttgt aatcattggt ttcatcatat cgattaatag tacagtcatc ttgagatttt
50041 caacacacta ttatagctag ctaagatact tttaaattat gacttatttt tcatcattta
50101 cgatacatca ttctcgataa cttactattt gttatggtat ttatagattt aaagaaagtg
50161 aaggagatta agcaatgtaa cttggcttaa tctcttttg attatattta ttaaggcaaa
50221 gcttcatttc ccttaaatca agtatatctt ctgaataagc aaatgaaatc gtgacgccaa
50281 ctacagtgat aacttcgcct aaaacacaat gagtgtggtg ttttcacttt tcataaactc
50341 ctctaagttt gtgtttttaa actgtgatgg atttgaatgt ttttgaatta cgttgtcgcg
50401 tccttgggat tgcaatccaa gaacgtcagc ataatctaaa tcaattttga tttggtagaa
50461 gacaagagaa gtacttttgc ctgtatcatc tttcgcattt ctttttgttg tctttcgatc

50521 ccgatcggaa actaattttc ctgcattatc caaagcatca ataacatttt tcgtgtcttc
50581 gaattgactg tctgttaaca tcttatcgaa tacatttcgg ataattttta cttcgatgta
50641 gtcatcttta agtgagagga taccaaagtt ttgaatcata ttagcaagac gtgtttgttg
50701 tgaaaacttg cccaggtttt ccgcaataaa ctgtacgatt gtatccatcg ctttatctgc
50761 aagagaacgt tctgacaccg tttcattatg atagcttaat aaatagttct ttatgctgct
50821 aacgtttaac tgtaggtcta gtgattgatt taaaatatgg gctgttgttg ttagagttgc
50881 ataatgttta atcattcgaa taccagtgtt actggattca gttgatagta attgataaaa
50941 aacactgtct tctttttgaa aaagggaagt aacatcatta gtgttgtcta ataggaattg
51001 agcaattata ggtaaaacat gaccattatt ttgactaacc acttttttta tagtatctga
51061 gttttgagca cttgatgtga atttttcaga cacctcaata gtacgaacac gcaaaccatc
51121 atttttagct ggatttttga aaatactatg ttcagaagtt gaaatcactg atgtatccta
51181 gtttttaggt gttttgacat caccatttat atttgctctt tgacgtcctt gtccctctgc
51241 aaatgaatat agaagacctg ttgtatcatg aaatgttgcc gctgataatt catctaatac
51301 tatgggtaca ccataatttc cgctaagata tccttctagt gcatttcttg ttgcattcca
51361 actccggaat aggttttcag taccttttgt tgggttgcca gctaccgatg cggctaacat
51421 agcggctgtc gattttcctg ttgatgattg gccagtgaaa gagaaaatag ttcctgaaaa
51481 ctcgacgtta ttatgatact taaggaaagc agtgactaat gaagatacgc caaacaacac
51541 atctagttct aaagataagt tcccatggac ttcttcttta tacatttgta accattctga
51601 taaattgcct tttggtatta aatcgtactt gttatcacat ataatagaag tgctattagt
51661 aatctcatta gaaaaataag gctggtctaa tgaaattaat ggtccaagta atgtatgaag
51721 cattcctaca cctgagtata ttgttgataa aggtgtggtg cttctcattt gttgcagtac
51781 gaaccctaaa tctctaatgt gcttttcatt aatggtaaat ccatattcaa ttaaggaggg
51841 taattgcaaa gcagtaagga ttgaagcctt ttcgacaagc tctatattat tcaaatccga
51901 aagaaatact ttttctttac gagtcgcggg gtctataaaa cgatctgtga aagcaatcgg
51961 tccagataac attatttcaa actccgtacc cccatctttt ttaggaggga tggttttgta
52021 ccatcccgaa ggtttgagct tgaattcgtc agtttgatat agaatttttt ccattaacag
52081 tgaccccctt ggttgtaaga ggaaattata atgatatgtc ggtacttaag tgggttagtt
52141 cctataataa tgaaagggat gcttggtttg tactttatcc accaagcaaa gcgtcttcct
52201 gcttcacgtt gatcgttgat gttgcactgt tctctgaata actgtaatta taaggtttag
52261 taatggcaat tttttattta aacaaatttt tacgaatgga ttgtctgttt attaattcag
52321 caacgtttat tgaagaaaaa taagtgtctt ctgataattg caaagggttt aattcaatgt
52381 aaactgaatt aagggtaggg taggtatgcc tgtttttttgg ttcttttaag ttcaatcgat
52441 taataaaatt gttgaacctt tgttctaagt catacaaaaa catcagtaat tcattaggag
52501 aatgatttat tatgcaatcc atattttcgt ttatgcttga agttatacat ctaaaaacag
52561 tattggtttc ttcgtataaa aataacttat taatagaggt aacattgatt aagactgagt
52621 atacgtataa taagagtgac tctgacatct catttagata aataagactg tgatcattct
52681 tatttacgat tattatatgt gattgaatca attcaataat gtctagaaga agacttgaaa
52741 agtcgctgtt aatcttggtc tttgaaaaat catcgtgacc atttgtctta ttacttagat
52801 gtagttgttc gtattttata gtttggcggt tgaggaatta agcatttgat tattataaac
52861 tatctcaaaa attaaattac ctcctttttt cttcctcgat gaaggtttat ctaatcttcc
52921 aatatacagg tttttgaagt aaattataga tatgataatc attaattcag taaatgtata
52981 cttattaagg ggtggagctt tagctaactt atcgatagta ctgccgatgc gtgctttgaa
53041 actattcaaa ttgtcgtgac tgtctatatc taaatcttca aatttactag caagggtttg
53101 caaagtagaa attacaactt cttcttttgt ataatattta ttgaaaatat catcttcttt

53161 atcatcgctt tggagtttta aagtgcctgg ttctaagatg tcaaattttt tgtttcgatt
53221 ggtatttttt taaaatgtgc cttgtatttt tggggttgtt ccaaatgtgt atttgctaga
53281 ctacctgtaa ttgatattgc tttatttagc atttggtgtg tattaactgt atctgattct
53341 tctggaaatt taaatctaaa agtaaaatct ctactaccga atgtactttt tacggctaca
53401 gttttgtact tatgctcagg caatgtaatt aaaccatctt gatgcaggag gaaaagtgtg
53461 tcatcattta atttctcaat tgtattaaat aagtaattat gcatagcctc tttatacatg
53521 gcgaattgat aatttagatt atcccattct attagttgat ttaaaataca atatagacct
53581 tcattcaatc ggtttacgaa tatcaataca gcttcttctc gtccaacaag gcttattaac
53641 tccttcaagt catcctttaa ttttaatttt tctccttcaa aattgtattg agtgaagcct
53701 atagcctttt gaaaagtaac ttccatgaca gagttggttt cagaatattc taacgattca
53761 tttattgcaa aaatcaaatt ttttataaat atggaattac tcatggtttg taattgcata
53821 tactacccaa ctttcttatt tactttaaaa caaatataag ctaaagtagt tattatcata
53881 aagggaacgt acgttcttat ttagctgatt tctatgaaga gaggttatgg atattgaatg
53941 aagaaatctt acgcgaagta gcagatattt ttataggtga tgacagagat agcatttatg
54001 attataaaac tgggaacgaa ttagtgaggt tttttaatca ttactttaat aaaggggaca
54061 tatatcaggc tccgtttcca tctagatggc tatatgttgt gaaacatttg caaactctga
54121 ttcaggagag aaagatcaat caatttttca cattaatttt aagcaatcac tatattaagt
54181 atgaactgaa aattgacgag gttgaagcag caaagcaggc tgctaaagca cttaagttgt
54241 tcaacaaaag attaaatcat tatgggtact acataacagg aactaacaat gctagatatt
54301 ttatggataa ggatgaagat acggagtcta ttggttatgg agggtatgcg aatatttatt
54361 tacagaagac tacaggtctt gctgtaaaaa aattgaaaga ggagtatctt actgattctt
54421 caattaaaag taggtttaag agagaatttg atctcactaa atcttttgat acaaatccat
54481 tgttcattaa tgtgtttgaa tttaatgaat cagattattc atacacgatg gagttagctg
54541 atgaaacttt gaaagattac attgaaagca agacaattag tgagctagaa aaagtaaaga
54601 taataatgaa aattttaaaa gcgatgagtc aagcacatag tgaaaataaa atacacagag
54661 atatcagttc taaaaatgta ttaatgttta gaggaaaagt caaaatatca gacttaggat
54721 taggtaaaaa ccttgatgaa attcattcgc atcaaacctt tgatacaaac ggtgtaggac
54781 aatataaata ctgtgcaccg gaacagatgt atagtttaaa acaagcagat aaacaatctg
54841 atgtttttag tttaggaaga ttgataaatt ttattatgac tggaaatgta gttaacaacc
54901 atcacctatt tagaggtgta tctgataagg ctacgaacag tagtaaagaa tacagatttg
54961 aagatgcaaa tgaaatgttg aaaatgctgc agagaatttt agagtatcac agtagtgcaa
55021 agcacgtcga aaaatgtcaa gaaaagctga aaagaggagt gtttgatgat gaaagcgaag
55081 aatttattat gacacgaagt gatgaacaat tatgtcaaat ggttctaagt tctaataata
55141 atgagcaagc gtgtttaatt cgttatatgc aaaaaaacga atcttcagca tgtgatttaa
55201 tagagagtat taatagaaag tatcaagagt tttgtggaag gtttgaagac tacgatcctt
55261 ttgctaaatt agcatatatg attttatgta ataacttcag ttatagagtg aatgaaacag
55321 cagctagagt gctaaattat gttgcttggt ctgtaaatag attttcggca caagacttaa
55381 ttaaaggttt aattaataga ggagtggagc ctttgattga agaaaaatta aaggacaatt
55441 aaaaaaacct catcattaac tgatacgcag aggcgtatca taagt

//

LOCUS Staphylococcus 41806 bp DNA linear UNK

DEFINITION Staphylococcus Staphylococcus aureus strain ZY05

ACCESSION Staphylococcus

KEYWORDS .

SOURCE Staphylococcus Staphylococcus aureus
ORGANISM Staphylococcus Staphylococcus aureus
Bacteria; Bacillati; Bacillota; Bacilli; Bacillales;
Staphylococcaceae; Staphylococcus; Staphylococcus aureus.
FEATURES Location/Qualifiers
source 1..41806
/organism="Staphylococcus Staphylococcus aureus"
/mol_type="genomic DNA"
/db_xref="taxon:703339"
/project="hwwang_703339"
/genome_id="703339.289"
/genome_md5=""
CDS 1..480
/translation="MKITILAVGKLKEKYWKQAIAEYEKRLGPYTKIDIIEVPDEKAP

ENMSDKEIEQVKEKEGQRILAKIKPQSTVITLEIQGKMLSSEGLAQELNQRMTQGQSD

FVFVIGGSNGLHKDVLQRSNYALSFSKMTFPHQMMRVVLIEQVYRAFKIMRGEAYHK"
/db_xref="SEED:fig|703339.289.peg.1"
/EC_number="2.1.1.177"
/product="23S rRNA
(pseudouridine(1915)-N(3))-methyltransferase (EC
2.1.1.177)"
/transl_table=11
CDS 726..1031
/transl_table=11
/translation="MKGNIQCPRLIKGSLLDFVLETVDVFVNGSKNDCELVNFNYKEG
KVVSRYSTKFESNPKLRKKQLISTVKVVRYVIFDFEKILWGIGKVLLKFIILRQCLL"
/db_xref="SEED:fig|703339.289.peg.2"
/product="hypothetical protein"
CDS 1230..2093
/transl_table=11
/product="hypothetical protein"
/db_xref="SEED:fig|703339.289.peg.3"

/translation="MKHINQFSESTLAQLEKDQQHVFYVYCLMDPRNDECFYVGKGKG
NRIFKHKQDAQKKVLYEDIFIEENKDNLKFNRINEICSNDLNVLGYIISYGLTESEAF

SAENVLINFLNLTNKTTLTNMINGHGSKAYLVEDLENEFGYDSINLENINTNELILAV

KIRDAFLLDKDESKEYPINESKRDRNNLKSRTLGSWIIGKDKIHKIKYIIGINTGANN

AVVSAYEVSFEQAESIETNNGRMRYAFIGLSERDATLKKLNLYKKALPDLRFGSGSAT
AYINNGTMKVD"

CDS 2201..3676
/translation="MPSDDIVKKLYSKEDIRLELGLTPHKFNKKMETIAKLFKIDMKI

FHNYKGQDKNNQYTFNGVAKELIKVLLKSVDYYPVDINSKKFKQNGKSKKEMIENIDN

SSYMKYIYQLMKSINEIQYKRLIADIHMKDVYQNTKAWLNNGESINKKEQELYQYMTI

LPLHKRVELQNEVLKSIDETIFQFLAKEHRNNQIEENNELEAYTKAIKEGRNPKNDYE

LNHLLYKKNQLPLDSLIEDIWDYEETEYTELDWLIADMLKRSQRADSNFIESLEKKNK

LRKNIHKDSIKSISKLIDHELVKNNRKWDVAEFYKNQQFWNNSTLRRSSQNFGILYYK

KNRLHTMNQSNIIKKIKIIEANIESANKQPSYLDFFLQAKFDLERLESELMKYSINPK

YFNKINSDYVRYAIEDVHNAIIKIDRYISNDKVKLNYSNVQMVVRNEITEQGSYFVTQ
ALNTLSKVEESGYSFDNFMVGPFVEDFKKAIKNRRFGSKKD"
/db_xref="SEED:fig|703339.289.peg.4"
/product="hypothetical protein"
/transl_table=11
CDS 3902..5002
/product="hypothetical protein"
/db_xref="SEED:fig|703339.289.peg.5"

/translation="METRDKLMSLTQSDKTQQWLMDKSSNQDDIQQLQQQFSQQLDQQ

YNALLADEKAKLDQYVEVHQGLESLKEEIESEPITLNIDKLPDIKATMLERAKNDEHS

DKIEKLFDRLEQALNGTNRLYTQLSLIGTRTHRITTKNFNLQGLPKAVQHTILPSKFK

KVYTVDFKSFEPSVAAYMTQDSKLIDLLNQKDGLYDALLSELGLSDELRVFVKRAFIG

SFLFGGNFKNPKFKLNQYVSEVQWLDAVSQFTKVIELKKHVEKSNSMPMPYSIEHDMS

AFQGSSIMAIYVQTVASYIFKHILLKVYKAQCDQKTFKIIVPIHDAIMIECEDEEIAQ
NVGQLMKDTANQLFNGEFAHVTVEEIGGVDHE"
/transl_table=11
CDS 4995..5366
/product="hypothetical protein"
/db_xref="SEED:fig|703339.289.peg.6"
/translation="MNNDRGQSLHIPSSTPIKENNIYVATLHSVIQTDFSGEIKHQFT

YEIEVNNQIVYANRNIPTKPSANQLSIHDWLKRHSNYSASHENYEPYIDQKHLILLGQ
YNGNYYVQDVASLDAFGGVLS"

/transl_table=11
CDS 5363..7006
/db_xref="SEED:fig|703339.289.peg.7"
/translation="MNHILQMLSKLLSVAKEAIDRQGLIAILTIPVNNNDEIEETAQG

ETVYNELIDQLRLNIPKDTDYQPNIYSYFGIKKNPNDTVLMEMMIKVFHIKRFNSELF

IFKVNGWQKINGDELQGLISKMIQVLLVDYKPSLSTLKNVVDGLQKSTDVEELVENER

YIGCGENMFDLNTFQVVKNSIDIFPKTRLNLSLSTNDVITDKIPPYFKQYMLQLANYD

DDLQYFLFQHTAVLLTADTKYRRGLILYGGAKNGKSVYIELVKSFFYSKDIVSKPLNE

LEGRFDKESLIDKSLMASHEIGQSRIQEKIVNDFKKLLSVESMHVDRKGKTQVEVILD
LKLIFSTNAILNFPPEHAKALERRINIIPCEYYVEKADTSLIDKLQSEKKEIFLYLMY

VYQQIVKADIEYLENSRVTEITHDWLNFGYEFVSSRSVSNANQKACINLLRKLIEIKS

GSRIKVSELNKVINEEIKVSSQVIKQLIQANFDTQTKLYNGYDYWIDLGWKEANKKEI
HDISEKDNIISLDKNENITDDEALDEENLDFDWEDFDDE"
/product="putative primase"
/transl_table=11
CDS 7232..8908
/transl_table=11
/product="Cassette chromosome recombinase B"
/translation="MKGKIALYSRVSTSEQSEHGYSIHEQEQVLIKEVVKNFPGYDYE

TYTDSGISGKNIEGRPAMKRLLQDVKDNKIEMVLSWKLNRISRSMRDVFNIIHEFKEH

DVGYKSISENIDTSNASGEVLVTMFGLIGSIERQTLISNVKLSMNAKARSGEAITGRV

LGYKLSLNPLTQKNDLVIDENEANIVREIFDLYLNHNKGLKAITTVLNQKGYRTINQK

PFSVYGVKYILNNPVYKGYVRFNNHQNWAVQRRSGKSDKNDVILVKGKHEAIISEEVF

DKVHEKLASKSFKPGRPIGGDFYLRGLIKCPECGNNMVCRRTYYKTKKSKERTIKRYY

ICSLFNRSGSSACHSNSINAEVVERVINVHLNRILSQPDIIKQIASNVIEELKQKHSN

QTEIKYDIDSLEKQKAKLKTQQERLLELFLDDQMDSEMLKAKQSQMNQQLEVLDQQIK

EAQQANQSQDEIPNFDKLKGRLILMITRFSVYLRKATPEAKNQLMKMLIDSIEITTDK
QVKLVRYKIDESLIPQSLKKDWGSFFMPKFQFEIYGQNDYFIDQITTFTT"
/db_xref="SEED:fig|703339.289.peg.8"

CDS 9014..9352
/product="FIG01108299: hypothetical protein"
/translation="MTLEQQLKHYITNLFNLPRDEVWHCESIEEIADDILPNQYVRLG

PLSNKTLQTNTYYSDTLHESNIYPFILYYQKQLIAIGYIDENHDMDFLYLHNTIMPLL
DQRYLLTGGQ"
/db_xref="SEED:fig|703339.289.peg.9"
/transl_table=11
CDS 9448..9759
/transl_table=11
/db_xref="SEED:fig|703339.289.peg.10"

/translation="MNRYITRGIANNLPNILQHQLWQLVSEREQEQTKDNTSVDYFHI

FQFNKHRNQLYIKHKQERPEYAKIHKANYSKAININKVYIIREDDVDLSYYVMLLPEE
Y"
/product="FIG01107894: hypothetical protein"
CDS 9775..10281
/db_xref="SEED:fig|703339.289.peg.11"
/translation="METIKSTLKTEAIFSDDKQHRYLLKKTWNSEKQSITIITMYPHY

DGILNIDLTTQLIMNKVSEMDAFGSINFVNLYSNITTPINLKHLENAYDKHTDIQIMK

AVKESDEVILAWGAYAKKPGVEARVNEVLEMLKPHKKKVKRLMNPETNEIMHPLNSKA
RQKWILKV"
/product="hypothetical protein"
/transl_table=11
CDS complement(10430..11104)
/transl_table=11
/db_xref="SEED:fig|703339.289.peg.12"
/translation="MNYFRYKQFNKDVITVAVGYYLRYTLSYRDISEILRERGVNVHH

STVYRWVQEYAPILYQIWKKKHKKAYYKWRIDETYIKIKGKWSYLYRAIDAEGHTLDI

WLRKQRDNHSAYAFIKRLIKQFGKPQKVITDQAPSTKVAMAKVIKAFKLKPDCHCTSK
YLNNLIEQDHRHIKVRKTRYQSINTAKNTLKGIECIYALYKKNRRSLQICGFLPCHEI
SIMLAS"
/product="Transposase for insertion sequence-like element
IS431mec"
CDS 11362..11529
/transl_table=11
/product="Hydroxymethylglutaryl-CoA synthase N-domain
homolog (EC 2.3.3.10)"
/EC_number="2.3.3.10"

/db_xref="SEED:fig|703339.289.peg.13"
/translation="MLESREQLSVEEYETFFNRFDNQEFDFERELTQDPYSKVYLYSI
EDHIRTYKIEK"
CDS 12486..13229
/product="Glycerophosphoryl diester phosphodiesterase (EC
3.1.4.46)"
/EC_number="3.1.4.46"
/translation="MLTVYGHRGLPSKAPENTIASFKAASEVEGINWLELDVAITKDE

QLIIIHDDYLERTTNMSGEITELNYDEIKDASAGSWFGEKFKDEHLPTFDDVVKIANE

YNMNLNVELKGITGPNGLALSKSMVKQVEEQLTNLNQNQEVLISSFNVVLVKLAEEIM

PQYNRAVIFHTTSFREDWRTLLDYCNAKIVNTEDAKLTKAKVKMVKEAGYELNVWTVN
KPARANQLANWGVDGIFTDNADKMVHLSQ"
/db_xref="SEED:fig|703339.289.peg.14"
/db_xref="GO:0008889"
/transl_table=11
CDS 13326..13754
/transl_table=11
/db_xref="SEED:fig|703339.289.peg.15"
/translation="MKYDDFIVGETFKTKSLHITEEEIIQFATTFDPQYMHIDKEKAE

QSRFKGIIASGMHTLSISFKLWVEEGKYGEEVVAGTQMNNVKFIKPVYPGNTLYVIAE
ITNKKSIKKENGLVTVSLSTYNENEEIVFKGEVTALINNS"
/product="MaoC domain protein"
CDS complement(13800..15806)
/transl_table=11
/db_xref="SEED:fig|703339.289.peg.16"
/db_xref="GO:0008658"
/db_xref="GO:0046677"
/translation="MKKIKIVPLILIVVVVGFGIYFYASKDKEINNTIDAIEDKNFKQ

VYKDSSYISKSDNGEVEMTERPIKIYNSLGVKDINIQDRKIKKVSKNKKRVDAQYKIK

TNYGNIDRNVQFNFVKEDGMWKLDWDHSVIIPGMQKDQSIHIENLKSERGKILDRNNV

ELANTGTAYEIGIVPKNVSKKDYKAIAKELSISEDYIKQQMDQNWVQDDTFVPLKTVK

KMDEYLRDFAKKFHLTTNETESRNYPLGKATSHLLGYVGPINSEELKQKEYKGYKDDA

VIGKKGLEKLYDKKLQHEDGYRVTIVDDNSNTIAHTLIEKKKKDGKDIQLTIDAKVQK

SIYNNMKNDYGSGTAIHPQTGELLALVSTPSYDVYPFMYGMSNEEYNKLTEDKKEPLL

NKFQITTSPGSTQKILTAMIGLNNKTLDDKTSYKIDGKGWQKDKSWGGYNVTRYEVVN

GNIDLKQAIESSDNIFFARVALELGSKKFEKGMKKLGVGEDIPSDYPFYNAQISNKNL

DNEILLADSGYGQGEILINPVQILSIYSALENNGNINAPHLLKDTKNKVWKKNIISKE

NINLLTDGMQQVVNKTHKEDIYRSYANLIGKSGTAELKMKQGETGRQIGWFISYDKDN
PNMMMAINVKDVQDKGMASYNAKISGKVYDELYENGNKKYDIDE"
/product="Penicillin-binding protein PBP2a, methicillin
resistance determinant MecA, transpeptidase"
CDS 16054..16197
/transl_table=11
/db_xref="SEED:fig|703339.289.peg.17"

/translation="MNYFRYKQFNKDVITVAVGYYLRYALSYRDMSEILRERGVNVHH
LTV"
/product="Mobile element protein"
CDS 16477..16728
/product="Mobile element protein"
/translation="MAKVIKAFKLKPDCHCTSKYLNNLIEQDHRHIKVRKTRYQSINT
AKNTLKGIECIYGLYKKNRRSLQICGFSPCHEISIMLTS"
/db_xref="SEED:fig|703339.289.peg.18"
/transl_table=11
CDS complement(16788..17216)
/product="PhnB protein; putative DNA binding
3-demethylubiquinone-9 3-methyltransferase domain
protein"
/translation="MKFQVSPYFLITQNAEGLKKLYEELFGAKTLYIQRIKDRPIDKK

LNIDENDLNKIDQCVLQFGDIKIMIADDTEGLPITQGNNISLCLTFDQVEETKHVYDQ
LIEKGSEVLKTFSPEFYTEGYGYVRDPYGISLHLFTKRKN"
/db_xref="SEED:fig|703339.289.peg.19"
/transl_table=11
CDS 17297..18226
/transl_table=11
/db_xref="SEED:fig|703339.289.peg.20"
/translation="MKKSVRLYNMIEYCNENRNFKLNDLMSEFNISRSTALRDIKEIE

ALGVPLYSNPGKNGGYTIIGNRDQTKIAISDEELKALVFTLSSISNVSNLPFQTEYQE
ILKKLYNNSNKKELINQYNDLFQYFNEDKYQFKSYKLFNEIIRLIIENKSFETCYSQN

YIKEQYKGIGIMYKNHQWYFVVANIESKLVNLLNISKIKELYEMGETQECNDITMQNF

QQFMVKNETAIDILIRSNVMGLNILKGYLWSDYMIENIDEETYLFKSKVNAKDIDFIA
KLIVTGGVNVKVETPNSLKNAVKVELTKIINMY"
/product="Transcriptional regulator, YafY family"
CDS 18388..18870
/transl_table=11
/translation="MNKNITALKSTENTTYMQYTVHIDDQIINNIKECTKKFKILPME
DKIPLSPLLQPEYAGEVQDFISTYEQFMINFGKVILDSQGIKIQFESESLSSIQRGIQ

EHCYLNERTNDIDVTKEWYLCKFSNQILEEDKNQLYNALKQLMNDSKNKKQAFWTDPN
"
/db_xref="SEED:fig|703339.289.peg.21"
/product="Similarity"
CDS 18938..19252
/transl_table=11
/product="Mobile element protein"

/translation="MKRVSYSVETKYKAVEMKIAGFSTKEIMKELNIKNRTQVETWWR

WYRNEESYRFSQHVGKQYTYGKGLEELSEIERLKLENKRKDIELDILKKYKALERKWY
QQ"
/db_xref="SEED:fig|703339.289.peg.22"
CDS 19237..20079
/translation="MVPAVVIDLVDQLKVKYSIKLILEVLNIPKSTYYRWKNKTYKND

TVTQKVIELCEANHYTYGYRKITALINQCYTSPINHKRVQRIMQKHHLNCRVRPKKTT

RIGKPYYKTDNLLQRQFKASCPMEVLTTDITYLPFGHSMLYLSSIMDIYNGEIVAYKI

DDKQDQSLVNDTLNQIDIPEGCILHSDQGSVYTSYAYYQLCEEKGIIRSMSRKGTPAD

NAPIESFHSSLKSETFYINNQLNSSNHIVIDIVEKYIKNYNNNRIQQKLGYLSPVKYR
ELAA"
/db_xref="SEED:fig|703339.289.peg.23"
/product="Mobile element protein"
/transl_table=11
CDS 20086..21651
/transl_table=11
/translation="MFLLSSRLRGSVPFQEVFKVHIDIYLYNKEKSEYKYQVSSYFNL

VKENPKITYKKRHLQEKQGVKGTTFTNKLKLLNKMYGVDVAKYQPFYNSNNPEYERGQ

FGERYISQRSNYEFNRLQYQIIDMLSKILDKHPLPKSDNNYKHIPTIEKAILSGDSHS

FYEYFEDIMKEIISMENSSLKEKLLTDFTYQSQCRWYSESEKLKLQLESFMHKVLESN

YYEGNKLYRMLSHAIEETINEADEDKVHFNYFKDYFLTDGGVKNWEQISEKITEFNGK

VINDIQNEYNKIQFNNANRNQKLNFNYLYHCFEFSNNLVKARVNGNRGYILYFVDKYK

IKMPFLDQLLINYLNQEIGQESINYNMQTLFEKERYDRSSTIEKLVATSKFKYEKDDS
DLFKQLFNDVENSIDRLGIYLLNNGINSNDENARYYRSFLKELSRIKSKLTPFSLEIS

KSSGREQHYPDDAIDDKDERRKNKEETYHAFDDKSDIDSKLKNKINVSIDNLLSVKIK
SNLYDISPNTKKQ"
/db_xref="SEED:fig|703339.289.peg.24"
/product="Similarity"
CDS 21846..22955
/transl_table=11
/product="hypothetical protein"

/translation="MYQANIRDLITKLPQSNKTEHFLMNKFSNQDKVQQLQRQISQQL

DQQYNELLANEKAKLDQYVEVHHNLEPLKKEIESESINLDTDKLPDIKATMLEKAKND

EHFDKIEQLFDRLDQSLNGTNRLYTQLSLIGTRTHRITTKRFNVQGLPKLVQQMILPS

QFKKVYTIDFKSFEPSVAAYMTQDEQLIDYLNHEEGLYDALLRDLSLSKEKRVSVKRA

FIGSFLFGGRYSSSKFKINQEVSEINWLQVMSKFKKVIEFKEQVEKYKTMPTPYGIEH

DMSAFQGSSIMAIYVQTVASYIFKHILLEVYKAQCEKKTFKIIVPIHDAIMIECNDKG
IAQNVAQLMKDTANQLFNGEFAHVTVEALGGIDNE"
/db_xref="SEED:fig|703339.289.peg.25"
CDS 22948..23316
/translation="MNNDRGKSLQIPQSTLLKEGSIYVATLHSVYEKNFSGDIKHQFT

YEVELNQETHYVNRNITVKSMSHQLSIADWIKRHSNYNVNHINYDPYIDRKHLVLVGQ
YNGNYYIQDVAPLDEFGGVL"
/db_xref="SEED:fig|703339.289.peg.26"
/product="hypothetical protein"
/transl_table=11
CDS 23316..24932
/translation="MNHILEMLIKLLKVGMEAIDRKGLIAILTSSIGNDEMDDSEQAV

MVYNELIDKLQLNIPKDVDYRPNIYSYFGIQKKPNDTILVEMMICIFHIKRFDSELFV

FKDKGWQKVSEDELQGLISKMIQVLLVDYKPSLSTLKNVVDGIQKSTDIEKLVEHRQY

IGCGRNMFNLKTFKVVDNDLEIFPKTRLDLELDINDTITDKIPPNFKQYMLELANYDH

DLQYFLFQHMAVLLTADTKLRRGLFLYGTAKNGKSVYIKLVKSFFYSNDIVSKTLNEL

GGRFDKESLIGKRIMASDEVGKANIDEATVNDFKKLLSVEPIHADRKGRTQVEVTLDL

KLIFNTNAVLNFPSSHAKALERRIAVIPCEYYVEKADPDLIEKLQDEKKEIFLYLMYV

YKQIVKNDIEYLQNDRVTEISHDWLNFGYEFVSSKSANIAHQKACINLLRKLIEIKPG

SRIKVSRLNEVIRDEIKVSSQVINDLVQANFNVQSRLNNGYKYWVDLGWKETDKKDDM
ISFDKNENVTDDEFLYEDDLNLGWEDFDDE"
/db_xref="SEED:fig|703339.289.peg.27"
/product="putative primase"
/transl_table=11
CDS 25157..26836
/transl_table=11
/product="Cassette chromosome recombinase B"
/db_xref="SEED:fig|703339.289.peg.28"
/translation="MKGKIALYSRVSTSEQSEHGYSEKEQEQLLIKEVMKNFPGYDYE

TYTDSGISGKNIEGRPAMKRLLQDVKDNKIEMVLSWKLNRISRSMRDVFNIIHEFKEH

DVGYKSISENIDTSNASGEVLVTMFGLIGSIERQTLISNVKLSMNAKARSGEAITGRV

LGYKLSLNPLTQKNDLVIDENEAHIVREIFDLYLNHNKGFKAITTILNQKGYRTINQK

PFSVFGVKYILNNPVYKGYVRFNNHQNWAVQRRSGKSDKNDVILVKGKHEAIISEDVF

DQVHEKLASKSFKPGRPIGGDFYLRGLIKCPECGNNMVCRRTYYKTKKSKERTIKRYY

ICSLFNRSGSSACHSNAINAEVVERVINVHLNRILSQPNVIKQIASSVIEELKQKHSK

QTEIKYDIDSLEKQKAKVKTQQERLLELFLDDEMDSEMLKAKQSEMNQQLEVLDQQIK

EAKQANQSQDDIPNFDKLKARLILMITRFSVYLRKATPEAKNQLMKMLIDSIEITTDK
QVKLVRYKIDESLIPQSLKKDWGSFFIPKFNFVINVTKKNRIENLSLLPLF"
CDS 26925..27263
/translation="MTLSKQLKTYITERFKLNYQETWACETIDAVAEDVLPEKYIKNS
PLEHKILNTFTYYNDELHEISIYPFLCYLDKELVAIGYLDNFDLDFIFLNDTHQVIID
ERYLLQKGGE"
/db_xref="SEED:fig|703339.289.peg.29"
/product="hypothetical protein"
/transl_table=11

CDS 27266..27355
/product="hypothetical protein"
/translation="MNWIKVAQLSVTVINEVIEIMKEKQNGGE"
/db_xref="SEED:fig|703339.289.peg.30"
/transl_table=11
CDS 27357..27668
/transl_table=11
/product="hypothetical protein"
/db_xref="SEED:fig|703339.289.peg.31"
/translation="MKINRYITRGVSEHLSLDLQILLWNMVKDRDNQLHTDYLHIFKL

QEDDNILSITHEQEQPAYKLEYHYTNYVKNQNALPKKVYVIREDDVDAFYYVMLLPEE
Y"
CDS 27683..28186
/transl_table=11
/product="hypothetical protein"
/translation="MNTIKNTIHTEAIFSDDEQHRYLLKKTWDEKKPACTVITMYPHL

DGVLSLDLTTVLILNQLANSEQYGAVYLVNLFSNIKTPENLKHIKEPYDKHTDIHLMK

AISESDTVILAYGAYAKRPVVVERVEQVMEMLKPHKKKVKKLINPATNEIMHPLNPKA
RQKWTLK"
/db_xref="SEED:fig|703339.289.peg.32"
CDS 28200..28421
/transl_table=11
/product="hypothetical protein"
/db_xref="SEED:fig|703339.289.peg.33"
/translation="MNHETKQSDWRTVASCLASQNYISIVKGLVHHFTAIEDEEILDK
IYDDFMNDDSITTVLNNDLQTIINHYLSK"
CDS complement(28496..31615)
/transl_table=11
/product="Type I restriction-modification system,
restriction subunit R (EC 3.1.21.3)"
/EC_number="3.1.21.3"
/db_xref="SEED:fig|703339.289.peg.34"

/translation="MNFQFNEDDLEQVALEWLQSLGYDYKKGNEISMTSLTPERKSDK
DVVLHERLEKALRKINSDIHPRFIEKALHELTLEKSPNLLENNLTFHENLINGIEIED
YDDEGQSIVEIVKIIDFEYPQNNDFLAVNQLTIVNGDYKKRPDIVLFINGLPIVVIEL

KNSTNETVGVEDGYHQLETYKMRIPQLFTFNEVLVTSDGINTKAGSLTANYDRFMTWR

SKDGQTESSPGLASLDVLIHGMLNPETLLDLIRYFVLFQDDGKGHISKILAAYHQYYA

VNKAVDRALLASSEQGDGKGGVIWHTQGSGKSLTMVFFSGKLIQMLNNPTLVVVTDRN

DLDNQLYSTFVKSKGRSGKGLLRQTPKQAETRKELKSLLSVESGGIVFTTMQKFEPEQ

NETTMAALTERKNVIVMADEAHRTQYGFNAKYDDKGEGIKYGYAKYLRDALPNATFVG

FTGTPVASTDKNTQMVFGNYIDVYDMTQAVADGSTVKIYYESRVIPLNLPQSLDLDEA

YNDITEDQEEDVKQRLKSKWSRIEALAGAKPRVEALAKDIIQHFETRQQAMKGKGMIV

TMSRRIAVDLYDEIIRLKPEWHSDDDDKGVIKVVMTGSSSDPTSFQRHIGPKKRRNLL

EKRMKDVDDELQLVIVRDMWLTGFDVPSMHTMYIDKPMKGHNLMQAIARVNRVFKDKP

GGLIVDYVGIAESLKEALKEYTESDQAQTAIDTDKAVELMLLKYDVIQDMLYNLDYSK

FNSDKKSERYYTISNTMDYVIGLGEDERQRFIKTVTELGKAFALCATEPTAQELNDEI

AFFKAVKAGLVKLLQPPKEGKTRKTPAEVEAEINQLVSQSVVTEDVIDVYQTLGLEQP

DLSILSDDFLKDVEGLKQKNVAVELLNRLLKGQVKSLMKTNATVSKRFSEMLGNSINK

YNSRSIETSKVIEELIQLAKDIKQEQQRGNELGLNSDEIAFYDALASHETAKEAMGDK

ELRAIAHELTKTVKENMGVDWSKRDSAKAKMRVAVRRLLKKYGYPPDLQKMAVEQVVE
QAELMANNQ"
CDS complement(31599..32900)
/transl_table=11
/translation="MEYNTIFFGDVAKIKNGYAFKSKEFQEKGIPVIKIKNIISPIVD

TKDSQKVSIKTYEKTKGFSLKKNDILISLTGSGVNQMSSAVGKVGRIEFDYPALQNQR
VGKFELKYSNSADLDFLFYYFLQPKITEYLVRNSTGSANQANINSKLIETVKIPNFSL

IKQKSISKFLNQITRKIETNQKMIANLKELSQTLFKHWFVDFEFPDEDGNPYKSSGGE

MIDSELGKIPSNWKIYKLKDIASHKKETFNPKKSEEVTVKHFSLPAYDNEEQAIEEEV

NKIKSNKWIINNNCVLFSKMNPDTKRIWLPVIDNKKLNVASSEFVVMESPNNKINSFI

YNICLNSQFIDYLKANTTGSTNSRQRVKPTIAVNYKLAIEDSIVKKYSEIITPYMEEM
KILRSEIGKLTQLRDTLLPKLMSGELEISDDIEVNSDELSI"
/db_xref="SEED:fig|703339.289.peg.35"
/product="Type I restriction-modification system,
specificity subunit S"

CDS complement(32890..34404)
/EC_number="2.1.1.72"
/product="Type I restriction-modification system, DNA-methyltransferase subunit M (EC 2.1.1.72)"
/db_xref="SEED:fig|703339.289.peg.36"

/translation="MATIGFEEKLWQAADKLRGSMDAAEYKNVALGLIFLKYVSDSFE

EKYEELKQDPYADEEDQDEYLAENIFWVPKEARWQYINDNAKKPEIGQIIDKAMIAIE

NENESLKGVLPKEYARPALDKEKLGDIIDLFTFKVGDTESRKQDVLGRVYEYFIAKFA

SAEGKNAGEFYTPSSIVKLLVEMIEPYKGRIYDPCCGSGGMFVQSERFVEKHQGRLDD

IAIYGQESNPTTWKLAKMNLAIRGIDNDLGERNADTFHNDLHKGLKADYILANPPFNA

SDWGQERLLDDYRWQFGIPPKGNANYAWIEHMISKLAPNGTAGFVLANGSMSTSGKDE

LEIRKNLIEQDLVECIVTLPGQLFYSTQIPVCLWFISNNKGQNGKKERENEILFIDAR

EIGHMVSRTLKEFSDEDIQKVAQTYHAWRGTNDKPYEDIAGFCKVANLEEVKNNEYIL
TPGRYVGLADVEEDEEPFEQKMERITSELSEQFAKSKELEDQIRKSLEGLGYGV"
/transl_table=11

CDS 34600..34746
/product="hypothetical protein"
/translation="MDGVTNVKIHILTFVKIVVIVDIVAIVNTRLDKLGIFNSINKKF
KCSQ"
/db_xref="SEED:fig|703339.289.peg.37"
/transl_table=11

CDS 34846..36909
/product="FIG131328: Predicted ATP-dependent endonuclease of the OLD family"
/translation="MYISSVKIKNFRNFDNIDIDFHEGVNVLIGHNNSGKSNLLRALS

LIFDGSVRKQLSVEDFNNSLTKESLKKEAPKIVISVHITQSENERLMSDELITVSNWL

TKLEEPYEARIQYEFFLPKDEEKNYIDLVKNIDEKEEIWNLIRSQFIRLYVNKIWVGN

PEHQIPIDNDSLNKFDFQFLDAIRDVERDMFSGKNTLLKSVIDFFIDYDIKSDETITE

EEQKEKLEERRKEFSDNSSDLIETIHKRLDSGNQKILSYTNGIGASYDKSTPDFKGNL

TESEIYTVLQLIIKHETGMTLPITHNGLGYNNLIFMALLLSKMQADSDGNFLGSNAKV
FPILAIEEPEAHLHPTMQNEFIKFLKNNIREKKVKQIFITTHSTHISSSTNIDDIICL

YTDDSKTNVSYPGKVFDKNNQSKKYVQRFLDATKSNMLFAEKVIFVEGIAEQLLLHIF

ADYLNKPLEKNHVAVINVGGRYFNHFLSLFDSNNKYAINRKVSCITDLDPMRKEKNEN

GQKNNFKACYPFEYEMDLDKFDYSTNNSLDKYLNNEHSNIRVFVQPNKYGKTFEYQLM

FDNPSLKLLLTDSISNSQELAELMDHYEENESLQKLIDTLSPSNENQRIIKSLKDTND

SWNEDNKKKALIASRYLNSIGKGENALELASVLKDNLELKGQSDYEDFVVPKYIEGAI
RWVCE"
/db_xref="SEED:fig|703339.289.peg.38"
/transl_table=11
CDS 36911..38659
/transl_table=11
/product="ATP-dependent DNA helicase UvrD/PcrA,
clostridial paralog 2"
/translation="MIINAQTKLNIEQDFKIQAGPGAGKTEFLVNHIKNVIQTSEKLE

RMKKIACITYTNTAAQTVLQRLGKSVSNRVDISTIHSFLYRNVVKPYCSFLPKEYGIC

VQKLKGHSDPIVINKYVNAWLKTEYFTKLKPPSNRNQLLKMPVLKKALQNWLLSMKLS

YENNDVQFICDNTKAQAIDKKSKKRMGINKSNLNILEKQLLNYKKIYWEKGIIDHDDV
LFFSYVLIRDYPFILTILCAKYPYFFIDEFQDTSQLQDFIIDKIRQKGCIVGVIGDKA
QAIYSFQGARVSLFEKFKVDLINSHTILENHRSSYQIVTFLNSIREDITQTVYSGIEN

DKVTILIGNRIRSYTKVVNICNKESLVTLSRDNITSNAMRIKLEGNNFDKKLLEKFQE
IDSNSKRRNTIISLIKAIELAKNTKYKDALKIVESIYKDENNPKKIALNSLINMINKY

DKYGNGTLMNFYNTLCKTLDIKLSGFRKGQVKDFYENTLYKNMVICTNIVEDTSNHIT

IHKAKGAEFENVFVIGNKDTLSLLLKPDLMNDEEHRIFYVAMSRAKKKLFLQFDYLDN
NEQKKIKERYDVSIIQ"
/db_xref="SEED:fig|703339.289.peg.39"
CDS 38878..39051
/translation="MQDVAINADHKVFFLMIIFNVIMLVSSIIQMILGKGRRITPAKP
TKDAKLHLDNQSS"
/db_xref="SEED:fig|703339.289.peg.40"
/product="Multidrug resistance protein"
/transl_table=11
CDS complement(39119..40543)
/transl_table=11
/db_xref="SEED:fig|703339.289.peg.41"

/translation="MEMEFNENNIDLETIIRDEVNKYLSRDIGDLPATQQAPLELREK

YEKMEVPNKGRDIYEVLKDLNNEVLNYLYRPNHPRSFSFIPGPASRLSWLGDILTTAN

NIHASNFANATLPINIERNLINYLVGKIGYEIKPAGGVFVSGGSMANLTAIVAARDAQ

VEMEDIKKATVYLTSQTHHSVGKALHVAGFLKNNIRRIEYNDDFTMNTDSLKAAIDKD

IEEGYKPAMVIATAGTTNTGSVDDFTTLGDTCDHYNLWLHVDGAYGLSHILSDKAQHL

FKGIERSDSASWDAHKLLFQTYSCAMVIVKEKNHLLQSFGEDAEYLDDIASDDDVIDP
EMLGIELTRPARALKLWITLQVIGEDEIIERIKYGEGLAEYAEEYVSGLDNWRIISHA

NLSIVNFRYENSDLTEAQNNQLNSMAAQKIADSGYAIAYTTVLNNQRVIRLCTTNPLT
THDDIRETINRLDQYIGDYDMKDA"
/product="Decarboxylase, pyridoxal-dependent"
CDS complement(40626..40838)
/transl_table=11
/product="D-3-phosphoglycerate dehydrogenase (EC
1.1.1.95)"
/EC_number="1.1.1.95"
/translation="MRSSSPVLRGSYRIPKDKHQYKLKITDDNLQVDYPRQFLKGAHT
PSVKAQHVIHWGDTFWVSSLWLAWFY"
/db_xref="SEED:fig|703339.289.peg.42"
/db_xref="GO:0004617"
CDS 41488..41655
/db_xref="SEED:fig|703339.289.peg.43"
/translation="MKFKSEKLIIANQTKVINYRIQVLLKPQTNIETQTQVKNNTLSE
TDEQSNADLVQ"
/product="hypothetical protein"
/transl_table=11
ORIGIN
1 atgaaaatca ccattttagc tgtagggaaa ctaaaagaga aatattggaa gcaagccata
61 gcagaatatg aaaaacgttt aggcccatac accaagatag acatcataga agttccagac
121 gaaaaagcac cagaaaatat gagcgacaaa gaaattgagc aagtaaaaga aaaagaaggc
181 caacgaatac tagccaaaat caaaccacaa tcaacagtca ttacattaga aatacaagga
241 aagatgctat cttccgaagg attggcacaa gaattgaacc aacgcatgac ccaagggcaa
301 agcgactttg tattcgtcat tggcggatca aacggcctgc acaaggacgt cttacaacgt
361 agtaactacg cactatcatt cagcaaaatg acattcccac atcaaatgat gcgggttgtg
421 ttaattgagc aagtgtatag agcgtttaag attatgcgtg gagaggctta tcataaataa
481 aactaaaaat tagattgtgt ataatttaaa aatttaatga gatgtggagg aattacatat
541 atgaaatatt ggagtatacc ttgcaatatc atacgatgtt tatagagtgt ttaataaacc
601 atttttcaac tattgatgat ctagaatata taataactgt acaaattata ttgattatgg
661 aactacaatt caattaagaa attgatgatg aaattttaaa tttaaactaa tggaatcaag

721 aaagaatgaa aggaaatata caatgcccac gattaataaa aggaagttta ttagattttg
781 tgttagaaac agtagatgtt tttgtaaatg gaagtaaaaa tgattgcgaa ttagtaaatt
841 ttaattataa agaaggaaaa gtagtttcac gttatagtac aaaatttgaa agcaatccta
901 aattaagaaa aaagcaattg atatcgacgg taaaagttgt aaggtatgtt atttttgatt
961 ttgagaaaat tttatggggc atagggaaag ttttattgaa gttcatcata ttaaggcaat
1021 gtttactata agaaagttga aatacactca gaaacagact ggattcccgt ttttttcaaa
1081 ttggcacaaa atgatacata ggcccaccta tatattgaag atttaagtta aacgataaat
1141 aatatttttc tcttttgtat agaaattttt ttgggaatga tattaattag tataaaaatg
1201 aatatgtcta caaagaagga gagattttat tgaaacacat taatcaattt tctgaatcga
1261 cactagcaca actagaaaaa gatcaacaac acgtatttta tgtatactgt ttgatggacc
1321 caagaaatga tgaatgcttt tatgttggta aaggtaaggg gaatagaata tttaaacata
1381 aacaagatgc gcagaaaaaa gtattgtacg aagatatatt tatagaagaa aataaagata
1441 acttgaaatt taatagaatt aacgaaattt gtagcaatga tttgaatgtt ttaggatata
1501 taatcagtta tggattaacc gaatctgaag cattttctgc agaaaatgtt cttataaact
1561 ttcttaacct aacaaataaa actacattaa caaatatgat taacggtcat ggatctaagg
1621 catacttagt tgaagattta gaaaatgaat ttggctacga ttcaattaat cttgaaaaca
1681 taaatacgaa tgaattaatt ttagcagtga aaatcagaga tgcatttcta ttagataagg
1741 atgaaagtaa agagtatcct attaatgaaa gtaaacgtga tagaaataac cttaaatcgc
1801 gtacattagg tagttggata ataggaaaag ataaaataca taaaataaaa tatattattg
1861 gtatcaatac aggtgctaat aatgcagttg tctcagctta tgaagtatca tttgaacaag
1921 cggagagtat cgaaacaaat aatggtagaa tgagatatgc gtttattggg ctttcagaaa
1981 gagatgctac tctaaaaaaa ttgaatttat acaaaaaagc actaccagat ttaagatttg
2041 gtagtggtag cgctacagca tatataaata atgggacaat gaaagttgat taaatgaata
2101 aaaacatcca attccttaaa ttagcctttg aaaattttat actcgtatat ataagtgaaa
2161 aacttaaaag aattagatgt taattaaagg agagaatgct atgccatcag atgatattgt
2221 aaaaaaacta tactcaaaag aggatattcg attagaatta ggacttactc cgcataagtt
2281 taataaaaaa atggaaacta ttgctaagct ttttaaaatt gatatgaaaa tttttcacaa
2341 ttacaaaggc caagataaaa ataatcagta tacatttaat ggtgtcgcaa aagaattaat
2401 caaagtgttg ctaaaaagtg ttgattatta cccagtggat atcaattcca aaaaatttaa
2461 acaaaatgga aaatctaaaa aagaaatgat tgaaaatata gataactcta gttacatgaa
2521 atatatttat cagttaatga aatctatcaa tgaaattcaa tacaaaaggt taattgctga
2581 tatacatatg aaagatgtgt accagaatac aaaagcatgg ttaaataatg gtgaatcaat
2641 taataaaaaa gaacaagagc tataccaata tatgacaatt ttaccattac ataaaagagt
2701 tgaactgcaa aatgaggtat taaaatctat agacgaaaca atttttcaat ttctcgcaaa
2761 agaacataga aataatcaaa ttgaagaaaa taatgaatta gaagcatata caaaagcaat
2821 aaaagaaggt agaaatccta aaaatgatta tgaattaaat catctcctat ataaaaaaaa
2881 tcaattaccc ttagatagtc taatagaaga tatatgggac tatgaggaaa cagagtatac
2941 tgagcttgat tggttaattg cagatatgct aaaacgttca caaagagctg atagtaattt
3001 tatcgaaagt ttagagaaga aaaacaaatt gagaaaaaat attcataaag attccattaa
3061 aagtatatct aagttgattg atcatgaatt agttaaaaat aatagaaagt gggacgtagc
3121 agaattttat aaaaatcaac aattttggaa taatagtaca ttaagacgta gctctcaaaa
3181 ttttggaatt ttatattaca aaaagaatag gttacataca atgaatcaaa gtaatattat
3241 taagaaaata aaaattatcg aagctaatat agagagtgca aataaacagc cgagctattt
3301 agatttcttt ttacaagcta agtttgattt agagaggtta gaaagtgaat taatgaaata

3361 tagcattaat ccgaaatatt ttaataagat aaatagtgat tacgtaagat atgctattga
3421 agatgtacat aatgcaatta ttaaaattga cagatatata agtaacgata aagtgaagtt
3481 aaactattca aatgtccaaa tggtagtgag aaacgaaata acagaacaag gaagctactt
3541 tgtaacccag gcattgaata cactgtcaaa ggtagaagag tctggctaca gctttgataa
3601 tttcatggta ggacctttcg tagaagactt taaaaaagca attaaaaatc gtagatttgg
3661 ttctaaaaaa gattaagatt gttttttaga gttaaacata agaataaatt taaataaaaa
3721 aataaaccca acaaaccttg ttatatcaat gtttgttggg tttatttttt tggtgatttt
3781 ttaataactc aattacatct taggataata tcgctaaatt atacattgtc gaggccaaga
3841 caaataaaaa attaaagcaa aagttagcgc gcaactttct gctataaagg agcaaattta
3901 tatggagaca agagacaaac tgatgtcatt gactcaaagt gacaaaacac agcaatggct
3961 aatggacaag tcatctaacc aagatgacat tcaacaattg cagcaacaat tcagtcagca
4021 gcttgatcaa caatataatg cacttttagc tgatgaaaaa gctaagttag accaatacgt
4081 ggaagtacat caaggattgg aatcattaaa ggaagagatt gaatcagaac ctattacgct
4141 taatatcgat aaattacccg atatcaaagc aacaatgctt gaaagagcca agaatgatga
4201 acattctgat aaaatcgaaa agctatttga taggttagaa caggcattaa atggtacgaa
4261 tcgattatat acgcaattat cgttgattgg tacacgaaca catcgaatta caactaaaaa
4321 ttttaatctt caaggcttac ctaaagcagt ccaacatacg attttacctt caaaatttaa
4381 gaaggtgtat acagtcgatt ttaaatcgtt tgaaccatca gttgcagcgt acatgactca
4441 agattcaaaa ctgattgact tgttaaatca gaaagacgga ctgtatgacg cattgctaag
4501 tgaattaggc ttatcagatg agctacgtgt atttgttaaa cgtgcattta ttggttcgtt
4561 tctatttgga ggtaacttca aaaatcctaa attcaagctg aatcaatatg taagtgaagt
4621 acaatggttg gatgcggtca gccaatttac aaaagtcatt gaacttaaga agcacgttga
4681 aaagagtaat tccatgccta tgccttatag tattgagcat gatatgagcg catttcaagg
4741 tagcagtatt atggcaatct acgtacaaac tgtagcgagt tatattttca agcacattct
4801 gctaaaagtg tacaaagcac agtgcgatca aaaaacgttc aagattatag tacctataca
4861 cgatgcgatt atgattgaat gtgaagatga agaaattgca caaaatgtgg gtcagttaat
4921 gaaagatacg gctaaccagt tgttcaatgg tgaatttgca catgtgacag tggaagaaat
4981 aggaggcgta gaccatgaat aatgatagag gacaaagcct acacatccca agtagtacac
5041 caatcaaaga aaataatata tatgtagcta cgttacattc tgtgatccaa acagatttct
5101 caggtgaaat aaagcaccaa ttcacgtatg aaattgaagt gaacaatcag attgtatatg
5161 cgaatcgtaa tattccaaca aaaccgagcg ctaatcagtt gtcaattcat gattggctga
5221 aacgtcatag caactatagc gcaagtcatg aaaactatga gccttatatt gatcagaaac
5281 atttaattct attaggtcaa tataacggta actattatgt acaagatgta gcatcgttag
5341 atgcgtttgg aggcgtatta tcatgaatca tatattacaa atgttatcta agctattaag
5401 tgtggccaag gaggcaatcg accgtcaagg tctgattgct atcctaacta ttcctgttaa
5461 taataacgat gaaatagaag aaacggctca aggtgaaacc gtgtataacg aacttatcga
5521 tcagttacga cttaatatcc caaaagatac ggattatcaa cctaacatct atagttattt
5581 tggtattaag aagaatccta atgacaccgt actcatggaa atgatgataa aggtttttca
5641 tatcaaacgc tttaattcag aactgtttat tttcaaagtt aacgggtggc aaaagataaa
5701 tggagatgaa ttacaagggt tgatatctaa aatgatacaa gtattgcttg tagattataa
5761 gccttcacta agcactctaa aaaatgtcgt agatggattg caaaaatcaa cagatgtaga
5821 agaacttgtt gagaatgagc gctatattgg ttgtggtgaa aatatgttcg atcttaatac
5881 gtttcaagtc gttaaaaatt caatcgatat ctttccaaaa acacgattga atttatcatt
5941 aagtacaaat gatgtaatta ctgataagat accgccttat tttaagcaat atatgttaca

6001 acttgcgaat tatgacgatg atttacaata ctttcttttc caacatacag cagtattact
6061 tacagctgat actaaatacc gtaggggtct catattatat ggtggagcta agaatggtaa
6121 atctgtatat attgaactag ttaaatcatt tttctatagt aaagatattg tgtctaagcc
6181 acttaatgag cttgaaggtc gttttgacaa agaaagttta attgacaaaa gtctaatggc
6241 aagtcatgaa attgggcaat ctaggattca agaaaagatc gtaaatgact tcaaaaagtt
6301 attatctgta gaatcaatgc atgttgatcg taaaggaaaa actcaagtgg aagtcatttt
6361 ggatttgaaa cttattttta gtacaaatgc gatacttaat tttcctcctg aacatgcgaa
6421 agctttggag cgtcgaatta atattattcc atgtgagtat tatgttgaaa aagcggacac
6481 ttcattaatt gataagctcc agagtgagaa gaaagaaatc tttctttact tgatgtatgt
6541 gtatcaacag attgtaaaag cagatatcga gtatcttgaa aatagccgtg tcactgaaat
6601 tactcacgat tggttaaatt ttggatatga atttgtttct agcaggtccg taagtaatgc
6661 aaatcagaaa gcatgtatta atttactcag aaaacttata gaaatcaaat caggatcacg
6721 aatcaaagta tccgagttaa ataaagttat taatgaagaa ataaaggtaa gttctcaggt
6781 tattaaacag ttaattcaag caaactttga tactcaaacc aaactataca atggctacga
6841 ttattggatt gatttaggtt ggaaagaagc caataaaaaa gagattcatg atatttcgga
6901 aaaagataat attatttcat tagataaaaa tgaaaatata acagacgatg aggcattaga
6961 tgaagagaat ttggactttg attgggagga ctttgacgat gaataatgaa caaattgaag
7021 catttgtaga agtgcttgta cctatcatag aagaacgtat caataaaggt aattaaggct
7081 aattacgtac tacaggcagt tgcctgtagt actcatatga ttaagtggta aaagtgataa
7141 aaatgaaacg aaattataaa tatatattat ctatatgttg ttacaagacc gatagtctgt
7201 agcaataatc taataaaagg agcggtatga tatgaagggt aaaattgcac tttattcacg
7261 cgttagtacg tcagagcagt cggagcatgg gtactcaatc catgagcagg aacaagtact
7321 catcaaagaa gttgtgaaaa atttcccagg ttatgactat gagacatata ctgactcagg
7381 catttcaggt aaaaatattg aaggtcgtcc ggcaatgaaa cgtctattac aagatgttaa
7441 ggataataaa atcgaaatgg tgttaagttg gaaattgaat cgtatctcac gatcaatgag
7501 agacgtgttt aatattattc atgaattcaa agaacatgat gtagggtata aatcgatttc
7561 tgagaatatt gatacatcca atgcttctgg agaagtactc gttacaatgt ttgggttaat
7621 aggatctata gaacgccaga ctttgatttc caatgtgaaa ctttctatga atgctaaggc
7681 aaggagcgga gaggcaatca ccggtcgtgt tttaggctac aaattatcac ttaatccact
7741 tacacagaaa aatgatttgg ttatcgatga aaatgaagct aatattgtac gtgaaatttt
7801 cgatttatat ttgaatcata ataaaggcct caaagccatt acaaccgtac ttaatcaaaa
7861 ggggtatcgt actattaatc aaaagccatt ttcagtgtat ggtgttaaat acattttgaa
7921 taatccagtc tataaaggct atgtcagatt caataaccat caaaactggg ctgtacagcg
7981 aagaagtggc aaaagtgata aaaatgatgt gatattggtc aaaggtaaac atgaagccat
8041 tataagtgaa gaggtatttg ataaagttca tgaaaaatta gcttctaaaa gttttaaacc
8101 gggcagacct attggtggag atttctactt acgtggcctt attaaatgcc cagaatgcgg
8161 aaataatatg gtatgtcgac ggacgtatta taaaacgaaa aagtccaaag aacggacaat
8221 caagcgttat tacatttgtt ccttattcaa tcgttcaggg agttctgcat gtcacagtaa
8281 ttccatcaat gctgaagtcg tcgagcgtgt aattaatgtt catttgaatc gtattctgtc
8341 tcaaccagat attatcaagc agattgcgtc aaatgtgata gaagaactga aacaaaagca
8401 tagtaaccaa acagaaatta aatatgacat tgatagttta gaaaaacaaa aagctaagct
8461 taaaacacaa caagaacgat tgttagaatt gttcttagat gatcagatgg atagcgaaat
8521 gttaaaagct aaacaaagtc aaatgaatca acagttagaa gtattagatc aacaaattaa
8581 agaagcgcaa caagcaaatc aatcacagga tgaaatacct aattttgata aattaaaagg

8641 acgactcatt ttgatgataa cacgattcag cgtgtactta agaaaggcta cacccgaagc
8701 taaaaatcaa cttatgaaaa tgttaattga ttcaattgaa attacgacag ataaacaagt
8761 aaaacttgta aggtataaaa ttgacgaaag tcttatccct caatctttga aaaaagattg
8821 ggggtctttt tttatgccca aattccaatt tgaaatatat ggtcaaaatg attatttcat
8881 cgaccaaatt accactttta ccacttagtt attagtgaca aaagtgagcg aaatgaaata
8941 aaaatcaaat atatattatc aaaatgatgt atcacatgca tacatcaatc aaatacatta
9001 ggaggtcata accatgacac tagaacaaca actcaagcac tatataacca acttattcaa
9061 tctgccaagg gacgaagtgt ggcactgcga atctatcgag gaaatcgctg atgatatctt
9121 acccaatcaa tatgtaagac ttggcccact cagtaataaa acacttcaga ctaataccta
9181 ctactctgac acacttcatg aaagtaatat ctatcctttc attctctact atcagaaaca
9241 actcatagcc atcggttata tcgacgaaaa tcacgatatg gatttcttat acctacacaa
9301 cactatcatg cctcttttgg atcaacgata cttactaaca ggaggacaat aaaatgcata
9361 aatacatcaa aattacacaa ttagtcatta caatactaag tgaaatcatc atttggatga
9421 aagagtcaga acgaaaggaa gtctcttatg aatagatata tcacccgggg tatcgccaac
9481 aacttaccta atatcttaca acaccaatta tggcaactcg tatctgagcg agaacaagaa
9541 caaaccaaag ataatacttc agtagattat tttcatatat tccagttcaa taagcatcgc
9601 aatcaattat atatcaaaca caaacaagaa cgacctgaat atgcgaaaat ccataaagct
9661 aattattcaa aagcaatcaa tatcaataag gtctacatta ttcgagaaga tgatgtagac
9721 ctttcttatt atgtcatgtt attacctgaa gaatactaga ggagtgaaaa ttatatggaa
9781 acaatcaaaa gtacattaaa aacagaagcc atattcagtg atgacaaaca acatcgctat
9841 ctacttaaga aaacatggaa tagtgaaaaa caatcaatca caatcattac aatgtatccg
9901 cattatgatg gcattctcaa tattgaccta acgacccaac tcatcatgaa caaagtttca
9961 gaaatggatg catttggttc aatcaatttt gtgaatctat actctaatat tacaacccct
10021 atcaatctca aacatttaga aaatgcgtat gataagcata cagatattca aattatgaag
10081 gcagtgaaag agtcagatga agtgatatta gcttggggcg cttacgctaa aaagcccggt
10141 gttgaagcac gtgttaatga agtattagag atgttgaaac cacataaaaa gaaagtaaaa
10201 cgactcatga atccagaaac caatgaaatc atgcatcccc ttaattcgaa agcacgtcaa
10261 aaatggatat taaaagtata gcaactaaaa tgtttattca ctatccatga aaataattga
10321 atttcatata tatcttttaa tctacaatat gattaaaaga tatattattt ggttctgttg
10381 caaagtaaaa aaatatagct aaccactaat ttatcatgtc agtgttcgct taacttgcta
10441 gcatgatgct aatttcatgg catggcaaaa atccgcagat ctgaagagac ctgcggttct
10501 ttttatatag agcgtaaata cattcaatac cttttaaagt attctttgct gtattgatac
10561 tttgatacct tgtctttctt actttaatat gacggtgatc ttgctcaatg aggttattca
10621 gatatttcga tgtacaatga cagtcaggtt taagtttaaa agctttaatt actttagcca
10681 ttgctacctt cgttgaaggt gcctgatctg taattacctt ttgaggttta ccaaattgtt
10741 taatgagacg tttgataaac gcatatgctg aatgattatc tcgttgctta cgcaaccaaa
10801 tatctaatgt atgtccctct gcatcaatgg cacgatataa atagctccat tttcctttta
10861 ttttgatgta cgtctcatca atacgccatt tgtaataagc ttttttatgc tttttcttcc
10921 aaatttgata caaaattggg gcatattctt gaacccaacg gtagaccgtt gaatgatgaa
10981 cgtttacacc acgttccctt aatatttcag atatatcacg ataactcaat gtatatctta
11041 gatagtagcc aacggctaca gtgataacat ccttgttaaa ttgtttatat ctgaaatagt
11101 tcatacagaa gactcctttt tgttaaaatt atactataaa ttcaactttg caacagaacc
11161 gtattatgga atagagatgt tggtaacatt tatacaggat cattatactt aagtttaatt
11221 tcgttattac agaaccacac attccaacca gaagagaaag tatgtctatt tagttatggt

11281 tcaggagcag taggagaaat ctttagtggt tcaatcgtta aaggatatga caaagcatta
11341 gataaagaga aacacttaaa tatgctagaa tctagagagc aattatcagt cgaagaatac
11401 gaaacattct ttaacagatt tgataatcaa gaatttgatt tcgaacgtga attgacacaa
11461 gatccatatt caaaagtata cttatacagt atagaagacc atatcagaac atataagata
11521 gagaaataaa ctagtggccg attgtgcttg atgagcttgg gacataaatc ctaactcgaa
11581 ataaataagc atatcactaa actgattttt taaagtttac agtgatatgc ttattttttt
11641 atcttacgat tttgtacgtg catgcttgcc taggggtatg gctcgagcca ttagtctctc
11701 gcacatacta ttccctcagg cgtcagcact tacaaaatcg gttgtaattt tcatttttat
11761 acgcattctt actgagatta tactaataag aggaatagta aaagcaattc taagtaaaat
11821 tgcagataag aggtttgtta aaagcagttc taagtaaaat tgcagataag aggtttgtta
11881 aaagcagttc taagtaaaat tgcagataag aggtttgtta aaagcagttc tcagtaaaat
11941 tacagataag aggtacgtta aaagcagttc taagtaaaat tgcagataag aggtttgtta
12001 aaagcagttc taagtaaaat tgcagataag aggtacgtta aaagcaattc catgcaaaat
12061 tgctgataag gggtaagtta aaagcagttc tcagtaaaat tgcagataag aggtacgtta
12121 aaagcagttc taggcaaaat tgcagataag aggtgcgtta aaagcagttc taagtaaaat
12181 tgctgataag gggtaagtta aaagcaatcc taagtaaaat tgcagataag aggtaagtta
12241 aaagcaatcc taagtaaaat tgcagataag gggtacagaa aaactagact tgattacaaa
12301 atggagcttg ggacataaat gattttttaa aaatgagatg agacgtagat taactccata
12361 atcaatacga atctatcgac ttctttattt atgatattca tctcttttta atggaaataa
12421 aagtgcgatt aatgtgataa tacagttacg ttaattaaaa aaataaaaat gcaaggagag
12481 gtaatatgct aactgtatat ggacatagag gattacctag taaagctccg gaaaatacaa
12541 ttgcatcatt taaagctgct tcagaagtag aaggtataaa ctggttggag ttagatgttg
12601 caattacaaa agatgaacaa ctgattatca ttcatgatga ttatttagaa cggactacaa
12661 atatgtccgg ggaaataact gaattgaatt atgatgaaat taaagatgct tctgcaggat
12721 cttggtttgg tgaaaaattc aaagatgaac atttgccaac tttcgatgat gtagtaaaaa
12781 tagcaaatga atataatatg aatttaaatg tagaattaaa aggtattact ggaccgaatg
12841 gactagcact ttctaaaagt atggttaagc aagtggaaga acaattaaca aacttaaatc
12901 agaatcaaga agtgctcatt tcaagcttta atgttgtgct tgttaaactt gcagaagaaa
12961 tcatgccaca atataacaga gcagttatat tccatacaac ttcgtttcgt gaagactgga
13021 gaacactttt agattactgt aatgctaaaa tagtaaacac tgaagatgcc aaacttacta
13081 aagcaaaagt aaaaatggta aaagaagcgg gttatgaatt gaacgtatgg actgtaaaca
13141 aaccagcacg tgcaaaccaa cttgctaatt ggggagttga tggtatcttt acagacaatg
13201 cagataaaat ggtgcatttg tctcaataga aagttagagg tgagtcttac gtttcagtga
13261 cggtagactt acctttaaca tgttacatac taaaaaatta atttgaataa gaaagagaga
13321 catatatgaa atacgatgat tttatagtag gagaaacatt caaaacaaaa agccttcata
13381 ttacagaaga agaaattatc caatttgcaa caacttttga tcctcaatat atgcatatag
13441 ataaagaaaa agcagaacaa agtagattta aaggtatcat tgcatctggc atgcatacac
13501 tttcaatatc atttaaatta tgggtagaag aaggtaaata cggagaagaa gttgtagcag
13561 gaacacaaat gaataacgtt aaatttatta aacctgtata cccaggtaat acattgtacg
13621 ttatcgctga aattacaaat aagaaatcca taaaaaaaga aaatggactc gttacagtgt
13681 cactttcaac atacaatgaa aatgaagaaa ttgtatttaa gggagaagta acagcactta
13741 ttaataattc ataataaaac agtgaagcaa ccatcgttac ggattgcttc actgttttgt
13801 tattcatcta tatcgtattt tttattaccg ttctcatata gctcatcata cactttacct
13861 gagattttgg cattgtagct agccattcct ttatcttgta catctttaac attaatagcc

13921 atcatcatgt ttggattatc tttatcatat gatataaacc acccaatttg tctgccagtt
13981 tctccttgtt tcattttgag ttctgcagta ccggatttgc caattaagtt tgcataagat
14041 ctataaatat cttctttatg tgttttattt acgacttgtt gcataccatc agttaataga
14101 ttgatatttt ctttggaaat aatatttttc ttccaaactt tgtttttcgt gtcttttaat
14161 aagtgaggtg cgttaatatt gccattattt tctaatgcgc tatagattga aaggatctgt
14221 actgggttaa tcagtatttc accttgtccg taacctgaat cagctaataa tatttcatta
14281 tctaaatttt tgtttgaaat ttgagcatta taaaatggat aatcacttgg tatatcttca
14341 ccaacaccta gtttttcat gcctttttca aatttcttac tgcctaattc gagtgctact
14401 ctagcaaaga aaatgttatc tgatgattct attgcttgtt ttaagtcgat attaccattt
14461 accacttcat atcttgtaac gttgtaacca ccccaagatt tatcttttttg ccaaccttta
14521 ccatcgattt tataacttgt tttatcgtct aatgttttgt tatttaaccc aatcattgct
14581 gttaatattt tttgagttga acctggtgaa gttgtaatct ggaacttgtt gagcagaggt
14641 tctttttat cttcggttaa tttattatat tcttcgttac tcatgccata cataaatgga
14701 tagacgtcat atgaaggtgt gcttacaagt gctaataatt cacctgtttg agggtggata
14761 gcagtacctg agccataatc atttttcatg ttgttataaa tactcttttg aactttagca
14821 tcaatagtta gttgaatatc tttgccatct tttttctttt tctctattaa tgtatgtgcg
14881 attgtattgc tattatcgtc aacgattgtg acacgatagc catcttcatg ttggagcttt
14941 ttatcgtaaa gtttttcgag tcccttttta ccaataactg catcatcttt atagccttta
15001 tattcttttt gttttaattc ttcagagtta atgggaccaa cataacctaa tagatgtgaa
15061 gtcgcttttc ctagaggata gttacgactt tctgtttcat tagttgtaag atgaaatttt
15121 tttgcgaaat ctcttaaata ttcatccatt tttttaacgg ttttaagtgg aacgaaggta
15181 tcatcttgta cccaattttg atccatttgt tgtttgatat agtcttcaga aatacttagt
15241 tctttagcga ttgctttata atcttttta gatacattct ttggaacgat gcctatctca
15301 tatgctgttc ctgtattggc caattccaca ttgtttcggt ctaaaatttt accacgttct
15361 gattttaaat tttcaatatg tatgctttgg tctttctgca ttcctggaat aatgacgcta
15421 tgatcccaat ctaacttcca cataccatct tctttaacaa aattaaattg aacgttgcga
15481 tcaatgttac cgtagtttgt tttaatttta tattgagcat ctactcgttt tttattttta
15541 gatacttttt ttattttacg atcctgaatg tttatatctt taacgcctaa actattatat
15601 atttttatcg gacgttcagt catttctact tcaccattat cgcttttaga aatataactg
15661 ctatctttat aaacttgttt gaaattttta tcttcaattg catcaatagt attattaatt
15721 tctttatctt ttgaagcata aaaatatata ccaaacccga caactacaac tattaaaata
15781 agtggaacaa tttttatctt tttcatcaat atcctcctta tataagacta catttgtaat
15841 atattacaaa tgtagtattt atgtcaaaat aatgttataa tttttgtgat atggaggtgt
15901 agaaggtgtt atcatctttt ttaatgttaa gtataatcag ttcattgctc acgatatgtg
15961 taattttttt agtgagaatg ctctatataa aatatacggt tctgttgcaa agttgaattt
16021 atagtataat tataaccaaa aggagtcttc tgtatgaact atttcagata taaacaattt
16081 aacaaggatg ttatcactgt agccgttggc tactatctaa gatatgcatt gagttatcgt
16141 gatatgtctg aaatattaag ggaacgtggt gtaaacgttc atcatttaac ggtctagcgt
16201 tgagttcaag aatatgcccc gattttatat caaatttgga agaaaaagca taaaaaagct
16261 tattacaaat ggcgtattga tgagacgtac atcaaaataa aaggaaaatg gaactattta
16321 tatcgtgcca ttgatacaga gggacataca ttagatattt ggttgcgtaa gcaacgagat
16381 aatcattcag catatgtatt tatcaaacgt ctcattaaac aatttggtaa acctcaaaag
16441 gtaattacag attaggcacc ttcaacgaag gtcgcaatgg ctaaagtcat taaagctttt
16501 aaacttaaac ctgactgtca ttgtacatcg aaatatctga ataacctcat tgagcaagat

16561 caccgtcata ttaaagtaag aaagacaaga tatcaaagta tcaatacggc aaagaatact
16621 ttaaaaggta ttgaatgtat ttacggtcta tataaaaaga accgcaggtc tcttcagatc
16681 tgcggatttt cgccatgcca tgaaattagc atcatgctaa caagttaaca caaagtatta
16741 ttttaaaatt gagattagac atttattttt caactttgca gcagaaccta attttttctc
16801 tttgtaaata aatgcaaaga gataccataa ggatctctta cataaccata accttcagta
16861 tagaattctg gactaaatgt tttcaatacc tcactgcctt tttctattaa ctggtcatat
16921 acatgtttag tttcttctac ttggtcaaaa gtgagacaaa gagatatatt attaccttgt
16981 gttatgggca aaccttcagt gtcatctgcg atcataattt ttatatctcc aaattgaagt
17041 acacattgat caattttatt taaatcattt tcgtcaatat taagtttctt atctataggt
17101 ctatctttaa tacgttgaat atacagtgtt ttagcgccaa acagctcttc atacaacttt
17161 tttaaaccct ctgcattttg agtgattaaa aaatatggac ttacttgaaa tttcatgttt
17221 tttcctcctt agatttgtta taaatagagt atatacttaa ttagtgtcat ctattgacac
17281 taaaaggaga ataataatga aaaaatctgt tagattatat aatatgattg aatattgtaa
17341 tgaaaatagg aacttcaaat taaatgattt aatgtcagaa tttaatattt ctcgtagtac
17401 cgctttaagg gatataaaag aaattgaagc attaggagta cctttatata gtaatccagg
17461 gaaaaatggt ggttatacga tcataggtaa tcgagaccaa acgaaaatag caatctcaga
17521 tgaagagttg aaagctttag tatttacact ttcgagtatt tcaaatgtga gtaatctacc
17581 ttttcaaaca gaatatcaag aaatattaaa aaaattatat aataactcga ataaaaaaga
17641 gttaataaac caatataatg atctatttca atattttaat gaagataagt atcagttcaa
17701 aagttataag ttatttaatg aaatcattag attgataatt gagaataagt cttttgaaac
17761 ctgttattca caaaactata ttaaagaaca atataaaggt attggcatta tgtataaaaa
17821 tcatcaatgg tattttgttg tagctaatat agaatcaaag ttagtgaatc tattaaatat
17881 ttcgaaaata aaagaactat atgaaatggg agagactcaa gagtgtaatg atataactat
17941 gcagaatttt caacagttca tggttaaaaa tgaaacagct attgatattc ttattagaag
18001 caatgttatg ggattgaata tcttgaaagg ctacctgtgg agtgactata tgattgaaaa
18061 tattgacgaa gagacatatt tatttaaatc aaaagtgaac gcgaaagata tagattttat
18121 agctaagtta attgtcacag gtggtgtcaa tgtaaaagta gagaccccta atagtttgaa
18181 aaatgctgtt aaagttgaat taactaaaat aataaacatg tattaatagt aatttaatca
18241 taattgtaat aacaaaaaga tgacaacatt aaacaattta aatttataca aaaaagcatt
18301 accaaattta aaattgggta gtggtagtgc tacagcatac ataaatagtt ataattaaat
18361 aaagatgggt caatgaaggt gaattaagtg aataaaaata ttacagctct aaaatctact
18421 gaaaatacta catatatgca gtatacggtt catatcgatg atcaaattat aaataatata
18481 aaagagtgta ccaaaaagtt taaaattttg cctatggaag ataaaattcc attatcaccg
18541 ttgttacaac cagaatatgc aggagaggta caagatttta ttagtacata tgagcagttt
18601 atgattaatt ttggtaaagt aatattggat agtcaaggca taaaaataca gtttgaaagt
18661 gaatcattaa gtagtattca acgaggcatt caagaacatt gttacttaaa tgagcgaaca
18721 aatgacattg atgtgactaa agaatggtat ttatgcaaat tttcaaatca aatattagaa
18781 gaggataaaa atcaattgta taatgcttta aagcaactca tgaatgattc taaaaataaa
18841 aagcaagctt tttggactga ccccaattag tgggaattat ataaaaacac tttcgttgaa
18901 ttcataataa aatgaatcat gcggaggtgt tttttctatg aaaagagttt cttattcagt
18961 agaaacaaag tataaagcag ttgaaatgaa aatagctggt ttttcgacaa aagaaattat
19021 gaaagaatta aatattaaaa atagaacaca agtggaaact tggtggcgat ggtatcgaaa
19081 tgaggaaagt tatagatttt cacaacacgt tgggaaacaa tatacctacg gtaaaggatt
19141 agaagaattg tcagaaatag aacgattaaa attagaaaat aagagaaaag atatagaatt

19201 ggatatttta aaaaagtaca aggcattgga aaggaagtgg taccagcagt agtcatagat
19261 ttagtggatc aattaaaagt aaaatattca atcaaattga tactagaagt attaaacata
19321 cctaaatcaa catattaccg atggaaaaac aaaacctata aaaatgatac tgtaacacaa
19381 aaagttattg aattatgtga agctaaccac tatacctacg gttatcgtaa gattacagca
19441 ctgattaatc aatgttatac atcaccaatt aatcataaga gagtacagag aattatgcag
19501 aagcatcatt tgaactgccg agttagacct aaaaagacga caaggatagg taaaccgtat
19561 tataaaactg acaatttatt acaaagacaa tttaaagcga gttgtcccat ggaagtatta
19621 acaaccgata ttacttattt accatttggt cattctatgt tgtatttatc ttcgataatg
19681 gatatttata acggagaaat tgtggcgtat aaaatagatg ataaacaaga ccaaagttta
19741 gttaatgata cattaaatca aatcgatata cccgaggggt gtatattaca tagtgatcaa
19801 ggcagcgttt atacatctta tgcttattat caattatgcg aagaaaaagg cattatcaga
19861 agtatgtccc gaaagggaac acctgctgat aacgccccga tagaaagttt tcattcctcg
19921 ctaaagtctg aaacttttta catcaataat cagcttaata gctctaatca tattgtaata
19981 gatattgtcg aaaaatacat taaaaactat aataataatc gaattcaaca aaaactaggc
20041 tacttatctc ctgtaaaata cagagaatta gcagcctaga acatagtgtt tttattaagt
20101 tcccgtttaa gggggtcagt accttttcaa gaggtgttta aggttcatat tgatatttat
20161 ctctacaata aagaaaaatc tgaatataag tatcaagtat ctagttattt caatcttgta
20221 aaagaaaatc ctaaaataac ttataaaaaa agacatttac aagagaaaca aggtgtcaag
20281 ggtacaactt tcacgaacaa attaaaatta ttgaacaaaa tgtatggtgt agatgttgca
20341 aaatatcagc cattttataa ttctaataat cctgaatatg aaagaggtca atttggtgaa
20401 agatacattt ctcaaagatc taactatgaa tttaatagac ttcaatatca aattatagat
20461 atgttatcga aaatacttga taagcatcca ttaccaaagt cagataataa ttataaacat
20521 attccaacta ttgaaaaagc aatattaagt ggggattctc atagttttta tgagtatttt
20581 gaagatataa tgaaagaaat tataagtatg gaaaattctt ctttaaagga aaaattattg
20641 actgatttta catatcaatc tcaatgtaga tggtactctg aaagtgaaaa attaaaattg
20701 caacttgaaa gttttatgca taaagttttg gagagcaatt attatgaggg taacaaatta
20761 tatagaatgt tgtctcatgc aattgaagaa acaatcaatg aggctgatga agataaggtt
20821 catttcaatt attttaaaga ttatttttg acagacggtg gggtgaagaa ttgggagcag
20881 attagcgaga aaattactga attcaatggt aaagttatta acgatatcca aaatgaatat
20941 aataagattc aatttaataa cgcaaatcga aatcagaaac taaactttaa ttatttatac
21001 cattgctttg agtttagtaa taacttagta aaagcaaggg taaatggaaa tagaggttac
21061 attttatatt ttgtagataa atataaaata aaaatgccct ttttggatca attattaatc
21121 aattatttga atcaagaaat aggtcaggaa agtattaatt ataatatgca aacattgttt
21181 gaaaaagaaa gatatgatag aagtagtacc attgaaaaat tagtagcaac aagcaaattt
21241 aagtatgaaa aagatgattc agatttattc aaacaacttt tcaatgatgt tgaaaattca
21301 atagacagat taggtattta cttactaaat aatggtataa attcgaatga tgaaaatgca
21361 agatattata gatcgttttt aaaggaactt agtagaataa aaagtaaatt aacgccattt
21421 tctcttgaaa taagtaagtc tagcggaaga gagcaacatt atcctgatga tgctattgat
21481 gataaagatg agagaagaaa aaataaagaa gaaacatatc atgcttttga tgataaaagc
21541 gatattgact ccaaattaaa gaataaaata aatgtttcta tcgataattt attgtcagtt
21601 aaaattaaga gcaatttgta tgatattagt ccaaatacaa aaaaacaata actataatga
21661 gccaactcaa gaatgagttg gctttttaat tgtaatttta cgcttccatt acaggaatca
21721 atgttatgga agccattaag caattaattt gagtcattta tagtataggt acaacgttgt
21781 gatgttcaca tgataatcag cccgggagat tattgtgtaa taacttatag aaggagcaaa

21841 tcaccatgta tcaagcaaat atacgtgatt taattacaaa attgcctcaa agcaataaaa
21901 cagaacactt tttaatgaac aaattttcaa atcaagataa agttcagcag ctacaaagac
21961 aaattagcca acagctagat caacaatata atgagctttt ggctaatgaa aaagctaagc
22021 tagaccaata cgtggaagta caccataatt tagaaccatt aaagaaagag attgaatcag
22081 aatctattaa ccttgatacc gataaattac ctgatatcaa agcgacaatg cttgaaaagg
22141 ctaagaacga tgaacatttt gataaaatcg aacagctatt tgatagatta gatcagtcat
22201 taaatggtac gaatcgatta tatacgcaat tatcgttgat tggcacacga acacatcgga
22261 tcacaacgaa aagatttaat gttcaaggct tgcctaaatt agtccaacaa atgattttac
22321 cttcgcaatt taaaaaggtt tatacaatag attttaaatc attcgaacca tcagttgctg
22381 cgtatatgac acaagacgaa caactgattg actacttgaa tcatgaagaa gggttatacg
22441 atgcattact gagagactta tctttgtcaa aagagaagcg cgtgagtgtg aaacgtgcat
22501 ttatagggtc atttcttttt ggcggtcgtt atagtagctc taaattcaaa atcaatcaag
22561 aggttagtga aattaactgg ctacaagtaa tgagcaaatt caagaaggtc attgaattta
22621 aggagcaagt cgaaaaatat aaaacaatgc ctacgcctta cggcattgaa catgatatga
22681 gcgcatttca aggtagtagt attatggcaa tttatgtaca aacggtagca agctatattt
22741 tcaagcacat tttgttggaa gtgtacaaag cacagtgcga aaaaaaaacg ttcaagatta
22801 tagtgccgat acacgatgcg attatgattg aatgtaatga taaggggatt gcacaaaatg
22861 tagcacagct catgaaagat acagctaatc aactgtttaa tggtgaattt gcacatgtga
22921 cagtggaagc tttaggaggt atagacaatg aataatgata gaggaaaaag tcttcaaatt
22981 ccccaaagta cattgttaaa agaaggatct atatacgttg ctacgttaca ttctgtgtac
23041 gagaagaact tctcaggtga tattaaacat cagtttacgt atgaagtaga acttaaccaa
23101 gaaacgcact atgtaaatcg caatattacc gtaaaatcta tgagtcacca attatcaatt
23161 gctgattgga ttaaacgtca cagtaactat aacgtaaacc acattaatta tgatccatac
23221 attgatcgaa aacatttggt ccttgtaggg caatataacg gaaattatta tattcaagat
23281 gtagcaccat tagatgaatt tggaggggta ttgtaatgaa tcatatttta gaaatgttaa
23341 taaaattatt aaaagtgggt atggaggcaa tcgaccgtaa aggtctgatt gccatcctaa
23401 caagtagtat tggaaatgat gaaatggatg attctgaaca agctgtaatg gtgtataacg
23461 agcttatcga taagctacag cttaacattc ctaaagatgt cgactataga cctaacatat
23521 acagttattt tggtattcaa aaaaagccaa atgacacaat attagtggaa atgatgatat
23581 gtatttttca tatcaagcgt tttgattcag agttgtttgt tttcaaagat aaaggttggc
23641 aaaaggtaag tgaagatgaa ttgcaagggt tgatatctaa aatgatacaa gtgttgctag
23701 ttgattataa accttcacta agtactttga aaaacgtagt agatggcata cagaaatcaa
23761 cggacataga aaaacttgtt gagcatagac agtacattgg ttgtggacga aatatgttca
23821 atctaaagac ctttaaagtg gttgataatg accttgaaat attccctaaa acacgcttag
23881 atttggaatt agatataaat gacacgatta cggacaagat acccccgaat ttcaaacaat
23941 atatgttaga gttggcgaat tatgaccatg atttacaata tttccttttc caacatatgg
24001 cagtgttatt gacggcagat actaaactac gtcgtggact ttttttgtat ggaactgcaa
24061 aaaatgggaa atcggtctat attaaattag ttaagtcatt cttttatagt aatgatatcg
24121 tatctaaaac acttaatgaa cttggcgggc gtttcgataa ggaaagtcta attggtaaac
24181 gaattatggc aagtgatgaa gtggggaaag ctaatattga tgaagcaact gtgaatgatt
24241 tcaaaaaatt actatctgtt gaaccaattc atgctgaccg taaaggaaga acgcaagtag
24301 aagttacttt agatttaaaa ctcattttta atacgaatgc tgtactcaat tttccatcat
24361 cacatgcaaa agcattagag cgtagaattg ctgttattcc atgtgaatat tatgttgaaa
24421 aagctgaccc tgacttaatt gaaaagttac aggatgaaaa gaaagaaatc tttctttact

24481 tgatgtatgt gtataagcaa attgtaaaaa atgatatcga gtacctccaa aatgatcgtg
24541 ttactgaaat ttctcatgat tggttaaatt ttggatatga atttgtttct agtaaatcag
24601 caaatattgc acatcagaaa gcgtgtatta atttactcag aaaacttata gaaatcaaac
24661 cagggtcacg tatcaaagtg tctaggctaa atgaggttat tagagatgaa attaaagtaa
24721 gctctcaagt tattaatgat ttggttcaag ctaactttaa tgtacaaagt agactaaata
24781 atggttataa gtattgggtc gatttaggat ggaaagaaac tgataaaaaa gatgacatga
24841 tttcattcga taaaaatgag aatgtaacag atgatgaatt cttatacgaa gatgatttga
24901 acttaggttg ggaggacttt gacgatgaat aatgaacaaa ttgaagcatt tgtagaagtg
24961 cttgtgccta tcatagaaga acgtatcaat aaaggtaagt aatctaatta cgtactacag
25021 gcagttgcct gtagtactca tatgattaag tggtaaaagt gataaaaatg aaacgaaatt
25081 ataaatatat attatctata tgttgttaca agaccgatgg tctgtagcaa taatctaata
25141 aaaggagcgg tatgatatga agggtaaaat tgcactttat tcacgtgtta gtacgtctga
25201 gcagtcagaa catggttatt ctgaaaagga gcaggaacaa ctactcatca aagaagttat
25261 gaaaaatttc ccaggttatg actatgagac atatactgac tcaggcattt caggtaaaaa
25321 tattgaaggt cgtccggcaa tgaaacgtct attacaagat gttaaggata ataaaatcga
25381 aatggtatta agttggaaat tgaatcgtat ttctcgctca atgagagacg tgtttaatat
25441 tattcatgaa ttcaaagaac atgacgtagg gtataaatcg atttctgaga atattgatac
25501 atccaatgct tctggagaag tactcgttac aatgtttgga ctaataggat ctatagaacg
25561 ccagactttg atttccaatg tgaaactttc tatgaatgct aaggcaagga gcggagaggc
25621 aatcaccggt cgtgttttag gctacaaatt atcacttaat ccattgacac agaaaaatga
25681 tttagttatt gatgaaaatg aagctcatat tgtacgggaa atctttgatt tatatttgaa
25741 tcacaataaa ggatttaaag caatcacgac aattctaaat caaaaaggat atcgtaccat
25801 taatcaaaaa ccattttcag tgtttggcgt gaaatacatt ttgaataatc cagtctataa
25861 aggctatgtc agattcaata atcatcaaaa ttgggctgtt cagcgaagaa gtggtaaaag
25921 tgataaaaat gatgtgatat tggtcaaagg taagcatgaa gccattataa gtgaagatgt
25981 atttgatcaa gttcatgaga aactagcttc taaaagtttt aaaccgggtc gacctattgg
26041 tggagatttc tacttacgtg gccttattaa atgcccagaa tgcggaaata atatggtatg
26101 tcgacggacg tattataaaa cgaaaaagtc caaagaacgc acaatcaaac gctattacat
26161 ttgttcatta ttcaatcgct caggaagttc tgcctgtcat agtaatgcga ttaatgctga
26221 agtcgtcgaa cgcgtaatca atgttcattt gaatcgtatt ctttcacaac ctaatgttat
26281 taagcagatt gcgtcaagtg tgatagaaga actgaaacaa aagcatagta aacaaacaga
26341 aataaaatat gatattgata gtctagaaaa acaaaaagca aaagttaaaa cacaacaaga
26401 acgattattg gaattgttct tagatgatga aatggatagc gaaatgttaa aagctaaaca
26461 aagtgaaatg aatcaacagt tagaagtatt agaccaacaa attaaagaag caaaacaagc
26521 aaatcaatca caggatgata tacccaattt tgataagtta aaagcacgac tcattttgat
26581 gataacacga ttcagtgtgt acttaagaaa ggctacaccc gaagctaaaa atcaacttat
26641 gaaaatgtta attgattcaa ttgaaattac gacagataaa caagtaaaac ttgtaaggta
26701 taaaattgat gaaagtctta tccctcaatc tttgaaaaaa gattgggggt cttttttat
26761 acctaaattt aactttgtga taaatgtcac aaagaaaaat aggattgaaa atttatcact
26821 tttaccactt ttttagagtg acaaaagtgg aggagttttg aaatatttta taaatatata
26881 ttttatttat ggagtacaca ttattaatta aggaggtcat tataatgacg ctaagcaaac
26941 aacttaaaac gtatatcact gaacgattta aattaaatta tcaagaaact tgggcttgtg
27001 aaaccataga tgcggtggct gaagatgtat tacctgaaaa atatattaaa aatagtccac
27061 ttgaacataa aattttaaat acttttacct attacaatga tgaattacat gaaatcagca

27121 tttacccttt tttatgttat ctagataagg aattagtagc aataggttat ttagataatt
27181 ttgatttaga ctttatattt ttaaatgaca ctcatcaagt cattattgat gaacgctact
27241 tgttacaaaa agggggcgag taattatgaa ttggatcaag gtcgctcaac tatctgtcac
27301 agttatcaat gaagtcattg agattatgaa agaaaaacaa aatggagggg aatagtatga
27361 aaatcaatcg atacattaca agaggtgtta gtgaacattt atctctagac cttcaaatct
27421 tactttggaa catggtaaaa gatcgagaca atcaacttca tacagattac ctacacattt
27481 ttaaactaca agaagatgat aatatactct caatcacaca tgaacaagaa caacccgcat
27541 acaaattgga atatcactat acaaactatg taaaaaatca aaatgcatta cctaagaaag
27601 tctacgtcat ccgagaagat gatgtagacg ctttttatta tgtgatgctt ttaccagaag
27661 aatactaaaa ggagtgacaa tgatgaatac aatcaaaaat acaatacaca cagaagcgat
27721 atttagcgat gatgaacaac accgctactt actcaaaaag acttgggatg aaaagaaacc
27781 tgcatgtacg gtaatcacca tgtaccctca tctagatggc gtattatcac tcgatcttac
27841 aactgttctt atcctcaatc aattagcgaa ttctgaacaa tatggcgctg tatatcttgt
27901 aaatctattt tctaatatta aaaccccaga gaaccttaaa catattaaag aaccttatga
27961 taaacacaca gacattcatt taatgaaagc gataagtgaa agtgacacag tgattttagc
28021 ctatggtgct tatgcgaagc gacccgttgt tgtcgaacgc gttgagcaag tgatggaaat
28081 gttaaaacct cataaaaaga aagtaaaaaa gctcataaat ccggcaacga atgaaattat
28141 gcatccgctt aatcctaaag cgcgtcaaaa atggacattg aaataaagga ggattatcta
28201 tgaaccatga aactaaacaa tcagattggc gaacggttgc tagttgttta gcatcacaaa
28261 attatatatc gattgtaaaa ggtttagtac atcattttac agcgatcgaa gatgaggaaa
28321 ttcttgataa aatctatgat gattttatga atgatgactc tataacaacg gtacttaaca
28381 atgatttaca gacaatcatt aaccattatc tatcaaaata aagactatac taattacaat
28441 aaaaacagag catccttcac ttttatggtg agggatgctc ttttaatttt ttttttatt
28501 gattattcgc cataagttct gcttgctcta caacctgctc tacagccatt ttttgtaaat
28561 ctggtggata gccatatttt ttaagcaatc gtctaacagc tacgcgcatt ttagcttttg
28621 cgctatcacg tttagaccaa tcaacaccca tgttttcttt cactgtttta gttagctcat
28681 gagcaatcgc acgtagttct ttatctccca tggcttcttt tgctgtttca tgtgaagcta
28741 aagcatcgta aaacgcaatc tcatctgaat tcaggcctaa ttcatttcct cgttgttgtt
28801 cttgtttaat atctttagcg agttgaataa gttcttcaat cactttagat gtttcaatgg
28861 aacgactatt atatttatta atcgaatttc ctaacatctc agagaaacgc ttagataccg
28921 ttgcattcgt tttcattaat gatttaactt gtcctttgag taatcgattt aataattcta
28981 ccgccacatt tttctgtttc aatccttcga catcttttaa aaagtcatca gataagattg
29041 ataaatcggg ttgttcaaga cctagcgttt gataaacatc aatgacatct tcagtcacaa
29101 cagattgtga cacaagttga ttaatctctg cttcaacttc tgcaggtgtt ttacgtgttt
29161 ttccttcttt tggcggttgt aacaatttaa caagtcctgc tttaactgct ttaaagaagg
29221 caatctcatc gttgagttct tgggctgtag gttcagtcgc acaaagagca aaggctttcc
29281 ctaattctgt gactgtttta ataaaacgtt gacgttcatc ttcacctaaa ccaattacat
29341 aatccatcgt attcgatatt gtataataac gttcagattt tttatctgaa ttaaatttag
29401 agtaatcaag attatataac atatcttgaa ttacatcata cttcaataac atcaattcaa
29461 ccgctttatc cgtatctatt gccgtttgag cttgatcaga ttctgtatat tctttaagtg
29521 cctcttttaa actttcggca atccccacat aatcgacaat taaaccaccc ggcttatctt
29581 taaacactcg attaactcga gcaattgctt gcatcaaatt atgccctttc atcggtttat
29641 caatatacat cgtatgcata gaaggaacat caaaccctgt cagccacata tctcgaacaa
29701 tcacgagttg taactcatca tccacatctt tcatacgttt ttctaataaa ttacggcgtt

29761 ttttaggacc aatgtgtctt tggaaagaag ttgggtcact agatgagcct gtcattacca
29821 ctttaatgac tcctttatca tcatcatctg aatgccattc tggttttaga cgaatgattt
29881 catcatataa atcaacagca attcgacgac tcatcgttac aatcattcct ttgcctttca
29941 tcgcttgttg acgtgtttca aagtgttgga tgatatcttt ggctagggct tctacacgag
30001 gttttgcgcc tgctaaagct tcaatacggg accattttga ttttaaacgc tgttttacat
30061 cctcttcttg atcttcagta atgtcattat acgcttcatc taaatctaaa ctttgaggta
30121 gatttaatgg aattacacga ctttcatagt aaattttaac ggtgcttcca tcagcgacgg
30181 cttgtgtcat atcataaaca tcgatatagt ttccgaaaac catttgcgta tttttatccg
30241 ttgaagctac gggtgtgcct gtaaatccta cgaatgttgc attcggtaaa gcatctctta
30301 aatatttggc ataaccatat ttaatgcctt cacctttatc atcgtatttt gcgttaaagc
30361 catattgtgt acgatgagct tcgtctgcca tgacaatcac attttttcgt tctgttaggg
30421 cagccatggt cgtttcgttt tgttcaggtt caaatttttg catcgttgta aatacaatac
30481 cacccgactc aacagataat aatgatttta attctttacg tgtttcagct tgttttggtg
30541 tttgtcttaa taatccttta ccagaacgtc cttttgattt aacaaacgta ctgtacagtt
30601 ggttatctaa atcattacga tccgttacga cgactaaggt agggttattc agcatttgaa
30661 ttaattttcc agagaaaaag accatggtta aacttttacc ggacccttgg gtatgccaaa
30721 taacgccgcc tttaccatca ccttgctcag atgaagctaa taaagctctg tcaacggctt
30781 tattaacggc ataatattga tgatacgctg ctagaatttt actgatatgt cctttgccat
30841 catcttgaaa taaaacaaaa tatcgaatta aatcaagtaa tgtttctgga tttaacatgc
30901 catgaatgag tacgtctaag ctagctaaac ctggtgatga ttcagtttga ccatctttac
30961 tgcgccaagt cataaaccga tcataatttg cggttagaga gccagccttt gtattaatac
31021 catcacttgt aacaagcact tcgttaaacg taaataattg tggaatacgc atcttatacg
31081 tttctaattg atgataaccg tcttcgacgc ccacggtttc atttgttgaa tttttaagtt
31141 caatcactac gataggcaag ccattgataa agagtacaat atcgggacgt tttttatagt
31201 ctccattaac gatagttaat tgattgactg ctaaaaaatc attgttttgt ggatattcaa
31261 aatcaataat tttaacaatt tctacaatcg attgtccttc atcatcatag tcttcgattt
31321 caataccatt aatcaaattt tcatgaaagg taaggttatt ttctaaaaga ttgggcgact
31381 tctctaaagt tagttcatgt agcgcttttt caataaaacg aggatggata tctgagttga
31441 tttttcttaa tgctttctct aaacgttcat gaaggacaac atctttatca cttttacgtt
31501 ctggtgttag acttgtcata ctaatctcat tacctttttt atagtcatag cctagtgatt
31561 gtagccattc taatgcgact tgttctaaat catcttcatt aaattgaaag ttcatcacta
31621 ttcacctcaa tatcatcaga tatttctagt tctccagaca ttagtttagg aagaagagta
31681 tctcgtaatt gagttaattt accaatttct gatcttaata ttttcatttc ttccatatat
31741 ggagtgatta tttcagaata tttttttaca atactatctt caattgctaa tttataatta
31801 acagctattg ttggttttac tctttgtcta ctatttgttg aaccggtagt attagctttt
31861 aaataatcta taaattgaga attaagacat atattatata tgaatgaatt aattttatta
31921 ttaggagatt ccattacaac aaattctgat gaagcaacat ttaatttttt attatctatt
31981 acaggtaacc aaatcctttt agtatcagga ttcatttttg aaaataatac gcaattgtta
32041 ttaataatcc atttattact ttttatttta ttgacttctt cctctatagc ttgttcttca
32101 ttatcataag caggtaaact aaaatgttta actgttactt cttcactctt tttaggatta
32161 aaagtttcct ttttatgact tgctatatct tttaatttat agattttcca attacttggt
32221 atcttaccta actcactatc aatcatttct cctccactag atttataagg attaccatct
32281 tcatctggaa attcaaaatc tacaaaccaa tgcttaaata gtgtttgtga aagttcttta
32341 aggtttgcta tcatttttttg gttagtttct atttttcttg tgatttgatt taaaaattta

32401 gaaatgcttt tttgttttat taaagaaaaa ttaggaattt tgacagtttc aataagttta
32461 gaatttatat tagcttgatt agcactccct gtagaatttc taactaaata ctcagttatt
32521 ttaggttgta gaaaataata aaataaaaaa tctaagtctg cactattact atattttaat
32581 tcgaattttc ccacacgttg attttgtaat gctggataat caaattcaat tcttccaact
32641 tttcctactg cagaagacat ttgattcacg cctgatccag ttaatgatat taatatatca
32701 tttttcttta atgaaaaacc tttagttttt tcatatgttt taatagagac tttttgagag
32761 tcttttgtat caacaatggg tgaaattata ttcttaattt taataaccgg tatacctttt
32821 tcttgaaatt cttttgattt aaaagcatat ccatttttaa ttttagctac atcaccgaaa
32881 aaaattgtat tatactccat atcctaaccc ctccaatgat tttcggattt ggtcttcaag
32941 ttcttttgat tttgcgaatt gttcacttaa ttcagaagta atacgttcca ttttttgttc
33001 aaaaggttct tcatcttctt caacgtcagc taaaccaaca tatcgtccag gtgttaaaat
33061 gtattcatta ttcttaactt cttctaaatt agctacttta caaaaaccag ctatatcttc
33121 ataaggttta tcgtttgtac ctctccacgc atgatacgtc tgtgctactt tttgaatatc
33181 ttcatctgaa aattctttta atgttctaga taccatatgg ccgatttcac gagcatcgat
33241 aaataaaatt tcattttcac gctctttttt accattttga cctttattat tactaatgaa
33301 ccaaagacat acaggtattt gggttgagta aaagagttgt ccaggtaaag taacaataca
33361 ttcaactaaa tcttgttcaa ttagattttt acgaatttct aattcatctt ttccgcttgt
33421 agacattgat ccgttggcta atacgaatcc tgctgtacca ttaggtgcta attttgaaat
33481 catatgttca atccatgcat agttagcatt accttttggt ggaataccaa attgccaacg
33541 gtaatcatca agtaatcgtt cttgacccca atcacttgcg ttaaaaggag gattggctaa
33601 tatgtaatct gcttttaatc ctttatgtaa atcattatgg aatgtatcag catttcgttc
33661 acctaagtca ttatcaatac cacgaatcgc taagttcatt tttgctaatt tccaagtcgt
33721 agggttagat tcttgtccgt aaatcgcaat atcgtctaat cgaccttgat gtttctcaac
33781 gaagcgttca ctttgtacaa acatcccacc tgaaccgcaa cacggatcgt aaatacggcc
33841 tttataaggc tcaatcatct caactaataa ctttacaatt gatgacgggg tatagaattc
33901 tccagcattt ttaccttcgg cacttgcgaa tttagcaata aagtactcat acacacgacc
33961 taacacatct tgtttacgac tctctgtgtc gccaacttta aatgtaaata aatcaataat
34021 atcgcctaat ttttctttat ctaatgcagg tcttgcatat tctttaggta atacaccttt
34081 gagtgattca ttttcatttt caattgcaat cattgcttta tcaataattt gtccaatttc
34141 tggtttttttc gcattatcat tgatatattg ccatcttgct tcttttggca cccagaaaat
34201 gttttctgct aagtattcat cttggtcctc ttcatctgca tatggatcct gcttcaattc
34261 ttcatacttt tcttcaaaag aatctgatac atattttaaa aagattaaac ctagtgctac
34321 atttttatac tcagcagcat ccatacttcc tctcaattta tcggcagctt gccataattt
34381 ttcttcaaaa cctatagtgg ccattaataa cacctcttaa aattaatgtt aaatcaacta
34441 tagcaaaaaa tagaagatga ttgtgtagtg ttttattaat atttaattta tctagactat
34501 caaaaaaata tagattatta ttatcttaaa ttaataaaca ggagtgaatt aaaatgaata
34561 aaaaatataa attaaaacga tacgataaaa gatttagagg tggatggtgt tacaaatgtg
34621 aagattcata ttcttacctt tgtgaagatt gtggttattg tagatattgt tgccattgtt
34681 aatacaagac tagataaatt gggtatcttt aactcaatta ataaaaaatt taaatgtagt
34741 caatagcttg ctaactttat ttgatgtaat ataattttgg cttttagcat tcagttaaaa
34801 agaagtaaat aattgacaga ttttttagag aactggagaa aaaagatgta catttctagt
34861 gttaaaatta agaattttag aaattttgat aatatcgata ttgatttcca tgagggagtt
34921 aatgtattaa tcggacataa taattcgggg aaatcaaatt tattacgtgc tttatcttta
34981 atttttgacg gttcagttag aaagcaactg tcagtggaag attttaataa ctctttaaca

35041 aaagaatctt taaaaaaaga ggctccaaaa atagtaattt cagtacatat aacacaatct
35101 gaaaacgaaa gactgatgtc tgatgagtta attacggtaa gtaattggct gactaaatta
35161 gaagaacctt atgaagctag aatacaatat gaattctttt taccaaaaga tgaggaaaaa
35221 aattacatag atttagtaaa aaatatagat gaaaaagaag aaatatggaa tttaattaga
35281 agccagttta ttaggttgta tgtcaataaa atctgggtgg gtaatccaga acatcagatt
35341 cctatagata atgatagttt aaataaattt gatttccaat ttttagacgc aataagagat
35401 gtagagagag atatgtttag tggtaaaaat acattattaa aaagtgtaat agattttttt
35461 atagattacg atataaaatc cgatgaaaca atcacagaag aagaacagaa agaaaaatta
35521 gaagaaagaa gaaaagaatt ttctgataat tctagtgatt tgattgaaac tattcataaa
35581 agattagata gtgggaatca aaaaattttg tcttatacaa atggcatagg agcatcttat
35641 gataaatcaa caccggattt taaaggtaat cttacagaaa gtgaaattta tactgtattg
35701 caattgataa ttaaacatga aactggaatg actttaccta taactcataa cgggttgggt
35761 tacaataatt taattttcat ggccttgcta ttgtctaaaa tgcaagctga ttctgatgga
35821 aattttctag gtagtaatgc aaaagtattt ccgatactag ctattgaaga accagaggct
35881 catctacatc ctactatgca aaatgaattt attaaatttt taaaaaataa cattagagaa
35941 aaaaaggtaa agcagatttt cataactact cattcaacac atatttcttc ttctacaaat
36001 attgatgata ttatttgtct ttacacagat gatagtaaaa ctaatgttag ttatcctgga
36061 aaagtttttg ataagaataa tcaaagtaag aagtacgtac aacggttttt agatgccact
36121 aagtcaaata tgctatttgc agaaaaggta atatttgtgg aaggtatagc agaacaatta
36181 ctattacata tatttgcaga ctatttaaat aaaccgttag aaaagaatca tgttgcagta
36241 ataaatgtag gcgggagata ttttaatcat tttttatctt tgtttgatag taataataaa
36301 tatgcaatta atcgtaaggt ttcttgtata acagatttag atccaatgcg aaaagaaaaa
36361 aatgaaaatg gacaaaaaaa taattttaaa gcatgctatc cttttgaata tgaaatggat
36421 ttagataaat ttgattattc aactaataat tctctagata aatatctaaa taatgagcac
36481 agtaatatac gagtatttgt tcaacctaat aaatatggaa aaacatttga gtaccaactt
36541 atgtttgaca atccttcatt aaaactgtta cttacagact cgatttctaa tagtcaagag
36601 ctagcagaat taatggatca ctatgaagaa aatgaatctt tacaaaaatt aattgataca
36661 ttatcaccat ctaatgaaaa tcagagaata ataaaatctt taaaagacac aaatgacagt
36721 tggaatgaag ataataaaaa gaaagcatta atagcatcaa gatacctaaa ttccatagga
36781 aaaggagaaa atgctctaga gctagcttca gtattaaaag ataatttaga gttaaaaggc
36841 cagagtgact atgaagattt tgtagtacca aaatatatag aaggtgcaat tcgatgggtg
36901 tgtgaataat atgattatta atgctcaaac gaaattaaat atagaacaag attttaaaat
36961 acaggctggt cctggtgctg gaaaaacaga atttttggtg aatcatatca aaaatgtaat
37021 acaaacttct gaaaagctcg aacgtatgaa gaaaatagcg tgtattactt atacgaatac
37081 ggctgctcag acagttttac aaagattagg aaaaagtgtt tcaaatagag tagatatatc
37141 tacaatacat agctttttgt atagaaacgt agttaaacca tattgttctt ttctacccaa
37201 agaatacgga atttgtgtgc aaaaattaaa aggacatagt gatccaattg tgattaacaa
37261 atatgtaaat gcatggttaa aaactgaata ttttacgaaa ttaaaaccgc ctagtaatag
37321 aaatcaacta ctaaaaatgc cagtactaaa gaaagctttg caaaactggc tactatctat
37381 gaaactttct tacgaaaata atgatgtgca atttatttgt gataatacca aagctcaagc
37441 aattgataaa aaaagtaaaa aaagaatggg aataaataaa tcaaacttaa atatactaga
37501 aaagcaatta ttaaattata aaaaaatata ttgggagaaa ggaattatcg atcatgatga
37561 tgttttgttt ttttcatatg tattaattag agattaccca ttcattctca ctattttatg
37621 tgctaaatat ccctactttt ttatagatga atttcaagat actagtcaat tacaagattt

37681 tattatagat aaaataagac agaaaggctg catagtgggg gttattggcg ataaggcaca
37741 ggcaatttac agctttcaag gtgcacgggt ttccttattt gagaagttta aagttgattt
37801 aataaattct cacactatct tagaaaatca tagaagttca tatcaaatag ttactttttt
37861 aaatagtatc agagaagata ttactcaaac agtatatagt ggtattgaaa atgataaagt
37921 tacaattttg attggaaatc ggattcgcag ttatactaag gtagttaata tttgtaacaa
37981 ggagtcactt gttaccttgt caagagataa tattacttct aatgcaatga gaataaagtt
38041 ggaaggaaat aattttgata aaaagttatt ggaaaaattt caagaaattg atagtaatag
38101 taaaagaagg aatactatta tttcgcttat aaaagctatt gaattagcta agaatactaa
38161 atataaagat gccttaaaaa tagtcgaatc gatttataaa gatgaaaata atcctaaaaa
38221 aatagctctt aattctctaa taaatatgat taataaatat gataaatacg gtaatggtac
38281 attaatgaat ttctacaata ctttatgtaa aactttagat attaaacttt cagggtttag
38341 aaaggggcaa gtaaaagatt tttacgaaaa cactttatat aaaaaatatgg tgatatgtac
38401 aaatatcgtt gaagacacaa gtaatcatat tactatccac aaagcgaaag gcgcagaatt
38461 cgaaaatgta tttgttatag gtaataaaga cactttaagt ctacttttga aaccagattt
38521 aatgaacgat gaagagcatc gtatattcta tgtagctatg agtcgagcaa agaagaaact
38581 ttttttacaa tttgattatc ttgataataa tgaacaaaag aaaattaagg aacgttatga
38641 tgtaagtatt atacaataac ctaatctaag aatatttaaa tccaatatat ttcttgatta
38701 taattcaata atagataaaa tctataaacg attaaaaaga aaatatattt gctaagactt
38761 ataaaaatga agagataata gattttccta aattacagtt aaaagaagta aatagattga
38821 acactttgaa agattctaat ccaaaatttg aaccactaaa attgaaagat gaagaagatg
38881 caagatgtag ccatcaacgc tgatcacaaa gtatttttct taatgattat ttttaatgtt
38941 atcatgctcg tttcttctat tattcaaatg attttaggaa aaggcagacg tatcacacca
39001 gcaaaaccta ctaaagatgc taagttacac ttagataatc aatcctcata aagagaaaaa
39061 agaaccccta aaaagttcaa ctttttgggg gttacttact attaagaagt atttttgatt
39121 aagcatcttt catatcgtaa tcaccaatat actgatcaag gcgatttatt gtttctctga
39181 tatcatcatg tgtcgttaat gggttcgttg tacataaacg aatgacacgt tggttattca
39241 atactgtggt ataagcaatg gcataaccag aatctgcaat tttttgcgca gccatgctat
39301 tgagttgatt attttgtgct tcagttaaat cactgttttc atatctgaag ttcacaatgg
39361 aaagattagc gtgtgagata atacgccagt tatcgagacc tgacacgtat tcttctgcat
39421 attctgctaa accttcgccg tatttaatgc gctcgataat ttcatcttca ccaatgactt
39481 gcagagtaat ccataatttt aaagcacgtg cagggcgtgt aagttcaatg cctaacattt
39541 ctggatcaat cacatcatca tctgaagcaa tatcatccaa gtactcagca tcttcgccaa
39601 agctttgcag cagatgattc ttttctttca cgattaccat agcgcaacta tatgtttgga
39661 acagtaattt gtgagcatcc caactggcac tatctgaacg ttcaatgcct ttaaataagt
39721 gttgtgcttt atcggataag atatgtgata gtccataagc accgtctaca tggagccata
39781 gattgtagtg atcacatgta tctccaagtg tggtgaaatc atcgacagaa cctgtattag
39841 tcgtgcctgc agtcgcaatc accatagcag gtttgtaacc ttcttcaata tctttatcaa
39901 ttgcggcttt taagctatct gtattcattg taaagtcatc gttgtactca attctgcgga
39961 tattattttt aaggaagcct gcaacatgca aggctttgcc tacagaatga tgcgtttgag
40021 atgtaagata aacggttgct tttttaatat cttccatttc tacctgtgca tctctagccg
40081 caacgatagc tgttaaattg gccattgaac cgccagatac aaagacacca ccggccggtt
40141 tgatctcata accgattttt cctaccaagt agttaattaa attacgctca atattaattg
40201 gcagcgtcgc attggcaaaa ttagaggcat ggatattatt tgctgttgtt aaaatgtctc
40261 caagccatga tagacgtgat gctggacctg gtataaatga gaatgaacgt ggatggttcg

40321 gacgatataa ataattcagt acttcattat ttaaatcttt gagtacttca tagatatctc
40381 gcccttattt cggcacttcc atcttttcat atttctctct caattcgagc ggtgcctgtt
40441 gtgtcgcagg taagtctcca atatctctag ataaatattt attaacttca tctctgataa
40501 tcgtctctaa gtcgatattg ttctcattga attccatttc catactctct ccttcaatta
40561 tatttttaat aaaagacaga cgtattaatt acgacgtttt actacaatat aaatgatagc
40621 agatattaat aaaaccacgc taaccataat gacgaaaccc agaaggtgtc gccccaatga
40681 ataacgtgct gtgctttaac actcggtgta tgtgcgcctt tcagaaattg acgagggtaa
40741 tcgacttgca gattatcatc tgtgattttc aatttgtatt gatgcttgtc ttttggaatt
40801 cgataactac cccgtagaac aggggacgag gacctcatct tgatgatccc aattggcttg
40861 gttaaaaaga aatcgataat cctcgtagaa tagagaaaaa gtattaaaaa tatatttgat
40921 ttgataaaat attttgcaaa aaagagaaac aaaaataact ttcatataaa taatttcttt
40981 gcgattaagt tggaattaga aatactaaat acaaataaca gcgaatggaa tgcatattat
41041 tgaggaattg gatgtgtagt gcaatttcga gagttactac ttaacttaag aaaataaaaa
41101 gaagaaaaca aacaataaga attaaatctg aagcaagata taaaagcaaa tatttttgct
41161 acaattatat tagtaaatac tttattaatt agcatacgac tgaaagcgaa gtaacagcta
41221 ataatgttcc agatatagac attagtgtta tgaaagaaca atattaccat ctagaaaatg
41281 taatttttaa aaacctgaag ataaagtaga ctattctgtg agttttcatg ttaagaattc
41341 tgaacttggg gatgtagtat aaatatggtt tagtagaaaa tgacgctgga gctgtagctg
41401 agattgacta tcaagaatta actaaagtag attattaccc atcaacaaaa atccaagatt
41461 taacactatc taataatagt gcgaataatg aagtttaaaa gtgaaaaact gataatagcg
41521 aatcaaacta aagtaatcaa ttacagaatt caagtactac tcaagcctca aacaaatatt
41581 gaaactcaaa cacaagttaa gaataataca ctgtcagaaa cagatgaaca gtctaatgcc
41641 gatttagtcc agtaatagcc tctgtattat tagccgcacg attattatca ataattaaat
41701 aaagttaata ttaaataata aaaatccttc aaattatttg ttgaaaaaat aataactttg
41761 aaggtttttt agcaaaatca ctgataggga gaagcgtatc ataaat
//
LOCUS Staphylococcus 39139 bp DNA linear UNK
DEFINITION Staphylococcus Staphylococcus aureus strain HL24830
ACCESSION Staphylococcus
KEYWORDS .
SOURCE Staphylococcus Staphylococcus aureus
ORGANISM Staphylococcus Staphylococcus aureus
Bacteria; Bacillati; Bacillota; Bacilli; Bacillales;
Staphylococcaceae; Staphylococcus; Staphylococcus aureus.
FEATURES Location/Qualifiers
source 1..39139
/organism="Staphylococcus Staphylococcus aureus"
/project="hwwang_703339"
/genome_id="703339.290"
/genome_md5=""
/db_xref="taxon:703339"
/mol_type="genomic DNA"
CDS 1..480
/product="23S rRNA

(pseudouridine(1915)-N(3))-methyltransferase (EC
2.1.1.177)"
/db_xref="SEED:fig|703339.290.peg.1"
/EC_number="2.1.1.177"
/translation="MKITILAVGKLKEKYWKQAIAEYEKRLGPYTKIDIIEVPDEKAP

ENMSDKEIEQVKEKEGQRILAKIKPQSTVITLEIQGKMLSSEGLAQELNQRMTQGQSD

FVFVIGGSNGLHKDVLQRSNYALSFSKMTFPHQMMRVVLIEQVYRAFKIMRGEAYHK"
/transl_table=11
CDS 726..1031
/transl_table=11
/translation="MKGNIQCPRLIKGSLLDFVLETVDVFVNGSKNDCELVNFNYKEG
KVVSRYSTKFESNPKLRKKQLISTVKVVRYVIFDFEKILWGIGKVLLKFIILRQCLL"
/db_xref="SEED:fig|703339.290.peg.2"
/product="hypothetical protein"
CDS 1230..2093

/translation="MKHINQFSESTLAQLEKDQQHVFYVYCLMDPRNDECFYVGKGKG
NRIFKHKQDAQKKVLYEDIFIEENKDNLKFNRINEICSNDLNVLGYIISYGLTESEAF

SAENVLINFLNLTNKTTLTNMINGHGSKAYLVEDLENEFGYDSINLENINTNELILAV

KIRDAFLLDKDESKEYPINESKRDRNNLKSRTLGSWIIGKDKIHKIKYIIGINTGANN

AVVSAYEVSFEQAESIETNNGRMRYAFIGLSERDATLKKLNLYKKALPDLRFGSGSAT
AYINNGTMKVD"
/product="hypothetical protein"
/db_xref="SEED:fig|703339.290.peg.3"
/transl_table=11
CDS 2201..3676
/db_xref="SEED:fig|703339.290.peg.4"
/product="hypothetical protein"
/translation="MPSDDIVKKLYSKEDIRLELGLTPHKFNKKMETIAKLFKIDMKI

FHNYKGQDKNNQYTFNGVAKELIKVLLKSVDYYPVDINSKKFKQNGKSKKEMIENIDN

SSYMKYIYQLMKSINEIQYKRLIADIHMKDVYQNTKAWLNNGESINKKEQELYQYMTI

LPLHKRVELQNEVLKSIDETIFQFLAKEHRNNQIEENNELEAYTKAIKEGRNPKNDYE

LNHLLYKKNQLPLDSLIEDIWDYEETEYTELDWLIADMLKRSQRADSNFIESLEKKNK

LRKNIHKDSIKSISKLIDHELVKNNRKWDVAEFYKNQQFWNNSTLRRSSQNFGILYYK

KNRLHTMNQSNIIKKIKIIEANIESANKQPSYLDFFLQAKFDLERLESELMKYSINPK

YFNKINSDYVRYAIEDVHNAIIKIDRYISNDKVKLNYSNVQMVVRNEITEQGSYFVTQ
ALNTLSKVEESGYSFDNFMVGPFVEDFKKAIKNRRFGSKKD"
/transl_table=11
CDS 3902..5002
/product="hypothetical protein"
/db_xref="SEED:fig|703339.290.peg.5"

/translation="METRDKLMSLTQSDKTQQWLMDKSSNQDDIQQLQQQFSQQLDQQ

YNALLADEKAKLDQYVEVHQGLESLKEEIESEPITLNIDKLPDIKATMLERAKNDEHS

DKIEKLFDRLEQALNGTNRLYTQLSLIGTRTHRITTKNFNLQGLPKAVQHTILPSKFK

KVYTVDFKSFEPSVAAYMTQDSKLIDLLNQKDGLYDALLSELGLSDELRVFVKRAFIG

SFLFGGNFKNPKFKLNQYVSEVQWLDAVSQFTKVIELKKHVEKSNSMPMPYSIEHDMS

AFQGSSIMAIYVQTVASYIFKHILLKVYKAQCDQKTFKIIVPIHDAIMIECEDEEIAQ
NVGQLMKDTANQLFNGEFAHVTVEEIGGVDHE"
/transl_table=11
CDS 4995..5366
/transl_table=11
/db_xref="SEED:fig|703339.290.peg.6"
/product="hypothetical protein"
/translation="MNNDRGQSLHIPSSTPIKENNIYVATLHSVIQTDFSGEIKHQFT

YEIEVNNQIVYANRNIPTKPSANQLSIHDWLKRHSNYSASHENYEPYIDQKHLILLGQ
YNGNYYVQDVASLDAFGGVLS"
CDS 5363..7006
/db_xref="SEED:fig|703339.290.peg.7"
/product="putative primase"
/translation="MNHILQMLSKLLSVAKEAIDRQGLIAILTIPVNNNDEIEETAQG

ETVYNELIDQLRLNIPKDTDYQPNIYSYFGIKKNPNDTVLMEMMIKVFHIKRFNSELF

IFKVNGWQKINGDELQGLISKMIQVLLVDYKPSLSTLKNVVDGLQKSTDVEELVENER

YIGCGENMFDLNTFQVVKNSIDIFPKTRLNLSLSTNDVITDKIPPYFKQYMLQLANYD

DDLQYFLFQHTAVLLTADTKYRRGLILYGGAKNGKSVYIELVKSFFYSKDIVSKPLNE

LEGRFDKESLIDKSLMASHEIGQSRIQEKIVNDFKKLLSVESMHVDRKGKTQVEVILD
LKLIFSTNAILNFPPEHAKALERRINIIPCEYYVEKADTSLIDKLQSEKKEIFLYLMY

VYQQIVKADIEYLENSRVTEITHDWLNFGYEFVSSRSVSNANQKACINLLRKLIEIKS

GSRIKVSELNKVINEEIKVSSQVIKQLIQANFDTQTKLYNGYDYWIDLGWKEANKKEI
HDISEKDNIISLDKNENITDDEALDEENLDFDWEDFDDE"
/transl_table=11
CDS 7232..8908
/transl_table=11
/translation="MKGKIALYSRVSTSEQSEHGYSIHEQEQVLIKEVVKNFPGYDYE

TYTDSGISGKNIEGRPAMKRLLQDVKDNKIEMVLSWKLNRISRSMRDVFNIIHEFKEH

DVGYKSISENIDTSNASGEVLVTMFGLIGSIERQTLISNVKLSMNAKARSGEAITGRV

LGYKLSLNPLTQKNDLVIDENEANIVREIFDLYLNHNKGLKAITTVLNQKGYRTINQK

PFSVYGVKYILNNPVYKGYVRFNNHQNWAVQRRSGKSDKNDVILVKGKHEAIISEEVF

DKVHEKLASKSFKPGRPIGGDFYLRGLIKCPECGNNMVCRRTYYKTKKSKERTIKRYY

ICSLFNRSGSSACHSNSINAEVVERVINVHLNRILSQPDIIKQIASNVIEELKQKHSN

QTEIKYDIDSLEKQKAKLKTQQERLLELFLDDQMDSEMLKAKQSQMNQQLEVLDQQIK

EAQQANQSQDEIPNFDKLKGRLILMITRFSVYLRKATPEAKNQLMKMLIDSIEITTDK
QVKLVRYKIDESLIPQSLKKDWGSFFMPKFQFEIYGQNDYFIDQITTFTT"
/product="Cassette chromosome recombinase B"
/db_xref="SEED:fig|703339.290.peg.8"
CDS 9014..9352
/db_xref="SEED:fig|703339.290.peg.9"
/product="FIG01108299: hypothetical protein"
/translation="MTLEQQLKHYITNLFNLPRDEVWHCESIEEIADDILPNQYVRLG

PLSNKTLQTNTYYSDTLHESNIYPFILYYQKQLIAIGYIDENHDMDFLYLHNTIMPLL
DQRYLLTGGQ"
/transl_table=11
CDS 9354..9455
/transl_table=11
/translation="MHKYIKITQLVITILSEIIIWMKESERKEVSYE"
/db_xref="SEED:fig|703339.290.peg.10"
/product="hypothetical protein"
CDS 9448..9759

/translation="MNRYITRGIANNLPNILQHQLWQLVSEREQEQTKDNTSVDYFHI

FQFNKHRNQLYIKHKQERPEYAKIHKANYSKAININKVYIIREDDVDLSYYVMLLPEE
Y"
/product="FIG01107894: hypothetical protein"
/db_xref="SEED:fig|703339.290.peg.11"
/transl_table=11
CDS 9775..10281
/db_xref="SEED:fig|703339.290.peg.12"
/product="hypothetical protein"
/translation="METIKSTLKTEAIFSDDKQHRYLLKKTWNSEKQSITIITMYPHY

DGILNIDLTTQLIMNKVSEMDAFGSINFVNLYSNITTPINLKHLENAYDKHTDIQIMK

AVKESDEVILAWGAYAKKPGVEARVNEVLEMLKPHKKKVKRLMNPETNEIMHPLNSKA
RQKWILKV"
/transl_table=11
CDS complement(10430..11104)
/translation="MNYFRYKQFNKDVITVAVGYYLRYTLSYRDISEILRERGVNVHH

STVYRWVQEYAPILYQIWKKKHKKAYYKWRIDETYIKIKGKWSYLYRAIDAEGHTLDI

WLRKQRDNHSAYAFIKRLIKQFGKPQKVITDQAPSTKVAMAKVIKAFKLKPDCHCTSK
YLNNLIEQDHRHIKVRKTRYQSINTAKNTLKGIECIYALYKKNRRSLQIYGFSPCHEI
SIMLAS"
/db_xref="SEED:fig|703339.290.peg.13"
/product="Transposase for insertion sequence-like element
IS431mec"
/transl_table=11
CDS 11352..13358
/db_xref="SEED:fig|703339.290.peg.14"
/db_xref="GO:0008658"
/db_xref="GO:0046677"
/product="Penicillin-binding protein PBP2a, methicillin
resistance determinant MecA, transpeptidase"
/translation="MKKIKIVPLILIVVVVGFGIYFYASKDKEINNTIDAIEDKNFKQ

VYKDSSYISKSDNGEVEMTERPIKIYNSLGVKDINIQDRKIKKVSKNKKRVDAQYKIK

TNYGNIDRNVQFNFVKEDGMWKLDWDHSVIIPGMQKDQSIHIENLKSERGKILDRNNV

ELANTGTAYEIGIVPKNVSKKDYKAIAKELSISEDYIKQQMDQNWVQDDTFVPLKTVK

KMDEYLRDFAKKFHLTTNETESRNYPLGKATSHLLGYVGPINSEELKQKEYKGYKDDA

VIGKKGLEKLYDKKLQHEDGYRVTIVDDNSNTIAHTLIEKKKKDGKDIQLTIDAKVQK

SIYNNMKNDYGSGTAIHPQTGELLALVSTPSYDVYPFMYGMSNEEYNKLTEDKKEPLL

NKFQITTSPGSTQKILTAMIGLNNKTLDDKTSYKIDGKGWQKDKSWGGYNVTRYEVVN

GNIDLKQAIESSDNIFFARVALELGSKKFEKGMKKLGVGEDIPSDYPFYNAQISNKNL

DNEILLADSGYGQGEILINPVQILSIYSALENNGNINAPHLLKDTKNKVWKKNIISKE

NINLLTDGMQQVVNKTHKEDIYRSYANLIGKSGTAELKMKQGETGRQIGWFISYDKDN
PNMMMAINVKDVQDKGMASYNAKISGKVYDELYENGNKKYDIDE"
/transl_table=11
CDS complement(13404..13832)
/translation="MKYDDFIVGETFKTKSLHITEEEIIQFATTFDPQYMHIDKEKAE

QSRFKGIIASGMHTLSISFKLWVEEGKYGEEVVAGTQMNNVKFIKPVYPGNTLYVIAE
ITNKKSIKKENGLVTVSLSTYNENEEIVFKGEVTALINNS"
/product="MaoC domain protein"
/db_xref="SEED:fig|703339.290.peg.15"
/transl_table=11
CDS complement(13929..14672)
/EC_number="3.1.4.46"
/translation="MLTVYGHRGLPSKAPENTIASFKAASEVEGINWLELDVAITKDE

QLIIIHDDYLERTTNMSGEITELNYDEIKDASAGSWFGEKFKDEHLPTFDDVVKIANE

YNMNLNVELKGITGPNGLALSKSMVKQVEEQLTNLNQNQEVLISSFNVVLVKLAEEIM

PQYNRAVIFHTTSFREDWRTLLDYCNAKIVNTEDAKLTKAKVKMVKEAGYELNVWTVN
KPARANQLANWGVDGIFTDNADKMVHLSQ"
/db_xref="SEED:fig|703339.290.peg.16"
/db_xref="GO:0008889"
/product="Glycerophosphoryl diester phosphodiesterase (EC
3.1.4.46)"
/transl_table=11
CDS complement(15092..15331)
/translation="MLIFLSYDFVRACLPRGMARAISLSHILFPQASALTKSVVIFIF
IRILTEIILIRGIVKAILSKIADKRYVKSNSMQNC"
/product="hypothetical protein"
/db_xref="SEED:fig|703339.290.peg.17"
/transl_table=11

CDS complement(15429..15596)
/product="Hydroxymethylglutaryl-CoA synthase N-domain
homolog (EC 2.3.3.10)"
/db_xref="SEED:fig|703339.290.peg.18"
/translation="MLESREQLSVEEYETFFNRFDNQEFDFERELTQDPYSKVYLYSI
EDHIRTYKIEK"
/EC_number="2.3.3.10"
/transl_table=11
CDS 15854..16528
/product="Transposase for insertion sequence-like element
IS431mec"
/db_xref="SEED:fig|703339.290.peg.19"
/translation="MNYFRYKQFNKDVITVAVGYYLRYTLSYRDISEILRERGVNVHH

STVYRWVQEYAPILYQIWKKKHKKAYYKWRIDETYIKIKGKWSYLYRAIDAEGHTLDI

WLRKQRDNHSAYAFIKRLIKQFGKPQKVITDQAPSTKVAMAKVIKAFKLKPDCHCTSK
YLNNLIEQDHRHIKVRKTRYQSINTAKNTLKGIECIYALYKKNRRSLQIYGFSPCHEI
SIMLAS"
/transl_table=11
CDS complement(16588..17016)
/translation="MKFQVSPYFLITQNAEGLKKLYEELFGAKTLYIQRIKDRPIDKK

LNIDENDLNKIDQCVLQFGDIKIMIADDTEGLPITQGNNISLCLTFDQVEETKHVYDQ
LIEKGSEVLKTFSPEFYTEGYGYVRDPYGISLHLFTKRKN"
/db_xref="SEED:fig|703339.290.peg.20"
/product="PhnB protein; putative DNA binding
3-demethylubiquinone-9 3-methyltransferase domain
protein"
/transl_table=11
CDS 17097..18026
/translation="MKKSVRLYNMIEYCNENRNFKLNDLMSEFNISRSTALRDIKEIE

ALGVPLYSNPGKNGGYTIIGNRDQTKIAISDEELKALVFTLSSISNVSNLPFQTEYQE
ILKKLYNNSNKKELINQYNDLFQYFNEDKYQFKSYKLFNEIIRLIIENKSFETCYSQN

YIKEQYKGIGIMYKNHQWYFVVANIESKLVNLLNISKIKELYEMGETQECNDITMQNF

QQFMVKNETAIDILIRSNVMGLNILKGYLWSDYMIENIDEETYLFKSKVNAKDIDFIA
KLIVTGGVNVKVETPNSLKNAVKVELTKIINMY"
/db_xref="SEED:fig|703339.290.peg.21"
/product="Transcriptional regulator, YafY family"
/transl_table=11
CDS 18188..20176

/product="Similarity"
/db_xref="SEED:fig|703339.290.peg.22"
/translation="MNKNITALKSTENTTYMQYTVHIDDQIINNIKECTKKFKILPME
DKIPLSPLLQPEYAGEVQDFISTYEQFMINFGKVILDSQGIKIQFESESLSSIQRGIQ

EHCYLNERTNDIDVTKEWYLCKFSNQILEEDKNQLYNALKQLMNDSKNKKQAFQEVFK

VHIDIYLYNKEKSEYKYQVSSYFNLVKENPKITYKKRHLQEKQGVKGTTFTNKLKLLN

KMYGVDVAKYQPFYNSNNPEYERGQFGERYISQRSNYEFNRLQYQIIDMLSKILDKHP
LPKSDNNYKHIPTIEKAILSGDSHSFYEYFEDIMKEIISMENSSLKEKLLTDFTYQSQ

CRWYSESEKLKLQLESFMHKVLESNYYEGNKLYRMLSHAIEETINEADEDKVHFNYFK

DYFLTDGGVKNWEQISEKITEFNGKVINDIQNEYNKIQFNNANRNQKLNFNYLYHCFE

FSNNLVKARVNGNRGYILYFVDKYKIKMPFLDQLLINYLNQEIGQESINYNMQTLFEK

ERYDRSSTIEKLVATSKFKYEKDDSDLFKQLFNDVENSIDRLGIYLLNNGINSNDENA

RYYRSFLKELSRIKSKLTPFSLEISKSSGREQHYPDDAIDDKDERRKNKEETYHAFDD
KSDIDSKLKNKINVSIDNLLSVKIKSNLYDISPNTKKQ"
/transl_table=11
CDS 20371..21480

/translation="MYQANIRDLITKLPQSNKTEHFLMNKFSNQDKVQQLQRQISQQL

DQQYNELLANEKAKLDQYVEVHHNLEPLKKEIESESINLDTDKLPDIKATMLEKAKND

EHFDKIEQLFDRLDQSLNGTNRLYTQLSLIGTRTHRITTKRFNVQGLPKLVQQMILPS

QFKKVYTIDFKSFEPSVAAYMTQDEQLIDYLNHEEGLYDALLRDLSLSKEKRVSVKRA

FIGSFLFGGRYSSSKFKINQEVSEINWLQVMSKFKKVIEFKEQVEKYKTMPTPYGIEH

DMSAFQGSSIMAIYVQTVASYIFKHILLEVYKAQCEKKTFKIIVPIHDAIMIECNDKG
IAQNVAQLMKDTANQLFNGEFAHVTVEALGGIDNE"
/db_xref="SEED:fig|703339.290.peg.23"
/product="hypothetical protein"
/transl_table=11
CDS 21473..21841
/transl_table=11
/translation="MNNDRGKSLQIPQSTLLKEGSIYVATLHSVYEKNFSGDIKHQFT

YEVELNQETHYVNRNITVKSMSHQLSIADWIKRHSNYNVNHINYDPYIDRKHLVLVGQ
YNGNYYIQDVAPLDEFGGVL"
/product="hypothetical protein"
/db_xref="SEED:fig|703339.290.peg.24"
CDS 21841..23457
/transl_table=11
/translation="MNHILEMLIKLLKVGMEAIDRKGLIAILTSSIGNDEMDDSEQAV

MVYNELIDKLQLNIPKDVDYRPNIYSYFGIQKKPNDTILVEMMICIFHIKRFDSELFV

FKDKGWQKVSEDELQGLISKMIQVLLVDYKPSLSTLKNVVDGIQKSTDIEKLVEHRQY

IGCGRNMFNLKTFKVVDNDLEIFPKTRLDLELDINDTITDKIPPNFKQYMLELANYDH

DLQYFLFQHMAVLLTADTKLRRGLFLYGTAKNGKSVYIKLVKSFFYSNDIVSKTLNEL

GGRFDKESLIGKRIMASDEVGKANIDEATVNDFKKLLSVEPIHADRKGRTQVEVTLDL

KLIFNTNAVLNFPSSHAKALERRIAVIPCEYYVEKADPDLIEKLQDEKKEIFLYLMYV

YKQIVKNDIEYLQNDRVTEISHDWLNFGYEFVSSKSANIAHQKACINLLRKLIEIKPG

SRIKVSRLNEVIRDEIKVSSQVINDLVQANFNVQSRLNNGYKYWVDLGWKETDKKDDM
ISFDKNENVTDDEFLYEDDLNLGWEDFDDE"
/db_xref="SEED:fig|703339.290.peg.25"
/product="putative primase"
CDS 23682..25361
/transl_table=11
/product="Cassette chromosome recombinase B"
/db_xref="SEED:fig|703339.290.peg.26"
/translation="MKGKIALYSRVSTSEQSEHGYSEKEQEQLLIKEVMKNFPGYDYE

TYTDSGISGKNIEGRPAMKRLLQDVKDNKIEIVLSWKLNRISRSMRDVFNIIHEFKEH

GVGYKSISENIDTSNASGEVLVTMFGLIGSIERSTLASNVKMSMNAKARSGEAITGRV

LGYKLSLNPLTQKNDLVIDENEAHIVREIFDLYLNHNKGLKAITTILNQKGYRTINQK

PFSVFGVKYILNNPVYKGYVRFNNHQNWAVQRRSGKSDKNDVILVKGKHEAIISEDVF

DQVHEKLASKSFKPGRPIGGDFYLRGLIKCPECGNNMVCRRTYYKTKKSKERTIKRYY

ICSLFNRSGSSACHSNAINAEVVERVINVHLNRILSQPNVIKQIASSVIEELKQKHSK

QTEIKYDIDSLEKQKAKVKTQQERLLELFLDDEMDSEMLKAKQSEMNQQLEVLDQQIK

EAKQANQSQGDIPNFDKLKARLILMITRFSLYLRKATPEAKNQLMKMLIDSIEITTDK
QVKLVRYKIDESLIPQSLKKDWGSFFMPKFNFVINVANENRIENLSLLPLF"
CDS 25450..25788
/transl_table=11
/product="hypothetical protein"
/db_xref="SEED:fig|703339.290.peg.27"
/translation="MTLSKQLKTYITERFKLNYQETWACETVDAVAEDVLPEKYIKNS
PLEHKILNTFTYYNDELHEISIYPFLCYLDKELVAIGYLDNFDLDFIFLNDTHQVIID
ERYLLQKGGE"
CDS 25791..25880
/translation="MNWIKVAQLSVTVINEVIEIMKEKQSGGK"
/db_xref="SEED:fig|703339.290.peg.28"
/product="FIG01108299: hypothetical protein"
/transl_table=11
CDS 25882..26193
/transl_table=11
/translation="MNINRYITRGINESIPLDLQILLWHMVEEKDNQPHTDYLHIFKL

QEDDNMLSIKHEQEQPAYKLEYHYTNYEKNQNALPKKVYVIREDGVDAFYYVMLLPEE
Y"
/db_xref="SEED:fig|703339.290.peg.29"
/product="hypothetical protein"
CDS 26211..26714
/transl_table=11
/product="hypothetical protein"
/db_xref="SEED:fig|703339.290.peg.30"
/translation="MNTIKNTIYTEAIFSKDEKHRYLLKKTWDEKKPACTVITMYPHL

DGVLSLDLTTVLILNQLANSEQYGSVYLVNLFSNIKTPENLKHIKEPYDEHTDIHLMK

AISESDTVILAYGVYAKRPVVVERVEQVMEMLKPHKKKVKRLINPATNDIMHPLNPKA
RQKWTLK"
CDS 26728..26949
/db_xref="SEED:fig|703339.290.peg.31"
/product="hypothetical protein"
/translation="MNHETKQSDWHTVANCLESQNYTSIVKGLVHHFTAIEDEEILDK
IYDDFMNDDSITTVLNNDLQIIINHYLSK"
/transl_table=11
CDS complement(27023..30142)
/EC_number="3.1.21.3"

/translation="MSFQFSEDDLEQVALEWLQSLGYDYKKGNEISMTGLAPERKSDK

DVVLHERLEKSLRKINSDIHHRFIEKAIHELTLEKSPNLLENNLTFHENLINGIEIED

YDDEGQSVVEIVKIVDFEHPQNNDFLAVNQFTVINGDYTKRPDIVLFINGLPIVVIEL

KNSTNETVGVEDGYHQLETYKMRIPQLFTFNEVLVTSDGINTKASSLTANYDRFMTWR

SKDGETESSSSLASLDVLIHGMLNPNTLLDLIRYFVLFQDDGKGHISKILAAYHQYYA

VNKAVDRALLASSGPGDGKGGVIWHTQGSGKSLTMVFFSGKLIQMLNNPTLVVVTDRN

DLDNQLYSTFVKSKGRSGKGLLRQTPKQAETRKELKSLLSVESGGIVFTTMQKFEPEQ

NETTMAALTERKNVIVMADEAHRTQYGFNAKYNDKGEGIKYGYAKYLRDALPNATFVG

FTGTPVASTDKNTQMVFGNYIDVYDMTQAVADGSTVKIYYESRVIPLNLPQNLDLDEA

YNDITEDQEEDVKQRLKSKWSRIEALAGAKPRIEALAKDIIQHFETRQQAMKGKGMIV

TMSRRIAVDLYDEIIRLKPEWHSDDDDKGVIKVVMTGSSSDPASFQRHIGPKKRRNLL

EKRMKDVNDELQLVIVRDMWLTGFDVPSMHTMYIDKPMKGHNLMQAIARVNRVFKDKP

GGLIVDYVGIAESLKEALKEYTESDQAQTAIDTDKAVELMLLKYDVIQDMLYNLDYSK

FNSNKKLERYYTISNTMDYVIGLGEDERQRFIKTVTELGKAFALCATEPTAQELNDEI

AFFKAVKAGLVKLLQPPKEGKIRKTPAEVEAEINQLVSQSVVTEDVIDVYQTLGLEQP

DLSILSDDFLKDVEGLKQKNVAVELLNRLLKGQVKSLMKTNATVSKRFSEMLGNSINK

YNSRSIETSKVIEELIQLAKDIKQEQQRGKELGLNSDEIAFYDALASHETAKEAMGDK

ELRAIAHELTKTVKENMGVDWSKRDSAKAKMRVAVRRLLKKYGYPPDLQKMAVEQVVE
QAELMASNQ"
/product="Type I restriction-modification system,
restriction subunit R (EC 3.1.21.3)"
/db_xref="SEED:fig|703339.290.peg.32"
/transl_table=11
CDS complement(30126..31283)
/translation="MEFKKYLLSEISDYKTGRLNSNAAIENGKYKFFTCAPNPLTINS

YSYDDDVILLAGNNANGIFHINRFNGKFDAYQRTYIIKPKFDVIDIDYLYYYLKFKLN
FLRSQSIGSATKYLTKPILDNLEIYSPKSKRKETIISSILKILDKKIELNKRIIANLE

ELSQTLFKRWFVDFEFPDENGNPYKSSGGEMIDSELGEIPSGWKVDELGNYIKIKSGK

RPKNKVDKKDIENVVPIIGASKIMGYTNDYLYNEKIIIIGRVGTHGVIQRFSTRTWPS
DNTFVIISDFESIIYQVLKSIDYISLNRGSTQPLLSQKDIKNTKVAMPTNETLLSTYQ
KENNHILKMMDQKNIENQKLTQLRDTLLPKLMSGEIEIPDDIEVNEDELSI"
/product="Type I restriction-modification system, specificity subunit S"
/db_xref="SEED:fig|703339.290.peg.33"
/transl_table=11
CDS complement(31273..32787)
/transl_table=11
/EC_number="2.1.1.72"

/translation="MATIGFEEKLWQAADKLRGSMDAAEYKNVALGLIFLKYVSDSFE

EKYEELKQDPYADEEDQDEYLAENIFWVPKEARWQYINDNAKKPEIGQIIDKAMIAIE

KENESLKGVLPKEYARPALDKEKLGDIIDLFTFKVGDTESRKQDVLGRVYEYFIAKFA

SAEGKNAGEFYTPSSIVKLLVEMIEPYKGRIYDPCCGSGGMFVQSERFVERHQGRLDD

IAIYGQESNPTTWKLAKMNLAIRGIDNDLGERNADTFHNDLHKGLKADYILANPPFNA

SDWGQERLLDDYRWQFGIPPKGNANYAWIEHMISKLAPNGTAGFVLANGSMSTSGKDE

LEIRKNLIEQDLVECIVTLPGQLFYSTQIPVCLWFISNNKGQNGKKERKNEILFIDAR

EIGHMVSRTLKEFSDEDIQKVAQTYHAWKGTNDKSYEDIAGFCKVANLEEIKNNEYIL
TPGRYVGLTDVEEDKEPFEQKMERITSELSEQFAKSKELENQIRKSLEGLGYGV"
/product="Type I restriction-modification system, DNA-methyltransferase subunit M (EC 2.1.1.72)"
/db_xref="SEED:fig|703339.290.peg.34"
CDS 32954..33847
/translation="MNGGANLLFTEEQLKLYSKPLSESEKEKCENAIRIIQESLESLG

YEIKKGIHRNNEDTLSYQIKMTNSSKDYELSIFVKGSYATNTNVRQNSDVDIAVVKES

EFFDKYREGKTRENYKFISSNKPPYYFKDEVEEALIEKFGRSEVRRGNKAIRINGNTY

RKETDCVPCFRYRDYSNDYMDDPNNFIGGITIYSDKGERIINYPEQHINNSVIKNNNT

NYKYKKMVRIIKEIRYQLIDSKNRNAEQTSSFGVEGLFWNIPDYKYSNDEMLGDTFNA
LIAFLIDNIDKLSEFKEPNDR"
/product="hypothetical protein"

/db_xref="SEED:fig|703339.290.peg.35"
/transl_table=11
CDS 33944..35116
/translation="MTQVHFTLKSEEIQSIIEYSVKDDVSKNILTTVFNQLMENQRTE

YIQAKEYERTENRQSQRNGYYERSFTTRVGTLELKVPRTRDGHFSPTVFERYQRNEKA

LMASMLEMYVSGVSTRKVSKIVEELCGKSVSKSFVSSLTEQLEPMVNEWQNRLLSEKN

YPYLMTDVLYIKVREENRVLSKSCHIAIGITKDGDREIIGFMIQSGESEETWTTFFEY

LKERGLQGTELVISDAHKGLVSAIRKSFTNVSWQRCQVHFLRNIFTTIPKKNSKSFRE

AVKGIFKFTDINLAREAKNRLIHDYIDQPKYSKACASLDDGFEDAFQYTVQGNSHNRL
KSTNLIERLNQEVRRREKIIRIFPNQTSANRLIGAVLMDLHDEWIYSSRKYINFDK"
/db_xref="SEED:fig|703339.290.peg.36"
/product="Mobile element protein"
/transl_table=11
CDS 35161..35565
/transl_table=11
/product="Acetyltransferase, GNAT family"
/db_xref="SEED:fig|703339.290.peg.37"
/translation="MTEFMEEVLIDDNMVFDIDNLKGFLNDTSSFGFIAKENNKIIGF

AYCYTLLRPDGKTMFYLHSIGMLPNYQDKGYGSKLLSFIKEYSKEIGCSEMFLITDKG
NPRACHVYEKLGGKNDYKDEIVYVYDYEKGDK"
CDS 35566..37005
/transl_table=11
/EC_number="2.7.1.190"
/EC_number="2.3.1.82"
/translation="MNIVENEICIRTLIDDDFPLMLKWLTDERVLEFYGGRDKKYTLE

SLKKHYTEPWEDEVFRVIIEYNNVPIGYGQIYKMYDELYTDYHYPKTDEIVYGMDQFI

GEPNYWSKGIGTRYIKLIFEFLKKERNANAVILDPHKNNPRAIRAYQKSGFRIIEDLP

EHELHEGKKEDCYLMEYRYDDNATNVKAMKYLIEHYFDNFKVDSIEIIGSGYDSVAYL
VNNEYIFKTKFSTNKKKGYAKEKAIYNFLNTNLETNVKIPNIEYSYISDELSILGYKE

IKGTFLTPEIYSTMSEEEQNLLKRDIASFLRQMHGLDYTDISECTIDNKQNVLEEYIL

LRETIYNDLTDIEKDYIESFMERLNATTVFEGKKCLCHNDFSCNHLLLDGNNRLTGII
DFGDSGIIDEYCDFIYLLEDSEEEIGTNFGEDILRMYGNIDIEKAKEYQDIVEEYYPI
ETIVYGIKNIKQEFIENGRKEIYKRTYKD"

/product="Aminoglycoside N(6')-acetyltransferase (EC
2.3.1.82) => AAC(6')-Ib/AAC(6')-II / Aminoglycoside
2''-phosphotransferase (EC 2.7.1.190) => APH(2'')"
/db_xref="SEED:fig|703339.290.peg.38"
CDS complement(37135..38307)
/db_xref="SEED:fig|703339.290.peg.39"
/product="Mobile element protein"
/translation="MTQVHFTLKSEEIQSIIEYSVKDDVSKNILTTVFNQLMENQRTE

YIQAKEYERTENRQSQRNGYYERSFTTRVGTLELKVPRTRDGHFSPTVFERYQRNEKA

LMASMLEMYVSGVSTRKVSKIVEELCGKSVSKSFVSSLTEQLEPMVNEWQNRLLSEKN

YPYLMTDVLYIKVREENRVLSKSCHIAIGITKDGDREIIGFMIQSGESEETWTTFFEY

LKERGLQGTELVISDAHKGLVSAIRKSFTNVSWQRCQVHFLRNIFTTIPKKNSKSFRE

AVKGIFKFTDINLAREAKNRLIHDYIDQPKYSKACASLDDGFEDAFQYTVQGNSHNRL
KSTNLIERLNQEVRRREKIIRIFPNQTSANRLIGAVLMDLHDEWIYSSRKYINFDK"
/transl_table=11
CDS 38326..39060
/transl_table=11
/translation="MFLSLKTLYRKRTVIWPFYFYTIIRTLSNDILYLCDSQEKNNVY

KNFILDVKNYFEYSGEKKMNDKINHLIKITGWFTGLFFLLLIIVSCTVWQIGWIDIIS

YTVTISTFITIGYERFMWKWKLFRIFNKQADISGDYEATLRHFYGEGGIKKVSVEIKQ
TFLTVNIEFRSDEITSNSITADIIEEHGKNILYYTYITNPKAEFEKKNPINRGTTRLI
VEENELTGKYWTGAETTGDLRLKKLK"
/db_xref="SEED:fig|703339.290.peg.40"
/product="hypothetical protein"
ORIGIN
1 atgaaaatca ccattttagc tgtagggaaa ctaaaagaga aatattggaa gcaagccata
61 gcagaatatg aaaaacgttt aggcccatac accaagatag acatcataga agttccagac
121 gaaaaagcac cagaaaatat gagcgacaaa gaaattgagc aagtaaaaga aaaagaaggc
181 caacgaatac tagccaaaat caaaccacaa tcaacagtca ttacattaga aatacaagga
241 aagatgctat cttccgaagg attggcacaa gaattgaacc aacgcatgac ccaagggcaa
301 agcgactttg tattcgtcat tggtggatca aacggcctgc acaaggacgt cttacaacgc
361 agtaactacg cactatcatt cagcaaaatg acatttccac atcaaatgat gcgggttgtg
421 ttaattgagc aagtgtatag agcatttaag attatgcgag gagaggctta tcataaataa
481 aactaaaaat tagattgtgt ataatttaaa aatttaatga gatgtggagg aattacatat
541 atgaaatatt ggagtatacc ttgcaatatc atacgatgtt tatagagtgt ttaataaacc
601 atttttcaac tattgatgat ctagaatata taataactgt acaaattata ttgattatgg
661 aactacaatt caattaagaa attgatgatg aaattttaaa tttaaactaa tggaatcaag

721 aaagaatgaa aggaaatata caatgcccac gattaataaa aggaagttta ttagattttg
781 tgttagaaac agtagatgtt tttgtaaatg gaagtaaaaa tgattgcgaa ttagtaaatt
841 ttaattataa agaaggaaaa gtagtttcac gttatagtac aaaatttgaa agcaatccta
901 aattaagaaa aaagcaattg atatcgacgg taaaagttgt aaggtatgtt atttttgatt
961 ttgagaaaat tttatggggc atagggaaag ttttattgaa gttcatcata ttaaggcaat
1021 gtttactata agaaagttga aatacactca gaaacagact ggattcccgt ttttttcaaa
1081 ttggcacaaa atgatacata ggcccaccta tatattgaag atttaagtta aacgataaat
1141 aatatttttc tcttttgtat agaaattttt ttgggaatga tattaattag tataaaaatg
1201 aatatgtcta caaagaagga gagattttat tgaaacacat taatcaattt tctgaatcga
1261 cactagcaca actagaaaaa gatcaacaac acgtatttta tgtatactgt ttgatggacc
1321 caagaaatga tgaatgcttt tatgttggta aaggtaaggg gaatagaata tttaaacata
1381 aacaagatgc gcagaaaaaa gtattgtacg aagatatatt tatagaagaa aataaagata
1441 acttgaaatt taatagaatt aacgaaattt gtagcaatga tttgaatgtt ttaggatata
1501 taatcagtta tggattaacc gaatctgaag cattttctgc agaaaatgtt cttataaact
1561 ttcttaacct aacaaataaa actacattaa caaatatgat taacggtcat ggatctaagg
1621 catacttagt tgaagattta gaaaatgaat ttggctacga ttcaattaat cttgaaaaca
1681 taaatacgaa tgaattaatt ttagcagtga aaatcagaga tgcatttcta ttagataagg
1741 atgaaagtaa agagtatcct attaatgaaa gtaaacgtga tagaaataac cttaaatcgc
1801 gtacattagg tagttggata ataggaaaag ataaaataca taaaataaaa tatattattg
1861 gtatcaatac aggtgctaat aatgcagttg tctcagctta tgaagtatca tttgaacaag
1921 cggagagtat cgaaacaaat aatggtagaa tgagatatgc gtttattggg ctttcagaaa
1981 gagatgctac tctaaaaaaa ttgaatttat acaaaaaagc actaccagat ttaagatttg
2041 gtagtggtag cgctacagca tatataaata atgggacaat gaaagttgat taaatgaata
2101 aaaacatcca attccttaaa ttagcctttg aaaattttat actcgtatat ataagtgaaa
2161 aacttaaaag aattagatgt taattaaagg agagaatgct atgccatcag atgatattgt
2221 aaaaaaacta tactcaaaag aggatattcg attagaatta ggacttactc cgcataagtt
2281 taataaaaaa atggaaacta ttgctaagct ttttaaaatt gatatgaaaa tttttcacaa
2341 ttacaaaggc caagataaaa ataatcagta tacatttaat ggtgtcgcaa aagaattaat
2401 caaagtgttg ctaaaaagtg ttgattatta cccagtggat atcaattcca aaaaatttaa
2461 acaaaatgga aaatctaaaa aagaaatgat tgaaaatata gataactcta gttacatgaa
2521 atatatttat cagttaatga aatctatcaa tgaaattcaa tacaaaaggt taattgctga
2581 tatacatatg aaagatgtgt accagaatac aaaagcatgg ttaaataatg gtgaatcaat
2641 taataaaaaa gaacaagagc tataccaata tatgacaatt ttaccattac ataaaagagt
2701 tgaactgcaa aatgaggtat taaaatctat agacgaaaca atttttcaat ttctcgcaaa
2761 agaacataga aataatcaaa ttgaagaaaa taatgaatta gaagcatata caaaagcaat
2821 aaaagaaggt agaaatccta aaaatgatta tgaattaaat catctcctat ataaaaaaaa
2881 tcaattaccc ttagatagtc taatagaaga tatatgggac tatgaggaaa cagagtatac
2941 tgagcttgat tggttaattg cagatatgct aaaacgttca caaagagctg atagtaattt
3001 tatcgaaagt ttagagaaga aaaacaaatt gagaaaaaat attcataaag attccattaa
3061 aagtatatct aagttgattg atcatgaatt agttaaaaat aatagaaagt gggacgtagc
3121 agaattttat aaaaatcaac aattttggaa taatagtaca ttaagacgta gctctcaaaa
3181 ttttggaatt ttatattaca aaaagaatag gttacataca atgaatcaaa gtaatattat
3241 taagaaaata aaaattatcg aagctaatat agagagtgca aataaacagc cgagctattt
3301 agatttcttt ttacaagcta agtttgattt agagaggtta gaaagtgaat taatgaaata

3361 tagcattaat ccgaaatatt ttaataagat aaatagtgat tacgtaagat atgctattga
3421 agatgtacat aatgcaatta ttaaaattga cagatatata agtaacgata aagtgaagtt
3481 aaactattca aatgtccaaa tggtagtgag aaacgaaata acagaacaag gaagctactt
3541 tgtaacccag gcattgaata cactgtcaaa ggtagaagag tctggctaca gctttgataa
3601 tttcatggta ggacctttcg tagaagactt taaaaaagca attaaaaatc gtagatttgg
3661 ttctaaaaaa gattaagatt gttttttaga gttaaacata agaataaatt taaataaaaa
3721 aataaaccca acaaaccttg ttatatcaat gtttgttggg tttatttttt tggtgatttt
3781 ttaataactc aattacatct taggataata tcgctaaatt atacattgtc gaggccaaga
3841 caaataaaaa attaaagcaa aagttagcgc gcaactttct gctataaagg agcaaattta
3901 tatggagaca agagacaaac tgatgtcatt gactcaaagt gacaaaacac agcaatggct
3961 aatggacaag tcatctaacc aagatgacat tcaacaattg cagcaacaat tcagtcagca
4021 gcttgatcaa caatataatg cacttttagc tgatgaaaaa gctaagttag accaatacgt
4081 ggaagtacat caaggattgg aatcattaaa ggaagagatt gaatcagaac ctattacgct
4141 taatatcgat aaattacccg atatcaaagc aacaatgctt gaaagagcca agaatgatga
4201 acattctgat aaaatcgaaa agctatttga taggttagaa caggcattaa atggtacgaa
4261 tcgattatat acgcaattat cgttgattgg tacacgaaca catcgaatta caactaaaaa
4321 ttttaatctt caaggcttac ctaaagcagt ccaacatacg attttacctt caaaatttaa
4381 gaaggtgtat acagtcgatt ttaaatcgtt tgaaccatca gttgcagcgt acatgactca
4441 agattcaaaa ctgattgact tgttaaatca gaaagacgga ctgtatgacg cattgctaag
4501 tgaattaggc ttatcagatg agctacgtgt atttgttaaa cgtgcattta ttggttcgtt
4561 tctatttgga ggtaacttca aaaatcctaa attcaagctg aatcaatatg taagtgaagt
4621 acaatggttg gatgcggtca gccaatttac aaaagtcatt gaacttaaga agcacgttga
4681 aaagagtaat tccatgccta tgccttatag tattgagcat gatatgagcg catttcaagg
4741 tagcagtatt atggcaatct acgtacaaac tgtagcgagt tatattttca agcacattct
4801 gctaaaagtg tacaaagcac agtgcgatca aaaaacgttc aagattatag tacctataca
4861 cgatgcgatt atgattgaat gtgaagatga agaaattgca caaaatgtgg gtcagttaat
4921 gaaagatacg gctaaccagt tgttcaatgg tgaatttgca catgtgacag tggaagaaat
4981 aggaggcgta gaccatgaat aatgatagag gacaaagcct acacatccca agtagtacac
5041 caatcaaaga aaataatata tatgtagcta cgttacattc tgtgatccaa acagatttct
5101 caggtgaaat aaagcaccaa ttcacgtatg aaattgaagt gaacaatcag attgtatatg
5161 cgaatcgtaa tattccaaca aaaccgagcg ctaatcagtt gtcaattcat gattggctga
5221 aacgtcatag caactatagc gcaagtcatg aaaactatga gccttatatt gatcagaaac
5281 atttaattct attaggtcaa tataacggta actattatgt acaagatgta gcatcgttag
5341 atgcgtttgg aggcgtatta tcatgaatca tatattacaa atgttatcta agctattaag
5401 tgtggccaag gaggcaatcg accgtcaagg tctgattgct atcctaacta ttcctgttaa
5461 taataacgat gaaatagaag aaacggctca aggtgaaacc gtgtataacg aacttatcga
5521 tcagttacga cttaatatcc caaaagatac ggattatcaa cctaacatct atagttattt
5581 tggtattaag aagaatccta atgacaccgt actcatggaa atgatgataa aggtttttca
5641 tatcaaacgc tttaattcag aactgtttat tttcaaagtt aacgggtggc aaaagataaa
5701 tggagatgaa ttacaagggt tgatatctaa aatgatacaa gtattgcttg tagattataa
5761 gccttcacta agcactctaa aaaatgtcgt agatggattg caaaaatcaa cagatgtaga
5821 agaacttgtt gagaatgagc gctatattgg ttgtggtgaa aatatgttcg atcttaatac
5881 gtttcaagtc gttaaaaatt caatcgatat ctttccaaaa acacgattga atttatcatt
5941 aagtacaaat gatgtaatta ctgataagat accgccttat tttaagcaat atatgttaca

6001 acttgcgaat tatgacgatg atttacaata ctttcttttc caacatacag cagtattact
6061 tacagctgat actaaatacc gtaggggtct catattatat ggtggagcta agaatggtaa
6121 atctgtatat attgaactag ttaaatcatt tttctatagt aaagatattg tgtctaagcc
6181 acttaatgag cttgaaggtc gttttgacaa agaaagttta attgacaaaa gtctaatggc
6241 aagtcatgaa attgggcaat ctaggattca agaaaagatc gtaaatgact tcaaaaagtt
6301 attatctgta gaatcaatgc atgttgatcg taaaggaaaa actcaagtgg aagtcatttt
6361 ggatttgaaa cttattttta gtacaaatgc gatacttaat tttcctcctg aacatgcgaa
6421 agctttggag cgtcgaatta atattattcc atgtgagtat tatgttgaaa aagcggacac
6481 ttcattaatt gataagctcc agagtgagaa gaaagaaatc tttctttact tgatgtatgt
6541 gtatcaacag attgtaaaag cagatatcga gtatcttgaa aatagccgtg tcactgaaat
6601 tactcacgat tggttaaatt ttggatatga atttgtttct agcaggtccg taagtaatgc
6661 aaatcagaaa gcatgtatta atttactcag aaaacttata gaaatcaaat caggatcacg
6721 aatcaaagta tccgagttaa ataaagttat taatgaagaa ataaaggtaa gttctcaggt
6781 tattaaacag ttaattcaag caaactttga tactcaaacc aaactataca atggctacga
6841 ttattggatt gatttaggtt ggaaagaagc caataaaaaa gagattcatg atatttcgga
6901 aaaagataat attatttcat tagataaaaa tgaaaatata acagacgatg aggcattaga
6961 tgaagagaat ttggactttg attgggagga ctttgacgat gaataatgaa caaattgaag
7021 catttgtaga agtgcttgta cctatcatag aagaacgtat caataaaggt aattaaggct
7081 aattacgtac tacaggcagt tgcctgtagt actcatatga ttaagtggta aaagtgataa
7141 aaatgaaacg aaattataaa tatatattat ctatatgttg ttacaagacc gatagtctgt
7201 agcaataatc taataaaagg agcggtatga tatgaagggt aaaattgcac tttattcacg
7261 cgttagtacg tcagagcagt cggagcatgg gtactcaatc catgagcagg aacaagtact
7321 catcaaagaa gttgtgaaaa atttcccagg ttatgactat gagacatata ctgactcagg
7381 catttcaggt aaaaatattg aaggtcgtcc ggcaatgaaa cgtctattac aagatgttaa
7441 ggataataaa atcgaaatgg tgttaagttg gaaattgaat cgtatctcac gatcaatgag
7501 agacgtgttt aatattattc atgaattcaa agaacatgat gtagggtata aatcgatttc
7561 tgagaatatt gatacatcca atgcttctgg agaagtactc gttacaatgt ttgggttaat
7621 aggatctata gaacgccaga ctttgatttc gaatgtgaaa ctttctatga atgctaaggc
7681 aaggagcgga gaggcaatca ccggtcgtgt tttaggctac aaattatcac ttaatccact
7741 tacacagaaa aatgatttgg ttatcgatga aaatgaagct aatattgtac gtgaaatttt
7801 cgatttatat ttgaatcata ataaaggcct caaagccatt acaaccgtac ttaatcaaaa
7861 ggggtatcgt actattaatc aaaagccatt ttcagtgtat ggtgttaaat acattttgaa
7921 taatccagtc tataaaggct atgtcagatt caataaccat caaaactggg ctgtacagcg
7981 aagaagtggc aaaagtgata aaaatgatgt gatattggtc aaaggtaaac atgaagccat
8041 tataagtgaa gaggtatttg ataaagttca tgaaaaatta gcttctaaaa gttttaaacc
8101 gggcagacct attggtggag atttctactt acgtggcctt attaaatgcc cagaatgcgg
8161 aaataatatg gtatgtcgac ggacgtatta taaaacgaaa aagtccaaag aacggacaat
8221 caagcgttat tacatttgtt ccttattcaa tcgttcaggg agttctgcat gtcacagtaa
8281 ttccatcaat gctgaagtcg tcgagcgtgt aattaatgtt catttgaatc gtattctgtc
8341 tcaaccagat attatcaagc agattgcgtc aaatgtgata gaagaactga aacaaaagca
8401 tagtaaccaa acagaaatta aatatgacat tgatagttta gaaaaacaaa aagctaagct
8461 taaaacacaa caagaacgat tgttagaatt gttcttagat gatcagatgg atagcgaaat
8521 gttaaaagct aaacaaagtc aaatgaatca acagttagaa gtattagatc aacaaattaa
8581 agaagcgcaa caagcaaatc aatcacagga tgaaatacct aattttgata aattaaaagg

8641 acgactcatt ttgatgataa cacgattcag cgtgtactta agaaaggcta cacccgaagc
8701 taaaaatcaa cttatgaaaa tgttaattga ttcaattgaa attacgacag ataaacaagt
8761 aaaacttgta aggtataaaa ttgacgaaag tcttatccct caatctttga aaaaagattg
8821 ggggtctttt tttatgccca aattccaatt tgaaatatat ggtcaaaatg attatttcat
8881 cgaccaaatt accactttta ccacttagtt attagtgaca aaagtgagcg aaatgaaata
8941 aaaatcaaat atatattatc aaaatgatgt atcacatgca tacatcaatc aaatacatta
9001 ggaggtcata accatgacac tagaacaaca actcaagcac tatataacca acttattcaa
9061 tctgccaagg gacgaagtgt ggcactgcga atctatcgag gaaatcgctg atgatatctt
9121 acccaatcaa tatgtaagac ttggcccact cagtaataaa acacttcaga ctaataccta
9181 ctactctgac acacttcatg aaagtaatat ctatcctttc attctctact atcagaaaca
9241 actcatagcc atcggttata tcgacgaaaa tcacgatatg gatttcttat acctacacaa
9301 cactatcatg cctcttttgg atcaacgata cttactaaca ggaggacaat aaaatgcata
9361 aatacatcaa aattacacaa ttagtcatta caatactaag tgaaatcatc atttggatga
9421 aagagtcaga acgaaaggaa gtctcttatg aatagatata tcacccgggg tatcgccaac
9481 aacttaccta atatcttaca acaccaatta tggcaactcg tatctgagcg agaacaagaa
9541 caaaccaaag ataatacttc agtagattat tttcatatat tccagttcaa taagcatcgc
9601 aatcaattat atatcaaaca caaacaagaa cgacctgaat atgcgaaaat ccataaagct
9661 aattattcaa aagcaatcaa tatcaataag gtctacatta ttcgagaaga tgatgtagac
9721 ctttcttatt atgtcatgtt attacctgaa gaatactaga ggagtgaaaa ttatatggaa
9781 acaatcaaaa gtacattaaa aacagaagcc atattcagtg atgacaaaca acatcgctat
9841 ctacttaaga aaacatggaa tagtgaaaaa caatcaatca caatcattac aatgtatccg
9901 cattatgatg gcattctcaa tattgaccta acgacccaac tcatcatgaa caaagtttca
9961 gaaatggatg catttggttc aatcaatttt gtgaatctat actctaatat tacaacccct
10021 atcaatctca aacatttaga aaatgcgtat gataagcata cagatattca aattatgaag
10081 gcagtgaaag agtcagatga agtgatatta gcttggggcg cttacgctaa aaagcccggt
10141 gttgaagcac gtgttaatga agtattagag atgttgaaac cacataaaaa gaaagtaaaa
10201 cgactcatga atccagaaac caatgaaatc atgcatcccc ttaattcgaa agcacgtcaa
10261 aaatggatat taaaagtata gcaactaaaa tgtttattca ctatccatga aaataattga
10321 atttcatata tatcttttaa tctacaatat gattaaaaga tatattattt ggttctgttg
10381 caaagtaaaa aaatatagct aaccactaat ttatcatgtc agtgttcgct taacttgcta
10441 gcatgatgct aatttcgtgg catggcgaaa atccgtagat ctgaagagac ctgcggttct
10501 ttttatatag agcgtaaata cattcaatac cttttaaagt attctttgct gtattgatac
10561 tttgatacct tgtctttctt actttaatat gacggtgatc ttgctcaatg aggttattca
10621 gatatttcga tgtacaatga cagtcaggtt taagtttaaa agctttaatt actttagcca
10681 ttgctacctt cgttgaaggt gcctgatctg taattacctt ttgaggttta ccaaattgtt
10741 taatgagacg tttgataaac gcatatgctg aatgattatc tcgttgctta cgcaaccaaa
10801 tatctaatgt atgtccctct gcatcaatgg cacgatataa atagctccat tttcctttta
10861 ttttgatgta cgtctcatca atacgccatt tgtaataagc ttttttatgc tttttcttcc
10921 aaatttgata caaaattggg gcatattctt gaacccaacg gtagaccgtt gaatgatgaa
10981 cgtttacacc acgttcccttt aatatttcag atatatcacg ataactcaat gtatatctta
11041 gatagtagcc aacggctaca gtgataacat ccttgttaaa ttgtttatat ctgaaatagt
11101 tcatacagaa gactcctttt tgttaaaatt atactataaa ttcaactttg caacagaacc
11161 ggatatttta tatagagcat tctcactaaa aaaattacac atatcgtgag caatgaactg
11221 attatactta acattaaaaa agatgataac accttctaca cctccatatc acaaaaatta

11281 taacattatt ttgacataaa tactacattt gtaatatatt acaaatgtag tcttatataa
11341 ggaggatatt gatgaaaaag ataaaaattg ttccacttat tttaatagtt gtagttgtcg
11401 ggtttggtat atatttttat gcttcaaaag ataaagaaat taataatact attgatgcaa
11461 ttgaagataa aaatttcaaa caagtttata aagatagcag ttatatttct aaaagcgata
11521 atggtgaagt agaaatgact gaacgtccga taaaaatata taatagttta ggcgttaaag
11581 atataaacat tcaggatcgt aaaataaaaa aagtatctaa aaataaaaaa cgagtagatg
11641 ctcaatataa aattaaaaca aactacggta acattgatcg caacgttcaa tttaattttg
11701 ttaaagaaga tggtatgtgg aagttagatt gggatcatag cgtcattatt ccaggaatgc
11761 agaaagacca aagcatacat attgaaaatt taaaatcaga acgtggtaaa attttagacc
11821 gaaacaatgt ggaattggcc aatacaggaa cagcatatga gataggcatc gttccaaaga
11881 atgtatctaa aaaagattat aaagcaatcg ctaaagaact aagtatttct gaagactata
11941 tcaaacaaca aatggatcaa aattgggtac aagatgatac cttcgttcca cttaaaaccg
12001 ttaaaaaaat ggatgaatat ttaagagatt tcgcaaaaaa atttcatctt acaactaatg
12061 aaacagaaag tcgtaactat cctctaggaa aagcgacttc acatctatta ggttatgttg
12121 gtcccattaa ctctgaagaa ttaaaacaaa aagaatataa aggctataaa gatgatgcag
12181 ttattggtaa aaagggactc gaaaaacttt acgataaaaa gctccaacat gaagatggct
12241 atcgtgtcac aatcgttgac gataatagca atacaatcgc acatacatta atagagaaaa
12301 agaaaaaaga tggcaaagat attcaactaa ctattgatgc taaagttcaa aagagtattt
12361 ataacaacat gaaaaatgat tatggctcag gtactgctat ccaccctcaa acaggtgaat
12421 tattagcact tgtaagcaca ccttcatatg acgtctatcc atttatgtat ggcatgagta
12481 acgaagaata taataaatta accgaagata aaaaagaacc tctgctcaac aagttccaga
12541 ttacaacttc accaggttca actcaaaaaa tattaacagc aatgattggg ttaaataaca
12601 aaacattaga cgataaaaca agttataaaa tcgatggtaa aggttggcaa aaagataaat
12661 cttggggtgg ttacaacgtt acaagatatg aagtggtaaa tggtaatatc gacttaaaac
12721 aagcaataga atcatcagat aacattttct ttgctagagt agcactcgaa ttaggcagta
12781 agaaatttga aaaaggcatg aaaaaactag gtgttggtga agatatacca agtgattatc
12841 cattttataa tgctcaaatt tcaaacaaaa atttagataa tgaaatatta ttagctgatt
12901 caggttacgg acaaggtgaa atactgatta acccagtaca gatcctttca atctatagcg
12961 cattagaaaa taatggcaat attaacgcac ctcacttatt aaaagacacg aaaaacaaag
13021 tttggaagaa aaatattatt tccaaagaaa atatcaatct attaactgat ggtatgcaac
13081 aagtcgtaaa taaaacacat aaagaagata tttatagatc ttatgcaaac ttaattggca
13141 aatccggtac tgcagaactc aaaatgaaac aaggagaaac tggcagacaa attgggtggt
13201 ttatatcata tgataaagat aatccaaaca tgatgatggc tattaatgtt aaagatgtac
13261 aagataaagg aatggctagc tacaatgcca aaatctcagg taaagtgtat gatgagctat
13321 atgagaacgg taataaaaaa tacgatatag atgaataaca aaacagtgaa gcaatccgta
13381 acgatggttg cttcactgtt ttattatgaa ttattaataa gtgctgttac ttctccctta
13441 aatacaattt cttcattttc attgtatgtt gaaagtgaca ctgtaacgag tccattttct
13501 tttttatgg atttcttatt tgtaatttca gcgataacgt acaatgtatt acctgggtat
13561 acaggtttaa taaatttaac gttattcatt tgtgttcctg ctacaacttc ttctccgtat
13621 ttaccttctt ctacccataa tttaaatgat attgaaagtg tatgcatgcc agatgcaatg
13681 atacctttaa atctactttg ttctgctttt tctttatcta tatgcatata ttgaggatca
13741 aaagttgttg caaattggat aatttcttct tctgtaatat gaaggctttt tgttttgaat
13801 gtttctccta ctataaaatc atcgtatttc atatatgtct ctctttctta ttcaaattaa
13861 ttttttagta tgtaacatgt taaaggtaag tctaccgtca ctgaaacgta agactcacct

13921 ctaactttct attgagacaa atgcaccatt ttatctgcat tgtctgtaaa gataccatca
13981 actccccaat tagcaagttg gtttgcacgt gctggtttgt ttacagtcca tacgttcaat
14041 tcataacccg cttcttttac catttttact tttgctttag taagtttggc atcttcagtg
14101 tttactattt tagcattaca gtaatctaaa agtgttctcc agtcttcacg aaacgaagtt
14161 gtatggaata taactgctct gttatattgt ggcatgattt cttctgcaag tttaacaagc
14221 acaacattaa agcttgaaat gagcacttct tgattctgat ttaagtttgt taattgttct
14281 tccacttgct taaccatact tttagaaagt gctagtccat tcggtccagt aatacctttt
14341 aattctacat ttaaattcat attatattca tttgctattt ttactacatc atcgaaagtt
14401 ggcaaatgtt catctttgaa tttttcacca aaccaagatc ctgcagaagc atctttaatt
14461 tcatcataat tcaattcagt tatttccccg gacatatttg tagtccgttc taaataatca
14521 tcatgaatga taatcagttg ttcatctttt gtaattgcaa catctaactc caaccagttt
14581 ataccttcta cttctgaagc agctttaaat gatgcaattg tattttccgg agctttacta
14641 ggtaatcctc tatgtccata tacagttagc atattacctc tccttgcatt tttatttttt
14701 taattaacgt aactgtatta tcacattaat cgcactttta tttccattaa aaagagatga
14761 atatcataaa taaagaagtc gatagattcg tattgattat ggagttaatc tacgtctcat
14821 ctcattttta aaaaatcatt tatgtcccaa gctccatttt gtaatcaagt ctagtttttc
14881 tgtacccctt atctgcaatt ttacttagga ttgcttttaa cttacctctt atctgcaatt
14941 ttacttagga ttgcttttaa cttacccctt atcagcaatt ttactgagaa ctgcttttaa
15001 cgcacctctt atctgcaatt ttgcctagaa ctgcttttaa cgtacctctt atctgcaatt
15061 ttactgagaa ctgcttttaa cttacccctt atcagcaatt ttgcatggaa ttgcttttaa
15121 cgtacctctt atctgcaatt ttacttagaa ttgcttttac tattcctctt attagtataa
15181 tctcagtaag aatgcgtata aaaatgaaaa ttacaaccga ttttgtaagt gctgacgcct
15241 gagggaatag tatgtgcgag agactaatgg ctcgagccat acccctaggc aagcatgcac
15301 gtacaaaatc gtaagataaa aaaataagca tatcactgta aactttaaaa aatcagttta
15361 gtgatatgct tatttatttc gagttaggat ttatgtccca agctcatcaa gcacaatcgg
15421 ccactagttt atttctctat cttatatgtt ctgatatggt cttctatact gtataagtat
15481 acttttgaat atggatcttg tgtcaattca cgttcgaaat caaattcttg attatcaaat
15541 ctgttaaaga atgtttcgta ttcttcgact gataattgct ctctagattc tagcatattt
15601 aagtgtttct ctttatctaa tgctttgtca tatcctttaa cgattgaacc actaaagatt
15661 tctcctactg ctcctgaacc ataactaaat agacatactt tctcttctgg ttggaatgtg
15721 tggttctgta ataacgaaat taaacttaag tataatgatc ctgtataaat gttaccaaca
15781 tctctattcc ataatacggt tctgttgcaa agttgaattt atagtataat tttaacaaaa
15841 aggagtcttc tgtatgaact atttcagata taaacaattt aacaaggatg ttatcactgt
15901 agccgttggc tactatctaa gatatacatt gagttatcgt gatatatctg aaatattaag
15961 ggaacgtggt gtaaacgttc atcattcaac ggtctaccgt tgggttcaag aatatgcccc
16021 aattttgtat caaatttgga agaaaaagca taaaaaagct tattacaaat ggcgtattga
16081 tgagacgtac atcaaaataa aaggaaaatg gagctattta tatcgtgcca ttgatgcaga
16141 gggacataca ttagatattt ggttgcgtaa gcaacgagat aatcattcag catatgcgtt
16201 tatcaaacgt ctcattaaac aatttggtaa acctcaaaag gtaattacag atcaggcacc
16261 ttcaacgaag gtagcaatgg ctaaagtaat taaagctttt aaacttaaac ctgactgtca
16321 ttgtacatcg aaatatctga ataacctcat tgagcaagat caccgtcata ttaaagtaag
16381 aaagacaagg tatcaaagta tcaatacagc aaagaatact ttaaaaggta ttgaatgtat
16441 ttacgctcta tataaaaaga accgcaggtc tcttcagatc tacggatttt cgccatgcca
16501 cgaaattagc atcatgctag caagttaaca caaagtatta ttttaaaatt gagattagac

16561 atttattttt caactttgca gcagaaccta attttttctc tttgtaaata aatgcaaaga
16621 gataccataa ggatctctta cataaccata accttcagta tagaattctg gactaaatgt
16681 tttcaatacc tcactgcctt tttctattaa ctggtcatat acatgtttag tttcttctac
16741 ttggtcaaaa gtgagacaaa gagatatatt attaccttgt gttatgggca aaccttcagt
16801 gtcatctgcg atcataattt ttatatctcc aaattgaagt acacattgat caattttatt
16861 taaatcattt tcgtcaatat taagtttctt atctataggt ctatctttaa tacgttgaat
16921 atacagtgtt ttagcgccaa acagctcttc atacaacttt tttaaaccct ctgcattttg
16981 agtgattaaa aaatatggac ttacttgaaa tttcatgttt tttcctcctt agatttgtta
17041 taaatagagt atatacttaa ttagtgtcat ctattgacac taaaaggaga ataataatga
17101 aaaaatctgt tagattatat aatatgattg aatattgtaa tgaaaatagg aacttcaaat
17161 taaatgattt aatgtcagaa tttaatattt ctcgtagtac cgctttaagg gatataaaag
17221 aaattgaagc attaggagta cctttatata gtaatccagg gaaaaatggt ggttatacga
17281 tcataggtaa tcgagaccaa acgaaaatag caatctcaga tgaagagttg aaagctttag
17341 tatttacact ttcgagtatt tcaaatgtga gtaatctacc ttttcaaaca gaatatcaag
17401 aaatattaaa aaaattatat aataactcga ataaaaaaga gttaataaac caatataatg
17461 atctatttca atattttaat gaagataagt atcagttcaa aagttataag ttatttaatg
17521 aaatcattag attgataatt gagaataagt cttttgaaac ctgttattca caaaactata
17581 ttaaagaaca atataaaggt attggcatta tgtataaaaa tcatcaatgg tattttgttg
17641 tagctaatat agaatcaaag ttagtgaatc tattaaatat ttcgaaaata aaagaactat
17701 atgaaatggg agagactcaa gagtgtaatg atataactat gcagaatttt caacagttca
17761 tggttaaaaa tgaaacagct attgatattc ttattagaag caatgttatg ggattgaata
17821 tcttgaaagg ctacctgtgg agtgactata tgattgaaaa tattgacgaa gagacatatt
17881 tatttaaatc aaaagtgaac gcgaaagata tagattttat agctaagtta attgtcacag
17941 gtggtgtcaa tgtaaaagta gagaccccta atagtttgaa aaatgctgtt aaagttgaat
18001 taactaaaat aataaacatg tattaatagt aatttaatca taattgtaat aacaaaaaga
18061 tgacaacatt aaacaattta aatttataca aaaaagcatt accaaattta aaattgggta
18121 gtggtagtgc tacagcatac ataaatagtt ataattaaat aaagatgggt caatgaaggt
18181 gaattaagtg aataaaaata ttacagctct aaaatctact gaaaatacta catatatgca
18241 gtatacggtt catatcgatg atcaaattat aaataatata aaagagtgta ccaaaaagtt
18301 taaaattttg cctatggaag ataaaattcc attatcaccg ttgttacaac cagaatatgc
18361 aggagaggta caagatttta ttagtacata tgagcagttt atgattaatt ttggtaaagt
18421 aatattggat agtcaaggca taaaaataca gtttgaaagt gaatcattaa gtagtattca
18481 acgaggcatt caagaacatt gttacttaaa tgagcgaaca aatgacattg atgtgactaa
18541 agaatggtat ttatgcaaat tttcaaatca aatattagaa gaggataaaa atcaattgta
18601 taatgcttta aagcaactca tgaatgattc taaaaataaa aagcaagctt ttcaagaggt
18661 gtttaaggtt catattgata tttatctcta caataaagaa aaatctgaat ataagtatca
18721 agtatctagt tatttcaatc ttgtaaaaga aaatcctaaa ataacttata aaaaaagaca
18781 tttacaagag aaacaaggtg tcaagggtac aactttcacg aacaaattaa aattattgaa
18841 caaaatgtat ggtgtagatg ttgcaaaata tcagccattt tataattcta ataatcctga
18901 atatgaaaga ggtcaatttg gtgaaagata catttctcaa agatctaact atgaatttaa
18961 tagacttcaa tatcaaatta tagatatgtt atcgaaaata cttgataagc atccattacc
19021 aaagtcagat aataattata aacatattcc aactattgaa aaagcaatat taagtgggga
19081 ttctcatagt ttttatgagt attttgaaga tataatgaaa gaaattataa gtatggaaaa
19141 ttcttcttta aaggaaaaat tattgactga ttttacatat caatctcaat gtagatggta

19201 ctctgaaagt gaaaaattaa aattgcaact tgaaagtttt atgcataaag ttttggagag
19261 caattattat gagggtaaca aattatatag aatgttgtct catgcaattg aagaaacaat
19321 caatgaggct gatgaagata aggttcattt caattatttt aaagattatt ttttgacaga
19381 cggtggggtg aagaattggg agcagattag cgagaaaatt actgaattca atggtaaagt
19441 tattaacgat atccaaaatg aatataataa gattcaattt aataacgcaa atcgaaatca
19501 gaaactaaac tttaattatt tataccattg ctttgagttt agtaataact tagtaaaagc
19561 aagggtaaat ggaaatagag gttacatttt atattttgta gataaatata aaataaaaat
19621 gcccttttttg gatcaattat taatcaatta tttgaatcaa gaaataggtc aggaaagtat
19681 taattataat atgcaaacat tgtttgaaaa agaaagatat gatagaagta gtaccattga
19741 aaaattagta gcaacaagca aatttaagta tgaaaaagat gattcagatt tattcaaaca
19801 acttttcaat gatgttgaaa attcaataga cagattaggt atttacttac taaataatgg
19861 tataaattcg aatgatgaaa atgcaagata ttatagatcg tttttaaagg aacttagtag
19921 aataaaaagt aaattaacgc cattttctct tgaaataagt aagtctagcg gaagagagca
19981 acattatcct gatgatgcta ttgatgataa agatgagaga agaaaaaata aagaagaaac
20041 atatcatgct tttgatgata aaagcgatat tgactccaaa ttaaagaata aaataaatgt
20101 ttctatcgat aatttattgt cagttaaaat taagagcaat ttgtatgata ttagtccaaa
20161 tacaaaaaaa caataactat aatgagccaa ctcaagaatg agttggcttt ttaattgtaa
20221 ttttacgctt ccattacagg aatcaatgtt atggaagcca ttaagcaatt aatttgagtc
20281 atttatagta taggtacaac gttgtgatgt tcacatgata atcagcccgg gagattattg
20341 tgtaataact tatagaagga gcaaatcacc atgtatcaag caaatatacg tgatttaatt
20401 acaaaattgc ctcaaagcaa taaaacagaa cactttttaa tgaacaaatt ttcaaatcaa
20461 gataaagttc agcagctaca aagacaaatt agccaacagc tagatcaaca atataatgag
20521 cttttggcta atgaaaaagc taagctagac caatacgtgg aagtacacca taatttagaa
20581 ccattaaaga aagagattga atcagaatct attaaccttg ataccgataa attacctgat
20641 atcaaagcga caatgcttga aaaggctaag aacgatgaac attttgataa aatcgaacag
20701 ctatttgata gattagatca gtcattaaat ggtacgaatc gattatatac gcaattatcg
20761 ttgattggca cacgaacaca tcggatcaca acgaaaagat ttaatgttca aggcttgcct
20821 aaattagtcc aacaaatgat tttaccttcg caatttaaaa aggtttatac aatagatttt
20881 aaatcattcg aaccatcagt tgctgcgtat atgacacaag acgaacaact gattgactac
20941 ttgaatcatg aagaagggtt atacgatgca ttactgagag acttatcttt gtcaaaagag
21001 aagcgcgtga gtgtgaaacg tgcatttata gggtcatttc tttttggcgg tcgttatagt
21061 agctctaaat tcaaaatcaa tcaagaggtt agtgaaatta actggctaca agtaatgagc
21121 aaattcaaga aggtcattga atttaaggag caagtcgaaa aatataaaac aatgcctacg
21181 ccttacggca ttgaacatga tatgagcgca tttcaaggta gtagtattat ggcaatttat
21241 gtacaaacgg tagcaagcta tattttcaag cacattttgt tggaagtgta caaagcacag
21301 tgcgaaaaaa aaacgttcaa gattatagtg ccgatacacg atgcgattat gattgaatgt
21361 aatgataagg ggattgcaca aaatgtagca cagctcatga aagatacagc taatcaactg
21421 tttaatggtg aatttgcaca tgtgacagtg gaagctttag gaggtataga caatgaataa
21481 tgatagagga aaaagtcttc aaattcccca aagtacattg ttaaaagaag gatctatata
21541 cgttgctacg ttacattctg tgtacgagaa gaacttctca ggtgatatta aacatcagtt
21601 tacgtatgaa gtagaactta accaagaaac gcactatgta aatcgcaata ttaccgtaaa
21661 atctatgagt caccaattat caattgctga ttggattaaa cgtcacagta actataacgt
21721 aaaccacatt aattatgatc catacattga tcgaaaacat ttggtccttg tagggcaata
21781 taacggaaat tattatattc aagatgtagc accattagat gaatttggag gggtattgta

21841 atgaatcata ttttagaaat gttaataaaa ttattaaaag tgggtatgga ggcaatcgac
21901 cgtaaaggtc tgattgccat cctaacaagt agtattggaa atgatgaaat ggatgattct
21961 gaacaagctg taatggtgta taacgagctt atcgataagc tacagcttaa cattcctaaa
22021 gatgtcgact atagacctaa catatacagt tattttggta ttcaaaaaaa gccaaatgac
22081 acaatattag tggaaatgat gatatgtatt tttcatatca agcgttttga ttcagagttg
22141 tttgttttca aagataaagg ttggcaaaag gtaagtgaag atgaattgca agggttgata
22201 tctaaaatga tacaagtgtt gctagttgat tataaacctt cactaagtac tttgaaaaac
22261 gtagtagatg gcatacagaa atcaacggac atagaaaaac ttgttgagca tagacagtac
22321 attggttgtg gacgaaatat gttcaatcta aagaccttta aagtggttga taatgacctt
22381 gaaatattcc ctaaaacacg cttagatttg gaattagata taaatgacac gattacggac
22441 aagatacccc cgaatttcaa acaatatatg ttagagttgg cgaattatga ccatgattta
22501 caatatttcc ttttccaaca tatggcagtg ttattgacgg cagatactaa actacgtcgt
22561 ggactttttt tgtatggaac tgcaaaaaat gggaaatcgg tctatattaa attagttaag
22621 tcattctttt atagtaatga tatcgtatct aaaacactta atgaacttgg cgggcgtttc
22681 gataaggaaa gtctaattgg taaacgaatt atggcaagtg atgaagtggg gaaagctaat
22741 attgatgaag caactgtgaa tgatttcaaa aaattactat ctgttgaacc aattcatgct
22801 gaccgtaaag gaagaacgca agtagaagtt actttagatt taaaactcat ttttaatacg
22861 aatgctgtac tcaattttcc atcatcacat gcaaaagcat tagagcgtag aattgctgtt
22921 attccatgtg aatattatgt tgaaaaagct gaccctgact taattgaaaa gttacaggat
22981 gaaaagaaag aaatctttct ttacttgatg tatgtgtata agcaaattgt aaaaaatgat
23041 atcgagtacc tccaaaatga tcgtgttact gaaatttctc atgattggtt aaattttgga
23101 tatgaatttg tttctagtaa atcagcaaat attgcacatc agaaagcgtg tattaattta
23161 ctcagaaaac ttatagaaat caaaccaggg tcacgtatca aagtgtctag gctaaatgag
23221 gttattagag atgaaattaa agtaagctct caagttatta atgatttggt tcaagctaac
23281 tttaatgtac aaagtagact aaataatggt tataagtatt gggtcgattt aggatggaaa
23341 gaaactgata aaaaagatga catgatttca ttcgataaaa atgagaatgt aacagatgat
23401 gaattcttat acgaagatga tttgaactta ggttgggagg actttgacga tgaataatga
23461 acaaattgaa gcatttgtag aagtgcttgt gcctatcata gaagaacgta tcaataaagg
23521 taagtaatct aattacgtac tacaggcagt tgcctgtagt actcatatga ttaagtggta
23581 aaagtgataa aaatgaaacg aaattataaa tatatattat ctatatgttg ttacaagacc
23641 gatggtctgt agcaataatc taataaaagg agcggtatga tatgaagggt aaaattgcac
23701 tttattcacg tgttagtacg tctgagcagt cagaacatgg ttattctgaa aaggagcagg
23761 aacaactact catcaaagaa gttatgaaaa atttcccagg ttatgactat gagacatata
23821 ctgactcagg catttcaggt aaaaatattg aaggtcgtcc ggcaatgaaa cgtctattac
23881 aagatgttaa ggataataaa atcgaaatag tgttaagttg gaaattgaat cgtatctcac
23941 gatcaatgag agacgtgttt aatattattc atgaattcaa agagcatggc gtaggttata
24001 aatcgatttc tgagaatatt gatacatcca atgcttctgg tgaagtttta gttacaatgt
24061 ttggattaat aggatcaatc gaacgttcaa cactggccag taatgtcaag atgtcgatga
24121 atgctaaagc acggagcgga gaggcaatca caggtcgtgt tttaggctac aaattatcac
24181 ttaatccatt gacacagaaa aatgatttag ttattgatga aaatgaagct catattgtac
24241 gggaaatctt tgatttatat ttgaatcaca ataaaggact taaagcaatc acgacaattc
24301 taaatcaaaa aggatatcgc accattaatc aaaaaccatt ttcagtgttt ggtgtgaaat
24361 atattttgaa taatccagtc tataaaggct atgtcagatt caataaccat caaaactggg
24421 ctgtacagcg aagaagtggt aaaagtgata aaaatgatgt gatattggtc aaaggtaagc

24481 atgaagccat tataagtgaa gatgtatttg atcaagttca tgagaaacta gcttcaaaaa
24541 gttttaaacc gggacgacct attggtggag atttctactt acgtggcctt attaaatgcc
24601 cagaatgcgg aaataatatg gtatgtcgac ggacgtatta taaaacgaaa aagtccaaag
24661 aacggacaat caaacgctat tacatttgtt cattattcaa ccgctcagga agttctgcct
24721 gtcatagtaa tgcgattaat gctgaagtcg tcgaacgcgt aatcaatgtt catttgaatc
24781 gtattctttc acaacctaat gttattaagc agattgcgtc aagtgtgata gaagaactga
24841 aacaaaagca tagtaaacaa acagaaataa aatatgatat tgatagtcta gaaaaacaaa
24901 aagcaaaagt taaaacacaa caagaacgat tattggaatt gttcttagat gatgaaatgg
24961 atagcgaaat gttaaaagct aaacaaagtg aaatgaatca acagttagaa gtattagacc
25021 aacaaattaa agaagcaaaa caagcaaatc aatcacaggg tgatataccc aattttgata
25081 agttaaaagc acgactcatt ttgatgataa cacgattcag tctgtactta agaaaggcta
25141 cacccgaagc taaaaatcaa cttatgaaaa tgttaattga ttcaattgaa attacgacag
25201 ataaacaagt aaaacttgta aggtataaaa ttgatgaaag tcttatccct caatctttga
25261 aaaaagattg ggggtctttt tttatgccta aatttaactt tgtgataaat gtcgcaaatg
25321 aaaataggat tgaaaattta tcacttttac cactttttta gagtgacaaa agtggaggaa
25381 ttttgaaata ttttataaat atatatttta tttatggagt acacattatt aattaaggag
25441 gtcattataa tgacgctaag caaacaactt aaaacgtata ttactgaacg atttaaatta
25501 aattatcaag aaacttgggc ttgtgaaacc gtagatgcgg tggctgaaga tgtattacct
25561 gaaaaatata ttaaaaatag tccacttgaa cataaaattt taaatacttt tacctattac
25621 aatgatgaat tacatgaaat cagcatttac cctttttat gttatttaga taaggaatta
25681 gtagcaatag gttatttaga taattttgat ttagacttta tatttttaaa tgacactcat
25741 caagtcatta ttgatgaacg ctacttgtta caaaaagggg gcgagtaatt atgaactgga
25801 tcaaggtcgc tcaactatct gtcacagtta tcaatgaagt cattgagatc atgaaagaaa
25861 aacagagtgg aggaaaatag tatgaacatc aatcgataca tcacaagagg aattaatgaa
25921 agtattccac tagaccttca aatcttactt tggcacatgg tagaagaaaa agataaccag
25981 cctcataccg attacctaca cattttcaaa ctacaagaag atgataatat gttgtcaatt
26041 aaacatgaac aagaacagcc cgcatacaag ttagaatatc actatacaaa ctatgaaaaa
26101 aatcaaaatg cattacctaa gaaagtctac gtcattcgtg aagatggcgt agacgctttt
26161 tattatgtga tgcttttacc agaagaatac taaaggaagt gattatatat atgaatacaa
26221 tcaaaaatac gatatacaca gaagctatat ttagcaagga tgaaaaacac cgctatttac
26281 tcaagaaaac atgggatgaa aagaaacctg cttgtacagt gataacgatg taccctcatt
26341 tagatggtgt attatcactc gatctcacaa ctgttcttat tcttaaccaa ttagcgaatt
26401 ctgaacaata tggttctgta tatcttgtaa atctattctc taatattaaa acaccagaga
26461 accttaaaca tattaaagaa ccttatgatg agcacacaga tatacactta atgaaagcga
26521 taagtgaaag tgacacagtg attttagcct atggtgttta tgcaaagcgt cccgttgttg
26581 tcgaacgtgt tgagcaagtg atggaaatgt taaaacctca taaaaagaaa gtaaaaaggc
26641 tcataaatcc agcaacgaat gatattatgc atccgcttaa tcctaaagcg cgtcaaaaat
26701 ggacattgaa ataaaggagg attatctatg aaccatgaaa ctaaacaatc agattggcat
26761 acggttgcta attgtttaga atcgcaaaat tatacatcga tcgtaaaagg attagtacat
26821 catttcacag cgattgaaga tgaagaaata cttgataaaa tctatgatga ttttatgaat
26881 gatgactcta taacaacggt acttaacaat gatttacaga taattattaa tcattaccta
26941 tcaaaatgaa cattatatta cttacaataa aaatagagca tccttcactt tgcggtgagg
27001 gatgctcttt tagtttattt ttttattggt tactcgccat aagctctgct tgttctacga
27061 cttgctctac agccattttt tgtaaatctg gtggatagcc atatttttta agcaatcttc

27121 taacagctac gcgcatttta gcttttgcgc tatcacgttt agaccaatca acacccatgt
27181 tttctttcac tgttttagtt agctcatgag caatcgcacg tagttcttta tctcccatgg
27241 cttcttttgc tgtttcatgt gaagctaaag catcgtaaaa cgcaatctca tctgaattca
27301 ggcctaattc ttttcctcgt tgttgttctt gtttgatatc tttagcgagt tgaataagtt
27361 cttcaatcac tttagatgtt tcaatggaac gactattata tttattaatc gagtttccta
27421 acatttcaga gaaacgctta gatactgtcg cgttcgtttt cattaatgat ttgacttgcc
27481 ctttgagtaa tcgatttaat aattctactg caacattttt ttgtttcaat ccttccacgt
27541 cttttaagaa atcatctgat aggattgata aatcgggttg ttcaagacct agcgtttgat
27601 aaacatcaat gacatcttca gtcacaacag attgtgacac aagttgatta atctctgctt
27661 caacttctgc aggtgtttta cgtatttttc cttcttttgg cggttgtaac aatttaacaa
27721 gtcctgcttt aaccgcttta aagaaggcaa tttcatcatt gagttcttgg gctgttggtt
27781 cagtcgcaca aagagcaaag gctttaccta actctgtgac cgttttaata aaacgttgac
27841 gttcatcttc acctaaacca atcacataat ccatcgtatt cgatattgta taataacgtt
27901 ccaatttttt atttgaatta aatttagaat aatcaagatt atataacata tcttgaatta
27961 catcatactt caataacatc aattcaaccg ctttatctgt atctatcgct gtttgagctt
28021 gatcagattc tgtatactct ttaagtgctt ctttcaaact ttcggcaata cccacataat
28081 cgacaatcaa tccacccggt ttatctttaa acactcgatt aactcgggca attgcttgca
28141 tcaaattatg gcctttcatt ggtttatcaa tatacatcgt atgcatagaa ggaacatcaa
28201 accctgtcag ccacatatct cgaacaatca cgagttgtaa ttcatcattc acatctttca
28261 tacgtttttc taataaatta cgacgttttt taggaccaat atgtctttgg aaagaagctg
28321 ggtcactaga ggagcccgtc attaccactt taatgacccc tttatcatca tcatctgaat
28381 gccattctgg ttttagacga atgatttcat catataaatc aacagcaatt cgacgactca
28441 tcgttacaat cattcctttg cctttcatcg cttgttgacg tgtttcaaag tgttggatga
28501 tatctttggc tagggcttcg atacgaggtt ttgcacctgc taaggcttca attcgtgacc
28561 attttgattt taaacgctgt tttacatcct cttcttggtc ttctgtaatg tcattatatg
28621 cttcatctaa atctaaattt tggggtagat ttaatggaat tacacgactt tcatagtaaa
28681 ttttaactgt acttccatca gctacagctt gtgtcatgtc ataaacatcg atatagtttc
28741 cgaaaaccat ttgtgtattt ttatccgttg aagctacggg tgtgcccgta aatccgacga
28801 atgttgcatt cggtaaagca tctcttaaat atttagcata accatattta atgccttcac
28861 ctttatcatt atactttgca ttaaagccat attgtgtacg atgagcctca tctgccataa
28921 caatcacatt tttacgttct gttagggcag ccatggtcgt ttcattttgt tcaggttcaa
28981 atttttgcat tgttgtaaat acaataccac ccgactcaac agataataac gattttaatt
29041 ctttacgtgt ttcagcttgt tttggcgttt gtcttaataa tcctttacca gaacgtcctt
29101 ttgatttaac aaatgtactg tatagttggt tatctaaatc attacgatct gtaacaacga
29161 ctaaggtagg attattcagc atttgaatta attttccaga gaaaaagacc atggttaaac
29221 ttttaccaga cccttgggta tgccaaataa cgccgccttt accatcaccc ggcccagatg
29281 aagctaataa agctctatca acagctttat taacagcata gtattgatga tatgctgcta
29341 gaattttact gatatgccct ttaccatcat cttggaataa tacaaaatat cgaattaaat
29401 caagtagagt attaggattt aacatcccat gaatcagtac gtctaagcta gctaaacttg
29461 acgaagattc tgtttctcca tctttagaac gccaagtcat aaaacgatca tagttcgcag
29521 ttagtgaact ggctttagta ttaataccat cacttgtaac aagcacttca ttaaatgtaa
29581 ataattgtgg aatacgcatc ttatacgttt ctaattgatg ataaccatct tcgacgccta
29641 cggtttcatt ggttgaattt ttaagttcga tcacaacgat aggcaagcca ttgataaaga
29701 ggacaatatc gggacgtttt gtatagtctc cgttaatgac agtgaattga ttgactgcta

29761 aaaaatcatt gttttgtgga tgttcaaaat caacgatttt aacaatttct actaccgatt
29821 gtccttcgtc atcatagtct tcgatttcaa taccattgat caaattttca tgaaaggtaa
29881 ggttattttc taaaagattg ggcgacttct ctaaagttag ttcatgtatg gctttttcga
29941 taaaacgatg atgaatatct gaattaatct ttcttaatga tttttctaat cgttcatgaa
30001 ggacaacatc tttatcactt ttacgttctg gcgctagacc tgtcatacta atctcattac
30061 cttttttgta gtcatagccc agtgattgta gccattctaa tgcgacttgt tctaaatcat
30121 cttcactaaa ttgaaagctc atcttcattc acctcgatat catcaggtat ctctatttcc
30181 cccgacatta attttggtag taaagtatct cttaattgtg ttagtttttg gttttctata
30241 ttttttggt ccatcatttt taaaatatga ttattttcct tttgatatgt tgatagcaaa
30301 gtttcattag ttggcattgc tactttagtg ttcttaatat ctttttgaga taatagaggt
30361 tgtgtagaac ctctattcaa tgatatatag tctatacttt taagtacttg atatataatg
30421 ctttcaaaat cactgattat tacaaaagta ttatctgatg gccatgttct tgtagaaaat
30481 ctttggatta caccatgagt tcctactctt ccaattataa taattttttc attatataaa
30541 taatcattgg tataacccat tattttacta gcaccaatta ttggcaccac attttctata
30601 tcttttttat caactttatt tttaggtctt ttaccacttt ttatttttat ataatttcct
30661 aattcatcaa ctttccaacc gctaggtatc tcacctagtt cactatcaat catctcgcca
30721 ccacttgatt tgtacggatt tccattttca tctgggaatt caaaatcaac aaaccaacgt
30781 ttgaacagtg tttgtgatag ttcctctagg tttgctatga ttcttttatt aagttctatt
30841 tttttatcta aaatttttaa aatagaactt ataatagttt cttttctttt gcttttagga
30901 gaataaattt ctaaattatc tagtataggt tttgtgagat atttagtagc actacctatt
30961 gattgcgaac gtaaaaaatt caatttaaat ttaagataat aatataaata atcaatatct
31021 atgacgtcaa atttaggttt tataatatat gtcctttgat aagcgtcaaa ttttccattg
31081 aatctattta tatggaaaat tccattagca ttattgccag caagtaagat tacatcatca
31141 tcatatgaat aagaattaat tgttaaagga ttaggagcac aagtgaaaaa tttatatttg
31201 ccattttcaa tcgcagcatt agaattaagt cgacctgtct tataatcgct tatttcagat
31261 aaaagatatt ttttaaactc catagcctaa tccctccaat gatttgcgga tttggttttc
31321 aagttctttt gatttcgcaa attgttcgct taattcagaa gtaatgcgtt ccattttttg
31381 ctcaaacggt tctttatctt cttcaacatc agttaaacct acataacgtc ctggcgttaa
31441 aatatattca ttattcttaa tttcttcaag gttagctact ttacaaaaac cagctatatc
31501 ttcataggat ttgtcgtttg tacctttcca cgcatgatac gtttgtgcta ctttttgaat
31561 atcttcatct gaaaattctt ttaatgttct agataccata tgaccgattt cacgagcatc
31621 aataaataaa atttcatttt tacgttcttt tttaccattt tgacctttat tattactaat
31681 gaaccaaaga catacaggta tttgtgttga ataaaagagt tgacctggta aggtaacaat
31741 acattccact aaatcttgtt cgataagatt ttttcggatt tctaattcat cttttccact
31801 tgtagacatt gaaccgttgg ctaatacaaa tcctgctgta ccattaggtg ctaattttga
31861 aatcatatgt tcaatccatg catagttggc attacctttt ggcggaatac caaattgcca
31921 acggtaatca tcaagtaatc gttcttgacc ccaatcactt gcgttaaaag gaggattggc
31981 taatatgtaa tctgctttta atcctttatg taaatcgtta tggaatgtat cagcatttcg
32041 ttcacctaag tcattatcaa taccacgaat cgctaagttc attttagcta atttccaagt
32101 tgtaggatta gattcttgcc cataaatcgc aatatcgtct aatcgacctt gatgtctttc
32161 aacgaagcgt tcactttgta caaacatccc acctgaacca cagcatggat cgtagatacg
32221 acctttataa ggttcaatca tctcaaccaa taattttaca attgatgagg gggtatagaa
32281 ctctccggca tttttccctt cagcgcttgc aaacttagca ataaaatact catacactcg
32341 gcctaataca tcttgcttac gactttcagt atcgcctacc ttaaaagtaa ataaatcaat

32401 aatatcgcct aatttttctt tatctaacgc agggcgcgca tattctttag gtaacacacc
32461 ttttaatgat tcattttctt tttcaatcgc aatcatggct ttatcaataa tttgtccaat
32521 ttctggtttt ttcgcattat cattaatata ttgccatctt gcttcttttg gcacccagaa
32581 aatattttct gctaaatatt catcttgatc ctcttcatca gcataaggat cttgcttcaa
32641 ttcttcatac ttttcttcaa aagaatctga tacatatttt aaaaagatta aacctaatgc
32701 tacgttctta tattcagcag catccatact tcctcttaat ttatcggcag cttgccataa
32761 tttttcttca aatccgattg tcgccattta tattacacct ctatattatc ctttatacaa
32821 atatagcaaa tattaaatct ttatgtgact tttcttgtga ttttctttgt gaaaaatatt
32881 gtttgttgta ttatttaagt aatattaagt agctaattat aaaagtacag attattatat
32941 agttaaactg agtatgaatg ggggagcaaa tttgttattt actgaagaac aattaaaatt
33001 atattctaaa ccattgtcag aatctgaaaa agaaaagtgt gaaaatgcaa taagaattat
33061 tcaagaatct ctggagtcat taggatatga aataaaaaag ggtatacata gaaacaatga
33121 agatacgcta tcatatcaaa ttaaaatgac taattcatcg aaagattatg aactaagtat
33181 atttgtgaaa ggttcgtatg caacaaatac caatgtaaga caaaatagtg acgttgatat
33241 tgcagtggta aaagaaagtg agttttttga taaatataga gaaggtaaaa ctagagaaaa
33301 ttataaattt atttctagta ataagcctcc gtattatttt aaagatgaag tagaagaagc
33361 tttgattgaa aaatttggaa gaagtgaggt aagaagaggt aataaagcaa ttagaatcaa
33421 tggcaatact taccgtaaag aaacagattg tgtaccttgt tttagatata gagattatag
33481 taatgattat atggatgatc caaataattt cattggagga atcacaattt attcagataa
33541 aggtgaacga attataaatt atccggaaca gcatataaat aatagtgtta taaaaaataa
33601 caatacaaat tataaatata aaaagatggt tagaataata aaggaaataa gatatcaatt
33661 aatagatagt aaaaatagaa acgcggaaca aacttcttca tttggagttg aaggtttgtt
33721 ttggaacata ccggattaca aatatagcaa tgatgaaatg ttaggtgata catttaatgc
33781 attaattgca tttttaatag ataatataga taaattaagt gaatttaaag aacctaatga
33841 cagataaagt ccgtataatt gtgtaaaagt aaaaaggcca tataacagtc cttttacggt
33901 acaatgtttt taacgacaaa aacataccca ggaggacttt tacatgaccc aagtacattt
33961 tacactgaaa agcgaagaga ttcaaagcat tattgaatat tctgtaaagg atgacgtttc
34021 taaaaatatt ttaacaacgg tatttaatca actaatggaa aatcaacgaa cagaatatat
34081 tcaagcaaaa gaatatgaac gaacagaaaa ccgacaaagt caacgaaatg gctattatga
34141 gcgcagcttt acgacacgtg taggcacgct agaattaaaa gtacccagaa cacgtgatgg
34201 ccatttttca cccacagtgt ttgaacgtta tcaacgaaac gaaaaagccc tcatggcttc
34261 aatgttggaa atgtatgtat caggcgtttc aactcgtaaa gtatcaaaaa ttgtggaaga
34321 actttgtggt aaatccgtct ctaagtcctt cgtttctagc ttaacagaac agctagaacc
34381 tatggttaac gagtggcaga atcgtttatt atcagaaaaa aattatcctt acttaatgac
34441 cgatgtactc tatataaaag tacgagaaga aaatcgagta ctctcaaaaa gctgtcatat
34501 agcgattgga ataaccaaag atggcgaccg tgaaattatc ggcttcatga ttcaaagtgg
34561 cgaaagcgaa gagacctgga caacattttt tgaataccta aaagaacgcg gtttacaagg
34621 tacggaactc gttatttctg atgcgcacaa aggattagtc tctgccatta gaaaatcctt
34681 caccaacgta agttggcaaa gatgccaagt tcacttccta agaaatatct ttaccaccat
34741 tcctaaaaaa aattcaaaat ctttcagaga agctgttaaa ggaattttta agttcacaga
34801 tattaactta gcgcgtgagg ctaaaaatcg attgattcat gattatatcg atcaaccaaa
34861 atattcaaaa gcttgcgcat cattggatga tggattcgaa gacgcctttc aatataccgt
34921 acaaggaaat tcccacaatc gactaaagag taccaatcta attgaacgac tgaatcaaga
34981 agtacgcaga agagaaaaga ttattcgcat cttccccaat caaacatcag ccaatcgctt

35041 aattggagcc gttcttatgg acctacatga tgaatggatt tattcttcaa gaaaatacat
35101 caattttgat aagtagaaat ggtaaaaaca ttgtatagca ttttacacag gagtctggac
35161 ttgactgagt ttatggaaga agttttaatt gatgataata tggtttttga tattgataat
35221 ttaaaaggat ttcttaatga taccagttca tttgggttta tagctaaaga aaataataaa
35281 attataggat ttgcatattg ctatacactt ttaagacctg atggaaaaac aatgttttat
35341 ttacactcaa taggaatgtt acctaactat caagacaaag gttatggttc aaaattatta
35401 tcttttatta aggaatattc taaagagatt ggttgttctg aaatgttttt aataactgat
35461 aaaggtaatc ctagagcttg ccatgtatat gaaaaattag gtggtaaaaa tgattataaa
35521 gatgaaatag tatatgtata tgattatgaa aaaggtgata aataaatgaa tatagttgaa
35581 aatgaaatat gtataagaac tttaatagat gatgattttc ctttgatgtt aaaatggtta
35641 actgatgaaa gagtattaga attttatggt ggtagagata aaaaatatac attagaatca
35701 ttaaaaaaac attatacaga gccttgggaa gatgaagttt ttagagtaat tattgaatat
35761 aacaatgttc ctattggata tggacaaata tataaaatgt atgatgagtt atatactgat
35821 tatcattatc caaaaactga tgagatagtc tatggtatgg atcaatttat aggagagcca
35881 aattattgga gtaaaggaat tggtacaaga tatattaaat tgatttttga atttttgaaa
35941 aaagaaagaa atgctaatgc agttatttta gaccctcata aaaataatcc aagagcaata
36001 agggcatacc aaaaatctgg ttttagaatt attgaagatt tgccagaaca tgaattacac
36061 gagggcaaaa aagaagattg ttatttaatg gaatatagat atgatgataa tgccacaaat
36121 gttaaggcaa tgaaatattt aattgagcat tactttgata atttcaaagt agatagtatt
36181 gaaataatcg gtagtggtta tgatagtgtg gcatatttag ttaataatga atacattttt
36241 aaaacaaaat ttagtactaa taagaaaaaa ggttatgcaa aagaaaaagc aatatataat
36301 tttttaaata caaatttaga aactaatgta aaaattccta atattgaata ttcgtatatt
36361 agtgatgaat tatctatact aggttataaa gaaattaaag gaactttttt aacaccagaa
36421 atttattcta ctatgtcaga agaagaacaa aatttgttaa aacgagatat tgccagtttt
36481 ttaagacaaa tgcacggttt agattataca gatattagtg aatgtactat tgataataaa
36541 caaaatgtat tagaagagta tatattgttg cgtgaaacta tttataatga tttaactgat
36601 atagaaaaag attatataga aagttttatg gaaagactaa atgcaacaac agtttttgag
36661 ggtaaaaagt gtttatgcca taatgatttt agttgtaatc atctattgtt agatggcaat
36721 aatagattaa ctggaataat tgattttgga gattctggaa ttatagatga atattgtgat
36781 tttatatact tacttgaaga tagtgaagaa gaaataggaa caaattttgg agaagatata
36841 ttaagaatgt atggaaatat agatattgag aaagcaaaag aatatcaaga tatagttgaa
36901 gaatattatc ctattgaaac tattgtttat ggaattaaaa atattaaaca ggaatttatc
36961 gaaaatggta gaaaagaaat ttataaaagg acttataaag attgattata taatatatga
37021 aaagctatta taaaagacat tagtattaaa tagtttaaaa aaatgaaaaa taataaagga
37081 agtgagtcaa gtccagactc ctgtgtaaaa tgctatacaa tgtttttacc atttctactt
37141 atcaaaattg atgtattttc ttgaagaata aatccattca tcatgtaggt ccataagaac
37201 ggctccaatt aagcgattgg ctgatgtttg attggggaag atgcgaataa tcttttctct
37261 tctgcgtact tcttgattca gtcgttcaat tagattggta ctctttagtc gattgtggga
37321 atttccttgt acggtatatt gaaaggcgtc ttcgaatcca tcatccaatg atgcgcaagc
37381 ttttgaatat tttggttgat cgatataatc atgaatcaat cgatttttag cctcacgcgc
37441 taagttaata tctgtgaact taaaaattcc tttaacagct tctctgaaag attttgaatt
37501 ttttttagga atggtggtaa agatatttct taggaagtga acttggcatc tttgccaact
37561 tacgttggtg aaggattttc taatggcaga gactaatcct ttgtgcgcat cagaaataac
37621 gagttccgta ccttgtaaac cgcgttcttt taggtattca aaaaatgttg tccaggtctc

37681 ttcgctttcg ccactttgaa tcatgaagcc gataatttca cggtcgccat ctttggttat
37741 tccaatcgct atatgacagc tttttgagag tactcgattt tcttctcgta cttttatata
37801 gagtacatcg gtcattaagt aaggataatt tttttctgat aataaacgat tctgccactc
37861 gttaaccata ggttctagct gttctgttaa gctagaaacg aaggacttag agacggattt
37921 accacaaagt tcttccacaa tttttgatac tttacgagtt gaaacgcctg atacatacat
37981 ttccaacatt gaagccatga gggctttttc gtttcgttga taacgttcaa acactgtggg
38041 tgaaaaatgg ccatcacgtg ttctgggtac ttttaattct agcgtgccta cacgtgtcgt
38101 aaagctgcgc tcataatagc catttcgttg actttgtcgg ttttctgttc gttcatattc
38161 ttttgcttga atatattctg ttcgttgatt ttccattagt tgattaaata ccgttgttaa
38221 aatattttta gaaacgtcat cctttacaga atattcaata atgctttgaa tctcttcgct
38281 tttcagtgta aaatgtactt gggtcatgta aaagtcctcc tgggtatgtt tttgtcgtta
38341 aaaacattgt accgtaaaag gactgttata tggccttttt acttttacac aattatacgg
38401 actttatcta atgacattct atatttatgc gattctcaag aaaaaaataa tgtttataaa
38461 aattttattc tggatgtaaa gaattatttt gaatatagtg gtgagaaaaa aatgaatgac
38521 aaaataaatc atttgattaa aataacagga tggtttacag gactcttttt cttgttacta
38581 attatagtat catgtacggt atggcaaata ggatggatag atatcataag ttatactgta
38641 acaatatcaa catttattac tatagggtat gaaaggttta tgtggaagtg gaaattgttt
38701 cgtatattta ataagcaagc tgatatatca ggagattatg aggcaacact cagacatttt
38761 tatggagaag gtggaattaa aaaggtaagt gtggaaataa aacaaacttt tttaacagta
38821 aatattgaat tcagaagcga tgaaattact agtaatagta ttacagcaga tattattgaa
38881 gaacatggta aaaatatatt gtactatact tatataacaa atccaaaagc cgaattcgaa
38941 aagaaaaatc ctattaatcg aggaacaaca agattaatag ttgaagaaaa tgaattgact
39001 ggaaagtatt ggactggagc cgaaaccaca ggtgatctaa gactcaaaaa actaaaataa
39061 agtattgata ccaaaataaa ttcaatttta ttttggtatt tttagcaata tcactaacaa
39121 ggggaggcgt atcataagt
//
LOCUS Staphylococcus 43312 bp DNA linear UNK
DEFINITION Staphylococcus Staphylococcus aureus strain HZW450
ACCESSION Staphylococcus
KEYWORDS .
SOURCE Staphylococcus Staphylococcus aureus
ORGANISM Staphylococcus Staphylococcus aureus
Bacteria; Bacillati; Bacillota; Bacilli; Bacillales;
Staphylococcaceae; Staphylococcus; Staphylococcus aureus.
FEATURES Location/Qualifiers
source 1..43312
/project="hwwang_703339"
/db_xref="taxon:703339"
/genome_id="703339.291"
/mol_type="genomic DNA"
/genome_md5=""
/organism="Staphylococcus Staphylococcus aureus"
CDS 1..480
/transl_table=11

/translation="MKITILAVGKLKEKYWKQAIAEYEKRLGPYTKIDIIEVPDEKAP

ENMSDKEIEQVKEKEGQRILAKIKPQSTVITLEIQGKMLSSEGLAQELNQRMTQGQSD

FVFVIGGSNGLHKDVLQRSNYALSFSKMTFPHQMMRVVLIEQVYRAFKIMRGEAYHK"
/product="23S rRNA
(pseudouridine(1915)-N(3))-methyltransferase (EC
2.1.1.177)"
/EC_number="2.1.1.177"
/db_xref="SEED:fig|703339.291.peg.1"
CDS 726..1031
/translation="MKGNIQCPRLIKGSLLDFVLETVDVFVNGSKNDCELVNFNYKEG
KVVSRYSTKFESNPKLRKKQLISTVKVVRYVIFDFEKILWGIGKVLLKFIILRQCLL"
/transl_table=11
/db_xref="SEED:fig|703339.291.peg.2"
/product="hypothetical protein"
CDS 1230..2093
/transl_table=11

/translation="MKHINQFSESTLAQLEKDQQHVFYVYCLMDPRNDECFYVGKGKG
NRIFKHKQDAQKKVLYEDIFIEENKDNLKFNRINEICSNDLNVLGYIISYGLTESEAF

SAENVLINFLNLTNKTTLTNMINGHGSKAYLVEDLENEFGYDSINLENINTNELILAV

KIRDAFLLDKDESKEYPINESKRDRNNLKSRTLGSWIIGKDKIHKIKYIIGINTGANN

AVVSAYEVSFEQAESIETNNGRMRYAFIGLSERDATLKKLNLYKKALPDLRFGSGSAT
AYINNGTMKVD"
/product="hypothetical protein"
/db_xref="SEED:fig|703339.291.peg.3"
CDS 2201..3676
/transl_table=11
/translation="MPSDDIVKKLYSKEDIRLELGLTPHKFNKKMETIAKLFKIDMKI

FHNYKGQDKNNQYTFNGVAKELIKVLLKSVDYYPVDINSKKFKQNGKSKKEMIENIDN

SSYMKYIYQLMKSINEIQYKRLIADIHMKDVYQNTKAWLNNGESINKKEQELYQYMTI

LPLHKRVELQNEVLKSIDETIFQFLAKEHRNNQIEENNELEAYTKAIKEGRNPKNDYE

LNHLLYKKNQLPLDSLIEDIWDYEETEYTELDWLIADMLKRSQRADSNFIESLEKKNK

LRKNIHKDSIKSISKLIDHELVKNNRKWDVAEFYKNQQFWNNSTLRRSSQNFGILYYK

KNRLHTMNQSNIIKKIKIIEANIESANKQPSYLDFFLQAKFDLERLESELMKYSINPK

YFNKINSDYVRYAIEDVHNAIIKIDRYISNDKVKLNYSNVQMVVRNEITEQGSYFVTQ
ALNTLSKVEESGYSFDNFMVGPFVEDFKKAIKNRRFGSKKD"
/product="hypothetical protein"
/db_xref="SEED:fig|703339.291.peg.4"
CDS 3902..5002
/db_xref="SEED:fig|703339.291.peg.5"
/product="hypothetical protein"

/translation="METRDKLMSLTQSDKTQQWLMDKSSNQDDIQQLQQQFSQQLDQQ

YNALLADEKAKLDQYVEVHQGLESLKEEIESEPITLNIDKLPDIKATMLERAKNDEHS

DKIEKLFDRLEQALNGTNRLYTQLSLIGTRTHRITTKNFNLQGLPKAVQHTILPSKFK

KVYTVDFKSFEPSVAAYMTQDSKLIDLLNQKDGLYDALLSELGLSDELRVFVKRAFIG

SFLFGGNFKNPKFKLNQYVSEVQWLDAVSQFTKVIELKKHVEKSNSMPMPYSIEHDMS

AFQGSSIMAIYVQTVASYIFKHILLKVYKAQCDQKTFKIIVPIHDAIMIECEDEEIAQ
NVGQLMKDTANQLFNGEFAHVTVEEIGGVDHE"
/transl_table=11
CDS 4995..5366
/translation="MNNDRGQSLHIPSSTPIKENNIYVATLHSVIQTDFSGEIKHQFT

YEIEVNNQIVYANRNIPTKPSANQLSIHDWLKRHSNYSASHENYEPYIDQKHLILLGQ
YNGNYYVQDVASLDAFGGVLS"
/transl_table=11
/db_xref="SEED:fig|703339.291.peg.6"
/product="hypothetical protein"
CDS 5363..7006
/db_xref="SEED:fig|703339.291.peg.7"
/product="putative primase"
/translation="MNHILQMLSKLLSVAKEAIDRQGLIAILTIPVNNNDEIEETAQG

ETVYNELIDQLRLNIPKDTDYQPNIYSYFGIKKNPNDTVLMEMMIKVFHIKRFNSELF

IFKVNGWQKINGDELQGLISKMIQVLLVDYKPSLSTLKNVVDGLQKSTDVEELVENER

YIGCGENMFDLNTFQVVKNSIDIFPKTRLNLSLSTNDVITDKIPPYFKQYMLQLANYD

DDLQYFLFQHTAVLLTADTKYRRGLILYGGAKNGKSVYIELVKSFFYSKDIVSKPLNE

LEGRFDKESLIDKSLMASHEIGQSRIQEKIVNDFKKLLSVESMHVDRKGKTQVEVILD
LKLIFSTNAILNFPPEHAKALERRINIIPCEYYVEKADTSLIDKLQSEKKEIFLYLMY

VYQQIVKADIEYLENSRVTEITHDWLNFGYEFVSSRSVSNANQKACINLLRKLIEIKS

GSRIKVSELNKVINEEIKVSSQVIKQLIQANFDTQTKLYNGYDYWIDLGWKEANKKEI
HDISEKDNIISLDKNENITDDEALDEENLDFDWEDFDDE"
/transl_table=11
CDS 7232..8908
/transl_table=11
/translation="MKGKIALYSRVSTSEQSEHGYSIHEQEQVLIKEVVKNFPGYDYE

TYTDSGISGKNIEGRPAMKRLLQDVKDNKIEMVLSWKLNRISRSMRDVFNIIHEFKEH

DVGYKSISENIDTSNASGEVLVTMFGLIGSIERQTLISNVKLSMNAKARSGEAITGRV

LGYKLSLNPLTQKNDLVIDENEANIVREIFDLYLNHNKGLKAITTVLNQKGYRTINQK

PFSVYGVKYILNNPVYKGYVRFNNHQNWAVQRRSGKSDKNDVILVKGKHEAIISEEVF

DKVHEKLASKSFKPGRPIGGDFYLRGLIKCPECGNNMVCRRTYYKTKKSKERTIKRYY

ICSLFNRSGSSACHSNSINAEVVERVINVHLNRILSQPDIIKQIASNVIEELKQKHSN

QTEIKYDIDSLEKQKAKLKTQQERLLELFLDDQMDSEMLKAKQSQMNQQLEVLDQQIK

EAQQANQSQDEIPNFDKLKGRLILMITRFSVYLRKATPEAKNQLMKMLIDSIEITTDK
QVKLVRYKIDESLIPQSLKKDWGSFFMPKFQFEIYGQNDYFIDQITTFTT"
/product="Cassette chromosome recombinase B"
/db_xref="SEED:fig|703339.291.peg.8"
CDS 9014..9352
/translation="MTLEQQLKHYITNLFNLPRDEVWHCESIEEIADDILPNQYVRLG

PLSNKTLQTNTYYSDTLHESNIYPFILYYQKQLIAIGYIDENHDMDFLYLHNTIMPLL
DQRYLLTGGQ"
/transl_table=11
/db_xref="SEED:fig|703339.291.peg.9"
/product="FIG01108299: hypothetical protein"
CDS 9448..9759

/translation="MNRYITRGIANNLPNILQHQLWQLVSEREQEQTKDNTSVDYFHI

FQFNKHRNQLYIKHKQERPEYAKIHKANYSKAININKVYIIREDDVDLSYYVMLLPEE
Y"

/transl_table=11
/db_xref="SEED:fig|703339.291.peg.10"
/product="FIG01107894: hypothetical protein"
CDS 9775..10281
/db_xref="SEED:fig|703339.291.peg.11"
/product="hypothetical protein"
/translation="METIKSTLKTEAIFSDDKQHRYLLKKTWNSEKQSITIITMYPHY

DGILNIDLTTQLIMNKVSEMDAFGSINFVNLYSNITTPINLKHLENAYDKHTDIQIMK

AVKESDEVILAWGAYAKKPGVEARVNEVLEMLKPHKKKVKRLMNPETNEIMHPLNSKA
RQKWILKV"
/transl_table=11
CDS 10606..12252
/translation="MYKNYNMTQLTLPIETSVRIPQNDISRYVNEIVETIPDSEFDEF

RHHRGATSYHPKMMLKIILYAYTQSVFSGRRIEKLLHDSIRMMWLAQDQTPSYKTINR

FRVNPNTDALIESLFIQFHSQCLKQNLIDDNSIFIDGTKVEASANRYTFVWKKSIQNH
ESKLNENSKALYRDLVEEKIIPEIKEDGDSDLTIEEIDLIGSHLDKEIEDLNHSIENE

DCTQIRKQTRKKRTEIKKFKKKFDDYSERKSKYEEQKSILKDRNSFSKTDHDATFMRM

KEDHMKNGQLKPGYNLQIATNSQFVLSYDLFQNPTDTRTLIPFLTMIQNTFGYLPEYI

VADAGYGSEQNYMAIIDDFNKTPLITYGMFIKDKTRKFKSDIFNTQNWKYDELNDEFI

CPNNKRIGFKRYAYRNDRYGFKRDFKLYECDDCSACSLRQQCMKPNSKSNKKIMKNYN

WEYFKAQINQKLSEPETKKIYSQRKIDVEPVFGFMKAILGFTRMSVRGINKVKRELGF
VLMALNIRKIVARRAVYYQIHLKKADFYQIINRNQLFYIA"
/transl_table=11
/db_xref="SEED:fig|703339.291.peg.12"
/product="Transposase, IS4 family"
CDS complement(12337..13011)
/db_xref="SEED:fig|703339.291.peg.13"
/product="Transposase for insertion sequence-like element
IS431mec"
/translation="MNYFRYKQFNKDVITVAVGYYLRYTLSYRDISEILRERGVNVHH

STVYRWVQEYAPILYQIWKKKHKKAYYKWRIDETYIKIKGKWSYLYRAIDAEGHTLDI

WLRKQRDNHSAYAFIKRLIKQFGKPQKVITDQAPSTKVAMAKVIKAFKLKPDCHCTSK
YLNNLIEQDHRHIKVRKTRYQSINTAKNTLKGIECIYALYKKNRRSLQICGFSPCHEI

SIMLAS"
/transl_table=11
CDS 13269..13436
/EC_number="2.3.3.10"
/product="Hydroxymethylglutaryl-CoA synthase N-domain
homolog (EC 2.3.3.10)"
/db_xref="SEED:fig|703339.291.peg.14"
/transl_table=11
/translation="MLESREQLSVEEYETFFNRFDNQEFDFERELTQDPYSKVYLYSI
EDHIRTYKIEK"
CDS 13993..14736
/translation="MLTVYGHRGLPSKAPENTIASFKAASEVEGINWLELDVAITKDE

QLIIIHDDYLERTTNMSGEITELNYDEIKDASAGSWFGEKFKDEHLPTFDDVVKIANE

YNMNLNVELKGITGPNGLALSKSMVKQVEEQLTNLNQNQEVLISSFNVVLVKLAEEIM

PQYNRAVIFHTTSFREDWRTLLDYCNAKIVNTEDAKLTKAKVKMVKEAGYELNVWTVN
KPARANQLANWGVDGIFTDNADKMVHLSQ"
/transl_table=11
/db_xref="SEED:fig|703339.291.peg.15"
/db_xref="GO:0008889"
/product="Glycerophosphoryl diester phosphodiesterase (EC
3.1.4.46)"
/EC_number="3.1.4.46"
CDS 14833..15261
/translation="MKYDDFIVGETFKTKSLHITEEEIIQFATTFDPQYMHIDKEKAE

QSRFKGIIASGMHTLSISFKLWVEEGKYGEEVVAGTQMNNVKFIKPVYPGNTLYVIAE
ITNKKSIKKENGLVTVSLSTYNENEEIVFKGEVTALINNS"
/transl_table=11
/db_xref="SEED:fig|703339.291.peg.16"
/product="MaoC domain protein"
CDS complement(15307..17313)
/db_xref="SEED:fig|703339.291.peg.17"
/db_xref="GO:0008658"
/db_xref="GO:0046677"
/product="Penicillin-binding protein PBP2a, methicillin
resistance determinant MecA, transpeptidase"
/translation="MKKIKIVPLILIVVVVGFGIYFYASKDKEINNTIDAIEDKNFKQ

VYKDSSYISKSDNGEVEMTERPIKIYNSLGVKDINIQDRKIKKVSKNKKRVDAQYKIK

TNYGNIDRNVQFNFVKEDGMWKLDWDHSVIIPGMQKDQSIHIENLKSERGKILDRNNV

ELANTGTAYEIGIVPKNVSKKDYKAIAKELSISEDYIKQQMDQNWVQDDTFVPLKTVK

KMDEYLRDFAKKFHLTTNETESRNYPLGKATSHLLGYVGPINSEELKQKEYKGYKDDA

VIGKKGLEKLYDKKLQHEDGYRVTIVDDNSNTIAHTLIEKKKKDGKDIQLTIDAKVQK

SIYNNMKNDYGSGTAIHPQTGELLALVSTPSYDVYPFMYGMSNEEYNKLTEDKKEPLL

NKFQITTSPGSTQKILTAMIGLNNKTLDDKTSYKIDGKGWQKDKSWGGYNVTRYEVVN

GNIDLKQAIESSDNIFFARVALELGSKKFEKGMKKLGVGEDIPSDYPFYNAQISNKNL

DNEILLADSGYGQGEILINPVQILSIYSALENNGNINAPHLLKDTKNKVWKKNIISKE

NINLLTDGMQQVVNKTHKEDIYRSYANLIGKSGTAELKMKQGETGRQIGWFISYDKDN
PNMMMAINVKDVQDKGMASYNAKISGKVYDELYENGNKKYDIDE"
/transl_table=11
CDS 17561..17704

/translation="MNYFRYKQFNKDVITVAVGYYLRYALSYRDMSEILRERGVNVHH
LTV"
/transl_table=11
/db_xref="SEED:fig|703339.291.peg.18"
/product="Mobile element protein"
CDS 17984..18235
/translation="MAKVIKAFKLKPDCHCTSKYLNNLIEQDHRHIKVRKTRYQSINT
AKNTLKGIECIYGLYKKNRRSLQICGFSPCHEISIMLTS"
/transl_table=11
/db_xref="SEED:fig|703339.291.peg.19"
/product="Mobile element protein"
CDS complement(18295..18723)
/db_xref="SEED:fig|703339.291.peg.20"
/product="PhnB protein; putative DNA binding
3-demethylubiquinone-9 3-methyltransferase domain
protein"
/translation="MKFQVSPYFLITQNAEGLKKLYEELFGAKTLYIQRIKDRPIDKK

LNIDENDLNKIDQCVLQFGDIKIMIADDTEGLPITQGNNISLCLTFDQVEETKHVYDQ
LIEKGSEVLKTFSPEFYTEGYGYVRDPYGISLHLFTKRKN"
/transl_table=11
CDS 18804..19733
/product="Transcriptional regulator, YafY family"
/db_xref="SEED:fig|703339.291.peg.21"

/transl_table=11
/translation="MKKSVRLYNMIEYCNENRNFKLNDLMSEFNISRSTALRDIKEIE

ALGVPLYSNPGKNGGYTIIGNRDQTKIAISDEELKALVFTLSSISNVSNLPFQTEYQE
ILKKLYNNSNKKELINQYNDLFQYFNEDKYQFKSYKLFNEIIRLIIENKSFETCYSQN

YIKEQYKGIGIMYKNHQWYFVVANIESKLVNLLNISKIKELYEMGETQECNDITMQNF

QQFMVKNETAIDILIRSNVMGLNILKGYLWSDYMIENIDEETYLFKSKVNAKDIDFIA
KLIVTGGVNVKVETPNSLKNAVKVELTKIINMY"
CDS 19895..20377
/transl_table=11
/translation="MNKNITALKSTENTTYMQYTVHIDDQIINNIKECTKKFKILPME
DKIPLSPLLQPEYAGEVQDFISTYEQFMINFGKVILDSQGIKIQFESESLSSIQRGIQ

EHCYLNERTNDIDVTKEWYLCKFSNQILEEDKNQLYNALKQLMNDSKNKKQAFWTDPN
"
/product="Similarity"
/db_xref="SEED:fig|703339.291.peg.22"
CDS 20445..20759

/translation="MKRVSYSVETKYKAVEMKIAGFSTKEIMKELNIKNRTQVETWWR

WYRNEESYRFSQHVGKQYTYGKGLEELSEIERLKLENKRKDIELDILKKYKALERKWY
QQ"
/transl_table=11
/db_xref="SEED:fig|703339.291.peg.23"
/product="Mobile element protein"
CDS 20744..21586
/transl_table=11
/translation="MVPAVVIDLVDQLKVKYSIKLILEVLNIPKSTYYRWKNKTYKND

TVTQKVIELCEANHYTYGYRKITALINQCYTSPINHKRVQRIMQKHHLNCRVRPKKTT

RIGKPYYKTDNLLQRQFKASCPMEVLTTDITYLPFGHSMLYLSSIMDIYNGEIVAYKI

DDKQDQSLVNDTLNQIDIPEGCILHSDQGSVYTSYAYYQLCEEKGIIRSMSRKGTPAD

NAPIESFHSSLKSETFYINNQLNSSNHIVIDIVEKYIKNYNNNRIQQKLGYLSPVKYR
ELAA"
/product="Mobile element protein"
/db_xref="SEED:fig|703339.291.peg.24"
CDS 21593..23158
/product="Similarity"

/db_xref="SEED:fig|703339.291.peg.25"
/transl_table=11
/translation="MFLLSSRLRGSVPFQEVFKVHIDIYLYNKEKSEYKYQVSSYFNL

VKENPKITYKKRHLQEKQGVKGTTFTNKLKLLNKMYGVDVAKYQPFYNSNNPEYERGQ

FGERYISQRSNYEFNRLQYQIIDMLSKILDKHPLPKSDNNYKHIPTIEKAILSGDSHS

FYEYFEDIMKEIISMENSSLKEKLLTDFTYQSQCRWYSESEKLKLQLESFMHKVLESN

YYEGNKLYRMLSHAIEETINEADEDKVHFNYFKDYFLTDGGVKNWEQISEKITEFNGK

VINDIQNEYNKIQFNNANRNQKLNFNYLYHCFEFSNNLVKARVNGNRGYILYFVDKYK

IKMPFLDQLLINYLNQEIGQESINYNMQTLFEKERYDRSSTIEKLVATSKFKYEKDDS
DLFKQLFNDVENSIDRLGIYLLNNGINSNDENARYYRSFLKELSRIKSKLTPFSLEIS

KSSGREQHYPDDAIDDKDERRKNKEETYHAFDDKSDIDSKLKNKINVSIDNLLSVKIK
SNLYDISPNTKKQ"
CDS 23353..24309

/translation="MYQANIRDLITKLPQSNKTEHFLMNKFSNQDKVQQLQRQISQQL

DQQYNELLANEKAKLDQYVEVHHNLEPLKKEIESESINLDTDKLPDIKATMLEKAKND

EHFDKIEQLFDRLDQSLNGTNRLYTQLSLIGTRTHRITTKRFNVQGLPKLVQQMILPS

QFKKVYTIDFKSFEPSVAAYMTQDEQLIDYLNHEEGLYDALLRDLSLSKEKRVSVKRA

FIGSFLFGGRYSSSKFKINQEVSEINWLQVMSKFKKVIEFKEQVEKYKTMPTPYGIEH
DMSAFQGSSIMAIYVQTVASYIFKHILLEVYKAQCEKKRSRL"
/transl_table=11
/db_xref="SEED:fig|703339.291.peg.26"
/product="hypothetical protein"
CDS 24309..24461
/product="hypothetical protein"
/db_xref="SEED:fig|703339.291.peg.27"
/transl_table=11

/translation="MPIHDAIMIECNDKGIAQNVAQLMKDTANQLFNGEFAHVTVEAL
GGIDNE"
CDS 24454..24822
/db_xref="SEED:fig|703339.291.peg.28"
/product="hypothetical protein"

/translation="MNNDRGKSLQIPQSTLLKEGSIYVATLHSVYEKNFSGDIKHQFT

YEVELNQETHYVNRNITVKSMSHQLSIADWIKRHSNYNVNHINYDPYIDRKHLVLVGQ
YNGNYYIQDVAPLDEFGGVL"
/transl_table=11
CDS 24822..26438
/transl_table=11
/translation="MNHILEMLIKLLKVGMEAIDRKGLIAILTSSIGNDEMDDSEQAV

MVYNELIDKLQLNIPKDVDYRPNIYSYFGIQKKPNDTILVEMMICIFHIKRFDSELFV

FKDKGWQKVSEDELQGLISKMIQVLLVDYKPSLSTLKNVVDGIQKSTDIEKLVEHRQY

IGCGRNMFNLKTFKVVDNDLEIFPKTRLDLELDINDTITDKIPPNFKQYMLELANYDH

DLQYFLFQHMAVLLTADTKLRRGLFLYGTAKNGKSVYIKLVKSFFYSNDIVSKTLNEL

GGRFDKESLIGKRIMASDEVGKANIDEATVNDFKKLLSVEPIHADRKGRTQVEVTLDL

KLIFNTNAVLNFPSSHAKALERRIAVIPCEYYVEKADPDLIEKLQDEKKEIFLYLMYV

YKQIVKNDIEYLQNDRVTEISHDWLNFGYEFVSSKSANIAHQKACINLLRKLIEIKPG

SRIKVSRLNEVIRDEIKVSSQVINDLVQANFNVQSRLNNGYKYWVDLGWKETDKKDDM
ISFDKNENVTDDEFLYEDDLNLGWEDFDDE"
/product="putative primase"
/db_xref="SEED:fig|703339.291.peg.29"
CDS 26663..28342
/transl_table=11
/translation="MKGKIALYSRVSTSEQSEHGYSEKEQEQLLIKEVMKNFPGYDYE

TYTDSGISGKNIEGRPAMKRLLQDVKDNKIEMVLSWKLNRISRSMRDVFNIIHEFKEH

DVGYKSISENIDTSNASGEVLVTMFGLIGSIERQTLISNVKLSMNAKARSGEAITGRV

LGYKLSLNPLTQKNDLVIDENEAHIVREIFDLYLNHNKGFKAITTILNQKGYRTINQK

PFSVFGVKYILNNPVYKGYVRFNNHQNWAVQRRSGKSDKNDVILVKGKHEAIISEDVF

DQVHEKLASKSFKPGRPIGGDFYLRGLIKCPECGNNMVCRRTYYKTKKSKERTIKRYY

ICSLFNRSGSSACHSNAINAEVVERVINVHLNRILSQPNVIKQIASSVIEELKQKHSK

QTEIKYDIDSLEKQKAKVKTQQERLLELFLDDEMDSEMLKAKQSEMNQQLEVLDQQIK

EAKQANQSQDDIPNFDKLKARLILMITRFSVYLRKATPEAKNQLMKMLIDSIEITTDK
QVKLVRYKIDESLIPQSLKKDWGSFFIPKFNFVINVTKKNRIENLSLLPLF"
/product="Cassette chromosome recombinase B"
/db_xref="SEED:fig|703339.291.peg.30"
CDS 28431..28769
/product="hypothetical protein"
/db_xref="SEED:fig|703339.291.peg.31"
/transl_table=11
/translation="MTLSKQLKTYITERFKLNYQETWACETIDAVAEDVLPEKYIKNS
PLEHKILNTFTYYNDELHEISIYPFLCYLDKELVAIGYLDNFDLDFIFLNDTHQVIID
ERYLLQKGGE"
CDS 28772..28861
/product="hypothetical protein"
/db_xref="SEED:fig|703339.291.peg.32"
/transl_table=11
/translation="MNWIKVAQLSVTVINEVIEIMKEKQNGGE"
CDS 28863..29174
/translation="MKINRYITRGVSEHLSLDLQILLWNMVKDRDNQLHTDYLHIFKL

QEDDNILSITHEQEQPAYKLEYHYTNYVKNQNALPKKVYVIREDDVDAFYYVMLLPEE
Y"
/transl_table=11
/db_xref="SEED:fig|703339.291.peg.33"
/product="hypothetical protein"
CDS 29189..29692
/transl_table=11
/translation="MNTIKNTIHTEAIFSDDEQHRYLLKKTWDEKKPACTVITMYPHL

DGVLSLDLTTVLILNQLANSEQYGAVYLVNLFSNIKTPENLKHIKEPYDKHTDIHLMK

AISESDTVILAYGAYAKRPVVVERVEQVMEMLKPHKKKVKKLINPATNEIMHPLNPKA
RQKWTLK"
/product="hypothetical protein"
/db_xref="SEED:fig|703339.291.peg.34"
CDS 29706..29927
/translation="MNHETKQSDWRTVASCLASQNYISIVKGLVHHFTAIEDEEILDK
IYDDFMNDDSITTVLNNDLQTIINHYLSK"
/transl_table=11
/db_xref="SEED:fig|703339.291.peg.35"
/product="hypothetical protein"
CDS complement(30002..33121)
/db_xref="SEED:fig|703339.291.peg.36"
/product="Type I restriction-modification system,

restriction subunit R (EC 3.1.21.3)"
/EC_number="3.1.21.3"

/translation="MNFQFNEDDLEQVALEWLQSLGYDYKKGNEISMTSLTPERKSDK
DVVLHERLEKALRKINSDIHPRFIEKALHELTLEKSPNLLENNLTFHENLINGIEIED
YDDEGQSIVEIVKIIDFEYPQNNDFLAVNQLTIVNGDYKKRPDIVLFINGLPIVVIEL

KNSTNETVGVEDGYHQLETYKMRIPQLFTFNEVLVTSDGINTKAGSLTANYDRFMTWR

SKDGQTESSPGLASLDVLIHGMLNPETLLDLIRYFVLFQDDGKGHISKILAAYHQYYA

VNKAVDRALLASSEQGDGKGGVIWHTQGSGKSLTMVFFSGKLIQMLNNPTLVVVTDRN

DLDNQLYSTFVKSKGRSGKGLLRQTPKQAETRKELKSLLSVESGGIVFTTMQKFEPEQ

NETTMAALTERKNVIVMADEAHRTQYGFNAKYDDKGEGIKYGYAKYLRDALPNATFVG

FTGTPVASTDKNTQMVFGNYIDVYDMTQAVADGSTVKIYYESRVIPLNLPQSLDLDEA

YNDITEDQEEDVKQRLKSKWSRIEALAGAKPRVEALAKDIIQHFETRQQAMKGKGMIV

TMSRRIAVDLYDEIIRLKPEWHSDDDDKGVIKVVMTGSSSDPTSFQRHIGPKKRRNLL

EKRMKDVDDELQLVIVRDMWLTGFDVPSMHTMYIDKPMKGHNLMQAIARVNRVFKDKP

GGLIVDYVGIAESLKEALKEYTESDQAQTAIDTDKAVELMLLKYDVIQDMLYNLDYSK

FNSDKKSERYYTISNTMDYVIGLGEDERQRFIKTVTELGKAFALCATEPTAQELNDEI

AFFKAVKAGLVKLLQPPKEGKTRKTPAEVEAEINQLVSQSVVTEDVIDVYQTLGLEQP

DLSILSDDFLKDVEGLKQKNVAVELLNRLLKGQVKSLMKTNATVSKRFSEMLGNSINK

YNSRSIETSKVIEELIQLAKDIKQEQQRGNELGLNSDEIAFYDALASHETAKEAMGDK

ELRAIAHELTKTVKENMGVDWSKRDSAKAKMRVAVRRLLKKYGYPPDLQKMAVEQVVE
QAELMANNQ"
/transl_table=11
CDS complement(33105..34406)
/product="Type I restriction-modification system,
specificity subunit S"
/db_xref="SEED:fig|703339.291.peg.37"
/transl_table=11
/translation="MEYNTIFFGDVAKIKNGYAFKSKEFQEKGIPVIKIKNIISPIVD

TKDSQKVSIKTYEKTKGFSLKKNDILISLTGSGVNQMSSAVGKVGRIEFDYPALQNQR
VGKFELKYSNSADLDFLFYYFLQPKITEYLVRNSTGSANQANINSKLIETVKIPNFSL

IKQKSISKFLNQITRKIETNQKMIANLKELSQTLFKHWFVDFEFPDEDGNPYKSSGGE

MIDSELGKIPSNWKIYKLKDIASHKKETFNPKKSEEVTVKHFSLPAYDNEEQAIEEEV

NKIKSNKWIINNNCVLFSKMNPDTKRIWLPVIDNKKLNVASSEFVVMESPNNKINSFI

YNICLNSQFIDYLKANTTGSTNSRQRVKPTIAVNYKLAIEDSIVKKYSEIITPYMEEM
KILRSEIGKLTQLRDTLLPKLMSGELEISDDIEVNSDELSI"
CDS complement(34396..35910)
/transl_table=11

/translation="MATIGFEEKLWQAADKLRGSMDAAEYKNVALGLIFLKYVSDSFE

EKYEELKQDPYADEEDQDEYLAENIFWVPKEARWQYINDNAKKPEIGQIIDKAMIAIE

NENESLKGVLPKEYARPALDKEKLGDIIDLFTFKVGDTESRKQDVLGRVYEYFIAKFA

SAEGKNAGEFYTPSSIVKLLVEMIEPYKGRIYDPCCGSGGMFVQSERFVEKHQGRLDD

IAIYGQESNPTTWKLAKMNLAIRGIDNDLGERNADTFHNDLHKGLKADYILANPPFNA

SDWGQERLLDDYRWQFGIPPKGNANYAWIEHMISKLAPNGTAGFVLANGSMSTSGKDE

LEIRKNLIEQDLVECIVTLPGQLFYSTQIPVCLWFISNNKGQNGKKERENEILFIDAR

EIGHMVSRTLKEFSDEDIQKVAQTYHAWRGTNDKPYEDIAGFCKVANLEEVKNNEYIL
TPGRYVGLADVEEDEEPFEQKMERITSELSEQFAKSKELEDQIRKSLEGLGYGV"
/product="Type I restriction-modification system,
DNA-methyltransferase subunit M (EC 2.1.1.72)"
/EC_number="2.1.1.72"
/db_xref="SEED:fig|703339.291.peg.38"
CDS 36106..36252
/product="hypothetical protein"
/db_xref="SEED:fig|703339.291.peg.39"
/transl_table=11
/translation="MDGVTNVKIHILTFVKIVVIVDIVAIVNTRLDKLGIFNSINKKF
KCSQ"
CDS 36352..38415
/product="FIG131328: Predicted ATP-dependent endonuclease
of the OLD family"

/db_xref="SEED:fig|703339.291.peg.40"
/transl_table=11
/translation="MYISSVKIKNFRNFDNIDIDFHEGVNVLIGHNNSGKSNLLRALS

LIFDGSVRKQLSVEDFNNSLTKESLKKEAPKIVISVHITQSENERLMSDELITVSNWL

TKLEEPYEARIQYEFFLPKDEEKNYIDLVKNIDEKEEIWNLIRSQFIRLYVNKIWVGN

PEHQIPIDNDSLNKFDFQFLDAIRDVERDMFSGKNTLLKSVIDFFIDYDIKSDETITE

EEQKEKLEERRKEFSDNSSDLIETIHKRLDSGNQKILSYTNGIGASYDKSTPDFKGNL

TESEIYTVLQLIIKHETGMTLPITHNGLGYNNLIFMALLLSKMQADSDGNFLGSNAKV
FPILAIEEPEAHLHPTMQNEFIKFLKNNIREKKVKQIFITTHSTHISSSTNIDDIICL

YTDDSKTNVSYPGKVFDKNNQSKKYVQRFLDATKSNMLFAEKVIFVEGIAEQLLLHIF

ADYLNKPLEKNHVAVINVGGRYFNHFLSLFDSNNKYAINRKVSCITDLDPMRKEKNEN

GQKNNFKACYPFEYEMDLDKFDYSTNNSLDKYLNNEHSNIRVFVQPNKYGKTFEYQLM

FDNPSLKLLLTDSISNSQELAELMDHYEENESLQKLIDTLSPSNENQRIIKSLKDTND

SWNEDNKKKALIASRYLNSIGKGENALELASVLKDNLELKGQSDYEDFVVPKYIEGAI
RWVCE"
CDS 38417..40165
/transl_table=11
/translation="MIINAQTKLNIEQDFKIQAGPGAGKTEFLVNHIKNVIQTSEKLE

RMKKIACITYTNTAAQTVLQRLGKSVSNRVDISTIHSFLYRNVVKPYCSFLPKEYGIC

VQKLKGHSDPIVINKYVNAWLKTEYFTKLKPPSNRNQLLKMPVLKKALQNWLLSMKLS

YENNDVQFICDNTKAQAIDKKSKKRMGINKSNLNILEKQLLNYKKIYWEKGIIDHDDV
LFFSYVLIRDYPFILTILCAKYPYFFIDEFQDTSQLQDFIIDKIRQKGCIVGVIGDKA
QAIYSFQGARVSLFEKFKVDLINSHTILENHRSSYQIVTFLNSIREDITQTVYSGIEN

DKVTILIGNRIRSYTKVVNICNKESLVTLSRDNITSNAMRIKLEGNNFDKKLLEKFQE
IDSNSKRRNTIISLIKAIELAKNTKYKDALKIVESIYKDENNPKKIALNSLINMINKY

DKYGNGTLMNFYNTLCKTLDIKLSGFRKGQVKDFYENTLYKNMVICTNIVEDTSNHIT

IHKAKGAEFENVFVIGNKDTLSLLLKPDLMNDEEHRIFYVAMSRAKKKLFLQFDYLDN
NEQKKIKERYDVSIIQ"

/product="ATP-dependent DNA helicase UvrD/PcrA,
clostridial paralog 2"
/db_xref="SEED:fig|703339.291.peg.41"
CDS 40384..40557
/db_xref="SEED:fig|703339.291.peg.42"
/product="Multidrug resistance protein"
/translation="MQDVAINADHKVFFLMIIFNVIMLVSSIIQMILGKGRRITPAKP
TKDAKLHLDNQSS"
/transl_table=11
CDS complement(40625..42049)
/transl_table=11
/translation="MEMEFNENNIDLETIIRDEVNKYLSRDIGDLPATQQAPLELREK

YEKMEVPNKGRDIYEVLKDLNNEVLNYLYRPNHPRSFSFIPGPASRLSWLGDILTTAN

NIHASNFANATLPINIERNLINYLVGKIGYEIKPAGGVFVSGGSMANLTAIVAARDAQ

VEMEDIKKATVYLTSQTHHSVGKALHVAGFLKNNIHRIEYNDDFTMNTDSLKAAIDKD

IEEGYKPAMVIATAGTTNTGSVDDFTTLGDTCDHYNLWLHVDGAYGLSHILSDKAQHL

FKGIERSDSASWDAHKLLFQTYSCAMVIVKEKNHLLQSFGEDAEYLDDIASDDDVIDP
EMLGIELTRPARALKLWITLQVIGEDEIIERIKYGEGLAEYAEEYVSGLDNWRIISHA

NLSIVNFRYENSDLTEAQNNQLNSMAAQKIADSGYAIAYTTVLNNQRVIRLCTTNPLT
THDDIRETINRLDQYIGDYDMKDA"
/product="Decarboxylase, pyridoxal-dependent"
/db_xref="SEED:fig|703339.291.peg.43"
CDS complement(42132..42344)
/transl_table=11
/translation="MRSSSPVLRGSYRIPKDKHQYKLKITDDNLQVDYPRQFLKGAHT
PSVKAQHVIHWGDTFWVSSLWLAWFY"
/EC_number="1.1.1.95"
/product="D-3-phosphoglycerate dehydrogenase (EC
1.1.1.95)"
/db_xref="SEED:fig|703339.291.peg.44"
/db_xref="GO:0004617"
CDS 42994..43161
/product="hypothetical protein"
/db_xref="SEED:fig|703339.291.peg.45"
/transl_table=11
/translation="MKFKSEKLIIANQTKVINYRIQVLLKPQTNIETQTQVKNNTLSE
TDEQSNADLVQ"
ORIGIN

1 atgaaaatca ccattttagc tgtagggaaa ctaaaagaga aatattggaa gcaagccata
61 gcagaatatg aaaaacgttt aggcccatac accaagatag acatcataga agttccagac
121 gaaaaagcac cagaaaatat gagcgacaaa gaaattgagc aagtaaaaga aaaagaaggc
181 caacgaatac tagccaaaat caaaccacaa tcaacagtca ttacattaga aatacaagga
241 aagatgctat cttccgaagg attggcacaa gaattgaacc aacgcatgac ccaagggcaa
301 agcgactttg tattcgtcat tggcggatca aacggcctgc acaaggacgt cttacaacgt
361 agtaactacg cactatcatt cagcaaaatg acattcccac atcaaatgat gcgggttgtg
421 ttaattgagc aagtgtatag agcgtttaag attatgcgtg gagaggctta tcataaataa
481 aactaaaaat tagattgtgt ataatttaaa aatttaatga gatgtggagg aattacatat
541 atgaaatatt ggagtatacc ttgcaatatc atacgatgtt tatagagtgt ttaataaacc
601 atttttcaac tattgatgat ctagaatata taataactgt acaaattata ttgattatgg
661 aactacaatt caattaagaa attgatgatg aaattttaaa tttaaactaa tggaatcaag
721 aaagaatgaa aggaaatata caatgcccac gattaataaa aggaagttta ttagattttg
781 tgttagaaac agtagatgtt tttgtaaatg gaagtaaaaa tgattgcgaa ttagtaaatt
841 ttaattataa agaaggaaaa gtagtttcac gttatagtac aaaatttgaa agcaatccta
901 aattaagaaa aaagcaattg atatcgacgg taaaagttgt aaggtatgtt atttttgatt
961 ttgagaaaat tttatggggc atagggaaag ttttattgaa gttcatcata ttaaggcaat
1021 gtttactata agaaagttga aatacactca gaaacagact ggattcccgt ttttttcaaa
1081 ttggcacaaa atgatacata ggcccaccta tatattgaag atttaagtta aacgataaat
1141 aatatttttc tcttttgtat agaaattttt ttgggaatga tattaattag tataaaaatg
1201 aatatgtcta caaagaagga gagattttat tgaaacacat taatcaattt tctgaatcga
1261 cactagcaca actagaaaaa gatcaacaac acgtatttta tgtatactgt ttgatggacc
1321 caagaaatga tgaatgcttt tatgttggta aaggtaaggg gaatagaata tttaaacata
1381 aacaagatgc gcagaaaaaa gtattgtacg aagatatatt tatagaagaa aataaagata
1441 acttgaaatt taatagaatt aacgaaattt gtagcaatga tttgaatgtt ttaggatata
1501 taatcagtta tggattaacc gaatctgaag cattttctgc agaaaatgtt cttataaact
1561 ttcttaacct aacaaataaa actacattaa caaatatgat taacggtcat ggatctaagg
1621 catacttagt tgaagattta gaaaatgaat ttggctacga ttcaattaat cttgaaaaca
1681 taaatacgaa tgaattaatt ttagcagtga aaatcagaga tgcatttcta ttagataagg
1741 atgaaagtaa agagtatcct attaatgaaa gtaaacgtga tagaaataac cttaaatcgc
1801 gtacattagg tagttggata ataggaaaag ataaaataca taaaataaaa tatattattg
1861 gtatcaatac aggtgctaat aatgcagttg tctcagctta tgaagtatca tttgaacaag
1921 cggagagtat cgaaacaaat aatggtagaa tgagatatgc gtttattggg ctttcagaaa
1981 gagatgctac tctaaaaaaa ttgaatttat acaaaaaagc actaccagat ttaagatttg
2041 gtagtggtag cgctacagca tatataaata atgggacaat gaaagttgat taaatgaata
2101 aaaacatcca attccttaaa ttagcctttg aaaattttat actcgtatat ataagtgaaa
2161 aacttaaaag aattagatgt taattaaagg agagaatgct atgccatcag atgatattgt
2221 aaaaaaacta tactcaaaag aggatattcg attagaatta ggacttactc cgcataagtt
2281 taataaaaaa atggaaacta ttgctaagct ttttaaaatt gatatgaaaa tttttcacaa
2341 ttacaaaggc caagataaaa ataatcagta tacatttaat ggtgtcgcaa aagaattaat
2401 caaagtgttg ctaaaaagtg ttgattatta cccagtggat atcaattcca aaaaatttaa
2461 acaaaatgga aaatctaaaa aagaaatgat tgaaaatata gataactcta gttacatgaa
2521 atatatttat cagttaatga aatctatcaa tgaaattcaa tacaaaaggt taattgctga
2581 tatacatatg aaagatgtgt accagaatac aaaagcatgg ttaaataatg gtgaatcaat

2641 taataaaaaa gaacaagagc tataccaata tatgacaatt ttaccattac ataaaagagt
2701 tgaactgcaa aatgaggtat taaaatctat agacgaaaca atttttcaat ttctcgcaaa
2761 agaacataga aataatcaaa ttgaagaaaa taatgaatta gaagcatata caaaagcaat
2821 aaaagaaggt agaaatccta aaaatgatta tgaattaaat catctcctat ataaaaaaaa
2881 tcaattaccc ttagatagtc taatagaaga tatatgggac tatgaggaaa cagagtatac
2941 tgagcttgat tggttaattg cagatatgct aaaacgttca caaagagctg atagtaattt
3001 tatcgaaagt ttagagaaga aaaacaaatt gagaaaaaat attcataaag attccattaa
3061 aagtatatct aagttgattg atcatgaatt agttaaaaat aatagaaagt gggacgtagc
3121 agaattttat aaaaatcaac aattttggaa taatagtaca ttaagacgta gctctcaaaa
3181 ttttggaatt ttatattaca aaaagaatag gttacataca atgaatcaaa gtaatattat
3241 taagaaaata aaaattatcg aagctaatat agagagtgca aataaacagc cgagctattt
3301 agatttcttt ttacaagcta agtttgattt agagaggtta gaaagtgaat taatgaaata
3361 tagcattaat ccgaaatatt ttaataagat aaatagtgat tacgtaagat atgctattga
3421 agatgtacat aatgcaatta ttaaaattga cagatatata agtaacgata aagtgaagtt
3481 aaactattca aatgtccaaa tggtagtgag aaacgaaata acagaacaag gaagctactt
3541 tgtaacccag gcattgaata cactgtcaaa ggtagaagag tctggctaca gctttgataa
3601 tttcatggta ggacctttcg tagaagactt taaaaaagca attaaaaatc gtagatttgg
3661 ttctaaaaaa gattaagatt gttttttaga gttaaacata agaataaatt taaataaaaa
3721 aataaaccca acaaaccttg ttatatcaat gtttgttggg tttatttttt tggtgatttt
3781 ttaataactc aattacatct taggataata tcgctaaatt atacattgtc gaggccaaga
3841 caaataaaaa attaaagcaa aagttagcgc gcaactttct gctataaagg agcaaattta
3901 tatggagaca agagacaaac tgatgtcatt gactcaaagt gacaaaacac agcaatggct
3961 aatggacaag tcatctaacc aagatgacat tcaacaattg cagcaacaat tcagtcagca
4021 gcttgatcaa caatataatg cacttttagc tgatgaaaaa gctaagttag accaatacgt
4081 ggaagtacat caaggattgg aatcattaaa ggaagagatt gaatcagaac ctattacgct
4141 taatatcgat aaattacccg atatcaaagc aacaatgctt gaaagagcca agaatgatga
4201 acattctgat aaaatcgaaa agctatttga taggttagaa caggcattaa atggtacgaa
4261 tcgattatat acgcaattat cgttgattgg tacacgaaca catcgaatta caactaaaaa
4321 ttttaatctt caaggcttac ctaaagcagt ccaacatacg attttacctt caaaatttaa
4381 gaaggtgtat acagtcgatt ttaaatcgtt tgaaccatca gttgcagcgt acatgactca
4441 agattcaaaa ctgattgact tgttaaatca gaaagacgga ctgtatgacg cattgctaag
4501 tgaattaggc ttatcagatg agctacgtgt atttgttaaa cgtgcattta ttggttcgtt
4561 tctatttgga ggtaacttca aaaatcctaa attcaagctg aatcaatatg taagtgaagt
4621 acaatggttg gatgcggtca gccaatttac aaaagtcatt gaacttaaga agcacgttga
4681 aaagagtaat tccatgccta tgccttatag tattgagcat gatatgagcg catttcaagg
4741 tagcagtatt atggcaatct acgtacaaac tgtagcgagt tatattttca agcacattct
4801 gctaaaagtg tacaaagcac agtgcgatca aaaaacgttc aagattatag tacctataca
4861 cgatgcgatt atgattgaat gtgaagatga agaaattgca caaaatgtgg gtcagttaat
4921 gaaagatacg gctaaccagt tgttcaatgg tgaatttgca catgtgacag tggaagaaat
4981 aggaggcgta gaccatgaat aatgatagag gacaaagcct acacatccca agtagtacac
5041 caatcaaaga aaataatata tatgtagcta cgttacattc tgtgatccaa acagatttct
5101 caggtgaaat aaagcaccaa ttcacgtatg aaattgaagt gaacaatcag attgtatatg
5161 cgaatcgtaa tattccaaca aaaccgagcg ctaatcagtt gtcaattcat gattggctga
5221 aacgtcatag caactatagc gcaagtcatg aaaactatga gccttatatt gatcagaaac

5281 atttaattct attaggtcaa tataacggta actattatgt acaagatgta gcatcgttag
5341 atgcgtttgg aggcgtatta tcatgaatca tatattacaa atgttatcta agctattaag
5401 tgtggccaag gaggcaatcg accgtcaagg tctgattgct atcctaacta ttcctgttaa
5461 taataacgat gaaatagaag aaacggctca aggtgaaacc gtgtataacg aacttatcga
5521 tcagttacga cttaatatcc caaaagatac ggattatcaa cctaacatct atagttattt
5581 tggtattaag aagaatccta atgacaccgt actcatggaa atgatgataa aggtttttca
5641 tatcaaacgc tttaattcag aactgtttat tttcaaagtt aacgggtggc aaaagataaa
5701 tggagatgaa ttacaagggt tgatatctaa aatgatacaa gtattgcttg tagattataa
5761 gccttcacta agcactctaa aaaatgtcgt agatggattg caaaaatcaa cagatgtaga
5821 agaacttgtt gagaatgagc gctatattgg ttgtggtgaa aatatgttcg atcttaatac
5881 gtttcaagtc gttaaaaatt caatcgatat ctttccaaaa acacgattga atttatcatt
5941 aagtacaaat gatgtaatta ctgataagat accgccttat tttaagcaat atatgttaca
6001 acttgcgaat tatgacgatg atttacaata ctttcttttc caacatacag cagtattact
6061 tacagctgat actaaatacc gtaggggtct catattatat ggtggagcta agaatggtaa
6121 atctgtatat attgaactag ttaaatcatt tttctatagt aaagatattg tgtctaagcc
6181 acttaatgag cttgaaggtc gttttgacaa agaaagttta attgacaaaa gtctaatggc
6241 aagtcatgaa attgggcaat ctaggattca agaaaagatc gtaaatgact tcaaaaagtt
6301 attatctgta gaatcaatgc atgttgatcg taaaggaaaa actcaagtgg aagtcatttt
6361 ggatttgaaa cttattttta gtacaaatgc gatacttaat tttcctcctg aacatgcgaa
6421 agctttggag cgtcgaatta atattattcc atgtgagtat tatgttgaaa aagcggacac
6481 ttcattaatt gataagctcc agagtgagaa gaaagaaatc tttctttact tgatgtatgt
6541 gtatcaacag attgtaaaag cagatatcga gtatcttgaa aatagccgtg tcactgaaat
6601 tactcacgat tggttaaatt ttggatatga atttgtttct agcaggtccg taagtaatgc
6661 aaatcagaaa gcatgtatta atttactcag aaaacttata gaaatcaaat caggatcacg
6721 aatcaaagta tccgagttaa ataaagttat taatgaagaa ataaaggtaa gttctcaggt
6781 tattaaacag ttaattcaag caaactttga tactcaaacc aaactataca atggctacga
6841 ttattggatt gatttaggtt ggaaagaagc caataaaaaa gagattcatg atatttcgga
6901 aaaagataat attatttcat tagataaaaa tgaaaatata acagacgatg aggcattaga
6961 tgaagagaat ttggactttg attgggagga ctttgacgat gaataatgaa caaattgaag
7021 catttgtaga agtgcttgta cctatcatag aagaacgtat caataaaggt aattaaggct
7081 aattacgtac tacaggcagt tgcctgtagt actcatatga ttaagtggta aaagtgataa
7141 aaatgaaacg aaattataaa tatatattat ctatatgttg ttacaagacc gatagtctgt
7201 agcaataatc taataaaagg agcggtatga tatgaagggt aaaattgcac tttattcacg
7261 cgttagtacg tcagagcagt cggagcatgg gtactcaatc catgagcagg aacaagtact
7321 catcaaagaa gttgtgaaaa atttcccagg ttatgactat gagacatata ctgactcagg
7381 catttcaggt aaaaatattg aaggtcgtcc ggcaatgaaa cgtctattac aagatgttaa
7441 ggataataaa atcgaaatgg tgttaagttg gaaattgaat cgtatctcac gatcaatgag
7501 agacgtgttt aatattattc atgaattcaa agaacatgat gtagggtata aatcgatttc
7561 tgagaatatt gatacatcca atgcttctgg agaagtactc gttacaatgt ttgggttaat
7621 aggatctata gaacgccaga ctttgatttc gaatgtgaaa ctttctatga atgctaaggc
7681 aaggagcgga gaggcaatca ccggtcgtgt tttaggctac aaattatcac ttaatccact
7741 tacacagaaa aatgatttgg ttatcgatga aaatgaagct aatattgtac gtgaaatttt
7801 cgatttatat ttgaatcata ataaaggcct caaagccatt acaaccgtac ttaatcaaaa
7861 ggggtatcgt actattaatc aaaagccatt ttcagtgtat ggtgttaaat acattttgaa

7921 taatccagtc tataaaggct atgtcagatt caataaccat caaaactggg ctgtacagcg
7981 aagaagtggc aaaagtgata aaaatgatgt gatattggtc aaaggtaaac atgaagccat
8041 tataagtgaa gaggtatttg ataaagttca tgaaaaatta gcttctaaaa gttttaaacc
8101 gggcagacct attggtggag atttctactt acgtggcctt attaaatgcc cagaatgcgg
8161 aaataatatg gtatgtcgac ggacgtatta taaaacgaaa aagtccaaag aacggacaat
8221 caagcgttat tacatttgtt ccttattcaa tcgttcaggg agttctgcat gtcacagtaa
8281 ttccatcaat gctgaagtcg tcgagcgtgt aattaatgtt catttgaatc gtattctgtc
8341 tcaaccagat attatcaagc agattgcgtc aaatgtgata gaagaactga aacaaaagca
8401 tagtaaccaa acagaaatta aatatgacat tgatagttta gaaaaacaaa aagctaagct
8461 taaaacacaa caagaacgat tgttagaatt gttcttagat gatcagatgg atagcgaaat
8521 gttaaaagct aaacaaagtc aaatgaatca acagttagaa gtattagatc aacaaattaa
8581 agaagcgcaa caagcaaatc aatcacagga tgaaatacct aattttgata aattaaaagg
8641 acgactcatt ttgatgataa cacgattcag cgtgtactta agaaaggcta cacccgaagc
8701 taaaaatcaa cttatgaaaa tgttaattga ttctattgaa attacgacag ataaacaagt
8761 aaaacttgta aggtataaaa ttgacgaaag tcttatccct caatctttga aaaaagattg
8821 ggggtctttt tttatgccca aattccaatt tgaaatatat ggtcaaaatg attatttcat
8881 cgaccaaatt accactttta ccacttagtt attagtgaca aaagtgagcg aaatgaaata
8941 aaaatcaaat atatattatc aaaatgatgt atcacatgca tacatcaatc aaatacatta
9001 ggaggtcata accatgacac tagaacaaca actcaagcac tatataacca acttattcaa
9061 tctgccaagg gacgaagtgt ggcactgcga atctatcgag gaaatcgctg atgatatctt
9121 acccaatcaa tatgtaagac ttggcccact cagtaataaa acacttcaga ctaataccta
9181 ctactctgac acacttcatg aaagtaatat ctatcctttc attctctact atcagaaaca
9241 actcatagcc atcggttata tcgacgaaaa tcacgatatg gatttcttat acctacacaa
9301 cactatcatg cctcttttgg atcaacgata cttactaaca ggaggacaat aaaatgcata
9361 aatacatcaa aattacacaa ttagtcatta caatactaag tgaaatcatc atttggatga
9421 aagagtcaga acgaaaggaa gtctcttatg aatagatata tcacccgggg tatcgccaac
9481 aacttaccta atatcttaca acaccaatta tggcaactcg tatctgagcg agaacaagaa
9541 caaaccaaag ataatacttc agtagattat tttcatatat tccagttcaa taagcatcgc
9601 aatcaattat atatcaaaca caaacaagaa cgacctgaat atgcgaaaat ccataaagct
9661 aattattcaa aagcaatcaa tatcaataag gtctacatta ttcgagaaga tgatgtagac
9721 ctttcttatt atgtcatgtt attacctgaa gaatactaga ggagtgaaaa ttatatggaa
9781 acaatcaaaa gtacattaaa aacagaagcc atattcagtg atgacaaaca acatcgctat
9841 ctacttaaga aaacatggaa tagtgaaaaa caatcaatca caatcattac aatgtatccg
9901 cattatgatg gcattctcaa tattgaccta acgacccaac tcatcatgaa caaagtttca
9961 gaaatggatg catttggttc aatcaatttt gtgaatctat actctaatat tacaacccct
10021 atcaatctca aacatttaga aaatgcgtat gataagcata cagatattca aattatgaag
10081 gcagtgaaag agtcagatga agtgatatta gcttggggcg cttacgctaa aaagcccggt
10141 gttgaagcac gtgttaatga agtattagag atgttgaaac cacataaaaa gaaagtaaaa
10201 cgactcatga atccagaaac caatgaaatc atgcatcccc ttaattcgaa agcacgtcaa
10261 aaatggatat taaaagtata gcaactaaaa tgtttattca ctatccatga aaataattga
10321 atttcatcag gctgggacat aaatccctaa aaaacagcag taagataatt ttcaattaga
10381 aaatatctta ctgctgttct ctatttatac aatacttcgt attgaatggc ttcgctttcc
10441 tagggtgccg tctcagcctc ggtcttcgac tggcactgct ccctcaggag tctcgccatt
10501 aatactacgt attaacatgt aattttactt tgaaatactt taaaaaaata agacactttc

10561 gtataattta ataaatacca ctaaactaaa ttaacgaggt gccttatgta taaaaattat
10621 aacatgaccc aacttacact accaatagaa acttctgtta gaattcctca aaatgatatt
10681 tcgcgatatg ttaatgaaat tgttgaaacg atacctgata gcgaattcga tgaattcaga
10741 catcatcgtg gcgcaacatc ctatcatcca aaaatgatgt taaaaatcat cttatatgca
10801 tatactcaat ctgtattttc tggtcgtaga atagagaaat tacttcatga cagtattcga
10861 atgatgtggt tagctcaaga tcaaacacct tcttataaaa ctattaatcg ttttagagtg
10921 aatcctaata ctgatgcgtt aattgaatct ttatttattc agttccatag tcaatgttta
10981 aagcaaaatc ttattgatga taattcaatt tttattgatg gtacaaaagt agaagctagt
11041 gccaatagat atacatttgt gtggaagaaa agtattcaaa atcacgaatc gaaattgaac
11101 gaaaattcaa aagcattata tcgtgactta gttgaagaaa aaataatacc agagataaaa
11161 gaagatggag atagcgattt aacaatagaa gaaatagatt taattggtag tcatttagat
11221 aaagaaatcg aagatttaaa tcattctatt gagaacgaag attgtactca aattagaaaa
11281 cagacccgta aaaaaagaac tgagattaag aagttcaaaa agaaatttga tgattattcc
11341 gaaagaaaaa gtaaatatga agaacaaaaa tcgattctta aagatagaaa tagcttttct
11401 aaaactgatc atgatgcaac ttttatgaga atgaaggaag accatatgaa aaatggtcaa
11461 cttaagccag gatacaattt acaaatagcg acaaattctc aatttgtttt atcctatgac
11521 ctatttcaaa acccgacaga tactagaact ttaattccat ttttaacaat gattcaaaat
11581 accttcggtt atttaccgga gtatattgta gctgatgcag gttatggtag tgagcaaaac
11641 tatatggcta ttatagatga ttttaataaa acgccactta ttacgtatgg catgtttatt
11701 aaagataaaa ctagaaagtt taaaagtgac atttttaaca ctcaaaattg gaaatatgac
11761 gaacttaatg atgaatttat atgtcctaat aacaaaagaa taggttttaa aagatatgca
11821 taccgtaatg atagatatgg ttttaaacgt gacttcaagc tatatgaatg cgatgactgt
11881 tcagcatgtt ctttgaggca acaatgcatg aagccaaatt cgaaatccaa taagaaaatc
11941 atgaagaatt ataattggga atactttaaa gcccaaatta atcaaaagct ttctgaacca
12001 gaaacgaaaa aaatctatag tcaaagaaaa attgatgtag agcctgtttt tggatttatg
12061 aaggctattt tgggtttcac tcgaatgtca gttcgaggaa taaataaagt taaacgagag
12121 ctaggttttg tattaatggc acttaatata aggaaaatag tagctcgacg agctgtatat
12181 tatcaaatac atttaaaaaa agctgatttc tatcaaataa ttaatagaaa tcagcttttt
12241 tacattgcct aagaacttaa tgtcccagcc tctatttggt tctgttgcaa agtaaaaaaa
12301 tatagctaac cactaattta tcatgtcagt gttcgcttaa cttgctagca tgatgctaat
12361 ttcatggcat ggcgaaaatc cgcagatctg aagagacctg cggttctttt tatatagagc
12421 gtaaatacat tcaatacctt ttaaagtatt ctttgctgta ttgatacttt gataccttgt
12481 ctttcttact ttaatatgac ggtgatcttg ctcaatgagg ttattcagat atttcgatgt
12541 acaatgacag tcaggtttaa gtttaaaagc tttaattact ttagccattg ctaccttcgt
12601 tgaaggtgcc tgatctgtaa ttaccttttg aggtttacca aattgtttaa tgagacgttt
12661 gataaacgca tatgctgaat gattatctcg ttgcttacgc aaccaaatat ctaatgtatg
12721 tccctctgca tcaatggcac gatataaata gctccatttt ccttttattt tgatgtacgt
12781 ctcatcaata cgccatttgt aataagcttt tttatgcttt ttcttccaaa tttgatacaa
12841 aattggggca tattcttgaa cccaacggta gaccgttgaa tgatgaacgt ttacaccacg
12901 ttcccttaat atttcagata tatcacgata actcaatgta tatcttagat agtagccaac
12961 ggctacagtg ataacatcct tgttaaattg tttatatctg aaatagttca tacagaagac
13021 tcctttttgt taaaattata ctataaattc aactttgcaa cagaaccgta ttatggaata
13081 gagatgttgg taacatttat acaggatcat tatacttaag tttaatttcg ttattacaga
13141 accacacatt ccaaccagaa gagaaagtat gtctatttag ttatggttca ggagcagtag

13201 gagaaatctt tagtggttca atcgttaaag gatatgacaa agcattagat aaagagaaac
13261 acttaaatat gctagaatct agagagcaat tatcagtcga agaatacgaa acattcttta
13321 acagatttga taatcaagaa tttgatttcg aacgtgaatt gacacaagat ccatattcaa
13381 aagtatactt atacagtata gaagaccata tcagaacata taagatagag aaataaacta
13441 gtggccgatt gtgcttgatg agcttgggac ataaatccta actcgaaata aataagcata
13501 tcactaaact gattttttaa agtttacagt gatatgctta tttttttatc ttacgatttt
13561 gtacgtgcat gcttgcctag gggtatggct cgagccatta gtctctcgca catactattc
13621 cctcaggcgt cagcacttac aaaatcggtt gtaattttca tttttatacg cattcttact
13681 gagattatac taataagagg aatagtaaaa gcaattctaa gtaaaattgc agataagagg
13741 taagttaaaa gcaatcctaa gtaaaattgc agataagggg tacagaaaaa ctagacttga
13801 ttacaaaatg gagcttggga cataaatgat tttttaaaaa tgagatgaga cgtagattaa
13861 ctccataatc aatacgaatc tatcgacttc tttatttatg atattcatct ctttttaatg
13921 gaaataaaag tgcgattaat gtgataatac agttacgtta attaaaaaaa taaaaatgca
13981 aggagaggta atatgctaac tgtatatgga catagaggat tacctagtaa agctccggaa
14041 aatacaattg catcatttaa agctgcttca gaagtagaag gtataaactg gttggagtta
14101 gatgttgcaa ttacaaaaga tgaacaactg attatcattc atgatgatta tttagaacgg
14161 actacaaata tgtccgggga aataactgaa ttgaattatg atgaaattaa agatgcttct
14221 gcaggatctt ggtttggtga aaaattcaaa gatgaacatt tgccaacttt cgatgatgta
14281 gtaaaaatag caaatgaata taatatgaat ttaaatgtag aattaaaagg tattactgga
14341 ccgaatggac tagcactttc taaaagtatg gttaagcaag tggaagaaca attaacaaac
14401 ttaaatcaga atcaagaagt gctcatttca agctttaatg ttgtgcttgt taaacttgca
14461 gaagaaatca tgccacaata taacagagca gttatattcc atacaacttc gtttcgtgaa
14521 gactggagaa cacttttaga ttactgtaat gctaaaatag taaacactga agatgccaaa
14581 cttactaaag caaaagtaaa aatggtaaaa gaagcgggtt atgaattgaa cgtatggact
14641 gtaaacaaac cagcacgtgc aaaccaactt gctaattggg gagttgatgg tatctttaca
14701 gacaatgcag ataaaatggt gcatttgtct caatagaaag ttagaggtga gtcttacgtt
14761 tcagtgacgg tagacttacc tttaacatgt tacatactaa aaaattaatt tgaataagaa
14821 agagagacat atatgaaata cgatgatttt atagtaggag aaacattcaa aacaaaaagc
14881 cttcatatta cagaagaaga aattatccaa tttgcaacaa cttttgatcc tcaatatatg
14941 catatagata aagaaaaagc agaacaaagt agatttaaag gtatcattgc atctggcatg
15001 catacacttt caatatcatt taaattatgg gtagaagaag gtaaatacgg agaagaagtt
15061 gtagcaggaa cacaaatgaa taacgttaaa tttattaaac ctgtataccc aggtaataca
15121 ttgtacgtta tcgctgaaat tacaaataag aaatccataa aaaaagaaaa tggactcgtt
15181 acagtgtcac tttcaacata caatgaaaat gaagaaattg tatttaaggg agaagtaaca
15241 gcacttatta ataattcata ataaaacagt gaagcaacca tcgttacgga ttgcttcact
15301 gttttgttat tcatctatat cgtatttttt attaccgttc tcatatagct catcatacac
15361 tttacctgag attttggcat tgtagctagc cattccttta tcttgtacat ctttaacatt
15421 aatagccatc atcatgtttg gattatcttt atcatatgat ataaaccacc caatttgtct
15481 gccagtttct ccttgtttca ttttgagttc tgcagtaccg gatttgccaa ttaagtttgc
15541 ataagatcta taaatatctt ctttatgtgt tttatttacg acttgttgca taccatcagt
15601 taatagattg atattttctt tggaaataat atttttcttc caaactttgt ttttcgtgtc
15661 ttttaataag tgaggtgcgt taatattgcc attattttct aatgcgctat agattgaaag
15721 gatctgtact gggttaatca gtatttcacc ttgtccgtaa cctgaatcag ctaataatat
15781 ttcattatct aaatttttgt ttgaaatttg agcattataa aatggataat cacttggtat

15841 atcttcacca acacctagtt ttttcatgcc tttttcaaat ttcttactgc ctaattcgag
15901 tgctactcta gcaaagaaaa tgttatctga tgattctatt gcttgtttta agtcgatatt
15961 accatttacc acttcatatc ttgtaacgtt gtaaccaccc caagatttat ctttttgcca
16021 acctttacca tcgattttat aacttgtttt atcgtctaat gttttgttat ttaacccaat
16081 cattgctgtt aatatttttt gagttgaacc tggtgaagtt gtaatctgga acttgttgag
16141 cagaggttct tttttatctt cggttaattt attatattct tcgttactca tgccatacat
16201 aaatggatag acgtcatatg aaggtgtgct tacaagtgct aataattcac ctgtttgagg
16261 gtggatagca gtacctgagc cataatcatt tttcatgttg ttataaatac tcttttgaac
16321 tttagcatca atagttagtt gaatatcttt gccatctttt ttctttttct ctattaatgt
16381 atgtgcgatt gtattgctat tatcgtcaac gattgtgaca cgatagccat cttcatgttg
16441 gagcttttta tcgtaaagtt tttcgagtcc ctttttacca ataactgcat catctttata
16501 gcctttatat tctttttgtt ttaattcttc agagttaatg ggaccaacat aacctaatag
16561 atgtgaagtc gcttttccta gaggatagtt acgactttct gtttcattag ttgtaagatg
16621 aaattttttt gcgaaatctc ttaaatattc atccattttt ttaacggttt taagtggaac
16681 gaaggtatca tcttgtaccc aattttgatc catttgttgt ttgatatagt cttcagaaat
16741 acttagttct ttagcgattg ctttataatc ttttttagat acattctttg gaacgatgcc
16801 tatctcatat gctgttcctg tattggccaa ttccacattg tttcggtcta aaattttacc
16861 acgttctgat tttaaatttt caatatgtat gctttggtct ttctgcattc ctggaataat
16921 gacgctatga tcccaatcta acttccacat accatcttct ttaacaaaat taaattgaac
16981 gttgcgatca atgttaccgt agtttgtttt aattttatat tgagcatcta ctcgtttttt
17041 atttttagat acttttttta ttttacgatc ctgaatgttt atatctttaa cgcctaaact
17101 attatatatt tttatcggac gttcagtcat ttctacttca ccattatcgc ttttagaaat
17161 ataactgcta tctttataaa cttgtttgaa atttttatct tcaattgcat caatagtatt
17221 attaatttct ttatcttttg aagcataaaa atatatacca aacccgacaa ctacaactat
17281 taaaataagt ggaacaattt ttatcttttt catcaatatc ctccttatat aagactacat
17341 ttgtaatata ttacaaatgt agtatttatg tcaaaataat gttataattt ttgtgatatg
17401 gaggtgtaga aggtgttatc atctttttta atgttaagta taatcagttc attgctcacg
17461 atatgtgtaa tttttttagt gagaatgctc tatataaaat atacggttct gttgcaaagt
17521 tgaatttata gtataattat aaccaaaagg agtcttctgt atgaactatt tcagatataa
17581 acaatttaac aaggatgtta tcactgtagc cgttggctac tatctaagat atgcattgag
17641 ttatcgtgat atgtctgaaa tattaaggga acgtggtgta aacgttcatc atttaacggt
17701 ctagcgttga gttcaagaat atgccccgat tttatatcaa atttggaaga aaaagcataa
17761 aaaagcttat tacaaatggc gtattgatga gacgtacatc aaaataaaag gaaaatggaa
17821 ctatttatat cgtgccattg atacagaggg acatacatta gatatttggt tgcgtaagca
17881 acgagataat cattcagcat atgtatttat caaacgtctc attaaacaat ttggtaaacc
17941 tcaaaaggta attacagatt aggcaccttc aacgaaggtc gcaatggcta aagtcattaa
18001 agcttttaaa cttaaacctg actgtcattg tacatcgaaa tatctgaata acctcattga
18061 gcaagatcac cgtcatatta aagtaagaaa gacaagatat caaagtatca atacggcaaa
18121 gaatacttta aaaggtattg aatgtattta cggtctatat aaaaagaacc gcaggtctct
18181 tcagatctgc ggattttcgc catgccatga aattagcatc atgctaacaa gttaacacaa
18241 agtattattt taaaattgag attagacatt tatttttcaa ctttgcagca gaacctaatt
18301 ttttctcttt gtaaataaat gcaaagagat accataagga tctcttacat aaccataacc
18361 ttcagtatag aattctggac taaatgtttt caatacctca ctgccttttt ctattaactg
18421 gtcatataca tgtttagttt cttctacttg gtcaaaagtg agacaaagag atatattatt

18481 accttgtgtt atgggcaaac cttcagtgtc atctgcgatc ataattttta tatctccaaa
18541 ttgaagtaca cattgatcaa ttttatttaa atcattttcg tcaatattaa gtttcttatc
18601 tataggtcta tctttaatac gttgaatata cagtgtttta gcgccaaaca gctcttcata
18661 caactttttt aaaccctctg cattttgagt gattaaaaaa tatggactta cttgaaattt
18721 catgtttttt cctccttaga tttgttataa atagagtata tacttaatta gtgtcatcta
18781 ttgacactaa aaggagaata ataatgaaaa aatctgttag attatataat atgattgaat
18841 attgtaatga aaataggaac ttcaaattaa atgatttaat gtcagaattt aatatttctc
18901 gtagtaccgc tttaagggat ataaaagaaa ttgaagcatt aggagtacct ttatatagta
18961 atccagggaa aaatggtggt tatacgatca taggtaatcg agaccaaacg aaaatagcaa
19021 tctcagatga agagttgaaa gctttagtat ttacactttc gagtatttca aatgtgagta
19081 atctaccttt tcaaacagaa tatcaagaaa tattaaaaaa attatataat aactcgaata
19141 aaaaagagtt aataaaccaa tataatgatc tatttcaata ttttaatgaa gataagtatc
19201 agttcaaaag ttataagtta tttaatgaaa tcattagatt gataattgag aataagtctt
19261 ttgaaacctg ttattcacaa aactatatta aagaacaata taaaggtatt ggcattatgt
19321 ataaaaatca tcaatggtat tttgttgtag ctaatataga atcaaagtta gtgaatctat
19381 taaatatttc gaaaataaaa gaactatatg aaatgggaga gactcaagag tgtaatgata
19441 taactatgca gaattttcaa cagttcatgg ttaaaaatga aacagctatt gatattctta
19501 ttagaagcaa tgttatggga ttgaatatct tgaaaggcta cctgtggagt gactatatga
19561 ttgaaaatat tgacgaagag acatatttat ttaaatcaaa agtgaacgcg aaagatatag
19621 attttatagc taagttaatt gtcacaggtg gtgtcaatgt aaaagtagag acccctaata
19681 gtttgaaaaa tgctgttaaa gttgaattaa ctaaaataat aaacatgtat taatagtaat
19741 ttaatcataa ttgtaataac aaaaagatga caacattaaa caatttaaat ttatacaaaa
19801 aagcattacc aaatttaaaa ttgggtagtg gtagtgctac agcatacata aatagttata
19861 attaaataaa gatgggtcaa tgaaggtgaa ttaagtgaat aaaaatatta cagctctaaa
19921 atctactgaa aatactacat atatgcagta tacggttcat atcgatgatc aaattataaa
19981 taatataaaa gagtgtacca aaaagtttaa aattttgcct atggaagata aaattccatt
20041 atcaccgttg ttacaaccag aatatgcagg agaggtacaa gattttatta gtacatatga
20101 gcagtttatg attaattttg gtaaagtaat attggatagt caaggcataa aaatacagtt
20161 tgaaagtgaa tcattaagta gtattcaacg aggcattcaa gaacattgtt acttaaatga
20221 gcgaacaaat gacattgatg tgactaaaga atggtattta tgcaaatttt caaatcaaat
20281 attagaagag gataaaaatc aattgtataa tgctttaaag caactcatga atgattctaa
20341 aaataaaaag caagcttttt ggactgaccc caattagtgg gaattatata aaaacacttt
20401 cgttgaattc ataataaaat gaatcatgcg gaggtgtttt ttctatgaaa agagtttctt
20461 attcagtaga aacaaagtat aaagcagttg aaatgaaaat agctggtttt tcgacaaaag
20521 aaattatgaa agaattaaat attaaaaata gaacacaagt ggaaacttgg tggcgatggt
20581 atcgaaatga ggaaagttat agattttcac aacacgttgg gaaacaatat acctacggta
20641 aaggattaga agaattgtca gaaatagaac gattaaaatt agaaaataag agaaaagata
20701 tagaattgga tattttaaaa aagtacaagg cattggaaag gaagtggtac cagcagtagt
20761 catagattta gtggatcaat taaaagtaaa atattcaatc aaattgatac tagaagtatt
20821 aaacatacct aaatcaacat attaccgatg gaaaaacaaa acctataaaa atgatactgt
20881 aacacaaaaa gttattgaat tatgtgaagc taaccactat acctacggtt atcgtaagat
20941 tacagcactg attaatcaat gttatacatc accaattaat cataagagag tacagagaat
21001 tatgcagaag catcatttga actgccgagt tagacctaaa aagacgacaa ggataggtaa
21061 accgtattat aaaactgaca atttattaca aagacaattt aaagcgagtt gtcccatgga

21121 agtattaaca accgatatta cttatttacc atttggtcat tctatgttgt atttatcttc
21181 gataatggat atttataacg gagaaattgt ggcgtataaa atagatgata aacaagacca
21241 aagtttagtt aatgatacat taaatcaaat cgatataccc gaggggtgta tattacatag
21301 tgatcaaggc agcgtttata catcttatgc ttattatcaa ttatgcgaag aaaaaggcat
21361 tatcagaagt atgtcccgaa agggaacacc tgctgataac gccccgatag aaagttttca
21421 ttcctcgcta aagtctgaaa ctttttacat caataatcag cttaatagct ctaatcatat
21481 tgtaatagat attgtcgaaa aatacattaa aaactataat aataatcgaa ttcaacaaaa
21541 actaggctac ttatctcctg taaaatacag agaattagca gcctagaaca tagtgttttt
21601 attaagttcc cgtttaaggg ggtcagtacc ttttcaagag gtgtttaagg ttcatattga
21661 tatttatctc tacaataaag aaaaatctga atataagtat caagtatcta gttatttcaa
21721 tcttgtaaaa gaaaatccta aaataactta taaaaaaaga catttacaag agaaacaagg
21781 tgtcaagggt acaactttca cgaacaaatt aaaattattg aacaaaatgt atggtgtaga
21841 tgttgcaaaa tatcagccat tttataattc taataatcct gaatatgaaa gaggtcaatt
21901 tggtgaaaga tacatttctc aaagatctaa ctatgaattt aatagacttc aatatcaaat
21961 tatagatatg ttatcgaaaa tacttgataa gcatccatta ccaaagtcag ataataatta
22021 taaacatatt ccaactattg aaaaagcaat attaagtggg gattctcata gtttttatga
22081 gtattttgaa gatataatga aagaaattat aagtatggaa aattcttctt taaaggaaaa
22141 attattgact gattttacat atcaatctca atgtagatgg tactctgaaa gtgaaaaatt
22201 aaaattgcaa cttgaaagtt ttatgcataa agttttggag agcaattatt atgagggtaa
22261 caaattatat agaatgttgt ctcatgcaat tgaagaaaca atcaatgagg ctgatgaaga
22321 taaggttcat ttcaattatt ttaaagatta ttttttgaca gacggtgggg tgaagaattg
22381 ggagcagatt agcgagaaaa ttactgaatt caatggtaaa gttattaacg atatccaaaa
22441 tgaatataat aagattcaat ttaataacgc aaatcgaaat cagaaactaa actttaatta
22501 tttataccat tgctttgagt ttagtaataa cttagtaaaa gcaagggtaa atggaaatag
22561 aggttacatt ttatattttg tagataaata taaaataaaa atgccctttt tggatcaatt
22621 attaatcaat tatttgaatc aagaaatagg tcaggaaagt attaattata atatgcaaac
22681 attgtttgaa aaagaaagat atgatagaag tagtaccatt gaaaaattag tagcaacaag
22741 caaatttaag tatgaaaaag atgattcaga tttattcaaa caacttttca atgatgttga
22801 aaattcaata gacagattag gtatttactt actaaataat ggtataaatt cgaatgatga
22861 aaatgcaaga tattatagat cgtttttaaa ggaacttagt agaataaaaa gtaaattaac
22921 gccattttct cttgaaataa gtaagtctag cggaagagag caacattatc ctgatgatgc
22981 tattgatgat aaagatgaga gaagaaaaaa taaagaagaa acatatcatg cttttgatga
23041 taaaagcgat attgactcca aattaaagaa taaaataaat gtttctatcg ataatttatt
23101 gtcagttaaa attaagagca atttgtatga tattagtcca aatacaaaaa aacaataact
23161 ataatgagcc aactcaagaa tgagttggct ttttaattgt aattttacgc ttccattaca
23221 ggaatcaatg ttatggaagc cattaagcaa ttaatttgag tcatttatag tataggtaca
23281 acgttgtgat gttcacatga taatcagccc gggagattat tgtgtaataa cttatagaag
23341 gagcaaatca ccatgtatca agcaaatata cgtgatttaa ttacaaaatt gcctcaaagc
23401 aataaaacag aacacttttt aatgaacaaa ttttcaaatc aagataaagt tcagcagcta
23461 caaagacaaa ttagccaaca gctagatcaa caatataatg agcttttggc taatgaaaaa
23521 gctaagctag accaatacgt ggaagtacac cataatttag aaccattaaa gaaagagatt
23581 gaatcagaat ctattaacct tgataccgat aaattacctg atatcaaagc gacaatgctt
23641 gaaaaggcta agaacgatga acattttgat aaaatcgaac agctatttga tagattagat
23701 cagtcattaa atggtacgaa tcgattatat acgcaattat cgttgattgg cacacgaaca

23761 catcggatca caacgaaaag atttaatgtt caaggcttgc ctaaattagt ccaacaaatg
23821 attttacctt cgcaatttaa aaaggtttat acaatagatt ttaaatcatt cgaaccatca
23881 gttgctgcgt atatgacaca agacgaacaa ctgattgact acttgaatca tgaagaaggg
23941 ttatacgatg cattactgag agacttatct ttgtcaaaag agaagcgcgt gagtgtgaaa
24001 cgtgcattta tagggtcatt tctttttggc ggtcgttata gtagctctaa attcaaaatc
24061 aatcaagagg ttagtgaaat taactggcta caagtaatga gcaaattcaa gaaggtcatt
24121 gaatttaagg agcaagtcga aaaatataaa acaatgccta cgccttacgg cattgaacat
24181 gatatgagcg catttcaagg tagtagtatt atggcaattt atgtacaaac ggtagcaagc
24241 tatattttca agcacatttt gttggaagtg tacaaagcac agtgcgaaaa aaaacgttca
24301 agattatagt gccgatacac gatgcgatta tgattgaatg taatgataag gggattgcac
24361 aaaatgtagc acagctcatg aaagatacag ctaatcaact gtttaatggt gaatttgcac
24421 atgtgacagt ggaagcttta ggaggtatag acaatgaata atgatagagg aaaaagtctt
24481 caaattcccc aaagtacatt gttaaaagaa ggatctatat acgttgctac gttacattct
24541 gtgtacgaga agaacttctc aggtgatatt aaacatcagt ttacgtatga agtagaactt
24601 aaccaagaaa cgcactatgt aaatcgcaat attaccgtaa aatctatgag tcaccaatta
24661 tcaattgctg attggattaa acgtcacagt aactataacg taaaccacat taattatgat
24721 ccatacattg atcgaaaaca tttggtcctt gtagggcaat ataacggaaa ttattatatt
24781 caagatgtag caccattaga tgaatttgga ggggtattgt aatgaatcat attttagaaa
24841 tgttaataaa attattaaaa gtgggtatgg aggcaatcga ccgtaaaggt ctgattgcca
24901 tcctaacaag tagtattgga aatgatgaaa tggatgattc tgaacaagct gtaatggtgt
24961 ataacgagct tatcgataag ctacagctta acattcctaa agatgtcgac tatagaccta
25021 acatatacag ttattttggt attcaaaaaa agccaaatga cacaatatta gtggaaatga
25081 tgatatgtat ttttcatatc aagcgttttg attcagagtt gtttgttttc aaagataaag
25141 gttggcaaaa ggtaagtgaa gatgaattgc aagggttgat atctaaaatg atacaagtgt
25201 tgctagttga ttataaacct tcactaagta ctttgaaaaa cgtagtagat ggcatacaga
25261 aatcaacgga catagaaaaa cttgttgagc atagacagta cattggttgt ggacgaaata
25321 tgttcaatct aaagaccttt aaagtggttg ataatgacct tgaaatattc cctaaaacac
25381 gcttagattt ggaattagat ataaatgaca cgattacgga caagataccc ccgaatttca
25441 aacaatatat gttagagttg gcgaattatg accatgattt acaatatttc cttttccaac
25501 atatggcagt gttattgacg gcagatacta aactacgtcg tggacttttt ttgtatggaa
25561 ctgcaaaaaa tgggaaatcg gtctatatta aattagttaa gtcattcttt tatagtaatg
25621 atatcgtatc taaaacactt aatgaacttg gcgggcgttt cgataaggaa agtctaattg
25681 gtaaacgaat tatggcaagt gatgaagtgg ggaaagctaa tattgatgaa gcaactgtga
25741 atgatttcaa aaaattacta tctgttgaac caattcatgc tgaccgtaaa ggaagaacgc
25801 aagtagaagt tactttagat ttaaaactca tttttaatac gaatgctgta ctcaattttc
25861 catcatcaca tgcaaaagca ttagagcgta gaattgctgt tattccatgt gaatattatg
25921 ttgaaaaagc tgaccctgac ttaattgaaa agttacagga tgaaaagaaa gaaatctttc
25981 tttacttgat gtatgtgtat aagcaaattg taaaaaatga tatcgagtac ctccaaaatg
26041 atcgtgttac tgaaatttct catgattggt taaattttgg atatgaattt gtttctagta
26101 aatcagcaaa tattgcacat cagaaagcgt gtattaattt actcagaaaa cttatagaaa
26161 tcaaaccagg gtcacgtatc aaagtgtcta ggctaaatga ggttattaga gatgaaatta
26221 aagtaagctc tcaagttatt aatgatttgg ttcaagctaa ctttaatgta caaagtagac
26281 taaataatgg ttataagtat tgggtcgatt taggatggaa agaaactgat aaaaaagatg
26341 acatgatttc attcgataaa aatgagaatg taacagatga tgaattctta tacgaagatg

26401 atttgaactt aggttgggag gactttgacg atgaataatg aacaaattga agcatttgta
26461 gaagtgcttg tgcctatcat agaagaacgt atcaataaag gtaagtaatc taattacgta
26521 ctacaggcag ttgcctgtag tactcatatg attaagtggt aaaagtgata aaaatgaaac
26581 gaaattataa atatatatta tctatatgtt gttacaagac cgatggtctg tagcaataat
26641 ctaataaaag gagcggtatg atatgaaggg taaaattgca ctttattcac gtgttagtac
26701 gtctgagcag tcagaacatg gttattctga aaaggagcag gaacaactac tcatcaaaga
26761 agttatgaaa aatttcccag gttatgacta tgagacatat actgactcag gcatttcagg
26821 taaaaatatt gaaggtcgtc cggcaatgaa acgtctatta caagatgtta aggataataa
26881 aatcgaaatg gtattaagtt ggaaattgaa tcgtatttct cgctcaatga gagacgtgtt
26941 taatattatt catgaattca aagaacatga cgtagggtat aaatcgattt ctgagaatat
27001 tgatacatcc aatgcttctg gagaagtact cgttacaatg tttggactaa taggatctat
27061 agaacgccag actttgattt ccaatgtgaa actttctatg aatgctaagg caaggagcgg
27121 agaggcaatc accggtcgtg ttttaggcta caaattatca cttaatccat tgacacagaa
27181 aaatgattta gttattgatg aaaatgaagc tcatattgta cgggaaatct ttgatttata
27241 tttgaatcac aataaaggat ttaaagcaat cacgacaatt ctaaatcaaa aaggatatcg
27301 taccattaat caaaaaccat tttcagtgtt tggcgtgaaa tacattttga ataatccagt
27361 ctataaaggc tatgtcagat tcaataatca tcaaaattgg gctgttcagc gaagaagtgg
27421 taaaagtgat aaaaatgatg tgatattggt caaaggtaag catgaagcca ttataagtga
27481 agatgtattt gatcaagttc atgagaaact agcttctaaa agttttaaac cgggtcgacc
27541 tattggtgga gatttctact tacgtggcct tattaaatgc ccagaatgcg gaaataatat
27601 ggtatgtcga cggacgtatt ataaaacgaa aaagtccaaa gaacgcacaa tcaaacgcta
27661 ttacatttgt tcattattca atcgctcagg aagttctgcc tgtcatagta atgcgattaa
27721 tgctgaagtc gtcgaacgcg taatcaatgt tcatttgaat cgtattcttt cacaacctaa
27781 tgttattaag cagattgcgt caagtgtgat agaagaactg aaacaaaagc atagtaaaca
27841 aacagaaata aaatatgata ttgatagtct agaaaaacaa aaagcaaaag ttaaaacaca
27901 acaagaacga ttattggaat tgttcttaga tgatgaaatg gatagcgaaa tgttaaaagc
27961 taaacaaagt gaaatgaatc aacagttaga agtattagac caacaaatta aagaagcaaa
28021 acaagcaaat caatcacagg atgatatacc caattttgat aagttaaaag cacgactcat
28081 tttgatgata acacgattca gtgtgtactt aagaaaggct acacccgaag ctaaaaatca
28141 acttatgaaa atgttaattg attcaattga aattacgaca gataaacaag taaaacttgt
28201 aaggtataaa attgatgaaa gtcttatccc tcaatctttg aaaaaagatt gggggtcttt
28261 ttttatacct aaatttaact ttgtgataaa tgtcacaaag aaaaatagga ttgaaaattt
28321 atcactttta ccactttttt agagtgacaa aagtggagga gttttgaaat attttataaa
28381 tatatatttt atttatggag tacacattat taattaagga ggtcattata atgacgctaa
28441 gcaaacaact taaaacgtat atcactgaac gatttaaatt aaattatcaa gaaacttggg
28501 cttgtgaaac catagatgcg gtggctgaag atgtattacc tgaaaaatat attaaaaata
28561 gtccacttga acataaaatt ttaaatactt ttacctatta caatgatgaa ttacatgaaa
28621 tcagcattta cccttttta tgttatctag ataaggaatt agtagcaata ggttatttag
28681 ataattttga tttagacttt atatttttaa atgacactca tcaagtcatt attgatgaac
28741 gctacttgtt acaaaaaggg ggcgagtaat tatgaattgg atcaaggtcg ctcaactatc
28801 tgtcacagtt atcaatgaag tcattgagat tatgaaagaa aaacaaaatg gaggggaata
28861 gtatgaaaat caatcgatac attacaagag gtgttagtga acatttatct ctagaccttc
28921 aaatcttact ttggaacatg gtaaaagatc gagacaatca acttcataca gattacctac
28981 acatttttaa actacaagaa gatgataata tactctcaat cacacatgaa caagaacaac

29041 ccgcatacaa attggaatat cactatacaa actatgtaaa aaatcaaaat gcattaccta
29101 agaaagtcta cgtcatccga gaagatgatg tagacgcttt ttattatgtg atgcttttac
29161 cagaagaata ctaaaaggag tgacaatgat gaatacaatc aaaaatacaa tacacacaga
29221 agcgatattt agcgatgatg aacaacaccg ctacttactc aaaaagactt gggatgaaaa
29281 gaaacctgca tgtacggtaa tcaccatgta ccctcatcta gatggcgtat tatcactcga
29341 tcttacaact gttcttatcc tcaatcaatt agcgaattct gaacaatatg gcgctgtata
29401 tcttgtaaat ctattttcta atattaaaac cccagagaac cttaaacata ttaaagaacc
29461 ttatgataaa cacacagaca ttcatttaat gaaagcgata agtgaaagtg acacagtgat
29521 tttagcctat ggtgcttatg cgaagcgacc cgttgttgtc gaacgcgttg agcaagtgat
29581 ggaaatgtta aaacctcata aaaagaaagt aaaaaagctc ataaatccgg caacgaatga
29641 aattatgcat ccgcttaatc ctaaagcgcg tcaaaaatgg acattgaaat aaaggaggat
29701 tatctatgaa ccatgaaact aaacaatcag attggcgaac ggttgctagt tgtttagcat
29761 cacaaaatta tatatcgatt gtaaaaggtt tagtacatca ttttacagcg atcgaagatg
29821 aggaaattct tgataaaatc tatgatgatt ttatgaatga tgactctata acaacggtac
29881 ttaacaatga tttacagaca atcattaacc attatctatc aaaataaaga ctatactaat
29941 tacaataaaa acagagcatc cttcactttt atggtgaggg atgctctttt aatttatttt
30001 tttattgatt attcgccata agttctgctt gctctacaac ctgctctaca gccatttttt
30061 gtaaatctgg tggatagcca tattttttaa gcaatcgtct aacagctacg cgcattttag
30121 cttttgcgct atcacgttta gaccaatcaa cacccatgtt ttctttcact gttttagtta
30181 gctcatgagc aatcgcacgt agttctttat ctcccatggc ttcttttgct gtttcatgtg
30241 aagctaaagc atcgtaaaac gcaatctcat ctgaattcag gcctaattca tttcctcgtt
30301 gttgttcttg tttaatatct ttagcgagtt gaataagttc ttcaatcact ttagatgttt
30361 caatggaacg actattatat ttattaatcg aatttcctaa catctcagag aaacgcttag
30421 ataccgttgc attcgttttc attaatgatt taacttgtcc tttgagtaat cgatttaata
30481 attctaccgc cacatttttc tgtttcaatc cttcgacatc ttttaaaaag tcatcagata
30541 agattgataa atcgggttgt tcaagaccta gcgtttgata aacatcaatg acatcttcag
30601 tcacaacaga ttgtgacaca agttgattaa tctctgcttc aacttctgca ggtgttttac
30661 gtgtttttcc ttcttttggc ggttgtaaca atttaacaag tcctgcttta actgctttaa
30721 agaaggcaat ctcatcgttg agttcttggg ctgtaggttc agtcgcacaa agagcaaagg
30781 ctttccctaa ttctgtgact gttttaataa aacgttgacg ttcatcttca cctaaaccaa
30841 ttacataatc catcgtattc gatattgtat aataacgttc agatttttta tctgaattaa
30901 atttagagta atcaagatta tataacatat cttgaattac atcatacttc aataacatca
30961 attcaaccgc tttatccgta tctattgccg tttgagcttg atcagattct gtatattctt
31021 taagtgcctc ttttaaactt tcggcaatcc ccacataatc gacaattaaa ccacccggct
31081 tatctttaaa cactcgatta actcgagcaa ttgcttgcat caaattatgc cctttcatcg
31141 gtttatcaat atacatcgta tgcatagaag gaacatcaaa ccctgtcagc cacatatctc
31201 gaacaatcac gagttgtaac tcatcatcca catctttcat acgtttttct aataaattac
31261 ggcgtttttt aggaccaatg tgtctttgga aagaagttgg gtcactagat gagcctgtca
31321 ttaccacttt aatgactcct ttatcatcat catctgaatg ccattctggt tttagacgaa
31381 tgatttcatc atataaatca acagcaattc gacgactcat cgttacaatc attcctttgc
31441 ctttcatcgc ttgttgacgt gtttcaaagt gttggatgat atctttggct agggcttcta
31501 cacgaggttt tgcgcctgct aaagcttcaa tacgggacca ttttgatttt aaacgctgtt
31561 ttacatcctc ttcttgatct tcagtaatgt cattatacgc ttcatctaaa tctaaacttt
31621 gaggtagatt taatggaatt acacgacttt catagtaaat tttaacggtg cttccatcag

31681 cgacggcttg tgtcatatca taaacatcga tatagtttcc gaaaaccatt tgcgtatttt
31741 tatccgttga agctacgggt gtgcctgtaa atcctacgaa tgttgcattc ggtaaagcat
31801 ctcttaaata tttggcataa ccatatttaa tgccttcacc tttatcatcg tattttgcgt
31861 taaagccata ttgtgtacga tgagcttcgt ctgccatgac aatcacattt tttcgttctg
31921 ttagggcagc catggtcgtt tcgttttgtt caggttcaaa tttttgcatc gttgtaaata
31981 caataccacc cgactcaaca gataataatg attttaattc tttacgtgtt tcagcttgtt
32041 ttggtgtttg tcttaataat cctttaccag aacgtccttt tgatttaaca aacgtactgt
32101 acagttggtt atctaaatca ttacgatccg ttacgacgac taaggtaggg ttattcagca
32161 tttgaattaa ttttccagag aaaaagacca tggttaaact tttaccggac ccttgggtat
32221 gccaaataac gccgccttta ccatcacctt gctcagatga agctaataaa gctctgtcaa
32281 cggctttatt aacggcataa tattgatgat acgctgctag aattttactg atatgtcctt
32341 tgccatcatc ttgaaataaa acaaaaatatc gaattaaatc aagtaatgtt tctggattta
32401 acatgccatg aatgagtacg tctaagctag ctaaacctgg tgatgattca gtttgaccat
32461 ctttactgcg ccaagtcata aaccgatcat aatttgcggt tagagagcca gcctttgtat
32521 taataccatc acttgtaaca agcacttcgt taaacgtaaa taattgtgga atacgcatct
32581 tatacgtttc taattgatga taaccgtctt cgacgcccac ggtttcattt gttgaatttt
32641 taagttcaat cactacgata ggcaagccat tgataaagag tacaatatcg ggacgttttt
32701 tatagtctcc attaacgata gttaattgat tgactgctaa aaaatcattg ttttgtggat
32761 attcaaaatc aataatttta acaatttcta caatcgattg tccttcatca tcatagtctt
32821 cgatttcaat accattaatc aaattttcat gaaaggtaag gttattttct aaaagattgg
32881 gcgacttctc taaagttagt tcatgtagcg ctttttcaat aaaacgagga tggatatctg
32941 agttgatttt tcttaatgct ttctctaaac gttcatgaag gacaacatct ttatcacttt
33001 tacgttctgg tgttagactt gtcatactaa tctcattacc ttttttatag tcatagccta
33061 gtgattgtag ccattctaat gcgacttgtt ctaaatcatc ttcattaaat tgaaagttca
33121 tcactattca cctcaatatc atcagatatt tctagttctc cagacattag tttaggaaga
33181 agagtatctc gtaattgagt taatttacca atttctgatc ttaatatttt catttcttcc
33241 atatatggag tgattatttc agaatatttt tttacaatac tatcttcaat tgctaattta
33301 taattaacag ctattgttgg ttttactctt tgtctactat ttgttgaacc ggtagtatta
33361 gcttttaaat aatctataaa ttgagaatta agacatatat tatatatgaa tgaattaatt
33421 ttattattag gagattccat tacaacaaat tctgatgaag caacatttaa tttttttatta
33481 tctattacag gtaaccaaat ccttttagta tcaggattca tttttgaaaa taatacgcaa
33541 ttgttattaa taatccattt attacttttt attttattga cttcttcctc tatagcttgt
33601 tcttcattat cataagcagg taaactaaaa tgtttaactg ttacttcttc actcttttta
33661 ggattaaaag tttccttttt atgacttgct atatctttta atttatagat tttccaatta
33721 cttggtatct tacctaactc actatcaatc atttctcctc cactagattt ataaggatta
33781 ccatcttcat ctggaaattc aaaatctaca aaccaatgct taaatagtgt ttgtgaaagt
33841 tctttaaggt ttgctatcat tttttggtta gtttctattt ttcttgtgat ttgatttaaa
33901 aatttagaaa tgcttttttg ttttattaaa gaaaaattag gaattttgac agtttcaata
33961 agtttagaat ttatattagc ttgattagca ctccctgtag aatttctaac taaatactca
34021 gttattttag gttgtagaaa ataataaaat aaaaaatcta agtctgcact attactatat
34081 tttaattcga attttcccac acgttgattt tgtaatgctg gataatcaaa ttcaattctt
34141 ccaacttttc ctactgcaga agacatttga ttcacgcctg atccagttaa tgatattaat
34201 atatcatttt tctttaatga aaaaccttta gttttttcat atgttttaat agagactttt
34261 tgagagtctt ttgtatcaac aatgggtgaa attatattct taattttaat aaccggtata

34321 cctttttctt gaaattcttt tgatttaaaa gcatatccat ttttaatttt agctacatca
34381 ccgaaaaaaa ttgtattata ctccatatcc taaccccctcc aatgattttc ggatttggtc
34441 ttcaagttct tttgattttg cgaattgttc acttaattca gaagtaatac gttccatttt
34501 ttgttcaaaa ggttcttcat cttcttcaac gtcagctaaa ccaacatatc gtccaggtgt
34561 taaaatgtat tcattattct taacttcttc taaattagct actttacaaa aaccagctat
34621 atcttcataa ggtttatcgt ttgtacctct ccacgcatga tacgtctgtg ctactttttg
34681 aatatcttca tctgaaaatt cttttaatgt tctagatacc atatggccga tttcacgagc
34741 atcgataaat aaaatttcat tttcacgctc ttttttacca ttttgacctt tattattact
34801 aatgaaccaa agacatacag gtatttgggt tgagtaaaag agttgtccag gtaaagtaac
34861 aatacattca actaaatctt gttcaattag atttttacga atttctaatt catctttttcc
34921 gcttgtagac attgatccgt tggctaatac gaatcctgct gtaccattag gtgctaattt
34981 tgaaatcata tgttcaatcc atgcatagtt agcattacct tttggtggaa taccaaattg
35041 ccaacggtaa tcatcaagta atcgttcttg accccaatca cttgcgttaa aaggaggatt
35101 ggctaatatg taatctgctt ttaatccttt atgtaaatca ttatggaatg tatcagcatt
35161 tcgttcacct aagtcattat caataccacg aatcgctaag ttcatttttg ctaatttcca
35221 agtcgtaggg ttagattctt gtccgtaaat cgcaatatcg tctaatcgac cttgatgttt
35281 ctcaacgaag cgttcacttt gtacaaacat cccacctgaa ccgcaacacg gatcgtaaat
35341 acggcccttta taaggctcaa tcatctcaac taataacttt acaattgatg acggggtata
35401 gaattctcca gcatttttac cttcggcact tgcgaattta gcaataaagt actcatacac
35461 acgacctaac acatcttgtt tacgactctc tgtgtcgcca actttaaatg taaataaatc
35521 aataatatcg cctaattttt ctttatctaa tgcaggtctt gcatattctt taggtaatac
35581 acctttgagt gattcatttt cattttcaat tgcaatcatt gctttatcaa taatttgtcc
35641 aatttctggt tttttcgcat tatcattgat atattgccat cttgcttctt ttggcaccca
35701 gaaaatgttt tctgctaagt attcatcttg gtcctcttca tctgcatatg gatcctgctt
35761 caattcttca tacttttctt caaaagaatc tgatacatat tttaaaaaga ttaaacctag
35821 tgctacattt ttatactcag cagcatccat acttcctctc aatttatcgg cagcttgcca
35881 taatttttct tcaaaaccta tagtggccat taataacacc tcttaaaatt aatgttaaat
35941 caactatagc aaaaaataga agatgattgt gtagtgtttt attaatattt aatttatcta
36001 gactatcaaa aaaatataga ttattattat cttagattaa taaacaggag tgaattaaaa
36061 tgaataaaaa atataaatta aaacgatacg ataaaagatt tagaggtgga tggtgttaca
36121 aatgtgaaga ttcatattct taccttttgtg aagattgtgg ttattgtaga tattgttgcc
36181 attgttaata caagactaga taaattgggt atctttaact caattaataa aaaatttaaa
36241 tgtagtcaat agcttgctaa ctttatttga tgtaatataa ttttggcttt tagcattcag
36301 ttaaaaagaa gtaaataatt gacagatttt ttagagaact ggagaaaaaa gatgtacatt
36361 tctagtgtta aaattaagaa ttttagaaat tttgataata tcgatattga tttccatgag
36421 ggagttaatg tattaatcgg acataataat tcggggaaat caaatttatt acgtgcttta
36481 tctttaattt ttgacggttc agttagaaag caactgtcag tggaagattt taataactct
36541 ttaacaaaag aatctttaaa aaaagaggct ccaaaaatag taatttcagt acatataaca
36601 caatctgaaa acgaaagact gatgtctgat gagttaatta cggtaagtaa ttggctgact
36661 aaattagaag aaccttatga agctagaata caatatgaat tctttttacc aaaagatgag
36721 gaaaaaaatt acatagattt agtaaaaaat atagatgaaa aagaagaaat atggaattta
36781 attagaagcc agtttattag gttgtatgtc aataaaatct gggtgggtaa tccagaacat
36841 cagattccta tagataatga tagtttaaat aaatttgatt tccaattttt agacgcaata
36901 agagatgtag agagagatat gtttagtggt aaaaatacat tattaaaaag tgtaatagat

36961 tttttatag attacgatat aaaatccgat gaaacaatca cagaagaaga acagaaagaa
37021 aaattagaag aaagaagaaa agaattttct gataattcta gtgatttgat tgaaactatt
37081 cataaaagat tagatagtgg gaatcaaaaa attttgtctt atacaaatgg cataggagca
37141 tcttatgata aatcaacacc ggattttaaa ggtaatctta cagaaagtga aatttatact
37201 gtattgcaat tgataattaa acatgaaact ggaatgactt tacctataac tcataacggg
37261 ttgggttaca ataatttaat tttcatggcc ttgctattgt ctaaaatgca agctgattct
37321 gatggaaatt ttctaggtag taatgcaaaa gtatttccga tactagctat tgaagaacca
37381 gaggctcatc tacatcctac tatgcaaaat gaatttatta aatttttaaa aaataacatt
37441 agagaaaaaa aggtaaagca gattttcata actactcatt caacacatat ttcttcttct
37501 acaaatattg atgatattat ttgtctttac acagatgata gtaaaactaa tgttagttat
37561 cctggaaaag tttttgataa gaataatcaa agtaagaagt acgtacaacg gtttttagat
37621 gccactaagt caaatatgct atttgcagaa aaggtaatat ttgtggaagg tatagcagaa
37681 caattactat tacatatatt tgcagactat ttaaataaac cgttagaaaa gaatcatgtt
37741 gcagtaataa atgtaggcgg gagatatttt aatcattttt tatctttgtt tgatagtaat
37801 aataaatatg caattaatcg taaggtttct tgtataacag atttagatcc aatgcgaaaa
37861 gaaaaaaatg aaaatggaca aaaaaataat tttaaagcat gctatccttt tgaatatgaa
37921 atggatttag ataaatttga ttattcaact aataattctc tagataaata tctaaataat
37981 gagcacagta atatacgagt atttgttcaa cctaataaat atggaaaaac atttgagtac
38041 caacttatgt ttgacaatcc ttcattaaaa ctgttactta cagactcgat ttctaatagt
38101 caagagctag cagaattaat ggatcactat gaagaaaatg aatctttaca aaaattaatt
38161 gatacattat caccatctaa tgaaaatcag agaataataa aatctttaaa agacacaaat
38221 gacagttgga atgaagataa taaaaagaaa gcattaatag catcaagata cctaaattcc
38281 ataggaaaag gagaaaatgc tctagagcta gcttcagtat taaaagataa tttagagtta
38341 aaaggccaga gtgactatga agattttgta gtaccaaaat atatagaagg tgcaattcga
38401 tgggtgtgtg aataatatga ttattaatgc tcaaacgaaa ttaaatatag aacaagattt
38461 taaaatacag gctggtcctg gtgctggaaa aacagaattt ttggtgaatc atatcaaaaa
38521 tgtaatacaa acttctgaaa agctcgaacg tatgaagaaa atagcgtgta ttacttatac
38581 gaatacggct gctcagacag ttttacaaag attaggaaaa agtgtttcaa atagagtaga
38641 tatatctaca atacatagct ttttgtatag aaacgtagtt aaaccatatt gttcttttct
38701 acccaaagaa tacggaattt gtgtgcaaaa attaaaagga catagtgatc caattgtgat
38761 taacaaatat gtaaatgcat ggttaaaaac tgaatatttt acgaaattaa aaccgcctag
38821 taatagaaat caactactaa aaatgccagt actaaagaaa gctttgcaaa actggctact
38881 atctatgaaa ctttcttacg aaaataatga tgtgcaattt atttgtgata ataccaaagc
38941 tcaagcaatt gataaaaaaa gtaaaaaaag aatgggaata aataaatcaa acttaaatat
39001 actagaaaag caattattaa attataaaaa aatatattgg gagaaaggaa ttatcgatca
39061 tgatgatgtt ttgttttttt catatgtatt aattagagat tacccattca ttctcactat
39121 tttatgtgct aaatatccct actttttat agatgaattt caagatacta gtcaattaca
39181 agattttatt atagataaaa taagacagaa aggctgcata gtgggggtta ttggcgataa
39241 ggcacaggca atttacagct ttcaaggtgc acgggtttcc ttatttgaga agtttaaagt
39301 tgatttaata aattctcaca ctatcttaga aaatcataga agttcatatc aaatagttac
39361 tttttaaat agtatcagag aagatattac tcaaacagta tatagtggta ttgaaaatga
39421 taaagttaca attttgattg gaaatcggat tcgcagttat actaaggtag ttaatatttg
39481 taacaaggag tcacttgtta ccttgtcaag agataatatt acttctaatg caatgagaat
39541 aaagttggaa ggaaataatt ttgataaaaa gttattggaa aaatttcaag aaattgatag

39601 taatagtaaa agaaggaata ctattatttc gcttataaaa gctattgaat tagctaagaa
39661 tactaaatat aaagatgcct taaaaatagt cgaatcgatt tataaagatg aaaataatcc
39721 taaaaaaata gctcttaatt ctctaataaa tatgattaat aaatatgata aatacggtaa
39781 tggtacatta atgaatttct acaatacttt atgtaaaact ttagatatta aactttcagg
39841 gtttagaaag gggcaagtaa aagattttta cgaaaacact ttatataaaa atatggtgat
39901 atgtacaaat atcgttgaag acacaagtaa tcatattact atccacaaag cgaaaggcgc
39961 agaattcgaa aatgtatttg ttataggtaa taaagacact ttaagtctac ttttgaaacc
40021 agatttaatg aacgatgaag agcatcgtat attctatgta gctatgagtc gagcaaagaa
40081 gaaacttttt ttacaatttg attatcttga taataatgaa caaaagaaaa ttaaggaacg
40141 ttatgatgta agtattatac aataacctaa tctaagaata tttaaatcca atatatttct
40201 tgattataat tcaataatag ataaaatcta taaacgatta aaaagaaaat atatttgcta
40261 agacttataa aaatgaagag ataatagatt ttcctaaatt acagttaaaa gaagtaaata
40321 gattgaacac tttgaaagat tctaatccaa aatttgaacc actaaaattg aaagatgaag
40381 aagatgcaag atgtagccat caacgctgat cacaaagtat ttttcttaat gattattttt
40441 aatgttatca tgctcgtttc ttctattatt caaatgattt taggaaaagg cagacgtatc
40501 acaccagcaa aacctactaa agatgctaag ttacacttag ataatcaatc ctcataaaga
40561 gaaaaaagaa cccctaaaaa gttcaacttt ttgggggtta cttactatta agaagtattt
40621 ttgattaagc atctttcata tcgtaatcac caatatactg atcaaggcga tttattgttt
40681 ctctgatatc atcatgtgtc gttaatgggt tcgttgtaca taaacgaatg acacgttggt
40741 tattcaatac tgtggtataa gcaatggcat aaccagaatc tgcaattttt tgcgcagcca
40801 tgctattgag ttgattattt tgtgcttcag ttaaatcact gttttcatat ctgaagttca
40861 caatggaaag attagcgtgt gagataatac gccagttatc gagacctgac acgtattctt
40921 ctgcatattc tgctaaacct tcgccgtatt taatgcgctc gataatttca tcttcaccaa
40981 tgacttgcag agtaatccat aattttaaag cacgtgcagg gcgtgtaagt tcaatgccta
41041 acatttctgg atcaatcaca tcatcatctg aagcaatatc atccaagtac tcagcatctt
41101 cgccaaagct ttgcagcaga tgattctttt ctttcacgat taccatagcg caactatatg
41161 tttggaacag taatttgtga gcatcccaac tggcactatc tgaacgttca atgcctttaa
41221 ataagtgttg tgctttatcg gataagatat gtgatagtcc ataagcaccg tctacatgga
41281 gccatagatt gtagtgatca catgtatctc caagtgtggt gaaatcatcg acagaacctg
41341 tattagtcgt gcctgcagtc gcaatcacca tagcaggttt gtaaccttct tcaatatctt
41401 tatcaattgc ggcttttaag ctatctgtat tcattgtaaa gtcatcgttg tactcaattc
41461 tgtggatatt atttttaagg aagcctgcaa catgcaaggc tttgcctaca gaatgatgcg
41521 tttgagatgt aagataaacg gttgcttttt taatatcttc catttctacc tgtgcatctc
41581 tagccgcaac gatagctgtt aaattggcca ttgaaccgcc agatacaaag acaccaccgg
41641 ccggtttgat ctcataaccg atttttccta ccaagtagtt aattaaatta cgctcaatat
41701 taattggcag cgtcgcattg gcaaaattag aggcatggat attatttgct gttgttaaaa
41761 tgtctccaag ccatgataga cgtgatgctg gacctggtat aaatgagaat gaacgtggat
41821 ggttcggacg atataaataa ttcagtactt cattatttaa atctttgagt acttcataga
41881 tatctcgccc tttattcggc acttccatct tttcatattt ctctctcaat tcgagcggtg
41941 cctgttgtgt cgcaggtaag tctccaatat ctctagataa atatttatta acttcatctc
42001 tgataatcgt ctctaagtcg atattgttct cattgaattc catttccata ctctctcctt
42061 caattatatt tttaataaaa gacagacgta ttaattacga cgttttacta caatataaat
42121 gatagcagat attaataaaa ccacgctaac cataatgacg aaacccagaa ggtgtcgccc
42181 caatgaataa cgtgctgtgc tttaacactc ggtgtatgtg cgcctttcag aaattgacga

42241 gggtaatcga cttgcagatt atcatctgtg attttcaatt tgtattgatg cttgtctttt
42301 ggaattcgat aactaccccg tagaacaggg gacgaggacc tcatcttgat gatcccaatt
42361 ggcttggtta aaaagaaatc gataatcctc gtagaataga gaaaaagtat taaaaatata
42421 tttgatttga taaaatattt tgcaaaaaag agaaacaaaa ataactttca tataaataat
42481 ttctttgcga ttaagttgga attagaaata ctaaatacaa ataacagcga atggaatgca
42541 tattattgag gaattggatg tgtagtgcaa tttcgagagt tactacttaa cttaagaaaa
42601 taaaaagaag aaaacaaaca ataagaatta aatctgaagc aagatataaa agcaaatatt
42661 tttgctacaa ttatattagt aaatacttta ttaattagca tacgactgaa agcgaagtaa
42721 cagctaataa tgttccagat atagacatta gtgttatgaa agaacaatat taccatctag
42781 aaaatgtaat ttttaaaaac ctgaagataa agtagactat tctgtgagtt ttcatgttaa
42841 gaattctgaa cttggggatg tagtataaat atggtttagt agaaaatgac gctggagctg
42901 tagctgagat tgactatcaa gaattaacta aagtagatta ttacccatca acaaaaaatcc
42961 aagatttaac actatctaat aatagtgcga ataatgaagt ttaaaagtga aaaactgata
43021 atagcgaatc aaactaaagt aatcaattac agaattcaag tactactcaa gcctcaaaca
43081 aatattgaaa ctcaaacaca agttaagaat aatacactgt cagaaacaga tgaacagtct
43141 aatgccgatt tagtccagta atagcctctg tattattagc cgcacgatta ttatcaataa
43201 ttaaataaag ttaatattaa ataataaaaa tccttcaaat tatttgttga aaaaataata
43261 actttgaagg ttttttagca aaatcactga tagggagaag cgtatcataa at

//

LOCUS   CP009828   41844 bp   DNA   linear   BCT 03-DEC-2015
DEFINITION Staphylococcus aureus strain MS4, complete genome.
ACCESSION   CP009828 REGION: 34078..75921
VERSION   CP009828.1 GI:959165045
DBLINK   BioProject: PRJNA263760
            BioSample: SAMN03145755
KEYWORDS   .
SOURCE   Staphylococcus aureus
  ORGANISM Staphylococcus aureus
            Bacteria; Bacillati; Bacillota; Bacilli; Bacillales;
            Staphylococcaceae; Staphylococcus.
REFERENCE   1 (bases 1 to 41844)
  AUTHORS   Yu,Z.
  TITLE   Genome sequence of Staphylococcus Aureus
  JOURNAL   Unpublished
REFERENCE   2 (bases 1 to 41844)
  AUTHORS   Yu,Z.
  TITLE   Direct Submission
  JOURNAL   Submitted (07-NOV-2014) Department of Infectious Diseases, Nanshan
            Hospital of Guangdong Medical College, No 89, Taoyuan Road, Nanshan
            District, Shenzhen 518052, China
COMMENT   Annotation was added by the NCBI Prokaryotic Genome Annotation
            Pipeline (released 2013). Information about the Pipeline can be
            found here: http://www.ncbi.nlm.nih.gov/genome/annotation_prok/

##Genome-Assembly-Data-START##
Assembly Method :: Celera Assembler v. Celera Assembler 8.0
Genome Representation :: Full
Expected Final Version :: Yes
Genome Coverage :: 308.0x
Sequencing Technology :: Illumina HiSeq; PacBio
##Genome-Assembly-Data-END##

##Genome-Annotation-Data-START##
Annotation Provider :: NCBI
Annotation Date :: 11/07/2014 15:39:30
Annotation Pipeline :: NCBI Prokaryotic Genome Annotation Pipeline
Annotation Method :: Best-placed reference protein set; GeneMarkS+
Annotation Software revision :: 2.8 (rev. 450396)
Features Annotated :: Gene; CDS; rRNA; tRNA; ncRNA; repeat_region
Genes :: 2,736
CDS :: 2,595
Pseudo Genes :: 68
rRNAs :: 16 (5S, 16S, 23S)
tRNAs :: 57
ncRNA :: 0
Frameshifted Genes :: 32
##Genome-Annotation-Data-END##

FEATURES Location/Qualifiers
source 1..41844
/organism="Staphylococcus aureus"
/mol_type="genomic DNA"
/strain="MS4"
/isolation_source="the wound exudate of bone fracture"
/host="Homo sapiens"
/db_xref="taxon:1280"
/geo_loc_name="China: Shenzhen"
/lat_lon="22.53128 N 113.92356 E"
/collection_date="05-Jul-2012"
/collected_by="Deng Qiwen"
gene 1..480
/locus_tag="NI36_00135"
CDS 1..480
/locus_tag="NI36_00135"
/inference="EXISTENCE: similar to AA sequence:RefSeq:WP_002445009.1"

/note="Derived by automated computational analysis using
gene prediction method: Protein Homology."
/codon_start=1
/transl_table=11
/product="50S rRNA methyltransferase"
/protein_id="ALQ98330.1"
/db_xref="GI:959165070"
/translation="MKITILAVGKLKEKYWKQAIAEYEKRLGPYTKIDIIEVPDEKAP

ENMSDKEIEQVKEKEGQRILAKIKPQSTVITLEIQGKMLSSEGLAQELNQRMTQGQSD

FVFVIGGSNGLHKDVLQRSNYALSFSKMTFPHQMMRVVLIEQVYRAFKIMRGEAYHK"
gene 726..1031
/locus_tag="NI36_00140"
CDS 726..1031
/locus_tag="NI36_00140"
/inference="EXISTENCE: similar to AA
sequence:RefSeq:WP_000677988.1"
/note="Derived by automated computational analysis using
gene prediction method: Protein Homology."
/codon_start=1
/transl_table=11
/product="hypothetical protein"
/protein_id="ALQ98331.1"
/db_xref="GI:959165071"
/translation="MKGNIQCPRLIKGSLLDFVLETVDVFVNGSKNDCELVNFNYKEG
KVVSRYSTKFESNPKLRKKQLISTVKVVRYVIFDFEKILWGIGKVLLKFIILRQCLL"
gene 1230..2093
/locus_tag="NI36_00145"
CDS 1230..2093
/locus_tag="NI36_00145"
/inference="EXISTENCE: similar to AA
sequence:RefSeq:WP_020220483.1"
/note="Derived by automated computational analysis using
gene prediction method: Protein Homology."
/codon_start=1
/transl_table=11
/product="hypothetical protein"
/protein_id="ALQ98332.1"
/db_xref="GI:959165072"

/translation="MKHINQFSESTLAQLEKDQQHVFYVYCLMDPRNDECFYVGKGKG
NRIFKHKQDAQKKVLYEDIFIEENKDNLKFNRINEICSNDLNVLGYIISYGLTESEAF

SAENVLINFLNLTNKTTLTNMINGHGSKAYLVEDLENEFGYDSINLENINTNELILAV

KIRDAFLLDKDESKEYPINESKRDRNNLKSRTLGSWIIGKDKIHKIKYIIGINTGANN

AVVSAYEVSFEQAESIETNNGRMRYAFIGLSERDATLKKLNLYKKALPDLRFGSGSAT
AYINNGTMKVD"
gene 2201..3676
/locus_tag="NI36_00150"
CDS 2201..3676
/locus_tag="NI36_00150"
/inference="EXISTENCE: similar to AA
sequence:RefSeq:WP_001138664.1"
/note="Derived by automated computational analysis using
gene prediction method: Protein Homology."
/codon_start=1
/transl_table=11
/product="hypothetical protein"
/protein_id="ALQ98333.1"
/db_xref="GI:959165073"
/translation="MPSDDIVKKLYSKEDIRLELGLTPHKFNKKMETIAKLFKIDMKI

FHNYKGQDKNNQYTFNGVAKELIKVLLKSVDYYPVDINSKKFKQNGKSKKEMIENIDN

SSYMKYIYQLMKSINEIQYKRLIADIHMKDVYQNTKAWLNNGESINKKEQELYQYMTI

LPLHKRVELQNEVLKSIDETIFQFLAKEHRNNQIEENNELEAYTKAIKEGRNPKNDYE

LNHLLYKKNQLPLDSLIEDIWDYEETEYTELDWLIADMLKRSQRADSNFIESLEKKNK

LRKNIHKDSIKSISKLIDHELVKNNRKWDVAEFYKNQQFWNNSTLRRSSQNFGILYYK

KNRLHTMNQSNIIKKIKIIEANIESANKQPSYLDFFLQAKFDLERLESELMKYSINPK

YFNKINSDYVVRYAIEDVHNAIIKIDRYISNDKVKLNYSNVQMVVRNEITEQGSYFVTQ
ALNTLSKVEESGYSFDNFMVGPFVEDFKKAIKNRRFGSKKD"
gene 3902..5002
/locus_tag="NI36_00155"
CDS 3902..5002
/locus_tag="NI36_00155"
/inference="EXISTENCE: similar to AA
sequence:RefSeq:WP_016187175.1"
/note="Derived by automated computational analysis using
gene prediction method: Protein Homology."
/codon_start=1

/transl_table=11
/product="hypothetical protein"
/protein_id="ALQ98334.1"
/db_xref="GI:959165074"

/translation="METRDKLMSLTQSDKTQQWLMDKSSNQDDIQQLQQQFSQQLDQQ

YNALLADEKAKLDQYVEVHQGLESLKEEIESEPITLNIDKLPDIKATMLERAKNDEHS

DKIEKLFDRLEQALNGTNRLYTQLSLIGTRTHRITTKNFNLQGLPKAVQHTILPSKFK

KVYTVDFKSFEPSVAAYMTQDSKLIDLLNQKDGLYDALLSELGLSDELRVFVKRAFIG

SFLFGGNFKNPKFKLNQYVSEVQWLDAVSQFTKVIELKKHVEKSNSMPMPYSIEHDMS

AFQGSSIMAIYVQTVASYIFKHILLKVYKAQCDQKTFKIIVPIHDAIMIECEDEEIAQ
NVGQLMKDTANQLFNGEFAHVTVEEIGGVDHE"
gene 4995..5366
/locus_tag="NI36_00160"
CDS 4995..5366
/locus_tag="NI36_00160"
/inference="EXISTENCE: similar to AA
sequence:RefSeq:WP_001058156.1"
/note="Derived by automated computational analysis using
gene prediction method: Protein Homology."
/codon_start=1
/transl_table=11
/product="hypothetical protein"
/protein_id="ALQ98335.1"
/db_xref="GI:959165075"
/translation="MNNDRGQSLHIPSSTPIKENNIYVATLHSVIQTDFSGEIKHQFT

YEIEVNNQIVYANRNIPTKPSANQLSIHDWLKRHSNYSASHENYEPYIDQKHLILLGQ
YNGNYYVQDVASLDAFGGVLS"
gene 5363..7006
/locus_tag="NI36_00165"
CDS 5363..7006
/locus_tag="NI36_00165"
/inference="EXISTENCE: similar to AA
sequence:RefSeq:WP_012086574.1"
/note="Derived by automated computational analysis using
gene prediction method: Protein Homology."
/codon_start=1
/transl_table=11

/product="DNA primase"
/protein_id="ALQ98336.1"
/db_xref="GI:959165076"
/translation="MNHILQMLSKLLSVAKEAIDRQGLIAILTIPVNNNDEIEETAQG

ETVYNELIDQLRLNIPKDTDYQPNIYSYFGIKKNPNDTVLMEMMIKVFHIKRFNSELF

IFKVNGWQKINGDELQGLISKMIQVLLVDYKPSLSTLKNVVDGLQKSTDVEELVENER

YIGCGENMFDLNTFQVVKNSIDIFPKTRLNLSLSTNDVITDKIPPYFKQYMLQLANYD

DDLQYFLFQHTAVLLTADTKYRRGLILYGGAKNGKSVYIELVKSFFYSKDIVSKPLNE

LEGRFDKESLIDKSLMASHEIGQSRIQEKIVNDFKKLLSVESMHVDRKGKTQVEVILD
LKLIFSTNAILNFPPEHAKALERRINIIPCEYYVEKADTSLIDKLQSEKKEIFLYLMY

VYQQIVKADIEYLENSRVTEITHDWLNFGYEFVSSRSVSNANQKACINLLRKLIEIKS

GSRIKVSELNKVINEEIKVSSQVIKQLIQANFDTQTKLYNGYDYWIDLGWKEANKKEI
HDISEKDNIISLDKNENITDDEALDEENLDFDWEDFDDE"
gene 7232..8908
/locus_tag="NI36_00170"
CDS 7232..8908
/locus_tag="NI36_00170"
/inference="EXISTENCE: similar to AA
sequence:RefSeq:WP_000676594.1"
/note="Derived by automated computational analysis using
gene prediction method: Protein Homology."
/codon_start=1
/transl_table=11
/product="recombinase RecB"
/protein_id="ALQ98337.1"
/db_xref="GI:959165077"
/translation="MKGKIALYSRVSTSEQSEHGYSIHEQEQVLIKEVVKNFPGYDYE

TYTDSGISGKNIEGRPAMKRLLQDVKDNKIEMVLSWKLNRISRSMRDVFNIIHEFKEH

DVGYKSISENIDTSNASGEVLVTMFGLIGSIERQTLISNVKLSMNAKARSGEAITGRV

LGYKLSLNPLTQKNDLVIDENEANIVREIFDLYLNHNKGLKAITTVLNQKGYRTINQK

PFSVYGVKYILNNPVYKGYVRFNNHQNWAVQRRSGKSDKNDVILVKGKHEAIISEEVF

DKVHEKLASKSFKPGRPIGGDFYLRGLIKCPECGNNMVCRRTYYKTKKSKERTIKRYY

ICSLFNRSGSSACHSNSINAEVVERVINVHLNRILSQPDIIKQIASNVIEELKQKHSN

QTEIKYDIDSLEKQKAKLKTQQERLLELFLDDQMDSEMLKAKQSQMNQQLEVLDQQIK

EAQQANQSQDEIPNFDKLKGRLILMITRFSVYLRKATPEAKNQLMKMLIDSIEITTDK
QVKLVRYKIDESLIPQSLKKDWGSFFMPKFQFEIYGQNDYFIDQITTFTT"
gene 9014..9352
/locus_tag="NI36_00175"
CDS 9014..9352
/locus_tag="NI36_00175"
/inference="EXISTENCE: similar to AA
sequence:RefSeq:WP_000171559.1"
/note="Derived by automated computational analysis using
gene prediction method: Protein Homology."
/codon_start=1
/transl_table=11
/product="hypothetical protein"
/protein_id="ALQ98338.1"
/db_xref="GI:959165078"
/translation="MTLEQQLKHYITNLFNLPRDEVWHCESIEEIADDILPNQYVRLG

PLSNKTLQTNTYYSDTLHESNIYPFILYYQKQLIAIGYIDENHDMDFLYLHNTIMPLL
DQRYLLTGGQ"
gene 9448..9759
/locus_tag="NI36_00180"
CDS 9448..9759
/locus_tag="NI36_00180"
/inference="EXISTENCE: similar to AA
sequence:RefSeq:WP_001083109.1"
/note="Derived by automated computational analysis using
gene prediction method: Protein Homology."
/codon_start=1
/transl_table=11
/product="hypothetical protein"
/protein_id="ALQ98339.1"
/db_xref="GI:959165079"

/translation="MNRYITRGIANNLPNILQHQLWQLVSEREQEQTKDNTSVDYFHI

FQFNKHRNQLYIKHKQERPEYAKIHKANYSKAININKVYIIREDDVDLSYYVMLLPEE
Y"
gene 9775..10281
/locus_tag="NI36_00185"

CDS 9775..10281
/locus_tag="NI36_00185"
/inference="EXISTENCE: similar to AA
sequence:RefSeq:WP_000447786.1"
/note="Derived by automated computational analysis using
gene prediction method: Protein Homology."
/codon_start=1
/transl_table=11
/product="hypothetical protein"
/protein_id="ALQ98340.1"
/db_xref="GI:959165080"
/translation="METIKSTLKTEAIFSDDKQHRYLLKKTWNSEKQSITIITMYPHY

DGILNIDLTTQLIMNKVSEMDAFGSINFVNLYSNITTPINLKHLENAYDKHTDIQIMK

AVKESDEVILAWGAYAKKPGVEARVNEVLEMLKPHKKKVKRLMNPETNEIMHPLNSKA
RQKWILKV"
gene complement(10430..11104)
/locus_tag="NI36_00190"
CDS complement(10430..11104)
/locus_tag="NI36_00190"
/inference="EXISTENCE: similar to AA
sequence:SwissProt:P0A043.1"
/note="Derived by automated computational analysis using
gene prediction method: Protein Homology."
/codon_start=1
/transl_table=11
/product="integrase"
/protein_id="ALQ98341.1"
/db_xref="GI:959165081"
/translation="MNYFRYKQFNKDVITVAVGYYLRYTLSYRDISEILRERGVNVHH

STVYRWVQEYAPILYQIWKKKHKKAYYKWRIDETYIKIKGKWSYLYRAIDAEGHTLDI

WLRKQRDNHSAYAFIKRLIKQFGKPQKVITDQAPSTKVAMAKVIKAFKLKPDCHCTSK
YLNNLIEQDHRHIKVRKTRYQSINTAKNTLKGIECIYALYKKNRRSLQICGFSPCHEI
SIMLAS"
gene 11362..11529
/locus_tag="NI36_00195"
CDS 11362..11529
/locus_tag="NI36_00195"
/inference="EXISTENCE: similar to AA
sequence:RefSeq:WP_000894158.1"
/note="Derived by automated computational analysis using

gene prediction method: Protein Homology."
/codon_start=1
/transl_table=11
/product="nucleoid-structuring protein H-NS"
/protein_id="ALQ98342.1"
/db_xref="GI:959165082"
/translation="MLESREQLSVEEYETFFNRFDNQEFDFERELTQDPYSKVYLYSI
EDHIRTYKIEK"
gene 12526..13269
/locus_tag="NI36_00200"
CDS 12526..13269
/locus_tag="NI36_00200"
/inference="EXISTENCE: similar to AA
sequence:RefSeq:WP_001830227.1"
/note="Derived by automated computational analysis using
gene prediction method: Protein Homology."
/codon_start=1
/transl_table=11
/product="glycerophosphodiester phosphodiesterase"
/protein_id="ALQ98343.1"
/db_xref="GI:959165083"
/translation="MLTVYGHRGLPSKAPENTIASFKAASEVEGINWLELDVAITKDE

QLIIIHDDYLERTTNMSGEITELNYDEIKDASAGSWFGEKFKDEHLPTFDDVVKIANE

YNMNLNVELKGITGPNGLALSKSMVKQVEEQLTNLNQNQEVLISSFNVVLVKLAEEIM

PQYNRAVIFHTTSFREDWRTLLDYCNAKIVNTEDAKLTKAKVKMVKEAGYELNVWTVN
KPARANQLANWGVDGIFTDNADKMVHLSQ"
gene 13366..13794
/locus_tag="NI36_00205"
CDS 13366..13794
/locus_tag="NI36_00205"
/inference="EXISTENCE: similar to AA
sequence:RefSeq:WP_000872606.1"
/note="Derived by automated computational analysis using
gene prediction method: Protein Homology."
/codon_start=1
/transl_table=11
/product="hypothetical protein"
/protein_id="ALQ98344.1"
/db_xref="GI:959165084"
/translation="MKYDDFIVGETFKTKSLHITEEEIIQFATTFDPQYMHIDKEKAE

QSRFKGIIASGMHTLSISFKLWVEEGKYGEEVVAGTQMNNVKFIKPVYPGNTLYVIAE
ITNKKSIKKENGLVTVSLSTYNENEEIVFKGEVTALINNS"
gene complement(13840..15846)
/locus_tag="NI36_00210"
CDS complement(13840..15846)
/locus_tag="NI36_00210"
/inference="EXISTENCE: similar to AA
sequence:RefSeq:WP_001801873.1"
/note="Derived by automated computational analysis using
gene prediction method: Protein Homology."
/codon_start=1
/transl_table=11
/product="peptidase"
/protein_id="ALQ98345.1"
/db_xref="GI:959165085"
/translation="MKKIKIVPLILIVVVVGFGIYFYASKDKEINNTIDAIEDKNFKQ

VYKDSSYISKSDNGEVEMTERPIKIYNSLGVKDINIQDRKIKKVSKNKKRVDAQYKIK

TNYGNIDRNVQFNFVKEDGMWKLDWDHSVIIPGMQKDQSIHIENLKSERGKILDRNNV

ELANTGTAYEIGIVPKNVSKKDYKAIAKELSISEDYIKQQMDQNWVQDDTFVPLKTVK

KMDEYLRDFAKKFHLTTNETESRNYPLGKATSHLLGYVGPINSEELKQKEYKGYKDDA

VIGKKGLEKLYDKKLQHEDGYRVTIVDDNSNTIAHTLIEKKKKDGKDIQLTIDAKVQK

SIYNNMKNDYGSGTAIHPQTGELLALVSTPSYDVYPFMYGMSNEEYNKLTEDKKEPLL

NKFQITTSPGSTQKILTAMIGLNNKTLDDKTSYKIDGKGWQKDKSWGGYNVTRYEVVN

GNIDLKQAIESSDNIFFARVALELGSKKFEKGMKKLGVGEDIPSDYPFYNAQISNKNL

DNEILLADSGYGQGEILINPVQILSIYSALENNGNINAPHLLKDTKNKVWKKNIISKE

NINLLTDGMQQVVNKTHKEDIYRSYANLIGKSGTAELKMKQGETGRQIGWFISYDKDN
PNMMMAINVKDVQDKGMASYNAKISGKVYDELYENGNKKYDIDE"
gene 16094..16768
/locus_tag="NI36_00215"
/note="integrase; disrupted; Derived by automated
computational analysis using gene prediction method:
Protein Homology."
/pseudo
gene complement(16828..17256)

/locus_tag="NI36_00220"
CDS complement(16828..17256)
/locus_tag="NI36_00220"
/inference="EXISTENCE: similar to AA
sequence:RefSeq:WP_000671997.1"
/note="Derived by automated computational analysis using
gene prediction method: Protein Homology."
/codon_start=1
/transl_table=11
/product="3-demethylubiquinone-9 3-methyltransferase"
/protein_id="ALQ98346.1"
/db_xref="GI:959165086"
/translation="MKFQVSPYFLITQNAEGLKKLYEELFGAKTLYIQRIKDRPIDKK

LNIDENDLNKIDQCVLQFGDIKIMIADDTEGLPITQGNNISLCLTFDQVEETKHVYDQ
LIEKGSEVLKTFSPEFYTEGYGYVRDPYGISLHLFTKRKN"
gene 17337..18266
/locus_tag="NI36_00225"
CDS 17337..18266
/locus_tag="NI36_00225"
/inference="EXISTENCE: similar to AA
sequence:RefSeq:WP_000750311.1"
/note="Derived by automated computational analysis using
gene prediction method: Protein Homology."
/codon_start=1
/transl_table=11
/product="transcriptional regulator"
/protein_id="ALQ98347.1"
/db_xref="GI:959165087"
/translation="MKKSVRLYNMIEYCNENRNFKLNDLMSEFNISRSTALRDIKEIE

ALGVPLYSNPGKNGGYTIIGNRDQTKIAISDEELKALVFTLSSISNVSNLPFQTEYQE
ILKKLYNNSNKKELINQYNDLFQYFNEDKYQFKSYKLFNEIIRLIIENKSFETCYSQN

YIKEQYKGIGIMYKNHQWYFVVANIESKLVNLLNISKIKELYEMGETQECNDITMQNF

QQFMVKNETAIDILIRSNVMGLNILKGYLWSDYMIENIDEETYLFKSKVNAKDIDFIA
KLIVTGGVNVKVETPNSLKNAVKVELTKIINMY"
gene 18428..18910
/locus_tag="NI36_00230"
CDS 18428..18910
/locus_tag="NI36_00230"
/inference="EXISTENCE: similar to AA
sequence:RefSeq:WP_001038847.1"

/note="Derived by automated computational analysis using
gene prediction method: Protein Homology."
/codon_start=1
/transl_table=11
/product="hypothetical protein"
/protein_id="ALQ98348.1"
/db_xref="GI:959165088"
/translation="MNKNITALKSTENTTYMQYTVHIDDQIINNIKECTKKFKILPME
DKIPLSPLLQPEYAGEVQDFISTYEQFMINFGKVILDSQGIKIQFESESLSSIQRGIQ

EHCYLNERTNDIDVTKEWYLCKFSNQILEEDKNQLYNALKQLMNDSKNKKQAFWTDPN
"
gene 18978..19292
/locus_tag="NI36_00235"
CDS 18978..19292
/locus_tag="NI36_00235"
/inference="EXISTENCE: similar to AA
sequence:RefSeq:WP_001832442.1"
/note="Derived by automated computational analysis using
gene prediction method: Protein Homology."
/codon_start=1
/transl_table=11
/product="transposase"
/protein_id="ALQ98349.1"
/db_xref="GI:959165089"

/translation="MKRVSYSVETKYKAVEMKIAGFSTKEIMKELNIKNRTQVETWWR

WYRNEESYRFSQHVGKQYTYGKGLEELSEIERLKLENKRKDIELDILKKYKALERKWY
QQ"
gene 19304..20119
/locus_tag="NI36_00240"
CDS 19304..20119
/locus_tag="NI36_00240"
/inference="EXISTENCE: similar to AA
sequence:RefSeq:WP_000372483.1"
/note="Derived by automated computational analysis using
gene prediction method: Protein Homology."
/codon_start=1
/transl_table=11
/product="transposase"
/protein_id="ALQ98350.1"
/db_xref="GI:959165090"
/translation="MDQLKVKYSIKLILEVLNIPKSTYYRWKNKTYKNDTVTQKVIEL

CEANHYTYGYRKITALINQCYTSPINHKRVQRIMQKHHLNCRVRPKKTTRIGKPYYKT

DNLLQRQFKASCPMEVLTTDITYLPFGHSMLYLSSIMDIYNGEIVAYKIDDKQDQSLV

NDTLNQIDIPEGCILHSDQGSVYTSYAYYQLCEEKGIIRSMSRKGTPADNAPIESFHS
SLKSETFYINNQLNSSNHIVIDIVEKYIKNYNNNRIQQKLGYLSPVKYRELAA"
gene 20174..21691
/locus_tag="NI36_00245"
CDS 20174..21691
/locus_tag="NI36_00245"
/inference="EXISTENCE: similar to AA
sequence:RefSeq:WP_000477412.1"
/note="Derived by automated computational analysis using
gene prediction method: Protein Homology."
/codon_start=1
/transl_table=11
/product="hypothetical protein"
/protein_id="ALQ98351.1"
/db_xref="GI:959165091"
/translation="MFKVHIDIYLYNKEKSEYKYQVSSYFNLVKENPKITYKKRHLQE

KQGVKGTTFTNKLKLLNKMYGVDVAKYQPFYNSNNPEYERGQFGERYISQRSNYEFNR
LQYQIIDMLSKILDKHPLPKSDNNYKHIPTIEKAILSGDSHSFYEYFEDIMKEIISME

NSSLKEKLLTDFTYQSQCRWYSESEKLKLQLESFMHKVLESNYYEGNKLYRMLSHAIE

ETINEADEDKVHFNYFKDYFLTDGGVKNWEQISEKITEFNGKVINDIQNEYNKIQFNN

ANRNQKLNFNYLYHCFEFSNNLVKARVNGNRGYILYFVDKYKIKMPFLDQLLINYLNQ

EIGQESINYNMQTLFEKERYDRSSTIEKLVATSKFKYEKDDSDLFKQLFNDVENSIDR
LGIYLLNNGINSNDENARYYRSFLKELSRIKSKLTPFSLEISKSSGREQHYPDDAIDD
KDERRKNKEETYHAFDDKSDIDSKLKNKINVSIDNLLSVKIKSNLYDISPNTKKQ"
gene 21886..22995
/locus_tag="NI36_00250"
CDS 21886..22995
/locus_tag="NI36_00250"
/inference="EXISTENCE: similar to AA
sequence:RefSeq:WP_002437509.1"
/note="Derived by automated computational analysis using
gene prediction method: Protein Homology."
/codon_start=1
/transl_table=11

/product="hypothetical protein"
/protein_id="ALQ98352.1"
/db_xref="GI:959165092"

/translation="MYQANIRDLITKLPQSNKTEHFLMNKFSNQDKVQQLQRQISQQL

DQQYNELLANEKAKLDQYVEVHHNLEPLKKEIESESINLDTDKLPDIKATMLEKAKND

EHFDKIEQLFDRLDQSLNGTNRLYTQLSLIGTRTHRITTKRFNVQGLPKLVQQMILPS

QFKKVYTIDFKSFEPSVAAYMTQDEQLIDYLNHEEGLYDALLRDLSLSKEKRVSVKRA

FIGSFLFGGRYSSSKFKINQEVSEINWLQVMSKFKKVIEFKEQVEKYKTMPTPYGIEH

DMSAFQGSSIMAIYVQTVASYIFKHILLEVYKAQCEKKTFKIIVPIHDAIMIECNDKG
IAQNVAQLMKDTANQLFNGEFAHVTVEALGGIDNE"
gene 22988..23356
/locus_tag="NI36_00255"
CDS 22988..23356
/locus_tag="NI36_00255"
/inference="EXISTENCE: similar to AA
sequence:RefSeq:WP_001058155.1"
/note="Derived by automated computational analysis using
gene prediction method: Protein Homology."
/codon_start=1
/transl_table=11
/product="hypothetical protein"
/protein_id="ALQ98353.1"
/db_xref="GI:959165093"
/translation="MNNDRGKSLQIPQSTLLKEGSIYVATLHSVYEKNFSGDIKHQFT

YEVELNQETHYVNRNITVKSMSHQLSIADWIKRHSNYNVNHINYDPYIDRKHLVLVGQ
YNGNYYIQDVAPLDEFGGVL"
gene 23356..24972
/locus_tag="NI36_00260"
CDS 23356..24972
/locus_tag="NI36_00260"
/inference="EXISTENCE: similar to AA
sequence:RefSeq:WP_001015575.1"
/note="Derived by automated computational analysis using
gene prediction method: Protein Homology."
/codon_start=1
/transl_table=11
/product="DNA primase"

/protein_id="ALQ98354.1"
/db_xref="GI:959165094"
/translation="MNHILEMLIKLLKVGMEAIDRKGLIAILTSSIGNDEMDDSEQAV

MVYNELIDKLQLNIPKDVDYRPNIYSYFGIQKKPNDTILVEMMICIFHIKRFDSELFV

FKDKGWQKVSEDELQGLISKMIQVLLVDYKPSLSTLKNVVDGIQKSTDIEKLVEHRQY

IGCGRNMFNLKTFKVVDNDLEIFPKTRLDLELDINDTITDKIPPNFKQYMLELANYDH

DLQYFLFQHMAVLLTADTKLRRGLFLYGTAKNGKSVYIKLVKSFFYSNDIVSKTLNEL

GGRFDKESLIGKRIMASDEVGKANIDEATVNDFKKLLSVEPIHADRKGRTQVEVTLDL

KLIFNTNAVLNFPSSHAKALERRIAVIPCEYYVEKADPDLIEKLQDEKKEIFLYLMYV

YKQIVKNDIEYLQNDRVTEISHDWLNFGYEFVSSKSANIAHQKACINLLRKLIEIKPG

SRIKVSRLNEVIRDEIKVSSQVINDLVQANFNVQSRLNNGYKYWVDLGWKETDKKDDM
ISFDKNENVTDDEFLYEDDLNLGWEDFDDE"

gene 25197..26876
/locus_tag="NI36_00265"

CDS 25197..26876
/locus_tag="NI36_00265"
/inference="EXISTENCE: similar to AA
sequence:RefSeq:WP_000676592.1"
/note="Derived by automated computational analysis using
gene prediction method: Protein Homology."
/codon_start=1
/transl_table=11
/product="recombinase RecB"
/protein_id="ALQ98355.1"
/db_xref="GI:959165095"
/translation="MKGKIALYSRVSTSEQSEHGYSEKEQEQLLIKEVMKNFPGYDYE

TYTDSGISGKNIEGRPAMKRLLQDVKDNKIEMVLSWKLNRISRSMRDVFNIIHEFKEH

DVGYKSISENIDTSNASGEVLVTMFGLIGSIERQTLISNVKLSMNAKARSGEAITGRV

LGYKLSLNPLTQKNDLVIDENEAHIVREIFDLYLNHNKGFKAITTILNQKGYRTINQK

PFSVFGVKYILNNPVYKGYVRFNNHQNWAVQRRSGKSDKNDVILVKGKHEAIISEDVF

DQVHEKLASKSFKPGRPIGGDFYLRGLIKCPECGNNMVCRRTYYKTKKSKERTIKRYY

ICSLFNRSGSSACHSNAINAEVVERVINVHLNRILSQPNVIKQIASSVIEELKQKHSK

QTEIKYDIDSLEKQKAKVKTQQERLLELFLDDEMDSEMLKAKQSEMNQQLEVLDQQIK

EAKQANQSQDDIPNFDKLKARLILMITRFSVYLRKATPEAKNQLMKMLIDSIEITTDK
QVKLVRYKIDESLIPQSLKKDWGSFFIPKFNFVINVTKKNRIENLSLLPLF"
gene 26965..27303
/locus_tag="NI36_00270"
CDS 26965..27303
/locus_tag="NI36_00270"
/inference="EXISTENCE: similar to AA
sequence:RefSeq:WP_000175889.1"
/note="Derived by automated computational analysis using
gene prediction method: Protein Homology."
/codon_start=1
/transl_table=11
/product="hypothetical protein"
/protein_id="ALQ98356.1"
/db_xref="GI:959165096"
/translation="MTLSKQLKTYITERFKLNYQETWACETIDAVAEDVLPEKYIKNS
PLEHKILNTFTYYNDELHEISIYPFLCYLDKELVAIGYLDNFDLDFIFLNDTHQVIID
ERYLLQKGGE"
gene 27397..27708
/locus_tag="NI36_00275"
CDS 27397..27708
/locus_tag="NI36_00275"
/inference="EXISTENCE: similar to AA
sequence:RefSeq:WP_002501910.1"
/note="Derived by automated computational analysis using
gene prediction method: Protein Homology."
/codon_start=1
/transl_table=11
/product="hypothetical protein"
/protein_id="ALQ98357.1"
/db_xref="GI:959165097"
/translation="MKINRYITRGVSEHLSLDLQILLWNMVKDRDNQLHTDYLHIFKL

QEDDNILSITHEQEQPAYKLEYHYTNYVKNQNALPKKVYVIREDDVDAFYYVMLLPEE
Y"
gene 27723..28226
/locus_tag="NI36_00280"
CDS 27723..28226
/locus_tag="NI36_00280"

/inference="EXISTENCE: similar to AA
sequence:RefSeq:WP_002506253.1"
/note="Derived by automated computational analysis using
gene prediction method: Protein Homology."
/codon_start=1
/transl_table=11
/product="hypothetical protein"
/protein_id="ALQ98358.1"
/db_xref="GI:959165098"
/translation="MNTIKNTIHTEAIFSDDEQHRYLLKKTWDEKKPACTVITMYPHL

DGVLSLDLTTVLILNQLANSEQYGAVYLVNLFSNIKTPENLKHIKEPYDKHTDIHLMK

AISESDTVILAYGAYAKRPVVVERVEQVMEMLKPHKKKVKKLINPATNEIMHPLNPKA
RQKWTLK"
gene 28240..28461
/locus_tag="NI36_00285"
CDS 28240..28461
/locus_tag="NI36_00285"
/inference="EXISTENCE: similar to AA
sequence:RefSeq:WP_002504703.1"
/note="Derived by automated computational analysis using
gene prediction method: Protein Homology."
/codon_start=1
/transl_table=11
/product="hypothetical protein"
/protein_id="ALQ98359.1"
/db_xref="GI:959165099"
/translation="MNHETKQSDWRTVASCLASQNYISIVKGLVHHFTAIEDEEILDK
IYDDFMNDDSITTVLNNDLQTIINHYLSK"
gene complement(28536..31655)
/locus_tag="NI36_00290"
CDS complement(28536..31655)
/locus_tag="NI36_00290"
/inference="EXISTENCE: similar to AA
sequence:RefSeq:WP_001010920.1"
/note="Derived by automated computational analysis using
gene prediction method: Protein Homology."
/codon_start=1
/transl_table=11
/product="DEAD/DEAH box helicase"
/protein_id="ALQ98360.1"
/db_xref="GI:959165100"

/translation="MNFQFNEDDLEQVALEWLQSLGYDYKKGNEISMTSLTPERKSDK
DVVLHERLEKALRKINSDIHPRFIEKALHELTLEKSPNLLENNLTFHENLINGIEIED
YDDEGQSIVEIVKIIDFEYPQNNDFLAVNQLTIVNGDYKKRPDIVLFINGLPIVVIEL

KNSTNETVGVEDGYHQLETYKMRIPQLFTFNEVLVTSDGINTKAGSLTANYDRFMTWR

SKDGQTESSPGLASLDVLIHGMLNPETLLDLIRYFVLFQDDGKGHISKILAAYHQYYA

VNKAVDRALLASSEQGDGKGGVIWHTQGSGKSLTMVFFSGKLIQMLNNPTLVVVTDRN

DLDNQLYSTFVKSKGRSGKGLLRQTPKQAETRKELKSLLSVESGGIVFTTMQKFEPEQ

NETTMAALTERKNVIVMADEAHRTQYGFNAKYDDKGEGIKYGYAKYLRDALPNATFVG

FTGTPVASTDKNTQMVFGNYIDVYDMTQAVADGSTVKIYYESRVIPLNLPQSLDLDEA

YNDITEDQEEDVKQRLKSKWSRIEALAGAKPRVEALAKDIIQHFETRQQAMKGKGMIV

TMSRRIAVDLYDEIIRLKPEWHSDDDDKGVIKVVMTGSSSDPTSFQRHIGPKKRRNLL

EKRMKDVDDELQLVIVRDMWLTGFDVPSMHTMYIDKPMKGHNLMQAIARVNRVFKDKP

GGLIVDYVGIAESLKEALKEYTESDQAQTAIDTDKAVELMLLKYDVIQDMLYNLDYSK

FNSDKKSERYYTISNTMDYVIGLGEDERQRFIKTVTELGKAFALCATEPTAQELNDEI

AFFKAVKAGLVKLLQPPKEGKTRKTPAEVEAEINQLVSQSVVTEDVIDVYQTLGLEQP

DLSILSDDFLKDVEGLKQKNVAVELLNRLLKGQVKSLMKTNATVSKRFSEMLGNSINK

YNSRSIETSKVIEELIQLAKDIKQEQQRGNELGLNSDEIAFYDALASHETAKEAMGDK

ELRAIAHELTKTVKENMGVDWSKRDSAKAKMRVAVRRLLKKYGYPPDLQKMAVEQVVE
QAELMANNQ"

gene complement(31639..32940)
/locus_tag="NI36_00295"

CDS complement(31639..32940)
/locus_tag="NI36_00295"
/inference="EXISTENCE: similar to AA
sequence:RefSeq:WP_000456234.1"
/note="Derived by automated computational analysis using
gene prediction method: Protein Homology."
/codon_start=1
/transl_table=11

/product="restriction endonuclease subunit S"
/protein_id="ALQ98361.1"
/db_xref="GI:959165101"
/translation="MEYNTIFFGDVAKIKNGYAFKSKEFQEKGIPVIKIKNIISPIVD

TKDSQKVSIKTYEKTKGFSLKKNDILISLTGSGVNQMSSAVGKVGRIEFDYPALQNQR
VGKFELKYSNSADLDFLFYYFLQPKITEYLVRNSTGSANQANINSKLIETVKIPNFSL

IKQKSISKFLNQITRKIETNQKMIANLKELSQTLFKHWFVDFEFPDEDGNPYKSSGGE

MIDSELGKIPSNWKIYKLKDIASHKKETFNPKKSEEVTVKHFSLPAYDNEEQAIEEEV

NKIKSNKWIINNNCVLFSKMNPDTKRIWLPVIDNKKLNVASSEFVVMESPNNKINSFI

YNICLNSQFIDYLKANTTGSTNSRQRVKPTIAVNYKLAIEDSIVKKYSEIITPYMEEM
KILRSEIGKLTQLRDTLLPKLMSGELEISDDIEVNSDELSI"
gene complement(32930..34444)
/locus_tag="NI36_00300"
CDS complement(32930..34444)
/locus_tag="NI36_00300"
/inference="EXISTENCE: similar to AA
sequence:RefSeq:WP_017175164.1"
/note="Derived by automated computational analysis using
gene prediction method: Protein Homology."
/codon_start=1
/transl_table=11
/product="N-6 DNA methylase"
/protein_id="ALQ98362.1"
/db_xref="GI:959165102"

/translation="MATIGFEEKLWQAADKLRGSMDAAEYKNVALGLIFLKYVSDSFE

EKYEELKQDPYADEEDQDEYLAENIFWVPKEARWQYINDNAKKPEIGQIIDKAMIAIE

NENESLKGVLPKEYARPALDKEKLGDIIDLFTFKVGDTESRKQDVLGRVYEYFIAKFA

SAEGKNAGEFYTPSSIVKLLVEMIEPYKGRIYDPCCGSGGMFVQSERFVEKHQGRLDD

IAIYGQESNPTTWKLAKMNLAIRGIDNDLGERNADTFHNDLHKGLKADYILANPPFNA

SDWGQERLLDDYRWQFGIPPKGNANYAWIEHMISKLAPNGTAGFVLANGSMSTSGKDE

LEIRKNLIEQDLVECIVTLPGQLFYSTQIPVCLWFISNNKGQNGKKERENEILFIDAR

EIGHMVSRTLKEFSDEDIQKVAQTYHAWRGTNDKPYEDIAGFCKVANLEEVKNNEYIL
TPGRYVGLADVEEDEEPFEQKMERITSELSEQFAKSKELEDQIRKSLEGLGYGV"
gene 34886..36949
/locus_tag="NI36_00305"
CDS 34886..36949
/locus_tag="NI36_00305"
/inference="EXISTENCE: similar to AA
sequence:RefSeq:WP_000275972.1"
/note="Derived by automated computational analysis using
gene prediction method: Protein Homology."
/codon_start=1
/transl_table=11
/product="chromosome segregation protein SMC"
/protein_id="ALQ98363.1"
/db_xref="GI:959165103"
/translation="MYISSVKIKNFRNFDNIDIDFHEGVNVLIGHNNSGKSNLLRALS

LIFDGSVRKQLSVEDFNNSLTKESLKKEAPKIVISVHITQSENERLMSDELITVSNWL

TKLEEPYEARIQYEFFLPKDEEKNYIDLVKNIDEKEEIWNLIRSQFIRLYVNKIWVGN

PEHQIPIDNDSLNKFDFQFLDAIRDVERDMFSGKNTLLKSVIDFFIDYDIKSDETITE

EEQKEKLEERRKEFSDNSSDLIETIHKRLDSGNQKILSYTNGIGASYDKSTPDFKGNL

TESEIYTVLQLIIKHETGMTLPITHNGLGYNNLIFMALLLSKMQADSDGNFLGSNAKV
FPILAIEEPEAHLHPTMQNEFIKFLKNNIREKKVKQIFITTHSTHISSSTNIDDIICL

YTDDSKTNVSYPGKVFDKNNQSKKYVQRFLDATKSNMLFAEKVIFVEGIAEQLLLHIF

ADYLNKPLEKNHVAVINVGGRYFNHFLSLFDSNNKYAINRKVSCITDLDPMRKEKNEN

GQKNNFKACYPFEYEMDLDKFDYSTNNSLDKYLNNEHSNIRVFVQPNKYGKTFEYQLM

FDNPSLKLLLTDSISNSQELAELMDHYEENESLQKLIDTLSPSNENQRIIKSLKDTND

SWNEDNKKKALIASRYLNSIGKGENALELASVLKDNLELKGQSDYEDFVVPKYIEGAI
RWVCE"
gene 36951..38699
/locus_tag="NI36_00310"
CDS 36951..38699
/locus_tag="NI36_00310"
/inference="EXISTENCE: similar to AA
sequence:RefSeq:WP_000585108.1"

/note="Derived by automated computational analysis using
gene prediction method: Protein Homology."
/codon_start=1
/transl_table=11
/product="DNA helicase II"
/protein_id="ALQ98364.1"
/db_xref="GI:959165104"
/translation="MIINAQTKLNIEQDFKIQAGPGAGKTEFLVNHIKNVIQTSEKLE

RMKKIACITYTNTAAQTVLQRLGKSVSNRVDISTIHSFLYRNVVKPYCSFLPKEYGIC

VQKLKGHSDPIVINKYVNAWLKTEYFTKLKPPSNRNQLLKMPVLKKALQNWLLSMKLS

YENNDVQFICDNTKAQAIDKKSKKRMGINKSNLNILEKQLLNYKKIYWEKGIIDHDDV
LFFSYVLIRDYPFILTILCAKYPYFFIDEFQDTSQLQDFIIDKIRQKGCIVGVIGDKA
QAIYSFQGARVSLFEKFKVDLINSHTILENHRSSYQIVTFLNSIREDITQTVYSGIEN

DKVTILIGNRIRSYTKVVNICNKESLVTLSRDNITSNAMRIKLEGNNFDKKLLEKFQE
IDSNSKRRNTIISLIKAIELAKNTKYKDALKIVESIYKDENNPKKIALNSLINMINKY

DKYGNGTLMNFYNTLCKTLDIKLSGFRKGQVKDFYENTLYKNMVICTNIVEDTSNHIT

IHKAKGAEFENVFVIGNKDTLSLLLKPDLMNDEEHRIFYVAMSRAKKKLFLQFDYLDN
NEQKKIKERYDVSIIQ"
gene 38918..39091
/locus_tag="NI36_00315"
CDS 38918..39091
/locus_tag="NI36_00315"
/inference="EXISTENCE: similar to AA
sequence:RefSeq:WP_001154379.1"
/note="Derived by automated computational analysis using
gene prediction method: Protein Homology."
/codon_start=1
/transl_table=11
/product="multidrug transporter"
/protein_id="ALQ98365.1"
/db_xref="GI:959165105"
/translation="MQDVAINADHKVFFLMIIFNVIMLVSSIIQMILGKGRRITPAKP
TKDAKLHLDNQSS"
gene complement(39159..40577)
/locus_tag="NI36_00320"
CDS complement(39159..40577)
/locus_tag="NI36_00320"
/inference="EXISTENCE: similar to AA

sequence:RefSeq:WP_002494529.1"
/note="Derived by automated computational analysis using
gene prediction method: Protein Homology."
/codon_start=1
/transl_table=11
/product="pyridoxal-dependent decarboxylase"
/protein_id="ALR00838.1"
/db_xref="GI:959167577"
/translation="MEFNENNIDLETIIRDEVNKYLSRDIGDLPATQQAPLELREKYE

KMEVPNKGRDIYEVLKDLNNEVLNYLYRPNHPRSFSFIPGPASRLSWLGDILTTANNI

HASNFANATLPINIERNLINYLVGKIGYEIKPAGGVFVSGGSMANLTAIVAARDAQVE

MEDIKKATVYLTSQTHHSVGKALHVAGFLKNNIRRIEYNDDFTMNTDSLKAAIDKDIE

EGYKPAMVIATAGTTNTGSVDDFTTLGDTCDHYNLWLHVDGAYGLSHILSDKAQHLFK

GIERSDSASWDAHKLLFQTYSCAMVIVKEKNHLLQSFGEDAEYLDDIASDDDVIDPEM
LGIELTRPARALKLWITLQVIGEDEIIERIKYGEGLAEYAEEYVSGLDNWRIISHANL

SIVNFRYENSDLTEAQNNQLNSMAAQKIADSGYAIAYTTVLNNQRVIRLCTTNPLTTH
DDIRETINRLDQYIGDYDMKDA"

ORIGIN
1 atgaaaatca ccattttagc tgtagggaaa ctaaaagaga aatattggaa gcaagccata
61 gcagaatatg aaaaacgttt aggcccatac accaagatag acatcataga agttccagac
121 gaaaaagcac cagaaaatat gagcgacaaa gaaattgagc aagtaaaaga aaaagaaggc
181 caacgaatac tagccaaaat caaaccacaa tcaacagtca ttacattaga aatacaagga
241 aagatgctat cttccgaagg attggcacaa gaattgaacc aacgcatgac ccaagggcaa
301 agcgactttg tattcgtcat tggcggatca aacggcctgc acaaggacgt cttacaacgt
361 agtaactacg cactatcatt cagcaaaatg acattcccac atcaaatgat gcgggttgtg
421 ttaattgagc aagtgtatag agcgtttaag attatgcgtg gagaggctta tcataaataa
481 aactaaaaat tagattgtgt ataatttaaa aatttaatga gatgtggagg aattacatat
541 atgaaatatt ggagtatacc ttgcaatatc atacgatgtt tatagagtgt ttaataaacc
601 atttttcaac tattgatgat ctagaatata taataactgt acaaattata ttgattatgg
661 aactacaatt caattaagaa attgatgatg aaattttaaa tttaaactaa tggaatcaag
721 aaagaatgaa aggaaatata caatgcccac gattaataaa aggaagttta ttagattttg
781 tgttagaaac agtagatgtt tttgtaaatg gaagtaaaaa tgattgcgaa ttagtaaatt
841 ttaattataa agaaggaaaa gtagtttcac gttatagtac aaaatttgaa agcaatccta
901 aattaagaaa aaagcaattg atatcgacgg taaaagttgt aaggtatgtt atttttgatt
961 ttgagaaaat tttatggggc atagggaaag ttttattgaa gttcatcata ttaaggcaat
1021 gtttactata agaaagttga aatacactca gaaacagact ggattcccgt ttttttcaaa
1081 ttggcacaaa atgatacata ggcccaccta tatattgaag atttaagtta aacgataaat
1141 aatatttttc tcttttgtat agaaattttt ttgggaatga tattaattag tataaaaatg

1201 aatatgtcta caaagaagga gagattttat tgaaacacat taatcaattt tctgaatcga
1261 cactagcaca actagaaaaa gatcaacaac acgtatttta tgtatactgt ttgatggacc
1321 caagaaatga tgaatgcttt tatgttggta aaggtaaggg gaatagaata tttaaacata
1381 aacaagatgc gcagaaaaaa gtattgtacg aagatatatt tatagaagaa aataaagata
1441 acttgaaatt taatagaatt aacgaaattt gtagcaatga tttgaatgtt ttaggatata
1501 taatcagtta tggattaacc gaatctgaag cattttctgc agaaaatgtt cttataaact
1561 ttcttaacct aacaaataaa actacattaa caaatatgat taacggtcat ggatctaagg
1621 catacttagt tgaagattta gaaaatgaat ttggctacga ttcaattaat cttgaaaaca
1681 taaatacgaa tgaattaatt ttagcagtga aaatcagaga tgcatttcta ttagataagg
1741 atgaaagtaa agagtatcct attaatgaaa gtaaacgtga tagaaataac cttaaatcgc
1801 gtacattagg tagttggata ataggaaaag ataaaataca taaaataaaa tatattattg
1861 gtatcaatac aggtgctaat aatgcagttg tctcagctta tgaagtatca tttgaacaag
1921 cggagagtat cgaaacaaat aatggtagaa tgagatatgc gtttattggg ctttcagaaa
1981 gagatgctac tctaaaaaaa ttgaatttat acaaaaaagc actaccagat ttaagatttg
2041 gtagtggtag cgctacagca tatataaata atgggacaat gaaagttgat taaatgaata
2101 aaaacatcca attccttaaa ttagcctttg aaaattttat actcgtatat ataagtgaaa
2161 aacttaaaag aattagatgt taattaaagg agagaatgct atgccatcag atgatattgt
2221 aaaaaaacta tactcaaaag aggatattcg attagaatta ggacttactc cgcataagtt
2281 taataaaaaa atggaaacta ttgctaagct ttttaaaatt gatatgaaaa tttttcacaa
2341 ttacaaaggc caagataaaa ataatcagta tacatttaat ggtgtcgcaa aagaattaat
2401 caaagtgttg ctaaaaagtg ttgattatta cccagtggat atcaattcca aaaaatttaa
2461 acaaaatgga aaatctaaaa aagaaatgat tgaaaatata gataactcta gttacatgaa
2521 atatatttat cagttaatga aatctatcaa tgaaattcaa tacaaaaggt taattgctga
2581 tatacatatg aaagatgtgt accagaatac aaaagcatgg ttaaataatg gtgaatcaat
2641 taataaaaaa gaacaagagc tataccaata tatgacaatt ttaccattac ataaaagagt
2701 tgaactgcaa aatgaggtat taaaatctat agacgaaaca atttttcaat ttctcgcaaa
2761 agaacataga aataatcaaa ttgaagaaaa taatgaatta gaagcatata caaaagcaat
2821 aaaagaaggt agaaatccta aaaatgatta tgaattaaat catctcctat ataaaaaaaa
2881 tcaattaccc ttagatagtc taatagaaga tatatgggac tatgaggaaa cagagtatac
2941 tgagcttgat tggttaattg cagatatgct aaaacgttca caaagagctg atagtaattt
3001 tatcgaaagt ttagagaaga aaaacaaatt gagaaaaaat attcataaag attccattaa
3061 aagtatatct aagttgattg atcatgaatt agttaaaaat aatagaaagt gggacgtagc
3121 agaattttat aaaaatcaac aattttggaa taatagtaca ttaagacgta gctctcaaaa
3181 ttttggaatt ttatattaca aaaagaatag gttacataca atgaatcaaa gtaatattat
3241 taagaaaata aaaattatcg aagctaatat agagagtgca aataaacagc cgagctattt
3301 agatttcttt ttacaagcta agtttgattt agagaggtta gaaagtgaat taatgaaata
3361 tagcattaat ccgaaatatt ttaataagat aaatagtgat tacgtaagat atgctattga
3421 agatgtacat aatgcaatta ttaaaattga cagatatata agtaacgata aagtgaagtt
3481 aaactattca aatgtccaaa tggtagtgag aaacgaaata acagaacaag gaagctactt
3541 tgtaacccag gcattgaata cactgtcaaa ggtagaagag tctggctaca gctttgataa
3601 tttcatggta ggacctttcg tagaagactt taaaaaagca attaaaaatc gtagatttgg
3661 ttctaaaaaa gattaagatt gttttttaga gttaaacata agaataaatt taaataaaaa
3721 aataaaccca acaaaccttg ttatatcaat gtttgttggg tttatttttt tggtgatttt
3781 ttaataactc aattacatct taggataata tcgctaaatt atacattgtc gaggccaaga

3841 caaataaaaa attaaagcaa aagttagcgc gcaactttct gctataaagg agcaaattta
3901 tatggagaca agagacaaac tgatgtcatt gactcaaagt gacaaaacac agcaatggct
3961 aatggacaag tcatctaacc aagatgacat tcaacaattg cagcaacaat tcagtcagca
4021 gcttgatcaa caatataatg cacttttagc tgatgaaaaa gctaagttag accaatacgt
4081 ggaagtacat caaggattgg aatcattaaa ggaagagatt gaatcagaac ctattacgct
4141 taatatcgat aaattacccg atatcaaagc aacaatgctt gaaagagcca agaatgatga
4201 acattctgat aaaatcgaaa agctatttga taggttagaa caggcattaa atggtacgaa
4261 tcgattatat acgcaattat cgttgattgg tacacgaaca catcgaatta caactaaaaa
4321 ttttaatctt caaggcttac ctaaagcagt ccaacatacg attttacctt caaaatttaa
4381 gaaggtgtat acagtcgatt ttaaatcgtt tgaaccatca gttgcagcgt acatgactca
4441 agattcaaaa ctgattgact tgttaaatca gaaagacgga ctgtatgacg cattgctaag
4501 tgaattaggc ttatcagatg agctacgtgt atttgttaaa cgtgcattta ttggttcgtt
4561 tctatttgga ggtaacttca aaaatcctaa attcaagctg aatcaatatg taagtgaagt
4621 acaatggttg gatgcggtca gccaatttac aaaagtcatt gaacttaaga agcacgttga
4681 aaagagtaat tccatgccta tgccttatag tattgagcat gatatgagcg catttcaagg
4741 tagcagtatt atggcaatct acgtacaaac tgtagcgagt tatattttca agcacattct
4801 gctaaaagtg tacaaagcac agtgcgatca aaaaacgttc aagattatag tacctataca
4861 cgatgcgatt atgattgaat gtgaagatga agaaattgca caaaatgtgg gtcagttaat
4921 gaaagatacg gctaaccagt tgttcaatgg tgaatttgca catgtgacag tggaagaaat
4981 aggaggcgta gaccatgaat aatgatagag gacaaagcct acacatccca agtagtacac
5041 caatcaaaga aaataatata tatgtagcta cgttacattc tgtgatccaa acagatttct
5101 caggtgaaat aaagcaccaa ttcacgtatg aaattgaagt gaacaatcag attgtatatg
5161 cgaatcgtaa tattccaaca aaaccgagcg ctaatcagtt gtcaattcat gattggctga
5221 aacgtcatag caactatagc gcaagtcatg aaaactatga gccttatatt gatcagaaac
5281 atttaattct attaggtcaa tataacggta actattatgt acaagatgta gcatcgttag
5341 atgcgtttgg aggcgtatta tcatgaatca tatattacaa atgttatcta agctattaag
5401 tgtggccaag gaggcaatcg accgtcaagg tctgattgct atcctaacta ttcctgttaa
5461 taataacgat gaaatagaag aaacggctca aggtgaaacc gtgtataacg aacttatcga
5521 tcagttacga cttaatatcc caaaagatac ggattatcaa cctaacatct atagttattt
5581 tggtattaag aagaatccta atgacaccgt actcatggaa atgatgataa aggtttttca
5641 tatcaaacgc tttaattcag aactgtttat tttcaaagtt aacgggtggc aaaagataaa
5701 tggagatgaa ttacaagggt tgatatctaa aatgatacaa gtattgcttg tagattataa
5761 gccttcacta agcactctaa aaaatgtcgt agatggattg caaaaatcaa cagatgtaga
5821 agaacttgtt gagaatgagc gctatattgg ttgtggtgaa aatatgttcg atcttaatac
5881 gtttcaagtc gttaaaaatt caatcgatat ctttccaaaa acacgattga atttatcatt
5941 aagtacaaat gatgtaatta ctgataagat accgccttat tttaagcaat atatgttaca
6001 acttgcgaat tatgacgatg atttacaata ctttcttttc caacatacag cagtattact
6061 tacagctgat actaaatacc gtaggggtct catattatat ggtggagcta agaatggtaa
6121 atctgtatat attgaactag ttaaatcatt tttctatagt aaagatattg tgtctaagcc
6181 acttaatgag cttgaaggtc gttttgacaa agaaagttta attgacaaaa gtctaatggc
6241 aagtcatgaa attgggcaat ctaggattca agaaaagatc gtaaatgact tcaaaaagtt
6301 attatctgta gaatcaatgc atgttgatcg taaaggaaaa actcaagtgg aagtcatttt
6361 ggatttgaaa cttattttta gtacaaatgc gatacttaat tttcctcctg aacatgcgaa
6421 agctttggag cgtcgaatta atattattcc atgtgagtat tatgttgaaa aagcggacac

6481 ttcattaatt gataagctcc agagtgagaa gaaagaaatc tttctttact tgatgtatgt
6541 gtatcaacag attgtaaaag cagatatcga gtatcttgaa aatagccgtg tcactgaaat
6601 tactcacgat tggttaaatt ttggatatga atttgtttct agcaggtccg taagtaatgc
6661 aaatcagaaa gcatgtatta atttactcag aaaacttata gaaatcaaat caggatcacg
6721 aatcaaagta tccgagttaa ataaagttat taatgaagaa ataaaggtaa gttctcaggt
6781 tattaaacag ttaattcaag caaactttga tactcaaacc aaactataca atggctacga
6841 ttattggatt gatttaggtt ggaaagaagc caataaaaaa gagattcatg atatttcgga
6901 aaaagataat attatttcat tagataaaaa tgaaaatata acagacgatg aggcattaga
6961 tgaagagaat ttggactttg attgggagga ctttgacgat gaataatgaa caaattgaag
7021 catttgtaga agtgcttgta cctatcatag aagaacgtat caataaaggt aattaaggct
7081 aattacgtac tacaggcagt tgcctgtagt actcatatga ttaagtggta aaagtgataa
7141 aaatgaaacg aaattataaa tatatattat ctatatgttg ttacaagacc gatagtctgt
7201 agcaataatc taataaaagg agcggtatga tatgaagggt aaaattgcac tttattcacg
7261 cgttagtacg tcagagcagt cggagcatgg gtactcaatc catgagcagg aacaagtact
7321 catcaaagaa gttgtgaaaa atttcccagg ttatgactat gagacatata ctgactcagg
7381 catttcaggt aaaaatattg aaggtcgtcc ggcaatgaaa cgtctattac aagatgttaa
7441 ggataataaa atcgaaatgg tgttaagttg gaaattgaat cgtatctcac gatcaatgag
7501 agacgtgttt aatattattc atgaattcaa agaacatgat gtagggtata aatcgatttc
7561 tgagaatatt gatacatcca atgcttctgg agaagtactc gttacaatgt ttgggttaat
7621 aggatctata gaacgccaga ctttgatttc gaatgtgaaa ctttctatga atgctaaggc
7681 aaggagcgga gaggcaatca ccggtcgtgt tttaggctac aaattatcac ttaatccact
7741 tacacagaaa aatgatttgg ttatcgatga aaatgaagct aatattgtac gtgaaatttt
7801 cgatttatat ttgaatcata ataaaggcct caaagccatt acaaccgtac ttaatcaaaa
7861 ggggtatcgt actattaatc aaaagccatt ttcagtgtat ggtgttaaat acattttgaa
7921 taatccagtc tataaaggct atgtcagatt caataaccat caaaactggg ctgtacagcg
7981 aagaagtggc aaaagtgata aaaatgatgt gatattggtc aaaggtaaac atgaagccat
8041 tataagtgaa gaggtatttg ataaagttca tgaaaaatta gcttctaaaa gttttaaacc
8101 gggcagacct attggtggag atttctactt acgtggcctt attaaatgcc cagaatgcgg
8161 aaataatatg gtatgtcgac ggacgtatta taaaacgaaa aagtccaaag aacggacaat
8221 caagcgttat tacatttgtt ccttattcaa tcgttcaggg agttctgcat gtcacagtaa
8281 ttccatcaat gctgaagtcg tcgagcgtgt aattaatgtt catttgaatc gtattctgtc
8341 tcaaccagat attatcaagc agattgcgtc aaatgtgata gaagaactga aacaaaagca
8401 tagtaaccaa acagaaatta aatatgacat tgatagttta gaaaaacaaa aagctaagct
8461 taaaacacaa caagaacgat tgttagaatt gttcttagat gatcagatgg atagcgaaat
8521 gttaaaagct aaacaaagtc aaatgaatca acagttagaa gtattagatc aacaaattaa
8581 agaagcgcaa caagcaaatc aatcacagga tgaaatacct aattttgata aattaaaagg
8641 acgactcatt ttgatgataa cacgattcag cgtgtactta agaaaggcta cacccgaagc
8701 taaaaatcaa cttatgaaaa tgttaattga ttcaattgaa attacgacag ataaacaagt
8761 aaaacttgta aggtataaaa ttgacgaaag tcttatccct caatctttga aaaaagattg
8821 ggggtctttt tttatgccca aattccaatt tgaaatatat ggtcaaaatg attatttcat
8881 cgaccaaatt accactttta ccacttagtt attagtgaca aaagtgagcg aaatgaaata
8941 aaaatcaaat atatattatc aaaatgatgt atcacatgca tacatcaatc aaatacatta
9001 ggaggtcata accatgacac tagaacaaca actcaagcac tatataacca acttattcaa
9061 tctgccaagg gacgaagtgt ggcactgcga atctatcgag gaaatcgctg atgatatctt

9121 acccaatcaa tatgtaagac ttggcccact cagtaataaa acacttcaga ctaataccta
9181 ctactctgac acacttcatg aaagtaatat ctatcctttc attctctact atcagaaaca
9241 actcatagcc atcggttata tcgacgaaaa tcacgatatg gatttcttat acctacacaa
9301 cactatcatg cctcttttgg atcaacgata cttactaaca ggaggacaat aaaatgcata
9361 aatacatcaa aattacacaa ttagtcatta caatactaag tgaaatcatc atttggatga
9421 aagagtcaga acgaaaggaa gtctcttatg aatagatata tcacccgggg tatcgccaac
9481 aacttaccta atatcttaca acaccaatta tggcaactcg tatctgagcg agaacaagaa
9541 caaaccaaag ataatacttc agtagattat tttcatatat tccagttcaa taagcatcgc
9601 aatcaattat atatcaaaca caaacaagaa cgacctgaat atgcgaaaat ccataaagct
9661 aattattcaa aagcaatcaa tatcaataag gtctacatta ttcgagaaga tgatgtagac
9721 ctttcttatt atgtcatgtt attacctgaa gaatactaga ggagtgaaaa ttatatggaa
9781 acaatcaaaa gtacattaaa aacagaagcc atattcagtg atgacaaaca acatcgctat
9841 ctacttaaga aaacatggaa tagtgaaaaa caatcaatca caatcattac aatgtatccg
9901 cattatgatg gcattctcaa tattgaccta acgacccaac tcatcatgaa caaagtttca
9961 gaaatggatg catttggttc aatcaatttt gtgaatctat actctaatat tacaacccct
10021 atcaatctca aacatttaga aaatgcgtat gataagcata cagatattca aattatgaag
10081 gcagtgaaag agtcagatga agtgatatta gcttggggcg cttacgctaa aaagcccggt
10141 gttgaagcac gtgttaatga agtattagag atgttgaaac cacataaaaa gaaagtaaaa
10201 cgactcatga atccagaaac caatgaaatc atgcatcccc ttaattcgaa agcacgtcaa
10261 aaatggatat taaaagtata gcaactaaaa tgtttattca ctatccatga aaataattga
10321 atttcatata tatcttttaa tctacaatat gattaaaaga tatattattt ggttctgttg
10381 caaagtaaaa aaatatagct aaccactaat ttatcatgtc agtgttcgct taacttgcta
10441 gcatgatgct aatttcatgg catggcgaaa atccgcagat ctgaagagac ctgcggttct
10501 ttttatatag agcgtaaata cattcaatac cttttaaagt attctttgct gtattgatac
10561 tttgatacct tgtctttctt actttaatat gacggtgatc ttgctcaatg aggttattca
10621 gatatttcga tgtacaatga cagtcaggtt taagtttaaa agctttaatt actttagcca
10681 ttgctacctt cgttgaaggt gcctgatctg taattacctt ttgaggttta ccaaattgtt
10741 taatgagacg tttgataaac gcatatgctg aatgattatc tcgttgctta cgcaaccaaa
10801 tatctaatgt atgtccctct gcatcaatgg cacgatataa atagctccat tttcctttta
10861 ttttgatgta cgtctcatca atacgccatt tgtaataagc ttttttatgc tttttcttcc
10921 aaatttgata caaaattggg gcatattctt gaacccaacg gtagaccgtt gaatgatgaa
10981 cgtttacacc acgttccctt aatatttcag atatatcacg ataactcaat gtatatctta
11041 gatagtagcc aacggctaca gtgataacat ccttgttaaa ttgtttatat ctgaaatagt
11101 tcatacagaa gactcctttt tgttaaaatt atactataaa ttcaactttg caacagaacc
11161 gtattatgga atagagatgt tggtaacatt tatacaggat cattatactt aagtttaatt
11221 tcgttattac agaaccacac attccaacca gaagagaaag tatgtctatt tagttatggt
11281 tcaggagcag taggagaaat ctttagtggt tcaatcgtta aaggatatga caaagcatta
11341 gataaagaga aacacttaaa tatgctagaa tctagagagc aattatcagt cgaagaatac
11401 gaaacattct ttaacagatt tgataatcaa gaatttgatt tcgaacgtga attgacacaa
11461 gatccatatt caaaagtata cttatacagt atagaagacc atatcagaac atataagata
11521 gagaaataaa ctagtggccg attgtgcttg atgagcttgg gacataaatc ctaactcgaa
11581 ataaataagc atatcactaa actgattttt taaagtttac agtgatatgc ttattttttt
11641 atcttacgat tttgtacgtg catgcttgcc taggggtatg gctcgagcca ttagtctctc
11701 gcacatacta ttccctcagg cgtcagcact tacaaaatcg gttgtaattt tcatttttat

11761 acgcattctt actgagatta tactaataag aggaatagta aaagcaattc taagtaaaat
11821 tgcagataag aggtttgtta aaagcagttc taagtaaaat tgcagataag aggtttgtta
11881 aaagcagttc taagtaaaat tgcagataag aggtttgtta aaagcagttc tcagtaaaat
11941 tacagataag aggtacgtta aaagcagttc taagtaaaat tgcagataag aggtttgtta
12001 aaagcagttc taagtaaaat tgcagataag aggtttgtta aaagcagttc taagtaaaat
12061 tgcagataag aggtacgtta aaagcaattc catgcaaaat tgctgataag gggtaagtta
12121 aaagcagttc tcagtaaaat tgcagataag aggtacgtta aaagcagttc taggcaaaat
12181 tgcagataag aggtgcgtta aaagcagttc taagtaaaat tgctgataag gggtaagtta
12241 aaagcaatcc taagtaaaat tgcagataag aggtaagtta aaagcaatcc taagtaaaat
12301 tgcagataag gggtacagaa aaactagact tgattacaaa atggagcttg ggacataaat
12361 gattttttaa aaatgagatg agacgtagat taactccata atcaatacga atctatcgac
12421 ttctttattt atgatattca tctcttttta atggaaataa aagtgcgatt aatgtgataa
12481 tacagttacg ttaattaaaa aaataaaaat gcaaggagag gtaatatgct aactgtatat
12541 ggacatagag gattacctag taaagctccg gaaaatacaa ttgcatcatt taaagctgct
12601 tcagaagtag aaggtataaa ctggttggag ttagatgttg caattacaaa agatgaacaa
12661 ctgattatca ttcatgatga ttatttagaa cggactacaa atatgtccgg ggaaataact
12721 gaattgaatt atgatgaaat taaagatgct tctgcaggat cttggtttgg tgaaaaattc
12781 aaagatgaac atttgccaac tttcgatgat gtagtaaaaa tagcaaatga atataatatg
12841 aatttaaatg tagaattaaa aggtattact ggaccgaatg gactagcact ttctaaaagt
12901 atggttaagc aagtggaaga acaattaaca aacttaaatc agaatcaaga agtgctcatt
12961 tcaagcttta atgttgtgct tgttaaactt gcagaagaaa tcatgccaca atataacaga
13021 gcagttatat tccatacaac ttcgtttcgt gaagactgga gaacactttt agattactgt
13081 aatgctaaaa tagtaaacac tgaagatgcc aaacttacta aagcaaaagt aaaaatggta
13141 aaagaagcgg gttatgaatt gaacgtatgg actgtaaaca aaccagcacg tgcaaaccaa
13201 cttgctaatt ggggagttga tggtatcttt acagacaatg cagataaaat ggtgcatttg
13261 tctcaataga aagttagagg tgagtcttac gtttcagtga cggtagactt acctttaaca
13321 tgttacatac taaaaaatta atttgaataa gaaagagaga catatatgaa atacgatgat
13381 tttatagtag gagaaacatt caaaacaaaa agccttcata ttacagaaga agaaattatc
13441 caatttgcaa caacttttga tcctcaatat atgcatatag ataaagaaaa agcagaacaa
13501 agtagattta aaggtatcat tgcatctggc atgcatacac tttcaatatc atttaaatta
13561 tgggtagaag aaggtaaata cggagaagaa gttgtagcag gaacacaaat gaataacgtt
13621 aaatttatta aacctgtata cccaggtaat acattgtacg ttatcgctga aattacaaat
13681 aagaaatcca taaaaaaaga aaatggactc gttacagtgt cactttcaac atacaatgaa
13741 aatgaagaaa ttgtatttaa gggagaagta acagcactta ttaataattc ataataaaac
13801 agtgaagcaa ccatcgttac ggattgcttc actgttttgt tattcatcta tatcgtattt
13861 tttattaccg ttctcatata gctcatcata cactttacct gagattttgg cattgtagct
13921 agccattcct ttatcttgta catcttttaac attaatagcc atcatcatgt ttggattatc
13981 tttatcatat gatataaacc acccaatttg tctgccagtt tctccttgtt tcattttgag
14041 ttctgcagta ccggatttgc caattaagtt tgcataagat ctataaatat cttctttatg
14101 tgttttattt acgacttgtt gcataccatc agttaataga ttgatatttt ctttggaaat
14161 aatatttttc ttccaaactt tgtttttcgt gtcttttaat aagtgaggtg cgttaatatt
14221 gccattattt tctaatgcgc tatagattga aaggatctgt actgggttaa tcagtatttc
14281 accttgtccg taacctgaat cagctaataa tatttcatta tctaaatttt tgtttgaaat
14341 ttgagcatta taaaatggat aatcacttgg tatatcttca ccaacaccta gtttttttcat

14401 gcctttttca aatttcttac tgcctaattc gagtgctact ctagcaaaga aaatgttatc
14461 tgatgattct attgcttgtt ttaagtcgat attaccattt accacttcat atcttgtaac
14521 gttgtaacca ccccaagatt tatctttttg ccaaccttta ccatcgattt tataacttgt
14581 tttatcgtct aatgttttgt tatttaaccc aatcattgct gttaatattt tttgagttga
14641 acctggtgaa gttgtaatct ggaacttgtt gagcagaggt tcttttttat cttcggttaa
14701 tttattatat tcttcgttac tcatgccata cataaatgga tagacgtcat atgaaggtgt
14761 gcttacaagt gctaataatt cacctgtttg agggtggata gcagtacctg agccataatc
14821 atttttcatg ttgttataaa tactcttttg aactttagca tcaatagtta gttgaatatc
14881 tttgccatct tttttctttt tctctattaa tgtatgtgcg attgtattgc tattatcgtc
14941 aacgattgtg acacgatagc catcttcatg ttggagcttt ttatcgtaaa gtttttcgag
15001 tccctttta ccaataactg catcatcttt atagccttta tattcttttt gttttaattc
15061 ttcagagtta atgggaccaa cataacctaa tagatgtgaa gtcgcttttc ctagaggata
15121 gttacgactt tctgtttcat tagttgtaag atgaaatttt tttgcgaaat ctcttaaata
15181 ttcatccatt tttttaacgg ttttaagtgg aacgaaggta tcatcttgta cccaattttg
15241 atccatttgt tgtttgatat agtcttcaga aatacttagt tctttagcga ttgctttata
15301 atctttttta gatacattct ttggaacgat gcctatctca tatgctgttc ctgtattggc
15361 caattccaca ttgtttcggt ctaaaatttt accacgttct gattttaaat tttcaatatg
15421 tatgctttgg tctttctgca ttcctggaat aatgacgcta tgatcccaat ctaacttcca
15481 cataccatct tctttaacaa aattaaattg aacgttgcga tcaatgttac cgtagtttgt
15541 tttaatttta tattgagcat ctactcgttt tttattttta gatacttttt ttattttacg
15601 atcctgaatg tttatatctt taacgcctaa actattatat atttttatcg gacgttcagt
15661 catttctact tcaccattat cgcttttaga aatataactg ctatctttat aaacttgttt
15721 gaaattttta tcttcaattg catcaatagt attattaatt tctttatctt ttgaagcata
15781 aaaatatata ccaaacccga caactacaac tattaaaata agtggaacaa tttttatctt
15841 tttcatcaat atcctcctta tataagacta catttgtaat atattacaaa tgtagtattt
15901 atgtcaaaat aatgttataa tttttgtgat atggaggtgt agaaggtgtt atcatctttt
15961 ttaatgttaa gtataatcag ttcattgctc acgatatgtg taattttttt agtgagaatg
16021 ctctatataa aatatacggt tctgttgcaa agttgaattt atagtataat tataaccaaa
16081 aggagtcttc tgtatgaact atttcagata taaacaattt aacaaggatg ttatcactgt
16141 agccgttggc tactatctaa gatatgcatt gagttatcgt gatatgtctg aaatattaag
16201 ggaacgtggt gtaaacgttc atcatttaac ggtctagcgt tgagttcaag aatatgcccc
16261 gattttatat caaatttgga agaaaaagca taaaaaagct tattacaaat ggcgtattga
16321 tgagacgtac atcaaaataa aaggaaaatg gaactattta tatcgtgcca ttgatacaga
16381 gggacataca ttagatattt ggttgcgtaa gcaacgagat aatcattcag catatgtatt
16441 tatcaaacgt ctcattaaac aatttggtaa acctcaaaag gtaattacag attaggcacc
16501 ttcaacgaag gtcgcaatgg ctaaagtcat taaagctttt aaacttaaac ctgactgtca
16561 ttgtacatcg aaatatctga ataacctcat tgagcaagat caccgtcata ttaaagtaag
16621 aaagacaaga tatcaaagta tcaatacggc aaagaatact ttaaaaggta ttgaatgtat
16681 ttacggtcta tataaaaaga accgcaggtc tcttcagatc tgcggatttt cgccatgcca
16741 tgaaattagc atcatgctaa caagttaaca caaagtatta ttttaaaatt gagattagac
16801 atttattttt caactttgca gcagaaccta attttttctc tttgtaaata aatgcaaaga
16861 gataccataa ggatctctta cataaccata accttcagta tagaattctg gactaaatgt
16921 tttcaatacc tcactgcctt tttctattaa ctggtcatat acatgtttag tttcttctac
16981 ttggtcaaaa gtgagacaaa gagatatatt attaccttgt gttatgggca aaccttcagt

17041 gtcatctgcg atcataattt ttatatctcc aaattgaagt acacattgat caattttatt
17101 taaatcattt tcgtcaatat taagtttctt atctataggt ctatctttaa tacgttgaat
17161 atacagtgtt ttagcgccaa acagctcttc atacaacttt tttaaaccct ctgcattttg
17221 agtgattaaa aaatatggac ttacttgaaa tttcatgttt tttcctcctt agatttgtta
17281 taaatagagt atatacttaa ttagtgtcat ctattgacac taaaaggaga ataataatga
17341 aaaaatctgt tagattatat aatatgattg aatattgtaa tgaaaatagg aacttcaaat
17401 taaatgattt aatgtcagaa tttaatattt ctcgtagtac cgctttaagg gatataaaag
17461 aaattgaagc attaggagta cctttatata gtaatccagg aaaaaatggt ggttatacga
17521 tcataggtaa tcgagaccaa acgaaaatag caatctcaga tgaagagttg aaagctttag
17581 tatttacact ttcgagtatt tcaaatgtga gtaatctacc ttttcaaaca gaatatcaag
17641 aaatattaaa aaaattatat aataactcga ataaaaaaga gttaataaac caatataatg
17701 atctatttca atattttaat gaagataagt atcagttcaa aagttataag ttatttaatg
17761 aaatcattag attgataatt gagaataagt cttttgaaac ctgttattca caaaactata
17821 ttaaagaaca atataaaggt attggcatta tgtataaaaa tcatcaatgg tattttgttg
17881 tagctaatat agaatcaaag ttagtgaatc tattaaatat ttcgaaaata aaagaactat
17941 atgaaatggg agagactcaa gagtgtaatg atataactat gcagaatttt caacagttca
18001 tggttaaaaa tgaaacagct attgatattc ttattagaag caatgttatg ggattgaata
18061 tcttgaaagg ctacctgtgg agtgactata tgattgaaaa tattgacgaa gagacatatt
18121 tatttaaatc aaaagtgaac gcgaaagata tagattttat agctaagtta attgtcacag
18181 gtggtgtcaa tgtaaaagta gagaccccta atagtttgaa aaatgctgtt aaagttgaat
18241 taactaaaat aataaacatg tattaatagt aatttaatca taattgtaat aacaaaaaga
18301 tgacaacatt aaacaattta aatttataca aaaaagcatt accaaattta aaattgggta
18361 gtggtagtgc tacagcatac ataaatagtt ataattaaat aaagatgggt caatgaaggt
18421 gaattaagtg aataaaaata ttacagctct aaaatctact gaaaatacta catatatgca
18481 gtatacggtt catatcgatg atcaaattat aaataatata aaagagtgta ccaaaaagtt
18541 taaaattttg cctatggaag ataaaattcc attatcaccg ttgttacaac cagaatatgc
18601 aggagaggta caagatttta ttagtacata tgagcagttt atgattaatt ttggtaaagt
18661 aatattggat agtcaaggca taaaaataca gtttgaaagt gaatcattaa gtagtattca
18721 acgaggcatt caagaacatt gttacttaaa tgagcgaaca aatgacattg atgtgactaa
18781 agaatggtat ttatgcaaat tttcaaatca aatattagaa gaggataaaa atcaattgta
18841 taatgcttta aagcaactca tgaatgattc taaaaataaa aagcaagctt tttggactga
18901 ccccaattag tgggaattat ataaaaacac tttcgttgaa ttcataataa aatgaatcat
18961 gcggaggtgt tttttctatg aaaagagttt cttattcagt agaaacaaag tataaagcag
19021 ttgaaatgaa aatagctggt ttttcgacaa aagaaattat gaaagaatta aatattaaaa
19081 atagaacaca agtggaaact tggtggcgat ggtatcgaaa tgaggaaagt tatagatttt
19141 cacaacacgt tgggaaacaa tatacctacg gtaaaggatt agaagaattg tcagaaatag
19201 aacgattaaa attagaaaat aagagaaaag atatagaatt ggatatttta aaaaagtaca
19261 aggcattgga aaggaagtgg taccagcagt agtcatagat ttagtggatc aattaaaagt
19321 aaaatattca atcaaattga tactagaagt attaaacata cctaaatcaa catattaccg
19381 atggaaaaac aaaacctata aaaatgatac tgtaacacaa aaagttattg aattatgtga
19441 agctaaccac tatacctacg gttatcgtaa gattacagca ctgattaatc aatgttatac
19501 atcaccaatt aatcataaga gagtacagag aattatgcag aagcatcatt tgaactgccg
19561 agttagacct aaaaagacga caaggatagg taaaccgtat tataaaactg acaatttatt
19621 acaaagacaa tttaaagcga gttgtcccat ggaagtatta acaaccgata ttacttattt

19681 accatttggt cattctatgt tgtatttatc ttcgataatg gatatttata acggagaaat
19741 tgtggcgtat aaaatagatg ataaacaaga ccaaagttta gttaatgata cattaaatca
19801 aatcgatata cccgaggggt gtatattaca tagtgatcaa ggcagcgttt atacatctta
19861 tgcttattat caattatgcg aagaaaaagg cattatcaga agtatgtccc gaaagggaac
19921 acctgctgat aacgccccga tagaaagttt tcattcctcg ctaaagtctg aaacttttta
19981 catcaataat cagcttaata gctctaatca tattgtaata gatattgtcg aaaaatacat
20041 taaaaactat aataataatc gaattcaaca aaaactaggc tacttatctc ctgtaaaata
20101 cagagaatta gcagcctaga acatagtgtt tttattaagt tcccgtttaa gggggtcagt
20161 accttttcaa gaggtgttta aggttcatat tgatatttat ctctacaata aagaaaaatc
20221 tgaatataag tatcaagtat ctagttattt caatcttgta aaagaaaatc ctaaaataac
20281 ttataaaaaa agacatttac aagagaaaca aggtgtcaag ggtacaactt tcacgaacaa
20341 attaaaatta ttgaacaaaa tgtatggtgt agatgttgca aaatatcagc cattttataa
20401 ttctaataat cctgaatatg aaagaggtca atttggtgaa agatacattt ctcaaagatc
20461 taactatgaa tttaatagac ttcaatatca aattatagat atgttatcga aaatacttga
20521 taagcatcca ttaccaaagt cagataataa ttataaacat attccaacta ttgaaaaagc
20581 aatattaagt ggggattctc atagttttta tgagtatttt gaagatataa tgaaagaaat
20641 tataagtatg gaaaattctt ctttaaagga aaaattattg actgatttta catatcaatc
20701 tcaatgtaga tggtactctg aaagtgaaaa attaaaattg caacttgaaa gttttatgca
20761 taaagttttg gagagcaatt attatgaggg taacaaatta tatagaatgt tgtctcatgc
20821 aattgaagaa acaatcaatg aggctgatga agataaggtt catttcaatt attttaaaga
20881 ttatttttttg acagacggtg gggtgaagaa ttgggagcag attagcgaga aaattactga
20941 attcaatggt aaagttatta acgatatcca aaatgaatat aataagattc aatttaataa
21001 cgcaaatcga aatcagaaac taaactttaa ttatttatac cattgctttg agtttagtaa
21061 taacttagta aaagcaaggg taaatggaaa tagaggttac attttatatt ttgtagataa
21121 atataaaata aaaatgccct ttttggatca attattaatc aattatttga atcaagaaat
21181 aggtcaggaa agtattaatt ataatatgca aacattgttt gaaaaagaaa gatatgatag
21241 aagtagtacc attgaaaaat tagtagcaac aagcaaattt aagtatgaaa aagatgattc
21301 agatttattc aaacaacttt tcaatgatgt tgaaaattca atagacagat taggtattta
21361 cttactaaat aatggtataa attcgaatga tgaaaatgca agatattata gatcgttttt
21421 aaaggaactt agtagaataa aaagtaaatt aacgccattt tctcttgaaa taagtaagtc
21481 tagcggaaga gagcaacatt atcctgatga tgctattgat gataaagatg agagaagaaa
21541 aaataaagaa gaaacatatc atgcttttga tgataaaagc gatattgact ccaaattaaa
21601 gaataaaata aatgtttcta tcgataattt attgtcagtt aaaattaaga gcaatttgta
21661 tgatattagt ccaaatacaa aaaaacaata actataatga gccaactcaa gaatgagttg
21721 gctttttaat tgtaatttta cgcttccatt acaggaatca atgttatgga agccattaag
21781 caattaattt gagtcattta tagtataggt acaacgttgt gatgttcaca tgataatcag
21841 cccgggagat tattgtgtaa taacttatag aaggagcaaa tcaccatgta tcaagcaaat
21901 atacgtgatt taattacaaa attgcctcaa agcaataaaa cagaacactt tttaatgaac
21961 aaattttcaa atcaagataa agttcagcag ctacaaagac aaattagcca acagctagat
22021 caacaatata atgagctttt ggctaatgaa aaagctaagc tagaccaata cgtggaagta
22081 caccataatt tagaaccatt aaagaaagag attgaatcag aatctattaa ccttgatacc
22141 gataaattac ctgatatcaa agcgacaatg cttgaaaagg ctaagaacga tgaacatttt
22201 gataaaatcg aacagctatt tgatagatta gatcagtcat taaatggtac gaatcgatta
22261 tatacgcaat tatcgttgat tggcacacga acacatcgga tcacaacgaa aagatttaat

22321 gttcaaggct tgcctaaatt agtccaacaa atgattttac cttcgcaatt taaaaaggtt
22381 tatacaatag attttaaatc attcgaacca tcagttgctg cgtatatgac acaagacgaa
22441 caactgattg actacttgaa tcatgaagaa gggttatacg atgcattact gagagactta
22501 tctttgtcaa aagagaagcg cgtgagtgtg aaacgtgcat ttatagggtc atttcttttt
22561 ggcggtcgtt atagtagctc taaattcaaa atcaatcaag aggttagtga aattaactgg
22621 ctacaagtaa tgagcaaatt caagaaggtc attgaattta aggagcaagt cgaaaaatat
22681 aaaacaatgc ctacgcctta cggcattgaa catgatatga gcgcatttca aggtagtagt
22741 attatggcaa tttatgtaca aacggtagca agctatattt tcaagcacat tttgttggaa
22801 gtgtacaaag cacagtgcga aaaaaaaacg ttcaagatta tagtgccgat acacgatgcg
22861 attatgattg aatgtaatga taaggggatt gcacaaaatg tagcacagct catgaaagat
22921 acagctaatc aactgtttaa tggtgaattt gcacatgtga cagtggaagc tttaggaggt
22981 atagacaatg aataatgata gaggaaaaag tcttcaaatt ccccaaagta cattgttaaa
23041 agaaggatct atatacgttg ctacgttaca ttctgtgtac gagaagaact tctcaggtga
23101 tattaaacat cagtttacgt atgaagtaga acttaaccaa gaaacgcact atgtaaatcg
23161 caatattacc gtaaaatcta tgagtcacca attatcaatt gctgattgga ttaaacgtca
23221 cagtaactat aacgtaaacc acattaatta tgatccatac attgatcgaa aacatttggt
23281 ccttgtaggg caatataacg gaaattatta tattcaagat gtagcaccat tagatgaatt
23341 tggaggggta ttgtaatgaa tcatatttta gaaatgttaa taaaattatt aaaagtgggt
23401 atggaggcaa tcgaccgtaa aggtctgatt gccatcctaa caagtagtat tggaaatgat
23461 gaaatggatg attctgaaca agctgtaatg gtgtataacg agcttatcga taagctacag
23521 cttaacattc ctaaagatgt cgactataga cctaacatat acagttattt tggtattcaa
23581 aaaaagccaa atgacacaat attagtggaa atgatgatat gtatttttca tatcaagcgt
23641 tttgattcag agttgtttgt tttcaaagat aaaggttggc aaaaggtaag tgaagatgaa
23701 ttgcaagggt tgatatctaa aatgatacaa gtgttgctag ttgattataa accttcacta
23761 agtactttga aaaacgtagt agatggcata cagaaatcaa cggacataga aaaacttgtt
23821 gagcatagac agtacattgg ttgtggacga aatatgttca atctaaagac ctttaaagtg
23881 gttgataatg accttgaaat attccctaaa acacgcttag atttggaatt agatataaat
23941 gacacgatta cggacaagat acccccgaat ttcaaacaat atatgttaga gttggcgaat
24001 tatgaccatg atttacaata tttccttttc caacatatgg cagtgttatt gacggcagat
24061 actaaactac gtcgtggact ttttttgtat ggaactgcaa aaaatgggaa atcggtctat
24121 attaaattag ttaagtcatt cttttatagt aatgatatcg tatctaaaac acttaatgaa
24181 cttggcgggc gtttcgataa ggaaagtcta attggtaaac gaattatggc aagtgatgaa
24241 gtggggaaag ctaatattga tgaagcaact gtgaatgatt tcaaaaaatt actatctgtt
24301 gaaccaattc atgctgaccg taaaggaaga acgcaagtag aagttacttt agatttaaaa
24361 ctcattttta atacgaatgc tgtactcaat tttccatcat cacatgcaaa agcattagag
24421 cgtagaattg ctgttattcc atgtgaatat tatgttgaaa aagctgaccc tgacttaatt
24481 gaaaagttac aggatgaaaa gaaagaaatc tttctttact tgatgtatgt gtataagcaa
24541 attgtaaaaa atgatatcga gtacctccaa aatgatcgtg ttactgaaat ttctcatgat
24601 tggttaaatt ttggatatga atttgtttct agtaaatcag caaatattgc acatcagaaa
24661 gcgtgtatta atttactcag aaaacttata gaaatcaaac cagggtcacg tatcaaagtg
24721 tctaggctaa atgaggttat tagagatgaa attaaagtaa gctctcaagt tattaatgat
24781 ttggttcaag ctaactttaa tgtacaaagt agactaaata atggttataa gtattgggtc
24841 gatttaggat ggaaagaaac tgataaaaaa gatgacatga tttcattcga taaaaatgag
24901 aatgtaacag atgatgaatt cttatacgaa gatgatttga acttaggttg ggaggacttt

24961 gacgatgaat aatgaacaaa ttgaagcatt tgtagaagtg cttgtgccta tcatagaaga
25021 acgtatcaat aaaggtaagt aatctaatta cgtactacag gcagttgcct gtagtactca
25081 tatgattaag tggtaaaagt gataaaaatg aaacgaaatt ataaatatat attatctata
25141 tgttgttaca agaccgatgg tctgtagcaa taatctaata aaaggagcgg tatgatatga
25201 agggtaaaat tgcactttat tcacgtgtta gtacgtctga gcagtcagaa catggttatt
25261 ctgaaaagga gcaggaacaa ctactcatca aagaagttat gaaaaatttc ccaggttatg
25321 actatgagac atatactgac tcaggcattt caggtaaaaa tattgaaggt cgtccggcaa
25381 tgaaacgtct attacaagat gttaaggata ataaaatcga aatggtatta agttggaaat
25441 tgaatcgtat ttctcgctca atgagagacg tgtttaatat tattcatgaa ttcaaagaac
25501 atgacgtagg gtataaatcg atttctgaga atattgatac atccaatgct tctggagaag
25561 tactcgttac aatgtttgga ctaataggat ctatagaacg ccagactttg atttccaatg
25621 tgaaactttc tatgaatgct aaggcaagga gcggagaggc aatcaccggt cgtgttttag
25681 gctacaaatt atcacttaat ccattgacac agaaaaatga tttagttatt gatgaaaatg
25741 aagctcatat tgtacgggaa atctttgatt tatatttgaa tcacaataaa ggatttaaag
25801 caatcacgac aattctaaat caaaaaggat atcgtaccat taatcaaaaa ccattttcag
25861 tgtttggcgt gaaatacatt ttgaataatc cagtctataa aggctatgtc agattcaata
25921 atcatcaaaa ttgggctgtt cagcgaagaa gtggtaaaag tgataaaaat gatgtgatat
25981 tggtcaaagg taagcatgaa gccattataa gtgaagatgt atttgatcaa gttcatgaga
26041 aactagcttc taaaagtttt aaaccgggtc gacctattgg tggagatttc tacttacgtg
26101 gccttattaa atgcccagaa tgcggaaata atatggtatg tcgacggacg tattataaaa
26161 cgaaaaagtc caaagaacgc acaatcaaac gctattacat ttgttcatta ttcaatcgct
26221 caggaagttc tgcctgtcat agtaatgcga ttaatgctga agtcgtcgaa cgcgtaatca
26281 atgttcattt gaatcgtatt ctttcacaac ctaatgttat taagcagatt gcgtcaagtg
26341 tgatagaaga actgaaacaa aagcatagta aacaaacaga aataaaatat gatattgata
26401 gtctagaaaa acaaaaagca aaagttaaaa cacaacaaga acgattattg gaattgttct
26461 tagatgatga aatggatagc gaaatgttaa aagctaaaca aagtgaaatg aatcaacagt
26521 tagaagtatt agaccaacaa attaaagaag caaaacaagc aaatcaatca caggatgata
26581 tacccaattt tgataagtta aaagcacgac tcattttgat gataacacga ttcagtgtgt
26641 acttaagaaa ggctacaccc gaagctaaaa atcaacttat gaaaatgtta attgattcaa
26701 ttgaaattac gacagataaa caagtaaaac ttgtaaggta taaaattgat gaaagtctta
26761 tccctcaatc tttgaaaaaa gattgggggt cttttttat acctaaattt aactttgtga
26821 taaatgtcac aaagaaaaat aggattgaaa atttatcact tttaccactt ttttagagtg
26881 acaaaagtgg aggagttttg aaatatttta taaatatata ttttatttat ggagtacaca
26941 ttattaatta aggaggtcat tataatgacg ctaagcaaac aacttaaaac gtatatcact
27001 gaacgattta aattaaatta tcaagaaact tgggcttgtg aaaccataga tgcggtggct
27061 gaagatgtat tacctgaaaa atatattaaa aatagtccac ttgaacataa aattttaaat
27121 acttttacct attacaatga tgaattacat gaaatcagca tttacccttt tttatgttat
27181 ctagataagg aattagtagc aataggttat ttagataatt ttgatttaga ctttatattt
27241 ttaaatgaca ctcatcaagt cattattgat gaacgctact tgttacaaaa agggggcgag
27301 taattatgaa ttggatcaag gtcgctcaac tatctgtcac agttatcaat gaagtcattg
27361 agattatgaa agaaaaacaa aatggagggg aatagtatga aaatcaatcg atacattaca
27421 agaggtgtta gtgaacattt atctctagac cttcaaatct tactttggaa catggtaaaa
27481 gatcgagaca atcaacttca tacagattac ctacacattt ttaaactaca agaagatgat
27541 aatatactct caatcacaca tgaacaagaa caacccgcat acaaattgga atatcactat

27601 acaaactatg taaaaaatca aaatgcatta cctaagaaag tctacgtcat ccgagaagat
27661 gatgtagacg ctttttatta tgtgatgctt ttaccagaag aatactaaaa ggagtgacaa
27721 tgatgaatac aatcaaaaat acaatacaca cagaagcgat atttagcgat gatgaacaac
27781 accgctactt actcaaaaag acttgggatg aaaagaaacc tgcatgtacg gtaatcacca
27841 tgtaccctca tctagatggc gtattatcac tcgatcttac aactgttctt atcctcaatc
27901 aattagcgaa ttctgaacaa tatggcgctg tatatcttgt aaatctattt tctaatatta
27961 aaaccccaga gaaccttaaa catattaaag aaccttatga taaacacaca gacattcatt
28021 taatgaaagc gataagtgaa agtgacacag tgattttagc ctatggtgct tatgcgaagc
28081 gacccgttgt tgtcgaacgc gttgagcaag tgatggaaat gttaaaacct cataaaaaga
28141 aagtaaaaaa gctcataaat ccggcaacga atgaaattat gcatccgctt aatcctaaag
28201 cgcgtcaaaa atggacattg aaataaagga ggattatcta tgaaccatga aactaaacaa
28261 tcagattggc gaacggttgc tagttgttta gcatcacaaa attatatatc gattgtaaaa
28321 ggtttagtac atcattttac agcgatcgaa gatgaggaaa ttcttgataa aatctatgat
28381 gattttatga atgatgactc tataacaacg gtacttaaca atgatttaca gacaatcatt
28441 aaccattatc tatcaaaata aagactatac taattacaat aaaaacagag catccttcac
28501 ttttatggtg agggatgctc ttttaattta tttttttatt gattattcgc cataagttct
28561 gcttgctcta caacctgctc tacagccatt ttttgtaaat ctggtggata gccatatttt
28621 ttaagcaatc gtctaacagc tacgcgcatt ttagcttttg cgctatcacg tttagaccaa
28681 tcaacaccca tgttttcttt cactgtttta gttagctcat gagcaatcgc acgtagttct
28741 ttatctccca tggcttcttt tgctgtttca tgtgaagcta aagcatcgta aaacgcaatc
28801 tcatctgaat tcaggcctaa ttcatttcct cgttgttgtt cttgtttaat atctttagcg
28861 agttgaataa gttcttcaat cactttagat gtttcaatgg aacgactatt atatttatta
28921 atcgaatttc ctaacatctc agagaaacgc ttagataccg ttgcattcgt tttcattaat
28981 gatttaactt gtcctttgag taatcgattt aataattcta ccgccacatt tttctgtttc
29041 aatccttcga catcttttaa aaagtcatca gataagattg ataaatcggg ttgttcaaga
29101 cctagcgttt gataaacatc aatgacatct tcagtcacaa cagattgtga cacaagttga
29161 ttaatctctg cttcaacttc tgcaggtgtt ttacgtgttt ttccttcttt tggcggttgt
29221 aacaatttaa caagtcctgc tttaactgct ttaaagaagg caatctcatc gttgagttct
29281 tgggctgtag gttcagtcgc acaaagagca aaggctttcc ctaattctgt gactgtttta
29341 ataaaacgtt gacgttcatc ttcacctaaa ccaattacat aatccatcgt attcgatatt
29401 gtataataac gttcagattt tttatctgaa ttaaatttag agtaatcaag attatataac
29461 atatcttgaa ttacatcata cttcaataac atcaattcaa ccgctttatc cgtatctatt
29521 gccgtttgag cttgatcaga ttctgtatat tctttaagtg cctcttttaa actttcggca
29581 atccccacat aatcgacaat taaaccaccc ggcttatctt taaacactcg attaactcga
29641 gcaattgctt gcatcaaatt atgccctttc atcggtttat caatatacat cgtatgcata
29701 gaaggaacat caaaccctgt cagccacata tctcgaacaa tcacgagttg taactcatca
29761 tccacatctt tcatacgttt ttctaataaa ttacggcgtt ttttaggacc aatgtgtctt
29821 tggaaagaag ttgggtcact agatgagcct gtcattacca ctttaatgac tcctttatca
29881 tcatcatctg aatgccattc tggttttaga cgaatgattt catcatataa atcaacagca
29941 attcgacgac tcatcgttac aatcattcct ttgcctttca tcgcttgttg acgtgtttca
30001 aagtgttgga tgatatcttt ggctagggct tctacacgag gttttgcgcc tgctaaagct
30061 tcaatacggg accattttga ttttaaacgc tgttttacat cctcttcttg atcttcagta
30121 atgtcattat acgcttcatc taaatctaaa ctttgaggta gatttaatgg aattacacga
30181 ctttcatagt aaattttaac ggtgcttcca tcagcgacgg cttgtgtcat atcataaaca

30241 tcgatatagt ttccgaaaac catttgcgta tttttatccg ttgaagctac gggtgtgcct
30301 gtaaatccta cgaatgttgc attcggtaaa gcatctctta aatatttggc ataaccatat
30361 ttaatgcctt cacctttatc atcgtatttt gcgttaaagc catattgtgt acgatgagct
30421 tcgtctgcca tgacaatcac attttttcgt tctgttaggg cagccatggt cgtttcgttt
30481 tgttcaggtt caaatttttg catcgttgta aatacaatac cacccgactc aacagataat
30541 aatgatttta attctttacg tgtttcagct tgttttggtg tttgtcttaa taatccttta
30601 ccagaacgtc cttttgattt aacaaacgta ctgtacagtt ggttatctaa atcattacga
30661 tccgttacga cgactaaggt agggttattc agcatttgaa ttaattttcc agagaaaaag
30721 accatggtta aacttttacc ggacccttgg gtatgccaaa taacgccgcc tttaccatca
30781 ccttgctcag atgaagctaa taaagctctg tcaacggctt tattaacggc ataatattga
30841 tgatacgctg ctagaatttt actgatatgt cctttgccat catcttgaaa taaaacaaaa
30901 tatcgaatta aatcaagtaa tgtttctgga tttaacatgc catgaatgag tacgtctaag
30961 ctagctaaac ctggtgatga ttcagtttga ccatctttac tgcgccaagt cataaaccga
31021 tcataatttg cggttagaga gccagccttt gtattaatac catcacttgt aacaagcact
31081 tcgttaaacg taaataattg tggaatacgc atcttatacg tttctaattg atgataaccg
31141 tcttcgacgc ccacggtttc atttgttgaa tttttaagtt caatcactac gataggcaag
31201 ccattgataa agagtacaat atcgggacgt tttttatagt ctccattaac gatagttaat
31261 tgattgactg ctaaaaaatc attgttttgt ggatattcaa aatcaataat tttaacaatt
31321 tctacaatcg attgtccttc atcatcatag tcttcgattt caataccatt aatcaaattt
31381 tcatgaaagg taaggttatt ttctaaaaga ttgggcgact tctctaaagt tagttcatgt
31441 agcgcttttt caataaaacg aggatggata tctgagttga tttttcttaa tgctttctct
31501 aaacgttcat gaaggacaac atctttatca cttttacgtt ctggtgttag acttgtcata
31561 ctaatctcat taccttttt atagtcatag cctagtgatt gtagccattc taatgcgact
31621 tgttctaaat catcttcatt aaattgaaag ttcatcacta ttcacctcaa tatcatcaga
31681 tatttctagt tctccagaca ttagtttagg aagaagagta tctcgtaatt gagttaattt
31741 accaatttct gatcttaata ttttcatttc ttccatatat ggagtgatta tttcagaata
31801 tttttttaca atactatctt caattgctaa tttataatta acagctattg ttggttttac
31861 tctttgtcta ctatttgttg aaccggtagt attagctttt aaataatcta taaattgaga
31921 attaagacat atattatata tgaatgaatt aattttatta ttaggagatt ccattacaac
31981 aaattctgat gaagcaacat ttaatttttt attatctatt acaggtaacc aaatcctttt
32041 agtatcagga ttcattttg aaaataatac gcaattgtta ttaataatcc atttattact
32101 ttttatttta ttgacttctt cctctatagc ttgttcttca ttatcataag caggtaaact
32161 aaaatgttta actgttactt cttcactctt tttaggatta aaagtttcct ttttatgact
32221 tgctatatct tttaatttat agattttcca attacttggt atcttaccta actcactatc
32281 aatcatttct cctccactag atttataagg attaccatct tcatctggaa attcaaaatc
32341 tacaaaccaa tgcttaaata gtgtttgtga aagttcttta aggtttgcta tcattttttg
32401 gttagtttct atttttcttg tgatttgatt taaaaattta gaaatgcttt tttgttttat
32461 taaagaaaaa ttaggaattt tgacagtttc aataagttta gaatttatat tagcttgatt
32521 agcactccct gtagaatttc taactaaata ctcagttatt ttaggttgta gaaaataata
32581 aaataaaaaa tctaagtctg cactattact atattttaat tcgaattttc ccacacgttg
32641 attttgtaat gctggataat caaattcaat tcttccaact tttcctactg cagaagacat
32701 ttgattcacg cctgatccag ttaatgatat taatatatca tttttcttta atgaaaaacc
32761 tttagttttt tcatatgttt taatagagac tttttgagag tcttttgtat caacaatggg
32821 tgaaattata ttcttaattt taataaccgg tataccttttt tcttgaaatt cttttgattt

32881 aaaagcatat ccatttttaa ttttagctac atcaccgaaa aaaattgtat tatactccat
32941 atcctaaccc ctccaatgat tttcggattt ggtcttcaag ttcttttgat tttgcgaatt
33001 gttcacttaa ttcagaagta atacgttcca ttttttgttc aaaaggttct tcatcttctt
33061 caacgtcagc taaaccaaca tatcgtccag gtgttaaaat gtattcatta ttcttaactt
33121 cttctaaatt agctacttta caaaaaccag ctatatcttc ataaggttta tcgtttgtac
33181 ctctccacgc atgatacgtc tgtgctactt tttgaatatc ttcatctgaa aattctttta
33241 atgttctaga taccatatgg ccgatttcac gagcatcgat aaataaaatt tcattttcac
33301 gctctttttt accattttga cctttattat tactaatgaa ccaaagacat acaggtattt
33361 gggttgagta aaagagttgt ccaggtaaag taacaataca ttcaactaaa tcttgttcaa
33421 ttagattttt acgaatttct aattcatctt ttccgcttgt agacattgat ccgttggcta
33481 atacgaatcc tgctgtacca ttaggtgcta attttgaaat catatgttca atccatgcat
33541 agttagcatt accttttggt ggaataccaa attgccaacg gtaatcatca agtaatcgtt
33601 cttgacccca atcacttgcg ttaaaaggag gattggctaa tatgtaatct gcttttaatc
33661 ctttatgtaa atcattatgg aatgtatcag catttcgttc acctaagtca ttatcaatac
33721 cacgaatcgc taagttcatt tttgctaatt tccaagtcgt agggttagat tcttgtccgt
33781 aaatcgcaat atcgtctaat cgaccttgat gtttctcaac gaagcgttca ctttgtacaa
33841 acatcccacc tgaaccgcaa cacggatcgt aaatacggcc tttataaggc tcaatcatct
33901 caactaataa ctttacaatt gatgacgggg tatagaattc tccagcattt ttaccttcgg
33961 cacttgcgaa tttagcaata aagtactcat acacacgacc taacacatct tgtttacgac
34021 tctctgtgtc gccaacttta aatgtaaata aatcaataat atcgcctaat ttttctttat
34081 ctaatgcagg tcttgcatat tctttaggta atacaccttt gagtgattca ttttcatttt
34141 caattgcaat cattgcttta tcaataattt gtccaatttc tggttttttc gcattatcat
34201 tgatatattg ccatcttgct tcttttggca cccagaaaat gttttctgct aagtattcat
34261 cttggtcctc ttcatctgca tatggatcct gcttcaattc ttcatacttt tcttcaaaag
34321 aatctgatac atattttaaa aagattaaac ctagtgctac atttttatac tcagcagcat
34381 ccatacttcc tctcaattta tcggcagctt gccataattt ttcttcaaaa cctatagtgg
34441 ccattaataa cacctcttaa aattaatgtt aaatcaacta tagcaaaaaa tagaagatga
34501 ttgtgtagtg ttttattaat atttaattta tctagactat caaaaaaata tagattatta
34561 ttatcttaaa ttaataaaca ggagtgaatt aaaatgaata aaaaatataa attaaaacga
34621 tacgataaaa gatttagagg tggatggtgt tacaaatgtg aagattcata ttcttacctt
34681 tgtgaagatt gtggttattg tagatattgt tgccattgtt aatacaagac tagataaatt
34741 gggtatcttt aactcaatta ataaaaaatt taaatgtagt caatagcttg ctaactttat
34801 ttgatgtaat ataattttgg cttttagcat tcagttaaaa agaagtaaat aattgacaga
34861 ttttttagag aactggagaa aaaagatgta catttctagt gttaaaatta agaattttag
34921 aaattttgat aatatcgata ttgatttcca tgagggagtt aatgtattaa tcggacataa
34981 taattcgggg aaatcaaatt tattacgtgc tttatcttta atttttgacg gttcagttag
35041 aaagcaactg tcagtggaag attttaataa ctctttaaca aaagaatctt taaaaaaaga
35101 ggctccaaaa atagtaattt cagtacatat aacacaatct gaaaacgaaa gactgatgtc
35161 tgatgagtta attacggtaa gtaattggct gactaaatta gaagaacctt atgaagctag
35221 aatacaatat gaattctttt taccaaaaga tgaggaaaaa aattacatag atttagtaaa
35281 aaatatagat gaaaaagaag aaatatggaa tttaattaga agccagttta ttaggttgta
35341 tgtcaataaa atctgggtgg gtaatccaga acatcagatt cctatagata atgatagttt
35401 aaataaattt gatttccaat ttttagacgc aataagagat gtagagagag atatgtttag
35461 tggtaaaaat acattattaa aaagtgtaat agattttttt atagattacg atataaaatc

35521 cgatgaaaca atcacagaag aagaacagaa agaaaaatta gaagaaagaa gaaaagaatt
35581 ttctgataat tctagtgatt tgattgaaac tattcataaa agattagata gtgggaatca
35641 aaaaattttg tcttatacaa atggcatagg agcatcttat gataaatcaa caccggattt
35701 taaaggtaat cttacagaaa gtgaaattta tactgtattg caattgataa ttaaacatga
35761 aactggaatg actttaccta taactcataa cgggttgggt tacaataatt taattttcat
35821 ggccttgcta ttgtctaaaa tgcaagctga ttctgatgga aattttctag gtagtaatgc
35881 aaaagtattt ccgatactag ctattgaaga accagaggct catctacatc ctactatgca
35941 aaatgaattt attaaatttt taaaaaataa cattagagaa aaaaaggtaa agcagatttt
36001 cataactact cattcaacac atatttcttc ttctacaaat attgatgata ttatttgtct
36061 ttacacagat gatagtaaaa ctaatgttag ttatcctgga aaagtttttg ataagaataa
36121 tcaaagtaag aagtacgtac aacggttttt agatgccact aagtcaaata tgctatttgc
36181 agaaaaggta atatttgtgg aaggtatagc agaacaatta ctattacata tatttgcaga
36241 ctatttaaat aaaccgttag aaaagaatca tgttgcagta ataaatgtag gcgggagata
36301 ttttaatcat tttttatctt tgtttgatag taataataaa tatgcaatta atcgtaaggt
36361 ttcttgtata acagatttag atccaatgcg aaaagaaaaa aatgaaaatg gacaaaaaaa
36421 taattttaaa gcatgctatc cttttgaata tgaaatggat ttagataaat ttgattattc
36481 aactaataat tctctagata aatatctaaa taatgagcac agtaatatac gagtatttgt
36541 tcaacctaat aaatatggaa aaacatttga gtaccaactt atgtttgaca atccttcatt
36601 aaaactgtta cttacagact cgatttctaa tagtcaagag ctagcagaat taatggatca
36661 ctatgaagaa aatgaatctt tacaaaaatt aattgataca ttatcaccat ctaatgaaaa
36721 tcagagaata ataaaatctt taaaagacac aaatgacagt tggaatgaag ataataaaaa
36781 gaaagcatta atagcatcaa gatacctaaa ttccatagga aaaggagaaa atgctctaga
36841 gctagcttca gtattaaaag ataatttaga gttaaaaggc cagagtgact atgaagattt
36901 tgtagtacca aaatatatag aaggtgcaat tcgatgggtg tgtgaataat atgattatta
36961 atgctcaaac gaaattaaat atagaacaag attttaaaat acaggctggt cctggtgctg
37021 gaaaaacaga atttttggtg aatcatatca aaaatgtaat acaaacttct gaaaagctcg
37081 aacgtatgaa gaaaatagcg tgtattactt atacgaatac ggctgctcag acagttttac
37141 aaagattagg aaaaagtgtt tcaaatagag tagatatatc tacaatacat agctttttgt
37201 atagaaacgt agttaaacca tattgttctt ttctacccaa agaatacgga atttgtgtgc
37261 aaaaattaaa aggacatagt gatccaattg tgattaacaa atatgtaaat gcatggttaa
37321 aaactgaata ttttacgaaa ttaaaaccgc ctagtaatag aaatcaacta ctaaaaatgc
37381 cagtactaaa gaaagctttg caaaactggc tactatctat gaaactttct tacgaaaata
37441 atgatgtgca atttatttgt gataatacca aagctcaagc aattgataaa aaaagtaaaa
37501 aaagaatggg aataaataaa tcaaacttaa atatactaga aaagcaatta ttaaattata
37561 aaaaaatata ttgggagaaa ggaattatcg atcatgatga tgttttgttt ttttcatatg
37621 tattaattag agattaccca ttcattctca ctattttatg tgctaaatat ccctactttt
37681 ttatagatga atttcaagat actagtcaat tacaagattt tattatagat aaaataagac
37741 agaaaggctg catagtgggg gttattggcg ataaggcaca ggcaatttac agctttcaag
37801 gtgcacgggt ttccttattt gagaagttta aagttgattt aataaattct cacactatct
37861 tagaaaatca tagaagttca tatcaaatag ttactttttt aaatagtatc agagaagata
37921 ttactcaaac agtatatagt ggtattgaaa atgataaagt tacaattttg attggaaatc
37981 ggattcgcag ttatactaag gtagttaata tttgtaacaa ggagtcactt gttaccttgt
38041 caagagataa tattacttct aatgcaatga gaataaagtt ggaaggaaat aattttgata
38101 aaaagttatt ggaaaaattt caagaaattg atagtaatag taaaagaagg aatactatta

38161 tttcgcttat aaaagctatt gaattagcta agaatactaa atataaagat gccttaaaaa
38221 tagtcgaatc gatttataaa gatgaaaata atcctaaaaa aatagctctt aattctctaa
38281 taaatatgat taataaatat gataaatacg gtaatggtac attaatgaat ttctacaata
38341 ctttatgtaa aactttagat attaaacttt cagggtttag aaaggggcaa gtaaaagatt
38401 tttacgaaaa cactttatat aaaaatatgg tgatatgtac aaatatcgtt gaagacacaa
38461 gtaatcatat tactatccac aaagcgaaag gcgcagaatt cgaaaatgta tttgttatag
38521 gtaataaaga cactttaagt ctacttttga aaccagattt aatgaacgat gaagagcatc
38581 gtatattcta tgtagctatg agtcgagcaa agaagaaact ttttttacaa tttgattatc
38641 ttgataataa tgaacaaaag aaaattaagg aacgttatga tgtaagtatt atacaataac
38701 ctaatctaag aatatttaaa tccaatatat ttcttgatta taattcaata atagataaaa
38761 tctataaacg attaaaaaga aaatatattt gctaagactt ataaaaatga agagataata
38821 gattttccta aattacagtt aaaagaagta aatagattga acactttgaa agattctaat
38881 ccaaaatttg aaccactaaa attgaaagat gaagaagatg caagatgtag ccatcaacgc
38941 tgatcacaaa gtatttttct taatgattat ttttaatgtt atcatgctcg tttcttctat
39001 tattcaaatg attttaggaa aaggcagacg tatcacacca gcaaaaccta ctaaagatgc
39061 taagttacac ttagataatc aatcctcata aagagaaaaa agaacccctа aaaagttcaa
39121 ctttttgggg gttacttact attaagaagt atttttgatt aagcatcttt catatcgtaa
39181 tcaccaatat actgatcaag gcgatttatt gtttctctga tatcatcatg tgtcgttaat
39241 gggttcgttg tacataaacg aatgacacgt tggttattca atactgtggt ataagcaatg
39301 gcataaccag aatctgcaat tttttgcgca gccatgctat tgagttgatt attttgtgct
39361 tcagttaaat cactgttttc atatctgaag ttcacaatgg aaagattagc gtgtgagata
39421 atacgccagt tatcgagacc tgacacgtat tcttctgcat attctgctaa accttcgccg
39481 tatttaatgc gctcgataat ttcatcttca ccaatgactt gcagagtaat ccataatttt
39541 aaagcacgtg cagggcgtgt aagttcaatg cctaacattt ctggatcaat cacatcatca
39601 tctgaagcaa tatcatccaa gtactcagca tcttcgccaa agctttgcag cagatgattc
39661 ttttctttca cgattaccat agcgcaacta tatgtttgga acagtaattt gtgagcatcc
39721 caactggcac tatctgaacg ttcaatgcct ttaaataagt gttgtgcttt atcggataag
39781 atatgtgata gtccataagc accgtctaca tggagccata gattgtagtg atcacatgta
39841 tctccaagtg tggtgaaatc atcgacagaa cctgtattag tcgtgcctgc agtcgcaatc
39901 accatagcag gtttgtaacc ttcttcaata tctttatcaa ttgcggcttt taagctatct
39961 gtattcattg taaagtcatc gttgtactca attctgcgga tattattttt aaggaagcct
40021 gcaacatgca aggctttgcc tacagaatga tgcgtttgag atgtaagata aacggttgct
40081 tttttaatat cttccatttc tacctgtgca tctctagccg caacgatagc tgttaaattg
40141 gccattgaac cgccagatac aaagacacca ccggccggtt tgatctcata accgattttt
40201 cctaccaagt agttaattaa attacgctca atattaattg gcagcgtcgc attggcaaaa
40261 ttagaggcat ggatattatt tgctgttgtt aaaatgtctc caagccatga tagacgtgat
40321 gctggacctg gtataaatga gaatgaacgt ggatggttcg gacgatataa ataattcagt
40381 acttcattat ttaaatcttt gagtacttca tagatatctc gcccttttatt cggcacttcc
40441 atcttttcat atttctctct caattcgagc ggtgcctgtt gtgtcgcagg taagtctcca
40501 atatctctag ataaatattt attaacttca tctctgataa tcgtctctaa gtcgatattg
40561 ttctcattga attccatttc catactctct ccttcaatta tatttttaat aaaagacaga
40621 cgtattaatt acgacgtttt actacaatat aaatgatagc agatattaat aaaaccacgc
40681 taaccataat gacgaaaccc agaaggtgtc gccccaatga ataacgtgct gtgctttaac
40741 actcggtgta tgtgcgcctt tcagaaattg acgagggtaa tcgacttgca gattatcatc

40801 tgtgattttc aatttgtatt gatgcttgtc ttttggaatt cgataactac cccgtagaac
40861 aggggacgag gacctcatct tgatgatccc aattggcttg gttaaaaaga aatcgataat
40921 cctcgtagaa tagagaaaaa gtattaaaaa tatatttgat ttgataaaat attttgcaaa
40981 aaagagaaac aaaaataact ttcatataaa taatttcttt gcgattaagt tggaattaga
41041 aatactaaat acaaataaca gcgaatggaa tgcatattat tgaggaattg gatgtgtagt
41101 gcaatttcga gagttactac ttaacttaag aaaataaaaa gaagaaaaca aacaataaga
41161 attaaatctg aagcaagata taaaagcaaa tatttttgct acaattatat tagtaaatac
41221 tttattaatt agcatacgac tgaaagcgaa gtaacagcta ataatgttcc agatatagac
41281 attagtgtta tgaaagaaca atattaccat ctagaaaatg taatttttaa aaacctgaag
41341 ataaagtaga ctattctgtg agttttcatg ttaagaattc tgaacttggg gatgtagtat
41401 aaatatggtt tagtagaaaa tgacgctgga gctgtagctg agattgacta tcaagaatta
41461 actaaagtag attattaccc atcaacaaaa atccaagatt taacactatc taataatagt
41521 gcgaataatg aagtttaaaa gtgaaaaact gataatagcg aatcaaacta aagtaatcaa
41581 ttacagaatt caagtactac tcaagcctca aacaaatatt gaaactcaaa cacaagttaa
41641 gaataataca ctgtcagaaa cagatgaaca gtctaatgcc gatttagtcc agtaatagcc
41701 tctgtattat tagccgcacg attattatca ataattaaat aaagttaata ttaaataata
41761 aaaatccttc aaattatttg ttgaaaaaat aataactttg aaggtttttt agcaaaatca
41821 ctgatatggga gaagcgtatc ataa

//

LOCUS Staphylococcus 65755 bp DNA linear UNK
DEFINITION Staphylococcus Staphylococcus aureus strain NCTC9944
ACCESSION Staphylococcus
KEYWORDS .
SOURCE Staphylococcus Staphylococcus aureus
ORGANISM Staphylococcus Staphylococcus aureus
Bacteria; Bacillati; Bacillota; Bacilli; Bacillales;
Staphylococcaceae; Staphylococcus; Staphylococcus aureus.
FEATURES Location/Qualifiers
source 1..65755
/genome_md5=""
/genome_id="703339.303"
/db_xref="taxon:703339"
/project="hwwang_703339"
/organism="Staphylococcus Staphylococcus aureus"
/mol_type="genomic DNA"
CDS 264..569
/db_xref="SEED:fig|703339.303.peg.1"
/product="hypothetical protein"
/translation="MKGNIQCLRLIKGSLLDFVLETVDVFVNGSKNDCELVNFNYKEG
KVVSRYSTKFESNPKLRKEQLISTVKVVRYVIFDFEKILWGIGKVLLKFIILRQCLL"
/transl_table=11
CDS 767..1630
/db_xref="SEED:fig|703339.303.peg.2"
/product="hypothetical protein"

/translation="MKHINHFSESTLAYLEKDHEHAFYVYCLIDPRNDECFYVGKGKG

NRIFKHKQDAQRQLLYEDIFREENKDNLKFNRINEICSNDLNVLGYIISYGLTESEAF

SAENVLINFLNLTNKTTLTNMINGHGSKAYLVEDLENEFGYDSINLENINTNELILAV

KIRDAFLLDKDESKEYPINESKRDRNNLKSRTLGSWIIGKDKIHKIKYIIGINTGANN

AVVSAYEVSFEQAESIETNNGRMRYAFIGLSERDATLKKLNLYKKALPDLRFGSGSAT

AYINNGTMKVD"
/transl_table=11
CDS 1738..3213
/product="hypothetical protein"
/db_xref="SEED:fig|703339.303.peg.3"
/transl_table=11
/translation="MPSDDIVKKLYSKEDIRLELGLTPHKFNKKMETIAKLFKIDMKI

FHNYKGQDKNNQYTFNGVAKELIKVLLKSVDYYPVDINSKKFKQNGKSKKEMIENIDN

SSYMKYIYQLMKSINEIQYKRLIADIHMKDVYQNTKAWLNNGESINKKEQELYQYMTI

LPLHKRVELQNEVLKSIDETIFQFLAKEHRNNQIEENNELEAYTKAIKEGRNPKNDYE

LNHLLYKKNQLPLDSLIEDIWDYEETEYTELDWLIADMLKRSQRADSNFIESLEKKNK

LRKNIHKDSIKSISKLIDHELVKNNRKWDVAEFYKNQQFWNNSTLRRSSQNFGILYYK

KNRLHTMNQSNIIKKIKIIEANIESANKQPSYLDFFLQAKFDLERLESELMKYSINPK

YFNKINSDYVRYAIEDVHNAIIKIDRYISNDKVKLNYSNVQMVVRNEITEQGSYFVTQ

ALNTLSKVEESGYSFDNFMVGPFVEDFKKAIKNRRFGSKKD"
CDS 3286..4539
/product="hypothetical protein"
/db_xref="SEED:fig|703339.303.peg.4"
/transl_table=11
/translation="MFVGFIFFGDFLITQLHLRIISLNYTLSRPRQIKIKAKVSAQLS

AIKEQIYMETRDKLMSLTQSDKTQQWLMDKSSNQDDIQQLQQQFSQQLDQQYNALLAD

EKAKLDQYVEVHQGLESLKEEIESEPITLNIDKLPDIKATMLERAKNDEHSDKIEKLF

DRLEQALNGTNRLYTQLSLIGTRTHRITTKNFNLQGLPKAVQHTILPSKFKKVYTVDF

KSFEPSVAAYMTQDSKLIDLLNQKDGLYDALLSELGLSDELRVFVKRAFIGSFLFGGN

FKNPKFKLNQYVSEVQWLDAVSQFTKVIELKKHVEKSNSMPMPYSIEHDMSAFQGSSI

MAIYVQTVASYIFKHILLKVYKAQCDQKTFKIIVPIHDAIMIECEDEEIAQNVGQLMK
DTANQLFNGEFAHVTVEEIGGVDHE"
CDS 4532..4903
/db_xref="SEED:fig|703339.303.peg.5"
/product="hypothetical protein"
/translation="MNNDRGQSLHIPSSTPIKENNIYVATLHSVIQTDFSGEIKHQFT

YEIEVNNQIVYANRNIPTKPSANQLSIHDWLKRHSNYSVGHDTYEPYIDRKHLIHIGQ
YSGNYYVQDVASLDAFGGVLS"
/transl_table=11
CDS 4900..6543
/transl_table=11
/translation="MNHILQMLSKLLSVAKEAIDRQGLIAILTIPVNNNDEIEETAQG

ETVYNELIDQLRLNIPKDTDYQPNIYSYFGIKKNPNDTVLMEMMIKVFHIKRFNSELF

IFKVNGWQKINGDELQGLISKMIQVLLVDYKPSLSTLKNVVDGLQKSTDVEELVENER

YIGCGENMFDLNTFQVVKNSIDIFPKTRLNLSLSTNDVITDKIPPYFKQYMLQLANYD

DDLQYFLFQHTAVLLTADTKYRRGLILYGGAKNGKSVYIELVKSFFYSKDIVSKPLNE

LEGRFDKESLIDKSLMASHEIGQSRIQEKIVNDFKKLLSVESMHVDRKGKTQVEVILD
LKLIFSTNAILNFPPEHAKALERRINIIPCEYYVEKADTSLIDKLQSEKKEIFLYLMY

VYQQIVKADIEYLENSRVTEITHDWLNFGYEFVSSRSVSNANQKACINLLRKLIEIKS

GSRIKVSELNKVINEEIKVSSQVINQLIQANFDTQTKLYNGYDYWIDLGWKEANKKEI
HDISEKDNIISLDKNENITDDEALDEENLDFDWEDFDDE"
/product="putative primase"
/db_xref="SEED:fig|703339.303.peg.6"
CDS 6768..8447
/transl_table=11
/translation="MKGKIALYSRVSTSEQSEHGYSVKEQEQVLIKEVVKNFPGYDYE

TYTDSGISGKNIEGRPAMKRLLQDVKDNKIEMVLSWKLNRISRSMRDVFNIIHEFKEH

DVGYKSISENIDTSNASGEVLVTMFGLIGSIERQTLISNVKLSMNAKARSGEAITGRV

LGYKLSLNPLTQKNDLVINENEAHIVREIFDLYLNHNKGLKAITTILNQKGYRTINQK

PFSVFGVKYILNNPVYKGYVRFNNHQNWAVQRRSGKSDKNDVILVKGKHEAIISEDVF

DQVHEKLASKSFKPGRPIGGDFYLRGLIKCPECGNNMVCRRTYYKTKKSKERTIKRYY

ICSLFNRSGSSACHSNSINAEVVERVINVHLNRILSQPNVIKQIASSVIEELKQKHSK

QTEIKYDIDSLDKQKAKVKTQQERLLELFLDDEMDSEMLKAKQSKMNQQLEVLDQQIK

EAKQANQSQDDIPNFDKLKARLILMITRFSVYLRKATPEAKNQLMKMLIDSIEITTDK
QVKLVRYKIDESLIPQSLKKDWGSFFMPKFNFVINVTNENRIENLSLLPLF"
/product="Cassette chromosome recombinase B"
/db_xref="SEED:fig|703339.303.peg.7"
CDS 8536..8874
/db_xref="SEED:fig|703339.303.peg.8"
/product="hypothetical protein"
/transl_table=11
/translation="MTLSKQLKTYITERFKLNYQETWARETVDAVAEDVLPEKYIKNS
PLENKILNTFTYYNDELHEISIYPFLCYLDKELVAIGYLDNFDLDFIFLNDTHQVIID
ERYLLQKGGE"
CDS 8877..8966
/transl_table=11
/translation="MNWIKVAQLSVTVINEVIEIMKEKQNGGE"
/db_xref="SEED:fig|703339.303.peg.9"
/product="hypothetical protein"
CDS 8968..9279
/transl_table=11
/translation="MQINRYITRGISEHLSLDLQILLWNMVKERDNQPDTDYLHIFRL

QEDENILSITHEQEQPAYKLEYHYTNYEKNQNALPKKVYVIREDDVDLSYYVMLLPEE
Y"
/product="hypothetical protein"
/db_xref="SEED:fig|703339.303.peg.10"
CDS 9295..9801
/product="hypothetical protein"
/db_xref="SEED:fig|703339.303.peg.11"
/transl_table=11
/translation="MKTIESTLETEAIFSDDQQHRYLLKKTWDSEKQKITIITMYPNY

TGLLRIDLTTQLIMNKVSEMDAFGSINFVNLYSNITTPINLKHLENAYDNHTDIQIMK

AVKESDEVILAWGAYAKKPVVESRVNEVLEMLKPHKKKVKRLMNPATNEIMHPLNPKA
RQKWTLKA"
CDS 9822..10139
/transl_table=11

/translation="MKEINIVSLQMIKTDTLSYLKNRISNPEDAAEILRSFIGNSDRE
HLILICMNSKNEPTHIQILSIGSINQTVIHPREIFKTAILSNANSIMLGHNHPSGDIL
TIV"
/db_xref="SEED:fig|703339.303.peg.12"
/product="DNA repair protein RadC truncated by transposon
Tn554"
CDS 10258..11343
/db_xref="SEED:fig|703339.303.peg.13"
/product="Transposase A from transposon Tn554"
/translation="MKVQRIEVENKPYPLYLLLDKEYQLIEPVMKFIKYLDNTGKSPN

TIKAYCYHLKLLYEFMEQRGVILNDINFELLADFVGWLRYPSASNVIDLQSKKAIREE

TTVNTILNVVMSFLDYLSRLGEFKSIDVFKQAKGRNFKGFLHHVNKGRYQKNVLKLRV

KKKQIRTLRSKEVKQIIDACHTKRDKLILMLMYEGGLRIGEVLSLRLEDIVTWDNQIH
LTPRDVNVNEAYIKLRKERTIHVSKELMSLYTDYLIYEYSEELEHDYVFISLKEGYFG

KPLKYQSVLDLVRRIVKRTGIEFTSHMLRHTHATQLIREGWDVAFVQKRLGHAHVQTT
LNTYVHLSDQDMKNEFNKYLERKEHKK"
/transl_table=11
CDS 11340..13232
/transl_table=11

/translation="MNASSKRKIISQSEISKKIAVMNEEMQGFWANNSWDIRKCPHPS

AIELSKNPALRNRWVRFERVKNLWLRTELKYFYFYHLNNGIWNAKTVWIRKGTVINKM
LDFLDLKYPSITSITEVPIEKAMTEYRTYLTKRGVRITTTNYKITANQEKTPVKANSY

YVTNLKQFMEFYENFYFDGEEWDKDVWDRRNLPLPDDKVNPTQYEYTINFKGFRNTYF

KQLVKRYCKLRLNVDSFSYVSDIAQRLKEFFNFLDMKFKRVQRVHQLTRVEIEAYLSE
LNMMGIKPSTITGRISILEGLFSTLLRLEWDDVPSKILIYSEDYPKIPRAKPRFIDEF

VLEQLNSHLDKLPEYIATMTMIVQECGMRISELCTLKKGCLLEDKDGDFFLKYYQWKM

KKEHIVPISKEVALLIKVREDKVSEEFPDSEYLFPRKDGSPLKQETFRGELNKLAYEQ

NIVDKSGEIYRFHAHAFRHTVGTRMINNGMPQHIVQKFLGHESPEMTSRYAHIFDETL

KNEFTKFQEKLVTNNGDVLDLDEDNEVDDVELQWFKKNINAQVLPNGYCRLPVVAGGC

PHANACLDCTHFCTSKQFLPQHEEQLERTEELLAIAKDKQWQRQVETNSRVKERLEQI
IGSLTG"

/db_xref="SEED:fig|703339.303.peg.14"
/product="Transposase B from transposon Tn554"
CDS 13239..13616
/db_xref="SEED:fig|703339.303.peg.15"
/product="Mobile element protein"
/transl_table=11
/translation="MDKQVRNTTEIVRLAKQKSKKTREKVDKAISKFSIEGKVINFNS

IAKEANVSKSWLYKEHDIRQRIESLRERQITANVVSKPKKSSRSEEILIKTLKRRVME
LEKENKKLQNQIQKLYGDLYNKE"
CDS 13767..14066
/transl_table=11
/translation="MSNLINGKIPNQAIQTLKIVKDLFGSSIVGVYLFGSAVNGGLRI

NSDVDVLVVVNHSLPQLTRKKLTERLMTISGKIGNTDSVRPLEVTVINRSEVVPW"
/product="Aminoglycoside 9-nucleotidyltransferase (EC
2.7.7.-) => ANT(9)-I"
/db_xref="SEED:fig|703339.303.peg.16"
CDS 14157..14549
/transl_table=11
/translation="MAIVLAQARKNSISLFGPDSSSILVSVPLTDIRRAIKDSLPELI

EGIKGDERNVILTLARMWQTVTTGEITSKDVAAEWAIPLLPKEHVTLLDIARKGYRGE
CDDKWEGLYSKVKALVKYMKNSIETSLN"
/db_xref="SEED:fig|703339.303.peg.17"
/product="Aminoglycoside 9-nucleotidyltransferase (EC
2.7.7.-) => ANT(9)-I"
CDS complement(14674..15405)
/translation="MNQKNPKDTQNFITSKKHVKEILNHTNISKQDNVIEIGSGKGHF
TKELVKMSRSVTAIEIDGGLCQVTKEAVNPSENIKVIQTDILKFSFPKHINYKIYGNI

PYNISTDIVKRITFESQAKYSYLIVEKGFAKRLQNLQRALGLLLMVEMDIKMLKKVPP

LYFHPKPSVDSVLIVLERHQPLISKKDYKKYRSFVYKWVNREYRVLFTKNQFRQALKH
ANVTNINKLSKEQFLSIFNSYKLFH"
/transl_table=11
/EC_number="2.1.1.184"
/db_xref="SEED:fig|703339.303.peg.18"
/product="23S rRNA
(adenine(2058)-N(6))-dimethyltransferase (EC 2.1.1.184)
=> Erm(A)"
CDS complement(15463..15594)
/transl_table=11

/translation="MHQYRSSRNYFISFIMKKWKGDKSMGTFSIFVINKVRYQPNQN"
/product="hypothetical protein"
/db_xref="SEED:fig|703339.303.peg.19"
CDS 15913..16575
/product="Mobile element protein"
/db_xref="SEED:fig|703339.303.peg.20"

/translation="MNTIESQFDKVAEDYDFVNELLNDYSFFVSNMSPKKGRALDIGC

GSGLLVEKLASYYDEVVGIDISNQMLDLAKSKRQLTNTVYLNMNAEQLNFNEKFDFIV

SRTTFHHLDDIASVIQQMKELLNEEGRIVILDNVSEVETPPTYVYKLGAIQEFLPHCF

KFGIKNAIRIYNHNTSKSWLEHLASDKYLSEQNYYDLYEKLLPGCQFHKMGWAMGVVW
TK"
/transl_table=11
CDS 17123..17995
/translation="MDRAFRILTIYNRLLQNKSVNKQSLTLELDTSPRTIQRDIDDIR
NFLYESKSWISTPKEITYDYKSESYKLEQDKSKNVHFMYNILTALYLTTPKLSHYFYQ

YLKLLIIKHHSNHRTTLLKYLERFEIDKNQTGISTSSLVVQAMNENKYLQYNQKILLP

LSMYYQMHAFHLVYRMNDEIYIDDINQMNLSFSEKSVDTHEKAKVQTYITFEISKDVW

SKMHRYYQIHVIEKYDAHYLIVTFKMTRIEAIQLCFMYRSNIRIISPPDLKEQVIDEL
LLLQTTYLKQQVQK"
/transl_table=11
/product="hypothetical protein"
/db_xref="SEED:fig|703339.303.peg.21"
CDS 18052..18342
/product="Predicted cell-wall-anchored protein SasC
(LPXTG motif)"
/db_xref="SEED:fig|703339.303.peg.22"
/db_xref="GO:0016020"
/transl_table=11
/translation="MSNIIKTIGKGIGKGVGAAAYYSVATVGAAIGAIWETTGDRGLM
RDALSDSSNQDLMEIYEIQKKDDGDYDTYIAAKSLLQERGFTYNHETNKWEK"
CDS 18358..18846
/translation="MEFTYYETKSLNDFENELKTLALNKKYTFSKETGVLKEDEILEK

SNSHERELLKVFKDNAQITTYHFKSHKSNYLIIMGETKAPNIIGLTVIFNKKPFKQMG
KIALGWLIALTSGGSILAIGAVFGILTSVARRTLVIKGPLGKEISQIIENELGEPIEI
KK"
/transl_table=11

/product="hypothetical protein"
/db_xref="SEED:fig|703339.303.peg.23"
CDS 18981..19508
/translation="MKKIFASLGVILLVAILGVAVVFAYGSYKDLELKKEEAKLEDKK

NKKIDKNKTTENENQDQINNQTITAPNEKAQYGNNLGVQEKSTDKNDPNGVLRFDTNG

DGFISTSEVTPEAQKLVDEGKLQPSSDRMVEEWTKNKNNVDNTYDYDESYKKQQEMYK
KAARGEVPEPGETIK"
/transl_table=11
/product="hypothetical protein"
/db_xref="SEED:fig|703339.303.peg.24"
CDS 19570..19986
/product="hypothetical protein"
/db_xref="SEED:fig|703339.303.peg.25"
/transl_table=11

/translation="MLQQISRSRIRKNKLQAKQKENEESNSQTTSETSNNENQSNQSQ

KNSQDQATTEENTNDEQQVTRDNMFDYAIAAVNEVGDADLLKFQEPEYNGSEWTINAN
NKSGAGANTIVVKDDGTVQIWNGPKTSMNHETKIEL"
CDS 20275..20439
/db_xref="SEED:fig|703339.303.peg.26"
/product="hypothetical protein"
/translation="MDEFELIYILISPYLLITKILKPHKQKVNWLIKPATNKIMHPLN
PKARQKWTLN"
/transl_table=11
CDS 20453..20674
/db_xref="SEED:fig|703339.303.peg.27"
/product="hypothetical protein"
/transl_table=11
/translation="MNQDSIQSDWQTVAKCLASQNYISIVKGLVHHFTNIDDEEILDK
TYDDFMNDDSITTVLNNDFQDIINRYLSK"
CDS 20824..21390
/db_xref="SEED:fig|703339.303.peg.28"
/product="hypothetical protein"
/translation="MKKYIISLVLLTSFLLSACTINIERDEENSEKNKQKENTHTDSN

NTSSNESDTSKQSSQENTINQTEQNQPANNNSSSQQNPQIITENEAIQKVKDEFPSTR

TGNDYRIDTTRTDNNVYAIKFTSQDAEGYPMQAAVIIDKRTGEFIDYIDDRSDEDKER
HVQHAKESTLYKGPYDAFRKDFSHKINE"
/transl_table=11
CDS complement(21470..23698)

/EC_number="3.1.21.3"
/product="Type I restriction-modification system,
restriction subunit R (EC 3.1.21.3)"
/db_xref="SEED:fig|703339.303.peg.29"
/transl_table=11

/translation="MQQNPQGSGKSLTMVFFSGKLIQMLNNPTLVVVTDRNDLDNQLY

STFVKSKGRSGKGLLRQTPKQAETRKELKSLLSVESGGIVFTTMQKFEPEQNETTMSA

LTERKNVIVMADEAHRTQYGFNAKYDDKGEGIKYGYAKYLRDALPNATFVGFTGTPVA

STDKNTQMVFGNYIDVYDMTQAVADGSTVKIYYESRIIPLNLPQNLDLDEAYNDITED

QEEDVKQRLKSKWSRIEALAGAKPRVEALAKDIIQHFETRQQAMKGKGMIVTMSRRIA

VDLYDEIIRLKPEWHSDDDDKGVIKVVMTGSSSDPTSFQRHIGPKKRRNLLEKRMKDM

NDELQLVIVRDMWLTGFDVPSMHTMYIDKPMKGHNLMQAIARVNRVFKDKPGGLIVDY

VGIAESLKEALKEYTESDQAQTAIDTDKAVELMLLKYDVIQDMLYNLDYSKFNSEKKS

ERYYAISDTMDYVIGLGEDERQRFIKTVTELGKAFALCATEPTAQELNDEIAFFKAVK

AGLVKLLQPPKEGKTRKTPAEVEAEINQLVSQSVVTEDVIDVYQTLGLEQPDLSILSD

DFLKDVEGLKQKNVAVELLNRLLKGQVKSLMKTNATVSKRFSEMLGNSINKYNSRSIE

TSKVIEELIQLAKDIKQEQQRGNELGLNSDEIAFYDALASHETAKEAMGDKELRAIAH

ELTKTVKENMGVDWSKRDSAKAKMRVAVRRLLKKYGYPPDLQKMAVEQVVEQAELMAS
NQ"

CDS complement(23745..24419)
/translation="MNYFRYKQFNKDVITVAVGYYLRYALSYRDISEILRERGVNVHH

STVYRWVQEYAPILYRIWKKKHKKAYYKWRIDETYIKIKGKWSYLYRAIDAEGHTLDI

WLRKQRDNHSAYAFIKRLIKQFGKPQKVITDQAPSTKVAMAKVIKAFKLKPDCHCTSK
YLNNLIEQDHRHIKVRKTRYQSINTAKNTLKGIECIYALYKKNRRSLQIYGFSPCHEI
SIMLAS"
/transl_table=11
/db_xref="SEED:fig|703339.303.peg.30"
/product="Mobile element protein"

CDS 24702..26048

/db_xref="SEED:fig|703339.303.peg.31"
/product="similar to glutathione reductase"

/translation="MTKKYDLIVIGTGSAGSITAAKCNKAGWNVAMVDDRPFGGTCAL

RGCDPKKVLHGAAELIDWNKRMVKNGVPSEVSINWKDLMNFKRTFTDDVPEKKEEALN

KQGIDTYHGNASFVSEDKLEVNKEVLEGSHFLIASGAKPTPLPIKGEEHLTYSDEFLE
LDELPQRIVFVGGGYISFEFAHIAARAGSEVHIIHRGQRPLENFDIDLVDILLEKSKE

IGIQVHLQHSVESIEKEQGKFHVYARKKEDITRFEADIVIHGAGRVPALDMNLEKGNI

ERKKHGVHVNEYLQSVSNPNVYAAGDAAATDGLPLTPVASADSHVVASNLLKGNSKKI

EYPVIPSAVFTVPKMASVGMSEEEAKNSGRNIKVKQKNISDWFTYKRTNEDFAAFKVL
IDEDSDQVVGAHLISNEADELINHFATAIRFGISTKELKQMIFAYPTAASDIAHML"
/transl_table=11
CDS 26348..26755
/product="FIG01110237: hypothetical protein"
/db_xref="SEED:fig|703339.303.peg.32"
/translation="MEMKISELAKACDVNKETVRYYERKGLIAGPPRNESGYRIYSEE

TADRVRFIKRMKELDFSLKEIHLLFGVVDQDGERCKDMYAFTVQKTKEIERKVQDLLR
IQRLLEELKEKCPDEKAIYTCPIIETLMGGPDK"
/transl_table=11
CDS 26772..27125
/product="FIG01109440: hypothetical protein"
/db_xref="SEED:fig|703339.303.peg.33"
/translation="MKKRISFTAIMTVLLIGLTACGAESDTANESKVQDIQGNPVSLP

NEKPTLIYFMATWCPSCIYNEEIFKEIHQLNPNDVQLITVSLDPNTDTKESFAKFKQD
YGGDWPHVLKIGVEL"
/transl_table=11
CDS 27122..27802
/translation="MSFSFLFILTAGMVAAFNPCGIALLPSYISYLIGGETKDHSFRY
AIFKGLGLGGAMTTGFLTIFVLAGLLIGGLGSALTGIFPILSLVMGILIALLGLGMLF

GKHLPIKIGSFQVKPGKWSIYFYGIAYAVTSLGCTLPAFMLVVSASLNDNSVTAVIIK

FIIYSLGMGIVVTAITMVSLISRQLVQKFLHNYMGSIQKIAAVVIFLSGLYMAYYWYF
GSGGICTF"
/transl_table=11
/db_xref="SEED:fig|703339.303.peg.34"
/product="FIG01108609: hypothetical protein"

CDS 27940..28275
/translation="MDENRSKGNRWGVWAFFGILLVPLLVPLLCCAGPILLVALGSTG

IGALFAGATGNWWLTGIFAALAIVMIALILSKLLKNKYNSPEGNGKTKNKTDCCTPPE
SVDRKHETR"
/transl_table=11
/db_xref="SEED:fig|703339.303.peg.35"
/product="hypothetical protein"
CDS 28333..29976
/product="Mercuric ion reductase (EC 1.16.1.1)"
/db_xref="SEED:fig|703339.303.peg.36"
/EC_number="1.16.1.1"
/transl_table=11

/translation="MTQNSYKIPIQGMTCTGCEEHVTEALEQAGAKDVSADFRRGEAI

FELSDDQIEKAKQNISAAGYQPGEEESQPSENSVDFNRDGDYDLLIIGSGGAAFSAAI

KANENGAKVAMVERGTVGGTCVNIGCVPSKTMLRAGEINGLAQNNPFTGLQTSTGAAD

LAQLTEQKDGLVSQMRQEKYIDLIEEYGFDLIRGEASFIDDKTIQVNGQNITSKSFLI

ATGASPAVPEIPGMNEVDYLTSTSALELKEVPQRLAVIGSGYIAAELGQMFHNLGTEV

TLMQRSERLFKTYDPEISEAIDESLTEQGLNLITGVTYQKVEQNGKSTSIYIEVNGQE

QVIEADQVLVATGRKPNTETLNLESAGVKTGKKGEVLTNEYLQTSNNRIYAAGDVTLG

PQFVYVAAYEGGIVANNALGLAKRKIDLRFVPGVTFTNPSIATVGLTEQQAKEKGYDV

KTSVLPLDAVPRALVNHETTGVYKLVVNAQTQKLIGAHIVSENAGDVIYAATLAVQFG
LTIEDLTDSFAPYLTMAEGLKLAALTFDKDVSKLSCCAG"
CDS 30058..30708
/db_xref="SEED:fig|703339.303.peg.37"
/db_xref="GO:0018836"
/product="Organomercurial lyase (EC 4.99.1.2)"
/EC_number="4.99.1.2"

/translation="MKNISEFSAQLNQTFDQGEAVSMEWLFRPLLKMLAEGDPVPVED

IAAETGKPVEEVKQVLQTLPSVELDEQGRVVGYGLTLVPTPHRFEVDGKQLYAWCALD

TLMFPALIGRTVHIASPCHGTGKSVRLTVEPDRVVSVEPSTAVVSIVTPDEMASVRSA

FCNNVHFFSSPSAAQDWLNQHPESSVLPVEDAFELGRHLGARYEESGPTNGSCCNI"
/transl_table=11
CDS complement(31011..31370)
/transl_table=11

/translation="MEGAKTIISKYGYPQEATMRRLIWYNSGPWKRTIVYKDPVPHNF

PTPHLDFLKQTIDYKVPVHLYDAIAAFDGSVYLDRTTGEASAKCHEEAMNFLSLNLLN
DIVTGKRDVQGAKAFYA"
/db_xref="SEED:fig|703339.303.peg.38"
/product="FIG01109191: hypothetical protein"
CDS complement(31469..32143)
/transl_table=11
/translation="MNYFRYKQFNKDVITVAVGYYLRYTLSYRDISEILRERGVNVHH

STVYRWVQEYAPILYQIWKKKHKKAYYKWRIDETYIKIKGKWSYLYRAIDAEGHTLDI

WLRKQRDNHSAYAFIKRLIKQFGKPQKVITDQAPSTKVAMAKVIKAFKLKPDCHCTSK
YLNNLIEQDHRHIKVRKTRYQSINTAKNTLKGIECIYALYKKNRRSHQIYGFSPCHEI
SIMLAS"
/db_xref="SEED:fig|703339.303.peg.39"
/product="Transposase for insertion sequence-like element
IS431mec"
CDS 32340..33719
/product="Tetracycline resistance, MFS efflux pump =>
Tet(K)"
/db_xref="SEED:fig|703339.303.peg.40"
/translation="MFSLYKKFKGLFYSVLFWLCILSFFSVLNEMVLNVSLPDIANHF
NTTPGITNWVNTAYMLTFSIGTAVYGKLSDYINIKKLLIIGISLSCLGSLIAFIGHNH

FFILIFGRLVQGVGSAAFPSLIMVVVARNITRKKQGKAFGFIGSIVALGEGLGPSIGG
IIAHYIHWSYLLILPMITIVTIPFLIKVMVPGKSTKNTLDIVGIVLMSISIICFMLFT
TNYNWTFLILFTIFFVIFIKHISRVSNPFINPKLGKNIPFMLGLFSGGLIFSIVAGFI

SMVPYMMKTIYHVNVATIGNSVIFPGTMSVIVFGYFGGFLVDRKGSLFVFILGSLSIS

ISFLTIAFFVEFSMWLTTFMFIFVMGGLSFTKTVISKIVSSSLSEEEVASGMSLLNFT
SFLSEGTGIAIVGGLLSLQLINRKLVLEFINYSSGVYSNILVAMAILIILCCLLTIIV
FKRSEKQFE"
/transl_table=11
CDS 33905..35146
/product="hypothetical protein"
/db_xref="SEED:fig|703339.303.peg.41"
/transl_table=11

/translation="MSYSIVRVSKVKSGTNTTGIQKHVQRENNNYENEDIDHSKTYLN

YDLVNANKQNFNNLIDEKIEQNYTGKRKIRTDAIKHIDGLITSDNDFFDNQTPEDTKQ

FFEYAKEFLEQEYGKDNLLYATVHMDEKTPHMHYGVVPITDDGRLSAKEVVGNKKALT

AFQDRFNEHVKQRGYDLERGQSRQVTNAKHEQISQYKQKTEYHKQEYERESQKTDHIK

QKNDKLMQEYQKSLNTLKKPINVPYEQETEKVGGLFSKEIQETGNVVISQKDFNEFQK

QIKAAQDISEDYEYIKSGRALDDKDKEIREKDDLLNKAVERIENADDNFNQLYENAKP

LKENIEIALKLLKILLKELERVLGRNTFAERVNKLTEDEPKLNGLAGNLDKKMNPELY
SEQEQQQEQQKNQKRDRGMHL"
CDS 35332..35433
/translation="MKFREAFENFITSKYVLGVLVVLTVYQIIQMLK"
/transl_table=11
/product="hypothetical protein"
/db_xref="SEED:fig|703339.303.peg.42"
CDS 35673..35765
/transl_table=11
/translation="MYKNNHANHSNHLENHDLDNFSKTGYSNSR"
/product="Replication initiation protein, topisomerase"
/db_xref="SEED:fig|703339.303.peg.43"
CDS 35773..36627
/product="Replication initiation protein, topisomerase"
/db_xref="SEED:fig|703339.303.peg.44"

/translation="MDAHTVCISDPKLSFDAMTIVGNLNRDNAQALSKFMSVEPQIRL

WDILQTKFKAKALQEKVYIEYDKVKADSWDRRNMRIEFNPNKLTRDEMIWLKQNIISY

MEDDGFTRLDLAFDFEDDLSDYYAMSDKAVKKTIFYGRNGKPETKYFGVRDSNRFIRI

YNKKQERKDNADAEVMSEHLWRVEIELKRDMVDYWNDCFSDLHILQPDWKTIQRTADR

AIVFMLLSDEEEWGKLHRNSRTKYKNLIKEISPVDLTDLMKSTLKANEKQLQKQIDFW
QHEFKFWK"
/transl_table=11
CDS complement(36716..37390)
/product="Transposase for insertion sequence-like element
IS431mec"
/db_xref="SEED:fig|703339.303.peg.45"

/transl_table=11
/translation="MNYFRYKQFNKDVITVAVGYYLRYTLSYRDISEILRERGVNVHH

STVYRWVQEYAPILYQIWKKKHKKAYYKWRIDETYIKIKGKWSYLYRAIDAEGHTLDI

WLRKQRDNHSAYAFIKRLIKQFGKPQKVITDQAPSTKVAMAKVIKAFKLKPDCHCTSK
YLNNLIEQDHRHIKVRKTRYQSINTAKNTLKGIECIYALYKKNRRSLQIYGFSPCHEI
SIMLAS"
CDS 37648..37815
/product="Hydroxymethylglutaryl-CoA synthase N-domain
homolog (EC 2.3.3.10)"
/db_xref="SEED:fig|703339.303.peg.46"
/EC_number="2.3.3.10"
/translation="MLESREQLSVEEYETFFNRFDNQEFDFERELTQDPYSKVYLYSI
EDHIRTYKIEK"
/transl_table=11
CDS 37913..38143
/translation="MLIFLSYDFVRACLPRGMARAISLSHILFPQASALTKSVVIFIF
IRILTEIILIRGIVKAILSKIADKRFVKSSSK"
/transl_table=11
/product="hypothetical protein"
/db_xref="SEED:fig|703339.303.peg.47"
CDS 38732..39475
/EC_number="3.1.4.46"
/db_xref="SEED:fig|703339.303.peg.48"
/db_xref="GO:0008889"
/product="Glycerophosphoryl diester phosphodiesterase (EC
3.1.4.46)"
/transl_table=11
/translation="MLTVYGHRGLPSKAPENTIASFKAASEVEGINWLELDVAITKDE

QLIIIHDDYLERTTNMSGEITELNYDEIKDASAGSWFGEKFKDEHLPTFDDVVKIANE

YNMNLNVELKGITGPNGLALSKSMVKQVEEQLTNLNQNQEVLISSFNVVLVKLAEEIM

PQYNRAVIFHTTSFREDWRTLLDYCNAKIVNTEDAKLTKAKVKMVKEAGYELNVWTVN
KPARANQLANWGVDGIFTDNADKMVHLSQ"
CDS 39572..40000
/transl_table=11
/translation="MKYDDFIVGETFKTKSLHITEEEIIQFATTFDPQYMHIDKEKAE

QSRFKGIIASGMHTLSISFKLWVEEGKYGEEVVAGTQMNNVKFIKPVYPGNTLYVIAE
ITNKKSIKKENGLVTVSLSTYNENEEIVFKGEVTALINNS"
/db_xref="SEED:fig|703339.303.peg.49"

/product="MaoC domain protein"
CDS complement(40046..42052)
/transl_table=11
/translation="MKKIKIVPLILIVVVVGFGIYFYASKDKEINNTIDAIEDKNFKQ

VYKDSSYISKSDNGEVEMTERPIKIYNSLGVKDINIQDRKIKKVSKNKKRVDAQYKIK

TNYGNIDRNVQFNFVKEDGMWKLDWDHSVIIPGMQKDQSIHIENLKSERGKILDRNNV

ELANTGTAYEIGIVPKNVSKKDYKAIAKELSISEDYIKQQMDQKWVQDDTFVPLKTVK

KMDEYLSDFAKKFHLTTNETESRNYPLEKATSHLLGYVGPINSEELKQKEYKGYKDDA

VIGKKGLEKLYDKKLQHEDGYRVTIVDDNSNTIAHTLIEKKKKDGKDIQLTIDAKVQK

SIYNNMKNDYGSGTAIHPQTGELLALVSTPSYDVYPFMYGMSNEEYNKLTEDKKEPLL

NKFQITTSPGSTQKILTAMIGLNNKTLDDKTSYKIDGKGWQKDKSWGGYNVTRYEVVN

GNIDLKQAIESSDNIFFARVALELGSKKFEKGMKKLGVGEDIPSDYPFYNAQISNKNL

DNEILLADSGYGQGEILINPVQILSIYSALENNGNINAPHLLKDTKNKVWKKNIISKE

NINLLTDGMQQVVNKTHKEDIYRSYANLIGKSGTAELKMKQGETGRQIGWFISYDKDN
PNMMMAINVKDVQDKGMASYNAKISGKVYDELYENGNKKYDIDE"
/product="Penicillin-binding protein PBP2a, methicillin
resistance determinant MecA, transpeptidase"
/db_xref="SEED:fig|703339.303.peg.50"
/db_xref="GO:0008658"
/db_xref="GO:0046677"
CDS 42152..43909
/db_xref="SEED:fig|703339.303.peg.51"
/db_xref="GO:0008658"
/product="Methicillin resistance regulatory
sensor-transducer MecR1"
/translation="MLSSFLMLSIISSLLTICVIFLVRMLYIKYTQNIMSHKIWLLVL

VSTLIPLIPFYKISNFTFSKDMMNRNVSDTTSSVSHMLDGQQSSVTKDLAINVNQFET

SNITYMILLIWVFGSLLCLFYMIKAFRQIDVIKSSSLESSYLNERLKVCQSKMQFYKK

HITISYSSNIDNPMVFGLVKSQIVLPTVVVETMNDKEIEYIILHELSHVKSHDLIFNQ

LYVVFKMIFWFNPALYISKTMMDNDCEKVCDRNVLKILNRHEHIRYGESILKCSILKS

QHINNVAAQYLLGFNSNIKERVKYIALYDSMPKPNRNKRIVAYIVCSISLLIQAPLLS

AHVQQDKYETNVSYKKLNQLAPYFKGFDGSFVLYNEREQAYSIYNEPESKQRYSPNST

YKIYLALMAFDQNLLSLNHTEQQWDKHQYPFKEWNQDQNLNSSMKYSVNWYYENLNKH

LRQDEVKSYLDLIEYGNEEISGNENYWNESSLKISAIEQVNLLKNMKQHNMHFDNKAI

EKVENSMTLKQKDTYKYVGKTGTGIVNHKEANGWFVGYVETKDNTYYFATHLKGEDNA
NGEKAQQISERILKEMELI"
/transl_table=11
CDS 43909..44112

/translation="MDNKTYEISSAEWEVMNIIWMKKYASANNIIEEIQMQKDWSPKT
IRTLITRLYKKGFIDRKKDNKIF"
/transl_table=11
/db_xref="SEED:fig|703339.303.peg.52"
/db_xref="GO:0003677"
/db_xref="GO:0016481"
/db_xref="GO:0016566"
/product="Methicillin resistance repressor MecI"
CDS 44753..45901
/translation="MENILNINDNEKRVLREIYNHHNISRTQISKNLEINKATISSIL

NKLKYKSLVNEVGEGDSTKSGGRKPILLKVNHLYGYFISLDLTYSSVEVMYNYFDGNV
IKHESYDLPDEKVSSILSIIKKHIDIQEKLDTYNGLLGVSVSIHGVVDNEQHVTYLPF

HETEGISIAKKIKEITNVPVVVENEANLSALYERNFNHNLSYNNLIALSIHKGIGAGL

IINNQLYRGANGEAGEIGKTLVSKVSDNVEIFHKIEDIFSQEALLHNLSNQLNEKMTL

SKLIQFYNEKNPVVVEEMEQFINKIAVLIHNLNTQFNPNAIYINCPLFNEMPEILEAI
KNQFKQYSRNEIQIKLTSNVKFATLLGGTLAIIQKVLQINDIYLDIKA"
/transl_table=11
/product="Putative ROK-family transcriptional regulator"
/db_xref="SEED:fig|703339.303.peg.53"
CDS complement(46015..46320)

/translation="MPQTNIVTSSIHSSEMTGEEHNVLDVRNDDEWRKGHLKQAIHIP

HGKLLNEDIPFDKNDLIYVHCQSGVRSSIAVGILEYKGFTNIVNVRGGYQHLPAFLK"
/transl_table=11
/product="Polysulfide binding protein"

/db_xref="SEED:fig|703339.303.peg.54"
CDS complement(46411..47349)
/translation="MFFKQFYEDNFSQASYLIGCQRTGEAMIIDPIRDLTKYLKVADK

EGFTITKAAETHIHADFASGIRDVANKLNASIYVSDEGKDEFNYKNMPKQTHFVKHQD
IIYLGKIKLEVLHTPGHPPESISFLLTDEGGSSSIPMGLFSGDFIFVGDVGRPDLLEK

AVNVEGSTKIGAKQMYQSIQFAATLPDYIQIWPGHGAGSPCGKALGSLPTTTLGYEKI

NNWAFNVKDETSFIETLTLNQPAPPHHFSQMKKINQFGMQMYQPYNVYPSSSNTQTAF
DLRSKEAFHGGHMHGTINIPFNKTFINQIGWYLDYD"
/transl_table=11
/product="Zn-dependent hydroxyacylglutathione hydrolase /
Polysulfide binding protein"
/db_xref="SEED:fig|703339.303.peg.55"
CDS complement(47382..48446)
/transl_table=11
/translation="MEQYKEVHINSLNKEELEKLAKMGQLIDVRTKEEYELGHINGST

LHPVEEIKSFNKNSNKTYYIHCKSGNRSTKACDYLAKQGYDVVNLKGGYKDYEAKNFN
SAPLIEKDIEIKENRKQFDFRGLQCPGPIVNISKEINNISTGEQIEVTVTDPGFNSDI

KSWAKQTGNTLVNLTEEANVINAIIQKEKPKEFEINDTATGTTIVLFSGELDKAVAAM

IIANGAKAAGKDVTIFFTFWGLNALKKAQSTRVKKKGISKMFDLMLPKDPIHMPLSKM

NMFGLGNIMMRYVMNKKNVDSLYSLIDQAIDQDIKLIACTMSMDVMGISKEELRDEVD
YGGVGTYIGHTEQANHNLFI"
/product="Disulfide bond regulator"
/db_xref="SEED:fig|703339.303.peg.56"
CDS 48582..48842

/translation="MTYDKKMINRINRIQGQLNGVVKMMEEEKDCKDIITQLSASKGS
IQRLMGIIISENLIECVKTAEENNESSQELINEAVNLLVKSK"
/transl_table=11
/product="Metal-sensitive transcriptional repressor"
/db_xref="SEED:fig|703339.303.peg.57"
CDS 48842..49486
/db_xref="SEED:fig|703339.303.peg.58"
/product="FIG003846: hypothetical protein"
/transl_table=11
/translation="MDIANMTIMLLIGVLGGFISGLIGIGGAIIIYPAILILPPLIGI

PAYSAYIASGLTSSQVFFSTLSGSLNARKQPAFSPKLVIYMGGGMLIGSMLGAILASL

FNAAFVNTVYVIIAILALILMFIKVKPTTQETKSKPLLFIIVGFGIGVISGIVGAGGA
FIIIPVLLALFKLPMNTVVNNSIAIAFISSVGAFFIKLMQGYIPVESAIFLIIE"
CDS complement(49754..50413)
/translation="MKKSPIPKIFGDDKMIATILTATAVYVATGIDYLVILILLFSQV

KKGQVKHIWIGQYIGTAIVIGASLLVAQGVVNLIPQQWVIGLLGLLPLYLGVKIWIKG
EEDEDESSILSLFSSGKFNQLFLTMIFIVLASSADDFSIYIPYFTTLSMSEIFIVTIV
FLIMVGVLCYVSYRLASFDFISETIEKYERWIVPIVFIGLGIYILFENGTFNALISFL
L"
/transl_table=11
/product="Cadmium resistance protein"
/db_xref="SEED:fig|703339.303.peg.59"
CDS complement(50452..52866)
/db_xref="SEED:fig|703339.303.peg.60"
/product="Cadmium-transporting ATPase (EC 3.6.3.3)"
/EC_number="3.6.3.3"

/translation="MDSSTKTLTEDKQVYRVEGFSCANCAGKFEKNVKELSGVHDAKV

NFGASKIDVFGSATVEDLEKAGAFENLKVAPEKARRRVEPVVTEEKNVYRVEGFSCAN

CAGKFEKNVKQLAGVQDAKVNFGASKIDVYGNASVEELEKAGAFENLKVIPEKLANPS
IQAVKEDTKAPKEEKIPFYKKHSTLLFATLLIAFGYLSHFVNGEDNLVTSMLFVSSIV

IGGYSLFKVGFQNLIRFDFDMKTLMTVAVIGAAIIGEWAEASIVVILFAISEALERFS

MDRARQSIRSLMDIAPKEALVRRNGQEIMIHVDDIAVGDIMIVKPGEKIAMDGIIING

VSAVNQAAITGESVPVAKTVDDEVFAGTLNEEGLLEVKITKYVEDTTISKIIHLVEEA

QGERAPAQAFVDKFAKYYTPIIMVIAALVAVVPPLFFGGSWDTWVYQGLAVLVVGCPC
ALVITTPISIVSAIGNAAKKGVLIKGGVYLEELGAIKAIAFDKTGTLTKGVPVVTDFK

VLNDQVEEKELFSIITALEYRSQHPLASAIMKKAEQDNITYSDVRVKDFTSITGRGIQ

GNIDGTTYYIGSPRLFKELNVSDFSLEFENKVKVLQNQGKTAMIIGTDQTILGVIAVA

DEVRETSKNVILKLHQLGIKQTIMLTGDNQGTAEAIGAHVGVSDIQSELLPQDKLDYI

KKMKAEHGNVAMIGDGVNDAPALAASTVGIAMGGAGTDTAIETADIALMGDDLSKLPF
AVRLSRKTLNIIKANITFAIGIKIIALLLVIPGWLTLWIAILSDMGATILVALNSLRL
MRVKDK"
/transl_table=11
CDS complement(52859..53224)

/db_xref="SEED:fig|703339.303.peg.61"
/product="Cadmium efflux system accessory protein"
/transl_table=11

/translation="MTKDMCEVTYIHEDKVNRAKKDLAKQNPMDVAKVFKALSDDTRV
KIAYVLSLEGELCVCDVANIIESSTATASHHLRLLKNLGIAKYRKEGKLVYYSLDDEH
VKQLVEKAFLHQREVASIG"
CDS 53345..53452
/db_xref="SEED:fig|703339.303.peg.62"
/product="hypothetical protein"
/translation="MLETNPFGDFNQILKYLKHNCPVSLSASFHNLSTD"
/transl_table=11
CDS complement(53462..53839)
/translation="MDKQVRNTTEIVRLAKQKSKKTREKVDKAISKFSIEGKVINFNS

IAKEANVSKSWLYKEHDIRQRIESLRERQITANVVSKPKKSSRSEEILIKTLKRRVME
LEKENKKLQNQIQKLYGDLYNKE"
/transl_table=11
/product="Mobile element protein"
/db_xref="SEED:fig|703339.303.peg.63"
CDS complement(53846..55738)

/translation="MNASSKRKIISQSEISKKIAVMNEEMQGFWANNSWDIRKCPHPS

AIELSKNPALRNRWVRFERVKNLWLRTELKYFYFYHLNNGIWNAKTVWIRKGTVINKM
LDFLDLKYPSITSITEVPIEKAMTEYRTYLTKRGVRITTTNYKITANQEKTPVKANSY

YVTNLKQFMEFYENFYFDGEEWDKDVWDRRNLPLPDDKVNPTQYEYTINFKGFRNTYF

KQLVKRYCKLRLNVDSFSYVSDIAQRLKEFFNFLDMKFKRVQRVHQLTRVEIEAYLSE
LNMMGIKPSTITGRISILEGLFSTLLRLEWDDVPSKILIYSEDYPKIPRAKPRFIDEF

VLEQLNSHLDKLPEYIATMTMIVQECGMRISELCTLKKGCLLEDKDGDFFLKYYQWKM

KKEHIVPISKEVALLIKVREDKVSEEFPDSEYLFPRKDGSPLKQETFRGELNKLAYEQ

NIVDKSGEIYRFHAHAFRHTVGTRMINNGMPQHIVQKFLGHESPEMTSRYAHIFDETL

KNEFTKFQEKLVTNNGDVLDLDEDNEVDDVELQWFKKNINAQVLPNGYCRLPVVAGGC

PHANACLDCTHFCTSKQFLPQHEEQLERTEELLAIAKDKQWQRQVETNSRVKERLEQI
IGSLTG"
/transl_table=11
/db_xref="SEED:fig|703339.303.peg.64"

/product="Transposase B from transposon Tn554"
CDS complement(55933..56256)
/transl_table=11

/translation="MSRKRIDVVKVQMVKEDTLWYLKRRIEEPKDAADIMRDFIGNAD
REHFILICLNSKNEPTHIETVSIGTINFAVIHPREIFKTAILSNATGMIIGHNHPSGD
ILTIV"
/db_xref="SEED:fig|703339.303.peg.65"
/product="DNA repair protein RadC truncated by transposon
Tn554"
CDS complement(56249..56455)
/db_xref="SEED:fig|703339.303.peg.66"
/product="FIG01110849: hypothetical protein"
/transl_table=11
/translation="MNQTWKEVYQYLMHQPHIDVIKGIIAFSSNKKNNSELDELYSIY
MNDDSITSIVHPDLIESIELIHNE"
CDS complement(56457..56978)
/translation="MKSIKSVLETEAIFSQDKMHRYLLKKTWDIDKEVLTIITMYPHY

DGVINVDLTTQLIVNKVAEKDEYGGVNFINLFSNIDTPINLKHIENSHDKHTDIHIMK

AVKEADSVLLAWGSYGKKPLVENRVNEVLDMLKPHSKKISILTNPQTNEIMHPLNPYA
RKAWTIKPLKETK"
/transl_table=11
/product="hypothetical protein"
/db_xref="SEED:fig|703339.303.peg.67"
CDS complement(56997..57308)
/db_xref="SEED:fig|703339.303.peg.68"
/product="FIG01107894: hypothetical protein"
/translation="MKRYITRSIANYLPAMLQQQLWKLVAQRENEQFKELEEIDYFHI

FQFNMHNSQLYIKHKQERPEYVKIHKANYSKAININKVYIIREDDVDLSYYVMLLPEE
Y"
/transl_table=11
CDS complement(57311..57400)
/translation="MNKWIHIAQISVTVITEVITIMKDIEKEK"
/transl_table=11
/db_xref="SEED:fig|703339.303.peg.69"
/product="hypothetical protein"
CDS complement(57393..57743)
/translation="MTLEKQLKEYITNLFNLPKDEKWECESIEEVADNILPDQYIRLG
PLTNKILHTYTYYSDTLHERHIYPFILYYQKQLIAIGYIDETNDMDFLYLHNTVMPLL
DQRHLLEKENYNNE"
/transl_table=11

/product="FIG01108299: hypothetical protein"
/db_xref="SEED:fig|703339.303.peg.70"
CDS complement(58214..59842)
/product="Site-specific recombinase"
/db_xref="SEED:fig|703339.303.peg.71"
/translation="MQQLKQKRVGIYVRVSTEIQSTEGYSIDGQINQIREYCDFNNFV

VVDVYADRGISGKSMNRPELQRLLKDANEGQIDSVMVYKTNRLARNTSDLLKIVEDLH

RQNVEFFSLSERMEVNTSSGKLMLQILASFSEFERNNIVENVFMGQTRRAQEGYYQGN

LPLGYDKIPDSKHELMINQHEANIVKYIFESYAKGHGYRKIANALNHKGYVTKKGKPF

SIGSVTYILSNPFYVGKIQFAKYKDWNEKRRKGLNDKPIIAEGKHSPIIIQDLWDKVQ

LRKKQVSQKPQVHGKGTNLLTGIVHCPQCGAPMAASNTTNTLKDGTKKRIRYYSCSNF

RNKGSKVCSANSVRADVIEKYVMDQILEIVKSDKVINQVLERVNQENKVDIGALNHDI

AYKQQQYDEVSGKLHNLVKTIEDNPDLTSALKATIHQYETQLNDITNQMNQLKQQQNQ

EKLSYDTKQIAALLQRIFQNIESMDKAQLKALYLTVIDRIDIRKDGNHKKQFYVTLKL
NNEIIKQLFNNTPLDEVLLSTSSLFLPQTLFLQI"
/transl_table=11
CDS complement(59863..61209)
/db_xref="SEED:fig|703339.303.peg.72"
/product="Cassette chromosome recombinase A"
/transl_table=11
/translation="MKRAIGYLRQSTTKQQSLPAQKQAIELLAPKHNIQNIQYISDKQ

SGRTDNRTGYQQVTERIQQRQCDVLCCYRLNRLHRNLKNALKLMKLCQKYHVHILSVH

DGYFDMDKAFDRLKLNIFMSLAELESDNIGEQVKNGLREKAKQGKLITTHAPFGYHYQ

NGTFIINNDESPTVKAVFNYYLQGYGYKKIAQYLEDDNKLITRKPYQVRNIIMNPNYC

GRVINQYGQYNNMVPPIVSATKYEHAQAIRNKKQLHCIPSENQLKQKIKCPCCDSTLT

NMTIRKKHTLRYYICPKNMNESRFVCSFKGINAQKLEVQVLATCQNFFQNQQLYSKIN

NAIHQRLKKQRVIEAKSTLTQEQLIDKLAKGMIDAESFRKQTHLMNQKHKTISSISDN
QLQTSLQKVIQKSFTLNMLHPYIDEIRITKNKALVGIYFKNEPLNIVNQTSQSSIA"
CDS complement(61402..61677)
/translation="MNTVALAHEIEDERFEYLESTPLDTVKECCKQEGRQISNTYTEE

YKLINDILEKVIKPTSIVAYGEYEDYIHLKKFAQRRISNSLLLLRCN"
/transl_table=11
/db_xref="SEED:fig|703339.303.peg.73"
/product="hypothetical protein"
CDS complement(61767..63554)
/product="hypothetical protein"
/db_xref="SEED:fig|703339.303.peg.74"
/transl_table=11
/translation="MTNIIFSEYPYWLTEDSWYEVIPPKGKNDQQKSIRLSSPILIKN
KFLDPKSGVEKLVISNGQNIERIETSDILTSQKLPSLIMYGFSINEKYIKSLSNALQL

MRQSLPLSSLYTGVGVLNTDKGVVISLDEPYLSKEITQSQADEIICETQYDLQPKGTF

EDWWQMYLDEVKGNLLLELAVVFGISSLVTAFLKTKHEVEFAGTIFSFTGNSSTGKST

AAALGVSITGNPTKGSNTLFRSWNGTRNALEGYLSNNYGVPIVLDELSSATFKDTTGL

LYSIAEGQGRQRSNIHGEVKSLKNWGTSVISTAEHSIFNNSARNDGLNVRTIEISEAF

TTNADNADTIKKATSANYGHIMPLVAEYLLNREQEVIKWFQNEHLRFKTQLEKETSNT
GIRMFKRYATITTSARILERVIATPVDLNAVRDYLINYHLDSVSERSLADKAIEVIVQ

FVAQNRGKFSDDTRLSTLIENYGMISLEDDHIQVKMIASVFKNMLKEHNFQDTNNVID

ALRDKDYIQSDANRKTTKRSVKDKEGKPKTIVFYHLKLDKSYAPIFGLSEKSKFNIPL
SFISEHQHPILRDMISKAREWDKNGDIDF"
CDS complement(63554..63838)
/db_xref="SEED:fig|703339.303.peg.75"
/product="FIG01108228: hypothetical protein"

/translation="MKEKEFYTLKRAVTSIAFNLPVGTMFMYHDLNVRANVLCSTEIQ
QDVGRWFAHFVKYTPNLPFIVVGKKGSSLLYLKTAPNPMNHQNNWKGGNN"
/transl_table=11
CDS 63975..65024
/product="ORFID:MW0042"
/db_xref="SEED:fig|703339.303.peg.76"
/transl_table=11
/translation="MIPTIEEIIDKYGSLVDCLKFDISGEVYEDLLLLRSLSDLNEHQ

KMHRVSFIQKHFNPNFDYLLDDSEFRSMNKIYRNLNTMTYINSQNNEFKLKPFINEEQ

LKSLLAIKKVDEHLSYDPSITSKALADINKTQKRLVTKPNILYNRSKKENEYVRLGGK

ISYTKMEKHWEYLHNEIQYYNLTNQLISYVALEKEYAWAFLNELFYLIELYLKAFKGQ

KKTGHEFEEGLNAFIQSYVIILLIDIRLPTVRLYIIRRIVDECEKESDNQKRIKLIEE

SIKKYRYVKEQIERFGNALKEELSIATLSIEQNMLEAKIDYYKNYYYPRKKTKHNNIE
YNVSMFFKAVDSLKK"
CDS 65077..65649

/translation="MFRLKKWYLKQKGYFCRVGEKWDNSNLSHENIHSYVDRVLEYET

ETRRNGQPTEEYILASSGEKINEDLRSGTNDHKEILEVFDKYKVKEDITTYRTVSPRI

YKLMEKNAKNLNGVDLFELGILHTSLIKGYESREEGYKLRVKVKKGTPAFYVGNLTGE
ESHYYEVIVVNNLKLKIISIEDYYINCEVV"
/transl_table=11
/db_xref="SEED:fig|703339.303.peg.77"
/product="FIG01109033: hypothetical protein"
ORIGIN
1 gaagcgtatc ataaataaaa ctaaaaatta ggttgtgtat aatttaaaaa tttaatgaga
61 tgtggaggaa ttacatatat gaaatattgg attatacctt gcaatatcat acgatgttta
121 tagagtgttt aataaaccat ttttcaacta ttgatgatct agaatatata ataactgtac
181 aaattatatt gattatggaa ctacaattaa attaagaaat tgatgatgaa attttaaatt
241 taaactaatg gaatcaagaa agaatgaaag gaaatataca atgcctacga ttaataaaag
301 gaagtttatt agattttgtg ttagaaacag tggatgtttt tgtaaatgga agtaaaaatg
361 attgtgaatt agtaaatttt aattataaag aaggaaaagt agtttcacgt tatagtacaa
421 aatttgaaag caatcctaaa ttaagaaaag agcaattgat atcgacggta aaagttgtaa
481 ggtatgttat ttttgatttt gagaaaattt tatggggcat agggaaagtt ttattgaagt
541 tcatcatatt aagacaatgt ttactataag aaagttgaaa taaactcaga aacagacttg
601 attcccgttt attcaaattg gcgcaaaatg atacatataa acacctatat attgaagatt
661 taagtcaaac ggtaaataat atttttcact tttaaacaga aactttttg gaaattatat
721 taattagtat aaaaatgaat atgttcacaa agaaggagag attttattga aacacattaa
781 tcatttttct gaatcgacac tagcatactt ggaaaaagac catgaacatg cattttatgt
841 atattgttta atagacccaa gaaatgatga atgcttttat gttggtaaag gtaaggggaa
901 tagaatattt aaacataaac aagatgcgca gagacaatta ttgtacgaag atatattcag
961 agaagaaaat aaagataact tgaaatttaa tagaattaac gaaatttgta gcaatgattt
1021 gaatgtttta ggatatataa tcagttatgg attaaccgaa tctgaagcat tttctgcaga
1081 aaatgttctt ataaactttc ttaacctaac aaataaaact acattaacaa atatgattaa
1141 cggtcatgga tctaaggcat acttagttga agatttagaa aatgaatttg gctacgattc
1201 aattaatctt gaaaacataa atacgaatga attaatttta gcagtgaaaa tcagagatgc
1261 atttctatta gataaggatg aaagtaaaga gtatcctatt aatgaaagta aacgtgatag
1321 aaataacctt aaatcgcgta cattaggtag ttggataata ggaaaagata aaatacataa
1381 aataaaatat attattggta tcaatacagg tgctaataat gcagttgtct cagcttatga
1441 agtatcattt gaacaagcgg agagtatcga aacaaataat ggtagaatga gatatgcgtt
1501 tattgggctt tcagaaagag atgctactct aaaaaaattg aatttataca aaaaagcact
1561 accagattta agatttggta gtggtagcgc tacagcatat ataaataatg ggacaatgaa
1621 agttgattaa atgaataaaa acatccaatt ccttaaatta gcctttgaaa attttatact

1681 cgtatatata agtgaaaaac ttaaaagaat tagatgttaa ttaaaggaga gaatgctatg
1741 ccatcagatg atattgtaaa aaaactatac tcaaaagagg atattcgatt agaattagga
1801 cttactccgc ataagtttaa taaaaaaatg gaaactattg ctaagctttt taaaattgat
1861 atgaaaattt ttcacaatta caaaggccaa gataaaaata atcagtatac atttaatggt
1921 gtcgcaaaag aattaatcaa agtgttgcta aaaagtgttg attattaccc agtggatatc
1981 aattccaaaa aatttaaaca aaatggaaaa tctaaaaaag aaatgattga aaatatagat
2041 aactctagtt acatgaaata tatttatcag ttaatgaaat ctatcaatga aattcaatac
2101 aaaaggttaa ttgctgatat acatatgaaa gatgtgtacc agaatacaaa agcatggtta
2161 aataatggtg aatcaattaa taaaaaagaa caagagctat accaatatat gacaatttta
2221 ccattacata aaagagttga actgcaaaat gaggtattaa aatctataga cgaaacaatt
2281 tttcaatttc tcgcaaaaga acatagaaat aatcaaattg aagaaaataa tgaattagaa
2341 gcatatacaa aagcaataaa agaaggtaga aatcctaaaa atgattatga attaaatcat
2401 ctcctatata aaaaaaatca attaccctta gatagtctaa tagaagatat atgggactat
2461 gaggaaacag agtatactga gcttgattgg ttaattgcag atatgctaaa acgttcacaa
2521 agagctgata gtaattttat cgaaagttta gagaagaaaa acaaattgag aaaaaatatt
2581 cataaagatt ccattaaaag tatatctaag ttgattgatc atgaattagt taaaaataat
2641 agaaagtggg acgtagcaga attttataaa aatcaacaat tttggaataa tagtacatta
2701 agacgtagct ctcaaaattt tggaatttta tattacaaaa agaataggtt acatacaatg
2761 aatcaaagta atattattaa gaaaataaaa attatcgaag ctaatataga gagtgcaaat
2821 aaacagccga gctatttaga tttcttttta caagctaagt ttgatttaga gaggttagaa
2881 agtgaattaa tgaaatatag cattaatccg aaatatttta ataagataaa tagtgattac
2941 gtaagatatg ctattgaaga tgtacataat gcaattatta aaattgacag atatataagt
3001 aacgataaag tgaagttaaa ctattcaaat gtccaaatgg tagtgagaaa cgaaataaca
3061 gaacaaggaa gctactttgt aacccaggca ttgaatacac tgtcaaaggt agaagagtct
3121 ggctacagct ttgataattt catggtagga cctttcgtag aagactttaa aaaagcaatt
3181 aaaaatcgta gatttggttc taaaaaagat taagattgtt ttttagagtt aaacataaga
3241 ataaatttaa ataaaaaaat aaacccaaca aacctcgtta tatcaatgtt tgttgggttt
3301 atttttttg gtgatttttt aataactcaa ttacatctta ggataatatc gctaaattat
3361 acattgtcga ggccaagaca aataaaaatt aaagcaaaag ttagcgcgca actttctgct
3421 ataaaggagc aaatttatat ggagacaaga gacaaactga tgtcattgac tcaaagtgac
3481 aaaacacagc aatggctaat ggacaagtca tctaaccaag atgacattca acaattgcag
3541 caacaattca gtcagcagct tgatcaacaa tataatgcac ttttagctga tgaaaaagct
3601 aagttagacc aatacgtgga agtacatcaa ggattggaat cattaaagga agagattgaa
3661 tcagaaccta ttacgcttaa tatcgataaa ttacccgata tcaaagcaac aatgcttgaa
3721 agagccaaga atgatgaaca ttctgataaa atcgaaaagc tatttgatag gttagaacag
3781 gcattaaatg gtacgaatcg attatatacg caattatcgt tgattggtac acgaacacat
3841 cgaattacaa ctaaaaattt taatcttcaa ggcttaccta aagcagtcca acatacgatt
3901 ttaccttcaa aatttaagaa ggtgtataca gtcgatttta aatcgtttga accatcagtt
3961 gcagcgtaca tgactcaaga ttcaaaactg attgacttgt taaatcagaa agacggactg
4021 tatgacgcat tgctaagtga attaggctta tcagatgagc tacgtgtatt tgttaaacgt
4081 gcatttattg gttcgtttct atttggaggt aacttcaaaa atcctaaatt caagctgaat
4141 caatatgtaa gtgaagtaca atggttggat gcggtcagcc aatttacaaa agtcattgaa
4201 cttaagaagc acgttgaaaa gagtaattcc atgcctatgc cttatagtat tgagcatgat
4261 atgagcgcat ttcaaggtag cagtattatg gcaatctacg tacaaactgt agcgagttat

4321 attttcaagc acattctgct aaaagtgtac aaagcacagt gcgatcaaaa aacgttcaag
4381 attatagtac ctatacacga tgcgattatg attgaatgtg aagatgaaga aattgcacaa
4441 aatgtgggtc agttaatgaa agatacggct aaccagttgt tcaatggtga atttgcacat
4501 gtgacagtgg aagaaatagg aggcgtagac catgaataat gatagaggac aaagcctaca
4561 catcccaagt agtacaccaa tcaaagaaaa taatatatat gtagctacgt tacattctgt
4621 gatccaaaca gatttctcag gtgaaataaa gcaccaattc acgtatgaaa ttgaagtgaa
4681 caatcagatt gtatatgcga atcgtaatat tccaacaaaa ccgagcgcta atcagttgtc
4741 aattcatgat tggctgaaac gtcatagcaa ctatagcgtg ggtcatgata cgtatgaacc
4801 ttatattgac cgtaagcatt taatccatat tggtcaatat agcggtaact attatgtaca
4861 agatgtagca tcgttagatg cgtttggagg cgtattatca tgaatcatat attacaaatg
4921 ttatctaagc tattaagtgt ggccaaggag gcaatcgacc gtcaaggtct gattgctatc
4981 ctaactattc ctgttaataa taacgatgaa atagaagaaa cggctcaagg tgaaaccgtg
5041 tataacgaac ttatcgatca gttacgactt aatatcccaa aagatacgga ttatcaacct
5101 aacatctata gttattttgg tattaagaag aatcctaatg acaccgtact catggaaatg
5161 atgataaagg tttttcatat caaacgcttt aattcagaac tgtttatttt caaagttaac
5221 gggtggcaaa agataaatgg agatgaatta caagggttga tatctaaaat gatacaagta
5281 ttgcttgtag attataagcc ttcactaagc actctaaaaa atgtcgtaga tggattgcaa
5341 aaatcaacag atgtagaaga acttgttgag aatgagcgct atattggttg tggtgaaaat
5401 atgttcgatc ttaatacgtt tcaagtcgtt aaaaattcaa tcgatatctt tccaaaaaca
5461 cgattgaatt tatcattaag tacaaatgat gtaattactg ataagatacc gccttatttt
5521 aagcaatata tgttacaact tgcgaattat gacgatgatt tacaatactt tcttttccaa
5581 catacagcag tattacttac agctgatact aaataccgta ggggtctcat attatatggt
5641 ggagctaaga atggtaaatc tgtatatatt gaactagtta aatcattttt ctatagtaaa
5701 gatattgtgt ctaagccact taatgagctt gaaggtcgtt ttgacaaaga aagtttaatt
5761 gacaaaagtc taatggcaag tcatgaaatt gggcaatcta ggattcaaga aaagatcgta
5821 aatgacttca aaaagttatt atctgtagaa tcaatgcatg ttgatcgtaa aggaaaaact
5881 caagtggaag tcattttgga tttgaaactt atttttagta caaatgcgat acttaatttt
5941 cctcctgaac atgcgaaagc tttggagcgt cgaattaata ttattccatg tgagtattat
6001 gttgaaaaag cggacacttc attaattgat aagctccaga gtgagaagaa agaaatcttt
6061 ctttacttga tgtatgtgta tcaacagatt gtaaaagcag atatcgagta tcttgaaaat
6121 agccgtgtca ctgaaattac tcacgattgg ttaaattttg gatatgaatt tgtttctagc
6181 aggtccgtaa gtaatgcaaa tcagaaagca tgtattaatt tactcagaaa acttatagaa
6241 atcaaatcag gatcacgaat caaagtatcc gagttaaata aagttattaa tgaagaaata
6301 aaggtgagtt ctcaggttat taatcagtta attcaagcaa actttgatac tcaaaccaaa
6361 ctatacaatg gctacgatta ttggattgat ttaggttgga aagaagccaa taaaaaagag
6421 attcatgata tttcggaaaa agataatatt atttcattag ataaaaatga aaatataaca
6481 gacgatgagg cattagatga agagaatttg gactttgatt gggaggactt tgacgatgaa
6541 taatgaacaa attgaagcat ttgtagaagt gcttgtgcct atcatagaag aacgtatcaa
6601 taaaggtaag taatctaatt acgtactaca ggcaactgcc tgtagtactc atataattaa
6661 gtggtaaaag tgataaaaat gaaacgaaat tataaatata tattatctat atgttgttac
6721 aagaccgata gtctgtagca ataatctaat aaaaggagcg gtatgaaatg aagggtaaaa
6781 ttgcacttta ttcacgtgtt agtacgtctg agcagtcaga acatggttat tctgtaaaag
6841 agcaggaaca agtactcatc aaagaagttg tgaaaaattt cccaggttat gactatgaga
6901 catatactga ctcaggcatt tcaggtaaaa atattgaagg tcgtccggca atgaaacgtc

6961 tattacaaga tgttaaggat aataaaatcg aaatggtgtt aagttggaaa ttgaatcgta
7021 tctcacgatc aatgagagac gtgtttaata ttattcatga attcaaagaa catgatgtag
7081 ggtataaatc gatttctgag aatattgata catccaatgc ttctggagaa gtactcgtta
7141 caatgtttgg gttaatagga tctatagaac gccagacttt gatttcgaat gtgaaacttt
7201 ctatgaatgc taaggcaagg agcggagagg caatcaccgg tcgtgtttta ggctacaaat
7261 tatcacttaa tccacttaca cagaaaaatg atttagttat taatgaaaat gaagctcata
7321 ttgtacggga aatctttgat ttatatttga atcacaataa aggacttaaa gcaatcacga
7381 caattctaaa tcaaaaagga tatcgcacca ttaatcaaaa accattttca gtgtttggcg
7441 tgaaatatat tttgaataat ccagtctata aaggctatgt cagattcaat aaccatcaaa
7501 attgggctgt tcagcgaaga agtggtaaaa gtgataaaaa tgatgtgata ttggtcaaag
7561 gtaagcatga agccattata agtgaagatg tatttgatca agttcatgag aaactagctt
7621 ctaaaagttt taaaccgggt cgacctattg gtggagattt ctacttacgt ggccttatta
7681 aatgcccaga atgcggaaat aatatggtat gtcgacggac gtattataaa acgaaaaagt
7741 ccaaagaacg aacaatcaag cgttattaca tttgttcctt attcaatcgt tcagggagtt
7801 ctgcatgtca cagtaattcc atcaatgctg aagtcgtcga acgcgtaatc aatgttcatt
7861 tgaatcgtat tctttcacaa cctaatgtta ttaagcagat tgcgtcaagt gtgatagaag
7921 aactgaaaca aaagcatagt aaacaaacag aaataaaata tgatattgat agtctagaca
7981 aacaaaaagc aaaagttaaa acacaacaag aacgattatt ggaattgttt ttagatgatg
8041 aaatggatag cgaaatgtta aaagctaaac aaagtaaaat gaatcaacag ttagaagtat
8101 tagaccaaca aattaaagaa gcaaaacaag caaatcaatc acaggatgat atacccaatt
8161 ttgataagtt aaaagcacga ctcattttga tgataacacg attcagcgtg tacttaagaa
8221 aggctacacc cgaagctaaa aatcaactta tgaaaatgtt aattgattca attgaaatta
8281 cgacagataa acaagtaaaa cttgtaaggt ataaaattga tgaaagtctt atccctcaat
8341 ctttgaaaaa agattggggg tcttttttta tgcctaaatt taactttgtg ataaatgtca
8401 caaatgaaaa taggattgaa aatttatcac ttttaccact tttttagagt gacaaaagtg
8461 gagggatttt gaaatatttt ataaatatat attttattta tggagtacac attattaatt
8521 aaggaggtca ttataatgac gctaagcaaa caacttaaaa cgtatattac tgaacgattt
8581 aaattaaatt atcaagaaac ttgggctcgt gaaaccgtag atgcggtggc tgaagatgta
8641 ttacctgaaa aatatattaa aaatagtcca cttgaaaata aaattttaaa tacttttacc
8701 tattacaatg atgaattaca tgaaatcagc atttaccctt ttttatgtta tctagataaa
8761 gaattagtag caataggtta tttagataat tttgatttag actttatatt tttaaatgac
8821 actcatcaag tcattattga tgaacgctac ttgttacaaa aagggggtga gtaattatga
8881 attggattaa ggtcgctcaa ctatctgtca cagttatcaa tgaagtcatt gagattatga
8941 aagaaaaaca aaatggaggg gaatagtatg caaatcaatc gatacattac acgaggcatt
9001 agtgaacacc tatctctaga ccttcaaatc ttactttgga atatggtaaa agaaagagat
9061 aatcaacctg atacagatta cctacacatt tttagactgc aagaagatga gaatatactc
9121 tcaatcacac atgaacaaga acaacctgca tacaaattgg aatatcacta tacaaactat
9181 gaaaaaaatc aaaacgcatt acctaagaaa gtctacgtca ttcgagaaga tgatgtagac
9241 ctttcttatt atgtcatgtt actaccagaa gaatactaga ggagtgaaaa ttacgtgaaa
9301 acaatcgaaa gtacattaga aaccgaagct atattcagtg atgaccaaca acatcgctat
9361 ctacttaaga aaacatggga cagtgaaaaa caaaagatca caatcatcac aatgtatcca
9421 aattatacgg gccttcttcg tatagattta actacccaat tgataatgaa caaagtttca
9481 gaaatggatg catttggttc catcaatttt gtgaatctat actctaatat tacaactcct
9541 atcaatctca aacatttaga aaacgcctat gataatcata cagatattca aattatgaaa

9601 gccgtgaaag agtcagatga agtgatacta gcttggggtg cttacgctaa aaagcccgtt
9661 gttgaatcac gcgtcaatga agtattagag atgttgaaac cacataagaa aaaagtgaaa
9721 cgactcatga accccgcaac caatgaaatc atgcatcccc ttaatccgaa agcgagacag
9781 aaatggactt tgaaagcata acctatatat ggaggtaaac gatgaaggaa atcaatattg
9841 tttcactaca aatgataaaa acagacacat taagttatct aaaaaatcgt atttcaaacc
9901 ctgaggatgc ggcagaaatc ttgcgttcat tcattggaaa cagtgaccga gaacatctca
9961 ttctcatatg tatgaatagt aaaaatgaac ctacacacat tcaaatacta tcgattggat
10021 ctattaacca aacagtgatt catcccagag aaatattcaa aacagcaata ctcagtaacg
10081 ccaacagtat tatgctcggt cataatcatc caagtgggga tatattaacc atagtataat
10141 aaataatagt agttaaagta gtttaagagg agatattaaa gggatatttt actttaaata
10201 aaaattaaca aggtttataa tttgtagtag ttaaagtatt taaagaggtg ggaacatgtg
10261 aaggttcaaa ggatagaagt ggagaataag ccgtatccat tgtatttatt actagataaa
10321 gaataccagc taatagaacc agtaatgaaa tttattaaat acttagataa tactggtaag
10381 tctcctaata ccattaaggc atactgctat catttaaagt tgctgtacga gttcatggaa
10441 cagagaggtg ttattcttaa tgatattaac tttgagttgt tagcagactt cgtaggttgg
10501 ttgagatatc cttcagcatc aaatgtaatt gatcttcagt caaaaaaagc cataagagaa
10561 gaaacgacag tgaatacaat tttaaatgta gttatgagtt ttcttgatta tttaagtaga
10621 ttaggagaat ttaaatcaat tgatgtattt aaacaagcaa agggaagaaa tttcaaagga
10681 tttttacatc atgttaataa gggtagatac caaaagaatg tcttaaagtt aagggttaaa
10741 aagaaacaga taagaacatt gagatcaaag gaagttaagc aaattattga tgcttgtcat
10801 acgaaaagag ataaattaat tttaatgctt atgtatgagg gtggtttaag aatcggtgaa
10861 gtgttatcgc ttaggcttga agatattgtc acttgggaca atcaaatcca tttaacacct
10921 agagatgtta atgttaatga agcttatatt aaattaagga aggaaagaac aatacatgtg
10981 agtaaagaac ttatgtcact ttatacagat tacttgatat atgagtatag tgaggaattg
11041 gagcatgatt atgtttttat ttccttaaaa gaaggctatt ttgggaaacc actaaagtac
11101 caaagtgttc ttgatctagt tagaagaata gttaaaagga ctggaataga atttacatca
11161 catatgcttc gccacactca cgcaacgcag ctaattaggg aaggatggga tgttgcgttc
11221 gttcaaaaga gattaggtca cgcacatgtt cagacaacgt taaataccta tgttcatctt
11281 tcagatcagg atatgaaaaa tgagtttaat aaatacctcg agagaaagga gcataagaaa
11341 tgaatgcttc tagtaaaagg aaaattatta gtcagagtga gattagcaaa aaaatagctg
11401 taatgaatga agaaatgcag gggttttggg ctaataatag ttgggatata agaaaatgtc
11461 cacatccttc tgccatagaa ttaagtaaga atcctgcttt aaggaatcgt tgggttcgtt
11521 ttgaacgtgt taaaaatctg tggttaagaa cagaattgaa atatttttat ttttaccatt
11581 taaacaatgg aatatggaat gcaaaaactg tctggattag aaaaggaaca gtaattaata
11641 aaatgttaga tttcttagat ttaaagtatc ctagcattac ttcaattact gaagttccta
11701 ttgaaaaagc aatgacggag tatagaactt atttgacaaa acggggtgtt agaattacca
11761 ctactaatta taagattact gctaatcaag aaaaaacacc tgtaaaagct aattcctact
11821 atgttactaa tctaaaacaa tttatggagt tttatgagaa cttttatttt gatggagagg
11881 agtgggataa agacgtttgg gatagacgta acttaccttt gccagatgat aaggttaacc
11941 caacacaata tgaatataca attaacttta aagggtttcg gaatacatat tttaaacaac
12001 ttgtaaaaag atattgtaag ttgagattga acgtggatag cttttcctat gtaagtgata
12061 ttgcccaaag acttaaagag ttctttaatt ttctggacat gaaatttaaa cgagttcaga
12121 gagtacacca attaacgaga gtggaaattg aagcatattt aagtgaacta aacatgatgg
12181 gaataaaacc tagtacaata actgggagga tctctatatt ggaaggacta tttagtaccc

12241 ttcttaggct agaatgggat gatgttcctt ccaaaatatt aatttattct gaggactatc
12301 cgaaaatacc aagagcaaaa cctcgcttta tagatgaatt cgtcctagag caattgaaca
12361 gtcatcttga taaattaccc gaatatatag ctacgatgac tatgattgtt caagaatgtg
12421 gaatgaggat aagtgaattg tgcaccttga aaaaaggctg tctattagag gacaaagatg
12481 gagatttctt tttaaagtat tatcaatgga aaatgaaaaa ggagcatata gttccaatat
12541 ctaaagaggt agctttactt attaaagttc gggaagataa agtttcagag gaatttccag
12601 atagtgaata cctctttcca agaaaagatg gatcgccatt aaaacaagaa acatttagag
12661 gtgagttaaa taaattagct tatgagcaaa atatagtgga taaatcaggt gagatttata
12721 gattccatgc ccatgccttt cgccatacag taggaacaag aatgattaac aacgggatgc
12781 cccagcatat tgtgcagaaa tttttggggc atgaaagccc agaaatgaca agcagatacg
12841 ctcatatctt tgatgaaact ctaaaaaatg aatttactaa atttcaggaa aaactggtta
12901 ccaataatgg agatgtgctt gatctagatg aagataatga agtcgatgat gtagagcttc
12961 aatggttcaa gaaaaatata aatgcacaag tgcttccaaa tggttattgt agattgccag
13021 tagtagcagg tggttgtcca catgcgaatg catgcttaga ttgcactcac ttctgtacca
13081 gtaagcaatt cttaccacag cacgaagaac agttagagcg tacagaagag ttattagcca
13141 tagctaagga taaacaatgg caaagacaag tagagactaa tagccgtgtt aaagagcgtt
13201 tagaacaaat cattggaagt ttgacggggt aattatcaat ggataaacaa gttagaaata
13261 caacagaaat tgtacgtttg gcgaagcaga aatcaaaaaa gacaagggaa aaagtagaca
13321 aagcgatttc taaattttcg attgaaggta aagttattaa ttttaattca atagcaaagg
13381 aagctaatgt ttctaaatca tggctttata aggaacacga tattaggcaa agaatcgaat
13441 cccttcgtga gcgtcaaata acagcaaatg tagtctcaaa acccaagaaa agttctcgtt
13501 cggaggaaat ccttattaaa accttaaaaa gaagagtaat ggaattagaa aaagaaaata
13561 aaaaattaca gaaccaaatt caaaaattat atggagatct gtataataaa gaataattat
13621 taatctgtag acaaattgtg aaaggatgta cttaaacgct aacggtcagc tttattgaac
13681 agtaatttaa gtatatgtcc aatctagggt aagtaaattg agtatcaata taaactttat
13741 atgaacataa tcaacgaggt gaaatcatga gcaatttgat taacggaaaa ataccaaatc
13801 aagcgattca aacattaaaa atcgtaaaag atttatttgg aagttcaata gttggagtat
13861 atctatttgg ttcagcagta aatggtggtt tacgcattaa cagcgatgta gatgttctag
13921 tcgtcgtgaa tcatagttta cctcaattaa ctcgaaaaaa actaacagaa agactaatga
13981 ctatatcagg aaagattgga aatacggatt ctgttagacc acttgaagtt acggttataa
14041 ataggagtga agttgtccct tggtaatatc ctccaaaaag agaatttata tacggtgagt
14101 ggctcagggg tgaatttgag aatggacaaa ttcaggaacc aagctatgat cctgatttgg
14161 ctattgtttt agcacaagca agaaagaata gtatttctct atttggtcct gattcttcaa
14221 gtatacttgt ctccgtacct ttgacagata ttcgaagagc aattaaggat tctttgccag
14281 aactaattga ggggataaaa ggtgatgagc gtaatgtaat tttaacccta gctcgaatgt
14341 ggcaaacagt gactactggt gaaattacct cgaaagatgt cgctgcagaa tgggctatac
14401 ctcttttacc taaagagcat gtaactttac tggatatagc tagaaaaggc tatcggggag
14461 agtgtgatga taagtgggaa ggactatatt caaaggtgaa agcactcgtt aagtatatga
14521 aaaattctat agaaacttct ctcaattagg ctaattttat tgcaataaca ggtgcttact
14581 tttaaaacta ctgatttatt gataaatatt gaacaatttt gggaagaata aagcgtcctc
14641 ttgtgaaatt agagaacgct ttattacttt aatttagtga aacaatttgt aactattgaa
14701 aatagaaaga aattgttcct tcgatagttt attaatatta gtgacatttg catgcttcaa
14761 agcctgtcgg aattggtttt tagtgaaaag aacacgatat tcacggttta cccacttata
14821 aacaaaagat cgatactttt tgtagtcctt ctttgaaatc aatggttgat gtcgttcaag

14881 aacaatcaat acagagtcta cacttggctt aggatgaaaa tatagtggtg gtactttttt
14941 gagcattttt atatccatct ccaccattaa tagtaaaccc aaagctcgtt gcagattttg
15001 caatcttttc gcaaatccct tctcaacgat aagatagcta tatttagcct gactttcaaa
15061 ggtaattctt ttgacaatat ccgtactgat gttataagga atattaccat atatcttata
15121 gtttatatgt tttgggaagg aaaattttag aatatccgtt tgaatcactt ttatattctc
15181 agaggggttt accgcttctt tagtcacttg acataagcct ccatcaattt ctatagcagt
15241 aactgatcga ctcattttga ctagctcttt ggtaaaatgt ccttttcctg atccgatttc
15301 tattacgttg tcttgtttac tgatattcgt gtgattcaat atttctttta catgcttttt
15361 agaagtaata aaattttgcg tgtctttagg gtttttctgg ttcattataa ccttctcctt
15421 actggttata atgaactgac attaagcgtt cattataacc aattaatttt gatttggttg
15481 ataacgaact ttattaataa caaatataga aaaagtaccc atacttttat ctcctttcca
15541 tttttcatt atgaatgaga taaagtaatt tctactactg cgatactggt gcacataatt
15601 gaaacctcct tatgtaactg aatatgattg tatcatcttt tcattttaga ataaaccttt
15661 atatgtaatt ttgcacttga cacttcagat ggacgttttc ttcaaaataa tatgttttga
15721 aacttgataa gagaagccac tgtctttact aaaaagtaaa ggctgcgata aaggacagtg
15781 actttaataa agttgcactg ttgtattgtc tttggataaa attgtctaaa gggaagtttt
15841 atccccaacg aaaaaacatc taaagtggta agtaatgaaa gaatgtcgat aaagtaggaa
15901 tgggggatct ttttgaatac aatagaatca cagtttgata aggttgcaga agattacgat
15961 ttcgtgaatg agcttttgaa tgattattca ttctttgtgt ctaatatgtc tccaaagaaa
16021 ggcagagcat tagatatcgg atgtggctcg ggtttgttag tggagaaatt agcaagttat
16081 tatgatgaag tggtagggat tgatatttct aatcaaatgc tcgatcttgc caaatctaaa
16141 cgtcaactaa caaatacggt ctatctgaat atgaatgcag aacaacttaa ttttaatgag
16201 aagtttgatt ttattgtaag ccgaacaacc tttcatcatt tggatgatat agccagcgta
16261 atacagcaaa tgaaggaact gttgaatgag gaaggaagaa tagttattct tgataatgta
16321 tctgaagtcg agacaccgcc tacctatgtt tataaattag gggcaatcca agagttttta
16381 ccgcattgtt tcaaatttgg aataaaaaat gcgattagaa tttataatca caacacatct
16441 aaatcatggc ttgagcattt agcttcagat aagtacctat ctgaacaaaa ttattacgat
16501 ttatatgaaa agttgttgcc tggatgccaa ttccataaaa tgggttgggc aatgggggtt
16561 gtctggacaa aataacttaa agctgtggct ttgaaaagtt gaatttgtga ttgttttcag
16621 agctatcggg tgctttagta taaataacaa tgaataaaaa atcagcctgt atcttatgtt
16681 actattaagt aatttaagat acaggtttta ctatttttga aataagtacc ttccacttca
16741 aatacatgta ttcaaagtga aagtattgat ttaaatgatt cttttgtaat ttactcttga
16801 taaagtttaa ccccctctcc tgaagatatc aatgtaaccg aacggttaat gctcatcagt
16861 gaaatgatgg gcattttatt ttatgatcat attatttttt cagatagtaa caactattcc
16921 attagacaac atgatttaga agtagaggta tagccgtatt ttgtcactac cgttatttct
16981 tcacttatca atatttataa aatcccttta taacaacatt ttcaactgaa aaatattttt
17041 tacaactttt attaaatata cgtaggattg acttattgtg acaaacctat tttgtaatat
17101 ataacttaaa ggaggtgaca tattggatcg tgcatttcga atacttacga tatacaatcg
17161 tttactacaa aataaatccg tcaacaagca atcactcaca ttagaactcg atacaagtcc
17221 tcggacgata caaagagata tcgacgatat aagaaacttc ttatatgaaa gtaaatcatg
17281 gataagtaca cctaaagaaa tcacgtacga ttataaatct gaaagttata agctcgaaca
17341 agataagagt aaaaatgtgc attttatgta taatattttg actgctttat acctgacgac
17401 acctaaatta agtcattact tttatcaata tcttaaatta ttaatcataa aacatcattc
17461 aaatcatcga actacattat taaaatatct tgagcgcttt gaaattgata agaatcaaac

17521 aggtatttcg acatcatcat tagttgttca agcaatgaac gaaaataaat atcttcaata
17581 caatcaaaaa atattattac ctttatcgat gtactatcaa atgcatgctt ttcacttggt
17641 atatcgaatg aacgatgaaa tttatatcga tgatattaat caaatgaatc tgagtttctc
17701 tgaaaaatct gtggacacac atgaaaaagc aaaggtacaa acctatatca cgtttgaaat
17761 ttctaaagat gtgtggtcaa aaatgcatcg atattatcaa attcatgtga tagaaaaata
17821 tgatgcgcat tatcttatcg tcacttttaa gatgacaagg atcgaagcga ttcaactctg
17881 ttttatgtat cgatccaata tacgtatcat ttcaccccct gatttgaagg aacaagtgat
17941 tgatgaatta cttttattac aaacaaccta tttaaaacaa caggttcaaa agtagaaata
18001 acatacaata tttaataaat aacattaata ataatgaaag gaatgggaat tatgagtaac
18061 atcataaaaa caataggcaa aggtattgga aaaggtgtcg gtgcagcagc atattattca
18121 gttgcaactg ttggcgcagc tatcggtgcg atttgggaaa caacaggcga tagaggttta
18181 atgagagatg ctttaagtga tagttctaat caagatttga tggaaatata tgagatacaa
18241 aagaaagatg atggggatta tgatacttat attgcagcta aatctttatt acaagagaga
18301 ggattcactt ataatcacga aacgaacaag tgggaaaaat agtaaggagt gaaataaatg
18361 gaatttacat attatgaaac gaaatcttta aatgattttg aaaatgaact gaaaacactt
18421 gctttaaata aaaaatatac attcagtaag gaaaccggag tccttaaaga agatgaaatc
18481 cttgaaaaat cgaatagcca tgagcgcgaa ctattaaaag tatttaaaga taatgcacag
18541 ataacaactt atcattttaa gagtcataaa agtaattatt taattattat gggagaaaca
18601 aaagcgccta atattattgg attaacagtc atatttaaca aaaaaccttt taaacaaatg
18661 ggcaaaattg cattaggatg gctaattgct ttgacttcag gtggaagtat attagccatt
18721 ggagcagtat ttggaatact tacctcagtt gcaagacgta cacttgtaat caaaggacca
18781 ttaggaaaag aaatttcaca aattatagaa aatgaattag gagaaccgat agaaattaaa
18841 aaataataga aagtaactat tttcaaagac acattgattt caaaatgtgt tctttttcaa
18901 ttattttaga ataaatatag aaaagcgtag caaaatagat taatataaaa ttaaaataaa
18961 tatagaatgg ggttagcaat gtgaagaaaa tttttgcaag cttaggtgtt attttattag
19021 ttgcaatatt gggagttgct gttgtatttg catatgggag ttacaaagat ttagaactaa
19081 aaaaagaaga agctaaacta gaggataaga agaataagaa aatcgataaa aacaaaacta
19141 cagaaaatga aaatcaagac cagattaata atcaaacaat tacagcacca aatgaaaaag
19201 cacaatatgg caataaccta ggagttcaag aaaaaagtac ggataaaaat gatcctaatg
19261 gtgtgcttag atttgataca aacggagatg gatttatctc cactagtgaa gtaacaccag
19321 aagctcaaaa attagttgat gaaggtaaac tacagccatc ttcagataga atggttgaag
19381 agtggactaa gaataaaaat aatgtagata atacatatga ctatgatgaa tcatataaaa
19441 aacaacaaga aatgtacaaa aaagctgctc gtggcgaagt tcctgaacct ggagaaacaa
19501 taaaataaaa aagcactagc tacaattgcg ataattataa tttttggcat attagcacct
19561 gttgtttttt tgttacaaca aatatcaaga agtagaatta gaaaaaataa attacaggct
19621 aagcaaaaag aaaacgaaga aagtaattct caaactacta gtgaaacatc taataatgaa
19681 aatcaatcga atcaaagtca aaagaatagt caagatcaag ctacaacaga agaaaataca
19741 aatgatgaac aacaagtgac tagagataat atgtttgatt acgctattgc tgctgtaaat
19801 gaagtagggg atgctgattt attaaaattc caagaaccgg aatacaatgg gtcagaatgg
19861 acaattaatg caaataataa atcaggtgct ggagctaaca ccatcgtagt taaagatgat
19921 ggaacagttc aaatatggaa tggacctaaa acatctatga atcatgaaac taaaattgaa
19981 ttatagtgaa agcgattaac aagctctaca tcacctttac gagtgatgta gagctctctt
20041 atcatatcaa gttactttgc catgaatact agtcgggtga aaaattatat gtaacattca
20101 aagcgtatta tgaaaataaa tgatattcat tcatcaccaa taaccacaac gcttacttaa

20161 aacaagatgg aaccatgaaa aataatttac cacagttatt attatgtaaa tgcattacga
20221 agagatcctt actctaagct taacgacaca attaattatg aacaaaattc agaaatggat
20281 gaatttgaat taatctatat tctgatttca ccctacttat taatcaccaa aattttgaaa
20341 ccgcataaac aaaaagttaa ctggctcatt aaaccagcaa ccaataaaat tatgcatcca
20401 ctcaacccta aagcacgtca aaaatggaca ttaaactaaa ggagaattat ctatgaacca
20461 agattcaatt caatcagatt ggcaaacagt tgctaagtgc ttagcgtcac aaaactatat
20521 ttcaattgtg aaaggtttag ttcatcattt cacaaacatc gatgatgagg aaatacttga
20581 taaaacctat gacgatttta tgaatgatga ctctataaca acggtactta ataatgattt
20641 tcaagatatt atcaatcgct acttatcaaa ataaaatgta tataatagta aaaccagagc
20701 atcccttcac ttttatagtg agggatgctc tctatttaat ttttttgcta tttaccatta
20761 tatatattta attttattta aaatgtattg tataatttaa aataatggag tgacatttaa
20821 tttatgaaaa aatatataat tagtttagta cttttaactt cttttttact atccgcttgt
20881 acaattaata ttgagcggga tgaggaaaat agtgagaaaa ataaacagaa agaaaacact
20941 catactgatt caaacaatac aagttcgaat gaatcagata cttctaaaca atcatctcaa
21001 gaaaatacta taaatcaaac agaacaaaat caaccagcta ataataattc aagttcacaa
21061 caaaatccgc aaataataac agaaaatgaa gctattcaaa aggtaaaaga tgagtttcca
21121 tctacacgaa caggaaatga ctatcgtata gatactacta gaactgataa taatgtgtac
21181 gctatcaaat ttacttctca agatgcagaa ggttatccta tgcaagctgc tgttattatt
21241 gataagcgaa caggagaatt tattgattat attgatgata gaagcgatga agataaagag
21301 cgtcatgtcc aacatgctaa agaaagtact ctttataaag gtccatacga tgctttcaga
21361 aaagatttta gtcataaaat aaatgaatga agtatatata tgaattatca tctttaatat
21421 aagagcatcc ctcactgcaa agtgaaggat gctcttttag tttatttttt tactgattac
21481 tcgccataag ttctgcttgc tctacaacct gctctacagc cattttttgt aaatctggtg
21541 gatagccata ttttttaagc aatcgtctaa cagctacgcg cattttagct tttgcgctat
21601 cgcgtttaga ccaatcaaca cccatgtttt ctttcactgt tttagttagc tcatgagcaa
21661 tcgcacgtag ttctttatct cccatggctt cttttgctgt ttcatgtgaa gctaaagcat
21721 cgtaaaacgc aatctcatct gaattcaggc ctaattcatt tcctcgttgt tgttcttgtt
21781 taatatcttt agcgagttga atgagttctt caatcacttt tgatgtttca atagaacggc
21841 tattatattt attaatcgaa tttcctaaca tttcagagaa acgcttagat actgtcgcgt
21901 tcgttttcat taatgatttt acttgccctt tgagtaatcg atttaataat tctaccgcaa
21961 cattttttg tttcaatcct tcgacatctt ttaggaagtc atctgatagg attgataaat
22021 cgggttgttc aagacctagt gtttggtaaa catcaatcac atcttctgtc acaacagatt
22081 gtgacacaag ttgattaatc tctgcttcaa cttctgcagg tgttttacgt gtttttcctt
22141 cttttggcgg ttgtaacaat ttaacaagtc ctgctttaac tgctttaaag aaggcaattt
22201 catcattgag ttcttgggct gtaggttcag tcgcacaaag cgcaaaagct ttccctaact
22261 ctgtgaccgt tttaataaaa cgctggcgtt catcttcgcc taaaccaatc acataatcca
22321 tcgtatctga aatcgcataa taacgttcag actttttctc tgaattaaac ttagaatagt
22381 caagattata taacatatct tgaatcacat catatttcaa taacatcaat tcaaccgctt
22441 tatctgtatc tatcgccgtt tgtgcttgat cagattctgt atattcttta agtgcctctt
22501 ttaaactttc agcgataccc acataatcga caatcaatcc acccggttta tctttaaaca
22561 ctcgattgac acgagcaatc gcttgcatta aattatgacc tttcatcggt ttatcgatat
22621 acatggtatg catggaaggt acatcaaatc ctgtgagcca catatctcga acaatcacga
22681 gttgtaattc atcattcata tctttcatac gtttttctaa taaattacga cgttttttag
22741 gaccaatatg tctttggaaa gaagttgggt cactagatga gccagtcatc accactttaa

22801 tgaccccttt atcatcatca tctgaatgcc attctggttt tagacgaatg atttcatcgt
22861 ataaatcaac agcaattcga cgactcatcg ttacgatcat tcctttacct ttcatcgctt
22921 gctgacgtgt ttcaaaatgt tggatgatgt ctttggctaa agcttctaca cgaggttttg
22981 cgcctgctaa ggcttcaatt cgtgaccatt ttgattttaa acgctgtttt acatcctctt
23041 cttgatcctc tgtaatgtca ttatatgctt cgtctaagtc taaattttga ggtaaattta
23101 atggaattat gcgactttca tagtaaattt tcaccgtact tccatcagct acggcttgtg
23161 tcatatcata aacatcgata tagttcccaa agaccatttg cgtattttta tccgttgaag
23221 ctacgggcgt gcccgtaaat cctacaaacg tcgcattcgg taaagcatct cttaaatact
23281 tcgcataacc atatttaatg ccttcccctt tatcatcgta ttttgcatta aaaccatatt
23341 gtgtacgatg cgcttcatct gccataacaa tcacattttt acgttctgtt agagcagaca
23401 tggtcgtttc gttttgttca ggttcaaatt tttgcatcgt tgtaaataca ataccgcccg
23461 actcaacaga taataacgat tttaattctt tacgtgtttc agcttgtttt ggtgtttgtc
23521 ttaataaacc tttaccagac cgtcctttcg atttaacaaa cgtactgtat agttggttat
23581 ctaaatcatt acgatctgtt acgacaacta aggtaggatt attaagcatt tgaattaatt
23641 ttccagagaa aaagaccatg gttaaacttt taccagaccc ttgagggttc tgttgcaaag
23701 taaaaaaata tagctaacca ctaatttatc atgtcagtgt tcgcttaact tgctagcatg
23761 atgctaattt cgtggcatgg cgaaaatccg tagatctgaa gagacctgcg gttcttttta
23821 tatagagcgt aaatacattc aatacctttt aaagtattct ttgctgtatt gatactttga
23881 taccttgtct ttcttacttt aatatgacgg tgatcttgct caatgaggtt attcagatat
23941 ttcgatgtac aatgacagtc aggtttaagt ttaaaagctt taattacttt agccattgct
24001 accttcgttg aaggtgcctg atctgtaatt accttttgag gtttaccaaa ttgtttaatg
24061 agacgtttga taaacgcata tgctgaatga ttatctcgtt gcttacgcaa ccaaatatct
24121 aatgtatgtc cctctgcatc aatggcacga tataaatagc tccattttcc ttttattttg
24181 atgtacgtct catcaatacg ccatttgtaa taagcttttt tatgcttttt cttccaaatt
24241 cgatataaaa ttggtgcata ttcttgaacc caacggtaga ccgttgaatg atgaacgttt
24301 acaccacgtt cccttaatat ttcagatata tcacgataac tcaatgcata tcttagatag
24361 tagccaacgg ctacagtgat aacatccttg ttaaattgtt tatatctgaa atagttcata
24421 cagaaggctc ctttttgtta aaattatact ataaattcaa ctttgcaaca gaaccatttg
24481 cgatacaacg aggcgacatt agccgttaat ttgcttaact gtagaagatg taaaagaacc
24541 ccttgccata tatttaacaa tgttgtccgg gataacaaca cagtgaatcc accgtttagt
24601 taagatgatg gattttttca aactgtggat gaggtacaga gtttcattag ataaaaacag
24661 ggaaaactta ttaaagagtg atttctgtga ggaggataat gatgacaaag aaatacgatc
24721 ttattgtgat tggtacaggt tctgcggggt caatcacagc agcaaaatgt aataaagccg
24781 gctggaatgt agctatggta gatgacaggc cgtttggagg cacgtgtgca ctacgtggat
24841 gtgacccaaa aaaggttcta catggtgctg ccgagcttat tgattggaac aaacggatgg
24901 ttaagaatgg agtaccgtct gaagtctcca tcaactggaa agatcttatg aattttaaaa
24961 gaacctttac agatgatgtc ccggaaaaaa aagaggaagc attgaataaa cagggaatcg
25021 atacgtatca tggcaatgca tctttcgtga gtgaggacaa acttgaagtg aataaagaag
25081 tgctcgaggg gagtcatttc ttgatcgcta gtggtgcgaa acctacccct ttacccataa
25141 aaggagaaga acatcttact tatagtgatg agtttttgga gttggatgaa ttaccacaaa
25201 gaatcgtctt tgttggtggt gggtatattt cgtttgaatt tgctcatatc gcggcacgtg
25261 ctggatcaga agttcatatt atccatcgag gtcaaagacc tttggagaat tttgatatag
25321 atcttgtaga tatcctttta gaaaagtcga aagagattgg catacaggta catcttcaac
25381 attccgttga atccattgaa aaagaacaag gaaagtttca tgtgtacgct cgaaaaaaag

25441 aggatataac gcggtttgaa gcagacattg ttattcatgg tgctggacgg gtccctgcct
25501 tagatatgaa tcttgaaaaa ggaaatatag aaaggaaaaa acatggtgtc catgttaatg
25561 agtatttgca aagtgtaagt aacccgaatg tctatgcagc tggagatgct gcagcaacgg
25621 atggcttgcc cctcacacct gtagccagtg cagattctca tgtcgtagca tctaatttat
25681 tgaaagggaa cagcaaaaaa attgaatatc ccgtgattcc atctgctgta tttaccgtac
25741 ctaaaatggc atcggtaggt atgagcgagg aggaagccaa aaactctggc cggaatatta
25801 aagtaaagca gaaaaacatc tccgactggt ttacgtataa acggacaaat gaggactttg
25861 ctgcgtttaa agtgctaatt gacgaggata gtgatcaagt tgtcggtgct catttgatta
25921 gtaatgaagc cgatgaacta attaaccact ttgcaacagc cattcgcttt ggaatttcaa
25981 ccaaagaatt gaaacaaatg atatttgcct atccaacggc agcttcggac attgcacaca
26041 tgttgtaagt ttgcgttttg tgagatgttt ataaaccact ttatcgtcta atcttgttat
26101 atgatcttag aatcaaaaag aaaataactt ttttgacagc cacatttttc aacggaagaa
26161 tgtggcttct tggcgtgaag agcagatatc tttattctta aacttctaaa aaagcttatg
26221 tgaacacttg ataaataagg ttttttatct gacctatttt ataagattat tctataaaag
26281 aaaaaataat gtatgacttg accgtgtact atggtacagg gtttatactt tttattgagg
26341 tgacaatatg gagatgaaaa tcagtgaatt ggctaaagcg tgtgatgtga ataaagaaac
26401 cgttcggtat tacgagcgga aaggattgat agccgggcct cccagaaacg aatcagggta
26461 tcgaatatat tcagaggaaa cagcagatcg ggtacggttt attaaacgaa tgaaggaatt
26521 ggatttctcg ctaaaggaaa tccacctgtt gtttggtgtg gttgatcaag atggggagag
26581 atgtaaagat atgtacgcct ttaccgttca aaaaaccaaa gaaatcgagc ggaaagtgca
26641 ggatttgtta cgaatccaac ggttattaga ggaattaaaa gaaaagtgtc cagatgaaaa
26701 ggcgatatat acctgtccta ttattgaaac gttaatggga gggcctgata aataaatggg
26761 ggaagatttg aatgaaaaaa cgcatttcct tcaccgctat aatgacggtg ttgttaatcg
26821 gtttaacagc ttgtggagca gaatctgata ccgcgaatga atcaaaggtt caagacattc
26881 aaggaaatcc agtcagccta cctaatgaga aacccacact catttatttt atggcaacct
26941 ggtgtccatc ttgtatatac aatgaggaaa tctttaagga aattcatcaa ctaaacccga
27001 acgatgttca attgatcaca gttagtttag accctaacac agatacaaaa gaaagctttg
27061 cgaaatttaa acaggattat ggcggggatt ggccccatgt tttaaagata ggagtggaat
27121 tatgagtttt tcgtttctct tcattctgac cgctggaatg gttgcggcct ttaatccttg
27181 tggtatcgcc ttgcttcctt cttatatctc ctatttaatt ggaggcgaaa caaaggatca
27241 ttcgttccgc tatgcaattt ttaaaggatt agggcttggt ggagcgatga ccacggggtt
27301 tttaacgatt tttgtattgg ctggtttgtt gataggagga ttgggaagcg cactaacagg
27361 gatttttccg attctttcat tggttatggg tatactcatt gctttattgg ggttgggcat
27421 gctatttggg aagcatttgc cgattaaaat aggatctttt caagtcaagc cagggaaatg
27481 gtctatctat ttttacggaa tagcctatgc cgtgacatca cttggttgta ccttgccagc
27541 ctttatgttg gtggtctctg catcgctgaa tgacaatagc gtaacggccg tgatcatcaa
27601 gttcatcatc tactcccttg gtatgggaat tgtggtgaca gcgatcacga tggtctcatt
27661 gatttcacga caattggtac agaagtttct gcacaactat atgggttcta tccaaaaaat
27721 agcagctgtt gtgattttcc tctccggttt gtacatggct tattactggt atttcggttc
27781 tggtggcatt tgtacatttt aaaatccaac cgatcaggtg taacaaaaaa tagatgaaag
27841 atgtaaaaaa gctaaacgaa caaatttcaa gttaggctat aggccatgta tgtaccttaa
27901 ccaaagaata aaattttttt agaaagagtg gtgagaatga tggatgagaa tcgctcgaaa
27961 ggtaaccgct ggggtgtttg ggcctttttc ggaatattat tggttccttt attggttcct
28021 ttactttgtt gcgcaggacc gattctcctt gttgcattag ggagcacagg tattggtgcc

28081 ctctttgctg gtgcgacagg aaattggtgg ttgaccggca tttttgcagc attggccatt
28141 gttatgattg ctctgatcct cagcaagttg ttgaagaaca aatacaattc tcctgaaggc
28201 aatggaaaaa caaaaaataa aacggattgc tgtacgcctc cagagagcgt ggatcggaaa
28261 catgagacaa gataaatatg agataaatgt acagaactgt ctatcaaact ttaaatagaa
28321 agagggattc aaatgactca aaattcatat aaaataccca ttcaaggcat gacatgcaca
28381 ggctgtgaag aacatgtaac cgaagcattg gaacaagccg gagctaaaga tgtttcggcc
28441 gatttccgac gcggtgaggc catttttgaa ctcagcgatg atcagatcga aaaggctaag
28501 caaaatattt cggcagccgg ctatcaaccc ggagaggaag aaagccagcc ctctgaaaac
28561 agtgtagatt tcaatcggga tggcgattac gatcttctga ttattggttc cggcggtgcg
28621 gcgttttctg cagctatcaa ggccaatgaa aacggggcga aagtggccat ggtagaacgg
28681 ggaaccgtcg gggggacctg cgttaacatc ggttgtgtac cgtcaaaaac catgcttcgt
28741 gccggtgaaa taaacggtct cgcccaaaac aatccgttta ccggacttca aacgagtacc
28801 ggtgctgcgg accttgccca attgaccgaa caaaaagatg gattagtcag tcaaatgcgt
28861 caagaaaaat atatagactt gatcgaagaa tatggatttg atctcattcg tggcgaggcc
28921 tcgtttatcg acgataagac gatacaagtg aatggacaaa acatcacgtc taaaagcttt
28981 ttaatcgcaa cgggggcttc tccggctgtt ccggaaatcc cgggaatgaa tgaggttgat
29041 tatttaacaa gtacatccgc actcgaatta aaagaggttc cacaacgatt ggcagtgatc
29101 ggttctggct atatcgcagc ggaattaggt caaatgtttc acaacctcgg aacagaagtg
29161 actctcatgc aaagaagcga gcgtctgttt aaaacctacg atcctgaaat ttccgaagcc
29221 atcgatgaat ccttaactga gcaaggactt aacctgatca ctggggtcac ttatcaaaag
29281 gttgagcaaa acggtaagtc gacaagcatt tatattgaag tgaacggtca agaacaagtc
29341 atcgaagccg atcaagtcct cgtggcaaca ggaagaaagc cgaacacaga gactttaaac
29401 cttgaatcag caggtgtgaa aacagggaaa aaaggcgaag tgctgaccaa tgaatatttg
29461 caaacgtcga ataaccgaat atatgccgcg ggcgatgtga ccctcggtcc gcaattcgtt
29521 tatgttgcag cttatgaagg cgggattgtg gcaaataatg cgttgggtct agcgaaacgc
29581 aaaatcgatc ttcgctttgt tcccggcgta accttcacca atccatcgat cgccacagtc
29641 ggcttgactg aacaacaggc aaaagaaaaa ggttacgatg tcaaaacatc ggtccttccg
29701 ttggacgctg taccgcgggc cttagtcaat cacgaaacaa caggggtcta taaacttgta
29761 gtcaacgccc aaacccagaa attgatcggg gcgcacattg tgagtgaaaa tgctggagat
29821 gtgatttatg cggcaacgtt agcggttcaa tttggattga ccattgaaga ccttacggat
29881 agctttgcgc cttatttaac gatggctgaa ggattaaagc ttgcagcctt gacgtttgat
29941 aaagacgtat cgaaattatc ttgttgtgca ggctaatggt tccttttgat tctaatgcta
30001 tgttgcagga ggcacttaaa acaatgaagt ggagaaaata taaggaggaa taagcttatg
30061 aaaaatattt cagaattctc agcccaactt aatcaaactt ttgatcaagg ggaagccgtc
30121 tctatggagt ggttattccg tccgttgcta aaaatgctgg cggagggcga tccagtcccc
30181 gttgaggaca tcgcggcgga gaccgggaag cccgtcgagg aagttaagca agtcctacag
30241 actctaccta gtgtggaact tgatgagcag ggccgtgtcg tcggttatgg cctcacactg
30301 gtccctaccc cccatcgctt cgaggttgat gggaagcaac tatatgcatg gtgcgccctt
30361 gacacactta tgtttccagc actcatcggc cggacggtcc acatcgcttc gccttgtcac
30421 ggcaccggta agtccgtacg gttgacggtg gaaccggacc gcgttgtaag cgtcgagcct
30481 tcaacagccg ttgtctcgat tgttacacca gatgaaatgg cctcggttcg gtcggccttc
30541 tgtaacaacg ttcacttttt cagttcaccg agtgcagccc aagactggct taaccaacac
30601 cctgagtcga gcgttttgcc cgttgaagat gcctttgaac tgggtcgcca tttgggagcg
30661 cgttatgagg agtcaggacc tactaatggg tcctgttgta acatttaatc ttaggcaatt

30721 acgcctgcgt caatcttggc cttacgatga tgtcctgtaa ccgaagctct atctagtgaa
30781 accaatttta tgggaaacca aataaataaa aagatcaatt gcttgtgcct atatcgggga
30841 gttaataaag cgagaaagtg gattcttgaa tataggagta aataaggtgc caaaatggca
30901 ccttatttaa aatatgtaac gccaaggtct gccgtattat attgcattgg aaaaagaaaa
30961 ccttctgtat aaggagatgt tatatgatat ttggtaaact gttcggcagt ttaggcgtag
31021 aaagcttttg ctccttgaac atcacgtttg ccagttacaa tgtcatttaa caagtttaaa
31081 gataaaaaat tcatcgcttc ctcatgacac tttgctgatg cctcacctgt agttcgatct
31141 aagtagacac ttccatcaaa agcggcaatc gcatcatata agtgtacggg cactttataa
31201 tcgatggtct gcttaaggaa atcaaggtga ggggtaggga agttatgagg gactggatcc
31261 ttatatacta tcgtgcgttt ccatggcccg ctattgtacc aaattaatcg tctcattgtt
31321 gcttcctggg gatatccata cttactaatt atggtttttg ccccttccaa cggtggtccc
31381 tgccaatagg acaaaatgtg atttaaatgg gttctgttgc aaagtaaaaa aatatagcta
31441 accactaatt tatcatgtca gtgttcgctt aacttgctag catgatgcta atttcgtggc
31501 atggcgaaaa tccgtagatc tgatgagacc tgcggttctt tttatataga gcgtaaatac
31561 attcaatacc ttttaaagta ttctttgctg tattgatact ttgatacctt gtctttctta
31621 ctttaatatg acggtgatct tgctcaatga ggttattcag atatttcgat gtacaatgac
31681 agtcaggttt aagtttaaaa gctttaatta ctttagccat tgctaccttc gttgaaggtg
31741 cctgatctgt aattaccttt tgaggtttac caaattgttt aatgagacgt ttgataaacg
31801 catatgctga atgattatct cgttgcttac gcaaccaaat atctaatgta tgtccctctg
31861 catcaatggc acgatataaa tagctccatt ttccttttat tttgatgtac gtctcatcaa
31921 tacgccattt gtaataagct tttttatgct ttttcttcca aatttgatac aaaattgggg
31981 catattcttg aacccaacgg tagaccgttg aatgatgaac gtttacacca cgttccctta
32041 atatttcaga tatatcacga taactcaatg tatatcttag atagtagcca acggctacag
32101 tgataacatc cttgttaaat tgtttatatc tgaaatagtt catacagaag actccttttt
32161 gttaaaatta tactataaat tcaactttgc aacagaacca caaaaatgat atatttaaac
32221 tattctaatt taggaggatt tttttatgaa gtgtctattt aaaaatttgg ggaatttata
32281 tgaggtgaaa gaataattta cccctataaa ctttagtcac ctcaagtaaa gaggtaaaat
32341 tgtttagttt atataaaaaa tttaaaggtt tgttttatag cgttttattt tggctttgta
32401 ttctttcatt ttttagtgta ttaaatgaaa tggttttaaa tgtttcttta cctgatattg
32461 caaatcattt taatactact cctggaatta caaactgggt aaacactgca tatatgttaa
32521 ctttttcgat aggaacagca gtatatggaa aattatctga ttatataaat ataaaaaaat
32581 tgttaattat tggtattagt ttgagctgtc ttggttcatt gattgctttt attggtcaca
32641 atcacttttt tattttgatt tttggtaggt tagtacaagg agtaggatct gctgcattcc
32701 cttcactgat tatggtggtt gtagctagaa atattacaag aaaaaaacaa ggcaaagcct
32761 ttggttttat aggatcaatt gtagctttag gtgaagggtt aggtccttca atagggggaa
32821 taatagcaca ttatattcat tggtcttacc tacttatact tcctatgatt acaatagtaa
32881 ctataccttt tcttattaaa gtaatggtac ctggtaaatc aacaaaaaat acattagata
32941 tcgtaggtat tgttttaatg tctataagta ttatatgttt tatgttattt acgacaaatt
33001 ataattggac ttttttaata ctcttcacaa tcttttttgt gatttttatt aaacatattt
33061 caagagtttc taaccctttt attaatccta aactagggaa aaacattccg tttatgcttg
33121 gtttgttttc tggtgggcta atattttcta tagtagctgg ttttatatca atggtgcctt
33181 atatgatgaa aactatttat catgtaaatg tagcgacaat aggtaatagt gttattttc
33241 ctggaaccat gagtgttatt gtttttggtt attttggtgg ttttttagtg gatagaaaag
33301 gatcattatt tgtttttatt ttaggatcat tgtctatctc tataagtttt ttaactattg

33361 catttttgt tgagtttagt atgtggttga ctacttttat gtttatattt gttatgggcg
33421 gattatcttt tactaaaaca gttatatcaa aaatagtatc aagtagtctt tctgaagaag
33481 aagttgcttc tggaatgagt ttgctaaatt tcacaagttt tttatcagag ggaacaggta
33541 tagcaattgt aggaggttta ttgtcactac aattgattaa tcgtaaacta gttctggaat
33601 ttataaatta ttcttctgga gtgtatagta atattcttgt agccatggct atccttatta
33661 ttttatgttg tcttttgacg attattgtat ttaaacgttc tgaaaagcag tttgaatagt
33721 tatattatat tttggtttag aactatgagt ggctagcatt ttgccactca ttttttgcgt
33781 tagcaaaaac aggtttaagc ctcgcagagc acacgtatta acgacttatt aaaaataagt
33841 ctagtgtctt agacttaaac tattaaatac acatgaaacc tttgtgctta ggagtgattt
33901 ttatatgtct tattccattg ttagagtttc aaaagttaaa tctggaacaa atacaacggg
33961 catacaaaaa catgttcaaa gagaaaataa taattatgaa aatgaagata tagaccatag
34021 taaaacttac ttaaattatg atttggtaaa tgctaataaa cagaatttta ataacttgat
34081 tgatgaaaaa atcgaacaga attatacagg caaaagaaaa attagaacag acgcgattaa
34141 acacattgat ggtttaatta catcagacaa tgatttcttt gataatcaaa cgccagaaga
34201 tacaaagcag ttttttgaat atgctaaaga gtttttagaa caagaatacg gtaaagataa
34261 tttattatat gcaacagttc acatggacga aaaaacacca catatgcatt atggcgttgt
34321 tccaataact gatgatggtc gtttaagtgc taaagaagtt gtaggtaata aaaaagcttt
34381 aacagcgttt caagatagat ttaatgagca tgttaaacaa cgaggatatg atttagaacg
34441 tgggcaatca agacaagtaa caaatgctaa acatgagcaa ataagtcagt ataaacaaaa
34501 aacagaatat cataagcaag aatatgaacg tgagagccaa aaaacagacc atataaagca
34561 aaagaacgat aaattaatgc aagagtacca aaaatcgtta aatacgctta aaaagcctat
34621 aaatgttccg tatgagcaag aaactgaaaa agtaggtggt ttatttagca aagaaataca
34681 agaaactgga aatgttgtaa taagccaaaa agatttcaat gaatttcaga aacagataaa
34741 agctgctcaa gatatttcgg aagattacga gtatataaag tctggtagag ccttagatga
34801 taaagataag gaaatacgag agaaagatga tttattaaat aaagcagttg agcgtattga
34861 aaacgcagac gataatttta accaacttta cgaaaatgca aagccactta aagagaatat
34921 agaaatagcg ttaaagcttt taaaaatctt actaaaagag ttagaacgag ttttaggaag
34981 aaataccttt gcggaaagag ttaataagtt aacagaagat gaaccaaaac taaatggttt
35041 agcaggaaac ttagataaaa aaatgaatcc agaattatat tcagaacagg aacagcaaca
35101 agaacaacaa aagaatcaaa aacgagatag aggtatgcac ttatagaaca tgcatttatg
35161 ccgagaaaac ttattggttg gaatgggcta tgtgttagct aacttgttag cgagttggtt
35221 ggacttgaat tgggattaat cccaagaaag taccaactca acaacacata aagccctgta
35281 ggttccgacc aataaggaaa ttggaataaa gcaataaaag gagttgaaga aatgaaattc
35341 agagaagcct ttgagaattt tataacaagt aagtatgtac ttggtgtttt agtagtttta
35401 actgtttacc agataataca aatgcttaaa taaaaaaaga cttgatctga ttagaccaag
35461 tcttttgata gtgttatatt aataacaaaa taaaaaggag tcgctcacgc cctgaccaaa
35521 gtttgtgaac gacatcattc aaagaaaaaa acactgagtt gtttttataa tcttgtatat
35581 ttagatatta aacgatattt aaatatacat caagatatat atttgggtga gcgattcctt
35641 aaacgaaatt gagattaagg agtcgatttt ttatgtataa aaacaatcat gcaaatcatt
35701 caaatcattt ggaaaatcac gatttagaca atttttctaa aaccggctac tctaatagcc
35761 ggtaatagcc ggttggacgc acatactgtg tgcatatctg atccaaaatt aagttttgat
35821 gcaatgacga tcgttggaaa tctcaaccga gacaacgctc aagccctttc taaatttatg
35881 agtgtagagc cccaaataag actttgggat attcttcaaa caaagtttaa agctaaagca
35941 cttcaagaaa aagtttatat tgaatatgac aaagtgaaag cagatagttg ggatagacgt

36001 aatatgcgta ttgaatttaa tccaaacaaa cttacacgag atgaaatgat ttggttaaaa
36061 caaaatataa taagctacat ggaagatgac ggttttacaa gattagattt agcctttgat
36121 tttgaagatg atttgagtga ctactatgca atgtctgata aagcagttaa gaaaactatt
36181 ttttatggtc gtaatggtaa gccagaaaca aaatattttg gcgtgagaga tagtaataga
36241 tttattagaa tttataataa aaagcaagaa cgtaaagata atgcagatgc tgaagttatg
36301 tctgaacatt tatggcgtgt agaaatcgaa cttaaaagag atatggtgga ttactggaat
36361 gattgcttta gtgatttaca tatcttgcaa ccagattgga aaactatcca acgcactgcg
36421 gatagagcaa tagtttttat gttattgagt gatgaagaag aatggggaaa gcttcacaga
36481 aattctagaa caaaatataa gaatttgata aaagaaattt cgccagtcga tttaacggac
36541 ttaatgaaat cgactttaaa agcgaacgaa aaacaattgc aaaaacaaat cgatttttgg
36601 caacatgaat ttaaattttg gaaatagtgt acatattaat attactgaac aaaaatggtt
36661 ctgttgcaaa gtaaaaaaat atagctaacc actaatttat catgtcagtg ttcgcttaac
36721 ttgctagcat gatgctaatt tcgtggcatg gcgaaaatcc gtagatctga agagacctgc
36781 ggttcttttt atatagagcg taaatacatt caataccttt taaagtattc tttgctgtat
36841 tgatactttg ataccttgtc tttcttactt taatatgacg gtgatcttgc tcaatgaggt
36901 tattcagata tttcgatgta caatgacagt caggtttaag tttaaaagct ttaattactt
36961 tagccattgc taccttcgtt gaaggtgcct gatctgtaat taccttttga ggtttaccaa
37021 attgtttaat gagacgtttg ataaacgcat atgctgaatg attatctcgt tgcttacgca
37081 accaaatatc taatgtatgt ccctctgcat caatggcacg atataaatag ctccattttc
37141 cttttatttt gatgtacgtc tcatcaatac gccatttgta ataagctttt ttatgctttt
37201 tcttccaaat ttgatacaaa attggggcat attcttgaac ccaacggtag accgttgaat
37261 gatgaacgtt tacaccacgt tcccttaata tttcagatat atcacgataa ctcaatgtat
37321 atcttagata gtagccaacg gctacagtga taacatcctt gttaaattgt ttatatctga
37381 aatagttcat acagaagact cctttttgtt aaaattatac tataaattca actttgcaac
37441 agaaccgtat tatggaatag agatgttggt aacatttata caggatcatt atacttaagt
37501 ttaatttcgt tattacagaa ccacacattc caaccagaag agaaagtatg tctatttagt
37561 tatggttcag gagcagtagg agaaatcttt agtggttcaa tcgttaaagg atatgacaaa
37621 gcattagata aagagaaaca cttaaatatg ctagaatcta gagagcaatt atcagtcgaa
37681 gaatacgaaa cattctttaa cagatttgat aatcaagaat ttgatttcga acgtgaattg
37741 acacaagatc catattcaaa agtatactta tacagtatag aagaccatat cagaacatat
37801 aagatagaga aataaactag tggccgattg tgcttgatga gcttgggaca taaatcctaa
37861 ctcgaaataa ataagcatat cactaaactg attttttaaa gtttacagtg atatgcttat
37921 ttttttatct tacgattttg tacgtgcatg cttgcctagg ggtatggctc gagccattag
37981 tctctcgcac atactattcc ctcaggcgtc agcacttaca aaatcggttg taattttcat
38041 ttttatacgc attcttactg agattatact aataagagga atagtaaaag caattctaag
38101 taaaattgca gataagaggt ttgttaaaag cagttctaag taaaattgca gataagaggt
38161 ttgttaaaag cagttctcag taaaattaca gataagaggt acgttaaaag cagttctaag
38221 taaaattgca gataagaggt ttgttaaaag cagttctaag taaaattgca gataagaggt
38281 acgttaaaag caattccatg caaaattgct gataaggggt aagttaaaag cagttctcag
38341 taaaattgca gataagaggt acgttaaaag cagttctagg caaaattgca gataagaggt
38401 gcgttaaaag cagttctcag taaaattgct gataaggggt aagttaaaag caatcctaag
38461 taaaattgca gataagaggt aagttaaaag caatcctaag taaaattgca gataaggggt
38521 acagaaaaac tagacttgat tacaaaatgg agcttgggac ataaatgatt ttttaaaaat
38581 gagatgagac gtagattaac tccataatca atacgaatct atcgacttct ttatttatga

38641 tattcatctc tttttaatgg aaataaaagt gcgattaatg tgataataca gttacgttaa
38701 ttaaaaaaat aaaaatgcaa ggagaggtaa tatgctaact gtatatggac atagaggatt
38761 acctagtaaa gctccggaaa atacaattgc atcatttaaa gctgcttcag aagtagaagg
38821 tataaactgg ttggagttag atgttgcaat tacaaaagat gaacaactga ttatcattca
38881 tgatgattat ttagaacgga ctacaaatat gtccggggaa ataactgaat tgaattatga
38941 tgaaattaaa gatgcttctg caggatcttg gtttggtgaa aaattcaaag atgaacattt
39001 gccaactttc gatgatgtag taaaaatagc aaatgaatat aatatgaatt taaatgtaga
39061 attaaaaggt attactggac cgaatggact agcactttct aaaagtatgg ttaagcaagt
39121 ggaagaacaa ttaacaaact taaatcagaa tcaagaagtg ctcatttcaa gctttaatgt
39181 tgtgcttgtt aaacttgcag aagaaatcat gccacaatat aacagagcag ttatattcca
39241 tacaacttcg tttcgtgaag actggagaac acttttagat tactgtaatg ctaaaatagt
39301 aaacactgaa gatgccaaac ttactaaagc aaaagtaaaa atggtaaaag aagcgggtta
39361 tgaattgaac gtatggactg taaacaaacc agcacgtgca aaccaacttg ctaattgggg
39421 agttgatggt atctttacag acaatgcaga taaaatggtg catttgtctc aatagaaagt
39481 tagaggtgag tcttacgttt cagtgacggt agacttacct ttaacatgtt acatactaaa
39541 aaattaattt gaataagaaa gagagacata tatgaaatac gatgatttta tagtaggaga
39601 aacattcaaa acaaaaagcc ttcatattac agaagaagaa attatccaat ttgcaacaac
39661 ttttgatcct caatatatgc atatagataa agaaaaagca gaacaaagta gatttaaagg
39721 tatcattgca tctggcatgc atacactttc aatatcattt aaattatggg tagaagaagg
39781 taaatacgga gaagaagttg tagcaggaac acaaatgaat aacgttaaat ttattaaacc
39841 tgtataccca ggtaatacat tgtacgttat cgctgaaatt acaaataaga aatccataaa
39901 aaaagaaaat ggactcgtta cagtgtcact ttcaacatac aatgaaaatg aagaaattgt
39961 atttaaggga gaagtaacag cacttattaa taattcataa taaaacagtg aagcaaccat
40021 cgttacggat tgcttcactg ttttgttatt catctatatc gtatttttta ttaccgttct
40081 catatagctc atcatacact ttacctgaga ttttggcatt gtagctagcc attcctttat
40141 cttgtacatc tttaacatta atagccatca tcatgtttgg attatcttta tcatatgata
40201 taaaccaccc aatttgtctg ccagtttctc cttgtttcat tttgagttct gcagtaccgg
40261 atttgccaat taagtttgca taagatctat aaatatcttc tttatgtgtt ttatttacga
40321 cttgttgcat accatcagtt aatagattga tattttcttt ggaaataata tttttcttcc
40381 aaactttgtt tttcgtgtct tttaataagt gaggtgcgtt aatattgcca ttattttcta
40441 atgcgctata gattgaaagg atctgtactg ggttaatcag tatttcacct tgtccgtaac
40501 ctgaatcagc taataatatt tcattatcta aatttttgtt tgaaatttga gcattataaa
40561 atggataatc acttggtata tcttcaccaa cacctagttt tttcatgcct ttttcaaatt
40621 tcttactgcc taattcgagt gctactctag caaagaaaat gttatctgat gattctattg
40681 cttgttttaa gtcgatatta ccatttacca cttcatatct tgtaacgttg taaccacccc
40741 aagatttatc tttttgccaa cctttaccat cgattttata acttgtttta tcgtctaatg
40801 ttttgttatt taacccaatc attgctgtta atattttttg agttgaacct ggtgaagttg
40861 taatctggaa cttgttgagc agaggttctt ttttatcttc ggttaattta ttatattctt
40921 cgttactcat gccatacata aatggataga cgtcatatga aggtgtgctt acaagtgcta
40981 ataattcacc tgtttgaggg tggatagcag tacctgagcc ataatcattt ttcatgttgt
41041 tataaatact cttttgaact ttagcatcaa tagttagttg aatatctttg ccatcttttt
41101 tctttttctc tattaatgta tgtgcgattg tattgctatt atcgtcaacg attgtgacac
41161 gatagccatc ttcatgttgg agctttttat cgtaaagttt ttcgagtccc tttttaccaa
41221 taactgcatc atctttatag cctttatatt ctttttgttt taattcttca gagttaatgg

41281 gaccaacata acctaataga tgtgaagtcg ctttttctag aggatagtta cgactttctg
41341 tttcattagt tgtaagatga aatttttttg cgaaatcact taaatattca tccatttttt
41401 taacggtttt aagtggaacg aaggtatcat cttgtaccca cttttgatcc atttgttgtt
41461 tgatatagtc ttcagaaata cttagttctt tagcgattgc tttataatct tttttagata
41521 cattctttgg aacgatgcct atctcatatg ctgttcctgt attggccaat tccacattgt
41581 ttcggtctaa aattttacca cgttctgatt ttaaattttc aatatgtatg ctttggtctt
41641 tctgcattcc tggaataatg acgctatgat cccaatctaa cttccacata ccatcttctt
41701 taacaaaatt aaattgaacg ttgcgatcaa tgttaccgta gtttgtttta attttatatt
41761 gagcatctac tcgtttttta tttttagata cttttttttat tttacgatcc tgaatgttta
41821 tatctttaac gcctaaacta ttatatattt ttatcggacg ttcagtcatt tctacttcac
41881 cattatcgct tttagaaata taactgctat ctttataaac ttgtttgaaa tttttatctt
41941 caattgcatc aatagtatta ttaatttctt tatcttttga agcataaaaa tatataccaa
42001 acccgacaac tacaactatt aaaataagtg gaacaatttt tatctttttc atcaatatcc
42061 tccttatata agactacatt tgtagtatat tacaaatgta gtatttatgt caaaataatg
42121 ttataatttt tgtgatatgg aggtgtagaa ggtgttatca tcttttttaa tgttaagtat
42181 aatcagttca ttgctcacga tatgtgtaat ttttttagtg agaatgctct atataaaata
42241 tactcaaaat attatgtcac ataagatttg gttattagtg ctcgtctcca cgttaattcc
42301 attaatacca ttttacaaaa tatcgaattt tacattttca aaagatatga tgaatcgaaa
42361 tgtatctgac acgacttctt cggttagtca tatgttagat ggtcaacaat catctgttac
42421 gaaagactta gcaattaatg ttaatcagtt tgagacctca aatataacgt atatgattct
42481 tttgatatgg gtatttggta gtttgttgtg cttattttat atgattaagg cattccgaca
42541 aattgatgtt attaaaagtt cgtcattgga atcgtcatat cttaatgaac gacttaaagt
42601 atgtcaaagt aagatgcagt tctacaaaaa gcatataaca attagttata gttcaaacat
42661 tgataatccg atggtatttg gtttagtgaa atcccaaatt gtactaccaa ctgtcgtagt
42721 cgaaaccatg aatgacaaag aaattgaata tattattcta catgaactat cacatgtgaa
42781 aagtcatgac ttaatattca accagcttta tgttgttttt aaaatgatat tctggtttaa
42841 tcctgcacta tatataagta aaacaatgat ggacaatgac tgtgaaaaag tatgtgatag
42901 aaacgtttta aaaattttga atcgccatga acatatacgt tatggtgaat cgatattaaa
42961 atgctctatt ttaaaatctc agcacataaa taatgtggca gcacaatatt tactaggttt
43021 taattcaaat attaaagaac gtgttaagta tattgcactt tatgattcaa tgcctaaacc
43081 taatcgaaac aagcgtattg ttgcgtatat tgtatgtagt atatcgcttt taatacaagc
43141 accgttacta tctgcacatg ttcaacaaga caaatatgaa acaaatgtat catataaaaa
43201 attaaatcaa ctagctccgt atttcaaagg atttgatgga agttttgtgc tttataatga
43261 acgggagcaa gcttattcta tttataatga accagaaagt aaacaacgat attcacctaa
43321 ttctacttac aaaatttatt tagcgttaat ggcattcgac caaaatttac tctcattaaa
43381 tcatactgaa caacaatggg ataaacatca atatccattt aaagaatgga accaagatca
43441 aaatttaaat tcttcaatga aatattcagt aaattggtat tacgaaaatt taaacaaaca
43501 tttaagacaa gatgaggtta aatcttattt agatctaatt gaatatggta atgaagaaat
43561 atcagggaat gaaaattatt ggaatgaatc ttcattaaaa atttctgcaa tagaacaggt
43621 taatttgttg aaaaatatga aacaacataa catgcatttt gataataagg ctattgaaaa
43681 agttgaaaat agtatgactt tgaaacaaaa agatacttat aaatatgtag gtaaaactgg
43741 aacaggaatc gtgaatcaca aagaagcaaa tggatggttc gtaggttatg ttgaaacgaa
43801 agataatacg tattattttg ctacacattt aaaaggcgaa gacaatgcga atggcgaaaa
43861 agcacaacaa atttctgagc gtattttaaa agaaatggag ttaatataat ggataataaa

43921 acgtatgaaa tatcatctgc agaatgggaa gttatgaata tcatttggat gaaaaaatat
43981 gcaagtgcga ataatataat agaagaaata caaatgcaaa aggactggag tccaaaaacc
44041 attcgtacac ttataacgag attgtataaa aagggattta tagatcgtaa aaaagacaat
44101 aaaatttttt aatattactc tcttgtagaa gaaagtgata taaaatataa aacatctaaa
44161 aactttatca ataaagtata caaaggcggt ttcaattcac ttgtcttaaa ctttgtagaa
44221 aaagaagatc tatcacaaga tgaaatagaa gaattgagaa atatattgaa taaaaaataa
44281 aattgttgtg tttacaacaa tacatagaaa acagaggaaa caatcaagtc gttgaatatt
44341 tcctctgttt tttagttgaa aaaattaacc gaaagcctga atgcaagtct tgattaaatc
44401 aataatgctt gtaataacac cagtgaaatc catatgcata ccctctttct atttaagata
44461 cattaagtat aatatcaaac aaataaaaaa tgttaaaaat tccctaattg gctatttaga
44521 ttgcataaat gtcaaaaatt tgaaaaacat acaacgactt tgcataaaaa atcgtcatat
44581 tggaaatacg taatttattg aaataataaa aaaaataaaa gaacgaagat gataacctaa
44641 gtgaggtttt aagttgttct aaggtttaat ttaatttatg ttaaaatagt tggtataaaa
44701 atacatgata aactataaac taaattcaaa ataacttatg gggtaggcaa ttatggaaaa
44761 tattttaaat ataaatgata atgaaaaaag agtgctaagg gaaatttata accatcataa
44821 tatttcgcgt actcaaatat ctaaaaatct tgagattaat aaggcaacga tttctagtat
44881 tttgaataag ttaaagtata aatctcttgt taatgaggtt ggtgagggtg atagcacgaa
44941 gagtggtggt agaaaaccta ttcttctgaa ggttaatcat ctttatggtt attttatttc
45001 tttggattta acttatagtt ctgttgaagt gatgtacaat tattttgatg gtaatgtcat
45061 taagcatgaa tcttatgatt tacctgatga aaaggttagt agtatattaa gcataataaa
45121 aaaacatatt gatattcagg agaaacttga tacttataac ggactattag gtgtgtctgt
45181 ttctatacat ggagttgtgg ataatgagca gcatgtgaca tatttaccat tccatgaaac
45241 tgaaggaatt tcaattgcta agaaaataaa agaaattact aatgttccag tcgtagttga
45301 aaatgaagcg aatctttcag cgttatatga acgtaatttt aatcataatt tatcctacaa
45361 taatcttatt gctttaagta tacataaagg tattggtgct gggcttatta ttaataatca
45421 attgtatcgt ggtgcaaatg gggaagcggg tgaaattgga aaaacacttg tctcaaaagt
45481 tagcgataat gtggagatct ttcataagat tgaagatatt ttttcacaag aagctttact
45541 gcataattta agtaatcaac taaatgagaa gatgacgctt agcaaattaa ttcaatttta
45601 taatgaaaaa aatccagtcg tagttgaaga aatggaacaa tttataaata aaattgctgt
45661 tttaatacat aatttaaaca cccagtttaa tccgaatgca atttacatta actgtccatt
45721 gttcaatgaa atgcctgaaa tattagaagc aattaagaac cagttcaaac aatattcacg
45781 taacgaaatt caaataaagt taacatctaa tgtcaaattt gcaactttgc taggtggtac
45841 attagcaatt atccaaaaag tactacagat taatgatatt tacttagata taaaagcata
45901 aaaaactaat tcaaatgaat aatcaaagtt cgtaattgtc tttataaaaa aatccctcaa
45961 tccgaattga attttcggat tgagggattt ttatagttct attgcagaag aaaactattt
46021 taaaaatgct ggtaaatgtt gatagccacc tctaacgtta acaatattcg taaatccttt
46081 atattctaat attcctaccg ctattgaact tctaacacct gattgacaat gtacataaat
46141 taggtcattt ttatcgaaag gtatatcttc atttaaaagt ttaccgtgag gaatatgaat
46201 tgcttgtttt aaatgacctt tacgccattc atcatcatta cgaacatcta atacattatg
46261 ttcttcacca gtcatttcag aactatgaat agatgatgtg acgatatttg tttgtggcaa
46321 acggtaactt tttacatttt caaaaccaat taattgtaaa gcatgaatag cttgttgaac
46381 ggtagattta tcgccaatta attcaatatc ttagtcataa tctaaatacc aaccaatttg
46441 atttataaaa gttttattaa aaggaatatt gatagttcca tgcatatgac caccatggaa
46501 tgcttcttta cttcgaagat caaaagcagt ttgtgtattg cttgaactag ggtaaacatt

46561 atatggttgg tacatttgca taccaaattg atttattttt ttcatttgtg aaaaatggtg
46621 tggtggagct ggctgattga gtgttaaagt ttcgataaat gaagtttcat ctttaacatt
46681 aaaagcccag ttgtttattt tctcataacc caaagtagtt gtaggtaatg aacctagcgc
46741 tttaccacaa ggactccctg caccatgacc tggccaaatt tgaatatagt ctggtaatgt
46801 tgcagcaaat tgtatggact gatacatttg ttttgctccg atttttgtag aaccttcaac
46861 atttacagct ttttctaata gatctggtct acctacatca ccaacaaaga tgaagtcacc
46921 gctaaataat cccattggta tactggaact cccaccttcg tcagtaagta aaaaactaat
46981 actctcaggg ggatggcctg gagtgtgtaa gacttctaat ttaatctttc ctaaatagat
47041 aatatcttga tgcttaacga aatgtgtttg tttaggcata tttttataat taaattcatc
47101 tttaccttca tcagatacgt atatacttgc attcaattta tttgccacat ctctaatacc
47161 tgaagcaaaa tcagcatgaa tatgtgtttc tgcagcttta gtaattgtga atccttcttt
47221 atctgcaact tttaaatatt ttgttaaatc tcgtataggg tcaataatca ttgcttctcc
47281 tgtacgttga catccaatta aataagatgc ttgtgaaaaa ttgtcttcat aaaattgttt
47341 gaaaaacaaa aaaaactcct ttttaaaata gattttattg attagataaa taagttatga
47401 tttgcttgct cagtatgtcc aatataagta cctacgccac cataatcgac ttcatctctt
47461 aattcttctt ttgaaattcc cataacatcc atactcatgg tacaagcaat taactttata
47521 tcttgatcga ttgcttgatc gataagtgag tataaagaat caacattttt cttgttcatt
47581 acataacgca tcataatatt acctagtcca aacatattca tttttgataa tggcatatgt
47641 attggatcct taggtaacat aaggtcaaac attttggaaa tacctttctt tttaacgcga
47701 gttgattgcg ctttttttaa tgcgttgagg ccccaaaaag taaagaaaat agttacatct
47761 ttacctgctg ctttagcgcc atttgcgatg atcattgctg ctactgcctt atctaactca
47821 ccgctaaata aaacaattgt tgtacctgta gcagtgtcat tgatttcaaa ttcttttggc
47881 ttttcttttt gaataattgc attaattaca tttgcttctt cagtaagatt tacaagggta
47941 ttccctgttt gtttcgccca actttttaata tcactattga aaccaggatc tgtaactgtt
48001 acctcgattt gctcacccgt tgaaatattg ttaatttctt tactgatatt aacaataggt
48061 ccagggcact gaagacctct aaaatcaaat tgtttacgat tctctttgat ttcaatatct
48121 ttttctatta aaggagcact attgaagttc tttgcttcat aatctttata tccacccttt
48181 aaattcacga catcataacc ttgtttggct aaataatcgc aagctttagt gcttcggtta
48241 ccgcttttac aatgtatata atacgttttg ttgctattct tattgaatga tttaatctct
48301 tctactgggt gtaaagttga accgttaatg tgtcctaatt catattcttc ttttgttcta
48361 acatcaatca attgacccat ttttgccaat ttttctaatt cttctttgtt taatgaatta
48421 atgtgtactt ctttgtattg ttccatactt acctcctata aatacctatg agggtataat
48481 aaaacggata gaatcatttg ccaaatacct ataggggtat ttgacaattt tgttttaatt
48541 tattattatt aacttaatca atttatgtgg aggaaatgaa tatgacttat gataaaaaaa
48601 tgattaatcg tataaataga atacaaggtc aattaaatgg tgtcgtaaaa atgatggaag
48661 aagaaaaaga ttgcaaagat ataattacgc aacttagtgc atctaaaggt tctatacaac
48721 gtttaatggg gattataatt agtgaaaatt taatagaatg cgttaaaaca gcagaagaaa
48781 ataatgaaag ttctcaagaa ttaattaatg aagcagttaa tttattagtt aaaagtaaat
48841 aatggatata gcaaatatga ctattatgtt gctaattggc gtactgggtg gatttatatc
48901 tggattaata ggtattgggg gcgcaattat tatttaccca gctattctta tattgccacc
48961 attaataggt atacctgcgt atagtgcata tattgcttcg ggacttacct ctagtcaagt
49021 atttttcagt acacttagtg gatcattaaa tgcaagaaaa caaccagctt tctctcctaa
49081 acttgttata tatatgggag ggggtatgtt gattggaagc atgttagggg caattttagc
49141 tagtttgttt aatgctgctt ttgtaaatac ggtatatgta ataatcgcca tacttgcttt

49201 aatattgatg tttattaaag ttaaacctac tacacaagag acgaaatcta aacctttgct
49261 atttattata gttggatttg gaattggtgt aatttcggga attgtgggtg caggtggagc
49321 atttatcatc attcctgtat tattagcatt atttaaatta ccaatgaata cggtagtgaa
49381 caatagcata gcaattgctt ttatatcttc agtaggggca ttttttataa aattaatgca
49441 aggatatata ccagtagaaa gtgcaatttt tttgataatt gaatgatgat cattttctga
49501 aaatattatg tggtcatata atataatgcc catcatttca ctaatctctt ttattctctg
49561 agttattttg atatctcctg gagaaggtgt cggatcttat caagagtaaa ttatgaaaaa
49621 aatggtttaa atcaatgttc ttactttaag tacatgtgtt taacgtaaat gataattagt
49681 taaatataaa aaggttatcc caatcaatag gataacctct ttatacttta ctttatggtt
49741 tggattcgcc acttcaaaga agaaatgaaa tcagagcatt gaatgttcca ttttcaaaca
49801 atatataaat acctagccca atgaatacaa ttggtacaat ccaacgttca tatttctcaa
49861 ttgtttccga tataaaatcg aaggaagcta gacggtaact gacatagcac aaaactccaa
49921 ccataattag aaagacaata gtgacaataa agatttcaga catacttaag gtcgtgaagt
49981 acggtatata aatggaaaag tcatccgcac tggaagccaa tacgatgaaa atcatcgtca
50041 aaaataactg attaaatttt ccagaggaga ataaagataa aatgctactt tcatcttcat
50101 cctcttctcc tttaatccat attttcacgc ctaggtaaag tggtaaaagt ccaagtagtc
50161 cgataaccca ttgctgagga attaaattta caaccccctg tgcaactaaa agacttgctc
50221 ctatcacaat tgcagtccct atatattgtc ctatccaaat atgttttacc tgaccttttt
50281 ttacttgcga aaacaaaaga attaatatga cgagataatc aattcctgtt gctacatata
50341 ccgcagtagc cgtcagtatc gtcgcgatca ttttatcatc tccaaatatt ttagggatag
50401 gacttttctt caaaatgaaa agtccttccg taaattgcac acatatagta cttatttatc
50461 cttcactctc atcagtcgca aactatttaa tgctaccaaa atagtggctc ccatatcgga
50521 aagaatcgca atccaaaggg ttagccagcc tggaataacc aatagtaagg caattatctt
50581 aattccgatg gcaaacgtga tgttcgcttt gatgatattt agcgttttcc tgctaagtct
50641 tactgcaaat ggaagcttac ttaaatcatc tcccattaat gcaatatcag ctgtctcgat
50701 ggcagtatct gttccagcac cgcccattgc aatgccaaca gtggatgcag caagtgcagg
50761 agcatcattg acgccatcgc caatcatagc tacattacca tgctcggctt tcattttttt
50821 aatatagtcc aacttatcct gtggcaacaa ttcggactga atatcagaaa cgcctacatg
50881 agcaccgatt gcttctgcgg taccttgatt atcacctgtc agcataattg tttgcttgat
50941 tcctaactga tgaagtttta gaatcacatt tttacttgtt tcgcggacct catctgctac
51001 agcaatcacg ccgaggattg tttggtccgt tccaataatc atggccgttt tcccttggtt
51061 ttgtaaaact ttcactttat tttcaaactc aaggctaaaa tcggaaacat ttaattcttt
51121 aaaaagcctt ggactgccaa tgtaataggt tgttccatct atattccctt gaatgccccg
51181 acctgtaata gaagtgaagt ccttcactct aacatcggaa taagtaatat tatcttgctc
51241 tgctttcttc attattgctg aagcaagtgg atgttgtgat cgatattcta aagctgtaat
51301 aatggaaaac agctcttttt cttccacttg atcatttaac actttaaaat ctgttaccac
51361 tggtacacct tttgtcagtg ttcctgtttt atcaaatgcg attgccttaa tggctcctaa
51421 ttcctctaga tagacaccgc ctttaatcaa cacacctttt ttagctgcat ttccaattgc
51481 cgagacaatc gagattggag tagtaataac taatgcacac ggacatccaa ctacaagtac
51541 cgctaatcct tgataaaccc aagtatccca acttccacca aagaataaag gtggaacgac
51601 tgcaacgagc gccgcaataa ccataatgat cggcgtataa tatttcgcaa atttatctac
51661 gaatgcttgc gctggagcgc gctccccttg tgcttcctca accagatgaa taatcttgga
51721 gatagttgta tcctctacgt atttggtgat ttttacttca agtagtccct cttcgttaag
51781 cgtgcctgca aatacttcat catctaccgt tttggcaaca gggacagatt ctcctgttat

51841 agcagcctgg ttgacagccg acacaccatt tataatgatc ccatccatgg caattttctc
51901 ccctggtttg acgatcataa tatcacccac ggcaatatcg tccacatgga tcattatttc
51961 ctgaccattc cgcctaacaa gtgcttcttt tggggcaata tccatcaatg aacgaatgga
52021 ctgtcttgct ctatccatag aaaaacgttc aagtgcttca ctgattgcaa agagaatgac
52081 aacaatggat gcctctgccc attcaccaat gatggcagct ccaataactg caacggtcat
52141 cagggttttc atgtcgaaat caaagcgtat caaattttga aaaccaactt taaatagtga
52201 atatccgcca attacaatcg aacttacaaa taacatggac gttacaaggt tatcttctcc
52261 atttacaaag tgagaaaggt aaccaaaagc aatcagtaat gtggcaaaca gcaatgtgct
52321 gtgtttttta taaaacggta ttttctcttc tttaggagcc ttagtgtctt ctttgaccgc
52381 ttgtatcgat ggattcgcca gtttttcagg aattacctta agattctcga aagcacctgc
52441 tttttcaagc tcttcaaccg atgcgtttcc atatacatca attttagaag cgccaaagtt
52501 cacttttgca tcctgaactc cagctagttg ttttacattt ttttcaaact tcccagcaca
52561 gtttgcgcaa gaaaatccct ccacacggta aacatttttc tcttctgtta ccactggttc
52621 gacccttctt ctagccttct ctggtgccac tttaagattc tcgaaagcac cagccttttc
52681 cagatcttca acggttgcac tgccaaagac atcaattttg gaagctccga aattgacttt
52741 agcatcatgc acccctgata gttcttttac atttttttca aacttcccag cacaattcgc
52801 acacgagaaa ccctccacac ggtaaacctg tttatcttct gttaatgttt ttgttgaact
52861 atccaatact agcaacctcc ctttgatgca agaaagcttt ttctacaagc tgtttaacat
52921 gctcatcatc tagtgaataa tagactaatt ttccttcttt acggtatttt gctataccta
52981 aatttttcaa taatcttaaa tgatgggatg ccgtagccgt tgaagattca atgatattag
53041 ctacatcaca aacacataac tctccctcta aagacaaaac ataagcaatt ttaactcttg
53101 tatcatctga tagagcctta aaaactttcg ctacatccat aggattctgt ttagcaaggt
53161 cttttttagc cctgtttacc ttatcttcat gaatataggt aacttcacac atatcttttg
53221 tcataattac cctccttatt caaatgactg tttgtttgat gattataata tatcccacta
53281 taaacaaata gtcaaatgat tgtttgaatg atatataatt aatattaaaa aggattggtt
53341 tctaatgtta gaaaccaatc ctttcggaga ttttaaccaa attttaaagt atcttaaaca
53401 taactgcccc gttagtttaa gtgcatcctt tcacaatctg tctacagatt aataataaaa
53461 actactcttt attatacaga tctccatata atttttgaat ttggttctgt aattttttat
53521 tttctttttc taattccata actcttcttt ttaaggtttt aataaggatt tcctccgaac
53581 gagaactttt cttgggtttt gagactacat ttgctgttat ttgacgctca cgaagggatt
53641 cgattctttg cctaatatcg tgttccttat aaagccatga tttagaaaca ttagcttcct
53701 ttgctattga attaaaatta ataactttac cttcaatcga aaatttagaa atcgctttgt
53761 ctactttttc ccttgtcttt tttgatttct gcttcgccaa acgtacaatt tctgttgtat
53821 ttctaacttg tttatccatt gataattacc ccgtcaaact tccaatgatt tgttctaaac
53881 gctctttaac acggctatta gtctctactt gtctttgcca ttgtttatcc ttagctatgg
53941 ctaataactc ttctgtacgc tctaactgtt cttcgtgctg tggtaagaat tgcttactgg
54001 tacagaagtg agtgcaatct aagcatgcat tcgcatgtgg acaaccacct gctactactg
54061 gcaatctaca ataaccattt ggaagcactt gtgcatttat atttttcttg aaccattgaa
54121 gctctacatc atcgacttca ttatcttcat ctagatcaag cacatctcca ttattggtaa
54181 ccagtttttc ctgaaattta gtaaattcat tttttagagt ttcatcaaag atatgagcgt
54241 atctgcttgt catttctggg ctttcatgcc ccaaaaattt ctgcacaata tgctggggca
54301 tcccgttgtt aatcattctt gttcctactg tatggcgaaa ggcatgggca tggaatctat
54361 aaatctcacc tgatttatcc actatatttt gctcataagc taatttattt aactcacctc
54421 taaatgtttc ttgttttaat ggcgatccat cttttcttgg aaagaggtat tcactatctg

54481 gaaattcctc tgaaacttta tcttcccgaa ctttaataag taaagctacc tctttagata
54541 ttggaactat atgctccttt ttcattttcc attgataata ctttaaaaag aaatctccat
54601 ctttgtcctc taatagacag cctttttca aggtgcacaa ttcacttatc ctcattccac
54661 attcttgaac aatcatagtc atcgtagcta tatattcggg taatttatca agatgactgt
54721 tcaattgctc taggacgaat tcatctataa agcgaggttt tgctcttggt attttcggat
54781 agtcctcaga ataaattaat attttggaag gaacatcatc ccattctagc ctaagaaggg
54841 tactaaatag tccttccaat atagagatcc tcccagttat tgtactaggt tttattccca
54901 tcatgtttag ttcacttaaa tatgcttcaa tttccactct cgttaattgg tgtactctct
54961 gaactcgttt aaatttcatg tccagaaaat taaagaactc tttaagtctt tgggcaatat
55021 cacttacata ggaaaagcta tccacgttca atctcaactt acaatatctt tttacaagtt
55081 gtttaaaata tgtattccga aaccctttaa agttaattgt atattcatat tgtgttgggt
55141 taaccttatc atctggcaaa ggtaagttac gtctatccca aacgtcttta tcccactcct
55201 ctccatcaaa ataaaagttc tcataaaact ccataaattg ttttagatta gtaacatagt
55261 aggaattagc ttttacaggt gttttttctt gattagcagt aatcttataa ttagtagtgg
55321 taattctaac accccgtttt gtcaaataag ttctatactc cgtcattgct ttttcaatag
55381 gaacttcagt aattgaagta atgctaggat actttaaatc taagaaatct aacattttat
55441 taattactgt tccttttcta atccagacag tttttgcatt ccatattcca ttgtttaaat
55501 ggtaaaaata aaaatatttc aattctgttc ttaaccacag atttttaaca cgttcaaaac
55561 gaacccaacg attccttaaa gcaggattct tacttaattc tatggcagaa ggatgtggac
55621 attttcttat atcccaacta ttattagccc aaaacccctg catttcttca ttcattacag
55681 ctattttttt gctgatctca ctctgactga taattttcct tttactagaa gcattcattt
55741 agctggtatt ctttatctag taataaatac aatggatacg gcttattctc cacttctatc
55801 ctttgaaccc tcacacgcgc ccccctcttt aaacacttta agtactacaa attataaacc
55861 ttgttaattt ttatttaaag taaaatctcc ctttaacatc tcctcctgaa ctactttaac
55921 tactattatt tattatacta tggttaatat gtctccactt gggtggttat ggccaataat
55981 catacccgtt gcgttactta atatggctgt tttaaatatt tctcgaggat gtataaccgc
56041 aaaattaatg gttcctatag agacggtttc aatatgtgta ggttcatttt tgctattcaa
56101 gcaaattaaa atgaagtgtt ctcggtcagc atttcctatg aagtcacgca taatatcagc
56161 agcgtctttt ggttcttcaa tacgtctttt taaataccaa agtgtatctt ctttaaccat
56221 ttggactttg acaacatcta ttctttttct actcattatg gataagctct atactttcta
56281 ttaagtcagg gtgaacaatg gatgtgatag aatcatcatt catataaatg gaatatagct
56341 catctaattc tgaattattt tttttgtttg atgaaaaggc gataatgcct ttaataacat
56401 cgatgtgtgg ttgatgcatt aaatattggt atacttcttt ccatgtttga ttcattctat
56461 ttagtctcct ttaatggttt aattgtccat gcctttctag cgtatggatt taatggatgc
56521 atgatttcat ttgtttgagg atttgttaat atactgattt tcttactgtg aggttttaac
56581 atgtctaata cttcattcac tcgattttcc acaagaggtt tttttccgta ggaaccccaa
56641 gccaataaaa cactatcggc ttctttgaca gctttcataa tgtgaatatc cgtgtgcttg
56701 tcatggctgt tttcgatatg ttttaaatta atgggggtat caatatttga aaaaagattt
56761 ataaaattaa cgccaccata ttcatctttt tcagctactt tattgacaat aagttgtgtt
56821 gtaaggtcaa cgttgataac accatcataa tgaggataca tcgttataat tgtaagtact
56881 tctttatcaa tatcccaagt tttcttgaga agataacgat gcattttatc ctgactaaat
56941 atcgcttcag tttctaatac acttttgatt gatttcattt tatctaactc ctttatttaa
57001 tattcttcag gtaacaacat gacgtaataa gaaaggtcta catcatcttc tcggataatg
57061 tagaccttat taatattgat agcttttgaa taatttgcct tatgaatttt tacgtactct

57121 ggacgttctt gcttatgttt gatatataat tgactattat gcatattgaa ctggaatata
57181 tgaaagtaat caatctcttc tagttccttg aattgttcgt tttcgcgttg cgctacaagt
57241 ttccataact gttgctgcaa cattgctggc aagtaattgg caatactacg tgtaatataa
57301 cgtttcatgc tcatttctcc ttttctatat ctttcattat tgtaataact tctgtaatga
57361 ctgtgacgct tatctgagct atgtgtatcc atttattcat tattataatt ctccttttca
57421 agtaagtgtc tttgatctag tagtggcata actgtgttat gaagatataa gaagtccata
57481 tcgtttgttt catcaatata accaatcgca ataagttgtt tttgatagta gaggataaag
57541 gggtatatat gcctttcatg aagtgtgtca gagtagtaag tatacgtatg aaggattttg
57601 ttggtaagcg gaccaagtct gatatattga tcaggaagga tattatctgc cacttcctcg
57661 atagattcac attcccattt ttcgtccttt ggcagattga ataagttagt tatatactct
57721 ttgagttgtt tttctagtgt catggttatg acctcctata atgtttttga tgtatacatg
57781 taatacacca tatagataat atatgcttca aaaaaatttt gtttcgtttg tgaatcccat
57841 tgttattaag aaaatatccc aatagaaaat tatcacaatt gttgatatga caaggaaatc
57901 ccattatccc gctcatttct caggaaatat agcgatatat ttatatatca tttggatttg
57961 gtggtgtcag tacgacttac tttttctttt tatattattt atttataaat aataatggga
58021 tttcgggatt acgcttgcgt aatccttatc ctacatgtat ttatcaaatc ccattgctat
58081 cccgttcata ttttattttg ggatgttctt tcgtcataca atataagctt cacattgagt
58141 tgtgacttgt tagtatgttg aaaagattcg ttagatgggt gttattgaat gacgaaagga
58201 cagtacatag atattagatt tgaagaaaga gcgtttgagg caaaaataaa gacgaagtgc
58261 tgaggagcac ttcgtcgaga ggggtattat tgaaaagttg tttaataatt tcattattga
58321 gttttagtgt aacgtagaac tgttttttat gattaccgtc tttacgaata tcaatacggt
58381 caatgactgt aagatataat gctttgagtt gtgctttatc cattgattct atattttgaa
58441 atattcgttg taatagggca gcgatttgtt tcgtatcata agatagtttc tcttgatttt
58501 gttgctgttt gagttgattc atttgatttg taatgtcatt gagttgtgtt tcatattgat
58561 gaatagttgc tttcaatgca gatgttaggt ccggattatc ttcaatggtt ttaactaaat
58621 tatggagttt cccgctgact tcatcgtatt gttgttgttt ataagcgata tcgtggttca
58681 atgcaccaat atcgacttta ttttcttgat tgacacgttc taagacttgg ttaatgactt
58741 tatcactttt gacaatttcg agtatttgat ccatgacgta tttctcaatc acatcagctc
58801 taacgctatt cgcagaacat acttttgagc ctttgtttcg gaagttactg caagaataat
58861 aacgtattcg cttcttggta ccatctttca atgtgttcgt tgtgttacta gctgccattg
58921 gtgcaccaca ttgtggacaa tgaacgatac ctgttaatag attagttcct ttaccgtgga
58981 cttgaggttt ttgactgact tgttttttac gtaattggac tttatcccat aagtcttgaa
59041 taataatagg ggaatgctta ccttcagcta ttattggttt atcattcagc cctttacgac
59101 gcttttcatt ccaatctttg tactttgcga attgaatttt accaacatag aatggattag
59161 ataagatata ggtcactgaa ccaatactga aaggctttcc ttttttagtc acgtatcctt
59221 tgtgattgag tgcattcgca attttacgat atccgtggcc tttagcatat gactcaaata
59281 tatatttgac aatattcgct tcatgttggt ttatcatgag ttcatgcttg ctatccggta
59341 ttttgtcata gcccagcggc aaattgcctt gataatagcc ttcttgagcg cgtcgggttt
59401 gacccatgaa tacattttcg acaatattat ttctttcaaa ttctgaaaaa ctcgctagaa
59461 tttgtagcat caatttacca ctgcttgtat tgacttccat acgctcagat aagctgaaga
59521 attcgacatt ttgacgatga aggtcttcaa caattttgag taagtcagaa gtgttacgtg
59581 ctagtcggtt tgttttgtag accataacag aatcaatctg accttcgttc gcatctttta
59641 acaaacgttg tagttctggt cggttcatag attttccaga gatacctcta tccgcgtata
59701 catctacaac aacaaagtta ttgaaatcac aatattctcg aatttgattg atttgtccat

59761 cgatactata gccttcagta ctttggattt ccgttgatac acgaacatag ataccgacac
59821 gtttttgttt gagttgttgc atgatttttt catcctttct gattaagcaa tcgatgattg
59881 cgaggtttgg ttcacaatgt tcaatggttc atttttgaaa tagatcccaa caagggcttt
59941 attttttgta atgcgaattt catcaatata gggatgcagc atgtttaacg tgaaactttt
60001 ctgtataacc ttttgtagtg atgtttgtaa ctgattatca cttatggagg atatggtttt
60061 gtgcttttga ttcatcaaat gagtctgttt tctgaatgat tcagcatcaa tcataccttt
60121 ggcaagttta tctatcagtt gttcttgagt tagcgtactt ttagcttcta tcactctttg
60181 ttttttgagg cgttgatgaa ttgcattatt aatttttgaa tagagctgtt ggttttgaaa
60241 gaagttctga catgtagcta agacttgaac ttctaatttt tgtgcattta ttcctttgaa
60301 tgaacagaca aagcgagatt cattcatatt tttaggacaa atataatatc gcaatgtatg
60361 tttttttctt attgtcatat ttgtcagtgt tgagtcacaa caaggacatt tgatcttttg
60421 tttcagctga ttctctgaag gtatacagtg aagttgcttc ttattacgga ttgcttgagc
60481 atgttcatat ttcgttgccg aaacaatagg tggtaccata ttgttatatt gaccatattg
60541 attgatgaca cgaccacaat aatttgggtt cataattata tttcgtacct gataaggctt
60601 gcgggtaata agtttattat cgtcttctaa atattgtgca atcttcttgt agccatatcc
60661 ttgaagataa taattgaata cagctttgac ggtaggtgat tcatcattat taatgatgaa
60721 agtaccattt tgatagtgat aaccgaaagg cgcatgggtc gttatgagtt taccttgttt
60781 tgccttttct ctaagtccat ttttgacttg ttctccaata ttatcggatt caagttcagc
60841 cagactcatg aatatattga gttttaggcg atcaaacgct ttatccatat caaaatagcc
60901 atcatgaaca cttagaatat gaacatgata tttttgacag agtttcatga gttttaatgc
60961 atttttcaag ttgcgatgaa gtcgattcaa gcgataacaa cataatacgt cacattgtct
61021 ttgttggatg cgttcggtga cttgttgata gcctgttcga ttatctgttc tgcctgattg
61081 cttatcacta atgtattgga tattttgaat attgtgcttt ggagctaata attctattgc
61141 ttgcttttga gctgggagtg attgttgttt cgttgtactt tggcgcaaat aaccaatggc
61201 tcgtttcata gtatttcctc ctttcaaaga gataatatat atttatgaac gaaattatag
61261 ataagcccaa caccagtcgg gtgttgggcg gtattattag tcatcagcgt gattgatttc
61321 ttcaataacc agatcggcta atagtatgat taattcgtcc atgtatatca cctcattatt
61381 catagtatta acaacaattc tttagttgca tctcaaaagt agtaacgaat tcgatattct
61441 tctttgagca aattttttta aatgaatgta atcttcatat tctccatatg caacaatgga
61501 cgtaggtttg attactttct ccaaaatgtc atttattaac ttgtactcct cagtgtaagt
61561 gttacttatt tgacgacctt cttgtttaca gcattccttc acagtatcta ggggtgtgct
61621 ttctaagtat tcaaatcttt catcttcaat ttcgtgagct agtgctactg tattcatagt
61681 acaattcctc cattgattta atatttgtgc tacaacacct ttaaataggt gttgtagcaa
61741 atttatttaa taacatattt taagctttaa aagtcaatat caccgttttt atcccattct
61801 cttgcttttg aaatcatgtc tcgtagtatt ggatgttggt gttcagagat aaatgataag
61861 ggtatattga atttactttt ttcagataga ccaaaaatag gagcgtatga tttatcaagc
61921 ttaagatgat aaaatacgat ggtttttggt ttgccttctt tgtctttgac actacgtttt
61981 gttgttttac gattggcgtc tgactggatg taatctttat ccctcagtgc atcaatgaca
62041 ttatttgtat cttggaaatt atgttctttt aacatatttt taaaaaccga ggcaatcatt
62101 ttcacctgga tatgatcatc ttcaagtgaa atcattccat aattttcaat aagcgttgat
62161 aacctagtgt cgtcagaaaa cttaccacga ttttgtgcta caaattggac aatgacttca
62221 attgctttat cagctaggct tcgctcacta acagaatcta aatgatagtt aataagatag
62281 tcccttacag cattcaaatc aacaggtgtg gcaatgacac gttccaatat acgtgctgat
62341 gttgtaatgg ttgcatagcg tttgaacatc cgaattcctg tattgctggt ttccttttct

62401 agttgagttt tgaaccttag gtgctcattt tggaaccatt taatgacttc ttgttcacga
62461 ttgagtagat attcagctac taatggcatg atatgaccat aattagctga tgttgctttt
62521 ttaatagtat ctgcgttgtc agcgttagtt gtaaaagctt cagatatttc aatggtacgt
62581 acattcaaac catcattacg tgcactatta ttgaaaatac tgtgttcggc agtactaatt
62641 actgaggttc cccagttttt tagtgacttg acttctccgt gtatattaga acgttgtctt
62701 ccttgacctt cggcaattga atagagcaaa ccagttgtat ctttaaaggt tgcagatgac
62761 agttcgtcca atacaatagg tacaccatag ttgttactta gatatccttc aagcgcgttt
62821 ctagtgccat tccatgatct aaacagcgta ttactacctt tagtagggtt gccggttatt
62881 gagactccta aagccgctgc agttgattta cccgtgcttg aatttcccgt aaatgagaaa
62941 atcgtacctg cgaattcaac ctcatgtttt gtttttaaaa acgcagttac taatgaagaa
63001 ataccaaaca ctacagcaag ttcgagtaac aagtttcctt ttacttcatc aagatacatt
63061 tgccaccagt cttcaaaagt gcctttaggt tgtagatcgt actgtgtttc acaaatgatt
63121 tcatccgcct gcgactgtgt aatttctttt gaaagatatg gttcatctaa tgaaatgacg
63181 acacctttat cagtatttaa cacaccgaca cccgtataca acgatgacag aggcagtgat
63241 tggcgcatta attgtagtgc attgcttaat gatttaatgt acttctcatt gatgctgaag
63301 ccatacatga ttagtgaagg taacttttgc gatgttaaaa tatctgatgt ttcaatacgt
63361 tcaatatttt gtccgttgga aataactagt ttctcaaccc cagatttggg gtcaagaaac
63421 ttgtttttaa ttaaaattgg actagataag cgtatggact tttgctgatc atttttacct
63481 ttcggtggta taacttcata ccatgaatct tcagtaagcc agtatggata ttcactaaag
63541 atgatgttag tcattagttg ttaccacctt tccagttatt ttgatgattc atcggatttg
63601 gtgcagtttt aaggtagagc aaagatgaac cttttttttcc aactacaatg aatggaaggt
63661 taggtgtata ttttacaaaa tgtgcaaacc aacgaccaac atcttgttga atttcagtcg
63721 agcacaaaac attagccctc acattcaaat cgtgatacat aaacatggtg cctacaggta
63781 aattaaatgc gatgcttgta acagcacgtt ttagtgtata aaattctttt tctttcatca
63841 taagttcctc caaataagtc ttattagtca atcaatactg tagatgctta acacctcaat
63901 ggttgattgc tataataagc ataaatgaag cattttaatt agacgcggaa caaaaaatag
63961 ggaggggggt atatatgata cctacgatag aagaaattat tgacaagtat ggcagtttag
64021 tagattgtct aaagtttgat atatcaggtg aggtttatga agacttattg ttattaagaa
64081 gtttaagcga tttaaatgaa caccaaaaga tgcatagagt ttcatttata caaaaacact
64141 ttaaccctaa ttttgattat ttgttagatg actcagaatt tagaagtatg aataaaattt
64201 atagaaattt aaatactatg acgtatatca atagtcaaaa caatgaattt aaacttaaac
64261 catttataaa tgaagaacaa ttaaaatcac tacttgcgat aaagaaagta gatgaacatt
64321 tgagttatga cccatctatt acctcaaaag cattagcaga tataaacaag acgcaaaaaa
64381 gattggttac aaagccaaat attctctata atcgatccaa aaaagaaaat gagtatgtac
64441 ggttaggagg aaaaatatca tatacaaaga tggagaagca ttgggaatat ttacataatg
64501 agattcagta ttataatctt actaatcaat taattagtta tgttgcttta gaaaaggaat
64561 atgcatgggc ttttttaaat gaattatttt atttaataga attatatttg aaggcattta
64621 aaggacaaaa gaaaactggc catgaatttg aagaaggatt gaacgcattt attcaatcat
64681 atgtcattat tttacttata gatataagat taccgactgt taggttatat attattaggc
64741 gtattgtgga tgaatgcgaa aaagaatcag ataatcaaaa aagaattaaa ctgatagagg
64801 aatccattaa aaagtacaga tatgtaaaag aacagattga aagatttgga aatgcgttga
64861 aagaagaatt atcaatcgcg actttgtcta ttgaacagaa tatgttagaa gctaaaattg
64921 attattataa gaattactac tatcctagaa aaaaaacaaa gcataacaat attgagtaca
64981 acgtatcaat gttttttaaa gctgtagatt ctttaaagaa atagtattcg acggacaata

65041 tttatagata ataattaatt agagaggttt taagttgtgt ttagattaaa aaaatggtat
65101 ttaaaacaaa aaggctattt ttgccgtgtc ggtgaaaagt gggacaatag taatttatcc
65161 catgaaaata tacatagtta tgtagataga gtacttgaat atgagactga aacacgtaga
65221 aacggccaac caactgaaga atatatatta gctagtagtg gggaaaaaat aaatgaagat
65281 ttaagaagtg gtactaatga tcacaaagaa attctagagg tatttgataa atataaagta
65341 aaagaggata taactactta tagaacggtt agtcctcgga tatacaaatt aatggagaaa
65401 aatgctaaaa acttaaatgg agtagatttg tttgaacttg gaattttaca cacttcatta
65461 ataaaagggt acgaatcaag agaagaaggt tacaaattac gggtaaaagt aaaaaaagga
65521 acaccagctt tctatgtagg taatttaaca ggcgaagaaa gccattatta tgaggtgatt
65581 gtagttaata atttaaaatt aaaaatcata tctattgaag attactatat aaattgtgaa
65641 gtagtataat tggaatcaat tctgttagtt gttcataata agccaattat ttttttaatg
65701 aacttattac ttaaaaaccg catcatcaac tgataagcag aagcgtatca taagt
//
LOCUS Staphylococcus 41805 bp DNA linear UNK
DEFINITION Staphylococcus Staphylococcus aureus strain VGC1
ACCESSION Staphylococcus
KEYWORDS .
SOURCE Staphylococcus Staphylococcus aureus
ORGANISM Staphylococcus Staphylococcus aureus
Bacteria; Bacillati; Bacillota; Bacilli; Bacillales;
Staphylococcaceae; Staphylococcus; Staphylococcus aureus.
FEATURES Location/Qualifiers
source 1..41805
/genome_md5=""
/mol_type="genomic DNA"
/genome_id="703339.330"
/organism="Staphylococcus Staphylococcus aureus"
/db_xref="taxon:703339"
/project="hwwang_703339"
CDS 1..480
/product="23S rRNA
(pseudouridine(1915)-N(3))-methyltransferase (EC
2.1.1.177)"
/EC_number="2.1.1.177"
/transl_table=11
/translation="MKITILAVGKLKEKYWKQAIAEYEKRLGPYTKIDIIEVPDEKAP

ENMSDKEIEQVKEKEGQRILAKIKPQSTVITLEIQGKMLSSEGLAQELNQRMTQGQSD

FVFVIGGSNGLHKDVLQRSNYALSFSKMTFPHQMMRVVLIEQVYRAFKIMRGEAYHK"
/db_xref="SEED:fig|703339.330.peg.1"
CDS 726..1031
/db_xref="SEED:fig|703339.330.peg.2"
/translation="MKGNIQCPRLIKGSLLDFVLETVDVFVNGSKNDCELVNFNYKEG

KVVSRYSTKFESNPKLRKKQLISTVKVVRYVIFDFEKILWGIGKVLLKFIILRQCLL"
/transl_table=11
/product="hypothetical protein"
CDS 1230..2093
/product="hypothetical protein"
/db_xref="SEED:fig|703339.330.peg.3"

/translation="MKHINQFSESTLAQLEKDQQHVFYVYCLMDPRNDECFYVGKGKG
NRIFKHKQDAQKKVLYEDIFIEENKDNLKFNRINEICSNDLNVLGYIISYGLTESEAF

SAENVLINFLNLTNKTTLTNMINGHGSKAYLVEDLENEFGYDSINLENINTNELILAV

KIRDAFLLDKDESKEYPINESKRDRNNLKSRTLGSWIIGKDKIHKIKYIIGINTGANN

AVVSAYEVSFEQAESIETNNGRMRYAFIGLSERDATLKKLNLYKKALPDLRFGSGSAT
AYINNGTMKVD"
/transl_table=11
CDS 2201..3676
/db_xref="SEED:fig|703339.330.peg.4"
/translation="MPSDDIVKKLYSKEDIRLELGLTPHKFNKKMETIAKLFKIDMKI

FHNYKGQDKNNQYTFNGVAKELIKVLLKSVDYYPVDINSKKFKQNGKSKKEMIENIDN

SSYMKYIYQLMKSINEIQYKRLIADIHMKDVYQNTKAWLNNGESINKKEQELYQYMTI

LPLHKRVELQNEVLKSIDETIFQFLAKEHRNNQIEENNELEAYTKAIKEGRNPKNDYE

LNHLLYKKNQLPLDSLIEDIWDYEETEYTELDWLIADMLKRSQRADSNFIESLEKKNK

LRKNIHKDSIKSISKLIDHELVKNNRKWDVAEFYKNQQFWNNSTLRRSSQNFGILYYK

KNRLHTMNQSNIIKKIKIIEANIESANKQPSYLDFFLQAKFDLERLESELMKYSINPK

YFNKINSDYVRYAIEDVHNAIIKIDRYISNDKVKLNYSNVQMVVRNEITEQGSYFVTQ
ALNTLSKVEESGYSFDNFMVGPFVEDFKKAIKNRRFGSKKD"
/transl_table=11
/product="hypothetical protein"
CDS 3902..5002
/product="hypothetical protein"

/translation="METRDKLMSLTQSDKTQQWLMDKSSNQDDIQQLQQQFSQQLDQQ

YNALLADEKAKLDQYVEVHQGLESLKEEIESEPITLNIDKLPDIKATMLERAKNDEHS

DKIEKLFDRLEQALNGTNRLYTQLSLIGTRTHRITTKNFNLQGLPKAVQHTILPSKFK

KVYTVDFKSFEPSVAAYMTQDSKLIDLLNQKDGLYDALLSELGLSDELRVFVKRAFIG

SFLFGGNFKNPKFKLNQYVSEVQWLDAVSQFTKVIELKKHVEKSNSMPMPYSIEHDMS

AFQGSSIMAIYVQTVASYIFKHILLKVYKAQCDQKTFKIIVPIHDAIMIECEDEEIAQ
NVGQLMKDTANQLFNGEFAHVTVEEIGGVDHE"
/db_xref="SEED:fig|703339.330.peg.5"
/transl_table=11
CDS 4995..5366
/translation="MNNDRGQSLHIPSSTPIKENNIYVATLHSVIQTDFSGEIKHQFT

YEIEVNNQIVYANRNIPTKPSANQLSIHDWLKRHSNYSASHENYEPYIDQKHLILLGQ
YNGNYYVQDVASLDAFGGVLS"
/db_xref="SEED:fig|703339.330.peg.6"
/transl_table=11
/product="hypothetical protein"
CDS 5363..7006
/transl_table=11
/db_xref="SEED:fig|703339.330.peg.7"
/translation="MNHILQMLSKLLSVAKEAIDRQGLIAILTIPVNNNDEIEETAQG

ETVYNELIDQLRLNIPKDTDYQPNIYSYFGIKKNPNDTVLMEMMIKVFHIKRFNSELF

IFKVNGWQKINGDELQGLISKMIQVLLVDYKPSLSTLKNVVDGLQKSTDVEELVENER

YIGCGENMFDLNTFQVVKNSIDIFPKTRLNLSLSTNDVITDKIPPYFKQYMLQLANYD

DDLQYFLFQHTAVLLTADTKYRRGLILYGGAKNGKSVYIELVKSFFYSKDIVSKPLNE

LEGRFDKESLIDKSLMASHEIGQSRIQEKIVNDFKKLLSVESMHVDRKGKTQVEVILD
LKLIFSTNAILNFPPEHAKALERRINIIPCEYYVEKADTSLIDKLQSEKKEIFLYLMY

VYQQIVKADIEYLENSRVTEITHDWLNFGYEFVSSRSVSNANQKACINLLRKLIEIKS

GSRIKVSELNKVINEEIKVSSQVIKQLIQANFDTQTKLYNGYDYWIDLGWKEANKKEI
HDISKKDNIISLDKNENITDDEALDEENLDFDWEDFDDE"
/product="putative primase"
CDS 7232..8908
/product="Cassette chromosome recombinase B"
/transl_table=11
/db_xref="SEED:fig|703339.330.peg.8"
/translation="MKGKIALYSRVSTSEQSEHGYSIHEQEQVLIKEVVKNFPGYDYE

TYTDSGISGKNIEGHPAMKRLLQDVKDNKIEMVLSWKLNRISRSMRDVFNIIHEFKEH

DVGYKSISENIDTSNASGEVLVTMFGLIGSIERQTLISNVKLSMNAKARSGEAITGRV

LGYKLSLNPLTQKNDLVIDENEANIVREIFDLYLNHNKGLKAITTVLNQKGYRTINQK

PFSVYGVKYILNNPVYKGYVRFNNHQNWAVQRRSGKSDKNDVILVKGKHEAIISEEVF

DKVHEKLASKSFKPGRPIGGDFYLRGLIKCPECGNNMVCRRTYYKTKKSKERTIKRYY

ICSLFNRSGSSACHSNSINAEVVERVINVHLNRILSQPDIIKQIASNVIEELKQKHSN

QTEIKYDIDSLEKQKAKLKTQQERLLELFLDDQMDSEMLKAKQSQMNQQLEVLDQQIK

EAQQANQSQDEIPNFDKLKGRLILMITRFSVYLRKATPEAKNQLMKMLIDSIEITTDK
QVKLVRYKIDESLIPQSLKKDWGSFFMPKFQFEIYGQNDYFIDQITTFTT"
CDS 9014..9352
/product="FIG01108299: hypothetical protein"
/db_xref="SEED:fig|703339.330.peg.9"
/translation="MTLEQQLKHYITNLFNLPRDEVWHCESIEEIADDILPNQYVRLG

PLSNKTLQTNTYYSDTLHESNIYPFILYYQKQLIAIGYIDENHDMDFLYLHNTIMPLL
DQRYLLTGGQ"
/transl_table=11
CDS 9448..9759
/transl_table=11
/db_xref="SEED:fig|703339.330.peg.10"

/translation="MNRYITRGIANNLPNILQHQLWQLVSEREQEQTKDNTSVDYFHI

FQFNKHRNQLYIKHKQERPEYAKIHKANYSKAININKVYIIREDDVDLSYYVMLLPEE
Y"
/product="FIG01107894: hypothetical protein"
CDS 9775..10281
/product="hypothetical protein"
/transl_table=11
/db_xref="SEED:fig|703339.330.peg.11"
/translation="METIKSTLKTEAIFSDDKQHRYLLKKTWNSEKQSITIITMYPHY

DGILNIDLTTQLIMNKVSEMDAFGSINFVNLYSNITTPINLKHLENAYDKHTDIQIMK

AVKESDEVILAWGAYAKKPGVEARVNEVLEMLKPHKKKVKRLMNPETNEIMHPLNSKA
RQKWILKV"

CDS complement(10430..11104)
/product="Transposase for insertion sequence-like element
IS431mec"
/translation="MNYFRYKQFNKDVITVAVGYYLRYTLSYRDISEILRERGVNVHH

STVYRWVQEYAPILYQIWKKKHKKAYYKWRIDETYIKIKGKWSYLYRAIDAEGHTLDI

WLRKQRDNHSAYAFIKRLIKQFGKPQKVITDQAPSTKVAMAKVIKAFKLKPDCHCTSK
YLNNLIEQDHRHIKVRKTRYQSINTAKNTLKGIECIYALYKKNRRSLQICGFSPCHEI
SIMLAS"
/db_xref="SEED:fig|703339.330.peg.12"
/transl_table=11
CDS 11362..11529
/db_xref="SEED:fig|703339.330.peg.13"
/translation="MLESREQLSVEEYETFFNRFDNQEFDFERELTQDPYSKVYLYSI
EDHIRTYKIEK"
/transl_table=11
/product="Hydroxymethylglutaryl-CoA synthase N-domain
homolog (EC 2.3.3.10)"
/EC_number="2.3.3.10"
CDS 12486..13229
/transl_table=11
/translation="MLTVYGHRGLPSKAPENTIASFKAASEVEGINWLELDVAITKDE

QLIIIHDDYLERTTNMSGEITELNYDEIKDASAGSWFGEKFKDEHLPTFDDVVKIANE

YNMNLNVELKGITGPNGLALSKSMVKQVEEQLTNLNQNQEVLISSFNVVLVKLAEEIM

PQYNRAVIFHTTSFREDWRTLLDYCNAKIVNTEDAKLTKAKVKMVKEAGYELNVWTVN
KPARANQLANWGVDGIFTDNADKMVHLSQ"
/db_xref="SEED:fig|703339.330.peg.14"
/db_xref="GO:0008889"
/product="Glycerophosphoryl diester phosphodiesterase (EC
3.1.4.46)"
/EC_number="3.1.4.46"
CDS 13326..13730
/db_xref="SEED:fig|703339.330.peg.15"
/translation="MKYDDFIVGETFKTKSLHITEEEIIQFATTFDPQYMHIDKEKAE

QSRFKGIIASGMHTLSISFKLWVEEGKYGEEVVAGTQMNNVKFIKPVYPGNTLYVIAE
ITNKKSIKKKMDSLQCHFQHTMKMKKLYLREK"
/transl_table=11
/product="MaoC domain protein"
CDS complement(13799..15805)

/product="Penicillin-binding protein PBP2a, methicillin
resistance determinant MecA, transpeptidase"
/db_xref="SEED:fig|703339.330.peg.16"
/db_xref="GO:0008658"
/db_xref="GO:0046677"
/translation="MKKIKIVPLILIVVVVGFGIYFYASKDKEINNTIDAIEDKNFKQ

VYKDSSYISKSDNGEVEMTERPIKIYNSLGVKDINIQDRKIKKVSKNKKRVDAQYKIK

TNYGNIDRNVQFNFVKEDGMWKLDWDHSVIIPGMQKDQSIHIENLKSERGKILDRNNV

ELANTGTAYEIGIVPKNVSKKDYKAIAKELSISEDYIKQQMDQNWVQDDTFVPLKTVK

KMDEYLRDFAKKFHLTTNETESRNYPLGKATSHLLGYVGPINSEELKQKEYKGYKDDA

VIGKKGLEKLYDKKLQHEDGYRVTIVDDNSNTIAHTLIEKKKKDGKDIQLTIDAKVQK

SIYNNMKNDYGSGTAIHPQTGELLALVSTPSYDVYPFMYGMSNEEYNKLTEDKKEPLL

NKFQITTSPGSTQKILTAMIGLNNKTLDDKTSYKIDGKGWQKDKSWGGYNVTRYEVVN

GNIDLKQAIESSDNIFFARVALELGSKKFEKGMKKLGVGEDIPSDYPFYNAQISNKNL

DNEILLADSGYGQGEILINPVQILSIYSALENNGNINAPHLLKDTKNKVWKKNIISKE

NINLLTDGMQQVVNKTHKEDIYRSYANLIGKSGTAELKMKQGETGRQIGWFISYDKDN
PNMMMAINVKDVQDKGMASYNAKISGKVYDELYENGNKKYDIDE"
/transl_table=11
CDS 16053..16196
/product="Mobile element protein"
/transl_table=11

/translation="MNYFRYKQFNKDVITVAVGYYLRYALSYRDMSEILRERGVNVHH
LTV"
/db_xref="SEED:fig|703339.330.peg.17"
CDS 16476..16727
/transl_table=11
/translation="MAKVIKAFKLKPDCHCTSKYLNNLIEQDHRHIKVRKTRYQSINT
AKNTLKGIECIYGLYKKNRRSLQICGFSPCHEISIMLTS"
/db_xref="SEED:fig|703339.330.peg.18"
/product="Mobile element protein"
CDS complement(16787..17215)
/transl_table=11
/db_xref="SEED:fig|703339.330.peg.19"

/translation="MKFQVSPYFLITQNAEGLKKLYEELFGAKTLYIQRIKDRPIDKK

LNIDENDLNKIDQCVLQFGDIKIMIADDTEGLPITQGNNISLCLTFDQVEETKHVYDQ
LIEKGSEVLKTFSPEFYTEGYGYVRDPYGISLHLFTKRKN"
/product="PhnB protein; putative DNA binding
3-demethylubiquinone-9 3-methyltransferase domain
protein"
CDS 17296..18225
/db_xref="SEED:fig|703339.330.peg.20"
/translation="MKKSVRLYNMIEYCNENRNFKLNDLMSEFNISRSTALRDIKEIE

ALGVPLYSNPGKNGGYTIIGNRDQTKIAISDEELKALVFTLSSISNVSNLPFQTEYQE
ILKKLYNNSNKKELINQYNDLFQYFNEDKYQFKSYKLFNEIIRLIIENKSFETCYSQN

YIKEQYKGIGIMYKNHQWYFVVANIESKLVNLLNISKIKELYEMGETQECNDITMQNF

QQFMVKNETAIDILIRSNVMGLNILKGYLWSDYMIENIDEETYLFKSKVNAKDIDFIA
KLIVTGGVNVKVETPNSLKNAVKVELTKIINMY"
/transl_table=11
/product="Transcriptional regulator, YafY family"
CDS 18387..18869
/product="Similarity"
/translation="MNKNITALKSTENTTYMQYTVHIDDQIINNIKECTKKFKILPME
DKIPLSPLLQPEYAGEVQDFISTYEQFMINFGKVILDSQGIKIQFESESLSSIQRGIQ

EHCYLNERTNDIDVTKEWYLCKFSNQILEEDKNQLYNALKQLMNDSKNKKQAFWTDPN
"
/db_xref="SEED:fig|703339.330.peg.21"
/transl_table=11
CDS 18937..19251
/product="Mobile element protein"

/translation="MKRVSYSVETKYKAVEMKIAGFSTKEIMKELNIKNRTQVETWWR

WYRNEESYRFSQHVGKQYTYGKGLEELSEIERLKLENKRKDIELDILKKYKALERKWY
QQ"
/db_xref="SEED:fig|703339.330.peg.22"
/transl_table=11
CDS 19236..20078
/db_xref="SEED:fig|703339.330.peg.23"
/translation="MVPAVVIDLVDQLKVKYSIKLILEVLNIPKSTYYRWKNKTYKND

TVTQKVIELCEANHYTYGYRKITALINQCYTSPINHKRVQRIMQKHHLNCRVRPKKTT

RIGKPYYKTDNLLQRQFKASCPMEVLTTDITYLPFGHSMLYLSSIMDIYNGEIVAYKI

DDKQDQSLVNDTLNQIDIPEGCILHSDQGSVYTSYAYYQLCEEKGIIRSMSRKGTPAD

NAPIESFHSSLKSETFYINNQLNSSNHIVIDIVEKYIKNYNNNRIQQKLGYLSPVKYR
ELAA"
/transl_table=11
/product="Mobile element protein"
CDS 20085..21650
/transl_table=11
/translation="MFLLSSRLRGSVPFQEVFKVHIDIYLYNKEKSEYKYQVSSYFNL

VKENPKITYKKRHLQEKQGVKGTTFTNKLKLLNKMYGVDVAKYQPFYNSNNPEYERGQ

FGERYISQRSNYEFNRLQYQIIDMLSKILDKHPLPKSDNNYKHIPTIEKAILSGDSHS

FYEYFEDIMKEIISMENSSLKEKLLTDFTYQSQCRWYSESEKLKLQLESFMHKVLESN

YYEGNKLYRMLSHAIEETINEADEDKVHFNYFKDYFLTDGGVKNWEQISEKITEFNGK

VINDIQNEYNKIQFNNANRNQKLNFNYLYHCFEFSNNLVKARVNGNRGYILYFVDKYK

IKMPFLDQLLINYLNQEIGQESINYNMQTLFEKERYDRSSTIEKLVATSKFKYEKDDS
DLFKQLFNDVENSIDRLGIYLLNNGINSNDENARYYRSFLKELSRIKSKLTPFSLEIS

KSSGREQHYPDDAIDDKDERRKNKEETYHAFDDKSDIDSKLKNKINVSIDNLLSVKIK
SNLYDISPNTKKQ"
/db_xref="SEED:fig|703339.330.peg.24"
/product="Similarity"
CDS 21845..22954
/product="hypothetical protein"
/transl_table=11
/db_xref="SEED:fig|703339.330.peg.25"

/translation="MYQANIRDLITKLPQSNKTEHFLMNKFSNQDKVQQLQRQISQQL

DQQYNELLANEKAKLDQYVEVHHNLEPLKKEIESESINLDTDKLPDIKATMLEKAKND

EHFDKIEQLFDRLDQSLNGTNRLYTQLSLIGTRTHRITTKRFNVQGLPKLVQQMILPS

QFKKVYTIDFKSFEPSVAAYMTQDEQLIDYLNHEEGLYDALLRDLSLSKEKRVSVKRA

FIGSFLFGGRYSSSKFKINQEVSEINWLQVMSKFKKVIEFKEQVEKYKTMPTPYGIEH

DMSAFQGSSIMAIYVQTVASYIFKHILLEVYKAQCEKKTFKIIVPIHDAIMIECNDKG
IAQNVAQLMKDTANQLFNGEFAHVTVEALGGIDNE"
CDS 22947..23315
/product="hypothetical protein"
/translation="MNNDRGKSLQIPQSTLLKEGSIYVATLHSVYEKNFSGDIKHQFT

YEVELNQETHYVNRNITVKSMSHQLSIADWIKRHSNYNVNHINYDPYIDRKHLVLVGQ
YNGNYYIQDVAPLDEFGGVL"
/db_xref="SEED:fig|703339.330.peg.26"
/transl_table=11
CDS 23315..24931
/translation="MNHILEMLIKLLKVGMEAIDRKGLIAILTSSIGNDEMDDSEQAV

MVYNELIDKLQLNIPKDVDYRPNIYSYFGIQKKPNDTILVEMMICIFHIKRFDSELFV

FKDKGWQKVSEDELQGLISKMIQVLLVDYKPSLSTLKNVVDGIQKSTDIEKLVEHRQY

IGCGRNMFNLKTFKVVDNDLEIFPKTRLDLELDINDTITDKIPPNFKQYMLELANYDH

DLQYFLFQHMAVLLTADTKLRRGLFLYGTAKNGKSVYIKLVKSFFYSNDIVSKTLNEL

GGRFDKESLIGKRIMASDEVGKANIDEATVNDFKKLLSVEPIHADRKGRTQVEVTLDL

KLIFNTNAVLNFPSSHAKALERRIAVIPCEYYVEKADPDLIEKLQDEKKEIFLYLMYV

YKQIVKNDIEYLQNDRVTEISHDWLNFGYEFVSSSKSANIAHQKACINLLRKLIEIKPG

SRIKVSRLNEVIRDEIKVSSQVINDLVQANFNVQSRLNNGYKYWVDLGWKETDKKDDM
ISFDKNENVTDDEFLYEDDLNLGWEDFDDE"
/db_xref="SEED:fig|703339.330.peg.27"
/transl_table=11
/product="putative primase"
CDS 25156..26835
/transl_table=11
/translation="MKGKIALYSRVSTSEQSEHGYSEKEQEQLLIKEVMKNFPGYDYE

TYTDSGISGKNIEGRPAMKRLLQDVKDNKIEMVLSWKLNRISRSMRDVFNIIHEFKEH

DVGYKSISENIDTSNASGEVLVTMFGLIGSIERQTLISNVKLSMNAKARSGEAITGRV

LGYKLSLNPLTQKNDLVIDENEAHIVREIFDLYLNHNKGFKAITTILNQKGYRTINQK

PFSVFGVKYILNNPVYKGYVRFNNHQNWAVQRRSGKSDKNDVILVKGKHEAIISEDVF

DQVHEKLASKSFKPGRPIGGDFYLRGLIKCPECGNNMVCRRTYYKTKKSKERTIKRYY

ICSLFNRSGSSACHSNAINAEVVERVINVHLNRILSQPNVIKQIASSVIEELKQKHSK

QTEIKYDIDSLEKQKAKVKTQQERLLELFLDDEMDSEMLKAKQSEMNQQLEVLDQQIK

EAKQANQSQDDIPNFDKLKARLILMITRFSVYLRKATPEAKNQLMKMLIDSIEITTDK
QVKLVRYKIDESLIPQSLKKDWGSFFIPKFNFVINVTKKNRIENLSLLPLF"
/db_xref="SEED:fig|703339.330.peg.28"
/product="Cassette chromosome recombinase B"
CDS 26924..27262
/product="hypothetical protein"
/db_xref="SEED:fig|703339.330.peg.29"
/translation="MTLSKQLKTYITERFKLNYQETWACETIDAVAEDVLPEKYIKNS
PLEHKILNTFTYYNDELHEISIYPFLCYLDKELVAIGYLDNFDLDFIFLNDTHQVIID
ERYLLQKGGE"
/transl_table=11
CDS 27265..27354
/product="hypothetical protein"
/transl_table=11
/translation="MNWIKVAQLSVTVINEVIEIMKEKQNGGE"
/db_xref="SEED:fig|703339.330.peg.30"
CDS 27356..27667
/product="hypothetical protein"
/translation="MKINRYITRGVSEHLSLDLQILLWNMVKDRDNQLHTDYLHIFKL

QEDDNILSITHEQEQPAYKLEYHYTNYVKNQNALPKKVYYVIREDDVDAFYYVMLLPEE
Y"
/db_xref="SEED:fig|703339.330.peg.31"
/transl_table=11
CDS 27682..28185
/product="hypothetical protein"
/transl_table=11
/translation="MNTIKNTIHTEAIFSDDEQHRYLLKKTWDEKKPACTVITMYPHL

DGVLSLDLTTVLILNQLANSEQYGAVYLVNLFSNIKTPENLKHIKEPYDKHTDIHLMK

AISESDTVILAYGAYAKRPVVVERVEQVMEMLKPHKKKVKKLINPATNEIMHPLNPKA
RQKWTLK"
/db_xref="SEED:fig|703339.330.peg.32"
CDS 28199..28420
/translation="MNHETKQSDWRTVASCLASQNYISIVKGLVHHFTAIEDEEILDK
IYDDFMNDDSITTVLNNDLQTIINHYLSK"
/db_xref="SEED:fig|703339.330.peg.33"

/transl_table=11
/product="hypothetical protein"
CDS complement(28495..31614)
/EC_number="3.1.21.3"
/product="Type I restriction-modification system, restriction subunit R (EC 3.1.21.3)"
/db_xref="SEED:fig|703339.330.peg.34"

/translation="MNFQFNEDDLEQVALEWLQSLGYDYKKGNEISMTSLTPERKSDK
DVVLHERLEKALRKINSDIHPRFIEKALHELTLEKSPNLLENNLTFHENLINGIEIED
YDDEGQSIVEIVKIIDFEYPQNNDFLAVNQLTIVNGDYKKRPDIVLFINGLPIVVIEL

KNSTNETVGVEDGYHQLETYKMRIPQLFTFNEVLVTSDGINTKAGSLTANYDRFMTWR

SKDGQTESSPGLASLDVLIHGMLNPETLLDLIRYFVLFQDDGKGHISKILAAYHQYYA

VNKAVDRALLASSEQGDGKGGVIWHTQGSGKSLTMVFFSGKLIQMLNNPTLVVVTDRN

DLDNQLYSTFVKSKGRSGKGLLRQTPKQAETRKELKSLLSVESGGIVFTTMQKFEPEQ

NETTMAALTERKNVIVMADEAHRTQYGFNAKYDDKGEGIKYGYAKYLRDALPNATFVG

FTGTPVASTDKNTQMVFGNYIDVYDMTQAVADGSTVKIYYESRVIPLNLPQSLDLDEA

YNDITEDQEEDVKQRLKSKWSRIEALAGAKPRVEALAKDIIQHFETRQQAMKGKGMIV

TMSRRIAVDLYDEIIRLKPEWHSDDDDKGVIKVVMTGSSSDPTSFQRHIGPKKRRNLL

EKRMKDVDDELQLVIVRDMWLTGFDVPSMHTMYIDKPMKGHNLMQAIARVNRVFKDKP

GGLIVDYVGIAESLKEALKEYTESDQAQTAIDTDKAVELMLLKYDVIQDMLYNLDYSK

FNSDKKSERYYTISNTMDYVIGLGEDERQRFIKTVTELGKAFALCATEPTAQELNDEI

AFFKAVKAGLVKLLQPPKEGKTRKTPAEVEAEINQLVSQSVVTEDVIDVYQTLGLEQP

DLSILSDDFLKDVEGLKQKNVAVELLNRLLKGQVKSLMKTNATVSKRFSEMLGNSINK

YNSRSIETSKVIEELIQLAKDIKQEQQRGNELGLNSDEIAFYDALASHETAKEAMGDK

ELRAIAHELTKTVKENMGVDWSKRDSAKAKMRVAVRRLLKKYGYPPDLQKMAVEQVVE
QAELMANNQ"
/transl_table=11
CDS complement(31598..32899)

/translation="MEYNTIFFGDVAKIKNGYAFKSKEFQEKGIPVIKIKNIISPIVD

TKDSQKVSIKTYEKTKGFSLKKNDILISLTGSGVNQMSSAVGKVGRIEFDYPALQNQR
VGKFELKYSNSADLDFLFYYFLQPKITEYLVRNSTGSANQANINSKLIETVKIPNFSL

IKQKSISKFLNQITRKIETNQKMIANLKELSQTLFKHWFVDFEFPDEDGNPYKSSGGE

MIDSELGKIPSNWKIYKLKDIASHKKETFNPKKSEEVTVKHFSLPAYDNEEQAIEEEV

NKIKSNKWIINNNCVLFSKMNPDTKRIWLPVIDNKKLNVASSEFVVMESPNNKINSFI

YNICLNSQFIDYLKANTTGSTNSRQRVKPTIAVNYKLAIEDSIVKKYSEIITPYMEEM
KILRSEIGKLTQLRDTLLPKLMSGELEISDDIEVNSDELSI"
/db_xref="SEED:fig|703339.330.peg.35"
/transl_table=11
/product="Type I restriction-modification system,
specificity subunit S"
CDS complement(32889..34403)
/EC_number="2.1.1.72"
/product="Type I restriction-modification system,
DNA-methyltransferase subunit M (EC 2.1.1.72)"
/transl_table=11

/translation="MATIGFEEKLWQAADKLRGSMDAAEYKNVALGLIFLKYVSDSFE

EKYEELKQDPYADEEDQDEYLAENIFWVPKEARWQYINDNAKKPEIGQIIDKAMIAIE

NENESLKGVLPKEYARPALDKEKLGDIIDLFTFKVGDTESRKQDVLGRVYEYFIAKFA

SAEGKNAGEFYTPSSIVKLLVEMIEPYKGRIYDPCCGSGGMFVQSERFVEKHQGRLDD

IAIYGQESNPTTWKLAKMNLAIRGIDNDLGERNADTFHNDLHKGLKADYILANPPFNA

SDWGQERLLDDYRWQFGIPPKGNANYAWIEHMISKLAPNGTAGFVLANGSMSTSGKDE

LEIRKNLIEQDLVECIVTLPGQLFYSTQIPVCLWFISNNKGQNGKKERENEILFIDAR

EIGHMVSRTLKEFSDEDIQKVAQTYHAWRGTNDKPYEDIAGFCKVANLEEVKNNEYIL
TPGRYVGLADVEEDEEPFEQKMERITSELSEQFAKSKELEDQIRKSLEGLGYGV"
/db_xref="SEED:fig|703339.330.peg.36"
CDS 34617..34745
/product="hypothetical protein"
/transl_table=11
/translation="MKIHILTFVKIVVIVDIVAIVNTRLDKLGIFNSINKKFKCSQ"

/db_xref="SEED:fig|703339.330.peg.37"
CDS 34845..36908
/transl_table=11
/db_xref="SEED:fig|703339.330.peg.38"
/translation="MYISSVKIKNFRNFDNIDIDFHEGVNVLIGHNNSGKSNLLRALS

LIFDGSVRKQLSVEDFNNSLTKESLKKEAPKIVISVHITQSENERLMSDELITVSNWL

TKLEEPYEARIQYEFFLPKDEEKNYIDLVKNIDEKEEIWNLIRSQFIRLYVNKIWVGN

PEHQIPIDNDSLNKFDFQFLDAIRDVERDMFSGKNTLLKSVIDFFIDYDIKSDETITE

EEQKEKLEERRKEFSDNSSDLIETIHKRLDSGNQKILSYTNGIGASYDKSTPDFKGNL

TESEIYTVLQLIIKHETGMTLPITHNGLGYNNLIFMALLLSKMQADSDGNFLGSNAKV
FPILAIEEPEAHLHPTMQNEFIKFLKNNIREKKVKQIFITTHSTHISSSTNIDDIICL

YTDDSKTNVSYPGKVFDKNNQSKKYVQRFLDATKSNMLFAEKVIFVEGIAEQLLLHIF

ADYLNKPLEKNHVAVINVGGRYFNHFLSLFDSNNKYAINRKVSCITDLDPMRKEKNEN

GQKNNFKACYPFEYEMDLDKFDYSTNNSLDKYLNNEHSNIRVFVQPNKYGKTFEYQLM

FDNPSLKLLLTDSISNSQELAELMDHYEENESLQKLIDTLSPSNENQRIIKSLKDTND

SWNEDNKKKALIASRYLNSIGKGENALELASVLKDNLELKGQSDYEDFVVPKYIEGAI
RWVCE"
/product="FIG131328: Predicted ATP-dependent endonuclease
of the OLD family"
CDS 36910..38658
/transl_table=11
/db_xref="SEED:fig|703339.330.peg.39"
/translation="MIINAQTKLNIEQDFKIQAGPGAGKTEFLVNHIKNVIQTSEKLE

RMKKIACITYTNTAAQTVLQRLGKSVSNRVDISTIHSFLYRNVVKPYCSFLPKEYGIC

VQKLKGHSDPIVINKYVNAWLKTEYFTKLKPPSNRNQLLKMPVLKKALQNWLLSMKLS

YENNDVQFICDNTKAQAIDKKSKKRMGINKSNLNILEKQLLNYKKIYWEKGIIDHDDV
LFFSYVLIRDYPFILTILCAKYPYFFIDEFQDTSQLQDFIIDKIRQKGCIVGVIGDKA
QAIYSFQGARVSLFEKFKVDLINSHTILENHRSSYQIVTFLNSIREDITQTVYSGIEN

DKVTILIGNRIRSYTKVVNICNKESLVTLSRDNITSNAMRIKLEGNNFDKKLLEKFQE
IDSNSKRRNTIISLIKAIELAKNTKYKDALKIVESIYKDENNPKKIALNSLINMINKY

DKYGNGTLMNFYNTLCKTLDIKLSGFRKGQVKDFYENTLYKNMVICTNIVEDTSNHIT

IHKAKGAEFENVFVIGNKDTLSLLLKPDLMNDEEHRIFYVAMSRAKKKLFLQFDYLDN
NEQKKIKERYDVSIIQ"
/product="ATP-dependent DNA helicase UvrD/PcrA,
clostridial paralog 2"
CDS 38922..39050
/product="Multidrug resistance protein"
/transl_table=11
/translation="MIIFNVIMLVSSIIQMILGKGRRITPAKPTKDAKLHLDNQSS"
/db_xref="SEED:fig|703339.330.peg.40"
CDS complement(39118..40542)
/product="Decarboxylase, pyridoxal-dependent"
/transl_table=11
/translation="MEMEFNENNIDLETIIRDEVNKYLSRDIGDLPATQQAPLELREK

YEKMEVPNKGRDIYEVLKDLNNEVLNYLYRPNHPRSFSFIPGPASRLSWLGDILTTAN

NIHASNFANATLPINIERNLINYLVGKIGYEIKPAGGVFVSGGSMANLTAIVAARDAQ

VEMEDIKKATVYLTSQTHHSVGKALHVAGFLKNNIRRIEYNDDFTMNTDSLKAAIDKD

IEEGYKPAMVIATAGTTNTGSVDDFTTLGDTCDHYNLWLHVDGAYGLSHILSDKAQHL

FKGIERSDSASWDAHKLLFQTYSCAMVIVKEKNHLLQSFGEDAEYLDDIASDDDVIDP
EMLGIELTRPARALKLWITLQVIGEDEIIERIKYGEGLAEYAEEYVSGLDNWRIISHA

NLSIVNFRYENSDLTEAQNNQLNSMAAQKIADSGYAIAYTTVLNNQRVIRLCTTNPLT
THDDIRETINRLDQYIGDYDMKDA"
/db_xref="SEED:fig|703339.330.peg.41"
CDS complement(40625..40837)
/transl_table=11
/db_xref="SEED:fig|703339.330.peg.42"
/db_xref="GO:0004617"
/translation="MRSSSPVLRGSYRIPKDKHQYKLKITDDNLQVDYPRQFLKGAHT
PSVKAQHVIHWGDTFWVSSLWLAWFY"
/product="D-3-phosphoglycerate dehydrogenase (EC
1.1.1.95)"
/EC_number="1.1.1.95"
CDS 41487..41654
/product="hypothetical protein"
/translation="MKFKSEKLIIANQTKVINYRIQVLLKPQTNIETQTQVKNNTLSE
TDEQSNADLVQ"

/db_xref="SEED:fig|703339.330.peg.43"
/transl_table=11
ORIGIN
1 atgaaaatca ccattttagc tgtagggaaa ctaaaagaga aatattggaa gcaagccata
61 gcagaatatg aaaaacgttt aggcccatac accaagatag acatcataga agttccagac
121 gaaaaagcac cagaaaatat gagcgacaaa gaaattgagc aagtaaaaga aaaagaaggc
181 caacgaatac tagccaaaat caaaccacaa tcaacagtca ttacattaga aatacaagga
241 aagatgctat cttccgaagg attggcacaa gaattgaacc aacgcatgac ccaagggcaa
301 agcgactttg tattcgtcat tggcggatca aacggcctgc acaaggacgt cttacaacgt
361 agtaactacg cactatcatt cagcaaaatg acattcccac atcaaatgat gcgggttgtg
421 ttaattgagc aagtgtatag agcgtttaag attatgcgtg gagaggctta tcataaataa
481 aactaaaaat tagattgtgt ataatttaaa aatttaatga gatgtggagg aattacatat
541 atgaaatatt ggagtatacc ttgcaatatc atacgatgtt tatagagtgt ttaataaacc
601 atttttcaac tattgatgat ctagaatata taataactgt acaaattata ttgattatgg
661 aactacaatt caattaagaa attgatgatg aaattttaaa tttaaactaa tggaatcaag
721 aaagaatgaa aggaaatata caatgcccac gattaataaa aggaagttta ttagattttg
781 tgttagaaac agtagatgtt tttgtaaatg gaagtaaaaa tgattgcgaa ttagtaaatt
841 ttaattataa agaaggaaaa gtagtttcac gttatagtac aaaatttgaa agcaatccta
901 aattaagaaa aaagcaattg atatcgacgg taaaagttgt aaggtatgtt atttttgatt
961 ttgagaaaat tttatggggc atagggaaag ttttattgaa gttcatcata ttaaggcaat
1021 gtttactata agaaagttga aatacactca gaaacagact ggattcccgt ttttttcaaa
1081 ttggcacaaa atgatacata ggcccaccta tatattgaag atttaagtta aacgataaat
1141 aatatttttc tcttttgtat agaaattttt ttgggaatga tattaattag tataaaaatg
1201 aatatgtcta caaagaagga gagattttat tgaaacacat taatcaattt tctgaatcga
1261 cactagcaca actagaaaaa gatcaacaac acgtatttta tgtatactgt ttgatggacc
1321 caagaaatga tgaatgcttt tatgttggta aaggtaaggg gaatagaata tttaaacata
1381 aacaagatgc gcagaaaaaa gtattgtacg aagatatatt tatagaagaa aataaagata
1441 acttgaaatt taatagaatt aacgaaattt gtagcaatga tttgaatgtt ttaggatata
1501 taatcagtta tggattaacc gaatctgaag cattttctgc agaaaatgtt cttataaact
1561 ttcttaacct aacaaataaa actacattaa caaatatgat taacggtcat ggatctaagg
1621 catacttagt tgaagattta gaaaatgaat ttggctacga ttcaattaat cttgaaaaca
1681 taaatacgaa tgaattaatt ttagcagtga aaatcagaga tgcatttcta ttagataagg
1741 atgaaagtaa agagtatcct attaatgaaa gtaaacgtga tagaaataac cttaaatcgc
1801 gtacattagg tagttggata ataggaaaag ataaaataca taaaataaaa tatattattg
1861 gtatcaatac aggtgctaat aatgcagttg tctcagctta tgaagtatca tttgaacaag
1921 cggagagtat cgaaacaaat aatggtagaa tgagatatgc gtttattggg ctttcagaaa
1981 gagatgctac tctaaaaaaa ttgaatttat acaaaaaagc actaccagat ttaagatttg
2041 gtagtggtag cgctacagca tatataaata atgggacaat gaaagttgat taaatgaata
2101 aaaacatcca attccttaaa ttagcctttg aaaattttat actcgtatat ataagtgaaa
2161 aacttaaaag aattagatgt taattaaagg agagaatgct atgccatcag atgatattgt
2221 aaaaaaacta tactcaaaag aggatattcg attagaatta ggacttactc cgcataagtt
2281 taataaaaaa atggaaacta ttgctaagct ttttaaaatt gatatgaaaa tttttcacaa
2341 ttacaaaggc caagataaaa ataatcagta tacatttaat ggtgtcgcaa aagaattaat
2401 caaagtgttg ctaaaaagtg ttgattatta cccagtggat atcaattcca aaaaatttaa

2461 acaaaatgga aaatctaaaa aagaaatgat tgaaaatata gataactcta gttacatgaa
2521 atatatttat cagttaatga aatctatcaa tgaaattcaa tacaaaaggt taattgctga
2581 tatacatatg aaagatgtgt accagaatac aaaagcatgg ttaaataatg gtgaatcaat
2641 taataaaaaa gaacaagagc tataccaata tatgacaatt ttaccattac ataaaagagt
2701 tgaactgcaa aatgaggtat taaaatctat agacgaaaca atttttcaat ttctcgcaaa
2761 agaacataga aataatcaaa ttgaagaaaa taatgaatta gaagcatata caaaagcaat
2821 aaaagaaggt agaaatccta aaaatgatta tgaattaaat catctcctat ataaaaaaaa
2881 tcaattaccc ttagatagtc taatagaaga tatatgggac tatgaggaaa cagagtatac
2941 tgagcttgat tggttaattg cagatatgct aaaacgttca caaagagctg atagtaattt
3001 tatcgaaagt ttagagaaga aaaacaaatt gagaaaaaat attcataaag attccattaa
3061 aagtatatct aagttgattg atcatgaatt agttaaaaat aatagaaagt gggacgtagc
3121 agaattttat aaaaatcaac aattttggaa taatagtaca ttaagacgta gctctcaaaa
3181 ttttggaatt ttatattaca aaaagaatag gttacataca atgaatcaaa gtaatattat
3241 taagaaaata aaaattatcg aagctaatat agagagtgca aataaacagc cgagctattt
3301 agatttcttt ttacaagcta agtttgattt agagaggtta gaaagtgaat taatgaaata
3361 tagcattaat ccgaaatatt ttaataagat aaatagtgat tacgtaagat atgctattga
3421 agatgtacat aatgcaatta ttaaaattga cagatatata agtaacgata aagtgaagtt
3481 aaactattca aatgtccaaa tggtagtgag aaacgaaata acagaacaag gaagctactt
3541 tgtaacccag gcattgaata cactgtcaaa ggtagaagag tctggctaca gctttgataa
3601 tttcatggta ggacctttcg tagaagactt taaaaaagca attaaaaatc gtagatttgg
3661 ttctaaaaaa gattaagatt gttttttaga gttaaacata agaataaatt taaataaaaa
3721 aataaaccca acaaaccttg ttatatcaat gtttgttggg tttatttttt tggtgatttt
3781 ttaataactc aattacatct taggataata tcgctaaatt atacattgtc gaggccaaga
3841 caaataaaaa attaaagcaa aagttagcgc gcaactttct gctataaagg agcaaattta
3901 tatggagaca agagacaaac tgatgtcatt gactcaaagt gacaaaacac agcaatggct
3961 aatggacaag tcatctaacc aagatgacat tcaacaattg cagcaacaat tcagtcagca
4021 gcttgatcaa caatataatg cacttttagc tgatgaaaaa gctaagttag accaatacgt
4081 ggaagtacat caaggattgg aatcattaaa ggaagagatt gaatcagaac ctattacgct
4141 taatatcgat aaattacccg atatcaaagc aacaatgctt gaaagagcca agaatgatga
4201 acattctgat aaaatcgaaa agctatttga taggttagaa caggcattaa atggtacgaa
4261 tcgattatat acgcaattat cgttgattgg tacacgaaca catcgaatta caactaaaaa
4321 ttttaatctt caaggcttac ctaaagcagt ccaacatacg attttacctt caaaatttaa
4381 gaaggtgtat acagtcgatt ttaaatcgtt tgaaccatca gttgcagcgt acatgactca
4441 agattcaaaa ctgattgact tgttaaatca gaaagacgga ctgtatgacg cattgctaag
4501 tgaattaggc ttatcagatg agctacgtgt atttgttaaa cgtgcattta ttggttcgtt
4561 tctatttgga ggtaacttca aaaatcctaa attcaagctg aatcaatatg taagtgaagt
4621 acaatggttg gatgcggtca gccaatttac aaaagtcatt gaacttaaga agcacgttga
4681 aaagagtaat tccatgccta tgccttatag tattgagcat gatatgagcg catttcaagg
4741 tagcagtatt atggcaatct acgtacaaac tgtagcgagt tatattttca agcacattct
4801 gctaaaagtg tacaaagcac agtgcgatca aaaaacgttc aagattatag tacctataca
4861 cgatgcgatt atgattgaat gtgaagatga agaaattgca caaaatgtgg gtcagttaat
4921 gaaagatacg gctaaccagt tgttcaatgg tgaatttgca catgtgacag tggaagaaat
4981 aggaggcgta gaccatgaat aatgatagag gacaaagcct acacatccca agtagtacac
5041 caatcaaaga aaataatata tatgtagcta cgttacattc tgtgatccaa acagatttct

5101 caggtgaaat aaagcaccaa ttcacgtatg aaattgaagt gaacaatcag attgtatatg
5161 cgaatcgtaa tattccaaca aaaccgagcg ctaatcagtt gtcaattcat gattggctga
5221 aacgtcatag caactatagc gcaagtcatg aaaactatga gccttatatt gatcagaaac
5281 atttaattct attaggtcaa tataacggta actattatgt acaagatgta gcatcgttag
5341 atgcgtttgg aggcgtatta tcatgaatca tatattacaa atgttatcta agctattaag
5401 tgtggccaag gaggcaatcg accgtcaagg tctgattgct atcctaacta ttcctgttaa
5461 taataacgat gaaatagaag aaacggctca aggtgaaacc gtgtataacg aacttatcga
5521 tcagttacga cttaatatcc caaaagatac ggattatcaa cctaacatct atagttattt
5581 tggtattaag aagaatccta atgacaccgt actcatggaa atgatgataa aggtttttca
5641 tatcaaacgc tttaattcag aactgtttat tttcaaagtt aacgggtggc aaaagataaa
5701 tggagatgaa ttacaagggt tgatatctaa aatgatacaa gtattgcttg tagattataa
5761 gccttcacta agcactctaa aaaatgtcgt agatggattg caaaaatcaa cagatgtaga
5821 agaacttgtt gagaatgagc gctatattgg ttgtggtgaa aatatgttcg atcttaatac
5881 gtttcaagtc gttaaaaatt caatcgatat ctttccaaaa acacgattga atttatcatt
5941 aagtacaaat gatgtaatta ctgataagat accgccttat tttaagcaat atatgttaca
6001 acttgcgaat tatgacgatg atttacaata ctttcttttc caacatacag cagtattact
6061 tacagctgat actaaatacc gtaggggtct catattatat ggtggagcta agaatggtaa
6121 atctgtatat attgaactag ttaaatcatt tttctatagt aaagatattg tgtctaagcc
6181 acttaatgag cttgaaggtc gttttgacaa agaaagttta attgacaaaa gtctaatggc
6241 aagtcatgaa attgggcaat ctaggattca agaaaagatc gtaaatgact tcaaaaagtt
6301 attatctgta gaatcaatgc atgttgatcg taaaggaaaa actcaagtgg aagtcatttt
6361 ggatttgaaa cttattttta gtacaaatgc gatacttaat tttcctcctg aacatgcgaa
6421 agctttggag cgtcgaatta atattattcc atgtgagtat tatgttgaaa aagcggacac
6481 ttcattaatt gataagctcc agagtgagaa gaaagaaatc tttctttact tgatgtatgt
6541 gtatcaacag attgtaaaag cagatatcga gtatcttgaa aatagccgtg tcactgaaat
6601 tactcacgat tggttaaatt ttggatatga atttgtttct agcaggtccg taagtaatgc
6661 aaatcagaaa gcatgtatta atttactcag aaaacttata gaaatcaaat caggatcacg
6721 aatcaaagta tccgagttaa ataaagttat taatgaagaa ataaaggtaa gttctcaggt
6781 tattaaacag ttaattcaag caaactttga tactcaaacc aaactataca atggctacga
6841 ttattggatt gatttaggtt ggaaagaagc caataaaaaa gagattcatg atatttcgaa
6901 aaaagataat attatttcat tagataaaaa tgaaaatata acagacgatg aggcattaga
6961 tgaagagaat ttggactttg attgggagga ctttgacgat gaataatgaa caaattgaag
7021 catttgtaga agtgcttgta cctatcatag aagaacgtat caataaaggt aattaaggct
7081 aattacgtac tacaggcagt tgcctgtagt actcatatga ttaagtggta aaagtgataa
7141 aaatgaaacg aaattataaa tatatattat ctatatgttg ttacaagacc gatagtctgt
7201 agcaataatc taataaaagg agcggtatga tatgaagggt aaaattgcac tttattcacg
7261 cgttagtacg tcagagcagt cggagcatgg gtactcaatc catgagcagg aacaagtact
7321 catcaaagaa gttgtgaaaa atttcccagg ttatgactat gagacatata ctgactcagg
7381 catttcaggt aaaaatattg aaggtcatcc ggcaatgaaa cgtctattac aagatgttaa
7441 ggataataaa atcgaaatgg tgttaagttg gaaattgaat cgtatctcac gatcaatgag
7501 agacgtgttt aatattattc atgaattcaa agaacatgat gtagggtata aatcgatttc
7561 tgagaatatt gatacatcca atgcttctgg agaagtactc gttacaatgt ttgggttaat
7621 aggatctata gaacgccaga ctttgatttc gaatgtgaaa ctttctatga atgctaaggc
7681 aaggagcgga gaggcaatca ccggtcgtgt tttaggctac aaattatcac ttaatccact

7741 tacacagaaa aatgatttgg ttatcgatga aaatgaagct aatattgtac gtgaaatttt
7801 cgatttatat ttgaatcata ataaaggcct caaagccatt acaaccgtac ttaatcaaaa
7861 ggggtatcgt actattaatc aaaagccatt ttcagtgtat ggtgttaaat acattttgaa
7921 taatccagtc tataaaggct atgtcagatt caataaccat caaaactggg ctgtacagcg
7981 aagaagtggc aaaagtgata aaaatgatgt gatattggtc aaaggtaaac atgaagccat
8041 tataagtgaa gaggtatttg ataaagttca tgaaaaatta gcttctaaaa gttttaaacc
8101 gggcagacct attggtggag atttctactt acgtggcctt attaaatgcc cagaatgcgg
8161 aaataatatg gtatgtcgac ggacgtatta taaaacgaaa aagtccaaag aacggacaat
8221 caagcgttat tacatttgtt ccttattcaa tcgttcaggg agttctgcat gtcacagtaa
8281 ttccatcaat gctgaagtcg tcgagcgtgt aattaatgtt catttgaatc gtattctgtc
8341 tcaaccagat attatcaagc agattgcgtc aaatgtgata gaagaactga aacaaaagca
8401 tagtaaccaa acagaaatta aatatgacat tgatagttta gaaaaacaaa aagctaagct
8461 taaaacacaa caagaacgat tgttagaatt gttcttagat gatcagatgg atagcgaaat
8521 gttaaaagct aaacaaagtc aaatgaatca acagttagaa gtattagatc aacaaattaa
8581 agaagcgcaa caagcaaatc aatcacagga tgaaatacct aattttgata aattaaaagg
8641 acgactcatt ttgatgataa cacgattcag cgtgtactta agaaaggcta cacccgaagc
8701 taaaaatcaa cttatgaaaa tgttaattga ttcaattgaa attacgacag ataaacaagt
8761 aaaacttgta aggtataaaa ttgacgaaag tcttatccct caatctttga aaaaagattg
8821 ggggtctttt tttatgccca aattccaatt tgaaatatat ggtcaaaatg attatttcat
8881 cgaccaaatt accactttta ccacttagtt attagtgaca aaagtgagcg aaatgaaata
8941 aaaatcaaat atatattatc aaaatgatgt atcacatgca tacatcaatc aaatacatta
9001 ggaggtcata accatgacac tagaacaaca actcaagcac tatataacca acttattcaa
9061 tctgccaagg gacgaagtgt ggcactgcga atctatcgag gaaatcgctg atgatatctt
9121 acccaatcaa tatgtaagac ttggcccact cagtaataaa acacttcaga ctaataccta
9181 ctactctgac acacttcatg aaagtaatat ctatcccttc attctctact atcagaaaca
9241 actcatagcc atcggttata tcgacgaaaa tcacgatatg gatttcttat acctacacaa
9301 cactatcatg cctcttttgg atcaacgata cttactaaca ggaggacaat aaaatgcata
9361 aatacatcaa aattacacaa ttagtcatta caatactaag tgaaatcatc atttggatga
9421 aagagtcaga acgaaaggaa gtctcttatg aatagatata tcacccgggg tatcgccaac
9481 aacttaccta atatcttaca acaccaatta tggcaactcg tatctgagcg agaacaagaa
9541 caaaccaaag ataatacttc agtagattat tttcatatat tccagttcaa taagcatcgc
9601 aatcaattat atatcaaaca caaacaagaa cgacctgaat atgcgaaaat ccataaagct
9661 aattattcaa aagcaatcaa tatcaataag gtctacatta ttcgagaaga tgatgtagac
9721 ctttcttatt atgtcatgtt attacctgaa gaatactaga ggagtgaaaa ttatatggaa
9781 acaatcaaaa gtacattaaa aacagaagcc atattcagtg atgacaaaca acatcgctat
9841 ctacttaaga aaacatggaa tagtgaaaaa caatcaatca caatcattac aatgtatccg
9901 cattatgatg gcattctcaa tattgaccta acgacccaac tcatcatgaa caaagtttca
9961 gaaatggatg catttggttc aatcaatttt gtgaatctat actctaatat tacaacccct
10021 atcaatctca aacatttaga aaatgcgtat gataagcata cagatattca aattatgaag
10081 gcagtgaaag agtcagatga agtgatatta gcttggggcg cttacgctaa aaagcccggt
10141 gttgaagcac gtgttaatga agtattagag atgttgaaac cacataaaaa gaaagtaaaa
10201 cgactcatga atccagaaac caatgaaatc atgcatcccc ttaattcgaa agcacgtcaa
10261 aaatggatat taaaagtata gcaactaaaa tgtttattca ctatccatga aaataattga
10321 atttcatata tatcttttaa tctacaatat gattaaaaga tatattattt ggttctgttg

10381 caaagtaaaa aaatatagct aaccactaat ttatcatgtc agtgttcgct taacttgcta
10441 gcatgatgct aatttcatgg catggcgaaa atccgcagat ctgaagagac ctgcggttct
10501 ttttatatag agcgtaaata cattcaatac cttttaaagt attctttgct gtattgatac
10561 tttgatacct tgtctttctt actttaatat gacggtgatc ttgctcaatg aggttattca
10621 gatatttcga tgtacaatga cagtcaggtt taagtttaaa agctttaatt actttagcca
10681 ttgctacctt cgttgaaggt gcctgatctg taattacctt ttgaggttta ccaaattgtt
10741 taatgagacg tttgataaac gcatatgctg aatgattatc tcgttgctta cgcaaccaaa
10801 tatctaatgt atgtccctct gcatcaatgg cacgatataa atagctccat tttcctttta
10861 ttttgatgta cgtctcatca atacgccatt tgtaataagc ttttttatgc tttttcttcc
10921 aaatttgata caaaattggg gcatattctt gaacccaacg gtagaccgtt gaatgatgaa
10981 cgtttacacc acgttccctt aatatttcag atatatcacg ataactcaat gtatatctta
11041 gatagtagcc aacggctaca gtgataacat ccttgttaaa ttgtttatat ctgaaatagt
11101 tcatacagaa gactcctttt tgttaaaatt atactataaa ttcaactttg caacagaacc
11161 gtattatgga atagagatgt tggtaacatt tatacaggat cattatactt aagtttaatt
11221 tcgttattac agaaccacac attccaacca gaagagaaag tatgtctatt tagttatggt
11281 tcaggagcag taggagaaat ctttagtggt tcaatcgtta aaggatatga caaagcatta
11341 gataaagaga aacacttaaa tatgctagaa tctagagagc aattatcagt cgaagaatac
11401 gaaacattct ttaacagatt tgataatcaa gaatttgatt tcgaacgtga attgacacaa
11461 gatccatatt caaaagtata cttatacagt atagaagacc atatcagaac atataagata
11521 gagaaataaa ctagtggccg attgtgcttg atgagcttgg gacataaatc ctaactcgaa
11581 ataaataagc atatcactaa actgattttt taaagtttac agtgatatgc ttattttttt
11641 atcttacgat tttgtacgtg catgcttgcc taggggtatg gctcgagcca ttagtctctc
11701 gcacatacta ttccctcagg cgtcagcact tacaaaatcg gttgtaattt tcatttttat
11761 acgcattctt actgagatta tactaataag aggaatagta aaagcaattc taagtaaaat
11821 tgcagataag aggtttgtta aaagcagttc taagtaaaat tgcagataag aggtttgtta
11881 aaagcagttc taagtaaaat tgcagataag aggtttgtta aaagcagttc tcagtaaaat
11941 tacagataag aggtacgtta aaagcagttc taagtaaaat tgcagataag aggtttgtta
12001 aaagcagttc taagtaaaat tgcagataag aggtacgtta aaagcaattc catgcaaaat
12061 tgctgataag gggtaagtta aaagcagttc tcagtaaaat tgcagataag aggtacgtta
12121 aaagcagttc taggcaaaat tgcagataag aggtgcgtta aaagcagttc taagtaaaat
12181 tgctgataag gggtaagtta aaagcaatcc taagtaaaat tgcagataag aggtaagtta
12241 aaagcaatcc taagtaaaat tgcagataag gggtacagaa aaactagact tgattacaaa
12301 atggagcttg ggacataaat gattttttaa aaatgagatg agacgtagat taactccata
12361 atcaatacga atctatcgac ttctttattt atgatattca tctcttttta atggaaataa
12421 aagtgcgatt aatgtgataa tacagttacg ttaattaaaa aaataaaaat gcaaggagag
12481 gtaatatgct aactgtatat ggacatagag gattacctag taaagctccg gaaaatacaa
12541 ttgcatcatt taaagctgct tcagaagtag aaggtataaa ctggttggag ttagatgttg
12601 caattacaaa agatgaacaa ctgattatca ttcatgatga ttatttagaa cggactacaa
12661 atatgtccgg ggaaataact gaattgaatt atgatgaaat taaagatgct tctgcaggat
12721 cttggtttgg tgaaaaattc aaagatgaac atttgccaac tttcgatgat gtagtaaaaa
12781 tagcaaatga atataatatg aatttaaatg tagaattaaa aggtattact ggaccgaatg
12841 gactagcact ttctaaaagt atggttaagc aagtggaaga acaattaaca aacttaaatc
12901 agaatcaaga agtgctcatt tcaagcttta atgttgtgct tgttaaactt gcagaagaaa
12961 tcatgccaca atataacaga gcagttatat tccatacaac ttcgtttcgt gaagactgga

13021 gaacactttt agattactgt aatgctaaaa tagtaaacac tgaagatgcc aaacttacta
13081 aagcaaaagt aaaaatggta aaagaagcgg gttatgaatt gaacgtatgg actgtaaaca
13141 aaccagcacg tgcaaaccaa cttgctaatt ggggagttga tggtatcttt acagacaatg
13201 cagataaaat ggtgcatttg tctcaataga aagttagagg tgagtcttac gtttcagtga
13261 cggtagactt acctttaaca tgttacatac taaaaaatta atttgaataa gaaagagaga
13321 catatatgaa atacgatgat tttatagtag gagaaacatt caaaacaaaa agccttcata
13381 ttacagaaga agaaattatc caatttgcaa caacttttga tcctcaatat atgcatatag
13441 ataaagaaaa agcagaacaa agtagattta aaggtatcat tgcatctggc atgcatacac
13501 tttcaatatc atttaaatta tgggtagaag aaggtaaata cggagaagaa gttgtagcag
13561 gaacacaaat gaataacgtt aaatttatta aacctgtata cccaggtaat acattgtacg
13621 ttatcgctga aattacaaat aagaaatcca taaaaaagaa aatggactcg ttacagtgtc
13681 actttcaaca tacaatgaaa atgaagaaat tgtatttaag ggagaagtaa cagcacttat
13741 taataattca taataaaaca gtgaagcaac catcgttacg gattgcttca ctgttttgtt
13801 attcatctat atcgtatttt ttattaccgt tctcatatag ctcatcatac actttacctg
13861 agattttggc attgtagcta gccattcctt tatcttgtac atctttaaca ttaatagcca
13921 tcatcatgtt tggattatct ttatcatatg atataaacca cccaatttgt ctgccagttt
13981 ctccttgttt cattttgagt tctgcagtac cggatttgcc aattaagttt gcataagatc
14041 tataaatatc ttctttatgt gttttattta cgacttgttg cataccatca gttaatagat
14101 tgatattttc tttggaaata atatttttct tccaaacttt gtttttcgtg tcttttaata
14161 agtgaggtgc gttaatattg ccattatttt ctaatgcgct atagattgaa aggatctgta
14221 ctgggttaat cagtatttca ccttgtccgt aacctgaatc agctaataat atttcattat
14281 ctaaattttt gtttgaaatt tgagcattat aaaatggata atcacttggt atatcttcac
14341 caacacctag tttttcatg cctttttcaa atttcttact gcctaattcg agtgctactc
14401 tagcaaagaa aatgttatct gatgattcta ttgcttgttt taagtcgata ttaccattta
14461 ccacttcata tcttgtaacg ttgtaaccac cccaagattt atctttttgc caacctttac
14521 catcgatttt ataacttgtt ttatcgtcta atgttttgtt atttaaccca atcattgctg
14581 ttaatatttt ttgagttgaa cctggtgaag ttgtaatctg gaacttgttg agcagaggtt
14641 cttttttatc ttcggttaat ttattatatt cttcgttact catgccatac ataaatggat
14701 agacgtcata tgaaggtgtg cttacaagtg ctaataattc acctgtttga gggtggatag
14761 cagtacctga gccataatca tttttcatgt tgttataaat actcttttga actttagcat
14821 caatagttag ttgaatatct ttgccatctt ttttcttttt ctctattaat gtatgtgcga
14881 ttgtattgct attatcgtca acgattgtga cacgatagcc atcttcatgt tggagctttt
14941 tatcgtaaag tttttcgagt cccttttttac caataactgc atcatcttta tagcctttat
15001 attctttttg ttttaattct tcagagttaa tgggaccaac ataacctaat agatgtgaag
15061 tcgcttttcc tagaggatag ttacgacttt ctgtttcatt agttgtaaga tgaaattttt
15121 ttgcgaaatc tcttaaatat tcatccattt ttttaacggt tttaagtgga acgaaggtat
15181 catcttgtac ccaattttga tccatttgtt gtttgatata gtcttcagaa atacttagtt
15241 ctttagcgat tgctttataa tcttttttag atacattctt tggaacgatg cctatctcat
15301 atgctgttcc tgtattggcc aattccacat tgtttcggtc taaaatttta ccacgttctg
15361 attttaaatt ttcaatatgt atgctttggt ctttctgcat tcctggaata atgacgctat
15421 gatcccaatc taacttccac ataccatctt ctttaacaaa attaaattga acgttgcgat
15481 caatgttacc gtagtttgtt ttaattttat attgagcatc tactcgtttt ttatttttag
15541 atactttttt tattttacga tcctgaatgt ttatatcttt aacgcctaaa ctattatata
15601 tttttatcgg acgttcagtc atttctactt caccattatc gcttttagaa atataactgc

15661 tatctttata aacttgtttg aaatttttat cttcaattgc atcaatagta ttattaattt
15721 ctttatcttt tgaagcataa aaatatatac caaacccgac aactacaact attaaaataa
15781 gtggaacaat ttttatcttt ttcatcaata tcctccttat ataagactac atttgtaata
15841 tactacaaat gtagtattta tgtcaaaata atgttataat ttttgtgata tggaggtgta
15901 gaaggtgtta tcatcttttt taatgttaag tataatcagt tcattgctca cgatatgtgt
15961 aattttttta gtgagaatgc tctatataaa atatacggtt ctgttgcaaa gttgaattta
16021 tagtataatt ataaccaaaa ggagtcttct gtatgaacta tttcagatat aaacaattta
16081 acaaggatgt tatcactgta gccgttggct actatctaag atatgcattg agttatcgtg
16141 atatgtctga aatattaagg gaacgtggtg taaacgttca tcatttaacg gtctagcgtt
16201 gagttcaaga atatgccccg attttatatc aaatttggaa gaaaaagcat aaaaaagctt
16261 attacaaatg gcgtattgat gagacgtaca tcaaaataaa aggaaaatgg aactatttat
16321 atcgtgccat tgatacagag ggacatacat tagatatttg gttgcgtaag caacgagata
16381 atcattcagc atatgtattt atcaaacgtc tcattaaaca atttggtaaa cctcaaaagg
16441 taattacaga ttaggcacct tcaacgaagg tcgcaatggc taaagtcatt aaagctttta
16501 aacttaaacc tgactgtcat tgtacatcga aatatctgaa taacctcatt gagcaagatc
16561 accgtcatat taaagtaaga aagacaagat atcaaagtat caatacggca aagaatactt
16621 taaaaggtat tgaatgtatt tacggtctat ataaaaagaa ccgcaggtct cttcagatct
16681 gcggattttc gccatgccat gaaattagca tcatgctaac aagttaacac aaagtattat
16741 tttaaaattg agattagaca tttatttttc aactttgcag cagaacctaa ttttttctct
16801 ttgtaaataa atgcaaagag ataccataag gatctcttac ataaccataa ccttcagtat
16861 agaattctgg actaaatgtt ttcaatacct cactgccttt ttctattaac tggtcatata
16921 catgtttagt ttcttctact tggtcaaaag tgagacaaag agatatatta ttaccttgtg
16981 ttatgggcaa accttcagtg tcatctgcga tcataatttt tatatctcca aattgaagta
17041 cacattgatc aattttattt aaatcatttt cgtcaatatt aagtttctta tctataggtc
17101 tatctttaat acgttgaata tacagtgttt tagcgccaaa cagctcttca tacaactttt
17161 ttaaaccctc tgcattttga gtgattaaaa aatatggact tacttgaaat ttcatgtttt
17221 ttcctcctta gatttgttat aaatagagta tatacttaat tagtgtcatc tattgacact
17281 aaaaggagaa taataatgaa aaaatctgtt agattatata atatgattga atattgtaat
17341 gaaaatagga acttcaaatt aaatgattta atgtcagaat ttaatatttc tcgtagtacc
17401 gctttaaggg atataaaaga aattgaagca ttaggagtac ctttatatag taatccaggg
17461 aaaaatggtg gttatacgat cataggtaat cgagaccaaa cgaaaatagc aatctcagat
17521 gaagagttga aagctttagt atttacactt tcgagtattt caaatgtgag taatctacct
17581 tttcaaacag aatatcaaga aatattaaaa aaattatata ataactcgaa taaaaaagag
17641 ttaataaacc aatataatga tctatttcaa tattttaatg aagataagta tcagttcaaa
17701 agttataagt tatttaatga aatcattaga ttgataattg agaataagtc ttttgaaacc
17761 tgttattcac aaaactatat taaagaacaa tataaaggta ttggcattat gtataaaaat
17821 catcaatggt attttgttgt agctaatata gaatcaaagt tagtgaatct attaaatatt
17881 tcgaaaataa aagaactata tgaaatggga gagactcaag agtgtaatga tataactatg
17941 cagaattttc aacagttcat ggttaaaaat gaaacagcta ttgatattct tattagaagc
18001 aatgttatgg gattgaatat cttgaaaggc tacctgtgga gtgactatat gattgaaaat
18061 attgacgaag agacatattt atttaaatca aaagtgaacg cgaaagatat agattttata
18121 gctaagttaa ttgtcacagg tggtgtcaat gtaaaagtag agacccctaa tagtttgaaa
18181 aatgctgtta aagttgaatt aactaaaata ataaacatgt attaatagta atttaatcat
18241 aattgtaata acaaaaagat gacaacatta aacaatttaa atttatacaa aaaagcatta

18301 ccaaatttaa aattgggtag tggtagtgct acagcataca taaatagtta taattaaata
18361 aagatgggtc aatgaaggtg aattaagtga ataaaaatat tacagctcta aaatctactg
18421 aaaatactac atatatgcag tatacggttc atatcgatga tcaaattata aataatataa
18481 aagagtgtac caaaaagttt aaaattttgc ctatggaaga taaaattcca ttatcaccgt
18541 tgttacaacc agaatatgca ggagaggtac aagattttat tagtacatat gagcagttta
18601 tgattaattt tggtaaagta atattggata gtcaaggcat aaaaatacag tttgaaagtg
18661 aatcattaag tagtattcaa cgaggcattc aagaacattg ttacttaaat gagcgaacaa
18721 atgacattga tgtgactaaa gaatggtatt tatgcaaatt ttcaaatcaa atattagaag
18781 aggataaaaa tcaattgtat aatgctttaa agcaactcat gaatgattct aaaaataaaa
18841 agcaagcttt ttggactgac cccaattagt gggaattata taaaaacact ttcgttgaat
18901 tcataataaa atgaatcatg cggaggtgtt ttttctatga aaagagtttc ttattcagta
18961 gaaacaaagt ataaagcagt tgaaatgaaa atagctggtt tttcgacaaa agaaattatg
19021 aaagaattaa atattaaaaa tagaacacaa gtggaaactt ggtggcgatg gtatcgaaat
19081 gaggaaagtt atagattttc acaacacgtt gggaaacaat atacctacgg taaaggatta
19141 gaagaattgt cagaaataga acgattaaaa ttagaaaata agagaaaaga tatagaattg
19201 gatattttaa aaaagtacaa ggcattggaa aggaagtggt accagcagta gtcatagatt
19261 tagtggatca attaaaagta aaatattcaa tcaaattgat actagaagta ttaaacatac
19321 ctaaatcaac atattaccga tggaaaaaca aaacctataa aaatgatact gtaacacaaa
19381 aagttattga attatgtgaa gctaaccact atacctacgg ttatcgtaag attacagcac
19441 tgattaatca atgttataca tcaccaatta atcataagag agtacagaga attatgcaga
19501 agcatcattt gaactgccga gttagaccta aaaagacgac aaggataggt aaaccgtatt
19561 ataaaactga caatttatta caaagacaat ttaaagcgag ttgtcccatg gaagtattaa
19621 caaccgatat tacttattta ccatttggtc attctatgtt gtatttatct tcgataatgg
19681 atatttataa cggagaaatt gtggcgtata aaatagatga taaacaagac caaagtttag
19741 ttaatgatac attaaatcaa atcgatatac ccgaggggtg tatattacat agtgatcaag
19801 gcagcgttta tacatcttat gcttattatc aattatgcga agaaaaaggc attatcagaa
19861 gtatgtcccg aaagggaaca cctgctgata acgccccgat agaaagtttt cattcctcgc
19921 taaagtctga aactttttac atcaataatc agcttaatag ctctaatcat attgtaatag
19981 atattgtcga aaaatacatt aaaaactata ataataatcg aattcaacaa aaactaggct
20041 acttatctcc tgtaaaatac agagaattag cagcctagaa catagtgttt ttattaagtt
20101 cccgtttaag gggtcagta ccttttcaag aggtgtttaa ggttcatatt gatatttatc
20161 tctacaataa agaaaaatct gaatataagt atcaagtatc tagttatttc aatcttgtaa
20221 aagaaaatcc taaaataact tataaaaaaa gacatttaca agagaaacaa ggtgtcaagg
20281 gtacaacttt cacgaacaaa ttaaaattat tgaacaaaat gtatggtgta gatgttgcaa
20341 aatatcagcc attttataat tctaataatc ctgaatatga aagaggtcaa tttggtgaaa
20401 gatacatttc tcaaagatct aactatgaat ttaatagact tcaatatcaa attatagata
20461 tgttatcgaa aatacttgat aagcatccat taccaaagtc agataataat tataaacata
20521 ttccaactat tgaaaaagca atattaagtg gggattctca tagtttttat gagtattttg
20581 aagatataat gaaagaaatt ataagtatgg aaaattcttc tttaaaggaa aaattattga
20641 ctgattttac atatcaatct caatgtagat ggtactctga aagtgaaaaa ttaaaattgc
20701 aacttgaaag ttttatgcat aaagttttgg agagcaatta ttatgagggt aacaaattat
20761 atagaatgtt gtctcatgca attgaagaaa caatcaatga ggctgatgaa gataaggttc
20821 atttcaatta ttttaaagat tattttttga cagacggtgg ggtgaagaat tgggagcaga
20881 ttagcgagaa aattactgaa ttcaatggta aagttattaa cgatatccaa aatgaatata

20941 ataagattca atttaataac gcaaatcgaa atcagaaact aaactttaat tatttatacc
21001 attgctttga gtttagtaat aacttagtaa aagcaagggt aaatggaaat agaggttaca
21061 ttttatattt tgtagataaa tataaaataa aaatgccctt tttggatcaa ttattaatca
21121 attatttgaa tcaagaaata ggtcaggaaa gtattaatta taatatgcaa acattgtttg
21181 aaaaagaaag atatgataga agtagtacca ttgaaaaatt agtagcaaca agcaaattta
21241 agtatgaaaa agatgattca gatttattca aacaactttt caatgatgtt gaaaattcaa
21301 tagacagatt aggtatttac ttactaaata atggtataaa ttcgaatgat gaaaatgcaa
21361 gatattatag atcgttttta aaggaactta gtagaataaa aagtaaatta acgccatttt
21421 ctcttgaaat aagtaagtct agcggaagag agcaacatta tcctgatgat gctattgatg
21481 ataaagatga gagaagaaaa aataaagaag aaacatatca tgcttttgat gataaaagcg
21541 atattgactc caaattaaag aataaaataa atgtttctat cgataattta ttgtcagtta
21601 aaattaagag caatttgtat gatattagtc caaatacaaa aaaacaataa ctataatgag
21661 ccaactcaag aatgagttgg ctttttaatt gtaattttac gcttccatta caggaatcaa
21721 tgttatggaa gccattaagc aattaatttg agtcatttat agtataggta caacgttgtg
21781 atgttcacat gataatcagc ccgggagatt attgtgtaat aacttataga aggagcaaat
21841 caccatgtat caagcaaata tacgtgattt aattacaaaa ttgcctcaaa gcaataaaac
21901 agaacacttt ttaatgaaca aattttcaaa tcaagataaa gttcagcagc tacaaagaca
21961 aattagccaa cagctagatc aacaatataa tgagcttttg gctaatgaaa aagctaagct
22021 agaccaatac gtggaagtac accataattt agaaccatta aagaaagaga ttgaatcaga
22081 atctattaac cttgataccg ataaattacc tgatatcaaa gcgacaatgc ttgaaaaggc
22141 taagaacgat gaacattttg ataaaatcga acagctattt gatagattag atcagtcatt
22201 aaatggtacg aatcgattat atacgcaatt atcgttgatt ggcacacgaa cacatcggat
22261 cacaacgaaa agatttaatg ttcaaggctt gcctaaatta gtccaacaaa tgattttacc
22321 ttcgcaattt aaaaaggttt atacaataga ttttaaatca ttcgaaccat cagttgctgc
22381 gtatatgaca caagacgaac aactgattga ctacttgaat catgaagaag ggttatacga
22441 tgcattactg agagacttat ctttgtcaaa agagaagcgc gtgagtgtga aacgtgcatt
22501 tatagggtca tttctttttg gcggtcgtta tagtagctct aaattcaaaa tcaatcaaga
22561 ggttagtgaa attaactggc tacaagtaat gagcaaattc aagaaggtca ttgaatttaa
22621 ggagcaagtc gaaaaatata aaacaatgcc tacgccttac ggcattgaac atgatatgag
22681 cgcatttcaa ggtagtagta ttatggcaat ttatgtacaa acggtagcaa gctatatttt
22741 caagcacatt ttgttggaag tgtacaaagc acagtgcgaa aaaaaaacgt tcaagattat
22801 agtgccgata cacgatgcga ttatgattga atgtaatgat aaggggattg cacaaaatgt
22861 agcacagctc atgaaagata cagctaatca actgtttaat ggtgaatttg cacatgtgac
22921 agtggaagct ttaggaggta tagacaatga ataatgatag aggaaaaagt cttcaaattc
22981 cccaaagtac attgttaaaa gaaggatcta tatacgttgc tacgttacat tctgtgtacg
23041 agaagaactt ctcaggtgat attaaacatc agtttacgta tgaagtagaa cttaaccaag
23101 aaacgcacta tgtaaatcgc aatattaccg taaaatctat gagtcaccaa ttatcaattg
23161 ctgattggat taaacgtcac agtaactata acgtaaacca cattaattat gatccataca
23221 ttgatcgaaa acatttggtc cttgtagggc aatataacgg aaattattat attcaagatg
23281 tagcaccatt agatgaattt ggaggggtat tgtaatgaat catattttag aaatgttaat
23341 aaaattatta aaagtgggta tggaggcaat cgaccgtaaa ggtctgattg ccatcctaac
23401 aagtagtatt ggaaatgatg aaatggatga ttctgaacaa gctgtaatgg tgtataacga
23461 gcttatcgat aagctacagc ttaacattcc taaagatgtc gactatagac ctaacatata
23521 cagttatttt ggtattcaaa aaaagccaaa tgacacaata ttagtggaaa tgatgatatg

23581 tatttttcat atcaagcgtt ttgattcaga gttgtttgtt ttcaaagata aaggttggca
23641 aaaggtaagt gaagatgaat tgcaagggtt gatatctaaa atgatacaag tgttgctagt
23701 tgattataaa ccttcactaa gtactttgaa aaacgtagta gatggcatac agaaatcaac
23761 ggacatagaa aaacttgttg agcatagaca gtacattggt tgtggacgaa atatgttcaa
23821 tctaaagacc tttaaagtgg ttgataatga ccttgaaata ttccctaaaa cacgcttaga
23881 tttggaatta gatataaatg acacgattac ggacaagata cccccgaatt tcaaacaata
23941 tatgttagag ttggcgaatt atgaccatga tttacaatat ttccttttcc aacatatggc
24001 agtgttattg acggcagata ctaaactacg tcgtggactt tttttgtatg gaactgcaaa
24061 aaatgggaaa tcggtctata ttaaattagt taagtcattc ttttatagta atgatatcgt
24121 atctaaaaca cttaatgaac ttggcgggcg tttcgataag gaaagtctaa ttggtaaacg
24181 aattatggca agtgatgaag tggggaaagc taatattgat gaagcaactg tgaatgattt
24241 caaaaaatta ctatctgttg aaccaattca tgctgaccgt aaaggaagaa cgcaagtaga
24301 agttacttta gatttaaaac tcatttttaa tacgaatgct gtactcaatt ttccatcatc
24361 acatgcaaaa gcattagagc gtagaattgc tgttattcca tgtgaatatt atgttgaaaa
24421 agctgaccct gacttaattg aaaagttaca ggatgaaaag aaagaaatct ttctttactt
24481 gatgtatgtg tataagcaaa ttgtaaaaaa tgatatcgag tacctccaaa atgatcgtgt
24541 tactgaaatt tctcatgatt ggttaaattt tggatatgaa tttgtttcta gtaaatcagc
24601 aaatattgca catcagaaag cgtgtattaa tttactcaga aaacttatag aaatcaaacc
24661 agggtcacgt atcaaagtgt ctaggctaaa tgaggttatt agagatgaaa ttaaagtaag
24721 ctctcaagtt attaatgatt tggttcaagc taactttaat gtacaaagta gactaaataa
24781 tggttataag tattgggtcg atttaggatg gaaagaaact gataaaaaag atgacatgat
24841 ttcattcgat aaaaatgaga atgtaacaga tgatgaattc ttatacgaag atgatttgaa
24901 cttaggttgg gaggactttg acgatgaata atgaacaaat tgaagcattt gtagaagtgc
24961 ttgtgcctat catagaagaa cgtatcaata aaggtaagta atctaattac gtactacagg
25021 cagttgcctg tagtactcat atgattaagt ggtaaaagtg ataaaaatga aacgaaatta
25081 taaatatata ttatctatat gttgttacaa gaccgatggt ctgtagcaat aatctaataa
25141 aaggagcggt atgatatgaa gggtaaaatt gcactttatt cacgtgttag tacgtctgag
25201 cagtcagaac atggttattc tgaaaaggag caggaacaac tactcatcaa agaagttatg
25261 aaaaatttcc caggttatga ctatgagaca tatactgact caggcatttc aggtaaaaat
25321 attgaaggtc gtccggcaat gaaacgtcta ttacaagatg ttaaggataa taaaatcgaa
25381 atggtattaa gttggaaatt gaatcgtatt tctcgctcaa tgagagacgt gtttaatatt
25441 attcatgaat tcaaagaaca tgacgtaggg tataaatcga tttctgagaa tattgataca
25501 tccaatgctt ctggagaagt actcgttaca atgtttggac taataggatc tatagaacgc
25561 cagactttga tttccaatgt gaaactttct atgaatgcta aggcaaggag cggagaggca
25621 atcaccggtc gtgttttagg ctacaaatta tcacttaatc cattgacaca gaaaaatgat
25681 ttagttattg atgaaaatga agctcatatt gtacgggaaa tctttgattt atatttgaat
25741 cacaataaag gatttaaagc aatcacgaca attctaaatc aaaaaggata tcgtaccatt
25801 aatcaaaaac cattttcagt gtttggcgtg aaatacattt tgaataatcc agtctataaa
25861 ggctatgtca gattcaataa tcatcaaaat tgggctgttc agcgaagaag tggtaaaagt
25921 gataaaaatg atgtgatatt ggtcaaaggt aagcatgaag ccattataag tgaagatgta
25981 tttgatcaag ttcatgagaa actagcttct aaaagtttta aaccgggtcg acctattggt
26041 ggagatttct acttacgtgg ccttattaaa tgcccagaat gcggaaataa tatggtatgt
26101 cgacggacgt attataaaac gaaaaagtcc aaagaacgca caatcaaacg ctattacatt
26161 tgttcattat tcaatcgctc aggaagttct gcctgtcata gtaatgcgat taatgctgaa

26221 gtcgtcgaac gcgtaatcaa tgttcatttg aatcgtattc tttcacaacc taatgttatt
26281 aagcagattg cgtcaagtgt gatagaagaa ctgaaacaaa agcatagtaa acaaacagaa
26341 ataaaatatg atattgatag tctagaaaaa caaaaagcaa aagttaaaac acaacaagaa
26401 cgattattgg aattgttctt agatgatgaa atggatagcg aaatgttaaa agctaaacaa
26461 agtgaaatga atcaacagtt agaagtatta gaccaacaaa ttaaagaagc aaaacaagca
26521 aatcaatcac aggatgatat acccaatttt gataagttaa aagcacgact cattttgatg
26581 ataacacgat tcagtgtgta cttaagaaag gctacacccg aagctaaaaa tcaacttatg
26641 aaaatgttaa ttgattcaat tgaaattacg acagataaac aagtaaaact tgtaaggtat
26701 aaaattgatg aaagtcttat ccctcaatct ttgaaaaaag attgggggtc tttttttata
26761 cctaaattta actttgtgat aaatgtcaca aagaaaaata ggattgaaaa tttatcactt
26821 ttaccacttt tttagagtga caaaagtgga ggagttttga aatattttat aaatatatat
26881 tttatttatg gagtacacat tattaattaa ggaggtcatt ataatgacgc taagcaaaca
26941 acttaaaacg tatatcactg aacgatttaa attaaattat caagaaactt gggcttgtga
27001 aaccatagat gcggtggctg aagatgtatt acctgaaaaa tatattaaaa atagtccact
27061 tgaacataaa attttaaata cttttaccta ttacaatgat gaattacatg aaatcagcat
27121 ttaccctttt ttatgttatc tagataagga attagtagca ataggttatt tagataattt
27181 tgatttagac tttatatttt taaatgacac tcatcaagtc attattgatg aacgctactt
27241 gttacaaaaa gggggcgagt aattatgaat tggatcaagg tcgctcaact atctgtcaca
27301 gttatcaatg aagtcattga gattatgaaa gaaaaacaaa atggaggggа atagtatgaa
27361 aatcaatcga tacattacaa gaggtgttag tgaacattta tctctagacc ttcaaatctt
27421 actttggaac atggtaaaag atcgagacaa tcaacttcat acagattacc tacacatttt
27481 taaactacaa gaagatgata atatactctc aatcacacat gaacaagaac aacccgcata
27541 caaattggaa tatcactata caaactatgt aaaaaatcaa aatgcattac ctaagaaagt
27601 ctacgtcatc cgagaagatg atgtagacgc tttttattat gtgatgcttt taccagaaga
27661 atactaaaag gagtgacaat gatgaataca atcaaaaata caatacacac agaagcgata
27721 tttagcgatg atgaacaaca ccgctactta ctcaaaaaga cttgggatga aaagaaacct
27781 gcatgtacgg taatcaccat gtaccctcat ctagatggcg tattatcact cgatcttaca
27841 actgttctta tcctcaatca attagcgaat tctgaacaat atggcgctgt atatcttgta
27901 aatctatttt ctaatattaa aaccccagag aaccttaaac atattaaaga accttatgat
27961 aaacacacag acattcattt aatgaaagcg ataagtgaaa gtgacacagt gattttagcc
28021 tatggtgctt atgcgaagcg acccgttgtt gtcgaacgcg ttgagcaagt gatggaaatg
28081 ttaaaacctc ataaaaagaa agtaaaaaag ctcataaatc cggcaacgaa tgaaattatg
28141 catccgctta atcctaaagc gcgtcaaaaa tggacattga aataaaggag gattatctat
28201 gaaccatgaa actaaacaat cagattggcg aacggttgct agttgtttag catcacaaaa
28261 ttatatatcg attgtaaaag gtttagtaca tcattttaca gcgatcgaag atgaggaaat
28321 tcttgataaa atctatgatg attttatgaa tgatgactct ataacaacgg tacttaacaa
28381 tgatttacag acaatcatta accattatct atcaaaataa agactatact aattacaata
28441 aaaacagagc atccttcact tttatggtga gggatgctct tttaatttat tttttattg
28501 attattcgcc ataagttctg cttgctctac aacctgctct acagccattt tttgtaaatc
28561 tggtggatag ccatattttt taagcaatcg tctaacagct acgcgcattt tagcttttgc
28621 gctatcacgt ttagaccaat caacacccat gttttctttc actgttttag ttagctcatg
28681 agcaatcgca cgtagttctt tatctcccat ggcttctttt gctgtttcat gtgaagctaa
28741 agcatcgtaa aacgcaatct catctgaatt caggcctaat tcatttcctc gttgttgttc
28801 ttgtttaata tctttagcga gttgaataag ttcttcaatc actttagatg tttcaatgga

28861 acgactatta tatttattaa tcgaatttcc taacatctca gagaaacgct tagataccgt
28921 tgcattcgtt ttcattaatg atttaacttg tcctttgagt aatcgattta ataattctac
28981 cgccacattt ttctgtttca atccttcgac atcttttaaa aagtcatcag ataagattga
29041 taaatcgggt tgttcaagac ctagcgtttg ataaacatca atgacatctt cagtcacaac
29101 agattgtgac acaagttgat taatctctgc ttcaacttct gcaggtgttt tacgtgtttt
29161 tccttctttt ggcggttgta acaatttaac aagtcctgct ttaactgctt taaagaaggc
29221 aatctcatcg ttgagttctt gggctgtagg ttcagtcgca caaagagcaa aggctttccc
29281 taattctgtg actgttttaa taaaacgttg acgttcatct tcacctaaac caattacata
29341 atccatcgta ttcgatattg tataataacg ttcagatttt ttatctgaat taaatttaga
29401 gtaatcaaga ttatataaca tatcttgaat tacatcatac ttcaataaca tcaattcaac
29461 cgctttatcc gtatctattg ccgtttgagc ttgatcagat tctgtatatt ctttaagtgc
29521 ctcttttaaa ctttcggcaa tccccacata atcgacaatt aaaccacccg gcttatcttt
29581 aaacactcga ttaactcgag caattgcttg catcaaatta tgccctttca tcggtttatc
29641 aatatacatc gtatgcatag aaggaacatc aaaccctgtc agccacatat ctcgaacaat
29701 cacgagttgt aactcatcat ccacatcttt catacgtttt tctaataaat tacggcgttt
29761 tttaggacca atgtgtcttt ggaaagaagt tgggtcacta gatgagcctg tcattaccac
29821 tttaatgact cctttatcat catcatctga atgccattct ggttttagac gaatgatttc
29881 atcatataaa tcaacagcaa ttcgacgact catcgttaca atcattcctt tgcctttcat
29941 cgcttgttga cgtgtttcaa agtgttggat gatatctttg gctagggctt ctacacgagg
30001 ttttgcgcct gctaaagctt caatacggga ccattttgat tttaaacgct gttttacatc
30061 ctcttcttga tcttcagtaa tgtcattata cgcttcatct aaatctaaac tttgaggtag
30121 atttaatgga attacacgac tttcatagta aattttaacg gtgcttccat cagcgacggc
30181 ttgtgtcata tcataaacat cgatatagtt tccgaaaacc atttgcgtat ttttatccgt
30241 tgaagctacg ggtgtgcctg taaatcctac gaatgttgca ttcggtaaag catctcttaa
30301 atatttggca taaccatatt taatgccttc acctttatca tcgtattttg cgttaaagcc
30361 atattgtgta cgatgagctt cgtctgccat gacaatcaca tttttcgtt ctgttagggc
30421 agccatggtc gtttcgtttt gttcaggttc aaatttttgc atcgttgtaa atacaatacc
30481 acccgactca acagataata atgattttaa ttctttacgt gtttcagctt gttttggtgt
30541 ttgtcttaat aatcctttac cagaacgtcc ttttgattta acaaacgtac tgtacagttg
30601 gttatctaaa tcattacgat ccgttacgac gactaaggta gggttattca gcatttgaat
30661 taattttcca gagaaaaaga ccatggttaa acttttaccg gacccttggg tatgccaaat
30721 aacgccgcct ttaccatcac cttgctcaga tgaagctaat aaagctctgt caacggcttt
30781 attaacggca taatattgat gatacgctgc tagaatttta ctgatatgtc ctttgccatc
30841 atcttgaaat aaaacaaaat atcgaattaa atcaagtaat gtttctggat ttaacatgcc
30901 atgaatgagt acgtctaagc tagctaaacc tggtgatgat tcagtttgac catctttact
30961 gcgccaagtc ataaaccgat cataatttgc ggttagagag ccagcctttg tattaatacc
31021 atcacttgta acaagcactt cgttaaacgt aaataattgt ggaatacgca tcttatacgt
31081 ttctaattga tgataaccgt cttcgacgcc cacggtttca tttgttgaat ttttaagttc
31141 aatcactacg ataggcaagc cattgataaa gagtacaata tcgggacgtt ttttatagtc
31201 tccattaacg atagttaatt gattgactgc taaaaaatca ttgttttgtg gatattcaaa
31261 atcaataatt ttaacaattt ctacaatcga ttgtccttca tcatcatagt cttcgatttc
31321 aataccatta atcaaatttt catgaaaggt aaggttattt tctaaaagat tgggcgactt
31381 ctctaaagtt agttcatgta gcgctttttc aataaaacga ggatggatat ctgagttgat
31441 ttttcttaat gctttctcta aacgttcatg aaggacaaca tctttatcac ttttacgttc

31501 tggtgttaga cttgtcatac taatctcatt accttttta tagtcatagc ctagtgattg
31561 tagccattct aatgcgactt gttctaaatc atcttcatta aattgaaagt tcatcactat
31621 tcacctcaat atcatcagat atttctagtt ctccagacat tagtttagga agaagagtat
31681 ctcgtaattg agttaattta ccaatttctg atcttaatat tttcatttct tccatatatg
31741 gagtgattat ttcagaatat ttttttacaa tactatcttc aattgctaat ttataattaa
31801 cagctattgt tggttttact ctttgtctac tatttgttga accggtagta ttagctttta
31861 aataatctat aaattgagaa ttaagacata tattatatat gaatgaatta attttattat
31921 taggagattc cattacaaca aattctgatg aagcaacatt taatttttta ttatctatta
31981 caggtaacca aatcctttta gtatcaggat tcatttttga aaataatacg caattgttat
32041 taataatcca tttattactt tttattttat tgacttcttc ctctatagct tgttcttcat
32101 tatcataagc aggtaaacta aaatgtttaa ctgttacttc ttcactcttt ttaggattaa
32161 aagtttcctt tttatgactt gctatatctt ttaatttata gattttccaa ttacttggta
32221 tcttacctaa ctcactatca atcatttctc ctccactaga tttataagga ttaccatctt
32281 catctggaaa ttcaaaatct acaaaccaat gcttaaatag tgtttgtgaa agttctttaa
32341 ggtttgctat catttttgg ttagtttcta tttttcttgt gatttgattt aaaaatttag
32401 aaatgctttt ttgttttatt aaagaaaaat taggaatttt gacagtttca ataagtttag
32461 aatttatatt agcttgatta gcactccctg tagaatttct aactaaatac tcagttattt
32521 taggttgtag aaaataataa aataaaaaat ctaagtctgc actattacta tattttaatt
32581 cgaattttcc cacacgttga ttttgtaatg ctggataatc aaattcaatt cttccaactt
32641 ttcctactgc agaagacatt tgattcacgc ctgatccagt taatgatatt aatatatcat
32701 ttttctttaa tgaaaaacct ttagtttttt catatgtttt aatagagact ttttgagagt
32761 cttttgtatc aacaatgggt gaaattatat tcttaatttt aataaccggt atacctttt
32821 cttgaaattc ttttgattta aaagcatatc catttttaat tttagctaca tcaccgaaaa
32881 aaattgtatt atactccata tcctaacccc tccaatgatt ttcggatttg gtcttcaagt
32941 tcttttgatt ttgcgaattg ttcacttaat tcagaagtaa tacgttccat tttttgttca
33001 aaaggttctt catcttcttc aacgtcagct aaaccaacat atcgtccagg tgttaaaatg
33061 tattcattat tcttaacttc ttctaaatta gctactttac aaaaaccagc tatatcttca
33121 taaggtttat cgtttgtacc tctccacgca tgatacgtct gtgctacttt ttgaatatct
33181 tcatctgaaa attcttttaa tgttctagat accatatggc cgatttcacg agcatcgata
33241 aataaaattt catttcacg ctctttttta ccattttgac ctttattatt actaatgaac
33301 caaagacata caggtatttg ggttgagtaa aagagttgtc caggtaaagt aacaatacat
33361 tcaactaaat cttgttcaat tagattttta cgaatttcta attcatcttt tccgcttgta
33421 gacattgatc cgttggctaa tacgaatcct gctgtaccat taggtgctaa ttttgaaatc
33481 atatgttcaa tccatgcata gttagcatta ccttttggtg gaataccaaa ttgccaacgg
33541 taatcatcaa gtaatcgttc ttgaccccaa tcacttgcgt taaaaggagg attggctaat
33601 atgtaatctg cttttaatcc tttatgtaaa tcattatgga atgtatcagc atttcgttca
33661 cctaagtcat tatcaatacc acgaatcgct aagttcattt ttgctaattt ccaagtcgta
33721 gggttagatt cttgtccgta aatcgcaata tcgtctaatc gaccttgatg tttctcaacg
33781 aagcgttcac tttgtacaaa catcccacct gaaccgcaac acggatcgta aatacggcct
33841 ttataaggct caatcatctc aactaataac tttacaattg atgacggggt atagaattct
33901 ccagcatttt taccttcggc acttgcgaat ttagcaataa agtactcata cacacgacct
33961 aacacatctt gtttacgact ctctgtgtcg ccaactttaa atgtaaataa atcaataata
34021 tcgcctaatt tttctttatc taatgcaggt cttgcatatt ctttaggtaa tacacctttg
34081 agtgattcat tttcattttc aattgcaatc attgctttat caataatttg tccaatttct

34141 ggttttttcg cattatcatt gatatattgc catcttgctt cttttggcac ccagaaaatg
34201 ttttctgcta agtattcatc ttggtcctct tcatctgcat atggatcctg cttcaattct
34261 tcatactttt cttcaaaaga atctgataca tattttaaaa agattaaacc tagtgctaca
34321 tttttatact cagcagcatc catacttcct ctcaatttat cggcagcttg ccataatttt
34381 tcttcaaaac ctatagtggc cattaataac acctcttaaa attaatgtta aatcaactat
34441 agcaaaaaat agaagatgat tgtgtagtgt tttattaata tttaatttat ctagactatc
34501 aaaaaaatat agattattat tatcttaaat taataaacag gagtgaatta aaatgaataa
34561 aaaatataaa ttaaaacgat acgataaaag atttagaggt ggatggtgtt acaaatgtga
34621 agattcatat tcttaccttt gtgaagattg tggttattgt agatattgtt gccattgtta
34681 atacaagact agataaattg ggtatcttta actcaattaa taaaaaattt aaatgtagtc
34741 aatagcttgc taactttatt tgatgtaata taattttggc ttttagcatt cagttaaaaa
34801 gaagtaaata attgacagat tttttagaga actggagaaa aaagatgtac atttctagtg
34861 ttaaaattaa gaattttaga aattttgata atatcgatat tgatttccat gagggagtta
34921 atgtattaat cggacataat aattcgggga aatcaaattt attacgtgct ttatctttaa
34981 tttttgacgg ttcagttaga aagcaactgt cagtggaaga ttttaataac tctttaacaa
35041 aagaatcttt aaaaaaagag gctccaaaaa tagtaatttc agtacatata acacaatctg
35101 aaaacgaaag actgatgtct gatgagttaa ttacggtaag taattggctg actaaattag
35161 aagaacctta tgaagctaga atacaatatg aattcttttt accaaaagat gaggaaaaaa
35221 attacataga tttagtaaaa aatatagatg aaaaagaaga aatatggaat ttaattagaa
35281 gccagtttat taggttgtat gtcaataaaa tctgggtggg taatccagaa catcagattc
35341 ctatagataa tgatagttta aataaatttg atttccaatt tttagacgca ataagagatg
35401 tagagagaga tatgtttagt ggtaaaaata cattattaaa aagtgtaata gattttttta
35461 tagattacga tataaaatcc gatgaaacaa tcacagaaga agaacagaaa gaaaaattag
35521 aagaaagaag aaaagaattt tctgataatt ctagtgattt gattgaaact attcataaaa
35581 gattagatag tgggaatcaa aaaattttgt cttatacaaa tggcatagga gcatcttatg
35641 ataaatcaac accggatttt aaaggtaatc ttacagaaag tgaaatttat actgtattgc
35701 aattgataat taaacatgaa actggaatga ctttacctat aactcataac gggttgggtt
35761 acaataattt aattttcatg gccttgctat tgtctaaaat gcaagctgat tctgatggaa
35821 attttctagg tagtaatgca aaagtatttc cgatactagc tattgaagaa ccagaggctc
35881 atctacatcc tactatgcaa aatgaattta ttaaattttt aaaaaataac attagagaaa
35941 aaaaggtaaa gcagattttc ataactactc attcaacaca tatttcttct tctacaaata
36001 ttgatgatat tatttgtctt tacacagatg atagtaaaac taatgttagt tatcctggaa
36061 aagtttttga taagaataat caaagtaaga agtacgtaca acggttttta gatgccacta
36121 agtcaaatat gctatttgca gaaaaggtaa tatttgtgga aggtatagca gaacaattac
36181 tattacatat atttgcagac tatttaaata aaccgttaga aaagaatcat gttgcagtaa
36241 taaatgtagg cgggagatat tttaatcatt ttttatcttt gtttgatagt aataataaat
36301 atgcaattaa tcgtaaggtt tcttgtataa cagatttaga tccaatgcga aaagaaaaaa
36361 atgaaaatgg acaaaaaaat aattttaaag catgctatcc ttttgaatat gaaatggatt
36421 tagataaatt tgattattca actaataatt ctctagataa atatctaaat aatgagcaca
36481 gtaatatacg agtatttgtt caacctaata aatatggaaa aacatttgag taccaactta
36541 tgtttgacaa tccttcatta aaactgttac ttacagactc gatttctaat agtcaagagc
36601 tagcagaatt aatggatcac tatgaagaaa atgaatcttt acaaaaatta attgatacat
36661 tatcaccatc taatgaaaat cagagaataa taaaatcttt aaaagacaca aatgacagtt
36721 ggaatgaaga taataaaaag aaagcattaa tagcatcaag atacctaaat tccataggaa

36781 aaggagaaaa tgctctagag ctagcttcag tattaaaaga taatttagag ttaaaaggcc
36841 agagtgacta tgaagatttt gtagtaccaa aatatataga aggtgcaatt cgatgggtgt
36901 gtgaataata tgattattaa tgctcaaacg aaattaaata tagaacaaga ttttaaaata
36961 caggctggtc ctggtgctgg aaaaacagaa tttttggtga atcatatcaa aaatgtaata
37021 caaacttctg aaaagctcga acgtatgaag aaaatagcgt gtattactta tacgaatacg
37081 gctgctcaga cagttttaca aagattagga aaaagtgttt caaatagagt agatatatct
37141 acaatacata gctttttgta tagaaacgta gttaaaccat attgttcttt tctacccaaa
37201 gaatacggaa tttgtgtgca aaaattaaaa ggacatagtg atccaattgt gattaacaaa
37261 tatgtaaatg catggttaaa aactgaatat tttacgaaat taaaaccgcc tagtaataga
37321 aatcaactac taaaaatgcc agtactaaag aaagctttgc aaaactggct actatctatg
37381 aaactttctt acgaaaataa tgatgtgcaa tttatttgtg ataataccaa agctcaagca
37441 attgataaaa aaagtaaaaa aagaatggga ataaataaat caaacttaaa tatactagaa
37501 aagcaattat taaattataa aaaaatatat tgggagaaag gaattatcga tcatgatgat
37561 gttttgtttt tttcatatgt attaattaga gattacccat tcattctcac tattttatgt
37621 gctaaatatc cctacttttt tatagatgaa tttcaagata ctagtcaatt acaagatttt
37681 attatagata aaataagaca gaaaggctgc atagtggggg ttattggcga taaggcacag
37741 gcaatttaca gctttcaagg tgcacgggtt tccttatttg agaagtttaa agttgattta
37801 ataaattctc acactatctt agaaaatcat agaagttcat atcaaatagt tactttttta
37861 aatagtatca gagaagatat tactcaaaca gtatatagtg gtattgaaaa tgataaagtt
37921 acaattttga ttggaaatcg gattcgcagt tatactaagg tagttaatat ttgtaacaag
37981 gagtcacttg ttaccttgtc aagagataat attacttcta atgcaatgag aataaagttg
38041 gaaggaaata attttgataa aaagttattg gaaaaatttc aagaaattga tagtaatagt
38101 aaaagaagga atactattat ttcgcttata aaagctattg aattagctaa gaatactaaa
38161 tataaagatg ccttaaaaat agtcgaatcg atttataaag atgaaaataa tcctaaaaaa
38221 atagctctta attctctaat aaatatgatt aataaatatg ataaatacgg taatggtaca
38281 ttaatgaatt tctacaatac tttatgtaaa actttagata ttaaactttc agggtttaga
38341 aaggggcaag taaaagattt ttacgaaaac actttatata aaaatatggt gatatgtaca
38401 aatatcgttg aagacacaag taatcatatt actatccaca aagcgaaagg cgcagaattc
38461 gaaaatgtat ttgttatagg taataaagac actttaagtc tacttttgaa accagattta
38521 atgaacgatg aagagcatcg tatattctat gtagctatga gtcgagcaaa gaagaaactt
38581 tttttacaat ttgattatct tgataataat gaacaaaaga aaattaagga acgttatgat
38641 gtaagtatta tacaataacc taatctaaga atatttaaat ccaatatatt tcttgattat
38701 aattcaataa tagataaaat ctataaacga ttaaaaagaa aatatatttg ctaagactta
38761 taaaaatgaa gagataatag attttcctaa attacagtta aaagaagtaa atagattgaa
38821 cactttgaaa gattctaatc caaaatttga accactaaaa ttgaaagatg aagaagatgc
38881 aagatgtagc catcaacgct gatcacaaag tatttttctt aatgattatt tttaatgtta
38941 tcatgctcgt ttcttctatt attcaaatga ttttaggaaa aggcagacgt atcacaccag
39001 caaaacctac taaagatgct aagttacact tagataatca atcctcataa agagaaaaaa
39061 gaacccctaa aaagttcaac tttttggggg ttacttacta ttaagaagta tttttgatta
39121 agcatctttc atatcgtaat caccaatata ctgatcaagg cgatttattg tttctctgat
39181 atcatcatgt gtcgttaatg ggttcgttgt acataaacga atgacacgtt ggttattcaa
39241 tactgtggta taagcaatgg cataaccaga atctgcaatt ttttgcgcag ccatgctatt
39301 gagttgatta ttttgtgctt cagttaaatc actgttttca tatctgaagt tcacaatgga
39361 aagattagcg tgtgagataa tacgccagtt atcgagacct gacacgtatt cttctgcata

39421 ttctgctaaa ccttcgccgt atttaatgcg ctcgataatt tcatcttcac caatgacttg
39481 cagagtaatc cataatttta aagcacgtgc agggcgtgta agttcaatgc ctaacatttc
39541 tggatcaatc acatcatcat ctgaagcaat atcatccaag tactcagcat cttcgccaaa
39601 gctttgcagc agatgattct tttctttcac gattaccata gcgcaactat atgtttggaa
39661 cagtaatttg tgagcatccc aactggcact atctgaacgt tcaatgcctt taaataagtg
39721 ttgtgcttta tcggataaga tatgtgatag tccataagca ccgtctacat ggagccatag
39781 attgtagtga tcacatgtat ctccaagtgt ggtgaaatca tcgacagaac ctgtattagt
39841 cgtgcctgca gtcgcaatca ccatagcagg tttgtaacct tcttcaatat ctttatcaat
39901 tgcggctttt aagctatctg tattcattgt aaagtcatcg ttgtactcaa ttctgcggat
39961 attattttta aggaagcctg caacatgcaa ggctttgcct acagaatgat gcgtttgaga
40021 tgtaagataa acggttgctt ttttaatatc ttccatttct acctgtgcat ctctagccgc
40081 aacgatagct gttaaattgg ccattgaacc gccagataca aagacaccac cggccggttt
40141 gatctcataa ccgatttttc ctaccaagta gttaattaaa ttacgctcaa tattaattgg
40201 cagcgtcgca ttggcaaaat tagaggcatg gatattattt gctgttgtta aaatgtctcc
40261 aagccatgat agacgtgatg ctggacctgg tataaatgag aatgaacgtg gatggttcgg
40321 acgatataaa taattcagta cttcattatt taaatctttg agtacttcat agatatctcg
40381 ccctttattc ggcacttcca tcttttcata tttctctctc aattcgagcg gtgcctgttg
40441 tgtcgcaggt aagtctccaa tatctctaga taaatattta ttaacttcat ctctgataat
40501 cgtctctaag tcgatattgt tctcattgaa ttccatttcc atactctctc cttcaattat
40561 atttttaata aaagacagac gtattaatta cgacgtttta ctacaatata aatgatagca
40621 gatattaata aaaccacgct aaccataatg acgaaaccca gaaggtgtcg ccccaatgaa
40681 taacgtgctg tgctttaaca ctcggtgtat gtgcgccttt cagaaattga cgagggtaat
40741 cgacttgcag attatcatct gtgattttca atttgtattg atgcttgtct tttggaattc
40801 gataactacc ccgtagaaca ggggacgagg acctcatctt gatgatccca attggcttgg
40861 ttaaaaagaa atcgataatc ctcgtagaat agagaaaaag tattaaaaat atatttgatt
40921 tgataaaata ttttgcaaaa aagagaaaca aaaataactt tcatataaat aatttctttg
40981 cgattaagtt ggaattagaa atactaaata caaataacag cgaatggaat gcatattatt
41041 gaggaattgg atgtgtagtg caatttcgag agttactact taacttaaga aaataaaaag
41101 aagaaaacaa acaataagaa ttaaatctga agcaagatat aaaagcaaat atttttgcta
41161 caattatatt agtaaatact ttattaatta gcatacgact gaaagcgaag taacagctaa
41221 taatgttcca gatatagaca ttagtgttat gaaagaacaa tattaccatc tagaaaatgt
41281 aatttttaaa aacctgaaga taaagtagac tattctgtga gttttcatgt taagaattct
41341 gaacttgggg atgtagtata aatatggttt agtagaaaat gacgctggag ctgtagctga
41401 gattgactat caagaattaa ctaaagtaga ttattaccca tcaacaaaaa tccaagattt
41461 aacactatct aataatagtg cgaataatga agtttaaaag tgaaaaactg ataatagcga
41521 atcaaactaa agtaatcaat tacagaattc aagtactact caagcctcaa acaaatattg
41581 aaactcaaac acaagttaag aataatacac tgtcagaaac agatgaacag tctaatgccg
41641 atttagtcca gtaatagcct ctgtattatt agccgcacga ttattatcaa taattaaata
41701 aagttaatat taaataataa aaatccttca aattatttgt tgaaaaaata ataactttga
41761 aggtttttta gcaaaatcac tgatagggag aagcgtatca taaat
//
LOCUS Staphylococcus 41806 bp DNA linear UNK
DEFINITION Staphylococcus Staphylococcus aureus
ACCESSION Staphylococcus

KEYWORDS .
SOURCE Staphylococcus Staphylococcus aureus
ORGANISM Staphylococcus Staphylococcus aureus
Bacteria; Bacillati; Bacillota; Bacilli; Bacillales;
Staphylococcaceae; Staphylococcus; Staphylococcus aureus.
FEATURES Location/Qualifiers
source 1..41806
/genome_id="703339.339"
/project="hwwang_703339"
/organism="Staphylococcus Staphylococcus aureus"
/mol_type="genomic DNA"
/genome_md5=""
/db_xref="taxon:703339"
CDS 1..480
/product="23S rRNA
(pseudouridine(1915)-N(3))-methyltransferase (EC
2.1.1.177)"
/EC_number="2.1.1.177"
/db_xref="SEED:fig|703339.339.peg.1"
/transl_table=11
/translation="MKITILAVGKLKEKYWKQAIAEYEKRLGPYTKIDIIEVPDEKAP

ENMSDKEIEQVKEKEGQRILAKIKPQSTVITLEIQGKMLSSEGLAQELNQRMTQGQSD

FVFVIGGSNGLHKDVLQRSNYALSFSKMTFPHQMMRVVLIEQVYRAFKIMRGEAYHK"
CDS 726..1031
/product="hypothetical protein"
/db_xref="SEED:fig|703339.339.peg.2"
/translation="MKGNIQCPRLIKGSLLDFVLETVDVFVNGSKNDCELVNFNYKEG
KVVSRYSTKFESNPKLRKKQLISTVKVVRYVIFDFEKILWGIGKVLLKFIILRQCLL"
/transl_table=11
CDS 1230..2093
/product="hypothetical protein"

/translation="MKHINQFSESTLAQLEKDQQHVFYVYCLMDPRNDECFYVGKGKG
NRIFKHKQDAQKKVLYEDIFIEENKDNLKFNRINEICSNDLNVLGYIISYGLTESEAF

SAENVLINFLNLTNKTTLTNMINGHGSKAYLVEDLENEFGYDSINLENINTNELILAV

KIRDAFLLDKDESKEYPINESKRDRNNLKSRTLGSWIIGKDKIHKIKYIIGINTGANN

AVVSAYEVSFEQAESIETNNGRMRYAFIGLSERDATLKKLNLYKKALPDLRFGSGSAT
AYINNGTMKVD"
/transl_table=11

/db_xref="SEED:fig|703339.339.peg.3"
CDS 2201..3676
/product="hypothetical protein"
/translation="MPSDDIVKKLYSKEDIRLELGLTPHKFNKKMETIAKLFKIDMKI

FHNYKGQDKNNQYTFNGVAKELIKVLLKSVDYYPVDINSKKFKQNGKSKKEMIENIDN

SSYMKYIYQLMKSINEIQYKRLIADIHMKDVYQNTKAWLNNGESINKKEQELYQYMTI

LPLHKRVELQNEVLKSIDETIFQFLAKEHRNNQIEENNELEAYTKAIKEGRNPKNDYE

LNHLLYKKNQLPLDSLIEDIWDYEETEYTELDWLIADMLKRSQRADSNFIESLEKKNK

LRKNIHKDSIKSISKLIDHELVKNNRKWDVAEFYKNQQFWNNSTLRRSSQNFGILYYK

KNRLHTMNQSNIIKKIKIIEANIESANKQPSYLDFFLQAKFDLERLESELMKYSINPK

YFNKINSDYVRYAIEDVHNAIIKIDRYISNDKVKLNYSNVQMVVRNEITEQGSYFVTQ
ALNTLSKVEESGYSFDNFMVGPFVEDFKKAIKNRRFGSKKD"
/transl_table=11
/db_xref="SEED:fig|703339.339.peg.4"
CDS 3902..5002
/db_xref="SEED:fig|703339.339.peg.5"
/transl_table=11

/translation="METRDKLMSLTQSDKTQQWLMDKSSNQDDIQQLQQQFSQQLDQQ

YNALLADEKAKLDQYVEVHQGLESLKEEIESEPITLNIDKLPDIKATMLERAKNDEHS

DKIEKLFDRLEQALNGTNRLYTQLSLIGTRTHRITTKNFNLQGLPKAVQHTILPSKFK

KVYTVDFKSFEPSVAAYMTQDSKLIDLLNQKDGLYDALLSELGLSDELRVFVKRAFIG

SFLFGGNFKNPKFKLNQYVSEVQWLDAVSQFTKVIELKKHVEKSNSMPMPYSIEHDMS

AFQGSSIMAIYVQTVASYIFKHILLKVYKAQCDQKTFKIIVPIHDAIMIECEDEEIAQ
NVGQLMKDTANQLFNGEFAHVTVEEIGGVDHE"
/product="hypothetical protein"
CDS 4995..5366
/product="hypothetical protein"
/translation="MNNDRGQSLHIPSSTPIKENNIYVATLHSVIQTDFSGEIKHQFT

YEIEVNNQIVYANRNIPTKPSANQLSIHDWLKRHSNYSASHENYEPYIDQKHLILLGQ
YNGNYYVQDVASLDAFGGVLS"

/transl_table=11
/db_xref="SEED:fig|703339.339.peg.6"
CDS 5363..7006
/translation="MNHILQMLSKLLSVAKEAIDRQGLIAILTIPVNNNDEIEETAQG

ETVYNELIDQLRLNIPKDTDYQPNIYSYFGIKKNPNDTVLMEMMIKVFHIKRFNSELF

IFKVNGWQKINGDELQGLISKMIQVLLVDYKPSLSTLKNVVDGLQKSTDVEELVENER

YIGCGENMFDLNTFQVVKNSIDIFPKTRLNLSLSTNDVITDKIPPYFKQYMLQLANYD

DDLQYFLFQHTAVLLTADTKYRRGLILYGGAKNGKSVYIELVKSFFYSKDIVSKPLNE

LEGRFDKESLIDKSLMASHEIGQSRIQEKIVNDFKKLLSVESMHVDRKGKTQVEVILD
LKLIFSTNAILNFPPEHAKALERRINIIPCEYYVEKADTSLIDKLQSEKKEIFLYLMY

VYQQIVKADIEYLENSRVTEITHDWLNFGYEFVSSRSVSNANQKACINLLRKLIEIKS

GSRIKVSELNKVINEEIKVSSQVIKQLIQANFDTQTKLYNGYDYWIDLGWKEANKKEI
HDISEKDNIISLDKNENITDDEALDEENLDFDWEDFDDE"
/transl_table=11
/db_xref="SEED:fig|703339.339.peg.7"
/product="putative primase"
CDS 7232..8908
/db_xref="SEED:fig|703339.339.peg.8"
/transl_table=11
/translation="MKGKIALYSRVSTSEQSEHGYSIHEQEQVLIKEVVKNFPGYDYE

TYTDSGISGKNIEGRPAMKRLLQDVKDNKIEMVLSWKLNRISRSMRDVFNIIHEFKEH

DVGYKSISENIDTSNASGEVLVTMFGLIGSIERQTLISNVKLSMNAKARSGEAITGRV

LGYKLSLNPLTQKNDLVIDENEANIVREIFDLYLNHNKGLKAITTVLNQKGYRTINQK

PFSVYGVKYILNNPVYKGYVRFNNHQNWAVQRRSGKSDKNDVILVKGKHEAIISEEVF

DKVHEKLASKSFKPGRPIGGDFYLRGLIKCPECGNNMVCRRTYYKTKKSKERTIKRYY

ICSLFNRSGSSACHSNSINAEVVERVINVHLNRILSQPDIIKQIASNVIEELKQKHSN

QTEIKYDIDSLEKQKAKLKTQQERLLELFLDDQMDSEMLKAKQSQMNQQLEVLDQQIK

EAQQANQSQDEIPNFDKLKGRLILMITRFSVYLRKATPEAKNQLMKMLIDSIEITTDK
QVKLVRYKIDESLIPQSLKKDWGSFFMPKFQFEIYGQNDYFIDQITTFTT"

/product="Cassette chromosome recombinase B"
CDS 9014..9352
/transl_table=11
/translation="MTLEQQLKHYITNLFNLPRDEVWHCESIEEIADDILPNQYVRLG

PLSNKTLQTNTYYSDTLHESNIYPFILYYQKQLIAIGYIDENHDMDFLYLHNTIMPLL
DQRYLLTGGQ"
/db_xref="SEED:fig|703339.339.peg.9"
/product="FIG01108299: hypothetical protein"
CDS 9448..9759
/product="FIG01107894: hypothetical protein"
/db_xref="SEED:fig|703339.339.peg.10"

/translation="MNRYITRGIANNLPNILQHQLWQLVSEREQEQTKDNTSVDYFHI

FQFNKHRNQLYIKHKQERPEYAKIHKANYSKAININKVYIIREDDVDLSYYVMLLPEE
Y"
/transl_table=11
CDS 9775..10281
/db_xref="SEED:fig|703339.339.peg.11"
/translation="METIKSTLKTEAIFSDDKQHRYLLKKTWNSEKQSITIITMYPHY

DGILNIDLTTQLIMNKVSEMDAFGSINFVNLYSNITTPINLKHLENAYDKHTDIQIMK

AVKESDEVILAWGAYAKKPGVEARVNEVLEMLKPHKKKVKRLMNPETNEIMHPLNSKA
RQKWILKV"
/transl_table=11
/product="hypothetical protein"
CDS complement(10430..11104)
/db_xref="SEED:fig|703339.339.peg.12"
/translation="MNYFRYKQFNKDVITVAVGYYLRYTLSYRDISEILRERGVNVHH

STVYRWVQEYAPILYQIWKKKHKKAYYKWRIDETYIKIKGKWSYLYRAIDAEGHTLDI

WLRKQRDNHSAYAFIKRLIKQFGKPQKVITDQAPSTKVAMAKVIKAFKLKPDCHCTSK
YLNNLIEQDHRHIKVRKTRYQSINTAKNTLKGIECIYALYKKNRRSLQICGFSPCHEI
SIMLAS"
/transl_table=11
/product="Transposase for insertion sequence-like element
IS431mec"
CDS 11362..11529
/db_xref="SEED:fig|703339.339.peg.13"
/transl_table=11
/translation="MLESREQLSVEEYETFFNRFDNQEFDFERELTQDPYSKVYLYSI

EDHIRTYKIEK"
/EC_number="2.3.3.10"
/product="Hydroxymethylglutaryl-CoA synthase N-domain
homolog (EC 2.3.3.10)"
CDS 12486..13229
/EC_number="3.1.4.46"
/product="Glycerophosphoryl diester phosphodiesterase (EC
3.1.4.46)"
/db_xref="SEED:fig|703339.339.peg.14"
/db_xref="GO:0008889"
/transl_table=11
/translation="MLTVYGHRGLPSKAPENTIASFKAASEVEGINWLELDVAITKDE

QLIIIHDDYLERTTNMSGEITELNYDEIKDASAGSWFGEKFKDEHLPTFDDVVKIANE

YNMNLNVELKGITGPNGLALSKSMVKQVEEQLTNLNQNQEVLISSFNVVLVKLAEEIM

PQYNRAVIFHTTSFREDWRTLLDYCNAKIVNTEDAKLTKAKVKMVKEAGYELNVWTVN
KPARANQLANWGVDGIFTDNADKMVHLSQ"
CDS 13326..13754
/db_xref="SEED:fig|703339.339.peg.15"
/translation="MKYDDFIVGETFKTKSLHITEEEIIQFATTFDPQYMHIDKEKAE

QSRFKGIIASGMHTLSISFKLWVEEGKYGEEVVAGTQMNNVKFIKPVYPGNTLYVIAE
ITNKKSIKKENGLVTVSLSTYNENEEIVFKGEVTALINNS"
/transl_table=11
/product="MaoC domain protein"
CDS complement(13800..15806)
/translation="MKKIKIVPLILIVVVVGFGIYFYASKDKEINNTIDAIEDKNFKQ

VYKDSSYISKSDNGEVEMTERPIKIYNSLGVKDINIQDRKIKKVSKNKKRVDAQYKIK

TNYGNIDRNVQFNFVKEDGMWKLDWDHSVIIPGMQKDQSIHIENLKSERGKILDRNNV

ELANTGTAYEIGIVPKNVSKKDYKAIAKELSISEDYIKQQMDQNWVQDDTFVPLKTVK

KMDEYLRDFAKKFHLTTNETESRNYPLGKATSHLLGYVGPINSEELKQKEYKGYKDDA

VIGKKGLEKLYDKKLQHEDGYRVTIVDDNSNTIAHTLIEKKKKDGKDIQLTIDAKVQK

SIYNNMKNDYGSGTAIHPQTGELLALVSTPSYDVYPFMYGMSNEEYNKLTEDKKEPLL

NKFQITTSPGSTQKILTAMIGLNNKTLDDKTSYKIDGKGWQKDKSWGGYNVTRYEVVN

GNIDLKQAIESSDNIFFARVALELGSKKFEKGMKKLGVGEDIPSDYPFYNAQISNKNL

DNEILLADSGYGQGEILINPVQILSIYSALENNGNINAPHLLKDTKNKVWKKNIISKE

NINLLTDGMQQVVNKTHKEDIYRSYANLIGKSGTAELKMKQGETGRQIGWFISYDKDN
PNMMMAINVKDVQDKGMASYNAKISGKVYDELYENGNKKYDIDE"
/transl_table=11
/db_xref="SEED:fig|703339.339.peg.16"
/db_xref="GO:0008658"
/db_xref="GO:0046677"
/product="Penicillin-binding protein PBP2a, methicillin
resistance determinant MecA, transpeptidase"
CDS 16054..16197
/db_xref="SEED:fig|703339.339.peg.17"
/transl_table=11

/translation="MNYFRYKQFNKDVITVAVGYYLRYALSYRDMSEILRERGVNVHH
LTV"
/product="Mobile element protein"
CDS 16477..16728
/db_xref="SEED:fig|703339.339.peg.18"
/transl_table=11
/translation="MAKVIKAFKLKPDCHCTSKYLNNLIEQDHRHIKVRKTRYQSINT
AKNTLKGIECIYGLYKKNRRSLQICGFSPCHEISIMLTS"
/product="Mobile element protein"
CDS complement(16788..17216)
/product="PhnB protein; putative DNA binding
3-demethylubiquinone-9 3-methyltransferase domain
protein"
/db_xref="SEED:fig|703339.339.peg.19"
/transl_table=11
/translation="MKFQVSPYFLITQNAEGLKKLYEELFGAKTLYIQRIKDRPIDKK

LNIDENDLNKIDQCVLQFGDIKIMIADDTEGLPITQGNNISLCLTFDQVEETKHVYDQ
LIEKGSEVLKTFSPEFYTEGYGYVRDPYGISLHLFTKRKN"
CDS 17297..18226
/db_xref="SEED:fig|703339.339.peg.20"
/translation="MKKSVRLYNMIEYCNENRNFKLNDLMSEFNISRSTALRDIKEIE

ALGVPLYSNPGKNGGYTIIGNRDQTKIAISDEELKALVFTLSSISNVSNLPFQTEYQE
ILKKLYNNSNKKELINQYNDLFQYFNEDKYQFKSYKLFNEIIRLIIENKSFETCYSQN

YIKEQYKGIGIMYKNHQWYFVVANIESKLVNLLNISKIKELYEMGETQECNDITMQNF

QQFMVKNETAIDILIRSNVMGLNILKGYLWSDYMIENIDEETYLFKSKVNAKDIDFIA
KLIVTGGVNVKVETPNSLKNAVKVELTKIINMY"
/transl_table=11
/product="Transcriptional regulator, YafY family"
CDS 18388..18870
/transl_table=11
/translation="MNKNITALKSTENTTYMQYTVHIDDQIINNIKECTKKFKILPME
DKIPLSPLLQPEYAGEVQDFISTYEQFMINFGKVILDSQGIKIQFESESLSSIQRGIQ

EHCYLNERTNDIDVTKEWYLCKFSNQILEEDKNQLYNALKQLMNDSKNKKQAFWTDPN
"
/db_xref="SEED:fig|703339.339.peg.21"
/product="Similarity"
CDS 18938..19252
/db_xref="SEED:fig|703339.339.peg.22"

/translation="MKRVSYSVETKYKAVEMKIAGFSTKEIMKELNIKNRTQVETWWR

WYRNEESYRFSQHVGKQYTYGKGLEELSEIERLKLENKRKDIELDILKKYKALERKWY
QQ"
/transl_table=11
/product="Mobile element protein"
CDS 19237..20079
/transl_table=11
/translation="MVPAVVIDLVDQLKVKYSIKLILEVLNIPKSTYYRWKNKTYKND

TVTQKVIELCEANHYTYGYRKITALINQCYTSPINHKRVQRIMQKHHLNCRVRPKKTT

RIGKPYYKTDNLLQRQFKASCPMEVLTTDITYLPFGHSMLYLSSIMDIYNGEIVAYKI

DDKQDQSLVNDTLNQIDIPEGCILHSDQGSVYTSYAYYQLCEEKGIIRSMSRKGTPAD

NAPIESFHSSLKSETFYINNQLNSSNHIVIDIVEKYIKNYNNNRIQQKLGYLSPVKYR
ELAA"
/db_xref="SEED:fig|703339.339.peg.23"
/product="Mobile element protein"
CDS 20086..21651
/product="Similarity"
/db_xref="SEED:fig|703339.339.peg.24"
/translation="MFLLSSRLRGSVPFQEVFKVHIDIYLYNKEKSEYKYQVSSYFNL

VKENPKITYKKRHLQEKQGVKGTTFTNKLKLLNKMYGVDVAKYQPFYNSNNPEYERGQ

FGERYISQRSNYEFNRLQYQIIDMLSKILDKHPLPKSDNNYKHIPTIEKAILSGDSHS

FYEYFEDIMKEIISMENSSLKEKLLTDFTYQSQCRWYSESEKLKLQLESFMHKVLESN

YYEGNKLYRMLSHAIEETINEADEDKVHFNYFKDYFLTDGGVKNWEQISEKITEFNGK

VINDIQNEYNKIQFNNANRNQKLNFNYLYHCFEFSNNLVKARVNGNRGYILYFVDKYK

IKMPFLDQLLINYLNQEIGQESINYNMQTLFEKERYDRSSTIEKLVATSKFKYEKDDS
DLFKQLFNDVENSIDRLGIYLLNNGINSNDENARYYRSFLKELSRIKSKLTPFSLEIS

KSSGREQHYPDDAIDDKDERRKNKEETYHAFDDKSDIDSKLKNKINVSIDNLLSVKIK
SNLYDISPNTKKQ"
/transl_table=11
CDS 21846..22955
/db_xref="SEED:fig|703339.339.peg.25"

/translation="MYQANIRDLITKLPQSNKTEHFLMNKFSNQDKVQQLQRQISQQL

DQQYNELLANEKAKLDQYVEVHHNLEPLKKEIESESINLDTDKLPDIKATMLEKAKND

EHFDKIEQLFDRLDQSLNGTNRLYTQLSLIGTRTHRITTKRFNVQGLPKLVQQMILPS

QFKKVYTIDFKSFEPSVAAYMTQDEQLIDYLNHEEGLYDALLRDLSLSKEKRVSVKRA

FIGSFLFGGRYSSSKFKINQEVSEINWLQVMSKFKKVIEFKEQVEKYKTMPTPYGIEH

DMSAFQGSSIMAIYVQTVASYIFKHILLEVYKAQCEKKTFKIIVPIHDAIMIECNDKG
IAQNVAQLMKDTANQLFNGEFAHVTVEALGGIDNE"
/transl_table=11
/product="hypothetical protein"
CDS 22948..23316
/db_xref="SEED:fig|703339.339.peg.26"
/translation="MNNDRGKSLQIPQSTLLKEGSIYVATLHSVYEKNFSGDIKHQFT

YEVELNQETHYVNRNITVKSMSHQLSIADWIKRHSNYNVNHINYDPYIDRKHLVLVGQ
YNGNYYIQDVAPLDEFGGVL"
/transl_table=11
/product="hypothetical protein"
CDS 23316..24932
/product="putative primase"
/db_xref="SEED:fig|703339.339.peg.27"
/translation="MNHILEMLIKLLKVGMEAIDRKGLIAILTSSIGNDEMDDSEQAV

MVYNELIDKLQLNIPKDVDYRPNIYSYFGIQKKPNDTILVEMMICIFHIKRFDSELFV

FKDKGWQKVSEDELQGLISKMIQVLLVDYKPSLSTLKNVVDGIQKSTDIEKLVEHRQY

IGCGRNMFNLKTFKVVDNDLEIFPKTRLDLELDINDTITDKIPPNFKQYMLELANYDH

DLQYFLFQHMAVLLTADTKLRRGLFLYGTAKNGKSVYIKLVKSFFYSNDIVSKTLNEL

GGRFDKESLIGKRIMASDEVGKANIDEATVNDFKKLLSVEPIHADRKGRTQVEVTLDL

KLIFNTNAVLNFPSSHAKALERRIAVIPCEYYVEKADPDLIEKLQDEKKEIFLYLMYV

YKQIVKNDIEYLQNDRVTEISHDWLNFGYEFVSSKSANIAHQKACINLLRKLIEIKPG

SRIKVSRLNEVIRDEIKVSSQVINDLVQANFNVQSRLNNGYKYWVDLGWKETDKKDDM
ISFDKNENVTDDEFLYEDDLNLGWEDFDDE"
/transl_table=11
CDS 25157..26836
/db_xref="SEED:fig|703339.339.peg.28"
/translation="MKGKIALYSRVSTSEQSEHGYSEKEQEQLLIKEVMKNFPGYDYE

TYTDSGISGKNIEGRPAMKRLLQDVKDNKIEMVLSWKLNRISRSMRDVFNIIHEFKEH

DVGYKSISENIDTSNASGEVLVTMFGLIGSIERQTLISNVKLSMNAKARSGEAITGRV

LGYKLSLNPLTQKNDLVIDENEAHIVREIFDLYLNHNKGFKAITTILNQKGYRTINQK

PFSVFGVKYILNNPVYKGYVRFNNHQNWAVQRRSGKSDKNDVILVKGKHEAIISEDVF

DQVHEKLASKSFKPGRPIGGDFYLRGLIKCPECGNNMVCRRTYYKTKKSKERTIKRYY

ICSLFNRSGSSACHSNAINAEVVERVINVHLNRILSQPNVIKQIASSVIEELKQKHSK

QTEIKYDIDSLEKQKAKVKTQQERLLELFLDDEMDSEMLKAKQSEMNQQLEVLDQQIK

EAKQANQSQDDIPNFDKLKARLILMITRFSVYLRKATPEAKNQLMKMLIDSIEITTDK
QVKLVRYKIDESLIPQSLKKDWGSFFIPKFNFVINVTKKNRIENLSLLPLF"
/transl_table=11
/product="Cassette chromosome recombinase B"
CDS 26925..27263
/translation="MTLSKQLKTYITERFKLNYQETWACETIDAVAEDVLPEKYIKNS
PLEHKILNTFTYYNDELHEISIYPFLCYLDKELVAIGYLDNFDLDFIFLNDTHQVIID
ERYLLQKGGE"
/transl_table=11
/db_xref="SEED:fig|703339.339.peg.29"

/product="hypothetical protein"
CDS 27266..27355
/db_xref="SEED:fig|703339.339.peg.30"
/translation="MNWIKVAQLSVTVINEVIEIMKEKQNGGE"
/transl_table=11
/product="hypothetical protein"
CDS 27357..27668
/product="hypothetical protein"
/transl_table=11
/translation="MKINRYITRGVSEHLSLDLQILLWNMVKDRDNQLHTDYLHIFKL

QEDDNILSITHEQEQPAYKLEYHYTNYVKNQNALPKKVYVIREDDVDAFYYVMLLPEE
Y"
/db_xref="SEED:fig|703339.339.peg.31"
CDS 27683..28186
/product="hypothetical protein"
/db_xref="SEED:fig|703339.339.peg.32"
/transl_table=11
/translation="MNTIKNTIHTEAIFSDDEQHRYLLKKTWDEKKPACTVITMYPHL

DGVLSLDLTTVLILNQLANSEQYGAVYLVNLFSNIKTPENLKHIKEPYDKHTDIHLMK

AISESDTVILAYGAYAKRPVVVERVEQVMEMLKPHKKKVKKLINPATNEIMHPLNPKA
RQKWTLK"
CDS 28200..28421
/translation="MNHETKQSDWRTVASCLASQNYISIVKGLVHHFTAIEDEEILDK
IYDDFMNDDSITTVLNNDLQTIINHYLSK"
/transl_table=11
/db_xref="SEED:fig|703339.339.peg.33"
/product="hypothetical protein"
CDS complement(28496..31615)
/transl_table=11

/translation="MNFQFNEDDLEQVALEWLQSLGYDYKKGNEISMTSLTPERKSDK
DVVLHERLEKALRKINSDIHPRFIEKALHELTLEKSPNLLENNLTFHENLINGIEIED
YDDEGQSIVEIVKIIDFEYPQNNDFLAVNQLTIVNGDYKKRPDIVLFINGLPIVVIEL

KNSTNETVGVEDGYHQLETYKMRIPQLFTFNEVLVTSDGINTKAGSLTANYDRFMTWR

SKDGQTESSPGLASLDVLIHGMLNPETLLDLIRYFVLFQDDGKGHISKILAAYHQYYA

VNKAVDRALLASSEQGDGKGGVIWHTQGSGKSLTMVFFSGKLIQMLNNPTLVVVTDRN

DLDNQLYSTFVKSKGRSGKGLLRQTPKQAETRKELKSLLSVESGGIVFTTMQKFEPEQ

NETTMAALTERKNVIVMADEAHRTQYGFNAKYDDKGEGIKYGYAKYLRDALPNATFVG

FTGTPVASTDKNTQMVFGNYIDVYDMTQAVADGSTVKIYYESRVIPLNLPQSLDLDEA

YNDITEDQEEDVKQRLKSKWSRIEALAGAKPRVEALAKDIIQHFETRQQAMKGKGMIV

TMSRRIAVDLYDEIIRLKPEWHSDDDDKGVIKVVMTGSSSDPTSFQRHIGPKKRRNLL

EKRMKDVDDELQLVIVRDMWLTGFDVPSMHTMYIDKPMKGHNLMQAIARVNRVFKDKP

GGLIVDYVGIAESLKEALKEYTESDQAQTAIDTDKAVELMLLKYDVIQDMLYNLDYSK

FNSDKKSERYYTISNTMDYVIGLGEDERQRFIKTVTELGKAFALCATEPTAQELNDEI

AFFKAVKAGLVKLLQPPKEGKTRKTPAEVEAEINQLVSQSVVTEDVIDVYQTLGLEQP

DLSILSDDFLKDVEGLKQKNVAVELLNRLLKGQVKSLMKTNATVSKRFSEMLGNSINK

YNSRSIETSKVIEELIQLAKDIKQEQQRGNELGLNSDEIAFYDALASHETAKEAMGDK

ELRAIAHELTKTVKENMGVDWSKRDSAKAKMRVAVRRLLKKYGYPPDLQKMAVEQVVE
QAELMANNQ"
/db_xref="SEED:fig|703339.339.peg.34"
/product="Type I restriction-modification system,
restriction subunit R (EC 3.1.21.3)"
/EC_number="3.1.21.3"
CDS complement(31599..32900)
/product="Type I restriction-modification system,
specificity subunit S"
/db_xref="SEED:fig|703339.339.peg.35"
/transl_table=11
/translation="MEYNTIFFGDVAKIKNGYAFKSKEFQEKGIPVIKIKNIISPIVD

TKDSQKVSIKTYEKTKGFSLKKNDILISLTGSGVNQMSSAVGKVGRIEFDYPALQNQR
VGKFELKYSNSADLDFLFYYFLQPKITEYLVRNSTGSANQANINSKLIETVKIPNFSL

IKQKSISKFLNQITRKIETNQKMIANLKELSQTLFKHWFVDFEFPDEDGNPYKSSGGE

MIDSELGKIPSNWKIYKLKDIASHKKETFNPKKSEEVTVKHFSLPAYDNEEQAIEEEV

NKIKSNKWIINNNCVLFSKMNPDTKRIWLPVIDNKKLNVASSEFVVMESPNNKINSFI

YNICLNSQFIDYLKANTTGSTNSRQRVKPTIAVNYKLAIEDSIVKKYSEIITPYMEEM

KILRSEIGKLTQLRDTLLPKLMSGELEISDDIEVNSDELSI"
CDS complement(32890..34404)
/db_xref="SEED:fig|703339.339.peg.36"
/transl_table=11

/translation="MATIGFEEKLWQAADKLRGSMDAAEYKNVALGLIFLKYVSDSFE

EKYEELKQDPYADEEDQDEYLAENIFWVPKEARWQYINDNAKKPEIGQIIDKAMIAIE

NENESLKGVLPKEYARPALDKEKLGDIIDLFTFKVGDTESRKQDVLGRVYEYFIAKFA

SAEGKNAGEFYTPSSIVKLLVEMIEPYKGRIYDPCCGSGGMFVQSERFVEKHQGRLDD

IAIYGQESNPTTWKLAKMNLAIRGIDNDLGERNADTFHNDLHKGLKADYILANPPFNA

SDWGQERLLDDYRWQFGIPPKGNANYAWIEHMISKLAPNGTAGFVLANGSMSTSGKDE

LEIRKNLIEQDLVECIVTLPGQLFYSTQIPVCLWFISNNKGQNGKKERENEILFIDAR

EIGHMVSRTLKEFSDEDIQKVAQTYHAWRGTNDKPYEDIAGFCKVANLEEVKNNEYIL
TPGRYVGLADVEEDEEPFEQKMERITSELSEQFAKSKELEDQIRKSLEGLGYGV"
/product="Type I restriction-modification system, DNA-methyltransferase subunit M (EC 2.1.1.72)"
/EC_number="2.1.1.72"
CDS 34600..34746
/product="hypothetical protein"
/translation="MDGVTNVKIHILTFVKIVVIVDIVAIVNTRLDKLGIFNSINKKF
KCSQ"
/transl_table=11
/db_xref="SEED:fig|703339.339.peg.37"
CDS 34846..36909
/translation="MYISSVKIKNFRNFDNIDIDFHEGVNVLIGHNNSGKSNLLRALS

LIFDGSVRKQLSVEDFNNSLTKESLKKEAPKIVISVHITQSENERLMSDELITVSNWL

TKLEEPYEARIQYEFFLPKDEEKNYIDLVKNIDEKEEIWNLIRSQFIRLYVNKIWVGN

PEHQIPIDNDSLNKFDFQFLDAIRDVERDMFSGKNTLLKSVIDFFIDYDIKSDETITE

EEQKEKLEERRKEFSDNSSDLIETIHKRLDSGNQKILSYTNGIGASYDKSTPDFKGNL

TESEIYTVLQLIIKHETGMTLPITHNGLGYNNLIFMALLLSKMQADSDGNFLGSNAKV
FPILAIEEPEAHLHPTMQNEFIKFLKNNIREKKVKQIFITTHSTHISSSTNIDDIICL

YTDDSKTNVSYPGKVFDKNNQSKKYVQRFLDATKSNMLFAEKVIFVEGIAEQLLLHIF

ADYLNKPLEKNHVAVINVGGRYFNHFLSLFDSNNKYAINRKVSCITDLDPMRKEKNEN

GQKNNFKACYPFEYEMDLDKFDYSTNNSLDKYLNNEHSNIRVFVQPNKYGKTFEYQLM

FDNPSLKLLLTDSISNSQELAELMDHYEENESLQKLIDTLSPSNENQRIIKSLKDTND

SWNEDNKKKALIASRYLNSIGKGENALELASVLKDNLELKGQSDYEDFVVPKYIEGAI
RWVCE"
/transl_table=11
/db_xref="SEED:fig|703339.339.peg.38"
/product="FIG131328: Predicted ATP-dependent endonuclease
of the OLD family"
CDS 36911..38659
/db_xref="SEED:fig|703339.339.peg.39"
/translation="MIINAQTKLNIEQDFKIQAGPGAGKTEFLVNHIKNVIQTSEKLE

RMKKIACITYTNTAAQTVLQRLGKSVSNRVDISTIHSFLYRNVVKPYCSFLPKEYGIC

VQKLKGHSDPIVINKYVNAWLKTEYFTKLKPPSNRNQLLKMPVLKKALQNWLLSMKLS

YENNDVQFICDNTKAQAIDKKSKKRMGINKSNLNILEKQLLNYKKIYWEKGIIDHDDV
LFFSYVLIRDYPFILTILCAKYPYFFIDEFQDTSQLQDFIIDKIRQKGCIVGVIGDKA
QAIYSFQGARVSLFEKFKVDLINSHTILENHRSSYQIVTFLNSIREDITQTVYSGIEN

DKVTILIGNRIRSYTKVVNICNKESLVTLSRDNITSNAMRIKLEGNNFDKKLLEKFQE
IDSNSKRRNTIISLIKAIELAKNTKYKDALKIVESIYKDENNPKKIALNSLINMINKY

DKYGNGTLMNFYNTLCKTLDIKLSGFRKGQVKDFYENTLYKNMVICTNIVEDTSNHIT

IHKAKGAEFENVFVIGNKDTLSLLLKPDLMNDEEHRIFYVAMSRAKKKLFLQFDYLDN
NEQKKIKERYDVSIIQ"
/transl_table=11
/product="ATP-dependent DNA helicase UvrD/PcrA,
clostridial paralog 2"
CDS 38878..39051
/product="Multidrug resistance protein"
/transl_table=11
/translation="MQDVAINADHKVFFLMIIFNVIMLVSSIIQMILGKGRRITPAKP
TKDAKLHLDNQSS"
/db_xref="SEED:fig|703339.339.peg.40"
CDS complement(39119..40543)
/db_xref="SEED:fig|703339.339.peg.41"

/transl_table=11
/translation="MEMEFNENNIDLETIIRDEVNKYLSRDIGDLPATQQAPLELREK

YEKMEVPNKGRDIYEVLKDLNNEVLNYLYRPNHPRSFSFIPGPASRLSWLGDILTTAN

NIHASNFANATLPINIERNLINYLVGKIGYEIKPAGGVFVSGGSMANLTAIVAARDAQ

VEMEDIKKATVYLTSQTHHSVGKALHVAGFLKNNIRRIEYNDDFTMNTDSLKAAIDKD

IEEGYKPAMVIATAGTTNTGSVDDFTTLGDTCDHYNLWLHVDGAYGLSHILSDKAQHL

FKGIERSDSASWDAHKLLFQTYSCAMVIVKEKNHLLQSFGEDAEYLDDIASDDDVIDP
EMLGIELTRPARALKLWITLQVIGEDEIIERIKYGEGLAEYAEEYVSGLDNWRIISHA

NLSIVNFRYENSDLTEAQNNQLNSMAAQKIADSGYAIAYTTVLNNQRVIRLCTTNPLT
THDDIRETINRLDQYIGDYDMKDA"
/product="Decarboxylase, pyridoxal-dependent"
CDS complement(40626..40838)
/product="D-3-phosphoglycerate dehydrogenase (EC
1.1.1.95)"
/EC_number="1.1.1.95"
/db_xref="SEED:fig|703339.339.peg.42"
/db_xref="GO:0004617"
/translation="MRSSSPVLRGSYRIPKDKHQYKLKITDDNLQVDYPRQFLKGAHT
PSVKAQHVIHWGDTFWVSSLWLAWFY"
/transl_table=11
CDS 41488..41655
/product="hypothetical protein"
/transl_table=11
/translation="MKFKSEKLIIANQTKVINYRIQVLLKPQTNIETQTQVKNNTLSE
TDEQSNADLVQ"
/db_xref="SEED:fig|703339.339.peg.43"
ORIGIN
1 atgaaaatca ccattttagc tgtagggaaa ctaaaagaga aatattggaa gcaagccata
61 gcagaatatg aaaaacgttt aggcccatac accaagatag acatcataga agttccagac
121 gaaaaagcac cagaaaatat gagcgacaaa gaaattgagc aagtaaaaga aaaagaaggc
181 caacgaatac tagccaaaat caaaccacaa tcaacagtca ttacattaga aatacaagga
241 aagatgctat cttccgaagg attggcacaa gaattgaacc aacgcatgac ccaagggcaa
301 agcgactttg tattcgtcat tggcggatca aacggcctgc acaaggacgt cttacaacgt
361 agtaactacg cactatcatt cagcaaaatg acattcccac atcaaatgat gcgggttgtg
421 ttaattgagc aagtgtatag agcgtttaag attatgcgtg gagaggctta tcataaataa
481 aactaaaaat tagattgtgt ataatttaaa aatttaatga gatgtggagg aattacatat
541 atgaaatatt ggagtatacc ttgcaatatc atacgatgtt tatagagtgt ttaataaacc
601 atttttcaac tattgatgat ctagaatata taataactgt acaaattata ttgattatgg

661 aactacaatt caattaagaa attgatgatg aaattttaaa tttaaactaa tggaatcaag
721 aaagaatgaa aggaaatata caatgcccac gattaataaa aggaagttta ttagattttg
781 tgttagaaac agtagatgtt tttgtaaatg gaagtaaaaa tgattgcgaa ttagtaaatt
841 ttaattataa agaaggaaaa gtagtttcac gttatagtac aaaatttgaa agcaatccta
901 aattaagaaa aaagcaattg atatcgacgg taaaagttgt aaggtatgtt atttttgatt
961 ttgagaaaat tttatggggc atagggaaag ttttattgaa gttcatcata ttaaggcaat
1021 gtttactata agaaagttga aatacactca gaaacagact ggattcccgt ttttttcaaa
1081 ttggcacaaa atgatacata ggcccaccta tatattgaag atttaagtta aacgataaat
1141 aatatttttc tcttttgtat agaaattttt ttgggaatga tattaattag tataaaaatg
1201 aatatgtcta caaagaagga gagattttat tgaaacacat taatcaattt tctgaatcga
1261 cactagcaca actagaaaaa gatcaacaac acgtatttta tgtatactgt ttgatggacc
1321 caagaaatga tgaatgcttt tatgttggta aaggtaaggg gaatagaata tttaaacata
1381 aacaagatgc gcagaaaaaa gtattgtacg aagatatatt tatagaagaa aataaagata
1441 acttgaaatt taatagaatt aacgaaattt gtagcaatga tttgaatgtt ttaggatata
1501 taatcagtta tggattaacc gaatctgaag cattttctgc agaaaatgtt cttataaact
1561 ttcttaacct aacaaataaa actacattaa caaatatgat taacggtcat ggatctaagg
1621 catacttagt tgaagattta gaaaatgaat ttggctacga ttcaattaat cttgaaaaca
1681 taaatacgaa tgaattaatt ttagcagtga aaatcagaga tgcatttcta ttagataagg
1741 atgaaagtaa agagtatcct attaatgaaa gtaaacgtga tagaaataac cttaaatcgc
1801 gtacattagg tagttggata ataggaaaag ataaaataca taaaataaaa tatattattg
1861 gtatcaatac aggtgctaat aatgcagttg tctcagctta tgaagtatca tttgaacaag
1921 cggagagtat cgaaacaaat aatggtagaa tgagatatgc gtttattggg ctttcagaaa
1981 gagatgctac tctaaaaaaa ttgaatttat acaaaaaagc actaccagat ttaagatttg
2041 gtagtggtag cgctacagca tatataaata atgggacaat gaaagttgat taaatgaata
2101 aaaacatcca attccttaaa ttagcctttg aaaattttat actcgtatat ataagtgaaa
2161 aacttaaaag aattagatgt taattaaagg agagaatgct atgccatcag atgatattgt
2221 aaaaaaacta tactcaaaag aggatattcg attagaatta ggacttactc cgcataagtt
2281 taataaaaaa atggaaacta ttgctaagct ttttaaaatt gatatgaaaa tttttcacaa
2341 ttacaaaggc caagataaaa ataatcagta tacatttaat ggtgtcgcaa aagaattaat
2401 caaagtgttg ctaaaaagtg ttgattatta cccagtggat atcaattcca aaaaatttaa
2461 acaaaatgga aaatctaaaa aagaaatgat tgaaaatata gataactcta gttacatgaa
2521 atatatttat cagttaatga aatctatcaa tgaaattcaa tacaaaaggt taattgctga
2581 tatacatatg aaagatgtgt accagaatac aaaagcatgg ttaaataatg gtgaatcaat
2641 taataaaaaa gaacaagagc tataccaata tatgacaatt ttaccattac ataaaagagt
2701 tgaactgcaa aatgaggtat taaaatctat agacgaaaca atttttcaat ttctcgcaaa
2761 agaacataga aataatcaaa ttgaagaaaa taatgaatta gaagcatata caaaagcaat
2821 aaaagaaggt agaaatccta aaaatgatta tgaattaaat catctcctat ataaaaaaaa
2881 tcaattaccc ttagatagtc taatagaaga tatatgggac tatgaggaaa cagagtatac
2941 tgagcttgat tggttaattg cagatatgct aaaacgttca caaagagctg atagtaattt
3001 tatcgaaagt ttagagaaga aaaacaaatt gagaaaaaat attcataaag attccattaa
3061 aagtatatct aagttgattg atcatgaatt agttaaaaat aatagaaagt gggacgtagc
3121 agaattttat aaaaatcaac aattttggaa taatagtaca ttaagacgta gctctcaaaa
3181 ttttggaatt ttatattaca aaaagaatag gttacataca atgaatcaaa gtaatattat
3241 taagaaaata aaaattatcg aagctaatat agagagtgca aataaacagc cgagctattt

3301 agatttcttt ttacaagcta agtttgattt agagaggtta gaaagtgaat taatgaaata
3361 tagcattaat ccgaaatatt ttaataagat aaatagtgat tacgtaagat atgctattga
3421 agatgtacat aatgcaatta ttaaaattga cagatatata agtaacgata aagtgaagtt
3481 aaactattca aatgtccaaa tggtagtgag aaacgaaata acagaacaag gaagctactt
3541 tgtaacccag gcattgaata cactgtcaaa ggtagaagag tctggctaca gctttgataa
3601 tttcatggta ggacctttcg tagaagactt taaaaaagca attaaaaatc gtagatttgg
3661 ttctaaaaaa gattaagatt gttttttaga gttaaacata agaataaatt taaataaaaa
3721 aataaaccca acaaaccttg ttatatcaat gtttgttggg tttatttttt tggtgatttt
3781 ttaataactc aattacatct taggataata tcgctaaatt atacattgtc gaggccaaga
3841 caaataaaaa attaaagcaa aagttagcgc gcaactttct gctataaagg agcaaattta
3901 tatggagaca agagacaaac tgatgtcatt gactcaaagt gacaaaacac agcaatggct
3961 aatggacaag tcatctaacc aagatgacat tcaacaattg cagcaacaat tcagtcagca
4021 gcttgatcaa caatataatg cacttttagc tgatgaaaaa gctaagttag accaatacgt
4081 ggaagtacat caaggattgg aatcattaaa ggaagagatt gaatcagaac ctattacgct
4141 taatatcgat aaattacccg atatcaaagc aacaatgctt gaaagagcca agaatgatga
4201 acattctgat aaaatcgaaa agctatttga taggttagaa caggcattaa atggtacgaa
4261 tcgattatat acgcaattat cgttgattgg tacacgaaca catcgaatta caactaaaaa
4321 ttttaatctt caaggcttac ctaaagcagt ccaacatacg attttacctt caaaatttaa
4381 gaaggtgtat acagtcgatt ttaaatcgtt tgaaccatca gttgcagcgt acatgactca
4441 agattcaaaa ctgattgact tgttaaatca gaaagacgga ctgtatgacg cattgctaag
4501 tgaattaggc ttatcagatg agctacgtgt atttgttaaa cgtgcattta ttggttcgtt
4561 tctatttgga ggtaacttca aaaatcctaa attcaagctg aatcaatatg taagtgaagt
4621 acaatggttg gatgcggtca gccaatttac aaaagtcatt gaacttaaga agcacgttga
4681 aaagagtaat tccatgccta tgccttatag tattgagcat gatatgagcg catttcaagg
4741 tagcagtatt atggcaatct acgtacaaac tgtagcgagt tatattttca agcacattct
4801 gctaaaagtg tacaaagcac agtgcgatca aaaaacgttc aagattatag tacctataca
4861 cgatgcgatt atgattgaat gtgaagatga agaaattgca caaaatgtgg gtcagttaat
4921 gaaagatacg gctaaccagt tgttcaatgg tgaatttgca catgtgacag tggaagaaat
4981 aggaggcgta gaccatgaat aatgatagag gacaaagcct acacatccca agtagtacac
5041 caatcaaaga aaataatata tatgtagcta cgttacattc tgtgatccaa acagatttct
5101 caggtgaaat aaagcaccaa ttcacgtatg aaattgaagt gaacaatcag attgtatatg
5161 cgaatcgtaa tattccaaca aaaccgagcg ctaatcagtt gtcaattcat gattggctga
5221 aacgtcatag caactatagc gcaagtcatg aaaactatga gccttatatt gatcagaaac
5281 atttaattct attaggtcaa tataacggta actattatgt acaagatgta gcatcgttag
5341 atgcgtttgg aggcgtatta tcatgaatca tatattacaa atgttatcta agctattaag
5401 tgtggccaag gaggcaatcg accgtcaagg tctgattgct atcctaacta ttcctgttaa
5461 taataacgat gaaatagaag aaacggctca aggtgaaacc gtgtataacg aacttatcga
5521 tcagttacga cttaatatcc caaaagatac ggattatcaa cctaacatct atagttattt
5581 tggtattaag aagaatccta atgacaccgt actcatggaa atgatgataa aggtttttca
5641 tatcaaacgc tttaattcag aactgtttat tttcaaagtt aacgggtggc aaaagataaa
5701 tggagatgaa ttacaagggt tgatatctaa aatgatacaa gtattgcttg tagattataa
5761 gccttcacta agcactctaa aaaatgtcgt agatggattg caaaaatcaa cagatgtaga
5821 agaacttgtt gagaatgagc gctatattgg ttgtggtgaa aatatgttcg atcttaatac
5881 gtttcaagtc gttaaaaatt caatcgatat ctttccaaaa acacgattga atttatcatt

5941 aagtacaaat gatgtaatta ctgataagat accgccttat tttaagcaat atatgttaca
6001 acttgcgaat tatgacgatg atttacaata ctttcttttc caacatacag cagtattact
6061 tacagctgat actaaatacc gtaggggtct catattatat ggtggagcta agaatggtaa
6121 atctgtatat attgaactag ttaaatcatt tttctatagt aaagatattg tgtctaagcc
6181 acttaatgag cttgaaggtc gttttgacaa agaaagttta attgacaaaa gtctaatggc
6241 aagtcatgaa attgggcaat ctaggattca agaaaagatc gtaaatgact tcaaaaagtt
6301 attatctgta gaatcaatgc atgttgatcg taaaggaaaa actcaagtgg aagtcatttt
6361 ggatttgaaa cttattttta gtacaaatgc gatacttaat tttcctcctg aacatgcgaa
6421 agctttggag cgtcgaatta atattattcc atgtgagtat tatgttgaaa aagcggacac
6481 ttcattaatt gataagctcc agagtgagaa gaaagaaatc tttctttact tgatgtatgt
6541 gtatcaacag attgtaaaag cagatatcga gtatcttgaa aatagccgtg tcactgaaat
6601 tactcacgat tggttaaatt ttggatatga atttgtttct agcaggtccg taagtaatgc
6661 aaatcagaaa gcatgtatta atttactcag aaaacttata gaaatcaaat caggatcacg
6721 aatcaaagta tccgagttaa ataaagttat taatgaagaa ataaaggtaa gttctcaggt
6781 tattaaacag ttaattcaag caaactttga tactcaaacc aaactataca atggctacga
6841 ttattggatt gatttaggtt ggaaagaagc caataaaaaa gagattcatg atatttcgga
6901 aaaagataat attatttcat tagataaaaa tgaaaatata acagacgatg aggcattaga
6961 tgaagagaat ttggactttg attgggagga ctttgacgat gaataatgaa caaattgaag
7021 catttgtaga agtgcttgta cctatcatag aagaacgtat caataaaggt aattaaggct
7081 aattacgtac tacaggcagt tgcctgtagt actcatatga ttaagtggta aaagtgataa
7141 aaatgaaacg aaattataaa tatatattat ctatatgttg ttacaagacc gatagtctgt
7201 agcaataatc taataaaagg agcggtatga tatgaagggt aaaattgcac tttattcacg
7261 cgttagtacg tcagagcagt cggagcatgg gtactcaatc catgagcagg aacaagtact
7321 catcaaagaa gttgtgaaaa atttcccagg ttatgactat gagacatata ctgactcagg
7381 catttcaggt aaaaatattg aaggtcgtcc ggcaatgaaa cgtctattac aagatgttaa
7441 ggataataaa atcgaaatgg tgttaagttg gaaattgaat cgtatctcac gatcaatgag
7501 agacgtgttt aatattattc atgaattcaa agaacatgat gtagggtata aatcgatttc
7561 tgagaatatt gatacatcca atgcttctgg agaagtactc gttacaatgt ttgggttaat
7621 aggatctata gaacgccaga ctttgatttc gaatgtgaaa ctttctatga atgctaaggc
7681 aaggagcgga gaggcaatca ccggtcgtgt tttaggctac aaattatcac ttaatccact
7741 tacacagaaa aatgatttgg ttatcgatga aaatgaagct aatattgtac gtgaaatttt
7801 cgatttatat ttgaatcata ataaaggcct caaagccatt acaaccgtac ttaatcaaaa
7861 ggggtatcgt actattaatc aaaagccatt ttcagtgtat ggtgttaaat acattttgaa
7921 taatccagtc tataaaggct atgtcagatt caataaccat caaaactggg ctgtacagcg
7981 aagaagtggc aaaagtgata aaaatgatgt gatattggtc aaaggtaaac atgaagccat
8041 tataagtgaa gaggtatttg ataaagttca tgaaaaatta gcttctaaaa gttttaaacc
8101 gggcagacct attggtggag atttctactt acgtggcctt attaaatgcc cagaatgcgg
8161 aaataatatg gtatgtcgac ggacgtatta taaaacgaaa aagtccaaag aacggacaat
8221 caagcgttat tacatttgtt ccttattcaa tcgttcaggg agttctgcat gtcacagtaa
8281 ttccatcaat gctgaagtcg tcgagcgtgt aattaatgtt catttgaatc gtattctgtc
8341 tcaaccagat attatcaagc agattgcgtc aaatgtgata gaagaactga aacaaaagca
8401 tagtaaccaa acagaaatta aatatgacat tgatagttta gaaaaacaaa aagctaagct
8461 taaaacacaa caagaacgat tgttagaatt gttcttagat gatcagatgg atagcgaaat
8521 gttaaaagct aaacaaagtc aaatgaatca acagttagaa gtattagatc aacaaattaa

8581 agaagcgcaa caagcaaatc aatcacagga tgaaatacct aattttgata aattaaaagg
8641 acgactcatt ttgatgataa cacgattcag cgtgtactta agaaaggcta cacccgaagc
8701 taaaaatcaa cttatgaaaa tgttaattga ttcaattgaa attacgacag ataaacaagt
8761 aaaacttgta aggtataaaa ttgacgaaag tcttatccct caatctttga aaaaagattg
8821 ggggtctttt tttatgccca aattccaatt tgaaatatat ggtcaaaatg attatttcat
8881 cgaccaaatt accactttta ccacttagtt attagtgaca aaagtgagcg aaatgaaata
8941 aaaatcaaat atatattatc aaaatgatgt atcacatgca tacatcaatc aaatacatta
9001 ggaggtcata accatgacac tagaacaaca actcaagcac tatataacca acttattcaa
9061 tctgccaagg gacgaagtgt ggcactgcga atctatcgag gaaatcgctg atgatatctt
9121 acccaatcaa tatgtaagac ttggcccact cagtaataaa acacttcaga ctaataccta
9181 ctactctgac acacttcatg aaagtaatat ctatcctttc attctctact atcagaaaca
9241 actcatagcc atcggttata tcgacgaaaa tcacgatatg gatttcttat acctacacaa
9301 cactatcatg cctcttttgg atcaacgata cttactaaca ggaggacaat aaaatgcata
9361 aatacatcaa aattacacaa ttagtcatta caatactaag tgaaatcatc atttggatga
9421 aagagtcaga acgaaaggaa gtctcttatg aatagatata tcacccgggg tatcgccaac
9481 aacttaccta atatcttaca acaccaatta tggcaactcg tatctgagcg agaacaagaa
9541 caaaccaaag ataatacttc agtagattat tttcatatat tccagttcaa taagcatcgc
9601 aatcaattat atatcaaaca caaacaagaa cgacctgaat atgcgaaaat ccataaagct
9661 aattattcaa aagcaatcaa tatcaataag gtctacatta ttcgagaaga tgatgtagac
9721 ctttcttatt atgtcatgtt attacctgaa gaatactaga ggagtgaaaa ttatatggaa
9781 acaatcaaaa gtacattaaa aacagaagcc atattcagtg atgacaaaca acatcgctat
9841 ctacttaaga aaacatggaa tagtgaaaaa caatcaatca caatcattac aatgtatccg
9901 cattatgatg gcattctcaa tattgaccta acgacccaac tcatcatgaa caaagtttca
9961 gaaatggatg catttggttc aatcaatttt gtgaatctat actctaatat tacaacccct
10021 atcaatctca aacatttaga aaatgcgtat gataagcata cagatattca aattatgaag
10081 gcagtgaaag agtcagatga agtgatatta gcttggggcg cttacgctaa aaagcccggt
10141 gttgaagcac gtgttaatga agtattagag atgttgaaac cacataaaaa gaaagtaaaa
10201 cgactcatga atccagaaac caatgaaatc atgcatcccc ttaattcgaa agcacgtcaa
10261 aaatggatat taaaagtata gcaactaaaa tgtttattca ctatccatga aaataattga
10321 atttcatata tatcttttaa tctacaatat gattaaaaga tatattattt ggttctgttg
10381 caaagtaaaa aaatatagct aaccactaat ttatcatgtc agtgttcgct taacttgcta
10441 gcatgatgct aatttcatgg catggcgaaa atccgcagat ctgaagagac ctgcggttct
10501 ttttatatag agcgtaaata cattcaatac cttttaaagt attctttgct gtattgatac
10561 tttgatacct tgtctttctt actttaatat gacggtgatc ttgctcaatg aggttattca
10621 gatatttcga tgtacaatga cagtcaggtt taagtttaaa agctttaatt actttagcca
10681 ttgctacctt cgttgaaggt gcctgatctg taattacctt ttgaggttta ccaaattgtt
10741 taatgagacg tttgataaac gcatatgctg aatgattatc tcgttgctta cgcaaccaaa
10801 tatctaatgt atgtccctct gcatcaatgg cacgatataa atagctccat tttcctttta
10861 ttttgatgta cgtctcatca atacgccatt tgtaataagc ttttttatgc tttttcttcc
10921 aaatttgata caaaattggg gcatattctt gaacccaacg gtagaccgtt gaatgatgaa
10981 cgtttacacc acgttccctt aatatttcag atatatcacg ataactcaat gtatatctta
11041 gatagtagcc aacggctaca gtgataacat ccttgttaaa ttgtttatat ctgaaatagt
11101 tcatacagaa gactcctttt tgttaaaatt atactataaa ttcaactttg caacagaacc
11161 gtattatgga atagagatgt tggtaacatt tatacaggat cattatactt aagtttaatt

11221 tcgttattac agaaccacac attccaacca gaagagaaag tatgtctatt tagttatggt
11281 tcaggagcag taggagaaat ctttagtggt tcaatcgtta aaggatatga caaagcatta
11341 gataaagaga aacacttaaa tatgctagaa tctagagagc aattatcagt cgaagaatac
11401 gaaacattct ttaacagatt tgataatcaa gaatttgatt tcgaacgtga attgacacaa
11461 gatccatatt caaaagtata cttatacagt atagaagacc atatcagaac atataagata
11521 gagaaataaa ctagtggccg attgtgcttg atgagcttgg gacataaatc ctaactcgaa
11581 ataaataagc atatcactaa actgattttt taaagtttac agtgatatgc ttattttttt
11641 atcttacgat tttgtacgtg catgcttgcc taggggtatg gctcgagcca ttagtctctc
11701 gcacatacta ttccctcagg cgtcagcact tacaaaatcg gttgtaattt tcatttttat
11761 acgcattctt actgagatta tactaataag aggaatagta aaagcaattc taagtaaaat
11821 tgcagataag aggtttgtta aaagcagttc taagtaaaat tgcagataag aggtttgtta
11881 aaagcagttc taagtaaaat tgcagataag aggtttgtta aaagcagttc tcagtaaaat
11941 tacagataag aggtacgtta aaagcagttc taagtaaaat tgcagataag aggtttgtta
12001 aaagcagttc taagtaaaat tgcagataag aggtacgtta aaagcaattc catgcaaaat
12061 tgctgataag gggtaagtta aaagcagttc taagtaaaat tgcagataag aggtacgtta
12121 aaagcagttc taggcaaaat tgcagataag aggtgcgtta aaagcagttc taagtaaaat
12181 tgctgataag gggtaagtta aaagcaatcc taagtaaaat tgcagataag aggtgcgtta
12241 aaagcaatcc taagtaaaat tgcagataag gggtacagaa aaactagact tgattacaaa
12301 atggagcttg ggacataaat gattttttaa aaatgagatg agacgtagat taactccata
12361 atcaatacga atctatcgac ttctttattt atgatattca tctcttttta atggaaataa
12421 aagtgcgatt aatgtgataa tacagttacg ttaattaaaa aaataaaaat gcaaggagag
12481 gtaatatgct aactgtatat ggacatagag gattacctag taaagctccg gaaaatacaa
12541 ttgcatcatt taaagctgct tcagaagtag aaggtataaa ctggttggag ttagatgttg
12601 caattacaaa agatgaacaa ctgattatca ttcatgatga ttatttagaa cggactacaa
12661 atatgtccgg ggaaataact gaattgaatt atgatgaaat taaagatgct tctgcaggat
12721 cttggtttgg tgaaaaattc aaagatgaac atttgccaac tttcgatgat gtagtaaaaa
12781 tagcaaatga atataatatg aatttaaatg tagaattaaa aggtattact ggaccgaatg
12841 gactagcact ttctaaaagt atggttaagc aagtggaaga acaattaaca aacttaaatc
12901 agaatcaaga agtgctcatt tcaagcttta atgttgtgct tgttaaactt gcagaagaaa
12961 tcatgccaca atataacaga gcagttatat tccatacaac ttcgtttcgt gaagactgga
13021 gaacactttt agattactgt aatgctaaaa tagtaaacac tgaagatgcc aaacttacta
13081 aagcaaaagt aaaaatggta aaagaagcgg gttatgaatt gaacgtatgg actgtaaaca
13141 aaccagcacg tgcaaaccaa cttgctaatt ggggagttga tggtatcttt acagacaatg
13201 cagataaaat ggtgcatttg tctcaataga aagttagagg tgagtcttac gtttcagtga
13261 cggtagactt acctttaaca tgttacatac taaaaaatta atttgaataa gaaagagaga
13321 catatatgaa atacgatgat tttatagtag gagaaacatt caaaacaaaa agccttcata
13381 ttacagaaga agaaattatc caatttgcaa caacttttga tcctcaatat atgcatatag
13441 ataaagaaaa agcagaacaa agtagattta aaggtatcat tgcatctggc atgcatacac
13501 tttcaatatc atttaaatta tgggtagaag aaggtaaata cggagaagaa gttgtagcag
13561 gaacacaaat gaataacgtt aaatttatta aacctgtata cccaggtaat acattgtacg
13621 ttatcgctga aattacaaat aagaaatcca taaaaaaaga aaatggactc gttacagtgt
13681 cactttcaac atacaatgaa aatgaagaaa ttgtatttaa gggagaagta acagcactta
13741 ttaataattc ataataaaac agtgaagcaa ccatcgttac ggattgcttc actgttttgt
13801 tattcatcta tatcgtattt tttattaccg ttctcatata gctcatcata cactttacct

13861 gagattttgg cattgtagct agccattcct ttatcttgta catctttaac attaatagcc
13921 atcatcatgt ttggattatc tttatcatat gatataaacc acccaatttg tctgccagtt
13981 tctccttgtt tcattttgag ttctgcagta ccggatttgc caattaagtt tgcataagat
14041 ctataaatat cttctttatg tgttttattt acgacttgtt gcataccatc agttaataga
14101 ttgatatttt ctttggaaat aatatttttc ttccaaactt tgtttttcgt gtcttttaat
14161 aagtgaggtg cgttaatatt gccattattt tctaatgcgc tatagattga aaggatctgt
14221 actgggttaa tcagtatttc accttgtccg taacctgaat cagctaataa tatttcatta
14281 tctaaatttt tgtttgaaat ttgagcatta taaaatggat aatcacttgg tatatcttca
14341 ccaacaccta gtttttcat gccttttca aatttcttac tgcctaattc gagtgctact
14401 ctagcaaaga aaatgttatc tgatgattct attgcttgtt ttaagtcgat attaccattt
14461 accacttcat atcttgtaac gttgtaacca ccccaagatt tatctttttg ccaaccttta
14521 ccatcgattt tataacttgt tttatcgtct aatgttttgt tatttaaccc aatcattgct
14581 gttaatattt tttgagttga acctggtgaa gttgtaatct ggaacttgtt gagcagaggt
14641 tcttttttat cttcggttaa tttattatat tcttcgttac tcatgccata cataaatgga
14701 tagacgtcat atgaaggtgt gcttacaagt gctaataatt cacctgtttg agggtggata
14761 gcagtacctg agccataatc atttttcatg ttgttataaa tactcttttg aactttagca
14821 tcaatagtta gttgaatatc tttgccatct tttttctttt tctctattaa tgtatgtgcg
14881 attgtattgc tattatcgtc aacgattgtg acacgatagc catcttcatg ttggagcttt
14941 ttatcgtaaa gtttttcgag tccctttta ccaataactg catcatcttt atagccttta
15001 tattcttttt gttttaattc ttcagagtta atgggaccaa cataacctaa tagatgtgaa
15061 gtcgcttttc ctagaggata gttacgactt tctgtttcat tagttgtaag atgaaatttt
15121 tttgcgaaat ctcttaaata ttcatccatt tttttaacgg ttttaagtgg aacgaaggta
15181 tcatcttgta cccaattttg atccatttgt tgtttgatat agtcttcaga aatacttagt
15241 tctttagcga ttgctttata atctttttta gatacattct ttggaacgat gcctatctca
15301 tatgctgttc ctgtattggc caattccaca ttgtttcggt ctaaaatttt accacgttct
15361 gattttaaat tttcaatatg tatgctttgg tctttctgca ttcctggaat aatgacgcta
15421 tgatcccaat ctaacttcca cataccatct tctttaacaa aattaaattg aacgttgcga
15481 tcaatgttac cgtagtttgt tttaatttta tattgagcat ctactcgttt tttattttta
15541 gatacttttt ttattttacg atcctgaatg tttatatctt taacgcctaa actattatat
15601 atttttatcg gacgttcagt catttctact tcaccattat cgcttttaga aatataactg
15661 ctatctttat aaacttgttt gaaattttta tcttcaattg catcaatagt attattaatt
15721 tctttatctt ttgaagcata aaaatatata ccaaacccga caactacaac tattaaaata
15781 agtggaacaa tttttatctt tttcatcaat atcctcctta tataagacta catttgtaat
15841 atattacaaa tgtagtattt atgtcaaaat aatgttataa tttttgtgat atggaggtgt
15901 agaaggtgtt atcatctttt ttaatgttaa gtataatcag ttcattgctc acgatatgtg
15961 taattttttt agtgagaatg ctctatataa aatatacggt tctgttgcaa agttgaattt
16021 atagtataat tataaccaaa aggagtcttc tgtatgaact atttcagata taaacaattt
16081 aacaaggatg ttatcactgt agccgttggc tactatctaa gatatgcatt gagttatcgt
16141 gatatgtctg aaatattaag ggaacgtggt gtaaacgttc atcatttaac ggtctagcgt
16201 tgagttcaag aatatgcccc gattttatat caaatttgga agaaaaagca taaaaaagct
16261 tattacaaat ggcgtattga tgagacgtac atcaaaataa aaggaaaatg gaactattta
16321 tatcgtgcca ttgatacaga gggacataca ttagatattt ggttgcgtaa gcaacgagat
16381 aatcattcag catatgtatt tatcaaacgt ctcattaaac aatttggtaa acctcaaaag
16441 gtaattacag attaggcacc ttcaacgaag gtcgcaatgg ctaaagtcat taaagctttt

16501 aaacttaaac ctgactgtca ttgtacatcg aaatatctga ataacctcat tgagcaagat
16561 caccgtcata ttaaagtaag aaagacaaga tatcaaagta tcaatacggc aaagaatact
16621 ttaaaaggta ttgaatgtat ttacggtcta tataaaaaga accgcaggtc tcttcagatc
16681 tgcggatttt cgccatgcca tgaaattagc atcatgctaa caagttaaca caaagtatta
16741 ttttaaaatt gagattagac atttattttt caactttgca gcagaaccta attttttctc
16801 tttgtaaata aatgcaaaga gataccataa ggatctctta cataaccata accttcagta
16861 tagaattctg gactaaatgt tttcaatacc tcactgcctt tttctattaa ctggtcatat
16921 acatgtttag tttcttctac ttggtcaaaa gtgagacaaa gagatatatt attaccttgt
16981 gttatgggca aaccttcagt gtcatctgcg atcataattt ttatatctcc aaattgaagt
17041 acacattgat caattttatt taaatcattt tcgtcaatat taagtttctt atctataggt
17101 ctatctttaa tacgttgaat atacagtgtt ttagcgccaa acagctcttc atacaacttt
17161 tttaaaccct ctgcattttg agtgattaaa aaatatggac ttacttgaaa tttcatgttt
17221 tttcctcctt agatttgtta taaatagagt atatacttaa ttagtgtcat ctattgacac
17281 taaaaggaga ataataatga aaaaatctgt tagattatat aatatgattg aatattgtaa
17341 tgaaaatagg aacttcaaat taaatgattt aatgtcagaa tttaatattt ctcgtagtac
17401 cgctttaagg gatataaaag aaattgaagc attaggagta cctttatata gtaatccagg
17461 aaaaaatggt ggttatacga tcataggtaa tcgagaccaa acgaaaatag caatctcaga
17521 tgaagagttg aaagctttag tatttacact ttcgagtatt tcaaatgtga gtaatctacc
17581 ttttcaaaca gaatatcaag aaatattaaa aaaattatat aataactcga ataaaaaaga
17641 gttaataaac caatataatg atctatttca atattttaat gaagataagt atcagttcaa
17701 aagttataag ttatttaatg aaatcattag attgataatt gagaataagt cttttgaaac
17761 ctgttattca caaaactata ttaaagaaca atataaaggt attggcatta tgtataaaaa
17821 tcatcaatgg tattttgttg tagctaatat agaatcaaag ttagtgaatc tattaaatat
17881 ttcgaaaata aaagaactat atgaaatggg agagactcaa gagtgtaatg atataactat
17941 gcagaatttt caacagttca tggttaaaaa tgaaacagct attgatattc ttattagaag
18001 caatgttatg ggattgaata tcttgaaagg ctacctgtgg agtgactata tgattgaaaa
18061 tattgacgaa gagacatatt tatttaaatc aaaagtgaac gcgaaagata tagattttat
18121 agctaagtta attgtcacag gtggtgtcaa tgtaaaagta gagaccccta atagtttgaa
18181 aaatgctgtt aaagttgaat taactaaaat aataaacatg tattaatagt aatttaatca
18241 taattgtaat aacaaaaaga tgacaacatt aaacaattta aatttataca aaaaagcatt
18301 accaaattta aaattgggta gtggtagtgc tacagcatac ataaatagtt ataattaaat
18361 aaagatgggt caatgaaggt gaattaagtg aataaaaata ttacagctct aaaatctact
18421 gaaaatacta catatatgca gtatacggtt catatcgatg atcaaattat aaataatata
18481 aaagagtgta ccaaaaagtt taaaattttg cctatggaag ataaaattcc attatcaccg
18541 ttgttacaac cagaatatgc aggagaggta caagatttta ttagtacata tgagcagttt
18601 atgattaatt ttggtaaagt aatattggat agtcaaggca taaaaataca gtttgaaagt
18661 gaatcattaa gtagtattca acgaggcatt caagaacatt gttacttaaa tgagcgaaca
18721 aatgacattg atgtgactaa agaatggtat ttatgcaaat tttcaaatca aatattagaa
18781 gaggataaaa atcaattgta taatgcttta aagcaactca tgaatgattc taaaaataaa
18841 aagcaagctt tttggactga ccccaattag tgggaattat ataaaaacac tttcgttgaa
18901 ttcataataa aatgaatcat gcggaggtgt tttttctatg aaaagagttt cttattcagt
18961 agaaacaaag tataaagcag ttgaaatgaa aatagctggt ttttcgacaa aagaaattat
19021 gaaagaatta aatattaaaa atagaacaca agtggaaact tggtggcgat ggtatcgaaa
19081 tgaggaaagt tatagatttt cacaacacgt tgggaaacaa tatacctacg gtaaaggatt

19141 agaagaattg tcagaaatag aacgattaaa attagaaaat aagagaaaag atatagaatt
19201 ggatatttta aaaaagtaca aggcattgga aaggaagtgg taccagcagt agtcatagat
19261 ttagtggatc aattaaaagt aaaatattca atcaaattga tactagaagt attaaacata
19321 cctaaatcaa catattaccg atggaaaaac aaaacctata aaaatgatac tgtaacacaa
19381 aaagttattg aattatgtga agctaaccac tatacctacg gttatcgtaa gattacagca
19441 ctgattaatc aatgttatac atcaccaatt aatcataaga gagtacagag aattatgcag
19501 aagcatcatt tgaactgccg agttagacct aaaaagacga caaggatagg taaaccgtat
19561 tataaaactg acaatttatt acaaagacaa tttaaagcga gttgtcccat ggaagtatta
19621 acaaccgata ttacttattt accatttggt cattctatgt tgtatttatc ttcgataatg
19681 gatatttata acggagaaat tgtggcgtat aaaatagatg ataaacaaga ccaaagttta
19741 gttaatgata cattaaatca aatcgatata cccgaggggt gtatattaca tagtgatcaa
19801 ggcagcgttt atacatctta tgcttattat caattatgcg aagaaaaagg cattatcaga
19861 agtatgtccc gaaagggaac acctgctgat aacgccccga tagaaagttt tcattcctcg
19921 ctaaagtctg aaacttttta catcaataat cagcttaata gctctaatca tattgtaata
19981 gatattgtcg aaaaatacat taaaaactat aataataatc gaattcaaca aaaactaggc
20041 tacttatctc ctgtaaaata cagagaatta gcagcctaga acatagtgtt tttattaagt
20101 tcccgtttaa gggggtcagt accttttcaa gaggtgttta aggttcatat tgatatttat
20161 ctctacaata aagaaaaatc tgaatataag tatcaagtat ctagttattt caatcttgta
20221 aaagaaaatc ctaaaataac ttataaaaaa agacatttac aagagaaaca aggtgtcaag
20281 ggtacaactt tcacgaacaa attaaaatta ttgaacaaaa tgtatggtgt agatgttgca
20341 aaatatcagc cattttataa ttctaataat cctgaatatg aaagaggtca atttggtgaa
20401 agatacattt ctcaaagatc taactatgaa tttaatagac ttcaatatca aattatagat
20461 atgttatcga aaatacttga taagcatcca ttaccaaagt cagataataa ttataaacat
20521 attccaacta ttgaaaaagc aatattaagt ggggattctc atagttttta tgagtatttt
20581 gaagatataa tgaaagaaat tataagtatg gaaaattctt ctttaaagga aaaattattg
20641 actgatttta catatcaatc tcaatgtaga tggtactctg aaagtgaaaa attaaaattg
20701 caacttgaaa gttttatgca taaagttttg gagagcaatt attatgaggg taacaaatta
20761 tatagaatgt tgtctcatgc aattgaagaa acaatcaatg aggctgatga agataaggtt
20821 catttcaatt attttaaaga ttattttttg acagacggtg gggtgaagaa ttgggagcag
20881 attagcgaga aaattactga attcaatggt aaagttatta acgatatcca aaatgaatat
20941 aataagattc aatttaataa cgcaaatcga aatcagaaac taaactttaa ttatttatac
21001 cattgctttg agtttagtaa taacttagta aaagcaaggg taaatggaaa tagaggttac
21061 attttatatt ttgtagataa atataaaata aaaatgccct ttttggatca attattaatc
21121 aattatttga atcaagaaat aggtcaggaa agtattaatt ataatatgca aacattgttt
21181 gaaaaagaaa gatatgatag aagtagtacc attgaaaaat tagtagcaac aagcaaattt
21241 aagtatgaaa aagatgattc agatttattc aaacaacttt tcaatgatgt tgaaaattca
21301 atagacagat taggtattta cttactaaat aatggtataa attcgaatga tgaaaatgca
21361 agatattata gatcgttttt aaaggaactt agtagaataa aaagtaaatt aacgccattt
21421 tctcttgaaa taagtaagtc tagcggaaga gagcaacatt atcctgatga tgctattgat
21481 gataaagatg agagaagaaa aaataaagaa gaaacatatc atgcttttga tgataaaagc
21541 gatattgact ccaaattaaa gaataaaata aatgtttcta tcgataattt attgtcagtt
21601 aaaattaaga gcaatttgta tgatattagt ccaaatacaa aaaaacaata actataatga
21661 gccaactcaa gaatgagttg gctttttaat tgtaatttta cgcttccatt acaggaatca
21721 atgttatgga agccattaag caattaattt gagtcattta tagtataggt acaacgttgt

21781 gatgttcaca tgataatcag cccgggagat tattgtgtaa taacttatag aaggagcaaa
21841 tcaccatgta tcaagcaaat atacgtgatt taattacaaa attgcctcaa agcaataaaa
21901 cagaacactt tttaatgaac aaattttcaa atcaagataa agttcagcag ctacaaagac
21961 aaattagcca acagctagat caacaatata atgagctttt ggctaatgaa aaagctaagc
22021 tagaccaata cgtggaagta caccataatt tagaaccatt aaagaaagag attgaatcag
22081 aatctattaa ccttgatacc gataaattac ctgatatcaa agcgacaatg cttgaaaagg
22141 ctaagaacga tgaacatttt gataaaatcg aacagctatt tgatagatta gatcagtcat
22201 taaatggtac gaatcgatta tatacgcaat tatcgttgat tggcacacga acacatcgga
22261 tcacaacgaa aagatttaat gttcaaggct tgcctaaatt agtccaacaa atgattttac
22321 cttcgcaatt taaaaaggtt tatacaatag attttaaatc attcgaacca tcagttgctg
22381 cgtatatgac acaagacgaa caactgattg actacttgaa tcatgaagaa gggttatacg
22441 atgcattact gagagactta tctttgtcaa aagagaagcg cgtgagtgtg aaacgtgcat
22501 ttatagggtc atttcttttt ggcggtcgtt atagtagctc taaattcaaa atcaatcaag
22561 aggttagtga aattaactgg ctacaagtaa tgagcaaatt caagaaggtc attgaattta
22621 aggagcaagt cgaaaaatat aaaacaatgc ctacgcctta cggcattgaa catgatatga
22681 gcgcatttca aggtagtagt attatggcaa tttatgtaca aacggtagca agctatattt
22741 tcaagcacat tttgttggaa gtgtacaaag cacagtgcga aaaaaaaacg ttcaagatta
22801 tagtgccgat acacgatgcg attatgattg aatgtaatga taaggggatt gcacaaaatg
22861 tagcacagct catgaaagat acagctaatc aactgtttaa tggtgaattt gcacatgtga
22921 cagtggaagc tttaggaggt atagacaatg aataatgata gaggaaaaag tcttcaaatt
22981 ccccaaagta cattgttaaa agaaggatct atatacgttg ctacgttaca ttctgtgtac
23041 gagaagaact tctcaggtga tattaaacat cagtttacgt atgaagtaga acttaaccaa
23101 gaaacgcact atgtaaatcg caatattacc gtaaaatcta tgagtcacca attatcaatt
23161 gctgattgga ttaaacgtca cagtaactat aacgtaaacc acattaatta tgatccatac
23221 attgatcgaa aacatttggt ccttgtaggg caatataacg gaaattatta tattcaagat
23281 gtagcaccat tagatgaatt tggaggggta ttgtaatgaa tcatatttta gaaatgttaa
23341 taaaattatt aaaagtgggt atggaggcaa tcgaccgtaa aggtctgatt gccatcctaa
23401 caagtagtat tggaaatgat gaaatggatg attctgaaca agctgtaatg gtgtataacg
23461 agcttatcga taagctacag cttaacattc ctaaagatgt cgactataga cctaacatat
23521 acagttattt tggtattcaa aaaaagccaa atgacacaat attagtggaa atgatgatat
23581 gtatttttca tatcaagcgt tttgattcag agttgtttgt tttcaaagat aaaggttggc
23641 aaaaggtaag tgaagatgaa ttgcaagggt tgatatctaa aatgatacaa gtgttgctag
23701 ttgattataa accttcacta agtactttga aaaacgtagt agatggcata cagaaatcaa
23761 cggacataga aaaacttgtt gagcatagac agtacattgg ttgtggacga aatatgttca
23821 atctaaagac ctttaaagtg gttgataatg accttgaaat attccctaaa acacgcttag
23881 atttggaatt agatataaat gacacgatta cggacaagat acccccgaat ttcaaacaat
23941 atatgttaga gttggcgaat tatgaccatg atttacaata tttccttttc caacatatgg
24001 cagtgttatt gacggcagat actaaactac gtcgtggact ttttttgtat ggaactgcaa
24061 aaaatgggaa atcggtctat attaaattag ttaagtcatt cttttatagt aatgatatcg
24121 tatctaaaac acttaatgaa cttggcgggc gtttcgataa ggaaagtcta attggtaaac
24181 gaattatggc aagtgatgaa gtggggaaag ctaatattga tgaagcaact gtgaatgatt
24241 tcaaaaaatt actatctgtt gaaccaattc atgctgaccg taaaggaaga acgcaagtag
24301 aagttacttt agatttaaaa ctcattttta atacgaatgc tgtactcaat tttccatcat
24361 cacatgcaaa agcattagag cgtagaattg ctgttattcc atgtgaatat tatgttgaaa

24421 aagctgaccc tgacttaatt gaaaagttac aggatgaaaa gaaagaaatc tttctttact
24481 tgatgtatgt gtataagcaa attgtaaaaa atgatatcga gtacctccaa aatgatcgtg
24541 ttactgaaat ttctcatgat tggttaaatt ttggatatga atttgtttct agtaaatcag
24601 caaatattgc acatcagaaa gcgtgtatta atttactcag aaaacttata gaaatcaaac
24661 cagggtcacg tatcaaagtg tctaggctaa atgaggttat tagagatgaa attaaagtaa
24721 gctctcaagt tattaatgat ttggttcaag ctaactttaa tgtacaaagt agactaaata
24781 atggttataa gtattgggtc gatttaggat ggaaagaaac tgataaaaaa gatgacatga
24841 tttcattcga taaaaatgag aatgtaacag atgatgaatt cttatacgaa gatgatttga
24901 acttaggttg ggaggacttt gacgatgaat aatgaacaaa ttgaagcatt tgtagaagtg
24961 cttgtgccta tcatagaaga acgtatcaat aaaggtaagt aatctaatta cgtactacag
25021 gcagttgcct gtagtactca tatgattaag tggtaaaagt gataaaaatg aaacgaaatt
25081 ataaatatat attatctata tgttgttaca agaccgatgg tctgtagcaa taatctaata
25141 aaaggagcgg tatgatatga agggtaaaat tgcactttat tcacgtgtta gtacgtctga
25201 gcagtcagaa catggttatt ctgaaaagga gcaggaacaa ctactcatca aagaagttat
25261 gaaaaatttc ccaggttatg actatgagac atatactgac tcaggcattt caggtaaaaa
25321 tattgaaggt cgtccggcaa tgaaacgtct attacaagat gttaaggata ataaaatcga
25381 aatggtatta agttggaaat tgaatcgtat ttctcgctca atgagagacg tgtttaatat
25441 tattcatgaa ttcaaagaac atgacgtagg gtataaatcg atttctgaga atattgatac
25501 atccaatgct tctggagaag tactcgttac aatgtttgga ctaataggat ctatagaacg
25561 ccagactttg atttccaatg tgaaactttc tatgaatgct aaggcaagga gcggagaggc
25621 aatcaccggt cgtgttttag gctacaaatt atcacttaat ccattgacac agaaaaatga
25681 tttagttatt gatgaaaatg aagctcatat tgtacgggaa atctttgatt tatatttgaa
25741 tcacaataaa ggatttaaag caatcacgac aattctaaat caaaaaggat atcgtaccat
25801 taatcaaaaa ccattttcag tgtttggcgt gaaatacatt ttgaataatc cagtctataa
25861 aggctatgtc agattcaata atcatcaaaa ttgggctgtt cagcgaagaa gtggtaaaag
25921 tgataaaaat gatgtgatat tggtcaaagg taagcatgaa gccattataa gtgaagatgt
25981 atttgatcaa gttcatgaga aactagcttc taaaagtttt aaaccgggtc gacctattgg
26041 tggagatttc tacttacgtg gccttattaa atgcccagaa tgcggaaata atatggtatg
26101 tcgacggacg tattataaaa cgaaaaagtc caaagaacgc acaatcaaac gctattacat
26161 ttgttcatta ttcaatcgct caggaagttc tgcctgtcat agtaatgcga ttaatgctga
26221 agtcgtcgaa cgcgtaatca atgttcattt gaatcgtatt ctttcacaac ctaatgttat
26281 taagcagatt gcgtcaagtg tgatagaaga actgaaacaa aagcatagta aacaaacaga
26341 aataaaatat gatattgata gtctagaaaa acaaaaagca aaagttaaaa cacaacaaga
26401 acgattattg gaattgttct tagatgatga aatggatagc gaaatgttaa aagctaaaca
26461 aagtgaaatg aatcaacagt tagaagtatt agaccaacaa attaaagaag caaaacaagc
26521 aaatcaatca caggatgata tacccaattt tgataagtta aaagcacgac tcattttgat
26581 gataacacga ttcagtgtgt acttaagaaa ggctacaccc gaagctaaaa atcaacttat
26641 gaaaatgtta attgattcaa ttgaaattac gacagataaa caagtaaaac ttgtaaggta
26701 taaaattgat gaaagtctta tccctcaatc tttgaaaaaa gattgggggt cttttttat
26761 acctaaattt aactttgtga taaatgtcac aaagaaaaat aggattgaaa atttatcact
26821 tttaccactt ttttagagtg acaaaagtgg aggagttttg aaatatttta taaatatata
26881 ttttatttat ggagtacaca ttattaatta aggaggtcat tataatgacg ctaagcaaac
26941 aacttaaaac gtatatcact gaacgattta aattaaatta tcaagaaact tgggcttgtg
27001 aaaccataga tgcggtggct gaagatgtat tacctgaaaa atatattaaa aatagtccac

27061 ttgaacataa aattttaaat acttttacct attacaatga tgaattacat gaaatcagca
27121 tttacccttt tttatgttat ctagataagg aattagtagc aataggttat ttagataatt
27181 ttgatttaga ctttatattt ttaaatgaca ctcatcaagt cattattgat gaacgctact
27241 tgttacaaaa agggggcgag taattatgaa ttggatcaag gtcgctcaac tatctgtcac
27301 agttatcaat gaagtcattg agattatgaa agaaaaacaa aatggagggg aatagtatga
27361 aaatcaatcg atacattaca agaggtgtta gtgaacattt atctctagac cttcaaatct
27421 tactttggaa catggtaaaa gatcgagaca atcaacttca tacagattac ctacacattt
27481 ttaaactaca agaagatgat aatatactct caatcacaca tgaacaagaa caacccgcat
27541 acaaattgga atatcactat acaaactatg taaaaaatca aaatgcatta cctaagaaag
27601 tctacgtcat ccgagaagat gatgtagacg ctttttatta tgtgatgctt ttaccagaag
27661 aatactaaaa ggagtgacaa tgatgaatac aatcaaaaat acaatacaca cagaagcgat
27721 atttagcgat gatgaacaac accgctactt actcaaaaag acttgggatg aaaagaaacc
27781 tgcatgtacg gtaatcacca tgtaccctca tctagatggc gtattatcac tcgatcttac
27841 aactgttctt atcctcaatc aattagcgaa ttctgaacaa tatggcgctg tatatcttgt
27901 aaatctattt tctaatatta aaaccccaga gaaccttaaa catattaaag aaccttatga
27961 taaacacaca gacattcatt taatgaaagc gataagtgaa agtgacacag tgattttagc
28021 ctatggtgct tatgcgaagc gacccgttgt tgtcgaacgc gttgagcaag tgatggaaat
28081 gttaaaacct cataaaaaga aagtaaaaaa gctcataaat ccggcaacga atgaaattat
28141 gcatccgctt aatcctaaag cgcgtcaaaa atggacattg aaataaagga ggattatcta
28201 tgaaccatga aactaaacaa tcagattggc gaacggttgc tagttgttta gcatcacaaa
28261 attatatatc gattgtaaaa ggtttagtac atcattttac agcgatcgaa gatgaggaaa
28321 ttcttgataa aatctatgat gattttatga atgatgactc tataacaacg gtacttaaca
28381 atgatttaca gacaatcatt aaccattatc tatcaaaata aagactatac taattacaat
28441 aaaaacagag catccttcac ttttatggtg agggatgctc ttttaattta tttttttatt
28501 gattattcgc cataagttct gcttgctcta caacctgctc tacagccatt ttttgtaaat
28561 ctggtggata gccatatttt ttaagcaatc gtctaacagc tacgcgcatt ttagcttttg
28621 cgctatcacg tttagaccaa tcaacaccca tgttttcttt cactgtttta gttagctcat
28681 gagcaatcgc acgtagttct ttatctccca tggcttcttt tgctgtttca tgtgaagcta
28741 aagcatcgta aaacgcaatc tcatctgaat tcaggcctaa ttcatttcct cgttgttgtt
28801 cttgtttaat atctttagcg agttgaataa gttcttcaat cactttagat gtttcaatgg
28861 aacgactatt atatttatta atcgaatttc ctaacatctc agagaaacgc ttagataccg
28921 ttgcattcgt tttcattaat gatttaactt gtcctttgag taatcgattt aataattcta
28981 ccgccacatt tttctgtttc aatccttcga catcttttaa aaagtcatca gataagattg
29041 ataaatcggg ttgttcaaga cctagcgttt gataaacatc aatgacatct tcagtcacaa
29101 cagattgtga cacaagttga ttaatctctg cttcaacttc tgcaggtgtt ttacgtgttt
29161 ttccttcttt tggcggttgt aacaatttaa caagtcctgc tttaactgct ttaaagaagg
29221 caatctcatc gttgagttct tgggctgtag gttcagtcgc acaaagagca aaggctttcc
29281 ctaattctgt gactgtttta ataaaacgtt gacgttcatc ttcacctaaa ccaattacat
29341 aatccatcgt attcgatatt gtataataac gttcagattt tttatctgaa ttaaatttag
29401 agtaatcaag attatataac atatcttgaa ttacatcata cttcaataac atcaattcaa
29461 ccgctttatc cgtatctatt gccgtttgag cttgatcaga ttctgtatat tctttaagtg
29521 cctcttttaa actttcggca atccccacat aatcgacaat taaaccaccc ggcttatctt
29581 taaacactcg attaactcga gcaattgctt gcatcaaatt atgccctttc atcggtttat
29641 caatatacat cgtatgcata gaaggaacat caaaccctgt cagccacata tctcgaacaa

29701 tcacgagttg taactcatca tccacatctt tcatacgttt ttctaataaa ttacggcgtt
29761 ttttaggacc aatgtgtctt tggaaagaag ttgggtcact agatgagcct gtcattacca
29821 ctttaatgac tcctttatca tcatcatctg aatgccattc tggttttaga cgaatgattt
29881 catcatataa atcaacagca attcgacgac tcatcgttac aatcattcct ttgcctttca
29941 tcgcttgttg acgtgtttca aagtgttgga tgatatcttt ggctagggct tctacacgag
30001 gttttgcgcc tgctaaagct tcaatacggg accattttga ttttaaacgc tgttttacat
30061 cctcttcttg atcttcagta atgtcattat acgcttcatc taaatctaaa ctttgaggta
30121 gatttaatgg aattacacga ctttcatagt aaattttaac ggtgcttcca tcagcgacgg
30181 cttgtgtcat atcataaaca tcgatatagt ttccgaaaac catttgcgta tttttatccg
30241 ttgaagctac gggtgtgcct gtaaatccta cgaatgttgc attcggtaaa gcatctctta
30301 aatatttggc ataaccatat ttaatgcctt cacctttatc atcgtatttt gcgttaaagc
30361 catattgtgt acgatgagct tcgtctgcca tgacaatcac attttttcgt tctgttaggg
30421 cagccatggt cgtttcgttt tgttcaggtt caaatttttg catcgttgta aatacaatac
30481 cacccgactc aacagataat aatgatttta attctttacg tgtttcagct tgttttggtg
30541 tttgtcttaa taatccttta ccagaacgtc cttttgattt aacaaacgta ctgtacagtt
30601 ggttatctaa atcattacga tccgttacga cgactaaggt agggttattc agcatttgaa
30661 ttaattttcc agagaaaaag accatggtta aacttttacc ggacccttgg gtatgccaaa
30721 taacgccgcc tttaccatca ccttgctcag atgaagctaa taaagctctg tcaacggctt
30781 tattaacggc ataatattga tgatacgctg ctagaatttt actgatatgt cctttgccat
30841 catcttgaaa taaaacaaaa tatcgaatta aatcaagtaa tgtttctgga tttaacatgc
30901 catgaatgag tacgtctaag ctagctaaac ctggtgatga ttcagtttga ccatctttac
30961 tgcgccaagt cataaaccga tcataatttg cggttagaga gccagccttt gtattaatac
31021 catcacttgt aacaagcact tcgttaaacg taaataattg tggaatacgc atcttatacg
31081 tttctaattg atgataaccg tcttcgacgc ccacggtttc atttgttgaa tttttaagtt
31141 caatcactac gataggcaag ccattgataa agagtacaat atcgggacgt tttttatagt
31201 ctccattaac gatagttaat tgattgactg ctaaaaaatc attgttttgt ggatattcaa
31261 aatcaataat tttaacaatt tctacaatcg attgtccttc atcatcatag tcttcgattt
31321 caataccatt aatcaaattt tcatgaaagg taaggttatt ttctaaaaga ttgggcgact
31381 tctctaaagt tagttcatgt agcgcttttt caataaaacg aggatggata tctgagttga
31441 tttttcttaa tgctttctct aaacgttcat gaaggacaac atctttatca cttttacgtt
31501 ctggtgttag acttgtcata ctaatctcat taccttttttt atagtcatag cctagtgatt
31561 gtagccattc taatgcgact tgttctaaat catcttcatt aaattgaaag ttcatcacta
31621 ttcacctcaa tatcatcaga tatttctagt tctccagaca ttagtttagg aagaagagta
31681 tctcgtaatt gagttaattt accaatttct gatcttaata ttttcatttc ttccatatat
31741 ggagtgatta tttcagaata tttttttaca atactatctt caattgctaa tttataatta
31801 acagctattg ttggttttac tctttgtcta ctatttgttg aaccggtagt attagctttt
31861 aaataatcta taaattgaga attaagacat atattatata tgaatgaatt aattttatta
31921 ttaggagatt ccattacaac aaattctgat gaagcaacat ttaatttttt attatctatt
31981 acaggtaacc aaatcctttt agtatcagga ttcatttttg aaaataatac gcaattgtta
32041 ttaataatcc atttattact ttttatttta ttgacttctt cctctatagc ttgttcttca
32101 ttatcataag caggtaaact aaaatgttta actgttactt cttcactctt tttaggatta
32161 aaagtttcct ttttatgact tgctatatct tttaatttat agattttcca attacttggt
32221 atcttaccta actcactatc aatcatttct cctccactag atttataagg attaccatct
32281 tcatctggaa attcaaaatc tacaaaccaa tgcttaaata gtgtttgtga aagttcttta

32341 aggtttgcta tcatttttg gttagtttct atttttcttg tgatttgatt taaaaattta
32401 gaaatgcttt tttgttttat taaagaaaaa ttaggaattt tgacagtttc aataagttta
32461 gaatttatat tagcttgatt agcactccct gtagaatttc taactaaata ctcagttatt
32521 ttaggttgta gaaaataata aaataaaaaa tctaagtctg cactattact atattttaat
32581 tcgaattttc ccacacgttg attttgtaat gctggataat caaattcaat tcttccaact
32641 tttcctactg cagaagacat ttgattcacg cctgatccag ttaatgatat taatatatca
32701 tttttcttta atgaaaaacc tttagttttt tcatatgttt taatagagac tttttgagag
32761 tcttttgtat caacaatggg tgaaattata ttcttaattt taataaccgg tatacctttt
32821 tcttgaaatt cttttgattt aaaagcatat ccatttttaa ttttagctac atcaccgaaa
32881 aaaattgtat tatactccat atcctaaccc ctccaatgat tttcggattt ggtcttcaag
32941 ttcttttgat tttgcgaatt gttcacttaa ttcagaagta atacgttcca ttttttgttc
33001 aaaaggttct tcatcttctt caacgtcagc taaaccaaca tatcgtccag gtgttaaaat
33061 gtattcatta ttcttaactt cttctaaatt agctacttta caaaaaccag ctatatcttc
33121 ataaggttta tcgtttgtac ctctccacgc atgatacgtc tgtgctactt tttgaatatc
33181 ttcatctgaa aattctttta atgttctaga taccatatgg ccgatttcac gagcatcgat
33241 aaataaaatt tcattttcac gctctttttt accattttga cctttattat tactaatgaa
33301 ccaaagacat acaggtattt gggttgagta aaagagttgt ccaggtaaag taacaataca
33361 ttcaactaaa tcttgttcaa ttagattttt acgaatttct aattcatctt ttccgcttgt
33421 agacattgat ccgttggcta atacgaatcc tgctgtacca ttaggtgcta attttgaaat
33481 catatgttca atccatgcat agttagcatt accttttggt ggaataccaa attgccaacg
33541 gtaatcatca agtaatcgtt cttgacccca atcacttgcg ttaaaaggag gattggctaa
33601 tatgtaatct gcttttaatc ctttatgtaa atcattatgg aatgtatcag catttcgttc
33661 acctaagtca ttatcaatac cacgaatcgc taagttcatt tttgctaatt tccaagtcgt
33721 agggttagat tcttgtccgt aaatcgcaat atcgtctaat cgaccttgat gtttctcaac
33781 gaagcgttca ctttgtacaa acatcccacc tgaaccgcaa cacggatcgt aaatacggcc
33841 tttataaggc tcaatcatct caactaataa ctttacaatt gatgacgggg tatagaattc
33901 tccagcattt ttaccttcgg cacttgcgaa tttagcaata aagtactcat acacacgacc
33961 taacacatct tgtttacgac tctctgtgtc gccaacttta aatgtaaata aatcaataat
34021 atcgcctaat ttttctttat ctaatgcagg tcttgcatat tctttaggta atacaccttt
34081 gagtgattca ttttcatttt caattgcaat cattgcttta tcaataattt gtccaatttc
34141 tggtttttc gcattatcat tgatatattg ccatcttgct tcttttggca cccagaaaat
34201 gttttctgct aagtattcat cttggtcctc ttcatctgca tatggatcct gcttcaattc
34261 ttcatacttt tcttcaaaag aatctgatac atattttaaa aagattaaac ctagtgctac
34321 atttttatac tcagcagcat ccatacttcc tctcaattta tcggcagctt gccataattt
34381 ttcttcaaaa cctatagtgg ccattaataa cacctcttaa aattaatgtt aaatcaacta
34441 tagcaaaaaa tagaagatga ttgtgtagtg ttttattaat atttaattta tctagactat
34501 caaaaaaata tagattatta ttatcttaaa ttaataaaca ggagtgaatt aaaatgaata
34561 aaaaatataa attaaaacga tacgataaaa gatttagagg tggatggtgt tacaaatgtg
34621 aagattcata ttcttacctt tgtgaagatt gtggttattg tagatattgt tgccattgtt
34681 aatacaagac tagataaatt gggtatcttt aactcaatta ataaaaaatt taaatgtagt
34741 caatagcttg ctaactttat ttgatgtaat ataattttgg cttttagcat tcagttaaaa
34801 agaagtaaat aattgacaga ttttttagag aactggagaa aaaagatgta catttctagt
34861 gttaaaatta agaattttag aaattttgat aatatcgata ttgatttcca tgagggagtt
34921 aatgtattaa tcggacataa taattcgggg aaatcaaatt tattacgtgc tttatcttta

34981 atttttgacg gttcagttag aaagcaactg tcagtggaag attttaataa ctctttaaca
35041 aaagaatctt taaaaaaaga ggctccaaaa atagtaattt cagtacatat aacacaatct
35101 gaaaacgaaa gactgatgtc tgatgagtta attacggtaa gtaattggct gactaaatta
35161 gaagaacctt atgaagctag aatacaatat gaattctttt taccaaaaga tgaggaaaaa
35221 aattacatag atttagtaaa aaatatagat gaaaaagaag aaatatggaa tttaattaga
35281 agccagttta ttaggttgta tgtcaataaa atctgggtgg gtaatccaga acatcagatt
35341 cctatagata atgatagttt aaataaattt gatttccaat ttttagacgc aataagagat
35401 gtagagagag atatgtttag tggtaaaaat acattattaa aaagtgtaat agattttttt
35461 atagattacg atataaaatc cgatgaaaca atcacagaag aagaacagaa agaaaaatta
35521 gaagaaagaa gaaaagaatt ttctgataat tctagtgatt tgattgaaac tattcataaa
35581 agattagata gtgggaatca aaaaattttg tcttatacaa atggcatagg agcatcttat
35641 gataaatcaa caccggattt taaaggtaat cttacagaaa gtgaaattta tactgtattg
35701 caattgataa ttaaacatga aactggaatg actttaccta taactcataa cgggttgggt
35761 tacaataatt taattttcat ggccttgcta ttgtctaaaa tgcaagctga ttctgatgga
35821 aattttctag gtagtaatgc aaaagtattt ccgatactag ctattgaaga accagaggct
35881 catctacatc ctactatgca aaatgaattt attaaatttt taaaaaataa cattagagaa
35941 aaaaaggtaa agcagatttt cataactact cattcaacac atatttcttc ttctacaaat
36001 attgatgata ttatttgtct ttacacagat gatagtaaaa ctaatgttag ttatcctgga
36061 aaagtttttg ataagaataa tcaaagtaag aagtacgtac aacggttttt agatgccact
36121 aagtcaaata tgctatttgc agaaaaggta atatttgtgg aaggtatagc agaacaatta
36181 ctattacata tatttgcaga ctatttaaat aaaccgttag aaaagaatca tgttgcagta
36241 ataaatgtag gcgggagata ttttaatcat tttttatctt tgtttgatag taataataaa
36301 tatgcaatta atcgtaaggt ttcttgtata acagatttag atccaatgcg aaaagaaaaa
36361 aatgaaaatg gacaaaaaaa taattttaaa gcatgctatc cttttgaata tgaaatggat
36421 ttagataaat ttgattattc aactaataat tctctagata aatatctaaa taatgagcac
36481 agtaatatac gagtatttgt tcaacctaat aaatatggaa aaacatttga gtaccaactt
36541 atgtttgaca atccttcatt aaaactgtta cttacagact cgatttctaa tagtcaagag
36601 ctagcagaat taatggatca ctatgaagaa aatgaatctt tacaaaaatt aattgataca
36661 ttatcaccat ctaatgaaaa tcagagaata ataaaatctt taaaagacac aaatgacagt
36721 tggaatgaag ataataaaaa gaaagcatta atagcatcaa gatacctaaa ttccatagga
36781 aaaggagaaa atgctctaga gctagcttca gtattaaaag ataatttaga gttaaaaggc
36841 cagagtgact atgaagattt tgtagtacca aaatatatag aaggtgcaat tcgatgggtg
36901 tgtgaataat atgattatta atgctcaaac gaaattaaat atagaacaag attttaaaat
36961 acaggctggt cctggtgctg gaaaaacaga atttttggtg aatcatatca aaaatgtaat
37021 acaaacttct gaaaagctcg aacgtatgaa gaaaatagcg tgtattactt atacgaatac
37081 ggctgctcag acagttttac aaagattagg aaaaagtgtt tcaaatagag tagatatatc
37141 tacaatacat agctttttgt atagaaacgt agttaaacca tattgttctt ttctacccaa
37201 agaatacgga atttgtgtgc aaaaattaaa aggacatagt gatccaattg tgattaacaa
37261 atatgtaaat gcatggttaa aaactgaata ttttacgaaa ttaaaaccgc ctagtaatag
37321 aaatcaacta ctaaaaatgc cagtactaaa gaaagctttg caaaactggc tactatctat
37381 gaaactttct tacgaaaata atgatgtgca atttatttgt gataatacca aagctcaagc
37441 aattgataaa aaaagtaaaa aaagaatggg aataaataaa tcaaacttaa atatactaga
37501 aaagcaatta ttaaattata aaaaaatata ttgggagaaa ggaattatcg atcatgatga
37561 tgttttgttt ttttcatatg tattaattag agattaccca ttcattctca ctattttatg

37621 tgctaaatat ccctactttt ttatagatga atttcaagat actagtcaat tacaagattt
37681 tattatagat aaaataagac agaaaggctg catagtgggg gttattggcg ataaggcaca
37741 ggcaatttac agctttcaag gtgcacgggt ttccttattt gagaagttta aagttgattt
37801 aataaattct cacactatct tagaaaatca tagaagttca tatcaaatag ttactttttt
37861 aaatagtatc agagaagata ttactcaaac agtatatagt ggtattgaaa atgataaagt
37921 tacaattttg attggaaatc ggattcgcag ttatactaag gtagttaata tttgtaacaa
37981 ggagtcactt gttaccttgt caagagataa tattacttct aatgcaatga gaataaagtt
38041 ggaaggaaat aattttgata aaaagttatt ggaaaaattt caagaaattg atagtaatag
38101 taaaagaagg aatactatta tttcgcttat aaaagctatt gaattagcta agaatactaa
38161 atataaagat gccttaaaaa tagtcgaatc gatttataaa gatgaaaata atcctaaaaa
38221 aatagctctt aattctctaa taaatatgat taataaatat gataaatacg gtaatggtac
38281 attaatgaat ttctacaata ctttatgtaa aactttagat attaaacttt cagggtttag
38341 aaaggggcaa gtaaaagatt tttacgaaaa cactttatat aaaaatatgg tgatatgtac
38401 aaatatcgtt gaagacacaa gtaatcatat tactatccac aaagcgaaag gcgcagaatt
38461 cgaaaatgta tttgttatag gtaataaaga cactttaagt ctacttttga aaccagattt
38521 aatgaacgat gaagagcatc gtatattcta tgtagctatg agtcgagcaa agaagaaact
38581 ttttttacaa tttgattatc ttgataataa tgaacaaaag aaaattaagg aacgttatga
38641 tgtaagtatt atacaataac ctaatctaag aatatttaaa tccaatatat ttcttgatta
38701 taattcaata atagataaaa tctataaacg attaaaaaga aaatatattt gctaagactt
38761 ataaaaatga agagataata gattttccta aattacagtt aaaagaagta aatagattga
38821 acactttgaa agattctaat ccaaaatttg aaccactaaa attgaaagat gaagaagatg
38881 caagatgtag ccatcaacgc tgatcacaaa gtatttttct taatgattat ttttaatgtt
38941 atcatgctcg tttcttctat tattcaaatg attttaggaa aaggcagacg tatcacacca
39001 gcaaaaccta ctaaagatgc taagttacac ttagataatc aatcctcata aagagaaaaa
39061 agaacccccta aaaagttcaa ctttttgggg gttacttact attaagaagt atttttgatt
39121 aagcatcttt catatcgtaa tcaccaatat actgatcaag gcgatttatt gtttctctga
39181 tatcatcatg tgtcgttaat gggttcgttg tacataaacg aatgacacgt tggttattca
39241 atactgtggt ataagcaatg gcataaccag aatctgcaat tttttgcgca gccatgctat
39301 tgagttgatt attttgtgct tcagttaaat cactgttttc atatctgaag ttcacaatgg
39361 aaagattagc gtgtgagata atacgccagt tatcgagacc tgacacgtat tcttctgcat
39421 attctgctaa accttcgccg tatttaatgc gctcgataat ttcatcttca ccaatgactt
39481 gcagagtaat ccataatttt aaagcacgtg cagggcgtgt aagttcaatg cctaacattt
39541 ctggatcaat cacatcatca tctgaagcaa tatcatccaa gtactcagca tcttcgccaa
39601 agctttgcag cagatgattc ttttctttca cgattaccat agcgcaacta tatgtttgga
39661 acagtaattt gtgagcatcc caactggcac tatctgaacg ttcaatgcct ttaaataagt
39721 gttgtgcttt atcggataag atatgtgata gtccataagc accgtctaca tggagccata
39781 gattgtagtg atcacatgta tctccaagtg tggtgaaatc atcgacagaa cctgtattag
39841 tcgtgcctgc agtcgcaatc accatagcag gtttgtaacc ttcttcaata tctttatcaa
39901 ttgcggcttt taagctatct gtattcattg taaagtcatc gttgtactca attctgcgga
39961 tattattttt aaggaagcct gcaacatgca aggctttgcc tacagaatga tgcgtttgag
40021 atgtaagata aacggttgct tttttaatat cttccatttc tacctgtgca tctctagccg
40081 caacgatagc tgttaaattg gccattgaac cgccagatac aaagacacca ccggccggtt
40141 tgatctcata accgattttt cctaccaagt agttaattaa attacgctca atattaattg
40201 gcagcgtcgc attggcaaaa ttagaggcat ggatattatt tgctgttgtt aaaatgtctc

40261 caagccatga tagacgtgat gctggacctg gtataaatga gaatgaacgt ggatggttcg
40321 gacgatataa ataattcagt acttcattat ttaaatcttt gagtacttca tagatatctc
40381 gccctttatt cggcacttcc atcttttcat atttctctct caattcgagc ggtgcctgtt
40441 gtgtcgcagg taagtctcca atatctctag ataaatattt attaacttca tctctgataa
40501 tcgtctctaa gtcgatattg ttctcattga attccatttc catactctct ccttcaatta
40561 tatttttaat aaaagacaga cgtattaatt acgacgtttt actacaatat aaatgatagc
40621 agatattaat aaaaccacgc taaccataat gacgaaaccc agaaggtgtc gccccaatga
40681 ataacgtgct gtgctttaac actcggtgta tgtgcgcctt tcagaaattg acgagggtaa
40741 tcgacttgca gattatcatc tgtgattttc aatttgtatt gatgcttgtc ttttggaatt
40801 cgataactac cccgtagaac aggggacgag gacctcatct tgatgatccc aattggcttg
40861 gttaaaaaga aatcgataat cctcgtagaa tagagaaaaa gtattaaaaa tatatttgat
40921 ttgataaaat attttgcaaa aaagagaaac aaaaataact ttcatataaa taatttcttt
40981 gcgattaagt tggaattaga aatactaaat acaaataaca gcgaatggaa tgcatattat
41041 tgaggaattg gatgtgtagt gcaatttcga gagttactac ttaacttaag aaaataaaaa
41101 gaagaaaaca aacaataaga attaaatctg aagcaagata taaaagcaaa tatttttgct
41161 acaattatat tagtaaatac tttattaatt agcatacgac tgaaagcgaa gtaacagcta
41221 ataatgttcc agatatagac attagtgtta tgaaagaaca atattaccat ctagaaaatg
41281 taatttttaa aaacctgaag ataaagtaga ctattctgtg agttttcatg ttaagaattc
41341 tgaacttggg gatgtagtat aaatatggtt tagtagaaaa tgacgctgga gctgtagctg
41401 agattgacta tcaagaatta actaaagtag attattaccc atcaacaaaa atccaagatt
41461 taacactatc taataatagt gcgaataatg aagtttaaaa gtgaaaaact gataatagcg
41521 aatcaaacta aagtaatcaa ttacagaatt caagtactac tcaagcctca aacaaatatt
41581 gaaactcaaa cacaagttaa gaataataca ctgtcagaaa cagatgaaca gtctaatgcc
41641 gatttagtcc agtaatagcc tctgtattat tagccgcacg attattatca ataattaaat
41701 aaagttaata ttaaataata aaaatccttc aaattatttg ttgaaaaaat aataactttg
41761 aaggtttttt agcaaaatca ctgataggga gaagcgtatc ataaat
//
LOCUS Staphylococcus 41724 bp DNA linear UNK
DEFINITION Staphylococcus Staphylococcus aureus subsp. aureus M013
ACCESSION Staphylococcus
KEYWORDS .
SOURCE Staphylococcus Staphylococcus aureus
ORGANISM Staphylococcus Staphylococcus aureus
Bacteria; Bacillati; Bacillota; Bacilli; Bacillales;
Staphylococcaceae; Staphylococcus; Staphylococcus aureus.
FEATURES Location/Qualifiers
source 1..41724
/project="hwwang_703339"
/organism="Staphylococcus Staphylococcus aureus"
/genome_id="703339.343"
/genome_md5=""
/db_xref="taxon:703339"
/mol_type="genomic DNA"
CDS 1..480

/transl_table=11
/db_xref="SEED:fig|703339.343.peg.1"
/product="23S rRNA
(pseudouridine(1915)-N(3))-methyltransferase (EC
2.1.1.177)"
/translation="MKITILAVGKLKEKYWKQAIAEYEKRLGPYTKIDIIEVPDEKAP

ENMSDKEIEQVKEKEGQRILAKIKPQSTVITLEIQGKMLSSEGLAQELNQRMTQGQSD

FVFVIGGSNGLHKDVLQRSNYALSFSKMTFPHQMMRVVLIEQVYRAFKIMRGEAYHK"
/EC_number="2.1.1.177"
CDS 726..1031
/translation="MKGNIQCPRLIKGSLLDFVLETVDVFVNGSKNDCELVNFNYKEG
KVVSRYSTKFESNPKLRKKQLISTVKVVRYVIFDFEKILWGIGKVLLKFIILRQCLL"
/transl_table=11
/product="hypothetical protein"
/db_xref="SEED:fig|703339.343.peg.2"
CDS 1230..2093
/db_xref="SEED:fig|703339.343.peg.3"
/product="hypothetical protein"
/transl_table=11

/translation="MKHINQFSESTLAQLEKDQQHVFYVYCLMDPRNDECFYVGKGKG
NRIFKHKQDAQKKVLYEDIFIEENKDNLKFNRINEICSNDLNVLGYIISYGLTESEAF

SAENVLINFLNLTNKTTLTNMINGHGSKAYLVEDLENEFGYDSINLENINTNELILAV

KIRDAFLLDKDESKEYPINESKRDRNNLKSRTLGSWIIGKDKIHKIKYIIGINTGANN

AVVSAYEVSFEQAESIETNNGRMRYAFIGLSERDATLKKLNLYKKALPDLRFGSGSAT
AYINNGTMKVD"
CDS 2201..2893
/translation="MPSDDIVKKLYSKEDIRLELGLTPHKFNKKMETIAKLFKIDMKI

FHNYKGQDKNNQYTFNGVAKELIKVLLKSVDYYPVDINSKKFKQNGKSKKEMIENIDN

SSYMKYIYQLMKSINEIQYKRLIADIHMKDVYQNTKAWLNNGESINKKEQELYQYMTI

LPLHKRVELQNEVLKSIDETIFQFLAKEHRNNQIEENNELEAYTKAIKEGRNPKNDYE
LNHLLYKKINYP"
/db_xref="SEED:fig|703339.343.peg.4"
/product="hypothetical protein"
/transl_table=11
CDS 2965..3675

/db_xref="SEED:fig|703339.343.peg.5"
/product="hypothetical protein"
/transl_table=11
/translation="MLKRSQRADSNFIESLEKKNKLRKNIHKDSIKSISKLIDHELVK

NNRKWDVAEFYKNQQFWNNSTLRRSSQNFGILYYKKNRLHTMNQSNIIKKIKIIEANI

ESANKQPSYLDFFLQAKFDLERLESELMKYSINPKYFNKINSDYVRYAIEDVHNAIIK

IDRYISNDKVKLNYSNVQMVVRNEITEQGSYFVTQALNTLSKVEESGYSFDNFMVGPF
VEDFKKAIKNRRFGSKKD"
CDS 3901..5001
/db_xref="SEED:fig|703339.343.peg.6"
/product="hypothetical protein"
/transl_table=11

/translation="METRDKLMSLTQSDKTQQWLMDKSSNQDDIQQLQQQFSQQLDQQ

YNALLADEKAKLDQYVEVHQGLESLKEEIESEPITLNIDKLPDIKATMLERAKNDEHS

DKIEKLFDRLEQALNGTNRLYTQLSLIGTRTHRITTKNFNLQGLPKAVQHTILPSKFK

KVYTVDFKSFEPSVAAYMTQDSKLIDLLNQKDGLYDALLSELGLSDELRVFVKRAFIG

SFLFGGNFKNPKFKLNQYVSEVQWLDAVSQFTKVIELKKHVEKSNSMPMPYSIEHDMS

AFQGSSIMAIYVQTVASYIFKHILLKVYKAQCDQKTFKIIVPIHDAIMIECEDEEIAQ
NVGQLMKDTANQLFNGEFAHVTVEEIGGVDHE"
CDS 4994..5365
/product="hypothetical protein"
/db_xref="SEED:fig|703339.343.peg.7"
/transl_table=11
/translation="MNNDRGQSLHIPSSTPIKENNIYVATLHSVIQTDFSGEIKHQFT

YEIEVNNQIVYANRNIPTKPSANQLSIHDWLKRHSNYSASHENYEPYIDQKHLILLGQ
YNGNYYVQDVASLDAFGGVLS"
CDS 5362..7005
/translation="MNHILQMLSKLLSVAKEAIDRQGLIAILTIPVNNNDEIEETAQG

ETVYNELIDQLRLNIPKDTDYQPNIYSYFGIKKNPNDTVLMEMMIKVFHIKRFNSELF

IFKVNGWQKINGDELQGLISKMIQVLLVDYKPSLSTLKNVVDGLQKSTDVEELVENER

YIGCGENMFDLNTFQVVKNSIDIFPKTRLNLSLSTNDVITDKIPPYFKQYMLQLANYD

DDLQYFLFQHTAVLLTADTKYRRGLILYGGAKNGKSVYIELVKSFFYSKDIVSKPLNE

LEGRFDKESLIDKSLMASHEIGQSRIQEKIVNDFKKLLSVESMHVDRKGKTQVEVILD
LKLIFSTNAILNFPPEHAKALERRINIIPCEYYVEKADTSLIDKLQSEKKEIFLYLMY

VYQQIVKADIEYLENSRVTEITHDWLNFGYEFVSSRSVSNANQKACINLLRKLIEIKS

GSRIKVSELNKVINEEIKVSSQVIKQLIQANFDTQTKLYNGYDYWIDLGWKEANKKEI
HDISEKDNIISLDKNENITDDEALDEENLDFDWEDFDDE"
/product="putative primase"
/db_xref="SEED:fig|703339.343.peg.8"
/transl_table=11
CDS 7231..8907
/translation="MKGKIALYSRVSTSEQSEHGYSIHEQEQVLIKEVVKNFPGYDYE

TYTDSGISGKNIEGRPAMKRLLQDVKDNKIEMVLSWKLNRISRSMRDVFNIIHEFKEH

DVGYKSISENIDTSNASGEVLVTMFGLIGSIERQTLISNVKLSMNAKARSGEAITGRV

LGYKLSLNPLTQKNDLVIDENEANIVREIFDLYLNHNKGLKAITTVLNQKGYRTINQK

PFSVYGVKYILNNPVYKGYVRFNNHQNWAVQRRSGKSDKNDVILVKGKHEAIISEEVF

DKVHEKLASKSFKPGRPIGGDFYLRGLIKCPECGNNMVCRRTYYKTKKSKERTIKRYY

ICSLFNRSGSSACHSNSINAEVVERVINVHLNRILSQPDIIKQIASNVIEELKQKHSN

QTEIKYDIDSLEKQKAKLKTQQERLLELFLDDQMDSEMLKAKQSQMNQQLEVLDQQIK

EAQQANQSQDEIPNFDKLKGRLILMITRFSVYLRKATPEAKNQLMKMLIDSIEITTDK
QVKLVRYKIDESLIPQSLKKDWGSFFMPKFQFEIYGQNDYFIDQITTFTT"
/db_xref="SEED:fig|703339.343.peg.9"
/product="Cassette chromosome recombinase B"
/transl_table=11
CDS 9013..9351
/translation="MTLEQQLKHYITNLFNLPRDEVWHCESIEEIADDILPNQYVRLG

PLSNKTLQTNTYYSDTLHESNIYPFILYYQKQLIAIGYIDENHDMDFLYLHNTIMPLL
DQRYLLTGGQ"
/db_xref="SEED:fig|703339.343.peg.10"
/product="FIG01108299: hypothetical protein"
/transl_table=11
CDS 9447..9758

/translation="MNRYITRGIANNLPNILQHQLWQLVSEREQEQTKDNTSVDYFHI

FQFNKHRNQLYIKHKQERPEYAKIHKANYSKAININKVYIIREDDVDLSYYVMLLPEE
Y"
/transl_table=11
/product="FIG01107894: hypothetical protein"
/db_xref="SEED:fig|703339.343.peg.11"
CDS 9774..10280
/transl_table=11
/product="hypothetical protein"
/db_xref="SEED:fig|703339.343.peg.12"
/translation="METIKSTLKTEAIFSDDKQHRYLLKKTWNSEKQSITIITMYPHY

DGILNIDLTTQLIMNKVSEMDAFGSINFVNLYSNITTPINLKHLENAYDKHTDIQIMK

AVKESDEVILAWGAYAKKPGVEARVNEVLEMLKPHKKKVKRLMNPETNEIMHPLNSKA
RQKWILKV"
CDS complement(10429..11103)
/translation="MNYFRYKQFNKDVITVAVGYYLRYTLSYRDISEILRERGVNVHH

STVYRWVQEYAPILYQIWKKKHKKAYYKWRIDETYIKIKGKWSYLYRAIDAEGHTLDI

WLRKQRDNHSAYAFIKRLIKQFGKPQKVITDQAPSTKVAMAKVIKAFKLKPDCHCTSK
YLNNLIEQDHRHIKVRKTRYQSINTAKNTLKGIECIYALYKKNRRSLQICGFSPCHEI
SIMLAS"
/db_xref="SEED:fig|703339.343.peg.13"
/product="Transposase for insertion sequence-like element
IS431mec"
/transl_table=11
CDS 11361..11528
/EC_number="2.3.3.10"
/product="Hydroxymethylglutaryl-CoA synthase N-domain
homolog (EC 2.3.3.10)"
/db_xref="SEED:fig|703339.343.peg.14"
/transl_table=11
/translation="MLESREQLSVEEYETFFNRFDNQEFDFERELTQDPYSKVYLYSI
EDHIRTYKIEK"
CDS 12404..13147
/EC_number="3.1.4.46"
/translation="MLTVYGHRGLPSKAPENTIASFKAASEVEGINWLELDVAITKDE

QLIIIHDDYLERTTNMSGEITELNYDEIKDASAGSWFGEKFKDEHLPTFDDVVKIANE

YNMNLNVELKGITGPNGLALSKSMVKQVEEQLTNLNQNQEVLISSFNVVLVKLAEEIM

PQYNRAVIFHTTSFREDWRTLLDYCNAKIVNTEDAKLTKAKVKMVKEAGYELNVWTVN
KPARANQLANWGVDGIFTDNADKMVHLSQ"
/transl_table=11
/db_xref="SEED:fig|703339.343.peg.15"
/db_xref="GO:0008889"
/product="Glycerophosphoryl diester phosphodiesterase (EC
3.1.4.46)"
CDS 13244..13672
/transl_table=11
/db_xref="SEED:fig|703339.343.peg.16"
/product="MaoC domain protein"
/translation="MKYDDFIVGETFKTKSLHITEEEIIQFATTFDPQYMHIDKEKAE

QSRFKGIIASGMHTLSISFKLWVEEGKYGEEVVAGTQMNNVKFIKPVYPGNTLYVIAE
ITNKKSIKKENGLVTVSLSTYNENEEIVFKGEVTALINNS"
CDS complement(13718..15724)
/product="Penicillin-binding protein PBP2a, methicillin
resistance determinant MecA, transpeptidase"
/db_xref="SEED:fig|703339.343.peg.17"
/db_xref="GO:0008658"
/db_xref="GO:0046677"
/transl_table=11
/translation="MKKIKIVPLILIVVVVGFGIYFYASKDKEINNTIDAIEDKNFKQ

VYKDSSYISKSDNGEVEMTERPIKIYNSLGVKDINIQDRKIKKVSKNKKRVDAQYKIK

TNYGNIDRNVQFNFVKEDGMWKLDWDHSVIIPGMQKDQSIHIENLKSERGKILDRNNV

ELANTGTAYEIGIVPKNVSKKDYKAIAKELSISEDYIKQQMDQNWVQDDTFVPLKTVK

KMDEYLRDFAKKFHLTTNETESRNYPLGKATSHLLGYVGPINSEELKQKEYKGYKDDA

VIGKKGLEKLYDKKLQHEDGYRVTIVDDNSNTIAHTLIEKKKKDGKDIQLTIDAKVQK

SIYNNMKNDYGSGTAIHPQTGELLALVSTPSYDVYPFMYGMSNEEYNKLTEDKKEPLL

NKFQITTSPGSTQKILTAMIGLNNKTLDDKTSYKIDGKGWQKDKSWGGYNVTRYEVVN

GNIDLKQAIESSDNIFFARVALELGSKKFEKGMKKLGVGEDIPSDYPFYNAQISNKNL

DNEILLADSGYGQGEILINPVQILSIYSALENNGNINAPHLLKDTKNKVWKKNIISKE

NINLLTDGMQQVVNKTHKEDIYRSYANLIGKSGTAELKMKQGETGRQIGWFISYDKDN
PNMMMAINVKDVQDKGMASYNAKISGKVYDELYENGNKKYDIDE"
CDS 15972..16115
/transl_table=11
/product="Mobile element protein"
/db_xref="SEED:fig|703339.343.peg.18"

/translation="MNYFRYKQFNKDVITVAVGYYLRYALSYRDMSEILRERGVNVHH
LTV"
CDS 16395..16646
/transl_table=11
/product="Mobile element protein"
/db_xref="SEED:fig|703339.343.peg.19"
/translation="MAKVIKAFKLKPDCHCTSKYLNNLIEQDHRHIKVRKTRYQSINT
AKNTLKGIECIYGLYKKNRRSLQICGFSPCHEISIMLTS"
CDS complement(16706..17134)
/translation="MKFQVSPYFLITQNAEGLKKLYEELFGAKTLYIQRIKDRPIDKK

LNIDENDLNKIDQCVLQFGDIKIMIADDTEGLPITQGNNISLCLTFDQVEETKHVYDQ
LIEKGSEVLKTFSPEFYTEGYGYVRDPYGISLHLFTKRKN"
/transl_table=11
/product="PhnB protein; putative DNA binding
3-demethylubiquinone-9 3-methyltransferase domain
protein"
/db_xref="SEED:fig|703339.343.peg.20"
CDS 17215..18144
/translation="MKKSVRLYNMIEYCNENRNFKLNDLMSEFNISRSTALRDIKEIE

ALGVPLYSNPGKNGGYTIIGNRDQTKIAISDEELKALVFTLSSISNVSNLPFQTEYQE
ILKKLYNNSNKKELINQYNDLFQYFNEDKYQFKSYKLFNEIIRLIIENKSFETCYSQN

YIKEQYKGIGIMYKNHQWYFVVANIESKLVNLLNISKIKELYEMGETQECNDITMQNF

QQFMVKNETAIDILIRSNVMGLNILKGYLWSDYMIENIDEETYLFKSKVNAKDIDFIA
KLIVTGGVNVKVETPNSLKNAVKVELTKIINMY"
/db_xref="SEED:fig|703339.343.peg.21"
/product="Transcriptional regulator, YafY family"
/transl_table=11
CDS 18306..18788
/transl_table=11
/product="Similarity"
/db_xref="SEED:fig|703339.343.peg.22"
/translation="MNKNITALKSTENTTYMQYTVHIDDQIINNIKECTKKFKILPME
DKIPLSPLLQPEYAGEVQDFISTYEQFMINFGKVILDSQGIKIQFESESLSSIQRGIQ

EHCYLNERTNDIDVTKEWYLCKFSNQILEEDKNQLYNALKQLMNDSKNKKQAFWTDPN
"
CDS 18856..19170

/translation="MKRVSYSVETKYKAVEMKIAGFSTKEIMKELNIKNRTQVETWWR

WYRNEESYRFSQHVGKQYTYGKGLEELSEIERLKLENKRKDIELDILKKYKALERKWY
QQ"
/transl_table=11
/db_xref="SEED:fig|703339.343.peg.23"
/product="Mobile element protein"
CDS 19155..19997
/transl_table=11
/db_xref="SEED:fig|703339.343.peg.24"
/product="Mobile element protein"
/translation="MVPAVVIDLVDQLKVKYSIKLILEVLNIPKSTYYRWKNKTYKND

TVTQKVIELCEANHYTYGYRKITALINQCYTSPINHKRVQRIMQKHHLNCRVRPKKTT

RIGKPYYKTDNLLQRQFKASCPMEVLTTDITYLPFGHSMLYLSSIMDIYNGEIVAYKI

DDKQDQSLVNDTLNQIDIPEGCILHSDQGSVYTSYAYYQLCEEKGIIRSMSRKGTPAD

NAPIESFHSSLKSETFYINNQLNSSNHIVIDIVEKYIKNYNNNRIQQKLGYLSPVKYR
ELAA"
CDS 20004..21569
/db_xref="SEED:fig|703339.343.peg.25"
/product="Similarity"
/transl_table=11
/translation="MFLLSSRLRGSVPFQEVFKVHIDIYLYNKEKSEYKYQVSSYFNL

VKENPKITYKKRHLQEKQGVKGTTFTNKLKLLNKMYGVDVAKYQPFYNSNNPEYERGQ

FGERYISQRSNYEFNRLQYQIIDMLSKILDKHPLPKSDNNYKHIPTIEKAILSGDSHS

FYEYFEDIMKEIISMENSSLKEKLLTDFTYQSQCRWYSESEKLKLQLESFMHKVLESN

YYEGNKLYRMLSHAIEETINEADEDKVHFNYFKDYFLTDGGVKNWEQISEKITEFNGK

VINDIQNEYNKIQFNNANRNQKLNFNYLYHCFEFSNNLVKARVNGNRGYILYFVDKYK

IKMPFLDQLLINYLNQEIGQESINYNMQTLFEKERYDRSSTIEKLVATSKFKYEKDDS
DLFKQLFNDVENSIDRLGIYLLNNGINSNDENARYYRSFLKELSRIKSKLTPFSLEIS

KSSGREQHYPDDAIDDKDERRKNKEETYHAFDDKSDIDSKLKNKINVSIDNLLSVKIK
SNLYDISPNTKKQ"
CDS 21764..22873

/translation="MYQANIRDLITKLPQSNKTEHFLMNKFSNQDKVQQLQRQISQQL

DQQYNELLANEKAKLDQYVEVHHNLEPLKKEIESESINLDTDKLPDIKATMLEKAKND

EHFDKIEQLFDRLDQSLNGTNRLYTQLSLIGTRTHRITTKRFNVQGLPKLVQQMILPS

QFKKVYTIDFKSFEPSVAAYMTQDEQLIDYLNHEEGLYDALLRDLSLSKEKRVSVKRA

FIGSFLFGGRYSSSKFKINQEVSEINWLQVMSKFKKVIEFKEQVEKYKTMPTPYGIEH

DMSAFQGSSIMAIYVQTVASYIFKHILLEVYKAQCEKKTFKIIVPIHDAIMIECNDKG
IAQNVAQLMKDTANQLFNGEFAHVTVEALGGIDNE"
/transl_table=11
/product="hypothetical protein"
/db_xref="SEED:fig|703339.343.peg.26"
CDS 22866..23234
/product="hypothetical protein"
/db_xref="SEED:fig|703339.343.peg.27"
/transl_table=11
/translation="MNNDRGKSLQIPQSTLLKEGSIYVATLHSVYEKNFSGDIKHQFT

YEVELNQETHYVNRNITVKSMSHQLSIADWIKRHSNYNVNHINYDPYIDRKHLVLVGQ
YNGNYYIQDVAPLDEFGGVL"
CDS 23234..24850
/db_xref="SEED:fig|703339.343.peg.28"
/product="putative primase"
/transl_table=11
/translation="MNHILEMLIKLLKVGMEAIDRKGLIAILTSSIGNDEMDDSEQAV

MVYNELIDKLQLNIPKDVDYRPNIYSYFGIQKKPNDTILVEMMICIFHIKRFDSELFV

FKDKGWQKVSEDELQGLISKMIQVLLVDYKPSLSTLKNVVDGIQKSTDIEKLVEHRQY

IGCGRNMFNLKTFKVVDNDLEIFPKTRLDLELDINDTITDKIPPNFKQYMLELANYDH

DLQYFLFQHMAVLLTADTKLRRGLFLYGTAKNGKSVYIKLVKSFFYSNDIVSKTLNEL

GGRFDKESLIGKRIMASDEVGKANIDEATVNDFKKLLSVEPIHADRKGRTQVEVTLDL

KLIFNTNAVLNFPSSHAKALERRIAVIPCEYYVEKADPDLIEKLQDEKKEIFLYLMYV

YKQIVKNDIEYLQNDRVTEISHDWLNFGYEFVSSKSANIAHQKACINLLRKLIEIKPG

SRIKVSRLNEVIRDEIKVSSQVINDLVQANFNVQSRLNNGYKYWVDLGWKETDKKDDM
ISFDKNENVTDDEFLYEDDLNLGWEDFDDE"
CDS 25075..26754
/translation="MKGKIALYSRVSTSEQSEHGYSEKEQEQLLIKEVMKNFPGYDYE

TYTDSGISGKNIEGRPAMKRLLQDVKDNKIEMVLSWKLNRISRSMRDVFNIIHEFKEH

DVGYKSISENIDTSNASGEVLVTMFGLIGSIERQTLISNVKLSMNAKARSGEAITGRV

LGYKLSLNPLTQKNDLVIDENEAHIVREIFDLYLNHNKGFKAITTILNQKGYRTINQK

PFSVFGVKYILNNPVYKGYVRFNNHQNWAVQRRSGKSDKNDVILVKGKHEAIISEDVF

DQVHEKLASKSFKPGRPIGGDFYLRGLIKCPECGNNMVCRRTYYKTKKSKERTIKRYY

ICSLFNRSGSSACHSNAINAEVVERVINVHLNRILSQPNVIKQIASSVIEELKQKHSK

QTEIKYDIDSLEKQKAKVKTQQERLLELFLDDEMDSEMLKAKQSEMNQQLEVLDQQIK

EAKQANQSQDDIPNFDKLKARLILMITRFSVYLRKATPEAKNQLMKMLIDSIEITTDK
QVKLVRYKIDESLIPQSLKKDWGSFFIPKFNFVINVTKKNRIENLSLLPLF"
/transl_table=11
/db_xref="SEED:fig|703339.343.peg.29"
/product="Cassette chromosome recombinase B"
CDS 26843..27181
/product="hypothetical protein"
/db_xref="SEED:fig|703339.343.peg.30"
/transl_table=11
/translation="MTLSKQLKTYITERFKLNYQETWACETIDAVAEDVLPEKYIKNS
PLEHKILNTFTYYNDELHEISIYPFLCYLDKELVAIGYLDNFDLDFIFLNDTHQVIID
ERYLLQKGGE"
CDS 27184..27273
/transl_table=11
/db_xref="SEED:fig|703339.343.peg.31"
/product="hypothetical protein"
/translation="MNWIKVAQLSVTVINEVIEIMKEKQNGGE"
CDS 27275..27586
/product="hypothetical protein"
/db_xref="SEED:fig|703339.343.peg.32"
/transl_table=11

/translation="MKINRYITRGVSEHLSLDLQILLWNMVKDRDNQLHTDYLHIFKL

QEDDNILSITHEQEQPAYKLEYHYTNYVKNQNALPKKVYVIREDDVDAFYYVMLLPEE
Y"
CDS 27601..28104
/translation="MNTIKNTIHTEAIFSDDEQHRYLLKKTWDEKKPACTVITMYPHL

DGVLSLDLTTVLILNQLANSEQYGAVYLVNLFSNIKTPENLKHIKEPYDKHTDIHLMK

AISESDTVILAYGAYAKRPVVVERVEQVMEMLKPHKKKVKKLINPATNEIMHPLNPKA
RQKWTLK"
/transl_table=11
/product="hypothetical protein"
/db_xref="SEED:fig|703339.343.peg.33"
CDS 28118..28339
/transl_table=11
/db_xref="SEED:fig|703339.343.peg.34"
/product="hypothetical protein"
/translation="MNHETKQSDWRTVASCLASQNYISIVKGLVHHFTAIEDEEILDK
IYDDFMNDDSITTVLNNDLQTIINHYLSK"
CDS complement(28414..31533)

/translation="MNFQFNEDDLEQVALEWLQSLGYDYKKGNEISMTSLTPERKSDK
DVVLHERLEKALRKINSDIHPRFIEKALHELTLEKSPNLLENNLTFHENLINGIEIED
YDDEGQSIVEIVKIIDFEYPQNNDFLAVNQLTIVNGDYKKRPDIVLFINGLPIVVIEL

KNSTNETVGVEDGYHQLETYKMRIPQLFTFNEVLVTSDGINTKAGSLTANYDRFMTWR

SKDGQTESSPGLASLDVLIHGMLNPETLLDLIRYFVLFQDDGKGHISKILAAYHQYYA

VNKAVDRALLASSEQGDGKGGVIWHTQGSGKSLTMVFFSGKLIQMLNNPTLVVVTDRN

DLDNQLYSTFVKSKGRSGKGLLRQTPKQAETRKELKSLLSVESGGIVFTTMQKFEPEQ

NETTMAALTERKNVIVMADEAHRTQYGFNAKYDDKGEGIKYGYAKYLRDALPNATFVG

FTGTPVASTDKNTQMVFGNYIDVYDMTQAVADGSTVKIYYESRVIPLNLPQSLDLDEA

YNDITEDQEEDVKQRLKSKWSRIEALAGAKPRVEALAKDIIQHFETRQQAMKGKGMIV

TMSRRIAVDLYDEIIRLKPEWHSDDDDKGVIKVVMTGSSSDPTSFQRHIGPKKRRNLL

EKRMKDVDDELQLVIVRDMWLTGFDVPSMHTMYIDKPMKGHNLMQAIARVNRVFKDKP

GGLIVDYVGIAESLKEALKEYTESDQAQTAIDTDKAVELMLLKYDVIQDMLYNLDYSK

FNSDKKSERYYTISNTMDYVIGLGEDERQRFIKTVTELGKAFALCATEPTAQELNDEI

AFFKAVKAGLVKLLQPPKEGKTRKTPAEVEAEINQLVSQSVVTEDVIDVYQTLGLEQP

DLSILSDDFLKDVEGLKQKNVAVELLNRLLKGQVKSLMKTNATVSKRFSEMLGNSINK

YNSRSIETSKVIEELIQLAKDIKQEQQRGNELGLNSDEIAFYDALASHETAKEAMGDK

ELRAIAHELTKTVKENMGVDWSKRDSAKAKMRVAVRRLLKKYGYPPDLQKMAVEQVVE
QAELMANNQ"
/transl_table=11
/db_xref="SEED:fig|703339.343.peg.35"
/product="Type I restriction-modification system,
restriction subunit R (EC 3.1.21.3)"
/EC_number="3.1.21.3"
CDS complement(31517..32818)
/db_xref="SEED:fig|703339.343.peg.36"
/product="Type I restriction-modification system,
specificity subunit S"
/transl_table=11
/translation="MEYNTIFFGDVAKIKNGYAFKSKEFQEKGIPVIKIKNIISPIVD

TKDSQKVSIKTYEKTKGFSLKKNDILISLTGSGVNQMSSAVGKVGRIEFDYPALQNQR
VGKFELKYSNSADLDFLFYYFLQPKITEYLVRNSTGSANQANINSKLIETVKIPNFSL

IKQKSISKFLNQITRKIETNQKMIANLKELSQTLFKHWFVDFEFPDEDGNPYKSSGGE

MIDSELGKIPSNWKIYKLKDIASHKKETFNPKKSEEVTVKHFSLPAYDNEEQAIEEEV

NKIKSNKWIINNNCVLFSKMNPDTKRIWLPVIDNKKLNVASSEFVVMESPNNKINSFI

YNICLNSQFIDYLKANTTGSTNSRQRVKPTIAVNYKLAIEDSIVKKYSEIITPYMEEM
KILRSEIGKLTQLRDTLLPKLMSGELEISDDIEVNSDELSI"
CDS complement(32808..34322)

/translation="MATIGFEEKLWQAADKLRGSMDAAEYKNVALGLIFLKYVSDSFE

EKYEELKQDPYADEEDQDEYLAENIFWVPKEARWQYINDNAKKPEIGQIIDKAMIAIE

NENESLKGVLPKEYARPALDKEKLGDIIDLFTFKVGDTESRKQDVLGRVYEYFIAKFA

SAEGKNAGEFYTPSSIVKLLVEMIEPYKGRIYDPCCGSGGMFVQSERFVEKHQGRLDD

IAIYGQESNPTTWKLAKMNLAIRGIDNDLGERNADTFHNDLHKGLKADYILANPPFNA

SDWGQERLLDDYRWQFGIPPKGNANYAWIEHMISKLAPNGTAGFVLANGSMSTSGKDE

LEIRKNLIEQDLVECIVTLPGQLFYSTQIPVCLWFISNNKGQNGKKERENEILFIDAR

EIGHMVSRTLKEFSDEDIQKVAQTYHAWRGTNDKPYEDIAGFCKVANLEEVKNNEYIL
TPGRYVGLADVEEDEEPFEQKMERITSELSEQFAKSKELEDQIRKSLEGLGYGV"
/transl_table=11
/product="Type I restriction-modification system,
DNA-methyltransferase subunit M (EC 2.1.1.72)"
/db_xref="SEED:fig|703339.343.peg.37"
/EC_number="2.1.1.72"
CDS 34518..34664
/transl_table=11
/db_xref="SEED:fig|703339.343.peg.38"
/product="hypothetical protein"
/translation="MDGVTNVKIHILTFVKIVVIVDIVAIVNTRLDKLGIFNSINKKF
KCSQ"
CDS 34764..36827
/product="FIG131328: Predicted ATP-dependent endonuclease
of the OLD family"
/db_xref="SEED:fig|703339.343.peg.39"
/transl_table=11
/translation="MYISSVKIKNFRNFDNIDIDFHEGVNVLIGHNNSGKSNLLRALS

LIFDGSVRKQLSVEDFNNSLTKESLKKEAPKIVISVHITQSENERLMSDELITVSNWL

TKLEEPYEARIQYEFFLPKDEEKNYIDLVKNIDEKEEIWNLIRSQFIRLYVNKIWVGN

PEHQIPIDNDSLNKFDFQFLDAIRDVERDMFSGKNTLLKSVIDFFIDYDIKSDETITE

EEQKEKLEERRKEFSDNSSDLIETIHKRLDSGNQKILSYTNGIGASYDKSTPDFKGNL

TESEIYTVLQLIIKHETGMTLPITHNGLGYNNLIFMALLLSKMQADSDGNFLGSNAKV
FPILAIEEPEAHLHPTMQNEFIKFLKNNIREKKVKQIFITTHSTHISSSTNIDDIICL

YTDDSKTNVSYPGKVFDKNNQSKKYVQRFLDATKSNMLFAEKVIFVEGIAEQLLLHIF

ADYLNKPLEKNHVAVINVGGRYFNHFLSLFDSNNKYAINRKVSCITDLDPMRKEKNEN

GQKNNFKACYPFEYEMDLDKFDYSTNNSLDKYLNNEHSNIRVFVQPNKYGKTFEYQLM

FDNPSLKLLLTDSISNSQELAELMDHYEENESLQKLIDTLSPSNENQRIIKSLKDTND

SWNEDNKKKALIASRYLNSIGKGENALELASVLKDNLELKGQSDYEDFVVPKYIEGAI
RWVCE"
CDS 36829..38577
/db_xref="SEED:fig|703339.343.peg.40"
/product="ATP-dependent DNA helicase UvrD/PcrA,
clostridial paralog 2"
/transl_table=11
/translation="MIINAQTKLNIEQDFKIQAGPGAGKTEFLVNHIKNVIQTSEKLE

RMKKIACITYTNTAAQTVLQRLGKSVSNRVDISTIHSFLYRNVVKPYCSFLPKEYGIC

VQKLKGHSDPIVINKYVNAWLKTEYFTKLKPPSNRNQLLKMPVLKKALQNWLLSMKLS

YENNDVQFICDNTKAQAIDKKSKKRMGINKSNLNILEKQLLNYKKIYWEKGIIDHDDV
LFFSYVLIRDYPFILTILCAKYPYFFIDEFQDTSQLQDFIIDKIRQKGCIVGVIGDKA
QAIYSFQGARVSLFEKFKVDLINSHTILENHRSSYQIVTFLNSIREDITQTVYSGIEN

DKVTILIGNRIRSYTKVVNICNKESLVTLSRDNITSNAMRIKLEGNNFDKKLLEKFQE
IDSNSKRRNTIISLIKAIELAKNTKYKDALKIVESIYKDENNPKKIALNSLINMINKY

DKYGNGTLMNFYNTLCKTLDIKLSGFRKGQVKDFYENTLYKNMVICTNIVEDTSNHIT

IHKAKGAEFENVFVIGNKDTLSLLLKPDLMNDEEHRIFYVAMSRAKKKLFLQFDYLDN
NEQKKIKERYDVSIIQ"
CDS 38796..38969
/translation="MQDVAINADHKVFFLMIIFNVIMLVSSIIQMILGKGRRITPAKP
TKDAKLHLDNQSS"
/transl_table=11
/product="Multidrug resistance protein"
/db_xref="SEED:fig|703339.343.peg.41"
CDS complement(39037..40461)
/product="Decarboxylase, pyridoxal-dependent"
/db_xref="SEED:fig|703339.343.peg.42"
/transl_table=11
/translation="MEMEFNENNIDLETIIRDEVNKYLSRDIGDLPATQQAPLELREK

YEKMEVPNKGRDIYEVLKDLNNEVLNYLYRPNHPRSFSFIPGPASRLSWLGDILTTAN

NIHASNFANATLPINIERNLINYLVGKIGYEIKPAGGVFVSGGSMANLTAIVAARDAQ

VEMEDIKKATVYLTSQTHHSVGKALHVAGFLKNNIRRIEYNDDFTMNTDSLKAAIDKD

IEEGYKPAMVIATAGTTNTGSVDDFTTLGDTCDHYNLWLHVDGAYGLSHILSDKAQHL

FKGIERSDSASWDAHKLLFQTYSCAMVIVKEKNHLLQSFGEDAEYLDDIASDDDVIDP
EMLGIELTRPARALKLWITLQVIGEDEIIERIKYGEGLAEYAEEYVSGLDNWRIISHA

NLSIVNFRYENSDLTEAQNNQLNSMAAQKIADSGYAIAYTTVLNNQRVIRLCTTNPLT
THDDIRETINRLDQYIGDYDMKDA"

CDS complement(40544..40756)
/EC_number="1.1.1.95"
/db_xref="SEED:fig|703339.343.peg.43"
/db_xref="GO:0004617"
/product="D-3-phosphoglycerate dehydrogenase (EC
1.1.1.95)"
/transl_table=11
/translation="MRSSSPVLRGSYRIPKDKHQYKLKITDDNLQVDYPRQFLKGAHT
PSVKAQHVIHWGDTFWVSSLWLAWFY"

CDS 41406..41573
/db_xref="SEED:fig|703339.343.peg.44"
/product="hypothetical protein"
/transl_table=11
/translation="MKFKSEKLIIANQTKVINYRIQVLLKPQTNIETQTQVKNNTLSE
TDEQSNADLVQ"

ORIGIN
1 atgaaaatca ccattttagc tgtagggaaa ctaaaagaga aatattggaa gcaagccata
61 gcagaatatg aaaaacgttt aggcccatac accaagatag acatcataga agttccagac
121 gaaaaagcac cagaaaatat gagcgacaaa gaaattgagc aagtaaaaga aaaagaaggc
181 caacgaatac tagccaaaat caaaccacaa tcaacagtca ttacattaga aatacaagga
241 aagatgctat cttccgaagg attggcacaa gaattgaacc aacgcatgac ccaagggcaa
301 agcgactttg tattcgtcat tggcggatca aacggcctgc acaaggacgt cttacaacgt
361 agtaactacg cactatcatt cagcaaaatg acattcccac atcaaatgat gcgggttgtg
421 ttaattgagc aagtgtatag agcgtttaag attatgcgtg gagaggctta tcataaataa
481 aactaaaaat tagattgtgt ataatttaaa aatttaatga gatgtggagg aattacatat
541 atgaaatatt ggagtatacc ttgcaatatc atacgatgtt tatagagtgt ttaataaacc
601 atttttcaac tattgatgat ctagaatata taataactgt acaaattata ttgattatgg
661 aactacaatt caattaagaa attgatgatg aaattttaaa tttaaactaa tggaatcaag
721 aaagaatgaa aggaaatata caatgcccac gattaataaa aggaagttta ttagattttg
781 tgttagaaac agtagatgtt tttgtaaatg gaagtaaaaa tgattgcgaa ttagtaaatt
841 ttaattataa agaaggaaaa gtagtttcac gttatagtac aaaatttgaa agcaatccta
901 aattaagaaa aaagcaattg atatcgacgg taaaagttgt aaggtatgtt atttttgatt
961 ttgagaaaat tttatggggc atagggaaag ttttattgaa gttcatcata ttaaggcaat
1021 gtttactata agaaagttga aatacactca gaaacagact ggattcccgt ttttttcaaa
1081 ttggcacaaa atgatacata ggcccaccta tatattgaag atttaagtta aacgataaat
1141 aatatttttc tcttttgtat agaaattttt ttgggaatga tattaattag tataaaaatg
1201 aatatgtcta caaagaagga gagattttat tgaaacacat taatcaattt tctgaatcga

1261 cactagcaca actagaaaaa gatcaacaac acgtatttta tgtatactgt ttgatggacc
1321 caagaaatga tgaatgcttt tatgttggta aaggtaaggg gaatagaata tttaaacata
1381 aacaagatgc gcagaaaaaa gtattgtacg aagatatatt tatagaagaa aataaagata
1441 acttgaaatt taatagaatt aacgaaattt gtagcaatga tttgaatgtt ttaggatata
1501 taatcagtta tggattaacc gaatctgaag cattttctgc agaaaatgtt cttataaact
1561 ttcttaacct aacaaataaa actacattaa caaatatgat taacggtcat ggatctaagg
1621 catacttagt tgaagattta gaaaatgaat ttggctacga ttcaattaat cttgaaaaca
1681 taaatacgaa tgaattaatt ttagcagtga aaatcagaga tgcatttcta ttagataagg
1741 atgaaagtaa agagtatcct attaatgaaa gtaaacgtga tagaaataac cttaaatcgc
1801 gtacattagg tagttggata ataggaaaag ataaaataca taaaataaaa tatattattg
1861 gtatcaatac aggtgctaat aatgcagttg tctcagctta tgaagtatca tttgaacaag
1921 cggagagtat cgaaacaaat aatggtagaa tgagatatgc gtttattggg ctttcagaaa
1981 gagatgctac tctaaaaaaa ttgaatttat acaaaaaagc actaccagat ttaagatttg
2041 gtagtggtag cgctacagca tatataaata atgggacaat gaaagttgat taaatgaata
2101 aaaacatcca attccttaaa ttagcctttg aaaattttat actcgtatat ataagtgaaa
2161 aacttaaaag aattagatgt taattaaagg agagaatgct atgccatcag atgatattgt
2221 aaaaaaacta tactcaaaag aggatattcg attagaatta ggacttactc cgcataagtt
2281 taataaaaaa atggaaacta ttgctaagct ttttaaaatt gatatgaaaa tttttcacaa
2341 ttacaaaggc caagataaaa ataatcagta tacatttaat ggtgtcgcaa aagaattaat
2401 caaagtgttg ctaaaaagtg ttgattatta cccagtggat atcaattcca aaaaatttaa
2461 acaaaatgga aaatctaaaa aagaaatgat tgaaaatata gataactcta gttacatgaa
2521 atatatttat cagttaatga aatctatcaa tgaaattcaa tacaaaaggt taattgctga
2581 tatacatatg aaagatgtgt accagaatac aaaagcatgg ttaaataatg gtgaatcaat
2641 taataaaaaa gaacaagagc tataccaata tatgacaatt ttaccattac ataaaagagt
2701 tgaactgcaa aatgaggtat taaaatctat agacgaaaca atttttcaat ttctcgcaaa
2761 agaacataga aataatcaaa ttgaagaaaa taatgaatta gaagcatata caaaagcaat
2821 aaaagaaggt agaaatccta aaaatgatta tgaattaaat catctcctat ataaaaaaat
2881 caattaccct tagatagtct aatagaagat atatgggact atgaggaaac agagtatact
2941 gagcttgatt ggttaattgc agatatgcta aaacgttcac aaagagctga tagtaatttt
3001 atcgaaagtt tagagaagaa aaacaaattg agaaaaaata ttcataaaga ttccattaaa
3061 agtatatcta agttgattga tcatgaatta gttaaaaata atagaaagtg ggacgtagca
3121 gaattttata aaaatcaaca attttggaat aatagtacat taagacgtag ctctcaaaat
3181 tttggaattt tatattacaa aaagaatagg ttacatacaa tgaatcaaag taatattatt
3241 aagaaaataa aaattatcga agctaatata gagagtgcaa ataaacagcc gagctattta
3301 gatttctttt tacaagctaa gtttgattta gagaggttag aaagtgaatt aatgaaatat
3361 agcattaatc cgaaatattt taataagata aatagtgatt acgtaagata tgctattgaa
3421 gatgtacata atgcaattat taaaattgac agatatataa gtaacgataa agtgaagtta
3481 aactattcaa atgtccaaat ggtagtgaga aacgaaataa cagaacaagg aagctacttt
3541 gtaacccagg cattgaatac actgtcaaag gtagaagagt ctggctacag ctttgataat
3601 ttcatggtag gacctttcgt agaagacttt aaaaaagcaa ttaaaaatcg tagatttggt
3661 tctaaaaaag attaagattg ttttttagag ttaaacataa gaataaattt aaataaaaaa
3721 ataaacccaa caaaccttgt tatatcaatg tttgttgggt ttattttttt ggtgattttt
3781 taataactca attacatctt aggataatat cgctaaatta tacattgtcg aggccaagac
3841 aaataaaaaa ttaaagcaaa agttagcgcg caactttctg ctataaagga gcaaatttat

3901 atggagacaa gagacaaact gatgtcattg actcaaagtg acaaaacaca gcaatggcta
3961 atggacaagt catctaacca agatgacatt caacaattgc agcaacaatt cagtcagcag
4021 cttgatcaac aatataatgc acttttagct gatgaaaaag ctaagttaga ccaatacgtg
4081 gaagtacatc aaggattgga atcattaaag gaagagattg aatcagaacc tattacgctt
4141 aatatcgata aattacccga tatcaaagca acaatgcttg aaagagccaa gaatgatgaa
4201 cattctgata aaatcgaaaa gctatttgat aggttagaac aggcattaaa tggtacgaat
4261 cgattatata cgcaattatc gttgattggt acacgaacac atcgaattac aactaaaaat
4321 tttaatcttc aaggcttacc taaagcagtc caacatacga ttttaccttc aaaatttaag
4381 aaggtgtata cagtcgattt taaatcgttt gaaccatcag ttgcagcgta catgactcaa
4441 gattcaaaac tgattgactt gttaaatcag aaagacggac tgtatgacgc attgctaagt
4501 gaattaggct tatcagatga gctacgtgta tttgttaaac gtgcatttat tggttcgttt
4561 ctatttggag gtaacttcaa aaatcctaaa ttcaagctga atcaatatgt aagtgaagta
4621 caatggttgg atgcggtcag ccaatttaca aaagtcattg aacttaagaa gcacgttgaa
4681 aagagtaatt ccatgcctat gccttatagt attgagcatg atatgagcgc atttcaaggt
4741 agcagtatta tggcaatcta cgtacaaact gtagcgagtt atattttcaa gcacattctg
4801 ctaaaagtgt acaaagcaca gtgcgatcaa aaaacgttca agattatagt acctatacac
4861 gatgcgatta tgattgaatg tgaagatgaa gaaattgcac aaaatgtggg tcagttaatg
4921 aaagatacgg ctaaccagtt gttcaatggt gaatttgcac atgtgacagt ggaagaaata
4981 ggaggcgtag accatgaata atgatagagg acaaagccta cacatcccaa gtagtacacc
5041 aatcaaagaa aataatatat atgtagctac gttacattct gtgatccaaa cagatttctc
5101 aggtgaaata aagcaccaat tcacgtatga aattgaagtg aacaatcaga ttgtatatgc
5161 gaatcgtaat attccaacaa aaccgagcgc taatcagttg tcaattcatg attggctgaa
5221 acgtcatagc aactatagcg caagtcatga aaactatgag ccttatattg atcagaaaca
5281 tttaattcta ttaggtcaat ataacggtaa ctattatgta caagatgtag catcgttaga
5341 tgcgtttgga ggcgtattat catgaatcat atattacaaa tgttatctaa gctattaagt
5401 gtggccaagg aggcaatcga ccgtcaaggt ctgattgcta tcctaactat tcctgttaat
5461 aataacgatg aaatagaaga aacggctcaa ggtgaaaccg tgtataacga acttatcgat
5521 cagttacgac ttaatatccc aaaagatacg gattatcaac ctaacatcta tagttatttt
5581 ggtattaaga agaatcctaa tgacaccgta ctcatggaaa tgatgataaa ggtttttcat
5641 atcaaacgct ttaattcaga actgtttatt ttcaaagtta acgggtggca aaagataaat
5701 ggagatgaat tacaagggtt gatatctaaa atgatacaag tattgcttgt agattataag
5761 ccttcactaa gcactctaaa aaatgtcgta gatggattgc aaaaatcaac agatgtagaa
5821 gaacttgttg agaatgagcg ctatattggt tgtggtgaaa atatgttcga tcttaatacg
5881 tttcaagtcg ttaaaaattc aatcgatatc tttccaaaaa cacgattgaa tttatcatta
5941 agtacaaatg atgtaattac tgataagata ccgccttatt ttaagcaata tatgttacaa
6001 cttgcgaatt atgacgatga tttacaatac tttcttttcc aacatacagc agtattactt
6061 acagctgata ctaaataccg taggggtctc atattatatg gtggagctaa gaatggtaaa
6121 tctgtatata ttgaactagt taaatcattt ttctatagta aagatattgt gtctaagcca
6181 cttaatgagc ttgaaggtcg ttttgacaaa gaaagtttaa ttgacaaaag tctaatggca
6241 agtcatgaaa ttgggcaatc taggattcaa gaaaagatcg taaatgactt caaaaagtta
6301 ttatctgtag aatcaatgca tgttgatcgt aaaggaaaaa ctcaagtgga agtcattttg
6361 gatttgaaac ttatttttag tacaaatgcg atacttaatt ttcctcctga acatgcgaaa
6421 gctttggagc gtcgaattaa tattattcca tgtgagtatt atgttgaaaa agcggacact
6481 tcattaattg ataagctcca gagtgagaag aaagaaatct ttctttactt gatgtatgtg

6541 tatcaacaga ttgtaaaagc agatatcgag tatcttgaaa atagccgtgt cactgaaatt
6601 actcacgatt ggttaaattt tggatatgaa tttgtttcta gcaggtccgt aagtaatgca
6661 aatcagaaag catgtattaa tttactcaga aaacttatag aaatcaaatc aggatcacga
6721 atcaaagtat ccgagttaaa taaagttatt aatgaagaaa taaaggtaag ttctcaggtt
6781 attaaacagt taattcaagc aaactttgat actcaaacca aactatacaa tggctacgat
6841 tattggattg atttaggttg gaaagaagcc aataaaaaag agattcatga tatttcggaa
6901 aaagataata ttatttcatt agataaaaat gaaaatataa cagacgatga ggcattagat
6961 gaagagaatt tggactttga ttgggaggac tttgacgatg aataatgaac aaattgaagc
7021 atttgtagaa gtgcttgtac ctatcataga agaacgtatc aataaaggta attaaggcta
7081 attacgtact acaggcagtt gcctgtagta ctcatatgat taagtggtaa aagtgataaa
7141 aatgaaacga aattataaat atatattatc tatatgttgt tacaagaccg atagtctgta
7201 gcaataatct aataaaagga gcggtatgat atgaagggta aaattgcact ttattcacgc
7261 gttagtacgt cagagcagtc ggagcatggg tactcaatcc atgagcagga acaagtactc
7321 atcaaagaag ttgtgaaaaa tttcccaggt tatgactatg agacatatac tgactcaggc
7381 atttcaggta aaaatattga aggtcgtccg gcaatgaaac gtctattaca agatgttaag
7441 gataataaaa tcgaaatggt gttaagttgg aaattgaatc gtatctcacg atcaatgaga
7501 gacgtgttta atattattca tgaattcaaa gaacatgatg tagggtataa atcgatttct
7561 gagaatattg atacatccaa tgcttctgga gaagtactcg ttacaatgtt tgggttaata
7621 ggatctatag aacgccagac tttgatttcg aatgtgaaac tttctatgaa tgctaaggca
7681 aggagcggag aggcaatcac cggtcgtgtt ttaggctaca aattatcact taatccactt
7741 acacagaaaa atgatttggt tatcgatgaa aatgaagcta atattgtacg tgaaattttc
7801 gatttatatt tgaatcataa taaaggcctc aaagccatta caaccgtact taatcaaaag
7861 gggtatcgta ctattaatca aaagccattt tcagtgtatg gtgttaaata cattttgaat
7921 aatccagtct ataaaggcta tgtcagattc aataaccatc aaaactgggc tgtacagcga
7981 agaagtggca aaagtgataa aaatgatgtg atattggtca aaggtaaaca tgaagccatt
8041 ataagtgaag aggtatttga taaagttcat gaaaaattag cttctaaaag ttttaaaccg
8101 ggcagaccta ttggtggaga tttctactta cgtggcctta ttaaatgccc agaatgcgga
8161 aataatatgg tatgtcgacg gacgtattat aaaacgaaaa agtccaaaga acggacaatc
8221 aagcgttatt acatttgttc cttattcaat cgttcaggga gttctgcatg tcacagtaat
8281 tccatcaatg ctgaagtcgt cgagcgtgta attaatgttc atttgaatcg tattctgtct
8341 caaccagata ttatcaagca gattgcgtca aatgtgatag aagaactgaa acaaaagcat
8401 agtaaccaaa cagaaattaa atatgacatt gatagtttag aaaaacaaaa agctaagctt
8461 aaaacacaac aagaacgatt gttagaattg ttcttagatg atcagatgga tagcgaaatg
8521 ttaaaagcta aacaaagtca aatgaatcaa cagttagaag tattagatca acaaattaaa
8581 gaagcgcaac aagcaaatca atcacaggat gaaataccta attttgataa attaaaagga
8641 cgactcattt tgatgataac acgattcagc gtgtacttaa gaaaggctac acccgaagct
8701 aaaaatcaac ttatgaaaat gttaattgat tcaattgaaa ttacgacaga taaacaagta
8761 aaacttgtaa ggtataaaat tgacgaaagt cttatccctc aatctttgaa aaaagattgg
8821 gggtcttttt ttatgcccaa attccaattt gaaatatatg gtcaaaatga ttatttcatc
8881 gaccaaatta ccacttttac cacttagtta ttagtgacaa aagtgagcga aatgaaataa
8941 aaatcaaata tatattatca aaatgatgta tcacatgcat acatcaatca aatacattag
9001 gaggtcataa ccatgacact agaacaacaa ctcaagcact atataaccaa cttattcaat
9061 ctgccaaggg acgaagtgtg gcactgcgaa tctatcgagg aaatcgctga tgatatctta
9121 cccaatcaat atgtaagact tggcccactc agtaataaaa cacttcagac taatacctac

9181 tactctgaca cacttcatga aagtaatatc tatcctttca ttctctacta tcagaaacaa
9241 ctcatagcca tcggttatat cgacgaaaat cacgatatgg atttcttata cctacacaac
9301 actatcatgc ctcttttgga tcaacgatac ttactaacag gaggacaata aaatgcataa
9361 atacatcaaa attacacaat tagtcattac aatactaagt gaaatcatca tttggatgaa
9421 agagtcagaa cgaaaggaag tctcttatga atagatatat cacccggggt atcgccaaca
9481 acttacctaa tatcttacaa caccaattat ggcaactcgt atctgagcga gaacaagaac
9541 aaaccaaaga taatacttca gtagattatt ttcatatatt ccagttcaat aagcatcgca
9601 atcaattata tatcaaacac aaacaagaac gacctgaata tgcgaaaatc cataaagcta
9661 attattcaaa agcaatcaat atcaataagg tctacattat tcgagaagat gatgtagacc
9721 tttcttatta tgtcatgtta ttacctgaag aatactagag gagtgaaaat tatatggaaa
9781 caatcaaaag tacattaaaa acagaagcca tattcagtga tgacaaacaa catcgctatc
9841 tacttaagaa aacatggaat agtgaaaaac aatcaatcac aatcattaca atgtatccgc
9901 attatgatgg cattctcaat attgacctaa cgacccaact catcatgaac aaagtttcag
9961 aaatggatgc atttggttca atcaattttg tgaatctata ctctaatatt acaaccccta
10021 tcaatctcaa acatttagaa aatgcgtatg ataagcatac agatattcaa attatgaagg
10081 cagtgaaaga gtcagatgaa gtgatattag cttggggcgc ttacgctaaa aagcccggtg
10141 ttgaagcacg tgttaatgaa gtattagaga tgttgaaacc acataaaaag aaagtaaaac
10201 gactcatgaa tccagaaacc aatgaaatca tgcatcccct taattcgaaa gcacgtcaaa
10261 aatggatatt aaaagtatag caactaaaat gtttattcac tatccatgaa aataattgaa
10321 tttcatatat atcttttaat ctacaatatg attaaaagat atattatttg gttctgttgc
10381 aaagtaaaaa aatatagcta accactaatt tatcatgtca gtgttcgctt aacttgctag
10441 catgatgcta atttcatggc atggcgaaaa tccgcagatc tgaagagacc tgcggttctt
10501 tttatataga gcgtaaatac attcaatacc ttttaaagta ttctttgctg tattgatact
10561 ttgatacctt gtctttctta ctttaatatg acggtgatct tgctcaatga ggttattcag
10621 atatttcgat gtacaatgac agtcaggttt aagtttaaaa gctttaatta ctttagccat
10681 tgctaccttc gttgaaggtg cctgatctgt aattaccttt tgaggtttac caaattgttt
10741 aatgagacgt ttgataaacg catatgctga atgattatct cgttgcttac gcaaccaaat
10801 atctaatgta tgtccctctg catcaatggc acgatataaa tagctccatt ttccttttat
10861 tttgatgtac gtctcatcaa tacgccattt gtaataagct tttttatgct ttttcttcca
10921 aatttgatac aaaattgggg catattcttg aacccaacgg tagaccgttg aatgatgaac
10981 gtttacacca cgttccctta atatttcaga tatatcacga taactcaatg tatatcttag
11041 atagtagcca acggctacag tgataacatc cttgttaaat tgtttatatc tgaaatagtt
11101 catacagaag actccttttt gttaaaatta tactataaat tcaactttgc aacagaaccg
11161 tattatggaa tagagatgtt ggtaacattt atacaggatc attatactta agtttaattt
11221 cgttattaca gaaccacaca ttccaaccag aagagaaagt atgtctattt agttatggtt
11281 caggagcagt aggagaaatc tttagtggtt caatcgttaa aggatatgac aaagcattag
11341 ataaagagaa acacttaaat atgctagaat ctagagagca attatcagtc gaagaatacg
11401 aaacattctt taacagattt gataatcaag aatttgattt cgaacgtgaa ttgacacaag
11461 atccatattc aaaagtatac ttatacagta tagaagacca tatcagaaca tataagatag
11521 agaaataaac tagtggccga ttgtgcttga tgagcttggg acataaatcc taactcgaaa
11581 taaataagca tatcactaaa ctgatttttt aaagtttaca gtgatatgct tattttttta
11641 tcttacgatt ttgtacgtgc atgcttgcct aggggtatgg ctcgagccat tagtctctcg
11701 cacatactat tccctcaggc gtcagcactt acaaaatcgg ttgtaatttt catttttata
11761 cgcattctta ctgagattat actaataaga ggaatagtaa aagcaattct aagtaaaatt

11821 gcagataaga ggtttgttaa aagcagttct aagtaaaatt gcagataaga ggtttgttaa
11881 aagcagttct aagtaaaatt gcagataaga ggtttgttaa aagcagttct cagtaaaatt
11941 acagataaga ggtacgttaa aagcagttct aagtaaaatt gcagataaga ggtttgttaa
12001 aagcagttct aagtaaaatt gcagataaga ggtacgttaa aagcagttct aggcaaaatt
12061 gcagataaga ggtgcgttaa aagcagttct aagtaaaatt gctgataagg ggtaagttaa
12121 aagcaatcct aagtaaaatt gcagataaga ggtaagttaa aagcaatcct aagtaaaatt
12181 gcagataagg ggtacagaaa aactagactt gattacaaaa tggagcttgg gacataaatg
12241 attttttaaa aatgagatga gacgtagatt aactccataa tcaatacgaa tctatcgact
12301 tctttattta tgatattcat ctctttttaa tggaaataaa agtgcgatta atgtgataat
12361 acagttacgt taattaaaaa ataaaaatgc aaggagaggt aatatgctaa ctgtatatgg
12421 acatagagga ttacctagta aagctccgga aaatacaatt gcatcattta aagctgcttc
12481 agaagtagaa ggtataaact ggttggagtt agatgttgca attacaaaag atgaacaact
12541 gattatcatt catgatgatt atttagaacg gactacaaat atgtccgggg aaataactga
12601 attgaattat gatgaaatta aagatgcttc tgcaggatct tggtttggtg aaaaattcaa
12661 agatgaacat ttgccaactt tcgatgatgt agtaaaaata gcaaatgaat ataatatgaa
12721 tttaaatgta gaattaaaag gtattactgg accgaatgga ctagcacttt ctaaaagtat
12781 ggttaagcaa gtggaagaac aattaacaaa cttaaatcag aatcaagaag tgctcatttc
12841 aagctttaat gttgtgcttg ttaaacttgc agaagaaatc atgccacaat ataacagagc
12901 agttatattc catacaactt cgtttcgtga agactggaga acacttttag attactgtaa
12961 tgctaaaata gtaaacactg aagatgccaa acttactaaa gcaaaagtaa aaatggtaaa
13021 agaagcgggt tatgaattga acgtatggac tgtaaacaaa ccagcacgtg caaaccaact
13081 tgctaattgg ggagttgatg gtatctttac agacaatgca gataaaatgg tgcatttgtc
13141 tcaatagaaa gttagaggtg agtcttacgt ttcagtgacg gtagacttac ctttaacatg
13201 ttacatacta aaaaattaat ttgaataaga aagagagaca tatatgaaat acgatgattt
13261 tatagtagga gaaacattca aaacaaaaag ccttcatatt acagaagaag aaattatcca
13321 atttgcaaca actttttgatc ctcaatatat gcatatagat aaagaaaaag cagaacaaag
13381 tagatttaaa ggtatcattg catctggcat gcatacactt tcaatatcat ttaaattatg
13441 ggtagaagaa ggtaaatacg gagaagaagt tgtagcagga acacaaatga ataacgttaa
13501 atttattaaa cctgtatacc caggtaatac attgtacgtt atcgctgaaa ttacaaataa
13561 gaaatccata aaaaaagaaa atggactcgt tacagtgtca ctttcaacat acaatgaaaa
13621 tgaagaaatt gtatttaagg gagaagtaac agcacttatt aataattcat aataaaacag
13681 tgaagcaacc atcgttacgg attgcttcac tgttttgtta ttcatctata tcgtattttt
13741 tattaccgtt ctcatatagc tcatcataca ctttacctga gattttggca ttgtagctag
13801 ccattccttt atcttgtaca tctttaacat taatagccat catcatgttt ggattatctt
13861 tatcatatga tataaaccac ccaatttgtc tgccagtttc tccttgtttc attttgagtt
13921 ctgcagtacc ggatttgcca attaagtttg cataagatct ataaatatct tctttatgtg
13981 ttttatttac gacttgttgc ataccatcag ttaatagatt gatattttct ttggaaataa
14041 tatttttctt ccaaactttg tttttcgtgt cttttaataa gtgaggtgcg ttaatattgc
14101 cattattttc taatgcgcta tagattgaaa ggatctgtac tgggttaatc agtatttcac
14161 cttgtccgta acctgaatca gctaataata tttcattatc taaatttttg tttgaaattt
14221 gagcattata aaatggataa tcacttggta tatcttcacc aacacctagt tttttcatgc
14281 ctttttcaaa tttcttactg cctaattcga gtgctactct agcaaagaaa atgttatctg
14341 atgattctat tgcttgtttt aagtcgatat taccatttac cacttcatat cttgtaacgt
14401 tgtaaccacc ccaagattta tctttttgcc aacctttacc atcgatttta taacttgttt

14461 tatcgtctaa tgttttgtta tttaacccaa tcattgctgt taatattttt tgagttgaac
14521 ctggtgaagt tgtaatctgg aacttgttga gcagaggttc ttttttatct tcggttaatt
14581 tattatattc ttcgttactc atgccataca taaatggata gacgtcatat gaaggtgtgc
14641 ttacaagtgc taataattca cctgtttgag ggtggatagc agtacctgag ccataatcat
14701 ttttcatgtt gttataaata ctcttttgaa ctttagcatc aatagttagt tgaatatctt
14761 tgccatcttt tttctttttc tctattaatg tatgtgcgat tgtattgcta ttatcgtcaa
14821 cgattgtgac acgatagcca tcttcatgtt ggagcttttt atcgtaaagt ttttcgagtc
14881 cctttttacc aataactgca tcatctttat agcctttata ttctttttgt tttaattctt
14941 cagagttaat gggaccaaca taacctaata gatgtgaagt cgcttttcct agaggatagt
15001 tacgactttc tgtttcatta gttgtaagat gaaatttttt tgcgaaatct cttaaatatt
15061 catccatttt tttaacggtt ttaagtggaa cgaaggtatc atcttgtacc caattttgat
15121 ccatttgttg tttgatatag tcttcagaaa tacttagttc tttagcgatt gctttataat
15181 cttttttaga tacattcttt ggaacgatgc ctatctcata tgctgttcct gtattggcca
15241 attccacatt gtttcggtct aaaattttac cacgttctga ttttaaattt tcaatatgta
15301 tgctttggtc tttctgcatt cctggaataa tgacgctatg atcccaatct aacttccaca
15361 taccatcttc tttaacaaaa ttaaattgaa cgttgcgatc aatgttaccg tagtttgttt
15421 taattttata ttgagcatct actcgttttt tatttttaga tacttttttt attttacgat
15481 cctgaatgtt tatatcttta acgcctaaac tattatatat ttttatcgga cgttcagtca
15541 tttctacttc accattatcg cttttagaaa tataactgct atctttataa acttgtttga
15601 aatttttatc ttcaattgca tcaatagtat tattaatttc tttatctttt gaagcataaa
15661 aatatatacc aaacccgaca actacaacta ttaaaataag tggaacaatt tttatctttt
15721 tcatcaatat cctccttata taagactaca tttgtaatat attacaaatg tagtatttat
15781 gtcaaaataa tgttataatt tttgtgatat ggaggtgtag aaggtgttat catctttttt
15841 aatgttaagt ataatcagtt cattgctcac gatatgtgta attttttttag tgagaatgct
15901 ctatataaaa tatacggttc tgttgcaaag ttgaatttat agtataatta taaccaaaag
15961 gagtcttctg tatgaactat ttcagatata aacaatttaa caaggatgtt atcactgtag
16021 ccgttggcta ctatctaaga tatgcattga gttatcgtga tatgtctgaa atattaaggg
16081 aacgtggtgt aaacgttcat catttaacgg tctagcgttg agttcaagaa tatgccccga
16141 ttttatatca aatttggaag aaaaagcata aaaaagctta ttacaaatgg cgtattgatg
16201 agacgtacat caaaataaaa ggaaaatgga actatttata tcgtgccatt gatacagagg
16261 gacatacatt agatatttgg ttgcgtaagc aacgagataa tcattcagca tatgtattta
16321 tcaaacgtct cattaaacaa tttggtaaac ctcaaaaggt aattacagat taggcacctt
16381 caacgaaggt cgcaatggct aaagtcatta aagcttttaa acttaaacct gactgtcatt
16441 gtacatcgaa atatctgaat aacctcattg agcaagatca ccgtcatatt aaagtaagaa
16501 agacaagata tcaaagtatc aatacggcaa agaatacttt aaaaggtatt gaatgtattt
16561 acggtctata taaaaagaac cgcaggtctc ttcagatctg cggattttcg ccatgccatg
16621 aaattagcat catgctaaca agttaacaca aagtattatt ttaaaattga gattagacat
16681 ttatttttca actttgcagc agaacctaat tttttctctt tgtaaataaa tgcaaagaga
16741 taccataagg atctcttaca taaccataac cttcagtata gaattctgga ctaaatgttt
16801 tcaatacctc actgcctttt tctattaact ggtcatatac atgtttagtt tcttctactt
16861 ggtcaaaagt gagacaaaga gatatattat taccttgtgt tatgggcaaa ccttcagtgt
16921 catctgcgat cataattttt atatctccaa attgaagtac acattgatca attttattta
16981 aatcattttc gtcaatatta agtttcttat ctataggtct atctttaata cgttgaatat
17041 acagtgtttt agcgccaaac agctcttcat acaacttttt taaaccctct gcattttgag

17101 tgattaaaaa atatggactt acttgaaatt tcatgttttt tcctccttag atttgttata
17161 aatagagtat atacttaatt agtgtcatct attgacacta aaaggagaat aataatgaaa
17221 aaatctgtta gattatataa tatgattgaa tattgtaatg aaaataggaa cttcaaatta
17281 aatgatttaa tgtcagaatt taatatttct cgtagtaccg ctttaaggga tataaaagaa
17341 attgaagcat taggagtacc tttatatagt aatccaggga aaaatggtgg ttatacgatc
17401 ataggtaatc gagaccaaac gaaaatagca atctcagatg aagagttgaa agctttagta
17461 tttacacttt cgagtatttc aaatgtgagt aatctacctt ttcaaacaga atatcaagaa
17521 atattaaaaa aattatataa taactcgaat aaaaaagagt taataaacca atataatgat
17581 ctatttcaat attttaatga agataagtat cagttcaaaa gttataagtt atttaatgaa
17641 atcattagat tgataattga gaataagtct tttgaaacct gttattcaca aaactatatt
17701 aaagaacaat ataaaggtat tggcattatg tataaaaatc atcaatggta ttttgttgta
17761 gctaatatag aatcaaagtt agtgaatcta ttaaatattt cgaaaataaa agaactatat
17821 gaaatgggag agactcaaga gtgtaatgat ataactatgc agaattttca acagttcatg
17881 gttaaaaatg aaacagctat tgatattctt attagaagca atgttatggg attgaatatc
17941 ttgaaaggct acctgtggag tgactatatg attgaaaata ttgacgaaga gacatattta
18001 tttaaatcaa aagtgaacgc gaaagatata gattttatag ctaagttaat tgtcacaggt
18061 ggtgtcaatg taaaagtaga gacccctaat agtttgaaaa atgctgttaa agttgaatta
18121 actaaaataa taaacatgta ttaatagtaa tttaatcata attgtaataa caaaaagatg
18181 acaacattaa acaatttaaa tttatacaaa aaagcattac caaatttaaa attgggtagt
18241 ggtagtgcta cagcatacat aaatagttat aattaaataa agatgggtca atgaaggtga
18301 attaagtgaa taaaaatatt acagctctaa aatctactga aaatactaca tatatgcagt
18361 atacggttca tatcgatgat caaattataa ataatataaa agagtgtacc aaaaagttta
18421 aaattttgcc tatggaagat aaaattccat tatcaccgtt gttacaacca gaatatgcag
18481 gagaggtaca agattttatt agtacatatg agcagtttat gattaatttt ggtaaagtaa
18541 tattggatag tcaaggcata aaaatacagt ttgaaagtga atcattaagt agtattcaac
18601 gaggcattca agaacattgt tacttaaatg agcgaacaaa tgacattgat gtgactaaag
18661 aatggtattt atgcaaattt tcaaatcaaa tattagaaga ggataaaaat caattgtata
18721 atgctttaaa gcaactcatg aatgattcta aaaataaaaa gcaagctttt tggactgacc
18781 ccaattagtg ggaattatat aaaaacactt tcgttgaatt cataataaaa tgaatcatgc
18841 ggaggtgttt tttctatgaa aagagtttct tattcagtag aaacaaagta taaagcagtt
18901 gaaatgaaaa tagctggttt ttcgacaaaa gaaattatga aagaattaaa tattaaaaat
18961 agaacacaag tggaaacttg gtggcgatgg tatcgaaatg aggaaagtta tagattttca
19021 caacacgttg ggaaacaata tacctacggt aaaggattag aagaattgtc agaaatagaa
19081 cgattaaaat tagaaaataa gagaaaagat atagaattgg atattttaaa aaagtacaag
19141 gcattggaaa ggaagtggta ccagcagtag tcatagattt agtggatcaa ttaaaagtaa
19201 aatattcaat caaattgata ctagaagtat taaacatacc taaatcaaca tattaccgat
19261 ggaaaaacaa aacctataaa aatgatactg taacacaaaa agttattgaa ttatgtgaag
19321 ctaaccacta tacctacggt tatcgtaaga ttacagcact gattaatcaa tgttatacat
19381 caccaattaa tcataagaga gtacagagaa ttatgcagaa gcatcatttg aactgccgag
19441 ttagacctaa aaagacgaca aggataggta aaccgtatta taaaactgac aatttattac
19501 aaagacaatt taaagcgagt tgtcccatgg aagtattaac aaccgatatt acttatttac
19561 catttggtca ttctatgttg tatttatctt cgataatgga tatttataac ggagaaattg
19621 tggcgtataa aatagatgat aaacaagacc aaagtttagt taatgataca ttaaatcaaa
19681 tcgatatacc cgaggggtgt atattacata gtgatcaagg cagcgtttat acatcttatg

19741 cttattatca attatgcgaa gaaaaaggca ttatcagaag tatgtcccga aagggaacac
19801 ctgctgataa cgccccgata gaaagttttc attcctcgct aaagtctgaa actttttaca
19861 tcaataatca gcttaatagc tctaatcata ttgtaataga tattgtcgaa aaatacatta
19921 aaaactataa taataatcga attcaacaaa aactaggcta cttatctcct gtaaaataca
19981 gagaattagc agcctagaac atagtgtttt tattaagttc ccgtttaagg gggtcagtac
20041 cttttcaaga ggtgtttaag gttcatattg atatttatct ctacaataaa gaaaaatctg
20101 aatataagta tcaagtatct agttatttca atcttgtaaa agaaaatcct aaaataactt
20161 ataaaaaaag acatttacaa gagaaacaag gtgtcaaggg tacaactttc acgaacaaat
20221 taaaattatt gaacaaaatg tatggtgtag atgttgcaaa atatcagcca ttttataatt
20281 ctaataatcc tgaatatgaa agaggtcaat ttggtgaaag atacatttct caaagatcta
20341 actatgaatt taatagactt caatatcaaa ttatagatat gttatcgaaa atacttgata
20401 agcatccatt accaaagtca gataataatt ataaacatat tccaactatt gaaaaagcaa
20461 tattaagtgg ggattctcat agtttttatg agtattttga agatataatg aaagaaatta
20521 taagtatgga aaattcttct ttaaaggaaa aattattgac tgattttaca tatcaatctc
20581 aatgtagatg gtactctgaa agtgaaaaat taaaattgca acttgaaagt tttatgcata
20641 aagttttgga gagcaattat tatgagggta acaaattata tagaatgttg tctcatgcaa
20701 ttgaagaaac aatcaatgag gctgatgaag ataaggttca tttcaattat tttaaagatt
20761 atttttgac agacggtggg gtgaagaatt gggagcagat tagcgagaaa attactgaat
20821 tcaatggtaa agttattaac gatatccaaa atgaatataa taagattcaa tttaataacg
20881 caaatcgaaa tcagaaacta aactttaatt atttatacca ttgctttgag tttagtaata
20941 acttagtaaa agcaagggta aatggaaata gaggttacat tttatatttt gtagataaat
21001 ataaaataaa aatgcccttt ttggatcaat tattaatcaa ttatttgaat caagaaatag
21061 gtcaggaaag tattaattat aatatgcaaa cattgtttga aaaagaaaga tatgatagaa
21121 gtagtaccat tgaaaaatta gtagcaacaa gcaaatttaa gtatgaaaaa gatgattcag
21181 atttattcaa acaacttttc aatgatgttg aaaattcaat agacagatta ggtatttact
21241 tactaaataa tggtataaat tcgaatgatg aaaatgcaag atattataga tcgtttttaa
21301 aggaacttag tagaataaaa agtaaattaa cgccattttc tcttgaaata agtaagtcta
21361 gcggaagaga gcaacattat cctgatgatg ctattgatga taaagatgag agaagaaaaa
21421 ataaagaaga aacatatcat gcttttgatg ataaaagcga tattgactcc aaattaaaga
21481 ataaaataaa tgtttctatc gataatttat tgtcagttaa aattaagagc aatttgtatg
21541 atattagtcc aaatacaaaa aaacaataac tataatgagc caactcaaga atgagttggc
21601 tttttaattg taattttacg cttccattac aggaatcaat gttatggaag ccattaagca
21661 attaatttga gtcatttata gtataggtac aacgttgtga tgttcacatg ataatcagcc
21721 cgggagatta ttgtgtaata acttatagaa ggagcaaatc accatgtatc aagcaaatat
21781 acgtgattta attacaaaat tgcctcaaag caataaaaca gaacactttt taatgaacaa
21841 attttcaaat caagataaag ttcagcagct acaaagacaa attagccaac agctagatca
21901 acaatataat gagcttttgg ctaatgaaaa agctaagcta gaccaatacg tggaagtaca
21961 ccataattta gaaccattaa agaaagagat tgaatcagaa tctattaacc ttgataccga
22021 taaattacct gatatcaaag cgacaatgct tgaaaaggct aagaacgatg aacattttga
22081 taaaatcgaa cagctatttg atagattaga tcagtcatta aatggtacga atcgattata
22141 tacgcaatta tcgttgattg gcacacgaac acatcggatc acaacgaaaa gatttaatgt
22201 tcaaggcttg cctaaattag tccaacaaat gattttacct tcgcaattta aaaaggttta
22261 tacaatagat tttaaatcat tcgaaccatc agttgctgcg tatatgacac aagacgaaca
22321 actgattgac tacttgaatc atgaagaagg gttatacgat gcattactga gagacttatc

22381 tttgtcaaaa gagaagcgcg tgagtgtgaa acgtgcattt atagggtcat ttctttttgg
22441 cggtcgttat agtagctcta aattcaaaat caatcaagag gttagtgaaa ttaactggct
22501 acaagtaatg agcaaattca agaaggtcat tgaatttaag gagcaagtcg aaaaatataa
22561 aacaatgcct acgccttacg gcattgaaca tgatatgagc gcatttcaag gtagtagtat
22621 tatggcaatt tatgtacaaa cggtagcaag ctatattttc aagcacattt tgttggaagt
22681 gtacaaagca cagtgcgaaa aaaaaacgtt caagattata gtgccgatac acgatgcgat
22741 tatgattgaa tgtaatgata aggggattgc acaaaatgta gcacagctca tgaaagatac
22801 agctaatcaa ctgtttaatg gtgaatttgc acatgtgaca gtggaagctt taggaggtat
22861 agacaatgaa taatgataga ggaaaaagtc ttcaaattcc ccaaagtaca ttgttaaaag
22921 aaggatctat atacgttgct acgttacatt ctgtgtacga gaagaacttc tcaggtgata
22981 ttaaacatca gtttacgtat gaagtagaac ttaaccaaga aacgcactat gtaaatcgca
23041 atattaccgt aaaatctatg agtcaccaat tatcaattgc tgattggatt aaacgtcaca
23101 gtaactataa cgtaaaccac attaattatg atccatacat tgatcgaaaa catttggtcc
23161 ttgtagggca atataacgga aattattata ttcaagatgt agcaccatta gatgaatttg
23221 gaggggtatt gtaatgaatc atattttaga aatgttaata aaattattaa aagtgggtat
23281 ggaggcaatc gaccgtaaag gtctgattgc catcctaaca agtagtattg gaaatgatga
23341 aatggatgat tctgaacaag ctgtaatggt gtataacgag cttatcgata agctacagct
23401 taacattcct aaagatgtcg actatagacc taacatatac agttattttg gtattcaaaa
23461 aaagccaaat gacacaatat tagtggaaat gatgatatgt atttttcata tcaagcgttt
23521 tgattcagag ttgtttgttt tcaaagataa aggttggcaa aaggtaagtg aagatgaatt
23581 gcaagggttg atatctaaaa tgatacaagt gttgctagtt gattataaac cttcactaag
23641 tactttgaaa aacgtagtag atggcataca gaaatcaacg gacatagaaa aacttgttga
23701 gcatagacag tacattggtt gtggacgaaa tatgttcaat ctaaagacct ttaaagtggt
23761 tgataatgac cttgaaatat tccctaaaac acgcttagat ttggaattag atataaatga
23821 cacgattacg gacaagatac ccccgaattt caaacaatat atgttagagt tggcgaatta
23881 tgaccatgat ttacaatatt tccttttcca acatatggca gtgttattga cggcagatac
23941 taaactacgt cgtggacttt ttttgtatgg aactgcaaaa aatgggaaat cggtctatat
24001 taaattagtt aagtcattct tttatagtaa tgatatcgta tctaaaacac ttaatgaact
24061 tggcgggcgt ttcgataagg aaagtctaat tggtaaacga attatggcaa gtgatgaagt
24121 ggggaaagct aatattgatg aagcaactgt gaatgatttc aaaaaattac tatctgttga
24181 accaattcat gctgaccgta aaggaagaac gcaagtagaa gttactttag atttaaaact
24241 catttttaat acgaatgctg tactcaattt tccatcatca catgcaaaag cattagagcg
24301 tagaattgct gttattccat gtgaatatta tgttgaaaaa gctgaccctg acttaattga
24361 aaagttacag gatgaaaaga aagaaatctt tctttacttg atgtatgtgt ataagcaaat
24421 tgtaaaaaat gatatcgagt acctccaaaa tgatcgtgtt actgaaattt ctcatgattg
24481 gttaaatttt ggatatgaat ttgtttctag taaatcagca aatattgcac atcagaaagc
24541 gtgtattaat ttactcagaa aacttataga aatcaaacca gggtcacgta tcaaagtgtc
24601 taggctaaat gaggttatta gagatgaaat taaagtaagc tctcaagtta ttaatgattt
24661 ggttcaagct aactttaatg tacaaagtag actaaataat ggttataagt attgggtcga
24721 tttaggatgg aaagaaactg ataaaaaaga tgacatgatt tcattcgata aaaatgagaa
24781 tgtaacagat gatgaattct tatacgaaga tgatttgaac ttaggttggg aggactttga
24841 cgatgaataa tgaacaaatt gaagcatttg tagaagtgct tgtgcctatc atagaagaac
24901 gtatcaataa aggtaagtaa tctaattacg tactacaggc agttgcctgt agtactcata
24961 tgattaagtg gtaaaagtga taaaaatgaa acgaaattat aaatatatat tatctatatg

25021 ttgttacaag accgatggtc tgtagcaata atctaataaa aggagcggta tgatatgaag
25081 ggtaaaattg cactttattc acgtgttagt acgtctgagc agtcagaaca tggttattct
25141 gaaaaggagc aggaacaact actcatcaaa gaagttatga aaaatttccc aggttatgac
25201 tatgagacat atactgactc aggcatttca ggtaaaaata ttgaaggtcg tccggcaatg
25261 aaacgtctat tacaagatgt taaggataat aaaatcgaaa tggtattaag ttggaaattg
25321 aatcgtattt ctcgctcaat gagagacgtg tttaatatta ttcatgaatt caaagaacat
25381 gacgtagggt ataaatcgat ttctgagaat attgatacat ccaatgcttc tggagaagta
25441 ctcgttacaa tgtttggact aataggatct atagaacgcc agactttgat ttccaatgtg
25501 aaactttcta tgaatgctaa ggcaaggagc ggagaggcaa tcaccggtcg tgttttaggc
25561 tacaaattat cacttaatcc attgacacag aaaaatgatt tagttattga tgaaaatgaa
25621 gctcatattg tacgggaaat ctttgattta tatttgaatc acaataaagg atttaaagca
25681 atcacgacaa ttctaaatca aaaaggatat cgtaccatta atcaaaaacc attttcagtg
25741 tttggcgtga aatacatttt gaataatcca gtctataaag gctatgtcag attcaataat
25801 catcaaaatt gggctgttca gcgaagaagt ggtaaaagtg ataaaaatga tgtgatattg
25861 gtcaaaggta agcatgaagc cattataagt gaagatgtat ttgatcaagt tcatgagaaa
25921 ctagcttcta aaagttttaa accgggtcga cctattggtg gagatttcta cttacgtggc
25981 cttattaaat gcccagaatg cggaaataat atggtatgtc gacggacgta ttataaaacg
26041 aaaaagtcca aagaacgcac aatcaaacgc tattacattt gttcattatt caatcgctca
26101 ggaagttctg cctgtcatag taatgcgatt aatgctgaag tcgtcgaacg cgtaatcaat
26161 gttcatttga atcgtattct ttcacaacct aatgttatta agcagattgc gtcaagtgtg
26221 atagaagaac tgaaacaaaa gcatagtaaa caaacagaaa taaaatatga tattgatagt
26281 ctagaaaaac aaaaagcaaa agttaaaaca caacaagaac gattattgga attgttctta
26341 gatgatgaaa tggatagcga aatgttaaaa gctaaacaaa gtgaaatgaa tcaacagtta
26401 gaagtattag accaacaaat taaagaagca aaacaagcaa atcaatcaca ggatgatata
26461 cccaattttg ataagttaaa agcacgactc attttgatga taacacgatt cagtgtgtac
26521 ttaagaaagg ctacacccga agctaaaaat caacttatga aaatgttaat tgattcaatt
26581 gaaattacga cagataaaca agtaaaactt gtaaggtata aaattgatga aagtcttatc
26641 cctcaatctt tgaaaaaaga ttgggggtct ttttttatac ctaaatttaa ctttgtgata
26701 aatgtcacaa agaaaaatag gattgaaaat ttatcacttt taccactttt ttagagtgac
26761 aaaagtggag gagttttgaa atattttata aatatatatt ttatttatgg agtacacatt
26821 attaattaag gaggtcatta taatgacgct aagcaaacaa cttaaaacgt atatcactga
26881 acgatttaaa ttaaattatc aagaaacttg ggcttgtgaa accatagatg cggtggctga
26941 agatgtatta cctgaaaaat atattaaaaa tagtccactt gaacataaaa ttttaaatac
27001 ttttacctat tacaatgatg aattacatga aatcagcatt tacccttttt tatgttatct
27061 agataaggaa ttagtagcaa taggttattt agataatttt gatttagact ttatattttt
27121 aaatgacact catcaagtca ttattgatga acgctacttg ttacaaaaag ggggcgagta
27181 attatgaatt ggatcaaggt cgctcaacta tctgtcacag ttatcaatga agtcattgag
27241 attatgaaag aaaaacaaaa tggaggggaa tagtatgaaa atcaatcgat acattacaag
27301 aggtgttagt gaacatttat ctctagacct tcaaatctta ctttggaaca tggtaaaaga
27361 tcgagacaat caacttcata cagattacct acacattttt aaactacaag aagatgataa
27421 tatactctca atcacacatg aacaagaaca acccgcatac aaattggaat atcactatac
27481 aaactatgta aaaaatcaaa atgcattacc taagaaagtc tacgtcatcc gagaagatga
27541 tgtagacgct ttttattatg tgatgctttt accagaagaa tactaaaagg agtgacaatg
27601 atgaatacaa tcaaaaatac aatacacaca gaagcgatat ttagcgatga tgaacaacac

27661 cgctacttac tcaaaaagac ttgggatgaa aagaaacctg catgtacggt aatcaccatg
27721 taccctcatc tagatggcgt attatcactc gatcttacaa ctgttcttat cctcaatcaa
27781 ttagcgaatt ctgaacaata tggcgctgta tatcttgtaa atctattttc taatattaaa
27841 accccagaga accttaaaca tattaaagaa ccttatgata aacacacaga cattcattta
27901 atgaaagcga taagtgaaag tgacacagtg attttagcct atggtgctta tgcgaagcga
27961 cccgttgttg tcgaacgcgt tgagcaagtg atggaaatgt taaaacctca taaaaagaaa
28021 gtaaaaaagc tcataaatcc ggcaacgaat gaaattatgc atccgcttaa tcctaaagcg
28081 cgtcaaaaat ggacattgaa ataaaggagg attatctatg aaccatgaaa ctaaacaatc
28141 agattggcga acggttgcta gttgtttagc atcacaaaat tatatatcga ttgtaaaagg
28201 tttagtacat cattttacag cgatcgaaga tgaggaaatt cttgataaaa tctatgatga
28261 ttttatgaat gatgactcta taacaacggt acttaacaat gatttacaga caatcattaa
28321 ccattatcta tcaaaataaa gactatacta attacaataa aaacagagca tccttcactt
28381 ttatggtgag ggatgctctt ttaatttatt tttttattga ttattcgcca taagttctgc
28441 ttgctctaca acctgctcta cagccatttt ttgtaaatct ggtggatagc catatttttt
28501 aagcaatcgt ctaacagcta cgcgcatttt agcttttgcg ctatcacgtt tagaccaatc
28561 aacacccatg ttttctttca ctgttttagt tagctcatga gcaatcgcac gtagttcttt
28621 atctcccatg gcttcttttg ctgtttcatg tgaagctaaa gcatcgtaaa acgcaatctc
28681 atctgaattc aggcctaatt catttcctcg ttgttgttct tgtttaatat ctttagcgag
28741 ttgaataagt tcttcaatca ctttagatgt ttcaatggaa cgactattat atttattaat
28801 cgaatttcct aacatctcag agaaacgctt agataccgtt gcattcgttt tcattaatga
28861 tttaacttgt cctttgagta atcgatttaa taattctacc gccacatttt tctgtttcaa
28921 tccttcgaca tcttttaaaa agtcatcaga taagattgat aaatcgggtt gttcaagacc
28981 tagcgtttga taaacatcaa tgacatcttc agtcacaaca gattgtgaca caagttgatt
29041 aatctctgct tcaacttctg caggtgtttt acgtgttttt ccttcttttg gcggttgtaa
29101 caatttaaca agtcctgctt taactgcttt aaagaaggca atctcatcgt tgagttcttg
29161 ggctgtaggt tcagtcgcac aaagagcaaa ggctttccct aattctgtga ctgttttaat
29221 aaaacgttga cgttcatctt cacctaaacc aattacataa tccatcgtat tcgatattgt
29281 ataataacgt tcagattttt tatctgaatt aaatttagag taatcaagat tatataacat
29341 atcttgaatt acatcatact tcaataacat caattcaacc gctttatccg tatctattgc
29401 cgtttgagct tgatcagatt ctgtatattc tttaagtgcc tcttttaaac tttcggcaat
29461 ccccacataa tcgacaatta aaccacccgg cttatcttta aacactcgat taactcgagc
29521 aattgcttgc atcaaattat gccctttcat cggtttatca atatacatcg tatgcataga
29581 aggaacatca aaccctgtca gccacatatc tcgaacaatc acgagttgta actcatcatc
29641 cacatctttc atacgttttt ctaataaatt acggcgtttt ttaggaccaa tgtgtctttg
29701 gaaagaagtt gggtcactag atgagcctgt cattaccact ttaatgactc ctttatcatc
29761 atcatctgaa tgccattctg gttttagacg aatgatttca tcatataaat caacagcaat
29821 tcgacgactc atcgttacaa tcattccttt gcctttcatc gcttgttgac gtgtttcaaa
29881 gtgttggatg atatctttgg ctagggcttc tacacgaggt tttgcgcctg ctaaagcttc
29941 aatacgggac cattttgatt ttaaacgctg ttttacatcc tcttcttgat cttcagtaat
30001 gtcattatac gcttcatcta aatctaaact ttgaggtaga tttaatggaa ttacacgact
30061 ttcatagtaa attttaacgg tgcttccatc agcgacggct tgtgtcatat cataaacatc
30121 gatatagttt ccgaaaacca tttgcgtatt tttatccgtt gaagctacgg gtgtgcctgt
30181 aaatcctacg aatgttgcat tcggtaaagc atctcttaaa tatttggcat aaccatattt
30241 aatgccttca cctttatcat cgtattttgc gttaaagcca tattgtgtac gatgagcttc

30301 gtctgccatg acaatcacat tttttcgttc tgttagggca gccatggtcg tttcgttttg
30361 ttcaggttca aatttttgca tcgttgtaaa tacaatacca cccgactcaa cagataataa
30421 tgattttaat tctttacgtg tttcagcttg ttttggtgtt tgtcttaata atcctttacc
30481 agaacgtcct tttgatttaa caaacgtact gtacagttgg ttatctaaat cattacgatc
30541 cgttacgacg actaaggtag ggttattcag catttgaatt aattttccag agaaaaagac
30601 catggttaaa cttttaccgg acccttgggt atgccaaata acgccgcctt taccatcacc
30661 ttgctcagat gaagctaata aagctctgtc aacggcttta ttaacggcat aatattgatg
30721 atacgctgct agaattttac tgatatgtcc tttgccatca tcttgaaata aaacaaaata
30781 tcgaattaaa tcaagtaatg tttctggatt taacatgcca tgaatgagta cgtctaagct
30841 agctaaacct ggtgatgatt cagtttgacc atctttactg cgccaagtca taaaccgatc
30901 ataatttgcg gttagagagc cagcctttgt attaatacca tcacttgtaa caagcacttc
30961 gttaaacgta aataattgtg gaatacgcat cttatacgtt tctaattgat gataaccgtc
31021 ttcgacgccc acggtttcat ttgttgaatt tttaagttca atcactacga taggcaagcc
31081 attgataaag agtacaatat cgggacgttt tttatagtct ccattaacga tagttaattg
31141 attgactgct aaaaaatcat tgttttgtgg atattcaaaa tcaataattt taacaatttc
31201 tacaatcgat tgtccttcat catcatagtc ttcgatttca ataccattaa tcaaattttc
31261 atgaaaggta aggttatttt ctaaaagatt gggcgacttc tctaaagtta gttcatgtag
31321 cgctttttca ataaaacgag gatggatatc tgagttgatt tttcttaatg ctttctctaa
31381 acgttcatga aggacaacat ctttatcact tttacgttct ggtgttagac ttgtcatact
31441 aatctcatta cctttttat agtcatagcc tagtgattgt agccattcta atgcgacttg
31501 ttctaaatca tcttcattaa attgaaagtt catcactatt cacctcaata tcatcagata
31561 tttctagttc tccagacatt agtttaggaa gaagagtatc tcgtaattga gttaatttac
31621 caatttctga tcttaatatt ttcatttctt ccatatatgg agtgattatt tcagaatatt
31681 tttttacaat actatcttca attgctaatt tataattaac agctattgtt ggttttactc
31741 tttgtctact atttgttgaa ccggtagtat tagcttttaa ataatctata aattgagaat
31801 taagacatat attatatatg aatgaattaa ttttattatt aggagattcc attacaacaa
31861 attctgatga agcaacattt aattttttat tatctattac aggtaaccaa atccttttag
31921 tatcaggatt catttttgaa aataatacgc aattgttatt aataatccat ttattacttt
31981 ttattttatt gacttcttcc tctatagctt gttcttcatt atcataagca ggtaaactaa
32041 aatgtttaac tgttacttct tcactctttt taggattaaa agtttccttt ttatgacttg
32101 ctatatcttt taatttatag attttccaat tacttggtat cttacctaac tcactatcaa
32161 tcatttctcc tccactagat ttataaggat taccatcttc atctggaaat tcaaaatcta
32221 caaaccaatg cttaaatagt gtttgtgaaa gttctttaag gtttgctatc attttttggt
32281 tagtttctat ttttcttgtg atttgattta aaaatttaga aatgcttttt tgttttatta
32341 aagaaaaatt aggaattttg acagtttcaa taagtttaga atttatatta gcttgattag
32401 cactccctgt agaatttcta actaaatact cagttatttt aggttgtaga aaataataaa
32461 ataaaaaatc taagtctgca ctattactat attttaattc gaattttccc acacgttgat
32521 tttgtaatgc tggataatca aattcaattc ttccaacttt tcctactgca gaagacattt
32581 gattcacgcc tgatccagtt aatgatatta atatatcatt tttctttaat gaaaaacctt
32641 tagttttttc atatgtttta atagagactt tttgagagtc ttttgtatca acaatgggtg
32701 aaattatatt cttaatttta ataaccggta taccttttttc ttgaaattct tttgatttaa
32761 aagcatatcc atttttaatt ttagctacat caccgaaaaa aattgtatta tactccatat
32821 cctaacccct ccaatgattt tcggatttgg tcttcaagtt cttttgattt tgcgaattgt
32881 tcacttaatt cagaagtaat acgttccatt ttttgttcaa aaggttcttc atcttcttca

32941 acgtcagcta aaccaacata tcgtccaggt gttaaaatgt attcattatt cttaacttct
33001 tctaaattag ctactttaca aaaaccagct atatcttcat aaggtttatc gtttgtacct
33061 ctccacgcat gatacgtctg tgctactttt tgaatatctt catctgaaaa ttcttttaat
33121 gttctagata ccatatggcc gatttcacga gcatcgataa ataaaatttc attttcacgc
33181 tcttttttac cattttgacc tttattatta ctaatgaacc aaagacatac aggtatttgg
33241 gttgagtaaa agagttgtcc aggtaaagta acaatacatt caactaaatc ttgttcaatt
33301 agatttttac gaatttctaa ttcatctttt ccgcttgtag acattgatcc gttggctaat
33361 acgaatcctg ctgtaccatt aggtgctaat tttgaaatca tatgttcaat ccatgcatag
33421 ttagcattac cttttggtgg aataccaaat tgccaacggt aatcatcaag taatcgttct
33481 tgaccccaat cacttgcgtt aaaaggagga ttggctaata tgtaatctgc ttttaatcct
33541 ttatgtaaat cattatggaa tgtatcagca tttcgttcac ctaagtcatt atcaatacca
33601 cgaatcgcta agttcatttt tgctaatttc caagtcgtag ggttagattc ttgtccgtaa
33661 atcgcaatat cgtctaatcg accttgatgt ttctcaacga agcgttcact ttgtacaaac
33721 atcccacctg aaccgcaaca cggatcgtaa atacggcctt tataaggctc aatcatctca
33781 actaataact ttacaattga tgacggggta tagaattctc cagcattttt accttcggca
33841 cttgcgaatt tagcaataaa gtactcatac acacgaccta acacatcttg tttacgactc
33901 tctgtgtcgc caactttaaa tgtaaataaa tcaataatat cgcctaattt ttctttatct
33961 aatgcaggtc ttgcatattc tttaggtaat acacctttga gtgattcatt ttcattttca
34021 attgcaatca ttgctttatc aataatttgt ccaatttctg gtttttcgc attatcattg
34081 atatattgcc atcttgcttc ttttggcacc cagaaaatgt tttctgctaa gtattcatct
34141 tggtcctctt catctgcata tggatcctgc ttcaattctt catacttttc ttcaaaagaa
34201 tctgatacat attttaaaaa gattaaacct agtgctacat ttttatactc agcagcatcc
34261 atacttcctc tcaatttatc ggcagcttgc cataattttt cttcaaaacc tatagtggcc
34321 attaataaca cctcttaaaa ttaatgttaa atcaactata gcaaaaaata gaagatgatt
34381 gtgtagtgtt ttattaatat ttaatttatc tagactatca aaaaaatata gattattatt
34441 atcttaaatt aataaacagg agtgaattaa aatgaataaa aaatataaat taaaacgata
34501 cgataaaaga tttagaggtg gatggtgtta caaatgtgaa gattcatatt cttacctttg
34561 tgaagattgt ggttattgta gatattgttg ccattgttaa tacaagacta gataaattgg
34621 gtatctttaa ctcaattaat aaaaaattta aatgtagtca atagcttgct aactttattt
34681 gatgtaatat aattttggct tttagcattc agttaaaaag aagtaaataa ttgacagatt
34741 ttttagagaa ctggagaaaa aagatgtaca tttctagtgt taaaattaag aattttagaa
34801 attttgataa tatcgatatt gatttccatg agggagttaa tgtattaatc ggacataata
34861 attcggggaa atcaaattta ttacgtgctt tatctttaat ttttgacggt tcagttagaa
34921 agcaactgtc agtggaagat tttaataact ctttaacaaa agaatcttta aaaaaagagg
34981 ctccaaaaat agtaatttca gtacatataa cacaatctga aaacgaaaga ctgatgtctg
35041 atgagttaat tacggtaagt aattggctga ctaaattaga agaaccttat gaagctagaa
35101 tacaatatga attcttttta ccaaaagatg aggaaaaaaa ttacatagat ttagtaaaaa
35161 atatagatga aaaagaagaa atatggaatt taattagaag ccagtttatt aggttgtatg
35221 tcaataaaat ctgggtgggt aatccagaac atcagattcc tatagataat gatagtttaa
35281 ataaatttga tttccaattt ttagacgcaa taagagatgt agagagagat atgtttagtg
35341 gtaaaaatac attattaaaa agtgtaatag atttttttat agattacgat ataaaatccg
35401 atgaaacaat cacagaagaa gaacagaaag aaaaattaga agaaagaaga aaagaatttt
35461 ctgataattc tagtgatttg attgaaacta ttcataaaag attagatagt gggaatcaaa
35521 aaattttgtc ttatacaaat ggcataggag catcttatga taaatcaaca ccggatttta

35581 aaggtaatct tacagaaagt gaaatttata ctgtattgca attgataatt aaacatgaaa
35641 ctggaatgac tttacctata actcataacg ggttgggtta caataattta attttcatgg
35701 ccttgctatt gtctaaaatg caagctgatt ctgatggaaa ttttctaggt agtaatgcaa
35761 aagtatttcc gatactagct attgaagaac cagaggctca tctacatcct actatgcaaa
35821 atgaatttat taaattttta aaaaataaca ttagagaaaa aaaggtaaag cagattttca
35881 taactactca ttcaacacat atttcttctt ctacaaatat tgatgatatt atttgtcttt
35941 acacagatga tagtaaaact aatgttagtt atcctggaaa agtttttgat aagaataatc
36001 aaagtaagaa gtacgtacaa cggtttttag atgccactaa gtcaaatatg ctatttgcag
36061 aaaaggtaat atttgtggaa ggtatagcag aacaattact attacatata tttgcagact
36121 atttaaataa accgttagaa aagaatcatg ttgcagtaat aaatgtaggc gggagatatt
36181 ttaatcattt tttatctttg tttgatagta ataataaata tgcaattaat cgtaaggttt
36241 cttgtataac agatttagat ccaatgcgaa aagaaaaaaa tgaaaatgga caaaaaaata
36301 attttaaagc atgctatcct tttgaatatg aaatggattt agataaattt gattattcaa
36361 ctaataattc tctagataaa tatctaaata atgagcacag taatatacga gtatttgttc
36421 aacctaataa atatggaaaa acatttgagt accaacttat gtttgacaat ccttcattaa
36481 aactgttact tacagactcg atttctaata gtcaagagct agcagaatta atggatcact
36541 atgaagaaaa tgaatcttta caaaaattaa ttgatacatt atcaccatct aatgaaaatc
36601 agagaataat aaaatcttta aaagacacaa atgacagttg gaatgaagat aataaaaaga
36661 aagcattaat agcatcaaga tacctaaatt ccataggaaa aggagaaaat gctctagagc
36721 tagcttcagt attaaaagat aatttagagt taaaaggcca gagtgactat gaagattttg
36781 tagtaccaaa atatatagaa ggtgcaattc gatgggtgtg tgaataatat gattattaat
36841 gctcaaacga aattaaatat agaacaagat tttaaaatac aggctggtcc tggtgctgga
36901 aaaacagaat ttttggtgaa tcatatcaaa aatgtaatac aaacttctga aaagctcgaa
36961 cgtatgaaga aaatagcgtg tattacttat acgaatacgg ctgctcagac agttttacaa
37021 agattaggaa aaagtgtttc aaatagagta gatatatcta caatacatag ctttttgtat
37081 agaaacgtag ttaaaccata ttgttctttt ctacccaaag aatacggaat ttgtgtgcaa
37141 aaattaaaag gacatagtga tccaattgtg attaacaaat atgtaaatgc atggttaaaa
37201 actgaatatt ttacgaaatt aaaaccgcct agtaatagaa atcaactact aaaaatgcca
37261 gtactaaaga aagctttgca aaactggcta ctatctatga aactttctta cgaaaataat
37321 gatgtgcaat ttatttgtga taataccaaa gctcaagcaa ttgataaaaa aagtaaaaaa
37381 agaatgggaa taaataaatc aaacttaaat atactagaaa agcaattatt aaattataaa
37441 aaaatatatt gggagaaagg aattatcgat catgatgatg ttttgttttt ttcatatgta
37501 ttaattagag attacccatt cattctcact attttatgtg ctaaatatcc ctactttttt
37561 atagatgaat ttcaagatac tagtcaatta caagatttta ttatagataa aataagacag
37621 aaaggctgca tagtgggggt tattggcgat aaggcacagg caatttacag ctttcaaggt
37681 gcacgggttt ccttatttga gaagtttaaa gttgatttaa taaattctca cactatctta
37741 gaaaatcata gaagttcata tcaaatagtt acttttttaa atagtatcag agaagatatt
37801 actcaaacag tatatagtgg tattgaaaat gataaagtta caattttgat tggaaatcgg
37861 attcgcagtt atactaaggt agttaatatt tgtaacaagg agtcacttgt taccttgtca
37921 agagataata ttacttctaa tgcaatgaga ataaagttgg aaggaaataa ttttgataaa
37981 aagttattgg aaaaatttca agaaattgat agtaatagta aaagaaggaa tactattatt
38041 tcgcttataa aagctattga attagctaag aatactaaat ataaagatgc cttaaaaata
38101 gtcgaatcga tttataaaga tgaaaataat cctaaaaaaa tagctcttaa ttctctaata
38161 aatatgatta ataaatatga taaatacggt aatggtacat taatgaattt ctacaatact

38221 ttatgtaaaa ctttagatat taaactttca gggtttagaa aggggcaagt aaaagatttt
38281 tacgaaaaca ctttatataa aaatatggtg atatgtacaa atatcgttga agacacaagt
38341 aatcatatta ctatccacaa agcgaaaggc gcagaattcg aaaatgtatt tgttataggt
38401 aataaagaca ctttaagtct acttttgaaa ccagatttaa tgaacgatga agagcatcgt
38461 atattctatg tagctatgag tcgagcaaag aagaaacttt ttttacaatt tgattatctt
38521 gataataatg aacaaaagaa aattaaggaa cgttatgatg taagtattat acaataacct
38581 aatctaagaa tatttaaatc caatatattt cttgattata attcaataat agataaaatc
38641 tataaacgat taaaaagaaa atatatttgc taagacttat aaaaatgaag agataataga
38701 ttttcctaaa ttacagttaa aagaagtaaa tagattgaac actttgaaag attctaatcc
38761 aaaatttgaa ccactaaaat tgaaagatga agaagatgca agatgtagcc atcaacgctg
38821 atcacaaagt atttttctta atgattattt ttaatgttat catgctcgtt tcttctatta
38881 ttcaaatgat tttaggaaaa ggcagacgta tcacaccagc aaaacctact aaagatgcta
38941 agttacactt agataatcaa tcctcataaa gagaaaaaag aacccctaaa aagttcaact
39001 ttttgggggt tacttactat taagaagtat ttttgattaa gcatctttca tatcgtaatc
39061 accaatatac tgatcaaggc gatttattgt ttctctgata tcatcatgtg tcgttaatgg
39121 gttcgttgta cataaacgaa tgacacgttg gttattcaat actgtggtat aagcaatggc
39181 ataaccagaa tctgcaattt tttgcgcagc catgctattg agttgattat tttgtgcttc
39241 agttaaatca ctgttttcat atctgaagtt cacaatggaa agattagcgt gtgagataat
39301 acgccagtta tcgagacctg acacgtattc ttctgcatat tctgctaaac cttcgccgta
39361 tttaatgcgc tcgataattt catcttcacc aatgacttgc agagtaatcc ataattttaa
39421 agcacgtgca gggcgtgtaa gttcaatgcc taacatttct ggatcaatca catcatcatc
39481 tgaagcaata tcatccaagt actcagcatc ttcgccaaag ctttgcagca gatgattctt
39541 ttctttcacg attaccatag cgcaactata tgtttggaac agtaatttgt gagcatccca
39601 actggcacta tctgaacgtt caatgccttt aaataagtgt tgtgctttat cggataagat
39661 atgtgatagt ccataagcac cgtctacatg gagccataga ttgtagtgat cacatgtatc
39721 tccaagtgtg gtgaaatcat cgacagaacc tgtattagtc gtgcctgcag tcgcaatcac
39781 catagcaggt ttgtaacctt cttcaatatc tttatcaatt gcggctttta agctatctgt
39841 attcattgta aagtcatcgt tgtactcaat tctgcggata ttatttttaa ggaagcctgc
39901 aacatgcaag gctttgccta cagaatgatg cgtttgagat gtaagataaa cggttgcttt
39961 tttaatatct tccatttcta cctgtgcatc tctagccgca acgatagctg ttaaattggc
40021 cattgaaccg ccagatacaa agacaccacc ggccggtttg atctcataac cgatttttcc
40081 taccaagtag ttaattaaat tacgctcaat attaattggc agcgtcgcat tggcaaaatt
40141 agaggcatgg atattatttg ctgttgttaa aatgtctcca agccatgata gacgtgatgc
40201 tggacctggt ataaatgaga atgaacgtgg atggttcgga cgatataaat aattcagtac
40261 ttcattattt aaatctttga gtacttcata gatatctcgc cctttattcg gcacttccat
40321 cttttcatat ttctctctca attcgagcgg tgcctgttgt gtcgcaggta agtctccaat
40381 atctctagat aaatatttat taacttcatc tctgataatc gtctctaagt cgatattgtt
40441 ctcattgaat tccatttcca tactctctcc ttcaattata tttttaataa aagacagacg
40501 tattaattac gacgttttac tacaatataa atgatagcag atattaataa aaccacgcta
40561 accataatga cgaaacccag aaggtgtcgc cccaatgaat aacgtgctgt gctttaacac
40621 tcggtgtatg tgcgcctttc agaaattgac gagggtaatc gacttgcaga ttatcatctg
40681 tgattttcaa tttgtattga tgcttgtctt ttggaattcg ataactaccc cgtagaacag
40741 gggacgagga cctcatcttg atgatcccaa ttggcttggt taaaaagaaa tcgataatcc
40801 tcgtagaata gagaaaaagt attaaaaata tatttgattt gataaaatat tttgcaaaaa

40861 agagaaacaa aaataacttt catataaata atttctttgc gattaagttg gaattagaaa
40921 tactaaatac aaataacagc gaatggaatg catattattg aggaattgga tgtgtagtgc
40981 aatttcgaga gttactactt aacttaagaa aataaaaaga agaaaacaaa caataagaat
41041 taaatctgaa gcaagatata aaagcaaata tttttgctac aattatatta gtaaatactt
41101 tattaattag catacgactg aaagcgaagt aacagctaat aatgttccag atatagacat
41161 tagtgttatg aaagaacaat attaccatct agaaaatgta atttttaaaa acctgaagat
41221 aaagtagact attctgtgag ttttcatgtt aagaattctg aacttgggga tgtagtataa
41281 atatggttta gtagaaaatg acgctggagc tgtagctgag attgactatc aagaattaac
41341 taaagtagat tattacccat caacaaaaat ccaagattta acactatcta ataatagtgc
41401 gaataatgaa gtttaaaagt gaaaaactga taatagcgaa tcaaactaaa gtaatcaatt
41461 acagaattca agtactactc aagcctcaaa caaatattga aactcaaaca caagttaaga
41521 ataatacact gtcagaaaca gatgaacagt ctaatgccga tttagtccag taatagcctc
41581 tgtattatta gccgcacgat tattatcaat aattaaataa agttaatatt aaataataaa
41641 aatccttcaa attatttgtt gaaaaaataa taactttgaa ggttttttag caaaatcact
41701 gatagggaga agcgtatcat aaat
//
LOCUS Staphylococcus 55111 bp DNA linear UNK
DEFINITION Staphylococcus Staphylococcus aureus subsp. aureus ST398
ACCESSION Staphylococcus
KEYWORDS .
SOURCE Staphylococcus Staphylococcus aureus
ORGANISM Staphylococcus Staphylococcus aureus
Bacteria; Bacillati; Bacillota; Bacilli; Bacillales;
Staphylococcaceae; Staphylococcus; Staphylococcus aureus.
FEATURES Location/Qualifiers
source 1..55111
/db_xref="taxon:703339"
/genome_md5=""
/project="hwwang_703339"
/genome_id="703339.347"
/mol_type="genomic DNA"
/organism="Staphylococcus Staphylococcus aureus"
CDS 1..480
/transl_table=11
/translation="MKITILAVGKLKEKYWKQAIAEYEKRLGPYTKIDIIEVPDEKAP

ENMSDKEIEQVKEKEGQRILAKIKPQSTVITLEIQGKMLSSEGLAQELNQRMTQGQSD

FVFVIGGSNGLHKDVLQRSNYALSFSKMTFPHQMMRVVLIEQVYRAFKIMRGEAYHK"
/product="23S rRNA
(pseudouridine(1915)-N(3))-methyltransferase (EC
2.1.1.177)"
/EC_number="2.1.1.177"
/db_xref="SEED:fig|703339.347.peg.1"

CDS 726..1031
/transl_table=11
/translation="MKGNIQCPRLIKGSLLDFVLETVDVFVNGSKNDCELVNFNYKEG
KVVSRYSTKFESNPKLRKKQLISTVKVVRYVIFDFEKILWGIGKVLLKFIILRQCLL"
/product="hypothetical protein"
/db_xref="SEED:fig|703339.347.peg.2"
CDS 1230..2093
/db_xref="SEED:fig|703339.347.peg.3"
/product="hypothetical protein"

/translation="MKHINQFSESTLAQLEKDQQHVFYVYCLMDPRNDECFYVGKGKG
NRIFKHKQDAQKKVLYEDIFIEENKDNLKFNRINEICSNDLNVLGYIISYGLTESEAF

SAENVLINFLNLTNKTTLTNMINGHGSKAYLVEDLENEFGYDSINLENINTNELILAV

KIRDAFLLDKDESKEYPINESKRDRNNLKSRTLGSWIIGKDKIHKIKYIIGINTGANN

AVVSAYEVSFEQAESIETNNGRMRYAFIGLSERDATLKKLNLYKKALPDLRFGSGSAT
AYINNGTMKVD"
/transl_table=11
CDS 2201..3676
/db_xref="SEED:fig|703339.347.peg.4"
/product="hypothetical protein"
/translation="MPSDDIVKKLYSKEDIRLELGLTPHKFNKKMETIAKLFKIDMKI

FHNYKGQDKNNQYTFNGVAKELIKVLLKSVDYYPVDINSKKFKQNGKSKKEMIENIDN

SSYMKYIYQLMKSINEIQYKRLIADIHMKDVYQNTKAWLNNGESINKKEQELYQYMTI

LPLHKRVELQNEVLKSIDETIFQFLAKEHRNNQIEENNELEAYTKAIKEGRNPKNDYE

LNHLLYKKNQLPLDSLIEDIWDYEETEYTELDWLIADMLKRSQRADSNFIESLEKKNK

LRKNIHKDSIKSISKLIDHELVKNNRKWDVAEFYKNQQFWNNSTLRRSSQNFGILYYK

KNRLHTMNQSNIIKKIKIIEANIESANKQPSYLDFFLQAKFDLERLESELMKYSINPK

YFNKINSDYVRYAIEDVHNAIIKIDRYISNDKVKLNYSNVQMVVRNEITEQGSYFVTQ
ALNTLSKVEESGYSFDNFMVGPFVEDFKKAIKNRRFGSKKD"
/transl_table=11
CDS 3901..5001
/product="hypothetical protein"
/db_xref="SEED:fig|703339.347.peg.5"
/transl_table=11

/translation="METRDKLMSLTQSDKTQQWLMDKSSNQDDIQQLQQQFSQQLDQQ

YNALLADEKAKLDQYVEVHQGLESLKEEIESEPITLNIDKLPDIKATMLERAKNDEHS

DKIEKLFDRLEQALNGTNRLYTQLSLIGTRTHRITTKNFNLQGLPKAVQHTILPSKFK

KVYTVDFKSFEPSVAAYMTQDSKLIDLLNQKDGLYDALLSELGLSDELRVFVKRAFIG

SFLFGGNFKNPKFKLNQYVSEVQWLDAVSQFTKVIELKKHVEKSNSMPMPYSIEHDMS

AFQGSSIMAIYVQTVASYIFKHILLKVYKAQCDQKTFKIIVPIHDAIMIECEDEEIAQ
NVGQLMKDTANQLFNGEFAHVTVEEIGGVDHE"
CDS 4994..5365
/product="hypothetical protein"
/db_xref="SEED:fig|703339.347.peg.6"
/transl_table=11
/translation="MNNDRGQSLHIPSSTPIKENNIYVATLHSVIQTDFSGEIKHQFT

YEIEVNNQIVYANRNIPTKPSANQLSIHDWLKRHSNYSASHENYEPYIDQKHLILLGQ
YNGNYYVQDVASLDAFGGVLS"
CDS 5362..7005
/db_xref="SEED:fig|703339.347.peg.7"
/product="putative primase"
/transl_table=11
/translation="MNHILQMLSKLLSVAKEAIDRQGLIAILTIPVNNNDEIEETAQG

ETVYNELIDQLRLNIPKDTDYQPNIYSYFGIKKNPNDTVLMEMMIKVFHIKRFNSELF

IFKVNGWQKINGDELQGLISKMIQVLLVDYKPSLSTLKNVVDGLQKSTDVEELVENER

YIGCGENMFDLNTFQVVKNSIDIFPKTRLNLSLSTNDVITDKIPPYFKQYMLQLANYD

DDLQYFLFQHTAVLLTADTKYRRGLILYGGAKNGKSVYIELVKSFFYSKDIVSKPLNE

LEGRFDKESLIDKSLMASHEIGQSRIQEKIVNDFKKLLSVESMHVDRKGKTQVEVILD
LKLIFSTNAILNFPPEHAKALERRINIIPCEYYVEKADTSLIDKLQSEKKEIFLYLMY

VYQQIVKADIEYLENSRVTEITHDWLNFGYEFVSSRSVSNANQKACINLLRKLIEIKS

GSRIKVSELNKVINEEIKVSSQVIKQLIQANFDTQTKLYNGYDYWIDLGWKEANKKEI
HDISEKDNIISLDKNENITDDEALDEENLDFDWEDFDDE"
CDS 7231..8907
/product="Cassette chromosome recombinase B"

/db_xref="SEED:fig|703339.347.peg.8"
/transl_table=11
/translation="MKGKIALYSRVSTSEQSEHGYSIHEQEQVLIKEVVKNFPGYDYE

TYTDSGISGKNIEGRPAMKRLLQDVKDNKIEMVLSWKLNRISRSMRDVFNIIHEFKEH

DVGYKSISENIDTSNASGEVLVTMFGLIGSIERQTLISNVKLSMNAKARSGEAITGRV

LGYKLSLNPLTQKNDLVIDENEANIVREIFDLYLNHNKGLKAITTVLNQKGYRTINQK

PFSVYGVKYILNNPVYKGYVRFNNHQNWAVQRRSGKSDKNDVILVKGKHEAIISEEVF

DKVHEKLASKSFKPGRPIGGDFYLRGLIKCPECGNNMVCRRTYYKTKKSKERTIKRYY

ICSLFNRSGSSACHSNSINAEVVERVINVHLNRILSQPDIIKQIASNVIEELKQKHSN

QTEIKYDIDSLEKQKAKLKTQQERLLELFLDDQMDSEMLKAKQSQMNQQLEVLDQQIK

EAQQANQSQDEIPNFDKLKGRLILMITRFSVYLRKATPEAKNQLMKMLIDSIEITTDK
QVKLVRYKIDESLIPQSLKKDWGSFFMPKFQFEIYGQNDYFIDQITTFTT"
CDS 9013..9351
/transl_table=11
/translation="MTLEQQLKHYITNLFNLPRDEVWHCESIEEIADDILPNQYVRLG

PLSNKTLQTNTYYSDTLHESNIYPFILYYQKQLIAIGYIDENHDMDFLYLHNTIMPLL
DQRYLLTGGQ"
/product="FIG01108299: hypothetical protein"
/db_xref="SEED:fig|703339.347.peg.9"
CDS 9447..9758

/translation="MNRYITRGIANNLPNILQHQLWQLVSEREQEQTKDNTSVDYFHI

FQFNKHRNQLYIKHKQERPEYAKIHKANYSKAININKVYIIREDDVDLSYYVMLLPEE
Y"
/transl_table=11
/db_xref="SEED:fig|703339.347.peg.10"
/product="FIG01107894: hypothetical protein"
CDS 9774..10280
/transl_table=11
/translation="METIKSTLKTEAIFSDDKQHRYLLKKTWNSEKQSITIITMYPHY

DGILNIDLTTQLIMNKVSEMDAFGSINFVNLYSNITTPINLKHLENAYDKHTDIQIMK

AVKESDEVILAWGAYAKKPGVEARVNEVLEMLKPHKKKVKRLMNPETNEIMHPLNSKA

RQKWILKV"
/product="hypothetical protein"
/db_xref="SEED:fig|703339.347.peg.11"
CDS complement(10429..11103)
/translation="MNYFRYKQFNKDVITVAVGYYLRYTLSYRDISEILRERGVNVHH

STVYRWVQEYAPILYQIWKKKHKKAYYKWRIDETYIKIKGKWSYLYRAIDAEGHTLDI

WLRKQRDNHSAYAFIKRLIKQFGKPQKVITDQAPSTKVAMAKVIKAFKLKPDCHCTSK
YLNNLIEQDHRHIKVRKTRYQSINTAKNTLKGIECIYALYKKNRRSLQICGFSPCHEI
SIMLAS"
/transl_table=11
/db_xref="SEED:fig|703339.347.peg.12"
/product="Transposase for insertion sequence-like element
IS431mec"
CDS 11361..11528
/product="Hydroxymethylglutaryl-CoA synthase N-domain
homolog (EC 2.3.3.10)"
/EC_number="2.3.3.10"
/db_xref="SEED:fig|703339.347.peg.13"
/translation="MLESREQLSVEEYETFFNRFDNQEFDFERELTQDPYSKVYLYSI
EDHIRTYKIEK"
/transl_table=11
CDS 11626..11856
/transl_table=11
/translation="MLIFLSYDFVRACLPRGMARAISLSHILFPQASALTKSVVIFIF
IRILTEIILIRGIVKAILSKIADKRFVKSSSK"
/product="hypothetical protein"
/db_xref="SEED:fig|703339.347.peg.14"
CDS 12405..13148
/db_xref="SEED:fig|703339.347.peg.15"
/db_xref="GO:0008889"
/EC_number="3.1.4.46"
/product="Glycerophosphoryl diester phosphodiesterase (EC
3.1.4.46)"
/translation="MLTVYGHRGLPSKAPENTIASFKAASEVEGINWLELDVAITKDE

QLIIIHDDYLERTTNMSGEITELNYDEIKDASAGSWFGEKFKDEHLPTFDDVVKIANE

YNMNLNVELKGITGPNGLALSKSMVKQVEEQLTNLNQNQEVLISSFNVVLVKLAEEIM

PQYNRAVIFHTTSFREDWRTLLDYCNAKIVNTEDAKLTKAKVKMVKEAGYELNVWTVN
KPARANQLANWGVDGIFTDNADKMVHLSQ"
/transl_table=11

CDS 13245..13673
/translation="MKYDDFIVGETFKTKSLHITEEEIIQFATTFDPQYMHIDKEKAE

QSRFKGIIASGMHTLSISFKLWVEEGKYGEEVVAGTQMNNVKFIKPVYPGNTLYVIAE
ITNKKSIKKENGLVTVSLSTYNENEEIVFKGEVTALINNS"
/transl_table=11
/product="MaoC domain protein"
/db_xref="SEED:fig|703339.347.peg.16"
CDS complement(13719..15725)
/product="Penicillin-binding protein PBP2a, methicillin
resistance determinant MecA, transpeptidase"
/db_xref="SEED:fig|703339.347.peg.17"
/db_xref="GO:0008658"
/db_xref="GO:0046677"
/transl_table=11
/translation="MKKIKIVPLILIVVVVGFGIYFYASKDKEINNTIDAIEDKNFKQ

VYKDSSYISKSDNGEVEMTERPIKIYNSLGVKDINIQDRKIKKVSKNKKRVDAQYKIK

TNYGNIDRNVQFNFVKEDGMWKLDWDHSVIIPGMQKDQSIHIENLKSERGKILDRNNV

ELANTGTAYEIGIVPKNVSKKDYKAIAKELSISEDYIKQQMDQNWVQDDTFVPLKTVK

KMDEYLRDFAKKFHLTTNETESRNYPLGKATSHLLGYVGPINSEELKQKEYKGYKDDA

VIGKKGLEKLYDKKLQHEDGYRVTIVDDNSNTIAHTLIEKKKKDGKDIQLTIDAKVQK

SIYNNMKNDYGSGTAIHPQTGELLALVSTPSYDVYPFMYGMSNEEYNKLTEDKKEPLL

NKFQITTSPGSTQKILTAMIGLNNKTLDDKTSYKIDGKGWQKDKSWGGYNVTRYEVVN

GNIDLKQAIESSDNIFFARVALELGSKKFEKGMKKLGVGEDIPSDYPFYNAQISNKNL

DNEILLADSGYGQGEILINPVQILSIYSALENNGNINAPHLLKDTKNKVWKKNIISKE

NINLLTDGMQQVVNKTHKEDIYRSYANLIGKSGTAELKMKQGETGRQIGWFISYDKDN
PNMMMAINVKDVQDKGMASYNAKISGKVYDELYENGNKKYDIDE"
CDS 15973..16116
/transl_table=11

/translation="MNYFRYKQFNKDVITVAVGYYLRYALSYRDMSEILRERGVNVHH
LTV"
/product="Mobile element protein"
/db_xref="SEED:fig|703339.347.peg.18"

CDS 16396..16647
/transl_table=11
/translation="MAKVIKAFKLKPDCHCTSKYLNNLIEQDHRHIKVRKTRYQSINT
AKNTLKGIECIYGLYKKNRRSLQICGFSPCHEISIMLTS"
/db_xref="SEED:fig|703339.347.peg.19"
/product="Mobile element protein"
CDS complement(16707..17135)
/translation="MKFQVSPYFLITQNAEGLKKLYEELFGAKTLYIQRIKDRPIDKK

LNIDENDLNKIDQCVLQFGDIKIMIADDTEGLPITQGNNISLCLTFDQVEETKHVYDQ
LIEKGSEVLKTFSPEFYTEGYGYVRDPYGISLHLFTKRKN"
/transl_table=11
/db_xref="SEED:fig|703339.347.peg.20"
/product="PhnB protein; putative DNA binding
3-demethylubiquinone-9 3-methyltransferase domain
protein"
CDS 17216..18145
/db_xref="SEED:fig|703339.347.peg.21"
/product="Transcriptional regulator, YafY family"
/translation="MKKSVRLYNMIEYCNENRNFKLNDLMSEFNISRSTALRDIKEIE

ALGVPLYSNPGKNGGYTIIGNRDQTKIAISDEELKALVFTLSSISNVSNLPFQTEYQE
ILKKLYNNSNKKELINQYNDLFQYFNEDKYQFKSYKLFNEIIRLIIENKSFETCYSQN

YIKEQYKGIGIMYKNHQWYFVVANIESKLVNLLNISKIKELYEMGETQECNDITMQNF

QQFMVKNETAIDILIRSNVMGLNILKGYLWSDYMIENIDEETYLFKSKVNAKDIDFIA
KLIVTGGVNVKVETPNSLKNAVKVELTKIINMY"
/transl_table=11
CDS 18307..20295
/db_xref="SEED:fig|703339.347.peg.22"
/product="Similarity"
/transl_table=11
/translation="MNKNITALKSTENTTYMQYTVHIDDQIINNIKECTKKFKILPME
DKIPLSPLLQPEYAGEVQDFISTYEQFMINFGKVILDSQGIKIQFESESLSSIQRGIQ

EHCYLNERTNDIDVTKEWYLCKFSNQILEEDKNQLYNALKQLMNDSKNKKQAFQEVFK

VHIDIYLYNKEKSEYKYQVSSYFNLVKENPKITYKKRHLQEKQGVKGTTFTNKLKLLN

KMYGVDVAKYQPFYNSNNPEYERGQFGERYISQRSNYEFNRLQYQIIDMLSKILDKHP
LPKSDNNYKHIPTIEKAILSGDSHSFYEYFEDIMKEIISMENSSLKEKLLTDFTYQSQ

CRWYSESEKLKLQLESFMHKVLESNYYEGNKLYRMLSHAIEETINEADEDKVHFNYFK

DYFLTDGGVKNWEQISEKITEFNGKVINDIQNEYNKIQFNNANRNQKLNFNYLYHCFE

FSNNLVKARVNGNRGYILYFVDKYKIKMPFLDQLLINYLNQEIGQESINYNMQTLFEK

ERYDRSSTIEKLVATSKFKYEKDDSDLFKQLFNDVENSIDRLGIYLLNNGINSNDENA

RYYRSFLKELSRIKSKLTPFSLEISKSSGREQHYPDDAIDDKDERRKNKEETYHAFDD
KSDIDSKLKNKINVSIDNLLSVKIKSNLYDISPNTKKQ"
CDS 20490..21599
/transl_table=11

/translation="MYQANIRDLITKLPQSNKTEHFLMNKFSNQDKVQQLQRQISQQL

DQQYNELLANEKAKLDQYVEVHHNLEPLKKEIESESINLDTDKLPDIKATMLEKAKND

EHFDKIEQLFDRLDQSLNGTNRLYTQLSLIGTRTHRITTKRFNVQGLPKLVQQMILPS

QFKKVYTIDFKSFEPSVAAYMTQDEQLIDYLNHEEGLYDALLRDLSLSKEKRVSVKRA

FIGSFLFGGRYSSSKFKINQEVSEINWLQVMSKFKKVIEFKEQVEKYKTMPTPYGIEH

DMSAFQGSSIMAIYVQTVASYIFKHILLEVYKAQCEKKTFKIIVPIHDAIMIECNDKG
IAQNVAQLMKDTANQLFNGEFAHVTVEALGGIDNE"
/product="hypothetical protein"
/db_xref="SEED:fig|703339.347.peg.23"
CDS 21592..21960
/translation="MNNDRGKSLQIPQSTLLKEGSIYVATLHSVYEKNFSGDIKHQFT

YEVELNQETHYVNRNITVKSMSHQLSIADWIKRHSNYNVNHINYDPYIDRKHLVLVGQ
YNGNYYIQDVAPLDEFGGVL"
/transl_table=11
/product="hypothetical protein"
/db_xref="SEED:fig|703339.347.peg.24"
CDS 21960..23576
/translation="MNHILEMLIKLLKVGMEAIDRKGLIAILTSSIGNDEMDDSEQAV

MVYNELIDKLQLNIPKDVDYRPNIYSYFGIQKKPNDTILVEMMICIFHIKRFDSELFV

FKDKGWQKVSEDELQGLISKMIQVLLVDYKPSLSTLKNVVDGIQKSTDIEKLVEHRQY

IGCGRNMFNLKTFKVVDNDLEIFPKTRLDLELDINDTITDKIPPNFKQYMLELANYDH

DLQYFLFQHMAVLLTADTKLRRGLFLYGTAKNGKSVYIKLVKSFFYSNDIVSKTLNEL

GGRFDKESLIGKRIMASDEVGKANIDEATVNDFKKLLSVEPIHADRKGRTQVEVTLDL

KLIFNTNAVLNFPSSHAKALERRIAVIPCEYYVEKADPDLIEKLQDEKKEIFLYLMYV

YKQIVKNDIEYLQNDRVTEISHDWLNFGYEFVSSKSANIAHQKACINLLRKLIEIKPG

SRIKVSRLNEVIRDEIKVSSQVINDLVQANFNVQSRLNNGYKYWVDLGWKETDKKDDM
ISFDKNENVTDDEFLYEDDLNLGWEDFDDE"
/transl_table=11
/product="putative primase"
/db_xref="SEED:fig|703339.347.peg.25"
CDS 23801..25480
/db_xref="SEED:fig|703339.347.peg.26"
/product="Cassette chromosome recombinase B"
/translation="MKGKIALYSRVSTSEQSEHGYSIHEQEQVLIKEVVKNFPGYDYE

TYTDSGISGKNIEGRPAMKRLLQDVKDNKIEMVLSWKLNRISRSMRDVFNIIHEFKEH

DVGYKSISENIDTSNASGEVLVTMFGLIGSIERQTLISNVKLSMNAKARSGEAITGRV

LGYKLSLNPLTQKNDLVIDENEAHIVREIFDLYLNHNKGLKAITTILNQKGYRTINQK

PFSVFGVKYILNNPVYKGYVRFNNHQNWAVQRRSGKSDKNDVILVKGKHEAIISEDVF

DQVHEKLASKSFKPGRPIGGDFYLRGLIKCPECGNNMVCRRTYYKTKKSKERTIKRYY

ICSLFNRSGSSACHSNAINAEVVERVINVHLNRILSQPNVIKQIASSVIEELKQKHSK

QTEIKYDIDSLEKQKAKVKTQQERLLELFLDDEMDSEMLKAKQSEMNQQLEVLDQQIK

EAKQANQSQDDIPNFDKLKARLILMITRFSVYLRKATPEAKNQLMKMLIDSIEITTDK
QVKLVRYKIDESLIPQSLKKDWGSFFIPKFNFVINVTKKNRIENLSLLPLF"
/transl_table=11
CDS 25569..25907
/transl_table=11
/translation="MTLSKQLKTYITERFKLNYQETWACETIDAVAEDVLPEKYIKNS
PLEHKILNTFTYYNDELHEISIYPFLCYLDKELVAIGYLDNFDLDFIFLNDTHQVIID
ERYLLQKGGQ"
/product="hypothetical protein"
/db_xref="SEED:fig|703339.347.peg.27"
CDS 26001..26312
/db_xref="SEED:fig|703339.347.peg.28"
/product="hypothetical protein"

/translation="MNINRYITRGINESIPLDLQILLWNMVKERDNQPHTDYLHIFRL

QEDENILSITHEQEQPAYKLEYHYINYVKNQNTLPKKVYVIRENDVDVFYYVMLLPEE
Y"
/transl_table=11
CDS 26327..26797
/db_xref="SEED:fig|703339.347.peg.29"
/product="hypothetical protein"
/translation="MNTIKSTIHTEAIFSDDEQHRYLLKKTWDEKKTACTVITMYPHL

DGVLSLDLTTVLILNQLANSEQYGAVYLVNLFSNIKTTENLKHIKEPYDKHTDIHLMK
AISESDAVILAYGAYAKRPVVVERVEQVMEMLKPHKKKVKKLINPATNEGSVAK"
/transl_table=11
CDS complement(26838..27512)
/db_xref="SEED:fig|703339.347.peg.30"
/product="Mobile element protein"
/transl_table=11
/translation="MNYFRYKQFNKDVITVAVGYYLRYALSYRDISEILRERGVNVHH

STVYRWVQEYAPILYRIWKKKHKKAYYKWRIDETYIKIKGKWSYLYRAIDAEGHTLDI

WLRKQRDNHSAYAFIKRLIKQFGKPQKVITDQAPSTKVAMAKVIKAFKLKPDCHCTSK
YLNNLIEQDHRHIKVRKTRYQSINTAKNTLKGIECIYALYKKNRRSLQIYGFSPCHEI
SIMLAS"
CDS 27528..27662
/product="hypothetical protein"
/db_xref="SEED:fig|703339.347.peg.31"
/transl_table=11
/translation="MLKLYYKFNFATEPMKEPIERTKRISKRVGNLEFVLFTNSAKLL
"
CDS 27670..28197
/db_xref="SEED:fig|703339.347.peg.32"
/db_xref="GO:0003917"
/product="DNA topoisomerase III (EC 5.99.1.2)"
/EC_number="5.99.1.2"
/transl_table=11
/translation="MSYDKREQFNFIKQLFNDSSIRTIVNGCDSDREGSNIFYSSYYM

TGAKNKEIKRLWINSLEVDEIRKGFNNLQDNKKDLLLYYEAKTRQISDWLVGMNGSRL

FTLLLQQKGFNDSLSIGRVQSSTVYLIYQRQKEIEQFVSTPFYEIEGSFTAKNGMYKG
KAKIKSETLKLQLML"
CDS complement(28474..29088)
/translation="MKDETNNTGIRMFKRYAAITTSAKILSHVLATDIDIAKIRDYFI

DYHGHTVSERSLADKAIEVITQFVAQNRGKFSDDTALKNMIENHGLIALKDDYIEVKI

IASVFKDMLIENHFQDVNNVVNALKDKGFIESDRDRITTKRTVKDDNGKKQSLVFYHL
KLDPEYASIFGLTKDAEPIKAEINLDNKKMLESWKKQKLLIFLM"
/transl_table=11
/db_xref="SEED:fig|703339.347.peg.33"
/product="hypothetical protein"
CDS 29175..29450
/transl_table=11
/translation="MIDKLAKGAIDAETFRKQSQSLLQQSKPTLSINEQQIQRSFENV
IQQHFTLSMLYRYIDEIHISKNKSLVGIYFKNEPLNIVNQATQSSIA"
/db_xref="SEED:fig|703339.347.peg.34"
/product="Cassette chromosome recombinase A"
CDS complement(29562..29921)
/transl_table=11
/translation="MDDRALASALGIGVGGGLAWIMEGFQSGHGFIYSLCAGLIIGLL
FFEMIPESIELGGWVILLGGIAIGLVLFQSIHLLMDKITIITNSHQKDIFVRSGVLLI
ISIAIHNFPVGNIPLFV"
/product="hypothetical protein"
/db_xref="SEED:fig|703339.347.peg.35"
CDS 30128..30454
/transl_table=11
/translation="MEEKKELEEVNNKDLDDETLFVVSQTFKALGDPTRIRILHLLFY

KEYSVNGIAETLHLRQSTVSHQLRFLKNLRLVKFRREGTTLFYSHDDEHTMNMLKQAI
DHACHH"
/db_xref="SEED:fig|703339.347.peg.36"
/product="Transcriptional repressor CzrA, ArsR family"
CDS 30784..32709
/db_xref="SEED:fig|703339.347.peg.37"
/db_xref="GO:0004008"
/product="Lead, cadmium, zinc and mercury transporting
ATPase (EC 3.6.3.3) (EC 3.6.3.5); Copper-translocating
P-type ATPase (EC 3.6.3.4)"
/EC_number="3.6.3.3"
/EC_number="3.6.3.4"
/transl_table=11
/translation="MKNIQEQQAHESHSHDHSHDHDHGKMPIISYFIGLVLAIIGLFL
SDANLLIQNILFSIATITAGYHVIILEGIGETVENTKLKGKFTPNSHILMGLAAIGAS
LIGSFWEGTLLILIFSGAHFLEDYAEGKSKREITKLLEMNPTTAKLILPDGNTKIVDV

SELKVGDQLQVLNGDQVPIDGIILSGTTSIDESSINGESIPKEKSKGDEVFGSTINGT

GTFTMEVTKENKDTVFSKILQLVSQNQDNQTKAASIIQKFEPKYVNIVLIAIPLVMLL

APFLFDWTWSQSVYRGLVLLVAASPCALAAATVSVTLSTTSNLAKKGVLSKGSTYLSQ
LADIDAIAFDKTGTLTNGEPKVTNYYFTHSVNEENIIDIIVALEKESNHPLANAILEK

FEVKNKIDIEVTNQIGKGLTGDYNGKNYRIGKPTSFESASEEYTQFNHDWASEGKTVV

YVAENEEVIGIIALMDIPNEHAKETINYFKKLGIHTTLITGDSEMTGKAVGEQLGIDE

VIANVMPEDKSRIIEEQKEKFGVTAMVGDGVNDAPALVNADVGIAMGGGTDVAVEVSD

LVLMQNNLSKLVQSHKISSNMGRVIRQNIIFSMAVVAFLVVISLLGLTDITISVIVHE
GSTLVVILNGLRLLRSK"
CDS complement(33527..33661)
/db_xref="SEED:fig|703339.347.peg.38"
/product="hypothetical protein"
/transl_table=11
/translation="MVLTFSKTKFTCRHIECVLLGSNLIKQKFKREIISTGFLTRRLF
"
CDS complement(33855..34415)
/db_xref="SEED:fig|703339.347.peg.39"
/product="Zn-dependent hydroxyacylglutathione hydrolase /
Polysulfide binding protein"
/translation="MNLYQPYTVYPATNTNRLTFDLRSKEAYHGGHIEGTINIPYDKN

FINQIGWYLNYDQEINLIGDYHLVSKATHTLQLIGYDDVAGYQLPQSKVQTRSIHSED

ITGNESHILDVRNDNEWNNGHLSQAVHVPHGKLLDTDLPFSKNDVIYVHCQSGIRSSI
AIGILEHKGYHNIINVNEGYKDIHLS"
/transl_table=11
CDS complement(34434..35180)
/translation="MFFKQFYDKHLSQASYLIGCQKTGEAMIIDPIRDLSSYIRVADE

EGLTITHAAETHIHADFASGIRDVAIKLNANIYVSGESDDTLGYKNMPNHTYFVQHND

DIYVGNIKLKVLHTPGHTPESISFLLTDEGAGAQVPMGLFSGDFIFVGDIGRPDLLEK

AVKVEGSSEIGAKQMFKSIESIKDLPDYIQIWPGHGAGSPCGKSLGAIPTSTLGYEKQ
TNWAFSENNEATFIDKLISDQPAPPHHLHK"
/transl_table=11
/db_xref="SEED:fig|703339.347.peg.40"
/product="Zn-dependent hydroxyacylglutathione hydrolase /
Polysulfide binding protein"
CDS complement(35199..36266)

/transl_table=11
/translation="MSQYKTKHINDFTKKELEELGSKDQLIDVRQPEEFELGHIKNAL

LHSVENIETFKQSKNKTYYIYCKSGNRSRKASQFLAQRGYDVVNLDGGYTAYEEQHHN

DNLSKVKEDREIKDNRKTFNYSNLQCPGPIVNISKEIKNIAIGDQIEVVVTDHGFLND

IKSWVKQTGHTLVRLNDFGNEIHAIIQKEENKNIEVTHTKSGTTIVLFSGELDKAVAA

MIIANGAKAAGRDVTIFCTFWGLNALKKIQSQRIKKKGIAKLFDFMLPNSPINMPISK

MNMFGLGNLMMRYVMKKKNVDTLPSLIDQAVEQGVKLIACTMSMDVMGITKEELRDDV
EYGGVGAYIGYTEQANHNLFI"
/product="Disulfide bond regulator"
/db_xref="SEED:fig|703339.347.peg.41"
CDS 36405..36665
/db_xref="SEED:fig|703339.347.peg.42"
/product="FIG007303: uncharacterized protein"
/transl_table=11

/translation="MEYNKKMINRIHRIQGQLNGVIKMMEEEKNCKNVISQLSASKSS
IQRLMGIIISENLVECVKMSEENSEDSQALINEAVELLIKSK"
CDS 36662..37420
/product="FIG003846: hypothetical protein"
/db_xref="SEED:fig|703339.347.peg.43"
/transl_table=11
/translation="MISITTIIVMLVIGILGGFISGLVGIGGAIVIYPALLLLPPLFG

LPTYSAYIASGLTSSQVFFSTLSGSLNAYKNKNFSRTLILNMGSGMVIGSMLGAILAN
LINVQFVNTVYIIIALLALILMFIKVTPSTSHIKFNRLLLITIGGIIGLVSGIVGAGG
AFIIIPVLLVIFKLPMNMVVTNSIVIAFISSIGAFMIKLLQGYIPINSAIPLILGSIL
FTPLGMKMGQKIPDYIQKTIICILIVIAIIKLIF"
CDS 37620..37799
/translation="MRKMVQNLWWGAGYNIVAVPLAAGILAFIGLILSPAIGAILMSL
STVIVAINAFTLKLK"
/transl_table=11
/EC_number="3.6.3.3"
/EC_number="3.6.3.4"
/product="Lead, cadmium, zinc and mercury transporting
ATPase (EC 3.6.3.3) (EC 3.6.3.5); Copper-translocating
P-type ATPase (EC 3.6.3.4)"
/db_xref="SEED:fig|703339.347.peg.44"
/db_xref="GO:0004008"
CDS 37817..38362

/translation="MIKKLFFMILGSLLILSACSNNDEKDKDTNDQKSESHMKHNDES

KVPEDMTSTNEGEFKVGDKVTITAGHMPGMKGAEATVKGAYKTYAYVVSYKPTNGNEK

VSNHKWVVNEEIKDAPKDGFSKGDTVKLEASHMSGMKGATANIDNVKKTTVYVVDYKS
KDNGKIIKNHKWMTGNELKAR"
/transl_table=11
/db_xref="SEED:fig|703339.347.peg.45"
/product="DUF1541 domain-containing protein"
CDS 38643..40433
/transl_table=11
/translation="MEKFKEKVNDLEAIKTTRYYTEFVEKFKKIRDWAKNENLKNEAK

LAQYEIEIFSLCEKNPILSKNKSEKRFVATISFDDGREWPDIQKFTNDQIKYYEQRLN
ETNNLFLKVRYSDFLFEHGNKKITKTKYEISKCLLSCLVEILTYYSDDFNYTSVLARL
VEVSLLMGDREKLEKAIELIYLKMDEFDYNNEYSYVYELSKLIREILKSKHKKIILEN
HLNKIIIVLEKAIKNNFEDKNYYLHRVFCEELSQYRKFDLISSERRSELKKEIGKSYE

LEAEYQQGRNNKSLLVKANFLEKAMEKYMEIGEREKSNKMKILVKWTYEEYENSNEMN
LIRIPIEFPKEEIDKIIEGFISSDVQISLDKIAYSNDLIPKITVIEDLVDKLSKEFPL

QGLISKGLLNDGKKVVETTTEEDNKTINFNSNYMHHLNINVELLLKAIFDKLISEFEI

STEDFMQKFNKWELLDNKNVPFIEHGISRFLEKDYLSALHILVPQFESTVRRMFSKAG

YSTTSIRKGNTQQEVTFNEFLLRDDVKSTLGNDVHKLIQIVMVEQSGLNLRNEIAHGL
IELSNINYTKCILVIYLFLILTRYSIEKND"
/db_xref="SEED:fig|703339.347.peg.46"
/product="hypothetical protein"
CDS 40474..41127
/db_xref="SEED:fig|703339.347.peg.47"
/product="hypothetical protein"
/transl_table=11
/translation="MKLEELLLITQQPLIEYSKNKKLLEGSRLFDIDERMDERKIPKI

LTNSDKYHVVEIANYDNLIIIDNEVINGNELIQVGQCIDFDSNVMSYLRNLIVNNKYE
DDFFKILTNIKKSKQQISCVPYLIENGNNIHRINKIISYETILSFSIFDRISEFDFEN

RNFSRYFNDSEVILDTDDRYYHMMNIQDSNILQFQAIYLLVLMAFFRQCVNRKMRIS"
CDS complement(41313..41762)
/translation="MLTIIERKTRYLYASRLNTRKSNVVCNEIINIMKPLLPKSITMD

RGKEFALFEQIEDELNCSIYFSDLGCPYQRGSIENANGLLRQYYPKGTDFANITQKSL

DEAVKQINARPRMIFDYKSSEEMLKYHVSTQNCEPILNDCVRHEPVN"
/transl_table=11
/db_xref="SEED:fig|703339.347.peg.48"
/product="Transposase"
CDS complement(41830..42333)
/translation="MKKRKKRKELDYKTRLAIAKYLNDGLNIIQISKILGRDDAVLYR

EVNKRSVDGVYNPLLAEEDAIKSRSISDEQVAKRRRLNDETKAYIEEKLSLKWSPRQI

ASQIEKDTGYYISYPTIYRYIRNGLVKVDVKRDMRQAGKKYNKSSEKRGKLDVGSRVI
KYRLKKY"
/transl_table=11
/db_xref="SEED:fig|703339.347.peg.49"
/product="FIG01108533: hypothetical protein"
CDS complement(42442..42798)
/transl_table=11
/translation="MRRWFVLILGLVILLSACGQKYDKEIDEVTKLEKESIQDVKNTK

KYKNVERSKSYYKIYNDGEVIIMTYMPFKDSNTKVSRVYKINQTSDKYEEDSNIDAEK
FEKDNKPVYEENNMKK"
/product="hypothetical protein transposon-related"
/db_xref="SEED:fig|703339.347.peg.50"
CDS complement(42854..43444)
/product="hypothetical protein transposon-related"
/db_xref="SEED:fig|703339.347.peg.51"
/translation="MKQKIIMFTLVTVILFCAVLIGYQIPKQQVKMKQNQIEDLQEEQ

RILRDKNGELNKLVKRQSKTVISDEEKQIREVSSNFVKQMFEMKKDSSFKSKAPQIKP

LVTKDYYDTLFKDSKDKYDLYDDITVNDIHVYFDTYDPKKDSYKVFVQFDERIETDGD
DKIEHRQTSAQLDLVRTAEGWRIDNLKRFNLKPLGR"
/transl_table=11
CDS complement(43451..44497)
/product="Secretory antigen SsaA-like protein
transposon-related"
/db_xref="SEED:fig|703339.347.peg.52"
/translation="MVNKKDIAKKVIGKTPIGVKLKLAKVIIILCVVAFFIFPVIIMF

LLAPDLNKGKDDAGCTVSGGNVSANGIDKFNENAKGGKLEGKGKEIQKIAEKNKVPVN

IFMAIIASESQWGKGENATRQNNPLSVMGSKSIHDSTYPTIEDGLNAGAKNLYDVYIS

KGLDTPKKIGPKYAPVGASNDPNNMNARWIPTVEKIMKDLGGSEAKTSCSNGKGKSIK

FNGKLPHWSNDDPGKGNLYTAGQCTWYAYGMRKKMGKPVSTYWHDAHKWNDRAKAEGY

KVDKNPEPGALFIAEQGAGGHDGHYGHVAVVIGVSDGGKTFRITEMNWEGAFKVNERT
LKMTDGYSFIHDKE"
/transl_table=11
CDS complement(44487..46355)
/product="hypothetical protein transposon-related"
/db_xref="SEED:fig|703339.347.peg.53"
/translation="MKHKSVYLLCFLILPILLLTSVSAYAVSNPVGEPSHAVKPKIEK

YDLSHYRSIYSEKGDWNPFGQEEISRQINNVSDFFFSMTKILAGVTDYAIENLFQLDV
INDFADKIGDFVGDIYQKLLSNLALTLFIIVCFNAFIIFSVQGNAREALKRAFLIMCL

IGFGVGILANAGNIIRGTNNIGKDLNNIIMNSTSSIDGNIDYIHENSGMNHIRNQLFD

MTIYRTYLVMNYGTVDEKEIKAKGKDRIDNILKLDYDKKSEKELDDIVKDEVEKKNNK
YMTQGYVFQKLAISVIGFIITLFMSIVFLSISFAKLIFSTFALFLFLFLVFSWIVSFI
PGFELSVFSAFAKTLGYIILSACMTFLFVIVGLCIKLANSFIEPDSQNAYFLNSIFII

VILFVMYKKRAQIINFVSRGNISFSPSAIGAGVMNRTQERFNKIRHEQAQNKKAKREN

QRDEPAPPLQNDNDLRRRQQDKPMPLFINKDNQKNGNKRREQQESMNGNDVKSASVEK

KICSVESNANNYSKQPQKASQQEHQVRETRQRKDIQRSPQVVNQPLNNENHSINRKEQ

KSVQTAYDTDVQKRQIQNATQNQQSRQSGNRNQPITRNSQSKDRLKEQKDINKHGK"
/transl_table=11
CDS complement(46360..47598)
/db_xref="SEED:fig|703339.347.peg.54"
/product="FIG01108595: hypothetical protein"
/translation="MFIDYKSVNKKLDIWTLFAYFKPHLLYLLIGTAIIFVICKLIAQ

LLIRFKTKDLAEMIETQGFHNRTVIKKTTEYIPIDYKQTEYDYYPTMFYKRGKKTFVI

HIKKDGSRFQDNYLELETIIEPMFNSELVEKKHIGRYLRYEFMPLKYKKRIVMNGEQE

SNTIFYDTKIAITHQITWDFVKAPHGLVTGITGGGKTYFLFYVIRELFRRHSEVRLLD

PKVSDLSFMKRVIGDDKVADTKGQILKQLREANNEMEERFRLMNDSSDYKIGNDFRNF

DMRPYFIIFDEVTAFTSTLDKKELQEMNDYLINIIMKGRQAGVFMFLTAQRPDADVIK

GNVRDQLGLRVSLGNLSNDGYRMTFGQTDKEFQTIHDSDIGRGYISILGQYNEPILFD
APLMEQYDFVEDVKQILNKE"

/transl_table=11
CDS complement(47601..47717)
/transl_table=11
/translation="MAMNEIALFKGVRIQPYQKYIDFMFASVLAFIFLVYRI"
/product="FIG01108595: hypothetical protein"
/db_xref="SEED:fig|703339.347.peg.55"
CDS complement(47771..50266)
/product="conjugal transfer protein transposon-related"
/db_xref="SEED:fig|703339.347.peg.56"
/translation="MNLKAPYAGLRDNLLLTKTGEVWAYYRVRSESISTANYDAKEES

KKRMRYFLESIKGYQDFHFEMYDHDLDLRNKFKEISKDFDDEAFNVAEYYSKETIDVL

EQELQMITHNSFVMGVKIRELADDAVTLKEILKSTLSDTAERIVSNFGFDVNLDDKIL
DKYKTFERDLADSFLGIGGERLTENELAYIIRKPFISNATHDVQEESSRRAITDIYNA

VIDPTRLSVLEVENDNEKFYTTTVVVDDFPLDMEYTHLFEKAQNMPFPVECHVKAKII

NNEKVKKKFDRAKINHKQQAKEKQDTGDSASEDVQDNLFVLNQMEREVTGSGVFIEWV

FMFVIKSDDYRDCKRKAKRLMKRLKETQIYAVNPLADQLQLFYQCLHGNDLFINKYWL
QRTTATGIAENLFGVSQSLGTKTGFYIGRIDKFIRSVSREEAVASSRDIILFSLLLAA

KGIKGAVSDSPHVLITGQTGKGKSFLAKLLWIYTSFFKGQMLYWDPKSEFAEWFDRVT

ESKEMQEKYPLFINHLKTFKYVTLNYEDSTNYGCLDPIVFLDGIEANEMLKSVFDEIK

SFENYHHIETAIYQAISDTVEERENGKKVGSLNVIEKLQNNEDEHVKNAGDLLFEKTQ

NNILKLVFSDGTNPALNIQEKATILQVKGLDMPKADDDTSSYSTSEKNGITLMLLIGK

FLEKFGSRRDVQTTIFIDEGWAFSASRQGKKVGKRIKRTGRSENNSLVFITQSVKDKA

DDDGGNFGCHFAFDEKDEREDILKSLGLEYSKESPENMEMLKDLKKGQCIFSDFYGRV
GKMVVHCPFEEMTEAFRTQEDSASSKAEEKFAM"
/transl_table=11
CDS complement(50301..50684)
/db_xref="SEED:fig|703339.347.peg.57"
/product="hypothetical protein transposon-related"
/transl_table=11
/translation="MDKKAYNLKRTFEQPIVMYEFTDKFRINKGFRLDFWATFLIVWF
ILFLLFWYLLQPVIMSIGGLMFIYFTVAPYYITKYIVKLKQDGKKLFFFLWDFVIFVF
NVQLRKVKLSYDEEVEYHDKKITFK"
CDS complement(50696..50956)

/db_xref="SEED:fig|703339.347.peg.58"
/product="hypothetical protein transposon-related"

/translation="MSGLFNFFVLGADRPSLGGVGDWISNEVGTGIGLVVLVVGIVKW
ASGKYGHMVILFVVGGFLFLVSKGPEQVFNAIAGVWKMIFGG"
/transl_table=11
CDS complement(50961..52016)
/product="hypothetical protein transposon-related"
/db_xref="SEED:fig|703339.347.peg.59"
/transl_table=11
/translation="MKIISKYILTRFSKNGKHFDIPKEQRRVIPKFKNRRKMIVICFY

SLLAILLILLLASFIKATRANNDSKEAVNKTNMIQKKYEDNANTVQYSPKLKLYADKF

IDTYMNISKDSKELESREKELLKYFPSDYKKPDEKISDTERKLNSKEFYNIKRKDKQT
IIQYIVNYDVNITEKKEVKVKKKKKSEKDKDEYETKTEEKQRKVNQNILINIPIKSEN

NKYVVVEYPYFTPIPDSQLNKAKMVEDNLKDNKREDNPKAKAFIEDFFNKYASSKSDD

MAYLMDNPEGLEGTREVSQIREIRLYPKGDDYVAKVEILMKDKDSPLENLEHYTLDIT
KKDGKYYVKNMTNSIGG"
CDS complement(52077..52856)
/transl_table=11
/translation="MNKTWQDFFDKCLSVNANFTRIDIAIDDYKTYLKVPLLIKKAEK

AECVSRFRAGSAINGFNLSDGRSKGATFYIGSKQSNLYCRFYEKNYEQAFKRHCDVED

IGLWNRYEIQMRKAYAVNCAKVLSRTDNISEIVKSILHNNLRFISPPKDGNDKNRKRW

PLYRPWALFIKDTEKLNLTTRPTLKSIEDNLDWLCKQVATTLDTVLTAESMAQSEGLL
TDTDFLDKILAHSQFNDEHTNRINHYLEALKQKKHLSKDKC"
/product="Transcriptional regulator, Cro/CI family
protein transposon-related"
/db_xref="SEED:fig|703339.347.peg.60"
CDS complement(52853..53167)
/db_xref="SEED:fig|703339.347.peg.61"
/product="Transcriptional regulator, Cro/CI family
protein transposon-related"
/translation="MTLKNQCTPLTNRGVERTNKSAVEAVVDWVQVTFHITPISAVIE

DVIGLPITLFKKRNSGIYFYNRGYEFSNIKLYYSSDDESMGIHLQLTGTGCREFEHHL
HS"
/transl_table=11
CDS complement(53342..53644)

/product="hypothetical protein transposon-related"
/db_xref="SEED:fig|703339.347.peg.62"
/transl_table=11
/translation="MDYYTADRLYRYTNSSNLSEPILNYVASRINWGDKVSLMTLAKE

IQSKFNDSYVKENTVKGRPKIYADLCLLCMSLSEAGHGRMLQVNLEDCIYIGDIDV"
CDS complement(53658..53978)
/transl_table=11
/translation="MKPVLDIKKTLGQLNFLGVDEKYKYENGERTDKTVYAYKLASQE

QGEQITVKTPNKVELDYLQECELVEPDVKMYVQMSGDFGTIAYSWNAEDIKVVGSNSA
LKQK"
/db_xref="SEED:fig|703339.347.peg.63"
/product="hypothetical protein transposon-related"
CDS complement(54129..54413)
/translation="MTEFTPFFDGSNQLNYLKFVILRFHGVSKRKICKMYNFAYFRIL
EVCEMAKKNDYRFPYKDYKFLKQYGVSNTFICKMYHISIEDLEFFEVMNR"
/transl_table=11
/db_xref="SEED:fig|703339.347.peg.64"
/product="hypothetical protein transposon-related"
CDS 54513..55007
/transl_table=11
/translation="MIQTFIKETENKLAYSELELSVIFDYINNGDNNIFKSTNLNSKN

LLSKLKGIAWDLFHIRTSEDQIALRNTNSKEVFLHSIFTADKGLSNVINNYSISRMLA
HEEKLYVYRDNNIFKKLSKDKATKIQELLEKDRNSRISNVREAKYNIQENIDIYEKYI
NEMI"
/product="hypothetical protein"
/db_xref="SEED:fig|703339.347.peg.65"
CDS complement(55019..55111)
/db_xref="SEED:fig|703339.347.peg.66"
/product="hypothetical protein"
/transl_table=11
/translation="IYDKPPLINDITKVTNRYNGLILYLLYNRL"
ORIGIN
1 atgaaaatca ccattttagc tgtagggaaa ctaaaagaga aatattggaa gcaagccata
61 gcagaatatg aaaaacgttt aggcccatac accaagatag acatcataga agttccagac
121 gaaaaagcac cagaaaatat gagcgacaaa gaaattgagc aagtaaaaga aaaagaaggc
181 caacgaatac tagccaaaat caaaccacaa tccacagtca ttacattaga aatacaagga
241 aagatgctat cttccgaagg attggcccaa gagttgaacc aacgcatgac ccaagggcaa
301 agcgactttg tattcgtcat tggcggatca aacggcctgc acaaggatgt cttacaacgc
361 agtaactacg cactatcatt cagcaaaatg acatttccac accaaatgat gcgggttgtg
421 ttaattgagc aagtgtatag agcatttaag attatgcgtg gagaggctta tcataaataa
481 aactaaaaat tagattgtgt ataatttaaa aatttaatga gatgtggagg aattacatat

541 atgaaatatt ggagtatacc ttgcaatatc atacgatgtt tatagagtgt ttaataaacc
601 atttttcaac tattgatgat ctagaatata taataactgt acaaattata ttgattatgg
661 aactacaatt caattaagaa attgatgatg aaattttaaa tttaaactaa tggaatcaag
721 aaagaatgaa aggaaatata caatgcccac gattaataaa aggaagttta ttagattttg
781 tgttagaaac agtagatgtt tttgtaaatg gaagtaaaaa tgattgcgaa ttagtaaatt
841 ttaattataa agaaggaaaa gtagtttcac gttatagtac aaaatttgaa agcaatccta
901 aattaagaaa aaagcaattg atatcgacgg taaaagttgt aaggtatgtt atttttgatt
961 ttgagaaaat tttatggggc atagggaaag ttttattgaa gttcatcata ttaaggcaat
1021 gtttactata agaaagttga aatacactca gaaacagact ggattcccgt ttttttcaaa
1081 ttggcacaaa atgatacata ggcccaccta tatattgaag atttaagtta aacgataaat
1141 aatatttttc tcttttgtat agaaattttt ttgggaatga tattaattag tataaaaatg
1201 aatatgtcta caaagaagga gagattttat tgaaacacat taatcaattt tctgaatcga
1261 cactagcaca actagaaaaa gatcaacaac acgtatttta tgtatactgt ttgatggacc
1321 caagaaatga tgaatgcttt tatgttggta aaggtaaggg gaatagaata tttaaacata
1381 aacaagatgc gcagaaaaaa gtattgtacg aagatatatt tatagaagaa aataaagata
1441 acttgaaatt taatagaatt aacgaaattt gtagcaatga tttgaatgtt ttaggatata
1501 taatcagtta tggattaacc gaatctgaag cattttctgc agaaaatgtt cttataaact
1561 ttcttaacct aacaaataaa actacattaa caaatatgat taacggtcat ggatctaagg
1621 catacttagt tgaagattta gaaaatgaat ttggctacga ttcaattaat cttgaaaaca
1681 taaatacgaa tgaattaatt ttagcagtga aaatcagaga tgcatttcta ttagataagg
1741 atgaaagtaa agagtatcct attaatgaaa gtaaacgtga tagaaataac cttaaatcgc
1801 gtacattagg tagttggata ataggaaaag ataaaataca taaaataaaa tatattattg
1861 gtatcaatac aggtgctaat aatgcagttg tctcagctta tgaagtatca tttgaacaag
1921 cggagagtat cgaaacaaat aatggtagaa tgagatatgc gtttattggg ctttcagaaa
1981 gagatgctac tctaaaaaaa ttgaatttat acaaaaaagc actaccagat ttaagatttg
2041 gtagtggtag cgctacagca tatataaata atgggacaat gaaagttgat taaatgaata
2101 aaaacatcca attccttaaa ttagcctttg aaaattttat actcgtatat ataagtgaaa
2161 aacttaaaag aattagatgt taattaaagg agagaatgct atgccatcag atgatattgt
2221 aaaaaaacta tactcaaaag aggatattcg attagaatta ggacttactc cgcataagtt
2281 taataaaaaa atggaaacta ttgctaagct ttttaaaatt gatatgaaaa tttttcacaa
2341 ttacaaaggc caagataaaa ataatcagta tacatttaat ggtgtcgcaa aagaattaat
2401 caaagtgttg ctaaaaagtg ttgattatta cccagtggat atcaattcca aaaaatttaa
2461 acaaaatgga aaatctaaaa aagaaatgat tgaaaatata gataactcta gttacatgaa
2521 atatatttat cagttaatga aatctatcaa tgaaattcaa tacaaaaggt taattgctga
2581 tatacatatg aaagatgtgt accagaatac aaaagcatgg ttaaataatg gtgaatcaat
2641 taataaaaaa gaacaagagc tataccaata tatgacaatt ttaccattac ataaaagagt
2701 tgaactgcaa aatgaggtat taaaatctat agacgaaaca atttttcaat ttctcgcaaa
2761 agaacataga aataatcaaa ttgaagaaaa taatgaatta gaagcatata caaaagcaat
2821 aaaagaaggt agaaatccta aaaatgatta tgaattaaat catctcctat ataaaaaaaa
2881 tcaattaccc ttagatagtc taatagaaga tatatgggac tatgaggaaa cagagtatac
2941 tgagcttgat tggttaattg cagatatgct aaaacgttca caaagagctg atagtaattt
3001 tatcgaaagt ttagagaaga aaaacaaatt gagaaaaaat attcataaag attccattaa
3061 aagtatatct aagttgattg atcatgaatt agttaaaaat aatagaaagt gggacgtagc
3121 agaattttat aaaaatcaac aattttggaa taatagtaca ttaagacgta gctctcaaaa

3181 ttttggaatt ttatattaca aaaagaatag gttacataca atgaatcaaa gtaatattat
3241 taagaaaata aaaattatcg aagctaatat agagagtgca aataaacagc cgagctattt
3301 agatttcttt ttacaagcta agtttgattt agagaggtta gaaagtgaat taatgaaata
3361 tagcattaat ccgaaatatt ttaataagat aaatagtgat tacgtaagat atgctattga
3421 agatgtacat aatgcaatta ttaaaattga cagatatata agtaacgata aagtgaagtt
3481 aaactattca aatgtccaaa tggtagtgag aaacgaaata acagaacaag gaagctactt
3541 tgtaacccag gcattgaata cactgtcaaa ggtagaagag tctggctaca gctttgataa
3601 tttcatggta ggacctttcg tagaagactt taaaaaagca attaaaaatc gtagatttgg
3661 ttctaaaaaa gattaagatt gtttttaga gttaaacata agaataaatt taaataaaaa
3721 aataaaccca acaaaccttg ttatatcaat gtttgttggg tttattttt ggtgattttt
3781 taataactca attacatctt aggataatat cgctaaatta tacattgtcg aggccaagac
3841 aaataaaaaa ttaaagcaaa agttagcgcg caactttctg ctataaagga gcaaatttat
3901 atggagacaa gagacaaact gatgtcattg actcaaagtg acaaaacaca gcaatggcta
3961 atggacaagt catctaacca agatgacatt caacaattgc agcaacaatt cagtcagcag
4021 cttgatcaac aatataatgc acttttagct gatgaaaaag ctaagttaga ccaatacgtg
4081 gaagtacatc aaggattgga atcattaaag gaagagattg aatcagaacc tattacgctt
4141 aatatcgata aattacccga tatcaaagca acaatgcttg aaagagccaa gaatgatgaa
4201 cattctgata aaatcgaaaa gctatttgat aggttagaac aggcattaaa tggtacgaat
4261 cgattatata cgcaattatc gttgattggt acacgaacac atcgaattac aactaaaaat
4321 tttaatcttc aaggcttacc taaagcagtc caacatacga ttttaccttc aaaatttaag
4381 aaggtgtata cagtcgattt taaatcgttt gaaccatcag ttgcagcgta catgactcaa
4441 gattcaaaac tgattgactt gttaaatcag aaagacggac tgtatgacgc attgctaagt
4501 gaattaggct tatcagatga gctacgtgta tttgttaaac gtgcatttat tggttcgttt
4561 ctatttggag gtaacttcaa aaatcctaaa ttcaagctga atcaatatgt aagtgaagta
4621 caatggttgg atgcggtcag ccaatttaca aaagtcattg aacttaagaa gcacgttgaa
4681 aagagtaatt ccatgcctat gccttatagt attgagcatg atatgagcgc atttcaaggt
4741 agcagtatta tggcaatcta cgtacaaact gtagcgagtt atattttcaa gcacattctg
4801 ctaaaagtgt acaaagcaca gtgcgatcaa aaaacgttca agattatagt acctatacac
4861 gatgcgatta tgattgaatg tgaagatgaa gaaattgcac aaaatgtggg tcagttaatg
4921 aaagatacgg ctaaccagtt gttcaatggt gaatttgcac atgtgacagt ggaagaaata
4981 ggaggcgtag accatgaata atgatagagg acaaagccta cacatcccaa gtagtacacc
5041 aatcaaagaa aataatatat atgtagctac gttacattct gtgatccaaa cagatttctc
5101 aggtgaaata aagcaccaat tcacgtatga aattgaagtg aacaatcaga ttgtatatgc
5161 gaatcgtaat attccaacaa aaccgagcgc taatcagttg tcaattcatg attggctgaa
5221 acgtcatagc aactatagcg caagtcatga aaactatgag ccttatattg atcagaaaca
5281 tttaattcta ttaggtcaat ataacggtaa ctattatgta caagatgtag catcgttaga
5341 tgcgtttgga ggcgtattat catgaatcat atattacaaa tgttatctaa gctattaagt
5401 gtggccaagg aggcaatcga ccgtcaaggt ctgattgcta tcctaactat tcctgttaat
5461 aataacgatg aaatagaaga aacggctcaa ggtgaaaccg tgtataacga acttatcgat
5521 cagttacgac ttaatatccc aaaagatacg gattatcaac ctaacatcta tagttatttt
5581 ggtattaaga agaatcctaa tgacaccgta ctcatggaaa tgatgataaa ggtttttcat
5641 atcaaacgct ttaattcaga actgtttatt ttcaaagtta acgggtggca aaagataaat
5701 ggagatgaat tacaagggtt gatatctaaa atgatacaag tattgcttgt agattataag
5761 ccttcactaa gcactctaaa aaatgtcgta gatggattgc aaaaatcaac agatgtagaa

5821 gaacttgttg agaatgagcg ctatattggt tgtggtgaaa atatgttcga tcttaatacg
5881 tttcaagtcg ttaaaaattc aatcgatatc tttccaaaaa cacgattgaa tttatcatta
5941 agtacaaatg atgtaattac tgataagata ccgccttatt ttaagcaata tatgttacaa
6001 cttgcgaatt atgacgatga tttacaatac tttcttttcc aacatacagc agtattactt
6061 acagctgata ctaaataccg taggggtctc atattatatg gtggagctaa gaatggtaaa
6121 tctgtatata ttgaactagt taaatcattt ttctatagta aagatattgt gtctaagcca
6181 cttaatgagc ttgaaggtcg ttttgacaaa gaaagtttaa ttgacaaaag tctaatggca
6241 agtcatgaaa ttgggcaatc taggattcaa gaaaagatcg taaatgactt caaaaagtta
6301 ttatctgtag aatcaatgca tgttgatcgt aaaggaaaaa ctcaagtgga agtcattttg
6361 gatttgaaac ttatttttag tacaaatgcg atacttaatt ttcctcctga acatgcgaaa
6421 gctttggagc gtcgaattaa tattattcca tgtgagtatt atgttgaaaa agcggacact
6481 tcattaattg ataagctcca gagtgagaag aaagaaatct ttctttactt gatgtatgtg
6541 tatcaacaga ttgtaaaagc agatatcgag tatcttgaaa atagccgtgt cactgaaatt
6601 actcacgatt ggttaaattt tggatatgaa tttgtttcta gcaggtccgt aagtaatgca
6661 aatcagaaag catgtattaa tttactcaga aaacttatag aaatcaaatc aggatcacga
6721 atcaaagtat ccgagttaaa taaagttatt aatgaagaaa taaaggtaag ttctcaggtt
6781 attaaacagt taattcaagc aaactttgat actcaaacca aactatacaa tggctacgat
6841 tattggattg atttaggttg gaaagaagcc aataaaaaag agattcatga tatttcggaa
6901 aaagataata ttatttcatt agataaaaat gaaaatataa cagacgatga ggcattagat
6961 gaagagaatt tggactttga ttgggaggac tttgacgatg aataatgaac aaattgaagc
7021 atttgtagaa gtgcttgtac ctatcataga agaacgtatc aataaaggta attaaggcta
7081 attacgtact acaggcagtt gcctgtagta ctcatatgat taagtggtaa aagtgataaa
7141 aatgaaacga aattataaat atatattatc tatatgttgt tacaagaccg atagtctgta
7201 gcaataatct aataaaagga gcggtatgat atgaagggta aaattgcact ttattcacgc
7261 gttagtacgt cagagcagtc ggagcatggg tactcaatcc atgagcagga acaagtactc
7321 atcaaagaag ttgtgaaaaa tttcccaggt tatgactatg agacatatac tgactcaggc
7381 atttcaggta aaaatattga aggtcgtccg gcaatgaaac gtctattaca agatgttaag
7441 gataataaaa tcgaaatggt gttaagttgg aaattgaatc gtatctcacg atcaatgaga
7501 gacgtgttta atattattca tgaattcaaa gaacatgatg tagggtataa atcgatttct
7561 gagaatattg atacatccaa tgcttctgga gaagtactcg ttacaatgtt tgggttaata
7621 ggatctatag aacgccagac tttgatttcg aatgtgaaac tttctatgaa tgctaaggca
7681 aggagcggag aggcaatcac cggtcgtgtt ttaggctaca aattatcact taatccactt
7741 acacagaaaa atgatttggt tatcgatgaa aatgaagcta atattgtacg tgaaattttc
7801 gatttatatt tgaatcataa taaaggcctc aaagccatta caaccgtact taatcaaaag
7861 gggtatcgta ctattaatca aaagccattt tcagtgtatg gtgttaaata cattttgaat
7921 aatccagtct ataaaggcta tgtcagattc aataaccatc aaaactgggc tgtacagcga
7981 agaagtggca aaagtgataa aaatgatgtg atattggtca aaggtaaaca tgaagccatt
8041 ataagtgaag aggtatttga taaagttcat gaaaaattag cttctaaaag ttttaaaccg
8101 ggcagaccta ttggtggaga tttctactta cgtggcctta ttaaatgccc agaatgcgga
8161 aataatatgg tatgtcgacg gacgtattat aaaacgaaaa agtccaaaga acggacaatc
8221 aagcgttatt acatttgttc cttattcaat cgttcaggga gttctgcatg tcacagtaat
8281 tccatcaatg ctgaagtcgt cgagcgtgta attaatgttc atttgaatcg tattctgtct
8341 caaccagata ttatcaagca gattgcgtca aatgtgatag aagaactgaa acaaaagcat
8401 agtaaccaaa cagaaattaa atatgacatt gatagtttag aaaaacaaaa agctaagctt

8461 aaaacacaac aagaacgatt gttagaattg ttcttagatg atcagatgga tagcgaaatg
8521 ttaaaagcta aacaaagtca aatgaatcaa cagttagaag tattagatca acaaattaaa
8581 gaagcgcaac aagcaaatca atcacaggat gaaatacctа attttgataa attaaaagga
8641 cgactcattt tgatgataac acgattcagc gtgtacttaa gaaaggctac acccgaagct
8701 aaaaatcaac ttatgaaaat gttaattgat tcaattgaaa ttacgacaga taaacaagta
8761 aaacttgtaa ggtataaaat tgacgaaagt cttatccctc aatctttgaa aaaagattgg
8821 gggtcttttt ttatgcccaa attccaattt gaaatatatg gtcaaaatga ttatttcatc
8881 gaccaaatta ccacttttac cacttagtta ttagtgacaa aagtgagcga aatgaaataa
8941 aaatcaaata tatattatca aaatgatgta tcacatgcat acatcaatca aatacattag
9001 gaggtcataa ccatgacact agaacaacaa ctcaagcact atataaccaa cttattcaat
9061 ctgccaaggg acgaagtgtg gcactgcgaa tctatcgagg aaatcgctga tgatatctta
9121 cccaatcaat atgtaagact tggcccactc agtaataaaa cacttcagac taatacctac
9181 tactctgaca cacttcatga aagtaatatc tatcctttca ttctctacta tcagaaacaa
9241 ctcatagcca tcggttatat cgacgaaaat cacgatatgg atttcttata cctacacaac
9301 actatcatgc ctcttttgga tcaacgatac ttactaacag gaggacaata aaatgcataa
9361 atacatcaaa attacacaat tagtcattac aatactaagt gaaatcatca tttggatgaa
9421 agagtcagaa cgaaaggaag tctcttatga atagatatat cacccggggt atcgccaaca
9481 acttacctaa tatcttacaa caccaattat ggcaactcgt atctgagcga gaacaagaac
9541 aaaccaaaga taatacttca gtagattatt ttcatatatt ccagttcaat aagcatcgca
9601 atcaattata tatcaaacac aaacaagaac gacctgaata tgcgaaaatc cataaagcta
9661 attattcaaa agcaatcaat atcaataagg tctacattat tcgagaagat gatgtagacc
9721 tttcttatta tgtcatgtta ttacctgaag aatactagag gagtgaaaat tatatggaaa
9781 caatcaaaag tacattaaaa acagaagcca tattcagtga tgacaaacaa catcgctatc
9841 tacttaagaa aacatggaat agtgaaaaac aatcaatcac aatcattaca atgtatccgc
9901 attatgatgg cattctcaat attgacctaa cgacccaact catcatgaac aaagtttcag
9961 aaatggatgc atttggttca atcaattttg tgaatctata ctctaatatt acaaccccta
10021 tcaatctcaa acatttagaa aatgcgtatg ataagcatac agatattcaa attatgaagg
10081 cagtgaaaga gtcagatgaa gtgatattag cttggggcgc ttacgctaaa aagcccggtg
10141 ttgaagcacg tgttaatgaa gtattagaga tgttgaaacc acataaaaag aaagtaaaac
10201 gactcatgaa tccagaaacc aatgaaatca tgcatcccct taattcgaaa gcacgtcaaa
10261 aatggatatt aaaagtatag caactaaaat gtttattcac tatccatgaa aataattgaa
10321 tttcatatat atcttttaat ctacaatatg attaaaagat atattatttg gttctgttgc
10381 aaagtaaaaa aatatagcta accactaatt tatcatgtca gtgttcgctt aacttgctag
10441 catgatgcta atttcatggc atggcgaaaa tccgcagatc tgaagagacc tgcggttctt
10501 tttatataga gcgtaaatac attcaatacc ttttaaagta ttctttgctg tattgatact
10561 ttgatacctt gtctttctta ctttaatatg acggtgatct tgctcaatga ggttattcag
10621 atatttcgat gtacaatgac agtcaggttt aagtttaaaa gctttaatta ctttagccat
10681 tgctaccttc gttgaaggtg cctgatctgt aattaccttt tgaggtttac caaattgttt
10741 aatgagacgt ttgataaacg catatgctga atgattatct cgttgcttac gcaaccaaat
10801 atctaatgta tgtccctctg catcaatggc acgatataaa tagctccatt ttccttttat
10861 tttgatgtac gtctcatcaa tacgccattt gtaataagct tttttatgct ttttcttcca
10921 aatttgatac aaaattgggg catattcttg aacccaacgg tagaccgttg aatgatgaac
10981 gtttacacca cgttccctta atatttcaga tatatcacga taactcaatg tatatcttag
11041 atagtagcca acggctacag tgataacatc cttgttaaat tgtttatatc tgaaatagtt

11101 catacagaag actccttttt gttaaaatta tactataaat tcaactttgc aacagaaccg
11161 tattatggaa tagagatgtt ggtaacattt atacaggatc attatactta agtttaattt
11221 cgttattaca gaaccacaca ttccaaccag aagagaaagt atgtctattt agttatggtt
11281 caggagcagt aggagaaatc tttagtggtt caatcgttaa aggatatgac aaagcattag
11341 ataaagagaa acacttaaat atgctagaat ctagagagca attatcagtc gaagaatacg
11401 aaacattctt taacagattt gataatcaag aatttgattt cgaacgtgaa ttgacacaag
11461 atccatattc aaaagtatac ttatacagta tagaagacca tatcagaaca tataagatag
11521 agaaataaac tagtggccga ttgtgcttga tgagcttggg acataaatcc taactcgaaa
11581 taaataagca tatcactaaa ctgatttttt aaagtttaca gtgatatgct tattttttta
11641 tcttacgatt ttgtacgtgc atgcttgcct aggggtatgg ctcgagccat tagtctctcg
11701 cacatactat tccctcaggc gtcagcactt acaaaatcgg ttgtaatttt catttttata
11761 cgcattctta ctgagattat actaataaga ggaatagtaa aagcaattct aagtaaaatt
11821 gcagataaga ggtttgttaa aagcagttct aagtaaaatt gcagataaga ggtttgttaa
11881 aagcagttct cagtaaaatt acagataaga ggtacgttaa aagcagttct aagtaaaatt
11941 gcagataaga ggtttgttaa aagcagttct aagtaaaatt gcagataaga ggtacgttaa
12001 aagcaattcc atgcaaaatt gctgataagg ggtaagttaa aagcagttct cagtaaaatt
12061 gcagataaga ggtacgttaa aagcagttct aggcaaaatt gcagataaga ggtgcgttaa
12121 aagcagttct cagtaaaatt gctgataagg ggtaagttaa aagcaatcct aagtaaaatt
12181 gcagataagg ggtacagaaa aactagactt gattacaaaa tggagcttgg gacataaatg
12241 attttttaaa aatgagatga gacgtagatt aactccataa tcaatacgaa tctatcgact
12301 tctttattta tgatattcat ctctttttaa tggaaataaa agtgcgatta atgtgataat
12361 acagttacgt taattaaaaa aataaaaatg caaggagagg taatatgcta actgtatatg
12421 gacatagagg attacctagt aaagctccgg aaaatacaat tgcatcattt aaagctgctt
12481 cagaagtaga aggtataaac tggttggagt tagatgttgc aattacaaaa gatgaacaac
12541 tgattatcat tcatgatgat tatttagaac ggactacaaa tatgtccggg gaaataactg
12601 aattgaatta tgatgaaatt aaagatgctt ctgcaggatc ttggtttggt gaaaaattca
12661 aagatgaaca tttgccaact ttcgatgatg tagtaaaaat agcaaatgaa tataatatga
12721 atttaaatgt agaattaaaa ggtattactg gaccgaatgg actagcactt tctaaaagta
12781 tggttaagca agtggaagaa caattaacaa acttaaatca gaatcaagaa gtgctcattt
12841 caagctttaa tgttgtgctt gttaaacttg cagaagaaat catgccacaa tataacagag
12901 cagttatatt ccatacaact tcgtttcgtg aagactggag aacactttta gattactgta
12961 atgctaaaat agtaaacact gaagatgcca aacttactaa agcaaaagta aaaatggtaa
13021 aagaagcggg ttatgaattg aacgtatgga ctgtaaacaa accagcacgt gcaaaccaac
13081 ttgctaattg gggagttgat ggtatcttta cagacaatgc agataaaatg gtgcatttgt
13141 ctcaatagaa agttagaggt gagtcttacg tttcagtgac ggtagactta cctttaacat
13201 gttacatact aaaaaattaa tttgaataag aaagagagac atatatgaaa tacgatgatt
13261 ttatagtagg agaaacattc aaaacaaaaa gccttcatat tacagaagaa gaaattatcc
13321 aatttgcaac aacttttgat cctcaatata tgcatataga taaagaaaaa gcagaacaaa
13381 gtagatttaa aggtatcatt gcatctggca tgcatacact ttcaatatca tttaaattat
13441 gggtagaaga aggtaaatac ggagaagaag ttgtagcagg aacacaaatg aataacgtta
13501 aatttattaa acctgtatac ccaggtaata cattgtacgt tatcgctgaa attacaaata
13561 agaaatccat aaaaaaagaa aatggactcg ttacagtgtc actttcaaca tacaatgaaa
13621 atgaagaaat tgtatttaag ggagaagtaa cagcacttat taataattca taataaaaca
13681 gtgaagcaac catcgttacg gattgcttca ctgttttgtt attcatctat atcgtatttt

13741 ttattaccgt tctcatatag ctcatcatac actttacctg agattttggc attgtagcta
13801 gccattcctt tatcttgtac atctttaaca ttaatagcca tcatcatgtt tggattatct
13861 ttatcatatg atataaacca cccaatttgt ctgccagttt ctccttgttt cattttgagt
13921 tctgcagtac cggatttgcc aattaagttt gcataagatc tataaatatc ttctttatgt
13981 gttttattta cgacttgttg cataccatca gttaatagat tgatattttc tttggaaata
14041 atatttttct tccaaacttt gtttttcgtg tcttttaata agtgaggtgc gttaatattg
14101 ccattatttt ctaatgcgct atagattgaa aggatctgta ctgggttaat cagtatttca
14161 ccttgtccgt aacctgaatc agctaataat atttcattat ctaaattttt gtttgaaatt
14221 tgagcattat aaaatggata atcacttggt atatcttcac caacacctag tttttcatg
14281 cctttttcaa atttcttact gcctaattcg agtgctactc tagcaaagaa aatgttatct
14341 gatgattcta ttgcttgttt taagtcgata ttaccattta ccacttcata tcttgtaacg
14401 ttgtaaccac cccaagattt atctttttgc caacctttac catcgatttt ataacttgtt
14461 ttatcgtcta atgttttgtt atttaaccca atcattgctg ttaatatttt ttgagttgaa
14521 cctggtgaag ttgtaatctg gaacttgttg agcagaggtt cttttttatc ttcggttaat
14581 ttattatatt cttcgttact catgccatac ataaatggat agacgtcata tgaaggtgtg
14641 cttacaagtg ctaataattc acctgtttga gggtggatag cagtacctga gccataatca
14701 tttttcatgt tgttataaat actcttttga actttagcat caatagttag ttgaatatct
14761 ttgccatctt ttttcttttt ctctattaat gtatgtgcga ttgtattgct attatcgtca
14821 acgattgtga cacgatagcc atcttcatgt tggagctttt tatcgtaaag tttttcgagt
14881 ccctttttac caataactgc atcatcttta tagcctttat attctttttg ttttaattct
14941 tcagagttaa tgggaccaac ataacctaat agatgtgaag tcgcttttcc tagaggatag
15001 ttacgacttt ctgtttcatt agttgtaaga tgaaattttt ttgcgaaatc tcttaaatat
15061 tcatccattt ttttaacggt tttaagtgga acgaaggtat catcttgtac ccaattttga
15121 tccatttgtt gtttgatata gtcttcagaa atacttagtt ctttagcgat tgctttataa
15181 tcttttttag atacattctt tggaacgatg cctatctcat atgctgttcc tgtattggcc
15241 aattccacat tgtttcggtc taaaatttta ccacgttctg attttaaatt ttcaatatgt
15301 atgctttggt ctttctgcat tcctggaata atgacgctat gatcccaatc taacttccac
15361 ataccatctt ctttaacaaa attaaattga acgttgcgat caatgttacc gtagtttgtt
15421 ttaattttat attgagcatc tactcgtttt ttatttttag atactttttt tattttacga
15481 tcctgaatgt ttatatcttt aacgcctaaa ctattatata tttttatcgg acgttcagtc
15541 atttctactt caccattatc gcttttagaa atataactgc tatctttata aacttgtttg
15601 aaatttttat cttcaattgc atcaatagta ttattaattt ctttatcttt tgaagcataa
15661 aaatatatac caaacccgac aactacaact attaaaataa gtggaacaat ttttatcttt
15721 ttcatcaata tcctccttat ataagactac atttgtaata tattacaaat gtagtattta
15781 tgtcaaaata atgttataat ttttgtgata tggaggtgta gaaggtgtta tcatcttttt
15841 taatgttaag tataatcagt tcattgctca cgatatgtgt aatttttta gtgagaatgc
15901 tctatataaa atatacggtt ctgttgcaaa gttgaattta tagtataatt ataaccaaaa
15961 ggagtcttct gtatgaacta tttcagatat aaacaattta acaaggatgt tatcactgta
16021 gccgttggct actatctaag atatgcattg agttatcgtg atatgtctga aatattaagg
16081 gaacgtggtg taaacgttca tcatttaacg gtctagcgtt gagttcaaga atatgccccg
16141 attttatatc aaatttggaa gaaaaagcat aaaaaagctt attacaaatg gcgtattgat
16201 gagacgtaca tcaaaataaa aggaaaatgg aactatttat atcgtgccat tgatacagag
16261 ggacatacat tagatatttg gttgcgtaag caacgagata atcattcagc atatgtattt
16321 atcaaacgtc tcattaaaca atttggtaaa cctcaaaagg taattacaga ttaggcacct

16381 tcaacgaagg tcgcaatggc taaagtcatt aaagctttta aacttaaacc tgactgtcat
16441 tgtacatcga aatatctgaa taacctcatt gagcaagatc accgtcatat taaagtaaga
16501 aagacaagat atcaaagtat caatacggca aagaatactt taaaaggtat tgaatgtatt
16561 tacggtctat ataaaaagaa ccgcaggtct cttcagatct gcggattttc gccatgccat
16621 gaaattagca tcatgctaac aagttaacac aaagtattat tttaaaattg agattagaca
16681 tttatttttc aactttgcag cagaacctaa ttttttctct ttgtaaataa atgcaaagag
16741 ataccataag gatctcttac ataaccataa ccttcagtat agaattctgg actaaatgtt
16801 ttcaatacct cactgccttt ttctattaac tggtcatata catgtttagt ttcttctact
16861 tggtcaaaag tgagacaaag agatatatta ttaccttgtg ttatgggcaa accttcagtg
16921 tcatctgcga tcataatttt tatatctcca aattgaagta cacattgatc aattttattt
16981 aaatcatttt cgtcaatatt aagtttctta tctataggtc tatctttaat acgttgaata
17041 tacagtgttt tagcgccaaa cagctcttca tacaactttt ttaaaccctc tgcattttga
17101 gtgattaaaa aatatggact tacttgaaat ttcatgtttt ttcctcctta gatttgttat
17161 aaatagagta tatacttaat tagtgtcatc tattgacact aaaaggagaa taataatgaa
17221 aaaatctgtt agattatata atatgattga atattgtaat gaaaatagga acttcaaatt
17281 aaatgattta atgtcagaat ttaatatttc tcgtagtacc gctttaaggg atataaaaga
17341 aattgaagca ttaggagtac ctttatatag taatccaggg aaaaatggtg gttatacgat
17401 cataggtaat cgagaccaaa cgaaaatagc aatctcagat gaagagttga aagctttagt
17461 atttacactt tcgagtattt caaatgtgag taatctacct tttcaaacag aatatcaaga
17521 aatattaaaa aaattatata ataactcgaa taaaaaagag ttaataaacc aatataatga
17581 tctatttcaa tattttaatg aagataagta tcagttcaaa agttataagt tatttaatga
17641 aatcattaga ttgataattg agaataagtc ttttgaaacc tgttattcac aaaactatat
17701 taaagaacaa tataaaggta ttggcattat gtataaaaat catcaatggt attttgttgt
17761 agctaatata gaatcaaagt tagtgaatct attaaatatt tcgaaaataa aagaactata
17821 tgaaatggga gagactcaag agtgtaatga tataactatg cagaattttc aacagttcat
17881 ggttaaaaat gaaacagcta ttgatattct tattagaagc aatgttatgg gattgaatat
17941 cttgaaaggc tacctgtgga gtgactatat gattgaaaat attgacgaag agacatattt
18001 atttaaatca aaagtgaacg cgaaagatat agattttata gctaagttaa ttgtcacagg
18061 tggtgtcaat gtaaaagtag agacccctaa tagtttgaaa aatgctgtta aagttgaatt
18121 aactaaaata ataaacatgt attaatagta atttaatcat aattgtaata acaaaaagat
18181 gacaacatta aacaatttaa atttatacaa aaaagcatta ccaaatttaa aattgggtag
18241 tggtagtgct acagcataca taaatagtta taattaaata aagatgggtc aatgaaggtg
18301 aattaagtga ataaaaatat tacagctcta aaatctactg aaaatactac atatatgcag
18361 tatacggttc atatcgatga tcaaattata aataatataa aagagtgtac caaaaagttt
18421 aaaattttgc ctatggaaga taaaattcca ttatcaccgt tgttacaacc agaatatgca
18481 ggagaggtac aagattttat tagtacatat gagcagttta tgattaattt tggtaaagta
18541 atattggata gtcaaggcat aaaaatacag tttgaaagtg aatcattaag tagtattcaa
18601 cgaggcattc aagaacattg ttacttaaat gagcgaacaa atgacattga tgtgactaaa
18661 gaatggtatt tatgcaaatt ttcaaatcaa atattagaag aggataaaaa tcaattgtat
18721 aatgctttaa agcaactcat gaatgattct aaaaataaaa agcaagcttt tcaagaggtg
18781 tttaaggttc atattgatat ttatctctac aataaagaaa aatctgaata taagtatcaa
18841 gtatctagtt atttcaatct tgtaaaagaa aatcctaaaa taacttataa aaaaagacat
18901 ttacaagaga aacaaggtgt caagggtaca actttcacga acaaattaaa attattgaac
18961 aaaatgtatg gtgtagatgt tgcaaaatat cagccatttt ataattctaa taatcctgaa

19021 tatgaaagag gtcaatttgg tgaaagatac atttctcaaa gatctaacta tgaatttaat
19081 agacttcaat atcaaattat agatatgtta tcgaaaatac ttgataagca tccattacca
19141 aagtcagata ataattataa acatattcca actattgaaa aagcaatatt aagtggggat
19201 tctcatagtt tttatgagta ttttgaagat ataatgaaag aaattataag tatggaaaat
19261 tcttctttaa aggaaaaatt attgactgat tttacatatc aatctcaatg tagatggtac
19321 tctgaaagtg aaaaattaaa attgcaactt gaaagtttta tgcataaagt tttggagagc
19381 aattattatg agggtaacaa attatataga atgttgtctc atgcaattga agaaacaatc
19441 aatgaggctg atgaagataa ggttcatttc aattatttta aagattattt tttgacagac
19501 ggtggggtga agaattggga gcagattagc gagaaaatta ctgaattcaa tggtaaagtt
19561 attaacgata tccaaaatga atataataag attcaattta ataacgcaaa tcgaaatcag
19621 aaactaaact ttaattattt ataccattgc tttgagttta gtaataactt agtaaaagca
19681 agggtaaatg gaaatagagg ttacatttta tattttgtag ataaatataa aataaaaatg
19741 cccttttggg atcaattatt aatcaattat ttgaatcaag aaataggtca ggaaagtatt
19801 aattataata tgcaaacatt gtttgaaaaa gaaagatatg atagaagtag taccattgaa
19861 aaattagtag caacaagcaa atttaagtat gaaaaagatg attcagattt attcaaacaa
19921 cttttcaatg atgttgaaaa ttcaatagac agattaggta tttacttact aaataatggt
19981 ataaattcga atgatgaaaa tgcaagatat tatagatcgt ttttaaagga acttagtaga
20041 ataaaaagta aattaacgcc attttctctt gaaataagta agtctagcgg aagagagcaa
20101 cattatcctg atgatgctat tgatgataaa gatgagagaa gaaaaaataa agaagaaaca
20161 tatcatgctt ttgatgataa aagcgatatt gactccaaat taaagaataa aataaatgtt
20221 tctatcgata atttattgtc agttaaaatt aagagcaatt tgtatgatat tagtccaaat
20281 acaaaaaaac aataactata atgagccaac tcaagaatga gttggctttt taattgtaat
20341 tttacgcttc cattacagga atcaatgtta tggaagccat taagcaatta atttgagtca
20401 tttatagtat aggtacaacg ttgtgatgtt cacatgataa tcagcccggg agattattgt
20461 gtaataactt atagaaggag caaatcacca tgtatcaagc aaatatacgt gatttaatta
20521 caaaattgcc tcaaagcaat aaaacagaac actttttaat gaacaaattt tcaaatcaag
20581 ataaagttca gcagctacaa agacaaatta gccaacagct agatcaacaa tataatgagc
20641 ttttggctaa tgaaaaagct aagctagacc aatacgtgga agtacaccat aatttagaac
20701 cattaaagaa agagattgaa tcagaatcta ttaaccttga taccgataaa ttacctgata
20761 tcaaagcgac aatgcttgaa aaggctaaga acgatgaaca ttttgataaa atcgaacagc
20821 tatttgatag attagatcag tcattaaatg gtacgaatcg attatatacg caattatcgt
20881 tgattggcac acgaacacat cggatcacaa cgaaaagatt taatgttcaa ggcttgccta
20941 aattagtcca acaaatgatt ttaccttcgc aatttaaaaa ggtttataca atagatttta
21001 aatcattcga accatcagtt gctgcgtata tgacacaaga cgaacaactg attgactact
21061 tgaatcatga agaagggtta tacgatgcat tactgagaga cttatctttg tcaaaagaga
21121 agcgcgtgag tgtgaaacgt gcatttatag ggtcatttct ttttggcggt cgttatagta
21181 gctctaaatt caaaatcaat caagaggtta gtgaaattaa ctggctacaa gtaatgagca
21241 aattcaagaa ggtcattgaa tttaaggagc aagtcgaaaa atataaaaca atgcctacgc
21301 cttacggcat tgaacatgat atgagcgcat ttcaaggtag tagtattatg gcaatttatg
21361 tacaaacggt agcaagctat attttcaagc acattttgtt ggaagtgtac aaagcacagt
21421 gcgaaaaaaa aacgttcaag attatagtgc cgatacacga tgcgattatg attgaatgta
21481 atgataaggg gattgcacaa aatgtagcac agctcatgaa agatacagct aatcaactgt
21541 ttaatggtga atttgcacat gtgacagtgg aagctttagg aggtatagac aatgaataat
21601 gatagaggaa aaagtcttca aattccccaa agtacattgt taaaagaagg atctatatac

21661 gttgctacgt tacattctgt gtacgagaag aacttctcag gtgatattaa acatcagttt
21721 acgtatgaag tagaacttaa ccaagaaacg cactatgtaa atcgcaatat taccgtaaaa
21781 tctatgagtc accaattatc aattgctgat tggattaaac gtcacagtaa ctataacgta
21841 aaccacatta attatgatcc atacattgat cgaaaacatt tggtccttgt agggcaatat
21901 aacggaaatt attatattca agatgtagca ccattagatg aatttggagg ggtattgtaa
21961 tgaatcatat tttagaaatg ttaataaaat tattaaaagt gggtatggag gcaatcgacc
22021 gtaaaggtct gattgccatc ctaacaagta gtattggaaa tgatgaaatg gatgattctg
22081 aacaagctgt aatggtgtat aacgagctta tcgataagct acagcttaac attcctaaag
22141 atgtcgacta tagacctaac atatacagtt attttggtat tcaaaaaaag ccaaatgaca
22201 caatattagt ggaaatgatg atatgtattt ttcatatcaa gcgttttgat tcagagttgt
22261 ttgttttcaa agataaaggt tggcaaaagg taagtgaaga tgaattgcaa gggttgatat
22321 ctaaaatgat acaagtgttg ctagttgatt ataaaccttc actaagtact ttgaaaaacg
22381 tagtagatgg catacagaaa tcaacggaca tagaaaaact tgttgagcat agacagtaca
22441 ttggttgtgg acgaaatatg ttcaatctaa agacctttaa agtggttgat aatgaccttg
22501 aaatattccc taaaacacgc ttagatttgg aattagatat aaatgacacg attacggaca
22561 agataccccc gaatttcaaa caatatatgt tagagttggc gaattatgac catgatttac
22621 aatatttcct tttccaacat atggcagtgt tattgacggc agatactaaa ctacgtcgtg
22681 gactttttt gtatggaact gcaaaaaatg ggaaatcggt ctatattaaa ttagttaagt
22741 cattctttta tagtaatgat atcgtatcta aaacacttaa tgaacttggc gggcgtttcg
22801 ataaggaaag tctaattggt aaacgaatta tggcaagtga tgaagtgggg aaagctaata
22861 ttgatgaagc aactgtgaat gatttcaaaa aattactatc tgttgaacca attcatgctg
22921 accgtaaagg aagaacgcaa gtagaagtta ctttagattt aaaactcatt tttaatacga
22981 atgctgtact caattttcca tcatcacatg caaaagcatt agagcgtaga attgctgtta
23041 ttccatgtga atattatgtt gaaaaagctg accctgactt aattgaaaag ttacaggatg
23101 aaaagaaaga aatctttctt tacttgatgt atgtgtataa gcaaattgta aaaaatgata
23161 tcgagtacct ccaaaatgat cgtgttactg aaatttctca tgattggtta aattttggat
23221 atgaatttgt ttctagtaaa tcagcaaata ttgcacatca gaaagcgtgt attaatttac
23281 tcagaaaact tatagaaatc aaaccagggt cacgtatcaa agtgtctagg ctaaatgagg
23341 ttattagaga tgaaattaaa gtaagctctc aagttattaa tgatttggtt caagctaact
23401 ttaatgtaca aagtagacta aataatggtt ataagtattg ggtcgattta ggatggaaag
23461 aaactgataa aaaagatgac atgatttcat tcgataaaaa tgagaatgta acagatgatg
23521 aattcttata cgaagatgat ttgaacttag gttgggagga ctttgacgat gaataatgaa
23581 caaattgaag catttgtaga agtgcttgtg cctatcatag aagaacgtat caataaaggt
23641 aagtaatcta attacgtact acaggcagtt gcctgtagta ctcatatgat taagtggtaa
23701 aagtgataaa aatgaaacga aattataaat atatattatc tatatgttgt tacaagaccg
23761 atggtctgta gcaataatct aataaaagga gcggtatgat atgaagggta aaattgcact
23821 ttattcacgc gttagtacgt cagagcaatc ggagcatggg tactcaatcc atgagcaaga
23881 acaagtactc atcaaagagg ttgtgaaaaa tttcccaggt tatgactatg agacatatac
23941 tgactcaggc atttcaggta aaaatattga aggtcgtccg gcaatgaaac gtctattaca
24001 agatgttaag gataataaaa tcgaaatggt attaagttgg aaattgaatc gtatttctcg
24061 ctcaatgaga gacgtgttta atattattca tgaattcaaa gaacatgacg tagggtataa
24121 atcaatttct gagaatattg acacatccaa tgcttctgga gaagtactcg ttacaatgtt
24181 tgggttaata ggatctatag aacgccagac tttgatttcc aatgtgaaac tttctatgaa
24241 tgctaaggca cggagcggag aggcaatcac cggtcgtgtt ttaggctaca aattatcact

24301 taatccattg acacagaaaa atgatttagt tattgatgaa aatgaagctc atattgtacg
24361 ggaaatcttt gatttatatt tgaatcacaa taaaggactt aaagcaatca cgacaattct
24421 aaatcaaaaa ggatatcgca ccattaatca aaaaccattt tcagtgtttg gcgtgaaata
24481 tattttgaat aatccagtct ataaaggcta tgtcagattc aataaccatc aaaattgggc
24541 tgttcagcga agaagtggta aaagtgataa aaatgatgtg atattggtca aaggtaagca
24601 tgaagccatt ataagtgaag atgtatttga tcaagttcat gagaaactag cttctaaaag
24661 ttttaaaccg ggtcgaccta ttggtggaga tttctactta cgtggcctta ttaaatgccc
24721 agaatgcgga aataatatgg tatgtcgacg gacgtattat aaaacgaaaa agtccaaaga
24781 acgcacaatc aaacgctatt acatttgttc attattcaat cgctcaggaa gttctgcctg
24841 tcatagtaat gcgattaatg ctgaagtcgt cgaacgcgta atcaatgttc atttgaatcg
24901 tattctttca caacctaatg ttattaagca gattgcgtca agtgtgatag aagaactgaa
24961 acaaaagcat agtaaacaaa cagaaataaa atatgatatt gatagtctag aaaaacaaaa
25021 agcaaaagtt aaaacacaac aagaacgatt attggaattg ttcttagatg atgaaatgga
25081 tagcgaaatg ttaaaagcta aacaaagtga aatgaatcaa cagttagaag tattagacca
25141 acaaattaaa gaagcaaaac aagcaaatca atcacaggat gatataccca attttgataa
25201 gttaaaagca cgactcattt tgatgataac acgattcagt gtgtacttaa gaaaggctac
25261 acccgaagct aaaaatcaac ttatgaaaat gttaattgat tcaattgaaa ttacgacaga
25321 taaacaagta aaacttgtaa ggtataaaat tgatgaaagt cttatccctc aatctttgaa
25381 aaaagattgg gggtcttttt ttatacctaa atttaacttt gtgataaatg tcacaaagaa
25441 aaataggatt gaaaatttat cacttttacc actttttag agtgacaaaa gtggaggagt
25501 tttgaaatat tttataaata tatattttat ttatggagta cacattatta attaaggagg
25561 tcattataat gacgctaagc aaacaactta aaacgtatat cactgaacga tttaaattaa
25621 attatcaaga aacttgggct tgtgaaacca tagatgcggt ggctgaagat gtattacctg
25681 aaaaatatat taaaaatagt ccacttgaac ataaaatttt aaatactttt acctattaca
25741 atgatgaatt acatgaaatc agcatttacc cttttttatg ttatctagat aaggaattag
25801 tagcaatagg ttatttagat aattttgatt tagactttat atttttaaat gacactcatc
25861 aagttattat tgatgaacgc tacttgttac aaaaaggggg ccagtaatta tgaattggat
25921 caaggtcgct caactatctg tcacagttat caatgaagtc attgagatca tgaaagaaaa
25981 acagaatgga gaaaaatagt atgaacatca atcgatacat cacaagagga attaatgaaa
26041 gtattccact agaccttcaa atcttacttt ggaatatggt aaaagaacgg gacaatcaac
26101 ctcatacaga ttacctacac atttttagac tacaagaaga tgagaatata ctctcaatca
26161 cacatgaaca agaacaaccc gcatataaat tggaatatca ctatataaac tatgtaaaaa
26221 atcaaaacac attacctaag aaagtctacg tcatacgaga aaatgatgta gatgtttttt
26281 attatgtaat gcttttacca gaagaatact aaaaggagtg acaatgatga atacaatcaa
26341 aagtacgata cacacagaag ctatatttag cgatgatgaa caacaccgct acttactcaa
26401 aaagacgtgg gatgaaaaga aaaccgcttg tacagtgata accatgtatc ctcatttgga
26461 tggcgtatta tcactcgatc ttacaactgt tcttatcctc aatcaattag cgaattctga
26521 acaatatggc gctgtatatc ttgtaaatct attctctaat attaaaacta cagagaatct
26581 taaacatatt aaagaacctt atgataaaca caccgacatt catttaatga aagcgataag
26641 tgaaagtgac gcagtgattt tagcctatgg tgcttatgcg aagcgtcccg ttgttgtcga
26701 acgcgttgag caagtgatgg aaatgttaaa acctcataaa aagaaagtaa aaaagctcat
26761 aaatccggca acaaatgaag gttctgttgc aaagtaaaaa atatagctaa ccactaattt
26821 atcatgtcag tgttcgctta acttgctagc atgatgctaa tttcgtggca tggcgaaaat
26881 ccgtagatct gaagagacct gcggttcttt ttatatagag cgtaaataca ttcaatacct

26941 tttaaagtat tctttgctgt attgatactt tgataccttg tctttcttac tttaatatga
27001 cggtgatctt gctcaatgag gttattcaga tatttcgatg tacaatgaca gtcaggttta
27061 agtttaaaag ctttaattac tttagccatt gctaccttcg ttgaaggtgc ctgatctgta
27121 attacctttt gaggtttacc aaattgttta atgagacgtt tgataaacgc atatgctgaa
27181 tgattatctc gttgcttacg caaccaaata tctaatgtat gtccctctgc atcaatggca
27241 cgatataaat agctccattt tccttttatt ttgatgtacg tctcatcaat acgccatttg
27301 taataagctt ttttatgctt tttcttccaa attcgatata aaattggggc atattcttga
27361 acccaacggt agaccgttga atgatgaacg tttacaccac gttcccttaa tatttcagat
27421 atatcacgat aactcaatgc atatcttaga tagtagccaa cggctacagt gataacatcc
27481 ttgttaaatt gtttatatct gaaatagttc atacagaagg ctcctttttg ttaaaattat
27541 actataaatt caactttgca acagaaccaa tgaaagaacc aattgaaaga accaaaagaa
27601 tatcgaaaag agtgggcaac ttggaatttg tcctctttac caattctgcc aaactactat
27661 gagtataagg tgtcttacga taagagagag cagttcaact ttattaaaca gctattcaat
27721 gactctagca ttagaacaat tgtaaacggg tgtgattcgg accgcgaggg gtccaatatt
27781 ttttattcat cctactacat gactggagca aaaaataagg aaattaaacg attatggatt
27841 aattcactag aggttgatga aataaggaaa ggattcaata atcttcaaga taacaaaaaa
27901 gacttattac tttattacga agcaaagaca cgtcaaataa gtgattggct tgttggaatg
27961 aacggaagcc ggttatttac cctcctctta caacaaaagg ggtttaatga cagtctctcg
28021 attggacggg tacagtcttc cacagtatac cttatttatc aacgtcaaaa agaaatagaa
28081 caatttgttt ctacaccttt ctatgaaatt gaaggaagct ttaccgccaa aaatggcatg
28141 tataaaggaa aagcaaaaat taaaagtgaa actctaaaat tgcagctgat gctctaactc
28201 gtgctaatta caagtgtgaa tacaatataa aagataaaat cttcgaaaga aaatctggta
28261 agggttatac agagccacat catttaatac cgattagtaa atataaagat ttcgattata
28321 aaaattgtag tttagataca atggagaaca tcgtttcact ttgtagtcat tgtcataatt
28381 tacttcatta tggtaaattc gaagataaaa aacctattct tgaaaaacta tttaatgaaa
28441 gaaaaaaagc gttaagtgct gttggcttag atattacatt agaaaaatta gaagcttctg
28501 ttttttccat gattcaagca tctttttgtt atccaagtta atttcagctt taataggttc
28561 agcatcttta gttagaccaa aaatagacgc atattctgga tctagcttta aatggtaaaa
28621 gacaagtgac tgtttttgc cattgtcatc tttgactgtt cgttttgtcg taattcgatc
28681 acggtcagat tcgataaatc ccttatcctt tagtgcatta acaacattat tgacatcttg
28741 gaagtgattc tctatcaaca tatctttaaa tacagatgca atgattttga cttcgatata
28801 atcatctttt aaggcaatta gtccatggtt ctcaatcata ttctttaacg ctgtatcatc
28861 agaaaactta ccacggtttt gtgctacaaa ttgcgtaatg acttcgatcg ctttatctgc
28921 cagtgaacgt tcagagactg tatgaccatg ataatcaata aagtagtctc ttattttagc
28981 gatatcaata tctgtagcta aaacatgact aagaatcttg gccgaagtag tgatagccgc
29041 atagcgctta aacatacgaa taccagtatt atttgtttca tccttcaatt tatctttaaa
29101 ccaatgatgt aattcaaaaa cggatcaaaa aacaaagggc cctagaaact aggagcacac
29161 ttacacaaga acaattgata gataaattag ctaaaggagc catcgatgca gaaacgttca
29221 gaaaacaatc tcaatcgtta cttcaacaat caaaaccaac actatcaata aatgagcaac
29281 aaattcaaag gtcttttgaa aatgtaattc aacaacactt cacgttaagc atgttatacc
29341 gatatattga tgaaattcat atttctaaaa acaaaagcct tgtgggaatc tatttcaaaa
29401 atgaaccgct aaacattgta aaccaagcta cgcaatcatc gattgcttaa ttaatgaaag
29461 aatgaaaaat atgaaagaaa tgaaaagaaa atctgtagga tgttatgtta gagtttcaac
29521 aatttctcaa gacattgata aatttagtat taatggtcaa attacacaaa taaaggaata

29581 ttgccaacag ggaaattatg aattgctata ctgataatta aaagtacccc cgatcgaacg
29641 aaaatatctt tttgatgact attagtgata attgtaattt tatccattaa tagatgaatg
29701 gattggaata atactaagcc tattgctatt ccaccgagta aaattaccca cccgcctaat
29761 tcaatacttt ccggaatcat ttcaaaaaat aataagccaa ttataaggcc tgcacataat
29821 gaataaataa agccatgacc actttgaaaa ccttccatta tccaagcgag tccaccgcca
29881 acaccaattc caagtgcgga agctaacgcc cgatcatcca tactgcactc atcccactat
29941 tcttaaaaag gctttgttta ttttactcta aaaaatcaaa acaaaatata tgaattcata
30001 ttaaatatta tttgactctt tattcagatg gttatatcat atatatgaac atatattaat
30061 atatgtagat aaatagtgat tattatatat aaggttttga attgaaacct aaagtgaggg
30121 aaggataatg gaagaaaaaa aggaattaga ggaagtaaat aataaggact tagatgatga
30181 aacattattt gtcgtatcgc aaacatttaa agcgttaggt gatcctacga gaatccggat
30241 tctccatttg ctcttttata aggagtattc ggtaaacggt attgctgaaa cgctacatct
30301 tagacaatca acagtttccc atcaattgcg gttcttgaaa aatttacggt tagtaaaatt
30361 ccgaagggaa ggcacaacat tgttttattc ccatgatgat gaacatacta tgaatatgct
30421 aaaacaggcg atcgatcacg cctgtcatca ctagtattcc gtaaaccagt aataagtgtt
30481 aataataatt cacgtcatta atattgttat atgatcatat aacaatatta taggcatatg
30541 aaaagggggt tttgcaatgg atatggaaaa caaaaaaaca gaatggaaag cgttgtatga
30601 tatctcgaag gaaagtgaga tgggtgtagc agagagggtt agtgaatacg gttgataaat
30661 tcataggatg tattattgga tacaaaaagt ttggatgtgg tcaattaacg tcctggttta
30721 gtaaagctta ctgcgaatat attggaaccc gatatatcat atttatttag aaaggtgata
30781 gaaatgaaaa atattcaaga gcaacaagca cacgaaagtc atagccacga tcatagtcat
30841 gatcatgatc acggaaaaat gccaattatt tcatatttta ttggcttagt gttggctata
30901 attgggcttt ttttaagtga tgcaaattta ttaatacaaa acatcttatt ttcaattgcc
30961 acaatcacag ccggctacca tgtaattatt ctcgaaggaa ttggagagac agttgaaaat
31021 actaaattaa agggaaaatt cactcctaat tctcatattc taatgggatt agctgcaatc
31081 ggggcttctc tgatagggag tttttgggaa ggaacccttt tgatacttat tttttccggc
31141 gctcattttc ttgaagatta cgctgaagga aaaagtaaaa gagaaattac taagctactc
31201 gaaatgaacc caacgacagc taaattaatc ctacctgatg gaaacacaaa aattgttgat
31261 gtcagtgaat taaaagttgg agatcaactc caagtgctga acggtgatca agttccaatt
31321 gatgggatta ttttatccgg tactacctca attgatgaat cttctattaa tggagaaagt
31381 ataccgaaag agaagtctaa gggtgacgaa gtttttggaa gtacgattaa tggaacaggt
31441 acttttacta tggaagtcac taaggaaaac aaggatactg tattctctaa aattttacaa
31501 ttagttagtc aaaaccaaga taatcaaaca aaagctgcca gtatcattca aaaattcgag
31561 cctaaatatg ttaatatagt tttaatcgca ataccattag taatgttact tgctcctttt
31621 ctatttgatt ggacatggtc gcaaagtgtt tacaggggat tagtgctttt agtcgcagct
31681 tcaccgtgtg ctttggcagc agctactgta tctgtaacat tgtctacaac atctaaccta
31741 gctaaaaaag gcgtgctttc aaaaggaagt acttacctat cacaattagc ggatatagat
31801 gcaattgcct tcgataaaac aggaaccctt acgaacggag aacctaaagt aacaaattac
31861 tatttcactc attctgtgaa cgaagaaaat attattgata ttatagtcgc ccttgaaaag
31921 gaatccaatc acccactcgc taatgctatt ttagaaaaat ttgaagttaa aaataaaata
31981 gacatcgaag ttactaatca aattggaaaa ggtctgacag gagattataa tggaaaaaat
32041 tatcgtattg gtaagcctac ttcttttgaa agtgcttctg aagagtatac ccagttcaat
32101 catgattggg catcagaagg aaagacggtt gtatacgtag cagaaaatga agaagttatt
32161 gggattatag ctctaatgga tattccgaat gagcatgcta aagaaacaat taattacttt

32221 aagaaacttg gtatccacac gactttaatt actggtgatt cggaaatgac gggaaaagct
32281 gtaggcgaac aattgggaat agacgaagtt atcgctaatg taatgcctga agataaatcc
32341 agaattatag aagaacaaaa agaaaaattt ggagttactg ccatggttgg agatggtgtg
32401 aacgatgcac cggcccttgt taatgctgat gttggtatag ctatgggggg cggtactgat
32461 gtggcagtag aagtatctga tttggtttta atgcagaaca atttatctaa attagtacag
32521 tctcataaaa tttcctcaaa tatgggtcgt gttattaggc aaaatattat tttttcaatg
32581 gcagttgttg cctttttagt tgtcattagt ttgttaggat taactgatat tacaatcagt
32641 gtaattgttc atgaaggaag tactttagtt gttatactaa atggacttcg attattaaga
32701 tctaaataat gaacgaatcg attgacatga atgaactttg aagtgtggat tctacaatgt
32761 tcccataaca ttggacacta aaaaacagag caatctaata aagatgttat gagtaaaaac
32821 aatgccttgc ataccgtttt tatcatacgg gcggtcaaat aagcaattaa agagcatggg
32881 aagtataaaa tcataaacag tgacttaagg cagccagttt acattcaaaa gttaaattga
32941 ctgtattaaa agtttcgaaa cgattaatat ccgtatgggg gcaagcgtcg agccaaagat
33001 aatgccagga cagagcgtct ttttccgttc ttttaagtgg gaaaggtttt accttctcta
33061 tgccaagaca gtcccagagc taaaagggcc agttgctctt tttgggcgtt tgtgaacggt
33121 aatcgcagga aggggatttt cccaaccgca cctggttttc catcacaata aagttggcgg
33181 catttgttct gatattcttg ggaatactta cactatgaat ggccgtatta agtgatacag
33241 gagctgctat tattgtaata ctgaatgctc tccgcctttt gagggtaaaa gaataaaagt
33301 aaggataact aggtaaagct gttcaatcaa aaattgaaca gcttattttt catcaaaatc
33361 aaaaacgttt attataatac ctacacttgt tctgttaaat gtaccgattt tttaacctta
33421 ttgtatcagt aatatcttga aacgaagtaa gcgactaaaa tttctctttt atcatacatt
33481 tataaaaata cacttttaag aacggtttga aaatttttga aatagatcaa aataatcttc
33541 tggttaaaaa acctgtagat atgatttctc tcttaaattt ttgtttgatt agattagacc
33601 ctaataagac gcattcaata tgtctacacg tgaatttagt ctttgaaaat gtaaggacca
33661 ttattattat aaaaacccca gtataaaacg atacgctgaa tacagcaata aataaaaaaa
33721 ggcatgtact gtgtaaaatg tttgttaaat tatataaata ttttttaaat agatatttat
33781 tcaataatat aaaaagggac tataagctat atctaactac ttatagtcct tcttcattag
33841 tataaatata attattaaga aagatgtata tctttgtaac cttcgtttac attaataatg
33901 ttgtgataac ctttgtgttc taaaatacca atagctatag aacttctaat gccagattga
33961 cagtgtacat aaataacatc atttttactg aaaggtaaat ctgtgtctaa aagtttgccg
34021 tgtggtacat gaaccgcttg agataagtgg ccattattcc attcattatc attacgtaca
34081 tccaatatat gtgattcgtt acctgtaata tcttcactat gaatggaacg tgtttgaact
34141 ttagattgag gtaattgata tccagcaaca tcatcgtatc caatgagttg caaggtgtgc
34201 gttgcttttg aaacaaggtg atagtctcca atcaagttaa tttcttgatc atagtttaga
34261 taccagccaa tttgattgat gaaattttta tcatatggaa tattgattgt accttcaata
34321 tgtccaccat gatatgcctc cttactgcgg agatcaaaag ttaatctgtt tgtatttgta
34381 gctggataaa ccgtataagg ttgatataaa ttcataccga attgataatt ttttcatttg
34441 tgcaaatgat gtggtggtgc aggttggtca gaaatgagtt tatcgataaa ggtagcttcg
34501 ttattttcag aaaaagccca gttcgtttgt ttttcatagc caagcgtaga tgttggaata
34561 gcacctaaag atttaccaca aggactacca gcgccatggc caggccaaat ttgaatgtaa
34621 tctggcaagt ctttaatact ttcaatcgat ttaaacattt gtttagcgcc tatttcagat
34681 gatccttcta ctttaaccgc tttttctagt aaatcaggtc taccgatatc tcctacaaaa
34741 ataaaatcac cactgaatag tcccattgga acttgtgctc cagcaccttc gtcagtaagt
34801 aaaaaactta tactttctgg cgtgtgaccc ggtgtatgaa gcacttttaa ttttatattt

34861 cctacataaa tatcgtcatt atgttgaaca aaataagtgt ggttaggcat atttttataa
34921 cctaatgtgt catcactttc acccgataca taaatattag catttaactt tatagcaaca
34981 tctctaattc ctgaagcaaa atctgcatgt atatgtgttt cagctgcatg agtaatggtt
35041 aaaccttctt catcagcaac tcgaatatat gaagataagt cacgaatagg atcaataatc
35101 atggcttctc cagttttttg acaaccgatt aaataagatg cttgagataa atgtttatca
35161 taaaattgtt taaaaaacat aatatcattc ctttctaatt atataaataa attgtgatta
35221 gcttgttctg tatatccaat atatgcacct acgccaccgt attctacatc atcgcgtagt
35281 tcttcttttg taatgcccat cacatccata ctcattgtac aagcgataag tttaacacct
35341 tgctcaactg cttgatcaat aagagaaggt aaagtatcga catttttctt tttcatgaca
35401 taacgcatca ttagattgcc taaaccaaac atattcattt tagaaatagg catgttaatt
35461 ggtgaatttg gaagcatgaa gtcaaaaagt tttgcaatac cttttttctt aatgcgttga
35521 ctttgaattt ttttaagtgc atttaagccc caaaatgtgc aaaagatggt tacatctcta
35581 cctgcagctt tagcaccatt agcaataatc attgctgcaa ctgctttatc tagctcgccg
35641 ctaaataaaa caattgttgt tccacttttc gtgtgtgtca cttctatatt tttattttct
35701 tctttttgaa taatagcgtg aatttcatta ccaaaatcgt tgagtctaac aagcgtatgt
35761 ccagtttgtt tgacccaact tttaatatca tttaaaaatc cgtgatcagt cacaacaact
35821 tctatttgat caccaatagc gatgtttttg atttctttac taatattaac aataggtcct
35881 ggacactgta gattactata attgaatgtc ttacgattat ctttaatttc tctatcttct
35941 tttactttag ataaattatc attatgatgt tgttcttcgt atgcggtata acctccgtct
36001 aaattcacca catcgtatcc gcgttgtgca agaaattgac tggcttttct acttctatta
36061 ccacttttac aatagatata gtaagtttta tttttgcttt gtttaaaagt ctctatattt
36121 tcaactgaat gtaataaagc atttttaatg tgtcctaatt caaattcttc aggctgtcga
36181 acgtcaatta attgatcctt agaacccaat tcttctaatt cctttttagt gaaatcatta
36241 atgtgtttcg ttttatattg tgacataatt acctcctttt aaatacctat gggggtatat
36301 tataacgaat tagatagttt gtcaaatacc tatgggggta tttgacaaaa taaaatttaa
36361 tcaatatgat gtaattatta aaaatgaatg tggagtgaaa tatattggaa tataataaaa
36421 agatgattaa tcgtattcat cgcatacaag gacagcttaa tggagtcatt aaaatgatgg
36481 aggaagaaaa aaattgtaaa aacgtcatta gtcaattaag tgcttctaaa agttctattc
36541 aacggttaat gggtattatc attagtgaaa acttagtaga atgcgtcaaa atgtctgaag
36601 aaaatagtga agattctcag gcactaatta atgaagcagt tgaattattg ataaaaagta
36661 aatgataagt ataaccacca ttatagtaat gttagttatt ggtatcttag gaggatttat
36721 ttctggatta gtcggtatag gggagcaat tgttatttac ccggctctct tattattacc
36781 accattattt ggtttaccta cttatagtgc ttatattgct tcaggattaa cttcaagtca
36841 ggtgtttttt agtacattga gtggatcttt aaacgcatat aaaaataaaa atttttctag
36901 aacgcttata cttaatatgg gaagtggaat ggtaataggt agtatgctag gtgcaatctt
36961 agcgaattta ataaatgttc agtttgttaa tacggtttat attattattg cattactagc
37021 tcttattcta atgtttataa aagttacacc atctactagt catattaagt ttaatcgtct
37081 acttttaatt acaataggtg gtatcatcgg tttggtatct ggtattgtag gagctggtgg
37141 cgcatttatt attattccag tactacttgt tatatttaaa ttaccaatga acatggttgt
37201 tacaaatagt attgtaattg cattcatttc ttctattgga gcatttatga ttaaattatt
37261 acagggttat attcctatta atagtgcaat accgttaatt ttaggtagta tactatttac
37321 gcctctaggt atgaaaatgg gtcagaaaat acctgactat attcaaaaaa cgataatatg
37381 tattttaatc gtcattgcaa ttattaagtt gatattttaa aaacatgggg gtgggacaac
37441 attcattttg aacttgattg aatatatagt atatttaata aaatgcagct attgtggcgt

37501 agaatttaag aattaaaaaa tgattaatat agtttgaaaa gactgaatat aaatactaga
37561 aaaatagcca gtaaatgagt ttattatgaa ttcatttctt gactctttca aataatacta
37621 tgagaaaaat ggtgcaaaac ttatggtggg gtgcaggtta taatattgtt gctgtacctt
37681 tagcagctgg tattttagca tttatcggct tgattttatc acctgcaata ggtgctattt
37741 taatgtcact aagtacggtt atcgttgcaa ttaatgcctt tacattaaaa ttaaaataaa
37801 agataggagt tttattatga ttaaaaaatt attttttatg atattaggat cattactaat
37861 attatcagct tgctccaata atgatgaaaa agataaagac actaatgacc aaaaaagtga
37921 gagccatatg aagcataatg atgaaagtaa agttcctgaa gatatgacat cgactaatga
37981 gggtgaattt aaagtaggag ataaagtaac gattacagca gggcatatgc caggtatgaa
38041 aggtgcagaa gctactgtaa aaggtgcgta taaaacatat gcctatgttg taagttataa
38101 acccacaaat ggaaatgaaa aagtaagcaa tcataaatgg gtcgtaaacg aagagatcaa
38161 agatgcacct aaagatggat ttagtaaggg cgatactgtt aaattagaag caagtcatat
38221 gtctggtatg aaaggtgcta cagccaatat agataacgtg aaaaagacga ctgtttacgt
38281 agttgattac aaatccaaag ataatggtaa aatcattaaa aatcataaat ggatgacagg
38341 aaatgaattg aaagcacgat aaaaatctag ttctagattg agaaataaat agatataaag
38401 atatcctcct taatcaataa tttaaataac ttattattgt taaggaggat attttttagt
38461 gtgtaaatta aaaagaattt tagaagaata acatttatca aaaaactgtt cattacctta
38521 ttaaatgaaa ttatataatt aaaaatcgca tcattaaccg atacgcagaa gcatatcata
38581 aataaaacta aaaattaaat ataacgatta atattttaat ttcaatgagg taagattaag
38641 gtatggaaaa atttaaagaa aaagtaaatg acttagaagc aattaagact actagatatt
38701 atacagaatt tgtggagaaa tttaaaaaaa ttcgagattg ggctaaaaat gaaaatctaa
38761 agaatgaagc gaaattggca caatatgaga ttgaaatttt ctctttatgt gagaaaaatc
38821 ccattttatc aaaaaaataaa agtgaaaaaa gatttgtagc gacaattagt tttgatgatg
38881 gtagagagtg gccagacatt caaaaattta caaatgatca aattaaatac tatgagcaaa
38941 gattaaacga gacaaataat ttgtttttaa aagtaagata ttctgatttc ttatttgaac
39001 atggtaataa gaaaataact aaaactaaat atgaaatttc aaaatgtttg ctatcgtgct
39061 tagttgagat tcttacatat tacagtgacg attttaatta tacttcagta ttagctagat
39121 tagttgaagt atctctttta atgggagata gggaaaaact cgaaaaagct atcgaattaa
39181 tttatctaaa aatggatgaa tttgactata ataatgagta tagttatgta tatgaacttt
39241 caaaacttat aagagaaatt ttaaaatcta agcacaaaaa aataatctta gagaatcatt
39301 taaataaaat tataattgtt ttagaaaaag ctattaaaaa caattttgaa gataaaaatt
39361 attacctaca tagagtattt tgtgaagaac ttagtcaata cagaaaattc gatttaattt
39421 cttctgaaag aagaagtgaa ttgaaaaaag aaattggaaa aagctacgag ttagaggcgg
39481 aatatcaaca aggaagaaat aataagagtt tattggtaaa agcaaatttt ttagaaaaag
39541 ctatggaaaa atatatggaa attggagaaa gagaaaaaag taataagatg aagattttag
39601 taaaatggac ctatgaagaa tacgaaaaca gtaatgaaat gaatctaata agaattccaa
39661 tagaatttcc aaaagaggaa attgataaaa ttattgaagg atttatttcc tcagatgtac
39721 agatttcgtt agataaaata gcatacagta atgacttaat tcctaaaatc acagtaattg
39781 aagatctagt tgataagtta agtaaagagt ttcctttaca aggtttaatt tcaaaaggtt
39841 tattgaatga tggaaaaaaa gtagtggaaa ctactacaga agaagataat aaaacaatca
39901 attttaatag taattatatg catcatttaa atattaatgt tgaattactt cttaaagcta
39961 tttttgataa attaatatca gaatttgaaa tttccactga agatttcatg caaaaattta
40021 ataagtggga actattagat aataaaaatg ttccttttat tgaacatgga atttctcgat
40081 ttttagaaaa ggattatttg agcgctttgc atattttagt ccctcaattt gaatctacag

40141 ttagaagaat gttttctaaa gctggctatt caactacatc tattaggaaa ggaaatacgc
40201 aacaagaagt aacatttaat gaatttttac tcagagatga tgtgaaatcg accttaggaa
40261 atgatgtgca taaactaatt caaattgtga tggttgaaca gtcggggttg aatttacgca
40321 atgaaatagc tcatggatta atagaattat caaatataaa ttatacgaaa tgtattttgg
40381 ttatttattt atttttgata ttaactagat atagtattga aaaaaatgac taagtgataa
40441 tttatattat acaagttttg tggaggacat atcatgaaat tagaagagtt gctactaatc
40501 acacaacaac cattaattga atattctaaa aataaaaaat tattagaagg atcacgttta
40561 tttgatattg atgaaagaat ggatgaaaga aaaatcccca aaattttaac taatagcgat
40621 aagtaccatg ttgttgaaat agccaactac gataatttaa taattattga taatgaagta
40681 ataaacggta atgaattaat tcaagtcggt caatgtattg actttgatag taacgtaatg
40741 agctatttac gaaacttgat tgtaaataat aaatatgagg atgatttttt taaaatttta
40801 actaatataa aaaaaagtaa gcaacaaata agctgcgtac cttatttaat agaaaatggg
40861 aataatattc ataggataaa taaaataatt tcatatgaaa caatattatc attttctata
40921 tttgatagaa taagcgaatt tgattttgaa aatcgcaatt tctcaagata ttttaatgat
40981 tcagaagtta ttttagatac agatgataga tactatcata tgatgaatat acaagattct
41041 aatatacttc aatttcaagc aatttatcta ttagttttaa tggcattttt taggcagtgt
41101 gtcaatagaa aaatgagaat ttcttaaatt tttttaataa atattactaa aagagtacag
41161 aagaagaaaa agaagcattt tactagtaat atctgtcaaa aaccacagat ttaaatagta
41221 tgcttctttt tagattatgc attttatata caaaatttat tcgtcctgtc cttccgcgct
41281 cattgtaaac cattcgctta gattaaccta tattaattga cgggttcgtg cctcacacag
41341 tcatttaata taggttcaca attttgtgtg cttacatggt actttagcat ttcctcactc
41401 gatttatagt caaatatcat tcgaggccgt gcattaattt gtttaacagc ttcatctaat
41461 gacttttgtg ttatatttgc aaaatccgta cctttaggat aatactgtct taaaagtcca
41521 tttgcatttt caatagaacc acgttgatat ggacaaccca aatcactaaa ataaatacta
41581 caattcaatt catcttctat ttgttcaaat agagcaaact ccttacctct atccatagtt
41641 atacttttgg gtaataatgg tttcataata tttataatct cattacaaac tacattggat
41701 ttacgtgtat ttaatctact agcatataaa tatcttgtct ttctttcaat aatagttaac
41761 aagcaatatg ttgatggtct aacactccaa acagtatcaa tttcataatg tccaaattct
41821 tcacgattat taatactttt tgagccgata ctttataaca cgagacccta cgtctaattt
41881 gccacgcttt tcagaagact tattatattt cttgcctgct tgtctcatat cacgcttaac
41941 atcaactttt accaatccat tgcgaatata tctatatata gtaggataac ttatataata
42001 ccctgtatct ttttcaattt gacttgctat ctgtctagga gaccatttta atgaaagttt
42061 ttcttcaata tatgcttttg tttcatcatt caaacgtcta cgtttagcaa cttgttcatc
42121 tgaaatacta cgcgatttta tagcatcttc ctcagctaat aaaggattat aaacaccatc
42181 aacagaacgc ttatttactt ctctataaag aactgcatca tcacgaccta aaatcttaga
42241 aatctgaatg atgtttaaac catcatttaa atactttgcg atagctagac gtgttttata
42301 gtctaattct ttacgttttt ttcttttttt catgtatatc ctcctttact tttaaaaagg
42361 ttatttataa aataagttat aagaggatat atgttcatca actcccgata ccaccgctaa
42421 agcgtatagg gagttgattc tttatttttt catattattt tcttcataaa ctggtttatt
42481 atctttctca aacttttcag catcaatatt agaatcttct tcatatttat cgctagtctg
42541 atttatttta tatactcttg aaacttttgt attactatct ttaaaaggca tataagtcat
42601 tataataacc tcaccatcat tataaatctt ataataactt tttgaccgct caacattttt
42661 atacttcttt gtatttttca catcttgaat actttctttt tctaactttg taacttcatc
42721 aatttcttta tcatatttct gaccacatgc agataataat ataactaatc ctaaaattaa

42781 tacaaaccat cgcctcatga taaattcact cctttttacg taaattatat cattgctatc
42841 gtcttgaaac aaactaacgt cctagaggtt ttaaattgaa acgttttaaa ttatcaatac
42901 gccaaccttc agctgtacga actaaatcca attgtgcaga tgtttgacga tgttcaattt
42961 tatcatctcc atctgtttca atacgttcgt cgaactgaac aaaaacttta taactatctt
43021 ttttagggtc ataagtatca aaataaacat gaatatcatt aaccgttata tcatcatata
43081 aatcatattt atctttagaa tccttaaata atgtatcgta ataatccttt gtaactaaag
43141 gctttatttg tggtgctttt gatttaaaag aagaatcttt tttcatttca aacatctgtt
43201 taacaaaatt tgaagaaact tcacgtattt gtttttcttc atcagaaata actgtttttg
43261 attgtctctt tactaattta tttaactctc catttttatc tcttaatata cgttgttctt
43321 cctgcaaatc ttcaatttga ttttgtttca ttttaacttg ttgtttagga atctgataac
43381 caattaaaac agcacaaaat aatattactg ttacaagagt aaacataata attttttgct
43441 tcatttattt tcactcctta tcatgaataa aactatatcc gtcagtcatt ttcaatgtac
43501 gttcattaac tttaaaagca ccttcccaat tcatttctgt tattctaaat gttttaccac
43561 catcacttac accaataaca acagccacat gaccataatg cccatcatga ccgcctgcac
43621 cttgttcagc aataaataat gcaccaggtt ccggattttt atcaacttta taaccctcag
43681 ctttagctct atcattccat ttatgtgcat catgccaata agtcgataca ggtttaccca
43741 tttttttacg cataccgtat gcataccaag tacattgacc agcagtgtat aaattacctt
43801 ttccagggtc atcatttgac caatgtggca atttaccatt aaactttata gactttcctt
43861 taccattact acatgacgtt ttagcttctg aaccgcctaa atctttcata attttttcaa
43921 ctgttggtat ccaacgggca ttcatattat tagggtcatt tgacgcacca acaggcgcat
43981 atttgggacc tatttttta ggagtatcta aacctttaga aatataaaca tcatacaaat
44041 ttttagctcc cgcattaaga ccatcttcaa tagttggata tgttgaatca tgaatcgatt
44101 ttgaacccat aacagacaaa ggattatttt gtcttgtcgc attttctcct ttaccccatt
44161 gtgattcact cgcaataatg gccataaaaa tattcacagg tactttattt ttttctgcaa
44221 ttttctgtat ttcttttcct ttaccctcta attttccacc tttagcattt tcgttaaact
44281 tatcaatacc atttgccgaa acattaccgc cactaacagt acaaccagca tcatctttac
44341 ctttatttaa atcaggcgct aacaaaaaca ttataattac tggaaatata aaaaaagcca
44401 ccacacataa aatgatgatg actttcgcta atttcaattt aacgcctatt ggcgttttac
44461 ctattacttt tttagcaata tcttttttat ttaccatgct tattgatatc cttttgctct
44521 tttaaacggt cttttgactg agaattacgc gtaataggtt gattacgatt tccagattgt
44581 ctactttgtt gattctgtgt cgcattttgt atttgtcgtt tttgaacatc agtatcataa
44641 gcagtttgaa ctgacttctg ttctttacga tttattgaat gattctcatt atttaatggc
44701 tgattaacca cttgtggcga cctttgtata tcttttcgct gtcttgtttc tcgtacttga
44761 tgttcttgct gtgatgcttt ttgtggttgt ttagaataat tatttgcatt tgattcaaca
44821 gagcagattt tcttttcaac agaagcagat tttacatcgt taccattcat tgactcttgt
44881 tgttctctgc gcttattacc atttttttga ttatctttat ttataaataa aggcattggt
44941 ttatcctgtt gtcttcttct taaatcatta tcattttgaa gtggtggggc gggttcatct
45001 ctttgatttt ctcgtttagc tttcttattt tgcgcttgtt catgacgaat cttattaaaa
45061 cgttcttgtg ttcgattcat cacaccagca ccaattgcac taggagaaaa actaatatta
45121 ccacgactta caaaattgat aatttgagcc cgttttttat acattacaaa caatattaca
45181 attataaata ttgaatttaa aaaataagca ttttgactat ccggttcaat aaaactattt
45241 gcaagcttga tacacaaacc aactatcaca aataagaatg tcatacaagc acttaaaata
45301 atatagccta acgtttttgc aaacgcagag aaaacagata attcaaatcc tggtataaaa
45361 ctcactatcc aactgaatac taaaaataag aataaaaata aagcaaatgt agaaaaaatc

45421 aacttcgcaa aacttattga taaaaatact atcgacataa aaagtgtaat aataaaacca
45481 atcactgaaa tagccaactt ttgaaataca taaccttgcg tcatatattt attatttttc
45541 ttttcaacct catctttaac aatatcatct aattcttttt ctgatttttt atcataatca
45601 agttttaaaa tgttatcaat acggtcttta ccttttgctt ttatctcttt ttcgtcgaca
45661 gttccataat tcattactaa atatgttcta tatatcgtca tatcaaataa ttgattacgt
45721 atatgattca tacctgaatt ctcatgaata taatcaatat taccatcaat agaagatgta
45781 ctattcataa ttatattatt taagtcctta ccaatattgt ttgtaccacg tataatatta
45841 cctgcattcg caagtatacc aacaccaaaa ccaattaaac acataattaa aaacgctcgt
45901 tttaatgctt ctcttgcatt accttgtaca ctaaaaataa taaatgcgtt aaaacaaaca
45961 ataataaata atgttaatgc taaattactc aataactttt gatatatatc gccaacaaaa
46021 tcgccgattt tatccgcaaa atcattaata acatctaatt gaaataaatt ttcaatcgca
46081 taatctgtaa cacctgctaa aatcttagtc atagaaaaga aaaaatctga cacattatta
46141 atttgacgtg aaatttcttc ttgaccaaac ggattccagt cgcctttttc actatatatt
46201 gaacgataat gtgataaatc atacttttca atttttggct taactgcatg tgagggctct
46261 ccaactggat tagaaacagc gtaagcactc acagatgtta aaagcagaat aggcaaaatt
46321 aaaaagcata aaagatacac acttttatgc ttcatcaaat cactccttat ttaatatttg
46381 tttcacatct tccacaaaat catattgttc cattaaaggt gcatcaaata aaattggttc
46441 attatattgt ccaagtatag aaatataacc tcgaccgatg tcactatcat gaatcgtttg
46501 aaattcttta tcagtttgac caaaggtcat acgataacca tcatttgaca aattacctaa
46561 agacacacgt aaccctaatt ggtcacgcac attaccttta atcacatctg catctggtct
46621 ttgtgctgtt aaaaacataa atacaccagc ttgacgacct ttcataataa tattaattaa
46681 ataatcattc atttcttgta attctttttt atctaaagtt gaagtaaaag ccgttacctc
46741 atcaaaaata atgaaatagg ggcgcatatc aaaattacgg aaatcattcc cgattttata
46801 atccgaacta tcattcatta atctaaaacg ctcttccatt tcattattag cttctctcaa
46861 ttgttttaat atttgacctt tagtatctgc aactttatca tcaccaataa cgcgcttcat
46921 aaagctcaaa tctgaaactt ttgggtctaa taaacgtact tctgaatgtc tacggaataa
46981 ctcacgaatc acataaaata aaaaatatgt tttaccacct ccggttatac ctgtaaccaa
47041 tccatgaggt gctttcacaa aatcccaagt aatttgatgc gtaatagcaa tttttgtatc
47101 ataaaaaatt gtatttgatt cttgttcgcc attcataaca atacgctttt tatatttaag
47161 tggcataaat tcatatctca aataacgacc aatatgcttt ttttctacta attcagaatt
47221 aaacattggt tcaataatcg tttctaactc taaataatta tcttgaaacc tcgaaccgtc
47281 tttttaata tgaataacaa aagttttttt accacgttta taaaacatcg ttggataata
47341 gtcatattca gtttgtttat aatcaatagg gatatactcg gttgttttct tgattactgt
47401 acgattatga aaaccttgtg tttcaatcat ttcagctaaa tcttttgttt taaaacgaat
47461 taaaagctga gcgataagct tacatataac aaatataatt gctgtaccaa ttaataaata
47521 caataaatgc ggtttaaaat atgcaaacaa cgtccatata tccaattttt tattaactga
47581 tttataatca ataaacaaaa ttatatacga tataccaaaa aaataaatgc taatacactt
47641 gcaaacataa aatcaatata cttctgataa ggctgtatac gtacgccttt aaataaagca
47701 atttcattca ttgccatcaa atccactcct ttaaaataaa aagcacatac aattttgtat
47761 gtgctaaaaa ttacatcgca aatttttctt cagctttaga gcttgcagaa tcttcttgtg
47821 ttctaaatgc ttcagtcatt tcctcaaatg gacaatgtac aaccattttt ccaacacgtc
47881 cataaaaatc actgaatata cattgacctt tcttcaaatc tttcaacatc tccatatttt
47941 caggagattc ttttgaatat tctaagccta aagattttaa aatatcctca cgttcatctt
48001 tttcatcaaa tgcaaaatga caaccaaagt taccaccatc atcatctgct ttatctttta

48061 ccgattgtgt aataaataca agtgaattat tctcagaacg tcccgttctt tttatacgct
48121 taccaacttt tttaccttgt cttgaagcac taaaagccca accctcatca ataaatatag
48181 tcgtttgaac atctctacga gaaccgaatt tctctaaaaa cttaccaatt aataacatta
48241 atgtaatccc atttttctca cttgttgaat aacttgatgt atcatcatct gctttaggca
48301 tatctaaacc tttaacctgt aaaatagttg ctttttcttg aatatttaaa gctggattcg
48361 taccatcact aaaaacaagt tttaaaatat tgttttgcgt tttttcaaat aataaatcgc
48421 ctgcattttt tacatgctca tcttcattat tctgtaattt ttcaattaca tttaatgaac
48481 caactttttt accattttca cgttcttcaa ccgtatcaga aatagcttga taaattgctg
48541 tttcaatatg atgataattt tcaaaagatt taatttcatc aaacactgat ttcaacatct
48601 cattagcttc aataccatct aaaaatacaa tagggtctag acaaccataa ttggttgaat
48661 cttcataatt caatgttaca tatttaaatg tttttaaatg attaataaat aggggatact
48721 tttcttgcat ttccttactt tctgttactc tgtcaaacca ttctgcaaac tcagattttg
48781 gatcccagta caacatttga cctttaaaaa atgatgtata aatccataat aattttgcta
48841 aaaatgattt acctttacca gtttgaccag taattaaaac atgcggacta tcactgacag
48901 ccccccttaat accttttgcg gccaacaata aactaaacaa aattatatcc cttgaacttg
48961 caacagcttc ctcacgtgaa acagaacgta taaatttatc aatacgccct atataaaaac
49021 ccgtttttgt tcccaaactt tgagacacac caaataaatt ctctgctata cctgttgccg
49081 ttgttctttg taaccaatac ttattaataa ataaatcatt accatgtaaa cattgataaa
49141 acaattgtaa ctggtcagct aatggattaa ctgcatatat ttgagtttcc tttaatcgtt
49201 tcatcaaacg ctttgcttta cgtttacaat ctctataatc atcactttta attacaaaca
49261 taaataccca ctcaataaat acacctgagc cagtcacttc tcgttccatt tgattcaata
49321 caaacaaatt atcttgaaca tcttccgaag cactgtcgcc agtatcttgc ttttcttttg
49381 cttgttgttt atgatttatt ttcgcacggt caaatttttt cttaactttc tcattattaa
49441 tgatttttgc ttttacatgg cattcaaccg gaaaaggcat attttgagct ttctcaaata
49501 aatgtgtata ttccatatct aacggaaaat catcaacaac aactgttgtt gtataaaatt
49561 tctcgttatc attttcaact tctaaaacac tcaagcgagt agggtcaatt actgcattat
49621 aaatatctgt aatagctcgt cttgaagatt cttcctgtac gtcatgtgtt gcatttgata
49681 taaatggctt ccgaataata taagccagtt cattctcagt taaacgttcg cctccaatac
49741 ctagaaaact atcagccaaa tctcgttcaa atgttttata cttatctaaa attttatcgt
49801 ctaaattcac atcaaaacca aaattcgaaa caatacgctc agccgtatct gataatgtac
49861 tttttaaaat ctctttaagt gtaactgcat cgtcagctaa ttcacgtatt ttaacaccca
49921 taacaaaaga attatgcgta atcatttgta attcttgttc taatacgtca atagtttctt
49981 ttgaataata ttcagctaca ttaaaagctt catcatcaaa atctttacta atttctttaa
50041 atttatttcg caaatctaaa tcgtggtcat acatttcaaa atgaaaatct tgataacctt
50101 taatcgactc taaaaaataa cgcatacgct tttttgattc ttcttttgca tcataatttg
50161 ctgttgatat agactcagaa cgaacacgat aatacgccca aacttcgcca gttttcgtta
50221 ataacaaatt atctcttaaa cctgcatacg gtgcttttaa attcatgtca tccctccaat
50281 aaaaataacc ccaatagggg ctatttaaat gttatttttt tatcatgata ttctacttct
50341 tcatcataac ttaatttaac ttttcttaac tgcacattaa aaacaaatat tacaaaatcc
50401 cataagaaga aaaataattt ctttccatct tgttttaact taacaatata tttcgttata
50461 taataaggtg caaccgtaaa gtaaataaac ataagtccac caatcgacat aattacaggc
50521 tgtaataaat accaaaataa caaaaataat ataaaccaca caatcaaaaa tgtagcccaa
50581 aaatctaaac gaaaaccctt attaatacga aacttatctg taaattcata cataactatc
50641 ggttgttcaa aagttcgctt taaattataa gctttttttat ccaaatggaa tcacctcaac

50701 cgccaaaaat cattttccaa acacctgcaa tcgcattaaa tacctgttca ggacctttag
50761 aaactaagaa taaaaagcca ccaacaacaa ataatataac catatgccca tatttaccac
50821 tagcccattt tacaatacca acaaccaata ctactaaacc aatacccgta cctacttcat
50881 tacttatcca gtcaccaact ccaccaagtg atggacggtc agcacctaaa acaaaaaaat
50941 taaataaacc actcataaca ttaacctcca atagaattag tcatattttt aacgtaatat
51001 ttaccatctt tttttgttat atctaatgtg taatgctcca aattctccaa aggagaatct
51061 ttatctttca tcaaaatttc aactttagca acataatcat cgccttttgg atataaccta
51121 atttctctta tctgagaaac ttctctagtt ccttctaagc cctcagggtt atccattaaa
51181 taggccatat catcagactt actagaagca tatttattaa agaaatcttc aataaaagct
51241 tttgctttcg gattgtcttc ccgtttattg tctttcaaat tatcctcaac cattttagct
51301 ttattcaatt gactatcagg gattggcgta aaataaggat attcaacaac aacatactta
51361 ttattttcac tcttaatagg tatatttatt aaaatgtttt gatttacttt acgttgcttt
51421 tcttctgttt ttgtttcata ctcatcttta tctttttcac ttttcttttt cttcttaact
51481 ttaacttctt tcttttcagt aatattcaca tcataattaa caatatactg tataattgtt
51541 tgtttatctt tacgtttaat attataaaat tctttactat tcaattttcg ttctgtatct
51601 gaaatttttt catcaggttt tttataatct gatggaaaat attttaacaa ttctttttct
51661 ctagattcca attcttttga atctttcgag atattcatat acgtatcaat aaacttatca
51721 gcatataact ttaatttagg actatactga actgtattcg cattatcttc atactttttt
51781 tgaatcatat tcgttttatt aactgcttct ttactgtcat tatttgctct tgtagcttta
51841 ataaaagaag ctaataaaag aattaacaaa atagctaata atgagtaaaa acaaattaca
51901 atcatcttac gacgattttt aaattttgga attactcggc gttgttcttt aggaatatca
51961 aaatgctttc cgtttttaga aaatcgtgtt aaaatgtact tgcttattat tttcataaac
52021 gattttctaa aatcctttac gaaattttta ataaaaatca catcccagtt atttttttaa
52081 cacttatctt ttgataagtg cttttttgt tttaaagcct ccaaataatg attaattcta
52141 ttcgtatgct catcattaaa ttgtgaatgc gctaaaattt tatctaaaaa atcagtatct
52201 gtaagcaacc cctcagattg cgccatactt tcggcagtta acactgtatc taaagtagta
52261 gcaacttgtt tacataacca atcaagatta tcctcaatgg actttaatgt tggtctagta
52321 gttaaattca atttttcagt atctttaata aataacgccc aaggtctata taatggccac
52381 cgcttacgat ttttatcatt tccatcttta ggaggagaga taaaacgcaa attattatgc
52441 aatatagact ttacaatttc acttatatta tctgttctac ttaaaacttt cgcacaatta
52501 actgcatatg cctttcgcat ttgtatttca taacgattcc atagcccaat gtcttcgaca
52561 tcacaatgac gtttaaacgc ttgttcataa ttcttttcat aaaaacgaca atataaattg
52621 ctttgtttag aaccaatata aaaagtagcg cctttacttc ttccgtctga caaattaaaa
52681 ccgtttattg cgcttccagc tctaaaacgt gaaacacact cagctttttc agccttttta
52741 attaataaag gcacttttaa atatgtttta taatcatcaa ttgcaatatc aattcttgta
52801 aaatttgcat taactgataa acatttgtca aaaaaatctt gccaagtttt attcaactgt
52861 gtaaatgatg ttcaaactct ctacaacctg taccagttaa ctgtaagtga atgcccattg
52921 attcatcatc acttgaataa tacaatttaa tgtttgaaaa ttcataaccg cgattataaa
52981 agtaaatgcc actatttcgt tttttgaaca acgttatagg caaaccgata acgtcctcaa
53041 ttactgctga aataggggtt atatggaaag tgacctgaac ccagtcaaca actgcctcca
53101 cggcactttt atttgtacgc tctacccccc tattagtaag gggggtacac tggtttttta
53161 aagtcatatt tatttatgcc tcctatcaaa tcgcaagcga tttgatacat gaccgttctc
53221 gcttttttaa cgcgtatttg tcgcaggctc cgcccaaatg cttaacgcac tccgtgcttg
53281 catttctgcg cctgcatacg ctaaaagcta cacggtcatg ttaaatcact aatactatat

53341 gttaaacatc aatatcgcca atatatatac aatcttccaa attaacttgt agcattctgc
53401 catgtcctgc ttcactcaag ctcatacaca atagacaaag gtcagcgtaa attttaggcc
53461 gaccttttac cgtattttct ttaacgtaac tatcattaaa tttagactgt atttctttag
53521 ctaaagtcat taatgaaact ttatcgcccc aatttatacg actagcaaca tagtttaaaa
53581 taggttcact caaatttgaa ctatttgtgt agcgatataa tctatctgct gtataataat
53641 ccatttacat cacctaatta tttttgttta agtgcactat tagaaccaac tactttaata
53701 tcttctgcat tccagctata tgcgatagtt ccaaaatcgc ctgacatttg aacatacatt
53761 ttaacgtctg gttcaactaa ttcgcattct tgtaaatagt ctaattccac tttattaggc
53821 gttttaacag tgatttgttc gccttgttct tgactcgcta atttatatgc ataaacagtt
53881 ttatcagtac gctctccatt ttcatattta tatttttcat ctacacctaa aaagtttaat
53941 tgtcctaaag tttttttaat atccaatact ggtttcataa tatatccatc ctttacaaaa
54001 tttttaataa aattaaacga cttctaaagg gtcgcacgaa taggaataac tcccgcaaat
54061 ccgtaccact gcatttttga taatggagtg tctatgcttc tcctgaccta tcatattttt
54121 aatcaaacct atcggttcat cacctcaaaa aattctaagt cttcaattga aatatgatac
54181 atcttacaaa taaatgtatt agacacacca tattgtttta aaaatttata atccttataa
54241 ggaaatcgat aatcattctt ttttgccatt tcacaaactt ctaatatacg aaaatatgca
54301 aaattataca ttttacaaat ttttctttta gaaacaccat gaaatcttaa aattacaaat
54361 ttaagataat ttaattgatt tgaaccatca aaaaaaggtg taaactcagt cataattatc
54421 aattcctttc ataagaatta actgttaact agagtttaca ccaccttata aaaaatagca
54481 gtaaaaaatc agcagaaaat aaaattgttt acttgattca aactttttatt aaagaaactg
54541 aaaataaatt agcttactca gagttagagt taagtgttat ttttgattac ataaataatg
54601 gtgataataa tatatttaaa tctaccaatt tgaatagtaa aaatttattg agtaaattaa
54661 aaggaatagc ttgggattta tttcatatta gaacctcgga agatcaaatc gctctaagaa
54721 ataccaatag caaagaagtt tttttacatt caatttttac agcagataaa ggcttgagta
54781 atgtgataaa taactattca ataagtagga tgctagcaca tgaagaaaaa ttatatgtat
54841 atagagataa taatatattt aaaaaattat caaaagataa agctactaaa atacaagaat
54901 tgttagaaaa agatagaaat tctagaattt ctaatgtaag agaagcaaag tacaatatac
54961 aagaaaatat tgatatatat gaaaaatata ttaatgaaat gatttaaaaa tagaattatt
55021 agagtcgatt atagagaaga taaagtatta agccattata acgatttgtt actttagtaa
55081 tatcattgat aaggggaggc ttatcataaa t

//

LOCUS Staphylococcus 47013 bp DNA linear UNK

DEFINITION Staphylococcus Staphylococcus aureus subsp. aureus strain ISU926

ACCESSION Staphylococcus

KEYWORDS .

SOURCE Staphylococcus Staphylococcus aureus

ORGANISM Staphylococcus Staphylococcus aureus
Bacteria; Bacillati; Bacillota; Bacilli; Bacillales;
Staphylococcaceae; Staphylococcus; Staphylococcus aureus.

FEATURES Location/Qualifiers
source 1..47013
/mol_type="genomic DNA"
/organism="Staphylococcus Staphylococcus aureus"
/genome_md5=""

/genome_id="703339.353"
/db_xref="taxon:703339"
/project="hwwang_703339"
CDS 1..480
/translation="MKITILAVGKLKEKYWKQAIAEYEKRLGPYTKIDIIEVPDEKAP

ENMSDKEIEQVKEKEGQRILAKIKPQSTVITLEIQGKMLSSEGLAQELNQRMTQGQSD

FVFVIGGSNGLHKDVLQRSNYALSFSKMTFPHQMMRVVLIEQVYRAFKIMRGEAYHK"
/EC_number="2.1.1.177"
/product="23S rRNA
(pseudouridine(1915)-N(3))-methyltransferase (EC
2.1.1.177)"
/db_xref="SEED:fig|703339.353.peg.1"
/transl_table=11
CDS 726..1031
/db_xref="SEED:fig|703339.353.peg.2"
/transl_table=11
/translation="MKGNIQCPRLIKGSLLDFVLETVDVFVNGSKNDCELVNFNYKEG
KVVSRYSTKFESNPKLRKKQLISTVKVVRYVIFDFEKILWGIGKVLLKFIILRQCLL"
/product="hypothetical protein"
CDS 1230..2093

/translation="MKHINQFSESTLAQLEKDQQHVFYVYCLMDPRNDECFYVGKGKG
NRIFKHKQDAQKKVLYEDIFIEENKDNLKFNRINEICSNDLNVLGYIISYGLTESEAF

SAENVLINFLNLTNKTTLTNMINGHGSKAYLVEDLENEFGYDSINLENINTNELILAV

KIRDAFLLDKDESKEYPINESKRDRNNLKSRTLGSWIIGKDKIHKIKYIIGINTGANN

AVVSAYEVSFEQAESIETNNGRMRYAFIGLSERDATLKKLNLYKKALPDLRFGSGSAT
AYINNGTMKVD"
/product="hypothetical protein"
/db_xref="SEED:fig|703339.353.peg.3"
/transl_table=11
CDS 2201..3676
/product="hypothetical protein"
/translation="MPSDDIVKKLYSKEDIRLELGLTPHKFNKKMETIAKLFKIDMKI

FHNYKGQDKNNQYTFNGVAKELIKVLLKSVDYYPVDINSKKFKQNGKSKKEMIENIDN

SSYMKYIYQLMKSINEIQYKRLIADIHMKDVYQNTKAWLNNGESINKKEQELYQYMTI

LPLHKRVELQNEVLKSIDETIFQFLAKEHRNNQIEENNELEAYTKAIKEGRNPKNDYE

LNHLLYKKNQLPLDSLIEDIWDYEETEYTELDWLIADMLKRSQRADSNFIESLEKKNK

LRKNIHKDSIKSISKLIDHELVKNNRKWDVAEFYKNQQFWNNSTLRRSSQNFGILYYK

KNRLHTMNQSNIIKKIKIIEANIESANKQPSYLDFFLQAKFDLERLESELMKYSINPK

YFNKINSDYVRYAIEDVHNAIIKIDRYISNDKVKLNYSNVQMVVRNEITEQGSYFVTQ
ALNTLSKVEESGYSFDNFMVGPFVEDFKKAIKNRRFGSKKD"
/transl_table=11
/db_xref="SEED:fig|703339.353.peg.4"
CDS 3902..5002
/transl_table=11
/db_xref="SEED:fig|703339.353.peg.5"
/product="hypothetical protein"

/translation="METRDKLMSLTQSDKTQQWLMDKSSNQDDIQQLQQQFSQQLDQQ

YNALLADEKAKLDQYVEVHQGLESLKEEIESEPITLNIDKLPDIKATMLERAKNDEHS

DKIEKLFDRLEQALNGTNRLYTQLSLIGTRTHRITTKNFNLQGLPKAVQHTILPSKFK

KVYTVDFKSFEPSVAAYMTQDSKLIDLLNQKDGLYDALLSELGLSDELRVFVKRAFIG

SFLFGGNFKNPKFKLNQYVSEVQWLDAVSQFTKVIELKKHVEKSNSMPMPYSIEHDMS

AFQGSSIMAIYVQTVASYIFKHILLKVYKAQCDQKTFKIIVPIHDAIMIECEDEEIAQ
NVGQLMKDTANQLFNGEFAHVTVEEIGGVDHE"
CDS 4995..5366
/product="hypothetical protein"
/translation="MNNDRGQSLHIPSSTPIKENNIYVATLHSVIQTDFSGEIKHQFT

YEIEVNNQIVYANRNIPTKPSANQLSIHDWLKRHSNYSASHENYEPYIDQKHLILLGQ
YNGNYYVQDVASLDAFGGVLS"
/transl_table=11
/db_xref="SEED:fig|703339.353.peg.6"
CDS 5363..7006
/transl_table=11
/db_xref="SEED:fig|703339.353.peg.7"
/product="putative primase"
/translation="MNHILQMLSKLLSVAKEAIDRQGLIAILTIPVNNNDEIEETAQG

ETVYNELIDQLRLNIPKDTDYQPNIYSYFGIKKNPNDTVLMEMMIKVFHIKRFNSELF

IFKVNGWQKINGDELQGLISKMIQVLLVDYKPSLSTLKNVVDGLQKSTDVEELVENER

YIGCGENMFDLNTFQVVKNSIDIFPKTRLNLSLSTNDVITDKIPPYFKQYMLQLANYD

DDLQYFLFQHTAVLLTADTKYRRGLILYGGAKNGKSVYIELVKSFFYSKDIVSKPLNE

LEGRFDKESLIDKSLMASHEIGQSRIQEKIVNDFKKLLSVESMHVDRKGKTQVEVILD
LKLIFSTNAILNFPPEHAKALERRINIIPCEYYVEKADTSLIDKLQSEKKEIFLYLMY

VYQQIVKADIEYLENSRVTEITHDWLNFGYEFVSSRSVSNANQKACINLLRKLIEIKS

GSRIKVSELNKVINEEIKVSSQVIKQLIQANFDTQTKLYNGYDYWIDLGWKEANKKEI
HDISEKDNIISLDKNENITDDEALDEENLDFDWEDFDDE"
CDS 7232..8908
/product="Cassette chromosome recombinase B"
/translation="MKGKIALYSRVSTSEQSEHGYSIHEQEQVLIKEVVKNFPGYDYE

TYTDSGISGKNIEGRPAMKRLLQDVKDNKIEMVLSWKLNRISRSMRDVFNIIHEFKEH

DVGYKSISENIDTSNASGEVLVTMFGLIGSIERQTLISNVKLSMNAKARSGEAITGRV

LGYKLSLNPLTQKNDLVIDENEANIVREIFDLYLNHNKGLKAITTVLNQKGYRTINQK

PFSVYGVKYILNNPVYKGYVRFNNHQNWAVQRRSGKSDKNDVILVKGKHEAIISEEVF

DKVHEKLASKSFKPGRPIGGDFYLRGLIKCPECGNNMVCRRTYYKTKKSKERTIKRYY

ICSLFNRSGSSACHSNSINAEVVERVINVHLNRILSQPDIIKQIASNVIEELKQKHSN

QTEIKYDIDSLEKQKAKLKTQQERLLELFLDDQMDSEMLKAKQSQMNQQLEVLDQQIK

EAQQANQSQDEIPNFDKLKGRLILMITRFSVYLRKATPEAKNQLMKMLIDSIEITTDK
QVKLVRYKIDESLIPQSLKKDWGSFFMPKFQFEIYGQNDYFIDQITTFTT"
/transl_table=11
/db_xref="SEED:fig|703339.353.peg.8"
CDS 9014..9352
/product="FIG01108299: hypothetical protein"
/translation="MTLEQQLKHYITNLFNLPRDEVWHCESIEEIADDILPNQYVRLG

PLSNKTLQTNTYYSDTLHESNIYPFILYYQKQLIAIGYIDENHDMDFLYLHNTIMPLL
DQRYLLTGGQ"
/transl_table=11
/db_xref="SEED:fig|703339.353.peg.9"
CDS 9448..9759

/transl_table=11
/db_xref="SEED:fig|703339.353.peg.10"
/product="FIG01107894: hypothetical protein"

/translation="MNRYITRGIANNLPNILQHQLWQLVSEREQEQTKDNTSVDYFHI

FQFNKHRNQLYIKHKQERPEYAKIHKANYSKAININKVYIIREDDVDLSYYVMLLPEE
Y"
CDS 9775..10281
/transl_table=11
/db_xref="SEED:fig|703339.353.peg.11"
/product="hypothetical protein"
/translation="METIKSTLKTEAIFSDDKQHRYLLKKTWNSEKQSITIITMYPHY

DGILNIDLTTQLIMNKVSEMDAFGSINFVNLYSNITTPINLKHLENAYDKHTDIQIMK

AVKESDEVILAWGAYAKKPGVEARVNEVLEMLKPHKKKVKRLMNPETNEIMHPLNSKA
RQKWILKV"
CDS complement(10430..11104)
/translation="MNYFRYKQFNKDVITVAVGYYLRYTLSYRDISEILRERGVNVHH

STVYRWVQEYAPILYQIWKKKHKKAYYKWRIDETYIKIKGKWSYLYRAIDAEGHTLDI

WLRKQRDNHSAYAFIKRLIKQFGKPQKVITDQAPSTKVAMAKVIKAFKLKPDCHCTSK
YLNNLIEQDHRHIKVRKTRYQSINTAKNTLKGIECIYALYKKNRRSLQICGFSPCHEI
SIMLAS"
/product="Transposase for insertion sequence-like element
IS431mec"
/db_xref="SEED:fig|703339.353.peg.12"
/transl_table=11
CDS 11362..11529
/transl_table=11
/db_xref="SEED:fig|703339.353.peg.13"
/EC_number="2.3.3.10"
/product="Hydroxymethylglutaryl-CoA synthase N-domain
homolog (EC 2.3.3.10)"
/translation="MLESREQLSVEEYETFFNRFDNQEFDFERELTQDPYSKVYLYSI
EDHIRTYKIEK"
CDS 11627..11857
/db_xref="SEED:fig|703339.353.peg.14"
/transl_table=11
/translation="MLIFLSYDFVRACLPRGMARAISLSHILFPQASALTKSVVIFIF
IRILTEIILIRGIVKAILSKIADKRFVKSSSK"
/product="hypothetical protein"

CDS 12446..13189
/db_xref="SEED:fig|703339.353.peg.15"
/db_xref="GO:0008889"
/transl_table=11
/translation="MLTVYGHRGLPSKAPENTIASFKAASEVEGINWLELDVAITKDE

QLIIIHDDYLERTTNMSGEITELNYDEIKDASAGSWFGEKFKDEHLPTFDDVVKIANE

YNMNLNVELKGITGPNGLALSKSMVKQVEEQLTNLNQNQEVLISSFNVVLVKLAEEIM

PQYNRAVIFHTTSFREDWRTLLDYCNAKIVNTEDAKLTKAKVKMVKEAGYELNVWTVN
KPARANQLANWGVDGIFTDNADKMVHLSQ"
/product="Glycerophosphoryl diester phosphodiesterase (EC 3.1.4.46)"
/EC_number="3.1.4.46"
CDS 13286..13714
/transl_table=11
/db_xref="SEED:fig|703339.353.peg.16"
/product="MaoC domain protein"
/translation="MKYDDFIVGETFKTKSLHITEEEIIQFATTFDPQYMHIDKEKAE

QSRFKGIIASGMHTLSISFKLWVEEGKYGEEVVAGTQMNNVKFIKPVYPGNTLYVIAE
ITNKKSIKKENGLVTVSLSTYNENEEIVFKGEVTALINNS"
CDS complement(13760..15766)
/translation="MKKIKIVPLILIVVVVGFGIYFYASKDKEINNTIDAIEDKNFKQ

VYKDSSYISKSDNGEVEMTERPIKIYNSLGVKDINIQDRKIKKVSKNKKRVDAQYKIK

TNYGNIDRNVQFNFVKEDGMWKLDWDHSVIIPGMQKDQSIHIENLKSERGKILDRNNV

ELANTGTAYEIGIVPKNVSKKDYKAIAKELSISEDYIKQQMDQNWVQDDTFVPLKTVK

KMDEYLRDFAKKFHLTTNETESRNYPLGKATSHLLGYVGPINSEELKQKEYKGYKDDA

VIGKKGLEKLYDKKLQHEDGYRVTIVDDNSNTIAHTLIEKKKKDGKDIQLTIDAKVQK

SIYNNMKNDYGSGTAIHPQTGELLALVSTPSYDVYPFMYGMSNEEYNKLTEDKKEPLL

NKFQITTSPGSTQKILTAMIGLNNKTLDDKTSYKIDGKGWQKDKSWGGYNVTRYEVVN

GNIDLKQAIESSDNIFFARVALELGSKKFEKGMKKLGVGEDIPSDYPFYNAQISNKNL

DNEILLADSGYGQGEILINPVQILSIYSALENNGNINAPHLLKDTKNKVWKKNIISKE

NINLLTDGMQQVVNKTHKEDIYRSYANLIGKSGTAELKMKQGETGRQIGWFISYDKDN
PNMMMAINVKDVQDKGMASYNAKISGKVYDELYENGNKKYDIDE"
/product="Penicillin-binding protein PBP2a, methicillin
resistance determinant MecA, transpeptidase"
/db_xref="SEED:fig|703339.353.peg.17"
/db_xref="GO:0008658"
/db_xref="GO:0046677"
/transl_table=11
CDS 16014..16157
/db_xref="SEED:fig|703339.353.peg.18"
/transl_table=11

/translation="MNYFRYKQFNKDVITVAVGYYLRYALSYRDMSEILRERGVNVHH
LTV"
/product="Mobile element protein"
CDS 16437..16688
/translation="MAKVIKAFKLKPDCHCTSKYLNNLIEQDHRHIKVRKTRYQSINT
AKNTLKGIECIYGLYKKNRRSLQICGFSPCHEISIMLTS"
/product="Mobile element protein"
/db_xref="SEED:fig|703339.353.peg.19"
/transl_table=11
CDS complement(16748..17176)
/transl_table=11
/db_xref="SEED:fig|703339.353.peg.20"
/product="PhnB protein; putative DNA binding
3-demethylubiquinone-9 3-methyltransferase domain
protein"
/translation="MKFQVSPYFLITQNAEGLKKLYEELFGAKTLYIQRIKDRPIDKK

LNIDENDLNKIDQCVLQFGDIKIMIADDTEGLPITQGNNISLCLTFDQVEETKHVYDQ
LIEKGSEVLKTFSPEFYTEGYGYVRDPYGISLHLFTKRKN"
CDS 17257..18186
/transl_table=11
/db_xref="SEED:fig|703339.353.peg.21"
/product="Transcriptional regulator, YafY family"
/translation="MKKSVRLYNMIEYCNENRNFKLNDLMSEFNISRSTALRDIKEIE

ALGVPLYSNPGKNGGYTIIGNRDQTKIAISDEELKALVFTLSSISNVSNLPFQTEYQE
ILKKLYNNSNKKELINQYNDLFQYFNEDKYQFKSYKLFNEIIRLIIENKSFETCYSQN

YIKEQYKGIGIMYKNHQWYFVVANIESKLVNLLNISKIKELYEMGETQECNDITMQNF

QQFMVKNETAIDILIRSNVMGLNILKGYLWSDYMIENIDEETYLFKSKVNAKDIDFIA
KLIVTGGVNVKVETPNSLKNAVKVELTKIINMY"

CDS 18348..20336
/translation="MNKNITALKSTENTTYMQYTVHIDDQIINNIKECTKKFKILPME
DKIPLSPLLQPEYAGEVQDFISTYEQFMINFGKVILDSQGIKIQFESESLSSIQRGIQ

EHCYLNERTNDIDVTKEWYLCKFSNQILEEDKNQLYNALKQLMNDSKNKKQAFQEVFK

VHIDIYLYNKEKSEYKYQVSSYFNLVKENPKITYKKRHLQEKQGVKGTTFTNKLKLLN

KMYGVDVAKYQPFYNSNNPEYERGQFGERYISQRSNYEFNRLQYQIIDMLSKILDKHP
LPKSDNNYKHIPTIEKAILSGDSHSFYEYFEDIMKEIISMENSSLKEKLLTDFTYQSQ

CRWYSESEKLKLQLESFMHKVLESNYYEGNKLYRMLSHAIEETINEADEDKVHFNYFK

DYFLTDGGVKNWEQISEKITEFNGKVINDIQNEYNKIQFNNANRNQKLNFNYLYHCFE

FSNNLVKARVNGNRGYILYFVDKYKIKMPFLDQLLINYLNQEIGQESINYNMQTLFEK

ERYDRSSTIEKLVATSKFKYEKDDSDLFKQLFNDVENSIDRLGIYLLNNGINSNDENA

RYYRSFLKELSRIKSKLTPFSLEISKSSGREQHYPDDAIDDKDERRKNKEETYHAFDD
KSDIDSKLKNKINVSIDNLLSVKIKSNLYDISPNTKKQ"
/product="Similarity"
/db_xref="SEED:fig|703339.353.peg.22"
/transl_table=11
CDS 20531..21640
/product="hypothetical protein"

/translation="MYQANIRDLITKLPQSNKTEHFLMNKFSNQDKVQQLQRQISQQL

DQQYNELLANEKAKLDQYVEVHHNLEPLKKEIESESINLDTDKLPDIKATMLEKAKND

EHFDKIEQLFDRLDQSLNGTNRLYTQLSLIGTRTHRITTKRFNVQGLPKLVQQMILPS

QFKKVYTIDFKSFEPSVAAYMTQDEQLIDYLNHEEGLYDALLRDLSLSKEKRVSVKRA

FIGSFLFGGRYSSSKFKINQEVSEINWLQVMSKFKKVIEFKEQVEKYKTMPTPYGIEH

DMSAFQGSSIMAIYVQTVASYIFKHILLEVYKAQCEKKTFKIIVPIHDAIMIECNDKG
IAQNVAQLMKDTANQLFNGEFAHVTVEALGGIDNE"
/transl_table=11
/db_xref="SEED:fig|703339.353.peg.23"
CDS 21633..22001
/product="hypothetical protein"
/translation="MNNDRGKSLQIPQSTLLKEGSIYVATLHSVYEKNFSGDIKHQFT

YEVELNQETHYVNRNITVKSMSHQLSIADWIKRHSNYNVNHINYDPYIDRKHLVLVGQ
YNGNYYIQDVAPLDEFGGVL"
/transl_table=11
/db_xref="SEED:fig|703339.353.peg.24"
CDS 22001..23617
/translation="MNHILEMLIKLLKVGMEAIDRKGLIAILTSSIGNDEMDDSEQAV

MVYNELIDKLQLNIPKDVDYRPNIYSYFGIQKKPNDTILVEMMICIFHIKRFDSELFV

FKDKGWQKVSEDELQGLISKMIQVLLVDYKPSLSTLKNVVDGIQKSTDIEKLVEHRQY

IGCGRNMFNLKTFKVVDNDLEIFPKTRLDLELDINDTITDKIPPNFKQYMLELANYDH

DLQYFLFQHMAVLLTADTKLRRGLFLYGTAKNGKSVYIKLVKSFFYSNDIVSKTLNEL

GGRFDKESLIGKRIMASDEVGKANIDEATVNDFKKLLSVEPIHADRKGRTQVEVTLDL

KLIFNTNAVLNFPSSHAKALERRIAVIPCEYYVEKADPDLIEKLQDEKKEIFLYLMYV

YKQIVKNDIEYLQNDRVTEISHDWLNFGYEFVSSKSANIAHQKACINLLRKLIEIKPG

SRIKVSRLNEVIRDEIKVSSQVINDLVQANFNVQSRLNNGYKYWVDLGWKETDKKDDM
ISFDKNENVTDDEFLYEDDLNLGWEDFDDE"
/product="putative primase"
/db_xref="SEED:fig|703339.353.peg.25"
/transl_table=11
CDS 23842..25521
/translation="MKGKIALYSRVSTSEQSEHGYSIHEQEQVLIKEVVKNFPGYDYE

TYTDSGISGKNIEGRPAMKRLLQDVKDNKIEMVLSWKLNRISRSMRDVFNIIHEFKEH

DVGYKSISENIDTSNASGEVLVTMFGLIGSIERQTLISNVKLSMNAKARSGEAITGRV

LGYKLSLNPLTQKNDLVIDENEAHIVREIFDLYLNHNKGLKAITTILNQKGYRTINQK

PFSVFGVKYILNNPVYKGYVRFNNHQNWAVQRRSGKSDKNDVILVKGKHEAIISEDVF

DQVHEKLASKSFKPGRPIGGDFYLRGLIKCPECGNNMVCRRTYYKTKKSKERTIKRYY

ICSLFNRSGSSACHSNAINAEVVERVINVHLNRILSQPNVIKQIASSVIEELKQKHSK

QTEIKYDIDSLEKQKAKVKTQQERLLELFLDDEMDSEMLKAKQSEMNQQLEVLDQQIK

EAKQANQSQDDIPNFDKLKARLILMITRFSVYLRKATPEAKNQLMKMLIDSIEITTDK
QVKLVRYKIDESLIPQSLKKDWGSFFIPKFNFVINVTKKNRIENLSLLPLF"
/product="Cassette chromosome recombinase B"
/db_xref="SEED:fig|703339.353.peg.26"
/transl_table=11
CDS 25610..25948
/db_xref="SEED:fig|703339.353.peg.27"
/transl_table=11
/translation="MTLSKQLKTYITERFKLNYQETWACETIDAVAEDVLPEKYIKNS
PLEHKILNTFTYYNDELHEISIYPFLCYLDKELVAIGYLDNFDLDFIFLNDTHQVIID
ERYLLQKGGQ"
/product="hypothetical protein"
CDS 25951..26040
/transl_table=11
/db_xref="SEED:fig|703339.353.peg.28"
/product="FIG01108299: hypothetical protein"
/translation="MNWIKVAQLSVTVINEVIEIMKEKQNGEK"
CDS 26042..26353
/transl_table=11
/db_xref="SEED:fig|703339.353.peg.29"
/product="hypothetical protein"
/translation="MNINRYITRGINESIPLDLQILLWNMVKERDNQPHTDYLHIFRL

QEDENILSITHEQEQPAYKLEYHYINYVKNQNTLPKKVYVIRENDVDVFYYVMLLPEE
Y"
CDS 26365..26838
/db_xref="SEED:fig|703339.353.peg.30"
/transl_table=11
/translation="MMNTIKSTIHTEAIFSDDEQHRYLLKKTWDEKKTACTVITMYPH

LDGVLSLDLTTVLILNQLANSEQYGAVYLVNLFSNIKTTENLKHIKEPYDKHTDIHLM

KAISESDAVILAYGAYAKRPVVVERVEQVMEMLKPHKKKVKKLINPATNEGSVAK"
/product="hypothetical protein"
CDS complement(26879..27553)
/translation="MNYFRYKQFNKDVITVAVGYYLRYALSYRDISEILRERGVNVHH

STVYRWVQEYAPILYRIWKKKHKKAYYKWRIDETYIKIKGKWSYLYRAIDAEGHTLDI

WLRKQRDNHSAYAFIKRLIKQFGKPQKVITDQAPSTKVAMAKVIKAFKLKPDCHCTSK
YLNNLIEQDHRHIKVRKTRYQSINTAKNTLKGIECIYALYKKNRRSLQIYGFSPCHEI
SIMLAS"
/product="Mobile element protein"
/db_xref="SEED:fig|703339.353.peg.31"

/transl_table=11
CDS 27750..29129
/transl_table=11
/db_xref="SEED:fig|703339.353.peg.32"
/product="Tetracycline resistance, MFS efflux pump =>
Tet(K)"
/translation="MFSLYKKFKGLFYSVLFWLCILSFFSVLNEMVLNVSLPDIANHF
NTTPGITNWVNTAYMLTFSIGTAVYGKLSDYINIKKLLIIGISLSCLGSLIAFIGHNH

FFILIFGRLVQGVGSAAFPSLIMVVVARNITRKKQGKAFGFIGSIVALGEGLGPSIGG
IIAHYIHWSYLLILPMITIVTIPFLIKVMVPGKSTKNTLDIVGIVLMSISIICFMLFT
TNYNWTFLILFTIFFVIFIKHISRVSNPFINPKLGKNIPFMLGLFSGGLIFSIVAGFI

SMVPYMMKTIYHVNVATIGNSVIFPGTMSVIVFGYFGGFLVDRKGSLFVFILGSLSIS

ISFLTIAFFVEFSMWLTTFMFIFVMGGLSFTKTVISKIVSSSLSEEEVASGMSLLNFT
SFLSEGTGIAIVGGLLSLQLINRKLVLEFINYSSGVYSNILVAMAILIILCCLLTIIV
FKRSEKQFE"
CDS 29315..30556
/transl_table=11
/db_xref="SEED:fig|703339.353.peg.33"
/product="hypothetical protein"

/translation="MSYSIVRVSKVKSGTNTTGIQKHVQRENNNYENEDIDHSKTYLN

YDLVNANKQNFNNLIDEKIEQNYTGKRKIRTDAIKHIDGLITSDNDFFDNQTPEDTKQ

FFEYAKEFLEQEYGKDNLLYATVHMDEKTPHMHYGVVPITDDGRLSAKEVVGNKKALT

AFQDRFNEHVKQRGYDLERGQSRQVTNAKHEQISQYKQKTEYHKQEYERESQKTDHIK

QKNDKLMQEYQKSLNTLKKPINVPYEQETEKVGGLFSKEIQETGNVVISQKDFNEFQK

QIKAAQDISEDYEYIKSGRALDDKDKEIREKDDLLNKAVERIENADDNFNQLYENAKP

LKENIEIALKLLKILLKELERVLGRNTFAERVNKLTEDEPKLNGLAGNLDKKMNPELY
SEQEQQQEQQKNQKRDRGMHL"
CDS 30742..30843
/transl_table=11
/db_xref="SEED:fig|703339.353.peg.34"
/product="hypothetical protein"
/translation="MKFREAFENFITSKYVLGVLVVLTVYQIIQMLK"
CDS 31083..31175
/product="Replication initiation protein, topisomerase"

/translation="MYKNNHANHSNHLENHDLDNFSKTGYSNSR"
/transl_table=11
/db_xref="SEED:fig|703339.353.peg.35"
CDS 31198..32037

/translation="MCISDPKLSFDAMTIGGNLNRDNAQALSKFMSVEPQIRLWDILQ

TKFKAKALQEKVYIEYDKVKADSWDRRNMRIEFNPNKLTRDEMIWLKQNIISYMEDDG

FTRLDLAFDFEDDLSDYYAMSDKAVKKTIFYGRNGKPETKYFGVRDSNRFIRIYNKKQ

ERKDNADAEVMSEHLWRVEIELKRDMVDYWNDCFSDLHILQPDWKTIQRTADRAIVFM

LLSDEEEWGKLHRNSRTKYKNLIKEISPVDLTDLMKSTLKANEKQLQKQIDFWQHEFK
FWK"
/product="Replication initiation protein, topisomerase"
/db_xref="SEED:fig|703339.353.peg.36"
/transl_table=11
CDS complement(32125..32799)
/db_xref="SEED:fig|703339.353.peg.37"
/transl_table=11
/translation="MNYFRYKQFNKDVITVAVGYYLRYALSYRDISEILRERGVNVHH

STVYRWVQEYAPILYRIWKKKHKKAYYKWRIDETYIKIKGKWSYLYRAIDAEGHTLDI

WLRKQRDNHSAYAFIKRLIKQFGKPQKVITDQAPSTKVAMAKVIKAFKLKPDCHCTSK
YLNNLIEQDHRHIKVRKTRYQSINTAKNTLKGIECIYALYKKNRRSLQIYGFSPCHEI
SIMLAS"
/product="Mobile element protein"
CDS 32945..33472
/transl_table=11
/db_xref="SEED:fig|703339.353.peg.38"
/db_xref="GO:0003917"
/product="DNA topoisomerase III (EC 5.99.1.2)"
/EC_number="5.99.1.2"
/translation="MSYDKREQFNFIKQLFNDSSIRTIVNGCDSDREGSNIFYSSYYM

TGAKNKEIKRLWINSLEVDEIRKGFNNLQDNKKDLLLYYEAKTRQISDWLVGMNGSRL

FTLLLQQKGFNDSLSIGRVQSSTVYLIYQRQKEIEQFVSTPFYEIEGSFTAKNGMYKG
KAKIKSETLKLQLML"
CDS complement(33749..34363)
/translation="MKDETNNTGIRMFKRYAAITTSAKILSHVLATDIDIAKIRDYFI

DYHGHTVSERSLADKAIEVITQFVAQNRGKFSDDTALKNMIENHGLIALKDDYIEVKI

IASVFKDMLIENHFQDVNNVVNALKDKGFIESDRDRITTKRTVKDDNGKKQSLVFYHL
KLDPEYASIFGLTKDAEPIKAEINLDNKKMLESWKKQKLLIFLM"
/product="hypothetical protein"
/db_xref="SEED:fig|703339.353.peg.39"
/transl_table=11
CDS 34450..34725
/translation="MIDKLAKGAIDAETFRKQSQSLLQQSKPTLSINEQQIQRSFENV
IQQHFTLSMLYRYIDEIHISKNKSLVGIYFKNEPLNIVNQATQSSIA"
/product="Cassette chromosome recombinase A"
/db_xref="SEED:fig|703339.353.peg.40"
/transl_table=11
CDS complement(34837..35196)
/transl_table=11
/db_xref="SEED:fig|703339.353.peg.41"
/product="hypothetical protein"
/translation="MDDRALASALGIGVGGGLAWIMEGFQSGHGFIYSLCAGLIIGLL
FFEMIPESIELGGWVILLGGIAIGLVLFQSIHLLMDKITIITNSHQKDIFVRSGVLLI
ISIAIHNFPVGNIPLFV"
CDS 35403..35729
/transl_table=11
/db_xref="SEED:fig|703339.353.peg.42"
/product="Transcriptional repressor CzrA, ArsR family"
/translation="MEEKKELEEVNNKDLDDETLFVVSQTFKALGDPTRIRILHLLFY

KEYSVNGIAETLHLRQSTVSHQLRFLKNLRLVKFRREGTTLFYSHDDEHTMNMLKQAI
DHACHH"
CDS 36059..37984
/db_xref="SEED:fig|703339.353.peg.43"
/db_xref="GO:0004008"
/transl_table=11
/translation="MKNIQEQQAHESHSHDHSHDHDHGKMPIISYFIGLVLAIIGLFL
SDANLLIQNILFSIATITAGYHVIILEGIGETVENTKLKGKFTPNSHILMGLAAIGAS
LIGSFWEGTLLILIFSGAHFLEDYAEGKSKREITKLLEMNPTTAKLILPDGNTKIVDV

SELKVGDQLQVLNGDQVPIDGIILSGTTSIDESSINGESIPKEKSKGDEVFGSTINGT

GTFTMEVTKENKDTVFSKILQLVSQNQDNQTKAASIIQKFEPKYVNIVLIAIPLVMLL

APFLFDWTWSQSVYRGLVLLVAASPCALAAATVSVTLSTTSNLAKKGVLSKGSTYLSQ
LADIDAIAFDKTGTLTNGEPKVTNYYFTHSVNEENIIDIIVALEKESNHPLANAILEK

FEVKNKIDIEVTNQIGKGLTGDYNGKNYRIGKPTSFESASEEYTQFNHDWASEGKTVV

YVAENEEVIGIIALMDIPNEHAKETINYFKKLGIHTTLITGDSEMTGKAVGEQLGIDE

VIANVMPEDKSRIIEEQKEKFGVTAMVGDGVNDAPALVNADVGIAMGGGTDVAVEVSD

LVLMQNNLSKLVQSHKISSNMGRVIRQNIIFSMAVVAFLVVISLLGLTDITISVIVHE
GSTLVVILNGLRLLRSK"
/product="Lead, cadmium, zinc and mercury transporting
ATPase (EC 3.6.3.3) (EC 3.6.3.5); Copper-translocating
P-type ATPase (EC 3.6.3.4)"
/EC_number="3.6.3.4"
/EC_number="3.6.3.3"
CDS complement(38802..38936)
/transl_table=11
/db_xref="SEED:fig|703339.353.peg.44"
/product="hypothetical protein"
/translation="MVLTFSKTKFTCRHIECVLLGSNLIKQKFKREIISTGFLTRRLF
"
CDS complement(39130..40458)
/db_xref="SEED:fig|703339.353.peg.45"
/transl_table=11
/translation="MFFKQFYDKHLSQASYLIGCQKTGEAMIIDPIRDLSSYIRVADE

EGLTITHAAETHIHADFASGIRDVAIKLNANIYVSGESDDTLGYKNMPNHTYFVQHND

DIYVGNIKLKVLHTPGHTPESISFLLTDEGAGAQVPMGLFSGDFIFVGDIGRPDLLEK

AVKVEGSSEIGAKQMFKSIESIKDLPDYIQIWPGHGAGSPCGKSLGAIPTSTLGYEKQ

TNWAFSENNEATFIDKLISDQPAPPHHFAQMKKINQFGMNLYQPYTVYPATNTNRLTF

DLRSKEAYHGGHIEGTINIPYDKNFINQIGWYLNYDQEINLIGDYHLVSKATHTLQLI

GYDDVAGYQLPQSKVQTRSIHSEDITGNESHILDVRNDNEWNNGHLSQAVHVPHGKLL
DTDLPFSKNDVIYVHCQSGIRSSIAIGILEHKGYHNIINVNEGYKDIHLS"
/product="Zn-dependent hydroxyacylglutathione hydrolase /
Polysulfide binding protein"
CDS complement(40477..41544)
/translation="MSQYKTKHINDFTKKELEELGSKDQLIDVRQPEEFELGHIKNAL

LHSVENIETFKQSKNKTYYIYCKSGNRSRKASQFLAQRGYDVVNLDGGYTAYEEQHHN

DNLSKVKEDREIKDNRKTFNYSNLQCPGPIVNISKEIKNIAIGDQIEVVVTDHGFLND

IKSWVKQTGHTLVRLNDFGNEIHAIIQKEENKNIEVTHTKSGTTIVLFSGELDKAVAA

MIIANGAKAAGRDVTIFCTFWGLNALKKIQSQRIKKKGIAKLFDFMLPNSPINMPISK

MNMFGLGNLMMRYVMKKKNVDTLPSLIDQAVEQGVKLIACTMSMDVMGITKEELRDDV
EYGGVGAYIGYTEQANHNLFI"
/product="Disulfide bond regulator"
/db_xref="SEED:fig|703339.353.peg.46"
/transl_table=11
CDS 41683..41943
/transl_table=11
/db_xref="SEED:fig|703339.353.peg.47"
/product="FIG007303: uncharacterized protein"

/translation="MEYNKKMINRIHRIQGQLNGVIKMMEEEKNCKNVISQLSASKSS
IQRLMGIIISENLVECVKMSEENSEDSQALINEAVELLIKSK"
CDS 41940..42698
/db_xref="SEED:fig|703339.353.peg.48"
/transl_table=11
/translation="MISITTIIVMLVIGILGGFISGLVGIGGAIVIYPALLLLPPLFG

LPTYSAYIASGLTSSQVFFSTLSGSLNAYKNKNFSRTLILNMGSGMVIGSMLGAILAN
LINVQFVNTVYIIIALLALILMFIKVTPSTSHIKFNRLLLITIGGIIGLVSGIVGAGG
AFIIIPVLLVIFKLPMNMVVTNSIVIAFISSIGAFMIKLLQGYIPINSAIPLILGSIL
FTPLGMKMGQKIPDYIQKTIICILIVIAIIKLIF"
/product="FIG003846: hypothetical protein"
CDS 42907..43077
/db_xref="SEED:fig|703339.353.peg.49"
/db_xref="GO:0004008"
/transl_table=11
/translation="MVQNLWWGAGYNIVAVPLAAGILAFIGLILSPAIGAILMSLSTV
IVAINAFTLKLK"
/product="Lead, cadmium, zinc and mercury transporting
ATPase (EC 3.6.3.3) (EC 3.6.3.5); Copper-translocating
P-type ATPase (EC 3.6.3.4)"
/EC_number="3.6.3.3"
/EC_number="3.6.3.4"
CDS 43095..43640
/db_xref="SEED:fig|703339.353.peg.50"
/transl_table=11

/translation="MIKKLFFMILGSLLILSACSNNDEKDKDTNDQKSESHMKHNDES

KVPEDMTSTNEGEFKVGDKVTITAGHMPGMKGAEATVKGAYKTYAYVVSYKPTNGNEK

VSNHKWVVNEEIKDAPKDGFSKGDTVKLEASHMSGMKGATANIDNVKKTTVYVVDYKS
KDNGKIIKNHKWMTGNELKAR"
/product="DUF1541 domain-containing protein"
CDS 43921..45711
/transl_table=11
/db_xref="SEED:fig|703339.353.peg.51"
/product="hypothetical protein"
/translation="MEKFKEKVNDLEAIKTTRYYTEFVEKFKKIRDWAKNENLKNEAK

LAQYEIEIFSLCEKNPILSKNKSEKRFVATISFDDGREWPDIQKFTNDQIKYYEQRLN
ETNNLFLKVRYSDFLFEHGNKKITKTKYEISKCLLSCLVEILTYYSDDFNYTSVLARL
VEVSLLMGDREKLEKAIELIYLKMDEFDYNNEYSYVYELSKLIREILKSKHKKIILEN
HLNKIIIVLEKAIKNNFEDKNYYLHRVFCEELSQYRKFDLISSERRSELKKEIGKSYE

LEAEYQQGRNNKSLLVKANFLEKAMEKYMEIGEREKSNKMKILVKWTYEEYENSNEMN
LIRIPIEFPKEEIDKIIEGFISSDVQISLDKIAYSNDLIPKITVIEDLVDKLSKEFPL

QGLISKGLLNDGKKVVETTTEEDNKTINFNSNYMHHLNINVELLLKAIFDKLISEFEI

STEDFMQKFNKWELLDNKNVPFIEHGISRFLEKDYLSALHILVPQFESTVRRMFSKAG

YSTTSIRKGNTQQEVTFNEFLLRDDVKSTLGNDVHKLIQIVMVEQSGLNLRNEIAHGL
IELSNINYTKCILVIYLFLILTRYSIEKND"
CDS 45752..46909
/translation="MKLEELLLITQQPLIEYSKNKKLLEGSRLFDIDERMDERKIPKI

LTNSDKYHVVEIANYDNLIIIDNEVINGNELIQVGQCIDFDSNVMSYLRNLIVNNKYE
DDFFKILTNIKKSKQQISCVPYLIENGNNIHRINKIISYETILSFSIFDRISEFDFEN

RNFSRYFNDSEVILDTDDRYYHMMNIQDSNILQFQAIYLLVLMAFFIKNSSKKSAENK
IVYLIQTFIKETENKLAYSELELSVIFDYINNGDNNIFKSTNLNSKNLLSKLKGIAWD

LFHIRTSEDQIALRNTNSKEVFLHSIFTADKGLSNVINNYSISRMLAHEEKLYVYRDN
NIFKKLSKDKATKIQELLEKDRNSRISNVREAKYNIQENIDIYEKYINEMI"
/product="hypothetical protein"
/db_xref="SEED:fig|703339.353.peg.52"
/transl_table=11
CDS complement(46921..47013)
/product="hypothetical protein"
/translation="IYDKPPLINDITKVTNRYNGLILYLLYNRL"
/transl_table=11
/db_xref="SEED:fig|703339.353.peg.53"
ORIGIN

1 atgaaaatca ccattttagc tgtagggaaa ctaaaagaga aatattggaa gcaagccata
61 gcagaatatg aaaaacgttt aggcccatac accaagatag acatcataga agttccagac
121 gaaaaagcac cagaaaatat gagcgacaaa gaaattgagc aagtaaaaga aaaagaaggc
181 caacgaatac tagccaaaat caaaccacaa tccacagtca ttacattaga aatacaagga
241 aagatgctat cttccgaagg attggcccaa gagttgaacc aacgcatgac ccaagggcaa
301 agcgactttg tattcgtcat tggcggatca aacggcctgc acaaggatgt cttacaacgc
361 agtaactacg cactatcatt cagcaaaatg acatttccac accaaatgat gcgggttgtg
421 ttaattgagc aagtgtatag agcatttaag attatgcgtg gagaggctta tcataaataa
481 aactaaaaat tagattgtgt ataatttaaa aatttaatga gatgtggagg aattacatat
541 atgaaatatt ggagtatacc ttgcaatatc atacgatgtt tatagagtgt ttaataaacc
601 atttttcaac tattgatgat ctagaatata taataactgt acaaattata ttgattatgg
661 aactacaatt caattaagaa attgatgatg aaattttaaa tttaaactaa tggaatcaag
721 aaagaatgaa aggaaatata caatgcccac gattaataaa aggaagttta ttagattttg
781 tgttagaaac agtagatgtt tttgtaaatg gaagtaaaaa tgattgcgaa ttagtaaatt
841 ttaattataa agaaggaaaa gtagtttcac gttatagtac aaaatttgaa agcaatccta
901 aattaagaaa aaagcaattg atatcgacgg taaaagttgt aaggtatgtt atttttgatt
961 ttgagaaaat tttatggggc atagggaaag ttttattgaa gttcatcata ttaaggcaat
1021 gtttactata agaaagttga aatacactca gaaacagact ggattcccgt ttttttcaaa
1081 ttggcacaaa atgatacata ggcccaccta tatattgaag atttaagtta aacgataaat
1141 aatatttttc tcttttgtat agaaattttt ttgggaatga tattaattag tataaaaatg
1201 aatatgtcta caaagaagga gagattttat tgaaacacat taatcaattt tctgaatcga
1261 cactagcaca actagaaaaa gatcaacaac acgtatttta tgtatactgt ttgatggacc
1321 caagaaatga tgaatgcttt tatgttggta aaggtaaggg gaatagaata tttaaacata
1381 aacaagatgc gcagaaaaaa gtattgtacg aagatatatt tatagaagaa aataaagata
1441 acttgaaatt taatagaatt aacgaaattt gtagcaatga tttgaatgtt ttaggatata
1501 taatcagtta tggattaacc gaatctgaag cattttctgc agaaaatgtt cttataaact
1561 ttcttaacct aacaaataaa actacattaa caaatatgat taacggtcat ggatctaagg
1621 catacttagt tgaagattta gaaaatgaat ttggctacga ttcaattaat cttgaaaaca
1681 taaatacgaa tgaattaatt ttagcagtga aaatcagaga tgcatttcta ttagataagg
1741 atgaaagtaa agagtatcct attaatgaaa gtaaacgtga tagaaataac cttaaatcgc
1801 gtacattagg tagttggata ataggaaaag ataaaataca taaaataaaa tatattattg
1861 gtatcaatac aggtgctaat aatgcagttg tctcagctta tgaagtatca tttgaacaag
1921 cggagagtat cgaaacaaat aatggtagaa tgagatatgc gtttattggg ctttcagaaa
1981 gagatgctac tctaaaaaaa ttgaatttat acaaaaaagc actaccagat ttaagatttg
2041 gtagtggtag cgctacagca tatataaata atgggacaat gaaagttgat taaatgaata
2101 aaaacatcca attccttaaa ttagcctttg aaaattttat actcgtatat ataagtgaaa
2161 aacttaaaag aattagatgt taattaaagg agagaatgct atgccatcag atgatattgt
2221 aaaaaaacta tactcaaaag aggatattcg attagaatta ggacttactc cgcataagtt
2281 taataaaaaa atggaaacta ttgctaagct ttttaaaatt gatatgaaaa tttttcacaa
2341 ttacaaaggc caagataaaa ataatcagta tacatttaat ggtgtcgcaa aagaattaat
2401 caaagtgttg ctaaaaagtg ttgattatta cccagtggat atcaattcca aaaaatttaa
2461 acaaaatgga aaatctaaaa aagaaatgat tgaaaatata gataactcta gttacatgaa
2521 atatatttat cagttaatga aatctatcaa tgaaattcaa tacaaaaggt taattgctga
2581 tatacatatg aaagatgtgt accagaatac aaaagcatgg ttaaataatg gtgaatcaat

2641 taataaaaaa gaacaagagc tataccaata tatgacaatt ttaccattac ataaaagagt
2701 tgaactgcaa aatgaggtat taaaatctat agacgaaaca atttttcaat ttctcgcaaa
2761 agaacataga aataatcaaa ttgaagaaaa taatgaatta gaagcatata caaaagcaat
2821 aaaagaaggt agaaatccta aaaatgatta tgaattaaat catctcctat ataaaaaaaa
2881 tcaattaccc ttagatagtc taatagaaga tatatgggac tatgaggaaa cagagtatac
2941 tgagcttgat tggttaattg cagatatgct aaaacgttca caaagagctg atagtaattt
3001 tatcgaaagt ttagagaaga aaaacaaatt gagaaaaaat attcataaag attccattaa
3061 aagtatatct aagttgattg atcatgaatt agttaaaaat aatagaaagt gggacgtagc
3121 agaattttat aaaaatcaac aattttggaa taatagtaca ttaagacgta gctctcaaaa
3181 ttttggaatt ttatattaca aaaagaatag gttacataca atgaatcaaa gtaatattat
3241 taagaaaata aaaattatcg aagctaatat agagagtgca aataaacagc cgagctattt
3301 agatttcttt ttacaagcta agtttgattt agagaggtta gaaagtgaat taatgaaata
3361 tagcattaat ccgaaatatt ttaataagat aaatagtgat tacgtaagat atgctattga
3421 agatgtacat aatgcaatta ttaaaattga cagatatata agtaacgata aagtgaagtt
3481 aaactattca aatgtccaaa tggtagtgag aaacgaaata acagaacaag gaagctactt
3541 tgtaacccag gcattgaata cactgtcaaa ggtagaagag tctggctaca gctttgataa
3601 tttcatggta ggacctttcg tagaagactt taaaaaagca attaaaaatc gtagatttgg
3661 ttctaaaaaa gattaagatt gttttttaga gttaaacata agaataaatt taaataaaaa
3721 aataaaccca acaaaccttg ttatatcaat gtttgttggg tttatttttt tggtgatttt
3781 ttaataactc aattacatct taggataata tcgctaaatt atacattgtc gaggccaaga
3841 caaataaaaa attaaagcaa aagttagcgc gcaactttct gctataaagg agcaaattta
3901 tatggagaca agagacaaac tgatgtcatt gactcaaagt gacaaaacac agcaatggct
3961 aatggacaag tcatctaacc aagatgacat tcaacaattg cagcaacaat tcagtcagca
4021 gcttgatcaa caatataatg cacttttagc tgatgaaaaa gctaagttag accaatacgt
4081 ggaagtacat caaggattgg aatcattaaa ggaagagatt gaatcagaac ctattacgct
4141 taatatcgat aaattacccg atatcaaagc aacaatgctt gaaagagcca agaatgatga
4201 acattctgat aaaatcgaaa agctatttga taggttagaa caggcattaa atggtacgaa
4261 tcgattatat acgcaattat cgttgattgg tacacgaaca catcgaatta caactaaaaa
4321 ttttaatctt caaggcttac ctaaagcagt ccaacatacg attttacctt caaaatttaa
4381 gaaggtgtat acagtcgatt ttaaatcgtt tgaaccatca gttgcagcgt acatgactca
4441 agattcaaaa ctgattgact tgttaaatca gaaagacgga ctgtatgacg cattgctaag
4501 tgaattaggc ttatcagatg agctacgtgt atttgttaaa cgtgcattta ttggttcgtt
4561 tctatttgga ggtaacttca aaaatcctaa attcaagctg aatcaatatg taagtgaagt
4621 acaatggttg gatgcggtca gccaatttac aaaagtcatt gaacttaaga agcacgttga
4681 aaagagtaat tccatgccta tgccttatag tattgagcat gatatgagcg catttcaagg
4741 tagcagtatt atggcaatct acgtacaaac tgtagcgagt tatattttca agcacattct
4801 gctaaaagtg tacaaagcac agtgcgatca aaaaacgttc aagattatag tacctataca
4861 cgatgcgatt atgattgaat gtgaagatga agaaattgca caaaatgtgg gtcagttaat
4921 gaaagatacg gctaaccagt tgttcaatgg tgaatttgca catgtgacag tggaagaaat
4981 aggaggcgta gaccatgaat aatgatagag gacaaagcct acacatccca agtagtacac
5041 caatcaaaga aaataatata tatgtagcta cgttacattc tgtgatccaa acagatttct
5101 caggtgaaat aaagcaccaa ttcacgtatg aaattgaagt gaacaatcag attgtatatg
5161 cgaatcgtaa tattccaaca aaaccgagcg ctaatcagtt gtcaattcat gattggctga
5221 aacgtcatag caactatagc gcaagtcatg aaaactatga gccttatatt gatcagaaac

5281 atttaattct attaggtcaa tataacggta actattatgt acaagatgta gcatcgttag
5341 atgcgtttgg aggcgtatta tcatgaatca tatattacaa atgttatcta agctattaag
5401 tgtggccaag gaggcaatcg accgtcaagg tctgattgct atcctaacta ttcctgttaa
5461 taataacgat gaaatagaag aaacggctca aggtgaaacc gtgtataacg aacttatcga
5521 tcagttacga cttaatatcc caaaagatac ggattatcaa cctaacatct atagttattt
5581 tggtattaag aagaatccta atgacaccgt actcatggaa atgatgataa aggtttttca
5641 tatcaaacgc tttaattcag aactgtttat tttcaaagtt aacgggtggc aaaagataaa
5701 tggagatgaa ttacaagggt tgatatctaa aatgatacaa gtattgcttg tagattataa
5761 gccttcacta agcactctaa aaaatgtcgt agatggattg caaaaatcaa cagatgtaga
5821 agaacttgtt gagaatgagc gctatattgg ttgtggtgaa aatatgttcg atcttaatac
5881 gtttcaagtc gttaaaaatt caatcgatat ctttccaaaa acacgattga atttatcatt
5941 aagtacaaat gatgtaatta ctgataagat accgccttat tttaagcaat atatgttaca
6001 acttgcgaat tatgacgatg atttacaata ctttcttttc caacatacag cagtattact
6061 tacagctgat actaaatacc gtaggggtct catattatat ggtggagcta agaatggtaa
6121 atctgtatat attgaactag ttaaatcatt tttctatagt aaagatattg tgtctaagcc
6181 acttaatgag cttgaaggtc gttttgacaa agaaagttta attgacaaaa gtctaatggc
6241 aagtcatgaa attgggcaat ctaggattca agaaaagatc gtaaatgact tcaaaaagtt
6301 attatctgta gaatcaatgc atgttgatcg taaaggaaaa actcaagtgg aagtcatttt
6361 ggatttgaaa cttattttta gtacaaatgc gatacttaat tttcctcctg aacatgcgaa
6421 agctttggag cgtcgaatta atattattcc atgtgagtat tatgttgaaa aagcggacac
6481 ttcattaatt gataagctcc agagtgagaa gaaagaaatc tttctttact tgatgtatgt
6541 gtatcaacag attgtaaaag cagatatcga gtatcttgaa aatagccgtg tcactgaaat
6601 tactcacgat tggttaaatt ttggatatga atttgtttct agcaggtccg taagtaatgc
6661 aaatcagaaa gcatgtatta atttactcag aaaacttata gaaatcaaat caggatcacg
6721 aatcaaagta tccgagttaa ataaagttat taatgaagaa ataaaggtaa gttctcaggt
6781 tattaaacag ttaattcaag caaactttga tactcaaacc aaactataca atggctacga
6841 ttattggatt gatttaggtt ggaaagaagc caataaaaaa gagattcatg atatttcgga
6901 aaaagataat attatttcat tagataaaaa tgaaaatata acagacgatg aggcattaga
6961 tgaagagaat ttggactttg attgggagga ctttgacgat gaataatgaa caaattgaag
7021 catttgtaga agtgcttgta cctatcatag aagaacgtat caataaaggt aattaaggct
7081 aattacgtac tacaggcagt tgcctgtagt actcatatga ttaagtggta aaagtgataa
7141 aaatgaaacg aaattataaa tatatattat ctatatgttg ttacaagacc gatggtctgt
7201 agcaataatc taataaaagg agcggtatga tatgaagggt aaaattgcac tttattcacg
7261 cgttagtacg tcagagcaat cggagcatgg gtactcaatc catgagcaag aacaagtact
7321 catcaaagag gttgtgaaaa atttcccagg ttatgactat gagacatata ctgactcagg
7381 catttcaggt aaaaatattg aaggtcgtcc ggcaatgaaa cgtctattac aagatgttaa
7441 ggataataaa atcgaaatgg tattaagttg gaaattgaat cgtatttctc gctcaatgag
7501 agacgtgttt aatattattc atgaattcaa agaacatgac gtagggtata aatcaatttc
7561 tgagaatatt gacacatcca atgcttctgg agaagtactc gttacaatgt ttgggttaat
7621 aggatctata gaacgccaga ctttgatttc caatgtgaaa ctttctatga atgctaaggc
7681 acggagcgga gaggcaatca ccggtcgtgt tttaggctac aaattatcac ttaatccact
7741 tacacagaaa aatgatttgg ttatcgatga aaatgaagct aatattgtac gtgaaatttt
7801 cgatttatat ttgaatcata ataaaggcct caaagccatt acaaccgtac ttaatcaaaa
7861 ggggtatcgt actattaatc aaaagccatt ttcagtgtat ggtgttaaat acattttgaa

7921 taatccagtc tataaaggct atgtcagatt caataaccat caaaactggg ctgtacagcg
7981 aagaagtggc aaaagtgata aaaatgatgt gatattggtc aaaggtaaac atgaagccat
8041 tataagtgaa gaggtatttg ataaagttca tgaaaaatta gcttctaaaa gttttaaacc
8101 gggcagacct attggtggag atttctactt acgtggcctt attaaatgcc cagaatgcgg
8161 aaataatatg gtatgtcgac ggacgtatta taaaacgaaa aagtccaaag aacggacaat
8221 caagcgttat tacatttgtt ccttattcaa tcgttcaggg agttctgcat gtcacagtaa
8281 ttccatcaat gctgaagtcg tcgagcgtgt aattaatgtt catttgaatc gtattctgtc
8341 tcaaccagat attatcaagc agattgcgtc aaatgtgata gaagaactga aacaaaagca
8401 tagtaaccaa acagaaatta aatatgacat tgatagttta gaaaaacaaa aagctaagct
8461 taaaacacaa caagaacgat tgttagaatt gttcttagat gatcagatgg atagcgaaat
8521 gttaaaagct aaacaaagtc aaatgaatca acagttagaa gtattagatc aacaaattaa
8581 agaagcgcaa caagcaaatc aatcacagga tgaaatacct aattttgata aattaaaagg
8641 acgactcatt ttgatgataa cacgattcag cgtgtactta agaaaggcta cacccgaagc
8701 taaaaatcaa cttatgaaaa tgttaattga ttcaattgaa attacgacag ataaacaagt
8761 aaaacttgta aggtataaaa ttgacgaaag tcttatccct caatctttga aaaaagattg
8821 ggggtctttt tttatgccca aattccaatt tgaaatatat ggtcaaaatg attatttcat
8881 cgaccaaatt accactttta ccacttagtt attagtgaca aaagtgagcg aaatgaaata
8941 aaaatcaaat atatattatc aaaatgatgt atcacatgca tacatcaatc aaatacatta
9001 ggaggtcata accatgacac tagaacaaca actcaagcac tatataacca acttattcaa
9061 tctgccaagg gacgaagtgt ggcactgcga atctatcgag gaaatcgctg atgatatctt
9121 acccaatcaa tatgtaagac ttggcccact cagtaataaa acacttcaga ctaataccta
9181 ctactctgac acacttcatg aaagtaatat ctatcctttc attctctact atcagaaaca
9241 actcatagcc atcggttata tcgacgaaaa tcacgatatg gatttcttat acctacacaa
9301 cactatcatg cctcttttgg atcaacgata cttactaaca ggaggacaat aaaatgcata
9361 aatacatcaa aattacacaa ttagtcatta caatactaag tgaaatcatc atttggatga
9421 aagagtcaga acgaaaggaa gtctcttatg aatagatata tcacccgggg tatcgccaac
9481 aacttaccta atatcttaca acaccaatta tggcaactcg tatctgagcg agaacaagaa
9541 caaaccaaag ataatacttc agtagattat tttcatatat tccagttcaa taagcatcgc
9601 aatcaattat atatcaaaca caaacaagaa cgacctgaat atgcgaaaat ccataaagct
9661 aattattcaa aagcaatcaa tatcaataag gtctacatta ttcgagaaga tgatgtagac
9721 ctttcttatt atgtcatgtt attacctgaa gaatactaga ggagtgaaaa ttatatggaa
9781 acaatcaaaa gtacattaaa aacagaagcc atattcagtg atgacaaaca acatcgctat
9841 ctacttaaga aaacatggaa tagtgaaaaa caatcaatca caatcattac aatgtatccg
9901 cattatgatg gcattctcaa tattgaccta acgacccaac tcatcatgaa caaagtttca
9961 gaaatggatg catttggttc aatcaatttt gtgaatctat actctaatat tacaacccct
10021 atcaatctca aacatttaga aaatgcgtat gataagcata cagatattca aattatgaag
10081 gcagtgaaag agtcagatga agtgatatta gcttggggcg cttacgctaa aaagcccggt
10141 gttgaagcac gtgttaatga agtattagag atgttgaaac cacataaaaa gaaagtaaaa
10201 cgactcatga atccagaaac caatgaaatc atgcatcccc ttaattcgaa agcacgtcaa
10261 aaatggatat taaaagtata gcaactaaaa tgtttattca ctatccatga aaataattga
10321 atttcatata tatcttttaa tctacaatat gattaaaaga tatattattt ggttctgttg
10381 caaagtaaaa aaatatagct aaccactaat ttatcatgtc agtgttcgct taacttgcta
10441 gcatgatgct aatttcatgg catggcgaaa atccgcagat ctgaagagac ctgcggttct
10501 ttttatatag agcgtaaata cattcaatac cttttaaagt attctttgct gtattgatac

10561 tttgatacct tgtctttctt actttaatat gacggtgatc ttgctcaatg aggttattca
10621 gatatttcga tgtacaatga cagtcaggtt taagtttaaa agctttaatt actttagcca
10681 ttgctacctt cgttgaaggt gcctgatctg taattacctt ttgaggttta ccaaattgtt
10741 taatgagacg tttgataaac gcatatgctg aatgattatc tcgttgctta cgcaaccaaa
10801 tatctaatgt atgtccctct gcatcaatgg cacgatataa atagctccat tttcctttta
10861 ttttgatgta cgtctcatca atacgccatt tgtaataagc ttttttatgc tttttcttcc
10921 aaatttgata caaaattggg gcatattctt gaacccaacg gtagaccgtt gaatgatgaa
10981 cgtttacacc acgttccctt aatatttcag atatatcacg ataactcaat gtatatctta
11041 gatagtagcc aacggctaca gtgataacat ccttgttaaa ttgtttatat ctgaaatagt
11101 tcatacagaa gactcctttt tgttaaaatt atactataaa ttcaactttg caacagaacc
11161 gtattatgga atagagatgt tggtaacatt tatacaggat cattatactt aagtttaatt
11221 tcgttattac agaaccacac attccaacca gaagagaaag tatgtctatt tagttatggt
11281 tcaggagcag taggagaaat ctttagtggt tcaatcgtta aaggatatga caaagcatta
11341 gataaagaga aacacttaaa tatgctagaa tctagagagc aattatcagt cgaagaatac
11401 gaaacattct ttaacagatt tgataatcaa gaatttgatt tcgaacgtga attgacacaa
11461 gatccatatt caaaagtata cttatacagt atagaagacc atatcagaac atataagata
11521 gagaaataaa ctagtggccg attgtgcttg atgagcttgg gacataaatc ctaactcgaa
11581 ataaataagc atatcactaa actgattttt taaagtttac agtgatatgc ttattttttt
11641 atcttacgat tttgtacgtg catgcttgcc taggggtatg gctcgagcca ttagtctctc
11701 gcacatacta ttccctcagg cgtcagcact tacaaaatcg gttgtaattt tcatttttat
11761 acgcattctt actgagatta tactaataag aggaatagta aaagcaattc taagtaaaat
11821 tgcagataag aggtttgtta aaagcagttc taagtaaaat tgcagataag aggtttgtta
11881 aaagcagttc tcagtaaaat tacagataag aggtacgtta aaagcagttc taagtaaaat
11941 tgcagataag aggtttgtta aaagcagttc taagtaaaat tgcagataag aggtacgtta
12001 aaagcaattc catgcaaaat tgctgataag gggtaagtta aaagcagttc tcagtaaaat
12061 tgcagataag aggtacgtta aaagcagttc taggcaaaat tgcagataag aggtgcgtta
12121 aaagcagttc tcagtaaaat tgctgataag gggtaagtta aaagcaatcc taagtaaaat
12181 tgcagataag aggtaagtta aaagcaatcc taagtaaaat tgcagataag gggtacagaa
12241 aaactagact tgattacaaa atggagcttg ggacataaat gattttttaa aaatgagatg
12301 agacgtagat taactccata atcaatacga atctatcgac ttctttattt atgatattca
12361 tctcttttta atggaaataa aagtgcgatt aatgtgataa tacagttacg ttaattaaaa
12421 aaataaaaat gcaaggagag gtaatatgct aactgtatat ggacatagag gattacctag
12481 taaagctccg gaaaatacaa ttgcatcatt taaagctgct tcagaagtag aaggtataaa
12541 ctggttggag ttagatgttg caattacaaa agatgaacaa ctgattatca ttcatgatga
12601 ttatttagaa cggactacaa atatgtccgg ggaaataact gaattgaatt atgatgaaat
12661 taaagatgct tctgcaggat cttggtttgg tgaaaaattc aaagatgaac atttgccaac
12721 tttcgatgat gtagtaaaaa tagcaaatga atataatatg aatttaaatg tagaattaaa
12781 aggtattact ggaccgaatg gactagcact ttctaaaagt atggttaagc aagtggaaga
12841 acaattaaca aacttaaatc agaatcaaga agtgctcatt tcaagcttta atgttgtgct
12901 tgttaaactt gcagaagaaa tcatgccaca atataacaga gcagttatat tccatacaac
12961 ttcgtttcgt gaagactgga gaacactttt agattactgt aatgctaaaa tagtaaacac
13021 tgaagatgcc aaacttacta aagcaaaagt aaaaatggta aaagaagcgg gttatgaatt
13081 gaacgtatgg actgtaaaca aaccagcacg tgcaaaccaa cttgctaatt ggggagttga
13141 tggtatcttt acagacaatg cagataaaat ggtgcatttg tctcaataga aagttagagg

13201 tgagtcttac gtttcagtga cggtagactt acctttaaca tgttacatac taaaaaatta
13261 atttgaataa gaaagagaga catatatgaa atacgatgat tttatagtag gagaaacatt
13321 caaaacaaaa agccttcata ttacagaaga agaaattatc caatttgcaa caacttttga
13381 tcctcaatat atgcatatag ataaagaaaa agcagaacaa agtagattta aaggtatcat
13441 tgcatctggc atgcatacac tttcaatatc atttaaatta tgggtagaag aaggtaaata
13501 cggagaagaa gttgtagcag gaacacaaat gaataacgtt aaatttatta aacctgtata
13561 cccaggtaat acattgtacg ttatcgctga aattacaaat aagaaatcca taaaaaaaga
13621 aaatggactc gttacagtgt cactttcaac atacaatgaa aatgaagaaa ttgtatttaa
13681 gggagaagta acagcactta ttaataattc ataataaaac agtgaagcaa ccatcgttac
13741 ggattgcttc actgttttgt tattcatcta tatcgtattt tttattaccg ttctcatata
13801 gctcatcata cactttacct gagattttgg cattgtagct agccattcct ttatcttgta
13861 catctttaac attaatagcc atcatcatgt ttggattatc tttatcatat gatataaacc
13921 acccaatttg tctgccagtt tctccttgtt tcattttgag ttctgcagta ccggatttgc
13981 caattaagtt tgcataagat ctataaatat cttctttatg tgttttattt acgacttgtt
14041 gcataccatc agttaataga ttgatatttt ctttggaaat aatatttttc ttccaaactt
14101 tgtttttcgt gtcttttaat aagtgaggtg cgttaatatt gccattattt tctaatgcgc
14161 tatagattga aaggatctgt actgggttaa tcagtatttc accttgtccg taacctgaat
14221 cagctaataa tatttcatta tctaaatttt tgtttgaaat ttgagcatta taaaatggat
14281 aatcacttgg tatatcttca ccaacaccta gttttttcat gcctttttca aatttcttac
14341 tgcctaattc gagtgctact ctagcaaaga aaatgttatc tgatgattct attgcttgtt
14401 ttaagtcgat attaccattt accacttcat atcttgtaac gttgtaacca ccccaagatt
14461 tatctttttg ccaaccttta ccatcgattt tataacttgt tttatcgtct aatgttttgt
14521 tatttaaccc aatcattgct gttaatattt tttgagttga acctggtgaa gttgtaatct
14581 ggaacttgtt gagcagaggt tctttttttat cttcggttaa tttattatat tcttcgttac
14641 tcatgccata cataaatgga tagacgtcat atgaaggtgt gcttacaagt gctaataatt
14701 cacctgtttg agggtggata gcagtacctg agccataatc atttttcatg ttgttataaa
14761 tactcttttg aactttagca tcaatagtta gttgaatatc tttgccatct tttttctttt
14821 tctctattaa tgtatgtgcg attgtattgc tattatcgtc aacgattgtg acacgatagc
14881 catcttcatg ttggagcttt ttatcgtaaa gtttttcgag tccctttta ccaataactg
14941 catcatcttt atagccttta tattcttttt gttttaattc ttcagagtta atgggaccaa
15001 cataacctaa tagatgtgaa gtcgcttttc ctagaggata gttacgactt tctgtttcat
15061 tagttgtaag atgaaatttt tttgcgaaat ctcttaaata ttcatccatt tttttaacgg
15121 ttttaagtgg aacgaaggta tcatcttgta cccaattttg atccatttgt tgtttgatat
15181 agtcttcaga aatacttagt tctttagcga ttgctttata atctttttta gatacattct
15241 ttggaacgat gcctatctca tatgctgttc ctgtattggc caattccaca ttgtttcggt
15301 ctaaaatttt accacgttct gattttaaat tttcaatatg tatgctttgg tctttctgca
15361 ttcctggaat aatgacgcta tgatcccaat ctaacttcca cataccatct tctttaacaa
15421 aattaaattg aacgttgcga tcaatgttac cgtagtttgt tttaatttta tattgagcat
15481 ctactcgttt tttattttta gatacttttt ttattttacg atcctgaatg tttatatctt
15541 taacgcctaa actattatat atttttatcg gacgttcagt catttctact tcaccattat
15601 cgcttttaga aatataactg ctatctttat aaacttgttt gaaattttta tcttcaattg
15661 catcaatagt attattaatt tctttatctt ttgaagcata aaaatatata ccaaacccga
15721 caactacaac tattaaaata agtggaacaa tttttatctt tttcatcaat atcctcctta
15781 tataagacta catttgtaat atattacaaa tgtagtattt atgtcaaaat aatgttataa

15841 tttttgtgat atggaggtgt agaaggtgtt atcatctttt ttaatgttaa gtataatcag
15901 ttcattgctc acgatatgtg taattttttt agtgagaatg ctctatataa aatatacggt
15961 tctgttgcaa agttgaattt atagtataat tataaccaaa aggagtcttc tgtatgaact
16021 atttcagata taaacaattt aacaaggatg ttatcactgt agccgttggc tactatctaa
16081 gatatgcatt gagttatcgt gatatgtctg aaatattaag ggaacgtggt gtaaacgttc
16141 atcatttaac ggtctagcgt tgagttcaag aatatgcccc gattttatat caaatttgga
16201 agaaaaagca taaaaaagct tattacaaat ggcgtattga tgagacgtac atcaaaataa
16261 aaggaaaatg gaactattta tatcgtgcca ttgatacaga gggacataca ttagatattt
16321 ggttgcgtaa gcaacgagat aatcattcag catatgtatt tatcaaacgt ctcattaaac
16381 aatttggtaa acctcaaaag gtaattacag attaggcacc ttcaacgaag gtcgcaatgg
16441 ctaaagtcat taaagctttt aaacttaaac ctgactgtca ttgtacatcg aaatatctga
16501 ataacctcat tgagcaagat caccgtcata ttaaagtaag aaagacaaga tatcaaagta
16561 tcaatacggc aaagaatact ttaaaaggta ttgaatgtat ttacggtcta tataaaaaga
16621 accgcaggtc tcttcagatc tgcggatttt cgccatgcca tgaaattagc atcatgctaa
16681 caagttaaca caaagtatta ttttaaaatt gagattagac atttattttt caactttgca
16741 gcagaaccta attttttctc tttgtaaata aatgcaaaga gataccataa ggatctctta
16801 cataaccata accttcagta tagaattctg gactaaatgt tttcaatacc tcactgcctt
16861 tttctattaa ctggtcatat acatgtttag tttcttctac ttggtcaaaa gtgagacaaa
16921 gagatatatt attaccttgt gttatgggca aaccttcagt gtcatctgcg atcataattt
16981 ttatatctcc aaattgaagt acacattgat caattttatt taaatcattt tcgtcaatat
17041 taagtttctt atctataggt ctatctttaa tacgttgaat atacagtgtt ttagcgccaa
17101 acagctcttc atacaacttt tttaaaccct ctgcattttg agtgattaaa aaatatggac
17161 ttacttgaaa tttcatgttt tttcctcctt agatttgtta taaatagagt atatacttaa
17221 ttagtgtcat ctattgacac taaaaggaga ataataatga aaaaatctgt tagattatat
17281 aatatgattg aatattgtaa tgaaaatagg aacttcaaat taaatgattt aatgtcagaa
17341 tttaatattt ctcgtagtac cgctttaagg gatataaaag aaattgaagc attaggagta
17401 cctttatata gtaatccagg gaaaaatggt ggttatacga tcataggtaa tcgagaccaa
17461 acgaaaatag caatctcaga tgaagagttg aaagctttag tatttacact ttcgagtatt
17521 tcaaatgtga gtaatctacc ttttcaaaca gaatatcaag aaatattaaa aaaattatat
17581 aataactcga ataaaaaaga gttaataaac caatataatg atctatttca atattttaat
17641 gaagataagt atcagttcaa aagttataag ttatttaatg aaatcattag attgataatt
17701 gagaataagt cttttgaaac ctgttattca caaaactata ttaaagaaca atataaaggt
17761 attggcatta tgtataaaaa tcatcaatgg tattttgttg tagctaatat agaatcaaag
17821 ttagtgaatc tattaaatat ttcgaaaata aaagaactat atgaaatggg agagactcaa
17881 gagtgtaatg atataactat gcagaatttt caacagttca tggttaaaaa tgaaacagct
17941 attgatattc ttattagaag caatgttatg ggattgaata tcttgaaagg ctacctgtgg
18001 agtgactata tgattgaaaa tattgacgaa gagacatatt tatttaaatc aaaagtgaac
18061 gcgaaagata tagattttat agctaagtta attgtcacag gtggtgtcaa tgtaaaagta
18121 gagaccccta atagtttgaa aaatgctgtt aaagttgaat taactaaaat aataaacatg
18181 tattaatagt aatttaatca taattgtaat aacaaaaaga tgacaacatt aaacaattta
18241 aatttataca aaaaagcatt accaaattta aaattgggta gtggtagtgc tacagcatac
18301 ataaatagtt ataattaaat aaagatgggt caatgaaggt gaattaagtg aataaaaata
18361 ttacagctct aaaatctact gaaaatacta catatatgca gtatacggtt catatcgatg
18421 atcaaattat aaataatata aaagagtgta ccaaaaagtt taaaattttg cctatggaag

18481 ataaaattcc attatcaccg ttgttacaac cagaatatgc aggagaggta caagatttta
18541 ttagtacata tgagcagttt atgattaatt ttggtaaagt aatattggat agtcaaggca
18601 taaaaataca gtttgaaagt gaatcattaa gtagtattca acgaggcatt caagaacatt
18661 gttacttaaa tgagcgaaca aatgacattg atgtgactaa agaatggtat ttatgcaaat
18721 tttcaaatca aatattagaa gaggataaaa atcaattgta taatgcttta aagcaactca
18781 tgaatgattc taaaaataaa aagcaagctt ttcaagaggt gtttaaggtt catattgata
18841 tttatctcta caataaagaa aaatctgaat ataagtatca agtatctagt tatttcaatc
18901 ttgtaaaaga aaatcctaaa ataacttata aaaaaagaca tttacaagag aaacaaggtg
18961 tcaagggtac aactttcacg aacaaattaa aattattgaa caaaatgtat ggtgtagatg
19021 ttgcaaaata tcagccattt tataattcta ataatcctga atatgaaaga ggtcaatttg
19081 gtgaaagata catttctcaa agatctaact atgaatttaa tagacttcaa tatcaaatta
19141 tagatatgtt atcgaaaata cttgataagc atccattacc aaagtcagat aataattata
19201 aacatattcc aactattgaa aaagcaatat taagtgggga ttctcatagt ttttatgagt
19261 attttgaaga tataatgaaa gaaattataa gtatggaaaa ttcttcttta aaggaaaaat
19321 tattgactga ttttacatat caatctcaat gtagatggta ctctgaaagt gaaaaattaa
19381 aattgcaact tgaaagtttt atgcataaag ttttggagag caattattat gagggtaaca
19441 aattatatag aatgttgtct catgcaattg aagaaacaat caatgaggct gatgaagata
19501 aggttcattt caattatttt aaagattatt ttttgacaga cggtggggtg aagaattggg
19561 agcagattag cgagaaaatt actgaattca atggtaaagt tattaacgat atccaaaatg
19621 aatataataa gattcaattt aataacgcaa atcgaaatca gaaactaaac tttaattatt
19681 tataccattg ctttgagttt agtaataact tagtaaaagc aagggtaaat ggaaatagag
19741 gttacatttt atattttgta gataaatata aaataaaaat gcccttttttg gatcaattat
19801 taatcaatta tttgaatcaa gaaataggtc aggaaagtat taattataat atgcaaacat
19861 tgtttgaaaa agaaagatat gatagaagta gtaccattga aaaattagta gcaacaagca
19921 aatttaagta tgaaaaagat gattcagatt tattcaaaca acttttcaat gatgttgaaa
19981 attcaataga cagattaggt atttacttac taaataatgg tataaattcg aatgatgaaa
20041 atgcaagata ttatagatcg tttttaaagg aacttagtag aataaaaagt aaattaacgc
20101 cattttctct tgaaataagt aagtctagcg gaagagagca acattatcct gatgatgcta
20161 ttgatgataa agatgagaga agaaaaaata aagaagaaac atatcatgct tttgatgata
20221 aaagcgatat tgactccaaa ttaaagaata aaataaatgt ttctatcgat aatttattgt
20281 cagttaaaat taagagcaat ttgtatgata ttagtccaaa tacaaaaaaa caataactat
20341 aatgagccaa ctcaagaatg agttggcttt ttaattgtaa ttttacgctt ccattacagg
20401 aatcaatgtt atggaagcca ttaagcaatt aatttgagtc atttatagta taggtacaac
20461 gttgtgatgt tcacatgata atcagcccgg gagattattg tgtaataact tatagaagga
20521 gcaaatcacc atgtatcaag caaatatacg tgatttaatt acaaaattgc ctcaaagcaa
20581 taaaacagaa cactttttaa tgaacaaatt ttcaaatcaa gataaagttc agcagctaca
20641 aagacaaatt agccaacagc tagatcaaca atataatgag cttttggcta atgaaaaagc
20701 taagctagac caatacgtgg aagtacacca taatttagaa ccattaaaga aagagattga
20761 atcagaatct attaaccttg ataccgataa attacctgat atcaaagcga caatgcttga
20821 aaaggctaag aacgatgaac attttgataa aatcgaacag ctatttgata gattagatca
20881 gtcattaaat ggtacgaatc gattatatac gcaattatcg ttgattggca cacgaacaca
20941 tcggatcaca acgaaaagat ttaatgttca aggcttgcct aaattagtcc aacaaatgat
21001 tttaccttcg caatttaaaa aggtttatac aatagatttt aaatcattcg aaccatcagt
21061 tgctgcgtat atgacacaag acgaacaact gattgactac ttgaatcatg aagaagggtt

21121 atacgatgca ttactgagag acttatcttt gtcaaaagag aagcgcgtga gtgtgaaacg
21181 tgcatttata gggtcatttc tttttggcgg tcgttatagt agctctaaat tcaaaatcaa
21241 tcaagaggtt agtgaaatta actggctaca agtaatgagc aaattcaaga aggtcattga
21301 atttaaggag caagtcgaaa aatataaaac aatgcctacg ccttacggca ttgaacatga
21361 tatgagcgca tttcaaggta gtagtattat ggcaatttat gtacaaacgg tagcaagcta
21421 tattttcaag cacattttgt tggaagtgta caaagcacag tgcgaaaaaa aaacgttcaa
21481 gattatagtg ccgatacacg atgcgattat gattgaatgt aatgataagg ggattgcaca
21541 aaatgtagca cagctcatga aagatacagc taatcaactg tttaatggtg aatttgcaca
21601 tgtgacagtg gaagctttag gaggtataga caatgaataa tgatagagga aaaagtcttc
21661 aaattcccca aagtacattg ttaaaagaag gatctatata cgttgctacg ttacattctg
21721 tgtacgagaa gaacttctca ggtgatatta aacatcagtt tacgtatgaa gtagaactta
21781 accaagaaac gcactatgta aatcgcaata ttaccgtaaa atctatgagt caccaattat
21841 caattgctga ttggattaaa cgtcacagta actataacgt aaaccacatt aattatgatc
21901 catacattga tcgaaaacat ttggtccttg tagggcaata taacggaaat tattatattc
21961 aagatgtagc accattagat gaatttggag gggtattgta atgaatcata ttttagaaat
22021 gttaataaaa ttattaaaag tgggtatgga ggcaatcgac cgtaaaggtc tgattgccat
22081 cctaacaagt agtattggaa atgatgaaat ggatgattct gaacaagctg taatggtgta
22141 taacgagctt atcgataagc tacagcttaa cattcctaaa gatgtcgact atagacctaa
22201 catatacagt tattttggta ttcaaaaaaa gccaaatgac acaatattag tggaaatgat
22261 gatatgtatt tttcatatca agcgttttga ttcagagttg tttgttttca aagataaagg
22321 ttggcaaaag gtaagtgaag atgaattgca agggttgata tctaaaatga tacaagtgtt
22381 gctagttgat tataaacctt cactaagtac tttgaaaaac gtagtagatg gcatacagaa
22441 atcaacggac atagaaaaac ttgttgagca tagacagtac attggttgtg gacgaaatat
22501 gttcaatcta aagaccttta aagtggttga taatgacctt gaaatattcc ctaaaacacg
22561 cttagatttg gaattagata taaatgacac gattacggac aagatacccc cgaatttcaa
22621 acaatatatg ttagagttgg cgaattatga ccatgattta caatatttcc ttttccaaca
22681 tatggcagtg ttattgacgg cagatactaa actacgtcgt ggactttttt tgtatggaac
22741 tgcaaaaaat gggaaatcgg tctatattaa attagttaag tcattctttt atagtaatga
22801 tatcgtatct aaaacactta atgaacttgg cgggcgtttc gataaggaaa gtctaattgg
22861 taaacgaatt atggcaagtg atgaagtggg gaaagctaat attgatgaag caactgtgaa
22921 tgatttcaaa aaattactat ctgttgaacc aattcatgct gaccgtaaag gaagaacgca
22981 agtagaagtt actttagatt taaaactcat ttttaatacg aatgctgtac tcaattttcc
23041 atcatcacat gcaaaagcat tagagcgtag aattgctgtt attccatgtg aatattatgt
23101 tgaaaaagct gaccctgact taattgaaaa gttacaggat gaaaagaaag aaatctttct
23161 ttacttgatg tatgtgtata agcaaattgt aaaaaatgat atcgagtacc tccaaaatga
23221 tcgtgttact gaaatttctc atgattggtt aaattttgga tatgaatttg tttctagtaa
23281 atcagcaaat attgcacatc agaaagcgtg tattaattta ctcagaaaac ttatagaaat
23341 caaaccaggg tcacgtatca aagtgtctag gctaaatgag gttattagag atgaaattaa
23401 agtaagctct caagttatta atgatttggt tcaagctaac tttaatgtac aaagtagact
23461 aaataatggt tataagtatt gggtcgattt aggatggaaa gaaactgata aaaaagatga
23521 catgatttca ttcgataaaa atgagaatgt aacagatgat gaattcttat acgaagatga
23581 tttgaactta ggttgggagg actttgacga tgaataatga acaaattgaa gcatttgtag
23641 aagtgcttgt gcctatcata gaagaacgta tcaataaagg taagtaatct aattacgtac
23701 tacaggcagt tgcctgtagt actcatatga ttaagtggta aaagtgataa aaatgaaacg

23761 aaattataaa tatatattat ctatatgttg ttacaagacc gatggtctgt agcaataatc
23821 taataaaagg agcggtatga tatgaagggt aaaattgcac tttattcacg cgttagtacg
23881 tcagagcaat cggagcatgg gtactcaatc catgagcaag aacaagtact catcaaagag
23941 gttgtgaaaa atttcccagg ttatgactat gagacatata ctgactcagg catttcaggt
24001 aaaaatattg aaggtcgtcc ggcaatgaaa cgtctattac aagatgttaa ggataataaa
24061 atcgaaatgg tattaagttg gaaattgaat cgtatttctc gctcaatgag agacgtgttt
24121 aatattattc atgaattcaa agaacatgac gtagggtata aatcaatttc tgagaatatt
24181 gacacatcca atgcttctgg agaagtactc gttacaatgt ttgggttaat aggatctata
24241 gaacgccaga ctttgatttc caatgtgaaa ctttctatga atgctaaggc acggagcgga
24301 gaggcaatca ccggtcgtgt tttaggctac aaattatcac ttaatccatt gacacagaaa
24361 aatgatttag ttattgatga aaatgaagct catattgtac gggaaatctt tgatttatat
24421 ttgaatcaca ataaaggact taaagcaatc acgacaattc taaatcaaaa aggatatcgc
24481 accattaatc aaaaaccatt ttcagtgttt ggcgtgaaat atattttgaa taatccagtc
24541 tataaaggct atgtcagatt caataaccat caaaattggg ctgttcagcg aagaagtggt
24601 aaaagtgata aaaatgatgt gatattggtc aaaggtaagc atgaagccat tataagtgaa
24661 gatgtatttg atcaagttca tgagaaacta gcttctaaaa gttttaaacc gggtcgacct
24721 attggtggag atttctactt acgtggcctt attaaatgcc cagaatgcgg aaataatatg
24781 gtatgtcgac ggacgtatta taaaacgaaa aagtccaaag aacgcacaat caaacgctat
24841 tacatttgtt cattattcaa tcgctcagga agttctgcct gtcatagtaa tgcgattaat
24901 gctgaagtcg tcgaacgcgt aatcaatgtt catttgaatc gtattctttc acaacctaat
24961 gttattaagc agattgcgtc aagtgtgata gaagaactga aacaaaagca tagtaaacaa
25021 acagaaataa aatatgatat tgatagtcta gaaaaacaaa aagcaaaagt taaaacacaa
25081 caagaacgat tattggaatt gttcttagat gatgaaatgg atagcgaaat gttaaaagct
25141 aaacaaagtg aaatgaatca acagttagaa gtattagacc aacaaattaa agaagcaaaa
25201 caagcaaatc aatcacagga tgatataccc aattttgata agttaaaagc acgactcatt
25261 ttgatgataa cacgattcag tgtgtactta agaaaggcta cacccgaagc taaaaatcaa
25321 cttatgaaaa tgttaattga ttcaattgaa attacgacag ataaacaagt aaaacttgta
25381 aggtataaaa ttgatgaaag tcttatccct caatctttga aaaaagattg ggggtctttt
25441 tttataccta aatttaactt tgtgataaat gtcacaaaga aaaataggat tgaaaattta
25501 tcacttttac cacttttttta gagtgacaaa agtggaggag ttttgaaata ttttataaat
25561 atatatttta tttatggagt acacattatt aattaaggag gtcattataa tgacgctaag
25621 caaacaactt aaaacgtata tcactgaacg atttaaatta aattatcaag aaacttgggc
25681 ttgtgaaacc atagatgcgg tggctgaaga tgtattacct gaaaaatata ttaaaaatag
25741 tccacttgaa cataaaattt taaatacttt tacctattac aatgatgaat tacatgaaat
25801 cagcatttac ccttttttat gttatctaga taaggaatta gtagcaatag gttatttaga
25861 taattttgat ttagacttta tatttttaaa tgacactcat caagttatta ttgatgaacg
25921 ctacttgtta caaaaagggg gccagtaatt atgaattgga tcaaggtcgc tcaactatct
25981 gtcacagtta tcaatgaagt cattgagatc atgaaagaaa aacagaatgg agaaaaatag
26041 tatgaacatc aatcgataca tcacaagagg aattaatgaa agtattccac tagaccttca
26101 aatcttactt tggaatatgg taaaagaacg ggacaatcaa cctcatacag attacctaca
26161 catttttaga ctacaagaag atgagaatat actctcaatc acacatgaac aagaacaacc
26221 cgcatataaa ttggaatatc actatataaa ctatgtaaaa aatcaaaaca cattacctaa
26281 gaaagtctac gtcatacgag aaaatgatgt agatgttttt tattatgtaa tgcttttacc
26341 agaagaatac taaaaggagt gacaatgatg aatacaatca aaagtacgat acacacagaa

26401 gctatattta gcgatgatga acaacaccgc tacttactca aaaagacgtg ggatgaaaag
26461 aaaaccgctt gtacagtgat aaccatgtat cctcatttgg atggcgtatt atcactcgat
26521 cttacaactg ttcttatcct caatcaatta gcgaattctg aacaatatgg cgctgtatat
26581 cttgtaaatc tattctctaa tattaaaact acagagaatc ttaaacatat taaagaacct
26641 tatgataaac acaccgacat tcatttaatg aaagcgataa gtgaaagtga cgcagtgatt
26701 ttagcctatg gtgcttatgc gaagcgtccc gttgttgtcg aacgcgttga gcaagtgatg
26761 gaaatgttaa aacctcataa aaagaaagta aaaaagctca taaatccggc aacaaatgaa
26821 ggttctgttg caaagtaaaa aatatagcta accactaatt tatcatgtca gtgttcgctt
26881 aacttgctag catgatgcta atttcgtggc atggcgaaaa tccgtagatc tgaagagacc
26941 tgcggttctt tttatataga gcgtaaatac attcaatacc ttttaaagta ttctttgctg
27001 tattgatact ttgatacctt gtctttctta ctttaatatg acggtgatct tgctcaatga
27061 ggttattcag atatttcgat gtacaatgac agtcaggttt aagtttaaaa gctttaatta
27121 ctttagccat tgctaccttc gttgaaggtg cctgatctgt aattaccttt tgaggtttac
27181 caaattgttt aatgagacgt ttgataaacg catatgctga atgattatct cgttgcttac
27241 gcaaccaaat atctaatgta tgtccctctg catcaatggc acgatataaa tagctccatt
27301 ttccttttat tttgatgtac gtctcatcaa tacgccattt gtaataagct tttttatgct
27361 ttttcttcca aattcgatat aaaattgggg catattcttg aacccaacgg tagaccgttg
27421 aatgatgaac gtttacacca cgttccctta atatttcaga tatatcacga taactcaatg
27481 catatcttag atagtagcca acggctacag tgataacatc cttgttaaat tgtttatatc
27541 tgaaatagtt catacagaag gctccttttt gttaaaatta tactataaat tcaactttgc
27601 aacagaacca caaaaatgat atatttaaac tattctaatt tcggaggatt tttttatgaa
27661 gtgtctattt aaaaatttgg ggaatttata tgaggtgaaa gaataattta cccctataaa
27721 ctttagtcac ctcaagtaaa gaggtaaaat tgtttagttt atataaaaaa tttaaaggtt
27781 tgttttatag cgttttattt tggctttgta ttctttcatt ttttagtgta ttaaatgaaa
27841 tggttttaaa tgtttcttta cctgatattg caaatcattt taatactact cctggaatta
27901 caaactgggt aaacactgca tatatgttaa ctttttcgat aggaacagca gtatatggaa
27961 aattatctga ttatataaat ataaaaaaat tgttaattat tggtattagt ttgagctgtc
28021 ttggttcatt gattgctttt attggtcaca atcacttttt tattttgatt tttggtaggt
28081 tagtacaagg agtaggatct gctgcattcc cttcactgat tatggtggtt gtagctagaa
28141 atattacaag aaaaaaacaa ggcaaagcct ttggttttat aggatcaatt gtagctttag
28201 gtgaagggtt aggtccttca atagggggaa taatagcaca ttatattcat tggtcttacc
28261 tacttatact tcctatgatt acaatagtaa ctatacccttt tcttattaaa gtaatggtac
28321 ctggtaaatc aacaaaaaat acattagata tcgtaggtat tgttttaatg tctataagta
28381 ttatatgttt tatgttattt acgacaaatt ataattggac ttttttaata ctcttcacaa
28441 tcttttttgt gatttttatt aaacatattt caagagtttc taaccctttt attaatccta
28501 aactagggaa aaacattccg tttatgcttg gtttgttttc tggtgggcta atattttcta
28561 tagtagctgg ttttatatca atggtgcctt atatgatgaa aactatttat catgtaaatg
28621 tagcgacaat aggtaatagt gttatttttc ctggaaccat gagtgttatt gtttttggtt
28681 attttggtgg ttttttagtg gatagaaaag gatcattatt tgtttttatt tttaggatcat
28741 tgtctatctc tataagtttt ttaactattg cattttttgt tgagtttagt atgtggttga
28801 ctacttttat gtttatattt gttatgggcg gattatcttt tactaaaaca gttatatcaa
28861 aaatagtatc aagtagtctt tctgaagaag aagttgcttc tggaatgagt ttgctaaatt
28921 tcacaagttt ttttatcagag ggaacaggta tagcaattgt aggaggttta ttgtcactac
28981 aattgattaa tcgtaaacta gttctggaat ttataaatta ttcttctgga gtgtatagta

29041 atattcttgt agccatggct atccttatta ttttatgttg tcttttgacg attattgtat
29101 ttaaacgttc tgaaaagcag tttgaatagt tatattatat tttggtttag aactatgagt
29161 ggctagcatt ttgccactca ttttttgcgt tagcaaaaac aggtttaagc ctcgcagagc
29221 acacgtatta acgacttatt aaaaataagt ctagtgtatt agacttaaac tattaaatac
29281 acatgaaacc tttgtgctta ggagtgattt ttatatgtct tattccattg ttagagtttc
29341 aaaagttaaa tctggaacaa atacaacggg catacaaaaa catgttcaaa gagaaaataa
29401 taattatgaa aatgaagata tagaccatag taaaacttac ttaaattatg atttggtaaa
29461 tgctaataaa cagaatttta ataacttgat tgatgaaaaa atcgaacaga attatacagg
29521 caaaagaaaa attagaacag acgcgattaa acacattgat ggtttaatta catcagacaa
29581 tgatttcttt gataatcaaa cgccagaaga tacaaagcag ttttttgaat atgctaaaga
29641 gtttttagaa caagaatacg gtaaagataa tttattatat gcaacagttc acatggacga
29701 aaaaacacca catatgcatt atggcgttgt tccaataact gatgatggtc gtttaagtgc
29761 taaagaagtt gtaggtaata aaaaagcttt aacagcgttt caagatagat ttaatgagca
29821 tgttaaacaa cgaggatatg atttagaacg tgggcaatca agacaagtaa caaatgctaa
29881 acatgagcaa ataagtcagt ataaacaaaa aacagaatat cataagcaag aatatgaacg
29941 tgagagccaa aaaacagacc atataaagca aaagaacgat aaattaatgc aagagtacca
30001 aaaatcgtta aatacgctta aaaagcctat aaatgttccg tatgagcaag aaactgaaaa
30061 agtaggtggt ttatttagca aagaaataca agaaactgga aatgttgtaa taagccaaaa
30121 agatttcaat gaatttcaga aacagataaa agctgctcaa gatatttcgg aagattacga
30181 gtatataaag tctggtagag ccttagatga taaagataag gaaatacgag agaaagatga
30241 tttattaaat aaagcagttg agcgtattga aaacgcagac gataatttta accaacttta
30301 cgaaaatgca aagccactta aagagaatat agaaatagcg ttaaagcttt taaaaatctt
30361 actaaaagag ttagaacgag ttttaggaag aaataccttt gcggaaagag ttaataagtt
30421 aacagaagat gaaccaaaac taaatggttt agcaggaaac ttagataaaa aaatgaatcc
30481 agaattatat tcagaacagg aacagcaaca agaacaacaa aagaatcaaa aacgagatag
30541 aggtatgcac ttatagaaca tgcatttatg ccgagaaaat ttattggttg gaatgggcta
30601 tgtgttagct aacttgttag cgagttggtt ggacttgaat tgggattaat cccaagaaag
30661 taccaactca acaacacata aagccctgta ggttccgacc aataaggaaa ttggaataaa
30721 gcaataaaag gagttgaaga aatgaaattc agagaagcct ttgagaattt tataacaagt
30781 aagtatgtac ttggtgtttt agtagtttta actgtttacc agataataca aatgcttaaa
30841 taaaaaaaga cttgatctga ttagaccaag tcttttgata gtgttatatt aataacaaaa
30901 taaaaaggag tcgctcacgc cctgaccaaa gtttgtgaac gacatcattc aaagaaaaaa
30961 acactgagtt gtttttataa tcttgtatat ttagatatta aacgatattt aaatatacat
31021 caagatatat atttgggtga gcgattcctt aaacgaaatt gagattaagg agtcgatttt
31081 ttatgtataa aaacaatcat gcaaatcatt caaatcattt ggaaaatcac gatttagaca
31141 atttttctaa aaccggctac tctaatagcc ggtaatagcc ggttggacgc acatactgtg
31201 tgcatatctg atccaaaatt aagttttgat gcaatgacga tcggtggaaa tctcaaccga
31261 gacaacgctc aagccctttc taaatttatg agtgtagagc cccaaataag actttgggat
31321 attcttcaaa caaagtttaa agctaaagca cttcaagaaa aagtttatat tgaatatgac
31381 aaagtgaaag cagatagttg ggatagacgt aatatgcgta ttgaatttaa tccaaacaaa
31441 cttacacgag atgaaatgat ttggttaaaa caaaatataa taagctacat ggaagatgac
31501 ggttttacaa gattagattt agcctttgat tttgaagatg atttgagtga ctactatgca
31561 atgtctgata aagcagttaa gaaaactatt ttttatggtc gtaatggtaa gccagaaaca
31621 aaatattttg gcgtgagaga tagtaataga tttattagaa tttataataa aaagcaagaa

31681 cgtaaagata atgcagatgc tgaagttatg tctgaacatt tatggcgtgt agaaatcgaa
31741 cttaaaagag atatggtgga ttactggaat gattgcttta gtgatttaca tatcttgcaa
31801 ccagattgga aaactatcca acgcactgcg gatagagcaa tagtttttat gttattgagt
31861 gatgaagaag aatggggaaa gcttcacaga aattctagaa caaaatataa gaatttgata
31921 aaagaaattt cgccagtcga tttaacggac ttaatgaaat cgactttaaa agcgaacgaa
31981 aaacaattgc aaaaacaaat cgatttttgg caacatgaat ttaaattttg gaaatagtgt
32041 acatattaat attactgaac aaaaatggtt ctgttgcaaa gtaaaaaata tagctaacca
32101 ctaatttatc atgtcagtgt tcgcttaact tgctagcatg atgctaattt cgtggcatgg
32161 cgaaaatccg tagatctgaa gagacctgcg gttcttttta tatagagcgt aaatacattc
32221 aatacctttt aaagtattct ttgctgtatt gatactttga taccttgtct ttcttacttt
32281 aatatgacgg tgatcttgct caatgaggtt attcagatat ttcgatgtac aatgacagtc
32341 aggtttaagt ttaaaagctt taattacttt agccattgct accttcgttg aaggtgcctg
32401 atctgtaatt accttttgag gtttaccaaa ttgtttaatg agacgtttga taaacgcata
32461 tgctgaatga ttatctcgtt gcttacgcaa ccaaatatct aatgtatgtc cctctgcatc
32521 aatggcacga tataaatagc tccattttcc ttttattttg atgtacgtct catcaatacg
32581 ccatttgtaa taagcttttt tatgcttttt cttccaaatt cgatataaaa ttggggcata
32641 ttcttgaacc caacggtaga ccgttgaatg atgaacgttt acaccacgtt cccttaatat
32701 ttcagatata tcacgataac tcaatgcata tcttagatag tagccaacgg ctacagtgat
32761 aacatccttg ttaaattgtt tatatctgaa atagttcata cagaaggctc ctttttgtta
32821 aaattatact ataaattcaa ctttgcaaca gaaccaattg aaagaaccaa aagaatatcg
32881 aaaagagtgg gcaacttgga atttgtcctc tttaccaatt ctgccaaact actatgagta
32941 taaggtgtct tacgataaga gagagcagtt caactttatt aaacagctat tcaatgactc
33001 tagcattaga acaattgtaa acgggtgtga ttcggaccgc gaggggtcca atattttta
33061 ttcatcctac tacatgactg gagcaaaaaa taaggaaatt aaacgattat ggattaattc
33121 actagaggtt gatgaaataa ggaaaggatt caataatctt caagataaca aaaaagactt
33181 attactttat tacgaagcaa agacacgtca aataagtgat tggcttgttg gaatgaacgg
33241 aagccggtta tttaccctcc tcttacaaca aaaggggttt aatgacagtc tctcgattgg
33301 acgggtacag tcttccacag tataccttat ttatcaacgt caaaaagaaa tagaacaatt
33361 tgtttctaca cctttctatg aaattgaagg aagctttacc gccaaaaatg gcatgtataa
33421 aggaaaagca aaaattaaaa gtgaaactct aaaattgcag ctgatgctct aactcgtgct
33481 aattacaagt gtgaatacaa tataaaagat aaaatcttcg aaagaaaatc tggtaagggt
33541 tatacagagc cacatcattt aataccgatt agtaaatata aagatttcga ttataaaaat
33601 tgtagtttag atacaatgga gaacatcgtt tcactttgta gtcattgtca taatttactt
33661 cattatggta aattcgaaga taaaaaacct attcttgaaa aactatttaa tgaaagaaaa
33721 aaagcgttaa gtgctgttgg cttagatatt acattagaaa aattagaagc ttctgttttt
33781 tccatgattc aagcatcttt ttgttatcca agttaatttc agctttaata ggttcagcat
33841 ctttagttag accaaaaata gacgcatatt ctggatctag ctttaaatgg taaaagacaa
33901 gtgactgttt tttgccattg tcatctttga ctgttcgttt tgtcgtaatt cgatcacggt
33961 cagattcgat aaatcccttta tcctttagtg cattaacaac attattgaca tcttggaagt
34021 gattctctat caacatatct ttaaatacag atgcaatgat tttgacttcg atataatcat
34081 cttttaaggc aattagtcca tggttctcaa tcatattctt taacgctgta tcatcagaaa
34141 acttaccacg gttttgtgct acaaattgcg taatgacttc gatcgcttta tctgccagtg
34201 aacgttcaga gactgtatga ccatgataat caataaagta gtctcttatt ttagcgatat
34261 caatatctgt agctaaaaca tgactaagaa tcttggccga agtagtgata gccgcatagc

34321 gcttaaacat acgaatacca gtattatttg tttcatcctt caatttatct ttaaaccaat
34381 gatgtaattc aaaaacggat caaaaaacaa agggccctag aaactaggag cacacttaca
34441 caagaacaat tgatagataa attagctaaa ggagccatcg atgcagaaac gttcagaaaa
34501 caatctcaat cgttacttca acaatcaaaa ccaacactat caataaatga gcaacaaatt
34561 caaaggtctt ttgaaaatgt aattcaacaa cacttcacgt taagcatgtt ataccgatat
34621 attgatgaaa ttcatatttc taaaaacaaa agccttgtgg gaatctattt caaaaatgaa
34681 ccgctaaaca ttgtaaacca agctacgcaa tcatcgattg cttaattaat gaaagaatga
34741 aaaatatgaa agaaatgaaa agaaaatctg taggatgtta tgttagagtt tcaacaattt
34801 ctcaagacat tgataaattt agtattaatg gtcaaattac acaaataaag gaatattgcc
34861 aacagggaaa ttatgaattg ctatactgat aattaaaagt acccccgatc gaacgaaaat
34921 atctttttga tgactattag tgataattgt aattttatcc attaatagat gaatggattg
34981 gaataatact aagcctattg ctattccacc gagtaaaatt acccacccgc ctaattcaat
35041 actttccgga atcatttcaa aaaataataa gccaattata aggcctgcac ataatgaata
35101 aataaagcca tgaccacttt gaaaaccttc cattatccaa gcgagtccac cgccaacacc
35161 aattccaagt gcggaagcta acgcccgatc atccatactg cactcatccc actattctta
35221 aaaaggcttt gtttatttta ctctaaaaaa tcaaaacaaa atatatgaat tcatattaaa
35281 tattatttga ctctttattc agatggttat atcatatata tgaacatata ttaatatatg
35341 tagataaata gtgattatta tatataaggt tttgaattga aacctaaagt gagggaagga
35401 taatggaaga aaaaaaggaa ttagaggaag taaataataa ggacttagat gatgaaacat
35461 tatttgtcgt atcgcaaaca tttaaagcgt taggtgatcc tacgagaatc cggattctcc
35521 atttgctctt ttataaggag tattcggtaa acggtattgc tgaaacgcta catcttagac
35581 aatcaacagt ttcccatcaa ttgcggttct tgaaaaattt acggttagta aaattccgaa
35641 gggaaggcac aacattgttt tattcccatg atgatgaaca tactatgaat atgctaaaac
35701 aggcgatcga tcacgcctgt catcactagt attccgtaaa ccagtaataa gtgttaataa
35761 taattcacgt cattaatatt gttatatgat catataacaa tattataggc atatgaaaag
35821 ggggttttgc aatggatatg gaaaacaaaa aaacagaatg gaaagcgttg tatgatatct
35881 cgaaggaaag tgagatgggt gtagcagaga gggttagtga atacggttga taaattcata
35941 ggatgtatta ttggatacaa aaagtttgga tgtggtcaat taacgtcctg gtttagtaaa
36001 gcttactgcg aatatattgg aacccgatat atcatattta tttagaaagg tgatagaaat
36061 gaaaaaatatt caagagcaac aagcacacga aagtcatagc cacgatcata gtcatgatca
36121 tgatcacgga aaaatgccaa ttatttcata ttttattggc ttagtgttgg ctataattgg
36181 gcttttttta agtgatgcaa atttattaat acaaaacatc ttattttcaa ttgccacaat
36241 cacagccggc taccatgtaa ttattctcga aggaattgga gagacagttg aaaatactaa
36301 attaaaggga aaattcactc ctaattctca tattctaatg ggattagctg caatcggggc
36361 ttctctgata gggagttttt gggaaggaac ccttttgata cttatttttt ccggcgctca
36421 ttttcttgaa gattacgctg aaggaaaaag taaaagagaa attactaagc tactcgaaat
36481 gaacccaacg acagctaaat taatcctacc tgatggaaac acaaaaattg ttgatgtcag
36541 tgaattaaaa gttggagatc aactccaagt gctgaacggt gatcaagttc caattgatgg
36601 gattatttta tccggtacta cctcaattga tgaatcttct attaatggag aaagtatacc
36661 gaaagagaag tctaagggtg acgaagtttt tggaagtacg attaatggaa caggtacttt
36721 tactatggaa gtcactaagg aaaacaagga tactgtattc tctaaaattt tacaattagt
36781 tagtcaaaac caagataatc aaacaaaagc tgccagtatc attcaaaaat tcgagcctaa
36841 atatgttaat atagttttaa tcgcaatacc attagtaatg ttacttgctc cttttctatt
36901 tgattggaca tggtcgcaaa gtgtttacag gggattagtg cttttagtcg cagcttcacc

36961 gtgtgctttg gcagcagcta ctgtatctgt aacattgtct acaacatcta acctagctaa
37021 aaaaggcgtg ctttcaaaag gaagtactta cctatcacaa ttagcggata tagatgcaat
37081 tgccttcgat aaaacaggaa cccttacgaa cggagaacct aaagtaacaa attactattt
37141 cactcattct gtgaacgaag aaaatattat tgatattata gtcgcccttg aaaaggaatc
37201 caatcaccca ctcgctaatg ctattttaga aaaatttgaa gttaaaaata aaatagacat
37261 cgaagttact aatcaaattg gaaaaggtct gacaggagat tataatggaa aaaattatcg
37321 tattggtaag cctacttctt ttgaaagtgc ttctgaagag tatacccagt tcaatcatga
37381 ttgggcatca gaaggaaaga cggttgtata cgtagcagaa aatgaagaag ttattgggat
37441 tatagctcta atggatattc cgaatgagca tgctaaagaa acaattaatt actttaagaa
37501 acttggtatc cacacgactt taattactgg tgattcggaa atgacgggaa aagctgtagg
37561 cgaacaattg ggaatagacg aagttatcgc taatgtaatg cctgaagata aatccagaat
37621 tatagaagaa caaaaagaaa aatttggagt tactgccatg gttggagatg gtgtgaacga
37681 tgcaccggcc cttgttaatg ctgatgttgg tatagctatg gggggcggta ctgatgtggc
37741 agtagaagta tctgatttgg ttttaatgca gaacaattta tctaaattag tacagtctca
37801 taaaatttcc tcaaatatgg gtcgtgttat taggcaaaat attatttttt caatggcagt
37861 tgttgccttt ttagttgtca ttagtttgtt aggattaact gatattacaa tcagtgtaat
37921 tgttcatgaa ggaagtactt tagttgttat actaaatgga cttcgattat taagatctaa
37981 ataatgaacg aatcgattga catgaatgaa ctttgaagtg tggattctac aatgttccca
38041 taacattgga cactaaaaaa cagagcaatc taataaagat gttatgagta aaaacaatgc
38101 cttgcatacc gtttttatca tacgggcggt caaataagca attaaagagc atgggaagta
38161 taaaatcata aacagtgact taaggcagcc agtttacatt caaaagttaa attgactgta
38221 ttaaaagttt cgaaacgatt aatatccgta tgggggcaag cgtcgagcca aagataatgc
38281 caggacagag cgtctttttc cgttctttta agtgggaaag gttttacctt ctctatgcca
38341 agacagtccc agagctaaaa gggccagttg ctctttttgg gcgtttgtga acggtaatcg
38401 caggaagggg attttcccaa ccgcacctgg ttttccatca caataaagtt ggcggcattt
38461 gttctgatat tcttgggaat acttacacta tgaatggccg tattaagtga tacaggagct
38521 gctattattg taatactgaa tgctctccgc cttttgaggg taaaagaata aaagtaagga
38581 taactaggta aagctgttca atcaaaaatt gaacagctta tttttcatca aaatcaaaaa
38641 cgtttattat aatacctaca cttgttctgt taaatgtacc gattttttaa ccttattgta
38701 tcagtaatat cttgaaacga agtaagcgac taaaatttct cttttatcat acatttataa
38761 aaatacactt ttaagaacgg tttgaaaatt tttgaaatag atcaaaataa tcttctggtt
38821 aaaaaaacctg tagatatgat ttctctctta aatttttgtt tgattagatt agaccctaat
38881 aagacgcatt caatatgtct acacgtgaat ttagtctttg aaaatgtaag gaccattatt
38941 attataaaaa ccccagtata aaacgatacg ctgaatacag caataaataa aaaaaggcat
39001 gtactgtgta aaatgtttgt taaattatat aaatattttt taaatagata tttattcaat
39061 aatataaaaa gggactataa gctatatcta actacttata gtccttcttc attagtataa
39121 atataattat taagaaagat gtatatcttt gtaaccttcg tttacattaa taatgttgtg
39181 ataacctttg tgttctaaaa taccaatagc tatagaactt ctaatgccag attgacagtg
39241 tacataaata acatcatttt tactgaaagg taaatctgtg tctaaaagtt tgccgtgtgg
39301 tacatgaacc gcttgagata agtggccatt attccattca ttatcattac gtacatccaa
39361 tatatgtgat tcgttacctg taatatcttc actatgaatg gaacgtgttt gaactttaga
39421 ttgaggtaat tgatatccag caacatcatc gtatccaatg agttgcaagg tgtgcgttgc
39481 ttttgaaaca aggtgatagt ctccaatcaa gttaatttct tgatcatagt ttagatacca
39541 gccaatttga ttgatgaaat ttttatcata tggaatattg attgtacctt caatatgtcc

39601 accatgatat gcctccttac tgcggagatc aaaagttaat ctgtttgtat ttgtagctgg
39661 ataaaccgta taaggttgat ataaattcat accgaattga ttaatttttt tcatttgtgc
39721 aaaatgatgt ggtggtgcag gttggtcaga aatgagttta tcgataaagg tagcttcgtt
39781 attttcagaa aaagcccagt tcgtttgttt ttcatagcca agcgtagatg ttggaatagc
39841 acctaaagat ttaccacaag gactaccagc gccatggcca ggccaaattt gaatgtaatc
39901 tggcaagtct ttaatacttt caatcgattt aaacatttgt ttagcgccta tttcagatga
39961 tccttctact ttaaccgctt tttctagtaa atcaggtcta ccgatatctc ctacaaaaat
40021 aaaatcacca ctgaatagtc ccattggaac ttgtgctcca gcaccttcgt cagtaagtaa
40081 aaaacttata ctttctggcg tgtgacccgg tgtatgaagc acttttaatt ttatatttcc
40141 tacataaata tcgtcattat gttgaacaaa ataagtgtgg ttaggcatat ttttataacc
40201 taatgtgtca tcactttcac ccgatacata aatattagca tttaacttta tagcaacatc
40261 tctaattcct gaagcaaaat ctgcatgtat atgtgtttca gctgcatgag taatggttaa
40321 accttcttca tcagcaactc gaatatatga agataagtca cgaataggat caataatcat
40381 ggcttctcca gttttttgac aaccgattaa ataagatgct tgagataaat gtttatcata
40441 aaattgttta aaaaacataa tatcattcct ttctaattat ataaataaat tgtgattagc
40501 ttgttctgta tatccaatat atgcacctac gccaccgtat tctacatcat cgcgtagttc
40561 ttcttttgta atgcccatca catccatact cattgtacaa gcgataagtt taacaccttg
40621 ctcaactgct tgatcaataa gagaaggtaa agtatcgaca tttttctttt tcatgacata
40681 acgcatcatt agattgccta aaccaaacat attcatttta gaaataggca tgttaattgg
40741 tgaatttgga agcatgaagt caaaaagttt tgcaatacct tttttcttaa tgcgttgact
40801 ttgaattttt ttaagtgcat ttaagcccca aaatgtgcaa aagatggtta catctctacc
40861 tgcagcttta gcaccattag caataatcat tgctgcaact gctttatcta gctcgccgct
40921 aaataaaaca attgttgttc cacttttcgt gtgtgtcact tctatatttt tattttcttc
40981 tttttgaata atagcgtgaa tttcattacc aaaatcgttg agtctaacaa gcgtatgtcc
41041 agtttgtttg acccaacttt taatatcatt taaaaatccg tgatcagtca caacaacttc
41101 tatttgatca ccaatagcga tgtttttgat ttctttacta atattaacaa taggtcctgg
41161 acactgtaga ttactataat tgaatgtctt acgattatct ttaatttctc tatcttcttt
41221 tactttagat aaattatcat tatgatgttg ttcttcgtat gcggtataac ctccgtctaa
41281 attcaccaca tcgtatccgc gttgtgcaag aaattgactg gcttttctac ttctattacc
41341 acttttacaa tagatatagt aagttttatt tttgctttgt ttaaaagtct ctatattttc
41401 aactgaatgt aataaagcat ttttaatgtg tcctaattca aattcttcag gctgtcgaac
41461 gtcaattaat tgatccttag aacccaattc ttctaattcc tttttagtga aatcattaat
41521 gtgtttcgtt ttatattgtg acataattac ctccttttaa atacctatgg gggtatatta
41581 taacgaatta gatagtttgt caaataccta tgggggtatt tgacaaaata aaatttaatc
41641 aatatgatgt aattattaaa aatgaatgtg gagtgaaata tattggaata taataaaaag
41701 atgattaatc gtattcatcg catacaagga cagcttaatg gagtcattaa aatgatggag
41761 gaagaaaaaa attgtaaaaa cgtcattagt caattaagtg cttctaaaag ttctattcaa
41821 cggttaatgg gtattatcat tagtgaaaac ttagtagaat gcgtcaaaat gtctgaagaa
41881 aatagtgaag attctcaggc actaattaat gaagcagttg aattattgat aaaaagtaaa
41941 tgataagtat aaccaccatt atagtaatgt tagttattgg tatcttagga ggatttattt
42001 ctggattagt cggtataggg ggagcaattg ttatttaccc ggctctctta ttattaccac
42061 cattatttgg tttacctact tatagtgctt atattgcttc aggattaact tcaagtcagg
42121 tgttttttag tacattgagt ggatctttaa acgcatataa aaataaaaat ttttctagaa
42181 cgcttatact taatatggga agtggaatgg taataggtag tatgctaggt gcaatcttag

42241 cgaatttaat aaatgttcag tttgttaata cggtttatat tattattgca ttactagctc
42301 ttattctaat gtttataaaa gttacaccat ctactagtca tattaagttt aatcgtctac
42361 ttttaattac aataggtggt atcatcggtt tggtatctgg tattgtagga gctggtggcg
42421 catttattat tattccagta ctacttgtta tatttaaatt accaatgaac atggttgtta
42481 caaatagtat tgtaattgca ttcatttctt ctattggagc atttatgatt aaattattac
42541 agggttatat tcctattaat agtgcaatac cgttaatttt aggtagtata ctatttacgc
42601 ctctaggtat gaaaatgggt cagaaaatac ctgactatat tcaaaaaacg ataatatgta
42661 ttttaatcgt cattgcaatt attaagttga tattttaaaa acatgggggt gggacaacat
42721 tcattttgaa cttgattgaa tatatagtat atttaataaa atgcagctat tgtggcgtag
42781 aatttaagaa ttaaaaaatg attaatatag tttgaaaaga ctgaatataa atactagaaa
42841 aatagccagt aaatgagttt attatgaatt catttcttga ctctttcaaa taatactatg
42901 agaaaaatgg tgcaaaactt atggtggggt gcaggttata atattgttgc tgtaccttta
42961 gcagctggta ttttagcatt tatcggcttg attttatcac ctgcaatagg tgctatttta
43021 atgtcactaa gtacggttat cgttgcaatt aatgccttta cattaaaatt aaaataaaag
43081 ataggagttt tattatgatt aaaaaattat tttttatgat attaggatca ttactaatat
43141 tatcagcttg ctccaataat gatgaaaaag ataaagacac taatgaccaa aaaagtgaga
43201 gccatatgaa gcataatgat gaaagtaaag ttcctgaaga tatgacatcg actaatgagg
43261 gtgaatttaa agtaggagat aaagtaacga ttacagcagg gcatatgcca ggtatgaaag
43321 gtgcagaagc tactgtaaaa ggtgcgtata aaacatatgc ctatgttgta agttataaac
43381 ccacaaatgg aaatgaaaaa gtaagcaatc ataaatgggt cgtaaacgaa gagatcaaag
43441 atgcacctaa agatggattt agtaagggcg atactgttaa attagaagca agtcatatgt
43501 ctggtatgaa aggtgctaca gccaatatag ataacgtgaa aaagacgact gtttacgtag
43561 ttgattacaa atccaaagat aatggtaaaa tcattaaaaa tcataaatgg atgacaggaa
43621 atgaattgaa agcacgataa aaatctagtt ctagattgag aaataaatag atataaagat
43681 atcctcctta atcaataatt taaataactt attattgtta aggaggatat tttttagtgt
43741 gtaaattaaa aagaatttta gaagaataac atttatcaaa aaactgttca ttaccttatt
43801 aaatgaaatt atataattaa aaatcgcatc attaaccgat acgcagaagc atatcataaa
43861 taaaactaaa aattaaatat aacgattaat attttaattt caatgaggta agattaaggt
43921 atggaaaaat ttaaagaaaa agtaaatgac ttagaagcaa ttaagactac tagatattat
43981 acagaatttg tggagaaatt taaaaaaatt cgagattggg ctaaaaatga aaatctaaag
44041 aatgaagcga aattggcaca atatgagatt gaaattttct ctttatgtga gaaaaatccc
44101 attttatcaa aaaataaaag tgaaaaaaga tttgtagcga caattagttt tgatgatggt
44161 agagagtggc cagacattca aaaatttaca aatgatcaaa ttaaatacta tgagcaaaga
44221 ttaaacgaga caaataattt gtttttaaaa gtaagatatt ctgatttctt atttgaacat
44281 ggtaataaga aaataactaa aactaaatat gaaatttcaa aatgtttgct atcgtgctta
44341 gttgagattc ttacatatta cagtgacgat tttaattata cttcagtatt agctagatta
44401 gttgaagtat ctcttttaat gggagatagg gaaaaactcg aaaaagctat cgaattaatt
44461 tatctaaaaa tggatgaatt tgactataat aatgagtata gttatgtata tgaactttca
44521 aaacttataa gagaaatttt aaaatctaag cacaaaaaaa taatcttaga gaatcattta
44581 aataaaatta taattgtttt agaaaaagct attaaaaaca attttgaaga taaaaattat
44641 tacctacata gagtattttg tgaagaactt agtcaataca gaaaattcga tttaatttct
44701 tctgaaagaa gaagtgaatt gaaaaaagaa attggaaaaa gctacgagtt agaggcggaa
44761 tatcaacaag gaagaaataa taagagttta ttggtaaaag caaatttttt agaaaaagct
44821 atggaaaaat atatggaaat tggagaaaga gaaaaaagta ataagatgaa gattttagta

44881 aaatggacct atgaagaata cgaaaacagt aatgaaatga atctaataag aattccaata
44941 gaatttccaa aagaggaaat tgataaaatt attgaaggat ttatttcctc agatgtacag
45001 atttcgttag ataaaatagc atacagtaat gacttaattc ctaaaatcac agtaattgaa
45061 gatctagttg ataagttaag taaagagttt cctttacaag gtttaatttc aaaaggttta
45121 ttgaatgatg gaaaaaaagt agtggaaact actacagaag aagataataa aacaatcaat
45181 tttaatagta attatatgca tcatttaaat attaatgttg aattacttct taaagctatt
45241 tttgataaat taatatcaga atttgaaatt tccactgaag atttcatgca aaaatttaat
45301 aagtgggaac tattagataa taaaaatgtt ccttttattg aacatggaat ttctcgattt
45361 ttagaaaagg attatttgag cgctttgcat attttagtcc ctcaatttga atctacagtt
45421 agaagaatgt tttctaaagc tggctattca actacatcta ttaggaaagg aaatacgcaa
45481 caagaagtaa catttaatga atttttactc agagatgatg tgaaatcgac cttaggaaat
45541 gatgtgcata aactaattca aattgtgatg gttgaacagt cggggttgaa tttacgcaat
45601 gaaatagctc atggattaat agaattatca aatataaatt atacgaaatg tattttggtt
45661 atttatttat ttttgatatt aactagatat agtattgaaa aaaatgacta agtgataatt
45721 tatattatac aagttttgtg gaggacatat catgaaatta gaagagttgc tactaatcac
45781 acaacaacca ttaattgaat attctaaaaa taaaaaatta ttagaaggat cacgtttatt
45841 tgatattgat gaaagaatgg atgaaagaaa aatccccaaa attttaacta atagcgataa
45901 gtaccatgtt gttgaaatag ccaactacga taatttaata attattgata atgaagtaat
45961 aaacggtaat gaattaattc aagtcggtca atgtattgac tttgatagta acgtaatgag
46021 ctatttacga aacttgattg taaataataa atatgaggat gattttttta aaattttaac
46081 taatataaaa aaaagtaagc aacaaataag ctgcgtacct tatttaatag aaaatgggaa
46141 taatattcat aggataaata aaataatttc atatgaaaca atattatcat tttctatatt
46201 tgatagaata agcgaatttg attttgaaaa tcgcaatttc tcaagatatt ttaatgattc
46261 agaagttatt ttagatacag atgatagata ctatcatatg atgaatatac aagattctaa
46321 tatacttcaa tttcaagcaa tttatctatt agttttaatg gcattttttta taaaaaatag
46381 cagtaaaaaa tcagcagaaa ataaaattgt ttacttgatt caaactttta ttaaagaaac
46441 tgaaaataaa ttagcttact cagagttaga gttaagtgtt atttttgatt acataaataa
46501 tggtgataat aatatattta aatctaccaa tttgaatagt aaaaatttat tgagtaaatt
46561 aaaaggaata gcttgggatt tatttcatat tagaacctcg gaagatcaaa tcgctctaag
46621 aaataccaat agcaaagaag ttttttaca ttcaattttt acagcagata aaggcttgag
46681 taatgtgata aataactatt caataagtag gatgctagca catgaagaaa aattatatgt
46741 atatagagat aataatatat ttaaaaaatt atcaaaagat aaagctacta aaatacaaga
46801 attgttagaa aaagatagaa attctagaat ttctaatgta agagaagcaa agtacaatat
46861 acaagaaaat attgatatat atgaaaaata tattaatgaa atgatttaaa aatagaatta
46921 ttagagtcga ttatagagaa gataaagtat taagccatta taacgatttg ttactttagt
46981 aatatcattg ataaggggag gcttatcata aat

//

LOCUS Staphylococcus 41242 bp DNA linear UNK

DEFINITION Staphylococcus Staphylococcus caprae strain JMUB145

ACCESSION Staphylococcus

KEYWORDS .

SOURCE Staphylococcus Staphylococcus caprae

ORGANISM Staphylococcus Staphylococcus caprae

Bacteria; Bacillati; Bacillota; Bacilli; Bacillales;

Staphylococcaceae; Staphylococcus; Staphylococcus caprae.
FEATURES Location/Qualifiers
source 1..41242
/project="hwwang_1255645"
/db_xref="taxon:1255645"
/genome_md5=""
/organism="Staphylococcus Staphylococcus caprae"
/genome_id="1255645.4"
/mol_type="genomic DNA"
CDS 13..492
/db_xref="SEED:fig|1255645.4.peg.1"
/transl_table=11
/EC_number="2.1.1.177"
/product="23S rRNA
(pseudouridine(1915)-N(3))-methyltransferase (EC
2.1.1.177)"
/translation="MKITILSVGKLKEKYWKQAIAEYEKRLGAYTKVEIIEVPDEKAP

ENMSDKEIEQVKEKEGQRLLAKIKPQSTVITLEIQGKMLSSEGLAKELQTRMTQGQSD

FVFVIGGSNGLHQDVLQRSNYALSFSKMTFPHQMMRVILIEQIYRAFKIMRGEAYHK"
CDS 738..1043
/translation="MKGNIQCPRLIKGSLLDFVLETVDVFVNGSKNDCELVNFNYKEG
KVVSRYSTKFESNPKLRKKQLISTVKVVRYVIFDFEKILWGIGKVLLKFIILRQCLL"
/product="hypothetical protein"
/transl_table=11
/db_xref="SEED:fig|1255645.4.peg.2"
CDS 1242..2105
/db_xref="SEED:fig|1255645.4.peg.3"

/translation="MKHINQFSESTLAQLEKDQQHVFYVYCLMDPRNDECFYVGKGKG
NRIFKHKQDAQKKVLYEDIFIEENKDNLKFNRINEICSNDLNVLGYIISYGLTESEAF

SAENVLINFLNLTNKTTLTNMINGHGSKAYLVEDLENEFGYDSINLENINTNELILAV

KIRDAFLLDKDESKEYPINESKRDRNNLKSRTLGSWIIGKDKIHKIKYIIGINTGANN

AVVSAYEVSFEQAESIETNNGRMRYAFIGLSERDATLKKLNLYKKALPDLRFGSGSAT
AYINNGTMKVD"
/transl_table=11
/product="hypothetical protein"
CDS 2213..3688
/transl_table=11
/product="hypothetical protein"

/translation="MPSDDIVKKLYSKEDIRLELGLTPHKFNKKMETIAKLFKIDMKI

FHNYKGQDKNNQYTFNGVAKELIKVLLKSVDYYPVDINSKKFKQNGKSKKEMIENIDN

SSYMKYIYQLMKSINEIQYKRLIADIHMKDVYQNTKAWLNNGESINKKEQELYQYMTI

LPLHKRVELQNEVLKSIDETIFQFLAKEHRNNQIEENNELEAYTKAIKEGRNPKNDYE

LNHLLYKKNQLPLDSLIEDIWDYEETEYTELDWLIADMLKRSQRADSNFIESLEKKNK

LRKNIHKDSIKSISKLIDHELVKNNRKWDVAEFYKNQQFWNNSTLRRSSQNFGILYYK

KNRLHTMNQSNIIKKIKIIEANIESANKQPSYLDFFLQAKFDLERLESELMKYSINPK

YFNKINSDYVRYAIEDVHNAIIKIDRYISNDKVKLNYSNVQMVVRNEITEQGSYFVTQ
ALNTLSKVEESGYSFDNFMVGPFVEDFKKAIKNRRFGSKKD"
/db_xref="SEED:fig|1255645.4.peg.4"
CDS 3914..5014
/transl_table=11
/product="hypothetical protein"

/translation="METRDKLMSLTQSDKTQQWLMDKSSNQDDIQQLQQQFSQQLDQQ

YNALLADEKAKLDQYVEVHQGLESLKEEIESEPITLNIDKLPDIKATMLERAKNDEHS

DKIEKLFDRLEQALNGTNRLYTQLSLIGTRTHRITTKNFNLQGLPKAVQHTILPSKFK

KVYTVDFKSFEPSVAAYMTQDSKLIDLLNQKDGLYDALLSELGLSDELRVFVKRAFIG

SFLFGGNFKNPKFKLNQYVSEVQWLDAVSQFTKVIELKKHVEKSNSMPMPYSIEHDMS

AFQGSSIMAIYVQTVASYIFKHILLKVYKAQCDQKTFKIIVPIHDAIMIECEDEEIAQ
NVGQLMKDTANQLFNGEFAHVTVEEIGGVDHE"
/db_xref="SEED:fig|1255645.4.peg.5"
CDS 5007..5378
/translation="MNNDRGQSLHIPSSTPIKENNIYVATLHSVIQTDFSGEIKHQFT

YEIEVNNQIVYANRNIPTKPSANQLSIHDWLKRHSNYSASHENYEPYIDQKHLILLGQ
YNGNYYVQDVASLDAFGGVLS"
/product="hypothetical protein"
/transl_table=11
/db_xref="SEED:fig|1255645.4.peg.6"
CDS 5375..7018
/product="putative primase"

/transl_table=11
/translation="MNHILQMLSKLLSVAKEAIDRQGLIAILTIPVNNNDEIEETAQG

ETVYNELIDQLRLNIPKDTDYQPNIYSYFGIKKNPNDTVLMEMMIKVFHIKRFNSELF

IFKVNGWQKINGDELQGLISKMIQVLLVDYKPSLSTLKNVVDGLQKSTDVEELVENER

YIGCGENMFDLNTFQVVKNSIDIFPKTRLNLSLSTNDVITDKIPPYFKQYMLQLANYD

DDLQYFLFQHTAVLLTADTKYRRGLILYGGAKNGKSVYIELVKSFFYSKDIVSKPLNE

LEGRFDKESLIDKSLMASHEIGQSRIQEKIVNDFKKLLSVESMHVDRKGKTQVEVILD
LKLIFSTNAILNFPPEHAKALERRINIIPCEYYVEKADTSLIDKLQSEKKEIFLYLMY

VYQQIVKADIEYLENSRVTEITHDWLNFGYEFVSSRSVSNANQKACINLLRKLIEIKS

GSRIKVSELNKVINEEIKVSSQVIKQLIQANFDTQTKLYNGYDYWIDLGWKEANKKEI
HDISEKDNIISLDKNENITDDEALDEENLDFDWEDFDDE"
/db_xref="SEED:fig|1255645.4.peg.7"
CDS 7244..8920
/db_xref="SEED:fig|1255645.4.peg.8"
/transl_table=11
/product="Cassette chromosome recombinase B"
/translation="MKGKIALYSRVSTSEQSEHGYSIHEQEQVLIKEVVKNFPGYDYE

TYTDSGISGKNIEGRPAMKRLLQDVKDNKIEMVLSWKLNRISRSMRDVFNIIHEFKEH

DVGYKSISENIDTSNASGEVLVTMFGLIGSIERQTLISNVKLSMNAKARSGEAITGRV

LGYKLSLNPLTQKNDLVIDENEANIVREIFDLYLNHNKGLKAITTVLNQKGYRTINQK

PFSVYGVKYILNNPVYKGYVRFNNHQNWAVQRRSGKSDKNDVILVKGKHEAIISEEVF

DKVHEKLASKSFKPGRPIGGDFYLRGLIKCPECGNNMVCRRTYYKTKKSKERTIKRYY

ICSLFNRSGSSACHSNSINAEVVERVINVHLNRILSQPDIIKQIASNVIEELKQKHSN

QTEIKYDIDSLEKQKAKLKTQQERLLELFLDDQMDSEMLKAKQSQMNQQLEVLDQQIK

EAQQANQSQDEIPNFDKLKGRLILMITRFSVYLRKATPEAKNQLMKMLIDSIEITTDK
QVKLVRYKIDESLIPQSLKKDWGSFFMPKFQFEIYGQNDYFIDQITTFTT"
CDS 9026..9364
/db_xref="SEED:fig|1255645.4.peg.9"
/transl_table=11

/product="FIG01108299: hypothetical protein"
/translation="MTLEQQLKHYITNLFNLPRDEVWHCESIEEIADDILPNQYVRLG

PLSNKTLQTNTYYSDTLHESNIYPFILYYQKQLIAIGYIDENHDMDFLYLHNTIMPLL
DQRYLLTGGQ"
CDS 9460..9771
/db_xref="SEED:fig|1255645.4.peg.10"
/transl_table=11
/product="FIG01107894: hypothetical protein"

/translation="MNRYITRGIANNLPNILQHQLWQLVSEREQEQTKDNTSVDYFHI

FQFNKHRNQLYIKHKQERPEYAKIHKANYSKAININKVYIIREDDVDLSYYVMLLPEE
Y"
CDS 9787..10293
/product="hypothetical protein"
/transl_table=11
/translation="METIKSTLKTEAIFSDDKQHRYLLKKTWNSEKQSITIITMYPHY

DGILNIDLTTQLIMNKVSEMDAFGSINFVNLYSNITTPINLKHLENAYDKHTDIQIMK

AVKESDEVILAWGAYAKKPGVEARVNEVLEMLKPHKKKVKRLMNPETNEIMHPLNSKA
RQKWILKV"
/db_xref="SEED:fig|1255645.4.peg.11"
CDS complement(10442..11116)
/translation="MNYFRYKQFNKDVITVAVGYYLRYTLSYRDISEILRERGVNVHH

STVYRWVQEYAPILYQIWKKKHKKAYYKWRIDETYIKIKGKWSYLYRAIDAEGHTLDI

WLRKQRDNHSAYAFIKRLIKQFGKPQKVITDQAPSTKVAMAKVIKAFKLKPDCHCTSK
YLNNLIEQDHRHIKVRKTRYQSINTAKNTLKGIECIYALYKKNRRSLQICGFSPCHEI
SIMLAS"
/product="Transposase for insertion sequence-like element
IS431mec"
/transl_table=11
/db_xref="SEED:fig|1255645.4.peg.12"
CDS 11374..11541
/db_xref="SEED:fig|1255645.4.peg.13"
/translation="MLESREQLSVEEYETFFNRFDNQEFDFERELTQDPYSKVYLYSI
EDHIRTYKIEK"
/transl_table=11
/product="Hydroxymethylglutaryl-CoA synthase N-domain
homolog (EC 2.3.3.10)"
/EC_number="2.3.3.10"

CDS 12458..13201
/db_xref="SEED:fig|1255645.4.peg.14"
/db_xref="GO:0008889"
/product="Glycerophosphoryl diester phosphodiesterase (EC 3.1.4.46)"
/EC_number="3.1.4.46"
/transl_table=11
/translation="MLTVYGHRGLPSKAPENTIASFKAASEVEGINWLELDVAITKDE

QLIIIHDDYLERTTNMSGEITELNYDEIKDASAGSWFGEKFKDEHLPTFDDVVKIANE

YNMNLNVELKGITGPNGLALSKSMVKQVEEQLTNLNQNQEVLISSFNVVLVKLAEEIM

PQYNRAVIFHTTSFREDWRTLLDYCNAKIVNTEDAKLTKAKVKMVKEAGYELNVWTVN
KPARANQLANWGVDGIFTDNADKMVHLSQ"

CDS 13298..13726
/db_xref="SEED:fig|1255645.4.peg.15"
/translation="MKYDDFIVGETFKTKSLHITEEEIIQFATTFDPQYMHIDKEKAE

QSRFKGIIASGMHTLSISFKLWVEEGKYGEEVVAGTQMNNVKFIKPVYPGNTLYVIAE
ITNKKSIKKENGLVTVSLSTYNENEEIVFKGEVTALINNS"
/transl_table=11
/product="MaoC domain protein"

CDS complement(13772..15778)
/translation="MKKIKIVPLILIVVVVGFGIYFYASKDKEINNTIDAIEDKNFKQ

VYKDSSYISKSDNGEVEMTERPIKIYNSLGVKDINIQDRKIKKVSKNKKRVDAQYKIK

TNYGNIDRNVQFNFVKEDGMWKLDWDHSVIIPGMQKDQSIHIENLKSERGKILDRNNV

ELANTGTAYEIGIVPKNVSKKDYKAIAKELSISEDYIKQQMDQNWVQDDTFVPLKTVK

KMDEYLRDFAKKFHLTTNETESRNYPLGKATSHLLGYVGPINSEELKQKEYKGYKDDA

VIGKKGLEKLYDKKLQHEDGYRVTIVDDNSNTIAHTLIEKKKKDGKDIQLTIDAKVQK

SIYNNMKNDYGSGTAIHPQTGELLALVSTPSYDVYPFMYGMSNEEYNKLTEDKKEPLL

NKFQITTSPGSTQKILTAMIGLNNKTLDDKTSYKIDGKGWQKDKSWGGYNVTRYEVVN

GNIDLKQAIESSDNIFFARVALELGSKKFEKGMKKLGVGEDIPSDYPFYNAQISNKNL

DNEILLADSGYGQGEILINPVQILSIYSALENNGNINAPHLLKDTKNKVWKKNIISKE

NINLLTDGMQQVVNKTHKEDIYRSYANLIGKSGTAELKMKQGETGRQIGWFISYDKDN
PNMMMAINVKDVQDKGMASYNAKISGKVYDELYENGNKKYDIDE"
/transl_table=11
/product="Penicillin-binding protein PBP2a, methicillin
resistance determinant MecA, transpeptidase"
/db_xref="SEED:fig|1255645.4.peg.16"
/db_xref="GO:0008658"
/db_xref="GO:0046677"
CDS 16026..16169
/product="Transposase for insertion sequence-like element
IS431mec"
/transl_table=11

/translation="MNYFRYKQFNKDVITVAVGYYLRYTLSYRDMSEILRERGVNVHH
LTV"
/db_xref="SEED:fig|1255645.4.peg.17"
CDS 16449..16700
/db_xref="SEED:fig|1255645.4.peg.18"
/translation="MAKVIKAFKLKPDCHCTSKYLNNLIEQDHRHIKVRKTRYQSINT
AKNTLKGIECIYGLYKKNRRSLQICGFSPCHEISIMLTS"
/product="Mobile element protein"
/transl_table=11
CDS complement(16760..17188)
/db_xref="SEED:fig|1255645.4.peg.19"
/product="PhnB protein; putative DNA binding
3-demethylubiquinone-9 3-methyltransferase domain
protein"
/transl_table=11
/translation="MKFQVSPYFLITQNAEGLKKLYEELFGAKTLYIQRIKDRPIDKK

LNIDENDLNKIDQCVLQFGDIKIMIADDTEGLPITQGNNISLCLTFDQVEETKHVYDQ
LIEKGSEVLKTFSPEFYTEGYGYVRDPYGISLHLFTKRKN"
CDS 17269..18198
/db_xref="SEED:fig|1255645.4.peg.20"
/product="Transcriptional regulator, YafY family"
/transl_table=11
/translation="MKKSVRLYNMIEYCNENRNFKLNDLMSEFNISRSTALRDIKEIE

ALGVPLYSNPGKNGGYTIIGNRDQTKIAISDEELKALVFTLSSISNVSNLPFQTEYQE
ILKKLYNNSNKKELINQYNDLFQYFNEDKYQFKSYKLFNEIIRLIIENKSFETCYSQN

YIKEQYKGIGIMYKNHQWYFVVANIESKLVNLLNISKIKELYEMGETQECNDITMQNF

QQFMVKNETAIDILIRSNVMGLNILKGYLWSDYMIENIDEETYLFKSKVNAKDIDFIA

KLIVTGGVNVKVETPNSLKNAVKVELTKIINMY"
CDS 18360..18842
/product="Similarity"
/transl_table=11
/translation="MNKNITALKSTENTTYMQYTVHIDDQIINNIKECTKKFKILPME
DKIPLSPLLQPEYAGEVQDFISTYEQFMINFGKVILDSQGIKIQFESESLSSIQRGIQ

EHCYLNERTNDIDVTKEWYLCKFSNQILEEDKNQLYNALKQLMNDSKNKKQAFWTDPN
"
/db_xref="SEED:fig|1255645.4.peg.21"
CDS 18910..19224
/transl_table=11
/product="Mobile element protein"

/translation="MKRVSYSVETKYKAVEMKIAGFSTKEIMKELNIKNRTQVETWWR

WYRNEESYRFSQHVGKQYTYGKGLEELSEIERLKLENKRKDIELDILKKYKALERKWY
QQ"
/db_xref="SEED:fig|1255645.4.peg.22"
CDS 19209..20051
/translation="MVPAVVIDLVDQLKVKYSIKLILEVLNIPKSTYYRWKNKTYKND

TVTQKVIELCEANHYTYGYRKITALINQCYTSPINHKRVQRIMQKHHLNCRVRPKKTT

RIGKPYYKTDNLLQRQFKASCPMEVLTTDITYLPFGHSMLYLSSIMDIYNGEIVAYKI

DDKQDQSLVNDTLNQIDIPEGCILHSDQGSVYTSYAYYQLCEEKGIIRSMSRKGTPAD

NAPIESFHSSLKSETFYINNQLNSSNHIVIDIVEKYIKNYNNNRIQQKLGYLSPVKYR
ELAA"
/transl_table=11
/product="Mobile element protein"
/db_xref="SEED:fig|1255645.4.peg.23"
CDS 20058..21623
/product="Similarity"
/transl_table=11
/translation="MFLLSSRLRGSVPFQEVFKVHIDIYLYNKEKSEYKYQVSSYFNL

VKENPKITYKKRHLQEKQGVKGTTFTNKLKLLNKMYGVDVAKYQPFYNSNNPEYERGQ

FGERYISQRSNYEFNRLQYQIIDMLSKILDKHPLPKSDNNYKHIPTIEKAILSGDSHS

FYEYFEDIMKEIISMENSSLKEKLLTDFTYQSQCRWYSESEKLKLQLESFMHKVLESN

YYEGNKLYRMLSHAIEETINEADEDKVHFNYFKDYFLTDGGVKNWEQISEKITEFNGK

VINDIQNEYNKIQFNNANRNQKLNFNYLYHCFEFSNNLVKARVNGNRGYILYFVDKYK

IKMPFLDQLLINYLNQEIGQESINYNMQTLFEKERYDRSSTIEKLVATSKFKYEKDDS
DLFKQLFNDVENSIDRLGIYLLNNGINSNDENARYYRSFLKELSRIKSKLTPFSLEIS

KSSGREQHYPDDAIDDKDERRKNKEETYHAFDDKSDIDSKLKNKINVSIDNLLSVKIK
SNLYDISPNTKKQ"
/db_xref="SEED:fig|1255645.4.peg.24"
CDS 21818..22927
/db_xref="SEED:fig|1255645.4.peg.25"

/translation="MYQANIRDLITKLPQSNKTEHFLMNKFSNQDKVQQLQRQISQQL

DQQYNELLANEKAKLDQYVEVHHNLEPLKKEIESESINLDTDKLPDIKATMLEKAKND

EHFDKIEQLFDRLDQSLNGTNRLYTQLSLIGTRTHRITTKRFNVQGLPKLVQQMILPS

QFKKVYTIDFKSFEPSVAAYMTQDEQLIDYLNHEEGLYDALLRDLSLSKEKRVSVKRA

FIGSFLFGGRYSSSKFKINQEVSEINWLQVMSKFKKVIEFKEQVEKYKTMPTPYGIEH

DMSAFQGSSIMAIYVQTVASYIFKHILLEVYKAQCEKKTFKIIVPIHDAIMIECNDKG
IAQNVAQLMKDTANQLFNGEFAHVTVEALGGIDNE"
/product="hypothetical protein"
/transl_table=11
CDS 22920..23288
/db_xref="SEED:fig|1255645.4.peg.26"
/transl_table=11
/product="hypothetical protein"
/translation="MNNDRGKSLQIPQSTLLKEGSIYVATLHSVYEKNFSGDIKHQFT

YEVELNQETHYVNRNITVKSMSHQLSIADWIKRHSNYNVNHINYDPYIDRKHLVLVGQ
YNGNYYIQDVAPLDEFGGVL"
CDS 23288..24904
/translation="MNHILEMLIKLLKVGMEAIDRKGLIAILTSSIGNDEMDDSEQAV

MVYNELIDKLQLNIPKDVDYRPNIYSYFGIQKKPNDTILVEMMICIFHIKRFDSELFV

FKDKGWQKVSEDELQGLISKMIQVLLVDYKPSLSTLKNVVDGIQKSTDIEKLVEHRQY

IGCGRNMFNLKTFKVVDNDLEIFPKTRLDLELDINDTITDKIPPNFKQYMLELANYDH

DLQYFLFQHMAVLLTADTKLRRGLFLYGTAKNGKSVYIKLVKSFFYSNDIVSKTLNEL

GGRFDKESLIGKRIMASDEVGKANIDEATVNDFKKLLSVEPIHADRKGRTQVEVTLDL

KLIFNTNAVLNFPSSHAKALERRIAVIPCEYYVEKADPDLIEKLQDEKKEIFLYLMYV

YKQIVKNDIEYLQNDRVTEISHDWLNFGYEFVSSKSANIAHQKACINLLRKLIEIKPG

SRIKVSRLNEVIRDEIKVSSQVINDLVQANFNVQSRLNNGYKYWVDLGWKETDKKDDM
ISFDKNENVTDDEFLYEDDLNLGWEDFDDE"
/transl_table=11
/product="putative primase"
/db_xref="SEED:fig|1255645.4.peg.27"
CDS 25129..26808
/db_xref="SEED:fig|1255645.4.peg.28"
/translation="MKGKIALYSRVSTSEQSEHGYSEKEQEQLLIKEVMKNFPGYDYE

TYTDSGISGKNIEGRPAMKRLLQDVKDNKIEMVLSWKLNRISRSMRDVFNIIHEFKEH

DVGYKSISENIDTSNASGEVLVTMFGLIGSIERQTLISNVKLSMNAKARSGEAITGRV

LGYKLSLNPLTQKNDLVIDENEAHIVREIFDLYLNHNKGFKAITTILNQKGYRTINQK

PFSVFGVKYILNNPVYKGYVRFNNHQNWAVQRRSGKSDKNDVILVKGKHEAIISEDVF

DQVHEKLASKSFKPGRPIGGDFYLRGLIKCPECGNNMVCRRTYYKTKKSKERTIKRYY

ICSLFNRSGSSACHSNAINAEVVERVINVHLNRILSQPNVIKQIASSVIEELKQKHSK

QTEIKYDIDSLEKQKAKVKTQQERLLELFLDDEMDSEMLKAKQSEMNQQLEVLDQQIK

EAKQANQSQDDIPNFDKLKARLILMITRFSVYLRKATPEAKNQLMKMLIDSIEITTDK
QVKLVRYKIDESLIPQSLKKDWGSFFIPKFNFVINVTKKNRIENLSLLPLF"
/product="Cassette chromosome recombinase B"
/transl_table=11
CDS 26897..27235
/db_xref="SEED:fig|1255645.4.peg.29"
/transl_table=11
/product="hypothetical protein"
/translation="MTLSKQLKTYITERFKLNYQETWACETIDAVAEDVLPEKYIKNS
PLEHKILNTFTYYNDELHEISIYPFLCYLDKELVAIGYLDNFDLDFIFLNDTHQVIID
ERYLLQKGGE"
CDS 27238..27327
/transl_table=11

/product="hypothetical protein"
/translation="MNWIKVAQLSVTVINEVIEIMKEKQNGGE"
/db_xref="SEED:fig|1255645.4.peg.30"
CDS 27329..27640
/db_xref="SEED:fig|1255645.4.peg.31"
/translation="MKINRYITRGVSEHLSLDLQILLWNMVKDRDNQLHTDYLHIFKL

QEDDNILSITHEQEQPAYKLEYHYTNYVKNQNALPKKVYVIREDDVDAFYYVMLLPEE
Y"
/transl_table=11
/product="hypothetical protein"
CDS 27655..28158
/transl_table=11
/product="hypothetical protein"
/translation="MNTIKNTIHTEAIFSDDEQHRYLLKKTWDEKKPACTVITMYPHL

DGVLSLDLTTVLILNQLANSEQYGAVYLVNLFSNIKTPENLKHIKEPYDKHTDIHLMK

AISESDTVILAYGAYAKRPVVVERVEQVMEMLKPHKKKVKKLINPATNEIMHPLNPKA
RQKWTLK"
/db_xref="SEED:fig|1255645.4.peg.32"
CDS 28172..28393
/db_xref="SEED:fig|1255645.4.peg.33"
/transl_table=11
/product="hypothetical protein"
/translation="MNHETKQSDWRTVASCLASQNYISIVKGLVHHFTAIEDEEILDK
IYDDFMNDDSITTVLNNDLQTIINHYLSK"
CDS complement(28468..31587)
/db_xref="SEED:fig|1255645.4.peg.34"
/EC_number="3.1.21.3"
/product="Type I restriction-modification system,
restriction subunit R (EC 3.1.21.3)"
/transl_table=11

/translation="MNFQFNEDDLEQVALEWLQSLGYDYKKGNEISMTSLTPERKSDK
DVVLHERLEKALRKINSDIHPRFIEKALHELTLEKSPNLLENNLTFHENLINGIEIED
YDDEGQSIVEIVKIIDFEYPQNNDFLAVNQLTIVNGDYKKRPDIVLFINGLPIVVIEL

KNSTNETVGVEDGYHQLETYKMRIPQLFTFNEVLVTSDGINTKAGSLTANYDRFMTWR

SKDGQTESSPGLASLDVLIHGMLNPETLLDLIRYFVLFQDDGKGHISKILAAYHQYYA

VNKAVDRALLASSEQGDGKGGVIWHTQGSGKSLTMVFFSGKLIQMLNNPTLVVVTDRN

DLDNQLYSTFVKSKGRSGKGLLRQTPKQAETRKELKSLLSVESGGIVFTTMQKFEPEQ

NETTMAALTERKNVIVMADEAHRTQYGFNAKYDDKGEGIKYGYAKYLRDALPNATFVG

FTGTPVASTDKNTQMVFGNYIDVYDMTQAVADGSTVKIYYESRVIPLNLPQSLDLDEA

YNDITEDQEEDVKQRLKSKWSRIEALAGAKPRVEALAKDIIQHFETRQQAMKGKGMIV

TMSRRIAVDLYDEIIRLKPEWHSDDDDKGVIKVVMTGSSSDPTSFQRHIGPKKRRNLL

EKRMKDVDDELQLVIVRDMWLTGFDVPSMHTMYIDKPMKGHNLMQAIARVNRVFKDKP

GGLIVDYVGIAESLKEALKEYTESDQAQTAIDTDKAVELMLLKYDVIQDMLYNLDYSK

FNSDKKSERYYTISNTMDYVIGLGEDERQRFIKTVTELGKAFALCATEPTAQELNDEI

AFFKAVKAGLVKLLQPPKEGKTRKTPAEVEAEINQLVSQSVVTEDVIDVYQTLGLEQP

DLSILSDDFLKDVEGLKQKNVAVELLNRLLKGQVKSLMKTNATVSKRFSEMLGNSINK

YNSRSIETSKVIEELIQLAKDIKQEQQRGNELGLNSDEIAFYDALASHETAKEAMGDK

ELRAIAHELTKTVKENMGVDWSKRDSAKAKMRVAVRRLLKKYGYPPDLQKMAVEQVVE
QAELMANNQ"
CDS complement(31571..32872)
/translation="MEYNTIFFGDVAKIKNGYAFKSKEFQEKGIPVIKIKNIISPIVD

TKDSQKVSIKTYEKTKGFSLKKNDILISLTGSGVNQMSSAVGKVGRIEFDYPALQNQR
VGKFELKYSNSADLDFLFYYFLQPKITEYLVRNSTGSANQANINSKLIETVKIPNFSL

IKQKSISKFLNQITRKIETNQKMIANLKELSQTLFKHWFVDFEFPDEDGNPYKSSGGE

MIDSELGKIPSNWKIYKLKDIASHKKETFNPKKSEEVTVKHFSLPAYDNEEQAIEEEV

NKIKSNKWIINNNCVLFSKMNPDTKRIWLPVIDNKKLNVASSEFVVMESPNNKINSFI

YNICLNSQFIDYLKANTTGSTNSRQRVKPTIAVNYKLAIEDSIVKKYSEIITPYMEEM
KILRSEIGKLTQLRDTLLPKLMSGELEISDDIEVNSDELSI"
/transl_table=11
/product="Type I restriction-modification system,
specificity subunit S"
/db_xref="SEED:fig|1255645.4.peg.35"
CDS complement(32862..34376)
/product="Type I restriction-modification system,

DNA-methyltransferase subunit M (EC 2.1.1.72)"
/EC_number="2.1.1.72"
/transl_table=11

/translation="MATIGFEEKLWQAADKLRGSMDAAEYKNVALGLIFLKYVSDSFE

EKYEELKQDPYADEEDQDEYLAENIFWVPKEARWQYINDNAKKPEIGQIIDKAMIAIE

NENESLKGVLPKEYARPALDKEKLGDIIDLFTFKVGDTESRKQDVLGRVYEYFIAKFA

SAEGKNAGEFYTPSSIVKLLVEMIEPYKGRIYDPCCGSGGMFVQSERFVEKHQGRLDD

IAIYGQESNPTTWKLAKMNLAIRGIDNDLGERNADTFHNDLHKGLKADYILANPPFNA

SDWGQERLLDDYRWQFGIPPKGNANYAWIEHMISKLAPNGTAGFVLANGSMSTSGKDE

LEIRKNLIEQDLVECIVTLPGQLFYSTQIPVCLWFISNNKGQNGKKERENEILFIDAR

EIGHMVSRTLKEFSDEDIQKVAQTYHAWRGTNDKPYEDIAGFCKVANLEEVKNNEYIL
TPGRYVGLADVEEDEEPFEQKMERITSELSEQFAKSKELEDQIRKSLEGLGYGV"
/db_xref="SEED:fig|1255645.4.peg.36"
CDS 34572..34718
/translation="MDGVTNVKIHILTFVKIVVIVDIVAIVNTRLDKLGIFNSINKKF
KCSQ"
/transl_table=11
/product="hypothetical protein"
/db_xref="SEED:fig|1255645.4.peg.37"
CDS 34818..36881
/translation="MYISSVKIKNFRNFDNIDIDFHEGVNVLIGHNNSGKSNLLRALS

LIFDGSVRKQLSVEDFNNSLTKESLKKEAPKIVISVHITQSENERLMSDELITVSNWL

TKLEEPYEARIQYEFFLPKDEEKNYIDLVKNIDEKEEIWNLIRSQFIRLYVNKIWVGN

PEHQIPIDNDSLNKFDFQFLDAIRDVERDMFSGKNTLLKSVIDFFIDYDIKSDETITE

EEQKEKLEERRKEFSDNSSDLIETIHKRLDSGNQKILSYTNGIGASYDKSTPDFKGNL

TESEIYTVLQLIIKHETGMTLPITHNGLGYNNLIFMALLLSKMQADSDGNFLGSNAKV
FPILAIEEPEAHLHPTMQNEFIKFLKNNIREKKVKQIFITTHSTHISSSTNIDDIICL

YTDDSKTNVSYPGKVFDKNNQSKKYVQRFLDATKSNMLFAEKVIFVEGIAEQLLLHIF

ADYLNKPLEKNHVAVINVGGRYFNHFLSLFDSNNKYAINRKVSCITDLDPMRKEKNEN

GQKNNFKACYPFEYEMDLDKFDYSTNNSLDKYLNNEHSNIRVFVQPNKYGKTFEYQLM

FDNPSLKLLLTDSISNSQELAELMDHYEENESLQKLIDTLSPSNENQRIIKSLKDTND

SWNEDNKKKALIASRYLNSIGKGENALELASVLKDNLELKGQSDYEDFVVPKYIEGAI
RWVCE"
/product="FIG131328: Predicted ATP-dependent endonuclease
of the OLD family"
/transl_table=11
/db_xref="SEED:fig|1255645.4.peg.38"
CDS 36883..38631
/db_xref="SEED:fig|1255645.4.peg.39"
/translation="MIINAQTKLNIEQDFKIQAGPGAGKTEFLVNHIKNVIQTSEKLE

RMKKIACITYTNTAAQTVLQRLGKSVSNRVDISTIHSFLYRNVVKPYCSFLPKEYGIC

VQKLKGHSDPIVINKYVNAWLKTEYFTKLKPPSNRNQLLKMPVLKKALQNWLLSMKLS

YENNDVQFICDNTKAQAIDKKSKKRMGINKSNLNILEKQLLNYKKIYWEKGIIDHDDV
LFFSYVLIRDYPFILTILCAKYPYFFIDEFQDTSQLQDFIIDKIRQKGCIVGVIGDKA
QAIYSFQGARVSLFEKFKVDLINSHTILENHRSSYQIVTFLNSIREDITQTVYSGIEN

DKVTILIGNRIRSYTKVVNICNKESLVTLSRDNITSNAMRIKLEGNNFDKKLLEKFQE
IDSNSKRRNTIISLIKAIELAKNTKYKDALKIVESIYKDENNPKKIALNSLINMINKY

DKYGNGTLMNFYNTLCKTLDIKLSGFRKGQVKDFYENTLYKNMVICTNIVEDTSNHIT

IHKAKGAEFENVFVIGNKDTLSLLLKPDLMNDEEHRIFYVAMSRAKKKLFLQFDYLDN
NEQKKIKERYDVSIIQ"
/product="ATP-dependent DNA helicase UvrD/PcrA,
clostridial paralog 2"
/transl_table=11
CDS 38895..39023
/transl_table=11
/product="Multidrug resistance protein"
/translation="MIIFNVIMLVSSIIQMILGKGRRITPAKPTKDAKLHLDNQSS"
/db_xref="SEED:fig|1255645.4.peg.40"
CDS complement(39091..40515)
/db_xref="SEED:fig|1255645.4.peg.41"
/transl_table=11
/product="Decarboxylase, pyridoxal-dependent"
/translation="MEMEFNENNIDLETIIRDEVNKYLSRDIGDLPATQQAPLELREK

YEKMEVPNKGRDIYEVLKDLNNEVLNYLYRPNHPRSFSFIPGPASRLSWLGDILTTAN

NIHASNFANATLPINIERNLINYLVGKIGYEIKPAGGVFVSGGSMANLTAIVAARDAQ

VEMEDIKKATVYLTSQTHHSVGKALHVAGFLKNNIRRIEYNDDFTMNTDSLKAAIDKD

IEEGYKPAMVIATAGTTNTGSVDDFTTLGDTCDHYNLWLHVDGAYGLSHILSDKAQHL

FKGIERSDSASWDAHKLLFQTYSCAMVIVKEKNHLLQSFGEDAEYLDDIASDDDVIDP
EMLGIELTRPARALKLWITLQVIGEDEIIERIKYGEGLAEYAEEYVSGLDNWRIISHA

NLSIVNFRYENSDLTEAQNNQLNSMAAQKIADSGYAIAYTTVLNNQRVIRLCTTNPLT
THDDIRETINRLDQYIGDYDMKDA"

```
     CDS             complement(40598..40810)
                     /translation="MRSSSPVLRGSYRIPKDKHQYKLKITDDNLQVDYPRQFLKGAHT
                     PSVKAQHVIHWGDTFWVSSLWLAWFY"
                     /EC_number="1.1.1.95"
                     /product="D-3-phosphoglycerate dehydrogenase (EC
                     1.1.1.95)"
                     /transl_table=11
                     /db_xref="SEED:fig|1255645.4.peg.42"
                     /db_xref="GO:0004617"
ORIGIN
        1 atggaggata aaatgaaaat tacaatcctg tcagttggaa aactaaaaga gaaatattgg
       61 aaacaagcca tagcagaata tgaaaaacga ctaggagctt ataccaaagt tgaaataata
      121 gaagttccag acgaaaaagc accagaaaat atgagtgaca aagaaataga acaagtcaaa
      181 gaaaaagaag gacaacgact actagctaaa attaaaccac aatccacagt catcacatta
      241 gaaattcaag gcaaaatgtt gtcatcagaa ggtctagcca aagaactaca aacacgtatg
      301 acccaaggac aaagcgactt tgtattcgtc attggaggat caaacggctt acatcaagac
      361 gtcttacaac gtagcaacta cgcactctcc ttcagcaaaa tgaccttccc acaccaaatg
      421 atgcgcgtca tcttaatcga acaaatctac cgcgccttca aaattatgcg cggcgaggct
      481 tatcataaat aaaactaaaa attagattgt gtataattta aaaatttaat gagatgtgga
      541 ggaattacat atatgaaata ttggagtata ccttgcaata tcatacgatg tttatagagt
      601 gtttaataaa ccatttttca actattgatg atctagaata tataataact gtacaaatta
      661 tattgattat ggaactacaa ttcaattaag aaattgatga tgaaatttta aatttaaact
      721 aatggaatca agaaagaatg aaaggaaata tacaatgccc acgattaata aaaggaagtt
      781 tattagattt tgtgttagaa acagtagatg tttttgtaaa tggaagtaaa aatgattgcg
      841 aattagtaaa ttttaattat aaagaaggaa aagtagtttc acgttatagt acaaaatttg
      901 aaagcaatcc taaattaaga aaaaagcaat tgatatcgac ggtaaaagtt gtaaggtatg
      961 ttatttttga ttttgagaaa attttatggg gcatagggaa agttttattg aagttcatca
     1021 tattaaggca atgtttacta taagaaagtt gaaatacact cagaaacaga ctggattccc
     1081 gttttttca aattggcaca aaatgataca taggcccacc tatatattga agatttaagt
     1141 taaacgataa ataatatttt tctcttttgt atagaaattt ttttgggaat gatattaatt
     1201 agtataaaaa tgaatatgtc tacaaagaag gagagatttt attgaaacac attaatcaat
```

1261 tttctgaatc gacactagca caactagaaa aagatcaaca acacgtattt tatgtatact
1321 gtttgatgga cccaagaaat gatgaatgct tttatgttgg taaaggtaag gggaatagaa
1381 tatttaaaca taaacaagat gcgcagaaaa aagtattgta cgaagatata tttatagaag
1441 aaaataaaga taacttgaaa tttaatagaa ttaacgaaat ttgtagcaat gatttgaatg
1501 ttttaggata tataatcagt tatggattaa ccgaatctga agcattttct gcagaaaatg
1561 ttcttataaa ctttcttaac ctaacaaata aaactacatt aacaaatatg attaacggtc
1621 atggatctaa ggcatactta gttgaagatt tagaaaatga atttggctac gattcaatta
1681 atcttgaaaa cataaatacg aatgaattaa ttttagcagt gaaaatcaga gatgcatttc
1741 tattagataa ggatgaaagt aaagagtatc ctattaatga aagtaaacgt gatagaaata
1801 accttaaatc gcgtacatta ggtagttgga taataggaaa agataaaata cataaaataa
1861 aatatattat tggtatcaat acaggtgcta ataatgcagt tgtctcagct tatgaagtat
1921 catttgaaca agcggagagt atcgaaacaa ataatggtag aatgagatat gcgtttattg
1981 ggctttcaga aagagatgct actctaaaaa aattgaattt atacaaaaaa gcactaccag
2041 atttaagatt tggtagtggt agcgctacag catatataaa taatgggaca atgaaagttg
2101 attaaatgaa taaaaacatc caattcctta aattagcctt tgaaaatttt atactcgtat
2161 atataagtga aaaacttaaa agaattagat gttaattaaa ggagagaatg ctatgccatc
2221 agatgatatt gtaaaaaaac tatactcaaa agaggatatt cgattagaat taggacttac
2281 tccgcataag tttaataaaa aaatggaaac tattgctaag ctttttaaaa ttgatatgaa
2341 aatttttcac aattacaaag gccaagataa aaataatcag tatacattta atggtgtcgc
2401 aaaagaatta atcaaagtgt tgctaaaaag tgttgattat tacccagtgg atatcaattc
2461 caaaaaattt aaacaaaatg gaaaatctaa aaaagaaatg attgaaaata tagataactc
2521 tagttacatg aaatatattt atcagttaat gaaatctatc aatgaaattc aatacaaaag
2581 gttaattgct gatatacata tgaaagatgt gtaccagaat acaaaagcat ggttaaataa
2641 tggtgaatca attaataaaa aagaacaaga gctataccaa tatatgacaa ttttaccatt
2701 acataaaaga gttgaactgc aaaatgaggt attaaaatct atagacgaaa caatttttca
2761 atttctcgca aaagaacata gaaataatca aattgaagaa aataatgaat tagaagcata
2821 tacaaaagca ataaaagaag gtagaaatcc taaaaatgat tatgaattaa atcatctcct
2881 atataaaaaa aatcaattac ccttagatag tctaatagaa gatatatggg actatgagga
2941 aacagagtat actgagcttg attggttaat tgcagatatg ctaaaacgtt cacaaagagc
3001 tgatagtaat tttatcgaaa gtttagagaa gaaaaacaaa ttgagaaaaa atattcataa
3061 agattccatt aaaagtatat ctaagttgat tgatcatgaa ttagttaaaa ataatagaaa
3121 gtgggacgta gcagaatttt ataaaaatca acaattttgg aataatagta cattaagacg
3181 tagctctcaa aattttggaa ttttatatta caaaaagaat aggttacata caatgaatca
3241 aagtaatatt attaagaaaa taaaaattat cgaagctaat atagagagtg caaataaaca
3301 gccgagctat ttagatttct ttttacaagc taagtttgat ttagagaggt tagaaagtga
3361 attaatgaaa tatagcatta atccgaaata ttttaataag ataaatagtg attacgtaag
3421 atatgctatt gaagatgtac ataatgcaat tattaaaatt gacagatata taagtaacga
3481 taaagtgaag ttaaactatt caaatgtcca aatggtagtg agaaacgaaa taacagaaca
3541 aggaagctac tttgtaaccc aggcattgaa tacactgtca aaggtagaag agtctggcta
3601 cagctttgat aatttcatgg taggaccttt cgtagaagac tttaaaaaag caattaaaaa
3661 tcgtagattt ggttctaaaa aagattaaga ttgtttttta gagttaaaca taagaataaa
3721 tttaaataaa aaaataaacc caacaaacct tgttatatca atgtttgttg ggtttatttt
3781 tttggtgatt ttttaataac tcaattacat cttaggataa tatcgctaaa ttatacattg
3841 tcgaggccaa gacaaataaa aaattaaagc aaaagttagc gcgcaacttt ctgctataaa

3901 ggagcaaatt tatatggaga caagagacaa actgatgtca ttgactcaaa gtgacaaaac
3961 acagcaatgg ctaatggaca agtcatctaa ccaagatgac attcaacaat tgcagcaaca
4021 attcagtcag cagcttgatc aacaatataa tgcactttta gctgatgaaa aagctaagtt
4081 agaccaatac gtggaagtac atcaaggatt ggaatcatta aaggaagaga ttgaatcaga
4141 acctattacg cttaatatcg ataaattacc cgatatcaaa gcaacaatgc ttgaaagagc
4201 caagaatgat gaacattctg ataaaatcga aaagctattt gataggttag aacaggcatt
4261 aaatggtacg aatcgattat atacgcaatt atcgttgatt ggtacacgaa cacatcgaat
4321 tacaactaaa aattttaatc ttcaaggctt acctaaagca gtccaacata cgattttacc
4381 ttcaaaattt aagaaggtgt atacagtcga ttttaaatcg tttgaaccat cagttgcagc
4441 gtacatgact caagattcaa aactgattga cttgttaaat cagaaagacg gactgtatga
4501 cgcattgcta agtgaattag gcttatcaga tgagctacgt gtatttgtta aacgtgcatt
4561 tattggttcg tttctatttg gaggtaactt caaaaatcct aaattcaagc tgaatcaata
4621 tgtaagtgaa gtacaatggt tggatgcggt cagccaattt acaaaagtca ttgaacttaa
4681 gaagcacgtt gaaaagagta attccatgcc tatgccttat agtattgagc atgatatgag
4741 cgcatttcaa ggtagcagta ttatggcaat ctacgtacaa actgtagcga gttatatttt
4801 caagcacatt ctgctaaaag tgtacaaagc acagtgcgat caaaaaacgt tcaagattat
4861 agtacctata cacgatgcga ttatgattga atgtgaagat gaagaaattg cacaaaatgt
4921 gggtcagtta atgaaagata cggctaacca gttgttcaat ggtgaatttg cacatgtgac
4981 agtggaagaa ataggaggcg tagaccatga ataatgatag aggacaaagc ctacacatcc
5041 caagtagtac accaatcaaa gaaaataata tatatgtagc tacgttacat tctgtgatcc
5101 aaacagattt ctcaggtgaa ataaagcacc aattcacgta tgaaattgaa gtgaacaatc
5161 agattgtata tgcgaatcgt aatattccaa caaaaccgag cgctaatcag ttgtcaattc
5221 atgattggct gaaacgtcat agcaactata gcgcaagtca tgaaaactat gagccttata
5281 ttgatcagaa acatttaatt ctattaggtc aatataacgg taactattat gtacaagatg
5341 tagcatcgtt agatgcgttt ggaggcgtat tatcatgaat catatattac aaatgttatc
5401 taagctatta agtgtggcca aggaggcaat cgaccgtcaa ggtctgattg ctatcctaac
5461 tattcctgtt aataataacg atgaaataga agaaacggct caaggtgaaa ccgtgtataa
5521 cgaacttatc gatcagttac gacttaatat cccaaaagat acggattatc aacctaacat
5581 ctatagttat tttggtatta agaagaatcc taatgacacc gtactcatgg aaatgatgat
5641 aaaggttttt catatcaaac gctttaattc agaactgttt attttcaaag ttaacgggtg
5701 gcaaaagata aatggagatg aattacaagg gttgatatct aaaatgatac aagtattgct
5761 tgtagattat aagccttcac taagcactct aaaaaatgtc gtagatggat tgcaaaaatc
5821 aacagatgta gaagaacttg ttgagaatga gcgctatatt ggttgtggtg aaaatatgtt
5881 cgatcttaat acgtttcaag tcgttaaaaa ttcaatcgat atctttccaa aaacacgatt
5941 gaatttatca ttaagtacaa atgatgtaat tactgataag ataccgcctt attttaagca
6001 atatatgtta caacttgcga attatgacga tgatttacaa tactttcttt tccaacatac
6061 agcagtatta cttacagctg atactaaata ccgtaggggt ctcatattat atggtggagc
6121 taagaatggt aaatctgtat atattgaact agttaaatca tttttctata gtaaagatat
6181 tgtgtctaag ccacttaatg agcttgaagg tcgttttgac aaagaaagtt taattgacaa
6241 aagtctaatg gcaagtcatg aaattgggca atctaggatt caagaaaaga tcgtaaatga
6301 cttcaaaaag ttattatctg tagaatcaat gcatgttgat cgtaaaggaa aaactcaagt
6361 ggaagtcatt ttggatttga aacttatttt tagtacaaat gcgatactta attttcctcc
6421 tgaacatgcg aaagctttgg agcgtcgaat taatattatt ccatgtgagt attatgttga
6481 aaaagcggac acttcattaa ttgataagct ccagagtgag aagaaagaaa tctttcttta

6541 cttgatgtat gtgtatcaac agattgtaaa agcagatatc gagtatcttg aaaatagccg
6601 tgtcactgaa attactcacg attggttaaa ttttggatat gaatttgttt ctagcaggtc
6661 cgtaagtaat gcaaatcaga aagcatgtat taatttactc agaaaactta tagaaatcaa
6721 atcaggatca cgaatcaaag tatccgagtt aaataaagtt attaatgaag aaataaaggt
6781 aagttctcag gttattaaac agttaattca agcaaacttt gatactcaaa ccaaactata
6841 caatggctac gattattgga ttgatttagg ttggaaagaa gccaataaaa aagagattca
6901 tgatatttcg gaaaaagata atattatttc attagataaa aatgaaaata taacagacga
6961 tgaggcatta gatgaagaga atttggactt tgattgggag gactttgacg atgaataatg
7021 aacaaattga agcatttgta gaagtgcttg tacctatcat agaagaacgt atcaataaag
7081 gtaattaagg ctaattacgt actacaggca gttgcctgta gtactcatat gattaagtgg
7141 taaaagtgat aaaaatgaaa cgaaattata aatatatatt atctatatgt tgttacaaga
7201 ccgatagtct gtagcaataa tctaataaaa ggagcggtat gatatgaagg gtaaaattgc
7261 actttattca cgcgttagta cgtcagagca gtcggagcat gggtactcaa tccatgagca
7321 ggaacaagta ctcatcaaag aagttgtgaa aaatttccca ggttatgact atgagacata
7381 tactgactca ggcatttcag gtaaaaatat tgaaggtcgt ccggcaatga aacgtctatt
7441 acaagatgtt aaggataata aaatcgaaat ggtgttaagt tggaaattga atcgtatctc
7501 acgatcaatg agagacgtgt ttaatattat tcatgaattc aaagaacatg atgtagggta
7561 taaatcgatt tctgagaata ttgatacatc caatgcttct ggagaagtac tcgttacaat
7621 gtttgggtta ataggatcta tagaacgcca gactttgatt tcgaatgtga aactttctat
7681 gaatgctaag gcaaggagcg gagaggcaat caccggtcgt gttttaggct acaaattatc
7741 acttaatcca cttacacaga aaaatgattt ggttatcgat gaaaatgaag ctaatattgt
7801 acgtgaaatt ttcgatttat atttgaatca taataaaggc ctcaaagcca ttacaaccgt
7861 acttaatcaa aaggggtatc gtactattaa tcaaaagcca ttttcagtgt atggtgttaa
7921 atacattttg aataatccag tctataaagg ctatgtcaga ttcaataacc atcaaaactg
7981 ggctgtacag cgaagaagtg gcaaaagtga taaaaatgat gtgatattgg tcaaaggtaa
8041 acatgaagcc attataagtg aagaggtatt tgataaagtt catgaaaaat tagcttctaa
8101 aagttttaaa ccgggcagac ctattggtgg agatttctac ttacgtggcc ttattaaatg
8161 cccagaatgc ggaaataata tggtatgtcg acggacgtat tataaaacga aaaagtccaa
8221 agaacggaca atcaagcgtt attacatttg ttccttattc aatcgttcag ggagttctgc
8281 atgtcacagt aattccatca atgctgaagt cgtcgagcgt gtaattaatg ttcatttgaa
8341 tcgtattctg tctcaaccag atattatcaa gcagattgcg tcaaatgtga tagaagaact
8401 gaaacaaaag catagtaacc aaacagaaat taaatatgac attgatagtt tagaaaaaca
8461 aaaagctaag cttaaaacac aacaagaacg attgttagaa ttgttcttag atgatcagat
8521 ggatagcgaa atgttaaaag ctaaacaaag tcaaatgaat caacagttag aagtattaga
8581 tcaacaaatt aaagaagcgc aacaagcaaa tcaatcacag gatgaaatac ctaattttga
8641 taaattaaaa ggacgactca ttttgatgat aacacgattc agcgtgtact taagaaaggc
8701 tacacccgaa gctaaaaatc aacttatgaa aatgttaatt gattcaattg aaattacgac
8761 agataaacaa gtaaaacttg taaggtataa aattgacgaa agtcttatcc ctcaatcttt
8821 gaaaaaagat tgggggtctt tttttatgcc caaattccaa tttgaaatat atggtcaaaa
8881 tgattatttc atcgaccaaa ttaccacttt taccacttag ttattagtga caaaagtgag
8941 cgaaatgaaa taaaaatcaa atatatatta tcaaaatgat gtatcacatg catacatcaa
9001 tcaaatacat taggaggtca taaccatgac actagaacaa caactcaagc actatataac
9061 caacttattc aatctgccaa gggacgaagt gtggcactgc gaatctatcg aggaaatcgc
9121 tgatgatatc ttacccaatc aatatgtaag acttggccca ctcagtaata aaacacttca

9181 gactaatacc tactactctg acacacttca tgaaagtaat atctatcctt tcattctcta
9241 ctatcagaaa caactcatag ccatcggtta tatcgacgaa aatcacgata tggatttctt
9301 atacctacac aacactatca tgcctctttt ggatcaacga tacttactaa caggaggaca
9361 ataaaatgca taaatacatc aaaattacac aattagtcat tacaatacta agtgaaatca
9421 tcatttggat gaaagagtca gaacgaaagg aagtctctta tgaatagata tatcacccgg
9481 ggtatcgcca acaacttacc taatatctta caacaccaat tatggcaact cgtatctgag
9541 cgagaacaag aacaaaccaa agataatact tcagtagatt attttcatat attccagttc
9601 aataagcatc gcaatcaatt atatatcaaa cacaaacaag aacgacctga atatgcgaaa
9661 atccataaag ctaattattc aaaagcaatc aatatcaata aggtctacat tattcgagaa
9721 gatgatgtag acctttctta ttatgtcatg ttattacctg aagaatacta gaggagtgaa
9781 aattatatgg aaacaatcaa aagtacatta aaaacagaag ccatattcag tgatgacaaa
9841 caacatcgct atctacttaa gaaaacatgg aatagtgaaa aacaatcaat cacaatcatt
9901 acaatgtatc cgcattatga tggcattctc aatattgacc taacgaccca actcatcatg
9961 aacaaagttt cagaaatgga tgcatttggt tcaatcaatt ttgtgaatct atactctaat
10021 attacaaccc ctatcaatct caaacattta gaaaatgcgt atgataagca tacagatatt
10081 caaattatga aggcagtgaa agagtcagat gaagtgatat tagcttgggg cgcttacgct
10141 aaaaagcccg gtgttgaagc acgtgttaat gaagtattag agatgttgaa accacataaa
10201 aagaaagtaa aacgactcat gaatccagaa accaatgaaa tcatgcatcc ccttaattcg
10261 aaagcacgtc aaaaatggat attaaaagta tagcaactaa aatgtttatt cactatccat
10321 gaaaataatt gaatttcata tatatctttt aatctacaat atgattaaaa gatatattat
10381 ttggttctgt tgcaaagtaa aaaaatatag ctaaccacta atttatcatg tcagtgttcg
10441 cttaacttgc tagcatgatg ctaatttcat ggcatggcga aaatccgcag atctgaagag
10501 acctgcggtt ctttttatat agagcgtaaa tacattcaat accttttaaa gtattctttg
10561 ctgtattgat actttgatac cttgtctttc ttactttaat atgacggtga tcttgctcaa
10621 tgaggttatt cagatatttc gatgtacaat gacagtcagg tttaagttta aaagctttaa
10681 ttactttagc cattgctacc ttcgttgaag gtgcctgatc tgtaattacc ttttgaggtt
10741 taccaaattg tttaatgaga cgtttgataa acgcatatgc tgaatgatta tctcgttgct
10801 tacgcaacca aatatctaat gtatgtccct ctgcatcaat ggcacgatat aaatagctcc
10861 attttccttt tattttgatg tacgtctcat caatacgcca tttgtaataa gcttttttat
10921 gctttttctt ccaaatttga tacaaaattg gggcatattc ttgaacccaa cggtagaccg
10981 ttgaatgatg aacgtttaca ccacgttccc ttaatatttc agatatatca cgataactca
11041 atgtatatct tagatagtag ccaacggcta cagtgataac atccttgtta aattgtttat
11101 atctgaaata gttcatacag aagactcctt tttgttaaaa ttatactata aattcaactt
11161 tgcaacagaa ccgtattatg gaatagagat gttggtaaca tttatacagg atcattatac
11221 ttaagtttaa tttcgttatt acagaaccac acattccaac cagaagagaa agtatgtcta
11281 tttagttatg gttcaggagc agtaggagaa atctttagtg gttcaatcgt taaaggatat
11341 gacaaagcat tagataaaga gaaacactta aatatgctag aatctagaga gcaattatca
11401 gtcgaagaat acgaaacatt ctttaacaga tttgataatc aagaatttga tttcgaacgt
11461 gaattgacac aagatccata ttcaaaagta tacttataca gtatagaaga ccatatcaga
11521 acatataaga tagagaaata aactagtggc cgattgtgct tgatgagctt gggacataaa
11581 tcctaactcg aaataaataa gcatatcact aaactgattt tttaaagttt acagtgatat
11641 gcttattttt ttatcttacg attttgtacg tgcatgcttg cctaggggta tggctcgagc
11701 cattagtctc tcgcacatac tattccctca ggcgtcagca cttacaaaat cggttgtaat
11761 tttcattttt atacgcattc ttactgagat tatactaata agaggaatag taaaagcaat

11821 tctaagtaaa attgcagata agaggtttgt taaaagcagt tctaagtaaa attgcagata
11881 agaggtttgt taaaagcagt tctcagtaaa attacagata agaggtacgt taaaagcagt
11941 tctaagtaaa attgcagata agaggtttgt taaaagcagt tctaagtaaa attgcagata
12001 agaggtacgt taaaagcaat tccatgcaaa attgctgata aggggtaagt taaaagcagt
12061 tctcagtaaa attgcagata agaggtacgt taaaagcagt tctaggcaaa attgcagata
12121 agaggtgcgt taaaagcagt tctaagtaaa attgctgata aggggtaagt taaaagcaat
12181 cctaagtaaa attgcagata agaggtaagt taaaagcaat cctaagtaaa attgcagata
12241 aggggtacag aaaaactaga cttgattaca aaatggagct tgggacataa atgatttttt
12301 aaaaatgaga tgagacgtag attaactcca taatcaatac gaatctatcg acttctttat
12361 ttatgatatt catctctttt taatggaaat aaaagtgcga ttaatgtgat aatacagtta
12421 cgttaattaa aaaaataaaa atgcaaggag aggtaatatg ctaactgtat atggacatag
12481 aggattacct agtaaagctc cggaaaatac aattgcatca tttaaagctg cttcagaagt
12541 agaaggtata aactggttgg agttagatgt tgcaattaca aaagatgaac aactgattat
12601 cattcatgat gattatttag aacggactac aaatatgtcc ggggaaataa ctgaattgaa
12661 ttatgatgaa attaaagatg cttctgcagg atcttggttt ggtgaaaaat tcaaagatga
12721 acatttgcca actttcgatg atgtagtaaa aatagcaaat gaatataata tgaatttaaa
12781 tgtagaatta aaaggtatta ctggaccgaa tggactagca ctttctaaaa gtatggttaa
12841 gcaagtggaa gaacaattaa caaacttaaa tcagaatcaa gaagtgctca tttcaagctt
12901 taatgttgtg cttgttaaac ttgcagaaga aatcatgcca caatataaca gagcagttat
12961 attccataca acttcgtttc gtgaagactg gagaacactt ttagattact gtaatgctaa
13021 aatagtaaac actgaagatg ccaaacttac taaagcaaaa gtaaaaatgg taaaagaagc
13081 gggttatgaa ttgaacgtat ggactgtaaa caaaccagca cgtgcaaacc aacttgctaa
13141 ttggggagtt gatggtatct ttacagacaa tgcagataaa atggtgcatt tgtctcaata
13201 gaaagttaga ggtgagtctt acgtttcagt gacggtagac ttacctttaa catgttacat
13261 actaaaaaat taatttgaat aagaaagaga gacatatatg aaatacgatg attttatagt
13321 aggagaaaca ttcaaaacaa aaagccttca tattacagaa gaagaaatta tccaatttgc
13381 aacaactttt gatcctcaat atatgcatat agataaagaa aaagcagaac aaagtagatt
13441 taaaggtatc attgcatctg gcatgcatac actttcaata tcatttaaat tatgggtaga
13501 agaaggtaaa tacggagaag aagttgtagc aggaacacaa atgaataacg ttaaatttat
13561 taaacctgta tacccaggta atacattgta cgttatcgct gaaattacaa ataagaaatc
13621 cataaaaaaa gaaaatggac tcgttacagt gtcactttca acatacaatg aaaatgaaga
13681 aattgtattt aagggagaag taacagcact tattaataat tcataataaa acagtgaagc
13741 aaccatcgtt acggattgct tcactgtttt gttattcatc tatatcgtat tttttattac
13801 cgttctcata tagctcatca tacactttac ctgagatttt ggcattgtag ctagccattc
13861 ctttatcttg tacatcttta acattaatag ccatcatcat gtttggatta tctttatcat
13921 atgatataaa ccacccaatt tgtctgccag tttctccttg tttcattttg agttctgcag
13981 taccggattt gccaattaag tttgcataag atctataaat atcttcttta tgtgttttat
14041 ttacgacttg ttgcatacca tcagttaata gattgatatt ttctttggaa ataatatttt
14101 tcttccaaac tttgtttttc gtgtctttta ataagtgagg tgcgttaata ttgccattat
14161 tttctaatgc gctatagatt gaaaggatct gtactgggtt aatcagtatt tcaccttgtc
14221 cgtaacctga atcagctaat aatatttcat tatctaaatt tttgtttgaa atttgagcat
14281 tataaaatgg ataatcactt ggtatatctt caccaacacc tagttttttc atgccttttt
14341 caaatttctt actgcctaat tcgagtgcta ctctagcaaa gaaaatgtta tctgatgatt
14401 ctattgcttg ttttaagtcg atattaccat ttaccacttc atatcttgta acgttgtaac

14461 caccccaaga tttatctttt tgccaacctt taccatcgat tttataactt gttttatcgt
14521 ctaatgtttt gttatttaac ccaatcattg ctgttaatat tttttgagtt gaacctggtg
14581 aagttgtaat ctggaacttg ttgagcagag gttctttttt atcttcggtt aatttattat
14641 attcttcgtt actcatgcca tacataaatg gatagacgtc atatgaaggt gtgcttacaa
14701 gtgctaataa ttcacctgtt tgagggtgga tagcagtacc tgagccataa tcatttttca
14761 tgttgttata aatactcttt tgaactttag catcaatagt tagttgaata tctttgccat
14821 ctttttcttt tttctctatt aatgtatgtg cgattgtatt gctattatcg tcaacgattg
14881 tgacacgata gccatcttca tgttggagct ttttatcgta aagtttttcg agtccctttt
14941 taccaataac tgcatcatct ttatagcctt tatattcttt ttgttttaat tcttcagagt
15001 taatgggacc aacataacct aatagatgtg aagtcgcttt tcctagagga tagttacgac
15061 tttctgtttc attagttgta agatgaaatt tttttgcgaa atctcttaaa tattcatcca
15121 ttttttttaac ggttttaagt ggaacgaagg tatcatcttg tacccaattt tgatccattt
15181 gttgtttgat atagtcttca gaaatactta gttctttagc gattgcttta taatcttttt
15241 tagatacatt ctttggaacg atgcctatct catatgctgt tcctgtattg gccaattcca
15301 cattgtttcg gtctaaaatt ttaccacgtt ctgattttaa attttcaata tgtatgcttt
15361 ggtctttctg cattcctgga ataatgacgc tatgatccca atctaacttc cacataccat
15421 cttctttaac aaaattaaat tgaacgttgc gatcaatgtt accgtagttt gttttaattt
15481 tatattgagc atctactcgt tttttatttt tagatacttt ttttatttta cgatcctgaa
15541 tgtttatatc tttaacgcct aaactattat atatttttat cggacgttca gtcatttcta
15601 cttcaccatt atcgctttta gaaatataac tgctatcttt ataaacttgt ttgaaatttt
15661 tatcttcaat tgcatcaata gtattattaa tttctttatc ttttgaagca taaaaatata
15721 taccaaaccc gacaactaca actattaaaa taagtggaac aatttttatc tttttcatca
15781 atatcctcct tatataagac tacatttgta atatattaca aatgtagtat ttatgtcaaa
15841 ataatgttat aatttttgtg atatggaggt gtagaaggtg ttatcatctt ttttaatgtt
15901 aagtataatc agttcattgc tcacgatatg tgtaattttt ttagtgagaa tgctctatat
15961 aaaatatacg gttctgttgc aaagttgaat ttatagtata attataacca aaaggagtct
16021 tctgtatgaa ctatttcaga tataaacaat ttaacaagga tgttatcact gtagccgttg
16081 gctactatct aagatataca ttgagttatc gtgatatgtc tgaaatatta agggaacgtg
16141 gtgtaaacgt tcatcattta acggtctagc gttgagttca agaatatgcc ccgattttat
16201 atcaaatttg gaagaaaaag cataaaaaag cttattacaa atggcgtatt gatgagacgt
16261 acatcaaaat aaaaggaaaa tggaactatt tatatcgtgc cattgataca gagggacata
16321 cattagatat ttggttgcgt aagcaacgag ataatcattc agcatatgta tttatcaaac
16381 gtctcattaa acaatttggt aaacctcaaa aggtaattac agattaggca ccttcaacga
16441 aggtcgcaat ggctaaagtc attaaagctt ttaaacttaa acctgactgt cattgtacat
16501 cgaaatatct gaataacctc attgagcaag atcaccgtca tattaaagta agaaagacaa
16561 gatatcaaag tatcaatacg gcaaagaata ctttaaaagg tattgaatgt atttacggtc
16621 tatataaaaa gaaccgcagg tctcttcaga tctgcggatt ttcgccatgc catgaaatta
16681 gcatcatgct aacaagttaa cacaaagtat tattttaaaa ttgagattag acatttattt
16741 ttcaactttg cagcagaacc taattttttc tctttgtaaa taaatgcaaa gagataccat
16801 aaggatctct tacataacca taaccttcag tatagaattc tggactaaat gttttcaata
16861 cctcactgcc tttttctatt aactggtcat atacatgttt agtttcttct acttggtcaa
16921 aagtgagaca aagagatata ttattacctt gtgttatggg caaaccttca gtgtcatctg
16981 cgatcataat ttttatatct ccaaattgaa gtacacattg atcaatttta tttaaatcat
17041 tttcgtcaat attaagtttc ttatctatag gtctatcttt aatacgttga atatacagtg

17101 ttttagcgcc aaacagctct tcatacaact tttttaaacc ctctgcattt tgagtgatta
17161 aaaaatatgg acttacttga aatttcatgt tttttcctcc ttagatttgt tataaataga
17221 gtatatactt aattagtgtc atctattgac actaaaagga gaataataat gaaaaaatct
17281 gttagattat ataatatgat tgaatattgt aatgaaaata ggaacttcaa attaaatgat
17341 ttaatgtcag aatttaatat ttctcgtagt accgctttaa gggatataaa agaaattgaa
17401 gcattaggag tacctttata tagtaatcca gggaaaaatg gtggttatac gatcataggt
17461 aatcgagacc aaacgaaaat agcaatctca gatgaagagt tgaaagcttt agtatttaca
17521 ctttcgagta tttcaaatgt gagtaatcta ccttttcaaa cagaatatca agaaatatta
17581 aaaaaattat ataataactc gaataaaaaa gagttaataa accaatataa tgatctattt
17641 caatatttta atgaagataa gtatcagttc aaaagttata agttatttaa tgaaatcatt
17701 agattgataa ttgagaataa gtcttttgaa acctgttatt cacaaaacta tattaaagaa
17761 caatataaag gtattggcat tatgtataaa aatcatcaat ggtattttgt tgtagctaat
17821 atagaatcaa agttagtgaa tctattaaat atttcgaaaa taaaagaact atatgaaatg
17881 ggagagactc aagagtgtaa tgatataact atgcagaatt ttcaacagtt catggttaaa
17941 aatgaaacag ctattgatat tcttattaga agcaatgtta tgggattgaa tatcttgaaa
18001 ggctacctgt ggagtgacta tatgattgaa aatattgacg aagagacata tttatttaaa
18061 tcaaaagtga acgcgaaaga tatagatttt atagctaagt taattgtcac aggtggtgtc
18121 aatgtaaaag tagagacccc taatagtttg aaaaatgctg ttaaagttga attaactaaa
18181 ataataaaca tgtattaata gtaatttaat cataattgta ataacaaaaa gatgacaaca
18241 ttaaacaatt taaatttata caaaaaagca ttaccaaatt taaaattggg tagtggtagt
18301 gctacagcat acataaatag ttataattaa ataaagatgg gtcaatgaag gtgaattaag
18361 tgaataaaaa tattacagct ctaaaatcta ctgaaaatac tacatatatg cagtatacgg
18421 ttcatatcga tgatcaaatt ataaataata taaaagagtg taccaaaaag tttaaaattt
18481 tgcctatgga agataaaatt ccattatcac cgttgttaca accagaatat gcaggagagg
18541 tacaagattt tattagtaca tatgagcagt ttatgattaa ttttggtaaa gtaatattgg
18601 atagtcaagg cataaaaata cagtttgaaa gtgaatcatt aagtagtatt caacgaggca
18661 ttcaagaaca ttgttactta aatgagcgaa caaatgacat tgatgtgact aaagaatggt
18721 atttatgcaa attttcaaat caaatattag aagaggataa aaatcaattg tataatgctt
18781 taaagcaact catgaatgat tctaaaaata aaaagcaagc tttttggact gaccccaatt
18841 agtgggaatt atataaaaac actttcgttg aattcataat aaaatgaatc atgcggaggt
18901 gttttttcta tgaaaagagt ttcttattca gtagaaacaa agtataaagc agttgaaatg
18961 aaaatagctg gtttttcgac aaaagaaatt atgaaagaat taaatattaa aaatagaaca
19021 caagtggaaa cttggtggcg atggtatcga aatgaggaaa gttatagatt ttcacaacac
19081 gttgggaaac aatataccta cggtaaagga ttagaagaat tgtcagaaat agaacgatta
19141 aaattagaaa ataagagaaa agatatagaa ttggatattt taaaaaagta caaggcattg
19201 gaaaggaagt ggtaccagca gtagtcatag atttagtgga tcaattaaaa gtaaaatatt
19261 caatcaaatt gatactagaa gtattaaaca tacctaaatc aacatattac cgatggaaaa
19321 acaaaaccta taaaaatgat actgtaacac aaaaagttat tgaattatgt gaagctaacc
19381 actataccta cggttatcgt aagattacag cactgattaa tcaatgttat acatcaccaa
19441 ttaatcataa gagagtacag agaattatgc agaagcatca tttgaactgc cgagttagac
19501 ctaaaaagac gacaaggata ggtaaaccgt attataaaac tgacaattta ttacaaagac
19561 aatttaaagc gagttgtccc atggaagtat taacaaccga tattacttat ttaccatttg
19621 gtcattctat gttgtattta tcttcgataa tggatattta taacggagaa attgtggcgt
19681 ataaaataga tgataaacaa gaccaaagtt tagttaatga tacattaaat caaatcgata

19741 tacccgaggg gtgtatatta catagtgatc aaggcagcgt ttatacatct tatgcttatt
19801 atcaattatg cgaagaaaaa ggcattatca gaagtatgtc ccgaaaggga acacctgctg
19861 ataacgcccc gatagaaagt tttcattcct cgctaaagtc tgaaactttt tacatcaata
19921 atcagcttaa tagctctaat catattgtaa tagatattgt cgaaaaatac attaaaaact
19981 ataataataa tcgaattcaa caaaaactag gctacttatc tcctgtaaaa tacagagaat
20041 tagcagccta gaacatagtg tttttattaa gttcccgttt aagggggtca gtaccttttc
20101 aagaggtgtt taaggttcat attgatattt atctctacaa taaagaaaaa tctgaatata
20161 agtatcaagt atctagttat ttcaatcttg taaaagaaaa tcctaaaata acttataaaa
20221 aaagacattt acaagagaaa caaggtgtca agggtacaac tttcacgaac aaattaaaat
20281 tattgaacaa aatgtatggt gtagatgttg caaaatatca gccattttat aattctaata
20341 atcctgaata tgaaagaggt caatttggtg aaagatacat ttctcaaaga tctaactatg
20401 aatttaatag acttcaatat caaattatag atatgttatc gaaaatactt gataagcatc
20461 cattaccaaa gtcagataat aattataaac atattccaac tattgaaaaa gcaatattaa
20521 gtggggattc tcatagtttt tatgagtatt ttgaagatat aatgaaagaa attataagta
20581 tggaaaattc ttctttaaag gaaaaattat tgactgattt tacatatcaa tctcaatgta
20641 gatggtactc tgaaagtgaa aaattaaaat tgcaacttga aagttttatg cataaagttt
20701 tggagagcaa ttattatgag ggtaacaaat tatatagaat gttgtctcat gcaattgaag
20761 aaacaatcaa tgaggctgat gaagataagg ttcatttcaa ttattttaaa gattattttt
20821 tgacagacgg tggggtgaag aattgggagc agattagcga gaaaattact gaattcaatg
20881 gtaaagttat taacgatatc caaaatgaat ataataagat tcaatttaat aacgcaaatc
20941 gaaatcagaa actaaacttt aattatttat accattgctt tgagtttagt aataacttag
21001 taaaagcaag ggtaaatgga aatagaggtt acattttata ttttgtagat aaatataaaa
21061 taaaaatgcc ctttttggat caattattaa tcaattattt gaatcaagaa ataggtcagg
21121 aaagtattaa ttataatatg caaacattgt ttgaaaaaga aagatatgat agaagtagta
21181 ccattgaaaa attagtagca acaagcaaat ttaagtatga aaaagatgat tcagatttat
21241 tcaaacaact tttcaatgat gttgaaaatt caatagacag attaggtatt tacttactaa
21301 ataatggtat aaattcgaat gatgaaaatg caagatatta tagatcgttt ttaaaggaac
21361 ttagtagaat aaaaagtaaa ttaacgccat tttctcttga aataagtaag tctagcggaa
21421 gagagcaaca ttatcctgat gatgctattg atgataaaga tgagagaaga aaaaataaag
21481 aagaaacata tcatgctttt gatgataaaa gcgatattga ctccaaatta aagaataaaa
21541 taaatgtttc tatcgataat ttattgtcag ttaaaattaa gagcaatttg tatgatatta
21601 gtccaaatac aaaaaaacaa taactataat gagccaactc aagaatgagt tggcttttta
21661 attgtaattt tacgcttcca ttacaggaat caatgttatg gaagccatta agcaattaat
21721 ttgagtcatt tatagtatag gtacaacgtt gtgatgttca catgataatc agcccgggag
21781 attattgtgt aataacttat agaaggagca aatcaccatg tatcaagcaa atatacgtga
21841 tttaattaca aaattgcctc aaagcaataa aacagaacac tttttaatga acaaattttc
21901 aaatcaagat aaagttcagc agctacaaag acaaattagc caacagctag atcaacaata
21961 taatgagctt ttggctaatg aaaaagctaa gctagaccaa tacgtggaag tacaccataa
22021 tttagaacca ttaaagaaag agattgaatc agaatctatt aaccttgata ccgataaatt
22081 acctgatatc aaagcgacaa tgcttgaaaa ggctaagaac gatgaacatt ttgataaaat
22141 cgaacagcta tttgatagat tagatcagtc attaaatggt acgaatcgat tatatacgca
22201 attatcgttg attggcacac gaacacatcg gatcacaacg aaaagattta atgttcaagg
22261 cttgcctaaa ttagtccaac aaatgatttt accttcgcaa tttaaaaagg tttatacaat
22321 agattttaaa tcattcgaac catcagttgc tgcgtatatg acacaagacg aacaactgat

22381 tgactacttg aatcatgaag aagggttata cgatgcatta ctgagagact tatctttgtc
22441 aaaagagaag cgcgtgagtg tgaaacgtgc atttataggg tcatttcttt ttggcggtcg
22501 ttatagtagc tctaaattca aaatcaatca agaggttagt gaaattaact ggctacaagt
22561 aatgagcaaa ttcaagaagg tcattgaatt taaggagcaa gtcgaaaaat ataaaacaat
22621 gcctacgcct tacggcattg aacatgatat gagcgcattt caaggtagta gtattatggc
22681 aatttatgta caaacggtag caagctatat tttcaagcac attttgttgg aagtgtacaa
22741 agcacagtgc gaaaaaaaaa cgttcaagat tatagtgccg atacacgatg cgattatgat
22801 tgaatgtaat gataagggga ttgcacaaaa tgtagcacag ctcatgaaag atacagctaa
22861 tcaactgttt aatggtgaat ttgcacatgt gacagtggaa gctttaggag gtatagacaa
22921 tgaataatga tagaggaaaa agtcttcaaa ttccccaaag tacattgtta aaagaaggat
22981 ctatatacgt tgctacgtta cattctgtgt acgagaagaa cttctcaggt gatattaaac
23041 atcagtttac gtatgaagta gaacttaacc aagaaacgca ctatgtaaat cgcaatatta
23101 ccgtaaaatc tatgagtcac caattatcaa ttgctgattg gattaaacgt cacagtaact
23161 ataacgtaaa ccacattaat tatgatccat acattgatcg aaaacatttg gtccttgtag
23221 ggcaatataa cggaaattat tatattcaag atgtagcacc attagatgaa tttggagggg
23281 tattgtaatg aatcatattt tagaaatgtt aataaaatta ttaaaagtgg gtatggaggc
23341 aatcgaccgt aaaggtctga ttgccatcct aacaagtagt attggaaatg atgaaatgga
23401 tgattctgaa caagctgtaa tggtgtataa cgagcttatc gataagctac agcttaacat
23461 tcctaaagat gtcgactata gacctaacat atacagttat tttggtattc aaaaaaagcc
23521 aaatgacaca atattagtgg aaatgatgat atgtattttt catatcaagc gttttgattc
23581 agagttgttt gttttcaaag ataaaggttg gcaaaaggta agtgaagatg aattgcaagg
23641 gttgatatct aaaatgatac aagtgttgct agttgattat aaaccttcac taagtacttt
23701 gaaaaacgta gtagatggca tacagaaatc aacggacata gaaaaacttg ttgagcatag
23761 acagtacatt ggttgtggac gaaatatgtt caatctaaag acctttaaag tggttgataa
23821 tgaccttgaa atattcccta aaacacgctt agatttggaa ttagatataa atgacacgat
23881 tacggacaag atacccccga atttcaaaca atatatgtta gagttggcga attatgacca
23941 tgatttacaa tatttccttt tccaacatat ggcagtgtta ttgacggcag atactaaact
24001 acgtcgtgga cttttttgt atggaactgc aaaaaatggg aaatcggtct atattaaatt
24061 agttaagtca ttcttttata gtaatgatat cgtatctaaa acacttaatg aacttggcgg
24121 gcgtttcgat aaggaaagtc taattggtaa acgaattatg gcaagtgatg aagtggggaa
24181 agctaatatt gatgaagcaa ctgtgaatga tttcaaaaaa ttactatctg ttgaaccaat
24241 tcatgctgac cgtaaaggaa gaacgcaagt agaagttact ttagatttaa aactcatttt
24301 taatacgaat gctgtactca attttccatc atcacatgca aaagcattag agcgtagaat
24361 tgctgttatt ccatgtgaat attatgttga aaaagctgac cctgacttaa ttgaaaagtt
24421 acaggatgaa aagaaagaaa tctttcttta cttgatgtat gtgtataagc aaattgtaaa
24481 aaatgatatc gagtacctcc aaaatgatcg tgttactgaa atttctcatg attggttaaa
24541 ttttggatat gaatttgttt ctagtaaatc agcaaatattt gcacatcaga aagcgtgtat
24601 taatttactc agaaaactta tagaaatcaa accagggtca cgtatcaaag tgtctaggct
24661 aaatgaggtt attagagatg aaattaaagt aagctctcaa gttattaatg atttggttca
24721 agctaacttt aatgtacaaa gtagactaaa taatggttat aagtattggg tcgatttagg
24781 atggaaagaa actgataaaa aagatgacat gatttcattc gataaaaatg agaatgtaac
24841 agatgatgaa ttcttatacg aagatgattt gaacttaggt tgggaggact ttgacgatga
24901 ataatgaaca aattgaagca tttgtagaag tgcttgtgcc tatcatagaa gaacgtatca
24961 ataaaggtaa gtaatctaat tacgtactac aggcagttgc ctgtagtact catatgatta

25021 agtggtaaaa gtgataaaaa tgaaacgaaa ttataaatat atattatcta tatgttgtta
25081 caagaccgat ggtctgtagc aataatctaa taaaaggagc ggtatgatat gaagggtaaa
25141 attgcacttt attcacgtgt tagtacgtct gagcagtcag aacatggtta ttctgaaaag
25201 gagcaggaac aactactcat caaagaagtt atgaaaaatt tcccaggtta tgactatgag
25261 acatatactg actcaggcat ttcaggtaaa aatattgaag gtcgtccggc aatgaaacgt
25321 ctattacaag atgttaagga taataaaatc gaaatggtat taagttggaa attgaatcgt
25381 atttctcgct caatgagaga cgtgtttaat attattcatg aattcaaaga acatgacgta
25441 gggtataaat cgatttctga gaatattgat acatccaatg cttctggaga agtactcgtt
25501 acaatgtttg gactaatagg atctatagaa cgccagactt tgatttccaa tgtgaaactt
25561 tctatgaatg ctaaggcaag gagcggagag gcaatcaccg gtcgtgtttt aggctacaaa
25621 ttatcactta atccattgac acagaaaaat gatttagtta ttgatgaaaa tgaagctcat
25681 attgtacggg aaatctttga tttatatttg aatcacaata aaggatttaa agcaatcacg
25741 acaattctaa atcaaaaagg atatcgtacc attaatcaaa aaccattttc agtgtttggc
25801 gtgaaataca ttttgaataa tccagtctat aaaggctatg tcagattcaa taatcatcaa
25861 aattgggctg ttcagcgaag aagtggtaaa agtgataaaa atgatgtgat attggtcaaa
25921 ggtaagcatg aagccattat aagtgaagat gtatttgatc aagttcatga gaaactagct
25981 tctaaaagtt ttaaaccggg tcgacctatt ggtggagatt tctacttacg tggccttatt
26041 aaatgcccag aatgcggaaa taatatggta tgtcgacgga cgtattataa aacgaaaaag
26101 tccaaagaac gcacaatcaa acgctattac atttgttcat tattcaatcg ctcaggaagt
26161 tctgcctgtc atagtaatgc gattaatgct gaagtcgtcg aacgcgtaat caatgttcat
26221 ttgaatcgta ttctttcaca acctaatgtt attaagcaga ttgcgtcaag tgtgatagaa
26281 gaactgaaac aaaagcatag taaacaaaca gaaataaaat atgatattga tagtctagaa
26341 aaacaaaaag caaaagttaa aacacaacaa gaacgattat tggaattgtt cttagatgat
26401 gaaatggata gcgaaatgtt aaaagctaaa caaagtgaaa tgaatcaaca gttagaagta
26461 ttagaccaac aaattaaaga agcaaaacaa gcaaatcaat cacaggatga tatacccaat
26521 tttgataagt taaaagcacg actcattttg atgataacac gattcagtgt gtacttaaga
26581 aaggctacac ccgaagctaa aaatcaactt atgaaaatgt taattgattc aattgaaatt
26641 acgacagata aacaagtaaa acttgtaagg tataaaattg atgaaagtct tatccctcaa
26701 tctttgaaaa aagattgggg gtcttttttt atacctaaat ttaactttgt gataaatgtc
26761 acaaagaaaa ataggattga aaatttatca cttttaccac ttttttagag tgacaaaagt
26821 ggaggagttt tgaaatattt tataaatata tattttattt atggagtaca cattattaat
26881 taaggaggtc attataatga cgctaagcaa acaacttaaa acgtatatca ctgaacgatt
26941 taaattaaat tatcaagaaa cttgggcttg tgaaaccata gatgcggtgg ctgaagatgt
27001 attacctgaa aaatatatta aaaatagtcc acttgaacat aaaattttaa atacttttac
27061 ctattacaat gatgaattac atgaaatcag catttaccct tttttatgtt atctagataa
27121 ggaattagta gcaataggtt atttagataa ttttgattta gactttatat ttttaaatga
27181 cactcatcaa gtcattattg atgaacgcta cttgttacaa aaagggggcg agtaattatg
27241 aattggatca aggtcgctca actatctgtc acagttatca atgaagtcat tgagattatg
27301 aaagaaaaac aaaatggagg ggaatagtat gaaaatcaat cgatacatta caagaggtgt
27361 tagtgaacat ttatctctag accttcaaat cttactttgg aacatggtaa aagatcgaga
27421 caatcaactt catacagatt acctacacat ttttaaacta caagaagatg ataatatact
27481 ctcaatcaca catgaacaag aacaacccgc atacaaattg gaatatcact atacaaacta
27541 tgtaaaaaat caaaatgcat tacctaagaa agtctacgtc atccgagaag atgatgtaga
27601 cgctttttat tatgtgatgc ttttaccaga agaatactaa aaggagtgac aatgatgaat

27661 acaatcaaaa atacaataca cacagaagcg atatttagcg atgatgaaca acaccgctac
27721 ttactcaaaa agacttggga tgaaaagaaa cctgcatgta cggtaatcac catgtaccct
27781 catctagatg gcgtattatc actcgatctt acaactgttc ttatcctcaa tcaattagcg
27841 aattctgaac aatatggcgc tgtatatctt gtaaatctat tttctaatat taaaacccca
27901 gagaacctta aacatattaa agaaccttat gataaacaca cagacattca tttaatgaaa
27961 gcgataagtg aaagtgacac agtgatttta gcctatggtg cttatgcgaa gcgacccgtt
28021 gttgtcgaac gcgttgagca agtgatggaa atgttaaaac ctcataaaaa gaaagtaaaa
28081 aagctcataa atccggcaac gaatgaaatt atgcatccgc ttaatcctaa agcgcgtcaa
28141 aaatggacat tgaaataaag gaggattatc tatgaaccat gaaactaaac aatcagattg
28201 gcgaacggtt gctagttgtt tagcatcaca aaattatata tcgattgtaa aaggtttagt
28261 acatcatttt acagcgatcg aagatgagga aattcttgat aaaatctatg atgattttat
28321 gaatgatgac tctataacaa cggtacttaa caatgattta cagacaatca ttaaccatta
28381 tctatcaaaa taaagactat actaattaca ataaaaacag agcatccttc acttttatgg
28441 tgagggatgc tcttttaatt tattttttta ttgattattc gccataagtt ctgcttgctc
28501 tacaacctgc tctacagcca ttttttgtaa atctggtgga tagccatatt ttttaagcaa
28561 tcgtctaaca gctacgcgca ttttagcttt tgcgctatca cgtttagacc aatcaacacc
28621 catgttttct ttcactgttt tagttagctc atgagcaatc gcacgtagtt ctttatctcc
28681 catggcttct tttgctgttt catgtgaagc taaagcatcg taaaacgcaa tctcatctga
28741 attcaggcct aattcatttc ctcgttgttg ttcttgttta atatctttag cgagttgaat
28801 aagttcttca atcactttag atgtttcaat ggaacgacta ttatatttat taatcgaatt
28861 tcctaacatc tcagagaaac gcttagatac cgttgcattc gttttcatta atgatttaac
28921 ttgtcctttg agtaatcgat ttaataattc taccgccaca tttttctgtt tcaatccttc
28981 gacatctttt aaaaagtcat cagataagat tgataaatcg ggttgttcaa gacctagcgt
29041 ttgataaaca tcaatgacat cttcagtcac aacagattgt gacacaagtt gattaatctc
29101 tgcttcaact tctgcaggtg ttttacgtgt ttttccttct tttggcggtt gtaacaattt
29161 aacaagtcct gctttaactg ctttaaagaa ggcaatctca tcgttgagtt cttgggctgt
29221 aggttcagtc gcacaaagag caaaggcttt ccctaattct gtgactgttt taataaaacg
29281 ttgacgttca tcttcaccta aaccaattac ataatccatc gtattcgata ttgtataata
29341 acgttcagat tttttatctg aattaaattt agagtaatca agattatata acatatcttg
29401 aattacatca tacttcaata acatcaattc aaccgcttta tccgtatcta ttgccgtttg
29461 agcttgatca gattctgtat attctttaag tgcctctttt aaactttcgg caatccccac
29521 ataatcgaca attaaaccac ccggcttatc tttaaacact cgattaactc gagcaattgc
29581 ttgcatcaaa ttatgccctt tcatcggttt atcaatatac atcgtatgca tagaaggaac
29641 atcaaaccct gtcagccaca tatctcgaac aatcacgagt tgtaactcat catccacatc
29701 tttcatacgt ttttctaata aattacggcg ttttttagga ccaatgtgtc tttggaaaga
29761 agttgggtca ctagatgagc ctgtcattac cactttaatg actcctttat catcatcatc
29821 tgaatgccat tctggtttta gacgaatgat ttcatcatat aaatcaacag caattcgacg
29881 actcatcgtt acaatcattc ctttgccttt catcgcttgt tgacgtgttt caaagtgttg
29941 gatgatatct ttggctaggg cttctacacg aggttttgcg cctgctaaag cttcaatacg
30001 ggaccatttt gattttaaac gctgttttac atcctcttct tgatcttcag taatgtcatt
30061 atacgcttca tctaaatcta aactttgagg tagatttaat ggaattacac gactttcata
30121 gtaaatttta acggtgcttc catcagcgac ggcttgtgtc atatcataaa catcgatata
30181 gtttccgaaa accatttgcg tatttttatc cgttgaagct acgggtgtgc ctgtaaatcc
30241 tacgaatgtt gcattcggta aagcatctct taaatatttg gcataaccat atttaatgcc

30301 ttcaccttta tcatcgtatt ttgcgttaaa gccatattgt gtacgatgag cttcgtctgc
30361 catgacaatc acattttttc gttctgttag ggcagccatg gtcgtttcgt tttgttcagg
30421 ttcaaatttt tgcatcgttg taaatacaat accacccgac tcaacagata ataatgattt
30481 taattcttta cgtgtttcag cttgttttgg tgtttgtctt aataatcctt taccagaacg
30541 tccttttgat ttaacaaacg tactgtacag ttggttatct aaatcattac gatccgttac
30601 gacgactaag gtagggttat tcagcatttg aattaatttt ccagagaaaa agaccatggt
30661 taaactttta ccggaccctt gggtatgcca aataacgccg cctttaccat caccttgctc
30721 agatgaagct aataaagctc tgtcaacggc tttattaacg gcataatatt gatgatacgc
30781 tgctagaatt ttactgatat gtcctttgcc atcatcttga aataaaacaa aatatcgaat
30841 taaatcaagt aatgtttctg gatttaacat gccatgaatg agtacgtcta agctagctaa
30901 acctggtgat gattcagttt gaccatcttt actgcgccaa gtcataaacc gatcataatt
30961 tgcggttaga gagccagcct ttgtattaat accatcactt gtaacaagca cttcgttaaa
31021 cgtaaataat tgtggaatac gcatcttata cgtttctaat tgatgataac cgtcttcgac
31081 gcccacggtt tcatttgttg aatttttaag ttcaatcact acgataggca agccattgat
31141 aaagagtaca atatcgggac gttttttata gtctccatta acgatagtta attgattgac
31201 tgctaaaaaa tcattgtttt gtggatattc aaaatcaata attttaacaa tttctacaat
31261 cgattgtcct tcatcatcat agtcttcgat ttcaatacca ttaatcaaat tttcatgaaa
31321 ggtaaggtta ttttctaaaa gattgggcga cttctctaaa gttagttcat gtagcgcttt
31381 ttcaataaaa cgaggatgga tatctgagtt gatttttctt aatgctttct ctaaacgttc
31441 atgaaggaca acatctttat cacttttacg ttctggtgtt agacttgtca tactaatctc
31501 attacctttt ttatagtcat agcctagtga ttgtagccat tctaatgcga cttgttctaa
31561 atcatcttca ttaaattgaa agttcatcac tattcacctc aatatcatca gatatttcta
31621 gttctccaga cattagttta ggaagaagag tatctcgtaa ttgagttaat ttaccaattt
31681 ctgatcttaa tattttcatt tcttccatat atggagtgat tatttcagaa tattttttta
31741 caatactatc ttcaattgct aatttataat taacagctat tgttggtttt actctttgtc
31801 tactatttgt tgaaccggta gtattagctt ttaaataatc tataaattga gaattaagac
31861 atatattata tatgaatgaa ttaattttat tattaggaga ttccattaca acaaattctg
31921 atgaagcaac atttaatttt ttattatcta ttacaggtaa ccaaatcctt ttagtatcag
31981 gattcatttt tgaaaataat acgcaattgt tattaataat ccatttatta ctttttattt
32041 tattgacttc ttcctctata gcttgttctt cattatcata agcaggtaaa ctaaaatgtt
32101 taactgttac ttcttcactc tttttaggat taaaagtttc ctttttatga cttgctatat
32161 cttttaattt atagattttc caattacttg gtatcttacc taactcacta tcaatcattt
32221 ctcctccact agatttataa ggattaccat cttcatctgg aaattcaaaa tctacaaacc
32281 aatgcttaaa tagtgtttgt gaaagttctt taaggtttgc tatcattttt tggttagttt
32341 ctatttttct tgtgatttga tttaaaaatt tagaaatgct tttttgtttt attaaagaaa
32401 aattaggaat tttgacagtt tcaataagtt tagaatttat attagcttga ttagcactcc
32461 ctgtagaatt tctaactaaa tactcagtta ttttaggttg tagaaaataa taaaataaaa
32521 aatctaagtc tgcactatta ctatatttta attcgaattt tcccacacgt tgattttgta
32581 atgctggata atcaaattca attcttccaa cttttcctac tgcagaagac atttgattca
32641 cgcctgatcc agttaatgat attaatatat catttttctt taatgaaaaa cctttagttt
32701 tttcatatgt tttaatagag actttttgag agtcttttgt atcaacaatg ggtgaaatta
32761 tattcttaat tttaataacc ggtatacctt tttcttgaaa ttcttttgat ttaaaagcat
32821 atccattttt aattttagct acatcaccga aaaaaattgt attatactcc atatcctaac
32881 ccctccaatg attttcggat ttggtcttca agttcttttg attttgcgaa ttgttcactt

32941 aattcagaag taatacgttc cattttttgt tcaaaaggtt cttcatcttc ttcaacgtca
33001 gctaaaccaa catatcgtcc aggtgttaaa atgtattcat tattcttaac ttcttctaaa
33061 ttagctactt tacaaaaacc agctatatct tcataaggtt tatcgtttgt acctctccac
33121 gcatgatacg tctgtgctac tttttgaata tcttcatctg aaaattcttt taatgttcta
33181 gataccatat ggccgatttc acgagcatcg ataaataaaa tttcattttc acgctctttt
33241 ttaccatttt gacctttatt attactaatg aaccaaagac atacaggtat ttgggttgag
33301 taaaagagtt gtccaggtaa agtaacaata cattcaacta aatcttgttc aattagattt
33361 ttacgaattt ctaattcatc ttttccgctt gtagacattg atccgttggc taatacgaat
33421 cctgctgtac cattaggtgc taattttgaa atcatatgtt caatccatgc atagttagca
33481 ttaccttttg gtggaatacc aaattgccaa cggtaatcat caagtaatcg ttcttgaccc
33541 caatcacttg cgttaaaagg aggattggct aatatgtaat ctgcttttaa tcctttatgt
33601 aaatcattat ggaatgtatc agcatttcgt tcacctaagt cattatcaat accacgaatc
33661 gctaagttca tttttgctaa tttccaagtc gtagggttag attcttgtcc gtaaatcgca
33721 atatcgtcta atcgaccttg atgtttctca acgaagcgtt cactttgtac aaacatccca
33781 cctgaaccgc aacacggatc gtaaatacgg cctttataag gctcaatcat ctcaactaat
33841 aactttacaa ttgatgacgg ggtatagaat tctccagcat ttttaccttc ggcacttgcg
33901 aatttagcaa taaagtactc atacacacga cctaacacat cttgtttacg actctctgtg
33961 tcgccaactt taaatgtaaa taaatcaata atatcgccta atttttcttt atctaatgca
34021 ggtcttgcat attctttagg taatacacct ttgagtgatt cattttcatt ttcaattgca
34081 atcattgctt tatcaataat ttgtccaatt tctggttttt tcgcattatc attgatatat
34141 tgccatcttg cttcttttgg cacccagaaa atgttttctg ctaagtattc atcttggtcc
34201 tcttcatctg catatggatc ctgcttcaat tcttcatact tttcttcaaa agaatctgat
34261 acatatttta aaaagattaa acctagtgct acatttttat actcagcagc atccatactt
34321 cctctcaatt tatcggcagc ttgccataat ttttcttcaa aacctatagt ggccattaat
34381 aacacctctt aaaattaatg ttaaatcaac tatagcaaaa aatagaagat gattgtgtag
34441 tgttttatta atatttaatt tatctagact atcaaaaaaa tatagattat tattatctta
34501 aattaataaa caggagtgaa ttaaaatgaa taaaaaatat aaattaaaac gatacgataa
34561 aagatttaga ggtggatggt gttacaaatg tgaagattca tattcttacc tttgtgaaga
34621 ttgtggttat tgtagatatt gttgccattg ttaatacaag actagataaa ttgggtatct
34681 ttaactcaat taataaaaaa tttaaatgta gtcaatagct tgctaacttt atttgatgta
34741 atataatttt ggcttttagc attcagttaa aaagaagtaa ataattgaca gattttttag
34801 agaactggag aaaaaagatg tacatttcta gtgttaaaat taagaatttt agaaattttg
34861 ataatatcga tattgatttc catgagggag ttaatgtatt aatcggacat aataattcgg
34921 ggaaatcaaa tttattacgt gctttatctt taatttttga cggttcagtt agaaagcaac
34981 tgtcagtgga agattttaat aactctttaa caaaagaatc tttaaaaaaa gaggctccaa
35041 aaatagtaat ttcagtacat ataacacaat ctgaaaacga aagactgatg tctgatgagt
35101 taattacggt aagtaattgg ctgactaaat tagaagaacc ttatgaagct agaatacaat
35161 atgaattctt tttaccaaaa gatgaggaaa aaaattacat agatttagta aaaaatatag
35221 atgaaaaaga agaaatatgg aatttaatta gaagccagtt tattaggttg tatgtcaata
35281 aaatctgggt gggtaatcca gaacatcaga ttcctataga taatgatagt ttaaataaat
35341 ttgatttcca atttttagac gcaataagag atgtagagag agatatgttt agtggtaaaa
35401 atacattatt aaaaagtgta atagattttt ttatagatta cgatataaaa tccgatgaaa
35461 caatcacaga agaagaacag aaagaaaaat tagaagaaag aagaaaagaa ttttctgata
35521 attctagtga tttgattgaa actattcata aaagattaga tagtgggaat caaaaaattt

35581 tgtcttatac aaatggcata ggagcatctt atgataaatc aacaccggat tttaaaggta
35641 atcttacaga aagtgaaatt tatactgtat tgcaattgat aattaaacat gaaactggaa
35701 tgactttacc tataactcat aacgggttgg gttacaataa tttaattttc atggccttgc
35761 tattgtctaa aatgcaagct gattctgatg gaaattttct aggtagtaat gcaaaagtat
35821 ttccgatact agctattgaa gaaccagagg ctcatctaca tcctactatg caaaatgaat
35881 ttattaaatt tttaaaaaat aacattagag aaaaaaaggt aaagcagatt ttcataacta
35941 ctcattcaac acatatttct tcttctacaa atattgatga tattatttgt ctttacacag
36001 atgatagtaa aactaatgtt agttatcctg gaaaagtttt tgataagaat aatcaaagta
36061 agaagtacgt acaacggttt ttagatgcca ctaagtcaaa tatgctattt gcagaaaagg
36121 taatatttgt ggaaggtata gcagaacaat tactattaca tatatttgca gactatttaa
36181 ataaaccgtt agaaaagaat catgttgcag taataaatgt aggcgggaga tattttaatc
36241 attttttatc tttgtttgat agtaataata aatatgcaat taatcgtaag gtttcttgta
36301 taacagattt agatccaatg cgaaaagaaa aaaatgaaaa tggacaaaaa aataatttta
36361 aagcatgcta tccttttgaa tatgaaatgg atttagataa atttgattat tcaactaata
36421 attctctaga taaatatcta aataatgagc acagtaatat acgagtattt gttcaaccta
36481 ataaatatgg aaaaacattt gagtaccaac ttatgtttga caatccttca ttaaaactgt
36541 tacttacaga ctcgatttct aatagtcaag agctagcaga attaatggat cactatgaag
36601 aaaatgaatc tttacaaaaa ttaattgata cattatcacc atctaatgaa aatcagagaa
36661 taataaaatc tttaaaagac acaaatgaca gttggaatga agataataaa aagaaagcat
36721 taatagcatc aagataccta aattccatag gaaaaggaga aaatgctcta gagctagctt
36781 cagtattaaa agataattta gagttaaaag gccagagtga ctatgaagat tttgtagtac
36841 caaaatatat agaaggtgca attcgatggg tgtgtgaata atatgattat taatgctcaa
36901 acgaaattaa atatagaaca agattttaaa atacaggctg gtcctggtgc tggaaaaaca
36961 gaatttttgg tgaatcatat caaaaatgta atacaaactt ctgaaaagct cgaacgtatg
37021 aagaaaatag cgtgtattac ttatacgaat acggctgctc agacagtttt acaaagatta
37081 ggaaaaagtg tttcaaatag agtagatata tctacaatac atagcttttt gtatagaaac
37141 gtagttaaac catattgttc ttttctaccc aaagaatacg gaatttgtgt gcaaaaatta
37201 aaaggacata gtgatccaat tgtgattaac aaatatgtaa atgcatggtt aaaaactgaa
37261 tattttacga aattaaaacc gcctagtaat agaaatcaac tactaaaaat gccagtacta
37321 aagaaagctt tgcaaaactg gctactatct atgaaacttt cttacgaaaa taatgatgtg
37381 caatttattt gtgataatac caaagctcaa gcaattgata aaaaaagtaa aaaaagaatg
37441 ggaataaata aatcaaactt aaatatacta gaaaagcaat tattaaatta taaaaaaata
37501 tattgggaga aaggaattat cgatcatgat gatgttttgt ttttttcata tgtattaatt
37561 agagattacc cattcattct cactatttta tgtgctaaat atccctactt ttttatagat
37621 gaatttcaag atactagtca attacaagat tttattatag ataaaataag acagaaaggc
37681 tgcatagtgg gggttattgg cgataaggca caggcaattt acagctttca aggtgcacgg
37741 gtttccttat ttgagaagtt taaagttgat ttaataaatt ctcacactat cttagaaaat
37801 catagaagtt catatcaaat agttactttt ttaaatagta tcagagaaga tattactcaa
37861 acagtatata gtggtattga aaatgataaa gttacaattt tgattggaaa tcggattcgc
37921 agttatacta aggtagttaa tatttgtaac aaggagtcac ttgttacctt gtcaagagat
37981 aatattactt ctaatgcaat gagaataaag ttggaaggaa ataattttga taaaaagtta
38041 ttggaaaaat ttcaagaaat tgatagtaat agtaaaagaa ggaatactat tatttcgctt
38101 ataaaagcta ttgaattagc taagaatact aaatataaag atgccttaaa aatagtcgaa
38161 tcgatttata aagatgaaaa taatcctaaa aaaatagctc ttaattctct aataaatatg

38221 attaataaat atgataaata cggtaatggt acattaatga atttctacaa tactttatgt
38281 aaaactttag atattaaact ttcagggttt agaaaggggc aagtaaaaga tttttacgaa
38341 aacactttat ataaaaatat ggtgatatgt acaaatatcg ttgaagacac aagtaatcat
38401 attactatcc acaaagcgaa aggcgcagaa ttcgaaaatg tatttgttat aggtaataaa
38461 gacactttaa gtctactttt gaaaccagat ttaatgaacg atgaagagca tcgtatattc
38521 tatgtagcta tgagtcgagc aaagaagaaa ctttttttac aatttgatta tcttgataat
38581 aatgaacaaa agaaaattaa ggaacgttat gatgtaagta ttatacaata acctaatcta
38641 agaatattta aatccaatat atttcttgat tataattcaa taatagataa aatctataaa
38701 cgattaaaaa gaaaatatat ttgctaagac ttataaaaat gaagagataa tagattttcc
38761 taaattacag ttaaaagaag taaatagatt gaacactttg aaagattcta atccaaaatt
38821 tgaaccacta aaattgaaag atgaagaaga tgcaagatgt agccatcaac gctgatcaca
38881 aagtattttt cttaatgatt atttttaatg ttatcatgct cgtttcttct attattcaaa
38941 tgattttagg aaaaggcaga cgtatcacac cagcaaaacc tactaaagat gctaagttac
39001 acttagataa tcaatcctca taaagagaaa aaagaacccc taaaaagttc aactttttgg
39061 gggttactta ctattaagaa gtatttttga ttaagcatct ttcatatcgt aatcaccaat
39121 atactgatca aggcgattta ttgtttctct gatatcatca tgtgtcgtta atgggttcgt
39181 tgtacataaa cgaatgacac gttggttatt caatactgtg gtataagcaa tggcataacc
39241 agaatctgca attttttgcg cagccatgct attgagttga ttattttgtg cttcagttaa
39301 atcactgttt tcatatctga agttcacaat ggaaagatta gcgtgtgaga taatacgcca
39361 gttatcgaga cctgacacgt attcttctgc atattctgct aaaccttcgc cgtatttaat
39421 gcgctcgata atttcatctt caccaatgac ttgcagagta atccataatt ttaaagcacg
39481 tgcagggcgt gtaagttcaa tgcctaacat ttctggatca atcacatcat catctgaagc
39541 aatatcatcc aagtactcag catcttcgcc aaagctttgc agcagatgat tcttttcttt
39601 cacgattacc atagcgcaac tatatgtttg gaacagtaat ttgtgagcat cccaactggc
39661 actatctgaa cgttcaatgc ctttaaataa gtgttgtgct ttatcggata agatatgtga
39721 tagtccataa gcaccgtcta catggagcca tagattgtag tgatcacatg tatctccaag
39781 tgtggtgaaa tcatcgacag aacctgtatt agtcgtgcct gcagtcgcaa tcaccatagc
39841 aggtttgtaa ccttcttcaa tatctttatc aattgcggct tttaagctat ctgtattcat
39901 tgtaaagtca tcgttgtact caattctgcg gatattattt ttaaggaagc ctgcaacatg
39961 caaggctttg cctacagaat gatgcgtttg agatgtaaga taaacggttg cttttttaat
40021 atcttccatt tctacctgtg catctctagc cgcaacgata gctgttaaat tggccattga
40081 accgccagat acaaagacac caccggccgg tttgatctca taaccgattt ttcctaccaa
40141 gtagttaatt aaattacgct caatattaat tggcagcgtc gcattggcaa aattagaggc
40201 atggatatta tttgctgttg ttaaaatgtc tccaagccat gatagacgtg atgctggacc
40261 tggtataaat gagaatgaac gtggatggtt cggacgatat aaataattca gtacttcatt
40321 atttaaatct ttgagtactt catagatatc tcgcccttta ttcggcactt ccatcttttc
40381 atatttctct ctcaattcga gcggtgcctg ttgtgtcgca ggtaagtctc caatatctct
40441 agataaatat ttattaactt catctctgat aatcgtctct aagtcgatat tgttctcatt
40501 gaattccatt tccatactct ctccttcaat tatattttta ataaaagaca gacgtattaa
40561 ttacgacgtt ttactacaat ataaatgata gcagatatta ataaaaccac gctaaccata
40621 atgacgaaac ccagaaggtg tcgccccaat gaataacgtg ctgtgcttta acactcggtg
40681 tatgtgcgcc tttcagaaat tgacgagggt aatcgacttg cagattatca tctgtgattt
40741 tcaatttgta ttgatgcttg tcttttggaa ttcgataact accccgtaga acaggggacg
40801 aggacctcat cttgatgata ccaattggct tggttaaaaa gaaatcgata atcctcgtag

40861 aatagagaaa aagtattaaa aatatatttg atttgataaa atattttgca aaaaagagaa
40921 acaaaaataa ctttcatata aataatttct ttgcgattaa gttggaatta gaaatactaa
40981 atacaaataa cagcgaatgg aatgcatatt attgaggaat tggatgtgta gtgcaatttc
41041 gagagttact acttaactta agaaaataaa aagaagaaaa caaacaataa gaattaaatc
41101 tgaagcaaga tataaaagca aatatttttg ctacaattat attagtaaat actttattaa
41161 ttagcatacg actgaaagcg aagtaacagc taataatgtt ccagatatag acattagtgt
41221 tatgaaagaa caatattacc at
//
LOCUS Staphylococcus 38493 bp DNA linear UNK
DEFINITION Staphylococcaceae Staphylococcus coagulans strain 1031373
ACCESSION Staphylococcus
KEYWORDS .
SOURCE Staphylococcaceae Staphylococcus
ORGANISM Staphylococcaceae Staphylococcus
Bacteria; Bacillati; Bacillota; Bacilli; Bacillales;
Staphylococcaceae; Staphylococcus.
FEATURES Location/Qualifiers
source 1..38493
/genome_id="74706.92"
/mol_type="genomic DNA"
/db_xref="taxon:74706"
/organism="Staphylococcaceae Staphylococcus"
/genome_md5=""
/project="hwwang_74706"
CDS 13..492
/transl_table=11
/translation="MKITVVAVGKLKEKYWKQAIAEYSKRLGAYTKLEMIEVPDEKAP

ETMSDKEVEQVKQKEGQRILSKIKPQSTVITLEIEGKMLSSEALATTLNQRMTQGASD

FTFVIGGSNGLHQDVLNRSNFALSFSKMTFPHQMMRVILLEQVYRAFKIIRGEAYHK"
/db_xref="SEED:fig|74706.92.peg.1"
/product="23S rRNA
(pseudouridine(1915)-N(3))-methyltransferase (EC
2.1.1.177)"
/EC_number="2.1.1.177"
CDS 738..1043
/translation="MKGNIQCPRLIKGSLLDFVLETVDVFVNGSKNDCELVNFNYKEG
KVVSRYSTKFESNPKLRKKQLISTVKVVRYVIFDFEKILWGIGKVLLKFIILRQCLL"
/db_xref="SEED:fig|74706.92.peg.2"
/product="hypothetical protein"
/transl_table=11
CDS 1242..2105
/transl_table=11

/product="hypothetical protein"

/translation="MKHINQFSESTLAQLEKDQQHVFYVYCLMDPRNDECFYVGKGKG
NRIFKHKQDAQKKVLYEDIFIEENKDNLKFNRINEICSNDLNVLGYIISYGLTESEAF

SAENVLINFLNLTNKTTLTNMINGHGSKAYLVEDLENEFGYDSINLENINTNELILAV

KIRDAFLLDKDESKEYPINESKRDRNNLKSRTLGSWIIGKDKIHKIKYIIGINTGANN

AVVSAYEVSFEQAESIETNNGRMRYAFIGLSERDATLKKLNLYKKALPDLRFGSGSAT
AYINNGTMKVD"
/db_xref="SEED:fig|74706.92.peg.3"
CDS 2213..3688
/translation="MPSDDIVKKLYSKEDIRLELGLTPHKFNKKMETIAKLFKIDMKI

FHNYKGQDKNNQYTFNGVAKELIKVLLKSVDYYPVDINSKKFKQNGKSKKEMIENIDN

SSYMKYIYQLMKSINEIQYKRLIADIHMKDVYQNTKAWLNNGESINKKEQELYQYMTI

LPLHKRVELQNEVLKSIDETIFQFLAKEHRNNQIEENNELEAYTKAIKEGRNPKNDYE

LNHLLYKKNQLPLDSLIEDIWDYEETEYTELDWLIADMLKRSQRADSNFIESLEKKNK

LRKNIHKDSIKSISKLIDHELVKNNRKWDVAEFYKNQQFWNNSTLRRSSQNFGILYYK

KNRLHTMNQSNIIKKIKIIEANIESANKQPSYLDFFLQAKFDLERLESELMKYSINPK

YFNKINSDYVRYAIEDVHNAIIKIDRYISNDKVKLNYSNVQMVVRNEITEQGSYFVTQ
ALNTLSKVEESGYSFDNFMVGPFVEDFKKAIKNRRFGSKKD"
/db_xref="SEED:fig|74706.92.peg.4"
/product="hypothetical protein"
/transl_table=11
CDS 3914..5014
/product="hypothetical protein"
/db_xref="SEED:fig|74706.92.peg.5"

/translation="METRDKLMSLTQSDKTQQWLMDKSSNQDDIQQLQQQFSQQLDQQ

YNALLADEKAKLDQYVEVHQGLESLKEEIESEPITLNIDKLPDIKATMLERAKNDEHS

DKIEKLFDRLEQALNGTNRLYTQLSLIGTRTHRITTKNFNLQGLPKAVQHTILPSKFK

KVYTVDFKSFEPSVAAYMTQDSKLIDLLNQKDGLYDALLSELGLSDELRVFVKRAFIG

SFLFGGNFKNPKFKLNQYVSEVQWLDAVSQFTKVIELKKHVEKSNSMPMPYSIEHDMS

AFQGSSIMAIYVQTVASYIFKHILLKVYKAQCDQKTFKIIVPIHDAIMIECEDEEIAQ
NVGQLMKDTANQLFNGEFAHVTVEEIGGVDHE"
/transl_table=11
CDS 5007..5378
/translation="MNNDRGQSLHIPSSTPIKENNIYVATLHSVIQTDFSGEIKHQFT

YEIEVNNQIVYANRNIPTKPSANQLSIHDWLKRHSNYSASHENYEPYIDQKHLILLGQ
YNGNYYVQDVASLDAFGGVLS"
/db_xref="SEED:fig|74706.92.peg.6"
/product="hypothetical protein"
/transl_table=11
CDS 5375..7018
/transl_table=11
/translation="MNHILQMLSKLLSVAKEAIDRQGLIAILTIPVNNNDEIEETAQG

ETVYNELIDQLRLNIPKDTDYQPNIYSYFGIKKNPNDTVLMEMMIKVFHIKRFNSELF

IFKVNGWQKINGDELQGLISKMIQVLLVDYKPSLSTLKNVVDGLQKSTDVEELVENER

YIGCGENMFDLNTFQVVKNSIDIFPKTRLNLSLSTNDVITDKIPPYFKQYMLQLANYD

DDLQYFLFQHTAVLLTADTKYRRGLILYGGAKNGKSVYIELVKSFFYSKDIVSKPLNE

LEGRFDKESLIDKSLMASHEIGQSRIQEKIVNDFKKLLSVESMHVDRKGKTQVEVILD
LKLIFSTNAILNFPPEHAKALERRINIIPCEYYVEKADTSLIDKLQSEKKEIFLYLMY

VYQQIVKADIEYLENSRVTEITHDWLNFGYEFVSSRSVSNANQKACINLLRKLIEIKS

GSRIKVSELNKVINEEIKVSSQVIKQLIQANFDTQTKLYNGYDYWIDLGWKEANKKEI
HDISEKDNIISLDKNENITDDEALDEENLDFDWEDFDDE"
/db_xref="SEED:fig|74706.92.peg.7"
/product="putative primase"
CDS 7244..8920
/transl_table=11
/db_xref="SEED:fig|74706.92.peg.8"
/translation="MKGKIALYSRVSTSEQSEHGYSIHEQEQVLIKEVVKNFPGYDYE

TYTDSGISGKNIEGRPAMKRLLQDVKDNKIEMVLSWKLNRISRSMRDVFNIIHEFKEH

DVGYKSISENIDTSNASGEVLVTMFGLIGSIERQTLISNVKLSMNAKARSGEAITGRV

LGYKLSLNPLTQKNDLVIDENEANIVREIFDLYLNHNKGLKAITTVLNQKGYRTINQK

PFSVYGVKYILNNPVYKGYVRFNNHQNWAVQRRSGKSDKNDVILVKGKHEAIISEEVF

DKVHEKLASKSFKPGRPIGGDFYLRGLIKCPECGNNMVCRRTYYKTKKSKERTIKRYY

ICSLFNRSGSSACHSNSINAEVVERVINVHLNRILSQPDIIKQIASNVIEELKQKHSN

QTEIKYDIDSLEKQKAKLKTQQERLLELFLDDQMDSEMLKAKQSQMNQQLEVLDQQIK

EAQQANQSQDEIPNFDKLKGRLILMITRFSVYLRKATPEAKNQLMKMLIDSIEITTDK
QVKLVRYKIDESLIPQSLKKDWGSFFMPKFQFEIYGQNDYFIDQITTFTT"
/product="Cassette chromosome recombinase B"
CDS 9026..9364
/transl_table=11
/product="hypothetical protein"
/translation="MTLEQQLKHYITNLFNLPRDEVWHCESIEEIADDILPNQYVRLG

PLSNKTLQTNTYYSDTLHESNIYPFILYYQKQLIAIGYIDENHDMDFLYLHNTIMPLL
DQRYLLTGGQ"
/db_xref="SEED:fig|74706.92.peg.9"
CDS 9460..9771
/transl_table=11

/translation="MNRYITRGIANNLPNILQHQLWQLVSEREQEQTKDNTSVDYFHI

FQFNKHRNQLYIKHKQERPEYAKIHKANYSKAININKVYIIREDDVDLSYYVMLLPEE
Y"
/db_xref="SEED:fig|74706.92.peg.10"
/product="hypothetical protein"
CDS 9787..10293
/translation="METIKSTLKTEAIFSDDKQHRYLLKKTWNSEKQSITIITMYPHY

DGILNIDLTTQLIMNKVSEMDAFGSINFVNLYSNITTPINLKHLENAYDKHTDIQIMK

AVKESDEVILAWGAYAKKPGVEARVNEVLEMLKPHKKKVKRLMNPETNEIMHPLNSKA
RQKWILKV"
/db_xref="SEED:fig|74706.92.peg.11"
/product="hypothetical protein"
/transl_table=11
CDS complement(10442..11116)
/transl_table=11
/product="Transposase for insertion sequence-like element
IS431mec"
/translation="MNYFRYKQFNKDVITVAVGYYLRYTLSYRDISEILRERGVNVHH

STVYRWVQEYAPILYQIWKKKHKKAYYKWRIDETYIKIKGKWSYLYRAIDAEGHTLDI

WLRKQRDNHSAYAFIKRLIKQFGKPQKVITDQAPSTKVAMAKVIKAFKLKPDCHCTSK
YLNNLIEQDHRHIKVRKTRYQSINTAKNTLKGIECIYALYKKNRRSLQICGFSPCHEI
SIMLAS"
/db_xref="SEED:fig|74706.92.peg.12"
CDS 11374..11541
/EC_number="2.3.3.10"
/product="Hydroxymethylglutaryl-CoA synthase N-domain
homolog (EC 2.3.3.10)"
/db_xref="SEED:fig|74706.92.peg.13"
/translation="MLESREQLSVEEYETFFNRFDNQEFDFERELTQDPYSKVYLYSI
EDHIRTYKIEK"
/transl_table=11
CDS 12418..13161
/transl_table=11
/EC_number="3.1.4.46"
/product="Glycerophosphoryl diester phosphodiesterase (EC
3.1.4.46)"
/db_xref="SEED:fig|74706.92.peg.14"
/db_xref="GO:0008889"
/translation="MLTVYGHRGLPSKAPENTIASFKAASEVEGINWLELDVAITKDE

QLIIIHDDYLERTTNMSGEITELNYDEIKDASAGSWFGEKFKDEHLPTFDDVVKIANE

YNMNLNVELKGITGPNGLALSKSMVKQVEEQLTNLNQNQEVLISSFNVVLVKLAEEIM

PQYNRAVIFHTTSFREDWRTLLDYCNAKIVNTEDAKLTKAKVKMVKEAGYELNVWTVN
KPARANQLANWGVDGIFTDNADKMVHLSQ"
CDS 13258..13686
/translation="MKYDDFIVGETFKTKSLHITEEEIIQFATTFDPQYMHIDKEKAE

QSRFKGIIASGMHTLSISFKLWVEEGKYGEEVVAGTQMNNVKFIKPVYPGNTLYVIAE
ITNKKSIKKENGLVTVSLSTYNENEEIVFKGEVTALINNS"
/db_xref="SEED:fig|74706.92.peg.15"
/product="MaoC domain protein"
/transl_table=11
CDS complement(13732..15738)
/transl_table=11
/db_xref="SEED:fig|74706.92.peg.16"
/db_xref="GO:0008658"
/db_xref="GO:0046677"
/translation="MKKIKIVPLILIVVVVGFGIYFYASKDKEINNTIDAIEDKNFKQ

VYKDSSYISKSDNGEVEMTERPIKIYNSLGVKDINIQDRKIKKVSKNKKRVDAQYKIK

TNYGNIDRNVQFNFVKEDGMWKLDWDHSVIIPGMQKDQSIHIENLKSERGKILDRNNV

ELANTGTAYEIGIVPKNVSKKDYKAIAKELSISEDYIKQQMDQNWVQDDTFVPLKTVK

KMDEYLRDFAKKFHLTTNETESRNYPLGKATSHLLGYVGPINSEELKQKEYKGYKDDA

VIGKKGLEKLYDKKLQHEDGYRVTIVDDNSNTIAHTLIEKKKKDGKDIQLTIDAKVQK

SIYNNMKNDYGSGTAIHPQTGELLALVSTPSYDVYPFMYGMSNEEYNKLTEDKKEPLL

NKFQITTSPGSTQKILTAMIGLNNKTLDDKTSYKIDGKGWQKDKSWGGYNVTRYEVVN

GNIDLKQAIESSDNIFFARVALELGSKKFEKGMKKLGVGEDIPSDYPFYNAQISNKNL

DNEILLADSGYGQGEILINPVQILSIYSALENNGNINAPHLLKDTKNKVWKKNIISKE

NINLLTDGMQQVVNKTHKEDIYRSYANLIGKSGTAELKMKQGETGRQIGWFISYDKDN
PNMMMAINVKDVQDKGMASYNAKISGKVYDELYENGNKKYDIDE"
/product="Penicillin-binding protein PBP2a, methicillin
resistance determinant MecA, transpeptidase"
CDS 15986..16129
/transl_table=11
/product="Mobile element protein"

/translation="MNYFRYKQFNKDVITVAVGYYLRYALSYRDMSEILRERGVNVHH
LTV"
/db_xref="SEED:fig|74706.92.peg.17"
CDS 16409..16660
/transl_table=11
/product="Mobile element protein"
/db_xref="SEED:fig|74706.92.peg.18"
/translation="MAKVIKAFKLKPDCHCTSKYLNNLIEQDHRHIKVRKTRYQSINT
AKNTLKGIECIYGLYKKNRRSLQICGFSPCHEISIMLTS"
CDS complement(16720..17148)
/translation="MKFQVSPYFLITQNAEGLKKLYEELFGAKTLYIQRIKDRPIDKK

LNIDENDLNKIDQCVLQFGDIKIMIADDTEGLPITQGNNISLCLTFDQVEETKHVYDQ
LIEKGSEVLKTFSPEFYTEGYGYVRDPYGISLHLFTKRKN"
/db_xref="SEED:fig|74706.92.peg.19"
/product="PhnB protein; putative DNA binding
3-demethylubiquinone-9 3-methyltransferase domain

protein"
/transl_table=11
CDS 17229..18158
/transl_table=11
/translation="MKKSVRLYNMIEYCNENRNFKLNDLMSEFNISRSTALRDIKEIE

ALGVPLYSNPGKNGGYTIIGNRDQTKIAISDEELKALVFTLSSISNVSNLPFQTEYQE
ILKKLYNNSNKKELINQYNDLFQYFNEDKYQFKSYKLFNEIIRLIIENKSFETCYSQN

YIKEQYKGIGIMYKNHQWYFVVANIESKLVNLLNISKIKELYEMGETQECNDITMQNF

QQFMVKNETAIDILIRSNVMGLNILKGYLWSDYMIENIDEETYLFKSKVNAKDIDFIA
KLIVTGGVNVKVETPNSLKNAVKVELTKIINMY"
/db_xref="SEED:fig|74706.92.peg.20"
/product="Transcriptional regulator, YafY family"
CDS 18320..19804
/db_xref="SEED:fig|74706.92.peg.21"
/translation="MNKNITALKSTENTTYMQYTVHIDDQIINNIKECTKKFKILPME
DKIPLSPLLQPEYAGEVQDFISTYEQFMINFGKVILDSQGIKIQFESESLSSIQRGIQ

EHCYLNERTNDIDVTKEWYLCKFSNQILEEDKNQLYNALKQLMNDSKNKKQAFQEVFK

VHIDIYLYNKEKSEYKYQVSSYFNLVKENPKITYKKRHLQEKQGVKGTTFTNKLKLLN

KMYGVDVAKYQPFYNSNNPEYERGQFGERYISQRSNYEFNRLQYQIIDMLSKILDKHP
LPKSDNNYKHIPTIEKAILSGDSHSFYEYFEDIMKEIISMENSSLKEKLLTDFTYQSQ

CRWYSESEKLKLQLESFMHKVLESNYYEGNKLYRMLSHAIEETINEADEDKVHFNYFK

DYFLTDGGVKNWEQISEKITEFNGKVINDIQNEYNKIQFNNANRNQKLNFNYLYHCFE
FSNNLVKARVNGNRGYILYFVDKYKIKMPFLDQLFESRNRSGKY"
/product="Similarity"
/transl_table=11
CDS 19773..20297
/translation="MNQEIGQESINYNMQTLFEKERYDRSSTIEKLVATSKFKYEKDD
SDLFKQLFNDVENSIDRLGIYLLNNGINSNDENARYYRSFLKELSRIKSKLTPFSLEI

SKSSGREQHYPDDAIDDKDERRKNKEETYHAFDDKSDIDSKLKNKINVSIDNLLSVKI
KSNLYDISPNTKKQ"
/db_xref="SEED:fig|74706.92.peg.22"
/product="Similarity"
/transl_table=11
CDS 20492..21601
/transl_table=11

/product="hypothetical protein"

/translation="MYQANIRDLITKLPQSNKTEHFLMNKFSNQDKVQQLQRQISQQL

DQQYNELLANEKAKLDQYVEVHHNLEPLKKEIESESINLDTDKLPDIKATMLEKAKND

EHFDKIEQLFDRLDQSLNGTNRLYTQLSLIGTRTHRITTKRFNVQGLPKLVQQMILPS

QFKKVYTIDFKSFEPSVAAYMTQDEQLIDYLNHEEGLYDALLRDLSLSKEKRVSVKRA

FIGSFLFGGRYSSSKFKINQEVSEINWLQVMSKFKKVIEFKEQVEKYKTMPTPYGIEH

DMSAFQGSSIMAIYVQTVASYIFKHILLEVYKAQCEKKTFKIIVPIHDAIMIECNDKG
IAQNVAQLMKDTANQLFNGEFAHVTVEALGGIDNE"
/db_xref="SEED:fig|74706.92.peg.23"
CDS 21594..21962
/db_xref="SEED:fig|74706.92.peg.24"
/translation="MNNDRGKSLQIPQSTLLKEGSIYVATLHSVYEKNFSGDIKHQFT

YEVELNQETHYVNRNITVKSMSHQLSIADWIKRHSNYNVNHINYDPYIDRKHLVLVGQ
YNGNYYIQDVAPLDEFGGVL"
/product="hypothetical protein"
/transl_table=11
CDS 21962..23578
/db_xref="SEED:fig|74706.92.peg.25"
/translation="MNHILEMLIKLLKVGMEAIDRKGLIAILTSSIGNDEMDDSEQAV

MVYNELIDKLQLNIPKDVDYRPNIYSYFGIQKKPNDTILVEMMICIFHIKRFDSELFV

FKDKGWQKVSEDELQGLISKMIQVLLVDYKPSLSTLKNVVDGIQKSTDIEKLVEHRQY

IGCGRNMFNLKTFKVVDNDLEIFPKTRLDLELDINDTITDKIPPNFKQYMLELANYDH

DLQYFLFQHMAVLLTADTKLRRGLFLYGTAKNGKSVYIKLVKSFFYSNDIVSKTLNEL

GGRFDKESLIGKRIMASDEVGKANIDEATVNDFKKLLSVEPIHADRKGRTQVEVTLDL

KLIFNTNAVLNFPSSHAKALERRIAVIPCEYYVEKADPDLIEKLQDEKKEIFLYLMYV

YKQIVKNDIEYLQNDRVTEISHDWLNFGYEFVSSKSANIAHQKACINLLRKLIEIKPG

SRIKVSRLNEVIRDEIKVSSQVINDLVQANFNVQSRLNNGYKYWVDLGWKETDKKDDM
ISFDKNENVTDDEFLYEDDLNLGWEDFDDE"
/product="putative primase"

/transl_table=11
CDS 23803..25482
/translation="MKGKIALYSRVSTSEQSEHGYSEKEQEQLLIKEVMKNFPGYDYE

TYTDSGISGKNIEGRPAMKRLLQDVKDNKIEMVLSWKLNRISRSMRDVFNIIHEFKEH

DVGYKSISENIDTSNASGEVLVTMFGLIGSIERQTLISNVKLSMNAKARSGEAITGRV

LGYKLSLNPLTQKNDLVIDENEAHIVREIFDLYLNHNKGFKAITTILNQKGYRTINQK

PFSVFGVKYILNNPVYKGYVRFNNHQNWAVQRRSGKSDKNDVILVKGKHEAIISEDVF

DQVHEKLASKSFKPGRPIGGDFYLRGLIKCPECGNNMVCRRTYYKTKKSKERTIKRYY

ICSLFNRSGSSACHSNAINAEVVERVINVHLNRILSQPNVIKQIASSVIEELKQKHSK

QTEIKYDIDSLEKQKAKVKTQQERLLELFLDDEMDSEMLKAKQSEMNQQLEVLDQQIK

EAKQANQSQDDIPNFDKLKARLILMITRFSVYLRKATPEAKNQLMKMLIDSIEITTDK
QVKLVRYKIDESLIPQSLKKDWGSFFIPKFNFVINVTKKNRIENLSLLPLF"
/db_xref="SEED:fig|74706.92.peg.26"
/product="Cassette chromosome recombinase B"
/transl_table=11
CDS 25571..25909
/product="hypothetical protein"
/translation="MTLSKQLKTYITERFKLNYQETWACETIDAVAEDVLPEKYIKNS
PLEHKILNTFTYYNDELHEISIYPFLCYLDKELVAIGYLDNFDLDFIFLNDTHQIIID
ERYLLQKGGE"
/db_xref="SEED:fig|74706.92.peg.27"
/transl_table=11
CDS 25912..26001
/product="hypothetical protein"
/translation="MNWIKVAQLSVTVINEVIEIMKEKQNGGK"
/db_xref="SEED:fig|74706.92.peg.28"
/transl_table=11
CDS 26003..26314
/product="hypothetical protein"
/translation="MNINRYITRGISEQLSLDLQILLWHMVEEKDNQPHTDYLHIFKL

QEDDNMLSITHEQEQPAYKLEYHYINYEKNQNALPKKVYVIREDDVDVFYYVMLLPEE
Y"
/db_xref="SEED:fig|74706.92.peg.29"
/transl_table=11
CDS 26329..26832

/transl_table=11
/translation="MNTIKSTIHTEAIFSDDEQHRYLLKKTWDEKKPVCTVITMYPHL

DGVLSLDLTTVLILNQLANSEQYGAVYLVNLFSNIKTPENLKHIKEPYDKHTDIHLMK

AISESDTVILAYGAYAKRPVVVERVEQVMEMLKPHKKKVKKLINPATNDIMHPLNPKA
RQKWILK"
/db_xref="SEED:fig|74706.92.peg.30"
/product="hypothetical protein"
CDS 26847..27068
/product="hypothetical protein"
/translation="MNHETTHSDWRTVANCLASQNYISIVKGLVHHFTAIEDEEILDK
IYEDFMNNGSITTVLNNDLQDIINYYLSK"
/db_xref="SEED:fig|74706.92.peg.31"
/transl_table=11
CDS complement(27232..27363)
/transl_table=11

/translation="MKQRKKGMEDEELRAIAHELTKKVKENMGVIGLNVIAQKLKCA"
/db_xref="SEED:fig|74706.92.peg.32"
/product="hypothetical protein"
CDS complement(27360..27635)
/transl_table=11
/product="Type I restriction-modification system,
restriction subunit R (EC 3.1.21.3)"
/EC_number="3.1.21.3"

/translation="MKQKNVAVELLNRLLKGQVKSLMKTNATVSKRFSEMLRNSINKY
NSRSIETSKVIEELIQLAKDIKQEQQRGNELGLNSDEIAFLRCFSFT"
/db_xref="SEED:fig|74706.92.peg.33"
CDS 28620..29525
/db_xref="SEED:fig|74706.92.peg.34"
/translation="MKDVIYVENHYFVTVKENSIKFRNVIDKSEKFYLFEEIEAIIFD
HYKSYFSHKLVIKCIENDIAIIFCDKKHSPLTQLISSYGMTHRLQRIQSQFQLSGRTR

DRIWKKIVVNKIINQSKCLENNLHNENVKLLVNLAKDVSSGDKSNKEAQAARIYFKDL

YGKQFKRGRYNDIINSGLNYGYSILRSFIKKELALHGFEMSLGINHRSKENPFNLADD
IIEVFRPFVDNIVYEIVFKKNINTFDVNEKKLLLNVLYEKCIIDKKVVRLLDSVKIVI
QSLIRCYEENTPTYLLLPKMIEVGN"
/product="CRISPR-associated protein Cas1"
/transl_table=11
CDS 29525..29830
/product="CRISPR-associated protein Cas2"

/translation="MYLLVSFDLPRDTKFERRVASKYRTRLLELGFSMKQFSLYERYV
SDVQKKDKILEILQQEIPDTGSITLYVLPDEVNNSQITILGKEVKVVVRKEPKLIFL"
/db_xref="SEED:fig|74706.92.peg.35"
/transl_table=11
CDS 29844..32117
/transl_table=11
/db_xref="SEED:fig|74706.92.peg.36"
/translation="MDKKTTLMYGSLLHDIGKIIYRSNDHAFARGTHSKLGYNFLSQF

SEFKDNEVLDSIAYHHYKELAKANLANDNTAYITYIADNIASGIDRRDVIEEGDEEYE

KQAFNFDKYTPLYSVFNIVNSEKLKQISGKFKFSNESNIEYPKTENIQYSSGNYTTLM

KDMSYDLEHKLSIKEDTFPSLLQWTESLWQYVPSSTNKNQLIDISLYDHSRITCAIAS

CIFDYLNENNIHNYKDELFTKYENTKEFYQKEAFLLLSMDMSGIQDFIYNISGSKALK

SLRSRSFYLEIMLEVIVDQLLEKLELTRANLLYTGGGHAYLLVSNTDKGKEKINQFNT

ELKNWFMSEFTTDLSLSIAFEKCSGNDLMNTSGNYRNIWRNVSSKLSDIKAHKYSSED
ILKLNHFHSYGDRECKECLRSDIDINDDGLCSICEGIINISNDLRDKSFFVLSETGKL

KMPFDKFISVIDYEEAERLAQNNNHIRIYSKNKPYIGVGISTNLWMCDYDYASQNKDM
REKGIGSYVEREEGIKRLGVVRADIDNLGATFISGIPEKYNSISRTATLSRQLSLFFK

YELNHLLENYQITAIYSGGDDLFLIGAWDDIIEVSVYINEKFKDFTLGKLTMSAGVGM

FSGKYPISKMAFETGLLEEAAKTDEKNQIALWVQEKVYNWDEFKKYILEEKLLVLQHG

FSQTDEHGKAFIYKMLALLRNNETINIARLAYLLARSKMTEEFTSKIFNWAQNDKDKN
QLITALEYYVYQIREVD"
/product="CRISPR-associated protein, Csm1 family"
CDS 32120..32545
/transl_table=11
/product="CRISPR-associated protein, Csm2 family"
/db_xref="SEED:fig|74706.92.peg.37"
/translation="MILAKTKSGKKIDLTFAHEVVKSNVKTVKDKRGKENQVLFNGLT

TSKLRNLMEQVNRLYTIAFNSTEDQLNEEFIDELEYLKIKFYYEAGREKSVDEFLKKT
LMFPIIDRVIQKESKKFFLDYCKYFEALVAYAKYYQKED"
CDS 32547..33191
/transl_table=11
/product="CRISPR-associated RAMP Csm3"
/db_xref="SEED:fig|74706.92.peg.38"

/translation="MYSKIKISGTIEVVTGLHIGGGGESSMIGAIDSPVVRDLQTKLP

IIPGSSIKGKMRSLLAKHFGLKMKQENHNQDDESVLRLFGSSEKGNIQRARLQISDAF

FSEKTKEHFAQNDIAYTETKFENTINRLTAVANPRQIERVTRGSEFDFVFIYNVDEES
QVENDFENIEKAIHLLENDYLGGGGTRGNGRIQFNDITIETVVGEYDSTNLKIK"
CDS 33202..34110
/product="CRISPR-associated RAMP protein, Csm4 family"
/translation="MTTKVFKLSFKTPVHFGKKRLSDGEMTITSDTLFSALFIEALQL

GIETDWLLNDLIISDTFPYENEIYYLPKPLIKIESKEEGNHKAFKKLKYVPVHHYNQY

LNGELSAEDATDLNDIFSIGHFSLQTKVSLSAQEIDSSADSEPYSVGTFTFEPEAGLY

FIAKGSEETLDQLKDIMTSLQYSGLGGKRNAGYGQFEYEIINNQQLFKLLNQNGEHSI

LLSTAMAKEDEIKSALKEARYILNKRSGFIQSTNYSEMLVKKSDFYSFSSGSVFKNIF
NGDVFNVGHNGKHPVYRYAKPLWLEV"
/db_xref="SEED:fig|74706.92.peg.39"
/transl_table=11
CDS 34113..35135

/translation="MTIKNYEVVIKTLGPVHIGSGQVMKKQDYIYDFYNSKVYMINGN

KLVKFLKRKNILDTYQNFLRYPPKNPRENGLKDYLDAQNVKQSEWKAFVSYSEKVNQG

KKYGNIRPKPLNDLHLMVRDGQNKVYLPGSSIKGAIKTALVSKYNNEKNTDVYSKIKV
SDSEPIDERHLAIYQKIDINKSEKPMPLYRECVDVDTEIKFKLTIEDEIYSINEIEQS

IQDFYKNYYDKWLVGFKETKGGRRFALEGGMPDVLNQNILFLGAGAGFVSKTTHYQLK

SREQAKRDSFDILTKKFRRTYGKMKEMPSNVPVALKGTTNQSLHVSYQQGMCKISFQE
LNNEVL"
/db_xref="SEED:fig|74706.92.peg.40"
/product="CRISPR-associated protein, Csm5 family"
/transl_table=11
CDS 35135..36403

/translation="MKVLFSPIGNSDPWSNDRDGAMLHIVRHYKPDVVVLFFTESIWN

GNRNIPGRKNFDWENIVSKVSSRTKVDIKVDSIKYENDFDSYKDIFHFYINEIRTKYS

DAEILLNVTSGTPQMESTLCLEYISNPHNMKCIQVSTPAPIEGPKRSFAKLETVTEDL

NKVNANEKMASNRSKSINIISFREVMVRSQIKSLVNNYDYEGALNLVSDQKSFRNGKL

LRKRLLELTNQIKTHEVFPEINDKYRSVALKKSLFHYLLLNMRYNRLDVAETLIRVKS
IAEFILKTYIVGHWPTLIIEKDDKPYLNAEDNLSFIYKYKLLLEKRRQNLDVSRILGL

PAFIDILTVLEPNSKLLKEVNAVNDINGLRNSIAHNLETLDLDKNKNYKKIMLSVEAI
KNMLHISFPEIEEKDYNYFERKNKEFRELL"
/db_xref="SEED:fig|74706.92.peg.41"
/product="CRISPR-associated protein Csm6"
/transl_table=11
CDS 36400..37134
/product="CRISPR-associated endoribonuclease Cas6"
/translation="MINKITVELDLPDSIRFQYLGSILHGVLMDYLPNDIADQLHHEF
AYSPLKQRIYHKNKKVIWEIVCMSDRLFNEIAELFTSKNRLFLKYYQVYIEIYSFNIE

KVNVQNIMNQLLETEELNRYVRINIQTPMSFKYQSNYMIFPEVKRFFRSIMIQFDAFF
EEYKMYDKETLDFLEKNINIVDYKLKSTRFNLEKVKIPSFTGEIVFKIKGPLPFLQLT
HFLLKFGEFSGSGMKTSLGMGKYSII"
/db_xref="SEED:fig|74706.92.peg.42"
/transl_table=11
CDS 38175..38333
/transl_table=11
/db_xref="SEED:fig|74706.92.peg.43"
/translation="MKFKSKQLVIANQTKVINYRIQVLLKPQANIETQTQVKNNTLPE
TDEQSNAD"
/product="hypothetical protein"
ORIGIN
1 atggaggtca aaatgaaaat tactgttgtc gcagttggaa aactgaaaga gaaatactgg
61 aagcaagcta ttgcagagta cagcaaaaga ctaggggcat acacaaaatt agaaatgatt
121 gaagttccag atgaaaaagc acctgaaacg atgagcgata aagaagtcga acaagtgaaa
181 caaaaagagg gccaacgtat tttaagtaaa atcaaaccac aatctactgt catcacatta
241 gaaatcgaag gcaaaatgct cagttctgaa gccttagcta caacactaaa tcaacgtatg
301 acgcaagggg cgagtgactt taccttgtg ataggaggat cgaatggctt acaccaggac
361 gtcttaaacc gcagcaactt cgccctgtca tttagtaaaa tgacatttcc acatcaaatg
421 atgcgcgtca tcttgttaga gcaagtatat cgagcattta aaataatacg gggagaggct
481 tatcataaat aaaactaaaa attagattgt gtataattta aaaatttaat gagatgtgga
541 ggaattacat atatgaaata ttggagtata ccttgcaata tcatacgatg tttatagagt
601 gtttaataaa ccatttttca actattgatg atctagaata tataataact gtacaaatta
661 tattgattat ggaactacaa ttcaattaag aaattgatga tgaaatttta aatttaaact
721 aatggaatca agaaagaatg aaaggaaata tacaatgccc acgattaata aaaggaagtt
781 tattagattt tgtgttagaa acagtagatg tttttgtaaa tggaagtaaa aatgattgcg
841 aattagtaaa ttttaattat aaagaaggaa aagtagtttc acgttatagt acaaaatttg
901 aaagcaatcc taaattaaga aaaaagcaat tgatatcgac ggtaaaagtt gtaaggtatg
961 ttatttttga ttttgagaaa attttatggg gcatagggaa agttttattg aagttcatca

1021 tattaaggca atgtttacta taagaaagtt gaaatacact cagaaacaga ctggattccc
1081 gtttttttca aattggcaca aaatgataca taggcccacc tatatattga agatttaagt
1141 taaacgataa ataatatttt tctcttttgt atagaaattt ttttgggaat gatattaatt
1201 agtataaaaa tgaatatgtc tacaaagaag gagagatttt attgaaacac attaatcaat
1261 tttctgaatc gacactagca caactagaaa aagatcaaca acacgtattt tatgtatact
1321 gtttgatgga cccaagaaat gatgaatgct tttatgttgg taaaggtaag gggaatagaa
1381 tatttaaaca taaacaagat gcgcagaaaa aagtattgta cgaagatata tttatagaag
1441 aaaataaaga taacttgaaa tttaatagaa ttaacgaaat ttgtagcaat gatttgaatg
1501 ttttaggata tataatcagt tatggattaa ccgaatctga agcattttct gcagaaaatg
1561 ttcttataaa ctttcttaac ctaacaaata aaactacatt aacaaatatg attaacggtc
1621 atggatctaa ggcatactta gttgaagatt tagaaaatga atttggctac gattcaatta
1681 atcttgaaaa cataaatacg aatgaattaa ttttagcagt gaaaatcaga gatgcatttc
1741 tattagataa ggatgaaagt aaagagtatc ctattaatga aagtaaacgt gatagaaata
1801 accttaaatc gcgtacatta ggtagttgga taataggaaa agataaaata cataaaataa
1861 aatatattat tggtatcaat acaggtgcta ataatgcagt tgtctcagct tatgaagtat
1921 catttgaaca agcggagagt atcgaaacaa ataatggtag aatgagatat gcgtttattg
1981 ggctttcaga aagagatgct actctaaaaa aattgaattt atacaaaaaa gcactaccag
2041 atttaagatt tggtagtggt agcgctacag catatataaa taatgggaca atgaaagttg
2101 attaaatgaa taaaaacatc caattcctta aattagcctt tgaaaatttt atactcgtat
2161 atataagtga aaaacttaaa agaattagat gttaattaaa ggagagaatg ctatgccatc
2221 agatgatatt gtaaaaaaac tatactcaaa agaggatatt cgattagaat taggacttac
2281 tccgcataag tttaataaaa aaatggaaac tattgctaag ctttttaaaa ttgatatgaa
2341 aatttttcac aattacaaag gccaagataa aaataatcag tatacattta atggtgtcgc
2401 aaaagaatta atcaaagtgt tgctaaaaag tgttgattat tacccagtgg atatcaattc
2461 caaaaaattt aaacaaaatg gaaaatctaa aaaagaaatg attgaaaata tagataactc
2521 tagttacatg aaatatattt atcagttaat gaaatctatc aatgaaattc aatacaaaag
2581 gttaattgct gatatacata tgaaagatgt gtaccagaat acaaaagcat ggttaaataa
2641 tggtgaatca attaataaaa aagaacaaga gctataccaa tatatgacaa ttttaccatt
2701 acataaaaga gttgaactgc aaaatgaggt attaaaatct atagacgaaa caatttttca
2761 atttctcgca aaagaacata gaaataatca aattgaagaa aataatgaat tagaagcata
2821 tacaaaagca ataaaagaag gtagaaatcc taaaaatgat tatgaattaa atcatctcct
2881 atataaaaaa aatcaattac ccttagatag tctaatagaa gatatatggg actatgagga
2941 aacagagtat actgagcttg attggttaat tgcagatatg ctaaaacgtt cacaaagagc
3001 tgatagtaat tttatcgaaa gtttagagaa gaaaaacaaa ttgagaaaaa atattcataa
3061 agattccatt aaaagtatat ctaagttgat tgatcatgaa ttagttaaaa ataatagaaa
3121 gtgggacgta gcagaatttt ataaaaatca acaattttgg aataatagta cattaagacg
3181 tagctctcaa aattttggaa ttttatatta caaaaagaat aggttacata caatgaatca
3241 aagtaatatt attaagaaaa taaaaattat cgaagctaat atagagagtg caaataaaca
3301 gccgagctat ttagatttct ttttacaagc taagtttgat ttagagaggt tagaaagtga
3361 attaatgaaa tatagcatta atccgaaata ttttaataag ataaatagtg attacgtaag
3421 atatgctatt gaagatgtac ataatgcaat tattaaaatt gacagatata taagtaacga
3481 taaagtgaag ttaaactatt caaatgtcca aatggtagtg agaaacgaaa taacagaaca
3541 aggaagctac tttgtaaccc aggcattgaa tacactgtca aaggtagaag agtctggcta
3601 cagctttgat aatttcatgg taggaccttt cgtagaagac tttaaaaaag caattaaaaa

3661 tcgtagattt ggttctaaaa aagattaaga ttgtttttta gagttaaaca taagaataaa
3721 tttaaataaa aaaataaacc caacaaacct tgttatatca atgtttgttg ggtttatttt
3781 tttggtgatt ttttaataac tcaattacat cttaggataa tatcgctaaa ttatacattg
3841 tcgaggccaa gacaaataaa aaattaaagc aaaagttagc gcgcaacttt ctgctataaa
3901 ggagcaaatt tatatggaga caagagacaa actgatgtca ttgactcaaa gtgacaaaac
3961 acagcaatgg ctaatggaca agtcatctaa ccaagatgac attcaacaat tgcagcaaca
4021 attcagtcag cagcttgatc aacaatataa tgcactttta gctgatgaaa aagctaagtt
4081 agaccaatac gtggaagtac atcaaggatt ggaatcatta aaggaagaga ttgaatcaga
4141 acctattacg cttaatatcg ataaattacc cgatatcaaa gcaacaatgc ttgaaagagc
4201 caagaatgat gaacattctg ataaaatcga aaagctattt gataggttag aacaggcatt
4261 aaatggtacg aatcgattat atacgcaatt atcgttgatt ggtacacgaa cacatcgaat
4321 tacaactaaa aattttaatc ttcaaggctt acctaaagca gtccaacata cgattttacc
4381 ttcaaaattt aagaaggtgt atacagtcga ttttaaatcg tttgaaccat cagttgcagc
4441 gtacatgact caagattcaa aactgattga cttgttaaat cagaaagacg gactgtatga
4501 cgcattgcta agtgaattag gcttatcaga tgagctacgt gtatttgtta aacgtgcatt
4561 tattggttcg tttctatttg gaggtaactt caaaaatcct aaattcaagc tgaatcaata
4621 tgtaagtgaa gtacaatggt tggatgcggt cagccaattt acaaaagtca ttgaacttaa
4681 gaagcacgtt gaaaagagta attccatgcc tatgccttat agtattgagc atgatatgag
4741 cgcatttcaa ggtagcagta ttatggcaat ctacgtacaa actgtagcga gttatatttt
4801 caagcacatt ctgctaaaag tgtacaaagc acagtgcgat caaaaaacgt tcaagattat
4861 agtacctata cacgatgcga ttatgattga atgtgaagat gaagaaattg cacaaaatgt
4921 gggtcagtta atgaaagata cggctaacca gttgttcaat ggtgaatttg cacatgtgac
4981 agtggaagaa ataggaggcg tagaccatga ataatgatag aggacaaagc ctacacatcc
5041 caagtagtac accaatcaaa gaaaataata tatatgtagc tacgttacat tctgtgatcc
5101 aaacagattt ctcaggtgaa ataaagcacc aattcacgta tgaaattgaa gtgaacaatc
5161 agattgtata tgcgaatcgt aatattccaa caaaaccgag cgctaatcag ttgtcaattc
5221 atgattggct gaaacgtcat agcaactata gcgcaagtca tgaaaactat gagccttata
5281 ttgatcagaa acatttaatt ctattaggtc aatataacgg taactattat gtacaagatg
5341 tagcatcgtt agatgcgttt ggaggcgtat tatcatgaat catatattac aaatgttatc
5401 taagctatta agtgtggcca aggaggcaat cgaccgtcaa ggtctgattg ctatcctaac
5461 tattcctgtt aataataacg atgaaataga agaaacggct caaggtgaaa ccgtgtataa
5521 cgaacttatc gatcagttac gacttaatat cccaaaagat acggattatc aacctaacat
5581 ctatagttat tttggtatta agaagaatcc taatgacacc gtactcatgg aaatgatgat
5641 aaaggttttt catatcaaac gctttaattc agaactgttt attttcaaag ttaacgggtg
5701 gcaaaagata aatggagatg aattacaagg gttgatatct aaaatgatac aagtattgct
5761 tgtagattat aagccttcac taagcactct aaaaaatgtc gtagatggat tgcaaaaatc
5821 aacagatgta gaagaacttg ttgagaatga gcgctatatt ggttgtggtg aaaatatgtt
5881 cgatcttaat acgtttcaag tcgttaaaaa ttcaatcgat atctttccaa aaacacgatt
5941 gaatttatca ttaagtacaa atgatgtaat tactgataag ataccgcctt attttaagca
6001 atatatgtta caacttgcga attatgacga tgatttacaa tactttcttt tccaacatac
6061 agcagtatta cttacagctg atactaaata ccgtaggggt ctcatattat atggtggagc
6121 taagaatggt aaatctgtat atattgaact agttaaatca tttttctata gtaaagatat
6181 tgtgtctaag ccacttaatg agcttgaagg tcgttttgac aaagaaagtt taattgacaa
6241 aagtctaatg gcaagtcatg aaattgggca atctaggatt caagaaaaga tcgtaaatga

6301 cttcaaaaag ttattatctg tagaatcaat gcatgttgat cgtaaaggaa aaactcaagt
6361 ggaagtcatt ttggatttga aacttatttt tagtacaaat gcgatactta attttcctcc
6421 tgaacatgcg aaagctttgg agcgtcgaat taatattatt ccatgtgagt attatgttga
6481 aaaagcggac acttcattaa ttgataagct ccagagtgag aagaaagaaa tctttcttta
6541 cttgatgtat gtgtatcaac agattgtaaa agcagatatc gagtatcttg aaaatagccg
6601 tgtcactgaa attactcacg attggttaaa ttttggatat gaatttgttt ctagcaggtc
6661 cgtaagtaat gcaaatcaga aagcatgtat taatttactc agaaaactta tagaaatcaa
6721 atcaggatca cgaatcaaag tatccgagtt aaataaagtt attaatgaag aaataaaggt
6781 aagttctcag gttattaaac agttaattca agcaaacttt gatactcaaa ccaaactata
6841 caatggctac gattattgga ttgatttagg ttggaaagaa gccaataaaa aagagattca
6901 tgatatttcg gaaaaagata atattatttc attagataaa aatgaaaata taacagacga
6961 tgaggcatta gatgaagaga atttggactt tgattgggag gactttgacg atgaataatg
7021 aacaaattga agcatttgta gaagtgcttg tacctatcat agaagaacgt atcaataaag
7081 gtaattaagg ctaattacgt actacaggca gttgcctgta gtactcatat gattaagtgg
7141 taaaagtgat aaaaatgaaa cgaaattata aatatatatt atctatatgt tgttacaaga
7201 ccgatagtct gtagcaataa tctaataaaa ggagcggtat gatatgaagg gtaaaattgc
7261 actttattca cgcgttagta cgtcagagca gtcggagcat gggtactcaa tccatgagca
7321 ggaacaagta ctcatcaaag aagttgtgaa aaatttccca ggttatgact atgagacata
7381 tactgactca ggcatttcag gtaaaaatat tgaaggtcgt ccggcaatga aacgtctatt
7441 acaagatgtt aaggataata aaatcgaaat ggtgttaagt tggaaattga atcgtatctc
7501 acgatcaatg agagacgtgt ttaatattat tcatgaattc aaagaacatg atgtagggta
7561 taaatcgatt tctgagaata ttgatacatc caatgcttct ggagaagtac tcgttacaat
7621 gtttgggtta ataggatcta tagaacgcca gactttgatt tcgaatgtga aactttctat
7681 gaatgctaag gcaaggagcg gagaggcaat caccggtcgt gttttaggct acaaattatc
7741 acttaatcca cttacacaga aaaatgattt ggttatcgat gaaaatgaag ctaatattgt
7801 acgtgaaatt ttcgatttat atttgaatca taataaaggc ctcaaagcca ttacaaccgt
7861 acttaatcaa aaggggtatc gtactattaa tcaaaagcca ttttcagtgt atggtgttaa
7921 atacattttg aataatccag tctataaagg ctatgtcaga ttcaataacc atcaaaactg
7981 ggctgtacag cgaagaagtg gcaaaagtga taaaaatgat gtgatattgg tcaaaggtaa
8041 acatgaagcc attataagtg aagaggtatt tgataaagtt catgaaaaat tagcttctaa
8101 aagttttaaa ccgggcagac ctattggtgg agatttctac ttacgtggcc ttattaaatg
8161 cccagaatgc ggaaataata tggtatgtcg acggacgtat tataaaacga aaaagtccaa
8221 agaacggaca atcaagcgtt attacatttg ttccttattc aatcgttcag ggagttctgc
8281 atgtcacagt aattccatca atgctgaagt cgtcgagcgt gtaattaatg ttcatttgaa
8341 tcgtattctg tctcaaccag atattatcaa gcagattgcg tcaaatgtga tagaagaact
8401 gaaacaaaag catagtaacc aaacagaaat taaatatgac attgatagtt tagaaaaaca
8461 aaaagctaag cttaaaacac aacaagaacg attgttagaa ttgttcttag atgatcagat
8521 ggatagcgaa atgttaaaag ctaaacaaag tcaaatgaat caacagttag aagtattaga
8581 tcaacaaatt aaagaagcgc aacaagcaaa tcaatcacag gatgaaatac ctaattttga
8641 taaattaaaa ggacgactca ttttgatgat aacacgattc agcgtgtact taagaaaggc
8701 tacacccgaa gctaaaaatc aacttatgaa aatgttaatt gattcaattg aaattacgac
8761 agataaacaa gtaaaacttg taaggtataa aattgacgaa agtcttatcc ctcaatcttt
8821 gaaaaaagat tgggggtctt tttttatgcc caaattccaa tttgaaatat atggtcaaaa
8881 tgattatttc atcgaccaaa ttaccacttt taccacttag ttattagtga caaaagtgag

8941 cgaaatgaaa taaaaatcaa atatatatta tcaaaatgat gtatcacatg catacatcaa
9001 tcaaatacat taggaggtca taaccatgac actagaacaa caactcaagc actatataac
9061 caacttattc aatctgccaa gggacgaagt gtggcactgc gaatctatcg aggaaatcgc
9121 tgatgatatc ttacccaatc aatatgtaag acttggccca ctcagtaata aaacacttca
9181 gactaatacc tactactctg acacacttca tgaaagtaat atctatcctt tcattctcta
9241 ctatcagaaa caactcatag ccatcggtta tatcgacgaa aatcacgata tggatttctt
9301 atacctacac aacactatca tgcctctttt ggatcaacga tacttactaa caggaggaca
9361 ataaaatgca taaatacatc aaaattacac aattagtcat tacaatacta agtgaaatca
9421 tcatttggat gaaagagtca gaacgaaagg aagtctctta tgaatagata tatcacccgg
9481 ggtatcgcca acaacttacc taatatctta caacaccaat tatggcaact cgtatctgag
9541 cgagaacaag aacaaaccaa agataatact tcagtagatt attttcatat attccagttc
9601 aataagcatc gcaatcaatt atatatcaaa cacaaacaag aacgacctga atatgcgaaa
9661 atccataaag ctaattattc aaaagcaatc aatatcaata aggtctacat tattcgagaa
9721 gatgatgtag acctttctta ttatgtcatg ttattacctg aagaatacta gaggagtgaa
9781 aattatatgg aaacaatcaa aagtacatta aaaacagaag ccatattcag tgatgacaaa
9841 caacatcgct atctacttaa gaaaacatgg aatagtgaaa aacaatcaat cacaatcatt
9901 acaatgtatc cgcattatga tggcattctc aatattgacc taacgaccca actcatcatg
9961 aacaaagttt cagaaatgga tgcatttggt tcaatcaatt ttgtgaatct atactctaat
10021 attacaaccc ctatcaatct caaacattta gaaaatgcgt atgataagca tacagatatt
10081 caaattatga aggcagtgaa agagtcagat gaagtgatat tagcttgggg cgcttacgct
10141 aaaaagcccg gtgttgaagc acgtgttaat gaagtattag agatgttgaa accacataaa
10201 aagaaagtaa aacgactcat gaatccagaa accaatgaaa tcatgcatcc ccttaattcg
10261 aaagcacgtc aaaaatggat attaaaagta tagcaactaa aatgtttatt cactatccat
10321 gaaaataatt gaatttcata tatatctttt aatctacaat atgattaaaa gatatattat
10381 ttggttctgt tgcaaagtaa aaaaatatag ctaaccacta atttatcatg tcagtgttcg
10441 cttaacttgc tagcatgatg ctaatttcat ggcatggcga aaatccgcag atctgaagag
10501 acctgcggtt ctttttatat agagcgtaaa tacattcaat accttttaaa gtattctttg
10561 ctgtattgat actttgatac cttgtctttc ttactttaat atgacggtga tcttgctcaa
10621 tgaggttatt cagatatttc gatgtacaat gacagtcagg tttaagttta aaagctttaa
10681 ttactttagc cattgctacc ttcgttgaag gtgcctgatc tgtaattacc ttttgaggtt
10741 taccaaattg tttaatgaga cgtttgataa acgcatatgc tgaatgatta tctcgttgct
10801 tacgcaacca aatatctaat gtatgtccct ctgcatcaat ggcacgatat aaatagctcc
10861 attttccttt tattttgatg tacgtctcat caatacgcca tttgtaataa gcttttttat
10921 gctttttctt ccaaatttga tacaaaattg gggcatattc ttgaacccaa cggtagaccg
10981 ttgaatgatg aacgtttaca ccacgttccc ttaatatttc agatatatca cgataactca
11041 atgtatatct tagatagtag ccaacggcta cagtgataac atccttgtta aattgtttat
11101 atctgaaata gttcatacag aagactcctt tttgttaaaa ttatactata aattcaactt
11161 tgcaacagaa ccgtattatg gaatagagat gttggtaaca tttatacagg atcattatac
11221 ttaagtttaa tttcgttatt acagaaccac acattccaac cagaagagaa agtatgtcta
11281 tttagttatg gttcaggagc agtaggagaa atctttagtg gttcaatcgt taaaggatat
11341 gacaaagcat tagataaaga gaaacactta aatatgctag aatctagaga gcaattatca
11401 gtcgaagaat acgaaacatt ctttaacaga tttgataatc aagaatttga tttcgaacgt
11461 gaattgacac aagatccata ttcaaaagta tacttataca gtatagaaga ccatatcaga
11521 acatataaga tagagaaata aactagtggc cgattgtgct tgatgagctt gggacataaa

11581 tcctaactcg aaataaataa gcatatcact aaactgattt tttaaagttt acagtgatat
11641 gcttattttt ttatcttacg attttgtacg tgcatgcttg cctaggggta tggctcgagc
11701 cattagtctc tcgcacatac tattccctca ggcgtcagca cttacaaaat cggttgtaat
11761 tttcattttt atacgcattc ttactgagat tatactaata agaggaatag taaaagcaat
11821 tctaagtaaa attgcagata agaggtttgt taaaagcagt tctaagtaaa attgcagata
11881 agaggtttgt taaaagcagt tctcagtaaa attacagata agaggtacgt taaaagcagt
11941 tctaagtaaa attgcagata agaggtttgt taaaagcagt tctcagtaaa attgcagata
12001 agaggtacgt taaaagcaat tccatgcaaa attgctgata aggggtaagt taaaagcagt
12061 tctcagtaaa attgcagata agaggtacgt taaaagcagt tctaggcaaa attgcagata
12121 agaggtgcgt taaaagcagt tctcagtaaa attgctgata aggggtaagt taaaagcaat
12181 cctaagtaaa attgcagata aggggtacag aaaaactaga cttgattaca aaatggagct
12241 tgggacataa atgatttttt aaaaatgaga tgagacgtag attaactcca taatcaatac
12301 gaatctatcg acttctttat ttatgatatt catctctttt taatggaaat aaaagtgcga
12361 ttaatgtgat aatacagtta cgttaattaa aaaaataaaa atgcaaggag aggtaatatg
12421 ctaactgtat atggacatag aggattacct agtaaagctc cggaaaatac aattgcatca
12481 tttaaagctg cttcagaagt agaaggtata aactggttgg agttagatgt tgcaattaca
12541 aaagatgaac aactgattat cattcatgat gattatttag aacggactac aaatatgtcc
12601 ggggaaataa ctgaattgaa ttatgatgaa attaaagatg cttctgcagg atcttggttt
12661 ggtgaaaaat tcaaagatga acatttgcca actttcgatg atgtagtaaa aatagcaaat
12721 gaatataata tgaatttaaa tgtagaatta aaaggtatta ctggaccgaa tggactagca
12781 ctttctaaaa gtatggttaa gcaagtggaa gaacaattaa caaacttaaa tcagaatcaa
12841 gaagtgctca tttcaagctt taatgttgtg cttgttaaac ttgcagaaga aatcatgcca
12901 caatataaca gagcagttat attccataca acttcgtttc gtgaagactg gagaacactt
12961 ttagattact gtaatgctaa aatagtaaac actgaagatg ccaaacttac taaagcaaaa
13021 gtaaaaaatgg taaaagaagc gggttatgaa ttgaacgtat ggactgtaaa caaaccagca
13081 cgtgcaaacc aacttgctaa ttggggagtt gatggtatct ttacagacaa tgcagataaa
13141 atggtgcatt tgtctcaata gaaagttaga ggtgagtctt acgtttcagt gacggtagac
13201 ttacctttaa catgttacat actaaaaaat taatttgaat aagaaagaga gacatatatg
13261 aaatacgatg attttatagt aggagaaaca ttcaaaacaa aaagccttca tattacagaa
13321 gaagaaatta tccaatttgc aacaactttt gatcctcaat atatgcatat agataaagaa
13381 aaagcagaac aaagtagatt taaaggtatc attgcatctg gcatgcatac actttcaata
13441 tcatttaaat tatgggtaga agaaggtaaa tacggagaag aagttgtagc aggaacacaa
13501 atgaataacg ttaaatttat taaacctgta tacccaggta atacattgta cgttatcgct
13561 gaaattacaa ataagaaatc cataaaaaaa gaaaatggac tcgttacagt gtcactttca
13621 acatacaatg aaaatgaaga aattgtattt aagggagaag taacagcact tattaataat
13681 tcataataaa acagtgaagc aaccatcgtt acggattgct tcactgtttt gttattcatc
13741 tatatcgtat tttttattac cgttctcata tagctcatca tacactttac ctgagatttt
13801 ggcattgtag ctagccattc ctttatcttg tacatcttta acattaatag ccatcatcat
13861 gtttggatta tctttatcat atgatataaa ccacccaatt tgtctgccag tttctccttg
13921 tttcattttg agttctgcag taccggattt gccaattaag tttgcataag atctataaat
13981 atcttcttta tgtgttttat ttacgacttg ttgcatacca tcagttaata gattgatatt
14041 ttctttggaa ataatatttt tcttccaaac tttgtttttc gtgtctttta ataagtgagg
14101 tgcgttaata ttgccattat tttctaatgc gctatagatt gaaaggatct gtactgggtt
14161 aatcagtatt tcaccttgtc cgtaacctga atcagctaat aatatttcat tatctaaatt

14221 tttgtttgaa atttgagcat tataaaatgg ataatcactt ggtatatctt caccaacacc
14281 tagttttttc atgccttttt caaatttctt actgcctaat tcgagtgcta ctctagcaaa
14341 gaaaatgtta tctgatgatt ctattgcttg ttttaagtcg atattaccat ttaccacttc
14401 atatcttgta acgttgtaac caccccaaga tttatctttt tgccaacctt taccatcgat
14461 tttataactt gttttatcgt ctaatgtttt gttatttaac ccaatcattg ctgttaatat
14521 tttttgagtt gaacctggtg aagttgtaat ctggaacttg ttgagcagag gttctttttt
14581 atcttcggtt aatttattat attcttcgtt actcatgcca tacataaatg gatagacgtc
14641 atatgaaggt gtgcttacaa gtgctaataa ttcacctgtt tgagggtgga tagcagtacc
14701 tgagccataa tcatttttca tgttgttata aatactcttt tgaactttag catcaatagt
14761 tagttgaata tctttgccat cttttttctt tttctctatt aatgtatgtg cgattgtatt
14821 gctattatcg tcaacgattg tgacacgata gccatcttca tgttggagct ttttatcgta
14881 aagtttttcg agtccctttt taccaataac tgcatcatct ttatagcctt tatattcttt
14941 ttgttttaat tcttcagagt taatgggacc aacataacct aatagatgtg aagtcgcttt
15001 tcctagagga tagttacgac tttctgtttc attagttgta agatgaaatt tttttgcgaa
15061 atctcttaaa tattcatcca tttttttaac ggttttaagt ggaacgaagg tatcatcttg
15121 tacccaattt tgatccattt gttgtttgat atagtcttca gaaatactta gttctttagc
15181 gattgcttta taatcttttt tagatacatt ctttggaacg atgcctatct catatgctgt
15241 tcctgtattg gccaattcca cattgtttcg gtctaaaatt ttaccacgtt ctgattttaa
15301 attttcaata tgtatgcttt ggtctttctg cattcctgga ataatgacgc tatgatccca
15361 atctaacttc cacataccat cttctttaac aaaattaaat tgaacgttgc gatcaatgtt
15421 accgtagttt gttttaattt tatattgagc atctactcgt tttttatttt tagatacttt
15481 ttttatttta cgatcctgaa tgtttatatc tttaacgcct aaactattat atatttttat
15541 cggacgttca gtcatttcta cttcaccatt atcgctttta gaaatataac tgctatcttt
15601 ataaacttgt ttgaaatttt tatcttcaat tgcatcaata gtattattaa tttctttatc
15661 ttttgaagca taaaaatata taccaaaccc gacaactaca actattaaaa taagtggaac
15721 aatttttatc tttttcatca atatcctcct tatataagac tacatttgta atatattaca
15781 aatgtagtat ttatgtcaaa ataatgttat aatttttgtg atatggaggt gtagaaggtg
15841 ttatcatctt ttttaatgtt aagtataatc agttcattgc tcacgatatg tgtaattttt
15901 ttagtgagaa tgctctatat aaaatatacg gttctgttgc aaagttgaat ttatagtata
15961 attataacca aaaggagtct tctgtatgaa ctatttcaga tataaacaat ttaacaagga
16021 tgttatcact gtagccgttg gctactatct aagatatgca ttgagttatc gtgatatgtc
16081 tgaaatatta agggaacgtg gtgtaaacgt tcatcattta acggtctagc gttgagttca
16141 agaatatgcc ccgattttat atcaaatttg gaagaaaaag cataaaaaag cttattacaa
16201 atggcgtatt gatgagacgt acatcaaaat aaaaggaaaa tggaactatt tatatcgtgc
16261 cattgataca gagggacata cattagatat ttggttgcgt aagcaacgag ataatcattc
16321 agcatatgta tttatcaaac gtctcattaa acaatttggt aaacctcaaa aggtaattac
16381 agattaggca ccttcaacga aggtcgcaat ggctaaagtc attaaagctt ttaaacttaa
16441 acctgactgt cattgtacat cgaaatatct gaataacctc attgagcaag atcaccgtca
16501 tattaaagta agaaagacaa gatatcaaag tatcaatacg gcaaagaata ctttaaaagg
16561 tattgaatgt atttacggtc tatataaaaa gaaccgcagg tctcttcaga tctgcggatt
16621 ttcgccatgc catgaaatta gcatcatgct aacaagttaa cacaaagtat tattttaaaa
16681 ttgagattag acatttattt ttcaactttg cagcagaacc taattttttc tctttgtaaa
16741 taaatgcaaa gagataccat aaggatctct tacataacca taaccttcag tatagaattc
16801 tggactaaat gttttcaata cctcactgcc tttttctatt aactggtcat atacatgttt

16861 agtttcttct acttggtcaa aagtgagaca aagagatata ttattacctt gtgttatggg
16921 caaaccttca gtgtcatctg cgatcataat ttttatatct ccaaattgaa gtacacattg
16981 atcaatttta tttaaatcat tttcgtcaat attaagtttc ttatctatag gtctatcttt
17041 aatacgttga atatacagtg ttttagcgcc aaacagctct tcatacaact tttttaaacc
17101 ctctgcattt tgagtgatta aaaaatatgg acttacttga aatttcatgt tttttcctcc
17161 ttagatttgt tataaataga gtatatactt aattagtgtc atctattgac actaaaagga
17221 gaataataat gaaaaaatct gttagattat ataatatgat tgaatattgt aatgaaaata
17281 ggaacttcaa attaaatgat ttaatgtcag aatttaatat ttctcgtagt accgctttaa
17341 gggatataaa agaaattgaa gcattaggag tacctttata tagtaatcca gggaaaaatg
17401 gtggttatac gatcataggt aatcgagacc aaacgaaaat agcaatctca gatgaagagt
17461 tgaaagcttt agtatttaca ctttcgagta tttcaaatgt gagtaatcta ccttttcaaa
17521 cagaatatca agaaatatta aaaaaattat ataataactc gaataaaaaa gagttaataa
17581 accaatataa tgatctattt caatatttta atgaagataa gtatcagttc aaaagttata
17641 agttatttaa tgaaatcatt agattgataa ttgagaataa gtcttttgaa acctgttatt
17701 cacaaaacta tattaaagaa caatataaag gtattggcat tatgtataaa aatcatcaat
17761 ggtattttgt tgtagctaat atagaatcaa agttagtgaa tctattaaat atttcgaaaa
17821 taaaagaact atatgaaatg ggagagactc aagagtgtaa tgatataact atgcagaatt
17881 ttcaacagtt catggttaaa aatgaaacag ctattgatat tcttattaga agcaatgtta
17941 tgggattgaa tatcttgaaa ggctacctgt ggagtgacta tatgattgaa aatattgacg
18001 aagagacata tttatttaaa tcaaaagtga acgcgaaaga tatagatttt atagctaagt
18061 taattgtcac aggtggtgtc aatgtaaaag tagagacccc taatagtttg aaaaatgctg
18121 ttaaagttga attaactaaa ataataaaca tgtattaata gtaatttaat cataattgta
18181 ataacaaaaa gatgacaaca ttaaacaatt taaatttata caaaaaagca ttaccaaatt
18241 taaaattggg tagtggtagt gctacagcat acataaatag ttataattaa ataaagatgg
18301 gtcaatgaag gtgaattaag tgaataaaaa tattacagct ctaaaatcta ctgaaaatac
18361 tacatatatg cagtatacgg ttcatatcga tgatcaaatt ataaataata taaaagagtg
18421 taccaaaaag tttaaaattt tgcctatgga agataaaatt ccattatcac cgttgttaca
18481 accagaatat gcaggagagg tacaagattt tattagtaca tatgagcagt ttatgattaa
18541 ttttggtaaa gtaatattgg atagtcaagg cataaaaata cagtttgaaa gtgaatcatt
18601 aagtagtatt caacgaggca ttcaagaaca ttgttactta aatgagcgaa caaatgacat
18661 tgatgtgact aaagaatggt atttatgcaa attttcaaat caaatattag aagaggataa
18721 aaatcaattg tataatgctt taaagcaact catgaatgat tctaaaaata aaaagcaagc
18781 ttttcaagag gtgtttaagg ttcatattga tatttatctc tacaataaag aaaaatctga
18841 atataagtat caagtatcta gttatttcaa tcttgtaaaa gaaaatccta aaataactta
18901 taaaaaaaga catttacaag agaaacaagg tgtcaagggt acaactttca cgaacaaatt
18961 aaaattattg aacaaaatgt atggtgtaga tgttgcaaaa tatcagccat tttataattc
19021 taataatcct gaatatgaaa gaggtcaatt tggtgaaaga tacatttctc aaagatctaa
19081 ctatgaattt aatagacttc aatatcaaat tatagatatg ttatcgaaaa tacttgataa
19141 gcatccatta ccaaagtcag ataataatta taaacatatt ccaactattg aaaaagcaat
19201 attaagtggg gattctcata gtttttatga gtattttgaa gatataatga aagaaattat
19261 aagtatggaa aattcttctt taaaggaaaa attattgact gattttacat atcaatctca
19321 atgtagatgg tactctgaaa gtgaaaaatt aaaattgcaa cttgaaagtt ttatgcataa
19381 agttttggag agcaattatt atgagggtaa caaattatat agaatgttgt ctcatgcaat
19441 tgaagaaaca atcaatgagg ctgatgaaga taaggttcat ttcaattatt ttaaagatta

19501 tttttgaca gacggtgggg tgaagaattg ggagcagatt agcgagaaaa ttactgaatt
19561 caatggtaaa gttattaacg atatccaaaa tgaatataat aagattcaat ttaataacgc
19621 aaatcgaaat cagaaactaa actttaatta tttataccat tgctttgagt ttagtaataa
19681 cttagtaaaa gcaagggtaa atggaaatag aggttacatt ttatattttg tagataaata
19741 taaaataaaa atgccctttt tggatcaatt atttgaatca agaaataggt caggaaagta
19801 ttaattataa tatgcaaaca ttgtttgaaa aagaaagata tgatagaagt agtaccattg
19861 aaaaattagt agcaacaagc aaatttaagt atgaaaaaga tgattcagat ttattcaaac
19921 aacttttcaa tgatgttgaa aattcaatag acagattagg tatttactta ctaaataatg
19981 gtataaattc gaatgatgaa aatgcaagat attatagatc gtttttaaag gaacttagta
20041 gaataaaaag taaattaacg ccattttctc ttgaaataag taagtctagc ggaagagagc
20101 aacattatcc tgatgatgct attgatgata aagatgagag aagaaaaaat aaagaagaaa
20161 catatcatgc ttttgatgat aaaagcgata ttgactccaa attaaagaat aaaataaatg
20221 tttctatcga taatttattg tcagttaaaa ttaagagcaa tttgtatgat attagtccaa
20281 atacaaaaaa acaataacta taatgagcca actcaagaat gagttggctt tttaattgta
20341 attttacgct tccattacag gaatcaatgt tatggaagcc attaagcaat taatttgagt
20401 catttatagt ataggtacaa cgttgtgatg ttcacatgat aatcagcccg ggagattatt
20461 gtgtaataac ttatagaagg agcaaatcac catgtatcaa gcaaatatac gtgatttaat
20521 tacaaaattg cctcaaagca ataaaacaga acacttttta atgaacaaat tttcaaatca
20581 agataaagtt cagcagctac aaagacaaat tagccaacag ctagatcaac aatataatga
20641 gcttttggct aatgaaaaag ctaagctaga ccaatacgtg gaagtacacc ataatttaga
20701 accattaaag aaagagattg aatcagaatc tattaacctt gataccgata aattacctga
20761 tatcaaagcg acaatgcttg aaaaggctaa gaacgatgaa cattttgata aaatcgaaca
20821 gctatttgat agattagatc agtcattaaa tggtacgaat cgattatata cgcaattatc
20881 gttgattggc acacgaacac atcggatcac aacgaaaaga tttaatgttc aaggcttgcc
20941 taaattagtc caacaaatga ttttaccttc gcaatttaaa aaggtttata caatagattt
21001 taaatcattc gaaccatcag ttgctgcgta tatgacacaa gacgaacaac tgattgacta
21061 cttgaatcat gaagaagggt tatacgatgc attactgaga gacttatctt tgtcaaaaga
21121 gaagcgcgtg agtgtgaaac gtgcatttat agggtcattt ctttttggcg gtcgttatag
21181 tagctctaaa ttcaaaatca atcaagaggt tagtgaaatt aactggctac aagtaatgag
21241 caaattcaag aaggtcattg aatttaagga gcaagtcgaa aaatataaaa caatgcctac
21301 gccttacggc attgaacatg atatgagcgc atttcaaggt agtagtatta tggcaattta
21361 tgtacaaacg gtagcaagct atattttcaa gcacattttg ttggaagtgt acaaagcaca
21421 gtgcgaaaaa aaaacgttca agattatagt gccgatacac gatgcgatta tgattgaatg
21481 taatgataag gggattgcac aaaatgtagc acagctcatg aaagatacag ctaatcaact
21541 gtttaatggt gaatttgcac atgtgacagt ggaagcttta ggaggtatag acaatgaata
21601 atgatagagg aaaaagtctt caaattcccc aaagtacatt gttaaaagaa ggatctatat
21661 acgttgctac gttacattct gtgtacgaga agaacttctc aggtgatatt aaacatcagt
21721 ttacgtatga agtagaactt aaccaagaaa cgcactatgt aaatcgcaat attaccgtaa
21781 aatctatgag tcaccaatta tcaattgctg attggattaa acgtcacagt aactataacg
21841 taaaccacat taattatgat ccatacattg atcgaaaaca tttggtcctt gtagggcaat
21901 ataacggaaa ttattatatt caagatgtag caccattaga tgaatttgga ggggtattgt
21961 aatgaatcat attttagaaa tgttaataaa attattaaaa gtgggtatgg aggcaatcga
22021 ccgtaaaggt ctgattgcca tcctaacaag tagtattgga aatgatgaaa tggatgattc
22081 tgaacaagct gtaatggtgt ataacgagct tatcgataag ctacagctta acattcctaa

22141 agatgtcgac tatagaccta acatatacag ttattttggt attcaaaaaa agccaaatga
22201 cacaatatta gtggaaatga tgatatgtat ttttcatatc aagcgttttg attcagagtt
22261 gtttgttttc aaagataaag gttggcaaaa ggtaagtgaa gatgaattgc aagggttgat
22321 atctaaaatg atacaagtgt tgctagttga ttataaacct tcactaagta ctttgaaaaa
22381 cgtagtagat ggcatacaga aatcaacgga catagaaaaa cttgttgagc atagacagta
22441 cattggttgt ggacgaaata tgttcaatct aaagaccttt aaagtggttg ataatgacct
22501 tgaaatattc cctaaaacac gcttagattt ggaattagat ataaatgaca cgattacgga
22561 caagataccc ccgaatttca aacaatatat gttagagttg gcgaattatg accatgattt
22621 acaatatttc cttttccaac atatggcagt gttattgacg gcagatacta aactacgtcg
22681 tggacttttt ttgtatggaa ctgcaaaaaa tgggaaatcg gtctatatta aattagttaa
22741 gtcattcttt tatagtaatg atatcgtatc taaaacactt aatgaacttg gcgggcgttt
22801 cgataaggaa agtctaattg gtaaacgaat tatggcaagt gatgaagtgg ggaaagctaa
22861 tattgatgaa gcaactgtga atgatttcaa aaaattacta tctgttgaac caattcatgc
22921 tgaccgtaaa ggaagaacgc aagtagaagt tactttagat ttaaaactca tttttaatac
22981 gaatgctgta ctcaattttc catcatcaca tgcaaaagca ttagagcgta gaattgctgt
23041 tattccatgt gaatattatg ttgaaaaagc tgaccctgac ttaattgaaa agttacagga
23101 tgaaaagaaa gaaatctttc tttacttgat gtatgtgtat aagcaaattg taaaaaatga
23161 tatcgagtac ctccaaaatg atcgtgttac tgaaatttct catgattggt taaattttgg
23221 atatgaattt gtttctagta aatcagcaaa tattgcacat cagaaagcgt gtattaattt
23281 actcagaaaa cttatagaaa tcaaaccagg gtcacgtatc aaagtgtcta ggctaaatga
23341 ggttattaga gatgaaatta aagtaagctc tcaagttatt aatgatttgg ttcaagctaa
23401 ctttaatgta caaagtagac taaataatgg ttataagtat tgggtcgatt taggatggaa
23461 agaaactgat aaaaaagatg acatgatttc attcgataaa aatgagaatg taacagatga
23521 tgaattctta tacgaagatg atttgaactt aggttgggag gactttgacg atgaataatg
23581 aacaaattga agcatttgta gaagtgcttg tgcctatcat agaagaacgt atcaataaag
23641 gtaagtaatc taattacgta ctacaggcag ttgcctgtag tactcatatg attaagtggt
23701 aaaagtgata aaaatgaaac gaaattataa atatatatta tctatatgtt gttacaagac
23761 cgatggtctg tagcaataat ctaataaaag gagcggtatg atatgaaggg taaaattgca
23821 ctttattcac gtgttagtac gtctgagcag tcagaacatg gttattctga aaaggagcag
23881 gaacaactac tcatcaaaga agttatgaaa aatttcccag gttatgacta tgagacatat
23941 actgactcag gcatttcagg taaaaatatt gaaggtcgtc cggcaatgaa acgtctatta
24001 caagatgtta aggataataa aatcgaaatg gtattaagtt ggaaattgaa tcgtatttct
24061 cgctcaatga gagacgtgtt taatattatt catgaattca aagaacatga cgtagggtat
24121 aaatcgattt ctgagaatat tgatacatcc aatgcttctg gagaagtact cgttacaatg
24181 tttggactaa taggatctat agaacgccag actttgattt ccaatgtgaa actttctatg
24241 aatgctaagg caaggagcgg agaggcaatc accggtcgtg ttttaggcta caaattatca
24301 cttaatccat tgacacagaa aaatgattta gttattgatg aaaatgaagc tcatattgta
24361 cgggaaatct ttgatttata tttgaatcac aataaaggat ttaaagcaat cacgacaatt
24421 ctaaatcaaa aaggatatcg taccattaat caaaaaccat tttcagtgtt tggcgtgaaa
24481 tacattttga ataatccagt ctataaaggc tatgtcagat tcaataatca tcaaaattgg
24541 gctgttcagc gaagaagtgg taaaagtgat aaaaatgatg tgatattggt caaaggtaag
24601 catgaagcca ttataagtga agatgtattt gatcaagttc atgagaaact agcttctaaa
24661 agttttaaac cgggtcgacc tattggtgga gatttctact tacgtggcct tattaaatgc
24721 ccagaatgcg gaaataatat ggtatgtcga cggacgtatt ataaaacgaa aaagtccaaa

24781 gaacgcacaa tcaaacgcta ttacatttgt tcattattca atcgctcagg aagttctgcc
24841 tgtcatagta atgcgattaa tgctgaagtc gtcgaacgcg taatcaatgt tcatttgaat
24901 cgtattcttt cacaacctaa tgttattaag cagattgcgt caagtgtgat agaagaactg
24961 aaacaaaagc atagtaaaca aacagaaata aaatatgata ttgatagtct agaaaaacaa
25021 aaagcaaaag ttaaaacaca acaagaacga ttattggaat tgttcttaga tgatgaaatg
25081 gatagcgaaa tgttaaaagc taaacaaagt gaaatgaatc aacagttaga agtattagac
25141 caacaaatta aagaagcaaa acaagcaaat caatcacagg atgatatacc caattttgat
25201 aagttaaaag cacgactcat tttgatgata acacgattca gtgtgtactt aagaaaggct
25261 acacccgaag ctaaaaatca acttatgaaa atgttaattg attcaattga aattacgaca
25321 gataaacaag taaaacttgt aaggtataaa attgatgaaa gtcttatccc tcaatctttg
25381 aaaaaagatt gggggtcttt ttttatacct aaatttaact ttgtgataaa tgtcacaaag
25441 aaaaatagga ttgaaaattt atcactttta ccactttttt agagtgacaa aagtggagga
25501 gttttgaaat attttataaa tatatatttt atttatggag tacacattat taattaagga
25561 ggtcattata atgacgctaa gcaaacaact taaaacgtat atcactgaac gatttaaatt
25621 aaattatcaa gaaacttggg cttgtgaaac catagatgcg gtggctgaag atgtattacc
25681 tgaaaaatat attaaaaata gtccacttga acataaaatt ttaaatactt ttacctatta
25741 caatgatgaa ttacatgaaa tcagcattta cccttttta tgttatctag ataaggaatt
25801 agtagcaata ggttatttag ataattttga tttagacttt atatttttaa atgacactca
25861 tcaaattatt attgatgaac gctacttgtt acaaaaaggg ggcgagtaat tatgaactgg
25921 atcaaggttg ctcagctatc tgttacagtc attaacgaag tgattgagat catgaaagaa
25981 aagcagaatg gaggaaaata gtatgaacat caatcgatac atcacgagag gcattagtga
26041 acaactatct ctagaccttc aaatcttact ttggcacatg gtagaagaaa aagataacca
26101 gcctcatacc gattacctac acattttcaa actacaagaa gatgataata tgttgtcaat
26161 tacacatgaa caagaacagc ccgcatacaa gttagaatat cactatataa actatgaaaa
26221 aaatcaaaat gcattaccta agaaagtcta cgtcattcgt gaagatgatg tagacgtttt
26281 ttattatgtg atgcttttac cagaagaata ctaaaaggag tgacgttgat gaatacaatc
26341 aaaagtacga tacacacaga agctatattt agcgatgatg aacaacaccg ctacttactc
26401 aaaaagactt gggatgaaaa gaaacctgtt tgtacagtga taacgatgta tcctcattta
26461 gatggcgtat tatcactcga tcttactact gttcttatcc tcaaccaatt agcgaattct
26521 gaacaatatg gcgctgtata tctagtgaat ctattctcta atattaaaac accagagaat
26581 cttaaacata ttaaagaacc ttatgataaa cacacagaca tccatttgat gaaagcaatt
26641 agtgagagtg atacagtaat tctagcttat ggcgcctatg caaagcggcc tgttgtcgtc
26701 gaacgtgttg agcaagtgat ggaaatgtta aaacctcata aaaagaaagt caaaaaactc
26761 ataaacccag caacaaatga cattatgcat ccacttaacc ctaaagcacg tcaaaaatgg
26821 attttgaaat aaaaggagga ttatctatga accatgaaac tacacactca gactggcgaa
26881 cggttgctaa ttgtttagca tcacaaaatt atatatcgat cgtaaaagga ttagtacatc
26941 atttcacagc gattgaagat gaagaaatac tagataaaat ctatgaagat tttatgaata
27001 atggctctat tacaacggta cttaacaatg atttacaaga tattattaat tattacctat
27061 caaaataaaa atatattact tttaataaaa ttagagcatc cttcactttg cggtgagaga
27121 tgctctttta gtttattttt ttattgatta ctcgccataa gttctgcttg ctctacagcc
27181 atttttataa atctggtgga tagctatatt ttttaagtaa tcgtctaaca gctacgcgca
27241 ttttagcttt tgcgctatca cgtttagacc aatcaccccc atattttctt tcactttttt
27301 agttagctca tgagcaatcg cacgtagttc ttcatcttcc attccttttt ttcgctgttt
27361 catgtgaaac taaagcatcg taaaaatgcg atttcatctg aatttaaacc taattcattt

27421 cctcgttgtt gttcttgttt aatatctttg gcgagttgaa tgagttcttc aatcactttt
27481 gatgtttcaa tggaacggct attatattta ttaatcgaat ttcttaacat ttcagagaaa
27541 cgtttagata ccgtcgcatt cgttttcatt aatgatttga cttgtccctt gagtaatcga
27601 tttaataatt ctaccgccac atttttctgt ttcaatcctt ctacatcttt taaaaagtca
27661 tcagatagga ttgataaatc gggttgttca agacctaaag tttaataaat gtcaagtcaa
27721 tgaaatcttc agttaatagt gtatatttga ttattattag tttaaatata ttaaaattaa
27781 atacttatta tatatggttc tttactattt tagaagtata ttttttattt tcctgaacag
27841 tagttattaa atttatctct tattttaaat tagaaaggaa gaaaaagtta aatttatatt
27901 aagttttata tatgattaat tattaacgta ttaaatgtag tatactctta atatatagaa
27961 aattatcata atatttgtca aaaaaagtga catttcatat agtagcatgg tgttagttgt
28021 cacagttttt gacagccaaa ataacacttg aacctttgtg gttatgctat ttattaaagt
28081 atctgatcga taactacccc gaataacagg ggacgagaat ttttctttaa ctgtttttac
28141 tgcccattta atagtgatcg ataactaccc cgaataacag gggacgagaa tacaacttcg
28201 tcatctttca tcatttctct tacatcagat cgataactac cccgaataac aggggacgag
28261 aatatatttc ttccatgaat aacaccctcc tttttctag atcgataact accccgaata
28321 acaggggacg agaataagtt aacggcatta cctaataaaa atattttagg gatcgataac
28381 taccccgaat aacaggggac gagaactcat ctttcatgtc actgattaat tcatttgtag
28441 atcgataact accccgaata acaggggacg agaacggtaa tagttgctca ataggtaata
28501 aaacgtcggt gatcgataac tatcccgaat aacaggggac agagtgtaaa tttaattaca
28561 ctctaaaatt tgtaaatttt taatggaata cgcattgatt aatttttagg ggatgaaaaa
28621 tgaaagatgt tatttatgta gaaaatcatt actttgttac cgtgaaagaa aatagtatta
28681 aatttagaaa tgtaatagat aaaagtgaga aattttattt gtttgaagaa atagaagcga
28741 ttatttttga tcattataaa agctattttt ctcataaatt agtaattaaa tgtatagaaa
28801 atgatatcgc tattattttt tgtgataaaa agcactctcc attaacgcaa cttatttctt
28861 cttatggtat gactcatcgt cttcaaagga ttcaaagtca gtttcaatta tctgggagaa
28921 ctagagatag aatttggaaa aagattgttg taaataaaat tattaatcaa tcaaaatgtt
28981 tagaaaacaa tttacataat gagaatgtga agttattagt aaacttagca aaagatgtta
29041 gttctggaga taaaagtaat aaagaagcac aggctgcaag aatttatttt aaagatttat
29101 acggtaaaca atttaaacgt ggacggtaca atgatattat taactcaggg ttgaattatg
29161 ggtattcgat acttagatct tttataaaaa aagaactagc tttacatgga ttcgaaatga
29221 gtttaggcat taatcatcgt tcgaaagaaa atccatttaa tttagcagat gatattattg
29281 aagtttttcg tccttttgta gataatatcg tgtacgagat agtttttaag aaaaatatta
29341 atacatttga tgtaaatgaa aagaaattat tgttaaatgt tttgtatgaa aagtgcatta
29401 tagataaaaa agtggtgagg ttacttgata gtgtgaagat agttattcaa tcacttatta
29461 gatgctatga agaaaacacc cctacttatt tattactacc taaaatgatt gaggtgggga
29521 actaatgtat ttattagtta gttttgactt acctagagat actaaatttg aacgtagagt
29581 cgcaagtaag tatcgtactc gattattaga actcggtttt agtatgaagc agtttagttt
29641 atacgagagg tatgtcagcg atgttcaaaa aaaagataag attttagaaa ttctgcaaca
29701 agaaattcct gatactggaa gtattacact atatgtctta cctgatgagg taaataatag
29761 tcagattacc atattaggaa aagaggttaa ggttgttgtg cgaaaagaac ctaagctaat
29821 ttttctataa agtggaggtt acaatggata aaaaaacgac attaatgtat ggttctttac
29881 tacatgatat aggtaaaatt atctatcgga gtaatgatca tgcatttgca agaggaacgc
29941 attcaaagtt aggatacaat tttttatctc aattttcaga atttaaagat aacgaagtgc
30001 tcgatagtat tgcttatcat cattataaag aacttgcaaa agctaattta gctaatgata

30061 atacagctta tattacctat attgccgata atattgcgag tggtattgat agacgagatg
30121 ttatagaaga gggcgacgaa gaatacgaaa aacaagcatt taattttgat aagtatacac
30181 ctctatatag tgtgtttaat attgtgaatt ctgaaaaatt gaaacaaata agtggaaagt
30241 ttaaattttc taatgaaagt aatattgagt atcctaaaac tgagaacatt caatattcaa
30301 gtggaaatta tacaacacta atgaaagata tgagttatga tttagagcac aaattaagta
30361 ttaaagaaga tacatttcct tcattattac aatggacaga aagtctatgg caatatgtgc
30421 caagttcgac aaataaaaat caattaattg atatttctct ttatgatcat agtcgtatta
30481 catgtgctat tgctagttgt atatttgatt atttaaatga aaataatata cataattaca
30541 aagatgaatt gtttacaaag tatgaaaata ccaaagaatt ttatcaaaaa gaagcttttt
30601 tactacttag tatggatatg agtggtattc aagattttat ttacaacata agtggttcta
30661 aagcattaaa aagtttaaga tctcgtagtt tttacttaga aatcatgctt gaggtaatcg
30721 ttgatcaatt actagaaaaa ttagaattaa cacgagcaaa tcttttatac actggtggag
30781 gccatgcata tttattagtc tctaatacag ataaagggaa agaaaaaata aatcaattta
30841 atactgaatt aaaaaattgg tttatgtcag aattcactac agatctctca ttatcaattg
30901 cttttgaaaa atgtagtggt aatgacttaa tgaatactag tggtaattat agaaatattt
30961 ggcgtaatgt cagcagtaag ctttctgata ttaaagcaca taagtattct tcagaagata
31021 tattaaaatt aaatcatttt cattcatatg gggatcgaga atgtaaagaa tgtttaagaa
31081 gtgacataga tattaatgat gatggattat gcagtatatg tgaaggaatc attaatatat
31141 caaatgactt aagagataaa tcattctttg tactgtcaga aactggaaaa ttaaaaatgc
31201 cattcgataa atttatatcg gttatcgatt atgaagaagc agaaaggtta gcacaaaata
31261 ataatcacat tcgtatttac agtaaaaata aaccttatat tggtgtagga atatcaacga
31321 atttatggat gtgtgactac gactatgcta gtcaaaataa agatatgaga gaaaaaggta
31381 ttggaagtta tgtagaaaga gaagaaggca ttaagcgttt aggcgttgta cgtgccgaca
31441 ttgataatct tggcgctaca tttatatcag gaattccaga aaaatataat tcaatttcaa
31501 gaacagctac attgtctcgt caattatcat tattttttaa atatgaatta aatcatttat
31561 tagaaaatta tcaaattaca gctatatatt caggtggcga cgatttattt ttgattggcg
31621 catgggatga cattatagaa gtaagcgttt atataaatga aaagtttaaa gattttactc
31681 ttggtaaatt aacaatgtct gctggtgttg ggatgttcag tggtaagtat ccaatttcta
31741 aaatggcttt tgagacaggt ctacttgaag aagcagctaa aactgatgaa aaaaatcaga
31801 tagcactttg ggtgcaagaa aaagtatata actgggatga gtttaaaaag tatatcttag
31861 aagaaaaact tctcgtttta caacatgggt tttcacaaac agatgaacac ggaaaagcct
31921 ttatttataa aatgttagct ttactgcgaa ataatgaaac tattaatatt gctcgcttag
31981 cctatttatt agcaagaagt aaaatgacag aggaatttac atctaaaatt tttaattggg
32041 ctcaaaacga caaagataaa aatcaattaa ttacagcttt agagtattat gtttatcaaa
32101 taagggaggt tgattgagta tgatattagc taaaacaaaa agtgggaaaa aaatagattt
32161 aacttttgca catgaagtag taaaaagtaa tgtaaagaca gttaaagata aaagaggtaa
32221 agaaaatcaa gttttattta acggtcttac aacaagcaag ttaagaaatt tgatggagca
32281 agtgaaccga ctttatacta ttgcatttaa ttcgactgaa gatcaattga atgaagaatt
32341 tatcgatgaa ttagagtatt taaaaattaa attttattat gaagcaggtc gagaaaaaag
32401 tgttgatgaa ttcttgaaaa aaacattgat gttcccaatt attgatagag tgatacaaaa
32461 agaatcaaaa aaatttttct tagattactg taaatacttt gaagctctag ttgcatacgc
32521 taaatattat caaaaggagg attaatatgt attcaaaaat taaaatttca ggaacaattg
32581 aagtagttac tggtttacac atcggcgggg gcggtgaatc tagtatgatt ggcgcaattg
32641 attcccctgt agttagagat ttacaaacta aattacctat catacctggc agttcaatca

32701 aaggaaaaat gagaagttta ttagcaaagc attttggctt gaaaatgaaa caagagaatc
32761 ataaccaaga cgatgaaagt gttttaagat tattcggttc aagtgaaaaa ggaaatattc
32821 aaagagctcg tctacaaatt tctgatgcat tcttttctga aaagacgaaa gagcattttg
32881 cacaaaatga tattgcttat acagaaacga aatttgagaa tacaattaat cgtttaactg
32941 cagttgcaaa tccaagacaa attgagagag taacaagagg atctgagttt gactttgtat
33001 ttatttacaa tgtcgatgaa gaatcacaag ttgagaatga ttttgagaat attgaaaaag
33061 caattcactt attagagaat gactaccttg gtggcggcgg taccagaggt aacggacgta
33121 tccaatttaa tgatataact atcgagacgg ttgttggaga atacgacagt acaaatctta
33181 aaattaagta ggtgaaagac attgacaaca aaagtattta aactttcttt taagactcct
33241 gttcattttg ggaaaaaaag gttgtcagat ggggaaatga caataacttc tgatactttg
33301 tttagtgcgt tatttattga ggcgcttcaa ttgggtatag aaactgattg gttattaaac
33361 gatttaatca ttagtgatac atttccttat gaaaatgaaa tttattatct tcctaaacct
33421 ttgataaaaa ttgaatctaa agaagaaggc aaccataaag catttaaaaa gttaaaatat
33481 gttccggttc atcactataa tcaatattta aatggtgagt taagcgctga agatgcgaca
33541 gatttaaatg atatttttag tattgggcat ttttctctac aaacaaaggt ttcattatca
33601 gcacaagaaa ttgattcaag tgctgacagt gaaccttatt cagtgggaac atttactttt
33661 gaacctgaag ctggtttata ttttattgca aaaggatcag aagaaaccct tgaccaatta
33721 aaagatatta tgacttcatt acagtactca ggtttaggtg gtaaacgtaa tgcaggatat
33781 ggacaatttg aatatgaaat aataaataat caacaattat ttaagttatt gaatcaaaat
33841 ggagaacatt ctattctttt atcaacggca atggctaaag aagatgagat aaagagtgct
33901 ttaaaagagg caagatatat tttgaataaa cgttctggtt tcatacaatc aacaaattat
33961 tctgaaatgc tagttaaaaa aagtgatttc tatagctttt cttctggttc agtttttaaa
34021 aatatcttta acggtgacgt ttttaatgtt ggacataatg gtaaacaccc agtctatcgt
34081 tatgcgaagc ctttatggtt ggaggtataa gtatgacaat aaaaaattac gaagttgtta
34141 ttaaaacttt aggtccagtt catattggta gtggtcaagt tatgaagaaa caagattaca
34201 tttatgattt ctacaattct aaagtttata tgattaatgg aaataaactg gttaagtttt
34261 taaaaagaaa aaatatactc gatacatatc aaaacttttt gagataccca ccaaaaaatc
34321 caagagaaaa tggactaaaa gactatttag acgctcaaaa tgttaagcaa agtgaatgga
34381 aagcatttgt gagttattct gaaaaagtta atcaaggtaa gaaatatgga aatatacggc
34441 ctaaaccgct aaatgattta catttaatgg taagagatgg acaaaataag gtgtatcttc
34501 cgggaagttc aatcaaggga gctattaaaa cagcacttgt ttcaaaatat aataatgaaa
34561 aaaatacaga tgtatatagc aaaattaaag tcagcgattc agagcctatt gatgaaagac
34621 atttagcaat ttatcaaaaa atagatatta ataaaagcga aaaaccaatg cctttatata
34681 gagaatgtgt agatgtagat actgaaataa aatttaaatt aaccatagaa gatgaaattt
34741 attctattaa tgaaattgaa caaagcatcc aagattttta caaaaactat tatgacaaat
34801 ggttggtcgg tttcaaagaa acaaaaggtg gaagacgatt tgcattagaa ggcggtatgc
34861 ccgatgttct aaaccaaaat attttattct taggagctgg cgcaggattt gttagtaaaa
34921 cgacacatta tcaattaaaa agtcgagaac aagcaaaacg agattctttt gatattttaa
34981 ctaaaaaatt tcgtagaact tatgggaaaa tgaaagaaat gccttctaac gtaccagttg
35041 ctctaaaggg tacaactaat caaagtcttc atgtttcata tcaacaaggt atgtgtaaga
35101 ttagttttca agagttaaat aatgaggtgc tataatgaaa gtactattta gtccaatagg
35161 taactcagat ccatggagta atgatagaga tggtgcaatg cttcatattg tgcgtcatta
35221 taaacctgat gtagtcgttt tattttttac tgaaagcatt tggaatggta atagaaatat
35281 acctggacgt aaaaacttcg attgggaaaa cattgtttca aaagtatcat cgagaacaaa

35341 agtggatata aaagtggata gtattaaata tgaaaatgat tttgatagct ataaagatat
35401 atttcacttt tatataaatg agattagaac taaatattct gatgcagaaa ttttattaaa
35461 tgttacaagc ggaacaccac aaatggagtc aactttgtgt ttagaatata tttctaatcc
35521 tcataatatg aaatgtatac aagtttcaac acctgctcct attgaaggac ctaaacgttc
35581 atttgcgaaa cttgaaactg tgactgaaga tttaaataaa gttaatgcta atgaaaaaat
35641 ggcttctaat agaagtaaat caatcaatat tattagtttt agagaagtga tggtgcgctc
35701 tcaaataaaa agtttagtga ataattatga ttatgaaggt gcactgaatt tagtaagtga
35761 tcaaaagtct tttcgtaatg gtaaactatt aaggaaaaga ttattggaat taacaaatca
35821 gattaaaacg catgaagttt ttccagaaat taatgataag tacagaagtg ttgctttaaa
35881 aaaatcatta ttccattatt tattattaaa tatgcgatat aatcgtcttg atgtagctga
35941 aacgttaata agagtgaaat ctattgctga gtttatactt aaaacatata ttgtgggtca
36001 ttggcctact ctaataattg aaaaagatga taaaccttat ttaaatgctg aagataattt
36061 atcttttatt tataaatata aattactatt agaaaaaaga agacagaatt tggatgtttc
36121 aaggattcta ggtctacctg cgtttataga tatacttaca gtattagaac ctaactctaa
36181 actattaaaa gaagttaatg ctgtaaacga tataaatggt ttaagaaatt ctatagctca
36241 caatttggaa acattagatt tggataaaaa taaaaattat aagaaaataa tgttatctgt
36301 tgaagcaata aagaatatgt tgcatatctc atttcccgag atagaggaaa aagactataa
36361 ttatttcgaa agaaaaaaca aggaatttag agaattatta tgataaataa aattacagta
36421 gaattagact taccagatag tattcgtttt caatatttag gaagtatttt acatggtgtg
36481 ttaatggact atcttcctaa tgatattgct gatcagctac atcatgaatt tgcttatagt
36541 ccattgaagc aaagaatata tcataaaaac aaaaaagtaa tttgggaaat tgtttgtatg
36601 tcagatcgat tatttaatga gatagccgag ctattcactt ctaaaaacag gctgttttta
36661 aaatattacc aagtttatat tgagatttat tcttttaata ttgagaaagt aaacgttcaa
36721 aatattatga atcaactctt agaaacagag gaattgaatc gatatgtaag aattaatata
36781 cagacaccta tgtcttttaa atatcagagt aattacatga tttttcctga ggttaaacgt
36841 ttctttagaa gtattatgat acaatttgat gcttttttcg aagaatataa aatgtacgat
36901 aaagaaacat tagatttttt agaaaaaaat attaatattg ttgactataa attgaaaagt
36961 acacgattta acttagaaaa agttaaaatc ccttcattta cgggagaaat agtgtttaaa
37021 attaaaggac ccttaccttt tctacaacta actcattttt tattgaagtt tggcgaattt
37081 tctggatcag gtatgaaaac aagcttaggc atggggaaat atagtataat ataactagag
37141 tatagctgaa atttagttgt caaaaaatgt gacattctgc acttatagta caagtgattt
37201 gtcactattt ttgacagtaa attttgcctt tgaactattg atttgatagg ctttaaagag
37261 ctatctaaat tcgataacta ccccgtagaa gaggggacga gaactcttct aagacgcgat
37321 atgattctaa ttggtcttca ttcgataact acccccgtag aagaggggac gagaactgat
37381 atactccttt accatgtatt aattctggac cactattcga taactacccc gtagaagagg
37441 ggacgaggat ctcatcttga tgatcccaat tggcttggtt aaaaagaaat cgataatcct
37501 cgtagaatag agaaaaagta ttaaaaatat atttgatttg ataaaatatt ctgcaaaaaa
37561 gaggaacaaa aaatcaagat ttaaactgta attcattatt attgaaatta atcgacattc
37621 aatttctaaa taaacagaaa caaaaataac tttcatataa ataatttctt tgcgattaag
37681 ttggaattag aaatactaaa tacaaataac agcgaatgaa atgcatatta ttgaggaatt
37741 ggatgtgtag tgcaatttcg agagttacta cttaagaaaa taaaaagaag aaaacaaaca
37801 ataagaatta aatctgaagc aagatataaa agcaaatatt tttgatacaa ccacattagt
37861 aaatacttta ttaattagta tacgactgaa agcgaagtaa cagctaacaa tgctccagat
37921 atagacatta gtgttacgaa agaataatat taccatctag aaaatgtaat tttttaaaaa

37981 tctgaagata aagtagatta ctttgtgagt tttcatgtta agaattctga acttggagac
38041 gtagtataaa tatagcttag tagaaaatgc caccggagct gtagctgaga ttgactatca
38101 agaattaact aaattaggtt attacccatc aacaaaaatc caagatttaa cactgtctaa
38161 taatagtgcg aataatgaag tttaaaagta aacaactggt aatagcgaat caaactaaag
38221 taatcaatta cagaattcaa gtactactca agcctcaagc aaatattgaa actcaaacac
38281 aagttaagaa taatacactg cctgaaacag atgaacagtc taatgccgat tgagtccaat
38341 aatagcctct gtattattag ccacacgatt attatcaata tttaaataaa gttaatatta
38401 aataataaaa atccttcaaa ttatttgttg aaaaattaat aactttgaag gtttttagca
38461 aaatcactga tagggagagg catatcataa atg
//
LOCUS Staphylococcus 44455 bp DNA linear UNK
DEFINITION Staphylococcus Staphylococcus epidermidis strain 7
ACCESSION Staphylococcus
KEYWORDS .
SOURCE Staphylococcus Staphylococcus epidermidis
ORGANISM Staphylococcus Staphylococcus epidermidis
Bacteria; Bacillati; Bacillota; Bacilli; Bacillales;
Staphylococcaceae; Staphylococcus; Staphylococcus epidermidis.
FEATURES Location/Qualifiers
source 1..44455
/genome_md5=""
/organism="Staphylococcus Staphylococcus epidermidis"
/project="hwwang_176279"
/genome_id="176279.62"
/mol_type="genomic DNA"
/db_xref="taxon:176279"
CDS 1..480
/product="23S rRNA
(pseudouridine(1915)-N(3))-methyltransferase (EC
2.1.1.177)"
/translation="MKITILSVGKLKEKYWKQAIAEYEKRLGPYTKIELIEVPDEKAP

ENMSDKEIEQVKEKEGQRLLNKIKPQSTVITLEIKGKMLSSEGLAKELQTRMTQGQSD

FTFVIGGSNGLHQDVLQRSNYALSFSTMTFPHQMMRVILIEQIYRSFKIMRGEAYHK"
/db_xref="SEED:fig|176279.62.peg.1"
/transl_table=11
/EC_number="2.1.1.177"
CDS 726..1031
/transl_table=11
/db_xref="SEED:fig|176279.62.peg.2"
/translation="MKGNIQCPRLIKGSLLDFVLETVDVFVNGSKNDCELVNFNYKEG
KVVSRYSTKFESNPKLRKKQLISTVKVVRYVIFDFEKILWGIGKVLLKFIILRQCLL"
/product="hypothetical protein"

CDS 1230..2093

/translation="MKHINQFSESTLAQLEKDQQHVFYVYCLMDPRNDECFYVGKGKG
NRIFKHKQDAQKKVLYEDIFIEENKDNLKFNRINEICSNDLNVLGYIISYGLTESEAF

SAENVLINFLNLTNKTTLTNMINGHGSKAYLVEDLENEFGYDSINLENINTNELILAV

KIRDAFLLDKDESKEYPINESKRDRNNLKSRTLGSWIIGKDKIHKIKYIIGINTGANN

AVVSAYEVSFEQAESIETNNGRMRYAFIGLSERDATLKKLNLYKKALPDLRFGSGSAT
AYINNGTMKVD"
/db_xref="SEED:fig|176279.62.peg.3"
/transl_table=11
/product="hypothetical protein"
CDS 2201..3460
/db_xref="SEED:fig|176279.62.peg.4"
/translation="MPSDDIVKKLYSKEDIRLELGLTPHKFNKKMETIAKLFKIDMKI

FHNYKGQDKNNQYTFNGVAKELIKVLLKSVDYYPVDINSKKFKQNGKSKKEMIENIDN

SSYMKYIYQLMKSINEIQYKRLIADIHMKDVYQNTKAWLNNGESINKKEQELYQYMTI

LPLHKRVELQNEVLKSIDETIFQFLAKEHRNNQIEENNELEAYTKAIKEGRNPKNDYE

LNHLLYKKNQLPLDSLIEDIWDYEETEYTELDWLIADMLKRSQRADSNFIESLEKKNK

LRKNIHKDSIKSISKLIDHELVKNNRKWDVAEFYKNQQFWNNSTLRRSSQNFGILYYK

KNRLHTMNQSNIIKKIKIIEANIESANKQPSYLDFFLQAKFDLERLESELMKYSINPK
YFNKINSDYVSMLLKMYIMQLLKLTDI"
/transl_table=11
/product="hypothetical protein"
CDS 3472..3675
/product="hypothetical protein"
/db_xref="SEED:fig|176279.62.peg.5"

/translation="MKLNYSNVQMVVRNEITEQGSYFVTQALNTLSKVEESGYSFDNF
MVGPFVEDFKKAIKNRRFGSKKD"
/transl_table=11
CDS 3902..5002
/product="hypothetical protein"
/db_xref="SEED:fig|176279.62.peg.6"

/translation="METRDKLMSLTQSDKTQQWLMDKSSNQDDIQQLQQQFSQQLDQQ

YNALLADEKAKLDQYVEVHQGLESLKEEIESEPITLNIDKLPDIKATMLERAKNDEHS

DKIEKLFDRLEQALNGTNRLYTQLSLIGTRTHRITTKNFNLQGLPKAVQHTILPSKFK

KVYTVDFKSFEPSVAAYMTQDSKLIDLLNQKDGLYDALLSELGLSDELRVFVKRAFIG

SFLFGGNFKNPKFKLNQYVSEVQWLDAVSQFTKVIELKKHVEKSNSMPMPYSIEHDMS

AFQGSSIMAIYVQTVASYIFKHILLKVYKAQCDQKTFKIIVPIHDAIMIECEDEEIAQ
NVGQLMKDTANQLFNGEFAHVTVEEIGGVDHE"
/transl_table=11
CDS 4995..5366
/transl_table=11
/translation="MNNDRGQSLHIPSSTPIKENNIYVATLHSVIQTDFSGEIKHQFT

YEIEVNNQIVYANRNIPTKPSANQLSIHDWLKRHSNYSVGHDTYEPYIDRKHLIHIGQ
YSGNYYVQDVASLDAFGGVLS"
/db_xref="SEED:fig|176279.62.peg.7"
/product="hypothetical protein"
CDS 5363..7006
/db_xref="SEED:fig|176279.62.peg.8"
/translation="MNHILQMLSKLLSVAKEAIDRQGLIAILTIPVNNDDEIEETAQG

ETVYNELIDQLRLNIPKDTDYRPNIYSYFGIKKNPNDTVLMEMMIKVFHIKRFNSELF

VFKANGWQKINGDELQGLISKMIQVLLVDYKPSLSTLKNVVDGLQKSTDVEELVENER

YIGCGENMFDLNTFQVVKNSIDIFPKTRLNLSLSTNDVITDKIPPYFKQYMLQLANYD

DDLQYFLFQHTAVLLTADTKYRRGLILYGGAKNGKSVYIELVKSFFYSKDIVSKPLNE

LEGRFDKESLIDKSLMASHEIGQSRIQEKIVNDFKKLLSVESMHVDRKGKTQVEVILD
LKLIFSTNAILNFPPEHAKALERRINIIPCEYYVEKADTSLIDKLQSEKKEIFLYLMY

VYQQIVKADIEYLENSRVTEITHDWLNFGYEFVSSRSVSNANQKACINLLRKLIEIKS

GSRIKVSELNKVINEEIKVSSQVIKQLIQANFDTQTKLYNGYDYWIDLGWKEANKKEI
HDISEKDNIISLDKNENITDDEALDEENLDFDWEDFDDE"
/transl_table=11
/product="putative primase"
CDS 7232..8908
/product="Cassette chromosome recombinase B"
/translation="MKGKIALYSRVSTSEQSEHGYSIHEQEQVLIKEVVKNFPGYDYE

TYTDSGISGKNIEGRPAMKRLLQDVKDNKIEMVLSWKLNRISRSMRDVFNIIHEFKEH

DVGYKSISENIDTSNASGEVLVTMFGLIGSIERQTLISNVKLSMNAKARSGEAITGRV

LGYKLSLNPLTQKNDLVIDENEANIVREIFDLYLNHNKGLKAITTVLNQKGYRTINQK

PFSVYGVKYILNNPVYKGYVRFNNHQNWAVQRRSGKSDKNDVILVKGKHEAIISEEVF

DKVHEKLASKSFKPGRPIGGDFYLRGLIKCPECGNNMVCRRTYYKTKKSKERTIKRYY

ICSLFNRSGSSACHSNSINAEVVERVINVHLNRILSQPDIIKQIASNVIEELKQKHSN

QTEIKYDIDSLEKQKAKLKTQQERLLELFLDDQMDSEMLKAKQSQMNQQLEVLDQQIK

EAQQANQSQDEIPNFDKLKGRLILMITRFSVYLRKATPEAKNQLMKMLIDSIEITTDK
QVKLVRYKIDESLIPQSLKKDWGSFFMPKFQFEIYGQNDYFIDQITTFTT"
/db_xref="SEED:fig|176279.62.peg.9"
/transl_table=11
CDS 9014..9352
/transl_table=11
/db_xref="SEED:fig|176279.62.peg.10"
/translation="MTLEQQLKHYITNLFNLPRDEVWHCESIEEIADDILPNQYVRLG

PLSNKTLQTNTYYSDTLHESNIYPFILYYQKQLIAIGYIDENHDMDFLYLHNTIMPLL
DQRYLLTGGQ"
/product="FIG01108299: hypothetical protein"
CDS 9448..9759
/transl_table=11
/db_xref="SEED:fig|176279.62.peg.11"

/translation="MNRYITRGIANNLPNILQHQLWQLVSEREQEQTKDNTSVDYFHI

FQFNKHRNQLYIKHKQERPEYAKIHKANYSKAININKVYIIREDDVDLSYYVMLLPEE
Y"
/product="FIG01107894: hypothetical protein"
CDS 9775..10281
/product="hypothetical protein"
/db_xref="SEED:fig|176279.62.peg.12"
/translation="METIKSTLKTEAIFSDDKQHRYLLKKTWNSEKQSITIITMYPHY

DGILNIDLTTQLIMNKVSEMDAFGSINFVNLYSNITTPINLKHLENAYDKHTDIQIMK

AVKESDEVILAWGAYAKKPGVEARVNEVLEMLKPHKKKVKRLMNPETNEIMHPLNSKA

RQKWILKV"
/transl_table=11
CDS complement(10430..11104)
/product="Transposase for insertion sequence-like element IS431mec"
/transl_table=11
/translation="MNYFRYKQFNKDVITVAVGYYLRYTLSYRDISEILRERGVNVHH

STVYRWVQEYAPILYQIWKKKHKKAYYKWRIDETYIKIKGKWSYLYRAIDAEGHTLDI

WLRKQRDNHSAYAFIKRLIKQFGKPQKVITDQAPSTKVAMAKVIKAFKLKPDCHCTSK
YLNNLIEQDHRHIKVRKTRYQSINTAKNTLKGIECIYALYKKNRRSLQICGFSPCHEI
SIMLAS"
/db_xref="SEED:fig|176279.62.peg.13"
CDS 11444..12187
/product="Glycerophosphoryl diester phosphodiesterase (EC 3.1.4.46)"
/translation="MLTVYGHRGLPSKAPENTIASFKAASEVEGINWLELDVAITKDE

QLIIIHDDYLERTTNMSGEITELNYDEIKDASAGSWFGEKFKDEHLPTFDDVVKIANE

YNMNLNVELKGITGPNGLALSKSMVKQVEEQLTNLNQNQEVLISSFNVVLVKLAEEIM

PQYNRAVIFHTTSFREDWRTLLDYCNAKIVNTEDAKLTKAKVKMVKEAGYELNVWTVN
KPARANQLANWGVDGIFTDNADKMVHLSQ"
/db_xref="SEED:fig|176279.62.peg.14"
/db_xref="GO:0008889"
/transl_table=11
/EC_number="3.1.4.46"
CDS 12284..12712
/transl_table=11
/db_xref="SEED:fig|176279.62.peg.15"
/translation="MKYDDFIVGETFKTKSLHITEEEIIQFATTFDPQYMHIDKEKAE

QSRFKGIIASGMHTLSISFKLWVEEGKYGEEVVAGTQMNNVKFIKPVYPGNTLYVIAE
ITNKKSIKKENGLVTVSLSTYNENEEIVFKGEVTALINNS"
/product="MaoC domain protein"
CDS complement(12758..14764)
/translation="MKKIKIVPLILIVVVVGFGIYFYASKDKEINNTIDAIEDKNFKQ

VYKDSSYISKSDNGEVEMTERPIKIYNSLGVKDINIQDRKIKKVSKNKKRVDAQYKIK

TNYGNIDRNVQFNFVKEDGMWKLDWDHSVIIPGMQKDQSIHIENLKSERGKILDRNNV

ELANTGTAYEIGIVPKNVSKKDYKAIAKELSISEDYIKQQMDQNWVQDDTFVPLKTVK

KMDEYLRDFAKKFHLTTNETESRNYPLGKATSHLLGYVGPINSEELKQKEYKGYKDDA

VIGKKGLEKLYDKKLQHEDGYRVTIVDDNSNTIAHTLIEKKKKDGKDIQLTIDAKVQK

SIYNNMKNDYGSGTAIHPQTGELLALVSTPSYDVYPFMYGMSNEEYNKLTEDKKEPLL

NKFQITTSPGSTQKILTAMIGLNNKTLDDKTSYKIDGKGWQKDKSWGGYNVTRYEVVN

GNIDLKQAIESSDNIFFARVALELGSKKFEKGMKKLGVGEDIPSDYPFYNAQISNKNL

DNEILLADSGYGQGEILINPVQILSIYSALENNGNINAPHLLKDTKNKVWKKNIISKE

NINLLTDGMQQVVNKTHKEDIYRSYANLIGKSGTAELKMKQGETGRQIGWFISYDKDN
PNMMMAINVKDVQDKGMASYNAKISGKVYDELYENGNKKYDIDE"
/db_xref="SEED:fig|176279.62.peg.16"
/db_xref="GO:0008658"
/db_xref="GO:0046677"
/transl_table=11
/product="Penicillin-binding protein PBP2a, methicillin
resistance determinant MecA, transpeptidase"
CDS 15012..15155
/transl_table=11
/db_xref="SEED:fig|176279.62.peg.17"

/translation="MNYFRYKQFNKDVITVAVGYYLRYALSYRDMSEILRERGVNVHH
LTV"
/product="Mobile element protein"
CDS 15435..15686
/product="Mobile element protein"
/db_xref="SEED:fig|176279.62.peg.18"
/translation="MAKVIKAFKLKPDCHCTSKYLNNLIEQDHRHIKVRKTRYQSINT
AKNTLKGIECIYGLYKKNRRSLQICGFSPCHEISIMLTS"
/transl_table=11
CDS complement(15746..16174)
/product="PhnB protein; putative DNA binding
3-demethylubiquinone-9 3-methyltransferase domain
protein"
/db_xref="SEED:fig|176279.62.peg.19"
/translation="MKFQVSPYFLITQNAEGLKKLYEELFGAKTLYIQRIKDRPIDKK

LNIDENDLNKIDQCVLQFGDIKIMIADDTEGLPITQGNNISLCLTFDQVEETKHVYDQ
LIEKGSEVLKTFSPEFYTEGYGYVRDPYGISLHLFTKRKN"

/transl_table=11
CDS 16255..17184
/product="Transcriptional regulator, YafY family"
/translation="MKKSVRLYNMIEYCNENRNFKLNDLMSEFNISRSTALRDIKEIE

ALGVPLYSNPGKNGGYTIIGNRDQTKIAISDEELKALVFTLSSISNVSNLPFQTEYQE
ILKKLYNNSNKKELINQYNDLFQYFNEDKYQFKSYKLFNEIIRLIIENKSFETCYSQN

YIKEQYKGIGIMYKNHQWYFVVANIESKLVNLLNISKIKELYEMGETQECNDITMQNF

QQFMVKNETAIDILIRSNVMGLNILKGYLWSDYMIENIDEETYLFKSKVNAKDIDFIA
KLIVTGGVNVKVETPNSLKNAVKVELTKIINMY"
/db_xref="SEED:fig|176279.62.peg.20"
/transl_table=11
CDS 17346..19358
/product="Similarity"
/transl_table=11
/db_xref="SEED:fig|176279.62.peg.21"
/translation="MNKNITALKSTENTTYMQYTVHIDDQIINNIKECTKKFEILPME
DKIPLSPLLQPEYAGEVQVFISTYEQFMINFGKVILDSQGIKIQFESESLSSIQRGIQ

EHCYLNKRTNDIDVTKEWYLCKFSNRTLEEDKNQLYNALKQLMNDSKNKKQAFQEVFK
VHIDIYLYNKEKSEYKYQASSYFYLVKENPKIIYKKRHLQEKQGIKGTTFTNKLKLLN

KMYGVDVAKYQPFYNSNNPEYERGQFGERYISQRSNYEFNRLQYQIIYILSKILDKHP

LPKSDNNYKHIPTIEKAILRDDSHNFYEYFEDIMKEIMNMDNSPLQEKLLTDFTYQSQ

CRWYSENKKLKLQLERFMHKVLESNYYEGNKLLRMMSNAIEETINEAKEDKVHFNYFK

DYFLTDGGVKNWEQISEKITEFNGKVINDIQHEYNKIQSKNTNQNQKLNFVYLYHCFE

FSNNIVKAKDNESKNYILFFIDKYKIKTPFLDQLLINCLNQEIDQESINYNMQTMFEK

ERYDRSSTIEKLVATRKFKYEKDDSDLFKQLFNDVENSIDRLGIYLLNNGINSNDENA

RYYRSFLKELSRIKSKLTPFSLEISKSSGREQHYPDDAVDDKDERRKNKEEAYHAFDD
KSDIDYKLKNKISDYIDNLLSVKIRNNLYDISPNTKSNNYNEPTQK"
CDS 19528..20637
/product="hypothetical protein"
/db_xref="SEED:fig|176279.62.peg.22"

/translation="MYQTNTRDLITKLPQSNKTEHFLMNKFSNQDKVQQLQRQISQQL

DQQYNELLSNEKAKLDQYVEVHHNLEPLKKEIESESINLDTDKLPDIKATMLEKAKND

EHSDKIEHLFDRLDQSLNGTNRLYTQLSLIGTRTHRITTKRFNVQGLPKLVQQTILPS

QFKKVYTIDFKSFEPSVAAYMTQDEQLIDYLNLEEGLYDALLRDLSLSKEKRVSVKRA

FIGSFLFGGRYSSSKFKINQEVSEINWLQVMSKFKKIIEFKEQVEKYKTMPTPYGIEH

DMSAFQGSSIMAIYVQTVASYIFKHILLEVYKAQCEQKTFKIIVPIHDAIMIECNDKE
IAQSVAQLMKDTANRLFNGEFAHVTVEALGGIDNE"
/transl_table=11
CDS 20630..21001
/translation="MNNDRGKSLQIPQSTSLKEGSIYVATLHSVTETDYSGDIKHQFT

YEIEIDQQIFYVNRSVVVNVTHQQLSINDWLERHSNYSASHENYEPYIDQKHLILLGQ
YNGNYYVQDVASLDAFGGVLS"
/db_xref="SEED:fig|176279.62.peg.23"
/transl_table=11
/product="hypothetical protein"
CDS 20998..22617
/product="putative primase"
/transl_table=11
/translation="MNHILQMLSKLLSVAKEAIDRQGLIAILTIPVNNDDEIEETAQG

ETVYNELIDQLRLNIPKDTDYRPNIYSYFGIKKNPNDTVLMEMMIKVFHIKRFNSELF

VFKANGWQKINGDELQGLISKMIQVLLVDYKPSLSTLKNVVDGLQKSTDVEELVENEH

YIGCGENMFDLNTFQVVKNSIDIFPKTRLNLSLSTNDVITDKIPPYFKQYMLQLANYD

DDLQYFLFQHTAVLLTADTKYRRGLILYGGAKNGKSVYIELVKSFFYSKDIVSKPLNE

LEGRFDKESLIDKSLMASHEIGQSRIQEKIVNDFKKLLSVESMHVDRKGKTQVEVILD
LKLIFSTNAILNFPPEHAKALERRVNIIPCEYYVEKADTSLIDKLQSEKKEIFLYLMY

VYQQIVKADIEYLENSRVTEITHDWLNFGYEFVSSRSVSIPNQKACINLLRKLTAIKP

GSRIKVSRLNEVIREEIKVSSQVINDLVQANFNVQSRLNNGYKYWVDLGWKETDKKDD
MILFDENENVTDDEFLYEDDLNLGWEDFDDE"
/db_xref="SEED:fig|176279.62.peg.24"
CDS 22844..24523
/db_xref="SEED:fig|176279.62.peg.25"
/translation="MKGKIALYSRVSTSEQSEHGYSVKEQEQVLIKEVVKNFPGYDYE

TYTDSGISGKNIEGRPAMKRLLQDVKDNKIEMVLSWKLNRISRSMRDVFNIIHEFKEH

DVGYKSISENIDTSNASGEVLVTMFGLIGSIERSTLISNVKMSMNAKARSGEAITGRV
LGYKLSFNPLTQKNDLVIEENEANIVREIFDLYLNHNKGFKAITTILNKKGYRTINKN

PFSVFGVKYILNNPVYKGFVRFNNHQNWSVQRRSGKSDENDVILVKGKHEAIISEEVF

DQVHEKLASKSFKPGRPIGGDFYLRSLIKCPECGNNMICRRTYYKTKKSKERTIKRYY
ICSLFNRSGSSACHSNSINAEVVERVINVHLNRILSQPDIIKQIASNVIEELKQKHSS

QPEIKYDIDSLEKQKAKIKTQQERLLELFLDDQMDSEMLKAKQSQMNEQLEVLDQQIK

EAQQANQSQDGIPNFDKLKGRLILMITRFSVYLRKATPEAKNQLMKMLIDSIEITTDK
QVKLVRYKIDESLIPQSLKKDWGSFFMPKFNFVINVANENRIENLSLLPLF"
/transl_table=11
/product="Cassette chromosome recombinase B"
CDS 24612..24950
/product="hypothetical protein"
/transl_table=11
/db_xref="SEED:fig|176279.62.peg.26"
/translation="MTLSKQLKTYITERFKLNYQENWACENVDAVAEDVLPEKYIKNS
PLEHKILNTFTYYNDELHEISIYPFLCYLDKELVAIGYLDNFDLDFIFLNDTHQIIID
ERYLLQKGGE"
CDS 24953..25042
/product="hypothetical protein"
/transl_table=11
/db_xref="SEED:fig|176279.62.peg.27"
/translation="MNWIKVAQLSVTVINEVIEIMKEKQNGGE"
CDS 25044..25355
/product="hypothetical protein"
/db_xref="SEED:fig|176279.62.peg.28"
/translation="MQINRYITRGISEHLSLDLQILLWNMVKERDNQPDTDYLHIFRL

QEDENILSITHEQEQPAYKLEYHYTNYEKNQNALPKKVYVIREDDVDLSYYVMLLPEE
Y"
/transl_table=11
CDS 25371..25877
/transl_table=11
/db_xref="SEED:fig|176279.62.peg.29"
/translation="MKTIESTLETEAIFSDDQQHRYLLKKTWDSEKQKITIITMYPNY

TGLLRIDLTTQLIMNKVSEMDAFGSINFVNLYSNITTPINLKHLENAYDNHTDIQIMK

AVKESDEVILAWGAYAKKPVVESRVNEVLEMLKPHKKKVKRLMNPATNEIMHPLNPKA

RQKWTLKA"
/product="hypothetical protein"
CDS 25898..26344
/translation="MKEINIVSLQMIKTDTLSYLKNRISNPEDAAEILRSFIGNSDRE
HLILICMNSKNEPTHIQILSIGSINQTVIHPREIFKTAILSNANSIMLGHNHPSGDIT
PSPEDINVTERLMLISEMMGILFYDHIIFSDSNTYSIRQHDLEVEV"
/db_xref="SEED:fig|176279.62.peg.30"
/transl_table=11
/product="DNA repair protein RadC truncated by transposon
Tn554"
CDS 26514..27386
/db_xref="SEED:fig|176279.62.peg.31"
/translation="MDRAFRILTIYNRLLQNKSVNKQSLTLELDTSPRTIQRDIDDIR
NFLYESKSWISTPKEITYDYKSESYKLEQDKSKNVHFMYNILTALYLTTPKLSHYFYQ

YLKLLIIKHHSNHRTTLLKYLERFEIDKNQTGISTSSLVVQAMNENKYLQYNQKILLP

LSMYYQMHAFHLVYRMNDEIYIDDINQMNLSFSEKSVDTHEKAKVQTYITFEISKDVW

SKMHRYYQIHVIEKYDAHYLIVTFKMTRIEAIQLCFMYRSNIRIISPPDLKEQVIDEL
LLLQTTYLKQQVQK"
/transl_table=11
/product="hypothetical protein"
CDS 27443..27733
/translation="MSNIIKTIGKGIGKGVGAAAYYSVATVGAAIGAIWETTGDRGLM
RDALSDSSNQDLMEIYEIQKKDDGDYDTYIAAKSLLQERGFTYNHETNKWEK"
/db_xref="SEED:fig|176279.62.peg.32"
/db_xref="GO:0016020"
/transl_table=11
/product="Predicted cell-wall-anchored protein SasC
(LPXTG motif)"
CDS 27749..28237
/product="hypothetical protein"
/db_xref="SEED:fig|176279.62.peg.33"
/translation="MEFTYYETKSLNDFENELKTLALNKKYTFSKETGVLKEDEILEK

SNSHERELLKVFKDNAQITTYHFKSHKSNYLIIMGETKAPNIIGLTVIFNKKPFKQMG
KIALGWLIALTSGGSILAIGAVFGILTSVARRTLVIKGPLGKEISQIIENELGEPIEI
KK"
/transl_table=11
CDS 28372..28899
/product="hypothetical protein"
/translation="MKKIFASLGVILLVAILGVAVVFAYGSYKDLELKKEEAKLEDKK

NKKIDKNKTTENENQDQINNQTITAPNEKAQYGNNLGVQEKSTDKNDPNGVLRFDTNG

DGFISTSEVTPEAQKLVDEGKLQPSSDRMVEEWTKNKNNVDNTYDYDESYKKQQEMYK
KAARGKVPEPGETIK"
/db_xref="SEED:fig|176279.62.peg.34"
/transl_table=11
CDS 29353..29574
/product="hypothetical protein"
/transl_table=11
/db_xref="SEED:fig|176279.62.peg.35"
/translation="MDHETKQSDWRTVASCLASQNYISLVKGLVHYFTAIEDEEILDK
IYDDFMNDDSITTVLNNDLQTIINHYLSK"
CDS complement(29648..32767)
/product="Type I restriction-modification system,
restriction subunit R (EC 3.1.21.3)"

/translation="MNFQFNEDDLEQVALEWLQSLGYDYKKGNEISVTGLAPERKSDK
EVVLHERLEKALRKINSDIHPRFIEKAIHELTLEKSPNLLENNLTFHENLINGIEVED

YDDEGQSVVEIVKIVDFEHPQNNDFLAVNQFTVVNGDYTKRPDIVLFINGLPIVVIEL

KNSTNETVGVEDGYHQLETYKMRIPQLFTFNEVLVTSDGINTKAGSLTANYDRFMTWR

SKDGETESSSSLASLDVLIHGMLNPETLLDLIRYFVLFQDDGKGHISKILAAYHQYYA

VNKAVDRALLASSGPGDGKGGVIWHTQGSGKSLTMVFFSGKLIQMLNNPTLVVVTDRN

DLDNQLYSTFVKSKGRSGKGLLRQTPKQAETRKELKSLLSVESGGIVFTTMQKFEPEQ

NETTMAALTERKNVIVMADEAHRTQYGFNAKYDDKGEGIKYGYAKYLRDALPNATFVG

FTGTPVASTDKNTQMVFGNYIDVYDMTQAVADGSTVKIYYESRVIPLNLPQNLDLDEA

YNDITEDQEEDIKQRLKSKWSRIEALAGAKPRVEALAKDIIQHFETRQQAMKGKGMIV

TMSRRIAVDLYDEIIRLKPEWHSDDDDKGVIKVVMTGSSSDPSSFQRHIGPKKRRNLL

EKRMKDVNDELQLVIVRDMWLTGFDVPSMHTMYIDKPMKGHNLMQAIARVNRVFKDKP

GGLIVDYVGIAESLKEALKEYTESDQAQTAIDTDKAVELMLLKYDVIQDMLYNLDYSK

FDSEKKSERYYAISDTMDYVIGLGEDERQRFIKTVTELGKAFALCATEPTAQELNDEI

AFFKAVKAGLVKLLQPPKEGKTRKTPAEVEAEINQLVSQSVVTEDVIDVYQTLGLEQP

DLSILSDDFLKDVEGLKQKNVAVELLNRLLKGQVKSLMKTNATVSKRFSEMLGNSINK

YNSRSIETSKVIEELIQLAKDIKQEQQRGNELGLNSDEIAFYDALASHETAKEAMGDK

ELRAIAHELTKTVKENMGVDWSKRDSAKAKMRVAVRRLLKKYGYPPDLQKMAVEQVVE
QAELMASNQ"
/db_xref="SEED:fig|176279.62.peg.36"
/transl_table=11
/EC_number="3.1.21.3"
CDS complement(32751..34013)
/product="Type I restriction-modification system, specificity subunit S"
/db_xref="SEED:fig|176279.62.peg.37"
/translation="MEYKISDLCDIRRGASPRPIKNYIVNSYDNSLPWVKIADATKDS

SRYIYDTKERVSLEGSKKSVEVLPGDLILSNSATPGLPKFMKVKACIHDGWLLLRNFK
NVRKEFLYYILLLNRQKLLQLSNGSVFNNLKTDIVKNYIVDIPSIKKQDKILKIIGNI

EDKLDTNNKLIANLEELSQTLFKHWFVDFEFPDKNGNPYKSSGGEMIDSELGKIPDSW

KIYKLKDVASHKKETFNPKKSEEVTVKHFSLPAYDNEEQAINEEVNKIKSNKWIINNN

CVLFSKMNPDTKRVWLPLIDNKKINVASSEFVVMESPNNKINSFVYNICLNSQFIDYL

KANTTGSTNSRQRVKPTIAVNYKLAIEDSIVKNYSEIITPYMEKMKILRSEIGKLTQL
RDTLLPKLMSGELEIPDDVEVNTDELSI"
/transl_table=11
CDS complement(34003..35517)
/product="Type I restriction-modification system, DNA-methyltransferase subunit M (EC 2.1.1.72)"
/db_xref="SEED:fig|176279.62.peg.38"

/translation="MATIGFEEKLWQAADKLRGSMDAAEYKNVALGLIFLKYVSDSFE

EKYEELKQDSYADEEDQDEYLAENIFWVPKEARWQYINDNAKKPEIGQMIDKAMIAIE

NENESLKGVLPKEYARPALDKEKLGDIIDLFTFKVGDTESRKQDVLGRVYEYFIAKFA

SAEGKNAGEFYTPSSIVKLLVEMIEPYKGRIYDPCCGSGGMFVQSERFVEKHQGRLDD

IAIYGQESNPTTWKLAKMNLAIRGIDNDLGERNADTFHNDLHKGLKADYILANPPFNA

SDWGQERLLDDYRWQFGIPPKGNANYAWIEHMISKLAPNGTAGFVLANGSMSTSGKDE

LEIRKNLIEQDLVECIVTLPGQLFYSTQIPVCLWFISNNKGQNGKKERKNEILFIDAR

EIGHMVTRTLKEFSDEDIQKVAKTYHAWRGTNNTPYKDVAGFCKVADLEEVKNNEYIL
TPGRYVGLADVEEEEEPFEQKMERITSELSEQFAKSKELEDQIRKSLEGLGYGV"
/transl_table=11
/EC_number="2.1.1.72"
CDS complement(36001..36138)
/transl_table=11
/translation="MNKSDKLQNVVKLLSSLGVNIKKTKSRFDIINTLPTTKTVTQEL
K"
/db_xref="SEED:fig|176279.62.peg.39"
/product="Hypothetical protein SAV2085"
CDS complement(36251..36460)
/transl_table=11

/translation="MLNSNIVFIDNIETKEQAQMLNERLLNMIGVHTVSIDIERNMVN
VEYETPTNLNSIEKEIYDAGFKVLS"
/db_xref="SEED:fig|176279.62.peg.40"
/product="Copper(I) chaperone CopZ"
CDS complement(36476..36769)
/transl_table=11
/db_xref="SEED:fig|176279.62.peg.41"

/translation="MENQNHAHHSEQVKLNLKSRLNRIEGQVKAINRMIDEDVYCDDV
LTQIRATRSALNSVATKLLEHHMKSCIMEKINQGTEEEAMEELLVTFQKLVKD"
/product="Repressor CsoR of the copZA operon"
CDS complement(36923..37075)
/transl_table=11
/db_xref="SEED:fig|176279.62.peg.42"
/translation="MKIMINAKIKFNIKAVINMMLIIKEECEKDKCEKDKCEKDNCTI
STIFIN"
/product="hypothetical protein"
CDS 37074..38450
/product="Cardiolipin synthase, bacterial type ClsA"
/transl_table=11
/translation="MERRSAGSIWAWLLVLVFLPIVGFILYLLLGRQIQRKHIFKLKK

EDRKGLAMIVDEQLNALEKDDFSKGNHQIVKFKEMVHMLLYNNAAFLTTDNSIKIFTD

GHAKFDALIEDIQNAKDYIHIQYYIFKSDGLGRRILKALEDRLEDGIEVKMLYDDMGS

RTLRKKDLKQFKDKGGRAEAFFPSKLPLINLRMNNRNHRKIVIIDGKIGYVGGFNVGD

EYLGKDKKFGYWRDTHLRLEGDSVNALELRFILDWNSQSTRDNIRYEDRYFPDVDSGG

EIGVQIASSGPDEDWEQIKYGYLKMIMSAKHSIYIQSPYFIPDQAFIDAIKIAALGGV

EVNLMIPCKPDHPFVYWATLKNAASLLDAGVKIYQYNNGFLHSKTLIVDDEIATVGTT

NMDNRSFTLNFEVNAFIYDEDISLQLKKAFIKDMKVSYELTKELYNQRSTWIKFKEGI
SQLLSPIL"
/db_xref="SEED:fig|176279.62.peg.43"
CDS 38485..39132
/db_xref="SEED:fig|176279.62.peg.44"

/translation="MNQAEQILLAKEYMYQFHKNDYSGHDIAHIERVTSLAKYIAKQE
HQGDFLTIVLSALLHDVIDDKLTDKHHALSELYQFFKKIKLDDSVQKNIIFIIKHLSY

QNGRNNDVTLPIEGQIVRDADRLDAIGAIGIARTFQFSGHFNEPMWTESPYHSIPSEK

DMVDLPPSAIRHFYDKLLKLKKLMHTQTGSQLAEERHQFMKLFLSQFYSEWHLEE"
/transl_table=11
/product="Metal-dependent phosphohydrolase"
CDS 39515..40588
/transl_table=11
/db_xref="SEED:fig|176279.62.peg.45"

/translation="MNDLDHPQLIFESTGIYSRGMERFCCVNQINYIQMNPLEAKFKT

SALRSWKTDQADAHKLACLGPTLKQTGSLPIHELIFFELRERVRFHLEIENEQNRLKF
QILELLHQTFPGLERLFSSRYSIIALNIAEIFTHPDMVLDIDKDVLITHIFNSTNKGM

SMDKATKYALQLRVIAQESYPNVDRHSFLVEKLRLLIQQLKQSIHHLKQLDDAMIQLA

QQLDYFENIHSIPGIGKLSTAMIIGEIGDIKRFKSNKQLNAFVGIDIKRYQSGHTHCR

DTINKRGNKKARKLLFWVIMNIIRGQHHYDNHVVDYYYKLRKQPNEKPHKTAIIACIN
RLLKTIHYLVMNHKLYDYQMSPH"
/product="Mobile element protein"
CDS 40970..42589
/product="Transposase, IS4 family"
/transl_table=11
/db_xref="SEED:fig|176279.62.peg.46"
/translation="MYKDYNMNQLTLPLETSVLIPTNDISRHVNDIVETIPKTEFDEF

RHHRGATSYHPKMMLKVILYAYTQSVFSGRKIEKLLNDSIRMMWLSQNQKPSYKTINR

FRVNPKVDALLESLFIQFHSQCLKQHLIDDQTIFIDGTKVEANANRYTFVWKKSIQNH

ETKMNEDSKALYHELVTNKIIPEIKKDHDNDLTKEEIDLIGSHLDKEIEDLNQHIDNE

KCTKIRKQIRLKRTKIKQYKKQINDYSGRKHKYDVQKSILKDRNSYSKTDHDATFMRM

KEDHMKNGQLKPGYNLQIATNSQFVLSYNVYQNPTDTRTMIPFLNSIQETYGHLPEYI

VADAGYGSEPNYMAIIDDFNRILLITYGMFIKDKTKKYKSDIFNTQNWDYDEINDEYI

CPNNKRLGFKRYAYRHDKYGFKRDFKLYECDDCSECPLRHQCMKYNSKTNKKIMKNYN

WEYFKAQINKKLSEPKIKTIYSQRKIDVEPVFGFMKAILGFTRMSVRGINKAKRELGF
VLMALNIRKVTAQRAENIQKNIKKTISILFQ"
CDS 42678..43397
/db_xref="SEED:fig|176279.62.peg.47"
/translation="MKEFLMIFFNKYELKAIIAPGMISIMSFFTTLIIYFPDSLNLGS
SLIVIILLIFYIYLFSIIARENGKMVEKKLFSQSSNVYPTTQILRYSDDTLNIKTKER

YHSFLQESIPCIELPSYEDARDKKENYFDDEYDSAIDWLLKNTREDYMTAQHNMEYGF
YRNLLGIKYIGIIIVLISIVFSVVVFILSQENDTIQLVLSSIINVFFLYFWIFKVTKT
QTKKSAKRYAQSLLANCDFKS"
/transl_table=11
/product="hypothetical protein"
CDS complement(43394..44290)
/transl_table=11
/translation="MSIIKSYSVGNGDMFYIKHGTDNFSIIDCCLYNDKKDIIIKEIL

EIASTKEIVRFISTHPDEDHIHGLELLNKNLNITNFYCVKNNAKKNDFSQSFEEYKKL

RDSNKSFYIHKDSQRKWMNISDESRGSSGINILWPDTDNEYFKNELQKAAEKISFNDI

SPIIQYKLDKGVTALWMGDLTTDFMEKIENEVNWEKVDILFAPHHSRKSGRVPSNILN

KLNPKLIVVGEANSKDLYYYSNYTTITQRKAGDIVFDCQTGTINIYVSNLNYEAKNLK
KDSNIPTIYDNLKYIGSVTTYN"
/db_xref="SEED:fig|176279.62.peg.48"
/product="MBL-fold metallo-hydrolase superfamily"
ORIGIN
1 atgaagatta ctatcttatc agtcggaaaa ctaaaagaaa aatattggaa gcaagccatt
61 gcagaatatg aaaaaagatt aggaccttac acgaaaatcg aattaataga agtaccagat
121 gaaaaagcac ctgaaaatat gagcgacaaa gaaatcgaac aagtcaaaga aaaagaaggc
181 caacgcctac tcaataagat taaaccccaa tctacagtaa ttacgttgga aatcaaaggg
241 aaaatgttgt cttcagaagg actcgctaaa gagctacaaa cacgcatgac acaaggtcaa

301 agcgacttta catttgtcat aggtggctcc aatggtttac atcaagacgt cttacaacgc
361 agcaattacg ctctatcatt cagcactatg accttcccgc accaaatgat gcgcgtcata
421 ttgatagaac aaatttatcg ctcatttaag ataatgagag gcgaagcgta tcataaataa
481 aactaaaaat tagattgtgt ataatttaaa aatttaatga gatgtggagg aattacatat
541 atgaaatatt ggagtatacc ttgcaatatc atacgatgtt tatagagtgt ttaataaacc
601 atttttcaac tattgatgat ctagaatata taataactgt acaaattata ttgattatgg
661 aactacaatt caattaagaa attgatgatg aaattttaaa tttaaactaa tggaatcaag
721 aaagaatgaa aggaaatata caatgcccac gattaataaa aggaagttta ttagattttg
781 tgttagaaac agtagatgtt tttgtaaatg gaagtaaaaa tgattgcgaa ttagtaaatt
841 ttaattataa agaaggaaaa gtagtttcac gttatagtac aaaatttgaa agcaatccta
901 aattaagaaa aaagcaattg atatcgacgg taaaagttgt aaggtatgtt atttttgatt
961 ttgagaaaat tttatggggc atagggaaag ttttattgaa gttcatcata ttaaggcaat
1021 gtttactata agaaagttga aatacactca gaaacagact ggattcccgt ttttttcaaa
1081 ttggcacaaa atgatacata ggcccaccta tatattgaag atttaagtta aacgataaat
1141 aatatttttc tcttttgtat agaaattttt ttgggaatga tattaattag tataaaaatg
1201 aatatgtcta caaagaagga gagattttat tgaaacacat taatcaattt tctgaatcga
1261 cactagcaca actagaaaaa gatcaacaac acgtatttta tgtatactgt ttgatggacc
1321 caagaaatga tgaatgcttt tatgttggta aaggtaaggg gaatagaata tttaaacata
1381 aacaagatgc gcagaaaaaa gtattgtacg aagatatatt tatagaagaa aataaagata
1441 acttgaaatt taatagaatt aacgaaattt gtagcaatga tttgaatgtt ttaggatata
1501 taatcagtta tggattaacc gaatctgaag cattttctgc agaaaatgtt cttataaact
1561 ttcttaacct aacaaataaa actacattaa caaatatgat taacggtcat ggatctaagg
1621 catacttagt tgaagattta gaaaatgaat ttggctacga ttcaattaat cttgaaaaca
1681 taaatacgaa tgaattaatt ttagcagtga aaatcagaga tgcatttcta ttagataagg
1741 atgaaagtaa agagtatcct attaatgaaa gtaaacgtga tagaaataac cttaaatcgc
1801 gtacattagg tagttggata ataggaaaag ataaaataca taaaataaaa tatattattg
1861 gtatcaatac aggtgctaat aatgcagttg tctcagctta tgaagtatca tttgaacaag
1921 cggagagtat cgaaacaaat aatggtagaa tgagatatgc gtttattggg ctttcagaaa
1981 gagatgctac tctaaaaaaa ttgaatttat acaaaaaagc actaccagat ttaagatttg
2041 gtagtggtag cgctacagca tatataaata atgggacaat gaaagttgat taaatgaata
2101 aaaacatcca attccttaaa ttagcctttg aaaattttat actcgtatat ataagtgaaa
2161 aacttaaaag aattagatgt taattaaagg agagaatgct atgccatcag atgatattgt
2221 aaaaaaacta tactcaaaag aggatattcg attagaatta ggacttactc cgcataagtt
2281 taataaaaaa atggaaacta ttgctaagct ttttaaaatt gatatgaaaa tttttcacaa
2341 ttacaaaggc caagataaaa ataatcagta tacatttaat ggtgtcgcaa aagaattaat
2401 caaagtgttg ctaaaaagtg ttgattatta cccagtggat atcaattcca aaaaatttaa
2461 acaaaatgga aaatctaaaa aagaaatgat tgaaaatata gataactcta gttacatgaa
2521 atatatttat cagttaatga aatctatcaa tgaaattcaa tacaaaaggt taattgctga
2581 tatacatatg aaagatgtgt accagaatac aaaagcatgg ttaaataatg gtgaatcaat
2641 taataaaaaa gaacaagagc tataccaata tatgacaatt ttaccattac ataaaagagt
2701 tgaactgcaa aatgaggtat taaaatctat agacgaaaca atttttcaat ttctcgcaaa
2761 agaacataga aataatcaaa ttgaagaaaa taatgaatta gaagcatata caaaagcaat
2821 aaaagaaggt agaaatccta aaaatgatta tgaattaaat catctcctat ataaaaaaaa
2881 tcaattaccc ttagatagtc taatagaaga tatatgggac tatgaggaaa cagagtatac

2941 tgagcttgat tggttaattg cagatatgct aaaacgttca caaagagctg atagtaattt
3001 tatcgaaagt ttagagaaga aaaacaaatt gagaaaaaat attcataaag attccattaa
3061 aagtatatct aagttgattg atcatgaatt agttaaaaat aatagaaagt gggacgtagc
3121 agaattttat aaaaatcaac aattttggaa taatagtaca ttaagacgta gctctcaaaa
3181 ttttggaatt ttatattaca aaaagaatag gttacataca atgaatcaaa gtaatattat
3241 taagaaaata aaaattatcg aagctaatat agagagtgca aataaacagc cgagctattt
3301 agatttcttt ttacaagcta agtttgattt agagaggtta gaaagtgaat taatgaaata
3361 tagcattaat ccgaaatatt ttaataagat aaatagtgat tacgtaagta tgctattgaa
3421 gatgtacata atgcaattat taaaattgac agatatataa gtaacgataa agtgaagtta
3481 aactattcaa atgtccaaat ggtagtgaga aacgaaataa cagaacaagg aagctacttt
3541 gtaacccagg cattgaatac actgtcaaag gtagaagagt ctggctacag ctttgataat
3601 ttcatggtag gacctttcgt agaagacttt aaaaaagcaa ttaaaaatcg tagatttggt
3661 tctaaaaaag attaagattg ttttttagag ttaaacataa gaataaattt aaataaaaaa
3721 ataaacccaa caaaccttgt tatatcaatg tttgttgggt ttattttttt tggtgatttt
3781 ttaataactc aattacatct taggataata tcgctaaatt atacattgtc gaggccaaga
3841 caaataaaaa attaaagcaa aagttagcgc gcaactttct gctataaagg agcaaattta
3901 tatggagaca agagacaaac tgatgtcatt gactcaaagt gacaaaacac agcaatggct
3961 aatggacaag tcatctaacc aagatgacat tcaacaattg cagcaacaat tcagtcagca
4021 gcttgatcaa caatataatg cacttttagc tgatgaaaaa gctaagttag accaatacgt
4081 ggaagtacat caaggattgg aatcattaaa ggaagagatt gaatcagaac ctattacgct
4141 taatatcgat aaattacccg atatcaaagc aacaatgctt gaaagagcca agaatgatga
4201 acattctgat aaaatcgaaa agctatttga taggttagaa caggcattaa atggtacgaa
4261 tcgattatat acgcaattat cgttgattgg tacacgaaca catcgaatta caactaaaaa
4321 ttttaatctt caaggcttac ctaaagcagt ccaacatacg attttacctt caaaatttaa
4381 gaaggtgtat acagtcgatt ttaaatcgtt tgaaccatca gttgcagcgt acatgactca
4441 agattcaaaa ctgattgact tgttaaatca gaaagacgga ctgtatgacg cattgctaag
4501 tgaattaggc ttatcagatg agctacgtgt atttgttaaa cgtgcattta ttggttcgtt
4561 tctatttgga ggtaacttca aaaatcctaa attcaagctg aatcaatatg taagtgaagt
4621 acaatggttg gatgcggtca gccaatttac aaaagtcatt gaacttaaga agcacgttga
4681 aaagagtaat tccatgccta tgccttatag tattgagcat gatatgagcg catttcaagg
4741 tagcagtatt atggcaatct acgtacaaac tgtagcgagt tatattttca agcacattct
4801 gctaaaagtg tacaaagctc agtgcgatca aaaaacgttc aagattatag tacctataca
4861 cgatgcgatt atgattgaat gtgaagatga agaaattgca caaaatgtgg gtcagttaat
4921 gaaagatacg gctaaccagt tgttcaatgg tgaatttgca catgtgacag tggaagaaat
4981 aggaggcgta gaccatgaat aatgatagag gacaaagcct acacatccca agtagtacac
5041 caatcaaaga aaataatata tatgtagcta cgttacattc tgtgatccaa acagatttct
5101 caggtgaaat aaagcaccaa ttcacgtatg aaattgaagt gaacaatcag attgtatatg
5161 cgaatcgtaa tattccaaca aaaccgagcg ctaatcagtt gtcaattcat gattggctga
5221 aacgtcatag caactatagc gtgggtcatg atacgtatga accttatatt gaccgtaagc
5281 atttaatcca tattggtcaa tatagcggta actattatgt acaagatgta gcatcgttag
5341 atgcgtttgg aggcgtatta tcatgaatca tatattacaa atgttatcta agctattaag
5401 tgtggccaag gaggcaatcg accgtcaagg tctgattgct atcctaacta ttcctgttaa
5461 taatgacgat gaaatagaag aaacggctca aggtgaaacc gtgtataacg aacttatcga
5521 tcagttacga cttaatatcc caaaagatac ggattatcga cctaacatct atagttattt

5581 tggtattaag aagaatccta atgacaccgt actcatggaa atgatgataa aggtttttca
5641 tatcaaacgc tttaattcag aactgtttgt tttcaaagct aacgggtggc aaaagataaa
5701 tggagatgaa ttacaagggt tgatatctaa aatgatacaa gtattgcttg tagattataa
5761 gccttcacta agcactctaa aaaatgtcgt agatggattg caaaaatcaa cagatgtaga
5821 agaacttgtt gagaatgagc gctatattgg ttgtggtgaa aatatgttcg atcttaatac
5881 gtttcaagtc gttaaaaatt caatcgatat ctttccaaaa acacgattga atttatcatt
5941 aagtacaaat gatgtaatta ctgataagat accgccttat tttaagcaat atatgttaca
6001 acttgcgaat tatgacgatg atttacaata ctttcttttc cagcatacag cagtattact
6061 tacagctgat actaaatacc gtaggggtct catattatat ggtggagcta agaatggtaa
6121 atctgtatat attgaactag ttaaatcatt tttctatagt aaagatattg tgtctaagcc
6181 acttaatgag cttgaaggtc gttttgacaa agaaagttta attgacaaaa gtctaatggc
6241 aagtcatgaa attgggcaat ctaggattca agaaaagatc gtaaatgact tcaaaaagtt
6301 attatctgta gaatcaatgc atgttgatcg taaaggaaaa actcaagtgg aagtcatttt
6361 ggatttgaaa cttattttta gtacaaatgc gatacttaat tttcctcctg aacatgcgaa
6421 agctttggag cgtcgaatta atattattcc atgtgagtat tatgttgaaa aagcggacac
6481 ttcattaatt gataagctcc agagtgagaa gaaagaaatc tttctttact tgatgtatgt
6541 gtatcaacag attgtaaaag cagatatcga gtatcttgaa aatagccgtg tcactgaaat
6601 tactcacgat tggttaaatt ttggatatga atttgtttct agcaggtccg taagtaatgc
6661 aaatcagaaa gcatgtatta atttactcag aaaacttata gaaatcaaat caggatcacg
6721 aatcaaagta tccgagttaa ataaagttat taatgaagaa ataaaggtaa gttctcaggt
6781 tattaaacag ttaattcaag caaactttga tactcaaacc aaactataca atggctacga
6841 ttattggatt gatttaggtt ggaaagaagc caataaaaaa gagattcatg atatttcgga
6901 aaaagataat attatttcat tagataaaaa tgaaaatata acagacgatg aggcattaga
6961 tgaagagaat ttggactttg attgggagga ctttgacgat gaataatgaa caaattgaag
7021 catttgtaga agtgcttgta cctatcatag aagaacgtat caataaaggt aattaaggct
7081 aattacgtac tacaggcagt tgcctgtagt actcatatga ttaagtggta aaagtgataa
7141 aaatgaaacg aaattataaa tatatattat ctatatgttg ttacaagacc gatagtctgt
7201 agcaataatc taataaaagg agcggtatga tatgaagggt aaaattgcac tttattcacg
7261 cgttagtacg tcagagcagt cggagcatgg gtactcaatc catgagcagg aacaagtact
7321 catcaaagaa gttgtgaaaa atttcccagg ttatgactat gagacatata ctgactcagg
7381 catttcaggt aaaaatattg aaggtcgtcc ggcaatgaaa cgtctattac aagatgttaa
7441 ggataataaa atcgaaatgg tgttaagttg gaaattgaat cgtatctcac gatcaatgag
7501 agacgtgttt aatattattc atgaattcaa agaacatgat gtagggtata aatcgatttc
7561 tgagaatatt gatacatcca atgcttctgg agaagtactc gttacaatgt ttgggttaat
7621 aggatctata gaacgccaga ctttgatttc gaatgtgaaa ctttctatga atgctaaggc
7681 aaggagcgga gaggcaatca ccggtcgtgt tttaggctac aaattatcac ttaatccact
7741 tacacagaaa aatgatttgg ttatcgatga aaatgaagct aatattgtac gtgaaatttt
7801 cgatttatat ttgaatcata ataaaggcct caaagccatt acaaccgtac ttaatcaaaa
7861 ggggtatcgt actattaatc aaaagccatt ttcagtgtat ggtgttaaat acattttgaa
7921 taatccagtc tataaaggct atgtcagatt caataaccat caaaactggg ctgtacagcg
7981 aagaagtggc aaaagtgata aaaatgatgt gatattggtc aaaggtaaac atgaagccat
8041 tataagtgaa gaggtatttg ataaagttca tgaaaaatta gcttctaaaa gttttaaacc
8101 gggcagacct attggtggag atttctactt acgtggcctt attaaatgcc cagaatgcgg
8161 aaataatatg gtatgtcgac ggacgtatta taaaacgaaa aagtccaaag aacggacaat

8221 caagcgttat tacatttgtt ccttattcaa tcgttcaggg agttctgcat gtcacagtaa
8281 ttccatcaat gctgaagtcg tcgagcgtgt aattaatgtt catttgaatc gtattctgtc
8341 tcaaccagat attatcaagc agattgcgtc aaatgtgata gaagaactga aacaaaagca
8401 tagtaaccaa acagaaatta aatatgacat tgatagttta gaaaaacaaa aagctaagct
8461 taaaacacaa caagaacgat tgttagaatt gttcttagat gatcagatgg atagcgaaat
8521 gttaaaagct aaacaaagtc aaatgaatca acagttagaa gtattagatc aacaaattaa
8581 agaagcgcaa caagcaaatc aatcacagga tgaaatacct aattttgata aattaaaagg
8641 acgactcatt ttgatgataa cacgattcag cgtgtactta agaaaggcta cacccgaagc
8701 taaaaatcaa cttatgaaaa tgttaattga ttcaattgaa attacgacag ataaacaagt
8761 aaaacttgta aggtataaaa ttgacgaaag tcttatccct caatctttga aaaaagattg
8821 ggggtctttt tttatgccca aattccaatt tgaaatatat ggtcaaaatg attatttcat
8881 cgaccaaatt accactttta ccacttagtt attagtgaca aaagtgagcg aaatgaaata
8941 aaaatcaaat atatattatc aaaatgatgt atcacatgca tacatcaatc aaatacatta
9001 ggaggtcata accatgacac tagaacaaca actcaagcac tatataacca acttattcaa
9061 tctgccaagg gacgaagtgt ggcactgcga atctatcgag gaaatcgctg atgatatctt
9121 acccaatcaa tatgtaagac ttggcccact cagtaataaa acacttcaga ctaataccta
9181 ctactctgac acacttcatg aaagtaatat ctatcctttc attctctact atcagaaaca
9241 actcatagcc atcggttata tcgacgaaaa tcacgatatg gatttcttat acctacacaa
9301 cactatcatg cctcttttgg atcaacgata cttactaaca ggaggacaat aaaatgcata
9361 aatacatcaa aattacacaa ttagtcatta caatactaag tgaaatcatc atttggatga
9421 aagagtcaga acgaaaggaa gtctcttatg aatagatata tcacccgggg tatcgccaac
9481 aacttaccta atatcttaca acaccaatta tggcaactcg tatctgagcg agaacaagaa
9541 caaaccaaag ataatacttc agtagattat tttcatatat tccagttcaa taagcatcgc
9601 aatcaattat atatcaaaca caaacaagaa cgacctgaat atgcgaaaat ccataaagct
9661 aattattcaa aagcaatcaa tatcaataag gtctacatta ttcgagaaga tgatgtagac
9721 ctttcttatt atgtcatgtt attacctgaa gaatactaga ggagtgaaaa ttatatggaa
9781 acaatcaaaa gtacattaaa aacagaagcc atattcagtg atgacaaaca acatcgctat
9841 ctacttaaga aaacatggaa tagtgaaaaa caatcaatca caatcattac aatgtatccg
9901 cattatgatg gcattctcaa tattgaccta acgacccaac tcatcatgaa caaagtttca
9961 gaaatggatg catttggttc aatcaatttt gtgaatctat actctaatat tacaacccct
10021 atcaatctca aacatttaga aaatgcgtat gataagcata cagatattca aattatgaag
10081 gcagtgaaag agtcagatga agtgatatta gcttggggcg cttacgctaa aaagcccggt
10141 gttgaagcac gtgttaatga agtattagag atgttgaaac cacataaaaa gaaagtaaaa
10201 cgactcatga atccagaaac caatgaaatc atgcatcccc ttaattcgaa agcacgtcaa
10261 aaatggatat taaaagtata gcaactaaaa tgtttattca ctatccatga aaataattga
10321 atttcatata tatcttttaa tctacaatat gattaaaaga tatattattt ggttctgttg
10381 caaagtaaaa aaatatagct aaccactaat ttatcatgtc agtgttcgct taacttgcta
10441 gcatgatgct aatttcatgg catggcgaaa atccgcagat ctgaagagac ctgcggttct
10501 ttttatatag agcgtaaata cattcaatac cttttaaagt attctttgct gtattgatac
10561 tttgatacct tgtctttctt actttaatat gacggtgatc ttgctcaatg aggttattca
10621 gatatttcga tgtacaatga cagtcaggtt taagtttaaa agctttaatt actttagcca
10681 ttgctacctt cgttgaaggt gcctgatctg taattacctt ttgaggttta ccaaattgtt
10741 taatgagacg tttgataaac gcatatgctg aatgattatc tcgttgctta cgcaaccaaa
10801 tatctaatgt atgtccctct gcatcaatgg cacgatataa atagctccat tttcctttta

10861 ttttgatgta cgtctcatca atacgccatt tgtaataagc ttttttatgc tttttcttcc
10921 aaatttgata caaaattggg gcatattctt gaacccaacg gtagaccgtt gaatgatgaa
10981 cgtttacacc acgttccctt aatatttcag atatatcacg ataactcaat gtatatctta
11041 gatagtagcc aacggctaca gtgataacat ccttgttaaa ttgtttatat ctgaaatagt
11101 tcatacagaa gactcctttt tgttaaaatt atactataaa ttcaactttg caacagaacc
11161 agcaatccta agtaaaattg cagataagag gtaagttaaa agcaatccta agtaaaattg
11221 cagataaggg gtacagaaaa actagacttg attacaaaat ggagcttggg acataaatga
11281 ttttttaaaa atgagatgag acgtagatta actccataat caatacgaat ctatcgactt
11341 ctttatttat gatattcatc tctttttaat ggaaataaaa gtgcgattaa tgtgataata
11401 cagttacgtt aattaaaaaa ataaaaatgc aaggagaggt aatatgctaa ctgtatatgg
11461 acatagagga ttacctagta aagctccgga aaatacaatt gcatcattta aagctgcttc
11521 agaagtagaa ggtataaact ggttggagtt agatgttgca attacaaaag atgaacaact
11581 gattatcatt catgatgatt atttagaacg gactacaaat atgtccgggg aaataactga
11641 attgaattat gatgaaatta aagatgcttc tgcaggatct tggtttggtg aaaaattcaa
11701 agatgaacat ttgccaactt tcgatgatgt agtaaaaata gcaaatgaat ataatatgaa
11761 tttaaatgta gaattaaaag gtattactgg accgaatgga ctagcacttt ctaaaagtat
11821 ggttaagcaa gtggaagaac aattaacaaa cttaaatcag aatcaagaag tgctcatttc
11881 aagctttaat gttgtgcttg ttaaacttgc agaagaaatc atgccacaat ataacagagc
11941 agttatattc catacaactt cgtttcgtga agactggaga acacttttag attactgtaa
12001 tgctaaaata gtaaacactg aagatgccaa acttactaaa gcaaaagtaa aaatggtaaa
12061 agaagcgggt tatgaattga acgtatggac tgtaaacaaa ccagcacgtg caaaccaact
12121 tgctaattgg ggagttgatg gtatctttac agacaatgca gataaaatgg tgcatttgtc
12181 tcaatagaaa gttagaggtg agtcttacgt ttcagtgacg gtagacttac ctttaacatg
12241 ttacatacta aaaaattaat ttgaataaga aagagagaca tatatgaaat acgatgattt
12301 tatagtagga gaaacattca aaacaaaaag ccttcatatt acagaagaag aaattatcca
12361 atttgcaaca acttttgatc ctcaatatat gcatatagat aaagaaaaag cagaacaaag
12421 tagatttaaa ggtatcattg catctggcat gcatacactt tcaatatcat ttaaattatg
12481 ggtagaagaa ggtaaatacg gagaagaagt tgtagcagga acacaaatga ataacgttaa
12541 atttattaaa cctgtatacc caggtaatac attgtacgtt atcgctgaaa ttacaaataa
12601 gaaatccata aaaaaagaaa atggactcgt tacagtgtca ctttcaacat acaatgaaaa
12661 tgaagaaatt gtatttaagg gagaagtaac agcacttatt aataattcat aataaaacag
12721 tgaagcaacc atcgttacgg attgcttcac tgttttgtta ttcatctata tcgtattttt
12781 tattaccgtt ctcatatagc tcatcataca ctttacctga gattttggca ttgtagctag
12841 ccattccttt atcttgtaca tctttaacat taatagccat catcatgttt ggattatctt
12901 tatcatatga tataaaccac ccaatttgtc tgccagtttc tccttgtttc attttgagtt
12961 ctgcagtacc ggatttgcca attaagtttg cataagatct ataaatatct tctttatgtg
13021 ttttatttac gacttgttgc ataccatcag ttaatagatt gatattttct ttggaaataa
13081 tatttttctt ccaaactttg tttttcgtgt cttttaataa gtgaggtgcg ttaatattgc
13141 cattattttc taatgcgcta tagattgaaa ggatctgtac tgggttaatc agtatttcac
13201 cttgtccgta acctgaatca gctaataata tttcattatc taaatttttg tttgaaattt
13261 gagcattata aaatggataa tcacttggta tatcttcacc aacacctagt tttttcatgc
13321 ctttttcaaa tttcttactg cctaattcga gtgctactct agcaaagaaa atgttatctg
13381 atgattctat tgcttgtttt aagtcgatat taccatttac cacttcatat cttgtaacgt
13441 tgtaaccacc ccaagattta tctttttgcc aacctttacc atcgatttta taacttgttt

13501 tatcgtctaa tgttttgtta tttaacccaa tcattgctgt taatattttt tgagttgaac
13561 ctggtgaagt tgtaatctgg aacttgttga gcagaggttc ttttttatct tcggttaatt
13621 tattatattc ttcgttactc atgccataca taaatggata gacgtcatat gaaggtgtgc
13681 ttacaagtgc taataattca cctgtttgag ggtggatagc agtacctgag ccataatcat
13741 ttttcatgtt gttataaata ctcttttgaa ctttagcatc aatagttagt tgaatatctt
13801 tgccatcttt tttctttttc tctattaatg tatgtgcgat tgtattgcta ttatcgtcaa
13861 cgattgtgac acgatagcca tcttcatgtt ggagcttttt atcgtaaagt ttttcgagtc
13921 cctttttacc aataactgca tcatctttat agcctttata ttctttttgt tttaattctt
13981 cagagttaat gggaccaaca taacctaata gatgtgaagt cgcttttcct agaggatagt
14041 tacgactttc tgtttcatta gttgtaagat gaaatttttt tgcgaaatct cttaaatatt
14101 catccatttt tttaacggtt ttaagtggaa cgaaggtatc atcttgtacc caattttgat
14161 ccatttgttg tttgatatag tcttcagaaa tacttagttc tttagcgatt gctttataat
14221 ctttttttaga tacattcttt ggaacgatgc ctatctcata tgctgttcct gtattggcca
14281 attccacatt gtttcggtct aaaattttac cacgttctga ttttaaattt tcaatatgta
14341 tgctttggtc tttctgcatt cctggaataa tgacgctatg atcccaatct aacttccaca
14401 taccatcttc tttaacaaaa ttaaattgaa cgttgcgatc aatgttaccg tagtttgttt
14461 taattttata ttgagcatct actcgttttt tatttttaga tacttttttt attttacgat
14521 cctgaatgtt tatatcttta acgcctaaac tattatatat ttttatcgga cgttcagtca
14581 tttctacttc accattatcg cttttagaaa tataactgct atctttataa acttgtttga
14641 aatttttatc ttcaattgca tcaatagtat tattaatttc tttatctttt gaagcataaa
14701 aatatatacc aaacccgaca actacaacta ttaaaataag tggaacaatt tttatctttt
14761 tcatcaatat cctccttata taagactaca tttgtaatat attacaaatg tagtatttat
14821 gtcaaaataa tgttataatt tttgtgatat ggaggtgtag aaggtgttat catctttttt
14881 aatgttaagt ataatcagtt cattgctcac gatatgtgta attttttttag tgagaatgct
14941 ctatataaaa tatacggttc tgttgcaaag ttgaatttat agtataatta taaccaaaag
15001 gagtcttctg tatgaactat ttcagatata aacaatttaa caaggatgtt atcactgtag
15061 ccgttggcta ctatctaaga tatgcattga gttatcgtga tatgtctgaa atattaaggg
15121 aacgtggtgt aaacgttcat catttaacgg tctagcgttg agttcaagaa tatgccccga
15181 ttttatatca aatttggaag aaaaagcata aaaaagctta ttacaaatgg cgtattgatg
15241 agacgtacat caaaataaaa ggaaaatgga actatttata tcgtgccatt gatacagagg
15301 gacatacatt agatatttgg ttgcgtaagc aacgagataa tcattcagca tatgtattta
15361 tcaaacgtct cattaaacaa tttggtaaac ctcaaaaggt aattacagat taggcacctt
15421 caacgaaggt cgcaatggct aaagtcatta aagcttttaa acttaaacct gactgtcatt
15481 gtacatcgaa atatctgaat aacctcattg agcaagatca ccgtcatatt aaagtaagaa
15541 agacaagata tcaaagtatc aatacggcaa agaatacttt aaaaggtatt gaatgtattt
15601 acggtctata taaaaagaac cgcaggtctc ttcagatctg cggattttcg ccatgccatg
15661 aaattagcat catgctaaca agttaacaca aagtattatt ttaaaattga gattagacat
15721 ttatttttca actttgcagc agaacctaat tttttctctt tgtaaataaa tgcaaagaga
15781 taccataagg atctcttaca taaccataac cttcagtata gaattctgga ctaaatgttt
15841 tcaatacctc actgcctttt tctattaact ggtcatatac atgtttagtt tcttctactt
15901 ggtcaaaagt gagacaaaga gatatattat taccttgtgt tatgggcaaa ccttcagtgt
15961 catctgcgat cataattttt atatctccaa attgaagtac acattgatca attttattta
16021 aatcattttc gtcaatatta agtttcttat ctataggtct atctttaata cgttgaatat
16081 acagtgtttt agcgccaaac agctcttcat acaacttttt taaaccctct gcattttgag

16141 tgattaaaaa atatggactt acttgaaatt tcatgttttt tcctccttag atttgttata
16201 aatagagtat atacttaatt agtgtcatct attgacacta aaaggagaat aataatgaaa
16261 aaatctgtta gattatataa tatgattgaa tattgtaatg aaaataggaa cttcaaatta
16321 aatgatttaa tgtcagaatt taatatttct cgtagtaccg ctttaaggga tataaaagaa
16381 attgaagcat taggagtacc tttatatagt aatccaggga aaaatggtgg ttatacgatc
16441 ataggtaatc gagaccaaac gaaaatagca atctcagatg aagagttgaa agctttagta
16501 tttacacttt cgagtatttc aaatgtgagt aatctacctt ttcaaacaga atatcaagaa
16561 atattaaaaa aattatataa taactcgaat aaaaaagagt taataaacca atataatgat
16621 ctatttcaat attttaatga agataagtat cagttcaaaa gttataagtt atttaatgaa
16681 atcattagat tgataattga gaataagtct tttgaaacct gttattcaca aaactatatt
16741 aaagaacaat ataaaggtat tggcattatg tataaaaatc atcaatggta ttttgttgta
16801 gctaatatag aatcaaagtt agtgaatcta ttaaatattt cgaaaataaa agaactatat
16861 gaaatgggag agactcaaga gtgtaatgat ataactatgc agaattttca acagttcatg
16921 gttaaaaatg aaacagctat tgatattctt attagaagca atgttatggg attgaatatc
16981 ttgaaaggct acctgtggag tgactatatg attgaaaata ttgacgaaga gacatattta
17041 tttaaatcaa aagtgaacgc gaaagatata gattttatag ctaagttaat tgtcacaggt
17101 ggtgtcaatg taaaagtaga gacccctaat agtttgaaaa atgctgttaa agttgaatta
17161 actaaaataa taaacatgta ttaatagtaa tttaatcata attgtaataa caaaaagatg
17221 acaacattaa acaatttaaa tttatacaaa aaagcattac caaatttaaa attgggtagt
17281 ggtagtgcta cagcatacat aaatagttat aattaaataa agatgggtca atgaaggtga
17341 attaagtgaa taaaaatatt acagctctaa aatctactga aaatactaca tatatgcagt
17401 atacggttca tatcgatgat caaattataa ataatataaa agagtgtacc aaaaagtttg
17461 aaattttgcc tatggaagat aaaattccac tatcaccgtt gttacaacca gaatatgcag
17521 gagaggtaca agtttttatt agtacatatg agcagtttat gattaatttt ggtaaagtaa
17581 tattggatag tcaaggcata aaaatacagt ttgaaagtga atcattaagt agtattcaac
17641 gaggcattca agaacattgt tacttaaata agcgaacaaa tgacattgat gtgactaaag
17701 aatggtattt atgcaaattt tcaaatcgaa cattagaaga ggataaaaat caactgtata
17761 atgctttaaa acaactcatg aatgattcta aaaataaaaa gcaagctttt caagaggtgt
17821 ttaaggttca tattgatatt tatctctaca ataaagaaaa atctgaatat aagtatcaag
17881 catctagtta tttctatctt gtaaaagaaa atcctaaaat aatttataaa aaaagacatt
17941 tacaagagaa acaagggatc aagggtacaa ctttcacgaa caaattaaaa ttattgaaca
18001 aaatgtatgg tgtagatgtt gcaaaaatatc agccatttta taattctaat aatcctgaat
18061 atgaaagagg tcaatttggt gaaagataca tttctcaaag atctaactat gaatttaata
18121 gacttcaata tcaaattata tatatattat cgaaaatact tgataagcat ccattaccaa
18181 agtcagataa taattataaa catattccaa ctattgaaaa agcaatatta agagatgatt
18241 cccataattt ttatgagtat tttgaagata taatgaaaga aattatgaat atggataatt
18301 ctcccttaca ggaaaaatta ttgactgatt ttacatatca atcccaatgt agatggtact
18361 ctgaaaataa aaaattaaaa ttgcaacttg aaagatttat gcataaagtt ttagagagta
18421 attattatga gggcaacaaa ttattgagaa tgatgtctaa cgcaattgaa gaaacaatca
18481 atgaggctaa agaagataag gttcatttca attattttaa agattatttt ttgacagacg
18541 gtggggtgaa gaattgggag caaattagcg agaaaattac tgaattcaat ggtaaagtta
18601 ttaacgatat ccaacatgaa tataataaga tacagtccaa gaatacaaat caaaatcaga
18661 aactaaactt tgtttattta tatcattgct ttgagtttag taataacata gtaaaagcaa
18721 aagataatga aagtaaaaat tatatcttgt tttttataga taaatataaa ataaagacac

18781 catttttgga tcaattatta ataaattgtt tgaatcaaga aatagatcag gaaagtatta
18841 actacaatat gcaaacaatg tttgaaaaag aaagatacga tagaagtagt accattgaaa
18901 aattagtagc aacaagaaaa tttaagtatg aaaaagatga ttcagattta ttcaaacaac
18961 ttttcaatga tgttgaaaat tcaatagaca gattaggtat ttacttacta aataatggta
19021 taaattcgaa tgatgaaaat gcaagatact atagatcgtt tttaaaggaa cttagtagaa
19081 taaaaagtaa attaacgcca ttttctcttg aaataagtaa gtctagcgga agagagcaac
19141 attatcctga tgatgctgtt gatgataaag atgagagaag aaaaaataaa gaagaagcat
19201 atcatgcttt tgacgataaa agcgatattg actacaaatt aaagaataaa ataagtgatt
19261 atatcgataa tttattgtca gttaaaatta ggaataattt gtatgatatt agtccaaata
19321 caaaaagcaa taattataat gagccaactc aaaaatgagt tggctctttt gttgtaattt
19381 taggcttcca ttatgaaaat gaatgtgatg gaagcaatta tgtaattaat tagagtcata
19441 tatattatag atacaacgtt gtgatgttca cacgataatt agcccgggag attgttgtgt
19501 aataacttat agaaggagca aaacactatg tatcaaacaa atacacgtga tttaattaca
19561 aaattacctc aaagcaataa aacagaacac tttttaatga acaaattttc aaatcaagat
19621 aaagttcagc agctacaaag acaaattagc caacagctag atcaacaata taatgagctt
19681 ttgtctaatg aaaaagctaa gctagaccaa tatgtggaag tacaccataa tttagaacca
19741 ttaaagaaag agattgaatc agaatctatt aaccttgata ccgataaatt acctgatatc
19801 aaagcgacaa tgcttgaaaa ggctaagaac gatgaacatt ctgataaaat cgaacaccta
19861 tttgatagat tagatcagtc attaaatggt acgaatcgat tatatacaca attatcgttg
19921 attggcacac gaacacatcg gataacaacg aaaagattta atgttcaagg cttacctaaa
19981 ttagtccaac aaacgatttt accttcgcaa tttaaaaagg tttatacaat agattttaaa
20041 tcattcgaac catcagttgc tgcgtatatg acacaagacg aacaactgat tgactacttg
20101 aatcttgaag aagggttata cgatgcatta ctgagagact tatctttgtc aaaagagaag
20161 cgcgtgagtg tgaaacgtgc atttataggg tcatttcttt ttggcggtcg ttatagtagc
20221 tctaaattca aaatcaatca agaggttagt gaaattaact ggctacaagt aatgagcaaa
20281 ttcaagaaga tcattgaatt taaagagcaa gtcgaaaaat ataaaacaat gcctacgcct
20341 tacggcattg aacatgatat gagcgcattt caaggtagta gtattatggc aatttacgta
20401 caaacggtag cgagctatat tttcaagcac attttgttgg aagtgtacaa agcacagtgc
20461 gaacaaaaaa cgttcaagat tatagtgccg atacacgatg cgattatgat tgaatgtaat
20521 gataaggaga ttgcacaaag tgtagcacag ctcatgaaag atacagctaa tcgactgttt
20581 aatggtgaat ttgcacatgt gacagtggaa gctttaggag gtatagacaa tgaataatga
20641 tagaggaaaa agtcttcaaa ttccccaaag tacatcgtta aaagaaggat ctatatacgt
20701 tgctacatta cattctgtga ctgagacaga ttactcaggc gacataaaac atcaatttac
20761 gtatgaaata gagatagatc aacaaatatt ctatgtgaac cgtagtgttg tggtcaatgt
20821 gacacatcaa caattatcaa ttaatgactg gctcgaacgt catagtaact atagcgcaag
20881 tcatgaaaac tatgagcctt atattgatca gaaacattta attctattag gtcaatataa
20941 cggtaactat tatgtacaag atgtagcatc gttagatgcg tttggaggcg tattatcatg
21001 aatcatatat tacaaatgtt atctaagcta ttaagtgtgg ctaaggaggc aatcgaccgt
21061 caaggtctga ttgctatcct aactattcct gttaataatg acgatgaaat agaagaaacg
21121 gctcaaggtg aaaccgtgta taacgaactt atcgatcagt tacgacttaa tatcccaaaa
21181 gatacggatt atcgacctaa catctatagt tattttggta ttaagaagaa tcctaatgac
21241 accgtactca tggaaatgat gataaaggtt tttcatatca aacgctttaa ttcagaactg
21301 tttgttttca aagctaacgg gtggcaaaag ataaatggag atgaattaca agggttgata
21361 tctaaaatga tacaagtatt gcttgtagat tataagcctt cactaagcac tctaaaaaat

21421 gtcgtagatg gattgcaaaa atcaacagat gtagaagaac ttgttgaaaa tgagcactat
21481 attggttgtg gtgaaaatat gttcgatctt aatacgtttc aagtcgttaa aaattcaatc
21541 gatatctttc ctaaaacacg attgaattta tcattaagta caaatgatgt aattactgat
21601 aagataccgc cttattttaa gcaatatatg ttacaacttg cgaattatga cgatgattta
21661 caatactttc ttttccagca tacagcagta ttacttacag ctgatactaa ataccgtaga
21721 ggtctcattt tgtatggtgg agctaagaat ggtaaatctg tatatattga actagttaaa
21781 tcatttttct atagtaaaga tattgtgtct aagccactta atgagcttga aggtcgtttt
21841 gacaaagaaa gtttaattga caaaagtcta atggcaagtc atgaaattgg acaatctagg
21901 attcaagaaa agatcgtaaa tgacttcaaa aagttattat ctgtagaatc aatgcatgtt
21961 gatcgtaaag gaaaaactca agtggaagtc attttggatt tgaaacttat ttttagtaca
22021 aatgcgatac ttaattttcc tcctgaacat gcgaaagctt tggagcgtcg agttaatatt
22081 attccatgtg agtattatgt tgaaaaagcg gacacttcat taattgataa gctccagagt
22141 gagaagaaag aaatctttct ttacttgatg tatgtgtatc aacagattgt aaaagcagat
22201 atcgagtacc ttgaaaatag ccgtgtcact gaaattactc acgattggtt aaattttgga
22261 tatgaatttg tttctagcag gtccgtaagt attccaaatc agaaagcatg tattaattta
22321 ctccgaaaac ttacagcaat taaaccaggg tcacgtatca aagtgtctag gctaaatgag
22381 gttattagag aggaaattaa agtaagctct caagttatta atgatttggt tcaagctaat
22441 tttaatgtac aaagtagact aaataatggt tataagtatt gggtcgattt aggatggaaa
22501 gaaactgata aaaaagatga catgatttta ttcgatgaaa atgagaatgt aacagatgat
22561 gagttcttat acgaagatga tttgaactta ggttgggagg actttgacga tgaataatga
22621 acaaattgaa gcatttgtag aagtgcttgt acctatcata gaagaacgta tcaataaagg
22681 taagtaatct aattacgtac tacaggcagt tgcctgtagt actcatatga ttaagtggta
22741 aaagtgataa aaatgaaacg aaattataaa tatatattat ctatatgttg ttacaagacc
22801 gatagtctgt agcaataatc taataaaagg agaacggtat gatatgaagg gtaaaattgc
22861 actttattca cgtgttagta cgtctgagca gtcagaacat ggttattctg taaaggagca
22921 ggaacaagta ctcatcaaag aagttgtgaa aaatttccca ggttatgact atgagacata
22981 tactgactca ggcatttcag gtaaaaatat tgaaggtcgt ccggcaatga aacgtctatt
23041 acaagatgtt aaggataata aaatagaaat ggtattaagt tggaaattga atcgtatctc
23101 acgatcaatg agagacgtgt ttaatattat tcatgaattc aaagaacatg acgtagggta
23161 taaatcgatt tctgagaata ttgatacatc caatgcttct ggagaagtac tcgttacaat
23221 gtttgggtta ataggatcta tagaacgttc aacgctaatc agcaatgtga aaatgtcaat
23281 gaacgcaaaa gcacgtagtg gagaagcaat cacaggtaga gttttaggct acaaattatc
23341 atttaatcca ttgacacaga aaaacgattt agtcatagaa gaaaatgaag ctaacattgt
23401 tcgagaaata ttcgatttgt acttaaatca taataaagga tttaaggcaa ttacaacaat
23461 attgaacaaa aaaggatatc gtaccatcaa caaaaatcca ttttcggtat ttggtgtcaa
23521 atatattctg aacaaccctg tttataaagg ttttgttaga ttcaataatc atcaaaactg
23581 gtcagttcaa cgaagaagtg gtaaaagtga cgaaaatgat gtgatattgg tcaaagggaa
23641 acatgaagcc attataagtg aagaagtatt tgatcaagtt catgagaaat tagcatctaa
23701 aagttttaaa ccaggcagac ctattggcgg agatttttac cttcgtagcc ttattaaatg
23761 tccagaatgc ggtaataata tgatatgtcg acggacttat tataaaacca aaaagtccaa
23821 agaacggaca atcaagcgtt attacatttg ttccttattc aatcgttccg gaagttcagc
23881 atgtcacagt aattccatca atgcagaagt agtcgagcgt gtaattaatg ttcatttgaa
23941 tcgtattctg tctcaaccag atattatcaa gcagattgcg tcaaatgtga tcgaagaatt
24001 gaaacaaaag catagtagcc aaccagaaat taaatatgac attgatagtt tggaaaaaca

24061 gaaagccaag attaaaacac aacaagaacg attgttagaa ttgttcttag atgatcaaat
24121 ggatagcgaa atgttaaaag ctaaacaaag tcaaatgaat gaacagttag aagtattaga
24181 tcaacaaatt aaagaagcgc aacaagcaaa tcaatcacag gatggaatac caaattttga
24241 taagttaaaa ggacgactca ttttgatgat aacacgattc agcgtgtact taagaaaggc
24301 tacacccgaa gctaaaaatc aacttatgaa aatgttaatt gattcaattg aaattacgac
24361 agataaacaa gtaaaacttg taaggtataa aattgatgaa agtcttatcc ctcaatcttt
24421 gaaaaaagat tgggggtctt tttttatgcc taaatttaac tttgtgataa atgtcgcaaa
24481 tgaaaatagg attgaaaatt tatcactttt accacttttt tagagtgaca aaagtggagg
24541 aattttgaaa tattttataa atatatattt tatttatgga gtacaaatta ttaattaagg
24601 aggtcattat aatgacgcta agcaaacaac ttaaaacgta tattactgaa cgatttaaat
24661 taaattatca agaaaattgg gcttgtgaaa acgtagatgc ggtggctgaa gatgtattac
24721 ctgaaaaata tattaaaaat agtccacttg aacataaaat tttaaatact tttacctatt
24781 acaatgatga attacatgaa atcagtattt atcctttttt atgttatcta gataaggaat
24841 tagtagcaat aggttattta gataattttg atttagactt tatattttta aatgacactc
24901 atcaaattat tattgatgaa cgctacttgt tacaaaaagg gggtgagtaa ttatgaattg
24961 gattaaggtc gctcaactat ctgtcacagt tatcaatgaa gtcattgaga ttatgaaaga
25021 aaaacaaaat ggaggggaat agtatgcaaa tcaatcgata cattacacga ggcattagtg
25081 aacacctatc tctagacctt caaatcttac tttggaatat ggtaaaagaa agagataatc
25141 aacctgatac agattaccta cacattttta gactgcaaga agatgagaat atactctcaa
25201 tcacacatga acaagaacaa cctgcataca aattggaata tcactataca aactatgaaa
25261 aaaatcaaaa cgcattacct aagaaagtct acgtcattcg agaagatgat gtagaccttt
25321 cttattatgt catgttacta ccagaagaat actagaggag tgaaaattac gtgaaaacaa
25381 tcgaaagtac attagaaacc gaagctatat tcagtgatga ccaacaacat cgctatctac
25441 ttaagaaaac atgggacagt gaaaaacaaa agatcacaat catcacaatg tatccaaatt
25501 atacgggcct tcttcgtata gatttaacta cccaattgat aatgaacaaa gtttcagaaa
25561 tggatgcatt tggttccatc aattttgtga atctatactc taatattaca actcctatca
25621 atctcaaaca tttagaaaac gcctatgata atcatacaga tattcaaatt atgaaagccg
25681 tgaaagagtc agatgaagtg atactagctt ggggtgctta cgctaaaaag cccgttgttg
25741 aatcacgcgt caatgaagta ttagagatgt tgaaaccaca taagaaaaaa gtgaaacgac
25801 tcatgaaccc cgcaaccaat gaaatcatgc atccccttaa tccgaaagcg agacagaaat
25861 ggactttgaa agcataacct atatatggag gtaaacgatg aaggaaatca atattgtttc
25921 actacaaatg ataaaaacag acacattaag ttatctaaaa aatcgtattt caaaccctga
25981 ggatgcggca gaaatcttgc gttcattcat tggaaacagt gaccgagaac atctcattct
26041 catatgtatg aatagtaaaa atgaacctac acacattcaa atactatcga ttggatctat
26101 taaccaaaca gtgattcatc ccagagaaat attcaaaaca gcaatactca gtaacgccaa
26161 cagtattatg ctcggtcata atcatccaag tggggatata accccctctc ctgaagatat
26221 caatgtaacc gaacggttaa tgctcatcag tgaaatgatg ggcattttat tttatgatca
26281 tattattttt tcagatagta acacctattc cattagacaa catgatttag aagtagaggt
26341 atagccgtat tttgtcacta ccgttatttc ttcacttatc aatatttata aaatcccttt
26401 ataacaacat tttcaactga aaaatatttt ttacaacttt tattaaatat acgtaggatt
26461 gacttattgt gacaaaccta ttttgtaata tataacttaa aggaggtgac atattggatc
26521 gtgcatttcg aatacttacg atatacaatc gtttactaca aaataaatcc gtcaacaagc
26581 aatcactcac attagaactc gatacaagtc ctcggacgat acaaagagat atcgacgata
26641 taagaaactt cttatatgaa agtaaatcat ggataagtac acctaaagaa atcacgtacg

26701 attataaatc tgaaagttat aagctcgaac aagataagag taaaaatgtg cattttatgt
26761 ataatatttt gactgcttta tacctgacga cacctaaatt aagtcattac ttttatcaat
26821 atcttaaatt attaatcata aaacatcatt caaatcatcg aactacatta ttaaaatatc
26881 ttgagcgctt tgaaattgat aagaatcaaa caggtatttc gacatcatca ttagttgttc
26941 aagcaatgaa cgaaaataaa tatcttcaat acaatcaaaa aatattatta cctttatcga
27001 tgtactatca aatgcatgct tttcacttgg tatatcgaat gaacgatgaa atttatatcg
27061 atgatattaa tcaaatgaat ctgagtttct ctgaaaaatc tgtggacaca catgaaaaag
27121 caaaggtaca aacctatatc acgtttgaaa tttctaaaga tgtgtggtca aaaatgcatc
27181 gatattatca aattcatgtg atagaaaaat atgatgcgca ttatcttatc gtcactttta
27241 agatgacaag gatcgaagcg attcaactct gttttatgta tcgatccaat atacgtatca
27301 tttcaccccc tgatttgaag gaacaagtga ttgatgaatt acttttatta caaacaacct
27361 atttaaaaca acaggttcaa aagtagaaat aacatacaat atttaataaa taacattaat
27421 aataatgaaa ggaatgggaa ttatgagtaa catcataaaa acaataggca aaggtattgg
27481 aaaaggtgtc ggtgcagcag catattattc agttgcaact gttggcgcag ctatcggtgc
27541 gatttgggaa acaacaggcg atagaggttt aatgagagat gctttaagtg atagttctaa
27601 tcaagatttg atggaaatat atgagataca aaagaaagat gatggggatt atgatactta
27661 tattgcagct aaatctttat tacaagagag aggattcact tataatcacg aaacgaacaa
27721 gtgggaaaaa tagtaaggag tgaaataaat ggaatttaca tattatgaaa cgaaatcttt
27781 aaatgatttt gaaaatgaac tgaaaacact tgctttaaat aaaaaatata cattcagtaa
27841 ggaaaccgga gtccttaaag aagatgaaat ccttgaaaaa tcgaatagcc atgagcgcga
27901 actattaaaa gtatttaaag ataatgcaca gataacaact tatcatttta agagtcataa
27961 aagtaattat ttaattatta tgggagaaac aaaagcgcct aatattattg gattaacagt
28021 catatttaac aaaaaacctt ttaaacaaat gggcaaaatt gcattaggat ggctaattgc
28081 tttgacttca ggtggaagta tattagccat tggagcagta tttggaatac ttacctcagt
28141 tgcaagacgt acacttgtaa tcaaaggacc attaggaaaa gaaatttcac aaattataga
28201 aaatgaatta ggagaaccga tagaaattaa aaaataatag aaagtaacta ttttcaaaga
28261 cacattgatt tcaaaatgtg ttctttttca attattttag aataaatata gaaaagcgta
28321 gaaaaataga ttaatataaa attaaaataa atatagaatg gggttagcaa tgtgaagaaa
28381 atttttgcaa gcttaggtgt tattttatta gttgcaatat tgggagttgc tgttgtattt
28441 gcatatggga gttacaaaga tttagaacta aaaaaagaag aagctaaact agaggataag
28501 aagaataaga aaatcgataa aaacaaaact acagaaaatg aaaatcaaga ccagattaat
28561 aatcaaacaa ttacagcacc aaatgaaaaa gcacaatatg gcaataacct aggagttcaa
28621 gaaaaaagta cggataaaaa tgatcctaat ggtgtgctta gatttgatac aaacggagat
28681 ggatttatct ccactagtga agtaacacca gaagctcaaa aattagttga tgaaggtaaa
28741 ctacagccat cttcagatag aatggttgaa gagtggacta agaataaaaa taatgtagat
28801 aatacatatg actatgatga atcatataaa aaacaacaag aaatgtacaa aaaagctgct
28861 cgtggcaaag ttcctgaacc tggagaaaca ataaaataaa aaagcactag ctacaattgc
28921 gataattata atttttggca tattagcacc tgttgttttt ttgttacaac aaatatcaag
28981 aagtagaatt agaaaaaata aattacaggc taagcaaaaa gaaaataatg gaagtaactc
29041 tcaaattatt agtgaaacat ctaataatat gaatccagag aatgaaagtt caagtaataa
29101 tcaagaaaaa acacaaacta caacccacga agctacaatg aatcaacaaa aggtaactag
29161 agaaaatgtg tttgattacg ctattgctgc tgtaaatgaa gtaggaaatg cagatttatt
29221 aaaatttcaa caacctgaac acaatggttc agaatggaca attaatgcaa ataataaatc
29281 aggtgctggg gctaacacca tcgtagttaa agatgatgaa acagttcaaa tatggatgga

29341 cctaaaacat ctatggatca tgaaactaaa caatcagatt ggcgaacggt tgctagttgt
29401 ttagcatcgc aaaattatat atcgctcgta aaaggattag tacattattt cacagcgatt
29461 gaagatgaag aaatacttga taaaatctat gatgatttta tgaatgatga ctctataaca
29521 acggtactta acaatgattt acagacaatc attaaccatt atctatcaaa ataaagacta
29581 tactaattac aataaaaaca gagcatcctt cactttgcgg tgagggatgc tcttttagtt
29641 tatttttta ctgattactc gccataagct ctgcttgttc tacgacttgt tctacagcca
29701 tttttgtaa atctggtgga tagccatatt ttttaagcaa tcgtctaaca gctacgcgca
29761 ttttagcttt tgcgctatca cgtttagacc aatcaacgcc catattttct ttcactgttt
29821 tagttagctc atgagcaatc gcacgtagtt ctttatctcc catggcttct tttgctgttt
29881 catgtgaagc taaagcatcg taaaacgcaa tctcatctga attcaggcct aattcatttc
29941 ctcgttgttg ttcttgttta atatctttag cgagttgaat gagttcttca atcacttttg
30001 atgtttcaat agaacggcta ttatatttat taatcgaatt tcctaacatt tcagagaaac
30061 gcttagatac tgtcgcgttc gttttcatta atgattttac ttgccctttg agtaatcgat
30121 ttaataattc taccgcaaca tttttttgtt tcaatccttc gacatctttt aggaagtcat
30181 ctgataggat tgataaatcg ggttgttcaa gacctagtgt ttggtaaaca tcaatcacat
30241 cttctgtcac aacagattgt gacacaagtt gattaatctc tgcttcaact tctgcaggtg
30301 ttttacgtgt ttttccttct tttggcggtt gtaacaattt aacaagtcct gctttaactg
30361 ctttaaagaa ggcaatttca tcattgagtt cttgggctgt aggttcagtc gcacaaagag
30421 caaaagcttt ccctaactct gtgaccgttt taataaaacg ctggcgttca tcttcgccta
30481 aaccaatcac ataatccatc gtatctgaaa tcgcataata acgttcagac tttttctctg
30541 aatcaaactt agaatagtca agattatata acatatcttg aatcacatca tatttcaata
30601 acatcaattc aaccgcttta tctgtatcta tcgccgtttg tgcttgatca gattctgtat
30661 attctttaag tgcctctttc aaactttcgg caatccccac ataatcgaca atcaacccac
30721 ccggtttatc tttaaacact cgattaactc gagcaattgc ttgcattaaa ttatggcctt
30781 tcatcggttt atcaatatac atcgtatgca tagaagggac atcaaaccct gtcagccaca
30841 tgtctcgaac aatcacgagt tgtaattcat cattcacatc tttcatacgt ttttctaata
30901 aattacgacg ttttttagga ccaatatgtc tttggaaaga agatgggtca ctagatgaac
30961 cagtcatcac tactttgatg actcctttat catcatcatc tgaatgccac tctggtttta
31021 gacgaatgat ttcatcgtat aaatcaacag caattcgacg gctcatcgtt acaatcattc
31081 ctttaccttt catcgcctgt tgacgtgttt caaaatgttg gatgatatct ttggctagag
31141 cttctacacg tggctttgcg cctgcaagtg cttcaattcg tgaccatttt gattttaaac
31201 gctgctttat atcctcttct tgatcttctg taatgtcatt atatgcttca tctaaatcta
31261 aattttgagg tagatttaat ggaattacac gactttcata gtaaatttta accgtacttc
31321 catcagctac agcttgtgtc atatcataaa catcaatata gttcccaaag accatttgcg
31381 tatttttatc cgttgaagct acgggtgtac ccgtaaatcc tacaaacgtt gcattcggta
31441 aagcatctct taaatatttg gcataaccat atttaatgcc ttcgccttta tcatcgtatt
31501 ttgcgttaaa gccatattgt gtacgatgtg cttcatctgc cataacaatc acatttttac
31561 gttcagttag ggcagccatg gtcgtttcat tttgttcagg ttcaaatttt tgcattgttg
31621 taaatacaat accgcccgac tcaacagata ataacgattt taattcttta cgtgtttcag
31681 cttgttttgg tgtttgcctt aataatcctt taccagatcg acctttcgat ttaacaaacg
31741 tactgtatag ttggttatct aaatcattac ggtctgttac gacaactaag gtaggattat
31801 taagcatttg aattaatttt ccagagaaaa agaccatggt taaactttta ccagaccctt
31861 gagtatgcca aataacgcca cctttaccat cacccggccc agatgaagct aataaagctc
31921 tatcaacagc tttattaaca gcatagtatt gatgatacgc tgctagaatt ttactgatat

31981 gtcctttacc atcatcttgg aataatacaa aatatcgaat taaatcaagt aaggtttctg
32041 gatttaacat cccatgaatc agtacgtcta agctagctaa acttgacgaa gattccgttt
32101 ctccatcttt agaacgccaa gtcataaaac gatcatagtt cgcagttagt gaaccggctt
32161 tggtattaat accatcactt gtaacaagca cttcattaaa tgtaaataat tgtggaatac
32221 gcatcttata cgtttctaat tgatgataac catcttcgac gcccacggtt tcattcgttg
32281 aatttttaag ttcaattaca acgataggca agccattgat aaagagtaca atatcgggac
32341 gttttgtata gtctccgtta acaacagtga attgattgac tgctaaaaaa tcattgtttt
32401 gtggatgttc aaaatcaacg attttaacaa tttctactac cgattgtcct tcgtcatcat
32461 aatcttcaac ttcaatacca ttgattaaat tttcatgaaa ggtaaggtta ttttctaaaa
32521 ggttgggtga cttttctaaa gttagttcat gtatcgcttt ttcaataaaa cgaggatgga
32581 tgtctgagtt gatctttctt aatgctttct ctaaacgttc atgaaggaca acttctttat
32641 cacttttacg ttctggtgct agacctgtta cactaatctc attacctttt ttatagtcgt
32701 agcctaatga ttgtaaccat tctagtgcaa cttgttctaa atcatcttca ttaaattgaa
32761 agttcatctg tattcacctc aacatcatca ggtatttcta gttctccaga cataagttta
32821 gggagaagcg tatctcgtaa ttgagttaat ttaccaattt ctgatcttaa tattttcatt
32881 ttttccatat atggagtaat tatttcagag taatttttta caatactatc ttcaattgct
32941 aatttataat taacagctat tgttggcttt actctctgtc tactatttgt tgaaccagta
33001 gtattggctt ttagataatc aataaattga gaattaagac atatattata tacgaatgaa
33061 ttaattttat tatttggaga ttccattaca acaaattctg atgaagcaac atttattttt
33121 ttattatcta ttaaaggtaa ccaaaccctt ttagtatcag gattcatttt tgaaaataat
33181 acgcaattgt tattaataat ccatttatta ctttttattt tattgacttc ttcatttata
33241 gcttgttctt cattatcata agctggtaaa ctaaaatgtt taactgttac ttcttcgctc
33301 tttttaggat tgaaagtttc ctttttatga cttgctacat cttttaattt atagattttc
33361 caactgtctg gtatcttacc taactcacta tcaatcattt ctccaccgct agatttataa
33421 ggattaccat ttttatctgg aaattcaaaa tctacaaacc aatgtttaaa caatgtttgt
33481 gatagttcct caaggtttgc tattaattta ttattagtat ccaatttatc ttctatattg
33541 cctataattt ttaatatttt atcttgtttc ttgatagaag gtatatctac gatataattt
33601 tttacaatat ctgtttttaa attattaaaa acgctaccat tagataattg taataatttt
33661 tgtctattca ataataatat ataatataaa aattcttttc taacattttt gaaatttctt
33721 agtagtaacc acccatcatg tatacatgct ttaactttca tgaatttagg aagtcctggt
33781 gtagcactgt tagataaaat taagtctcct ggtaaaacct ctactgattt tttgcttcct
33841 tcaagagaaa ctcgctcttt agtatcataa atatatctac ttgagtcttt tgttgcatcg
33901 gcaattttca cccaaggcaa actattatca taactattta cgatataatt tttaatagga
33961 cgtggagaag cgcctcttct tatatcacat aaatcactga ttttatactc cataacccaa
34021 tccctccaat gatttacgga tttggtcttc aagctcttta gatttagcaa attgttcact
34081 taattcagaa gtaatgcgct ccattttttg ttcaaatggt tcttcttctt cttcaacatc
34141 agctaaacca acataacgtc caggtgtcaa aatatattca ttattcttaa cttcttctaa
34201 atcagctact ttacaaaaac cagctacatc tttataaggt gtattgtttg tacctctcca
34261 agcatgatat gtttttgcta ctttttgaat atcttcatct gaaaattctt ttaacgttct
34321 agttaccata tgaccgattt cacgagcatc aataaataaa atttcatttt tacgttcttt
34381 tttaccattt tgacctttgt tattgctaat gaaccaaagg catactggaa tttgtgtaga
34441 ataaaagagt tgaccaggta aagtaacaat acattccact aaatcttgtt caataagatt
34501 tttacgaatt tctaattcgt ctttaccact ggtagacata gatccattag ctaatacaaa
34561 tcctgctgta ccgttaggtg ctaattttga aatcatatgt tcaatccatg catagttggc

34621 attacctttt ggaggtattc caaattgcca acgataatca tcaagtaatc gttcttgtcc
34681 ccaatcactt gcattaaaag gaggattggc taatatgtaa tctgccttta atcctttatg
34741 tagatcgtta tggaatgtat cagcatttcg ttcacctaag tcattatcaa taccacggat
34801 agctaagttc attttagcta atttccaagt cgtagggtta gattcttgtc cgtaaatcgc
34861 aatatcgtct aatcgacctt gatgtttctc aacgaagcgt tcactttgta caaacatgcc
34921 ccctgaacca caacatggat cgtaaatgcg tcctttataa ggttcaatca tctcaactaa
34981 taactttaca attgatgatg gagtatagaa ttctccagca tttttacctt ctgcacttgc
35041 gaatttagca ataaaatact catacacacg gcctaacaca tcttgtttac gactctctgt
35101 gtcgccgact ttaaatgtaa ataaatcaat aatatcgcct aatttttctt tatctaatgc
35161 aggtcttgca tattctttag gtaatacacc tttgagtgat tcattttcat tttcaattgc
35221 aatcattgct ttatcaatca tttgtccaat ttctggtttt ttcgcattat cattgatata
35281 ttgccatctt gcttcttttg gaacccagaa aatgttttct gctaaatatt catcttgatc
35341 ctcttcatca gcataagaat cttgcttcaa ttcttcatat ttttcttcaa aagaatctga
35401 tacatatttt aaaaagatta gacctaatgc tacattttta tactcagcag catccatgct
35461 tcctcttaat ttatcggcag cttgccataa cttttcttca aaacctatag tggccattaa
35521 taacacctct taaatttaat gttaaatcaa ctatagcaaa aaatagaaga tgattgtgta
35581 gtattttatt aatatttatt ttatctagac tatcaaaaaa atatagatta ttattatctt
35641 aaattagtaa acaggagtga attaaaatga ataaaaaata taaattaaaa cgatacgata
35701 aaagatttag aggtggatgg tgttacaagt gtgaagattc atattcttac ctttgtgaag
35761 attgtggtta ttgtagatat tgttgccatt gttaattgaa aaaattttta tgaatattag
35821 gtcaaaattt ttctgtatca aaaacaccat ggtaaataat aaatatttat tgaaaacaaa
35881 tagaaaaaga gaatgggaca aaactcattt tgactttatc gttccacttc gataatgtat
35941 ctccaacata aaaaaagtct gagacaacta ataatgtctc agactttttt caatcacata
36001 ttatttaagt tcttgagtaa cggtttttgt ggtaggcaaa gtattaataa tgtcgaaacg
36061 agattttgtt tttttgatat tgacgcctaa agatgataat aatttgacta cattttgtaa
36121 cttatctgat ttgttcatgg tatctcacct cgttttttga ccttcaatga tatactcccc
36181 taattagtca tgctttacta ccatatttga aaattatttt aaattattta agttacacta
36241 aagcttttta ttatgaaagt actttaaatc cagcgtcgta aatttctttt tcaatactat
36301 ttaaattagt aggtgtttca tattcaacat taaccatatt tctttctata tcgattgata
36361 ccgtatgtac tccaatcata tttaacaaac gttcatttaa catttgtgct tgttcctttg
36421 tttcaatgtt atctataaat acaatattac tgtttaacat attacaatct cctaattaat
36481 ctttcactag tttttgaaat gtcactagta gttcttccat tgcttcttct tcagtacctt
36541 gatttatttt ttccataata caacttttca tatgatgttc tagtaactta gtagcaacac
36601 tattaagtgc tgaacgtgtt gctcttatct gagttaatac atcatcacag tagacgtctt
36661 catcaatcat tcgattaatt gctttaactt gtccttctat tcgatttaat cgagatttta
36721 aatttaattt aacttgctca gaatgatggg catgattttg attttccaac taattcaccc
36781 tttcaatagc ttctatatca tcgtataatc ttctctttat tacgtcaatt atacattttt
36841 attttaaaaa ttaatcatac catttattat agtgcatggt ataatatttg ttagtacatt
36901 aattttaaac gatttaggag gttcaattaa tgaatatggt agagatcgtg caattatctt
36961 tctcacattt atctttctca catttatctt tctcacattc ttctttaatt attaacatca
37021 tattaatcac tgcctttata ttaaacttga ttttcgcatt tatcattatc ttcatggaac
37081 gtcgtagtgc aggttcaatt tgggcgtggc ttttagttct tgtatttcta ccgatagtag
37141 gatttatttt atatctatta ttagggcgtc aaatacaacg caaacatatt tttaaattaa
37201 aaaaagaaga tcgtaaaggt ttagctatga tagttgatga gcaattaaat gcgcttgaaa

37261 aagatgattt ttctaaaggt aatcatcaaa ttgttaagtt taaagaaatg gtgcacatgt
37321 tactctataa taatgctgca ttcttaacta ctgataacag tataaaaatc tttacagatg
37381 gtcatgctaa atttgacgca ttaattgaag atattcagaa tgctaaagat tatatacata
37441 ttcaatacta tattttcaaa agtgatggtc ttggacgtcg tattttaaag gcactcgaag
37501 acagacttga agatggtata gaagttaaaa tgttatatga tgatatgggt tcaagaacat
37561 tacgcaagaa agatttgaaa cagtttaaag acaaaggtgg acgcgcagaa gccttcttcc
37621 cttccaaatt gcccttaatc aatttaagaa tgaataaccg taatcataga aaaattgtaa
37681 ttatagatgg caagattggg tatgttgggg gttttaatgt tggtgatgaa taccttggta
37741 aagataaaaa atttggttat tggagagata ctcatttaag acttgaaggt gattctgtca
37801 atgcattaga attaagattc atcttagatt ggaattcaca atcaacacgt gacaatatca
37861 gatatgaaga tagatatttt cctgatgtag attcaggcgg cgagattggt gttcaaatcg
37921 cttcaagtgg tcctgatgaa gactgggaac aaattaaata tggctattta aagatgatta
37981 tgtcagcaaa acattctatt tatattcaat caccttattt tattcctgat caagctttta
38041 ttgatgccat aaaaattgcc gctttaggtg gcgttgaagt taatttaatg attccatgta
38101 aacccgatca tccttttgtt tattgggcta cattaaaaaa tgccgcttct ttattagacg
38161 caggtgttaa gatttatcaa tataataatg gatttctaca ttcaaaaaca ctcattgtcg
38221 atgatgaaat cgctactgta ggtacaacaa atatggataa tcgtagtttt acattaaatt
38281 tcgaagtgaa tgcgttcatt tatgacgaag atatttctct tcaacttaaa aaagcattta
38341 ttaaagatat gaaagtctca tatgagttga ctaaagaatt atataatcaa cgtagtactt
38401 ggattaagtt taaagaaggc atatctcaat tattatcacc aattctttaa actcattcaa
38461 tacatgtttt taaaggagta gtttatgaat caagcagaac aaatattatt agctaaggaa
38521 tatatgtacc aatttcataa aaatgattac tctggacatg atattgcaca tattgaacgt
38581 gtcacatcat tagctaaata tattgccaaa caagaacatc aaggcgattt tcttacaata
38641 gttttgtcag ctttattaca tgacgtcata gatgataaat tgactgataa acaccatgct
38701 ttaagcgaat tatatcaatt ttttaaaaaa ataaaattgg atgattctgt tcaaaagaat
38761 attattttta ttatcaaaca tttgagttat caaaacggta gaaataacga tgttacactt
38821 ccaatcgaag gacaaatcgt tagagatgct gatcgcttag atgcaattgg tgctataggt
38881 atcgcaagaa cgtttcaatt ttctggacat ttcaatgagc caatgtggac agaatctccc
38941 tatcactcaa ttccttcaga aaaagatatg gttgatctac ctccttctgc tattagacac
39001 ttctatgata agttattaaa attaaaaaaa ttaatgcata cacaaacagg atctcaactg
39061 gctgaagaac gtcatcaatt tatgaaacta tttttatctc aattttatag cgaatggcac
39121 ttagaagaat aaaaaaaaca tactaagatt agctatgaag aaatctatga cgatagattt
39181 tttcatagct attttttata gttatagaga ggagtagact gtccagactc ttggatttta
39241 aatccgtaaa aaaaacaagt cagctttact ctcgcctttt gaaattcgtt tgtagtatgt
39301 tgggttcttg aaaccgtgta taggaaaatg aaatgagaaa ggttaagtaa agtttttagc
39361 ttctcaacta ttcaaaggag gttttttat cgattactta ggtgttgata ttagtaaaag
39421 gagtagtgta attgcacatt ataaaaatgc aaaattccaa aaagagtttt tcatccaaaa
39481 taataaaaat ggctacaatt atttactcaa gtatttgaat gacttagacc acccacaact
39541 catttttgaa tctacaggta tctattcaag aggtatggaa cgattttgtt gtgtaaatca
39601 aattaactat attcaaatga atccgttaga agccaaattt aaaacgagcg ctctaagatc
39661 atggaaaact gatcaggcag atgctcataa gcttgcttgt ttaggaccga cgcttaaaca
39721 aacaggcagc ttacctatac atgagttaat attctttgaa ttaagagaac gcgtccgttt
39781 tcatctagaa atcgagaatg aacaaaatcg acttaaattt cagatccttg aattactcca
39841 tcaaacattc cctggtttag aaagattatt tagtagtcga tattcaatca ttgcactcaa

39901 catcgcagaa atctttactc atccagacat ggttcttgat atcgacaagg atgtacttat
39961 tacacatata ttcaattcta caaataaggg aatgtcaatg gataaagcta caaaatatgc
40021 acttcaatta agagtgattg ctcaagaaag ctatcctaat gtcgatagac attcctttct
40081 agtcgaaaaa ttacgcttac ttattcaaca attaaaacaa tctattcatc atctcaaaca
40141 attagatgat gccatgattc aattagcaca acaactcgat tattttgaaa atattcattc
40201 gatacctggt attggtaagc taagcacagc tatgattatt ggggagattg gtgatattaa
40261 gcgatttaaa tcaaataaac aactcaacgc ttttgtaggc attgatatca aacgatatca
40321 atcaggtcat acacactgta gagataccat caacaagcgt ggtaataaaa aagcgagaaa
40381 acttttattt tgggtgatta tgaatataat aagagggcag catcattatg acaatcatgt
40441 cgtcgattat tactacaaac taagaaagca gcctaatgag aaacctcata agactgccat
40501 cattgcttgt ataaatcgat tattaaaaac gattcattat ctggtaatga atcataaatt
40561 gtacgattat caaatgtcac cacattagcc aaacgtacaa tcaaatacag tttaacacct
40621 tattcaaaaa aattaaaatg aacggtttag ttaagtaatg cttattttaa ttataagtac
40681 ttgactaatc gtaggaaaga gggtgggaca taattcctaa caaattagcc agtaaatgag
40741 tttattataa attcatttac tggcttcttt atttacaata ctacgtattg ttggctcgct
40801 ttcttagggg acagcttcag cctgtagtct tcagcttgtc ctgttccctc aagagtctcg
40861 ccaaaatact ttgtatttat atgtaatttt acattgaaat actttaaaaa aataaggcac
40921 tttcgtataa tttaataaac gtcactaaac taaattaacg aggtgcctta tgtataaaga
40981 ttataacatg aatcaactta ctctaccatt ggaaacttca gttcttatcc ccacaaatga
41041 tatttcacga catgtcaatg atattgtaga aacaattcct aaaactgaat tcgatgaatt
41101 cagacatcat cgtggtgcaa cctcgtacca tcccaaaatg atgttaaaag tcattctata
41161 tgcctacaca caatctgtgt tttcaggacg taagatagaa aaattactta atgatagcat
41221 ccggatgatg tggttatcac aaaatcaaaa gccttcttat aaaacgatta atcgatttag
41281 agtcaatcca aaagtagatg ctttattaga atcattattt attcaatttc atagtcagtg
41341 tttgaaacaa catcttatag atgatcaaac catttttatt gatggtacga aagttgaggc
41401 gaatgccaat cgatatacat ttgtatggaa aaagagtatt caaaaccatg agacaaagat
41461 gaatgaagat tctaaagcac tctatcatga attggtaaca aataaaatca tacctgagat
41521 taaaaaagat cacgataatg acttaacaaa ggaagaaata gatttgattg gtagtcattt
41581 ggataaagaa atcgaagatt taaatcaaca tatcgataat gaaaaatgta ctaaaataag
41641 aaaacaaata cggctcaaaa gaactaaaat taaacaatat aaaaagcaaa ttaatgatta
41701 ctctgggcga aagcataaat acgacgtcca aaaatctatt ttaaaggata gaaatagtta
41761 ttctaaaaca gatcatgatg ccacatttat gagaatgaaa gaagatcata tgaaaaatgg
41821 acaactcaag ccaggttata atttacaaat agcgacaaat tctcaattcg ttttatctta
41881 taatgtgtat caaaatccga ctgatacaag aacgatgata ccatttttaa attcaattca
41941 agagacctac ggtcatttac ctgaatatat tgtagctgat gcaggttatg gtagcgaacc
42001 caattatatg gcaattatag atgattttaa tcgaatacta cttataacct acggtatgtt
42061 tataaaagat aaaactaaaa aatataaaag cgacatcttt aacactcaaa attgggatta
42121 tgacgaaatt aacgatgaat atatttgtcc aaacaataag cgattgggtt ttaaaagata
42181 tgcctatcgc catgataaat atgggtttaa gagagatttc aaattatatg aatgtgatga
42241 ttgttcagaa tgtcctctga gacaccaatg catgaaatac aattcaaaaa cgaataaaaa
42301 aataatgaaa aattataatt gggaatattt taaagcccaa attaataaaa agctttcaga
42361 accaaaaata aaaaccatct acagtcaaag aaaaattgat gtggagcctg tttttggatt
42421 tatgaaggct attttgggtt tcactcgaat gtccgttcga gggataaata aagccaaaag
42481 agaactagga tttgtgctaa tggcacttaa tataagaaaa gtaacagctc aacgagctga

42541 aaatattcaa aaaaatataa aaaagacaat ttctatatta tttcaataga aattgtcttt
42601 tttcacttat ccagatactt tatgtcccgg actcttttaa ctttaaataa tatttaataa
42661 tgggagagaa aaatattatg aaggaatttt taatgatatt ttttaataaa tatgaattaa
42721 aagctattat agctccagga atgataagta tcatgtcttt ctttactacc ttgataatat
42781 attttcctga tagtttaaat ttaggttcta gtctaattgt aataattcta ttgatttttt
42841 atatctattt attttcaatc atagcaagag aaaatggcaa aatggttgaa aaaaagttgt
42901 tttctcaatc atcaaatgtt tatcccacta cacaaatttt aagatattca gacgacactt
42961 taaatataaa aaccaaagaa agatatcact cttttttaca agaaagcatt ccttgtatag
43021 aacttcctag ctacgaagat gctagagaca aaaaagagaa ttattttgat gatgagtacg
43081 actcagcaat tgattggtta ttaaaaaata caagagaaga ttatatgact gctcaacata
43141 atatggaata tggcttctat cgtaatttat taggaattaa atatattggt attataattg
43201 ttttaatatc tatagtattc agtgttgttg tttttatact atctcaagaa aatgatacga
43261 ttcaactagt actttctagt attattaatg tgtttttttt gtatttttgg atttttaaag
43321 ttactaaaac acagactaaa aaatctgcaa aacgttatgc tcaatcttta ttagctaatt
43381 gtgattttaa aagttaattg tatgtagtta cacttccaat atattttaaa ttatcgtaaa
43441 ttgtgggaat gttagagtct ttttttagat ttttagcctc atagtttaaa ttactcacat
43501 atatattgat agtaccagtt tgacagtcaa atactatatc gcctgctttt ctttgagtaa
43561 ttgtagtata attactataa tagtataaat cttttgaatt agcttctcct acaacaataa
43621 gcttagggtt taatttattt aagatattac tcggtactct tccacttttt ctagaatggt
43681 gaggtgcaaa taatatatct actttttccc agttaacttc attttcgatt ttttccataa
43741 aatcagtagt caagtctccc atccacaaag cagtcactcc tttatctaat ttgtactgaa
43801 tgataggaga tatatcatta aaactgattt tttctgctgc tttttgtaat tcatttttaa
43861 aatactcatt gtctgtatca ggccataata tattaatacc ggaactacct cgtgattcat
43921 cgctaatatt catccatttt ctttgactat ctttatgtat gtaaaaactt ttgttagaat
43981 ctctcaattt tttatattct tcaaaacttt gactaaaatc atttttttta gcattatttt
44041 ttacgcaata aaaattagtg atatttaaat ttttatttaa taactcaagt ccatgaatat
44101 gatcttcatc aggatgagta gatataaatc taacaatttc ttttgtagaa gctatctcta
44161 atatttcttt tattataata tcttttttat cattatataa acagcaatct ataatagaga
44221 aattatctgt accgtgttta atgtaaaaca tatcgccatt tcctactgaa tacgatttaa
44281 ttatactcaa attagtaatc ctcctttaat taataacaaa ttgtattaag aatatattaa
44341 gtaatgtaat tataagcgtt tattacttaa agtaaaagaa tatataatat tacttaaaat
44401 gacgtaaaaa taatttttta gtaaaaccac taataagggg aagcatatca taaat
//
LOCUS Staphylococcus 60554 bp DNA linear UNK
DEFINITION staphylococcus Staphylococcus epidermidis strain B1230143
ACCESSION Staphylococcus
KEYWORDS .
SOURCE staphylococcus Staphylococcus epidermidis
ORGANISM staphylococcus Staphylococcus epidermidis
Bacteria; Bacillati; Bacillota; Bacilli; Bacillales;
Staphylococcaceae; Staphylococcus; Staphylococcus epidermidis.
FEATURES Location/Qualifiers
source 1..60554
/project="hwwang_12142587"

/genome_id="12142587.3"
/mol_type="genomic DNA"
/db_xref="taxon:12142587"
/organism="staphylococcus Staphylococcus epidermidis"
/genome_md5=""
CDS complement(8..175)
/product="UTP--glucose-1-phosphate uridylyltransferase
(EC 2.7.7.9)"
/db_xref="SEED:fig|12142587.3.peg.1"
/db_xref="GO:0003983"
/transl_table=11
/translation="MNQYETTGKSIIRVQQINENETHRYGIVDPKESQNELFSVNKFV
ENQKLILPPPI"
/EC_number="2.7.7.9"
CDS complement(342..536)
/translation="MPKEMLPILNNPTIQYIVEEAVEAGIEDIIIVTGKHKRTIENHF
DNQKELEMTFEEKVSLIYFN"
/EC_number="2.7.7.9"
/product="UTP--glucose-1-phosphate uridylyltransferase
(EC 2.7.7.9)"
/db_xref="SEED:fig|12142587.3.peg.2"
/db_xref="GO:0003983"
/transl_table=11
CDS complement(1020..1151)

/translation="MKVNRDNVFDYVIAAVNQNNGVDASLIKFQQPEFSTQENAYGE"
/transl_table=11
/product="hypothetical protein"
/db_xref="SEED:fig|12142587.3.peg.3"
CDS complement(1448..1945)
/transl_table=11
/db_xref="SEED:fig|12142587.3.peg.4"
/product="hypothetical protein"
/translation="MKFNFFNSKNLDIFEKQIKQLALDKDHKFRKKEEILKEEEILNH
SSPKKRELLKVFNDKAKITFYHFNGRNSNYEIIIGETKFENVIGLEIKFSRKPFRQIG

KLLLAGAIFSIPVGGLLMGSVFLAGSGLVVSSGVRSLQIKGPLKNDFEEIISDTLGNP
ITIKE"
CDS complement(1952..2230)

/translation="MKDVFKKIGVGAGKVAAVPFVIAGAAISAVFETTGGRGIMRDAL
KDSSDQKLMEIYNIHDTADGDHDTCIACKSLLQERGYEYNYKTKKWEK"
/transl_table=11
/db_xref="SEED:fig|12142587.3.peg.5"

/product="hypothetical protein"
CDS complement(2528..3031)
/transl_table=11
/db_xref="SEED:fig|12142587.3.peg.6"
/product="hypothetical protein"
/translation="MNTIKSTIHTEAIFSDDEQHRYLLKKTWDEKKAACTVITMYPHL

DGVLSLDLTTVLILNQLANSERYGAVYLVNLFSNIKTPENLKHIKEPYDEHTDIHLMK

AISESDTVILAYGAYAKRPVVVERVAQVKEMLKPHKKKVKKLINPATNEIMHPLNPRA
RQKWTLK"
CDS complement(3046..3357)
/db_xref="SEED:fig|12142587.3.peg.7"
/product="hypothetical protein"
/transl_table=11
/translation="MKINRYITRGINESIPLDLQILLWHMVEKKDNQPHTDYLHIFKL

QEDENILSITHEQEQPTYKLEYHYTNYEKNQNALPKKVYVIREDDVDAFYYVMLLPEE
Y"
CDS complement(3444..3794)
/translation="MKTITQELKQYITHLFQLSNNEAWECEALEEAAENILPERFIND
SPLVHLTLETYTYYNDELHELSIYPFLMYANNQLISVGYLDHFDMDFLYLTDTQNIII
DERHLLKQGGQDHE"
/product="hypothetical protein"
/db_xref="SEED:fig|12142587.3.peg.8"
/transl_table=11
CDS complement(4295..5923)

/translation="MKQLKKKRIGLYARVSTEMQTDGYSIQGQLNQLTEYCQFQGYEV

VNEYTDRGISGKTMQRPELQRMLKDANEGKLDCIMVYKTNRLARNTSDLLNIVEELYK

INVEFFSLTEKIEIASSTGKLMLQILASFAEFERNTIVENVYNGQRQRAIEGYYQGNL

PLGYDKVPDSKKELMINQHEANIVKYIFDSYAKGHGYRKIANALNHKGYVTKKGKPFS

ISSITYIISNPFYIGKIQFAKYRHWSDKRRKGLNEEPIIADGKHAPIIDRNLWDKVQF

KRQESRKKPQVHGKGTNLLTGIVKCPKCGAAMAASNTTNTLKDGTKKRIRYYSCSNFR

NKGSKVCSANSVRADVLEKYVMDQILEIIKSKKVLKQLVEKVNEKSQIDVSSLNHDIA

YKQSQCEEIKAKMHTLTKTIEDSPDLDSVLKPTILSYQDELNQINNQIYQLEQDKQAE

APHYDADMIANVLQAIFKDIDKLEKSQLKSLYLTIIDRIDIRKDEHHKKQFYVTLKLN
NDIIKQLFNNHPLDEVLPSTSSLFLPQTLYITIE"
/transl_table=11
/product="Site-specific recombinase"
/db_xref="SEED:fig|12142587.3.peg.9"
CDS complement(5920..7281)
/db_xref="SEED:fig|12142587.3.peg.10"
/product="Cassette chromosome recombinase A"
/transl_table=11

/translation="MEEAIAYVRQSTLKQQSLATQKSLIMDTAKQYGWSNVAFYDDKK

TGRHTKRSGYQKMVEIITSGKCKVLCCYRLNRLHRNLKNAIQFFEICKKHHITIISVN

DGYFDLSKEFDCFRLNILMSLAEMESNNISEQTKNGIREKAKQGKLITTHAPFGYRYR

KGCFVVQEEEAYTVKSVYRWYLQGLGYKKISQHLDSNPNLIPRKPYQVRNILLNPNYC

GRVINKYGTFNDIVPPIIDIDTFEEAQERRIHKQLNRSNSRNKLKRRIKCPYCQSTLT

NLTIKKENYSLRYYVCPKNMNDAYRTCPFKSINAPELESEVLDTCKKYFEEQSFHKRL

NNTILKVLKQQQMKHKETHLTQEQLIEKLAQNRIDVKTFKRLSASCKSEENYSTYSTY
QIDDKLKYVIKNKITLQDIAPFIDCIKITQTKQLQGIYFKNSPLNIVEQSHLITPERN
EVI"
CDS complement(7468..8082)
/product="hypothetical protein"
/db_xref="SEED:fig|12142587.3.peg.11"
/transl_table=11
/translation="MKDETNNTGIRMFKRYAAITTSAKILSHVLSTDIDIAKIRDYFI

DYHAHTVSERSLADKAIEVITQFVAQNRGKFSDDTALKNMIENYGLIALKDDYIEVKI

IASVFKNMLIEHHFQDVNNVVNALRDKGFIESDRDRITTKRAVKDDYGKKQSLVFYHL
KLDSKYASIFGLTKDAEPIKAEINLDNKKILESWKKQNDDLIDF"
CDS 8169..8444
/db_xref="SEED:fig|12142587.3.peg.12"
/product="Cassette chromosome recombinase A"
/transl_table=11
/translation="MIDKLAKGAIDAETFRKQSQSLLQQSKPTLSINEQQIQRSFENV
IQQHFTLSMLYRYIDEIHISKNKSLVGIYFKNEPLNIVNQATQSSIA"
CDS complement(8556..8915)
/transl_table=11
/db_xref="SEED:fig|12142587.3.peg.13"

/product="hypothetical protein"
/translation="MDDRALASALGIGVGGGLAWIMEGFQSGHGFIYSLCAGLIIGLL
FFEMIPESIELGGWVILLGGIAIGLVLFQSIHLLMDKITIITNSHQKDIFVRSGVLLI
ISIAIHNFPVGNIPLFV"
CDS 9122..9448
/translation="MEEKKELEEVNNKDLDDETLFVVSQTFKALGDPTRIRILHLLFY

KEYSVNGIAETLHLRQSTVSHQLRFLKNLRLVKFRREGTTLFYSHDDEHTMNMLKQAI
DHACHH"
/db_xref="SEED:fig|12142587.3.peg.14"
/product="Transcriptional repressor CzrA, ArsR family"
/transl_table=11
CDS 9551..9649
/translation="MDMENKKTEWKALYDISKESEMGVAERVSEYG"
/transl_table=11
/db_xref="SEED:fig|12142587.3.peg.15"
/product="hypothetical protein"
CDS 9778..11703
/EC_number="3.6.3.4"
/EC_number="3.6.3.3"
/translation="MKNIQEQQAHESHSHDHSHDHDHGKMPIISYFIGLVLAIIGLFL
SDANLLIQNILFSIATITAGYHVIILEGIGETVENTKLKGKFTPNSHILMGLAAIGAS
LIGSFWEGTLLILIFSGAHFLEDYAEGKSKREITKLLEMNPTTAKLILPDGNTKIVDV

SELKVGDQLQVLNGDQVPIDGIILSGTTSIDESSINGESIPKEKSKGDEVFGSTINGT

GTFTMEVTKENKDTVFSKILQLVSQNQDNQTKAASIIQKFEPKYVNIVLIAIPLVMLL

APFLFDWTWSQSVYRGLVLLVAASPCALAAATVSVTLSTTSNLAKKGVLSKGSTYLSQ
LADIDAIAFDKTGTLTNGEPKVTNYYFTHSVNEENIIDIIVALEKESNHPLANAILEK

FEVKNKIDIEVTNQIGKGLTGDYNGKNYRIGKPTSFESASEEYTQFNHDWASEGKTVV

YVAENEEVIGIIALMDIPNEHAKETINYFKKLGIHTTLITGDSEMTGKAVGEQLGIDE

VIANVMPEDKSRIIEEQKEKFGVTAMVGDGVNDAPALVNADVGIAMGGGTDVAVEVSD

LVLMQNNLSKLVQSHKISSNMGRVIRQNIIFSMAVVAFLVVVSLLGLTDITISVIVHE
GSTLVVILNGLRLLRSK"
/transl_table=11
/product="Lead, cadmium, zinc and mercury transporting
ATPase (EC 3.6.3.3) (EC 3.6.3.5); Copper-translocating
P-type ATPase (EC 3.6.3.4)"
/db_xref="SEED:fig|12142587.3.peg.16"

/db_xref="GO:0004008"
CDS 12955..13260
/translation="MKGNIQCLRLIKGSLLDFVLETVDVFVNGSKNDCELVNFNYKEG
KVVSRYSTKFESNPKLRKEQLISTVKVVRYVIFDFEKILWGIGKVLLKFIILRQCLL"
/transl_table=11
/product="hypothetical protein"
/db_xref="SEED:fig|12142587.3.peg.17"
CDS 13458..14321
/translation="MKHINHFSESTLAYLEKDHEHAFYVYCLIDPRNDECFYVGKGKG

NRIFKHKQDAQRQLLYEDIFREENKDNLKFNRINEICSNDLNVLGYIISYGLTESEAF

SAENVLINFLNLTNKTTLTNMINGHGSKAYLVEDLENEFGYDSINLENINTNELILAV

KIRDAFLLDKDESKEYPINESKRDRNNLKSRTLGSWIIGKDKIHKIKYIIGINTGANN

AVVSAYEVSFEQAESIETNNGRMRYAFIGLSERDATLKKLNLYKKALPDLRFGSGSAT
AYINNGTMKVD"
/db_xref="SEED:fig|12142587.3.peg.18"
/product="hypothetical protein"
/transl_table=11
CDS 14429..15904
/translation="MPSDDIVKKLYSKEDIRLELGLTPHKFNKKMETIAKLFKIDMKI

FHNYKGQDKNNQYTFNGVAKELIKVLLKSVDYYPVDINSKKFKQNGKSKKEMIENIDN

SSYMKYIYQLMKSINEIQYKRLIADIHMKDVYQNTKAWLNNGESINKKEQELYQYMTI

LPLHKRVELQNEVLKSIDETIFQFLAKEHRNNQIEENNELEAYTKAIKEGRNPKNDYE

LNHLLYKKNQLPLDSLIEDIWDYEETEYTELDWLIADMLKRSQRADSNFIESLEKKNK

LRKNIHKDSIKSISKLIDHELVKNNRKWDVAEFYKNQQFWNNSTLRRSSQNFGILYYK

KNRLHTMNQSNIIKKIKIIEANIESANKQPSYLDFFLQAKFDLERLESELMKYSINPK

YFNKINSDYVRYAIEDVHNAIIKIDRYISNDKVKLNYSNVQMVVRNEITEQGSYFVTQ
ALNTLSKVEESGYSFDNFMVGPFVEDFKKAIKNRRFGSKKD"
/product="hypothetical protein"
/db_xref="SEED:fig|12142587.3.peg.19"
/transl_table=11
CDS 16131..17231

/translation="METRDKLMSLTQSDKTQQWLMDKSSNQDDIQQLQQQFSQQLDQQ

YNALLADEKAKLDQYVEVHQGLESLKEEIESEPITLNIDKLPDIKATMLERAKNDEHS

DKIEKLFDRLEQALNGTNRLYTQLSLIGTRTHRITTKNFNLQGLPKAVQHTILPSKFK

KVYTVDFKSFEPSVAAYMTQDSKLIDLLNQKDGLYDALLSELGLSDELRVFVKRAFIG

SFLFGGNFKNPKFKLNQYVSEVQWLDAVSQFTKVIELKKHVEKSNSMPMPYSIEHDMS

AFQGSSIMAIYVQTVASYIFKHILLKVYKAQCDQKTFKIIVPIHDAIMIECEDEEIAQ
NVGQLMKDTANQLFNGEFAHVTVEEIGGVDHE"
/transl_table=11
/db_xref="SEED:fig|12142587.3.peg.20"
/product="hypothetical protein"
CDS 17224..17595
/translation="MNNDRGQSLHIPSSTPIKENNIYVATLHSVIQTDFSGEIKHQFT

YEIEVNNQIVYANRNIPTKPSANQLSIHDWLKRHSNYSVGHDTYEPYIDRKHLIHIGQ
YSGNYYVQDVASLDAFGGVLS"
/db_xref="SEED:fig|12142587.3.peg.21"
/product="hypothetical protein"
/transl_table=11
CDS 17592..19235
/product="putative primase"
/db_xref="SEED:fig|12142587.3.peg.22"
/transl_table=11
/translation="MNHILQMLSKLLSVAKEAIDRQGLIAILTIPVNNNDEIEETAQG

ETVYNELIDQLRLNIPKDTDYQPNIYSYFGIKKNPNDTVLMEMMIKVFHIKRFNSELF

IFKVNGWQKINGDELQGLISKMIQVLLVDYKPSLSTLKNVVDGLQKSTDVEELVENER

YIGCGENMFDLNTFQVVKNSIDIFPKTRLNLSLSTNDVITDKIPPYFKQYMLQLANYD

DDLQYFLFQHTAVLLTADTKYRRGLILYGGAKNGKSVYIELVKSFFYSKDIVSKPLNE

LEGRFDKESLIDKSLMASHEIGQSRIQEKIVNDFKKLLSVESMHVDRKGKTQVEVILD
LKLIFSTNAILNFPPEHAKALERRINIIPCEYYVEKADTSLIDKLQSEKKEIFLYLMY

VYQQIVKADIEYLENSRVTEITHDWLNFGYEFVSSRSVSIANQKACINLLRKLIEIKS

GSRIKVSELNKVINEEIKVSSQVINQLIQANFDTQTKLYNGYDYWIDLGWKEANKKEI
HDISEKDNIISLDKNENITDDEALDEENLDFDWEDFDDE"
CDS 19462..21138

/translation="MKGKIALYSRVSTSEQSEHGYSVKEQEQVLIKEVVKNFPGYDYE

TYTDSGISGKNIEGRPAMKRLLQDVKDNKIEMVLSWKLNRISRSMRDVFNIIHEFKEH

DVGYKSISENIDTSNASGEVLVTMFGLIGSIERQTLISNVKLSMNAKARSGEAITGRV

LGYKLSLNPLTQKNDLVIDENEANIVREIFDLYLNHNKGLKAITTVLNQKGYRTINQK

PFSVYGVKYILNNPVYKGYVRFNNHQNWAVQRRSGKSDKNDVILVKGKHEAIISEEVF

DKVHEKLASKSFKPGRPIGGDFYLRGLIKCPECGNNMVCRRTYYKTKKSKERTIKRYY

ICSLFNRSGSSACHSNSINAEVVERVINVHLNRILSQPDIIKQIASNVIEELKQKHSN

QTEIKYDIDSLEKQKAKLKTQQERLLELFLDDQMDSEMLKAKQSQMNQQLEVLDQQIK

EAQQANQSQDEIPNFDKLKGRLILMITRFSVYLRKATPEAKNQLMKMLIDSIEITTDK
QVKLVRYKIDESLIPQSLKKDWGSFFMPKFQFEIYGQNDYFIDQITTFTT"
/product="Cassette chromosome recombinase B"
/db_xref="SEED:fig|12142587.3.peg.23"
/transl_table=11
CDS 21241..21582
/translation="MMTLEQQLKHYITNLFNLPKDEVWHCESIEEIADDILPNQYVRL

GPLSNKTLQTNTYYSDTLHESNIYPFILYYQKQLIAIGYIDENHDMDFLYLHNTIMPL
LDQRYLLTGGQ"
/product="FIG01108299: hypothetical protein"
/db_xref="SEED:fig|12142587.3.peg.24"
/transl_table=11
CDS 21584..21685
/translation="MHKYIKITQLVITILSEIIIWMKESERKEVSYE"
/transl_table=11
/db_xref="SEED:fig|12142587.3.peg.25"
/product="hypothetical protein"
CDS 21678..21989

/translation="MNRYITRGIANNLPNILQHQLWQLVSEREQEQTKDNTSVDYFHI

FQFNKHRNQLYIKHKQERPEYAKIHKANYSKAININKVYIIREDDVDLSYYVMLLPEE
Y"
/product="FIG01107894: hypothetical protein"
/db_xref="SEED:fig|12142587.3.peg.26"
/transl_table=11
CDS 22005..22511

/translation="METIKSTLKTEAIFSDDKQHRYLLKKTWNSEKQSITIITMYPHY

DGILNIDLTTQLIMNKISEMDAFGSIHFVNLYSNITTPINLKHLENAYDNHTDIQIMK

AVKESDEVILAWGAYAKKPIVEARVNEILEMLKTHKKKVKQLMNPATNEIMHPLNPKA
RQKWTLKA"
/product="hypothetical protein"
/db_xref="SEED:fig|12142587.3.peg.27"
/transl_table=11
CDS 22539..22985
/translation="MKEINIVSLQMIKTDTLSYLKARISNPEDATEIMRSFIGNSDRE

HLILICMNSKNEPTHIQTLSIGSINQTVIHPREIFKTAILSNANSIMLGHNHPSGDVT
PSPEDINVTKRLMLISEMMGILFYDHIIFSDSNTYSIRQHNLEVEV"
/transl_table=11
/db_xref="SEED:fig|12142587.3.peg.28"
/product="DNA repair protein RadC truncated by transposon
Tn554"
CDS complement(23040..23153)
/translation="MSPPLSYILQNGFVTISQSYVYLIKIVKNIFHLKLLL"
/transl_table=11
/product="hypothetical protein"
/db_xref="SEED:fig|12142587.3.peg.29"
CDS 23155..24027
/transl_table=11
/db_xref="SEED:fig|12142587.3.peg.30"
/product="FIG01110112: hypothetical protein"
/translation="MDRAFRILTIYNRLLQNKSVNKQSLTLELDTSPRTIQRDIDDIR

NFLYESKSWISTPKEITYDYKSESYKLEQDKNENVHFMYNILTALYLTTPQLSHYFYQ

YLKLLIIKHHSNHRTTLLKYLERFEIDKNQTGISTSSLVVQAMNENKYLQYNQNLLLA

LSMYYQMHAFHLVYRMNDEIYIDDINQMNLSFSEKTVDTHEKSKVQTYITFEISKDVW

SKMHRYYQIHVIEKYDVHYLIVTFKMTRPEAIQLCFMYRSNIRIISPPDLREQVIDEL
LLLQSTYLKQQVQE"
CDS 24079..24369

/translation="MGNIIKTIGKGIGKGVGAAAYYSVATVGAAIGAIWETTGDRGLM
RDALSDSSNKDLMEIYEIQKKDDGDYDTYIAAKSLLQERGFTYNHETNKWEK"
/product="hypothetical protein"
/db_xref="SEED:fig|12142587.3.peg.31"
/transl_table=11

CDS 24385..24747
/translation="MEFAYYEAKSLNDFENELKTLALNKKYTFSKETEVLKEEEILEK

SNSHERELLKVFKDNAQITTYHFKSSQLSASKSSIQRLMGIIISENLVECVKMSEENS
EDSQALINEAVELLIKSK"
/transl_table=11
/product="FIG007303: uncharacterized protein"
/db_xref="SEED:fig|12142587.3.peg.32"
CDS 24744..25502
/translation="MISITTIIVMLVIGILGGFISGLVGIGGAIVIYPALLLLPPLFG

LPTYSAYIASGLTSSQVFFSTLSGSLNAYKNKNFSRTLILNMGSGMVIGSMLGAILAN
LINVQFVNTVYIIIALLALILMFIKVTPSTSHIKFNRLLLITIGGIIGLVSGIVGAGG
AFIIIPVLLVIFKLPMNMVVTNSIVIAFISSIGAFMIKLLQGYIPINSAIPLILGSIL
FTPLGMKMGQKIPDYIQKTIICILIVIAIIKLIF"
/product="FIG003846: hypothetical protein"
/db_xref="SEED:fig|12142587.3.peg.33"
/transl_table=11
CDS 25711..25881
/translation="MVQNLWWGAGYNIVAVPLAAGILAFIGLILSPAIGAILMSLSTV
IVAINAFTLKLK"
/EC_number="3.6.3.4"
/EC_number="3.6.3.3"
/db_xref="SEED:fig|12142587.3.peg.34"
/db_xref="GO:0004008"
/product="Lead, cadmium, zinc and mercury transporting
ATPase (EC 3.6.3.3) (EC 3.6.3.5); Copper-translocating
P-type ATPase (EC 3.6.3.4)"
/transl_table=11
CDS 25899..26444

/translation="MIKKLFFMILGSLLILSACSNNDEKDKDTNDQKSESHMKHNDES

KVPEDMTSTNEGEFKVGDKVTITAGHMPGMKGAEATVKGAYKTYAYVVSYKPTNGNEK

VNNHKWVVNEEIKDAPKDGFSKGDTVKLEASHMSGMKGATANIDNVKKTTVYVVDYKS
KDNGKIIKNHKWMTGNELKAR"
/transl_table=11
/product="DUF1541 domain-containing protein"
/db_xref="SEED:fig|12142587.3.peg.35"
CDS 26887..28143

/translation="MIDNFMQVLKLIKEKRTNNVVKKSDWDKGDLYKTLVHDKLPKQL

KVHIKEDKYSVVGKVATGNYSKVPWISIYDENITKETKDGYYLVYLFHPEGEGIYLSL

NQGWSKISDMFPRDKNAAKQRALTLSSELNKYITSNEFNTGRFYYAENKDSSYDLKND

YPSGYSHGSIRFKYYDLNEGFTEEDMLEDLKKFLELFNELASKVTKTSYDSLVNSIDE
IQEDSEIEEIRTAQKDKTLKEVEAPKGIIPKYKKGVSKTTKNDSEIEKSNKENKLTGK

VGEKLALNYFNELIDNKIDEDKKEQFRNILNDNPGSQHGHGYDLVAFDPTNTDKAVEK
FIEIKTSTSSSIEEPFFMSLNEMFAMKEYKQKYLILRIFNVSGKEPQFYFIDPYANYS
EFKDVDDLIDKVFNVEAIQYKVFGEK"
/transl_table=11
/db_xref="SEED:fig|12142587.3.peg.36"
/product="hypothetical protein"
CDS complement(28588..29262)
/translation="MNYFRYKQFNKDVITVAVGYYLRYTLSYRDISEILRERGVNVHH

STVYRWVQEYAPILYQIWKKKHKKAYYKWRIDETYIKIKGKWSYLYRAIDAEGHTLDI

WLRKQRDNHSAYAFIKRLIKQFGKPQKVITDQAPSTKVAMAKVIKAFKLKPDCHCTSK
YLNNLIEQDHRHIKVRKTRYQSINTAKNTLKGIECIYALYKKNRRSLQIYGFSPCHEI
SIMLAS"
/transl_table=11
/db_xref="SEED:fig|12142587.3.peg.37"
/product="Transposase for insertion sequence-like element
IS431mec"
CDS 29520..29687
/transl_table=11
/product="Hydroxymethylglutaryl-CoA synthase N-domain
homolog (EC 2.3.3.10)"
/db_xref="SEED:fig|12142587.3.peg.38"
/EC_number="2.3.3.10"
/translation="MLESREQLSVEEYETFFNRFDNQEFDFERELTQDPYSKVYLYSI
EDHIRTYKIEK"
CDS 29785..30015
/product="hypothetical protein"
/db_xref="SEED:fig|12142587.3.peg.39"
/transl_table=11
/translation="MLIFLSYDFVRACLPRGMARAISLSHILFPQASALTKSVVIFIF
IRILTEIILIRGIVKAILSKIADKRFVKSSSK"
CDS 30484..31227
/EC_number="3.1.4.46"
/translation="MLTVYGHRGLPSKAPENTIASFKAASEVEGINWLELDVAITKDE

QLIIIHDDYLERTTNMSGEITELNYDEIKDASAGSWFGEKFKDEHLPTFDDVVKIANE

YNMNLNVELKGITGPNGLALSKSMVKQVEEQLTNLNQNQEVLISSFNVVLVKLAEEIM

PQYNRAVIFHTTSFREDWRTLLDYCNAKIVNTEDAKLTKAKVKMVKEAGYELNVWTVN
KPARANQLANWGVDGIFTDNADKMVHLSQ"
/transl_table=11
/db_xref="SEED:fig|12142587.3.peg.40"
/db_xref="GO:0008889"
/product="Glycerophosphoryl diester phosphodiesterase (EC 3.1.4.46)"
CDS 31324..31752
/db_xref="SEED:fig|12142587.3.peg.41"
/product="MaoC domain protein"
/transl_table=11
/translation="MKYDDFIVGETFKTKSLHITEEEIIQFATTFDPQYMHIDKEKAE

QSRFKGIIASGMHTLSISFKLWVEEGKYGEEVVAGTQMNNVKFIKPVYPGNTLYVIAE
ITNKKSIKKENGLVTVSLSTYNENEEIVFKGEVTALINNS"
CDS complement(31798..33804)
/translation="MKKIKIVPLILIVVVVGFGIYFYASKDKEINNTIDAIEDKNFKQ

VYKDSSYISKSDNGEVEMTERPIKIYNSLGVKDINIQDRKIKKVSKNKKRVDAQYKIK

TNYGNIDRNVQFNFVKEDGMWKLDWDHSVIIPGMQKDQSIHIENLKSERGKILDRNNV

ELANTGTAYEIGIVPKNVSKKDYKAIAKELSISEDYIKQQMDQNWVQDDTFVPLKTVK

KMDEYLSDFAKKFHLTTNETESRNYPLGKATSHLLGYVGPINSEELKQKEYKGYKDDA

VIGKKGLEKLYDKKLQHEDGYRVTIVDDNSNTIAHTLIEKKKKDGKDIQLTIDAKVQK

SIYNNMKNDYGSGTAIHPQTGELLALVSTPSYDVYPFMYGMSNEEYNKLTEDKKEPLL

NKFQITTSPGSTQKILTAMIGLNNKTLDDKTSYKIDGKGWQKDKSWGGYNVTRYEVVN

GNIDLKQAIESSDNIFFARVALELGSKKFEKGMKKLGVGEDIPSDYPFYNAQISNKNL

DNEILLADSGYGQGEILINPVQILSIYSALENNGNINAPHLLKDTKNKVWKKNIISKE

NINLLTDGMQQVVNKTHKEDIYRSYANLIGKSGTAELKMKQGETGRQIGWFISYDKDN
PNMMMAINVKDVQDKGMASYNAKISGKVYDELYENGNKKYDIDE"
/product="Penicillin-binding protein PBP2a, methicillin resistance determinant MecA, transpeptidase"
/db_xref="SEED:fig|12142587.3.peg.42"

/db_xref="GO:0008658"
/db_xref="GO:0046677"
/transl_table=11
CDS 33904..35661
/transl_table=11
/product="Methicillin resistance regulatory
sensor-transducer MecR1"
/db_xref="SEED:fig|12142587.3.peg.43"
/db_xref="GO:0008658"
/translation="MLSSFLMLSIISSLLTICVIFLVRMLYIKYTQNIMSHKIWLLVL

VSTLIPLIPFYKISNFTFSKDMMNRNVSDTTSSVSHMLDGQQSSVTKDLAINVNQFET

SNITYMILLIWVFGSLLCLFYMIKAFRQIDVIKSSSLESSYLNERLKVCQSKMQFYKK

HITISYSSNIDNPMVFGLVKSQIVLPTVVVETMNDKEIEYIILHELSHVKSHDLIFNQ

LYVVFKMIFWFNPALYISKTMMDNDCEKVCDRNVLKILNRHEHIRYGESILKCSILKS

QHINNVAAQYLLGFNSNIKERVKYIALYDSMPKPNRNKRIVAYIVCSISLLIQAPLLS

AHVQQDKYETNVSYKKLNQLAPYFKGFDGSFVLYNEREQAYSIYNEPESKQRYSPNST

YKIYLALMAFDQNLLSLNHTEQQWDKHQYPFKEWNQDQNLNSSMKYSVNWYYENLNKH

LRQDEVKSYLDLIEYGNEEISGNENYWNESSLKISAIEQVNLLKNMKQHNMHFDNKAI

EKVENSMTLKQKDTYKYVGKTGTGIVNHKEANGWFVGYVETKDNTYYFATHLKGEDNA
NGEKAQQISERILKEMELI"
CDS 35661..36032

/translation="MDNKTYEISSAEWEVMNIIWMKKYASANNIIEEIQMQKDWSPKT
IRTLITRLYKKGFIDRKKDNKIFQYYSLVEESDIKYKTSKNFINKVYKGGFNSLVLNF
VEKEDLSQDEIEELRNILNKK"
/transl_table=11
/product="Methicillin resistance repressor MecI"
/db_xref="SEED:fig|12142587.3.peg.44"
/db_xref="GO:0003677"
/db_xref="GO:0016481"
/db_xref="GO:0016566"
CDS 36540..37649
/transl_table=11
/product="Putative ROK-family transcriptional regulator"
/db_xref="SEED:fig|12142587.3.peg.45"

/translation="MLREIYNHHNISRTQISKNLEINKATISSILNKLKYKSLVNEVG

EGDSTKSGGRKPILLKVNHLYGYFISLDLTYSSVEVMYNYFDGNVIKHESYDLPDEKV
SSILSIIKKHIDIQEKLDTYNGLLGVSVSIHGVVDNEQHVTYLPFHETEGISIAKKIK

EITNVPVVVENEANLSALYERNFNHNLSYNNLIALSIHKGIGAGLIINNQLYRGANGE

AGEIGKTLVSKVSDNVEIFHKIEDIFSQEALLHNLSNQLNEKMTLSKLIQFYNEKNPV

VVEEMEQFINKIAVLIHNLNTQFNPNAIYINCPLFNEMPEILEAIKNQFKQYSRNEIQ
IKLTSNVKFATLLGGTLAIIQKVLQINDIYLDIKA"
CDS complement(37763..38068)

/translation="MPQTNIVTSSIHSSEMTGEEHNVLDVRNDDEWRKGHLKQAIHIP

HGKLLNEDIPFDKNDLIYVHCQSGVRSSIAVGILEYKGFTNIVNVRGGYQHLPAFLK"
/transl_table=11
/db_xref="SEED:fig|12142587.3.peg.46"
/product="Polysulfide binding protein"
CDS complement(38159..39097)
/transl_table=11
/product="Zn-dependent hydroxyacylglutathione hydrolase / Polysulfide binding protein"
/db_xref="SEED:fig|12142587.3.peg.47"
/translation="MFFKQFYEDNFSQASYLIGCQRTGEAMIIDPIRDLTKYLKVADK

EGFTITKAAETHIHADFASGIRDVANKLNASIYVSDEGKDEFNYKNMPKQTHFVKHQD
IIYLGKIKLEVLHTPGHPPESISFLLTDEGGGSSIPMGLFSGDFIFVGDVGRPDLLEK

AVNVEGSTKIGAKQMYQSIQFAATLPDYIQIWPGHGAGSPCGKALGSLPTTTLGYEKI

NNWAFNVKDETSFIETLTLNQPAPPHHFSQMKKINQFGMQMYQPYNVYPSSSNTQTAF
DLRSKEAFHGGHMHGTINIPFNKTFINQIGWYLDYD"
CDS complement(39130..40194)
/transl_table=11
/db_xref="SEED:fig|12142587.3.peg.48"
/product="Disulfide bond regulator"
/translation="MEQYKEVHINSLNKEELEKLAKMGQLIDVRTKEEYELGHINGST

LHPVEEIKSFNKNSNKTYYIHCKSGNRSTKACDYLAKQGYDVVNLKGGYKDYEAKNFN
SAPLIEKDIEIKENRKQFDFRGLQCPGPIVNISKEINNISTGEQIEVTVTDPGFNSDI

KSWAKQTGNTLVNLTEEANVINAIIQKEKPKEFEINDTATGTTIVLFSGELDKAVAAM

IIANGAKAAGKDVTIFFTFWGLNALKKAQSTRVKKKGISKMFDLMLPKDPIHMPLSKM

NMFGLGNIMMRYVMNKKNVDSLYSLIDQAIDQDIKLIACTMSMDVMGISKEELRDEVD
YGGVGTYIGHTEQANHNLFI"
CDS 40330..40590
/transl_table=11
/db_xref="SEED:fig|12142587.3.peg.49"
/product="Metal-sensitive transcriptional repressor"

/translation="MTYDKKMINRINRIQGQLNGVVKMMEEEKDCKDIITQLSASKGS
IQRLMGIIISENLIECVKTAEENNESSQELINEAVNLLVKSK"
CDS 40590..41234
/db_xref="SEED:fig|12142587.3.peg.50"
/product="FIG003846: hypothetical protein"
/transl_table=11
/translation="MDIANMTIMLLIGVLGGFISGLIGIGGAIIIYPAILILPPLIGI

PAYSAYIASGLTSSQVFFSTLSGSLNARKQPAFSPKLVIYMGGGMLIGSMLGAILASL
FNATFVNTVYVIIAILALILMFIKVKPTTQETKSKPLLFIIVGFGIGVISGIVGAGGA
FIIIPVLLALFKLPMNTVVNNSIAIAFISSVGAFFIKLMQGYIPVESAIFLIIE"
CDS complement(41502..41975)
/translation="MIGASLLVAQGVVNLIPQQWVIGLLGLLPLYLGVKIWIKGEEDE
DESSILSLFSSGKFNQLFLTMIFIVLASSADDFSIYIPYFTTLSMSEIFIVTIVFLIM
VGVLCYVSYRLASFDFISETIEKYERWIVPIVFIGLGIYILFENGTFNALISFLL"
/transl_table=11
/product="Cadmium resistance protein"
/db_xref="SEED:fig|12142587.3.peg.51"
CDS complement(42014..42187)
/translation="MQFTEGLFILKKSPIPKIFGDDKMIATILTATAVYVATGIDYLV
ILILLFSQVKKVR"
/transl_table=11
/db_xref="SEED:fig|12142587.3.peg.52"
/product="Cadmium resistance protein"
CDS complement(42199..44613)

/translation="MDSSTKTLTEDKQVYRVEGFSCANCAGKFEKNVKELSGVHDAKV

NFGASKIDVFGSATVEDLEKAGAFENLKVAPEKARRRVEPVVTEDKNVYRVEGFSCAN

CAGKFEKNVKQLAGVQDAKVNFGASKIDVYGNASVEELEKAGAFENLKVIPEKLANPS
IQAVKEDTKAPKEEKIPFYKKHSTLLFATLLIAFGYLSHFVNGEDNLVTSMLFVSSIV

IGGYSLFKVGFQNLIRFDFDMKTLMTVAVIGAAIIGEWAEASIVVILFAISEALERFS

MDRARQSIRSLMDIAPKEALVRRNGQEIMIHVDDIAVGDIMIVKPGEKIAMDGIIING

VSAVNQAAITGESVPVAKTVDDEVFAGTLNEEGLLEVKITKYVEDTTISKIIHLVEEA

QGERAPAQAFVDKFAKYYTPIIMVIAALVAVVPPLFFGGSWDTWVYQGLAVLVVGCPC
ALVITTPISIVSAIGNAAKKGVLIKGGVYLEELGAIKAIAFDKTGTLTKGVPVVTDFK

VLNDQVEEKELFSIITALEYRSQHPLASAIMKKAEQDNITYSDVRVKDFTSITGRGIQ

GNIDGTTYYIGSPRLFKELNVSDFSLEFENKVKVLQNQGKTAMIIGTDQTILGVIAVA

DEVRETSKNVILKLHQLGIKQTIMLTGDNQGTAEAIGAHVGVSDIQSELLPQDKLDYI

KKMKAEHGNVAMIGDGVNDAPALAASTVGIAMGGAGTDTAIETADIALMGDDLSKLPF
AVRLSRKTLNIIKANITFAIGIKIIALLLVIPGWLTLWIAILSDMGATILVALNSLRL
MRVKDK"
/EC_number="3.6.3.3"
/db_xref="SEED:fig|12142587.3.peg.53"
/product="Cadmium-transporting ATPase (EC 3.6.3.3)"
/transl_table=11
CDS complement(44606..44971)
/transl_table=11
/db_xref="SEED:fig|12142587.3.peg.54"
/product="Cadmium efflux system accessory protein"

/translation="MTKDMCEVTYIHEDKVNRAKKDLAKQNPMDVAKVFKALSDDTRV
KIAYVLSLEGELCVCDVANIIESSTATASHHLRLLKNLGIAKYRKEGKLVYYSLDDEH
VKQLVEKAFLHQREVASIG"
CDS 45092..45199
/translation="MLETNPFGDFNQILKYLKHNCPVSLSASFHNLSTD"
/product="hypothetical protein"
/db_xref="SEED:fig|12142587.3.peg.55"
/transl_table=11
CDS complement(45209..45586)
/translation="MNKQVRNTTEIVRLAKQKSQKTREKVDKAISKFSIEGKAINFNS

IAKEANVSKSWLYKEHDIRQRIESLRERQITSNVVSKPKKSSRSEEILIKTLKRRVME
LEKENKKLQNQIQKLYGDLYNKE"
/transl_table=11
/product="Mobile element protein"
/db_xref="SEED:fig|12142587.3.peg.56"
CDS complement(45593..47485)

/translation="MNASSKRKIISQSEISKKIAVMNEEMQGFWANNSWDIRKCPHPS

AIELSKNPALRNRWVRFERVKNLWLRTELKYFYFYHLNNGIWNAKTVWIRKGTVINKM

LDFLDLKYPSITSITEVPIDKAMTEYRTYLTKQGVRITTTNYKITANQEKTPVKANSY

YVTNLKQFIEFYEDFYFDGEEWDKDVWDRRNLPLPDDKVNPTQYEYTINFKGFRNTYF

KQLVKRYCKLRLNMDSFSYVSDIAQKLKEFFNFLDIKFKHVQRVHQLTRVEIEAYLSE
LNMMGIKPSTITGRISILEGLFSTLHRLEWDDVPSKLLIYPEDYPKIPRAKPRFIDEF

VLEQLNSHLDKLPEYIATMTMIVQECGMRISELCTLKKGCLLEDKDGDYFLKYYQWKM

KKEHIVPISKEVVLLIKVREDKVSEEFPDSEYLFPRKDGSPLKQETFRGELNKLAYEQ

NIVDKLGEIYRFHAHAFRHSVGTRMINNGVPQHIVQKFLGHESPEMTSRYAHIFDETL

KNEFTKFQEKLVTNNGDVLNLDDDSEVDDVELQWFKKNINAQVLPNGYCRLPVIAGGC

PHANACLDCTHFCTSKQFLPQHEEQLERTEELLTIAKDKQWQRQIETNSRVKERLEQI
IGSLTG"
/transl_table=11
/product="Transposase B from transposon Tn554"
/db_xref="SEED:fig|12142587.3.peg.57"
CDS complement(47680..48003)
/transl_table=11
/product="DNA repair protein RadC truncated by transposon
Tn554"
/db_xref="SEED:fig|12142587.3.peg.58"

/translation="MSRKRIDVVKVQMVKEDTLWYLKRRIEEPKDAADIMRDFIGNAD
REHFILICLNSKNEPTHIETVSIGTINFAVIHPREIFKTAILSNATGMIIGHNHPSGD
ILTIV"
CDS complement(47996..48202)
/db_xref="SEED:fig|12142587.3.peg.59"
/product="FIG01110849: hypothetical protein"
/transl_table=11
/translation="MNQTWKEVYQYLMHQPHIDVIKGIIAFSSNKKNNSELDELYSIY
MNDDSITSIVHPDLIESIELIHNE"
CDS complement(48204..48725)
/db_xref="SEED:fig|12142587.3.peg.60"
/product="hypothetical protein"
/transl_table=11
/translation="MKSIKSVLETEAIFSQDKMHRYLLKKTWDIDKEVLTIITMYPHY

DGVINVDLTTQLIVNKVAEKDEYGGVNFINLFSNIDTPINLKHIENSHDKHTDIHIMK

AVKEADSVLLAWGSYGKKPLVENRVNEVLDMLKPHSKKISILTNPQTNEIMHPLNPYA
RKAWTIKPLKETK"
CDS complement(48744..49055)
/translation="MKRYITRSIANYLPAMLQQQLWKLVAQRENEQFKELEEIDYFHI

FQFNMHNSQLYIKHKQERPEYVKIHKANYSKAININKVYIIREDDVDLSYYVMLLPEE
Y"
/transl_table=11
/product="FIG01107894: hypothetical protein"
/db_xref="SEED:fig|12142587.3.peg.61"
CDS complement(49058..49147)
/transl_table=11
/product="hypothetical protein"
/db_xref="SEED:fig|12142587.3.peg.62"
/translation="MNKWIHIARISVTVITEVITIMKDIEKEK"
CDS complement(49140..49490)
/translation="MTLEKQLKEYITNLFNLPKDEKWECESIEEVADNILPDQYIRLG
PLTNKILHTYTYYSDTLHERHIYPFILYYQKQLIAIGYIDETNDMDFLYLHNTVMPLL
DQRHLLEKENYNNE"
/db_xref="SEED:fig|12142587.3.peg.63"
/product="FIG01108299: hypothetical protein"
/transl_table=11
CDS complement(49961..51589)
/translation="MQQLKQKRVGIYVRVSTEIQSTEGYSIDGQINQIREYCDFNNFV

VVDVYADRGISGKSMNRPELQRLLKDANEGQIDSVMVYKTNRLARNTSDLLKIVEDLH

RQNVEFFSLSERMEVNTSSGKLMLQILASFSEFERNNIVENVFMGQTRRAQEGYYQGN

LPLGYDKIPDSKHELMINQHEANIVKYIFESYAKGHGYRKIANALNHKGYVTKKGKPF

SIGSVTYILSNPFYVGKIQFAKYKDWNEKRRKGLNDKPIIAEGKHSPIIIQDLWDKVQ

LRKKQVSQKPQVHGKGTNLLTGIVHCPQCGAPMAASNTTNTLKDGTKKRIRYYSCSNF

RNKGSKVCSANSVRADVIEKYVMDQILEIVKSDKVINQVLERVNQENKVDIGALNHDI

AYKQQQYDEVSGKLHNLVKTIEDNPDLTSALKATIHQYETQLNDITNQMNQLKQQQNQ

EKLSYDTKQIAALLQRIFQNIESMDKAQLKALYLTVIDRIDIRKDGNHKKQFYVTLKL
NNEIIKQLFNNTPLDEVLLSTSSLFLPQTLFLQI"
/db_xref="SEED:fig|12142587.3.peg.64"

/product="Site-specific recombinase"
/transl_table=11
CDS complement(51610..52956)
/translation="MKRAIGYLRQSTTKQQSLPAQKQAIELLAPKHNIQNIQYISDKQ

SGRTDNRTGYQQVTERIQQRQCDVLCCYRLNRLHRNLKNALKLMKLCQKYHVHILSVH

DGYFDMDKAFDRLKLNIFMSLAELESDNIGEQVKNGLREKAKQGKLITTHAPFGYHYQ

NGTFIINNDESPTVKAVFNYYLQGYGYKKIAQYLEDDNKLITRKPYQVRNIIMNPNYC

GRVINQYGQYNNMVPPIVSATKYEHAQAIRNKKQLHCIPSENQLKQKIKCPCCDSTLT

NMTIRKKHTLRYYICPKNMNESRFVCSFKGINAQKLEVQVLATCQNFFQNQQLYSKIN

NAIHQRLKKQRVIEAKSTLTQEQLIDKLAKGMIDAESFRKQTHLMNQKHKTISSISDN
QLQTSLQKVIQKSFTLNMLHPYIDEIRITKNKALVGIYFKNEPLNIVNQTSQSSIA"
/transl_table=11
/db_xref="SEED:fig|12142587.3.peg.65"
/product="Cassette chromosome recombinase A"
CDS complement(53149..53424)
/db_xref="SEED:fig|12142587.3.peg.66"
/product="hypothetical protein"
/transl_table=11
/translation="MNTVALAHEIEDERFEYLESTPLDTVKECCKQEGRQISNTYTEE
YKLINDILEKVIKPTSIVAYGEYEDYIHLKKFAQRRISNSLLLLRCN"
CDS complement(53514..55301)
/db_xref="SEED:fig|12142587.3.peg.67"
/product="hypothetical protein"
/transl_table=11
/translation="MTNIIFSEYPYWLTEDSWYEVIPPKGKNDQQKSIRLSSPILIKN
KFLDPKSGVEKLVISNGQNIERIETSDILTSQKLPSLIMYGFSINEKYIKSLSNALQL

MRQSLPLSSLYTGVGVLNTDKGVVISLDEPYLSKEITQSQADEIICETQYDLQPKGTF

EDWWQMYLDEVKGNLLLELAVVFGISSLVTAFLKTKHEVEFAGTIFSFTGNSSTGKST

AAALGVSITGNPTKGSNTLFRSWNGTRNALEGYLSNNYGVPIVLDELSSATFKDTTGL

LYSIAEGQGRQRSNIHGEVKSLKNWGTSVISTAEHSIFNNSARNDGLNVRTIEISEAF

TTNADNADTIKKATSANYGHIMPLVAEYLLNREQEVIKWFQNEHLRFKTQLEKETSNT
GIRMFKRYATITTSARILERVIATPVDLNAVRDYLINYHLDSVSERSLADKAIEVIVQ

FVAQNRGKFSDDTRLSTLIENYGMISLEDDHIQVKMIASVFKNMLKEHNFQDTNNVID

ALRDKDYIQSDANRKTTKRSVKDKEGKPKTIVFYHLKLDKSYAPIFGLSEKSKFNIPL
SFISEHQHPILRDMISKAREWDKNGDIDF"
CDS complement(55301..55585)
/product="FIG01108228: hypothetical protein"
/db_xref="SEED:fig|12142587.3.peg.68"
/transl_table=11

/translation="MKEKEFYTLKRAVTSIAFNLPVGTMFMYHDLNVRANVLCSTEIQ
QDVGRWFAHFVKYTPNLPFIVVGKKGSSLLYLKTAPNPMNHQNNWKGGNN"
CDS 55723..56772
/translation="MIPTIEEIIDKYGSLVDCLKFDISGEVYEDLLLLRSLSDLNEHQ

KMHRVSFIQKHFNPNFDYLLDDSEFRSMNKIYRNLNTMTYINSQNNEFKLKPFINEEQ

LKSLLAIKKVDEHLSYDPSITSKALADINKTQKRLVTKPNILYNRSKKENEYVRLGGK

ISYTKMEKHWEYLHNEIQYYNLTNQLISYVALEKEYAWAFLNELFYLIELYLKAFKGQ
KKTGHEFEEGLNAFIQSYVIILLIDIRLPTVRLYIIRRIVDECEKESDNQKRIKLIEE

SIKKYRYVKEQIERFGNALKEELSIATLSIEQNMLEAKIDYYKNYYYPRKKTKHNNIE
YNVSMFFKAVDSLKK"
/transl_table=11
/product="ORFID:MW0042"
/db_xref="SEED:fig|12142587.3.peg.69"
CDS 56825..57397
/transl_table=11
/db_xref="SEED:fig|12142587.3.peg.70"
/product="FIG01109033: hypothetical protein"

/translation="MFRLKKWYLKQKGYFCRVGEKWDNSNLSHENIHSYVDRVLEYET

ETRRNGQPTEEYILASSGEKINEDLRSGTNDHKEILEVFDKYKVKEDITTYRTVSPRI

YKLMEKNAKNLNGVDLFELGILHTSLIKGYESREEGYKLRVKVKKGTPAFYVGNLTGE
ESHYYEVIVVNNLKLKIISIEDYYINCEVV"
CDS 57729..58055
/product="Transcriptional regulator, Xre family"
/db_xref="SEED:fig|12142587.3.peg.71"
/transl_table=11
/translation="MISKQIKDLRKQHNYTQEELAEKLNTSRQTISKWEQGISEPDLI

MLMQLSQLFSVSTDYLITGSDNIIKKDNKSYYEMNFWAFMSEKWWVIIIIVIIICGTI

GQIFSN"
CDS 58092..58925
/transl_table=11
/product="hypothetical protein"
/db_xref="SEED:fig|12142587.3.peg.72"
/translation="MKIKKLLKTLLIILLCFVLSVIVQNISMLWHIVSIWSVEYILHA
LTYIVLSYFSVKWFIEIVLKSNMKDYRIIPVKFFKSCFLIGLILILVIDLIYLLFIPG
KLIIPHYSTSVGFMEMFFSAFLITGIAAPIFEEMVFRGILMRYFEKQYGILFGIIIPS

ILFSLVHLFNGELKGENLLLLLIGGSIAGIMYAVTAWTFNSIWASAILHMLWNINGLL

NITTQDDHWGIYQYILDTKNVLITGGNYGMDTSLLSICSYIAVIIVMIVTKRHFNFLL
K"
CDS 59130..59294
/db_xref="SEED:fig|12142587.3.peg.73"
/db_xref="GO:0003684"
/db_xref="GO:0005524"
/db_xref="GO:0006298"
/product="MutS-related protein, family 1"
/transl_table=11
/translation="MNIYQTYACLVIGIIVNSTTTILKRKKLISNIKQLWKSKKTLEE
FIRSNSRFDY"
CDS 59611..60384
/product="hypothetical protein"
/db_xref="SEED:fig|12142587.3.peg.74"
/transl_table=11
/translation="MNDLLKIYEKLKKDIENRWLIPFHYVVFGLALVVYFLEIPIYKL
VNNLDKELVDKVLYAFSLVYDHIVLIFILIIIIILIVYLFFDVFNMNRFVPSPTTYVD
GSESSINYVSAIKRLINFMILIITKYWITYFIVNLIFHNDKLLYLNNDSKHLYKCLLF
LNICIFIVHILKSIFIIKIMADDSLRLINENDLTDFYITLNKNDEYRMVKPKYRKIDN
FIIIKKSEDFTNSNEYKVINKSKNFEEIIYHFDNLTSNH"
ORIGIN
1 tttattgcta aattggaggg ggcagtatca atttctggtt ttctacaaat ttatttacac
61 taaacaactc attttgcgat tctttaggat caacaatacc gtatctatgt gtttcatttt
121 catttatttg ttgtactctt ataattgatt tacctgtcgt ttcatactgg ttcatcaatt
181 gttttatcgc cggagtgttt gcttgtcaat gtcatctcct aataaaacag caaatgattc
241 atttcctata aattgtctag cggttcatat agcatgactt aaaccctttt gctctctttg
301 tcttacataa aacatatttg ctaggttgtt tgagtatttt actaattgaa gtaaatcaga
361 cttacttttt cttcaaaagt catttccaat tctttttgat tgtcaaaatg attttcgatt
421 gttcttttat gcttacctgt aactataata atatcttcaa ttcctgcttc tactgcttct
481 tctactatat attggatagt aggattattt aaaataggta acatttcttt aggcatctct
541 ttagttgcag gtaaaatctg gctctaaacc tgctgcaggt attatagctt tcttaatttt
601 cataagttac atccttcctc taatatttca aatcattgat aaagctccta tacttttatc
661 aatagatttt tagaatagta ctaaataaaa cacactttaa aatgtactag tataaaatca

721 cacttatttt agtataacaa aacttaatag caaaaatata gagacaccca atatatttta
781 aacaattttt aatagcaaaa taatataaag aagtatgcta tatgtattat tcctttaaat
841 tgggaataat tttcattttt taattaaaat atatttcttt aaatttttgt attacctata
901 gtttaatgta actttttgtt attcaatttt tttgctcatt agaccattct aaatttgaac
961 tgccccatcc tggtctacaa taaaagtata tgcactttgg tcatttttat tatttgatgc
1021 tattctccat aagcattttc ttgtgttgaa aattcaggtt gttgaaattt tataagagaa
1081 gcgtctacgc cattgttttg attgacagct gctataacgt aatcaaacac attatctcta
1141 tttactttca aatcagaaaa ttcattttta ttagaaagta taagaaaagt ttatgatttt
1201 atttcaaaga aaaatttaga cataagttct tttctaatcc ttttctataa tgagctatta
1261 gtatacacta gtagtgagaa tgaacgtcgc ttttagacga ttgctaagaa gcatagttat
1321 ccgcctgact tacaaaaaac ggctgtagaa caagttgttg agcaagcaaa acctatggtg
1381 agtaatcagt aaaacataaa atataagagc attcctcacc tttaaaggga aggatgctct
1441 taatatttta ttcttttata gtaataggat ttcctaatgt atcagatata atctcttcaa
1501 aatcattttt taaaggtcct ttaatttgca aagatctcac cccacttgat acaactaaac
1561 cagaaccagc caaaaacact gatcccatca ataatcctcc tactggaatc gaaaaaatcg
1621 cgccagcaag caataatttg cctatttgtc taaaaggctt acggctgaat tttatttcta
1681 gtccaataac attttcaaac ttagtttcac ctattattat ctcataatta ctatttcttc
1741 cattaaaatg ataaaaagtt atcttagctt tatcattaaa aacttttaat aattctcttt
1801 tttttggact tgaatgattt aaaatttctt cttctttcaa aatttcttct tttttacgaa
1861 atttgtggtc tttatccaat gctaattgtt taatttgttt ttcaaaaata tctaaatttt
1921 tactattaaa aaaattaaat ttcataaaat actatttctc ccacttttta gtcttgtaat
1981 tatattcata tcctctttct tgcaatagag acttacaagc aatgcaagta tcatggtcac
2041 catcagcagt atcatgaata ttataaattt ccatcaattt ttggtccgaa gaatctttta
2101 aagcgtctct cataatacct ctaccgccag ttgtttcaaa tacagcactg atagcagctc
2161 ctgctattac aaatggaaca gcagctactt taccagctcc aactccaatt tttttaaaaa
2221 catctttcat aatgtattgc ctccattata atacttaaaa tcaataatta gaattactaa
2281 tttaattata atttttattt ttgaattttc aaacattttt aacgttttat tacgctttta
2341 aataatacaa ttattaatag taactttatg agagtattca aaaagtttat cgtcaatttt
2401 ttcgattttg attcctttta atagtacatt aagattttta ctattgaatc gttattgtac
2461 gatactaaag aattagcaaa ttttgctagt ccaattgtat aatattatat ttcgtagata
2521 atccttctta tttcaatgtc catttttgac gtgctctagg gttaagtgga tgcataattt
2581 catttgttgc tggattaatg agctttttga ctttcttttt atgaggtttt aacatttcct
2641 ttacttgcgc tacacgttcg acaacaactg gccgcttcgc ataggcgcca taagctagaa
2701 tgactgtgtc actttcgctt atcgctttca tcaagtggat atctgtgtgc tcatcgtatg
2761 gctctttgat atgtttaaga ttctctggag ttttaatatt agagaataga tttacaagat
2821 atacagcacc gtatcgttct gaattggcta attggttgag gataagaaca gttgtgagat
2881 cgagtgataa tacaccgtct aaatgaggat acatcgttat cactgtgcat gcagctttct
2941 tttcatccca tgttttcttg agtaaatagc ggtgctgttc atcatcgcta aatatggctt
3001 ctgtgtgtat cgtacttttg attgtattca tcatcgtcac ttcctttagt attcttctgg
3061 taaaagcatc acataataaa aagcgtccac gtcatcttca cgaatgacgt agactttctt
3121 aggtaatgca ttttgatttt tttcatagtt tgtatagtga tattccaatt tgtatgtggg
3181 ttgttcttgt tcatgtgtga ttgagagtat attctcatct tcttgtaatt taaaaatgtg
3241 taggtaatct gtatgaggct ggttatcttt tttttctacc atgtgccaaa gtaagatttg
3301 aaggtctagt ggaatacttt cattaattcc tcttgtgatg tatcgattga ttttcatgct

3361 atttccctcc cttctgcttt tctttcatga tgtcgatgat ttcgttgata actgtgacgg
3421 ataattgagc aactatgatc caattattca tggtcctgac ctccttgttt tagtaaatga
3481 cgttcatcaa taatgatatt ttgagtatct gtaaggtaca gaaagtccat gtcaaaatgg
3541 tctaagtatc cgacactgat gagttggtta ttggcataca ttagaaatgg atagatactt
3601 agctcatgta gttcatcatt atagtaggta taagtctcga gtgtgagatg taccagtgga
3661 gaatcattaa taaaacgttc cggtagaata ttttctgctg cttcctccag cgcttcacat
3721 tcccaagctt cgttattaga tagttggaat aggtgggtta tatattgttt gagttcttga
3781 gtgatggttt tcatattatt gcctcctaga tagtgtaata gtgatgtagt tcatatacat
3841 cattgagata atatatattt gatttgtcat ttattacgaa tcccggtggg aataagagaa
3901 aattccatat aaaaacccgc tacaaacgtt ggtatgccaa ggaaatcctg aaattccgcc
3961 tattttgaca aacaatcaac tcattattta taagtattga tgatagggtt gtgtctctgc
4021 ttccttatat atattattta tttataaaaa gtaacgggat tttgggattg tgcttgcaca
4081 atccttctgt ttcttcgaat ctgcaaatcc cattcctttc ccggtaaaaa atcattgtgg
4141 gatgttcttt agcaatttca atataagcat tgtgtagtca tgaaaaaaat gacggcaatg
4201 actgtttcat tagataagtg ttattgaaat tgataaagag aattctaaaa atggttagat
4261 aaaataagtg aaagaatagc agtgtagtta ttgtttattc aatagttata tataaagttt
4321 gtggcaaaaa taaagacgaa gtgctaggga gcacttcgtc gagtggatgg ttattaaata
4381 gttgtttgat tatatcatta tttaacttga gcgtaacata aaattgcttc ttatgatgct
4441 catcttttct tatgtcaatg cgatcgatga ttgttagata tagtgatttc aactgagatt
4501 tctctaattt gtctatatct ttgaatattg cttgtagtac attcgcaatc atatcagcat
4561 cataatgtgg agcttctgct tgtttatctt gttctagttg atagatttga ttatttattt
4621 gattcaattc atcttggtag ctaagtattg ttggttttaa tacgctatct aagtcaggtg
4681 agtcttcaat agttttcgtt aatgtatgca tttttgcttt tatttcttca cattgtgact
4741 gtttataggc aatatcatgg ttcaaagaag agacgtctat ttgacttttt tcatttacct
4801 tttcaaccaa ttgcttcaat acctttttgc ttttgataat ttccaatatc tgatccataa
4861 catatttttc tagtacatct gctctaacgc tattggcaga acaaactttt gaacctttat
4921 ttctaaaatt gctacatgag taatatctga ttcttttctt agtgccatct tttaatgtat
4981 tagttgtatt acttgccgcc attgctgcac cacatttcgg acattttaca atcccagtca
5041 gtaggtttgt tcctttgccg tgaacttgtg gtttcttgcg actctcttga cgtttaaact
5101 gtactttatc ccataggttt ctatcaataa taggcgcatg tttaccatca gcgataatcg
5161 gttcctcatt gagtcctttt cgtcttttat cgctccagtg tctatacttc gcaaactgta
5221 tctttccaat gtaaaaaggg tttgagatga tgtaggtaat ggacgaaata ctaaaaggtt
5281 tccctttctt agtcacataa cctttatgat tcaatgcgtt cgcaatctta cgataaccat
5341 gacctttagc gtatgaatca aaaatatatt taacaatatt cgcttcatgt tgattgatca
5401 tgagctcttt tttactgtca ggtactttat cgtagcctag aggtaaatta ccttgataat
5461 aaccttcaat agcacgttgt ctttggccat tgtagacatt ctctacaatc gtattacgtt
5521 caaattctgc gaagctggct aaaatttgga gcatcaattt acctgttgaa ctggcaattt
5581 ctattttttc agttagacta aaaaattcga cattaatctt atacaattcc tcgacaatat
5641 ttaacaaatc tgaggtattt ctagctaaac gatttgtttt gtagaccata atacaatcta
5701 atttaccttc gttggcatct tttaacatac gttgtaattc tggacgttgc attgttttac
5761 ctgatatacc acgatcggtg tattcattga cgacttcata gccttgaaat tgacaatact
5821 ctgtaagttg attcaattga ccttgaatac tgtaaccatc cgtttgcatt tcagtcgata
5881 cacgtgcata taatccaata cgtttctttt tgagttgttt catattactt cattcctttc
5941 aggagttatt aaatgtgatt gttcaacgat attgagtgga ctgtttttaa agtagattcc

6001 ttgtaattgt ttagtttgag ttattttgat gcaatcgata aaaggtgcta tatcctgtaa
6061 agttatttta ttttttatga catattttaa tttgtcatcg atttggtagg ttgaataagt
6121 agagtaattc tcttcacttt tacaactagc agataaacgt ttgaacgttt ttacatcaat
6181 acgattttga gctagctttt cgataagttg ttcttgtgtt agatgggttt ctttatgctt
6241 catttgttgc tgttttagga ctttgagtat tgtgttattc aatcgtttat gaaacgattg
6301 ttcttcaaaa tactttttac aagtgtcgag cacttcactt tcaagttctg gtgcattgat
6361 acttttaaat gggcatgtac gataagcgtc attcatattt ttaggacaaa catagtaacg
6421 tagagaatag ttctcttttt ttatcgttaa gtttgttaat gttgattgac agtaaggaca
6481 tttgattcgt cgctttagct tatttctaga attggatcga ttgagttgtt tatgaatacg
6541 acgctcttgt gcttcttcaa atgtatcaat atcaataata ggtggaacga tatcattaaa
6601 cgtgccatat ttattgatga cacgaccgca atagttaggg ttcaagagaa tatttctaac
6661 ttgatagggc ttacgaggaa taaggttagg attactatcc aaatgttggg aaatcttttt
6721 gtagcctaga ccttgtaagt accagcgata aaccgattta actgtatacg cttcttcttc
6781 ttgtacaaca aaacaacctt ttctataacg atagccaaac ggagcatgag ttgtgattag
6841 cttaccttgt ttggcttttt ctctaatccc attttttgtt tgttcgctga tattgtttga
6901 ttccatttcc gctagactca taagtatgtt caaacgaaag caatcaaact ctttagacaa
6961 atcaaaatat ccatcgttaa cactgatgat tgtgatgtga tgctttttac aaatttcaaa
7021 gaattgtatg gcatttttca aattacgatg tagtcggttc aagcgatagc aacacaatac
7081 tttacatttt ccagacgtaa ttatttctac catttttga taacctgaac gttttgtatg
7141 tcgacctgtt ttcttatcat cataaaacgc cacattagac catccatatt gcttagcggt
7201 atccataatg agcgattttt gagtagctaa gctttgttgt ttgagtgtac tttgacgtac
7261 ataagcaatc gcttcttcca tgttatacac ctccaaaaag ataatatata tttgtgagta
7321 aattagaatg aaaggtccaa cgtgctttta acacgttgga ccgtcgtgat tagttatcgt
7381 tctgtagctc ttcaacgact aaatcagcta gtaattcaat caattcatcc attttatcta
7441 ctcctgtacg atttcatctt taagttatta aaaatcaatt aaatcatcgt tctgtttttt
7501 ccatgattca agtatctttt tgttatccaa gttaatttca gctttaatag gttcagcatc
7561 tttagttaga ccaaaaatag acgcatattt tgaatctagc tttaaatggt aaaagacaag
7621 tgactgtttc ttgccatagt catctttaac tgctcttttc gtagttattc gatcacggtc
7681 agattcgata aatcctttat cccttagtgc attcacaaca ttgttgacat cttggaaatg
7741 atgctctatc aacatattct taaatacaga tgcaatgatt ttaacttcga tataatcatc
7801 ttttaaggca attagtccat agttctcaat catattcttt aatgctgtat catcagaaaa
7861 cttaccacgg ttttgtgcta caaattgcgt aatgacttca atcgctttat cagccagtga
7921 tcgttcagag actgtatgag catgataatc aataaagtag tctcttattt tagcgatatc
7981 aatatctgta gataaaacat gactaagaat cttggccgaa gtagtgatag ccgcatagcg
8041 cttaaacata cgaataccag tattatttgt ttcatccttc aatttatctt taaaccaatg
8101 atgtaattca aaaacggatc aaaaaacaaa aggccctaga aactaggagc acacttacac
8161 aagaacaatt gatagataaa ttagctaaag gagccatcga tgcagaaacg ttcagaaaac
8221 aatctcaatc gttacttcaa caatcaaaac caacactatc aataaatgag caacaaattc
8281 aaaggtcttt tgaaaatgta attcaacaac acttcacgtt aagcatgtta taccgatata
8341 ttgatgaaat tcatatttct aaaaacaaaa gccttgtggg aatttatttc aaaaatgaac
8401 cgctaaacat tgtaaaccaa gctacgcaat catcgattgc ttaattaatg aaaggatgaa
8461 aaatatgaaa gaaatgaaaa gaaaatctgt aggatgttat gttagagttt caacaatttc
8521 tcaagacatt gataaattta gtattaatgg tcaaattaca caaataaagg aatattgcca
8581 acagggaaat tatgaattgc tatactgata attaaaagta cccccgatcg aacgaaaata

8641 tctttttgat gactattagt gataattgta attttatcca ttaatagatg aatggattgg
8701 aataatacta agcctattgc tattccaccg agtaaaatta cccacccgcc taattcaata
8761 ctttccggaa tcatttcaaa aaataataag ccaattataa ggcctgcaca taatgaataa
8821 ataaagccat gaccactttg aaaaccttcc attatccaag cgagtccacc gccaacacca
8881 attccaagtg cggaagctaa cgcccgatca tccatactgc actcatccca ctattcttaa
8941 aaaggctttg tttattttac tctaaaaaat caaaacaaaa tatatgaatt catattaaat
9001 attatttgac tctttattca gatggttata tcatatatat gaacatatat taatacatgt
9061 agataaatag tgattattat atataaggtt ttgaattgaa acctaaagtg agggaaggat
9121 aatggaagaa aaaaaggaat tagaggaagt aaataataag gacttagatg atgaaacatt
9181 atttgtcgta tcgcaaacat ttaaagcgtt aggtgatcct acgagaatcc ggattctcca
9241 tttgctcttt tataaggagt attcggtaaa cggtattgct gaaacgctac atcttagaca
9301 atcaacagtt tcccatcaat tgcggttctt gaaaaattta cggttagtaa aattccgaag
9361 ggaaggcaca acattgtttt attcccatga tgatgaacat actatgaata tgctaaaaca
9421 ggcgatcgat cacgcctgtc atcactagta ttccgtaaac cagtaatgag tgttaataat
9481 aattcacgtc attaatattg ttatatgatc atataaaaat attataggca tatgaaaagg
9541 gggttttgca atggatatgg aaaacaaaaa aacagaatgg aaagcgttgt atgatatctc
9601 gaaggaaagt gagatgggtg tagcagagag ggttagtgaa tacggttgat aaattcatag
9661 gatgtattat tggatacaaa aagtttggat gtggtcaatt aacgtcctgg tttagtaaag
9721 cttactgcga atatattgga acccgatata tcatatttat ttagaaaggt gatagaaatg
9781 aaaaatattc aagagcaaca agcacacgaa agtcatagcc acgatcatag tcatgatcat
9841 gatcacggaa aaatgccaat tatttcatat tttattggct tagtgttggc tataattggg
9901 cttttttaa gtgatgcaaa tttattaata caaaacatct tattttcaat tgccacaatc
9961 acagccggct accatgtaat tattctcgaa ggaattggag agacagttga aaatactaaa
10021 ttaaagggaa aattcactcc taattctcat attctaatgg gattagctgc aatcggggct
10081 tctctgatag ggagtttttg ggaaggaacc cttttgatac ttattttttc cggcgctcat
10141 tttcttgaag attacgctga aggaaaaagt aaaagagaaa ttactaagct actcgaaatg
10201 aacccaacga cagctaaatt aatcctacct gatggaaaca caaaaattgt tgatgtcagt
10261 gaattaaaag ttggagatca actccaagtg ctgaacggtg atcaagttcc aattgatggg
10321 attattttat ccggtactac ctcaattgat gaatcttcta ttaatggaga aagtataccg
10381 aaagagaagt ctaagggtga cgaagttttt ggaagtacga ttaatggaac aggtactttt
10441 actatggaag tcactaagga aaacaaggat actgtattct ctaaaatttt acaattagtt
10501 agtcaaaacc aagataatca aacaaaagct gccagtatca ttcaaaaatt cgagcctaaa
10561 tatgttaata tagttttaat cgcaatacca ttagtaatgt tacttgctcc ttttctattt
10621 gattggacat ggtcgcaaag tgtatacagg ggattagtgc ttttagtcgc agcttcaccg
10681 tgtgctttgg cagcagctac tgtatctgta acattgtcta caacatctaa cctagctaaa
10741 aaaggcgtgc tttcaaaagg aagtacttac ctatcacaat tagcggatat agatgcaatt
10801 gccttcgata aaacaggaac ccttacgaac ggagaaccta aagtaacaaa ttactatttc
10861 actcattctg tgaacgaaga aaatattatt gatattatag tcgcccttga aaaggaatcc
10921 aatcacccac tcgctaatgc tattttagaa aaatttgaag ttaaaaataa aatagacatc
10981 gaagttacta atcaaattgg aaaaggtctg acaggagatt ataatggaaa aaattatcgt
11041 attggtaagc ctacttcttt tgaaagtgct tctgaagagt atacccagtt caatcatgat
11101 tgggcatcag aaggaaagac ggttgtatac gtagcagaaa atgaagaagt tattgggatt
11161 atagctctaa tggatattcc gaatgagcat gctaaagaaa caattaatta ctttaagaaa
11221 cttggtatcc acacgacttt aattactggt gattcggaaa tgacgggaaa agctgtaggc

11281 gaacaattgg gaatagacga agttatcgct aatgtaatgc ctgaagataa atccagaatt
11341 atagaagaac aaaaagaaaa atttggagtt actgccatgg ttggagatgg tgtgaacgat
11401 gcaccggccc ttgttaatgc tgatgttggt atagctatgg ggggcggtac tgatgtggca
11461 gtagaagtat ctgatttggt tttaatgcag aacaatttat ctaaattagt acagtctcat
11521 aaaatttcct caaatatggg tcgtgttatt aggcaaaata ttattttttc aatggcagtt
11581 gttgcctttt tagttgtcgt tagtttgtta ggattaactg atattacaat cagtgtaatt
11641 gttcatgaag gaagtacttt agttgttata ctaaatggac ttcgattatt aagatctaaa
11701 taatgaacga atcgattgac atgaatgaac tttgaagtgt ggattctaca atgttcccat
11761 aacattggac actaaaaaac agagcaatct aataaagatg ttatgagtaa aaacaatgcc
11821 ttgcataccg tttttatcat acgggcggtc aaataagcaa ttaaagagca tgggaagcat
11881 aaaatcataa acagtgactt aaggcagcca gtttacattc aaaagttaaa ttgactgtat
11941 taaaagtttc gaaacgatta atatccgtat gggggcaagc gtcgagccaa agataatgcc
12001 aggacagagc gtctttttcc gttcttttaa gtgggaaagg ttttaccttc tctatgccaa
12061 gacagtccca gagctaaaag ggccagttgc tctttttggg cgtttgtgaa cggtaatcgc
12121 aggaagggga ttttcccaac cgcacctggt tttccatcac aataaagttg gcggcatttg
12181 ttctgatatt cttgggaata cttacactat gaatggccgt attaagtgat acaggagctg
12241 ctattattgt aatactgaat gctctccgcc ttttgagggt aaaagaataa aagtaaggat
12301 aactaggtaa agctgttcaa tcaaaaattg aacagcttat ttttcatcaa aatcaaaaac
12361 gtttattata atacctacac ttgttctgtt aaatgtaccg attttttaac cttattgtat
12421 cagtaatatc ttgaaacgaa gtaagcgact aaaatttctc ttttatcata cgtttataaa
12481 aatacacttt taagaacggt ttgaaaattt ttgaaataga tcaaaataat cttctggtta
12541 aaaaacctgt agatatgatt tctctcttaa atttttgttt gattagatta gaccctaata
12601 agacgcattc aatatgtcta cacgtgaatt tagtctttga aaatgtaagg accattatta
12661 ttataaaaac cccagtataa aacgatacgc tgaagcgtat cataaataaa actaaaaatt
12721 aggttgtgta taatttaaaa atttaatgag atgtggagga attacatata tgaaatattg
12781 gattatacct tgcaatatca tacgatgttt atagagtgtt taataaacca tttttcaact
12841 attgatgatc tagaatatat aataactgta caaattatat tgattatgga actacaatta
12901 aattaagaaa ttgatgatga aattttaaat ttaaactaat ggaatcaaga aagaatgaaa
12961 ggaaatatac aatgcctacg attaataaaa ggaagtttat tagattttgt gttagaaaca
13021 gtggatgttt ttgtaaatgg aagtaaaaat gattgtgaat tagtaaattt taattataaa
13081 gaaggaaaag tagtttcacg ttatagtaca aaatttgaaa gcaatcctaa attaagaaaa
13141 gagcaattga tatcgacggt aaaagttgta aggtatgtta tttttgattt tgagaaaatt
13201 ttatggggca tagggaaagt tttattgaag ttcatcatat taagacaatg tttactataa
13261 gaaagttgaa ataaactcag aaacagactt gattcccgtt tattcaaatt ggcgcaaaat
13321 gatacatata aacacctata tattgaagat ttaagtcaaa cggtaaataa tatttttcac
13381 ttttaaacag aaactttttt ggaaattata ttaattagta taaaaatgaa tatgttcaca
13441 aagaaggaga gattttattg aaacacatta atcatttttc tgaatcgaca ctagcatact
13501 tggaaaaaga ccatgaacat gcattttatg tatattgttt aatagaccca agaaatgatg
13561 aatgctttta tgttggtaaa ggtaagggga atagaatatt taaacataaa caagatgcgc
13621 agagacaatt attgtacgaa gatatattca gagaagaaaa taaagataac ttgaaattta
13681 atagaattaa cgaaatttgt agcaatgatt tgaatgtttt aggatatata atcagttatg
13741 gattaaccga atctgaagca ttttctgcag aaaatgttct tataaacttt cttaacctaa
13801 caaataaaac tacattaaca aatatgatta acggtcatgg atctaaggca tacttagttg
13861 aagatttaga aaatgaattt ggctacgatt caattaatct tgaaaacata aatacgaatg

13921 aattaatttt agcagtgaaa atcagagatg catttctatt agataaggat gaaagtaaag
13981 agtatcctat taatgaaagt aaacgtgata gaaataacct taaatcgcgt acattaggta
14041 gttggataat aggaaaagat aaaatacata aaataaaata tattattggt atcaatacag
14101 gtgctaataa tgcagttgtc tcagcttatg aagtatcatt tgaacaagcg gagagtatcg
14161 aaacaaataa tggtagaatg agatatgcgt ttattgggct ttcagaaaga gatgctactc
14221 taaaaaaatt gaatttatac aaaaaagcac taccagattt aagatttggt agtggtagcg
14281 ctacagcata tataaataat gggacaatga aagttgatta aatgaataaa aacatccaat
14341 tccttaaatt agcctttgaa aattttatac tcgtatatat aagtgaaaaa cttaaaagaa
14401 ttagatgtta attaaaggag agaatgctat gccatcagat gatattgtaa aaaaactata
14461 ctcaaaagag gatattcgat tagaattagg acttactccg cataagttta ataaaaaaat
14521 ggaaactatt gctaagcttt ttaaaattga tatgaaaatt tttcacaatt acaaaggcca
14581 agataaaaat aatcagtata catttaatgg tgtcgcaaaa gaattaatca aagtgttgct
14641 aaaaagtgtt gattattacc cagtggatat caattccaaa aaatttaaac aaaatggaaa
14701 atctaaaaaa gaaatgattg aaaatataga taactctagt tacatgaaat atatttatca
14761 gttaatgaaa tctatcaatg aaattcaata caaaaggtta attgctgata tacatatgaa
14821 agatgtgtac cagaatacaa aagcatggtt aaataatggt gaatcaatta ataaaaaaga
14881 acaagagcta taccaatata tgacaatttt accattacat aaaagagttg aactgcaaaa
14941 tgaggtatta aaatctatag acgaaacaat ttttcaattt ctcgcaaaag aacatagaaa
15001 taatcaaatt gaagaaaata atgaattaga agcatataca aaagcaataa aagaaggtag
15061 aaatcctaaa aatgattatg aattaaatca tctcctatat aaaaaaaatc aattaccctt
15121 agatagtcta atagaagata tatgggacta tgaggaaaca gagtatactg agcttgattg
15181 gttaattgca gatatgctaa aacgttcaca aagagctgat agtaatttta tcgaaagttt
15241 agagaagaaa aacaaattga gaaaaaatat tcataaagat tccattaaaa gtatatctaa
15301 gttgattgat catgaattag ttaaaaataa tagaaagtgg gacgtagcag aattttataa
15361 aaatcaacaa ttttggaata atagtacatt aagacgtagc tctcaaaatt ttggaatttt
15421 atattacaaa aagaataggt tacatacaat gaatcaaagt aatattatta agaaaataaa
15481 aattatcgaa gctaatatag agagtgcaaa taaacagccg agctatttag atttcttttt
15541 acaagctaag tttgatttag agaggttaga aagtgaatta atgaaatata gcattaatcc
15601 gaaatatttt aataagataa atagtgatta cgtaagatat gctattgaag atgtacataa
15661 tgcaattatt aaaattgaca gatatataag taacgataaa gtgaagttaa actattcaaa
15721 tgtccaaatg gtagtgagaa acgaaataac agaacaagga agctactttg taacccaggc
15781 attgaataca ctgtcaaagg tagaagagtc tggctacagc tttgataatt tcatggtagg
15841 acctttcgta gaagacttta aaaaagcaat taaaaatcgt agatttggtt ctaaaaaaga
15901 ttaagattgt tttttagagt taaacataag aataaattta aataaaaaaa taaacccaac
15961 aaaccttgtt atatcaatgt ttgttgggtt tatttttttt ggtgattttt taataactca
16021 attacatctt aggataatat cgctaaatta tacattgtcg aggccaagac aaataaaaaa
16081 ttaaagcaaa agttagcgcg caactttctg ctataaagga gcaaatttat atggagacaa
16141 gagacaaact gatgtcattg actcaaagtg acaaaacaca gcaatggcta atggacaagt
16201 catctaacca agatgacatt caacaattgc agcaacaatt cagtcagcag cttgatcaac
16261 aatataatgc acttttagct gatgaaaaag ctaagttaga ccaatacgtg gaagtacatc
16321 aaggattgga atcattaaag gaagagattg aatcagaacc tattacgctt aatatcgata
16381 aattacccga tatcaaagca acaatgcttg aaagagccaa gaatgatgaa cattctgata
16441 aaatcgaaaa gctatttgat aggttagaac aggcattaaa tggtacgaat cgattatata
16501 cgcaattatc gttgattggt acacgaacac atcgaattac aactaaaaat tttaatcttc

16561 aaggcttacc taaagcagtc caacatacga ttttaccttc aaaatttaag aaggtgtata
16621 cagtcgattt taaatcgttt gaaccatcag ttgcagcgta catgactcaa gattcaaaac
16681 tgattgactt gttaaatcag aaagacggac tgtatgacgc attgctaagt gaattaggct
16741 tatcagatga gctacgtgta tttgttaaac gtgcatttat tggttcgttt ctatttggag
16801 gtaacttcaa aaatcctaaa ttcaagctga atcaatatgt aagtgaagta caatggttgg
16861 atgcggtcag ccaatttaca aaagtcattg aacttaagaa gcacgttgaa aagagtaatt
16921 ccatgcctat gccttatagt attgagcatg atatgagcgc atttcaaggt agcagtatta
16981 tggcaatcta cgtacaaact gtagcgagtt atattttcaa gcacattctg ctaaaagtgt
17041 acaaagctca gtgcgatcaa aaaacgttca agattatagt acctatacac gatgcgatta
17101 tgattgaatg tgaagatgaa gaaattgcac aaaatgtggg tcagttaatg aaagatacgg
17161 ctaaccagtt gttcaatggt gaatttgcac atgtgacagt ggaagaaata ggaggcgtag
17221 accatgaata atgatagagg acaaagccta cacatcccaa gtagtacacc aatcaaagaa
17281 aataatatat atgtagctac gttacattct gtgatccaaa cagatttctc aggtgaaata
17341 aagcaccaat tcacgtatga aattgaagtg aacaatcaga ttgtatatgc gaatcgtaat
17401 attccaacaa aaccgagcgc taatcagttg tcaattcatg attggctgaa acgtcatagc
17461 aactatagcg tgggtcatga tacgtatgaa ccttatattg accgtaagca tttaatccat
17521 attggtcaat atagcggtaa ctattatgta caagatgtag catcgttaga tgcgtttgga
17581 ggcgtattat catgaatcat atattacaaa tgttatctaa gctattaagt gtggccaagg
17641 aggcaatcga ccgtcaaggt ctgattgcta tcctaactat tcctgttaat aataacgatg
17701 aaatagaaga aacggctcaa ggtgaaaccg tgtataacga acttatcgat cagttacgac
17761 ttaatatccc aaaagatacg gattatcaac ctaacatcta tagttatttt ggtattaaga
17821 agaatcctaa tgacaccgta ctcatggaaa tgatgataaa ggtttttcat atcaaacgct
17881 ttaattcaga actgtttatt ttcaaagtta acgggtggca aaagataaat ggagatgaat
17941 tacaagggtt gatatctaaa atgatacaag tattgcttgt agattataag ccttcactaa
18001 gcactctaaa aaatgtcgta gatggattgc aaaaatcaac agatgtagaa gaacttgttg
18061 agaatgagcg ctatattggt tgtggtgaaa atatgttcga tcttaatacg tttcaagtcg
18121 ttaaaaattc aatcgatatc tttccaaaaa cacgattgaa tttatcatta agtacaaatg
18181 atgtaattac tgataagata ccgccttatt ttaagcaata tatgttacaa cttgcgaatt
18241 atgacgatga tttacaatac tttcttttcc aacatacagc agtattactt acagctgata
18301 ctaaataccg taggggtctc atattatatg gcggagctaa gaatggtaaa tctgtatata
18361 ttgaactagt taaatcattt ttctatagta aagatattgt gtctaagcca cttaatgagc
18421 ttgaaggtcg ttttgacaaa gaaagtttaa ttgacaaaag tctaatggca agtcatgaaa
18481 ttgggcaatc taggattcaa gaaaagattg taaatgactt caaaaagtta ttatctgtag
18541 aatcaatgca tgttgatcgt aaaggaaaaa ctcaagtgga agtcattttg gatttgaaac
18601 ttatttttag tacaaatgcg atacttaatt ttcctcctga acatgcgaaa gctttggagc
18661 gtcgaattaa tattattcca tgtgagtatt atgttgaaaa agcggacact tcattaattg
18721 ataagctcca gagtgagaag aaagaaatct ttctttactt gatgtatgtg tatcaacaga
18781 ttgtaaaagc agatatcgag taccttgaaa atagccgtgt cactgaaatt actcacgatt
18841 ggttaaattt tggatatgaa tttgtttcta gcaggtccgt aagtattgca aatcagaaag
18901 catgtattaa tttactcaga aaacttatag aaatcaaatc aggatcacga atcaaagtat
18961 ccgagttaaa taaagttatt aatgaagaaa taaaggtgag ttctcaggtt attaatcagt
19021 taattcaagc aaactttgat actcaaacca aactatacaa tggctacgat tattggattg
19081 atttaggttg gaaagaagcc aataaaaaag agattcatga tatttcggaa aaagataata
19141 ttatttcatt agataaaaat gaaaatataa cagacgatga ggcattagat gaagagaatt

19201 tggattttga ttgggaggac tttgacgatg aataatgaac aaattgaagc atttgtagaa
19261 gtgcttgtgc ctatcataga agaacgtatc aataaaggta agtaatctaa ttacgtacta
19321 caggcaactg cctgtagtac tcatataatt aagtggtaaa agtgataaaa atgaaacgaa
19381 attataaata tatattatct atatgttgtt acaagaccga tagtctgtag caataatcta
19441 ataaaaggag aacggtatga tatgaagggt aaaattgcac tttattcacg tgttagtacg
19501 tctgagcagt cagaacatgg ttattctgta aaggagcagg aacaagtact catcaaagaa
19561 gttgtgaaaa atttcccagg ttatgactat gagacatata ctgactcagg catttcaggt
19621 aaaaatattg aaggtcgtcc ggcaatgaaa cgtctattac aagatgttaa ggataataaa
19681 atagaaatgg tattaagttg gaaattgaat cgtatctcac gatcaatgag agacgtgttt
19741 aatattattc atgaattcaa agaacatgac gtagggtata aatcgatttc tgagaatatt
19801 gatacatcca atgcttctgg agaagtactc gttacaatgt ttgggttaat aggatctata
19861 gaacgccaga ctttgatttc caatgtgaaa ctttctatga atgctaaggc aaggagcgga
19921 gaggcaatca ccggtcgtgt tttaggctac aaattatcac ttaatccact tacacagaaa
19981 aatgatttgg ttatcgatga aaatgaagct aatattgtac gtgaaatttt cgatttatat
20041 ttgaatcata ataaaggcct caaagccatt acaaccgtac ttaatcaaaa ggggtatcgt
20101 actattaatc aaaagccatt ttcagtgtat ggtgttaaat acattttgaa taatccagtc
20161 tataaaggct atgtcagatt caataaccat caaaactggg ctgtacagcg aagaagtggc
20221 aaaagtgata aaaatgatgt gatattggtc aaaggtaaac atgaagccat tataagtgaa
20281 gaggtatttg acaaagttca tgaaaaatta gcctctaaaa gttttaaacc gggcagacct
20341 attggtggag atttctactt acgtggtctt attaaatgcc cagaatgcgg aaataatatg
20401 gtatgtcgac ggacgtatta taaaacgaaa aagtccaaag aacggacaat caagcgttat
20461 tacatttgtt ccttattcaa tcgttcaggg agttctgcat gtcacagtaa ttccatcaat
20521 gctgaagtcg tcgagcgtgt aattaatgtt catttgaatc gtattctgtc tcaaccagat
20581 attatcaagc agattgcgtc aaatgtgata gaagaactga aacaaaagca tagtaaccaa
20641 acagaaatta aatatgacat tgatagttta gaaaaacaaa aagctaagct taaaacacaa
20701 caagaacgat tgttagaatt gttcttagat gatcagatgg atagcgaaat gttaaaagct
20761 aaacaaagtc aaatgaatca acagttagaa gtattagatc aacaaattaa agaagcgcaa
20821 caagcaaatc aatcacagga tgaaatacct aattttgata aattaaaagg acgactcatt
20881 ttgatgataa cacgattcag cgtgtactta agaaaggcta cacccgaagc taaaaatcaa
20941 cttatgaaaa tgttaattga ttcaattgaa attacgacag ataaacaagt aaaacttgta
21001 aggtataaaa ttgacgaaag tcttatccct caatctttga aaaaagattg ggggtctttt
21061 tttatgccca aattccaatt tgaaatatat ggtcaaaatg attatttcat cgaccaaatt
21121 accactttta ccacttagtt attagtgaca aaagtgagcg aaatgaaata aaaatcaaat
21181 atatattatc aaaatgatgt atcacgtaca tacatcaatc aaatacatta ggaggtcata
21241 atgatgacat tagaacaaca actcaagcac tatataacca acttattcaa tctgccaaag
21301 gacgaagtgt ggcactgcga atctatcgag gaaatcgctg atgatatctt acccaatcaa
21361 tatgtaagac ttggcccact cagtaataaa acacttcaga ctaataccta ctactctgac
21421 acacttcacg aaagtaatat ctatcctttc attctctact atcagaaaca actcatagcc
21481 atcggttata tcgacgaaaa tcacgatatg gatttcttat acctacacaa cactatcatg
21541 cctcttttgg atcaacgata cttactaaca ggaggacaat aaaatgcata aatacatcaa
21601 aattacacaa ttagtcatta caatactaag tgaaatcatc atttggatga aagagtcaga
21661 acgaaaggaa gtctcttatg aatagatata tcacccgggg tatcgccaac aacttaccta
21721 atatcttaca acaccaatta tggcaactcg tatctgagcg agaacaagaa caaaccaaag
21781 ataatacttc agtagattat tttcatatat tccagttcaa taagcatcgc aatcaattat

21841 atatcaaaca caaacaagaa cgacctgaat atgcgaaaat ccataaagct aattattcaa
21901 aagcaatcaa tatcaataag gtctacatta ttcgagaaga tgatgtagac ctttcttatt
21961 atgtcatgtt attacctgaa gaatactaga ggagtgaaaa ttatatggaa acaatcaaaa
22021 gtacattaaa aacagaagct atattcagtg atgacaaaca acatcgctat ctacttaaga
22081 aaacatggaa tagtgaaaaa caatcaatca caatcattac aatgtatccg cattatgatg
22141 gcattctcaa tattgactta acgacccaac tcattatgaa caaaatttca gaaatggatg
22201 catttggttc aatccatttt gtgaatctgt actctaatat tacgacacct atcaacttaa
22261 aacatttaga aaatgcctat gataaccata cagatattca aattatgaaa gcagtgaaag
22321 aatcggatga agtgatatta gcatggggtg cttacgctaa aaaaccgatt gttgaagcgc
22381 gtgtcaatga aatattagag atgttgaaaa cacataaaaa gaaagtgaag caactcatga
22441 accccgcaac taatgaaatc atgcatcccc ttaatccaaa agcaagacag aaatggactt
22501 tgaaagcgta acctatatat gaagggtgga gataaattat gaaagaaatc aatatcgttt
22561 cactacaaat gataaaaaca gatacattaa gttatctaaa agctcgtatt tcaaaccctg
22621 aggatgcgac agaaatcatg cgttcattca ttggaaacag tgaccgagaa catctcattc
22681 tcatatgtat gaacagtaaa aatgaaccta cacatattca aacactatcg attggatcta
22741 ttaaccaaac agtgattcac cctagagaaa tattcaaaac agcgatactc agtaacgcaa
22801 atagtataat gctcggtcat aatcatccaa gtggagatgt aacaccctct cctgaagata
22861 tcaatgtaac caagcgatta atgctcatca gtgaaatgat gggcatttta ttttatgacc
22921 atattatttt ctcagatagt aacacctatt ccattagaca acataattta gaagtagagg
22981 tatagccgta ttttgtcact accgttattt cttcacttat caatatttat aaaatccctt
23041 tataacaaca atttcaaatg aaaaatattt tttacaattt ttattaaata tacgtaggat
23101 tgacttattg tgacaaaccc attttgtaat atataactta aaggaggtga catattggat
23161 cgtgcatttc gaatacttac gatatacaat cgtttactac aaaataaatc cgttaacaag
23221 caatcactca cattagaact cgatacaagt cctcggacga tacaaagaga tatcgacgat
23281 ataagaaact tcttatatga aagtaaatca tggataagta caccaaaaga aatcacgtac
23341 gattataaat ctgaaagtta taagctcgaa caagataaga atgaaaatgt gcattttatg
23401 tataatattt tgactgcttt atacctgacg acacctcaat taagtcatta cttttatcaa
23461 tatcttaaat tattaatcat caaacatcat tcaaatcatc gaactacatt attaaaatat
23521 cttgagcgct ttgaaattga taagaatcaa acaggtattt cgacatcatc attagttgtt
23581 caagcaatga acgaaaataa atatcttcaa tacaatcaaa atctattatt agctttatcg
23641 atgtactatc aaatgcatgc ttttcacttg gtatatcgaa tgaacgatga aatttatatc
23701 gatgatatta atcaaatgaa tctgagtttc tctgaaaaaa ctgtggacac acatgaaaaa
23761 tcaaaggtac aaacctatat cacgtttgaa atttctaaag atgtgtggtc aaaaatgcat
23821 cgatattatc aaattcatgt gatagagaaa tatgatgtac attatcttat cgtcactttt
23881 aagatgacaa gacccgaagc gattcaactc tgttttatgt atcgatccaa tatacgtatc
23941 atttcacccc ctgatttgag agaacaagtg attgatgagt tacttttatt gcagtcaaca
24001 tatttaaaac aacaggttca agaataagca taatttacaa tattttataa attattaaat
24061 acgaaaggga ttggaattat gggcaacata ataaaaacga tagggaaagg tattgggaaa
24121 ggtgtcggtg cagcagcata ttattcagtt gcgactgttg gcgctgccat cggtgcgatt
24181 tgggaaacaa caggcgatag aggtttgatg agagatgctt taagtgatag ttctaataaa
24241 gatttgatgg aaatatatga gatacagaag aaagatgatg gtgattatga tacttatatt
24301 gcggctaaat ccttattaca agagagagga ttcacttata atcacgaaac gaataaatgg
24361 gaaaaataac agggagtgaa ataaatggaa tttgcatatt atgaagcgaa atctttaaat
24421 gattttgaaa atgaactaaa aacacttgct ttaaataaaa aatatacatt tagtaaagaa

24481 accgaagtcc ttaaagaaga agaaattctt gaaaaatcga atagccatga gcgcgaacta
24541 ttaaaagtat ttaaagataa tgcgcagata acaacgtatc attttaagag tagtcaatta
24601 agtgcctcta aaagttctat tcaacggtta atgggtatta tcattagtga aaacttagta
24661 gaatgcgtca aaatgtctga agaaaatagt gaagattctc aggcactaat taatgaagca
24721 gttgaattat tgataaaaag taaatgataa gtataaccac cattatagta atgttagtta
24781 ttggtatctt aggaggattt atttctggat tagtcggtat agggggagcc attgttattt
24841 acccggctct cttattatta ccaccattat ttggtttacc tacttatagt gcttatattg
24901 cttcaggatt aacttcaagt caggtgtttt ttagtacatt gagtggatct ttaaacgcat
24961 ataaaaataa aaatttttct agaacgctta tacttaatat gggaagtgga atggtaatag
25021 gtagtatgct aggtgcaatc ttagcgaatt taataaatgt tcagtttgtt aatacggttt
25081 atattattat tgcattacta gctcttattc taatgtttat aaaagttaca ccatctacta
25141 gtcatattaa gtttaatcgt ctacttttaa ttacaatagg tggtatcatc ggtttagtat
25201 ctggtattgt aggagctggt ggcgcattta ttattattcc agtactactt gttatattta
25261 aattaccaat gaacatggta gttacaaata gtattgtaat tgcattcatt tcttctattg
25321 gagcatttat gattaaatta ttacagggtt atattcctat taatagtgca ataccgttaa
25381 ttttaggtag tatactattt acgcctctag gtatgaaaat gggtcagaaa atacctgact
25441 atattcaaaa aacgataata tgtattttaa tcgtcattgc aattattaag ttgatatttt
25501 aaaaaaatgg gagtgggaca acattcattt tgaacttgat tgaatatata gtatatttaa
25561 taaaatgcag ctattgtggc gtagaattta agaatcaaaa aatgattaat atagtttgaa
25621 aagactgaat ataaatacta gaaaaatagc cagtaaatga gtttattatg aattcatttc
25681 ttgactcttt caaataatac tatgagaaaa atggtgcaaa acttatggtg gggtgcaggt
25741 tataatattg ttgctgtacc tttagcagct ggtattttag catttatcgg cttgatttta
25801 tcacctgcaa taggtgctat tttaatgtca ctaagtacgg ttatcgttgc aattaatgcc
25861 tttacattaa aattaaaata aaagatagga gttttattat gattaaaaaa ttatttttta
25921 tgatattagg atcattacta atattatcag cttgttccaa taatgatgaa aaagataaag
25981 acactaatga ccaaaaaagt gagagccata tgaagcataa tgatgaaagt aaagttcctg
26041 aagatatgac atcgactaat gagggtgaat ttaaagtagg agataaagta acgattacag
26101 caggacatat gccaggtatg aaaggtgcag aagctactgt aaaaggtgcg tataaaacat
26161 atgcctatgt tgtaagttat aaacccacaa atggaaatga aaaagtaaac aatcataaat
26221 gggtcgtaaa cgaagagatc aaagatgcac ctaaagatgg atttagtaag ggcgatactg
26281 ttaaattaga agcaagtcat atgtctggta tgaaaggtgc tacagccaat atagataacg
26341 tgaaaaagac gactgtttac gtagttgatt acaaatccaa agataatggt aaaatcatta
26401 aaaatcataa atggatgaca ggaaatgaac tgaaagcacg ataaaaatct agttctagat
26461 tgagaaataa atagatataa agatatcctc cttaatcaat aatttaaata acttattatt
26521 gttaaggagg atatttttta gtgtgtaaat taaaaagaat tttagaagaa taacatttat
26581 caaaaaactg ttcattacct tattaaatga aattatataa ttaaaaaccg catcattaac
26641 cgatacgcag aagcttatca taagtaatga ggttcatgat ttttgacata gttagcctcc
26701 gcagtctttc atttcaagta aataatagcg aaatattctt tatactgaat acttatagtg
26761 aagcaaagtt ctagctttga gaaaattctt tctgcaacta aatatagtaa attacggtaa
26821 aatataaata agtacatatt gaagaaaatg agacataata tattttataa taggagggaa
26881 tttcaaatga tagacaactt tatgcaggtc cttaaattaa ttaaagagaa acgtaccaat
26941 aatgtagtta aaaaatctga ttgggataaa ggtgatctat ataaaacttt agtccatgat
27001 aagttaccca agcagttaaa agtgcatata aaagaagata aatattcagt tgtagggaag
27061 gttgctactg ggaactatag taaagttcct tggatttcaa tatatgatga gaatataaca

27121 aaagaaacaa aggatggata ttatttggta tatctttttc atccggaagg agaaggcata
27181 tacttatctt tgaatcaagg atggtcaaag ataagtgata tgtttccgcg ggataaaaat
27241 gctgcaaaac aaagagcatt aactttatct tccgaactca ataaatatat tacatcaaat
27301 gaatttaata ctggaagatt ttattacgca gaaaataaag attcatctta tgatttaaaa
27361 aatgattatc catcaggata ttctcatgga tcaataagat tcaaatatta tgatttgaat
27421 gaaggattca cagaagaaga tatgctagag gatttaaaga aatttttaga actatttaat
27481 gaattagctt caaaagttac aaaaacatcc tatgatagct tggtcaatag catagacgaa
27541 atacaggaag acagcgaaat tgaagaaatt agaacagcac aaaaagataa gacactcaag
27601 gaagtggaag cacctaaagg aataattcca aaatataaaa aaggtgtatc aaagactact
27661 aaaaatgatt cagaaattga aaaatcaaat aaagagaata aattaaccgg taaagttgga
27721 gaaaaattag cgctaaatta ctttaatgag ctaattgata ataaaataga cgaagataag
27781 aaagaacagt ttaggaatat tttaaatgat aatccaggct ctcaacacgg tcatggctat
27841 gatttagtag cttttgatcc aacaaataca gataaagctg tagaaaaatt tattgaaatt
27901 aaaacatcta catcttctag tattgaggaa ccatttttta tgtcgctaaa tgaaatgttt
27961 gctatgaaag aatataagca gaaatattta atattaagaa tatttaatgt ttccggtaaa
28021 gaaccacaat tttattttat agatccatat gcaaattatt ctgaatttaa agatgtagat
28081 gatctcattg acaaagtatt taatgtagaa gctattcagt ataaagtttt tggcgaaaaa
28141 tgattacttg aacaagagct aaaataaaat tgtgatctaa taaaaataga aactgtaatt
28201 taaataaaac tttctaaata agctaactga taaaaaatca gtttgtccac agtctgaaac
28261 aagattccta tattctttag gaatcttgtt ttttctattt ttatggtgat aaagagcaga
28321 taagataatg tgtaataatc acaaaaaagt taaatatttt aaggcttgtt taattattaa
28381 tgattttata tataaagagc agtataataa agttgttaat atattatgaa taatattcaa
28441 gtaattttat tgttttttaa tttgtcgata tttaagttga gttaaattta aagggtgtaa
28501 tttgttttac aatgatgaag ataattaggg ttctgttgca aagtaaaaaa atatagctaa
28561 ccactaattt atcatgtcag tgttcgctta acttgctagc atgatgctaa tttcgtggca
28621 tggcgaaaat ccgtagatct gaagagacct gcggttcttt ttatatagag cgtaaataca
28681 ttcaatacct tttaaagtat tctttgctgt attgatactt tgataccttg tctttcttac
28741 tttaatatga cggtgatctt gctcaatgag gttattcaga tatttcgatg tacaatgaca
28801 gtcaggttta agtttaaaag ctttaattac tttagccatt gctaccttcg ttgaaggtgc
28861 ctgatctgta attacctttt gaggtttacc aaattgttta atgagacgtt tgataaacgc
28921 atatgctgaa tgattatctc gttgcttacg caaccaaata tctaatgtat gtccctctgc
28981 atcaatggca cgatataaat agctccattt tccttttatt ttgatgtacg tctcatcaat
29041 acgccatttg taataagctt ttttatgctt tttcttccaa atttgataca aaattggggc
29101 atattcttga acccaacggt agaccgttga atgatgaacg tttacaccac gttcccttaa
29161 tatttcagat atatcacgat aactcaatgt atatcttaga tagtagccaa cggctacagt
29221 gataacatcc ttgttaaatt gtttatatct gaaatagttc atacagaaga ctcctttttg
29281 ttaaaattat actataaatt caactttgca acagaaccgt attatggaat agagatgttg
29341 gtaacattta tacaggatca ttatacttaa gtttaatttc gttattacag aaccacacat
29401 tccaaccaga agagaaagta tgtctattta gttatggttc aggagcagta ggagaaatct
29461 ttagtggttc aatcgttaaa ggatatgaca aagcattaga taaagagaaa cacttaaata
29521 tgctagaatc tagagagcaa ttatcagtcg aagaatacga aacattcttt aacagatttg
29581 ataatcaaga atttgatttc gaacgtgaat tgacacaaga tccatattca aaagtatact
29641 tatacagtat agaagaccat atcagaacat ataagataga gaaataaact agtggccgat
29701 tgtgcttgat gagcttggga cataaatcct aactcgaaat aaataagcat atcactaaac

29761 tgatttttta aagtttacag tgatatgctt atttttttat cttacgattt tgtacgtgca
29821 tgcttgccta ggggtatggc tcgagccatt agtctctcgc acatactatt ccctcaggcg
29881 tcagcactta caaaatcggt tgtaattttc atttttatac gcattcttac tgagattata
29941 ctaataagag gaatagtaaa agcaattcta agtaaaattg cagataagag gtttgttaaa
30001 agcagttcta agtaaaattg cagataagag gtttgttaaa agcagttcta agtaaaattg
30061 cagataagag gtttgttaaa agcagttctc agtaaaatta cagataagag gtacgttaaa
30121 agcagttcta agtaaaattg cagataagag gtacgttaaa agcagttcta ggcaaaattg
30181 cagataagag gtgcgttaaa agcagttctc agtaaaattg ctgataaggg gtaagttaaa
30241 agcaatccta agtaaaattg cagataaggg gtacagaaaa actagacttg attacaaaat
30301 ggagcttggg acataaatga ttttttaaaa atgagatgag acgtagatta actccataat
30361 caatacgaat ctatcgactt ctttatttat gatattcatc tctttttaat ggaaataaaa
30421 gtgcgattaa tgtgataata cagttacgtt aattaaaaaa ataaaaatgc aaggagaggt
30481 aatatgctaa ctgtatatgg acatagagga ttacctagta aagctccgga aaatacaatt
30541 gcatcattta aagctgcttc agaagtagaa ggtataaact ggttggagtt agatgttgca
30601 attacaaaag atgaacaact gattatcatt catgatgatt atttagaacg gactacaaat
30661 atgtccgggg aaataactga attgaattat gatgaaatta aagatgcttc tgcaggatct
30721 tggtttggtg aaaaattcaa agatgaacat ttgccaactt tcgatgatgt agtaaaaata
30781 gcaaatgaat ataatatgaa tttaaatgta gaattaaaag gtattactgg accgaatgga
30841 ctagcacttt ctaaaagtat ggttaagcaa gtggaagaac aattaacaaa cttaaatcag
30901 aatcaagaag tgctcatttc aagctttaat gttgtgcttg ttaaacttgc agaagaaatc
30961 atgccacaat ataacagagc agttatattc catacaactt cgtttcgtga agactggaga
31021 acacttttag attactgtaa tgctaaaata gtaaacactg aagatgccaa acttactaaa
31081 gcaaaagtaa aaatggtaaa agaagcgggt tatgaattga acgtatggac tgtaaacaaa
31141 ccagcacgtg caaaccaact tgctaattgg ggagttgatg gtatctttac agacaatgca
31201 gataaaatgg tgcatttgtc tcaatagaaa gttagaggtg agtcttacgt ttcagtgacg
31261 gtagacttac ctttaacatg ttacatacta aaaaattaat ttgaataaga aagagagaca
31321 tatatgaaat acgatgattt tatagtagga gaaacattca aaacaaaaag ccttcatatt
31381 acagaagaag aaattatcca atttgcaaca acttttgatc ctcaatatat gcatatagat
31441 aaagaaaaag cagaacaaag tagatttaaa ggtatcattg catctggcat gcatacactt
31501 tcaatatcat ttaaattatg ggtagaagaa ggtaaatacg gagaagaagt tgtagcagga
31561 acacaaatga ataacgttaa atttattaaa cctgtatacc caggtaatac attgtacgtt
31621 atcgctgaaa ttacaaataa gaaatccata aaaaaagaaa atggactcgt tacagtgtca
31681 ctttcaacat acaatgaaaa tgaagaaatt gtatttaagg gagaagtaac agcacttatt
31741 aataattcat aataaaacag tgaagcaacc atcgttacgg attgcttcac tgttttgtta
31801 ttcatctata tcgtattttt tattaccgtt ctcatatagc tcatcataca ctttacctga
31861 gattttggca ttgtagctag ccattccttt atcttgtaca tctttaacat taatagccat
31921 catcatgttt ggattatctt tatcatatga tataaaccac ccaatttgtc tgccagtttc
31981 tccttgtttc attttgagtt ctgcagtacc ggatttgcca attaagtttg cataagatct
32041 ataaatatct tctttatgtg ttttatttac gacttgttgc ataccatcag ttaatagatt
32101 gatattttct ttggaaataa tatttttctt ccaaactttg tttttcgtgt cttttaataa
32161 gtgaggtgcg ttaatattgc cattattttc taatgcgcta tagattgaaa ggatctgtac
32221 tgggttaatc agtatttcac cttgtccgta acctgaatca gctaataata tttcattatc
32281 taaatttttg tttgaaattt gagcattata aaatggataa tcacttggta tatcttcacc
32341 aacacctagt tttttcatgc ctttttcaaa tttcttactg cctaattcga gtgctactct

32401 agcaaagaaa atgttatctg atgattctat tgcttgtttt aagtcgatat taccatttac
32461 cacttcatat cttgtaacgt tgtaaccacc ccaagattta tctttttgcc aacctttacc
32521 atcgatttta taacttgttt tatcgtctaa tgttttgtta tttaacccaa tcattgctgt
32581 taatattttt tgagttgaac ctggtgaagt tgtaatctgg aacttgttga gcagaggttc
32641 ttttttatct tcggttaatt tattatattc ttcgttactc atgccataca taaatggata
32701 gacgtcatat gaaggtgtgc ttacaagtgc taataattca cctgtttgag ggtggatagc
32761 agtacctgag ccataatcat ttttcatgtt gttataaata ctcttttgaa ctttagcatc
32821 aatagttagt tgaatatctt tgccatcttt tttctttttc tctattaatg tatgtgcgat
32881 tgtattgcta ttatcgtcaa cgattgtgac acgatagcca tcttcatgtt ggagcttttt
32941 atcgtaaagt ttttcgagtc cctttttacc aataactgca tcatctttat agcctttata
33001 ttctttttgt tttaattctt cagagttaat gggaccaaca taacctaata gatgtgaagt
33061 cgcttttcct agaggatagt tacgactttc tgtttcatta gttgtaagat gaaatttttt
33121 tgcgaaatca cttaaatatt catccatttt tttaacggtt ttaagtggaa cgaaggtatc
33181 atcttgtacc caattttgat ccatttgttg tttgatatag tcttcagaaa tacttagttc
33241 tttagcgatt gctttataat cttttttaga tacattcttt ggaacgatgc ctatctcata
33301 tgctgttcct gtattggcca attccacatt gtttcggtct aaaattttac cacgttctga
33361 ttttaaattt tcaatatgta tgctttggtc tttctgcatt cctggaataa tgacgctatg
33421 atcccaatct aacttccaca taccatcttc tttaacaaaa ttaaattgaa cgttgcgatc
33481 aatgttaccg tagtttgttt taattttata ttgagcatct actcgttttt tatttttaga
33541 tacttttttt attttacgat cctgaatgtt tatatcttta acgcctaaac tattatatat
33601 ttttatcgga cgttcagtca tttctacttc accattatcg cttttagaaa tataactgct
33661 atctttataa acttgtttga aatttttatc ttcaattgca tcaatagtat tattaatttc
33721 tttatctttt gaagcataaa aatatatacc aaacccgaca actacaacta ttaaaataag
33781 tggaacaatt tttatctttt tcatcaatat cctccttata taagactaca tttgtagtat
33841 attacaaatg tagtatttat gtcaaaataa tgttataatt tttgtgatat ggaggtgtag
33901 aaggtgttat catctttttt aatgttaagt ataatcagtt cattgctcac gatatgtgta
33961 attttttag tgagaatgct ctatataaaa tatactcaaa atattatgtc acataagatt
34021 tggttattag tgctcgtctc cacgttaatt ccattaatac cattttacaa aatatcgaat
34081 tttacatttt caaaagatat gatgaatcga aatgtatctg acacgacttc ttcggttagt
34141 catatgttag atggtcaaca atcatctgtt acgaaagact tagcaattaa tgttaatcag
34201 tttgagacct caaatataac gtatatgatt cttttgatat gggtatttgg tagtttgttg
34261 tgcttatttt atatgattaa ggcattccga caaattgatg ttattaaaag ttcgtcattg
34321 gaatcgtcat atcttaatga acgacttaaa gtatgtcaaa gtaagatgca gttctacaaa
34381 aagcatataa caattagtta tagttcaaac attgataatc cgatggtatt tggtttagtg
34441 aaatcccaaa ttgtactacc aactgtcgta gtcgaaacca tgaatgacaa agaaattgaa
34501 tatattattc tacatgaact atcacatgtg aaaagtcatg acttaatatt caaccagctt
34561 tatgttgttt ttaaaatgat attctggttt aatcctgcac tatatataag taaaacaatg
34621 atggacaatg actgtgaaaa agtatgtgat agaaacgttt taaaaatttt gaatcgccat
34681 gaacatatac gttatggtga atcgatatta aaatgctcta ttttaaaatc tcagcacata
34741 aataatgtgg cagcacaata tttactaggt tttaattcaa atattaaaga acgtgttaag
34801 tatattgcac tttatgattc aatgcctaaa cctaatcgaa acaagcgtat tgttgcgtat
34861 attgtatgta gtatatcgct tttaatacaa gcaccgttac tatctgcaca tgttcaacaa
34921 gacaaatatg aaacaaatgt atcatataaa aaattaaatc aactagctcc gtatttcaaa
34981 ggatttgatg gaagttttgt gctttataat gaacgggagc aagcttattc tatttataat

35041 gaaccagaaa gtaaacaacg atattcacct aattctactt acaaaattta tttagcgtta
35101 atggcattcg accaaaattt actctcatta aatcatactg aacaacaatg ggataaacat
35161 caatatccat ttaaagaatg gaaccaagat caaaatttaa attcttcaat gaaatattca
35221 gtaaattggt attacgaaaa tttaaacaaa catttaagac aagatgaggt taaatcttat
35281 ttagatctaa ttgaatatgg taatgaagaa atatcaggga atgaaaatta ttggaatgaa
35341 tcttcattaa aaatttctgc aatagaacag gttaatttgt tgaaaaatat gaaacaacat
35401 aacatgcatt ttgataataa ggctattgaa aaagttgaaa atagtatgac tttgaaacaa
35461 aaagatactt ataaatatgt aggtaaaact ggaacaggaa tcgtgaatca caaagaagca
35521 aatggatggt tcgtaggtta tgttgaaacg aaagataata cgtattattt tgctacacat
35581 ttaaaaggcg aagacaatgc gaatggcgaa aaagcacaac aaatttctga gcgtatttta
35641 aaagaaatgg agttaatata atggataata aaacgtatga aatatcatct gcagaatggg
35701 aagttatgaa tatcatttgg atgaaaaaat atgcaagtgc gaataatata atagaagaaa
35761 tacaaatgca aaaggactgg agtccaaaaa ccattcgtac acttataacg agattgtata
35821 aaaagggatt tatagatcgt aaaaaagaca ataaaatttt tcaatattac tctcttgtag
35881 aagaaagtga tataaaatat aaaacatcta aaaactttat caataaagta tacaaaggcg
35941 gtttcaattc acttgtctta aactttgtag aaaaagaaga tctatcacaa gatgaaatag
36001 aagaattgag aaatatattg aataaaaaat aaaattgttg tgtttacaac aatacataga
36061 aaacagagga aacaatcaag tcgttgaata tttcctctgt tttttagttg aaaaaattaa
36121 ccgaaagcct gaatgcaagt cttgattaaa tcaataatgc ttgtaataac accagtgaaa
36181 tccatatgca taccctcttt ctatttaaga tacattaagt ataatatcaa acaaataaaa
36241 aatgttaaaa attccctaat tggctattta gattgcataa atgtcaaaaa tttgaaaaac
36301 atacaacgac tttgcataaa aaatcgtcat attggaaata cgtaatttat tgaaataata
36361 aaaaaaataa aagaacgaag atgataacct aagtgaggtt ttaagttgtt ctaaggttta
36421 atttaattta tgttaaaata gttggtataa aaatacatga taaactataa actaaattca
36481 aaataactta tggggtaggc aattatggaa aataaaatat aaatgataat gaaaaaagag
36541 tgctaaggga aatttataac catcataata tttcgcgtac tcaaatatct aaaaatcttg
36601 agattaataa ggcaacgatt tctagtattt tgaataagtt aaagtataaa tctcttgtta
36661 atgaggttgg tgagggtgat agcacgaaga gtggtggtag aaaacctatt cttctgaagg
36721 ttaatcatct ttatggttat tttatttctt tggatttaac ttatagttct gttgaagtga
36781 tgtacaatta ttttgatggt aatgtcatta agcatgaatc ttatgattta cctgatgaaa
36841 aggttagtag tatattaagc ataataaaaa aacatattga tattcaggag aaacttgata
36901 cttataacgg actattaggt gtgtctgttt ctatacatgg agttgtggat aatgagcagc
36961 atgtgacata tttaccattc catgaaactg aaggaatttc aattgctaag aaaataaaag
37021 aaattactaa tgttccagtc gtagttgaaa atgaagcgaa tctttcagcg ttatatgaac
37081 gtaattttaa tcataattta tcctacaata atcttattgc tttaagtata cataaaggta
37141 ttggtgctgg gcttattatt aataatcaat tgtatcgtgg tgcaaatggg gaagcgggtg
37201 aaattggaaa aacacttgtc tcaaaagtta gcgataatgt ggagatcttt cataagattg
37261 aagatatttt ttcacaagaa gctttactgc ataatttaag taatcaacta aatgagaaga
37321 tgacgcttag caaattaatt caattttata atgaaaaaaa tccagtcgta gttgaagaaa
37381 tggaacaatt tataaataaa attgctgttt taatacataa tttaaacacc cagtttaatc
37441 cgaatgcaat ttacattaac tgtccattgt tcaatgaaat gcctgaaata ttagaagcaa
37501 ttaagaacca gttcaaacaa tattcacgta acgaaattca aataaagtta acatctaatg
37561 tcaaatttgc aactttgcta ggtggtacat tagcaattat ccaaaaagta ctacagatta
37621 atgatattta cttagatata aaagcataaa aaactaattc aaatgaataa tcaaagttcg

37681 taattgtctt tataaaaaaa tccctcaatc cgaattgaat tttcggattg agggattttt
37741 atagttctat tgcagaagaa aactatttta aaaatgctgg taaatgttga tagccacctc
37801 taacgttaac aatattcgta aatcctttat attctaatat tcctaccgct attgaacttc
37861 taacacctga ttgacaatgt acataaatta ggtcattttt atcgaaaggt atatcttcat
37921 ttaaaagttt accgtgagga atatgaattg cttgttttaa atgaccttta cgccattcat
37981 catcattacg aacatctaat acattatgtt cttcaccagt catttcagaa ctatgaatag
38041 atgatgtgac gatatttgtt tgtggcaaac ggtaactttt tacattttca aaaccaatta
38101 attgtaaagc atgaatagct tgttgaacgg tagatttatc gccaattaat tcaatatctt
38161 agtcataatc taaataccaa ccaatttgat ttataaaagt tttattaaaa ggaatattga
38221 tagttccatg catatgacca ccatggaatg cttctttact tcgaagatca aaagcagttt
38281 gtgtattgct tgaactaggg taaacattat atggttggta catttgcata ccaaattgat
38341 ttattttttt catttgtgaa aaatggtgtg gtggagctgg ctgattgagt gttaaagttt
38401 cgataaatga agtttcatct ttaacattaa aagcccagtt gtttattttc tcataaccca
38461 aagtagttgt aggtaatgaa cctagcgctt taccacaagg actccctgca ccatgacctg
38521 gccaaatttg aatatagtct ggtaatgttg cagcaaattg tatggactga tacatttgtt
38581 ttgctccgat ttttgtagaa ccttcaacat ttacagcttt ttctaataga tctggtctac
38641 ctacatcacc aacaaagatg aagtcaccgc taaataatcc cattggtata ctggaacccc
38701 caccttcgtc agtaagtaaa aaactaatac tctcaggggg atggcctgga gtgtgtaaga
38761 cttctaattt aatctttcct aaatagataa tatcttgatg cttaacgaaa tgtgtttgtt
38821 taggcatatt tttataatta aattcatctt taccttcatc agatacgtat atacttgcat
38881 tcaatttatt tgccacatct ctaatacctg aagcaaaatc agcatgaata tgtgtttctg
38941 cagctttagt aattgtgaat ccttctttat ctgcaacttt taaatatttt gttaaatctc
39001 gtatagggtc aataatcatt gcttctcctg tacgttgaca tccaattaaa taagatgctt
39061 gtgaaaaatt gtcttcataa aattgtttga aaaacaaaaa aaactccttt ttaaaataga
39121 ttttattgat tagataaata agttatgatt tgcttgctca gtatgtccaa tataagtacc
39181 tacgccacca taatcgactt catctcttaa ttcttctttt gaaattccca taacatccat
39241 actcatggta caagcaatta actttatatc ttgatcgatt gcttgatcga taagtgagta
39301 taaagaatca acatttttct tgttcattac ataacgcatc ataatattac ctagtccaaa
39361 catattcatt tttgataatg gcatatgtat tggatcctta ggtaacataa ggtcaaacat
39421 tttggaaata cctttctttt taacgcgagt tgattgcgct ttttttaatg cgttgaggcc
39481 ccaaaaagta aagaaaatag ttacatcttt acctgctgct ttagcgccat ttgcgatgat
39541 cattgctgct actgccttat ctaactcacc gctaaataaa acaattgttg tacctgtagc
39601 agtgtcattg atttcaaatt cttttggctt ttctttttga ataattgcat taattacatt
39661 tgcttcttca gtaagattta caagggtatt ccctgtttgt ttcgcccaac ttttaatatc
39721 actattgaaa ccaggatctg taactgttac ctcgatttgc tcacccgttg aaatattgtt
39781 aatttcttta ctgatattaa caataggtcc agggcactga agacctctaa aatcaaattg
39841 tttacgattc tctttgattt caatatcttt ttctattaaa ggagcactat tgaagttctt
39901 tgcttcataa tctttatatc cacccttttaa attcacgaca tcataacctt gtttggctaa
39961 ataatcgcaa gctttagtgc ttcggttacc gcttttacaa tgtatataat acgttttgtt
40021 gctattctta ttgaatgatt taatctcttc tactgggtgt aaagttgaac cgttaatgtg
40081 tcctaattca tattcttctt ttgttctaac atcaatcaat tgacccattt ttgccaattt
40141 ttctaattct tctttgttta atgaattaat gtgtacttct ttgtattgtt ccatacttac
40201 ctcctataaa tacctatgag ggtataataa aacggataga atcatttgcc aaatacctat
40261 aggggtattt gacaattttg ttttaattta ttattattaa cttaatcaat ttatgtggag

40321 gaaatgaata tgacttatga taaaaaaatg attaatcgta taaatagaat acaaggtcaa
40381 ttaaatggtg tcgtaaaaat gatggaagaa gaaaaagatt gcaaagatat aattacgcaa
40441 cttagtgcat ctaaaggttc tatacaacgt ttaatgggga ttataattag tgaaaattta
40501 atagaatgcg ttaaaacagc agaagaaaat aatgaaagtt ctcaagaatt aattaatgaa
40561 gcagttaatt tattagttaa aagtaaataa tggatatagc aaatatgact attatgttgc
40621 taattggcgt actgggtgga tttatatctg gattaatagg tattgggggc gcaattatta
40681 tttacccagc tattcttata ttgccaccat taataggtat acctgcgtat agtgcatata
40741 ttgcttcggg acttacctct agtcaagtat ttttcagtac acttagtgga tcattaaatg
40801 caagaaaaca accagctttc tctcctaaac ttgttatata tatgggaggg ggtatgttga
40861 ttggaagcat gttaggggca attttagcta gtttgtttaa tgctactttt gtaaatacgg
40921 tatatgtaat aatcgccata cttgctttaa tattgatgtt tattaaagtt aaacctacta
40981 cacaagagac gaaatctaaa cctttgctat ttattatagt tggatttgga attggtgtaa
41041 tttcgggaat tgtgggtgca ggtggagcat ttatcatcat tcctgtatta ttagcattat
41101 ttaaattacc aatgaatacg gtagtgaaca atagcatagc aattgctttt atatcttcag
41161 taggggcatt ttttataaaa ttaatgcaag gatatatacc agtagaaagt gcaatttttt
41221 tgataattga atgatgatca ttttctgaaa atattatgtg gtcatataat ataatgccca
41281 tcatttcact aatctctttt attctctgag ttattttgat atctcctgga gaaggtgtcg
41341 gatcttatca agagtaaatt atgaaaaaaa tggtttaaat caatgttctt actttaagta
41401 catgtgttta acgtaaatga taattagtta aatataaaaa ggttatccca atcaatagga
41461 taacctcttt atactttact ttatggtttg gattcgccac ttcaaagaag aaatgaaatc
41521 agagcattga atgttccatt ttcaaacaat atataaatac ctagcccaat gaatacaatt
41581 ggtacaatcc aacgttcata tttctcaatt gtttccgata taaaatcgaa ggaagctaga
41641 cggtaactga catagcacaa aactccaacc ataattagaa agacaatagt gacaataaag
41701 atttcagaca tacttaaggt cgtgaagtac ggtatataaa tggaaaagtc atccgcactg
41761 gaagccaata cgatgaaaat catcgtcaaa aataactgat taaattttcc agaggagaat
41821 aaagataaaa tgctactttc atcttcatcc tcttctcctt taatccatat tttcacgcct
41881 aggtaaagtg gtaaaagtcc aagtagtccg ataacccatt gctgaggaat taaatttaca
41941 accccctgtg caactaaaag acttgctcct atcacaattg cagtccctat atattgtcct
42001 atccaaatat gttttacctg accttttta cttgcgaaaa caaaagaatt aatatgacga
42061 gataatcaat tcctgttgct acatataccg cagtagccgt cagtatcgtc gcgatcattt
42121 tatcatctcc aaatatttta gggataggac ttttcttcaa aatgaaaagt ccttccgtaa
42181 attgcacaca tatagtacct atttatcctt cactctcatc agtcgcaaac tatttaatgc
42241 taccaaaata gtggctccca tatcggaaag aatcgcaatc caaagggtta gccagcctgg
42301 aataaccaat agtaaggcaa ttatcttaat tccgatggca aacgtgatgt tcgctttgat
42361 gatatttagc gttttcctgc taagtcttac tgcaaatgga agcttactta aatcatctcc
42421 cattaatgca atatcagctg tctcgatggc agtatctgtt ccagcaccgc ccattgcaat
42481 gccaacagtg gatgcagcaa gtgcaggagc atcattgacg ccatcgccaa tcatagctac
42541 attaccatgc tcggctttca tttttttaat atagtccaac ttatcctgtg gcaacaattc
42601 ggactgaata tcagaaacgc ctacatgagc accgattgct tctgcggtac cttgattatc
42661 acctgtcagc ataattgttt gcttgattcc taactgatga agttttagaa tcacattttt
42721 acttgtttcg cggacctcat ctgctacagc aatcacgccg aggattgttt ggtccgttcc
42781 aataatcatg gccgttttcc cttggttttg taaaactttc actttatttt caaactcaag
42841 gctaaaatcg gaaacattta attctttaaa aagccttgga ctgccaatgt aataggttgt
42901 tccatctata ttcccttgaa tgccccgacc tgtaatagaa gtgaagtcct tcactctaac

42961 atcggaataa gtaatattat cttgctctgc tttcttcatt attgctgaag caagtggatg
43021 ttgtgatcga tattctaaag ctgtaataat ggaaaacagc tctttttctt ccacttgatc
43081 atttaacact ttaaaatctg ttaccactgg tacacctttt gtcagtgttc ctgttttatc
43141 aaatgcgatt gccttaatgg ctcctaattc ctctagatag acaccgcctt taatcaacac
43201 acctttttta gctgcatttc caattgccga gacaatcgag attggagtag taataactaa
43261 tgcacacgga catccaacta caagtaccgc taatccttga taaacccaag tatcccaact
43321 tccaccaaag aataaaggtg gaacgactgc aacgagcgcc gcaataacca taatgatcgg
43381 cgtataatat ttcgcaaatt tatctacgaa tgcttgcgct ggagcgcgct ccccttgtgc
43441 ttcctcaacc agatgaataa tcttggagat agttgtatcc tctacgtatt tggtgatttt
43501 tacttcaagt agtccctctt cgttaagcgt gcctgcaaat acttcatcat ctaccgtttt
43561 ggcaacaggg acagattctc ctgttatagc agcctggttg acagccgaca caccatttat
43621 aatgatccca tccatggcaa ttttctcccc tggtttgacg atcataatat cacccacggc
43681 aatatcgtcc acatggatca ttatttcctg accattccgc ctaacaagtg cttcttttgg
43741 ggcaatatcc atcaatgaac gaatggactg tcttgctcta tccatagaaa aacgttcaag
43801 tgcttcactg attgcaaaga gaatgacaac aatggatgcc tctgcccatt caccaatgat
43861 ggcagctcca ataactgcaa cggtcatcag ggttttcatg tcgaaatcaa agcgtatcaa
43921 attttgaaaa ccaactttaa atagtgaata tccgccaatt acaatcgaac ttacaaataa
43981 catggacgtt acaaggttat cttctccatt tacaaagtga gaaaggtaac caaaagcaat
44041 cagtaatgtg gcaaacagca atgtgctgtg ttttttataa aacggtattt tctcttcttt
44101 aggagcctta gtgtcttctt tgaccgcttg tatcgatgga ttcgccagtt tttcaggaat
44161 taccttaaga ttctcgaaag cacctgcttt ttcaagctct tcaaccgatg cgtttccata
44221 tacatcaatt ttagaagcgc caaagttcac ttttgcatcc tgaactccag ctagttgttt
44281 tacatttttt tcaaacttcc cagcacagtt tgcgcaagaa aatccctcca cacggtaaac
44341 atttttatct tctgttacca ctggttcgac ccttcttcta gccttctctg gtgccacttt
44401 aagattctcg aaagcaccag ccttttccag atcttcaacg gttgcactgc caaagacatc
44461 aattttggaa gctccgaaat tgactttagc atcatgcacc cctgatagtt cttttacatt
44521 tttttcaaac ttcccagcac aattcgcaca cgagaaaccc tccacacggt aaacctgttt
44581 atcttctgtt aatgtttttg ttgaactatc caatactagc aacctccctt tgatgcaaga
44641 aagctttttc tacaagctgt ttaacatgct catcatctag tgaataatag actaattttc
44701 cttctttacg gtattttgct atacctaaat ttttcaataa tcttaaatga tgggatgccg
44761 tagccgttga agattcaatg atattagcta catcacaaac acataactct ccctctaaag
44821 acaaaacata agcaatttta actcttgtat catctgatag agccttaaaa actttcgcta
44881 catccatagg attctgttta gcaaggtctt ttttagccct gtttacctta tcttcatgaa
44941 tataggtaac ttcacacata tcttttgtca taattaccct ccttattcaa atgactgttt
45001 gtttgatgat tataatatat cccactataa acaaatagtc aaatgattgt ttgaatgata
45061 tataattaat attaaaaagg attggtttct aatgttagaa accaatcctt tcggagattt
45121 taaccaaatt ttaaagtatc ttaaacataa ctgccccgtt agtttaagtg catcctttca
45181 caatctgtct acagattaat aataaaaact actctttatt atacagatct ccatataatt
45241 tttgaatttg gttctgtaat tttttatttt ctttttctaa ttccataact cttcttttta
45301 aggtttttat aaggatttcc tccgaacgag aacttttctt gggttttgag actacatttg
45361 atgttatttg acgctcacga agggattcga ttctttgcct aatatcgtgt tccttataaa
45421 gccatgattt agaaacatta gcttcctttg ctattgaatt aaaattaata gctttacctt
45481 caatcgaaaa tttagaaatc gctttgtcta ctttttctct tgtcttttgt gatttctgct
45541 tcgccaaacg tacaatttct gttgtatttc taacttgttt attcattgat aattaccccg

45601 ttaaacttcc aatgatttgt tctaaacgct ctttaacacg gctattagtc tctatttgtc
45661 tttgccattg tttatccttt gctatggtta ataactcttc tgtacgctct aactgttctt
45721 cgtgctgtgg taagaattgc ttactggtac agaagtgagt gcaatctaag catgcattcg
45781 catgtggaca accacctgct attactggca atctacaata accatttgga agcacttgtg
45841 catttatatt tttcttgaac cattgaagct ctacatcatc gacttcacta tcatcatcta
45901 gattgagcac atctccatta ttggtaacca gtttttcttg aaatttagta aattcatttt
45961 ttagagtttc atcaaagata tgagcgtatc tgcttgtcat ttctgggctt tcatgtccca
46021 aaaatttctg cacaatatgc tggggcaccc cgttgttaat cattcttgtt cctactgaat
46081 ggcgaaaggc atgggcatgg aatctataaa tctcgcctaa tttatccact atattttgct
46141 cataagctaa tttatttaat tcgcctctaa atgtttcttg ttttaatggc gatccatctt
46201 ttcttggaaa gaggtattca ctatctggaa attcctctga aactttatct tcccgaactt
46261 taataagtaa aactacctct ttagatattg gaactatatg ctcctttttc attttccatt
46321 gataatactt taaaaagtaa tctccatctt tgtcctctaa taaacagcct tttttcaagg
46381 tgcacaattc acttatcctc attccacatt cttgaacaat catagtcatc gtagctatat
46441 attcgggtaa tttatcaaga tgactgttca attgctctag aacgaattcg tctataaagc
46501 gaggttttgc tcttggtatt ttcggatagt cctcgggata aattaataat ttggaaggaa
46561 catcatccca ttctagccta tgaagggtac taaatagtcc ttccaatata gagatcctcc
46621 cagttattgt actaggtttt atccccatca tgtttagttc acttaaatat gcttcaattt
46681 ccactctcgt taattggtgt actctctgaa cgtgtttaaa ctttatgtcc agaaaattaa
46741 agaactcttt aagtttttgg gcaatatcac ttacatagga aaagctatcc atgttcaatc
46801 ttaacttaca atatcttttt acaagctgtt taaaatatgt attccgaaac cctttaaagt
46861 taattgtata ttcatattgt gttgggttaa ccttatcatc tggcaaaggt aagtttcgtc
46921 tatcccaaac gtctttatcc cactcctctc catcaaaata aaaatcctca taaaactcaa
46981 taaattgttt aagattagta acatagtaag aattagcttt tacaggtgtt ttttcttgat
47041 tagcagtaat cttataatta gtggtggtaa ttctaacacc ctgttttgtc aaatatgttc
47101 tatactccgt cattgcttta tcaataggaa cttcagtaat tgaagtaatg ctgggatact
47161 ttaaatctaa aaaatccaac attttattaa ttactgttcc ttttctaatc cagacagttt
47221 ttgcattcca tattccattg tttaaatggt aaaaataaaa atatttcaat tctgttctta
47281 accacagatt tttaacacgt tcaaaacgaa cccaacgatt tcttaaagca ggattcttac
47341 ttaattctat ggcagaagga tgtggacatt ttcttatatc ccaactatta ttagcccaaa
47401 acccttgcat ttcttcattc attacagcta ttttttgct gatctcactc tgactgataa
47461 ttttcctttt actagaagca ttcatttagc tggtattctt tatctagtaa taaatacaat
47521 ggatacggct tattctccac ttctatcctt tgaaccctca cacgcgcccc cctctttaaa
47581 cactttaagt actacaaatt ataaaccttg ttaattttta tttaaagtaa aatctccctt
47641 taacatctcc tcctgaacta ctttaactac tattatttat tatactatgg ttaatatgtc
47701 tccacttggg tggttatggc caataatcat acccgttgcg ttacttaata tggctgtttt
47761 aaatatttct cgaggatgta taaccgcaaa attaatggtt cctatagaga cggtttcaat
47821 atgtgtaggt tcatttttgc tattcaagca aattaaaatg aagtgttctc ggtcagcatt
47881 tcctatgaag tcacgcataa tatcagcagc gtcttttggt tcttcaatac gtctttttaa
47941 ataccaaagt gtatcttctt taaccatttg gactttgaca acatctattc tttttctact
48001 cattatggat aagctctata ctttctatta agtcagggtg aacaatggat gtgatagaat
48061 catcattcat ataaatggaa tatagctcat ctaattctga attatttttt ttgtttgatg
48121 aaaaggcgat aatgccttta ataacatcga tgtgtggttg atgcattaaa tattggtata
48181 cttctttcca tgtttgattc attctattta gtctccttta atggtttaat tgtccatgcc

48241 tttctagcgt atggatttaa tggatgcatg atttcatttg tttgaggatt tgttaatata
48301 ctgattttct tactgtgagg ttttaacatg tctaatactt cattcactcg attttccaca
48361 agaggttttt ttccgtagga accccaagcc aataaaacac tatcggcttc tttgacagct
48421 ttcataatgt gaatatccgt gtgcttgtca tggctgtttt cgatatgttt taaattaatg
48481 ggggtatcaa tatttgaaaa aagatttata aaattaacgc caccatattc atctttttca
48541 gctactttat tgacaataag ttgtgttgta aggtcaacgt tgataacacc atcataatga
48601 ggatacatcg ttataattgt aagtacttct ttatcaatat cccaagtttt cttgagaaga
48661 taacgatgca ttttatcctg actaaatatc gcttcagttt ctaatacact tttgattgat
48721 ttcattttat ctaactcctt tatttaatat tcttcaggta acaacatgac gtaataagaa
48781 aggtctacat catcttctcg gataatgtag accttattaa tattgatagc ttttgaataa
48841 tttgccttat gaatttttac gtactctgga cgttcttgct tatgtttgat atataattga
48901 ctattatgca tattgaactg gaatatatga aagtaatcaa tctcttctag ttccttgaat
48961 tgttcgtttt cgcgttgcgc tacaagtttc cataactgtt gctgcaacat tgctggcaag
49021 taattggcaa tactacgtgt aatataacgt ttcatgctca tttctccttt tctatatctt
49081 tcattattgt aataacttct gtaatgactg tgacgcttat ccgagctatg tgtatccatt
49141 tattcattat tataattctc cttttcaagt aagtgtcttt gatctagtag tggcataact
49201 gtgttatgaa gatataagaa gtccatatcg tttgtttcat caatataacc aatcgcaata
49261 agttgttttt gatagtagag gataaagggg tatatatgcc tttcatgaag tgtgtcagag
49321 tagtaagtat acgtatgaag gattttgttg gtaagcggac caagtctgat atattgatca
49381 ggaaggatat tatctgccac ttcctcgata gattcacatt cccatttttc gtcctttggc
49441 agattgaata agttagttat atactctttg agttgttttt ctagtgtcat ggttatgacc
49501 tcctataatg tttttgatgt atacatgtaa tacaccatat agataatata tgcttcaaaa
49561 aaattttgtt tcgtttgtga atcccattgt tattaagaaa atatcccaat agaaaattat
49621 cacaattgtt gatatgacaa ggaaatccca ttatcccgct catttctcag gaaatatagc
49681 gatatattta tatatcattt ggatttggtg gtgtcagtac gacttacttt ttctttttat
49741 attatttatt tataaataat aatgggattt cgggattacg cttgcgtaat ccttatccta
49801 catgtattta tcaaatccca ttgctatccc gttcatattt tattttggga tgttctttcg
49861 tcatacaata taagcttcac attgagttgt gacttgttag tatgttgaaa agattcgtta
49921 gatgggtgtt attgaatgac gaaaggacag tacatagata ttagatttga agaaagagcg
49981 tttgaggcaa aaataaagac gaagtgctga ggagcacttc gtcgagaggg gtattattga
50041 aaagttgttt aataatttca ttattgagtt ttagtgtaac gtagaactgt tttttatgat
50101 taccgtcttt acgaatatca atacggtcaa tgactgtaag atataatgct ttgagttgtg
50161 ctttatccat tgattctata ttttgaaata ttcgttgtaa tagggcagcg atttgtttcg
50221 tatcataaga tagtttctct tgattttgtt gctgtttgag ttgattcatt tgatttgtaa
50281 tgtcattgag ttgtgtttca tattgatgaa tagttgcttt caatgcagat gttaggtccg
50341 gattatcttc aatggtttta actaaattat ggagtttccc gctgacttca tcgtattgtt
50401 gttgtttata agcgatatcg tggttcaatg caccaatatc gactttattt tcttgattga
50461 cacgttctaa gacttggtta atgactttat cacttttgac aatttcgagt atttgatcca
50521 tgacgtattt ctcaatcaca tcagctctaa cgctattcgc agaacatact tttgagcctt
50581 tgtttcggaa gttactgcaa gaataataac gtattcgctt cttggtacca tctttcaatg
50641 tgttcgttgt gttactagct gccattggtg caccacattg tggacaatga acgatacctg
50701 ttaatagatt agttccttta ccgtggactt gaggtttttg actgacttgt tttttacgta
50761 attggacttt atcccataag tcttgaataa taatagggga atgcttacct tcagctatta
50821 ttggtttatc attcagccct ttacgacgct tttcattcca atctttgtac tttgcgaatt

50881 gaattttacc aacatagaat ggattagata agatataggt cactgaacca atactgaaag
50941 gctttccttt tttagtcacg tatcctttgt gattgagtgc attcgcaatt ttacgatatc
51001 cgtggccttt agcatatgac tcaaatatat atttgacaat attcgcttca tgttggttta
51061 tcatgagttc atgcttgcta tccggtattt tgtcatagcc cagcggcaaa ttgccttgat
51121 aatagccttc ttgagcgcgt cgggtttgac ccatgaatac attttcgaca atattatttc
51181 tttcaaattc tgaaaaactc gctagaattt gtagcatcaa tttaccactg cttgtattga
51241 cttccatacg ctcagataag ctgaagaatt cgacattttg acgatgaagg tcttcaacaa
51301 ttttgagtaa gtcagaagtg ttacgtgcta gtcggtttgt tttgtagacc ataacagaat
51361 caatctgacc ttcgttcgca tcttttaaca aacgttgtag ttctggtcgg ttcatagatt
51421 ttccagagat acctctatcc gcgtatacat ctacaacaac aaagttattg aaatcacaat
51481 attctcgaat ttgattgatt tgtccatcga tactatagcc ttcagtactt tggatttccg
51541 ttgatacacg aacatagata ccgacacgtt tttgtttgag ttgttgcatg attttttcat
51601 cctttctgat taagcaatcg atgattgcga ggtttggttc acaatgttca atggttcatt
51661 tttgaaatag atcccaacaa gggctttatt ttttgtaatg cgaatttcat caatataggg
51721 atgcagcatg tttaacgtga aacttttctg tataaccttt tgtagtgatg tttgtaactg
51781 attatcactt atggaggata tggttttgtg cttttgattc atcaaatgag tctgttttct
51841 gaatgattca gcatcaatca tacctttggc aagtttatct atcagttgtt cttgagttag
51901 cgtactttta gcttctatca ctctttgttt tttgaggcgt tgatgaattg cattattaat
51961 ttttgaatag agctgttggt tttgaaagaa gttctgacat gtagctaaga cttgaacttc
52021 taattttttgt gcatttattc ctttgaatga acagacaaag cgagattcat tcatattttt
52081 aggacaaata taatatcgca atgtatgttt ttttcttatt gtcatatttg tcagtgttga
52141 gtcacaacaa ggacatttga tcttttgttt cagctgattc tctgaaggta tacagtgaag
52201 ttgcttctta ttacggattg cttgagcatg ttcatatttc gttgccgaaa caataggtgg
52261 taccatattg ttatattgac catattgatt gatgacacga ccacaataat ttgggttcat
52321 aattatattt cgtacctgat aaggcttgcg ggtaataagt ttattatcgt cttctaaata
52381 ttgtgcaatc ttcttgtagc catatccttg aagataataa ttgaatacag ctttgacggt
52441 aggtgattca tcattattaa tgatgaaagt accattttga tagtgataac cgaaaggcgc
52501 atgggtcgtt atgagtttac cttgttttgc cttttctcta agtccatttt tgacttgttc
52561 tccaatatta tcggattcaa gttcagccag actcatgaat atattgagtt ttaggcgatc
52621 aaacgcttta tccatatcaa aatagccatc atgaacactt agaatatgaa catgatattt
52681 ttgacagagt ttcatgagtt ttaatgcatt tttcaagttg cgatgaagtc gattcaagcg
52741 ataacaacat aatacgtcac attgtctttg ttggatgcgt tcggtgactt gttgatagcc
52801 tgttcgatta tctgttctgc ctgattgctt atcactaatg tattggatat tttgaatatt
52861 gtgctttgga gctaataatt ctattgcttg cttttgagct gggagtgatt gttgtttcgt
52921 tgtactttgg cgcaaataac caatggctcg tttcatagta tttcctcctt tcaaagagat
52981 aatatatatt tatgaacgaa attatagata agcccaacac cagtcgggtg ttgggcggta
53041 ttattagtca tcagcgtgat tgatttcttc aataaccaga tcggctaata gtatgattaa
53101 ttcgtccatg tatatcacct cattattcat agtattaaca acaattcttt agttgcatct
53161 caaaagtagt aacgaattcg atattcttct ttgagcaaat ttttttaaat gaatgtaatc
53221 ttcatattct ccatatgcaa caatggacgt aggtttgatt actttctcca aaatgtcatt
53281 tattaacttg tactcctcag tgtaagtgtt acttatttga cgaccttctt gtttacagca
53341 ttccttcaca gtatctaggg gtgtgctttc taagtattca aatctttcat cttcaatttc
53401 gtgagctagt gctactgtat tcatagtaca attcctccat tgatttaata tttgtgctac
53461 aacaccttta aataggtgtt gtagcaaatt tatttaataa catattttaa gctttaaaag

53521 tcaatatcac cgtttttatc ccattctctt gcttttgaaa tcatgtctcg tagtattgga
53581 tgttggtgtt cagagataaa tgataagggt atattgaatt tacttttttc agatagacca
53641 aaaataggag cgtatgattt atcaagctta agatgataaa atacgatggt ttttggtttg
53701 ccttctttgt ctttgacact acgttttgtt gttttacgat tggcgtctga ctggatgtaa
53761 tctttatccc tcagtgcatc aatgacatta tttgtatctt ggaaattatg ttcttttaac
53821 atatttttaa aaaccgaggc aatcattttc acctggatat gatcatcttc aagtgaaatc
53881 attccataat tttcaataag cgttgataac ctagtgtcgt cagaaaactt accacgattt
53941 tgtgctacaa attggacaat gacttcaatt gctttatcag ctaggcttcg ctcactaaca
54001 gaatctaaat gatagttaat aagatagtcc cttacagcat tcaaatcaac aggtgtggca
54061 atgacacgtt ccaatatacg tgctgatgtt gtaatggttg catagcgttt gaacatccga
54121 attcctgtat tgctggtttc cttttctagt tgagttttga accttaggtg ctcattttgg
54181 aaccatttaa tgacttcttg ttcacgattg agtagatatt cagctactaa tggcatgata
54241 tgaccataat tagctgatgt tgctttttta atagtatctg cgttgtcagc gttagttgta
54301 aaagcttcag atatttcaat ggtacgtaca ttcaaaccat cattacgtgc actattattg
54361 aaaatactgt gttcggcagt actaattact gaggttcccc agtttttttag tgacttgact
54421 tctccgtgta tattagaacg ttgtcttcct tgaccttcgg caattgaata gagcaaacca
54481 gttgtatctt taaaggttgc agatgacagt tcgtccaata caataggtac accatagttg
54541 ttacttagat atccttcaag cgcgtttcta gtgccattcc atgatctaaa cagcgtatta
54601 ctacctttag tagggttgcc ggttattgag actcctaaag ccgctgcagt tgatttaccc
54661 gtgcttgaat ttcccgtaaa tgagaaaatc gtacctgcga attcaacctc atgttttgtt
54721 tttaaaaacg cagttactaa tgaagaaata ccaaacacta cagcaagttc gagtaacaag
54781 tttcctttta cttcatcaag atacatttgc caccagtctt caaaagtgcc tttaggttgt
54841 agatcgtact gtgtttcaca aatgatttca tccgcctgcg actgtgtaat ttcttttgaa
54901 agatatggtt catctaatga aatgacgaca cctttatcag tatttaacac accgacaccc
54961 gtatacaacg atgacagagg cagtgattgg cgcattaatt gtagtgcatt gcttaatgat
55021 ttaatgtact tctcattgat gctgaagcca tacatgatta gtgaaggtaa cttttgcgat
55081 gttaaaatat ctgatgtttc aatacgttca atattttgtc cgttggaaat aactagtttc
55141 tcaaccccag atttggggtc aagaaacttg tttttaatta aaattggact agataagcgt
55201 atggactttt gctgatcatt tttacctttc ggtggtataa cttcatacca tgaatcttca
55261 gtaagccagt atggatattc actaaagatg atgttagtca ttagttgtta ccacctttcc
55321 agttattttg atgattcatc ggatttggtg cagttttaag gtagagcaaa gatgaacctt
55381 tttttccaac tacaatgaat ggaaggttag gtgtatattt tacaaaatgt gcaaaccaac
55441 gaccaacatc ttgttgaatt tcagtcgagc acaaaacatt agccctcaca ttcaaatcgt
55501 gatacataaa catggtgcct acaggtaaat taaatgcgat gcttgtaaca gcacgtttta
55561 gtgtataaaa ttctttttct ttcatcataa gttcctccaa ataagtctta ttagtcaatc
55621 aatactgtag atgcttaaca cctcaatggt tgattgctat aataagcata aatgaagcat
55681 tttaattaga cgcggaacaa aaaataggga gggggggtat atatgatacc tacgatagaa
55741 gaaattattg acaagtatgg cagtttagta gattgtctaa agtttgatat atcaggtgag
55801 gtttatgaag acttattgtt attaagaagt ttaagcgatt taaatgaaca ccaaaagatg
55861 catagagttt catttataca aaaacacttt aaccctaatt ttgattattt gttagatgac
55921 tcagaattta gaagtatgaa taaaatttat agaaatttaa atactatgac gtatatcaat
55981 agtcaaaaca atgaatttaa acttaaacca tttataaatg aagaacaatt aaaatcacta
56041 cttgcgataa agaaagtaga tgaacatttg agttatgacc catctattac ctcaaaagca
56101 ttagcagata taaacaagac gcaaaaaaga ttggttacaa agccaaatat tctctataat

56161 cgatccaaaa aagaaaatga gtatgtacgg ttaggaggaa aaatatcata tacaaagatg
56221 gagaagcatt gggaatattt acataatgag attcagtatt ataatcttac taatcaatta
56281 attagttatg ttgctttaga aaaggaatat gcatgggctt ttttaaatga attattttat
56341 ttaatagaat tatatttgaa ggcatttaaa ggacaaaaga aaactggcca tgaatttgaa
56401 gaaggattga acgcatttat tcaatcatat gtcattattt tacttataga tataagatta
56461 ccgactgtta ggttatatat tattaggcgt attgtggatg aatgcgaaaa agaatcagat
56521 aatcaaaaaa gaattaaact gatagaggaa tccattaaaa agtacagata tgtaaaagaa
56581 cagattgaaa gatttggaaa tgcgttgaaa gaagaattat caatcgcgac tttgtctatt
56641 gaacagaata tgttagaagc taaaattgat tattataaga attactacta tcctagaaaa
56701 aaaacaaagc ataacaatat tgagtacaac gtatcaatgt tttttaaagc tgtagattct
56761 ttaaagaaat agtattcgac ggacaatatt tatatataat aattaattag agaggtttta
56821 agttgtgttt agattaaaaa aatggtattt aaaacaaaaa ggctattttt gccgtgtcgg
56881 tgaaaagtgg gacaatagta atttatccca tgaaaatata catagttatg tagatagagt
56941 acttgaatat gagactgaaa cacgtagaaa cggccaacca actgaagaat atatattagc
57001 tagtagtggg gaaaaaataa atgaagattt aagaagtggt actaatgatc acaaagaaat
57061 tctagaggta tttgataaat ataaagtaaa agaggatata actacttata gaacggttag
57121 tcctcggata tacaaattaa tggagaaaaa tgctaaaaac ttaaatggag tagatttgtt
57181 tgaacttgga attttacaca cttcattaat aaaagggtac gaatcaagag aagaaggtta
57241 caaattacgg gtaaaagtaa aaaaaggaac accagctttc tatgtaggta atttaacagg
57301 cgaagaaagc cattattatg aggtgattgt agttaataat ttaaaattaa aaatcatatc
57361 tattgaagat tactatataa attgtgaagt agtataattg gaatcaattc tgttagttgt
57421 tcataataag ccaattattt ttttaatgaa cttattactt aaaaaccgca tcatcaactg
57481 ataagcagaa gcgtatcaca aataaaacta aaaaataagt tgtatataac ttattttgaa
57541 attggttaag tatatagtat ctccaataaa atgtagttaa cttacgataa tgctgaacta
57601 tagctttgta aactaaaatg taaataatta caatcaaatt gcaacaatat agttcaagaa
57661 tgctacaatt tgaggacaga ttgatagcat taatcccttt aaaatgaagc taggagataa
57721 cttacattat gattagtaaa caaataaagg atttacgaaa gcaacataat tatactcaag
57781 aagagctagc tgaaaaatta aatacttcaa gacaaacaat ttctaaatgg gaacaaggta
57841 tttcagaacc agacttaatt atgcttatgc aattgtcaca attattttct gttagtacag
57901 actatctcat tacaggaagt gacaatatta ttaaaaaaga taataaaagc tattatgaaa
57961 tgaatttttg ggcatttatg tctgaaaaat ggtgggtaat tattattata gtaatcataa
58021 tttgtggaac aataggacaa attttttcaa actaatgtaa gtatctctca aatattttgg
58081 gaggttttat tatgaaaatc aaaaaattat taaagacatt attaattatt ttattatgtt
58141 ttgtattgtc tgttattgtg caaaatattt caatgctatg gcatattgtg agcatttgga
58201 gtgttgaata tattttacat gcattaactt atatagtact ttcatacttt tcagtaaaat
58261 ggttcattga aattgtgctt aaaagcaata tgaaagatta tagaataata ccggtgaaat
58321 ttttcaagtc ttgttttctt ataggattaa ttttgatatt agtaattgat ttaatatatc
58381 tattatttat tcctggtaaa ttgataatac ctcattattc aacaagcgtt ggatttatgg
58441 agatgttctt tagcgcattt ttaattactg gtattgctgc gcctatattt gaagagatgg
58501 tatttcgagg aatactaatg agatactttg aaaaacaata tggaatttta tttggaatta
58561 taataccctc gattttattt tctcttgttc atctatttaa tggagagtta aagggagaaa
58621 atttattatt actacttata ggtggctcaa ttgctggaat aatgtatgca gtgactgcat
58681 ggacgtttaa tagtatatgg gcctctgcta tattacatat gctttggaat ataaatggtt
58741 tattaaatat tacaactcaa gatgatcatt ggggaatata tcagtatata ttagatacaa

58801 aaaatgtact aattacaggt ggaaactatg gtatggatac atcattactt tcaatatgta
58861 gttatattgc agttattata gttatgattg ttactaaaag acactttaac tttttactaa
58921 agtaattata gaatagtgtg gttaatcctg acgactagtc ggatacaatt taaagagtaa
58981 taaaaagtgt attattaact gatatgcaaa agtatcccac taaagaaata aaaaaacaga
59041 tggtagaata tgttagcatt taatatacaa attagttttt aacttacatg aaaactagtt
59101 caataaaaaa gttaaattag gagcttatta tgaatatcta tcaaacctat gcatgcttag
59161 tcataggaat aatcgttaat agtactacta ctatactaaa aagaaaaaaa ttaatttcaa
59221 atattaaaca actttggaaa agtaaaaaga ctttagaaga atttatacgt tcaaattcga
59281 gatttgatta ttagtttaat cttcgacgta agaattattc tgatacatta gtagatgata
59341 aaacatggat agacttagat atggatactt tgcttcataa aattaatttt aactatacag
59401 ttatcggaga aatgaactcc ctataatcta attgagaaaa tataagaaga aaaaatttaa
59461 ggttttagag tcccatgtat tattccgttc tttcttgata gatgtgtaat attttagtgg
59521 atttaataat tagtaaattt ttaacaggca taataactta caattaagaa aagattaaaa
59581 cttattaata atagagaggt tatgtgaata atgaatgatt tgttaaaaat ttatgaaaaa
59641 ttgaaaaaag atattgaaaa caggtggtta ataccatttc attatgtggt ttttggatta
59701 gcattagttg tatattttct ggaaattcct atttataaat tggtgaataa cttagataaa
59761 gagcttgttg ataaggtttt atatgctttt tcattagtgt atgatcacat tgtattgata
59821 tttatattaa ttataattat cattttgata gtttatttat tctttgatgt gtttaatatg
59881 aatcgttttg ttccatcgcc tacaacttat gtagatggta gtgaaagttc tataaactat
59941 gtaagtgcta ttaaaagatt gataaacttt atgatattaa ttatcacaaa gtactggata
60001 acttacttta ttgtaaactt aatatttcat aatgataaat tactgtattt aaacaatgat
60061 tcaaaacatt tgtataagtg tctcttgttt ttaaatatct gtatctttat agtgcatata
60121 ttaaaatcaa tttttataat aaaaattatg gcagatgatt cgctaagact tattaatgaa
60181 aatgatttaa ctgattttta cataacatta aataaaaatg atgaatatcg tatggtaaaa
60241 cctaaatata gaaaaataga taattttatt attattaaaa aaagtgaaga tttcactaat
60301 tcaaatgagt ataaagttat taataaaagt aaaaattttg aggaaataat ttatcatttt
60361 gacaatctta cttcaaatca ttgatgattt ataaattatt attctaagtt taactagaaa
60421 ttaaattttt atgcaggcca atcgatttct ttatcgatta ataaaaatgg ttatattaat
60481 aacagttttg atgcttcgcg agatgtcatt agaaacatcg aagtaagcat cttagactat
60541 aaataaagtc attg

//

LOCUS Staphylococcus 38508 bp DNA linear UNK
DEFINITION Staphylococcus Staphylococcus epidermidis strain B1265603
ACCESSION Staphylococcus
KEYWORDS .
SOURCE Staphylococcus Staphylococcus epidermidis
ORGANISM Staphylococcus Staphylococcus epidermidis
Bacteria; Bacillati; Bacillota; Bacilli; Bacillales;
Staphylococcaceae; Staphylococcus; Staphylococcus epidermidis.
FEATURES Location/Qualifiers
source 1..38508
/db_xref="taxon:176279"
/genome_id="176279.67"
/organism="Staphylococcus Staphylococcus epidermidis"

/mol_type="genomic DNA"
/genome_md5=""
/project="hwwang_176279"
CDS complement(1..252)
/transl_table=11
/product="Mobile element protein"
/db_xref="SEED:fig|176279.67.peg.1"
/translation="MAKVIKAFKLKPDCHCTSKYLNNLIEQDHRHIKVRKTRYQSINT
AKNTLKGIECIYGLYKKNRKSLQICGFSPCHEISIMLTS"
CDS complement(532..675)
/product="Mobile element protein"
/db_xref="SEED:fig|176279.67.peg.2"

/translation="MNYFRYKQFNKDVITVAVGYYLRYALSYRDMSETLRERGVNVHH
LTV"
/transl_table=11
CDS 886..2076
/transl_table=11
/db_xref="SEED:fig|176279.67.peg.3"

/translation="MKKHAYKQLLLGMIAMLIVMSISRFAYTPILPFMQQDTSMDNQE
AGFLATWNYLGYLVGAVIPIFYIYKTKVIDLKIYLALNIISTILMGFTEQYLIWSIFR
LISGITSGTVFVLTSNIVLDSLKKIQREGISGVLYSAVGLGIFLSSIYLYFFASVDSW

KMVWFVLGIVSLVLGLIVLVFMKENTNTIINFKEEKMKINNRLNKKFVIFFSIAYFFE
GAGYIITGTFLVAIVKSIPSVAEYATLSWMFVGLGAIPSTLIWSLIAEKISYKKAIYG
AFILQIISVCLPVFTHEIFSLVISSVLFGGTFLGLTTLFISKGQSLMYKTDDPLNLVS
LLTVIYSLGQMLAPMFAGILIGKSNNYNIALIFATVLLILGLISTIFSYKQNESIGEK
YELK"
/product="Uncharacterized MFS-type transporter"
CDS 2063..2929
/product="Transcriptional regulator, LysR family"
/db_xref="SEED:fig|176279.67.peg.4"

/translation="MNLNDLEAFYKVCENRSFTKAAQELNYVQSNVTAKIQRLESYYQ
VKLLYRDKKGIKPTPEGIELLKYIKRIFTILHEAEHNVSNNKKLNINIGTIETIAAVH

LPKLLNEYKRKNPKIQINLKTDNSIKLIEHVKNREMDCAFISGNINDKDLICTNIFNE

KLVILSQKSNFKQNINENKSIIMFGEGCFYRKIFEDWLNYHDINIENKMTLNTLDGIL

GCVEAGLGIAFLPMSVLNKGNSDGIEITKVEESFQTISLNIIYRQDNKNNNVINQLKQ
VAFEKMKSNIIK"
/transl_table=11

CDS 2942..4012
/transl_table=11
/translation="MNLTTQITKMLGIKYPIIQAGMAGSTTPELVSTVSKQGGLGMIG

AGYFDSHQLSKEILEVQKLTQAPFAVNLFTPNDIKYDKKQIEQMNTKLKPYREALGLS
TPKNSTVKEKEKFEDAIEVIESLKVPIIAFTFGIPNQNIIKRLHNAGKILIGTATSVE

EAVENENAGMDIVVAQGYEAGGHRGSFTTINGEFPLVGTLSLVPQIVDNVSIPVIAAG

GIMDGRGLVASLALGAGAAQLGTAYLTTNESGADDKIKNEIIESSETDTILTNVFSGK

LARGIMNEFVHNMNLYSKQVPPYPLQNQLTTQIRKSALEKGYTEWTHIWSGQSTRLAD
TVDAAQLTKNIINDAVKIINNK"
/db_xref="SEED:fig|176279.67.peg.5"
/product="Putative oxidoreductase YrpB, nitronate
monooxygenase family"
CDS 4177..4536
/db_xref="SEED:fig|176279.67.peg.6"
/translation="MENTNSNMHKNDNSYVLSALSYLSIFFAPVILPLFVWILADEPT
SGHGKKALGNHIMTWISFCVGRGIFIFSKGVFDKPFDHQLLIFVIALIIAIVFFLIAL
ILYILNIIRGIKILLKK"
/product="hypothetical protein"
/transl_table=11
CDS complement(4774..5547)
/transl_table=11
/translation="MNDLLKIYEKLKKDIENRWLIPFHYVVFGLALVVYFLEIPIYKL
VNNLDKELVDKVLYAFSLVYDHIVLIFILIIIIILIVYLFFDVFNMNRFVPSPTTYVD
GSESSINYVSAIKRLINFMILIITKYWITYFIVNLIFHNDKLLYLNNDSKHLYKCLLF
LNICIFIVHILKSIFIIKIMADDSLRLINENDLTDFYITLNKNDEYRMVKPKYRKIDN
FIIIKKSEDFTNSNEYKVINKSKNFEEIIYHFDNLTSNH"
/db_xref="SEED:fig|176279.67.peg.7"
/product="hypothetical protein"
CDS complement(5864..6028)
/product="MutS-related protein, family 1"
/db_xref="SEED:fig|176279.67.peg.8"
/db_xref="GO:0003684"
/db_xref="GO:0005524"
/db_xref="GO:0006298"
/translation="MNIYQTYACLVIGIIVNSTTTILKRKKLISNIKQLWKSKKTLEE
FIRSNSRFDY"
/transl_table=11
CDS complement(6233..7066)
/translation="MKIKKLLKTLLIILLCFVLSVIVQNISMLWHIVSIWSVEYILHA
LTYIVLSYFSVKWFIEIVLKSNMKDYRIIPVKFFKSCFLIGLILILVIDLIYLLFIPG

KLIIPHYSTSVGFMEMFFSAFLITGIAAPIFEEMVFRGILMRYFEKQYGILFGIIIPS

ILFSLVHLFNGELKGENLLLLLIGGSIAGIMYAVTAWTFNSIWASAILHMLWNINGLL

NITTQDDHWGIYQYILDTKNVLITGGNYGMDTSLLSICSYIAVIIVMIVTKRHFNFLL
K"
/db_xref="SEED:fig|176279.67.peg.9"
/product="hypothetical protein"
/transl_table=11
CDS complement(7103..7429)
/transl_table=11
/product="Transcriptional regulator, Xre family"
/translation="MISKQIKDLRKQHNYTQEELAEKLNTSRQTISKWEQGISEPDLI

MLMQLSQLFSVSTDYLITGSDNIIKKDNKSYYEMNFWAFMSEKWWVIIIIVIIICGTI
GQIFSN"
/db_xref="SEED:fig|176279.67.peg.10"
CDS complement(7761..8333)
/product="FIG01109033: hypothetical protein"
/db_xref="SEED:fig|176279.67.peg.11"

/translation="MFRLKKWYLKQKGYFCRVGEKWDNSNLSHENIHSYVDRVLEYET

ETRRNGQPTEEYILASSGEKINEDLRSGTNDHKEILEVFDKYKVKEDITTYRTVSPRI

YKLMEKNAKNLNGVDLFELGILHTSLIKGYESREEGYKLRVKVKKGTPAFYVGNLTGE
ESHYYEVIVVNNLKLKIISIEDYYINCEVV"
/transl_table=11
CDS complement(8386..9435)
/product="ORFID:MW0042"
/db_xref="SEED:fig|176279.67.peg.12"
/translation="MIPTIEEIIDKYGSLVDCLKFDISGEVYEDLLLLRSLSDLNEHQ

KMHRVSFIQKHFNPNFDYLLDDSEFRSMNKIYRNLNTMTYINSQNNEFKLKPFINEEQ

LKSLLAIKKVDEHLSYDPSITSKALADINKTQKRLVTKPNILYNRSKKENEYVRLGGK

ISYTKMEKHWEYLHNEIQYYNLTNQLISYVALEKEYAWAFLNELFYLIELYLKAFKGQ
KKTGHEFEEGLNAFIQSYVIILLIDIRLPTVRLYIIRRIVDECEKESDNQKRIKLIEE

SIKKYRYVKEQIERFGNALKEELSIATLSIEQNMLEAKIDYYKNYYYPRKKTKHNNIE
YNVSMFFKAVDSLKK"
/transl_table=11
CDS 9573..9857

/translation="MKEKEFYTLKRAVTSIAFNLPVGTMFMYHDLNVRANVLCSTEIQ
QDVGRWFAHFVKYTPNLPFIVVGKKGSSLLYLKTAPNPMNHQNNWKGGNN"
/db_xref="SEED:fig|176279.67.peg.13"
/product="FIG01108228: hypothetical protein"
/transl_table=11
CDS 9857..11644
/product="hypothetical protein"
/db_xref="SEED:fig|176279.67.peg.14"
/translation="MTNIIFSEYPYWLTEDSWYEVIPPKGKNDQQKSIRLSSPILIKN
KFLDPKSGVEKLVISNGQNIERIETSDILTSQKLPSLIMYGFSINEKYIKSLSNALQL

MRQSLPLSSLYTGVGVLNTDKGVVISLDEPYLSKEITQSQADEIICETQYDLQPKGTF

EDWWQMYLDEVKGNLLLELAVVFGISSLVTAFLKTKHEVEFAGTIFSFTGNSSTGKST

AAALGVSITGNPTKGSNTLFRSWNGTRNALEGYLSNNYGVPIVLDELSSATFKDTTGL

LYSIAEGQGRQRSNIHGEVKSLKNWGTSVISTAEHSIFNNSARNDGLNVRTIEISEAF

TTNADNADTIKKATSANYGHIMPLVAEYLLNREQEVIKWFQNEHLRFKTQLEKETSNT
GIRMFKRYATITTSARILERVIATPVDLNAVRDYLINYHLDSVSERSLADKAIEVIVQ

FVAQNRGKFSDDTRLSTLIENYGMISLEDDHIQVKMIASVFKNMLKEHNFQDTNNVID

ALRDKDYIQSDANRKTTKRSVKDKEGKPKTIVFYHLKLDKSYAPIFGLSEKSKFNIPL
SFISEHQHPILRDMISKAREWDKNGDIDF"
/transl_table=11
CDS 11734..12009
/transl_table=11
/db_xref="SEED:fig|176279.67.peg.15"
/translation="MNTVALAHEIEDERFEYLESTPLDTVKECCKQEGRQISNTYTEE
YKLINDILEKVIKPTSIVAYGEYEDYIHLKKFAQRRISNSLLLLRCN"
/product="hypothetical protein"
CDS 12202..13548
/transl_table=11
/translation="MKRAIGYLRQSTTKQQSLPAQKQAIELLAPKHNIQNIQYISDKQ

SGRTDNRTGYQQVTERIQQRQCDVLCCYRLNRLHRNLKNALKLMKLCQKYHVHILSVH

DGYFDMDKAFDRLKLNIFMSLAELESDNIGEQVKNGLREKAKQGKLITTHAPFGYHYQ

NGTFIINNDESPTVKAVFNYYLQGYGYKKIAQYLEDDNKLITRKPYQVRNIIMNPNYC

GRVINQYGQYNNMVPPIVSATKYEHAQAIRNKKQLHCIPSENQLKQKIKCPCCDSTLT

NMTIRKKHTLRYYICPKNMNESRFVCSFKGINAQKLEVQVLATCQNFFQNQQLYSKIN

NAIHQRLKKQRVIEAKSTLTQEQLIDKLAKGMIDAESFRKQTHLMNQKHKTISSISDN
QLQTSLQKVIQKSFTLNMLHPYIDEIRITKNKALVGIYFKNEPLNIVNQTSQSSIA"
/db_xref="SEED:fig|176279.67.peg.16"
/product="Cassette chromosome recombinase A"
CDS 13569..15197
/product="Site-specific recombinase"
/translation="MQQLKQKRVGIYVRVSTEIQSTEGYSIDGQINQIREYCDFNNFV

VVDVYADRGISGKSMNRPELQRLLKDANEGQIDSVMVYKTNRLARNTSDLLKIVEDLH

RQNVEFFSLSERMEVNTSSGKLMLQILASFSEFERNNIVENVFMGQTRRAQEGYYQGN

LPLGYDKIPDSKHELMINQHEANIVKYIFESYAKGHGYRKIANALNHKGYVTKKGKPF

SIGSVTYILSNPFYVGKIQFAKYKDWNEKRRKGLNDKPIIAEGKHSPIIIQDLWDKVQ

LRKKQVSQKPQVHGKGTNLLTGIVHCPQCGAPMAASNTTNTLKDGTKKRIRYYSCSNF

RNKGSKVCSANSVRADVIEKYVMDQILEIVKSDKVINQVLERVNQENKVDIGALNHDI

AYKQQQYDEVSGKLHNLVKTIEDNPDLTSALKATIHQYETQLNDITNQMNQLKQQQNQ

EKLSYDTKQIAALLQRIFQNIESMDKAQLKALYLTVIDRIDIRKDGNHKKQFYVTLKL
NNEIIKQLFNNTPLDEVLLSTSSLFLPQTLFLQI"
/db_xref="SEED:fig|176279.67.peg.17"
/transl_table=11
CDS 15668..16018
/translation="MTLEKQLKEYITNLFNLPKDEKWECESIEEVADNILPDQYIRLG
PLTNKILHTYTYYSDTLHERHIYPFILYYQKQLIAIGYIDETNDMDFLYLHNTVMPLL
DQRHLLEKENYNNE"
/db_xref="SEED:fig|176279.67.peg.18"
/product="FIG01108299: hypothetical protein"
/transl_table=11
CDS 16011..16100
/product="hypothetical protein"
/translation="MNKWIHIARISVTVITEVITIMKDIEKEK"
/db_xref="SEED:fig|176279.67.peg.19"
/transl_table=11
CDS 16103..16414
/db_xref="SEED:fig|176279.67.peg.20"

/translation="MKRYITRSIANYLPAMLQQQLWKLVAQRENEQFKELEEIDYFHI

FQFNMHNSQLYIKHKQERPEYVKIHKANYSKAININKVYIIREDDVDLSYYVMLLPEE
Y"
/product="FIG01107894: hypothetical protein"
/transl_table=11
CDS 16433..16954
/transl_table=11
/db_xref="SEED:fig|176279.67.peg.21"
/translation="MKSIKSVLETEAIFSQDKMHRYLLKKTWDIDKEVLTIITMYPHY

DGVINVDLTTQLIVNKVAEKDEYGGVNFINLFSNIDTPINLKHIENSHDKHTDIHIMK

AVKEADSVLLAWGSYGKKPLVENRVNEVLDMLKPHSKKISILTNPQTNEIMHPLNPYA
RKAWTIKPLKETK"
/product="hypothetical protein"
CDS 16956..17162
/product="FIG01110849: hypothetical protein"
/db_xref="SEED:fig|176279.67.peg.22"
/translation="MNQTWKEVYQYLMHQPHIDVIKGIIAFSSNKKNNSELDELYSIY
MNDDSITSIVHPDLIESIELIHNE"
/transl_table=11
CDS 17155..17478
/transl_table=11

/translation="MSRKRIDVVKVQMVKEDTLWYLKRRIEEPKDAADIMRDFIGNAD
REHFILICLNSKNEPTHIETVSIGTINFAVIHPREIFKTAILSNATGMIIGHNHPSGD
ILTIV"
/db_xref="SEED:fig|176279.67.peg.23"
/product="DNA repair protein RadC truncated by transposon
Tn554"
CDS 17673..19565
/transl_table=11

/translation="MNASSKRKIISQSEISKKIAVMNEEMQGFWANNSWDIRKCPHPS

AIELSKNPALRNRWVRFERVKNLWLRTELKYFYFYHLNNGIWNAKTVWIRKGTVINKM

LDFLDLKYPSITSITEVPIDKAMTEYRTYLTKQGVRITTTNYKITANQEKTPVKANSY

YVTNLKQFIEFYEDFYFDGEEWDKDVWDRRNLPLPDDKVNPTQYEYTINFKGFRNTYF

KQLVKRYCKLRLNMDSFSYVSDIAQKLKEFFNFLDIKFKHVQRVHQLTRVEIEAYLSE
LNMMGIKPSTITGRISILEGLFSTLHRLEWDDVPSKLLIYPEDYPKIPRAKPRFIDEF

VLEQLNSHLDKLPEYIATMTMIVQECGMRISELCTLKKGCLLEDKDGDYFLKYYQWKM

KKEHIVPISKEVVLLIKVREDKVSEEFPDSEYLFPRKDGSPLKQETFRGELNKLAYEQ

NIVDKLGEIYRFHAHAFRHSVGTRMINNGVPQHIVQKFLGHESPEMTSRYAHIFDETL

KNEFTKFQEKLVTNNGDVLNLDDDSEVDDVELQWFKKNINAQVLPNGYCRLPVIAGGC

PHANACLDCTHFCTSKQFLPQHEEQLERTEELLTIAKDKQWQRQIETNSRVKERLEQI
IGSLTG"
/db_xref="SEED:fig|176279.67.peg.24"
/product="Transposase B from transposon Tn554"
CDS 19572..19949
/transl_table=11
/db_xref="SEED:fig|176279.67.peg.25"
/translation="MNKQVRNTTEIVRLAKQKSQKTREKVDKAISKFSIEGKAINFNS

IAKEANVSKSWLYKEHDIRQRIESLRERQITSNVVSKPKKSSRSEEILIKTLKRRVME
LEKENKKLQNQIQKLYGDLYNKE"
/product="Mobile element protein"
CDS complement(19959..20066)
/db_xref="SEED:fig|176279.67.peg.26"
/translation="MLETNPFGDFNQILKYLKHNCPVSLSASFHNLSTD"
/product="hypothetical protein"
/transl_table=11
CDS 20187..20552

/translation="MTKDMCEVTYIHEDKVNRAKKDLAKQNPMDVAKVFKALSDDTRV
KIAYVLSLEGELCVCDVANIIESSTATASHHLRLLKNLGIAKYRKEGKLVYYSLDDEH
VKQLVEKAFLHQREVASIG"
/db_xref="SEED:fig|176279.67.peg.27"
/product="Cadmium efflux system accessory protein"
/transl_table=11
CDS 20545..22959
/db_xref="SEED:fig|176279.67.peg.28"

/translation="MDSSTKTLTEDKQVYRVEGFSCANCAGKFEKNVKELSGVHDAKV

NFGASKIDVFGSATVEDLEKAGAFENLKVAPEKARRRVEPVVTEDKNVYRVEGFSCAN

CAGKFEKNVKQLAGVQDAKVNFGASKIDVYGNASVEELEKAGAFENLKVIPEKLANPS
IQAVKEDTKAPKEEKIPFYKKHSTLLFATLLIAFGYLSHFVNGEDNLVTSMLFVSSIV

IGGYSLFKVGFQNLIRFDFDMKTLMTVAVIGAAIIGEWAEASIVVILFAISEALERFS

MDRARQSIRSLMDIAPKEALVRRNGQEIMIHVDDIAVGDIMIVKPGEKIAMDGIIING

VSAVNQAAITGESVPVAKTVDDEVFAGTLNEEGLLEVKITKYVEDTTISKIIHLVEEA

QGERAPAQAFVDKFAKYYTPIIMVIAALVAVVPPLFFGGSWDTWVYQGLAVLVVGCPC
ALVITTPISIVSAIGNAAKKGVLIKGGVYLEELGAIKAIAFDKTGTLTKGVPVVTDFK

VLNDQVEEKELFSIITALEYRSQHPLASAIMKKAEQDNITYSDVRVKDFTSITGRGIQ

GNIDGTTYYIGSPRLFKELNVSDFSLEFENKVKVLQNQGKTAMIIGTDQTILGVIAVA

DEVRETSKNVILKLHQLGIKQTIMLTGDNQGTAEAIGAHVGVSDIQSELLPQDKLDYI

KKMKAEHGNVAMIGDGVNDAPALAASTVGIAMGGAGTDTAIETADIALMGDDLSKLPF
AVRLSRKTLNIIKANITFAIGIKIIALLLVIPGWLTLWIAILSDMGATILVALNSLRL
MRVKDK"
/product="Cadmium-transporting ATPase (EC 3.6.3.3)"
/transl_table=11
/EC_number="3.6.3.3"
CDS 22971..23144
/translation="MQFTEGLFILKKSPIPKIFGDDKMIATILTATAVYVATGIDYLV
ILILLFSQVKKVR"
/db_xref="SEED:fig|176279.67.peg.29"
/product="Cadmium resistance protein"
/transl_table=11
CDS 23183..23656
/product="Cadmium resistance protein"
/translation="MIGASLLVAQGVVNLIPQQWVIGLLGLLPLYLGVKIWIKGEEDE
DESSILSLFSSGKFNQLFLTMIFIVLASSADDFSIYIPYFTTLSMSEIFIVTIVFLIM
VGVLCYVSYRLASFDFISETIEKYERWIVPIVFIGLGIYILFENGTFNALISFLL"
/db_xref="SEED:fig|176279.67.peg.30"
/transl_table=11
CDS complement(23924..24568)
/transl_table=11
/translation="MDIANMTIMLLIGVLGGFISGLIGIGGAIIIYPAILILPPLIGI

PAYSAYIASGLTSSQVFFSTLSGSLNARKQPAFSPKLVIYMGGGMLIGSMLGAILASL
FNATFVNTVYVIIAILALILMFIKVKPTTQETKSKPLLFIIVGFGIGVISGIVGAGGA
FIIIPVLLALFKLPMNTVVNNSIAIAFISSVGAFFIKLMQGYIPVESAIFLIIE"
/db_xref="SEED:fig|176279.67.peg.31"
/product="FIG003846: hypothetical protein"
CDS complement(24568..24828)

/transl_table=11

/translation="MTYDKKMINRINRIQGQLNGVVKMMEEEKDCKDIITQLSASKGS
IQRLMGIIISENLIECVKTAEENNESSQELINEAVNLLVKSK"
/db_xref="SEED:fig|176279.67.peg.32"
/product="Metal-sensitive transcriptional repressor"
CDS 24964..26028
/translation="MEQYKEVHINSLNKEELEKLAKMGQLIDVRTKEEYELGHINGST

LHPVEEIKSFNKNSNKTYYIHCKSGNRSTKACDYLAKQGYDVVNLKGGYKDYEAKNFN
SAPLIEKDIEIKENRKQFDFRGLQCPGPIVNISKEINNISTGEQIEVTVTDPGFNSDI

KSWAKQTGNTLVNLTEEANVINAIIQKEKPKEFEINDTATGTTIVLFSGELDKAVAAM

IIANGAKAAGKDVTIFFTFWGLNALKKAQSTRVKKKGISKMFDLMLPKDPIHMPLSKM

NMFGLGNIMMRYVMNKKNVDSLYSLIDQAIDQDIKLIACTMSMDVMGISKEELRDEVD
YGGVGTYIGHTEQANHNLFI"
/db_xref="SEED:fig|176279.67.peg.33"
/product="Disulfide bond regulator"
/transl_table=11
CDS 26061..26999
/product="Zn-dependent hydroxyacylglutathione hydrolase /
Polysulfide binding protein"
/db_xref="SEED:fig|176279.67.peg.34"
/translation="MFFKQFYEDNFSQASYLIGCQRTGEAMIIDPIRDLTKYLKVADK

EGFTITKAAETHIHADFASGIRDVANKLNASIYVSDEGKDEFNYKNMPKQTHFVKHQD
IIYLGKIKLEVLHTPGHPPESISFLLTDEGGGSSIPMGLFSGDFIFVGDVGRPDLLEK

AVNVEGSTKIGAKQMYQSIQFAATLPDYIQIWPGHGAGSPCGKALGSLPTTTLGYEKI

NNWAFNVKDETSFIETLTLNQPAPPHHFSQMKKINQFGMQMYQPYNVYPSSSNTQTAF
DLRSKEAFHGGHMHGTINIPFNKTFINQIGWYLDYD"
/transl_table=11
CDS 27090..27395
/transl_table=11
/product="Polysulfide binding protein"

/translation="MPQTNIVTSSIHSSEMTGEEHNVLDVRNDDEWRKGHLKQAIHIP

HGKLLNEDIPFDKNDLIYVHCQSGVRSSIAVGILEYKGFTNIVNVRGGYQHLPAFLK"
/db_xref="SEED:fig|176279.67.peg.35"
CDS complement(27509..28618)

/product="Putative ROK-family transcriptional regulator"
/db_xref="SEED:fig|176279.67.peg.36"
/translation="MLREIYNHHNISRTQISKNLEINKATISSILNKLKYKSLVNEVG

EGDSTKSGGRKPILLKVNHLYGYFISLDLTYSSVEVMYNYFDGNVIKHESYDLPDEKV
SSILSIIKKHIDIQEKLDTYNGLLGVSVSIHGVVDNEQHVTYLPFHETEGISIAKKIK

EITNVPVVVENEANLSALYERNFNHNLSYNNLIALSIHKGIGAGLIINNQLYRGANGE

AGEIGKTLVSKVSDNVEIFHKIEDIFSQEALLHNLSNQLNEKMTLSKLIQFYNEKNPV

VVEEMEQFINKIAVLIHNLNTQFNPNAIYINCPLFNEMPEILEAIKNQFKQYSRNEIQ
IKLTSNVKFATLLGGTLAIIQKVLQINDIYLDIKA"
/transl_table=11
CDS complement(29126..29497)
/product="Methicillin resistance repressor MecI"

/translation="MDNKTYEISSAEWEVMNIIWMKKYASANNIIEEIQMQKDWSPKT
IRTLITRLYKKGFIDRKKDNKIFQYYSLVEESDIKYKTSKNFINKVYKGGFNSLVLNF
VEKEDLSQDEIEELRNILNKK"
/db_xref="SEED:fig|176279.67.peg.37"
/db_xref="GO:0003677"
/db_xref="GO:0016481"
/db_xref="GO:0016566"
/transl_table=11
CDS complement(29497..31254)
/transl_table=11
/product="Methicillin resistance regulatory
sensor-transducer MecR1"
/translation="MLSSFLMLSIISSLLTICVIFLVRMLYIKYTQNIMSHKIWLLVL

VSTLIPLIPFYKISNFTFSKDMMNRNVSDTTSSVSHMLDGQQSSVTKDLAINVNQFET

SNITYMILLIWVFGSLLCLFYMIKAFRQIDVIKSSSLESSYLNERLKVCQSKMQFYKK

HITISYSSNIDNPMVFGLVKSQIVLPTVVVETMNDKEIEYIILHELSHVKSHDLIFNQ

LYVVFKMIFWFNPALYISKTMMDNDCEKVCDRNVLKILNRHEHIRYGESILKCSILKS

QHINNVAAQYLLGFNSNIKERVKYIALYDSMPKPNRNKRIVAYIVCSISLLIQAPLLS

AHVQQDKYETNVSYKKLNQLAPYFKGFDGSFVLYNEREQAYSIYNEPESKQRYSPNST

YKIYLALMAFDQNLLSLNHTEQQWDKHQYPFKEWNQDQNLNSSMKYSVNWYYENLNKH

LRQDEVKSYLDLIEYGNEEISGNENYWNESSLKISAIEQVNLLKNMKQHNMHFDNKAI

EKVENSMTLKQKDTYKYVGKTGTGIVNHKEANGWFVGYVETKDNTYYFATHLKGEDNA
NGEKAQQISERILKEMELI"
/db_xref="SEED:fig|176279.67.peg.38"
/db_xref="GO:0008658"
CDS 31354..33360
/product="Penicillin-binding protein PBP2a, methicillin resistance determinant MecA, transpeptidase"
/translation="MKKIKIVPLILIVVVVGFGIYFYASKDKEINNTIDAIEDKNFKQ

VYKDSSYISKSDNGEVEMTERPIKIYNSLGVKDINIQDRKIKKVSKNKKRVDAQYKIK

TNYGNIDRNVQFNFVKEDGMWKLDWDHSVIIPGMQKDQSIHIENLKSERGKILDRNNV

ELANTGTAYEIGIVPKNVSKKDYKAIAKELSISEDYIKQQMDQNWVQDDTFVPLKTVK

KMDEYLSDFAKKFHLTTNETESRNYPLGKATSHLLGYVGPINSEELKQKEYKGYKDDA

VIGKKGLEKLYDKKLQHEDGYRVTIVDDNSNTIAHTLIEKKKKDGKDIQLTIDAKVQK

SIYNNMKNDYGSGTAIHPQTGELLALVSTPSYDVYPFMYGMSNEEYNKLTEDKKEPLL

NKFQITTSPGSTQKILTAMIGLNNKTLDDKTSYKIDGKGWQKDKSWGGYNVTRYEVVN

GNIDLKQAIESSDNIFFARVALELGSKKFEKGMKKLGVGEDIPSDYPFYNAQISNKNL

DNEILLADSGYGQGEILINPVQILSIYSALENNGNINAPHLLKDTKNKVWKKNIISKE

NINLLTDGMQQVVNKTHKEDIYRSYANLIGKSGTAELKMKQGETGRQIGWFISYDKDN
PNMMMAINVKDVQDKGMASYNAKISGKVYDELYENGNKKYDIDE"
/db_xref="SEED:fig|176279.67.peg.39"
/db_xref="GO:0008658"
/db_xref="GO:0046677"
/transl_table=11
CDS complement(33406..33834)
/transl_table=11
/db_xref="SEED:fig|176279.67.peg.40"
/translation="MKYDDFIVGETFKTKSLHITEEEIIQFATTFDPQYMHIDKEKAE

QSRFKGIIASGMHTLSISFKLWVEEGKYGEEVVAGTQMNNVKFIKPVYPGNTLYVIAE
ITNKKSIKKENGLVTVSLSTYNENEEIVFKGEVTALINNS"
/product="MaoC domain protein"

CDS complement(33931..34674)
/transl_table=11
/EC_number="3.1.4.46"
/translation="MLTVYGHRGLPSKAPENTIASFKAASEVEGINWLELDVAITKDE

QLIIIHDDYLERTTNMSGEITELNYDEIKDASAGSWFGEKFKDEHLPTFDDVVKIANE

YNMNLNVELKGITGPNGLALSKSMVKQVEEQLTNLNQNQEVLISSFNVVLVKLAEEIM

PQYNRAVIFHTTSFREDWRTLLDYCNAKIVNTEDAKLTKAKVKMVKEAGYELNVWTVN
KPARANQLANWGVDGIFTDNADKMVHLSQ"
/db_xref="SEED:fig|176279.67.peg.41"
/db_xref="GO:0008889"
/product="Glycerophosphoryl diester phosphodiesterase (EC 3.1.4.46)"
CDS complement(35143..35373)
/product="hypothetical protein"
/translation="MLIFLSYDFVRACLPRGMARAISLSHILFPQASALTKSVVIFIF
IRILTEIILIRGIVKAILSKIADKRFVKSSSK"
/db_xref="SEED:fig|176279.67.peg.42"
/transl_table=11
CDS complement(35471..35638)
/transl_table=11
/EC_number="2.3.3.10"
/translation="MLESREQLSVEEYETFFNRFDNQEFDFERELTQDPYSKVYLYSI
EDHIRTYKIEK"
/db_xref="SEED:fig|176279.67.peg.43"
/product="Hydroxymethylglutaryl-CoA synthase N-domain homolog (EC 2.3.3.10)"
CDS 35896..36570
/product="Transposase for insertion sequence-like element IS431mec"
/translation="MNYFRYKQFNKDVITVAVGYYLRYTLSYRDISEILRERGVNVHH

STVYRWVQEYAPILYQIWKKKHKKAYYKWRIDETYIKIKGKWSYLYRAIDAEGHTLDI

WLRKQRDNHSAYAFIKRLIKQFGKPQKVITDQAPSTKVAMAKVIKAFKLKPDCHCTSK
YLNNLIEQDHRHIKVRKTRYQSINTAKNTLKGIECIYALYKKNRRSLQIYGFSPCHEI
SIMLAS"
/db_xref="SEED:fig|176279.67.peg.44"
/transl_table=11
CDS complement(37015..38271)
/transl_table=11
/product="hypothetical protein"

/db_xref="SEED:fig|176279.67.peg.45"

/translation="MIDNFMQVLKLIKEKRTNNVVKKSDWDKGDLYKTLVHDKLPKQL

KVHIKEDKYSVVGKVATGNYSKVPWISIYDENITKETKDGYYLVYLFHPEGEGIYLSL

NQGWSKISDMFPRDKNAAKQRALTLSSELNKYITSNEFNTGRFYYAENKDSSYDLKND

YPSGYSHGSIRFKYYDLNEGFTEEDMLEDLKKFLELFNELASKVTKTSYDSLVNSIDE
IQEDSEIEEIRTAQKDKTLKEVEAPKGIIPKYKKGVSKTTKNDSEIEKSNKENKLTGK

VGEKLALNYFNELIDNKIDEDKKEQFRNILNDNPGSQHGHGYDLVAFDPTNTDKAVEK
FIEIKTSTSSSIEEPFFMSLNEMFAMKEYKQKYLILRIFNVSGKEPQFYFIDPYANYS
EFKDVDDLIDKVFNVEAIQYKVFGEK"

ORIGIN

1 ttaacttgtt agcatgatgc taatttcatg gcatggcgaa aatccgcaga tctgaagaga
61 cttgcggttc tttttatata gaccgtaaat acattcaata ccttttaaag tattctttgc
121 cgtattgata ctttgatatc ttgtctttct tactttaata tgacggtgat cttgctcaat
181 gaggttattc agatatttcg atgtacaatg acagtcaggt ttaagtttaa aagctttaat
241 gactttagcc attgcgacct tcgttgaagg tgcctaatct gtaattacct tttgaggttt
301 accaaattgt ttaatgagac gtttgataaa tacatatgct gaatgattat ctcgttgctt
361 acgcaaccaa atatctaatg tatgtccctc tgcatcaatg gcacgatata aatagttcca
421 ttttcctttt attttgatgt acgtctcatc aatacgccat ttgtaataag cttttttatg
481 ctttttcttc caaatttgat ataaaatcgg ggcatattct tgaactcaac gctagaccgt
541 taaatgatga acgtttacac cacgttccct taatgtttca gacatatcac gataactcaa
601 tgcatatctt agatagtagc caacggctac agtgataaca tccttgttaa attgtttata
661 tctgaaatag ttcatacaaa agactccttt ttattaaaat tatactataa attcaacttt
721 gcaacagaac cacttaaaga catgtttgtc atacatattt gtaaaaatca aaaaatcatg
781 gttatataca tcattatttt tgataataat tatcttaaat aatcattttt attaatataa
841 caaattgact atactaaaat taagttgaaa tgaaaggata attatatgaa aaaacatgca
901 tataaacaac ttttattagg tatgatagca atgttaatcg ttatgtctat cagtaggttt
961 gcttatacac ctatattacc atttatgcaa caagatacat ctatggacaa tcaagaagcg
1021 gggtttttag ctacatggaa ttatttaggg tatttagttg gcgctgttat tccaatattt
1081 tatatttata aaactaaagt aatagatttg aaaatttacc ttgctttaaa tattatttct
1141 actatcttaa tgggatttac agaacaatat ttaatttggt caatttttcg attgatttcc
1201 gggataacaa gcgggacggt atttgtatta acttcaaaca tagtactaga ttcattaaag
1261 aaaatacaaa gagaggggat atcaggtgta ttatatagtg ctgttggatt aggtattttt
1321 ttaagtagta tttatcttta ttttttgcc agtgtggatt catggaaaat ggtttggttt
1381 gttttaggaa tagtttcact ggttttgggc ttaattgtat tggtttttat gaaagaaaat
1441 acaaatacaa taataaattt taaagaagaa aaaatgaaaa ttaataatag actaaacaaa
1501 aagtttgtta tcttttttc tattgcttac ttctttgaag gggcaggtta tattattact
1561 ggtacttttc tagttgctat agttaaatca attccttctg ttgctgaata tgcgacttta
1621 agctggatgt ttgttggatt aggtgcaatt ccttctacat taatttggtc acttatagct
1681 gaaaaaatca gttataaaaa agccatttat ggtgcattta ttttacaaat tataagcgtt

1741 tgtctacctg tttttactca tgaaattttt agtttagtta ttagttcagt tctatttgga
1801 gggacttttt taggtttaac tacattattt atttctaaag ggcaaagttt aatgtacaaa
1861 acagatgacc ccttaaattt agtctcatta ctgactgtaa tatacagttt aggacaaatg
1921 ttagctccta tgtttgcggg aatcttaatt ggtaaatcga ataattataa cattgcttta
1981 atatttgcaa ctgttttatt aatactgggt cttatttcta caatatttag ctataaacaa
2041 aacgaaagta ttggtgaaaa atatgaactt aaatgactta gaagcttttt ataaagtctg
2101 cgaaaatcgt agctttacta aagcagcgca agaactgaat tatgttcaat caaatgtaac
2161 agctaaaatt caaagattag agtcttatta tcaagtgaaa ttactatata gagataaaaa
2221 aggaattaaa ccgacaccag aaggtattga attactaaaa tatattaaga gaatattcac
2281 aattttacat gaagcagaac ataatgtaag caacaataaa aaattaaata ttaacatagg
2341 gacaattgaa acaatagctg cagttcattt acctaaatta ttgaatgaat ataagaggaa
2401 aaatccaaaa attcaaatta atttaaaaac agataatagt attaaattaa ttgaacatgt
2461 aaagaataga gaaatggatt gtgcttttat atctgggaat attaacgata aagatcttat
2521 ttgcacaaat atctttaatg aaaaattagt gattttatct caaaagagca actttaaaca
2581 gaatataaac gaaaataagt ctattataat gtttggcgaa ggttgttttt atagaaaaat
2641 ttttgaagat tggcttaatt atcatgatat taatatagaa aataaaatga cattaaatac
2701 tttagatggc attttggggt gtgttgaagc tggattaggt atagcatttt tacctatgtc
2761 agtattaaat aaaggaaata gcgatggtat agaaataacc aaagtagagg aatcatttca
2821 aacaatatca ctaaatatca tttatcgaca agataataaa aataataatg tgataaatca
2881 attgaaacaa gttgcttttg agaaaatgaa atcaaatata ataaaataga aggtgttaat
2941 tatgaattta actactcaaa tcacaaaaat gttaggtatt aaatatccta ttatacaagc
3001 tggaatggca ggaagtacta cgccagaact agtttctact gttagtaaac aaggaggctt
3061 aggtatgata ggagcaggtt attttgattc acatcaactt tcgaaagaga ttttagaagt
3121 tcaaaaactg acacaagctc cttttgcagt aaatctattt acacccaatg atattaagta
3181 cgataaaaaa caaattgagc agatgaatac aaaactcaaa ccatatagag aagcgcttgg
3241 attatctact cccaaaaata gtacggttaa agaaaaagaa aagtttgaag atgcaataga
3301 agttattgaa tcattaaaag tacctatcat tgcttttact tttggtattc caaatcaaaa
3361 tattattaaa cgacttcata atgcaggaaa aattttaatt ggaactgcaa catcagtcga
3421 agaagcagtt gaaaatgaaa atgctggtat ggatatagtt gtagcccagg gctatgaagc
3481 aggtggccac agaggatcat ttacaacaat caatggtgaa tttccattgg tcggtacttt
3541 atctttagta ccacaaattg ttgataatgt gtctatacca gttattgcag ctggtggtat
3601 tatggatgga agaggtttag tagctagttt agctttgggg gctggagcag cgcaattagg
3661 tactgcttat ttaacaacga atgaaagtgg cgctgatgac aaaatcaaaa atgaaattat
3721 tgaaagctct gaaacagata caatactgac aaatgtattt agtggcaaat tggctcgcgg
3781 aattatgaat gaatttgtac acaatatgaa tttatattca aaacaggtgc ctccttatcc
3841 tttacaaaat caattaacaa cgcaaatacg aaaatctgct ttggagaaag gatatactga
3901 gtggacccat atttggagtg gacaaagtac tagattagct gacacagtcg atgcagctca
3961 attaacaaaa aatattatta atgatgcagt caaaattatc aataataagt gataaagctt
4021 ttgaggctgg ttatcttttt cttctaccat gtgccaaagt aagatttgaa ggtctagtgg
4081 aatactttca ttattgtaag tttctcttat aactgttgta atagctcgct attaatgttt
4141 ttataataat aaagaaaagt aaagaaggtg aaaaaagtgg aaaatacaaa tagtaatatg
4201 cataaaaatg acaatagtta cgttttatca gcattgagtt atttaagtat ctttttcgca
4261 cctgtcattt tacctttatt tgtatggata cttgctgatg agccaacatc aggacatggt
4321 aaaaaagcat tagggaatca tattatgaca tggattagtt tttgtgtagg cagaggaatt

4381 tttatttttt caaaaggagt ctttgataaa ccgtttgatc atcaattatt aatatttgtt
4441 attgctttaa tcattgctat tgtattcttt ttaattgctt taatattata tattctcaac
4501 attatcagag gaattaaaat tttattaaaa aaataagtga cttatatatc aacttattaa
4561 gggagttcta tactcccttt tttaatgcaa ttgtttttat aaacaatgac tttatttata
4621 gtctaagatg cttacttcga tgtttctaat gacatctcgc gaagcatcaa aactgttatt
4681 aatataacca tttttattaa tcgataaaga aatcgattgg cctgcataaa aatttaattt
4741 ctagttaaac ttagaataat aatttataaa tcatcaatga tttgaagtaa gattgtcaaa
4801 atgataaatt atttcctcaa aatttttact tttattaata actttatact catttgaatt
4861 agtgaaatct tcactttttt taataataat aaaattatct atttttctat atttaggttt
4921 taccatacga tattcatcat ttttatttaa tgttatgtaa aaatcagtta aatcattttc
4981 attaataagt cttagcgaat catctgccat aatttttatt ataaaaattg attttaatat
5041 atgcactata aagatacaga tatttaaaaa caagagacac ttatacaaat gttttgaatc
5101 attgtttaaa tacagtaatt tatcattatg aaatattaag tttacaataa agtaagttat
5161 ccagtacttt gtgataatta atatcataaa gtttatcaat cttttaatag cacttacata
5221 gtttatagaa ctttcactac catctacata agttgtaggc gatggaacaa aacgattcat
5281 attaaacaca tcaaagaata aataaactat caaaatgata attataatta atataaatat
5341 caatacaatg tgatcataca ctaatgaaaa agcatataaa accttatcaa caagctcttt
5401 atctaagtta ttcaccaatt tataaatagg aatttccaga aaatatacaa ctaatgctaa
5461 tccaaaaacc acataatgaa atggtattaa ccacctgttt tcaatatctt ttttcaattt
5521 ttcataaatt tttaacaaat cattcattat tcacataacc tctctattat taataagttt
5581 taatcttttc ttaattgtaa gttattatgc ctgttaaaaa tttactaatt attaaatcca
5641 ctaaaatatt acacatctat caagaaagaa cggaataata catgggactc taaaacctta
5701 aattttttct tcttatattt tctcaattag attataggga gttcatttct ccgataactg
5761 tatagttaaa attaatttta tgaagcaaag tatccatatc taagtctatc catgttttat
5821 catctactaa tgtatcagaa taattcttac gtcgaagatt aaactaataa tcaaatctcg
5881 aatttgaacg tataaattct tctaaagtct ttttactttt ccaaagttgt ttaatatttg
5941 aaattaattt ttttcttttt agtatagtag tagtactatt aacgattatt cctatgacta
6001 agcatgcata ggtttgatag atattcataa taagctccta atttaacttt tttattgaac
6061 tagttttcat gtaagttaaa aactaatttg tatattaaat gctaacatat tctaccatct
6121 gtttttttat ttctttagtg ggatactttt gcatatcagt taataataca ctttttatta
6181 ctctttaaat tgtatccgac tagtcgtcag gattaaccac actattctat aattacttta
6241 gtaaaaagtt aaagtgtctt ttagtaacaa tcataactat aataactgca atataactac
6301 atattgaaag taatgatgta tccataccat agtttccacc tgtaattagt acatttttttg
6361 tatctaatat atactgatat attcccccaat gatcatcttg agttgtaata tttaataaac
6421 catttatatt ccaaagcata tgtaatatag cagaggccca tatactatta aacgtccatg
6481 cagtcactgc atacattatt ccagcaattg agccacctat aagtagtaat aataaatttt
6541 ctccctttaa ctctccatta aatagatgaa caagagaaaa taaaatcgag ggtattataa
6601 ttccaaataa aattccatat tgtttttcaa agtatctcat tagtattcct cgaaatacca
6661 tctcttcaaa tataggcgca gcaataccag taattaaaaa tgcgctaaag aacatctcca
6721 taaatccaac gcttgttgaa taatgaggta ttatcaattt accaggaata aataatagat
6781 atattaaatc aattactaat atcaaaatta atcctataag aaaacaagac ttgaaaaatt
6841 tcaccggtat tattctataa tctttcatat tgcttttaag cacaatttca atgaaccatt
6901 ttactgaaaa gtatgaaagt actatataag ttaatgcatg taaaatatat tcaacactcc
6961 aaatgctcac aatatgccat agcattgaaa tattttgcac aataacagac aatacaaaac

7021 ataataaaat aattaataat gtctttaata atttttgat tttcataata aaacctccca
7081 aaatatttga gagatactta cattagtttg aaaaaatttg tcctattgtt ccacaaatta
7141 tgattactat aataataatt acccaccatt tttcagacat aaatgcccaa aaattcattt
7201 cataatagct tttattatct tttttaataa tattgtcact tcctgtaatg agatagtctg
7261 tactaacaga aaataattgt gacaattgca taagcataat taagtctggt tctgaaatac
7321 cttgttccca tttagaaatt gtttgtcttg aagtatttaa tttttcagct agctcttctt
7381 gagtataatt atgttgcttt cgtaaatcct ttatttgttt actaatcata atgtaagtta
7441 tctcctagct tcattttaaa gggattaatg ctatcaatct gtcctcaaat tgtagcattc
7501 ttgaactata ttgttgcaat ttgattgtaa ttatttacat tttagtttac aaagctatag
7561 ttcagcatta tcgtaagtta actacatttt attggagata ctatatactt aaccaatttc
7621 aaaataagtt atatacaact tattttttag ttttatttgt gatacgcttc tgcttatcag
7681 ttgatgatgc ggtttttaag taataagttc attaaaaaaa taattggctt attatgaaca
7741 actaacagaa ttgattccaa ttatactact tcacaattta tatagtaatc ttcaatagat
7801 atgattttta attttaaatt attaactaca atcacctcat aataatggct ttcttcgcct
7861 gttaaattac ctacatagaa agctggtgtt cctttttta cttttacccg taatttgtaa
7921 ccttcttctc ttgattcgta cccttttatt aatgaagtgt gtaaaattcc aagttcaaac
7981 aaatctactc catttaagtt tttagcattt ttctccatta atttgtatat ccgaggacta
8041 accgttctat aagtagttat atcctctttt actttatatt tatcaaatac ctctagaatt
8101 tctttgtgat cattagtacc acttcttaaa tcttcattta tttttcccc actactagct
8161 aatatatatt cttcagttgg ttggccgttt ctacgtgttt cagtctcata ttcaagtact
8221 ctatctacat aactatgtat attttcatgg gataaattac tattgtccca cttttcaccg
8281 acacggcaaa aatagccttt ttgttttaaa taccattttt ttaatctaaa cacaacttaa
8341 aacctctcta attaattatt atatataaat attgtccgtc gaatactatt tctttaaaga
8401 atctacagct ttaaaaaaca ttgatacgtt gtactcaata ttgttatgct ttgttttttt
8461 tctaggatag tagtaattct tataataatc aattttagct tctaacatat tctgttcaat
8521 agacaaagtc gcgattgata attcttcttt caacgcattt ccaaatcttt caatctgttc
8581 ttttacatat ctgtactttt taatggattc ctctatcagt ttaattcttt tttgattatc
8641 tgattctttt tcgcattcat ccacaatacg cctaataata tataacctaa cagtcggtaa
8701 tcttatatct ataagtaaaa taatgacata tgattgaata aatgcgttca atccttcttc
8761 aaattcatgg ccagttttct tttgtccttt aaatgccttc aaatataatt ctattaaata
8821 aaataattca tttaaaaaag cccatgcata ttccttttct aaagcaacat aactaattaa
8881 ttgattagta agattataat actgaatctc attatgtaaa tattcccaat gcttctccat
8941 ctttgtatat gatatttttc ctcctaaccg tacatactca ttttcttttt tggatcgatt
9001 atagagaata tttggctttg taaccaatct tttttgcgtc ttgtttatat ctgctaatgc
9061 ttttgaggta atagatgggt cataactcaa atgttcatct actttcttta tcgcaagtag
9121 tgattttaat tgttcttcat ttataaatgg tttaagttta aattcattgt tttgactatt
9181 gatatacgtc atagtattta aatttctata aattttattc atacttctaa attctgagtc
9241 atctaacaaa taatcaaaat tagggttaaa gtgtttttgt ataaatgaaa ctctatgcat
9301 cttttggtgt tcatttaaat cgcttaaact tcttaataac aataagtctt cataaacctc
9361 acctgatata tcaaacttta gacaatctac taaactgcca tacttgtcaa taatttcttc
9421 tatcgtaggt atcatatata cccccctcc ctatttttg ttccgcgtct aattaaaatg
9481 cttcatttat gcttattata gcaatcaacc attgaggtgt taagcatcta cagtattgat
9541 tgactaataa gacttatttg gaggaactta tgatgaaaga aaaagaattt tatacactaa
9601 aacgtgctgt tacaagcatc gcatttaatt tacctgtagg caccatgttt atgtatcacg

9661 atttgaatgt gagggctaat gttttgtgct cgactgaaat tcaacaagat gttggtcgtt
9721 ggtttgcaca ttttgtaaaa tatacaccta accttccatt cattgtagtt ggaaaaaaag
9781 gttcatcttt gctctacctt aaaactgcac caaatccgat gaatcatcaa aataactgga
9841 aaggtggtaa caactaatga ctaacatcat ctttagtgaa tatccatact ggcttactga
9901 agattcatgg tatgaagtta taccaccgaa aggtaaaaat gatcagcaaa agtccatacg
9961 cttatctagt ccaattttaa ttaaaaacaa gtttcttgac cccaaatctg gggttgagaa
10021 actagttatt tccaacggac aaaatattga acgtattgaa acatcagata ttttaacatc
10081 gcaaaagtta ccttcactaa tcatgtatgg cttcagcatc aatgagaagt acattaaatc
10141 attaagcaat gcactacaat taatgcgcca atcactgcct ctgtcatcgt tgtatacggg
10201 tgtcggtgtg ttaaatactg ataaaggtgt cgtcatttca ttagatgaac catatctttc
10261 aaaagaaatt acacagtcgc aggcggatga aatcatttgt gaaacacagt acgatctaca
10321 acctaaaggc acttttgaag actggtggca aatgtatctt gatgaagtaa aaggaaactt
10381 gttactcgaa cttgctgtag tgtttggtat ttcttcatta gtaactgcgt ttttaaaaac
10441 aaaacatgag gttgaattcg caggtacgat tttctcattt acgggaaatt caagcacggg
10501 taaatcaact gcagcggctt taggagtctc aataaccggc aaccctacta aaggtagtaa
10561 tacgctgttt agatcatgga atggcactag aaacgcgctt gaaggatatc taagtaacaa
10621 ctatggtgta cctattgtat tggacgaact gtcatctgca acctttaaag atacaactgg
10681 tttgctctat tcaattgccg aaggtcaagg aagacaacgt tctaatatac acggagaagt
10741 caagtcacta aaaaactggg gaacctcagt aattagtact gccgaacaca gtattttcaa
10801 taatagtgca cgtaatgatg gtttgaatgt acgtaccatt gaaatatctg aagcttttac
10861 aactaacgct gacaacgcag atactattaa aaaagcaaca tcagctaatt atggtcatat
10921 catgccatta gtagctgaat atctactcaa tcgtgaacaa gaagtcatta aatggttcca
10981 aaatgagcac ctaaggttca aaactcaact agaaaaggaa accagcaata caggaattcg
11041 gatgttcaaa cgctatgcaa ccattacaac atcagcacgt atattggaac gtgtcattgc
11101 cacacctgtt gatttgaatg ctgtaaggga ctatcttatt aactatcatt tagattctgt
11161 tagtgagcga agcctagctg ataaagcaat tgaagtcatt gtccaatttg tagcacaaaa
11221 tcgtggtaag ttttctgacg acactaggtt atcaacgctt attgaaaatt atggaatgat
11281 ttcacttgaa gatgatcata tccaggtgaa aatgattgcc tcggttttta aaaatatgtt
11341 aaaagaacat aatttccaag atacaaataa tgtcattgat gcactgaggg ataaagatta
11401 catccagtca gacgccaatc gtaaaacaac aaaacgtagt gtcaaagaca aagaaggcaa
11461 accaaaaacc atcgtatttt atcatcttaa gcttgataaa tcatacgctc ctatttttgg
11521 tctatctgaa aaaagtaaat tcaatatacc cttatcattt atctctgaac accaacatcc
11581 aatactacga gacatgattt caaaagcaag agaatgggat aaaaacggtg atattgactt
11641 ttaaagctta aaatatgtta ttaaataaat ttgctacaac acctatttaa aggtgttgta
11701 gcacaaatat taaatcaatg gaggaattgt actatgaata cagtagcact agctcacgaa
11761 attgaagatg aaagatttga atacttagaa agcacacccc tagatactgt gaaggaatgc
11821 tgtaaacaag aaggtcgtca aataagtaac acttacactg aggagtacaa gttaataaat
11881 gacattttgg agaaagtaat caaacctacg tccattgttg catatggaga atatgaagat
11941 tacattcatt taaaaaaatt tgctcaaaga agaatatcga attcgttact acttttgaga
12001 tgcaactaaa gaattgttgt taatactatg aataatgagg tgatatacat ggacgaatta
12061 atcatactat tagccgatct ggttattgaa gaaatcaatc acgctgatga ctaataatac
12121 cgcccaacac ccgactggtg ttgggcttat ctataatttc gttcataaat atatattatc
12181 tctttgaaag gaggaaatac tatgaaacga gccattggtt atttgcgcca aagtacaacg
12241 aaacaacaat cactcccagc tcaaaagcaa gcaatagaat tattagctcc aaagcacaat

12301 attcaaaata tccaatacat tagtgataag caatcaggca gaacagataa tcgaacaggc
12361 tatcaacaag tcaccgaacg catccaacaa agacaatgtg acgtattatg ttgttatcgc
12421 ttgaatcgac ttcatcgcaa cttgaaaaat gcattaaaac tcatgaaact ctgtcaaaaa
12481 tatcatgttc atattctaag tgttcatgat ggctattttg atatggataa agcgtttgat
12541 cgcctaaaac tcaatatatt catgagtctg gctgaacttg aatccgataa tattggagaa
12601 caagtcaaaa atggacttag agaaaaggca aaacaaggta aactcataac gacccatgcg
12661 cctttcggtt atcactatca aaatggtact ttcatcatta ataatgatga atcacctacc
12721 gtcaaagctg tattcaatta ttatcttcaa ggatatggct acaagaagat tgcacaatat
12781 ttagaagacg ataataaact tattacccgc aagccttatc aggtacgaaa tataattatg
12841 aacccaaatt attgtggtcg tgtcatcaat caatatggtc aatataacaa tatggtacca
12901 cctattgttt cggcaacgaa atatgaacat gctcaagcaa tccgtaataa gaagcaactt
12961 cactgtatac cttcagagaa tcagctgaaa caaaagatca aatgtccttg ttgtgactca
13021 acactgacaa atatgacaat aagaaaaaaa catacattgc gatattatat ttgtcctaaa
13081 aatatgaatg aatctcgctt tgtctgttca ttcaaaggaa taaatgcaca aaaattagaa
13141 gttcaagtct tagctacatg tcagaacttc tttcaaaacc aacagctcta ttcaaaaatt
13201 aataatgcaa ttcatcaacg cctcaaaaaa caaagagtga tagaagctaa aagtacgcta
13261 actcaagaac aactgataga taaacttgcc aaaggtatga ttgatgctga atcattcaga
13321 aaacagactc atttgatgaa tcaaaagcac aaaaccatat cctccataag tgataatcag
13381 ttacaaacat cactacaaaa ggttatacag aaaagtttca cgttaaacat gctgcatccc
13441 tatattgatg aaattcgcat tacaaaaaat aaagcccttg ttgggatcta tttcaaaaat
13501 gaaccattga acattgtgaa ccaaacctcg caatcatcga ttgcttaatc agaaaggatg
13561 aaaaaatcat gcaacaactc aaacaaaaac gtgtcggtat ctatgttcgt gtatcaacgg
13621 aaatccaaag tactgaaggc tatagtatcg atggacaaat caatcaaatt cgagaatatt
13681 gtgatttcaa taactttgtt gttgtagatg tatacgcgga tagaggtatc tctggaaaat
13741 ctatgaaccg accagaacta caacgtttgt taaaagatgc gaacgaaggt cagattgatt
13801 ctgttatggt ctacaaaaca aaccgactag cacgtaacac ttctgactta ctcaaaattg
13861 ttgaagacct tcatcgtcaa aatgtcgaat tcttcagctt atctgagcgt atggaagtca
13921 atacaagcag tggtaaattg atgctacaaa ttctagcgag tttttcagaa tttgaaagaa
13981 ataatattgt cgaaaatgta ttcatgggtc aaacccgacg cgctcaagaa ggctattatc
14041 aaggcaattt gccgctgggc tatgacaaaa taccggatag caagcatgaa ctcatgataa
14101 accaacatga agcgaatatt gtcaaatata tatttgagtc atatgctaaa ggccacggat
14161 atcgtaaaat tgcgaatgca ctcaatcaca aaggatacgt gactaaaaaa ggaaagcctt
14221 tcagtattgg ttcagtgacc tatatcttat ctaatccatt ctatgttggt aaaattcaat
14281 tcgcaaagta caaagattgg aatgaaaagc gtcgtaaagg gctgaatgat aaaccaataa
14341 tagctgaagg taagcattcc cctattatta ttcaagactt atgggataaa gtccaattac
14401 gtaaaaaaca agtcagtcaa aaacctcaag tccacggtaa aggaactaat ctattaacag
14461 gtatcgttca ttgtccacaa tgtggtgcac caatggcagc tagtaacaca acgaacacat
14521 tgaaagatgg taccaagaag cgaatacgtt attattcttg cagtaacttc cgaaacaaag
14581 gctcaaaagt atgttctgcg aatagcgtta gagctgatgt gattgagaaa tacgtcatgg
14641 atcaaatact cgaaattgtc aaaagtgata aagtcattaa ccaagtctta gaacgtgtca
14701 atcaagaaaa taaagtcgat attggtgcat tgaaccacga tatcgcttat aaacaacaac
14761 aatacgatga agtcagcggg aaactccata atttagttaa aaccattgaa gataatccgg
14821 acctaacatc tgcattgaaa gcaactattc atcaatatga aacacaactc aatgacatta
14881 caaatcaaat gaatcaactc aaacagcaac aaaatcaaga gaaactatct tatgatacga

14941 aacaaatcgc tgccctatta caacgaatat ttcaaaatat agaatcaatg gataaagcac
15001 aactcaaagc attatatctt acagtcattg accgtattga tattcgtaaa gacggtaatc
15061 ataaaaaaca gttctacgtt acactaaaac tcaataatga aattattaaa caacttttca
15121 ataatacccc tctcgacgaa gtgctcctca gcacttcgtc tttatttttg cctcaaacgc
15181 tctttcttca aatctaatat ctatgtactg tcctttcgtc attcaataac acccatctaa
15241 cgaatctttt caacatacta acaagtcaca actcaatgtg aagcttatat tgtatgacga
15301 aagaacatcc caaaataaaa tatgaacggg atagcaatgg gatttgataa atacatgtag
15361 gataaggatt acgcaagcgt aatcccgaaa tcccattatt atttataaat aaataatata
15421 aaaagaaaaa gtaagtcgta ctgacaccac caaatccaaa tgatatataa atatatcgct
15481 atatttcctg agaaatgagc gggataatgg gatttccttg tcatatcaac aattgtgata
15541 attttctatt gggatatttt cttaataaca atgggattca caaacgaaac aaaatttttt
15601 tgaagcatat attatctata tggtgtatta catgtataca tcaaaaacat tataggaggt
15661 cataaccatg acactagaaa aacaactcaa agagtatata actaacttat tcaatctgcc
15721 aaaggacgaa aaatgggaat gtgaatctat cgaggaagtg gcagataata tccttcctga
15781 tcaatatatc agacttggtc cgcttaccaa caaaatcctt catacgtata cttactactc
15841 tgacacactt catgaaaggc atatataccc ctttatcctc tactatcaaa aacaacttat
15901 tgcgattggt tatattgatg aaacaaacga tatggacttc ttatatcttc ataacacagt
15961 tatgccacta ctagatcaaa gacacttact tgaaaaggag aattataata atgaataaat
16021 ggatacacat agctcggata agcgtcacag tcattacaga agttattaca ataatgaaag
16081 atatagaaaa ggagaaatga gcatgaaacg ttatattaca cgtagtattg ccaattactt
16141 gccagcaatg ttgcagcaac agttatggaa acttgtagcg caacgcgaaa acgaacaatt
16201 caaggaacta gaagagattg attactttca tatattccag ttcaatatgc ataatagtca
16261 attatatatc aaacataagc aagaacgtcc agagtacgta aaaattcata aggcaaatta
16321 ttcaaaagct atcaatatta ataaggtcta cattatccga gaagatgatg tagacctttc
16381 ttattacgtc atgttgttac ctgaagaata ttaaataaag gagttagata aaatgaaatc
16441 aatcaaaagt gtattagaaa ctgaagcgat atttagtcag gataaaatgc atcgttatct
16501 tctcaagaaa acttgggata ttgataaaga agtacttaca attataacga tgtatcctca
16561 ttatgatggt gttatcaacg ttgaccttac aacacaactt attgtcaata aagtagctga
16621 aaaagatgaa tatggtggcg ttaattttat aaatcttttt tcaaatattg ataccccat
16681 taatttaaaa catatcgaaa acagccatga caagcacacg gatattcaca ttatgaaagc
16741 tgtcaaagaa gccgatagtg ttttattggc ttggggttcc tacggaaaaa aacctcttgt
16801 ggaaaatcga gtgaatgaag tattagacat gttaaaacct cacagtaaga aaatcagtat
16861 attaacaaat cctcaaacaa atgaaatcat gcatccatta aatccatacg ctagaaaggc
16921 atggacaatt aaaccattaa aggagactaa atagaatgaa tcaaacatgg aaagaagtat
16981 accaatattt aatgcatcaa ccacacatcg atgttattaa aggcattatc gccttttcat
17041 caaacaaaaa aaataattca gaattagatg agctatattc catttatatg aatgatgatt
17101 ctatcacatc cattgttcac cctgacttaa tagaaagtat agagcttatc cataatgagt
17161 agaaaaagaa tagatgttgt caaagtccaa atggttaaag aagatacact ttggtattta
17221 aaaagacgta ttgaagaacc aaaagacgct gctgatatta tgcgtgactt cataggaaat
17281 gctgaccgag aacacttcat tttaatttgc ttgaatagca aaaatgaacc tacacatatt
17341 gaaaccgtct ctataggaac cattaatttt gcggttatac atcctcgaga aatatttaaa
17401 acagccatat taagtaacgc aacgggtatg attattggcc ataaccaccc aagtggagac
17461 atattaacca tagtataata aataatagta gttaaagtag ttcaggagga gatgttaaag
17521 ggagatttta ctttaaataa aaattaacaa ggtttataat ttgtagtact taaagtgttt

17581 aaagaggggg gcgcgtgtga gggttcaaag gatagaagtg gagaataagc cgtatccatt
17641 gtatttatta ctagataaag aataccagct aaatgaatgc ttctagtaaa aggaaaatta
17701 tcagtcagag tgagatcagc aaaaaaatag ctgtaatgaa tgaagaaatg caagggtttt
17761 gggctaataa tagttgggat ataagaaaat gtccacatcc ttctgccata gaattaagta
17821 agaatcctgc tttaagaaat cgttgggttc gttttgaacg tgttaaaaat ctgtggttaa
17881 gaacagaatt gaaatatttt tatttttacc atttaaacaa tggaatatgg aatgcaaaaa
17941 ctgtctggat tagaaaagga acagtaatta ataaaatgtt ggatttttta gatttaaagt
18001 atcccagcat tacttcaatt actgaagttc ctattgataa agcaatgacg gagtatagaa
18061 catatttgac aaaacagggt gttagaatta ccaccactaa ttataagatt actgctaatc
18121 aagaaaaaac acctgtaaaa gctaattctt actatgttac taatcttaaa caatttattg
18181 agttttatga ggatttttat tttgatggag aggagtggga taaagacgtt tgggatagac
18241 gaaacttacc tttgccagat gataaggtta acccaacaca atatgaatat acaattaact
18301 ttaaagggtt tcggaataca tattttaaac agcttgtaaa aagatattgt aagttaagat
18361 tgaacatgga tagcttttcc tatgtaagtg atattgccca aaaacttaaa gagttcttta
18421 attttctgga cataaagttt aaacacgttc agagagtaca ccaattaacg agagtggaaa
18481 ttgaagcata tttaagtgaa ctaaacatga tggggataaa acctagtaca ataactggga
18541 ggatctctat attggaagga ctatttagta cccttcatag gctagaatgg gatgatgttc
18601 cttccaaatt attaatttat cccgaggact atccgaaaat accaagagca aaacctcgct
18661 ttatagacga attcgttcta gagcaattga acagtcatct tgataaatta cccgaatata
18721 tagctacgat gactatgatt gttcaagaat gtggaatgag gataagtgaa ttgtgcacct
18781 tgaaaaaagg ctgtttatta gaggacaaag atggagatta ctttttaaag tattatcaat
18841 ggaaaatgaa aaaggagcat atagttccaa tatctaaaga ggtagtttta cttattaaag
18901 ttcgggaaga taaagtttca gaggaatttc cagatagtga atacctcttt ccaagaaaag
18961 atggatcgcc attaaaacaa gaaacattta gaggcgaatt aaataaatta gcttatgagc
19021 aaaatatagt ggataaatta ggcgagattt atagattcca tgcccatgcc tttcgccatt
19081 cagtaggaac aagaatgatt aacaacgggg tgccccagca tattgtgcag aaatttttgg
19141 gacatgaaag cccagaaatg acaagcagat acgctcatat ctttgatgaa actctaaaaa
19201 atgaatttac taaatttcaa gaaaaactgg ttaccaataa tggagatgtg ctcaatctag
19261 atgatgatag tgaagtcgat gatgtagagc ttcaatggtt caagaaaaat ataaatgcac
19321 aagtgcttcc aaatggttat tgtagattgc cagtaatagc aggtggttgt ccacatgcga
19381 atgcatgctt agattgcact cacttctgta ccagtaagca attcttacca cagcacgaag
19441 aacagttaga gcgtacagaa gagttattaa ccatagcaaa ggataaacaa tggcaaagac
19501 aaatagagac taatagccgt gttaaagagc gtttagaaca aatcattgga agtttaacgg
19561 ggtaattatc aatgaataaa caagttagaa atacaacaga aattgtacgt ttggcgaagc
19621 agaaatcaca aaagacaaga gaaaaagtag acaaagcgat ttctaaattt tcgattgaag
19681 gtaaagctat taattttaat tcaatagcaa aggaagctaa tgtttctaaa tcatggcttt
19741 ataaggaaca cgatattagg caaagaatcg aatcccttcg tgagcgtcaa ataacatcaa
19801 atgtagtctc aaaacccaag aaaagttctc gttcggagga aatccttata aaaaccttaa
19861 aaagaagagt tatggaatta gaaaaagaaa ataaaaaatt acagaaccaa attcaaaaat
19921 tatatggaga tctgtataat aaagagtagt ttttattatt aatctgtaga cagattgtga
19981 aaggatgcac ttaaactaac ggggcagtta tgtttaagat actttaaaat ttggttaaaa
20041 tctccgaaag gattggtttc taacattaga aaccaatcct ttttaatatt aattatatat
20101 cattcaaaca atcatttgac tatttgttta tagtgggata tattataatc atcaaacaaa
20161 cagtcatttg aataaggagg gtaattatga caaaagatat gtgtgaagtt acctatattc

20221 atgaagataa ggtaaacagg gctaaaaaag accttgctaa acagaatcct atggatgtag
20281 cgaaagtttt taaggctcta tcagatgata caagagttaa aattgcttat gttttgtctt
20341 tagagggaga gttatgtgtt tgtgatgtag ctaatatcat tgaatcttca acggctacgg
20401 catcccatca tttaagatta ttgaaaaatt taggtatagc aaaataccgt aaagaaggaa
20461 aattagtcta ttattcacta gatgatgagc atgttaaaca gcttgtagaa aaagctttct
20521 tgcatcaaag ggaggttgct agtattggat agttcaacaa aaacattaac agaagataaa
20581 caggtttacc gtgtggaggg tttctcgtgt gcgaattgtg ctgggaagtt tgaaaaaaat
20641 gtaaaagaac tatcaggggt gcatgatgct aaagtcaatt tcggagcttc caaaattgat
20701 gtctttggca gtgcaaccgt tgaagatctg gaaaaggctg gtgctttcga gaatcttaaa
20761 gtggcaccag agaaggctag aagaagggtc gaaccagtgg taacagaaga taaaaatgtt
20821 taccgtgtgg agggattttc ttgcgcaaac tgtgctggga agtttgaaaa aaatgtaaaa
20881 caactagctg gagttcagga tgcaaaagtg aactttggcg cttctaaaat tgatgtatat
20941 ggaaacgcat cggttgaaga gcttgaaaaa gcaggtgctt tcgagaatct taaggtaatt
21001 cctgaaaaac tggcgaatcc atcgatacaa gcggtcaaag aagacactaa ggctcctaaa
21061 gaagagaaaa taccgtttta taaaaaacac agcacattgc tgtttgccac attactgatt
21121 gcttttggtt acctttctca ctttgtaaat ggagaagata accttgtaac gtccatgtta
21181 tttgtaagtt cgattgtaat tggcggatat tcactattta aagttggttt tcaaaatttg
21241 atacgctttg atttcgacat gaaaaccctg atgaccgttg cagttattgg agctgccatc
21301 attggtgaat gggcagaggc atccattgtt gtcattctct ttgcaatcag tgaagcactt
21361 gaacgttttt ctatggatag agcaagacag tccattcgtt cattgatgga tattgcccca
21421 aaagaagcac ttgttaggcg gaatggtcag gaaataatga tccatgtgga cgatattgcc
21481 gtgggtgata ttatgatcgt caaaccaggg gagaaaattg ccatggatgg gatcattata
21541 aatggtgtgt cggctgtcaa ccaggctgct ataacaggag aatctgtccc tgttgccaaa
21601 acggtagatg atgaagtatt tgcaggcacg cttaacgaag agggactact tgaagtaaaa
21661 atcaccaaat acgtagagga tacaactatc tccaagatta ttcatctggt tgaggaagca
21721 caaggggagc gcgctccagc gcaagcattc gtagataaat ttgcgaaata ttatacgccg
21781 atcattatgg ttattgcggc gctcgttgca gtcgttccac ctttattctt tggtggaagt
21841 tgggatactt gggtttatca aggattagcg gtacttgtag ttggatgtcc gtgtgcatta
21901 gttattacta ctccaatctc gattgtctcg gcaattggaa atgcagctaa aaaaggtgtg
21961 ttgattaaag gcggtgtcta tctagaggaa ttaggagcca ttaaggcaat cgcatttgat
22021 aaaacaggaa cactgacaaa aggtgtacca gtggtaacag attttaaagt gttaaatgat
22081 caagtggaag aaaaagagct gttttccatt attacagctt tagaatatcg atcacaacat
22141 ccacttgctt cagcaataat gaagaaagca gagcaagata atattactta ttccgatgtt
22201 agagtgaagg acttcacttc tattacaggt cggggcattc aagggaatat agatggaaca
22261 acctattaca ttggcagtcc aaggcttttt aaagaattaa atgtttccga ttttagcctt
22321 gagtttgaaa ataaagtgaa agttttacaa aaccaaggga aaacggccat gattattgga
22381 acggaccaaa caatcctcgg cgtgattgct gtagcagatg aggtccgcga aacaagtaaa
22441 aatgtgattc taaaacttca tcagttagga atcaagcaaa caattatgct gacaggtgat
22501 aatcaaggta ccgcagaagc aatcggtgct catgtaggcg tttctgatat tcagtccgaa
22561 ttgttgccac aggataagtt ggactatatt aaaaaaatga aagccgagca tggtaatgta
22621 gctatgattg gcgatggcgt caatgatgct cctgcacttg ctgcatccac tgttggcatt
22681 gcaatgggcg gtgctggaac agatactgcc atcgagacag ctgatattgc attaatggga
22741 gatgatttaa gtaagcttcc atttgcagta agacttagca ggaaaacgct aaatatcatc
22801 aaagcgaaca tcacgtttgc catcggaatt aagataattg ccttactatt ggttattcca

22861 ggctggctaa ccctttggat tgcgattctt tccgatatgg gagccactat tttggtagca
22921 ttaaatagtt tgcgactgat gagagtgaag gataaatagg tactatatgt gtgcaattta
22981 cggaaggact tttcattttg aagaaaagtc ctatccctaa aatatttgga gatgataaaa
23041 tgatcgcgac gatactgacg gctactgcgg tatatgtagc aacaggaatt gattatctcg
23101 tcatattaat tcttttgttt tcgcaagtaa aaaaggtcag gtaaaacata tttggatagg
23161 acaatatata gggactgcaa ttgtgatagg agcaagtctt ttagttgcac agggggttgt
23221 aaatttaatt cctcagcaat gggttatcgg actacttgga cttttaccac tttacctagg
23281 cgtgaaaata tggattaaag gagaagagga tgaagatgaa agtagcattt tatctttatt
23341 ctcctctgga aaatttaatc agttattttt gacgatgatt ttcatcgtat tggcttccag
23401 tgcggatgac ttttccattt atataccgta cttcacgacc ttaagtatgt ctgaaatctt
23461 tattgtcact attgtctttc taattatggt tggagttttg tgctatgtca gttaccgtct
23521 agcttccttc gattttatat cggaaacaat tgagaaatat gaacgttgga ttgtaccaat
23581 tgtattcatt gggctaggta tttatatatt gtttgaaaat ggaacattca atgctctgat
23641 ttcatttctt ctttgaagtg gcgaatccaa accataaagt aaagtataaa gaggttatcc
23701 tattgattgg gataaccttt ttatatttaa ctaattatca tttacgttaa acacatgtac
23761 ttaaagtaag aacattgatt taaaccattt ttttcataat ttactcttga taagatccga
23821 caccttctcc aggagatatc aaaataactc agagaataaa agagattagt gaaatgatgg
23881 gcattatatt atatgaccac ataatatttt cagaaaatga tcatcattca attatcaaaa
23941 aaattgcact ttctactggt atatatcctt gcattaattt tataaaaaat gcccctactg
24001 aagatataaa agcaattgct atgctattgt tcactaccgt attcattggt aatttaaata
24061 atgctaataa tacaggaatg atgataaatg ctccacctgc acccacaatt cccgaaatta
24121 caccaattcc aaatccaact ataataaata gcaaaggttt agatttcgtc tcttgtgtag
24181 taggtttaac tttaataaac atcaatatta aagcaagtat ggcgattatt acatataccg
24241 tatttacaaa agtagcatta aacaaactag ctaaaattgc ccctaacatg cttccaatca
24301 acatacccc tcccatatat ataacaagtt taggagagaa agctggttgt tttcttgcat
24361 ttaatgatcc actaagtgta ctgaaaaata cttgactaga ggtaagtccc gaagcaatat
24421 atgcactata cgcaggtata cctattaatg gtggcaatat aagaatagct gggtaaataa
24481 taattgcgcc cccaatacct attaatccag atataaatcc acccagtacg ccaattagca
24541 acataatagt catatttgct atatccatta tttactttta actaataaat taactgcttc
24601 attaattaat tcttgagaac tttcattatt ttcttctgct gttttaacgc attctattaa
24661 attttcacta attataatcc ccattaaacg ttgtatagaa cctttagatg cactaagttg
24721 cgtaattata tctttgcaat ctttttcttc ttccatcatt tttacgacac catttaattg
24781 accttgtatt ctatttatac gattaatcat ttttttatca taagtcatat tcatttcctc
24841 cacataaatt gattaagtta ataataataa attaaaacaa aattgtcaaa tacccctata
24901 ggtatttggc aaatgattct atccgtttta ttataccctc ataggtattt ataggaggta
24961 agtatggaac aatacaaaga agtacacatt aattcattaa acaaagaaga attagaaaaa
25021 ttggcaaaaa tgggtcaatt gattgatgtt agaacaaaag aagaatatga attaggacac
25081 attaacggtt caactttaca cccagtagaa gagattaaat cattcaataa gaatagcaac
25141 aaaacgtatt atatacattg taaaagcggt aaccgaagca ctaaagcttg cgattattta
25201 gccaaacaag gttatgatgt cgtgaattta aagggtggat ataaagatta tgaagcaaag
25261 aacttcaata gtgctccttt aatagaaaaa gatattgaaa tcaaagagaa tcgtaaacaa
25321 tttgatttta gaggtcttca gtgccctgga cctattgtta atatcagtaa agaaattaac
25381 aatatttcaa cgggtgagca aatcgaggta acagttacag atcctggttt caatagtgat
25441 attaaaagtt gggcgaaaca aacagggaat acccttgtaa atcttactga agaagcaaat

25501 gtaattaatg caattattca aaaagaaaag ccaaaagaat ttgaaatcaa tgacactgct
25561 acaggtacaa caattgtttt atttagcggt gagttagata aggcagtagc agcaatgatc
25621 atcgcaaatg gcgctaaagc agcaggtaaa gatgtaacta ttttctttac tttttggggc
25681 ctcaacgcat taaaaaaagc gcaatcaact cgcgttaaaa agaaaggtat ttccaaaatg
25741 tttgacctta tgttacctaa ggatccaata catatgccat tatcaaaaat gaatatgttt
25801 ggactaggta atattatgat gcgttatgta atgaacaaga aaaatgttga ttctttatac
25861 tcacttatcg atcaagcaat cgatcaagat ataaagttaa ttgcttgtac catgagtatg
25921 gatgttatgg gaatttcaaa agaagaatta agagatgaag tcgattatgg tggcgtaggt
25981 acttatattg gacatactga gcaagcaaat cataacttat ttatctaatc aataaaatct
26041 attttaaaaa ggagtttttt ttgtttttca aacaatttta tgaagacaat ttttcacaag
26101 catcttattt aattggatgt caacgtacag gagaagcaat gattattgac cctatacgag
26161 atttaacaaa atatttaaaa gttgcagata aagaaggatt cacaattact aaagctgcag
26221 aaacacatat tcatgctgat tttgcttcag gtattagaga tgtggcaaat aaattgaatg
26281 caagtatata cgtatctgat gaaggtaaag atgaatttaa ttataaaaat atgcctaaac
26341 aaacacattt cgttaagcat caagatatta tctatttagg aaagattaaa ttagaagtct
26401 tacacactcc aggccatccc cctgagagta ttagtttttt acttactgac gaaggtgggg
26461 gttccagtat accaatggga ttatttagcg gtgacttcat ctttgttggt gatgtaggta
26521 gaccagatct attagaaaaa gctgtaaatg ttgaaggttc tacaaaaatc ggagcaaaac
26581 aaatgtatca gtccatacaa tttgctgcaa cattaccaga ctatattcaa atttggccag
26641 gtcatggtgc agggagtcct tgtggtaaag cgctaggttc attacctaca actactttgg
26701 gttatgagaa aataaacaac tgggctttta atgttaaaga tgaaacttca tttatcgaaa
26761 ctttaacact caatcagcca gctccaccac accatttttc acaaatgaaa aaaataaatc
26821 aatttggtat gcaaatgtac caaccatata atgtttaccc tagttcaagc aatacacaaa
26881 ctgcttttga tcttcgaagt aaagaagcat tccatggtgg tcatatgcat ggaactatca
26941 atattccttt taataaaact tttataaatc aaattggttg gtatttagat tatgactaag
27001 atattgaatt aattggcgat aaatctaccg ttcaacaagc tattcatgct ttacaattaa
27061 ttggttttga aaatgtaaaa agttaccgtt tgccacaaac aaatatcgtc acatcatcta
27121 ttcatagttc tgaaatgact ggtgaagaac ataatgtatt agatgttcgt aatgatgatg
27181 aatggcgtaa aggtcattta aaacaagcaa ttcatattcc tcacggtaaa cttttaaatg
27241 aagatatacc tttcgataaa aatgacctaa tttatgtaca ttgtcaatca ggtgttagaa
27301 gttcaatagc ggtaggaata ttagaatata aaggatttac gaatattgtt aacgttagag
27361 gtggctatca acatttacca gcatttttaa aatagttttc ttctgcaata gaactataaa
27421 aatccctcaa tccgaaaatt caattcggat tgagggattt ttttataaag acaattacga
27481 actttgatta ttcatttgaa ttagtttttt atgcttttat atctaagtaa atatcattaa
27541 tctgtagtac tttttggata attgctaatg taccacctag caaagttgca aatttgacat
27601 tagatgttaa ctttatttga atttcgttac gtgaatattg tttgaactgg ttcttaattg
27661 cttctaatat ttcaggcatt tcattgaaca atggacagtt aatgtaaatt gcattcggat
27721 taaactgggt gtttaaatta tgtattaaaa cagcaatttt atttataaat tgttccattt
27781 cttcaactac gactggattt ttttcattat aaaattgaat taatttgcta agcgtcatct
27841 tctcatttag ttgattactt aaattatgca gtaaagcttc ttgtgaaaaa atatcttcaa
27901 tcttatgaaa gatctccaca ttatcgctaa cttttgagac aagtgttttt ccaatttcac
27961 ccgcttcccc atttgcacca cgatacaatt gattattaat aataagccca gcaccaatac
28021 ctttatgtat acttaaagca ataagattat tgtaggataa attatgatta aaattacgtt
28081 catataacgc tgaaagattc gcttcatttt caactacgac tggaacatta gtaatttctt

28141 ttattttctt agcaattgaa attccttcag tttcatggaa tggtaaatat gtcacatgct
28201 gctcattatc cacaactcca tgtatagaaa cagacacacc taatagtccg ttataagtat
28261 caagtttctc ctgaatatca atatgttttt ttattatgct taatatacta ctaacctttt
28321 catcaggtaa atcataagat tcatgcttaa tgacattacc atcaaaataa ttgtacatca
28381 cttcaacaga actataagtt aaatccaaag aaataaaata accataaaga tgattaacct
28441 tcagaagaat aggttttcta ccaccactct tcgtgctatc accctcacca acctcattaa
28501 caagagattt atactttaac ttattcaaaa tactagaaat cgttgcctta ttaatctcaa
28561 gatttttaga tatttgagta cgcgaaatat tatgatggtt ataaatttcc cttagcactc
28621 ttttttcatt atcatttata ttttattttc cataattgcc taccccataa gttattttga
28681 atttagttta tagtttatca tgtattttta taccaactat tttaacataa attaaattaa
28741 accttagaac aacttaaaac ctcacttagg ttatcatctt cgttctttta ttttttttat
28801 tatttcaata aattacgtat ttccaatatg acgatttttt atgcaaagtc gttgtatgtt
28861 tttcaaattt ttgacattta tgcaatctaa atagccaatt agggaatttt taacattttt
28921 tatttgtttg atattatact taatgtatct taaatagaaa gagggtatgc atatggattt
28981 cactggtgtt attacaagca ttattgattt aatcaagact tgcattcagg ctttcggtta
29041 atttttttcaa ctaaaaaaca gaggaaatat tcaacgactt gattgtttcc tctgttttct
29101 atgtattgtt gtaaacacaa caattttatt ttttattcaa tatatttctc aattcttcta
29161 tttcatcttg tgatagatct tctttttcta caaagtttaa gacaagtgaa ttgaaaccgc
29221 ctttgtatac tttattgata aagtttttag atgttttata ttttatatca ctttcttcta
29281 caagagagta atattgaaaa attttattgt cttttttacg atctataaat ccctttttat
29341 acaatctcgt tataagtgta cgaatggttt ttggactcca gtccttttgc atttgtattt
29401 cttctattat attattcgca cttgcatatt ttttcatcca aatgatattc ataacttccc
29461 attctgcaga tgatatttca tacgttttat tatccattat attaactcca tttcttttaa
29521 aatacgctca gaaatttgtt gtgctttttc gccattcgca ttgtcttcgc cttttaaatg
29581 tgtagcaaaa taatacgtat tatctttcgt ttcaacataa cctacgaacc atccatttgc
29641 ttctttgtga ttcacgattc ctgttccagt tttacctaca tatttataag tatctttttg
29701 tttcaaagtc atactatttt caactttttc aatagcctta ttatcaaaat gcatgttatg
29761 ttgtttcata tttttcaaca aattaacctg ttctattgca gaaattttta atgaagattc
29821 attccaataa ttttcattcc ctgatatttc ttcattacca tattcaatta gatctaaata
29881 agatttaacc tcatcttgtc ttaaatgttt gtttaaattt tcgtaatacc aatttactga
29941 atatttcatt gaagaattta aattttgatc ttggttccat tctttaaatg gatattgatg
30001 tttatcccat tgttgttcag tatgatttaa tgagagtaaa ttttggtcga atgccattaa
30061 cgctaaataa attttgtaag tagaattagg tgaatatcgt tgtttacttt ctggttcatt
30121 ataaatagaa taagcttgct cccgttcatt ataaagcaca aaacttccat caaatccttt
30181 gaaatacgga gctagttgat ttaatttttt atatgataca tttgtttcat atttgtcttg
30241 ttgaacatgt gcagatagta acggtgcttg tattaaaagc gatatactac atacaatata
30301 cgcaacaata cgcttgtttc gattaggttt aggcattgaa tcataaagtg caatatactt
30361 aacacgttct ttaatatttg aattaaaacc tagtaaatat tgtgctgcca cattatttat
30421 gtgctgagat tttaaaatag agcattttaa tatcgattca ccataacgta tatgttcatg
30481 gcgattcaaa atttttaaaa cgtttctatc acatactttt tcacagtcat tgtccatcat
30541 tgttttactt atatatagtg caggattaaa ccagaatatc attttaaaaa caacataaag
30601 ctggttgaat attaagtcat gacttttcac atgtgatagt tcatgtagaa taatatattc
30661 aatttctttg tcattcatgg tttcgactac gacagttggt agtacaattt gggatttcac
30721 taaaccaaat accatcggat tatcaatgtt tgaactataa ctaattgtta tatgcttttt

30781 gtagaactgc atcttacttt gacatacttt aagtcgttca ttaagatatg acgattccaa
30841 tgacgaactt ttaataacat caatttgtcg gaatgcctta atcatataaa ataagcacaa
30901 caaactacca aatacccata tcaaaagaat catatacgtt atatttgagg tctcaaactg
30961 attaacatta attgctaagt ctttcgtaac agatgattgt tgaccatcta acatatgact
31021 aaccgaagaa gtcgtgtcag atacatttcg attcatcata tcttttgaaa atgtaaaatt
31081 cgatattttg taaaatggta ttaatggaat taacgtggag acgagcacta ataaccaaat
31141 cttatgtgac ataatatttt gagtatattt tatatagagc attctcacta aaaaaattac
31201 acatatcgtg agcaatgaac tgattatact taacattaaa aaagatgata acaccttcta
31261 cacctccata tcacaaaaat tataacatta ttttgacata aatactacat ttgtaatata
31321 ctacaaatgt agtcttatat aaggaggata ttgatgaaaa agataaaaat tgttccactt
31381 attttaatag ttgtagttgt cgggtttggt atatattttt atgcttcaaa agataaagaa
31441 attaataata ctattgatgc aattgaagat aaaaaatttca aacaagttta taaagatagc
31501 agttatattt ctaaaagcga taatggtgaa gtagaaatga ctgaacgtcc gataaaaata
31561 tataatagtt taggcgttaa agatataaac attcaggatc gtaaaataaa aaaagtatct
31621 aaaaataaaa aacgagtaga tgctcaatat aaaattaaaa caaactacgg taacattgat
31681 cgcaacgttc aatttaattt tgttaaagaa gatggtatgt ggaagttaga ttgggatcat
31741 agcgtcatta ttccaggaat gcagaaagac caaagcatac atattgaaaa tttaaaatca
31801 gaacgtggta aaattttaga ccgaaacaat gtggaattgg ccaatacagg aacagcatat
31861 gagataggca tcgttccaaa gaatgtatct aaaaaagatt ataaagcaat cgctaaagaa
31921 ctaagtattt ctgaagacta tatcaaacaa caaatggatc aaaattgggt acaagatgat
31981 accttcgttc cacttaaaac cgttaaaaaa atggatgaat atttaagtga tttcgcaaaa
32041 aaatttcatc ttacaactaa tgaaacagaa agtcgtaact atcctctagg aaaagcgact
32101 tcacatctat taggttatgt tggtcccatt aactctgaag aattaaaaca aaaagaatat
32161 aaaggctata aagatgatgc agttattggt aaaaagggac tcgaaaaact ttacgataaa
32221 aagctccaac atgaagatgg ctatcgtgtc acaatcgttg acgataatag caatacaatc
32281 gcacatacat taatagagaa aaagaaaaaa gatggcaaag atattcaact aactattgat
32341 gctaaagttc aaaagagtat ttataacaac atgaaaaatg attatggctc aggtactgct
32401 atccaccctc aaacaggtga attattagca cttgtaagca caccttcata tgacgtctat
32461 ccatttatgt atggcatgag taacgaagaa tataataaat taaccgaaga taaaaaagaa
32521 cctctgctca acaagttcca gattacaact tcaccaggtt caactcaaaa aatattaaca
32581 gcaatgattg ggttaaataa caaaacatta gacgataaaa caagttataa aatcgatggt
32641 aaaggttggc aaaaagataa atcttggggt ggttacaacg ttacaagata tgaagtggta
32701 aatggtaata tcgacttaaa acaagcaata gaatcatcag ataacatttt ctttgctaga
32761 gtagcactcg aattaggcag taagaaattt gaaaaaggca tgaaaaaact aggtgttggt
32821 gaagatatac caagtgatta tccattttat aatgctcaaa tttcaaacaa aaatttagat
32881 aatgaaatat tattagctga ttcaggttac ggacaaggtg aaatactgat taacccagta
32941 cagatccttt caatctatag cgcattagaa aataatggca atattaacgc acctcactta
33001 ttaaaagaca cgaaaaacaa agtttggaag aaaaatatta tttccaaaga aaatatcaat
33061 ctattaactg atggtatgca acaagtcgta aataaaacac ataaagaaga tatttataga
33121 tcttatgcaa acttaattgg caaatccggt actgcagaac tcaaaatgaa acaaggagaa
33181 actggcagac aaattgggtg gtttatatca tatgataaag ataatccaaa catgatgatg
33241 gctattaatg ttaaagatgt acaagataaa ggaatggcta gctacaatgc caaaatctca
33301 ggtaaagtgt atgatgagct atatgagaac ggtaataaaa aatacgatat agatgaataa
33361 caaaacagtg aagcaatccg taacgatggt tgcttcactg ttttattatg aattattaat

33421 aagtgctgtt acttctccct taaatacaat ttcttcattt tcattgtatg ttgaaagtga
33481 cactgtaacg agtccatttt ctttttttat ggatttctta tttgtaattt cagcgataac
33541 gtacaatgta ttacctgggt atacaggttt aataaattta acgttattca tttgtgttcc
33601 tgctacaact tcttctccgt atttaccttc ttctacccat aatttaaatg atattgaaag
33661 tgtatgcatg ccagatgcaa tgataccttt aaatctactt tgttctgctt tttctttatc
33721 tatatgcata tattgaggat caaaagttgt tgcaaattgg ataatttctt cttctgtaat
33781 atgaaggctt tttgttttga atgtttctcc tactataaaa tcatcgtatt tcatatatgt
33841 ctctctttct tattcaaatt aattttttag tatgtaacat gttaaaggta agtctaccgt
33901 cactgaaacg taagactcac ctctaacttt ctattgagac aaatgcacca ttttatctgc
33961 attgtctgta aagataccat caactcccca attagcaagt tggtttgcac gtgctggttt
34021 gtttacagtc catacgttca attcataacc cgcttctttt accattttta cttttgcttt
34081 agtaagtttg gcatcttcag tgtttactat tttagcatta cagtaatcta aaagtgttct
34141 ccagtcttca cgaaacgaag ttgtatggaa tataactgct ctgttatatt gtggcatgat
34201 ttcttctgca agtttaacaa gcacaacatt aaagcttgaa atgagcactt cttgattctg
34261 atttaagttt gttaattgtt cttccacttg cttaaccata cttttagaaa gtgctagtcc
34321 attcggtcca gtaatacctt ttaattctac atttaaattc atattatatt catttgctat
34381 ttttactaca tcatcgaaag ttggcaaatg ttcatctttg aatttttcac caaaccaaga
34441 tcctgcagaa gcatctttaa tttcatcata attcaattca gttatttccc cggacatatt
34501 tgtagtccgt tctaaataat catcatgaat gataatcagt tgttcatctt ttgtaattgc
34561 aacatctaac tccaaccagt ttataccttc tacttctgaa gcagctttaa atgatgcaat
34621 tgtattttcc ggagctttac taggtaatcc tctatgtcca tatacagtta gcatattacc
34681 tctccttgca tttttatttt tttaattaac gtaactgtat tatcacatta atcgcacttt
34741 tatttccatt aaaaagagat gaatatcata aataaagaag tcgatagatt cgtattgatt
34801 atggagttaa tctacgtctc atctcatttt taaaaaatca tttatgtccc aagctccatt
34861 ttgtaatcaa gtctagtttt tctgtacccc ttatctgcaa ttttacttag gattgctttt
34921 aacttacccc ttatcagcaa ttttactgag aactgctttt aacgcacctc ttatctgcaa
34981 ttttgcctag aactgctttt aacgtacctc ttatctgcaa ttttacttag aactgctttt
35041 aacgtacctc ttatctgtaa ttttactgag aactgctttt aacaaacctc ttatctgcaa
35101 ttttacttag aactgctttt aacaaacctc ttatctgcaa ttttacttag aactgctttt
35161 aacaaacctc ttatctgcaa ttttacttag aattgctttt actattcctc ttattagtat
35221 aatctcagta agaatgcgta taaaaatgaa aattacaacc gattttgtaa gtgctgacgc
35281 ctgagggaat agtatgtgcg agagactaat ggctcgagcc atacccctag gcaagcatgc
35341 acgtacaaaa tcgtaagata aaaaaataag catatcactg taaactttaa aaaatcagtt
35401 tagtgatatg cttatttatt tcgagttagg atttatgtcc caagctcatc aagcacaatc
35461 ggccactagt ttatttctct atcttatatg ttctgatatg gtcttctata ctgtataagt
35521 atacttttga atatggatct tgtgtcaatt cacgttcgaa atcaaattct tgattatcaa
35581 atctgttaaa gaatgtttcg tattcttcga ctgataattg ctctctagat tctagcatat
35641 ttaagtgttt ctctttatct aatgctttgt catatccttt aacgattgaa ccactaaaga
35701 tttctcctac tgctcctgaa ccataactaa atagacatac tttctcttct ggttggaatg
35761 tgtggttctg taataacgaa attaaactta agtataatga tcctgtataa atgttaccaa
35821 catctctatt ccataatacg gttctgttgc aaagttgaat ttatagtata attttaacaa
35881 aaaggagtct tctgtatgaa ctatttcaga tataaacaat ttaacaagga tgttatcact
35941 gtagccgttg gctactatct aagatataca ttgagttatc gtgatatatc tgaaatatta
36001 agggaacgtg gtgtaaacgt tcatcattca acggtctacc gttgggttca agaatatgcc

36061 ccaattttgt atcaaatttg gaagaaaaag cataaaaaag cttattacaa atggcgtatt
36121 gatgagacgt acatcaaaat aaaaggaaaa tggagctatt tatatcgtgc cattgatgca
36181 gagggacata cattagatat ttggttgcgt aagcaacgag ataatcattc agcatatgcg
36241 tttatcaaac gtctcattaa acaatttggt aaacctcaaa aggtaattac agatcaggca
36301 ccttcaacga aggtagcaat ggctaaagta attaaagctt ttaaacttaa acctgactgt
36361 cattgtacat cgaaatatct gaataacctc attgagcaag atcaccgtca tattaaagta
36421 agaaagacaa ggtatcaaag tatcaataca gcaaagaata ctttaaaagg tattgaatgt
36481 atttacgctc tatataaaaa gaaccgcagg tctcttcaga tctacggatt ttcgccatgc
36541 cacgaaatta gcatcatgct agcaagttaa gcgaacactg acatgataaa ttagtggtta
36601 gctatatttt tttactttgc aacagaaccc taattatctt catcattgta aaacaaatta
36661 cacccttaa atttaactca acttaaatat cgacaaatta aaaaacaata aaattacttg
36721 aatattattc ataatatatt aacaacttta ttatactgct ctttatatat aaaatcatta
36781 ataattaaac aagccttaaa atatttaact tttttgtgat tattacacat tatcttatct
36841 gctctttatc accataaaaa tagaaaaaac aagattccta aagaatatag gaatcttgtt
36901 tcagactgtg gacaaactga ttttttatca gttagcttat ttagaaagtt ttatttaaat
36961 tacagtttct atttttatta gatcacaatt ttattttagc tcttgttcaa gtaatcattt
37021 ttcgccaaaa actttatact gaatagcttc tacattaaat actttgtcaa tgagatcatc
37081 tacatcttta aattcagaat aatttgcata tggatctata aaataaaatt gtggttcttt
37141 accggaaaca ttaaatattc ttaatattaa atatttctgc ttatattctt tcatagcaaa
37201 catttcattt agcgacataa aaaatggttc ctcaatacta gaagatgtag atgttttaat
37261 ttcaataaat ttttctacag ctttatctgt atttgttgga tcaaaagcta ctaaatcata
37321 gccatgaccg tgttgagagc ctggattatc atttaaaata ttcctaaact gttctttctt
37381 atcttcgtct attttattat caattagctc attaaagtaa tttagcgcta atttttctcc
37441 aactttaccg gttaatttat tctctttatt tgatttttca atttctgaat catttttagt
37501 agtctttgat acaccttttt tatattttgg aattattcct ttaggtgctt ccacttcctt
37561 gagtgtctta tctttttgtg ctgttctaat ttcttcaatt tcgctgtctt cctgtatttc
37621 gtctatgcta ttgaccaagc tatcatagga tgtttttgta acttttgaag ctaattcatt
37681 aaatagttct aaaaatttct ttaaatcctc tagcatatct tcttctgtga atccttcatt
37741 caaatcataa tatttgaatc ttattgatcc atgagaatat cctgatggat aatcattttt
37801 taaatcataa gatgaatctt tattttctgc gtaataaaat cttccagtat taaattcatt
37861 tgatgtaata tatttattga gttcggaaga taaagttaat gctctttgtt ttgcagcatt
37921 tttatcccgc ggaaacatat cacttatctt tgaccatcct tgattcaaag ataagtatat
37981 gccttctcct tccggatgaa aaagatatac caaataatat ccatcctttg tttcttttgt
38041 tatattctca tcatatattg aaatccaagg aactttacta tagttcccag tagcaacctt
38101 ccctacaact gaatatttat cttcttttat atgcactttt aactgcttgg gtaacttatc
38161 atggactaaa gttttatata gatcaccttt atcccaatca gattttttaa ctacattatt
38221 ggtacgtttc tctttaatta atttaaggac ctgcataaag ttgtctatca tttgaaattc
38281 cctcctatta taaaatatat tatgtctcat tttcttcaat atgtacttat ttatatttta
38341 ccgtaattta ctatatttag ttgcagaaag aattttctca aagctagaac tttgcttcac
38401 tataagtatt cagtataaag aatatttcgc tattatttac ttgaaatgaa agactgcgga
38461 ggctaactat gtcaaaaatc atgaacctca ttacttatga taagcttc
//
LOCUS Staphylococcus 52466 bp DNA linear UNK
DEFINITION Staphylococcus Staphylococcus epidermidis strain B1276296

ACCESSION Staphylococcus
KEYWORDS .
SOURCE Staphylococcus Staphylococcus epidermidis
ORGANISM Staphylococcus Staphylococcus epidermidis
Bacteria; Bacillati; Bacillota; Bacilli; Bacillales;
Staphylococcaceae; Staphylococcus; Staphylococcus epidermidis.
FEATURES Location/Qualifiers
source 1..52466
/genome_md5=""
/db_xref="taxon:176279"
/mol_type="genomic DNA"
/project="hwwang_176279"
/organism="Staphylococcus Staphylococcus epidermidis"
/genome_id="176279.68"
CDS 264..569
/translation="MKGNIQCLRLIKGSLLDFVLETVDVFVNGSKNDCELVNFNYKEG
KVVSRYSTKFESNPKLRKEQLISTVKVVRYVIFDFEKILWGIGKVLLKFIILRQCLL"
/product="hypothetical protein"
/db_xref="SEED:fig|176279.68.peg.1"
/transl_table=11
CDS 767..1630
/transl_table=11
/db_xref="SEED:fig|176279.68.peg.2"
/product="hypothetical protein"
/translation="MKHINHFSESTLAYLEKDHEHAFYVYCLIDPRNDECFYVGKGKG

NRIFKHKQDAQRQLLYEDIFREENKDNLKFNRINEICSNDLNVLGYIISYGLTESEAF

SAENVLINFLNLTNKTTLTNMINGHGSKAYLVEDLENEFGYDSINLENINTNELILAV

KIRDAFLLDKDESKEYPINESKRDRNNLKSRTLGSWIIGKDKIHKIKYIIGINTGANN

AVVSAYEVSFEQAESIETNNGRMRYAFIGLSERDATLKKLNLYKKALPDLRFGSGSAT
AYINNGTMKVD"
CDS 1738..3213
/transl_table=11
/db_xref="SEED:fig|176279.68.peg.3"
/translation="MPSDDIVKKLYSKEDIRLELGLTPHKFNKKMETIAKLFKIDMKI

FHNYKGQDKNNQYTFNGVAKELIKVLLKSVDYYPVDINSKKFKQNGKSKKEMIENIDN

SSYMKYIYQLMKSINEIQYKRLIADIHMKDVYQNTKAWLNNGESINKKEQELYQYMTI

LPLHKRVELQNEVLKSIDETIFQFLAKEHRNNQIEENNELEAYTKAIKEGRNPKNDYE

LNHLLYKKNQLPLDSLIEDIWDYEETEYTELDWLIADMLKRSQRADSNFIESLEKKNK

LRKNIHKDSIKSISKLIDHELVKNNRKWDVAEFYKNQQFWNNSTLRRSSQNFGILYYK

KNRLHTMNQSNIIKKIKIIEANIESANKQPSYLDFFLQAKFDLERLESELMKYSINPK

YFNKINSDYVRYAIEDVHNAIIKIDRYISNDKVKLNYSNVQMVVRNEITEQGSYFVTQ
ALNTLSKVEESGYSFDNFMVGPFVEDFKKAIKNRRFGSKKD"
/product="hypothetical protein"
CDS 3440..4540
/db_xref="SEED:fig|176279.68.peg.4"
/transl_table=11
/product="hypothetical protein"

/translation="METRDKLMSLTQSDKTQQWLMDKSSNQDDIQQLQQQFSQQLDQQ

YNALLADEKAKLDQYVEVHQGLESLKEEIESEPITLNIDKLPDIKATMLERAKNDEHS

DKIEKLFDRLEQALNGTNRLYTQLSLIGTRTHRITTKNFNLQGLPKAVQHTILPSKFK

KVYTVDFKSFEPSVAAYMTQDSKLIDLLNQKDGLYDALLSELGLSDELRVFVKRAFIG

SFLFGGNFKNPKFKLNQYVSEVQWLDAVSQFTKVIELKKHVEKSNSMPMPYSIEHDMS

AFQGSSIMAIYVQTVASYIFKHILLKVYKAQCDQKTFKIIVPIHDAIMIECEDEEIAQ
NVGQLMKDTANQLFNGEFAHVTVEEIGGVDHE"
CDS 4533..4904
/product="hypothetical protein"
/translation="MNNDRGQSLHIPSSTPIKENNIYVATLHSVIQTDFSGEIKHQFT

YEIEVNNQIVYANRNIPTKPSANQLSIHDWLKRHSNYSVGHDTYEPYIDRKHLIHIGQ
YSGNYYVQDVASLDAFGGVLS"
/db_xref="SEED:fig|176279.68.peg.5"
/transl_table=11
CDS 4901..6544
/product="putative primase"
/translation="MNHILQMLSKLLSVAKEAIDRQGLIAILTIPVNNNDEIEETAQG

ETVYNELIDQLRLNIPKDTDYQPNIYSYFGIKKNPNDTVLMEMMIKVFHIKRFNSELF

IFKVNGWQKINGDELQGLISKMIQVLLVDYKPSLSTLKNVVDGLQKSTDVEELVENER

YIGCGENMFDLNTFQVVKNSIDIFPKTRLNLSLSTNDVITDKIPPYFKQYMLQLANYD

DDLQYFLFQHTAVLLTADTKYRRGLILYGGAKNGKSVYIELVKSFFYSKDIVSKPLNE

LEGRFDKESLIDKSLMASHEIGQSRIQEKIVNDFKKLLSVESMHVDRKGKTQVEVILD
LKLIFSTNAILNFPPEHAKALERRINIIPCEYYVEKADTSLIDKLQSEKKEIFLYLMY

VYQQIVKADIEYLENSRVTEITHDWLNFGYEFVSSRSVSIANQKACINLLRKLIEIKS

GSRIKVSELNKVINEEIKVSSQVINQLIQANFDTQTKLYNGYDYWIDLGWKEANKKEI
HDISEKDNIISLDKNENITDDEALDEENLDFDWEDFDDE"
/transl_table=11
/db_xref="SEED:fig|176279.68.peg.6"
CDS 6771..8447
/translation="MKGKIALYSRVSTSEQSEHGYSVKEQEQVLIKEVVKNFPGYDYE

TYTDSGISGKNIEGRPAMKRLLQDVKDNKIEMVLSWKLNRISRSMRDVFNIIHEFKEH

DVGYKSISENIDTSNASGEVLVTMFGLIGSIERQTLISNVKLSMNAKARSGEAITGRV

LGYKLSLNPLTQKNDLVIDENEANIVREIFDLYLNHNKGLKAITTVLNQKGYRTINQK

PFSVYGVKYILNNPVYKGYVRFNNHQNWAVQRRSGKSDKNDVILVKGKHEAIISEEVF

DKVHEKLASKSFKPGRPIGGDFYLRGLIKCPECGNNMVCRRTYYKTKKSKERTIKRYY

ICSLFNRSGSSACHSNSINAEVVERVINVHLNRILSQPDIIKQIASNVIEELKQKHSN

QTEIKYDIDSLEKQKAKLKTQQERLLELFLDDQMDSEMLKAKQSQMNQQLEVLDQQIK

EAQQANQSQDEIPNFDKLKGRLILMITRFSVYLRKATPEAKNQLMKMLIDSIEITTDK
QVKLVRYKIDESLIPQSLKKDWGSFFMPKFQFEIYGQNDYFIDQITTFTT"
/product="Cassette chromosome recombinase B"
/transl_table=11
/db_xref="SEED:fig|176279.68.peg.7"
CDS 8550..8891
/translation="MMTLEQQLKHYITNLFNLPKDEVWHCESIEEIADDILPNQYVRL

GPLSNKTLQTNTYYSDTLHESNIYPFILYYQKQLIAIGYIDENHDMDFLYLHNTIMPL
LDQRYLLTGGQ"
/product="FIG01108299: hypothetical protein"
/transl_table=11
/db_xref="SEED:fig|176279.68.peg.8"
CDS 8893..8994
/product="hypothetical protein"

/translation="MHKYIKITQLVITILSEIIIWMKESERKEVSYE"
/db_xref="SEED:fig|176279.68.peg.9"
/transl_table=11
CDS 8987..9298
/db_xref="SEED:fig|176279.68.peg.10"
/transl_table=11
/product="FIG01107894: hypothetical protein"

/translation="MNRYITRGIANNLPNILQHQLWQLVSEREQEQTKDNTSVDYFHI

FQFNKHRNQLYIKHKQERPEYAKIHKANYSKAININKVYIIREDDVDLSYYVMLLPEE
Y"
CDS 9314..9820
/product="hypothetical protein"
/translation="METIKSTLKTEAIFSDDKQHRYLLKKTWNSEKQSITIITMYPHY

DGILNIDLTTQLIMNKISEMDAFGSIHFVNLYSNITTPINLKHLENAYDNHTDIQIMK

AVKESDEVILAWGAYAKKPIVEARVNEILEMLKTHKKKVKQLMNPATNEIMHPLNPKA
RQKWTLKA"
/db_xref="SEED:fig|176279.68.peg.11"
/transl_table=11
CDS 9848..10294
/translation="MKEINIVSLQMIKTDTLSYLKARISNPEDATEIMRSFIGNSDRE

HLILICMNSKNEPTHIQTLSIGSINQTVIHPREIFKTAILSNANSIMLGHNHPSGDVT
PSPEDINVTKRLMLISEMMGILFYDHIIFSDSNTYSIRQHNLEVEV"
/product="DNA repair protein RadC truncated by transposon
Tn554"
/db_xref="SEED:fig|176279.68.peg.12"
/transl_table=11
CDS 10464..11336
/db_xref="SEED:fig|176279.68.peg.13"
/transl_table=11
/product="FIG01110112: hypothetical protein"
/translation="MDRAFRILTIYNRLLQNKSVNKQSLTLELDTSPRTIQRDIDDIR

NFLYESKSWISTPKEITYDYKSESYKLEQDKNENVHFMYNILTALYLTTPQLSHYFYQ

YLKLLIIKHHSNHRTTLLKYLERFEIDKNQTGISTSSLVVQAMNENKYLQYNQNLLLA

LSMYYQMHAFHLVYRMNDEIYIDDINQMNLSFSEKTVDTHEKSKVQTYITFEISKDVW

SKMHRYYQIHVIEKYDVHYLIVTFKMTRPEAIQLCFMYRSNIRIISPPDLREQVIDEL

LLLQSTYLKQQVQE"
CDS 11388..11678
/transl_table=11
/db_xref="SEED:fig|176279.68.peg.14"

/translation="MGNIIKTIGKGIGKGVGAAAYYSVATVGAAIGAIWETTGDRGLM
RDALSDSSNKDLMEIYEIQKKDDGDYDTYIAAKSLLQERGFTYNHETNKWEK"
/product="hypothetical protein"
CDS 11694..12056
/db_xref="SEED:fig|176279.68.peg.15"
/transl_table=11
/product="FIG007303: uncharacterized protein"
/translation="MEFAYYEAKSLNDFENELKTLALNKKYTFSKETEVLKEEEILEK

SNSHERELLKVFKDNAQITTYHFKSSQLSASKSSIQRLMGIIISENLVECVKMSEENS
EDSQALINEAVELLIKSK"
CDS 12053..12811
/transl_table=11
/db_xref="SEED:fig|176279.68.peg.16"
/translation="MISITTIIVMLVIGILGGFISGLVGIGGAIVIYPALLLLPPLFG

LPTYSAYIASGLTSSQVFFSTLSGSLNAYKNKNFSRTLILNMGSGMVIGSMLGAILAN
LINVQFVNTVYIIIALLALILMFIKVTPSTSHIKFNRLLLITIGGIIGLVSGIVGAGG
AFIIIPVLLVIFKLPMNMVVTNSIVIAFISSIGAFMIKLLQGYIPINSAIPLILGSIL
FTPLGMKMGQKIPDYIQKTIICILIVIAIIKLIF"
/product="FIG003846: hypothetical protein"
CDS 13020..13190
/EC_number="3.6.3.4"
/EC_number="3.6.3.3"
/db_xref="SEED:fig|176279.68.peg.17"
/db_xref="GO:0004008"
/transl_table=11
/product="Lead, cadmium, zinc and mercury transporting
ATPase (EC 3.6.3.3) (EC 3.6.3.5); Copper-translocating
P-type ATPase (EC 3.6.3.4)"
/translation="MVQNLWWGAGYNIVAVPLAAGILAFIGLILSPAIGAILMSLSTV
IVAINAFTLKLK"
CDS 13208..13753
/transl_table=11
/db_xref="SEED:fig|176279.68.peg.18"
/product="DUF1541 domain-containing protein"

/translation="MIKKLFFMILGSLLILSACSNNDEKDKDTNDQKSESHMKHNDES

KVPEDMTSTNEGEFKVGDKVTITAGHMPGMKGAEATVKGAYKTYAYVVSYKPTNGNEK

VNNHKWVVNEEIKDAPKDGFSKGDTVKLEASHMSGMKGATANIDNVKKTTVYVVDYKS
KDNGKIIKNHKWMTGNELKAR"
CDS 14196..15452
/product="hypothetical protein"

/translation="MIDNFMQVLKLIKEKRTNNVVKKSDWDKGDLYKTLVHDKLPKQL

KVHIKEDKYSVVGKVATGNYSKVPWISIYDENITKETKDGYYLVYLFHPEGEGIYLSL

NQGWSKISDMFPRDKNAAKQRALTLSSELNKYITSNEFNTGRFYYAENKDSSYDLKND

YPSGYSHGSIRFKYYDLNEGFTEEDMLEDLKKFLELFNELASKVTKTSYDSLVNSIDE
IQEDSEIEEIRTAQKDKTLKEVEAPKGIIPKYKKGVSKTTKNDSEIEKSNKENKLTGK

VGEKLALNYFNELIDNKIDEDKKEQFRNILNDNPGSQHGHGYDLVAFDPTNTDKAVEK
FIEIKTSTSSSIEEPFFMSLNEMFAMKEYKQKYLILRIFNVSGKEPQFYFIDPYANYS
EFKDVDDLIDKVFNVEAIQYKVFGEK"
/transl_table=11
/db_xref="SEED:fig|176279.68.peg.19"
CDS complement(15897..16571)
/product="Transposase for insertion sequence-like element
IS431mec"
/translation="MNYFRYKQFNKDVITVAVGYYLRYTLSYRDISEILRERGVNVHH

STVYRWVQEYAPILYQIWKKKHKKAYYKWRIDETYIKIKGKWSYLYRAIDAEGHTLDI

WLRKQRDNHSAYAFIKRLIKQFGKPQKVITDQAPSTKVAMAKVIKAFKLKPDCHCTSK
YLNNLIEQDHRHIKVRKTRYQSINTAKNTLKGIECIYALYKKNRRSLQIYGFSPCHEI
SIMLAS"
/transl_table=11
/db_xref="SEED:fig|176279.68.peg.20"
CDS 16829..16996
/translation="MLESREQLSVEEYETFFNRFDNQEFDFERELTQDPYSKVYLYSI
EDHIRTYKIEK"
/product="Hydroxymethylglutaryl-CoA synthase N-domain
homolog (EC 2.3.3.10)"
/EC_number="2.3.3.10"
/transl_table=11
/db_xref="SEED:fig|176279.68.peg.21"
CDS 17094..17324
/transl_table=11
/db_xref="SEED:fig|176279.68.peg.22"

/translation="MLIFLSYDFVRACLPRGMARAISLSHILFPQASALTKSVVIFIF
IRILTEIILIRGIVKAILSKIADKRFVKSSSK"
/product="hypothetical protein"
CDS 17793..18536
/transl_table=11
/db_xref="SEED:fig|176279.68.peg.23"
/db_xref="GO:0008889"
/EC_number="3.1.4.46"
/translation="MLTVYGHRGLPSKAPENTIASFKAASEVEGINWLELDVAITKDE

QLIIIHDDYLERTTNMSGEITELNYDEIKDASAGSWFGEKFKDEHLPTFDDVVKIANE

YNMNLNVELKGITGPNGLALSKSMVKQVEEQLTNLNQNQEVLISSFNVVLVKLAEEIM

PQYNRAVIFHTTSFREDWRTLLDYCNAKIVNTEDAKLTKAKVKMVKEAGYELNVWTVN
KPARANQLANWGVDGIFTDNADKMVHLSQ"
/product="Glycerophosphoryl diester phosphodiesterase (EC
3.1.4.46)"
CDS 18633..19061
/transl_table=11
/db_xref="SEED:fig|176279.68.peg.24"
/product="MaoC domain protein"
/translation="MKYDDFIVGETFKTKSLHITEEEIIQFATTFDPQYMHIDKEKAE

QSRFKGIIASGMHTLSISFKLWVEEGKYGEEVVAGTQMNNVKFIKPVYPGNTLYVIAE
ITNKKSIKKENGLVTVSLSTYNENEEIVFKGEVTALINNS"
CDS complement(19107..21113)
/transl_table=11
/db_xref="SEED:fig|176279.68.peg.25"
/db_xref="GO:0008658"
/db_xref="GO:0046677"
/product="Penicillin-binding protein PBP2a, methicillin
resistance determinant MecA, transpeptidase"
/translation="MKKIKIVPLILIVVVVGFGIYFYASKDKEINNTIDAIEDKNFKQ

VYKDSSYISKSDNGEVEMTERPIKIYNSLGVKDINIQDRKIKKVSKNKKRVDAQYKIK

TNYGNIDRNVQFNFVKEDGMWKLDWDHSVIIPGMQKDQSIHIENLKSERGKILDRNNV

ELANTGTAYEIGIVPKNVSKKDYKAIAKELSISEDYIKQQMDQNWVQDDTFVPLKTVK

KMDEYLSDFAKKFHLTTNETESRNYPLGKATSHLLGYVGPINSEELKQKEYKGYKDDA

VIGKKGLEKLYDKKLQHEDGYRVTIVDDNSNTIAHTLIEKKKKDGKDIQLTIDAKVQK

SIYNNMKNDYGSGTAIHPQTGELLALVSTPSYDVYPFMYGMSNEEYNKLTEDKKEPLL

NKFQITTSPGSTQKILTAMIGLNNKTLDDKTSYKIDGKGWQKDKSWGGYNVTRYEVVN

GNIDLKQAIESSDNIFFARVALELGSKKFEKGMKKLGVGEDIPSDYPFYNAQISNKNL

DNEILLADSGYGQGEILINPVQILSIYSALENNGNINAPHLLKDTKNKVWKKNIISKE

NINLLTDGMQQVVNKTHKEDIYRSYANLIGKSGTAELKMKQGETGRQIGWFISYDKDN
PNMMMAINVKDVQDKGMASYNAKISGKVYDELYENGNKKYDIDE"
CDS 21213..22970
/transl_table=11
/db_xref="SEED:fig|176279.68.peg.26"
/db_xref="GO:0008658"
/translation="MLSSFLMLSIISSLLTICVIFLVRMLYIKYTQNIMSHKIWLLVL

VSTLIPLIPFYKISNFTFSKDMMNRNVSDTTSSVSHMLDGQQSSVTKDLAINVNQFET

SNITYMILLIWVFGSLLCLFYMIKAFRQIDVIKSSSLESSYLNERLKVCQSKMQFYKK

HITISYSSNIDNPMVFGLVKSQIVLPTVVVETMNDKEIEYIILHELSHVKSHDLIFNQ

LYVVFKMIFWFNPALYISKTMMDNDCEKVCDRNVLKILNRHEHIRYGESILKCSILKS

QHINNVAAQYLLGFNSNIKERVKYIALYDSMPKPNRNKRIVAYIVCSISLLIQAPLLS

AHVQQDKYETNVSYKKLNQLAPYFKGFDGSFVLYNEREQAYSIYNEPESKQRYSPNST

YKIYLALMAFDQNLLSLNHTEQQWDKHQYPFKEWNQDQNLNSSMKYSVNWYYENLNKH

LRQDEVKSYLDLIEYGNEEISGNENYWNESSLKISAIEQVNLLKNMKQHNMHFDNKAI

EKVENSMTLKQKDTYKYVGKTGTGIVNHKEANGWFVGYVETKDNTYYFATHLKGEDNA
NGEKAQQISERILKEMELI"
/product="Methicillin resistance regulatory
sensor-transducer MecR1"
CDS 22970..23341
/transl_table=11
/db_xref="SEED:fig|176279.68.peg.27"
/db_xref="GO:0003677"
/db_xref="GO:0016481"
/db_xref="GO:0016566"

/translation="MDNKTYEISSAEWEVMNIIWMKKYASANNIIEEIQMQKDWSPKT
IRTLITRLYKKGFIDRKKDNKIFQYYSLVEESDIKYKTSKNFINKVYKGGFNSLVLNF
VEKEDLSQDEIEELRNILNKK"
/product="Methicillin resistance repressor MecI"
CDS 23849..24958
/db_xref="SEED:fig|176279.68.peg.28"
/transl_table=11
/translation="MLREIYNHHNISRTQISKNLEINKATISSILNKLKYKSLVNEVG

EGDSTKSGGRKPILLKVNHLYGYFISLDLTYSSVEVMYNYFDGNVIKHESYDLPDEKV
SSILSIIKKHIDIQEKLDTYNGLLGVSVSIHGVVDNEQHVTYLPFHETEGISIAKKIK

EITNVPVVVENEANLSALYERNFNHNLSYNNLIALSIHKGIGAGLIINNQLYRGANGE

AGEIGKTLVSKVSDNVEIFHKIEDIFSQEALLHNLSNQLNEKMTLSKLIQFYNEKNPV

VVEEMEQFINKIAVLIHNLNTQFNPNAIYINCPLFNEMPEILEAIKNQFKQYSRNEIQ
IKLTSNVKFATLLGGTLAIIQKVLQINDIYLDIKA"
/product="Putative ROK-family transcriptional regulator"
CDS complement(25072..25377)
/db_xref="SEED:fig|176279.68.peg.29"
/transl_table=11
/product="Polysulfide binding protein"

/translation="MPQTNIVTSSIHSSEMTGEEHNVLDVRNDDEWRKGHLKQAIHIP

HGKLLNEDIPFDKNDLIYVHCQSGVRSSIAVGILEYKGFTNIVNVRGGYQHLPAFLK"
CDS complement(25468..26406)
/transl_table=11
/db_xref="SEED:fig|176279.68.peg.30"
/translation="MFFKQFYEDNFSQASYLIGCQRTGEAMIIDPIRDLTKYLKVADK

EGFTITKAAETHIHADFASGIRDVANKLNASIYVSDEGKDEFNYKNMPKQTHFVKHQD
IIYLGKIKLEVLHTPGHPPESISFLLTDEGGGSSIPMGLFSGDFIFVGDVGRPDLLEK

AVNVEGSTKIGAKQMYQSIQFAATLPDYIQIWPGHGAGSPCGKALGSLPTTTLGYEKI

NNWAFNVKDETSFIETLTLNQPAPPHHFSQMKKINQFGMQMYQPYNVYPSSSNTQTAF
DLRSKEAFHGGHMHGTINIPFNKTFINQIGWYLDYD"
/product="Zn-dependent hydroxyacylglutathione hydrolase /
Polysulfide binding protein"
CDS complement(26439..27503)
/db_xref="SEED:fig|176279.68.peg.31"
/transl_table=11

/translation="MEQYKEVHINSLNKEELEKLAKMGQLIDVRTKEEYELGHINGST

LHPVEEIKSFNKNSNKTYYIHCKSGNRSTKACDYLAKQGYDVVNLKGGYKDYEAKNFN
SAPLIEKDIEIKENRKQFDFRGLQCPGPIVNISKEINNISTGEQIEVTVTDPGFNSDI

KSWAKQTGNTLVNLTEEANVINAIIQKEKPKEFEINDTATGTTIVLFSGELDKAVAAM

IIANGAKAAGKDVTIFFTFWGLNALKKAQSTRVKKKGISKMFDLMLPKDPIHMPLSKM

NMFGLGNIMMRYVMNKKNVDSLYSLIDQAIDQDIKLIACTMSMDVMGISKEELRDEVD
YGGVGTYIGHTEQANHNLFI"
/product="Disulfide bond regulator"
CDS 27639..27899
/product="Metal-sensitive transcriptional repressor"

/translation="MTYDKKMINRINRIQGQLNGVVKMMEEEKDCKDIITQLSASKGS
IQRLMGIIISENLIECVKTAEENNESSQELINEAVNLLVKSK"
/db_xref="SEED:fig|176279.68.peg.32"
/transl_table=11
CDS 27899..28543
/translation="MDIANMTIMLLIGVLGGFISGLIGIGGAIIIYPAILILPPLIGI

PAYSAYIASGLTSSQVFFSTLSGSLNARKQPAFSPKLVIYMGGGMLIGSMLGAILASL
FNATFVNTVYVIIAILALILMFIKVKPTTQETKSKPLLFIIVGFGIGVISGIVGAGGA
FIIIPVLLALFKLPMNTVVNNSIAIAFISSVGAFFIKLMQGYIPVESAIFLIIE"
/product="FIG003846: hypothetical protein"
/transl_table=11
/db_xref="SEED:fig|176279.68.peg.33"
CDS complement(28811..29284)
/translation="MIGASLLVAQGVVNLIPQQWVIGLLGLLPLYLGVKIWIKGEEDE
DESSILSLFSSGKFNQLFLTMIFIVLASSADDFSIYIPYFTTLSMSEIFIVTIVFLIM
VGVLCYVSYRLASFDFISETIEKYERWIVPIVFIGLGIYILFENGTFNALISFLL"
/product="Cadmium resistance protein"
/transl_table=11
/db_xref="SEED:fig|176279.68.peg.34"
CDS complement(29323..29496)
/transl_table=11
/db_xref="SEED:fig|176279.68.peg.35"
/product="Cadmium resistance protein"
/translation="MQFTEGLFILKKSPIPKIFGDDKMIATILTATAVYYVATGIDYLV
ILILLFSQVKKVR"
CDS complement(29508..31922)
/transl_table=11
/db_xref="SEED:fig|176279.68.peg.36"

/EC_number="3.6.3.3"

/translation="MDSSTKTLTEDKQVYRVEGFSCANCAGKFEKNVKELSGVHDAKV

NFGASKIDVFGSATVEDLEKAGAFENLKVAPEKARRRVEPVVTEDKNVYRVEGFSCAN

CAGKFEKNVKQLAGVQDAKVNFGASKIDVYGNASVEELEKAGAFENLKVIPEKLANPS
IQAVKEDTKAPKEEKIPFYKKHSTLLFATLLIAFGYLSHFVNGEDNLVTSMLFVSSIV

IGGYSLFKVGFQNLIRFDFDMKTLMTVAVIGAAIIGEWAEASIVVILFAISEALERFS

MDRARQSIRSLMDIAPKEALVRRNGQEIMIHVDDIAVGDIMIVKPGEKIAMDGIIING

VSAVNQAAITGESVPVAKTVDDEVFAGTLNEEGLLEVKITKYVEDTTISKIIHLVEEA

QGERAPAQAFVDKFAKYYTPIIMVIAALVAVVPPLFFGGSWDTWVYQGLAVLVVGCPC
ALVITTPISIVSAIGNAAKKGVLIKGGVYLEELGAIKAIAFDKTGTLTKGVPVVTDFK

VLNDQVEEKELFSIITALEYRSQHPLASAIMKKAEQDNITYSDVRVKDFTSITGRGIQ

GNIDGTTYYIGSPRLFKELNVSDFSLEFENKVKVLQNQGKTAMIIGTDQTILGVIAVA

DEVRETSKNVILKLHQLGIKQTIMLTGDNQGTAEAIGAHVGVSDIQSELLPQDKLDYI

KKMKAEHGNVAMIGDGVNDAPALAASTVGIAMGGAGTDTAIETADIALMGDDLSKLPF
AVRLSRKTLNIIKANITFAIGIKIIALLLVIPGWLTLWIAILSDMGATILVALNSLRL
MRVKDK"
/product="Cadmium-transporting ATPase (EC 3.6.3.3)"
CDS complement(31915..32280)
/product="Cadmium efflux system accessory protein"

/translation="MTKDMCEVTYIHEDKVNRAKKDLAKQNPMDVAKVFKALSDDTRV
KIAYVLSLEGELCVCDVANIIESSTATASHHLRLLKNLGIAKYRKEGKLVYYSLDDEH
VKQLVEKAFLHQREVASIG"
/db_xref="SEED:fig|176279.68.peg.37"
/transl_table=11
CDS 32401..32508
/product="hypothetical protein"
/translation="MLETNPFGDFNQILKYLKHNCPVSLSASFHNLSTD"
/transl_table=11
/db_xref="SEED:fig|176279.68.peg.38"
CDS complement(32518..32895)
/translation="MNKQVRNTTEIVRLAKQKSQKTREKVDKAISKFSIEGKAINFNS

IAKEANVSKSWLYKEHDIRQRIESLRERQITSNVVSKPKKSSRSEEILIKTLKRRVME
LEKENKKLQNQIQKLYGDLYNKE"
/product="Mobile element protein"
/db_xref="SEED:fig|176279.68.peg.39"
/transl_table=11
CDS complement(32902..34794)
/transl_table=11
/db_xref="SEED:fig|176279.68.peg.40"

/translation="MNASSKRKIISQSEISKKIAVMNEEMQGFWANNSWDIRKCPHPS

AIELSKNPALRNRWVRFERVKNLWLRTELKYFYFYHLNNGIWNAKTVWIRKGTVINKM

LDFLDLKYPSITSITEVPIDKAMTEYRTYLTKQGVRITTTNYKITANQEKTPVKANSY

YVTNLKQFIEFYEDFYFDGEEWDKDVWDRRNLPLPDDKVNPTQYEYTINFKGFRNTYF

KQLVKRYCKLRLNMDSFSYVSDIAQKLKEFFNFLDIKFKHVQRVHQLTRVEIEAYLSE
LNMMGIKPSTITGRISILEGLFSTLHRLEWDDVPSKLLIYPEDYPKIPRAKPRFIDEF

VLEQLNSHLDKLPEYIATMTMIVQECGMRISELCTLKKGCLLEDKDGDYFLKYYQWKM

KKEHIVPISKEVVLLIKVREDKVSEEFPDSEYLFPRKDGSPLKQETFRGELNKLAYEQ

NIVDKLGEIYRFHAHAFRHSVGTRMINNGVPQHIVQKFLGHESPEMTSRYAHIFDETL

KNEFTKFQEKLVTNNGDVLNLDDDSEVDDVELQWFKKNINAQVLPNGYCRLPVIAGGC

PHANACLDCTHFCTSKQFLPQHEEQLERTEELLTIAKDKQWQRQIETNSRVKERLEQI
IGSLTG"
/product="Transposase B from transposon Tn554"
CDS complement(34989..35312)

/translation="MSRKRIDVVKVQMVKEDTLWYLKRRIEEPKDAADIMRDFIGNAD
REHFILICLNSKNEPTHIETVSIGTINFAVIHPREIFKTAILSNATGMIIGHNHPSGD
ILTIV"
/product="DNA repair protein RadC truncated by transposon
Tn554"
/db_xref="SEED:fig|176279.68.peg.41"
/transl_table=11
CDS complement(35305..35511)
/translation="MNQTWKEVYQYLMHQPHIDVIKGIIAFSSNKKNNSELDELYSIY
MNDDSITSIVHPDLIESIELIHNE"
/product="FIG01110849: hypothetical protein"

/transl_table=11
/db_xref="SEED:fig|176279.68.peg.42"
CDS complement(35513..36034)
/product="hypothetical protein"
/translation="MKSIKSVLETEAIFSQDKMHRYLLKKTWDIDKEVLTIITMYPHY

DGVINVDLTTQLIVNKVAEKDEYGGVNFINLFSNIDTPINLKHIENSHDKHTDIHIMK

AVKEADSVLLAWGSYGKKPLVENRVNEVLDMLKPHSKKISILTNPQTNEIMHPLNPYA
RKAWTIKPLKETK"
/transl_table=11
/db_xref="SEED:fig|176279.68.peg.43"
CDS complement(36053..36364)
/transl_table=11
/db_xref="SEED:fig|176279.68.peg.44"
/translation="MKRYITRSIANYLPAMLQQQLWKLVAQRENEQFKELEEIDYFHI

FQFNMHNSQLYIKHKQERPEYVKIHKANYSKAININKVYIIREDDVDLSYYVMLLPEE
Y"
/product="FIG01107894: hypothetical protein"
CDS complement(36367..36456)
/transl_table=11
/db_xref="SEED:fig|176279.68.peg.45"
/product="hypothetical protein"
/translation="MNKWIHIARISVTVITEVITIMKDIEKEK"
CDS complement(36449..36799)
/transl_table=11
/db_xref="SEED:fig|176279.68.peg.46"
/translation="MTLEKQLKEYITNLFNLPKDEKWECESIEEVADNILPDQYIRLG
PLTNKILHTYTYYSDTLHERHIYPFILYYQKQLIAIGYIDETNDMDFLYLHNTVMPLL
DQRHLLEKENYNNE"
/product="FIG01108299: hypothetical protein"
CDS complement(37270..38898)
/product="Site-specific recombinase"
/translation="MQQLKQKRVGIYVRVSTEIQSTEGYSIDGQINQIREYCDFNNFV

VVDVYADRGISGKSMNRPELQRLLKDANEGQIDSVMVYKTNRLARNTSDLLKIVEDLH

RQNVEFFSLSERMEVNTSSGKLMLQILASFSEFERNNIVENVFMGQTRRAQEGYYQGN

LPLGYDKIPDSKHELMINQHEANIVKYIFESYAKGHGYRKIANALNHKGYVTKKGKPF

SIGSVTYILSNPFYVGKIQFAKYKDWNEKRRKGLNDKPIIAEGKHSPIIIQDLWDKVQ

LRKKQVSQKPQVHGKGTNLLTGIVHCPQCGAPMAASNTTNTLKDGTKKRIRYYSCSNF

RNKGSKVCSANSVRADVIEKYVMDQILEIVKSDKVINQVLERVNQENKVDIGALNHDI

AYKQQQYDEVSGKLHNLVKTIEDNPDLTSALKATIHQYETQLNDITNQMNQLKQQQNQ

EKLSYDTKQIAALLQRIFQNIESMDKAQLKALYLTVIDRIDIRKDGNHKKQFYVTLKL
NNEIIKQLFNNTPLDEVLLSTSSLFLPQTLFLQI"
/transl_table=11
/db_xref="SEED:fig|176279.68.peg.47"
CDS complement(38919..40265)
/translation="MKRAIGYLRQSTTKQQSLPAQKQAIELLAPKHNIQNIQYISDKQ

SGRTDNRTGYQQVTERIQQRQCDVLCCYRLNRLHRNLKNALKLMKLCQKYHVHILSVH

DGYFDMDKAFDRLKLNIFMSLAELESDNIGEQVKNGLREKAKQGKLITTHAPFGYHYQ

NGTFIINNDESPTVKAVFNYYLQGYGYKKIAQYLEDDNKLITRKPYQVRNIIMNPNYC

GRVINQYGQYNNMVPPIVSATKYEHAQAIRNKKQLHCIPSENQLKQKIKCPCCDSTLT

NMTIRKKHTLRYYICPKNMNESRFVCSFKGINAQKLEVQVLATCQNFFQNQQLYSKIN

NAIHQRLKKQRVIEAKSTLTQEQLIDKLAKGMIDAESFRKQTHLMNQKHKTISSISDN
QLQTSLQKVIQKSFTLNMLHPYIDEIRITKNKALVGIYFKNEPLNIVNQTSQSSIA"
/product="Cassette chromosome recombinase A"
/transl_table=11
/db_xref="SEED:fig|176279.68.peg.48"
CDS complement(40458..40733)
/db_xref="SEED:fig|176279.68.peg.49"
/transl_table=11
/translation="MNTVALAHEIEDERFEYLESTPLDTVKECCKQEGRQISNTYTEE
YKLINDILEKVIKPTSIVAYGEYEDYIHLKKFAQRRISNSLLLLRCN"
/product="hypothetical protein"
CDS complement(40823..42610)
/transl_table=11
/db_xref="SEED:fig|176279.68.peg.50"
/product="hypothetical protein"
/translation="MTNIIFSEYPYWLTEDSWYEVIPPKGKNDQQKSIRLSSPILIKN
KFLDPKSGVEKLVISNGQNIERIETSDILTSQKLPSLIMYGFSINEKYIKSLSNALQL

MRQSLPLSSLYTGVGVLNTDKGVVISLDEPYLSKEITQSQADEIICETQYDLQPKGTF

EDWWQMYLDEVKGNLLLELAVVFGISSLVTAFLKTKHEVEFAGTIFSFTGNSSTGKST

AAALGVSITGNPTKGSNTLFRSWNGTRNALEGYLSNNYGVPIVLDELSSATFKDTTGL

LYSIAEGQGRQRSNIHGEVKSLKNWGTSVISTAEHSIFNNSARNDGLNVRTIEISEAF

TTNADNADTIKKATSANYGHIMPLVAEYLLNREQEVIKWFQNEHLRFKTQLEKETSNT
GIRMFKRYATITTSARILERVIATPVDLNAVRDYLINYHLDSVSERSLADKAIEVIVQ

FVAQNRGKFSDDTRLSTLIENYGMISLEDDHIQVKMIASVFKNMLKEHNFQDTNNVID

ALRDKDYIQSDANRKTTKRSVKDKEGKPKTIVFYHLKLDKSYAPIFGLSEKSKFNIPL
SFISEHQHPILRDMISKAREWDKNGDIDF"
CDS complement(42610..42894)
/db_xref="SEED:fig|176279.68.peg.51"
/transl_table=11

/translation="MKEKEFYTLKRAVTSIAFNLPVGTMFMYHDLNVRANVLCSTEIQ
QDVGRWFAHFVKYTPNLPFIVVGKKGSSLLYLKTAPNPMNHQNNWKGGNN"
/product="FIG01108228: hypothetical protein"
CDS 43032..44081
/product="ORFID:MW0042"
/translation="MIPTIEEIIDKYGSLVDCLKFDISGEVYEDLLLLRSLSDLNEHQ

KMHRVSFIQKHFNPNFDYLLDDSEFRSMNKIYRNLNTMTYINSQNNEFKLKPFINEEQ

LKSLLAIKKVDEHLSYDPSITSKALADINKTQKRLVTKPNILYNRSKKENEYVRLGGK

ISYTKMEKHWEYLHNEIQYYNLTNQLISYVALEKEYAWAFLNELFYLIELYLKAFKGQ
KKTGHEFEEGLNAFIQSYVIILLIDIRLPTVRLYIIRRIVDECEKESDNQKRIKLIEE

SIKKYRYVKEQIERFGNALKEELSIATLSIEQNMLEAKIDYYKNYYYPRKKTKHNNIE
YNVSMFFKAVDSLKK"
/db_xref="SEED:fig|176279.68.peg.52"
/transl_table=11
CDS 44134..44706
/transl_table=11
/db_xref="SEED:fig|176279.68.peg.53"
/product="FIG01109033: hypothetical protein"

/translation="MFRLKKWYLKQKGYFCRVGEKWDNSNLSHENIHSYVDRVLEYET

ETRRNGQPTEEYILASSGEKINEDLRSGTNDHKEILEVFDKYKVKEDITTYRTVSPRI

YKLMEKNAKNLNGVDLFELGILHTSLIKGYESREEGYKLRVKVKKGTPAFYVGNLTGE

ESHYYEVIVVNNLKLKIISIEDYYINCEVV"
CDS 45038..45364
/transl_table=11
/db_xref="SEED:fig|176279.68.peg.54"
/translation="MISKQIKDLRKQHNYTQEELAEKLNTSRQTISKWEQGISEPDLI

MLMQLSQLFSVSTDYLITGSDNIIKKDNKSYYEMNFWAFMSEKWWVIIIIVIIICGTI
GQIFSN"
/product="Transcriptional regulator, Xre family"
CDS 45401..46234
/db_xref="SEED:fig|176279.68.peg.55"
/transl_table=11
/product="hypothetical protein"
/translation="MKIKKLLKTLLIILLCFVLSVIVQNISMLWHIVSIWSVEYILHA
LTYIVLSYFSVKWFIEIVLKSNMKDYRIIPVKFFKSCFLIGLILILVIDLIYLLFIPG
KLIIPHYSTSVGFMEMFFSAFLITGIAAPIFEEMVFRGILMRYFEKQYGILFGIIIPS

ILFSLVHLFNGELKGENLLLLLIGGSIAGIMYAVTAWTFNSIWASAILHMLWNINGLL

NITTQDDHWGIYQYILDTKNVLITGGNYGMDTSLLSICSYIAVIIVMIVTKRHFNFLL
K"
CDS 46439..46603
/db_xref="SEED:fig|176279.68.peg.56"
/db_xref="GO:0003684"
/db_xref="GO:0005524"
/db_xref="GO:0006298"
/transl_table=11
/translation="MNIYQTYACLVIGIIVNSTTTILKRKKLISNIKQLWKSKKTLEE
FIRSNSRFDY"
/product="MutS-related protein, family 1"
CDS 46920..47693
/db_xref="SEED:fig|176279.68.peg.57"
/transl_table=11
/translation="MNDLLKIYEKLKKDIENRWLIPFHYVVFGLALVVYFLEIPIYKL
VNNLDKELVDKVLYAFSLVYDHIVLIFILIIIIILIVYLFFDVFNMNRFVPSPTTYVD
GSESSINYVSAIKRLINFMILIITKYWITYFIVNLIFHNDKLLYLNNDSKHLYKCLLF
LNICIFIVHILKSIFIIKIMADDSLRLINENDLTDFYITLNKNDEYRMVKPKYRKIDN
FIIIKKSEDFTNSNEYKVINKSKNFEEIIYHFDNLTSNH"
/product="hypothetical protein"
CDS complement(47931..48290)
/transl_table=11
/db_xref="SEED:fig|176279.68.peg.58"
/translation="MENTNSNMHKNDNSYVLSALSYLSIFFAPVILPLFVWILADEPT
SGHGKKALGNHIMTWISFCVGRGIFIFSKGVFDKPFDHQLLIFVIALIIAIVFFLIAL

ILYILNIIRGIKILLKK"
/product="hypothetical protein"
CDS complement(48455..49525)
/transl_table=11
/db_xref="SEED:fig|176279.68.peg.59"
/translation="MNLTTQITKMLGIKYPIIQAGMAGSTTPELVSTVSKQGGLGMIG

AGYFDSHQLSKEILEVQKLTQAPFAVNLFTPNDIKYDKKQIEQMNTKLKPYREALGLS
TPKNSTVKEKEKFEDAIEVIESLKVPIIAFTFGIPNQNIIKRLHNAGKILIGTATSVE

EAVENENAGMDIVVAQGYEAGGHRGSFTTINGEFPLVGTLSLVPQIVDNVSIPVIAAG

GIMDGRGLVASLALGAGAAQLGTAYLTTNESGADDKIKNEIIESSETDTILTNVFSGK

LARGIMNEFVHNMNLYSKQVPPYPLQNQLTTQIRKSALEKGYTEWTHIWSGQSTRLAD
TVDAAQLTKNIINDAVKIINNK"
/product="Putative oxidoreductase YrpB, nitronate
monooxygenase family"
CDS complement(49538..50404)
/product="Transcriptional regulator, LysR family"

/translation="MNLNDLEAFYKVCENRSFTKAAQELNYVQSNVTAKIQRLESYYQ
VKLLYRDKKGIKPTPEGIELLKYIKRIFTILHEAEHNVSNNKKLNINIGTIETIAAVH

LPKLLNEYKRKNPKIQINLKTDNSIKLIEHVKNREMDCAFISGNINDKDLICTNIFNE

KLVILSQKSNFKQNINENKSIIMFGEGCFYRKIFEDWLNYHDINIENKMTLNTLDGIL

GCVEAGLGIAFLPMSVLNKGNSDGIEITKVEESFQTISLNIIYRQDNKNNNVINQLKQ
VAFEKMKSNIIK"
/transl_table=11
/db_xref="SEED:fig|176279.68.peg.60"
CDS complement(50391..51581)
/db_xref="SEED:fig|176279.68.peg.61"
/transl_table=11
/product="Uncharacterized MFS-type transporter"

/translation="MKKHAYKQLLLGMIAMLIVMSISRFAYTPILPFMQQDTSMDNQE
AGFLATWNYLGYLVGAVIPIFYIYKTKVIDLKIYLALNIISTILMGFTEQYLIWSIFR
LISGITSGTVFVLTSNIVLDSLKKIQREGISGVLYSAVGLGIFLSSIYLYFFASVDSW

KMVWFVLGIVSLVLGLIVLVFMKENTNTIINFKEEKMKINNRLNKKFVIFFSIAYFFE
GAGYIITGTFLVAIVKSIPSVAEYATLSWMFVGLGAIPSTLIWSLIAEKISYKKAIYG
AFILQIISVCLPVFTHEIFSLVISSVLFGGTFLGLTTLFISKGQSLMYKTDDPLNLVS

LLTVIYSLGQMLAPMFAGILIGKSNNYNIALIFATVLLILGLISTIFSYKQNESIGEK
YELK"
CDS 51792..51935
/db_xref="SEED:fig|176279.68.peg.62"
/transl_table=11

/translation="MNYFRYKQFNKDVITVAVGYYLRYALSYRDMSETLRERGVNVHH
LTV"
/product="Mobile element protein"
CDS 52215..52466
/transl_table=11
/db_xref="SEED:fig|176279.68.peg.63"
/product="Mobile element protein"
/translation="MAKVIKAFKLKPDCHCTSKYLNNLIEQDHRHIKVRKTRYQSINT
AKNTLKGIECIYGLYKKNRKSLQICGFSPCHEISIMLTS"
ORIGIN
1 gaagcgtatc ataaataaaa ctaaaaatta ggttgtgtat aatttaaaaa tttaatgaga
61 tgtggaggaa ttacatatat gaaatattgg attataccttt gcaatatcat acgatgttta
121 tagagtgttt aataaaccat ttttcaacta ttgatgatct agaatatata ataactgtac
181 aaattatatt gattatggaa ctacaattaa attaagaaat tgatgatgaa attttaaatt
241 taaactaatg gaatcaagaa agaatgaaag gaaatataca atgcctacga ttaataaaag
301 gaagtttatt agattttgtg ttagaaacag tggatgtttt tgtaaatgga agtaaaaatg
361 attgtgaatt agtaaatttt aattataaag aaggaaaagt agtttcacgt tatagtacaa
421 aatttgaaag caatcctaaa ttaagaaaag agcaattgat atcgacggta aaagttgtaa
481 ggtatgttat ttttgatttt gagaaaattt tatggggcat agggaaagtt ttattgaagt
541 tcatcatatt aagacaatgt ttactataag aaagttgaaa taaactcaga aacagacttg
601 attcccgttt attcaaattg gcgcaaaatg atacatataa acacctatat attgaagatt
661 taagtcaaac ggtaaataat atttttcact tttaaacaga aacttttttg gaaattatat
721 taattagtat aaaaatgaat atgttcacaa agaaggagag attttattga aacacattaa
781 tcatttttct gaatcgacac tagcatactt ggaaaaagac catgaacatg cattttatgt
841 atattgttta atagacccaa gaaatgatga atgcttttat gttggtaaag gtaaggggaa
901 tagaatattt aaacataaac aagatgcgca gagacaatta ttgtacgaag atatattcag
961 agaagaaaat aaagataact tgaaatttaa tagaattaac gaaatttgta gcaatgattt
1021 gaatgtttta ggatatataa tcagttatgg attaaccgaa tctgaagcat tttctgcaga
1081 aaatgttctt ataaactttc ttaacctaac aaataaaact acattaacaa atatgattaa
1141 cggtcatgga tctaaggcat acttagttga agatttagaa aatgaatttg gctacgattc
1201 aattaatctt gaaaacataa atacgaatga attaatttta gcagtgaaaa tcagagatgc
1261 atttctatta gataaggatg aaagtaaaga gtatcctatt aatgaaagta aacgtgatag
1321 aaataacctt aaatcgcgta cattaggtag ttggataata ggaaaagata aaatacataa
1381 aataaaatat attattggta tcaatacagg tgctaataat gcagttgtct cagcttatga
1441 agtatcattt gaacaagcgg agagtatcga aacaaataat ggtagaatga gatatgcgtt
1501 tattgggctt tcagaaagag atgctactct aaaaaaattg aatttataca aaaaagcact
1561 accagattta agatttggta gtggtagcgc tacagcatat ataaataatg ggacaatgaa
1621 agttgattaa atgaataaaa acatccaatt ccttaaatta gcctttgaaa attttatact

1681 cgtatatata agtgaaaaac ttaaaagaat tagatgttaa ttaaaggaga gaatgctatg
1741 ccatcagatg atattgtaaa aaaactatac tcaaaagagg atattcgatt agaattagga
1801 cttactccgc ataagtttaa taaaaaaatg gaaactattg ctaagctttt taaaattgat
1861 atgaaaattt ttcacaatta caaaggccaa gataaaaata atcagtatac atttaatggt
1921 gtcgcaaaag aattaatcaa agtgttgcta aaaagtgttg attattaccc agtggatatc
1981 aattccaaaa aatttaaaca aaatggaaaa tctaaaaaag aaatgattga aaatatagat
2041 aactctagtt acatgaaata tatttatcag ttaatgaaat ctatcaatga aattcaatac
2101 aaaaggttaa ttgctgatat acatatgaaa gatgtgtacc agaatacaaa agcatggtta
2161 aataatggtg aatcaattaa taaaaaagaa caagagctat accaatatat gacaatttta
2221 ccattacata aaagagttga actgcaaaat gaggtattaa aatctataga cgaaacaatt
2281 tttcaatttc tcgcaaaaga acatagaaat aatcaaattg aagaaaataa tgaattagaa
2341 gcatatacaa aagcaataaa agaaggtaga aatcctaaaa atgattatga attaaatcat
2401 ctcctatata aaaaaaatca attaccctta gatagtctaa tagaagatat atgggactat
2461 gaggaaacag agtatactga gcttgattgg ttaattgcag atatgctaaa acgttcacaa
2521 agagctgata gtaattttat cgaaagttta gagaagaaaa acaaattgag aaaaaatatt
2581 cataaagatt ccattaaaag tatatctaag ttgattgatc atgaattagt taaaaataat
2641 agaaagtggg acgtagcaga attttataaa aatcaacaat tttggaataa tagtacatta
2701 agacgtagct ctcaaaattt tggaatttta tattacaaaa agaataggtt acatacaatg
2761 aatcaaagta atattattaa gaaaataaaa attatcgaag ctaatataga gagtgcaaat
2821 aaacagccga gctatttaga tttcttttta caagctaagt ttgatttaga gaggttagaa
2881 agtgaattaa tgaaatatag cattaatccg aaatatttta ataagataaa tagtgattac
2941 gtaagatatg ctattgaaga tgtacataat gcaattatta aaattgacag atatataagt
3001 aacgataaag tgaagttaaa ctattcaaat gtccaaatgg tagtgagaaa cgaaataaca
3061 gaacaaggaa gctactttgt aacccaggca ttgaatacac tgtcaaaggt agaagagtct
3121 ggctacagct ttgataattt catggtagga cctttcgtag aagactttaa aaaagcaatt
3181 aaaaatcgta gatttggttc taaaaaagat taagattgtt ttttagagtt aaacataaga
3241 ataaatttaa ataaaaaaat aaacccaaca aaccttgtta tatcaatgtt tgttgggttt
3301 atttttttg gtgatttttt aataactcaa ttacatctta ggataatatc gctaaattat
3361 acattgtcga ggccaagaca aataaaaaat taaagcaaaa gttagcgcgc aactttctgc
3421 tataaaggag caaatttata tggagacaag agacaaactg atgtcattga ctcaaagtga
3481 caaaacacag caatggctaa tggacaagtc atctaaccaa gatgacattc aacaattgca
3541 gcaacaattc agtcagcagc ttgatcaaca atataatgca cttttagctg atgaaaaagc
3601 taagttagac caatacgtgg aagtacatca aggattggaa tcattaaagg aagagattga
3661 atcagaacct attacgctta atatcgataa attacccgat atcaaagcaa caatgcttga
3721 aagagccaag aatgatgaac attctgataa aatcgaaaag ctatttgata ggttagaaca
3781 ggcattaaat ggtacgaatc gattatatac gcaattatcg ttgattggta cacgaacaca
3841 tcgaattaca actaaaaatt ttaatcttca aggcttacct aaagcagtcc aacatacgat
3901 tttaccttca aaatttaaga aggtgtatac agtcgatttt aaatcgtttg aaccatcagt
3961 tgcagcgtac atgactcaag attcaaaact gattgacttg ttaaatcaga aagacggact
4021 gtatgacgca ttgctaagtg aattaggctt atcagatgag ctacgtgtat ttgttaaacg
4081 tgcatttatt ggttcgtttc tatttggagg taacttcaaa aatcctaaat tcaagctgaa
4141 tcaatatgta agtgaagtac aatggttgga tgcggtcagc caatttacaa aagtcattga
4201 acttaagaag cacgttgaaa agagtaattc catgcctatg ccttatagta ttgagcatga
4261 tatgagcgca tttcaaggta gcagtattat ggcaatctac gtacaaactg tagcgagtta

4321 tattttcaag cacattctgc taaaagtgta caaagctcag tgcgatcaaa aaacgttcaa
4381 gattatagta cctatacacg atgcgattat gattgaatgt gaagatgaag aaattgcaca
4441 aaatgtgggt cagttaatga aagatacggc taaccagttg ttcaatggtg aatttgcaca
4501 tgtgacagtg gaagaaatag gaggcgtaga ccatgaataa tgatagagga caaagcctac
4561 acatcccaag tagtacacca atcaaagaaa ataatatata tgtagctacg ttacattctg
4621 tgatccaaac agatttctca ggtgaaataa agcaccaatt cacgtatgaa attgaagtga
4681 acaatcagat tgtatatgcg aatcgtaata ttccaacaaa accgagcgct aatcagttgt
4741 caattcatga ttggctgaaa cgtcatagca actatagcgt gggtcatgat acgtatgaac
4801 cttatattga ccgtaagcat ttaatccata ttggtcaata tagcggtaac tattatgtac
4861 aagatgtagc atcgttagat gcgtttggag gcgtattatc atgaatcata tattacaaat
4921 gttatctaag ctattaagtg tggccaagga ggcaatcgac cgtcaaggtc tgattgctat
4981 cctaactatt cctgttaata ataacgatga aatagaagaa acggctcaag gtgaaaccgt
5041 gtataacgaa cttatcgatc agttacgact taatatccca aaagatacgg attatcaacc
5101 taacatctat agttattttg gtattaagaa gaatcctaat gacaccgtac tcatggaaat
5161 gatgataaag gtttttcata tcaaacgctt taattcagaa ctgtttattt tcaaagttaa
5221 cgggtggcaa aagataaatg gagatgaatt acaagggttg atatctaaaa tgatacaagt
5281 attgcttgta gattataagc cttcactaag cactctaaaa aatgtcgtag atggattgca
5341 aaaatcaaca gatgtagaag aacttgttga gaatgagcgc tatattggtt gtggtgaaaa
5401 tatgttcgat cttaatacgt ttcaagtcgt taaaaattca atcgatatct ttccaaaaac
5461 acgattgaat ttatcattaa gtacaaatga tgtaattact gataagatac cgccttattt
5521 taagcaatat atgttacaac ttgcgaatta tgacgatgat ttacaatact ttcttttcca
5581 acatacagca gtattactta cagctgatac taaataccgt aggggtctca tattatatgg
5641 cggagctaag aatggtaaat ctgtatatat tgaactagtt aaatcatttt tctatagtaa
5701 agatattgtg tctaagccac ttaatgagct tgaaggtcgt tttgacaaag aaagtttaat
5761 tgacaaaagt ctaatggcaa gtcatgaaat tgggcaatct aggattcaag aaaagattgt
5821 aaatgacttc aaaaagttat tatctgtaga atcaatgcat gttgatcgta aaggaaaaac
5881 tcaagtggaa gtcattttgg atttgaaact tatttttagt acaaatgcga tacttaattt
5941 tcctcctgaa catgcgaaag ctttggagcg tcgaattaat attattccat gtgagtatta
6001 tgttgaaaaa gcggacactt cattaattga taagctccag agtgagaaga aagaaatctt
6061 tctttacttg atgtatgtgt atcaacagat tgtaaaagca gatatcgagt accttgaaaa
6121 tagccgtgtc actgaaatta ctcacgattg gttaaatttt ggatatgaat ttgtttctag
6181 caggtccgta agtattgcaa atcagaaagc atgtattaat ttactcagaa aacttataga
6241 aatcaaatca ggatcacgaa tcaaagtatc cgagttaaat aaagttatta atgaagaaat
6301 aaaggtgagt tctcaggtta ttaatcagtt aattcaagca aactttgata ctcaaaccaa
6361 actatacaat ggctacgatt attggattga tttaggttgg aaagaagcca ataaaaaaga
6421 gattcatgat atttcggaaa aagataatat tatttcatta gataaaaatg aaaatataac
6481 agacgatgag gcattagatg aagagaattt ggattttgat tgggaggact ttgacgatga
6541 ataatgaaca aattgaagca tttgtagaag tgcttgtgcc tatcatagaa gaacgtatca
6601 ataaaggtaa gtaatctaat tacgtactac aggcaactgc ctgtagtact catataatta
6661 agtggtaaaa gtgataaaaa tgaaacgaaa ttataaatat atattatcta tatgttgtta
6721 caagaccgat agtctgtagc aataatctaa taaaaggaga acggtatgat atgaagggta
6781 aaattgcact ttattcacgt gttagtacgt ctgagcagtc agaacatggt tattctgtaa
6841 aggagcagga acaagtactc atcaaagaag ttgtgaaaaa tttcccaggt tatgactatg
6901 agacatatac tgactcaggc atttcaggta aaaatattga aggtcgtccg gcaatgaaac

6961 gtctattaca agatgttaag gataataaaa tagaaatggt attaagttgg aaattgaatc
7021 gtatctcacg atcaatgaga gacgtgttta atattattca tgaattcaaa gaacatgacg
7081 tagggtataa atcgatttct gagaatattg atacatccaa tgcttctgga gaagtactcg
7141 ttacaatgtt tgggttaata ggatctatag aacgccagac tttgatttcc aatgtgaaac
7201 tttctatgaa tgctaaggca aggagcggag aggcaatcac cggtcgtgtt ttaggctaca
7261 aattatcact taatccactt acacagaaaa atgatttggt tatcgatgaa aatgaagcta
7321 atattgtacg tgaaattttc gatttatatt tgaatcataa taaaggcctc aaagccatta
7381 caaccgtact taatcaaaag gggtatcgta ctattaatca aaagccattt tcagtgtatg
7441 gtgttaaata cattttgaat aatccagtct ataaaggcta tgtcagattc aataaccatc
7501 aaaactgggc tgtacagcga agaagtggca aaagtgataa aaatgatgtg atattggtca
7561 aaggtaaaca tgaagccatt ataagtgaag aggtatttga caaagttcat gaaaaattag
7621 cctctaaaag ttttaaaccg ggcagaccta ttggtggaga tttctactta cgtggtctta
7681 ttaaatgccc agaatgcgga aataatatgg tatgtcgacg gacgtattat aaaacgaaaa
7741 agtccaaaga acggacaatc aagcgttatt acatttgttc cttattcaat cgttcaggga
7801 gttctgcatg tcacagtaat tccatcaatg ctgaagtcgt cgagcgtgta attaatgttc
7861 atttgaatcg tattctgtct caaccagata ttatcaagca gattgcgtca aatgtgatag
7921 aagaactgaa acaaaagcat agtaaccaaa cagaaattaa atatgacatt gatagtttag
7981 aaaaacaaaa agctaagctt aaaacacaac aagaacgatt gttagaattg ttcttagatg
8041 atcagatgga tagcgaaatg ttaaaagcta aacaaagtca aatgaatcaa cagttagaag
8101 tattagatca acaaattaaa gaagcgcaac aagcaaatca atcacaggat gaaataccta
8161 attttgataa attaaaagga cgactcattt tgatgataac acgattcagc gtgtacttaa
8221 gaaaggctac acccgaagct aaaaatcaac ttatgaaaat gttaattgat tcaattgaaa
8281 ttacgacaga taaacaagta aaacttgtaa ggtataaaat tgacgaaagt cttatccctc
8341 aatctttgaa aaaagattgg gggtcttttt ttatgcccaa attccaattt gaaatatatg
8401 gtcaaaatga ttatttcatc gaccaaatta ccacttttac cacttagtta ttagtgacaa
8461 aagtgagcga aatgaaataa aaatcaaata tatattatca aaatgatgta tcacgtacat
8521 acatcaatca aatacattag gaggtcataa tgatgacatt agaacaacaa ctcaagcact
8581 atataaccaa cttattcaat ctgccaaagg acgaagtgtg gcactgcgaa tctatcgagg
8641 aaatcgctga tgatatctta cccaatcaat atgtaagact tggcccactc agtaataaaa
8701 cacttcagac taatacctac tactctgaca cacttcacga aagtaatatc tatcctttca
8761 ttctctacta tcagaaacaa ctcatagcca tcggttatat cgacgaaaat cacgatatgg
8821 atttcttata cctacacaac actatcatgc ctcttttgga tcaacgatac ttactaacag
8881 gaggacaata aaatgcataa atacatcaaa attacacaat tagtcattac aatactaagt
8941 gaaatcatca tttggatgaa agagtcagaa cgaaaggaag tctcttatga atagatatat
9001 cacccggggt atcgccaaca acttacctaa tatcttacaa caccaattat ggcaactcgt
9061 atctgagcga gaacaagaac aaaccaaaga taatacttca gtagattatt ttcatatatt
9121 ccagttcaat aagcatcgca atcaattata tatcaaacac aaacaagaac gacctgaata
9181 tgcgaaaatc cataaagcta attattcaaa agcaatcaat atcaataagg tctacattat
9241 tcgagaagat gatgtagacc tttcttatta tgtcatgtta ttacctgaag aatactagag
9301 gagtgaaaat tatatggaaa caatcaaaag tacattaaaa acagaagcta tattcagtga
9361 tgacaaacaa catcgctatc tacttaagaa aacatggaat agtgaaaaac aatcaatcac
9421 aatcattaca atgtatccgc attatgatgg cattctcaat attgacttaa cgacccaact
9481 cattatgaac aaaatttcag aaatggatgc atttggttca atccattttg tgaatctgta
9541 ctctaatatt acgacaccta tcaacttaaa acatttagaa aatgcctatg ataaccatac

9601 agatattcaa attatgaaag cagtgaaaga atcggatgaa gtgatattag catggggtgc
9661 ttacgctaaa aaaccgattg ttgaagcgcg tgtcaatgaa atattagaga tgttgaaaac
9721 acataaaaag aaagtgaagc aactcatgaa ccccgcaact aatgaaatca tgcatcccct
9781 taatccaaaa gcaagacaga aatggacttt gaaagcgtaa cctatatatg aagggtggag
9841 ataaattatg aaagaaatca atatcgtttc actacaaatg ataaaaacag atacattaag
9901 ttatctaaaa gctcgtattt caaaccctga ggatgcgaca gaaatcatgc gttcattcat
9961 tggaaacagt gaccgagaac atctcattct catatgtatg aacagtaaaa atgaacctac
10021 acatattcaa acactatcga ttggatctat taaccaaaca gtgattcacc ctagagaaat
10081 attcaaaaca gcgatactca gtaacgcaaa tagtataatg ctcggtcata atcatccaag
10141 tggagatgta acaccctctc ctgaagatat caatgtaacc aagcgattaa tgctcatcag
10201 tgaaatgatg ggcattttat tttatgacca tattattttc tcagatagta acacctattc
10261 cattagacaa cataatttag aagtagaggt atagccgtat tttgtcacta ccgttatttc
10321 ttcacttatc aatatttata aaatcccttt ataacaacaa tttcaaatga aaaatatttt
10381 ttacaatttt tattaaatat acgtaggatt gacttattgt gacaaaccca ttttgtaata
10441 tataacttaa aggaggtgac atattggatc gtgcatttcg aatacttacg atatacaatc
10501 gtttactaca aaataaatcc gttaacaagc aatcactcac attagaactc gatacaagtc
10561 ctcggacgat acaaagagat atcgacgata taagaaactt cttatatgaa agtaaatcat
10621 ggataagtac accaaaagaa atcacgtacg attataaatc tgaaagttat aagctcgaac
10681 aagataagaa tgaaaatgtg cattttatgt ataatatttt gactgcttta tacctgacga
10741 cacctcaatt aagtcattac ttttatcaat atcttaaatt attaatcatc aaacatcatt
10801 caaatcatcg aactacatta ttaaaatatc ttgagcgctt tgaaattgat aagaatcaaa
10861 caggtatttc gacatcatca ttagttgttc aagcaatgaa cgaaaataaa tatcttcaat
10921 acaatcaaaa tctattatta gctttatcga tgtactatca aatgcatgct tttcacttgg
10981 tatatcgaat gaacgatgaa atttatatcg atgatattaa tcaaatgaat ctgagtttct
11041 ctgaaaaaac tgtggacaca catgaaaaat caaaggtaca aacctatatc acgtttgaaa
11101 tttctaaaga tgtgtggtca aaaatgcatc gatattatca aattcatgtg atagagaaat
11161 atgatgtaca ttatcttatc gtcactttta agatgacaag acccgaagcg attcaactct
11221 gttttatgta tcgatccaat atacgtatca tttcaccccc tgatttgaga gaacaagtga
11281 ttgatgagtt acttttattg cagtcaacat atttaaaaca acaggttcaa gaataagcat
11341 aatttacaat attttataaa ttattaaata cgaaagggat tggaattatg ggcaacataa
11401 taaaaacgat agggaaaggt attgggaaag gtgtcggtgc agcagcatat tattcagttg
11461 cgactgttgg cgctgccatc ggtgcgattt gggaaacaac aggcgataga ggtttgatga
11521 gagatgcttt aagtgatagt tctaataaag atttgatgga aatatatgag atacagaaga
11581 aagatgatgg tgattatgat acttatattg cggctaaatc cttattacaa gagagaggat
11641 tcacttataa tcacgaaacg aataaatggg aaaaataaca gggagtgaaa taaatggaat
11701 ttgcatatta tgaagcgaaa tctttaaatg attttgaaaa tgaactaaaa acacttgctt
11761 taaataaaaa atatacattt agtaaagaaa ccgaagtcct taaagaagaa gaaattcttg
11821 aaaaatcgaa tagccatgag cgcgaactat taaaagtatt taaagataat gcgcagataa
11881 caacgtatca ttttaagagt agtcaattaa gtgcctctaa aagttctatt caacggttaa
11941 tgggtattat cattagtgaa aacttagtag aatgcgtcaa aatgtctgaa gaaaatagtg
12001 aagattctca ggcactaatt aatgaagcag ttgaattatt gataaaaagt aaatgataag
12061 tataaccacc attatagtaa tgttagttat tggtatctta ggaggattta tttctggatt
12121 agtcggtata gggggagcca ttgttattta cccggctctc ttattattac caccattatt
12181 tggtttacct acttatagtg cttatattgc ttcaggatta acttcaagtc aggtgttttt

12241 tagtacattg agtggatctt taaacgcata taaaaataaa aatttttcta gaacgcttat
12301 acttaatatg ggaagtggaa tggtaatagg tagtatgcta ggtgcaatct tagcgaattt
12361 aataaatgtt cagtttgtta atacggttta tattattatt gcattactag ctcttattct
12421 aatgtttata aaagttacac catctactag tcatattaag tttaatcgtc tacttttaat
12481 tacaataggt ggtatcatcg gtttagtatc tggtattgta ggagctggtg gcgcatttat
12541 tattattcca gtactacttg ttatatttaa attaccaatg aacatggtag ttacaaatag
12601 tattgtaatt gcattcattt cttctattgg agcatttatg attaaattat tacagggtta
12661 tattcctatt aatagtgcaa taccgttaat tttaggtagt atactattta cgcctctagg
12721 tatgaaaatg ggtcagaaaa tacctgacta tattcaaaaa acgataatat gtattttaat
12781 cgtcattgca attattaagt tgatatttta aaaaaatggg agtgggacaa cattcatttt
12841 gaacttgatt gaatatatag tatatttaat aaaatgcagc tattgtggcg tagaatttaa
12901 gaatcaaaaa atgattaata tagtttgaaa agactgaata taaatactag aaaaatagcc
12961 agtaaatgag tttattatga attcatttct tgactctttc aaataatact atgagaaaaa
13021 tggtgcaaaa cttatggtgg ggtgcaggtt ataatattgt tgctgtacct ttagcagctg
13081 gtattttagc atttatcggc ttgattttat cacctgcaat aggtgctatt ttaatgtcac
13141 taagtacggt tatcgttgca attaatgcct ttacattaaa attaaaataa aagataggag
13201 ttttattatg attaaaaaat tattttttat gatattagga tcattactaa tattatcagc
13261 ttgttccaat aatgatgaaa aagataaaga cactaatgac caaaaaagtg agagccatat
13321 gaagcataat gatgaaagta aagttcctga agatatgaca tcgactaatg agggtgaatt
13381 taaagtagga gataaagtaa cgattacagc aggacatatg ccaggtatga aaggtgcaga
13441 agctactgta aaaggtgcgt ataaaacata tgcctatgtt gtaagttata aacccacaaa
13501 tggaaatgaa aaagtaaaca atcataaatg ggtcgtaaac gaagagatca aagatgcacc
13561 taaagatgga tttagtaagg gcgatactgt taaattagaa gcaagtcata tgtctggtat
13621 gaaaggtgct acagccaata tagataacgt gaaaaagacg actgtttacg tagttgatta
13681 caaatccaaa gataatggta aaatcattaa aaatcataaa tggatgacag gaaatgaact
13741 gaaagcacga taaaaatcta gttctagatt gagaaataaa tagatataaa gatatcctcc
13801 ttaatcaata atttaaataa cttattattg ttaaggagga tattttttag tgtgtaaatt
13861 aaaaagaatt ttagaagaat aacatttatc aaaaaactgt tcattacctt attaaatgaa
13921 attatataat taaaaaccgc atcattaacc gatacgcaga agcttatcat aagtaatgag
13981 gttcatgatt tttgacatag ttagcctccg cagtctttca tttcaagtaa ataatagcga
14041 aatattcttt atactgaata cttatagtga agcaaagttc tagctttgag aaaattcttt
14101 ctgcaactaa atatagtaaa ttacggtaaa atataaataa gtacatattg aagaaaatga
14161 gacataatat attttataat aggagggaat ttcaaatgat agacaacttt atgcaggtcc
14221 ttaaattaat taaagagaaa cgtaccaata atgtagttaa aaaatctgat tgggataaag
14281 gtgatctata taaaacttta gtccatgata agttacccaa gcagttaaaa gtgcatataa
14341 aagaagataa atattcagtt gtagggaagg ttgctactgg gaactatagt aaagttcctt
14401 ggatttcaat atatgatgag aatataacaa aagaaacaaa ggatggatat tatttggtat
14461 atctttttca tccggaagga gaaggcatat acttatcttt gaatcaagga tggtcaaaga
14521 taagtgatat gtttccgcgg gataaaaatg ctgcaaaaca aagagcatta actttatctt
14581 ccgaactcaa taaatatatt acatcaaatg aatttaatac tggaagattt tattacgcag
14641 aaaataaaga ttcatcttat gatttaaaaa atgattatcc atcaggatat tctcatggat
14701 caataagatt caaatattat gatttgaatg aaggattcac agaagaagat atgctagagg
14761 atttaaagaa atttttagaa ctatttaatg aattagcttc aaaagttaca aaaacatcct
14821 atgatagctt ggtcaatagc atagacgaaa tacaggaaga cagcgaaatt gaagaaatta

14881 gaacagcaca aaaagataag acactcaagg aagtggaagc acctaaagga ataattccaa
14941 aatataaaaa aggtgtatca aagactacta aaaatgattc agaaattgaa aaatcaaata
15001 aagagaataa attaaccggt aaagttggag aaaaattagc gctaaattac tttaatgagc
15061 taattgataa taaaatagac gaagataaga aagaacagtt taggaatatt ttaaatgata
15121 atccaggctc tcaacacggt catggctatg atttagtagc ttttgatcca acaaatacag
15181 ataaagctgt agaaaaattt attgaaatta aaacatctac atcttctagt attgaggaac
15241 catttttat gtcgctaaat gaaatgtttg ctatgaaaga atataagcag aaatatttaa
15301 tattaagaat atttaatgtt tccggtaaag aaccacaatt ttattttata gatccatatg
15361 caaattattc tgaatttaaa gatgtagatg atctcattga caaagtattt aatgtagaag
15421 ctattcagta taaagttttt ggcgaaaaat gattacttga acaagagcta aaataaaatt
15481 gtgatctaat aaaaatagaa actgtaattt aaataaaact ttctaaataa gctaactgat
15541 aaaaaatcag tttgtccaca gtctgaaaca agattcctat attctttagg aatcttgttt
15601 tttctatttt tatggtgata aagagcagat aagataatgt gtaataatca caaaaaagtt
15661 aaatatttta aggcttgttt aattattaat gattttatat ataaagagca gtataataaa
15721 gttgttaata tattatgaat aatattcaag taattttatt gtttttaat ttgtcgatat
15781 ttaagttgag ttaaatttaa agggtgtaat ttgttttaca atgatgaaga taattagggt
15841 tctgttgcaa agtaaaaaaa tatagctaac cactaattta tcatgtcagt gttcgcttaa
15901 cttgctagca tgatgctaat ttcgtggcat ggcgaaaatc cgtagatctg aagagacctg
15961 cggttctttt tatatagagc gtaaatacat tcaatacctt ttaaagtatt ctttgctgta
16021 ttgatacttt gataccttgt ctttcttact ttaatatgac ggtgatcttg ctcaatgagg
16081 ttattcagat atttcgatgt acaatgacag tcaggtttaa gtttaaaagc tttaattact
16141 ttagccattg ctaccttcgt tgaaggtgcc tgatctgtaa ttaccttttg aggtttacca
16201 aattgtttaa tgagacgttt gataaacgca tatgctgaat gattatctcg ttgcttacgc
16261 aaccaaatat ctaatgtatg tccctctgca tcaatggcac gatataaata gctccatttt
16321 ccttttattt tgatgtacgt ctcatcaata cgccatttgt aataagcttt tttatgcttt
16381 ttcttccaaa tttgatacaa aattggggca tattcttgaa cccaacggta gaccgttgaa
16441 tgatgaacgt ttacaccacg ttcccttaat atttcagata tatcacgata actcaatgta
16501 tatcttagat agtagccaac ggctacagtg ataacatcct tgttaaattg tttatatctg
16561 aaatagttca tacagaagac tcctttttgt taaaattata ctataaattc aactttgcaa
16621 cagaaccgta ttatggaata gagatgttgg taacatttat acaggatcat tatacttaag
16681 tttaatttcg ttattacaga accacacatt ccaaccagaa gagaaagtat gtctatttag
16741 ttatggttca ggagcagtag gagaaatctt tagtggttca atcgttaaag gatatgacaa
16801 agcattagat aaagagaaac acttaaatat gctagaatct agagagcaat tatcagtcga
16861 agaatacgaa acattcttta acagatttga taatcaagaa tttgatttcg aacgtgaatt
16921 gacacaagat ccatattcaa aagtatactt atacagtata gaagaccata tcagaacata
16981 taagatagag aaataaacta gtggccgatt gtgcttgatg agcttgggac ataaatccta
17041 actcgaaata aataagcata tcactaaact gattttttaa agtttacagt gatatgctta
17101 ttttttatc ttacgatttt gtacgtgcat gcttgcctag gggtatggct cgagccatta
17161 gtctctcgca catactattc cctcaggcgt cagcacttac aaaatcggtt gtaattttca
17221 tttttatacg cattcttact gagattatac taataagagg aatagtaaaa gcaattctaa
17281 gtaaaattgc agataagagg tttgttaaaa gcagttctaa gtaaaattgc agataagagg
17341 tttgttaaaa gcagttctaa gtaaaattgc agataagagg tttgttaaaa gcagttctca
17401 gtaaaattac agataagagg tacgttaaaa gcagttctaa gtaaaattgc agataagagg
17461 tacgttaaaa gcagttctag gcaaaattgc agataagagg tgcgttaaaa gcagttctca

17521 gtaaaattgc tgataagggg taagttaaaa gcaatcctaa gtaaaattgc agataagggg
17581 tacagaaaaa ctagacttga ttacaaaatg gagcttggga cataaatgat tttttaaaaa
17641 tgagatgaga cgtagattaa ctccataatc aatacgaatc tatcgacttc tttatttatg
17701 atattcatct ctttttaatg gaaataaaag tgcgattaat gtgataatac agttacgtta
17761 attaaaaaaa taaaaatgca aggagaggta atatgctaac tgtatatgga catagaggat
17821 tacctagtaa agctccggaa aatacaattg catcatttaa agctgcttca gaagtagaag
17881 gtataaactg gttggagtta gatgttgcaa ttacaaaaga tgaacaactg attatcattc
17941 atgatgatta tttagaacgg actacaaata tgtccgggga aataactgaa ttgaattatg
18001 atgaaattaa agatgcttct gcaggatctt ggtttggtga aaaattcaaa gatgaacatt
18061 tgccaacttt cgatgatgta gtaaaaaatag caaatgaata taatatgaat ttaaatgtag
18121 aattaaaagg tattactgga ccgaatggac tagcactttc taaaagtatg gttaagcaag
18181 tggaagaaca attaacaaac ttaaatcaga atcaagaagt gctcatttca agctttaatg
18241 ttgtgcttgt taaacttgca gaagaaatca tgccacaata taacagagca gttatattcc
18301 atacaacttc gtttcgtgaa gactggagaa cacttttaga ttactgtaat gctaaaatag
18361 taaacactga agatgccaaa cttactaaag caaaagtaaa aatggtaaaa gaagcgggtt
18421 atgaattgaa cgtatggact gtaaacaaac cagcacgtgc aaaccaactt gctaattggg
18481 gagttgatgg tatctttaca gacaatgcag ataaaatggt gcatttgtct caatagaaag
18541 ttagaggtga gtcttacgtt tcagtgacgg tagacttacc tttaacatgt tacatactaa
18601 aaaattaatt tgaataagaa agagagacat atatgaaata cgatgatttt atagtaggag
18661 aaacattcaa aacaaaaagc cttcatatta cagaagaaga aattatccaa tttgcaacaa
18721 cttttgatcc tcaatatatg catatagata aagaaaaagc agaacaaagt agatttaaag
18781 gtatcattgc atctggcatg catacacttt caatatcatt taaattatgg gtagaagaag
18841 gtaaatacgg agaagaagtt gtagcaggaa cacaaatgaa taacgttaaa tttattaaac
18901 ctgtataccc aggtaataca ttgtacgtta tcgctgaaat tacaaataag aaatccataa
18961 aaaaagaaaa tggactcgtt acagtgtcac tttcaacata caatgaaaat gaagaaattg
19021 tatttaaggg agaagtaaca gcacttatta ataattcata ataaaacagt gaagcaacca
19081 tcgttacgga ttgcttcact gttttgttat tcatctatat cgtatttttt attaccgttc
19141 tcatatagct catcatacac tttacctgag attttggcat tgtagctagc cattccttta
19201 tcttgtacat ctttaacatt aatagccatc atcatgtttg gattatcttt atcatatgat
19261 ataaaccacc caatttgtct gccagtttct ccttgtttca ttttgagttc tgcagtaccg
19321 gatttgccaa ttaagtttgc ataagatcta taaatatctt ctttatgtgt tttatttacg
19381 acttgttgca taccatcagt taatagattg atattttctt tggaaataat atttttcttc
19441 caaactttgt ttttcgtgtc ttttaataag tgaggtgcgt taatattgcc attattttct
19501 aatgcgctat agattgaaag gatctgtact gggttaatca gtatttcacc ttgtccgtaa
19561 cctgaatcag ctaataatat ttcattatct aaatttttgt ttgaaatttg agcattataa
19621 aatggataat cacttggtat atcttcacca acacctagtt ttttcatgcc tttttcaaat
19681 ttcttactgc ctaattcgag tgctactcta gcaaagaaaa tgttatctga tgattctatt
19741 gcttgtttta agtcgatatt accatttacc acttcatatc ttgtaacgtt gtaaccaccc
19801 caagatttat ctttttgcca acctttacca tcgattttat aacttgtttt atcgtctaat
19861 gttttgttat ttaacccaat cattgctgtt aatatttttt gagttgaacc tggtgaagtt
19921 gtaatctgga acttgttgag cagaggttct tttttatctt cggttaattt attatattct
19981 tcgttactca tgccatacat aaatggatag acgtcatatg aaggtgtgct tacaagtgct
20041 aataattcac ctgtttgagg gtggatagca gtacctgagc cataatcatt tttcatgttg
20101 ttataaatac tcttttgaac tttagcatca atagttagtt gaatatcttt gccatctttt

20161 ttctttttct ctattaatgt atgtgcgatt gtattgctat tatcgtcaac gattgtgaca
20221 cgatagccat cttcatgttg gagcttttta tcgtaaagtt tttcgagtcc ctttttacca
20281 ataactgcat catctttata gcctttatat tctttttgtt ttaattcttc agagttaatg
20341 ggaccaacat aacctaatag atgtgaagtc gcttttccta gaggatagtt acgactttct
20401 gtttcattag ttgtaagatg aaattttttt gcgaaatcac ttaaatattc atccattttt
20461 ttaacggttt taagtggaac gaaggtatca tcttgtaccc aattttgatc catttgttgt
20521 ttgatatagt cttcagaaat acttagttct ttagcgattg ctttataatc ttttttagat
20581 acattctttg gaacgatgcc tatctcatat gctgttcctg tattggccaa ttccacattg
20641 tttcggtcta aaattttacc acgttctgat tttaaatttt caatatgtat gctttggtct
20701 ttctgcattc ctggaataat gacgctatga tcccaatcta acttccacat accatcttct
20761 ttaacaaaat taaattgaac gttgcgatca atgttaccgt agtttgtttt aattttatat
20821 tgagcatcta ctcgtttttt atttttagat actttttta ttttacgatc ctgaatgttt
20881 atatctttaa cgcctaaact attatatatt tttatcggac gttcagtcat ttctacttca
20941 ccattatcgc ttttagaaat ataactgcta tctttataaa cttgtttgaa atttttatct
21001 tcaattgcat caatagtatt attaatttct ttatcttttg aagcataaaa atatatacca
21061 aacccgacaa ctacaactat taaaataagt ggaacaattt ttatcttttt catcaatatc
21121 ctccttatat aagactacat ttgtagtata ttacaaatgt agtatttatg tcaaaataat
21181 gttataattt ttgtgatatg gaggtgtaga aggtgttatc atctttttta atgttaagta
21241 taatcagttc attgctcacg atatgtgtaa ttttttagt gagaatgctc tatataaaat
21301 atactcaaaa tattatgtca cataagattt ggttattagt gctcgtctcc acgttaattc
21361 cattaatacc attttacaaa atatcgaatt ttacattttc aaaagatatg atgaatcgaa
21421 atgtatctga cacgacttct tcggttagtc atatgttaga tggtcaacaa tcatctgtta
21481 cgaaagactt agcaattaat gttaatcagt ttgagacctc aaatataacg tatatgattc
21541 ttttgatatg ggtatttggt agtttgttgt gcttatttta tatgattaag gcattccgac
21601 aaattgatgt tattaaaagt tcgtcattgg aatcgtcata tcttaatgaa cgacttaaag
21661 tatgtcaaag taagatgcag ttctacaaaa agcatataac aattagttat agttcaaaca
21721 ttgataatcc gatggtattt ggtttagtga aatcccaaat tgtactacca actgtcgtag
21781 tcgaaaccat gaatgacaaa gaaattgaat atattattct acatgaacta tcacatgtga
21841 aaagtcatga cttaatattc aaccagcttt atgttgtttt taaaatgata ttctggttta
21901 atcctgcact atatataagt aaaacaatga tggacaatga ctgtgaaaaa gtatgtgata
21961 gaaacgtttt aaaaattttg aatcgccatg aacatatacg ttatggtgaa tcgatattaa
22021 aatgctctat tttaaaatct cagcacataa ataatgtggc agcacaatat ttactaggtt
22081 ttaattcaaa tattaaagaa cgtgttaagt atattgcact ttatgattca atgcctaaac
22141 ctaatcgaaa caagcgtatt gttgcgtata ttgtatgtag tatatcgctt ttaatacaag
22201 caccgttact atctgcacat gttcaacaag acaaatatga aacaaatgta tcatataaaa
22261 aattaaatca actagctccg tatttcaaag gatttgatgg aagttttgtg ctttataatg
22321 aacgggagca agcttattct atttataatg aaccagaaag taaacaacga tattcaccta
22381 attctactta caaaatttat ttagcgttaa tggcattcga ccaaaattta ctctcattaa
22441 atcatactga acaacaatgg gataaacatc aatatccatt taaagaatgg aaccaagatc
22501 aaaatttaaa ttcttcaatg aaatattcag taaattggta ttacgaaaat ttaaacaaac
22561 atttaagaca agatgaggtt aaatcttatt tagatctaat tgaatatggt aatgaagaaa
22621 tatcagggaa tgaaaattat tggaatgaat cttcattaaa aatttctgca atagaacagg
22681 ttaatttgtt gaaaaatatg aaacaacata acatgcattt tgataataag gctattgaaa
22741 aagttgaaaa tagtatgact ttgaaacaaa aagatactta taaatatgta ggtaaaactg

22801 gaacaggaat cgtgaatcac aaagaagcaa atggatggtt cgtaggttat gttgaaacga
22861 aagataatac gtattatttt gctacacatt taaaaggcga agacaatgcg aatggcgaaa
22921 aagcacaaca aatttctgag cgtattttaa aagaaatgga gttaatataa tggataataa
22981 aacgtatgaa atatcatctg cagaatggga agttatgaat atcatttgga tgaaaaaata
23041 tgcaagtgcg aataatataa tagaagaaat acaaatgcaa aaggactgga gtccaaaaac
23101 cattcgtaca cttataacga gattgtataa aaagggattt atagatcgta aaaaagacaa
23161 taaaattttt caatattact ctcttgtaga agaaagtgat ataaaatata aaacatctaa
23221 aaactttatc aataaagtat acaaaggcgg tttcaattca cttgtcttaa actttgtaga
23281 aaaagaagat ctatcacaag atgaaataga agaattgaga aatatattga ataaaaaata
23341 aaattgttgt gtttacaaca atacatagaa aacagaggaa acaatcaagt cgttgaatat
23401 ttcctctgtt ttttagttga aaaaattaac cgaaagcctg aatgcaagtc ttgattaaat
23461 caataatgct tgtaataaca ccagtgaaat ccatatgcat accctctttc tatttaagat
23521 acattaagta taatatcaaa caaataaaaa atgttaaaaa ttccctaatt ggctatttag
23581 attgcataaa tgtcaaaaat ttgaaaaaca tacaacgact ttgcataaaa aatcgtcata
23641 ttggaaatac gtaatttatt gaaataataa aaaaaataaa agaacgaaga tgataaccta
23701 agtgaggttt taagttgttc taaggtttaa tttaatttat gttaaaatag ttggtataaa
23761 aatacatgat aaactataaa ctaaattcaa aataacttat ggggtaggca attatggaaa
23821 ataaaatata aatgataatg aaaaaagagt gctaagggaa atttataacc atcataatat
23881 ttcgcgtact caaatatcta aaaatcttga gattaataag gcaacgattt ctagtatttt
23941 gaataagtta aagtataaat ctcttgttaa tgaggttggt gagggtgata gcacgaagag
24001 tggtggtaga aaacctattc ttctgaaggt taatcatctt tatggttatt ttatttcttt
24061 ggatttaact tatagttctg ttgaagtgat gtacaattat tttgatggta atgtcattaa
24121 gcatgaatct tatgatttac ctgatgaaaa ggttagtagt atattaagca taataaaaaa
24181 acatattgat attcaggaga aacttgatac ttataacgga ctattaggtg tgtctgtttc
24241 tatacatgga gttgtggata atgagcagca tgtgacatat ttaccattcc atgaaactga
24301 aggaatttca attgctaaga aaataaaaga aattactaat gttccagtcg tagttgaaaa
24361 tgaagcgaat ctttcagcgt tatatgaacg taattttaat cataatttat cctacaataa
24421 tcttattgct ttaagtatac ataaaggtat tggtgctggg cttattatta ataatcaatt
24481 gtatcgtggt gcaaatgggg aagcgggtga aattggaaaa acacttgtct caaaagttag
24541 cgataatgtg gagatctttc ataagattga agatattttt tcacaagaag ctttactgca
24601 taatttaagt aatcaactaa atgagaagat gacgcttagc aaattaattc aattttataa
24661 tgaaaaaaat ccagtcgtag ttgaagaaat ggaacaattt ataaataaaa ttgctgtttt
24721 aatacataat ttaaacaccc agtttaatcc gaatgcaatt tacattaact gtccattgtt
24781 caatgaaatg cctgaaatat tagaagcaat taagaaccag ttcaaacaat attcacgtaa
24841 cgaaattcaa ataaagttaa catctaatgt caaatttgca actttgctag gtggtacatt
24901 agcaattatc caaaaagtac tacagattaa tgatatttac ttagatataa aagcataaaa
24961 aactaattca aatgaataat caaagttcgt aattgtcttt ataaaaaaat ccctcaatcc
25021 gaattgaatt ttcggattga gggattttta tagttctatt gcagaagaaa actattttaa
25081 aaatgctggt aaatgttgat agccacctct aacgttaaca atattcgtaa atcctttata
25141 ttctaatatt cctaccgcta ttgaacttct aacacctgat tgacaatgta cataaattag
25201 gtcattttta tcgaaaggta tatcttcatt taaaagttta ccgtgaggaa tatgaattgc
25261 ttgttttaaa tgacctttac gccattcatc atcattacga acatctaata cattatgttc
25321 ttcaccagtc atttcagaac tatgaataga tgatgtgacg atatttgttt gtggcaaacg
25381 gtaacttttt acattttcaa aaccaattaa ttgtaaagca tgaatagctt gttgaacggt

25441 agatttatcg ccaattaatt caatatctta gtcataatct aaataccaac caatttgatt
25501 tataaaagtt ttattaaaag gaatattgat agttccatgc atatgaccac catggaatgc
25561 ttctttactt cgaagatcaa aagcagtttg tgtattgctt gaactagggt aaacattata
25621 tggttggtac atttgcatac caaattgatt tatttttttc atttgtgaaa aatggtgtgg
25681 tggagctggc tgattgagtg ttaaagtttc gataaatgaa gtttcatctt taacattaaa
25741 agcccagttg tttattttct cataacccaa agtagttgta ggtaatgaac ctagcgcttt
25801 accacaagga ctccctgcac catgacctgg ccaaatttga atatagtctg gtaatgttgc
25861 agcaaattgt atggactgat acatttgttt tgctccgatt tttgtagaac cttcaacatt
25921 tacagctttt tctaatagat ctggtctacc tacatcacca acaaagatga agtcaccgct
25981 aaataatccc attggtatac tggaaccccc accttcgtca gtaagtaaaa aactaatact
26041 ctcaggggga tggcctggag tgtgtaagac ttctaattta atctttccta aatagataat
26101 atcttgatgc ttaacgaaat gtgtttgttt aggcatattt ttataattaa attcatcttt
26161 accttcatca gatacgtata tacttgcatt caatttattt gccacatctc taatacctga
26221 agcaaaatca gcatgaatat gtgtttctgc agctttagta attgtgaatc cttctttatc
26281 tgcaactttt aaatattttg ttaaatctcg tatagggtca ataatcattg cttctcctgt
26341 acgttgacat ccaattaaat aagatgcttg tgaaaaattg tcttcataaa attgtttgaa
26401 aaacaaaaaa aactcctttt taaaatagat tttattgatt agataaataa gttatgattt
26461 gcttgctcag tatgtccaat ataagtacct acgccaccat aatcgacttc atctcttaat
26521 tcttcttttg aaattcccat aacatccata ctcatggtac aagcaattaa ctttatatct
26581 tgatcgattg cttgatcgat aagtgagtat aaagaatcaa catttttctt gttcattaca
26641 taacgcatca taatattacc tagtccaaac atattcattt ttgataatgg catatgtatt
26701 ggatccttag gtaacataag gtcaaacatt ttggaaatac ctttcttttt aacgcgagtt
26761 gattgcgctt tttttaatgc gttgaggccc caaaaagtaa agaaaatagt tacatcttta
26821 cctgctgctt tagcgccatt tgcgatgatc attgctgcta ctgccttatc taactcaccg
26881 ctaaataaaa caattgttgt acctgtagca gtgtcattga tttcaaattc ttttggcttt
26941 tctttttgaa taattgcatt aattacattt gcttcttcag taagatttac aagggtattc
27001 cctgtttgtt tcgcccaact tttaatatca ctattgaaac caggatctgt aactgttacc
27061 tcgatttgct cacccgttga aatattgtta atttctttac tgatattaac aataggtcca
27121 gggcactgaa gacctctaaa atcaaattgt ttacgattct ctttgatttc aatatctttt
27181 tctattaaag gagcactatt gaagttcttt gcttcataat ctttatatcc accctttaaa
27241 ttcacgacat cataaccttg tttggctaaa taatcgcaag ctttagtgct tcggttaccg
27301 cttttacaat gtatataata cgttttgttg ctattcttat tgaatgattt aatctcttct
27361 actgggtgta aagttgaacc gttaatgtgt cctaattcat attcttcttt tgttctaaca
27421 tcaatcaatt gacccatttt tgccaatttt tctaattctt ctttgtttaa tgaattaatg
27481 tgtacttctt tgtattgttc catacttacc tcctataaat acctatgagg gtataataaa
27541 acggatagaa tcatttgcca aatacctata ggggtatttg acaattttgt tttaatttat
27601 tattattaac ttaatcaatt tatgtggagg aaatgaatat gacttatgat aaaaaaatga
27661 ttaatcgtat aaatagaata caaggtcaat taaatggtgt cgtaaaaatg atggaagaag
27721 aaaaagattg caaagatata attacgcaac ttagtgcatc taaaggttct atacaacgtt
27781 taatggggat tataattagt gaaaatttaa tagaatgcgt taaaacagca gaagaaaata
27841 atgaaagttc tcaagaatta attaatgaag cagttaattt attagttaaa agtaaataat
27901 ggatatagca aatatgacta ttatgttgct aattggcgta ctgggtggat ttatatctgg
27961 attaataggt attgggggcg caattattat ttacccagct attcttatat tgccaccatt
28021 aataggtata cctgcgtata gtgcatatat tgcttcggga cttacctcta gtcaagtatt

28081 tttcagtaca cttagtggat cattaaatgc aagaaaacaa ccagctttct ctcctaaact
28141 tgttatatat atgggagggg gtatgttgat tggaagcatg ttaggggcaa ttttagctag
28201 tttgtttaat gctacttttg taaatacggt atatgtaata atcgccatac ttgctttaat
28261 attgatgttt attaaagtta aacctactac acaagagacg aaatctaaac ctttgctatt
28321 tattatagtt ggatttggaa ttggtgtaat ttcgggaatt gtgggtgcag gtggagcatt
28381 tatcatcatt cctgtattat tagcattatt taaattacca atgaatacgg tagtgaacaa
28441 tagcatagca attgctttta tatcttcagt aggggcattt tttataaaat taatgcaagg
28501 atatatacca gtagaaagtg caattttttt gataattgaa tgatgatcat tttctgaaaa
28561 tattatgtgg tcatataata taatgcccat catttcacta atctctttta ttctctgagt
28621 tattttgata tctcctggag aaggtgtcgg atcttatcaa gagtaaatta tgaaaaaaat
28681 ggtttaaatc aatgttctta ctttaagtac atgtgtttaa cgtaaatgat aattagttaa
28741 atataaaaag gttatcccaa tcaataggat aacctcttta tactttactt tatggtttgg
28801 attcgccact tcaaagaaga aatgaaatca gagcattgaa tgttccattt tcaaacaata
28861 tataaatacc tagcccaatg aatacaattg gtacaatcca acgttcatat ttctcaattg
28921 tttccgatat aaaatcgaag gaagctagac ggtaactgac atagcacaaa actccaacca
28981 taattagaaa gacaatagtg acaataaaga tttcagacat acttaaggtc gtgaagtacg
29041 gtatataaat ggaaaagtca tccgcactgg aagccaatac gatgaaaatc atcgtcaaaa
29101 ataactgatt aaattttcca gaggagaata aagataaaat gctactttca tcttcatcct
29161 cttctccttt aatccatatt ttcacgccta ggtaaagtgg taaaagtcca agtagtccga
29221 taacccattg ctgaggaatt aaatttacaa ccccctgtgc aactaaaaga cttgctccta
29281 tcacaattgc agtccctata tattgtccta tccaaatatg ttttacctga ccttttttac
29341 ttgcgaaaac aaaagaatta atatgacgag ataatcaatt cctgttgcta catataccgc
29401 agtagccgtc agtatcgtcg cgatcatttt atcatctcca aatattttag ggataggact
29461 tttcttcaaa atgaaaagtc cttccgtaaa ttgcacacat atagtaccta tttatccttc
29521 actctcatca gtcgcaaact atttaatgct accaaaatag tggctcccat atcggaaaga
29581 atcgcaatcc aaagggttag ccagcctgga ataaccaata gtaaggcaat tatcttaatt
29641 ccgatggcaa acgtgatgtt cgctttgatg atatttagcg ttttcctgct aagtcttact
29701 gcaaatggaa gcttacttaa atcatctccc attaatgcaa tatcagctgt ctcgatggca
29761 gtatctgttc cagcaccgcc cattgcaatg ccaacagtgg atgcagcaag tgcaggagca
29821 tcattgacgc catcgccaat catagctaca ttaccatgct cggctttcat ttttttaata
29881 tagtccaact tatcctgtgg caacaattcg gactgaatat cagaaacgcc tacatgagca
29941 ccgattgctt ctgcggtacc ttgattatca cctgtcagca taattgtttg cttgattcct
30001 aactgatgaa gttttagaat cacattttta cttgtttcgc ggacctcatc tgctacagca
30061 atcacgccga ggattgtttg gtccgttcca ataatcatgg ccgttttccc ttggttttgt
30121 aaaactttca ctttattttc aaactcaagg ctaaaatcgg aaacatttaa ttctttaaaa
30181 agccttggac tgccaatgta ataggttgtt ccatctatat tcccttgaat gccccgacct
30241 gtaatagaag tgaagtcctt cactctaaca tcggaataag taatattatc ttgctctgct
30301 ttcttcatta ttgctgaagc aagtggatgt tgtgatcgat attctaaagc tgtaataatg
30361 gaaaacagct ctttttcttc cacttgatca tttaacactt taaaatctgt taccactggt
30421 acaccttttg tcagtgttcc tgttttatca aatgcgattg ccttaatggc tcctaattcc
30481 tctagataga caccgccttt aatcaacaca cctttttttag ctgcatttcc aattgccgag
30541 acaatcgaga ttggagtagt aataactaat gcacacggac atccaactac aagtaccgct
30601 aatccttgat aaacccaagt atcccaactt ccaccaaaga ataaaggtgg aacgactgca
30661 acgagcgccg caataaccat aatgatcggc gtataatatt tcgcaaattt atctacgaat

30721 gcttgcgctg gagcgcgctc cccttgtgct tcctcaacca gatgaataat cttggagata
30781 gttgtatcct ctacgtattt ggtgattttt acttcaagta gtccctcttc gttaagcgtg
30841 cctgcaaata cttcatcatc taccgttttg gcaacaggga cagattctcc tgttatagca
30901 gcctggttga cagccgacac accatttata atgatcccat ccatggcaat tttctcccct
30961 ggtttgacga tcataatatc acccacggca atatcgtcca catggatcat tatttcctga
31021 ccattccgcc taacaagtgc ttcttttggg gcaatatcca tcaatgaacg aatggactgt
31081 cttgctctat ccatagaaaa acgttcaagt gcttcactga ttgcaaagag aatgacaaca
31141 atggatgcct ctgcccattc accaatgatg gcagctccaa taactgcaac ggtcatcagg
31201 gttttcatgt cgaaatcaaa gcgtatcaaa ttttgaaaac caactttaaa tagtgaatat
31261 ccgccaatta caatcgaact tacaaataac atggacgtta caaggttatc ttctccattt
31321 acaaagtgag aaaggtaacc aaaagcaatc agtaatgtgg caaacagcaa tgtgctgtgt
31381 tttttataaa acggtatttt ctcttcttta ggagccttag tgtcttcttt gaccgcttgt
31441 atcgatggat tcgccagttt ttcaggaatt accttaagat tctcgaaagc acctgctttt
31501 tcaagctctt caaccgatgc gtttccatat acatcaattt tagaagcgcc aaagttcact
31561 tttgcatcct gaactccagc tagttgtttt acattttttt caaacttccc agcacagttt
31621 gcgcaagaaa atccctccac acggtaaaca tttttatctt ctgttaccac tggttcgacc
31681 cttcttctag ccttctctgg tgccacttta agattctcga aagcaccagc cttttccaga
31741 tcttcaacgg ttgcactgcc aaagacatca attttggaag ctccgaaatt gactttagca
31801 tcatgcaccc ctgatagttc ttttacattt ttttcaaact tcccagcaca attcgcacac
31861 gagaaaccct ccacacggta aacctgttta tcttctgtta atgtttttgt tgaactatcc
31921 aatactagca acctcccttt gatgcaagaa agctttttct acaagctgtt taacatgctc
31981 atcatctagt gaataataga ctaattttcc ttctttacgg tattttgcta tacctaaatt
32041 tttcaataat cttaaatgat gggatgccgt agccgttgaa gattcaatga tattagctac
32101 atcacaaaca cataactctc cctctaaaga caaaacataa gcaattttaa ctcttgtatc
32161 atctgataga gccttaaaaa ctttcgctac atccatagga ttctgtttag caaggtcttt
32221 tttagccctg tttaccttat cttcatgaat ataggtaact tcacacatat cttttgtcat
32281 aattaccctc cttattcaaa tgactgtttg tttgatgatt ataatatatc ccactataaa
32341 caaatagtca aatgattgtt tgaatgatat ataattaata ttaaaaagga ttggtttcta
32401 atgttagaaa ccaatccttt cggagatttt aaccaaattt taaagtatct taaacataac
32461 tgccccgtta gtttaagtgc atcctttcac aatctgtcta cagattaata ataaaaacta
32521 ctctttatta tacagatctc catataattt ttgaatttgg ttctgtaatt ttttattttc
32581 tttttctaat tccataactc ttctttttaa ggtttttata aggatttcct ccgaacgaga
32641 acttttcttg ggttttgaga ctacatttga tgttatttga cgctcacgaa gggattcgat
32701 tctttgccta atatcgtgtt ccttataaag ccatgattta gaaacattag cttcctttgc
32761 tattgaatta aaattaatag ctttaccttc aatcgaaaat ttagaaatcg ctttgtctac
32821 tttttctctt gtcttttgtg atttctgctt cgccaaacgt acaatttctg ttgtatttct
32881 aacttgttta ttcattgata attaccccgt taaacttcca atgatttgtt ctaaacgctc
32941 tttaacacgg ctattagtct ctatttgtct ttgccattgt ttatcctttg ctatggttaa
33001 taactcttct gtacgctcta actgttcttc gtgctgtggt aagaattgct tactggtaca
33061 gaagtgagtg caatctaagc atgcattcgc atgtggacaa ccacctgcta ttactggcaa
33121 tctacaataa ccatttggaa gcacttgtgc atttatattt ttcttgaacc attgaagctc
33181 tacatcatcg acttcactat catcatctag attgagcaca tctccattat tggtaaccag
33241 tttttcttga aatttagtaa attcattttt tagagtttca tcaaagatat gagcgtatct
33301 gcttgtcatt tctgggcttt catgtcccaa aaatttctgc acaatatgct ggggcacccc

33361 gttgttaatc attcttgttc ctactgaatg gcgaaaggca tgggcatgga atctataaat
33421 ctcgcctaat ttatccacta tattttgctc ataagctaat ttatttaatt cgcctctaaa
33481 tgtttcttgt tttaatggcg atccatcttt tcttggaaag aggtattcac tatctggaaa
33541 ttcctctgaa actttatctt cccgaacttt aataagtaaa actacctctt tagatattgg
33601 aactatatgc tcctttttca ttttccattg ataatacttt aaaaagtaat ctccatcttt
33661 gtcctctaat aaacagcctt ttttcaaggt gcacaattca cttatcctca ttccacattc
33721 ttgaacaatc atagtcatcg tagctatata ttcgggtaat ttatcaagat gactgttcaa
33781 ttgctctaga acgaattcgt ctataaagcg aggttttgct cttggtattt tcggatagtc
33841 ctcgggataa attaataatt tggaaggaac atcatcccat tctagcctat gaagggtact
33901 aaatagtcct tccaatatag agatcctccc agttattgta ctaggtttta tccccatcat
33961 gtttagttca cttaaatatg cttcaatttc cactctcgtt aattggtgta ctctctgaac
34021 gtgtttaaac tttatgtcca gaaaattaaa gaactcttta agtttttggg caatatcact
34081 tacataggaa aagctatcca tgttcaatct taacttacaa tatcttttta caagctgttt
34141 aaaatatgta ttccgaaacc ctttaaagtt aattgtatat tcatattgtg ttgggttaac
34201 cttatcatct ggcaaaggta agtttcgtct atcccaaacg tctttatccc actcctctcc
34261 atcaaaataa aaatcctcat aaaactcaat aaattgttta agattagtaa catagtaaga
34321 attagctttt acaggtgttt tttcttgatt agcagtaatc ttataattag tggtggtaat
34381 tctaacaccc tgttttgtca aatatgttct atactccgtc attgctttat caataggaac
34441 ttcagtaatt gaagtaatgc tgggatactt taaatctaaa aaatccaaca ttttattaat
34501 tactgttcct tttctaatcc agacagtttt tgcattccat attccattgt ttaaatggta
34561 aaaataaaaa tatttcaatt ctgttcttaa ccacagattt ttaacacgtt caaaacgaac
34621 ccaacgattt cttaaagcag gattcttact taattctatg gcagaaggat gtggacattt
34681 tcttatatcc caactattat tagcccaaaa cccttgcatt tcttcattca ttacagctat
34741 ttttttgctg atctcactct gactgataat tttcctttta ctagaagcat tcatttagct
34801 ggtattcttt atctagtaat aaatacaatg gatacggctt attctccact tctatccttt
34861 gaaccctcac acgcgccccc ctctttaaac actttaagta ctacaaatta taaaccttgt
34921 taatttttat ttaaagtaaa atctcccttt aacatctcct cctgaactac tttaactact
34981 attatttatt atactatggt taatatgtct ccacttgggt ggttatggcc aataatcata
35041 cccgttgcgt tacttaatat ggctgtttta aatatttctc gaggatgtat aaccgcaaaa
35101 ttaatggttc ctatagagac ggtttcaata tgtgtaggtt catttttgct attcaagcaa
35161 attaaaatga agtgttctcg gtcagcattt cctatgaagt cacgcataat atcagcagcg
35221 tcttttggtt cttcaatacg tctttttaaa taccaaagtg tatcttcttt aaccatttgg
35281 actttgacaa catctattct ttttctactc attatggata agctctatac tttctattaa
35341 gtcagggtga acaatggatg tgatagaatc atcattcata taaatggaat atagctcatc
35401 taattctgaa ttattttttt tgtttgatga aaaggcgata atgcctttaa taacatcgat
35461 gtgtggttga tgcattaaat attggtatac ttctttccat gtttgattca ttctatttag
35521 tctcctttaa tggtttaatt gtccatgcct ttctagcgta tggatttaat ggatgcatga
35581 tttcatttgt ttgaggattt gttaatatac tgattttctt actgtgaggt tttaacatgt
35641 ctaatacttc attcactcga ttttccacaa gaggtttttt tccgtaggaa ccccaagcca
35701 ataaaacact atcggcttct ttgacagctt tcataatgtg aatatccgtg tgcttgtcat
35761 ggctgttttc gatatgtttt aaattaatgg gggtatcaat atttgaaaaa agatttataa
35821 aattaacgcc accatattca tctttttcag ctactttatt gacaataagt tgtgttgtaa
35881 ggtcaacgtt gataacacca tcataatgag gatacatcgt tataattgta agtacttctt
35941 tatcaatatc ccaagttttc ttgagaagat aacgatgcat tttatcctga ctaaatatcg

36001 cttcagtttc taatacactt ttgattgatt tcattttatc taactccttt atttaatatt
36061 cttcaggtaa caacatgacg taataagaaa ggtctacatc atcttctcgg ataatgtaga
36121 ccttattaat attgatagct tttgaataat ttgccttatg aatttttacg tactctggac
36181 gttcttgctt atgtttgata tataattgac tattatgcat attgaactgg aatatatgaa
36241 agtaatcaat ctcttctagt tccttgaatt gttcgttttc gcgttgcgct acaagtttcc
36301 ataactgttg ctgcaacatt gctggcaagt aattggcaat actacgtgta atataacgtt
36361 tcatgctcat ttctcctttt ctatatcttt cattattgta ataacttctg taatgactgt
36421 gacgcttatc cgagctatgt gtatccattt attcattatt ataattctcc ttttcaagta
36481 agtgtctttg atctagtagt ggcataactg tgttatgaag atataagaag tccatatcgt
36541 ttgtttcatc aatataacca atcgcaataa gttgtttttg atagtagagg ataaaggggt
36601 atatatgcct ttcatgaagt gtgtcagagt agtaagtata cgtatgaagg attttgttgg
36661 taagcggacc aagtctgata tattgatcag gaaggatatt atctgccact tcctcgatag
36721 attcacattc ccatttttcg tcctttggca gattgaataa gttagttata tactctttga
36781 gttgtttttc tagtgtcatg gttatgacct cctataatgt ttttgatgta tacatgtaat
36841 acaccatata gataatatat gcttcaaaaa aattttgttt cgtttgtgaa tcccattgtt
36901 attaagaaaa tatcccaata gaaaattatc acaattgttg atatgacaag gaaatcccat
36961 tatcccgctc atttctcagg aaatatagcg atatatttat atatcatttg gatttggtgg
37021 tgtcagtacg acttactttt tctttttata ttatttattt ataaataata atgggatttc
37081 gggattacgc ttgcgtaatc cttatcctac atgtatttat caaatcccat tgctatcccg
37141 ttcatatttt attttgggat gttctttcgt catacaatat aagcttcaca ttgagttgtg
37201 acttgttagt atgttgaaaa gattcgttag atgggtgtta ttgaatgacg aaaggacagt
37261 acatagatat tagatttgaa gaaagagcgt ttgaggcaaa aataaagacg aagtgctgag
37321 gagcacttcg tcgagagggg tattattgaa aagttgttta ataatttcat tattgagttt
37381 tagtgtaacg tagaactgtt ttttatgatt accgtcttta cgaatatcaa tacggtcaat
37441 gactgtaaga tataatgctt tgagttgtgc tttatccatt gattctatat tttgaaatat
37501 tcgttgtaat agggcagcga tttgtttcgt atcataagat agtttctctt gattttgttg
37561 ctgtttgagt tgattcattt gatttgtaat gtcattgagt tgtgtttcat attgatgaat
37621 agttgctttc aatgcagatg ttaggtccgg attatcttca atggttttaa ctaaattatg
37681 gagtttcccg ctgacttcat cgtattgttg ttgtttataa gcgatatcgt ggttcaatgc
37741 accaatatcg actttatttt cttgattgac acgttctaag acttggttaa tgactttatc
37801 acttttgaca atttcgagta tttgatccat gacgtatttc tcaatcacat cagctctaac
37861 gctattcgca gaacatactt ttgagccttt gtttcggaag ttactgcaag aataataacg
37921 tattcgcttc ttggtaccat ctttcaatgt gttcgttgtg ttactagctg ccattggtgc
37981 accacattgt ggacaatgaa cgatacctgt taatagatta gttcctttac cgtggacttg
38041 aggtttttga ctgacttgtt ttttacgtaa ttggacttta tcccataagt cttgaataat
38101 aataggggaa tgcttacctt cagctattat tggtttatca ttcagccctt tacgacgctt
38161 ttcattccaa tctttgtact ttgcgaattg aattttacca acatagaatg gattagataa
38221 gatataggtc actgaaccaa tactgaaagg ctttcctttt ttagtcacgt atcctttgtg
38281 attgagtgca ttcgcaattt tacgatatcc gtggccttta gcatatgact caaatatata
38341 tttgacaata ttcgcttcat gttggtttat catgagttca tgcttgctat ccggtatttt
38401 gtcatagccc agcggcaaat tgccttgata atagccttct tgagcgcgtc gggtttgacc
38461 catgaataca ttttcgacaa tattatttct ttcaaattct gaaaaactcg ctagaatttg
38521 tagcatcaat ttaccactgc ttgtattgac ttccatacgc tcagataagc tgaagaattc
38581 gacattttga cgatgaaggt cttcaacaat tttgagtaag tcagaagtgt tacgtgctag

38641 tcggtttgtt ttgtagacca taacagaatc aatctgacct tcgttcgcat cttttaacaa
38701 acgttgtagt tctggtcggt tcatagattt tccagagata cctctatccg cgtatacatc
38761 tacaacaaca aagttattga aatcacaata ttctcgaatt tgattgattt gtccatcgat
38821 actatagcct tcagtacttt ggatttccgt tgatacacga acatagatac cgacacgttt
38881 ttgtttgagt tgttgcatga ttttttcatc ctttctgatt aagcaatcga tgattgcgag
38941 gtttggttca caatgttcaa tggttcattt ttgaaataga tcccaacaag ggctttattt
39001 tttgtaatgc gaatttcatc aatataggga tgcagcatgt ttaacgtgaa acttttctgt
39061 ataacctttt gtagtgatgt ttgtaactga ttatcactta tggaggatat ggttttgtgc
39121 ttttgattca tcaaatgagt ctgttttctg aatgattcag catcaatcat acctttggca
39181 agtttatcta tcagttgttc ttgagttagc gtacttttag cttctatcac tctttgtttt
39241 ttgaggcgtt gatgaattgc attattaatt tttgaataga gctgttggtt ttgaaagaag
39301 ttctgacatg tagctaagac ttgaacttct aatttttgtg catttattcc tttgaatgaa
39361 cagacaaagc gagattcatt catattttta ggacaaatat aatatcgcaa tgtatgtttt
39421 tttcttattg tcatatttgt cagtgttgag tcacaacaag gacatttgat cttttgtttc
39481 agctgattct ctgaaggtat acagtgaagt tgcttcttat tacggattgc ttgagcatgt
39541 tcatatttcg ttgccgaaac aataggtggt accatattgt tatattgacc atattgattg
39601 atgacacgac cacaataatt tgggttcata attatatttc gtacctgata aggcttgcgg
39661 gtaataagtt tattatcgtc ttctaaatat tgtgcaatct tcttgtagcc atatccttga
39721 agataataat tgaatacagc tttgacggta ggtgattcat cattattaat gatgaaagta
39781 ccattttgat agtgataacc gaaaggcgca tgggtcgtta tgagtttacc ttgttttgcc
39841 ttttctctaa gtccattttt gacttgttct ccaatattat cggattcaag ttcagccaga
39901 ctcatgaata tattgagttt taggcgatca aacgctttat ccatatcaaa atagccatca
39961 tgaacactta gaatatgaac atgatatttt tgacagagtt tcatgagttt taatgcattt
40021 ttcaagttgc gatgaagtcg attcaagcga taacaacata atacgtcaca ttgtctttgt
40081 tggatgcgtt cggtgacttg ttgatagcct gttcgattat ctgttctgcc tgattgctta
40141 tcactaatgt attggatatt ttgaatattg tgctttggag ctaataattc tattgcttgc
40201 ttttgagctg ggagtgattg ttgtttcgtt gtactttggc gcaaataacc aatggctcgt
40261 ttcatagtat ttcctccttt caaagagata atatatattt atgaacgaaa ttatagataa
40321 gcccaacacc agtcgggtgt tgggcggtat tattagtcat cagcgtgatt gatttcttca
40381 ataaccagat cggctaatag tatgattaat tcgtccatgt atatcacctc attattcata
40441 gtattaacaa caattcttta gttgcatctc aaaagtagta acgaattcga tattcttctt
40501 tgagcaaatt tttttaaatg aatgtaatct tcatattctc catatgcaac aatggacgta
40561 ggtttgatta ctttctccaa aatgtcattt attaacttgt actcctcagt gtaagtgtta
40621 cttatttgac gaccttcttg tttacagcat tccttcacag tatctagggg tgtgcttttct
40681 aagtattcaa atctttcatc ttcaatttcg tgagctagtg ctactgtatt catagtacaa
40741 ttcctccatt gatttaatat ttgtgctaca acacctttaa ataggtgttg tagcaaattt
40801 atttaataac atattttaag ctttaaaagt caatatcacc gtttttatcc cattctcttg
40861 cttttgaaat catgtctcgt agtattggat gttggtgttc agagataaat gataagggta
40921 tattgaattt acttttttca gatagaccaa aaataggagc gtatgattta tcaagcttaa
40981 gatgataaaa tacgatggtt tttggtttgc cttctttgtc tttgacacta cgttttgttg
41041 ttttacgatt ggcgtctgac tggatgtaat ctttatccct cagtgcatca atgacattat
41101 ttgtatcttg gaaattatgt tcttttaaca tatttttaaa aaccgaggca atcattttca
41161 cctggatatg atcatcttca agtgaaatca ttccataatt ttcaataagc gttgataacc
41221 tagtgtcgtc agaaaactta ccacgatttt gtgctacaaa ttggacaatg acttcaattg

41281 ctttatcagc taggcttcgc tcactaacag aatctaaatg atagttaata agatagtccc
41341 ttacagcatt caaatcaaca ggtgtggcaa tgacacgttc caatatacgt gctgatgttg
41401 taatggttgc atagcgtttg aacatccgaa ttcctgtatt gctggtttcc ttttctagtt
41461 gagttttgaa ccttaggtgc tcattttgga accatttaat gacttcttgt tcacgattga
41521 gtagatattc agctactaat ggcatgatat gaccataatt agctgatgtt gcttttttaa
41581 tagtatctgc gttgtcagcg ttagttgtaa aagcttcaga tatttcaatg gtacgtacat
41641 tcaaaccatc attacgtgca ctattattga aaatactgtg ttcggcagta ctaattactg
41701 aggttcccca gttttttagt gacttgactt ctccgtgtat attagaacgt tgtcttcctt
41761 gaccttcggc aattgaatag agcaaaccag ttgtatcttt aaaggttgca gatgacagtt
41821 cgtccaatac aataggtaca ccatagttgt tacttagata tccttcaagc gcgtttctag
41881 tgccattcca tgatctaaac agcgtattac tacctttagt agggttgccg gttattgaga
41941 ctcctaaagc cgctgcagtt gatttacccg tgcttgaatt tcccgtaaat gagaaaatcg
42001 tacctgcgaa ttcaacctca tgttttgttt ttaaaaacgc agttactaat gaagaaatac
42061 caaacactac agcaagttcg agtaacaagt ttccttttac ttcatcaaga tacatttgcc
42121 accagtcttc aaaagtgcct ttaggttgta gatcgtactg tgtttcacaa atgatttcat
42181 ccgcctgcga ctgtgtaatt tcttttgaaa gatatggttc atctaatgaa atgacgacac
42241 ctttatcagt atttaacaca ccgacacccg tatacaacga tgacagaggc agtgattggc
42301 gcattaattg tagtgcattg cttaatgatt taatgtactt ctcattgatg ctgaagccat
42361 acatgattag tgaaggtaac ttttgcgatg ttaaaatatc tgatgtttca atacgttcaa
42421 tattttgtcc gttggaaata actagtttct caaccccaga tttggggtca agaaacttgt
42481 ttttaattaa aattggacta gataagcgta tggacttttg ctgatcattt ttacctttcg
42541 gtggtataac ttcataccat gaatcttcag taagccagta tggatattca ctaaagatga
42601 tgttagtcat tagttgttac cacctttcca gttattttga tgattcatcg gatttggtgc
42661 agttttaagg tagagcaaag atgaaccttt ttttccaact acaatgaatg gaaggttagg
42721 tgtatatttt acaaaatgtg caaaccaacg accaacatct tgttgaattt cagtcgagca
42781 caaaacatta gccctcacat tcaaatcgtg atacataaac atggtgccta caggtaaatt
42841 aaatgcgatg cttgtaacag cacgttttag tgtataaaat tctttttctt tcatcataag
42901 ttcctccaaa taagtcttat tagtcaatca atactgtaga tgcttaacac ctcaatggtt
42961 gattgctata ataagcataa atgaagcatt ttaattagac gcggaacaaa aaatagggag
43021 ggggggtata tatgatacct acgatagaag aaattattga caagtatggc agtttagtag
43081 attgtctaaa gtttgatata tcaggtgagg tttatgaaga cttattgtta ttaagaagtt
43141 taagcgattt aaatgaacac caaaagatgc atagagtttc atttatacaa aaacacttta
43201 accctaattt tgattatttg ttagatgact cagaatttag aagtatgaat aaaatttata
43261 gaaatttaaa tactatgacg tatatcaata gtcaaaacaa tgaatttaaa cttaaaccat
43321 ttataaatga agaacaatta aaatcactac ttgcgataaa gaaagtagat gaacatttga
43381 gttatgaccc atctattacc tcaaaagcat tagcagatat aaacaagacg caaaaaagat
43441 tggttacaaa gccaaatatt ctctataatc gatccaaaaa agaaaatgag tatgtacggt
43501 taggaggaaa aatatcatat acaaagatgg agaagcattg ggaatattta cataatgaga
43561 ttcagtatta taatcttact aatcaattaa ttagttatgt tgctttagaa aaggaatatg
43621 catgggcttt tttaaatgaa ttattttatt taatagaatt atatttgaag gcatttaaag
43681 gacaaaagaa aactggccat gaatttgaag aaggattgaa cgcatttatt caatcatatg
43741 tcattatttt acttatagat ataagattac cgactgttag gttatatatt attaggcgta
43801 ttgtggatga atgcgaaaaa gaatcagata atcaaaaaag aattaaactg atagaggaat
43861 ccattaaaaa gtacagatat gtaaaagaac agattgaaag atttggaaat gcgttgaaag

43921 aagaattatc aatcgcgact ttgtctattg aacagaatat gttagaagct aaaattgatt
43981 attataagaa ttactactat cctagaaaaa aaacaaagca taacaatatt gagtacaacg
44041 tatcaatgtt ttttaaagct gtagattctt taaagaaata gtattcgacg gacaatattt
44101 atatataata attaattaga gaggttttaa gttgtgttta gattaaaaaa atggtattta
44161 aaacaaaaag gctatttttg ccgtgtcggt gaaaagtggg acaatagtaa tttatcccat
44221 gaaaatatac atagttatgt agatagagta cttgaatatg agactgaaac acgtagaaac
44281 ggccaaccaa ctgaagaata tatattagct agtagtgggg aaaaaataaa tgaagattta
44341 agaagtggta ctaatgatca caaagaaatt ctagaggtat ttgataaata taaagtaaaa
44401 gaggatataa ctacttatag aacggttagt cctcggatat acaaattaat ggagaaaaat
44461 gctaaaaact taaatggagt agatttgttt gaacttggaa ttttacacac ttcattaata
44521 aaagggtacg aatcaagaga agaaggttac aaattacggg taaaagtaaa aaaaggaaca
44581 ccagctttct atgtaggtaa tttaacaggc gaagaaagcc attattatga ggtgattgta
44641 gttaataatt taaaattaaa aatcatatct attgaagatt actatataaa ttgtgaagta
44701 gtataattgg aatcaattct gttagttgtt cataataagc caattatttt tttaatgaac
44761 ttattactta aaaaccgcat catcaactga taagcagaag cgtatcacaa ataaaactaa
44821 aaaataagtt gtatataact tattttgaaa ttggttaagt atatagtatc tccaataaaa
44881 tgtagttaac ttacgataat gctgaactat agctttgtaa actaaaatgt aaataattac
44941 aatcaaattg caacaatata gttcaagaat gctacaattt gaggacagat tgatagcatt
45001 aatcccttta aaatgaagct aggagataac ttacattatg attagtaaac aaataaagga
45061 tttacgaaag caacataatt atactcaaga agagctagct gaaaaattaa atacttcaag
45121 acaaacaatt tctaaatggg aacaaggtat ttcagaacca gacttaatta tgcttatgca
45181 attgtcacaa ttattttctg ttagtacaga ctatctcatt acaggaagtg acaatattat
45241 taaaaaagat aataaaagct attatgaaat gaatttttgg gcatttatgt ctgaaaaatg
45301 gtgggtaatt attattatag taatcataat ttgtggaaca ataggacaaa ttttttcaaa
45361 ctaatgtaag tatctctcaa atattttggg aggttttatt atgaaaatca aaaaattatt
45421 aaagacatta ttaattattt tattatgttt tgtattgtct gttattgtgc aaaatatttc
45481 aatgctatgg catattgtga gcatttggag tgttgaatat attttacatg cattaactta
45541 tatagtactt tcatactttt cagtaaaatg gttcattgaa attgtgctta aaagcaatat
45601 gaaagattat agaataatac cggtgaaatt tttcaagtct tgttttctta taggattaat
45661 tttgatatta gtaattgatt taatatatct attatttatt cctggtaaat tgataatacc
45721 tcattattca acaagcgttg gatttatgga gatgttcttt agcgcatttt taattactgg
45781 tattgctgcg cctatatttg aagagatggt atttcgagga atactaatga gatactttga
45841 aaaacaatat ggaattttat ttggaattat aataccctcg attttatttt ctcttgttca
45901 tctatttaat ggagagttaa agggagaaaa tttattatta ctacttatag gtggctcaat
45961 tgctggaata atgtatgcag tgactgcatg gacgtttaat agtatatggg cctctgctat
46021 attacatatg ctttggaata taaatggttt attaaatatt acaactcaag atgatcattg
46081 gggaatatat cagtatatat tagatacaaa aaatgtacta attacaggtg gaaactatgg
46141 tatggataca tcattacttt caatatgtag ttatattgca gttattatag ttatgattgt
46201 tactaaaaga cactttaact ttttactaaa gtaattatag aatagtgtgg ttaatcctga
46261 cgactagtcg gatacaattt aaagagtaat aaaaagtgta ttattaactg atatgcaaaa
46321 gtatcccact aaagaaataa aaaaacagat ggtagaatat gttagcattt aatatacaaa
46381 ttagttttta acttacatga aaactagttc aataaaaaag ttaaattagg agcttattat
46441 gaatatctat caaacctatg catgcttagt catagaaata atcgttaata gtactactac
46501 tatactaaaa agaaaaaaat taatttcaaa tattaaacaa ctttggaaaa gtaaaaagac

46561 tttagaagaa tttatacgtt caaattcgag atttgattat tagtttaatc ttcgacgtaa
46621 gaattattct gatacattag tagatgataa aacatggata gacttagata tggatacttt
46681 gcttcataaa attaatttta actatacagt tatcggagaa atgaactccc tataatctaa
46741 ttgagaaaat ataagaagaa aaaatttaag gttttagagt cccatgtatt attccgttct
46801 ttcttgatag atgtgtaata ttttagtgga tttaataatt agtaaatttt taacaggcat
46861 aataacttac aattaagaaa agattaaaac ttattaataa tagagaggtt atgtgaataa
46921 tgaatgattt gttaaaaatt tatgaaaaat tgaaaaaaga tattgaaaac aggtggttaa
46981 taccatttca ttatgtggtt tttggattag cattagttgt atattttctg gaaattccta
47041 tttataaatt ggtgaataac ttagataaag agcttgttga taaggtttta tatgcttttt
47101 cattagtgta tgatcacatt gtattgatat ttatattaat tataattatc attttgatag
47161 tttatttatt ctttgatgtg tttaatatga atcgttttgt tccatcgcct acaacttatg
47221 tagatggtag tgaaagttct ataaactatg taagtgctat taaaagattg ataaacttta
47281 tgatattaat tatcacaaag tactggataa cttactttat tgtaaactta atatttcata
47341 atgataaatt actgtattta aacaatgatt caaaacattt gtataagtgt ctcttgtttt
47401 taaatatctg tatctttata gtgcatatat taaaatcaat ttttataata aaaattatgg
47461 cagatgattc gctaagactt attaatgaaa atgatttaac tgatttttac ataacattaa
47521 ataaaaatga tgaatatcgt atggtaaaac ctaaatatag aaaaatagat aattttatta
47581 ttattaaaaa aagtgaagat ttcactaatt caaatgagta taaagttatt aataaaagta
47641 aaaattttga ggaaataatt tatcattttg acaatcttac ttcaaatcat tgatgattta
47701 taaattatta ttctaagttt aactagaaat taaattttta tgcaggccaa tcgatttctt
47761 tatcgattaa taaaaatggt tatattaata acagttttga tgcttcgcga gatgtcatta
47821 gaaacatcga agtaagcatc ttagactata aataaagtca ttgtttataa aaacaattgc
47881 attaaaaaag ggagtataga actcccttaa taagttgata tataagtcac ttattttttt
47941 aataaaattt taattcctct gataatgttg agaatatata atattaaagc aattaaaaag
48001 aatacaatag caatgattaa agcaataaca aatattaata attgatgatc aaacggttta
48061 tcaaagactc cttttgaaaa aataaaaatt cctctgccta cacaaaaact aatccatgtc
48121 ataatatgat tccctaatgc ttttttacca tgtcctgatg ttggctcatc agcaagtatc
48181 catacaaata aaggtaaaat gacaggtgcg aaaaagatac ttaaataact caatgctgat
48241 aaaacgtaac tattgtcatt tttatgcata ttactatttg tattttccac ttttttcacc
48301 ttctttactt ttctttatta ttataaaaac attaatagcg agctattaca acagttataa
48361 gagaaactta caataatgaa agtattccac tagaccttca aatcttactt tggcacatgg
48421 tagaagaaaa agataaccag cctcaaaagc tttatcactt attattgata attttgactg
48481 catcattaat aatatttttt gttaattgag ctgcatcgac tgtgtcagct aatctagtac
48541 tttgtccact ccaaatatgg gtccactcag tatatccttt ctccaaagca gattttcgta
48601 tttgcgttgt taattgattt tgtaaaggat aaggaggcac ctgttttgaa tataaattca
48661 tattgtgtac aaattcattc ataattccgc gagccaattt gccactaaat acatttgtca
48721 gtattgtatc tgtttcagag ctttcaataa tttcattttt gattttgtca tcagcgccac
48781 tttcattcgt tgttaaataa gcagtaccta attgcgctgc tccagccccc aaagctaaac
48841 tagctactaa acctcttcca tccataatac caccagctgc aataactggt atagacacat
48901 tatcaacaat ttgtggtact aaagataaag taccgaccaa tggaaattca ccattgattg
48961 ttgtaaatga tcctctgtgg ccacctgctt catagccctg ggctacaact atatccatac
49021 cagcattttc attttcaact gcttcttcga ctgatgttgc agttccaatt aaaatttttc
49081 ctgcattatg aagtcgttta ataatatttt gatttggaat accaaaagta aaagcaatga
49141 taggtacttt taatgattca ataacttcta ttgcatcttc aaacttttct ttttctttaa

49201 ccgtactatt tttgggagta gataatccaa gcgcttctct atatggtttg agttttgtat
49261 tcatctgctc aatttgtttt ttatcgtact taatatcatt gggtgtaaat agatttactg
49321 caaaaggagc ttgtgtcagt ttttgaactt ctaaaatctc tttcgaaagt tgatgtgaat
49381 caaaataacc tgctcctatc atacctaagc ctccttgttt actaacagta gaaactagtt
49441 ctggcgtagt acttcctgcc attccagctt gtataatagg atatttaata cctaacattt
49501 ttgtgatttg agtagttaaa ttcataatta acaccttcta ttttattata tttgatttca
49561 ttttctcaaa agcaacttgt ttcaattgat ttatcacatt attattttta ttatcttgtc
49621 gataaatgat atttagtgat attgtttgaa atgattcctc tactttggtt atttctatac
49681 catcgctatt tcctttattt aatactgaca taggtaaaaa tgctatacct aatccagctt
49741 caacacaccc caaaatgcca tctaaagtat ttaatgtcat tttattttct atattaatat
49801 catgataatt aagccaatct tcaaaaattt ttctataaaa acaaccttcg ccaaacatta
49861 taatagactt attttcgttt atattctgtt taaagttgct cttttgagat aaaatcacta
49921 atttttcatt aaagatattt gtgcaaataa gatctttatc gttaatattc ccagatataa
49981 aagcacaatc catttctcta ttctttacat gttcaattaa tttaatacta ttatctgttt
50041 ttaaattaat ttgaattttt ggatttttcc tcttatattc attcaataat ttaggtaaat
50101 gaactgcagc tattgtttca attgtcccta tgttaatatt taatttttta ttgttgctta
50161 cattatgttc tgcttcatgt aaaattgtga atattctctt aatatatttt agtaattcaa
50221 taccttctgg tgtcggttta attccttttt tatctctata tagtaatttc acttgataat
50281 aagactctaa tctttgaatt ttagctgtta catttgattg aacataattc agttcttgcg
50341 ctgctttagt aaagctacga ttttcgcaga ctttataaaa agcttctaag tcatttaagt
50401 tcatattttt caccaatact ttcgttttgt ttatagctaa atattgtaga aataagaccc
50461 agtattaata aaacagttgc aaatattaaa gcaatgttat aattattcga tttaccaatt
50521 aagattcccg caaacatagg agctaacatt tgtcctaaac tgtatattac agtcagtaat
50581 gagactaaat ttaaggggtc atctgttttg tacattaaac tttgcccttt agaaataaat
50641 aatgtagtta aacctaaaaa agtccctcca aatagaactg aactaataac taaactaaaa
50701 atttcatgag taaaaacagg tagacaaacg cttataattt gtaaaataaa tgcaccataa
50761 atggcttttt tataactgat tttttcagct ataagtgacc aaattaatgt agaaggaatt
50821 gcacctaatc caacaaacat ccagcttaaa gtcgcatatt cagcaacaga aggaattgat
50881 ttaactatag caactagaaa agtaccagta ataatataac ctgccccttc aaagaagtaa
50941 gcaatagaaa aaaagataac aaactttttg tttagtctat tattaatttt cattttttct
51001 tctttaaaat ttattattgt atttgtattt tctttcataa aaaccaatac aattaagccc
51061 aaaaccagtg aaactattcc taaaacaaac caaaccattt tccatgaatc cacactggca
51121 aaaaaaataaa gataaatact acttaaaaaa atacctaatc caacagcact atataataca
51181 cctgatatcc cctctctttg tattttcttt aatgaatcta gtactatgtt tgaagttaat
51241 acaaataccg tcccgcttgt tatcccggaa atcaatcgaa aaattgacca aattaaatat
51301 tgttctgtaa atcccattaa gatagtagaa ataatattta aagcaaggta aattttcaaa
51361 tctattactt tagttttata aatataaaat attggaataa cagcgccaac taaataccct
51421 aaataattcc atgtagctaa aaaccccgct tcttgattgt ccatagatgt atcttgttgc
51481 ataaatggta atataggtgt ataagcaaac ctactgatag acataacgat taacattgct
51541 atcataccta ataaaagttg tttatatgca tgttttttca tataattatc ctttcatttc
51601 aacttaattt tagtatagtc aatttgttat attaataaaa atgattattt aagataatta
51661 ttatcaaaaa taatgatgta tataaccatg attttttgat ttttacaaat atgtatgaca
51721 aacatgtctt taagtggttc tgttgcaaag ttgaatttat agtataattt taataaaaag
51781 gagtcttttg tatgaactat ttcagatata aacaatttaa caaggatgtt atcactgtag

51841 ccgttggcta ctatctaaga tatgcattga gttatcgtga tatgtctgaa acattaaggg
51901 aacgtggtgt aaacgttcat catttaacgg tctagcgttg agttcaagaa tatgccccga
51961 ttttatatca aatttggaag aaaaagcata aaaaagctta ttacaaatgg cgtattgatg
52021 agacgtacat caaaataaaa ggaaaatgga actatttata tcgtgccatt gatgcagagg
52081 gacatacatt agatatttgg ttgcgtaagc aacgagataa tcattcagca tatgtattta
52141 tcaaacgtct cattaaacaa tttggtaaac ctcaaaaggt aattacagat taggcacctt
52201 caacgaaggt cgcaatggct aaagtcatta aagcttttaa acttaaacct gactgtcatt
52261 gtacatcgaa atatctgaat aacctcattg agcaagatca ccgtcatatt aaagtaagaa
52321 agacaagata tcaaagtatc aatacggcaa agaatacttt aaaaggtatt gaatgtattt
52381 acggtctata taaaaagaac cgcaagtctc ttcagatctg cggattttcg ccatgccatg
52441 aaattagcat catgctaaca agttaa
//
LOCUS Staphylococcus 59504 bp DNA linear UNK
DEFINITION Staphylococcus Staphylococcus epidermidis strain C101
ACCESSION Staphylococcus
KEYWORDS .
SOURCE Staphylococcus Staphylococcus epidermidis
ORGANISM Staphylococcus Staphylococcus epidermidis
Bacteria; Bacillati; Bacillota; Bacilli; Bacillales;
Staphylococcaceae; Staphylococcus; Staphylococcus epidermidis.
FEATURES Location/Qualifiers
source 1..59504
/genome_id="176279.71"
/project="hwwang_176279"
/genome_md5=""
/mol_type="genomic DNA"
/organism="Staphylococcus Staphylococcus epidermidis"
/db_xref="taxon:176279"
CDS 1..480
/translation="MKITILSVGKLKEKYWKQAIAEYEKRLGPYTKIELIEVPDEKAP

ENMSDKEIEQVKEKEGQRLLNKIKPQSTVITLEIKGKMLSSEGLAKELQTRMTQGQSD

FTFVIGGSNGLHQDVLQRSNYALSFSNMTFPHQMMRVILIEQIYRAFKIMRGEAYHK"
/db_xref="SEED:fig|176279.71.peg.1"
/transl_table=11
/product="23S rRNA
(pseudouridine(1915)-N(3))-methyltransferase (EC
2.1.1.177)"
/EC_number="2.1.1.177"
CDS 533..2692
/db_xref="SEED:fig|176279.71.peg.2"

/translation="MLDKLKNVSHKSFDKYTNNDQLTKVNIFFGRNGSGKSSLSEWLK

LNNEESVIFNTRYLKNNIEQVEEIDGVSLVIGEKSINHSDQIKNLSDAINNLENFINR

KDNEISTSKKRIFDKMNSTLNEARKRFEITSNMVKQKRNADNDPINAFYSWKKDIRDV
NEEITIESLDELEKRIESNQILLNNIKIPILSFEYDEFNNMLKILMKEVIKPKSSVSY

KVSEWIKDGLKIHNMQSKEETCEFCGNSFDVELVKESIDARIENEFNYLMENLNNYKN

KVENSLSNMENLNIDVDSVQFFKQKEELFKLLQLLEDKEKKTYEPLNYPIDNWESIIK

VDKIIINYIDKLNEDIQNDKKIRDSITDFVKSWIGKKLKDDEEIQMEIYNINTNSEQI

KKAEECKKANNDWITNLKTDSSNLKQFSEIVNKRFEILDINIELEPNIETKSYRVLHR
VTGEKLNLVDLSEGEKRLIGFLHFYYNLFMIADSEMKENIEHIIIDDPITSLDTENRY
HLTKIINQFIELSKSENKQIFVFTHSSLDFHNFAFKGKNNTKETSHWYIEKIDGISKV

RKLSKEELNNYSDYYSSVFNEVMSFALKGKRKIAYINDYIKYANKMRFILESHARSHY
KIEYATATSVPELSKYYDIEKERMKDLEKSLDIINSLSHGISFIDNHNEELSVIEVHE
AIRKVIGIIYKKDKQHVSAMCSGVLTKENKEELQKWI"
/product="hypothetical protein"
/transl_table=11
CDS complement(3079..3246)
/translation="MKFKSEKLIIANQTKVINYRIQVLLKPQTNIETQTQVKNNTLSE
TDEQSNADLVQ"
/db_xref="SEED:fig|176279.71.peg.3"
/transl_table=11
/product="hypothetical protein"
CDS 3886..4098
/db_xref="SEED:fig|176279.71.peg.4"
/db_xref="GO:0004617"

/translation="MRSSSPVQRGSYRIPKDKHQYKLKITDDNLQVDYPRQFLKGAHA
PSVKAQHVIHWGATFWVSSLWLAWFY"
/EC_number="1.1.1.95"
/product="D-3-phosphoglycerate dehydrogenase (EC
1.1.1.95)"
/transl_table=11
CDS 4181..5176
/transl_table=11
/product="Decarboxylase, pyridoxal-dependent"
/db_xref="SEED:fig|176279.71.peg.5"
/translation="MEMEFNENNIDLETIIRDEVNKYLSRDIGDLPATQQAPLELREK

YEKMEVPNKGRDIYEVLKDLNNEVLNYLYRPNHPRSFSFIPGPASRLSWLGDILTTAN

NIHASNFANATLPINIERNLINYLVGKIGYEIKPAGGVFVSGGSMANLTAIVAARDAQ

VEMEDIKKATVYLTSQTHHSVGKALHVAGFLKNNIRRIEYNDDFTMNTDSLKAAIDKD

IEEDYKPAMVIATAGTTNTGSVDDFTTLGDICDHYNLWLHVDGAYGLSHILSDKAQHL

FKGIERSDSASWDAHKLLFQTYSCAMVIVKEKNHLFCKALAKMLSTWMILLQMMM"

CDS 5146..5607

/translation="MDDIASDDDVIDPEMLGIELTRPARALKLWITLQVIGEDEIIER

IKYGEGLAEYAEEYVSSFDNWRIISHANLSIVNFRYENSDLTEAQNNQLNSMAAQKIA

DSGYAIAYTTVLNNQRVIRLCTTNPLTTHDDIRETINRFDQYIGDYDMKDA"

/db_xref="SEED:fig|176279.71.peg.6"

/transl_table=11

/product="Decarboxylase, pyridoxal-dependent"

CDS complement(5675..5857)

/translation="MKKMQDVAINADHKVFFLMIIFNVIMLVSSIIQMILGKGRRITP

AKPAKDAKLHLDNQSS"

/db_xref="SEED:fig|176279.71.peg.7"

/transl_table=11

/product="Multidrug resistance protein"

CDS 6329..6643

/product="Transposase"

/transl_table=11

/db_xref="SEED:fig|176279.71.peg.8"

/translation="MKRVSYSVETKYKAVEMKAAGFSTKEIMKELNIRNRTQVKTWWR

WYRNGESYRFSQQVGKQYTYGKGLKELSEVEQLKLENKRKDIELDILKKYKALERKWY

QQ"

CDS 6628..7470

/db_xref="SEED:fig|176279.71.peg.9"

/translation="MVPTVVIDLVDQLKVKYSIKLILEVLNIPKSTYYRWKNKTHKND

TVTQKVIELCKANHYTYGYRKITALINQCYTSPINHKRVQRMMQKHHLNCRVRPKKTT

RIGKPYYKTDNLLQRQFKASCPMEVLTTDITYLPFGHSMLYLSSIMDIYNGEIVAYKI

DDKQDQSLVNDTLNQIDIPESCILHSDQSSVYTSYAYYQLCEEKGIIRSMSRKGTPAD

NAPIESFHSSLKSETFYINNELNRSNHIVIDIVEKYIKNYNNNRIQQKLGYLSPVKYR

KLIA"

/product="Mobile element protein"

/transl_table=11
CDS complement(7570..8499)
/translation="MSKIVVALGGNALGQSPKEQLDLLKSTSKSLVSLIDKGYEIVIS

HGNGPQVGSINLGLNYAAEHKQGPPFPFPECGAMSQAYIGYQMQESLQNELHSMGIDK

QVVTLVTQVQVASDDSAFNNPTKPIGLFYTKEQADKFTKEKGYTFVEDSGRGYRRVVP
SPQPISIVELDSIETLITHGTLVIAAGGGGIPVIKENEVYTGVDAVIDKDKTSALLAA

HLQSDQLIILTAVDHVYINYGKENQRGLDEVSVDEMKKHISDGQFAKGSMLPKVEAAL
QFLEKNTKGSVLITSLAGLGDALDGKIGTLIKN"
/db_xref="SEED:fig|176279.71.peg.10"
/db_xref="GO:0008804"
/transl_table=11
/product="Carbamate kinase (EC 2.7.2.2)"
/EC_number="2.7.2.2"
CDS complement(8519..9517)
/EC_number="2.1.3.3"
/product="Ornithine carbamoyltransferase (EC 2.1.3.3)"
/transl_table=11
/db_xref="SEED:fig|176279.71.peg.11"
/db_xref="GO:0004585"

/translation="MKNLRNRNFLTLLDFTQKEMEFLLNLSEDLKRAKYAGIEQQKMK

GKNIALLFEKDSTRTRCAFETAAYDQGAHVTYLGPTGSQMGKKESTKDTARVLGGMYD

GIEYRGFSQRVVEDLAKYSGVPVWNGLTDEDHPTQVLADFLTAKEVLKKPYNEINFTY

VGDGRNNVANALMQGAAIMGMTFHLVCPKELNPTDELLNRCNNIAEKNGGEILVTDDI

DEGVKGSDVIYTDVWVSMGEPDEVWEKRIKLLEPYRVTKELMKKTGNPNTIFEHCLPS

FHDTETKIGKQIQEKYGLNEMEVTNEVFESEQSVVFQEAENRAHTIKAVMVATLGE"
CDS complement(9555..10244)
/product="hypothetical protein"
/transl_table=11
/db_xref="SEED:fig|176279.71.peg.12"
/translation="MYEENIYIKNSEYEFDNNLKQLASYLNIPVSIVRPYKEDLTLYQ

YKKGQVIYHSTDQIKFVYFLVNGCILHESSNITGDNYLRLSKDENIFPMNFIFNETPA
PYEICTALTDCKILTLPKDLLEYLCRKHNEIFESLFKKLNETIQFQVEYIMALRANSA
KERIERILQILCLSIGDDNGEFYELKQIMTVQLISNLSGLNRKTTGEIIRELKIENII
YQDKRNWIIKK"

CDS complement(10286..11707)
/product="Arginine/ornithine antiporter ArcD"
/transl_table=11
/db_xref="SEED:fig|176279.71.peg.13"
/translation="MDENKLGKTSLIGLVIGSMIGGGAFNIISDMGGQAGGLAIIIGW

IITAIGMISLAFVFQNLTNERPDLDGGIYSYAQTGFGDFIGFSSAWGYWFAAFLGNVA
YATLLMSAVGNFFPIFKGGNTLPSIIIASILLWGVHFLILRGVETAAFINSIVTVAKL

IPIFLVIICMIVVFNFSTFKSGFYGMTSGSVGVFSWGDTMAQVKSTMLVTVWVFTGIE

GAVVFSGRAKSKKDVGTATVIGLISVLVIYFLMTVLAQGVIQQNQISKLANPSMAQVL

EHIVGHWGSVLVNIGLIISVLGAWLGWTLLAGELPFIVAKDGLFPKWFAKENKNKAPV
NALIITNILVQLFLISMLFTDSAYQFAFSLASSAILIPYTLSAFYQVKYTIQNKSKAN
LKQWIIGIIASIYTIWLVYAAGLDYLLLTMLLYIPGLLVYSYVQRDNNKHLTKLDYTL
FIFIIVLAIIGIVRLITGNISVF"
CDS complement(11793..13028)
/EC_number="3.5.3.6"
/transl_table=11
/product="Arginine deiminase (EC 3.5.3.6)"
/db_xref="SEED:fig|176279.71.peg.14"
/db_xref="GO:0016990"
/translation="MVQGPIQVNSEIGKLKTVLLKRPGKELENLVPDHLSGLLFDDIP
YLKVAQEEHDKFAQTLRDEGIEVVYLEKLAAESITEPEVRENFINDILTESKKTILGH

ETEIKEFFSKLSDQELVNKIMAGVRKEEIQLETTHLVEYMDDRYPFYLDPMPNLYFTR

DPQASIGRGMTINRMYWRARRRESIFMTYILKHHPRFKDKDVPVWLDRNSPFNIEGGD
ELVLSKDVLAIGISERTSAQAIEKLARNIFKDANTSFKKIVAIEIPNTRTFMHLDTVL

TMIDYDKFTVHAAIFKEENNMNIFTIEQNDGKDDIKITRSSKLRETLAEVLEVEKVDF

IPTGNGDVIDGAREQWNDGSNTLCIRPGVVVTYDRNYVSNQLLRDKGIKVIEITGSEL
VRGRGGPRCMSQPLFREDI"
CDS complement(13297..13743)
/transl_table=11
/product="Arginine pathway regulatory protein ArgR,
repressor of arg regulon"
/translation="MKKEKRLNLILTVIQQNQFNKKQQIVDYMAKHFGVYYSLTTISR
DLQELEIYKIPVENKKYIYKKINQTNQLSAKKQLEIFSDEIIEFITLNNYVLIKTSPG
FAQSISYYIDQLQMKEILGIIGGNDTLMILTSSNEIAEFVCYQLFP"
/db_xref="SEED:fig|176279.71.peg.15"
CDS 14543..14815

/transl_table=11
/product="Type I restriction-modification system,
restriction subunit R (EC 3.1.21.3)"
/EC_number="3.1.21.3"
/translation="MDYVISLGEDERQRFIKTVTELGKAFAFCDTEPKVQKLNDEIAF
FKAVKAGLVKLLQPSKEGKIYKTLAEVEAEINHLVSQSVVTEEVKN"
/db_xref="SEED:fig|176279.71.peg.16"
CDS 14941..15096
/transl_table=11
/product="type I restriction-modification system
endonuclease"
/db_xref="SEED:fig|176279.71.peg.17"
/translation="MTVDCDKKSSSRVNKRRSVKRLLKKYGYPSDLETIAINQVVEQA
EMMASNQ"
CDS complement(15581..15796)
/translation="MFKNILLPYDFENDFSAIPDYLEKVIDEDSVVVIYHVVTENDLA
ISVKYYNKHKEDIIREKEKNSLHFYVN"
/db_xref="SEED:fig|176279.71.peg.18"
/transl_table=11
/product="Universal stress protein family"
CDS complement(16678..17004)
/db_xref="SEED:fig|176279.71.peg.19"
/translation="MLKLYYKFNFATEPDNDYLINQKVFDAFYKALKGKQVLVYFGLF

KDARKKLKNGELDYLKEVDPTEYIYQIFYTWNKKEYLASEIFDLTESEEQEINHQMID
EIEEEK"
/product="Replication protein"
/transl_table=11
CDS 17020..17694
/product="Mobile element protein"
/transl_table=11
/db_xref="SEED:fig|176279.71.peg.20"
/translation="MNYFRYKQFNKDVITVDVSYYLRYALSYRDISEILRERGVNVHH

LTVYRWVQEYAPILYQIWKKKYQKAYYKWHIDETYIKIKGKWSYLYRAIDAEGHTLDI

WLRKQRDNHSAYAFIKRLIKQFGKPQKVVTDQAPSTKVAMAKVIKVFKLKPDCHCTSK
YLNNLIEQDHRHIKVRKTRYQSINTAKNTLKGIECIYALYKKNRRSLQIYGFSSCHEI
SIMLAS"
CDS 18150..18455
/product="hypothetical protein"
/transl_table=11
/translation="MKGNIQCLRLIKGSLLDFVLETVDVFVNGSKNDCELVNFNYKEG
KVVSRYSTKFESNPKLRKEQLISTVKVVRYVIFDFEKILWGIGKVLLKFIILRQCLL"

/db_xref="SEED:fig|176279.71.peg.21"
CDS 18653..19516
/translation="MKHINHFSESTLAYLEKDHEHAFYVYCLIDPRNDECFYVGKGKG

NRIFKHKQDAQRQLLYEDIFREENKDNLKFNRINEICSNDLNVLGYIISYGLTESEAF

SAENVLINFLNLTNKTTLTNMINGHGSKAYLVEDLENEFGYDSINLENINTNELILAV

KIRDAFLLDKDESKEYPINESKRDRNNLKSRTLGSWIIGKDKIHKIKYIIGINTGANN

AVVSAYEVSFEQAESIETNNGRMRYAFIGLSERDATLKKLNLYKKALPDLRFGSGSAT
AYINNGTMKVD"
/db_xref="SEED:fig|176279.71.peg.22"
/transl_table=11
/product="hypothetical protein"
CDS 19624..21099
/product="hypothetical protein"
/transl_table=11
/db_xref="SEED:fig|176279.71.peg.23"
/translation="MPSDDIVKKLYSKEDIRLELGLTPHKFNKKMETIAKLFKIDMKI

FHNYKGQDKNNQYTFNGVAKELIKVLLKSVDYYPVDINSKKFKQNGKSKKEMIENIDN

SSYMKYIYQLMKSINEIQYKRLIADIHMKDVYQNTKAWLNNGESINKKEQELYQYMTI

LPLHKRVELQNEVLKSIDETIFQFLAKEHRNNQIEENNELEAYTKAIKEGRNPKNDYE

LNHLLYKKNQLPLDSLIEDIWDYEETEYTELDWLIADMLKRSQRADSNFIESLEKKNK

LRKNIHKDSIKSISKLIDHELVKNNRKWDVAEFYKNQQFWNNSTLRRSSQNFGILYYK

KNRLHTMNQSNIIKKIKIIEANIESANKQPSYLDFFLQAKFDLERLESELMKYSINPK

YFNKINSDYVRYAIEDVHNAIIKIDRYISNDKVKLNYSNVQMVVRNEITEQGSYFVTQ
ALNTLSKVEESGYSFDNFMVGPFVEDFKKAIKNRRFGSKKD"
CDS 21326..22426

/translation="METRDKLMSLTQSDKTQQWLMDKSSNQDDIQQLQQQFSQQLDQQ

YNALLADEKAKLDQYVEVHQGLESLKEEIESEPITLNIDKLPDIKATMLERAKNDEHS

DKIEKLFDRLEQALNGTNRLYTQLSLIGTRTHRITTKNFNLQGLPKAVQHTILPSKFK

KVYTVDFKSFEPSVAAYMTQDSKLIDLLNQKDGLYDALLSELGLSDELRVFVKRAFIG

SFLFGGNFKNPKFKLNQYVSEVQWLDAVSQFTKVIELKKHVEKSNSMPMPYSIEHDMS

AFQGSSIMAIYVQTVASYIFKHILLKVYKAQCDQKTFKIIVPIHDAIMIECEDEEIAQ
NVGQLMKDTANQLFNGEFAHVTVEEIGGVDHE"
/db_xref="SEED:fig|176279.71.peg.24"
/transl_table=11
/product="hypothetical protein"
CDS 22419..22790
/transl_table=11
/product="hypothetical protein"
/translation="MNNDRGQSLHIPSSTPIKENNIYVATLHSVIQTDFSGEIKHQFT

YEIEVNNQIVYANRNIPTKPSANQLSIHDWLKRHSNYSVGHDTYEPYIDRKHLIHIGQ
YSGNYYVQDVASLDAFGGVLS"
/db_xref="SEED:fig|176279.71.peg.25"
CDS 22787..24430
/db_xref="SEED:fig|176279.71.peg.26"
/translation="MNHILQMLSKLLSVAKEAIDRQGLIAILTIPVNNNDEIEETAQG

ETVYNELIDQLRLNIPKDTDYQPNIYSYFGIKKNPNDTVLMEMMIKVFHIKRFNSELF

IFKVNGWQKINGDELQGLISKMIQVLLVDYKPSLSTLKNVVDGLQKSTDVEELVENER

YIGCGENMFDLNTFQVVKNSIDIFPKTRLNLSLSTNDVITDKIPPYFKQYMLQLANYD

DDLQYFLFQHTAVLLTADTKYRRGLILYGGAKNGKSVYIELVKSFFYSKDIVSKPLNE

LEGRFDKESLIDKSLMASHEIGQSRIQEKIVNDFKKLLSVESMHVDRKGKTQVEVILD
LKLIFSTNAILNFPPEHAKALERRINIIPCEYYVEKADTSLIDKLQSEKKEIFLYLMY

VYQQIVKADIEYLENSRVTEITHDWLNFGYEFVSSRSVSNANQKACINLLRKLIEIKS

GSRIKVSELNKVINEEIKVSSQVIKQLIQANFDTQTKLYNGYDYWIDLGWKEANKKEI
HDISEKDNIISLDKNENITDDEALDEENLDFDWEDFDDE"
/transl_table=11
/product="putative primase"
CDS 24658..26334
/translation="MKGKIALYSRVSTSEQSEHGYSVKEQEQVLIKEVVKNFPGYDYE

TYTDSGISGKNIEGRPAMKRLLQDVKDNKIEMVLSWKLNRISRSMRDVFNIIHEFKEH

DVGYKSISENIDTSNASGEVLVTMFGLIGSIERQTLISNVKLSMNAKARSGEAITGRV

LGYKLSLNPLTQKNDLVIDENEANIVREIFDLYLNHNKGLKAITTVLNQKGYRTINQK

PFSVYGVKYILNNPVYKGYVRFNNHQNWAVQRRSGKSDKNDVILVKGKHEAIISEEVF

DKVHEKLASKSFKPGRPIGGDFYLRGLIKCPECGNNMVCRRTYYKTKKSKERTIKRYY

ICSLFNRSGSSACHSNSINAEVVERVINVHLNRILSQPDIIKQIASNVIEELKQKHSN

QTEIKYDIDSLEKQKAKLKTQQERLLELFLDDQMDSEMLKAKQSQMNQQLEVLDQQIK

EAQQANQSQDEIPNFDKLKGRLILMITRFSVYLRKATPEAKNQLMKMLIDSIEITTDK
QVKLVRYKIDESLIPQSLKKDWGSFFMPKFQFEIYGQNDYFIDQITTFTT"
/db_xref="SEED:fig|176279.71.peg.27"
/product="Cassette chromosome recombinase B"
/transl_table=11
CDS 26437..26778
/transl_table=11
/product="FIG01108299: hypothetical protein"
/translation="MMTLEQQLKHYITNLFNLPKDEVWHCESIEEIADDILPNQYVRL

GPLSNKTLQTNTYYSDTLHESNIYPFILYYQKQLIAIGYIDENHDMDFLYLHNTIMPL
LDQRYLLTGGQ"
/db_xref="SEED:fig|176279.71.peg.28"
CDS 26780..26881
/translation="MHKYIKITQLVITILSEIIIWMKESERKEVSYE"
/db_xref="SEED:fig|176279.71.peg.29"
/transl_table=11
/product="hypothetical protein"
CDS 26874..27185
/transl_table=11
/product="FIG01107894: hypothetical protein"

/translation="MNRYITRGIANNLPNILQHQLWQLVSEREQEQTKDNTSVDYFHI

FQFNKHRNQLYIKHKQERPEYAKIHKANYSKAININKVYIIREDDVDLSYYVMLLPEE
Y"
/db_xref="SEED:fig|176279.71.peg.30"
CDS 27201..27707
/transl_table=11
/product="hypothetical protein"
/translation="METIKSTLKTEAIFSDDKQHRYLLKKTWNSEKQSITIITMYPHY

DGILNIDLTTQLIMNKVSEMDAFGSINFVNLYSNITTPINLKHLENAYDKHTDIQIMK

AVKESDEVILAWGAYAKKPGVEARVNEVLEMLKPHKKKVKRLMNPETNEIMHPLNSKA
RQKWILKV"
/db_xref="SEED:fig|176279.71.peg.31"
CDS complement(27856..28530)
/product="Transposase for insertion sequence-like element IS431mec"
/transl_table=11
/db_xref="SEED:fig|176279.71.peg.32"
/translation="MNYFRYKQFNKDVITVAVGYYLRYTLSYRDISEILRERGVNVHH

STVYRWVQEYAPILYQIWKKKHKKAYYKWRIDETYIKIKGKWSYLYRAIDAEGHTLDI

WLRKQRDNHSAYAFIKRLIKQFGKPQKVITDQAPSTKVAMAKVIKAFKLKPDCHCTSK
YLNNLIEQDHRHIKVRKTRYQSINTAKNTLKGIECIYALYKKNRRSLQICGFSPCHEI
SIMLAS"
CDS 28788..28955
/db_xref="SEED:fig|176279.71.peg.33"
/translation="MLESREQLSVEEYETFFNRFDNQEFDFERELTQDPYSKVYLYSI
EDHIRTYKIEK"
/EC_number="2.3.3.10"
/transl_table=11
/product="Hydroxymethylglutaryl-CoA synthase N-domain homolog (EC 2.3.3.10)"
CDS 29053..29283
/transl_table=11
/product="hypothetical protein"
/db_xref="SEED:fig|176279.71.peg.34"
/translation="MLIFLSYDFVRACLPRGMARAISLSHILFPQASALTKSVVIFIF
IRILTEIILIRGIVKAILSKIADKRFVKSSSK"
CDS 29792..30535
/translation="MLTVYGHRGLPSKAPENTIASFKAASEVEGINWLELDVAITKDE

QLIIIHDDYLERTTNMSGEITELNYDEIKDASAGSWFGEKFKDEHLPTFDDVVKIANE

YNMNLNVELKGITGPNGLALSKSMVKQVEEQLTNLNQNQEVLISSFNVVLVKLAEEIM

PQYNRAVIFHTTSFREDWRTLLDYCNAKIVNTEDAKLTKAKVKMVKEAGYELNVWTVN
KPARANQLANWGVDGIFTDNADKMVHLSQ"
/db_xref="SEED:fig|176279.71.peg.35"
/db_xref="GO:0008889"
/product="Glycerophosphoryl diester phosphodiesterase (EC 3.1.4.46)"
/transl_table=11
/EC_number="3.1.4.46"

CDS 30632..31060
/db_xref="SEED:fig|176279.71.peg.36"
/translation="MKYDDFIVGETFKTKSLHITEEEIIQFATTFDPQYMHIDKEKAE

QSRFKGIIASGMHTLSISFKLWVEEGKYGEEVVAGTQMNNVKFIKPVYPGNTLYVIAE
ITNKKSIKKENGLVTVSLSTYNENEEIVFKGEVTALINNS"
/transl_table=11
/product="MaoC domain protein"
CDS complement(31106..33112)
/transl_table=11
/product="Penicillin-binding protein PBP2a, methicillin
resistance determinant MecA, transpeptidase"
/db_xref="SEED:fig|176279.71.peg.37"
/db_xref="GO:0008658"
/db_xref="GO:0046677"
/translation="MKKIKIVPLILIVVVVGFGIYFYASKDKEINNTIDAIEDKNFKQ

VYKDSSYISKSDNGEVEMTERPIKIYNSLGVKDINIQDRKIKKVSKNKKRVDAQYKIK

TNYGNIDRNVQFNFVKEDGMWKLDWDHSVIIPGMQKDQSIHIENLKSERGKILDRNNV

ELANTGTAYEIGIVPKNVSKKDYKAIAKELSISEDYIKQQMDQNWVQDDTFVPLKTVK

KMDEYLRDFAKKFHLTTNETESRNYPLGKATSHLLGYVGPINSEELKQKEYKGYKDDA

VIGKKGLEKLYDKKLQHEDGYRVTIVDDNSNTIAHTLIEKKKKDGKDIQLTIDAKVQK

SIYNNMKNDYGSGTAIHPQTGELLALVSTPSYDVYPFMYGMSNEEYNKLTEDKKEPLL

NKFQITTSPGSTQKILTAMIGLNNKTLDDKTSYKIDGKGWQKDKSWGGYNVTRYEVVN

GNIDLKQAIESSDNIFFARVALELGSKKFEKGMKKLGVGEDIPSDYPFYNAQISNKNL

DNEILLADSGYGQGEILINPVQILSIYSALENNGNINAPHLLKDTKNKVWKKNIISKE

NINLLTDGMQQVVNKTHKEDIYRSYANLIGKSGTAELKMKQGETGRQIGWFISYDKDN
PNMMMAINVKDVQDKGMASYNAKISGKVYDELYENGNKKYDIDE"
CDS 33360..33503
/product="Mobile element protein"
/transl_table=11

/translation="MNYFRYKQFNKDVITVAVGYYLRYALSYRDMSEILRERGVNVHH
LTV"
/db_xref="SEED:fig|176279.71.peg.38"

CDS 33783..34034
/transl_table=11
/product="Mobile element protein"
/db_xref="SEED:fig|176279.71.peg.39"
/translation="MAKVIKAFKLKPDCHCTSKYLNNLIEQDHRHIKVRKTRYQSINT
AKNTLKGIECIYGLYKKNRRSLQICGFSPCHEISIMLTS"
CDS complement(34094..34522)
/db_xref="SEED:fig|176279.71.peg.40"
/translation="MKFQVSPYFLITQNAEGLKKLYEELFGAKTLYIQRIKDRPIDKQ

LNIDENDLNKIDQCVLQFGDIKIMIADDTEGLPITQGNNISLCLTFDQVEETKHVYDQ
LIEKNSEVLKTFSPEFYTEGYGYVRDPYGISLHLFTKRKN"
/product="PhnB protein; putative DNA binding
3-demethylubiquinone-9 3-methyltransferase domain
protein"
/transl_table=11
CDS 34603..35532
/db_xref="SEED:fig|176279.71.peg.41"
/translation="MKKSVRLYNMIEYCNENRNFKLNDLMSEFNISRSTALRDIKEIE

ALGVPLYSNPGKNGGYTIIGNRDQTKIAISDEELKALVFTLSSISNVSNLPFQTEYQE
ILKKLYNNSNKKELINQYNDLFQYFNEDKYQFKSYKLFNEIIRLIIENKSFEICYSQN

YIKEQYKGIGIMYKNHQWYFVVANIESKLVNLLNISKIKELYEMGETQECNDITMQNF

QQFMVKNETAIDILIRSNVMGLNILKGYLWSDYMIENIDEETYLFKSKVNAKDIDFIA
KLIVTGGVNVKVETPNSLKNAVKVELTKIINMY"
/transl_table=11
/product="Transcriptional regulator, YafY family"
CDS 35694..37706
/transl_table=11
/product="Similarity"
/db_xref="SEED:fig|176279.71.peg.42"
/translation="MNKNITALKSTENTTYMQYTVHIDDQIINNIKECTKKFEILPME
DKIPLSPLLQPEYAGEVQVFISTYEQFMINFGKVILDSQGIKIQFESESLSSIQRGIQ

EHCYLNKRTNDIDVTKEWYLCKFSNRTLEEDKNQLYNALKQLMNDSKNKKQAFQEVFK
VHIDIYLYNKEKSEYKYQASSYFYLVKENPKIIYKKRHLQEKQGIKGTTFTNKLKLLN

KMYGVDVAKYQPFYNSNNPEYERGQFGERYISQRSNYEFNRLQYQIIYILSKILDKHP

LPKSDNNYKHIPTIEKAILRDDSHNFYEYFEDIMKEIMNMDNSPLQEKLLTDFTYQSQ

CRWYSENKKLKLQLERFMHKVLESNYYEGNKLLRMMSNAIEETINEAKEDKVHFNYFK

DYFLTDGGVKNWEQISEKITEFNGKVINDIQHEYNKIQSKNTNQNQKLNFVYLYHCFE

FSNNIVKAKDNESKNYILFFIDKYKIKTPFLDQLLINCLNQEIDQESINYNMQTMFEK

ERYDRSSTIEKLVATRKFKYEKDDSDLFKQLFNDVENSIDRLGIYLLNNGINSNDENA

RYYRSFLKELSRIKSKLTPFSLEISKSSGREQHYPDDAVDDKDERRKNKEEAYHAFDD
KSDIDYKLKNKISDYIDNLLSVKIRNNLYDISPNTKSNNYNEPTQK"
CDS 37876..38985

/translation="MYQANIRDLITKLPQSNKTEHFLMNKFSNQDKVQQLQRQISQQL

DQQYNELLSNEKAKLDQYVEVHHNLEPLKKEIESESINLDTDKLPDIKATMLEKAKND

EHSDKIEHLFDRLDQSLNGTNRLYTQLSLIGTRTHRITTKRFNVQGLPKLVQQTILPS

QFKKVYTIDFKSFEPSVAAYMTQDEQLIDYLNHEEGLYDALLRDLSLSKEKRVSVKRA

FIGSFLFGGRYSSSKFKINQEVSEINWLQVMSKFKKVIEFKEQVEKYKTMPTPYGIEH

DMSAFQGSSIMAIYVQTVASYIFKYILLEVYKAQCEQKMFKIIVPIHDAIMIECNDKE
IAQSVAQLMKDTANQLFNGEFAHVTVEALRGIDNE"
/db_xref="SEED:fig|176279.71.peg.43"
/transl_table=11
/product="hypothetical protein"
CDS 38978..39349
/product="hypothetical protein"
/transl_table=11
/db_xref="SEED:fig|176279.71.peg.44"
/translation="MNNDRGKSLQIPQSTSLKEGSIYVATLHSVTETDYSGDIKHQFT

YEIEIDQQIFYVNRSVVVNVTHQQLSINDWLERHSNYSASHENYEPYIDQKHLILLGQ
YNGNYYVQDVASLDAFGGVLS"
CDS 39346..40989
/product="putative primase"
/transl_table=11
/translation="MNHILQMLSKLLSVAKEAIDRQGLIAILTIPVNNDDEIEETAQG

ETVYNELIDQLRLNIPKDTDYRPNIYSYFGIKKNPNDTVLMEMMIKVFHIKRFNSELF

VFKANGWQKINGDELQGLISKMIQVLLVDYKPSLSTLKNVVDGLQKSTDVEELVENER

YIGCGENMFDLNTFQVVKNSIDIFPKTRLNLSLSTNDVITDKIPPYFKQYMLQLANYD

DDLQYFLFQHTAVLLTADTKYRRGLILYGGAKNGKSVYIELVKSFFYSKDIVSKPLNE

LEGRFDKESLIDKSLMASHEIGQSRIQEKIVNDFKKLLSVESMHVDRKGKTQVEVILD
LKLIFSTNAILNFPPEHAKALERRINIIPCEYYVEKADTSLIDKLQSEKKEIFLYLMY

VYQQIVKADIEYLENSRVTEITHDWLNFGYEFVSSRSVSNANQKACINLLRKLIEIKS

GSRIKVSELNKVINEEIKVSSQVIKQLIQANFDTQTKLYNGYDYWIDLGWKEANKKEI
HDISEKDNIISLDKNENITDDEALDEENLDFDWEDFDDE"
/db_xref="SEED:fig|176279.71.peg.45"
CDS 41215..42894
/translation="MKGKIALYSRVSTSEQSEHGYSIHEQEQVLIKEVVKNFPGYDYE

TYTDSGISGKNIEGRPAMKRLLQDVKDNKIEMVLSWKLNRISRSMRDVFNIIHEFKEH

DVGYKSISENIDTSNASGEVLVTMFGLIGSIERQTLISNVKLSMNAKARSGEAITGRV

LGYKLSLNPLTQKNDLVIDENEAHIVREIFDLYLNHNKGLKAITTILNQKGYRTINQK

PFSVFGVKYILNNPVYKGYVRFNNHQNWAVQRRSGKSDKNDVILVKGKHEAIISEDVF

DQVHEKLASKSFKPGRPIGGDFYLRGLIKCPECGNNMVCRRTYYKTKKSKERTIKRYY

ICSLFNRSGSSACHSNAINAEVVERVINVHLNRILSQPNVIKQIASSVIEELKQKHSK

QTEIKYDIDSLEKQKAKVKTQQERLLELFLDDEMDSEMLKAKQSEMNQQLEVLDQQIK

EAKQANQSQDDIPNFDKLKAQLILMITRFSVYLRKATPEAKNQLMKMLIDSIEITTDK
QVKLVRYKIDESLIPQSLKKDWGSFFMPKFNFVINVTNENRIENLSLLPLF"
/db_xref="SEED:fig|176279.71.peg.46"
/transl_table=11
/product="Cassette chromosome recombinase B"
CDS 42983..43321
/translation="MTLSKQLKTYITERFKLNYQETWACETVDAVAEDVLPEKYIKNS
PLEHKILNTFTYYNDELHEISIYPFLCYLDKELVAIGYLDNFDLDFIFLNDTHQVIID
ERYLLQKGGE"
/db_xref="SEED:fig|176279.71.peg.47"
/transl_table=11
/product="hypothetical protein"
CDS 43324..43413
/db_xref="SEED:fig|176279.71.peg.48"
/translation="MNWIKVAQLSVTVINEVIEIMKEKQNGGE"
/product="hypothetical protein"

/transl_table=11
CDS 43415..43726
/product="hypothetical protein"
/transl_table=11
/db_xref="SEED:fig|176279.71.peg.49"
/translation="MKINRYITRGVSEHLSLDLQILLWNMVKDRDNQLHTDYLHIFKL

QEDDNILSITHEQEQPAYKLEYHYTNYVKNQNALPKKVYVIREDDVDAFYYVMLLPEE
Y"
CDS 43738..44244
/transl_table=11
/product="hypothetical protein"

/translation="MMNTIKNTIHTEAIFSDDEQHRYLLKKTWDEKKPACTVITMYPH

LDGVLSLDLTTVLILNQLANSEQYGAVYLVNLFSNIKTPENLKHIKEPYDKHTDIHLM

KAISESDTVILAYGAYAKRPVVVERVEQVMEMLKPHKKKVKKLINPATNEIMHPLNPK
ARQKWTLK"
/db_xref="SEED:fig|176279.71.peg.50"
CDS 44258..44479
/product="hypothetical protein"
/transl_table=11
/db_xref="SEED:fig|176279.71.peg.51"
/translation="MNHETKQSDWRTVASCLASQNYISIVKGLVHHFTAIEDEEILDK
IYDDFMNDDSITTVLNNDLQTIINHYLSK"
CDS complement(44554..47673)

/translation="MNFQFNEDDLEQVALEWLQSLGYDYKKGNEISMTSLTPERKSDK
DVVLHERLEKALRKINSDIHPRFIEKALHELTLEKSPNLLENNLTFHENLINGIEIED
YDDEGQSIVEIVKIIDFEYPQNNDFLAVNQLTIVNGDYKKRPDIVLFINGLPIVVIEL

KNSTNETVGVEDGYHQLETYKMRIPQLFTFNEVLVTSDGINTKAGSLTANYDRFMTWR

SKDGQTESSPGLASLDVLIHGMLNPETLLDLIRYFVLFQDDGKGHISKILAAYHQYYA

VNKAVDRALLASSEQGDGKGGVIWHTQGSGKSLTMVFFSGKLIQMLNNPTLVVVTDRN

DLDNQLYSTFVKSKGRSGKGLLRQTPKQAETRKELKSLLSVESGGIVFTTMQKFEPEQ

NETTMAALTERKNVIVMADEAHRTQYGFNAKYDDKGEGIKYGYAKYLRDALPNATFVG

FTGTPVASTDKNTQMVFGNYIDVYDMTQAVADGSTVKIYYESRVIPLNLPQSLDLDEA

YNDITEDQEEDVKQRLKSKWSRIEALAGAKPRVEALAKDIIQHFETRQQAMKGKGMIV

TMSRRIAVDLYDEIIRLKPEWHSDDDDKGVIKVVMTGSSSDPTSFQRHIGPKKRRNLL

EKRMKDVDDELQLVIVRDMWLTGFDVPSMHTMYIDKPMKGHNLMQAIARVNRVFKDKP

GGLIVDYVGIAESLKEALKEYTESDQAQTAIDTDKAVELMLLKYDVIQDMLYNLDYSK

FNSDKKSERYYTISNTMDYVIGLGEDERQRFIKTVTELGKAFALCATEPTAQELNDEI

AFFKAVKAGLVKLLQPPKEGKTRKTPAEVEAEINQLVSQSVVTEDVIDVYQTLGLEQP

DLSILSDDFLKDVEGLKQKNVAVELLNRLLKGQVKSLMKTNATVSKRFSEMLGNSINK

YNSRSIETSKVIEELIQLAKDIKQEQQRGNELGLNSDEIAFYDALASHETAKEAMGDK

ELRAIAHELTKTVKENMGVDWSKRDSAKAKMRVAVRRLLKKYGYPPDLQKMAVEQVVE
QAELMANNQ"
/db_xref="SEED:fig|176279.71.peg.52"
/product="Type I restriction-modification system,
restriction subunit R (EC 3.1.21.3)"
/transl_table=11
/EC_number="3.1.21.3"
CDS complement(47657..48958)
/transl_table=11
/product="Type I restriction-modification system,
specificity subunit S"
/translation="MEYNTIFFGDVAKIKNGYAFKSKEFQEKGIPVIKIKNIISPIVD

TKDSQKVSIKTYEKTKGFSLKKNDILISLTGSGVNQMSSAVGKVGRIEFDYPALQNQR
VGKFELKYSNSADLDFLFYYFLQPKITEYLVRNSTGSANQANINSKLIETVKIPNFSL

IKQKSISKFLNQITRKIETNQKMIANLKELSQTLFKHWFVDFEFPDEDGNPYKSSGGE

MIDSELGKIPSNWKIYKLKDIASHKKETFNPKKSEEVTVKHFSLPAYDNEEQAIEEEV

NKIKSNKWIINNNCVLFSKMNPDTKRIWLPVIDNKKLNVASSEFVVMESPNNKINSFI

YNICLNSQFIDYLKANTTGSTNSRQRVKPTIAVNYKLAIEDSIVKKYSEIITPYMEEM
KILRSEIGKLTQLRDTLLPKLMSGELEISDDIEVNSDELSI"
/db_xref="SEED:fig|176279.71.peg.53"
CDS complement(48948..50462)
/transl_table=11
/product="Type I restriction-modification system,

DNA-methyltransferase subunit M (EC 2.1.1.72)"
/EC_number="2.1.1.72"

/translation="MATIGFEEKLWQAADKLRGSMDAAEYKNVALGLIFLKYVSDSFE

EKYEELKQDPYADEEDQDEYLAENIFWVPKEARWQYINDNAKKPEIGQIIDKAMIAIE

NENESLKGVLPKEYARPALDKEKLGDIIDLFTFKVGDTESRKQDVLGRVYEYFIAKFA

SAEGKNAGEFYTPSSIVKLLVEMIEPYKGRIYDPCCGSGGMFVQSERFVEKHQGRLDD

IAIYGQESNPTTWKLAKMNLAIRGIDNDLGERNADTFHNDLHKGLKADYILANPPFNA

SDWGQERLLDDYRWQFGIPPKGNANYAWIEHMISKLAPNGTAGFVLANGSMSTSGKDE

LEIRKNLIEQDLVECIVTLPGQLFYSTQIPVCLWFISNNKGQNGKKERENEILFIDAR

EIGHMVSRTLKEFSDEDIQKVAQTYHAWRGTNDKPYEDIAGFCKVANLEEVKNNEYIL
TPGRYVGLADVEEDEEPFEQKMERITSELSEQFAKSKELEDQIRKSLEGLGYGV"
/db_xref="SEED:fig|176279.71.peg.54"
CDS 50658..50804
/transl_table=11
/product="hypothetical protein"
/translation="MDGVTNVKIHILTFVKIVVIVDIVAIVNTRLDKLGIFNSINKKF
KCSQ"
/db_xref="SEED:fig|176279.71.peg.55"
CDS 50904..52967
/db_xref="SEED:fig|176279.71.peg.56"
/translation="MYISSVKIKNFRNFDNIDIDFHEGVNVLIGHNNSGKSNLLRALS

LIFDGSVRKQLSVEDFNNSLTKESLKKEAPKIVISVHITQSENERLMSDELITVSNWL

TKLEEPYEARIQYEFFLPKDEEKNYIDLVKNIDEKEEIWNLIRSQFIRLYVNKIWVGN

PEHQIPIDNDSLNKFDFQFLDAIRDVERDMFSGKNTLLKSVIDFFIDYDIKSDETITE

EEQKEKLEERRKEFSDNSSDLIETIHKRLDSGNQKILSYTNGIGASYDKSTPDFKGNL

TESEIYTVLQLIIKHETGMTLPITHNGLGYNNLIFMALLLSKMQADSDGNFLGSNAKV
FPILAIEEPEAHLHPTMQNEFIKFLKNNIREKKVKQIFITTHSTHISSSTNIDDIICL

YTDDSKTNVSYPGKVFDKNNQSKKYVQRFLDATKSNMLFAEKVIFVEGIAEQLLLHIF

ADYLNKPLEKNHVAVINVGGRYFNHFLSLFDSNNKYAINRKVSCITDLDPMRKEKNEN

GQKNNFKVCYPFEYEMDLDKFDYSTNNSLDKYLNNEHSNIRVFVQPNKYGKTFEYQLM

FDNPSLKLLLTDSISNSQELAELMDHYEENESLQKLIDTLSPSNENQRIIKSLKDTND

SWNEDNKKKALIASRYLNSIGKGENALELASVLKDNLELKGQSDYEDFVVPKYIEGAI
RWVCE"
/transl_table=11
/product="FIG131328: Predicted ATP-dependent endonuclease
of the OLD family"
CDS 52969..54717
/product="ATP-dependent DNA helicase UvrD/PcrA,
clostridial paralog 2"
/transl_table=11
/db_xref="SEED:fig|176279.71.peg.57"
/translation="MIINAQTKLNIEQDFKIQAGPGAGKTEFLVNHIKNVIQTSEKLE

RMKKIACITYTNTAAQTVLQRLGKSVSNRVDISTIHSFLYRNVVKPYCSFLPKEYGIC

VQKLKGHSDPIVINKYVNAWLKTEYFTKLKPPSNRNQLLKMPVLKKALQNWLLSMKLS

YENNDVQFICDNTKAQAIDKKSKKRMGINKSNLNILEKQLLNYKKIYWEKGIIDHDDV
LFFSYVLIRDYPFILTILCAKYPYFFIDEFQDTSQLQDFIIDKIRQKGCIVGVIGDKA
QAIYSFQGARVSLFEKFKVDLINSHTILENHRSSYQIVTFLNSIREDITQTVYSGIEN

DKVTILIGNRIRSYTKVVNICNKESLVTLSRDNITSNAMRIKLEGNNFDKKLLEKFQE
IDSNSKRRNTIISLIKAIELAKNTKYKDALKIVESIYKDENNPKKIALNSLINMINKY

DKYGNGTLMNFYNTLCKTLDIKLSGFRKGQVKDFYENTLYKNMVICTNIVEDTSNHIT

IHKAKGAEFENVFVIGNKDTLSLLLKPDLMNDEEHRIFYVAMSRAKKKLFLQFDYLDN
NEQKKIKERYDVSIIQ"
CDS 54927..55109
/translation="MKKMQDVAINADHKVFFLMIIFNVIMLVSSIIQMILGKGRRITP
AKPTKDAKLHLDNQSS"
/db_xref="SEED:fig|176279.71.peg.58"
/product="Multidrug resistance protein"
/transl_table=11
CDS complement(55177..56601)
/transl_table=11
/product="Decarboxylase, pyridoxal-dependent"
/db_xref="SEED:fig|176279.71.peg.59"
/translation="MEMEFNENNIDLETIIRDEVNKYLSRDIGDLPATQQAPLELREK

YEKMEVPNKGRDIYEVLKDLNNEVLNYLYRPNHPRSFSFIPGPASRLSWLGDILTTAN

NIHASNFANATLPINIERNLINYLVGKIGYEIKPAGGVFVSGGSMANLTAIVAARDAQ

VEMEDIKKATVYLTSQTHHSVGKALHVAGFLKNNIRRIEYNDDFTMNTDSLKAAIDKD

IEEGYKPAMVIATAGTTNTGSVDDFTTLGDTCDHYNLWLHVDGAYGLSHILSDKAQHL

FKGIERSDSASWDAHKLLFQTYSCAMVIVKEKNHLLQSFGEDAEYLDDIASDDDVIDP
EMLGIELTRPARALKLWITLQVIGEDEIIERIKYGEGLAEYAEEYVSGLDNWRIISHA

NLSIVNFRYENSDLTEAQNNQLNSMAAQKIADSGYAIAYTTVLNNQRVIRLCTTNPLT
THDDIRETINRLDQYIGDYDMKDA"
CDS complement(56684..56896)
/translation="MRSSSPVLRGSYRIPKDKHQYKLKITDDNLQVDYPRQFLKGAHT
PSVKAQHVIHWGDTFWVSSLWLAWFY"
/db_xref="SEED:fig|176279.71.peg.60"
/db_xref="GO:0004617"
/transl_table=11
/product="D-3-phosphoglycerate dehydrogenase (EC
1.1.1.95)"
/EC_number="1.1.1.95"
CDS 57546..57713
/product="hypothetical protein"
/transl_table=11
/db_xref="SEED:fig|176279.71.peg.61"
/translation="MKFKSEKLIIANQTKVINYRIQVLLKPQTNIETQTQVKNNTLSE
TDEQSNADLVQ"
CDS 58151..58849
/product="hypothetical protein"
/transl_table=11
/db_xref="SEED:fig|176279.71.peg.62"
/translation="MNKDESKETEIKKSFNKTLSMYPIKNLEDLYDKEGYRDEEFNKG

DKGMWIVHSEMNIQKKGKALESRGMVLYMDRNTRTTHGYFILTKIKDTGKVKTDDSEK

KYPVKLENNKIIPTKQIKDEKLKKEIENFKFFSQYGNFKDLKDYKDGEVSYNPNAPNY

SAKYQLSNNDYNVKQIRKRYDIPTEQAPKLLLKGTGDLKGSSTGSKNIEFTFVEKKGE
NIYFTDSVEYTPSG"
CDS complement(59109..59504)
/translation="MKYKYVSNVIDNLKRLFNELLEIRYYVDEEKTYKENNLSVSFTL
TNKCNLSCSHCALSASPLSQDILSTNDVKYAIDKIIDVNPNTLILTGGEPFIRKDILE
IISYIRTNFKNKLVIMTNGMLIRKKFIPF"

/db_xref="SEED:fig|176279.71.peg.63"
/product="hypothetical protein"
/transl_table=11

ORIGIN

1 atgaagatta ctatcttatc agttggaaaa ctaaaagaaa aatattggaa gcaagccatt
61 gcagaatatg aaaaaagatt aggaccttat acgaaaatcg aattaataga agtaccagat
121 gaaaaagcac ctgaaaatat gagcgacaaa gaaattgaac aagtcaaaga aaaagaaggc
181 caacgcctac tcaataagat taaaccccaa tccacagtaa tcacgttgga aatcaaaggg
241 aaaatgttgt cttcagaagg actcgctaaa gaactacaaa cacgcatgac acaaggtcaa
301 agcgacttta catttgtcat aggtggctcc aatggtctac accaagacgt cttacaacgc
361 agcaactacg cactatcatt cagcaacatg acctttccac accaaatgat gcgcgtcata
421 ttaatcgaac aaatttatcg cgcattcaag atcatgagag gcgaagcgta tcataaataa
481 aattaaaatt gattgaaaat aaaatattat tacttattta aggggggcta tgatgttaga
541 taaattaaaa aatgttagtc ataaatcatt tgataaatat actaataatg atcaattgac
601 taaagtaaat atattttttg gaaggaatgg aagtgggaaa agttcattaa gtgagtggtt
661 aaaattaaat aatgaagaaa gtgtcatttt taatactcgt tacttaaaaa ataatattga
721 acaagttgaa gaaattgatg gtgtgagttt ggttataggt gaaaaatcta taaatcacag
781 tgaccaaatc aagaatttaa gtgatgctat aaataattta gaaaatttta ttaatcgaaa
841 agataatgaa attagtactt cgaaaaaaag aatatttgat aaaatgaatt ctacacttaa
901 tgaagctaga aagagatttg aaattactag taatatggtt aaacaaaaaa gaaatgcaga
961 caatgatccc atcaatgctt tttatagttg gaagaaagat attagagatg taaatgaaga
1021 gataactatt gaatctttag atgaattaga gaaaagaata gaaagcaacc aaatcttatt
1081 gaataatata aaaattccta ttttgtcttt tgaatatgat gaatttaata atatgttaaa
1141 aatattaatg aaagaagtta tcaaacctaa gagtagtgtg agttataaag tttctgaatg
1201 gataaaagat ggtttgaaaa ttcataatat gcaatcaaaa gaggaaacat gcgagttttg
1261 cggtaattca tttgatgtag aattggtgaa agaaagtata gatgcaagaa tagaaaatga
1321 atttaattat ttgatggaaa atctaaataa ttataaaaat aaagtagaga attcattaag
1381 taatatggaa aatttaaata ttgatgtaga ttcagtacaa ttttttaaac aaaaagaaga
1441 actatttaaa ctattacaat tattagaaga taaggaaaaa aagacatatg aaccacttaa
1501 ttatcctatc gataattggg aatcaataat taaagtagat aaaattatta tcaattatat
1561 tgacaaacta aacgaagaca ttcaaaacga taaaaaaatt agagattcaa ttactgactt
1621 tgtgaaatcg tggattggta agaagttaaa agatgatgaa gaaattcaaa tggaaattta
1681 caatattaat actaatagtg aacaaataaa aaaagcagaa gaatgtaaaa aagccaataa
1741 tgattggata acaaatttaa aaacagattc ctctaatttg aaacagttta gtgaaatagt
1801 aaataaaagg tttgaaatac ttgatataaa tattgaatta gaacctaata ttgaaactaa
1861 gagttataga gtattacata gagtaacagg agaaaaactt aacttagttg atttaagtga
1921 aggtgaaaaa cgattaatag gtttcttaca cttttattat aacttattta tgatagcaga
1981 tagtgagatg aaagaaaata ttgaacatat aatcatagat gatcccataa ctagtttaga
2041 tacagaaaat agataccatt taactaaaat tattaaccaa tttattgagc tttctaaatc
2101 tgaaaataaa caaatttttg tatttacgca ttcgtcgctt gatttccata attttgcttt
2161 taaaggaaag aataatacta aggaaacttc tcattggtat attgaaaaaa ttgatgggat
2221 tagtaaagtt agaaaattat ctaaagaaga gttaaataat tatagtgatt attatagctc
2281 agtttttaac gaagtaatgt cttttgcttt aaagggtaaa agaaagattg cttatataaa
2341 tgattatatt aaatatgcta ataaaatgag atttatttta gaatctcatg caagaagtca

2401 ttataaaata gaatatgcaa cggctacttc ggttcctgaa ttatcaaaat attatgatat
2461 agaaaaggaa agaatgaaag acttagaaaa gagcttagac atcattaata gtctctctca
2521 cggaatatca tttatagata atcataatga agaattgtct gttatagaag ttcatgaggc
2581 aataagaaaa gtgattggaa taatttataa gaaagataaa cagcatgtaa gtgcgatgtg
2641 tagcggggtt ttaactaagg aaaataaaga agaacttcaa aaatggattt aataaaattt
2701 ttatattgac tttatatagg ttaatcaaat aattaaacta gataaattac ttgtaaattt
2761 atgggctctt ctttcaaaaa ggattaattt attggtacgt gagtagtaag caagattcaa
2821 tataaaatta tttgttaatt gaagatgaga aacgataagt attaacttct tacttttaaa
2881 aaaccgcatc attaatcgat acgcagaagc atatcataaa tgatgcggtt tttgataagg
2941 ttctccttat cagtgatttt gctaaaaaac cttcaaagtt attatttttt caacaaataa
3001 tttgaaggat ttttattatt taatattaac tttatttaat tattgataat aatcgtgcgg
3061 ctaataatac agaggctatt actggactaa atcggcatta gactgttcat ctgtttctga
3121 cagtgtatta ttcttaactt gtgtttgagt ttcaatattt gtttgaggct tgagtagtac
3181 ttgaattctg taattgatta ctttagtttg attcgctatt atcagttttt cacttttaaa
3241 cttcattatt cgcactatta ttagatagtg ttaaatcttg gatttttgtt gatgggtaat
3301 aatctacttt agttaattct tgatagtcaa tctcagctcc agcgtcattt tctactaaac
3361 catatttata ctacatcccc aagttcagaa ttcttaacat gaaaactcac agaatagtct
3421 actttatctt caggttttta aaaaattaca ttttctagat ggtaatattg ttctttcata
3481 acactaatgt ctatatctgg aacattatta gctgttactt cgctttcagt cgtatgctaa
3541 ttaataaagt atttactaat ataattgtag caaaaatatt tgcttttata tcttgcttca
3601 gatttaattc ttattgtttg ttttcttctt tttattttct taagtagtaa ctctcgaaat
3661 tgcactacac atccaattcc tcaataatat gcattccatt cgctgttatt tgtatttagt
3721 atttctaatt ccaacttaat cgcaaagaaa ttatttatat gaaagttatt tttgtttctc
3781 tttttgcag aatattttat caaatcaaat atatttttaa tactttttct ctattctacg
3841 aggattatcg atttcttttt aaccaagcca attgggatca tcaagatgag gtcctcgtcc
3901 cctgttcaac ggggtagtta tcgaattcca aaagacaagc atcaatacaa attgaaaatc
3961 acagatgata atctgcaagt cgattaccct cgtcaatttc tgaaaggcgc acatgcaccg
4021 agtgttaaag cacagcacgt tattcattgg ggcgccacct tctgggtttc gtcgttatgg
4081 ttagcgtggt tttattaata tctgctatta tttatattgt agtaaaacgt cgtaattaat
4141 acgtctgtct tttattaaaa atataattga aggagagagt atggaaatgg aattcaatga
4201 gaacaatatc gacttagaga cgattatcag agatgaagtt aataaatatt tatctagaga
4261 tattggagac ttacctgcga cacaacaggc accgctcgag ttgagagaga aatatgaaaa
4321 gatggaagtg ccgaataaag ggcgagatat ctatgaagta ctcaaagatt taaataatga
4381 agtgctgaat tatttatatc gtccgaacca tccacgttca ttctcattta taccaggtcc
4441 agcatcacgt ctatcatggc ttggagacat tttaacaaca gcaaataata tccatgcctc
4501 taattttgcc aatgcgacgc tgccaattaa tattgagcgt aatttaatta actacttggt
4561 aggaaaaatc ggttatgaga tcaaaccggc cggtggtgtc tttgtatctg gcggttcaat
4621 ggccaattta acagctatcg ttgcggctag agatgcacag gtagaaatgg aagatattaa
4681 aaaagcaacc gtttatctta catctcaaac gcatcattct gtaggcaaag ccttgcatgt
4741 tgcaggcttc cttaaaaata atatccgcag aattgagtac aacgatgact ttacaatgaa
4801 tacagatagc ttaaaagccg caattgataa agatattgaa gaagattata aacctgctat
4861 ggtgattgcg actgcaggca cgactaatac aggttctgtc gatgatttca ccacacttgg
4921 agatatttgt gatcactaca atctatggct ccatgtagac ggtgcttatg gactatcaca
4981 tatcttatcc gataaagcac aacacttatt taaaggcatt gaacgttcag atagtgccag

5041 ttgggatgct cacaaattac tgttccaaac atatagttgc gctatggtaa tcgtgaaaga
5101 aaagaatcat ctcttttgca aagctttggc gaagatgctg agtacttgga tgatattgct
5161 tcagatgatg atgtgattga tccagaaatg ttaggcattg aacttacacg ccctgcacgt
5221 gctttaaaat tatggattac tctgcaagtc attggtgaag atgaaattat cgagcgcatt
5281 aaatacggcg aaggtttagc agaatatgca gaagaatacg tgtcaagttt cgataactgg
5341 cgtattatct cacacgctaa tctttccatt gtgaacttca gatatgaaaa cagtgattta
5401 actgaagcac aaaataatca actcaatagc atggctgcgc aaaaaattgc agattctggt
5461 tatgccattg cttataccac agtattgaat aaccaacgtg tcattcgttt atgtacaacg
5521 aatccattaa cgacacatga tgatatcaga gaaacaataa atcgctttga tcagtatatt
5581 ggtgattacg atatgaaaga tgcttaatca aaaatacttc ttaatagtaa gtaacaccca
5641 aaaagttgaa ctttttaggg gttctttttt ctctttatga ggattgatta tctaagtgta
5701 acttagcatc tttagcaggt tttgctggtg tgatacgtct accttttcct aaaatcattt
5761 gaataataga agaaacgagc atgataacat taaaaataat cattaagaaa aatactttgt
5821 gatcagcgtt gatggctaca tcttgcatct tcttcatctt tcaattttaa aggattgatt
5881 caaatgaacg aaagaaatct aatgttctcg tcccctgttc ttcggggtag ttatcgatcc
5941 gacactttaa taaatagcat aaccacaagg tttcaagtgt tattttggct gtcaaaaact
6001 gtgacaacta acattatgct actatatgaa atgtcacttt ttttgacaaa tattatgata
6061 attttctata tattaagagt atactacatt taatacgtta ataattaatc atatataaaa
6121 cttaatataa atttaacttt ttcttccttt ctaatttaaa ataagagata aatttaataa
6181 ctactgttca ggaaagtaaa aatatacttc tatattaaaa taataaaaaa agccatacac
6241 aattgaactg accccaatta gtgggaatta tataaaaaca ctttcgttga attcatatta
6301 aaatgaatca tacggaggtg ttttttctat gaaaagagtt tcttattcag tagaaacaaa
6361 gtataaagca gttgaaatga aagcagcagg attttcaaca aaagaaatta tgaaagaatt
6421 aaatattaga aatagaacac aagtgaaaac ttggtggcga tggtatcgaa atggggaaag
6481 ttatagattt tcacaacaag ttggtaaaca atatacctac ggtaaaggat taaaagagct
6541 gtcagaagta gaacaattaa aattagaaaa taagagaaaa gatatagaat tggatatttt
6601 aaaaaagtac aaggcattgg aaaggaagtg gtaccaacag tagtcataga tttagtggat
6661 caattaaaag taaaatattc aatcaaattg atactagaag tattaaacat acctaaatca
6721 acatattacc gatggaaaaa caaaacccat aaaaatgata ccgtaacaca aaaagtcatt
6781 gaattatgta aagctaacca ctatacctac ggttatcgta agattacagc attgattaat
6841 caatgttata catcaccaat taatcataag agagtacaga gaatgatgca gaagcatcat
6901 ttaaactgcc gagttagacc taaaaagacg acaagaatag gtaaaccgta ttataaaacg
6961 gacaatttat tacaaagaca atttaaagcg agttgtccaa tggaagtatt aacaaccgat
7021 attacttatt taccatttgg tcattctatg ttgtatttat cttcgataat ggatatttat
7081 aacggagaaa ttgtggcgta taaaatagat gataaacaag accaaagttt agttaatgat
7141 acattaaatc aaatcgatat acctgagagt tgtatattac atagtgatca aagcagcgtt
7201 tatacatctt atgcttatta tcaattgtgc gaagaaaaag gcattatcag aagtatgtcc
7261 cgaaagggaa cacctgccga taacgccccg atagaaagtt tccattcctc gctaaagtct
7321 gaaacttttt acatcaataa tgagcttaat cgctctaatc atattgtaat agatattgtc
7381 gaaaagtaca ttaaaaacta taataataat cgaattcaac aaaaactagg ctacttatcc
7441 cctgtaaaat acagaaaatt aatagcctag aacatggtgt ttttattaag ttcccgtttt
7501 aagggttcag tgccctagga ttataggctc ttttgtttat aaaggtaatt gaactaaagt
7561 attataattt caattcttaa ttaatgttcc tattttaccg tctaaagcgt cccctaatcc
7621 tgctagagat gtaatcaaaa cactgccttt agtatttttt tcaagaaatt gaagtgcagc

7681 ttcaactttt ggaagcatac ttcctttagc aaattgacca tcagagatat gttttttcat
7741 ttcatccaca gacacttcat cgagacctct ttggttttct tttccatagt taatgtaaac
7801 atggtccaca gcagttaata tgattaattg atcagattgt aaatgtgctg ctaataaagc
7861 actcgtttta tctttatcaa taactgcatc aacacctgta taaacttcat tttccttaat
7921 tactggaatt ccaccaccgc cggcagcgat aactagtgtt ccatgagtga ttaatgtttc
7981 tatactatct aactcaacta tacttatagg ttgtggggaa gggacaacgc gacgatagcc
8041 gcgtccagaa tcttctacaa aagtataacc tttttctttc gtaaatttat cagcctgctc
8101 ttttgtgtaa aataatccaa ttggtttagt agggttattg aaagcagaat catcgcttgc
8161 aacttgaact tgtgttacta gcgtaacaac ttgtttatct atacccattg aatgaagttc
8221 gttttgtaaa ctttcttgca tctgatagcc aatataagct tgactcatag caccacattc
8281 agggaaagga aaaggaggac cttgtttgtg ttccgccgca taatttaaac ctaagttaat
8341 acttccaact tgtggaccat taccatgact aattacaatt tcgtatcctt tatcgattaa
8401 acttactaga gatttagatg tactttttaa taaatctaat tgttctttag gcgattgtcc
8461 taaagcgtta ccacccaaag ctacgacgat tttagacata tttatatcct cctttcattt
8521 actctcctaa tgttgctacc atgactgctt ttattgtatg tgctctattc tctgcttctt
8581 ggaaaacaac tgattgttca ctttcaaata cttcatttgt tacttccatt tcatttaatc
8641 catacttttc ttgaatttgt ttaccgattt ttgtttcagt atcatggaat gatggtaggc
8701 aatgttcaaa aatagtattt gggtttcctg ttttttcat taattcttta gttacacgat
8761 atggttctaa taatttgatg cgtttttccc aaacttcatc aggttccccc atagataccc
8821 aaacatctgt ataaattaca tcagatcctt tgactccttc atcgatatca tcagttacaa
8881 ggatttcacc accatttttt tcagctatat tattacaacg gtttaataac tcgtcagttg
8941 gatttagttc ttttggacat acgagatgga aagtcatacc cataattgct gctccttgca
9001 ttaaggcatt tgcaacatta ttacgtccat ctccaacgta agtgaaatta atttcattat
9061 atggtttttt taatacttct ttagctgtta aaaagtcagc aagtacttgt gttggatgat
9121 cttcgtcagt taaaccattc catactggta cgccagaata ttttgccaag tcctcaacta
9181 ctcgttgtga gaatccacga tattctatgc catcatacat tccacctaaa acacgagcag
9241 tatctttggt agactctttt ttacccattt gagaacctgt tggcccaagg tatgttacat
9301 gcgcaccttg atcataagcc gctgtttcaa atgcacatcg agtgcgtgtt gaatcttttt
9361 caaaaagtag agcgatattt ttacctttca tttttgttg ttctattcct gcatattttg
9421 cgcgtttaag atcttcagat aaattaagta aaaattccat ttctttttgt gtgaagtcta
9481 acaaagttaa aaagtttcta tttcttaaat ttttcatttt gaatatctcc ttttcaatta
9541 atattttatt catgttattt ttttataatc caatttcttt tatcttgata tataatattt
9601 tctattttta attctctgat tatttcacca gtagtttttc tgttaagtcc agataaatta
9661 cttattaatt gaacagtcat aatttgtttt aattcataga attctccatt atcatcccca
9721 attgaaaggc ataaaatttg tagtattctt tcaattcttt ctttagctga attagctctt
9781 aacgccataa tatattctac ttgaaattga atagtctcat taagtttctt gaagagactt
9841 tcaaatattt cattatgctt tctacataaa tactcaagta aatctttcgg taaagttaat
9901 attttacaat ctgtcaaagc tgtacatatt tcatatggtg caggggtttc attaaatatg
9961 aagttcattg gaaatatatt ttcgtcttta cttaatctta aataattgtc accagtaata
10021 ttagaagatt catgtaaaat acaaccattt actaaaaagt atacaaattt tatttgatca
10081 gttgaatgat atatgacttg tcctttttta tattgataaa gtgttaaatc ctctttataa
10141 ggtctaacaa tactaacagg aatatttaag tatgatgcta attgtttaag attattatca
10201 aattcatatt ctgaattttt aatataaata ttttcttcat acataactat aaacccctta
10261 aaaattactt agtttaaatc ataacttaaa atacagaaat attacctgta atcaaacgaa

10321 ctattcctat tattgcaagt acaatgatga atatgaataa cgtataatcc aattttgtca
10381 aatgtttgtt attatccctt tgtacgtagc tgtatacgag taatccaggt atatataaca
10441 acatcgttaa tagtaaataa tctaatccag ctgcataaac caaccaaatt gtgtaaatag
10501 atgcaataat tcctattatc cattgtttta aattagcttt agatttattt tgaatagtat
10561 atttaacctg gtaaaaagca ctgagtgtat atggaattaa gattgcactt gatgcaagtg
10621 aaaacgcaaa ctgataggca ctatctgtaa acaacatact aattaaaaat aactgaacta
10681 atatattagt aataattaaa gcgttgaccg gagctttatt cttattttct ttagcaaacc
10741 atttcgggaa aagtccatct ttagctacaa tgaatggtaa ttcaccagct agtaatgtcc
10801 atcctaacca agctcctaaa acagagataa ttaagcctat attaactaac actgaacccc
10861 aatgacctac aatatgttct aatacttgtg ccattgatgg attagcaagt tttgaaattt
10921 ggttctgctg aatgacacct tgggctagta cagtcattaa gaaataaatg actagcacag
10981 aaatcaaacc aataacggta gcagttccta catccttttt agactttgca cgtccagaaa
11041 agacaacggc tccttcaatc cctgtgaata cccatacagt tactaacata gtacttttta
11101 cttgtgccat tgtatctccc caactaaaaa cgccaacact tccactagtc ataccataaa
11161 aaccggattt aaaagtactg aagttgaata caactatcat gcatataata actagaaata
11221 taggtattaa tttagctact gtaacaatac tatttataaa cgctgcagtt tctacacctc
11281 taagtattaa aaaatgtaca ccccataata aaattgatgc tatgataata cttggaagtg
11341 tgttacctcc tttaaatata gggaaaaagt tacccacagc tgacattaat agggttgcat
11401 aagccacatt acctagaaat gctgcaaacc aatatcccca agcacttgaa aaaccaataa
11461 aatctccaaa ccctgtttga gcataactat aaattcctcc atcaagatct ggtcgctcat
11521 ttgttaaatt ttgaaatacg aaagcaagag aaatcatacc aatagcagtt attatccaac
11581 cgataattat tgcaagtcca ccagcttggc cacccatatc tgagatgata ttgaatgcac
11641 caccgcctat catagagcct atgactaaac caattaagga agttttacct aatttatttt
11701 catccatatt aatctcccct aataaaggtg gtaaaaatct tgttatttga tttaatgaat
11761 gaattacttg tgacttttac cacctttcaa atttaaatat cttctctaaa taacggctga
11821 ctcatacatc ttgggcctcc gcgtccacgt acaagttcac taccagtaat ttcaatcact
11881 ttaattcctt tgtcgcgtaa aagttggttt gatacatagt tgcgatcgta tgtcaccaca
11941 acccctggtc gaatacataa tgtgtttgag ccatcattcc attgttcacg tgcaccatca
12001 ataacgtcgc cattacctgt tggaataaag tccacttttt ctacttctaa aacttcagca
12061 agtgtttcac gtaacttgct agaacgagta atttttatat cgtccttacc atcattttgt
12121 tctatggtaa atatattcat attattttct tctttaaata ttgctgcatg tactgtaaac
12181 ttatcgtagt caatcatagt tagtactgta tctaggtgca taaatgtacg tgtattaggt
12241 atttcaatag ctacgatttt tttaaaactt gtgtttgcat ctttgaaaat attacgtgct
12301 aacttttcta ttgcttgagc tgatgtacgt tctgatatac caatagctaa aacatctttc
12361 gataatacta attcatctcc accttcaata ttaaatggtg agttacgatc taaccatact
12421 ggtacatctt tatctttaaa tcttggatga tgtttcagta tatatgtcat aaaaatagat
12481 tctctacgtc gtgctctcca atacattctg ttaattgtca ttcctctacc aattgaagct
12541 tggggatctc ttgtaaaata aaggttgggc attggatcta agtaaaatgg atatctatca
12601 tccatatatt ctactaaatg agttgtttca agttgaattt cttctttacg tacgccagcc
12661 atgattttat ttacaagttc ttggtcagat aactttgaaa agaattcttt aatttcagtt
12721 tcatgaccta atattgtctt tttagattct gttaatatat cgtttatgaa gttctcgcgt
12781 acttctggct cagtaataga ttctgctgca agtttttcta aataaactac ttcgattcct
12841 tcatctctca aagtttgagc aaatttgtca tgctcttctt gtgcaacttt taagtaggga
12901 atatcatcga ataataaacc acttaaatga tcaggtacta aattttctaa ttcttttcct

12961 ggtcttttta acaacacagt tttcaatttg cctatttcac tatttacttg aatgggtcct
13021 tgtaccattt caaattcctc ctttgcttta ttacattcac attataaaac gctttcaaaa
13081 aacatcgtgt gatttaattc acaattgttt agggagggtt ttcacatata tagatttatt
13141 caatgcaaaa ttttgtatga atatgtattt tgtactatat aatagtgaat aatttgaaaa
13201 atctcattgt taaaacctta tcttattcaa gcaaaaagat tttttactca tttttttgta
13261 ttagtaatca ttcctattaa tataatcatt taatttttaa gggaataatt gataacaaac
13321 aaattctgct atttcatttg aagaagtcaa aatcatcaaa gtatcgttac ctccaataat
13381 tcctaatatt tctttcattt gtaattgatc tatgtaataa cttatacttt gagcaaagcc
13441 aggagatgtt tttattaaga catagttatt tagcgttata aattcaataa tctcatcact
13501 aaatatttct aattgttttt ttgcacttaa ttgatttgtt tgatttattt tcttgtaaat
13561 atacttttta ttttcaacag ggattttgta aatttctaat tcttgtaagt cacgagaaat
13621 agttgtcaag ctatagtaaa ctccaaaatg ttttgccatg taatccacta tttgttgttt
13681 tttattaaac tgattctgtt gtataacagt taagataaga tttaaacgtt tttcttttttt
13741 catttttatt acccccttta ttatttttga ttatgaaata gattttaaat aactgtataa
13801 aaatcactta taaatatcaa tttacaaata attttgaaat agtggatgct ttaattataa
13861 gtagattatt ttacgcctaa aaggagagat tgattagaat agattatagt catcataatt
13921 ataaaatcaa aactttaagt aacattaatg aaatataata aaaataattt gcgtaataat
13981 agcgaaatgt gtaaattgta aatttcaggt gataacaaaa catgttaggt gagaatgtct
14041 tacagttaca tttactatgt ataaataaaa taataacatt aatagtgaga gttaggtaat
14101 aaccacaata tctcataaaa gaagtttgga aaaaggcatt gaaataaaaa gtagtttata
14161 agttgaagat atttaggtat gtattagagc gctttataaa taaataggtt tatacataga
14221 attacaaaaa taatataaaa aatagaatct aatacatgat aaagaaagga ttaaaataat
14281 ttagaaaatg tatttagagt gatattgtta tagagattgg ttaacttgtc acataaattg
14341 gaatgaggaa gagaactaaa taataagaaa ttaaaataat ctaattcaac ttttggatta
14401 gtgcttgtat tttaacttga tacagactat tctatttttt ctaaatgatg tgattcaaga
14461 tatgctatat cattttgatt aatctaaatt taattcagag aaaaagtctg aacgttatta
14521 taattatacg atttcaaata cgatggatta tgtgattagt ttaggtgaag atgaacgtca
14581 acgttttatt aaaacggtca cagaattagg aaaagccttt gcgttttgtg atacagaacc
14641 gaaagtgcaa aagctcaatg atgaaattgc cttctttaaa gccgttaaag caggacttgt
14701 taaattgtta caaccatcaa aagaaggaaa aatatataag acacttgctg aagttgaagc
14761 agagattaat catcttgtgt cacaatctgt ggttacggaa gaggtaaaga attagggtta
14821 aattctgaca aaattgattt ttacgatact ttaatctcac atgaaacaga aaaagaagtt
14881 ataggtgata aaaatcttag agcaattgcc catgaattaa caaaaattgt gaaagaaaat
14941 atgactgtag attgtgataa gaaatcaagt agtagagtta ataagaggag atcagtaaaa
15001 agattattga agaaatacgg atatccatct gacttagaaa caatagcaat caatcaagtg
15061 gttgaacaag cagagatgat ggctagtaat cagtaagaaa aactaataaa acatactaag
15121 attagctatg aaaaaatcta tcgtcataga tttcttcata gctaatctta gtatgttttt
15181 agaaaagaaa attagtgaaa aaactttact tatcatttat tagttcggtg tgctcatatc
15241 acatcataat ataagctatt atgccgagaa aattttaata aaaaaataaa aaaacctaaa
15301 tactctctcg atttgtgaga aagtatttag gctttttgaa ttaataaatt tcttacttct
15361 tatgtttatt taacaattag tactgggaca gaacttcttt tagcaatctt atgtgtaaca
15421 tctcctaaaa catgtttaat attcaaatcg actctatgat tactcataat tactaaatca
15481 aattcaccat tatttatatc tccagaagta attttttcta agattgtatc tttaatatgc
15541 ccaaaatcta catctatttt atattgaata tctctttttt ctaattcacg taaaaatgga

15601 gtgagttttt ttctttttct ctaataatat cttctttatg cttattataa tatttgacac
15661 taattgcaag atcattttct gttacaacgt gataaattac aacaactgaa tcttcatcga
15721 tgactttttc taaatagtca gggatagcac taaaatcatt ttcgaaatca tagggtaata
15781 atatattttt gaacatctac accagcctcc tattacttat ataccctgaa tatatatgct
15841 tattctgatt ctgttgcaaa gtaaaaaata tagctaacca ctatttgaaa ttttaagata
15901 ttttttaga gcatttagag atatttatag aataataaac aggttaaaaa gtaataggat
15961 taataggatt tggataaaaa aatcaatcct atttatagga ttgacgtcga ctaaagccta
16021 tgaaatcaac aaaaataata gtattcagac aatttaaagt ggtcttgtaa caattgtaaa
16081 tagtatgcaa tattagaggt tatagtatcg atcaattctt atttaatatt aactataagt
16141 attgttatat cagcgtttat aataatttta aaaagagcat gtaatcaagg cgagcataag
16201 ataatccttc agctatgctg aataccactt aaggtggtac tggatttcct tatgctcatg
16261 caaaatccta ttttgtttgt aatatttcaa aaagtaaaac atacattgtt aaaaaatagg
16321 ttattctttt cttcactttt tactttaatt tcttattttt atttccaaaa tagtcattgt
16381 taataaaaat tatagtaccc ttatttgcaa ttttgagata ctttttagag catttagggg
16441 tgttttatat attatttatt taatggctct taaaagacaa atatgaacta aatttaaaaa
16501 tcagaaagac aaaacaaaat tttttgctta accagtgtcg aattttttag acactgccca
16561 tctatatgca aattaaaaat tggcataaaa tagcttaaaa atttatcaac tttttagttc
16621 ctgcaggggc aatttctttc aaaaaaacgc tagctttttg ctaacgttta agaaaattta
16681 tttttcttct tcaatttcat caatcatttg atgattgatt tcttgttctt cactttctgt
16741 taaatcaaaa atttcactcg ctaaatactc ttttttattc caagtataaa aaatttgata
16801 aatatattcg gtcggatcaa cttcttttaa gtaatctaat tctccatttt ttaatttctt
16861 tcttgcgtct ttaaatagtc caaaataaac aagaacttgt tttcctttta acgctttata
16921 aaatgcatcg aacacttttt gattaattaa atagtcgtta tccggttctg ttgcaaagtt
16981 gaatttatag tataatttta acaaaaagga gtcttctgta tgaactattt cagatataaa
17041 caatttaaca aggatgttat cactgtagac gttagctact atctaagata tgcattgagt
17101 tatcgtgata tatctgaaat attaagggaa cgtggtgtaa acgttcatca tttaacggtc
17161 taccgttggg ttcaagaata tgccccaatt ttatatcaaa tttggaagaa aaagtatcaa
17221 aaagcttatt acaaatggca tattgatgag acgtacatca aaataaaagg aaaatggagc
17281 tatttatatc gtgccattga tgcagaggga catacattag atatttggtt gcgtaagcaa
17341 cgagataatc attcagcata tgcgtttatc aaacgtctca ttaaacaatt tggtaaacct
17401 caaaaggtag ttacagatca ggcaccttca acgaaggtag caatggctaa agtaattaaa
17461 gtttttaaac ttaaacctga ctgtcattgt acatcgaaat atctgaataa cctcattgag
17521 caagatcacc gtcatattaa agtaagaaag acaagatatc aaagtatcaa tacggcaaag
17581 aatactttaa aaggtattga atgtatttat gctctatata aaaagaaccg caggtctctt
17641 cagatctacg gattttcgtc atgccacgaa attagcatca tgctagcaag ttaagcgaac
17701 actgacatga taaattagtg gttagctata ttttttttact ttgcaacaga accttattac
17761 ttatataggt gagagtatga taagtgagaa tgtaccattt aaataagttg ttaaatatga
17821 gcattagcga ttattaaaaa agtagaatta tgtttaggga atttttagca atatcattga
17881 taaggggaag cgtatcataa ataaaactaa aaattaggtt gtgtataatt taaaaattta
17941 atgagatgtg gaggaattac atatatgaaa tattggatta taccttgcaa tatcatacga
18001 tgtttataga gtgtttaata aaccattttt caactattga tgatctagaa tatataataa
18061 ctgtacaaat tatattgatt atggaactac aattaaatta agaaattgat gatgaaattt
18121 taaatttaaa ctaatggaat caagaaagaa tgaaaggaaa tatacaatgc ctacgattaa
18181 taaaaggaag tttattagat tttgtgttag aaacagtgga tgtttttgta aatggaagta

18241 aaaatgattg tgaattagta aattttaatt ataaagaagg aaaagtagtt tcacgttata
18301 gtacaaaatt tgaaagcaat cctaaattaa gaaaagagca attgatatcg acggtaaaag
18361 ttgtaaggta tgttattttt gattttgaga aaattttatg gggcataggg aaagttttat
18421 tgaagttcat catattaaga caatgtttac tataagaaag ttgaaataaa ctcagaaaca
18481 gacttgattc ccgtttattc aaattggcgc aaaatgatac atataaacac ctatatattg
18541 aagatttaag tcaaacggta aataatattt ttcactttta aacagaaact tttttggaaa
18601 ttatattaat tagtataaaa atgaatatgt tcacaaagaa ggagagattt tattgaaaca
18661 cattaatcat ttttctgaat cgacactagc atacttggaa aaagaccatg aacatgcatt
18721 ttatgtatat tgtttaatag acccaagaaa tgatgaatgc ttttatgttg gtaaaggtaa
18781 ggggaataga atatttaaac ataaacaaga tgcgcagaga caattattgt acgaagatat
18841 attcagagaa gaaaataaag ataacttgaa atttaataga attaacgaaa tttgtagcaa
18901 tgatttgaat gttttaggat atataatcag ttatggatta accgaatctg aagcattttc
18961 tgcagaaaat gttcttataa actttcttaa cctaacaaat aaaactacat taacaaatat
19021 gattaacggt catggatcta aggcatactt agttgaagat ttagaaaatg aatttggcta
19081 cgattcaatt aatcttgaaa acataaatac gaatgaatta attttagcag tgaaaatcag
19141 agatgcattt ctattagata aggatgaaag taaagagtat cctattaatg aaagtaaacg
19201 tgatagaaat aaccttaaat cgcgtacatt aggtagttgg ataataggaa aagataaaat
19261 acataaaata aaatatatta ttggtatcaa tacaggtgct aataatgcag ttgtctcagc
19321 ttatgaagta tcatttgaac aagcggagag tatcgaaaca aataatggta gaatgagata
19381 tgcgtttatt gggctttcag aaagagatgc tactctaaaa aaattgaatt tatacaaaaa
19441 agcactacca gatttaagat ttggtagtgg tagcgctaca gcatatataa ataatgggac
19501 aatgaaagtt gattaaatga ataaaaacat ccaattcctt aaattagcct ttgaaaattt
19561 tatactcgta tatataagtg aaaaacttaa aagaattaga tgttaattaa aggagagaat
19621 gctatgccat cagatgatat tgtaaaaaaa ctatactcaa aagaggatat tcgattagaa
19681 ttaggactta ctccgcataa gtttaataaa aaaatggaaa ctattgctaa gctttttaaa
19741 attgatatga aaatttttca caattacaaa ggccaagata aaaataatca gtatacattt
19801 aatggtgtcg caaaagaatt aatcaaagtg ttgctaaaaa gtgttgatta ttacccagtg
19861 gatatcaatt ccaaaaaatt taaacaaaat ggaaaatcta aaaaagaaat gattgaaaat
19921 atagataact ctagttacat gaaatatatt tatcagttaa tgaaatctat caatgaaatt
19981 caatacaaaa ggttaattgc tgatatacat atgaaagatg tgtaccagaa tacaaaagca
20041 tggttaaata atggtgaatc aattaataaa aaagaacaag agctatacca atatatgaca
20101 attttaccat tacataaaag agttgaactg caaaatgagg tattaaaatc tatagacgaa
20161 acaatttttc aatttctcgc aaaagaacat agaaataatc aaattgaaga aaataatgaa
20221 ttagaagcat atacaaaagc aataaaagaa ggtagaaatc ctaaaaatga ttatgaatta
20281 aatcatctcc tatataaaaa aaatcaatta cccttagata gtctaataga agatatatgg
20341 gactatgagg aaacagagta tactgagctt gattggttaa ttgcagatat gctaaaacgt
20401 tcacaaagag ctgatagtaa ttttatcgaa agtttagaga agaaaaacaa attgagaaaa
20461 aatattcata aagattccat taaaagtata tctaagttga ttgatcatga attagttaaa
20521 aataatagaa agtgggacgt agcagaattt tataaaaatc aacaattttg gaataatagt
20581 acattaagac gtagctctca aaattttgga attttatatt acaaaaagaa taggttacat
20641 acaatgaatc aaagtaatat tattaagaaa ataaaaatta tcgaagctaa tatagagagt
20701 gcaaataaac agccgagcta tttagatttc tttttacaag ctaagtttga tttagagagg
20761 ttagaaagtg aattaatgaa atatagcatt aatccgaaat attttaataa gataaatagt
20821 gattacgtaa gatatgctat tgaagatgta cataatgcaa ttattaaaat tgacagatat

20881 ataagtaacg ataaagtgaa gttaaactat tcaaatgtcc aaatggtagt gagaaacgaa
20941 ataacagaac aaggaagcta ctttgtaacc caggcattga atacactgtc aaaggtagaa
21001 gagtctggct acagctttga taatttcatg gtaggacctt tcgtagaaga ctttaaaaaa
21061 gcaattaaaa atcgtagatt tggttctaaa aaagattaag attgtttttt agagttaaac
21121 ataagaataa atttaaataa aaaaataaac ccaacaaacc ttgttatatc aatgtttgtt
21181 gggtttattt tttttggtga ttttttaata actcaattac atcttaggat aatatcgcta
21241 aattatacat tgtcgaggcc aagacaaata aaaaattaaa gcaaaagtta gcgcgcaact
21301 ttctgctata aaggagcaaa tttatatgga gacaagagac aaactgatgt cattgactca
21361 aagtgacaaa acacagcaat ggctaatgga caagtcatct aaccaagatg acattcaaca
21421 attgcagcaa caattcagtc agcagcttga tcaacaatat aatgcacttt tagctgatga
21481 aaaagctaag ttagaccaat acgtggaagt acatcaagga ttggaatcat taaaggaaga
21541 gattgaatca gaacctatta cgcttaatat cgataaatta cccgatatca aagcaacaat
21601 gcttgaaaga gccaagaatg atgaacattc tgataaaatc gaaaagctat ttgataggtt
21661 agaacaggca ttaaatggta cgaatcgatt atatacgcaa ttatcgttga ttggtacacg
21721 aacacatcga attacaacta aaaattttaa tcttcaaggc ttacctaaag cagtccaaca
21781 tacgatttta ccttcaaaat ttaagaaggt gtatacagtc gattttaaat cgtttgaacc
21841 atcagttgca gcgtacatga ctcaagattc aaaactgatt gacttgttaa atcagaaaga
21901 cggactgtat gacgcattgc taagtgaatt aggcttatca gatgagctac gtgtatttgt
21961 taaacgtgca tttattggtt cgtttctatt tggaggtaac ttcaaaaatc ctaaattcaa
22021 gctgaatcaa tatgtaagtg aagtacaatg gttggatgcg gtcagccaat ttacaaaagt
22081 cattgaactt aagaagcacg ttgaaaagag taattccatg cctatgcctt atagtattga
22141 gcatgatatg agcgcatttc aaggtagcag tattatggca atctacgtac aaactgtagc
22201 gagttatatt ttcaagcaca ttctgctaaa agtgtacaaa gctcagtgcg atcaaaaaac
22261 gttcaagatt atagtaccta tacacgatgc gattatgatt gaatgtgaag atgaagaaat
22321 tgcacaaaat gtgggtcagt taatgaaaga tacggctaac cagttgttca atggtgaatt
22381 tgcacatgtg acagtggaag aaataggagg cgtagaccat gaataatgat agaggacaaa
22441 gcctacacat cccaagtagt acaccaatca aagaaaataa tatatatgta gctacgttac
22501 attctgtgat ccaaacagat ttctcaggtg aaataaagca ccaattcacg tatgaaattg
22561 aagtgaacaa tcagattgta tatgcgaatc gtaatattcc aacaaaaccg agcgctaatc
22621 agttgtcaat tcatgattgg ctgaaacgtc atagcaacta tagcgtgggt catgatacgt
22681 atgaacctta tattgaccgt aagcatttaa tccatattgg tcaatatagc ggtaactatt
22741 atgtacaaga tgtagcatcg ttagatgcgt ttggaggcgt attatcatga atcatatatt
22801 acaaatgtta tctaagctat taagtgtggc caaggaggca atcgaccgtc aaggtctgat
22861 tgctatccta actattcctg ttaataataa cgatgaaata gaagaaacgg ctcaaggtga
22921 aaccgtgtat aacgaactta tcgatcagtt acgacttaat atcccaaaag atacggatta
22981 tcaacctaac atctatagtt attttggtat taagaagaat cctaatgaca ccgtactcat
23041 ggaaatgatg ataaaggttt ttcatatcaa acgctttaat tcagaactgt ttattttcaa
23101 agttaacggg tggcaaaaga taaatggaga tgaattacaa gggttgatat ctaaaatgat
23161 acaagtattg cttgtagatt ataagccttc actaagcact ctaaaaaatg tcgtagatgg
23221 attgcaaaaa tcaacagatg tagaagaact tgttgagaat gagcgctata ttggttgtgg
23281 tgaaaatatg ttcgatctta atacgtttca agtcgttaaa aattcaatcg atatctttcc
23341 aaaaacacga ttgaatttat cattaagtac aaatgatgta attactgata agataccgcc
23401 ttattttaag caatatatgt tacaacttgc gaattatgac gatgatttac aatactttct
23461 tttccaacat acagcagtat tacttacagc tgatactaaa taccgtaggg gtctcatatt

23521 atatggtgga gctaagaatg gtaaatctgt atatattgaa ctagttaaat catttttcta
23581 tagtaaagat attgtgtcta agccacttaa tgagcttgaa ggtcgttttg acaaagaaag
23641 tttaattgac aaaagtctaa tggcaagtca tgaaattggg caatctagga ttcaagaaaa
23701 gatcgtaaat gacttcaaaa agttattatc tgtagaatca atgcatgttg atcgtaaagg
23761 aaaaactcaa gtggaagtca ttttggattt gaaacttatt tttagtacaa atgcgatact
23821 taattttcct cctgaacatg cgaaagcttt ggagcgtcga attaatatta ttccatgtga
23881 gtattatgtt gaaaaagcgg acacttcatt aattgataag ctccagagtg agaagaaaga
23941 aatctttctt tacttgatgt atgtgtatca acagattgta aaagcagata tcgagtatct
24001 tgaaaatagc cgtgtcactg aaattactca cgattggtta aattttggat atgaatttgt
24061 ttctagcagg tccgtaagta atgcaaatca gaaagcatgt attaatttac tcagaaaact
24121 tatagaaatc aaatcaggat cacgaatcaa agtatccgag ttaaataaag ttattaatga
24181 agaaataaag gtaagttctc aggttattaa acagttaatt caagcaaact ttgatactca
24241 aaccaaacta tacaatggct acgattattg gattgattta ggttggaaag aagccaataa
24301 aaaagagatt catgatattt cggaaaaaga taatattatt tcattagata aaaatgaaaa
24361 tataacagac gatgaggcat tagatgaaga gaatttggac tttgattggg aggactttga
24421 cgatgaataa tgaacaaatt gaagcatttg tagaagtgct tgtacctatc atagaagaac
24481 gtatcaataa aggtaattaa ggctaattac gtactacagg cagttgcctg tagtactcat
24541 atgattaagt ggtaaaagtg ataaaaatga aacgaaatta taaatatata ttatctatat
24601 gttgttacaa gaccgatagt ctgtagcaat aatctaataa aaggagaacg gtatgatatg
24661 aagggtaaaa ttgcacttta ttcacgtgtt agtacgtctg agcagtcaga acatggttat
24721 tctgtaaagg agcaggaaca agtactcatc aaagaagttg tgaaaaattt cccaggttat
24781 gactatgaga catatactga ctcaggcatt tcaggtaaaa atattgaagg tcgtccggca
24841 atgaaacgtc tattacaaga tgttaaggat aataaaatag aaatggtatt aagttggaaa
24901 ttgaatcgta tctcacgatc aatgagagac gtgtttaata ttattcatga attcaaagaa
24961 catgacgtag ggtataaatc gatttctgag aatattgata catccaatgc ttctggagaa
25021 gtactcgtta caatgtttgg gttaatagga tctatagaac gccagacttt gatttccaat
25081 gtgaaacttt ctatgaatgc taaggcaagg agcggagagg ccatcaccgg tcgtgtttta
25141 ggctacaaat tatcacttaa tccacttaca cagaaaaatg atttggttat cgatgaaaat
25201 gaagctaata ttgtacgtga aattttcgat ttatatttga atcataataa aggcctcaaa
25261 gccattacaa ccgtacttaa tcaaaagggg tatcgtacta ttaatcaaaa gccattttca
25321 gtgtatggtg ttaaatacat tttgaataat ccagtctata aaggctatgt cagattcaat
25381 aaccatcaaa actgggctgt acagcgaaga agtggcaaaa gtgataaaaa tgatgtgata
25441 ttggtcaaag gtaaacatga agccattata agtgaagagg tatttgacaa agttcatgaa
25501 aaattagctt ctaaaagttt taaaccgggc agacctattg gtggagattt ctacttacgt
25561 ggtcttatta aatgcccaga atgcggaaat aatatggtat gtcgacggac gtattataaa
25621 acgaaaaagt ccaaagaacg gacaatcaag cgttattaca tttgttcctt attcaatcgt
25681 tcagggagtt ctgcatgtca cagtaattcc atcaatgctg aagtcgtcga gcgtgtaatt
25741 aatgttcatt tgaatcgtat tctgtctcaa ccagatatta tcaagcagat tgcgtcaaat
25801 gtgatagaag aactgaaaca aaagcatagt aaccaaacag aaattaaata tgacattgat
25861 agtttagaaa aacaaaaagc taagcttaaa acacaacaag aacgattgtt agaattgttc
25921 ttagatgatc agatggatag cgaaatgtta aaagctaaac aaagtcaaat gaatcaacag
25981 ttagaagtat tagatcaaca aattaaagaa gcgcaacaag caaatcaatc acaggatgaa
26041 atacctaatt ttgataaatt aaaaggacga ctcattttga tgataacacg attcagcgtg
26101 tacttaagaa aggctacacc cgaagctaaa aatcaactta tgaaaatgtt aattgattca

26161 attgaaatta cgacagataa acaagtaaaa cttgtaaggt ataaaattga cgaaagtctt
26221 atccctcaat ctttgaaaaa agattggggg tcttttttta tgcccaaatt ccaatttgaa
26281 atatatggtc aaaatgatta tttcatcgac caaattacca cttttaccac ttagttatta
26341 gtgacaaaag tgagcgaaat gaaataaaaa tcaaatatat attatcaaaa tgatgtatca
26401 cgtacataca tcaatcaaat acattaggag gtcataatga tgacattaga acaacaactc
26461 aagcactata taaccaactt attcaatctg ccaaaggacg aagtgtggca ctgcgaatct
26521 atcgaggaaa tcgctgatga tatcttaccc aatcaatatg taagacttgg cccactcagt
26581 aataaaacac ttcagactaa tacctactac tctgacacac ttcatgaaag taatatctat
26641 cctttcattc tctactatca gaaacaactc atagccatcg gttatatcga cgaaaatcac
26701 gatatggatt tcttatacct acacaacact atcatgcctc ttttggatca acgatactta
26761 ctaacaggag gacaataaaa tgcataaata catcaaaatt acacaattag tcattacaat
26821 actaagtgaa atcatcattt ggatgaaaga gtcagaacga aaggaagtct cttatgaata
26881 gatatatcac ccggggtatc gccaacaact tacctaatat cttacaacac caattatggc
26941 aactcgtatc tgagcgagaa caagaacaaa ccaaagataa tacttcagta gattattttc
27001 atatattcca gttcaataag catcgcaatc aattatatat caaacacaaa caagaacgac
27061 ctgaatatgc gaaaatccat aaagctaatt attcaaaagc aatcaatatc aataaggtct
27121 acattattcg agaagatgat gtagaccttt cttattatgt catgttatta cctgaagaat
27181 actagaggag tgaaaattat atggaaacaa tcaaaagtac attaaaaaca gaagccatat
27241 tcagtgatga caaacaacat cgctatctac ttaagaaaac atggaatagt gaaaaacaat
27301 caatcacaat cattacaatg tatccgcatt atgatggcat tctcaatatt gacctaacga
27361 cccaactcat catgaacaaa gtttcagaaa tggatgcatt tggttcaatc aattttgtga
27421 atctatactc taatattaca acccctatca atctcaaaca tttagaaaat gcgtatgata
27481 agcatacaga tattcaaatt atgaaggcag tgaaagagtc agatgaagtg atattagctt
27541 ggggcgctta cgctaaaaag cccggtgttg aagcacgtgt taatgaagta ttagagatgt
27601 tgaaaccaca taaaaagaaa gtaaaacgac tcatgaatcc agaaaccaat gaaatcatgc
27661 atccccttaa ttcgaaagca cgtcaaaaat ggatattaaa agtatagcaa ctaaaatgtt
27721 tattcactat ccatgaaaat aattgaattt catatatatc ttttaatcta caatatgatt
27781 aaaagatata ttatttggtt ctgttgcaaa gtaaaaaaat atagctaacc actaatttat
27841 catgtcagtg ttcgcttaac ttgctagcat gatgctaatt tcatggcatg gcgaaaatcc
27901 gcagatctga agagacctgc ggttcttttt atatagagcg taaatacatt caataccttt
27961 taaagtattc tttgctgtat tgatactttg ataccttgtc tttcttactt taatatgacg
28021 gtgatcttgc tcaatgaggt tattcagata tttcgatgta caatgacagt caggtttaag
28081 tttaaaagct ttaattactt tagccattgc taccttcgtt gaaggtgcct gatctgtaat
28141 taccttttga ggtttaccaa attgtttaat gagacgtttg ataaacgcat atgctgaatg
28201 attatctcgt tgcttacgca accaaatatc taatgtatgt ccctctgcat caatggcacg
28261 atataaatag ctccattttc cttttatttt gatgtacgtc tcatcaatac gccatttgta
28321 ataagctttt ttatgctttt tcttccaaat ttgatacaaa attggggcat attcttgaac
28381 ccaacggtag accgttgaat gatgaacgtt tacaccacgt tcccttaata tttcagatat
28441 atcacgataa ctcaatgtat atcttagata gtagccaacg gctacagtga taacatcctt
28501 gttaaattgt ttatatctga aatagttcat acagaagact cctttttgtt aaaattatac
28561 tataaattca actttgcaac agaaccgtat tatggaatag agatgttggt aacatttata
28621 caggatcatt atacttaagt ttaatttcgt tattacagaa ccacacattc caaccagaag
28681 agaaagtatg tctatttagt tatggttcag gagcagtagg agaaatcttt agtggttcaa
28741 tcgttaaagg atatgacaaa gcattagata aagagaaaca cttaaatatg ctagaatcta

28801 gagagcaatt atcagtcgaa gaatacgaaa cattctttaa cagatttgat aatcaagaat
28861 ttgatttcga acgtgaattg acacaagatc catattcaaa agtatactta tacagtatag
28921 aagaccatat cagaacatat aagatagaga aataaactag tggccgattg tgcttgatga
28981 gcttgggaca taaatcctaa ctcgaaataa ataagcatat cactaaactg attttttaaa
29041 gtttacagtg atatgcttat ttttttatct tacgattttg tacgtgcatg cttgcctagg
29101 ggtatggctc gagccattag tctctcgcac atactattcc ctcaggcgtc agcacttaca
29161 aaatcggttg taattttcat ttttatacgc attcttactg agattatact aataagagga
29221 atagtaaaag caattctaag taaaattgca gataagaggt ttgttaaaag cagttctaag
29281 taaaattgca gataagaggt ttgttaaaag cagttctcag taaaattaca gataagaggt
29341 acgttaaaag cagttctaag taaaattgca gataagaggt ttgttaaaag cagttctaag
29401 taaaattgca gataagaggt acgttaaaag cagttctagg caaaattgca gataagaggt
29461 gcgttaaaag cagttctaag taaaattgct gataaggggt aagttaaaag caatcctaag
29521 taaaattgca gataagaggt gcgttaaaag caatcctaag taaaattgca gataaggggt
29581 acagaaaaac tagacttgat tacaaaatgg agcttgggac ataaatgatt ttttaaaaat
29641 gagatgagac gtagattaac tccataatca atacgaatct atcgacttct ttatttatga
29701 tattcatctc tttttaatgg aaataaaagt gcgattaatg tgataataca gttacgttaa
29761 ttaaaaaaat aaaaatgcaa ggagaggtaa tatgctaact gtatatggac atagaggatt
29821 acctagtaaa gctccggaaa atacaattgc atcatttaaa gctgcttcag aagtagaagg
29881 tataaactgg ttggagttag atgttgcaat tacaaaagat gaacaactga ttatcattca
29941 tgatgattat ttagaacgga ctacaaatat gtccggggaa ataactgaat tgaattatga
30001 tgaaattaaa gatgcttctg caggatcttg gtttggtgaa aaattcaaag atgaacattt
30061 gccaactttc gatgatgtag taaaaatagc aaatgaatat aatatgaatt taaatgtaga
30121 attaaaaggt attactggac cgaatggact agcactttct aaaagtatgg ttaagcaagt
30181 ggaagaacaa ttaacaaact taaatcagaa tcaagaagtg ctcatttcaa gctttaatgt
30241 tgtgcttgtt aaacttgcag aagaaatcat gccacaatat aacagagcag ttatattcca
30301 tacaacttcg tttcgtgaag actggagaac acttttagat tactgtaatg ctaaaatagt
30361 aaacactgaa gatgccaaac ttactaaagc aaaagtaaaa atggtaaaag aagcgggtta
30421 tgaattgaac gtatggactg taaacaaacc agcacgtgca aaccaacttg ctaattgggg
30481 agttgatggt atctttacag acaatgcaga taaaatggtg catttgtctc aatagaaagt
30541 tagaggtgag tcttacgttt cagtgacggt agacttacct ttaacatgtt acatactaaa
30601 aaattaattt gaataagaaa gagagacata tatgaaatac gatgatttta tagtaggaga
30661 aacattcaaa acaaaaagcc ttcatattac agaagaagaa attatccaat ttgcaacaac
30721 ttttgatcct caatatatgc atatagataa agaaaaagca gaacaaagta gatttaaagg
30781 tatcattgca tctggcatgc atacactttc aatatcattt aaattatggg tagaagaagg
30841 taaatacgga gaagaagttg tagcaggaac acaaatgaat aacgttaaat ttattaaacc
30901 tgtataccca ggtaatacat tgtacgttat cgctgaaatt acaaataaga aatccataaa
30961 aaaagaaaat ggactcgtta cagtgtcact ttcaacatac aatgaaaatg aagaaattgt
31021 atttaaggga gaagtaacag cacttattaa taattcataa taaaacagtg aagcaaccat
31081 cgttacggat tgcttcactg ttttgttatt catctatatc gtatttttta ttaccgttct
31141 catatagctc atcatacact ttacctgaga ttttggcatt gtagctagcc attcctttat
31201 cttgtacatc tttaacatta atagccatca tcatgtttgg attatcttta tcatatgata
31261 taaaccaccc aatttgtctg ccagtttctc cttgtttcat tttgagttct gcagtaccgg
31321 atttgccaat taagtttgca taagatctat aaatatcttc tttatgtgtt ttatttacga
31381 cttgttgcat accatcagtt aatagattga tattttcttt ggaaataata tttttcttcc

31441 aaactttgtt tttcgtgtct tttaataagt gaggtgcgtt aatattgcca ttattttcta
31501 atgcgctata gattgaaagg atctgtactg ggttaatcag tatttcacct tgtccgtaac
31561 ctgaatcagc taataatatt tcattatcta aatttttgtt tgaaatttga gcattataaa
31621 atggataatc acttggtata tcttcaccaa cacctagttt tttcatgcct ttttcaaatt
31681 tcttactgcc taattcgagt gctactctag caaagaaaat gttatctgat gattctattg
31741 cttgttttaa gtcgatatta ccatttacca cttcatatct tgtaacgttg taaccacccc
31801 aagatttatc tttttgccaa cctttaccat cgattttata acttgtttta tcgtctaatg
31861 ttttgttatt taacccaatc attgctgtta atattttttg agttgaacct ggtgaagttg
31921 taatctggaa cttgttgagc agaggttctt ttttatcttc ggttaattta ttatattctt
31981 cgttactcat gccatacata aatggataga cgtcatatga aggtgtgctt acaagtgcta
32041 ataattcacc tgtttgaggg tggatagcag tacctgagcc ataatcattt ttcatgttgt
32101 tataaatact cttttgaact ttagcatcaa tagttagttg aatatctttg ccatcttttt
32161 tctttttctc tattaatgta tgtgcgattg tattgctatt atcgtcaacg attgtgacac
32221 gatagccatc ttcatgttgg agctttttat cgtaaagttt ttcgagtccc tttttaccaa
32281 taactgcatc atctttatag cctttatatt ctttttgttt taattcttca gagttaatgg
32341 gaccaacata acctaataga tgtgaagtcg cttttcctag aggatagtta cgactttctg
32401 tttcattagt tgtaagatga aattttttttg cgaaatctct taaatattca tccatttttt
32461 taacggtttt aagtggaacg aaggtatcat cttgtaccca attttgatcc atttgttgtt
32521 tgatatagtc ttcagaaata cttagttctt tagcgattgc tttataatct tttttagata
32581 cattctttgg aacgatgcct atctcatatg ctgttcctgt attggccaat tccacattgt
32641 ttcggtctaa aattttacca cgttctgatt ttaaattttc aatatgtatg ctttggtctt
32701 tctgcattcc tggaataatg acgctatgat cccaatctaa cttccacata ccatcttctt
32761 taacaaaatt aaattgaacg ttgcgatcaa tgttaccgta gtttgtttta attttatatt
32821 gagcatctac tcgtttttta tttttagata cttttttttat tttacgatcc tgaatgttta
32881 tatctttaac gcctaaacta ttatatattt ttatcggacg ttcagtcatt tctacttcac
32941 cattatcgct tttagaaata taactgctat ctttataaac ttgtttgaaa tttttatctt
33001 caattgcatc aatagtatta ttaatttctt tatcttttga agcataaaaa tatataccaa
33061 acccgacaac tacaactatt aaaataagtg gaacaatttt tatctttttc atcaatatcc
33121 tccttatata agactacatt tgtaatatat tacaaatgta gtatttatgt caaaataatg
33181 ttataatttt tgtgatatgg aggtgtagaa ggtgttatca tcttttttaa tgttaagtat
33241 aatcagttca ttgctcacga tatgtgtaat ttttttagtg agaatgctct atataaaata
33301 tacggttctg ttgcaaagtt gaatttatag tataattata accaaaagga gtcttctgta
33361 tgaactattt cagatataaa caatttaaca aggatgttat cactgtagcc gttggctact
33421 atctaagata tgcattgagt tatcgtgata tgtctgaaat attaagggaa cgtggtgtaa
33481 acgttcatca tttaacggtc tagcgttgag ttcaagaata tgccccgatt ttatatcaaa
33541 tttggaagaa aaagcataaa aaagcttatt acaaatggcg tattgatgag acgtacatca
33601 aaataaaagg aaaatggaac tatttatatc gtgccattga tacagaggga catacattag
33661 atatttggtt gcgtaagcaa cgagataatc attcagcata tgtatttatc aaacgtctca
33721 ttaaacaatt tggtaaacct caaaaggtaa ttacagatta ggcaccttca acgaaggtcg
33781 caatggctaa agtcattaaa gcttttaaac ttaaacctga ctgtcattgt acatcgaaat
33841 atctgaataa cctcattgag caagatcacc gtcatattaa agtaagaaag acaagatatc
33901 aaagtatcaa tacggcaaag aatactttaa aaggtattga atgtatttac ggtctatata
33961 aaaagaaccg caggtctctt cagatctgcg gattttcgcc atgccatgaa attagcatca
34021 tgctaacaag ttaacacaaa gtattatttt aaaattgaga ttagacattt atttttcaac

34081 tttgcagcag aacctaattt tttctctttg taaataaatg caaagagata ccataaggat
34141 ctcttacata accataacct tcagtataga attctggact aaatgttttc aatacctcac
34201 tgtttttttc tattaactgg tcatatacat gtttagtttc ttctacttgg tcaaaagtga
34261 gacaaagaga tatattatta ccttgtgtta tgggcaaacc ttcagtgtca tctgcgatca
34321 taatttttat atctccaaat tgaagtacac attgatcaat tttatttaaa tcattttcgt
34381 caatattaag ttgcttatct ataggtctat ctttaatacg ttgaatatac agtgttttag
34441 cgccaaacag ctcttcatac aactttttta aaccctctgc attttgagtg attaaaaaat
34501 atggacttac ttgaaatttc atgtttttc ctccttagat ttgttataaa tagagtatat
34561 acttaattag tgtcatctat tgacactaaa aggagaataa taatgaaaaa atctgttaga
34621 ttatataata tgattgaata ttgtaatgaa aataggaact tcaaattaaa tgatttaatg
34681 tcagaattta atatttctcg tagtaccgct ttaagggata taaaagaaat tgaagcatta
34741 ggagtacctt tatatagtaa tccagggaaa aatggtggtt atacgatcat aggtaatcga
34801 gaccaaacga aaatagcaat ctcagatgaa gagttgaaag ctttagtatt tacactttcg
34861 agtatttcaa atgtgagtaa tctacctttt caaacagaat atcaagaaat attaaaaaaa
34921 ttatataata actcgaataa aaaagagtta ataaaccaat ataatgatct atttcaatat
34981 tttaatgaag ataagtatca gttcaaaagt tataagttat ttaatgaaat cattagattg
35041 ataattgaga ataagtcttt tgaaatctgt tattcacaaa actatattaa agaacaatat
35101 aaaggtattg gcattatgta taaaaatcat caatggtatt ttgttgtagc taatatagaa
35161 tcaaagttag tgaatctatt aaatatttcg aaaataaaag aactatatga aatgggagag
35221 actcaagagt gtaatgatat aactatgcag aattttcaac agttcatggt taaaaatgaa
35281 actgctattg atattcttat tagaagcaat gttatgggat tgaatatctt gaaaggctac
35341 ctgtggagtg actatatgat tgaaaatatt gacgaagaga catatttatt taaatcaaaa
35401 gtgaacgcga aagatataga tttcatagct aagttaattg tcacaggtgg tgtcaatgta
35461 aaagtagaga cccctaatag tttgaaaaat gctgttaaag ttgaattaac taaaataata
35521 aacatgtatt aatagtaata taatcataat tgtaataaca aaaagatgac aacattaaac
35581 aatttaaatt tatacaaaaa agcattatca aatttaaaat ttggtagtgg tagtgctaca
35641 gcatacataa atagttataa ttaaataaag atgggtcaat gaaggtgaat taagtgaata
35701 aaaatattac agctctaaaa tctactgaaa atactacata tatgcagtat acggttcata
35761 tcgatgatca aattataaat aatataaaag agtgtaccaa aaagtttgaa attttgccta
35821 tggaagataa aattccacta tcaccgttgt tacaaccaga atatgcagga gaggtacaag
35881 tttttattag tacatatgag cagtttatga ttaattttgg taaagtaata ttggatagtc
35941 aaggcataaa aatacagttt gaaagtgaat cattaagtag tattcaacga ggcattcaag
36001 aacattgtta cttaaataag cgaacaaatg acattgatgt gactaaagaa tggtatttat
36061 gcaaattttc aaatcgaaca ttagaagagg ataaaaatca actgtataat gctttaaaac
36121 aactcatgaa tgattctaaa aataaaaagc aagcttttca agaggtgttt aaggttcata
36181 ttgatattta tctctacaat aaagaaaaat ctgaatataa gtatcaagca tctagttatt
36241 tctatcttgt aaaagaaaat cctaaaataa tttataaaaa aagacattta caagagaaac
36301 aagggatcaa gggtacaact ttcacgaaca aattaaaatt attgaacaaa atgtatggtg
36361 tagatgttgc aaaatatcag ccattttata attctaataa tcctgaatat gaaagaggtc
36421 aatttggtga aagatacatt tctcaaagat ctaactatga atttaataga cttcaatatc
36481 aaattatata tatattatcg aaaatacttg ataagcatcc attaccaaag tcagataata
36541 attataaaca tattccaact attgaaaaag caatattaag agatgattcc cataattttt
36601 atgagtattt tgaagatata atgaaagaaa ttatgaatat ggataattct cccttacagg
36661 aaaaattatt gactgatttt acatatcaat cccaatgtag atggtactct gaaaataaaa

36721 aattaaaatt gcaacttgaa agatttatgc ataaagtttt agagagtaat tattatgagg
36781 gcaacaaatt attgagaatg atgtctaacg caattgaaga aacaatcaat gaggctaaag
36841 aagataaggt tcatttcaat tattttaaag attatttttt gacagacggt ggggtgaaga
36901 attgggagca aattagcgag aaaattactg aattcaatgg taaagttatt aacgatatcc
36961 aacatgaata taataagata cagtccaaga atacaaatca aaatcagaaa ctaaactttg
37021 tttatttata tcattgcttt gagtttagta ataacatagt aaaagcaaaa gataatgaaa
37081 gtaaaaatta tatcttgttt tttatagata aatataaaat aaagacacca tttttggatc
37141 aattattaat aaattgtttg aatcaagaaa tagatcagga aagtattaac tacaatatgc
37201 aaacaatgtt tgaaaaagaa agatacgata gaagtagtac cattgaaaaa ttagtagcaa
37261 caagaaaatt taagtatgaa aaagatgatt cagatttatt caaacaactt ttcaatgatg
37321 ttgaaaattc aatagacaga ttaggtattt acttactaaa taatggtata aattcgaatg
37381 atgaaaatgc aagatactat agatcgtttt taaaggaact tagtagaata aaaagtaaat
37441 taacgccatt ttctcttgaa ataagtaagt ctagcggaag agagcaacat tatcctgatg
37501 atgctgttga tgataaagat gagagaagaa aaaataaaga agaagcatat catgcttttg
37561 acgataaaag cgatattgac tacaaattaa agaataaaat aagtgattat atcgataatt
37621 tattgtcagt taaaattagg aataatttgt atgatattag tccaaataca aaaagcaata
37681 attataatga gccaactcaa aaatgagttg gctcttttgt tgtaatttta cgcttccatt
37741 acaggaatca atgttatgga agccattaag taattaattt gagtcatata tattatagat
37801 acaacgttgt gatgttcaca cgataatcag cccgggagat tgttgtgtaa taacttatag
37861 aaggagcaaa tcaccatgta tcaagcaaat atacgtgatt taattacaaa attgcctcaa
37921 agcaataaaa cagaacactt tttaatgaat aaattttcaa atcaagataa agttcagcag
37981 ctacaaagac aaattagcca acagctagat caacaatata atgagctttt gtctaatgaa
38041 aaagctaagc tagaccaata tgtggaagta caccataatt tagaaccatt aaagaaagag
38101 attgaatcag aatctattaa ccttgatacc gataaattac ctgatatcaa agcgacaatg
38161 cttgaaaagg ctaagaacga tgaacattct gataaaatcg aacacctatt tgatagatta
38221 gatcagtcat taaatggtac gaatcgatta tatacgcaat tatcgttgat tggcacacga
38281 acacatcgga ttacaacgaa aagatttaat gttcaaggct tacctaaatt agtccaacaa
38341 acgattttac cttcgcaatt taaaaaggtt tatacaatag attttaaatc attcgaacca
38401 tcagttgctg cgtatatgac acaagacgaa caactgattg actacttgaa tcatgaagaa
38461 gggttatacg atgcattact gagagactta tctttgtcaa aagagaagcg cgtgagtgtg
38521 aaacgtgcat ttatagggtc attccttttt ggcggtcgtt atagtagctc taaattcaaa
38581 atcaatcaag aggttagtga aattaactgg ctacaagtaa tgagcaaatt caagaaggtc
38641 attgaattta aggagcaagt cgaaaaatat aaaacaatgc ctacgcctta cggcattgaa
38701 catgatatga gcgcatttca aggtagtagt attatggcaa tttacgtaca aacggtagcg
38761 agctatattt tcaagtacat tttgttggaa gtgtacaaag cacagtgcga acaaaaaatg
38821 ttcaagatta tagtgccgat acacgatgcg attatgattg aatgtaatga taaggagatt
38881 gcacaaagtg tagcacagct catgaaagat acagctaatc aactgtttaa tggtgaattt
38941 gcacatgtga cagtggaagc tttaagaggt atagacaatg aataatgata gaggaaaaag
39001 tcttcaaatt ccccaaagta catcgttaaa agaaggatct atatacgttg ctacattaca
39061 ttctgtgact gagacagatt actcaggcga cataaaacat caatttacgt atgaaataga
39121 gatagatcaa caaatattct atgtgaaccg tagtgttgtg gtcaatgtga cacatcaaca
39181 attatcaatt aatgactggc tcgaacgtca tagtaactat agcgcaagtc atgaaaacta
39241 tgagccttat attgatcaga aacatttaat tctattaggt caatataacg gtaactatta
39301 tgtacaagat gtagcatcgt tagatgcgtt tggaggcgta ttatcatgaa tcatatatta

39361 caaatgttat ctaagctatt aagtgtggct aaggaggcaa tcgaccgtca aggtctgatt
39421 gctatcctaa ctattcctgt taataatgac gatgaaatag aagaaacggc tcaaggtgaa
39481 accgtgtata acgaacttat cgatcagtta cgacttaata tcccaaaaga tacggattat
39541 cgacctaaca tctatagtta ttttggtatt aagaagaatc ctaatgacac cgtactcatg
39601 gaaatgatga taaaggtttt tcatatcaaa cgctttaatt cagaactgtt tgttttcaaa
39661 gctaacgggt ggcaaaagat aaatggagat gaattacaag ggttgatatc taaaatgata
39721 caagtattgc ttgtagatta taagccttca ctaagcactc taaaaaatgt cgtagatgga
39781 ttgcaaaaat caacagatgt agaagaactt gttgagaatg agcgctatat tggttgtggt
39841 gaaaatatgt tcgatcttaa tacgtttcaa gtcgttaaaa attcaatcga tatctttcca
39901 aaaacacgat tgaatttatc attaagtaca aatgatgtaa ttactgataa gataccgcct
39961 tattttaagc aatatatgtt acaacttgcg aattatgacg atgatttaca atactttctt
40021 ttccaacata cagcagtatt acttacagct gatactaaat accgtagggg tctcatatta
40081 tatggtggag ctaagaatgg taaatctgta tatattgaac tagttaaatc atttttctat
40141 agtaaagata ttgtgtctaa gccacttaat gagcttgaag gtcgttttga caaagaaagt
40201 ttaattgaca aaagtctaat ggcaagtcat gaaattgggc aatctaggat tcaagaaaag
40261 atcgtaaatg acttcaaaaa gttattatct gtagaatcaa tgcatgttga tcgtaaagga
40321 aaaactcaag tggaagtcat tttggatttg aaacttattt ttagtacaaa tgcgatactt
40381 aattttcctc ctgaacatgc gaaagctttg gagcgtcgaa ttaatattat tccatgtgag
40441 tattatgttg aaaaagcgga cacttcatta attgataagc tccagagtga gaagaaagaa
40501 atctttcttt acttgatgta tgtgtatcaa cagattgtaa aagcagatat cgagtatctt
40561 gaaaatagcc gtgtcactga aattactcac gattggttaa attttggata tgaatttgtt
40621 tctagcaggt ccgtaagtaa tgcaaatcag aaagcatgta ttaatttact cagaaaactt
40681 atagaaatca aatcaggatc acgaatcaaa gtatccgagt taaataaagt tattaatgaa
40741 gaaataaagg taagttctca ggttattaaa cagttaattc aagcaaactt tgatactcaa
40801 accaaactat acaatggcta cgattattgg attgatttag gttggaaaga agccaataaa
40861 aaagagattc atgatatttc ggaaaaagat aatattattt cattagataa aaatgaaaat
40921 ataacagacg atgaggcatt agatgaagag aatttggact ttgattggga ggactttgac
40981 gatgaataat gaacaaattg aagcatttgt agaagtgctt gtacctatca tagaagaacg
41041 tatcaataaa ggtaattaag gctaattacg tactacaggc agttgcctgt agtactcata
41101 tgattaagtg gtaaaagtga taaaaatgaa acgaaattat aaatatatat tatccatatg
41161 ttgttacaag accgatagtc tgtagcaata atctaataaa aggagcggta tgatatgaag
41221 ggtaaaattg cactttattc acgcgttagt acgtcagagc agtcggagca tgggtactca
41281 atccatgagc aggaacaagt actcatcaaa gaagttgtga aaaatttccc aggttatgac
41341 tatgagacat atactgactc aggcatttca ggtaaaaata ttgaaggtcg tccggcaatg
41401 aaacgtctat tacaagatgt taaggataat aaaatagaaa tggtattaag ttggaaattg
41461 aatcgtatct cacgatcaat gagagacgtg tttaatatta ttcatgaatt caaagaacat
41521 gacgtagggt ataaatcgat ttctgagaat attgatacat ccaatgcttc tggagaagta
41581 ctcgttacaa tgtttgggtt aataggatct atagaacgcc agactttgat ttccaatgtg
41641 aaactttcta tgaatgctaa ggcacggagc ggagaggcca tcaccggtcg tgttttaggc
41701 tacaaattat cacttaatcc attgacacag aaaaatgatt tagttattga tgaaaatgaa
41761 gctcatattg tacgggaaat ctttgattta tatttgaatc acaataaagg acttaaagca
41821 atcacgacaa ttctaaatca aaaaggatat cgcaccatta atcaaaaacc attttcagtg
41881 tttggtgtga aatatatttt gaataatcca gtctataaag gctatgtcag attcaataac
41941 catcaaaatt gggctgttca gcgaagaagt ggtaaaagtg ataaaaatga tgtgatattg

42001 gtcaaaggta agcatgaagc cattataagt gaagatgtat ttgatcaagt tcatgagaaa
42061 ctagcttcta aaagttttaa accgggtcga cctattggtg gagatttcta cttacgtggc
42121 cttattaaat gcccagaatg cggaaataat atggtatgtc gacggacgta ttataaaacg
42181 aaaaagtcga aagaacggac aatcaagcgt tattacattt gttcattatt caaccgctca
42241 ggaagttctg cctgtcatag taatgcgatt aatgctgaag tcgtcgaacg cgtaatcaat
42301 gttcatttga atcgtattct ttcacaacct aatgttatta agcagattgc gtcaagtgtg
42361 atagaagaac tgaaacaaaa gcatagtaaa caaacagaaa taaaatatga tattgatagt
42421 ctagaaaaac aaaaagcaaa agttaaaaca caacaagaac gattattgga attgttctta
42481 gatgatgaaa tggatagcga aatgttaaaa gctaaacaaa gtgaaatgaa tcaacagtta
42541 gaagtattag accaacaaat taaagaagca aaacaagcaa atcaatcaca ggatgatata
42601 cccaattttg ataagttaaa agcacaactc attttgatga taacacgatt cagtgtgtac
42661 ttaagaaagg ctacacccga agctaaaaat caacttatga aaatgttaat tgattcaatt
42721 gaaattacga cagataaaca agtaaaactt gtaaggtata aaattgatga aagtcttatc
42781 cctcaatctt tgaaaaaaga ttgggggtct ttttttatgc ctaaatttaa ctttgtgata
42841 aatgtcacaa atgaaaatag gattgaaaat ttatcacttt taccactttt ttagagtgac
42901 aaaagtggag gaattttgaa atattttata aatatatatt ttatttatgg agtacacatt
42961 ataaattaag gaggtcatta taatgacgct aagcaaacaa cttaaaacgt atattactga
43021 acgatttaaa ttaaattatc aagaaacttg ggcttgtgaa accgtagatg cggtggctga
43081 agatgtatta cctgaaaaat atattaaaaa tagtccactt gaacataaaa ttttaaatac
43141 ttttacctat tacaatgatg aattacatga aatcagcatt taccctttttt tatgttatct
43201 agataaggaa ttagtagcaa taggttattt agataatttt gatttagact ttatattttt
43261 aaatgacact catcaagtca ttattgatga acgctacttg ttacaaaaag ggggcgagta
43321 attatgaatt ggatcaaggt cgctcaacta tctgtcacag ttatcaatga agtcattgag
43381 attatgaaag aaaaacaaaa tggaggggaa tagtatgaaa atcaatcgat acattacaag
43441 aggtgttagt gaacatttat ctctagacct tcaaatctta ctttggaaca tggtaaaaga
43501 tcgagacaat caacttcata cagattacct acacattttt aaactacaag aagatgataa
43561 tatactctca atcacacatg aacaagaaca acccgcatac aaattggaat atcactatac
43621 aaactatgta aaaaatcaaa atgcattacc taagaaagtc tacgtcatcc gagaagatga
43681 tgtagacgct ttttattatg tgatgctttt accagaagaa tactaaaagg agtgacaatg
43741 atgaatacaa tcaaaaatac aatacacaca gaagcgatat ttagcgatga tgaacaacac
43801 cgctacttac tcaaaaagac ttgggatgaa aagaaacctg catgtacggt aatcaccatg
43861 taccctcatc tagatggcgt attatcactc gatcttacaa ctgttcttat cctcaatcaa
43921 ttagcgaatt ctgaacaata tggcgctgta tatcttgtaa atctattttc taatattaaa
43981 accccagaga accttaaaca tattaaagaa ccttatgata aacacacaga cattcattta
44041 atgaaagcga taagtgaaag tgacacagtg attttagcct atggtgctta tgcgaagcga
44101 cccgttgttg tcgaacgcgt tgagcaagtg atggaaatgt taaaacctca taaaaagaaa
44161 gtaaaaaagc tcataaatcc ggcaacgaat gaaattatgc atccgcttaa tcctaaagcg
44221 cgtcaaaaat ggacattgaa ataaaggagg attatctatg aaccatgaaa ctaaacaatc
44281 agattggcga acggttgcta gttgtttagc atcacaaaat tatatatcga ttgtaaaagg
44341 tttagtacat cattttacag cgatcgaaga tgaggaaatt cttgataaaa tctatgatga
44401 ttttatgaat gatgactcta taacaacggt acttaacaat gatttacaga caatcattaa
44461 ccattatcta tcaaaataaa gactatacta attacaataa aaacagagca tccttcactt
44521 ttatggtgag ggatgctctt ttaatttatt tttttattga ttattcgcca taagttctgc
44581 ttgctctaca acctgctcta cagccatttt ttgtaaatct ggtggatagc catatttttt

44641 aagcaatcgt ctaacagcta cgcgcatttt agcttttgcg ctatcacgtt tagaccaatc
44701 aacacccatg ttttctttca ctgttttagt tagctcatga gcaatcgcac gtagttcttt
44761 atctcccatg gcttcttttg ctgtttcatg tgaagctaaa gcatcgtaaa acgcaatctc
44821 atctgaattc aggcctaatt catttcctcg ttgttgttct tgtttaatat ctttagcgag
44881 ttgaataagt tcttcaatca ctttagatgt ttcaatggaa cgactattat atttattaat
44941 cgaatttcct aacatctcag agaaacgctt agataccgtt gcattcgttt tcattaatga
45001 tttaacttgt cctttgagta atcgatttaa taattctacc gccacatttt tctgtttcaa
45061 tccttcgaca tcttttaaaa agtcatcaga taagattgat aaatcgggtt gttcaagacc
45121 tagcgtttga taaacatcaa tgacatcttc agtcacaaca gattgtgaca caagttgatt
45181 aatctctgct tcaacttctg caggtgtttt acgtgttttt ccttcttttg gcggttgtaa
45241 caatttaaca agtcctgctt taactgcttt aaagaaggca atctcatcgt tgagttcttg
45301 ggctgtaggt tcagtcgcac aaagagcaaa ggctttccct aattctgtga ctgttttaat
45361 aaaacgttga cgttcatctt cacctaaacc aattacataa tccatcgtat tcgatattgt
45421 ataataacgt tcagattttt tatctgaatt aaatttagag taatcaagat tatataacat
45481 atcttgaatt acatcatact tcaataacat caattcaacc gctttatccg tatctattgc
45541 cgtttgagct tgatcagatt ctgtatattc tttaagtgcc tcttttaaac tttcggcaat
45601 ccccacataa tcgacaatta aaccacccgg cttatcttta aacactcgat taactcgagc
45661 aattgcttgc atcaaattat gccctttcat cggtttatca atatacatcg tatgcataga
45721 aggaacatca aaccctgtca gccacatatc tcgaacaatc acgagttgta actcatcatc
45781 cacatctttc atacgttttt ctaataaatt acggcgtttt ttaggaccaa tgtgtctttg
45841 gaaagaagtt gggtcactag atgagcctgt cattaccact ttaatgactc ctttatcatc
45901 atcatctgaa tgccattctg gttttagacg aatgatttca tcatataaat caacagcaat
45961 tcgacgactc atcgttacaa tcattccttt gcctttcatc gcttgttgac gtgtttcaaa
46021 gtgttggatg atatctttgg ctagggcttc tacacgaggt tttgcgcctg ctaaagcttc
46081 aatacgggac cattttgatt ttaaacgctg ttttacatcc tcttcttgat cttcagtaat
46141 gtcattatac gcttcatcta aatctaaact ttgaggtaga tttaatggaa ttacacgact
46201 ttcatagtaa attttaacgg tgcttccatc agcgacggct tgtgtcatat cataaacatc
46261 gatatagttt ccgaaaacca tttgcgtatt tttatccgtt gaagctacgg gtgtgcctgt
46321 aaatcctacg aatgttgcat tcggtaaagc atctcttaaa tatttggcat aaccatattt
46381 aatgccttca cctttatcat cgtattttgc gttaaagcca tattgtgtac gatgagcttc
46441 gtctgccatg acaatcacat tttttcgttc tgttagggca gccatggtcg tttcgttttg
46501 ttcaggttca aatttttgca tcgttgtaaa tacaatacca cccgactcaa cagataataa
46561 tgattttaat tctttacgtg tttcagcttg ttttggtgtt tgtcttaata atcctttacc
46621 agaacgtcct tttgatttaa caaacgtact gtacagttgg ttatctaaat cattacgatc
46681 cgttacgacg actaaggtag ggttattcag catttgaatt aattttccag agaaaaagac
46741 catggttaaa cttttaccgg acccttgggt atgccaaata acgccgcctt taccatcacc
46801 ttgctcagat gaagctaata aagctctgtc aacggcttta ttaacggcat aatattgatg
46861 atacgctgct agaattttac tgatatgtcc tttgccatca tcttgaaata aaacaaaata
46921 tcgaattaaa tcaagtaatg tttctggatt taacatgcca tgaatgagta cgtctaagct
46981 agctaaacct ggtgatgatt cagtttgacc atctttactg cgccaagtca taaaccgatc
47041 ataatttgcg gttagagagc cagcctttgt attaatacca tcacttgtaa caagcacttc
47101 gttaaacgta aataattgtg gaatacgcat cttatacgtt tctaattgat gataaccgtc
47161 ttcgacgccc acggtttcat ttgttgaatt tttaagttca atcactacga taggcaagcc
47221 attgataaag agtacaatat cgggacgttt tttatagtct ccattaacga tagttaattg

47281 attgactgct aaaaaatcat tgttttgtgg atattcaaaa tcaataattt taacaatttc
47341 tacaatcgat tgtccttcat catcatagtc ttcgatttca ataccattaa tcaaattttc
47401 atgaaaggta aggttatttt ctaaaagatt gggcgacttc tctaaagtta gttcatgtag
47461 cgctttttca ataaaacgag gatggatatc tgagttgatt tttcttaatg ctttctctaa
47521 acgttcatga aggacaacat ctttatcact tttacgttct ggtgttagac ttgtcatact
47581 aatctcatta cctttttat agtcatagcc tagtgattgt agccattcta atgcgacttg
47641 ttctaaatca tcttcattaa attgaaagtt catcactatt cacctcaata tcatcagata
47701 tttctagttc tccagacatt agtttaggaa gaagagtatc tcgtaattga gttaatttac
47761 caatttctga tcttaatatt ttcatttctt ccatatatgg agtgattatt tcagaatatt
47821 tttttacaat actatcttca attgctaatt tataattaac agctattgtt ggttttactc
47881 tttgtctact atttgttgaa ccggtagtat tagcttttaa ataatctata aattgagaat
47941 taagacatat attatatatg aatgaattaa ttttattatt aggagattcc attacaacaa
48001 attctgatga agcaacattt aattttttat tatctattac aggtaaccaa atccttttag
48061 tatcaggatt catttttgaa aataatacgc aattgttatt aataatccat ttattacttt
48121 ttattttatt gacttcttcc tctatagctt gttcttcatt atcataagca ggtaaactaa
48181 aatgtttaac tgttacttct tcactctttt taggattaaa agtttccttt ttatgacttg
48241 ctatatcttt taatttatag attttccaat tacttggtat cttacctaac tcactatcaa
48301 tcatttctcc tccactagat ttataaggat taccatcttc atctggaaat tcaaaatcta
48361 caaaccaatg cttaaatagt gtttgtgaaa gttctttaag gtttgctatc attttttggt
48421 tagtttctat ttttcttgtg atttgattta aaaatttaga aatgcttttt tgttttatta
48481 aagaaaaatt aggaattttg acagtttcaa taagtttaga atttatatta gcttgattag
48541 cactccctgt agaatttcta actaaatact cagttatttt aggttgtaga aaataataaa
48601 ataaaaaatc taagtctgca ctattactat attttaattc gaattttccc acacgttgat
48661 tttgtaatgc tggataatca aattcaattc ttccaacttt tcctactgca gaagacattt
48721 gattcacgcc tgatccagtt aatgatatta atatatcatt tttctttaat gaaaaacctt
48781 tagttttttc atatgtttta atagagactt tttgagagtc ttttgtatca acaatgggtg
48841 aaattatatt cttaatttta ataaccggta taccttttc ttgaaattct tttgatttaa
48901 aagcatatcc atttttaatt ttagctacat caccgaaaaa aattgtatta tactccatat
48961 cctaacccct ccaatgattt tcggatttgg tcttcaagtt cttttgattt tgcgaattgt
49021 tcacttaatt cagaagtaat acgttccatt ttttgttcaa aaggttcttc atcttcttca
49081 acgtcagcta aaccaacata tcgtccaggt gttaaaatgt attcattatt cttaacttct
49141 tctaaattag ctactttaca aaaaccagct atatcttcat aaggtttatc gtttgtacct
49201 ctccacgcat gatacgtctg tgctactttt tgaatatctt catctgaaaa ttcttttaat
49261 gttctagata ccatatggcc gatttcacga gcatcgataa ataaaatttc attttcacgc
49321 tcttttttac cattttgacc tttattatta ctaatgaacc aaagacatac aggtatttgg
49381 gttgagtaaa agagttgtcc aggtaaagta acaatacatt caactaaatc ttgttcaatt
49441 agatttttac gaatttctaa ttcatctttt ccgcttgtag acattgatcc gttggctaat
49501 acgaatcctg ctgtaccatt aggtgctaat tttgaaatca tatgttcaat ccatgcatag
49561 ttagcattac cttttggtgg aataccaaat tgccaacggt aatcatcaag taatcgttct
49621 tgaccccaat cacttgcgtt aaaaggagga ttggctaata tgtaatctgc ttttaatcct
49681 ttatgtaaat cattatggaa tgtatcagca tttcgttcac ctaagtcatt atcaatacca
49741 cgaatcgcta agttcatttt tgctaatttc caagtcgtag ggttagattc ttgtccgtaa
49801 atcgcaatat cgtctaatcg accttgatgt ttctcaacga agcgttcact ttgtacaaac
49861 atcccacctg aaccgcaaca cggatcgtaa atacggcctt tataaggctc aatcatctca

49921 actaataact ttacaattga tgacggggta tagaattctc cagcattttt accttcggca
49981 cttgcgaatt tagcaataaa gtactcatac acacgaccta acacatcttg tttacgactc
50041 tctgtgtcgc caactttaaa tgtaaataaa tcaataatat cgcctaattt ttctttatct
50101 aatgcaggtc ttgcatattc tttaggtaat acacctttga gtgattcatt ttcattttca
50161 attgcaatca ttgctttatc aataatttgt ccaatttctg gttttttcgc attatcattg
50221 atatattgcc atcttgcttc ttttggcacc cagaaaatgt tttctgctaa gtattcatct
50281 tggtcctctt catctgcata tggatcctgc ttcaattctt catacttttc ttcaaaagaa
50341 tctgatacat attttaaaaa gattaaacct agtgctacat ttttatactc agcagcatcc
50401 atacttcctc tcaatttatc ggcagcttgc cataattttt cttcaaaacc tatagtggcc
50461 attaataaca cctcttaaaa ttaatgttaa atcaactata gcaaaaaata gaagatgatt
50521 gtgtagtgtt ttattaatat ttaatttatc tagactatca aaaaaatata gattattatt
50581 atcttaaatt aataaacagg agtgaattaa aatgaataaa aaatataaat taaaacgata
50641 cgataaaaga tttagaggtg gatggtgtta caaatgtgaa gattcatatt cttacctttg
50701 tgaagattgt ggttattgta gatattgttg ccattgttaa tacaagacta gataaattgg
50761 gtatctttaa ctcaattaat aaaaaattta aatgtagtca atagcttgct aactttattt
50821 gatgtaatat aattttggct tttagcattc agttaaaaag aagtaaataa ttgacagatt
50881 ttttagagaa ctggagaaaa aagatgtaca tttctagtgt taaaattaag aattttagaa
50941 attttgataa tatcgatatt gatttccatg agggagttaa tgtattaatc ggacataata
51001 attcggggaa atcaaattta ttacgtgctt tatctttaat ttttgacggt tcagttagaa
51061 agcaactgtc agtggaagat tttaataact ctttaacaaa agaatcttta aaaaaagagg
51121 ctccaaaaat agtaatttca gtacatataa cacaatctga aaacgaaaga ctgatgtctg
51181 atgagttaat tacggtaagt aattggctga ctaaattaga agaaccttat gaagctagaa
51241 tacaatatga attcttttta ccaaaagatg aggaaaaaaa ttacatagat ttagtaaaaa
51301 atatagatga aaaagaagaa atatggaatt taattagaag ccagtttatt aggttgtatg
51361 tcaataaaat ctgggtgggt aatccagaac atcagattcc tatagataat gatagtttaa
51421 ataaatttga tttccaattt ttagacgcaa taagagatgt agagagagat atgtttagtg
51481 gtaaaaatac attattaaaa agtgtaatag attttttat agattacgat ataaaatccg
51541 atgaaacaat cacagaagaa gaacagaaag aaaaattaga agaaagaaga aaagaatttt
51601 ctgataattc tagtgatttg attgaaacta ttcataaaag attagatagt gggaatcaaa
51661 aaattttgtc ttatacaaat ggcataggag catcttatga taaatcaaca ccggatttta
51721 aaggtaatct tacagaaagt gaaatttata ctgtattgca attgataatt aaacatgaaa
51781 ctggaatgac tttacctata actcataacg ggttgggtta caataattta attttcatgg
51841 ccttgctatt gtctaaaatg caagctgatt ctgatggaaa ttttctaggt agtaatgcaa
51901 aagtatttcc gatactagct attgaagaac cagaggctca tctacatcct actatgcaaa
51961 atgaatttat taaattttta aaaaataaca ttagagaaaa aaaggtaaag cagattttca
52021 taactactca ttcaacacat atttcttctt ctacaaatat tgatgatatt atttgtcttt
52081 acacagatga tagtaaaact aatgttagtt atcctggaaa agtttttgat aagaataatc
52141 aaagtaagaa gtacgtacaa cggtttttag atgccactaa gtcaaatatg ctatttgcag
52201 aaaaggtaat atttgtggaa ggtatagcag aacaattact attacatata tttgcagact
52261 atttaaataa accgttagaa aagaatcatg ttgcagtaat aaatgtaggc gggagatatt
52321 ttaatcattt tttatctttg tttgatagta ataataaata tgcaattaat cgtaaggttt
52381 cttgtataac agatttagat ccaatgcgaa aagaaaaaaa tgaaaatgga caaaaaaata
52441 attttaaagt atgctatcct tttgaatatg aaatggattt agataaattt gattattcaa
52501 ctaataattc tctagataaa tatctaaata atgagcacag taatatacga gtatttgttc

52561 aacctaataa atatggaaaa acatttgagt accaacttat gtttgacaat ccttcattaa
52621 aactgttact tacagactcg atttctaata gtcaagagct agcagaatta atggatcact
52681 atgaagaaaa tgaatcttta caaaaattaa ttgatacatt atcaccatct aatgaaaatc
52741 agagaataat aaaatcttta aaagacacaa atgacagttg gaatgaagat aataaaaaga
52801 aagcattaat agcatcaaga tacctaaatt ccataggaaa aggagaaaat gctctagagc
52861 tagcttcagt attaaaagat aatttagagt taaaaggcca gagtgactat gaagattttg
52921 tagtaccaaa atatatagaa ggtgcaattc gatgggtgtg tgaataatat gattattaat
52981 gctcaaacga aattaaatat agaacaagat tttaaaatac aggctggtcc tggtgctgga
53041 aaaacagaat ttttggtgaa tcatatcaaa aatgtaatac aaacttctga aaagctcgaa
53101 cgtatgaaga aaatagcgtg tattacttat acgaatacgg ctgctcagac agttttacaa
53161 agattaggaa aaagtgtttc aaatagagta gatatatcta caatacatag ctttttgtat
53221 agaaacgtag ttaaaccata ttgttctttt ctacccaaag aatacggaat ttgtgtgcaa
53281 aaattaaaag gacatagtga tccaattgtg attaacaaat atgtaaatgc atggttaaaa
53341 actgaatatt ttacgaaatt aaaaccgcct agtaatagaa atcaactact aaaaatgcca
53401 gtactaaaga aagctttgca aaactggcta ctatctatga aactttctta cgaaaataat
53461 gatgtgcaat ttatttgtga taataccaaa gctcaagcaa ttgataaaaa aagtaaaaaa
53521 agaatgggaa taaataaatc aaacttaaat atactagaaa agcaattatt aaattataaa
53581 aaaatatatt gggagaaagg aattatcgat catgatgatg ttttgttttt ttcatatgta
53641 ttaattagag attacccatt cattctcact attttatgtg ctaaatatcc ctactttttt
53701 atagatgaat ttcaagatac tagtcaatta caagatttta ttatagataa aataagacag
53761 aaaggctgca tagtgggggt tattggcgat aaggcacagg caatttacag ctttcaaggt
53821 gcacgggttt ccttatttga gaagtttaaa gttgatttaa taaattctca cactatctta
53881 gaaaatcata gaagttcata tcaaatagtt actttttaa atagtatcag agaagatatt
53941 actcaaacag tatatagtgg tattgaaaat gataaagtta caattttgat tggaaatcgg
54001 attcgcagtt atactaaggt agttaatatt tgtaacaagg agtcacttgt taccttgtca
54061 agagataata ttacttctaa tgcaatgaga ataaagttgg aaggaaataa ttttgataaa
54121 aagttattgg aaaaatttca agaaattgat agtaatagta aaagaaggaa tactattatt
54181 tcgcttataa aagctattga attagctaag aatactaaat ataaagatgc cttaaaaata
54241 gtcgaatcga tttataaaga tgaaaataat cctaaaaaaa tagctcttaa ttctctaata
54301 aatatgatta ataaatatga taaatacggt aatggtacat taatgaattt ctacaatact
54361 ttatgtaaaa ctttagatat taaactttca gggtttagaa aggggcaagt aaaagatttt
54421 tacgaaaaca ctttatataa aaatatggtg atatgtacaa atatcgttga agacacaagt
54481 aatcatatta ctatccacaa agcgaaaggc gcagaattcg aaaatgtatt tgttataggt
54541 aataaagaca ctttaagtct acttttgaaa ccagatttaa tgaacgatga agagcatcgt
54601 atattctatg tagctatgag tcgagcaaag aagaaacttt ttttacaatt tgattatctt
54661 gataataatg aacaaaagaa aattaaggaa cgttatgatg taagtattat acaataacct
54721 aatctaagaa tatttaaatc caatatattt cttgattata attcaataat agataaaatc
54781 tataaacgat taaaaagaaa atatatttgc taagacttat aaaaatgaag agataataga
54841 ttttcctaaa ttacagttaa aagaagtaaa tagattgaac actttgaaag attctaatcc
54901 aaaatttgaa ccactaaaat tgaaagatga agaagatgca agatgtagcc atcaacgctg
54961 atcacaaagt atttttctta atgattattt ttaatgttat catgctcgtt tcttctatta
55021 ttcaaatgat tttaggaaaa ggcagacgta tcacaccagc aaaacctact aaagatgcta
55081 agttacactt agataatcaa tcctcataaa gagaaaaaag aacccctaaa aagttcaact
55141 ttttgggggt tacttactat taagaagtat ttttgattaa gcatctttca tatcgtaatc

55201 accaatatac tgatcaaggc gatttattgt ttctctgata tcatcatgtg tcgttaatgg
55261 gttcgttgta cataaacgaa tgacacgttg gttattcaat actgtggtat aagcaatggc
55321 ataaccagaa tctgcaattt tttgcgcagc catgctattg agttgattat tttgtgcttc
55381 agttaaatca ctgttttcat atctgaagtt cacaatggaa agattagcgt gtgagataat
55441 acgccagtta tcgagacctg acacgtattc ttctgcatat tctgctaaac cttcgccgta
55501 tttaatgcgc tcgataattt catcttcacc aatgacttgc agagtaatcc ataattttaa
55561 agcacgtgca gggcgtgtaa gttcaatgcc taacatttct ggatcaatca catcatcatc
55621 tgaagcaata tcatccaagt actcagcatc ttcgccaaag ctttgcagca gatgattctt
55681 ttctttcacg attaccatag cgcaactata tgtttggaac agtaatttgt gagcatccca
55741 actggcacta tctgaacgtt caatgccttt aaataagtgt tgtgctttat cggataagat
55801 atgtgatagt ccataagcac cgtctacatg gagccataga ttgtagtgat cacatgtatc
55861 tccaagtgtg gtgaaatcat cgacagaacc tgtattagtc gtgcctgcag tcgcaatcac
55921 catagcaggt ttgtaacctt cttcaatatc tttatcaatt gcggctttta agctatctgt
55981 attcattgta aagtcatcgt tgtactcaat tctgcggata ttatttttaa ggaagcctgc
56041 aacatgcaag gctttgccta cagaatgatg cgtttgagat gtaagataaa cggttgcttt
56101 tttaatatct tccatttcta cctgtgcatc tctagccgca acgatagctg ttaaattggc
56161 cattgaaccg ccagatacaa agacaccacc ggccggtttg atctcataac cgatttttcc
56221 taccaagtag ttaattaaat tacgctcaat attaattggc agcgtcgcat tggcaaaatt
56281 agaggcatgg atattatttg ctgttgttaa aatgtctcca agccatgata gacgtgatgc
56341 tggacctggt ataaatgaga atgaacgtgg atggttcgga cgatataaat aattcagtac
56401 ttcattattt aaatctttga gtacttcata gatatctcgc cctttattcg gcacttccat
56461 cttttcatat ttctctctca attcgagcgg tgcctgttgt gtcgcaggta agtctccaat
56521 atctctagat aaatatttat taacttcatc tctgataatc gtctctaagt cgatattgtt
56581 ctcattgaat tccatttcca tactctctcc ttcaattata tttttaataa aagacagacg
56641 tattaattac gacgttttac tacaatataa atgatagcag atattaataa aaccacgcta
56701 accataatga cgaaacccag aaggtgtcgc cccaatgaat aacgtgctgt gctttaacac
56761 tcggtgtatg tgcgcctttc agaaattgac gagggtaatc gacttgcaga ttatcatctg
56821 tgattttcaa tttgtattga tgcttgtctt ttggaattcg ataactaccc cgtagaacag
56881 gggacgagga cctcatcttg atgatcccaa ttggcttggt taaaaagaaa tcgataatcc
56941 tcgtagaata gagaaaaagt attaaaaata tatttgattt gataaaatat tttgcaaaaa
57001 agagaaacaa aaataacttt catataaata atttctttgc gattaagttg gaattagaaa
57061 tactaaatac aaataacagc gaatggaatg catattattg aggaattgga tgtgtagtgc
57121 aatttcgaga gttactactt aacttaagaa aataaaaaga agaaaacaaa caataagaat
57181 taaatctgaa gcaagatata aaagcaaata tttttgctac aattatatta gtaaatactt
57241 tattaattag catacgactg aaagcgaagt aacagctaat aatgttccag atatagacat
57301 tagtgttatg aaagaacaat attaccatct agaaaatgta atttttaaaa acctgaagat
57361 aaagtagact attctgtgag ttttcatgtt aagaattctg aacttgggga tgtagtataa
57421 atatggttta gtagaaaatg acgctggagc tgtagctgag attgactatc aagaattaac
57481 taaagtagat tattacccat caacaaaaat ccaagattta acactatcta ataatagtgc
57541 gaataatgaa gtttaaaagt gaaaaactga taatagcgaa tcaaactaaa gtaatcaatt
57601 acagaattca agtactactc aagcctcaaa caaatattga aactcaaaca caagttaaga
57661 ataatacact gtcagaaaca gatgaacagt ctaatgccga tttagtccag taatagcctc
57721 tgtattatta gccgcacgat tattatcaat aattaaataa agttaatatt aaataataaa
57781 aatccttcaa attatttgtt gaaaaaataa taactttgaa ggttttttag caaaatcact

57841 gatagggaga agcgtataat aagtaaaaat aattgatgaa ccacgcagtt atacataaat
57901 atgactatgt ggtttttata ctatataacc ataacaatta tatacaaaaa taatttaaaa
57961 tgaatgggtg tttattgttt ggtaatataa aagagagatt tgtattataa ctcaatattt
58021 atatgtagat accatgactt taaaaaagaa ttcaaaaaca gagagatgtg aaaatgtgaa
58081 acattcgaaa aagttatttt tatgcgtcag ttttttatta ataacgattt ttattggtgg
58141 aggtggattt atgaataaag acgagagtaa agaaacggaa atcaaaaaaa gttttaataa
58201 aacgttaagt atgtatccta ttaaaaactt agaagatcta tacgataaag aaggatatcg
58261 agacgaagaa tttaataaag gcgataaagg aatgtggata gttcactctg aaatgaatat
58321 acaaaaaaag gggaaagcat tggaatcaag agggatggtt ttatatatgg atagaaatac
58381 aagaaccact cacggctatt ttattcttac caaaataaaa gatacaggta aagtcaaaac
58441 agatgacagt gaaaagaaat atccagtaaa gctagaaaat aataaaataa ttccaacaaa
58501 acaaattaaa gatgaaaagt taaaaaaaga aatagaaaac tttaagtttt tctcacaata
58561 tggcaatttt aaagatttaa aagattataa agacggtgag gtatcttata atcctaatgc
58621 gcctaattat tcagctaaat atcaactcag taataatgat tataatgtaa aacaaataag
58681 aaaaagatat gatattccaa ccgaacaagc ccctaagttg ttattaaaag gtacaggtga
58741 tttaaaaggt tcatccacag gttctaaaaa tattgaattt acttttgtag agaaaaaagg
58801 agaaaatatt tattttactg acagcgttga atacacgcca agtgggtaag aaaagctcaa
58861 aaaaattatc atccatactc ataaaaaata gaactgatga gattattacg ctcatgagtt
58921 ctattttat gtcaacaact ttatttccaa catcacccta aatattgcat ttgatatccg
58981 attctcggag gaggataatt agttccttat ccataccatc aaaattttca ctataattta
59041 acttctacga cgccatgtgc acacgtaagg atgccatttc gcacttattg acgtatacga
59101 ttcttttttt aaaatggaat gaattttttt ctgattaaca taccgttagt cataatcacc
59161 aacttattct taaagttagt tcttatatag gaaataattt ctaatatatc ttttctaata
59221 aagggttctc cgccagttag aataagcgta ttcgggttga cgtcaataat cttatcaatt
59281 gcatatttaa catcatttgt acttaaaata tcttgtgata aaggagaagc agatagagca
59341 caatgactac aagaaagatt gcatttattt gtaagagtaa aagacacact taaattattt
59401 tctttataag ttttttcttc atctacgtag tatcgtattt caagcaattc gttaaaaagc
59461 cgctttaagt tatctatcac attacttaca tatttatact tcat
//
LOCUS Staphylococcus 71907 bp DNA linear UNK
DEFINITION Staphylococcus Staphylococcus epidermidis strain C99
ACCESSION Staphylococcus
KEYWORDS .
SOURCE Staphylococcus Staphylococcus epidermidis
ORGANISM Staphylococcus Staphylococcus epidermidis
Bacteria; Bacillati; Bacillota; Bacilli; Bacillales;
Staphylococcaceae; Staphylococcus; Staphylococcus epidermidis.
FEATURES Location/Qualifiers
source 1..71907
/genome_id="176279.70"
/project="hwwang_176279"
/organism="Staphylococcus Staphylococcus epidermidis"
/genome_md5=""
/db_xref="taxon:176279"

/mol_type="genomic DNA"
CDS 1..480
/product="23S rRNA
(pseudouridine(1915)-N(3))-methyltransferase (EC
2.1.1.177)"
/db_xref="SEED:fig|176279.70.peg.1"
/transl_table=11
/EC_number="2.1.1.177"
/translation="MKITILSVGKLKEKYWKQAIAEYEKRLGPYTKIELIEVPDEKAP

ENMSDKEIEQVKEKEGQRLLNKIKPQSTVITLEIKGKMLSSEGLAKELQTRMTQGQSD

FTFVIGGSNGLHQDVLQRSNYALSFSNMTFPHQMMRVILIEQIYRAFKIMRGEAYHK"
CDS 533..2692

/translation="MLDKLKNVSHKSFDKYTNNDQLTKVNIFFGRNGSGKSSLSEWLK

LNNEESVIFNTRYLKNNIEQVEEIDGVSLVIGEKSINHSDQIKNLSDAINNLENFINR

KDNEISTSKKRIFDKMNSTLNEARKRFEITSNMVKQKRNADNDPINAFYSWKKDIRDV
NEEITIESLDELEKRIESNQILLNNIKIPILSFEYDEFNNMLKILMKEVIKPKSSVSY

KVSEWIKDGLKIHNMQSKEETCEFCGNSFDVELVKESIDARIENEFNYLMENLNNYKN

KVENSLSNMENLNIDVDSVQFFKQKEELFKLLQLLEDKEKKTYEPLNYPIDNWESIIK

VDKIIINYIDKLNEDIQNDKKIRDSITDFVKSWIGKKLKDDEEIQMEIYNINTNSEQI

KKAEECKKANNDWITNLKTDSSNLKQFSEIVNKRFEILDINIELEPNIETKSYRVLHR
VTGEKLNLVDLSEGEKRLIGFLHFYYNLFMIADSEMKENIEHIIIDDPITSLDTENRY
HLTKIINQFIELSKSENKQIFVFTHSSLDFHNFAFKGKNNTKETSHWYIEKIDGISKV

RKLSKEELNNYSDYYSSVFNEVMSFALKGKRKIAYINDYIKYANKMRFILESHARSHY
KIEYATATSVPELSKYYDIEKERMKDLEKSLDIINSLSHGISFIDNHNEELSVIEVHE
AIRKVIGIIYKKDKQHVSAMCSGVLTKENKEELQKWI"
/transl_table=11
/product="hypothetical protein"
/db_xref="SEED:fig|176279.70.peg.2"
CDS complement(3079..3246)
/product="hypothetical protein"
/db_xref="SEED:fig|176279.70.peg.3"
/transl_table=11
/translation="MKFKSEKLIIANQTKVINYRIQVLLKPQTNIETQTQVKNNTLSE
TDEQSNADLVQ"

CDS 3886..4098
/EC_number="1.1.1.95"

/translation="MRSSSPVQRGSYRIPKDKHQYKLKITDDNLQVDYPRQFLKGAHA
PSVKAQHVIHWGATFWVSSLWLAWFY"
/db_xref="SEED:fig|176279.70.peg.4"
/db_xref="GO:0004617"
/product="D-3-phosphoglycerate dehydrogenase (EC
1.1.1.95)"
/transl_table=11
CDS 4181..5605
/translation="MEMEFNENNIDLETIIRDEVNKYLSRDIGDLPATQQAPLELREK

YEKMEVPNKGRDIYEVLKDLNNEVLNYLYRPNHPRSFSFIPGPASRLSWLGDILTTAN

NIHASNFANATLPINIERNLINYLVGKIGYEIKPAGGVFVSGGSMANLTAIVAARDAQ

VEMEDIKKATVYLTSQTHHSVGKALHVAGFLKNNIRRIEYNDDFTMNTDSLKAAIDKD

IEEDYKPAMVIATAGTTNTGSVDDFTTLGDICDHYNLWLHVDGAYGLSHILSDKAQHL

FKGIERSDSASWDAHKLLFQTYSCAMVIVKEKNHLLQSFGEDAEYLDDIASDDDVIDP
EMLGIELTRPARALKLWITLQVIGEDEIIERIKYGEGLAEYAEEYVSSFDNWRIISHA

NLSIVNFRYENSDLTEAQNNQLNSMAAQKIADSGYAIAYTTVLNNQRVIRLCTTNPLT
THDDIRETINRFDQYIGDYDMKDA"
/db_xref="SEED:fig|176279.70.peg.5"
/product="Decarboxylase, pyridoxal-dependent"
/transl_table=11
CDS complement(5673..5855)
/translation="MKKMQDVAINADHKVFFLMIIFNVIMLVSSIIQMILGKGRRITP
AKPAKDAKLHLDNQSS"
/product="Multidrug resistance protein"
/db_xref="SEED:fig|176279.70.peg.6"
/transl_table=11
CDS 6327..6641

/translation="MKRVSYSVETKYKAVEMKAAGFSTKEIMKELNIRNRTQVKTWWR

WYRNGESYRFSQQVGKQYTYGKGLKELSEVEQLKLENKRKDIELDILKKYKALERKWY
QQ"
/product="Transposase"
/db_xref="SEED:fig|176279.70.peg.7"
/transl_table=11

CDS 6626..7468
/db_xref="SEED:fig|176279.70.peg.8"
/product="Mobile element protein"
/transl_table=11
/translation="MVPTVVIDLVDQLKVKYSIKLILEVLNIPKSTYYRWKNKTHKND

TVTQKVIELCKANHYTYGYRKITALINQCYTSPINHKRVQRMMQKHHLNCRVRPKKTT

RIGKPYYKTDNLLQRQFKASCPMEVLTTDITYLPFGHSMLYLSSIMDIYNGEIVAYKI

DDKQDQSLVNDTLNQIDIPESCILHSDQSSVYTSYAYYQLCEEKGIIRSMSRKGTPAD

NAPIESFHSSLKSETFYINNELNRSNHIVIDIVEKYIKNYNNNRIQQKLGYLSPVKYR
KLIA"
CDS complement(7568..8497)
/transl_table=11
/product="Carbamate kinase (EC 2.7.2.2)"
/db_xref="SEED:fig|176279.70.peg.9"
/db_xref="GO:0008804"
/translation="MSKIVVALGGNALGQSPKEQLDLLKSTSKSLVSLIDKGYEIVIS

HGNGPQVGSINLGLNYAAEHKQGPPFPFPECGAMSQAYIGYQMQESLQNELHSMGIDK

QVVTLVTQVQVASDDSAFNNPTKPIGLFYTKEQADKFTKEKGYTFVEDSGRGYRRVVP
SPQPISIVELDSIETLITHGTLVIAAGGGGIPVIKENEVYTGVDAVIDKDKTSALLAA

HLQSDQLIILTAVDHVYINYGKENQRGLDEVSVDEMKKHISDGQFAKGSMLPKVEAAL
QFLEKNTKGSVLITSLAGLGDALDGKIGTLIKN"
/EC_number="2.7.2.2"
CDS complement(8517..9515)
/product="Ornithine carbamoyltransferase (EC 2.1.3.3)"
/db_xref="SEED:fig|176279.70.peg.10"
/db_xref="GO:0004585"
/transl_table=11
/EC_number="2.1.3.3"

/translation="MKNLRNRNFLTLLDFTQKEMEFLLNLSEDLKRAKYAGIEQQKMK

GKNIALLFEKDSTRTRCAFETAAYDQGAHVTYLGPTGSQMGKKESTKDTARVLGGMYD

GIEYRGFSQRVVEDLAKYSGVPVWNGLTDEDHPTQVLADFLTAKEVLKKPYNEINFTY

VGDGRNNVANALMQGAAIMGMTFHLVCPKELNPTDELLNRCNNIAEKNGGEILVTDDI

DEGVKGSDVIYTDVWVSMGEPDEVWEKRIKLLEPYRVTKELMKKTGNPNTIFEHCLPS

FHDTETKIGKQIQEKYGLNEMEVTNEVFESEQSVVFQEAENRAHTIKAVMVATLGE"
CDS complement(9553..10242)
/transl_table=11
/product="hypothetical protein"
/db_xref="SEED:fig|176279.70.peg.11"
/translation="MYEENIYIKNSEYEFDNNLKQLASYLNIPVSIVRPYKEDLTLYQ

YKKGQVIYHSTDQIKFVYFLVNGCILHESSNITGDNYLRLSKDENIFPMNFIFNETPA
PYEICTALTDCKILTLPKDLLEYLCRKHNEIFESLFKKLNETIQFQVEYIMALRANSA
KERIERILQILCLSIGDDNGEFYELKQIMTVQLISNLSGLNRKTTGEIIRELKIENII
YQDKRNWIIKK"
CDS complement(10284..11705)
/db_xref="SEED:fig|176279.70.peg.12"
/product="Arginine/ornithine antiporter ArcD"
/transl_table=11
/translation="MDENKLGKTSLIGLVIGSMIGGGAFNIISDMGGQAGGLAIIIGW

IITAIGMISLAFVFQNLTNERPDLDGGIYSYAQTGFGDFIGFSSAWGYWFAAFLGNVA
YATLLMSAVGNFFPIFKGGNTLPSIIIASILLWGVHFLILRGVETAAFINSIVTVAKL

IPIFLVIICMIVVFNFSTFKSGFYGMTSGSVGVFSWGDTMAQVKSTMLVTVWVFTGIE

GAVVFSGRAKSKKDVGTATVIGLISVLVIYFLMTVLAQGVIQQNQISKLANPSMAQVL

EHIVGHWGSVLVNIGLIISVLGAWLGWTLLAGELPFIVAKDGLFPKWFAKENKNKAPV
NALIITNILVQLFLISMLFTDSAYQFAFSLASSAILIPYTLSAFYQVKYTIQNKSKAN
LKQWIIGIIASIYTIWLVYAAGLDYLLLTMLLYIPGLLVYSYVQRDNNKHLTKLDYTL
FIFIIVLAIIGIVRLITGNISVF"
CDS complement(11791..13026)
/transl_table=11
/product="Arginine deiminase (EC 3.5.3.6)"
/db_xref="SEED:fig|176279.70.peg.13"
/db_xref="GO:0016990"
/translation="MVQGPIQVNSEIGKLKTVLLKRPGKELENLVPDHLSGLLFDDIP
YLKVAQEEHDKFAQTLRDEGIEVVYLEKLAAESITEPEVRENFINDILTESKKTILGH

ETEIKEFFSKLSDQELVNKIMAGVRKEEIQLETTHLVEYMDDRYPFYLDPMPNLYFTR

DPQASIGRGMTINRMYWRARRRESIFMTYILKHHPRFKDKDVPVWLDRNSPFNIEGGD
ELVLSKDVLAIGISERTSAQAIEKLARNIFKDANTSFKKIVAIEIPNTRTFMHLDTVL

TMIDYDKFTVHAAIFKEENNMNIFTIEQNDGKDDIKITRSSKLRETLAEVLEVEKVDF

IPTGNGDVIDGAREQWNDGSNTLCIRPGVVVTYDRNYVSNQLLRDKGIKVIEITGSEL
VRGRGGPRCMSQPLFREDI"
/EC_number="3.5.3.6"
CDS complement(13295..13741)
/translation="MKKEKRLNLILTVIQQNQFNKKQQIVDYMAKHFGVYYSLTTISR
DLQELEIYKIPVENKKYIYKKINQTNQLSAKKQLEIFSDEIIEFITLNNYVLIKTSPG
FAQSISYYIDQLQMKEILGIIGGNDTLMILTSSNEIAEFVCYQLFP"
/transl_table=11
/product="Arginine pathway regulatory protein ArgR,
repressor of arg regulon"
/db_xref="SEED:fig|176279.70.peg.14"
CDS 14541..14813
/db_xref="SEED:fig|176279.70.peg.15"
/product="Type I restriction-modification system,
restriction subunit R (EC 3.1.21.3)"
/transl_table=11
/EC_number="3.1.21.3"
/translation="MDYVISLGEDERQRFIKTVTELGKAFAFCDTEPKVQKLNDEIAF
FKAVKAGLVKLLQPSKEGKIYKTLAEVEAEINHLVSQSVVTEEVKN"
CDS 14939..15094
/transl_table=11
/db_xref="SEED:fig|176279.70.peg.16"
/product="type I restriction-modification system
endonuclease"
/translation="MTVDCDKKSSSRVNKRRSVKRLLKKYGYPSDLETIAINQVVEQA
EMMASNQ"
CDS complement(15364..15795)
/transl_table=11
/product="Universal stress protein family"
/db_xref="SEED:fig|176279.70.peg.17"
/translation="MFKNILLPYDFENDFSAIPDYLEKVIDEDSVVVIYHVVTENDLA
ISVKYYNKHKEDIIREKEKKLTPFLRELEKRDIQYKIDVDFGHIKDTILEKITSGDIN
NGEFDLVIMSNHRVDLNIKHVLGDVTHKIAKRSSVPVLIVK"
CDS complement(16677..17003)
/product="Replication protein"
/db_xref="SEED:fig|176279.70.peg.18"
/transl_table=11
/translation="MLKLYYKFNFATEPDNDYLINQKVFDAFYKALKGKQVLVYFGLF

KDARKKLKNGELDYLKEVDPTEYIYQIFYTWNKKEYLASEIFDLTESEEQEINHQMID
EIEEEK"
CDS 17019..17693
/transl_table=11

/product="Mobile element protein"
/db_xref="SEED:fig|176279.70.peg.19"
/translation="MNYFRYKQFNKDVITVDVSYYLRYALSYRDISEILRERGVNVHH

LTVYRWVQEYAPILYQIWKKKYQKAYYKWHIDETYIKIKGKWSYLYRAIDAEGHTLDI

WLRKQRDNHSAYAFIKRLIKQFGKPQKVVTDQAPSTKVAMAKVIKVFKLKPDCHCTSK
YLNNLIEQDHRHIKVRKTRYQSINTAKNTLKGIECIYALYKKNRRSLQIYGFSSCHEI
SIMLAS"
CDS 18149..18454
/translation="MKGNIQCLRLIKGSLLDFVLETVDVFVNGSKNDCELVNFNYKEG
KVVSRYSTKFESNPKLRKEQLISTVKVVRYVIFDFEKILWGIGKVLLKFIILRQCLL"
/transl_table=11
/db_xref="SEED:fig|176279.70.peg.20"
/product="hypothetical protein"
CDS 18652..19515
/db_xref="SEED:fig|176279.70.peg.21"
/product="hypothetical protein"
/transl_table=11
/translation="MKHINHFSESTLAYLEKDHEHAFYVYCLIDPRNDECFYVGKGKG

NRIFKHKQDAQRQLLYEDIFREENKDNLKFNRINEICSNDLNVLGYIISYGLTESEAF

SAENVLINFLNLTNKTTLTNMINGHGSKAYLVEDLENEFGYDSINLENINTNELILAV

KIRDAFLLDKDESKEYPINESKRDRNNLKSRTLGSWIIGKDKIHKIKYIIGINTGANN

AVVSAYEVSFEQAESIETNNGRMRYAFIGLSERDATLKKLNLYKKALPDLRFGSGSAT
AYINNGTMKVD"
CDS 19623..21098
/translation="MPSDDIVKKLYSKEDIRLELGLTPHKFNKKMETIAKLFKIDMKI

FHNYKGQDKNNQYTFNGVAKELIKVLLKSVDYYPVDINSKKFKQNGKSKKEMIENIDN

SSYMKYIYQLMKSINEIQYKRLIADIHMKDVYQNTKAWLNNGESINKKEQELYQYMTI

LPLHKRVELQNEVLKSIDETIFQFLAKEHRNNQIEENNELEAYTKAIKEGRNPKNDYE

LNHLLYKKNQLPLDSLIEDIWDYEETEYTELDWLIADMLKRSQRADSNFIESLEKKNK

LRKNIHKDSIKSISKLIDHELVKNNRKWDVAEFYKNQQFWNNSTLRRSSQNFGILYYK

KNRLHTMNQSNIIKKIKIIEANIESANKQPSYLDFFLQAKFDLERLESELMKYSINPK

YFNKINSDYVRYAIEDVHNAIIKIDRYISNDKVKLNYSNVQMVVRNEITEQGSYFVTQ
ALNTLSKVEESGYSFDNFMVGPFVEDFKKAIKNRRFGSKKD"
/product="hypothetical protein"
/db_xref="SEED:fig|176279.70.peg.22"
/transl_table=11
CDS 21325..22425

/translation="METRDKLMSLTQSDKTQQWLMDKSSNQDDIQQLQQQFSQQLDQQ

YNALLADEKAKLDQYVEVHQGLESLKEEIESEPITLNIDKLPDIKATMLERAKNDEHS

DKIEKLFDRLEQALNGTNRLYTQLSLIGTRTHRITTKNFNLQGLPKAVQHTILPSKFK

KVYTVDFKSFEPSVAAYMTQDSKLIDLLNQKDGLYDALLSELGLSDELRVFVKRAFIG

SFLFGGNFKNPKFKLNQYVSEVQWLDAVSQFTKVIELKKHVEKSNSMPMPYSIEHDMS

AFQGSSIMAIYVQTVASYIFKHILLKVYKAQCDQKTFKIIVPIHDAIMIECEDEEIAQ
NVGQLMKDTANQLFNGEFAHVTVEEIGGVDHE"
/transl_table=11
/product="hypothetical protein"
/db_xref="SEED:fig|176279.70.peg.23"
CDS 22418..22789
/transl_table=11
/db_xref="SEED:fig|176279.70.peg.24"
/product="hypothetical protein"
/translation="MNNDRGQSLHIPSSTPIKENNIYVATLHSVIQTDFSGEIKHQFT

YEIEVNNQIVYANRNIPTKPSANQLSIHDWLKRHSNYSVGHDTYEPYIDRKHLIHIGQ
YSGNYYVQDVASLDAFGGVLS"
CDS 22786..24429
/translation="MNHILQMLSKLLSVAKEAIDRQGLIAILTIPVNNNDEIEETAQG

ETVYNELIDQLRLNIPKDTDYQPNIYSYFGIKKNPNDTVLMEMMIKVFHIKRFNSELF

IFKVNGWQKINGDELQGLISKMIQVLLVDYKPSLSTLKNVVDGLQKSTDVEELVENER

YIGCGENMFDLNTFQVVKNSIDIFPKTRLNLSLSTNDVITDKIPPYFKQYMLQLANYD

DDLQYFLFQHTAVLLTADTKYRRGLILYGGAKNGKSVYIELVKSFFYSKDIVSKPLNE

LEGRFDKESLIDKSLMASHEIGQSRIQEKIVNDFKKLLSVESMHVDRKGKTQVEVILD
LKLIFSTNAILNFPPEHAKALERRINIIPCEYYVEKADTSLIDKLQSEKKEIFLYLMY

VYQQIVKADIEYLENSRVTEITHDWLNFGYEFVSSRSVSNANQKACINLLRKLIEIKS

GSRIKVSELNKVINEEIKVSSQVIKQLIQANFDTQTKLYNGYDYWIDLGWKEANKKEI
HDISEKDNIISLDKNENITDDEALDEENLDFDWEDFDDE"
/transl_table=11
/product="putative primase"
/db_xref="SEED:fig|176279.70.peg.25"
CDS 24657..26333
/product="Cassette chromosome recombinase B"
/db_xref="SEED:fig|176279.70.peg.26"
/transl_table=11
/translation="MKGKIALYSRVSTSEQSEHGYSVKEQEQVLIKEVVKNFPGYDYE

TYTDSGISGKNIEGRPAMKRLLQDVKDNKIEMVLSWKLNRISRSMRDVFNIIHEFKEH

DVGYKSISENIDTSNASGEVLVTMFGLIGSIERQTLISNVKLSMNAKARSGEAITGRV

LGYKLSLNPLTQKNDLVIDENEANIVREIFDLYLNHNKGLKAITTVLNQKGYRTINQK

PFSVYGVKYILNNPVYKGYVRFNNHQNWAVQRRSGKSDKNDVILVKGKHEAIISEEVF

DKVHEKLASKSFKPGRPIGGDFYLRGLIKCPECGNNMVCRRTYYKTKKSKERTIKRYY

ICSLFNRSGSSACHSNSINAEVVERVINVHLNRILSQPDIIKQIASNVIEELKQKHSN

QTEIKYDIDSLEKQKAKLKTQQERLLELFLDDQMDSEMLKAKQSQMNQQLEVLDQQIK

EAQQANQSQDEIPNFDKLKGRLILMITRFSVYLRKATPEAKNQLMKMLIDSIEITTDK
QVKLVRYKIDESLIPQSLKKDWGSFFMPKFQFEIYGQNDYFIDQITTFTT"
CDS 26436..26777
/translation="MMTLEQQLKHYITNLFNLPKDEVWHCESIEEIADDILPNQYVRL

GPLSNKTLQTNTYYSDTLHESNIYPFILYYQKQLIAIGYIDENHDMDFLYLHNTIMPL
LDQRYLLTGGQ"
/transl_table=11
/product="FIG01108299: hypothetical protein"
/db_xref="SEED:fig|176279.70.peg.27"
CDS 26779..26880
/translation="MHKYIKITQLVITILSEIIIWMKESERKEVSYE"
/transl_table=11
/product="hypothetical protein"
/db_xref="SEED:fig|176279.70.peg.28"
CDS 26873..27184

/translation="MNRYITRGIANNLPNILQHQLWQLVSEREQEQTKDNTSVDYFHI

FQFNKHRNQLYIKHKQERPEYAKIHKANYSKAININKVYIIREDDVDLSYYVMLLPEE
Y"
/transl_table=11
/db_xref="SEED:fig|176279.70.peg.29"
/product="FIG01107894: hypothetical protein"
CDS 27200..27706
/transl_table=11
/product="hypothetical protein"
/db_xref="SEED:fig|176279.70.peg.30"
/translation="METIKSTLKTEAIFSDDKQHRYLLKKTWNSEKQSITIITMYPHY

DGILNIDLTTQLIMNKVSEMDAFGSINFVNLYSNITTPINLKHLENAYDKHTDIQIMK

AVKESDEVILAWGAYAKKPGVEARVNEVLEMLKPHKKKVKRLMNPETNEIMHPLNSKA
RQKWILKV"
CDS complement(27855..28529)
/db_xref="SEED:fig|176279.70.peg.31"
/product="Transposase for insertion sequence-like element
IS431mec"
/transl_table=11
/translation="MNYFRYKQFNKDVITVAVGYYLRYTLSYRDISEILRERGVNVHH

STVYRWVQEYAPILYQIWKKKHKKAYYKWRIDETYIKIKGKWSYLYRAIDAEGHTLDI

WLRKQRDNHSAYAFIKRLIKQFGKPQKVITDQAPSTKVAMAKVIKAFKLKPDCHCTSK
YLNNLIEQDHRHIKVRKTRYQSINTAKNTLKGIECIYALYKKNRRSLQICGFSPCHEI
SIMLAS"
CDS 28787..28954
/translation="MLESREQLSVEEYETFFNRFDNQEFDFERELTQDPYSKVYLYSI
EDHIRTYKIEK"
/EC_number="2.3.3.10"
/transl_table=11
/db_xref="SEED:fig|176279.70.peg.32"
/product="Hydroxymethylglutaryl-CoA synthase N-domain
homolog (EC 2.3.3.10)"
CDS 29791..30534
/db_xref="SEED:fig|176279.70.peg.33"
/db_xref="GO:0008889"
/product="Glycerophosphoryl diester phosphodiesterase (EC
3.1.4.46)"
/transl_table=11
/EC_number="3.1.4.46"

/translation="MLTVYGHRGLPSKAPENTIASFKAASEVEGINWLELDVAITKDE

QLIIIHDDYLERTTNMSGEITELNYDEIKDASAGSWFGEKFKDEHLPTFDDVVKIANE

YNMNLNVELKGITGPNGLALSKSMVKQVEEQLTNLNQNQEVLISSFNVVLVKLAEEIM

PQYNRAVIFHTTSFREDWRTLLDYCNAKIVNTEDAKLTKAKVKMVKEAGYELNVWTVN
KPARANQLANWGVDGIFTDNADKMVHLSQ"
CDS 30631..31059
/product="MaoC domain protein"
/db_xref="SEED:fig|176279.70.peg.34"
/transl_table=11
/translation="MKYDDFIVGETFKTKSLHITEEEIIQFATTFDPQYMHIDKEKAE

QSRFKGIIASGMHTLSISFKLWVEEGKYGEEVVAGTQMNNVKFIKPVYPGNTLYVIAE
ITNKKSIKKENGLVTVSLSTYNENEEIVFKGEVTALINNS"
CDS complement(31105..33111)
/translation="MKKIKIVPLILIVVVVGFGIYFYASKDKEINNTIDAIEDKNFKQ

VYKDSSYISKSDNGEVEMTERPIKIYNSLGVKDINIQDRKIKKVSKNKKRVDAQYKIK

TNYGNIDRNVQFNFVKEDGMWKLDWDHSVIIPGMQKDQSIHIENLKSERGKILDRNNV

ELANTGTAYEIGIVPKNVSKKDYKAIAKELSISEDYIKQQMDQNWVQDDTFVPLKTVK

KMDEYLRDFAKKFHLTTNETESRNYPLGKATSHLLGYVGPINSEELKQKEYKGYKDDA

VIGKKGLEKLYDKKLQHEDGYRVTIVDDNSNTIAHTLIEKKKKDGKDIQLTIDAKVQK

SIYNNMKNDYGSGTAIHPQTGELLALVSTPSYDVYPFMYGMSNEEYNKLTEDKKEPLL

NKFQITTSPGSTQKILTAMIGLNNKTLDDKTSYKIDGKGWQKDKSWGGYNVTRYEVVN

GNIDLKQAIESSDNIFFARVALELGSKKFEKGMKKLGVGEDIPSDYPFYNAQISNKNL

DNEILLADSGYGQGEILINPVQILSIYSALENNGNINAPHLLKDTKNKVWKKNIISKE

NINLLTDGMQQVVNKTHKEDIYRSYANLIGKSGTAELKMKQGETGRQIGWFISYDKDN
PNMMMAINVKDVQDKGMASYNAKISGKVYDELYENGNKKYDIDE"
/product="Penicillin-binding protein PBP2a, methicillin
resistance determinant MecA, transpeptidase"
/db_xref="SEED:fig|176279.70.peg.35"
/db_xref="GO:0008658"
/db_xref="GO:0046677"

/transl_table=11
CDS 33359..33502
/db_xref="SEED:fig|176279.70.peg.36"
/product="Mobile element protein"
/transl_table=11

/translation="MNYFRYKQFNKDVITVAVGYYLRYALSYRDMSEILRERGVNVHH
LTV"
CDS 33782..34033
/db_xref="SEED:fig|176279.70.peg.37"
/product="Mobile element protein"
/transl_table=11
/translation="MAKVIKAFKLKPDCHCTSKYLNNLIEQDHRHIKVRKTRYQSINT
AKNTLKGIECIYGLYKKNRRSLQICGFSPCHEISIMLTS"
CDS complement(34093..34521)
/translation="MKFQVSPYFLITQNAEGLKKLYEELFGAKTLYIQRIKDRPIDKQ

LNIDENDLNKIDQCVLQFGDIKIMIADDTEGLPITQGNNISLCLTFDQVEETKHVYDQ
LIEKNSEVLKTFSPEFYTEGYGYVRDPYGISLHLFTKRKN"
/db_xref="SEED:fig|176279.70.peg.38"
/product="PhnB protein; putative DNA binding
3-demethylubiquinone-9 3-methyltransferase domain
protein"
/transl_table=11
CDS 34602..35531
/product="Transcriptional regulator, YafY family"
/db_xref="SEED:fig|176279.70.peg.39"
/transl_table=11
/translation="MKKSVRLYNMIEYCNENRNFKLNDLMSEFNISRSTALRDIKEIE

ALGVPLYSNPGKNGGYTIIGNRDQTKIAISDEELKALVFTLSSISNVSNLPFQTEYQE
ILKKLYNNSNKKELINQYNDLFQYFNEDKYQFKSYKLFNEIIRLIIENKSFEICYSQN

YIKEQYKGIGIMYKNHQWYFVVANIESKLVNLLNISKIKELYEMGETQECNDITMQNF

QQFMVKNETAIDILIRSNVMGLNILKGYLWSDYMIENIDEETYLFKSKVNAKDIDFIA
KLIVTGGVNVKVETPNSLKNAVKVELTKIINMY"
CDS 35693..37705
/product="Similarity"
/db_xref="SEED:fig|176279.70.peg.40"
/transl_table=11
/translation="MNKNITALKSTENTTYMQYTVHIDDQIINNIKECTKKFEILPME
DKIPLSPLLQPEYAGEVQVFISTYEQFMINFGKVILDSQGIKIQFESESLSSIQRGIQ

EHCYLNKRTNDIDVTKEWYLCKFSNRTLEEDKNQLYNALKQLMNDSKNKKQAFQEVFK
VHIDIYLYNKEKSEYKYQASSYFYLVKENPKIIYKKRHLQEKQGIKGTTFTNKLKLLN

KMYGVDVAKYQPFYNSNNPEYERGQFGERYISQRSNYEFNRLQYQIIYILSKILDKHP

LPKSDNNYKHIPTIEKAILRDDSHNFYEYFEDIMKEIMNMDNSPLQEKLLTDFTYQSQ

CRWYSENKKLKLQLERFMHKVLESNYYEGNKLLRMMSNAIEETINEAKEDKVHFNYFK

DYFLTDGGVKNWEQISEKITEFNGKVINDIQHEYNKIQSKNTNQNQKLNFVYLYHCFE

FSNNIVKAKDNESKNYILFFIDKYKIKTPFLDQLLINCLNQEIDQESINYNMQTMFEK

ERYDRSSTIEKLVATRKFKYEKDDSDLFKQLFNDVENSIDRLGIYLLNNGINSNDENA

RYYRSFLKELSRIKSKLTPFSLEISKSSGREQHYPDDAVDDKDERRKNKEEAYHAFDD
KSDIDYKLKNKISDYIDNLLSVKIRNNLYDISPNTKSNNYNEPTQK"
CDS 37875..38984
/transl_table=11
/product="hypothetical protein"
/db_xref="SEED:fig|176279.70.peg.41"

/translation="MYQANIRDLITKLPQSNKTEHFLMNKFSNQDKVQQLQRQISQQL

DQQYNELLSNEKAKLDQYVEVHHNLEPLKKEIESESINLDTDKLPDIKATMLEKAKND

EHSDKIEHLFDRLDQSLNGTNRLYTQLSLIGTRTHRITTKRFNVQGLPKLVQQTILPS

QFKKVYTIDFKSFEPSVAAYMTQDEQLIDYLNHEEGLYDALLRDLSLSKEKRVSVKRA

FIGSFLFGGRYSSSKFKINQEVSEINWLQVMSKFKKVIEFKEQVEKYKTMPTPYGIEH

DMSAFQGSSIMAIYVQTVASYIFKYILLEVYKAQCEQKMFKIIVPIHDAIMIECNDKE
IAQSVAQLMKDTANQLFNGEFAHVTVEALRGIDNE"
CDS 38977..39348
/translation="MNNDRGKSLQIPQSTSLKEGSIYVATLHSVTETDYSGDIKHQFT

YEIEIDQQIFYVNRSVVVNVTHQQLSINDWLERHSNYSASHENYEPYIDQKHLILLGQ
YNGNYYVQDVASLDAFGGVLS"
/transl_table=11
/product="hypothetical protein"
/db_xref="SEED:fig|176279.70.peg.42"
CDS 39345..40988
/transl_table=11

/db_xref="SEED:fig|176279.70.peg.43"
/product="putative primase"
/translation="MNHILQMLSKLLSVAKEAIDRQGLIAILTIPVNNDDEIEETAQG

ETVYNELIDQLRLNIPKDTDYRPNIYSYFGIKKNPNDTVLMEMMIKVFHIKRFNSELF

VFKANGWQKINGDELQGLISKMIQVLLVDYKPSLSTLKNVVDGLQKSTDVEELVENER

YIGCGENMFDLNTFQVVKNSIDIFPKTRLNLSLSTNDVITDKIPPYFKQYMLQLANYD

DDLQYFLFQHTAVLLTADTKYRRGLILYGGAKNGKSVYIELVKSFFYSKDIVSKPLNE

LEGRFDKESLIDKSLMASHEIGQSRIQEKIVNDFKKLLSVESMHVDRKGKTQVEVILD
LKLIFSTNAILNFPPEHAKALERRINIIPCEYYVEKADTSLIDKLQSEKKEIFLYLMY

VYQQIVKADIEYLENSRVTEITHDWLNFGYEFVSSRSVSNANQKACINLLRKLIEIKS

GSRIKVSELNKVINEEIKVSSQVIKQLIQANFDTQTKLYNGYDYWIDLGWKEANKKEI
HDISEKDNIISLDKNENITDDEALDEENLDFDWEDFDDE"

CDS 41214..42893
/translation="MKGKIALYSRVSTSEQSEHGYSIHEQEQVLIKEVVKNFPGYDYE

TYTDSGISGKNIEGRPAMKRLLQDVKDNKIEMVLSWKLNRISRSMRDVFNIIHEFKEH

DVGYKSISENIDTSNASGEVLVTMFGLIGSIERQTLISNVKLSMNAKARSGEAITGRV

LGYKLSLNPLTQKNDLVIDENEAHIVREIFDLYLNHNKGLKAITTILNQKGYRTINQK

PFSVFGVKYILNNPVYKGYVRFNNHQNWAVQRRSGKSDKNDVILVKGKHEAIISEDVF

DQVHEKLASKSFKPGRPIGGDFYLRGLIKCPECGNNMVCRRTYYKTKKSKERTIKRYY

ICSLFNRSGSSACHSNAINAEVVERVINVHLNRILSQPNVIKQIASSVIEELKQKHSK

QTEIKYDIDSLEKQKAKVKTQQERLLELFLDDEMDSEMLKAKQSEMNQQLEVLDQQIK

EAKQANQSQDDIPNFDKLKAQLILMITRFSVYLRKATPEAKNQLMKMLIDSIEITTDK
QVKLVRYKIDESLIPQSLKKDWGSFFMPKFNFVINVTNENRIENLSLLPLF"
/transl_table=11
/product="Cassette chromosome recombinase B"
/db_xref="SEED:fig|176279.70.peg.44"

CDS 42982..43320
/product="hypothetical protein"
/db_xref="SEED:fig|176279.70.peg.45"

/transl_table=11
/translation="MTLSKQLKTYITERFKLNYQETWACETVDAVAEDVLPEKYIKNS
PLEHKILNTFTYYNDELHEISIYPFLCYLDKELVAIGYLDNFDLDFIFLNDTHQVIID
ERYLLQKGGE"
CDS 43323..43412
/product="hypothetical protein"
/db_xref="SEED:fig|176279.70.peg.46"
/transl_table=11
/translation="MNWIKVAQLSVTVINEVIEIMKEKQNGGE"
CDS 43414..43725
/transl_table=11
/product="hypothetical protein"
/db_xref="SEED:fig|176279.70.peg.47"
/translation="MKINRYITRGVSEHLSLDLQILLWNMVKDRDNQLHTDYLHIFKL

QEDDNILSITHEQEQPAYKLEYHYTNYVKNQNALPKKVYVIREDDVDAFYYVMLLPEE
Y"
CDS 43737..44243

/translation="MMNTIKNTIHTEAIFSDDEQHRYLLKKTWDEKKPACTVITMYPH

LDGVLSLDLTTVLILNQLANSEQYGAVYLVNLFSNIKTPENLKHIKEPYDKHTDIHLM

KAISESDTVILAYGAYAKRPVVVERVEQVMEMLKPHKKKVKKLINPATNEIMHPLNPK
ARQKWTLK"
/product="hypothetical protein"
/db_xref="SEED:fig|176279.70.peg.48"
/transl_table=11
CDS 44257..44478
/transl_table=11
/product="hypothetical protein"
/db_xref="SEED:fig|176279.70.peg.49"
/translation="MNHETKQSDWRTVASCLASQNYISIVKGLVHHFTAIEDEEILDK
IYDDFMNDDSITTVLNNDLQTIINHYLSK"
CDS complement(44553..47672)
/transl_table=11
/product="Type I restriction-modification system,
restriction subunit R (EC 3.1.21.3)"
/db_xref="SEED:fig|176279.70.peg.50"

/translation="MNFQFNEDDLEQVALEWLQSLGYDYKKGNEISMTSLTPERKSDK
DVVLHERLEKALRKINSDIHPRFIEKALHELTLEKSPNLLENNLTFHENLINGIEIED
YDDEGQSIVEIVKIIDFEYPQNNDFLAVNQLTIVNGDYKKRPDIVLFINGLPIVVIEL

KNSTNETVGVEDGYHQLETYKMRIPQLFTFNEVLVTSDGINTKAGSLTANYDRFMTWR

SKDGQTESSPGLASLDVLIHGMLNPETLLDLIRYFVLFQDDGKGHISKILAAYHQYYA

VNKAVDRALLASSEQGDGKGGVIWHTQGSGKSLTMVFFSGKLIQMLNNPTLVVVTDRN

DLDNQLYSTFVKSKGRSGKGLLRQTPKQAETRKELKSLLSVESGGIVFTTMQKFEPEQ

NETTMAALTERKNVIVMADEAHRTQYGFNAKYDDKGEGIKYGYAKYLRDALPNATFVG

FTGTPVASTDKNTQMVFGNYIDVYDMTQAVADGSTVKIYYESRVIPLNLPQSLDLDEA

YNDITEDQEEDVKQRLKSKWSRIEALAGAKPRVEALAKDIIQHFETRQQAMKGKGMIV

TMSRRIAVDLYDEIIRLKPEWHSDDDDKGVIKVVMTGSSSDPTSFQRHIGPKKRRNLL

EKRMKDVDDELQLVIVRDMWLTGFDVPSMHTMYIDKPMKGHNLMQAIARVNRVFKDKP

GGLIVDYVGIAESLKEALKEYTESDQAQTAIDTDKAVELMLLKYDVIQDMLYNLDYSK

FNSDKKSERYYTISNTMDYVIGLGEDERQRFIKTVTELGKAFALCATEPTAQELNDEI

AFFKAVKAGLVKLLQPPKEGKTRKTPAEVEAEINQLVSQSVVTEDVIDVYQTLGLEQP

DLSILSDDFLKDVEGLKQKNVAVELLNRLLKGQVKSLMKTNATVSKRFSEMLGNSINK

YNSRSIETSKVIEELIQLAKDIKQEQQRGNELGLNSDEIAFYDALASHETAKEAMGDK

ELRAIAHELTKTVKENMGVDWSKRDSAKAKMRVAVRRLLKKYGYPPDLQKMAVEQVVE
QAELMANNQ"
/EC_number="3.1.21.3"
CDS complement(47656..48957)
/transl_table=11
/db_xref="SEED:fig|176279.70.peg.51"
/product="Type I restriction-modification system, specificity subunit S"
/translation="MEYNTIFFGDVAKIKNGYAFKSKEFQEKGIPVIKIKNIISPIVD

TKDSQKVSIKTYEKTKGFSLKKNDILISLTGSGVNQMSSAVGKVGRIEFDYPALQNQR
VGKFELKYSNSADLDFLFYYFLQPKITEYLVRNSTGSANQANINSKLIETVKIPNFSL

IKQKSISKFLNQITRKIETNQKMIANLKELSQTLFKHWFVDFEFPDEDGNPYKSSGGE

MIDSELGKIPSNWKIYKLKDIASHKKETFNPKKSEEVTVKHFSLPAYDNEEQAIEEEV

NKIKSNKWIINNNCVLFSKMNPDTKRIWLPVIDNKKLNVASSEFVVMESPNNKINSFI

YNICLNSQFIDYLKANTTGSTNSRQRVKPTIAVNYKLAIEDSIVKKYSEIITPYMEEM
KILRSEIGKLTQLRDTLLPKLMSGELEISDDIEVNSDELSI"
CDS complement(48947..50461)
/EC_number="2.1.1.72"

/translation="MATIGFEEKLWQAADKLRGSMDAAEYKNVALGLIFLKYVSDSFE

EKYEELKQDPYADEEDQDEYLAENIFWVPKEARWQYINDNAKKPEIGQIIDKAMIAIE

NENESLKGVLPKEYARPALDKEKLGDIIDLFTFKVGDTESRKQDVLGRVYEYFIAKFA

SAEGKNAGEFYTPSSIVKLLVEMIEPYKGRIYDPCCGSGGMFVQSERFVEKHQGRLDD

IAIYGQESNPTTWKLAKMNLAIRGIDNDLGERNADTFHNDLHKGLKADYILANPPFNA

SDWGQERLLDDYRWQFGIPPKGNANYAWIEHMISKLAPNGTAGFVLANGSMSTSGKDE

LEIRKNLIEQDLVECIVTLPGQLFYSTQIPVCLWFISNNKGQNGKKERENEILFIDAR

EIGHMVSRTLKEFSDEDIQKVAQTYHAWRGTNDKPYEDIAGFCKVANLEEVKNNEYIL
TPGRYVGLADVEEDEEPFEQKMERITSELSEQFAKSKELEDQIRKSLEGLGYGV"
/product="Type I restriction-modification system,
DNA-methyltransferase subunit M (EC 2.1.1.72)"
/db_xref="SEED:fig|176279.70.peg.52"
/transl_table=11
CDS 50675..50803
/product="hypothetical protein"
/db_xref="SEED:fig|176279.70.peg.53"
/transl_table=11
/translation="MKIHILTFVKIVVIVDIVAIVNTRLDKLGIFNSINKKFKCSQ"
CDS 50903..52966
/translation="MYISSVKIKNFRNFDNIDIDFHEGVNVLIGHNNSGKSNLLRALS

LIFDGSVRKQLSVEDFNNSLTKESLKKEAPKIVISVHITQSENERLMSDELITVSNWL

TKLEEPYEARIQYEFFLPKDEEKNYIDLVKNIDEKEEIWNLIRSQFIRLYVNKIWVGN

PEHQIPIDNDSLNKFDFQFLDAIRDVERDMFSGKNTLLKSVIDFFIDYDIKSDETITE

EEQKEKLEERRKEFSDNSSDLIETIHKRLDSGNQKILSYTNGIGASYDKSTPDFKGNL

TESEIYTVLQLIIKHETGMTLPITHNGLGYNNLIFMALLLSKMQADSDGNFLGSNAKV
FPILAIEEPEAHLHPTMQNEFIKFLKNNIREKKVKQIFITTHSTHISSSTNIDDIICL

YTDDSKTNVSYPGKVFDKNNQSKKYVQRFLDATKSNMLFAEKVIFVEGIAEQLLLHIF

ADYLNKPLEKNHVAVINVGGRYFNHFLSLFDSNNKYAINRKVSCITDLDPMRKEKNEN

GQKNNFKVCYPFEYEMDLDKFDYSTNNSLDKYLNNEHSNIRVFVQPNKYGKTFEYQLM

FDNPSLKLLLTDSISNSQELAELMDHYEENESLQKLIDTLSPSNENQRIIKSLKDTND

SWNEDNKKKALIASRYLNSIGKGENALELASVLKDNLELKGQSDYEDFVVPKYIEGAI
RWVCE"
/db_xref="SEED:fig|176279.70.peg.54"
/product="FIG131328: Predicted ATP-dependent endonuclease
of the OLD family"
/transl_table=11
CDS 52968..54716
/db_xref="SEED:fig|176279.70.peg.55"
/product="ATP-dependent DNA helicase UvrD/PcrA,
clostridial paralog 2"
/transl_table=11
/translation="MIINAQTKLNIEQDFKIQAGPGAGKTEFLVNHIKNVIQTSEKLE

RMKKIACITYTNTAAQTVLQRLGKSVSNRVDISTIHSFLYRNVVKPYCSFLPKEYGIC

VQKLKGHSDPIVINKYVNAWLKTEYFTKLKPPSNRNQLLKMPVLKKALQNWLLSMKLS

YENNDVQFICDNTKAQAIDKKSKKRMGINKSNLNILEKQLLNYKKIYWEKGIIDHDDV
LFFSYVLIRDYPFILTILCAKYPYFFIDEFQDTSQLQDFIIDKIRQKGCIVGVIGDKA
QAIYSFQGARVSLFEKFKVDLINSHTILENHRSSYQIVTFLNSIREDITQTVYSGIEN

DKVTILIGNRIRSYTKVVNICNKESLVTLSRDNITSNAMRIKLEGNNFDKKLLEKFQE
IDSNSKRRNTIISLIKAIELAKNTKYKDALKIVESIYKDENNPKKIALNSLINMINKY

DKYGNGTLMNFYNTLCKTLDIKLSGFRKGQVKDFYENTLYKNMVICTNIVEDTSNHIT

IHKAKGAEFENVFVIGNKDTLSLLLKPDLMNDEEHRIFYVAMSRAKKKLFLQFDYLDN
NEQKKIKERYDVSIIQ"
CDS 54926..55108
/translation="MKKMQDVAINADHKVFFLMIIFNVIMLVSSIIQMILGKGRRITP
AKPTKDAKLHLDNQSS"
/product="Multidrug resistance protein"
/db_xref="SEED:fig|176279.70.peg.56"

/transl_table=11
CDS complement(55176..56600)
/translation="MEMEFNENNIDLETIIRDEVNKYLSRDIGDLPATQQAPLELREK

YEKMEVPNKGRDIYEVLKDLNNEVLNYLYRPNHPRSFSFIPGPASRLSWLGDILTTAN

NIHASNFANATLPINIERNLINYLVGKIGYEIKPAGGVFVSGGSMANLTAIVAARDAQ

VEMEDIKKATVYLTSQTHHSVGKALHVAGFLKNNIRRIEYNDDFTMNTDSLKAAIDKD

IEEGYKPAMVIATAGTTNTGSVDDFTTLGDTCDHYNLWLHVDGAYGLSHILSDKAQHL

FKGIERSDSASWDAHKLLFQTYSCAMVIVKEKNHLLQSFGEDAEYLDDIASDDDVIDP
EMLGIELTRPARALKLWITLQVIGEDEIIERIKYGEGLAEYAEEYVSGLDNWRIISHA

NLSIVNFRYENSDLTEAQNNQLNSMAAQKIADSGYAIAYTTVLNNQRVIRLCTTNPLT
THDDIRETINRLDQYIGDYDMKDA"
/product="Decarboxylase, pyridoxal-dependent"
/db_xref="SEED:fig|176279.70.peg.57"
/transl_table=11
CDS complement(56683..56895)
/transl_table=11
/db_xref="SEED:fig|176279.70.peg.58"
/db_xref="GO:0004617"
/product="D-3-phosphoglycerate dehydrogenase (EC
1.1.1.95)"
/translation="MRSSSPVLRGSYRIPKDKHQYKLKITDDNLQVDYPRQFLKGAHT
PSVKAQHVIHWGDTFWVSSLWLAWFY"
/EC_number="1.1.1.95"
CDS 57545..57712
/transl_table=11
/product="hypothetical protein"
/db_xref="SEED:fig|176279.70.peg.59"
/translation="MKFKSEKLIIANQTKVINYRIQVLLKPQTNIETQTQVKNNTLSE
TDEQSNADLVQ"
CDS 58150..58848
/translation="MNKDESKETEIKKSFNKTLSMYPIKNLEDLYDKEGYRDEEFNKG

DKGMWIVHSEMNIQKKGKALESRGMVLYMDRNTRTTHGYFILTKIKDTGKVKTDDSEK

KYPVKLENNKIIPTKQIKDEKLKKEIENFKFFSQYGNFKDLKDYKDGEVSYNPNAPNY

SAKYQLSNNDYNVKQIRKRYDIPTEQAPKLLLKGTGDLKGSSTGSKNIEFTFVEKKGE
NIYFTDSVEYTPSG"

/product="hypothetical protein"
/db_xref="SEED:fig|176279.70.peg.60"
/transl_table=11
CDS complement(59108..59503)
/translation="MKYKYVSNVIDNLKRLFNELLEIRYYVDEEKTYKENNLSVSFTL
TNKCNLSCSHCALSASPLSQDILSTNDVKYAIDKIIDVNPNTLILTGGEPFIRKDILE
IISYIRTNFKNKLVIMTNGMLIRKKFIPF"
/db_xref="SEED:fig|176279.70.peg.61"
/product="hypothetical protein"
/transl_table=11
CDS complement(59516..59653)

/translation="MYIKNTNVHEVYFKNKVLLVNLNNGQWVRTSRKYFEVLNKLVES
N"
/transl_table=11
/db_xref="SEED:fig|176279.70.peg.62"
/product="hypothetical protein"
CDS 59814..60719
/product="ABC transporter, ATP-binding protein"
/db_xref="SEED:fig|176279.70.peg.63"
/transl_table=11
/translation="MNEIVIKGLSKSYDGKKDALNSLDLVIPNGMFGILGRNGSGKST

LMNIIATILQPSKGTVTINGIEIKNSQKIRQMIGYLPQDFDFYPNMKVSEVLYYLGFL

SKINQKDFDKYVDLILRKVNLIDYKNKKVKSLSGGMKKRLGIAQAILHDPKVIIVDEP
TAGLDPEERVRLRNLLSDLAENKIVIISTHIVSDIESTCNRIAILDKGSLVYKGDIPS

LIQQSDDHIYEINLSPSELESFRENDLFITKTQEIDNKLKVRFISETKVANAKKVKAD
FEDAYMYFLKKQQGGLNHASIYNGI"
CDS 60694..61815
/translation="MRLFIMEFKHLMFSKTFLFYCLLIIIFTVLNCQQVLKHPINSDV

YQPQNMTMYSTDKNLIMSNEIKELKNEVHQNNFKTYQFGFLKQKKLNDQQISKIKKLI

SKMEKTNNFHQFKNYSNSVSHVVGWGSQYSKNNISLFGIKNMSKSKYVKEKNIIKNKE
YYYGAYSRYFSDIMGVVLGILPFFLGAQMILQDKKSNAIKTIHSKSISSYQLISTRVF
TILVLAYLPVLLVSIYFVTCLSMIHQISMFSLFIFYKILILWTLPTLIFTIAMGFFVT
IILNSYLGVMIQIVIWFINLNIGSRAIEGCYGSLLIPRHNTLFNAVFFYNHFNQILAN
RFTYFGLGLLLILLSTYIFDLKRREVGFNAKIFTNRYQN"
/transl_table=11
/product="hypothetical protein"
/db_xref="SEED:fig|176279.70.peg.64"
CDS 61781..62263

/transl_table=11
/product="FIG01109362: hypothetical protein"
/db_xref="SEED:fig|176279.70.peg.65"
/translation="MPKFLLIDIKTNLVKLYMGILILLFGGMFIFGVSNLNHERVTQY
IELIFTILGILLFTAIYSVDQNKKSKLTISAKCHDYQFVFMLRLVITIIYILFLITIN
LFIFYLFNSNIIVLRDMFIALTSTLFIGSICLLTSILSGNTLNGLMFGLVYFLFSLRI
"
CDS 62474..63139
/translation="MYNIMIVDDEEKIVSFIEESLYLEGFNTIVAYNGRDVLKQIDDK
IDLIILDIKMPYIDGFEVAQSLKESNIPIIFLTAKDNLDTRLKCFSLGAKDYLPKPFY
MEELIIRIKNVLNQSHSQSLTPNIKKTKELIIYYDTYRIDVNNMTIDITKKEFEIIKL

LSLNPQYYFSKEQIYDSINLNKTGNTQVVAEHIRKIRKKLSCYSRFEYIDTKWGVGYK
WLE"
/transl_table=11
/db_xref="SEED:fig|176279.70.peg.66"
/product="Two-component transcriptional response
regulator, LuxR family"
CDS 63127..64218
/translation="MARVESLKRKMTRLIIAIIVVSVGLVFLLQILLNILEFSNFGKS
LYTNFWKQPHYIFSFTSACISIMTPLLIIYVFTQFFYKKYITTSIDYLNDTIQGLKSN

RTDVEYKKMSISEFNKLIQEVEHIHTYLANSIKDQLSIQNNFKRKLEILEHDLKTPLT
VLQGHIELLRKINCSNLSQEEIHFKINREAVIALQSIDRINNQLQIHIKNINLLPNHS
DTIHVDKVIKIIKESYNQQYILKERELIIDVDHDFPYYTLSINNQVLYHVIDNLINNA

LKYAEKQISIKFEVINDHLLNFTITNDGRIFTEDELNSAKEWGTKGKESNGSGIGLYF
ANIVLQQYNSELILENENNHAKVSFNIPV"
/transl_table=11
/db_xref="SEED:fig|176279.70.peg.67"
/product="two-component sensor histidine kinase"
CDS 65152..66513
/transl_table=11
/product="Uncharacterized MFS-type transporter"
/db_xref="SEED:fig|176279.70.peg.68"
/translation="MNDTDRTHKENKLEQDITLPYLKKAVIFSITFGFFGVNMAFALQ

SAMMGRIFQTLGANPNNLGWFFILPPLVGMIVQPLIGYYSDRTWTRFGRRMPYLLIAG

PIGAIVLVLLPNAGSFNFGYASLMALSFGAVMTLLMDLTSNACMQPYKMIIGDMVNEK

QRDMAWSWQQIFSNLGGIIATVLPFLLTMLGMSNVAPKGEVPMTVKLAYYIAAAVLLI

ASIWTVISVREYEPKTYNYYHGINENNRNNKLSLLQLLKKAPKQFWQISVVQFFNWFA

LMYLWTYSTGAIAKNVWNVTDPSSAGYQAAGNWYGVLYCIQFLSSVIFGFVIARSKPS

QRKFWYSFGLVFGGIGLISIFFIHNQWLLIFSFVLIGINNLTMNTQPFTLLTEAIDGE

NSGAYLGLFNTSICLPQIVASIVSFGLYPLLGSSMPAMLLLAGIMMLLGAVSVKFINA
KFE"
CDS 67052..67537
/translation="MKKVSKFILASSLILGFSVTPELFNVTQAHAQESVKPYYNYSGY

TAHQSNFILDKNFKTSLKADNFKINGYKITQNESAKNDKDVYDQTYYGVSNHKANGVF

FSLDGKSVSEKELLNHYGEPKYTSTSAWGKEYTYKIGNKDVRFYVNRNGYVIKGQIIS
E"
/product="Immunodominant antigen B"
/db_xref="SEED:fig|176279.70.peg.69"
/transl_table=11
CDS 67936..69009
/db_xref="SEED:fig|176279.70.peg.70"
/product="Mobile element protein"
/transl_table=11

/translation="MNDLDHPQLIFESTGIYSRGMERFCCVNQINYIQMNPLEAKFKT

SALRSWKTDQADAHKLACLGPTLKQTDSLPIHELIFFELRERVRFHLEIENEQNRLKF
QILELLHQTFPGLERLFSSRYSIIALNIAEIFTHSDMVLDIDKEVLITHIFNSTDKGM

SMDKATKYALQLRVIAQESYPNVDRHSFLVEKLRLLIQQLKQSIHHLKQLDDAMIQLA

QQLDYFENIHSIPGIGKLSTAMIIGEIGDIKRFKSNKQLNAFVGIDIKRYQSGHTHCR

DTINKRGNKKARKLLFWVIMNIIRGQHHYDNHVVDYYYKLRKQPNEKPHKTAIIACIN
RLLKTIHYLVMNHKLYDYQMSPH"
CDS 69512..71572
/product="Lead, cadmium, zinc and mercury transporting
ATPase (EC 3.6.3.3) (EC 3.6.3.5); Copper-translocating
P-type ATPase (EC 3.6.3.4)"
/db_xref="SEED:fig|176279.70.peg.71"
/db_xref="GO:0004008"
/transl_table=11
/EC_number="3.6.3.4"
/EC_number="3.6.3.3"

/translation="MNNNGEEHNHQNHMNHSNQMHHDNHASHHHSGHAHHHGNFKVKF

FVSLIFAIPIILLSPLMGVNLPFQFTFPGSEWVVLILSTILFFYGGKPFLSGGKDEIA

TKKPGMMTLVALGISVAYIYSLYAFYMNNFSSATGHTMDFFWELATLILIMLLGHWIE

MNAVGNAGDALKKMAELLPNSAIKVMDNGQREEVKISDIMTDDIVEVKAGESIPTDGI

IVQGQTSIDESLVTGESKKVQKNQNDNVIGGSINGSGTIQVKVTAVGEDGYLSQVMGL

VNQAQNDKSSAELLSDKVAGYLFYFAVSVGVISFIVWMLIQNDVDFALERLVTVLVIA

CPHALGLAIPLVTARSTSIGAHNGLIIKNRESVEIAQHIDYVMMDKTGTLTEGNFSVN

HYESFKNDLSNDTILSLFASLESQSNHPLAISIVDFAKSKNVSFTNTQDVNNIPGVGL

EGLIDNKTYKITNVSYLDKHKLNYDDDLFTKLAQQGNSISYLIEDQQVIGMIAQGDQI

KESSKQMVADLLSRNITPVMLTGDNNEVAHAVAKELGISDVHAQLMPEDKESIIKDYQ

SNGNKVMMVGDGINDAPSLIRADIGIAIGAGTDVAVDSGDIILVKSNPSDIIHFLTLS

NNTMRKMVQNLWWGAGYNIVAVPLAAGALAFVGLILSPAVGAILMSLSTVIVAINAFT
LKLK"
CDS 71590..71907

/translation="MIKKLFFMILGSLLILSACSNNDEKDKDTNDQKSESHMKHNDES

KVPEDMKSTNEGEFKVGDKVTITAGHMPGMKGAEATVKGAYKTYAYVVSYKPTNGNEK
VNNH"
/transl_table=11
/db_xref="SEED:fig|176279.70.peg.72"
/product="DUF1541 domain-containing protein"
ORIGIN
1 atgaagatta ctatcttatc agttggaaaa ctaaaagaaa aatattggaa gcaagccatt
61 gcagaatatg aaaaaagatt aggaccttat acgaaaatcg aattaataga agtaccagat
121 gaaaaagcac ctgaaaatat gagcgacaaa gaaattgaac aagtcaaaga aaaagaaggc
181 caacgcctac tcaataagat taaaccccaa tccacagtaa tcacgttgga aatcaaaggg
241 aaaatgttgt cttcagaagg actcgctaaa gaactacaaa cacgcatgac acaaggtcaa
301 agcgacttta catttgtcat aggtggctcc aatggtctac accaagacgt cttacaacgc
361 agcaactacg cactatcatt cagcaacatg acctttccac accaaatgat gcgcgtcata
421 ttaatcgaac aaatttatcg cgcattcaag atcatgagag gcgaagcgta tcataaataa
481 aattaaaatt gattgaaaat aaaatattat tacttattta aggggggcta tgatgttaga
541 taaattaaaa aatgttagtc ataaatcatt tgataaatat actaataatg atcaattgac
601 taaagtaaat atattttttg gaaggaatgg aagtgggaaa agttcattaa gtgagtggtt
661 aaaattaaat aatgaagaaa gtgtcatttt taatactcgt tacttaaaaa ataatattga

721 acaagttgaa gaaattgatg gtgtgagttt ggttataggt gaaaaatcta taaatcacag
781 tgaccaaatc aagaatttaa gtgatgctat aaataattta gaaaatttta ttaatcgaaa
841 agataatgaa attagtactt cgaaaaaaag aatatttgat aaaatgaatt ctacacttaa
901 tgaagctaga aagagatttg aaattactag taatatggtt aaacaaaaaa gaaatgcaga
961 caatgatccc atcaatgctt tttatagttg gaagaaagat attagagatg taaatgaaga
1021 gataactatt gaatctttag atgaattaga gaaaagaata gaaagcaacc aaatcttatt
1081 gaataatata aaaattccta ttttgtcttt tgaatatgat gaatttaata atatgttaaa
1141 aatattaatg aaagaagtta tcaaacctaa gagtagtgtg agttataaag tttctgaatg
1201 gataaaagat ggtttgaaaa ttcataatat gcaatcaaaa gaggaaacat gcgagttttg
1261 cggtaattca tttgatgtag aattggtgaa agaaagtata gatgcaagaa tagaaaatga
1321 atttaattat ttgatggaaa atctaaataa ttataaaaat aaagtagaga attcattaag
1381 taatatggaa aatttaaata ttgatgtaga ttcagtacaa ttttttaaac aaaaagaaga
1441 actatttaaa ctattacaat tattagaaga taaggaaaaa aagacatatg aaccacttaa
1501 ttatcctatc gataattggg aatcaataat taaagtagat aaaattatta tcaattatat
1561 tgacaaacta aacgaagaca ttcaaaacga taaaaaaatt agagattcaa ttactgactt
1621 tgtgaaatcg tggattggta agaagttaaa agatgatgaa gaaattcaaa tggaaattta
1681 caatattaat actaatagtg aacaaataaa aaaagcagaa gaatgtaaaa aagccaataa
1741 tgattggata acaaatttaa aaacagattc ctctaatttg aaacagttta gtgaaatagt
1801 aaataaaagg tttgaaatac ttgatataaa tattgaatta gaacctaata ttgaaactaa
1861 gagttataga gtattacata gagtaacagg agaaaaactt aacttagttg atttaagtga
1921 aggtgaaaaa cgattaatag gtttcttaca cttttattat aacttattta tgatagcaga
1981 tagtgagatg aaagaaaata ttgaacatat aatcatagat gatcccataa ctagtttaga
2041 tacagaaaat agataccatt taactaaaat tattaaccaa tttattgagc tttctaaatc
2101 tgaaaataaa caaatttttg tatttacgca ttcgtcgctt gatttccata attttgcttt
2161 taaaggaaag aataatacta aggaaacttc tcattggtat attgaaaaaa ttgatgggat
2221 tagtaaagtt agaaaattat ctaaagaaga gttaaataat tatagtgatt attatagctc
2281 agtttttaac gaagtaatgt cttttgcttt aaagggtaaa agaaagattg cttatataaa
2341 tgattatatt aaatatgcta ataaaatgag atttatttta gaatctcatg caagaagtca
2401 ttataaaata gaatatgcaa cggctacttc ggttcctgaa ttatcaaaat attatgatat
2461 agaaaaggaa agaatgaaag acttagaaaa gagcttagac atcattaata gtctctctca
2521 cggaatatca tttatagata atcataatga agaattgtct gttatagaag ttcatgaggc
2581 aataagaaaa gtgattggaa taatttataa gaaagataaa cagcatgtaa gtgcgatgtg
2641 tagcggggtt ttaactaagg aaaataaaga agaacttcaa aaatggattt aataaaattt
2701 ttatattgac tttatatagg ttaatcaaat aattaaacta gataaattac ttgtaaattt
2761 atgggctctt ctttcaaaaa ggattaattt attggtacgt gagtagtaag caagattcaa
2821 tataaaatta tttgttaatt gaagatgaga aacgataagt attaacttct tacttttaaa
2881 aaaccgcatc attaatcgat acgcagaagc atatcataaa tgatgcggtt tttgataagg
2941 ttctccttat cagtgatttt gctaaaaaac cttcaaagtt attatttttt caacaaataa
3001 tttgaaggat ttttattatt taatattaac tttatttaat tattgataat aatcgtgcgg
3061 ctaataatac agaggctatt actggactaa atcggcatta gactgttcat ctgtttctga
3121 cagtgtatta ttcttaactt gtgtttgagt ttcaatattt gtttgaggct tgagtagtac
3181 ttgaattctg taattgatta ctttagtttg attcgctatt atcagttttt cacttttaaa
3241 cttcattatt cgcactatta ttagatagtg ttaaatcttg gatttttgtt gatgggtaat
3301 aatctacttt agttaattct tgatagtcaa tctcagctcc agcgtcattt tctactaaac

3361 catatttata ctacatcccc aagttcagaa ttcttaacat gaaaactcac agaatagtct
3421 actttatctt caggttttta aaaaattaca ttttctagat ggtaatattg ttctttcata
3481 acactaatgt ctatatctgg aacattatta gctgttactt cgctttcagt cgtatgctaa
3541 ttaataaagt atttactaat ataattgtag caaaaatatt tgcttttata tcttgcttca
3601 gatttaattc ttattgtttg ttttcttctt tttattttct taagtagtaa ctctcgaaat
3661 tgcactacac atccaattcc tcaataatat gcattccatt cgctgttatt tgtatttagt
3721 atttctaatt ccaacttaat cgcaaagaaa ttatttatat gaaagttatt tttgtttctc
3781 tttttgcag aatattttat caaatcaaat atatttttaa tactttttct ctattctacg
3841 aggattatcg atttcttttt aaccaagcca attgggatca tcaagatgag gtcctcgtcc
3901 cctgttcaac ggggtagtta tcgaattcca aaagacaagc atcaatacaa attgaaaatc
3961 acagatgata atctgcaagt cgattaccct cgtcaatttc tgaaaggcgc acatgcaccg
4021 agtgttaaag cacagcacgt tattcattgg ggcgccacct tctgggtttc gtcgttatgg
4081 ttagcgtggt tttattaata tctgctatta tttatattgt agtaaaacgt cgtaattaat
4141 acgtctgtct tttattaaaa atataattga aggagagagt atggaaatgg aattcaatga
4201 gaacaatatc gacttagaga cgattatcag agatgaagtt aataaatatt tatctagaga
4261 tattggagac ttacctgcga cacaacaggc accgctcgag ttgagagaga aatatgaaaa
4321 gatggaagtg ccgaataaag ggcgagatat ctatgaagta ctcaaagatt taaataatga
4381 agtgctgaat tatttatatc gtccgaacca tccacgttca ttctcattta taccaggtcc
4441 agcatcacgt ctatcatggc ttggagacat tttaacaaca gcaaataata tccatgcctc
4501 taattttgcc aatgcgacgc tgccaattaa tattgagcgt aatttaatta actacttggt
4561 aggaaaaatc ggttatgaga tcaaaccggc cggtggtgtc tttgtatctg gcggttcaat
4621 ggccaattta acagctatcg ttgcggctag agatgcacag gtagaaatgg aagatattaa
4681 aaaagcaacc gtttatctta catctcaaac gcatcattct gtaggcaaag ccttgcatgt
4741 tgcaggcttc cttaaaaata atatccgcag aattgagtac aacgatgact ttacaatgaa
4801 tacagatagc ttaaaagccg caattgataa agatattgaa gaagattata aacctgctat
4861 ggtgattgcg actgcaggca cgactaatac aggttctgtc gatgatttca ccacacttgg
4921 agatatttgt gatcactaca atctatggct ccatgtagac ggtgcttatg gactatcaca
4981 tatcttatcc gataaagcac aacacttatt taaaggcatt gaacgttcag atagtgccag
5041 ttgggatgct cacaaattac tgttccaaac atatagttgc gctatggtaa tcgtgaaaga
5101 aaagaatcat ctgctgcaaa gctttggcga agatgctgag tacttggatg atattgcttc
5161 agatgatgat gtgattgatc cagaaatgtt aggcattgaa cttacacgcc ctgcacgtgc
5221 tttaaaatta tggattactc tgcaagtcat tggtgaagat gaaattatcg agcgcattaa
5281 atacggcgaa ggtttagcag aatatgcaga agaatacgtg tcaagtttcg ataactggcg
5341 tattatctca cacgctaatc tttccattgt gaacttcaga tatgaaaaca gtgatttaac
5401 tgaagcacaa aataatcaac tcaatagcat ggctgcgcaa aaaattgcag attctggtta
5461 tgccattgct tataccacag tattgaataa ccaacgtgtc attcgtttat gtacaacgaa
5521 tccattaacg acacatgatg atatcagaga aacaataaat cgctttgatc agtatattgg
5581 tgattacgat atgaaagatg cttaatcaaa aatacttctt aatagtaagt aacacccaaa
5641 aagttgaact ttttaggggt tcttttttct ctttatgagg attgattatc taagtgtaac
5701 ttagcatctt tagcaggttt tgctggtgtg atacgtctac cttttcctaa aatcatttga
5761 ataatagaag aaacgagcat gataacatta aaaataatca ttaagaaaaa tactttgtga
5821 tcagcgttga tggctacatc ttgcatcttc ttcatctttc aattttaaag gattgattca
5881 aatgaacgaa agaaatctaa tgttctcgtc ccctgttctt cggggtagtt atcgatccga
5941 cactttaata aatagcataa ccacaaggtt tcaagtgtta ttttggctgt caaaaactgt

6001 gacaactaac attatgctac tatatgaaat gtcacttttt ttgacaaata ttatgataat
6061 tttctatata ttaagagtat actacattta atacgttaat aattaatcat atataaaact
6121 taatataaat ttaacttttt cttcctttct aatttaaaat aagagataaa tttaataact
6181 actgttcagg aaagtaaaaa tatacttcta tattaaaata ataaaaaaag ccatacacaa
6241 ttgaactgac cccaattagt gggaattata taaaaacact ttcgttgaat tcatattaaa
6301 atgaatcata cggaggtgtt ttttctatga aaagagtttc ttattcagta gaaacaaagt
6361 ataaagcagt tgaaatgaaa gcagcaggat tttcaacaaa agaaattatg aaagaattaa
6421 atattagaaa tagaacacaa gtgaaaactt ggtggcgatg gtatcgaaat ggggaaagtt
6481 atagattttc acaacaagtt ggtaaacaat atacctacgg taaaggatta aaagagctgt
6541 cagaagtaga acaattaaaa ttagaaaata agagaaaaga tatagaattg gatattttaa
6601 aaaagtacaa ggcattggaa aggaagtggt accaacagta gtcatagatt tagtggatca
6661 attaaaagta aaatattcaa tcaaattgat actagaagta ttaaacatac ctaaatcaac
6721 atattaccga tggaaaaaca aaacccataa aaatgatacc gtaacacaaa aagtcattga
6781 attatgtaaa gctaaccact atacctacgg ttatcgtaag attacagcat tgattaatca
6841 atgttataca tcaccaatta atcataagag agtacagaga atgatgcaga agcatcattt
6901 aaactgccga gttagaccta aaaagacgac aagaataggt aaaccgtatt ataaaacgga
6961 caatttatta caaagacaat ttaaagcgag ttgtccaatg gaagtattaa caaccgatat
7021 tacttattta ccatttggtc attctatgtt gtatttatct tcgataatgg atatttataa
7081 cggagaaatt gtggcgtata aaatagatga taaacaagac caaagtttag ttaatgatac
7141 attaaatcaa atcgatatac ctgagagttg tatattacat agtgatcaaa gcagcgttta
7201 tacatcttat gcttattatc aattgtgcga agaaaaaggc attatcagaa gtatgtcccg
7261 aaagggaaca cctgccgata acgccccgat agaaagtttc cattcctcgc taaagtctga
7321 aactttttac atcaataatg agcttaatcg ctctaatcat attgtaatag atattgtcga
7381 aaagtacatt aaaaactata ataataatcg aattcaacaa aaactaggct acttatcccc
7441 tgtaaaatac agaaaattaa tagcctagaa catggtgttt ttattaagtt cccgttttaa
7501 gggttcagtg ccctaggatt ataggctctt ttgtttataa aggtaattga actaaagtat
7561 tataatttca attcttaatt aatgttccta ttttaccgtc taaagcgtcc cctaatcctg
7621 ctagagatgt aatcaaaaca ctgcctttag tatttttttc aagaaattga agtgcagctt
7681 caacttttgg aagcatactt cctttagcaa attgaccatc agagatatgt tttttcattt
7741 catccacaga cacttcatcg agacctcttt ggttttcttt tccatagtta atgtaaacat
7801 ggtccacagc agttaatatg attaattgat cagattgtaa atgtgctgct aataaagcac
7861 tcgttttatc tttatcaata actgcatcaa cacctgtata aacttcattt tccttaatta
7921 ctggaattcc accaccgccg gcagcgataa ctagtgttcc atgagtgatt aatgtttcta
7981 tactatctaa ctcaactata cttataggtt gtggggaagg gacaacgcga cgatagccgc
8041 gtccagaatc ttctacaaaa gtataacctt tttctttcgt aaatttatca gcctgctctt
8101 ttgtgtaaaa taatccaatt ggtttagtag ggttattgaa agcagaatca tcgcttgcaa
8161 cttgaacttg tgttactagc gtaacaactt gtttatctat acccattgaa tgaagttcgt
8221 tttgtaaact ttcttgcatc tgatagccaa tataagcttg actcatagca ccacattcag
8281 ggaaaggaaa aggaggacct tgtttgtgtt ccgccgcata atttaaacct aagttaatac
8341 ttccaacttg tggaccatta ccatgactaa ttacaatttc gtatccttta tcgattaaac
8401 ttactagaga tttagatgta ctttttaata aatctaattg ttctttaggc gattgtccta
8461 aagcgttacc acccaaagct acgacgattt tagacatatt tatatcctcc tttcatttac
8521 tctcctaatg ttgctaccat gactgctttt attgtatgtg ctctattctc tgcttcttgg
8581 aaaacaactg attgttcact ttcaaatact tcatttgtta cttccatttc atttaatcca

8641 tacttttctt gaatttgttt accgattttt gtttcagtat catggaatga tggtaggcaa
8701 tgttcaaaaa tagtatttgg gtttcctgtt tttttcatta attctttagt tacacgatat
8761 ggttctaata atttgatgcg tttttcccaa acttcatcag gttcccccat agatacccaa
8821 acatctgtat aaattacatc agatcctttg actccttcat cgatatcatc agttacaagg
8881 atttcaccac cattttttc agctatatta ttacaacggt ttaataactc gtcagttgga
8941 tttagttctt ttggacatac gagatggaaa gtcataccca taattgctgc tccttgcatt
9001 aaggcatttg caacattatt acgtccatct ccaacgtaag tgaaattaat ttcattatat
9061 ggttttttta atacttcttt agctgttaaa aagtcagcaa gtacttgtgt tggatgatct
9121 tcgtcagtta aaccattcca tactggtacg ccagaatatt ttgccaagtc ctcaactact
9181 cgttgtgaga atccacgata ttctatgcca tcatacattc cacctaaaac acgagcagta
9241 tctttggtag actctttttt acccatttga gaacctgttg gcccaaggta tgttacatgc
9301 gcaccttgat cataagccgc tgtttcaaat gcacatcgag tgcgtgttga atctttttca
9361 aaaagtagag cgatattttt acctttcatt ttttgttgtt ctattcctgc atattttgcg
9421 cgtttaagat cttcagataa attaagtaaa aattccattt ctttttgtgt gaagtctaac
9481 aaagttaaaa agtttctatt tcttaaattt ttcattttga atatctcctt ttcaattaat
9541 attttattca tgttattttt ttataatcca atttctttta tcttgatata taatattttc
9601 tattttttaat tctctgatta tttcaccagt agtttttctg ttaagtccag ataaattact
9661 tattaattga acagtcataa tttgttttaa ttcatagaat tctccattat catccccaat
9721 tgaaaggcat aaaatttgta gtattctttc aattctttct ttagctgaat tagctcttaa
9781 cgccataata tattctactt gaaattgaat agtctcatta agtttcttga agagactttc
9841 aaatatttca ttatgctttc tacataaata ctcaagtaaa tctttcggta aagttaatat
9901 tttacaatct gtcaaagctg tacatatttc atatggtgca ggggtttcat taaatatgaa
9961 gttcattgga aatatatttt cgtctttact taatcttaaa taattgtcac cagtaatatt
10021 agaagattca tgtaaaatac aaccatttac taaaaagtat acaaatttta tttgatcagt
10081 tgaatgatat atgacttgtc ctttttata ttgataaagt gttaaatcct ctttataagg
10141 tctaacaata ctaacaggaa tatttaagta tgatgctaat tgtttaagat tattatcaaa
10201 ttcatattct gaatttttaa tataaatatt ttcttcatac ataactataa accccttaaa
10261 aattacttag tttaaatcat aacttaaaat acagaaatat tacctgtaat caaacgaact
10321 attcctatta ttgcaagtac aatgatgaat atgaataacg tataatccaa ttttgtcaaa
10381 tgtttgttat tatccctttg tacgtagctg tatacgagta atccaggtat atataacaac
10441 atcgttaata gtaaataatc taatccagct gcataaacca accaaattgt gtaaatagat
10501 gcaataattc ctattatcca ttgttttaaa ttagctttag atttattttg aatagtatat
10561 ttaacctggt aaaaagcact gagtgtatat ggaattaaga ttgcacttga tgcaagtgaa
10621 aacgcaaact gataggcact atctgtaaac aacatactaa ttaaaaataa ctgaactaat
10681 atattagtaa taattaaagc gttgaccgga gctttattct tattttcttt agcaaaccat
10741 ttcgggaaaa gtccatcttt agctacaatg aatggtaatt caccagctag taatgtccat
10801 cctaaccaag ctcctaaaac agagataatt aagcctatat taactaacac tgaaccccaa
10861 tgacctacaa tatgttctaa tacttgtgcc attgatggat tagcaagttt tgaaatttgg
10921 ttctgctgaa tgacaccttg ggctagtaca gtcattaaga aataaatgac tagcacagaa
10981 atcaaaccaa taacggtagc agttcctaca tcctttttag actttgcacg tccagaaaag
11041 acaacggctc cttcaatccc tgtgaatacc catacagtta ctaacatagt actttttact
11101 tgtgccattg tatctcccca actaaaaacg ccaacacttc cactagtcat accataaaaa
11161 ccggatttaa aagtactgaa gttgaataca actatcatgc atataataac tagaaatata
11221 ggtattaatt tagctactgt aacaatacta tttataaacg ctgcagtttc tacacctcta

11281 agtattaaaa aatgtacacc ccataataaa attgatgcta tgataatact tggaagtgtg
11341 ttacctcctt taaatatagg gaaaaagtta cccacagctg acattaatag ggttgcataa
11401 gccacattac ctagaaatgc tgcaaaccaa tatccccaag cacttgaaaa accaataaaa
11461 tctccaaacc ctgtttgagc ataactataa attcctccat caagatctgg tcgctcattt
11521 gttaaatttt gaaatacgaa agcaagagaa atcataccaa tagcagttat tatccaaccg
11581 ataattattg caagtccacc agcttggcca cccatatctg agatgatatt gaatgcacca
11641 ccgcctatca tagagcctat gactaaacca attaaggaag ttttacctaa tttattttca
11701 tccatattaa tctcccctaa taaaggtggt aaaaatcttg ttatttgatt taatgaatga
11761 attacttgtg acttttacca cctttcaaat ttaaatatct tctctaaata acggctgact
11821 catacatctt gggcctccgc gtccacgtac aagttcacta ccagtaattt caatcacttt
11881 aattcctttg tcgcgtaaaa gttggtttga tacatagttg cgatcgtatg tcaccacaac
11941 ccctggtcga atacataatg tgtttgagcc atcattccat tgttcacgtg caccatcaat
12001 aacgtcgcca ttacctgttg gaataaagtc cactttttct acttctaaaa cttcagcaag
12061 tgtttcacgt aacttgctag aacgagtaat ttttatatcg tccttaccat cattttgttc
12121 tatggtaaat atattcatat tattttcttc tttaaatatt gctgcatgta ctgtaaactt
12181 atcgtagtca atcatagtta gtactgtatc taggtgcata aatgtacgtg tattaggtat
12241 ttcaatagct acgatttttt taaaacttgt gtttgcatct ttgaaaatat tacgtgctaa
12301 cttttctatt gcttgagctg atgtacgttc tgatatacca atagctaaaa catctttcga
12361 taatactaat tcatctccac cttcaatatt aaatggtgag ttacgatcta accatactgg
12421 tacatcttta tctttaaatc ttggatgatg tttcagtata tatgtcataa aaatagattc
12481 tctacgtcgt gctctccaat acattctgtt aattgtcatt cctctaccaa ttgaagcttg
12541 gggatctctt gtaaaataaa ggttgggcat tggatctaag taaaatggat atctatcatc
12601 catatattct actaaatgag ttgtttcaag ttgaatttct tctttacgta cgccagccat
12661 gattttattt acaagttctt ggtcagataa ctttgaaaag aattctttaa tttcagtttc
12721 atgacctaat attgtctttt tagattctgt taatatatcg tttatgaagt tctcgcgtac
12781 ttctggctca gtaatagatt ctgctgcaag tttttctaaa taaactactt cgattccttc
12841 atctctcaaa gtttgagcaa atttgtcatg ctcttcttgt gcaactttta agtagggaat
12901 atcatcgaat aataaaccac ttaaatgatc aggtactaaa ttttctaatt cttttcctgg
12961 tctttttaac aacacagttt tcaatttgcc tatttcacta tttacttgaa tgggtccttg
13021 taccatttca aattcctcct ttgctttatt acattcacat tataaaacgc tttcaaaaaa
13081 catcgtgtga tttaattcac aattgtttag ggagggtttt cacatatata gatttattca
13141 atgcaaaatt ttgtatgaat atgtattttg tactatataa tagtgaataa tttgaaaaat
13201 ctcattgtta aaaccttatc ttattcaagc aaaaagattt tttactcatt tttttgtatt
13261 agtaatcatt cctattaata taatcattta atttttaagg gaataattga taacaaacaa
13321 attctgctat ttcatttgaa gaagtcaaaa tcatcaaagt atcgttacct ccaataattc
13381 ctaatatttc tttcatttgt aattgatcta tgtaataact tatactttga gcaaagccag
13441 gagatgtttt tattaagaca tagttattta gcgttataaa ttcaataatc tcatcactaa
13501 atatttctaa ttgttttttt gcacttaatt gatttgtttg atttattttc ttgtaaatat
13561 actttttatt ttcaacaggg attttgtaaa tttctaattc ttgtaagtca cgagaaatag
13621 ttgtcaagct atagtaaact ccaaaatgtt ttgccatgta atccactatt tgttgttttt
13681 tattaaactg attctgttgt ataacagtta agataagatt taaacgtttt tcttttttca
13741 tttttattac cccctttatt atttttgatt atgaaataga ttttaaataa ctgtataaaa
13801 atcacttata aatatcaatt tacaaataat tttgaaatag tggatgcttt aattataagt
13861 agattatttt acgcctaaaa ggagagattg attagaatag attatagtca tcataattat

13921 aaaatcaaaa ctttaagtaa cattaatgaa atataataaa aataatttgc gtaataatag
13981 cgaaatgtgt aaattgtaaa tttcaggtga taacaaaaca tgttaggtga gaatgtctta
14041 cagttacatt tactatgtat aaataaaata ataacattaa tagtgagagt taggtaataa
14101 ccacaatatc tcataaaaga agtttggaaa aaggcattga aataaaaagt agtttataag
14161 ttgaagatat ttaggtatgt attagagcgc tttataaata aataggttta tacatagaat
14221 tacaaaaata atataaaaaa tagaatctaa tacatgataa agaaaggatt aaaataattt
14281 agaaaatgta tttagagtga tattgttata gagattggtt aacttgtcac ataaattgga
14341 atgaggaaga gaactaaata ataagaaatt aaaataatct aattcaactt ttggattagt
14401 gcttgtattt taacttgata cagactattc tattttttct aaatgatgtg attcaagata
14461 tgctatatca ttttgattaa tctaaattta attcagagaa aaagtctgaa cgttattata
14521 attatacgat ttcaaatacg atggattatg tgattagttt aggtgaagat gaacgtcaac
14581 gttttattaa aacggtcaca gaattaggaa aagcctttgc gttttgtgat acagaaccga
14641 aagtgcaaaa gctcaatgat gaaattgcct tctttaaagc cgttaaagca ggacttgtta
14701 aattgttaca accatcaaaa gaaggaaaaa tatataagac acttgctgaa gttgaagcag
14761 agattaatca tcttgtgtca caatctgtgg ttacggaaga ggtaaagaat tagggttaaa
14821 ttctgacaaa attgattttt acgatacttt aatctcacat gaaacagaaa aagaagttat
14881 aggtgataaa aatcttagag caattgccca tgaattaaca aaaattgtga aagaaaatat
14941 gactgtagat tgtgataaga aatcaagtag tagagttaat aagaggagat cagtaaaaag
15001 attattgaag aaatacggat atccatctga cttagaaaca atagcaatca atcaagtggt
15061 tgaacaagca gagatgatgg ctagtaatca gtaagaaaaa ctaataaaac atactaagat
15121 tagctatgaa aaaatctatc gtcatagatt tcttcatagc taatcttagt atgtttttag
15181 aaaagaaaat tagtgaaaaa actttactta tcatttatta gttcggtgtg ctcatatcac
15241 atcataatat aagctattat gccgagaaaa ttttaataaa aaaataaaaa aacctaaata
15301 ctctctcgat ttgtgagaaa gtatttaggc tttttgaatt aataaatttc ttacttctta
15361 tgtttattta acaattagta ctgggacaga acttctttta gcaatcttat gtgtaacatc
15421 tcctaaaaca tgtttaatat tcaaatcgac tctatgatta ctcataatta ctaaatcaaa
15481 ttcaccatta tttatatctc cagaagtaat tttttctaag attgtatctt taatatgccc
15541 aaaatctaca tctattttat attgaatatc tcttttttct aattcacgta aaaatggagt
15601 gagttttttt tctttttctc taataatatc ttctttatgc ttattataat atttgacact
15661 aattgcaaga tcattttctg ttacaacgtg ataaattaca acaactgaat cttcatcgat
15721 gactttttct aaatagtcag ggatagcact aaaatcattt tcgaaatcat agggtaataa
15781 tatatttttg aacatctaca ccagcctcct attacttata taccctgaat atatatgctt
15841 attctgattc tgttgcaaag taaaaaatat agctaaccac tatttgaaat tttaagatat
15901 ttttttagag catttagaga tatttataga ataataaaca ggttaaaaag taataggatt
15961 aataggattt ggataaaaaa atcaatccta tttataggat tgacgtcgac taaagcctat
16021 gaaatcaaca aaaataatag tattcagaca atttaaagtg gtcttgtaac aattgtaaat
16081 agtatgcaat attagaggtt atagtatcga tcaattctta tttaatatta actataagta
16141 ttgttatatc agcgtttata ataattttaa aaagagcatg taatcaaggc gagcataaga
16201 taatccttca gctatgctga ataccactta aggtggtact ggatttcctt atgctcatgc
16261 aaaatcctat tttgtttgta atatttcaaa aagtaaaaca tacattgtta aaaaataggt
16321 tattcttttc ttcacttttt actttaattt cttattttta tttccaaaat agtcattgtt
16381 aataaaaatt atagtaccct tatttgcaat tttgagatac tttttagagc atttaggggt
16441 gttttatata ttatttattt aatggctctt aaaagacaaa tatgaactaa atttaaaaat
16501 cagaaagaca aaacaaaatt ttttgcttaa ccagtgtcga attttttaga cactgcccat

16561 ctatatgcaa attaaaaatt ggcataaaat agcttaaaaa tttatcaact ttttagttcc
16621 tgcaggggca atttctttca aaaaaacgct agctttttgc taacgtttaa gaaaatttat
16681 ttttcttctt caatttcatc aatcatttga tgattgattt cttgttcttc actttctgtt
16741 aaatcaaaaa tttcactcgc taaatactct tttttattcc aagtataaaa aatttgataa
16801 atatattcgg tcggatcaac ttcttttaag taatctaatt ctccattttt taatttcttt
16861 cttgcgtctt taaatagtcc aaaataaaca agaacttgtt ttccttttaa cgctttataa
16921 aatgcatcga acactttttg attaattaaa tagtcgttat ccggttctgt tgcaaagttg
16981 aatttatagt ataattttaa caaaaaggag tcttctgtat gaactatttc agatataaac
17041 aatttaacaa ggatgttatc actgtagacg ttagctacta tctaagatat gcattgagtt
17101 atcgtgatat atctgaaata ttaagggaac gtggtgtaaa cgttcatcat ttaacggtct
17161 accgttgggt tcaagaatat gccccaattt tatatcaaat ttggaagaaa aagtatcaaa
17221 aagcttatta caaatggcat attgatgaga cgtacatcaa aataaaagga aaatggagct
17281 atttatatcg tgccattgat gcagagggac atacattaga tatttggttg cgtaagcaac
17341 gagataatca ttcagcatat gcgtttatca aacgtctcat taaacaattt ggtaaacctc
17401 aaaaggtagt tacagatcag gcaccttcaa cgaaggtagc aatggctaaa gtaattaaag
17461 tttttaaact taaacctgac tgtcattgta catcgaaata tctgaataac ctcattgagc
17521 aagatcaccg tcatattaaa gtaagaaaga caagatatca aagtatcaat acggcaaaga
17581 atactttaaa aggtattgaa tgtatttatg ctctatataa aaagaaccgc aggtctcttc
17641 agatctacgg attttcgtca tgccacgaaa ttagcatcat gctagcaagt taagcgaaca
17701 ctgacatgat aaattagtgg ttagctatat ttttttactt tgcaacagaa ccttattact
17761 tatataggtg agagtatgat aagtgagaat gtaccattta aataagttgt taaatatgag
17821 cattagcgat tattaaaaaa gtagaattat gtttagggaa tttttagcaa tatcattgat
17881 aaggggaagc gtatcataaa taaaactaaa aattaggttg tgtataattt aaaaatttaa
17941 tgagatgtgg aggaattaca tatatgaaat attggattat accttgcaat atcatacgat
18001 gtttatagag tgtttaataa accatttttc aactattgat gatctagaat atataataac
18061 tgtacaaatt atattgatta tggaactaca attaaattaa gaaattgatg atgaaatttt
18121 aaatttaaac taatggaatc aagaaagaat gaaaggaaat atacaatgcc tacgattaat
18181 aaaaggaagt ttattagatt ttgtgttaga aacagtggat gtttttgtaa atggaagtaa
18241 aaatgattgt gaattagtaa attttaatta taaagaagga aaagtagttt cacgttatag
18301 tacaaaattt gaaagcaatc ctaaattaag aaaagagcaa ttgatatcga cggtaaaagt
18361 tgtaaggtat gttatttttg attttgagaa aattttatgg ggcataggga aagttttatt
18421 gaagttcatc atattaagac aatgtttact ataagaaagt tgaaataaac tcagaaacag
18481 acttgattcc cgtttattca aattggcgca aaatgataca tataaacacc tatatattga
18541 agatttaagt caaacggtaa ataatatttt tcacttttaa acagaaactt ttttggaaat
18601 tatattaatt agtataaaaa tgaatatgtt cacaaagaag gagagatttt attgaaacac
18661 attaatcatt tttctgaatc gacactagca tacttggaaa aagaccatga acatgcattt
18721 tatgtatatt gtttaataga cccaagaaat gatgaatgct tttatgttgg taaaggtaag
18781 gggaatagaa tatttaaaca taaacaagat gcgcagagac aattattgta cgaagatata
18841 ttcagagaag aaaataaaga taacttgaaa tttaatagaa ttaacgaaat ttgtagcaat
18901 gatttgaatg ttttaggata tataatcagt tatggattaa ccgaatctga agcattttct
18961 gcagaaaatg ttcttataaa ctttcttaac ctaacaaata aaactacatt aacaaatatg
19021 attaacggtc atggatctaa ggcatactta gttgaagatt tagaaaatga atttggctac
19081 gattcaatta atcttgaaaa cataaatacg aatgaattaa ttttagcagt gaaaatcaga
19141 gatgcatttc tattagataa ggatgaaagt aaagagtatc ctattaatga aagtaaacgt

19201 gatagaaata accttaaatc gcgtacatta ggtagttgga taataggaaa agataaaata
19261 cataaaataa aatatattat tggtatcaat acaggtgcta ataatgcagt tgtctcagct
19321 tatgaagtat catttgaaca agcggagagt atcgaaacaa ataatggtag aatgagatat
19381 gcgtttattg ggctttcaga aagagatgct actctaaaaa aattgaattt atacaaaaaa
19441 gcactaccag atttaagatt tggtagtggt agcgctacag catatataaa taatgggaca
19501 atgaaagttg attaaatgaa taaaaacatc caattcctta aattagcctt tgaaaatttt
19561 atactcgtat atataagtga aaaacttaaa agaattagat gttaattaaa ggagagaatg
19621 ctatgccatc agatgatatt gtaaaaaaac tatactcaaa agaggatatt cgattagaat
19681 taggacttac tccgcataag tttaataaaa aaatggaaac tattgctaag ctttttaaaa
19741 ttgatatgaa aatttttcac aattacaaag gccaagataa aaataatcag tatacattta
19801 atggtgtcgc aaaagaatta atcaaagtgt tgctaaaaag tgttgattat tacccagtgg
19861 atatcaattc caaaaaattt aaacaaaatg gaaaatctaa aaaagaaatg attgaaaata
19921 tagataactc tagttacatg aaatatattt atcagttaat gaaatctatc aatgaaattc
19981 aatacaaaag gttaattgct gatatacata tgaaagatgt gtaccagaat acaaaagcat
20041 ggttaaataa tggtgaatca attaataaaa aagaacaaga gctataccaa tatatgacaa
20101 ttttaccatt acataaaaga gttgaactgc aaaatgaggt attaaaatct atagacgaaa
20161 caatttttca atttctcgca aaagaacata gaaataatca aattgaagaa aataatgaat
20221 tagaagcata tacaaaagca ataaaagaag gtagaaatcc taaaaatgat tatgaattaa
20281 atcatctcct atataaaaaa aatcaattac ccttagatag tctaatagaa gatatatggg
20341 actatgagga aacagagtat actgagcttg attggttaat tgcagatatg ctaaaacgtt
20401 cacaaagagc tgatagtaat tttatcgaaa gtttagagaa gaaaaacaaa ttgagaaaaa
20461 atattcataa agattccatt aaaagtatat ctaagttgat tgatcatgaa ttagttaaaa
20521 ataatagaaa gtgggacgta gcagaatttt ataaaaatca acaattttgg aataatagta
20581 cattaagacg tagctctcaa aattttggaa ttttatatta caaaaagaat aggttacata
20641 caatgaatca aagtaatatt attaagaaaa taaaaattat cgaagctaat atagagagtg
20701 caaataaaca gccgagctat ttagatttct ttttacaagc taagtttgat ttagagaggt
20761 tagaaagtga attaatgaaa tatagcatta atccgaaata ttttaataag ataaatagtg
20821 attacgtaag atatgctatt gaagatgtac ataatgcaat tattaaaatt gacagatata
20881 taagtaacga taaagtgaag ttaaactatt caaatgtcca aatggtagtg agaaacgaaa
20941 taacagaaca aggaagctac tttgtaaccc aggcattgaa tacactgtca aaggtagaag
21001 agtctggcta cagctttgat aatttcatgg taggaccttt cgtagaagac tttaaaaaag
21061 caattaaaaa tcgtagattt ggttctaaaa aagattaaga ttgtttttta gagttaaaca
21121 taagaataaa tttaaataaa aaaataaacc caacaaacct tgttatatca atgtttgttg
21181 ggtttatttt ttttggtgat tttttaataa ctcaattaca tcttaggata atatcgctaa
21241 attatacatt gtcgaggcca agacaaataa aaaattaaag caaaagttag cgcgcaactt
21301 tctgctataa aggagcaaat ttatatggag acaagagaca aactgatgtc attgactcaa
21361 agtgacaaaa cacagcaatg gctaatggac aagtcatcta accaagatga cattcaacaa
21421 ttgcagcaac aattcagtca gcagcttgat caacaatata atgcactttt agctgatgaa
21481 aaagctaagt tagaccaata cgtggaagta catcaaggat tggaatcatt aaaggaagag
21541 attgaatcag aacctattac gcttaatatc gataaattac ccgatatcaa agcaacaatg
21601 cttgaaagag ccaagaatga tgaacattct gataaaatcg aaaagctatt tgataggtta
21661 gaacaggcat taaatggtac gaatcgatta tatacgcaat tatcgttgat tggtacacga
21721 acacatcgaa ttacaactaa aaattttaat cttcaaggct tacctaaagc agtccaacat
21781 acgattttac cttcaaaatt taagaaggtg tatacagtcg attttaaatc gtttgaacca

21841 tcagttgcag cgtacatgac tcaagattca aaactgattg acttgttaaa tcagaaagac
21901 ggactgtatg acgcattgct aagtgaatta ggcttatcag atgagctacg tgtatttgtt
21961 aaacgtgcat ttattggttc gtttctattt ggaggtaact tcaaaaatcc taaattcaag
22021 ctgaatcaat atgtaagtga agtacaatgg ttggatgcgg tcagccaatt tacaaaagtc
22081 attgaactta agaagcacgt tgaaaagagt aattccatgc ctatgcctta tagtattgag
22141 catgatatga gcgcatttca aggtagcagt attatggcaa tctacgtaca aactgtagcg
22201 agttatattt tcaagcacat tctgctaaaa gtgtacaaag ctcagtgcga tcaaaaaacg
22261 ttcaagatta tagtacctat acacgatgcg attatgattg aatgtgaaga tgaagaaatt
22321 gcacaaaatg tgggtcagtt aatgaaagat acggctaacc agttgttcaa tggtgaattt
22381 gcacatgtga cagtggaaga aataggaggc gtagaccatg aataatgata gaggacaaag
22441 cctacacatc ccaagtagta caccaatcaa agaaaataat atatatgtag ctacgttaca
22501 ttctgtgatc caaacagatt tctcaggtga aataaagcac caattcacgt atgaaattga
22561 agtgaacaat cagattgtat atgcgaatcg taatattcca acaaaaccga gcgctaatca
22621 gttgtcaatt catgattggc tgaaacgtca tagcaactat agcgtgggtc atgatacgta
22681 tgaaccttat attgaccgta agcatttaat ccatattggt caatatagcg gtaactatta
22741 tgtacaagat gtagcatcgt tagatgcgtt tggaggcgta ttatcatgaa tcatatatta
22801 caaatgttat ctaagctatt aagtgtggcc aaggaggcaa tcgaccgtca aggtctgatt
22861 gctatcctaa ctattcctgt taataataac gatgaaatag aagaaacggc tcaaggtgaa
22921 accgtgtata acgaacttat cgatcagtta cgacttaata tcccaaaaga tacggattat
22981 caacctaaca tctatagtta ttttggtatt aagaagaatc ctaatgacac cgtactcatg
23041 gaaatgatga taaaggtttt tcatatcaaa cgctttaatt cagaactgtt tattttcaaa
23101 gttaacgggt ggcaaaagat aaatggagat gaattacaag ggttgatatc taaaatgata
23161 caagtattgc ttgtagatta taagccttca ctaagcactc taaaaaatgt cgtagatgga
23221 ttgcaaaaat caacagatgt agaagaactt gttgagaatg agcgctatat tggttgtggt
23281 gaaaatatgt tcgatcttaa tacgtttcaa gtcgttaaaa attcaatcga tatctttcca
23341 aaaacacgat tgaatttatc attaagtaca aatgatgtaa ttactgataa gataccgcct
23401 tattttaagc aatatatgtt acaacttgcg aattatgacg atgatttaca atactttctt
23461 ttccaacata cagcagtatt acttacagct gatactaaat accgtagggg tctcatatta
23521 tatggtggag ctaagaatgg taaatctgta tatattgaac tagttaaatc atttttctat
23581 agtaaagata ttgtgtctaa gccacttaat gagcttgaag gtcgttttga caaagaaagt
23641 ttaattgaca aaagtctaat ggcaagtcat gaaattgggc aatctaggat tcaagaaaag
23701 atcgtaaatg acttcaaaaa gttattatct gtagaatcaa tgcatgttga tcgtaaagga
23761 aaaactcaag tggaagtcat tttggatttg aaacttattt ttagtacaaa tgcgatactt
23821 aattttcctc ctgaacatgc gaaagctttg gagcgtcgaa ttaatattat tccatgtgag
23881 tattatgttg aaaaagcgga cacttcatta attgataagc tccagagtga gaagaaagaa
23941 atctttcttt acttgatgta tgtgtatcaa cagattgtaa aagcagatat cgagtatctt
24001 gaaaatagcc gtgtcactga aattactcac gattggttaa attttggata tgaatttgtt
24061 tctagcaggt ccgtaagtaa tgcaaatcag aaagcatgta ttaatttact cagaaaactt
24121 atagaaatca aatcaggatc acgaatcaaa gtatccgagt taaataaagt tattaatgaa
24181 gaaataaagg taagttctca ggttattaaa cagttaattc aagcaaactt tgatactcaa
24241 accaaactat acaatggcta cgattattgg attgatttag gttggaaaga agccaataaa
24301 aaagagattc atgatatttc ggaaaaagat aatattattt cattagataa aaatgaaaat
24361 ataacagacg atgaggcatt agatgaagag aatttggact ttgattggga ggactttgac
24421 gatgaataat gaacaaattg aagcatttgt agaagtgctt gtacctatca tagaagaacg

24481 tatcaataaa ggtaattaag gctaattacg tactacaggc agttgcctgt agtactcata
24541 tgattaagtg gtaaaagtga taaaaatgaa acgaaattat aaatatatat tatctatatg
24601 ttgttacaag accgatagtc tgtagcaata atctaataaa aggagaacgg tatgatatga
24661 agggtaaaat tgcactttat tcacgtgtta gtacgtctga gcagtcagaa catggttatt
24721 ctgtaaagga gcaggaacaa gtactcatca aagaagttgt gaaaaatttc ccaggttatg
24781 actatgagac atatactgac tcaggcattt caggtaaaaa tattgaaggt cgtccggcaa
24841 tgaaacgtct attacaagat gttaaggata ataaaataga aatggtatta agttggaaat
24901 tgaatcgtat ctcacgatca atgagagacg tgtttaatat tattcatgaa ttcaaagaac
24961 atgacgtagg gtataaatcg atttctgaga atattgatac atccaatgct tctggagaag
25021 tactcgttac aatgtttggg ttaataggat ctatagaacg ccagactttg atttccaatg
25081 tgaaactttc tatgaatgct aaggcaagga gcggagaggc catcaccggt cgtgttttag
25141 gctacaaatt atcacttaat ccacttacac agaaaaatga tttggttatc gatgaaaatg
25201 aagctaatat tgtacgtgaa attttcgatt tatatttgaa tcataataaa ggcctcaaag
25261 ccattacaac cgtacttaat caaaaggggt atcgtactat taatcaaaag ccattttcag
25321 tgtatggtgt taaatacatt ttgaataatc cagtctataa aggctatgtc agattcaata
25381 accatcaaaa ctgggctgta cagcgaagaa gtggcaaaag tgataaaaat gatgtgatat
25441 tggtcaaagg taaacatgaa gccattataa gtgaagaggt atttgacaaa gttcatgaaa
25501 aattagcttc taaaagtttt aaaccgggca gacctattgg tggagatttc tacttacgtg
25561 gtcttattaa atgcccagaa tgcggaaata atatggtatg tcgacggacg tattataaaa
25621 cgaaaaagtc caaagaacgg acaatcaagc gttattacat ttgttcctta ttcaatcgtt
25681 cagggagttc tgcatgtcac agtaattcca tcaatgctga agtcgtcgag cgtgtaatta
25741 atgttcattt gaatcgtatt ctgtctcaac cagatattat caagcagatt gcgtcaaatg
25801 tgatagaaga actgaaacaa aagcatagta accaaacaga aattaaatat gacattgata
25861 gtttagaaaa acaaaaagct aagcttaaaa cacaacaaga acgattgtta gaattgttct
25921 tagatgatca gatggatagc gaaatgttaa aagctaaaca aagtcaaatg aatcaacagt
25981 tagaagtatt agatcaacaa attaaagaag cgcaacaagc aaatcaatca caggatgaaa
26041 tacctaattt tgataaatta aaaggacgac tcattttgat gataacacga ttcagcgtgt
26101 acttaagaaa ggctacaccc gaagctaaaa atcaacttat gaaaatgtta attgattcaa
26161 ttgaaattac gacagataaa caagtaaaac ttgtaaggta taaaattgac gaaagtctta
26221 tccctcaatc tttgaaaaaa gattgggggt cttttttat gcccaaattc caatttgaaa
26281 tatatggtca aaatgattat ttcatcgacc aaattaccac ttttaccact tagttattag
26341 tgacaaaagt gagcgaaatg aaataaaaat caaatatata ttatcaaaat gatgtatcac
26401 gtacatacat caatcaaata cattaggagg tcataatgat gacattagaa caacaactca
26461 agcactatat aaccaactta ttcaatctgc caaaggacga agtgtggcac tgcgaatcta
26521 tcgaggaaat cgctgatgat atcttaccca atcaatatgt aagacttggc ccactcagta
26581 ataaaacact tcagactaat acctactact ctgacacact tcatgaaagt aatatctatc
26641 ctttcattct ctactatcag aaacaactca tagccatcgg ttatatcgac gaaaatcacg
26701 atatggattt cttataccta cacaacacta tcatgcctct tttggatcaa cgatacttac
26761 taacaggagg acaataaaat gcataaatac atcaaaatta cacaattagt cattacaata
26821 ctaagtgaaa tcatcatttg gatgaaagag tcagaacgaa aggaagtctc ttatgaatag
26881 atatatcacc cggggtatcg ccaacaactt acctaatatc ttacaacacc aattatggca
26941 actcgtatct gagcgagaac aagaacaaac caaagataat acttcagtag attattttca
27001 tatattccag ttcaataagc atcgcaatca attatatatc aaacacaaac aagaacgacc
27061 tgaatatgcg aaaatccata aagctaatta ttcaaaagca atcaatatca ataaggtcta

27121 cattattcga gaagatgatg tagacctttc ttattatgtc atgttattac ctgaagaata
27181 ctagaggagt gaaaattata tggaaacaat caaaagtaca ttaaaaacag aagccatatt
27241 cagtgatgac aaacaacatc gctatctact taagaaaaca tggaatagtg aaaaacaatc
27301 aatcacaatc attacaatgt atccgcatta tgatggcatt ctcaatattg acctaacgac
27361 ccaactcatc atgaacaaag tttcagaaat ggatgcattt ggttcaatca attttgtgaa
27421 tctatactct aatattacaa cccctatcaa tctcaaacat ttagaaaatg cgtatgataa
27481 gcatacagat attcaaatta tgaaggcagt gaaagagtca gatgaagtga tattagcttg
27541 gggcgcttac gctaaaaagc ccggtgttga agcacgtgtt aatgaagtat tagagatgtt
27601 gaaaccacat aaaaagaaag taaaacgact catgaatcca gaaaccaatg aaatcatgca
27661 tccccttaat tcgaaagcac gtcaaaaatg gatattaaaa gtatagcaac taaaatgttt
27721 attcactatc catgaaaata attgaatttc atatatatct tttaatctac aatatgatta
27781 aaagatatat tatttggttc tgttgcaaag taaaaaaata tagctaacca ctaatttatc
27841 atgtcagtgt tcgcttaact tgctagcatg atgctaattt catggcatgg cgaaaatccg
27901 cagatctgaa gagacctgcg gttcttttta tatagagcgt aaatacattc aatacctttt
27961 aaagtattct ttgctgtatt gatactttga taccttgtct ttcttacttt aatatgacgg
28021 tgatcttgct caatgaggtt attcagatat ttcgatgtac aatgacagtc aggtttaagt
28081 ttaaaagctt taattacttt agccattgct accttcgttg aaggtgcctg atctgtaatt
28141 accttttgag gtttaccaaa ttgtttaatg agacgtttga taaacgcata tgctgaatga
28201 ttatctcgtt gcttacgcaa ccaaatatct aatgtatgtc cctctgcatc aatggcacga
28261 tataaatagc tccattttcc ttttattttg atgtacgtct catcaatacg ccatttgtaa
28321 taagcttttt tatgcttttt cttccaaatt tgatacaaaa ttggggcata ttcttgaacc
28381 caacggtaga ccgttgaatg atgaacgttt acaccacgtt cccttaatat ttcagatata
28441 tcacgataac tcaatgtata tcttagatag tagccaacgg ctacagtgat aacatccttg
28501 ttaaattgtt tatatctgaa atagttcata cagaagactc ctttttgtta aaattatact
28561 ataaattcaa ctttgcaaca gaaccgtatt atggaataga gatgttggta acatttatac
28621 aggatcatta tacttaagtt taatttcgtt attacagaac cacacattcc aaccagaaga
28681 gaaagtatgt ctatttagtt atggttcagg agcagtagga gaaatcttta gtggttcaat
28741 cgttaaagga tatgacaaag cattagataa agagaaacac ttaaatatgc tagaatctag
28801 agagcaatta tcagtcgaag aatacgaaac attctttaac agatttgata atcaagaatt
28861 tgatttcgaa cgtgaattga cacaagatcc atattcaaaa gtatacttat acagtataga
28921 agaccatatc agaacatata agatagagaa ataaactagt ggccgattgt gcttgatgag
28981 cttgggacat aaatcctaac tcgaaataaa taagcatatc actaaactga ttttttaaag
29041 tttacagtga tatgcttatt tttttatctt acgattttgt acgtgcatgc ttgcctaggg
29101 gtatggctcg agccattagt ctctcgcaca tactattccc tcaggcgtca gcacttacaa
29161 aatcggttgt aattttcatt tttatacgca ttcttactga gattatacta ataagaggaa
29221 tagtaaaagc aattctaagt aaaattgcag ataagaggtt tgttaaaagc agttctaagt
29281 aaaattgcag ataagaggtt tgttaaaagc agttctcagt aaaattacag ataagaggta
29341 cgttaaaagc agttctaagt aaaattgcag ataagaggtt tgttaaaagc agttctaagt
29401 aaaattgcag ataagaggta cgttaaaagc agttctaggc aaaattgcag ataagaggtg
29461 cgttaaaagc agttctaagt aaaattgctg ataaggggta agttaaaagc aatcctaagt
29521 aaaattgcag ataagaggtg cgttaaaagc aatcctaagt aaaattgcag ataaggggta
29581 cagaaaaact agacttgatt acaaaatgga gcttgggaca taaatgattt tttaaaaatg
29641 agatgagacg tagattaact ccataatcaa tacgaatcta tcgacttctt tatttatgat
29701 attcatctct ttttaatgga aataaaagtg cgattaatgt gataatacag ttacgttaat

29761 taaaaaaata aaaatgcaag gagaggtaat atgctaactg tatatggaca tagaggatta
29821 cctagtaaag ctccggaaaa tacaattgca tcatttaaag ctgcttcaga agtagaaggt
29881 ataaactggt tggagttaga tgttgcaatt acaaaagatg aacaactgat tatcattcat
29941 gatgattatt tagaacggac tacaaatatg tccggggaaa taactgaatt gaattatgat
30001 gaaattaaag atgcttctgc aggatcttgg tttggtgaaa aattcaaaga tgaacatttg
30061 ccaactttcg atgatgtagt aaaaatagca aatgaatata atatgaattt aaatgtagaa
30121 ttaaaaggta ttactggacc gaatggacta gcactttcta aaagtatggt taagcaagtg
30181 gaagaacaat taacaaactt aaatcagaat caagaagtgc tcatttcaag ctttaatgtt
30241 gtgcttgtta aacttgcaga agaaatcatg ccacaatata acagagcagt tatattccat
30301 acaacttcgt ttcgtgaaga ctggagaaca cttttagatt actgtaatgc taaaatagta
30361 aacactgaag atgccaaact tactaaagca aaagtaaaaa tggtaaaaga agcgggttat
30421 gaattgaacg tatggactgt aaacaaacca gcacgtgcaa accaacttgc taattgggga
30481 gttgatggta tctttacaga caatgcagat aaaatggtgc atttgtctca atagaaagtt
30541 agaggtgagt cttacgtttc agtgacggta gacttacctt taacatgtta catactaaaa
30601 aattaatttg aataagaaag agagacatat atgaaatacg atgattttat agtaggagaa
30661 acattcaaaa caaaaagcct tcatattaca gaagaagaaa ttatccaatt tgcaacaact
30721 tttgatcctc aatatatgca tatagataaa gaaaaagcag aacaaagtag atttaaaggt
30781 atcattgcat ctggcatgca tacactttca atatcattta aattatgggt agaagaaggt
30841 aaatacggag aagaagttgt agcaggaaca caaatgaata acgttaaatt tattaaacct
30901 gtatacccag gtaatacatt gtacgttatc gctgaaatta caaataagaa atccataaaa
30961 aaagaaaatg gactcgttac agtgtcactt tcaacataca atgaaaatga agaaattgta
31021 tttaagggag aagtaacagc acttattaat aattcataat aaaacagtga agcaaccatc
31081 gttacggatt gcttcactgt tttgttattc atctatatcg tattttttat taccgttctc
31141 atatagctca tcatacactt tacctgagat tttggcattg tagctagcca ttcctttatc
31201 ttgtacatct ttaacattaa tagccatcat catgtttgga ttatctttat catatgatat
31261 aaaccaccca atttgtctgc cagtttctcc ttgtttcatt ttgagttctg cagtaccgga
31321 tttgccaatt aagtttgcat aagatctata aatatcttct ttatgtgttt tatttacgac
31381 ttgttgcata ccatcagtta atagattgat attttctttg gaaataatat ttttcttcca
31441 aactttgttt ttcgtgtctt ttaataagtg aggtgcgtta atattgccat tattttctaa
31501 tgcgctatag attgaaagga tctgtactgg gttaatcagt atttcacctt gtccgtaacc
31561 tgaatcagct aataatattt cattatctaa atttttgttt gaaatttgag cattataaaa
31621 tggataatca cttggtatat cttcaccaac acctagtttt ttcatgcctt tttcaaattt
31681 cttactgcct aattcgagtg ctactctagc aaagaaaatg ttatctgatg attctattgc
31741 ttgttttaag tcgatattac catttaccac ttcatatctt gtaacgttgt aaccacccca
31801 agatttatct ttttgccaac ctttaccatc gattttataa cttgttttat cgtctaatgt
31861 tttgttattt aacccaatca ttgctgttaa tattttttga gttgaacctg gtgaagttgt
31921 aatctggaac ttgttgagca gaggttcttt tttatcttcg gttaatttat tatattcttc
31981 gttactcatg ccatacataa atggatagac gtcatatgaa ggtgtgctta caagtgctaa
32041 taattcacct gtttgagggt ggatagcagt acctgagcca taatcatttt tcatgttgtt
32101 ataaatactc ttttgaactt tagcatcaat agttagttga atatctttgc catctttttt
32161 ctttttctct attaatgtat gtgcgattgt attgctatta tcgtcaacga ttgtgacacg
32221 atagccatct tcatgttgga gctttttatc gtaaagtttt tcgagtccct ttttaccaat
32281 aactgcatca tctttatagc ctttatattc tttttgtttt aattcttcag agttaatggg
32341 accaacataa cctaatagat gtgaagtcgc ttttcctaga ggatagttac gactttctgt

32401 ttcattagtt gtaagatgaa attttttgc gaaatctctt aaatattcat ccattttttt
32461 aacggtttta agtggaacga aggtatcatc ttgtacccaa ttttgatcca tttgttgttt
32521 gatatagtct tcagaaatac ttagttcttt agcgattgct ttataatctt ttttagatac
32581 attctttgga acgatgccta tctcatatgc tgttcctgta ttggccaatt ccacattgtt
32641 tcggtctaaa attttaccac gttctgattt taaattttca atatgtatgc tttggtcttt
32701 ctgcattcct ggaataatga cgctatgatc ccaatctaac ttccacatac catcttcttt
32761 aacaaaatta aattgaacgt tgcgatcaat gttaccgtag tttgttttaa ttttatattg
32821 agcatctact cgttttttat ttttagatac tttttttatt ttacgatcct gaatgtttat
32881 atctttaacg cctaaactat tatatatttt tatcggacgt tcagtcattt ctacttcacc
32941 attatcgctt ttagaaatat aactgctatc tttataaact tgtttgaaat ttttatcttc
33001 aattgcatca atagtattat taatttcttt atcttttgaa gcataaaaat atataccaaa
33061 cccgacaact acaactatta aaataagtgg aacaattttt atctttttca tcaatatcct
33121 ccttatataa gactacattt gtaatatatt acaaatgtag tatttatgtc aaaataatgt
33181 tataattttt gtgatatgga ggtgtagaag gtgttatcat cttttttaat gttaagtata
33241 atcagttcat tgctcacgat atgtgtaatt tttttagtga gaatgctcta tataaaatat
33301 acggttctgt tgcaaagttg aatttatagt ataattataa ccaaaaggag tcttctgtat
33361 gaactatttc agatataaac aatttaacaa ggatgttatc actgtagccg ttggctacta
33421 tctaagatat gcattgagtt atcgtgatat gtctgaaata ttaagggaac gtggtgtaaa
33481 cgttcatcat ttaacggtct agcgttgagt tcaagaatat gccccgattt tatatcaaat
33541 ttggaagaaa aagcataaaa aagcttatta caaatggcgt attgatgaga cgtacatcaa
33601 aataaaagga aaatggaact atttatatcg tgccattgat acagagggac atacattaga
33661 tatttggttg cgtaagcaac gagataatca ttcagcatat gtatttatca aacgtctcat
33721 taaacaattt ggtaaacctc aaaaggtaat tacagattag gcaccttcaa cgaaggtcgc
33781 aatggctaaa gtcattaaag cttttaaact taaacctgac tgtcattgta catcgaaata
33841 tctgaataac ctcattgagc aagatcaccg tcatattaaa gtaagaaaga caagatatca
33901 aagtatcaat acggcaaaga atactttaaa aggtattgaa tgtatttacg gtctatataa
33961 aaagaaccgc aggtctcttc agatctgcgg attttcgcca tgccatgaaa ttagcatcat
34021 gctaacaagt taacacaaag tattatttta aaattgagat tagacattta tttttcaact
34081 ttgcagcaga acctaatttt ttctctttgt aaataaatgc aaagagatac cataaggatc
34141 tcttacataa ccataacctt cagtatagaa ttctggacta aatgttttca atacctcact
34201 gtttttttct attaactggt catatacatg tttagtttct tctacttggt caaaagtgag
34261 acaaagagat atattattac cttgtgttat gggcaaacct tcagtgtcat ctgcgatcat
34321 aatttttata tctccaaatt gaagtacaca ttgatcaatt ttatttaaat cattttcgtc
34381 aatattaagt tgcttatcta taggtctatc tttaatacgt tgaatataca gtgttttagc
34441 gccaaacagc tcttcataca actttttttaa accctctgca ttttgagtga ttaaaaaaata
34501 tggacttact tgaaatttca tgttttttcc tccttagatt tgttataaat agagtatata
34561 cttaattagt gtcatctatt gacactaaaa ggagaataat aatgaaaaaa tctgttagat
34621 tatataatat gattgaatat tgtaatgaaa ataggaactt caaattaaat gatttaatgt
34681 cagaatttaa tatttctcgt agtaccgctt taagggatat aaaagaaatt gaagcattag
34741 gagtaccttt atatagtaat ccagggaaaa atggtggtta tacgatcata ggtaatcgag
34801 accaaacgaa aatagcaatc tcagatgaag agttgaaagc tttagtattt acactttcga
34861 gtatttcaaa tgtgagtaat ctaccttttc aaacagaata tcaagaaata ttaaaaaaat
34921 tatataataa ctcgaataaa aaagagttaa taaaccaata taatgatcta tttcaatatt
34981 ttaatgaaga taagtatcag ttcaaaagtt ataagttatt taatgaaatc attagattga

35041 taattgagaa taagtctttt gaaatctgtt attcacaaaa ctatattaaa gaacaatata
35101 aaggtattgg cattatgtat aaaaatcatc aatggtattt tgttgtagct aatatagaat
35161 caaagttagt gaatctatta aatatttcga aaataaaaga actatatgaa atgggagaga
35221 ctcaagagtg taatgatata actatgcaga attttcaaca gttcatggtt aaaaatgaaa
35281 ctgctattga tattcttatt agaagcaatg ttatgggatt gaatatcttg aaaggctacc
35341 tgtggagtga ctatatgatt gaaaatattg acgaagagac atatttattt aaatcaaaag
35401 tgaacgcgaa agatatagat ttcatagcta agttaattgt cacaggtggt gtcaatgtaa
35461 aagtagagac ccctaatagt ttgaaaaatg ctgttaaagt tgaattaact aaaataataa
35521 acatgtatta atagtaatat aatcataatt gtaataacaa aaagatgaca acattaaaca
35581 atttaaattt atacaaaaaa gcattatcaa atttaaaatt tggtagtggt agtgctacag
35641 catacataaa tagttataat taaataaaga tgggtcaatg aaggtgaatt aagtgaataa
35701 aaatattaca gctctaaaat ctactgaaaa tactacatat atgcagtata cggttcatat
35761 cgatgatcaa attataaata atataaaaga gtgtaccaaa aagtttgaaa ttttgcctat
35821 ggaagataaa attccactat caccgttgtt acaaccagaa tatgcaggag aggtacaagt
35881 ttttattagt acatatgagc agtttatgat taattttggt aaagtaatat tggatagtca
35941 aggcataaaa atacagtttg aaagtgaatc attaagtagt attcaacgag gcattcaaga
36001 acattgttac ttaaataagc gaacaaatga cattgatgtg actaaagaat ggtatttatg
36061 caaattttca aatcgaacat tagaagagga taaaaatcaa ctgtataatg ctttaaaaca
36121 actcatgaat gattctaaaa ataaaaagca agcttttcaa gaggtgttta aggttcatat
36181 tgatatttat ctctacaata aagaaaaatc tgaatataag tatcaagcat ctagttattt
36241 ctatcttgta aaagaaaatc ctaaaataat ttataaaaaa agacatttac aagagaaaca
36301 agggatcaag ggtacaactt tcacgaacaa attaaaatta ttgaacaaaa tgtatggtgt
36361 agatgttgca aaatatcagc cattttataa ttctaataat cctgaatatg aaagaggtca
36421 atttggtgaa agatacattt ctcaaagatc taactatgaa tttaatagac ttcaatatca
36481 aattatatat atattatcga aaatacttga taagcatcca ttaccaaagt cagataataa
36541 ttataaacat attccaacta ttgaaaaagc aatattaaga gatgattccc ataattttta
36601 tgagtatttt gaagatataa tgaaagaaat tatgaatatg gataattctc ccttacagga
36661 aaaattattg actgatttta catatcaatc ccaatgtaga tggtactctg aaaataaaaa
36721 attaaaattg caacttgaaa gatttatgca taaagtttta gagagtaatt attatgaggg
36781 caacaaatta ttgagaatga tgtctaacgc aattgaagaa acaatcaatg aggctaaaga
36841 agataaggtt catttcaatt attttaaaga ttattttttg acagacggtg gggtgaagaa
36901 ttgggagcaa attagcgaga aaattactga attcaatggt aaagttatta acgatatcca
36961 acatgaatat aataagatac agtccaagaa tacaaatcaa aatcagaaac taaactttgt
37021 ttatttatat cattgctttg agtttagtaa taacatagta aaagcaaaag ataatgaaag
37081 taaaaattat atcttgtttt ttatagataa atataaaata aagacaccat ttttggatca
37141 attattaata aattgtttga atcaagaaat agatcaggaa agtattaact acaatatgca
37201 aacaatgttt gaaaaagaaa gatacgatag aagtagtacc attgaaaaat tagtagcaac
37261 aagaaaattt aagtatgaaa aagatgattc agatttattc aaacaacttt tcaatgatgt
37321 tgaaaattca atagacagat taggtattta cttactaaat aatggtataa attcgaatga
37381 tgaaaatgca agatactata gatcgttttt aaaggaactt agtagaataa aaagtaaatt
37441 aacgccattt tctcttgaaa taagtaagtc tagcggaaga gagcaacatt atcctgatga
37501 tgctgttgat gataaagatg agagaagaaa aaataaagaa gaagcatatc atgcttttga
37561 cgataaaagc gatattgact acaaattaaa gaataaaata agtgattata tcgataattt
37621 attgtcagtt aaaattagga ataatttgta tgatattagt ccaaatacaa aaagcaataa

37681 ttataatgag ccaactcaaa aatgagttgg ctcttttgtt gtaattttac gcttccatta
37741 caggaatcaa tgttatggaa gccattaagt aattaatttg agtcatatat attatagata
37801 caacgttgtg atgttcacac gataatcagc ccgggagatt gttgtgtaat aacttataga
37861 aggagcaaat caccatgtat caagcaaata tacgtgattt aattacaaaa ttgcctcaaa
37921 gcaataaaac agaacacttt ttaatgaata aattttcaaa tcaagataaa gttcagcagc
37981 tacaaagaca aattagccaa cagctagatc aacaatataa tgagcttttg tctaatgaaa
38041 aagctaagct agaccaatat gtggaagtac accataattt agaaccatta aagaaagaga
38101 ttgaatcaga atctattaac cttgataccg ataaattacc tgatatcaaa gcgacaatgc
38161 ttgaaaaggc taagaacgat gaacattctg ataaaatcga acacctattt gatagattag
38221 atcagtcatt aaatggtacg aatcgattat atacgcaatt atcgttgatt ggcacacgaa
38281 cacatcggat tacaacgaaa agatttaatg ttcaaggctt acctaaatta gtccaacaaa
38341 cgattttacc ttcgcaattt aaaaaggttt atacaataga ttttaaatca ttcgaaccat
38401 cagttgctgc gtatatgaca caagacgaac aactgattga ctacttgaat catgaagaag
38461 ggttatacga tgcattactg agagacttat ctttgtcaaa agagaagcgc gtgagtgtga
38521 aacgtgcatt tatagggtca ttcctttttg gcggtcgtta tagtagctct aaattcaaaa
38581 tcaatcaaga ggttagtgaa attaactggc tacaagtaat gagcaaattc aagaaggtca
38641 ttgaatttaa ggagcaagtc gaaaaatata aaacaatgcc tacgccttac ggcattgaac
38701 atgatatgag cgcatttcaa ggtagtagta ttatggcaat ttacgtacaa acggtagcga
38761 gctatatttt caagtacatt ttgttggaag tgtacaaagc acagtgcgaa caaaaaatgt
38821 tcaagattat agtgccgata cacgatgcga ttatgattga atgtaatgat aaggagattg
38881 cacaaagtgt agcacagctc atgaaagata cagctaatca actgtttaat ggtgaatttg
38941 cacatgtgac agtggaagct ttaagaggta tagacaatga ataatgatag aggaaaaagt
39001 cttcaaattc cccaaagtac atcgttaaaa gaaggatcta tatacgttgc tacattacat
39061 tctgtgactg agacagatta ctcaggcgac ataaaacatc aatttacgta tgaaatagag
39121 atagatcaac aaatattcta tgtgaaccgt agtgttgtgg tcaatgtgac acatcaacaa
39181 ttatcaatta atgactggct cgaacgtcat agtaactata gcgcaagtca tgaaaactat
39241 gagccttata ttgatcagaa acatttaatt ctattaggtc aatataacgg taactattat
39301 gtacaagatg tagcatcgtt agatgcgttt ggaggcgtat tatcatgaat catatattac
39361 aaatgttatc taagctatta agtgtggcta aggaggcaat cgaccgtcaa ggtctgattg
39421 ctatcctaac tattcctgtt aataatgacg atgaaataga agaaacggct caaggtgaaa
39481 ccgtgtataa cgaacttatc gatcagttac gacttaatat cccaaaagat acggattatc
39541 gacctaacat ctatagttat tttggtatta agaagaatcc taatgacacc gtactcatgg
39601 aaatgatgat aaaggttttt catatcaaac gctttaattc agaactgttt gttttcaaag
39661 ctaacgggtg gcaaaagata aatggagatg aattacaagg gttgatatct aaaatgatac
39721 aagtattgct tgtagattat aagccttcac taagcactct aaaaaatgtc gtagatggat
39781 tgcaaaaatc aacagatgta gaagaacttg ttgagaatga gcgctatatt ggttgtggtg
39841 aaaatatgtt cgatcttaat acgtttcaag tcgttaaaaa ttcaatcgat atctttccaa
39901 aaacacgatt gaatttatca ttaagtacaa atgatgtaat tactgataag ataccgcctt
39961 attttaagca atatatgtta caacttgcga attatgacga tgatttacaa tactttcttt
40021 tccaacatac agcagtatta cttacagctg atactaaata ccgtaggggt ctcatattat
40081 atggtggagc taagaatggt aaatctgtat atattgaact agttaaatca tttttctata
40141 gtaaagatat tgtgtctaag ccacttaatg agcttgaagg tcgttttgac aaagaaagtt
40201 taattgacaa aagtctaatg gcaagtcatg aaattgggca atctaggatt caagaaaaga
40261 tcgtaaatga cttcaaaaag ttattatctg tagaatcaat gcatgttgat cgtaaaggaa

40321 aaactcaagt ggaagtcatt ttggatttga aacttatttt tagtacaaat gcgatactta
40381 attttcctcc tgaacatgcg aaagctttgg agcgtcgaat taatattatt ccatgtgagt
40441 attatgttga aaaagcggac acttcattaa ttgataagct ccagagtgag aagaaagaaa
40501 tctttcttta cttgatgtat gtgtatcaac agattgtaaa agcagatatc gagtatcttg
40561 aaaatagccg tgtcactgaa attactcacg attggttaaa ttttggatat gaatttgttt
40621 ctagcaggtc cgtaagtaat gcaaatcaga aagcatgtat taatttactc agaaaactta
40681 tagaaatcaa atcaggatca cgaatcaaag tatccgagtt aaataaagtt attaatgaag
40741 aaataaaggt aagttctcag gttattaaac agttaattca agcaaacttt gatactcaaa
40801 ccaaactata caatggctac gattattgga ttgatttagg ttggaaagaa gccaataaaa
40861 aagagattca tgatatttcg gaaaaagata atattatttc attagataaa aatgaaaata
40921 taacagacga tgaggcatta gatgaagaga atttggactt tgattgggag gactttgacg
40981 atgaataatg aacaaattga agcatttgta gaagtgcttg tacctatcat agaagaacgt
41041 atcaataaag gtaattaagg ctaattacgt actacaggca gttgcctgta gtactcatat
41101 gattaagtgg taaaagtgat aaaaatgaaa cgaaattata aatatatatt atccatatgt
41161 tgttacaaga ccgatagtct gtagcaataa tctaataaaa ggagcggtat gatatgaagg
41221 gtaaaattgc actttattca cgcgttagta cgtcagagca gtcggagcat gggtactcaa
41281 tccatgagca ggaacaagta ctcatcaaag aagttgtgaa aaatttccca ggttatgact
41341 atgagacata tactgactca ggcatttcag gtaaaaatat tgaaggtcgt ccggcaatga
41401 aacgtctatt acaagatgtt aaggataata aaatagaaat ggtattaagt tggaaattga
41461 atcgtatctc acgatcaatg agagacgtgt ttaatattat tcatgaattc aaagaacatg
41521 acgtagggta taaatcgatt tctgagaata ttgatacatc caatgcttct ggagaagtac
41581 tcgttacaat gtttgggtta ataggatcta tagaacgcca gactttgatt tccaatgtga
41641 aactttctat gaatgctaag gcacggagcg gagaggccat caccggtcgt gttttaggct
41701 acaaattatc acttaatcca ttgacacaga aaaatgattt agttattgat gaaaatgaag
41761 ctcatattgt acgggaaatc tttgatttat atttgaatca caataaagga cttaaagcaa
41821 tcacgacaat tctaaatcaa aaaggatatc gcaccattaa tcaaaaacca ttttcagtgt
41881 ttggtgtgaa atatattttg aataatccag tctataaagg ctatgtcaga ttcaataacc
41941 atcaaaattg ggctgttcag cgaagaagtg gtaaaagtga taaaaatgat gtgatattgg
42001 tcaaaggtaa gcatgaagcc attataagtg aagatgtatt tgatcaagtt catgagaaac
42061 tagcttctaa aagttttaaa ccgggtcgac ctattggtgg agatttctac ttacgtggcc
42121 ttattaaatg cccagaatgc ggaaataata tggtatgtcg acggacgtat tataaaacga
42181 aaaagtcgaa agaacggaca atcaagcgtt attacatttg ttcattattc aaccgctcag
42241 gaagttctgc ctgtcatagt aatgcgatta atgctgaagt cgtcgaacgc gtaatcaatg
42301 ttcatttgaa tcgtattctt tcacaaccta atgttattaa gcagattgcg tcaagtgtga
42361 tagaagaact gaaacaaaag catagtaaac aaacagaaat aaaatatgat attgatagtc
42421 tagaaaaaca aaaagcaaaa gttaaaacac aacaagaacg attattggaa ttgttcttag
42481 atgatgaaat ggatagcgaa atgttaaaag ctaaacaaag tgaaatgaat caacagttag
42541 aagtattaga ccaacaaatt aaagaagcaa aacaagcaaa tcaatcacag gatgatatac
42601 ccaattttga taagttaaaa gcacaactca ttttgatgat aacacgattc agtgtgtact
42661 taagaaaggc tacacccgaa gctaaaaatc aacttatgaa aatgttaatt gattcaattg
42721 aaattacgac agataaacaa gtaaaacttg taaggtataa aattgatgaa agtcttatcc
42781 ctcaatcttt gaaaaaagat tgggggtctt tttttatgcc taaatttaac tttgtgataa
42841 atgtcacaaa tgaaaatagg attgaaaatt tatcactttt accacttttt tagagtgaca
42901 aaagtggagg aattttgaaa tattttataa atatatattt tatttatgga gtacacatta

42961 taaattaagg aggtcattat aatgacgcta agcaaacaac ttaaaacgta tattactgaa
43021 cgatttaaat taaattatca agaaacttgg gcttgtgaaa ccgtagatgc ggtggctgaa
43081 gatgtattac ctgaaaaata tattaaaaat agtccacttg aacataaaat tttaaatact
43141 tttacctatt acaatgatga attacatgaa atcagcattt accctttttt atgttatcta
43201 gataaggaat tagtagcaat aggttattta gataattttg atttagactt tatattttta
43261 aatgacactc atcaagtcat tattgatgaa cgctacttgt tacaaaaagg gggcgagtaa
43321 ttatgaattg gatcaaggtc gctcaactat ctgtcacagt tatcaatgaa gtcattgaga
43381 ttatgaaaga aaaacaaaat ggaggggaat agtatgaaaa tcaatcgata cattacaaga
43441 ggtgttagtg aacatttatc tctagacctt caaatcttac tttggaacat ggtaaaagat
43501 cgagacaatc aacttcatac agattaccta cacattttta aactacaaga agatgataat
43561 atactctcaa tcacacatga acaagaacaa cccgcataca aattggaata tcactataca
43621 aactatgtaa aaaatcaaaa tgcattacct aagaaagtct acgtcatccg agaagatgat
43681 gtagacgctt tttattatgt gatgctttta ccagaagaat actaaaagga gtgacaatga
43741 tgaatacaat caaaaataca atacacacag aagcgatatt tagcgatgat gaacaacacc
43801 gctacttact caaaaagact tgggatgaaa agaaacctgc atgtacggta atcaccatgt
43861 accctcatct agatggcgta ttatcactcg atcttacaac tgttcttatc ctcaatcaat
43921 tagcgaattc tgaacaatat ggcgctgtat atcttgtaaa tctattttct aatattaaaa
43981 ccccagagaa ccttaaacat attaaagaac cttatgataa acacacagac attcatttaa
44041 tgaaagcgat aagtgaaagt gacacagtga ttttagccta tggtgcttat gcgaagcgac
44101 ccgttgttgt cgaacgcgtt gagcaagtga tggaaatgtt aaaacctcat aaaaagaaag
44161 taaaaaagct cataaatccg gcaacgaatg aaattatgca tccgcttaat cctaaagcgc
44221 gtcaaaaatg gacattgaaa taaaggagga ttatctatga accatgaaac taaacaatca
44281 gattggcgaa cggttgctag ttgtttagca tcacaaaatt atatatcgat tgtaaaaggt
44341 ttagtacatc attttacagc gatcgaagat gaggaaattc ttgataaaat ctatgatgat
44401 tttatgaatg atgactctat aacaacggta cttaacaatg atttacagac aatcattaac
44461 cattatctat caaaataaag actatactaa ttacaataaa aacagagcat ccttcacttt
44521 tatggtgagg gatgctcttt taatttattt ttttattgat tattcgccat aagttctgct
44581 tgctctacaa cctgctctac agccattttt tgtaaatctg gtggatagcc atattttta
44641 agcaatcgtc taacagctac gcgcatttta gcttttgcgc tatcacgttt agaccaatca
44701 acacccatgt tttctttcac tgttttagtt agctcatgag caatcgcacg tagttcttta
44761 tctcccatgg cttcttttgc tgtttcatgt gaagctaaag catcgtaaaa cgcaatctca
44821 tctgaattca ggcctaattc atttcctcgt tgttgttctt gtttaatatc tttagcgagt
44881 tgaataagtt cttcaatcac tttagatgtt tcaatggaac gactattata tttattaatc
44941 gaatttccta acatctcaga gaaacgctta gataccgttg cattcgtttt cattaatgat
45001 ttaacttgtc ctttgagtaa tcgatttaat aattctaccg ccacattttt ctgtttcaat
45061 ccttcgacat cttttaaaaa gtcatcagat aagattgata aatcgggttg ttcaagacct
45121 agcgtttgat aaacatcaat gacatcttca gtcacaacag attgtgacac aagttgatta
45181 atctctgctt caacttctgc aggtgtttta cgtgtttttc cttcttttgg cggttgtaac
45241 aatttaacaa gtcctgcttt aactgcttta aagaaggcaa tctcatcgtt gagttcttgg
45301 gctgtaggtt cagtcgcaca aagagcaaag gctttcccta attctgtgac tgttttaata
45361 aaacgttgac gttcatcttc acctaaacca attacataat ccatcgtatt cgatattgta
45421 taataacgtt cagatttttt atctgaatta aatttagagt aatcaagatt atataacata
45481 tcttgaatta catcatactt caataacatc aattcaaccg ctttatccgt atctattgcc
45541 gtttgagctt gatcagattc tgtatattct ttaagtgcct cttttaaact ttcggcaatc

45601 cccacataat cgacaattaa accacccggc ttatctttaa acactcgatt aactcgagca
45661 attgcttgca tcaaattatg ccctttcatc ggtttatcaa tatacatcgt atgcatagaa
45721 ggaacatcaa accctgtcag ccacatatct cgaacaatca cgagttgtaa ctcatcatcc
45781 acatctttca tacgtttttc taataaatta cggcgttttt taggaccaat gtgtctttgg
45841 aaagaagttg ggtcactaga tgagcctgtc attaccactt taatgactcc tttatcatca
45901 tcatctgaat gccattctgg ttttagacga atgatttcat catataaatc aacagcaatt
45961 cgacgactca tcgttacaat cattcctttg cctttcatcg cttgttgacg tgtttcaaag
46021 tgttggatga tatctttggc tagggcttct acacgaggtt ttgcgcctgc taaagcttca
46081 atacgggacc attttgattt taaacgctgt tttacatcct cttcttgatc ttcagtaatg
46141 tcattatacg cttcatctaa atctaaactt tgaggtagat ttaatggaat tacacgactt
46201 tcatagtaaa ttttaacggt gcttccatca gcgacggctt gtgtcatatc ataaacatcg
46261 atatagtttc cgaaaaccat ttgcgtattt ttatccgttg aagctacggg tgtgcctgta
46321 aatcctacga atgttgcatt cggtaaagca tctcttaaat atttggcata accatattta
46381 atgccttcac ctttatcatc gtattttgcg ttaaagccat attgtgtacg atgagcttcg
46441 tctgccatga caatcacatt ttttcgttct gttagggcag ccatggtcgt ttcgttttgt
46501 tcaggttcaa atttttgcat cgttgtaaat acaataccac ccgactcaac agataataat
46561 gattttaatt ctttacgtgt ttcagcttgt tttggtgttt gtcttaataa tcctttacca
46621 gaacgtcctt ttgatttaac aaacgtactg tacagttggt tatctaaatc attacgatcc
46681 gttacgacga ctaaggtagg gttattcagc atttgaatta attttccaga gaaaaagacc
46741 atggttaaac ttttaccgga cccttgggta tgccaaataa cgccgccttt accatcacct
46801 tgctcagatg aagctaataa agctctgtca acggctttat taacggcata atattgatga
46861 tacgctgcta gaattttact gatatgtcct ttgccatcat cttgaaataa aacaaaatat
46921 cgaattaaat caagtaatgt ttctggattt aacatgccat gaatgagtac gtctaagcta
46981 gctaaacctg gtgatgattc agtttgacca tctttactgc gccaagtcat aaaccgatca
47041 taatttgcgg ttagagagcc agcctttgta ttaataccat cacttgtaac aagcacttcg
47101 ttaaacgtaa ataattgtgg aatacgcatc ttatacgttt ctaattgatg ataaccgtct
47161 tcgacgccca cggtttcatt tgttgaattt ttaagttcaa tcactacgat aggcaagcca
47221 ttgataaaga gtacaatatc gggacgtttt ttatagtctc cattaacgat agttaattga
47281 ttgactgcta aaaaatcatt gttttgtgga tattcaaaat caataatttt aacaatttct
47341 acaatcgatt gtccttcatc atcatagtct tcgatttcaa taccattaat caaattttca
47401 tgaaaggtaa ggttattttc taaaagattg ggcgacttct ctaaagttag ttcatgtagc
47461 gctttttcaa taaaacgagg atggatatct gagttgattt ttcttaatgc tttctctaaa
47521 cgttcatgaa ggacaacatc tttatcactt ttacgttctg gtgttagact tgtcatacta
47581 atctcattac cttttttata gtcatagcct agtgattgta gccattctaa tgcgacttgt
47641 tctaaatcat cttcattaaa ttgaaagttc atcactattc acctcaatat catcagatat
47701 ttctagttct ccagacatta gtttaggaag aagagtatct cgtaattgag ttaatttacc
47761 aatttctgat cttaatattt tcatttcttc catatatgga gtgattattt cagaatattt
47821 ttttacaata ctatcttcaa ttgctaattt ataattaaca gctattgttg gttttactct
47881 ttgtctacta tttgttgaac cggtagtatt agcttttaaa taatctataa attgagaatt
47941 aagacatata ttatatatga atgaattaat tttattatta ggagattcca ttacaacaaa
48001 ttctgatgaa gcaacattta attttttatt atctattaca ggtaaccaaa tccttttagt
48061 atcaggattc atttttgaaa ataatacgca attgttatta ataatccatt tattactttt
48121 tattttattg acttcttcct ctatagcttg ttcttcatta tcataagcag gtaaactaaa
48181 atgtttaact gttacttctt cactcttttt aggattaaaa gtttcctttt tatgacttgc

48241 tatatctttt aatttataga ttttccaatt acttggtatc ttacctaact cactatcaat
48301 catttctcct ccactagatt tataaggatt accatcttca tctggaaatt caaaatctac
48361 aaaccaatgc ttaaatagtg tttgtgaaag ttctttaagg tttgctatca ttttttggtt
48421 agtttctatt tttcttgtga tttgatttaa aaatttagaa atgctttttt gttttattaa
48481 agaaaaatta ggaattttga cagtttcaat aagtttagaa tttatattag cttgattagc
48541 actccctgta gaatttctaa ctaaatactc agttatttta ggttgtagaa aataataaaa
48601 taaaaaatct aagtctgcac tattactata ttttaattcg aattttccca cacgttgatt
48661 ttgtaatgct ggataatcaa attcaattct tccaactttt cctactgcag aagacatttg
48721 attcacgcct gatccagtta atgatattaa tatatcattt ttctttaatg aaaaaccttt
48781 agttttttca tatgttttaa tagagacttt ttgagagtct tttgtatcaa caatgggtga
48841 aattatattc ttaattttaa taaccggtat acctttttct tgaaattctt ttgatttaaa
48901 agcatatcca tttttaattt tagctacatc accgaaaaaa attgtattat actccatatc
48961 ctaacccctc caatgatttt cggatttggt cttcaagttc ttttgatttt gcgaattgtt
49021 cacttaattc agaagtaata cgttccattt tttgttcaaa aggttcttca tcttcttcaa
49081 cgtcagctaa accaacatat cgtccaggtg ttaaaatgta ttcattattc ttaacttctt
49141 ctaaattagc tactttacaa aaaccagcta tatcttcata aggtttatcg tttgtacctc
49201 tccacgcatg atacgtctgt gctacttttt gaatatcttc atctgaaaat tcttttaatg
49261 ttctagatac catatggccg atttcacgag catcgataaa taaaatttca ttttcacgct
49321 ctttttttacc attttgacct ttattattac taatgaacca aagacataca ggtatttggg
49381 ttgagtaaaa gagttgtcca ggtaaagtaa caatacattc aactaaatct tgttcaatta
49441 gatttttacg aatttctaat tcatcttttc cgcttgtaga cattgatccg ttggctaata
49501 cgaatcctgc tgtaccatta ggtgctaatt ttgaaatcat atgttcaatc catgcatagt
49561 tagcattacc ttttggtgga ataccaaatt gccaacggta atcatcaagt aatcgttctt
49621 gaccccaatc acttgcgtta aaaggaggat tggctaatat gtaatctgct tttaatcctt
49681 tatgtaaatc attatggaat gtatcagcat ttcgttcacc taagtcatta tcaataccac
49741 gaatcgctaa gttcattttt gctaatttcc aagtcgtagg gttagattct tgtccgtaaa
49801 tcgcaatatc gtctaatcga ccttgatgtt tctcaacgaa gcgttcactt tgtacaaaca
49861 tcccacctga accgcaacac ggatcgtaaa tacggccttt ataaggctca atcatctcaa
49921 ctaataactt tacaattgat gacggggtat agaattctcc agcattttta ccttcggcac
49981 ttgcgaattt agcaataaag tactcataca cacgacctaa cacatcttgt ttacgactct
50041 ctgtgtcgcc aactttaaat gtaaataaat caataatatc gcctaatttt tctttatcta
50101 atgcaggtct tgcatattct ttaggtaata cacctttgag tgattcattt tcattttcaa
50161 ttgcaatcat tgctttatca ataatttgtc caatttctgg ttttttcgca ttatcattga
50221 tatattgcca tcttgcttct tttggcaccc agaaaatgtt ttctgctaag tattcatctt
50281 ggtcctcttc atctgcatat ggatcctgct tcaattcttc atacttttct tcaaaagaat
50341 ctgatacata ttttaaaaag attaaaccta gtgctacatt tttatactca gcagcatcca
50401 tacttcctct caatttatcg gcagcttgcc ataatttttc ttcaaaacct atagtggcca
50461 ttaataacac ctcttaaaat taatgttaaa tcaactatag caaaaaatag aagatgattg
50521 tgtagtgttt tattaatatt taatttatct agactatcaa aaaaatatag attattatta
50581 tcttaaatta ataaacagga gtgaattaaa atgaataaaa aatataaatt aaaacgatac
50641 gataaaagat ttagaggtgg atggtgttac aaatgtgaag attcatattc ttacctttgt
50701 gaagattgtg gttattgtag atattgttgc cattgttaat acaagactag ataaattggg
50761 tatctttaac tcaattaata aaaaatttaa atgtagtcaa tagcttgcta actttatttg
50821 atgtaatata attttggctt ttagcattca gttaaaaaga agtaaataat tgacagattt

50881 tttagagaac tggagaaaaa agatgtacat ttctagtgtt aaaattaaga attttagaaa
50941 ttttgataat atcgatattg atttccatga gggagttaat gtattaatcg gacataataa
51001 ttcggggaaa tcaaatttat tacgtgcttt atctttaatt tttgacggtt cagttagaaa
51061 gcaactgtca gtggaagatt ttaataactc tttaacaaaa gaatctttaa aaaaagaggc
51121 tccaaaaata gtaatttcag tacatataac acaatctgaa aacgaaagac tgatgtctga
51181 tgagttaatt acggtaagta attggctgac taaattagaa gaaccttatg aagctagaat
51241 acaatatgaa ttctttttac caaaagatga ggaaaaaaat tacatagatt tagtaaaaaa
51301 tatagatgaa aaagaagaaa tatggaattt aattagaagc cagtttatta ggttgtatgt
51361 caataaaatc tgggtgggta atccagaaca tcagattcct atagataatg atagtttaaa
51421 taaatttgat ttccaatttt tagacgcaat aagagatgta gagagagata tgtttagtgg
51481 taaaaataca ttattaaaaa gtgtaataga tttttttata gattacgata taaaatccga
51541 tgaaacaatc acagaagaag aacagaaaga aaaattagaa gaaagaagaa aagaattttc
51601 tgataattct agtgatttga ttgaaactat tcataaaaga ttagatagtg ggaatcaaaa
51661 aattttgtct tatacaaatg gcataggagc atcttatgat aaatcaacac cggattttaa
51721 aggtaatctt acagaaagtg aaatttatac tgtattgcaa ttgataatta aacatgaaac
51781 tggaatgact ttacctataa ctcataacgg gttgggttac aataatttaa ttttcatggc
51841 cttgctattg tctaaaatgc aagctgattc tgatggaaat tttctaggta gtaatgcaaa
51901 agtatttccg atactagcta ttgaagaacc agaggctcat ctacatccta ctatgcaaaa
51961 tgaatttatt aaatttttaa aaaataacat tagagaaaaa aaggtaaagc agattttcat
52021 aactactcat tcaacacata tttcttcttc tacaaatatt gatgatatta tttgtcttta
52081 cacagatgat agtaaaacta atgttagtta tcctggaaaa gtttttgata agaataatca
52141 aagtaagaag tacgtacaac ggtttttaga tgccactaag tcaaatatgc tatttgcaga
52201 aaaggtaata tttgtggaag gtatagcaga acaattacta ttacatatat ttgcagacta
52261 tttaaataaa ccgttagaaa agaatcatgt tgcagtaata aatgtaggcg ggagatattt
52321 taatcatttt ttatctttgt ttgatagtaa taataaatat gcaattaatc gtaaggtttc
52381 ttgtataaca gatttagatc caatgcgaaa agaaaaaaat gaaaatggac aaaaaaataa
52441 ttttaaagta tgctatcctt ttgaatatga aatggattta gataaatttg attattcaac
52501 taataattct ctagataaat atctaaataa tgagcacagt aatatacgag tatttgttca
52561 acctaataaa tatggaaaaa catttgagta ccaacttatg tttgacaatc cttcattaaa
52621 actgttactt acagactcga tttctaatag tcaagagcta gcagaattaa tggatcacta
52681 tgaagaaaat gaatctttac aaaaattaat tgatacatta tcaccatcta atgaaaatca
52741 gagaataata aaatctttaa aagacacaaa tgacagttgg aatgaagata ataaaaagaa
52801 agcattaata gcatcaagat acctaaattc cataggaaaa ggagaaaatg ctctagagct
52861 agcttcagta ttaaaagata atttagagtt aaaaggccag agtgactatg aagattttgt
52921 agtaccaaaa tatatagaag gtgcaattcg atgggtgtgt gaataatatg attattaatg
52981 ctcaaacgaa attaaatata gaacaagatt ttaaaataca ggctggtcct ggtgctggaa
53041 aaacagaatt tttggtgaat catatcaaaa atgtaataca aacttctgaa aagctcgaac
53101 gtatgaagaa aatagcgtgt attacttata cgaatacggc tgctcagaca gttttacaaa
53161 gattaggaaa aagtgtttca aatagagtag atatatctac aatacatagc tttttgtata
53221 gaaacgtagt taaaccatat tgttcttttc tacccaaaga atacggaatt tgtgtgcaaa
53281 aattaaaagg acatagtgat ccaattgtga ttaacaaata tgtaaatgca tggttaaaaa
53341 ctgaatattt tacgaaatta aaaccgccta gtaatagaaa tcaactacta aaaatgccag
53401 tactaaagaa agctttgcaa aactggctac tatctatgaa actttcttac gaaaataatg
53461 atgtgcaatt tatttgtgat aataccaaag ctcaagcaat tgataaaaaa agtaaaaaaa

53521 gaatgggaat aaataaatca aacttaaata tactagaaaa gcaattatta aattataaaa
53581 aaatatattg ggagaaagga attatcgatc atgatgatgt tttgtttttt tcatatgtat
53641 taattagaga ttacccattc attctcacta ttttatgtgc taaatatccc tactttttta
53701 tagatgaatt tcaagatact agtcaattac aagattttat tatagataaa ataagacaga
53761 aaggctgcat agtgggggtt attggcgata aggcacaggc aatttacagc tttcaaggtg
53821 cacgggtttc cttatttgag aagtttaaag ttgatttaat aaattctcac actatcttag
53881 aaaatcatag aagttcatat caaatagtta ctttttttaaa tagtatcaga gaagatatta
53941 ctcaaacagt atatagtggt attgaaaatg ataaagttac aattttgatt ggaaatcgga
54001 ttcgcagtta tactaaggta gttaatattt gtaacaagga gtcacttgtt accttgtcaa
54061 gagataatat tacttctaat gcaatgagaa taaagttgga aggaaataat tttgataaaa
54121 agttattgga aaaatttcaa gaaattgata gtaatagtaa aagaaggaat actattattt
54181 cgcttataaa agctattgaa ttagctaaga atactaaata taaagatgcc ttaaaaatag
54241 tcgaatcgat ttataaagat gaaaataatc ctaaaaaaat agctcttaat tctctaataa
54301 atatgattaa taaatatgat aaatacggta atggtacatt aatgaatttc tacaatactt
54361 tatgtaaaac tttagatatt aaactttcag ggtttagaaa ggggcaagta aaagattttt
54421 acgaaaacac tttatataaa aatatggtga tatgtacaaa tatcgttgaa gacacaagta
54481 atcatattac tatccacaaa gcgaaaggcg cagaattcga aaatgtattt gttataggta
54541 ataaagacac tttaagtcta cttttgaaac cagatttaat gaacgatgaa gagcatcgta
54601 tattctatgt agctatgagt cgagcaaaga agaaactttt tttacaattt gattatcttg
54661 ataataatga acaaaagaaa attaaggaac gttatgatgt aagtattata caataaccta
54721 atctaagaat atttaaatcc aatatatttc ttgattataa ttcaataata gataaaatct
54781 ataaacgatt aaaaagaaaa tatatttgct aagacttata aaaatgaaga gataatagat
54841 tttcctaaat tacagttaaa agaagtaaat agattgaaca ctttgaaaga ttctaatcca
54901 aaatttgaac cactaaaatt gaaagatgaa gaagatgcaa gatgtagcca tcaacgctga
54961 tcacaaagta tttttcttaa tgattatttt taatgttatc atgctcgttt cttctattat
55021 tcaaatgatt ttaggaaaag gcagacgtat cacaccagca aaacctacta aagatgctaa
55081 gttacactta gataatcaat cctcataaag agaaaaaaga acccctaaaa agttcaactt
55141 tttgggggtt acttactatt aagaagtatt tttgattaag catctttcat atcgtaatca
55201 ccaatatact gatcaaggcg atttattgtt tctctgatat catcatgtgt cgttaatggg
55261 ttcgttgtac ataaacgaat gacacgttgg ttattcaata ctgtggtata agcaatggca
55321 taaccagaat ctgcaatttt ttgcgcagcc atgctattga gttgattatt ttgtgcttca
55381 gttaaatcac tgttttcata tctgaagttc acaatggaaa gattagcgtg tgagataata
55441 cgccagttat cgagacctga cacgtattct tctgcatatt ctgctaaacc ttcgccgtat
55501 ttaatgcgct cgataatttc atcttcacca atgacttgca gagtaatcca taattttaaa
55561 gcacgtgcag ggcgtgtaag ttcaatgcct aacatttctg gatcaatcac atcatcatct
55621 gaagcaatat catccaagta ctcagcatct tcgccaaagc tttgcagcag atgattcttt
55681 tctttcacga ttaccatagc gcaactatat gtttggaaca gtaatttgtg agcatcccaa
55741 ctggcactat ctgaacgttc aatgccttta aataagtgtt gtgctttatc ggataagata
55801 tgtgatagtc cataagcacc gtctacatgg agccatagat tgtagtgatc acatgtatct
55861 ccaagtgtgg tgaaatcatc gacagaacct gtattagtcg tgcctgcagt cgcaatcacc
55921 atagcaggtt tgtaaccttc ttcaatatct ttatcaattg cggcttttaa gctatctgta
55981 ttcattgtaa agtcatcgtt gtactcaatt ctgcggatat tatttttaag gaagcctgca
56041 acatgcaagg ctttgcctac agaatgatgc gtttgagatg taagataaac ggttgctttt
56101 ttaatatctt ccatttctac ctgtgcatct ctagccgcaa cgatagctgt taaattggcc

56161 attgaaccgc cagatacaaa gacaccaccg gccggtttga tctcataacc gatttttcct
56221 accaagtagt taattaaatt acgctcaata ttaattggca gcgtcgcatt ggcaaaatta
56281 gaggcatgga tattatttgc tgttgttaaa atgtctccaa gccatgatag acgtgatgct
56341 ggacctggta taaatgagaa tgaacgtgga tggttcggac gatataaata attcagtact
56401 tcattattta aatctttgag tacttcatag atatctcgcc ctttattcgg cacttccatc
56461 ttttcatatt tctctctcaa ttcgagcggt gcctgttgtg tcgcaggtaa gtctccaata
56521 tctctagata aatatttatt aacttcatct ctgataatcg tctctaagtc gatattgttc
56581 tcattgaatt ccatttccat actctctcct tcaattatat ttttaataaa agacagacgt
56641 attaattacg acgttttact acaatataaa tgatagcaga tattaataaa accacgctaa
56701 ccataatgac gaaacccaga aggtgtcgcc ccaatgaata acgtgctgtg ctttaacact
56761 cggtgtatgt gcgcctttca gaaattgacg agggtaatcg acttgcagat tatcatctgt
56821 gattttcaat ttgtattgat gcttgtcttt tggaattcga taactacccc gtagaacagg
56881 ggacgaggac ctcatcttga tgatcccaat tggcttggtt aaaaagaaat cgataatcct
56941 cgtagaatag agaaaaagta ttaaaaatat atttgatttg ataaaatatt ttgcaaaaaa
57001 gagaaacaaa aataactttc atataaataa tttctttgcg attaagttgg aattagaaat
57061 actaaataca aataacagcg aatggaatgc atattattga ggaattggat gtgtagtgca
57121 atttcgagag ttactactta acttaagaaa ataaaaagaa gaaaacaaac aataagaatt
57181 aaatctgaag caagatataa aagcaaatat ttttgctaca attatattag taaatacttt
57241 attaattagc atacgactga aagcgaagta acagctaata atgttccaga tatagacatt
57301 agtgttatga aagaacaata ttaccatcta gaaaatgtaa tttttaaaaa cctgaagata
57361 aagtagacta ttctgtgagt tttcatgtta agaattctga acttggggat gtagtataaa
57421 tatggtttag tagaaaatga cgctggagct gtagctgaga ttgactatca agaattaact
57481 aaagtagatt attacccatc aacaaaaatc caagatttaa cactatctaa taatagtgcg
57541 aataatgaag tttaaaagtg aaaaactgat aatagcgaat caaactaaag taatcaatta
57601 cagaattcaa gtactactca agcctcaaac aaatattgaa actcaaacac aagttaagaa
57661 taatacactg tcagaaacag atgaacagtc taatgccgat ttagtccagt aatagcctct
57721 gtattattag ccgcacgatt attatcaata attaaataaa gttaatatta aataataaaa
57781 atccttcaaa ttatttgttg aaaaaataat aactttgaag gttttttagc aaaatcactg
57841 atagggagaa gcgtataata agtaaaaata attgatgaac cacgcagtta tacataaata
57901 tgactatgtg gtttttatac tatataacca taacaattat atacaaaaat aatttaaaat
57961 gaatgggtgt ttattgtttg gtaatataaa agagagattt gtattataac tcaatattta
58021 tatgtagata ccatgacttt aaaaaagaat tcaaaaacag agagatgtga aaatgtgaaa
58081 cattcgaaaa agttattttt atgcgtcagt tttttattaa taacgatttt tattggtgga
58141 ggtggattta tgaataaaga cgagagtaaa gaaacggaaa tcaaaaaaag ttttaataaa
58201 acgttaagta tgtatcctat taaaaactta gaagatctat acgataaaga aggatatcga
58261 gacgaagaat ttaataaagg cgataaagga atgtggatag ttcactctga aatgaatata
58321 caaaaaaagg ggaaagcatt ggaatcaaga gggatggttt tatatatgga tagaaataca
58381 agaaccactc acggctattt tattcttacc aaaataaaag atacaggtaa agtcaaaaca
58441 gatgacagtg aaaagaaata tccagtaaag ctagaaaata ataaaataat tccaacaaaa
58501 caaattaaag atgaaaagtt aaaaaaagaa atagaaaact ttaagttttt ctcacaatat
58561 ggcaatttta aagatttaaa agattataaa gacggtgagg tatcttataa tcctaatgcg
58621 cctaattatt cagctaaata tcaactcagt aataatgatt ataatgtaaa acaaataaga
58681 aaaagatatg atattccaac cgaacaagcc cctaagttgt tattaaaagg tacaggtgat
58741 ttaaaaggtt catccacagg ttctaaaaat attgaattta cttttgtaga gaaaaaagga

58801 gaaaatattt attttactga cagcgttgaa tacacgccaa gtgggtaaga aaagctcaaa
58861 aaaattatca tccatactca taaaaaatag aactgatgag attattacgc tcatgagttc
58921 tatttttatg tcaacaactt tatttccaac atcaccctaa atattgcatt tgatatccga
58981 ttctcggagg aggataatta gttccttatc cataccatca aaattttcac tataatttaa
59041 cttctacgac gccatgtgca cacgtaagga tgccatttcg cacttattga cgtatacgat
59101 tcttttttta aaatggaatg aattttttc tgattaacat accgttagtc ataatcacca
59161 acttattctt aaagttagtt cttatatagg aaataatttc taatatatct tttctaataa
59221 agggttctcc gccagttaga ataagcgtat tcgggttgac gtcaataatc ttatcaattg
59281 catatttaac atcatttgta cttaaaatat cttgtgataa aggagaagca gatagagcac
59341 aatgactaca agaaagattg catttatttg taagagtaaa agacacactt aaattatttt
59401 ctttataagt tttttcttca tctacgtagt atcgtatttc aagcaattcg ttaaaaagcc
59461 gctttaagtt atctatcaca ttacttacat atttatactt cataaaatct ccaatttaat
59521 tagattcaac caatttgttt aaaacttcaa aatattttct acttgttctt acccattgtc
59581 cattgtttaa gttgactagt aaaactttat ttttaaagta aacttcatgt acatttgtat
59641 ttttgatata catctaatca cctcgatatc aagttatcat tactttatca attttctatc
59701 aatttaatta atattgacca aaccttcata attgtatatt ttgtgaattt cctaaaattg
59761 atatattatt gatttctttt tgatatgttg atataaaagg aggctaattt tggatgaatg
59821 aaatagttat caaaggtttg agtaaatcat atgatggaaa gaaagatgcg cttaattcat
59881 tggatttagt aattcccaat ggtatgtttg gtattttagg aagaaacgga tctggtaaat
59941 ctacattaat gaatatcatt gcaacaattc ttcaaccaag taagggaaca gtaacaatta
60001 atggtattga aattaaaaat tcacaaaaga ttagacaaat gattggttac ttacctcaag
60061 attttgattt ttatcctaat atgaaagtct cggaagtttt atactattta ggttttttat
60121 ctaaaataaa tcaaaaggat ttcgataaat atgtagattt aattcttcgt aaggttaatc
60181 taattgatta taaaaataaa aaggttaaaa gtttatctgg tggaatgaaa aaaagattag
60241 gtatagcgca agccatttta catgacccca aagtaattat tgtcgatgaa ccaacggcgg
60301 gattagatcc agaagaaaga gtaaggctac gcaatttact atctgattta gctgaaaaca
60361 aaattgtgat tatatcaacg cacattgtga gcgacattga atcaacttgt aataggattg
60421 ctattttaga taaagggagt cttgtatata aaggtgacat accgtcgttg atacaacaaa
60481 gtgatgatca tatttatgaa atcaatctat ctccttcgga attagaatca tttagagaaa
60541 acgacttatt tattactaaa actcaagaaa ttgataataa attaaaagtt aggtttattt
60601 ctgaaacaaa agttgcaaac gctaaaaaag ttaaagctga ttttgaagat gcttatatgt
60661 attttttaaa aaaacaacaa ggaggtttga atcatgcgtc tatttataat ggaatttaaa
60721 catttaatgt ttagtaagac atttctattt tattgtttat tgattattat attcacggta
60781 ttgaattgtc aacaagttct caaacatcct attaattcag atgtatatca acctcaaaat
60841 atgactatgt attctactga taaaaactta ataatgtcaa atgaaattaa agaattaaag
60901 aacgaagtac atcaaaataa ctttaaaacg tatcaatttg gatttttaaa gcaaaaaaag
60961 ttaaatgacc aacaaatttc taaaataaag aaactaatat caaaaatgga aaaaacgaat
61021 aattttcatc agtttaaaaa ttattctaat agcgtatcac atgttgttgg atggggatct
61081 caatattcta aaaataatat ctcattattt ggaattaaga atatgagtaa gagtaagtat
61141 gttaaagaga agaatattat aaaaaataaa gagtactatt atggagcata tagtagatat
61201 ttttcagata ttatgggggt agtgttagga atacttccat tctttttggg agctcaaatg
61261 attcttcaag acaaaaaaag taatgccatt aaaacgattc attctaaatc tatatcatca
61321 taccaattaa tttctacacg tgtatttact attcttgttt tggcatattt accagtatta
61381 ttagtatcaa tatattttgt tacttgttta tcgatgatac atcaaatatc gatgttttct

61441 ttatttattt tttataaaat tttaatcttg tggacattac ccactttaat atttactatc
61501 gcgatgggat tttttgtcac aattatattg aattcttatt taggtgtaat gatacaaata
61561 gtaatttggt ttatcaattt aaatataggt tctcgtgcca ttgaggggtg ctatggttct
61621 ctattgattc ctcgacataa tactttattt aacgcagttt ttttctataa tcacttcaat
61681 caaattttag ctaatagatt tacatacttt ggcttaggtt tacttttaat tctacttagt
61741 acatatatat ttgacctgaa aagaagagag gtaggtttta atgccaaaat ttttactaat
61801 agatatcaaa actaatttag tcaaacttta catgggaatt ttaattctat tatttggtgg
61861 aatgtttatt tttggtgtat ctaatcttaa tcatgagaga gttacacaat acattgagtt
61921 aatatttact atactaggca tattgttatt tactgctatc tattctgtag atcaaaacaa
61981 gaagtctaag ttaactatta gtgcaaagtg tcatgactac caattcgtat ttatgcttag
62041 attagttata acaatcatct atatactgtt cttaattaca atcaatttat ttatatttta
62101 tttatttaac tcaaatatca ttgttttgag agatatgttt attgctttaa ccagtacttt
62161 atttatagga tcaatatgtt tattaacaag tattcttagt ggtaatacat taaatggctt
62221 aatgtttgga cttgtatatt tcttattctc gcttaggatt tagaaatcta ggctttatgt
62281 atttattctc agaaaccttt aactttccat tttatataaa aatatttcaa tttttattag
62341 gaattgtatt attaattctt agttacacta ttatcaaaaa agtacaaaat taatacgttt
62401 tggaatcaag cattgtcatt tataatttct agcgtaacat gaatactata aaaacaagct
62461 taggtgattt gtgatgtata acataatgat tgtcgacgat gaagaaaaaa ttgtgtcatt
62521 tatagaagaa agtttatact tagaaggttt taacactatt gtcgcttata atggacgtga
62581 cgttttaaaa caaatcgatg acaaaattga tttaatcatt ttagatatta aaatgccgta
62641 tatagatggg tttgaagttg ctcaatcact taaagagagt aatataccta ttatttttct
62701 tactgctaaa gataatcttg atactagact gaaatgtttc tcgcttggtg caaaggatta
62761 cttacctaag cctttctata tggaagaact tataattcga attaaaaatg tacttaatca
62821 aagtcattct caaagtttga ctcccaatat aaaaaaaact aaagaattaa tcatttatta
62881 tgatacatat agaattgatg taaataacat gacgatagat attacaaaaa aggaatttga
62941 aattataaaa ttactatctt taaatcctca atattatttc agtaaagaac agatatatga
63001 ttcgattaat ttaaataaga caggcaatac tcaagttgtg gctgaacata tacgtaaaat
63061 aagaaaaaag ttaagttgtt atagtcgttt tgaatacatc gatacaaagt ggggagttgg
63121 atataaatgg ctagagtaga aagtttaaaa agaaaaatga cacgtcttat catagcaatt
63181 atagttgtca gtgttggatt agtattttta ctacaaattt tacttaatat attagaattt
63241 tcaaattttg gaaagtcttt atacactaat ttttggaaac aacctcatta cattttctct
63301 tttactagcg catgtatcag tattatgact cctttgttaa tcatttacgt gttcacacaa
63361 ttttttata aaaagtacat tacaactagt attgattatc ttaatgatac gattcaagga
63421 ttaaaaagta accgtactga tgttgaatat aagaaaatga gtatttcaga atttaataaa
63481 ttgattcaag aagtagaaca tatacatact tatcttgcta atagtattaa agatcaatta
63541 tctattcaaa ataattttaa aagaaaactc gaaattttag agcatgattt aaaaaccccg
63601 ttaaccgtcc ttcaaggtca tattgaatta ttaagaaaga ttaattgttc taatctgtct
63661 caggaggaaa tacattttaa aataaacagg gaagcagtca ttgcgttaca atctattgat
63721 agaataaata atcaattaca aatacatatt aaaaacatca acttattgcc aaaccattca
63781 gatacaattc atgttgataa ggtaataaaa atcattaaag agagctataa tcaacaatac
63841 atattaaagg agagagaact gattattgat gttgatcatg atttcccta ttatacctta
63901 agcattaaca atcaagttct ataccacgtc atcgataatc taattaataa cgccttgaaa
63961 tatgctgaaa aacaaatatc tataaagttt gaagttatca acgaccattt actaaacttc
64021 accataacca atgatggaag aatatttact gaagatgaat tgaattctgc caaagagtgg

64081 ggaacaaaag gtaaagaatc aaatgggtct ggtattggtc tatattttgc taatattgtg
64141 cttcaacaat ataatagtga acttatttta gaaaatgaga ataatcacgc aaaagtaagt
64201 ttcaatattc ctgtttaaca atgtttatta atttaaagaa taaagtttgt atttattgct
64261 aagatttaaa attataaaca catctgaatg attaattatc gtatagacat ctggagttga
64321 tatcttgatt cctattacat cgctattatt attgtgattg aatttgatat ttttttaatt
64381 agcaacataa ttttgagaat atataaagtc atttaaagat tattgaaaac aaaaagctaa
64441 agtaattatt agtttttaat cctttattgt tacccttggt tattaactac taaaaggact
64501 aagtgaccgt aatgtgtcgc tcagtccttt ttataatccc ttctccaact tctttaatta
64561 agaaatatat ccattagtcg tccactgtct actcagacta ttccgtctat tattcaattg
64621 tctattctcc accgctcggt caaggctcag tgattgttgt tcaattaagt cgatataggt
64681 tctttctttc aattaagact tctaggtttg tacgcaatat ggtcgcactg gtgaaatgat
64741 tatagcaatt atgataacaa agccaagaat attacgcttt gttccaggtg gtttttttatt
64801 atttgaattt aaactcacgt ataccaaaca tttctcaaca atactatatc atttcatttg
64861 ggtaagtaat gattaatgat atcttgaaaa aagtcgtatc tgtatcactc tttaaaaatc
64921 actgaaatag cttactcaag aatctcttca tctttgattg ctttaaaata ttgatgtcga
64981 taaattcaag tttaaaaata aaatttattc aaaaaaggta tcaaagcttg cttaaaaatt
65041 gtggagtgtt aaaattatta ttgaaaacgg ttccaatgaa ttgttgtttg ctataggtag
65101 ttaatcgttg aattaattaa ctatatcgag taataaatgg gagagataga aatgaatgat
65161 acagatagaa cacataagga aaataagtta gaacaagata ttacactacc ttatttaaaa
65221 aaagcagtta ttttttcaat tacttttggt ttctttggtg taaatatggc ttttgcactt
65281 caatcagcaa tgatggggag aatattccaa acgttaggag ctaatcctaa taatttagga
65341 tggtttttta ttttgccacc tttagtagga atgattgttc aacccccttat tggatactac
65401 tctgacagaa cttggaccag atttggacgt cgtatgccgt atctgttgat agctggtccg
65461 attggagcaa tcgtattagt cttgttacct aatgcaggct catttaattt tggatatgct
65521 tcactcatgg ctttatcctt tggtgcggtg atgactttac ttatggattt gacttcaaat
65581 gcatgtatgc aaccatacaa aatgattatt ggcgatatgg taaacgaaaa acaaagagat
65641 atggcatggt cttggcaaca aattttttca aatttaggtg gtattattgc tacagtatta
65701 ccatttttat taacaatgtt gggaatgagt aatgtagcac ctaaaggtga agtaccaatg
65761 actgtaaaat tagcttatta tattgcagca gcagtattat taattgcttc tatatggaca
65821 gttatctccg ttagggaata tgagcctaaa acgtataatt attatcatgg aatcaatgag
65881 aacaatagaa ataataaatt aagcttatta cagttattaa aaaaagcccc taaacaattt
65941 tggcaaatta gcgtagttca attttttaac tggtttgcat tgatgtatct ttggacatat
66001 tcaacaggtg caatagcaaa gaatgtttgg aatgtgacag atccttcttc agccggatat
66061 caagcagcgg gaaattggta tggcgtatta tactgtatac aatttttgag ttcagttatt
66121 tttggttttg tcattgcaag atctaaacca tcccaaagaa aattttggta tagctttgga
66181 ttagttttcg gtggaatagg tcttatttca atattcttta tccataatca atggttactc
66241 attttttcat ttgtattgat tggtattaat aatttaacga tgaatacaca accatttaca
66301 ttattaactg aggcaatcga tggagaaaat tcaggggcat atttaggttt atttaataca
66361 agtatttgtc taccacaaat tgtagcgtct atagtcagtt ttggactgta tccattattg
66421 ggaagttcaa tgcctgcgat gctactgctt gcaggaatca tgatgctttt aggtgcagtt
66481 tcagttaaat ttattaatgc aaaatttgaa tagactagta aaaaaactta ataatattaa
66541 taagagcgga aatagagtgt ttttcgctct ttttttataa aaaattatta gaaatatgca
66601 ttaaacaaag aaatgatgta ttagtaatta aagtagttga gatcttacca tcattgataa
66661 gactctgaaa tatcttatca ataatttctt cattttcatt ggctgtgaaa tgacgtatta

66721 aaccttttat aaatgataaa aatattgtaa tttcaagcaa ctgtgcacta atctgattgt
66781 attgattcag gtctatagct aatatttatt taaaaagaca tttctgtctc gctttagcat
66841 tgaatggata tgtgacctca tgcatttctg gactatttaa agtattgcaa ttaataaatt
66901 taaaaaactt tatttatttc tctttatatg gcacaagtaa atgtaaaagt gcgataattg
66961 gtaaaattaa atttatttat gtaatgttac tgaaatattg taacattaat gtaataatgt
67021 ggcctgtaag acaaatatga ggagggtttt tatgaaaaaa gtttctaaat ttattttagc
67081 tagtagttta attttgggct tttccgtaac gcccgaatta tttaatgtca ctcaagcaca
67141 tgcgcaagaa tctgtgaaac cttattataa ttatagtggt tatacagcac atcaaagtaa
67201 ctttattcta gataagaatt ttaaaacatc tttaaaagct gataacttca agattaatgg
67261 ttacaagatt actcaaaacg aatcagctaa aaatgataaa gatgtatatg atcaaacata
67321 ttacggggtt tctaatcata aagcgaatgg tgttttcttc tcattagacg gtaagtcagt
67381 gagcgaaaaa gaattattga atcattacgg tgaaccaaag tatacgtcta catcagcatg
67441 gggcaaagaa tatacgtata aaattggaaa taaagatgta agattttatg ttaatagaaa
67501 tggatatgtc attaaaggac aaatcattag tgaatagagt ggggtatcaa taaagcaaac
67561 atactaagat tagctatgaa gaaatctatg acgatagatt ttttcatagc tattttttat
67621 agttatagag agaagtagac tgtccagact cttggatttt aaatccgtat aaaaaacaag
67681 tcagctttac tctcgccttt tgaaattcgt ttgtagtatg ttgggttctt gaaaccgtgt
67741 ataggaaaat gaaatgagaa aggttaagta aagtttttag cttctcaatt attcaaagga
67801 ggttttttta tcgattactt aggtgttgat attagtaaaa gaagtagtgt agttgcacat
67861 tataaaaatg gaaaattcca aaaagagttt ttcatccaga ataataaaaa tggttacaat
67921 tatttactca agtatttgaa tgacttagac cacccacaac tcatttttga atctacaggt
67981 atctattcaa gaggtatgga acgattttgt tgtgtaaatc aaattaacta tattcaaatg
68041 aatccgttag aagccaaatt taaaacgagc gctctaagat catggaaaac tgatcaggca
68101 gatgctcata agcttgcttg tttaggaccg acgcttaaac aaacagacag cttacctata
68161 catgagttaa tattctttga attaagagaa cgcgtccgtt ttcatctaga aatcgagaat
68221 gaacaaaatc gacttaaatt tcagatcctt gaattactcc atcaaacatt ccctggttta
68281 gaaagattgt ttagtagtcg atattcaatc attgcactca acatcgcaga aatctttact
68341 cattcagaca tggttcttga tatcgacaag gaggtactga ttacacatat attcaattct
68401 acagataagg gaatgtcaat ggataaagct acaaaatatg cacttcaatt aagggtgatt
68461 gctcaagaaa gctatcctaa tgtcgataga cattcctttc tagtcgaaaa attacgctta
68521 cttattcaac aattaaaaca atctattcat catctcaaac aattagatga tgccatgatt
68581 caattagcac aacaactcga ttattttgaa aatattcatt cgatacctgg tattggtaag
68641 ctaagcacag ctatgattat tggggagatt ggtgatatta agcgatttaa atcaaataaa
68701 caactcaatg cttttgttgg cattgatatc aaacgatatc aatcaggtca tacacactgt
68761 agagatacca tcaacaagcg tggtaataaa aaagcgagaa aacttttatt ttgggtgatt
68821 atgaatataa taagagggca gcatcattat gacaatcatg tcgtcgatta ttactacaaa
68881 ctaagaaagc agcctaatga gaaacctcat aagactgcca tcattgcttg tataaatcga
68941 ttattaaaaa caattcatta tcttgtaatg aatcataaat tgtacgatta tcaaatgtca
69001 ccacattagc caaacgtaca atcaaatata gtttaacacc ttattcaaaa aaattaaaat
69061 gaacggttta gttaagtaat gcttatttta attataagta cttgactaat cgtaggaaga
69121 cgaggctggg acataattcc tagcaaaata gccagtaaat gagtttacta taaactcatt
69181 tactggcttc tctaataatt atcaagacaa tttgcgtttc taggcatact ttgaaatgcg
69241 ctattttctt taagaatatt aatatatgac ttgtgttggt aatgataacc ttggttagag
69301 tgtattgtca tgtgatagtt aagattagat cttttaatta aaacattgtt ttaaatatcg

69361 atgacaaggt ctaatgtagg acgtgtagat ataaggttcg gaattatata aatccataaa
69421 aggagataaa tcgaaaacat ttataccccct atggggtata tgttatagta taagtagctt
69481 tactataaca ttttcattag gaggggttaa tttgaataat aatggtgaag agcataatca
69541 tcaaaatcac atgaatcatt ccaatcaaat gcatcatgat aaccatgcct cacatcatca
69601 tagtggccat gcacatcatc atggaaattt taaagttaag ttttttgttt cattaatttt
69661 tgcaatacct atcattcttt tatcgccact gatgggtgtt aacttacctt ttcaattcac
69721 atttccaggt tctgaatggg tagtgttaat attaagtaca attttattct tttatggtgg
69781 taaaccgttc ttgtctggtg gtaaagatga aattgctaca aaaaaaccag gtatgatgac
69841 cttagttgcc ctaggtattt cagtagctta tatttatagc ttgtatgctt tttatatgaa
69901 taactttagt agtgcaactg gtcatacaat ggactttttc tgggaattag caaccttaat
69961 tttaattatg ctattaggac attggataga aatgaatgct gtcggaaatg ctggagatgc
70021 tttaaagaaa atggcagaac tgttacctaa tagtgctatt aaagttatgg ataatggcca
70081 acgcgaagaa gttaaaatat cagacatcat gactgatgat atcgtcgaag taaaagccgg
70141 agaaagcatt ccaacagatg gtattatcgt tcaaggacaa acatctatag atgaatccct
70201 agtcactgga gaatctaaaa aagtacaaaa aaatcaaaat gacaacgtca tcgggggttc
70261 tattaatggg tctggaacaa tacaagtcaa ggttacagct gtgggagaag atggatatct
70321 ttctcaagtt atgggacttg ttaatcaagc acaaaatgat aaatctagtg ctgaattgtt
70381 atctgataaa gtagcgggtt atttattcta ctttgctgta agtgttggcg tgatttcttt
70441 tattgtctgg atgctcattc aaaatgatgt tgattttgca ttagaacgtc ttgtaactgt
70501 gttagtcatt gcttgtccac atgctttagg cttggcaata cctttagtca ctgcacgttc
70561 tacttcaatt ggtgcacata atggtttaat tattaaaaat agagagtctg tagaaatagc
70621 tcaacatatc gattatgtaa tgatggataa aactggtact ttaactgagg gtaacttttc
70681 tgtgaatcat tatgagagct ttaaaaatga tttgagtaat gatacaatat taagcctttt
70741 cgcctcatta gaaagtcaat ctaatcaccc attagctata agtattgttg attttgcgaa
70801 aagtaaaaat gtttcattta ctaatacaca agacgttaat aatattccag gtgtcggatt
70861 agaaggtcta attgataata aaacatataa aataacaaat gtctcttatc ttgataaaca
70921 taaacttaat tatgacgatg acttgtttac taaattagct caacaaggta attcaatcag
70981 ctatttaatt gaggatcaac aagtcattgg catgattgct caaggagatc aaattaaaga
71041 aagctcaaaa caaatggtag ctgatttact atcaagaaat attacaccag tcatgcttac
71101 aggtgacaat aatgaagtgg cacacgctgt cgcaaaagaa ttaggtatta gtgatgtcca
71161 tgcacaactc atgccagaag ataaggaaag cattataaaa gattatcaaa gtaacggtaa
71221 taaagtcatg atggtcggag acggtatcaa cgatgcgccg agccttataa gagccgatat
71281 tggtatagca attggtgcag gcacagatgt tgctgtggat tcaggtgata tcatacttgt
71341 taaaagtaat ccatcagata tcattcattt cttgaccctt tcaaataata ctatgagaaa
71401 aatggttcaa aacttatggt ggggtgcagg ttataatatt gttgctgtac ctttagcagc
71461 tggcgcatta gcttttgtcg ggttaatatt atcaccagct gtaggagcaa tattaatgtc
71521 tttaagtaca gttatagtag cgattaatgc ttttacatta aaattgaaat aaaagatagg
71581 agttttatta tgattaaaaa attatttttt atgatattag gatcattact aatattatca
71641 gcttgctcca ataatgatga aaaagataaa gacactaatg atcaaaaaag tgagagccat
71701 atgaagcata atgatgaaag taaagttcct gaagatatga aatcgactaa tgagggtgaa
71761 tttaaagtag gagataaagt aacgattaca gcagggcata tgccaggtat gaaaggtgca
71821 gaagctactg taaaaggtgc gtataaaaca tatgcctatg ttgtaagtta taaacccaca
71881 aatggaaatg aaaaagtaaa caatcat

//

LOCUS Staphylococcus 41606 bp DNA linear UNK
DEFINITION Staphylococcus Staphylococcus epidermidis strain FDAARGOS_529
ACCESSION Staphylococcus
KEYWORDS .
SOURCE Staphylococcus Staphylococcus epidermidis
ORGANISM Staphylococcus Staphylococcus epidermidis
Bacteria; Bacillati; Bacillota; Bacilli; Bacillales;
Staphylococcaceae; Staphylococcus; Staphylococcus epidermidis.
FEATURES Location/Qualifiers
source 1..41606
/organism="Staphylococcus Staphylococcus epidermidis"
/genome_id="176279.73"
/db_xref="taxon:176279"
/project="hwwang_176279"
/mol_type="genomic DNA"
/genome_md5=""
CDS 1..480
/translation="MKITILSVGKLKEKYWKQAIAEYEKRLGPYTKIELIEVPDEKAP

ENMSDKEIEQVKEKEGQRLLNKIKPQSTVITLEIKGKMLSSEGLAKELQTRMTQGQSD

FTFVIGGSNGLHQDVLQRSNYALSFSNMTFPHQMIRVILIEQIYRAFKIMRGEAYHK"
/db_xref="SEED:fig|176279.73.peg.1"
/transl_table=11
/product="23S rRNA
(pseudouridine(1915)-N(3))-methyltransferase (EC
2.1.1.177)"
/EC_number="2.1.1.177"
CDS 726..1031
/transl_table=11
/db_xref="SEED:fig|176279.73.peg.2"
/translation="MKGNIQCPRLIKGSLLDFVLETVDVFVNGSKNDCELVNFNYKEG
KVVSRYSTKFESNPKLRKKQLISTVKVVRYVIFDFEKILWGIGKVLLKFIILRQCLL"
/product="hypothetical protein"
CDS 1230..2093
/product="hypothetical protein"

/translation="MKHINQFSESTLAQLEKDQQHVFYVYCLMDPRNDECFYVGKGKG
NRIFKHKQDAQKKVLYEDIFIEENKDNLKFNRINEICSNDLNVLGYIISYGLTESEAF

SAENVLINFLNLTNKTTLTNMINGHGSKAYLVEDLENEFGYDSINLENINTNELILAV

KIRDAFLLDKDESKEYPINESKRDRNNLKSRTLGSWIIGKDKIHKIKYIIGINTGANN

AVVSAYEVSFEQAESIETNNGRMRYAFIGLSERDATLKKLNLYKKALPDLRFGSGSAT
AYINNGTMKVD"
/transl_table=11
/db_xref="SEED:fig|176279.73.peg.3"
CDS 2201..3676
/product="hypothetical protein"
/translation="MPSDDIVKKLYSKEDIRLELGLTPHKFNKKMETIAKLFKIDMKI

FHNYKGQDKNNQYTFNGVAKELIKVLLKSVDYYPVDINSKKFKQNGKSKKEMIENIDN

SSYMKYIYQLMKSINEIQYKRLIADIHMKDVYQNTKAWLNNGESINKKEQELYQYMTI

LPLHKRVELQNEVLKSIDETIFQFLAKEHRNNQIEENNELEAYTKAIKEGRNPKNDYE

LNHLLYKKNQLPLDSLIEDIWDYEETEYTELDWLIADMLKRSQRADSNFIESLEKKNK

LRKNIHKDSIKSISKLIDHELVKNNRKWDVAEFYKNQQFWNNSTLRRSSQNFGILYYK

KNRLHTMNQSNIIKKIKIIEANIESANKQPSYLDFFLQAKFDLERLESELMKYSINPK

YFNKINSDYVRYAIEDVHNAIIKIDRYISNDKVKLNYSNVQMVVRNEITEQGSYFVTQ
ALNTLSKVEESGYSFDNFMVGPFVEDFKKAIKNRRFGSKKD"
/transl_table=11
/db_xref="SEED:fig|176279.73.peg.4"
CDS 3902..5002
/product="hypothetical protein"

/translation="METRDKLMSLTQSDKTQQWLMDKSSNQDDIQQLQQQFSQQLDQQ

YNALLADEKAKLDQYVEVHQGLESLKEEIESEPITLNIDKLPDIKATMLERAKNDEHS

DKIEKLFDRLEQALNGTNRLYTQLSLIGTRTHRITTKNFNLQGLPKAVQHTILPSKFK

KVYTVDFKSFEPSVAAYMTQDSKLIDLLNQKDGLYDALLSELGLSDELRVFVKRAFIG

SFLFGGNFKNPKFKLNQYVSEVQWLDAVSQFTKVIELKKHVEKSNSMPMPYSIEHDMS

AFQGSSIMAIYVQTVASYIFKHILLKVYKAQCDQKTFKIIVPIHDAIMIECEDEEIAQ
NVGQLMKDTANQLFNGEFAHVTVEEIGGVDHE"
/transl_table=11
/db_xref="SEED:fig|176279.73.peg.5"
CDS 4995..5366
/transl_table=11
/db_xref="SEED:fig|176279.73.peg.6"

/translation="MNNDRGQSLHIPSSTPIKENNIYVATLHSVIQTDFSGEIKHQFT

YEIEVNNQIVYANRNIPTKPSANQLSIHDWLKRHSNYSASHENYEPYIDQKHLILLGQ
YNGNYYVQDVASLDAFGGVLS"
/product="hypothetical protein"
CDS 5363..7006
/translation="MNHILQMLSKLLSVAKEAIDRQGLIAILTIPVNNNDEIEETAQG

ETVYNELIDQLRLNIPKDTDYQPNIYSYFGIKKNPNDTVLMEMMIKVFHIKRFNSELF

IFKVNGWQKINGDELQGLISKMIQVLLVDYKPSLSTLKNVVDGLQKSTDVEELVENER

YIGCGENMFDLNTFQVVKNSIDIFPKTRLNLSLSTNDVITDKIPPYFKQYMLQLANYD

DDLQYFLFQHTAVLLTADTKYRRGLILYGGAKNGKSVYIELVKSFFYSKDIVSKPLNE

LEGRFDKESLIDKSLMASHEIGQSRIQEKIVNDFKKLLSVESMHVDRKGKTQVEVILD
LKLIFSTNAILNFPPEHAKALERRINIIPCEYYVEKADTSLIDKLQSEKKEIFLYLMY

VYQQIVKADIEYLENSRVTEITHDWLNFGYEFVSSRSVSNANQKACINLLRKLIEIKS

GSRIKVSELNKVINEEIKVSSQVIKQLIQANFDTQTKLYNGYDYWIDLGWKEANKKEI
HDISEKDNIISLDKNENITDDEALDEENLDFDWEDFDDE"
/transl_table=11
/db_xref="SEED:fig|176279.73.peg.7"
/product="putative primase"
CDS 7232..8908
/product="Cassette chromosome recombinase B"
/translation="MKGKIALYSRVSTSEQSEHGYSIHEQEQVLIKEVVKNFPGYDYE

TYTDSGISGKNIEGRPAMKRLLQDVKDNKIEMVLSWKLNRISRSMRDVFNIIHEFKEH

DVGYKSISENIDTSNASGEVLVTMFGLIGSIERQTLISNVKLSMNAKARSGEAITGRV

LGYKLSLNPLTQKNDLVIDENEANIVREIFDLYLNHNKGLKAITTVLNQKGYRTINQK

PFSVYGVKYILNNPVYKGYVRFNNHQNWAVQRRSGKSDKNDVILVKGKHEAIISEEVF

DKVHEKLASKSFKPGRPIGGDFYLRGLIKCPECGNNMVCRRTYYKTKKSKERTIKRYY

ICSLFNRSGSSACHSNSINAEVVERVINVHLNRILSQPDIIKQIASNVIEELKQKHSN

QTEIKYDIDSLEKQKAKLKTQQERLLELFLDDQMDSEMLKAKQSQMNQQLEVLDQQIK

EAQQANQSQDEIPNFDKLKGRLILMITRFSVYLRKATPEAKNQLMKMLIDSIEITTDK
QVKLVRYKIDESLIPQSLKKDWGSFFMPKFQFEIYGQNDYFIDQITTFTT"
/transl_table=11
/db_xref="SEED:fig|176279.73.peg.8"
CDS 9014..9352
/translation="MTLEQQLKHYITNLFNLPRDEVWHCESIEEIADDILPNQYVRLG

PLSNKTLQTNTYYSDTLHESNIYPFILYYQKQLIAIGYIDENHDMDFLYLHNTIMPLL
DQRYLLTGGQ"
/db_xref="SEED:fig|176279.73.peg.9"
/transl_table=11
/product="FIG01108299: hypothetical protein"
CDS 9448..9759

/translation="MNRYITRGIANNLPNILQHQLWQLVSEREQEQTKDNTSVDYFHI

FQFNKHRNQLYIKHKQERPEYAKIHKANYSKAININKVYIIREDDVDLSYYVMLLPEE
Y"
/transl_table=11
/db_xref="SEED:fig|176279.73.peg.10"
/product="FIG01107894: hypothetical protein"
CDS 9775..10281
/product="hypothetical protein"
/translation="METIKSTLKTEAIFSDDKQHRYLLKKTWNSEKQSITIITMYPHY

DGILNIDLTTQLIMNKVSEMDAFGSINFVNLYSNITTPINLKHLENAYDKHTDIQIMK

AVKESDEVILAWGAYAKKPGVEARVNEVLEMLKPHKKKVKRLMNPETNEIMHPLNSKA
RQKWILKV"
/transl_table=11
/db_xref="SEED:fig|176279.73.peg.11"
CDS complement(10430..11104)
/translation="MNYFRYKQFNKDVITVAVGYYLRYTLSYRDISEILRERGVNVHH

STVYRWVQEYAPILYQIWKKKHKKAYYKWRIDETYIKIKGKWSYLYRAIDAEGHTLDI

WLRKQRDNHSAYAFIKRLIKQFGKPQKVITDQAPSTKVAMAKVIKAFKLKPDCHCTSK
YLNNLIEQDHRHIKVRKTRYQSINTAKNTLKGIECIYALYKKNRRSLQICGFSPCHEI
SIMLAS"
/db_xref="SEED:fig|176279.73.peg.12"
/transl_table=11
/product="Transposase for insertion sequence-like element
IS431mec"
CDS 11362..11529

/translation="MLESREQLSVEEYETFFNRFDNQEFDFERELTQDPYSKVYLYSI
EDHIRTYKIEK"
/transl_table=11
/db_xref="SEED:fig|176279.73.peg.13"
/EC_number="2.3.3.10"
/product="Hydroxymethylglutaryl-CoA synthase N-domain
homolog (EC 2.3.3.10)"

CDS 11627..11857
/translation="MLIFLSYDFVRACLPRGMARAISLSHILFPQASALTKSVVIFIF
IRILTEIILIRGIVKAILSKIADKRFVKSSSK"
/transl_table=11
/db_xref="SEED:fig|176279.73.peg.14"
/product="hypothetical protein"

CDS 12286..13029
/db_xref="SEED:fig|176279.73.peg.15"
/db_xref="GO:0008889"
/transl_table=11
/translation="MLTVYGHRGLPSKAPENTIASFKAASEVEGINWLELDVAITKDE

QLIIIHDDYLERTTNMSGEITELNYDEIKDASAGSWFGEKFKDEHLPTFDDVVKIANE

YNMNLNVELKGITGPNGLALSKSMVKQVEEQLTNLNQNQEVLISSFNVVLVKLAEEIM

PQYNRAVIFHTTSFREDWRTLLDYCNAKIVNTEDAKLTKAKVKMVKEAGYELNVWTVN
KPARANQLANWGVDGIFTDNADKMVHLSQ"
/EC_number="3.1.4.46"
/product="Glycerophosphoryl diester phosphodiesterase (EC
3.1.4.46)"

CDS 13126..13554
/db_xref="SEED:fig|176279.73.peg.16"
/transl_table=11
/translation="MKYDDFIVGETFKTKSLHITEEEIIQFATTFDPQYMHIDKEKAE

QSRFKGIIASGMHTLSISFKLWVEEGKYGEEVVAGTQMNNVKFIKPVYPGNTLYVIAE
ITNKKSIKKENGLVTVSLSTYNENEEIVFKGEVTALINNS"
/product="MaoC domain protein"

CDS complement(13600..15606)
/translation="MKKIKIVPLILIVVVVGFGIYFYASKDKEINNTIDAIEDKNFKQ

VYKDSSYISKSDNGEVEMTERPIKIYNSLGVKDINIQDRKIKKVSKNKKRVDAQYKIK

TNYGNIDRNVQFNFVKEDGMWKLDWDHSVIIPGMQKDQSIHIENLKSERGKILDRNNV

ELANTGTAYEIGIVPKNVSKKDYKAIAKELSISEDYIKQQMDQNWVQDDTFVPLKTVK

KMDEYLRDFAKKFHLTTNETESRNYPLGKATSHLLGYVGPINSEELKQKEYKGYKDDA

VIGKKGLEKLYDKKLQHEDGYRVTIVDDNSNTIAHTLIEKKKKDGKDIQLTIDAKVQK

SIYNNMKNDYGSGTAIHPQTGELLALVSTPSYDVYPFMYGMSNEEYNKLTEDKKEPLL

NKFQITTSPGSTQKILTAMIGLNNKTLDDKTSYKIDGKGWQKDKSWGGYNVTRYEVVN

GNIDLKQAIESSDNIFFARVALELGSKKFEKGMKKLGVGEDIPSDYPFYNAQISNKNL

DNEILLADSGYGQGEILINPVQILSIYSALENNGNINAPHLLKDTKNKVWKKNIISKE

NINLLTDGMQQVVNKTHKEDIYRSYANLIGKSGTAELKMKQGETGRQIGWFISYDKDN
PNMMMAINVKDVQDKGMASYNAKISGKVYDELYENGNKKYDIDE"
/transl_table=11
/db_xref="SEED:fig|176279.73.peg.17"
/db_xref="GO:0008658"
/db_xref="GO:0046677"
/product="Penicillin-binding protein PBP2a, methicillin
resistance determinant MecA, transpeptidase"
CDS 15854..15997
/product="Mobile element protein"
/db_xref="SEED:fig|176279.73.peg.18"
/transl_table=11

/translation="MNYFRYKQFNKDVITVAVGYYLRYALSYRDMSEILRERGVNVHH
LTV"
CDS 16277..16528
/transl_table=11
/db_xref="SEED:fig|176279.73.peg.19"
/translation="MAKVIKAFKLKPDCHCTSKYLNNLIEQDHRHIKVRKTRYQSINT
AKNTLKGIECIYGLYKKNRRSLQICGFSPCHEISIMLTS"
/product="Mobile element protein"
CDS complement(16588..17016)
/transl_table=11
/db_xref="SEED:fig|176279.73.peg.20"
/translation="MKFQVSPYFLITQNAEGLKKLYEELFGAKTLYIQRIKDRPIDKK

LNIDENDLNKIDQCVLQFGDIKIMIADDTEGLPITQGNNISLCLTFDQVEETKHVYDQ
LIEKGSEVLKTFSPEFYTEGYGYVRDPYGISLHLFTKRKN"
/product="PhnB protein; putative DNA binding
3-demethylubiquinone-9 3-methyltransferase domain
protein"

CDS 17097..18026
/product="Transcriptional regulator, YafY family"
/translation="MKKSVRLYNMIEYCNENRNFKLNDLMSEFNISRSTALRDIKEIE

ALGVPLYSNPGKNGGYTIIGNRDQTKIAISDEELKALVFTLSSISNVSNLPFQTEYQE
ILKKLYNNSNKKELINQYNDLFQYFNEDKYQFKSYKLFNEIIRLIIENKSFETCYSQN

YIKEQYKGIGIMYKNHQWYFVVANIESKLVNLLNISKIKELYEMGETQECNDITMQNF

QQFMVKNETAIDILIRSNVMGLNILKGYLWSDYMIENIDEETYLFKSKVNAKDIDFIA
KLIVTGGVNVKVETPNSLKNAVKVELTKIINMY"
/transl_table=11
/db_xref="SEED:fig|176279.73.peg.21"
CDS 18188..18670
/transl_table=11
/db_xref="SEED:fig|176279.73.peg.22"
/translation="MNKNITALKSTENTTYMQYTVHIDDQIINNIKECTKKFKILPME
DKIPLSPLLQPEYAGEVQDFISTYEQFMINFGKVILDSQGIKIQFESESLSSIQRGIQ

EHCYLNERTNDIDVTKEWYLCKFSNQILEEDKNQLYNALKQLMNDSKNKKQAFWTDPN
"
/product="Similarity"
CDS 18738..19052
/product="Mobile element protein"

/translation="MKRVSYSVETKYKAVEMKIAGFSTKEIMKELNIKNRTQVETWWR

WYRNEESYRFSQHVGKQYTYGKGLEELSEIERLKLENKRKDIELDILKKYKALERKWY
QQ"
/db_xref="SEED:fig|176279.73.peg.23"
/transl_table=11
CDS 19037..19879
/product="Mobile element protein"
/translation="MVPAVVIDLVDQLKVKYSIKLILEVLNIPKSTYYRWKNKTYKND

TVTQKVIELCEANHYTYGYRKITALINQCYTSPINHKRVQRIMQKHHLNCRVRPKKTT

RIGKPYYKTDNLLQRQFKASCPMEVLTTDITYLPFGHSMLYLSSIMDIYNGEIVAYKI

DDKQDQSLVNDTLNQIDIPEGCILHSDQGSVYTSYAYYQLCEEKGIIRSMSRKGTPAD

NAPIESFHSSLKSETFYINNQLNSSNHIVIDIVEKYIKNYNNNRIQQKLGYLSPVKYR
ELAA"
/transl_table=11

/db_xref="SEED:fig|176279.73.peg.24"
CDS 19886..21451
/product="Similarity"
/db_xref="SEED:fig|176279.73.peg.25"
/transl_table=11
/translation="MFLLSSRLRGSVPFQEVFKVHIDIYLYNKEKSEYKYQVSSYFNL

VKENPKITYKKRHLQEKQGVKGTTFTNKLKLLNKMYGVDVAKYQPFYNSNNPEYERGQ

FGERYISQRSNYEFNRLQYQIIDMLSKILDKHPLPKSDNNYKHIPTIEKAILSGDSHS

FYEYFEDIMKEIISMENSSLKEKLLTDFTYQSQCRWYSESEKLKLQLESFMHKVLESN

YYEGNKLYRMLSHAIEETINEADEDKVHFNYFKDYFLTDGGVKNWEQISEKITEFNGK

VINDIQNEYNKIQFNNANRNQKLNFNYLYHCFEFSNNLVKARVNGNRGYILYFVDKYK

IKMPFLDQLLINYLNQEIGQESINYNMQTLFEKERYDRSSTIEKLVATSKFKYEKDDS
DLFKQLFNDVENSIDRLGIYLLNNGINSNDENARYYRSFLKELSRIKSKLTPFSLEIS

KSSGREQHYPDDAIDDKDERRKNKEETYHAFDDKSDIDSKLKNKINVSIDNLLSVKIK
SNLYDISPNTKKQ"
CDS 21646..22755
/product="hypothetical protein"
/db_xref="SEED:fig|176279.73.peg.26"
/transl_table=11

/translation="MYQANIRDLITKLPQSNKTEHFLMNKFSNQDKVQQLQRQISQQL

DQQYNELLANEKAKLDQYVEVHHNLEPLKKEIESESINLDTDKLPDIKATMLEKAKND

EHFDKIEQLFDRLDQSLNGTNRLYTQLSLIGTRTHRITTKRFNVQGLPKLVQQMILPS

QFKKVYTIDFKSFEPSVAAYMTQDEQLIDYLNHEEGLYDALLRDLSLSKEKRVSVKRA

FIGSFLFGGRYSSSKFKINQEVSEINWLQVMSKFKKVIEFKEQVEKYKTMPTPYGIEH

DMSAFQGSSIMAIYVQTVASYIFKHILLEVYKAQCEKKTFKIIVPIHDAIMIECNDKG
IAQNVAQLMKDTANQLFNGEFAHVTVEALGGIDNE"
CDS 22748..23116
/product="hypothetical protein"
/translation="MNNDRGKSLQIPQSTLLKEGSIYVATLHSVYEKNFSGDIKHQFT

YEVELNQETHYVNRNITVKSMSHQLSIADWIKRHSNYNVNHINYDPYIDRKHLVLVGQ

YNGNYYIQDVAPLDEFGGVL"
/db_xref="SEED:fig|176279.73.peg.27"
/transl_table=11
CDS 23116..24732
/product="putative primase"
/db_xref="SEED:fig|176279.73.peg.28"
/transl_table=11
/translation="MNHILEMLIKLLKVGMEAIDRKGLIAILTSSIGNDEMDDSEQAV

MVYNELIDKLQLNIPKDVDYRPNIYSYFGIQKKPNDTILVEMMICIFHIKRFDSELFV

FKDKGWQKVSEDELQGLISKMIQVLLVDYKPSLSTLKNVVDGIQKSTDIEKLVEHRQY

IGCGRNMFNLKTFKVVDNDLEIFPKTRLDLELDINDTITDKIPPNFKQYMLELANYDH

DLQYFLFQHMAVLLTADTKLRRGLFLYGTAKNGKSVYIKLVKSFFYSNDIVSKTLNEL

GGRFDKESLIGKRIMASDEVGKANIDEATVNDFKKLLSVEPIHADRKGRTQVEVTLDL

KLIFNTNAVLNFPSSHAKALERRIAVIPCEYYVEKADPDLIEKLQDEKKEIFLYLMYV

YKQIVKNDIEYLQNDRVTEISHDWLNFGYEFVSSKSANIAHQKACINLLRKLIEIKPG

SRIKVSRLNEVIRDEIKVSSQVINDLVQANFNVQSRLNNGYKYWVDLGWKETDKKDDM
ISFDKNENVTDDEFLYEDDLNLGWEDFDDE"
CDS 24957..26636
/translation="MKGKIALYSRVSTSEQSEHGYSEKEQEQLLIKEVMKNFPGYDYE

TYTDSGISGKNIEGRPAMKRLLQDVKDNKIEMVLSWKLNRISRSMRDVFNIIHEFKEH

DVGYKSISENIDTSNASGEVLVTMFGLIGSIERQTLISNVKLSMNAKARSGEAITGRV

LGYKLSLNPLTQKNDLVIDENEAHIVREIFDLYLNHNKGFKAITTILNQKGYRTINQK

PFSVFGVKYILNNPVYKGYVRFNNHQNWAVQRRSGKSDKNDVILVKGKHEAIISEDVF

DQVHEKLASKSFKPGRPIGGDFYLRGLIKCPECGNNMVCRRTYYKTKKSKERTIKRYY

ICSLFNRSGSSACHSNAINAEVVERVINVHLNRILSQPNVIKQIASSVIEELKQKHSK

QTEIKYDIDSLEKQKAKVKTQQERLLELFLDDEMDSEMLKAKQSEMNQQLEVLDQQIK

EAKQANQSQDDIPNFDKLKARLILMITRFSVYLRKATPEAKNQLMKMLIDSIEITTDK
QVKLVRYKIDESLIPQSLKKDWGSFFIPKFNFVINVTKKNRIENLSLLPLF"

/db_xref="SEED:fig|176279.73.peg.29"
/transl_table=11
/product="Cassette chromosome recombinase B"
CDS 26725..27063
/db_xref="SEED:fig|176279.73.peg.30"
/transl_table=11
/translation="MTLSKQLKTYITERFKLNYQETWACETIDAVAEDVLPEKYIKNS
PLEHKILNTFTYYNDELHEISIYPFLCYLDKELVAIGYLDNFDLDFIFLNDTHQVIID
ERYLLQKGGE"
/product="hypothetical protein"
CDS 27066..27155
/transl_table=11
/db_xref="SEED:fig|176279.73.peg.31"
/translation="MNWIKVAQLSVTVINEVIEIMKEKQNGGE"
/product="hypothetical protein"
CDS 27157..27468
/product="hypothetical protein"
/translation="MKINRYITRGVSEHLSLDLQILLWNMVKDRDNQLHTDYLHIFKL

QEDDNILSITHEQEQPAYKLEYHYTNYVKNQNALPKKVYVIREDDVDAFYYVMLLPEE
Y"
/transl_table=11
/db_xref="SEED:fig|176279.73.peg.32"
CDS 27483..27986
/translation="MNTIKNTIHTEAIFSDDEQHRYLLKKTWDEKKPACTVITMYPHL

DGVLSLDLTTVLILNQLANSEQYGAVYLVNLFSNIKTPENLKHIKEPYDKHTDIHLMK

AISESDTVILAYGAYAKRPVVVERVEQVMEMLKPHKKKVKKLINPATNEIMHPLNPKA
RQKWTLK"
/transl_table=11
/db_xref="SEED:fig|176279.73.peg.33"
/product="hypothetical protein"
CDS 28000..28221
/product="hypothetical protein"
/db_xref="SEED:fig|176279.73.peg.34"
/transl_table=11
/translation="MNHETKQSDWRTVASCLASQNYISIVKGLVHHFTAIEDEEILDK
IYDDFMNDDSITTVLNNDLQTIINHYLSK"
CDS complement(28296..31415)
/EC_number="3.1.21.3"
/product="Type I restriction-modification system,
restriction subunit R (EC 3.1.21.3)"
/db_xref="SEED:fig|176279.73.peg.35"

/transl_table=11

/translation="MNFQFNEDDLEQVALEWLQSLGYDYKKGNEISMTSLTPERKSDK
DVVLHERLEKALRKINSDIHPRFIEKALHELTLEKSPNLLENNLTFHENLINGIEIED
YDDEGQSIVEIVKIIDFEYPQNNDFLAVNQLTIVNGDYKKRPDIVLFINGLPIVVIEL

KNSTNETVGVEDGYHQLETYKMRIPQLFTFNEVLVTSDGINTKAGSLTANYDRFMTWR

SKDGQTESSPGLASLDVLIHGMLNPETLLDLIRYFVLFQDDGKGHISKILAAYHQYYA

VNKAVDRALLASSEQGDGKGGVIWHTQGSGKSLTMVFFSGKLIQMLNNPTLVVVTDRN

DLDNQLYSTFVKSKGRSGKGLLRQTPKQAETRKELKSLLSVESGGIVFTTMQKFEPEQ

NETTMAALTERKNVIVMADEAHRTQYGFNAKYDDKGEGIKYGYAKYLRDALPNATFVG

FTGTPVASTDKNTQMVFGNYIDVYDMTQAVADGSTVKIYYESRVIPLNLPQSLDLDEA

YNDITEDQEEDVKQRLKSKWSRIEALAGAKPRVEALAKDIIQHFETRQQAMKGKGMIV

TMSRRIAVDLYDEIIRLKPEWHSDDDDKGVIKVVMTGSSSDPTSFQRHIGPKKRRNLL

EKRMKDVDDELQLVIVRDMWLTGFDVPSMHTMYIDKPMKGHNLMQAIARVNRVFKDKP

GGLIVDYVGIAESLKEALKEYTESDQAQTAIDTDKAVELMLLKYDVIQDMLYNLDYSK

FNSDKKSERYYTISNTMDYVIGLGEDERQRFIKTVTELGKAFALCATEPTAQELNDEI

AFFKAVKAGLVKLLQPPKEGKTRKTPAEVEAEINQLVSQSVVTEDVIDVYQTLGLEQP

DLSILSDDFLKDVEGLKQKNVAVELLNRLLKGQVKSLMKTNATVSKRFSEMLGNSINK

YNSRSIETSKVIEELIQLAKDIKQEQQRGNELGLNSDEIAFYDALASHETAKEAMGDK

ELRAIAHELTKTVKENMGVDWSKRDSAKAKMRVAVRRLLKKYGYPPDLQKMAVEQVVE
QAELMANNQ"
CDS complement(31399..32700)
/product="Type I restriction-modification system,
specificity subunit S"
/translation="MEYNTIFFGDVAKIKNGYAFKSKEFQEKGIPVIKIKNIISPIVD

TKDSQKVSIKTYEKTKGFSLKKNDILISLTGSGVNQMSSAVGKVGRIEFDYPALQNQR
VGKFELKYSNSADLDFLFYYFLQPKITEYLVRNSTGSANQANINSKLIETVKIPNFSL

IKQKSISKFLNQITRKIETNQKMIANLKELSQTLFKHWFVDFEFPDEDGNPYKSSGGE

MIDSELGKIPSNWKIYKLKDIASHKKETFNPKKSEEVTVKHFSLPAYDNEEQAIEEEV

NKIKSNKWIINNNCVLFSKMNPDTKRIWLPVIDNKKLNVASSEFVVMESPNNKINSFI

YNICLNSQFIDYLKANTTGSTNSRQRVKPTIAVNYKLAIEDSIVKKYSEIITPYMEEM
KILRSEIGKLTQLRDTLLPKLMSGELEISDDIEVNSDELSI"
/transl_table=11
/db_xref="SEED:fig|176279.73.peg.36"
CDS complement(32690..34204)
/EC_number="2.1.1.72"
/product="Type I restriction-modification system, DNA-methyltransferase subunit M (EC 2.1.1.72)"
/db_xref="SEED:fig|176279.73.peg.37"
/transl_table=11

/translation="MATIGFEEKLWQAADKLRGSMDAAEYKNVALGLIFLKYVSDSFE

EKYEELKQDPYADEEDQDEYLAENIFWVPKEARWQYINDNAKKPEIGQIIDKAMIAIE

NENESLKGVLPKEYARPALDKEKLGDIIDLFTFKVGDTESRKQDVLGRVYEYFIAKFA

SAEGKNAGEFYTPSSIVKLLVEMIEPYKGRIYDPCCGSGGMFVQSERFVEKHQGRLDD

IAIYGQESNPTTWKLAKMNLAIRGIDNDLGERNADTFHNDLHKGLKADYILANPPFNA

SDWGQERLLDDYRWQFGIPPKGNANYAWIEHMISKLAPNGTAGFVLANGSMSTSGKDE

LEIRKNLIEQDLVECIVTLPGQLFYSTQIPVCLWFISNNKGQNGKKERENEILFIDAR

EIGHMVSRTLKEFSDEDIQKVAQTYHAWRGTNDKPYEDIAGFCKVANLEEVKNNEYIL
TPGRYVGLADVEEDEEPFEQKMERITSELSEQFAKSKELEDQIRKSLEGLGYGV"
CDS 34400..34546
/translation="MDGVTNVKIHILTFVKIVVIVDIVAIVNTRLDKLGIFNSINKKF
KCSQ"
/transl_table=11
/db_xref="SEED:fig|176279.73.peg.38"
/product="hypothetical protein"
CDS 34646..36709
/product="FIG131328: Predicted ATP-dependent endonuclease of the OLD family"
/translation="MYISSVKIKNFRNFDNIDIDFHEGVNVLIGHNNSGKSNLLRALS

LIFDGSVRKQLSVEDFNNSLTKESLKKEAPKIVISVHITQSENERLMSDELITVSNWL

TKLEEPYEARIQYEFFLPKDEEKNYIDLVKNIDEKEEIWNLIRSQFIRLYVNKIWVGN

PEHQIPIDNDSLNKFDFQFLDAIRDVERDMFSGKNTLLKSVIDFFIDYDIKSDETITE

EEQKEKLEERRKEFSDNSSDLIETIHKRLDSGNQKILSYTNGIGASYDKSTPDFKGNL

TESEIYTVLQLIIKHETGMTLPITHNGLGYNNLIFMALLLSKMQADSDGNFLGSNAKV
FPILAIEEPEAHLHPTMQNEFIKFLKNNIREKKVKQIFITTHSTHISSSTNIDDIICL

YTDDSKTNVSYPGKVFDKNNQSKKYVQRFLDATKSNMLFAEKVIFVEGIAEQLLLHIF

ADYLNKPLEKNHVAVINVGGRYFNHFLSLFDSNNKYAINRKVSCITDLDPMRKEKNEN

GQKNNFKACYPFEYEMDLDKFDYSTNNSLDKYLNNEHSNIRVFVQPNKYGKTFEYQLM

FDNPSLKLLLTDSISNSQELAELMDHYEENESLQKLIDTLSPSNENQRIIKSLKDTND

SWNEDNKKKALIASRYLNSIGKGENALELASVLKDNLELKGQSDYEDFVVPKYIEGAI
RWVCE"
/db_xref="SEED:fig|176279.73.peg.39"
/transl_table=11
CDS 36711..38459
/transl_table=11
/db_xref="SEED:fig|176279.73.peg.40"
/translation="MIINAQTKLNIEQDFKIQAGPGAGKTEFLVNHIKNVIQTSEKLE

RMKKIACITYTNTAAQTVLQRLGKSVSNRVDISTIHSFLYRNVVKPYCSFLPKEYGIC

VQKLKGHSDPIVINKYVNAWLKTEYFTKLKPPSNRNQLLKMPVLKKALQNWLLSMKLS

YENNDVQFICDNTKAQAIDKKSKKRMGINKSNLNILEKQLLNYKKIYWEKGIIDHDDV
LFFSYVLIRDYPFILTILCAKYPYFFIDEFQDTSQLQDFIIDKIRQKGCIVGVIGDKA
QAIYSFQGARVSLFEKFKVDLINSHTILENHRSSYQIVTFLNSIREDITQTVYSGIEN

DKVTILIGNRIRSYTKVVNICNKESLVTLSRDNITSNAMRIKLEGNNFDKKLLEKFQE
IDSNSKRRNTIISLIKAIELAKNTKYKDALKIVESIYKDENNPKKIALNSLINMINKY

DKYGNGTLMNFYNTLCKTLDIKLSGFRKGQVKDFYENTLYKNMVICTNIVEDTSNHIT

IHKAKGAEFENVFVIGNKDTLSLLLKPDLMNDEEHRIFYVAMSRAKKKLFLQFDYLDN
NEQKKIKERYDVSIIQ"
/product="ATP-dependent DNA helicase UvrD/PcrA,

clostridial paralog 2"
CDS 38678..38851
/product="Multidrug resistance protein"
/translation="MQDVAINADHKVFFLMIIFNVIMLVSSIIQMILGKGRRITPAKP
TKDAKLHLDNQSS"
/transl_table=11
/db_xref="SEED:fig|176279.73.peg.41"
CDS complement(38919..40343)
/product="Decarboxylase, pyridoxal-dependent"
/transl_table=11
/db_xref="SEED:fig|176279.73.peg.42"
/translation="MEMEFNENNIDLETIIRDEVNKYLSRDIGDLPATQQAPLELREK

YEKMEVPNKGRDIYEVLKDLNNEVLNYLYRPNHPRSFSFIPGPASRLSWLGDILTTAN

NIHASNFANATLPINIERNLINYLVGKIGYEIKPAGGVFVSGGSMANLTAIVAARDAQ

VEMEDIKKATVYLTSQTHHSVGKALHVAGFLKNNIRRIEYNDDFTMNTDSLKAAIDKD

IEEGYKPAMVIATAGTTNTGSVDDFTTLGDTCDHYNLWLHVDGAYGLSHILSDKAQHL

FKGIERSDSASWDAHKLLFQTYSCAMVIVKEKNHLLQSFGEDAEYLDDIASDDDVIDP
EMLGIELTRPARALKLWITLQVIGEDEIIERIKYGEGLAEYAEEYVSGLDNWRIISHA

NLSIVNFRYENSDLTEAQNNQLNSMAAQKIADSGYAIAYTTVLNNQRVIRLCTTNPLT
THDDIRETINRLDQYIGDYDMKDA"
CDS complement(40426..40638)
/EC_number="1.1.1.95"
/product="D-3-phosphoglycerate dehydrogenase (EC
1.1.1.95)"
/translation="MRSSSPVLRGSYRIPKDKHQYKLKITDDNLQVDYPRQFLKGAHT
PSVKAQHVIHWGDTFWVSSLWLAWFY"
/transl_table=11
/db_xref="SEED:fig|176279.73.peg.43"
/db_xref="GO:0004617"
CDS 41288..41455
/product="hypothetical protein"
/db_xref="SEED:fig|176279.73.peg.44"
/transl_table=11
/translation="MKFKSEKLIIANQTKVINYRIQVLLKPQTNIETQTQVKNNTLSE
TDEQSNADLVQ"
ORIGIN
1 atgaaaatta ctattttatc agttggaaaa ctaaaagaaa aatattggaa gcaagccatt
61 gcagaatatg aaaaaagatt aggaccttac acgaaaatcg aattaataga agtaccagat

121 gaaaaagcac ctgaaaacat gagcgacaaa gaaatcgaac aagtcaaaga aaaagaaggc
181 caacgcctac tcaataagat taaaccccaa tctacagtaa tcacgttgga aatcaaaggg
241 aaaatgttgt cttcagaagg actagctaaa gaactacaaa cacgcatgac acaaggtcaa
301 agcgacttta catttgtcat aggtggctcc aatggtttac accaagacgt cttacaacgt
361 agcaattacg ctttatcatt cagcaatatg accttcccac accaaatgat acgcgtcata
421 ttgatcgaac aaatttatcg cgcatttaag atcatgagag gcgaggctta tcataaataa
481 aactaaaaat tagattgtgt ataatttaaa aatttaatga gatgtggagg aattacatat
541 atgaaatatt ggagtatacc ttgcaatatc atacgatgtt tatagagtgt ttaataaacc
601 atttttcaac tattgatgat ctagaatata taataactgt acaaattata ttgattatgg
661 aactacaatt caattaagaa attgatgatg aaattttaaa tttaaactaa tggaatcaag
721 aaagaatgaa aggaaatata caatgcccac gattaataaa aggaagttta ttagattttg
781 tgttagaaac agtagatgtt tttgtaaatg gaagtaaaaa tgattgcgaa ttagtaaatt
841 ttaattataa agaaggaaaa gtagtttcac gttatagtac aaaatttgaa agcaatccta
901 aattaagaaa aaagcaattg atatcgacgg taaaagttgt aaggtatgtt atttttgatt
961 ttgagaaaat tttatggggc atagggaaag ttttattgaa gttcatcata ttaaggcaat
1021 gtttactata agaaagttga aatacactca gaaacagact ggattcccgt ttttttcaaa
1081 ttggcacaaa atgatacata ggcccaccta tatattgaag atttaagtta aacgataaat
1141 aatatttttc tcttttgtat agaaattttt ttgggaatga tattaattag tataaaaatg
1201 aatatgtcta caaagaagga gagattttat tgaaacacat taatcaattt tctgaatcga
1261 cactagcaca actagaaaaa gatcaacaac acgtatttta tgtatactgt ttgatggacc
1321 caagaaatga tgaatgcttt tatgttggta aaggtaaggg gaatagaata tttaaacata
1381 aacaagatgc gcagaaaaaa gtattgtacg aagatatatt tatagaagaa aataaagata
1441 acttgaaatt taatagaatt aacgaaattt gtagcaatga tttgaatgtt ttaggatata
1501 taatcagtta tggattaacc gaatctgaag cattttctgc agaaaatgtt cttataaact
1561 ttcttaacct aacaaataaa actacattaa caaatatgat taacggtcat ggatctaagg
1621 catacttagt tgaagattta gaaaatgaat ttggctacga ttcaattaat cttgaaaaca
1681 taaatacgaa tgaattaatt ttagcagtga aaatcagaga tgcatttcta ttagataagg
1741 atgaaagtaa agagtatcct attaatgaaa gtaaacgtga tagaaataac cttaaatcgc
1801 gtacattagg tagttggata ataggaaaag ataaaataca taaaataaaa tatattattg
1861 gtatcaatac aggtgctaat aatgcagttg tctcagctta tgaagtatca tttgaacaag
1921 cggagagtat cgaaacaaat aatggtagaa tgagatatgc gtttattggg ctttcagaaa
1981 gagatgctac tctaaaaaaa ttgaatttat acaaaaaagc actaccagat ttaagatttg
2041 gtagtggtag cgctacagca tatataaata atgggacaat gaaagttgat taaatgaata
2101 aaaacatcca attccttaaa ttagcctttg aaaattttat actcgtatat ataagtgaaa
2161 aacttaaaag aattagatgt taattaaagg agagaatgct atgccatcag atgatattgt
2221 aaaaaaacta tactcaaaag aggatattcg attagaatta ggacttactc cgcataagtt
2281 taataaaaaa atggaaacta ttgctaagct ttttaaaatt gatatgaaaa tttttcacaa
2341 ttacaaaggc caagataaaa ataatcagta tacatttaat ggtgtcgcaa aagaattaat
2401 caaagtgttg ctaaaaagtg ttgattatta cccagtggat atcaattcca aaaaatttaa
2461 acaaaatgga aaatctaaaa aagaaatgat tgaaaatata gataactcta gttacatgaa
2521 atatatttat cagttaatga aatctatcaa tgaaattcaa tacaaaaggt taattgctga
2581 tatacatatg aaagatgtgt accagaatac aaaagcatgg ttaaataatg gtgaatcaat
2641 taataaaaaa gaacaagagc tataccaata tatgacaatt ttaccattac ataaaagagt
2701 tgaactgcaa aatgaggtat taaaatctat agacgaaaca atttttcaat ttctcgcaaa

2761 agaacataga aataatcaaa ttgaagaaaa taatgaatta gaagcatata caaaagcaat
2821 aaaagaaggt agaaatccta aaaatgatta tgaattaaat catctcctat ataaaaaaaa
2881 tcaattaccc ttagatagtc taatagaaga tatatgggac tatgaggaaa cagagtatac
2941 tgagcttgat tggttaattg cagatatgct aaaacgttca caaagagctg atagtaattt
3001 tatcgaaagt ttagagaaga aaaacaaatt gagaaaaaat attcataaag attccattaa
3061 aagtatatct aagttgattg atcatgaatt agttaaaaat aatagaaagt gggacgtagc
3121 agaattttat aaaaatcaac aattttggaa taatagtaca ttaagacgta gctctcaaaa
3181 ttttggaatt ttatattaca aaaagaatag gttacataca atgaatcaaa gtaatattat
3241 taagaaaata aaaattatcg aagctaatat agagagtgca aataaacagc cgagctattt
3301 agatttcttt ttacaagcta agtttgattt agagaggtta gaaagtgaat taatgaaata
3361 tagcattaat ccgaaatatt ttaataagat aaatagtgat tacgtaagat atgctattga
3421 agatgtacat aatgcaatta ttaaaattga cagatatata agtaacgata aagtgaagtt
3481 aaactattca aatgtccaaa tggtagtgag aaacgaaata acagaacaag gaagctactt
3541 tgtaacccag gcattgaata cactgtcaaa ggtagaagag tctggctaca gctttgataa
3601 tttcatggta ggacctttcg tagaagactt taaaaaagca attaaaaatc gtagatttgg
3661 ttctaaaaaa gattaagatt gttttttaga gttaaacata agaataaatt taaataaaaa
3721 aataaaccca acaaaccttg ttatatcaat gtttgttggg tttatttttt tggtgatttt
3781 ttaataactc aattacatct taggataata tcgctaaatt atacattgtc gaggccaaga
3841 caaataaaaa attaaagcaa aagttagcgc gcaactttct gctataaagg agcaaattta
3901 tatggagaca agagacaaac tgatgtcatt gactcaaagt gacaaaacac agcaatggct
3961 aatggacaag tcatctaacc aagatgacat tcaacaattg cagcaacaat tcagtcagca
4021 gcttgatcaa caatataatg cacttttagc tgatgaaaaa gctaagttag accaatacgt
4081 ggaagtacat caaggattgg aatcattaaa ggaagagatt gaatcagaac ctattacgct
4141 taatatcgat aaattacccg atatcaaagc aacaatgctt gaaagagcca agaatgatga
4201 acattctgat aaaatcgaaa agctatttga taggttagaa caggcattaa atggtacgaa
4261 tcgattatat acgcaattat cgttgattgg tacacgaaca catcgaatta caactaaaaa
4321 ttttaatctt caaggcttac ctaaagcagt ccaacatacg attttacctt caaaatttaa
4381 gaaggtgtat acagtcgatt ttaaatcgtt tgaaccatca gttgcagcgt acatgactca
4441 agattcaaaa ctgattgact tgttaaatca gaaagacgga ctgtatgacg cattgctaag
4501 tgaattaggc ttatcagatg agctacgtgt atttgttaaa cgtgcattta ttggttcgtt
4561 tctatttgga ggtaacttca aaaatcctaa attcaagctg aatcaatatg taagtgaagt
4621 acaatggttg gatgcggtca gccaatttac aaaagtcatt gaacttaaga agcacgttga
4681 aaagagtaat tccatgccta tgccttatag tattgagcat gatatgagcg catttcaagg
4741 tagcagtatt atggcaatct acgtacaaac tgtagcgagt tatattttca agcacattct
4801 gctaaaagtg tacaaagcac agtgcgatca aaaaacgttc aagattatag tacctataca
4861 cgatgcgatt atgattgaat gtgaagatga agaaattgca caaaatgtgg gtcagttaat
4921 gaaagatacg gctaaccagt tgttcaatgg tgaatttgca catgtgacag tggaagaaat
4981 aggaggcgta gaccatgaat aatgatagag gacaaagcct acacatccca agtagtacac
5041 caatcaaaga aaataatata tatgtagcta cgttacattc tgtgatccaa acagatttct
5101 caggtgaaat aaagcaccaa ttcacgtatg aaattgaagt gaacaatcag attgtatatg
5161 cgaatcgtaa tattccaaca aaaccgagcg ctaatcagtt gtcaattcat gattggctga
5221 aacgtcatag caactatagc gcaagtcatg aaaactatga gccttatatt gatcagaaac
5281 atttaattct attaggtcaa tataacggta actattatgt acaagatgta gcatcgttag
5341 atgcgtttgg aggcgtatta tcatgaatca tatattacaa atgttatcta agctattaag

5401 tgtggccaag gaggcaatcg accgtcaagg tctgattgct atcctaacta ttcctgttaa
5461 taataacgat gaaatagaag aaacggctca aggtgaaacc gtgtataacg aacttatcga
5521 tcagttacga cttaatatcc caaaagatac ggattatcaa cctaacatct atagttattt
5581 tggtattaag aagaatccta atgacaccgt actcatggaa atgatgataa aggtttttca
5641 tatcaaacgc tttaattcag aactgtttat tttcaaagtt aacgggtggc aaaagataaa
5701 tggagatgaa ttacaagggt tgatatctaa aatgatacaa gtattgcttg tagattataa
5761 gccttcacta agcactctaa aaaatgtcgt agatggattg caaaaatcaa cagatgtaga
5821 agaacttgtt gagaatgagc gctatattgg ttgtggtgaa aatatgttcg atcttaatac
5881 gtttcaagtc gttaaaaatt caatcgatat ctttccaaaa acacgattga atttatcatt
5941 aagtacaaat gatgtaatta ctgataagat accgccttat tttaagcaat atatgttaca
6001 acttgcgaat tatgacgatg atttacaata ctttcttttc caacatacag cagtattact
6061 tacagctgat actaaatacc gtaggggtct catattatat ggtggagcta agaatggtaa
6121 atctgtatat attgaactag ttaaatcatt tttctatagt aaagatattg tgtctaagcc
6181 acttaatgag cttgaaggtc gttttgacaa agaaagttta attgacaaaa gtctaatggc
6241 aagtcatgaa attgggcaat ctaggattca agaaaagatc gtaaatgact tcaaaaagtt
6301 attatctgta gaatcaatgc atgttgatcg taaaggaaaa actcaagtgg aagtcatttt
6361 ggatttgaaa cttattttta gtacaaatgc gatacttaat tttcctcctg aacatgcgaa
6421 agctttggag cgtcgaatta atattattcc atgtgagtat tatgttgaaa aagcggacac
6481 ttcattaatt gataagctcc agagtgagaa gaaagaaatc tttctttact tgatgtatgt
6541 gtatcaacag attgtaaaag cagatatcga gtatcttgaa aatagccgtg tcactgaaat
6601 tactcacgat tggttaaatt ttggatatga atttgtttct agcaggtccg taagtaatgc
6661 aaatcagaaa gcatgtatta atttactcag aaaacttata gaaatcaaat caggatcacg
6721 aatcaaagta tccgagttaa ataaagttat taatgaagaa ataaaggtaa gttctcaggt
6781 tattaaacag ttaattcaag caaactttga tactcaaacc aaactataca atggctacga
6841 ttattggatt gatttaggtt ggaaagaagc caataaaaaa gagattcatg atatttcgga
6901 aaaagataat attatttcat tagataaaaa tgaaaatata acagacgatg aggcattaga
6961 tgaagagaat ttggactttg attgggagga ctttgacgat gaataatgaa caaattgaag
7021 catttgtaga agtgcttgta cctatcatag aagaacgtat caataaaggt aattaaggct
7081 aattacgtac tacaggcagt tgcctgtagt actcatatga ttaagtggta aaagtgataa
7141 aaatgaaacg aaattataaa tatatattat ctatatgttg ttacaagacc gatagtctgt
7201 agcaataatc taataaaagg agcggtatga tatgaagggt aaaattgcac tttattcacg
7261 cgttagtacg tcagagcagt cggagcatgg gtactcaatc catgagcagg aacaagtact
7321 catcaaagaa gttgtgaaaa atttcccagg ttatgactat gagacatata ctgactcagg
7381 catttcaggt aaaaatattg aaggtcgtcc ggcaatgaaa cgtctattac aagatgttaa
7441 ggataataaa atcgaaatgg tgttaagttg gaaattgaat cgtatctcac gatcaatgag
7501 agacgtgttt aatattattc atgaattcaa agaacatgat gtagggtata aatcgatttc
7561 tgagaatatt gatacatcca atgcttctgg agaagtactc gttacaatgt ttgggttaat
7621 aggatctata gaacgccaga ctttgatttc gaatgtgaaa ctttctatga atgctaaggc
7681 aaggagcgga gaggcaatca ccggtcgtgt tttaggctac aaattatcac ttaatccact
7741 tacacagaaa aatgatttgg ttatcgatga aaatgaagct aatattgtac gtgaaatttt
7801 cgatttatat ttgaatcata ataaaggcct caaagccatt acaaccgtac ttaatcaaaa
7861 ggggtatcgt actattaatc aaaagccatt ttcagtgtat ggtgttaaat acattttgaa
7921 taatccagtc tataaaggct atgtcagatt caataaccat caaaactggg ctgtacagcg
7981 aagaagtggc aaaagtgata aaaatgatgt gatattggtc aaaggtaaac atgaagccat

8041 tataagtgaa gaggtatttg ataaagttca tgaaaaatta gcttctaaaa gttttaaacc
8101 gggcagacct attggtggag atttctactt acgtggcctt attaaatgcc cagaatgcgg
8161 aaataatatg gtatgtcgac ggacgtatta taaaacgaaa aagtccaaag aacggacaat
8221 caagcgttat tacatttgtt ccttattcaa tcgttcaggg agttctgcat gtcacagtaa
8281 ttccatcaat gctgaagtcg tcgagcgtgt aattaatgtt catttgaatc gtattctgtc
8341 tcaaccagat attatcaagc agattgcgtc aaatgtgata gaagaactga aacaaaagca
8401 tagtaaccaa acagaaatta aatatgacat tgatagttta gaaaaacaaa aagctaagct
8461 taaaacacaa caagaacgat tgttagaatt gttcttagat gatcagatgg atagcgaaat
8521 gttaaaagct aaacaaagtc aaatgaatca acagttagaa gtattagatc aacaaattaa
8581 agaagcgcaa caagcaaatc aatcacagga tgaaatacct aattttgata aattaaaagg
8641 acgactcatt ttgatgataa cacgattcag cgtgtactta agaaaggcta cacccgaagc
8701 taaaaatcaa cttatgaaaa tgttaattga ttcaattgaa attacgacag ataaacaagt
8761 aaaacttgta aggtataaaa ttgacgaaag tcttatccct caatctttga aaaaagattg
8821 ggggtctttt tttatgccca aattccaatt tgaaatatat ggtcaaaatg attatttcat
8881 cgaccaaatt accactttta ccacttagtt attagtgaca aaagtgagcg aaatgaaata
8941 aaaatcaaat atatattatc aaaatgatgt atcacatgca tacatcaatc aaatacatta
9001 ggaggtcata accatgacac tagaacaaca actcaagcac tatataacca acttattcaa
9061 tctgccaagg gacgaagtgt ggcactgcga atctatcgag gaaatcgctg atgatatctt
9121 acccaatcaa tatgtaagac ttggcccact cagtaataaa acacttcaga ctaataccta
9181 ctactctgac acacttcatg aaagtaatat ctatcctttc attctctact atcagaaaca
9241 actcatagcc atcggttata tcgacgaaaa tcacgatatg gatttcttat acctacacaa
9301 cactatcatg cctcttttgg atcaacgata cttactaaca ggaggacaat aaaatgcata
9361 aatacatcaa aattacacaa ttagtcatta caatactaag tgaaatcatc atttggatga
9421 aagagtcaga acgaaaggaa gtctcttatg aatagatata tcacccgggg tatcgccaac
9481 aacttaccta atatcttaca acaccaatta tggcaactcg tatctgagcg agaacaagaa
9541 caaaccaaag ataatacttc agtagattat tttcatatat tccagttcaa taagcatcgc
9601 aatcaattat atatcaaaca caaacaagaa cgacctgaat atgcgaaaat ccataaagct
9661 aattattcaa aagcaatcaa tatcaataag gtctacatta ttcgagaaga tgatgtagac
9721 ctttcttatt atgtcatgtt attacctgaa gaatactaga ggagtgaaaa ttatatggaa
9781 acaatcaaaa gtacattaaa aacagaagcc atattcagtg atgacaaaca acatcgctat
9841 ctacttaaga aaacatggaa tagtgaaaaa caatcaatca caatcattac aatgtatccg
9901 cattatgatg gcattctcaa tattgaccta acgacccaac tcatcatgaa caaagtttca
9961 gaaatggatg catttggttc aatcaatttt gtgaatctat actctaatat tacaacccct
10021 atcaatctca aacatttaga aaatgcgtat gataagcata cagatattca aattatgaag
10081 gcagtgaaag agtcagatga agtgatatta gcttggggcg cttacgctaa aaagcccggt
10141 gttgaagcac gtgttaatga agtattagag atgttgaaac cacataaaaa gaaagtaaaa
10201 cgactcatga atccagaaac caatgaaatc atgcatcccc ttaattcgaa agcacgtcaa
10261 aaatggatat taaaagtata gcaactaaaa tgtttattca ctatccatga aaataattga
10321 atttcatata tatcttttaa tctacaatat gattaaaaga tatattattt ggttctgttg
10381 caaagtaaaa aaatatagct aaccactaat ttatcatgtc agtgttcgct taacttgcta
10441 gcatgatgct aatttcatgg catggcgaaa atccgcagat ctgaagagac ctgcggttct
10501 ttttatatag agcgtaaata cattcaatac cttttaaagt attctttgct gtattgatac
10561 tttgatacct tgtctttctt actttaatat gacggtgatc ttgctcaatg aggttattca
10621 gatatttcga tgtacaatga cagtcaggtt taagtttaaa agctttaatt actttagcca

10681 ttgctacctt cgttgaaggt gcctgatctg taattacctt ttgaggttta ccaaattgtt
10741 taatgagacg tttgataaac gcatatgctg aatgattatc tcgttgctta cgcaaccaaa
10801 tatctaatgt atgtccctct gcatcaatgg cacgatataa atagctccat tttcctttta
10861 ttttgatgta cgtctcatca atacgccatt tgtaataagc ttttttatgc tttttcttcc
10921 aaatttgata caaaattggg gcatattctt gaacccaacg gtagaccgtt gaatgatgaa
10981 cgtttacacc acgttccctt aatatttcag atatatcacg ataactcaat gtatatctta
11041 gatagtagcc aacggctaca gtgataacat ccttgttaaa ttgtttatat ctgaaatagt
11101 tcatacagaa gactcctttt tgttaaaatt atactataaa ttcaactttg caacagaacc
11161 gtattatgga atagagatgt tggtaacatt tatacaggat cattatactt aagtttaatt
11221 tcgttattac agaaccacac attccaacca gaagagaaag tatgtctatt tagttatggt
11281 tcaggagcag taggagaaat ctttagtggt tcaatcgtta aaggatatga caaagcatta
11341 gataaagaga aacacttaaa tatgctagaa tctagagagc aattatcagt cgaagaatac
11401 gaaacattct ttaacagatt tgataatcaa gaatttgatt tcgaacgtga attgacacaa
11461 gatccatatt caaaagtata cttatacagt atagaagacc atatcagaac atataagata
11521 gagaaataaa ctagtggccg attgtgcttg atgagcttgg gacataaatc ctaactcgaa
11581 ataaataagc atatcactaa actgattttt taaagtttac agtgatatgc ttattttttt
11641 atcttacgat tttgtacgtg catgcttgcc taggggtatg gctcgagcca ttagtctctc
11701 gcacatacta ttccctcagg cgtcagcact tacaaaatcg gttgtaattt tcatttttat
11761 acgcattctt actgagatta tactaataag aggaatagta aaagcaattc taagtaaaat
11821 tgcagataag aggtttgtta aaagcagttc taagtaaaat tgctgataag gggtaagtta
11881 aaagcagttc tcagtaaaat tgcagataag aggtacgtta aaagcagttc taggcaaaat
11941 tgcagataag aggtgcgtta aaagcagttc taagtaaaat tgctgataag gggtaagtta
12001 aaagcaatcc taagtaaaat tgcagataag aggtaagtta aaagcaatcc taagtaaaat
12061 tgcagataag ggtacagaa aaactagact tgattacaaa atggagcttg ggacataaat
12121 gattttttaa aaatgagatg agacgtagat taactccata atcaatacga atctatcgac
12181 ttctttattt atgatattca tctcttttta atggaaataa aagtgcgatt aatgtgataa
12241 tacagttacg ttaattaaaa aaataaaaat gcaaggagag gtaatatgct aactgtatat
12301 ggacatagag gattacctag taaagctccg gaaaatacaa ttgcatcatt taaagctgct
12361 tcagaagtag aaggtataaa ctggttggag ttagatgttg caattacaaa agatgaacaa
12421 ctgattatca ttcatgatga ttatttagaa cggactacaa atatgtccgg ggaaataact
12481 gaattgaatt atgatgaaat taaagatgct tctgcaggat cttggtttgg tgaaaaattc
12541 aaagatgaac atttgccaac tttcgatgat gtagtaaaaa tagcaaatga atataatatg
12601 aatttaaatg tagaattaaa aggtattact ggaccgaatg gactagcact ttctaaaagt
12661 atggttaagc aagtggaaga acaattaaca aacttaaatc agaatcaaga agtgctcatt
12721 tcaagcttta atgttgtgct tgttaaactt gcagaagaaa tcatgccaca atataacaga
12781 gcagttatat tccatacaac ttcgtttcgt gaagactgga gaacactttt agattactgt
12841 aatgctaaaa tagtaaacac tgaagatgcc aaacttacta aagcaaaagt aaaaatggta
12901 aaagaagcgg gttatgaatt gaacgtatgg actgtaaaca aaccagcacg tgcaaaccaa
12961 cttgctaatt ggggagttga tggtatcttt acagacaatg cagataaaat ggtgcatttg
13021 tctcaataga aagttagagg tgagtcttac gtttcagtga cggtagactt acctttaaca
13081 tgttacatac taaaaaatta atttgaataa gaaagagaga catatatgaa atacgatgat
13141 tttatagtag gagaaacatt caaaacaaaa agccttcata ttacagaaga agaaattatc
13201 caatttgcaa caacttttga tcctcaatat atgcatatag ataaagaaaa agcagaacaa
13261 agtagattta aaggtatcat tgcatctggc atgcatacac tttcaatatc atttaaatta

13321 tgggtagaag aaggtaaata cggagaagaa gttgtagcag gaacacaaat gaataacgtt
13381 aaatttatta aacctgtata cccaggtaat acattgtacg ttatcgctga aattacaaat
13441 aagaaatcca taaaaaaaga aaatggactc gttacagtgt cactttcaac atacaatgaa
13501 aatgaagaaa ttgtatttaa gggagaagta acagcactta ttaataattc ataataaaac
13561 agtgaagcaa ccatcgttac ggattgcttc actgttttgt tattcatcta tatcgtattt
13621 tttattaccg ttctcatata gctcatcata cactttacct gagattttgg cattgtagct
13681 agccattcct ttatcttgta catctttaac attaatagcc atcatcatgt ttggattatc
13741 tttatcatat gatataaacc acccaatttg tctgccagtt tctccttgtt tcattttgag
13801 ttctgcagta ccggatttgc caattaagtt tgcataagat ctataaatat cttctttatg
13861 tgttttattt acgacttgtt gcataccatc agttaataga ttgatatttt ctttggaaat
13921 aatatttttc ttccaaactt tgtttttcgt gtcttttaat aagtgaggtg cgttaatatt
13981 gccattattt tctaatgcgc tatagattga aaggatctgt actgggttaa tcagtatttc
14041 accttgtccg taacctgaat cagctaataa tatttcatta tctaaatttt tgtttgaaat
14101 ttgagcatta taaaatggat aatcacttgg tatatcttca ccaacaccta gtttttcat
14161 gcctttttca aatttcttac tgcctaattc gagtgctact ctagcaaaga aaatgttatc
14221 tgatgattct attgcttgtt ttaagtcgat attaccattt accacttcat atcttgtaac
14281 gttgtaacca ccccaagatt tatctttttg ccaaccttta ccatcgattt tataacttgt
14341 tttatcgtct aatgttttgt tatttaaccc aatcattgct gttaatattt tttgagttga
14401 acctggtgaa gttgtaatct ggaacttgtt gagcagaggt tctttttat cttcggttaa
14461 tttattatat tcttcgttac tcatgccata cataaatgga tagacgtcat atgaaggtgt
14521 gcttacaagt gctaataatt cacctgtttg agggtggata gcagtacctg agccataatc
14581 atttttcatg ttgttataaa tactcttttg aactttagca tcaatagtta gttgaatatc
14641 tttgccatct tttttctttt tctctattaa tgtatgtgcg attgtattgc tattatcgtc
14701 aacgattgtg acacgatagc catcttcatg ttggagcttt ttatcgtaaa gtttttcgag
14761 tccctttta ccaataactg catcatcttt atagccttta tattcttttt gttttaattc
14821 ttcagagtta atgggaccaa cataacctaa tagatgtgaa gtcgcttttc ctagaggata
14881 gttacgactt tctgtttcat tagttgtaag atgaaatttt tttgcgaaat ctcttaaata
14941 ttcatccatt tttttaacgg ttttaagtgg aacgaaggta tcatcttgta cccaattttg
15001 atccatttgt tgtttgatat agtcttcaga aatacttagt tctttagcga ttgctttata
15061 atcttttta gatacattct ttggaacgat gcctatctca tatgctgttc ctgtattggc
15121 caattccaca ttgtttcggt ctaaaatttt accacgttct gattttaaat tttcaatatg
15181 tatgctttgg tctttctgca ttcctggaat aatgacgcta tgatcccaat ctaacttcca
15241 cataccatct tctttaacaa aattaaattg aacgttgcga tcaatgttac cgtagtttgt
15301 tttaatttta tattgagcat ctactcgttt tttattttta gatacttttt ttattttacg
15361 atcctgaatg tttatatctt taacgcctaa actattatat atttttatcg gacgttcagt
15421 catttctact tcaccattat cgcttttaga aatataactg ctatctttat aaacttgttt
15481 gaaattttta tcttcaattg catcaatagt attattaatt tctttatctt ttgaagcata
15541 aaaatatata ccaaacccga caactacaac tattaaaata agtggaacaa tttttatctt
15601 tttcatcaat atcctcctta tataagacta catttgtaat atattacaaa tgtagtattt
15661 atgtcaaaat aatgttataa tttttgtgat atggaggtgt agaaggtgtt atcatctttt
15721 ttaatgttaa gtataatcag ttcattgctc acgatatgtg taattttttt agtgagaatg
15781 ctctatataa aatatacggt tctgttgcaa agttgaattt atagtataat tataaccaaa
15841 aggagtcttc tgtatgaact atttcagata taaacaattt aacaaggatg ttatcactgt
15901 agccgttggc tactatctaa gatatgcatt gagttatcgt gatatgtctg aaatattaag

15961 ggaacgtggt gtaaacgttc atcatttaac ggtctagcgt tgagttcaag aatatgcccc
16021 gattttatat caaatttgga agaaaaagca taaaaaagct tattacaaat ggcgtattga
16081 tgagacgtac atcaaaataa aaggaaaatg gaactattta tatcgtgcca ttgatacaga
16141 gggacataca ttagatattt ggttgcgtaa gcaacgagat aatcattcag catatgtatt
16201 tatcaaacgt ctcattaaac aatttggtaa acctcaaaag gtaattacag attaggcacc
16261 ttcaacgaag gtcgcaatgg ctaaagtcat taaagctttt aaacttaaac ctgactgtca
16321 ttgtacatcg aaatatctga ataacctcat tgagcaagat caccgtcata ttaaagtaag
16381 aaagacaaga tatcaaagta tcaatacggc aaagaatact ttaaaaggta ttgaatgtat
16441 ttacggtcta tataaaaaga accgcaggtc tcttcagatc tgcggatttt cgccatgcca
16501 tgaaattagc atcatgctaa caagttaaca caaagtatta ttttaaaatt gagattagac
16561 atttattttt caactttgca gcagaaccta attttttctc tttgtaaata aatgcaaaga
16621 gataccataa ggatctctta cataaccata accttcagta tagaattctg gactaaatgt
16681 tttcaatacc tcactgcctt tttctattaa ctggtcatat acatgtttag tttcttctac
16741 ttggtcaaaa gtgagacaaa gagatatatt attaccttgt gttatgggca aaccttcagt
16801 gtcatctgcg atcataattt ttatatctcc aaattgaagt acacattgat caattttatt
16861 taaatcattt tcgtcaatat taagtttctt atctataggt ctatctttaa tacgttgaat
16921 atacagtgtt ttagcgccaa acagctcttc atacaacttt tttaaaccct ctgcattttg
16981 agtgattaaa aaatatggac ttacttgaaa tttcatgttt tttcctcctt agatttgtta
17041 taaatagagt atatacttaa ttagtgtcat ctattgacac taaaaggaga ataataatga
17101 aaaaatctgt tagattatat aatatgattg aatattgtaa tgaaaatagg aacttcaaat
17161 taaatgattt aatgtcagaa tttaatattt ctcgtagtac cgctttaagg gatataaaag
17221 aaattgaagc attaggagta cctttatata gtaatccagg gaaaaatggt ggttatacga
17281 tcataggtaa tcgagaccaa acgaaaatag caatctcaga tgaagagttg aaagctttag
17341 tatttacact ttcgagtatt tcaaatgtga gtaatctacc ttttcaaaca gaatatcaag
17401 aaatattaaa aaaattatat aataactcga ataaaaaaga gttaataaac caatataatg
17461 atctatttca atattttaat gaagataagt atcagttcaa aagttataag ttatttaatg
17521 aaatcattag attgataatt gagaataagt cttttgaaac ctgttattca caaaactata
17581 ttaaagaaca atataaaggt attggcatta tgtataaaaa tcatcaatgg tattttgttg
17641 tagctaatat agaatcaaag ttagtgaatc tattaaatat ttcgaaaata aaagaactat
17701 atgaaatggg agagactcaa gagtgtaatg atataactat gcagaatttt caacagttca
17761 tggttaaaaa tgaaacagct attgatattc ttattagaag caatgttatg ggattgaata
17821 tcttgaaagg ctacctgtgg agtgactata tgattgaaaa tattgacgaa gagacatatt
17881 tatttaaatc aaaagtgaac gcgaaagata tagattttat agctaagtta attgtcacag
17941 gtggtgtcaa tgtaaaagta gagaccccta atagtttgaa aaatgctgtt aaagttgaat
18001 taactaaaat aataaacatg tattaatagt aatttaatca taattgtaat aacaaaaaga
18061 tgacaacatt aaacaattta aatttataca aaaaagcatt accaaattta aaattgggta
18121 gtggtagtgc tacagcatac ataaatagtt ataattaaat aaagatgggt caatgaaggt
18181 gaattaagtg aataaaaata ttacagctct aaaatctact gaaaatacta catatatgca
18241 gtatacggtt catatcgatg atcaaattat aaataatata aaagagtgta ccaaaaagtt
18301 taaaattttg cctatggaag ataaaattcc attatcaccg ttgttacaac cagaatatgc
18361 aggagaggta caagatttta ttagtacata tgagcagttt atgattaatt ttggtaaagt
18421 aatattggat agtcaaggca taaaaataca gtttgaaagt gaatcattaa gtagtattca
18481 acgaggcatt caagaacatt gttacttaaa tgagcgaaca aatgacattg atgtgactaa
18541 agaatggtat ttatgcaaat tttcaaatca aatattagaa gaggataaaa atcaattgta

18601 taatgcttta aagcaactca tgaatgattc taaaaataaa aagcaagctt tttggactga
18661 ccccaattag tgggaattat ataaaaacac tttcgttgaa ttcataataa aatgaatcat
18721 gcggaggtgt tttttctatg aaaagagttt cttattcagt agaaacaaag tataaagcag
18781 ttgaaatgaa aatagctggt ttttcgacaa aagaaattat gaaagaatta aatattaaaa
18841 atagaacaca agtggaaact tggtggcgat ggtatcgaaa tgaggaaagt tatagatttt
18901 cacaacacgt tgggaaacaa tatacctacg gtaaaggatt agaagaattg tcagaaatag
18961 aacgattaaa attagaaaat aagagaaaag atatagaatt ggatatttta aaaaagtaca
19021 aggcattgga aaggaagtgg taccagcagt agtcatagat ttagtggatc aattaaaagt
19081 aaaatattca atcaaattga tactagaagt attaaacata cctaaatcaa catattaccg
19141 atggaaaaac aaaacctata aaaatgatac tgtaacacaa aaagttattg aattatgtga
19201 agctaaccac tatacctacg gttatcgtaa gattacagca ctgattaatc aatgttatac
19261 atcaccaatt aatcataaga gagtacagag aattatgcag aagcatcatt tgaactgccg
19321 agttagacct aaaaagacga caaggatagg taaaccgtat tataaaactg acaatttatt
19381 acaaagacaa tttaaagcga gttgtcccat ggaagtatta acaaccgata ttacttattt
19441 accatttggt cattctatgt tgtatttatc ttcgataatg gatatttata acggagaaat
19501 tgtggcgtat aaaatagatg ataaacaaga ccaaagttta gttaatgata cattaaatca
19561 aatcgatata cccgaggggt gtatattaca tagtgatcaa ggcagcgttt atacatctta
19621 tgcttattat caattatgcg aagaaaaagg cattatcaga agtatgtccc gaaagggaac
19681 acctgctgat aacgccccga tagaaagttt tcattcctcg ctaaagtctg aaacttttta
19741 catcaataat cagcttaata gctctaatca tattgtaata gatattgtcg aaaaatacat
19801 taaaaactat aataataatc gaattcaaca aaaactaggc tacttatctc ctgtaaaata
19861 cagagaatta gcagcctaga acatagtgtt tttattaagt tcccgtttaa gggggtcagt
19921 accttttcaa gaggtgttta aggttcatat tgatatttat ctctacaata aagaaaaatc
19981 tgaatataag tatcaagtat ctagttattt caatcttgta aaagaaaatc ctaaaataac
20041 ttataaaaaa agacatttac aagagaaaca aggtgtcaag ggtacaactt tcacgaacaa
20101 attaaaatta ttgaacaaaa tgtatggtgt agatgttgca aaatatcagc cattttataa
20161 ttctaataat cctgaatatg aaagaggtca atttggtgaa agatacattt ctcaaagatc
20221 taactatgaa tttaatagac ttcaatatca aattatagat atgttatcga aaatacttga
20281 taagcatcca ttaccaaagt cagataataa ttataaacat attccaacta ttgaaaaagc
20341 aatattaagt ggggattctc atagttttta tgagtatttt gaagatataa tgaaagaaat
20401 tataagtatg gaaaattctt ctttaaagga aaaattattg actgatttta catatcaatc
20461 tcaatgtaga tggtactctg aaagtgaaaa attaaaattg caacttgaaa gttttatgca
20521 taaagttttg gagagcaatt attatgaggg taacaaatta tatagaatgt tgtctcatgc
20581 aattgaagaa acaatcaatg aggctgatga agataaggtt catttcaatt attttaaaga
20641 ttatttttg acagacggtg gggtgaagaa ttgggagcag attagcgaga aaattactga
20701 attcaatggt aaagttatta acgatatcca aaatgaatat aataagattc aatttaataa
20761 cgcaaatcga aatcagaaac taaactttaa ttatttatac cattgctttg agtttagtaa
20821 taacttagta aaagcaaggg taaatggaaa tagaggttac attttatatt ttgtagataa
20881 atataaaata aaaatgccct ttttggatca attattaatc aattatttga atcaagaaat
20941 aggtcaggaa agtattaatt ataatatgca aacattgttt gaaaaagaaa gatatgatag
21001 aagtagtacc attgaaaaat tagtagcaac aagcaaattt aagtatgaaa aagatgattc
21061 agatttattc aaacaacttt tcaatgatgt tgaaaattca atagacagat taggtattta
21121 cttactaaat aatggtataa attcgaatga tgaaaatgca agatattata gatcgttttt
21181 aaaggaactt agtagaataa aaagtaaatt aacgccattt tctcttgaaa taagtaagtc

21241 tagcggaaga gagcaacatt atcctgatga tgctattgat gataaagatg agagaagaaa
21301 aaataaagaa gaaacatatc atgcttttga tgataaaagc gatattgact ccaaattaaa
21361 gaataaaata aatgtttcta tcgataattt attgtcagtt aaaattaaga gcaatttgta
21421 tgatattagt ccaaatacaa aaaaacaata actataatga gccaactcaa gaatgagttg
21481 gctttttaat tgtaatttta cgcttccatt acaggaatca atgttatgga agccattaag
21541 caattaattt gagtcattta tagtataggt acaacgttgt gatgttcaca tgataatcag
21601 cccgggagat tattgtgtaa taacttatag aaggagcaaa tcaccatgta tcaagcaaat
21661 atacgtgatt taattacaaa attgcctcaa agcaataaaa cagaacactt tttaatgaac
21721 aaattttcaa atcaagataa agttcagcag ctacaaagac aaattagcca acagctagat
21781 caacaatata atgagctttt ggctaatgaa aaagctaagc tagaccaata cgtggaagta
21841 caccataatt tagaaccatt aaagaaagag attgaatcag aatctattaa ccttgatacc
21901 gataaattac ctgatatcaa agcgacaatg cttgaaaagg ctaagaacga tgaacatttt
21961 gataaaatcg aacagctatt tgatagatta gatcagtcat taaatggtac gaatcgatta
22021 tatacgcaat tatcgttgat tggcacacga acacatcgga tcacaacgaa aagatttaat
22081 gttcaaggct tgcctaaatt agtccaacaa atgattttac cttcgcaatt taaaaaggtt
22141 tatacaatag attttaaatc attcgaacca tcagttgctg cgtatatgac acaagacgaa
22201 caactgattg actacttgaa tcatgaagaa gggttatacg atgcattact gagagactta
22261 tctttgtcaa aagagaagcg cgtgagtgtg aaacgtgcat ttatagggtc atttcttttt
22321 ggcggtcgtt atagtagctc taaattcaaa atcaatcaag aggttagtga aattaactgg
22381 ctacaagtaa tgagcaaatt caagaaggtc attgaattta aggagcaagt cgaaaaatat
22441 aaaacaatgc ctacgcctta cggcattgaa catgatatga gcgcatttca aggtagtagt
22501 attatggcaa tttatgtaca aacggtagca agctatattt tcaagcacat tttgttggaa
22561 gtgtacaaag cacagtgcga aaaaaaaacg ttcaagatta tagtgccgat acacgatgcg
22621 attatgattg aatgtaatga taaggggatt gcacaaaatg tagcacagct catgaaagat
22681 acagctaatc aactgtttaa tggtgaattt gcacatgtga cagtggaagc tttaggaggt
22741 atagacaatg aataatgata gaggaaaaag tcttcaaatt ccccaaagta cattgttaaa
22801 agaaggatct atatacgttg ctacgttaca ttctgtgtac gagaagaact tctcaggtga
22861 tattaaacat cagtttacgt atgaagtaga acttaaccaa gaaacgcact atgtaaatcg
22921 caatattacc gtaaaatcta tgagtcacca attatcaatt gctgattgga ttaaacgtca
22981 cagtaactat aacgtaaacc acattaatta tgatccatac attgatcgaa aacatttggt
23041 ccttgtaggg caatataacg gaaattatta tattcaagat gtagcaccat tagatgaatt
23101 tggaggggta ttgtaatgaa tcatatttta gaaatgttaa taaaattatt aaaagtgggt
23161 atggaggcaa tcgaccgtaa aggtctgatt gccatcctaa caagtagtat tggaaatgat
23221 gaaatggatg attctgaaca agctgtaatg gtgtataacg agcttatcga taagctacag
23281 cttaacattc ctaaagatgt cgactataga cctaacatat acagttattt tggtattcaa
23341 aaaaagccaa atgacacaat attagtggaa atgatgatat gtatttttca tatcaagcgt
23401 tttgattcag agttgtttgt tttcaaagat aaaggttggc aaaaggtaag tgaagatgaa
23461 ttgcaagggt tgatatctaa aatgatacaa gtgttgctag ttgattataa accttcacta
23521 agtactttga aaaacgtagt agatggcata cagaaatcaa cggacataga aaaacttgtt
23581 gagcatagac agtacattgg ttgtggacga aatatgttca atctaaagac ctttaaagtg
23641 gttgataatg accttgaaat attccctaaa acacgcttag atttggaatt agatataaat
23701 gacacgatta cggacaagat acccccgaat ttcaaacaat atatgttaga gttggcgaat
23761 tatgaccatg atttacaata tttccttttc caacatatgg cagtgttatt gacggcagat
23821 actaaactac gtcgtggact ttttttgtat ggaactgcaa aaaatgggaa atcggtctat

23881 attaaattag ttaagtcatt cttttatagt aatgatatcg tatctaaaac acttaatgaa
23941 cttggcgggc gtttcgataa ggaaagtcta attggtaaac gaattatggc aagtgatgaa
24001 gtggggaaag ctaatattga tgaagcaact gtgaatgatt tcaaaaaatt actatctgtt
24061 gaaccaattc atgctgaccg taaaggaaga acgcaagtag aagttacttt agatttaaaa
24121 ctcattttta atacgaatgc tgtactcaat tttccatcat cacatgcaaa agcattagag
24181 cgtagaattg ctgttattcc atgtgaatat tatgttgaaa aagctgaccc tgacttaatt
24241 gaaaagttac aggatgaaaa gaaagaaatc tttctttact tgatgtatgt gtataagcaa
24301 attgtaaaaa atgatatcga gtacctccaa aatgatcgtg ttactgaaat ttctcatgat
24361 tggttaaatt ttggatatga atttgtttct agtaaatcag caaatattgc acatcagaaa
24421 gcgtgtatta atttactcag aaaacttata gaaatcaaac cagggtcacg tatcaaagtg
24481 tctaggctaa atgaggttat tagagatgaa attaaagtaa gctctcaagt tattaatgat
24541 ttggttcaag ctaactttaa tgtacaaagt agactaaata atggttataa gtattgggtc
24601 gatttaggat ggaaagaaac tgataaaaaa gatgacatga tttcattcga taaaaatgag
24661 aatgtaacag atgatgaatt cttatacgaa gatgatttga acttaggttg ggaggacttt
24721 gacgatgaat aatgaacaaa ttgaagcatt tgtagaagtg cttgtgccta tcatagaaga
24781 acgtatcaat aaaggtaagt aatctaatta cgtactacag gcagttgcct gtagtactca
24841 tatgattaag tggtaaaagt gataaaaatg aaacgaaatt ataaatatat attatctata
24901 tgttgttaca agaccgatgg tctgtagcaa taatctaata aaaggagcgg tatgatatga
24961 agggtaaaat tgcactttat tcacgtgtta gtacgtctga gcagtcagaa catggttatt
25021 ctgaaaagga gcaggaacaa ctactcatca aagaagttat gaaaaatttc ccaggttatg
25081 actatgagac atatactgac tcaggcattt caggtaaaaa tattgaaggt cgtccggcaa
25141 tgaaacgtct attacaagat gttaaggata ataaaatcga aatggtatta agttggaaat
25201 tgaatcgtat ttctcgctca atgagagacg tgtttaatat tattcatgaa ttcaaagaac
25261 atgacgtagg gtataaatcg atttctgaga atattgatac atccaatgct tctggagaag
25321 tactcgttac aatgtttgga ctaataggat ctatagaacg ccagactttg atttccaatg
25381 tgaaactttc tatgaatgct aaggcaagga gcggagaggc aatcaccggt cgtgttttag
25441 gctacaaatt atcacttaat ccattgacac agaaaaatga tttagttatt gatgaaaatg
25501 aagctcatat tgtacgggaa atctttgatt tatatttgaa tcacaataaa ggatttaaag
25561 caatcacgac aattctaaat caaaaaggat atcgtaccat taatcaaaaa ccattttcag
25621 tgtttggcgt gaaatacatt ttgaataatc cagtctataa aggctatgtc agattcaata
25681 atcatcaaaa ttgggctgtt cagcgaagaa gtggtaaaag tgataaaaat gatgtgatat
25741 tggtcaaagg taagcatgaa gccattataa gtgaagatgt atttgatcaa gttcatgaga
25801 aactagcttc taaaagtttt aaaccgggtc gacctattgg tggagatttc tacttacgtg
25861 gccttattaa atgcccagaa tgcggaaata atatggtatg tcgacggacg tattataaaa
25921 cgaaaaagtc caaagaacgc acaatcaaac gctattacat ttgttcatta ttcaatcgct
25981 caggaagttc tgcctgtcat agtaatgcga ttaatgctga agtcgtcgaa cgcgtaatca
26041 atgttcattt gaatcgtatt ctttcacaac ctaatgttat taagcagatt gcgtcaagtg
26101 tgatagaaga actgaaacaa aagcatagta aacaaacaga aataaaatat gatattgata
26161 gtctagaaaa acaaaaagca aaagttaaaa cacaacaaga acgattattg gaattgttct
26221 tagatgatga aatggatagc gaaatgttaa aagctaaaca aagtgaaatg aatcaacagt
26281 tagaagtatt agaccaacaa attaaagaag caaaacaagc aaatcaatca caggatgata
26341 tacccaattt tgataagtta aaagcacgac tcattttgat gataacacga ttcagtgtgt
26401 acttaagaaa ggctacaccc gaagctaaaa atcaacttat gaaaatgtta attgattcaa
26461 ttgaaattac gacagataaa caagtaaaac ttgtaaggta taaaattgat gaaagtctta

26521 tccctcaatc tttgaaaaaa gattgggggt cttttttat acctaaattt aactttgtga
26581 taaatgtcac aaagaaaaat aggattgaaa atttatcact tttaccactt ttttagagtg
26641 acaaaagtgg aggagttttg aaatatttta taaatatata ttttatttat ggagtacaca
26701 ttattaatta aggaggtcat tataatgacg ctaagcaaac aacttaaaac gtatatcact
26761 gaacgattta aattaaatta tcaagaaact tgggcttgtg aaaccataga tgcggtggct
26821 gaagatgtat tacctgaaaa atatattaaa aatagtccac ttgaacataa aattttaaat
26881 acttttacct attacaatga tgaattacat gaaatcagca tttacccttt tttatgttat
26941 ctagataagg aattagtagc aataggttat ttagataatt ttgatttaga ctttatattt
27001 ttaaatgaca ctcatcaagt cattattgat gaacgctact tgttacaaaa agggggcgag
27061 taattatgaa ttggatcaag gtcgctcaac tatctgtcac agttatcaat gaagtcattg
27121 agattatgaa agaaaaacaa aatggagggg aatagtatga aaatcaatcg atacattaca
27181 agaggtgtta gtgaacattt atctctagac cttcaaatct tactttggaa catggtaaaa
27241 gatcgagaca atcaacttca tacagattac ctacacattt ttaaactaca agaagatgat
27301 aatatactct caatcacaca tgaacaagaa caacccgcat acaaattgga atatcactat
27361 acaaactatg taaaaaatca aaatgcatta cctaagaaag tctacgtcat ccgagaagat
27421 gatgtagacg ctttttatta tgtgatgctt ttaccagaag aatactaaaa ggagtgacaa
27481 tgatgaatac aatcaaaaat acaatacaca cagaagcgat atttagcgat gatgaacaac
27541 accgctactt actcaaaaag acttgggatg aaaagaaacc tgcatgtacg gtaatcacca
27601 tgtaccctca tctagatggc gtattatcac tcgatcttac aactgttctt atcctcaatc
27661 aattagcgaa ttctgaacaa tatggcgctg tatatcttgt aaatctattt tctaatatta
27721 aaaccccaga gaaccttaaa catattaaag aaccttatga taaacacaca gacattcatt
27781 taatgaaagc gataagtgaa agtgacacag tgattttagc ctatggtgct tatgcgaagc
27841 gacccgttgt tgtcgaacgc gttgagcaag tgatggaaat gttaaaacct cataaaaaga
27901 aagtaaaaaa gctcataaat ccggcaacga atgaaattat gcatccgctt aatcctaaag
27961 cgcgtcaaaa atggacattg aaataaagga ggattatcta tgaaccatga aactaaacaa
28021 tcagattggc gaacggttgc tagttgttta gcatcacaaa attatatatc gattgtaaaa
28081 ggtttagtac atcattttac agcgatcgaa gatgaggaaa ttcttgataa aatctatgat
28141 gattttatga atgatgactc tataacaacg gtacttaaca atgatttaca gacaatcatt
28201 aaccattatc tatcaaaata aagactatac taattacaat aaaaacagag catccttcac
28261 ttttatggtg agggatgctc ttttaattta tttttttatt gattattcgc cataagttct
28321 gcttgctcta caacctgctc tacagccatt ttttgtaaat ctggtggata gccatatttt
28381 ttaagcaatc gtctaacagc tacgcgcatt ttagcttttg cgctatcacg tttagaccaa
28441 tcaacaccca tgttttcttt cactgtttta gttagctcat gagcaatcgc acgtagttct
28501 ttatctccca tggcttcttt tgctgtttca tgtgaagcta aagcatcgta aaacgcaatc
28561 tcatctgaat tcaggcctaa ttcatttcct cgttgttgtt cttgtttaat atctttagcg
28621 agttgaataa gttcttcaat cactttagat gtttcaatgg aacgactatt atatttatta
28681 atcgaatttc ctaacatctc agagaaacgc ttagataccg ttgcattcgt tttcattaat
28741 gatttaactt gtcctttgag taatcgattt aataattcta ccgccacatt tttctgtttc
28801 aatccttcga catcttttaa aaagtcatca gataagattg ataaatcggg ttgttcaaga
28861 cctagcgttt gataaacatc aatgacatct tcagtcacaa cagattgtga cacaagttga
28921 ttaatctctg cttcaacttc tgcaggtgtt ttacgtgttt ttccttcttt tggcggttgt
28981 aacaatttaa caagtcctgc tttaactgct ttaaagaagg caatctcatc gttgagttct
29041 tgggctgtag gttcagtcgc acaaagagca aaggctttcc ctaattctgt gactgtttta
29101 ataaaacgtt gacgttcatc ttcacctaaa ccaattacat aatccatcgt attcgatatt

29161 gtataataac gttcagattt tttatctgaa ttaaatttag agtaatcaag attatataac
29221 atatcttgaa ttacatcata cttcaataac atcaattcaa ccgctttatc cgtatctatt
29281 gccgtttgag cttgatcaga ttctgtatat tctttaagtg cctcttttaa actttcggca
29341 atccccacat aatcgacaat taaaccaccc ggcttatctt taaacactcg attaactcga
29401 gcaattgctt gcatcaaatt atgccctttc atcggtttat caatatacat cgtatgcata
29461 gaaggaacat caaaccctgt cagccacata tctcgaacaa tcacgagttg taactcatca
29521 tccacatctt tcatacgttt ttctaataaa ttacggcgtt ttttaggacc aatgtgtctt
29581 tggaaagaag ttgggtcact agatgagcct gtcattacca ctttaatgac tcctttatca
29641 tcatcatctg aatgccattc tggttttaga cgaatgattt catcatataa atcaacagca
29701 attcgacgac tcatcgttac aatcattcct ttgcctttca tcgcttgttg acgtgtttca
29761 aagtgttgga tgatatcttt ggctagggct tctacacgag gttttgcgcc tgctaaagct
29821 tcaatacggg accattttga ttttaaacgc tgttttacat cctcttcttg atcttcagta
29881 atgtcattat acgcttcatc taaatctaaa ctttgaggta gatttaatgg aattacacga
29941 ctttcatagt aaattttaac ggtgcttcca tcagcgacgg cttgtgtcat atcataaaca
30001 tcgatatagt ttccgaaaac catttgcgta tttttatccg ttgaagctac gggtgtgcct
30061 gtaaatccta cgaatgttgc attcggtaaa gcatctctta aatatttggc ataaccatat
30121 ttaatgcctt cacctttatc atcgtatttt gcgttaaagc catattgtgt acgatgagct
30181 tcgtctgcca tgacaatcac attttttcgt tctgttaggg cagccatggt cgtttcgttt
30241 tgttcaggtt caaatttttg catcgttgta aatacaatac cacccgactc aacagataat
30301 aatgatttta attctttacg tgtttcagct tgttttggtg tttgtcttaa taatccttta
30361 ccagaacgtc cttttgattt aacaaacgta ctgtacagtt ggttatctaa atcattacga
30421 tccgttacga cgactaaggt agggttattc agcatttgaa ttaattttcc agagaaaaag
30481 accatggtta aacttttacc ggacccttgg gtatgccaaa taacgccgcc tttaccatca
30541 ccttgctcag atgaagctaa taaagctctg tcaacggctt tattaacggc ataatattga
30601 tgatacgctg ctagaatttt actgatatgt cctttgccat catcttgaaa taaaacaaaa
30661 tatcgaatta aatcaagtaa tgtttctgga tttaacatgc catgaatgag tacgtctaag
30721 ctagctaaac ctggtgatga ttcagtttga ccatctttac tgcgccaagt cataaaccga
30781 tcataatttg cggttagaga gccagccttt gtattaatac catcacttgt aacaagcact
30841 tcgttaaacg taaataattg tggaatacgc atcttatacg tttctaattg atgataaccg
30901 tcttcgacgc ccacggtttc atttgttgaa tttttaagtt caatcactac gataggcaag
30961 ccattgataa agagtacaat atcgggacgt tttttatagt ctccattaac gatagttaat
31021 tgattgactg ctaaaaaatc attgttttgt ggatattcaa aatcaataat tttaacaatt
31081 tctacaatcg attgtccttc atcatcatag tcttcgattt caataccatt aatcaaattt
31141 tcatgaaagg taaggttatt ttctaaaaga ttgggcgact tctctaaagt tagttcatgt
31201 agcgcttttt caataaaacg aggatggata tctgagttga tttttcttaa tgctttctct
31261 aaacgttcat gaaggacaac atctttatca cttttacgtt ctggtgttag acttgtcata
31321 ctaatctcat taccttttt atagtcatag cctagtgatt gtagccattc taatgcgact
31381 tgttctaaat catcttcatt aaattgaaag ttcatcacta ttcacctcaa tatcatcaga
31441 tatttctagt tctccagaca ttagtttagg aagaagagta tctcgtaatt gagttaattt
31501 accaatttct gatcttaata ttttcatttc ttccatatat ggagtgatta tttcagaata
31561 tttttttaca atactatctt caattgctaa tttataatta acagctattg ttggttttac
31621 tctttgtcta ctatttgttg aaccggtagt attagctttt aaataatcta taaattgaga
31681 attaagacat atattatata tgaatgaatt aattttatta ttaggagatt ccattacaac
31741 aaattctgat gaagcaacat ttaatttttt attatctatt acaggtaacc aaatcctttt

31801 agtatcagga ttcatttttg aaaataatac gcaattgtta ttaataatcc atttattact
31861 ttttatttta ttgacttctt cctctatagc ttgttcttca ttatcataag caggtaaact
31921 aaaatgttta actgttactt cttcactctt tttaggatta aaagtttcct ttttatgact
31981 tgctatatct tttaatttat agattttcca attacttggt atcttaccta actcactatc
32041 aatcatttct cctccactag atttataagg attaccatct tcatctggaa attcaaaatc
32101 tacaaaccaa tgcttaaata gtgtttgtga aagttcttta aggtttgcta tcattttttg
32161 gttagtttct atttttcttg tgatttgatt taaaaattta gaaatgcttt tttgttttat
32221 taaagaaaaa ttaggaattt tgacagtttc aataagttta gaatttatat tagcttgatt
32281 agcactccct gtagaatttc taactaaata ctcagttatt ttaggttgta gaaaataata
32341 aaataaaaaa tctaagtctg cactattact atattttaat tcgaattttc ccacacgttg
32401 attttgtaat gctggataat caaattcaat tcttccaact tttcctactg cagaagacat
32461 ttgattcacg cctgatccag ttaatgatat taatatatca tttttcttta atgaaaaacc
32521 tttagttttt tcatatgttt taatagagac tttttgagag tcttttgtat caacaatggg
32581 tgaaattata ttcttaattt taataaccgg tataccttttt tcttgaaatt cttttgattt
32641 aaaagcatat ccatttttaa ttttagctac atcaccgaaa aaaattgtat tatactccat
32701 atcctaaccc ctccaatgat tttcggattt ggtcttcaag ttcttttgat tttgcgaatt
32761 gttcacttaa ttcagaagta atacgttcca ttttttgttc aaaaggttct tcatcttctt
32821 caacgtcagc taaaccaaca tatcgtccag gtgttaaaat gtattcatta ttcttaactt
32881 cttctaaatt agctacttta caaaaaccag ctatatcttc ataaggttta tcgtttgtac
32941 ctctccacgc atgatacgtc tgtgctactt tttgaatatc ttcatctgaa aattctttta
33001 atgttctaga taccatatgg ccgatttcac gagcatcgat aaataaaatt tcattttcac
33061 gctctttttt accattttga cctttattat tactaatgaa ccaaagacat acaggtattt
33121 gggttgagta aaagagttgt ccaggtaaag taacaataca ttcaactaaa tcttgttcaa
33181 ttagattttt acgaatttct aattcatctt ttccgcttgt agacattgat ccgttggcta
33241 atacgaatcc tgctgtacca ttaggtgcta attttgaaat catatgttca atccatgcat
33301 agttagcatt acctttttggt ggaataccaa attgccaacg gtaatcatca agtaatcgtt
33361 cttgacccca atcacttgcg ttaaaaggag gattggctaa tatgtaatct gcttttaatc
33421 ctttatgtaa atcattatgg aatgtatcag catttcgttc acctaagtca ttatcaatac
33481 cacgaatcgc taagttcatt tttgctaatt tccaagtcgt agggttagat tcttgtccgt
33541 aaatcgcaat atcgtctaat cgaccttgat gtttctcaac gaagcgttca ctttgtacaa
33601 acatcccacc tgaaccgcaa cacggatcgt aaatacggcc tttataaggc tcaatcatct
33661 caactaataa ctttacaatt gatgacgggg tatagaattc tccagcattt ttaccttcgg
33721 cacttgcgaa tttagcaata aagtactcat acacacgacc taacacatct tgtttacgac
33781 tctctgtgtc gccaacttta aatgtaaata aatcaataat atcgcctaat ttttctttat
33841 ctaatgcagg tcttgcatat tctttaggta atacaccttt gagtgattca ttttcatttt
33901 caattgcaat cattgcttta tcaataattt gtccaatttc tggttttttc gcattatcat
33961 tgatatattg ccatcttgct tcttttggca cccagaaaat gttttctgct aagtattcat
34021 cttggtcctc ttcatctgca tatggatcct gcttcaattc ttcatacttt tcttcaaaag
34081 aatctgatac atattttaaa aagattaaac ctagtgctac atttttatac tcagcagcat
34141 ccatacttcc tctcaattta tcggcagctt gccataattt ttcttcaaaa cctatagtgg
34201 ccattaataa cacctcttaa aattaatgtt aaatcaacta tagcaaaaaa tagaagatga
34261 ttgtgtagtg ttttattaat atttaattta tctagactat caaaaaaata tagattatta
34321 ttatcttaaa ttaataaaca ggagtgaatt aaaatgaata aaaaatataa attaaaacga
34381 tacgataaaa gatttagagg tggatggtgt tacaaatgtg aagattcata ttcttacctt

34441 tgtgaagatt gtggttattg tagatattgt tgccattgtt aatacaagac tagataaatt
34501 gggtatcttt aactcaatta ataaaaaatt taaatgtagt caatagcttg ctaactttat
34561 ttgatgtaat ataattttgg cttttagcat tcagttaaaa agaagtaaat aattgacaga
34621 ttttttagag aactggagaa aaaagatgta catttctagt gttaaaatta agaattttag
34681 aaattttgat aatatcgata ttgatttcca tgagggagtt aatgtattaa tcggacataa
34741 taattcgggg aaatcaaatt tattacgtgc tttatcttta atttttgacg gttcagttag
34801 aaagcaactg tcagtggaag attttaataa ctctttaaca aaagaatctt taaaaaaaga
34861 ggctccaaaa atagtaattt cagtacatat aacacaatct gaaaacgaaa gactgatgtc
34921 tgatgagtta attacggtaa gtaattggct gactaaatta gaagaacctt atgaagctag
34981 aatacaatat gaattctttt taccaaaaga tgaggaaaaa aattacatag atttagtaaa
35041 aaatatagat gaaaaagaag aaatatggaa tttaattaga agccagttta ttaggttgta
35101 tgtcaataaa atctgggtgg gtaatccaga acatcagatt cctatagata atgatagttt
35161 aaataaattt gatttccaat ttttagacgc aataagagat gtagagagag atatgtttag
35221 tggtaaaaat acattattaa aaagtgtaat agattttttt atagattacg atataaaatc
35281 cgatgaaaca atcacagaag aagaacagaa agaaaaatta gaagaaagaa gaaaagaatt
35341 ttctgataat tctagtgatt tgattgaaac tattcataaa agattagata gtgggaatca
35401 aaaaattttg tcttatacaa atggcatagg agcatcttat gataaatcaa caccggattt
35461 taaaggtaat cttacagaaa gtgaaattta tactgtattg caattgataa ttaaacatga
35521 aactggaatg actttaccta taactcataa cgggttgggt tacaataatt taattttcat
35581 ggccttgcta ttgtctaaaa tgcaagctga ttctgatgga aattttctag gtagtaatgc
35641 aaaagtattt ccgatactag ctattgaaga accagaggct catctacatc ctactatgca
35701 aaatgaattt attaaatttt taaaaaataa cattagagaa aaaaaggtaa agcagatttt
35761 cataactact cattcaacac atatttcttc ttctacaaat attgatgata ttatttgtct
35821 ttacacagat gatagtaaaa ctaatgttag ttatcctgga aaagtttttg ataagaataa
35881 tcaaagtaag aagtacgtac aacggttttt agatgccact aagtcaaata tgctatttgc
35941 agaaaaggta atatttgtgg aaggtatagc agaacaatta ctattacata tatttgcaga
36001 ctatttaaat aaaccgttag aaaagaatca tgttgcagta ataaatgtag gcgggagata
36061 ttttaatcat tttttatctt tgtttgatag taataataaa tatgcaatta atcgtaaggt
36121 ttcttgtata acagatttag atccaatgcg aaaagaaaaa aatgaaaatg gacaaaaaaa
36181 taattttaaa gcatgctatc cttttgaata tgaaatggat ttagataaat ttgattattc
36241 aactaataat tctctagata aatatctaaa taatgagcac agtaatatac gagtatttgt
36301 tcaacctaat aaatatggaa aaacatttga gtaccaactt atgtttgaca atccttcatt
36361 aaaactgtta cttacagact cgatttctaa tagtcaagag ctagcagaat taatggatca
36421 ctatgaagaa aatgaatctt tacaaaaatt aattgataca ttatcaccat ctaatgaaaa
36481 tcagagaata ataaaatctt taaaagacac aaatgacagt tggaatgaag ataataaaaa
36541 gaaagcatta atagcatcaa gatacctaaa ttccatagga aaaggagaaa atgctctaga
36601 gctagcttca gtattaaaag ataatttaga gttaaaaggc cagagtgact atgaagattt
36661 tgtagtacca aaatatatag aaggtgcaat tcgatgggtg tgtgaataat atgattatta
36721 atgctcaaac gaaattaaat atagaacaag attttaaaat acaggctggt cctggtgctg
36781 gaaaaacaga atttttggtg aatcatatca aaaatgtaat acaaacttct gaaaagctcg
36841 aacgtatgaa gaaaatagcg tgtattactt atacgaatac ggctgctcag acagttttac
36901 aaagattagg aaaaagtgtt tcaaatagag tagatatatc tacaatacat agctttttgt
36961 atagaaacgt agttaaacca tattgttctt ttctacccaa agaatacgga atttgtgtgc
37021 aaaaattaaa aggacatagt gatccaattg tgattaacaa atatgtaaat gcatggttaa

37081 aaactgaata ttttacgaaa ttaaaaccgc ctagtaatag aaatcaacta ctaaaaatgc
37141 cagtactaaa gaaagctttg caaaactggc tactatctat gaaactttct tacgaaaata
37201 atgatgtgca atttatttgt gataatacca aagctcaagc aattgataaa aaaagtaaaa
37261 aaagaatggg aataaataaa tcaaacttaa atatactaga aaagcaatta ttaaattata
37321 aaaaaatata ttgggagaaa ggaattatcg atcatgatga tgttttgttt ttttcatatg
37381 tattaattag agattaccca ttcattctca ctattttatg tgctaaatat ccctactttt
37441 ttatagatga atttcaagat actagtcaat tacaagattt tattatagat aaaataagac
37501 agaaaggctg catagtgggg gttattggcg ataaggcaca ggcaatttac agctttcaag
37561 gtgcacgggt ttccttattt gagaagttta aagttgattt aataaattct cacactatct
37621 tagaaaatca tagaagttca tatcaaatag ttactttttt aaatagtatc agagaagata
37681 ttactcaaac agtatatagt ggtattgaaa atgataaagt tacaattttg attggaaatc
37741 ggattcgcag ttatactaag gtagttaata tttgtaacaa ggagtcactt gttaccttgt
37801 caagagataa tattacttct aatgcaatga gaataaagtt ggaaggaaat aattttgata
37861 aaaagttatt ggaaaaattt caagaaattg atagtaatag taaaagaagg aatactatta
37921 tttcgcttat aaaagctatt gaattagcta agaatactaa atataaagat gccttaaaaa
37981 tagtcgaatc gatttataaa gatgaaaata atcctaaaaa aatagctctt aattctctaa
38041 taaatatgat taataaatat gataaatacg gtaatggtac attaatgaat ttctacaata
38101 ctttatgtaa aactttagat attaaacttt cagggtttag aaaggggcaa gtaaaagatt
38161 tttacgaaaa cactttatat aaaaatatgg tgatatgtac aaatatcgtt gaagacacaa
38221 gtaatcatat tactatccac aaagcgaaag gcgcagaatt cgaaaatgta tttgttatag
38281 gtaataaaga cactttaagt ctacttttga aaccagattt aatgaacgat gaagagcatc
38341 gtatattcta tgtagctatg agtcgagcaa agaagaaact ttttttacaa tttgattatc
38401 ttgataataa tgaacaaaag aaaattaagg aacgttatga tgtaagtatt atacaataac
38461 ctaatctaag aatatttaaa tccaatatat ttcttgatta taattcaata atagataaaa
38521 tctataaacg attaaaaaga aaatatattt gctaagactt ataaaaatga agagataata
38581 gattttccta aattacagtt aaaagaagta aatagattga acactttgaa agattctaat
38641 ccaaaatttg aaccactaaa attgaaagat gaagaagatg caagatgtag ccatcaacgc
38701 tgatcacaaa gtatttttct taatgattat ttttaatgtt atcatgctcg tttcttctat
38761 tattcaaatg attttaggaa aaggcagacg tatcacacca gcaaaaccta ctaaagatgc
38821 taagttacac ttagataatc aatcctcata aagagaaaaa agaacccccta aaaagttcaa
38881 ctttttgggg gttacttact attaagaagt atttttgatt aagcatcttt catatcgtaa
38941 tcaccaatat actgatcaag gcgatttatt gtttctctga tatcatcatg tgtcgttaat
39001 gggttcgttg tacataaacg aatgacacgt tggttattca atactgtggt ataagcaatg
39061 gcataaccag aatctgcaat tttttgcgca gccatgctat tgagttgatt attttgtgct
39121 tcagttaaat cactgttttc atatctgaag ttcacaatgg aaagattagc gtgtgagata
39181 atacgccagt tatcgagacc tgacacgtat tcttctgcat attctgctaa accttcgccg
39241 tatttaatgc gctcgataat ttcatcttca ccaatgactt gcagagtaat ccataatttt
39301 aaagcacgtg cagggcgtgt aagttcaatg cctaacattt ctggatcaat cacatcatca
39361 tctgaagcaa tatcatccaa gtactcagca tcttcgccaa agctttgcag cagatgattc
39421 ttttctttca cgattaccat agcgcaacta tatgtttgga acagtaattt gtgagcatcc
39481 caactggcac tatctgaacg ttcaatgcct ttaaataagt gttgtgcttt atcggataag
39541 atatgtgata gtccataagc accgtctaca tggagccata gattgtagtg atcacatgta
39601 tctccaagtg tggtgaaatc atcgacagaa cctgtattag tcgtgcctgc agtcgcaatc
39661 accatagcag gtttgtaacc ttcttcaata tctttatcaa ttgcggcttt taagctatct

39721 gtattcattg taaagtcatc gttgtactca attctgcgga tattattttt aaggaagcct
39781 gcaacatgca aggctttgcc tacagaatga tgcgtttgag atgtaagata aacggttgct
39841 tttttaatat cttccatttc tacctgtgca tctctagccg caacgatagc tgttaaattg
39901 gccattgaac cgccagatac aaagacacca ccggccggtt tgatctcata accgattttt
39961 cctaccaagt agttaattaa attacgctca atattaattg gcagcgtcgc attggcaaaa
40021 ttagaggcat ggatattatt tgctgttgtt aaaatgtctc caagccatga tagacgtgat
40081 gctggacctg gtataaatga gaatgaacgt ggatggttcg gacgatataa ataattcagt
40141 acttcattat ttaaatcttt gagtacttca tagatatctc gccctttatt cggcacttcc
40201 atcttttcat atttctctct caattcgagc ggtgcctgtt gtgtcgcagg taagtctcca
40261 atatctctag ataaatattt attaacttca tctctgataa tcgtctctaa gtcgatattg
40321 ttctcattga attccatttc catactctct ccttcaatta tatttttaat aaaagacaga
40381 cgtattaatt acgacgtttt actacaatat aaatgatagc agatattaat aaaaccacgc
40441 taaccataat gacgaaaccc agaaggtgtc gccccaatga ataacgtgct gtgctttaac
40501 actcggtgta tgtgcgcctt tcagaaattg acgagggtaa tcgacttgca gattatcatc
40561 tgtgattttc aatttgtatt gatgcttgtc ttttggaatt cgataactac cccgtagaac
40621 aggggacgag gacctcatct tgatgatccc aattggcttg gttaaaaaga aatcgataat
40681 cctcgtagaa tagagaaaaa gtattaaaaa tatatttgat ttgataaaat attttgcaaa
40741 aaagagaaac aaaaataact ttcatataaa taatttcttt gcgattaagt tggaattaga
40801 aatactaaat acaaataaca gcgaatggaa tgcatattat tgaggaattg gatgtgtagt
40861 gcaatttcga gagttactac ttaacttaag aaaataaaaa gaagaaaaca aacaataaga
40921 attaaatctg aagcaagata taaaagcaaa tatttttgct acaattatat tagtaaatac
40981 tttattaatt agcatacgac tgaaagcgaa gtaacagcta ataatgttcc agatatagac
41041 attagtgtta tgaaagaaca atattaccat ctagaaaatg taatttttaa aaacctgaag
41101 ataaagtaga ctattctgtg agttttcatg ttaagaattc tgaacttggg gatgtagtat
41161 aaatatggtt tagtagaaaa tgacgctgga gctgtagctg agattgacta tcaagaatta
41221 actaaagtag attattaccc atcaacaaaa atccaagatt taacactatc taataatagt
41281 gcgaataatg aagtttaaaa gtgaaaaact gataatagcg aatcaaacta aagtaatcaa
41341 ttacagaatt caagtactac tcaagcctca aacaaatatt gaaactcaaa cacaagttaa
41401 gaataataca ctgtcagaaa cagatgaaca gtctaatgcc gatttagtcc agtaatagcc
41461 tctgtattat tagccgcacg attattatca ataattaaat aaagttaata ttaaataata
41521 aaaatccttc aaattatttg ttgaaaaaat aataactttg aaggtttttt agcaaaatca
41581 ctgataggga gaagcatatc ataaat
//
LOCUS Staphylococcus 36840 bp DNA linear UNK
DEFINITION Staphylococcus Staphylococcus epidermidis strain HD21-2
ACCESSION Staphylococcus
KEYWORDS .
SOURCE Staphylococcus Staphylococcus epidermidis
ORGANISM Staphylococcus Staphylococcus epidermidis
Bacteria; Bacillati; Bacillota; Bacilli; Bacillales;
Staphylococcaceae; Staphylococcus; Staphylococcus epidermidis.
FEATURES Location/Qualifiers
source 1..36840
/genome_md5=""

/project="hwwang_176279"
/db_xref="taxon:176279"
/genome_id="176279.78"
/mol_type="genomic DNA"
/organism="Staphylococcus Staphylococcus epidermidis"
CDS 1..480
/db_xref="SEED:fig|176279.78.peg.1"
/EC_number="2.1.1.177"
/translation="MKITILSVGKLKEKYWKQAIAEYEKRLGPYTKIELIEVPDEKAP

KNMSDKEIEQVKEKEGQRLLNKIKPQSTVITLEIKGKMLSSEGLAKELQTRMTQGQSD

FTFVIGGSNGLHQDVLQRSNYALSFSTMTFPHQMMRVILIEQIYRSFKIMRGEAYHK"
/transl_table=11
/product="23S rRNA
(pseudouridine(1915)-N(3))-methyltransferase (EC
2.1.1.177)"
CDS 700..1956
/db_xref="SEED:fig|176279.78.peg.2"

/translation="MIDNFMQVLKLIKEKRTNNVVKKSDWDKGDLYKTLVHDKLPKQL

KVHIKEDKYSVVGKVATGNYSKVPWISIYDENITKETKDGYYLVYLFHPEGEGIYLSL

NQGWSKISDMFPRDKNAAKQRALTLSSELNKYITSNEFNTGRFYYAENKDSSYDLKND

YPSGYSHGSIRFKYYDLNEGFTEEDMLEDLKKFLELFNELASKVTKTSYDSLVNSIDE
IQEDSEIEEIRTAQKDKTLKEVEAPKGIIPKYKKGVSKTTKNDSEIEKSNKENKLTGK

VGEKLALNYFNELIDNKIDEDKKEQFRNILNDNPGSQHGHGYDLVAFDPTNTDKAVEK
FIEIKTSTSSSIEEPFFMSLNEMFAMKEYKQKYLILRIFNVSGKEPQFYFIDPYANYS
EFKDVDDLIDKVFNVEAIQYKVFGEK"
/transl_table=11
/product="hypothetical protein"
CDS 2371..2610
/product="hypothetical protein"
/transl_table=11
/translation="MSDNLSLFIDYINDNIIYGSEIKREKLENLFNQFAIKNVEKNIV
YDELKSLDITIIESQDSYKNKLKRLFSVLLQSKKI"
/db_xref="SEED:fig|176279.78.peg.3"
CDS complement(2642..3316)
/db_xref="SEED:fig|176279.78.peg.4"
/product="Mobile element protein"
/translation="MNYFRYKQFNKDVITVAVGYYLRYALSYRDISEILRGRGVNVHH

STVYRWVQEYAPILYQIWKKKHKKAYYKWRIDETYIKIKGKWSYLYRAIDAEGHTLDI

WLRKQRDNHSAYAFIKRLIKQFGKPQKVITDQAPSTKVAMVKVIKAFKLKPDCHCTSK
YLNNLIEQDHRHIKVRKTRYQSINTAKNTLKGIECIYALYKKNRRSLQIYGFSPCHEI
SIMLAS"
/transl_table=11
CDS complement(3407..3613)
/db_xref="SEED:fig|176279.78.peg.5"
/product="Truncated replication protein for plasmid"
/translation="MSDLEEGLHRKRLISYGGLLKEIHKKLNLDDTEEGDLIHTDDDE
KADEDGFSIIAMWNWERKNYFIKE"
/transl_table=11
CDS complement(3617..4390)
/translation="MCPNSSIYSDEKSRVLVDKTKSGKVRPWREKKIANVDYFELLHI

LEFKKAERVKDCAEILEYKQNRETGERKLYRVWFCKSRLCPMCNWRRAMKHGIQSQKV

VAEVIKQKPTVRWLFLTLTVKNVYDGEELNKSLSDMAQGFRRMMQYKKINKNLVGFMR

ATEVTINNKDNSYNQHMHVLVCVEPTYFKNTENYVNQKQWIQFWKKAMKLDYDPNVKV
QMIRPKNKYKSDIQSAIDETAKYPVKDTDFMTDDEEKNL"
/transl_table=11
/product="Truncated replication protein for pUB110
plasmid"
/db_xref="SEED:fig|176279.78.peg.6"
CDS complement(4635..5897)
/db_xref="SEED:fig|176279.78.peg.7"
/transl_table=11

/translation="MSYAVCRMQKVKSAGLKGMQFHNQRERKSRTNDDIDHERTRENY

DLKNDKNIDYNERVKEIIESQKTGTRKTRKDAVLVNELLVTSDRDFFEQLDPGEQKRF

FEESYKLFSERYGKQNIAYATVHNDEQTPHMHLGVVPMRDGKLQGKNVFNRQELLWLQ

DKFPEHMKKQGFELKRGERGSDRKHIETAKFKKQTLEKEIDFLEKNLAVKKDEWTAYS

DKVKSDLEVPAKRHMKSVEVPTGEKSMFGLGKEIMKTEKKPTKNVVISERDYKNLVTA

ARDNDRLKQHVRNLMSTDMAREYKKLSKEHGQVKEKYSGLVERFNENVNDYNELLEEN

KSLKSKISDLKRDVSLIYESTKEFLKERTDGLKAFKNVFKGFVDKVKDKTAQFQEKHD
LEPKKNEFELTHNREVKKERSRDQGMSL"

/product="Plasmid recombination enzyme"
CDS complement(6407..6811)
/db_xref="SEED:fig|176279.78.peg.8"
/transl_table=11
/translation="MRMLQSIPALPVGDIKKSIGFYCDKLGFTLVHHEDGFAVLMCNE

VRIHLWEASDEGWRSRSNDSPVCTGAESFIAGTASCRIEVEGIDELYQHIKPLGILHP
NTSLKDQWWDERDFAVIDPDNNLISFFQQIKS"
/product="Bleomycin resistance protein"
CDS complement(7028..7789)
/product="Aminoglycoside 4'-nucleotidyltransferase (EC
2.7.7.-) => ANT(4')-Ia"
/translation="MNGPIIMTREERMKIVHEIKERILDKYGDDVKAIGVYGSLGRQT

DGPYSDIEMMCVMSTEEAEFSHEWTTGEWKVEVNFDSEEILLDYASQVESDWPLTHGQ

FFSILPIYDSGGYLEKVYQTAKSVEAQTFHDAICALIVEELFEYAGKWRNIRVQGPTT

FLPSLTVQVAMAGAMLIGLHHRICYTTSASVLTEAVKQSDLPSGYDHLCQFVMSGQLS
DSEKLLESLENFWNGIQEWTERHGYIVDVSKRIPF"
/transl_table=11
/db_xref="SEED:fig|176279.78.peg.9"
CDS complement(7991..8665)
/product="Mobile element protein"
/transl_table=11
/translation="MNYFRYKQFNKDVITVAVGYYLRYALSYRDISEILRGRGVNVHH

STVYRWVQEYAPILYQIWKKKHKKAYYKWRIDETYIKIKGKWSYLYRAIDAEGHTLDI

WLRKQRDNHSAYAFIKRLIKQFGKPQKVITDQAPSTKVAMVKVIKAFKLKPDCHCTSK
YLNNLIEQDHRHIKVRKTRYQSINTAKNTLKGIECIYALYKKNRRSLQIYGFSPCHEI
SIMLAS"
/db_xref="SEED:fig|176279.78.peg.10"
CDS 8923..9090
/EC_number="2.3.3.10"
/db_xref="SEED:fig|176279.78.peg.11"
/translation="MLESREQLSVEEYETFFNRFDNQEFDFERELTQDPYSKVYLYSI
EDHIRTYKIEK"
/transl_table=11
/product="Hydroxymethylglutaryl-CoA synthase N-domain
homolog (EC 2.3.3.10)"
CDS 9927..10670
/product="Glycerophosphoryl diester phosphodiesterase (EC
3.1.4.46)"

/transl_table=11
/translation="MLTVYGHRGLPSKAPENTIASFKAASEVEGINWLELDVAITKDE

QLIIIHDDYLERTTNMSGEITELNYDEIKDASAGSWFGEKFKDEHLPTFDDVVKIANE

YNMNLNVELKGITGPNGLALSKSMVKQVEEQLTNLNQNQEVLISSFNVVLVKLAEEIM

PQYNRAVIFHTTSFREDWRTLLDYCNAKIVNTEDAKLTKAKVKMVKEAGYELNVWTVN
KPARANQLANWGVDGIFTDNADKMVHLSQ"
/EC_number="3.1.4.46"
/db_xref="SEED:fig|176279.78.peg.12"
/db_xref="GO:0008889"
CDS 10767..11195
/product="MaoC domain protein"
/translation="MKYDDFIVGETFKTKSLHITEEEIIQFATTFDPQYMHIDKEKAE

QSRFKGIIASGMHTLSISFKLWVEEGKYGEEVVAGTQMNNVKFIKPVYPGNTLYVIAE
ITNKKSIKKENGLVTVSLSTYNENEEIVFKGEVTALINNS"
/transl_table=11
/db_xref="SEED:fig|176279.78.peg.13"
CDS complement(11241..13247)
/transl_table=11
/translation="MKKIKIVPLILIVVVVGFGIYFYASKDKEINNTIDAIEDKNFKQ

VYKDSSYISKSDNGEVEMTERPIKIYNSLGVKDINIQDRKIKKVSKNKKRVDAQYKIK

TNYGNIDRNVQFNFVKEDGMWKLDWDHSVIIPGMQKDQSIHIENLKSERGKILDRNNV

ELANTGTAYEIGIVPKNVSKKDYKAIAKELSISEDYIKQQMDQNWVQDDTFVPLKTVK

KMDEYLSDFAKKFHLTTNETESRNYPLGKATSHLLGYVGPINSEELKQKEYKGYKDDA

VIGKKGLEKLYDKKLQHEDGYRVTIVDDNSNTIAHTLIEKKKKDGKDIQLTIDAKVQK

SIYNNMKNDYGSGTAIHPQTGELLALVSTPSYDVYPFMYGMSNEEYNKLTEDKKEPLL

NKFQITTSPGSTQKILTAMIGLNNKTLDDKTSYKIDGKGWQKDKSWGGYNVTRYEVVN

GNIDLKQAIESSDNIFFARVALELGSKKFEKGMKKLGVGEDIPSDYPFYNAQISNKNL

DNEILLADSGYGQGEILINPVQILSIYSALENNGNINAPHLLKDTKNKVWKKNIISKE

NINLLTDGMQQVVNKTHKEDIYRSYANLIGKSGTAELKMKQGETGRQIGWFISYDKDN
PNMMMAINVKDVQDKGMASYNAKISGKVYDELYENGNKKYDIDE"

/product="Penicillin-binding protein PBP2a, methicillin
resistance determinant MecA, transpeptidase"
/db_xref="SEED:fig|176279.78.peg.14"
/db_xref="GO:0008658"
/db_xref="GO:0046677"
CDS 13419..15104
/translation="MLYIKYTQNIMSHKIWLLVLVSTLIPLIPFYKISNFTFSKDMMN

RNVSDTTSSVSHMLDGQQSSVTKDLAINVNQFETSNITYMILLIWVFGSLLCLFYMIK

AFRQIDVIKSSSLESSYLNERLKVCQSKMQFYKKHITISYSSNIDNPMVFGLVKSQIV

LPTVVVETMNDKEIEYIILHELSHVKSHDLIFNQLYVVFKMIFWFNPALYISKTMMDN

DCEKVCDRNVLKILNRHEHIRYGESILKCSILKSQHINNVAAQYLLGFNSNIKERVKY

IALYDSMPKPNRNKRIVAYIVCSISLLIQAPLLSAHVQQDKYETNVSYKKLNQLAPYF

KGFDGSFVLYNEREQAYSIYNEPESKQRYSPNSTYKIYLALMAFDQNLLSLNHTEQQW

DKHQYPFKEWNQDQNLNSSMKYSVNWYYENLNKHLRQDEVKSYLDLIEYGNEEISGNE

NYWNESSLKISAIEQVNLLKNMKQHNMHFDNKAIEKVENSMTLKQKDTYKYVGKTGTG
IVNHKEANGWFVGYVETKDNTYYFATHLKGEDNANGEKAQQISERILKEMELI"
/transl_table=11
/product="Methicillin resistance regulatory
sensor-transducer MecR1"
/db_xref="SEED:fig|176279.78.peg.15"
/db_xref="GO:0008658"
CDS 15104..15475

/translation="MDNKTYEISSAEWEVMNIIWMKKYASANNIIEEIQMQKDWSPKT
IRTLITRLYKKGFIDRKKDNKIFQYYSLVEESDIKYKTSKNFINKVYKGGFNSLVLNF
VEKEDLSQDEIEELRNILNKK"
/transl_table=11
/product="Methicillin resistance repressor MecI"
/db_xref="SEED:fig|176279.78.peg.16"
/db_xref="GO:0003677"
/db_xref="GO:0016481"
/db_xref="GO:0016566"
CDS 15576..15788
/product="hypothetical protein"
/translation="MQVLIKSIMLVITPVKSICIPSFYLRYIKYNIKQIKNVKNSLIG
YLDCINVKNLKNIQRLCIKNRHIGNT"

/transl_table=11
/db_xref="SEED:fig|176279.78.peg.17"
CDS 15983..17092
/db_xref="SEED:fig|176279.78.peg.18"
/product="Putative ROK-family transcriptional regulator"
/translation="MLREIYNHHNISRTQISKNLEINKATISSILNKLKYKSLVNEVG

EGDSTKSGGRKPILLKVNHLYGYFISLDLTYSSVEVMYNYFDGNVIKHESYDLPDEKV
SSILSIIKKHIDIQEKLDTYNGLLGVSVSIHGVVDNEQHVTYLPFHETEGISIAKKIK

EITNVPVVVENEANLSALYERNFNHNLSYNNLIALSIHKGIGAGLIINNQLYRGANGE

AGEIGKTLVSKVSDNVEIFHKIEDIFSQEALLHNLSNQLNEKMTLSKLIQFYNEKNPV

VVEEMEQFINKIAVLIHNLNTQFNPNAIYINCPLFNEMPEILEAIKNQFKQYSRNEIQ
IKLTSNVKFATLLGGTLAIIQKVLQINDIYLDIKA"
/transl_table=11
CDS complement(17206..17511)
/product="Polysulfide binding protein"

/translation="MPQTNIVTSSIHSSEMTGEEHNVLDVRNDDEWRKGHLKQAIHIP

HGKLLNEDIPFDKNDLIYVHCQSGVRSSIAVGILEYKGFTNIVNVRGGYQHLPAFLK"
/transl_table=11
/db_xref="SEED:fig|176279.78.peg.19"
CDS complement(17602..18540)
/translation="MFFKQFYEDNFSQASYLIGCQRTGEAMIIDPIRDLTKYLKVADK

EGFTITKAAETHIHADFASGIRDVANKLNASIYVSDEGKDEFNYKNMPKQTHFVKHQD
IIYLGKIKLEVLHTPGHPPESISFLLTDEGGGSSIPMGLFSGDFIFVGDVGRPDLLEK

AVNVEGSTKIGAKQMYQSIQFAATLPDYIQIWPGHGAGSPCGKALGSLPTTTLGYEKI

NNWAFNVKDETSFIETLTLNQPAPPHHFSQMKKINQFGMQMYQPYNVYPSSSNTQTAF
DLRSKEAFHGGHMHGTINIPFNKTFINQIGWYLDYD"
/transl_table=11
/product="Zn-dependent hydroxyacylglutathione hydrolase /
Polysulfide binding protein"
/db_xref="SEED:fig|176279.78.peg.20"
CDS complement(18573..19637)
/translation="MEQYKEVHINSLNKEELEKLAKMGQLIDVRTKEEYELGHINGST

LHPVEEIKSFNKNSNKTYYIHCKSGNRSTKACDYLAKQGYDVVNLKGGYKDYEAKNFN
SAPLIEKDIEIKENRKQFDFRGLQCPGPIVNISKEINNISTGEQIEVTVTDPGFNSDI

KSWAKQTGNTLVNLTEEANVINAIIQKEKPKEFEINDTATGTTIVLFSGELDKAVAAM

IIANGAKAAGKDVTIFFTFWGLNALKKAQSTRVKKKGISKMFDLMLPKDPIHMPLSKM

NMFGLGNIMMRYVMNKKNVDSLYSLIDQAIDQDIKLIACTMSMDVMGISKEELRDEVD
YGGVGTYIGHTEQANHNLFI"
/transl_table=11
/product="Disulfide bond regulator"
/db_xref="SEED:fig|176279.78.peg.21"
CDS 19767..20033
/db_xref="SEED:fig|176279.78.peg.22"
/product="Metal-sensitive transcriptional repressor"

/translation="MNMTYDKKMINRINRIQGQLNGVVKMMEEEKDCKDIITQLSASK
GSIQRLMGIIISENLIECVKTAEENNESSQELINEAVNLLVKSK"
/transl_table=11
CDS 20048..20677
/product="FIG003846: hypothetical protein"
/transl_table=11
/translation="MTIMLLIGVLGGFISGLIGIGGAIIIYPAILILPPLIGIPAYSA

YIASGLTSSQVFFSTLSGSLNARKQPAFSPKLVIYMGGGMLIGSMLGAILASLFNATF
VNTVYVIIAILALILMFIKVKPTTQETKSKPLLFIIVGFGIGVISGIVGAGGAFIIIP
VLLALFKLPMNTVVNNSIAIAFISSVGAFFIKLMQGYIPVESAIFLIIE"
/db_xref="SEED:fig|176279.78.peg.23"
CDS complement(20945..21418)
/db_xref="SEED:fig|176279.78.peg.24"
/transl_table=11
/translation="MIGASLLVAQGVVNLIPQQWVIGLLGLLPLYLGVKIWIKGEEDE
DESSILSLFSSGKFNQLFLTMIFIVLASSADDFSIYIPYFTTLSMSEIFIVTIVFLIM
VGVLCYVSYRLASFDFISETIEKYERWIVPIVFIGLGIYILFENGTFNALISFLL"
/product="Cadmium resistance protein"
CDS complement(21457..21630)
/product="Cadmium resistance protein"
/translation="MQFTEGLFILKKSPIPKIFGDDKMIATILTATAVYYVATGIDYLV
ILILLFSQVKKVR"
/transl_table=11
/db_xref="SEED:fig|176279.78.peg.25"
CDS complement(21642..24056)
/db_xref="SEED:fig|176279.78.peg.26"
/EC_number="3.6.3.3"
/transl_table=11

/translation="MDSSTKTLTEDKQVYRVEGFSCANCAGKFEKNVKELSGVHDAKV

NFGASKIDVFGSATVEDLEKAGAFENLKVAPEKARRRVEPVVTEDKNVYRVEGFSCAN

CAGKFEKNVKQLAGVQDAKVNFGASKIDVYGNASVEELEKAGAFENLKVIPEKLANPS
IQAVKEDTKAPKEEKIPFYKKHSTLLFATLLIAFGYLSHFVNGEDNLVTSMLFVSSIV

IGGYSLFKVGFQNLIRFDFDMKTLMTVAVIGAAIIGEWAEASIVVILFAISEALERFS

MDRARQSIRSLMDIAPKEALVRRNGQEIMIHVDDIAVGDIMIVKPGEKIAMDGIIING

VSAVNQAAITGESVPVAKTVDDEVFAGTLNEEGLLEVKITKYVEDTTISKIIHLVEEA

QGERAPAQAFVDKFAKYYTPIIMVIAALVAVVPPLFFGGSWDTWVYQGLAVLVVGCPC
ALVITTPISIVSAIGNAAKKGVLIKGGVYLEELGAIKAIAFDKTGTLTKGVPVVTDFK

VLNDQVEEKELFSIITALEYRSQHPLASAIMKKAEQDNITYSDVRVKDFTSITGRGIQ

GNIDGTTYYIGSPRLFKELNVSDFSLEFENKVKVLQNQGKTAMIIGTDQTILGVIAVA

DEVRETSKNVILKLHQLGIKQTIMLTGDNQGTAEAIGAHVGVSDIQSELLPQDKLDYI

KKMKAEHGNVAMIGDGVNDAPALAASTVGIAMGGAGTDTAIETADIALMGDDLSKLPF
AVRLSRKTLNIIKANITFAIGIKIIALLLVIPGWLTLWIAILSDMGATILVALNSLRL
MRVKDK"
/product="Cadmium-transporting ATPase (EC 3.6.3.3)"
CDS complement(24049..24414)
/db_xref="SEED:fig|176279.78.peg.27"

/translation="MTKDMCEVTYIHEDKVNRAKKDLAKQNPMDVAKVFKALSDDTRV
KIAYVLSLEGELCVCDVANIIESSTATASHHLRLLKNLGIAKYRKEGKLVYYSLDDEH
VKQLVEKAFLHQREVASIG"
/transl_table=11
/product="Cadmium efflux system accessory protein"
CDS 24535..24642
/translation="MLETNPFGDFNQILKYLKHNCPVSLSASFHNLSTD"
/transl_table=11
/product="hypothetical protein"
/db_xref="SEED:fig|176279.78.peg.28"
CDS complement(24652..25029)
/translation="MNKQVRNTTEIVRLAKQKSQKTREKVDKAISKFSIEGKAINFNS

IAKEANVSKSWLYKEHDIRQRIESLRERQITSNVVSKPKKSSRSEEILIKTLKRRVME
LEKENKKLQNQIQKLYGDLYNKE"

/transl_table=11
/product="Mobile element protein"
/db_xref="SEED:fig|176279.78.peg.29"
CDS complement(25036..26928)
/db_xref="SEED:fig|176279.78.peg.30"
/product="Transposase B from transposon Tn554"

/translation="MNASSKRKIISQSEISKKIAVMNEEMQGFWANNSWDIRKCPHPS

AIELSKNPALRNRWVRFERVKNLWLRTELKYFYFYHLNNGIWNAKTVWIRKGTVINKM

LDFLDLKYPSITSITEVPIDKAMTEYRTYLTKQGVRITTTNYKITANQEKTPVKANSY

YVTNLKQFIEFYEDFYFDGEEWDKDVWDRRNLPLPDDKVNPTQYEYTINFKGFRNTYF

KQLVKRYCKLRLNMDSFSYVSDIAQKLKEFFNFLDIKFKHVQRVHQLTRVEIEAYLSE
LNMMGIKPSTITGRISILEGLFSTLHRLEWDDVPSKLLIYPEDYPKIPRAKPRFIDEF

VLEQLNSHLDKLPEYIATMTMIVQECGMRISELCTLKKGCLLEDKDGDYFLKYYQWKM

KKEHIVPISKEVVLLIKVREDKVSEEFPDSEYLFPRKDGSPLKQETFRGELNKLAYEQ

NIVDKLGEIYRFHAHAFRHSVGTRMINNGVPQHIVQKFLGHESPEMTSRYAHIFDETL

KNEFTKFQEKLVTNNGDVLNLDDDSEVDDVELQWFKKNINAQVLPNGYCRLPVIAGGC

PHANACLDCTHFCTSKQFLPQHEEQLERTEELLTIAKDKQWQRQIETNSRVKERLEQI
IGSLTG"
/transl_table=11
CDS complement(27123..27446)
/db_xref="SEED:fig|176279.78.peg.31"
/transl_table=11

/translation="MSRKRIDVVKVQMVKEDTLWYLKRRIEEPKDAADIMRDFIGNAD
REHFILICLNSKNEPTHIETVSIGTINFAVIHPREIFKTAILSNATGMIIGHNHPSGD
ILTIV"
[truncated: 444,883 more chars]
